# Heterogeneous viral-host response at single-cell resolution and viral adaptive mutational atlas in birds and mammals upon H5 influenza virus infection

**DOI:** 10.1093/nsr/nwag380

**Published:** 2026-06-25

**Authors:** Jiahao Zhang, Huaiyuan Cai, Tingting Man, Lihong Huang, Letian Li, Yu Chen, Haixing Yang, Shiping Ding, Yiqun Chen, Ruixuan Zhu, Zhengze Wang, Qian Zhang, Jinjun Liu, Xiaona Chen, He Zhang, Xinyu Zhuang, Xiaoshu Zhou, Hui Guo, Tao Zhang, Yi Liu, Junlong Xiong, Jiangtao Zhou, Weize Xu, Xiaofeng Wu, Weixin Jia, Zhangyong Ning, Ke Xiao, Jinxia Dai, Weifeng Shi, Wenbao Qi, Gang Cao, Ming Liao

**Affiliations:** Key Laboratory of Zoonoses, Ministry of Agriculture and Rural Affairs, College of Veterinary Medicine, South China Agricultural University, Guangzhou 510642, China; State Key Laboratory for Animal Disease Control and Prevention, South China Agricultural University, Guangzhou 510642, China; State Key Laboratory of Agricultural Microbiology, Huazhong Agricultural University, Wuhan 430070, China; College of Veterinary Medicine, Huazhong Agricultural University, Wuhan 430070, China; National Avian Influenza Para-Reference Laboratory, Guangzhou 510642, China; National and Regional Joint Engineering Laboratory for Medicament of Zoonoses Prevention and Control, Guangzhou 510642, China; State Key Laboratory of Agricultural Microbiology, Huazhong Agricultural University, Wuhan 430070, China; College of Veterinary Medicine, Huazhong Agricultural University, Wuhan 430070, China; State Key Laboratory of Agricultural Microbiology, Huazhong Agricultural University, Wuhan 430070, China; College of Veterinary Medicine, Huazhong Agricultural University, Wuhan 430070, China; Key Laboratory of Zoonoses, Ministry of Agriculture and Rural Affairs, College of Veterinary Medicine, South China Agricultural University, Guangzhou 510642, China; State Key Laboratory for Animal Disease Control and Prevention, South China Agricultural University, Guangzhou 510642, China; National Avian Influenza Para-Reference Laboratory, Guangzhou 510642, China; National and Regional Joint Engineering Laboratory for Medicament of Zoonoses Prevention and Control, Guangzhou 510642, China; Changchun Veterinary Research Institute, Chinese Academy of Agricultural Sciences, Changchun 130122, China; State Key Laboratory of Pathogen and Biosecurity, Key Laboratory of Jilin Province for Zoonosis Prevention and Control, Changchun 130122, China; State Key Laboratory of Agricultural Microbiology, Huazhong Agricultural University, Wuhan 430070, China; College of Life Science and Technology, Huazhong Agricultural University, Wuhan 430070, China; State Key Laboratory of Agricultural Microbiology, Huazhong Agricultural University, Wuhan 430070, China; College of Veterinary Medicine, Huazhong Agricultural University, Wuhan 430070, China; Key Laboratory of Zoonoses, Ministry of Agriculture and Rural Affairs, College of Veterinary Medicine, South China Agricultural University, Guangzhou 510642, China; Key Laboratory of Zoonoses, Ministry of Agriculture and Rural Affairs, College of Veterinary Medicine, South China Agricultural University, Guangzhou 510642, China; State Key Laboratory of Agricultural Microbiology, Huazhong Agricultural University, Wuhan 430070, China; College of Veterinary Medicine, Huazhong Agricultural University, Wuhan 430070, China; Lingang Laboratory, Shanghai Tech University, Shanghai 200031, China; College of Bioinformatics, Huazhong Agricultural University, Wuhan 430070, China; State Key Laboratory of Agricultural Microbiology, Huazhong Agricultural University, Wuhan 430070, China; College of Life Science and Technology, Huazhong Agricultural University, Wuhan 430070, China; Key Laboratory of Zoonoses, Ministry of Agriculture and Rural Affairs, College of Veterinary Medicine, South China Agricultural University, Guangzhou 510642, China; Changchun Veterinary Research Institute, Chinese Academy of Agricultural Sciences, Changchun 130122, China; State Key Laboratory of Pathogen and Biosecurity, Key Laboratory of Jilin Province for Zoonosis Prevention and Control, Changchun 130122, China; Changchun Veterinary Research Institute, Chinese Academy of Agricultural Sciences, Changchun 130122, China; State Key Laboratory of Pathogen and Biosecurity, Key Laboratory of Jilin Province for Zoonosis Prevention and Control, Changchun 130122, China; College of Biomedicine and Health, Huazhong Agricultural University, Wuhan 430070, China; State Key Laboratory of Agricultural Microbiology, Huazhong Agricultural University, Wuhan 430070, China; College of Veterinary Medicine, Huazhong Agricultural University, Wuhan 430070, China; Key Laboratory of Zoonoses, Ministry of Agriculture and Rural Affairs, College of Veterinary Medicine, South China Agricultural University, Guangzhou 510642, China; Key Laboratory of Zoonoses, Ministry of Agriculture and Rural Affairs, College of Veterinary Medicine, South China Agricultural University, Guangzhou 510642, China; Key Laboratory of Zoonoses, Ministry of Agriculture and Rural Affairs, College of Veterinary Medicine, South China Agricultural University, Guangzhou 510642, China; Key Laboratory of Zoonoses, Ministry of Agriculture and Rural Affairs, College of Veterinary Medicine, South China Agricultural University, Guangzhou 510642, China; State Key Laboratory of Agricultural Microbiology, Huazhong Agricultural University, Wuhan 430070, China; College of Veterinary Medicine, Huazhong Agricultural University, Wuhan 430070, China; State Key Laboratory of Agricultural Microbiology, Huazhong Agricultural University, Wuhan 430070, China; College of Veterinary Medicine, Huazhong Agricultural University, Wuhan 430070, China; Key Laboratory of Zoonoses, Ministry of Agriculture and Rural Affairs, College of Veterinary Medicine, South China Agricultural University, Guangzhou 510642, China; State Key Laboratory for Animal Disease Control and Prevention, South China Agricultural University, Guangzhou 510642, China; National Avian Influenza Para-Reference Laboratory, Guangzhou 510642, China; National and Regional Joint Engineering Laboratory for Medicament of Zoonoses Prevention and Control, Guangzhou 510642, China; Key Laboratory of Zoonoses Prevention and Control of Guangdong Province, Guangzhou 510642, China; Key Laboratory of Zoonoses, Ministry of Agriculture and Rural Affairs, College of Veterinary Medicine, South China Agricultural University, Guangzhou 510642, China; State Key Laboratory for Animal Disease Control and Prevention, South China Agricultural University, Guangzhou 510642, China; Shenzhen Institute of Advanced Technology, Chinese Academy of Sciences, Shenzhen 518107, China; State Key Laboratory of Agricultural Microbiology, Huazhong Agricultural University, Wuhan 430070, China; College of Veterinary Medicine, Huazhong Agricultural University, Wuhan 430070, China; Ruijin Hospital, Shanghai Jiao Tong University School of Medicine, Shanghai 200025, China; Shanghai Institute of Virology, Shanghai Jiao Tong University School of Medicine, Shanghai 200025, China; Key Laboratory of Zoonoses, Ministry of Agriculture and Rural Affairs, College of Veterinary Medicine, South China Agricultural University, Guangzhou 510642, China; State Key Laboratory for Animal Disease Control and Prevention, South China Agricultural University, Guangzhou 510642, China; National Avian Influenza Para-Reference Laboratory, Guangzhou 510642, China; National and Regional Joint Engineering Laboratory for Medicament of Zoonoses Prevention and Control, Guangzhou 510642, China; Key Laboratory of Zoonoses Prevention and Control of Guangdong Province, Guangzhou 510642, China; State Key Laboratory of Agricultural Microbiology, Huazhong Agricultural University, Wuhan 430070, China; College of Veterinary Medicine, Huazhong Agricultural University, Wuhan 430070, China; Faculty of Life and Health Sciences, Shenzhen University of Advanced Technology, Shenzhen 518107, China; Shenzhen Institute of Advanced Technology, Chinese Academy of Sciences, Shenzhen 518107, China; Key Laboratory of Zoonoses, Ministry of Agriculture and Rural Affairs, College of Veterinary Medicine, South China Agricultural University, Guangzhou 510642, China; National Avian Influenza Para-Reference Laboratory, Guangzhou 510642, China; National and Regional Joint Engineering Laboratory for Medicament of Zoonoses Prevention and Control, Guangzhou 510642, China; Key Laboratory of Zoonoses Prevention and Control of Guangdong Province, Guangzhou 510642, China; College of Animal Science and Technology, Zhongkai University of Agriculture and Engineering, Guangzhou 510225, China; Key Laboratory for Prevention and Control of Avian Influenza and Other Major Poultry Diseases, Ministry of Agriculture and Rural Affairs, Institute of Animal Health, Guangdong Academy of Agricultural Sciences, Guangzhou 510640, China

**Keywords:** H5 subtype, highly pathogenic avian influenza virus, scRNA-seq, lung microenvironment, cross-species, adaptive mutations, MiP-seq

## Abstract

H5 highly pathogenic avian influenza viruses (HPAIVs) are prevalent in birds, causing infection in over 60 mammalian species worldwide. However, species-specific viral-host response at the single-cell level and within-host evolution upon H5 HPAIV infection are poorly understood. Here, by integrating spatial transcriptomics and single-cell genomics technologies, we present a comprehensive single-cell transcriptomic landscape of 1499 669 cells in the lungs from birds (chickens, ducks, and pigeons) and mammals (mice and pigs) upon H5 HPAIV infection. H5 HPAIV primarily infected avian endothelial cells and mammalian epithelial cells, respectively. Among the single-cell transcriptomic lung landscape, H5 HPAIV infection induced significant inflammation in fibroblasts and the shedding of alveolar epithelial cells of chickens upon infection, while the expression levels of cytokine- and inflammation-related genes slightly decreased in fibroblasts and endothelial cells of infected ducks and pigeons, respectively. However, mice only exhibited significantly antiviral response in macrophages and neutrophils, while antiviral response was elevated in most subtype cells of pigs upon infection. Notably, we identified 19 viral non-synonymous intrahost single nucleotide variants in the infected birds and mammals and assessed their molecular characterizations *in vitro*. Three of them were identified to increase polymerase activity and viral replication *in situ*, among which the PB2-E627K mutation was enriched in the alveolar structure of lungs, accompanied by elevated expression of interferon-stimulated genes. Collectively, we elucidated distinct viral replication, host response, and viral adaptation in the lungs across diverse birds and mammals upon H5 HPAIV infection.

## INTRODUCTION

First identified in Guangdong province, China in 1996, the H5 subtype highly pathogenic avian influenza virus (HPAIV) has caused mass mortality in poultry and wild birds, as well as incidental infection in over 60 mammalian species, and ∼1000 sporadic human cases [1,2], with the mortality rate of ∼50%, posing a great threat to the poultry

industry and public health. During the global dissemination, the H5 subtype HPAIV has evolved into multiple clades, among which clade 2.3.4.4 has become dominant worldwide [2–4]. Since 2024, clade 2.3.4.4b H5 HPAIV has maintained enzootic circulation in wild birds across multiple continents, with documented spillover to marine mammals, carnivores, and rodents [4–7]. Strikingly, in the spring of 2024, an unprecedented outbreak of 2.3.4.4b H5N1 HPAIV occurred in dairy cattle herds in the USA, leading to multidirectional interspecies transmissions [8–10]. Mammal-to-mammal transmission has also been observed in farmed mink and sea lions, underscoring ongoing viral adaptation to mammalian hosts [11]. Concurrently, human infections have escalated, with 70 laboratory-confirmed cases reported in the United States between March 2024 and May 2025 [12,13], highlighting its capacity to cross species barriers.

Chickens and ducks are the world’s two most significant sources of poultry meat. Ducks are the natural reservoirs of avian influenza viruses, which frequently come into contact with wild and domestic birds, accelerating the generation of novel influenza variants [14–16]. Feral pigeons occupy synanthropic niches, facilitating viral bridging between wild birds, domestic poultry, and human settlements [17]. Mice serve as a good model for evaluating the pathogenicity of influenza viruses in mammals. Pigs have always been spotlighted in the context of avian influenza virus infection due to their role as a ‘mixing vessel’, accelerating the generation of novel avian influenza variants, which play an important role in the interspecies transmission of avian influenza viruses [18,19]. Although several recent studies compared the pathogenicity of 2.3.4.4b H5 HPAIV in birds and mammals [8,19–21], the understanding of the cellular and molecular variations in host responses and within-host evolution across diverse species infected with H5 HPAIV remains very limited.

HPAIVs, particularly the H5 subtype, cause severe respiratory disease in humans. These infections frequently progress to acute respiratory distress syndrome, with immune-mediated lung damage being a primary driver of mortality [22,23]. The lung damage is caused by the infiltration of immune cells that attack both infected and bystander cells [24]. Although recent studies have reported the host response heterogeneity in lungs infected with seasonal influenza virus using single-cell RNA sequencing (scRNA-seq) and spatial transcriptomics [25–28], a holistic lung immune microenvironment atlas across diverse species upon H5 HPAIV infection is poorly understood. In addition, viral adaptive mutations have been found to facilitate influenza viruses crossing the species barrier to infect mammals. Therefore, deciphering the correlation between key adaptive mutations of H5 HPAIV and lung immune microenvironment is highly warranted.

Herein, we mapped the lung transcriptomic atlas of representative birds (chickens, ducks, and pigeons) and mammals (mice and pigs) upon 2.3.4.4b H5 HPAIV infection, which unveils the dynamic viral-host immune response. Importantly, we also identified 19 viral intrahost single-nucleotide variants (iSNVs) and assessed their molecular characterizations *in vitro*, and three of them were identified to increase virus replication *in situ*. Our results provide a comprehensive resource elucidating heterogeneous viral-host response at the single-cell level and within-host evolution across different species following H5 HPAIV infection.

## RESULTS

### Species-specific tissue tropism of H5 HPAIV across avian and mammalian hosts

**Figure 1.**
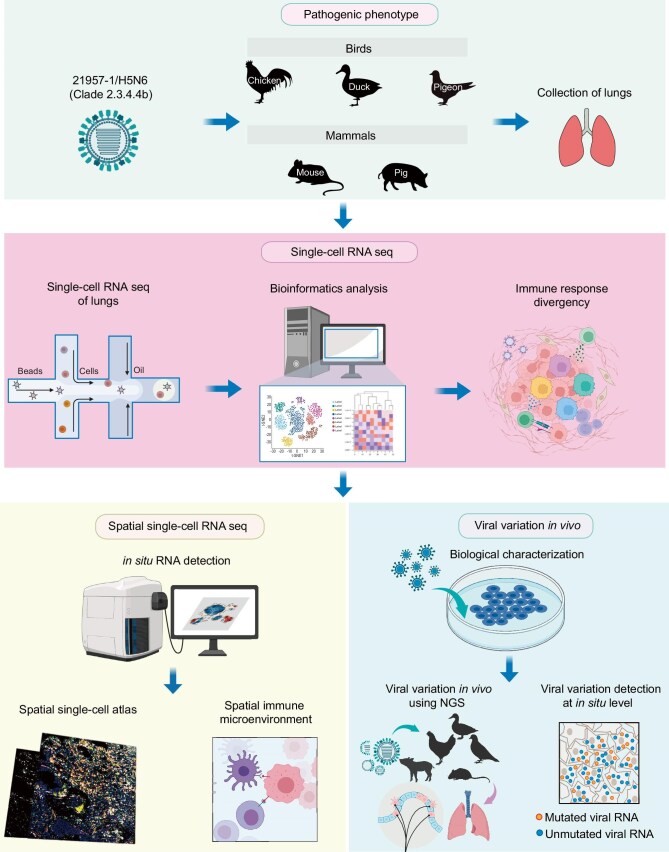
Schematic illustration of the experiment design in this study. Single-cell RNA sequencing, spatial single-cell atlas, and viral infection of lungs in chickens, ducks, pigeons, mice, and pigs infected with H5N6 influenza virus. The scRNA-seq and *in situ* hybridization (MiP-seq) were applied to obtain visualization of spatial single-cell transcriptomic profiles in the lungs of chickens, ducks, pigeons, mice, and pigs throughout H5N6 influenza virus infection.

**Figure 2.**
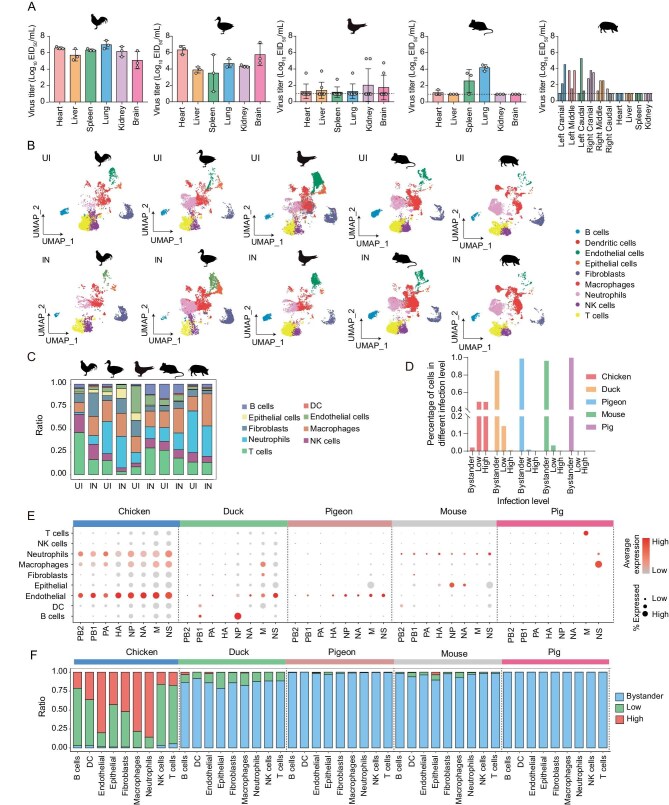
Single-cell profiling reveals viral-load heterogeneity in specific cell types in different species. (A) Viral titers in the heart, liver, spleen, lung, kidney, and brain of infected chickens, ducks, pigeons, mice, and pigs. The viral titers are presented as the mean ± SD. The dashed lines indicate the lower limit of detection. EID_50_, 50% egg infective dose. (B) UMAP plots of integrated cell types of uninfected (UI) and infected (IN) lungs across different species. (C) The proportions of different cell types in the lungs of different species were compared between UI and IN samples. (D) The number of infected cells with different infection levels across different species. Bystander represents non-infected cells (UMI counts of viral transcripts = 0), low represents lowly infected cells (0 < UMI counts of viral transcripts < 8), and high represents highly infected cells (UMI counts of viral transcripts ≥ 8). (E) A dot plot revealing the expression levels of eight gene segments across different species. (F) The ratios of different subtype cells with different infection levels across various species.

To investigate the divergent pathogenicity of H5 HPAIV infection across different species, we used a duck-origin H5 HPAIV to evaluate its pathogenicity in chickens, ducks, pigeons, mice, and pigs (Fig. 1). It belonged to clade 2.3.4.4b and was closely related to recently emerged wild bird- and bovine-origin H5 HPAIV (Fig. S1). Significant interspecies differences in viral replication efficiency were observed across organs (Fig. 2A). The average viral titers in the heart, liver, spleen, lung, kidney, and brain of chickens were 6.58, 5.71, 6.33, 6.97, 6.17, and 5.08 lgEID_50_/g, respectively, while the average viral titers in the counterparts of ducks were 6.33, 3.89, 3.50, 4.67, 4.33, and 5.75 lgEID_50_/g, respectively. However, in pigeons, virus replication was low in most tested organs (Fig. 2A), and the average viral titers in heart, liver, spleen, lung, kidney, and brain of pigeons were only 1.27, 1.4, 1.18, 1.28, 2.03, and 1.75 lgEID_50_/g, respectively. Further, the virus only efficiently replicated in the lungs of the three mice, with the average viral titer of 4.17 lgEID_50_/g. One of the infected pig exhibited clear clinical signs such as wheezing, dyspnea, and coughing, and the virus was detected in the lungs with viral titers ranging from 1.25 to 5.25 lgEID_50_/g.

Histopathological examination of lungs across different species upon infection revealed distinct pathological alterations. Chickens developed severe peribronchial hemorrhage, capillary congestion, and perivascular edema (Fig. S2), indicating acute vascular injury. Lymphocytic infiltration and cellular debris accumulation were observed in localized areas, indicating an inflammatory response. Ducks showed relatively mild pathological changes, with intact parabronchial architecture, moderate vascular congestion, and only scattered lymphocytic infiltration (Fig. S3). In pigeons, the lung structure was largely preserved, with minimal histological alterations, consistent with restricted viral replication (Fig. S4). In contrast, mammalian species developed differential pulmonary lesions. In mammals, mice displayed diffuse alveolar damage with alveolar collapse, septal thickening, and neutrophil-dominated inflammation (Fig. S5). In pigs, the lungs displayed severe alveolar wall thickening and interstitial broadening, along with collapsed alveolar spaces and accumulation of eosinophilic exudates (Fig. S6). Collectively, these results highlight significant inter-species differences in lung pathology upon H5 subtype HPAIV infection. Avian species, especially chickens, showed inflammatory responses, while mammalian species, particularly pigs and mice, developed severe lesions.

### Single-cell atlas of H5 HPAIV cellular tropism across avian and mammalian hosts

We conducted scRNA-seq to comprehensively examine the cellular and molecular alterations in the lungs in five uninfected and infected species (Fig. 1). A total of 98 316 cells were obtained in the lungs of uninfected chickens (*n* = 9747), ducks (*n* = 9834), pigeons (*n* = 17 156), mice (*n* = 9315), and pigs (*n* = 11 284), while the cell counts in the lungs of infected chickens, ducks, pigeons, mice, and pigs were 3511, 9370, 7703, 10 683, 9713, respectively. Nine cell types were annotated, including B cells, T cells, NK cells, dendritic cells, neutrophils, macrophages, endothelial cells, epithelial cells, and fibroblasts (Fig. 2B, Fig. S7 and Data S1).

Post infection, we found the proportion of T cells decreased from 46.45% to 16.66% in the lung of chickens, while an increased infiltration (from 10.85% to 21.32%) was found in macrophages (Fig. 2C). In contrast, the proportions of macrophages and neutrophils remained stable after infection in ducks, while T cells decreased from 15.71% to 3.67%. Pigeons had an increase in the proportions of T cells (from 8.68% to 29.56%) and B cells (from 1.61% to 11.48%) after infection, while an increased proportion of macrophages was observed in the infected mice (from 18.29% to 27.31%) and pigs (from 16.14% to 35.36%) (Fig. 2C).

We investigated the presence of H5 HPAIV poly-adenylated mRNA in the infected cells. According to the expression counts of influenza virus transcripts, the cells susceptible to H5 HPAIV infection could be divided into highly infected cells, lowly infected cells, and uninfected cells. We found that 48.85% of the cells from chicken were highly infected cells, ranking first (Fig. 2D and E), while only 0.58%, 0.10%, 0.26%, and 0.02% of the cells were highly infected in ducks, pigeons, mice, and pigs, respectively. Besides, the lowly infected cells were abundant in chickens (49.07%) and ducks (14.49%), while only 0.87%, 3.27%, and 0.25% of the cells in pigeons, mice, and pigs were lowly infected cells (Fig. 2D).

We then analyzed the viral infection levels among various cell types. In detail, the viral mRNA can be detected in all the examined cell types in the chicken lungs. Particularly, 85.71% of the neutrophils, 80.00% of endothelial cells, and 78.13% of macrophages were highly infected cells (Fig. 2F). In contrast, only 2.01% and 0.75% of the endothelial cells in ducks and pigeons were highly infected (Fig. 2F). Within the mammalian hosts, 10.53% of epithelial cells and 6.72% of macrophages in mice can be infected, whereas in pigs, the infection ability was limited in almost all cell types (Fig. 2F). Overall, H5 HPAIV exhibits extensive cell type tropism in chickens, particularly neutrophils and endothelial cells, in contrast to a predominance of infection in epithelial cells and macrophages in mice.

### Single-cell resolution of species-divergent immune responses to H5 HPAIV infection

We identified up-regulated differentially expressed genes (DEGs) in cell-type-specific transcriptomes across species upon infection (Figs S8–S11). Gene ontology (GO) enrichment analysis of these DEGs revealed species-divergent immune pathways (Figs S12–S16). Our results suggested that the cytokine-mediated signaling pathway was enriched in the up-regulated DEGs of endothelial cells and fibroblasts in the infected chickens, while the up-regulated DEGs in macrophages and neutrophils of the infected ducks were enriched in the ATP metabolic process. In infected mice, the up-regulated DEGs were enriched in response to interferon-beta and defense response to virus in most cell types. In infected pigs, the up-regulated DEGs were enriched in response to cell–cell adhesion in most cell types.

Then, we compared the expression levels of inflammation-related genes, interferon stimulated genes (ISGs), cytokine-related genes, ATP metabolic process, and cell–cell adhesion molecules in all cell types across five species upon infection (Fig. S17). In infected chickens, the expression levels of inflammation-related genes were significantly up-regulated (3.46-fold) in fibroblasts upon infection, while those of the inflammatory genes significantly decreased (2.83-fold) in fibroblasts of pigeons upon infection. In the infected ducks, the expression levels of cell–cell adhesion-associated genes significantly decreased in most cell types (i.e. fibroblasts, macrophages, neutrophils, NK cells, and T cells). Notably, the expression levels of the ISGs were significantly up-regulated 1.92- and 2.01-fold in macrophages and neutrophils of mice upon infection, respectively. Pigs showed broad ISG induction in endothelial cells, fibroblasts, macrophages, neutrophils, and T cells (Fig. S17), indicating robust antiviral states despite limited viral replication.

Together, these results suggested that H5 HPAIV infection induced significant inflammation in fibroblasts of chickens upon infection, and the expression levels of cytokine- and inflammation-related genes significantly decreased in fibroblasts of the infected ducks and pigeons, respectively. However, mice only exhibited significantly antiviral response in macrophages and neutrophils, while antiviral response elevated in most immune cell types of pigs upon infection.

### Subtype-specific host responses in endothelial cells and fibroblasts to H5 HPAIV

**Figure 3.**
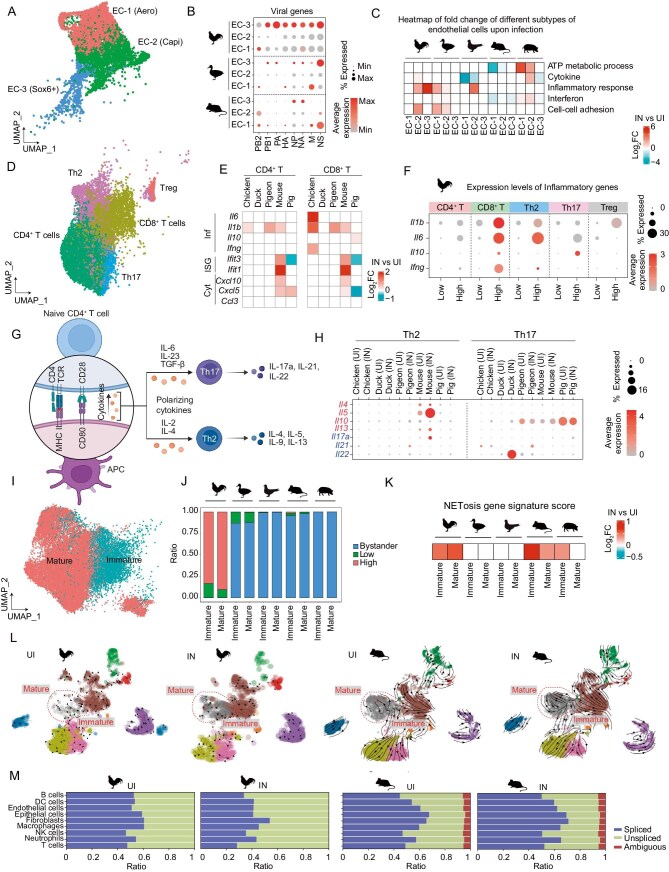
Distinct immune response of endothelial cells, T cells, and neutrophils in different species during infection. (A) UMAP plot for three subtypes of endothelial cells in lungs of different species. EC represents endothelial cells, Aero represents aerocytes, and Capi represents capillary. (B) Dot plot revealing the viral gene expression level in endothelial cells of infected lungs in chickens, ducks, and mice. (C) Gene-level heatmaps showing log_2_ fold changes relative to uninfected species in different gene scores. (D) UMAP plot of different subtypes of T cells of lungs in different species. (E) Gene-level heatmaps showing log_2_ fold changes in *Il1b, Il6, Il10*, and *Ifng* genes of CD8^+^ T cells with different infection levels across all species. (F) Gene-level heatmaps showing log_2_ fold changes relative to UI species in interferon stimulated genes, inflammatory-related genes, and chemokine receptors of CD4^+^ and CD8^+^ T cells of different species. (G) T cell differentiation flow chart of Th2 and Th17. (H) Gene-level heatmaps showing log_2_ fold changes relative to UI species in *Il4, Il5, Il10, Il13, Il17a, Il21*, and *Il22* genes of Th2 and Th17 cells of different species. (I) UMAP plot of mature and immature neutrophils of lungs in different species. (J) Proportions of mature and immature neutrophils with different infection levels of different species. (K) Heatmaps showing log_2_ fold changes relative to UI species for NETosis gene module scores in neutrophils of different species. (L) UMAP plots of RNA velocity of different cell types in UI and IN chickens and mice. Arrow direction and length indicate qualitative and quantitative changes, respectively. (M) The proportions of spliced and unspliced RNA in different cell types of UI and IN chickens and mice.

Transcriptomes of fibroblasts and endothelial cells from five infected species were compared to uninfected species, focusing on immune pathway activation. Here, we divided the endothelial cells into three subtypes according to the maker genes of previously described, including endothelial aerocytes, capillary endothelial cells, and aerocytes *Sox6*^high^. High viral load was observed solely in aerocytes *Sox6*^high^ of infected chickens and ducks. However, in infected mice, aerocytes are the main infection subtype of endothelial cells (Fig. 3A and B). Next, we examined gene module scores of different subtype endothelial cells upon infection. In chickens, EC-3 (aerocytes *Sox6*^high^) showed highest infection rates and pro-inflammatory gene upregulation (32-fold), suggesting infection-driven hyperinflammation (Fig. 3A–C). However, the expression levels of cytokine-related genes significantly decreased 12.73- and 2.50-fold in EC-1 (endothelial aerocytes) and EC-2 (capillary endothelial cells) of pigeons upon infection, respectively. Mammalian endothelial subtypes showed no consistent pro-inflammatory gene induction (Fig. 3C).

In fibroblasts, H5 HPAIV primarily infected Fibro-2 and Fibro-3 of chickens, while mainly infecting Fibro-2 and Fibro-3 of mice and ducks (Fig. S18A and B). Notably, we also found that the expression levels of inflammatory response-related genes significantly increased 112.99-, 10.13-, and 3.39-fold in Fibro-1, Fibro-2, and Fibro-3 of chickens upon infection (Fig. S20C), respectively. The expression levels of interferon response-related genes significantly decreased 1.95-fold in mice upon infection, while significantly elevated in pigs (Fibro-1 and Fibro-2) upon infection (Fig. S18C). Together, these results suggest that non-immune cells in chickens exhibited infection-proportional hyperinflammation, while pigs showed infection-independent ISG induction in fibroblasts.

### CD8^+^ T cell hyperinflammation drives immunopathology in H5 HPAIV-infected chickens

Herein, we identified five T cell clusters based on the expression levels of marker genes, including CD4^+^, CD8^+^, Th2, Th17, and Treg (Fig. 3D and Fig. S19A). A notable increase in the CD4^+^ T cells (from 25.06% to 43.58%) and a decrease in the CD8^+^ T cells (from 22.98% to 8.10%) were found in the infected chickens. The proportion of CD4^+^ T cells slightly increased (from 26.56% to 37.21%), but that of CD8^+^ T cells (from 22.98% to 8.10%) decreased in ducks after infection. In contrast, a notable increase was found in CD8^+^ T cells (from 8.95% to 29.33%) after infection in pigeons, but not in mice after infection. Nonetheless, a notable increase in the CD4^+^ T cells (from 29.94% to 47.43%) and a decrease in the CD8^+^ T cells (from 49.92% to 26.02%) were found in the infected pigs, similar to infected chickens (Fig. S19B).

CD4^+^ T cells infected with H5 HPAIV were involved in the inflammation-related pathways and were associated with oxidative stress and cell death [29], while increased CD8^+^ T cell response after influenza virus infection can result in the airway cytokine and chemokine expression [30]. Here, inflammatory genes *Il1b* and *Il6*, indicators of the severity of viral infection, increased 2.89- and 3.94-fold in the CD8^+^ T cells in chickens after infection, respectively. *Il1b* also increased 2-fold in CD8^+^ T cells of pigeons after infection, while the expression levels of inflammatory genes did not show significant differences in mammals (Fig. 3E). Notably, the Pearson correlation coefficients between inflammatory genes (*Il1b* and *Il6*) and viral genes in the CD8^+^ T cells in chickens were 0.36 (*P* = 4e-12) and 0.64 (*P* = 2.2e-4), indicating a high correlation (Fig. 3F). Furthermore, the expression levels of ISGs (*Ifit1 and Ifit3*) increased 3.92- and 1.97-fold in the CD4*^+^*T cells in the infected mice, respectively, and they increased 3.78- and 1.92-fold of the CD8*^+^*T cells, respectively. However, a down-regulation of 1.37- and 1.35-fold in *Ifit3* was found in the CD4*^+^*and CD8^+^ T cells in the infected pigs (Fig. 3E). Th2 cells, which are influenced by *Il4* and *Ifnγ*, produce signature cytokines such as *Il4, Il5, Il-13*, and *Il10*, crucial for regulating immune responses during viral infection (Fig. 3G). Here, we found that the expression levels of *Il4* and *Il5* genes increased 1.78- and 3.87-fold in mice following infection, respectively (Fig. 3H). Furthermore, a marked up-regulation of the *Il22* gene in the Th17 cells, promoting inflammatory pathology, was specifically observed in ducks after infection (Fig. 3H).

### Divergent neutrophil responses to H5 HPAIV infection across species

During acute influenza virus infection, neutrophils are already active in the early phase of inflammation [31,32]. Here, we divided the neutrophils into immature and mature neutrophils using transcriptional markers (Fig. 3I). 83.57% of the immature neutrophils and 90.63% of the mature neutrophils were highly infected in chickens (Fig. 3J). In contrast, 33.33% of immature neutrophils were highly infected cells in mice, while 11.54% of mature neutrophils were highly infected cells (Fig. 3J). We then performed RNA velocity analysis to analyze the RNA dynamics of different cell types in chickens and mice after infection, examining the time-resolved RNA transcription and splicing pattern [33]. After infection, the proportion of spliced RNA of neutrophils noticeably decreased from 54% to 44% in chickens, while it increased from 57% to 65% in mice (Figs 3L and M). These results suggest that the development from immature to mature neutrophils is crucial for mice after infection.

Neutrophils have the potential to mitigate lung injury during influenza virus infections. However, the excessive release of neutrophil extracellular traps (NETs) has been associated with severe lung inflammation and fatal outcomes [31,32,34]. Our results suggested that the expression levels of NETosis signature gene (i.e. *Akt1, Akt2, Atg7, Bst1, Cd93, Csf3, Csf3r, Cybb, Entpd4, F3, Fpr1, Hmgb1, Hpse, Il1b, Il6, Irak4, Itgam, Itgb2, Padi4, Pde4b, Pik3ca, Ptafr, Ripk1, Selplg, Tlr2, Tlr4*, and *Tnf*) score significantly increased 1.41- and 1.52-fold in immature and mature neutrophils of chickens upon infection, while only significantly increased 1.64-fold in immature neutrophils of mice upon infection, but exhibited no significant differences in the expression levels of NETosis signature gene score in ducks, pigeons, and pigs (Fig. S20). Together, these results suggested that heightened NETosis signatures in chickens and mice correlate with immunopathology, whereas absent induction in ducks and pigs suggests a protective restraint mechanism.

### Heterogeneous kinetics of macrophages in the infected avian and mammalian species

**Figure 4.**
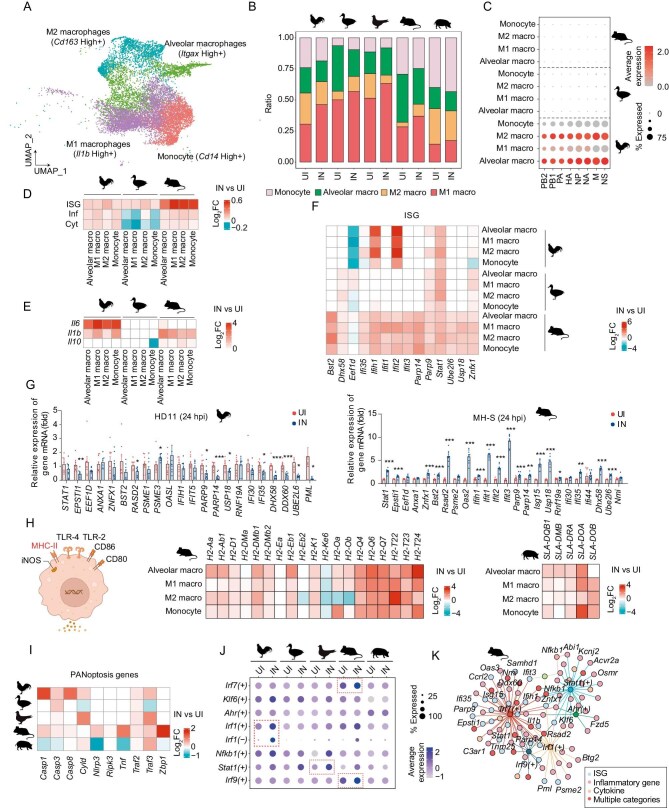
Distinct immune response in macrophages in different species during infection. (A) UMAP plots for three subtypes of macrophages in the lungs of different species. (B) The proportions of different subtypes of macrophages in uninfected (UI) and infected (IN) species. Macro represents macrophages. (C) Dot plot revealing the viral gene expression level in macrophages of infected lungs in chickens, ducks, and mice. (D) Gene-level heatmaps showing log_2_ fold changes relative to UI species in cytokine-related genes, inflammatory-related genes, and interferon-stimulated genes of macrophages in chickens, ducks, and mice. (E) Gene-level heatmaps showing log_2_ fold changes relative to UI species in *Il1b, Il6*, and *Il10* genes of macrophages in chickens, ducks, and mice. Macro represents macrophages. (F) Gene-level heatmaps showing log_2_ fold changes relative to UI species in interferon stimulated genes of macrophages in chickens, ducks, and mice. (G) The expression level changes of immune-related genes in HD11 cells and MH-S cells upon infection at 24 hpi. Data are represented as means ± SEM. The independent-sample (*n* = 9) *t*-test was used for analysis. (H) Gene-level heatmaps showing log_2_ fold changes relative to UI species in MHC-II related genes of macrophages across mice and pigs. (I) Gene-level heatmaps showing log_2_ fold changes relative to UI species in PANoptosis genes of macrophages across different species. (J) Dot plot of the RSS score of TFs in macrophages across different species. The size of the dot represents the proportion of the cells in which genes are detected. The color of the dot indicates the gene expression level. (K) TF regulation network of mice between given TFs and target interferon-stimulated genes, inflammatory-related genes, and cytokine-related genes. Multiple categories represent the genes that belong to up to two gene sets.

Here, we performed a sub-clustering analysis of macrophages (Fig. 4A). Four subtype macrophages were defined based on the marker genes, including monocyte-derived macrophages (i.e. *Cd14* and *Adgre1*), M1 macrophages (i.e. *Il1b* and *Nos2*), M2 macrophages (i.e. *Cd163* and *Il10*), and alveolar macrophages (i.e. *Csf2rb* and *Itgax*). The proportion of each subtype underwent distinctive changes (Fig. 4B). The sub-population of M1 macrophages increased in all species after infection, while the sub-population of M2 macrophages varied across animals. Chickens showed M2 macrophage reduction (from 25.0% to 18.6%), while mice exhibited M2 expansion (from 3.7% to 9.6%). Viral RNA was predominantly detected in chicken M2 and tissue-resident macrophage (Fig. 4C), suggesting tropism for immunosuppressive subsets.

We then compared the expression level of three gene modules in macrophages (cytokine-related genes, inflammatory-related genes, and ISGs) across species. A significant up-regulation of ISGs was found in all subtype macrophages in the infected mice (Fig. 4D and F). In chickens, the expression levels of ISGs slightly increased after infection. However, some ISGs, such as *Ifih1* and *Ifit2*, increased from 4.96- to 21.41-fold in macrophages of infected chickens, while the expression level of *Eef1d* gene significant decreased from 4.06- to 6.96-fold in macrophages of infected chickens (Fig. 4F). In addition, a significant reduction in cytokine- and inflammatory-related genes was observed in the infected ducks (Fig. 4E). Notably, the expression level of *Il6* significantly increased 16.55-, 13.27-, 10.70-, and 8.28-fold in the M1 macrophages, M2 macrophages, monocyte, and alveolar macrophages of the infected chickens, respectively (Fig. 4E). *In vitro* studies demonstrated that the mRNA expression levels of *Rsad2, Oas2, Ifit1, Ifit3*, and *Isg15* genes significantly increased over 5-fold in the mouse-origin MH-S cells after infection 24 h post-infection (hpi) (Fig. 4G; Fig. S21). Similarly, the mRNA expression levels of *Ifit2, Ifit3, Parp9, Parp14, Isg15, Dhx58*, and *Ube2l6* genes significantly increased in the pig-origin 3D4/21 cells (Fig. 4G; Fig. S21). Conversely, the expression levels of *Epstl1, Parp9, Parp14, Dhx58*, and *Ddx60* genes significantly decreased in the chicken-origin HD11 cells after infection (Fig. 4G; Fig. S21).

Since macrophages exhibit the role of antigen presentation, we then investigated the expression levels of the major histocompatibility complex class II (MHC-II) in the macrophages of different species after infection (Fig. 4H). We compared the expression level of MHC-II genes in macrophages of mice (i.e. *H2-Aa, H2-Ab1, H2-D1, H2-DMa, H2-DMb1, H2DMb2, H2-Ea, H2Eb1, H2-K1, H2-Ke6, H2-Oa, H2-Ob, H2-Q4, H2-Q6, H2-Q7, H2-T22, H2-T23, T2-T24*), and pigs (i.e. *SLA-DQB1, SLA-DMB, SLA-DRA, SLA-DOA, SLA-DOB*), and found that the expression levels of MHC-II related genes significantly increased in macrophages of mice and pigs upon infection (Fig. 4H).

PANoptosis is a newly defined programmed cell death triggered by constant stimuli, which can cause excessive inflammation and tissue damage [35]. Here, we found that the PANoptosis genes, *Casp1* and *Casp8*, were significantly up-regulated (2.36- and 2.89-fold) in the macrophages of chickens after infection, but slightly decreased in the infected pigs (Fig. 4I). Mice exhibited pan-macrophage *Zbp1* upregulation (from 2.69- to 3.48-fold), whereas pigs showed *Nlrp3* down-regulation (1.96-fold) (Fig. 4I). These findings suggest that *Zbp1*-mediated PANoptosis activation in murine macrophages correlates with disease severity, while porcine *Nlrp3* suppression suggests inflammasome evasion, which was consistent with species-specific pathology outcomes.

Transcriptional regulatory network analysis was performed using the single-cell regulatory network inference and clustering pipeline [36]. The expression level of inflammation-related transcriptional factors (TFs), such as *Irf1* and *Nfkb1*, significantly increased in chickens after infection. In the infected mice, the regulon specificity score (RSS) of *Irf7* and *Irf9* increased (Fig. 4J), indicating strengthened interferon regulatory networks. Regulatory network visualization revealed that most ISGs were regulated by the core regulator *Irf7* in mice (Fig. 4K), while *Klf6* and *Irf1* were the key hub regulons modulating most of the inflammatory-related genes in chickens.

### Distinct alterations of lung immune microenvironments in different species upon H5 HPAIV infection

**Figure 5.**
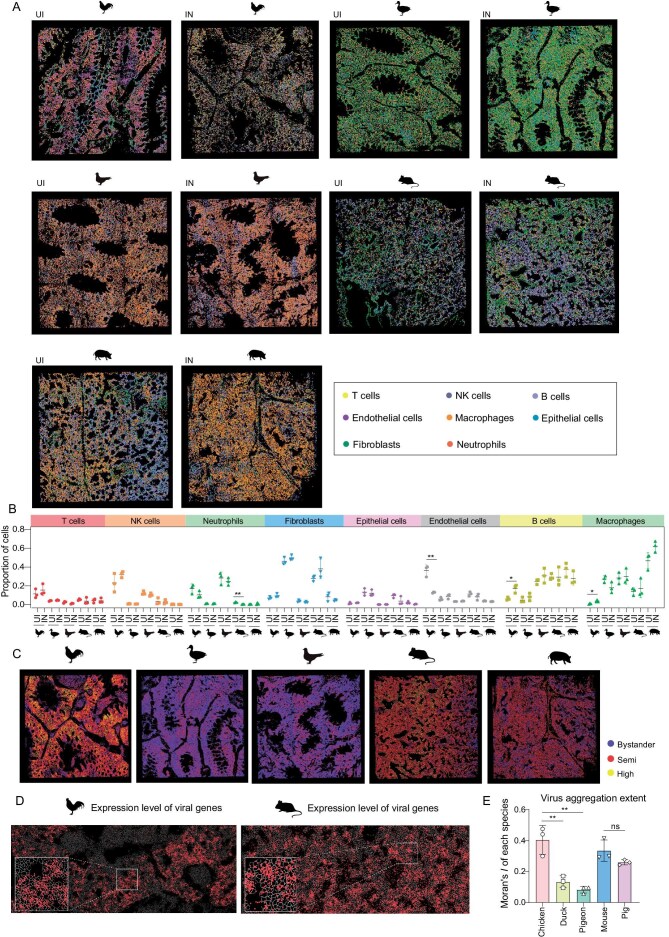
Spatial single-cell atlas of lungs in different species during infection. (A) Spatial localization of different cell types in uninfected (UI) and infected (IN) lungs in chickens, ducks, pigeons, mice, and pigs. (B) The proportions of different cell types in the lungs of different species were compared between UI and IN species. (C) Overlay of spatial localization of different viral infection levels in chickens, ducks, pigeons, mice, and pigs. (D) Spatial single-cell atlas showing the location of RNA of viral genes in chickens and mice. (E) Changes of Moran’s *I* for spatial localization of viral genes in UI and IN species.

To delineate the dynamic single-cell spatial transcriptomic architectures within lungs infected with H5 HPAIV, we leveraged the target spatial-omics method, multi-omics *in situ* pairwise sequencing (MiP-seq) [37], to map the spatial expression patterns of 149 genes (cell type-specific marker genes, viral genes, and immunity-related genes) (Fig. 1; Data S3). All of the cell type-specific marker genes and immunity-related genes across different species used in the spatial single-cell atlas were obtained from scRNA-seq data. A total of 1401 353 cells were imaged, including 590 493 cells from uninfected animals (chickens, 147 499; ducks, 165 802; pigeons, 126 554; mice, 65 038; pigs, 85 600) and 810 860 cells from infected animals (chickens, 213 477; ducks, 184 826; pigeons, 212 159; mice, 90 361; pigs, 110 037), respectively. We obtained a single-cell spatial transcriptomic atlas of lungs both in uninfected and infected animals (Fig. 5A; Figs S22, S28, and S29). The spatial expression levels of the marker genes measured by MiP-seq were available in Figs S23–S27.

Then, we quantified the cell proportion of each cell type during H5 HPAIV infection using spatial single-cell transcriptome data. The abundance of B cells significantly increased 3.43-fold (from 5.57% to 19.15%) in chickens after infection, suggesting intensive germinal center formation and antibody production, while the proportion of endothelial cells significantly decreased 4.11-fold (from 37.11% to 9.03%) in chickens upon infection, respectively, suggestive of the widespread vascular damage in chickens upon infection. However, no significant changes in the proportions of cell types were found in ducks and pigeons after infection (Fig. 5B). The absence of proportional changes in ducks and pigeons highlights refined viral tolerance mechanisms in these reservoir species, where homeostasis is preserved despite infection. In mammals, the proportion of neutrophils significantly decreased 4.49-fold (from 2.92% to 0.65%) in mice after infection, suggestive of neutrophil depletion. However, no significant changes in the proportions of cell types were found in pigs after infection (Fig. 5B).

In addition, spatial proximity analysis within each cell type exhibited species-specific alterations in the lungs after H5 HPAIV infection using Sopa [38]. Por data showed that the spatial proximity of epithelial cells to other cell types significantly increased 2.27- and 2.01-fold in the lungs of infected chickens and pigeons, respectively. Concurrently, the 1.39-fold decrease in endothelial cell proximity in infected chickens points to vascular dysfunction or altered leukocyte trafficking, potentially contributing to edema or impaired immune cell extravasation. In infected ducks, no significant differences in the spatial proximity within different cell types (Fig. S30). Conversely, mammalian responses diverged markedly. The significant decrease in neutrophil proximity (2.91-fold) in mice upon infection indicates disrupted tissue integrity and immune dysregulation (Fig. S30), which might reflect aberrant chemotaxis, impaired retention within inflamed foci.

Moran’s *I* is a statistical measure used to assess the spatial autocorrelation of data, indicating the degree of clustering or dispersion of a phenomenon in space. Here, we found the Moran’s *I* of endothelial cells, epithelial cells, and fibroblasts significantly decreased 1.61-, 1.2-, and 1.26-fold in chickens upon infection, respectively, while that of macrophages significantly increased 1.18-fold in chickens after infection (Fig. S30). This was accompanied by the shedding of alveolar epithelial cells, the damage of endothelial cells and fibroblasts in chickens. However, in infected ducks, pigeons, mice, and pigs, no significant changes in the Moran’s *I* were found in each cell type (Fig. S30). These results suggest that revealing a disordered structure of the injured lung was found in the infected chickens, but not observed in ducks, pigeons, mice, and pigs.

### Correlation of viral infection and spatial immune microenvironment

The pulmonary immune microenvironment plays a crucial role in coordinating the host response to viral infections. Here, we used *in situ* hybridization to detect viral RNA expression levels in the infected lungs of different species. Our analysis revealed that highly infected cells were primarily located within chickens’ pulmonary parenchyma and mice’s alveolar structures (Fig. 5C; Fig. S29). In addition, the spatial expression level of viral genes were aggregative in chickens, followed by mice, and pigs, whereas ducks, and pigeons displayed the lowest levels (Figs 5D and E). These results suggest that H5 HPAIV might infect different histological structures of the lungs in birds and mammals.

We then compared the expression levels of several immunity-related genes across cell types with different infection levels using the spatial single-cell lung atlas in five species (Fig. S31). In chickens, *Dhx58* and *Stat1* genes were found to be increased 2.84- and 2.16-fold in the highly infected cells compared to the non-infected cells, while *Dhx58* gene increased 18.11-fold in the highly infected cells compared to the lowly infected cells of ducks (Fig. S31). The expression levels of *Dhx58* and *Isg15* genes elevated 4.50- and 1.86-fold in the highly infected cells compared to the bystander cells in mice. However, the expression level of the *Samhd1* gene increased 3.21-fold in the highly infected cells compared to the bystander cells in pigs, respectively (Fig. S31).

To better understand the distinct immune microenvironment of the infected lungs, we analyzed the dynamics in cell distribution and transcriptome in the highly infected areas. Because chicken and mice lungs showed noticeable spatial infection differences, we selected them for analysis. For each slice, we identified highly infected areas and drew concentric circles with varying radii from these areas to divide them into three circles. We observed that the proportion of T cells increased 1.32-fold in the inner layer of the infected chicken lungs compared with the outer layers, while the proportion of macrophages increased 1.36-fold in the inner layer of the infected mice lungs compared with the outer layers. Furthermore, the expression level of *Ifit2* decreased from the inner to the outer layers in mice, suggesting the higher expression level of *Ifit2* in the highly infected spatial location of the lungs.

### Viral adaptive mutations in the lung microenvironment across different species

**Figure 6.**
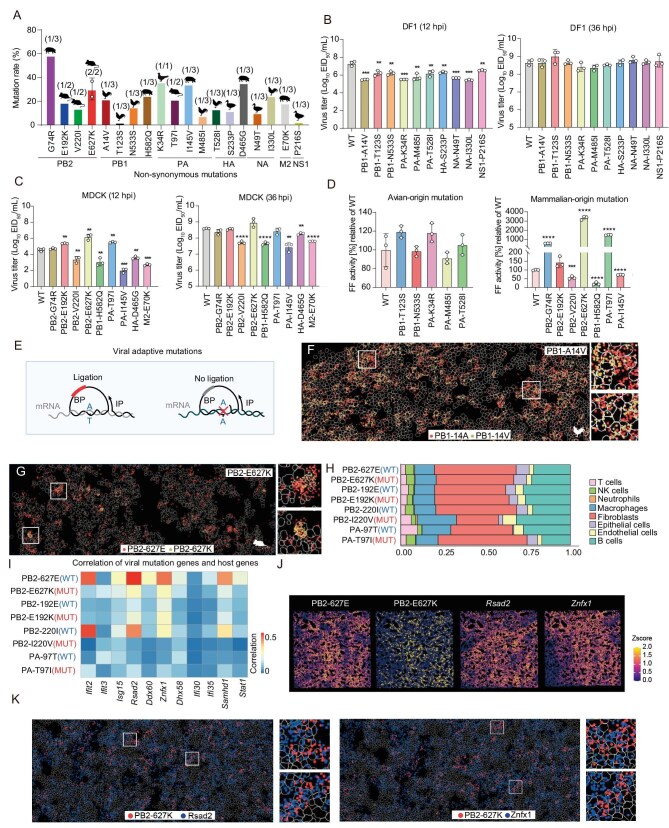
Characterization of viral adaptive mutations in birds and mammals. (A) Mutation rates of 19 non-synonymous viral mutations in the lungs of different species after infection. Viral titers after the inoculation of wild-type virus and each mutant into DF1 cells (B) and MDCK cells at 12 and 36 hpi (C). The supernatants of infected cells were collected at 12 and 36 hpi. (D) Fold changes of polymerase activity of different mutations relative to wild-type virus in avian DF1 cells and human HEK293T cells. Each point on the curve is the mean ± standard deviation from three independent experiments. The independent samples *t*-test was used for analysis. (E) The mode chart of Mip-seq is used in the detection of adaptive mutation. (F) Spatial single-cell atlas showing the location of viral mRNA of the PB1 gene with amino acid A or V at position 14 in chicken lung. The red color represents amino acid A at position 14 of PB1 protein, and the yellow color represents amino acid V at position 14 of the PB1 protein. (G) Spatial single-cell atlas showing the location of mRNA of the PB2 gene with amino acid E or K at position 627 in mouse lung. The red color represents amino acid E at position 627, and the yellow color represents amino acid K at position 627. (H) The proportions of adaptive mutations in different cell types of infected mice lungs. (I) The correlation of spatial distributions between viral genes with adaptive mutations and immunity-related genes in mice. (J) Overall spatial single-cell atlas of PB2 protein with amino acid E or K at position 627 and host genes (*Rsad2* and *Znfx1*) in mice lungs. (K) Spatial single-cell atlas showing the location of viral mRNA of the PB2 gene with amino acid K at position 627 and host genes (*Rsad2* and *Znfx1*) in mice lung.

To analyze the adaptation of H5 HPAIV in birds and mammals, we sequenced the full-length genome sequences of the infected lung samples (chicken: *n* = 3; duck: *n* = 3; pigeon: *n* = 1; mice: *n* = 2; pigs: *n* = 3) at 3 dpi (Fig. 1). A total of 19 non-synonymous iSNVs were identified, including 6 in chickens, 3 in ducks, 1 in pigeons, 4 in mice, and 5 in pigs (Fig. 6A; Data S6). Specifically, the A14V, T123S, and N533S mutations in the PB1 protein, M485I and T528I mutations in the PA protein, and I330L mutation in the NA protein were found in one infected chicken. The S223P mutation in the HA protein was found in one infected duck, while the N49T mutation in the NA protein and the P216S mutation in the NS1 protein were found in another duck. However, only the K34R mutation in the PA protein was found in one infected pigeon. In mammals, the E192K and E627K mutations in the PB2 protein and the T97I mutation in the PA protein were found in one infected mouse, while the V220I and E627K mutations in the PB2 protein were found in another infected mouse. In pigs, the H562Q mutation in the PB1 protein and the D465G mutation in the HA protein were found in one infected pig, while the I145V mutation in the PA protein and the D465G mutation in the HA protein were found in another infected pig. Importantly, eight non-synonymous mutations were observed in the polymerase genes in the infected mammals, including G74R (58%), E192K (18%), V220I (13%), and E627K (30%) in the PB2 protein, H562Q (24%) in the PB1 protein, and T97I (0.21%) and I145V (34%) in the PA protein (Fig. 6A).

We also conducted sequence analysis from GISAID’s Epiflu Database in each viral gene of H5N6 viruses derived from birds and humans. Most amino acid mutations identified in this study were conserved in nature (Fig. S32). Avian-origin mutation A14V in PB2 protein, N49K in NA protein, and P216T in NS1 protein were both prevalent in birds and humans, while mutation E627K in PB2 protein was specifically prevalent in humans (Fig. S32). To further assess the biological significance of the 19 non-synonymous iSNVs, we introduced them into the 21957-1/H5N6 virus using reverse genetics and constructed 19 mutant 21957-1/H5N6 strains, each carrying one non-synonymous iSNV. We then measured the viral replication kinetics of these 10 mutants in avian cell line Douglas Foster-1 Chicken cell (DF1), and no significant difference in viral replication was observed 36 hpi (Fig. 6B). Similarly, we further measured the viral replication kinetics of these mutants in the mammalian cell line Madin-Darby Canine Kidney (MDCK). PB2-E192K, PB2-E627K, and PA-T97I mutants showed significantly higher virus titers than the wild-type (WT) virus at 12 hpi, whereas the viral replication decreased for the mutants with PB2-V220I, PB1-H562Q, PA-I145V, HA-D465G, and M2-E70K both at 12 and 36 hpi (Fig. 6C; Data S7 and S8).

We conducted a mini-genome assay to gain further insights into the effects of the mentioned mutations on polymerase activity of the virus. Again, no significant differences in the polymerase activity were observed for the H5N6 viruses carrying the avian-origin non-synonymous mutations in avian cells (Fig. 6D; Data S9 and S10). However, in mammalian cells, the higher virus titers were detected for the mutants with higher polymerase activity in the minireplicon assays, including PB2-E627K and PA-T97I (Fig. 6D; Data S9 and S10). The polymerase activity of the PB2-G74R mutation increased 4.75-fold without enhancing viral replication in MDCK cells, suggesting post-replication constraints (Fig. 6D; Data S9 and S10). These results suggest that PB2-E627K and PA-T97I mutations increase the viral replication and polymerase activity of H5 HPAIV in mammals.

### Spatial atlas of viral mutations and the correlation with immune responses in the lungs

By applying *in situ* hybridization (MiP-seq), we were able to detect the target RNA with non-synonymous mutations *in situ* at the single-cell level (Fig. 6E). Since the viral infection ability is high in chickens and mice based on single-cell spatial atlas compared with other species, we mainly focused on the adaptive mutations of infected chickens and mice (Figs 6F and G). Our results suggested that mammalian adaptive mutations PB2-E627K, PB2-E192K, and PA-T97I were enriched in fibroblasts and macrophages in mice (Fig. 6H). In the spatial single-cell atlas, the mutation PB2-E627K was mainly observed around the alveolar walls in mice (Fig. 6J).

Since PB2-E627K, PB2-E192K, and PA-T97I significantly increased the polymerase activity and viral replication, we then investigated the local immune response genes around the viral genes with specific adaptive mutations in mice. We found that some ISGs, such as *Rsad2* and *Znfx1*, were highly correlated with the spatial expression of the PB2 gene with the E627K mutation, which is mainly located around the alveoli structure of mouse lungs (Figs 6J–L). MH-S and NIH/3T3 cells were then infected with the WT virus and the mutants carrying these adaptive mutations. Our results showed that compared with WT, the expression level of *Ifit2, Ifit3, Ifih1, Isg15, Rsad2*, and *Dhx58* genes significantly increased in NIH/3T3 cells after being infected with PA-T97I mutant, while *Ifit3, Ifih1, Ifi35, Isg15*, and *Dhx58* genes significantly increased in NIH/3T3 cells after being infected with PB2-E192K mutant. Notably, the expression level of *Ifit3, Isg15*, and *Dhx58* genes significantly increased in NIH/3T3 cells after being infected with PB2-E627K mutant (Fig. S33), indicating that these adaptive mutations of H5 HPAIV promote the induction of ISGs.

## DISCUSSION

H5 HPAIV is endemic in domestic and wild birds worldwide [2,39,40]. Notably, it has frequently crossed the barriers to infect mammals, such as marine mammals, pigs, dogs, ferrets, minks, dairy cows, and humans [3–6,41,42], which raises substantial concerns regarding the potential risks of H5 HPAIV to public health. Therefore, understanding the cellular and molecular variations in host responses and within-host evolution across diverse species infected with 2.3.4.4b H5 HPAIV is highly warranted. Here, by integrating scRNA-seq and *in situ* MiP-seq, we mapped a comprehensive single-cell transcriptomic atlas of the lungs in various birds (chickens, ducks, pigeons) and mammals (mice and pigs) infected with H5 HPAIV. This reveals species-specific viral tropism, immune landscape remodeling, and within-host adaptation mechanisms.

Influenza virus infection can induce a strong infiltration of immune cells into the human lung, with potential detrimental effects on the integrity of the lung tissue [43]. Here, the infiltration of B cells was found in chickens upon infection, while endothelial cells significantly decreased in chickens upon infection. In mammals, the proportion of neutrophils significantly decreased in mice after infection, suggestive of distinct changes in the proportion of cell types after infection. Epithelial cells showed the highest mRNA expression levels of viral genes in infected mice, but the mRNA was mainly found in the macrophages of infected pigs. Previous studies indicated that the highest percentage of virus-infected cells were epithelial cells of the mice infected with H1N1 influenza viruses [26]. Similarly, our findings suggest that epithelial cells and macrophages in mammals are the main susceptible cell types for H5 HPAIV infection.

Severity of respiratory viral diseases is associated with virus-driven perturbations in the immune system and resultant tissue injury [25,31,44]. Aberrant interferon-related responses could lead to alterations in cytokine elaboration that deplete resident immune cells, while simultaneously recruiting hyperactive macrophages and functionally altering neutrophils [31,45], thereby disrupting the balance between pro-inflammation and antiviral immune response [46]. Our results showed that a broad and intense inflammation in fibroblasts and the infiltration in T cells of infected chickens, while the expression levels of cytokine- and inflammation-related genes significantly decreased in fibroblasts of infected ducks and pigeons, respectively. However, H5 HPAIV can obviously activate interferon-related responses in macrophages and neutrophils of mice, while antiviral response elevated in broader subtype cells of pigs upon infection. Previous studies have showed that *IL6* and *IL10* are highly expressed in severe patients infected with the influenza virus [47,48]. Herein, an up-regulation of the *Il6* and *Il10* genes was only found in CD8^+^ T cells of the infected chickens. These findings suggested that chickens induced severe pro-inflammation after infection, while antiviral response in immune cells cannot be activated. Mice exhibited a balanced pro-inflammation and antiviral response after infection, while pigs showed limited infectivity and replication of the virus, which represents a limitation and potential contributor to the overall modest transcriptional changes.

Humans infected with multiple emerging subtype avian influenza viruses with diverse adaptive mutations were frequently reported in recent years [3,40,49–52]. Recent research reported that a single glutamine to leucine mutation at residue 226 of H5N1 hemagglutinin can enact the change from avian to human specificity, which could be an indicator of human pandemic risk [53]. Herein, we identified 19 non-synonymous viral iSNVs in H5 HPAIV-infected birds and mammals. Most of them were enriched in polymerase proteins in mammals. Mutations PB2-E192K, PB2-E627K, and PA-T97I significantly increased polymerase activity and viral replication in mammalian cells, suggesting that they might increase the risk of H5 HPAIV infection in humans. Previous studies also demonstrated that avian influenza viruses typically possess amino acid E at position 627 of PB2, and the substitution by K or V at this position is a well-recognized mammalian adaptation signature [54–56]. Here, we delineated the *in situ* viral adaptive mutation map and lung microenvironment using Mip-seq [37]. We found that mutations PB2-E627K, PB2-E192K, and PA-T97I were enriched in fibroblasts and epithelial cells. Importantly, we revealed some ISGs (i.e. *Rsad2*) significantly highly expressed in PB2-E627K mutated lung areas around alveolar walls, which may partially lead to more pronounced immune responses. Here, we found that some adaptive mutations reducing *in vitro* replication yet enhance ISG coupling, suggestive of an alternative immune evasion strategy. Influenza polymerase proteins concurrently catalyze RNA synthesis and antagonize RIG-I signaling [57,58]. We hypothesize these mutations attenuate replicative output while preserving immune antagonist functions, thereby limiting immunostimulatory RNA production and preventing ISG-driven immunopathology [59,60].

This study has several limitations. The changes in the proportion of some cell types in scRNA-seq and MiP-seq data following infection were not consistent. The primary reason for these observed differences lies in the fundamental technical limitations of each platform. Cell capture of scRNA-seq technology can differentially affect cell viability and recovery for certain fragile or adherent populations, which could decrease the efficiency of cell capture. However, MiP-seq technique analyzes intact tissue sections, preserving the native spatial context and capturing a significantly larger number of cells across a broader tissue area. Flow cytometric analysis will be performed to examine alterations in cellular composition in future studies.

In summary, we mapped a comprehensive molecular and cellular single-cell landscape of the lung microenvironment in five avian and mammalian species upon H5 HPAIV infection, which provides a valuable resource to elucidate the intricate single-cell immune responses and cross-species risk assessment of various species upon H5 HPAIV infection.

## MATERIALS AND METHODS

### Study design

The purpose of this study was to investigate the divergent pathogenicity, within-host evolution, and host response at single-cell level across birds (chickens, ducks, and pigeons) and mammals (mice and pigs) upon H5 HPAIV infection. First, we investigate the pathogenicity of H5 HPAIV infection across different species. Second, we mapped the single-cell and spatial lung transcriptomic atlas across different species upon infection. Last, we identified viral iSNVs and assessed their molecular characterizations *in vitro* and *in vivo.*

### Sample source and ethical statement

Six-week-old specific pathogen-free (SPF) chickens (*Gallus gallus domesticus*) were purchased from Guangdong Dahuanong Animal Health Products, China. Six-week-old SPF ducks (*Tadorna*) were purchased from Harbin Weike Biotechonology Co., Ltd., Harbin, China. Six-week-old pigeons (*Columba livia domestica*) were purchased from pigeon farm from Guangzhou, China. Six-week-old bama pigs (*Sus scrofa)* were purchased from Hubei Aofei Biotechnology, Wuhan, China. Three 6-week-old mice (*Mus musculus*) were purchased from Vital River, China. All animal experiments were carried out in ABSL-3 facilities in compliance with the approved protocol by the biosafety committee of South China Agriculture University (SCAU). All animals involved in experiments were reviewed and approved by the Institution Animal Care and Use Committee at SCAU and treated in accordance with its guidelines (2017A002). The animals were monitored twice per day for clinical signs, and we provided sufficient feed and water for experimental animals every day.

The detailed materials and methods are available in the Supplementary data.

## Supplementary Material

nwag380_Supplemental_Files
