# Supplementary material for: Heterogeneous viral-host response at single-cell resolution and viral adaptive mutational atlas in birds and mammals upon H5 influenza virus infection
Source: Natl Sci Rev. 2026 Jun 25;13(14):nwag380. doi: 10.1093/nsr/nwag380 (PMC13458601; doi:10.1093/nsr/nwag380)
Supplement: Supplementary file 1 [file nwag380_supplemental_files.zip › Materials and Methods.docx]

**Materials and Methods**

**Ethical statement**

All animal experiments were carried out in ABSL-3 facilities in compliance with the approved protocol by the biosafety committee of South China Agriculture University (SCAU). All animals involved in experiments were reviewed and approved by the Institution Animal Care and Use Committee at SCAU and treated in accordance with its guidelines (2017A002). The animals were monitored twice per day for clinical signs, and we provided sufficient feed and water for experimental animals every day.

**Virus isolation and cell lines**

The H5N6 HPAIV, namely A/duck/Sichuan/21957-1/2021 (H5N6) (21957-1/H5N6), was isolated from an infected duck [1]. All viruses were propagated in 10-day-old SPF embryonated eggs at 37°C and frozen at −80°C. All cells used, including human embryonic kidney 293T (HEK293T), Madin-Darby Canine Kidney (MDCK), HD11, MH-S, 3D4/21, NIH/3T3, and DF1, were provided by the National Avian Influenza Para-Reference Laboratory at SCAU. HEK293T, MDCK, HD11, MH-S, 3D4/21, NIH/3T3, and DF1 cells were cultured in Dulbecco’s modified Eagle’s medium (DMEM) (Gibco, USA) supplemented with 10% fetal bovine serum (ExCell Bio, China), 100 U/mL of penicillin-streptomycin (Gibco, USA). They were incubated at 37℃ in a 5% CO_2_ incubator.

**Phylogenetic analysis**

The corresponding reference sequences of the HA gene were downloaded from GISAID (https://www.gisaid.org). The sequence datasets together with 21957-1/H5N6 were aligned using MAFFT [2], then the most appropriate alternative model was conducted using ModelFinder [3]. Phylogenetic trees were constructed using the maximum likelihood (ML) method in the IQ-TREE with 1,000 bootstraps [4]. Figtree v1.4.4 were used to complete the annotation of the phylogenetic tree.

**Animal experiments**

Groups of three 6-week-old specific pathogen-free (SPF) chickens (*Gallus gallus domesticus*, sourced from Guangdong Dahuanong Animal Health Products, China), three 6-week-old SPF ducks (*Tadorna*, provided by Harbin Weike Biotechonology Co., Ltd., Harbin, China), and eight 6-week-old pigeons (*Columba livia domestica*, *s*ourced from a pigeon farm in Guangzhou, China) were intranasally inoculated with 10^6^ EID_50_ in 0.2 mL of the 21957-1/H5N6 virus. Three 6-week-old bama pigs (*Sus scrofa),* obtained from Hubei Aofei Biotechnology, Wuhan, China, were intranasally inoculated with 10^6^ EID_50_ in 2 mL of the 21957-1/H5N6 virus. Three 6-week-old mice (*Mus musculus,* acquired from Vital River, China) were inoculated intranasally with 10^6^ EID_50_ in 50 µL of the 21957-1/H5N6 virus. A control group (n=3) for each species was inoculated with phosphate buffered saline (PBS). All of the bama pigs tested negative for African swine fever virus. All of the serums of bama pigs used in this study were negative for H1, H5, H7, and H9 subtype influenza viruses. All of the species used in this study were inbred. At 3 days post-infection (dpi), ducks, pigeons, and mice were dissected to evaluate virus replication in organs, including the heart, liver, spleen, lung, kidney, and brain. The organs of infected chickens were collected at the moribund stage (~3 dpi) due to lethal infection. Pigs were also assessed for virus replication in organs at 3 dpi, including the heart, liver, spleen, lung, and kidney, and the lungs were further divided into five parts to detect viral titers. Tissue samples were collected for virus titration by an EID_50_ assay. The lung samples in different species were divided into four parts at the same location. The first part was used for the detection of viral titers, the second part was used for scRNA-seq, the third part was used for the detection of viral variation using NGS, and the fourth part was used for the spatial single-cell transcriptome.

**Deep sequencing**

Total RNA was isolated from lung samples collected from three infected chickens (n=3), ducks (n=3), pigeon (n=1), mice (n=2), and pigs (n=3) at 3 dpi using RNeasy Mini Kit (Qiagen, USA). The gene segments were sequenced on an Illumina NovaSeq 6000 platform (Gene Denovo, China). The raw NGS reads were processed by filtering out low-quality reads, adapter-contaminated reads, poly-Ns, duplication, and host contaminated reads. The quality-filtered reads were aligned to the genomes of 21957-1/H5N6 using the Burrows-Wheeler Aligner (BWA, version 0.7.12) and SAMtools (version 1.4) for reference-based assembly [5,6]. Finally, the adaptive mutation calling of each sample was calculated using GATK4.

**scRNA-seq sample preparation**

The collected lung samples of infected chickens, ducks, pigeons, mice, and pigs, together with uninfected chickens, ducks, pigeons, mice, and pigs were rinsed using PBS, then stored in RPMI 1640 (10-040-CVR, Corning, USA) with 0.04% bovine serum albumin (BSA). Each lung sample of three uninfected and infected species was pooled together. Due to viral-load heterogeneity in the lungs of pigs, we selected the pulmonary congestion area of samples for single-cell preparation. Cells of each tissue were separated by mechanical extraction. Briefly, the tissues were first cut into several pieces and then chopped thoroughly into smaller pieces. The smaller pieces of each sample were added to fresh collagenase, and incubated at 37℃ for 30 min. Then, cells were centrifuged at 500 × g for 5 min at 4°C. After filtration with a 30 µm strainer, the cells were resuspended in 0.04% BSA containing 0.2 U/µL RNase inhibitor and centrifuged at 500 × g for 10 min at 4°C to discard cellular impurities within the supernatant. This step was repeated twice, and the cell viability was above 90%. Subsequently, the cell density was adjusted to 1 × 10^6^ cells/mL. The high-quality single-cell suspension was subjected to encapsulation using a 10× Genomics v.3 kit (10× Genomics, USA). The library preparation and RNA sequencing were performed by Gene Denovo company.

**Alignment, quantification, quality control, and scRNA-seq data processing**

scRNA-seq reads were processed with the Cell Ranger v3.0.2 (10× Genomics) using chickens, ducks, pigeons, mice, and pigs reference genomes from NCBI Assembly. To facilitate the integration of the cross-species single-cell lung data sets, we mapped genes from other species to the mouse homologs, and downloaded the homologs of the five species (chickens, ducks, pigeons, and pigs) using Ensembl from BioMart. Since we mainly focused on the expression levels of the homologous genes of each species, we used one-to-one orthologs for analysis. If there was a 1:1 match between a non-mouse and mouse gene, the non-mouse gene name was converted to the mouse gene name. If the non-mouse gene and mouse gene were not matched, the non-mouse gene name was deleted.

For each dataset from scRNA-seq, the cells containing genes expressed less than 300 and exceeding than 5000 as well as less than 1000 unique molecular identifiers (UMIs) were removed from the gene expression matrix. In addition, cells with mitochondrial gene fraction of < 20% were kept for subsequent analysis, and potential doublet cells predicted by DoubleFinder were further filtered out. Then, we conducted log normalization of each filtered gene expression matrix. Variable genes were determined using the FindVariableGenes function of Seurat [7] with default parameters (selection.method=“vst”, nfeatures = 2000). Clusters were identified using the FindClusters function (resolution =1) and visualized using the RunUMAP function (reduction = “pca”).

We applied Seurat’s SCTransform-based integration workflow to harmonize the scRNA-seq data across species and conditions. We used SCTransform() to analyze each sample independently, regressing out mitochondrial gene expression (mitoRatio) to account for technical variation. We selected the top 2000 variable features using SelectIntegrationFeatures() and prepared the data with PrepSCTIntegration(). Integration anchors were identified with FindIntegrationAnchors() using the SCT normalization method, followed by data integration with IntegrateData(). This pipeline effectively mitigated batch effects while preserving biological variability. Finally, all differential expression genes (DEGs) for each Seurat object were identified using the FindMarkers function (only.pos = T, min.pct = 0.1, logfc.threshold = 0.5). The list of DEGs of each cell type across five species upon infection were available from Supplementary Data 11-19.

**Cluster marker analysis and cell type annotation**

Firstly, we identified cell types based on the SingleR and the top 10 expressed genes in each cluster and classical marker genes of each species. The top 10 expressed genes in each cluster of different species were available from Supplementary Data 1. Secondly, the identity of each cluster was manually annotated based on the specific expression of the marker genes that had orthologous genes as commonly known markers published in the CellMarker database ([http://iocc.hrbmu.edu.cn/CellMarker/](http://iocc.hrbmu.edu.cn/CellMarker/)).

**Viral infection level annotation**

According to the expression counts of avian influenza virus (AIV) transcripts, the cells in the clusters susceptible to influenza virus infection could be divided into highly infected cells (High, total UMI counts of viral transcripts ≥ 8), low infected cells (Low, total UMI counts of viral transcripts ≥ 1), and non-infected cells (Bystander, UMI counts of viral transcripts = 0).

**Transcription factor (TF) analysis**

Python package pySCENIC was used for gene regulatory network inference [8]. We input the mouse TF list as a reference to identify co-expression modules by the GRNBoost2 algorithm. Then, we obtained the regulons by detecting the genes directly targeted by the TF and removing other genes based on the enrichment of motifs around the transcription start site using cisTarget databases. The mouse TF list was downloaded from (https://github.com/aertslab/pySCENIC/blob/master/resources/mm_mgi_tfs.txt), and the cisTarget database was downloaded from (<https://resources.aertslab.org/cistarget>). Using AUCell, the regulon activity score was measured as the area under the recovery curve. The RSS scores were used to detect the cell type-specific regulons [9]. The TFs with RSS scores exceeding 0.1 were identified as specific for illustrating the picture. Subsequent visualizations take place in R, where the transcription factor regulatory network is plotted through the igraph package (version 1.6.0).

**MiP-seq probe design**

MiP-seq probe includes a set of padlock probes with RCA initiator primer probe for target-dependent RCA, and query probes, as detailed in our prior research [10]. The process begins with using a self-developed probe-searching program to screen the specific target sequence without any off-target signal. We choose the exon close to the 5’ end, and select 2 to 7 target sites for each gene. Both ends of the padlock probe are complementary to the target sequence (12-16 bp). The probes for *in situ* hybridization include four query probes (seq-1, se-2, seq-3, and seq-4) conjugated respectively with Alexa Fluor 488, Alexa Fluor 546, Alexa Fluor 594, and Alexa Fluor 647. Probes were synthesized by Sangon Biotech Company (Shanghai, China). All probe sequences are listed in Supplementary Data 3.

**MiP-seq for *in situ* hybridization**

The slices of collected lung samples of infected chickens (n=3), ducks (n=3), pigeons (n=3), mice (n=3), and pigs (n=3), together with uninfected chickens (n=3), ducks (n=3), pigeons (n=3), mice (n=3), and pigs (n=3) were obtained for MiP-seq analysis. The padlock probes were phosphorylated at the 5’ end with T4 Polynucleotide Kinase (N102-01, Vazyme, China) before MiP-seq. Phosphorylated padlock probes (1 nM/probe) and equimolar RCA initiator (1 nM/probe) in hybridization buffer (2×SSC (B548109, Sangon, China) with 10% formamide (A100606, Sangon, China) and 20 mM RVC (Beyotime, R0108)) were added to the hybridization chamber of pretreated lung slices and incubated for 2 h at 37°C. After annealing of probes to RNA targets, samples were washed twice with DEPC-PBSTR (0.1% Tween-20, 0.1 U/µL RRI in DEPC-PBS) for 10 min each time, followed by washing with 4×SSC in DEPC-PBSTR once. The ligase reaction mixture (1 U/μL SplintR ligase (M0375L, NEB, USA), 1× buffer, 0.2U/μL RRI) was then added to the hybridization chamber for 2 h incubation at 25°C. Following ligation, samples were washed with DEPC-PBSTR twice for 10 min each time and incubated with rolling circle amplification reaction solution (1 U/μL Phi29 (N106-01, Vazyme, China), 1×RCA buffer, 0.25 μM dNTP, 0.2 μg/μL BSA, 5% Glyceryl) at 30°C overnight. After double washing with DEPC-PBSTR (10 mins each time), the samples were ready for direct detection with a fluorescent probe, and imaged by Leica TCS SP8 confocal microscope with 63× water lens and 20× lens (NA 1.2). Channels of DAPI, Alexa Fluor 488, Alexa Fluor 546, Alexa Fluor 594, and Alexa Fluor 647 were scanned. After each round of imaging, signals were stripped using stripping buffer (60% formamide in 2×SSC) 10 times at 40°C. The mixtures for the next rounds of RNA detection were then added to perform multiple round RNA detection for high-throughput readout.

**Image processing and gene decoding**

Image registration was performed using the BigWarp function within ImageJ (version 2.9.0, Fiji). Cell segmentation was primarily conducted using the Cellpose package (version 2.0.1). When manual cell segmentation was difficult, we selected to use the Napari software. Upon completion of these steps, all cell masks and positional information were obtained. To balance the intensity of all channels, deep learning mode U-FISH (https://github.com/UFISH-Team/U-FISH) (version 0.1.1) was employed to identify signal points [11]. Then, each channel with the mean image intensity at these signal spots was scaled. The mean image of these normalized channel images was used for further analysis. U-FISH was used for candidate spot calling, and the intensity threshold was set to 0.5 for the mean images. The single channels were combined to analyze the intensity of the dual barcode spots. For each spot, all the codes from each round were combined to generate its barcode. The gene can be deciphered by matching the barcode with the gene codebook.

**Annotation of spatial cell types in different groups**

We annotated cell types based on the expression of marker genes by calculating the MGEscore. To determine the cell types, we first calculated a ranking score for the expression of each marker gene. This was done by ranking the gene expression levels in descending order, where ties were assigned the minimum rank. The score for each gene was then derived by dividing the maximum rank by the rank of the gene expression in each cell. Subsequently, we calculated the weighted average score for each cell type, based on the marker genes associated with that cell type. For each cell type, we identified its specific marker genes and computed their individual scores. The weighted average score for a cell type was obtained by averaging these individual scores. Finally, we assigned cell types by comparing the weighted average scores across all cell types for each cell. A cell was annotated as a specific cell type if its score for that cell type was significantly higher than the scores for other cell types. If no cell type showed a markedly higher score, the cell was classified as 'undefined'.

**Spatial distance of each cell type**

For the statistical analysis of mean hop distance between different cell clusters in spatial single-cell transcriptome data, we employed Sopa (codes available at <https://github.com/gustaveroussy/sopa>) (version 1.0.6). This process began by calculating the spatial locations of cells to construct Delaunay graphs, which connect each cell to its nearest neighbors, forming a network of triangles covering the spatial domain without overlapping edges. To ensure realistic cell-cell interactions, we pruned long edges, typically those longer than 50 microns, that were unlikely to correspond to physical interactions. Using the pruned Delaunay graph, we calculated the mean hop distance between different cell clusters by determining the number of edges in the shortest path connecting them within the graph. For each pair of cell clusters, we computed the average hop distance by considering all pairwise distances between cells of the two clusters. The calculated mean hop distances were then recorded and analyzed to understand the spatial relationships between different cell clusters, providing insights into the cellular architecture and potential interactions within the tissue.

**Statistical analysis of spatial gene expression correlation**

We employed the KernelDensity function from the *sklearn.neighbors* module to compute the probability density distributions of all gene expressions. Subsequently, we utilized the correlation function from *scipy.spatial.distance* to evaluate the correlation between the spatial expression distributions of host and viral genes. The resulting correlation values were scaled to a range of -1 to 1, where values greater than 0 indicate a positive correlation, and values less than 0 indicate a negative correlation. To facilitate the reproducibility of our results, we encapsulated this process into the Spatialspots package, which includes test data and a comprehensive tutorial. This ensures that other researchers can easily replicate our analysis and apply the methodology to their own datasets.

**Moran's I analysis of spatial gene expression**

To evaluate the spatial autocorrelation of cell distributions, we calculated Moran's I index. Moran's I is a statistical measure used to assess the spatial autocorrelation of data, indicating the degree of clustering or dispersion of a phenomenon in space. Firstly, cell masks were obtained, and the coordinates of the cells were extracted from these masks. These coordinates were then used to construct a grid over the spatial domain of the cell coordinates. A kernel density estimate (KDE) of the cell distribution was computed, representing the cell density at each grid point. To quantify spatial relationships, a distance matrix between all grid points was constructed. A spatial weight matrix was defined based on a threshold distance, assigning weights to grid points within this distance. The weight matrix was row-normalized to ensure comparability. Moran's I statistic was calculated using the density values and the weight matrix. This involved computing the mean density and the deviations from this mean. The numerator was obtained by summing the product of the weighted differences from the mean density for all pairs of points, while the denominator was the sum of the squared differences from the mean density. Moran's I was then derived to assess the degree of spatial clustering or dispersion among the cells. Codes are available at <https://github.com/wikk-chy/Spatialspots.>

**Spatial immune microenvironment analysis**

Data points were divided into regions using K-means clustering (KMeans from sklearn). Kernel Density Estimation (KDE) was then performed on each cluster to identify areas with high gene density. The top density points from the KDE results were further filtered using DBSCAN clustering (DBSCAN from sklearn) to ensure a minimum distance between density centers. Representative points were selected as the mean coordinates of the DBSCAN clusters. The results of the KDE and the top density centers were visualized using matplotlib. Based on the field of view and imaging data, three radii were defined: 120 pixels (circle1), 240 pixels (circle2), and 420 pixels (circle3). Euclidean distances between center cells and surrounding cells were calculated, and the counts of surrounding cell types within these radii were determined. Differences in cell counts between consecutive radii were calculated to understand the distribution of cell types around the density centers. Gene expression statistics were calculated within the specified radii around each density center. The number of cells expressing specific genes was determined for each radius. To account for the area differences among the three regions, gene expression densities were weighted at 15, 5, and 3 for the 120 pixels (circle1), 240 pixels (circle2), and 420 pixels (circle3) regions, respectively. The code for this analysis is available at [https://github.com/wikk-chy/SpatialImmunoCircle](https://github.com/wikk-chy/SpatialImmunoCircle).

**Quantitative reverse transcription PCR in different cell lines infected with H5N6 HPAIV**

In the first experiment, the HD11, MH-S, and 3D4/21 cells were infected with H5N6 HPAIV for 1 h at an MOI of 0.01, 0.01, and 0.0001, respectively. Subsequently, the cells were rinsed to eliminate any unbound viruses and then cultured in DMEM supplemented with 0.2% BSA at 37℃ in a 5% CO_2_ environment for 6 h and 24 h. In the second experiment, the MH-S and NIH/3T3 cells were infected with 21957-1/H5N6 virus and different mutants (PB2-E192K, PB2-E627K, PB2-I220V, and PA-T97I) for 1 h at an MOI of 1. Subsequently, the cells were rinsed to eliminate any unbound viruses and then cultured in DMEM supplemented with 0.2% BSA at 37℃ in a 5% CO_2_ environment for 24 h. The mRNA levels of specific genes were detected using qPCR. First, total RNA was extracted using the RNeasy Mini Kit (Qiagen) following the manufacturer’s protocols. Then, reverse transcription (RT) was performed using the PrimeScript RT Reagent Kit (Takara, Japan) according to the manufacturer’s instructions. The qRT-PCR mixture for each reaction sample consisted of 10 μL of 2×SYBR PCR master mix (Vazyme, China), 7 μL of nuclease-free water, 0.5 μL of each primer, and 2 μL of cDNA template. The expression levels of the genes were detected using the 7500 Real-Time PCR System (Applied Biosystems, USA), initiating with a cycle at 95℃ for 10 min, followed by 40 cycles of 95℃ for 15 s and 60℃ for 1 min. The relative expression level of each gene, normalized to GAPDH.

**Plasmid construction and reverse genetics**

All eight gene segments of the 21957-1/H5N6 virus were amplified by RT-PCR, and were individually cloned into the dual-promoter plasmid pHW2000 for academic purpose [12,13]. The primers were designed to include the desired change for the site-directed mutagenesis method. The PCR products were subsequently cloned into a pHW2000 plasmid system. The cloning was verified through DNA sequencing. For the generation of recombinant viruses, HEK293T cells were co-transfected with eight pHW2000 plasmids derived either from 21957-1/H5N6 (either WT or mutant) using polyethyleneimine (Polysciences, USA). Following a 2-hour incubation period in Opti-MEM (Gibco, USA) at 37℃, the supernatant was discarded, and the cells were incubated with infection medium (Opti-MEM supplemented with 0.2% BSA and 0.08 μg/mL TPCK-trypsin) for 48 h at 37℃. All viruses were then propagated in 10-day-old SPF chicken embryos, and the recombinant virus sequences were confirmed before use.

**Virus growth kinetics**

Confluent MDCK or DF1 cells were exposed to a starting virus load with an MOI of 0.001 and 0.001 for 1 h in 12-well plates at 37℃, respectively. After two PBS washes, the cells were then cultured in DMEM supplemented with 0.2% BSA at 37℃ in a 5% CO_2_ incubator. Virus titers at 12- and 36-hours post-infection were determined using the 50% tissue culture infective dose (TCID_50_) assay in MDCK cells.

**Luciferase reporter assay**

HEK293T or DF1 cells were co-transfected with 100 ng of pHW2000 plasmids expressing PB2, PB1, PA (either WT or mutant), along with 200 ng of pHW2000-NP plasmids, 20 ng of the thymidine kinase promoter-Renilla luciferase reporter plasmids (pRL-TK), and 100 ng of the luciferase reporter plasmids (pol I-NP-Luc). Lipofectamine^TM^ 3000 (Invitrogen, USA) was used for transfection according to the manufacturer's protocol. After 24 h post-transfection, the cells were lysed, and the relative polymerase activity was examined using the Dual-Luciferase Reporter Gene Assay Kit (Yeasen, China). Reporter gene expression was determined by measuring luminescence using a GloMax® 96 Microplate Luminometer (Promega, USA). The activity of firefly luciferase was standardized by calculating the ratio of firefly luciferase activity to that of the transfection control, Renilla luciferase.

**Quantification and statistical analysis**

The data of the studies were presented as mean ± standard error of mean (SEM). Student’s t-test was utilized to compare the differences between the two groups. Statistical significance was indicated as ∗ (p < 0.05), ∗∗ (p < 0.01), ∗∗∗ (p < 0.001), ∗∗∗∗ (p < 0.0001). The statistical analyses and calculations of the studies were performed using GraphPad Prism 7. To assess the statistical significance of differences in gene expression between sample groups of scRNA-seq data, we employed the Wilcoxon rank-sum test. The significance levels were denoted as follows: ∗ (p < 0.05), ∗∗ (p < 0.01), ∗∗∗ (p < 0.001), ∗∗∗∗ (p < 0.0001). The R package ggpubr (version 0.6.0) was used to create violin plots. All data analyses were performed on a Linux-based workstation using R (version R.4.2.2) and Python (version 3.9.12).

**References**

1.	Zhang J, Ye H, Liu Y, Liao M, Qi W. Resurgence of H5N6 avian influenza virus in 2021 poses new threat to public health. *Lancet Microbe*. Aug 2022;3(8):e558. doi:10.1016/S2666-5247(22)00148-3

2.	Katoh K, Misawa K, Kuma K, Miyata T. MAFFT: a novel method for rapid multiple sequence alignment based on fast Fourier transform. *Nucleic Acids Res*. Jul 15 2002;30(14):3059–66. doi:10.1093/nar/gkf436

3.	Kalyaanamoorthy S, Minh BQ, Wong TKF, von Haeseler A, Jermiin LS. ModelFinder: fast model selection for accurate phylogenetic estimates. *Nat Methods*. Jun 2017;14(6):587–589. doi:10.1038/nmeth.4285

4.	Nguyen LT, Schmidt HA, von Haeseler A, Minh BQ. IQ-TREE: a fast and effective stochastic algorithm for estimating maximum-likelihood phylogenies. *Mol Biol Evol*. Jan 2015;32(1):268–74. doi:10.1093/molbev/msu300

5.	Li H, Durbin R. Fast and accurate short read alignment with Burrows-Wheeler transform. *Bioinformatics*. Jul 15 2009;25(14):1754–60. doi:10.1093/bioinformatics/btp324

6.	Li H, Handsaker B, Wysoker A, et al. The Sequence Alignment/Map format and SAMtools. *Bioinformatics*. Aug 15 2009;25(16):2078–9. doi:10.1093/bioinformatics/btp352

7.	Hao Y, Hao S, Andersen-Nissen E, et al. Integrated analysis of multimodal single-cell data. *Cell*. Jun 24 2021;184(13):3573–3587 e29. doi:10.1016/j.cell.2021.04.048

8.	Aibar S, Gonzalez-Blas CB, Moerman T, et al. SCENIC: single-cell regulatory network inference and clustering. *Nat Methods*. Nov 2017;14(11):1083–1086. doi:10.1038/nmeth.4463

9.	Suo S, Zhu Q, Saadatpour A, Fei L, Guo G, Yuan GC. Revealing the Critical Regulators of Cell Identity in the Mouse Cell Atlas. *Cell Rep*. Nov 6 2018;25(6):1436–1445 e3. doi:10.1016/j.celrep.2018.10.045

10.	Wu X, Xu W, Deng L, et al. Spatial multi-omics at subcellular resolution via high-throughput in situ pairwise sequencing. *Nat Biomed Eng*. May 14 2024;doi:10.1038/s41551-024-01205-7

11.	Xu W, Cai H, Zhang Q, et al. U-FISH: a fluorescent spot detector for imaging-based spatial-omics analysis and AI-assisted FISH diagnosis. *Genome Biol*. Sep 1 2025;26(1):261. doi:10.1186/s13059-025-03736-x

12.	Hoffmann E, Neumann G, Kawaoka Y, Hobom G, Webster RG. A DNA transfection system for generation of influenza A virus from eight plasmids. *Proc Natl Acad Sci U S A*. May 23 2000;97(11):6108–13. doi:10.1073/pnas.100133697

13.	Hoffmann E, Stech J, Guan Y, Webster RG, Perez DR. Universal primer set for the full-length amplification of all influenza A viruses. *Arch Virol*. Dec 2001;146(12):2275–89. doi:10.1007/s007050170002
