# Supplementary material for: Heterogeneous viral-host response at single-cell resolution and viral adaptive mutational atlas in birds and mammals upon H5 influenza virus infection
Source: Natl Sci Rev. 2026 Jun 25;13(14):nwag380. doi: 10.1093/nsr/nwag380 (PMC13458601; doi:10.1093/nsr/nwag380)
Supplement: Supplementary file 1 [file nwag380_supplemental_files.zip › Supplementary Data 1.pdf]

Supplementary Data 1. The marker genes of different cell types in this study.

Gene	Avg_log$_2$FC	Cell type
Ebf1	4.481607	B cells
Igkc	3.779754	B cells
Ighm	3.593437	B cells
Cd79a	3.346135	B cells
H2-Aa	3.245109	B cells
Pax5	3.083079	B cells
Bank1	2.992565	B cells
Aff3	2.841724	B cells
H2-Eb1	2.727843	B cells
Smc6	2.721883	B cells
Tcf4	3.212958	Dendritic cells
Irf8	2.507576	Dendritic cells
P2ry14	2.425003	Dendritic cells
Cyb561a3	2.264763	Dendritic cells
Cadm1	2.314113	Dendritic cells
Plac8	2.848777	Dendritic cells
Ltb	2.208053	Dendritic cells
H2-Ab1	2.173679	Dendritic cells
Cst3	4.36969	Dendritic cells
H2-Aa	2.273509	Dendritic cells
Magi1	3.240961	Endothelial cells
Epas1	3.233472	Endothelial cells
Ptprm	3.124368	Endothelial cells
Calcrl	3.112663	Endothelial cells
Ldb2	3.066887	Endothelial cells
Mcf2l	3.060592	Endothelial cells
Tmem100	3.036797	Endothelial cells
Rapgef5	3.030207	Endothelial cells
Adgrl4	3.005136	Endothelial cells
Sftpc	3.535273	Endothelial cells
Mgp	5.259861	Fibroblasts
Dcn	4.927551	Fibroblasts
Apoa1	4.756124	Fibroblasts
Col1a1	4.641308	Fibroblasts
Col1a2	4.545605	Fibroblasts
Col3a1	4.348999	Fibroblasts
Gsn	3.972218	Fibroblasts
Clec3b	3.821842	Fibroblasts
Sparcl1	3.615627	Fibroblasts
Ccn1	3.428505	Fibroblasts

Jchain	4.398445	Macrophages
Saa3	3.605985	Macrophages
C1qb	3.165215	Macrophages
C1qa	3.137648	Macrophages
Lyz2	2.807307	Macrophages
Bpi	2.789379	Macrophages
Apoe	2.747809	Macrophages
Ctss	2.679491	Macrophages
C1qc	2.434603	Macrophages
Lgals3	2.298768	Macrophages
S100a8	4.514006	Neutrophils
Sds	3.578653	Neutrophils
G0s2	3.531777	Neutrophils
Cda	3.25012	Neutrophils
Sorl1	3.108171	Neutrophils
Mctp1	3.10165	Neutrophils
Lrrk2	2.965986	Neutrophils
Notch2	2.96255	Neutrophils
Ifit3	2.907339	Neutrophils
S100a9	4.659976	Neutrophils
Ccl5	3.374745	NK cells
Gzma	3.192436	NK cells
Il12rb2	2.603011	NK cells
Il2rb	2.353143	NK cells
Nkg7	2.268783	NK cells
Klrk1	2.226782	NK cells
Runx3	2.193239	NK cells
Cblb	2.178381	NK cells
Ctsw	2.156009	NK cells
Ikzf2	1.921948	NK cells
Tcf7	3.223306	T cells
Tox	2.836231	T cells
Bcl11b	2.645649	T cells
Itk	2.639588	T cells
Satb1	2.513511	T cells
Lef1	2.470458	T cells
Cd247	2.46465	T cells
Camk4	2.433599	T cells
Grap2	2.414038	T cells
Calcb	2.389841	T cells
