# Supplementary material for: Heterogeneous viral-host response at single-cell resolution and viral adaptive mutational atlas in birds and mammals upon H5 influenza virus infection
Source: Natl Sci Rev. 2026 Jun 25;13(14):nwag380. doi: 10.1093/nsr/nwag380 (PMC13458601; doi:10.1093/nsr/nwag380)
Supplement: Supplementary file 1 [file nwag380_supplemental_files.zip › Supplementary Data 10.pdf]

Supplementary Data 10. The raw data of the firefly luciferase activities and Renilla luciferase activities in HEK293T cells after transfected with plasmids. FI represents firefly luciferase activities, and TK represents Renilla luciferase activities.

Mutations	WT			PB2-G74R		
FI	452157	405100	465068	1809448	2235024	2391868
TK	118582	115332	101203	87803	92751	79319
FI/TK	3.813	3.512	4.595	20.60804	24.09703	30.15504
FI	418809	426117	441291	2108099	2596372	2484143
TK	118772	115463	102538	109867	106879	96604
FI/TK	3.526	3.691	4.304	19.18774	24.29263	25.7147
FI	404271	432611	410380	2403704	2645663	2619698
TK	119191	115674	100455	95839	105388	105982
FI/TK	3.391791	3.739916	4.085212	25.08065	25.10403	24.71833
Mutations	PB2-E192K			PB2-V220I		
FI	528332	402984	586367	219616	175847	208905
TK	47913	49651	56211	62414	64844	61270
FI/TK	11.0269	8.116332	10.43153	3.518698	2.711847	3.409581
FI	527289	399894	588443	218141	190811	204900
TK	63237	66327	73265	76466	78391	70781
FI/TK	8.338299	6.029128	8.031707	2.852784	2.434093	2.894845
FI	519213	424991	565077	213950	193349	217596
TK	68505	69278	78919	81186	81605	73450
FI/TK	7.579199	6.134574	7.160215	2.635307	2.369328	2.962505
Mutations	PB2-E627K			PB1-H562Q		
FI	31664202	30443544	31992866	109954	98387	47503
TK	351814	325378	282010	63728	66123	44263
FI/TK	90.00268	93.56362	113.4459	1.725364	1.487939	1.073199
FI	29840426	32913202	34006904	105051	94635	46679
TK	240094	504487	259419	63778	66582	46726
FI/TK	124.2864	65.24093	131.0887	1.647135	1.42133	0.998994
FI	37692888	32227074	38365628	109131	93783	46346
TK	595847	319801	710269	65199	65691	45706
FI/TK	63.25934	100.7723	54.01563	1.673814	1.427638	1.014003
Mutations	PA-I145V			PA-T97I		
FI	141014	143002	184323	5022405	4416392	4160169
TK	53852	49596	61138	107921	101071	88753
FI/TK	2.618547	2.883337	3.014868	46.53779	43.69594	46.87356
FI	134429	136464	175569	4809266	4694677	4012014

TK	54671	49355	59258	92906	98610	83492
FI/TK	2.458872	2.764948	2.96279	51.76486	47.60853	48.05268
FI	141716	157114	177878	5141018	5016632	3871020
TK	54594	51685	57867	86214	104652	84996
FI/TK	2.595816	3.039837	3.073911	59.6309	47.93632	45.54355
