# Supplementary material for: Heterogeneous viral-host response at single-cell resolution and viral adaptive mutational atlas in birds and mammals upon H5 influenza virus infection
Source: Natl Sci Rev. 2026 Jun 25;13(14):nwag380. doi: 10.1093/nsr/nwag380 (PMC13458601; doi:10.1093/nsr/nwag380)
Supplement: Supplementary file 1 [file nwag380_supplemental_files.zip › Supplementary Data 11.pdf]

ementary Data 11. Differentially expressed genes of macrophages across different species upon H5 HPAIV inf

gene	log2FoldChange	p value	host
Hpgd	3.14	0.00000000	chicken
Ifit2	3.99	0.00000000	chicken
Il6	3.75	0.00000000	chicken
Trak1	2.95	0.00000000	chicken
Tpt1	-2.43	0.00000000	chicken
Oasl2	2.17	0.00000000	chicken
Ccl9	2.55	0.00000000	chicken
Smarcal1	-2.21	0.00000000	chicken
H3f3a	-2.41	0.00000000	chicken
Dusp1	2.89	0.00000000	chicken
Rack1	-2.36	0.00000000	chicken
Angpt1	2.01	0.00000000	chicken
Nos2	3.80	0.00000000	chicken
Tgm4	3.13	0.00000000	chicken
Eef1b2	-2.35	0.00000000	chicken
Irf1	2.39	0.00000000	chicken
Tnip3	2.15	0.00000000	chicken
Sgk1	2.41	0.00000000	chicken
Ifih1	3.04	0.00000000	chicken
Dusp16	2.14	0.00000000	chicken
Prdm1	2.22	0.00000000	chicken
Igdcc4	2.66	0.00000000	chicken
Lpgat1	2.25	0.00000000	chicken
Eef2	-1.83	0.00000000	chicken
Vmp1	1.97	0.00000000	chicken
Htr7	1.20	0.00000000	chicken
Bst1	1.79	0.00000000	chicken
Tnip2	2.45	0.00000000	chicken
Birc2	2.20	0.00000000	chicken
Pabpc1	-1.30	0.00000000	chicken
Eef1d	-2.27	0.00000000	chicken
Csta1	2.27	0.00000000	chicken
Xdh	1.90	0.00000000	chicken
Rgs1	2.03	0.00000000	chicken
Il10ra	1.80	0.00000000	chicken
F3	2.69	0.00000000	chicken
Tasl	2.03	0.00000000	chicken
Lta4h	-2.52	0.00000000	chicken
Cd74	-2.61	0.00000000	chicken
Cst3	-2.82	0.00000000	chicken

Snx10	1.76	0.00000000	chicken
Susd1	2.10	0.00000000	chicken
Spi1	1.43	0.00000000	chicken
Grb10	1.83	0.00000000	chicken
Osbpl5	1.94	0.00000000	chicken
Ptprj	1.68	0.00000000	chicken
Socs3	1.52	0.00000000	chicken
Fau	-1.59	0.00000000	chicken
Rasgef1a	2.42	0.00000000	chicken
Rsad2	1.48	0.00000000	chicken
Itm2a	-1.77	0.00000000	chicken
Ckap2l	2.21	0.00000000	chicken
Runx3	-1.70	0.00000000	chicken
Zdhhc20	1.60	0.00000000	chicken
Plin2	1.59	0.00000000	chicken
Lrp6	1.54	0.00000000	chicken
Acod1	1.90	0.00000000	chicken
Atf3	1.52	0.00000000	chicken
Uvrag	1.63	0.00000000	chicken
Stat1	1.49	0.00000000	chicken
Tnfaip3	1.56	0.00000000	chicken
Zfp352	1.96	0.00000000	chicken
Nampt	1.53	0.00000000	chicken
Slc44a3	2.27	0.00000000	chicken
Hmgb1	-1.73	0.00000000	chicken
Tfec	2.05	0.00000000	chicken
Glipr2	1.27	0.00000000	chicken
Cflar	2.07	0.00000000	chicken
Lao1	1.64	0.00000000	chicken
Arxes2	-1.69	0.00000000	chicken
Pla2g4a	1.92	0.00000000	chicken
Zfp593	-1.60	0.00000000	chicken
Dpp8	1.32	0.00000000	chicken
Arap2	1.90	0.00000000	chicken
Spidr	2.24	0.00000000	chicken
Csrnp1	1.53	0.00000000	chicken
Btg2	1.41	0.00000000	chicken
Il1b	1.61	0.00000000	chicken
Thsd4	1.33	0.00000000	chicken
Trank1	1.64	0.00000000	chicken
BC016579	2.13	0.00000000	chicken
Nsmce2	1.54	0.00000000	chicken
Baz2b	1.63	0.00000000	chicken
Dst	2.90	0.00000000	chicken
Rnf19b	1.26	0.00000000	chicken
Dio2	2.83	0.00000000	chicken

Ifi30	-2.12	0.00000000	chicken
Gab1	1.82	0.00000000	chicken
Pdgfa	1.44	0.00000000	chicken
Nme2	-1.51	0.00000000	chicken
Ybx1	-1.40	0.00000000	chicken
Cd3g	-1.38	0.00000000	chicken
Elmo1	-1.80	0.00000000	chicken
Klf15	0.88	0.00000000	chicken
Slc39a13	1.00	0.00000000	chicken
Fry	1.86	0.00000000	chicken
Btg1b	-1.37	0.00000000	chicken
Rnf213	1.06	0.00000000	chicken
Npas2	1.42	0.00000000	chicken
Ndnf	0.95	0.00000000	chicken
Bend6	1.81	0.00000000	chicken
Ly86	-2.41	0.00000000	chicken
Hivep1	1.54	0.00000000	chicken
Sema3c	2.33	0.00000000	chicken
Spata5	2.00	0.00000000	chicken
Atp6v0d2	1.30	0.00000000	chicken
Laptm5	-1.13	0.00000000	chicken
Actg1	-0.81	0.00000000	chicken
Tln2	2.90	0.00000000	chicken
Tle5	-1.51	0.00000000	chicken
Erc1	1.55	0.00000000	chicken
Ints14	1.12	0.00000000	chicken
Fos	1.43	0.00000000	chicken
Basp1	1.28	0.00000000	chicken
Pdlim5	1.48	0.00000000	chicken
Samsn1	1.45	0.00000000	chicken
Dll1	1.80	0.00000000	chicken
Etv6	1.53	0.00000000	chicken
Wnk1	1.40	0.00000000	chicken
Urm1	-1.40	0.00000000	chicken
Ahrr	-2.17	0.00000000	chicken
Gbp8	1.52	0.00000000	chicken
Ly75	1.68	0.00000000	chicken
Dgkh	1.63	0.00000000	chicken
F13a1	1.91	0.00000000	chicken
Casp1	1.55	0.00000000	chicken
Ulbp1	-1.39	0.00000000	chicken
Nadk	1.19	0.00000000	chicken
Zbtb46	1.71	0.00000000	chicken
Ddx60	1.13	0.00000000	chicken
Pou2f1	1.30	0.00000000	chicken
Eif3m	-1.31	0.00000000	chicken

Lsmem1	1.30	0.00000000	chicken
Atxn7l1	1.25	0.00000000	chicken
Rad51b	1.40	0.00000000	chicken
Ly6e	1.39	0.00000000	chicken
Tnfsf15	1.21	0.00000000	chicken
Bzw2	1.14	0.00000000	chicken
Arhgap11a	1.47	0.00000000	chicken
Hbegf	1.29	0.00000000	chicken
Tomm20	-1.44	0.00000000	chicken
Jun	1.03	0.00000000	chicken
Atp6v1c2	0.84	0.00000000	chicken
Dennd4a	1.24	0.00000000	chicken
Sh3bgrl3	-1.37	0.00000000	chicken
Nsa2	-1.21	0.00000000	chicken
Svbp	-1.25	0.00000000	chicken
Wdfy2	0.90	0.00000000	chicken
Eif3l	-1.14	0.00000000	chicken
Dennd1a	2.09	0.00000000	chicken
Helz2	1.60	0.00000000	chicken
Rngtt	1.28	0.00000000	chicken
Inhbb	0.88	0.00000000	chicken
Tax1bp1	1.30	0.00000000	chicken
Plod2	1.49	0.00000000	chicken
B3gnt5	1.05	0.00000000	chicken
Pla2r1	1.37	0.00000000	chicken
Eif3e	-1.36	0.00000000	chicken
Ctsz	-1.62	0.00000000	chicken
Chac1	0.97	0.00000000	chicken
Ddx5	-0.88	0.00000000	chicken
Jarid2	-1.19	0.00000000	chicken
G0s2	2.02	0.00000000	chicken
Arid5b	1.08	0.00000000	chicken
Lsp1	-1.36	0.00000000	chicken
Ppa2	1.30	0.00000000	chicken
Dock7	0.99	0.00000000	chicken
Coro1c	-0.90	0.00000000	chicken
Eif3f	-1.19	0.00000000	chicken
Rhoa	-0.94	0.00000000	chicken
Aif1l	-1.56	0.00000000	chicken
Serbp1	-0.98	0.00000000	chicken
Bank1	1.21	0.00000000	chicken
Ctsa	-1.52	0.00000000	chicken
Slc35e2	0.96	0.00000000	chicken
Eya3	1.05	0.00000000	chicken
Csf3	1.24	0.00000000	chicken
Ccl20	3.12	0.00000000	chicken

Itpr2	-1.50	0.00000000	chicken
Egr1	0.67	0.00000000	chicken
B2m	-0.80	0.00000000	chicken
Fam135a	-1.39	0.00000000	chicken
Alpk1	1.20	0.00000000	chicken
Hnrnpa1	-1.30	0.00000000	chicken
Notch1	-1.45	0.00000000	chicken
Arsg	0.94	0.00000000	chicken
Grid1	1.32	0.00000000	chicken
Add3	-0.97	0.00000000	chicken
Fgf2	0.91	0.00000000	chicken
Adap2	0.67	0.00000000	chicken
Zpld1	1.42	0.00000000	chicken
Tifa	0.62	0.00000000	chicken
Septin10	1.08	0.00000000	chicken
Cox7b	-1.11	0.00000000	chicken
Bcl2a1d	0.90	0.00000000	chicken
Rybp	1.10	0.00000000	chicken
Plac8	1.41	0.00000000	chicken
Slc37a1	0.92	0.00000000	chicken
Ammecr1	1.29	0.00000000	chicken
Aldh1a3	1.77	0.00000000	chicken
Ssr2	-1.30	0.00000000	chicken
Sh3kbp1	-1.56	0.00000000	chicken
St3gal6	1.51	0.00000000	chicken
Tmed10	-0.93	0.00000000	chicken
Dstn	-0.82	0.00000000	chicken
Tnpo1	1.19	0.00000000	chicken
Sbf2	1.20	0.00000000	chicken
Tgfbi	-1.45	0.00000000	chicken
Htra1	1.32	0.00000000	chicken
Pgls	-1.20	0.00000000	chicken
Arf6	-1.05	0.00000000	chicken
Ssu72	1.00	0.00000000	chicken
Tspan13	0.70	0.00000000	chicken
Fbxo11	1.47	0.00000000	chicken
Asah1	-1.23	0.00000000	chicken
Hsp90b1	-1.66	0.00000000	chicken
Grn	-1.60	0.00000000	chicken
Tmem123	1.11	0.00000000	chicken
Eif2s3y	-1.03	0.00000000	chicken
4932438A13Rik	1.05	0.00000000	chicken
Jak2	0.94	0.00000000	chicken
Sgms1	1.12	0.00000000	chicken
Vgll4	1.39	0.00000000	chicken
Ptpn2	0.93	0.00000000	chicken

Dennd4c	1.06	0.00000000	chicken
Eif3d	-1.03	0.00000000	chicken
Haus6	1.17	0.00000000	chicken
Ralb	1.64	0.00000000	chicken
Itga4	-1.29	0.00000000	chicken
Pim3	0.98	0.00000000	chicken
Ppib	-1.27	0.00000000	chicken
Cblb	-1.32	0.00000000	chicken
Rabgap1l	-1.50	0.00000000	chicken
Ddx3y	-0.84	0.00000000	chicken
Slc25a3	-0.95	0.00000000	chicken
Tcf7	-2.18	0.00000000	chicken
Dip2b	0.90	0.00000000	chicken
Cd83	1.23	0.00000000	chicken
Pdia3	-1.10	0.00000000	chicken
Slc8a1	1.05	0.00000000	chicken
Tbl1xr1	1.06	0.00000000	chicken
Cpne8	1.66	0.00000000	chicken
Ptgs2	2.33	0.00000000	chicken
Cdk11b	0.88	0.00000000	chicken
Csf1r	-1.58	0.00000000	chicken
Zfand3	1.12	0.00000000	chicken
Syt16	0.93	0.00000000	chicken
Plxnc1	-1.20	0.00000000	chicken
Ttc39a	0.89	0.00000000	chicken
Rcor1	0.96	0.00000000	chicken
Cox6a1	-1.06	0.00000000	chicken
Casp8	1.18	0.00000000	chicken
Eif1	-0.97	0.00000000	chicken
Dram1	0.97	0.00000000	chicken
Cd200r1	0.65	0.00000000	chicken
Zc3hav1l	0.98	0.00000000	chicken
Tet2	1.05	0.00000000	chicken
Hnrnph2	-0.90	0.00000000	chicken
Itgb2l	-0.98	0.00000000	chicken
Cyba	-1.19	0.00000000	chicken
Cdk6	1.14	0.00000000	chicken
Rubcnl	1.03	0.00000000	chicken
Rcc1l	0.73	0.00000000	chicken
Tmem266	0.87	0.00000000	chicken
Cmtm3	-0.92	0.00000000	chicken
Cxadr	0.67	0.00000000	chicken
Zdhhc14	1.08	0.00000000	chicken
Fnbp1	-1.21	0.00000000	chicken
Wnt5b	1.59	0.00000000	chicken
Tsc22d3	1.22	0.00000000	chicken

Babam2	0.90	0.00000000	chicken
Pgs1	0.59	0.00000000	chicken
Alcam	1.38	0.00000000	chicken
Klf5	0.71	0.00000000	chicken
Tbc1d1	1.24	0.00000000	chicken
Nt5dc2	-1.08	0.00000000	chicken
Lpp	0.94	0.00000000	chicken
Ankrd39	-0.80	0.00000000	chicken
Rnaset2b	1.35	0.00000000	chicken
Kcnq5	-2.06	0.00000000	chicken
Creb5	0.95	0.00000000	chicken
H2az2	-0.81	0.00000000	chicken
Tbxas1	1.16	0.00000000	chicken
Cd200	0.75	0.00000000	chicken
Arhgef28	0.45	0.00000000	chicken
Eif4b	-1.05	0.00000000	chicken
Lrrk1	1.43	0.00000000	chicken
Socs1	0.89	0.00000000	chicken
Sash1	0.77	0.00000000	chicken
Ybx3	-0.95	0.00000000	chicken
Creg1	-1.21	0.00000000	chicken
Ifnar1	1.01	0.00000000	chicken
S100a4	-1.89	0.00000000	chicken
Calm2	-0.90	0.00000000	chicken
Itga2	1.04	0.00000000	chicken
Txnip	0.44	0.00000000	chicken
Bmpr1b	1.03	0.00000000	chicken
Fermt1	1.03	0.00000000	chicken
Samd4	0.69	0.00000000	chicken
Sntb1	0.96	0.00000000	chicken
Gab2	1.13	0.00000000	chicken
Stat2	0.59	0.00000000	chicken
Vav3	1.04	0.00000000	chicken
Nckipsd	-1.06	0.00000000	chicken
Prkg2	1.32	0.00000000	chicken
Bcl6	1.11	0.00000000	chicken
Tiparp	-0.91	0.00000000	chicken
Acsl5	0.57	0.00000000	chicken
Nmi	0.77	0.00000000	chicken
Golga4	1.00	0.00000000	chicken
Ankrd42	0.44	0.00000000	chicken
Ppfibp1	-1.00	0.00000000	chicken
Klf6	0.77	0.00000000	chicken
Pwwp2b	0.76	0.00000000	chicken
Glo1	-0.84	0.00000000	chicken
Crip1	-1.58	0.00000000	chicken

Il27ra	-0.88	0.00000000	chicken
Tmsb4x	-0.44	0.00000000	chicken
Alas1	0.85	0.00000000	chicken
Ctps2	1.07	0.00000000	chicken
Trio	0.98	0.00000000	chicken
Hopx	0.81	0.00000000	chicken
Pparg	1.18	0.00000000	chicken
Gsn	-0.98	0.00000000	chicken
Rhob	0.88	0.00000000	chicken
Eno1b	-0.67	0.00000000	chicken
Ncf4	0.90	0.00000000	chicken
Tmcc1	0.71	0.00000000	chicken
Tcf7l2	1.67	0.00000000	chicken
Dusp5	0.84	0.00000000	chicken
Cox5a	-0.81	0.00000000	chicken
Hif1a	0.76	0.00000000	chicken
Zfand2a	0.87	0.00000000	chicken
Hibadh	0.65	0.00000000	chicken
Susd6	-0.95	0.00000000	chicken
Hnrnpa3	-0.74	0.00000000	chicken
Cask	-1.21	0.00000000	chicken
Tia1	0.91	0.00000000	chicken
Gpr68	0.60	0.00000000	chicken
Gpx1	-0.96	0.00000000	chicken
Tm4sf19	1.01	0.00000000	chicken
Pdia4	-1.39	0.00000000	chicken
Cib1	-0.75	0.00000000	chicken
Sfxn1	-0.84	0.00000000	chicken
Batf3	0.74	0.00000000	chicken
Arhgap12	0.83	0.00000000	chicken
Klf13	-1.01	0.00000000	chicken
Cubn	0.76	0.00000000	chicken
Cd3e	-1.08	0.00000000	chicken
Cstf3	0.70	0.00000000	chicken
Arg2	0.68	0.00000000	chicken
Sptbn1	-1.19	0.00000000	chicken
Calm1	-0.67	0.00000000	chicken
Rbm6	0.72	0.00000000	chicken
Itm2b	-0.85	0.00000000	chicken
Nfkbiz	0.79	0.00000000	chicken
Riok3	0.72	0.00000000	chicken
Tex2	0.50	0.00000000	chicken
Ptpre	1.72	0.00000000	chicken
Ccnd2	-1.04	0.00000000	chicken
Chsy1	0.85	0.00000000	chicken
Tut4	0.76	0.00000000	chicken

Ccnl1	-0.96	0.00000000	chicken
Emb	-0.74	0.00000000	chicken
Fkbp1a	-0.69	0.00000000	chicken
Ramp2	-0.70	0.00000000	chicken
Fam171b	0.51	0.00000000	chicken
Anxa6	-0.83	0.00000000	chicken
Zdhhc8	0.65	0.00000000	chicken
Epb41	-0.86	0.00000000	chicken
Pde4b	0.75	0.00000000	chicken
Trib1	0.48	0.00000000	chicken
Insr	0.73	0.00000000	chicken
Rnf19a	0.76	0.00000000	chicken
Anxa2	0.70	0.00000000	chicken
Ndufb10	-0.78	0.00000000	chicken
Pacsin2	0.77	0.00000000	chicken
Rpn1	-0.84	0.00000000	chicken
Zfp704	-1.25	0.00000000	chicken
Ubxn2a	0.54	0.00000000	chicken
Ccni	-0.70	0.00000000	chicken
Slbp	0.42	0.00000000	chicken
Ctss	-1.09	0.00000000	chicken
Lpxn	-0.71	0.00000000	chicken
Nr4a3	1.10	0.00000000	chicken
Tasor2	0.71	0.00000000	chicken
Sla	0.76	0.00000000	chicken
Ip6k2	0.86	0.00000000	chicken
Zfp706	-0.92	0.00000000	chicken
Prdx4	-1.17	0.00000000	chicken
Dock2	-1.27	0.00000000	chicken
Rab30	0.84	0.00000000	chicken
Eif4a2	-0.71	0.00000000	chicken
Plcb2	0.71	0.00000000	chicken
Niban1	-0.92	0.00000000	chicken
Slc39a12	0.56	0.00000000	chicken
Nin	0.48	0.00000000	chicken
Kank1	0.39	0.00000000	chicken
Hectd1	0.81	0.00000000	chicken
Golm2	-0.90	0.00000000	chicken
Eif2b3	-1.24	0.00000000	chicken
Piga	0.44	0.00000000	chicken
Helz	0.77	0.00000000	chicken
Bri3	-1.18	0.00000000	chicken
Utrn	1.00	0.00000000	chicken
Dock11	-1.01	0.00000000	chicken
Zfc3h1	0.65	0.00000000	chicken
Tmed5	-0.70	0.00000000	chicken

Atf7ip	0.86	0.00000000	chicken
Hyou1	-0.97	0.00000000	chicken
Ankrd13a	-1.07	0.00000000	chicken
Zranb1	0.52	0.00000000	chicken
Ctsc	0.83	0.00000000	chicken
Cd63	-1.00	0.00000000	chicken
Cd86	0.39	0.00000000	chicken
Oat	-0.81	0.00000000	chicken
Ppia	-0.50	0.00000000	chicken
Als2cl	0.79	0.00000000	chicken
Ubtd2	0.80	0.00000000	chicken
Tpp1	-0.97	0.00000000	chicken
Rhot1	0.67	0.00000000	chicken
Pfkfb3	0.63	0.00000000	chicken
Ndfip1	-0.87	0.00000000	chicken
Ranbp10	0.59	0.00000000	chicken
Net1	0.90	0.00000000	chicken
Adss	0.66	0.00000000	chicken
Calml3	0.35	0.00000000	chicken
Bcl11b	-1.42	0.00000000	chicken
Pcmtd1	-1.02	0.00000000	chicken
Akap6	0.77	0.00000000	chicken
Ddrgk1	-0.75	0.00000000	chicken
Themis2	0.78	0.00000000	chicken
Canx	-0.90	0.00000000	chicken
Fmnl1	-0.76	0.00000000	chicken
Hectd2	0.70	0.00000000	chicken
Ppp1r10	0.60	0.00000000	chicken
Scg5	0.66	0.00000000	chicken
Hivep3	0.93	0.00000000	chicken
Vps50	0.70	0.00000000	chicken
Sumo3	-0.80	0.00000000	chicken
Foxo1	-1.00	0.00000000	chicken
Adgrd1	0.74	0.00000000	chicken
Ankrd44	-1.24	0.00000000	chicken
Utp6	0.67	0.00000000	chicken
Arpc2	-0.54	0.00000000	chicken
Arih2	0.54	0.00000000	chicken
Arid1b	-1.06	0.00000000	chicken
Rab38	0.56	0.00000000	chicken
Idh2	-0.71	0.00000000	chicken
Ube2w	0.87	0.00000000	chicken
St6gal1	-0.73	0.00000000	chicken
Osbpl6	0.52	0.00000000	chicken
Ostf1	-0.71	0.00000000	chicken
Cox4i1	-0.77	0.00000000	chicken

Atp6v1g3	0.49	0.00000000	chicken
Ttc7b	0.64	0.00000000	chicken
Bsg	-0.78	0.00000000	chicken
Fbln5	0.59	0.00000000	chicken
Atp5d	-0.63	0.00000000	chicken
Tle1	-1.09	0.00000000	chicken
Il18rap	0.84	0.00000000	chicken
Crot	0.56	0.00000000	chicken
Eps8	1.00	0.00000000	chicken
Fdft1	-0.83	0.00000000	chicken
Slc17a6	0.58	0.00000000	chicken
Ints6	0.60	0.00000000	chicken
Kcnb1	0.73	0.00000000	chicken
Il1rap	-1.04	0.00000000	chicken
Esd	-0.61	0.00000000	chicken
Steap4	0.97	0.00000000	chicken
Bcl2l14	0.28	0.00000000	chicken
Stab1	-1.21	0.00000000	chicken
Ppp2r5e	1.09	0.00000000	chicken
H3f3b	-0.70	0.00000000	chicken
Ndufb2	-0.82	0.00000000	chicken
Nek10	0.72	0.00000000	chicken
Eda	-1.23	0.00000000	chicken
Garem1	0.63	0.00000000	chicken
Nabp1	0.56	0.00000000	chicken
Gpr183	0.95	0.00000000	chicken
Rarb	0.39	0.00000000	chicken
Snap47	0.77	0.00000000	chicken
Ttc28	0.61	0.00000000	chicken
Itgav	0.87	0.00000000	chicken
Fbxl5	0.74	0.00000000	chicken
Hmgn3	-0.79	0.00000000	chicken
Fam53a	0.54	0.00000000	chicken
Sdcbp	-1.06	0.00000000	chicken
Nap1l1	-0.94	0.00000000	chicken
Rcn2	-0.71	0.00000000	chicken
Ets2	0.64	0.00000000	chicken
Elmo3	0.38	0.00000000	chicken
Pstpip2	0.48	0.00000000	chicken
P2ry4	-0.53	0.00000000	chicken
Spats2l	0.50	0.00000000	chicken
Vti1a	0.86	0.00000000	chicken
Vegfa	0.73	0.00000000	chicken
Acadl	-0.66	0.00000000	chicken
Apbb1ip	-0.84	0.00000000	chicken
Samd8	0.56	0.00000000	chicken

Prkce	-1.15	0.00000000	chicken
Uqcrc2	-0.62	0.00000000	chicken
Def6	-0.77	0.00000000	chicken
Zeb2	0.70	0.00000000	chicken
Tmbim6	-0.66	0.00000000	chicken
Il19	0.25	0.00000000	chicken
Kazn	0.33	0.00000000	chicken
Csf2ra	0.65	0.00000000	chicken
Snapc1l	0.50	0.00000000	chicken
Taok1	0.68	0.00000000	chicken
Tnfsf13b	0.57	0.00000000	chicken
Pfdn1	0.55	0.00000000	chicken
Gdpd5	-1.01	0.00000000	chicken
Ets1	-1.08	0.00000000	chicken
Fnip1	0.72	0.00000000	chicken
Phtf2	-0.69	0.00000000	chicken
Daw1	1.88	0.00000000	chicken
Btbd11	0.78	0.00000000	chicken
Cdh13	0.85	0.00000000	chicken
Tmprss7	0.36	0.00000000	chicken
Carhsp1	-0.67	0.00000000	chicken
Sdc4	1.25	0.00000000	chicken
Btf3	-0.76	0.00000000	chicken
Ndufa4	-0.80	0.00000000	chicken
Foxk2	0.76	0.00000000	chicken
Fnip2	0.79	0.00000000	chicken
Rras2	-0.88	0.00000000	chicken
Slco2b1	-1.05	0.00000000	chicken
Qk	0.72	0.00000000	chicken
Ccdc141	0.59	0.00000000	chicken
Tspan9	0.68	0.00000000	chicken
Eif5a2	-0.88	0.00000000	chicken
Arhgef12	-0.73	0.00000000	chicken
Csnk1d	0.62	0.00000000	chicken
Tbc1d7	0.70	0.00000000	chicken
Il18r1	0.63	0.00000000	chicken
Ndufb1	-0.72	0.00000000	chicken
Ndufaf8	-0.54	0.00000000	chicken
Pde3b	-0.83	0.00000000	chicken
Atp6v0c	-0.83	0.00000000	chicken
S100a11	-0.78	0.00000000	chicken
Zup1	0.37	0.00000000	chicken
Usp25	0.64	0.00000000	chicken
Gstm5	-0.56	0.00000000	chicken
Cmpk2	0.38	0.00000000	chicken
Gch1	0.43	0.00000000	chicken

Shtn1	-0.98	0.00000000	chicken
Sh3bgrl	-0.63	0.00000000	chicken
P2ry13	0.23	0.00000000	chicken
Adgrg2	1.22	0.00000000	chicken
Dnajb9	0.56	0.00000000	chicken
Sbk2	0.36	0.00000000	chicken
Eif3h	-0.73	0.00000000	chicken
Ndufc2	-0.74	0.00000000	chicken
Ttll1	0.49	0.00000000	chicken
Zzz3	0.59	0.00000000	chicken
Sorl1	-0.45	0.00000000	chicken
Dnah12	0.75	0.00000000	chicken
Zfhx3	-0.83	0.00000000	chicken
Rasgef1b	1.13	0.00000000	chicken
Cacnb4	0.62	0.00000000	chicken
Ptafr	0.57	0.00000000	chicken
Flnb	1.01	0.00000000	chicken
Sap18	-0.58	0.00000000	chicken
Dnajb12	0.49	0.00000000	chicken
Dnase2b	-0.91	0.00000000	chicken
Slc24a4	-1.00	0.00000000	chicken
Plk3	0.62	0.00000000	chicken
Gstp3	-0.56	0.00000000	chicken
Abhd6	0.66	0.00000000	chicken
Ndufb5	-0.67	0.00000000	chicken
Tox	-1.29	0.00000000	chicken
Fgd4	0.62	0.00000000	chicken
Phf21a	0.68	0.00000000	chicken
Cfap45	0.29	0.00000000	chicken
Prkn	-1.42	0.00000000	chicken
Stat4	-0.75	0.00000000	chicken
Ralbp1	-0.77	0.00000000	chicken
Neurl1b	0.61	0.00000000	chicken
Chp1	0.60	0.00000000	chicken
Polk	0.56	0.00000000	chicken
Tagap1	0.92	0.00000000	chicken
Gng10	-0.47	0.00000000	chicken
Lamp2	-0.85	0.00000000	chicken
Tgfb1	-0.71	0.00000000	chicken
Slco4a1	-0.69	0.00000000	chicken
Ckap4	-0.69	0.00000000	chicken
Tpmt	-0.54	0.00000000	chicken
Abhd17c	0.53	0.00000000	chicken
Adipor2	0.65	0.00000000	chicken
Emc3	-0.54	0.00000000	chicken
Dpys	0.33	0.00000000	chicken

Igf1r	-1.19	0.00000000	chicken
Eif4ebp1	-0.73	0.00000000	chicken
Hk2	0.49	0.00000000	chicken
Nptx2	0.59	0.00000000	chicken
Ifng	1.11	0.00000000	chicken
Arhgap30	-0.76	0.00000000	chicken
Sec62	-0.71	0.00000000	chicken
Abhd14b	-0.53	0.00000000	chicken
Brsk2	-1.47	0.00000000	chicken
Slc2a3	-0.64	0.00000000	chicken
Adamts3	0.42	0.00000000	chicken
Nlrc5	0.33	0.00000000	chicken
Tmed2	-0.79	0.00000000	chicken
Lrmda	0.61	0.00000000	chicken
Slc9a3r1	-0.59	0.00000000	chicken
Nupl1	0.71	0.00000000	chicken
Acta2	0.71	0.00000000	chicken
Cyp1a2	-0.55	0.00000000	chicken
Gnai2	-0.62	0.00000000	chicken
Unc13d	-0.66	0.00000000	chicken
Pkib	-1.23	0.00000000	chicken
Dpp10	0.72	0.00000000	chicken
Arf1	-0.58	0.00000000	chicken
Cep85l	0.87	0.00000000	chicken
Pcdh10	0.80	0.00000000	chicken
Dnmt3a	-0.77	0.00000000	chicken
Wwox	-1.22	0.00000000	chicken
Ndrg1	0.78	0.00000000	chicken
Azin2	0.30	0.00000000	chicken
Adamtsl2	0.63	0.00000000	chicken
Cdh2	0.56	0.00000000	chicken
Tasp1	0.52	0.00000000	chicken
Arpc1b	-0.48	0.00000000	chicken
Fosl2	0.65	0.00000000	chicken
Ccny	0.77	0.00000000	chicken
Abcc4	0.94	0.00000000	chicken
Tulp4	0.44	0.00000000	chicken
Galnt9	0.68	0.00000000	chicken
Sema7a	0.28	0.00000000	chicken
Slc25a26	0.51	0.00000000	chicken
Anxa5	-0.94	0.00000000	chicken
Inpp5k	0.59	0.00000000	chicken
Ndufa13	-0.51	0.00000000	chicken
Rapsn	0.35	0.00000000	chicken
Il2ra	-1.07	0.00000000	chicken
Prkag2	0.68	0.00000000	chicken

Bmp2	0.40	0.00000000	chicken
Grk3	-0.64	0.00000000	chicken
Fsbp	0.49	0.00000000	chicken
Ndufb4	-0.48	0.00000000	chicken
Gstm4	-0.70	0.00000000	chicken
Hsbp1	-0.62	0.00000000	chicken
Grap2	-0.84	0.00000000	chicken
Cab39l	-0.87	0.00000000	chicken
Il20ra	-1.19	0.00000000	chicken
Plxna1	0.65	0.00000000	chicken
Tln1	-0.71	0.00000000	chicken
Ncf1	0.55	0.00000000	chicken
Slc38a6	-0.75	0.00000000	chicken
Braf	0.52	0.00000000	chicken
Dennd3	0.92	0.00000000	chicken
Eif3g	-0.67	0.00000000	chicken
Ube2q2	0.69	0.00000000	chicken
Rgs19	-0.50	0.00000000	chicken
Ubl3	-0.74	0.00000000	chicken
Ackr2	0.34	0.00000000	chicken
Atp5j	-0.56	0.00000000	chicken
Ripk2	0.55	0.00000000	chicken
Spred2	0.55	0.00000000	chicken
Tsc22d2	0.34	0.00000000	chicken
Romo1	-0.78	0.00000000	chicken
Rel	0.63	0.00000000	chicken
Nme7	0.90	0.00000000	chicken
Prpsap1	0.45	0.00000000	chicken
Nf1	1.18	0.00000000	chicken
Phactr3	0.24	0.00000000	chicken
Fbn1	0.50	0.00000000	chicken
Lrrfip2	0.59	0.00000000	chicken
Dad1	-0.89	0.00000000	chicken
Hnrnpa2b1	-0.59	0.00000000	chicken
Zswim5	0.49	0.00000000	chicken
Atp5pb	-0.50	0.00000000	chicken
Fndc3a	0.73	0.00000000	chicken
G3bp1	-0.63	0.00000000	chicken
Dap	-0.67	0.00000000	chicken
Fbxo15	0.43	0.00000000	chicken
Atic	-0.61	0.00000000	chicken
Tomm6	-0.62	0.00000000	chicken
Ccser1	-1.39	0.00000000	chicken
Atp8a1	0.50	0.00000000	chicken
P4hb	-0.60	0.00000000	chicken
Sap30	-0.45	0.00000000	chicken

Cops7a	-0.73	0.00000000	chicken
Ktn1	-0.83	0.00000000	chicken
Inhba	1.56	0.00000000	chicken
Tmcc3	-1.34	0.00000000	chicken
Lrpap1	-0.53	0.00000000	chicken
Rgs2	0.96	0.00000000	chicken
Sestd1	0.72	0.00000000	chicken
Arhgap45	-0.62	0.00000000	chicken
Ints6l	0.42	0.00000000	chicken
Cox7a2	-0.63	0.00000000	chicken
Dek	-0.77	0.00000000	chicken
Necab2	0.73	0.00000000	chicken
Fbp1	-0.40	0.00000000	chicken
Atg7	0.62	0.00000000	chicken
Aco1	0.63	0.00000000	chicken
Csde1	-0.78	0.00000000	chicken
Ifngr1	-0.79	0.00000000	chicken
Lemd3	0.55	0.00000000	chicken
Rere	-0.80	0.00000000	chicken
Arfgef1	0.70	0.00000000	chicken
Appl1	0.56	0.00000000	chicken
Zcchc10	0.23	0.00000000	chicken
Ifrd1	0.83	0.00000000	chicken
Atp2b4	-0.94	0.00000000	chicken
Lamtor2	-0.61	0.00000000	chicken
Reep2	0.73	0.00000000	chicken
Bhlhe41	-0.70	0.00000000	chicken
Nbn	0.40	0.00000000	chicken
Zmiz1	-1.15	0.00000000	chicken
Ptp4a2	-0.53	0.00000000	chicken
Cirbp	-0.66	0.00000000	chicken
Calcb	-0.98	0.00000000	chicken
Ttc7	0.64	0.00000000	chicken
Bend5	0.50	0.00000000	chicken
Xylt1	0.96	0.00000000	chicken
Erp29	-0.56	0.00000000	chicken
Sh3gl1	0.40	0.00000000	chicken
Slc25a25	0.45	0.00000000	chicken
Tubb4b	0.51	0.00000000	chicken
Cd28	-0.91	0.00000000	chicken
Dpp4	1.28	0.00000000	chicken
Eif2ak2	0.35	0.00000000	chicken
Csf3r	-0.56	0.00000000	chicken
Fam102a	-0.83	0.00000000	chicken
Chd7	0.55	0.00000000	chicken
Rbm43	0.33	0.00000000	chicken

Akr1c13	-0.44	0.00000000	chicken
Cln6	-0.42	0.00000000	chicken
Tm2d2	-0.47	0.00000000	chicken
Ywhag	0.52	0.00000000	chicken
Ergic1	0.55	0.00000000	chicken
Bnip3	-0.63	0.00000000	chicken
Cdk5rap1	-0.86	0.00000000	chicken
Sting1	0.32	0.00000000	chicken
Hnrnpr	-0.72	0.00000000	chicken
Ndufb6	-0.65	0.00000000	chicken
5031439G07Rik	0.65	0.00000000	chicken
Prcp	-0.62	0.00000000	chicken
Gpc5	0.47	0.00000000	chicken
Hhex	0.73	0.00000000	chicken
Tmem35b	-0.41	0.00000000	chicken
Atp5j2	-0.69	0.00000000	chicken
Tapbp	-0.63	0.00000000	chicken
Dock5	-0.66	0.00000000	chicken
Lars2	0.36	0.00000000	chicken
Sec24b	0.48	0.00000000	chicken
Itga8	0.74	0.00000000	chicken
Hmgcs1	0.53	0.00000000	chicken
Hal	-0.39	0.00000000	chicken
Xpo6	-0.66	0.00000000	chicken
Zfp423	0.44	0.00000000	chicken
Plekhb2	-0.81	0.00000000	chicken
Lrba	-1.11	0.00000000	chicken
Jchain	-4.31	0.00000000	chicken
Arpc4	-0.40	0.00000000	chicken
Csnk2a2	-0.73	0.00000000	chicken
Pigyl	-0.28	0.00000000	chicken
Zfp36l1	0.40	0.00000000	chicken
Traf3	0.60	0.00000000	chicken
Smim4	-0.52	0.00000000	chicken
Gadd45b	0.82	0.00000000	chicken
Svil	0.59	0.00000000	chicken
Rnf24	0.28	0.00000000	chicken
Pik3ap1	0.67	0.00000000	chicken
Aff2	-0.71	0.00000000	chicken
Gpr34	-1.29	0.00000000	chicken
Umad1	-0.75	0.00000000	chicken
Irf7	1.02	0.00000000	chicken
Hesx1	0.39	0.00000000	chicken
Slc1a6	-0.45	0.00000000	chicken
Pdia6	-0.85	0.00000000	chicken
Phlda2	0.45	0.00000000	chicken

Ccdc88a	0.67	0.00000000	chicken
Ccdc122	0.47	0.00000000	chicken
Sik1	0.64	0.00000001	chicken
Ggps1	-0.74	0.00000001	chicken
Slamf8	-0.44	0.00000001	chicken
Sergef	0.67	0.00000001	chicken
Rims2	0.53	0.00000001	chicken
Atp5a1	-0.47	0.00000001	chicken
Hpcal1	-0.76	0.00000001	chicken
Tkt	0.61	0.00000001	chicken
Spred1	0.58	0.00000001	chicken
Pan3	-0.75	0.00000001	chicken
Capn3	0.91	0.00000001	chicken
Cyrib	-0.63	0.00000001	chicken
Arid4b	-0.76	0.00000001	chicken
Zfp438	0.36	0.00000001	chicken
Bach2	-1.08	0.00000001	chicken
Eaf2	0.35	0.00000001	chicken
Hsf2bp	0.52	0.00000001	chicken
Cenpu	0.79	0.00000001	chicken
Nacc2	0.39	0.00000001	chicken
Actb	-0.05	0.00000001	chicken
Ch25h	0.25	0.00000001	chicken
Slc16a1	-0.36	0.00000001	chicken
Rasa3	-0.81	0.00000001	chicken
Yipf1	-0.42	0.00000001	chicken
Tgif1	0.53	0.00000001	chicken
Abcc5	0.80	0.00000001	chicken
Ddx19a	-0.62	0.00000001	chicken
Eloa	0.44	0.00000001	chicken
Tnks	0.55	0.00000001	chicken
Elk3	-0.66	0.00000001	chicken
Igsf3	0.66	0.00000001	chicken
Atp6v0e	-0.80	0.00000001	chicken
Tmem120a	0.46	0.00000001	chicken
Atp1b1	0.52	0.00000001	chicken
Prmt7	0.38	0.00000001	chicken
Scube2	0.35	0.00000002	chicken
Ap4e1	0.33	0.00000002	chicken
Rbbp6	0.44	0.00000002	chicken
Park7	-0.67	0.00000002	chicken
Id3	0.68	0.00000002	chicken
Arid2	0.80	0.00000002	chicken
Rab3gap2	0.50	0.00000002	chicken
Tmem59	-0.66	0.00000002	chicken
Sh3bp2	0.55	0.00000002	chicken

Hao1	0.45	0.00000002	chicken
Tll2	0.44	0.00000002	chicken
E2f3	0.66	0.00000002	chicken
Epc1	0.56	0.00000002	chicken
Ncoa3	0.43	0.00000002	chicken
Flvcr2	0.91	0.00000002	chicken
Rad54b	0.29	0.00000002	chicken
Card11	-0.65	0.00000002	chicken
She	-0.26	0.00000002	chicken
Amn	0.35	0.00000002	chicken
Epcam	0.39	0.00000002	chicken
Ldha	-0.61	0.00000003	chicken
Ptpn12	0.52	0.00000003	chicken
Faf2	0.46	0.00000003	chicken
Slc46a2	0.53	0.00000003	chicken
Faf1	0.50	0.00000003	chicken
Trappc9	0.43	0.00000003	chicken
Stat3	-0.56	0.00000003	chicken
Pi4ka	-0.81	0.00000003	chicken
Usp34	0.48	0.00000003	chicken
Ddost	-0.52	0.00000003	chicken
Ndel1	0.56	0.00000003	chicken
Hexa	-0.42	0.00000003	chicken
Il6ra	-0.60	0.00000003	chicken
Klhl6	-0.64	0.00000003	chicken
Ampd3	0.49	0.00000004	chicken
Larp4	0.40	0.00000004	chicken
Ikzf3	-0.57	0.00000004	chicken
Pdxdc1	0.43	0.00000004	chicken
Thra	0.41	0.00000004	chicken
Dcun1d4	-0.60	0.00000004	chicken
Pag1	-0.94	0.00000005	chicken
Cidea	0.24	0.00000005	chicken
Actr3	-0.49	0.00000005	chicken
Zbtb49	0.21	0.00000005	chicken
Numb	0.90	0.00000005	chicken
Ttc3	0.57	0.00000005	chicken
Eif3a	-0.67	0.00000006	chicken
Lhfp	-1.14	0.00000006	chicken
Pde7b	-1.49	0.00000006	chicken
Tomm7	-0.59	0.00000006	chicken
Asb8	0.35	0.00000006	chicken
Derl3	-0.52	0.00000006	chicken
Tesmin	0.31	0.00000007	chicken
Septin9	-0.48	0.00000007	chicken
Txnrd2	0.39	0.00000007	chicken

Slc23a2	0.68	0.00000007	chicken
Taldo1	-0.77	0.00000007	chicken
Igdcc3	0.52	0.00000008	chicken
Erlin2	-0.51	0.00000008	chicken
Rnf144a	0.45	0.00000008	chicken
Cndp2	0.41	0.00000008	chicken
Cd2ap	0.30	0.00000008	chicken
Nr2c2	0.43	0.00000008	chicken
Golgb1	-0.60	0.00000008	chicken
Iws1	-0.74	0.00000008	chicken
Gnai3	-0.63	0.00000008	chicken
Jak1	-0.61	0.00000008	chicken
Tbc1d9b	0.49	0.00000008	chicken
Lbr	-0.73	0.00000009	chicken
Ptrh2	-0.44	0.00000009	chicken
Timp4	0.41	0.00000009	chicken
Slc12a4	0.73	0.00000009	chicken
Tanc1	0.22	0.00000009	chicken
Pcf11	0.41	0.00000010	chicken
Ube2j1	0.61	0.00000010	chicken
Wdsub1	0.65	0.00000010	chicken
Tspan18	0.54	0.00000011	chicken
Ndufa10	-0.53	0.00000011	chicken
Gdi2	-0.64	0.00000011	chicken
Scp2	-0.45	0.00000011	chicken
Zswim8	0.35	0.00000012	chicken
Sytl1	-0.58	0.00000012	chicken
Eif3k	0.31	0.00000012	chicken
Slc66a1	0.38	0.00000013	chicken
Ndufb8	-0.55	0.00000014	chicken
Dbi	-0.52	0.00000014	chicken
Fndc3b	-0.90	0.00000015	chicken
Tmed3	-0.48	0.00000016	chicken
Rilpl2	-0.47	0.00000016	chicken
Ywhab	-0.40	0.00000017	chicken
Itgb3	0.62	0.00000019	chicken
Rhoq	0.29	0.00000019	chicken
Tdpoz8	0.35	0.00000020	chicken
Tex264	-0.33	0.00000022	chicken
Dglucy	0.63	0.00000022	chicken
Abraxas2	0.39	0.00000022	chicken
Fmc1	-0.58	0.00000024	chicken
Sat1	0.47	0.00000024	chicken
Zmynd11	-0.65	0.00000025	chicken
Cap2	0.32	0.00000026	chicken
Rimbp2	0.58	0.00000026	chicken

Ginm1	-0.56	0.00000026	chicken
Cacnb2	0.95	0.00000028	chicken
Insc	0.61	0.00000029	chicken
Lpcat1	-0.45	0.00000032	chicken
Xrcc4	0.44	0.00000032	chicken
Bhlhe40	0.63	0.00000033	chicken
Tmem9b	-0.46	0.00000034	chicken
Ccdc88c	0.63	0.00000035	chicken
Ap1s2	-0.79	0.00000035	chicken
Pxn	0.30	0.00000036	chicken
Prkar2b	0.86	0.00000036	chicken
Rnf220	0.50	0.00000037	chicken
Ciao2a	-0.35	0.00000037	chicken
Rnf166	-0.45	0.00000040	chicken
Rmc1	0.24	0.00000041	chicken
Coro7	-0.65	0.00000041	chicken
Gabrr2	0.24	0.00000041	chicken
Rasd1	0.50	0.00000042	chicken
Usp15	0.48	0.00000044	chicken
Prelid1	-0.45	0.00000046	chicken
Ccdc127	0.21	0.00000051	chicken
Ppp6r3	0.73	0.00000051	chicken
Stt3a	-0.43	0.00000054	chicken
Sccpdh	-0.36	0.00000056	chicken
Unc93b1	-0.34	0.00000056	chicken
H13	-0.65	0.00000056	chicken
Sorbs1	0.44	0.00000056	chicken
Rdx	-0.65	0.00000056	chicken
Ube3c	0.65	0.00000060	chicken
Ephb3	-0.43	0.00000061	chicken
Colec12	-1.04	0.00000062	chicken
C9orf72	0.40	0.00000063	chicken
Pdcd4	-0.46	0.00000068	chicken
Tst	-0.54	0.00000068	chicken
Acot10	0.41	0.00000070	chicken
Tab2	0.30	0.00000070	chicken
Lgmn	0.53	0.00000073	chicken
Nudt3	0.26	0.00000080	chicken
Fam160a1	0.60	0.00000081	chicken
Dusp23	-0.45	0.00000081	chicken
Lef1	-0.66	0.00000082	chicken
Hsp90ab1	-0.72	0.00000090	chicken
Pccb	-0.49	0.00000093	chicken
Ptpra	-0.56	0.00000094	chicken
Zbtb8b	0.26	0.00000094	chicken
Arfgap3	0.51	0.00000094	chicken

Foxn3	0.46	0.00000095	chicken
Ppp4r3a	0.60	0.00000097	chicken
Tmem164	0.51	0.00000098	chicken
Evi2a	-0.59	0.00000104	chicken
Cytip	-0.57	0.00000104	chicken
Dyrk3	0.21	0.00000108	chicken
Pard3b	0.67	0.00000108	chicken
Kcnq1	0.72	0.00000112	chicken
Nfkb1	0.54	0.00000113	chicken
Zfand6	0.48	0.00000114	chicken
Ppp1ca	-0.51	0.00000126	chicken
Ranbp3	0.40	0.00000126	chicken
Hmces	-0.36	0.00000132	chicken
Skap2	0.59	0.00000153	chicken
Gsto1	-0.31	0.00000157	chicken
Icos	-0.81	0.00000158	chicken
Ssbp1	-0.51	0.00000160	chicken
Cyp2r1	0.35	0.00000175	chicken
Gimap5	0.25	0.00000179	chicken
Akr1e1	-0.59	0.00000180	chicken
Ostc	-0.56	0.00000191	chicken
Leprot	-0.48	0.00000195	chicken
Gns	-0.59	0.00000195	chicken
Pm20d2	0.43	0.00000201	chicken
Itgb1bp1	0.18	0.00000207	chicken
Cab39	-0.60	0.00000213	chicken
Cox7c	-0.41	0.00000218	chicken
Pak2	-0.64	0.00000222	chicken
Rfxap	-0.55	0.00000236	chicken
Dnajc3	-0.75	0.00000241	chicken
Gnai1	-0.76	0.00000248	chicken
Pacc1	0.24	0.00000253	chicken
Ugcg	0.49	0.00000259	chicken
Slx4ip	0.65	0.00000261	chicken
Fgl2	-0.80	0.00000262	chicken
Usp14	0.44	0.00000269	chicken
Ndufa6	-0.45	0.00000272	chicken
Rad18	-0.41	0.00000274	chicken
Acaa1b	0.20	0.00000286	chicken
Zfyve9	0.43	0.00000290	chicken
Bach1	0.45	0.00000308	chicken
Wdr25	0.34	0.00000311	chicken
Acsl3	-0.76	0.00000341	chicken
Nrp1	-0.64	0.00000341	chicken
Dhrs3	0.56	0.00000348	chicken
Naga	-0.32	0.00000350	chicken

Dpep2	0.59	0.00000359	chicken
Tnfrsf9	-0.68	0.00000370	chicken
Tbc1d15	0.46	0.00000381	chicken
Ankdd1b	0.19	0.00000388	chicken
Dnaja1	0.30	0.00000392	chicken
Cyb561a3	-0.33	0.00000423	chicken
Dis3l	-0.39	0.00000423	chicken
Bcor	-0.67	0.00000449	chicken
Churc1	-0.57	0.00000451	chicken
Eps15	0.50	0.00000457	chicken
Tmem245	-0.65	0.00000460	chicken
Capn10	0.46	0.00000470	chicken
Nus1	0.63	0.00000473	chicken
Eif3b	-0.48	0.00000484	chicken
Acox1	0.62	0.00000484	chicken
Reps2	-0.43	0.00000489	chicken
Tubd1	0.23	0.00000525	chicken
Atp5c1	-0.35	0.00000526	chicken
Foxk1	0.49	0.00000547	chicken
Gclc	-0.50	0.00000592	chicken
Wbp1	-0.47	0.00000599	chicken
Hs6st1	-0.89	0.00000607	chicken
Nr3c2	-0.70	0.00000619	chicken
Cox7a2l	-0.49	0.00000680	chicken
Pgrmc1	-0.27	0.00000686	chicken
Camkmt	0.52	0.00000692	chicken
Tomm40l	-0.38	0.00000696	chicken
Gtf3a	-0.41	0.00000708	chicken
Pbxip1	-0.31	0.00000713	chicken
Rbks	0.39	0.00000719	chicken
Phf2	0.37	0.00000738	chicken
Abca2	-0.36	0.00000741	chicken
Dnm1	0.38	0.00000767	chicken
Syde2	0.35	0.00000793	chicken
Suclg1	-0.40	0.00000848	chicken
Slc26a5	-0.44	0.00000851	chicken
Hipk2	0.27	0.00000873	chicken
Timm13	-0.44	0.00000878	chicken
Zc3h12d	-0.72	0.00000955	chicken
H2ac20	0.29	0.00000959	chicken
Unk	0.19	0.00000974	chicken
Ubr7	-0.53	0.00000987	chicken
Slc43a2	-0.62	0.00001001	chicken
Sinhcaf	0.41	0.00001015	chicken
Fgd6	-0.43	0.00001061	chicken
Lonrf1	0.32	0.00001064	chicken

Ago3	-0.83	0.00001068	chicken
Lama3	0.47	0.00001069	chicken
Efhd1	-0.65	0.00001113	chicken
Stard3nl	0.36	0.00001169	chicken
Azin1	0.42	0.00001177	chicken
Rapgef5	0.39	0.00001185	chicken
Pcsk7	-0.59	0.00001186	chicken
Cd44	0.90	0.00001188	chicken
Hvcn1	-0.56	0.00001269	chicken
Ube2e1	0.55	0.00001303	chicken
Anp32e	-0.54	0.00001336	chicken
Kars	-0.45	0.00001363	chicken
Gatd1	0.45	0.00001409	chicken
Atp7a	0.34	0.00001424	chicken
Orc1	0.39	0.00001460	chicken
Zdhhc7	0.36	0.00001475	chicken
Rap2c	0.24	0.00001482	chicken
Anxa11	-0.46	0.00001548	chicken
Nucks1	-0.62	0.00001566	chicken
Gcg	0.99	0.00001655	chicken
Npepps	0.32	0.00001658	chicken
Lsamp	0.46	0.00001712	chicken
Il1r2	-1.25	0.00001727	chicken
Ndufs5	-0.54	0.00001807	chicken
Ikzf1	-0.73	0.00001838	chicken
Tprgl	-0.46	0.00001847	chicken
Ssbp3	-0.57	0.00001865	chicken
Ncf2	0.44	0.00001990	chicken
Bicd2	0.59	0.00002043	chicken
Syne1	-0.84	0.00002050	chicken
Dgkd	-0.71	0.00002084	chicken
Ccpg1	-0.55	0.00002093	chicken
Srgap1	0.38	0.00002126	chicken
Nmt1	0.27	0.00002187	chicken
Zfp516	0.46	0.00002296	chicken
Actr2	-0.43	0.00002321	chicken
Ogt	-0.59	0.00002333	chicken
Rpp30	0.20	0.00002406	chicken
Rab29	-0.61	0.00002477	chicken
Cebpb	0.29	0.00002509	chicken
Clpx	0.36	0.00002554	chicken
Hook1	0.44	0.00002560	chicken
Pibf1	0.34	0.00002594	chicken
Lamc1	-0.53	0.00002599	chicken
Clta	-0.37	0.00002617	chicken
Aco2	-0.44	0.00002664	chicken

Blm	0.21	0.00002665	chicken
Camk2d	0.42	0.00002723	chicken
Parp12	0.24	0.00002744	chicken
Slc45a4	0.32	0.00002758	chicken
Atad2b	0.50	0.00002869	chicken
Chrna5	0.18	0.00002970	chicken
Atp2a3	-0.61	0.00002988	chicken
Tnfrsf11a	-0.71	0.00003016	chicken
Bzw1	-0.61	0.00003060	chicken
Ik	-0.36	0.00003137	chicken
Isg20	0.28	0.00003339	chicken
Rbx1	-0.56	0.00003349	chicken
Naxe	-0.27	0.00003519	chicken
Supv3l1	0.25	0.00003535	chicken
Lclat1	0.43	0.00003564	chicken
Chmp4b	-0.52	0.00003573	chicken
Api5	-0.62	0.00003982	chicken
Cars	0.35	0.00004075	chicken
Iqcb1	0.32	0.00004151	chicken
Dido1	0.27	0.00004259	chicken
Atp1b3	0.47	0.00004364	chicken
Rpia	-0.36	0.00004386	chicken
Tpr	-0.57	0.00004471	chicken
Arhgdib	-0.17	0.00004515	chicken
Irf4	-0.64	0.00004629	chicken
Gnpda1	-0.41	0.00004680	chicken
Naca	-0.53	0.00005029	chicken
Srp72	-0.45	0.00005105	chicken
Adgrg6	-1.20	0.00005135	chicken
Top1	-0.60	0.00005185	chicken
Dot1l	0.39	0.00005221	chicken
Eci3	-0.35	0.00005381	chicken
Nfil3	0.48	0.00005569	chicken
Rab27a	-0.33	0.00005645	chicken
Tmem135	0.64	0.00005941	chicken
Cyfip1	0.49	0.00005968	chicken
Pdxk	-0.43	0.00006400	chicken
Smad1	0.19	0.00006533	chicken
Dctn2	-0.38	0.00006606	chicken
Nt5c3	0.87	0.00006687	chicken
Ivns1abp	-0.67	0.00006825	chicken
Nqo2	0.35	0.00006827	chicken
1600014C10Rik	0.27	0.00006835	chicken
Prpsap2	-0.47	0.00006898	chicken
Fyco1	1.05	0.00007192	chicken
Asns	-0.45	0.00007287	chicken

Dlgap1	0.37	0.00007421	chicken
Samhd1	0.49	0.00007460	chicken
Ovch2	0.19	0.00007595	chicken
Slc22a3	-0.76	0.00007630	chicken
Ppm1h	0.63	0.00007709	chicken
Hes1	-0.38	0.00007749	chicken
Hspa8	0.14	0.00007928	chicken
Vegfc	0.74	0.00008062	chicken
Pcdhgb4	-0.50	0.00008381	chicken
Wsb2	-0.38	0.00008633	chicken
Eif5	0.34	0.00008817	chicken
E2f5	0.22	0.00009039	chicken
Scin	-0.89	0.00009046	chicken
Creb3l2	-0.63	0.00009211	chicken
Kmt2e	0.27	0.00009582	chicken
Ccser2	-0.45	0.00009684	chicken
Ehmt1	0.34	0.00009812	chicken
Arhgap31	0.42	0.00010107	chicken
Arhgdia	-0.26	0.00010490	chicken
Smg1	0.38	0.00010544	chicken
Sipa1l2	0.40	0.00010923	chicken
Ncald	-0.42	0.00010945	chicken
Pgd	-0.54	0.00011000	chicken
Arrdc4	0.17	0.00011199	chicken
Tcn2	-0.43	0.00011432	chicken
Cdc73	-0.72	0.00011457	chicken
Cnot2	0.36	0.00012493	chicken
Lmo2	-0.52	0.00012496	chicken
Ssh2	-0.56	0.00013016	chicken
Kcnmb1	0.63	0.00013265	chicken
Nusap1	0.27	0.00013575	chicken
Rnd3	0.32	0.00013935	chicken
Pkn2	-0.52	0.00015099	chicken
Armc9	0.28	0.00015118	chicken
Lonrf2	0.40	0.00015254	chicken
Ano5	0.51	0.00015405	chicken
Stxbp1	-0.50	0.00015447	chicken
Tdrd3	0.45	0.00015451	chicken
Tlr7	-0.19	0.00015797	chicken
Irf5	0.28	0.00015993	chicken
Lipa	0.68	0.00016062	chicken
Washc2	-0.61	0.00016082	chicken
Cyth4	0.27	0.00016085	chicken
Atp5g3	-0.39	0.00016103	chicken
Vps33b	-0.46	0.00016240	chicken
Nat1	-0.29	0.00016400	chicken

Eif1ad12	-0.36	0.00016605	chicken
Psmd14	0.43	0.00016654	chicken
Gpalpp1	-0.46	0.00016660	chicken
Git2	-0.57	0.00016843	chicken
Polr1f	-0.48	0.00016925	chicken
Asb5	-0.52	0.00017057	chicken
Snrnp25	-0.40	0.00017218	chicken
Gna13	0.46	0.00017425	chicken
Tfrc	-0.83	0.00018376	chicken
Endov	0.22	0.00020520	chicken
Dip2a	0.53	0.00020725	chicken
Eml4	-0.54	0.00021330	chicken
Otulinl	-0.51	0.00021485	chicken
Cdc42ep4	0.29	0.00021830	chicken
Arl6ip1	-0.53	0.00022386	chicken
Arpp19	-0.50	0.00022590	chicken
Hipk3	0.25	0.00023308	chicken
Sec63	-0.57	0.00023823	chicken
Rbm33	0.32	0.00025570	chicken
Cyld	0.30	0.00025946	chicken
Cttnbp2nl	-0.56	0.00026006	chicken
Selenot	-0.56	0.00027124	chicken
Tlk1	-0.66	0.00027597	chicken
Ptprs	0.20	0.00028665	chicken
Hadh	-0.56	0.00028709	chicken
Tdrd5	0.17	0.00028868	chicken
Slco3a1	-0.49	0.00029079	chicken
Nudt16l1	-0.43	0.00029177	chicken
Brip1	0.59	0.00029331	chicken
Hspe1-rs1	-0.26	0.00029574	chicken
Ppp1cb	0.41	0.00030105	chicken
Cmklr1	-0.40	0.00030129	chicken
Snx1	-0.44	0.00030573	chicken
Snrpd1	-0.49	0.00030643	chicken
Frmd4a	0.13	0.00031484	chicken
Oxct1	-0.39	0.00033272	chicken
Vrk2	0.30	0.00033387	chicken
Exog	-0.32	0.00034794	chicken
Lamp1	-0.45	0.00035984	chicken
Lrp12	0.39	0.00037050	chicken
Oxr1	0.41	0.00037769	chicken
Slc2a5	0.38	0.00038923	chicken
Exoc6	0.49	0.00039499	chicken
Txnrd1	1.24	0.00039890	chicken
Cracr2b	0.20	0.00040299	chicken
Elp2	0.26	0.00041203	chicken

Cbfa2t3	-0.42	0.00041621	chicken
Papss1	-0.53	0.00042244	chicken
Lman2	-0.41	0.00042266	chicken
Ndufb9	-0.43	0.00042662	chicken
Commd3	-0.36	0.00042811	chicken
Spty2d1	-0.66	0.00043244	chicken
Eif4ebp2	-0.27	0.00043693	chicken
Snn	-0.37	0.00046297	chicken
Tmem50a	-0.36	0.00047349	chicken
Chd1	0.30	0.00047428	chicken
Klhl18	0.37	0.00047916	chicken
Eif3i	-0.47	0.00048191	chicken
Psap	-0.55	0.00048298	chicken
Slc40a1	-1.16	0.00048784	chicken
Stk26	0.43	0.00048859	chicken
C1galt1	-0.27	0.00049819	chicken
Syncrip	-0.53	0.00049953	chicken
Nup210	-0.36	0.00051634	chicken
Rara	-0.48	0.00052181	chicken
Czib	-0.28	0.00052415	chicken
D430041D05Rik	0.31	0.00052651	chicken
St3gal2	-0.41	0.00053791	chicken
Slc3a1	0.27	0.00054832	chicken
Emilin2	0.33	0.00055359	chicken
Elovl5	-0.33	0.00055688	chicken
Dnajc13	0.45	0.00055775	chicken
Pde10a	0.79	0.00056036	chicken
Fkbp3	-0.35	0.00056840	chicken
Sec13	-0.48	0.00057325	chicken
Septin2	-0.38	0.00058455	chicken
Nck2	0.42	0.00059703	chicken
Rbis	-0.51	0.00060816	chicken
Foxn2	0.30	0.00061280	chicken
Serp1	-0.58	0.00063556	chicken
Ndufv2	-0.31	0.00063882	chicken
R3hdm1	-0.60	0.00064549	chicken
2900026A02Rik	-0.63	0.00066739	chicken
Apoa1	0.13	0.00067599	chicken
Qpctl	-0.36	0.00068123	chicken
Trim59	0.17	0.00069792	chicken
Parvb	0.53	0.00070099	chicken
Pmepa1	-0.44	0.00070840	chicken
Snrpe	-0.30	0.00071032	chicken
Cd82	-0.70	0.00071378	chicken
Smchd1	0.37	0.00071768	chicken
Vps4b	-0.40	0.00073992	chicken

Psmg4	-0.26	0.00074418	chicken
Snx25	-0.51	0.00076642	chicken
Rac2	-0.23	0.00077480	chicken
Ubap1	0.07	0.00079033	chicken
Shld1	0.28	0.00080193	chicken
Sdhd	-0.56	0.00081533	chicken
Irf8	0.11	0.00081717	chicken
Trak2	0.27	0.00081979	chicken
Uqcrfs1	-0.34	0.00083819	chicken
Nt5c3b	0.29	0.00084272	chicken
Gsk3b	0.41	0.00085252	chicken
Smap1	-0.52	0.00092166	chicken
Glcci1	-0.54	0.00095241	chicken
Nedd9	0.03	0.00099010	chicken
Pter	-0.36	0.00099331	chicken
Cd72	-0.22	0.00101136	chicken
Cat	-0.42	0.00101435	chicken
Enah	0.41	0.00101853	chicken
Rnf214	-0.65	0.00103795	chicken
Creb1	0.17	0.00104304	chicken
Zc3h6	0.22	0.00105039	chicken
Fbxl2	-0.59	0.00105692	chicken
Tssc4	0.27	0.00109633	chicken
Rdh10	0.49	0.00109773	chicken
Add1	0.63	0.00110427	chicken
Dtx2	-0.71	0.00112188	chicken
Ckap5	0.41	0.00112742	chicken
Satb1	-0.71	0.00113912	chicken
C1qtnf12	0.20	0.00114305	chicken
Rev3l	0.31	0.00115508	chicken
Parp9	0.32	0.00116156	chicken
Tedc1	0.23	0.00116798	chicken
Commd4	-0.40	0.00116957	chicken
Ppp2ca	0.35	0.00120166	chicken
Tomm22	-0.38	0.00122621	chicken
Saraf	0.49	0.00123573	chicken
Ppt1	-0.36	0.00127172	chicken
Phlpp1	-0.50	0.00127646	chicken
Atp6v0a1	0.32	0.00128352	chicken
Uqcrh	-0.42	0.00132748	chicken
Hmgb2	-0.39	0.00135377	chicken
Cnot6	0.24	0.00135632	chicken
Rpn2	-0.47	0.00138666	chicken
Cd38	0.24	0.00139714	chicken
Coq9	-0.30	0.00144106	chicken
Capzb	-0.39	0.00145656	chicken

Errfi1	0.34	0.00146776	chicken
Ipmk	-0.56	0.00152540	chicken
Lpcat2b	-0.42	0.00153385	chicken
Cyfip2	-0.37	0.00159896	chicken
Uxs1	-0.51	0.00160720	chicken
Suco	0.27	0.00162552	chicken
Actr10	-0.26	0.00162725	chicken
Zfp292	-0.47	0.00174551	chicken
Snx30	-0.45	0.00175194	chicken
B3gnt7	0.19	0.00180685	chicken
Commd1b	0.35	0.00181131	chicken
Trpm7	0.78	0.00182264	chicken
F10	-0.75	0.00183190	chicken
Abcb4	0.32	0.00188645	chicken
Alox5ap	-0.32	0.00191097	chicken
Polr2l	-0.26	0.00196252	chicken
Fuca2	-0.22	0.00199536	chicken
Ppp1r12a	0.30	0.00200435	chicken
Hp1bp3	-0.41	0.00203265	chicken
Sdk1	-0.58	0.00203670	chicken
Uri1	0.26	0.00214741	chicken
Tmed7	-0.35	0.00215709	chicken
Lmf2	-0.21	0.00219510	chicken
Cd164	-0.30	0.00223277	chicken
Lpin2	-0.63	0.00224302	chicken
Ankrd46	0.23	0.00226585	chicken
Spats2	-0.45	0.00228130	chicken
Cds2	0.32	0.00232234	chicken
Scaf4	0.35	0.00233998	chicken
Antxr2	0.45	0.00241076	chicken
Spcs1	-0.51	0.00251066	chicken
Klhl7	-0.29	0.00254760	chicken
Tubb1	0.33	0.00268131	chicken
Hexb	-0.18	0.00274691	chicken
Ndufa8	-0.43	0.00277224	chicken
Gabarapl2	-0.35	0.00279921	chicken
Sugt1	0.40	0.00283537	chicken
Stx8	0.34	0.00284517	chicken
Abca1	-0.30	0.00284871	chicken
Xpa	0.29	0.00288306	chicken
Tcp11l2	-0.39	0.00295799	chicken
Txndc5	-1.40	0.00299759	chicken
Lrmp	0.45	0.00299759	chicken
Kcnma1	-0.48	0.00300932	chicken
Ssbp2	-0.55	0.00310078	chicken
Sec23b	-0.42	0.00315172	chicken

Bambi	-0.38	0.00317659	chicken
Rab8b	-0.40	0.00319245	chicken
B4galt5	0.21	0.00323023	chicken
Luc7l	-0.57	0.00328858	chicken
1810058I24Rik	-0.22	0.00329089	chicken
Ndufab1	-0.37	0.00334019	chicken
Cpne4	-1.47	0.00341276	chicken
Dtx3l	0.21	0.00344891	chicken
Chst13	0.29	0.00345138	chicken
Supt3	-0.52	0.00346028	chicken
Efhd2	-0.38	0.00360704	chicken
Secisbp2l	0.30	0.00360940	chicken
Crispld2	0.20	0.00361036	chicken
Itpr1	-0.65	0.00365907	chicken
Jag2	-0.40	0.00373023	chicken
Dync1h1	-0.49	0.00391286	chicken
Ociad1	-0.37	0.00404011	chicken
Sirt2	-0.26	0.00419273	chicken
Crtam	0.21	0.00435047	chicken
Pikfyve	0.61	0.00445823	chicken
Ddx46	-0.62	0.00446648	chicken
Abcd2	-0.49	0.00455020	chicken
Rbm39	-0.55	0.00458718	chicken
Cd247	-0.57	0.00460919	chicken
Srsf11	-0.59	0.00462582	chicken
Tnfsf8	-0.53	0.00465549	chicken
Cep192	0.30	0.00471711	chicken
Pi4k2b	-0.19	0.00473572	chicken
Zdhhc9	-0.24	0.00474417	chicken
Cdk14	-0.75	0.00499744	chicken
Khdc4	0.20	0.00502373	chicken
Yme1l1	-0.42	0.00503960	chicken
Cdc42bpb	-0.35	0.00519081	chicken
Napa	0.20	0.00519436	chicken
Yipf3	-0.27	0.00520906	chicken
Eif4e2	0.34	0.00523846	chicken
Csf1	0.32	0.00529337	chicken
Aldh18a1	-0.41	0.00538003	chicken
Tcerg1	-0.55	0.00541372	chicken
Uhrf2	-0.40	0.00541407	chicken
Hdgf	-0.27	0.00551626	chicken
Celf2	0.40	0.00551790	chicken
1110004F10Rik	-0.37	0.00560695	chicken
Txn1	-0.46	0.00561924	chicken
Cstb	0.17	0.00564910	chicken
Ppp4r1	-0.62	0.00567516	chicken

Inpp5d	-0.62	0.00567946	chicken
Klhl20	0.14	0.00569511	chicken
Cdc14b	0.27	0.00572885	chicken
Slc9a8	0.29	0.00588262	chicken
Anapc16	-0.56	0.00591070	chicken
Zfand5	0.26	0.00592732	chicken
Ess2	0.26	0.00593893	chicken
Sh3pxd2b	0.43	0.00599667	chicken
Ilk	-0.31	0.00609550	chicken
Ndufa5	-0.46	0.00616160	chicken
Pea15a	0.20	0.00625719	chicken
Plcd1	0.35	0.00627149	chicken
Dis3	0.20	0.00631172	chicken
Trim24	0.37	0.00638247	chicken
Hnrnpu	-0.56	0.00638816	chicken
Paxbp1	-0.52	0.00640416	chicken
Prpf19	-0.36	0.00649620	chicken
AA467197	0.67	0.00670543	chicken
Taf4	0.17	0.00672609	chicken
Dusp7	0.40	0.00673713	chicken
Eny2	-0.33	0.00674106	chicken
Grap	-0.26	0.00704719	chicken
Trmt11	-0.29	0.00705219	chicken
Pebp1	-0.37	0.00706027	chicken
Fitm2	0.26	0.00720497	chicken
Znrf2	-0.66	0.00741669	chicken
Eaf1	0.28	0.00743795	chicken
Rab5if	-0.41	0.00744465	chicken
U2surp	-0.50	0.00747229	chicken
Phactr2	-0.87	0.00749512	chicken
Slc2a6	0.34	0.00759187	chicken
Kbtbd8	0.18	0.00759564	chicken
Dock9	0.32	0.00807139	chicken
Bnip3l	-0.53	0.00807229	chicken
Dnah10	0.20	0.00814673	chicken
Smim20	-0.35	0.00817605	chicken
Htra2	-0.31	0.00840785	chicken
AU040320	0.31	0.00844622	chicken
Pdlim1	0.29	0.00847091	chicken
Jade2	0.24	0.00856272	chicken
Pnpla6	0.17	0.00879128	chicken
Nav3	0.73	0.00887860	chicken
Pdzd8	-0.52	0.00893069	chicken
Sik3	0.28	0.00899880	chicken
Ust	-0.75	0.00906585	chicken
Rlf	0.16	0.00910508	chicken

Dpagt1	-0.26	0.00938563	chicken
Was	-0.39	0.00954103	chicken
Timm17a	-0.32	0.00971640	chicken
Stk24	0.32	0.00974075	chicken
Plekhf2	0.13	0.00982204	chicken
Ifngr2	0.30	0.00987452	chicken
Nkd1	-1.43	0.01000603	chicken
Tirap	0.30	0.01001172	chicken
Uck2	0.17	0.01003453	chicken
Fggy	0.37	0.01008029	chicken
Atp5k	-0.32	0.01022499	chicken
Hspa9	-0.40	0.01025484	chicken
Ift22	0.16	0.01035446	chicken
Sec61a1	-0.40	0.01056872	chicken
Rnf11	-0.49	0.01061268	chicken
Efr3a	0.41	0.01063329	chicken
Jund	0.18	0.01078911	chicken
Cnot11	-0.32	0.01082678	chicken
Raph1	-0.63	0.01089829	chicken
Cnnm2	0.41	0.01100433	chicken
Ogfrl1	0.40	0.01151268	chicken
Rassf5	0.44	0.01154207	chicken
Dcun1d5	-0.36	0.01163589	chicken
Pgam1	-0.27	0.01164357	chicken
Pdcd6	0.27	0.01170613	chicken
Pik3r4	0.36	0.01178846	chicken
Nop58	-0.58	0.01217579	chicken
Esrrg	0.55	0.01220905	chicken
Ndufv3	-0.34	0.01305326	chicken
Parp4	-0.56	0.01334818	chicken
Fam204a	-0.39	0.01345081	chicken
Plekhm1	0.25	0.01361219	chicken
Cul1	0.26	0.01365055	chicken
Vapb	-0.46	0.01374913	chicken
Zc3h15	-0.51	0.01393408	chicken
Ciao2b	-0.23	0.01441664	chicken
Gng2	-0.42	0.01454481	chicken
Bin2	-0.15	0.01456634	chicken
Ap3m2	0.29	0.01480376	chicken
Herpud2	-0.33	0.01525295	chicken
Abcg1	0.57	0.01540685	chicken
Fap	0.59	0.01546389	chicken
Slk	-0.46	0.01547828	chicken
Spz1	-0.49	0.01554856	chicken
Smarca2	-0.43	0.01596952	chicken
Sirt5	-0.29	0.01602910	chicken

Rangap1	-0.34	0.01614458	chicken
Peli2	0.49	0.01649233	chicken
Invs	0.14	0.01694777	chicken
Hbp1	0.17	0.01702242	chicken
Lgr4	0.28	0.01725830	chicken
Plekhd1	0.22	0.01777521	chicken
Fahd2a	-0.36	0.01800623	chicken
Itgb5	-0.31	0.01821499	chicken
Rnls	0.25	0.01827446	chicken
Gipc2	0.15	0.01830932	chicken
Rasal2	-0.87	0.01836055	chicken
Prom1	0.25	0.01858595	chicken
Serinc3	-0.48	0.01862979	chicken
Slc16a3	-0.57	0.01879707	chicken
Tctn3	-0.38	0.01915645	chicken
Pigx	-0.24	0.01939467	chicken
Abtb1	0.27	0.01953674	chicken
Ubl5b	-0.37	0.01953904	chicken
Slc6a6	0.54	0.01965122	chicken
Snrpd3	-0.29	0.02021759	chicken
Atp10d	0.13	0.02035481	chicken
Tmem208	-0.31	0.02053566	chicken
2510002D24Rik	-0.29	0.02130804	chicken
Tmx3	-0.41	0.02160705	chicken
Pfdn2	-0.40	0.02231668	chicken
Ss18l1	0.27	0.02236912	chicken
Snx24	0.15	0.02258126	chicken
Bms1	-0.44	0.02274979	chicken
Rnaseh2b	-0.39	0.02289379	chicken
Gls	0.36	0.02312513	chicken
Fam241a	-0.33	0.02362092	chicken
Fth1	-0.35	0.02379982	chicken
Vnn3	-0.38	0.02457401	chicken
Gramd4	-0.50	0.02467774	chicken
Xbp1	-0.62	0.02480363	chicken
Usp33	0.22	0.02496076	chicken
Vps37a	0.05	0.02579157	chicken
Wdr1	-0.30	0.02602880	chicken
Rgl1	-0.52	0.02613812	chicken
Hscb	-0.32	0.02676397	chicken
Bcap31	-0.37	0.02695047	chicken
Trafd1	0.21	0.02757343	chicken
Pdcd5	-0.38	0.02758354	chicken
Atp5h	-0.28	0.02832843	chicken
Cwc15	-0.30	0.02862963	chicken
Nbeal1	0.32	0.02909579	chicken

Gda	0.12	0.02989868	chicken
Abce1	-0.49	0.03026232	chicken
Tada1	0.24	0.03117806	chicken
Sipa1l1	-0.09	0.03201621	chicken
Zfp451	0.23	0.03228086	chicken
Ugp2	0.35	0.03345955	chicken
Slc37a2	-0.28	0.03347611	chicken
Phkb	-0.29	0.03355623	chicken
Ift20	-0.52	0.03384274	chicken
Tmem115	-0.25	0.03415332	chicken
Sik2	0.15	0.03418356	chicken
B230219D22Rik	-0.61	0.03424881	chicken
Kcnj2	0.15	0.03443003	chicken
Gpatch3	0.16	0.03446022	chicken
Xpo5	-0.36	0.03509136	chicken
Sgsh	-0.27	0.03547292	chicken
Igf2bp3	0.30	0.03564344	chicken
Ptbp1	-0.44	0.03577599	chicken
Cep76	0.20	0.03584262	chicken
Qdpr	-0.29	0.03729300	chicken
BC004004	0.35	0.03770693	chicken
Hsd17b10	-0.26	0.03774241	chicken
Cxcr2	-0.11	0.03782650	chicken
Tmem243	0.17	0.03810415	chicken
Cpt1a	0.52	0.03910173	chicken
Tasor	0.24	0.03930195	chicken
Sumo1	-0.40	0.04018818	chicken
Trim25	0.18	0.04019250	chicken
Inip	-0.22	0.04029422	chicken
Fam126a	-0.63	0.04066399	chicken
Pknox1	0.14	0.04167742	chicken
Cndp1	0.21	0.04184930	chicken
Aup1	-0.29	0.04228723	chicken
Gtf2h5	-0.43	0.04426296	chicken
Npc2	-0.44	0.04426355	chicken
Usp22	0.38	0.04591404	chicken
Prkcb	-0.41	0.04599597	chicken
Smpd1	-0.46	0.04600905	chicken
Zfp608	-0.35	0.04650116	chicken
Akr7a5	0.16	0.04731827	chicken
Pls3	-0.28	0.04755425	chicken
Gnpat	-0.28	0.04759585	chicken
Lat2	-0.23	0.04769856	chicken
Dhx36	-0.51	0.04845381	chicken
Ly96	0.37	0.04910197	chicken
Rab14	-0.40	0.04967942	chicken

Cln5	0.29	0.05092900	chicken
Psmc2	-0.30	0.05105730	chicken
Rbm14	-0.40	0.05153007	chicken
Atp6v1a	0.32	0.05205251	chicken
Ilrun	0.29	0.05267329	chicken
Dock10	-0.22	0.05324233	chicken
Smarca1	-0.47	0.05330554	chicken
Celf1	0.38	0.05417826	chicken
Fam13a	0.50	0.05474640	chicken
Gcnt4	-0.44	0.05488440	chicken
Aftph	0.10	0.05580797	chicken
Agfg1	0.36	0.05611821	chicken
Polr2i	-0.27	0.05769671	chicken
Lnpep	-0.39	0.05782836	chicken
Upf2	0.23	0.05918797	chicken
Ghdc	-0.32	0.06022441	chicken
Ppm1d	0.42	0.06081638	chicken
Ptger4	0.04	0.06111205	chicken
Klf7	0.29	0.06128949	chicken
Acss2	-0.33	0.06204566	chicken
Golga7	-0.21	0.06215425	chicken
Parp1	-0.34	0.06217506	chicken
Prss45	0.30	0.06266475	chicken
9130409I23Rik	-0.32	0.06303066	chicken
Rfx2	-0.03	0.06389219	chicken
Brd8	0.23	0.06408831	chicken
Usp49	0.18	0.06482173	chicken
Rab12	-0.49	0.06562956	chicken
Usp16	0.31	0.06655962	chicken
Tpd52l2	0.30	0.06713221	chicken
Ube2e3	-0.36	0.06752084	chicken
Ppil4	-0.43	0.06817802	chicken
Atf2	0.20	0.06828480	chicken
Baiap2l1	0.41	0.06917946	chicken
Fkbp8	-0.22	0.06929694	chicken
Rmnd1	-0.35	0.07060876	chicken
Nars2	0.18	0.07175953	chicken
Tom1l2	0.19	0.07258279	chicken
Sdhc	-0.19	0.07282925	chicken
Fgf18	0.15	0.07355313	chicken
Fes	-0.29	0.07534144	chicken
Ap3s1	0.19	0.07538101	chicken
Chst11	0.35	0.07580088	chicken
Cdc42	-0.31	0.07585054	chicken
Slc2a8	-0.39	0.07657490	chicken
Atp5b	-0.11	0.07705819	chicken

Slc39a10	-0.48	0.07721834	chicken
Lamtor5	-0.41	0.07770275	chicken
Pecam1	0.13	0.07826043	chicken
Ostm1	0.19	0.07870558	chicken
Tm9sf2	0.27	0.07871668	chicken
Ttc17	0.34	0.07905393	chicken
Ndfip2	0.20	0.08093832	chicken
Ctsh	-0.34	0.08156242	chicken
Pptc7	0.23	0.08171313	chicken
Qsox1	-0.36	0.08186133	chicken
Zcchc7	0.17	0.08217232	chicken
Ube2q1	-0.50	0.08262188	chicken
Gpatch2l	0.26	0.08437027	chicken
Arglu1	-0.58	0.08513848	chicken
Qpct	-0.33	0.08558696	chicken
Fosb	0.28	0.08573011	chicken
Sdf4	-0.43	0.08678436	chicken
Hsd17b12	0.26	0.08828710	chicken
Hnrnpll	-0.41	0.08898725	chicken
Gnas	0.46	0.09102330	chicken
Slc35b1	-0.40	0.09422792	chicken
Tacc1	-0.34	0.09435965	chicken
Rcor3	-0.24	0.09458895	chicken
Eml6	0.30	0.09505462	chicken
Pbrm1	-0.44	0.09560853	chicken
Glb1	-0.28	0.09593058	chicken
Galnt7	-0.36	0.09612642	chicken
Btk	-0.35	0.09689551	chicken
C4b	-0.15	0.09961580	chicken
Nfkbie	0.20	0.10052325	chicken
B3gnt2	-0.53	0.10111837	chicken
Gatd3a	-0.22	0.10228890	chicken
Pgm1	-0.31	0.10538010	chicken
Smdt1	-0.33	0.10547652	chicken
Slc9a9	-0.49	0.10606707	chicken
Pinx1	0.19	0.10638177	chicken
Rab7	-0.46	0.10749300	chicken
Acot7	-0.30	0.10923685	chicken
Fkbp11	-0.96	0.11394469	chicken
Slc25a5	-0.43	0.11568976	chicken
Runx1	-0.39	0.11820236	chicken
Ube2a	-0.36	0.11845038	chicken
Irf2	-0.36	0.11982966	chicken
Hsf3	-0.53	0.12326198	chicken
Dlg1	0.20	0.12400418	chicken
Gtf2f2	-0.55	0.12447394	chicken

Rab5a	-0.44	0.12557466	chicken
Sms	-0.31	0.12914244	chicken
Slc7a5	-0.38	0.13023277	chicken
Emp1	0.47	0.13057350	chicken
Nt5dc1	-0.56	0.13187110	chicken
Kcnk13	-0.81	0.13282531	chicken
Arf4	0.33	0.13347054	chicken
Nol9	-0.50	0.13865677	chicken
Sil1	-0.25	0.13976075	chicken
Ndufa2	-0.40	0.13981941	chicken
Chm	-0.41	0.14215747	chicken
Actn1	-0.26	0.14389295	chicken
Wdr37	0.21	0.14393883	chicken
Apoo	0.32	0.14829837	chicken
Casp3	0.42	0.14994489	chicken
Ston2	0.06	0.15191326	chicken
Nrbp1	0.15	0.15225069	chicken
Pdia5	-0.26	0.15271629	chicken
Ccdc6	-0.54	0.15725270	chicken
Erc2	0.81	0.15797063	chicken
Usf3	0.29	0.15804301	chicken
Cst7	0.21	0.16174187	chicken
Bmpr2	-0.49	0.16226439	chicken
Uqcr11	-0.37	0.16288617	chicken
Gfpt1	0.36	0.16413408	chicken
Lmbr1	-0.38	0.16439324	chicken
Arpc5	-0.11	0.16648201	chicken
Soat1	0.16	0.16670195	chicken
Rassf3	0.44	0.16815430	chicken
Dennd5a	0.30	0.16836651	chicken
Tob1	-0.23	0.16917802	chicken
Adprh	-0.29	0.17247488	chicken
B4galt2	-0.22	0.17409915	chicken
Aldh3a2	-0.13	0.17421052	chicken
Pak3	0.10	0.17742673	chicken
Psme3	-0.42	0.17858105	chicken
Sec11c	-0.31	0.17900042	chicken
Casp8ap2	0.24	0.18304496	chicken
Sult1b1	-0.15	0.18313052	chicken
Hnrnpd	-0.26	0.19025786	chicken
Champ1	-0.35	0.19344632	chicken
Nfkbib	-0.25	0.19486830	chicken
Inpp5a	-0.59	0.19656358	chicken
Psmd3	0.29	0.19670595	chicken
Tnfrsf26	0.05	0.19691907	chicken
Adsl	-0.27	0.19693466	chicken

Kat6b	-0.64	0.19881304	chicken
Abcb8	-0.28	0.20074917	chicken
Dnajb2	-0.35	0.20382182	chicken
Pm20d1	-0.19	0.20843468	chicken
Top2b	-0.34	0.21002449	chicken
Strn3	0.40	0.21024503	chicken
Ndufs3	-0.43	0.21048489	chicken
Gfod1	0.47	0.21165527	chicken
Edem1	-0.32	0.21290281	chicken
Kctd2	-0.21	0.21360898	chicken
Ube2d2a	0.27	0.21621040	chicken
Cc2d1b	0.21	0.21721231	chicken
Ptpn9	-0.35	0.21867910	chicken
Prex1	-0.37	0.22746352	chicken
Dag1	-0.30	0.22761177	chicken
Srp14	-0.45	0.22987800	chicken
Amdhd2	0.13	0.23418684	chicken
Sdhb	-0.39	0.24035698	chicken
Pnn	-0.54	0.24152808	chicken
1700034I23Rik	0.16	0.24251494	chicken
Hbs1l	0.22	0.24255553	chicken
Khdrbs1	-0.24	0.24988683	chicken
Sav1	-0.29	0.25177440	chicken
Fbxo34	-0.36	0.25364708	chicken
Osgin2	-0.28	0.25415774	chicken
Setx	0.27	0.25970812	chicken
Dmxl1	-0.41	0.26182451	chicken
Zfp207	0.17	0.26402943	chicken
Ctcf	-0.44	0.26888707	chicken
Hdac2	-0.43	0.27658902	chicken
Sdr16c5	-0.31	0.27803835	chicken
Usp6nl	0.13	0.28948109	chicken
Traf2	0.14	0.28981799	chicken
Glrx3	-0.25	0.29636005	chicken
Ssr3	-0.65	0.29925517	chicken
Ywhaq	-0.24	0.30233478	chicken
Chuk	-0.42	0.31384801	chicken
Psma3	-0.37	0.31597101	chicken
Clcn6	0.22	0.31796554	chicken
Ahcyl2	-0.30	0.32226271	chicken
Tal1	0.07	0.32351933	chicken
Samm50	-0.33	0.32429546	chicken
Sde2	-0.46	0.33334924	chicken
Bpnt2	-0.26	0.33524403	chicken
Sec61g	-0.46	0.33584489	chicken
Klf12	-0.55	0.33586672	chicken

Arid3a	-0.08	0.33618448	chicken
Zcchc14	0.10	0.34006758	chicken
Polr2h	-0.29	0.34338023	chicken
Sptlc1	0.33	0.34345636	chicken
St13	-0.49	0.34559928	chicken
Fam174a	-0.19	0.34777469	chicken
Ubn2	0.15	0.35226164	chicken
Grpel2	0.15	0.35696527	chicken
Kansl1l	0.13	0.35938118	chicken
Traf7	-0.21	0.36071346	chicken
Agpat5	-0.33	0.36286558	chicken
Dnajc1	-0.62	0.36294899	chicken
Ube2l3	0.25	0.36365677	chicken
Gramd1b	-0.24	0.36810450	chicken
Crim1	-0.63	0.36941794	chicken
Psma2	0.29	0.37427250	chicken
Upf1	-0.49	0.37629526	chicken
Sf3b6	-0.47	0.37795552	chicken
Ppard	0.26	0.38339268	chicken
Ewsr1	-0.46	0.38713390	chicken
Vcan	0.20	0.38973741	chicken
Cdkal1	0.29	0.39005480	chicken
Galnt2	-0.37	0.39618609	chicken
Trabd	0.34	0.39937425	chicken
Pxk	0.38	0.40136713	chicken
Lamp3	0.30	0.40460526	chicken
Ube2cbp	-0.49	0.40553414	chicken
Reep5	-0.34	0.40967600	chicken
Ugt8a	0.10	0.41302516	chicken
Capn11	-0.19	0.41317476	chicken
Snf8	-0.13	0.42204198	chicken
Trp53bp2	0.24	0.42436301	chicken
Exoc4	-0.60	0.42998738	chicken
Cxcr4	-0.23	0.43402784	chicken
Ccng2	-0.27	0.43435268	chicken
Snap91	-0.69	0.44421807	chicken
Erp44	0.24	0.44486520	chicken
Tmem106b	-0.32	0.44660558	chicken
Sf3b1	-0.48	0.44736399	chicken
Ubn1	-0.35	0.44758329	chicken
Siah2	-0.20	0.44927765	chicken
Nelfe	-0.25	0.44960912	chicken
Stt3b	-0.37	0.44989616	chicken
Atp11c	0.14	0.45110574	chicken
Ndufa9	-0.37	0.45468661	chicken
Cox5b-ps	-0.31	0.45535658	chicken

Jade1	0.18	0.46045073	chicken
Phf11a	0.23	0.47145984	chicken
Usp36	-0.60	0.47165454	chicken
Use1	0.11	0.47244587	chicken
Vamp3	-0.18	0.47251997	chicken
Gpatch11	-0.25	0.48010347	chicken
Gypc	0.23	0.48449042	chicken
Cry1	-0.59	0.48751704	chicken
Timm10	-0.18	0.49584731	chicken
Ergic2	-0.34	0.49908260	chicken
Smad3	-0.22	0.50396719	chicken
Sap30l	-0.29	0.50429960	chicken
Slc29a4	-0.47	0.51557428	chicken
Usp44	0.21	0.51798844	chicken
Cnot7	-0.48	0.52303227	chicken
Per3	0.14	0.52425057	chicken
Ppp2r5c	0.25	0.52881853	chicken
Zbtb21	0.23	0.53993130	chicken
Rassf1	-0.57	0.53997211	chicken
Slc35a5	0.12	0.54607910	chicken
Syngr3	-0.35	0.55496011	chicken
Heca	0.05	0.56397512	chicken
Epha4	0.59	0.56572927	chicken
Armh1	0.15	0.59874199	chicken
Tial1	-0.47	0.60053791	chicken
Vps29	-0.41	0.60755226	chicken
Cttn	0.14	0.60826420	chicken
Ep400	-0.40	0.61491392	chicken
Npm3	0.19	0.61610399	chicken
Sucnr1	0.10	0.62541141	chicken
Agbl1	0.44	0.63132695	chicken
Serpinb8	-0.26	0.63773406	chicken
Unc119b	-0.20	0.64169431	chicken
Osbpl9	0.22	0.64451103	chicken
BC005624	-0.34	0.64511241	chicken
Atf1	0.10	0.65223018	chicken
Hint1	-0.15	0.65366054	chicken
Glrx	0.44	0.65556462	chicken
Psmb2	-0.25	0.65636747	chicken
Pnpla2	0.09	0.65798294	chicken
Pdgfb	-0.56	0.66152319	chicken
Smc4	-0.48	0.66682021	chicken
Pafah1b2	-0.36	0.67147423	chicken
Snx16	0.27	0.67327695	chicken
Grcc10	-0.31	0.67461849	chicken
Grk2	0.07	0.67725496	chicken

Lsm14a	-0.53	0.71115149	chicken
Ercc3	0.27	0.71496915	chicken
Fus	0.10	0.73368449	chicken
Thoc7	-0.41	0.73428255	chicken
Appl2	0.42	0.74224395	chicken
Tulp1	0.12	0.74401792	chicken
Cyth3	0.25	0.74800630	chicken
Stk38l	-0.29	0.75469120	chicken
Nmrk1	0.24	0.77351835	chicken
Fam120b	0.28	0.77474609	chicken
Kpna1	0.21	0.78712794	chicken
Sirt1	-0.44	0.79041083	chicken
Ssr1	-0.47	0.81307217	chicken
Rab3a	-0.26	0.81377298	chicken
Il1r1	-0.17	0.82408051	chicken
B3gntl1	-0.02	0.83755842	chicken
Nucb2	-0.41	0.84289210	chicken
Tmpo	-0.25	0.86072535	chicken
Hacd3	-0.36	0.86472050	chicken
Kmt5b	-0.39	0.88532566	chicken
Atp10a	0.27	0.89226087	chicken
Ankrd13d	-0.38	0.90719559	chicken
Ski	-0.44	0.91622305	chicken
Galnt1	-0.34	0.92101434	chicken
Fyn	0.46	0.93135612	chicken
Nfatc1	-0.42	0.93387958	chicken
Eif1b	-0.40	0.93441204	chicken
Hras	-0.49	0.93548898	chicken
Rad23b	-0.50	0.95235000	chicken
Ifnar2	0.12	0.95546018	chicken
Dcaf13	-0.31	0.96971011	chicken
Rheb	-0.43	0.99508726	chicken
Psmd1	0.29	1.00000000	chicken
Cyria	-0.58	1.00000000	chicken
Serpine2	-0.17	1.00000000	chicken
Ei24	-0.24	1.00000000	chicken
B3gnt4	-0.18	1.00000000	chicken
Plrg1	0.18	1.00000000	chicken
Cacul1	-0.47	1.00000000	chicken
Csnk1g1	0.21	1.00000000	chicken
Ninj1	0.08	1.00000000	chicken
Pik3r6	0.26	1.00000000	chicken
Akap8	-0.20	1.00000000	chicken
Evi5	-0.48	1.00000000	chicken
Rbbp5	0.19	1.00000000	chicken
Cdk7	0.08	1.00000000	chicken

Inpp4b	-0.38	1.00000000	chicken
Eif2ak3	0.35	1.00000000	chicken
Lap3	-0.30	1.00000000	chicken
Pcca	0.29	1.00000000	chicken
Gigyf2	0.32	1.00000000	chicken
Klf10	0.34	1.00000000	chicken
Dsn1	0.20	1.00000000	chicken
Sdf2	-0.15	1.00000000	chicken
Itgb1	0.24	1.00000000	chicken
Dkc1	-0.38	1.00000000	chicken
Sptssa	-0.40	1.00000000	chicken
Gnal	-0.47	1.00000000	chicken
Cdk1	0.10	1.00000000	chicken
Brf1	-0.65	1.00000000	chicken
Srpk2	-0.41	1.00000000	chicken
Pef1	-0.28	1.00000000	chicken
Arhgef9	-0.18	1.00000000	chicken
Aebp2	0.45	1.00000000	chicken
Prkcsh	-0.19	1.00000000	chicken
Fhod1	0.28	1.00000000	chicken
Cabin1	0.17	1.00000000	chicken
Taf3	-0.43	1.00000000	chicken
Dnajb4	0.17	1.00000000	chicken
Fbxw2	-0.25	1.00000000	chicken
Tent2	-0.40	1.00000000	chicken
Slc30a4	-0.18	1.00000000	chicken
Ntng1	-0.33	1.00000000	chicken
Syf2	-0.40	1.00000000	chicken
Kctd6	0.12	1.00000000	chicken
Pid1	0.11	1.00000000	chicken
Vdr	0.31	1.00000000	chicken
Xpr1	0.39	1.00000000	chicken
Ago1	-0.25	1.00000000	chicken
Bbs7	-0.33	1.00000000	chicken
P2rx4	-0.19	1.00000000	chicken
Gpr107	0.23	1.00000000	chicken
Gltp	0.29	1.00000000	chicken
Cnpy3	-0.30	1.00000000	chicken
Rab11fip2	0.15	1.00000000	chicken
Pomp	-0.32	1.00000000	chicken
Lrrc1	0.18	1.00000000	chicken
Tmem250-ps	-0.12	1.00000000	chicken
C1qbp	-0.27	1.00000000	chicken
Ndufs7	-0.42	1.00000000	chicken
Fer	0.17	1.00000000	chicken
Kdm4c	0.27	1.00000000	chicken

Snhg4	-0.43	1.00000000	chicken
Aga	-0.22	1.00000000	chicken
Traf5	-0.36	1.00000000	chicken
Nkiras2	-0.21	1.00000000	chicken
Gnb1l	0.15	1.00000000	chicken
Trim35	-0.23	1.00000000	chicken
Sqstm1	0.44	1.00000000	chicken
1110059E24Rik	0.10	1.00000000	chicken
Tmem131	-0.40	1.00000000	chicken
Thoc5	-0.09	1.00000000	chicken
Cerk	-0.36	1.00000000	chicken
Lmnb1	0.24	1.00000000	chicken
Ireb2	-0.30	1.00000000	chicken
Dars	-0.56	1.00000000	chicken
Plxnd1	0.37	1.00000000	chicken
Bclaf1	-0.41	1.00000000	chicken
Tiam2	0.22	1.00000000	chicken
Cops8	-0.23	1.00000000	chicken
Bin1	0.28	1.00000000	chicken
Akt1	0.24	1.00000000	chicken
Cep164	-0.47	1.00000000	chicken
Opa1	-0.37	1.00000000	chicken
Erh	-0.33	1.00000000	chicken
9930021J03Rik	-0.34	1.00000000	chicken
Setd5	0.23	1.00000000	chicken
Phlpp2	0.16	1.00000000	chicken
Stx18	-0.30	1.00000000	chicken
Banp	0.24	1.00000000	chicken
Ccdc93	-0.40	1.00000000	chicken
Tsen2	0.12	1.00000000	chicken
Akap11	-0.22	1.00000000	chicken
Atg4b	0.14	1.00000000	chicken
Pcnt	-0.34	1.00000000	chicken
Edc3	-0.47	1.00000000	chicken
Dab2	0.35	1.00000000	chicken
Vldlr	0.13	1.00000000	chicken
Herc3	0.20	1.00000000	chicken
Tmem87a	-0.38	1.00000000	chicken
Slc15a4	0.25	1.00000000	chicken
Slc1a4	-0.22	1.00000000	chicken
Gars	-0.39	1.00000000	chicken
Commd7	-0.16	1.00000000	chicken
Nup93	0.62	1.00000000	chicken
Pds5b	-0.52	1.00000000	chicken
Arl2bp	0.18	1.00000000	chicken
Cog7	0.23	1.00000000	chicken

Frmd4b	0.32	1.00000000	chicken
Cap1	-0.32	1.00000000	chicken
Usp3	0.21	1.00000000	chicken
Prpf40a	-0.19	1.00000000	chicken
Rab1a	-0.25	1.00000000	chicken
Rer1	-0.27	1.00000000	chicken
Chmp6	-0.27	1.00000000	chicken
Ppm1b	0.25	1.00000000	chicken
Tgfbrap1	0.20	1.00000000	chicken
Tmem19	0.09	1.00000000	chicken
Naa20	-0.31	1.00000000	chicken
Chn1	0.25	1.00000000	chicken
Wac	0.20	1.00000000	chicken
Arl5b	-0.15	1.00000000	chicken
Fyb	0.48	1.00000000	chicken
Prmt9	0.11	1.00000000	chicken
AI597479	0.02	1.00000000	chicken
Erap1	-0.18	1.00000000	chicken
Rab18	0.27	1.00000000	chicken
Akap9	0.10	1.00000000	chicken
Agpat2	0.19	1.00000000	chicken
Creld1	-0.20	1.00000000	chicken
Hsd17b4	-0.21	1.00000000	chicken
Dnajc19	-0.40	1.00000000	chicken
Cnot8	-0.31	1.00000000	chicken
Fbxo28	0.03	1.00000000	chicken
Tgfbr2	-0.30	1.00000000	chicken
Pdss1	0.15	1.00000000	chicken
Phf20	0.27	1.00000000	chicken
Hic2	0.04	1.00000000	chicken
Rbm22	-0.50	1.00000000	chicken
Tpd52	-0.25	1.00000000	chicken
Laptm4a	-0.31	1.00000000	chicken
Ank	-0.59	1.00000000	chicken
Lbh	-0.50	1.00000000	chicken
Actr1a	-0.36	1.00000000	chicken
Arpc1a	-0.19	1.00000000	chicken
Fkbp5	-0.33	1.00000000	chicken
Ilf3	-0.42	1.00000000	chicken
Trpc4ap	-0.35	1.00000000	chicken
Kif13a	0.48	1.00000000	chicken
Ndrg3	-0.16	1.00000000	chicken
Zfp654	0.24	1.00000000	chicken
Kcna3	0.05	1.00000000	chicken
Pms2	0.13	1.00000000	chicken
Cd40	0.25	1.00000000	chicken

Chpt1	-0.18	1.00000000	chicken
Cebpz	0.24	1.00000000	chicken
Thada	0.31	1.00000000	chicken
Limd2	-0.04	1.00000000	chicken
Ccdc25	-0.30	1.00000000	chicken
Smap2	-0.34	1.00000000	chicken
Adcy7	-0.50	1.00000000	chicken
Lcp2	0.15	1.00000000	chicken
Tpst1	-0.34	1.00000000	chicken
Ankrd10	-0.48	1.00000000	chicken
Slc9a1	-0.29	1.00000000	chicken
Disp1	0.26	1.00000000	chicken
Wdr77	-0.19	1.00000000	chicken
Rnf128	0.06	1.00000000	chicken
D5Ertd579e	0.05	1.00000000	chicken
Elof1	-0.32	1.00000000	chicken
Hibch	-0.26	1.00000000	chicken
Pcbd2	-0.21	1.00000000	chicken
Neurl1a	0.08	1.00000000	chicken
Phkg1	-0.18	1.00000000	chicken
Ccdc32	-0.18	1.00000000	chicken
Paip1	-0.39	1.00000000	chicken
Adarb1	-0.16	1.00000000	chicken
Gnptab	-0.35	1.00000000	chicken
Wtap	-0.42	1.00000000	chicken
Pdhb	-0.23	1.00000000	chicken
Tram1l1	-0.55	1.00000000	chicken
Trpv2	0.32	1.00000000	chicken
Arl1	-0.51	1.00000000	chicken
Zfp36l2	-0.04	1.00000000	chicken
Sar1a	-0.32	1.00000000	chicken
Setdb2	0.29	1.00000000	chicken
Fbxo22	0.12	1.00000000	chicken
Pld1	-0.33	1.00000000	chicken
Tmtc1	-0.59	1.00000000	chicken
Ippk	0.10	1.00000000	chicken
Enpp6	0.25	1.00000000	chicken
Ddhd1	-0.39	1.00000000	chicken
Txndc17	-0.34	1.00000000	chicken
Reck	0.21	1.00000000	chicken
Dazap2	-0.19	1.00000000	chicken
Slc41a1	-0.16	1.00000000	chicken
Amd2	0.16	1.00000000	chicken
Pim1	0.11	1.00000000	chicken
Hsph1	0.34	1.00000000	chicken
Smad6	-0.41	1.00000000	chicken

Prkag1	-0.22	1.00000000	chicken
Gne	-0.29	1.00000000	chicken
Tmem165	-0.33	1.00000000	chicken
Epb41l3	-0.39	1.00000000	chicken
Nup153	0.17	1.00000000	chicken
Ciao1	-0.19	1.00000000	chicken
Wbp4	-0.55	1.00000000	chicken
Ghitm	-0.36	1.00000000	chicken
Lin54	0.20	1.00000000	chicken
Osbpl1a	-0.52	1.00000000	chicken
Arl15	0.06	1.00000000	chicken
Uqcrb	-0.49	1.00000000	chicken
Zc3h12a	0.25	1.00000000	chicken
Wipf1	-0.44	1.00000000	chicken
Snx12	-0.35	1.00000000	chicken
Pmf1	-0.33	1.00000000	chicken
Fmnl2	0.23	1.00000000	chicken
Ccnd1	-0.54	1.00000000	chicken
Uqcrc1	-0.26	1.00000000	chicken
Armh3	0.29	1.00000000	chicken
Cmtm4	0.08	1.00000000	chicken
Sp3	-0.53	1.00000000	chicken
Pde8a	-0.26	1.00000000	chicken
Ptpn6	-0.30	1.00000000	chicken
Apaf1	0.19	1.00000000	chicken
Utp18	-0.25	1.00000000	chicken
Tfdp2	-0.47	1.00000000	chicken
Rbm17	0.37	1.00000000	chicken
Dync1li2	0.20	1.00000000	chicken
Usp38	-0.33	1.00000000	chicken
Prkar1a	-0.36	1.00000000	chicken
Acap1	-0.21	1.00000000	chicken
Sf3b5	-0.42	1.00000000	chicken
Wasl	-0.24	1.00000000	chicken
Ccnl2	0.08	1.00000000	chicken
Hnrnpm	-0.37	1.00000000	chicken
Rnpep	-0.26	1.00000000	chicken
Nsd3	-0.39	1.00000000	chicken
Vim	-0.44	1.00000000	chicken
Vps26a	0.29	1.00000000	chicken
Smarcc1	-0.27	1.00000000	chicken
Prkd1	-0.38	1.00000000	chicken
Ube2v2	-0.47	1.00000000	chicken
Irak2	0.21	1.00000000	chicken
Tcf20	-0.39	1.00000000	chicken
Hmgb3	-0.39	1.00000000	chicken

Exoc3	0.19	1.00000000	chicken
Sun1	0.19	1.00000000	chicken
Arfgef3	0.87	1.00000000	chicken
Atrx	0.13	1.00000000	chicken
Srsf10	-0.45	1.00000000	chicken
Nfatc2	-0.41	1.00000000	chicken
Sufu	0.12	1.00000000	chicken
Srm	-0.13	1.00000000	chicken
Zfp598	-0.21	1.00000000	chicken
Becn2	-0.26	1.00000000	chicken
Jmy	-0.28	1.00000000	chicken
Lyrm4	0.21	1.00000000	chicken
Tesk2	0.11	1.00000000	chicken
Psmf1	-0.22	1.00000000	chicken
Tram2	-0.24	1.00000000	chicken
Lztr1	0.22	1.00000000	chicken
Wwp1	0.37	1.00000000	chicken
Usp20	0.17	1.00000000	chicken
Pin4	-0.26	1.00000000	chicken
H2az1	-0.46	1.00000000	chicken
Anapc2	-0.20	1.00000000	chicken
Rnf126	-0.33	1.00000000	chicken
Zmat2	-0.29	1.00000000	chicken
Trip11	0.15	1.00000000	chicken
Wdr12	-0.26	1.00000000	chicken
Grk4	-0.56	1.00000000	chicken
Rin2	0.10	1.00000000	chicken
Ppfia1	-0.15	1.00000000	chicken
Adk	-0.62	1.00000000	chicken
Nras	-0.29	1.00000000	chicken
Pik3r1	-0.38	1.00000000	chicken
Impa1	-0.21	1.00000000	chicken
Cnbp	-0.37	1.00000000	chicken
Kdm4b	-0.42	1.00000000	chicken
Rb1cc1	0.39	1.00000000	chicken
Pgk1	-0.20	1.00000000	chicken
Hexim1	0.15	1.00000000	chicken
Rbms3	0.31	1.00000000	chicken
Cep112	0.13	1.00000000	chicken
Slc20a2	0.10	1.00000000	chicken
Ctdspl	-0.37	1.00000000	chicken
Gsr	-0.26	1.00000000	chicken
Edf1	-0.42	1.00000000	chicken
Tlk2	0.25	1.00000000	chicken
Zdhhc5	-0.22	1.00000000	chicken
Bmp2k	0.30	1.00000000	chicken

Ppm1k	-0.28	1.00000000	chicken
Edem3	-0.31	1.00000000	chicken
Acsbg2	0.17	1.00000000	chicken
Psma4	-0.45	1.00000000	chicken
Flot2	-0.20	1.00000000	chicken
Rnpepl1	0.30	1.00000000	chicken
Ndufa12	-0.38	1.00000000	chicken
Xiap	0.14	1.00000000	chicken
Paqr8	-0.23	1.00000000	chicken
Ass1	-0.26	1.00000000	chicken
Ube2k	0.26	1.00000000	chicken
Rap1gds1	-0.35	1.00000000	chicken
Pfkp	0.30	1.00000000	chicken
Dnajc2	-0.53	1.00000000	chicken
Spg21	-0.29	1.00000000	chicken
Slc31a2	-0.18	1.00000000	chicken
Tacc3	0.34	1.00000000	chicken
Synj1	0.13	1.00000000	chicken
Herpud1	0.21	1.00000000	chicken
Ndufs8	-0.07	1.00000000	chicken
Dr1	-0.06	1.00000000	chicken
Stau1	-0.29	1.00000000	chicken
Smc3	0.66	1.00000000	chicken
Rabep1	0.14	1.00000000	chicken
Eci1	-0.21	1.00000000	chicken
Atxn7	0.18	1.00000000	chicken
Arid4a	-0.63	1.00000000	chicken
Pnpla8	0.20	1.00000000	chicken
Sptan1	-0.33	1.00000000	chicken
Lpin1	-0.27	1.00000000	chicken
Cryl1	-0.29	1.00000000	chicken
Nup85	-0.23	1.00000000	chicken
Clip1	0.22	1.00000000	chicken
Reps1	-0.51	1.00000000	chicken
Sh3bp5	0.21	1.00000000	chicken
Rab2a	-0.37	1.00000000	chicken
Golt1b	0.02	1.00000000	chicken
Lars	-0.26	1.00000000	chicken
Nsl1	0.05	1.00000000	chicken
Phf14	-0.34	1.00000000	chicken
Cope	-0.30	1.00000000	chicken
Brwd1	-0.41	1.00000000	chicken
Xrn2	-0.32	1.00000000	chicken
Herc1	0.14	1.00000000	chicken
Rnf144b	-0.78	1.00000000	chicken
Crlf3	-0.29	1.00000000	chicken

Ugdh	-0.20	1.00000000	chicken
Tnrc6b	-0.41	1.00000000	chicken
Uspl1	-0.38	1.00000000	chicken
2700049A03Rik	0.03	1.00000000	chicken
Stard8	0.18	1.00000000	chicken
Bcl2l15	0.08	1.00000000	chicken
Tmem181a	0.22	1.00000000	chicken
Gpatch1	0.21	1.00000000	chicken
Scamp2	-0.09	1.00000000	chicken
Tmem30a	-0.24	1.00000000	chicken
Atp6v1h	0.17	1.00000000	chicken
Nr2c1	-0.36	1.00000000	chicken
Atp6v0b	-0.16	1.00000000	chicken
Iqgap2	-0.46	1.00000000	chicken
Smarce1	-0.24	1.00000000	chicken
Cldnd1	-0.24	1.00000000	chicken
Ogdh	-0.37	1.00000000	chicken
Stk3	-0.43	1.00000000	chicken
Eprs	-0.37	1.00000000	chicken
Cpsf2	0.12	1.00000000	chicken
Xpo1	0.24	1.00000000	chicken
Snx9	-0.54	1.00000000	chicken
Zbtb1	-0.25	1.00000000	chicken
Ndufb7	-0.09	1.00000000	chicken
Otud6b	-0.29	1.00000000	chicken
Prdx3	-0.35	1.00000000	chicken
Arrdc2	-0.07	1.00000000	chicken
Txndc9	-0.30	1.00000000	chicken
Tnrc6c	-0.39	1.00000000	chicken
Fkbp15	0.20	1.00000000	chicken
Spsb1	0.16	1.00000000	chicken
Cdc25b	-0.32	1.00000000	chicken
Ofd1	0.16	1.00000000	chicken
Sike1	-0.17	1.00000000	chicken
Lrch1	0.07	1.00000000	chicken
Fmn1	0.42	1.00000000	chicken
Jazf1	0.07	1.00000000	chicken
Fam120a	-0.33	1.00000000	chicken
Akirin2	0.21	1.00000000	chicken
Dera	0.13	1.00000000	chicken
Cltb	-0.32	1.00000000	chicken
Parvg	0.24	1.00000000	chicken
Ndufs2	-0.14	1.00000000	chicken
Nfyc	-0.42	1.00000000	chicken
Plekho1	-0.35	1.00000000	chicken
Stmn1	-0.43	1.00000000	chicken

Copg1	-0.26	1.00000000	chicken
Cers5	0.07	1.00000000	chicken
Dohh	-0.14	1.00000000	chicken
Cdv3	-0.36	1.00000000	chicken
Dus1l	-0.18	1.00000000	chicken
Tshz2	-0.75	1.00000000	chicken
Creld2	-0.39	1.00000000	chicken
Rabgap1	0.28	1.00000000	chicken
Borcs5	0.23	1.00000000	chicken
Tmem259	-0.19	1.00000000	chicken
Gemin4	-0.20	1.00000000	chicken
Psmd12	-0.20	1.00000000	chicken
Gosr1	0.27	1.00000000	chicken
Tfg	-0.31	1.00000000	chicken
Cep170	0.11	1.00000000	chicken
Adam19	-0.39	1.00000000	chicken
Rufy3	-0.39	1.00000000	chicken
Gtf2a2	-0.33	1.00000000	chicken
Zfp644	-0.41	1.00000000	chicken
Il13ra1	0.11	1.00000000	chicken
Ccn1	0.22	1.00000000	chicken
Ube2r2	0.17	1.00000000	chicken
Ppp1r21	0.16	1.00000000	chicken
Yipf4	-0.26	1.00000000	chicken
Zbtb38	-0.29	1.00000000	chicken
Sbno2	-0.33	1.00000000	chicken
Timmdc1	-0.20	1.00000000	chicken
Acsl1	-0.01	1.00000000	chicken
Septin14	-0.28	1.00000000	chicken
Rnf103	0.07	1.00000000	chicken
Cnot1	-0.46	1.00000000	chicken
Ttl	-0.42	1.00000000	chicken
Csnk1e	0.07	1.00000000	chicken
Kansl1	0.16	1.00000000	chicken
Ssb	-0.32	1.00000000	chicken
Ncbp2	0.23	1.00000000	chicken
Setd1b	0.16	1.00000000	chicken
Sap130	0.20	1.00000000	chicken
Galns	0.27	1.00000000	chicken
Slc49a4	0.20	1.00000000	chicken
Polr2e	-0.11	1.00000000	chicken
Dnlz	-0.34	1.00000000	chicken
Nup50	-0.38	1.00000000	chicken
Oit3	0.10	1.00000000	chicken
Rbm24	-0.04	1.00000000	chicken
Calr	-0.33	1.00000000	chicken

Zbtb2	0.07	1.00000000	chicken
Vezf1	0.06	1.00000000	chicken
Cul5	0.24	1.00000000	chicken
Cdc26	-0.25	1.00000000	chicken
Uba3	0.21	1.00000000	chicken
Coa5	-0.19	1.00000000	chicken
Kidins220	0.27	1.00000000	chicken
Tbc1d22a	-0.47	1.00000000	chicken
Rab7b	-0.41	1.00000000	chicken
Tpp2	-0.38	1.00000000	chicken
Vkorc1l1	0.14	1.00000000	chicken
Zfand2b	-0.08	1.00000000	chicken
Zfp512b	-0.23	1.00000000	chicken
Vps54	0.01	1.00000000	chicken
Ubtf	-0.26	1.00000000	chicken
Zfp609	0.18	1.00000000	chicken
Pigk	-0.24	1.00000000	chicken
Lonp1	-0.21	1.00000000	chicken
Dhx37	-0.23	1.00000000	chicken
Hmox1	0.14	1.00000000	chicken
Cnot9	-0.16	1.00000000	chicken
Ikbke	-0.25	1.00000000	chicken
Impa2	0.18	1.00000000	chicken
Prelid3b	-0.41	1.00000000	chicken
Atp6v1e2	-0.27	1.00000000	chicken
Gbe1	-0.28	1.00000000	chicken
Wdr7	0.16	1.00000000	chicken
Tmem258	-0.46	1.00000000	chicken
Copb2	-0.32	1.00000000	chicken
Bdh1	-0.21	1.00000000	chicken
Pnrc2	-0.32	1.00000000	chicken
Hspa13	0.29	1.00000000	chicken
Prr5l	-0.36	1.00000000	chicken
Txndc12	-0.19	1.00000000	chicken
Ndufb3	-0.23	1.00000000	chicken
Arhgef7	-0.35	1.00000000	chicken
Cpne3	-0.06	1.00000000	chicken
Snx14	0.21	1.00000000	chicken
Heatr5b	0.21	1.00000000	chicken
Uhmk1	-0.12	1.00000000	chicken
Osbpl3	-0.32	1.00000000	chicken
Lsm12	0.09	1.00000000	chicken
Wdr61	-0.11	1.00000000	chicken
Cbx3	-0.37	1.00000000	chicken
Ythdc1	-0.42	1.00000000	chicken
Stoml2	-0.16	1.00000000	chicken

Wdr13	-0.24	1.00000000	chicken
Src	0.11	1.00000000	chicken
Rcsd1	0.00	1.00000000	chicken
Cyth1	-0.35	1.00000000	chicken
Tm9sf5	-0.18	1.00000000	chicken
Agtpbp1	-0.25	1.00000000	chicken
Tmem141	-0.06	1.00000000	chicken
Tenm3	-0.40	1.00000000	chicken
Gas7	0.29	1.00000000	chicken
Cd47	0.20	1.00000000	chicken
Ticam1	0.21	1.00000000	chicken
Dync1i2	-0.35	1.00000000	chicken
Plagl1	-0.32	1.00000000	chicken
Tnfsf11	0.54	1.00000000	chicken
Adh5	-0.07	1.00000000	chicken
Lasp1	-0.24	1.00000000	chicken
Tpi1	-0.15	1.00000000	chicken
Lman1	-0.74	1.00000000	chicken
Scfd2	0.12	1.00000000	chicken
Dazap1	-0.47	1.00000000	chicken
D16Ertd472e	-0.02	1.00000000	chicken
Psma6	0.18	1.00000000	chicken
Gpatch4	0.16	1.00000000	chicken
Uba2	0.15	1.00000000	chicken
Gtf2a1	0.26	1.00000000	chicken
Sft2d1	0.21	1.00000000	chicken
Pfdn4	-0.51	1.00000000	chicken
Alg5	-0.06	1.00000000	chicken
Ipo5	-0.25	1.00000000	chicken
Rundc3b	-0.46	1.00000000	chicken
Ube2g2	-0.35	1.00000000	chicken
Hsp90aa1	-0.47	1.00000000	chicken
Ythdf1	-0.32	1.00000000	chicken
Naa50	0.24	1.00000000	chicken
Socs6	0.09	1.00000000	chicken
Sh3glb1	-0.05	1.00000000	chicken
Cradd	0.11	1.00000000	chicken
Prdm8	-0.28	1.00000000	chicken
Taz	-0.19	1.00000000	chicken
Tcf3	-0.47	1.00000000	chicken
Higd1a	-0.24	1.00000000	chicken
Crebrf	0.08	1.00000000	chicken
Dnajc18	-0.27	1.00000000	chicken
Slc30a7	-0.22	1.00000000	chicken
Hnrnpab	-0.47	1.00000000	chicken
Dock8	-0.24	1.00000000	chicken

Rab5c	-0.33	1.00000000	chicken
Cfap20	-0.24	1.00000000	chicken
Urb1	0.08	1.00000000	chicken
Cisd2	0.14	1.00000000	chicken
Plscr1	0.06	1.00000000	chicken
Rgs18	0.02	1.00000000	chicken
Kif3a	0.01	1.00000000	chicken
Ahi1	-0.35	1.00000000	chicken
Plekha8	-0.14	1.00000000	chicken
Tmem263	-0.26	1.00000000	chicken
Rexo1	-0.37	1.00000000	chicken
Txlng	0.08	1.00000000	chicken
Ccnd3	-0.17	1.00000000	chicken
Arrdc1	-0.11	1.00000000	chicken
Rcbtb1	-0.20	1.00000000	chicken
Septin8	0.04	1.00000000	chicken
Ripk1	0.27	1.00000000	chicken
Tmem183a	-0.22	1.00000000	chicken
Nedd8	-0.26	1.00000000	chicken
Cnn2	-0.26	1.00000000	chicken
Rab9	-0.26	1.00000000	chicken
Prep	-0.19	1.00000000	chicken
Psip1	-0.31	1.00000000	chicken
Ldlrad4	0.25	1.00000000	chicken
Fhit	-0.54	1.00000000	chicken
Lysmd2	0.09	1.00000000	chicken
Rtraf	-0.36	1.00000000	chicken
Ryk	-0.42	1.00000000	chicken
Hikeshi	-0.18	1.00000000	chicken
Flii	-0.19	1.00000000	chicken
Atp11a	0.44	1.00000000	chicken
Card9	-0.43	1.00000000	chicken
Rpp14	-0.25	1.00000000	chicken
Taf2	-0.13	1.00000000	chicken
Arhgap15	-0.18	1.00000000	chicken
Wdr41	-0.20	1.00000000	chicken
Fkbp4	-0.41	1.00000000	chicken
Dyrk1a	0.09	1.00000000	chicken
Tmem33	-0.22	1.00000000	chicken
Arnt	-0.29	1.00000000	chicken
Gga3	0.12	1.00000000	chicken
Fubp3	-0.36	1.00000000	chicken
Lrch3	-0.35	1.00000000	chicken
Hdac4	0.15	1.00000000	chicken
Oser1	0.13	1.00000000	chicken
Naa35	-0.30	1.00000000	chicken

Atp6ap2	-0.28	1.00000000	chicken
Fut8	-0.20	1.00000000	chicken
Slc25a36	0.28	1.00000000	chicken
Iah1	-0.09	1.00000000	chicken
Rhog	0.12	1.00000000	chicken
Cxxc4	-0.24	1.00000000	chicken
Itpk1	-0.27	1.00000000	chicken
Sdhaf2	-0.15	1.00000000	chicken
Rad54l2	0.16	1.00000000	chicken
Tgs1	-0.29	1.00000000	chicken
Dusp6	-0.20	1.00000000	chicken
Slc10a7	0.36	1.00000000	chicken
Cul4a	0.15	1.00000000	chicken
Atf4	0.13	1.00000000	chicken
Naa25	0.08	1.00000000	chicken
Sco1	-0.23	1.00000000	chicken
Ahr	-0.13	1.00000000	chicken
Cert1	0.44	1.00000000	chicken
Ttll12	-0.13	1.00000000	chicken
Dapp1	0.07	1.00000000	chicken
Tbcd	0.21	1.00000000	chicken
Erlec1	-0.49	1.00000000	chicken
Klf11	0.05	1.00000000	chicken
Gpatch2	0.23	1.00000000	chicken
Sesn2	0.13	1.00000000	chicken
Nfic	0.13	1.00000000	chicken
Slc2a13	0.12	1.00000000	chicken
Zdhhc17	0.11	1.00000000	chicken
Rtkn2	-0.04	1.00000000	chicken
Ero1a	-0.24	1.00000000	chicken
Prkar2a	-0.38	1.00000000	chicken
Ppm1a	-0.27	1.00000000	chicken
Lrrfip1	-0.25	1.00000000	chicken
Virma	-0.12	1.00000000	chicken
Fam168b	-0.22	1.00000000	chicken
Hk1	-0.26	1.00000000	chicken
Chmp7	-0.25	1.00000000	chicken
Blcap	-0.24	1.00000000	chicken
Elovl1	0.23	1.00000000	chicken
Oaz2	-0.27	1.00000000	chicken
Nek6	0.12	1.00000000	chicken
C2cd5	-0.34	1.00000000	chicken
Emc1	0.09	1.00000000	chicken
Crtc1	-0.25	1.00000000	chicken
Itpripl2	-0.29	1.00000000	chicken
Ubap2	0.23	1.00000000	chicken

Dpf3	-0.43	1.00000000	chicken
Ntan1	-0.34	1.00000000	chicken
Wdr45b	0.15	1.00000000	chicken
Tor4a	-0.27	1.00000000	chicken
Chmp2b	-0.25	1.00000000	chicken
Smad5	-0.36	1.00000000	chicken
Rnf217	-0.39	1.00000000	chicken
Tec	0.01	1.00000000	chicken
Sox5	0.15	1.00000000	chicken
Atp2c1	-0.13	1.00000000	chicken
Rsrc2	0.04	1.00000000	chicken
Lrp8	0.12	1.00000000	chicken
Tlr4	0.07	1.00000000	chicken
Aspm	-0.04	1.00000000	chicken
Ubash3a	-0.28	1.00000000	chicken
Limd1	0.11	1.00000000	chicken
Birc6	-0.43	1.00000000	chicken
Bcl2l13	-0.13	1.00000000	chicken
Dcps	0.11	1.00000000	chicken
Bnip2	-0.35	1.00000000	chicken
Tcp1	-0.30	1.00000000	chicken
Clptm1l	-0.20	1.00000000	chicken
Stxbp5	-0.37	1.00000000	chicken
Abhd5	0.11	1.00000000	chicken
Kpnb1	-0.39	1.00000000	chicken
Acsl4	0.27	1.00000000	chicken
Eif4g2	-0.20	1.00000000	chicken
Ascc1	0.15	1.00000000	chicken
Rnf141	0.15	1.00000000	chicken
Prdx1	0.05	1.00000000	chicken
Smu1	-0.23	1.00000000	chicken
Hebp1	0.44	1.00000000	chicken
Clint1	-0.31	1.00000000	chicken
Stx17	0.07	1.00000000	chicken
Ankrd52	0.11	1.00000000	chicken
Eea1	-0.26	1.00000000	chicken
Tbk1	-0.30	1.00000000	chicken
Fam107b	0.28	1.00000000	chicken
Ilf2	-0.21	1.00000000	chicken
Taf13	-0.30	1.00000000	chicken
Pxdc1	-0.38	1.00000000	chicken
Snx3	-0.21	1.00000000	chicken
Cdc42se1	-0.38	1.00000000	chicken
Caprin1	-0.46	1.00000000	chicken
E2f4	-0.14	1.00000000	chicken
Dapk1	0.33	1.00000000	chicken

Crnkl1	-0.20	1.00000000	chicken
Kctd20	0.21	1.00000000	chicken
Cybc1	-0.25	1.00000000	chicken
Vcp	0.23	1.00000000	chicken
Smyd3	-0.40	1.00000000	chicken
Rars	-0.19	1.00000000	chicken
Tbc1d2b	-0.15	1.00000000	chicken
Psmb1	-0.17	1.00000000	chicken
Ibtk	-0.07	1.00000000	chicken
Fxr1	-0.06	1.00000000	chicken
Fadd	-0.27	1.00000000	chicken
Taf5	-0.19	1.00000000	chicken
Scamp1	-0.26	1.00000000	chicken
Iqgap1	-0.19	1.00000000	chicken
Desi2	-0.22	1.00000000	chicken
Axin1	0.12	1.00000000	chicken
Ptpn1	0.25	1.00000000	chicken
Ap3m1	-0.34	1.00000000	chicken
Commd5	-0.16	1.00000000	chicken
Nr1h3	0.17	1.00000000	chicken
Pip4k2a	-0.30	1.00000000	chicken
Pphln1	0.11	1.00000000	chicken
Rev1	-0.32	1.00000000	chicken
Nelfb	-0.25	1.00000000	chicken
Homer1	-0.33	1.00000000	chicken
Trappc11	-0.24	1.00000000	chicken
Hcls1	-0.07	1.00000000	chicken
Zc3h7a	-0.35	1.00000000	chicken
Ubqln1	0.13	1.00000000	chicken
Napepld	-0.15	1.00000000	chicken
Atp6v1c1	-0.43	1.00000000	chicken
Nono	-0.24	1.00000000	chicken
Cct5	-0.25	1.00000000	chicken
Uhrf1bp1l	0.09	1.00000000	chicken
Rchy1	-0.16	1.00000000	chicken
Nbas	-0.23	1.00000000	chicken
Gpi1	-0.22	1.00000000	chicken
Tmem223	-0.20	1.00000000	chicken
Ndufa1	-0.23	1.00000000	chicken
Ano10	-0.54	1.00000000	chicken
Nptn	0.12	1.00000000	chicken
Golga5	0.13	1.00000000	chicken
Tmem214	-0.13	1.00000000	chicken
Nckap1l	-0.21	1.00000000	chicken
Ddx27	-0.24	1.00000000	chicken
Trerf1	-0.48	1.00000000	chicken

Htatsf1	-0.37	1.00000000	chicken
Rell1	-0.26	1.00000000	chicken
Ccz1	-0.22	1.00000000	chicken
Ptdss1	0.06	1.00000000	chicken
Derl1	-0.29	1.00000000	chicken
Ttf1	-0.27	1.00000000	chicken
Eri3	0.07	1.00000000	chicken
Dynlrb1	-0.12	1.00000000	chicken
Harbi1	0.03	1.00000000	chicken
Slc38a2	0.45	1.00000000	chicken
Wdr43	0.05	1.00000000	chicken
Srpr	-0.21	1.00000000	chicken
Erbin	0.23	1.00000000	chicken
Rabl6	-0.32	1.00000000	chicken
Rcc2	-0.21	1.00000000	chicken
Hdgfl2	-0.17	1.00000000	chicken
Rbsn	-0.31	1.00000000	chicken
Polg2	0.03	1.00000000	chicken
Elmo2	-0.11	1.00000000	chicken
Chka	0.20	1.00000000	chicken
Btbd1	-0.23	1.00000000	chicken
Bcap29	-0.12	1.00000000	chicken
Wdr36	-0.25	1.00000000	chicken
Blmh	-0.17	1.00000000	chicken
Tns3	-0.25	1.00000000	chicken
Dnajb6	0.13	1.00000000	chicken
Wdfy1	0.07	1.00000000	chicken
Wdr74	-0.16	1.00000000	chicken
Runx2	-0.30	1.00000000	chicken
Cbfb	0.27	1.00000000	chicken
Prdm15	0.07	1.00000000	chicken
Pias1	-0.46	1.00000000	chicken
2210016F16Rik	-0.13	1.00000000	chicken
Cdk9	0.02	1.00000000	chicken
Usp47	0.09	1.00000000	chicken
Itfg1	-0.18	1.00000000	chicken
Bud31	-0.13	1.00000000	chicken
Tmem192	-0.16	1.00000000	chicken
Eif4h	-0.31	1.00000000	chicken
Impdh2	-0.30	1.00000000	chicken
Rac1	-0.28	1.00000000	chicken
Rbm26	-0.19	1.00000000	chicken
Hadha	-0.08	1.00000000	chicken
Anapc10	0.08	1.00000000	chicken
Wbp11	-0.28	1.00000000	chicken
Prdm10	0.04	1.00000000	chicken

Oxsr1	0.05	1.00000000	chicken
Ppil1	-0.25	1.00000000	chicken
Uqcr10	-0.22	1.00000000	chicken
Kif2a	-0.32	1.00000000	chicken
Atp6v0d1	0.08	1.00000000	chicken
Urb2	-0.11	1.00000000	chicken
Wdr20	0.15	1.00000000	chicken
Itprid2	-0.03	1.00000000	chicken
Pmm2	-0.19	1.00000000	chicken
Clcn7	0.09	1.00000000	chicken
Sub1	-0.23	1.00000000	chicken
Nr4a2	-0.38	1.00000000	chicken
Sp4	-0.28	1.00000000	chicken
Ppid	0.04	1.00000000	chicken
Adnp	-0.37	1.00000000	chicken
Senp1	0.05	1.00000000	chicken
Lpcat3	-0.16	1.00000000	chicken
Smarcd2	-0.24	1.00000000	chicken
Chd2	0.17	1.00000000	chicken
Rrbp1	-0.37	1.00000000	chicken
Gabpa	-0.42	1.00000000	chicken
Polr2m	-0.14	1.00000000	chicken
Rab22a	-0.09	1.00000000	chicken
Sbno1	0.19	1.00000000	chicken
Rnf114	-0.22	1.00000000	chicken
Pno1	0.08	1.00000000	chicken
Vcl	0.06	1.00000000	chicken
Kdm3b	0.00	1.00000000	chicken
Ctdp1	-0.38	1.00000000	chicken
Arih1	-0.41	1.00000000	chicken
Skp1	-0.03	1.00000000	chicken
Gpbp1l1	-0.34	1.00000000	chicken
Kat2b	-0.20	1.00000000	chicken
Nsfl1c	-0.12	1.00000000	chicken
Immt	-0.25	1.00000000	chicken
Pdpk1	0.07	1.00000000	chicken
St8sia4	-0.42	1.00000000	chicken
Nfyb	-0.24	1.00000000	chicken
Zfp827	-0.28	1.00000000	chicken
Sesn1	-0.47	1.00000000	chicken
Sbf1	-0.22	1.00000000	chicken
Ecpas	-0.16	1.00000000	chicken
Rbbp4	-0.29	1.00000000	chicken
Btbd9	0.11	1.00000000	chicken
Nrbf2	-0.34	1.00000000	chicken
Borcs6	-0.27	1.00000000	chicken

Rmdn3	-0.01	1.00000000	chicken
Ccnt1	0.13	1.00000000	chicken
Gabpb1	0.10	1.00000000	chicken
Larp1	0.07	1.00000000	chicken
Kpna6	0.07	1.00000000	chicken
Rlim	0.03	1.00000000	chicken
Phb2	-0.16	1.00000000	chicken
Sqle	-0.09	1.00000000	chicken
Ankrd17	-0.40	1.00000000	chicken
St14	-0.33	1.00000000	chicken
Exd2	0.18	1.00000000	chicken
Psme3ip1	-0.25	1.00000000	chicken
Hlx	-0.02	1.00000000	chicken
Hlcs	-0.25	1.00000000	chicken
Dip2c	-0.70	1.00000000	chicken
Aars	-0.14	1.00000000	chicken
Tmtc3	-0.24	1.00000000	chicken
Bcl2	-0.56	1.00000000	chicken
Tcea1	-0.25	1.00000000	chicken
Adam9	-0.10	1.00000000	chicken
Usp4	-0.06	1.00000000	chicken
Etnk1	-0.39	1.00000000	chicken
Rhoh	-0.21	1.00000000	chicken
Gnl3	-0.33	1.00000000	chicken
Aacs	0.24	1.00000000	chicken
Phf12	-0.37	1.00000000	chicken
Dvl1	0.10	1.00000000	chicken
Polr2b	-0.29	1.00000000	chicken
Gusb	-0.21	1.00000000	chicken
Leo1	0.16	1.00000000	chicken
Uchl5	-0.21	1.00000000	chicken
Pdap1	-0.20	1.00000000	chicken
Pakap	-0.22	1.00000000	chicken
Zfpm1	0.09	1.00000000	chicken
Psmc5	-0.17	1.00000000	chicken
Ngly1	0.06	1.00000000	chicken
Atrn	0.15	1.00000000	chicken
Ipo7	0.10	1.00000000	chicken
Ube2j2	-0.24	1.00000000	chicken
Clk1	-0.07	1.00000000	chicken
Nudt14	-0.31	1.00000000	chicken
Atad5	0.02	1.00000000	chicken
Snx6	-0.27	1.00000000	chicken
Cct8	-0.11	1.00000000	chicken
Trim33	0.06	1.00000000	chicken
Sos2	0.20	1.00000000	chicken

Selenof	-0.15	1.00000000	chicken
Kat7	-0.07	1.00000000	chicken
Scap	-0.26	1.00000000	chicken
Nubp1	-0.21	1.00000000	chicken
Dgkz	-0.22	1.00000000	chicken
Abracl	-0.18	1.00000000	chicken
Lrif1	-0.22	1.00000000	chicken
Tmem41b	0.05	1.00000000	chicken
Zfp143	0.12	1.00000000	chicken
Ebag9	-0.31	1.00000000	chicken
Amfr	-0.21	1.00000000	chicken
Tpm1	0.20	1.00000000	chicken
Sec11a	-0.14	1.00000000	chicken
Atg12	-0.24	1.00000000	chicken
Phpt1	-0.15	1.00000000	chicken
Car2	0.21	1.00000000	chicken
Stam2	0.03	1.00000000	chicken
Rufy1	-0.24	1.00000000	chicken
Pds5a	0.24	1.00000000	chicken
Heatr5a	-0.45	1.00000000	chicken
C1qa	0.18	1.00000000	chicken
Pard3	0.22	1.00000000	chicken
Stim2	-0.32	1.00000000	chicken
Hnrnpk	-0.27	1.00000000	chicken
Acap3	0.23	1.00000000	chicken
Arcn1	-0.18	1.00000000	chicken
Dpyd	0.22	1.00000000	chicken
Clns1a	-0.14	1.00000000	chicken
Ppif	0.24	1.00000000	chicken
Eif4a3l1	-0.29	1.00000000	chicken
Scfd1	-0.34	1.00000000	chicken
Phf10	-0.34	1.00000000	chicken
Ttc4	-0.20	1.00000000	chicken
Yaf2	0.05	1.00000000	chicken
Prkcd	0.13	1.00000000	chicken
Snu13	-0.28	1.00000000	chicken
Vma21	-0.30	1.00000000	chicken
Ddx17	0.11	1.00000000	chicken
Nasp	-0.13	1.00000000	chicken
Cnot4	0.15	1.00000000	chicken
Nab1	-0.20	1.00000000	chicken
Zcchc2	-0.23	1.00000000	chicken
Hps3	-0.26	1.00000000	chicken
Jagn1	-0.13	1.00000000	chicken
Timp2	-0.40	1.00000000	chicken
Lin52	0.16	1.00000000	chicken

Drg1	-0.21	1.00000000	chicken
Rtf1	-0.16	1.00000000	chicken
Ptges3	0.12	1.00000000	chicken
Crk	0.02	1.00000000	chicken
Ear1	0.43	1.00000000	chicken
Cmtr1	0.22	1.00000000	chicken
Eif6	0.29	1.00000000	chicken
Rad23a	-0.38	1.00000000	chicken
Epn2	0.16	1.00000000	chicken
Nup205	-0.14	1.00000000	chicken
Ppp1r1c	0.16	1.00000000	chicken
Rnf4	0.06	1.00000000	chicken
Lsm4	0.06	1.00000000	chicken
Tent4b	-0.29	1.00000000	chicken
Hira	-0.22	1.00000000	chicken
Ino80	0.04	1.00000000	chicken
Klhl13	0.33	1.00000000	chicken
Dhx38	-0.23	1.00000000	chicken
Atg101	0.08	1.00000000	chicken
Nsf	-0.17	1.00000000	chicken
Ppp2r2d	0.19	1.00000000	chicken
Ecd	-0.23	1.00000000	chicken
Wdr4	0.11	1.00000000	chicken
Elf1	0.15	1.00000000	chicken
Luc7l3	-0.03	1.00000000	chicken
Grpel1	-0.11	1.00000000	chicken
Rusc2	0.14	1.00000000	chicken
Abhd12	0.11	1.00000000	chicken
Eif4e	-0.39	1.00000000	chicken
Lonrf3	0.22	1.00000000	chicken
Pdss2	-0.26	1.00000000	chicken
Chic2	0.08	1.00000000	chicken
Clec16a	0.10	1.00000000	chicken
Ttc19	0.08	1.00000000	chicken
Arhgef6	-0.37	1.00000000	chicken
Tef	-0.25	1.00000000	chicken
Tspan5	-0.29	1.00000000	chicken
Rin3	0.15	1.00000000	chicken
Dpp7	-0.14	1.00000000	chicken
Wdr82	0.10	1.00000000	chicken
Itm2c	-0.06	1.00000000	chicken
Trps1	-0.41	1.00000000	chicken
Sf3a1	-0.20	1.00000000	chicken
Znfx1	0.10	1.00000000	chicken
Amot	-0.16	1.00000000	chicken
Dnajc15	-0.05	1.00000000	chicken

Ago4	-0.19	1.00000000	chicken
Fxyd6	-0.28	1.00000000	chicken
Nuak1	-0.24	1.00000000	chicken
Rnf216	0.03	1.00000000	chicken
Cnot6l	-0.14	1.00000000	chicken
Strada	0.09	1.00000000	chicken
Slc7a11	0.07	1.00000000	chicken
Ttc39b	0.07	1.00000000	chicken
Rad21	-0.28	1.00000000	chicken
Stau2	0.02	1.00000000	chicken
Pnrc1	-0.05	1.00000000	chicken
Cited4	0.32	1.00000000	chicken
Plod1	-0.13	1.00000000	chicken
Scyl2	-0.31	1.00000000	chicken
Wapl	-0.29	1.00000000	chicken
Fbxl17	0.02	1.00000000	chicken
Zmynd8	-0.18	1.00000000	chicken
Nol11	-0.25	1.00000000	chicken
Arntl	0.24	1.00000000	chicken
Arl6ip4	0.05	1.00000000	chicken
Sod2	-0.27	1.00000000	chicken
Stx12	0.18	1.00000000	chicken
Rbl1	-0.23	1.00000000	chicken
Eftud2	-0.13	1.00000000	chicken
Rab39b	-0.22	1.00000000	chicken
Ints2	0.19	1.00000000	chicken
Plekha1	-0.25	1.00000000	chicken
Camk2g	-0.14	1.00000000	chicken
Pmp22	-0.13	1.00000000	chicken
Ccdc126	-0.21	1.00000000	chicken
Cpeb2	0.24	1.00000000	chicken
Nup107	0.12	1.00000000	chicken
Pgrmc2	-0.23	1.00000000	chicken
Hdac1	-0.14	1.00000000	chicken
Eml3	-0.17	1.00000000	chicken
Pstpip1	-0.31	1.00000000	chicken
Dyrk2	0.07	1.00000000	chicken
Polr1e	-0.28	1.00000000	chicken
Rab31	-0.27	1.00000000	chicken
Itch	0.02	1.00000000	chicken
Ufl1	-0.26	1.00000000	chicken
Ppp1cc	-0.14	1.00000000	chicken
Arl5a	0.03	1.00000000	chicken
Hprt	-0.12	1.00000000	chicken
Supt4a	-0.14	1.00000000	chicken
Tbc1d12	-0.20	1.00000000	chicken

Cpne2	0.02	1.00000000	chicken
Lzic	-0.20	1.00000000	chicken
Gorab	0.13	1.00000000	chicken
Naa15	-0.34	1.00000000	chicken
4932411N23Rik	-0.06	1.00000000	chicken
Cebpd	0.14	1.00000000	chicken
Pex13	0.12	1.00000000	chicken
Rp2	0.07	1.00000000	chicken
Dbr1	-0.32	1.00000000	chicken
Crem	-0.18	1.00000000	chicken
Vsig4	-0.09	1.00000000	chicken
Tob2	-0.28	1.00000000	chicken
Snrpf	-0.28	1.00000000	chicken
Ubl7	-0.13	1.00000000	chicken
Trib2	0.06	1.00000000	chicken
Haus2	-0.20	1.00000000	chicken
Sertad2	0.22	1.00000000	chicken
Il15	-0.01	1.00000000	chicken
Smim19	-0.21	1.00000000	chicken
Slc16a10	-0.53	1.00000000	chicken
Tgfbr1	0.20	1.00000000	chicken
Tfpi2	0.17	1.00000000	chicken
Paip2	-0.26	1.00000000	chicken
Etf1	-0.31	1.00000000	chicken
Tnfaip8	-0.08	1.00000000	chicken
Atad1	-0.14	1.00000000	chicken
Bend7	0.31	1.00000000	chicken
Nxt2	-0.20	1.00000000	chicken
Bbx	-0.40	1.00000000	chicken
Fam177a	-0.25	1.00000000	chicken
Rpf2	0.11	1.00000000	chicken
N4bp1	-0.50	1.00000000	chicken
Sp1	0.06	1.00000000	chicken
Snx5	0.09	1.00000000	chicken
Akap1	-0.19	1.00000000	chicken
Bcl9l	-0.12	1.00000000	chicken
Tor1a	-0.11	1.00000000	chicken
Ltf	-0.09	1.00000000	chicken
5730455P16Rik	-0.17	1.00000000	chicken
Slc2a9	0.32	1.00000000	chicken
Chd5	-0.08	1.00000000	chicken
Ccnc	-0.08	1.00000000	chicken
Rab11fip4	-0.30	1.00000000	chicken
Naa40	-0.02	1.00000000	chicken
Helb	0.00	1.00000000	chicken
Ccnyl1	0.08	1.00000000	chicken

Hdac7	-0.22	1.00000000	chicken
Psme4	0.28	1.00000000	chicken
Pcgf6	-0.18	1.00000000	chicken
Pcna	-0.11	1.00000000	chicken
Vps53	0.02	1.00000000	chicken
Gcc2	0.02	1.00000000	chicken
Vapa	-0.31	1.00000000	chicken
Dlgap4	0.05	1.00000000	chicken
Cotl1	0.37	1.00000000	chicken
Ikbkb	-0.01	1.00000000	chicken
Pbx1	0.08	1.00000000	chicken
Nfat5	0.06	1.00000000	chicken
Serinc1	0.18	1.00000000	chicken
Btf3l4	-0.21	1.00000000	chicken
C2cd2l	-0.17	1.00000000	chicken
Pcnp	-0.23	1.00000000	chicken
Btbd10	-0.17	1.00000000	chicken
Adam10	-0.21	1.00000000	chicken
Kdm3a	-0.02	1.00000000	chicken
Nploc4	0.07	1.00000000	chicken
Ncor2	-0.21	1.00000000	chicken
Vps37b	0.06	1.00000000	chicken
Rabggtb	0.02	1.00000000	chicken
Smc6	0.07	1.00000000	chicken
Hivep2	0.15	1.00000000	chicken
Lyn	0.12	1.00000000	chicken
Ddx1	-0.22	1.00000000	chicken
Lactb	-0.19	1.00000000	chicken
Pcm1	0.00	1.00000000	chicken
Stx7	-0.30	1.00000000	chicken
Rbpj	-0.22	1.00000000	chicken
Kif21a	-0.01	1.00000000	chicken
Ptpn11	-0.22	1.00000000	chicken
Eef1e1	-0.24	1.00000000	chicken
Id2	0.05	1.00000000	chicken
Lrp1	-0.22	1.00000000	chicken
Rmrp	0.01	1.00000000	chicken
Oga	-0.04	1.00000000	chicken
Sars	-0.24	1.00000000	chicken
Crygn	0.09	1.00000000	chicken
Rbm34	-0.26	1.00000000	chicken
Eif3j2	0.02	1.00000000	chicken
Dynlt1a	0.14	1.00000000	chicken
Nolc1	-0.18	1.00000000	chicken
Ezr	0.12	1.00000000	chicken
Poldip3	0.14	1.00000000	chicken

Sel1l3	-0.15	1.00000000	chicken
Gspt1	-0.37	1.00000000	chicken
Drg2	-0.11	1.00000000	chicken
Srrm1	-0.29	1.00000000	chicken
Slc9a6	-0.21	1.00000000	chicken
Dctn3	-0.13	1.00000000	chicken
Rsbn1l	-0.06	1.00000000	chicken
Rc3h1	-0.29	1.00000000	chicken
Rnf150	-0.21	1.00000000	chicken
Ddx42	-0.16	1.00000000	chicken
Cebpg	-0.32	1.00000000	chicken
Notch2	-0.23	1.00000000	chicken
Wfs1	0.08	1.00000000	chicken
Tmem229b	0.07	1.00000000	chicken
Cdkn1b	-0.45	1.00000000	chicken
Nmt2	-0.21	1.00000000	chicken
Sem1	0.23	1.00000000	chicken
Phip	-0.04	1.00000000	chicken
Tnks1bp1	0.06	1.00000000	chicken
Srsf3	0.09	1.00000000	chicken
Rap2b	0.20	1.00000000	chicken
Rgs13	0.09	1.00000000	chicken
Stx2	0.02	1.00000000	chicken
Ep300	-0.32	1.00000000	chicken
Zbed4	0.01	1.00000000	chicken
Snx13	-0.15	1.00000000	chicken
Prpf6	-0.21	1.00000000	chicken
Tardbp	0.06	1.00000000	chicken
Uqcc2	0.10	1.00000000	chicken
Syap1	0.17	1.00000000	chicken
Srgap3	-0.24	1.00000000	chicken
Psmc3	-0.20	1.00000000	chicken
Ppie	-0.13	1.00000000	chicken
Fam13b	0.02	1.00000000	chicken
Sec22a	0.02	1.00000000	chicken
Atp11b	-0.36	1.00000000	chicken
Papola	0.04	1.00000000	chicken
Dym	0.08	1.00000000	chicken
Ulk2	0.07	1.00000000	chicken
Eif2ak1	-0.02	1.00000000	chicken
Ptgdr	-0.10	1.00000000	chicken
Srp19	-0.16	1.00000000	chicken
Tiam1	0.33	1.00000000	chicken
Bdh2	0.05	1.00000000	chicken
Acbd6	-0.08	1.00000000	chicken
Kif1b	-0.06	1.00000000	chicken

Wdfy3	-0.09	1.00000000	chicken
Cpsf3	-0.01	1.00000000	chicken
Rprd1b	-0.27	1.00000000	chicken
Ankrd28	-0.05	1.00000000	chicken
Dtd1	-0.15	1.00000000	chicken
Vps41	-0.17	1.00000000	chicken
Cnot10	0.00	1.00000000	chicken
Cfdp1	0.04	1.00000000	chicken
Rnaseh1	-0.25	1.00000000	chicken
Cwc25	0.02	1.00000000	chicken
Rgs14	-0.06	1.00000000	chicken
Gk5	-0.18	1.00000000	chicken
Itsn1	-0.09	1.00000000	chicken
Rbmxl1	-0.41	1.00000000	chicken
Glrx5	-0.24	1.00000000	chicken
Adrb2	-0.22	1.00000000	chicken
Rab20	-0.41	1.00000000	chicken
Ropn1l	0.01	1.00000000	chicken
Plin4	-0.13	1.00000000	chicken
Sclt1	-0.31	1.00000000	chicken
Emc6	-0.10	1.00000000	chicken
Srsf2	-0.01	1.00000000	chicken
1110059G10Rik	0.06	1.00000000	chicken
Txlna	-0.28	1.00000000	chicken
Atl2	-0.34	1.00000000	chicken
Lhfpl2	0.23	1.00000000	chicken
Ccndbp1	-0.21	1.00000000	chicken
Cyb5a	0.01	1.00000000	chicken
Polr2k	0.03	1.00000000	chicken
Alox5	0.06	1.00000000	chicken
Tcf25	-0.35	1.00000000	chicken
Eif2b4	-0.19	1.00000000	chicken
Eepd1	-0.25	1.00000000	chicken
Usp24	0.05	1.00000000	chicken
Polr2c	-0.11	1.00000000	chicken
Asap1	-0.17	1.00000000	chicken
Wsb1	0.02	1.00000000	chicken
Sod1	0.12	1.00000000	chicken
Dnajc7	-0.21	1.00000000	chicken
Smad2	-0.43	1.00000000	chicken
Tspan3	-0.29	1.00000000	chicken
Scaper	-0.07	1.00000000	chicken
Armc8	0.06	1.00000000	chicken
Elp4	-0.01	1.00000000	chicken
N4bp2l2	0.00	1.00000000	chicken
Slc13a1	0.24	1.00000000	chicken

Prdm2	0.03	1.00000000	chicken
Commd2	-0.37	1.00000000	chicken
Cplane2	-0.04	1.00000000	chicken
Zdhhc3	-0.19	1.00000000	chicken
Gnpnat1	-0.10	1.00000000	chicken
Efhc1	-0.24	1.00000000	chicken
Pus7l	-0.04	1.00000000	chicken
Acbd3	-0.18	1.00000000	chicken
Tut7	-0.04	1.00000000	chicken
Ttll5	0.54	1.00000000	chicken
Eloc	0.00	1.00000000	chicken
Wipi1	-0.12	1.00000000	chicken
Zfyve21	-0.20	1.00000000	chicken
Sf3a2	-0.11	1.00000000	chicken
Pfdn6	0.06	1.00000000	chicken
Smndc1	-0.14	1.00000000	chicken
Tbp	-0.20	1.00000000	chicken
Fastk	-0.14	1.00000000	chicken
Ppp1r13b	0.01	1.00000000	chicken
Ndufs6	-0.25	1.00000000	chicken
Yeats2	-0.25	1.00000000	chicken
Ctr9	-0.17	1.00000000	chicken
Ormdl2	-0.07	1.00000000	chicken
Rfk	-0.13	1.00000000	chicken
Sar1b	-0.01	1.00000000	chicken
Extl3	-0.09	1.00000000	chicken
Tmod3	-0.24	1.00000000	chicken
Trip12	0.01	1.00000000	chicken
Usp19	-0.11	1.00000000	chicken
Smim7	-0.31	1.00000000	chicken
Nkrf	-0.10	1.00000000	chicken
Rpa1	-0.10	1.00000000	chicken
8030462N17Rik	0.07	1.00000000	chicken
Fbxo45	-0.21	1.00000000	chicken
Ppp1r16b	-0.11	1.00000000	chicken
Sacm1l	0.08	1.00000000	chicken
Pgap2	0.14	1.00000000	chicken
Tfdp1	-0.17	1.00000000	chicken
Spg11	-0.16	1.00000000	chicken
Ankhd1	0.14	1.00000000	chicken
Timm50	0.07	1.00000000	chicken
Arfgef2	-0.17	1.00000000	chicken
Dld	-0.11	1.00000000	chicken
Fh1	-0.16	1.00000000	chicken
Dcun1d3	0.06	1.00000000	chicken
Optn	0.10	1.00000000	chicken

Ppp2r5a	0.25	1.00000000	chicken
Slc35b2	-0.30	1.00000000	chicken
Acaca	0.20	1.00000000	chicken
Il18	0.17	1.00000000	chicken
Frs2	0.10	1.00000000	chicken
Tuba1b	-0.34	1.00000000	chicken
Ddx52	0.02	1.00000000	chicken
Nhsl1	0.03	1.00000000	chicken
Sltm	-0.15	1.00000000	chicken
Ptprc	-0.13	1.00000000	chicken
Txn2	-0.13	1.00000000	chicken
Exoc2	0.12	1.00000000	chicken
Dcaf5	-0.08	1.00000000	chicken
Slc25a13	-0.01	1.00000000	chicken
Pola1	-0.49	1.00000000	chicken
Stat5b	-0.08	1.00000000	chicken
Nr3c1	0.08	1.00000000	chicken
Ahctf1	0.03	1.00000000	chicken
Tns1	0.10	1.00000000	chicken
Sfpq	-0.30	1.00000000	chicken
Itpkb	-0.05	1.00000000	chicken
Zfp618	-0.31	1.00000000	chicken
Tubb5	0.03	1.00000000	chicken
Nifk	-0.07	1.00000000	chicken
Ap1g1	0.01	1.00000000	chicken
Atg3	-0.17	1.00000000	chicken
Rpap2	0.07	1.00000000	chicken
Pwwp2a	0.18	1.00000000	chicken
Usp37	-0.18	1.00000000	chicken
BC051665	0.20	1.00000000	chicken
Acp2	0.18	1.00000000	chicken
Pus10	-0.04	1.00000000	chicken
Prpf8	-0.19	1.00000000	chicken
Nop2	-0.07	1.00000000	chicken
Tax1bp3	-0.28	1.00000000	chicken
Ldah	-0.26	1.00000000	chicken
Ttc27	0.14	1.00000000	chicken
Rpa3	-0.14	1.00000000	chicken
Vti1b	0.09	1.00000000	chicken
Rrp15	0.01	1.00000000	chicken
Dusp10	0.06	1.00000000	chicken
Tmem167	-0.14	1.00000000	chicken
Lrrc59	-0.18	1.00000000	chicken
Atg5	-0.09	1.00000000	chicken
Chchd10	-0.44	1.00000000	chicken
Fbxw7	-0.44	1.00000000	chicken

Strap	-0.06	1.00000000	chicken
Trappc12	-0.23	1.00000000	chicken
Rab33b	-0.15	1.00000000	chicken
Pcid2	-0.24	1.00000000	chicken
Nhp2	-0.05	1.00000000	chicken
Pafah1b1	0.07	1.00000000	chicken
Spire1	-0.15	1.00000000	chicken
Nup98	-0.25	1.00000000	chicken
Pten	-0.15	1.00000000	chicken
Arhgef18	0.01	1.00000000	chicken
Ufd1	-0.25	1.00000000	chicken
Rnh1	-0.35	1.00000000	chicken
P4ha1	-0.09	1.00000000	chicken
Cemip2	0.04	1.00000000	chicken
Cul2	0.01	1.00000000	chicken
Thoc1	-0.24	1.00000000	chicken
Iars	-0.27	1.00000000	chicken
Etfa	-0.16	1.00000000	chicken
Aqr	-0.18	1.00000000	chicken
Ppp6r2	0.00	1.00000000	chicken
Utp11	-0.22	1.00000000	chicken
Adrm1	0.09	1.00000000	chicken
Bdp1	-0.01	1.00000000	chicken
Gpat3	-0.01	1.00000000	chicken
Prnp	-0.14	1.00000000	chicken
Cramp1	-0.01	1.00000000	chicken
Dhx8	-0.09	1.00000000	chicken
Adipor1	-0.17	1.00000000	chicken
Get4	-0.19	1.00000000	chicken
Fez2	0.17	1.00000000	chicken
Phax	-0.15	1.00000000	chicken
Lypla1	-0.13	1.00000000	chicken
Carnmt1	0.11	1.00000000	chicken
Sppl2a	-0.07	1.00000000	chicken
Txnrd3	-0.10	1.00000000	chicken
Eif2b5	0.07	1.00000000	chicken
Alg12	-0.08	1.00000000	chicken
Ufm1	-0.17	1.00000000	chicken
Exoc8	-0.14	1.00000000	chicken
Klhl5	0.02	1.00000000	chicken
Arfip1	-0.24	1.00000000	chicken
Arl8b	-0.10	1.00000000	chicken
Arfgap2	-0.09	1.00000000	chicken
Psen1	0.03	1.00000000	chicken
Aldoc	0.21	1.00000000	chicken
Rexo2	-0.51	1.00000000	chicken

Ccdc186	0.06	1.00000000	chicken
Tmbim1	0.09	1.00000000	chicken
Jade3	-0.02	1.00000000	chicken
Tex10	0.05	1.00000000	chicken
Ubap2l	0.13	1.00000000	chicken
Irf2bp2	-0.22	1.00000000	chicken
Gyg	-0.04	1.00000000	chicken
Brix1	-0.05	1.00000000	chicken
Fancd2	0.02	1.00000000	chicken
Fbxo8	-0.02	1.00000000	chicken
Cd99l2	-0.18	1.00000000	chicken
Rab43	-0.24	1.00000000	chicken
Strn	-0.13	1.00000000	chicken
Snrpb2	-0.18	1.00000000	chicken
Psmg2	-0.04	1.00000000	chicken
Dok1	-0.24	1.00000000	chicken
Tnks2	0.10	1.00000000	chicken
Naif1	-0.19	1.00000000	chicken
Ahdc1	-0.18	1.00000000	chicken
Tmem70	-0.10	1.00000000	chicken
Siah1b	-0.35	1.00000000	chicken
Nrf1	-0.20	1.00000000	chicken
Snrpg	0.12	1.00000000	chicken
Tubb2a	-0.07	1.00000000	chicken
Senp7	-0.44	1.00000000	chicken
Pank3	0.10	1.00000000	chicken
Cbwd1	0.14	1.00000000	chicken
Pfdn5	-0.22	1.00000000	chicken
Aimp1	-0.20	1.00000000	chicken
Twf2	-0.30	1.00000000	chicken
Eif2ak4	-0.16	1.00000000	chicken
Acvrl1	-0.11	1.00000000	chicken
Ctsk	-0.42	1.00000000	chicken
Arsb	-0.08	1.00000000	chicken
Lin7c	-0.07	1.00000000	chicken
Hsf1	-0.13	1.00000000	chicken
Hgs	0.05	1.00000000	chicken
Nol7	-0.20	1.00000000	chicken
Senp6	0.01	1.00000000	chicken
Ap2a1	-0.21	1.00000000	chicken
Ubox5	0.07	1.00000000	chicken
Btaf1	0.01	1.00000000	chicken
Pnpt1	-0.16	1.00000000	chicken
Fbxl22	-0.01	1.00000000	chicken
Plxnb2	0.35	1.00000000	chicken
Cntrl	-0.38	1.00000000	chicken

Tmem230	-0.15	1.00000000	chicken
Dctn6	-0.05	1.00000000	chicken
Vamp4	-0.11	1.00000000	chicken
Tmem53	-0.10	1.00000000	chicken
Bud13	0.04	1.00000000	chicken
Eif2s1	-0.24	1.00000000	chicken
Tubgcp3	0.00	1.00000000	chicken
Abi1	-0.03	1.00000000	chicken
Plcl2	0.01	1.00000000	chicken
Lcor	0.12	1.00000000	chicken
Eapp	-0.05	1.00000000	chicken
Edc4	0.12	1.00000000	chicken
Clasp1	0.15	1.00000000	chicken
Syngr2	-0.02	1.00000000	chicken
Ern1	-0.06	1.00000000	chicken
BC048403	-0.15	1.00000000	chicken
Agps	0.09	1.00000000	chicken
Abcd3	0.08	1.00000000	chicken
Ecsit	0.08	1.00000000	chicken
Commd10	0.18	1.00000000	chicken
Slc25a1	-0.12	1.00000000	chicken
Gnb4	-0.04	1.00000000	chicken
Ap1ar	-0.20	1.00000000	chicken
Whamm	0.00	1.00000000	chicken
Ran	-0.03	1.00000000	chicken
Arl8a	-0.20	1.00000000	chicken
Slc35e3	-0.18	1.00000000	chicken
Paxip1	0.03	1.00000000	chicken
Cct2	-0.20	1.00000000	chicken
Fbrsl1	-0.11	1.00000000	chicken
Rsu1	-0.23	1.00000000	chicken
Abcf2	-0.16	1.00000000	chicken
Ctsb	-0.06	1.00000000	chicken
Pdk4	0.27	1.00000000	chicken
Washc4	-0.13	1.00000000	chicken
Hmgcr	-0.31	1.00000000	chicken
Ube2d1	-0.01	1.00000000	chicken
Gpcpd1	-0.08	1.00000000	chicken
Atxn2	0.01	1.00000000	chicken
Tbck	-0.16	1.00000000	chicken
Pxylp1	0.38	1.00000000	chicken
Psmd9	-0.18	1.00000000	chicken
Steep1	0.03	1.00000000	chicken
Rit1	-0.22	1.00000000	chicken
Cops4	0.11	1.00000000	chicken
Pogz	0.07	1.00000000	chicken

Usp7	-0.29	1.00000000	chicken
Zmpste24	-0.11	1.00000000	chicken
Sppl3	-0.07	1.00000000	chicken
Cdca4	-0.25	1.00000000	chicken
Wdr44	-0.21	1.00000000	chicken
Far1	-0.31	1.00000000	chicken
Il2rb	-0.18	1.00000000	chicken
Akr1a1	0.05	1.00000000	chicken
Ubac2	-0.08	1.00000000	chicken
Cmip	0.08	1.00000000	chicken
Trmt10c	-0.24	1.00000000	chicken
Tk2	0.00	1.00000000	chicken
Sec22b	-0.15	1.00000000	chicken
Surf6	0.14	1.00000000	chicken
Arhgef3	-0.33	1.00000000	chicken
Nipal3	0.10	1.00000000	chicken
Akap7	0.09	1.00000000	chicken
Ctnnb1	-0.14	1.00000000	chicken
Snx4	-0.09	1.00000000	chicken
Rnf185	-0.04	1.00000000	chicken
Cyb5r2	0.05	1.00000000	chicken
Smurf2	0.06	1.00000000	chicken
Nudcd3	-0.10	1.00000000	chicken
Glg1	-0.21	1.00000000	chicken
Eefsec	0.16	1.00000000	chicken
Trrap	0.03	1.00000000	chicken
Arfip2	-0.13	1.00000000	chicken
Fermt2	-0.13	1.00000000	chicken
Btrc	-0.17	1.00000000	chicken
Yars	-0.07	1.00000000	chicken
Vps11	-0.28	1.00000000	chicken
Ppp1r9a	-0.22	1.00000000	chicken
Plek	0.19	1.00000000	chicken
Commd8	0.00	1.00000000	chicken
Eif2s2	-0.05	1.00000000	chicken
Hdac3	-0.06	1.00000000	chicken
Cpne1	0.09	1.00000000	chicken
Nipsnap2	-0.04	1.00000000	chicken
Abhd2	0.15	1.00000000	chicken
Nbr1	-0.03	1.00000000	chicken
Ing4	0.00	1.00000000	chicken
Zfp639	0.03	1.00000000	chicken
Sart3	0.17	1.00000000	chicken
Wdr26	-0.07	1.00000000	chicken
Bap1	-0.11	1.00000000	chicken
Igf2r	-0.22	1.00000000	chicken

Usp10	0.06	1.00000000	chicken
Exoc5	0.13	1.00000000	chicken
Ppp1r8	0.05	1.00000000	chicken
Psmd7	0.02	1.00000000	chicken
Srbd1	0.12	1.00000000	chicken
Ptpn22	-0.38	1.00000000	chicken
E2f6	-0.09	1.00000000	chicken
Plcxd1	-0.14	1.00000000	chicken
Creb3	0.17	1.00000000	chicken
Pfn2	-0.18	1.00000000	chicken
Arhgap24	0.36	1.00000000	chicken
Srp9	-0.06	1.00000000	chicken
Psmb7	-0.21	1.00000000	chicken
Rbm31y	-0.21	1.00000000	chicken
Aig1	-0.14	1.00000000	chicken
Nap1l4	-0.26	1.00000000	chicken
Naa30	-0.26	1.00000000	chicken
Clk2	-0.03	1.00000000	chicken
Rnf6	-0.03	1.00000000	chicken
Tuba1a	-0.39	1.00000000	chicken
Zfp236	-0.05	1.00000000	chicken
Cdc42se2	0.01	1.00000000	chicken
Klhl24	-0.26	1.00000000	chicken
Upf3b	-0.24	1.00000000	chicken
Nr4a1	-0.05	1.00000000	chicken
Polr1d	-0.02	1.00000000	chicken
Tlr2	-0.02	1.00000000	chicken
Gtf2h1	0.02	1.00000000	chicken
Kif5c	-0.31	1.00000000	chicken
Brox	0.04	1.00000000	chicken
Cass4	-0.09	1.00000000	chicken
Vps35	-0.02	1.00000000	chicken
Dnajc5	0.03	1.00000000	chicken
Pik3ca	-0.19	1.00000000	chicken
Fbxo30	0.02	1.00000000	chicken
Fubp1	0.10	1.00000000	chicken
Pemt	-0.08	1.00000000	chicken
Slc22a23	-0.19	1.00000000	chicken
A1bg	-0.09	1.00000000	chicken
Phf3	-0.26	1.00000000	chicken
Ctnna1	0.00	1.00000000	chicken
Sarnp	-0.13	1.00000000	chicken
Slc4a2	-0.23	1.00000000	chicken
Carmil1	0.21	1.00000000	chicken
Casc3	0.06	1.00000000	chicken
Ctnnbl1	-0.15	1.00000000	chicken

Ptpn4	-0.28	1.00000000	chicken
4921524J17Rik	-0.03	1.00000000	chicken
Ate1	-0.18	1.00000000	chicken
Arpc3	-0.10	1.00000000	chicken
Iars2	-0.11	1.00000000	chicken
Atp6v0a2	-0.20	1.00000000	chicken
Slirp	-0.04	1.00000000	chicken
Spopl	-0.20	1.00000000	chicken
Tmem185a	-0.27	1.00000000	chicken
Cdk8	-0.06	1.00000000	chicken
Spag9	-0.20	1.00000000	chicken
Stub1	-0.05	1.00000000	chicken
Glt1d1	-0.34	1.00000000	chicken
Cbl	0.02	1.00000000	chicken
Kansl3	-0.13	1.00000000	chicken
Psmb3	-0.03	1.00000000	chicken
Thoc2	-0.25	1.00000000	chicken
Ercc6	-0.25	1.00000000	chicken
Ythdf2	-0.20	1.00000000	chicken
Lemd2	-0.17	1.00000000	chicken
Wdr91	-0.21	1.00000000	chicken
Gzf1	-0.17	1.00000000	chicken
Rap1b	0.10	1.00000000	chicken
Cyp3a57	0.27	1.00000000	chicken
Zfand4	-0.16	1.00000000	chicken
Pik3cg	-0.03	1.00000000	chicken
Wbp2nl	-0.22	1.00000000	chicken
Lcp1	-0.21	1.00000000	chicken
Isca1	-0.29	1.00000000	chicken
Hipk1	0.06	1.00000000	chicken
Ost4	0.30	1.00000000	chicken
Rftn1	0.15	1.00000000	chicken
Scpep1	-0.26	1.00000000	chicken
Tubb6	-0.24	1.00000000	chicken
Pcbp2	-0.25	1.00000000	chicken
Gadd45a	0.15	1.00000000	chicken
Gtdc1	0.03	1.00000000	chicken
Cul4b	-0.29	1.00000000	chicken
Pcnx	0.05	1.00000000	chicken
Litaf	0.01	1.00000000	chicken
Acvr2a	0.14	1.00000000	chicken
Nfe2l1	-0.02	1.00000000	chicken
Acot8	-0.09	1.00000000	chicken
Tollip	-0.13	1.00000000	chicken
Chchd1	-0.14	1.00000000	chicken
Cops5	-0.18	1.00000000	chicken

Arhgap10	0.21	1.00000000	chicken
Kdm7a	-0.32	1.00000000	chicken
Pdk3	-0.09	1.00000000	chicken
Snrpb	-0.09	1.00000000	chicken
Npepl1	-0.10	1.00000000	chicken
Klhl36	0.00	1.00000000	chicken
Hook3	-0.10	1.00000000	chicken
Esyt2	-0.23	1.00000000	chicken
Ola1	0.12	1.00000000	chicken
Ncoa6	0.02	1.00000000	chicken
Azi2	-0.27	1.00000000	chicken
Phb	-0.06	1.00000000	chicken
Sdha	-0.10	1.00000000	chicken
Cyb5b	-0.05	1.00000000	chicken
Cmss1	-0.12	1.00000000	chicken
Ftsj3	0.23	1.00000000	chicken
Dclk3	0.38	1.00000000	chicken
Rwdd1	-0.16	1.00000000	chicken
Nob1	-0.15	1.00000000	chicken
Agap1	0.04	1.00000000	chicken
Capza2	-0.11	1.00000000	chicken
Xrn1	-0.10	1.00000000	chicken
Tbc1d25	0.05	1.00000000	chicken
Dop1b	-0.07	1.00000000	chicken
Riok1	-0.06	1.00000000	chicken
Nfs1	0.04	1.00000000	chicken
St7	-0.59	1.00000000	chicken
U2af1	-0.21	1.00000000	chicken
Rptor	0.04	1.00000000	chicken
Zeb1	-0.02	1.00000000	chicken
Snx2	-0.16	1.00000000	chicken
Dcp2	-0.27	1.00000000	chicken
Prr5	0.17	1.00000000	chicken
Brd7	-0.22	1.00000000	chicken
Ubr5	-0.03	1.00000000	chicken
Parp8	0.05	1.00000000	chicken
Cilk1	0.03	1.00000000	chicken
Rbm45	-0.09	1.00000000	chicken
S100a10	-0.08	1.00000000	chicken
Ankrd11	0.09	1.00000000	chicken
Atp13a3	0.01	1.00000000	chicken
Zcrb1	-0.25	1.00000000	chicken
Brd1	-0.30	1.00000000	chicken
Poldip2	-0.07	1.00000000	chicken
Stk40	-0.82	1.00000000	chicken
Ccdc91	0.00	1.00000000	chicken

Fn1	-0.83	1.00000000	chicken
Qser1	-0.18	1.00000000	chicken
Fam8a1	-0.25	1.00000000	chicken
Fbxw11	-0.06	1.00000000	chicken
Smpdl3a	0.13	1.00000000	chicken
Hspa5	0.11	1.00000000	chicken
Luc7l2	0.01	1.00000000	chicken
Srebf1	0.07	1.00000000	chicken
Ell	-0.04	1.00000000	chicken
Hic1	0.21	1.00000000	chicken
Timp3	-0.03	1.00000000	chicken
Tnfrsf1a	-0.10	1.00000000	chicken
Stard5	0.02	1.00000000	chicken
Dcp1a	-0.06	1.00000000	chicken
Rcan1	-0.03	1.00000000	chicken
Rnf13	0.11	1.00000000	chicken
Klhdc4	-0.04	1.00000000	chicken
Kpna4	-0.20	1.00000000	chicken
Cop1	-0.28	1.00000000	chicken
Thap4	0.03	1.00000000	chicken
Dcun1d1	-0.05	1.00000000	chicken
Tcirg1	0.15	1.00000000	chicken
Sucla2	-0.06	1.00000000	chicken
Slc7a3	0.16	1.00000000	chicken
Cpeb4	-0.02	1.00000000	chicken
Arl4a	-0.11	1.00000000	chicken
Rab6a	0.05	1.00000000	chicken
Esf1	0.12	1.00000000	chicken
Il13ra2	-0.08	1.00000000	chicken
Csnk1a1	0.18	1.00000000	chicken
Qars	-0.08	1.00000000	chicken
Rimklb	-0.19	1.00000000	chicken
Arhgap5	-0.27	1.00000000	chicken
C1qc	-0.11	1.00000000	chicken
Szrd1	0.00	1.00000000	chicken
Ezh2	-0.20	1.00000000	chicken
Cbll1	-0.18	1.00000000	chicken
Ric1	-0.17	1.00000000	chicken
Bclaf3	-0.26	1.00000000	chicken
Tsn	-0.17	1.00000000	chicken
Rsrp1	-0.28	1.00000000	chicken
Blnk	-0.10	1.00000000	chicken
Hnrnph3	-0.30	1.00000000	chicken
Zzef1	0.01	1.00000000	chicken
Tes	0.09	1.00000000	chicken
Prmt3	0.04	1.00000000	chicken

Ddx24	-0.10	1.00000000	chicken
Cyb5r4	-0.03	1.00000000	chicken
Arhgap27	0.08	1.00000000	chicken
Stk11	-0.23	1.00000000	chicken
Cnih1	-0.09	1.00000000	chicken
Gab3	0.09	1.00000000	chicken
Pkm	0.28	1.00000000	chicken
Ing3	-0.08	1.00000000	chicken
Srf	-0.13	1.00000000	chicken
Prpf4b	-0.15	1.00000000	chicken
Psmc1	-0.17	1.00000000	chicken
Arhgap25	0.02	1.00000000	chicken
Yeats4	-0.16	1.00000000	chicken
Ube2o	-0.13	1.00000000	chicken
Kpna3	-0.13	1.00000000	chicken
Aoah	0.12	1.00000000	chicken
Pudp	0.11	1.00000000	chicken
Fars2	-0.25	1.00000000	chicken
Nol10	-0.19	1.00000000	chicken
Ints13	-0.11	1.00000000	chicken
Cpsf4l	-0.04	1.00000000	chicken
Cactin	-0.24	1.00000000	chicken
Stag2	-0.20	1.00000000	chicken
Lrpprc	-0.14	1.00000000	chicken
BC048507	0.24	1.00000000	chicken
Dph3	-0.21	1.00000000	chicken
Tnip1	0.08	1.00000000	chicken
Nek9	0.06	1.00000000	chicken
Paf1	-0.02	1.00000000	chicken
Atad3a	0.00	1.00000000	chicken
Acbd5	-0.18	1.00000000	chicken
Psmb6	-0.19	1.00000000	chicken
Crcp	-0.09	1.00000000	chicken
Imp4	0.11	1.00000000	chicken
Herc2	-0.41	1.00000000	chicken
Ncbp3	0.06	1.00000000	chicken
Hnrnpul1	-0.04	1.00000000	chicken
Fli1	0.28	1.00000000	chicken
Ext1	-0.23	1.00000000	chicken
Sptlc2	-0.21	1.00000000	chicken
Slu7	-0.18	1.00000000	chicken
Tra2a	-0.07	1.00000000	chicken
Hspa4	-0.16	1.00000000	chicken
Decr1	0.06	1.00000000	chicken
Hif1an	-0.18	1.00000000	chicken
Napg	-0.13	1.00000000	chicken

Zyx	-0.03	1.00000000	chicken
Copz1	-0.24	1.00000000	chicken
Chfr	0.04	1.00000000	chicken
Anapc13	-0.10	1.00000000	chicken
Cmtm7	0.15	1.00000000	chicken
Sdc1	-0.16	1.00000000	chicken
Crebbp	-0.19	1.00000000	chicken
Tdp2	-0.01	1.00000000	chicken
Limk1	-0.07	1.00000000	chicken
Fabp3	0.71	1.00000000	chicken
Il16	0.03	1.00000000	chicken
Fyttd1	-0.11	1.00000000	chicken
Nxpe3	-0.02	1.00000000	chicken
Plbd1	-0.07	1.00000000	chicken
Abhd18	0.00	1.00000000	chicken
Dis3l2	-0.22	1.00000000	chicken
Sec16a	-0.23	1.00000000	chicken
Sacs	0.12	1.00000000	chicken
Tspan14	0.13	1.00000000	chicken
Hspa4l	-0.10	1.00000000	chicken
Il6st	-0.03	1.00000000	chicken
Cdc40	-0.17	1.00000000	chicken
Efl1	-0.27	1.00000000	chicken
Phaf1	-0.04	1.00000000	chicken
Uso1	-0.14	1.00000000	chicken
Eif2a	-0.05	1.00000000	chicken
Nrp2	0.17	1.00000000	chicken
Zfp318	-0.16	1.00000000	chicken
Smg7	0.03	1.00000000	chicken
Rreb1	-0.04	1.00000000	chicken
St8sia1	-0.51	1.00000000	chicken
Bcl2l1	-0.17	1.00000000	chicken
Ifi35	0.09	1.00000000	chicken
Actr6	-0.20	1.00000000	chicken
Ppp2r2a	-0.01	1.00000000	chicken
Blk	-0.19	1.00000000	chicken
Rnmt	-0.14	1.00000000	chicken
Fam133b	-0.32	1.00000000	chicken
Eipr1	-0.27	1.00000000	chicken
Cep43	-0.01	1.00000000	chicken
Srxn1	0.04	1.00000000	chicken
Rundc1	-0.21	1.00000000	chicken
Vac14	0.08	1.00000000	chicken
Asxl2	0.01	1.00000000	chicken
Arhgap18	-0.21	1.00000000	chicken
Dnajc16	-0.09	1.00000000	chicken

Zfp407	-0.25	1.00000000	chicken
Wdr70	-0.10	1.00000000	chicken
Taf15	0.04	1.00000000	chicken
Sgms2	-0.27	1.00000000	chicken
Dalrd3	0.07	1.00000000	chicken
Ide	-0.06	1.00000000	chicken
Prpf3	0.03	1.00000000	chicken
Dapk2	-0.19	1.00000000	chicken
Rsl24d1	0.15	1.00000000	chicken
Dnaja2	-0.22	1.00000000	chicken
S100a6	-0.36	1.00000000	chicken
Ttyh3	-0.37	1.00000000	chicken
Necap2	0.06	1.00000000	chicken
Arid5a	-0.10	1.00000000	chicken
Jmjd1c	0.15	1.00000000	chicken
Inpp4a	-0.17	1.00000000	chicken
Snapin	-0.02	1.00000000	chicken
Gtpbp4	0.02	1.00000000	chicken
Slc39a9	-0.24	1.00000000	chicken
Strip1	-0.07	1.00000000	chicken
Fbxo9	-0.12	1.00000000	chicken
Nudc	0.18	1.00000000	chicken
Prkrip1	-0.10	1.00000000	chicken
Atp2a2	-0.10	1.00000000	chicken
C3	-0.06	1.00000000	chicken
Ube2g1	-0.02	1.00000000	chicken
Lyrm1	0.06	1.00000000	chicken
Tbc1d5	-0.13	1.00000000	chicken
Cdk19	-0.19	1.00000000	chicken
Brpf3	0.00	1.00000000	chicken
Trappc8	-0.17	1.00000000	chicken
Polr2f	0.04	1.00000000	chicken
Ppa1	-0.09	1.00000000	chicken
Lmna	-0.20	1.00000000	chicken
Picalm	-0.07	1.00000000	chicken
Pik3c2a	0.09	1.00000000	chicken
Atp1a1	0.04	1.00000000	chicken
Vps39	-0.13	1.00000000	chicken
Ranbp9	0.01	1.00000000	chicken
Prox1	-0.43	1.00000000	chicken
Fam172a	-0.22	1.00000000	chicken
Ylpm1	-0.19	1.00000000	chicken
Nanp	-0.25	1.00000000	chicken
Ttc33	0.01	1.00000000	chicken
Raf1	-0.08	1.00000000	chicken
Slc66a2	-0.22	1.00000000	chicken

C1qb	-0.20	1.00000000	chicken
Pik3cd	0.01	1.00000000	chicken
Ap3b1	-0.29	1.00000000	chicken
Tmem104	-0.28	1.00000000	chicken
Fem1b	-0.07	1.00000000	chicken
Fam104a	-0.13	1.00000000	chicken
Fbxo33	-0.11	1.00000000	chicken
Fgfr1op2	-0.18	1.00000000	chicken
Pde4d	-0.32	1.00000000	chicken
Fam3c	-0.08	1.00000000	chicken
Zfp830	-0.06	1.00000000	chicken
Slain2	-0.16	1.00000000	chicken
Vezt	0.06	1.00000000	chicken
Bmt2	-0.01	1.00000000	chicken
Snx29	-0.08	1.00000000	chicken
Gtf2e1	0.03	1.00000000	chicken
Setd3	-0.18	1.00000000	chicken
Tom1	-0.12	1.00000000	chicken
Disc1	-0.21	1.00000000	chicken
Itga1	-0.04	1.00000000	chicken
Bub3	-0.19	1.00000000	chicken
Nelfcd	-0.03	1.00000000	chicken
Ss18	-0.03	1.00000000	chicken
Rfc1	-0.11	1.00000000	chicken
Tspo	-0.01	1.00000000	chicken
Ncoa7	-0.26	1.00000000	chicken
Gpr65	-0.34	1.00000000	chicken
9530068E07Rik	-0.16	1.00000000	chicken
Osbpl8	-0.19	1.00000000	chicken
Rbm19	-0.16	1.00000000	chicken
Nemf	-0.08	1.00000000	chicken
Dctn1	-0.09	1.00000000	chicken
Csrp1	-0.12	1.00000000	chicken
Cog5	-0.16	1.00000000	chicken
Acvr1	-0.13	1.00000000	chicken
Kat6a	-0.07	1.00000000	chicken
Fbxo32	-0.03	1.00000000	chicken
Cherp	-0.09	1.00000000	chicken
Pcgf5	-0.13	1.00000000	chicken
Gpr137b	-0.20	1.00000000	chicken
Lgals3	0.09	1.00000000	chicken
Gbf1	-0.13	1.00000000	chicken
Nde1	-0.04	1.00000000	chicken
Psmb4	-0.03	1.00000000	chicken
1810013L24Rik	0.02	1.00000000	chicken
Ube2v1	-0.18	1.00000000	chicken

Aven	0.08	1.00000000	chicken
Icosl	-0.22	1.00000000	chicken
Kif5b	-0.27	1.00000000	chicken
Nbeal2	-0.17	1.00000000	chicken
Abcc1	0.07	1.00000000	chicken
Sec24a	-0.18	1.00000000	chicken
Anks1	-0.21	1.00000000	chicken
Thumpd3	-0.12	1.00000000	chicken
Rft1	-0.04	1.00000000	chicken
Nub1	0.03	1.00000000	chicken
Gtf2e2	0.03	1.00000000	chicken
Cul3	-0.15	1.00000000	chicken
Idh3a	-0.02	1.00000000	chicken
Ahcyl1	0.04	1.00000000	chicken
Ube2i	-0.15	1.00000000	chicken
Diaph2	-0.06	1.00000000	chicken
Chst15	-0.29	1.00000000	chicken
Nip7	0.03	1.00000000	chicken
Vwa8	-0.11	1.00000000	chicken
Psma7	0.22	1.00000000	chicken
Rnf20	-0.04	1.00000000	chicken
Rab21	-0.20	1.00000000	chicken
Ubr1	-0.23	1.00000000	chicken
Dnm1l	0.03	1.00000000	chicken
Snip1	-0.04	1.00000000	chicken
Parg	-0.16	1.00000000	chicken
Nubpl	-0.24	1.00000000	chicken
Emc2	-0.09	1.00000000	chicken
Tbc1d14	-0.36	1.00000000	chicken
Slc16a7	-0.19	1.00000000	chicken
Tubg1	-0.07	1.00000000	chicken
Osbp	-0.05	1.00000000	chicken
App	0.19	1.00000000	chicken
Igsf6	0.03	1.00000000	chicken
Osbpl2	-0.05	1.00000000	chicken
Hcfc2	0.06	1.00000000	chicken
Snx8	0.01	1.00000000	chicken
Ncln	0.03	1.00000000	chicken
Atp2b1	0.23	1.00000000	chicken
F5	-0.70	1.00000000	chicken
Dcaf1	-0.20	1.00000000	chicken
Rilp	0.07	1.00000000	chicken
Gpr155	-0.05	1.00000000	chicken
Lims2	-0.13	1.00000000	chicken
Cct7	-0.06	1.00000000	chicken
Dnaaf9	-0.05	1.00000000	chicken

Afg3l2	-0.06	1.00000000	chicken
Fundc1	-0.13	1.00000000	chicken
Foxj2	-0.04	1.00000000	chicken
Wasf2	0.00	1.00000000	chicken
Timm44	-0.09	1.00000000	chicken
Gosr2	-0.20	1.00000000	chicken
Ptp4a1	-0.11	1.00000000	chicken
Arrdc3	-0.24	1.00000000	chicken
Srsf7	-0.21	1.00000000	chicken
Slc44a1	-0.03	1.00000000	chicken
Pmpcb	-0.05	1.00000000	chicken
Cir1	-0.05	1.00000000	chicken
Slc4a7	-0.23	1.00000000	chicken
Smarca4	-0.13	1.00000000	chicken
Stom	0.14	1.00000000	chicken
Clcn5	0.24	1.00000000	chicken
Snrpc	-0.09	1.00000000	chicken
Snap23	0.18	1.00000000	chicken
Zdhhc6	-0.11	1.00000000	chicken
Hsf2	-0.02	1.00000000	chicken
Seh1l	-0.18	1.00000000	chicken
Il17ra	0.02	1.00000000	chicken
Oaz1	0.00	1.00000000	chicken
Glyr1	-0.18	1.00000000	chicken
Pik3r5	0.02	1.00000000	chicken
G3bp2	-0.17	1.00000000	chicken
Rnft1	-0.04	1.00000000	chicken
Prkd3	-0.03	1.00000000	chicken
C1d	-0.18	1.00000000	chicken
Suclg2	-0.12	1.00000000	chicken
Tspan4	-0.29	1.00000000	chicken
Pdha1	-0.11	1.00000000	chicken
Ypel5	-0.15	1.00000000	chicken
Fgr	-0.06	1.00000000	chicken
Npl	0.02	1.00000000	chicken
Lfng	-0.01	1.00000000	chicken
Rictor	0.01	1.00000000	chicken
Bcar3	0.14	1.00000000	chicken
Nipa2	-0.03	1.00000000	chicken
Ganc	-0.05	1.00000000	chicken
Rgs9	-0.16	1.00000000	chicken
Glipr1l1	-0.14	1.00000000	chicken
Tnik	0.08	1.00000000	chicken
Uggt1	-0.19	1.00000000	chicken
Nedd4	0.17	1.00000000	chicken
Comt	0.02	1.00000000	chicken

Fmr1	-0.01	1.00000000	chicken
Nop56	0.01	1.00000000	chicken
Irf2bpl	-0.02	1.00000000	chicken
Pik3c3	-0.13	1.00000000	chicken
Uqcrq	-0.10	1.00000000	chicken
Elf2	-0.16	1.00000000	chicken
Arhgap29	-0.24	1.00000000	chicken
Smarca5	0.07	1.00000000	chicken
Brd2	-0.07	1.00000000	chicken
Utp3	-0.17	1.00000000	chicken
Lamtor3	-0.01	1.00000000	chicken
Baz1b	-0.26	1.00000000	chicken
Denr	-0.09	1.00000000	chicken
Pspc1	-0.18	1.00000000	chicken
Fam214a	0.05	1.00000000	chicken
Rnf146	-0.03	1.00000000	chicken
Lmbrd1	-0.08	1.00000000	chicken
Ppp3ca	-0.05	1.00000000	chicken
Slc2a1	-0.14	1.00000000	chicken
Snrk	-0.07	1.00000000	chicken
Evl	-0.08	1.00000000	chicken
Lnpk	-0.07	1.00000000	chicken
Pes1	0.07	1.00000000	chicken
Ccdc171	-0.08	1.00000000	chicken
Zc3h13	-0.06	1.00000000	chicken
Zranb2	-0.18	1.00000000	chicken
Vps13a	0.14	1.00000000	chicken
Rock1	-0.06	1.00000000	chicken
Nod1	0.05	1.00000000	chicken
Kifbp	-0.15	1.00000000	chicken
Swap70	-0.03	1.00000000	chicken
Nup88	-0.13	1.00000000	chicken
Dtnbp1	-0.03	1.00000000	chicken
Srebf2	-0.14	1.00000000	chicken
Igf2bp2	-0.06	1.00000000	chicken
Prkx	-0.18	1.00000000	chicken
Tm9sf3	-0.01	1.00000000	chicken
Rnf139	-0.12	1.00000000	chicken
Galk2	-0.02	1.00000000	chicken
Dync1li1	-0.16	1.00000000	chicken
Atp6v1b2	-0.21	1.00000000	chicken
Xpo4	-0.18	1.00000000	chicken
Nfu1	-0.05	1.00000000	chicken
Pwp1	-0.05	1.00000000	chicken
Nkap	-0.19	1.00000000	chicken
Lgals8	-0.04	1.00000000	chicken

Tmf1	-0.06	1.00000000	chicken
Eif4g1	-0.19	1.00000000	chicken
Fam32a	-0.12	1.00000000	chicken
Tapt1	-0.02	1.00000000	chicken
Cadm1	0.13	1.00000000	chicken
Prr14l	-0.20	1.00000000	chicken
Siva1	0.02	1.00000000	chicken
Alg2	-0.01	1.00000000	chicken
Ddx6	-0.20	1.00000000	chicken
Rasgrp1	-0.05	1.00000000	chicken
Aak1	-0.11	1.00000000	chicken
Acad9	0.01	1.00000000	chicken
Washc1	-0.12	1.00000000	chicken
Rilpl1	-0.13	1.00000000	chicken
Lats1	-0.11	1.00000000	chicken
Heph	0.02	1.00000000	chicken
Cast	0.10	1.00000000	chicken
Dennd1b	-0.02	1.00000000	chicken
Tmem65	-0.09	1.00000000	chicken
Psmd13	0.10	1.00000000	chicken
Acap2	-0.08	1.00000000	chicken
Prdx6	-0.26	1.00000000	chicken
Rapgef1	-0.09	1.00000000	chicken
Fasn	-0.08	1.00000000	chicken
Syk	-0.16	1.00000000	chicken
Arhgap26	-0.35	1.00000000	chicken
Narf	-0.06	1.00000000	chicken
Sbds	-0.19	1.00000000	chicken
Ywhae	-0.26	1.00000000	chicken
Ywhah	-0.20	1.00000000	chicken
Abcb7	-0.05	1.00000000	chicken
Tmem39a	0.00	1.00000000	chicken
Stim1	-0.20	1.00000000	chicken
Ndufs4	-0.12	1.00000000	chicken
Chchd5	0.03	1.00000000	chicken
Arfgap1	-0.09	1.00000000	chicken
Setd2	-0.17	1.00000000	chicken
Gpatch8	-0.19	1.00000000	chicken
Camsap2	-0.15	1.00000000	chicken
Batf	-0.05	1.00000000	chicken
Osbpl11	-0.12	1.00000000	chicken
Ccrl2	-0.01	1.00000000	chicken
Psmc6	0.02	1.00000000	chicken
Syne3	0.01	1.00000000	chicken
Ppp3cb	-0.04	1.00000000	chicken
Ubr2	-0.24	1.00000000	chicken

Noc3l	0.13	1.00000000	chicken
Gfm1	-0.01	1.00000000	chicken
Sin3a	0.01	1.00000000	chicken
Shisa5	-0.08	1.00000000	chicken
Pepd	-0.04	1.00000000	chicken
Atp9b	-0.29	1.00000000	chicken
Trmt1l	-0.19	1.00000000	chicken
Ube4b	0.18	1.00000000	chicken
Dhdds	-0.05	1.00000000	chicken
Pnisr	-0.30	1.00000000	chicken
Hadhb	-0.11	1.00000000	chicken
Ppig	-0.07	1.00000000	chicken
Yy2	-0.11	1.00000000	chicken
Ap2b1	0.01	1.00000000	chicken
Ss18l2	-0.18	1.00000000	chicken
Ube2f	0.03	1.00000000	chicken
Ahsa2	-0.28	1.00000000	chicken
Rnf10	-0.15	1.00000000	chicken
U2af2	0.01	1.00000000	chicken
Vrk1	-0.09	1.00000000	chicken
Gabarapl1	-0.20	1.00000000	chicken
Ciapin1	-0.07	1.00000000	chicken
Cdk17	0.04	1.00000000	chicken
Nelfa	-0.04	1.00000000	chicken
Ckb	-0.16	1.00000000	chicken
Pum3	-0.09	1.00000000	chicken
Sec14l1	0.05	1.00000000	chicken
Nufip1	-0.01	1.00000000	chicken
Fgd3	0.08	1.00000000	chicken
Rftn2	-0.02	1.00000000	chicken
Peli1	0.07	1.00000000	chicken
Bend3	-0.24	1.00000000	chicken
Nsrp1	-0.14	1.00000000	chicken
Hectd4	-0.12	1.00000000	chicken
Selenok	-0.08	1.00000000	chicken
Ebna1bp2	-0.17	1.00000000	chicken
Bcat1	-0.06	1.00000000	chicken
Garnl3	0.00	1.00000000	chicken
Slc25a20	-0.02	1.00000000	chicken
Nav1	-0.12	1.00000000	chicken
Rprd2	-0.07	1.00000000	chicken
Ppp1r2	-0.10	1.00000000	chicken
Appbp2	-0.02	1.00000000	chicken
Clcn3	-0.13	1.00000000	chicken
Rab35	-0.14	1.00000000	chicken
Tpk1	-0.26	1.00000000	chicken

Tsg101	-0.21	1.00000000	chicken
Dhx30	0.00	1.00000000	chicken
Ube2e2	-0.04	1.00000000	chicken
Esco1	-0.02	1.00000000	chicken
Lsm1	-0.11	1.00000000	chicken
Pitpnb	0.06	1.00000000	chicken
Atp6v1d	-0.04	1.00000000	chicken
Bin3	-0.15	1.00000000	chicken
Kdm5b	-0.08	1.00000000	chicken
Trim8	-0.21	1.00000000	chicken
Alkbh5	-0.09	1.00000000	chicken
Cript	-0.09	1.00000000	chicken
Thrap3	-0.14	1.00000000	chicken
Cltc	0.00	1.00000000	chicken
Nfatc3	-0.30	1.00000000	chicken
Cybb	0.15	1.00000000	chicken
Rspry1	-0.06	1.00000000	chicken
Dedd	0.04	1.00000000	chicken
Ywhaz	0.11	1.00000000	chicken
Hypk	-0.15	1.00000000	chicken
Cdyl2	0.02	1.00000000	chicken
Wwp2	0.00	1.00000000	chicken
Ppm1g	-0.19	1.00000000	chicken
Ahsa1	0.05	1.00000000	chicken
Wdr3	-0.12	1.00000000	chicken
Ino80d	-0.09	1.00000000	chicken
Ist1	-0.19	1.00000000	chicken
Bfar	-0.05	1.00000000	chicken
Smim14	-0.09	1.00000000	chicken
Ssrp1	-0.13	1.00000000	chicken
Dpysl2	-0.16	1.00000000	chicken
Atp13a2	0.02	1.00000000	chicken
Nrros	0.10	1.00000000	chicken
Secisbp2	0.01	1.00000000	chicken
Ap2m1	0.04	1.00000000	chicken
4933400A11Rik	-0.05	1.00000000	chicken
Lmbrd2	-0.19	1.00000000	chicken
Washc5	-0.02	1.00000000	chicken
Zfp592	-0.07	1.00000000	chicken
Rxra	-0.18	1.00000000	chicken
Setd7	-0.12	1.00000000	chicken
Tra2b	-0.12	1.00000000	chicken
Farp1	-0.18	1.00000000	chicken
Numa1	-0.02	1.00000000	chicken
Skil	-0.16	1.00000000	chicken
Aldh9a1	-0.06	1.00000000	chicken

Ttc1	-0.10	1.00000000	chicken
Larp4b	-0.09	1.00000000	chicken
Zc3h11a	-0.16	1.00000000	chicken
Sel1l	-0.17	1.00000000	chicken
Clasp2	-0.13	1.00000000	chicken
Grb2	-0.17	1.00000000	chicken
Zfp148	0.01	1.00000000	chicken
Abhd13	-0.06	1.00000000	chicken
Vps26c	-0.18	1.00000000	chicken
Ankib1	-0.24	1.00000000	chicken
Ptpa	-0.07	1.00000000	chicken
Rnpc3	-0.11	1.00000000	chicken
Rab3gap1	-0.15	1.00000000	chicken
Snap29	-0.05	1.00000000	chicken
Tmtc2	-0.52	1.00000000	chicken
Jpt2	0.16	1.00000000	chicken
Aff4	-0.13	1.00000000	chicken
Atg2b	-0.11	1.00000000	chicken
Llph	-0.05	1.00000000	chicken
Ttc13	0.04	1.00000000	chicken
Znhit6	-0.14	1.00000000	chicken
Phf5a	-0.12	1.00000000	chicken
Phka2	-0.08	1.00000000	chicken
Tcof1	0.07	1.00000000	chicken
Zcchc17	-0.04	1.00000000	chicken
Zc3h18	-0.17	1.00000000	chicken
Tmx4	-0.06	1.00000000	chicken
Calcrl	-0.67	1.00000000	chicken
Orai2	-0.11	1.00000000	chicken
Ppat	0.02	1.00000000	chicken
Tmem131l	-0.24	1.00000000	chicken
Gid8	-0.18	1.00000000	chicken
Tmem241	0.05	1.00000000	chicken
Nfkb2	-0.05	1.00000000	chicken
Shoc2	-0.03	1.00000000	chicken
Per2	-0.20	1.00000000	chicken
Bcas3	0.02	1.00000000	chicken
Usp48	-0.09	1.00000000	chicken
Gphn	-0.14	1.00000000	chicken
Gxylt1	-0.17	1.00000000	chicken
Ppp6c	-0.12	1.00000000	chicken
Wrn	-0.16	1.00000000	chicken
Vdac2	0.09	1.00000000	chicken
Cep350	-0.13	1.00000000	chicken
Ccdc43	-0.16	1.00000000	chicken
Phf20l1	-0.17	1.00000000	chicken

Rb1	-0.15	1.00000000	chicken
Rsf1	-0.12	1.00000000	chicken
Ptch1	0.04	1.00000000	chicken
Adam17	-0.13	1.00000000	chicken
Ltn1	0.06	1.00000000	chicken
Tfeb	-0.10	1.00000000	chicken
Taok3	-0.10	1.00000000	chicken
Tnfrsf21	-0.06	1.00000000	chicken
Ddx23	-0.06	1.00000000	chicken
Tnrc6a	0.05	1.00000000	chicken
Smox	0.14	1.00000000	chicken
Ascc3	-0.14	1.00000000	chicken
Ubash3b	0.07	1.00000000	chicken
Slmap	-0.28	1.00000000	chicken
Srsf5	-0.15	1.00000000	chicken
Rbm12	-0.13	1.00000000	chicken
Specc1l	0.02	1.00000000	chicken
Cct4	-0.06	1.00000000	chicken
Nop53	-0.05	1.00000000	chicken
Sos1	-0.10	1.00000000	chicken
Fryl	-0.06	1.00000000	chicken
Arl6ip5	-0.08	1.00000000	chicken
Guk1	0.13	1.00000000	chicken
Ypel1	-0.07	1.00000000	chicken
Ric8b	-0.15	1.00000000	chicken
Rsrc1	0.03	1.00000000	chicken
Yod1	-0.07	1.00000000	chicken
Ube2b	-0.09	1.00000000	chicken
Stk17b	-0.23	1.00000000	chicken
Ralgapa1	-0.02	1.00000000	chicken
Lrrc8d	-0.01	1.00000000	chicken
Surf4	-0.09	1.00000000	chicken
Immp2l	-0.11	1.00000000	chicken
Npc1	-0.14	1.00000000	chicken
Nop16	-0.08	1.00000000	chicken
Psmd2	-0.05	1.00000000	chicken
Taf6	-0.16	1.00000000	chicken
Tdg	-0.15	1.00000000	chicken
Spast	0.00	1.00000000	chicken
Psma5	-0.09	1.00000000	chicken
Zfp711	-0.14	1.00000000	chicken
Rab40b	-0.12	1.00000000	chicken
Itsn2	-0.04	1.00000000	chicken
Nrd1	-0.06	1.00000000	chicken
Ccdc47	-0.12	1.00000000	chicken
Unc45a	-0.02	1.00000000	chicken

Slc37a3	-0.06	1.00000000	chicken
Dph6	-0.09	1.00000000	chicken
Aktip	-0.02	1.00000000	chicken
Irs4	-0.12	1.00000000	chicken
Eif5b	-0.02	1.00000000	chicken
Golga1	-0.09	1.00000000	chicken
Dock1	0.07	1.00000000	chicken
Gorasp2	0.07	1.00000000	chicken
Rdm1	-0.08	1.00000000	chicken
Capn15	-0.13	1.00000000	chicken
Jpt1	-0.18	1.00000000	chicken
Camk1d	-0.37	1.00000000	chicken
Rnf145	-0.16	1.00000000	chicken
Nktr	-0.12	1.00000000	chicken
Tmem101	-0.03	1.00000000	chicken
Pdcl3	-0.08	1.00000000	chicken
Pus7	-0.14	1.00000000	chicken
Dnajb14	0.07	1.00000000	chicken
Nr1d2	-0.09	1.00000000	chicken
Uchl3	-0.07	1.00000000	chicken
Tcf12	0.00	1.00000000	chicken
Arhgap6	-0.37	1.00000000	chicken
Ensa	-0.10	1.00000000	chicken
Rapgef2	0.21	1.00000000	chicken
Otulin	-0.12	1.00000000	chicken
Dock4	-0.09	1.00000000	chicken
Ythdc2	-0.01	1.00000000	chicken
Zc3h12c	0.03	1.00000000	chicken
Traf3ip3	-0.17	1.00000000	chicken
Relch	-0.16	1.00000000	chicken
Golim4	-0.01	1.00000000	chicken
Ptbp2	0.05	1.00000000	chicken
Dctn4	0.06	1.00000000	chicken
Psmd6	-0.03	1.00000000	chicken
Rgs10	-0.27	1.00000000	chicken
Ube2h	-0.05	1.00000000	chicken
Otub1	-0.19	1.00000000	chicken
Atp6v1g1	-0.20	1.00000000	chicken
Dicer1	-0.24	1.00000000	chicken
Golga3	-0.14	1.00000000	chicken
Srpk1	-0.17	1.00000000	chicken
Smn1	-0.07	1.00000000	chicken
Exoc6b	-0.30	1.00000000	chicken
Chd9	-0.10	1.00000000	chicken
Qrich1	-0.14	1.00000000	chicken
Aspg	0.09	1.00000000	chicken

Auh	0.07	1.00000000	chicken
Cd300c	0.00	1.00000000	chicken
Itpka	-0.04	1.00000000	chicken
Nutf2	-0.10	1.00000000	chicken
Scaf8	-0.17	1.00000000	chicken
Hemk1	-0.13	1.00000000	chicken
Derl2	0.05	1.00000000	chicken
Vcpip1	-0.13	1.00000000	chicken
Wdr81	-0.08	1.00000000	chicken
Prpf39	-0.13	1.00000000	chicken
Sf3a3	0.18	1.00000000	chicken
Cox10	-0.08	1.00000000	chicken
Idh1	0.01	1.00000000	chicken
Gtf3c1	-0.05	1.00000000	chicken
2410004B18Rik	0.04	1.00000000	chicken
Rnps1	-0.19	1.00000000	chicken
Exoc7	-0.03	1.00000000	chicken
Lrrc8a	-0.14	1.00000000	chicken
Cops2	0.12	1.00000000	chicken
Fbxo25	0.02	1.00000000	chicken
Dnaja4	-0.16	1.00000000	chicken
Jdp2	0.01	1.00000000	chicken
Ptk2	0.12	1.00000000	chicken
Snw1	-0.04	1.00000000	chicken
Insig1	-0.02	1.00000000	chicken
Ntmt1	-0.07	1.00000000	chicken
Usp1	-0.10	1.00000000	chicken
Hspd1	-0.17	1.00000000	chicken
Htt	-0.14	1.00000000	chicken
Dus3l	-0.07	1.00000000	chicken
Cwc22	-0.13	1.00000000	chicken
Zfp131	-0.08	1.00000000	chicken
Rrs1	0.06	1.00000000	chicken
Hps5	-0.15	1.00000000	chicken
Cct6b	0.03	1.00000000	chicken
Zrsr1	-0.09	1.00000000	chicken
Psma1	-0.05	1.00000000	chicken
Vps18	-0.03	1.00000000	chicken
Cdk12	-0.13	1.00000000	chicken
Sgsm2	-0.21	1.00000000	chicken
Stk10	-0.02	1.00000000	chicken
Kif13b	-0.20	1.00000000	chicken
Sgpl1	-0.19	1.00000000	chicken
Ube3a	-0.10	1.00000000	chicken
Ubqln4	-0.04	1.00000000	chicken
Slc38a1	-0.01	1.00000000	chicken

Zfp326	0.01	1.00000000	chicken
4933434E20Rik	-0.01	1.00000000	chicken
Cars2	-0.01	1.00000000	chicken
Nlk	-0.08	1.00000000	chicken
Tfip11	-0.08	1.00000000	chicken
Sap30bp	0.01	1.00000000	chicken
Lsm5	0.10	1.00000000	chicken
Armc1	-0.03	1.00000000	chicken
Anxa1	-0.48	1.00000000	chicken
Cog2	-0.13	1.00000000	chicken
Zbtb41	-0.20	1.00000000	chicken
Prkca	-0.13	1.00000000	chicken
Zfyve1	-0.19	1.00000000	chicken
Socs5	-0.12	1.00000000	chicken
Poll	0.05	1.00000000	chicken
Nav2	-0.06	1.00000000	chicken
Rab11a	-0.11	1.00000000	chicken
Sri	-0.23	1.00000000	chicken
Wbp2	-0.09	1.00000000	chicken
Gcn1	-0.10	1.00000000	chicken
Nfe2l2	-0.19	1.00000000	chicken
Thyn1	-0.10	1.00000000	chicken
Srp68	0.04	1.00000000	chicken
Ehbp1	-0.01	1.00000000	chicken
Radx	-0.12	1.00000000	chicken
Ctbp1	-0.22	1.00000000	chicken
Ythdf3	-0.18	1.00000000	chicken
Rassf2	-0.02	1.00000000	chicken
Clk4	-0.20	1.00000000	chicken
Rae1	-0.04	1.00000000	chicken
Tvp23b	-0.04	1.00000000	chicken
Adora2b	0.02	1.00000000	chicken
Stk4	-0.04	1.00000000	chicken
Zfat	-0.09	1.00000000	chicken
Slc25a24	-0.13	1.00000000	chicken
Vps8	-0.10	1.00000000	chicken
Otud4	-0.21	1.00000000	chicken
Ccnk	-0.08	1.00000000	chicken
Arhgap17	-0.17	1.00000000	chicken
Osbp2	-0.02	1.00000000	chicken
Sae1	-0.13	1.00000000	chicken
Ralgps2	0.06	1.00000000	chicken
Copa	-0.12	1.00000000	chicken
Rragd	-0.07	1.00000000	chicken
Gapvd1	0.21	1.00000000	chicken
Traf6	-0.01	1.00000000	chicken

Hace1	0.01	1.00000000	chicken
Slc30a9	-0.10	1.00000000	chicken
Rtn4	0.11	1.00000000	chicken
Btbd7	0.00	1.00000000	chicken
Aplp2	-0.22	1.00000000	chicken
Arl4c	-0.10	1.00000000	chicken
Ccdc59	-0.15	1.00000000	chicken
Atxn1	-0.05	1.00000000	chicken
Csnk2a1	-0.08	1.00000000	chicken
Ogfod3	-0.06	1.00000000	chicken
Iqsec1	0.03	1.00000000	chicken
Gsta1	0.29	1.00000000	chicken
Rock2	-0.17	1.00000000	chicken
Pmm1	0.00	1.00000000	chicken
Slc25a16	-0.20	1.00000000	chicken
Lancl2	-0.07	1.00000000	chicken
Angel1	-0.07	1.00000000	chicken
Cse1l	-0.11	1.00000000	chicken
Srp54a	0.00	1.00000000	chicken
Sdf2l1	-0.11	1.00000000	chicken
Agpat4	-0.04	1.00000000	chicken
Wipi2	-0.13	1.00000000	chicken
Fas	-0.12	1.00000000	chicken
Nrip1	0.21	1.00000000	chicken
Copb1	-0.17	1.00000000	chicken
Bcr	-0.05	1.00000000	chicken
Gnb1	0.00	1.00000000	chicken
Eif4g3	-0.10	1.00000000	chicken
Zfp384	-0.10	1.00000000	chicken
Pitpna	0.00	1.00000000	chicken
Psmd4	-0.03	1.00000000	chicken
Srsf1	-0.04	1.00000000	chicken
Aagab	-0.07	1.00000000	chicken
Acp1	0.06	1.00000000	chicken
Reep3	-0.06	1.00000000	chicken
Nop14	-0.03	1.00000000	chicken
Ldhb	0.31	1.00000000	chicken
Nsun2	-0.05	1.00000000	chicken
Dcp1b	-0.05	1.00000000	chicken
Katnbl1	-0.08	1.00000000	chicken
Supt6	-0.20	1.00000000	chicken
Larp7	0.04	1.00000000	chicken
Yipf5	-0.09	1.00000000	chicken
Chmp2a	-0.02	1.00000000	chicken
Tomm70a	0.03	1.00000000	chicken
Vps35l	-0.07	1.00000000	chicken

Txndc11	-0.07	1.00000000	chicken
Xpot	-0.13	1.00000000	chicken
Lrch2	0.00	1.00000000	chicken
Cstf1	-0.08	1.00000000	chicken
Pum1	-0.07	1.00000000	chicken
Chmp5	-0.10	1.00000000	chicken
Anapc15-ps	-0.11	1.00000000	chicken
Kdm1a	-0.01	1.00000000	chicken
Ggnbp2	-0.05	1.00000000	chicken
Rab3il1	0.08	1.00000000	chicken
Rnf34	-0.15	1.00000000	chicken
Fbxw8	-0.05	1.00000000	chicken
Agtrap	-0.14	1.00000000	chicken
Ppil2	-0.11	1.00000000	chicken
Nars	-0.04	1.00000000	chicken
Por	0.02	1.00000000	chicken
Obp2a	-0.94	1.00000000	chicken
Ncoa2	-0.18	1.00000000	chicken
Nisch	-0.11	1.00000000	chicken
Kmt2c	-0.08	1.00000000	chicken
Kras	-0.17	1.00000000	chicken
Htr2a	0.25	1.00000000	chicken
Chchd3	0.01	1.00000000	chicken
Foxp1	-0.01	1.00000000	chicken
Bak1	-0.12	1.00000000	chicken
Ogfr	-0.07	1.00000000	chicken
Kdelr2	-0.18	1.00000000	chicken
Ubxn7	-0.11	1.00000000	chicken
Lamtor1	-0.04	1.00000000	chicken
Sec31a	-0.01	1.00000000	chicken
Sfswap	-0.18	1.00000000	chicken
Slc30a5	-0.08	1.00000000	chicken
Rnf111	-0.12	1.00000000	chicken
Synrg	-0.13	1.00000000	chicken
Zfp638	-0.14	1.00000000	chicken
Smg6	-0.06	1.00000000	chicken
Prrc2c	-0.12	1.00000000	chicken
Cd81	-0.09	1.00000000	chicken
Krit1	-0.09	1.00000000	chicken
Srfbp1	-0.06	1.00000000	chicken
Anxa7	0.00	1.00000000	chicken
Prkaa1	0.07	1.00000000	chicken
Tent5a	-0.27	1.00000000	chicken
Wdr48	-0.07	1.00000000	chicken
BC005537	-0.07	1.00000000	chicken
Pla2g7	0.06	1.00000000	chicken

Pacs2	-0.21	1.00000000	chicken
Dhx15	-0.06	1.00000000	chicken
Sf3b3	-0.07	1.00000000	chicken
Colgalt1	-0.14	1.00000000	chicken
Rbm5	-0.10	1.00000000	chicken
Tmem268	-0.06	1.00000000	chicken
Odc1	-0.11	1.00000000	chicken
Bag1	-0.12	1.00000000	chicken
Tpm3-rs7	-0.33	1.00000000	chicken
Vdac1	-0.14	1.00000000	chicken
Plekhm2	-0.08	1.00000000	chicken
Ash1l	-0.13	1.00000000	chicken
Ube4a	-0.04	1.00000000	chicken
Pde7a	-0.07	1.00000000	chicken
Fnbp1l	-0.12	1.00000000	chicken
Plekhm3	-0.07	1.00000000	chicken
Atf6	-0.01	1.00000000	chicken
Vps13d	-0.17	1.00000000	chicken
Akap8l	-0.11	1.00000000	chicken
Ak2	-0.02	1.00000000	chicken
Srsf6	0.02	1.00000000	chicken
Zbtb37	0.01	1.00000000	chicken
Necap1	-0.12	1.00000000	chicken
Chid1	0.02	1.00000000	chicken
Zfyve26	-0.11	1.00000000	chicken
Cnp	0.01	1.00000000	chicken
Saal1	-0.12	1.00000000	chicken
Zcchc8	-0.10	1.00000000	chicken
Ncoa4	-0.21	1.00000000	chicken
Susd3	0.05	1.00000000	chicken
Trdmt1	-0.08	1.00000000	chicken
Chchd2	-0.15	1.00000000	chicken
Rbm47	-0.59	1.00000000	chicken
Nnt	-0.02	1.00000000	chicken
Vps13c	-0.06	1.00000000	chicken
Cacybp	-0.03	1.00000000	chicken
Pum2	-0.14	1.00000000	chicken
Ilkap	-0.09	1.00000000	chicken
Ppp1r7	-0.17	1.00000000	chicken
Rtf2	-0.13	1.00000000	chicken
Lig3	-0.14	1.00000000	chicken
Hmbs	-0.05	1.00000000	chicken
Slc9a7	-0.15	1.00000000	chicken
Prcc	-0.08	1.00000000	chicken
Ankmy2	-0.04	1.00000000	chicken
Rasa2	-0.16	1.00000000	chicken

Spen	-0.11	1.00000000	chicken
Gpd2	-0.17	1.00000000	chicken
Cdc37	-0.08	1.00000000	chicken
Pik3cb	-0.10	1.00000000	chicken
D630045J12Rik	0.02	1.00000000	chicken
Eps15l1	-0.17	1.00000000	chicken
Ncoa1	0.09	1.00000000	chicken
Pdcd10	-0.12	1.00000000	chicken
Tle4	0.06	1.00000000	chicken
Spg7	-0.10	1.00000000	chicken
Fam160b1	-0.06	1.00000000	chicken
Exoc1	-0.15	1.00000000	chicken
Gtf2b	-0.14	1.00000000	chicken
Tab3	-0.05	1.00000000	chicken
Cdc27	-0.14	1.00000000	chicken
Ehd3	-0.13	1.00000000	chicken
Vbp1	0.02	1.00000000	chicken
Pisd	0.00	1.00000000	chicken
Gatad2a	-0.09	1.00000000	chicken
Letm1	0.02	1.00000000	chicken
Arhgap21	-0.39	1.00000000	chicken
Unc50	-0.05	1.00000000	chicken
B4galt1	0.02	1.00000000	chicken
Flot1	0.04	1.00000000	chicken
Fzd6	-0.04	1.00000000	chicken
Rrp1b	-0.17	1.00000000	chicken
Aurkaip1	-0.09	1.00000000	chicken
Kdm5a	-0.03	1.00000000	chicken
Rab8a	0.04	1.00000000	chicken
Noc2l	-0.05	1.00000000	chicken
Naxd	-0.11	1.00000000	chicken
Plcg2	-0.01	1.00000000	chicken
Bcas2	-0.18	1.00000000	chicken
Rpa2	0.01	1.00000000	chicken
Epc2	-0.07	1.00000000	chicken
Csl	-0.05	1.00000000	chicken
Efcab14	-0.13	1.00000000	chicken
Ranbp1	-0.20	1.00000000	chicken
Gnl2	-0.10	1.00000000	chicken
Rffl	-0.05	1.00000000	chicken
Sgk3	-0.04	1.00000000	chicken
Tmco1	-0.05	1.00000000	chicken
Prrc2b	-0.19	1.00000000	chicken
Tmem184b	-0.08	1.00000000	chicken
Slx4	0.02	1.00000000	chicken
Ubp1	-0.09	1.00000000	chicken

Atxn3	-0.12	1.00000000	chicken
Usp8	-0.15	1.00000000	chicken
Rnf130	0.02	1.00000000	chicken
Ambra1	-0.18	1.00000000	chicken
Pggt1b	-0.03	1.00000000	chicken
Riox1	-0.02	1.00000000	chicken
Exosc10	-0.08	1.00000000	chicken
Vta1	-0.05	1.00000000	chicken
Ppp2cb	-0.13	1.00000000	chicken
Elavl1	-0.07	1.00000000	chicken
Ing1	-0.10	1.00000000	chicken
Senp5	0.02	1.00000000	chicken
Gpx4	-0.11	1.00000000	chicken
Xpo7	-0.12	1.00000000	chicken
Poc1b	-0.10	1.00000000	chicken
Rab10	-0.05	1.00000000	chicken
Fam174c	-0.08	1.00000000	chicken
Cops3	-0.07	1.00000000	chicken
Adar	-0.07	1.00000000	chicken
Rbms1	-0.07	1.00000000	chicken
Slc12a7	-0.06	1.00000000	chicken
Ppp3r1	-0.02	1.00000000	chicken
Usp45	-0.05	1.00000000	chicken
Suds3	0.08	1.00000000	chicken
Scaf11	-0.04	1.00000000	chicken
Snx10	2.48	0.00000000	duck
Plac8	1.97	0.00000000	duck
Sox6	-4.72	0.00000000	duck
Nos2	4.06	0.00000000	duck
Samhd1	1.98	0.00000000	duck
Rac2	2.00	0.00000000	duck
S100a11	1.87	0.00000000	duck
F13a1	2.50	0.00000000	duck
Sh3bgrl3	1.62	0.00000000	duck
Stat1	1.57	0.00000000	duck
Abtb2	2.04	0.00000000	duck
Bri3	1.44	0.00000000	duck
Synm	-3.94	0.00000000	duck
Optn	1.85	0.00000000	duck
Laptm5	1.42	0.00000000	duck
Dop1b	1.74	0.00000000	duck
Npc2	1.53	0.00000000	duck
Nampt	1.47	0.00000000	duck
Arl6ip5	1.37	0.00000000	duck
Acod1	2.99	0.00000000	duck
Ly96	1.54	0.00000000	duck

Rnf19b	1.52	0.00000000	duck
Oat	1.37	0.00000000	duck
Fyn	1.71	0.00000000	duck
Abcg1	1.68	0.00000000	duck
Aspg	2.39	0.00000000	duck
Akap13	1.19	0.00000000	duck
Anxa1	1.44	0.00000000	duck
Txn1	1.33	0.00000000	duck
Klf15	1.65	0.00000000	duck
Rundc1	1.32	0.00000000	duck
Hpgds	1.49	0.00000000	duck
Anxa11	1.22	0.00000000	duck
Wdr37	1.59	0.00000000	duck
Nfil3	1.33	0.00000000	duck
Smpd1	1.22	0.00000000	duck
Fry	1.59	0.00000000	duck
Rnf130	1.21	0.00000000	duck
Psmb2	1.09	0.00000000	duck
Fabp3	1.25	0.00000000	duck
Ncf1	1.32	0.00000000	duck
Fzd6	1.22	0.00000000	duck
Limd2	1.31	0.00000000	duck
Pgd	1.29	0.00000000	duck
Lrmda	1.75	0.00000000	duck
Dach2	1.53	0.00000000	duck
Ctsb	1.37	0.00000000	duck
Epsti1	1.38	0.00000000	duck
Fbrsl1	1.16	0.00000000	duck
Crot	1.15	0.00000000	duck
Lmnb1	1.13	0.00000000	duck
Nfam1	1.15	0.00000000	duck
Reep5	1.21	0.00000000	duck
Ptgs2	1.40	0.00000000	duck
Arhgdib	1.20	0.00000000	duck
Itsn1	1.24	0.00000000	duck
Zfand2a	1.41	0.00000000	duck
Lims2	1.31	0.00000000	duck
Dot1l	1.20	0.00000000	duck
Csrp1	1.22	0.00000000	duck
Aopep	1.39	0.00000000	duck
Ncor2	1.11	0.00000000	duck
Fuca2	1.09	0.00000000	duck
Blnk	1.48	0.00000000	duck
Nadk	1.00	0.00000000	duck
Ica1	1.26	0.00000000	duck
Ostm1	1.16	0.00000000	duck

Angpt1	1.87	0.00000000	duck
Acss1	0.98	0.00000000	duck
Arsb	1.22	0.00000000	duck
Atf4	1.02	0.00000000	duck
Vdac1	0.92	0.00000000	duck
Atf3	0.92	0.00000000	duck
Pnrc1	0.89	0.00000000	duck
Kcnk5	1.10	0.00000000	duck
Actb	0.98	0.00000000	duck
Ogfr	0.90	0.00000000	duck
Ero1a	1.08	0.00000000	duck
Ntng1	1.53	0.00000000	duck
Atp6v1h	0.92	0.00000000	duck
Dctn3	1.03	0.00000000	duck
Tcirg1	1.19	0.00000000	duck
Fnip1	0.86	0.00000000	duck
Fchsd2	0.91	0.00000000	duck
Psma7	0.90	0.00000000	duck
Cpb2	1.47	0.00000000	duck
Vsir	1.05	0.00000000	duck
Arpc3	0.98	0.00000000	duck
Atp6v1b2	0.93	0.00000000	duck
Atp6ap2	1.01	0.00000000	duck
Rhog	1.01	0.00000000	duck
Dgkh	1.45	0.00000000	duck
Rnf19a	0.85	0.00000000	duck
Zdhhc15	1.00	0.00000000	duck
Plcg2	1.27	0.00000000	duck
Palld	-3.52	0.00000000	duck
Gch1	1.03	0.00000000	duck
Itgb1	0.92	0.00000000	duck
Calm1	0.87	0.00000000	duck
Rfesd	-3.11	0.00000000	duck
Atp6v1a	0.86	0.00000000	duck
Prok2	1.37	0.00000000	duck
Vmp1	0.88	0.00000000	duck
Atp6v1g3	1.13	0.00000000	duck
Zfp706	0.84	0.00000000	duck
Wdfy3	2.35	0.00000000	duck
Eif2ak2	0.81	0.00000000	duck
Gabarapl2	0.84	0.00000000	duck
Taldo1	0.91	0.00000000	duck
Cnot9	0.92	0.00000000	duck
Nfkbiz	0.92	0.00000000	duck
Pid1	1.29	0.00000000	duck
Ttc39b	0.75	0.00000000	duck

Frmd4b	1.19	0.00000000	duck
Prkcd	0.91	0.00000000	duck
Cert1	1.19	0.00000000	duck
Zeb2	1.28	0.00000000	duck
5031439G07Rik	1.00	0.00000000	duck
Trim25	0.76	0.00000000	duck
Atp5b	0.90	0.00000000	duck
Ypel5	0.85	0.00000000	duck
Atp6v0e	0.87	0.00000000	duck
Lcp2	0.90	0.00000000	duck
Tubb5	0.93	0.00000000	duck
Cebpa	0.94	0.00000000	duck
Atp11c	0.81	0.00000000	duck
Gpx1	0.93	0.00000000	duck
Rnf166	0.78	0.00000000	duck
Pla2g4a	0.92	0.00000000	duck
Tnrc6b	-3.34	0.00000000	duck
Ski	0.82	0.00000000	duck
St8sia4	1.33	0.00000000	duck
Sntb1	1.45	0.00000000	duck
Trappc3	0.83	0.00000000	duck
Celf2	1.26	0.00000000	duck
Ston2	1.25	0.00000000	duck
Glrx	1.05	0.00000000	duck
Syap1	0.75	0.00000000	duck
Lasp1	0.87	0.00000000	duck
Rab20	0.97	0.00000000	duck
Gpr137b	0.80	0.00000000	duck
Ilk	0.78	0.00000000	duck
Yipf3	0.77	0.00000000	duck
Sh3kbp1	0.82	0.00000000	duck
Apobec1	1.03	0.00000000	duck
Cask	0.95	0.00000000	duck
Appl2	1.07	0.00000000	duck
Phactr4	0.84	0.00000000	duck
Tgds	0.86	0.00000000	duck
Zfand5	0.88	0.00000000	duck
Sos1	-1.83	0.00000000	duck
Tmem250-ps	0.78	0.00000000	duck
Ccdc71l	0.96	0.00000000	duck
Slc9a3r1	0.82	0.00000000	duck
Ppp1r12a	0.78	0.00000000	duck
Syngr2	0.76	0.00000000	duck
Tgm4	2.38	0.00000000	duck
Zfp217	1.08	0.00000000	duck
Xxylt1	0.88	0.00000000	duck

Rnf213	0.66	0.00000000	duck
Ubl5b	0.68	0.00000000	duck
Ndufs4	0.71	0.00000000	duck
Cbfb	0.90	0.00000000	duck
Trak1	0.99	0.00000000	duck
Grk4	0.75	0.00000000	duck
Fam214a	0.98	0.00000000	duck
Depdc1b	0.95	0.00000000	duck
Sec61g	1.02	0.00000000	duck
Abca3	0.86	0.00000000	duck
Usp10	0.52	0.00000000	duck
Cdc42se2	0.89	0.00000000	duck
Gadd45g	1.14	0.00000000	duck
Kif15	1.07	0.00000000	duck
Sacm1l	0.65	0.00000000	duck
Stom	0.69	0.00000000	duck
Diablo	0.83	0.00000000	duck
Acsbg2	0.76	0.00000000	duck
Rsu1	0.86	0.00000000	duck
Panx2	0.83	0.00000000	duck
Sema3d	-2.40	0.00000000	duck
Arg2	0.83	0.00000000	duck
Tspan14	0.48	0.00000000	duck
Fhl3	0.75	0.00000000	duck
Cdk5rap1	0.68	0.00000000	duck
Gas7	0.96	0.00000000	duck
Romo1	0.72	0.00000000	duck
Klhdc1	0.82	0.00000000	duck
Sub1	0.71	0.00000000	duck
Ndfip1	0.72	0.00000000	duck
Znfx1	0.55	0.00000000	duck
Rxra	0.76	0.00000000	duck
Tpi1	0.80	0.00000000	duck
Fam13a	1.13	0.00000000	duck
Atp5g3	0.80	0.00000000	duck
Klf6	0.67	0.00000000	duck
Igsf9	0.76	0.00000000	duck
Ppp3ca	0.79	0.00000000	duck
Spats2	0.72	0.00000000	duck
Jund	0.82	0.00000000	duck
Numb	0.77	0.00000000	duck
Lsp1	0.73	0.00000000	duck
Ktn1	0.42	0.00000000	duck
Peli1	0.75	0.00000000	duck
Als2cl	0.73	0.00000000	duck
Tfdp2	0.89	0.00000000	duck

Dram1	1.18	0.00000000	duck
Trank1	0.20	0.00000000	duck
Bud31	0.64	0.00000000	duck
Vps54	0.68	0.00000000	duck
Zmiz1	0.81	0.00000000	duck
Srgn	0.67	0.00000000	duck
Dync1i2	0.50	0.00000000	duck
Dtwd1	0.78	0.00000000	duck
Camk2g	0.55	0.00000000	duck
Ost4	0.99	0.00000000	duck
Ssr2	0.72	0.00000000	duck
Zswim6	0.74	0.00000000	duck
Sash3	0.76	0.00000000	duck
Ap2m1	0.55	0.00000000	duck
Ralb	0.67	0.00000000	duck
Plekhb2	0.57	0.00000000	duck
Eif3g	0.63	0.00000000	duck
Chst12	0.76	0.00000000	duck
Gng7	0.68	0.00000000	duck
Sppl3	0.72	0.00000000	duck
Gnpda1	0.70	0.00000000	duck
Pef1	0.69	0.00000000	duck
Dcun1d2	-2.00	0.00000000	duck
Bcl6	0.77	0.00000000	duck
Abca1	0.61	0.00000000	duck
Foxk1	0.64	0.00000000	duck
Arhgef26	0.86	0.00000000	duck
Sh3bgrl	0.66	0.00000000	duck
Pdrg1	0.58	0.00000000	duck
Cdc34	0.61	0.00000000	duck
Herc4	0.25	0.00000000	duck
Ctss	0.96	0.00000000	duck
Lrpap1	0.82	0.00000000	duck
Coro1c	0.67	0.00000000	duck
Gnai2	0.67	0.00000000	duck
Rcsd1	0.72	0.00000000	duck
Btf3	0.70	0.00000000	duck
Ctbs	0.74	0.00000000	duck
Riok3	0.61	0.00000000	duck
Dctn6	0.76	0.00000000	duck
Pgm1	0.71	0.00000000	duck
Dtx3l	0.49	0.00000000	duck
Dcaf6	0.56	0.00000000	duck
Plek	0.58	0.00000000	duck
Ccdc93	0.72	0.00000000	duck
Fem1c	0.62	0.00000000	duck

Sf3a2	0.67	0.00000000	duck
Pkm	0.63	0.00000000	duck
Tmem258	0.66	0.00000000	duck
Ptprm	1.41	0.00000000	duck
Zdhhc14	0.70	0.00000000	duck
Alcam	1.23	0.00000000	duck
Osbpl9	0.43	0.00000000	duck
Dbnl	0.69	0.00000000	duck
Hibadh	0.61	0.00000000	duck
Bcap29	0.37	0.00000000	duck
Arid4a	0.55	0.00000000	duck
Slc7a6	0.36	0.00000000	duck
Acp2	0.64	0.00000000	duck
Cux1	0.65	0.00000000	duck
Wdr82	0.52	0.00000000	duck
Bmp2k	0.61	0.00000000	duck
Ulk2	0.41	0.00000000	duck
Supt4a	0.58	0.00000000	duck
Ccdc12	0.70	0.00000000	duck
Ubap2	0.60	0.00000000	duck
Txnrd1	1.11	0.00000000	duck
Fam3c	0.73	0.00000000	duck
Nrp2	1.23	0.00000000	duck
Pik3r6	0.77	0.00000000	duck
Heatr5a	0.51	0.00000000	duck
Nr4a3	0.63	0.00000000	duck
H13	0.65	0.00000000	duck
Psmd2	0.51	0.00000000	duck
Lamtor5	0.74	0.00000000	duck
Sestd1	0.87	0.00000000	duck
Rock2	0.78	0.00000000	duck
Dnajc3	0.47	0.00000000	duck
Polr2h	0.54	0.00000000	duck
Ndufb6	0.55	0.00000000	duck
Arpc1b	0.68	0.00000000	duck
Rab27a	0.65	0.00000000	duck
Dock10	0.93	0.00000000	duck
Pold2	0.60	0.00000000	duck
Suclg1	0.58	0.00000000	duck
Cib1	0.70	0.00000000	duck
Prex1	0.59	0.00000000	duck
Lamp1	0.54	0.00000000	duck
Nceh1	0.67	0.00000000	duck
Bambi	1.07	0.00000000	duck
Atp5a1	0.70	0.00000000	duck
Pard3	1.27	0.00000000	duck

Tmem35b	0.69	0.00000000	duck
Hmox1	0.99	0.00000000	duck
Kctd10	0.64	0.00000000	duck
Cables1	0.72	0.00000000	duck
Agtpbp1	0.67	0.00000000	duck
Eif4ebp1	0.67	0.00000000	duck
Psmb1	0.67	0.00000000	duck
Atp1b3	0.51	0.00000000	duck
Abhd17a	0.55	0.00000000	duck
Ripor3	-2.72	0.00000000	duck
Hsbp1	0.57	0.00000000	duck
Prdm1	0.75	0.00000000	duck
Ndufb10	0.53	0.00000000	duck
Ier5	0.74	0.00000000	duck
Fzr1	0.48	0.00000000	duck
Endov	0.57	0.00000000	duck
Pla2g15	0.61	0.00000000	duck
Hspa8	0.68	0.00000000	duck
Pak3	1.00	0.00000000	duck
Zbtb44	0.40	0.00000000	duck
Dazap2	0.50	0.00000000	duck
Pam	0.67	0.00000000	duck
Erap1	0.53	0.00000000	duck
Polr2e	0.65	0.00000000	duck
Ndufs5	0.58	0.00000000	duck
Ctsc	0.62	0.00000000	duck
Lyrm7	0.72	0.00000000	duck
Klf10	0.55	0.00000000	duck
Ptpn1	0.53	0.00000000	duck
Psma3	0.55	0.00000000	duck
Atp13a3	0.61	0.00000000	duck
Gab1	0.58	0.00000000	duck
Epb41	-2.57	0.00000000	duck
Arf1	0.59	0.00000000	duck
Atp5j	0.54	0.00000000	duck
Cobll1	1.13	0.00000000	duck
Atp6v0c	0.56	0.00000000	duck
Gpi1	0.62	0.00000000	duck
Sptan1	-2.28	0.00000000	duck
Osbp2	0.89	0.00000000	duck
Sdhb	0.46	0.00000000	duck
Kif13a	0.49	0.00000000	duck
Larp1b	-2.19	0.00000000	duck
Snrk	-1.42	0.00000000	duck
Edem3	0.58	0.00000000	duck
Kif21a	0.63	0.00000000	duck

Timp2	0.64	0.00000000	duck
Ythdf2	0.50	0.00000000	duck
Tbc1d1	0.83	0.00000000	duck
Arpc5	0.58	0.00000000	duck
Srp19	0.60	0.00000000	duck
Rab1a	0.44	0.00000000	duck
Atp1a1	0.73	0.00000000	duck
Atp23	0.62	0.00000000	duck
Ptafr	0.60	0.00000000	duck
Snrpd3	0.68	0.00000000	duck
Pdcd10	0.53	0.00000000	duck
Afg3l2	0.45	0.00000000	duck
Atrn	0.61	0.00000000	duck
Ube3c	0.49	0.00000000	duck
Srp68	0.55	0.00000000	duck
Elovl6	-2.11	0.00000000	duck
Atg2b	0.31	0.00000000	duck
Tax1bp1	0.54	0.00000000	duck
Btf3l4	0.41	0.00000000	duck
Paxip1	-1.67	0.00000000	duck
Nus1	0.60	0.00000000	duck
Supt5	0.45	0.00000000	duck
Ppara	0.60	0.00000000	duck
Wnt5b	0.49	0.00000000	duck
Cish	-1.51	0.00000000	duck
Id2	0.91	0.00000000	duck
B2m	0.57	0.00000000	duck
Slc22a3	0.75	0.00000000	duck
Actr3	0.51	0.00000000	duck
Edem1	0.48	0.00000000	duck
Tpmt	0.62	0.00000000	duck
Chmp5	0.40	0.00000000	duck
Itm2b	0.54	0.00000000	duck
Aplf	0.66	0.00000000	duck
Ostf1	0.53	0.00000000	duck
Hdac1	0.60	0.00000000	duck
Vgll4	0.57	0.00000000	duck
Rictor	0.48	0.00000000	duck
Parp9	0.35	0.00000000	duck
Fosb	0.26	0.00000000	duck
Clta	0.77	0.00000000	duck
Atp6v1d	0.57	0.00000000	duck
Lpcat3	0.46	0.00000000	duck
Nab1	0.58	0.00000000	duck
Slc6a6	-0.98	0.00000000	duck
Rel	0.61	0.00000000	duck

Cpne3	0.45	0.00000000	duck
Smarcd2	0.57	0.00000000	duck
Dda1	0.24	0.00000000	duck
Nap1l1	0.39	0.00000000	duck
Tspo	0.55	0.00000000	duck
Capg	0.63	0.00000000	duck
Sri	0.55	0.00000000	duck
Kif1b	0.37	0.00000000	duck
Skap2	0.55	0.00000000	duck
Sipa1l1	-1.94	0.00000000	duck
Adap2	0.64	0.00000000	duck
Acadm	0.41	0.00000000	duck
Arhgap15	0.75	0.00000000	duck
Atf6	0.33	0.00000000	duck
Tst	0.63	0.00000000	duck
Gpd2	0.30	0.00000000	duck
Cd86	0.71	0.00000000	duck
Nudt21	0.52	0.00000000	duck
Tbc1d2	0.54	0.00000000	duck
Lgmn	0.32	0.00000000	duck
Sema4a	0.67	0.00000000	duck
Rhag	-1.56	0.00000000	duck
Lipa	0.78	0.00000000	duck
Canx	0.36	0.00000000	duck
Tkt	0.55	0.00000000	duck
Arhgdia	0.53	0.00000000	duck
Susd3	0.60	0.00000000	duck
Hipk3	0.50	0.00000000	duck
Sucnr1	0.63	0.00000000	duck
Ankrd28	0.28	0.00000000	duck
Dcp2	0.44	0.00000000	duck
Rab10	0.28	0.00000000	duck
Nsa2	0.51	0.00000000	duck
Ap1s2	0.44	0.00000000	duck
Coro1b	0.48	0.00000000	duck
Nr4a1	0.73	0.00000000	duck
Ank1	-1.51	0.00000000	duck
Dennd4a	-1.17	0.00000000	duck
Lpxn	0.58	0.00000000	duck
Rbx1	0.54	0.00000000	duck
Slc4a2	0.60	0.00000000	duck
Urm1	0.47	0.00000000	duck
Clcn7	0.59	0.00000000	duck
Tmem230	0.45	0.00000000	duck
Ptbp3	0.37	0.00000000	duck
Grpel1	0.50	0.00000000	duck

Xpo6	-1.75	0.00000000	duck
Paqr7	0.49	0.00000000	duck
Rlim	0.49	0.00000000	duck
Smdt1	0.60	0.00000000	duck
Psma5	0.52	0.00000000	duck
Spred2	0.46	0.00000000	duck
Ormdl2	0.52	0.00000000	duck
Rubcnl	0.50	0.00000000	duck
Dnajc5	0.41	0.00000000	duck
Tnip3	1.46	0.00000000	duck
Tmem241	0.62	0.00000000	duck
Usp25	0.38	0.00000000	duck
Snx17	0.63	0.00000000	duck
Taf13	0.54	0.00000000	duck
Aff1	0.33	0.00000000	duck
Lamtor2	0.52	0.00000000	duck
Eif3i	0.51	0.00000000	duck
Ugp2	0.55	0.00000000	duck
Xpa	0.45	0.00000000	duck
Dynlrb1	0.42	0.00000000	duck
Tulp1	0.55	0.00000000	duck
Adora2b	0.63	0.00000000	duck
Wdr89	0.47	0.00000000	duck
Fam174a	0.51	0.00000000	duck
F3	1.09	0.00000000	duck
Fli1	0.33	0.00000000	duck
Rsbn1l	0.10	0.00000000	duck
Polr2d	0.40	0.00000000	duck
Atp6v0d1	0.64	0.00000000	duck
Dusp16	0.68	0.00000000	duck
Jarid2	0.59	0.00000000	duck
Lsm8	0.44	0.00000000	duck
Hsd17b12	0.54	0.00000000	duck
Nabp1	0.47	0.00000000	duck
Eef1a1	0.33	0.00000000	duck
Nt5m	0.43	0.00000000	duck
Erp29	0.42	0.00000000	duck
Larp4b	0.32	0.00000000	duck
Chordc1	0.71	0.00000000	duck
Larp7	0.42	0.00000000	duck
Wdr45b	0.36	0.00000000	duck
Zdhhc6	0.53	0.00000000	duck
Zc3h3	0.58	0.00000000	duck
Arl8b	0.42	0.00000000	duck
Atp5j2	0.53	0.00000000	duck
Ndufb2	0.68	0.00000000	duck

Wdr81	0.55	0.00000000	duck
Ube2q1	0.36	0.00000000	duck
Rasgef1a	0.43	0.00000000	duck
Aco1	0.63	0.00000000	duck
Prelid3a	0.42	0.00000000	duck
Casp3	0.49	0.00000000	duck
Dnajb9	0.23	0.00000000	duck
Cept1	0.33	0.00000000	duck
Cyrib	0.31	0.00000000	duck
Flii	0.52	0.00000000	duck
Tm2d2	0.57	0.00000000	duck
Psmb4	0.54	0.00000000	duck
Actn1	0.40	0.00000000	duck
Zfp516	0.43	0.00000000	duck
Aoah	0.60	0.00000000	duck
Pim3	0.39	0.00000000	duck
Nono	0.55	0.00000000	duck
Adcy9	-2.32	0.00000000	duck
Baiap2l1	0.60	0.00000000	duck
Shisa5	0.42	0.00000000	duck
Kdelr2	0.58	0.00000000	duck
Fam76a	0.43	0.00000000	duck
Slc39a11	0.51	0.00000000	duck
Zdhhc17	0.28	0.00000000	duck
Smad7	0.64	0.00000000	duck
Tm6sf1	0.52	0.00000000	duck
Chmp2b	0.54	0.00000000	duck
Serp1	0.59	0.00000000	duck
Ncapd2	0.50	0.00000000	duck
Acox1	0.38	0.00000000	duck
Pou2f1	0.19	0.00000000	duck
Bcas3	0.48	0.00000000	duck
Nmnat2	0.51	0.00000000	duck
Amfr	0.37	0.00000000	duck
Sft2d1	0.30	0.00000000	duck
Adm	0.24	0.00000000	duck
Commd4	0.55	0.00000000	duck
Abcc5	0.57	0.00000000	duck
Rangap1	0.42	0.00000000	duck
Snrpg	0.37	0.00000000	duck
Phf21a	0.59	0.00000000	duck
Ciao2b	0.52	0.00000000	duck
Stxbp1	0.51	0.00000000	duck
Palb2	0.54	0.00000000	duck
Rnf11	0.37	0.00000000	duck
Septin12	0.46	0.00000000	duck

Trib1	0.40	0.00000000	duck
Glod4	0.56	0.00000000	duck
Pik3cb	0.49	0.00000000	duck
Ppp1ca	0.44	0.00000000	duck
Stt3a	0.65	0.00000000	duck
Srpk2	0.52	0.00000000	duck
Slc16a6	-1.27	0.00000000	duck
Ssbp2	-1.98	0.00000000	duck
Synrg	0.41	0.00000000	duck
Gper1	0.59	0.00000000	duck
Soat1	0.73	0.00000000	duck
Ppib	1.00	0.00000000	duck
Hook3	0.24	0.00000000	duck
Ric1	0.38	0.00000000	duck
Usp47	0.19	0.00000000	duck
Parp10	0.35	0.00000000	duck
Lrrc8d	0.66	0.00000000	duck
Serpine2	0.39	0.00000000	duck
Setx	0.26	0.00000000	duck
Ubap1	0.40	0.00000000	duck
Chmp4b	0.40	0.00000000	duck
Ap3b1	0.26	0.00000000	duck
Selenof	0.44	0.00000000	duck
Snx2	0.26	0.00000000	duck
Psmd6	0.38	0.00000000	duck
Azin1	0.42	0.00000000	duck
Ssbp1	0.48	0.00000000	duck
Diaph2	0.50	0.00000000	duck
Chka	0.61	0.00000000	duck
Actr2	0.41	0.00000000	duck
Snx8	0.51	0.00000000	duck
Psmd7	0.37	0.00000000	duck
Nipbl	0.24	0.00000000	duck
G0s2	0.39	0.00000000	duck
Sumo1	0.38	0.00000000	duck
Esr1	0.55	0.00000000	duck
Ppp1cb	-1.14	0.00000000	duck
Cnot4	0.25	0.00000000	duck
Gpalpp1	0.13	0.00000000	duck
Erlec1	0.41	0.00000000	duck
Wbp2nl	0.35	0.00000000	duck
Jmjd1c	-1.13	0.00000000	duck
Cemip2	0.48	0.00000000	duck
Ccnh	0.40	0.00000000	duck
Ndufaf3	0.31	0.00000000	duck
Pxn	0.49	0.00000000	duck

Slc23a2	0.06	0.00000000	duck
Prdx1	0.35	0.00000000	duck
Aup1	0.41	0.00000000	duck
Cdc42se1	0.44	0.00000000	duck
Emb	0.51	0.00000000	duck
Anxa2	0.60	0.00000000	duck
Jmjd8	0.43	0.00000000	duck
Psma6	0.45	0.00000000	duck
Tc2n	0.63	0.00000000	duck
Cenpu	0.35	0.00000000	duck
N4bp1	0.16	0.00000000	duck
Prkab1	0.36	0.00000000	duck
Rpain	0.46	0.00000000	duck
Fgl2	0.43	0.00000000	duck
Ahsa1	0.36	0.00000000	duck
Neurl1b	0.32	0.00000000	duck
Thoc7	0.36	0.00000000	duck
Tbc1d14	0.36	0.00000000	duck
Slc25a36	0.15	0.00000000	duck
2700049A03Rik	0.53	0.00000000	duck
Haus8	0.48	0.00000000	duck
Psmd14	0.43	0.00000000	duck
Gramd3	0.53	0.00000000	duck
Tmem65	0.38	0.00000000	duck
Tubd1	0.32	0.00000000	duck
Gpatch2l	0.04	0.00000000	duck
Oser1	0.32	0.00000000	duck
Gtf2b	0.22	0.00000000	duck
Eif5b	0.21	0.00000000	duck
Pigx	0.41	0.00000000	duck
Yaf2	0.55	0.00000000	duck
Unc13d	0.46	0.00000000	duck
Nek9	0.40	0.00000000	duck
Adar	0.19	0.00000000	duck
Rer1	0.27	0.00000000	duck
Rapgef1	0.48	0.00000000	duck
Phc2	0.38	0.00000000	duck
Cdc123	0.38	0.00000000	duck
Ilvbl	0.38	0.00000000	duck
Cd47	0.39	0.00000000	duck
Cpt1a	0.35	0.00000000	duck
Helq	0.41	0.00000000	duck
Epn2	-1.22	0.00000000	duck
Ikzf2	0.47	0.00000000	duck
Eprs	0.29	0.00000000	duck
Tnrc6c	0.12	0.00000000	duck

Rgs19	0.50	0.00000000	duck
Tbc1d16	0.45	0.00000000	duck
Nsfl1c	0.43	0.00000000	duck
Ssr1	0.48	0.00000000	duck
Ssr3	0.53	0.00000000	duck
Zfyve28	0.35	0.00000000	duck
Acsf2	0.44	0.00000000	duck
Ran	0.47	0.00000000	duck
Gab3	0.52	0.00000000	duck
Helz	-0.02	0.00000000	duck
Wdfy2	0.59	0.00000000	duck
Scamp2	0.44	0.00000000	duck
Rab7	0.43	0.00000000	duck
C3	0.47	0.00000000	duck
Abracl	0.50	0.00000000	duck
Luc7l	0.25	0.00000000	duck
Txnrd3	0.32	0.00000000	duck
AU040320	0.25	0.00000000	duck
Prpf39	0.15	0.00000000	duck
AW554918	0.66	0.00000000	duck
Il17ra	0.35	0.00000000	duck
Kctd2	0.32	0.00000000	duck
Fdx1	0.31	0.00000000	duck
Tomm22	0.48	0.00000000	duck
Ndufb3	0.26	0.00000000	duck
Rexo2	0.48	0.00000000	duck
Kcnab2	0.42	0.00000000	duck
Errfi1	0.26	0.00000000	duck
Ankrd13a	0.32	0.00000000	duck
Zfp503	0.40	0.00000000	duck
Rrbp1	0.25	0.00000000	duck
Tmbim6	0.38	0.00000000	duck
Eef1d	0.20	0.00000000	duck
Il6ra	0.61	0.00000000	duck
Zc3h13	0.35	0.00000000	duck
Npc1	0.54	0.00000000	duck
Psmb3	0.50	0.00000000	duck
Tomm7	0.60	0.00000000	duck
Pgap4	0.39	0.00000000	duck
Echdc2	0.44	0.00000000	duck
Slc43a2	0.56	0.00000000	duck
Itpkb	0.46	0.00000000	duck
Adss	0.20	0.00000000	duck
Fbxl4	0.06	0.00000000	duck
Spi1	0.19	0.00000000	duck
Rbms1	0.12	0.00000000	duck

Helz2	0.20	0.00000000	duck
Vamp3	0.34	0.00000000	duck
Il18	0.57	0.00000000	duck
Imp3	0.37	0.00000000	duck
B4galt1	0.41	0.00000000	duck
Sh3glb1	0.21	0.00000000	duck
Slc35f5	0.45	0.00000000	duck
Rnf216	0.45	0.00000000	duck
Sec62	0.31	0.00000000	duck
Asap1	0.39	0.00000000	duck
Rnf20	0.31	0.00000000	duck
Rab2a	0.24	0.00000000	duck
Zzz3	0.08	0.00000000	duck
Prelid1	0.48	0.00000000	duck
Gtf2h5	0.30	0.00000000	duck
Slc30a9	0.10	0.00000000	duck
Lman2	0.40	0.00000000	duck
Ube2i	0.13	0.00000000	duck
Pamr1	0.44	0.00000000	duck
Cdv3	0.32	0.00000000	duck
Ehd4	0.34	0.00000000	duck
Hmgcr	0.38	0.00000000	duck
Sf3b6	0.35	0.00000000	duck
Prkar1b	0.37	0.00000000	duck
Rpa1	0.26	0.00000000	duck
Capn15	0.30	0.00000000	duck
Arhgap25	0.50	0.00000000	duck
Alpk1	0.43	0.00000000	duck
Clip1	0.33	0.00000000	duck
Wdr26	0.00	0.00000000	duck
Cdk17	-1.46	0.00000000	duck
Osbpl6	0.83	0.00000000	duck
Hras	0.29	0.00000000	duck
Ubn2	0.21	0.00000000	duck
Donson	0.38	0.00000000	duck
Puf60	0.18	0.00000000	duck
Zmat2	0.39	0.00000000	duck
Tmem33	0.42	0.00000000	duck
Psen1	0.29	0.00000000	duck
Rbm31y	0.35	0.00000000	duck
Ndufb9	0.49	0.00000000	duck
Tmem50a	0.40	0.00000000	duck
Samm50	0.18	0.00000000	duck
Wipi1	0.30	0.00000000	duck
Plcl1	0.26	0.00000000	duck
Ldah	0.39	0.00000000	duck

Stag1	0.36	0.00000000	duck
Plekha2	0.34	0.00000000	duck
Lgals3	0.33	0.00000000	duck
E2f2	0.36	0.00000000	duck
Ppp1r14c	0.64	0.00000000	duck
Pde8a	-1.40	0.00000000	duck
Alox5	-2.19	0.00000000	duck
Tdg	0.34	0.00000000	duck
Atp2a2	0.23	0.00000000	duck
Fam32a	0.36	0.00000000	duck
Cst7	0.43	0.00000000	duck
Chmp1a	0.36	0.00000000	duck
Psme3	0.34	0.00000000	duck
Nifk	0.37	0.00000000	duck
Raph1	0.72	0.00000000	duck
Cebpd	0.27	0.00000000	duck
Ccng2	-1.26	0.00000000	duck
Rpp38	0.34	0.00000000	duck
Oaz1	0.38	0.00000000	duck
Atad2b	0.30	0.00000000	duck
Dph5	0.34	0.00000000	duck
Prdx6	0.45	0.00000000	duck
Dcaf12	0.23	0.00000000	duck
Sf3b4	0.39	0.00000000	duck
Vapa	0.17	0.00000000	duck
Exosc3	0.34	0.00000000	duck
Efhd2	0.50	0.00000000	duck
Vma21	0.09	0.00000000	duck
Dhx58	0.29	0.00000000	duck
Tom1	0.35	0.00000000	duck
Psd3	0.51	0.00000000	duck
Sgsm3	0.37	0.00000000	duck
Caprin1	0.02	0.00000000	duck
Ywhae	0.23	0.00000000	duck
Eps15	0.26	0.00000000	duck
Nsmce2	0.47	0.00000000	duck
Psmd12	0.16	0.00000000	duck
Slc15a4	0.16	0.00000000	duck
Tpm3-rs7	0.38	0.00000000	duck
Vps13b	-2.00	0.00000000	duck
Sstr4	0.50	0.00000000	duck
Ano10	0.41	0.00000000	duck
Taf12	0.38	0.00000000	duck
Phactr2	0.90	0.00000000	duck
Chpf2	0.35	0.00000000	duck
Lrrk2	0.36	0.00000000	duck

Bzw2	0.21	0.00000000	duck
Tmem203	0.35	0.00000000	duck
Ei24	0.44	0.00000000	duck
Ampd3	0.26	0.00000000	duck
Ubqln1	0.06	0.00000000	duck
Rbm22	0.33	0.00000000	duck
Rtn4	0.25	0.00000000	duck
Itga4	-1.12	0.00000000	duck
Chd1	0.09	0.00000000	duck
Atp1b1	0.32	0.00000000	duck
Rngtt	0.82	0.00000000	duck
Zcchc7	0.08	0.00000000	duck
Gpr155	-0.16	0.00000000	duck
Slc7a11	0.43	0.00000000	duck
Cers5	0.19	0.00000000	duck
Aig1	0.47	0.00000000	duck
Golim4	0.40	0.00000000	duck
Phlpp2	0.09	0.00000000	duck
Lsm4	0.26	0.00000000	duck
Snrpc	0.44	0.00000000	duck
Ppargc1a	0.48	0.00000000	duck
Atg7	0.09	0.00000000	duck
Sbno2	0.11	0.00000000	duck
Snrpb	0.51	0.00000000	duck
Derl3	0.45	0.00000000	duck
Csnk1a1	0.22	0.00000000	duck
Extl3	0.28	0.00000000	duck
Rnf144a	0.27	0.00000000	duck
Ttf1	0.38	0.00000000	duck
Ankrd10	0.33	0.00000000	duck
Sidt2	0.33	0.00000000	duck
Slc26a4	0.33	0.00000000	duck
Slc7a3	0.45	0.00000000	duck
Shc1	0.34	0.00000000	duck
Bub3	0.38	0.00000000	duck
Rbpj	0.16	0.00000000	duck
Taok1	0.07	0.00000000	duck
Sec11a	0.45	0.00000000	duck
As3mt	0.38	0.00000000	duck
Fbxl20	0.19	0.00000000	duck
Esd	0.25	0.00000000	duck
Dram2	0.41	0.00000000	duck
Psmd4	0.11	0.00000000	duck
Ufm1	0.36	0.00000000	duck
Fth1	0.71	0.00000000	duck
Nudt12	0.30	0.00000000	duck

Kars	0.23	0.00000000	duck
Rnf13	0.25	0.00000000	duck
Poldip3	0.26	0.00000000	duck
Cct8	0.43	0.00000000	duck
Tax1bp3	0.29	0.00000000	duck
Pdss1	0.41	0.00000000	duck
Fem1a	0.27	0.00000000	duck
Cidea	0.51	0.00000000	duck
Commd1b	0.39	0.00000000	duck
Tfrc	-1.49	0.00000000	duck
Cops7a	0.38	0.00000000	duck
Adgrg3	0.39	0.00000000	duck
Smad5	0.24	0.00000000	duck
Pgs1	0.29	0.00000000	duck
Lemd2	0.19	0.00000000	duck
Kif2a	0.46	0.00000000	duck
Eef1b2	0.10	0.00000000	duck
Ensa	0.23	0.00000000	duck
Ntmt1	0.37	0.00000000	duck
Crispld2	0.53	0.00000000	duck
Pfn2	0.25	0.00000000	duck
Reep2	0.17	0.00000000	duck
Hvcn1	0.51	0.00000000	duck
Sgpp1	-0.02	0.00000000	duck
Sdc2	0.87	0.00000000	duck
Yeats4	0.42	0.00000000	duck
Zmpste24	0.11	0.00000000	duck
Usp14	0.15	0.00000000	duck
Jazf1	0.48	0.00000000	duck
Lamp2	0.17	0.00000000	duck
Gsr	0.32	0.00000000	duck
Bclaf3	0.42	0.00000000	duck
Ppm1k	0.39	0.00000000	duck
Ext1	0.76	0.00000000	duck
Blzf1	0.31	0.00000000	duck
Tgif1	0.48	0.00000000	duck
Stx8	-0.05	0.00000000	duck
Snap29	0.12	0.00000000	duck
Eif3j2	0.02	0.00000000	duck
Klf13	-1.13	0.00000000	duck
Snrpd1	0.40	0.00000000	duck
Eif3d	0.09	0.00000000	duck
Cpa6	0.48	0.00000000	duck
Appl1	0.32	0.00000000	duck
Tcf25	0.23	0.00000000	duck
Pum1	0.03	0.00000000	duck

Rb1cc1	-0.20	0.00000000	duck
Sucla2	0.28	0.00000000	duck
Arhgap29	0.09	0.00000000	duck
9130409I23Rik	0.23	0.00000000	duck
Emc3	0.35	0.00000000	duck
Zfp326	0.10	0.00000000	duck
Commd8	0.36	0.00000000	duck
Ngf	0.43	0.00000000	duck
Rnf141	0.22	0.00000000	duck
Ptpn6	0.39	0.00000000	duck
Psmd13	0.29	0.00000000	duck
Ndufa8	0.21	0.00000000	duck
Cope	0.44	0.00000000	duck
Ppargc1b	0.44	0.00000000	duck
Gxylt1	0.39	0.00000000	duck
Trerf1	0.31	0.00000000	duck
Satb1	0.41	0.00000000	duck
Krr1	0.37	0.00000000	duck
Pygl	0.34	0.00000000	duck
Itpka	0.04	0.00000000	duck
Exoc1	0.32	0.00000000	duck
Lsg1	0.44	0.00000000	duck
Ndufb4	0.55	0.00000000	duck
Jak2	-1.80	0.00000000	duck
Ikbke	0.40	0.00000000	duck
Fam107b	0.08	0.00000000	duck
Pgap2	0.46	0.00000000	duck
Tmed5	0.21	0.00000000	duck
Jade1	-1.73	0.00000000	duck
Avl9	0.24	0.00000000	duck
Gramd1c	0.36	0.00000000	duck
Il17b	0.31	0.00000000	duck
Ckap5	0.17	0.00000000	duck
Etv3	0.13	0.00000000	duck
Hk2	0.29	0.00000000	duck
Ndufb1	0.34	0.00000000	duck
Atxn1	0.28	0.00000000	duck
Iws1	0.15	0.00000000	duck
Cisd2	0.29	0.00000000	duck
Fbxo3	-2.04	0.00000000	duck
Trim23	0.21	0.00000000	duck
Traf2	0.21	0.00000000	duck
Wdr24	0.31	0.00000000	duck
Cfd	0.58	0.00000000	duck
Abhd6	0.45	0.00000000	duck
Cmas	0.35	0.00000000	duck

Eepd1	-1.51	0.00000000	duck
Fgd3	0.18	0.00000000	duck
Vps35l	0.03	0.00000000	duck
Zfp131	0.23	0.00000000	duck
Gns	0.26	0.00000000	duck
Naga	0.43	0.00000000	duck
Pbx1	0.22	0.00000000	duck
Rpn1	0.43	0.00000000	duck
Echs1	0.38	0.00000000	duck
Slc9a5	0.35	0.00000000	duck
Arhgef6	0.32	0.00000000	duck
Tcerg1	0.13	0.00000000	duck
Ubxn2a	0.29	0.00000000	duck
Klhl24	0.04	0.00000000	duck
Git2	0.16	0.00000000	duck
Dusp15	0.27	0.00000000	duck
Caap1	0.20	0.00000000	duck
Cyria	0.29	0.00000000	duck
Adipor1	0.23	0.00000000	duck
Ipmk	-1.62	0.00000000	duck
Fxn	0.22	0.00000000	duck
Rtf2	0.00	0.00000000	duck
Nipa2	0.13	0.00000000	duck
Tob2	0.28	0.00000000	duck
Asxl2	-0.19	0.00000000	duck
Hypk	0.37	0.00000000	duck
Bin2	0.31	0.00000000	duck
Atic	0.37	0.00000000	duck
Glipr2	0.21	0.00000000	duck
Leprot	0.37	0.00000000	duck
Rhot1	0.33	0.00000000	duck
Tmx3	0.28	0.00000000	duck
Grb2	0.21	0.00000000	duck
Galnt11	0.42	0.00000000	duck
Garem1	0.13	0.00000000	duck
Kras	0.22	0.00000000	duck
Eef2	0.11	0.00000000	duck
Cep112	0.44	0.00000000	duck
Actr10	0.30	0.00000000	duck
Lta4h	0.29	0.00000000	duck
Plekhm2	0.42	0.00000000	duck
Gzf1	0.28	0.00000000	duck
Nudt5	0.38	0.00000000	duck
Tspan4	0.56	0.00000000	duck
Babam2	0.23	0.00000000	duck
Bbx	0.45	0.00000000	duck

Arhgap1	0.27	0.00000000	duck
Prdx3	0.35	0.00000000	duck
Zbtb21	0.27	0.00000000	duck
Ulk4	-0.60	0.00000000	duck
Tor4a	0.08	0.00000000	duck
Raver2	0.37	0.00000000	duck
Tnni2	0.30	0.00000000	duck
Asah1	0.29	0.00000000	duck
Taar1	0.45	0.00000000	duck
Jade2	-1.41	0.00000000	duck
Sirt6	0.29	0.00000000	duck
Trdmt1	0.05	0.00000000	duck
Fbxo8	0.06	0.00000000	duck
Nme7	0.33	0.00000000	duck
Eif3a	-0.11	0.00000000	duck
Polr2l	0.48	0.00000000	duck
Bin3	0.30	0.00000000	duck
Golph3	0.19	0.00000000	duck
Slc44a2	0.34	0.00000000	duck
Pstpip1	0.12	0.00000000	duck
Dusp1	0.54	0.00000000	duck
Zfand6	0.03	0.00000000	duck
Dennd4c	0.50	0.00000000	duck
Ppp6c	0.31	0.00000000	duck
Fhod1	0.33	0.00000000	duck
Arxes2	0.36	0.00000000	duck
Golga7	0.23	0.00000000	duck
Sh3pxd2a	0.28	0.00000000	duck
Ppt1	0.46	0.00000000	duck
Vps4b	0.22	0.00000000	duck
Ncoa2	0.17	0.00000000	duck
Psmc2	0.36	0.00000000	duck
Prkaa1	0.15	0.00000000	duck
Tusc2	0.31	0.00000000	duck
Cstf3	-0.04	0.00000000	duck
Ppif	0.45	0.00000000	duck
Itgav	0.27	0.00000000	duck
Scarb1	0.31	0.00000000	duck
Nfkb2	0.39	0.00000000	duck
Cep83	0.29	0.00000000	duck
Csf3r	0.36	0.00000000	duck
Cmss1	0.70	0.00000000	duck
Cdc42bpb	0.24	0.00000000	duck
Rad21	0.02	0.00000000	duck
Brip1	0.27	0.00000000	duck
Tes	0.33	0.00000000	duck

Arfgef2	0.05	0.00000000	duck
Ephb1	0.94	0.00000000	duck
Btd	0.25	0.00000000	duck
Nup98	-0.20	0.00000000	duck
Wrn	-1.44	0.00000000	duck
Fbxl5	-0.88	0.00000000	duck
Pdxk	0.12	0.00000000	duck
Gpr75	0.29	0.00000000	duck
Nrip1	-0.07	0.00000000	duck
Slc2a13	0.53	0.00000000	duck
Rnps1	0.24	0.00000000	duck
Derl1	0.05	0.00000000	duck
Zc3h18	0.01	0.00000000	duck
Nudt14	0.32	0.00000000	duck
Rragc	0.28	0.00000000	duck
Cdyl2	-0.07	0.00000000	duck
Nhsl2	0.36	0.00000000	duck
Rpf2	0.30	0.00000000	duck
Ifitm10	0.26	0.00000000	duck
Rhbdf2	0.24	0.00000000	duck
Papss1	0.45	0.00000000	duck
Dhx36	0.12	0.00000000	duck
Fmnl1	0.30	0.00000000	duck
Ip6k2	0.08	0.00000000	duck
Psmc3	0.21	0.00000000	duck
Bcor	-1.16	0.00000000	duck
Arntl2	0.07	0.00000000	duck
Cnih1	0.32	0.00000000	duck
Tnfaip8	0.37	0.00000000	duck
Emc6	0.30	0.00000000	duck
Llph	0.13	0.00000000	duck
Notch2	0.36	0.00000000	duck
Naxd	0.46	0.00000000	duck
Brca2	0.45	0.00000000	duck
Tmem199	0.31	0.00000000	duck
Rnf128	0.36	0.00000000	duck
Fmr1	-0.17	0.00000000	duck
Dctn4	0.23	0.00000000	duck
Atp5c1	0.22	0.00000000	duck
Vps72	0.29	0.00000000	duck
Epc2	-0.04	0.00000000	duck
Tmem42	0.38	0.00000000	duck
Nudc	0.25	0.00000000	duck
Hpx	1.26	0.00000000	duck
Dipk1b	0.34	0.00000000	duck
Sh3gl3	0.35	0.00000000	duck

Acot7	0.39	0.00000000	duck
Bhlhe41	0.39	0.00000000	duck
Pepd	0.51	0.00000000	duck
Exoc6	0.17	0.00000000	duck
Pla2r1	1.11	0.00000000	duck
Orc4	0.30	0.00000000	duck
Znrf2	0.11	0.00000000	duck
Ddx17	-1.35	0.00000000	duck
Ppm1d	0.10	0.00000000	duck
Insig1	0.49	0.00000000	duck
Ranbp3	-0.20	0.00000000	duck
Washc5	0.35	0.00000000	duck
Ttc33	0.06	0.00000000	duck
Top3a	0.28	0.00000000	duck
Ccnk	0.16	0.00000000	duck
Nxt2	-1.36	0.00000000	duck
Ric8a	0.30	0.00000000	duck
Septin7	0.10	0.00000000	duck
Psmc1	0.29	0.00000000	duck
Ube2j1	0.14	0.00000000	duck
Rnf115	0.25	0.00000000	duck
Papln	0.38	0.00000000	duck
Rufy1	0.24	0.00000000	duck
Zdhhc9	0.34	0.00000000	duck
Sec63	-1.57	0.00000000	duck
Sec14l1	0.11	0.00000000	duck
Sort1	0.34	0.00000000	duck
Sec61a1	0.37	0.00000000	duck
Herc2	-1.97	0.00000000	duck
Polr1e	0.24	0.00000000	duck
Gdi2	0.42	0.00000000	duck
Spg21	0.25	0.00000000	duck
Tnfaip8l1	0.30	0.00000000	duck
Ppp2r2a	0.03	0.00000000	duck
Slc37a1	-0.03	0.00000000	duck
Plxnb2	2.04	0.00000000	duck
Spsb1	0.36	0.00000000	duck
Slco4c1	0.31	0.00000000	duck
P4hb	0.27	0.00000000	duck
Ndufa4	0.46	0.00000000	duck
Cd9	0.21	0.00000000	duck
Ggcx	0.30	0.00000000	duck
Dnaja1	0.29	0.00000000	duck
Htr4	-1.07	0.00000000	duck
Osbpl1a	-1.78	0.00000000	duck
Gda	0.28	0.00000000	duck

Sf3a1	0.13	0.00000000	duck
Tmem183a	-0.13	0.00000000	duck
Rab22a	-0.02	0.00000000	duck
Asb3	0.21	0.00000000	duck
Hmces	0.22	0.00000000	duck
Csf1r	0.22	0.00000000	duck
Ccpg1	-0.02	0.00000000	duck
Strip1	0.14	0.00000000	duck
Tmem268	0.21	0.00000000	duck
Abhd2	0.18	0.00000000	duck
Rilpl2	0.35	0.00000000	duck
Tpm2	0.35	0.00000000	duck
Ern1	0.04	0.00000000	duck
Ptbp2	0.30	0.00000000	duck
Rab9	0.03	0.00000000	duck
Kdm7a	-0.80	0.00000000	duck
Exoc5	0.05	0.00000000	duck
Usp34	-0.10	0.00000000	duck
Psmb7	0.06	0.00000000	duck
Dtx2	0.20	0.00000000	duck
Ubqln4	0.26	0.00000000	duck
Abca4	0.37	0.00000000	duck
Antxr2	-1.14	0.00000000	duck
Pum2	-1.22	0.00000000	duck
Polr2f	0.42	0.00000000	duck
Ap3d1	-0.10	0.00000000	duck
B4galt2	0.27	0.00000000	duck
Ugdh	0.31	0.00000000	duck
Slc25a3	0.16	0.00000000	duck
Usp8	-0.03	0.00000000	duck
Coro2a	0.40	0.00000000	duck
Usp33	-0.04	0.00000000	duck
Tifa	0.29	0.00000000	duck
Smim5	0.29	0.00000000	duck
Trappc9	0.46	0.00000000	duck
Usp4	0.12	0.00000000	duck
Tmem222	0.28	0.00000000	duck
Tspan3	0.05	0.00000000	duck
Plekhm3	0.35	0.00000000	duck
Disc1	0.71	0.00000000	duck
Paip1	0.05	0.00000000	duck
Relch	-0.03	0.00000000	duck
Adpgk	0.33	0.00000000	duck
Fkbp3	0.12	0.00000000	duck
Pithd1	0.30	0.00000000	duck
Kif5c	0.37	0.00000000	duck

Hfm1	-0.46	0.00000000	duck
Gnai3	-1.15	0.00000000	duck
Srsf4	0.29	0.00000000	duck
Gnb4	0.14	0.00000000	duck
Kidins220	0.07	0.00000000	duck
Coq5	0.35	0.00000000	duck
Tmem167	0.14	0.00000000	duck
Hlf	0.49	0.00000000	duck
N4bp2	0.34	0.00000000	duck
Ppp1r3c	0.27	0.00000000	duck
Creb3l2	0.32	0.00000000	duck
Nlrc5	-0.01	0.00000000	duck
Atp6v1c1	0.26	0.00000000	duck
Fam53a	0.10	0.00000000	duck
Rcc1l	0.22	0.00000000	duck
Brd7	0.04	0.00000000	duck
Aak1	0.07	0.00000000	duck
Trappc4	0.27	0.00000000	duck
Nptn	0.16	0.00000000	duck
Pdia4	0.49	0.00000000	duck
Pou1f1	0.42	0.00000000	duck
Aimp1	0.03	0.00000000	duck
Slc2a6	0.39	0.00000000	duck
Tank	0.24	0.00000000	duck
Rhoa	0.36	0.00000000	duck
Zcchc14	0.29	0.00000000	duck
Nek7	0.10	0.00000000	duck
Exoc2	0.08	0.00000000	duck
Slc1a2	0.16	0.00000000	duck
Prkag1	0.28	0.00000000	duck
Guk1	0.27	0.00000000	duck
Rab11fip4	0.31	0.00000000	duck
Cdkn1a	0.45	0.00000000	duck
Fyb	0.37	0.00000000	duck
Parp16	0.28	0.00000000	duck
Arpp19	0.01	0.00000000	duck
Tasor	0.00	0.00000000	duck
DPCD	0.26	0.00000000	duck
Tnfaip3	0.17	0.00000000	duck
Tpra1	0.07	0.00000000	duck
Srf	0.18	0.00000000	duck
Polr2k	0.26	0.00000000	duck
Gga2	0.29	0.00000000	duck
Rnf41	0.21	0.00000000	duck
Tut7	0.12	0.00000000	duck
Rassf5	0.42	0.00000000	duck

Eif3m	0.18	0.00000000	duck
Lpar1	0.85	0.00000000	duck
Tbc1d15	-0.23	0.00000000	duck
Zfp36l2	0.18	0.00000000	duck
Zdhhc12	0.13	0.00000000	duck
Kdm2a	0.00	0.00000000	duck
Traf7	0.20	0.00000000	duck
Rab21	0.05	0.00000000	duck
Wdr91	0.04	0.00000000	duck
Acbd3	0.27	0.00000000	duck
Stim2	0.47	0.00000000	duck
Smu1	0.10	0.00000000	duck
Ubr2	-1.58	0.00000000	duck
Casp8ap2	0.14	0.00000000	duck
Igf2bp3	0.53	0.00000000	duck
Tfpi2	0.37	0.00000000	duck
Gabarapl1	0.19	0.00000000	duck
Vdac2	0.27	0.00000000	duck
Lactb	0.35	0.00000000	duck
Pgm2	0.19	0.00000000	duck
Gatad2a	0.14	0.00000000	duck
Slc44a1	0.48	0.00000000	duck
Wipi2	0.01	0.00000000	duck
Pnpla2	0.14	0.00000000	duck
Smarce1	0.11	0.00000000	duck
Serinc1	-0.11	0.00000000	duck
Serinc3	0.21	0.00000000	duck
Acvr2a	0.17	0.00000000	duck
Cmtm3	0.32	0.00000000	duck
Pde6d	0.35	0.00000000	duck
Aftph	0.06	0.00000000	duck
Anxa4	0.23	0.00000000	duck
Atp6v1c2	0.36	0.00000000	duck
Surf4	0.30	0.00000000	duck
Ak2	-0.41	0.00000000	duck
Grid1	0.27	0.00000000	duck
Fam114a2	0.14	0.00000000	duck
Trmu	0.26	0.00000000	duck
Nprl3	-1.30	0.00000000	duck
Plk2	0.30	0.00000000	duck
Hsdl1	0.22	0.00000000	duck
Pdap1	0.19	0.00000000	duck
Trp53inp1	0.11	0.00000000	duck
Ywhaz	0.14	0.00000000	duck
Tmem67	0.33	0.00000000	duck
Blmh	0.20	0.00000000	duck

Fbxw11	0.01	0.00000000	duck
Lmbr1	0.27	0.00000000	duck
Prpf40a	0.10	0.00000000	duck
Gpr160	0.30	0.00000000	duck
Vapb	-0.06	0.00000000	duck
Sde2	-0.06	0.00000000	duck
Dennd11	0.29	0.00000000	duck
Ptpa	0.18	0.00000000	duck
Capn7	-1.40	0.00000000	duck
Elovl1	0.37	0.00000000	duck
Tcp11l2	0.03	0.00000000	duck
Kdm1b	0.17	0.00000000	duck
Tex264	0.29	0.00000000	duck
Akt1	0.31	0.00000000	duck
Faf2	0.04	0.00000000	duck
Ubl7	0.25	0.00000000	duck
Cmtm6	0.21	0.00000000	duck
Aff4	-0.95	0.00000000	duck
Themis2	0.32	0.00000000	duck
Nav1	0.20	0.00000000	duck
Vta1	-0.04	0.00000000	duck
Xbp1	0.05	0.00000000	duck
Stk17b	-1.32	0.00000000	duck
Vamp4	0.31	0.00000000	duck
Ctif	0.39	0.00000000	duck
Fbxw7	0.24	0.00000000	duck
Tpm1	-0.06	0.00000000	duck
Slc10a7	0.24	0.00000000	duck
B4galt3	0.19	0.00000000	duck
Sdhaf4	0.28	0.00000000	duck
Cnpy3	0.35	0.00000000	duck
Zbtb7b	0.28	0.00000000	duck
Ap1g1	-0.08	0.00000000	duck
Usp7	-0.13	0.00000000	duck
Nr1h3	0.10	0.00000000	duck
9930021J03Rik	0.03	0.00000000	duck
Zc3h12a	0.18	0.00000000	duck
Ehd3	0.15	0.00000000	duck
Fnbp1l	0.37	0.00000000	duck
Klhl36	0.23	0.00000000	duck
Fmn1	0.40	0.00000000	duck
Tlnrd1	0.30	0.00000000	duck
Ifnar1	-1.53	0.00000000	duck
Cldn18	0.72	0.00000000	duck
Sirt5	0.13	0.00000000	duck
Cnot8	0.16	0.00000000	duck

Hivep1	0.01	0.00000000	duck
Tmem251	0.20	0.00000000	duck
Copa	0.15	0.00000000	duck
Slc22a23	0.05	0.00000000	duck
Ankmy2	0.06	0.00000000	duck
Cnot2	-0.18	0.00000000	duck
Tbc1d9b	0.06	0.00000000	duck
Lrp12	0.17	0.00000000	duck
Lrrc8a	0.16	0.00000000	duck
Ncln	0.28	0.00000000	duck
Psmd1	-0.15	0.00000000	duck
G3bp2	0.02	0.00000000	duck
Lpgat1	0.04	0.00000000	duck
Fxr1	-1.73	0.00000000	duck
Susd6	-1.38	0.00000000	duck
Txn2	0.26	0.00000000	duck
Galk1	0.29	0.00000000	duck
Slc17a5	0.05	0.00000000	duck
Phc1	0.21	0.00000000	duck
Ap3m1	0.20	0.00000000	duck
Rsrc1	0.28	0.00000000	duck
Tet2	0.26	0.00000000	duck
Abcc4	-1.34	0.00000000	duck
Idh3a	0.43	0.00000000	duck
Brix1	0.17	0.00000000	duck
Acsl3	0.27	0.00000000	duck
Shoc2	0.11	0.00000000	duck
Snx12	0.15	0.00000000	duck
Btg1b	0.09	0.00000000	duck
Lzic	0.27	0.00000000	duck
Capzb	0.17	0.00000000	duck
Foxj2	0.08	0.00000000	duck
Gpx3	0.44	0.00000000	duck
Bnc2	-0.81	0.00000000	duck
Ripk1	0.28	0.00000000	duck
Lrch3	-0.07	0.00000000	duck
Garre1	0.05	0.00000000	duck
Gnptg	0.13	0.00000000	duck
Zhx1	0.07	0.00000000	duck
Cdk6	0.66	0.00000000	duck
Slc39a9	-1.07	0.00000000	duck
Trio	0.65	0.00000000	duck
Unc119b	0.20	0.00000000	duck
Samd4	0.25	0.00000000	duck
Clint1	-0.01	0.00000000	duck
Cwc15	-0.09	0.00000000	duck

Phf5a	0.15	0.00000000	duck
Purb	0.24	0.00000000	duck
Fbxl17	0.36	0.00000000	duck
Wasf2	0.17	0.00000000	duck
Adrm1	0.00	0.00000000	duck
Fam204a	0.00	0.00000000	duck
Lbr	-0.23	0.00000000	duck
Cog7	0.32	0.00000000	duck
Clp1	0.24	0.00000000	duck
Tbl1xr1	0.08	0.00000000	duck
Vwa8	0.31	0.00000000	duck
Idh1	0.15	0.00000000	duck
Amacr	0.31	0.00000000	duck
Txndc12	0.23	0.00000000	duck
Tmem164	0.06	0.00000000	duck
Wbp4	-1.50	0.00000000	duck
Eri1	0.15	0.00000000	duck
Spcs2	0.33	0.00000000	duck
Hsdl2	0.32	0.00000000	duck
Parp11	0.30	0.00000000	duck
Jpt1	0.09	0.00000000	duck
Eif3b	0.10	0.00000000	duck
Stxbp5	0.00	0.00000000	duck
Gla	0.23	0.00000000	duck
Fbxo25	-0.10	0.00000000	duck
Rnf149	0.09	0.00000000	duck
Ttc39a	0.19	0.00000000	duck
Slc30a5	0.04	0.00000000	duck
Far1	-0.08	0.00000000	duck
Impa1	0.03	0.00000000	duck
Pdcd6	0.14	0.00000000	duck
Zfat	-0.11	0.00000000	duck
Zswim8	-0.10	0.00000000	duck
Polr2b	0.21	0.00000000	duck
Psmf1	0.12	0.00000000	duck
Secisbp2l	-0.14	0.00000000	duck
Ascc2	0.09	0.00000000	duck
Tars	0.31	0.00000000	duck
St8sia1	0.43	0.00000000	duck
Golph3l	0.26	0.00000000	duck
Dsn1	0.21	0.00000000	duck
Vcl	-0.04	0.00000000	duck
Zfp654	0.02	0.00000000	duck
Nsd1	-0.09	0.00000000	duck
Tmem248	0.20	0.00000000	duck
Cers2	0.15	0.00000000	duck

Pbx4	-1.27	0.00000000	duck
Zcrb1	0.15	0.00000000	duck
Pelo	0.16	0.00000000	duck
Lcp1	0.24	0.00000000	duck
Psmg3	0.28	0.00000000	duck
Dapk1	0.62	0.00000000	duck
Carhsp1	0.15	0.00000000	duck
Smarca5	0.01	0.00000000	duck
Atg12	0.28	0.00000000	duck
Yars	0.27	0.00000000	duck
Phb2	0.14	0.00000000	duck
Stx11	0.18	0.00000000	duck
Pafah1b1	-0.02	0.00000000	duck
Oit3	-1.03	0.00000000	duck
Tsc22d3	-0.93	0.00000000	duck
Bola3	0.18	0.00000000	duck
Plin2	0.10	0.00000000	duck
Fig4	0.06	0.00000000	duck
Kif5b	-0.22	0.00000000	duck
Sp3	-1.09	0.00000000	duck
Lysmd4	0.09	0.00000000	duck
Cactin	0.09	0.00000000	duck
Eif2s2	-0.02	0.00000000	duck
Ncstn	0.22	0.00000000	duck
Hdac4	0.12	0.00000000	duck
Zfhx3	0.24	0.00000000	duck
Taf3	-0.27	0.00000000	duck
Aptx	0.20	0.00000000	duck
Gramd2	0.28	0.00000000	duck
Nfkbie	0.19	0.00000000	duck
Edc3	0.11	0.00000000	duck
Uprt	0.25	0.00000000	duck
Dusp7	0.24	0.00000000	duck
Phb	0.35	0.00000000	duck
Klhl21	0.15	0.00000000	duck
Atp11a	-1.13	0.00000000	duck
Plekha1	0.33	0.00000000	duck
Rtraf	0.22	0.00000000	duck
Agap1	0.97	0.00000000	duck
Pin1	0.20	0.00000000	duck
Josd1	0.10	0.00000000	duck
Slc44a3	0.23	0.00000000	duck
Abcc1	-1.70	0.00000000	duck
Rab39b	0.22	0.00000000	duck
Bag5	-1.13	0.00000000	duck
Gusb	0.23	0.00000000	duck

Filip1l	0.26	0.00000000	duck
Dntt	0.37	0.00000000	duck
Grb10	0.36	0.00000000	duck
Ndufa6	0.25	0.00000000	duck
Ifih1	-0.06	0.00000000	duck
Rheb	-0.07	0.00000000	duck
Cog1	0.28	0.00000000	duck
Ralgapa2	-1.55	0.00000000	duck
Tor2a	0.18	0.00000000	duck
Stoml1	0.23	0.00000000	duck
Smyd2	0.28	0.00000000	duck
Aebp2	-0.34	0.00000000	duck
Rab14	0.02	0.00000000	duck
Fam20c	0.26	0.00000000	duck
Tcf7l2	0.25	0.00000000	duck
Trim8	0.24	0.00000000	duck
Gnas	-0.36	0.00000000	duck
Ppp1cc	0.16	0.00000000	duck
Eif4a3l1	0.09	0.00000000	duck
Rac1	0.11	0.00000000	duck
Kbtbd4	0.20	0.00000000	duck
Rnh1	0.26	0.00000000	duck
Camta1	-0.09	0.00000000	duck
Rnf220	-0.07	0.00000000	duck
Bend5	0.10	0.00000000	duck
Lmf2	0.37	0.00000000	duck
Tut4	-0.27	0.00000000	duck
Rbm17	0.04	0.00000000	duck
Psmg4	0.22	0.00000000	duck
Slc38a10	0.13	0.00000000	duck
Nfatc3	-0.15	0.00000000	duck
Isca2	0.07	0.00000000	duck
Creb5	0.65	0.00000000	duck
Snx13	0.02	0.00000000	duck
Lcor	0.17	0.00000000	duck
Rsl24d1	0.07	0.00000000	duck
Klf7	0.12	0.00000000	duck
Prkd3	0.14	0.00000000	duck
Ccny	0.05	0.00000000	duck
Tanc1	0.09	0.00000000	duck
Gpatch2	0.22	0.00000000	duck
Ubash3b	0.32	0.00000000	duck
Trappc2l	0.19	0.00000000	duck
Ppp2r5e	-0.20	0.00000000	duck
Zc3h15	-0.15	0.00000000	duck
Ddost	0.38	0.00000000	duck

Bysl	0.28	0.00000000	duck
Ptger4	0.32	0.00000000	duck
Rbm47	0.57	0.00000000	duck
Tmem242	0.25	0.00000000	duck
Sat1	0.07	0.00000000	duck
Plbd2	0.30	0.00000000	duck
Ssbp3	-1.37	0.00000000	duck
Lrrfip2	0.16	0.00000000	duck
Arl6ip4	0.14	0.00000000	duck
Tmem165	0.13	0.00000000	duck
Slc25a47	0.25	0.00000000	duck
Rassf2	0.22	0.00000000	duck
Crem	0.22	0.00000000	duck
Txndc17	0.38	0.00000000	duck
Wsb2	0.26	0.00000000	duck
Ccdc134	0.28	0.00000000	duck
Gpbp1l1	-0.31	0.00000000	duck
Slc39a13	-0.07	0.00000000	duck
Snw1	-0.09	0.00000000	duck
Slc35b3	0.02	0.00000000	duck
Snrnp200	0.10	0.00000000	duck
Hmox2	0.10	0.00000000	duck
Rc3h1	-0.38	0.00000000	duck
Slc66a1	0.34	0.00000000	duck
Nmt1	0.30	0.00000000	duck
Cry2	0.28	0.00000000	duck
Ndufa5	0.11	0.00000000	duck
Swap70	0.17	0.00000000	duck
Vangl1	0.25	0.00000000	duck
Vps18	0.11	0.00000000	duck
C1qa	1.66	0.00000000	duck
Nfe2l1	0.16	0.00000000	duck
Cldnd1	0.17	0.00000000	duck
Emc8	0.07	0.00000000	duck
Thrap3	-0.22	0.00000000	duck
Eif3h	-0.17	0.00000000	duck
Gab2	0.37	0.00000000	duck
Hadh	0.29	0.00000000	duck
Picalm	-0.21	0.00000000	duck
Vti1b	0.08	0.00000000	duck
Ctcf	-0.09	0.00000000	duck
Chsy1	-0.18	0.00000000	duck
Chchd3	0.01	0.00000000	duck
Sh2b2	0.30	0.00000000	duck
Tmx4	-0.05	0.00000000	duck
Sirt7	0.15	0.00000000	duck

Dapk2	0.04	0.00000000	duck
Creg1	-1.93	0.00000000	duck
Nrp1	1.00	0.00000000	duck
Trim3	0.23	0.00000000	duck
Rnf10	-0.06	0.00000000	duck
Irf2bpl	0.28	0.00000000	duck
Dusp10	-0.10	0.00000000	duck
Sptlc1	0.13	0.00000000	duck
Tshz2	0.37	0.00000000	duck
Arnt	-0.17	0.00000000	duck
Kat6a	-0.10	0.00000000	duck
Etnk1	-0.04	0.00000000	duck
Ankrd12	-1.24	0.00000000	duck
Zcchc17	0.10	0.00000000	duck
Upf1	-0.02	0.00000000	duck
Tbc1d22a	0.09	0.00000000	duck
Lbh	0.12	0.00000000	duck
Uhmk1	0.14	0.00000000	duck
Vps37a	-0.01	0.00000000	duck
Anapc13	0.23	0.00000000	duck
Dnaja2	0.05	0.00000000	duck
Vps13d	-1.37	0.00000000	duck
Slc3a1	0.12	0.00000000	duck
Ogdh	-0.90	0.00000000	duck
Tcea1	0.11	0.00000000	duck
Tubb4b	0.20	0.00000000	duck
Gyg	0.09	0.00000000	duck
Plekha7	0.44	0.00000000	duck
Arl1	0.20	0.00000000	duck
Nup50	-0.04	0.00000000	duck
Rspry1	0.00	0.00000000	duck
Atp2a3	0.17	0.00000000	duck
Snrnp40	0.27	0.00000000	duck
Plcb4	0.37	0.00000000	duck
Nt5dc2	-0.99	0.00000000	duck
Slc46a2	0.54	0.00000000	duck
Natd1	0.25	0.00000000	duck
Fut8	0.10	0.00000000	duck
Ugcg	-1.26	0.00000000	duck
Yipf4	-0.08	0.00000000	duck
Furin	0.00	0.00000000	duck
Ostc	0.27	0.00000000	duck
Spag9	-0.04	0.00000000	duck
Smim20	0.23	0.00000000	duck
Kat2b	-0.14	0.00000000	duck
D5Ertd579e	-1.52	0.00000000	duck

Tlcd1	0.33	0.00000000	duck
Insr	-0.04	0.00000000	duck
Cct4	0.13	0.00000000	duck
Sod1	-0.20	0.00000000	duck
Stx7	0.18	0.00000000	duck
B3gnt2	-0.01	0.00000000	duck
Zyx	0.15	0.00000000	duck
Disp1	-1.49	0.00000000	duck
Acadl	0.16	0.00000000	duck
Clic4	0.12	0.00000000	duck
Elovl7	0.32	0.00000000	duck
Pstpip2	0.02	0.00000000	duck
Tmf1	-0.10	0.00000000	duck
Sdf2l1	0.26	0.00000000	duck
Vash1	-1.34	0.00000000	duck
Rasa2	-1.05	0.00000000	duck
C1qb	1.46	0.00000000	duck
Rpn2	0.04	0.00000000	duck
Noct	0.16	0.00000000	duck
Hadhb	0.16	0.00000000	duck
Hipk2	0.61	0.00000000	duck
Dip2b	0.21	0.00000000	duck
Vps37c	0.03	0.00000000	duck
Arf6	0.12	0.00000000	duck
Atp2c1	-0.04	0.00000000	duck
Crnkl1	0.08	0.00000000	duck
Ube2a	-0.10	0.00000000	duck
Atrx	-1.34	0.00000000	duck
Lysmd2	0.23	0.00000000	duck
Slc25a5	0.38	0.00000000	duck
Zfyve9	-0.09	0.00000000	duck
Galnt17	0.31	0.00000000	duck
Rab11fip2	0.12	0.00000000	duck
Ammecr1l	0.12	0.00000000	duck
Zfp513	0.16	0.00000000	duck
Vps51	0.21	0.00000000	duck
Ube2k	-0.28	0.00000000	duck
Fam193a	-0.05	0.00000000	duck
Ndufs3	0.12	0.00000000	duck
Hivep2	0.17	0.00000000	duck
Cdc26	0.17	0.00000000	duck
Rbks	0.20	0.00000000	duck
Bach1	0.08	0.00000000	duck
Gpatch4	0.20	0.00000000	duck
Prune1	0.14	0.00000000	duck
Thap1	0.06	0.00000000	duck

Smg1	-0.22	0.00000000	duck
Cass4	0.02	0.00000000	duck
Tmco1	0.04	0.00000000	duck
Pdia3	0.13	0.00000000	duck
Odc1	0.18	0.00000000	duck
Srebf2	0.30	0.00000000	duck
Scamp3	0.22	0.00000000	duck
Gcc2	-0.23	0.00000000	duck
Tepsin	0.16	0.00000000	duck
Trmt10a	0.16	0.00000000	duck
Fam114a1	0.29	0.00000000	duck
Il13ra1	0.20	0.00000000	duck
Hdac7	0.25	0.00000000	duck
Rbbp7	-0.85	0.00000000	duck
Itga1	0.17	0.00000000	duck
Sypl2	0.24	0.00000000	duck
Prkx	-1.05	0.00000000	duck
Ociad1	0.14	0.00000000	duck
Cyfip2	0.09	0.00000000	duck
Dnajc19	0.08	0.00000000	duck
Prpf19	0.23	0.00000000	duck
Pigt	0.17	0.00000000	duck
Tnfsf11	0.18	0.00000000	duck
Slc49a4	0.21	0.00000000	duck
Rab35	-0.01	0.00000000	duck
Hebp1	0.14	0.00000000	duck
Dnajb2	0.20	0.00000000	duck
Brms1l	0.02	0.00000000	duck
Pitpnb	0.05	0.00000000	duck
Nisch	-0.24	0.00000000	duck
Sec11c	0.14	0.00000000	duck
Ralbp1	-0.09	0.00000000	duck
Crim1	-1.64	0.00000000	duck
Herpud1	0.14	0.00000000	duck
Eif4a2	0.16	0.00000000	duck
Tmem87a	-0.04	0.00000000	duck
Sdf2	0.19	0.00000000	duck
Kctd5	-0.16	0.00000000	duck
Zbtb7a	0.09	0.00000000	duck
Larp4	-0.10	0.00000000	duck
Sh2b3	-1.26	0.00000000	duck
Bmp2	0.49	0.00000000	duck
Gosr1	-0.11	0.00000000	duck
Zfyve27	0.14	0.00000000	duck
Smad3	0.27	0.00000000	duck
Ppat	-0.56	0.00000000	duck

Derl2	0.09	0.00000000	duck
Zranb2	-1.15	0.00000000	duck
Scaf4	-0.10	0.00000000	duck
Ptp4a2	0.14	0.00000000	duck
Glyr1	0.01	0.00000000	duck
Eml4	0.37	0.00000000	duck
Chd9	-0.33	0.00000000	duck
Dpy30	0.17	0.00000000	duck
Tmtc3	-0.23	0.00000000	duck
Papolg	0.08	0.00000000	duck
Dtl	0.01	0.00000000	duck
Ndufv2	0.03	0.00000000	duck
Desi1	-0.10	0.00000000	duck
Senp5	0.05	0.00000000	duck
Ctnna1	0.30	0.00000000	duck
Usp15	-0.09	0.00000000	duck
Decr2	0.15	0.00000000	duck
Ergic1	-1.16	0.00000000	duck
Vps41	0.09	0.00000000	duck
Fam210b	0.16	0.00000000	duck
Zfyve21	0.07	0.00000000	duck
Shld1	0.15	0.00000000	duck
Lfng	0.21	0.00000000	duck
Gbe1	0.41	0.00000000	duck
Tmc7	0.17	0.00000000	duck
Ppm1f	0.08	0.00000000	duck
Btbd1	0.23	0.00000000	duck
Cdk5rap3	0.21	0.00000000	duck
Ahcyl1	0.11	0.00000000	duck
Naa35	0.17	0.00000000	duck
Polr2c	0.18	0.00000000	duck
Pank2	0.12	0.00000000	duck
Crkl	-0.02	0.00000000	duck
Eci3	0.12	0.00000000	duck
Amd2	0.09	0.00000000	duck
Pdia5	0.27	0.00000000	duck
Rtf1	-0.09	0.00000000	duck
Capza2	-0.07	0.00000000	duck
Bend7	0.26	0.00000000	duck
Neto2	0.28	0.00000000	duck
Fbxo32	-0.02	0.00000000	duck
Suds3	-0.05	0.00000000	duck
Cdk12	0.14	0.00000000	duck
Sap130	0.13	0.00000000	duck
Nkiras2	0.18	0.00000000	duck
Tnfsf15	0.36	0.00000000	duck

Ccnyl1	0.28	0.00000000	duck
Tm9sf3	0.16	0.00000000	duck
Smarca2	-0.24	0.00000000	duck
Sdf4	-0.07	0.00000000	duck
Bak1	0.20	0.00000000	duck
Gpbp1	-0.33	0.00000000	duck
L3mbtl3	0.13	0.00000000	duck
Pafah1b2	0.10	0.00000000	duck
Kbtbd8	-0.02	0.00000000	duck
Rbm25	-0.05	0.00000000	duck
Irf7	0.13	0.00000000	duck
Isca1	-0.04	0.00000000	duck
Dhrs3	0.08	0.00000000	duck
Ddx23	0.10	0.00000000	duck
Stk11	0.18	0.00000000	duck
Ppip5k2	0.19	0.00000000	duck
Larp1	0.01	0.00000000	duck
Pak2	0.09	0.00000000	duck
Zbtb16	0.55	0.00000000	duck
Fosl2	0.21	0.00000000	duck
Ripk2	0.37	0.00000000	duck
Frs2	-0.07	0.00000000	duck
Pcgf5	0.13	0.00000000	duck
Ywhab	0.11	0.00000000	duck
Elf1	-0.15	0.00000000	duck
Susd1	-0.87	0.00000000	duck
Dcun1d5	0.06	0.00000000	duck
Tbc1d8b	0.08	0.00000000	duck
Aldoc	0.26	0.00000000	duck
Nkd1	0.17	0.00000000	duck
Tns1	0.39	0.00000000	duck
Galns	0.00	0.00000000	duck
Syn2	0.05	0.00000000	duck
Ddx60	-0.58	0.00000000	duck
Slc25a37	-1.19	0.00000000	duck
Selenoi	0.22	0.00000000	duck
Rmi2	0.20	0.00000000	duck
Slc7a5	0.13	0.00000000	duck
Dgkd	-0.06	0.00000000	duck
Lrp8	0.18	0.00000000	duck
Ybx1	-0.08	0.00000000	duck
Ubxn2b	0.10	0.00000000	duck
Swi5	0.06	0.00000000	duck
Cwc25	0.02	0.00000000	duck
Appbp2	0.02	0.00000000	duck
Ranbp1	0.14	0.00000000	duck

Hnrnpa0	0.22	0.00000001	duck
Srpk1	-0.16	0.00000001	duck
Hdac2	0.11	0.00000001	duck
Smndc1	-0.01	0.00000001	duck
Ube4b	-0.12	0.00000001	duck
Dennd2a	0.27	0.00000001	duck
Serac1	0.14	0.00000001	duck
Fam185a	0.08	0.00000001	duck
Aurkaip1	0.25	0.00000001	duck
Lrmp	0.16	0.00000001	duck
Cited4	0.19	0.00000001	duck
Tmem223	0.25	0.00000001	duck
Emc2	0.17	0.00000001	duck
Plxna2	0.18	0.00000001	duck
Tia1	-0.11	0.00000001	duck
Rrm2b	0.19	0.00000001	duck
Lsm5	0.24	0.00000001	duck
Thyn1	0.19	0.00000001	duck
Cdc40	-0.18	0.00000001	duck
Lin52	0.21	0.00000001	duck
Pex13	0.07	0.00000001	duck
Pdhx	0.17	0.00000001	duck
Tacc1	0.18	0.00000001	duck
Ube2r2	-0.28	0.00000001	duck
Itpr1	0.16	0.00000001	duck
Triap1	0.19	0.00000001	duck
Get4	0.09	0.00000001	duck
Clcc1	-0.22	0.00000001	duck
Ambra1	-0.04	0.00000001	duck
Adgrg2	-0.66	0.00000001	duck
Tmem9b	0.14	0.00000001	duck
Cpne8	0.45	0.00000001	duck
Atxn1l	0.06	0.00000001	duck
Rcor1	0.03	0.00000001	duck
Ppp4r3a	-0.36	0.00000001	duck
Itgb6	0.22	0.00000001	duck
Cryl1	0.15	0.00000001	duck
Cndp2	0.20	0.00000001	duck
Tmem39a	0.23	0.00000001	duck
Adap1	0.20	0.00000001	duck
Slc35b1	0.24	0.00000001	duck
Rwdd1	0.26	0.00000001	duck
Ric8b	-0.14	0.00000002	duck
Vegfc	-0.21	0.00000002	duck
Pgpep1	0.18	0.00000002	duck
Tnip1	-0.01	0.00000002	duck

Snrpf	0.30	0.00000002	duck
Tom1l2	-0.02	0.00000002	duck
Ube2g2	0.16	0.00000002	duck
Ccnt1	0.09	0.00000002	duck
Ubn1	-0.17	0.00000002	duck
Crlf3	0.14	0.00000002	duck
Zmym3	0.14	0.00000002	duck
Acvr1b	0.06	0.00000002	duck
Snx30	0.24	0.00000002	duck
Tet1	-1.20	0.00000002	duck
Fars2	0.09	0.00000002	duck
Fnbp1	0.17	0.00000002	duck
Osbpl8	-0.12	0.00000002	duck
Ap2b1	0.03	0.00000002	duck
Supt3	0.25	0.00000002	duck
Plekhj1	0.14	0.00000002	duck
Usp48	-1.58	0.00000003	duck
Lemd3	-0.19	0.00000003	duck
Ccdc47	0.00	0.00000003	duck
Siah1b	-0.20	0.00000003	duck
Psd	0.19	0.00000003	duck
Gars	0.18	0.00000003	duck
Nde1	-0.05	0.00000003	duck
Specc1	0.14	0.00000003	duck
Rbl2	-0.23	0.00000003	duck
Klf5	0.20	0.00000003	duck
Naa60	0.00	0.00000003	duck
Zpr1	0.15	0.00000003	duck
Exosc7	0.04	0.00000003	duck
Acvr1	0.07	0.00000003	duck
Impact	0.17	0.00000003	duck
Cwf19l2	-0.03	0.00000003	duck
Suclg2	0.29	0.00000003	duck
Wsb1	-0.09	0.00000003	duck
Ttc1	0.00	0.00000003	duck
Pdlim5	0.16	0.00000003	duck
Wbp11	-0.19	0.00000003	duck
Dicer1	-1.23	0.00000003	duck
Zfp292	-0.19	0.00000004	duck
Eea1	0.14	0.00000004	duck
Sema7a	0.11	0.00000004	duck
Chn1	0.13	0.00000004	duck
Nfyc	-0.10	0.00000004	duck
Nipsnap1	0.21	0.00000004	duck
Pars2	0.22	0.00000004	duck
Nras	-0.01	0.00000004	duck

Ap3m2	0.09	0.00000004	duck
Ppie	0.16	0.00000005	duck
Rnf139	0.14	0.00000005	duck
Gtpbp10	-2.00	0.00000005	duck
Bnip2	-0.16	0.00000005	duck
Dcun1d1	-0.05	0.00000005	duck
Nfyb	0.15	0.00000005	duck
Cops4	-0.06	0.00000005	duck
Gtf2a2	0.15	0.00000005	duck
Tdrd3	-0.42	0.00000005	duck
Dock9	-0.80	0.00000005	duck
Braf	-0.27	0.00000006	duck
B3gnt4	0.21	0.00000006	duck
Rap2a	0.16	0.00000006	duck
Hsd17b4	-0.14	0.00000006	duck
Tmed2	0.12	0.00000006	duck
Ap3s1	0.04	0.00000006	duck
Ctsh	0.28	0.00000007	duck
Tm9sf4	0.19	0.00000007	duck
Rnaseh2b	0.15	0.00000007	duck
Zbtb10	0.24	0.00000007	duck
Stim1	0.08	0.00000007	duck
Bcar1	0.24	0.00000007	duck
Ppp1r8	0.07	0.00000007	duck
Pacsin2	0.02	0.00000008	duck
Nip7	0.20	0.00000008	duck
Slx4	0.08	0.00000008	duck
St6galnac4	0.26	0.00000009	duck
Vps36	0.06	0.00000009	duck
Tmem138	0.03	0.00000009	duck
Trp53bp2	0.12	0.00000009	duck
Ncf2	0.02	0.00000009	duck
Id3	0.34	0.00000010	duck
Rfc5	0.21	0.00000010	duck
Itpr2	0.02	0.00000010	duck
Areg	-0.91	0.00000010	duck
Rgp1	0.09	0.00000010	duck
Pcgf3	0.27	0.00000010	duck
Rnf34	-0.77	0.00000011	duck
G3bp1	-0.11	0.00000011	duck
Smcr8	0.15	0.00000011	duck
Rnf214	0.00	0.00000011	duck
Strn3	-0.38	0.00000012	duck
Heph	0.18	0.00000013	duck
Wdr53	0.07	0.00000013	duck
Tomm20	0.02	0.00000013	duck

Cdkal1	0.10	0.00000013	duck
Gsn	0.15	0.00000013	duck
Slc9a8	0.00	0.00000013	duck
Thoc5	0.09	0.00000013	duck
Pdzd8	-0.20	0.00000013	duck
Cul1	-0.31	0.00000013	duck
Tma16	0.23	0.00000013	duck
Retreg1	0.22	0.00000013	duck
Pmepa1	0.27	0.00000014	duck
Hes6	0.21	0.00000014	duck
Txnl1	-0.35	0.00000015	duck
Rnft1	0.13	0.00000015	duck
Csnk1g2	0.00	0.00000015	duck
Itprid2	0.31	0.00000015	duck
Kmt5b	-0.28	0.00000016	duck
Nfya	-0.27	0.00000016	duck
Ino80d	-0.21	0.00000016	duck
Ube2g1	-0.19	0.00000016	duck
Tpcn1	0.24	0.00000017	duck
Tspan8	0.29	0.00000017	duck
Plag1	-1.28	0.00000017	duck
Cop1	-1.09	0.00000018	duck
Fst	0.16	0.00000018	duck
Fyttd1	-0.07	0.00000018	duck
Ccdc77	-0.49	0.00000019	duck
Pak4	0.17	0.00000019	duck
Ccnl2	-0.06	0.00000019	duck
Zfyve26	-0.10	0.00000020	duck
Tcp1	0.05	0.00000021	duck
Rabepk	0.12	0.00000022	duck
Prkrip1	0.03	0.00000022	duck
Zmynd11	-1.34	0.00000023	duck
Stambpl1	-0.84	0.00000023	duck
Ccdc59	0.05	0.00000023	duck
Bsdc1	0.00	0.00000024	duck
Srrm1	-0.18	0.00000026	duck
Rpf1	0.08	0.00000026	duck
Acaca	0.21	0.00000027	duck
Taf15	0.21	0.00000027	duck
Nmt2	0.24	0.00000028	duck
Pwp1	0.27	0.00000029	duck
Spg11	0.06	0.00000029	duck
Dek	0.01	0.00000029	duck
Dars	-0.02	0.00000029	duck
Tbc1d5	0.14	0.00000030	duck
St13	-0.07	0.00000030	duck

Gk5	0.20	0.00000030	duck
4933400A11Rik	0.12	0.00000032	duck
Strada	-0.07	0.00000032	duck
Snx20	-0.10	0.00000034	duck
Pole4	0.20	0.00000034	duck
Six4	0.08	0.00000034	duck
Tfec	0.83	0.00000036	duck
Nt5e	0.11	0.00000036	duck
Hmg20b	0.18	0.00000037	duck
Plaa	-0.07	0.00000038	duck
Sec22a	0.12	0.00000038	duck
Tnip2	-1.25	0.00000038	duck
Ssrp1	0.29	0.00000038	duck
Atmin	-0.12	0.00000039	duck
Itpripl2	0.25	0.00000039	duck
Dtx4	0.20	0.00000041	duck
Slc19a2	0.09	0.00000042	duck
Hnrnpm	-0.21	0.00000043	duck
Tmem98	0.25	0.00000043	duck
Pdia6	0.38	0.00000043	duck
Abl1	-0.06	0.00000045	duck
Prrc1	0.07	0.00000048	duck
Srp72	0.03	0.00000049	duck
Ncoa6	-0.24	0.00000049	duck
Naa50	-0.15	0.00000051	duck
Arrdc4	0.02	0.00000052	duck
Slc30a1	0.11	0.00000052	duck
Gcn1	0.15	0.00000054	duck
Slc7a10	-0.55	0.00000055	duck
Ppfia1	-0.21	0.00000055	duck
Rapsn	0.16	0.00000059	duck
Prpf3	0.04	0.00000060	duck
Ctnnb1	-0.02	0.00000062	duck
Yy2	-0.28	0.00000064	duck
Ube2e3	-0.14	0.00000064	duck
Xpo4	0.04	0.00000068	duck
Ilf2	0.01	0.00000074	duck
Smap1	0.16	0.00000074	duck
Cxcr4	0.38	0.00000075	duck
Tln2	-0.39	0.00000077	duck
A4galt	0.20	0.00000079	duck
Opa1	-0.36	0.00000080	duck
Qars	0.15	0.00000080	duck
Timm13	0.24	0.00000080	duck
Ttc17	-0.23	0.00000081	duck
Rabgap1l	-0.18	0.00000083	duck

Hlcs	-0.16	0.00000086	duck
Ephx1	0.04	0.00000087	duck
Klf11	0.15	0.00000089	duck
Nr4a2	0.05	0.00000089	duck
Xpr1	-0.22	0.00000090	duck
Igf2r	-0.17	0.00000092	duck
Vps26a	0.07	0.00000096	duck
Pcyt2	-0.10	0.00000096	duck
Dpm2	0.14	0.00000096	duck
Arih1	-1.39	0.00000097	duck
Tpd52l2	0.01	0.00000098	duck
Ndufs7	0.10	0.00000098	duck
Kif13b	0.01	0.00000099	duck
Snx24	0.18	0.00000100	duck
Svil	-0.10	0.00000100	duck
Abhd13	-0.05	0.00000100	duck
Dcps	0.01	0.00000101	duck
Scaf11	-0.26	0.00000102	duck
Raf1	-0.15	0.00000107	duck
Atl2	-0.28	0.00000108	duck
Arcn1	-0.10	0.00000108	duck
Snu13	0.21	0.00000110	duck
Zdhhc21	0.22	0.00000111	duck
Alg2	0.10	0.00000113	duck
Copb2	0.06	0.00000116	duck
Rsrp1	-1.28	0.00000121	duck
Saraf	-0.03	0.00000126	duck
Abca2	0.13	0.00000127	duck
P2ry13	-0.02	0.00000129	duck
Cacna1i	0.11	0.00000132	duck
Tssc4	0.19	0.00000133	duck
Flnb	-0.43	0.00000134	duck
Nkrf	0.01	0.00000135	duck
Cript	0.12	0.00000136	duck
Jun	0.39	0.00000140	duck
Ginm1	0.00	0.00000140	duck
Zdhhc7	-0.12	0.00000143	duck
Plekha5	0.33	0.00000146	duck
Pcif1	-0.01	0.00000148	duck
Atp5pb	0.09	0.00000149	duck
Clpx	-0.10	0.00000153	duck
Trp53inp2	0.17	0.00000158	duck
Litaf	0.50	0.00000160	duck
Ddx49	0.03	0.00000161	duck
Aco2	0.12	0.00000166	duck
Kin	-0.09	0.00000175	duck

Ttc28	0.23	0.00000176	duck
Tmem41b	-0.20	0.00000182	duck
Foxn2	0.06	0.00000184	duck
Ubfd1	-0.16	0.00000189	duck
Tln1	0.03	0.00000190	duck
Faf1	-0.27	0.00000193	duck
Trafd1	0.01	0.00000202	duck
Eif3f	0.23	0.00000205	duck
Klhdc2	-0.02	0.00000205	duck
Igf2bp2	0.24	0.00000210	duck
Nat10	0.05	0.00000219	duck
Cnot11	-0.03	0.00000222	duck
Ibtk	-0.18	0.00000235	duck
Arf4	0.05	0.00000244	duck
Necap1	0.05	0.00000246	duck
Rnpepl1	0.04	0.00000246	duck
Zfp511	0.06	0.00000247	duck
Vcpkmt	0.15	0.00000250	duck
Slc25a25	0.02	0.00000251	duck
Kdsr	0.00	0.00000277	duck
Eipr1	-1.14	0.00000277	duck
Washc4	0.07	0.00000281	duck
Crebl2	0.01	0.00000296	duck
Rasa3	-0.08	0.00000296	duck
Kdm1a	-0.24	0.00000296	duck
Vti1a	0.00	0.00000300	duck
Eftud2	0.09	0.00000304	duck
Pno1	0.13	0.00000308	duck
Cars	-0.11	0.00000315	duck
Cnot10	-0.11	0.00000318	duck
Api5	-0.16	0.00000324	duck
Celf1	-0.37	0.00000334	duck
Trip12	-1.25	0.00000334	duck
Arfip2	0.09	0.00000336	duck
Xrn1	-0.21	0.00000341	duck
Ints2	0.04	0.00000344	duck
Fez2	0.06	0.00000346	duck
Ddx46	-0.29	0.00000360	duck
Esrrg	0.20	0.00000362	duck
Eif3l	-0.02	0.00000369	duck
Ube3b	0.04	0.00000370	duck
Cpeb4	-0.45	0.00000375	duck
Syf2	-0.06	0.00000394	duck
Tle4	0.24	0.00000415	duck
Fam8a1	-0.21	0.00000417	duck
Bptf	-0.43	0.00000420	duck

Ppp2ca	-0.98	0.00000424	duck
Fam172a	0.09	0.00000434	duck
Nop14	0.14	0.00000453	duck
Bhlhe40	0.01	0.00000459	duck
Adarb1	0.16	0.00000474	duck
Nelfcd	0.02	0.00000493	duck
Adgrg6	0.32	0.00000544	duck
Zranb1	-0.26	0.00000549	duck
Stx6	0.22	0.00000554	duck
Dusp22	-1.17	0.00000557	duck
Tatdn1	0.14	0.00000558	duck
Hnrnpk	-0.16	0.00000558	duck
Arid1a	-0.13	0.00000570	duck
Dcaf5	-0.53	0.00000636	duck
Arfip1	0.05	0.00000666	duck
Ascc1	0.16	0.00000669	duck
Zfr	-0.38	0.00000698	duck
Sap30	0.04	0.00000718	duck
Hmgcs1	0.16	0.00000719	duck
Arfgap2	0.14	0.00000723	duck
Lclat1	-0.19	0.00000739	duck
Adora2a	-0.18	0.00000748	duck
Ddx1	-0.20	0.00000776	duck
Ppil1	0.19	0.00000797	duck
Dlat	0.06	0.00000798	duck
Nfat5	-0.26	0.00000813	duck
Vps29	0.25	0.00000820	duck
Lrp6	0.14	0.00000822	duck
Stard5	-0.50	0.00000837	duck
Primpol	-0.10	0.00000838	duck
Rnf185	-0.04	0.00000839	duck
Tmem30a	-0.12	0.00000842	duck
Epas1	-1.54	0.00000850	duck
Hscb	0.14	0.00000863	duck
Ino80c	0.11	0.00000864	duck
Prdm15	-0.06	0.00000868	duck
Cwc27	-0.22	0.00000879	duck
Eya3	-0.08	0.00000880	duck
Elovl5	0.03	0.00000900	duck
Agpat4	0.19	0.00000902	duck
Stat3	0.05	0.00000965	duck
Tdrd7	-0.03	0.00000989	duck
Lsm14a	-0.28	0.00000995	duck
Vat1	0.21	0.00001009	duck
Ctbp1	-0.12	0.00001018	duck
Slc25a44	0.12	0.00001064	duck

Hspa9	0.02	0.00001083	duck
Htra1	0.19	0.00001112	duck
Sgsh	0.27	0.00001165	duck
Smg7	-0.19	0.00001254	duck
Syne3	-0.17	0.00001275	duck
Nelfa	-0.16	0.00001278	duck
Dennd1a	-0.36	0.00001291	duck
Sppl2a	-0.14	0.00001307	duck
Vrk2	0.26	0.00001516	duck
Kdm5b	-0.03	0.00001539	duck
Scp2	-0.09	0.00001553	duck
Fes	0.17	0.00001600	duck
Dbi	0.31	0.00001612	duck
Erlin1	-0.11	0.00001621	duck
Tle3	0.08	0.00001637	duck
Hexb	0.12	0.00001841	duck
Ist1	0.01	0.00001923	duck
Prmt7	-0.16	0.00001938	duck
Cep44	0.04	0.00001944	duck
Ccnb2	0.18	0.00001971	duck
1700034I23Rik	0.14	0.00002031	duck
Selenok	0.12	0.00002034	duck
Gtdc1	0.31	0.00002042	duck
Smim19	0.15	0.00002072	duck
Nucb2	-0.74	0.00002262	duck
Hbegf	-1.02	0.00002310	duck
Fam13b	0.01	0.00002334	duck
Wwox	0.22	0.00002377	duck
Nuak1	-0.21	0.00002383	duck
Qrich1	-0.05	0.00002401	duck
Tmem59	0.05	0.00002416	duck
Golga4	-0.33	0.00002428	duck
Pxylp1	0.11	0.00002517	duck
Abi1	-1.04	0.00002564	duck
Pinx1	-0.20	0.00002600	duck
Paxbp1	-0.46	0.00002632	duck
Rab5a	-0.09	0.00002743	duck
Tcea2	0.04	0.00002816	duck
Zrsr1	-0.22	0.00002851	duck
Ccdc186	-0.37	0.00002894	duck
Pmvk	0.17	0.00002901	duck
Tsr3	0.01	0.00002919	duck
Denr	0.12	0.00002922	duck
Timp3	0.22	0.00002982	duck
Kctd7	0.05	0.00002992	duck
Abraxas2	-0.03	0.00003038	duck

Abcb7	-0.32	0.00003098	duck
Nqo2	0.09	0.00003119	duck
Vps35	-0.02	0.00003126	duck
Trpm7	-0.13	0.00003128	duck
Thoc2	-0.29	0.00003131	duck
Dcaf1	-0.92	0.00003164	duck
Tmem70	-0.13	0.00003190	duck
Ecpas	-0.16	0.00003288	duck
Ywhaq	0.17	0.00003291	duck
Sec23b	0.12	0.00003312	duck
Pip4k2a	-0.29	0.00003358	duck
Taf10	0.16	0.00003442	duck
Gpn2	0.10	0.00003483	duck
Sf3a3	0.09	0.00003513	duck
Ikbkb	0.01	0.00003533	duck
Surf6	0.07	0.00003590	duck
Ints13	-0.26	0.00003636	duck
Rapgef2	0.67	0.00003653	duck
Bzw1	-0.15	0.00003693	duck
Prdm10	-0.24	0.00003774	duck
Arhgap21	0.28	0.00003794	duck
Taf2	0.07	0.00003842	duck
Prkcb	-0.10	0.00003859	duck
Tnks2	-0.33	0.00003906	duck
Arl15	-0.04	0.00004201	duck
Kbtbd2	-0.16	0.00004271	duck
Stk3	-1.37	0.00004348	duck
Rarres1	0.70	0.00004511	duck
Chchd5	0.03	0.00004514	duck
Dapk3	0.17	0.00004571	duck
Cotl1	0.08	0.00004713	duck
Fbxo45	-0.15	0.00004715	duck
Sgms1	0.01	0.00004802	duck
Rap2c	0.02	0.00004951	duck
Pdcl3	0.17	0.00004966	duck
Commd2	0.00	0.00005015	duck
Anapc1	0.11	0.00005023	duck
Nr3c1	-0.48	0.00005072	duck
Insig2	0.09	0.00005110	duck
Rassf3	0.14	0.00005241	duck
Npepl1	0.02	0.00005490	duck
Sccpdh	-0.20	0.00005512	duck
Akt3	-0.88	0.00005864	duck
Dynlt3	0.03	0.00005865	duck
Csgalnact2	0.02	0.00006043	duck
Inpp5a	0.06	0.00006063	duck

Clk2	0.10	0.00006138	duck
Brox	0.06	0.00006192	duck
Usp20	0.01	0.00006444	duck
Ptpn12	-0.13	0.00006549	duck
Snx1	0.10	0.00006586	duck
Etf1	0.01	0.00006642	duck
Chfr	-0.05	0.00006691	duck
Tfg	0.08	0.00006703	duck
Pcna	0.11	0.00006713	duck
Bcat1	0.17	0.00006742	duck
Eif4e2	0.02	0.00006781	duck
Sh3bp2	-0.09	0.00006815	duck
Eloc	-0.03	0.00006951	duck
Wdr61	0.05	0.00007019	duck
Pfdn4	0.18	0.00007055	duck
Csnk2a2	-0.83	0.00007213	duck
Runx2	-0.89	0.00007694	duck
Snorc	-0.02	0.00007758	duck
C1qc	1.22	0.00008013	duck
Atf2	-0.44	0.00008291	duck
Pnpla7	-1.06	0.00008373	duck
Bcas2	0.02	0.00008398	duck
Cnot7	-0.30	0.00008452	duck
Nsmaf	-0.11	0.00008698	duck
Acbd6	-0.13	0.00009715	duck
Ptbp1	-0.09	0.00009867	duck
Sulf2	0.29	0.00010110	duck
Akap10	-0.21	0.00010666	duck
Arid5b	0.05	0.00010745	duck
Rasa1	-0.17	0.00010872	duck
Phkb	-0.43	0.00011171	duck
Nfx1	-0.23	0.00011246	duck
Gclc	-0.18	0.00011471	duck
Vps37b	0.08	0.00011663	duck
Axin1	-1.12	0.00012000	duck
Pten	-0.19	0.00012076	duck
Eif5a2	-1.01	0.00012215	duck
Rsrc2	-0.46	0.00012397	duck
Plcl2	-0.05	0.00012855	duck
Lrrc15	0.24	0.00013241	duck
Utp18	-0.07	0.00013304	duck
Bcl2l11	0.13	0.00013305	duck
Cnih4	0.33	0.00013440	duck
Irak4	0.00	0.00013458	duck
Nol9	0.01	0.00013644	duck
Cdc14a	-1.64	0.00013791	duck

Supt7l	-0.08	0.00014103	duck
Stx2	-0.23	0.00014500	duck
Ptprs	0.18	0.00014654	duck
Fjx1	0.16	0.00016023	duck
Agfg1	-0.05	0.00016166	duck
Eif2s1	0.12	0.00016317	duck
Clk4	-0.31	0.00016589	duck
Pank4	-0.08	0.00017032	duck
Phf14	0.20	0.00017046	duck
Lmnb2	-0.02	0.00017096	duck
Wwp1	-0.28	0.00017343	duck
Bcl2	-0.02	0.00017506	duck
Rdh10	-0.45	0.00017815	duck
Etv6	-0.21	0.00017922	duck
Rab11fip1	0.04	0.00018185	duck
Gpsm1	0.15	0.00018563	duck
Ppil3	-0.04	0.00018610	duck
Parp4	0.00	0.00018809	duck
Suox	-0.27	0.00019366	duck
Dnajc7	-0.03	0.00019435	duck
Ncoa1	0.24	0.00019519	duck
Kdm3b	-0.28	0.00019848	duck
Abraxas1	0.05	0.00020109	duck
Tasp1	-0.20	0.00021350	duck
Rnd3	0.27	0.00021390	duck
Kcnj2	0.28	0.00022500	duck
Cct2	0.02	0.00022741	duck
Pik3c2a	-0.16	0.00023057	duck
Sod2	0.03	0.00024558	duck
Dtnbp1	-0.36	0.00024738	duck
Hdac9	-0.30	0.00024917	duck
Rad54l2	-0.05	0.00024988	duck
Hs6st3	-1.30	0.00025417	duck
Tbpl1	-0.04	0.00026335	duck
Supt6	-0.26	0.00026454	duck
Eps8	0.31	0.00026966	duck
Stau2	0.24	0.00027521	duck
Smg5	-0.09	0.00027614	duck
Plod2	-0.03	0.00027636	duck
Stk40	0.01	0.00027798	duck
Pop4	-0.03	0.00027959	duck
Unc50	-0.02	0.00029164	duck
Uba2	-0.27	0.00030459	duck
Glcci1	-1.17	0.00030582	duck
Crtc1	-0.14	0.00031175	duck
Rabggtb	-0.01	0.00031186	duck

Arrdc3	-0.22	0.00031248	duck
Rai2	0.11	0.00032188	duck
Cct7	0.25	0.00032620	duck
Bloc1s4	-0.15	0.00033253	duck
Spg7	0.02	0.00033423	duck
Ube2j2	-0.12	0.00034729	duck
Sh3gl1	0.11	0.00035526	duck
Fbxo9	-0.07	0.00035591	duck
Pds5a	-0.11	0.00036208	duck
Top1	-0.72	0.00037258	duck
Rnmt	-0.05	0.00037461	duck
Lrrc59	0.30	0.00037799	duck
Dynll2	0.02	0.00037866	duck
Plcb2	-0.92	0.00038308	duck
Lrif1	-0.14	0.00039800	duck
Ago3	-0.18	0.00040377	duck
Actn4	0.11	0.00041217	duck
Sel1l	-0.33	0.00042463	duck
Asb7	0.02	0.00043442	duck
Fcho2	-0.54	0.00043472	duck
Ivd	0.08	0.00044151	duck
Sfxn1	0.17	0.00044300	duck
Nap1l4	-0.36	0.00044721	duck
Anapc2	0.20	0.00045536	duck
Fer	0.01	0.00048009	duck
Dcaf13	0.14	0.00048137	duck
Plagl1	0.09	0.00049075	duck
Trps1	-0.58	0.00049095	duck
Anxa7	0.15	0.00049297	duck
Prepl	-0.01	0.00049929	duck
St6gal1	-0.90	0.00050011	duck
Pde4b	-0.80	0.00050137	duck
B4galt5	-0.10	0.00051432	duck
Zcchc8	-0.07	0.00054708	duck
Vps26c	0.11	0.00057494	duck
Kpnb1	0.13	0.00058089	duck
Cables2	-0.19	0.00059100	duck
Trmt10c	0.05	0.00059972	duck
Uhrf1bp1l	0.14	0.00060173	duck
Cul2	-0.22	0.00061325	duck
Evi5	-1.45	0.00062279	duck
Rit1	-0.37	0.00063645	duck
Ap1b1	0.13	0.00063925	duck
Tapt1	-1.49	0.00064375	duck
Foxo4	-0.09	0.00064860	duck
Tmem104	-0.05	0.00065015	duck

Wdr1	-0.02	0.00065417	duck
Cmtr1	-0.23	0.00068741	duck
Ska2	-0.06	0.00069557	duck
Prkce	0.02	0.00070629	duck
Poldip2	0.12	0.00070789	duck
Rnf138	0.19	0.00070973	duck
Ppp3cc	-0.07	0.00071586	duck
Pik3ca	-1.47	0.00072324	duck
Tsc22d2	-0.29	0.00073150	duck
Abhd5	-0.08	0.00074863	duck
Arfgap1	0.04	0.00075293	duck
Klf4	0.29	0.00075774	duck
Whamm	0.04	0.00076614	duck
Traf6	0.02	0.00082255	duck
Ttc19	0.02	0.00083371	duck
Fuca1	0.13	0.00083483	duck
Gtf2h1	-0.24	0.00083834	duck
Smurf1	0.03	0.00083969	duck
Gtf2a1	-0.37	0.00085072	duck
Slc25a29	0.13	0.00086871	duck
Slc9a4	-0.09	0.00088843	duck
Tent5a	0.33	0.00089252	duck
Prpf6	-0.34	0.00092161	duck
Gga3	-0.02	0.00092957	duck
Lpin2	-0.40	0.00092995	duck
Rgs9	-0.63	0.00093337	duck
Stx17	-0.13	0.00096401	duck
Rnf170	-0.01	0.00096606	duck
Smarcb1	0.13	0.00096626	duck
Rasgrp1	0.11	0.00099315	duck
2610507B11Rik	0.06	0.00101538	duck
Usf3	-0.02	0.00102911	duck
Cfap20	0.08	0.00105273	duck
Wdr48	-0.44	0.00106007	duck
Arhgap12	0.13	0.00106730	duck
Aqr	-0.32	0.00112352	duck
Exosc10	0.02	0.00113897	duck
Atxn7	-0.53	0.00115036	duck
Uchl3	-0.20	0.00115280	duck
Slc4a3	0.19	0.00115491	duck
Cramp1	-0.21	0.00124518	duck
Nup205	0.06	0.00133755	duck
Srp14	-0.08	0.00139228	duck
Ralgps1	-0.38	0.00140296	duck
Cul4b	-0.42	0.00142009	duck
Stard4	0.05	0.00142736	duck

Ptpn4	-1.30	0.00146132	duck
Bicd2	0.03	0.00153384	duck
Kdm4b	-0.31	0.00153951	duck
Snrpb2	-0.13	0.00154275	duck
Tlk2	-0.24	0.00156083	duck
Il6st	0.14	0.00158817	duck
Herpud2	-0.16	0.00160227	duck
Ankrd13c	-0.23	0.00171309	duck
Slc41a3	-0.11	0.00171951	duck
Ppil4	-0.03	0.00173225	duck
Sar1a	-0.41	0.00174246	duck
Idh2	0.15	0.00178320	duck
Ddx27	0.00	0.00182044	duck
Atp2b1	-0.09	0.00186723	duck
Luzp1	0.12	0.00190010	duck
Rnf121	0.14	0.00190314	duck
Il16	-1.11	0.00194006	duck
Kdm4c	-0.35	0.00201736	duck
Srp54a	-0.12	0.00213518	duck
Lrp3	-0.96	0.00214208	duck
Atp9b	-0.20	0.00220480	duck
Gnl2	0.00	0.00222129	duck
Scmh1	0.15	0.00222175	duck
Gstk1	0.11	0.00228131	duck
Pdcl	-0.06	0.00244624	duck
Sgms2	0.24	0.00245234	duck
Oga	-0.42	0.00246205	duck
Pag1	0.04	0.00252741	duck
Grk2	0.08	0.00255181	duck
Sirt1	-0.47	0.00267159	duck
Nrros	0.13	0.00271157	duck
Slc40a1	-0.24	0.00279864	duck
Traf3	-0.12	0.00280494	duck
Stk38	-0.20	0.00286273	duck
Clasp2	-0.32	0.00298277	duck
Dhx30	-0.08	0.00300469	duck
Arap2	0.10	0.00312983	duck
Cnot6	0.07	0.00320791	duck
Srek1ip1	-0.07	0.00328977	duck
Scaper	-0.22	0.00329430	duck
Iqsec1	0.08	0.00334093	duck
Pitpnm2	-0.92	0.00335193	duck
Golga5	-0.13	0.00336706	duck
Vps50	-0.03	0.00337683	duck
Polk	-0.34	0.00340021	duck
Zdhhc3	-0.20	0.00350147	duck

Acot10	-0.19	0.00364281	duck
Bcl7b	-0.08	0.00366707	duck
Zcchc10	-0.02	0.00368109	duck
Foxk2	-0.38	0.00385124	duck
Phtf2	0.09	0.00388739	duck
Cubn	-0.16	0.00405881	duck
Nae1	0.01	0.00411075	duck
Slc4a7	-0.38	0.00423177	duck
Ndufa10	0.26	0.00425228	duck
Prpf38a	0.07	0.00427596	duck
Fbxo33	0.00	0.00436076	duck
Fgfbp1	0.64	0.00442073	duck
Slc35a3	0.09	0.00447584	duck
Ccdc88a	-0.09	0.00452190	duck
Hnrnpd	0.02	0.00461938	duck
Katnbl1	0.13	0.00465716	duck
Ppid	0.07	0.00472384	duck
Ptpn11	-0.18	0.00480907	duck
Smc6	-0.20	0.00495334	duck
Commd9	0.08	0.00497546	duck
Wnk1	-1.06	0.00498150	duck
Stk10	-0.03	0.00498396	duck
Eny2	0.04	0.00499113	duck
Kpna3	-0.14	0.00501005	duck
Stk4	-0.15	0.00517088	duck
Plxnd1	0.20	0.00518643	duck
Zfp687	0.04	0.00522871	duck
Lmbrd2	-0.10	0.00534829	duck
Slc26a5	-0.05	0.00542257	duck
Ap1ar	0.12	0.00548769	duck
Ap1m1	0.02	0.00563834	duck
Arsk	-0.93	0.00569331	duck
Rpia	0.13	0.00573813	duck
Osbpl2	-0.04	0.00584582	duck
Dpp9	0.00	0.00587610	duck
Btbd10	-0.22	0.00612519	duck
Nudt3	-0.26	0.00628693	duck
Gpr107	-0.23	0.00633951	duck
Pqlc3	0.11	0.00638066	duck
Bclaf1	-0.38	0.00649410	duck
Zfand3	-0.04	0.00649633	duck
Atm	0.07	0.00655119	duck
Rbbp4	0.04	0.00664013	duck
Hsf5	0.13	0.00668221	duck
Sema4d	-0.28	0.00671245	duck
Setd5	-0.19	0.00710031	duck

Zfp36l1	-0.09	0.00718470	duck
Eif4e	-0.39	0.00752038	duck
Pes1	0.18	0.00756008	duck
Rbm34	-0.23	0.00756175	duck
Lmbrd1	-0.09	0.00757216	duck
Eif2ak3	-0.17	0.00778244	duck
Ttc39c	0.02	0.00785638	duck
Tcf3	-0.71	0.00794011	duck
Hecw2	-0.74	0.00804426	duck
Selenoo	-1.07	0.00820214	duck
Hmg20a	-0.07	0.00835423	duck
Pfkfb3	-0.73	0.00838902	duck
Plxnc1	-0.03	0.00847940	duck
Sdc1	0.19	0.00870640	duck
Setd3	-0.20	0.00872117	duck
Naa30	-0.37	0.00885446	duck
Trim33	-0.36	0.00888684	duck
Samd8	0.09	0.00889569	duck
Spred1	-0.08	0.00939546	duck
Cpsf2	-0.16	0.00944655	duck
Arid3b	0.02	0.00951745	duck
Ankfy1	0.09	0.00963929	duck
Nf2	-0.21	0.00980587	duck
Fbxo28	-0.14	0.00992219	duck
Cab39	-0.21	0.01009161	duck
Slc39a6	0.08	0.01012782	duck
Cggbp1	-0.03	0.01033684	duck
Dctn5	0.09	0.01051212	duck
Rnf145	-0.18	0.01097940	duck
Ap4s1	0.10	0.01118142	duck
Chpt1	0.14	0.01131908	duck
Dync1h1	-0.16	0.01147532	duck
Ing3	-0.50	0.01148865	duck
Eri3	0.02	0.01173377	duck
Tmem208	0.11	0.01232034	duck
Calm2	-0.14	0.01235825	duck
Ptp4a1	0.04	0.01248650	duck
Prpf8	-0.19	0.01249377	duck
Pspc1	-0.47	0.01299702	duck
Dnajb12	-0.06	0.01310434	duck
Ythdf3	-0.28	0.01316770	duck
Tlcd3a	-0.48	0.01333364	duck
Lrrk1	0.10	0.01340940	duck
Tmem231	0.05	0.01386638	duck
Hat1	-0.08	0.01464662	duck
Borcs6	0.07	0.01506488	duck

Adam19	0.11	0.01513623	duck
Tnpo1	-0.41	0.01519203	duck
Dstn	0.12	0.01527629	duck
Ccdc127	-0.05	0.01532425	duck
Cops8	-0.06	0.01571707	duck
Unk	-0.09	0.01585062	duck
Ranbp10	-0.17	0.01663612	duck
Uhrf1bp1	-0.76	0.01676130	duck
Cpd	-0.10	0.01722388	duck
Slc16a3	-0.01	0.01727918	duck
Pcnt	-0.83	0.01741056	duck
Srp9	-0.09	0.01780133	duck
Fn1	-2.33	0.01784351	duck
Zc3h7b	-0.18	0.01798786	duck
Zfp532	-0.24	0.01843796	duck
Atp13a1	-0.03	0.01923746	duck
Uba3	-0.08	0.01977558	duck
Prkar1a	-0.06	0.02053875	duck
Nsrp1	-0.26	0.02068446	duck
Ggnbp2	-0.09	0.02077974	duck
Hectd1	0.07	0.02092597	duck
Slc9a1	0.12	0.02106645	duck
Kdm5a	-0.88	0.02205280	duck
Thumpd3	-0.04	0.02283885	duck
Wdr33	-0.53	0.02339563	duck
Nlk	-0.41	0.02490333	duck
Aida	0.01	0.02511968	duck
Nars	-0.05	0.02513013	duck
Ncoa4	-0.90	0.02530164	duck
Ppig	-0.37	0.02532610	duck
Rap2b	-0.08	0.02631193	duck
St7l	-0.09	0.02668905	duck
Prkg2	0.03	0.02682222	duck
Ube2v2	-0.30	0.02858801	duck
Dhx38	-0.07	0.02882537	duck
Actr6	-0.14	0.02888796	duck
Btbd7	-0.12	0.02940923	duck
Entpd1	0.07	0.02948351	duck
Zbtb8os	0.06	0.03038268	duck
Sparc	0.19	0.03049614	duck
Leprotl1	-0.01	0.03113756	duck
Nufip1	-0.16	0.03145321	duck
Cd81	-0.75	0.03184314	duck
Ino80	-0.44	0.03225764	duck
Acad9	-0.14	0.03296925	duck
Eif3e	-0.24	0.03384947	duck

Slc45a4	-0.05	0.03402962	duck
Nol7	0.12	0.03508180	duck
Haus2	-0.09	0.03557261	duck
Yipf5	-0.13	0.03588803	duck
Gtpbp1	0.02	0.03636145	duck
Phrf1	-0.10	0.03684505	duck
Pde7b	0.01	0.03787432	duck
Dvl1	-0.27	0.03832072	duck
Itch	-0.49	0.03867210	duck
Stxbp3	-0.21	0.03991725	duck
Ninj1	-0.08	0.04042066	duck
Wdr44	-0.19	0.04085452	duck
Ankrd54	0.01	0.04146203	duck
Gnat2	0.09	0.04163371	duck
Abtb1	-0.19	0.04205766	duck
Ptdss1	-0.05	0.04211786	duck
Jmjd4	0.02	0.04218735	duck
Eif2a	-0.12	0.04221879	duck
Hint1	0.22	0.04255883	duck
Gpcpd1	-1.12	0.04266922	duck
Cdc27	-0.22	0.04290377	duck
Jade3	-0.16	0.04290951	duck
Hp1bp3	-0.19	0.04364340	duck
Psmc6	-0.40	0.04367607	duck
Galk2	-0.02	0.04429155	duck
Ranbp9	-0.58	0.04542245	duck
Atg13	-0.18	0.04595279	duck
Ybx3	-0.73	0.04622280	duck
Tab3	-0.23	0.04683348	duck
Gna12	-0.01	0.04850412	duck
Pola1	-0.01	0.05014552	duck
Pnpla8	-0.61	0.05088020	duck
Trappc8	-0.42	0.05123993	duck
Slc4a9	-0.74	0.05212894	duck
Acsl4	-0.74	0.05226578	duck
Tnfsf8	0.02	0.05248148	duck
Ubl3	-0.11	0.05336559	duck
Hace1	0.11	0.05421765	duck
Ahcyl2	-0.01	0.05554338	duck
Snx14	-0.38	0.05791572	duck
Orai2	0.10	0.05815056	duck
Pip5k1c	-0.12	0.06052928	duck
Niban1	-0.06	0.06071056	duck
Hnrnpu	-0.25	0.06074292	duck
Upb1	-0.14	0.06094310	duck
Katnip	0.03	0.06252698	duck

Pdcd5	0.03	0.06273214	duck
Trabd	0.00	0.06371681	duck
Rreb1	-0.37	0.06466141	duck
Elp1	0.04	0.06558806	duck
Ube3a	-0.31	0.06585128	duck
Hspa2	-1.12	0.06662684	duck
Ndrg3	-1.00	0.06686373	duck
Odf2	0.00	0.06744861	duck
Ext2	0.16	0.07031569	duck
Snx18	-0.07	0.07486494	duck
Pofut2	-0.09	0.07500742	duck
Ep300	-0.48	0.07571162	duck
Slc25a46	0.03	0.07720674	duck
Wars	-0.22	0.07932491	duck
Tiam1	-0.83	0.07950026	duck
Aplp2	-0.21	0.07997472	duck
Usp19	0.01	0.08079380	duck
Hbp1	-0.45	0.08168506	duck
Hadha	0.01	0.08219761	duck
Cdk7	-0.12	0.08273315	duck
Thada	0.03	0.08453981	duck
Ube2h	-0.57	0.08697819	duck
Zbtb20	0.36	0.08714604	duck
Dusp8	0.28	0.08931403	duck
Dcun1d3	-0.49	0.09024047	duck
Sh3bp5	-0.19	0.09235511	duck
Slu7	-0.42	0.09241858	duck
Cebpg	-0.08	0.09560045	duck
Bicral	-1.00	0.09802245	duck
Itm2a	-0.06	0.09821352	duck
Siah2	0.11	0.10312361	duck
Apoo	-0.14	0.10336010	duck
Rab8b	-0.18	0.10385902	duck
Slc38a1	-0.07	0.10597846	duck
Trappc12	0.06	0.10985393	duck
Ereg	-1.12	0.10987571	duck
Rprd1a	-0.32	0.11017448	duck
Dynlt1a	0.08	0.11153828	duck
Wbp2	-0.11	0.11210205	duck
Pisd	0.00	0.11248836	duck
Tcf4	-0.52	0.11501122	duck
Chic2	-0.62	0.11898714	duck
Pde10a	0.53	0.11918915	duck
Dnajc16	0.07	0.13992006	duck
Dock4	0.28	0.14366934	duck
Dusp5	-0.10	0.14424118	duck

Prdx4	0.26	0.15081419	duck
Hbs1l	-0.47	0.15683878	duck
Hif1an	0.02	0.16365839	duck
Omg	0.08	0.16373889	duck
P4ha1	-0.80	0.16407439	duck
Sec13	-0.08	0.17039447	duck
Ndfip2	-0.33	0.17289596	duck
Ago4	-0.16	0.17298341	duck
Srsf11	-0.29	0.17444823	duck
Srpr	-0.02	0.17734460	duck
Rcbtb1	0.07	0.17744485	duck
Ppard	0.00	0.17910484	duck
Pank3	-0.34	0.18588499	duck
Borcs5	-0.36	0.18782383	duck
Septin9	0.02	0.18927396	duck
Slk	-0.76	0.18996974	duck
Lman1	0.26	0.19127983	duck
Pias2	-0.11	0.19382802	duck
Htt	-0.70	0.19425564	duck
Stam	0.11	0.19686585	duck
Ddx55	0.01	0.19721194	duck
Nutf2	0.13	0.19812621	duck
Uxs1	-0.27	0.19892019	duck
Tfdp1	-0.89	0.20122085	duck
Usb1	0.01	0.20389313	duck
Ccnf	0.01	0.20419006	duck
Inhba	-1.57	0.20472769	duck
Erlin2	-0.25	0.21951535	duck
Shprh	-0.57	0.22965256	duck
Nup54	-0.41	0.23500839	duck
Arl9	0.16	0.23566964	duck
Cd247	-0.19	0.23624032	duck
Abcd3	-0.09	0.23677870	duck
Zfp598	-0.11	0.23879712	duck
Zc3h12d	-0.63	0.23903046	duck
Ccnl1	-0.45	0.23982615	duck
Lmo2	-0.97	0.24484412	duck
Galnt1	-0.26	0.24492485	duck
Pnrc2	-0.15	0.25901385	duck
Rabgap1	-0.13	0.27170904	duck
Rbm33	-0.68	0.27279514	duck
Exoc7	-0.19	0.27676839	duck
Slc35e1	-0.14	0.27694359	duck
Gramd1b	-0.19	0.27781307	duck
Esco1	-0.61	0.28054559	duck
Gpc5	0.13	0.28323710	duck

Clip2	0.13	0.28738642	duck
Emsy	-0.31	0.29359301	duck
Psmd10	-0.08	0.29951136	duck
Smap2	-0.09	0.30437435	duck
Hexim1	-0.53	0.30461102	duck
Angptl4	-0.03	0.30499570	duck
Saal1	-0.03	0.30804300	duck
Dgkq	-0.08	0.31638618	duck
Gpr34	0.20	0.31820632	duck
Fbxl22	-0.12	0.31918837	duck
Cdk9	-0.01	0.32299419	duck
Acot11	0.07	0.32742650	duck
Uri1	-0.53	0.33489037	duck
Abcd2	-0.12	0.33653830	duck
Tmx1	-0.37	0.33790873	duck
Cebpb	-0.16	0.33956593	duck
Rab12	0.20	0.34184700	duck
Pld1	0.09	0.36408102	duck
Rmdn3	-0.29	0.37666634	duck
Jak1	0.06	0.38091405	duck
Clk1	-1.21	0.38392714	duck
Lactb2	-0.13	0.39026412	duck
Nupl1	-0.23	0.40382980	duck
Fam91a1	-0.18	0.41858298	duck
Ddx5	-0.35	0.42432825	duck
Tra2b	-0.21	0.43211815	duck
Ywhag	-0.05	0.43781416	duck
Camsap2	-0.98	0.43951718	duck
Tpp2	-0.25	0.44183568	duck
Cnep1r1	-0.34	0.44903277	duck
Fndc3b	-0.18	0.45173122	duck
Tm9sf2	-0.21	0.45301912	duck
Herc1	-1.00	0.45589562	duck
Nub1	-0.21	0.45720239	duck
Vdac3	-0.05	0.46262219	duck
Aprt	0.11	0.46821179	duck
Vps8	-0.27	0.46986024	duck
Gfod2	-0.03	0.48608468	duck
Psme3ip1	-0.09	0.49201663	duck
Arl3	0.00	0.49204853	duck
Gga1	0.05	0.50518383	duck
Peak1	-0.25	0.51496511	duck
Arih2	-0.20	0.53041713	duck
Prkdc	-0.88	0.53240409	duck
Slc30a7	-0.34	0.53811245	duck
Rap1b	-0.24	0.54031910	duck

Nf1	-0.40	0.54685528	duck
Pik3c3	-0.24	0.55713714	duck
Rnf217	-0.27	0.56128631	duck
Atp11b	-0.19	0.56143731	duck
Arsg	-0.80	0.56168858	duck
Ubap2l	-0.10	0.58473724	duck
Ubp1	-0.32	0.58736210	duck
Zfp438	-0.68	0.59332217	duck
Gorasp2	-0.09	0.59414378	duck
Akap8l	-0.25	0.60666898	duck
Rufy3	0.24	0.61089664	duck
Nr1d2	-0.39	0.61686178	duck
Scrib	-0.33	0.62272657	duck
Zfp384	-0.22	0.62810216	duck
Gucd1	-0.22	0.63573666	duck
Ate1	-0.44	0.64361922	duck
Zfand4	-0.37	0.67059815	duck
Emc7	-0.26	0.67149365	duck
Slc27a1	0.03	0.67797030	duck
Crk	-0.20	0.68435169	duck
Gpr137c	0.05	0.68445255	duck
Ing4	-0.12	0.69156885	duck
Smarcad1	-0.13	0.70062236	duck
Bod1l	-1.20	0.70530441	duck
Tent4b	-0.40	0.70674068	duck
Tnrc18	0.08	0.72010384	duck
Ppp1r3b	-0.05	0.72032189	duck
Acss2	0.08	0.72801015	duck
Plpp1	-0.32	0.72928515	duck
Cul3	-0.54	0.73685548	duck
Scube2	-0.09	0.73905809	duck
Tmem51	0.19	0.75413085	duck
Plcxd1	-0.09	0.75901053	duck
Tbc1d23	0.04	0.77584423	duck
Rala	-0.29	0.78962616	duck
Nr2c2	-0.62	0.81049071	duck
Adnp2	-0.11	0.81491663	duck
Adat1	-0.26	0.81675808	duck
Rnf4	-0.34	0.81743239	duck
Nedd4	0.10	0.83188947	duck
Tbxas1	-0.53	0.84860873	duck
Sar1b	0.01	0.85246570	duck
Entpd5	-0.91	0.85309921	duck
Sec23a	-0.87	0.86324340	duck
Lyn	-0.66	0.87379401	duck
Fgd4	-0.12	0.90785710	duck

Klhdc10	-0.05	0.92644247	duck
Drg1	-0.15	0.93056268	duck
Lcmt1	-0.01	0.94005605	duck
Rdx	-0.62	0.94718084	duck
Sel1l3	0.11	0.95122423	duck
Irf2	-0.29	0.96154617	duck
Rassf6	-0.22	0.97526119	duck
Atg14	-0.04	0.97867572	duck
Pdgfb	0.02	0.99470211	duck
Rcc2	0.09	0.99499233	duck
Ints8	-0.63	1.00000000	duck
Gnptab	0.00	1.00000000	duck
Sec22b	0.04	1.00000000	duck
Rmc1	-0.27	1.00000000	duck
Fkbp5	-0.72	1.00000000	duck
Xrn2	-0.42	1.00000000	duck
Vcpip1	-1.13	1.00000000	duck
Ttc7	-0.19	1.00000000	duck
Ptpn2	-1.09	1.00000000	duck
Pwwp2a	-0.08	1.00000000	duck
Serbp1	-0.07	1.00000000	duck
Tec	-0.89	1.00000000	duck
Glrx5	-0.43	1.00000000	duck
Wiz	-0.31	1.00000000	duck
Btg2	-0.64	1.00000000	duck
Cwf19l1	-0.18	1.00000000	duck
Nt5c3	-0.46	1.00000000	duck
Angptl2	-0.76	1.00000000	duck
Rbm12b2	-0.30	1.00000000	duck
Carnmt1	-0.21	1.00000000	duck
Rexo4	-0.10	1.00000000	duck
Arhgef12	-1.29	1.00000000	duck
Srsf6	-0.21	1.00000000	duck
Vdr	-0.25	1.00000000	duck
Arglu1	-0.71	1.00000000	duck
Ppm1a	-0.43	1.00000000	duck
Slc35e4	0.16	1.00000000	duck
Ralgapa1	-0.36	1.00000000	duck
Fgd6	0.25	1.00000000	duck
C1d	-0.15	1.00000000	duck
Spty2d1	-0.08	1.00000000	duck
Acbd5	-0.61	1.00000000	duck
Pnisr	-0.70	1.00000000	duck
Ndufs6	-0.04	1.00000000	duck
Sppl2b	-0.12	1.00000000	duck
Rnf168	-0.09	1.00000000	duck

Pdp1	-0.31	1.00000000	duck
Sos2	-0.48	1.00000000	duck
Dcun1d4	-0.33	1.00000000	duck
Impa2	0.03	1.00000000	duck
Wdr70	-0.45	1.00000000	duck
Birc6	-0.58	1.00000000	duck
Lancl2	-0.15	1.00000000	duck
Vps39	-0.27	1.00000000	duck
Cgrrf1	-0.17	1.00000000	duck
Armc1	-0.59	1.00000000	duck
Tpm4	0.41	1.00000000	duck
Tacc3	-0.69	1.00000000	duck
Usp24	-0.34	1.00000000	duck
Rasal2	0.34	1.00000000	duck
Zfp618	-0.31	1.00000000	duck
Fermt1	0.25	1.00000000	duck
Cdc42	-0.51	1.00000000	duck
Dync1li1	-0.06	1.00000000	duck
Pogz	-0.10	1.00000000	duck
Ikzf5	-0.16	1.00000000	duck
Golga1	-0.28	1.00000000	duck
Bend6	-0.38	1.00000000	duck
Letm1	-0.15	1.00000000	duck
Ints4	-0.06	1.00000000	duck
Rab8a	-0.14	1.00000000	duck
Slc8a1	0.13	1.00000000	duck
Rest	-0.24	1.00000000	duck
Cnst	-0.19	1.00000000	duck
Ulk1	-1.15	1.00000000	duck
Smim14	0.12	1.00000000	duck
Ralgapb	-0.28	1.00000000	duck
Rap1gds1	-0.22	1.00000000	duck
Smarcc1	-0.46	1.00000000	duck
Arhgef7	-0.13	1.00000000	duck
Lgals8	-0.47	1.00000000	duck
Prkar2a	-0.85	1.00000000	duck
Septin6	-0.04	1.00000000	duck
Cyld	-0.23	1.00000000	duck
Oard1	-0.36	1.00000000	duck
Card9	0.07	1.00000000	duck
Ppp4r1	0.01	1.00000000	duck
Slc46a3	-0.99	1.00000000	duck
Fbxl14	0.13	1.00000000	duck
Fbxw2	0.01	1.00000000	duck
Cap1	-0.08	1.00000000	duck
Rpa2	0.21	1.00000000	duck

Alas1	-0.03	1.00000000	duck
Bend3	0.14	1.00000000	duck
Ippk	-0.17	1.00000000	duck
Qser1	-0.02	1.00000000	duck
Scarb2	-0.60	1.00000000	duck
Rab3il1	-0.18	1.00000000	duck
Ppp2r5a	-0.32	1.00000000	duck
Ypel1	-0.05	1.00000000	duck
Rasgef1b	-0.10	1.00000000	duck
Crybg3	-0.55	1.00000000	duck
Slf2	-0.21	1.00000000	duck
Fem1b	-0.17	1.00000000	duck
Wdr47	0.00	1.00000000	duck
Dnajb14	-0.05	1.00000000	duck
Tra2a	-1.13	1.00000000	duck
Phf2	-0.33	1.00000000	duck
Zfp512	-0.01	1.00000000	duck
Tnfrsf21	0.04	1.00000000	duck
Elp3	-0.02	1.00000000	duck
Fnip2	0.09	1.00000000	duck
Adamtsl3	0.02	1.00000000	duck
Naa16	-0.84	1.00000000	duck
Tm2d1	-0.32	1.00000000	duck
Ints1	0.07	1.00000000	duck
2510039O18Rik	-0.14	1.00000000	duck
Hmbox1	-0.50	1.00000000	duck
Arl4c	0.17	1.00000000	duck
Ss18	-0.31	1.00000000	duck
Entpd4	-0.61	1.00000000	duck
Nectin3	0.12	1.00000000	duck
Ogt	-0.71	1.00000000	duck
Usp45	-0.46	1.00000000	duck
Npl	-0.21	1.00000000	duck
Rbis	-0.10	1.00000000	duck
Rab3gap2	-0.43	1.00000000	duck
Zbtb37	-0.71	1.00000000	duck
Rrp1b	-0.54	1.00000000	duck
Paip2	-0.19	1.00000000	duck
Vav3	-0.17	1.00000000	duck
Pfdn1	-0.05	1.00000000	duck
Fryl	-1.02	1.00000000	duck
Nol11	-0.16	1.00000000	duck
Strn	-0.33	1.00000000	duck
Lmtk2	-0.59	1.00000000	duck
Park7	0.21	1.00000000	duck
Pik3cg	0.05	1.00000000	duck

Efl1	-0.01	1.00000000	duck
Tmem11	-0.17	1.00000000	duck
Fam117a	-0.13	1.00000000	duck
Hmgxb4	-0.02	1.00000000	duck
Txlna	-0.11	1.00000000	duck
Plbd1	0.22	1.00000000	duck
Fubp1	-0.54	1.00000000	duck
Uap1l1	0.04	1.00000000	duck
Golm2	-0.20	1.00000000	duck
Copg1	0.07	1.00000000	duck
Bcl11a	-0.14	1.00000000	duck
Rassf8	-0.17	1.00000000	duck
Add3	-0.72	1.00000000	duck
Efcab14	-0.19	1.00000000	duck
Uggt1	-0.19	1.00000000	duck
Usp38	-0.41	1.00000000	duck
Kpna6	-0.30	1.00000000	duck
Tmem184b	-0.87	1.00000000	duck
Inpp5k	-0.43	1.00000000	duck
R3hdm1	-0.39	1.00000000	duck
Tns3	1.15	1.00000000	duck
Phospho1	-0.21	1.00000000	duck
Bsg	-0.59	1.00000000	duck
Atxn2	-0.53	1.00000000	duck
Prrc2c	-0.34	1.00000000	duck
Ythdf1	-0.16	1.00000000	duck
Gna11	-0.14	1.00000000	duck
Arfrp1	-0.21	1.00000000	duck
Gna13	-0.68	1.00000000	duck
Rab31	0.11	1.00000000	duck
Cblb	-0.63	1.00000000	duck
Vezt	-0.24	1.00000000	duck
Akap8	-0.39	1.00000000	duck
Gnpnat1	0.11	1.00000000	duck
Ipo9	-0.19	1.00000000	duck
Sf3b3	-0.01	1.00000000	duck
Ppme1	-0.07	1.00000000	duck
Cnot6l	-0.82	1.00000000	duck
Dtymk	0.03	1.00000000	duck
Klhl5	-0.10	1.00000000	duck
Adk	-0.27	1.00000000	duck
Dph3	-0.07	1.00000000	duck
Pigf	-0.04	1.00000000	duck
Usp6nl	-1.02	1.00000000	duck
Fbxo11	-0.49	1.00000000	duck
Pdk3	-1.08	1.00000000	duck

Uck2	-0.21	1.00000000	duck
Sephs1	0.01	1.00000000	duck
Ssb	0.04	1.00000000	duck
Gpatch11	-0.15	1.00000000	duck
Gabpa	-0.27	1.00000000	duck
Cops3	-0.43	1.00000000	duck
Gabra1	-0.08	1.00000000	duck
Plrg1	-0.02	1.00000000	duck
Ccng1	-0.02	1.00000000	duck
Napg	-0.37	1.00000000	duck
Traf5	-0.43	1.00000000	duck
Bcl2l13	-0.35	1.00000000	duck
Setd7	-0.16	1.00000000	duck
Hgs	-0.19	1.00000000	duck
Clasp1	-0.28	1.00000000	duck
Cstf1	-0.22	1.00000000	duck
Snx9	-0.84	1.00000000	duck
Trip11	-0.86	1.00000000	duck
Hmgb2	0.01	1.00000000	duck
Sec31a	-0.18	1.00000000	duck
Copb1	0.01	1.00000000	duck
Nedd4l	-0.40	1.00000000	duck
Auh	-0.14	1.00000000	duck
Fbxo46	0.03	1.00000000	duck
Noc2l	-0.16	1.00000000	duck
Tecpr2	-0.28	1.00000000	duck
Pi4kb	-0.14	1.00000000	duck
Slc48a1	-0.12	1.00000000	duck
Ncbp3	-0.21	1.00000000	duck
Wac	-0.53	1.00000000	duck
Arhgap45	-0.09	1.00000000	duck
Scml2	-0.40	1.00000000	duck
Cltc	-0.25	1.00000000	duck
Ube2o	-0.39	1.00000000	duck
Dmxl1	-0.90	1.00000000	duck
Znrf3	-0.47	1.00000000	duck
Rprd1b	-0.41	1.00000000	duck
Caml	0.00	1.00000000	duck
Wdr20	-0.63	1.00000000	duck
Csnk1g1	-0.27	1.00000000	duck
Scd2	-0.39	1.00000000	duck
Tnfrsf11a	-0.77	1.00000000	duck
Txlng	-0.37	1.00000000	duck
Rsf1	-0.63	1.00000000	duck
Aff2	-0.34	1.00000000	duck
Ubxn7	-0.22	1.00000000	duck

Atp6v0a2	-0.13	1.00000000	duck
Dnajc1	-0.12	1.00000000	duck
Smad4	-0.33	1.00000000	duck
Zfp148	-0.51	1.00000000	duck
Ankra2	0.02	1.00000000	duck
Hnrnpa3	-0.44	1.00000000	duck
Rfwd3	-0.24	1.00000000	duck
Ash2l	-0.07	1.00000000	duck
Lmna	0.05	1.00000000	duck
Hps3	0.00	1.00000000	duck
Tmem161b	-0.31	1.00000000	duck
Efr3a	-0.51	1.00000000	duck
Hnrnpr	-0.54	1.00000000	duck
Sbno1	-0.24	1.00000000	duck
Fbxo34	-0.34	1.00000000	duck
Ppm1g	-0.16	1.00000000	duck
Scfd1	-0.42	1.00000000	duck
Bpnt2	-0.13	1.00000000	duck
Hdx	-0.06	1.00000000	duck
Rev1	-0.79	1.00000000	duck
Ppp1r21	-0.75	1.00000000	duck
Hyou1	0.10	1.00000000	duck
Cdca4	-0.17	1.00000000	duck
Zfp407	-0.21	1.00000000	duck
Rbbp6	-0.43	1.00000000	duck
S1pr4	-0.03	1.00000000	duck
Nup210	-0.03	1.00000000	duck
Tle5	0.08	1.00000000	duck
Camk1d	-0.65	1.00000000	duck
Tent2	-0.30	1.00000000	duck
Pitpna	-0.14	1.00000000	duck
Hsp90b1	-0.10	1.00000000	duck
Phf12	-0.42	1.00000000	duck
Scpep1	0.29	1.00000000	duck
Dhx8	-0.10	1.00000000	duck
Tsc1	-0.76	1.00000000	duck
Smc3	-0.36	1.00000000	duck
B3gnt5	-0.21	1.00000000	duck
Cbl	-0.34	1.00000000	duck
Camkmt	-0.17	1.00000000	duck
Zc3h7a	-1.01	1.00000000	duck
Uso1	-0.33	1.00000000	duck
Fbxo22	-0.21	1.00000000	duck
Cir1	-0.42	1.00000000	duck
Atxn7l2	-0.37	1.00000000	duck
Uvrag	-0.12	1.00000000	duck

Ubr3	-1.35	1.00000000	duck
Chd7	-0.13	1.00000000	duck
Ube4a	-0.10	1.00000000	duck
Atf1	-0.59	1.00000000	duck
Ppp6r2	-0.35	1.00000000	duck
Tsn	-0.13	1.00000000	duck
Stk26	-0.63	1.00000000	duck
Cog5	-0.32	1.00000000	duck
Slc38a2	-0.32	1.00000000	duck
Tasor2	0.03	1.00000000	duck
Arl4a	-0.16	1.00000000	duck
Samsn1	-0.47	1.00000000	duck
Ergic2	-0.26	1.00000000	duck
Nlrx1	0.11	1.00000000	duck
Slc7a6os	-0.24	1.00000000	duck
Iqgap1	-0.64	1.00000000	duck
Lamc1	0.16	1.00000000	duck
Dmap1	-0.31	1.00000000	duck
Sertad2	-0.36	1.00000000	duck
Ppp2r2d	-0.77	1.00000000	duck
Ell2	-0.81	1.00000000	duck
Tmem120a	-0.04	1.00000000	duck
Ano5	-0.26	1.00000000	duck
Btbd2	-0.50	1.00000000	duck
Sla2	-0.27	1.00000000	duck
Snx6	-0.14	1.00000000	duck
Fasn	0.20	1.00000000	duck
Cln6	0.03	1.00000000	duck
Socs3	0.13	1.00000000	duck
Cry1	-0.62	1.00000000	duck
Cpsf7	-0.15	1.00000000	duck
Phf20	-0.64	1.00000000	duck
Ccdc117	-0.66	1.00000000	duck
Ankrd11	-0.14	1.00000000	duck
Ago2	-1.32	1.00000000	duck
Parp1	-0.72	1.00000000	duck
Ubtd2	-0.35	1.00000000	duck
Psmg2	-0.14	1.00000000	duck
Rock1	-0.54	1.00000000	duck
Exoc6b	0.03	1.00000000	duck
Arid4b	-0.89	1.00000000	duck
Lrba	-0.77	1.00000000	duck
Pmm2	0.01	1.00000000	duck
Cd82	-0.03	1.00000000	duck
Cog2	-0.63	1.00000000	duck
Ubac1	-0.13	1.00000000	duck

Kifbp	-0.14	1.00000000	duck
Usp22	-0.54	1.00000000	duck
Dym	-1.29	1.00000000	duck
Sms	-0.21	1.00000000	duck
Hibch	-0.18	1.00000000	duck
Csrnp1	-0.47	1.00000000	duck
Sesn1	-0.50	1.00000000	duck
Zfp318	-0.30	1.00000000	duck
Kansl1	-0.68	1.00000000	duck
Lonp2	-0.66	1.00000000	duck
Nucks1	-0.24	1.00000000	duck
Ipo8	-0.31	1.00000000	duck
Plekhh3	0.09	1.00000000	duck
Tk2	-0.22	1.00000000	duck
Timm44	-0.03	1.00000000	duck
Phf3	-0.71	1.00000000	duck
Copg2	-0.38	1.00000000	duck
Uchl5	-0.07	1.00000000	duck
Gipc2	-0.25	1.00000000	duck
Reep3	-0.44	1.00000000	duck
Dnaja4	-0.84	1.00000000	duck
Nup214	-0.14	1.00000000	duck
Coq2	-0.35	1.00000000	duck
Tmem185a	-0.30	1.00000000	duck
Nbeal2	-0.50	1.00000000	duck
Arhgap5	-0.82	1.00000000	duck
Sin3a	-0.18	1.00000000	duck
Lsm6	0.17	1.00000000	duck
Deaf1	-0.18	1.00000000	duck
Sltm	-0.17	1.00000000	duck
Ubac2	0.10	1.00000000	duck
Sf3b1	-0.33	1.00000000	duck
Scaf8	-1.12	1.00000000	duck
Pmp22	-0.03	1.00000000	duck
Arhgap6	-0.16	1.00000000	duck
Azi2	-0.90	1.00000000	duck
Smad2	-0.37	1.00000000	duck
Pcnx	-0.66	1.00000000	duck
Cilk1	-0.49	1.00000000	duck
Csl	0.06	1.00000000	duck
Glb1	0.01	1.00000000	duck
Itfg1	-0.65	1.00000000	duck
Gnpda2	-0.08	1.00000000	duck
Ints10	-0.15	1.00000000	duck
Gse1	-0.01	1.00000000	duck
Tada1	-0.33	1.00000000	duck

Srsf5	-0.51	1.00000000	duck
Scyl2	-0.33	1.00000000	duck
Agps	-0.19	1.00000000	duck
Pik3r1	-0.48	1.00000000	duck
Agl	-0.47	1.00000000	duck
Nr2c1	-0.38	1.00000000	duck
Pdss2	-0.21	1.00000000	duck
Dhdds	-0.22	1.00000000	duck
Smarca1	-0.07	1.00000000	duck
Rab30	-0.05	1.00000000	duck
Zeb1	-0.40	1.00000000	duck
Ccdc28a	-0.30	1.00000000	duck
Upf2	-0.30	1.00000000	duck
Arhgef18	-0.20	1.00000000	duck
Snx4	-0.22	1.00000000	duck
Btrc	-0.58	1.00000000	duck
Lrrc1	-0.33	1.00000000	duck
Xpot	-0.31	1.00000000	duck
Erp44	-0.68	1.00000000	duck
Basp1	-0.37	1.00000000	duck
Pcm1	-0.62	1.00000000	duck
Clptm1l	-0.20	1.00000000	duck
Igf1r	0.03	1.00000000	duck
Slain2	-0.35	1.00000000	duck
Hagh	-0.57	1.00000000	duck
Pi4ka	-0.60	1.00000000	duck
Arhgef3	-0.22	1.00000000	duck
Gsg1	-0.59	1.00000000	duck
Pknox1	-1.25	1.00000000	duck
Dusp6	0.14	1.00000000	duck
Rnf146	-0.40	1.00000000	duck
Pip5k1a	-0.20	1.00000000	duck
Sorbs1	0.04	1.00000000	duck
Dazap1	-0.59	1.00000000	duck
Cdk5r1	-0.53	1.00000000	duck
Galc	0.06	1.00000000	duck
Suco	-0.51	1.00000000	duck
Cc2d2a	-0.58	1.00000000	duck
Cracr2a	-0.24	1.00000000	duck
Hnrnpab	-0.54	1.00000000	duck
Eif1	0.05	1.00000000	duck
Rere	-0.22	1.00000000	duck
Pxk	-0.51	1.00000000	duck
Nubp1	-0.30	1.00000000	duck
Parg	-0.66	1.00000000	duck
Snx5	-0.06	1.00000000	duck

Cdk19	-0.75	1.00000000	duck
Xylt1	-0.90	1.00000000	duck
Rcn2	-0.02	1.00000000	duck
Fbxw8	-0.25	1.00000000	duck
Cd74	-0.37	1.00000000	duck
Zbtb43	-0.90	1.00000000	duck
Phf20l1	-0.67	1.00000000	duck
Rnf114	-0.25	1.00000000	duck
Pls3	0.15	1.00000000	duck
Iffo2	0.01	1.00000000	duck
Fndc3a	-0.43	1.00000000	duck
Eif5	-0.35	1.00000000	duck
Osbpl5	-0.10	1.00000000	duck
Fos	0.31	1.00000000	duck
Ebag9	-0.38	1.00000000	duck
Otud7b	-0.09	1.00000000	duck
Usp16	-0.05	1.00000000	duck
Eif4h	-0.31	1.00000000	duck
Gpn3	-0.10	1.00000000	duck
Tubb1	-0.59	1.00000000	duck
Tef	-0.47	1.00000000	duck
Cds2	-0.17	1.00000000	duck
Plec	-0.86	1.00000000	duck
Cflar	-0.95	1.00000000	duck
St3gal6	-0.79	1.00000000	duck
Bnip3l	-0.67	1.00000000	duck
C2cd2	-0.90	1.00000000	duck
Eps15l1	-0.48	1.00000000	duck
Rgs1	-0.45	1.00000000	duck
Btaf1	-0.80	1.00000000	duck
Fam168b	-0.49	1.00000000	duck
Zfp236	-0.57	1.00000000	duck
Hacd3	-0.07	1.00000000	duck
Cd83	-0.16	1.00000000	duck
Anxa6	-0.11	1.00000000	duck
Tmem50b	-0.18	1.00000000	duck
Orc3	-0.86	1.00000000	duck
Rhof	-0.14	1.00000000	duck
Ccser2	0.07	1.00000000	duck
Fgfr1op2	-0.46	1.00000000	duck
Armh3	-1.15	1.00000000	duck
Taok3	-0.83	1.00000000	duck
Plod1	-0.32	1.00000000	duck
Naa15	-0.43	1.00000000	duck
Fam102b	-0.43	1.00000000	duck
Ahctf1	-0.65	1.00000000	duck

Immt	-0.06	1.00000000	duck
Zbtb34	-0.88	1.00000000	duck
Ube2b	-0.59	1.00000000	duck
Tnrc6a	-0.58	1.00000000	duck
Cyth1	-0.20	1.00000000	duck
Ankrd52	-0.11	1.00000000	duck
Ccni	-0.40	1.00000000	duck
Rab6a	-0.32	1.00000000	duck
Lonrf3	-0.75	1.00000000	duck
Gpatch1	-0.49	1.00000000	duck
Nrbf2	-0.02	1.00000000	duck
Sec24b	-0.83	1.00000000	duck
Per2	-0.72	1.00000000	duck
Dok2	-0.05	1.00000000	duck
Usp1	-0.12	1.00000000	duck
Ipo7	-0.53	1.00000000	duck
Eif4g3	-0.30	1.00000000	duck
Rc3h2	-0.40	1.00000000	duck
Pkn2	-0.51	1.00000000	duck
Epc1	-0.68	1.00000000	duck
Brd3	-0.84	1.00000000	duck
Exd1	-0.07	1.00000000	duck
Ehmt1	-0.26	1.00000000	duck
Arhgap17	-0.21	1.00000000	duck
Osbpl3	-0.82	1.00000000	duck
Ncoa3	-0.36	1.00000000	duck
Dock11	-0.35	1.00000000	duck
Bst1	-0.69	1.00000000	duck
Pofut1	-0.12	1.00000000	duck
Fa2h	-0.54	1.00000000	duck
Atg16l1	-0.38	1.00000000	duck
Hnrnpa2b1	-0.66	1.00000000	duck
Etfrf1	-0.28	1.00000000	duck
Fubp3	-0.06	1.00000000	duck
Zfyve1	-0.16	1.00000000	duck
Itgb2l	-0.09	1.00000000	duck
Gnb1	-0.19	1.00000000	duck
Sap30l	-0.27	1.00000000	duck
Klhl15	-0.43	1.00000000	duck
Zc3h12c	-0.24	1.00000000	duck
Zbtb38	-0.35	1.00000000	duck
Senp7	-0.75	1.00000000	duck
Nktr	-0.94	1.00000000	duck
Slc25a16	-0.76	1.00000000	duck
Rnf126	-0.11	1.00000000	duck
Cdc37l1	-0.63	1.00000000	duck

Txndc9	-1.07	1.00000000	duck
Hip1	-0.10	1.00000000	duck
Slc12a4	-1.05	1.00000000	duck
Prpsap1	-0.07	1.00000000	duck
Brd1	-0.91	1.00000000	duck
Fgf10	-0.20	1.00000000	duck
Ppp1r13b	-0.03	1.00000000	duck
Nedd9	-0.39	1.00000000	duck
Adcy7	-0.39	1.00000000	duck
Lhfpl2	0.13	1.00000000	duck
Spast	-0.48	1.00000000	duck
Khdrbs1	-0.20	1.00000000	duck
Ireb2	-0.46	1.00000000	duck
Nyx	-0.54	1.00000000	duck
Styk1	-0.52	1.00000000	duck
Tnks	-0.49	1.00000000	duck
Ascc3	-0.82	1.00000000	duck
Ggps1	-0.38	1.00000000	duck
Upp1	-0.30	1.00000000	duck
Ppa2	-0.33	1.00000000	duck
Tmem168	-0.21	1.00000000	duck
Dlgap4	-0.23	1.00000000	duck
Adam8	-0.27	1.00000000	duck
Pitpnm3	-0.21	1.00000000	duck
Hnrnph3	-0.45	1.00000000	duck
Slc35a1	0.04	1.00000000	duck
Pcmtd1	-1.33	1.00000000	duck
Lats1	-0.20	1.00000000	duck
Tiam2	-0.55	1.00000000	duck
Setd1b	-0.78	1.00000000	duck
Ankrd22	-1.25	1.00000000	duck
Add1	-0.58	1.00000000	duck
Pdpk1	-0.53	1.00000000	duck
Ube2e2	0.32	1.00000000	duck
Anxa5	-0.05	1.00000000	duck
Runx1	-0.07	1.00000000	duck
Cct3	-0.42	1.00000000	duck
Dip2a	-0.76	1.00000000	duck
Ankrd44	-0.80	1.00000000	duck
Smox	-0.43	1.00000000	duck
Slc25a24	-0.42	1.00000000	duck
Rb1	-0.45	1.00000000	duck
Stag2	-0.74	1.00000000	duck
Cdc73	-1.08	1.00000000	duck
Lonp1	-0.16	1.00000000	duck
Bnip3	-0.43	1.00000000	duck

Ampd2	-0.22	1.00000000	duck
Zfp711	-0.65	1.00000000	duck
Ppp2cb	-0.32	1.00000000	duck
Casd1	-0.65	1.00000000	duck
Adprh	-0.11	1.00000000	duck
Sart3	-0.25	1.00000000	duck
Rbms3	0.27	1.00000000	duck
Flot2	-0.32	1.00000000	duck
Dhx15	-0.57	1.00000000	duck
Pgrmc1	0.05	1.00000000	duck
Laptm4a	-0.18	1.00000000	duck
Arhgap31	0.06	1.00000000	duck
Gpr180	-0.31	1.00000000	duck
Ets1	-0.29	1.00000000	duck
Hprt	-0.66	1.00000000	duck
Sphk1	-0.35	1.00000000	duck
Ppp3cb	-0.56	1.00000000	duck
Arhgap26	-0.54	1.00000000	duck
Daglb	-0.52	1.00000000	duck
Selenop	0.02	1.00000000	duck
Klhdc3	-0.18	1.00000000	duck
Tent4a	-0.16	1.00000000	duck
Limk2	-0.37	1.00000000	duck
Baz1a	-0.23	1.00000000	duck
Snap23	-0.36	1.00000000	duck
Cep350	-0.80	1.00000000	duck
Tmem123	-0.58	1.00000000	duck
Jdp2	-0.03	1.00000000	duck
Nfrkb	-0.07	1.00000000	duck
Cttnbp2nl	0.07	1.00000000	duck
Pikfyve	-0.54	1.00000000	duck
Fbxo30	-0.26	1.00000000	duck
Gfpt1	-0.74	1.00000000	duck
C1galt1	-0.15	1.00000000	duck
Socs7	-0.06	1.00000000	duck
Cog6	-0.17	1.00000000	duck
Nup107	-0.15	1.00000000	duck
Phf6	-0.40	1.00000000	duck
Rexo1	-0.52	1.00000000	duck
Tmed10	-0.25	1.00000000	duck
Lrig2	-0.66	1.00000000	duck
Enpp4	-0.52	1.00000000	duck
Scamp1	-0.32	1.00000000	duck
Ddb1	-0.30	1.00000000	duck
Gan	-0.31	1.00000000	duck
Dennd6a	-0.65	1.00000000	duck

Rai1	-0.18	1.00000000	duck
Adam10	-0.43	1.00000000	duck
Htatsf1	-0.66	1.00000000	duck
Skp1	-0.48	1.00000000	duck
Ccdc6	-0.65	1.00000000	duck
Tob1	-0.37	1.00000000	duck
Prpf4b	-0.49	1.00000000	duck
Trim59	-0.32	1.00000000	duck
Scn8a	-0.12	1.00000000	duck
Cntrl	-0.43	1.00000000	duck
Zmym2	-0.53	1.00000000	duck
Lin7c	-0.09	1.00000000	duck
Tlr4	-0.22	1.00000000	duck
Psma4	-0.42	1.00000000	duck
Zbtb41	-0.52	1.00000000	duck
Fam120a	-0.67	1.00000000	duck
Pdxdc1	-0.27	1.00000000	duck
Gnal	-0.75	1.00000000	duck
Tsc2	-0.35	1.00000000	duck
Cnot1	-0.38	1.00000000	duck
Tspan2	-0.20	1.00000000	duck
Taf5	-0.21	1.00000000	duck
Gfod1	-0.72	1.00000000	duck
Klhl12	-0.37	1.00000000	duck
Ifngr1	-0.40	1.00000000	duck
Arhgap10	-0.54	1.00000000	duck
Kcnq5	-0.43	1.00000000	duck
Ash1l	-0.99	1.00000000	duck
Rabl6	-0.31	1.00000000	duck
Rp2	-0.57	1.00000000	duck
Leo1	-0.65	1.00000000	duck
Arl5a	-0.16	1.00000000	duck
Top2b	-0.88	1.00000000	duck
Lipc	-0.12	1.00000000	duck
Ccdc126	-0.36	1.00000000	duck
Rad51b	-0.55	1.00000000	duck
Ercc3	-0.20	1.00000000	duck
Hivep3	-0.01	1.00000000	duck
Vps13c	-0.29	1.00000000	duck
Btbd9	-1.41	1.00000000	duck
Akirin2	-0.30	1.00000000	duck
Ppp2r5c	-0.75	1.00000000	duck
Bdh1	-0.21	1.00000000	duck
Ero1b	-0.60	1.00000000	duck
Siva1	-0.06	1.00000000	duck
Grk3	-0.18	1.00000000	duck

Tmem131l	-1.14	1.00000000	duck
Stub1	-0.34	1.00000000	duck
Cep170	-0.67	1.00000000	duck
Tgfbr2	0.17	1.00000000	duck
Irak2	-0.37	1.00000000	duck
Tpr	-0.72	1.00000000	duck
Npepps	-0.27	1.00000000	duck
Dusp11	-0.21	1.00000000	duck
Gak	-0.43	1.00000000	duck
Sufu	-0.65	1.00000000	duck
Ywhah	-0.22	1.00000000	duck
Cops5	-0.23	1.00000000	duck
Cd44	-0.23	1.00000000	duck
Clcn3	-0.70	1.00000000	duck
Sesn2	-0.14	1.00000000	duck
Cpeb2	-0.70	1.00000000	duck
Setdb1	-0.38	1.00000000	duck
Ndufaf2	-0.64	1.00000000	duck
Ell	-0.17	1.00000000	duck
Fam214b	-0.18	1.00000000	duck
Rfx2	-0.70	1.00000000	duck
Esf1	-0.69	1.00000000	duck
Rpa3	-0.28	1.00000000	duck
Tmtc1	-0.08	1.00000000	duck
Cdk5rap2	-0.77	1.00000000	duck
Senp6	-0.42	1.00000000	duck
Kdm2b	-0.17	1.00000000	duck
Dennd1b	-0.33	1.00000000	duck
Rlf	-0.61	1.00000000	duck
Sptlc2	-0.41	1.00000000	duck
Prkag2	-0.48	1.00000000	duck
Casp9	-0.13	1.00000000	duck
Dcp1b	-1.37	1.00000000	duck
Tab2	-0.76	1.00000000	duck
Rbm5	-0.87	1.00000000	duck
Hook1	-0.26	1.00000000	duck
Septin11	-0.14	1.00000000	duck
Rhoq	-0.03	1.00000000	duck
Inpp4a	-0.41	1.00000000	duck
Papola	-0.69	1.00000000	duck
Nop58	-0.15	1.00000000	duck
Trim24	-0.86	1.00000000	duck
Abce1	-0.64	1.00000000	duck
Reps1	-0.82	1.00000000	duck
Sgsm2	-0.42	1.00000000	duck
Eif4g2	-0.09	1.00000000	duck

Hpse	-0.55	1.00000000	duck
Ppp6r3	-0.81	1.00000000	duck
Bcl7a	-0.46	1.00000000	duck
Hspa4	-0.60	1.00000000	duck
Spry1	-0.09	1.00000000	duck
Fam222b	-0.77	1.00000000	duck
Kmt2e	-0.63	1.00000000	duck
Dync1li2	-0.45	1.00000000	duck
Cluh	-0.37	1.00000000	duck
Pard3b	-0.32	1.00000000	duck
Ngly1	-0.57	1.00000000	duck
Ttc37	-0.55	1.00000000	duck
Nrbp1	-0.57	1.00000000	duck
Tulp4	-0.60	1.00000000	duck
Psip1	-0.17	1.00000000	duck
Adam17	-0.47	1.00000000	duck
Zzef1	-0.90	1.00000000	duck
Dnajb4	-0.75	1.00000000	duck
Btbd11	-0.64	1.00000000	duck
Rhoh	-0.08	1.00000000	duck
Supv3l1	-0.29	1.00000000	duck
Veph1	-0.67	1.00000000	duck
Nbr1	-0.62	1.00000000	duck
Csde1	-0.55	1.00000000	duck
Pip5k1b	-0.70	1.00000000	duck
Cbx1	-0.23	1.00000000	duck
Sync	-0.06	1.00000000	duck
Erc1	-0.71	1.00000000	duck
Sema3c	-0.29	1.00000000	duck
Tmcc1	-0.98	1.00000000	duck
Cacna1c	-0.35	1.00000000	duck
Ano9	-0.30	1.00000000	duck
Prdm2	-0.19	1.00000000	duck
Nfkb1	-0.13	1.00000000	duck
Ly86	-1.03	1.00000000	duck
Nrbp2	-0.64	1.00000000	duck
Foxo1	-0.56	1.00000000	duck
Hectd4	-0.65	1.00000000	duck
Itpk1	-1.19	1.00000000	duck
Srsf10	-0.49	1.00000000	duck
Anp32e	-0.38	1.00000000	duck
Pan3	-0.80	1.00000000	duck
Atxn7l1	-0.71	1.00000000	duck
Dnm1l	-0.15	1.00000000	duck
Nfia	-0.59	1.00000000	duck
Acap2	-0.47	1.00000000	duck

Septin2	-0.11	1.00000000	duck
Ifngr2	-0.28	1.00000000	duck
Arid2	-0.94	1.00000000	duck
Clec16a	-0.51	1.00000000	duck
Dyrk1a	-0.56	1.00000000	duck
Slc30a4	-0.40	1.00000000	duck
Sugt1	-0.67	1.00000000	duck
Suz12	-0.38	1.00000000	duck
Ide	-0.78	1.00000000	duck
Hmgn3	-0.06	1.00000000	duck
Atp2b4	-0.01	1.00000000	duck
Ctnnbl1	-0.49	1.00000000	duck
Yme1l1	-0.41	1.00000000	duck
Dhx40	-0.38	1.00000000	duck
Gtf2f2	-0.87	1.00000000	duck
C2cd5	-0.44	1.00000000	duck
Ptpre	-0.32	1.00000000	duck
Kansl1l	-0.75	1.00000000	duck
Slc35a5	-0.50	1.00000000	duck
Atp7a	-0.71	1.00000000	duck
Kpna4	-0.56	1.00000000	duck
Emp1	-0.73	1.00000000	duck
Pparg	-0.93	1.00000000	duck
Hspa5	-0.37	1.00000000	duck
Ugt8a	-0.71	1.00000000	duck
Atg5	-0.88	1.00000000	duck
Erh	-0.20	1.00000000	duck
Elavl1	-0.45	1.00000000	duck
Cpeb3	-0.79	1.00000000	duck
Rabep1	-0.41	1.00000000	duck
Rab11a	-0.33	1.00000000	duck
Gpatch8	-0.57	1.00000000	duck
Rbm6	-0.52	1.00000000	duck
Stt3b	-0.34	1.00000000	duck
Ing1	-0.15	1.00000000	duck
Bcl2l1	-0.69	1.00000000	duck
Fam126b	-1.02	1.00000000	duck
Zmynd8	-0.42	1.00000000	duck
Ccdc174	-0.42	1.00000000	duck
Rbm26	-0.78	1.00000000	duck
Vps53	-0.19	1.00000000	duck
Kctd20	-0.79	1.00000000	duck
Tbcd	-0.64	1.00000000	duck
Gabpb1	-0.50	1.00000000	duck
Crebrf	-1.19	1.00000000	duck
Stam2	-0.53	1.00000000	duck

Tmem131	-0.66	1.00000000	duck
Dpysl2	-0.11	1.00000000	duck
Ndufs1	-0.32	1.00000000	duck
Brwd3	-0.76	1.00000000	duck
Lars	-0.40	1.00000000	duck
Naaa	-0.10	1.00000000	duck
Lpcat2b	-0.37	1.00000000	duck
Atad1	-0.56	1.00000000	duck
Ufd1	-0.39	1.00000000	duck
Ankle2	-0.22	1.00000000	duck
Chuk	-0.70	1.00000000	duck
Ice2	-0.46	1.00000000	duck
Ciao2a	-0.30	1.00000000	duck
Tfam	-0.08	1.00000000	duck
Dcp1a	-0.40	1.00000000	duck
Uhrf2	-0.68	1.00000000	duck
Pptc7	-0.53	1.00000000	duck
Tesk2	-0.53	1.00000000	duck
Oxr1	-0.82	1.00000000	duck
Bmpr2	-0.57	1.00000000	duck
Abhd12	-0.21	1.00000000	duck
Rftn1	-0.70	1.00000000	duck
Spopl	-0.31	1.00000000	duck
Tenm3	-0.32	1.00000000	duck
Ctdspl	-0.92	1.00000000	duck
Rfng	-0.24	1.00000000	duck
Fam135a	-0.20	1.00000000	duck
Ppil2	-0.71	1.00000000	duck
Ptx3	-0.90	1.00000000	duck
Ogfrl1	-0.33	1.00000000	duck
Rragd	-0.41	1.00000000	duck
Arl5b	-0.51	1.00000000	duck
Arntl	-0.69	1.00000000	duck
Ghitm	-0.74	1.00000000	duck
Vps13a	-0.82	1.00000000	duck
Gpr141	-0.35	1.00000000	duck
Wapl	-0.47	1.00000000	duck
Ilrun	-1.11	1.00000000	duck
Evl	-0.29	1.00000000	duck
Ammecr1	-0.69	1.00000000	duck
Sgpl1	-0.31	1.00000000	duck
Fam126a	-0.24	1.00000000	duck
Gigyf2	-0.49	1.00000000	duck
Cul5	-0.67	1.00000000	duck
Pcmt1	-0.81	1.00000000	duck
Gsk3b	-0.23	1.00000000	duck

Rfx3	-0.43	1.00000000	duck
Cfl2	-0.40	1.00000000	duck
Gapvd1	-0.53	1.00000000	duck
Parvg	-0.45	1.00000000	duck
Cpne1	-0.62	1.00000000	duck
Lin54	-0.49	1.00000000	duck
Wdr7	-0.70	1.00000000	duck
Nod1	0.00	1.00000000	duck
Bdp1	-0.84	1.00000000	duck
Nav3	0.14	1.00000000	duck
Steep1	-0.35	1.00000000	duck
Dpp7	-0.04	1.00000000	duck
Traf3ip3	-0.51	1.00000000	duck
Spen	-0.67	1.00000000	duck
Stk38l	-0.16	1.00000000	duck
Hs6st1	-0.13	1.00000000	duck
Bach2	-0.32	1.00000000	duck
Ola1	-0.25	1.00000000	duck
Nvl	-0.25	1.00000000	duck
Ctdp1	-0.66	1.00000000	duck
Kpna1	-0.57	1.00000000	duck
Ptges3	-0.90	1.00000000	duck
Fbxo38	-0.45	1.00000000	duck
Ube2q2	-0.44	1.00000000	duck
Nbas	-0.35	1.00000000	duck
Sec16a	-0.50	1.00000000	duck
Slc12a2	-0.47	1.00000000	duck
Golga3	-0.42	1.00000000	duck
Clock	-0.59	1.00000000	duck
Chst13	-0.30	1.00000000	duck
Epb41l2	-0.11	1.00000000	duck
Xpo7	-0.67	1.00000000	duck
Zfp592	-0.59	1.00000000	duck
Eaf1	-0.28	1.00000000	duck
Pik3ap1	-0.38	1.00000000	duck
Elf2	-0.38	1.00000000	duck
Hipk1	-0.77	1.00000000	duck
Cops2	-0.43	1.00000000	duck
Prelid3b	-0.39	1.00000000	duck
Otulinl	-0.07	1.00000000	duck
Ints6	-0.66	1.00000000	duck
Rnf111	-0.92	1.00000000	duck
Pds5b	-0.39	1.00000000	duck
Lypla1	-0.18	1.00000000	duck
Nek6	-0.65	1.00000000	duck
Dnase2b	0.21	1.00000000	duck

Vezf1	-1.09	1.00000000	duck
Dennd5a	-0.48	1.00000000	duck
Golgb1	-0.51	1.00000000	duck
Fam78a	-0.14	1.00000000	duck
Ptpn9	-0.13	1.00000000	duck
Ldlrad3	-0.67	1.00000000	duck
Pbrm1	-0.79	1.00000000	duck
Lonrf1	-0.91	1.00000000	duck
Rbsn	-0.41	1.00000000	duck
Cdk8	-0.78	1.00000000	duck
Tial1	-0.74	1.00000000	duck
Prrc2b	-0.55	1.00000000	duck
Tmem154	-0.43	1.00000000	duck
Nsmce1	-0.51	1.00000000	duck
Gid8	-0.50	1.00000000	duck
1110059G10Rik	-0.43	1.00000000	duck
Adipor2	-0.38	1.00000000	duck
Erbin	-0.78	1.00000000	duck
Ctr9	-0.44	1.00000000	duck
Rbm39	-0.27	1.00000000	duck
Rell1	-0.63	1.00000000	duck
Hnrnpll	-0.63	1.00000000	duck
Inpp5d	-0.74	1.00000000	duck
Edc4	-0.20	1.00000000	duck
Sla	-0.65	1.00000000	duck
Pcgf6	-0.49	1.00000000	duck
Pik3r5	-0.28	1.00000000	duck
Cerk	-0.54	1.00000000	duck
Nnt	-0.15	1.00000000	duck
Gpn1	-0.58	1.00000000	duck
Ssh2	-0.39	1.00000000	duck
Hspa4l	-0.52	1.00000000	duck
Dapp1	-0.78	1.00000000	duck
Srgap3	-0.59	1.00000000	duck
Lrch1	-0.57	1.00000000	duck
Zcchc2	-0.72	1.00000000	duck
Baz1b	-0.83	1.00000000	duck
Atg4c	-0.68	1.00000000	duck
Fam122b	-0.33	1.00000000	duck
Apaf1	-0.63	1.00000000	duck
Ddx6	-0.43	1.00000000	duck
Txk	-0.35	1.00000000	duck
Uba5	-0.09	1.00000000	duck
Arid1b	-0.64	1.00000000	duck
Creb1	-0.27	1.00000000	duck
Anapc7	-0.24	1.00000000	duck

Hormad1	-0.32	1.00000000	duck
Dnajb6	-0.28	1.00000000	duck
Ehbp1	-0.43	1.00000000	duck
Cacul1	-0.65	1.00000000	duck
Osbpl11	-0.51	1.00000000	duck
Nolc1	-0.17	1.00000000	duck
Parn	-0.66	1.00000000	duck
Efhd1	0.03	1.00000000	duck
Rpap2	-0.55	1.00000000	duck
Nhlrc2	-0.44	1.00000000	duck
Trappc11	-0.39	1.00000000	duck
Invs	-0.52	1.00000000	duck
Cep85l	-0.69	1.00000000	duck
Ssu72	-0.42	1.00000000	duck
Smurf2	-0.88	1.00000000	duck
Luc7l3	-0.75	1.00000000	duck
Nfe2l2	-0.53	1.00000000	duck
Edrf1	-0.65	1.00000000	duck
Khdc4	-0.30	1.00000000	duck
Kat6b	-0.60	1.00000000	duck
Elk3	-0.26	1.00000000	duck
Fam199x	-0.44	1.00000000	duck
Cbx3	-0.37	1.00000000	duck
Eif4g1	-0.16	1.00000000	duck
Lpar6	-0.18	1.00000000	duck
Ube2d1	-0.43	1.00000000	duck
Acaa2	-0.30	1.00000000	duck
Gphn	-0.81	1.00000000	duck
Ezh2	-0.45	1.00000000	duck
Cdkn1b	-0.53	1.00000000	duck
Cct5	-0.28	1.00000000	duck
Tbc1d12	-0.46	1.00000000	duck
Pex14	-0.34	1.00000000	duck
Pbdc1	-0.17	1.00000000	duck
Dnttip2	-0.40	1.00000000	duck
Immp1l	-0.68	1.00000000	duck
Ggh	-0.61	1.00000000	duck
Gtf2e1	-0.63	1.00000000	duck
Drd4	-0.55	1.00000000	duck
Ankrd27	-0.56	1.00000000	duck
Irf1	-0.55	1.00000000	duck
Reps2	-0.96	1.00000000	duck
Qk	-0.33	1.00000000	duck
Tlk1	-0.81	1.00000000	duck
Zfp638	-0.60	1.00000000	duck
Sun1	-1.09	1.00000000	duck

Esyt2	-0.30	1.00000000	duck
Ankrd17	-0.58	1.00000000	duck
Cdk13	-0.79	1.00000000	duck
Ythdc1	-0.78	1.00000000	duck
Ikzf1	-0.84	1.00000000	duck
Usp44	-0.48	1.00000000	duck
Zfp644	-0.82	1.00000000	duck
Epg5	-0.38	1.00000000	duck
Fam98a	-0.29	1.00000000	duck
Fkbp4	0.03	1.00000000	duck
Pfpl	-0.11	1.00000000	duck
Chp1	-0.50	1.00000000	duck
Prr5l	-0.37	1.00000000	duck
Smchd1	-0.97	1.00000000	duck
Parp8	-0.46	1.00000000	duck
Acsl1	-0.36	1.00000000	duck
Nck2	-0.64	1.00000000	duck
Hif1a	-0.37	1.00000000	duck
Narf	-0.75	1.00000000	duck
Zfp207	-0.87	1.00000000	duck
Zfp652	-0.70	1.00000000	duck
Ndrg1	-0.16	1.00000000	duck
Pcf11	2.88	0.00000000	pigeons
App	4.15	0.00000000	pigeons
Tpt1	4.99	0.00000000	pigeons
Aqp1	-4.90	0.00000000	pigeons
Ptprm	-4.83	0.00000000	pigeons
Sdcbp	2.44	0.00000000	pigeons
Vim	4.42	0.00000000	pigeons
Cyria	5.17	0.00000000	pigeons
Cd2ap	3.77	0.00000000	pigeons
Rab33a	6.29	0.00000000	pigeons
Igfbp7	-4.26	0.00000000	pigeons
Lnpep	2.64	0.00000000	pigeons
Arpc2	3.07	0.00000000	pigeons
Rgs2	3.47	0.00000000	pigeons
Plscr1	2.52	0.00000000	pigeons
Il1rap	4.41	0.00000000	pigeons
Lpp	2.87	0.00000000	pigeons
Dido1	2.27	0.00000000	pigeons
Il1b	6.27	0.00000000	pigeons
Cfd	-5.17	0.00000000	pigeons
Fos	-2.68	0.00000000	pigeons
F10	3.27	0.00000000	pigeons
Tollip	2.32	0.00000000	pigeons
Cmip	3.08	0.00000000	pigeons

Epas1	-3.92	0.00000000	pigeons
Timp3	-3.99	0.00000000	pigeons
Niban1	2.72	0.00000000	pigeons
Apbb1ip	2.10	0.00000000	pigeons
Gpat3	2.59	0.00000000	pigeons
Cklf	2.10	0.00000000	pigeons
Wtap	2.00	0.00000000	pigeons
Atf7ip	2.71	0.00000000	pigeons
Ankhd1	2.14	0.00000000	pigeons
Txnip	2.45	0.00000000	pigeons
Dapp1	2.75	0.00000000	pigeons
Adss	2.14	0.00000000	pigeons
Fyb	2.12	0.00000000	pigeons
Rbms3	-4.14	0.00000000	pigeons
Gadd45b	2.41	0.00000000	pigeons
Tgfbr1	2.29	0.00000000	pigeons
Vsig4	2.17	0.00000000	pigeons
Atp5b	2.15	0.00000000	pigeons
Trib1	2.42	0.00000000	pigeons
Tmbim1	1.96	0.00000000	pigeons
Sap18	1.89	0.00000000	pigeons
Far1	2.08	0.00000000	pigeons
Calcrl	-3.85	0.00000000	pigeons
Steap4	3.35	0.00000000	pigeons
Rapgef5	-3.59	0.00000000	pigeons
Rasal2	-3.72	0.00000000	pigeons
Pecam1	-3.20	0.00000000	pigeons
U2surp	1.74	0.00000000	pigeons
Cnbp	1.81	0.00000000	pigeons
Fgd4	2.20	0.00000000	pigeons
Slc12a7	1.92	0.00000000	pigeons
Oga	1.63	0.00000000	pigeons
Tmtc1	-3.85	0.00000000	pigeons
Crim1	-3.16	0.00000000	pigeons
Tjp1	-3.73	0.00000000	pigeons
Ldb2	-3.73	0.00000000	pigeons
Fa2h	1.98	0.00000000	pigeons
Fnip1	2.51	0.00000000	pigeons
Pfkfb1	1.98	0.00000000	pigeons
Arhgap15	2.12	0.00000000	pigeons
Adnp	1.57	0.00000000	pigeons
Ddit4	-3.02	0.00000000	pigeons
Tiparp	1.96	0.00000000	pigeons
Adgrl4	-3.57	0.00000000	pigeons
Adgrl2	-3.68	0.00000000	pigeons
Syncrip	1.59	0.00000000	pigeons

Phip	1.89	0.00000000	pigeons
Lpgat1	1.77	0.00000000	pigeons
Cars	1.61	0.00000000	pigeons
Vcp	1.44	0.00000000	pigeons
Arid5b	2.11	0.00000000	pigeons
4932411N23Rik	-3.18	0.00000000	pigeons
B4galt5	1.67	0.00000000	pigeons
Hecw2	-3.53	0.00000000	pigeons
Pla2g4a	2.19	0.00000000	pigeons
Pank3	1.96	0.00000000	pigeons
Strap	1.38	0.00000000	pigeons
Pparg	2.43	0.00000000	pigeons
Tmem268	1.98	0.00000000	pigeons
Scaper	2.86	0.00000000	pigeons
Rxra	1.65	0.00000000	pigeons
Grb2	1.73	0.00000000	pigeons
Adora2b	1.75	0.00000000	pigeons
Adgrf5	-3.43	0.00000000	pigeons
Sptbn1	-2.57	0.00000000	pigeons
Spi1	1.82	0.00000000	pigeons
Wipf1	1.63	0.00000000	pigeons
Tmem100	-3.44	0.00000000	pigeons
Ube2j1	1.69	0.00000000	pigeons
Samsn1	1.70	0.00000000	pigeons
Tpd52	1.66	0.00000000	pigeons
Zfp516	2.11	0.00000000	pigeons
Atp5g3	1.75	0.00000000	pigeons
Sdk1	-3.38	0.00000000	pigeons
Arap2	1.75	0.00000000	pigeons
Lrrfip1	2.20	0.00000000	pigeons
Pstpip1	2.00	0.00000000	pigeons
Ppm1b	1.86	0.00000000	pigeons
Nars	1.26	0.00000000	pigeons
Sun2	1.51	0.00000000	pigeons
Sparc	-3.27	0.00000000	pigeons
Nova1	-3.33	0.00000000	pigeons
Syne2	-3.33	0.00000000	pigeons
Pik3r5	1.59	0.00000000	pigeons
Rin3	1.86	0.00000000	pigeons
Asap1	2.11	0.00000000	pigeons
Stom	1.56	0.00000000	pigeons
Fbh1	1.58	0.00000000	pigeons
Rab11fip4	1.68	0.00000000	pigeons
Nsmce2	2.00	0.00000000	pigeons
Nedd4	1.84	0.00000000	pigeons
Hbegf	1.83	0.00000000	pigeons

Snx20	1.52	0.00000000	pigeons
Pfkp	1.48	0.00000000	pigeons
Akap12	-3.26	0.00000000	pigeons
Pomp	1.32	0.00000000	pigeons
Zdhhc20	1.33	0.00000000	pigeons
Rmdn2	2.15	0.00000000	pigeons
Ptprb	-3.27	0.00000000	pigeons
Dars	1.61	0.00000000	pigeons
Sdhc	1.21	0.00000000	pigeons
Rasa2	1.64	0.00000000	pigeons
Ptpn6	1.35	0.00000000	pigeons
Htr1f	2.72	0.00000000	pigeons
Dock11	1.64	0.00000000	pigeons
Plcb1	-3.03	0.00000000	pigeons
Gpx3	-3.11	0.00000000	pigeons
Dock2	1.65	0.00000000	pigeons
Itga4	1.68	0.00000000	pigeons
Trpc4ap	1.22	0.00000000	pigeons
Svil	2.46	0.00000000	pigeons
Sec23ip	1.19	0.00000000	pigeons
Rbm6	1.36	0.00000000	pigeons
Ndufa1	1.35	0.00000000	pigeons
Cdk5r1	1.46	0.00000000	pigeons
Ube2w	1.29	0.00000000	pigeons
Ovch2	1.89	0.00000000	pigeons
Dot1l	1.64	0.00000000	pigeons
Nfia	-3.11	0.00000000	pigeons
Clec16a	1.61	0.00000000	pigeons
Zfp352	-2.55	0.00000000	pigeons
Itgb2l	1.19	0.00000000	pigeons
Rtf2	1.16	0.00000000	pigeons
Pik3cg	1.38	0.00000000	pigeons
Acsbg2	1.34	0.00000000	pigeons
Alox5ap	1.16	0.00000000	pigeons
Angpt1	2.60	0.00000000	pigeons
Hsp90aa1	-1.29	0.00000000	pigeons
Als2cl	1.79	0.00000000	pigeons
Cbfb	1.82	0.00000000	pigeons
Cavin2	-3.10	0.00000000	pigeons
Tmem154	1.11	0.00000000	pigeons
Rp2	1.41	0.00000000	pigeons
Braf	1.38	0.00000000	pigeons
Pknox1	1.54	0.00000000	pigeons
Afdn	-3.13	0.00000000	pigeons
Ptpre	1.93	0.00000000	pigeons
Cacybp	1.26	0.00000000	pigeons

Rfwd3	1.49	0.00000000	pigeons
Dock9	-2.72	0.00000000	pigeons
Antxr2	1.56	0.00000000	pigeons
Stard13	-3.02	0.00000000	pigeons
Kdm7a	1.61	0.00000000	pigeons
Xpo6	1.27	0.00000000	pigeons
Nek6	1.31	0.00000000	pigeons
Slc2a6	1.34	0.00000000	pigeons
Plin2	1.45	0.00000000	pigeons
Prep	1.29	0.00000000	pigeons
Appl2	1.43	0.00000000	pigeons
Ptgs2	2.61	0.00000000	pigeons
Enox2	1.09	0.00000000	pigeons
Lonrf3	1.76	0.00000000	pigeons
Sox6	-2.52	0.00000000	pigeons
Oxr1	1.74	0.00000000	pigeons
Nadk	1.32	0.00000000	pigeons
Plcg2	1.26	0.00000000	pigeons
Pcnp	1.06	0.00000000	pigeons
Chst13	1.22	0.00000000	pigeons
Bcl7a	1.20	0.00000000	pigeons
Btbd11	1.69	0.00000000	pigeons
Actn1	1.64	0.00000000	pigeons
Rnf19b	1.18	0.00000000	pigeons
Pfdn1	1.22	0.00000000	pigeons
Lrch3	1.31	0.00000000	pigeons
Eprs	1.23	0.00000000	pigeons
Dtx2	1.39	0.00000000	pigeons
Ptafr	1.21	0.00000000	pigeons
Ptprc	1.19	0.00000000	pigeons
Ezr	1.26	0.00000000	pigeons
Notch2	1.20	0.00000000	pigeons
Egr1	-2.14	0.00000000	pigeons
Acsl1	1.56	0.00000000	pigeons
Ergic1	1.24	0.00000000	pigeons
Kctd12	1.35	0.00000000	pigeons
Dap	1.26	0.00000000	pigeons
Slc38a2	1.51	0.00000000	pigeons
Fam189a1	-2.97	0.00000000	pigeons
Syk	1.17	0.00000000	pigeons
Anapc15-ps	1.10	0.00000000	pigeons
Rasgrp1	1.66	0.00000000	pigeons
Sat1	1.17	0.00000000	pigeons
Greb1	1.22	0.00000000	pigeons
Ets2	1.69	0.00000000	pigeons
Atp5k	1.08	0.00000000	pigeons

Rac2	1.02	0.00000000	pigeons
Tnfaip3	1.27	0.00000000	pigeons
Dll4	-2.84	0.00000000	pigeons
Slc16a3	1.15	0.00000000	pigeons
Rgcc	-2.50	0.00000000	pigeons
Raf1	1.24	0.00000000	pigeons
Cers5	1.04	0.00000000	pigeons
Rnaset2b	1.28	0.00000000	pigeons
Il16	1.16	0.00000000	pigeons
Kifbp	1.02	0.00000000	pigeons
Bmp2k	1.15	0.00000000	pigeons
Cyld	0.99	0.00000000	pigeons
Podxl	-2.85	0.00000000	pigeons
Tagap1	1.44	0.00000000	pigeons
Col4a1	-2.78	0.00000000	pigeons
Ccdc88a	1.19	0.00000000	pigeons
Rap2c	1.05	0.00000000	pigeons
Atp1b1	1.20	0.00000000	pigeons
Lpcat2b	1.05	0.00000000	pigeons
Rgs18	1.20	0.00000000	pigeons
Lcp1	1.09	0.00000000	pigeons
Cd74	-1.15	0.00000000	pigeons
Arhgdib	1.09	0.00000000	pigeons
Irak2	1.31	0.00000000	pigeons
Trpm1	1.46	0.00000000	pigeons
Cd9	1.49	0.00000000	pigeons
Arl6ip5	0.86	0.00000000	pigeons
Fgl2	1.10	0.00000000	pigeons
Rnf38	1.17	0.00000000	pigeons
Coro1c	1.07	0.00000000	pigeons
Sla	1.43	0.00000000	pigeons
Tnfaip8l1	0.98	0.00000000	pigeons
Kars	1.08	0.00000000	pigeons
Pik3cb	1.40	0.00000000	pigeons
Cnn2	0.92	0.00000000	pigeons
Brd8	0.98	0.00000000	pigeons
Ppard	1.01	0.00000000	pigeons
Pgs1	0.97	0.00000000	pigeons
Ccz1	0.96	0.00000000	pigeons
Arhgef9	-2.61	0.00000000	pigeons
C3	1.01	0.00000000	pigeons
Cdc37	0.94	0.00000000	pigeons
Stat1	1.07	0.00000000	pigeons
Vav3	1.37	0.00000000	pigeons
Capn3	1.63	0.00000000	pigeons
Snap23	0.83	0.00000000	pigeons

Nrbf2	1.04	0.00000000	pigeons
Sfswap	0.98	0.00000000	pigeons
Tut7	1.00	0.00000000	pigeons
Slc9a9	1.55	0.00000000	pigeons
Trrap	0.92	0.00000000	pigeons
Lrrk2	1.12	0.00000000	pigeons
Snrpd2	0.99	0.00000000	pigeons
Sema6a	-2.78	0.00000000	pigeons
Zc3h12a	1.22	0.00000000	pigeons
Tardbp	1.01	0.00000000	pigeons
Eif2ak3	1.05	0.00000000	pigeons
Elovl7	1.20	0.00000000	pigeons
Heg1	-2.73	0.00000000	pigeons
Evi2a	1.03	0.00000000	pigeons
Tiam1	1.22	0.00000000	pigeons
Rassf2	1.03	0.00000000	pigeons
Prokr2	1.09	0.00000000	pigeons
Dram1	1.04	0.00000000	pigeons
Sh3bp5	1.23	0.00000000	pigeons
Kcne3	-2.73	0.00000000	pigeons
Plekhg1	-2.68	0.00000000	pigeons
Slirp	0.93	0.00000000	pigeons
Prelid3b	1.04	0.00000000	pigeons
Hacd2	0.88	0.00000000	pigeons
Dennd1b	1.44	0.00000000	pigeons
Rassf5	0.90	0.00000000	pigeons
Lyn	1.28	0.00000000	pigeons
Bcat1	0.93	0.00000000	pigeons
Acox1	0.92	0.00000000	pigeons
Fbxl21	0.90	0.00000000	pigeons
Ivns1abp	0.92	0.00000000	pigeons
Grk4	0.96	0.00000000	pigeons
Snx4	0.92	0.00000000	pigeons
Tial1	0.82	0.00000000	pigeons
Atp5c1	0.89	0.00000000	pigeons
Il1rl1	1.55	0.00000000	pigeons
Adcy7	0.99	0.00000000	pigeons
Pias1	1.17	0.00000000	pigeons
Hspa8	-1.91	0.00000000	pigeons
Trpm8	1.15	0.00000000	pigeons
Arhgap30	0.83	0.00000000	pigeons
Hps5	1.01	0.00000000	pigeons
Apip	0.88	0.00000000	pigeons
Tnip2	1.35	0.00000000	pigeons
Ssh2	0.97	0.00000000	pigeons
Fermt2	-2.63	0.00000000	pigeons

Rab29	0.85	0.00000000	pigeons
Tnfaip8	1.32	0.00000000	pigeons
Rlf	0.85	0.00000000	pigeons
Phax	0.92	0.00000000	pigeons
Slc15a4	0.88	0.00000000	pigeons
Tmem71	0.98	0.00000000	pigeons
Add3	1.14	0.00000000	pigeons
Ptk2b	0.82	0.00000000	pigeons
Ccng2	1.01	0.00000000	pigeons
Stk17b	1.02	0.00000000	pigeons
Sinhcaf	1.06	0.00000000	pigeons
Zbtb20	-1.66	0.00000000	pigeons
Ppa2	1.16	0.00000000	pigeons
Dock8	0.85	0.00000000	pigeons
Arvcf	-2.60	0.00000000	pigeons
Slc45a4	1.17	0.00000000	pigeons
Anxa11	0.92	0.00000000	pigeons
Btg2	1.22	0.00000000	pigeons
Spcs1	0.85	0.00000000	pigeons
Polr1d	0.92	0.00000000	pigeons
Nfkb2	0.73	0.00000000	pigeons
Arl4a	-2.14	0.00000000	pigeons
Blcap	0.92	0.00000000	pigeons
Npr3	-2.61	0.00000000	pigeons
Slmap	0.91	0.00000000	pigeons
Tmem63a	0.88	0.00000000	pigeons
Arhgap31	-2.31	0.00000000	pigeons
Eaf1	0.99	0.00000000	pigeons
Snx10	1.37	0.00000000	pigeons
Ccdc25	0.83	0.00000000	pigeons
Kdm3a	0.85	0.00000000	pigeons
Syne1	1.22	0.00000000	pigeons
Igf1r	1.10	0.00000000	pigeons
Sorbs1	-2.43	0.00000000	pigeons
Rasgef1a	1.86	0.00000000	pigeons
Nucb2	0.83	0.00000000	pigeons
Atxn7l1	1.20	0.00000000	pigeons
Disc1	1.41	0.00000000	pigeons
Unc13d	0.83	0.00000000	pigeons
Nfatc3	0.93	0.00000000	pigeons
Ap1s2	0.79	0.00000000	pigeons
Ankrd44	1.03	0.00000000	pigeons
Laptm5	0.78	0.00000000	pigeons
Pstpip2	0.77	0.00000000	pigeons
Chgb	1.02	0.00000000	pigeons
Cbl	0.71	0.00000000	pigeons

Limd2	0.73	0.00000000	pigeons
Znfx1	0.81	0.00000000	pigeons
Fam107b	1.21	0.00000000	pigeons
Rlim	0.76	0.00000000	pigeons
Eepd1	1.18	0.00000000	pigeons
Lrmp	1.14	0.00000000	pigeons
Lpl	-2.48	0.00000000	pigeons
Atp11b	0.82	0.00000000	pigeons
Dok2	0.71	0.00000000	pigeons
Pik3ap1	1.05	0.00000000	pigeons
Prok2	1.14	0.00000000	pigeons
Dusp10	1.11	0.00000000	pigeons
Helz	0.75	0.00000000	pigeons
Anxa6	0.77	0.00000000	pigeons
Cstf3	0.73	0.00000000	pigeons
Cdh5	-2.47	0.00000000	pigeons
Rbm33	0.97	0.00000000	pigeons
Zyx	0.77	0.00000000	pigeons
Cpne8	0.88	0.00000000	pigeons
Plekhb2	0.68	0.00000000	pigeons
Ctdspl	-1.89	0.00000000	pigeons
Gabrr2	0.95	0.00000000	pigeons
Ppm1a	0.77	0.00000000	pigeons
Itch	0.71	0.00000000	pigeons
Spopl	0.84	0.00000000	pigeons
Galc	1.01	0.00000000	pigeons
Dock1	-2.44	0.00000000	pigeons
Rock1	1.05	0.00000000	pigeons
Evl	1.36	0.00000000	pigeons
Ube2v1	0.71	0.00000000	pigeons
Sri	0.76	0.00000000	pigeons
Eif4b	0.76	0.00000000	pigeons
Trim36	1.01	0.00000000	pigeons
Itprip	0.75	0.00000000	pigeons
Trpv2	0.76	0.00000000	pigeons
Serpinb1a	0.82	0.00000000	pigeons
Corin	-2.69	0.00000000	pigeons
Pmp22	-2.48	0.00000000	pigeons
Golm2	0.73	0.00000000	pigeons
Ski	-1.60	0.00000000	pigeons
Hck	0.77	0.00000000	pigeons
Rad23b	0.80	0.00000000	pigeons
Tmem204	-2.36	0.00000000	pigeons
Pgam1	0.69	0.00000000	pigeons
Trio	-1.93	0.00000000	pigeons
Cdc42	0.71	0.00000000	pigeons

Foxn2	0.62	0.00000000	pigeons
Sucnr1	0.79	0.00000000	pigeons
Cog3	0.76	0.00000000	pigeons
Sap30bp	0.71	0.00000000	pigeons
Rapgef2	-2.30	0.00000000	pigeons
Pitpna	0.77	0.00000000	pigeons
Gna13	0.87	0.00000000	pigeons
Fyn	0.97	0.00000000	pigeons
Atp6v0e	0.79	0.00000000	pigeons
Abtb1	0.86	0.00000000	pigeons
Nfkbiz	1.10	0.00000000	pigeons
Trdmt1	1.09	0.00000000	pigeons
G0s2	1.02	0.00000000	pigeons
Shisa5	0.72	0.00000000	pigeons
Dennd4c	1.27	0.00000000	pigeons
Skap2	1.25	0.00000000	pigeons
Susd1	1.09	0.00000000	pigeons
Glrx3	0.70	0.00000000	pigeons
Ugp2	0.71	0.00000000	pigeons
Ino80c	1.10	0.00000000	pigeons
Stx11	1.01	0.00000000	pigeons
Dapk1	0.97	0.00000000	pigeons
Igf2r	0.91	0.00000000	pigeons
Gse1	0.99	0.00000000	pigeons
Klf15	1.05	0.00000000	pigeons
Pcgf5	1.10	0.00000000	pigeons
Banp	0.99	0.00000000	pigeons
S1pr1	-2.34	0.00000000	pigeons
Atp13a3	0.92	0.00000000	pigeons
Ehd3	0.80	0.00000000	pigeons
Tmem106b	0.86	0.00000000	pigeons
Actr2	0.71	0.00000000	pigeons
Prex2	-2.39	0.00000000	pigeons
Sarnp	0.64	0.00000000	pigeons
Jcad	-2.32	0.00000000	pigeons
Vps37b	0.77	0.00000000	pigeons
Atp6v1c1	0.75	0.00000000	pigeons
Arpc5	0.69	0.00000000	pigeons
Lcp2	0.76	0.00000000	pigeons
Synm	-2.03	0.00000000	pigeons
Cav1	-2.29	0.00000000	pigeons
Chsy1	1.27	0.00000000	pigeons
Ccnyl1	0.71	0.00000000	pigeons
Fnbp1l	-2.30	0.00000000	pigeons
Tdpoz8	0.68	0.00000000	pigeons
Ugt8a	0.87	0.00000000	pigeons

Acod1	1.67	0.00000000	pigeons
Celf2	1.51	0.00000000	pigeons
Noct	0.70	0.00000000	pigeons
Pnpla8	0.96	0.00000000	pigeons
Smtnl2	0.98	0.00000000	pigeons
Capza2	0.70	0.00000000	pigeons
Atp6v1a	0.73	0.00000000	pigeons
Ptp4a1	0.73	0.00000000	pigeons
Ptpra	0.83	0.00000000	pigeons
Arntl	1.14	0.00000000	pigeons
Cotl1	0.83	0.00000000	pigeons
Ttl	0.62	0.00000000	pigeons
Ccm2	0.65	0.00000000	pigeons
Efnb2	-2.29	0.00000000	pigeons
Nsd3	0.79	0.00000000	pigeons
Pgd	0.69	0.00000000	pigeons
Tars	0.63	0.00000000	pigeons
Dgat2	0.96	0.00000000	pigeons
Stk26	0.63	0.00000000	pigeons
Apmap	0.78	0.00000000	pigeons
Agps	0.59	0.00000000	pigeons
Akirin2	0.82	0.00000000	pigeons
Cipc	0.67	0.00000000	pigeons
Pde4b	0.76	0.00000000	pigeons
Nop58	0.72	0.00000000	pigeons
Sec63	0.64	0.00000000	pigeons
Naa20	0.62	0.00000000	pigeons
C1qbp	0.78	0.00000000	pigeons
Itpr1	0.95	0.00000000	pigeons
Lfng	1.09	0.00000000	pigeons
Parn	0.75	0.00000000	pigeons
Gpx1	1.17	0.00000000	pigeons
Ndufaf1	0.97	0.00000000	pigeons
Xpnpep1	0.65	0.00000000	pigeons
Yrdc	0.57	0.00000000	pigeons
Traf3	0.89	0.00000000	pigeons
Chd9	0.78	0.00000000	pigeons
Neo1	-2.19	0.00000000	pigeons
Fabp3	0.71	0.00000000	pigeons
Clpx	0.66	0.00000000	pigeons
Cast	0.83	0.00000000	pigeons
Ubtd2	0.79	0.00000000	pigeons
Atxn1	0.79	0.00000000	pigeons
Iqgap1	0.87	0.00000000	pigeons
Tcf12	-1.76	0.00000000	pigeons
Plek	0.47	0.00000000	pigeons

Wdfy3	1.54	0.00000000	pigeons
Zdhhc2	0.91	0.00000000	pigeons
Tnks	0.90	0.00000000	pigeons
Crip2	0.62	0.00000000	pigeons
Atp6v0d1	0.55	0.00000000	pigeons
Dcn	-2.54	0.00000000	pigeons
Il17ra	0.77	0.00000000	pigeons
Ifngr1	0.86	0.00000000	pigeons
Itm2b	-1.77	0.00000000	pigeons
Nin	0.78	0.00000000	pigeons
Socs7	0.67	0.00000000	pigeons
Thbs1	-2.70	0.00000000	pigeons
Csnk1d	0.69	0.00000000	pigeons
Efl1	0.80	0.00000000	pigeons
Arpc1b	0.83	0.00000000	pigeons
Jun	-1.02	0.00000000	pigeons
Emc2	0.57	0.00000000	pigeons
Bccip	0.55	0.00000000	pigeons
Gpr65	0.80	0.00000000	pigeons
Cndp2	0.71	0.00000000	pigeons
Ints13	0.56	0.00000000	pigeons
Kbtbd8	0.65	0.00000000	pigeons
Herpud2	0.58	0.00000000	pigeons
Ube3a	0.75	0.00000000	pigeons
Bicd1	-2.27	0.00000000	pigeons
Sgk1	1.23	0.00000000	pigeons
Fdx1	0.65	0.00000000	pigeons
Csf3r	0.68	0.00000000	pigeons
Atp6v1h	0.82	0.00000000	pigeons
Arhgef18	0.60	0.00000000	pigeons
Rars	0.56	0.00000000	pigeons
Plekha8	0.69	0.00000000	pigeons
Qk	-1.16	0.00000000	pigeons
Rell1	0.70	0.00000000	pigeons
Hif1a	0.66	0.00000000	pigeons
Psme3ip1	0.56	0.00000000	pigeons
Wipi1	0.61	0.00000000	pigeons
Atp6ap2	0.68	0.00000000	pigeons
Cebpb	0.69	0.00000000	pigeons
Klhl22	0.64	0.00000000	pigeons
Slc9a3r2	-2.17	0.00000000	pigeons
Borcs5	0.82	0.00000000	pigeons
Ints7	0.71	0.00000000	pigeons
Ptdss1	0.67	0.00000000	pigeons
Hs1bp3	0.76	0.00000000	pigeons
Pla2g15	0.75	0.00000000	pigeons

Skap1	-2.14	0.00000000	pigeons
Washc5	0.72	0.00000000	pigeons
Rhof	0.61	0.00000000	pigeons
Il22ra2	0.77	0.00000000	pigeons
Gnb1	0.82	0.00000000	pigeons
Edem1	0.53	0.00000000	pigeons
Oaz1	0.73	0.00000000	pigeons
Osbpl9	0.69	0.00000000	pigeons
Tmem123	0.68	0.00000000	pigeons
Spg21	0.66	0.00000000	pigeons
Setd2	0.51	0.00000000	pigeons
Dedd	0.55	0.00000000	pigeons
Ly96	0.64	0.00000000	pigeons
Tm6sf1	0.68	0.00000000	pigeons
Tab2	0.65	0.00000000	pigeons
Gyg	0.59	0.00000000	pigeons
Tkt	0.59	0.00000000	pigeons
Kcnc1	-2.12	0.00000000	pigeons
Gpr27	0.67	0.00000000	pigeons
Icosl	0.91	0.00000000	pigeons
Pard3b	-2.20	0.00000000	pigeons
Rbfox2	-2.18	0.00000000	pigeons
Gbf1	0.98	0.00000000	pigeons
Rab10	0.49	0.00000000	pigeons
Galnt18	-2.06	0.00000000	pigeons
Zfp36l1	0.97	0.00000000	pigeons
Tbc1d24	0.72	0.00000000	pigeons
Zfand3	0.90	0.00000000	pigeons
Tapbpl	0.57	0.00000000	pigeons
Sgms1	0.71	0.00000000	pigeons
Ncoa2	1.01	0.00000000	pigeons
Kctd10	0.51	0.00000000	pigeons
Rassf9	-2.02	0.00000000	pigeons
Vmp1	0.67	0.00000000	pigeons
Ttc21b	0.60	0.00000000	pigeons
Psme4	0.77	0.00000000	pigeons
Pip5k1b	0.85	0.00000000	pigeons
Klhl5	0.66	0.00000000	pigeons
Usp4	0.55	0.00000000	pigeons
Fryl	-1.60	0.00000000	pigeons
Tgfbr3	-2.10	0.00000000	pigeons
Nudt3	0.54	0.00000000	pigeons
Dclk1	-2.27	0.00000000	pigeons
Cxadr	0.98	0.00000000	pigeons
Pggt1b	0.72	0.00000000	pigeons
Efemp1	-2.10	0.00000000	pigeons

Foxk2	0.76	0.00000000	pigeons
Sbno2	0.45	0.00000000	pigeons
Stambp	0.57	0.00000000	pigeons
Frs2	0.71	0.00000000	pigeons
Slc6a6	1.36	0.00000000	pigeons
Fgd3	0.68	0.00000000	pigeons
Bsg	-1.95	0.00000000	pigeons
Capzb	0.68	0.00000000	pigeons
Fhl3	0.58	0.00000000	pigeons
Slc25a16	0.69	0.00000000	pigeons
Zfp777	0.53	0.00000000	pigeons
Ssu72	0.43	0.00000000	pigeons
Smap2	0.54	0.00000000	pigeons
Hnrnpll	0.51	0.00000000	pigeons
Tec	0.83	0.00000000	pigeons
Efcab14	0.46	0.00000000	pigeons
Ssbp2	-1.90	0.00000000	pigeons
Phf20l1	0.55	0.00000000	pigeons
Cntrl	0.61	0.00000000	pigeons
Smad6	-1.98	0.00000000	pigeons
Cd3g	1.19	0.00000000	pigeons
Oat	0.44	0.00000000	pigeons
Ptprn2	-2.07	0.00000000	pigeons
Auts2	-1.97	0.00000000	pigeons
Gpatch8	0.65	0.00000000	pigeons
Cdkn2aip	0.54	0.00000000	pigeons
Chmp4b	0.64	0.00000000	pigeons
Ncf2	0.60	0.00000000	pigeons
Ehbp1	0.78	0.00000000	pigeons
Zc3h7a	0.45	0.00000000	pigeons
Fgd5	-2.04	0.00000000	pigeons
Arl14ep	0.54	0.00000000	pigeons
Ostf1	0.46	0.00000000	pigeons
Nde1	0.55	0.00000000	pigeons
Tbx3	-1.99	0.00000000	pigeons
Ccdc85a	-1.99	0.00000000	pigeons
Plekhf2	0.47	0.00000000	pigeons
Gpcpd1	0.74	0.00000000	pigeons
Arhgef38	0.83	0.00000000	pigeons
Txndc11	0.65	0.00000000	pigeons
Rassf3	-1.95	0.00000000	pigeons
Actb	0.74	0.00000000	pigeons
Npas3	-2.14	0.00000000	pigeons
Noc3l	0.60	0.00000000	pigeons
Itpr2	0.63	0.00000000	pigeons
Gtf2h1	0.49	0.00000000	pigeons

Vps4b	0.41	0.00000000	pigeons
Polr1f	0.53	0.00000000	pigeons
Cnn3	-2.08	0.00000000	pigeons
Lpxn	0.50	0.00000000	pigeons
Acaca	0.47	0.00000000	pigeons
Sdc2	-1.91	0.00000000	pigeons
Ganc	0.66	0.00000000	pigeons
Prkce	-1.26	0.00000000	pigeons
Ywhaz	0.69	0.00000000	pigeons
Tfec	0.84	0.00000000	pigeons
Tab3	0.50	0.00000000	pigeons
Arfip1	0.58	0.00000000	pigeons
Arrdc4	0.73	0.00000000	pigeons
Apcdd1	-1.97	0.00000000	pigeons
Basp1	0.89	0.00000000	pigeons
Rpf2	0.61	0.00000000	pigeons
Ankrd22	0.65	0.00000000	pigeons
Limk1	0.51	0.00000000	pigeons
Bst1	0.49	0.00000000	pigeons
Kidins220	0.78	0.00000000	pigeons
Wapl	0.75	0.00000000	pigeons
Plxnc1	0.45	0.00000000	pigeons
Zc3h18	0.40	0.00000000	pigeons
Wdr44	0.45	0.00000000	pigeons
Tank	0.50	0.00000000	pigeons
Rnf216	0.65	0.00000000	pigeons
Iqgap2	0.43	0.00000000	pigeons
Rab11fip2	0.64	0.00000000	pigeons
Fam53a	0.79	0.00000000	pigeons
Scap	0.69	0.00000000	pigeons
Fam104a	0.45	0.00000000	pigeons
Ephb1	-1.94	0.00000000	pigeons
Nbeal2	0.43	0.00000000	pigeons
Ppp1cc	0.38	0.00000000	pigeons
Rsu1	0.69	0.00000000	pigeons
Optn	0.51	0.00000000	pigeons
Inip	0.46	0.00000000	pigeons
Nop56	0.54	0.00000000	pigeons
Irak4	0.56	0.00000000	pigeons
Zfp148	0.59	0.00000000	pigeons
Csrnp1	0.54	0.00000000	pigeons
Zbtb21	0.48	0.00000000	pigeons
Dok1	0.49	0.00000000	pigeons
Ube2d2a	0.39	0.00000000	pigeons
Atp5j2	0.46	0.00000000	pigeons
Rhoj	-1.84	0.00000000	pigeons

Rev3l	1.00	0.00000000	pigeons
F5	1.15	0.00000000	pigeons
Rab5a	0.36	0.00000000	pigeons
Pak2	0.52	0.00000000	pigeons
Ndel1	0.34	0.00000000	pigeons
Spcs2	0.50	0.00000000	pigeons
Ap1g1	0.30	0.00000000	pigeons
Sec24d	0.66	0.00000000	pigeons
Serpine2	0.50	0.00000000	pigeons
Ppp6r2	0.40	0.00000000	pigeons
Opa1	0.52	0.00000000	pigeons
Jmjd1c	0.74	0.00000000	pigeons
Rhob	0.70	0.00000000	pigeons
Rin2	0.52	0.00000000	pigeons
Nhsl2	0.45	0.00000000	pigeons
Ninj1	0.65	0.00000000	pigeons
Anxa2	0.77	0.00000000	pigeons
Rab11a	0.42	0.00000000	pigeons
Papss1	0.56	0.00000000	pigeons
Nup214	0.42	0.00000000	pigeons
Slc4a7	0.80	0.00000000	pigeons
Zfp521	-1.92	0.00000000	pigeons
Aff4	0.45	0.00000000	pigeons
Eapp	0.53	0.00000000	pigeons
Slc25a17	0.47	0.00000000	pigeons
Gsk3b	-1.15	0.00000000	pigeons
Runx1t1	-1.93	0.00000000	pigeons
Id2	0.76	0.00000000	pigeons
Eno1b	0.68	0.00000000	pigeons
Swap70	0.59	0.00000000	pigeons
Gorasp1	0.55	0.00000000	pigeons
Usp44	0.41	0.00000000	pigeons
Cd47	0.52	0.00000000	pigeons
Jchain	3.57	0.00000000	pigeons
Gnpda2	0.45	0.00000000	pigeons
Atf2	0.41	0.00000000	pigeons
B3gnt2	0.54	0.00000000	pigeons
Sars	0.39	0.00000000	pigeons
Tbp	0.50	0.00000000	pigeons
Rnf13	0.40	0.00000000	pigeons
Trabd	0.51	0.00000000	pigeons
Pou2f1	0.80	0.00000000	pigeons
Rap1gds1	0.63	0.00000000	pigeons
Sytl1	0.53	0.00000000	pigeons
Nfe2l1	0.39	0.00000000	pigeons
Rcor1	0.60	0.00000000	pigeons

Tmem167	0.46	0.00000000	pigeons
Stk40	0.47	0.00000000	pigeons
Rab25	0.44	0.00000000	pigeons
Get4	0.35	0.00000000	pigeons
Ralgps2	-1.97	0.00000000	pigeons
Phf12	0.45	0.00000000	pigeons
Atad1	0.37	0.00000000	pigeons
Eif2b4	0.41	0.00000000	pigeons
S100a11	0.64	0.00000000	pigeons
Secisbp2l	0.31	0.00000000	pigeons
Nus1	0.55	0.00000000	pigeons
Zfp207	0.31	0.00000000	pigeons
Ube2l3	0.39	0.00000000	pigeons
Arid5a	0.51	0.00000000	pigeons
Ncstn	0.39	0.00000000	pigeons
Tmem30a	0.39	0.00000000	pigeons
Zfp366	-1.90	0.00000000	pigeons
Vcl	0.88	0.00000000	pigeons
Cep128	0.81	0.00000000	pigeons
Rilpl2	0.42	0.00000000	pigeons
Ankrd11	0.67	0.00000000	pigeons
Srpr	0.29	0.00000000	pigeons
Ahsa2	0.60	0.00000000	pigeons
Osbpl10	-1.84	0.00000000	pigeons
Ap3m1	0.39	0.00000000	pigeons
Csf1r	0.58	0.00000000	pigeons
Etf1	0.35	0.00000000	pigeons
Cd44	0.53	0.00000000	pigeons
Cul1	0.37	0.00000000	pigeons
Odf2	0.39	0.00000000	pigeons
Pfkfb3	0.84	0.00000000	pigeons
Psmg2	0.42	0.00000000	pigeons
Ptpn14	-1.94	0.00000000	pigeons
Edem3	0.59	0.00000000	pigeons
Stk38	0.44	0.00000000	pigeons
Ankfy1	0.38	0.00000000	pigeons
Spred2	-2.01	0.00000000	pigeons
Impa2	0.56	0.00000000	pigeons
Abi1	0.76	0.00000000	pigeons
Akt1	0.37	0.00000000	pigeons
Tmem164	0.57	0.00000000	pigeons
Cep112	0.64	0.00000000	pigeons
Nfkb1	0.63	0.00000000	pigeons
Stk11ip	0.44	0.00000000	pigeons
Wbp2	0.37	0.00000000	pigeons
Stx7	0.49	0.00000000	pigeons

Dlc1	-1.78	0.00000000	pigeons
Cdc42bpa	-1.96	0.00000000	pigeons
Gnai1	0.50	0.00000000	pigeons
Dohh	0.43	0.00000000	pigeons
Arhgap21	-1.76	0.00000000	pigeons
Ugcg	0.70	0.00000000	pigeons
Rybp	0.68	0.00000000	pigeons
Pde10a	-1.61	0.00000000	pigeons
Fgfr2	-1.83	0.00000000	pigeons
Net1	0.74	0.00000000	pigeons
Gpd2	0.58	0.00000000	pigeons
Arl3	0.35	0.00000000	pigeons
Nup210	0.38	0.00000000	pigeons
Rfx2	0.50	0.00000000	pigeons
Rabgap1	0.39	0.00000000	pigeons
Il27ra	0.31	0.00000000	pigeons
Polr2i	0.44	0.00000000	pigeons
Copa	0.31	0.00000000	pigeons
Dach1	-1.76	0.00000000	pigeons
Osbpl8	0.37	0.00000000	pigeons
Ap2a1	0.31	0.00000000	pigeons
Sec16a	0.27	0.00000000	pigeons
Gnai3	0.43	0.00000000	pigeons
Lamtor3	0.42	0.00000000	pigeons
Serinc5	0.53	0.00000000	pigeons
Hmox1	0.67	0.00000000	pigeons
Bri3bp	0.40	0.00000000	pigeons
Sh3bgrl3	0.35	0.00000000	pigeons
Ubr5	0.52	0.00000000	pigeons
Arid4a	0.54	0.00000000	pigeons
Narf	0.54	0.00000000	pigeons
Tek	-1.80	0.00000000	pigeons
Sec61a2	0.39	0.00000000	pigeons
Plcl2	0.82	0.00000000	pigeons
Ell	0.39	0.00000000	pigeons
Wdr1	0.52	0.00000000	pigeons
Aak1	0.44	0.00000000	pigeons
Spidr	0.97	0.00000000	pigeons
Hook3	0.34	0.00000000	pigeons
Rnf111	0.43	0.00000000	pigeons
Ccdc40	0.49	0.00000000	pigeons
D5Ertd579e	0.65	0.00000000	pigeons
Pdpk1	0.18	0.00000000	pigeons
Jak1	0.47	0.00000000	pigeons
Cul4a	0.52	0.00000000	pigeons
Atp11a	0.92	0.00000000	pigeons

Thumpd3	0.35	0.00000000	pigeons
Slc35a5	0.49	0.00000000	pigeons
Nfil3	0.64	0.00000000	pigeons
Stc1	0.79	0.00000000	pigeons
Abhd2	0.38	0.00000000	pigeons
Chd7	0.99	0.00000000	pigeons
Rhbdf2	0.48	0.00000000	pigeons
Pkn2	0.28	0.00000000	pigeons
Ubap1	0.33	0.00000000	pigeons
Clic4	-1.98	0.00000000	pigeons
Pdcd10	0.30	0.00000000	pigeons
Ewsr1	0.23	0.00000000	pigeons
Ncoa6	0.32	0.00000000	pigeons
Ttc17	0.29	0.00000000	pigeons
Pdzd8	0.38	0.00000000	pigeons
5031439G07Rik	0.50	0.00000000	pigeons
Arhgap45	0.37	0.00000000	pigeons
Ltn1	0.33	0.00000000	pigeons
Agtpbp1	0.62	0.00000000	pigeons
LTO1	0.42	0.00000000	pigeons
Tsnax	0.59	0.00000000	pigeons
Nfkbie	0.36	0.00000000	pigeons
Adamtsl2	0.76	0.00000000	pigeons
Exoc2	0.59	0.00000000	pigeons
Ginm1	0.34	0.00000000	pigeons
Emc3	0.29	0.00000000	pigeons
Tcirg1	0.37	0.00000000	pigeons
Adam17	0.35	0.00000000	pigeons
Yap1	-1.79	0.00000000	pigeons
Clk4	0.25	0.00000000	pigeons
Plcb2	0.39	0.00000000	pigeons
Hpgd	-1.74	0.00000000	pigeons
Cap1	0.32	0.00000000	pigeons
Tdg	0.35	0.00000000	pigeons
Slc40a1	0.49	0.00000000	pigeons
Nckap1	-1.84	0.00000000	pigeons
Cnot6l	0.42	0.00000000	pigeons
Dnajc13	0.31	0.00000000	pigeons
Hace1	0.48	0.00000000	pigeons
Sbno1	0.23	0.00000000	pigeons
Helz2	0.25	0.00000000	pigeons
Kdm5a	0.38	0.00000000	pigeons
Chfr	0.51	0.00000000	pigeons
Rnf6	0.45	0.00000000	pigeons
Brap	0.40	0.00000000	pigeons
Rasgrf2	-1.70	0.00000000	pigeons

Rcsd1	0.20	0.00000000	pigeons
Golim4	0.38	0.00000000	pigeons
Pim3	1.03	0.00000000	pigeons
Ptprj	0.48	0.00000000	pigeons
Klhdc10	0.39	0.00000000	pigeons
Unc93b1	0.43	0.00000000	pigeons
Eef1e1	0.32	0.00000000	pigeons
Osbpl1a	-1.57	0.00000000	pigeons
Azi2	0.40	0.00000000	pigeons
Kif2a	0.47	0.00000000	pigeons
Tmed10	0.32	0.00000000	pigeons
Elovl5	0.25	0.00000000	pigeons
Stx3	0.46	0.00000000	pigeons
Ifnar1	0.47	0.00000000	pigeons
Agap1	-1.86	0.00000000	pigeons
Fry	0.70	0.00000000	pigeons
Ccnd2	0.41	0.00000000	pigeons
Nfe2l2	0.46	0.00000000	pigeons
Zfp706	0.37	0.00000000	pigeons
Trps1	-1.45	0.00000000	pigeons
Aamp	0.46	0.00000000	pigeons
Dph5	0.50	0.00000000	pigeons
Akt3	-1.73	0.00000000	pigeons
Stau2	0.58	0.00000000	pigeons
Tead4	-1.89	0.00000000	pigeons
Sftpc	-2.15	0.00000000	pigeons
Fli1	-1.29	0.00000000	pigeons
Il31ra	0.66	0.00000000	pigeons
Wdr20	0.46	0.00000000	pigeons
Sppl3	0.41	0.00000000	pigeons
Snx18	0.47	0.00000000	pigeons
Vta1	0.36	0.00000000	pigeons
Epn2	0.47	0.00000000	pigeons
Klf8	0.39	0.00000000	pigeons
Nasp	0.39	0.00000000	pigeons
Arpc3	0.49	0.00000000	pigeons
Yipf4	0.22	0.00000000	pigeons
Prkag2	0.48	0.00000000	pigeons
Agpat2	0.40	0.00000000	pigeons
Ube2k	0.57	0.00000000	pigeons
Csnk2a2	0.29	0.00000000	pigeons
Vps26a	0.39	0.00000000	pigeons
Rad54l2	0.35	0.00000000	pigeons
Lamc1	-1.92	0.00000000	pigeons
Gpbp1l1	0.21	0.00000000	pigeons
Acp1	0.28	0.00000000	pigeons

Ablim1	-1.91	0.00000000	pigeons
Cd28	1.13	0.00000000	pigeons
Ppp1cb	0.31	0.00000000	pigeons
Smcr8	0.35	0.00000000	pigeons
Lonp1	0.27	0.00000000	pigeons
Atp11c	0.35	0.00000000	pigeons
Ap2b1	0.26	0.00000000	pigeons
Tnpo3	0.32	0.00000000	pigeons
Bach1	0.49	0.00000000	pigeons
Rab20	0.44	0.00000000	pigeons
Ostm1	0.33	0.00000000	pigeons
N4bp1	0.41	0.00000000	pigeons
Parp6	0.36	0.00000000	pigeons
E2f3	0.42	0.00000000	pigeons
Nipa2	0.26	0.00000000	pigeons
Ccdc93	0.46	0.00000000	pigeons
Rbm34	0.25	0.00000000	pigeons
Dtd1	0.42	0.00000000	pigeons
Clk1	0.27	0.00000000	pigeons
Sash3	0.46	0.00000000	pigeons
Tfpi2	-2.03	0.00000000	pigeons
Neurl1b	-1.69	0.00000000	pigeons
Vps35	0.26	0.00000000	pigeons
Atp6v0b	0.34	0.00000000	pigeons
Rai14	-1.73	0.00000000	pigeons
Trappc3	0.33	0.00000000	pigeons
Cald1	-1.90	0.00000000	pigeons
Rnf103	0.37	0.00000000	pigeons
Ubtf	0.38	0.00000000	pigeons
Senp1	0.36	0.00000000	pigeons
Ube2v2	0.29	0.00000000	pigeons
Ccnk	0.26	0.00000000	pigeons
Prkcz	0.68	0.00000000	pigeons
Gab3	0.70	0.00000000	pigeons
Ccnl2	0.27	0.00000000	pigeons
Vdac2	0.32	0.00000000	pigeons
Adipor2	0.45	0.00000000	pigeons
Wdr37	0.42	0.00000000	pigeons
Tns1	-1.89	0.00000000	pigeons
Nxpe3	0.45	0.00000000	pigeons
Ric8b	0.40	0.00000000	pigeons
Chst15	0.62	0.00000000	pigeons
Gars	0.35	0.00000000	pigeons
Capn7	0.30	0.00000000	pigeons
Atf3	1.02	0.00000000	pigeons
Ascc2	0.26	0.00000000	pigeons

Btbd9	0.72	0.00000000	pigeons
Ptpn9	0.37	0.00000000	pigeons
Rexo1	0.30	0.00000000	pigeons
Cnot1	0.18	0.00000000	pigeons
Arih2	0.36	0.00000000	pigeons
Gsr	0.28	0.00000000	pigeons
Naa15	0.31	0.00000000	pigeons
Becn2	0.35	0.00000000	pigeons
Rnpep	0.34	0.00000000	pigeons
Atp2b1	0.40	0.00000000	pigeons
Rnf213	0.30	0.00000000	pigeons
Trim28	0.33	0.00000000	pigeons
Kmt2c	0.61	0.00000000	pigeons
Zfp644	0.35	0.00000000	pigeons
Kdr	-1.67	0.00000000	pigeons
Ankrd13a	0.34	0.00000000	pigeons
Pspc1	0.40	0.00000000	pigeons
Phf20	0.41	0.00000000	pigeons
4933400A11Rik	0.31	0.00000000	pigeons
Ogdh	0.29	0.00000000	pigeons
Kansl1	0.30	0.00000000	pigeons
Eya3	0.36	0.00000000	pigeons
Gapvd1	0.35	0.00000000	pigeons
Cobll1	-1.64	0.00000000	pigeons
Pafah1b1	0.17	0.00000000	pigeons
Klhl6	0.45	0.00000000	pigeons
Sulf2	0.60	0.00000000	pigeons
Rspry1	0.43	0.00000000	pigeons
Tmem41b	0.33	0.00000000	pigeons
Slc45a3	0.60	0.00000000	pigeons
Rad21	0.26	0.00000000	pigeons
Stam2	0.41	0.00000000	pigeons
Rheb	0.31	0.00000000	pigeons
Bzw2	0.46	0.00000000	pigeons
Cnot6	0.18	0.00000000	pigeons
Col22a1	-1.62	0.00000000	pigeons
Klhl15	0.34	0.00000000	pigeons
Tjp2	-1.71	0.00000000	pigeons
Sfxn1	0.41	0.00000000	pigeons
Tbc1d1	0.83	0.00000000	pigeons
Klhl18	0.34	0.00000000	pigeons
Zfand1	0.34	0.00000000	pigeons
Zdhhc9	0.21	0.00000000	pigeons
Aars	0.40	0.00000000	pigeons
Ankrd42	0.39	0.00000000	pigeons
Stk10	0.23	0.00000000	pigeons

Hpse	0.27	0.00000000	pigeons
Ntan1	0.27	0.00000000	pigeons
Smad3	0.82	0.00000000	pigeons
Cgnl1	-1.59	0.00000000	pigeons
Tent4a	0.38	0.00000000	pigeons
Cherp	0.31	0.00000000	pigeons
Crot	0.32	0.00000000	pigeons
Slc37a3	0.35	0.00000000	pigeons
Scamp2	0.25	0.00000000	pigeons
Ptpn1	0.27	0.00000000	pigeons
Tmem181a	0.34	0.00000000	pigeons
Fndc3a	0.43	0.00000000	pigeons
Cdc14a	0.53	0.00000000	pigeons
Ndfip2	0.40	0.00000000	pigeons
Rnf149	0.28	0.00000000	pigeons
Qars	0.32	0.00000000	pigeons
U2af1	0.21	0.00000000	pigeons
Hp1bp3	0.18	0.00000000	pigeons
Acp2	0.31	0.00000000	pigeons
Fkbp1a	-1.57	0.00000000	pigeons
Abcc4	0.76	0.00000000	pigeons
Sfmbt1	0.64	0.00000000	pigeons
Atg3	0.33	0.00000000	pigeons
Trafd1	0.32	0.00000000	pigeons
Ythdf3	0.25	0.00000000	pigeons
Sdha	0.24	0.00000000	pigeons
Nipal3	0.32	0.00000000	pigeons
Usp33	0.33	0.00000000	pigeons
Dock10	0.58	0.00000000	pigeons
Ippk	0.46	0.00000000	pigeons
Pten	0.13	0.00000000	pigeons
Yme1l1	0.19	0.00000000	pigeons
Nrap	0.34	0.00000000	pigeons
Glyr1	0.15	0.00000000	pigeons
Acvr1b	0.36	0.00000000	pigeons
Btaf1	0.22	0.00000000	pigeons
Tmem87a	0.26	0.00000000	pigeons
Srp68	0.27	0.00000000	pigeons
Orc3	0.38	0.00000000	pigeons
Csgalnact2	0.50	0.00000000	pigeons
Ctss	0.18	0.00000000	pigeons
Gulp1	-1.65	0.00000000	pigeons
Kdm4c	0.59	0.00000000	pigeons
Sppl2a	0.14	0.00000000	pigeons
Card9	0.29	0.00000000	pigeons
Atxn2	0.25	0.00000000	pigeons

Pde8a	1.37	0.00000000	pigeons
Hivep1	0.76	0.00000000	pigeons
Lifr	-1.55	0.00000000	pigeons
Thoc5	0.32	0.00000000	pigeons
Atp6v1e2	0.28	0.00000000	pigeons
Sytl3	0.76	0.00000000	pigeons
Rel	0.48	0.00000000	pigeons
Nf1	0.19	0.00000000	pigeons
Acvrl1	-1.70	0.00000000	pigeons
Capn15	0.38	0.00000000	pigeons
Ddhd1	0.40	0.00000000	pigeons
Vps53	0.36	0.00000000	pigeons
Cst7	0.48	0.00000000	pigeons
Pcid2	0.37	0.00000000	pigeons
Idh3b	0.38	0.00000000	pigeons
Hdac3	0.34	0.00000000	pigeons
Fmnl1	0.39	0.00000000	pigeons
Dgkd	-1.81	0.00000000	pigeons
Crlf3	0.27	0.00000000	pigeons
Gucd1	0.34	0.00000000	pigeons
Dnajc3	0.30	0.00000000	pigeons
Traf2	0.20	0.00000000	pigeons
Agbl1	-1.58	0.00000000	pigeons
Grsf1	0.34	0.00000000	pigeons
Bckdhb	0.59	0.00000000	pigeons
Zfp654	0.21	0.00000000	pigeons
Sync	0.38	0.00000000	pigeons
Ddx17	0.19	0.00000000	pigeons
Wdfy1	0.27	0.00000000	pigeons
Eml4	0.77	0.00000000	pigeons
Arhgef3	-1.68	0.00000000	pigeons
Colec10	-1.50	0.00000000	pigeons
Eif4h	0.11	0.00000000	pigeons
Sec22a	0.33	0.00000000	pigeons
Atp2a3	0.16	0.00000000	pigeons
Gsn	0.30	0.00000000	pigeons
Dpyd	-1.63	0.00000000	pigeons
Anxa7	0.34	0.00000000	pigeons
Traf6	0.24	0.00000000	pigeons
Psmd1	0.22	0.00000000	pigeons
Stx8	0.42	0.00000000	pigeons
Vdr	0.35	0.00000000	pigeons
Fbxl5	0.29	0.00000000	pigeons
Ogfr	0.27	0.00000000	pigeons
Eif2s3y	0.21	0.00000000	pigeons
Wdr82	0.18	0.00000000	pigeons

Fyttd1	0.14	0.00000000	pigeons
Igsf6	0.34	0.00000000	pigeons
Syf2	0.18	0.00000000	pigeons
Crkl	0.30	0.00000000	pigeons
Srebf2	0.23	0.00000000	pigeons
Wnt5b	0.42	0.00000000	pigeons
Rab8b	0.25	0.00000000	pigeons
Tmcc3	-1.51	0.00000000	pigeons
Pld4	0.28	0.00000000	pigeons
Zfp518a	0.38	0.00000000	pigeons
Acap2	0.30	0.00000000	pigeons
Lta4h	0.27	0.00000000	pigeons
Lrrc8b	0.31	0.00000000	pigeons
Tmem248	0.12	0.00000000	pigeons
Psmd14	0.44	0.00000000	pigeons
Kpnb1	0.22	0.00000000	pigeons
Atp6v1b2	0.31	0.00000000	pigeons
Trib2	-1.60	0.00000000	pigeons
Gcnt4	0.35	0.00000000	pigeons
Zdhhc3	0.27	0.00000000	pigeons
Gdap2	0.29	0.00000000	pigeons
Cyfip2	0.38	0.00000000	pigeons
Ide	0.23	0.00000000	pigeons
Sec24a	0.21	0.00000000	pigeons
Ralb	0.29	0.00000000	pigeons
Prpf8	0.16	0.00000000	pigeons
Larp7	0.19	0.00000000	pigeons
Pdcd6	0.14	0.00000000	pigeons
Ogt	0.12	0.00000000	pigeons
Cadps2	-1.50	0.00000000	pigeons
Ankle2	0.21	0.00000000	pigeons
Gnai2	0.29	0.00000000	pigeons
Ubxn6	0.28	0.00000000	pigeons
Zfp512b	0.25	0.00000000	pigeons
Exoc3	0.39	0.00000000	pigeons
Cacnb4	0.55	0.00000000	pigeons
Pcnx	0.33	0.00000000	pigeons
Atp13a1	0.26	0.00000000	pigeons
Tmem42	0.26	0.00000000	pigeons
G3bp1	0.14	0.00000000	pigeons
Rapgef1	0.32	0.00000000	pigeons
Ssrp1	0.23	0.00000000	pigeons
Ccni	0.38	0.00000000	pigeons
Prpf3	0.21	0.00000000	pigeons
Apaf1	0.43	0.00000000	pigeons
Il18	0.46	0.00000000	pigeons

Tax1bp3	0.23	0.00000000	pigeons
Ube2a	0.13	0.00000000	pigeons
Hbs1l	0.23	0.00000000	pigeons
Arhgap5	-1.64	0.00000000	pigeons
Stxbp1	0.32	0.00000000	pigeons
Sik3	0.24	0.00000000	pigeons
Stat3	0.22	0.00000000	pigeons
Otulin	0.20	0.00000000	pigeons
Sgpp1	0.32	0.00000000	pigeons
Wipi2	0.28	0.00000000	pigeons
Hectd1	0.58	0.00000000	pigeons
Lcor	0.37	0.00000000	pigeons
Fes	0.30	0.00000000	pigeons
Xrcc4	0.80	0.00000000	pigeons
AU040320	0.24	0.00000000	pigeons
Cct6b	0.24	0.00000000	pigeons
Anapc1	0.22	0.00000000	pigeons
Abce1	0.10	0.00000000	pigeons
Caskin2	-1.44	0.00000000	pigeons
Surf4	0.10	0.00000000	pigeons
Rab21	0.05	0.00000000	pigeons
Pik3cd	0.30	0.00000000	pigeons
Smarcal1	-0.53	0.00000000	pigeons
Tacc1	0.24	0.00000000	pigeons
Rc3h2	0.20	0.00000000	pigeons
Tmem59	0.11	0.00000000	pigeons
Nt5c3	0.31	0.00000000	pigeons
Tax1bp1	0.59	0.00000000	pigeons
Tmem120a	0.33	0.00000000	pigeons
Erlin1	0.18	0.00000000	pigeons
Sec62	0.12	0.00000000	pigeons
Ythdf2	0.08	0.00000000	pigeons
Ogfrl1	0.33	0.00000000	pigeons
Ypel5	0.09	0.00000000	pigeons
Taok3	0.12	0.00000000	pigeons
Vapa	0.20	0.00000000	pigeons
Stard3nl	0.13	0.00000000	pigeons
Agfg1	0.17	0.00000000	pigeons
Atp6v0a4	0.25	0.00000000	pigeons
Tspan14	0.30	0.00000000	pigeons
Unc45a	0.36	0.00000000	pigeons
Nmi	0.19	0.00000000	pigeons
Tle3	0.19	0.00000000	pigeons
Npepps	0.23	0.00000000	pigeons
Enah	-1.39	0.00000000	pigeons
Ube2h	0.23	0.00000000	pigeons

Cab39	0.19	0.00000000	pigeons
Lnpk	0.35	0.00000000	pigeons
Zfp384	0.26	0.00000000	pigeons
Rab14	0.14	0.00000000	pigeons
Ech1	0.23	0.00000000	pigeons
Srsf11	0.12	0.00000000	pigeons
Srp9	0.11	0.00000000	pigeons
Vps37a	0.19	0.00000000	pigeons
Rnmt	0.13	0.00000000	pigeons
Nrp1	-1.57	0.00000000	pigeons
Idh1	0.23	0.00000000	pigeons
Ddrgk1	0.20	0.00000000	pigeons
Rbm12	0.11	0.00000000	pigeons
Scaf4	0.19	0.00000000	pigeons
Enpp4	0.38	0.00000000	pigeons
Dnajb12	0.06	0.00000000	pigeons
Abcg1	0.51	0.00000000	pigeons
Dnajc7	0.15	0.00000000	pigeons
Rab22a	0.28	0.00000000	pigeons
Ncbp1	0.22	0.00000000	pigeons
Twsg1	-1.40	0.00000000	pigeons
Flii	0.13	0.00000000	pigeons
Tcerg1	0.18	0.00000000	pigeons
Arhgap25	0.27	0.00000000	pigeons
Kdm2a	0.30	0.00000000	pigeons
Btf3l4	0.16	0.00000000	pigeons
Ppp3cc	0.26	0.00000000	pigeons
Fam168b	0.13	0.00000000	pigeons
Appbp2	0.23	0.00000000	pigeons
Srp14	0.24	0.00000000	pigeons
Dnase2b	0.36	0.00000000	pigeons
Ttc13	0.26	0.00000000	pigeons
Dcaf1	0.18	0.00000000	pigeons
Cnot2	0.39	0.00000000	pigeons
Pde1c	-1.58	0.00000000	pigeons
Runx1	0.34	0.00000000	pigeons
Fbxo42	0.19	0.00000000	pigeons
4833439L19Rik	0.18	0.00000000	pigeons
Pias2	0.30	0.00000000	pigeons
Strn	0.27	0.00000000	pigeons
Bud31	0.06	0.00000000	pigeons
Fam76a	0.22	0.00000000	pigeons
Ikbke	0.31	0.00000000	pigeons
Gns	0.33	0.00000000	pigeons
Zfp335	0.17	0.00000000	pigeons
Ppp3r1	0.17	0.00000000	pigeons

Ttc39b	0.53	0.00000000	pigeons
Zfhx3	0.20	0.00000000	pigeons
Prrg1	-1.32	0.00000000	pigeons
Ikzf1	0.57	0.00000000	pigeons
Foxf1	-1.33	0.00000000	pigeons
Unc5c	-1.40	0.00000000	pigeons
Stox2	-1.58	0.00000000	pigeons
Lrrc56	0.31	0.00000000	pigeons
Zdhhc13	0.30	0.00000000	pigeons
Il10	0.43	0.00000000	pigeons
Etl4	-1.10	0.00000000	pigeons
Bmp6	-1.34	0.00000000	pigeons
Pdlim1	-1.43	0.00000000	pigeons
Acsl3	0.36	0.00000000	pigeons
Tecpr2	0.24	0.00000000	pigeons
Tmem222	0.22	0.00000000	pigeons
Ywhab	0.00	0.00000000	pigeons
Pum1	0.13	0.00000000	pigeons
Lrif1	0.14	0.00000000	pigeons
Tmem230	0.12	0.00000000	pigeons
Haao	0.42	0.00000000	pigeons
Ddx6	0.06	0.00000000	pigeons
Setd5	0.14	0.00000000	pigeons
Trank1	0.37	0.00000000	pigeons
Cdk12	0.09	0.00000000	pigeons
Dr1	-0.01	0.00000000	pigeons
Xrn2	0.17	0.00000000	pigeons
Egfl7	-1.31	0.00000000	pigeons
Ncln	0.26	0.00000000	pigeons
Tbc1d9b	0.20	0.00000000	pigeons
Cttnbp2nl	-1.72	0.00000000	pigeons
Phldb2	-1.42	0.00000000	pigeons
Rnf10	0.09	0.00000000	pigeons
Hexim1	0.33	0.00000000	pigeons
Sbf2	0.58	0.00000000	pigeons
Thoc2	0.16	0.00000000	pigeons
Pcsk2	-1.30	0.00000000	pigeons
Ass1	0.56	0.00000000	pigeons
Cdc42se2	0.52	0.00000000	pigeons
Utp3	0.24	0.00000000	pigeons
Erlec1	0.18	0.00000000	pigeons
Rer1	0.15	0.00000000	pigeons
Zfp711	0.15	0.00000000	pigeons
Hagh	0.30	0.00000000	pigeons
Chpt1	0.27	0.00000000	pigeons
Vrk1	0.29	0.00000000	pigeons

Eif3e	0.15	0.00000000	pigeons
Dusp16	0.53	0.00000000	pigeons
Kif11	0.34	0.00000000	pigeons
Osbpl11	0.18	0.00000000	pigeons
Ramp3	-1.36	0.00000000	pigeons
Add1	0.10	0.00000000	pigeons
Cct4	0.12	0.00000000	pigeons
Celf1	0.28	0.00000000	pigeons
Dynlt1a	0.26	0.00000000	pigeons
B2m	-0.31	0.00000000	pigeons
Derl1	0.17	0.00000000	pigeons
Pcif1	0.05	0.00000000	pigeons
Pim1	0.26	0.00000000	pigeons
Atg2b	0.18	0.00000000	pigeons
Aagab	0.32	0.00000000	pigeons
Api5	0.09	0.00000000	pigeons
Sos2	0.09	0.00000000	pigeons
Nr2c1	0.28	0.00000000	pigeons
Tbc1d16	0.33	0.00000000	pigeons
Sox18	-1.29	0.00000000	pigeons
Ubqln1	0.18	0.00000000	pigeons
Aftph	0.04	0.00000000	pigeons
Nfu1	0.20	0.00000000	pigeons
Nr3c2	-1.50	0.00000000	pigeons
Wdr25	0.36	0.00000000	pigeons
Paip1	0.19	0.00000000	pigeons
Ly75	0.18	0.00000000	pigeons
Esrp2	0.18	0.00000000	pigeons
Ppp4r3a	0.16	0.00000000	pigeons
Cul2	0.29	0.00000000	pigeons
Tmed2	0.10	0.00000000	pigeons
Serpind1	-1.43	0.00000000	pigeons
Sf3a2	0.18	0.00000000	pigeons
Gzf1	0.16	0.00000000	pigeons
Abcc5	0.20	0.00000000	pigeons
Wwc2	-1.33	0.00000000	pigeons
Pip4k2a	0.51	0.00000000	pigeons
Copg1	0.18	0.00000000	pigeons
Spg11	0.31	0.00000000	pigeons
Washc4	0.21	0.00000000	pigeons
Psmc1	0.08	0.00000000	pigeons
Irf7	0.27	0.00000000	pigeons
Angptl2	0.46	0.00000000	pigeons
Prkcb	0.32	0.00000000	pigeons
Slc25a43	0.31	0.00000000	pigeons
Bzw1	0.21	0.00000000	pigeons

Ranbp3	0.20	0.00000000	pigeons
Reep5	0.18	0.00000000	pigeons
Cabin1	0.10	0.00000000	pigeons
Rpa1	0.17	0.00000000	pigeons
Cenpu	0.41	0.00000000	pigeons
Yeats4	0.18	0.00000000	pigeons
Slc25a24	0.12	0.00000000	pigeons
Ik	0.06	0.00000000	pigeons
Zcchc17	0.18	0.00000000	pigeons
Bag3	0.53	0.00000000	pigeons
Pdlim5	0.27	0.00000000	pigeons
Fbxw7	0.48	0.00000000	pigeons
Zfp831	0.39	0.00000000	pigeons
Herc1	0.21	0.00000000	pigeons
Setd3	0.24	0.00000000	pigeons
Nup50	0.13	0.00000000	pigeons
Lrpprc	0.39	0.00000000	pigeons
Eif3d	0.12	0.00000000	pigeons
Cpsf2	0.12	0.00000000	pigeons
Tmem184b	0.09	0.00000000	pigeons
Dusp6	0.30	0.00000000	pigeons
Cpsf3	0.17	0.00000000	pigeons
Apoa1	-2.26	0.00000000	pigeons
R3hdm4	0.24	0.00000000	pigeons
Ubap2	0.15	0.00000000	pigeons
Gnptab	0.18	0.00000000	pigeons
Bpnt1	0.23	0.00000000	pigeons
Phospho1	0.28	0.00000000	pigeons
Psmd9	0.05	0.00000000	pigeons
Nsun2	0.17	0.00000000	pigeons
Rragd	0.30	0.00000000	pigeons
Ptbp1	0.35	0.00000000	pigeons
Psme3	0.28	0.00000000	pigeons
Fh1	0.11	0.00000000	pigeons
Sdf2l1	0.34	0.00000000	pigeons
Dnm1l	0.23	0.00000000	pigeons
Thada	0.32	0.00000000	pigeons
Rictor	0.17	0.00000000	pigeons
Fkbp8	0.16	0.00000000	pigeons
Zbtb43	0.18	0.00000000	pigeons
Klf10	0.18	0.00000000	pigeons
Stk3	0.19	0.00000000	pigeons
Ankrd13d	0.14	0.00000000	pigeons
Gab1	0.90	0.00000000	pigeons
Tbc1d23	0.10	0.00000000	pigeons
Rangap1	0.20	0.00000000	pigeons

Fat4	-1.22	0.00000000	pigeons
Wdr26	0.13	0.00000000	pigeons
Pts	0.24	0.00000000	pigeons
Sucla2	0.10	0.00000000	pigeons
Csde1	0.03	0.00000000	pigeons
Ppil3	0.19	0.00000000	pigeons
Ccndbp1	0.12	0.00000000	pigeons
Csnk2a1	0.08	0.00000000	pigeons
Arid3b	0.25	0.00000000	pigeons
Pmepa1	0.20	0.00000000	pigeons
Sf3a3	0.14	0.00000000	pigeons
Uba3	0.22	0.00000000	pigeons
Arcn1	0.07	0.00000000	pigeons
Reep1	-1.26	0.00000000	pigeons
Slc35f5	0.20	0.00000000	pigeons
Ube4b	0.15	0.00000000	pigeons
Ilf3	0.03	0.00000000	pigeons
Rint1	0.16	0.00000000	pigeons
Nr1h3	-0.03	0.00000000	pigeons
Dda1	0.16	0.00000000	pigeons
Pex13	0.13	0.00000000	pigeons
Srsf5	-0.01	0.00000000	pigeons
Gps1	0.19	0.00000000	pigeons
Psma1	0.08	0.00000000	pigeons
Spred1	-1.13	0.00000000	pigeons
Kdm5b	0.12	0.00000000	pigeons
P2rx1	0.28	0.00000000	pigeons
Pole4	0.24	0.00000000	pigeons
Nr2c2	0.14	0.00000000	pigeons
Phf2	0.17	0.00000000	pigeons
Rsbn1l	0.22	0.00000000	pigeons
Ppp2r2d	0.14	0.00000000	pigeons
Dynlrb1	-0.01	0.00000000	pigeons
Zfand5	0.14	0.00000000	pigeons
Taldo1	0.12	0.00000000	pigeons
Lasp1	0.08	0.00000000	pigeons
Dennd5a	0.22	0.00000000	pigeons
Gpr176	0.38	0.00000000	pigeons
Klf7	0.24	0.00000000	pigeons
Lbr	0.02	0.00000000	pigeons
Entpd4	0.25	0.00000000	pigeons
Slc3a1	0.42	0.00000000	pigeons
Uri1	0.15	0.00000000	pigeons
S100a13	-1.64	0.00000000	pigeons
Paqr3	0.25	0.00000000	pigeons
Asb3	0.48	0.00000000	pigeons

Zbtb49	0.31	0.00000000	pigeons
Cul4b	0.04	0.00000000	pigeons
Sf3a1	0.02	0.00000000	pigeons
Adgrg1	-1.29	0.00000000	pigeons
Ei24	0.17	0.00000000	pigeons
Ncam1	0.44	0.00000000	pigeons
Slc39a9	0.11	0.00000000	pigeons
Tspan18	-1.43	0.00000000	pigeons
Vwa8	0.25	0.00000000	pigeons
Rnf34	0.03	0.00000000	pigeons
Smg1	0.07	0.00000000	pigeons
Pgm2	0.21	0.00000000	pigeons
Plrg1	0.04	0.00000000	pigeons
Dazap1	-0.03	0.00000000	pigeons
Cltc	-0.01	0.00000000	pigeons
Tmx1	0.01	0.00000000	pigeons
Fgfr1op2	0.08	0.00000000	pigeons
Rc3h1	0.17	0.00000000	pigeons
Ntn4	-1.21	0.00000000	pigeons
Rbx1	0.12	0.00000000	pigeons
Adar	0.24	0.00000000	pigeons
Plekhm2	0.10	0.00000000	pigeons
Tpm1	0.08	0.00000000	pigeons
Pdgfb	-1.31	0.00000000	pigeons
Ep400	0.01	0.00000000	pigeons
Dlst	0.14	0.00000000	pigeons
Hdac2	0.10	0.00000000	pigeons
Serpini1	0.14	0.00000000	pigeons
Prpsap2	0.10	0.00000000	pigeons
Nlk	0.08	0.00000000	pigeons
Farp1	-1.26	0.00000000	pigeons
Sh3glb1	0.10	0.00000000	pigeons
Ireb2	0.00	0.00000000	pigeons
Etfa	0.08	0.00000000	pigeons
Pabpc1	0.40	0.00000000	pigeons
Tpi1	0.22	0.00000000	pigeons
Usp15	0.10	0.00000000	pigeons
Asxl3	-1.37	0.00000000	pigeons
Afg3l2	-0.01	0.00000000	pigeons
Kif1b	0.39	0.00000000	pigeons
Dennd2a	-1.50	0.00000000	pigeons
Rab8a	0.14	0.00000000	pigeons
Sec23b	0.15	0.00000000	pigeons
Errfi1	0.23	0.00000000	pigeons
Gpn3	0.08	0.00000000	pigeons
Ripor3	-1.24	0.00000000	pigeons

Sash1	-1.28	0.00000000	pigeons
Zdhhc7	0.09	0.00000000	pigeons
Ube3c	0.05	0.00000000	pigeons
Blzf1	0.16	0.00000000	pigeons
Nsrp1	0.09	0.00000000	pigeons
Tnfrsf11b	-1.18	0.00000000	pigeons
Helq	0.23	0.00000000	pigeons
Tgm4	0.91	0.00000000	pigeons
Lims2	-1.49	0.00000000	pigeons
Txlna	0.27	0.00000000	pigeons
Zcrb1	0.25	0.00000000	pigeons
Zfat	0.10	0.00000000	pigeons
Srf	0.18	0.00000000	pigeons
Gatad2a	0.06	0.00000000	pigeons
Fosl2	0.23	0.00000000	pigeons
Gtf2a2	0.15	0.00000000	pigeons
Tcp1	0.12	0.00000000	pigeons
Ndufv3	0.14	0.00000000	pigeons
Arg2	0.35	0.00000000	pigeons
Psma2	0.08	0.00000000	pigeons
Zc3h12d	1.47	0.00000000	pigeons
Gcc2	0.14	0.00000000	pigeons
Trnt1	0.19	0.00000000	pigeons
Vps29	0.11	0.00000000	pigeons
Cpsf4l	0.12	0.00000000	pigeons
Smg7	0.19	0.00000000	pigeons
Clcn7	0.20	0.00000000	pigeons
Atp1b3	-1.38	0.00000000	pigeons
Togaram1	0.26	0.00000000	pigeons
Slc25a5	0.21	0.00000000	pigeons
Trp53i11	-1.25	0.00000000	pigeons
Copb2	0.07	0.00000000	pigeons
Slc35b3	0.22	0.00000000	pigeons
Ywhae	-0.03	0.00000000	pigeons
Zfand6	0.13	0.00000000	pigeons
Twf2	0.17	0.00000000	pigeons
Apba2	-1.24	0.00000000	pigeons
Stat2	0.20	0.00000000	pigeons
Erp44	0.11	0.00000000	pigeons
Copb1	0.05	0.00000000	pigeons
Tnrc6b	-1.35	0.00000000	pigeons
Hbp1	-0.02	0.00000000	pigeons
Ap3b1	0.18	0.00000000	pigeons
Exoc1	0.22	0.00000000	pigeons
Eif3m	0.21	0.00000000	pigeons
Grk3	0.16	0.00000000	pigeons

Npepl1	0.22	0.00000000	pigeons
Rab9	0.16	0.00000000	pigeons
Slc25a22	0.17	0.00000000	pigeons
Derl3	0.31	0.00000000	pigeons
Grb10	0.42	0.00000000	pigeons
Ercc6	0.26	0.00000000	pigeons
Slc9a3r1	0.24	0.00000000	pigeons
Lrp1	0.28	0.00000000	pigeons
Sptlc2	0.08	0.00000000	pigeons
Slc9a8	0.26	0.00000000	pigeons
Rhoh	0.28	0.00000000	pigeons
Reep2	0.25	0.00000000	pigeons
Alkbh5	0.09	0.00000000	pigeons
F2r	-1.24	0.00000000	pigeons
Wbp2nl	0.11	0.00000000	pigeons
Pgpep1	0.23	0.00000000	pigeons
Ccdc88c	0.66	0.00000000	pigeons
Pdrg1	0.12	0.00000000	pigeons
Ddx27	0.08	0.00000000	pigeons
Ptk2	-1.38	0.00000000	pigeons
Rabggtb	0.23	0.00000000	pigeons
Emc6	0.12	0.00000000	pigeons
Chordc1	0.01	0.00000000	pigeons
Abca1	0.37	0.00000000	pigeons
Prrc2c	0.14	0.00000000	pigeons
Slc16a6	0.51	0.00000000	pigeons
Trappc11	0.12	0.00000000	pigeons
Prpf40a	0.00	0.00000000	pigeons
Ibtk	0.16	0.00000000	pigeons
Wdr81	0.20	0.00000000	pigeons
Pphln1	0.22	0.00000000	pigeons
Hyou1	0.22	0.00000000	pigeons
Galnt1	0.27	0.00000000	pigeons
Usp30	0.18	0.00000000	pigeons
Amotl2	-1.21	0.00000000	pigeons
Sf3b1	0.02	0.00000000	pigeons
Smarca5	0.10	0.00000000	pigeons
Il20ra	0.29	0.00000000	pigeons
Adipor1	0.11	0.00000000	pigeons
Farsb	0.06	0.00000000	pigeons
Cebpg	0.22	0.00000000	pigeons
Spsb4	-1.18	0.00000000	pigeons
Dicer1	0.09	0.00000000	pigeons
Rbm26	0.15	0.00000000	pigeons
Phc3	0.25	0.00000000	pigeons
Saal1	0.54	0.00000000	pigeons

Thoc7	0.08	0.00000000	pigeons
Suclg1	0.16	0.00000000	pigeons
Cish	0.30	0.00000000	pigeons
Pam	0.42	0.00000000	pigeons
Atp7a	0.02	0.00000000	pigeons
Bbs2	0.14	0.00000000	pigeons
Pde4d	0.67	0.00000000	pigeons
Tlk2	0.05	0.00000000	pigeons
Rab18	0.04	0.00000000	pigeons
Dffb	0.22	0.00000000	pigeons
Kcnj5	-1.49	0.00000000	pigeons
Arsb	0.30	0.00000000	pigeons
Trim33	0.05	0.00000000	pigeons
Zfp622	0.12	0.00000000	pigeons
Ripk1	0.13	0.00000000	pigeons
Pmm2	0.20	0.00000000	pigeons
Ep300	0.00	0.00000000	pigeons
Gabpa	0.06	0.00000000	pigeons
Cdh23	0.24	0.00000000	pigeons
Tmem33	0.12	0.00000000	pigeons
Ankib1	0.25	0.00000000	pigeons
Adora2a	0.17	0.00000000	pigeons
Denr	0.15	0.00000000	pigeons
Abracl	0.01	0.00000000	pigeons
Strada	0.20	0.00000000	pigeons
Ric1	0.10	0.00000000	pigeons
Taf2	-0.04	0.00000000	pigeons
Acadl	0.16	0.00000000	pigeons
Exoc6	0.13	0.00000000	pigeons
Ldlrad4	0.72	0.00000000	pigeons
Fmr1	-0.04	0.00000000	pigeons
Atp6v1g1	0.06	0.00000000	pigeons
Pak3	0.37	0.00000000	pigeons
Snx2	0.13	0.00000000	pigeons
Gata3	-1.38	0.00000000	pigeons
Eif3f	0.12	0.00000000	pigeons
Zbtb26	0.11	0.00000000	pigeons
Bcl2l1	0.12	0.00000000	pigeons
Bcr	0.01	0.00000000	pigeons
Psmc2	-0.01	0.00000000	pigeons
Dis3l2	0.09	0.00000000	pigeons
Jade3	0.57	0.00000000	pigeons
Gpalpp1	-0.02	0.00000000	pigeons
Ralgapa1	0.05	0.00000000	pigeons
Eif3g	0.11	0.00000000	pigeons
Phf3	0.32	0.00000000	pigeons

Fez2	0.06	0.00000000	pigeons
Lrp12	0.12	0.00000000	pigeons
Rprd1b	0.00	0.00000000	pigeons
Pus7l	0.25	0.00000000	pigeons
Adamts2	-1.35	0.00000000	pigeons
Hhex	0.09	0.00000000	pigeons
Psma7	0.08	0.00000000	pigeons
Chmp2b	0.10	0.00000000	pigeons
Psma3	-0.04	0.00000000	pigeons
Laptm4a	-1.27	0.00000000	pigeons
Psma6	0.04	0.00000000	pigeons
Faf2	-0.04	0.00000000	pigeons
Tie1	-1.08	0.00000000	pigeons
Fancm	0.25	0.00000000	pigeons
Nap1l4	0.01	0.00000000	pigeons
Prdm15	0.19	0.00000000	pigeons
Soga1	0.09	0.00000000	pigeons
Psmc3	-0.02	0.00000000	pigeons
Commd7	0.15	0.00000000	pigeons
Cmtr1	0.12	0.00000000	pigeons
Snx27	0.23	0.00000000	pigeons
Lpcat3	0.02	0.00000000	pigeons
Gnl2	0.07	0.00000000	pigeons
Sin3a	-0.01	0.00000000	pigeons
Atl2	-0.06	0.00000000	pigeons
Dynlt3	0.08	0.00000000	pigeons
Parp9	0.03	0.00000000	pigeons
Il10ra	0.21	0.00000000	pigeons
Luc7l	-0.07	0.00000000	pigeons
Rgs6	0.86	0.00000000	pigeons
Pdap1	-0.06	0.00000000	pigeons
Pcsk7	0.14	0.00000000	pigeons
Tmem47	-1.07	0.00000000	pigeons
Dip2b	0.13	0.00000000	pigeons
Aup1	0.16	0.00000000	pigeons
Lama4	-1.18	0.00000000	pigeons
Slc10a7	0.32	0.00000000	pigeons
Adnp2	0.07	0.00000000	pigeons
Ppp1r2	0.03	0.00000000	pigeons
Necap1	0.17	0.00000000	pigeons
Pes1	0.15	0.00000000	pigeons
Tbc1d15	0.03	0.00000000	pigeons
Cdc27	0.08	0.00000000	pigeons
Umad1	0.30	0.00000000	pigeons
Hipk3	0.14	0.00000000	pigeons
Ubr2	0.00	0.00000000	pigeons

Immt	0.11	0.00000000	pigeons
Skil	0.15	0.00000000	pigeons
Uba2	0.03	0.00000000	pigeons
Slc38a10	0.14	0.00000000	pigeons
Ndufs4	0.23	0.00000000	pigeons
Hlf	-1.28	0.00000000	pigeons
Zc3h15	-0.05	0.00000000	pigeons
Zbtb7a	-0.02	0.00000000	pigeons
Ndufs1	0.15	0.00000000	pigeons
Dhx38	0.06	0.00000000	pigeons
Sar1b	0.10	0.00000000	pigeons
Prmt9	0.02	0.00000000	pigeons
Cmtm3	0.10	0.00000000	pigeons
Exoc6b	-1.54	0.00000000	pigeons
Syt11	-0.03	0.00000000	pigeons
Esyt2	0.04	0.00000000	pigeons
Gpr107	0.03	0.00000000	pigeons
Vps18	0.18	0.00000000	pigeons
Trip12	0.21	0.00000000	pigeons
Srsf1	-0.11	0.00000000	pigeons
Smarce1	0.02	0.00000000	pigeons
Usp38	-0.04	0.00000000	pigeons
Cmtm8	-1.06	0.00000000	pigeons
Cul3	0.00	0.00000000	pigeons
Fbxw2	0.12	0.00000000	pigeons
Ammecr1	0.16	0.00000000	pigeons
Synrg	0.24	0.00000000	pigeons
Cttnbp2	-1.16	0.00000000	pigeons
Eif2ak1	0.14	0.00000000	pigeons
Hnrnpm	-0.09	0.00000000	pigeons
Tia1	0.22	0.00000000	pigeons
Pag1	0.32	0.00000000	pigeons
Pcmtd2	-0.01	0.00000000	pigeons
Upf3b	-0.06	0.00000000	pigeons
Kpna6	0.11	0.00000000	pigeons
Itfg2	-0.06	0.00000000	pigeons
Kin	0.07	0.00000000	pigeons
Nedd9	0.32	0.00000000	pigeons
Pnisr	0.09	0.00000000	pigeons
Actr8	0.13	0.00000000	pigeons
Arpc1a	-0.03	0.00000000	pigeons
Ncoa7	0.64	0.00000000	pigeons
Virma	0.12	0.00000000	pigeons
Tmem258	0.14	0.00000000	pigeons
Cnot7	-0.01	0.00000000	pigeons
Prkar2a	0.13	0.00000000	pigeons

Endog	0.12	0.00000000	pigeons
Dock4	-1.25	0.00000000	pigeons
Rnf146	-0.05	0.00000000	pigeons
Eng	-1.03	0.00000000	pigeons
Hsp90b1	-0.59	0.00000000	pigeons
Snw1	0.06	0.00000000	pigeons
Ndufb9	0.18	0.00000000	pigeons
Usp14	-0.02	0.00000000	pigeons
Kcnq5	0.43	0.00000000	pigeons
Scube3	-1.09	0.00000000	pigeons
Bub3	0.14	0.00000000	pigeons
Slc35a1	0.24	0.00000000	pigeons
Gtf2f2	0.15	0.00000000	pigeons
Gpi1	-0.03	0.00000000	pigeons
Stxbp5	0.19	0.00000000	pigeons
Dpp9	0.13	0.00000000	pigeons
Tcf7l2	-1.12	0.00000000	pigeons
Rab6a	-0.05	0.00000000	pigeons
Hs6st1	-1.55	0.00000000	pigeons
Snrpd3	-0.03	0.00000000	pigeons
Nt5c3b	0.25	0.00000000	pigeons
Psmd2	0.09	0.00000000	pigeons
Psmc5	-0.01	0.00000000	pigeons
Btbd10	0.06	0.00000000	pigeons
Elf1	0.23	0.00000000	pigeons
Supt6	-0.01	0.00000000	pigeons
Lrrfip2	0.13	0.00000000	pigeons
Lig4	0.22	0.00000000	pigeons
Zhx2	0.27	0.00000000	pigeons
Usp48	0.00	0.00000000	pigeons
Eif2b3	-0.04	0.00000000	pigeons
Baiap2l1	0.18	0.00000000	pigeons
Atp2a2	0.06	0.00000000	pigeons
Nr2f2	-1.19	0.00000000	pigeons
Srp54a	-0.01	0.00000000	pigeons
Sh3bgrl	-0.02	0.00000000	pigeons
Hvcn1	0.56	0.00000000	pigeons
Hsd17b12	-0.91	0.00000000	pigeons
Cript	0.08	0.00000000	pigeons
Erg	-1.26	0.00000000	pigeons
Rab3gap2	0.18	0.00000000	pigeons
Rffl	0.27	0.00000000	pigeons
Pank2	0.16	0.00000000	pigeons
Apool	0.09	0.00000000	pigeons
Ppil4	-0.02	0.00000000	pigeons
Ifnar2	-0.06	0.00000000	pigeons

Larp4	0.06	0.00000000	pigeons
Crk	-0.06	0.00000000	pigeons
Ndrg3	0.20	0.00000000	pigeons
Slc25a46	0.15	0.00000000	pigeons
Sirt1	-0.03	0.00000000	pigeons
Nup88	0.07	0.00000000	pigeons
Dync1li1	-0.03	0.00000000	pigeons
Rnf138	0.17	0.00000000	pigeons
Plpp3	-1.06	0.00000000	pigeons
Ubfd1	0.08	0.00000000	pigeons
Klhl24	0.22	0.00000000	pigeons
Grpel1	0.17	0.00000000	pigeons
Alas1	0.14	0.00000000	pigeons
Lsm14a	-0.07	0.00000000	pigeons
Psmb1	-0.04	0.00000000	pigeons
Slc30a5	0.07	0.00000000	pigeons
Ppm1e	0.24	0.00000000	pigeons
Blmh	-0.02	0.00000000	pigeons
Tnip3	0.28	0.00000000	pigeons
Slc35c2	0.04	0.00000000	pigeons
Ptp4a2	0.06	0.00000000	pigeons
Ube2f	0.16	0.00000000	pigeons
Elavl1	-0.01	0.00000000	pigeons
Bmt2	0.11	0.00000000	pigeons
Nfyc	0.10	0.00000000	pigeons
Vti1b	0.15	0.00000000	pigeons
Tbpl1	0.10	0.00000000	pigeons
Kcnq1	-1.04	0.00000000	pigeons
Lonp2	-0.11	0.00000000	pigeons
Gpr171	0.47	0.00000000	pigeons
Fyco1	0.20	0.00000000	pigeons
Jakmip3	-1.03	0.00000000	pigeons
Rae1	0.03	0.00000000	pigeons
Ctbs	0.14	0.00000000	pigeons
Aqr	-0.03	0.00000000	pigeons
Ccnc	0.06	0.00000000	pigeons
Gpr155	0.14	0.00000000	pigeons
Btbd2	0.13	0.00000000	pigeons
Scarb2	-1.46	0.00000000	pigeons
Azin1	0.05	0.00000000	pigeons
Wsb1	-0.02	0.00000000	pigeons
Lrrc59	0.17	0.00000000	pigeons
Flt1	-1.15	0.00000000	pigeons
Tspan4	-1.14	0.00000000	pigeons
Slc23a2	0.10	0.00000000	pigeons
Eya2	0.32	0.00000000	pigeons

Ccnd3	0.20	0.00000000	pigeons
Rgs9	0.11	0.00000000	pigeons
Wasf2	-0.10	0.00000000	pigeons
Dcun1d5	0.12	0.00000000	pigeons
Atg13	0.19	0.00000000	pigeons
Sdad1	0.10	0.00000000	pigeons
Rexo2	0.02	0.00000000	pigeons
Tmtc4	0.24	0.00000000	pigeons
Cpt1a	0.14	0.00000000	pigeons
Ppp2r2a	-0.12	0.00000000	pigeons
Kdm1a	-0.04	0.00000000	pigeons
Tmem131	0.16	0.00000000	pigeons
Zfp292	0.13	0.00000000	pigeons
Sike1	0.05	0.00000000	pigeons
F13a1	-1.49	0.00000000	pigeons
Brox	0.05	0.00000000	pigeons
Snip1	-0.03	0.00000000	pigeons
Leo1	0.04	0.00000000	pigeons
Khdrbs1	-0.01	0.00000000	pigeons
Pdhx	0.06	0.00000000	pigeons
Nrbp2	0.23	0.00000000	pigeons
Creb1	0.14	0.00000000	pigeons
Arf4	0.02	0.00000000	pigeons
Gpr137b	0.11	0.00000000	pigeons
Ggnbp2	-0.06	0.00000000	pigeons
Tspan2	0.26	0.00000000	pigeons
Btbd1	0.10	0.00000000	pigeons
Ints11	0.13	0.00000000	pigeons
Rbm38	0.26	0.00000000	pigeons
Atg12	0.18	0.00000000	pigeons
Siah2	0.23	0.00000000	pigeons
Ptges3	-0.01	0.00000000	pigeons
Tor1b	-0.03	0.00000000	pigeons
Gtf2a1	-0.06	0.00000000	pigeons
Poldip3	0.02	0.00000000	pigeons
Bend5	0.10	0.00000000	pigeons
Rasa3	0.38	0.00000000	pigeons
Usp7	0.03	0.00000000	pigeons
Dnajc5	0.04	0.00000000	pigeons
Comtd1	0.24	0.00000000	pigeons
Slc15a1	-1.10	0.00000000	pigeons
Ngly1	-0.03	0.00000000	pigeons
H13	0.01	0.00000000	pigeons
Tes	0.14	0.00000000	pigeons
Ndufb1	-0.06	0.00000000	pigeons
Nufip2	-0.10	0.00000000	pigeons

Cltb	-0.10	0.00000000	pigeons
Rab2a	-0.01	0.00000000	pigeons
Tsen2	0.20	0.00000000	pigeons
Dlgap4	-0.09	0.00000000	pigeons
Ash1l	-0.14	0.00000000	pigeons
Usp16	0.05	0.00000000	pigeons
Cdk17	-1.41	0.00000000	pigeons
Arl8a	0.02	0.00000000	pigeons
Ramp2	-1.49	0.00000000	pigeons
Npc1	0.20	0.00000000	pigeons
Qrich1	-0.03	0.00000000	pigeons
Apoo	-0.04	0.00000000	pigeons
Llph	0.00	0.00000000	pigeons
Sec61a1	0.13	0.00000000	pigeons
Atp2c1	-0.12	0.00000000	pigeons
Atp6v1d	-0.08	0.00000000	pigeons
Fgr	0.03	0.00000000	pigeons
Polr1e	0.12	0.00000000	pigeons
Hsbp1	-0.07	0.00000000	pigeons
Bcl2l13	-0.05	0.00000000	pigeons
Cmas	0.25	0.00000000	pigeons
Smarcc1	-0.04	0.00000000	pigeons
Exosc7	0.18	0.00000000	pigeons
Rasgrp3	-1.24	0.00000000	pigeons
Ppm1f	-1.46	0.00000000	pigeons
Btk	0.16	0.00000000	pigeons
Aplf	0.02	0.00000000	pigeons
Hspa13	0.17	0.00000000	pigeons
Ralgapb	-0.07	0.00000000	pigeons
Setd7	0.00	0.00000000	pigeons
Nup98	-0.08	0.00000000	pigeons
Tnfrsf21	0.20	0.00000000	pigeons
Sf3b3	-0.06	0.00000000	pigeons
Cbx7	0.23	0.00000000	pigeons
Nelfcd	-0.03	0.00000000	pigeons
Xpo7	-0.03	0.00000000	pigeons
Lrrc8d	0.09	0.00000000	pigeons
Gabpb1	0.15	0.00000000	pigeons
Rad17	0.10	0.00000000	pigeons
Rnaseh1	0.04	0.00000000	pigeons
Cnppd1	0.05	0.00000000	pigeons
2510039O18Rik	0.10	0.00000000	pigeons
Uhrf1bp1l	0.07	0.00000000	pigeons
Vill	-1.02	0.00000000	pigeons
B4galt2	0.11	0.00000000	pigeons
Sec61g	0.49	0.00000000	pigeons

Atg5	0.06	0.00000000	pigeons
Nrd1	0.02	0.00000000	pigeons
Erbin	0.11	0.00000000	pigeons
Xpot	0.01	0.00000000	pigeons
Sdf4	-0.14	0.00000000	pigeons
Riok1	0.13	0.00000000	pigeons
Scaf11	-0.07	0.00000000	pigeons
Ostc	0.09	0.00000000	pigeons
Zrsr1	-0.07	0.00000000	pigeons
Osbpl2	0.01	0.00000000	pigeons
Exosc10	0.10	0.00000000	pigeons
Adgrl3	0.60	0.00000000	pigeons
Ctu2	0.15	0.00000000	pigeons
Fbxw11	-0.03	0.00000000	pigeons
Lmbrd1	0.11	0.00000000	pigeons
Lin54	0.04	0.00000000	pigeons
Rragc	0.12	0.00000000	pigeons
Sugt1	0.04	0.00000000	pigeons
Ppargc1a	-1.15	0.00000000	pigeons
Edn1	-1.09	0.00000000	pigeons
Cnot8	0.06	0.00000000	pigeons
Anxa5	-1.20	0.00000000	pigeons
Vcpip1	0.03	0.00000000	pigeons
Tbk1	0.03	0.00000000	pigeons
Nop14	-0.01	0.00000000	pigeons
Rai2	0.05	0.00000000	pigeons
Pik3c2a	0.09	0.00000000	pigeons
Setx	-0.10	0.00000000	pigeons
Tbrg4	0.19	0.00000000	pigeons
Ppp2r5d	0.14	0.00000000	pigeons
Sod2	0.09	0.00000000	pigeons
Plagl1	0.31	0.00000000	pigeons
Vldlr	0.25	0.00000000	pigeons
Nono	-0.03	0.00000000	pigeons
Smad1	-1.32	0.00000000	pigeons
Gigyf2	0.17	0.00000000	pigeons
Tbl1xr1	0.18	0.00000000	pigeons
Nrbp1	0.12	0.00000000	pigeons
Senp5	0.11	0.00000000	pigeons
Tasp1	0.16	0.00000000	pigeons
Sacm1l	-0.08	0.00000000	pigeons
Tc2n	0.18	0.00000000	pigeons
Zzz3	-0.07	0.00000000	pigeons
Sec11a	0.02	0.00000000	pigeons
Eif3k	-1.05	0.00000000	pigeons
Nfya	0.15	0.00000000	pigeons

Fggy	0.39	0.00000000	pigeons
Ebag9	-0.02	0.00000000	pigeons
Ahcyl1	0.18	0.00000000	pigeons
Fuca2	0.14	0.00000000	pigeons
Crbn	0.16	0.00000000	pigeons
Snrpa1	0.06	0.00000000	pigeons
Ambra1	-0.02	0.00000000	pigeons
Prelid1	0.17	0.00000000	pigeons
Sccpdh	0.08	0.00000000	pigeons
Axin2	-1.15	0.00000000	pigeons
Tcea1	-0.04	0.00000000	pigeons
Larp1	-0.02	0.00000000	pigeons
Kdm4b	-0.10	0.00000000	pigeons
Prpf6	-0.07	0.00000000	pigeons
Crlf2	0.42	0.00000000	pigeons
Gnb4	0.11	0.00000000	pigeons
Wwp2	0.11	0.00000000	pigeons
Wdr36	0.18	0.00000000	pigeons
Luzp1	-1.48	0.00000000	pigeons
Slk	0.26	0.00000000	pigeons
Nr3c1	0.00	0.00000000	pigeons
Setdb1	-0.02	0.00000000	pigeons
Uggt1	-0.06	0.00000000	pigeons
Pigt	0.11	0.00000000	pigeons
Trerf1	0.09	0.00000000	pigeons
Comt	0.57	0.00000000	pigeons
Atl1	0.03	0.00000000	pigeons
Ctso	0.15	0.00000000	pigeons
Tmem104	0.24	0.00000000	pigeons
Nbn	0.07	0.00000000	pigeons
Usp20	0.16	0.00000000	pigeons
Uhmk1	0.13	0.00000000	pigeons
Ctsb	0.18	0.00000000	pigeons
Ssbp1	0.07	0.00000000	pigeons
Tmem9b	0.04	0.00000000	pigeons
Atg14	0.15	0.00000000	pigeons
Lactb	-0.11	0.00000000	pigeons
Zfp318	0.05	0.00000000	pigeons
Fas	0.06	0.00000000	pigeons
Tgfb2	-1.03	0.00000000	pigeons
Sgsm3	0.13	0.00000000	pigeons
Rgs1	1.96	0.00000000	pigeons
Birc6	0.16	0.00000000	pigeons
Numa1	0.11	0.00000000	pigeons
Taok1	-0.05	0.00000000	pigeons
Psmd3	0.06	0.00000000	pigeons

Anapc7	0.17	0.00000000	pigeons
Esco1	-0.01	0.00000000	pigeons
Inhba	-0.81	0.00000000	pigeons
Klhl7	0.12	0.00000000	pigeons
Ikzf2	0.18	0.00000000	pigeons
Tfg	-0.02	0.00000000	pigeons
Emc8	0.15	0.00000000	pigeons
Sema7a	-1.30	0.00000000	pigeons
Ppil1	0.01	0.00000000	pigeons
Psmb7	-0.05	0.00000000	pigeons
Ckb	-1.14	0.00000000	pigeons
Adam22	0.38	0.00000000	pigeons
Cope	0.06	0.00000000	pigeons
Upf1	-0.11	0.00000000	pigeons
Rbfox1	-1.12	0.00000000	pigeons
Cwc15	-0.23	0.00000000	pigeons
Secisbp2	-0.16	0.00000000	pigeons
Htra1	-1.11	0.00000000	pigeons
Zcchc8	-0.06	0.00000000	pigeons
Tm9sf3	-0.10	0.00000000	pigeons
Phf13	0.09	0.00000000	pigeons
Pacsin2	0.16	0.00000000	pigeons
Phka2	0.08	0.00000000	pigeons
Nfyb	0.12	0.00000000	pigeons
Casp3	0.17	0.00000000	pigeons
Traf3ip3	0.17	0.00000000	pigeons
Fem1c	0.24	0.00000000	pigeons
Pdhb	0.04	0.00000000	pigeons
Zeb1	0.33	0.00000000	pigeons
Itgb1	-1.06	0.00000000	pigeons
Ptpn2	-0.04	0.00000000	pigeons
Cflar	-0.02	0.00000000	pigeons
Ubp1	-0.07	0.00000000	pigeons
Hopx	0.16	0.00000000	pigeons
St3gal1	-1.06	0.00000000	pigeons
Bcl2l11	0.16	0.00000000	pigeons
Dnajb14	0.14	0.00000000	pigeons
Zfc3h1	0.03	0.00000000	pigeons
Ube3b	0.13	0.00000000	pigeons
Zbtb38	0.12	0.00000000	pigeons
Ube2j2	-0.07	0.00000000	pigeons
Btf3	0.12	0.00000000	pigeons
Xpa	-0.12	0.00000000	pigeons
Ola1	-0.03	0.00000000	pigeons
Esd	0.04	0.00000000	pigeons
Actr10	-0.01	0.00000000	pigeons

Upf2	-0.06	0.00000000	pigeons
Tk2	-0.07	0.00000000	pigeons
Rpia	-0.10	0.00000000	pigeons
Eci1	0.16	0.00000000	pigeons
Epb41l3	0.83	0.00000000	pigeons
Cmpk2	0.17	0.00000000	pigeons
Clk3	0.02	0.00000000	pigeons
Supt20	-0.04	0.00000000	pigeons
Stam	0.07	0.00000000	pigeons
Wdr45b	0.12	0.00000000	pigeons
Necap2	0.01	0.00000000	pigeons
Stx18	0.12	0.00000000	pigeons
Ddx11	0.22	0.00000000	pigeons
Dnmt1	0.09	0.00000000	pigeons
Sfpq	-0.14	0.00000000	pigeons
Nhlrc2	0.06	0.00000000	pigeons
Vil1	0.11	0.00000000	pigeons
Copg2	0.05	0.00000000	pigeons
Stag2	0.24	0.00000000	pigeons
Armc1	-0.04	0.00000000	pigeons
Cdca7l	-1.23	0.00000000	pigeons
Ube2q1	-0.21	0.00000000	pigeons
Nelfb	-0.01	0.00000000	pigeons
Naaa	0.15	0.00000000	pigeons
Hcfc2	-0.03	0.00000000	pigeons
Oard1	-0.02	0.00000000	pigeons
Eif2ak2	0.00	0.00000000	pigeons
Eif3b	-0.01	0.00000000	pigeons
Rab35	-0.18	0.00000000	pigeons
Nfatc1	-1.05	0.00000000	pigeons
Ndufs3	0.01	0.00000000	pigeons
Zbtb11	-0.02	0.00000000	pigeons
Hif1an	0.11	0.00000000	pigeons
Irf2bpl	-0.01	0.00000000	pigeons
Dnaja2	-0.21	0.00000000	pigeons
Hmg20b	-0.06	0.00000000	pigeons
Nolc1	0.11	0.00000000	pigeons
Champ1	0.12	0.00000000	pigeons
Ppp1r8	-0.06	0.00000000	pigeons
Rit1	0.04	0.00000000	pigeons
Rbpj	0.13	0.00000000	pigeons
Eif4g1	0.07	0.00000000	pigeons
Nceh1	0.16	0.00000000	pigeons
Ppp3cb	-0.06	0.00000000	pigeons
Commd6	0.00	0.00000000	pigeons
Clint1	-0.06	0.00000000	pigeons

Slc12a2	-1.25	0.00000000	pigeons
Spry2	-1.10	0.00000000	pigeons
Cpeb4	0.01	0.00000000	pigeons
Nf2	0.05	0.00000000	pigeons
Hip1	0.15	0.00000000	pigeons
Gtpbp4	0.14	0.00000000	pigeons
Rbbp4	0.05	0.00000000	pigeons
Nsa2	-0.04	0.00000000	pigeons
Fam172a	0.06	0.00000000	pigeons
Smchd1	-0.14	0.00000000	pigeons
Cct2	-0.08	0.00000000	pigeons
Uso1	-0.08	0.00000000	pigeons
Ankrd50	-1.03	0.00000000	pigeons
Hddc2	0.09	0.00000000	pigeons
Wdtc1	0.03	0.00000000	pigeons
Slc25a34	0.09	0.00000000	pigeons
Ndst4	0.66	0.00000000	pigeons
Vopp1	-0.02	0.00000000	pigeons
Crtc1	0.13	0.00000000	pigeons
Emp2	-0.99	0.00000000	pigeons
Fbxo28	0.05	0.00000000	pigeons
Pls3	-1.47	0.00000000	pigeons
Elovl1	-0.07	0.00000000	pigeons
Ano5	0.36	0.00000000	pigeons
Arnt	-0.12	0.00000000	pigeons
Naa35	0.06	0.00000000	pigeons
Bbx	0.11	0.00000000	pigeons
G3bp2	-0.11	0.00000000	pigeons
4932438A13Rik	-0.09	0.00000000	pigeons
Ap2m1	-0.14	0.00000000	pigeons
Elmo2	-0.09	0.00000000	pigeons
Srp72	-0.06	0.00000000	pigeons
Git2	0.17	0.00000000	pigeons
Aspscr1	0.02	0.00000000	pigeons
Pik3r4	0.05	0.00000000	pigeons
Sec14l1	-1.36	0.00000000	pigeons
Ddx23	-0.02	0.00000000	pigeons
Prkca	0.15	0.00000000	pigeons
Lrmda	-0.86	0.00000000	pigeons
Lamtor2	0.00	0.00000000	pigeons
Sh3kbp1	0.21	0.00000000	pigeons
Ufm1	-0.07	0.00000000	pigeons
Rbl2	0.02	0.00000000	pigeons
Gid8	0.07	0.00000000	pigeons
Fam168a	-1.26	0.00000000	pigeons
Cnnm2	-1.11	0.00000000	pigeons

Ccser1	-1.28	0.00000000	pigeons
Fancd2	-0.13	0.00000000	pigeons
Ifrd1	0.10	0.00000000	pigeons
Nek7	-0.02	0.00000000	pigeons
Csf1	0.39	0.00000000	pigeons
Ctr9	-0.02	0.00000000	pigeons
Tnrc6c	0.06	0.00000000	pigeons
Cnih1	0.03	0.00000000	pigeons
Cdc40	0.10	0.00000000	pigeons
Bcl11a	0.34	0.00000000	pigeons
Caps2	0.21	0.00000000	pigeons
Sdhaf2	0.08	0.00000000	pigeons
Btbd7	-0.01	0.00000000	pigeons
Ankmy2	0.01	0.00000000	pigeons
Slit3	-1.05	0.00000000	pigeons
Tnip1	-0.11	0.00000000	pigeons
Fam91a1	0.11	0.00000000	pigeons
Kdm1b	0.04	0.00000000	pigeons
Pnrc2	-0.02	0.00000000	pigeons
Hikeshi	-0.04	0.00000000	pigeons
Psmb2	-0.02	0.00000000	pigeons
Itgb1bp1	0.10	0.00000000	pigeons
Spen	0.07	0.00000000	pigeons
Snap47	0.16	0.00000000	pigeons
Faap100	0.09	0.00000000	pigeons
Nfs1	0.10	0.00000000	pigeons
Ppp1r27	0.09	0.00000000	pigeons
Rsl24d1	-0.06	0.00000000	pigeons
Fam120a	0.02	0.00000000	pigeons
Ndufa8	-0.03	0.00000000	pigeons
Satb1	0.15	0.00000000	pigeons
Tgfbr2	-1.25	0.00000000	pigeons
Ing4	0.01	0.00000000	pigeons
Supt4a	0.00	0.00000000	pigeons
Cenpt	0.11	0.00000000	pigeons
Gas7	0.05	0.00000000	pigeons
Rbm39	0.09	0.00000000	pigeons
Riok3	-0.10	0.00000000	pigeons
Cops7a	-0.12	0.00000000	pigeons
L3mbtl3	-0.06	0.00000000	pigeons
Lrba	-1.16	0.00000000	pigeons
Park7	-0.02	0.00000000	pigeons
Ccnh	0.20	0.00000000	pigeons
Tbc1d5	0.13	0.00000000	pigeons
Cog2	0.00	0.00000000	pigeons
Tnrc6a	-0.14	0.00000000	pigeons

Yipf3	-0.01	0.00000000	pigeons
Unc119b	-0.06	0.00000000	pigeons
Ptbp2	0.05	0.00000000	pigeons
Polr2f	0.04	0.00000000	pigeons
Pafah1b2	-0.08	0.00000000	pigeons
Arih1	-0.19	0.00000000	pigeons
Tm9sf2	-0.18	0.00000000	pigeons
Brd7	-0.01	0.00000000	pigeons
Tbxas1	0.11	0.00000000	pigeons
Tor2a	0.10	0.00000000	pigeons
Ss18	-0.03	0.00000000	pigeons
Ripor1	-1.21	0.00000000	pigeons
Ddx1	-0.08	0.00000000	pigeons
Atrx	-0.08	0.00000000	pigeons
Khdrbs3	-0.99	0.00000000	pigeons
Cln5	0.11	0.00000000	pigeons
Ciapin1	-0.03	0.00000000	pigeons
Fam114a2	0.03	0.00000000	pigeons
Eml1	-0.87	0.00000000	pigeons
Actr1a	-0.16	0.00000000	pigeons
Rfc1	-0.09	0.00000000	pigeons
Clcn3	0.09	0.00000000	pigeons
Dytn	0.37	0.00000000	pigeons
Clk2	0.10	0.00000000	pigeons
Tm9sf4	0.05	0.00000000	pigeons
Rprd2	-0.01	0.00000000	pigeons
Ythdf1	-0.09	0.00000000	pigeons
Ppp1ca	-0.03	0.00000000	pigeons
Tfpi	-1.27	0.00000000	pigeons
Sub1	-0.07	0.00000000	pigeons
Yipf5	0.04	0.00000000	pigeons
Sntb1	0.30	0.00000000	pigeons
1700034I23Rik	0.02	0.00000000	pigeons
Poldip2	0.01	0.00000000	pigeons
Cds2	-0.04	0.00000000	pigeons
Csnk1e	-0.10	0.00000000	pigeons
Iqsec1	0.14	0.00000000	pigeons
Rpap3	0.03	0.00000000	pigeons
Pigf	0.14	0.00000000	pigeons
Bicral	-0.03	0.00000000	pigeons
Tpr	-0.13	0.00000000	pigeons
Nab1	0.08	0.00000000	pigeons
Tubb6	-1.31	0.00000000	pigeons
Rpn1	-0.15	0.00000000	pigeons
Rnf114	-0.04	0.00000000	pigeons
Fkbp3	-0.04	0.00000000	pigeons

Ncoa5	-0.02	0.00000000	pigeons
Ndufs2	-0.10	0.00000000	pigeons
Atp23	0.02	0.00000000	pigeons
Elmo1	0.42	0.00000000	pigeons
Gstcd	0.12	0.00000000	pigeons
Rabgap1l	0.21	0.00000000	pigeons
Stk4	0.10	0.00000000	pigeons
Fbxo25	0.07	0.00000000	pigeons
Scyl2	-0.11	0.00000000	pigeons
Ywhaq	0.01	0.00000000	pigeons
Armt1	0.08	0.00000000	pigeons
Pofut1	0.05	0.00000000	pigeons
Asxl2	-0.09	0.00000000	pigeons
Rabepk	-0.09	0.00000000	pigeons
Tom1	0.17	0.00000000	pigeons
Ndufa10	-0.04	0.00000000	pigeons
Usp10	0.02	0.00000000	pigeons
Invs	0.03	0.00000000	pigeons
Ipo8	0.03	0.00000000	pigeons
Ube4a	0.04	0.00000000	pigeons
Eif3l	-0.02	0.00000000	pigeons
Hax1	0.03	0.00000000	pigeons
Herpud1	-0.01	0.00000000	pigeons
Fem1a	-0.05	0.00000000	pigeons
Tmcc1	-0.02	0.00000000	pigeons
Erh	0.11	0.00000000	pigeons
Polr2d	0.02	0.00000000	pigeons
Puf60	-0.05	0.00000000	pigeons
R3hdm1	0.02	0.00000000	pigeons
Dgkz	0.10	0.00000000	pigeons
Ipo11	0.15	0.00000000	pigeons
Snx12	0.01	0.00000000	pigeons
Golph3l	0.09	0.00000000	pigeons
Ero1a	0.11	0.00000000	pigeons
Usp54	-1.23	0.00000000	pigeons
Dcaf13	0.04	0.00000000	pigeons
Eif4a3l1	0.03	0.00000000	pigeons
Colgalt2	-1.16	0.00000000	pigeons
Zfp507	0.11	0.00000000	pigeons
Fam185a	0.12	0.00000000	pigeons
Ttc39a	0.25	0.00000000	pigeons
Atp6v0a2	0.03	0.00000000	pigeons
Ptger4	0.41	0.00000000	pigeons
Shoc2	-0.04	0.00000000	pigeons
Alg12	0.01	0.00000000	pigeons
Snx19	-0.01	0.00000000	pigeons

Snhg4	-0.12	0.00000000	pigeons
Eif4e	-0.11	0.00000000	pigeons
Srgap3	0.12	0.00000000	pigeons
Psmd6	0.03	0.00000000	pigeons
Fkbp15	-0.01	0.00000000	pigeons
Tspan32	0.26	0.00000000	pigeons
Npnt	0.20	0.00000000	pigeons
Vps41	0.03	0.00000000	pigeons
Vamp4	0.07	0.00000000	pigeons
Caprin1	-0.12	0.00000000	pigeons
Creg1	-1.18	0.00000000	pigeons
Sp4	0.13	0.00000000	pigeons
Pdcd5	0.11	0.00000000	pigeons
Bclaf1	-0.14	0.00000000	pigeons
Tacc3	-0.08	0.00000000	pigeons
Epc1	0.04	0.00000000	pigeons
Kras	-0.06	0.00000000	pigeons
Wnk1	0.19	0.00000000	pigeons
Xylt1	0.07	0.00000000	pigeons
Bhlhe40	0.31	0.00000000	pigeons
Serac1	0.09	0.00000000	pigeons
Psmb3	0.11	0.00000000	pigeons
Srsf2	-0.13	0.00000000	pigeons
Eif2s2	0.06	0.00000000	pigeons
Kat2b	-0.11	0.00000000	pigeons
Klhl2	0.20	0.00000000	pigeons
Iars2	-0.01	0.00000000	pigeons
Cep170	-0.02	0.00000000	pigeons
Gnb5	-1.10	0.00000000	pigeons
Cnih4	0.02	0.00000000	pigeons
Ppil2	0.09	0.00000000	pigeons
Spag9	-0.10	0.00000000	pigeons
Arf6	-0.06	0.00000000	pigeons
Dnmt3a	-0.04	0.00000000	pigeons
Rbm15	0.06	0.00000000	pigeons
Kdelr2	-0.09	0.00000000	pigeons
Rnf19a	0.01	0.00000000	pigeons
Tmem11	0.11	0.00000000	pigeons
Ext2	-0.06	0.00000000	pigeons
Ints6	-1.47	0.00000000	pigeons
Rpn2	-0.10	0.00000000	pigeons
Prex1	0.05	0.00000000	pigeons
Lrrc57	0.11	0.00000000	pigeons
Atad2b	-0.04	0.00000000	pigeons
Tmem128	0.07	0.00000000	pigeons
Ptges2	0.00	0.00000000	pigeons

Smc6	0.01	0.00000000	pigeons
Sec24b	-0.03	0.00000000	pigeons
Suds3	-0.18	0.00000000	pigeons
Tlk1	-0.06	0.00000000	pigeons
Spty2d1	-0.81	0.00000000	pigeons
Ap1m1	0.02	0.00000000	pigeons
Zcchc7	0.18	0.00000000	pigeons
Ssr2	0.11	0.00000000	pigeons
Tbc1d22b	0.06	0.00000000	pigeons
Tmem245	0.14	0.00000000	pigeons
Lamp2	-0.18	0.00000000	pigeons
Ranbp17	-1.08	0.00000000	pigeons
Haus2	0.11	0.00000000	pigeons
Szrd1	0.08	0.00000000	pigeons
Slc25a3	-0.17	0.00000000	pigeons
Amd2	-0.01	0.00000000	pigeons
Agl	0.00	0.00000000	pigeons
Tmco1	-0.07	0.00000000	pigeons
Ypel2	0.08	0.00000000	pigeons
Ftsj3	0.02	0.00000000	pigeons
Stx16	0.06	0.00000000	pigeons
Yod1	0.01	0.00000000	pigeons
Fxyd6	-1.16	0.00000000	pigeons
Pabpc4	-0.02	0.00000000	pigeons
Stx12	-0.16	0.00000000	pigeons
Bcas2	0.00	0.00000000	pigeons
Zmynd8	-0.02	0.00000000	pigeons
Cxcr4	1.16	0.00000000	pigeons
Clip1	-0.05	0.00000000	pigeons
Abr	0.09	0.00000000	pigeons
Tmem39a	0.03	0.00000000	pigeons
Slc39a6	0.04	0.00000000	pigeons
Prkar1a	-0.15	0.00000000	pigeons
Smu1	-0.17	0.00000000	pigeons
Sin3b	0.05	0.00000000	pigeons
Phb	-0.01	0.00000000	pigeons
Def6	0.07	0.00000000	pigeons
Urm1	-0.10	0.00000000	pigeons
Utp23	0.06	0.00000000	pigeons
Rbm22	-0.19	0.00000000	pigeons
Usp24	-0.10	0.00000000	pigeons
Xpo4	0.10	0.00000000	pigeons
Pdcl3	-0.07	0.00000000	pigeons
Nacc2	0.13	0.00000000	pigeons
Cep83	-1.42	0.00000000	pigeons
Fam3c	-0.01	0.00000000	pigeons

Lyrm1	0.07	0.00000000	pigeons
Tmem35b	-0.08	0.00000000	pigeons
Hoxa5	-1.16	0.00000000	pigeons
Zmat2	0.11	0.00000000	pigeons
Polr2l	-0.14	0.00000000	pigeons
Itfg1	-0.08	0.00000000	pigeons
Rmnd5a	-0.11	0.00000000	pigeons
Cep350	-0.10	0.00000000	pigeons
Cstf2	0.01	0.00000000	pigeons
Pxk	-0.09	0.00000000	pigeons
Eif3j2	-0.23	0.00000000	pigeons
Aimp1	-0.10	0.00000000	pigeons
Psmd13	-0.15	0.00000000	pigeons
Surf2	0.04	0.00000000	pigeons
Cul5	-0.03	0.00000000	pigeons
Tmem168	-0.01	0.00000000	pigeons
Cd83	0.40	0.00000000	pigeons
Fubp3	-0.13	0.00000000	pigeons
Selenot	-0.14	0.00000000	pigeons
Ube2g1	-0.24	0.00000000	pigeons
B3gntl1	0.15	0.00000000	pigeons
Phb2	-0.12	0.00000000	pigeons
Eps8	-0.94	0.00000000	pigeons
Negr1	0.46	0.00000000	pigeons
Nsfl1c	-0.01	0.00000000	pigeons
Slc16a10	0.54	0.00000000	pigeons
Ift20	0.02	0.00000000	pigeons
Usp28	-1.11	0.00000000	pigeons
Zhx1	-0.11	0.00000000	pigeons
Ndufb8	-0.02	0.00000000	pigeons
Prrc1	0.06	0.00000000	pigeons
Akap10	-0.05	0.00000000	pigeons
Camk2g	-0.07	0.00000000	pigeons
Hadha	0.04	0.00000000	pigeons
Cbll1	-0.02	0.00000000	pigeons
Plk2	-1.00	0.00000000	pigeons
Rabl6	-0.06	0.00000000	pigeons
Abcd3	0.05	0.00000000	pigeons
Ppt1	-0.05	0.00000000	pigeons
Kdelr3	0.08	0.00000000	pigeons
Zfp410	0.07	0.00000000	pigeons
Dnajb2	-0.02	0.00000000	pigeons
Rpp30	0.06	0.00000000	pigeons
Hadh	-0.06	0.00000000	pigeons
Gpx4	-0.10	0.00000000	pigeons
Rmdn3	-0.06	0.00000000	pigeons

Chaf1a	0.02	0.00000000	pigeons
Zfp830	-0.03	0.00000000	pigeons
Tprgl	-0.02	0.00000000	pigeons
Katnbl1	0.01	0.00000000	pigeons
Orai1	0.18	0.00000000	pigeons
Gtpbp10	0.05	0.00000000	pigeons
Dcp2	-0.01	0.00000000	pigeons
Tada1	-0.08	0.00000000	pigeons
Amn1	0.12	0.00000000	pigeons
Seh1l	-0.12	0.00000000	pigeons
Nop16	0.05	0.00000000	pigeons
Hipk1	-0.22	0.00000000	pigeons
Cops4	-0.01	0.00000000	pigeons
Psmd12	-0.08	0.00000000	pigeons
Per2	0.19	0.00000000	pigeons
Polk	-0.10	0.00000000	pigeons
Vps13d	0.01	0.00000000	pigeons
Tsr3	0.06	0.00000000	pigeons
Sel1l	-0.09	0.00000000	pigeons
Fam199x	0.05	0.00000000	pigeons
Dcp1a	-0.04	0.00000000	pigeons
Smurf1	0.06	0.00000000	pigeons
Cdc16	0.02	0.00000000	pigeons
Aco1	0.05	0.00000000	pigeons
Ppp1r12a	0.19	0.00000000	pigeons
Zbtb48	0.09	0.00000000	pigeons
Hps3	0.02	0.00000000	pigeons
Sdhd	-0.16	0.00000000	pigeons
Adam19	0.06	0.00000000	pigeons
Dffa	0.07	0.00000000	pigeons
Ino80d	-0.12	0.00000000	pigeons
Ctnna1	-0.03	0.00000000	pigeons
Ufl1	-0.13	0.00000000	pigeons
Tln1	-0.29	0.00000000	pigeons
Ddb1	-0.05	0.00000000	pigeons
Ikbkb	-0.07	0.00000000	pigeons
Impa1	0.01	0.00000000	pigeons
Wac	-0.16	0.00000000	pigeons
Tmem208	-0.11	0.00000000	pigeons
Rnf4	-0.04	0.00000000	pigeons
Rab5c	-0.20	0.00000000	pigeons
Gpatch2l	-0.07	0.00000000	pigeons
Gosr1	-0.07	0.00000000	pigeons
Nhp2	-0.04	0.00000000	pigeons
Pyroxd1	0.01	0.00000000	pigeons
Cd63	-0.12	0.00000000	pigeons

Srpk1	-0.17	0.00000000	pigeons
Hsf2	-0.03	0.00000000	pigeons
Avl9	0.06	0.00000000	pigeons
Trappc2	0.03	0.00000000	pigeons
Nudt16l1	-0.01	0.00000000	pigeons
Dtx3l	-0.12	0.00000000	pigeons
Tmem192	0.09	0.00000000	pigeons
Paxbp1	-0.18	0.00000000	pigeons
Pigl	0.24	0.00000000	pigeons
Rbm18	-0.13	0.00000000	pigeons
Parg	-0.12	0.00000000	pigeons
Fgd6	-1.07	0.00000000	pigeons
Ica1	-1.03	0.00000000	pigeons
Aebp2	0.18	0.00000000	pigeons
Slc35b1	-0.05	0.00000000	pigeons
Psmc6	-0.15	0.00000000	pigeons
Ssr1	-0.15	0.00000000	pigeons
Ppp2r5c	-0.02	0.00000000	pigeons
Nipsnap2	-0.06	0.00000000	pigeons
Pisd	-0.09	0.00000000	pigeons
Atxn1l	0.01	0.00000000	pigeons
Arl8b	-0.17	0.00000000	pigeons
Snx13	-0.07	0.00000000	pigeons
Eif4g3	0.07	0.00000000	pigeons
Tbc1d12	-0.12	0.00000000	pigeons
Polr2e	-0.16	0.00000000	pigeons
Stx17	-0.23	0.00000000	pigeons
Tomm70a	-0.18	0.00000000	pigeons
Atp9a	0.12	0.00000000	pigeons
Ublcp1	-0.09	0.00000000	pigeons
Rmnd1	0.09	0.00000000	pigeons
Socs4	0.07	0.00000000	pigeons
Pin4	-0.06	0.00000000	pigeons
Cdk8	0.06	0.00000000	pigeons
Pnpt1	0.01	0.00000000	pigeons
Slc41a1	0.02	0.00000000	pigeons
Fam8a1	0.00	0.00000000	pigeons
Prkrip1	-0.08	0.00000000	pigeons
Parvb	-0.45	0.00000000	pigeons
Polr2k	-0.07	0.00000000	pigeons
Crebl2	0.03	0.00000000	pigeons
Dstn	0.25	0.00000000	pigeons
Zbtb24	0.10	0.00000000	pigeons
Luc7l3	-0.18	0.00000000	pigeons
Ints4	0.01	0.00000000	pigeons
Trappc8	-0.10	0.00000000	pigeons

Smpd2	-0.06	0.00000000	pigeons
Ttbk1	0.14	0.00000000	pigeons
Samd8	0.08	0.00000000	pigeons
Ndufb6	-0.10	0.00000000	pigeons
Ccdc6	-0.06	0.00000000	pigeons
Ipo7	-0.28	0.00000000	pigeons
Gphn	-1.03	0.00000000	pigeons
Stim2	-1.31	0.00000000	pigeons
Txndc17	-0.22	0.00000000	pigeons
Aprt	0.09	0.00000000	pigeons
Zranb2	-0.15	0.00000000	pigeons
Nudc	-0.09	0.00000000	pigeons
Pdss1	0.05	0.00000000	pigeons
Kctd20	0.23	0.00000000	pigeons
Ube2g2	-0.11	0.00000000	pigeons
Nsmaf	0.06	0.00000000	pigeons
Psmd4	-0.05	0.00000000	pigeons
Larp4b	-0.20	0.00000000	pigeons
Lats1	-0.18	0.00000000	pigeons
Psma4	-0.05	0.00000000	pigeons
Lonrf1	0.00	0.00000000	pigeons
Rbm15b	-0.10	0.00000000	pigeons
Stoml2	0.01	0.00000000	pigeons
Hspa5	-0.82	0.00000000	pigeons
Ascc3	0.16	0.00000000	pigeons
Bcap29	-0.22	0.00000000	pigeons
Ankrd17	0.07	0.00000000	pigeons
Aimp2	-0.08	0.00000000	pigeons
Samhd1	-0.27	0.00000000	pigeons
Ttll12	0.03	0.00000000	pigeons
Tmf1	-0.15	0.00000000	pigeons
Cd81	-0.93	0.00000000	pigeons
Arv1	0.05	0.00000000	pigeons
Jarid2	-0.46	0.00000000	pigeons
Cpsf4	-0.02	0.00000000	pigeons
Gpatch1	-0.13	0.00000000	pigeons
Rnpc3	-0.03	0.00000000	pigeons
Sec13	-0.04	0.00000000	pigeons
Ubqln4	-0.11	0.00000000	pigeons
Arfgap2	-0.02	0.00000000	pigeons
Eny2	-0.12	0.00000000	pigeons
Pla2r1	-0.06	0.00000000	pigeons
Rsl1d1	-0.06	0.00000000	pigeons
Camk2d	-1.01	0.00000000	pigeons
Rnaseh2b	-0.13	0.00000000	pigeons
Kifap3	-0.03	0.00000000	pigeons

Ppp6r3	0.00	0.00000000	pigeons
Ghdc	0.06	0.00000000	pigeons
Ndufa2	-0.11	0.00000000	pigeons
Polr2c	-0.06	0.00000000	pigeons
Adrm1	-0.14	0.00000000	pigeons
Iws1	-0.28	0.00000000	pigeons
Kctd9	0.04	0.00000000	pigeons
Arl5a	-0.13	0.00000000	pigeons
Pus10	-0.08	0.00000000	pigeons
Ankrd10	-0.50	0.00000000	pigeons
Hsdl2	0.10	0.00000000	pigeons
Zswim7	-0.09	0.00000000	pigeons
Fmnl2	-1.25	0.00000000	pigeons
Tmem242	-0.20	0.00000000	pigeons
Clp1	0.05	0.00000000	pigeons
Emc7	-0.18	0.00000000	pigeons
Actr6	-0.03	0.00000000	pigeons
Drg2	0.02	0.00000000	pigeons
Lrpap1	-0.09	0.00000000	pigeons
Ttc1	-0.06	0.00000000	pigeons
Zmpste24	-0.05	0.00000000	pigeons
Erp29	0.15	0.00000000	pigeons
Nr4a3	0.38	0.00000000	pigeons
Taf5	0.04	0.00000000	pigeons
Inpp5a	0.39	0.00000000	pigeons
Capn11	-0.09	0.00000000	pigeons
Slu7	-0.23	0.00000000	pigeons
Rwdd4a	-0.04	0.00000000	pigeons
Sbds	-0.04	0.00000000	pigeons
Cdk7	-0.02	0.00000000	pigeons
Vezt	0.02	0.00000000	pigeons
Rpa3	0.03	0.00000000	pigeons
Baz1b	-0.17	0.00000000	pigeons
Rbm45	0.00	0.00000000	pigeons
Letm1	-0.13	0.00000000	pigeons
Taf13	-0.22	0.00000000	pigeons
Taf15	0.00	0.00000000	pigeons
Gosr2	-0.07	0.00000000	pigeons
Fer	0.09	0.00000000	pigeons
Ipo5	-0.14	0.00000000	pigeons
Rbm12b2	0.00	0.00000000	pigeons
Lemd2	-0.05	0.00000000	pigeons
Eif2a	0.02	0.00000000	pigeons
Ndufa13	-0.08	0.00000000	pigeons
Prrc2b	-0.17	0.00000000	pigeons
Znhit6	-0.09	0.00000000	pigeons

Actg1	0.36	0.00000000	pigeons
Rcbtb1	-0.01	0.00000000	pigeons
Edc3	-0.06	0.00000000	pigeons
Arhgdia	-0.02	0.00000000	pigeons
Glipr2	-0.11	0.00000000	pigeons
Tango6	0.05	0.00000000	pigeons
Cops3	-0.23	0.00000000	pigeons
Nxt2	0.01	0.00000000	pigeons
Hat1	-0.06	0.00000000	pigeons
Tpd52l2	-0.20	0.00000000	pigeons
Acer2	-0.99	0.00000000	pigeons
Shtn1	-0.73	0.00000000	pigeons
Cdv3	-0.26	0.00000000	pigeons
Carhsp1	-0.11	0.00000000	pigeons
Gemin4	-0.01	0.00000000	pigeons
Nup93	-0.04	0.00000000	pigeons
Flot2	0.00	0.00000000	pigeons
Rrp1b	-0.08	0.00000000	pigeons
Ndufs8	-0.16	0.00000000	pigeons
Ncor2	-0.13	0.00000000	pigeons
Bcl6	0.11	0.00000000	pigeons
Zfp609	-0.01	0.00000000	pigeons
Bdp1	-0.11	0.00000000	pigeons
Tatdn1	0.10	0.00000000	pigeons
Vezf1	-0.18	0.00000000	pigeons
Heatr6	0.07	0.00000000	pigeons
Rab7b	-0.02	0.00000000	pigeons
Pwwp2a	-0.13	0.00000000	pigeons
Srrm1	-0.13	0.00000000	pigeons
Rbfa	-0.99	0.00000000	pigeons
Ints10	0.06	0.00000000	pigeons
Sf3b6	-0.21	0.00000000	pigeons
Ndufb3	-0.12	0.00000000	pigeons
Gtf2h5	-0.21	0.00000000	pigeons
Ubac2	0.02	0.00000000	pigeons
Kdm3b	-0.24	0.00000000	pigeons
Chic2	-0.12	0.00000000	pigeons
Bnip2	-0.26	0.00000000	pigeons
Prkaa1	-0.06	0.00000000	pigeons
E2f6	-0.06	0.00000000	pigeons
Akr1a1	-0.03	0.00000000	pigeons
Tnks2	-0.16	0.00000000	pigeons
Glt1d1	0.10	0.00000000	pigeons
Noc2l	-0.09	0.00000000	pigeons
Ppme1	0.03	0.00000000	pigeons
Rpa2	-0.05	0.00000000	pigeons

Xpr1	-0.02	0.00000000	pigeons
Suclg2	0.11	0.00000000	pigeons
Prpf38a	0.02	0.00000000	pigeons
Cfdp1	-0.09	0.00000000	pigeons
Rtf1	-0.20	0.00000000	pigeons
Hectd4	-0.18	0.00000000	pigeons
Kpna3	-0.25	0.00000000	pigeons
9130409I23Rik	0.01	0.00000000	pigeons
Cep44	-0.06	0.00000000	pigeons
Hdac1	0.02	0.00000000	pigeons
Cops2	-0.11	0.00000000	pigeons
Itgav	-0.12	0.00000000	pigeons
Auh	0.06	0.00000000	pigeons
Carmil1	-0.97	0.00000000	pigeons
Crebrf	-0.13	0.00000000	pigeons
Blvra	0.15	0.00000000	pigeons
Psen1	-0.22	0.00000000	pigeons
Acss2	0.05	0.00000000	pigeons
Cog7	-0.04	0.00000000	pigeons
Gk5	-1.23	0.00000000	pigeons
Stx2	-0.12	0.00000000	pigeons
4933427D14Rik	0.04	0.00000000	pigeons
Zfp592	-0.10	0.00000000	pigeons
Ube2i	-0.14	0.00000000	pigeons
Zbtb2	-0.26	0.00000000	pigeons
Nifk	-0.18	0.00000000	pigeons
Klhl36	0.02	0.00000000	pigeons
St7l	-0.07	0.00000000	pigeons
Ubr1	-0.19	0.00000000	pigeons
Cir1	-0.03	0.00000000	pigeons
Rnf128	0.13	0.00000000	pigeons
Gorasp2	-0.02	0.00000000	pigeons
Kbtbd2	-0.11	0.00000001	pigeons
Xrn1	-0.30	0.00000001	pigeons
Naa50	-0.15	0.00000001	pigeons
Acad9	-0.18	0.00000001	pigeons
Rab27a	-0.21	0.00000001	pigeons
Abcd2	-0.22	0.00000001	pigeons
B4galt1	-0.02	0.00000001	pigeons
Zbtb33	-0.07	0.00000001	pigeons
Eef2	-0.06	0.00000001	pigeons
Kpna1	-0.19	0.00000001	pigeons
Ddx52	-0.01	0.00000001	pigeons
Nkrf	-0.04	0.00000001	pigeons
Prr5	0.37	0.00000001	pigeons
Slc7a5	-0.08	0.00000001	pigeons

Ppp1r7	-0.05	0.00000001	pigeons
Xpo5	-0.01	0.00000001	pigeons
Abcb7	-0.03	0.00000001	pigeons
Aco2	-0.15	0.00000001	pigeons
Dtnbp1	-0.05	0.00000001	pigeons
Rnasel	0.13	0.00000001	pigeons
Zfp276	-0.09	0.00000001	pigeons
Trmt10c	0.09	0.00000001	pigeons
Insig2	0.04	0.00000001	pigeons
Cacna2d2	-0.08	0.00000001	pigeons
Srsf3	-0.19	0.00000001	pigeons
Fbxw8	-0.03	0.00000001	pigeons
Dip2c	-0.93	0.00000001	pigeons
Smndc1	-0.30	0.00000001	pigeons
Dpy30	0.02	0.00000001	pigeons
Nsl1	-0.08	0.00000001	pigeons
Zzef1	-0.16	0.00000001	pigeons
Reps1	-0.23	0.00000001	pigeons
Atrn	-1.15	0.00000001	pigeons
Sec31b	-0.04	0.00000001	pigeons
Nudcd3	-0.09	0.00000001	pigeons
Fktn	-0.07	0.00000001	pigeons
Slc20a1	0.05	0.00000002	pigeons
Ruvbl1	-0.09	0.00000002	pigeons
Dnajc10	0.06	0.00000002	pigeons
Uchl5	-0.08	0.00000002	pigeons
Vwf	0.33	0.00000002	pigeons
Naa30	-0.18	0.00000002	pigeons
Tcof1	-0.18	0.00000002	pigeons
Unc119	-0.06	0.00000002	pigeons
Angel1	0.07	0.00000002	pigeons
Ube2b	-0.13	0.00000002	pigeons
Sdhaf4	0.01	0.00000002	pigeons
Synj1	-0.21	0.00000002	pigeons
Sipa1l2	0.27	0.00000002	pigeons
Tgfbrap1	-0.04	0.00000002	pigeons
Cct7	-0.12	0.00000002	pigeons
Ankrd28	0.10	0.00000002	pigeons
Cebpz	-0.08	0.00000003	pigeons
Pcyt1a	-0.02	0.00000003	pigeons
Trappc4	-0.03	0.00000003	pigeons
Gramd4	-0.06	0.00000003	pigeons
Armc8	-0.15	0.00000003	pigeons
St3gal6	-0.22	0.00000003	pigeons
Bloc1s2	0.01	0.00000003	pigeons
Ciao1	-0.02	0.00000003	pigeons

Htra2	-0.03	0.00000003	pigeons
Ypel1	-1.12	0.00000003	pigeons
Ikzf3	0.17	0.00000003	pigeons
Psen2	-0.01	0.00000003	pigeons
Ssb	-0.10	0.00000003	pigeons
Aurkaip1	-0.07	0.00000003	pigeons
Lrrc1	-0.02	0.00000003	pigeons
Cant1	-0.10	0.00000003	pigeons
Hspa9	-0.09	0.00000003	pigeons
Fem1b	-0.07	0.00000003	pigeons
Commd1b	-0.11	0.00000003	pigeons
Top1	-0.12	0.00000003	pigeons
Tbc1d2b	-0.11	0.00000004	pigeons
Ext1	-0.63	0.00000004	pigeons
Arfrp1	-0.02	0.00000004	pigeons
Ints1	-0.04	0.00000004	pigeons
Pitrm1	0.00	0.00000004	pigeons
Shroom4	-0.92	0.00000004	pigeons
Yipf1	-0.08	0.00000004	pigeons
Ctcf	-0.26	0.00000004	pigeons
Rsrc2	-0.21	0.00000004	pigeons
Tnfrsf18	0.56	0.00000004	pigeons
Ppie	-0.09	0.00000004	pigeons
Naa25	-0.03	0.00000005	pigeons
Phf21a	0.09	0.00000005	pigeons
Fbxo9	-0.08	0.00000005	pigeons
Erc1	-0.99	0.00000005	pigeons
Alg2	0.00	0.00000005	pigeons
Yaf2	-0.10	0.00000005	pigeons
Desi1	-0.01	0.00000005	pigeons
Ccpg1	-0.24	0.00000006	pigeons
Atf6	-0.27	0.00000006	pigeons
Sde2	-0.27	0.00000006	pigeons
Trim25	-0.06	0.00000006	pigeons
Fbxo11	-0.13	0.00000006	pigeons
Fam13b	-0.24	0.00000006	pigeons
Anks1	-1.04	0.00000006	pigeons
Cdc73	-0.17	0.00000006	pigeons
Ank2	-0.91	0.00000006	pigeons
Ndufv2	0.04	0.00000006	pigeons
Rpap1	0.07	0.00000007	pigeons
Ddx31	-0.04	0.00000007	pigeons
Aven	0.14	0.00000007	pigeons
Emp1	-0.74	0.00000007	pigeons
Usp22	-1.19	0.00000007	pigeons
Rab30	0.22	0.00000007	pigeons

Sdc4	-0.41	0.00000007	pigeons
Prdx3	0.00	0.00000007	pigeons
Rnf20	-0.21	0.00000008	pigeons
Trp53bp2	-1.09	0.00000008	pigeons
Ppp2ca	-0.14	0.00000008	pigeons
Cep85l	-0.15	0.00000008	pigeons
Dck	0.00	0.00000008	pigeons
Fbxl3	0.01	0.00000009	pigeons
Acat1	-0.11	0.00000009	pigeons
Stxbp6	-0.88	0.00000009	pigeons
Golga1	-0.07	0.00000009	pigeons
Nudt21	-0.08	0.00000010	pigeons
Sgpl1	-0.08	0.00000010	pigeons
Fam214a	-0.22	0.00000010	pigeons
Rab7	-0.19	0.00000010	pigeons
Pfdn4	-0.07	0.00000010	pigeons
Etnk1	-0.15	0.00000010	pigeons
Aff1	-0.53	0.00000011	pigeons
Exoc4	-0.05	0.00000012	pigeons
Dnaja3	-0.03	0.00000012	pigeons
Arpp19	-0.14	0.00000012	pigeons
Smurf2	-1.11	0.00000012	pigeons
Impdh2	-0.07	0.00000013	pigeons
Sacs	0.01	0.00000013	pigeons
Usp6nl	-0.28	0.00000013	pigeons
Gga3	-0.08	0.00000013	pigeons
Rufy3	-0.11	0.00000014	pigeons
Prpf4b	-0.27	0.00000014	pigeons
Cnot4	-0.15	0.00000014	pigeons
Sptlc1	-0.02	0.00000015	pigeons
Zfp469	0.22	0.00000015	pigeons
Slc25a36	-0.11	0.00000015	pigeons
Psip1	-0.18	0.00000015	pigeons
Ranbp9	-0.24	0.00000015	pigeons
Dvl1	-0.05	0.00000015	pigeons
Pank4	-0.02	0.00000016	pigeons
Exoc5	-0.24	0.00000016	pigeons
Dera	-0.01	0.00000017	pigeons
Lrig2	-0.11	0.00000017	pigeons
Rsrc1	-0.15	0.00000017	pigeons
Ufsp2	-0.10	0.00000017	pigeons
Zfp143	-0.13	0.00000017	pigeons
Nusap1	-0.32	0.00000017	pigeons
Prdm10	0.00	0.00000018	pigeons
Slain2	-0.15	0.00000018	pigeons
Thyn1	0.13	0.00000018	pigeons

Atxn7	-0.10	0.00000019	pigeons
Sft2d1	-0.27	0.00000019	pigeons
Fbxl14	-0.23	0.00000020	pigeons
Ifi35	-0.24	0.00000020	pigeons
Zfp330	0.01	0.00000021	pigeons
Dusp15	-0.01	0.00000021	pigeons
Washc3	-0.11	0.00000022	pigeons
Naxd	-0.16	0.00000023	pigeons
Ddx42	-0.23	0.00000023	pigeons
Creld2	0.21	0.00000023	pigeons
Slx4	-0.08	0.00000024	pigeons
Pdcd4	-0.08	0.00000025	pigeons
Gnl3	-0.03	0.00000026	pigeons
Zdhhc6	-0.30	0.00000027	pigeons
Slc43a2	0.03	0.00000028	pigeons
Fam32a	-0.30	0.00000028	pigeons
Gng2	-0.07	0.00000028	pigeons
Nbas	-0.17	0.00000029	pigeons
Ddx28	0.03	0.00000030	pigeons
Acaa2	-0.10	0.00000030	pigeons
Tmem183a	-0.17	0.00000030	pigeons
Tomm20	-0.19	0.00000031	pigeons
Rnf130	-0.15	0.00000031	pigeons
Dmap1	-1.09	0.00000033	pigeons
Nek9	-0.10	0.00000034	pigeons
Fbxo33	-0.24	0.00000035	pigeons
Acot10	-0.01	0.00000035	pigeons
Nans	-0.16	0.00000036	pigeons
Zfp598	-0.15	0.00000036	pigeons
Coq9	0.05	0.00000037	pigeons
Nol10	-0.08	0.00000038	pigeons
Arhgef6	-0.49	0.00000042	pigeons
Ranbp1	-0.25	0.00000043	pigeons
Ing3	-0.03	0.00000045	pigeons
Pi4ka	-0.28	0.00000046	pigeons
Snrpb2	-0.21	0.00000047	pigeons
Idh3a	-0.06	0.00000048	pigeons
Guk1	0.01	0.00000048	pigeons
Tpk1	-1.05	0.00000051	pigeons
Wdr91	-0.06	0.00000051	pigeons
Cpeb3	0.07	0.00000051	pigeons
Dnajc17	-0.02	0.00000052	pigeons
Ctnnbl1	-0.04	0.00000058	pigeons
Kpna4	-0.25	0.00000059	pigeons
Limk2	0.16	0.00000060	pigeons
Kcnma1	-1.18	0.00000061	pigeons

Eif1	0.21	0.00000064	pigeons
Surf6	-0.08	0.00000065	pigeons
Ap1ar	0.00	0.00000071	pigeons
Pogz	-0.01	0.00000072	pigeons
Zfyve26	-0.10	0.00000076	pigeons
Pno1	-0.05	0.00000079	pigeons
Eea1	-0.21	0.00000081	pigeons
Txndc12	-0.12	0.00000087	pigeons
Heatr5a	-0.22	0.00000088	pigeons
Ran	-0.18	0.00000090	pigeons
Vps11	-0.09	0.00000091	pigeons
Ccng1	-0.06	0.00000092	pigeons
Eif6	0.03	0.00000094	pigeons
Creb5	-0.56	0.00000094	pigeons
Pex7	-0.14	0.00000094	pigeons
Eef1b2	-0.32	0.00000097	pigeons
Ate1	-0.14	0.00000098	pigeons
St8sia4	0.01	0.00000103	pigeons
Pusl1	0.05	0.00000104	pigeons
Glo1	-0.26	0.00000104	pigeons
Dnajb9	-0.09	0.00000105	pigeons
Amdhd2	-0.14	0.00000107	pigeons
Lmo2	-0.96	0.00000108	pigeons
Ercc3	-0.13	0.00000108	pigeons
Phlpp2	-0.17	0.00000109	pigeons
Rnf141	-0.18	0.00000109	pigeons
Lzic	-0.10	0.00000110	pigeons
Ttc3	-0.03	0.00000112	pigeons
Tnfsf15	0.12	0.00000112	pigeons
Ddx46	-0.30	0.00000113	pigeons
Usf3	-0.02	0.00000117	pigeons
Golga5	-0.11	0.00000122	pigeons
Arid2	-0.08	0.00000123	pigeons
Dcun1d1	-0.12	0.00000124	pigeons
Dusp23	-0.08	0.00000125	pigeons
Ndufb10	-0.27	0.00000125	pigeons
Ints8	-0.08	0.00000131	pigeons
Pdia6	-0.03	0.00000137	pigeons
Aig1	-0.01	0.00000147	pigeons
Ndrg1	-0.28	0.00000150	pigeons
Ern1	-0.16	0.00000159	pigeons
Ppara	0.07	0.00000168	pigeons
Trmt11	-0.02	0.00000170	pigeons
Dhx36	-0.36	0.00000178	pigeons
Lin52	-0.05	0.00000182	pigeons
Txnrd3	-0.07	0.00000187	pigeons

Pign	0.09	0.00000193	pigeons
Jagn1	-0.11	0.00000199	pigeons
Taf3	-0.19	0.00000201	pigeons
Nup85	-0.09	0.00000203	pigeons
Gcn1	-0.21	0.00000204	pigeons
Pum2	-0.22	0.00000206	pigeons
Selenof	-0.29	0.00000223	pigeons
Eif2b1	-0.07	0.00000232	pigeons
Dym	-0.20	0.00000232	pigeons
Ube2q2	-0.27	0.00000237	pigeons
Gtf3c1	-0.03	0.00000269	pigeons
Ampd3	-0.05	0.00000275	pigeons
Pbrm1	-0.33	0.00000277	pigeons
Chmp5	-0.28	0.00000290	pigeons
Rprd1a	0.03	0.00000298	pigeons
Skp1	-0.09	0.00000299	pigeons
Tapt1	-0.24	0.00000308	pigeons
Pigb	-0.05	0.00000322	pigeons
Raph1	-1.04	0.00000323	pigeons
Ndufaf7	0.02	0.00000331	pigeons
Slc25a37	0.09	0.00000347	pigeons
Tcf25	-0.17	0.00000352	pigeons
Eif1b	0.06	0.00000358	pigeons
Ndufaf2	-0.01	0.00000379	pigeons
Ankrd49	-0.01	0.00000397	pigeons
Cbx6	-0.16	0.00000400	pigeons
Usp25	-0.19	0.00000400	pigeons
Thrap3	-0.25	0.00000403	pigeons
Usp45	-0.24	0.00000403	pigeons
Dync1li2	-0.32	0.00000404	pigeons
Ppp3ca	0.04	0.00000408	pigeons
Aplp2	-1.26	0.00000412	pigeons
Zbtb41	-0.17	0.00000417	pigeons
Slc39a8	0.06	0.00000422	pigeons
Fam126a	0.04	0.00000423	pigeons
Plxnb2	-0.71	0.00000429	pigeons
Atf4	-0.68	0.00000429	pigeons
Csnk1a1	0.02	0.00000434	pigeons
Pdxdc1	-0.10	0.00000446	pigeons
Scamp1	-0.04	0.00000465	pigeons
Hnrnpr	-0.21	0.00000467	pigeons
C2cd2l	-0.12	0.00000468	pigeons
Specc1l	-0.91	0.00000475	pigeons
Slc22a23	-0.94	0.00000500	pigeons
Eftud2	-0.11	0.00000506	pigeons
Klf3	-1.25	0.00000517	pigeons

Nutf2	-0.12	0.00000519	pigeons
Ugdh	-0.10	0.00000562	pigeons
AW554918	0.15	0.00000570	pigeons
Uap1	-0.06	0.00000590	pigeons
Rftn1	0.15	0.00000610	pigeons
Slc25a20	-0.13	0.00000612	pigeons
Appl1	-0.14	0.00000622	pigeons
Rnf145	-0.12	0.00000629	pigeons
Sart3	-0.12	0.00000650	pigeons
Psmf1	-0.15	0.00000669	pigeons
Gpatch2	-0.18	0.00000672	pigeons
Krr1	-0.22	0.00000695	pigeons
Hmbs	-0.14	0.00000697	pigeons
Dcaf5	-0.23	0.00000745	pigeons
Eri1	-0.12	0.00000771	pigeons
Dus1l	-0.05	0.00000772	pigeons
Htatsf1	-0.17	0.00000773	pigeons
Foxk1	-0.22	0.00000776	pigeons
Ctsk	-1.09	0.00000782	pigeons
Cygb	-0.84	0.00000799	pigeons
Fasn	-0.19	0.00000800	pigeons
Fam135a	-0.31	0.00000800	pigeons
Dpy19l4	-0.09	0.00000807	pigeons
Phkb	-0.14	0.00000822	pigeons
Glg1	-0.27	0.00000836	pigeons
Eif4a2	-0.45	0.00000883	pigeons
Tma16	-0.16	0.00000892	pigeons
Gtpbp1	-0.16	0.00000921	pigeons
Cwc27	-0.13	0.00000925	pigeons
Sav1	-0.11	0.00000949	pigeons
Drc7	-0.01	0.00000959	pigeons
Dennd1a	-0.06	0.00000970	pigeons
Ssr3	-0.19	0.00000980	pigeons
Xbp1	-0.08	0.00001018	pigeons
Adcy9	-1.11	0.00001073	pigeons
Edrf1	-0.02	0.00001130	pigeons
Gjd4	-0.17	0.00001172	pigeons
Sertad2	0.25	0.00001179	pigeons
Osbpl3	-0.13	0.00001233	pigeons
Dync1i2	-0.22	0.00001255	pigeons
Vav2	-0.92	0.00001255	pigeons
Pin1	-0.17	0.00001281	pigeons
Urb1	-0.13	0.00001288	pigeons
D630045J12Rik	-0.75	0.00001337	pigeons
Nubp1	-0.22	0.00001354	pigeons
Ctsc	-0.17	0.00001434	pigeons

Exoc7	-0.11	0.00001440	pigeons
Alpk1	0.22	0.00001489	pigeons
Srfbp1	-0.08	0.00001697	pigeons
Tiprl	-0.16	0.00001754	pigeons
Slc25a28	-0.08	0.00001798	pigeons
Slc25a25	-0.20	0.00001816	pigeons
Tcf3	-0.17	0.00001835	pigeons
Ncoa3	-0.26	0.00001841	pigeons
Cops8	-0.30	0.00001858	pigeons
Clns1a	-0.13	0.00001920	pigeons
Bet1	-0.12	0.00002022	pigeons
Sec11c	-0.11	0.00002108	pigeons
Immp2l	-0.85	0.00002141	pigeons
Bcl11b	0.62	0.00002177	pigeons
Epc2	-0.28	0.00002427	pigeons
Fam162a	-0.15	0.00002430	pigeons
Drg1	-0.12	0.00002457	pigeons
Bag1	-0.13	0.00002483	pigeons
Baz2b	-1.16	0.00002495	pigeons
Crnkl1	-0.15	0.00002496	pigeons
Tmem165	-0.19	0.00002646	pigeons
Tecpr1	-0.03	0.00002651	pigeons
Hadhb	-0.08	0.00002686	pigeons
Chmp6	-0.28	0.00002730	pigeons
Kmt5b	-0.29	0.00002814	pigeons
Cwc25	-0.03	0.00002857	pigeons
Tob1	-0.34	0.00002957	pigeons
Trim35	-0.13	0.00002971	pigeons
Serbp1	-0.04	0.00002975	pigeons
Ctps2	0.02	0.00003093	pigeons
Commd4	-0.08	0.00003211	pigeons
Nfxl1	-0.19	0.00003232	pigeons
Ifi30	-0.19	0.00003261	pigeons
Sf3b5	-0.23	0.00003286	pigeons
Fbxo22	-0.08	0.00003318	pigeons
Rap1b	-0.25	0.00003490	pigeons
Klf13	-0.13	0.00003594	pigeons
Pptc7	-0.15	0.00003743	pigeons
Snrpd1	-0.16	0.00003864	pigeons
Lonrf2	-0.23	0.00003977	pigeons
Samm50	-0.02	0.00003993	pigeons
Dcun1d2	-0.80	0.00004101	pigeons
Hmgb2	-0.07	0.00004407	pigeons
Cdk6	-0.68	0.00004704	pigeons
Saraf	-0.16	0.00005007	pigeons
Taf12	-0.17	0.00005010	pigeons

Lrrc8a	-0.19	0.00005044	pigeons
Ldlrad3	-0.31	0.00005225	pigeons
Smc3	-0.27	0.00005366	pigeons
Eef1d	-0.57	0.00005588	pigeons
Sap30l	-0.28	0.00005950	pigeons
Cpne3	-0.30	0.00006053	pigeons
Cstf1	-0.16	0.00006299	pigeons
Rnf214	-0.26	0.00006386	pigeons
Ly86	-1.13	0.00006565	pigeons
Ubn2	-0.26	0.00006686	pigeons
Sap30	-0.17	0.00006714	pigeons
Ppid	-0.14	0.00006807	pigeons
Brwd1	-0.35	0.00006980	pigeons
Arhgap12	-0.08	0.00006994	pigeons
Ndufa6	-0.31	0.00007547	pigeons
Lrp3	-0.29	0.00007914	pigeons
Rasgef1b	-0.02	0.00007955	pigeons
Rab3gap1	-0.05	0.00008145	pigeons
Nabp1	-0.19	0.00008176	pigeons
Atg101	-0.26	0.00008305	pigeons
Sap130	-0.22	0.00008641	pigeons
Nptn	-0.32	0.00008969	pigeons
Fn1	-0.34	0.00009104	pigeons
Hsd17b4	-0.19	0.00009110	pigeons
Ndufa5	-0.10	0.00009614	pigeons
Tpra1	-0.13	0.00009856	pigeons
Pdia4	0.05	0.00009880	pigeons
Commd2	-0.16	0.00009897	pigeons
Frmd4a	-0.96	0.00010171	pigeons
Ranbp10	-0.22	0.00010864	pigeons
Zdhhc21	-0.11	0.00010914	pigeons
Desi2	-0.13	0.00011808	pigeons
Chp1	-0.29	0.00012329	pigeons
Tada3	-0.13	0.00012580	pigeons
Bmpr2	-0.64	0.00012852	pigeons
Gab2	-0.01	0.00012937	pigeons
Aacs	-0.96	0.00013137	pigeons
Ip6k1	-0.27	0.00014381	pigeons
Zbtb37	-0.08	0.00014488	pigeons
Commd9	-0.20	0.00014501	pigeons
Dctn1	-0.18	0.00014620	pigeons
Casp7	-0.27	0.00014974	pigeons
Sumo1	-0.25	0.00015093	pigeons
Gabarapl2	-0.28	0.00016191	pigeons
Gtdc1	0.04	0.00016321	pigeons
Rbbp6	-0.08	0.00016906	pigeons

Ckap5	-0.27	0.00017034	pigeons
Prdm2	-0.32	0.00017239	pigeons
Ifih1	-0.07	0.00017873	pigeons
Arrdc3	-0.35	0.00017954	pigeons
Zeb2	0.55	0.00019434	pigeons
Tmem185a	-0.18	0.00019790	pigeons
Dynll2	-0.11	0.00020216	pigeons
Zswim8	-0.22	0.00020445	pigeons
Cbx3	-0.27	0.00020672	pigeons
Nav3	-1.07	0.00021965	pigeons
Nr4a2	0.14	0.00023814	pigeons
Dus2	-0.01	0.00024539	pigeons
Ripk2	-0.11	0.00024702	pigeons
Psma5	-0.22	0.00025818	pigeons
Dpm2	-0.05	0.00025886	pigeons
Snx17	-0.44	0.00026348	pigeons
Hipk2	-1.02	0.00028013	pigeons
Pi4k2b	-0.13	0.00028332	pigeons
Etv6	-0.59	0.00028649	pigeons
Zfp438	0.10	0.00028871	pigeons
Ttc37	-0.10	0.00029066	pigeons
Kctd3	-0.13	0.00029350	pigeons
Kat6a	-0.39	0.00029716	pigeons
Cops5	-0.17	0.00030123	pigeons
Trpm7	-0.22	0.00030512	pigeons
Sqstm1	-0.22	0.00031005	pigeons
Eif1ad12	-0.31	0.00031332	pigeons
Abhd13	-0.15	0.00031463	pigeons
Snrpe	-0.25	0.00031648	pigeons
Exosc1	-0.13	0.00032815	pigeons
Rbsn	-0.15	0.00033309	pigeons
Gnas	-0.25	0.00033546	pigeons
Ncoa4	0.07	0.00033643	pigeons
Brd3	-0.17	0.00033860	pigeons
Pard3	0.30	0.00035093	pigeons
Ctsh	-0.26	0.00035453	pigeons
Tulp3	-0.10	0.00035576	pigeons
Vti1a	0.15	0.00036032	pigeons
Paxip1	-0.07	0.00037854	pigeons
Sh3bp4	-0.84	0.00039744	pigeons
Tmx3	-0.23	0.00040217	pigeons
Ppib	0.25	0.00040512	pigeons
Nrf1	-0.21	0.00041790	pigeons
Usp8	-0.31	0.00041898	pigeons
Rfx7	0.05	0.00042148	pigeons
Cass4	-0.11	0.00044609	pigeons

Immp1l	-0.18	0.00045638	pigeons
Zranb1	-0.37	0.00049510	pigeons
Pcmt1	-0.18	0.00050318	pigeons
Zbed4	-0.28	0.00050974	pigeons
Arxes2	-0.20	0.00051047	pigeons
Rgl1	-0.95	0.00052649	pigeons
Pebp1	-0.15	0.00053311	pigeons
Syne3	-0.26	0.00053574	pigeons
Taf1l	-0.18	0.00053979	pigeons
Oser1	-0.28	0.00054044	pigeons
Alcam	-0.54	0.00054416	pigeons
Cramp1	-0.37	0.00054779	pigeons
Hmbox1	-0.25	0.00056590	pigeons
Stau1	-0.16	0.00057321	pigeons
Nudcd1	-0.10	0.00057650	pigeons
Nod1	-0.16	0.00060091	pigeons
Fbxl4	-0.16	0.00060297	pigeons
Cnot11	-0.13	0.00061026	pigeons
Helb	-0.05	0.00062273	pigeons
Lrch2	-0.09	0.00064875	pigeons
Zfp106	-0.15	0.00065545	pigeons
Zfp131	-0.08	0.00067018	pigeons
Dkc1	-0.02	0.00067556	pigeons
Atm	-0.12	0.00067972	pigeons
Adgrg6	0.14	0.00069357	pigeons
Spryd7	-0.12	0.00069666	pigeons
Eif4e2	-0.14	0.00069832	pigeons
Twf1	-0.89	0.00071406	pigeons
Tet2	0.14	0.00073552	pigeons
Ttf1	-0.12	0.00075679	pigeons
Ehd4	-0.41	0.00076631	pigeons
Epb41	-0.36	0.00081234	pigeons
Tbc1d22a	-0.29	0.00081552	pigeons
Cmtm7	-0.01	0.00085164	pigeons
Rbm17	-0.20	0.00085856	pigeons
Phf10	-0.20	0.00086638	pigeons
Ilf2	-0.28	0.00088729	pigeons
Ppp1r16b	-0.87	0.00088983	pigeons
Bap1	-0.13	0.00091611	pigeons
Thoc1	-0.15	0.00095614	pigeons
Bnip3l	-0.37	0.00096211	pigeons
Sos1	-0.43	0.00096392	pigeons
Pwwp2b	-0.36	0.00098648	pigeons
Lamp3	-0.03	0.00100679	pigeons
Tmem70	-0.19	0.00105087	pigeons
Taf5l	-1.16	0.00105802	pigeons

Edf1	-0.15	0.00115465	pigeons
Ulk1	-0.31	0.00118133	pigeons
Rgs3	-1.10	0.00119124	pigeons
Tmsb4x	-0.04	0.00120919	pigeons
Tex2	0.02	0.00121761	pigeons
Farp2	-0.23	0.00121940	pigeons
Pla2g7	-0.39	0.00132908	pigeons
Txndc9	-0.33	0.00135127	pigeons
Pdia3	-0.15	0.00136776	pigeons
Per3	-0.17	0.00140483	pigeons
Tfip11	-0.10	0.00148987	pigeons
Pitpnb	-0.33	0.00151188	pigeons
Dlg1	-1.00	0.00151386	pigeons
Arfgap1	-0.22	0.00153995	pigeons
Eif2s1	-0.28	0.00154682	pigeons
Ppp1r13b	-1.13	0.00163368	pigeons
Camk1d	0.32	0.00164102	pigeons
Wdr3	-0.20	0.00165564	pigeons
Ndufs7	-0.18	0.00166214	pigeons
Diaph2	0.30	0.00170547	pigeons
Prkcd	-0.21	0.00174041	pigeons
Fbxo34	-0.15	0.00188919	pigeons
Jakmip1	0.04	0.00201787	pigeons
Itm2a	-0.43	0.00202533	pigeons
Usp3	-0.11	0.00203573	pigeons
Dync1h1	-0.02	0.00214322	pigeons
Golga4	-1.01	0.00225895	pigeons
Nol9	-0.30	0.00245191	pigeons
Ddost	-0.04	0.00248837	pigeons
Trak1	-1.05	0.00268841	pigeons
Bin1	-1.01	0.00271754	pigeons
Nr4a1	-0.20	0.00271916	pigeons
Chd2	-0.09	0.00290848	pigeons
Glul	-1.07	0.00302364	pigeons
Zcchc14	-0.15	0.00306032	pigeons
Cc2d1b	-0.11	0.00318894	pigeons
Kcnk1	-0.20	0.00326188	pigeons
Rrn3	-0.16	0.00329857	pigeons
Slc2a8	-0.12	0.00341149	pigeons
Eif3h	-0.14	0.00341331	pigeons
Rrbp1	-0.20	0.00349555	pigeons
Wwp1	-0.14	0.00351675	pigeons
Ndufb5	-0.31	0.00352141	pigeons
Nub1	-0.30	0.00363158	pigeons
Zcchc2	-0.34	0.00376422	pigeons
Tmem141	-0.13	0.00383347	pigeons

Arl1	-0.18	0.00386421	pigeons
Zdhhc17	-0.33	0.00413621	pigeons
Rsf1	-0.35	0.00431560	pigeons
Tm2d1	-0.15	0.00442721	pigeons
Inpp5k	0.21	0.00450659	pigeons
Cask	-0.63	0.00450852	pigeons
Oxsr1	-0.37	0.00464188	pigeons
Polr2b	-0.22	0.00465642	pigeons
Rufy1	-0.24	0.00468888	pigeons
Poc1b	-0.21	0.00471209	pigeons
Irf2	-0.34	0.00474457	pigeons
Rac1	-0.28	0.00479184	pigeons
Lmo3	-1.03	0.00499913	pigeons
Vps36	-0.22	0.00500648	pigeons
Ptpn11	-0.25	0.00519979	pigeons
Arhgef7	-1.00	0.00522020	pigeons
Chid1	-0.18	0.00554968	pigeons
Slc13a1	0.13	0.00566793	pigeons
Pik3ca	-1.18	0.00566834	pigeons
Fbxo32	-0.08	0.00570378	pigeons
Acvr1	-0.19	0.00575823	pigeons
Ago2	-0.34	0.00577516	pigeons
Slc2a13	0.05	0.00587916	pigeons
Prkd3	-0.25	0.00665130	pigeons
Tbc1d14	-0.35	0.00674362	pigeons
Abcb10	-0.84	0.00676343	pigeons
Polr3b	-0.88	0.00723593	pigeons
Hook1	-0.22	0.00729647	pigeons
Wdr61	-0.16	0.00748195	pigeons
Pcm1	-0.30	0.00760691	pigeons
Clta	-0.14	0.00790914	pigeons
Dcbld2	-0.15	0.00810428	pigeons
Vgll4	-0.09	0.00837212	pigeons
Atp6v0c	0.01	0.00882109	pigeons
Tmem50b	-0.16	0.00909425	pigeons
Tra2b	-0.35	0.00914673	pigeons
Wdr47	-0.22	0.00920594	pigeons
Dnajc2	-0.18	0.00938331	pigeons
Vapb	-0.40	0.00947208	pigeons
Aff2	-0.30	0.00970818	pigeons
Clptm1l	-0.32	0.00971847	pigeons
Reps2	-0.87	0.00983112	pigeons
Cct5	-0.34	0.01001565	pigeons
Pds5a	-0.40	0.01009765	pigeons
Ice2	-0.12	0.01042595	pigeons
Pinx1	-0.17	0.01061164	pigeons

Pdxk	-0.18	0.01124808	pigeons
Stt3b	-0.37	0.01136679	pigeons
Ing5	-0.28	0.01194411	pigeons
Dennd4a	-0.26	0.01226145	pigeons
Nnt	-0.11	0.01237032	pigeons
Ndufa4	-0.27	0.01238073	pigeons
Ubl3	-0.07	0.01309500	pigeons
Supt3	-0.04	0.01314752	pigeons
Pde3b	-0.40	0.01329038	pigeons
Hoxa3	-1.02	0.01358709	pigeons
Adam23	-0.11	0.01514922	pigeons
Angel2	-0.24	0.01521331	pigeons
Slc30a9	-0.38	0.01574197	pigeons
Snx14	-1.03	0.01600975	pigeons
Cct8	-0.29	0.01621767	pigeons
Ubac1	-0.22	0.01655029	pigeons
Kif5c	0.17	0.01680779	pigeons
Chmp1a	-0.30	0.01681280	pigeons
Slc30a7	-0.20	0.01712680	pigeons
Arhgap29	-0.21	0.01770010	pigeons
Uspl1	-0.27	0.01794037	pigeons
Senp7	-0.26	0.01911481	pigeons
Ergic2	-0.22	0.01925468	pigeons
Proser1	-0.30	0.01957528	pigeons
Hspa4	-0.37	0.02104013	pigeons
Hnrnph3	-0.32	0.02195622	pigeons
Fam177a	-1.10	0.02211166	pigeons
Ttc7b	-0.17	0.02241965	pigeons
Tmem50a	-0.41	0.02247868	pigeons
Rcan1	-1.05	0.02285228	pigeons
Syap1	-0.34	0.02296865	pigeons
Adam10	-0.43	0.02433970	pigeons
Dctn3	-0.22	0.02661810	pigeons
Lbh	-0.82	0.02730369	pigeons
Pibf1	-0.13	0.02881786	pigeons
Btrc	-0.32	0.02882884	pigeons
Pepd	-0.10	0.02992785	pigeons
Tmem131l	-0.16	0.03116073	pigeons
Tshz2	-0.79	0.03148596	pigeons
Zhx3	-0.22	0.03334106	pigeons
Snx3	-0.34	0.03337486	pigeons
Ybx1	-0.46	0.03453629	pigeons
Scfd1	-0.25	0.03572058	pigeons
Suco	-0.32	0.03617506	pigeons
Kdm2b	-0.16	0.03657140	pigeons
Vps45	-0.23	0.03781797	pigeons

Psmb4	-0.44	0.03920409	pigeons
Nfat5	-0.26	0.03921455	pigeons
Alox5	-0.15	0.03936130	pigeons
Txnrd1	-0.46	0.04120933	pigeons
Fxr1	-0.47	0.04139571	pigeons
Dpysl2	-1.10	0.04290092	pigeons
Ttyh3	-0.51	0.04299822	pigeons
Cpne1	-0.29	0.04420895	pigeons
Ppfibp1	-0.96	0.04539305	pigeons
Prr14l	-0.33	0.04547196	pigeons
Dnajc15	-0.20	0.04570299	pigeons
Lysmd2	-0.11	0.04931417	pigeons
Elp4	-0.17	0.05014763	pigeons
Rbm19	-0.19	0.05107283	pigeons
Pde6d	-0.19	0.05120229	pigeons
Bend7	-0.23	0.05343759	pigeons
Lrrc28	-0.25	0.05692782	pigeons
Bfar	-0.36	0.05828657	pigeons
Tnpo1	-0.39	0.06054451	pigeons
Pdcd7	-0.19	0.06067092	pigeons
Pik3r1	-0.07	0.06726461	pigeons
Ttc33	-0.10	0.06749211	pigeons
Ldah	-0.17	0.06887499	pigeons
Smyd3	-0.76	0.06991537	pigeons
Rora	0.05	0.07987543	pigeons
Hdgfl3	-0.15	0.08110609	pigeons
Parvg	-0.17	0.08212929	pigeons
Sun1	-0.35	0.08221004	pigeons
Herc3	-0.22	0.08306951	pigeons
Kif5b	-0.34	0.08369612	pigeons
Ppm1g	-0.20	0.08854918	pigeons
Tcn2	-0.33	0.08896253	pigeons
Fam204a	-0.31	0.09174464	pigeons
Fzd6	-0.31	0.09338547	pigeons
Hdac4	-0.86	0.09589495	pigeons
Hpcal1	-1.16	0.10572993	pigeons
Lgals8	-0.06	0.10822346	pigeons
Snap29	-0.38	0.11251856	pigeons
Timm13	-0.32	0.11804657	pigeons
Irf1	-0.30	0.11894414	pigeons
Efr3a	-0.22	0.12301897	pigeons
Srp19	-0.24	0.12527937	pigeons
Tspo	-0.28	0.12584985	pigeons
Foxn3	-0.68	0.13061237	pigeons
Pdk3	-0.31	0.13319253	pigeons
Heatr5b	-0.24	0.13398404	pigeons

Spata5	-0.12	0.13734227	pigeons
Fkbp11	0.39	0.14094798	pigeons
Rbms1	-0.32	0.14104167	pigeons
Ppm1k	-0.22	0.14175017	pigeons
Gpatch11	-0.18	0.14286738	pigeons
Kat7	-0.33	0.14337410	pigeons
Napg	-0.32	0.14883388	pigeons
Spz1	-0.43	0.14957667	pigeons
Scaf8	-0.28	0.15279227	pigeons
Shroom2	-0.89	0.15476366	pigeons
Plekha1	-0.39	0.15562333	pigeons
Cdc42bpb	-1.07	0.15648199	pigeons
Nemf	-0.23	0.15932415	pigeons
Zc3h13	-0.43	0.16492835	pigeons
Slc9a6	-0.31	0.16837091	pigeons
Camsap2	-1.08	0.17303474	pigeons
Zbtb46	-0.24	0.18602657	pigeons
Qser1	-0.37	0.18787929	pigeons
Fndc3b	0.02	0.19505548	pigeons
Rpap2	-0.09	0.19660073	pigeons
Ghitm	-0.47	0.19997020	pigeons
Strn3	-0.43	0.20597014	pigeons
Tm4sf19	0.06	0.22141029	pigeons
Rbm31y	-0.79	0.22267472	pigeons
Napepld	-0.34	0.22439843	pigeons
Rabep1	-0.28	0.22489927	pigeons
Sgip1	-0.81	0.22803743	pigeons
Rev1	-0.31	0.23199189	pigeons
Scpep1	-0.33	0.24955291	pigeons
Itgb3	-0.40	0.25548865	pigeons
Psmd7	-0.43	0.27645117	pigeons
Sh3pxd2a	-0.16	0.28067495	pigeons
Ercc4	-0.27	0.28724502	pigeons
Rb1	-0.11	0.28789611	pigeons
Scp2	-0.32	0.29852859	pigeons
Eif5b	-0.41	0.30022349	pigeons
Dusp7	-0.65	0.31270591	pigeons
Ascc1	-0.35	0.33778335	pigeons
Aif1l	-0.15	0.33914360	pigeons
Agpat5	-0.39	0.34233001	pigeons
Vps39	-0.19	0.35380996	pigeons
Kat6b	-0.93	0.37107666	pigeons
Klf6	-0.33	0.38337061	pigeons
Arl15	-0.64	0.40581426	pigeons
Tdrd3	-0.48	0.42371946	pigeons
Slf2	-0.39	0.44481364	pigeons

Isca1	-0.32	0.46292096	pigeons
Il6st	-0.87	0.46365818	pigeons
Cog5	-0.24	0.47664936	pigeons
Usp47	-0.41	0.49009127	pigeons
Esf1	-0.23	0.49673154	pigeons
Vps25	-0.21	0.49947667	pigeons
Acbd3	-0.40	0.49997059	pigeons
Usp34	-0.41	0.53321429	pigeons
Nol11	-0.33	0.54939507	pigeons
Hmgcr	-0.30	0.55968169	pigeons
Utp18	-0.24	0.58183520	pigeons
Ccdc117	-0.23	0.58925145	pigeons
Exosc9	-0.21	0.60533857	pigeons
Bms1	-0.30	0.61628452	pigeons
Kmt2e	-0.04	0.61877765	pigeons
Gclc	-0.33	0.62039696	pigeons
Ndufa12	-0.37	0.62649243	pigeons
Abcf2	-0.46	0.64692348	pigeons
Aida	-0.26	0.65521819	pigeons
Inpp4a	-0.12	0.66022894	pigeons
Ulk2	-0.34	0.68604007	pigeons
Zfp236	-0.26	0.72620029	pigeons
Lamp1	-0.22	0.73728534	pigeons
Stat4	0.06	0.73830981	pigeons
Dgkh	-0.77	0.76601716	pigeons
Prelid3a	-0.53	0.79991982	pigeons
Acbd5	-0.33	0.83980600	pigeons
Pik3c3	-0.23	0.86089527	pigeons
Zfp618	-0.24	0.86438492	pigeons
Tcf7	1.05	0.86479888	pigeons
Sp3	-0.37	0.87190384	pigeons
Tmbim6	-0.46	0.87847338	pigeons
Sh2d1a	-0.69	0.88113393	pigeons
Hnrnpa2b1	-0.53	0.90712707	pigeons
Fth1	0.17	0.93825558	pigeons
Vcan	0.52	0.94664447	pigeons
Dennd6a	-0.25	1.00000000	pigeons
Lmbrd2	-0.25	1.00000000	pigeons
Prdx4	-0.22	1.00000000	pigeons
Smc4	-0.26	1.00000000	pigeons
Phactr2	-0.27	1.00000000	pigeons
Ndufab1	-0.24	1.00000000	pigeons
Phlpp1	-0.16	1.00000000	pigeons
Zc3h12c	-0.13	1.00000000	pigeons
Usp12	-0.85	1.00000000	pigeons
Snx25	-0.73	1.00000000	pigeons

Ctsz	-0.22	1.00000000	pigeons
Susd6	-0.33	1.00000000	pigeons
Cbfa2t3	-0.83	1.00000000	pigeons
Smad5	-0.79	1.00000000	pigeons
Abca4	-0.16	1.00000000	pigeons
Dhx15	-0.46	1.00000000	pigeons
Dgkq	0.08	1.00000000	pigeons
Ahsa1	-0.39	1.00000000	pigeons
Hnrnpa3	-0.03	1.00000000	pigeons
Nisch	-0.44	1.00000000	pigeons
Ctnnb1	-1.06	1.00000000	pigeons
Itpkb	-0.45	1.00000000	pigeons
Bloc1s6	-0.44	1.00000000	pigeons
Lman2	-0.42	1.00000000	pigeons
Srsf10	-0.29	1.00000000	pigeons
Decr1	-0.19	1.00000000	pigeons
Pnn	-0.44	1.00000000	pigeons
Dek	-0.36	1.00000000	pigeons
Cd3e	0.03	1.00000000	pigeons
Sesn1	-0.07	1.00000000	pigeons
Snrnp25	-0.26	1.00000000	pigeons
Tob2	-0.44	1.00000000	pigeons
Sltm	-0.47	1.00000000	pigeons
Cdkal1	-0.01	1.00000000	pigeons
Acsl4	-0.32	1.00000000	pigeons
Acvr2a	-0.31	1.00000000	pigeons
Dnajc1	-0.26	1.00000000	pigeons
Hscb	-0.22	1.00000000	pigeons
Nktr	-0.90	1.00000000	pigeons
Ehmt1	-0.39	1.00000000	pigeons
Larp1b	-0.44	1.00000000	pigeons
Ncbp3	-0.24	1.00000000	pigeons
Gbe1	-0.05	1.00000000	pigeons
Trip11	-0.32	1.00000000	pigeons
Eif5a2	-0.18	1.00000000	pigeons
Plbd1	-0.16	1.00000000	pigeons
Atp9b	-0.32	1.00000000	pigeons
Lclat1	-0.39	1.00000000	pigeons
Tdp2	-0.29	1.00000000	pigeons
Cd82	-0.95	1.00000000	pigeons
Nap1l1	-0.77	1.00000000	pigeons
Cyth3	-0.90	1.00000000	pigeons
Cry1	-0.87	1.00000000	pigeons
Chmp7	-0.28	1.00000000	pigeons
Ythdc1	-0.53	1.00000000	pigeons
Eif5	-0.24	1.00000000	pigeons

Bag5	-0.47	1.00000000	pigeons
Creb3l2	-0.06	1.00000000	pigeons
Cactin	-0.30	1.00000000	pigeons
Pank1	-0.19	1.00000000	pigeons
Tspan3	-1.05	1.00000000	pigeons
Ano10	-0.68	1.00000000	pigeons
Tspan9	-0.60	1.00000000	pigeons
Rnft1	-0.29	1.00000000	pigeons
Cisd2	-0.26	1.00000000	pigeons
Ppp1r9a	-0.28	1.00000000	pigeons
Brms1l	-0.30	1.00000000	pigeons
Zbtb1	-0.22	1.00000000	pigeons
Ndfip1	-0.50	1.00000000	pigeons
Wdr35	-0.72	1.00000000	pigeons
Ube2d1	-0.33	1.00000000	pigeons
Uvrag	-0.41	1.00000000	pigeons
Aoah	-0.32	1.00000000	pigeons
Akap11	-0.51	1.00000000	pigeons
Ccnd1	-0.62	1.00000000	pigeons
Amot	-0.48	1.00000000	pigeons
Tnik	-0.26	1.00000000	pigeons
Eif4g2	-0.02	1.00000000	pigeons
Atp2b4	-0.74	1.00000000	pigeons
Picalm	-0.22	1.00000000	pigeons
Bcor	-0.68	1.00000000	pigeons
Ccser2	-0.15	1.00000000	pigeons
Foxo1	-0.83	1.00000000	pigeons
Dnaja4	-0.08	1.00000000	pigeons
Idh2	-0.33	1.00000000	pigeons
Rcc1	-0.21	1.00000000	pigeons
Ylpm1	-0.52	1.00000000	pigeons
Kif13b	-0.42	1.00000000	pigeons
Abca3	-0.43	1.00000000	pigeons
Pef1	-0.39	1.00000000	pigeons
Cnpy3	-0.40	1.00000000	pigeons
Gna11	-0.49	1.00000000	pigeons
Pikfyve	-0.47	1.00000000	pigeons
Prdm1	-0.13	1.00000000	pigeons
Hspa4l	-0.36	1.00000000	pigeons
Bin3	-0.62	1.00000000	pigeons
Esrrg	-0.20	1.00000000	pigeons
Ino80	-0.51	1.00000000	pigeons
Sms	-0.41	1.00000000	pigeons
Arhgap6	-0.54	1.00000000	pigeons
Pcmtd1	-0.24	1.00000000	pigeons
Bend3	-0.32	1.00000000	pigeons

Hdac7	-0.94	1.00000000	pigeons
Wbp11	-0.30	1.00000000	pigeons
Stk24	-0.67	1.00000000	pigeons
Trim24	-0.14	1.00000000	pigeons
Arglu1	-0.54	1.00000000	pigeons
Cog6	-0.34	1.00000000	pigeons
Rnf144a	-0.89	1.00000000	pigeons
Nup107	-1.02	1.00000000	pigeons
Utrn	-0.23	1.00000000	pigeons
Nbeal1	-0.43	1.00000000	pigeons
Ero1b	-0.25	1.00000000	pigeons
Sgms2	-0.27	1.00000000	pigeons
Tspan13	-0.72	1.00000000	pigeons
Amfr	-0.48	1.00000000	pigeons
Jak2	-0.38	1.00000000	pigeons
Inpp5d	-0.96	1.00000000	pigeons
Dram2	-0.36	1.00000000	pigeons
Fut8	-0.09	1.00000000	pigeons
Tra2a	-0.50	1.00000000	pigeons
Atg16l1	-0.49	1.00000000	pigeons
Rreb1	-0.43	1.00000000	pigeons
Notch1	-0.53	1.00000000	pigeons
Bicd2	-0.42	1.00000000	pigeons
Tpp2	-0.56	1.00000000	pigeons
Asah1	-0.60	1.00000000	pigeons
Btg1b	0.24	1.00000000	pigeons
Cmss1	-0.70	1.00000000	pigeons
Zdhhc14	-0.86	1.00000000	pigeons
Naca	-0.38	1.00000000	pigeons
Ctdp1	-0.19	1.00000000	pigeons
Sec31a	-1.01	1.00000000	pigeons
Ebf1	-0.05	1.00000000	pigeons
Pde7b	0.28	1.00000000	pigeons
Klhl8	-0.30	1.00000000	pigeons
Sipa1l1	-0.23	1.00000000	pigeons
Brf1	-0.46	1.00000000	pigeons
Tmed5	-0.61	1.00000000	pigeons
Arhgap8	-0.08	1.00000000	pigeons
Nucks1	-0.97	1.00000000	pigeons
Nrp2	-0.64	1.00000000	pigeons
Parp1	-0.32	1.00000000	pigeons
Eif2ak4	-0.44	1.00000000	pigeons
Ctbp1	-0.05	1.00000000	pigeons
Ankrd46	-0.29	1.00000000	pigeons
Rubcnl	-0.43	1.00000000	pigeons
Rfx3	-0.68	1.00000000	pigeons

Ccnl1	-0.50	1.00000000	pigeons
Rad51b	0.06	1.00000000	pigeons
Nr1d2	-0.76	1.00000000	pigeons
Clcc1	-0.28	1.00000000	pigeons
Timp2	-0.54	1.00000000	pigeons
Atp8a1	-0.07	1.00000000	pigeons
Tmpo	-0.82	1.00000000	pigeons
Rusc2	-0.34	1.00000000	pigeons
Nfatc2	-0.17	1.00000000	pigeons
Scfd2	-0.20	1.00000000	pigeons
Lsm8	-0.32	1.00000000	pigeons
Zfp326	-0.50	1.00000000	pigeons
Hmgb3	-0.27	1.00000000	pigeons
Cmtm4	-0.48	1.00000000	pigeons
Tiam2	-0.35	1.00000000	pigeons
Pbx1	-0.92	1.00000000	pigeons
Arl6ip1	-0.60	1.00000000	pigeons
Serinc3	-0.65	1.00000000	pigeons
N4bp2l2	-0.42	1.00000000	pigeons
Smarca2	-0.40	1.00000000	pigeons
Cisd1	-0.41	1.00000000	pigeons
Eif4ebp1	-0.43	1.00000000	pigeons
Tor4a	-0.28	1.00000000	pigeons
Jade1	-0.43	1.00000000	pigeons
Plxdc2	-0.21	1.00000000	pigeons
Ndufs6	-0.50	1.00000000	pigeons
Lpin2	-0.47	1.00000000	pigeons
Rnf11	-0.59	1.00000000	pigeons
Oxct1	-0.39	1.00000000	pigeons
Cwf19l2	-0.25	1.00000000	pigeons
Itpk1	-0.78	1.00000000	pigeons
Chst14	-0.37	1.00000000	pigeons
Fnbp1	-0.43	1.00000000	pigeons
Ywhah	-0.78	1.00000000	pigeons
Htt	-0.48	1.00000000	pigeons
Rere	-0.18	1.00000000	pigeons
Gtf2b	-0.43	1.00000000	pigeons
Sec23a	-0.46	1.00000000	pigeons
Srpk2	-0.44	1.00000000	pigeons
Ap3s1	-0.71	1.00000000	pigeons
Clock	-0.54	1.00000000	pigeons
Ppp2r5e	-0.67	1.00000000	pigeons
Cerk	-0.32	1.00000000	pigeons
Pds5b	-0.81	1.00000000	pigeons
C2cd5	-0.50	1.00000000	pigeons
Atp10a	-0.05	1.00000000	pigeons

Commd10	-0.31	1.00000000	pigeons
Clasp1	-0.43	1.00000000	pigeons
Wbp4	-0.49	1.00000000	pigeons
Npc2	-0.86	1.00000000	pigeons
Usp53	-0.50	1.00000000	pigeons
S100a10	0.13	1.00000000	pigeons
Ppm1d	-0.44	1.00000000	pigeons
Nvl	-0.42	1.00000000	pigeons
Plekha3	-0.41	1.00000000	pigeons
Crip1	0.10	1.00000000	pigeons
Crem	0.05	1.00000000	pigeons
Fubp1	-0.58	1.00000000	pigeons
Leprot	-0.47	1.00000000	pigeons
Rbm47	-0.18	1.00000000	pigeons
Zfyve1	-0.74	1.00000000	pigeons
Lrch1	-0.39	1.00000000	pigeons
Dctn6	-0.41	1.00000000	pigeons
Snx9	-0.35	1.00000000	pigeons
Odc1	-0.37	1.00000000	pigeons
Klhdc1	-0.40	1.00000000	pigeons
Rcbtb2	-0.36	1.00000000	pigeons
Dnaja1	-0.38	1.00000000	pigeons
Vps50	-0.47	1.00000000	pigeons
Rab37	-0.39	1.00000000	pigeons
Tlnrd1	-0.80	1.00000000	pigeons
Insr	-0.63	1.00000000	pigeons
C1qb	-0.67	1.00000000	pigeons
Gnal	-0.14	1.00000000	pigeons
Ifngr2	-0.57	1.00000000	pigeons
Znrf1	-0.58	1.00000000	pigeons
Arfgap3	-0.43	1.00000000	pigeons
Prkn	-0.07	1.00000000	pigeons
Fam133b	-0.50	1.00000000	pigeons
Cacul1	-0.69	1.00000000	pigeons
Ak2	-0.28	1.00000000	pigeons
Dbi	-0.45	1.00000000	pigeons
Fkbp4	-0.54	1.00000000	pigeons
Casp8ap2	-0.67	1.00000000	pigeons
Dst	0.00	1.00000000	pigeons
Ppia	-0.05	1.00000000	pigeons
Unk	-0.51	1.00000000	pigeons
Rwdd1	-0.51	1.00000000	pigeons
Ipmk	-0.72	1.00000000	pigeons
Tox2	-0.16	1.00000000	pigeons
Tanc1	-0.37	1.00000000	pigeons
Cnot10	-0.52	1.00000000	pigeons

Nrip1	-0.29	1.00000000	pigeons
Dnajb6	-0.59	1.00000000	pigeons
Ppfia1	-0.48	1.00000000	pigeons
Tmem266	-0.29	1.00000000	pigeons
Rnf220	-0.66	1.00000000	pigeons
Ddx3y	-0.21	1.00000000	pigeons
Ccny	-0.75	1.00000000	pigeons
Tcf20	-0.30	1.00000000	pigeons
Tns3	-0.39	1.00000000	pigeons
Ets1	-0.35	1.00000000	pigeons
Fbrsl1	-0.01	1.00000000	pigeons
Zfp639	-0.41	1.00000000	pigeons
Arhgef12	-0.80	1.00000000	pigeons
Ybx3	-0.86	1.00000000	pigeons
Runx3	-0.23	1.00000000	pigeons
Fchsd2	-0.62	1.00000000	pigeons
Dmxl1	-0.47	1.00000000	pigeons
Arid1b	-0.21	1.00000000	pigeons
Sh3gl1	-0.79	1.00000000	pigeons
Cd99l2	-0.45	1.00000000	pigeons
Top2b	-0.82	1.00000000	pigeons
Hsph1	-0.53	1.00000000	pigeons
Ppp4r1	-0.59	1.00000000	pigeons
Acaa1b	-0.48	1.00000000	pigeons
Ahi1	-0.34	1.00000000	pigeons
Rala	-0.41	1.00000000	pigeons
Smap1	-0.43	1.00000000	pigeons
Rb1cc1	-0.57	1.00000000	pigeons
Usp37	-0.45	1.00000000	pigeons
Snx29	-0.51	1.00000000	pigeons
Pip5k1a	-0.91	1.00000000	pigeons
Hspd1	-0.62	1.00000000	pigeons
Cyfip1	-0.10	1.00000000	pigeons
Lemd3	-0.57	1.00000000	pigeons
Glcci1	-0.33	1.00000000	pigeons
Hivep2	0.03	1.00000000	pigeons
Cdkn1b	-0.69	1.00000000	pigeons
Rftn2	-0.41	1.00000000	pigeons
Atp1a1	-0.56	1.00000000	pigeons
Tmem135	-0.76	1.00000000	pigeons
Tfdp1	-0.42	1.00000000	pigeons
Lgmn	-0.71	1.00000000	pigeons
Erlin2	-0.42	1.00000000	pigeons
Rassf6	-0.51	1.00000000	pigeons
Zfp407	-0.05	1.00000000	pigeons
Jade2	-0.67	1.00000000	pigeons

Ddx5	-0.01	1.00000000	pigeons
Cdk19	-0.51	1.00000000	pigeons
Cpeb2	-0.51	1.00000000	pigeons
Fto	-0.34	1.00000000	pigeons
Tfrc	-0.47	1.00000000	pigeons
Smim14	-0.73	1.00000000	pigeons
Prdx1	0.06	1.00000000	pigeons
Trappc12	-0.51	1.00000000	pigeons
Sh3bp2	-0.57	1.00000000	pigeons
Ttc28	-0.69	1.00000000	pigeons
Ptpn12	-0.56	1.00000000	pigeons
Polr3a	-0.70	1.00000000	pigeons
Ccdc50	-0.70	1.00000000	pigeons
Lmbr1	-0.48	1.00000000	pigeons
Homer1	-0.27	1.00000000	pigeons
Peak1	-0.60	1.00000000	pigeons
Bach2	-0.02	1.00000000	pigeons
Hmgb1	-0.57	1.00000000	pigeons
Fbxl17	-0.20	1.00000000	pigeons
Elf2	-0.75	1.00000000	pigeons
Abcc1	-0.44	1.00000000	pigeons
Brd1	-0.65	1.00000000	pigeons
Rhoa	-0.15	1.00000000	pigeons
Samd4	-0.60	1.00000000	pigeons
Flnb	-0.26	1.00000000	pigeons
Pxdc1	-0.77	1.00000000	pigeons
Slc25a13	-0.50	1.00000000	pigeons
Arhgap26	-0.67	1.00000000	pigeons
Slc4a4	-0.58	1.00000000	pigeons
Lcmt2	-0.42	1.00000000	pigeons
Tox	-0.20	1.00000000	pigeons
Sod1	-0.43	1.00000000	pigeons
Nfx1	-0.60	1.00000000	pigeons
Trim8	-0.78	1.00000000	pigeons
Pcna	-0.73	1.00000000	pigeons
Rgs10	-0.36	1.00000000	pigeons
Canx	-0.45	1.00000000	pigeons
Ralbp1	-0.64	1.00000000	pigeons
Shprh	-0.74	1.00000000	pigeons
Nme7	-0.94	1.00000000	pigeons
Ssbp3	-0.43	1.00000000	pigeons
Lyar	-0.49	1.00000000	pigeons
Csrp1	-0.70	1.00000000	pigeons
Eif3i	-0.71	1.00000000	pigeons
Ppp2r5a	-0.80	1.00000000	pigeons
Wdr7	-0.60	1.00000000	pigeons

Slc12a4	-0.75	1.00000000	pigeons
Fermt1	-0.39	1.00000000	pigeons
Ppig	-0.56	1.00000000	pigeons
Atp6v0a1	-0.68	1.00000000	pigeons
Hlcs	-0.49	1.00000000	pigeons
Wasl	-0.77	1.00000000	pigeons
Akap9	-0.65	1.00000000	pigeons
Tef	-0.68	1.00000000	pigeons
Disp1	-0.47	1.00000000	pigeons
Ccdc186	-0.52	1.00000000	pigeons
Tmem65	-0.51	1.00000000	pigeons
Vps54	-0.58	1.00000000	pigeons
Plxna1	-0.62	1.00000000	pigeons
Slc30a1	-0.60	1.00000000	pigeons
Swi5	-0.62	1.00000000	pigeons
Hdx	-0.68	1.00000000	pigeons
Rsrp1	-0.65	1.00000000	pigeons
Tgif1	-0.59	1.00000000	pigeons
Plk3	-0.27	1.00000000	pigeons
Ggps1	-0.69	1.00000000	pigeons
Ptpn4	-0.58	1.00000000	pigeons
Dnajb4	-0.84	1.00000000	pigeons
P4ha1	-0.81	1.00000000	pigeons
Kansl1l	-0.41	1.00000000	pigeons
Numb	-0.13	1.00000000	pigeons
Uxs1	-0.67	1.00000000	pigeons
Adk	-0.41	1.00000000	pigeons
Itsn1	-0.67	1.00000000	pigeons
Sptan1	-0.48	1.00000000	pigeons
Rock2	-0.57	1.00000000	pigeons
Oaz2	-0.60	1.00000000	pigeons
Lgals3	-0.47	1.00000000	pigeons
Dusp1	0.25	1.00000000	pigeons
Itsn2	-0.59	1.00000000	pigeons
Kif13a	-0.51	1.00000000	pigeons
Arhgap10	-0.48	1.00000000	pigeons
Clasp2	-0.35	1.00000000	pigeons
Zfp652	-0.65	1.00000000	pigeons
Phtf2	-0.50	1.00000000	pigeons
Xpo1	-0.75	1.00000000	pigeons
Cdca4	-0.66	1.00000000	pigeons
Tbc1d4	-0.49	1.00000000	pigeons
Tpm4	-0.64	1.00000000	pigeons
Fars2	-0.41	1.00000000	pigeons
Cd247	0.36	1.00000000	pigeons
2900026A02Rik	-0.50	1.00000000	pigeons

Nup153	-0.63	1.00000000	pigeons
Cnst	-0.90	1.00000000	pigeons
Rcn2	-0.76	1.00000000	pigeons
Rtn4	-0.47	1.00000000	pigeons
Hoxa7	-0.57	1.00000000	pigeons
Gfod1	-0.57	1.00000000	pigeons
Zmynd11	-0.69	1.00000000	pigeons
Anp32e	-0.85	1.00000000	pigeons
Heca	-0.71	1.00000000	pigeons
Arid4b	-0.61	1.00000000	pigeons
Pola1	-0.50	1.00000000	pigeons
Hnrnpu	-0.54	1.00000000	pigeons
Snrk	-0.48	1.00000000	pigeons
Faf1	-0.55	1.00000000	pigeons
Smg6	-0.68	1.00000000	pigeons
Hnrnpab	-0.69	1.00000000	pigeons
Rab12	-0.64	1.00000000	pigeons
Plbd2	-0.61	1.00000000	pigeons
Srebf1	-0.57	1.00000000	pigeons
Dyrk1a	-0.50	1.00000000	pigeons
Rdx	-0.69	1.00000000	pigeons
Senp6	-0.67	1.00000000	pigeons
Limd1	-0.56	1.00000000	pigeons
Plcb4	-0.51	1.00000000	pigeons
C1qa	-0.52	1.00000000	pigeons
Sik1	-0.46	1.00000000	pigeons
Scoc	-0.60	1.00000000	pigeons
Ip6k2	-0.77	1.00000000	pigeons
Pan3	-0.80	1.00000000	pigeons
Rsad2	3.35	0.00000000	mice
Irf7	2.89	0.00000000	mice
B2m	1.52	0.00000000	mice
Oasl2	1.74	0.00000000	mice
Rnf213	2.26	0.00000000	mice
Zbp1	1.85	0.00000000	mice
Ifi44	2.43	0.00000000	mice
Isg15	2.96	0.00000000	mice
Oasl1	2.45	0.00000000	mice
Bst2	2.25	0.00000000	mice
Ifi27l2a	2.52	0.00000000	mice
Parp14	2.13	0.00000000	mice
H2-K1	1.64	0.00000000	mice
Rtp4	1.46	0.00000000	mice
Irgm1	1.48	0.00000000	mice
Slfn4	2.52	0.00000000	mice
Ly6e	1.84	0.00000000	mice

Ifih1	1.79	0.00000000	mice
Ifi202b	1.76	0.00000000	mice
Xaf1	1.34	0.00000000	mice
Ifit3	1.79	0.00000000	mice
Ifi204	1.89	0.00000000	mice
H2-D1	1.19	0.00000000	mice
Trim30a	1.63	0.00000000	mice
Tap1	1.18	0.00000000	mice
Lgals3bp	1.46	0.00000000	mice
Cxcl10	3.19	0.00000000	mice
Ifit2	2.04	0.00000000	mice
Cfb	2.08	0.00000000	mice
Oas3	1.21	0.00000000	mice
Phf11b	1.74	0.00000000	mice
Slfn5	1.82	0.00000000	mice
Ccl4	2.59	0.00000000	mice
Stat1	1.44	0.00000000	mice
Ifi203	1.71	0.00000000	mice
Ddx60	1.54	0.00000000	mice
Herc6	1.79	0.00000000	mice
Ifit1	1.88	0.00000000	mice
Ifi44l	1.20	0.00000000	mice
Phf11d	1.33	0.00000000	mice
Fcgr1	1.82	0.00000000	mice
Stat2	1.21	0.00000000	mice
Nlrc5	1.25	0.00000000	mice
Gbp2b	1.65	0.00000000	mice
Usp18	1.34	0.00000000	mice
Ifi209	1.53	0.00000000	mice
Samd9l	1.23	0.00000000	mice
H2-T23	1.16	0.00000000	mice
Ifi47	1.27	0.00000000	mice
Ly6a	2.71	0.00000000	mice
Oas1a	0.87	0.00000000	mice
Parp9	1.12	0.00000000	mice
Slfn8	1.16	0.00000000	mice
Psmb8	1.21	0.00000000	mice
Psmb9	1.22	0.00000000	mice
Gbp2	1.39	0.00000000	mice
Ly6c2	2.49	0.00000000	mice
Sp100	1.19	0.00000000	mice
Trim30d	1.18	0.00000000	mice
Znfx1	1.15	0.00000000	mice
Slfn1	1.19	0.00000000	mice
Eif2ak2	1.04	0.00000000	mice
Daxx	1.15	0.00000000	mice

Ifi211	1.22	0.00000000	mice
Pnp	1.34	0.00000000	mice
Ddx58	1.08	0.00000000	mice
Gbp7	1.27	0.00000000	mice
H2-Q7	1.09	0.00000000	mice
H2-T22	1.08	0.00000000	mice
Nedd9	-1.19	0.00000000	mice
Psme1	1.11	0.00000000	mice
Tspo	1.46	0.00000000	mice
Gbp5	1.43	0.00000000	mice
Klf9	-0.87	0.00000000	mice
Lgals9	0.96	0.00000000	mice
Trafd1	1.05	0.00000000	mice
Isg20	1.39	0.00000000	mice
Igtp	0.79	0.00000000	mice
Klrk1	0.87	0.00000000	mice
Spon1	1.60	0.00000000	mice
Gbp3	1.03	0.00000000	mice
Oas2	0.80	0.00000000	mice
Sp110	0.92	0.00000000	mice
Dtx3l	0.83	0.00000000	mice
Irf1	1.26	0.00000000	mice
Tor3a	1.02	0.00000000	mice
Ly6c1	1.49	0.00000000	mice
Cd52	1.29	0.00000000	mice
Ube2l6	0.92	0.00000000	mice
Epsti1	1.14	0.00000000	mice
Trf	-0.93	0.00000000	mice
Ctsb	0.99	0.00000000	mice
Ifi214	0.89	0.00000000	mice
Npc2	1.13	0.00000000	mice
A930037H05Rik	0.85	0.00000000	mice
Ifi35	0.87	0.00000000	mice
Inpp4b	1.33	0.00000000	mice
Helz2	0.72	0.00000000	mice
Parp12	0.78	0.00000000	mice
Ccl2	2.09	0.00000000	mice
Cd40	1.15	0.00000000	mice
Sp140	0.89	0.00000000	mice
Ifitm3	1.07	0.00000000	mice
H3f3b	0.85	0.00000000	mice
Acod1	0.63	0.00000000	mice
Phf11a	0.75	0.00000000	mice
Dhx58	0.65	0.00000000	mice
Pde7b	1.49	0.00000000	mice
Xylt1	-1.17	0.00000000	mice

Ass1	0.99	0.00000000	mice
Nmi	0.75	0.00000000	mice
Ctsc	1.15	0.00000000	mice
Slamf7	1.23	0.00000000	mice
AY036118	0.93	0.00000000	mice
Gpcpd1	-0.78	0.00000000	mice
Glul	-0.79	0.00000000	mice
Fkbp5	-0.62	0.00000000	mice
Il1b	1.39	0.00000000	mice
Eepd1	-1.17	0.00000000	mice
Ifit3b	0.87	0.00000000	mice
Slfn2	0.77	0.00000000	mice
Gbp4	0.83	0.00000000	mice
4930430E12Rik	1.12	0.00000000	mice
Nampt	0.92	0.00000000	mice
Aif1	1.58	0.00000000	mice
Cxcl16	1.36	0.00000000	mice
Cmpk2	0.90	0.00000000	mice
Ccrl2	1.13	0.00000000	mice
Ascc3	1.02	0.00000000	mice
9930111J21Rik2	0.79	0.00000000	mice
Lpin1	-0.96	0.00000000	mice
Tapbp	0.77	0.00000000	mice
Cytip	-0.75	0.00000000	mice
Gadd45b	1.07	0.00000000	mice
Ifi207	0.94	0.00000000	mice
Ccl6	-0.49	0.00000000	mice
Irf9	0.59	0.00000000	mice
Il1rn	1.24	0.00000000	mice
Ifi205	0.80	0.00000000	mice
Psme2	0.90	0.00000000	mice
Calhm6	0.82	0.00000000	mice
Oas1g	0.42	0.00000000	mice
Ifngr1	-0.83	0.00000000	mice
Gab2	-0.79	0.00000000	mice
Klf13	-0.70	0.00000000	mice
Lst1	1.06	0.00000000	mice
Psmb10	0.86	0.00000000	mice
Ccr5	1.07	0.00000000	mice
Ctsz	0.78	0.00000000	mice
Rassf3	-0.73	0.00000000	mice
C130026I21Rik	0.66	0.00000000	mice
Svil	-0.83	0.00000000	mice
Ctss	0.75	0.00000000	mice
Stk10	-0.90	0.00000000	mice
Grn	1.03	0.00000000	mice

Ssh2	-0.84	0.00000000	mice
Resf1	0.93	0.00000000	mice
Gbp6	0.54	0.00000000	mice
Ccnd3	-0.90	0.00000000	mice
Il18bp	0.66	0.00000000	mice
Stxbp6	-1.41	0.00000000	mice
Irgm2	0.51	0.00000000	mice
Csrnp1	0.80	0.00000000	mice
Zmiz1	-0.77	0.00000000	mice
Runx2	-0.83	0.00000000	mice
Hdac4	-0.74	0.00000000	mice
Ifi208	0.47	0.00000000	mice
Pml	0.63	0.00000000	mice
Plac8	1.24	0.00000000	mice
Sgms1	-0.71	0.00000000	mice
Socs3	0.83	0.00000000	mice
Rasa4	0.68	0.00000000	mice
Stk24	-0.71	0.00000000	mice
Clec2d	0.58	0.00000000	mice
Nt5c3	0.72	0.00000000	mice
Traf1	0.95	0.00000000	mice
Casp4	0.78	0.00000000	mice
Celf2	-0.67	0.00000000	mice
H2-T24	0.43	0.00000000	mice
Lilr4b	0.68	0.00000000	mice
Dck	0.69	0.00000000	mice
Ogfr	0.64	0.00000000	mice
Sh3bp5	-0.79	0.00000000	mice
Serpina3g	0.95	0.00000000	mice
Trim30c	0.87	0.00000000	mice
Ext1	-0.92	0.00000000	mice
Pde4d	-0.84	0.00000000	mice
Tor1aip1	0.69	0.00000000	mice
Fcer1g	0.65	0.00000000	mice
Fndc3b	-0.67	0.00000000	mice
Rnf114	0.57	0.00000000	mice
Gbp9	0.49	0.00000000	mice
Chil3	-1.15	0.00000000	mice
Dmxl2	-1.10	0.00000000	mice
Zup1	0.76	0.00000000	mice
Pnpla8	-0.64	0.00000000	mice
Fam107b	-0.81	0.00000000	mice
Ccl12	1.46	0.00000000	mice
Aoah	1.09	0.00000000	mice
Zfp608	-0.81	0.00000000	mice
Fam129a	-0.65	0.00000000	mice

Ccl5	2.46	0.00000000	mice
Lilrb4a	0.60	0.00000000	mice
Cidec	-0.58	0.00000000	mice
AW112010	1.75	0.00000000	mice
Fam117b	-0.67	0.00000000	mice
Ly6i	0.67	0.00000000	mice
Sdc3	0.73	0.00000000	mice
Tsc22d3	-0.68	0.00000000	mice
Agap1	-1.40	0.00000000	mice
Tbc1d2	-0.73	0.00000000	mice
Gbp8	0.49	0.00000000	mice
Cyth1	-0.52	0.00000000	mice
Irf8	0.95	0.00000000	mice
Iigp1	0.85	0.00000000	mice
Adam22	-0.49	0.00000000	mice
Ifi206	0.44	0.00000000	mice
Ndufb1-ps	0.67	0.00000000	mice
Rin3	-0.68	0.00000000	mice
Fcgrt	-0.62	0.00000000	mice
Bnip3l	-0.52	0.00000000	mice
Peli2	-0.46	0.00000000	mice
Chmp4b	0.58	0.00000000	mice
Prkch	-0.85	0.00000000	mice
Cd180	0.65	0.00000000	mice
Arhgap26	-0.74	0.00000000	mice
Asxl1	-0.67	0.00000000	mice
Ptafr	0.64	0.00000000	mice
Eef1b2	-0.51	0.00000000	mice
1600014C10Rik	0.76	0.00000000	mice
Ier5	0.74	0.00000000	mice
Pag1	-0.67	0.00000000	mice
Arhgap25	-0.67	0.00000000	mice
Adar	0.47	0.00000000	mice
Smap2	-0.62	0.00000000	mice
Btg2	0.80	0.00000000	mice
Saa3	1.38	0.00000000	mice
BC147527	0.36	0.00000000	mice
Acaa1b	-0.67	0.00000000	mice
Pde3b	-0.85	0.00000000	mice
Ttll5	-1.02	0.00000000	mice
Ggta1	-0.69	0.00000000	mice
Ccpg1	-0.53	0.00000000	mice
Scimp	0.72	0.00000000	mice
Wfdc17	1.14	0.00000000	mice
Deptor	-0.48	0.00000000	mice
Parp11	0.49	0.00000000	mice

Flvcr2	-0.90	0.00000000	mice
A330040F15Rik	0.56	0.00000000	mice
Wdfy3	-0.68	0.00000000	mice
Vps13b	-0.58	0.00000000	mice
Slc15a3	0.66	0.00000000	mice
Sort1	-0.84	0.00000000	mice
Adipor2	-0.86	0.00000000	mice
Irf2bp2	-0.55	0.00000000	mice
Siglec1	0.49	0.00000000	mice
Snx24	-0.86	0.00000000	mice
H2-Q6	0.41	0.00000000	mice
Kdm6b	0.54	0.00000000	mice
Alox5	-0.37	0.00000000	mice
Gsk3b	-0.57	0.00000000	mice
Clic1	0.60	0.00000000	mice
Parp10	0.40	0.00000000	mice
Cd72	1.17	0.00000000	mice
Ltc4s	-0.75	0.00000000	mice
Net1	-0.69	0.00000000	mice
Nupr1	0.70	0.00000000	mice
Cxcl9	1.36	0.00000000	mice
Clec16a	-0.88	0.00000000	mice
G530011O06Rik	0.39	0.00000000	mice
Tns1	-0.82	0.00000000	mice
Hk3	0.58	0.00000000	mice
Ccl7	1.48	0.00000000	mice
Rock2	-0.62	0.00000000	mice
Samhd1	0.81	0.00000000	mice
Ccny	-0.64	0.00000000	mice
Pygl	-0.50	0.00000000	mice
Cd274	0.86	0.00000000	mice
Fcho2	-0.58	0.00000000	mice
Srsf5	-0.43	0.00000000	mice
H2-Q4	0.40	0.00000000	mice
Actb	0.53	0.00000000	mice
Rapgef1	-0.59	0.00000000	mice
Socs1	0.46	0.00000000	mice
Tsc22d1	-0.60	0.00000000	mice
Fgl2	0.70	0.00000000	mice
Sla	-0.60	0.00000000	mice
Sppl2a	0.62	0.00000000	mice
Lncpint	-0.61	0.00000000	mice
App	-0.63	0.00000000	mice
Sdc4	0.76	0.00000000	mice
Prkar2b	-0.55	0.00000000	mice
Itsn2	-0.57	0.00000000	mice

Lgals1	1.02	0.00000000	mice
Aplp2	-0.58	0.00000000	mice
Tmsb10	1.04	0.00000000	mice
Il2rg	0.55	0.00000000	mice
Stk17b	-0.61	0.00000000	mice
Slfn9	0.38	0.00000000	mice
Sgk1	-0.79	0.00000000	mice
Ifit1bl1	0.46	0.00000000	mice
Zfas1	0.69	0.00000000	mice
Foxn3	-0.65	0.00000000	mice
Sh3tc1	-0.53	0.00000000	mice
Dip2b	-0.64	0.00000000	mice
Cflar	0.67	0.00000000	mice
Ubc	0.56	0.00000000	mice
Nfkbie	0.60	0.00000000	mice
Apol8	-0.29	0.00000000	mice
Tap2	0.49	0.00000000	mice
Adarb1	-0.65	0.00000000	mice
Tmem106a	0.53	0.00000000	mice
Tmem87b	-0.43	0.00000000	mice
Abtb2	0.71	0.00000000	mice
Csnk1g3	-0.50	0.00000000	mice
Inhba	1.51	0.00000000	mice
Pnpla7	-0.56	0.00000000	mice
Nfkbib	0.51	0.00000000	mice
Cpne8	-0.73	0.00000000	mice
Tmsb4x	-0.30	0.00000000	mice
Uba7	0.36	0.00000000	mice
Psma7	0.58	0.00000000	mice
Olfr56	0.37	0.00000000	mice
Ldlrad4	-0.72	0.00000000	mice
Tank	0.76	0.00000000	mice
Egfem1	-1.21	0.00000000	mice
Arl5c	0.78	0.00000000	mice
Pcnx	-0.65	0.00000000	mice
Slc36a4	-0.36	0.00000000	mice
Fap	0.89	0.00000000	mice
Cstb	0.83	0.00000000	mice
Clic4	0.63	0.00000000	mice
Notch1	-0.47	0.00000000	mice
Dock10	-0.73	0.00000000	mice
Dock5	-0.63	0.00000000	mice
Cd83	0.71	0.00000000	mice
Pon2	-0.43	0.00000000	mice
Ddit3	0.49	0.00000000	mice
Fli1	-0.64	0.00000000	mice

Kdr	0.56	0.00000000	mice
Slc28a2	0.35	0.00000000	mice
Casp1	0.47	0.00000000	mice
Tgfbr2	-0.71	0.00000000	mice
Tmcc1	-0.59	0.00000000	mice
Chmp2a	0.52	0.00000000	mice
Sass6	0.32	0.00000000	mice
Rassf5	-0.46	0.00000000	mice
Grk5	-0.70	0.00000000	mice
Actn1	-0.55	0.00000000	mice
Fam120a	-0.46	0.00000000	mice
Ramp1	-0.35	0.00000000	mice
Sema4d	0.53	0.00000000	mice
Cast	-0.50	0.00000000	mice
A930015D03Rik	0.49	0.00000000	mice
Tpst1	0.40	0.00000000	mice
1190005I06Rik	-0.21	0.00000000	mice
Prkca	-0.59	0.00000000	mice
Ptpn12	-0.65	0.00000000	mice
Kcnq1ot1	-0.76	0.00000000	mice
Tmem154	-0.32	0.00000000	mice
Calm1	0.52	0.00000000	mice
Ddx3x	-0.43	0.00000000	mice
Zfp36l2	-0.50	0.00000000	mice
Lima1	-0.77	0.00000000	mice
Tgfbr1	-0.54	0.00000000	mice
Scgb1a1	-0.13	0.00000000	mice
Tnf	1.29	0.00000000	mice
Gpx4	-0.44	0.00000000	mice
C3ar1	0.65	0.00000000	mice
Iqsec1	-0.66	0.00000000	mice
Sirpa	-0.57	0.00000000	mice
Kmt2c	-0.49	0.00000000	mice
Esyt2	-0.52	0.00000000	mice
Usp48	-0.38	0.00000000	mice
Nfkbia	0.57	0.00000000	mice
Per2	-0.26	0.00000000	mice
Luc7l2	-0.42	0.00000000	mice
Ppp3ca	-0.47	0.00000000	mice
Tspan5	-0.72	0.00000000	mice
Lrrc58	-0.46	0.00000000	mice
Selenow	0.68	0.00000000	mice
Insr	-0.38	0.00000000	mice
Hectd1	-0.51	0.00000000	mice
Tacc2	-0.26	0.00000000	mice
Ubb	0.44	0.00000000	mice

Cox17	0.44	0.00000000	mice
Ptgs2	0.95	0.00000000	mice
Batf2	0.25	0.00000000	mice
Cd9	-0.74	0.00000000	mice
Ankrd44	-0.54	0.00000000	mice
Xpo7	-0.35	0.00000000	mice
Cox7a2l	-0.38	0.00000000	mice
Klhl9	-0.39	0.00000000	mice
A530032D15Rik	0.30	0.00000000	mice
Tgfbi	1.07	0.00000000	mice
Uqcrh	-0.41	0.00000000	mice
Smox	0.56	0.00000000	mice
Dip2c	-0.83	0.00000000	mice
Dock7	-0.57	0.00000000	mice
Egr1	0.91	0.00000000	mice
Brd1	-0.42	0.00000000	mice
Zc3hav1	0.55	0.00000000	mice
Cdc42bpb	-0.50	0.00000000	mice
Klf7	-0.53	0.00000000	mice
Abhd12	-0.88	0.00000000	mice
Kcnn3	-0.41	0.00000000	mice
Pak1	0.62	0.00000000	mice
Ifrd1	0.50	0.00000000	mice
Abca1	-0.63	0.00000000	mice
Nus1	-0.50	0.00000000	mice
Asap1	-0.51	0.00000000	mice
Rapgef2	1.03	0.00000000	mice
Dusp2	0.78	0.00000000	mice
Slc9a9	-0.52	0.00000000	mice
Rack1	-0.37	0.00000000	mice
Treml2	0.28	0.00000000	mice
Pitpna	-0.41	0.00000000	mice
Adap2	0.68	0.00000000	mice
Prep	-0.38	0.00000000	mice
Lgmn	1.05	0.00000000	mice
Hip1	-0.51	0.00000000	mice
Npepps	-0.72	0.00000000	mice
Itm2b	0.49	0.00000000	mice
Tmem140	0.41	0.00000000	mice
Hck	0.56	0.00000000	mice
Tob1	-0.43	0.00000000	mice
Fabp1	-0.65	0.00000000	mice
Hook3	-0.41	0.00000000	mice
Ypel3	-0.40	0.00000000	mice
Gramd4	-0.41	0.00000000	mice
Cycs	0.66	0.00000000	mice

Fndc3a	0.76	0.00000000	mice
Acox1	-0.56	0.00000000	mice
Vim	-0.53	0.00000000	mice
Dnajc3	-0.40	0.00000000	mice
Cdk6	-0.64	0.00000000	mice
Ski	-0.50	0.00000000	mice
Ahnak	-0.57	0.00000000	mice
Sh2d4b	-0.40	0.00000000	mice
Slirp	0.38	0.00000000	mice
Eif4b	-0.37	0.00000000	mice
Cd300lf	0.45	0.00000000	mice
Atrn	-0.37	0.00000000	mice
Ppa1	0.33	0.00000000	mice
Bcar3	-0.89	0.00000000	mice
Csprs	0.19	0.00000000	mice
Itgal	-0.40	0.00000000	mice
Nfam1	-0.45	0.00000000	mice
Nmral1	0.26	0.00000000	mice
St6galnac4	0.39	0.00000000	mice
Arrb1	-0.33	0.00000000	mice
Tspan14	-0.58	0.00000000	mice
Perp	-0.25	0.00000000	mice
Slc9a4	-0.39	0.00000000	mice
Lonrf1	-0.30	0.00000000	mice
Rxra	-0.24	0.00000000	mice
Ncf2	-0.46	0.00000000	mice
Cd33	-0.38	0.00000000	mice
Glud1	-0.40	0.00000000	mice
Atf3	0.85	0.00000000	mice
Cab39	-0.43	0.00000000	mice
Etnk1	0.50	0.00000000	mice
Rab2a	-0.36	0.00000000	mice
Pik3r6	-0.52	0.00000000	mice
Eef1a1	-0.31	0.00000000	mice
Arl15	-0.62	0.00000000	mice
Il15ra	0.35	0.00000000	mice
Psma5	0.49	0.00000000	mice
Amz1	-0.34	0.00000000	mice
Smarca2	-0.39	0.00000000	mice
Slc29a3	0.48	0.00000000	mice
Jund	-0.44	0.00000000	mice
Smyd3	-0.55	0.00000000	mice
Ccr2	0.68	0.00000000	mice
Tgtp2	0.19	0.00000000	mice
Tbc1d15	-0.37	0.00000000	mice
Cnot6l	-0.52	0.00000000	mice

Nln	-0.47	0.00000000	mice
Sulf2	-0.34	0.00000000	mice
Vps13d	-0.41	0.00000000	mice
Arhgap39	-0.40	0.00000000	mice
Rfx7	-0.40	0.00000000	mice
Rnf19b	0.47	0.00000000	mice
Pald1	-0.25	0.00000000	mice
Plau	0.75	0.00000000	mice
Lrp1	-0.43	0.00000000	mice
Dot1l	0.51	0.00000000	mice
Slco3a1	0.41	0.00000000	mice
Ankrd11	-0.34	0.00000000	mice
Ddi2	-0.42	0.00000000	mice
Xpr1	-0.47	0.00000000	mice
Adgrl2	-0.42	0.00000000	mice
Ift140	-0.25	0.00000000	mice
Cd69	0.53	0.00000000	mice
Jak2	0.54	0.00000000	mice
Sec61g	0.47	0.00000000	mice
Pttg1	0.29	0.00000000	mice
Vav2	-0.39	0.00000000	mice
Tm6sf1	-0.45	0.00000000	mice
Slco2b1	-0.22	0.00000000	mice
Peli1	0.63	0.00000000	mice
Fry	-0.44	0.00000000	mice
Adap2os	0.32	0.00000000	mice
Zbtb16	-0.35	0.00000000	mice
Il15	0.72	0.00000000	mice
Elmsan1	-0.56	0.00000000	mice
Top2b	-0.34	0.00000000	mice
Lcor	-0.50	0.00000000	mice
Ncoa1	-0.48	0.00000000	mice
Tbc1d30	-0.21	0.00000000	mice
Prkar2a	-0.30	0.00000000	mice
Gvin1	0.23	0.00000000	mice
Cdk13	-0.41	0.00000000	mice
Cramp1l	-0.31	0.00000000	mice
Plaur	-0.40	0.00000000	mice
Herpud1	-0.35	0.00000000	mice
Cib2	-0.24	0.00000000	mice
Arhgef6	-0.47	0.00000000	mice
Shtn1	-0.89	0.00000000	mice
Pex14	-0.49	0.00000000	mice
Atp2c1	-0.48	0.00000000	mice
Plk2	0.69	0.00000000	mice
Tor1aip2	0.44	0.00000000	mice

Tacc1	-0.37	0.00000000	mice
Psma4	0.47	0.00000000	mice
Cpeb2	-0.44	0.00000000	mice
Ttyh2	-0.50	0.00000000	mice
Nfia	-0.42	0.00000000	mice
Themis2	0.39	0.00000000	mice
4930599N23Rik	0.40	0.00000000	mice
Plekhg1	-0.67	0.00000000	mice
Eif2ak3	-0.33	0.00000000	mice
Serinc3	-0.40	0.00000000	mice
Patj	-0.39	0.00000000	mice
Exoc5	-0.38	0.00000000	mice
Pten	-0.42	0.00000000	mice
Ciao2b	0.35	0.00000000	mice
Bckdhb	-0.70	0.00000000	mice
Lepr	-0.45	0.00000000	mice
Atp6v0c	0.51	0.00000000	mice
Ggnbp2	-0.44	0.00000000	mice
Coro2a	-0.39	0.00000000	mice
Clcn3	-0.33	0.00000000	mice
Pycard	0.42	0.00000000	mice
Kif13a	-0.37	0.00000000	mice
Add3	-0.42	0.00000000	mice
Selenop	-0.28	0.00000000	mice
Dapk1	-1.15	0.00000000	mice
Gpr157	-0.16	0.00000000	mice
Evi5	-0.35	0.00000000	mice
Abcg1	-0.99	0.00000000	mice
Large1	-0.55	0.00000000	mice
Kat6b	-0.40	0.00000000	mice
Pim1	0.36	0.00000000	mice
Pik3cg	-0.36	0.00000000	mice
Ddx24	0.44	0.00000000	mice
Chp1	-0.40	0.00000000	mice
Shisa5	0.40	0.00000000	mice
Rab44	-0.34	0.00000000	mice
Cebpa	-0.43	0.00000000	mice
B4galnt1	-0.32	0.00000000	mice
Strn3	-0.44	0.00000000	mice
Idh1	-0.37	0.00000000	mice
Ly75	-0.69	0.00000000	mice
Dennd4c	-0.62	0.00000000	mice
Sdcbp	0.41	0.00000000	mice
Pde4dip	-0.56	0.00000000	mice
Bcl3	0.40	0.00000000	mice
Ptbp3	-0.35	0.00000000	mice

Tmem245	-0.36	0.00000000	mice
Sptbn1	-0.37	0.00000000	mice
Nfe2l2	0.54	0.00000000	mice
Ybx1	-0.29	0.00000000	mice
Siglecf	-0.26	0.00000000	mice
Tnfsf10	0.23	0.00000000	mice
Litaf	0.41	0.00000000	mice
Gna13	0.50	0.00000000	mice
Fgd4	-0.42	0.00000000	mice
Tcf12	-0.44	0.00000000	mice
Gdi2	-0.38	0.00000000	mice
Atp5md	0.43	0.00000000	mice
Scfd2	-0.37	0.00000000	mice
Id3	0.62	0.00000000	mice
Rora	-0.35	0.00000000	mice
Heatr5a	-0.39	0.00000000	mice
Pid1	-0.90	0.00000000	mice
Ear1	-0.49	0.00000000	mice
Pdlim1	-0.30	0.00000000	mice
Ikzf1	-0.47	0.00000000	mice
Sap30	0.36	0.00000000	mice
Slamf8	0.24	0.00000000	mice
Slc12a6	-0.52	0.00000000	mice
Prkce	-0.48	0.00000000	mice
Adgrl3	-0.71	0.00000000	mice
Il6ra	-0.37	0.00000000	mice
Xist	-0.37	0.00000000	mice
Ywhah	-0.38	0.00000000	mice
Atp5k	0.41	0.00000000	mice
Fam53b	-0.18	0.00000000	mice
Klhl6	-0.41	0.00000000	mice
Clec4a3	0.61	0.00000000	mice
Rabac1	-0.32	0.00000000	mice
Rb1	-0.43	0.00000000	mice
Usf2	-0.32	0.00000000	mice
Vav3	-0.68	0.00000000	mice
Rptor	-0.43	0.00000000	mice
Kcnip4	-1.19	0.00000000	mice
Hspa4l	-0.65	0.00000000	mice
Clec4n	-0.44	0.00000000	mice
Ptbp2	-0.37	0.00000000	mice
Picalm	-0.33	0.00000000	mice
Celf4	-0.26	0.00000000	mice
Ogdh	-0.38	0.00000000	mice
Prrc2b	-0.33	0.00000000	mice
Snrk	-0.29	0.00000000	mice

Gnaq	-0.36	0.00000000	mice
Kmt2e	-0.36	0.00000000	mice
Soga1	-0.26	0.00000000	mice
Psma2	0.40	0.00000000	mice
Lacc1	0.31	0.00000000	mice
Klhl24	-0.36	0.00000000	mice
Ncor2	-0.32	0.00000000	mice
Slc6a6	-0.48	0.00000000	mice
Capn15	-0.30	0.00000000	mice
Atp13a3	-0.39	0.00000000	mice
Retreg1	-0.55	0.00000000	mice
Tapt1	-0.36	0.00000000	mice
Adk	-0.47	0.00000000	mice
Akap13	-0.49	0.00000000	mice
Id2	-0.50	0.00000000	mice
Nek1	-0.21	0.00000000	mice
B3glct	-0.23	0.00000000	mice
Nt5c2	-0.37	0.00000000	mice
Phf3	-0.35	0.00000000	mice
Stard3	0.28	0.00000000	mice
Apbb1ip	-0.31	0.00000000	mice
Nsd1	-0.37	0.00000000	mice
Cdc42bpg	-0.34	0.00000000	mice
Qk	-0.33	0.00000000	mice
Spns1	-0.34	0.00000000	mice
Iqgap1	-0.32	0.00000000	mice
Lat2	0.36	0.00000000	mice
Atxn10	-0.38	0.00000000	mice
Cbl	-0.41	0.00000000	mice
Ccnd1	0.41	0.00000000	mice
Pbrm1	-0.37	0.00000000	mice
Tgm2	0.56	0.00000000	mice
Eif1	-0.34	0.00000000	mice
Evi2a	0.31	0.00000000	mice
Esd	0.48	0.00000000	mice
Pitpnc1	-0.47	0.00000000	mice
Ecpas	-0.43	0.00000000	mice
Dock1	-0.33	0.00000000	mice
Lgals8	0.43	0.00000000	mice
Wdfy2	-0.36	0.00000000	mice
Pvt1	-0.46	0.00000000	mice
Lipg	0.38	0.00000000	mice
Arf4	0.43	0.00000000	mice
Timeless	0.17	0.00000000	mice
Rnase6	0.39	0.00000000	mice
Senp6	-0.34	0.00000000	mice

Ccl3	1.00	0.00000000	mice
Trerf1	-0.40	0.00000000	mice
Vkorc1l1	-0.39	0.00000000	mice
Atox1	0.44	0.00000000	mice
Zzef1	-0.39	0.00000000	mice
Rcbtb2	-0.33	0.00000000	mice
Cyb5a	-0.39	0.00000000	mice
Pan3	-0.34	0.00000000	mice
Basp1	0.76	0.00000000	mice
Tfeb	-0.32	0.00000000	mice
Tpt1	-0.25	0.00000000	mice
Glrx5	-0.25	0.00000000	mice
Ezr	-0.36	0.00000000	mice
Samd8	-0.53	0.00000000	mice
Rapgef5	-0.61	0.00000000	mice
Rhou	0.36	0.00000000	mice
Fam241a	0.44	0.00000000	mice
Icam1	0.52	0.00000000	mice
Ccdc88a	-0.46	0.00000000	mice
Scaf8	-0.35	0.00000000	mice
Ucp2	-0.28	0.00000000	mice
Inpp5d	-0.55	0.00000000	mice
Gsto1	-0.28	0.00000000	mice
Nod2	0.35	0.00000000	mice
Unc119	-0.40	0.00000000	mice
Snx2	0.50	0.00000000	mice
Ndufa13	0.41	0.00000000	mice
Tle1	-0.28	0.00000000	mice
Lrrfip1	-0.35	0.00000000	mice
Fmnl2	0.57	0.00000000	mice
Ptpn6	0.42	0.00000000	mice
Sema3e	-0.46	0.00000000	mice
Lamp2	0.34	0.00000000	mice
Rrbp1	0.44	0.00000000	mice
Syne3	-0.27	0.00000000	mice
Cox5b	0.39	0.00000000	mice
Tdrd7	0.37	0.00000000	mice
Psmb3	0.44	0.00000000	mice
A530040E14Rik	0.15	0.00000000	mice
Cluh	-0.22	0.00000000	mice
Itga5	0.49	0.00000000	mice
Qdpr	-0.26	0.00000000	mice
Hmox1	-0.66	0.00000000	mice
Rap1gds1	-0.38	0.00000000	mice
Gstm1	-0.26	0.00000000	mice
Hpse	0.32	0.00000000	mice

Slc39a12	-0.48	0.00000000	mice
Nav2	-0.45	0.00000000	mice
Sod2	0.50	0.00000000	mice
Pros1	-0.29	0.00000000	mice
Rnf135	0.24	0.00000000	mice
Vat1	-0.45	0.00000000	mice
Cox7c	0.40	0.00000000	mice
Ttc7b	-0.38	0.00000000	mice
Tnfrsf21	-0.34	0.00000000	mice
Ergic1	-0.37	0.00000000	mice
Sos2	-0.35	0.00000000	mice
Crip1	-0.39	0.00000000	mice
Rmnd5a	-0.34	0.00000000	mice
Tpd52	-0.37	0.00000000	mice
Dhfr	0.52	0.00000000	mice
Comt	-0.31	0.00000000	mice
Ube2q2	-0.31	0.00000000	mice
Arid5b	0.61	0.00000000	mice
Setdb2	0.31	0.00000000	mice
Arl13b	-0.31	0.00000000	mice
Bzw2	-0.28	0.00000000	mice
Herc1	-0.39	0.00000000	mice
Chd6	-0.26	0.00000000	mice
C1qc	0.84	0.00000000	mice
Tent4b	-0.39	0.00000000	mice
Bhlhe41	-0.30	0.00000000	mice
Psmd4	0.38	0.00000000	mice
S100a1	-0.29	0.00000000	mice
Prdm1	-0.33	0.00000000	mice
Atf7ip	0.46	0.00000000	mice
Pabpc1	-0.29	0.00000000	mice
Colgalt1	-0.44	0.00000000	mice
Usp25	0.46	0.00000000	mice
Itpr2	-0.41	0.00000000	mice
Ncor1	-0.29	0.00000000	mice
Eif3a	-0.30	0.00000000	mice
Abcc5	-0.77	0.00000000	mice
Cdc42ep4	0.29	0.00000000	mice
Fbxw17	0.22	0.00000000	mice
Ffar4	-0.22	0.00000000	mice
Ubr1	-0.27	0.00000000	mice
Bmpr2	-0.38	0.00000000	mice
Ftl1	-0.24	0.00000000	mice
Sde2	0.37	0.00000000	mice
Rab40c	-0.24	0.00000000	mice
Apobec1	0.49	0.00000000	mice

Ncoa4	-0.44	0.00000000	mice
Atp6v0d2	-0.60	0.00000000	mice
Zfp292	-0.28	0.00000000	mice
Kansl1	-0.37	0.00000000	mice
Flna	-0.32	0.00000000	mice
Adipor1	-0.35	0.00000000	mice
Jade1	-0.21	0.00000000	mice
Ppp2r5c	-0.39	0.00000000	mice
Actr3	0.30	0.00000000	mice
Tmem173	0.23	0.00000000	mice
Plcb1	-0.53	0.00000000	mice
Tcirg1	0.43	0.00000000	mice
Ilrun	0.45	0.00000000	mice
1700017B05Rik	-0.35	0.00000000	mice
Cyb561a3	-0.31	0.00000000	mice
Tmem219	0.33	0.00000000	mice
Lonp2	-0.35	0.00000000	mice
C1qb	0.97	0.00000000	mice
A530064D06Rik	0.17	0.00000000	mice
Sept11	-0.47	0.00000000	mice
Clec12a	0.44	0.00000000	mice
Tagln2	-0.35	0.00000000	mice
Vopp1	-0.23	0.00000000	mice
Slmap	-0.36	0.00000000	mice
Vav1	0.44	0.00000000	mice
Dstn	-0.40	0.00000000	mice
Cd2	-0.28	0.00000000	mice
Afap1	-0.25	0.00000000	mice
Rbm6	-0.36	0.00000000	mice
Exoc6b	-0.37	0.00000000	mice
Dock4	-0.40	0.00000000	mice
Cmtr1	0.29	0.00000000	mice
Cd68	0.45	0.00000000	mice
Nek6	-0.30	0.00000000	mice
Lpar1	0.50	0.00000000	mice
Tbl1xr1	-0.40	0.00000000	mice
Phip	-0.40	0.00000000	mice
Pdlim5	-0.30	0.00000000	mice
Tlr1	0.16	0.00000000	mice
Ivns1abp	-0.26	0.00000000	mice
Naca	-0.28	0.00000000	mice
C5ar1	-0.39	0.00000000	mice
Spred1	0.41	0.00000000	mice
Vps37b	0.47	0.00000000	mice
Nfat5	0.55	0.00000000	mice
Nup210	-0.24	0.00000000	mice

Nrg4	-0.38	0.00000000	mice
Stx11	0.28	0.00000000	mice
Nr3c1	-0.36	0.00000000	mice
P2rx4	0.52	0.00000000	mice
Krt79	-0.34	0.00000000	mice
Lilra5	-0.28	0.00000000	mice
Ppp1r12b	-0.45	0.00000000	mice
Creb3l2	-0.22	0.00000000	mice
Ank2	0.61	0.00000000	mice
Raf1	-0.36	0.00000000	mice
Sipa1l2	-0.49	0.00000000	mice
Tm9sf3	-0.31	0.00000000	mice
Zfp644	-0.32	0.00000000	mice
Cd81	-0.21	0.00000000	mice
Fam168a	-0.39	0.00000000	mice
Bbs9	-0.24	0.00000000	mice
Itprid2	-0.41	0.00000000	mice
Tnfrsf1b	0.37	0.00000000	mice
Pdcd4	-0.32	0.00000000	mice
Rras2	-0.40	0.00000000	mice
Grina	0.33	0.00000000	mice
Hnrnpa0	-0.26	0.00000000	mice
AA467197	0.54	0.00000000	mice
Fam189a2	-0.10	0.00000000	mice
Tgif1	0.41	0.00000000	mice
Sema4b	-0.22	0.00000000	mice
Grasp	0.44	0.00000000	mice
Gpr137b	-0.45	0.00000000	mice
Adam10	-0.31	0.00000000	mice
H2afy	0.48	0.00000000	mice
Ncf4	0.34	0.00000000	mice
Sept9	-0.48	0.00000000	mice
Cask	-0.33	0.00000000	mice
Cyp2ab1	-0.20	0.00000000	mice
Lyz2	-0.41	0.00000000	mice
Dennd1a	0.67	0.00000000	mice
Cers6	0.72	0.00000000	mice
Vstm2a	-0.14	0.00000000	mice
Gsr	-0.32	0.00000000	mice
Rasa1	-0.30	0.00000000	mice
Notch2	-0.30	0.00000000	mice
Aopep	-0.55	0.00000000	mice
Zfp326	-0.30	0.00000000	mice
Eef2	-0.29	0.00000000	mice
Ckb	0.45	0.00000000	mice
Itpripl2	-0.36	0.00000000	mice

Rufy4	-0.13	0.00000000	mice
Hat1	0.41	0.00000000	mice
Lmna	-0.37	0.00000000	mice
Ube3c	-0.30	0.00000000	mice
Bcas3	-0.46	0.00000000	mice
Cdadc1	-0.20	0.00000000	mice
Rpn1	-0.26	0.00000000	mice
Atp2b1	-0.34	0.00000000	mice
Sec24d	-0.25	0.00000000	mice
Pbxip1	-0.32	0.00000000	mice
Herc4	-0.46	0.00000000	mice
Ppp1r9a	-0.17	0.00000000	mice
Ppp1r14b	-0.31	0.00000000	mice
Fcgr4	0.64	0.00000000	mice
Smad1	-0.22	0.00000000	mice
Xdh	0.46	0.00000000	mice
Smpdl3a	-0.41	0.00000000	mice
Ctnnd1	-0.32	0.00000000	mice
Larp4	-0.31	0.00000000	mice
Car13	0.24	0.00000000	mice
Ell	-0.36	0.00000000	mice
Ahctf1	-0.25	0.00000000	mice
C1qa	1.11	0.00000000	mice
Pcgf5	0.46	0.00000000	mice
Hvcn1	-0.30	0.00000000	mice
C3	0.56	0.00000000	mice
Jmjd1c	-0.34	0.00000000	mice
P2rx7	0.75	0.00000000	mice
Cxcl1	1.02	0.00000000	mice
Txn1	0.40	0.00000000	mice
St6gal1	-0.28	0.00000000	mice
Agps	-0.29	0.00000000	mice
Sestd1	0.41	0.00000000	mice
Rab20	0.41	0.00000000	mice
Tnfsf9	0.31	0.00000000	mice
Arhgef12	-0.30	0.00000000	mice
Yeats4	-0.30	0.00000000	mice
Ear2	-0.65	0.00000000	mice
Kcnh4	-0.15	0.00000000	mice
Socs2	-0.21	0.00000000	mice
Trim56	0.29	0.00000000	mice
Psme2b	0.17	0.00000000	mice
Acp2	0.34	0.00000000	mice
Otud4	-0.26	0.00000000	mice
Wdr26	-0.26	0.00000000	mice
Arhgef11	-0.28	0.00000000	mice

Tprgl	-0.26	0.00000000	mice
Tcof1	0.29	0.00000000	mice
Naa50	-0.34	0.00000000	mice
Syne2	-0.20	0.00000000	mice
Adrb1	-0.16	0.00000000	mice
Helz	-0.29	0.00000000	mice
Crtc3	-0.35	0.00000000	mice
Acbd5	-0.28	0.00000000	mice
Stag1	-0.31	0.00000000	mice
Pard3b	-0.36	0.00000000	mice
Tbc1d5	-0.36	0.00000000	mice
Zfhx3	-0.37	0.00000000	mice
Fto	-0.35	0.00000000	mice
Zfp64	-0.29	0.00000000	mice
Slc25a36	-0.23	0.00000000	mice
Tbc1d1	0.45	0.00000000	mice
Lrp12	-0.46	0.00000000	mice
Dock2	-0.26	0.00000000	mice
Ttc3	-0.21	0.00000000	mice
Tmem164	-0.40	0.00000000	mice
Ehd4	0.40	0.00000000	mice
Sec11c	-0.34	0.00000000	mice
Foxk1	-0.21	0.00000000	mice
Trim12c	0.21	0.00000000	mice
Pde7a	-0.38	0.00000000	mice
Ly96	0.23	0.00000000	mice
Ncoa7	0.33	0.00000000	mice
Ier3	0.45	0.00000000	mice
Abca9	-0.17	0.00000000	mice
Ipmk	-0.28	0.00000000	mice
Nod1	0.41	0.00000000	mice
Syne1	-0.52	0.00000000	mice
Srp9	-0.29	0.00000000	mice
Tram1	-0.25	0.00000000	mice
Lpl	-0.39	0.00000000	mice
Kazald1	-0.15	0.00000000	mice
Junb	0.42	0.00000000	mice
Ch25h	0.48	0.00000000	mice
Hspd1	-0.21	0.00000000	mice
Prune2	-0.19	0.00000000	mice
Gda	-0.41	0.00000000	mice
Lsr	-0.16	0.00000000	mice
Ostf1	-0.28	0.00000000	mice
Psmd8	0.40	0.00000000	mice
Usp24	-0.25	0.00000000	mice
Rnf34	0.24	0.00000000	mice

Trps1	-0.47	0.00000000	mice
Htr7	0.34	0.00000000	mice
Vwf	-0.20	0.00000000	mice
Sell	0.52	0.00000000	mice
Slc6a4	-0.14	0.00000000	mice
Slc9a7	-0.33	0.00000000	mice
Ppp1r15a	0.45	0.00000000	mice
Ppm1a	-0.25	0.00000000	mice
Cep120	-0.28	0.00000000	mice
Dcp2	0.26	0.00000000	mice
Il21r	0.29	0.00000000	mice
Psmb4	0.37	0.00000000	mice
Igkc	-0.92	0.00000000	mice
Gltp	-0.25	0.00000000	mice
Dad1	-0.27	0.00000000	mice
Hira	-0.19	0.00000000	mice
Nol4l	-0.42	0.00000000	mice
Col3a1	0.24	0.00000000	mice
Agap3	-0.17	0.00000000	mice
C2cd2l	-0.16	0.00000000	mice
Lrrc8d	-0.33	0.00000000	mice
Pltp	-0.23	0.00000000	mice
Ppfibp1	-0.26	0.00000000	mice
Slc49a4	-0.34	0.00000000	mice
Gclc	-0.21	0.00000000	mice
Fnbp1	-0.34	0.00000000	mice
Sh3bgrl2	-0.18	0.00000000	mice
Rasal2	-0.35	0.00000000	mice
Nsf	-0.34	0.00000000	mice
Srsf7	0.38	0.00000000	mice
Creg1	-0.28	0.00000000	mice
Ube2l3	0.30	0.00000000	mice
Nek7	-0.31	0.00000000	mice
Rnf180	-0.43	0.00000000	mice
Tmem64	-0.19	0.00000000	mice
Ptov1	-0.11	0.00000000	mice
Dst	-0.86	0.00000000	mice
Spire1	-0.19	0.00000000	mice
Fmnl1	-0.29	0.00000000	mice
Arhgap21	-0.27	0.00000000	mice
Zbtb7a	-0.28	0.00000000	mice
Def6	-0.24	0.00000000	mice
Huwe1	-0.25	0.00000000	mice
Cyba	0.32	0.00000000	mice
Polr1d	-0.27	0.00000000	mice
Pomp	0.35	0.00000000	mice

Gdf15	-0.30	0.00000000	mice
Chsy1	-0.24	0.00000000	mice
Rnf13	-0.22	0.00000000	mice
Zfp704	-0.27	0.00000000	mice
Tmed2	-0.21	0.00000000	mice
Atrx	-0.27	0.00000000	mice
Ptgfrn	-0.16	0.00000000	mice
Fbxl17	-0.42	0.00000000	mice
Arsb	-0.26	0.00000000	mice
Senp2	-0.31	0.00000000	mice
Fmn1	-0.43	0.00000000	mice
Taldo1	-0.27	0.00000000	mice
Scaf4	-0.28	0.00000000	mice
Nudt3	-0.21	0.00000000	mice
Scarb2	-0.32	0.00000000	mice
Klhl21	-0.14	0.00000000	mice
Ankfy1	0.32	0.00000000	mice
Cacna1d	0.74	0.00000000	mice
Siglece	-0.18	0.00000000	mice
Syncrip	-0.26	0.00000000	mice
Snrnp27	0.25	0.00000000	mice
Ifnar1	0.34	0.00000000	mice
Adcy3	-0.20	0.00000000	mice
Nfkb1	0.35	0.00000000	mice
Nin	-0.35	0.00000000	mice
Serpinb6a	-0.22	0.00000000	mice
Phgdh	-0.17	0.00000000	mice
Slc7a8	0.54	0.00000000	mice
Atp11c	-0.31	0.00000000	mice
Pip4k2a	-0.36	0.00000000	mice
Ubp1	-0.24	0.00000000	mice
Cerk	-0.34	0.00000000	mice
Aph1c	-0.37	0.00000000	mice
Stk39	0.25	0.00000000	mice
Tjp2	-0.15	0.00000000	mice
Hsd3b7	-0.24	0.00000000	mice
Pttg1ip	-0.24	0.00000000	mice
Wdr91	-0.22	0.00000000	mice
Rab14	-0.25	0.00000000	mice
Spty2d1	0.40	0.00000000	mice
Sfxn3	-0.23	0.00000000	mice
Kazn	-0.10	0.00000000	mice
Akt3	0.46	0.00000000	mice
Sbf2	-0.32	0.00000000	mice
AW011738	0.16	0.00000000	mice
Fn1	-0.71	0.00000000	mice

Krt19	-0.16	0.00000000	mice
Snrpf	0.34	0.00000000	mice
Stt3b	-0.29	0.00000000	mice
Tfdp2	-0.15	0.00000000	mice
Aco1	-0.18	0.00000000	mice
Il20rb	-0.18	0.00000000	mice
Il4ra	0.31	0.00000000	mice
Atp5j2	0.36	0.00000000	mice
Rsrp1	0.43	0.00000000	mice
Uggt1	-0.26	0.00000000	mice
Ppp3r1	-0.23	0.00000000	mice
Prr13	-0.32	0.00000000	mice
Kdm4c	-0.28	0.00000000	mice
Slc35e4	-0.16	0.00000000	mice
Pacs1	-0.32	0.00000000	mice
Wdfy4	-0.37	0.00000000	mice
Prkcg	-0.19	0.00000000	mice
Cd24a	-0.27	0.00000000	mice
Pcbp2	-0.23	0.00000000	mice
Zcchc14	-0.15	0.00000000	mice
Ybx3	-0.22	0.00000000	mice
Nap1l1	-0.23	0.00000000	mice
Riok3	-0.26	0.00000000	mice
Osgin2	0.33	0.00000000	mice
Nop10	-0.22	0.00000000	mice
Sfpq	-0.22	0.00000000	mice
B430306N03Rik	0.10	0.00000000	mice
Fbxl20	-0.20	0.00000000	mice
Tbc1d4	-0.18	0.00000000	mice
Cop1	-0.27	0.00000000	mice
Ercc6	-0.24	0.00000000	mice
Pgap2	0.30	0.00000000	mice
Zbtb20	-0.38	0.00000000	mice
Aldh1b1	0.13	0.00000000	mice
Ctps2	-0.23	0.00000000	mice
Acss1	-0.18	0.00000000	mice
Rel	0.36	0.00000000	mice
Rnf150	-0.37	0.00000000	mice
Trim30b	0.10	0.00000000	mice
Gch1	0.32	0.00000000	mice
Plet1	-0.34	0.00000000	mice
Pvr	0.26	0.00000000	mice
Dstyk	-0.20	0.00000000	mice
Lonrf3	-0.19	0.00000000	mice
Tmem135	-0.35	0.00000000	mice
Fgfr2	-0.24	0.00000000	mice

Atrnl1	-0.46	0.00000000	mice
Atg2b	-0.18	0.00000000	mice
Slc29a1	-0.22	0.00000000	mice
Htr2c	-0.33	0.00000000	mice
Dpp4	0.21	0.00000000	mice
Card11	-0.44	0.00000000	mice
Cul1	-0.24	0.00000000	mice
Ddx46	-0.25	0.00000000	mice
Oga	-0.27	0.00000000	mice
Cdyl	-0.26	0.00000000	mice
Arg2	0.29	0.00000000	mice
Cnp	0.22	0.00000000	mice
Pla2g12a	-0.10	0.00000000	mice
Sec31a	-0.23	0.00000000	mice
Gtf2f1	0.25	0.00000000	mice
Dipk1a	-0.21	0.00000000	mice
Kif1b	-0.29	0.00000000	mice
Cers5	-0.25	0.00000000	mice
Egln1	-0.16	0.00000000	mice
Pepd	-0.24	0.00000000	mice
Akt1	-0.23	0.00000000	mice
Yaf2	-0.24	0.00000000	mice
Carhsp1	0.26	0.00000000	mice
Elmo1	-0.27	0.00000000	mice
Rara	-0.26	0.00000000	mice
Flrt3	0.31	0.00000000	mice
Npm1	-0.20	0.00000000	mice
Dek	0.36	0.00000000	mice
Zbtb4	-0.14	0.00000000	mice
Exoc3	-0.28	0.00000000	mice
Trem1	-0.21	0.00000000	mice
Sppl3	-0.30	0.00000000	mice
Csde1	-0.22	0.00000000	mice
Zfand6	-0.27	0.00000000	mice
Lrmp	-0.26	0.00000000	mice
Dedd2	-0.17	0.00000000	mice
Ikbke	0.23	0.00000000	mice
Ksr1	-0.20	0.00000000	mice
Acaca	-0.27	0.00000000	mice
Nfatc1	-0.28	0.00000000	mice
Clint1	-0.32	0.00000000	mice
Igf1r	-0.64	0.00000000	mice
Ost4	-0.24	0.00000000	mice
Cd53	0.21	0.00000000	mice
Imp3	-0.14	0.00000000	mice
Plaat3	0.37	0.00000000	mice

Gsdmd	0.22	0.00000000	mice
Tshz1	-0.20	0.00000000	mice
Camk2g	-0.28	0.00000000	mice
Canx	-0.23	0.00000000	mice
Ppp4r1	-0.24	0.00000000	mice
Enthd1	0.19	0.00000000	mice
Csf3r	0.07	0.00000000	mice
Rbm44	-0.16	0.00000000	mice
Smchd1	0.39	0.00000000	mice
Etv3	0.45	0.00000000	mice
Usb1	0.24	0.00000000	mice
Hadhb	-0.24	0.00000000	mice
Rb1cc1	-0.31	0.00000000	mice
Pygo2	-0.10	0.00000000	mice
Hexb	0.43	0.00000000	mice
Cgas	0.25	0.00000000	mice
Foxp1	-0.35	0.00000000	mice
Ddx6	-0.27	0.00000000	mice
Tmem189	-0.30	0.00000000	mice
Xbp1	-0.22	0.00000000	mice
Atp5g2	-0.22	0.00000000	mice
Fpr1	-0.25	0.00000000	mice
Snd1	-0.28	0.00000000	mice
Cd302	-0.23	0.00000000	mice
Optn	0.24	0.00000000	mice
Arhgap10	0.44	0.00000000	mice
Dlst	-0.24	0.00000000	mice
Pgam1	-0.22	0.00000000	mice
Hspa12a	-0.11	0.00000000	mice
Tmbim1	-0.27	0.00000000	mice
Brwd1	-0.29	0.00000000	mice
Coro1c	-0.31	0.00000000	mice
Fkbp1a	-0.16	0.00000000	mice
Psmb6	0.40	0.00000000	mice
Sephs2	-0.17	0.00000000	mice
Usp9x	-0.25	0.00000000	mice
Sun2	-0.18	0.00000000	mice
Eif4g1	-0.23	0.00000000	mice
Degs1	-0.30	0.00000000	mice
A330023F24Rik	-0.24	0.00000000	mice
Tlnrd1	-0.16	0.00000000	mice
Far1	-0.24	0.00000000	mice
Hnrnph1	-0.22	0.00000000	mice
C77080	-0.12	0.00000000	mice
Gab3	-0.22	0.00000000	mice
Lmnb1	0.14	0.00000000	mice

Zswim6	-0.30	0.00000000	mice
Zbtb11	-0.32	0.00000000	mice
Slk	-0.23	0.00000000	mice
Nipbl	-0.28	0.00000000	mice
Uqcrb	0.33	0.00000000	mice
Snx13	-0.28	0.00000000	mice
Prkag2	-0.26	0.00000000	mice
Arhgap31	-0.40	0.00000000	mice
Eloc	0.33	0.00000000	mice
Trim21	0.12	0.00000000	mice
Tapbpl	0.17	0.00000000	mice
Pgd	-0.29	0.00000000	mice
Hexa	-0.28	0.00000000	mice
4930595D18Rik	0.14	0.00000000	mice
Clcn7	0.34	0.00000000	mice
Adrb2	-0.17	0.00000000	mice
Dbnl	0.30	0.00000000	mice
Ccbe1	-0.15	0.00000000	mice
Dop1a	-0.19	0.00000000	mice
Fnta	-0.16	0.00000000	mice
Eif2ak4	-0.15	0.00000000	mice
Rasa3	-0.47	0.00000000	mice
Hdlbp	-0.25	0.00000000	mice
Irs2	-0.24	0.00000000	mice
Arel1	0.18	0.00000000	mice
Arl5b	0.30	0.00000000	mice
Sftpc	0.26	0.00000000	mice
Psmb2	0.29	0.00000000	mice
Gpr132	0.29	0.00000000	mice
Unc93b1	0.33	0.00000000	mice
Slc9a3r2	-0.08	0.00000000	mice
Kdm6a	-0.29	0.00000000	mice
Hmgn1	-0.13	0.00000000	mice
Dcaf5	-0.28	0.00000000	mice
Cnnm2	-0.21	0.00000000	mice
Pstpip1	0.19	0.00000000	mice
Igflr1	-0.08	0.00000000	mice
Lyrm4	-0.11	0.00000000	mice
Vegfa	0.40	0.00000000	mice
Pnpt1	0.19	0.00000000	mice
Tiam2	-0.25	0.00000000	mice
Gla	0.35	0.00000000	mice
Atxn1	-0.63	0.00000000	mice
2010109A12Rik	-0.13	0.00000000	mice
Wwp1	-0.24	0.00000000	mice
Ppp4r2	0.39	0.00000000	mice

Fkbp4	-0.18	0.00000000	mice
Plcg2	-0.27	0.00000000	mice
Baz1a	-0.39	0.00000000	mice
Chd8	-0.23	0.00000000	mice
Fzd7	-0.10	0.00000000	mice
Prpf38a	0.21	0.00000000	mice
Ankib1	-0.22	0.00000000	mice
Rusc2	-0.14	0.00000000	mice
Il6st	-0.25	0.00000000	mice
Rnf216	-0.26	0.00000000	mice
Tpm3	0.25	0.00000000	mice
Setbp1	-0.74	0.00000000	mice
Usp3	-0.28	0.00000000	mice
Cds2	-0.17	0.00000000	mice
Bmx	-0.12	0.00000000	mice
Cd80	-0.44	0.00000000	mice
Pet100	0.21	0.00000000	mice
Rnf38	-0.28	0.00000000	mice
Ampd3	-0.22	0.00000000	mice
Dok1	0.16	0.00000000	mice
Ppp2ca	-0.24	0.00000000	mice
Hspa1a	0.38	0.00000000	mice
Rybp	0.29	0.00000000	mice
Gorasp2	-0.21	0.00000000	mice
Slc7a11	-0.44	0.00000000	mice
Kctd9	-0.12	0.00000000	mice
Adora2b	0.29	0.00000000	mice
Pcyox1	-0.14	0.00000000	mice
Cox6b2	-0.10	0.00000000	mice
Camsap2	-0.23	0.00000000	mice
Svbp	0.29	0.00000000	mice
Pabpc4	-0.12	0.00000000	mice
Rnh1	0.41	0.00000000	mice
Serinc1	-0.24	0.00000000	mice
Ppp1cc	-0.24	0.00000000	mice
Nmt2	-0.27	0.00000000	mice
Zdhhc9	-0.14	0.00000000	mice
Ptpn18	0.33	0.00000000	mice
Sik2	-0.27	0.00000000	mice
Atp5e	0.35	0.00000000	mice
Arid5a	0.27	0.00000000	mice
Cd36	-0.26	0.00000000	mice
Ubtf	-0.22	0.00000000	mice
Nr4a1	0.54	0.00000000	mice
Kmt5b	-0.22	0.00000000	mice
Crlf3	0.43	0.00000000	mice

Rcc2	-0.19	0.00000000	mice
Nrp2	-0.35	0.00000000	mice
AB124611	0.21	0.00000000	mice
Uqcrq	0.33	0.00000000	mice
Hipk2	-0.44	0.00000000	mice
Cfap43	-0.21	0.00000000	mice
Ubash3b	-0.35	0.00000000	mice
Dcaf6	-0.26	0.00000000	mice
Nceh1	-0.46	0.00000000	mice
Cox6c	0.34	0.00000000	mice
Vps13c	-0.20	0.00000000	mice
Rfx2	-0.13	0.00000000	mice
Fam89a	-0.10	0.00000000	mice
Relb	0.27	0.00000000	mice
Pdzd8	-0.27	0.00000000	mice
Pld4	0.33	0.00000000	mice
Arhgef18	-0.23	0.00000000	mice
Scp2	-0.20	0.00000000	mice
Pdcd6ip	-0.26	0.00000000	mice
Hcls1	0.28	0.00000000	mice
Pi4ka	-0.31	0.00000000	mice
Dip2a	-0.14	0.00000000	mice
Cd44	-0.28	0.00000000	mice
Arih1	-0.20	0.00000000	mice
Snx7	-0.26	0.00000000	mice
Wwtr1	-0.38	0.00000000	mice
Phf2	-0.17	0.00000000	mice
Orai1	-0.28	0.00000000	mice
Prkn	-0.30	0.00000000	mice
Hnrnpll	-0.35	0.00000000	mice
Jmy	-0.14	0.00000000	mice
Cmc2	-0.10	0.00000000	mice
Klf3	-0.32	0.00000000	mice
Irf5	0.25	0.00000000	mice
Camk1	-0.22	0.00000000	mice
Trim69	0.23	0.00000000	mice
Uri1	-0.20	0.00000000	mice
Dym	-0.26	0.00000000	mice
Scamp1	-0.26	0.00000000	mice
Prkag1	-0.26	0.00000000	mice
Ddx50	-0.23	0.00000000	mice
Laptm5	-0.27	0.00000000	mice
Il12rb2	-0.16	0.00000000	mice
Ube2k	-0.22	0.00000000	mice
Elob	0.32	0.00000000	mice
Klhdc4	-0.20	0.00000000	mice

Sptssa	-0.16	0.00000000	mice
Tnks	-0.27	0.00000000	mice
Ehmt1	-0.18	0.00000000	mice
Zfp398	-0.12	0.00000000	mice
Ggct	0.22	0.00000000	mice
Ift172	0.18	0.00000000	mice
Lnx2	-0.15	0.00000000	mice
Faf1	-0.27	0.00000000	mice
Usp15	-0.24	0.00000000	mice
Usp34	-0.21	0.00000000	mice
BC028528	0.24	0.00000000	mice
Gabbr1	-0.08	0.00000000	mice
Tgfb1	-0.27	0.00000000	mice
Rgmb	-0.11	0.00000000	mice
Rhof	-0.21	0.00000000	mice
Cdc73	-0.20	0.00000000	mice
Ubl3	-0.26	0.00000000	mice
Cox7a2	0.32	0.00000000	mice
Cd86	0.48	0.00000000	mice
Dtnb	-0.21	0.00000000	mice
Clca3a1	0.13	0.00000000	mice
Hebp1	-0.34	0.00000000	mice
6530409C15Rik	0.12	0.00000000	mice
St6galnac6	0.27	0.00000000	mice
Reep3	-0.29	0.00000000	mice
Thbs1	-1.04	0.00000000	mice
Denr	0.32	0.00000000	mice
Prpf39	-0.25	0.00000000	mice
Ccdc25	0.21	0.00000000	mice
Setd7	-0.19	0.00000000	mice
Rbm26	-0.27	0.00000000	mice
Zfyve9	-0.42	0.00000000	mice
Spen	-0.18	0.00000000	mice
Clec4a1	0.29	0.00000000	mice
Pgls	-0.18	0.00000000	mice
Plec	-0.27	0.00000000	mice
Stk4	-0.24	0.00000000	mice
Arid1b	-0.30	0.00000000	mice
Zcchc2	0.32	0.00000000	mice
Sel1l	-0.21	0.00000000	mice
Cpt1a	-0.24	0.00000000	mice
Akr1b3	-0.24	0.00000000	mice
Rlim	-0.24	0.00000000	mice
Jag1	-0.18	0.00000000	mice
Trappc8	-0.26	0.00000000	mice
Asb13	0.16	0.00000000	mice

Nt5e	-0.19	0.00000000	mice
Odf2	-0.19	0.00000000	mice
Klra2	0.31	0.00000000	mice
Cnot1	-0.24	0.00000000	mice
Trim29	-0.07	0.00000000	mice
Sorbs1	-0.23	0.00000000	mice
Slc25a4	-0.01	0.00000000	mice
Rrad	0.24	0.00000000	mice
Atxn7	-0.28	0.00000000	mice
Serpinb9	0.22	0.00000000	mice
Ank	-0.40	0.00000000	mice
Ap2a2	-0.24	0.00000000	mice
Purb	-0.17	0.00000000	mice
Eif4ebp2	-0.27	0.00000000	mice
Tlk1	-0.27	0.00000000	mice
Ctsf	-0.07	0.00000000	mice
Tbl1x	-0.29	0.00000000	mice
Haus2	-0.21	0.00000000	mice
Rab8a	0.25	0.00000000	mice
Tcaf1	-0.08	0.00000000	mice
Ptpn22	-0.35	0.00000000	mice
Cep170	-0.24	0.00000000	mice
Col4a3bp	-0.28	0.00000000	mice
Aak1	-0.26	0.00000000	mice
Nr4a3	0.26	0.00000000	mice
Nbeal1	-0.21	0.00000000	mice
Gcnt1	-0.28	0.00000000	mice
Lrp6	-0.17	0.00000000	mice
Ubr5	-0.30	0.00000000	mice
Rbms1	-0.22	0.00000000	mice
Cmklr1	0.18	0.00000000	mice
Per1	-0.16	0.00000000	mice
Zbtb38	-0.13	0.00000000	mice
Arntl	-0.16	0.00000000	mice
Tcf4	0.34	0.00000000	mice
Ulk2	-0.18	0.00000000	mice
Dubr	-0.10	0.00000000	mice
Uxs1	-0.15	0.00000000	mice
Eif4e	0.42	0.00000000	mice
Abhd17c	-0.23	0.00000000	mice
Gsap	-0.36	0.00000000	mice
Aff1	0.48	0.00000000	mice
Fgr	0.30	0.00000000	mice
Tbc1d22a	-0.24	0.00000000	mice
Parp4	-0.25	0.00000000	mice
Kctd6	-0.11	0.00000000	mice

Hk2	0.49	0.00000000	mice
Ypel2	-0.17	0.00000000	mice
Edem3	-0.17	0.00000000	mice
Hnrnpul1	-0.26	0.00000000	mice
Hgf	-0.31	0.00000000	mice
Eif2s2	0.34	0.00000000	mice
Itga4	-0.38	0.00000000	mice
Tmpo	0.29	0.00000000	mice
Appbp2	-0.25	0.00000000	mice
Tecpr1	-0.16	0.00000000	mice
Rasgrp1	0.21	0.00000000	mice
Lyst	-0.44	0.00000000	mice
Brk1	-0.18	0.00000000	mice
Cul2	-0.16	0.00000000	mice
Kif5b	-0.23	0.00000000	mice
Cmss1	0.71	0.00000000	mice
Arhgap5	-0.14	0.00000000	mice
Tuba1b	0.41	0.00000000	mice
B3gnt7	-0.12	0.00000000	mice
Dicer1	-0.17	0.00000000	mice
Psma1	0.29	0.00000000	mice
Slc7a1	-0.26	0.00000000	mice
Lrrc25	0.23	0.00000000	mice
Rnpep	0.32	0.00000000	mice
F630040K05Rik	0.13	0.00000000	mice
Acvr1b	-0.15	0.00000000	mice
Tmem11	-0.14	0.00000000	mice
Usp32	-0.28	0.00000000	mice
F5	-0.48	0.00000000	mice
Clmn	-0.11	0.00000000	mice
Chpt1	0.26	0.00000000	mice
Sowahc	0.29	0.00000000	mice
Ndufa3	0.26	0.00000000	mice
Rassf4	-0.21	0.00000000	mice
Cd93	-0.29	0.00000000	mice
Dhrs3	-0.34	0.00000000	mice
G3bp1	-0.26	0.00000000	mice
Rnf169	-0.21	0.00000000	mice
Tulp4	-0.33	0.00000000	mice
Rasa2	-0.34	0.00000000	mice
Supt3	-0.24	0.00000000	mice
Fam13b	-0.24	0.00000000	mice
Cln3	-0.25	0.00000000	mice
Kif16b	-0.21	0.00000000	mice
Cdkn1a	0.35	0.00000000	mice
Capzb	-0.20	0.00000000	mice

Emb	-0.39	0.00000000	mice
Sgms2	-0.35	0.00000000	mice
Rab5if	0.25	0.00000000	mice
Gak	-0.22	0.00000000	mice
Efhd2	0.31	0.00000000	mice
Pld2	-0.08	0.00000000	mice
Srpk2	-0.21	0.00000000	mice
Anxa1	-0.23	0.00000000	mice
Snrnp70	-0.21	0.00000000	mice
Serpinb1a	-0.13	0.00000000	mice
Gfod1	-0.37	0.00000000	mice
Zfp706	-0.20	0.00000000	mice
Plxdc2	-0.24	0.00000000	mice
Tfec	-0.19	0.00000000	mice
Rbpms	-0.28	0.00000000	mice
Abce1	-0.14	0.00000000	mice
Cd74	0.10	0.00000000	mice
Gtf2i	-0.10	0.00000000	mice
Tent4a	0.23	0.00000000	mice
Gng12	0.25	0.00000000	mice
Cd22	-0.10	0.00000000	mice
Csrp1	0.27	0.00000000	mice
Psmc4	0.24	0.00000000	mice
Wwox	-0.53	0.00000000	mice
Fars2	-0.29	0.00000000	mice
Atad2	-0.16	0.00000000	mice
Ist1	0.28	0.00000000	mice
Cdc14b	-0.18	0.00000000	mice
Alcam	-0.50	0.00000000	mice
Slc3a2	0.33	0.00000000	mice
Psen1	-0.26	0.00000000	mice
Qsox1	-0.23	0.00000000	mice
Abcd2	-0.17	0.00000000	mice
Snx14	-0.17	0.00000000	mice
Slc48a1	-0.16	0.00000000	mice
Pfdn5	-0.17	0.00000000	mice
Psmc3	0.31	0.00000000	mice
Spata5	-0.21	0.00000000	mice
Golga4	-0.18	0.00000000	mice
Plcb2	-0.14	0.00000000	mice
Fcgr2b	0.43	0.00000000	mice
Larp4b	-0.22	0.00000000	mice
Inpp4a	-0.21	0.00000000	mice
Cdc42bpa	-0.14	0.00000000	mice
Josd1	-0.12	0.00000000	mice
Trpm7	-0.21	0.00000000	mice

Tnfsf13	-0.16	0.00000000	mice
Parvg	-0.10	0.00000000	mice
Tom1	-0.22	0.00000000	mice
Kpna1	-0.22	0.00000000	mice
Crtam	-0.12	0.00000000	mice
Il6	0.25	0.00000000	mice
Wwc2	-0.17	0.00000000	mice
Itgav	0.54	0.00000000	mice
Dgka	-0.16	0.00000000	mice
Usp6nl	-0.25	0.00000000	mice
Fos	0.71	0.00000000	mice
4931406C07Rik	-0.14	0.00000000	mice
Tnfrsf13b	0.12	0.00000000	mice
S100a11	-0.22	0.00000000	mice
Tanc2	-0.19	0.00000000	mice
Naa20	0.33	0.00000000	mice
Katna1	0.23	0.00000000	mice
Atg5	-0.23	0.00000000	mice
Rock1	-0.22	0.00000000	mice
Ebf1	-0.54	0.00000000	mice
Plekhb2	-0.20	0.00000000	mice
9930021J03Rik	-0.28	0.00000000	mice
Pik3r1	-0.16	0.00000000	mice
Zfp568	-0.17	0.00000000	mice
Dock11	-0.32	0.00000000	mice
H2afv	-0.15	0.00000000	mice
Pparg	-0.08	0.00000000	mice
Plxnb2	0.20	0.00000000	mice
Plekhm3	-0.23	0.00000000	mice
Arfgef1	-0.20	0.00000000	mice
Sem1	0.20	0.00000000	mice
Acadm	-0.18	0.00000000	mice
Nosip	0.16	0.00000000	mice
Zfp516	0.27	0.00000000	mice
Ppp2r5e	-0.23	0.00000000	mice
Osbpl11	-0.22	0.00000000	mice
Nfatc3	-0.24	0.00000000	mice
Slc25a22	0.13	0.00000000	mice
Clasp2	-0.21	0.00000000	mice
Hivep1	0.36	0.00000000	mice
Ano6	-0.23	0.00000000	mice
Endov	0.15	0.00000000	mice
Tmed10	-0.19	0.00000000	mice
Tmcc3	-0.47	0.00000000	mice
Dgkd	-0.34	0.00000000	mice
Rbm39	-0.19	0.00000000	mice

Tomm20	-0.19	0.00000000	mice
Xpot	-0.12	0.00000000	mice
Ubald2	0.32	0.00000000	mice
Cebpb	-0.25	0.00000000	mice
Ptpn4	-0.17	0.00000000	mice
Lmo4	-0.22	0.00000000	mice
Psmd12	0.26	0.00000000	mice
Dusp4	0.20	0.00000000	mice
Ly86	0.26	0.00000000	mice
Prickle2	-0.13	0.00000000	mice
Poc1b	-0.19	0.00000000	mice
Trio	-0.41	0.00000000	mice
Polr2l	0.30	0.00000000	mice
Itpr1	0.38	0.00000000	mice
Rp9	-0.22	0.00000000	mice
Akap8	-0.15	0.00000000	mice
Rap2c	0.27	0.00000000	mice
Emc8	-0.15	0.00000000	mice
Ece1	0.30	0.00000000	mice
D5Ertd579e	-0.15	0.00000000	mice
2310001H17Rik	0.17	0.00000000	mice
Gpi1	-0.20	0.00000000	mice
Prag1	-0.11	0.00000000	mice
Dab2	-0.36	0.00000000	mice
Anpep	0.19	0.00000000	mice
Emc6	-0.17	0.00000000	mice
Il1a	1.07	0.00000000	mice
Cebpz	-0.20	0.00000000	mice
Rfc2	0.15	0.00000000	mice
Setx	-0.22	0.00000000	mice
Cdc42ep3	-0.26	0.00000000	mice
Acot1	-0.07	0.00000000	mice
Guk1	-0.15	0.00000000	mice
Cnr2	-0.23	0.00000000	mice
Pdgfc	-0.12	0.00000000	mice
Cd164	-0.41	0.00000000	mice
Higd2a	-0.19	0.00000000	mice
Ak8	-0.10	0.00000000	mice
Thbd	-0.18	0.00000000	mice
Dnmt3a	-0.31	0.00000000	mice
Cbfb	-0.21	0.00000000	mice
Vcpip1	0.26	0.00000000	mice
Tusc3	-0.15	0.00000000	mice
Reps2	-0.25	0.00000000	mice
Znrf1	-0.24	0.00000000	mice
Fam199x	-0.09	0.00000000	mice

Zfp503	-0.10	0.00000000	mice
Uhrf2	-0.22	0.00000000	mice
Foxk2	-0.13	0.00000000	mice
Noc4l	0.16	0.00000000	mice
Runx1	-0.23	0.00000000	mice
Trem2	-0.11	0.00000000	mice
Galnt1	-0.20	0.00000000	mice
Top1	-0.37	0.00000000	mice
Fbxo22	-0.16	0.00000000	mice
Arhgap45	-0.29	0.00000000	mice
Bak1	0.23	0.00000000	mice
Klhl7	-0.19	0.00000000	mice
Spcs3	-0.22	0.00000000	mice
Atp5j	0.28	0.00000000	mice
Satb1	-0.42	0.00000000	mice
Eif3d	0.18	0.00000000	mice
3110082I17Rik	-0.17	0.00000000	mice
Herpud2	-0.18	0.00000000	mice
Slc8a1	0.51	0.00000000	mice
Snx9	-0.20	0.00000000	mice
Smdt1	0.30	0.00000000	mice
Hsp90b1	-0.13	0.00000000	mice
Susd1	-0.21	0.00000000	mice
Elovl5	-0.17	0.00000000	mice
Tnks2	-0.22	0.00000000	mice
Serpine1	-0.23	0.00000000	mice
Diaph1	-0.20	0.00000000	mice
Zfp148	-0.24	0.00000000	mice
Ythdf3	-0.22	0.00000000	mice
Slc25a37	0.24	0.00000000	mice
Capg	0.54	0.00000000	mice
Taok3	-0.25	0.00000000	mice
Tmem223	-0.11	0.00000000	mice
Ywhag	-0.20	0.00000000	mice
Ptpn9	-0.24	0.00000000	mice
Asxl2	-0.21	0.00000000	mice
Dnajb9	-0.20	0.00000000	mice
Cdkn2d	0.17	0.00000000	mice
Taco1	-0.06	0.00000000	mice
Tnfaip1	-0.08	0.00000000	mice
Ogt	-0.22	0.00000000	mice
Erbin	-0.19	0.00000000	mice
Sypl	-0.19	0.00000000	mice
Impdh1	-0.11	0.00000000	mice
Crim1	-0.21	0.00000000	mice
Usp22	-0.14	0.00000000	mice

Stag2	-0.22	0.00000000	mice
Clock	-0.22	0.00000000	mice
Kctd12b	-0.07	0.00000000	mice
Csnk1g1	-0.24	0.00000000	mice
Clec5a	0.06	0.00000000	mice
B230307C23Rik	0.18	0.00000000	mice
Clec4d	-0.47	0.00000000	mice
Desi2	-0.23	0.00000000	mice
Wdfy1	0.32	0.00000000	mice
Zmym2	-0.16	0.00000000	mice
Cdc42se2	-0.24	0.00000000	mice
Dhx9	-0.15	0.00000000	mice
Galnt3	-0.15	0.00000000	mice
Tifa	0.25	0.00000000	mice
Rarres1	-0.09	0.00000000	mice
Clec2g	0.14	0.00000000	mice
Klhl2	-0.21	0.00000000	mice
Cpne2	0.02	0.00000000	mice
Exoc2	-0.21	0.00000000	mice
Lmo2	0.21	0.00000000	mice
Rassf1	-0.20	0.00000000	mice
Prkacb	-0.22	0.00000000	mice
Fam168b	-0.19	0.00000000	mice
Atg16l1	-0.22	0.00000000	mice
Ankrd12	-0.20	0.00000000	mice
Stra6l	0.17	0.00000000	mice
Cracr2b	-0.05	0.00000000	mice
Tnrc6c	-0.24	0.00000000	mice
Flot1	0.13	0.00000000	mice
Atp2a2	-0.08	0.00000000	mice
Timm10b	0.25	0.00000000	mice
Gas2l1	-0.12	0.00000000	mice
Cpq	0.21	0.00000000	mice
Rprd2	-0.19	0.00000000	mice
Rad54l2	-0.16	0.00000000	mice
Tmem65	-0.23	0.00000000	mice
Ccni	-0.17	0.00000000	mice
Gsdme	-0.20	0.00000000	mice
Sgsm2	-0.11	0.00000000	mice
Relch	-0.22	0.00000000	mice
Dpagt1	-0.08	0.00000000	mice
Cysltr1	0.10	0.00000000	mice
Rab11b	-0.21	0.00000000	mice
Cdc42se1	-0.19	0.00000000	mice
Sertad1	0.25	0.00000000	mice
Lcorl	-0.21	0.00000000	mice

Ddb1	-0.15	0.00000000	mice
Sgcb	0.12	0.00000000	mice
Pdcd6	-0.17	0.00000000	mice
Ndufs4	0.23	0.00000000	mice
Chd2	-0.24	0.00000000	mice
Ttll3	-0.11	0.00000000	mice
Cenpv	-0.09	0.00000000	mice
Erc1	-0.21	0.00000000	mice
Scand1	0.25	0.00000000	mice
Dram1	0.29	0.00000000	mice
Tnpo3	-0.20	0.00000000	mice
Cspp1	-0.21	0.00000000	mice
Yars	-0.17	0.00000000	mice
Slc43a2	-0.18	0.00000000	mice
Tmed3	-0.12	0.00000000	mice
Gatad2b	-0.25	0.00000000	mice
Dag1	-0.14	0.00000000	mice
Sh2d1b1	-0.18	0.00000000	mice
Ubn2	-0.22	0.00000000	mice
Srsf6	-0.18	0.00000000	mice
Hnrnpf	-0.19	0.00000000	mice
Pfn1	0.20	0.00000000	mice
Aprt	-0.20	0.00000000	mice
Galk2	-0.14	0.00000000	mice
Zbtb44	-0.11	0.00000000	mice
Zbtb1	-0.13	0.00000000	mice
Plekho2	0.26	0.00000000	mice
Tpi1	0.27	0.00000000	mice
Ets1	-0.30	0.00000000	mice
Nrg1	0.52	0.00000000	mice
Pin4	0.15	0.00000000	mice
S100a10	-0.21	0.00000000	mice
Aup1	0.19	0.00000000	mice
Dclre1c	0.37	0.00000000	mice
Aamp	-0.13	0.00000000	mice
Foxj3	-0.19	0.00000000	mice
Cat	-0.19	0.00000000	mice
Snx30	-0.20	0.00000000	mice
Gpat3	-0.26	0.00000000	mice
Rnf24	0.25	0.00000000	mice
Tbc1d14	-0.20	0.00000000	mice
Scaf11	-0.21	0.00000000	mice
Btbd1	-0.17	0.00000000	mice
Wdr3	-0.19	0.00000000	mice
Rere	-0.26	0.00000000	mice
Psmc2	0.20	0.00000000	mice

Hadh	-0.10	0.00000000	mice
Igfbp7	0.38	0.00000000	mice
Itgax	-0.24	0.00000000	mice
Rnf157	0.17	0.00000000	mice
E330009J07Rik	0.18	0.00000000	mice
Pccb	-0.06	0.00000000	mice
Aven	-0.15	0.00000000	mice
Sun1	-0.12	0.00000000	mice
Itga1	-0.27	0.00000000	mice
Lrp8	-0.20	0.00000000	mice
Casp8	0.29	0.00000000	mice
Sdha	-0.21	0.00000000	mice
Rbsn	-0.11	0.00000000	mice
Twistnb	-0.23	0.00000000	mice
Spata6	-0.22	0.00000000	mice
Tmem86a	0.20	0.00000000	mice
C130050O18Rik	-0.10	0.00000000	mice
Cabin1	-0.17	0.00000000	mice
Ptpre	-0.28	0.00000000	mice
Dynlt3	-0.15	0.00000000	mice
Zfp800	0.33	0.00000000	mice
Slc16a6	-0.25	0.00000000	mice
Tet2	-0.17	0.00000000	mice
Emilin1	-0.07	0.00000000	mice
Xpo4	-0.17	0.00000000	mice
Smurf2	-0.26	0.00000000	mice
Itk	-0.40	0.00000000	mice
N4bp2l1	-0.18	0.00000000	mice
Ermp1	-0.13	0.00000000	mice
Chd7	-0.22	0.00000000	mice
Ndfip1	-0.24	0.00000000	mice
Psmc5	0.23	0.00000000	mice
Tnrc6a	-0.20	0.00000000	mice
Dusp16	0.28	0.00000000	mice
Pak2	-0.23	0.00000000	mice
Plekho1	0.36	0.00000000	mice
Hsp90aa1	0.33	0.00000000	mice
Fam126a	-0.17	0.00000000	mice
Ralgapa1	-0.28	0.00000000	mice
Cdk17	-0.34	0.00000000	mice
Fam162a	-0.08	0.00000000	mice
Smc6	-0.28	0.00000000	mice
Ngly1	-0.18	0.00000000	mice
Hax1	0.18	0.00000000	mice
Plekhm1	-0.17	0.00000000	mice
Rbx1	0.32	0.00000000	mice

Kmt2a	-0.17	0.00000000	mice
Usp47	-0.18	0.00000000	mice
Gna15	-0.09	0.00000000	mice
Dpy19l4	-0.12	0.00000000	mice
Ppm1b	-0.17	0.00000000	mice
Wipf1	-0.18	0.00000000	mice
Heca	-0.23	0.00000000	mice
Bptf	-0.17	0.00000000	mice
Dnajc5	-0.20	0.00000000	mice
Ubald1	-0.13	0.00000000	mice
Tex261	-0.20	0.00000000	mice
Snx20	0.24	0.00000000	mice
Pianp	-0.11	0.00000000	mice
Paip2	-0.20	0.00000000	mice
Tex264	-0.12	0.00000000	mice
Itga9	0.26	0.00000000	mice
Tmem43	-0.13	0.00000000	mice
Rnf130	-0.22	0.00000000	mice
F7	-0.06	0.00000000	mice
Pigt	-0.12	0.00000001	mice
Pbx1	-0.29	0.00000001	mice
Dnah17	-0.15	0.00000001	mice
Tmc6	-0.21	0.00000001	mice
Smc4	-0.13	0.00000001	mice
Zranb1	-0.19	0.00000001	mice
Shprh	-0.14	0.00000001	mice
Cyth3	-0.16	0.00000001	mice
S1pr1	-0.19	0.00000001	mice
Psap	-0.17	0.00000001	mice
Vgll4	-0.20	0.00000001	mice
Ylpm1	-0.14	0.00000001	mice
Pld1	-0.16	0.00000001	mice
Tdrd3	-0.13	0.00000001	mice
Ctnnb1	-0.33	0.00000001	mice
Ninj1	0.17	0.00000001	mice
Srgn	0.03	0.00000001	mice
Fam222b	-0.16	0.00000001	mice
Lamtor2	-0.17	0.00000001	mice
Snhg6	0.14	0.00000001	mice
Acly	-0.15	0.00000001	mice
H2-Eb1	0.63	0.00000001	mice
Pik3ca	-0.23	0.00000001	mice
Heatr3	-0.08	0.00000001	mice
Rai1	-0.14	0.00000001	mice
Nfix	-0.14	0.00000001	mice
Snrpb2	0.24	0.00000001	mice

Ccdc85b	-0.10	0.00000001	mice
Ripk2	0.34	0.00000001	mice
Fgd3	-0.55	0.00000001	mice
Ndufs5	0.25	0.00000001	mice
Herc2	-0.16	0.00000001	mice
Arhgap22	-0.10	0.00000001	mice
Cnnm3	-0.09	0.00000001	mice
Zfand2a	-0.23	0.00000001	mice
Tbc1d16	-0.12	0.00000001	mice
Rnf7	-0.09	0.00000001	mice
Ipo7	-0.18	0.00000001	mice
Slc11a1	0.24	0.00000001	mice
Sacm1l	-0.14	0.00000001	mice
Tlr3	0.12	0.00000001	mice
Oat	-0.16	0.00000001	mice
0610010F05Rik	-0.14	0.00000001	mice
Azi2	0.22	0.00000001	mice
Aldh9a1	-0.14	0.00000001	mice
Selenoi	-0.12	0.00000001	mice
Hpcal1	-0.14	0.00000001	mice
Ubqln4	-0.05	0.00000001	mice
Cst3	0.19	0.00000001	mice
Rbm8a	0.22	0.00000001	mice
Rab31	-0.25	0.00000001	mice
Gpd2	-0.14	0.00000001	mice
Add1	-0.24	0.00000001	mice
Pld3	-0.21	0.00000001	mice
Gpd1l	-0.09	0.00000001	mice
Pabpc1l	-0.12	0.00000001	mice
Ywhaq	-0.19	0.00000001	mice
Ncoa6	-0.19	0.00000001	mice
Gas7	-0.42	0.00000001	mice
S1pr2	0.11	0.00000001	mice
Cct6a	-0.13	0.00000001	mice
Api5	-0.17	0.00000001	mice
Atxn2l	-0.15	0.00000001	mice
Tmem131	0.60	0.00000001	mice
Tbc1d12	-0.16	0.00000001	mice
Efl1	-0.15	0.00000001	mice
Epc2	-0.18	0.00000001	mice
Atp6v0a2	0.20	0.00000001	mice
Pbx3	-0.23	0.00000001	mice
Sptan1	-0.17	0.00000001	mice
Ubl5	0.21	0.00000001	mice
Limd2	0.15	0.00000001	mice
Lcn2	0.36	0.00000001	mice

Tnrc6b	-0.28	0.00000001	mice
Rexo2	-0.18	0.00000002	mice
Kidins220	-0.20	0.00000002	mice
Lpxn	0.28	0.00000002	mice
L1cam	-0.16	0.00000002	mice
Itgb5	-0.16	0.00000002	mice
Tsc22d2	-0.14	0.00000002	mice
Wbp11	-0.26	0.00000002	mice
Asph	-0.18	0.00000002	mice
Pxn	0.28	0.00000002	mice
Homer1	0.34	0.00000002	mice
Nr2c2	-0.19	0.00000002	mice
Cybb	0.37	0.00000002	mice
Pkig	-0.18	0.00000002	mice
Lztfl1	0.28	0.00000002	mice
Frat2	-0.12	0.00000002	mice
Gk	0.38	0.00000002	mice
Nme1	-0.19	0.00000002	mice
Pgm2l1	-0.10	0.00000002	mice
Rftn1	-0.32	0.00000002	mice
Usp49	-0.12	0.00000002	mice
Gtf3c2	-0.16	0.00000002	mice
Phf14	-0.16	0.00000002	mice
Sorl1	-0.68	0.00000002	mice
Sae1	-0.14	0.00000002	mice
Scap	-0.10	0.00000002	mice
Ift22	0.15	0.00000002	mice
Setd1b	-0.15	0.00000002	mice
Cdc42	0.16	0.00000002	mice
Iars	-0.10	0.00000002	mice
Tagap	0.18	0.00000002	mice
Tsix	0.19	0.00000002	mice
Dlg1	-0.23	0.00000002	mice
Syk	-0.21	0.00000002	mice
Hist1h1c	-0.13	0.00000003	mice
Dnm2	-0.23	0.00000003	mice
Ipo8	-0.13	0.00000003	mice
Hace1	-0.13	0.00000003	mice
Snx27	-0.20	0.00000003	mice
Washc2	-0.21	0.00000003	mice
Psmd7	0.29	0.00000003	mice
Panx1	-0.11	0.00000003	mice
Bach2	-0.51	0.00000003	mice
Osbp	-0.18	0.00000003	mice
Brpf1	-0.13	0.00000003	mice
Usp4	-0.13	0.00000003	mice

E330020D12Rik	-0.12	0.00000003	mice
Rsl1d1	-0.13	0.00000004	mice
Ecm1	0.19	0.00000004	mice
Phf12	-0.15	0.00000004	mice
Ehbp1l1	-0.21	0.00000004	mice
Trappc9	-0.29	0.00000005	mice
Fam118b	-0.13	0.00000005	mice
Stxbp3	0.58	0.00000005	mice
Il10rb	0.21	0.00000005	mice
Dnajc1	-0.21	0.00000005	mice
Papss1	-0.17	0.00000005	mice
Ccdc88c	-0.23	0.00000005	mice
Zfp69	-0.07	0.00000005	mice
Hmox2	0.23	0.00000005	mice
Slc22a5	-0.09	0.00000006	mice
Safb2	-0.18	0.00000006	mice
Golim4	-0.13	0.00000006	mice
Nr1d2	-0.11	0.00000006	mice
Shld1	-0.07	0.00000006	mice
Arhgef10	-0.09	0.00000006	mice
Capn7	-0.20	0.00000006	mice
Slc31a1	0.15	0.00000006	mice
Gab1	-0.31	0.00000007	mice
Fam3c	-0.14	0.00000007	mice
Nbdy	-0.10	0.00000007	mice
Kmt5c	-0.04	0.00000007	mice
Znhit1	0.22	0.00000007	mice
Kif13b	-0.18	0.00000007	mice
Trim44	-0.17	0.00000007	mice
Rbm15b	-0.09	0.00000007	mice
Cd63	0.32	0.00000007	mice
Lnpep	-0.16	0.00000007	mice
Tctex1d2	0.20	0.00000007	mice
Numa1	-0.15	0.00000007	mice
Xrn2	0.23	0.00000008	mice
Syvn1	-0.10	0.00000008	mice
F630028O10Rik	-0.22	0.00000008	mice
Anxa3	-0.12	0.00000008	mice
Rnf146	-0.14	0.00000008	mice
Emp3	0.19	0.00000008	mice
Klf10	-0.16	0.00000009	mice
Ccdc115	-0.11	0.00000009	mice
Ptp4a2	-0.15	0.00000009	mice
Anxa4	0.29	0.00000010	mice
Paxip1	-0.08	0.00000010	mice
Trim65	-0.07	0.00000010	mice

Ss18	-0.17	0.00000010	mice
Jpt1	0.25	0.00000010	mice
Txndc11	-0.13	0.00000010	mice
Cndp2	0.29	0.00000010	mice
Pqlc1	-0.16	0.00000011	mice
Runx3	0.31	0.00000011	mice
Tmx4	-0.20	0.00000011	mice
Ndufaf4	-0.09	0.00000011	mice
Gna12	-0.13	0.00000011	mice
Arpc2	-0.14	0.00000012	mice
Ercc4	-0.11	0.00000012	mice
Thra	-0.09	0.00000012	mice
Tcp11l2	-0.14	0.00000012	mice
Ndufaf3	0.13	0.00000012	mice
Slain2	-0.20	0.00000013	mice
Lrrc8b	-0.16	0.00000013	mice
Kpnb1	-0.14	0.00000013	mice
Wdr36	-0.13	0.00000013	mice
Rlf	-0.23	0.00000013	mice
Fbxw7	-0.17	0.00000013	mice
Apc	-0.19	0.00000013	mice
Lipa	-0.28	0.00000014	mice
Acap2	-0.19	0.00000014	mice
Calu	-0.12	0.00000014	mice
Anapc1	-0.11	0.00000014	mice
Clec4e	0.28	0.00000014	mice
Dhx33	-0.08	0.00000014	mice
Ube3a	-0.20	0.00000015	mice
Cd300lb	-0.26	0.00000015	mice
H2afj	-0.21	0.00000015	mice
Arid1a	-0.20	0.00000015	mice
Atf7	-0.20	0.00000015	mice
Psma6	0.26	0.00000016	mice
Il17ra	-0.19	0.00000016	mice
Snhg15	0.20	0.00000016	mice
Akap9	-0.18	0.00000017	mice
Csnk1e	-0.14	0.00000017	mice
Uba52	0.21	0.00000017	mice
Terf2ip	-0.07	0.00000018	mice
Vrk2	0.27	0.00000018	mice
Sec63	-0.17	0.00000019	mice
Adrm1	0.21	0.00000019	mice
Gpr65	0.22	0.00000019	mice
Hopx	-0.34	0.00000019	mice
Hnrnpl	-0.17	0.00000020	mice
Bhlhe40	-0.28	0.00000020	mice

Rnft1	-0.14	0.00000020	mice
Entpd1	-0.57	0.00000020	mice
Ctdsp2	-0.17	0.00000021	mice
Tent5a	0.31	0.00000021	mice
G6pc3	-0.08	0.00000021	mice
Tmem131l	-0.32	0.00000022	mice
Kat14	-0.08	0.00000022	mice
Kmt5a	-0.14	0.00000022	mice
Ubr3	-0.15	0.00000022	mice
Slc12a7	-0.10	0.00000022	mice
Smarcc1	-0.19	0.00000023	mice
Gns	-0.22	0.00000023	mice
I830077J02Rik	0.16	0.00000023	mice
Sipa1l3	-0.11	0.00000024	mice
Iffo1	-0.06	0.00000024	mice
Pebp1	-0.11	0.00000024	mice
Slc2a6	0.13	0.00000026	mice
Arhgap9	-0.19	0.00000027	mice
Tcf7l2	-0.46	0.00000027	mice
Pla2g15	-0.30	0.00000027	mice
Sik3	0.21	0.00000027	mice
Ubxn6	-0.11	0.00000029	mice
Tnpo2	-0.12	0.00000030	mice
Dusp3	-0.18	0.00000031	mice
Ppp1r11	0.19	0.00000031	mice
Zdhhc18	0.30	0.00000031	mice
Pja2	-0.14	0.00000032	mice
Aida	0.17	0.00000033	mice
Ubtd2	-0.10	0.00000034	mice
Pcm1	-0.17	0.00000035	mice
Ep300	-0.23	0.00000035	mice
Ankrd10	-0.14	0.00000035	mice
Eea1	-0.30	0.00000036	mice
Pdpr	-0.09	0.00000036	mice
Ap2b1	-0.21	0.00000036	mice
Cdv3	-0.15	0.00000037	mice
Asah1	-0.13	0.00000037	mice
Cdkl2	-0.08	0.00000038	mice
Bend3	-0.08	0.00000039	mice
Fryl	-0.24	0.00000040	mice
Hyou1	-0.12	0.00000040	mice
Sidt2	-0.16	0.00000040	mice
Cyfip1	0.31	0.00000040	mice
Rab33b	-0.10	0.00000040	mice
Zdhhc20	-0.12	0.00000041	mice
Ralgapa2	-0.27	0.00000041	mice

Hgsnat	-0.21	0.00000041	mice
Hipk3	-0.18	0.00000041	mice
Ddt	0.31	0.00000041	mice
Celf1	-0.18	0.00000042	mice
Tubgcp3	-0.11	0.00000043	mice
Aph1b	-0.10	0.00000043	mice
Srsf10	-0.16	0.00000044	mice
Rmdn1	-0.12	0.00000045	mice
Cep83	-0.23	0.00000046	mice
Stau2	-0.10	0.00000046	mice
Igf2bp3	-0.10	0.00000047	mice
Rims3	-0.08	0.00000048	mice
Glrx3	-0.16	0.00000048	mice
Glis3	-0.49	0.00000048	mice
Togaram1	-0.13	0.00000049	mice
Rev3l	-0.17	0.00000049	mice
Blvrb	0.25	0.00000050	mice
Gapvd1	-0.16	0.00000050	mice
Tbc1d23	-0.21	0.00000050	mice
Ghitm	0.23	0.00000050	mice
Gpbp1l1	-0.16	0.00000053	mice
Abcg2	-0.07	0.00000054	mice
S100a6	0.24	0.00000058	mice
Hspbp1	-0.09	0.00000058	mice
Kat7	-0.13	0.00000059	mice
Ssr1	-0.19	0.00000059	mice
Ptprc	-0.18	0.00000060	mice
Setd2	-0.18	0.00000061	mice
Selenok	-0.23	0.00000061	mice
Pilra	-0.21	0.00000061	mice
Psd4	0.15	0.00000062	mice
Rbbp8	0.28	0.00000062	mice
Tsc1	-0.17	0.00000063	mice
Oaz1	-0.14	0.00000063	mice
Ctbp2	-0.09	0.00000064	mice
Ap1s2	-0.24	0.00000066	mice
Elovl6	-0.05	0.00000067	mice
Ltn1	-0.14	0.00000067	mice
Slc9a1	0.29	0.00000067	mice
Fam193b	-0.10	0.00000067	mice
Tesk1	0.16	0.00000069	mice
Arhgef1	-0.17	0.00000069	mice
Casd1	-0.12	0.00000071	mice
Batf	0.14	0.00000072	mice
Scarb1	-0.15	0.00000074	mice
Git2	-0.16	0.00000074	mice

Txlna	-0.08	0.00000075	mice
Trim14	0.21	0.00000076	mice
Dph3	-0.17	0.00000079	mice
Cul3	-0.16	0.00000080	mice
Usp10	-0.13	0.00000083	mice
Polr2e	-0.19	0.00000084	mice
Hltf	-0.13	0.00000087	mice
Tmbim4	0.17	0.00000088	mice
Hspa1b	0.27	0.00000090	mice
Tmem238	-0.07	0.00000097	mice
Egln2	-0.11	0.00000101	mice
Gtf3c6	0.17	0.00000103	mice
Ubqln2	-0.11	0.00000103	mice
Ncbp1	-0.09	0.00000103	mice
H2afx	-0.08	0.00000104	mice
Ddx31	-0.10	0.00000110	mice
Kdm5b	-0.26	0.00000110	mice
Cd300e	0.23	0.00000110	mice
Ndufb11	-0.13	0.00000112	mice
Rhot1	-0.11	0.00000113	mice
Unkl	-0.10	0.00000113	mice
Zeb2os	0.16	0.00000114	mice
Trip11	-0.14	0.00000126	mice
Ndufc1	0.27	0.00000130	mice
Tomm6	-0.17	0.00000130	mice
Anxa2	-0.15	0.00000130	mice
Alg9	-0.10	0.00000133	mice
Zfp667	0.22	0.00000137	mice
Reep5	-0.23	0.00000137	mice
Ext2	-0.10	0.00000137	mice
Utp20	-0.10	0.00000138	mice
1810013L24Rik	-0.16	0.00000141	mice
Zmym5	-0.10	0.00000143	mice
Scai	-0.15	0.00000145	mice
Nsmce2	-0.21	0.00000146	mice
Crlf2	0.15	0.00000147	mice
Ppib	0.18	0.00000152	mice
Ly9	0.11	0.00000154	mice
Srek1	-0.18	0.00000161	mice
Pnpla2	-0.15	0.00000161	mice
Gxylt1	-0.13	0.00000162	mice
Lrrfip2	-0.20	0.00000165	mice
Slc7a2	-0.10	0.00000167	mice
Bicra	-0.14	0.00000168	mice
Wdr7	-0.16	0.00000187	mice
Pcmtd2	-0.08	0.00000188	mice

Slc25a26	-0.11	0.00000190	mice
Cnn3	0.13	0.00000200	mice
Igf2r	0.13	0.00000201	mice
Slc23a2	-0.15	0.00000206	mice
Eno3	0.35	0.00000208	mice
Amfr	-0.13	0.00000209	mice
Kdm5a	-0.20	0.00000212	mice
Baz2b	-0.19	0.00000215	mice
Nufip2	-0.16	0.00000216	mice
Hbp1	-0.15	0.00000221	mice
Abca3	-0.14	0.00000221	mice
Retreg2	-0.10	0.00000232	mice
Jun	0.65	0.00000234	mice
Cpsf6	-0.14	0.00000234	mice
Actg1	-0.16	0.00000249	mice
Cd48	0.17	0.00000254	mice
Leng8	-0.09	0.00000255	mice
Tpm1	0.67	0.00000260	mice
Coq10b	0.25	0.00000262	mice
Ocrl	-0.10	0.00000271	mice
Tuba1c	0.18	0.00000272	mice
Ern1	0.50	0.00000274	mice
Pds5a	-0.17	0.00000274	mice
Aftph	0.26	0.00000277	mice
Eif2s3x	-0.15	0.00000280	mice
Ankle2	0.15	0.00000287	mice
Pkd1l2	0.08	0.00000289	mice
Ifitm6	0.19	0.00000304	mice
Kdm2a	-0.19	0.00000313	mice
Ptdss1	-0.13	0.00000314	mice
Strn	-0.20	0.00000315	mice
Cgnl1	-0.17	0.00000317	mice
Rragc	-0.23	0.00000317	mice
Ripk3	0.09	0.00000319	mice
Lrpprc	-0.14	0.00000328	mice
Calm2	-0.17	0.00000341	mice
Hsf1	-0.12	0.00000346	mice
Irak1	-0.13	0.00000353	mice
Nat10	-0.10	0.00000354	mice
Cdk16	-0.09	0.00000359	mice
Usp31	-0.11	0.00000360	mice
Pacs2	-0.10	0.00000366	mice
Ranbp9	-0.17	0.00000367	mice
Ifnar2	0.16	0.00000388	mice
Pla2g4a	0.23	0.00000392	mice
Insyn2b	0.13	0.00000394	mice

Nup153	-0.15	0.00000396	mice
Castor2	0.12	0.00000401	mice
Syngr2	0.31	0.00000402	mice
Gnl3	-0.12	0.00000403	mice
Siah1a	-0.16	0.00000408	mice
Fmnl3	-0.15	0.00000411	mice
Kmt2d	-0.14	0.00000418	mice
AU020206	-0.37	0.00000421	mice
Atxn7l3b	-0.16	0.00000432	mice
Dennd1c	0.12	0.00000439	mice
Zfx	-0.16	0.00000441	mice
Smim11	-0.10	0.00000463	mice
Calcoco1	-0.07	0.00000488	mice
Tnip1	0.19	0.00000491	mice
Sfr1	-0.21	0.00000492	mice
Polr2k	0.19	0.00000512	mice
Inpp5k	-0.15	0.00000517	mice
Acvr2a	-0.12	0.00000529	mice
Zmym4	-0.19	0.00000531	mice
Hps3	-0.14	0.00000542	mice
Il1rl2	-0.06	0.00000543	mice
Xrcc5	-0.06	0.00000547	mice
Itm2c	-0.13	0.00000548	mice
Pdxdc1	-0.13	0.00000557	mice
Nrd1	-0.17	0.00000561	mice
Atp6ap2	-0.17	0.00000561	mice
Tpk1	-0.13	0.00000562	mice
Hist1h2bc	-0.10	0.00000573	mice
Fam129b	-0.20	0.00000588	mice
Arhgap18	-0.17	0.00000608	mice
Cwc25	0.17	0.00000614	mice
Kdelr1	-0.13	0.00000627	mice
Prmt9	-0.11	0.00000628	mice
Swap70	0.33	0.00000635	mice
Dtx3	-0.05	0.00000638	mice
Uqcc2	0.17	0.00000653	mice
Uqcrfs1	-0.21	0.00000654	mice
Capza2	0.21	0.00000659	mice
Eif3e	-0.20	0.00000669	mice
Rnf103	-0.12	0.00000670	mice
Pced1b	-0.15	0.00000692	mice
Opa3	0.19	0.00000692	mice
Idh2	-0.12	0.00000698	mice
Wsb2	-0.15	0.00000715	mice
Ppp1cb	-0.12	0.00000716	mice
Oaf	-0.10	0.00000719	mice

Spast	-0.14	0.00000730	mice
Gnas	-0.13	0.00000751	mice
Atp5b	0.25	0.00000765	mice
Prrc1	-0.13	0.00000774	mice
Trpc4ap	-0.15	0.00000781	mice
Kifap3	-0.05	0.00000785	mice
Rhoa	-0.17	0.00000803	mice
Pigs	-0.09	0.00000807	mice
Tcf20	-0.16	0.00000808	mice
Arhgap17	-0.14	0.00000830	mice
Elp1	-0.08	0.00000832	mice
Rbl2	-0.12	0.00000840	mice
Sirpb1c	0.11	0.00000850	mice
Uso1	-0.13	0.00000855	mice
Pkn1	-0.15	0.00000895	mice
R3hdm1	-0.24	0.00000896	mice
Prdx5	0.38	0.00000898	mice
Fam135a	-0.09	0.00000914	mice
Aldoc	-0.06	0.00000931	mice
Hic2	-0.10	0.00000942	mice
Tmed5	-0.16	0.00000958	mice
Tnfrsf12a	0.21	0.00000975	mice
Hccs	-0.16	0.00000978	mice
Il3ra	0.11	0.00000983	mice
Nars2	-0.24	0.00000988	mice
Nsd3	0.31	0.00000998	mice
Ppp4r3a	-0.19	0.00001049	mice
Eif4g2	-0.16	0.00001064	mice
Atp6ap1	-0.18	0.00001071	mice
Kif23	-0.12	0.00001074	mice
Cebpg	-0.13	0.00001074	mice
Dbi	-0.12	0.00001087	mice
Ikbkb	-0.20	0.00001088	mice
Cpsf7	-0.13	0.00001102	mice
Nbn	0.18	0.00001157	mice
Por	0.24	0.00001158	mice
Gclm	-0.11	0.00001210	mice
Zhx1	-0.15	0.00001228	mice
Taf3	-0.19	0.00001235	mice
Psme3	-0.14	0.00001263	mice
Dynlt1f	0.10	0.00001265	mice
Akr7a5	-0.06	0.00001311	mice
Pdap1	0.21	0.00001330	mice
Hmgb1	-0.10	0.00001333	mice
Dera	0.09	0.00001346	mice
Zyg11b	-0.13	0.00001357	mice

Foxn2	-0.16	0.00001362	mice
Fam160b1	-0.10	0.00001390	mice
Scoc	-0.07	0.00001445	mice
Ppp2r1a	-0.14	0.00001474	mice
Agpat5	-0.16	0.00001555	mice
Ctdp1	-0.16	0.00001568	mice
Pank3	-0.11	0.00001613	mice
Endod1	0.13	0.00001620	mice
Tfb2m	-0.06	0.00001654	mice
Kansl1l	0.17	0.00001669	mice
Anapc11	-0.11	0.00001678	mice
Hmbox1	-0.13	0.00001697	mice
Camk4	-0.41	0.00001709	mice
Scd2	-0.10	0.00001741	mice
Rnf166	-0.13	0.00001741	mice
Tbxas1	-0.30	0.00001742	mice
Prpf18	-0.12	0.00001755	mice
Dynlrb1	0.21	0.00001773	mice
Ptprj	-0.18	0.00001782	mice
Zfp597	-0.05	0.00001794	mice
Ndufa11	0.24	0.00001894	mice
Brd9	-0.10	0.00001907	mice
Hipk1	-0.15	0.00001946	mice
Cox7b	0.27	0.00001971	mice
Specc1l	0.26	0.00001982	mice
Crebrf	-0.21	0.00001998	mice
Rgl1	0.47	0.00002016	mice
Esr1	0.10	0.00002028	mice
Setd5	-0.18	0.00002039	mice
Taf7	0.16	0.00002063	mice
Zbtb10	-0.06	0.00002071	mice
Jdp2	-0.28	0.00002132	mice
Kif2a	-0.15	0.00002153	mice
Tpm3-rs7	0.19	0.00002221	mice
Pa2g4	-0.12	0.00002304	mice
Lef1	-0.33	0.00002328	mice
Ids	-0.15	0.00002367	mice
Fas	0.17	0.00002385	mice
Ndufc2	0.28	0.00002391	mice
AI837181	0.10	0.00002407	mice
Cd84	-0.19	0.00002424	mice
Rcl1	-0.12	0.00002508	mice
Rrp1b	-0.17	0.00002533	mice
Slc16a7	-0.11	0.00002547	mice
Bcap29	-0.13	0.00002633	mice
Snx25	-0.11	0.00002643	mice

Acsl4	-0.18	0.00002728	mice
Lmbr1l	-0.15	0.00002729	mice
Ctcf	-0.17	0.00002735	mice
Ctnna1	-0.13	0.00002762	mice
Wnk1	-0.15	0.00002772	mice
Ier2	0.32	0.00002817	mice
Lamtor4	-0.15	0.00002832	mice
Ppargc1b	-0.07	0.00002903	mice
Kcnk13	-0.18	0.00002904	mice
Nfkbiz	0.18	0.00003035	mice
R3hdm4	-0.13	0.00003037	mice
Taf4	-0.10	0.00003041	mice
Arhgdia	-0.10	0.00003170	mice
Atp5a1	-0.16	0.00003192	mice
Nfkb2	0.16	0.00003218	mice
Apool	0.16	0.00003256	mice
5430427O19Rik	0.12	0.00003275	mice
Apaf1	0.32	0.00003299	mice
Hectd4	-0.09	0.00003502	mice
Cmtm3	-0.10	0.00003612	mice
Tmem170b	-0.15	0.00003612	mice
Apobec3	0.09	0.00003622	mice
Cope	0.17	0.00003681	mice
Apip	0.09	0.00003727	mice
Usp33	-0.16	0.00003769	mice
Coq10a	-0.09	0.00003805	mice
Csnk2b	0.17	0.00003836	mice
Zscan26	-0.11	0.00003909	mice
Ugcg	0.29	0.00003920	mice
Atf6	-0.24	0.00003948	mice
Arhgap24	-0.23	0.00004027	mice
Pgs1	0.19	0.00004048	mice
Atp5mpl	0.21	0.00004076	mice
Rangap1	-0.10	0.00004095	mice
Casp3	0.13	0.00004238	mice
Sec24a	-0.17	0.00004370	mice
Yeats2	-0.10	0.00004446	mice
Fuca1	-0.15	0.00004492	mice
Ice1	-0.13	0.00004549	mice
Ntpcr	0.15	0.00004713	mice
Fyttd1	-0.18	0.00004852	mice
Casp7	0.14	0.00004958	mice
Rhov	0.14	0.00005014	mice
Txndc5	-0.11	0.00005017	mice
Ifitm10	-0.19	0.00005059	mice
Vkorc1	-0.07	0.00005103	mice

Dhx15	-0.13	0.00005204	mice
Rpp21	0.14	0.00005332	mice
Rit1	-0.10	0.00005478	mice
Elof1	0.16	0.00005623	mice
Smarca5	-0.15	0.00005629	mice
Rreb1	-0.15	0.00005662	mice
Ireb2	-0.17	0.00005676	mice
Epc1	-0.12	0.00005697	mice
Slc31a2	0.20	0.00005705	mice
Psmd14	0.21	0.00005780	mice
Polr1a	-0.11	0.00005788	mice
Dido1	-0.13	0.00005893	mice
Zzz3	-0.15	0.00005937	mice
Plk3	-0.29	0.00005951	mice
Foxj2	-0.09	0.00005980	mice
Sesn1	-0.13	0.00006068	mice
Fbrsl1	-0.14	0.00006081	mice
Traf3	0.34	0.00006090	mice
Lhfpl2	-0.17	0.00006144	mice
C2cd5	-0.12	0.00006156	mice
Lpar6	-0.22	0.00006162	mice
Il10	0.25	0.00006184	mice
Rhob	-0.13	0.00006269	mice
Rictor	-0.14	0.00006493	mice
Tmem165	-0.15	0.00006521	mice
Pink1	-0.04	0.00006591	mice
Nfkbid	0.19	0.00006673	mice
Dcaf12	-0.19	0.00006684	mice
F11r	-0.03	0.00006703	mice
Cwc27	-0.18	0.00006711	mice
Zmynd11	-0.25	0.00006718	mice
Tmtc2	0.30	0.00006750	mice
Pgap1	-0.09	0.00006901	mice
Klhl18	-0.12	0.00007251	mice
Uvrag	-0.23	0.00007295	mice
Nrm	-0.12	0.00007298	mice
Irf2	0.25	0.00007420	mice
St7	-0.13	0.00007490	mice
Nlrp3	0.30	0.00007560	mice
Hbs1l	-0.14	0.00007593	mice
Rsu1	-0.15	0.00007659	mice
Lsp1	-0.09	0.00007768	mice
Rap2a	-0.09	0.00007788	mice
3830406C13Rik	0.14	0.00007803	mice
Brd3	-0.09	0.00007825	mice
4933432I03Rik	0.03	0.00007906	mice

Slc25a20	0.18	0.00008120	mice
Dapp1	-0.19	0.00008195	mice
Adss	-0.11	0.00008205	mice
Tom1l2	-0.15	0.00008284	mice
Fbxo8	-0.08	0.00008537	mice
Ccdc186	-0.11	0.00008723	mice
Amdhd2	0.18	0.00008900	mice
Ccdc6	-0.08	0.00009010	mice
Hnrnpd	0.20	0.00009140	mice
Birc6	0.02	0.00009200	mice
Pmaip1	0.26	0.00009235	mice
Rbm7	0.21	0.00009379	mice
Polr3e	-0.07	0.00009506	mice
Zfp110	-0.11	0.00009553	mice
Sept7	-0.19	0.00009579	mice
Slc9a6	-0.08	0.00009623	mice
Edf1	0.21	0.00009795	mice
Rbm42	0.16	0.00009933	mice
Rasgef1b	0.49	0.00009953	mice
Prxl2c	-0.12	0.00009956	mice
Slamf9	0.49	0.00010082	mice
Nf2	-0.12	0.00010152	mice
Gpr155	-0.06	0.00010318	mice
Fut11	-0.07	0.00010617	mice
Vwa5a	0.19	0.00010850	mice
Synj2	-0.09	0.00010862	mice
Fam217b	-0.08	0.00010954	mice
Ywhaz	-0.15	0.00011158	mice
Dynll1	0.31	0.00011190	mice
Lamtor5	0.19	0.00011223	mice
Ehbp1	-0.12	0.00011231	mice
Smc3	-0.08	0.00011233	mice
Trim16	-0.08	0.00011278	mice
Phf8	-0.15	0.00011464	mice
Ccdc57	-0.09	0.00011506	mice
Pip5k1c	-0.16	0.00011622	mice
Tmem30a	-0.15	0.00011624	mice
BC029722	-0.06	0.00011685	mice
Lats1	-0.12	0.00011932	mice
Uhmk1	-0.10	0.00012090	mice
Txnrd1	0.30	0.00012232	mice
Taok1	-0.17	0.00012392	mice
Thumpd1	-0.06	0.00012610	mice
Sumo3	0.16	0.00012612	mice
Ints6l	-0.09	0.00012663	mice
Fam102a	-0.08	0.00012796	mice

Vps13a	-0.15	0.00013325	mice
Eif3l	-0.12	0.00013379	mice
Sh3kbp1	-0.18	0.00013700	mice
Eif4g3	-0.23	0.00013759	mice
Ebna1bp2	0.14	0.00013786	mice
Rest	0.22	0.00013852	mice
Trpv2	-0.15	0.00013928	mice
4930469K13Rik	0.16	0.00014006	mice
Gpr146	-0.07	0.00014170	mice
Nomo1	-0.07	0.00014222	mice
Wdr33	-0.19	0.00014314	mice
Cisd1	-0.12	0.00014337	mice
Ssbp1	0.18	0.00014429	mice
Zc3h14	-0.10	0.00014564	mice
Atp9b	-0.16	0.00014584	mice
Wdr11	-0.10	0.00014717	mice
Klf6	0.30	0.00014775	mice
Adora2a	0.15	0.00014841	mice
Bcor	-0.17	0.00014878	mice
Acox3	-0.10	0.00014971	mice
Gapdh	-0.13	0.00015006	mice
Atg4c	-0.09	0.00015390	mice
Arf5	-0.13	0.00015564	mice
Cep128	-0.18	0.00015665	mice
Ssbp3	-0.11	0.00015802	mice
Chmp5	0.19	0.00015847	mice
Tsc22d4	0.19	0.00016641	mice
Tmem106c	-0.05	0.00016689	mice
Inpp5a	-0.17	0.00017004	mice
Acvr1	-0.13	0.00017060	mice
Pdzd11	0.12	0.00017162	mice
Zfat	-0.12	0.00017182	mice
Pptc7	-0.10	0.00017335	mice
Dpep2	-0.10	0.00017461	mice
Eps15l1	-0.12	0.00017644	mice
Arl6ip1	0.22	0.00017908	mice
Cnbp	-0.14	0.00018015	mice
Arrdc3	-0.23	0.00018218	mice
Atg16l2	-0.15	0.00018664	mice
Zfp280d	-0.15	0.00018894	mice
Arl8a	-0.14	0.00019354	mice
Nab2	-0.07	0.00019763	mice
Arhgap1	-0.09	0.00020158	mice
Zfp36	0.47	0.00020975	mice
Ddx39	0.12	0.00021271	mice
Itgb7	0.16	0.00022241	mice

2310022A10Rik	0.17	0.00022445	mice
Chd1l	-0.11	0.00022488	mice
Immp2l	-0.27	0.00022647	mice
Flad1	0.08	0.00022879	mice
Pirb	0.23	0.00023424	mice
Smim10l1	-0.08	0.00023806	mice
Rasgrp4	0.08	0.00024070	mice
Appl1	-0.13	0.00024186	mice
Proser1	-0.09	0.00024467	mice
Pigp	-0.07	0.00024489	mice
Ttc7	-0.22	0.00025278	mice
Hivep2	0.32	0.00025530	mice
Rad50	-0.08	0.00026078	mice
Prkdc	-0.08	0.00026271	mice
Slc7a7	0.11	0.00026454	mice
Pum2	-0.16	0.00026657	mice
Angptl4	-0.08	0.00027696	mice
Usp7	-0.11	0.00027889	mice
Edrf1	-0.09	0.00028885	mice
Fus	-0.12	0.00029220	mice
Nek9	-0.13	0.00029823	mice
Tmem109	-0.10	0.00030054	mice
Rnasel	-0.11	0.00030203	mice
Bag4	-0.08	0.00030519	mice
Prr5l	0.24	0.00031492	mice
Eif4e2	0.22	0.00031537	mice
Hspa9	-0.12	0.00031933	mice
Sept8	-0.06	0.00032148	mice
Cdkn1b	-0.14	0.00032508	mice
B4galt5	0.24	0.00032612	mice
Xpo6	-0.21	0.00032867	mice
Paf1	0.18	0.00032932	mice
Uqcc3	-0.08	0.00033084	mice
Fbxo32	-0.03	0.00033345	mice
Akirin2	-0.15	0.00033416	mice
Ino80d	-0.13	0.00033616	mice
Adgrg5	0.10	0.00033785	mice
Csnk2a1	-0.12	0.00033975	mice
Srgap2	0.34	0.00034165	mice
Pkd2	-0.10	0.00034229	mice
Capn2	-0.15	0.00034454	mice
Csk	0.20	0.00034520	mice
Phactr2	-0.21	0.00034917	mice
Cdk9	-0.11	0.00035068	mice
Sfswap	-0.17	0.00035130	mice
Dnajb14	-0.15	0.00035553	mice

Trim28	-0.11	0.00035874	mice
Hexim1	-0.09	0.00035930	mice
Dpp8	-0.18	0.00036662	mice
Nlk	-0.13	0.00037040	mice
Cmc1	0.17	0.00037668	mice
Cotl1	-0.16	0.00037762	mice
Foxo1	-0.19	0.00037859	mice
Arhgap23	-0.12	0.00038156	mice
Itpkb	0.39	0.00038267	mice
Dok2	-0.10	0.00038336	mice
Carmil1	0.13	0.00038582	mice
Trim11	-0.12	0.00038672	mice
Dcaf1	-0.10	0.00038960	mice
Entr1	-0.09	0.00039504	mice
Tax1bp3	-0.08	0.00039768	mice
AW554918	-0.17	0.00040497	mice
Slf2	-0.17	0.00041194	mice
Chfr	-0.10	0.00044483	mice
Eri3	-0.10	0.00044559	mice
Stk38	-0.26	0.00044640	mice
Ppp1r12a	-0.17	0.00045071	mice
Pde1b	-0.09	0.00045184	mice
Vipas39	0.08	0.00045436	mice
Ttll4	-0.06	0.00045499	mice
Srp72	-0.16	0.00046062	mice
Flnb	0.31	0.00046180	mice
Nrf1	-0.15	0.00047731	mice
Uhrf1bp1l	-0.21	0.00047973	mice
Vsir	-0.13	0.00048017	mice
Idnk	0.20	0.00048500	mice
S100a9	-0.43	0.00049009	mice
Atp6v1b2	-0.23	0.00049054	mice
Gpbp1	0.19	0.00049081	mice
Phlpp2	-0.11	0.00049104	mice
Gle1	-0.08	0.00049680	mice
Psmc6	0.19	0.00051029	mice
Fig4	-0.11	0.00052288	mice
Parvb	-0.10	0.00052627	mice
Hip1r	-0.15	0.00052663	mice
Cpped1	-0.10	0.00052747	mice
Iqgap2	-0.15	0.00053642	mice
Glrx	0.22	0.00054900	mice
Agfg2	-0.08	0.00055023	mice
Btf3	-0.14	0.00055978	mice
Firre	-0.06	0.00056929	mice
Lztr1	-0.05	0.00056946	mice

Pkd1	-0.06	0.00057735	mice
Pxmp4	-0.06	0.00057748	mice
Smad3	0.17	0.00057958	mice
Tnfaip2	0.51	0.00058295	mice
Nck1	-0.13	0.00058452	mice
Psmd11	0.23	0.00058506	mice
Npc1	-0.17	0.00058736	mice
Fam91a1	-0.11	0.00059246	mice
Ptk2b	-0.15	0.00059936	mice
Puf60	-0.13	0.00061557	mice
Nfyc	-0.15	0.00061572	mice
Coq2	0.16	0.00061574	mice
Ints8	-0.10	0.00061749	mice
Stk38l	0.23	0.00062175	mice
Csgalnact2	-0.18	0.00062521	mice
Cyp20a1	-0.13	0.00065673	mice
Klf16	-0.07	0.00065899	mice
Psmb7	0.17	0.00065982	mice
Trim41	-0.07	0.00068229	mice
Krr1	-0.08	0.00069721	mice
Cacybp	-0.15	0.00071029	mice
Naa25	0.15	0.00072072	mice
Frrs1	-0.16	0.00073078	mice
Ndrg3	-0.08	0.00073946	mice
P4hb	-0.13	0.00075883	mice
Snhg8	-0.07	0.00076198	mice
Ocel1	0.15	0.00077211	mice
Ifi27	-0.14	0.00078410	mice
Pdgfrl	-0.05	0.00081245	mice
Cmas	-0.17	0.00082018	mice
Aff3	-0.27	0.00082821	mice
Golt1b	-0.09	0.00083365	mice
Xpnpep1	-0.10	0.00084377	mice
Dctn4	-0.13	0.00084583	mice
Ppt2	-0.11	0.00086383	mice
Pdss2	-0.13	0.00086540	mice
Pecam1	0.15	0.00088197	mice
Pacsin2	-0.20	0.00089443	mice
Slc25a24	-0.11	0.00089446	mice
Pwwp3a	-0.05	0.00089958	mice
H2-Aa	0.33	0.00090318	mice
Tm9sf2	-0.16	0.00093160	mice
Spic	0.19	0.00094097	mice
Pfdn1	0.17	0.00094436	mice
Acot8	0.07	0.00094713	mice
Ebpl	-0.11	0.00094773	mice

Tubb2a	0.13	0.00098088	mice
Filip1l	0.29	0.00098716	mice
Fbxo6	0.11	0.00099274	mice
Eapp	0.19	0.00102615	mice
Rbm14	-0.05	0.00104063	mice
Gde1	-0.03	0.00104605	mice
Fam174a	-0.15	0.00104944	mice
Ddrgk1	-0.09	0.00106899	mice
Lrp4	-0.05	0.00106983	mice
Traf2	0.13	0.00107319	mice
Ccdc88b	0.19	0.00107393	mice
Cep85l	0.25	0.00109936	mice
Plxnd1	-0.04	0.00109979	mice
Eprs	-0.12	0.00111270	mice
Sdhaf1	-0.11	0.00111707	mice
Senp5	-0.12	0.00112907	mice
Capn1	-0.12	0.00116294	mice
Wars	0.13	0.00118494	mice
Naa16	-0.13	0.00118694	mice
Otud6b	-0.06	0.00118815	mice
Dtnbp1	-0.21	0.00118974	mice
Fbxo34	0.22	0.00119865	mice
Rapgef6	-0.15	0.00120352	mice
Nr1h3	0.20	0.00121690	mice
Ltb	0.03	0.00123592	mice
Larp1b	-0.17	0.00124248	mice
Spint2	-0.05	0.00124594	mice
Tomm7	0.18	0.00124758	mice
Eif3f	-0.23	0.00125006	mice
St7l	-0.11	0.00125126	mice
Ctns	-0.07	0.00125546	mice
Shld2	-0.10	0.00126044	mice
Cyren	-0.06	0.00127136	mice
Ric8b	-0.08	0.00127644	mice
Skap1	-0.17	0.00128471	mice
Sdhb	0.14	0.00128586	mice
Zfyve1	-0.10	0.00129682	mice
Wdr83os	-0.04	0.00130967	mice
Gtf2e2	-0.23	0.00131832	mice
Gphn	0.00	0.00134501	mice
Sf1	-0.16	0.00140217	mice
Klhl5	-0.13	0.00141631	mice
Ube4b	-0.11	0.00142165	mice
Slc22a15	-0.21	0.00142437	mice
Crat	-0.07	0.00143765	mice
Sord	-0.05	0.00144291	mice

Anp32b	-0.09	0.00144713	mice
Klf4	-0.14	0.00145576	mice
Sumo1	0.15	0.00149208	mice
Lap3	0.20	0.00150976	mice
Trrap	-0.10	0.00151346	mice
H2-Ke6	-0.09	0.00152672	mice
Arf3	-0.14	0.00153014	mice
Prdx4	0.16	0.00153306	mice
Serinc5	-0.07	0.00153906	mice
Xpc	-0.07	0.00157797	mice
Cxcl2	0.58	0.00159641	mice
Zfp871	-0.10	0.00160324	mice
H2-Ab1	0.50	0.00160893	mice
Fam32a	0.16	0.00161000	mice
Btbd9	-0.23	0.00164162	mice
Cntrl	-0.13	0.00164441	mice
Dnaja2	0.21	0.00164599	mice
Zadh2	-0.08	0.00167364	mice
Slc17a5	-0.10	0.00169382	mice
Ash2l	-0.09	0.00170147	mice
Tob2	-0.08	0.00170472	mice
Rnf144a	0.11	0.00171164	mice
Sys1	0.18	0.00171260	mice
Evi2	0.06	0.00171666	mice
Paip2b	-0.08	0.00172193	mice
Ubr2	-0.12	0.00173515	mice
2410002F23Rik	-0.08	0.00175455	mice
Cryl1	-0.05	0.00177021	mice
Ccdc82	-0.11	0.00177119	mice
Pspc1	-0.13	0.00180293	mice
Gfpt1	-0.17	0.00181216	mice
Uqcc1	-0.09	0.00182315	mice
Ube2g1	-0.18	0.00185939	mice
Oxct1	-0.16	0.00187528	mice
Ak3	-0.10	0.00189820	mice
Fam120b	-0.12	0.00190951	mice
Pilrb2	-0.12	0.00195692	mice
Tmem138	-0.05	0.00197115	mice
Heatr1	-0.10	0.00198696	mice
Tmem141	-0.06	0.00198886	mice
Abcb1a	0.13	0.00201444	mice
Dhrs7	-0.12	0.00203334	mice
Naip2	-0.18	0.00203353	mice
Adam9	0.27	0.00204004	mice
Agl	-0.07	0.00207107	mice
Tmem156	-0.13	0.00210635	mice

Gem	0.11	0.00222916	mice
Suco	-0.19	0.00223678	mice
C1qbp	-0.07	0.00230526	mice
Spryd7	0.11	0.00230760	mice
Vamp3	-0.14	0.00240533	mice
Tubb4b	0.29	0.00245136	mice
Ap3b1	-0.15	0.00245893	mice
Klrb1b	-0.06	0.00246379	mice
Fyco1	0.24	0.00248033	mice
Btk	-0.11	0.00248659	mice
Sav1	-0.14	0.00259752	mice
Nap1l4	-0.09	0.00260229	mice
Slc25a13	-0.08	0.00260705	mice
Cd82	-0.14	0.00265337	mice
Pfkfb3	-0.16	0.00265538	mice
Dusp1	0.41	0.00266739	mice
Cnot10	-0.07	0.00266857	mice
Selenos	-0.11	0.00267999	mice
Slc25a5	-0.15	0.00268671	mice
Rab24	0.11	0.00269633	mice
Hdac1	0.16	0.00272903	mice
Dpm2	0.11	0.00273505	mice
Taf4b	-0.10	0.00275416	mice
Zfp451	-0.14	0.00283958	mice
Gnptab	-0.18	0.00286915	mice
Smad4	-0.17	0.00296039	mice
Pex11a	-0.05	0.00297922	mice
Ero1l	-0.15	0.00299040	mice
Emc1	-0.06	0.00299536	mice
Sltm	-0.12	0.00303923	mice
Jtb	-0.11	0.00308662	mice
Gpr35	0.09	0.00308811	mice
Htra2	0.12	0.00325253	mice
Noc2l	-0.07	0.00326993	mice
Spi1	0.14	0.00327982	mice
Sirpb1b	0.04	0.00331800	mice
Zmpste24	-0.09	0.00334972	mice
Tlr8	-0.06	0.00338270	mice
Vapa	-0.15	0.00339894	mice
Polr2m	-0.11	0.00339976	mice
Alpk2	0.12	0.00341226	mice
Fam53c	0.07	0.00346491	mice
Pla2g7	0.24	0.00350744	mice
Tbcd	-0.09	0.00351031	mice
Rufy3	0.25	0.00354080	mice
Qser1	-0.08	0.00354773	mice

Tbca	-0.12	0.00358996	mice
Ash1l	-0.09	0.00364771	mice
Rasgrp2	-0.16	0.00365495	mice
Vps50	-0.13	0.00366167	mice
Cep68	0.09	0.00366703	mice
Pmepa1	0.24	0.00367584	mice
Daglb	-0.08	0.00369132	mice
Gba	0.13	0.00376251	mice
2510039O18Rik	-0.15	0.00377802	mice
Casz1	-0.09	0.00386208	mice
Thap4	-0.09	0.00387191	mice
Fbxo33	-0.07	0.00389772	mice
Snx4	-0.16	0.00390253	mice
Cnksr3	-0.12	0.00390784	mice
Ndufs7	0.20	0.00395163	mice
Ctsk	0.01	0.00396697	mice
Ncl	-0.09	0.00400057	mice
Fam214a	-0.08	0.00402466	mice
Stard7	-0.06	0.00406962	mice
Esco1	-0.11	0.00416250	mice
Epas1	-0.14	0.00417594	mice
Acbd6	-0.09	0.00420932	mice
Nsmce1	0.09	0.00422593	mice
Ttc27	-0.06	0.00422951	mice
Zfp52	-0.07	0.00431563	mice
Oip5os1	-0.07	0.00441594	mice
Commd4	0.10	0.00441982	mice
Tfdp1	-0.09	0.00456729	mice
Eif3k	-0.15	0.00458314	mice
Sec16a	-0.10	0.00459270	mice
Cul5	-0.10	0.00464049	mice
Brap	-0.10	0.00471524	mice
Gtf3c1	-0.08	0.00483401	mice
Zfp652	-0.17	0.00486776	mice
Bbip1	0.13	0.00493856	mice
Lpin2	-0.21	0.00499705	mice
Dmxl1	-0.06	0.00512364	mice
Ppan	-0.05	0.00516949	mice
Twf2	0.17	0.00529596	mice
Igsf6	0.10	0.00532076	mice
Bin2	0.18	0.00533417	mice
Cplane1	-0.12	0.00539454	mice
Stat3	0.19	0.00540067	mice
Nob1	-0.09	0.00540469	mice
4932438A13Rik	-0.20	0.00549084	mice
Zfp609	-0.16	0.00549567	mice

Pelo	-0.06	0.00561957	mice
Sumo2	0.23	0.00562748	mice
Hdac8	-0.16	0.00563690	mice
Chd1	-0.14	0.00571045	mice
Tecr	-0.14	0.00580888	mice
Znrf3	-0.26	0.00581550	mice
Kctd20	-0.14	0.00586263	mice
Khsrp	-0.11	0.00592056	mice
Tsta3	0.10	0.00599401	mice
Golph3l	-0.08	0.00604965	mice
Actr2	0.14	0.00620013	mice
Sec23a	-0.09	0.00623201	mice
St3gal5	-0.24	0.00645933	mice
Zfp655	-0.11	0.00646017	mice
Gls	-0.11	0.00650469	mice
Pnrc1	-0.15	0.00656897	mice
Acer3	0.18	0.00662389	mice
5330438D12Rik	0.13	0.00663452	mice
Khk	-0.08	0.00666360	mice
Blmh	-0.12	0.00677207	mice
Arid2	-0.13	0.00682609	mice
Fam102b	-0.08	0.00685511	mice
Pcnt	-0.12	0.00698416	mice
Commd1	0.14	0.00715097	mice
Bmp2k	-0.20	0.00728596	mice
Ophn1	-0.06	0.00732496	mice
Cltc	-0.16	0.00736230	mice
Phf6	0.15	0.00742684	mice
Chst11	-0.20	0.00752519	mice
Wrnip1	-0.04	0.00759513	mice
Enpp1	-0.03	0.00765242	mice
Cyc1	0.15	0.00766585	mice
Arl11	-0.13	0.00768942	mice
Slc38a6	-0.09	0.00771246	mice
Paxbp1	-0.07	0.00794791	mice
Aldh3a2	-0.10	0.00805255	mice
Ebi3	0.11	0.00810591	mice
Apba1	-0.23	0.00821112	mice
Cep63	-0.09	0.00824096	mice
Pdhb	-0.09	0.00826285	mice
Gimap6	-0.15	0.00827067	mice
Nxf1	-0.04	0.00836610	mice
4833420G17Rik	-0.12	0.00845006	mice
AU022793	-0.03	0.00855510	mice
Slc30a5	-0.11	0.00856392	mice
Nipa2	-0.11	0.00868523	mice

Tbck	-0.12	0.00872459	mice
Tmem273	-0.03	0.00875349	mice
Pcnx4	-0.08	0.00888397	mice
Fbxl14	-0.06	0.00894403	mice
Pum1	-0.10	0.00895480	mice
Arid3a	-0.10	0.00906634	mice
Tmem243	0.14	0.00964441	mice
Arl6ip6	-0.14	0.00981016	mice
Stom	-0.09	0.00982190	mice
Phf10	-0.09	0.00991026	mice
Nktr	-0.10	0.00999276	mice
Smad6	-0.14	0.01006527	mice
Tmod3	-0.13	0.01020317	mice
Ino80	-0.14	0.01029863	mice
Sh3pxd2b	-0.14	0.01042746	mice
E130309D02Rik	-0.05	0.01045977	mice
Sh2b3	-0.10	0.01052030	mice
Ctbp1	-0.05	0.01063354	mice
Plscr1	-0.10	0.01085063	mice
Klc1	-0.08	0.01130318	mice
Trmt1	-0.07	0.01167672	mice
Ctsh	0.31	0.01168324	mice
Styx	-0.08	0.01170073	mice
Vdr	-0.06	0.01170777	mice
Slc39a11	-0.10	0.01201701	mice
Znrf2	-0.07	0.01208610	mice
Luc7l	-0.07	0.01248570	mice
Fam78b	-0.07	0.01265270	mice
Rab4b	0.11	0.01302153	mice
Ndufa7	0.13	0.01314664	mice
Npepl1	-0.06	0.01315172	mice
Slc25a16	-0.06	0.01316366	mice
Tmem168	-0.10	0.01328510	mice
Fam89b	-0.09	0.01344096	mice
Cd37	-0.20	0.01348018	mice
Plbd1	-0.34	0.01350075	mice
Sptlc2	-0.26	0.01354187	mice
Yy1	-0.08	0.01367460	mice
Slc25a25	-0.07	0.01379207	mice
Tab2	-0.10	0.01385155	mice
Appl2	-0.06	0.01385330	mice
Ppp1r2	-0.17	0.01388805	mice
Spr	-0.04	0.01429963	mice
Stx16	0.19	0.01476547	mice
U2af1l4	0.07	0.01483055	mice
Abtb1	-0.09	0.01487029	mice

Nol7	0.14	0.01487696	mice
Necap2	0.18	0.01494078	mice
Crybg3	-0.10	0.01503072	mice
Tlr2	-0.14	0.01507052	mice
Nedd8	0.19	0.01507744	mice
Taf2	-0.12	0.01513175	mice
9430038I01Rik	-0.11	0.01534160	mice
Suclg2	-0.09	0.01545153	mice
Sparc	0.19	0.01561335	mice
Tmco4	-0.10	0.01575103	mice
Paip1	-0.09	0.01587057	mice
Eva1b	-0.06	0.01591064	mice
Amn1	0.04	0.01595363	mice
Agfg1	-0.14	0.01596381	mice
Pdpk1	-0.09	0.01631873	mice
Rsf1	-0.17	0.01638182	mice
A530013C23Rik	-0.13	0.01650710	mice
Cd244a	-0.22	0.01660344	mice
Csnk1a1	-0.10	0.01669137	mice
Uba5	-0.07	0.01674219	mice
Wiz	-0.07	0.01686492	mice
Gse1	-0.12	0.01691739	mice
Il16	-0.10	0.01701024	mice
Ipo9	-0.07	0.01705236	mice
Eif2b2	0.10	0.01718546	mice
Ddx17	-0.12	0.01766161	mice
Abl1	-0.13	0.01780320	mice
Hsd17b4	-0.10	0.01783226	mice
Rab6a	-0.12	0.01789058	mice
Nup210l	-0.25	0.01789170	mice
Phtf1	-0.05	0.01798811	mice
Farp2	-0.05	0.01818973	mice
Edem1	-0.14	0.01821724	mice
Rpia	-0.07	0.01822767	mice
Adnp	-0.10	0.01864080	mice
Slc22a4	-0.10	0.01866647	mice
Fgd2	0.07	0.01944813	mice
Ppm1h	-0.18	0.01952247	mice
Snw1	0.16	0.01963111	mice
Noct	0.31	0.01966074	mice
Glg1	-0.12	0.01966871	mice
Usf3	-0.19	0.01967019	mice
Rtcb	-0.09	0.01967081	mice
Rbis	0.16	0.01978323	mice
Zfp942	-0.08	0.02048616	mice
Hpgds	-0.06	0.02074933	mice

Hsph1	-0.06	0.02085662	mice
Elf4	-0.15	0.02126604	mice
Gtpbp4	-0.10	0.02155325	mice
Taf5l	-0.09	0.02169618	mice
Hspa8	0.21	0.02195644	mice
Tsn	-0.09	0.02209712	mice
Trim8	-0.11	0.02260976	mice
Tpp2	-0.10	0.02288090	mice
Fbl	-0.14	0.02292579	mice
Rnf220	-0.06	0.02301917	mice
Rhog	0.16	0.02306025	mice
Rtf2	0.17	0.02326042	mice
Fam111a	0.16	0.02358795	mice
Supt16	-0.09	0.02375018	mice
Nme2	-0.09	0.02381241	mice
Pea15a	0.19	0.02384208	mice
Rabep1	-0.19	0.02414804	mice
Ndrg1	0.27	0.02438295	mice
Rnf20	-0.08	0.02438312	mice
Nlrx1	-0.06	0.02498532	mice
Cnn2	0.05	0.02502710	mice
Rcor3	-0.02	0.02527507	mice
Ints3	-0.04	0.02532123	mice
Cdk2ap2	-0.19	0.02535505	mice
Slc4a2	-0.07	0.02535603	mice
Ctnnbip1	-0.11	0.02553708	mice
Ctsd	-0.50	0.02572148	mice
Abhd4	-0.07	0.02603148	mice
Slc41a2	-0.10	0.02626762	mice
Cnih1	-0.07	0.02627116	mice
Phrf1	-0.13	0.02633829	mice
Dse	-0.09	0.02645914	mice
Atp5g3	-0.12	0.02651665	mice
Nenf	0.00	0.02745222	mice
Elmo2	-0.09	0.02783194	mice
Immt	-0.16	0.02792030	mice
Vps54	0.25	0.02823838	mice
Bclaf3	0.17	0.02866346	mice
Rbmx	-0.06	0.02947671	mice
Erg28	0.10	0.02950297	mice
Il1rap	-0.47	0.02962405	mice
Srpk1	-0.09	0.03077390	mice
Epn1	-0.10	0.03092370	mice
Clcn6	-0.07	0.03121950	mice
Sin3a	-0.12	0.03157683	mice
Rnf11	-0.10	0.03167263	mice

Gps2	0.14	0.03193618	mice
Setd3	-0.09	0.03197952	mice
Gatm	0.51	0.03265349	mice
Creb5	0.27	0.03278768	mice
Dtx4	-0.11	0.03282173	mice
Phb2	-0.10	0.03289132	mice
Arap2	-0.31	0.03305824	mice
Ptpro	0.12	0.03321777	mice
Hnrnpm	-0.10	0.03387878	mice
Stk35	-0.08	0.03398581	mice
Grk6	-0.12	0.03440951	mice
Rabggtb	0.09	0.03447563	mice
Lair1	0.20	0.03489649	mice
Prkx	0.23	0.03507760	mice
Wasl	-0.16	0.03634189	mice
Arhgap6	-0.10	0.03706380	mice
Klhdc10	-0.14	0.03712066	mice
BC005537	-0.09	0.03743951	mice
Oxr1	-0.16	0.03751254	mice
Cradd	-0.10	0.03752617	mice
Pde6d	0.06	0.03798580	mice
Hspb11	0.06	0.03807656	mice
Prps1	-0.07	0.03824244	mice
Ptpn1	-0.06	0.03844034	mice
Auh	-0.19	0.03845361	mice
Coa5	0.16	0.03868528	mice
Gbf1	-0.10	0.03880855	mice
Safb	-0.11	0.03892415	mice
Rmc1	-0.08	0.03904693	mice
Tmem229b	0.09	0.03912638	mice
Ift57	-0.10	0.03923966	mice
Parg	-0.12	0.03933367	mice
Tasp1	-0.08	0.03968805	mice
Cav2	-0.07	0.04039828	mice
Zxdc	-0.08	0.04050337	mice
G0s2	0.21	0.04069444	mice
Fdps	0.15	0.04072975	mice
Pfdn4	0.09	0.04076881	mice
Atxn3	-0.09	0.04118250	mice
Exoc4	-0.06	0.04177042	mice
Sec24b	-0.15	0.04197454	mice
Vps29	0.17	0.04227960	mice
Dcaf8	-0.05	0.04240448	mice
Nrcam	-0.11	0.04309714	mice
Tab3	-0.06	0.04310348	mice
Hmgb2	0.23	0.04333916	mice

Copg2	0.10	0.04341237	mice
Cpox	-0.06	0.04447940	mice
Shc1	-0.02	0.04498593	mice
BC004004	-0.11	0.04520443	mice
Nfe2l1	-0.07	0.04526657	mice
Naip5	-0.08	0.04605910	mice
Tfip11	0.08	0.04696970	mice
Cln8	-0.08	0.04718716	mice
Chic2	-0.12	0.04748261	mice
Fpr2	-0.12	0.04753250	mice
Dnttip1	0.07	0.04802219	mice
Tiam1	-0.42	0.04820205	mice
Cog5	-0.14	0.04825968	mice
Lsm6	-0.07	0.04833486	mice
Hprt	-0.11	0.04851832	mice
Fhod1	0.15	0.04875985	mice
Atp6v0b	-0.10	0.04955238	mice
Glb1	-0.10	0.04996637	mice
Specc1	-0.15	0.05014381	mice
Nmt1	0.23	0.05018096	mice
Atp11b	0.29	0.05176088	mice
Fem1c	0.13	0.05246675	mice
Ttc5	-0.06	0.05265678	mice
Sos1	-0.09	0.05317252	mice
Tmem147	-0.09	0.05326570	mice
E2f1	-0.04	0.05347553	mice
Thap11	-0.06	0.05456283	mice
Pou2f1	-0.11	0.05473960	mice
Cnih4	0.17	0.05477316	mice
Cited2	0.17	0.05497112	mice
Aifm2	-0.11	0.05520225	mice
Dnmbp	-0.13	0.05597833	mice
Cnot7	-0.11	0.05662839	mice
Slc35b4	-0.05	0.05697245	mice
Psmd10	0.10	0.05720742	mice
Avpi1	-0.11	0.05723989	mice
Acbd3	-0.10	0.05771826	mice
Otud7b	-0.09	0.05779485	mice
Stam2	0.32	0.05836698	mice
Saraf	-0.13	0.05866572	mice
Alox5ap	-0.17	0.05905480	mice
Pgp	-0.05	0.05985134	mice
Zpr1	-0.10	0.05991077	mice
Atraid	-0.11	0.06000410	mice
Nup54	-0.13	0.06031810	mice
Itfg1	-0.14	0.06054021	mice

Adcy7	-0.13	0.06078580	mice
4933406I18Rik	-0.07	0.06086498	mice
Ifi30	0.33	0.06144291	mice
Ipo11	-0.05	0.06151771	mice
Crybg1	0.11	0.06213103	mice
Chn2	-0.10	0.06290544	mice
Psma3	0.18	0.06297621	mice
Xxylt1	-0.09	0.06328908	mice
Ube2h	-0.12	0.06419240	mice
Ppp6r3	-0.11	0.06422749	mice
Clip1	0.27	0.06424137	mice
Scmh1	-0.13	0.06504191	mice
1700016P03Rik	0.09	0.06526331	mice
Wdr13	-0.05	0.06556203	mice
Antxr2	-0.46	0.06568778	mice
Enoph1	-0.04	0.06590353	mice
Dnm1l	-0.09	0.06624429	mice
Pprc1	-0.07	0.06627163	mice
Tmem50a	-0.10	0.06644028	mice
Ing3	-0.13	0.06675519	mice
Rrp12	-0.03	0.06693741	mice
Stx18	-0.12	0.06776413	mice
Alas1	0.07	0.06808488	mice
Akr1a1	0.23	0.06855167	mice
Ptger2	-0.08	0.06861080	mice
Zfp954	-0.07	0.06953791	mice
Kbtbd2	-0.08	0.07031373	mice
Cox10	-0.08	0.07040973	mice
Tnip3	0.27	0.07045197	mice
Ddx10	-0.10	0.07087755	mice
Zbtb34	-0.05	0.07106739	mice
Brf1	-0.05	0.07123604	mice
Ube2w	-0.13	0.07221363	mice
Sat1	0.25	0.07221727	mice
Sp1	-0.10	0.07235520	mice
Glipr2	-0.08	0.07246156	mice
Ago4	-0.07	0.07320856	mice
Fosl1	0.12	0.07372774	mice
Zdhhc3	-0.09	0.07376861	mice
Zfp518a	-0.08	0.07382574	mice
Cirbp	-0.12	0.07416924	mice
Klhdc2	-0.06	0.07427107	mice
Cyb5r3	-0.05	0.07427517	mice
Tm2d2	-0.11	0.07434841	mice
Aff4	-0.12	0.07603718	mice
Rc3h1	-0.16	0.07604062	mice

Uck2	-0.13	0.07651268	mice
Nono	0.14	0.07758769	mice
Fbxl3	-0.10	0.07772635	mice
Kdm4b	-0.06	0.07820418	mice
B4galt1	-0.14	0.07841515	mice
Ccdc59	0.14	0.07934817	mice
Lrp10	0.14	0.07965387	mice
Rbm43	0.06	0.07979550	mice
Rnpc3	-0.09	0.08047151	mice
Rnf19a	-0.20	0.08076627	mice
Rnf8	-0.08	0.08119369	mice
Glo1	-0.07	0.08120008	mice
Ric8a	-0.04	0.08387041	mice
Tsen34	-0.07	0.08414196	mice
Rgcc	-0.14	0.08455748	mice
Uspl1	-0.06	0.08589437	mice
Itpk1	-0.09	0.08656660	mice
Dop1b	0.17	0.08705200	mice
Usp36	-0.09	0.08723809	mice
Gstcd	-0.04	0.08738928	mice
Atp1a1	-0.11	0.08787039	mice
Nom1	-0.08	0.09011423	mice
Bbx	-0.08	0.09136439	mice
Cks1b	-0.02	0.09224382	mice
Smg5	-0.07	0.09252341	mice
Gnai3	-0.14	0.09333729	mice
Lsamp	-0.41	0.09379490	mice
Cped1	-0.07	0.09441437	mice
Pde12	-0.04	0.09613957	mice
Tasor	-0.09	0.09697016	mice
Ddx5	-0.10	0.09698185	mice
Keap1	0.11	0.09707191	mice
Emp1	-0.18	0.09796798	mice
Chst15	-0.06	0.09936034	mice
C87436	-0.07	0.09977623	mice
Ppp1r18	-0.11	0.10098117	mice
Wdr43	-0.10	0.10241138	mice
Hes6	-0.06	0.10272289	mice
Ndufb7	0.19	0.10325930	mice
Spred2	-0.12	0.10389168	mice
Slc35a3	-0.07	0.10468149	mice
Numb	-0.11	0.10576727	mice
Atp8b4	-0.06	0.10613119	mice
Fes	0.13	0.10705205	mice
Tbc1d10b	-0.08	0.10715739	mice
Washc3	0.10	0.10803769	mice

Pisd	-0.10	0.10865066	mice
Tmem160	0.18	0.10934508	mice
Slc41a1	-0.06	0.10944701	mice
Tor2a	0.07	0.10957477	mice
Pkib	0.25	0.11083667	mice
Strip1	-0.08	0.11182562	mice
Grk3	-0.10	0.11199404	mice
Usp28	-0.04	0.11243941	mice
Pmm2	0.10	0.11296818	mice
Imp4	-0.09	0.11311173	mice
Aars	0.17	0.11324877	mice
Ibtk	-0.08	0.11365156	mice
Kti12	-0.06	0.11389469	mice
Sar1b	0.10	0.11421889	mice
Igf2bp2	0.12	0.11450417	mice
BC031181	0.09	0.11492573	mice
Stard5	-0.08	0.11958724	mice
Cxcr4	-0.05	0.11993359	mice
Tnfaip8	-0.16	0.11996346	mice
Emc4	0.10	0.12057372	mice
Bax	0.28	0.12270518	mice
Ifitm1	0.05	0.12296825	mice
Hdc	-0.38	0.12316366	mice
Vps8	-0.08	0.12340977	mice
Zfp277	-0.12	0.12422635	mice
Slc25a3	0.14	0.12462731	mice
H2afz	-0.13	0.12562359	mice
Ankrd17	-0.15	0.12748573	mice
Ralgapb	-0.09	0.13243961	mice
Eif2b3	-0.07	0.13526358	mice
Ankrd13c	-0.12	0.13654493	mice
Rsrc2	0.17	0.13792844	mice
Fbxo9	-0.06	0.13881492	mice
Cdc26	0.11	0.13922218	mice
Tram2	-0.06	0.13932563	mice
Nop16	-0.08	0.14148276	mice
Errfi1	0.21	0.14214490	mice
Ccdc22	-0.07	0.14742275	mice
Slc33a1	-0.06	0.14758422	mice
Bloc1s1	0.06	0.15131294	mice
Hspa4	-0.09	0.15167184	mice
Ppp3cb	-0.07	0.15244373	mice
Eaf1	-0.13	0.15315114	mice
Wwp2	-0.11	0.15447393	mice
Ipo5	-0.11	0.15508369	mice
Vwa8	-0.07	0.15543809	mice

Zfyve26	-0.15	0.15715621	mice
Gusb	-0.05	0.16064740	mice
Phyh	0.13	0.16295771	mice
Rnf31	0.05	0.16298575	mice
Prelid2	-0.04	0.16339288	mice
Timm8a1	-0.07	0.16351367	mice
Serf2	-0.07	0.16387559	mice
Gspt1	-0.10	0.16574597	mice
Strn4	-0.06	0.16691985	mice
Rnaseh2b	-0.07	0.16756345	mice
Lrrk2	-0.19	0.16915098	mice
Zfp746	-0.07	0.16927624	mice
Bcl7c	-0.07	0.17046056	mice
Vps35	-0.12	0.17159842	mice
Zfp467	0.07	0.17211451	mice
Zc3h12d	0.09	0.17340462	mice
Cfap20	-0.04	0.17481419	mice
Tspan31	0.13	0.17620747	mice
Plin3	-0.05	0.17785761	mice
Armcx3	0.09	0.18165985	mice
Rilpl2	-0.13	0.18187784	mice
Uqcrc1	0.15	0.18243079	mice
Vta1	0.09	0.18267488	mice
Tollip	-0.11	0.18290315	mice
Snx6	0.13	0.18398657	mice
Srxn1	0.16	0.18419939	mice
Cbfa2t3	0.13	0.18617949	mice
Ttc1	0.12	0.18625528	mice
Prdm4	-0.08	0.18639290	mice
Pphln1	-0.09	0.18718421	mice
Tmed7	-0.11	0.18721104	mice
Stub1	-0.09	0.18927316	mice
Ell2	0.24	0.19052369	mice
Lipo3	-0.05	0.19152808	mice
Phldb1	0.11	0.19197662	mice
Ccdc28b	0.07	0.19353514	mice
Cav1	-0.05	0.19420466	mice
Echdc1	-0.04	0.19570891	mice
Synrg	-0.09	0.19646221	mice
Sms	-0.07	0.19796017	mice
Usp14	-0.06	0.19837946	mice
Gramd1a	-0.08	0.19945720	mice
Jup	-0.07	0.20059910	mice
Zdhhc14	-0.26	0.20071140	mice
Emsy	-0.08	0.20478485	mice
Pip4k2c	-0.06	0.20548244	mice

Psip1	-0.12	0.20603846	mice
Hs6st1	-0.08	0.20686559	mice
Txn2	-0.07	0.20727128	mice
Rab8b	-0.11	0.20922715	mice
Pon3	-0.04	0.20944761	mice
Kctd12	-0.20	0.21134060	mice
Eed	-0.18	0.21357395	mice
Bmt2	-0.10	0.21527671	mice
Vti1a	-0.16	0.21636589	mice
Crk	-0.10	0.21749880	mice
Prkrip1	0.08	0.21799680	mice
Tusc1	-0.08	0.21978739	mice
Ppard	-0.12	0.22126157	mice
Sertad2	0.26	0.22151459	mice
Tmem268	-0.06	0.22506979	mice
Qrich1	-0.13	0.22551141	mice
Ambra1	-0.12	0.22564748	mice
Zc3h12a	-0.15	0.22696079	mice
Atp6v1d	0.17	0.22855953	mice
Dennd5a	-0.14	0.22863252	mice
Dcaf7	-0.11	0.23145909	mice
Ilf2	-0.09	0.23226000	mice
Dcaf17	-0.06	0.23287051	mice
Itch	-0.12	0.23293170	mice
Fam126b	-0.08	0.23592544	mice
Cog3	-0.10	0.23610493	mice
Slc25a19	0.10	0.23791516	mice
Anapc13	0.11	0.24050777	mice
Nabp1	-0.12	0.24073315	mice
Cand1	-0.07	0.24315124	mice
Psmg4	0.13	0.24388724	mice
Gas5	0.26	0.24389404	mice
Pfdn6	0.14	0.24588408	mice
Otub1	0.15	0.24740436	mice
Ganc	-0.07	0.24773110	mice
Dnajc17	0.06	0.25222998	mice
Bin1	0.16	0.25393730	mice
Idh3g	-0.04	0.25404813	mice
Ralbp1	-0.09	0.25425703	mice
Tmem88	0.06	0.25541340	mice
Stim1	-0.15	0.25542125	mice
Cox5a	-0.05	0.25593471	mice
Crl1	-0.13	0.25638196	mice
Nudt4	-0.32	0.25920874	mice
E130308A19Rik	-0.09	0.25944634	mice
Kdm3b	-0.12	0.26145771	mice

Arcn1	-0.07	0.26266261	mice
Hk1	0.12	0.26454602	mice
Clec4b1	-0.07	0.26648624	mice
Htatsf1	-0.02	0.26829887	mice
Nisch	-0.10	0.27007093	mice
Tmf1	-0.10	0.27241961	mice
Dok3	-0.15	0.27338700	mice
Acat1	-0.09	0.27377392	mice
Slpi	-0.11	0.27972394	mice
Tle5	-0.09	0.28191345	mice
Sdad1	-0.07	0.28404300	mice
Glyr1	-0.11	0.28619715	mice
Rnf141	-0.14	0.28648784	mice
Vps4a	-0.05	0.28703567	mice
Tm9sf1	-0.07	0.28720143	mice
Plxdc1	-0.05	0.28822319	mice
Ubqln1	-0.07	0.28990882	mice
Pfdn2	-0.08	0.29096330	mice
N4bp2	-0.12	0.29263479	mice
Cluap1	-0.06	0.29275764	mice
Stt3a	-0.06	0.29412600	mice
Hectd3	-0.06	0.30275297	mice
Sh2b2	0.11	0.30593518	mice
Sergef	-0.10	0.31222056	mice
Kdm3a	-0.13	0.31291862	mice
Cox20	0.09	0.31835427	mice
Pou2f2	0.06	0.32583501	mice
Atg7	-0.25	0.33048204	mice
Zfp869	-0.10	0.33279563	mice
Wac	-0.09	0.33798974	mice
Tes	-0.20	0.33810025	mice
Gtf2b	0.16	0.33998823	mice
Vac14	-0.10	0.34083159	mice
Zeb2	-0.07	0.34443073	mice
Dpysl2	0.15	0.34671428	mice
Polr2i	0.11	0.34802988	mice
Vrk1	-0.13	0.34948495	mice
Hpf1	0.15	0.35105131	mice
Cdyl2	-0.08	0.35665555	mice
Vamp5	-0.10	0.35730037	mice
Xrn1	0.20	0.35969377	mice
Cttnbp2nl	-0.08	0.36182426	mice
Gpr141	0.03	0.36525211	mice
Suclg1	0.10	0.36867505	mice
Klhl15	-0.06	0.37038960	mice
Bag3	-0.11	0.37257438	mice

Pikfyve	-0.12	0.37396665	mice
Gas2l3	0.24	0.37521264	mice
Esyt1	0.13	0.37715450	mice
Cnpy2	-0.07	0.38155397	mice
Tmem106b	-0.06	0.38182210	mice
Cct5	-0.08	0.38244955	mice
Kctd13	0.08	0.38323962	mice
Tbc1d10a	-0.10	0.38442986	mice
Nup160	-0.07	0.38474230	mice
Phc3	-0.06	0.38512592	mice
Rxrb	-0.08	0.38512764	mice
Rpf2	0.07	0.38566448	mice
Hnrnpul2	-0.12	0.38637922	mice
Kiz	-0.09	0.39255467	mice
Dyrk1a	-0.09	0.39308709	mice
0610012G03Rik	-0.05	0.39933095	mice
Amd1	-0.10	0.40214011	mice
Shq1	-0.06	0.40311317	mice
Zfp654	-0.08	0.40624527	mice
Bod1	-0.04	0.40889841	mice
Aldh3b1	-0.06	0.41487770	mice
Pi4k2a	-0.09	0.41528547	mice
Cept1	0.17	0.41804014	mice
Hdgf	-0.06	0.42301592	mice
Dazap2	-0.12	0.42487830	mice
Rnd3	-0.03	0.42724992	mice
1110038F14Rik	0.15	0.42770156	mice
Clpb	-0.05	0.43705123	mice
Rabl6	-0.12	0.44123991	mice
Wdr81	-0.07	0.44504846	mice
Prdx2	0.14	0.44835315	mice
Zfp24	-0.07	0.45346804	mice
Brd4	-0.09	0.45796267	mice
Rfc3	0.07	0.45874854	mice
Pcbp1	-0.12	0.46377863	mice
Pip5k1b	-0.15	0.46951388	mice
N4bp2l2	-0.15	0.47430243	mice
Rhoc	0.04	0.47713808	mice
Nfx1	-0.06	0.48271610	mice
Aip	0.07	0.48453198	mice
Bclaf1	-0.08	0.48816422	mice
Cox8a	-0.06	0.49497417	mice
Prrc2c	0.18	0.50605460	mice
Iars2	-0.08	0.50969914	mice
Ddhd2	-0.07	0.51172923	mice
Stk25	-0.01	0.51886876	mice

Ndufa2	0.17	0.52137195	mice
Zranb2	0.14	0.52604776	mice
Dnpep	0.07	0.52990562	mice
Snx12	-0.09	0.53169876	mice
Clk4	-0.10	0.53791769	mice
Fam219a	-0.17	0.54179508	mice
Zfp180	-0.07	0.54956204	mice
Cfl1	0.14	0.54978480	mice
Rbks	0.10	0.55253848	mice
Rin2	-0.05	0.55345748	mice
Rtn3	-0.05	0.56037029	mice
Arfgap3	-0.08	0.56169200	mice
Alyref	-0.05	0.56359316	mice
Zfp560	-0.02	0.56498216	mice
Rad23b	-0.12	0.56629283	mice
Nadk	-0.14	0.56663526	mice
Ddx39b	-0.11	0.57266165	mice
Szrd1	0.15	0.58228119	mice
Pcmtd1	-0.07	0.58481846	mice
Vasp	0.10	0.59195369	mice
Gigyf1	-0.05	0.59366266	mice
Olr1	0.11	0.59401357	mice
P2ry10b	-0.01	0.59740568	mice
Cd200r1	-0.07	0.59848850	mice
Sufu	-0.08	0.60117774	mice
Plekha1	-0.07	0.60256136	mice
Slc35a4	-0.06	0.60468408	mice
Ptpmt1	-0.05	0.60615800	mice
Gdi1	-0.10	0.60916478	mice
Phf13	-0.04	0.61036832	mice
Lipe	0.05	0.61276414	mice
Abcb7	-0.10	0.61612161	mice
Cd14	-0.44	0.61636231	mice
Tmem60	-0.05	0.61829628	mice
Psmd3	0.15	0.62512631	mice
Slc11a2	0.14	0.63550563	mice
Smg7	0.15	0.63694156	mice
Snx10	-0.12	0.63904943	mice
Tnfaip3	0.23	0.64305006	mice
Hsd11b1	-0.08	0.64609157	mice
Bsdc1	-0.13	0.65319631	mice
Phlpp1	0.40	0.66204602	mice
Pdcl	-0.06	0.66293595	mice
Hmces	-0.07	0.67131793	mice
Rbbp7	-0.06	0.68091983	mice
Ckap2l	0.18	0.68520585	mice

Dcaf10	-0.12	0.68737006	mice
Ubn1	-0.09	0.68755850	mice
Fance	-0.07	0.68877986	mice
Slc16a10	0.31	0.71313441	mice
Thap12	-0.03	0.71780137	mice
Zc3h3	-0.08	0.71933427	mice
Nsmaf	0.06	0.72365713	mice
Ythdc1	-0.10	0.72399362	mice
Fam49a	0.30	0.72463286	mice
Ankrd33b	0.00	0.72808585	mice
Osbpl2	-0.06	0.74009592	mice
Slc39a7	-0.07	0.74166020	mice
1110032A03Rik	-0.04	0.74215017	mice
Cbfa2t2	-0.07	0.74620096	mice
Tomm34	-0.07	0.74958649	mice
Nol11	-0.05	0.75550914	mice
Rnf145	-0.11	0.76400310	mice
Naa15	-0.06	0.76524282	mice
Slc20a1	-0.04	0.77590399	mice
Gabpb2	-0.08	0.78043493	mice
Atf2	-0.10	0.78658886	mice
Dnajc14	-0.04	0.79414522	mice
Pds5b	-0.08	0.80223090	mice
1810058I24Rik	-0.14	0.81348350	mice
Adgre5	-0.29	0.81848135	mice
Pdia4	-0.07	0.82250435	mice
B3galnt2	-0.06	0.82717270	mice
Txndc9	0.13	0.82765964	mice
Rcc1l	-0.02	0.83312862	mice
Creld2	-0.03	0.83785605	mice
Nup155	-0.07	0.83848744	mice
Tmem203	-0.03	0.83891825	mice
Tec	0.01	0.84643904	mice
Atg101	-0.07	0.84664689	mice
Gpr183	-0.08	0.84780688	mice
Brd7	0.19	0.84837533	mice
Wdr5	-0.05	0.85202151	mice
Smim19	-0.07	0.85649071	mice
Sod1	0.13	0.86334289	mice
Lasp1	0.08	0.86577717	mice
Sh3bp2	-0.07	0.86661363	mice
Smc5	0.15	0.87562320	mice
Nagk	-0.01	0.89655948	mice
Tbce	-0.11	0.90889316	mice
Acads	-0.05	0.92154884	mice
Gpaa1	-0.05	0.93713308	mice

Anp32e	-0.06	0.94626198	mice
Ctdspl	-0.04	0.94870965	mice
8030462N17Rik	-0.13	0.94910585	mice
Lmbrd2	-0.06	0.95002211	mice
Stmn1	-0.05	0.95043282	mice
Cdk14	-0.12	0.95309273	mice
Yipf3	-0.07	0.96363487	mice
Rmdn3	0.03	0.97573493	mice
Patl1	-0.08	0.97903114	mice
Rnf111	-0.10	0.97921927	mice
E230029C05Rik	0.24	1.00000000	mice
E2f4	0.09	1.00000000	mice
Lyrm1	0.06	1.00000000	mice
Gng5	-0.09	1.00000000	mice
Lcp2	0.19	1.00000000	mice
Ppp1r8	-0.03	1.00000000	mice
Dbf4	-0.07	1.00000000	mice
Pik3c3	-0.07	1.00000000	mice
Akt1s1	-0.02	1.00000000	mice
Plekhg3	-0.19	1.00000000	mice
Phkb	-0.09	1.00000000	mice
Ube2b	-0.11	1.00000000	mice
Zbtb25	-0.04	1.00000000	mice
Lrpap1	0.12	1.00000000	mice
Atxn2	-0.11	1.00000000	mice
Wdr70	-0.12	1.00000000	mice
Zfp653	-0.05	1.00000000	mice
Itpkc	0.07	1.00000000	mice
Vdac3	0.18	1.00000000	mice
Wars2	-0.11	1.00000000	mice
Tnfrsf11a	0.16	1.00000000	mice
Snap29	0.06	1.00000000	mice
Dlat	-0.06	1.00000000	mice
Lrrc4	0.11	1.00000000	mice
Sdhd	-0.10	1.00000000	mice
Rbm33	-0.08	1.00000000	mice
Pde8a	-0.07	1.00000000	mice
Spata1	-0.11	1.00000000	mice
Pygb	-0.03	1.00000000	mice
Banp	-0.07	1.00000000	mice
1700109H08Rik	-0.06	1.00000000	mice
Abi1	0.18	1.00000000	mice
Gdap2	-0.10	1.00000000	mice
Cd177	-0.14	1.00000000	mice
Eif3h	-0.12	1.00000000	mice
Plbd2	0.08	1.00000000	mice

Rgs1	0.59	1.00000000	mice
4930444A19Rik	0.08	1.00000000	mice
Sqstm1	0.20	1.00000000	mice
Napg	-0.09	1.00000000	mice
Pam	0.10	1.00000000	mice
Cmip	-0.12	1.00000000	mice
Kansl2	-0.07	1.00000000	mice
Rusc1	-0.02	1.00000000	mice
Hp	0.23	1.00000000	mice
Rac2	0.14	1.00000000	mice
Smc1a	-0.08	1.00000000	mice
1810037I17Rik	0.17	1.00000000	mice
Timm23	-0.08	1.00000000	mice
Zbtb2	-0.12	1.00000000	mice
4921524J17Rik	-0.04	1.00000000	mice
Prr14l	-0.11	1.00000000	mice
Slc38a10	-0.07	1.00000000	mice
Ccm2	-0.17	1.00000000	mice
Cdc42ep2	0.15	1.00000000	mice
Tnfaip6	0.09	1.00000000	mice
Bicral	-0.06	1.00000000	mice
Smim13	-0.08	1.00000000	mice
Wbp4	0.09	1.00000000	mice
Wdr1	-0.11	1.00000000	mice
Chchd7	-0.06	1.00000000	mice
Rpf1	-0.11	1.00000000	mice
Tep1	0.15	1.00000000	mice
Srpr	0.11	1.00000000	mice
Golm1	-0.05	1.00000000	mice
Stap1	0.24	1.00000000	mice
Ptrh2	0.06	1.00000000	mice
Acvrl1	0.10	1.00000000	mice
Abi3	0.14	1.00000000	mice
Sprtn	-0.05	1.00000000	mice
Zfp384	-0.08	1.00000000	mice
Kdsr	-0.10	1.00000000	mice
Ceacam1	-0.17	1.00000000	mice
Osgep	-0.05	1.00000000	mice
Tcp11l1	0.08	1.00000000	mice
Ide	-0.06	1.00000000	mice
Dusp5	0.18	1.00000000	mice
Thrap3	-0.07	1.00000000	mice
Fbxl12	-0.07	1.00000000	mice
Afg3l1	-0.04	1.00000000	mice
Smad5	-0.05	1.00000000	mice
Hs2st1	-0.06	1.00000000	mice

Trmt112	-0.07	1.00000000	mice
Ppm1g	-0.09	1.00000000	mice
Vps18	-0.11	1.00000000	mice
Paics	-0.05	1.00000000	mice
Zfp592	-0.08	1.00000000	mice
Slc12a9	0.08	1.00000000	mice
Colec12	-0.33	1.00000000	mice
Utrn	-0.18	1.00000000	mice
Wapl	-0.08	1.00000000	mice
Galnt2	-0.07	1.00000000	mice
Nudt21	-0.11	1.00000000	mice
Slc35f5	-0.09	1.00000000	mice
Bckdha	-0.10	1.00000000	mice
Ecd	-0.08	1.00000000	mice
Polr2f	0.12	1.00000000	mice
Higd1a	-0.09	1.00000000	mice
Txndc16	-0.07	1.00000000	mice
Glipr1	-0.11	1.00000000	mice
Eif1b	-0.09	1.00000000	mice
Sntb2	0.22	1.00000000	mice
Bag5	-0.08	1.00000000	mice
Faf2	0.11	1.00000000	mice
Twf1	-0.06	1.00000000	mice
Rnmt	-0.07	1.00000000	mice
Ptar1	-0.05	1.00000000	mice
Phf1	-0.05	1.00000000	mice
Ccnc	-0.04	1.00000000	mice
Srsf4	-0.07	1.00000000	mice
Eif2a	-0.08	1.00000000	mice
Snx29	-0.10	1.00000000	mice
Cxcl14	0.06	1.00000000	mice
Dnajb6	-0.08	1.00000000	mice
Lin54	0.18	1.00000000	mice
Ube2r2	-0.08	1.00000000	mice
Osm	0.27	1.00000000	mice
Carnmt1	-0.09	1.00000000	mice
Stat5b	-0.22	1.00000000	mice
Cry1	-0.09	1.00000000	mice
Cpeb4	-0.23	1.00000000	mice
Gemin7	-0.11	1.00000000	mice
Vps26b	-0.05	1.00000000	mice
Araf	-0.07	1.00000000	mice
Taf1	-0.07	1.00000000	mice
Susd6	0.12	1.00000000	mice
Snrpc	0.13	1.00000000	mice
Dexi	-0.04	1.00000000	mice

Nfrkb	-0.09	1.00000000	mice
Rrs1	-0.04	1.00000000	mice
Adprhl2	-0.07	1.00000000	mice
Pus10	0.07	1.00000000	mice
Clcn4	-0.07	1.00000000	mice
Akap11	-0.06	1.00000000	mice
Taf12	0.06	1.00000000	mice
Rexo1	-0.02	1.00000000	mice
Hnrnpr	-0.05	1.00000000	mice
Snx15	-0.02	1.00000000	mice
Eif2b5	-0.06	1.00000000	mice
Bcl2a1d	0.28	1.00000000	mice
Cog7	-0.02	1.00000000	mice
Arhgap11a	-0.04	1.00000000	mice
Slc25a28	-0.07	1.00000000	mice
Exosc4	0.06	1.00000000	mice
Nup205	-0.04	1.00000000	mice
Rbm28	-0.04	1.00000000	mice
Polk	-0.06	1.00000000	mice
Cir1	-0.07	1.00000000	mice
Cse1l	-0.10	1.00000000	mice
Elp2	-0.04	1.00000000	mice
Selenon	-0.14	1.00000000	mice
Serpinb8	-0.12	1.00000000	mice
Tmem87a	-0.11	1.00000000	mice
Cdc40	-0.11	1.00000000	mice
Gng10	-0.11	1.00000000	mice
Pex1	-0.07	1.00000000	mice
Zfp593	0.07	1.00000000	mice
Leng1	0.10	1.00000000	mice
Fbxw8	-0.05	1.00000000	mice
Ddx41	0.11	1.00000000	mice
Ttc39c	0.23	1.00000000	mice
Nol6	-0.04	1.00000000	mice
Sap18	0.14	1.00000000	mice
Hars	-0.06	1.00000000	mice
Kynu	-0.03	1.00000000	mice
Plxna1	-0.03	1.00000000	mice
Ube2d2a	-0.09	1.00000000	mice
Rsbn1l	-0.11	1.00000000	mice
Rnf214	0.09	1.00000000	mice
Atf1	-0.11	1.00000000	mice
Hsdl1	-0.02	1.00000000	mice
Phka2	-0.05	1.00000000	mice
Cuedc2	0.11	1.00000000	mice
Aimp1	0.13	1.00000000	mice

Galnt7	-0.10	1.00000000	mice
Slc38a1	0.21	1.00000000	mice
Rnf167	-0.10	1.00000000	mice
Sec13	0.09	1.00000000	mice
Abr	-0.14	1.00000000	mice
Btbd10	-0.08	1.00000000	mice
Nhlrc2	-0.05	1.00000000	mice
Tmx3	-0.09	1.00000000	mice
Vps39	-0.03	1.00000000	mice
Sin3b	-0.10	1.00000000	mice
Cx3cr1	0.16	1.00000000	mice
Ticam1	0.09	1.00000000	mice
S100pbp	-0.08	1.00000000	mice
Prdx1	0.14	1.00000000	mice
Elk3	0.20	1.00000000	mice
Upf3b	-0.06	1.00000000	mice
Hmgxb3	-0.06	1.00000000	mice
Vps45	-0.03	1.00000000	mice
Daam1	-0.08	1.00000000	mice
Cdh1	-0.08	1.00000000	mice
Crem	0.07	1.00000000	mice
Pqlc3	-0.04	1.00000000	mice
Ccdc167	0.07	1.00000000	mice
BC005561	0.11	1.00000000	mice
Tnfsf12	-0.01	1.00000000	mice
Tmem104	-0.06	1.00000000	mice
Plpp1	-0.03	1.00000000	mice
Nelfb	-0.06	1.00000000	mice
Ncoa2	-0.08	1.00000000	mice
Phc2	0.13	1.00000000	mice
C9orf72	0.15	1.00000000	mice
Srebf1	-0.07	1.00000000	mice
Tmem120a	-0.05	1.00000000	mice
Snrpg	0.15	1.00000000	mice
Cd300ld	0.14	1.00000000	mice
Arap1	0.15	1.00000000	mice
Runx2os1	-0.06	1.00000000	mice
Lypla2	-0.06	1.00000000	mice
Dnajc9	-0.07	1.00000000	mice
Gngt2	0.31	1.00000000	mice
Dnajc21	-0.11	1.00000000	mice
2810013P06Rik	-0.07	1.00000000	mice
Rab5b	-0.05	1.00000000	mice
Lrp5	-0.03	1.00000000	mice
Pcyt1a	-0.11	1.00000000	mice
Usp37	-0.08	1.00000000	mice

Rab11fip5	-0.03	1.00000000	mice
Acyp2	-0.04	1.00000000	mice
Dusp11	-0.08	1.00000000	mice
Gopc	-0.04	1.00000000	mice
Golgb1	-0.06	1.00000000	mice
Eci1	0.05	1.00000000	mice
Wls	-0.04	1.00000000	mice
Ube2z	-0.04	1.00000000	mice
Zfp740	-0.07	1.00000000	mice
Snapc3	-0.07	1.00000000	mice
Rpa1	-0.06	1.00000000	mice
Sec24c	-0.05	1.00000000	mice
Pithd1	-0.01	1.00000000	mice
Napsa	0.18	1.00000000	mice
Fam210a	-0.04	1.00000000	mice
Prpf31	0.11	1.00000000	mice
Il1r1	-0.13	1.00000000	mice
Eif2s1	0.16	1.00000000	mice
Actr10	0.10	1.00000000	mice
Ccl24	-0.17	1.00000000	mice
Poglut1	-0.05	1.00000000	mice
Crebbp	-0.07	1.00000000	mice
Rnaseh2c	-0.02	1.00000000	mice
Prdx6	-0.09	1.00000000	mice
Ttc37	-0.08	1.00000000	mice
Gpr18	0.04	1.00000000	mice
Rbm47	-0.09	1.00000000	mice
Slamf6	0.06	1.00000000	mice
Snf8	0.09	1.00000000	mice
Las1l	-0.07	1.00000000	mice
Brpf3	-0.03	1.00000000	mice
Dnajb11	0.14	1.00000000	mice
Cct7	0.11	1.00000000	mice
Tcp1	-0.07	1.00000000	mice
Anapc2	-0.06	1.00000000	mice
Pex16	-0.04	1.00000000	mice
Polr2h	-0.05	1.00000000	mice
Zfp821	0.07	1.00000000	mice
Alkbh5	-0.06	1.00000000	mice
F10	0.41	1.00000000	mice
Os9	0.09	1.00000000	mice
Rdx	-0.06	1.00000000	mice
Gzf1	-0.03	1.00000000	mice
Isyna1	0.06	1.00000000	mice
Siah2	-0.08	1.00000000	mice
Zcchc8	-0.06	1.00000000	mice

Ddx21	-0.01	1.00000000	mice
Plcl2	0.17	1.00000000	mice
Rnf170	-0.04	1.00000000	mice
Ndufs8	0.06	1.00000000	mice
Plp2	-0.10	1.00000000	mice
Eif4a2	-0.02	1.00000000	mice
Fyn	-0.14	1.00000000	mice
Cmtm7	0.09	1.00000000	mice
Olfm1	0.12	1.00000000	mice
Clcf1	0.05	1.00000000	mice
Unc50	-0.07	1.00000000	mice
Nop56	0.11	1.00000000	mice
Gpnmb	0.12	1.00000000	mice
Serbp1	-0.06	1.00000000	mice
Psmc1	0.12	1.00000000	mice
Zfand5	-0.10	1.00000000	mice
Sgsh	-0.03	1.00000000	mice
Zswim8	-0.08	1.00000000	mice
Ammecr1	-0.08	1.00000000	mice
Smim3	-0.21	1.00000000	mice
Smad7	0.09	1.00000000	mice
1110008P14Rik	-0.04	1.00000000	mice
Dctpp1	-0.02	1.00000000	mice
Bcas2	-0.05	1.00000000	mice
Il11ra1	-0.03	1.00000000	mice
Pde2a	-0.16	1.00000000	mice
Lpcat2	-0.19	1.00000000	mice
Smarcal1	-0.03	1.00000000	mice
Brd2	0.15	1.00000000	mice
Ppig	0.13	1.00000000	mice
Kcnab2	-0.06	1.00000000	mice
Tcf3	-0.04	1.00000000	mice
Pin1	0.10	1.00000000	mice
Arhgap35	-0.08	1.00000000	mice
Hlcs	-0.08	1.00000000	mice
Fkbp1b	-0.05	1.00000000	mice
Hint1	-0.07	1.00000000	mice
Fam8a1	-0.03	1.00000000	mice
Slc35c2	-0.07	1.00000000	mice
Nmrk1	-0.05	1.00000000	mice
Lbr	-0.16	1.00000000	mice
Arl8b	-0.16	1.00000000	mice
Pogz	-0.05	1.00000000	mice
Fnip1	-0.10	1.00000000	mice
Fdx2	-0.06	1.00000000	mice
Ttc13	-0.03	1.00000000	mice

Cxcl3	0.43	1.00000000	mice
Tsnax	-0.02	1.00000000	mice
Spidr	-0.10	1.00000000	mice
Nol8	-0.03	1.00000000	mice
Psme4	-0.09	1.00000000	mice
Rbm38	0.03	1.00000000	mice
Ogfod1	-0.06	1.00000000	mice
Ints11	0.05	1.00000000	mice
Chchd3	-0.09	1.00000000	mice
N4bp1	0.17	1.00000000	mice
Tbcel	-0.05	1.00000000	mice
Naxe	0.09	1.00000000	mice
Rcor1	-0.11	1.00000000	mice
Prex1	-0.09	1.00000000	mice
AC149090.1	0.10	1.00000000	mice
Dhdds	-0.06	1.00000000	mice
Fam110a	0.04	1.00000000	mice
U2af2	-0.06	1.00000000	mice
1600012H06Rik	-0.06	1.00000000	mice
Spata13	-0.32	1.00000000	mice
Rac1	-0.06	1.00000000	mice
Elf2	-0.08	1.00000000	mice
Fopnl	0.07	1.00000000	mice
Gid4	-0.06	1.00000000	mice
Cdkn2aipnl	-0.05	1.00000000	mice
Tmem259	-0.06	1.00000000	mice
Trim27	-0.04	1.00000000	mice
Timm44	0.07	1.00000000	mice
Dctn3	0.13	1.00000000	mice
Copa	-0.10	1.00000000	mice
Hcfc1r1	-0.07	1.00000000	mice
Axin1	-0.11	1.00000000	mice
Rnf115	0.17	1.00000000	mice
Stx4a	0.12	1.00000000	mice
Atg12	0.09	1.00000000	mice
Copb1	-0.12	1.00000000	mice
Zfp422	-0.04	1.00000000	mice
Tpr	-0.08	1.00000000	mice
Fbxo45	-0.06	1.00000000	mice
Ubxn7	-0.07	1.00000000	mice
Tbrg4	-0.01	1.00000000	mice
Eid1	-0.02	1.00000000	mice
Hcfc1	-0.06	1.00000000	mice
Apoe	-0.49	1.00000000	mice
Dennd4b	-0.05	1.00000000	mice
Urb1	-0.03	1.00000000	mice

Lsg1	-0.04	1.00000000	mice
Yme1l1	-0.07	1.00000000	mice
Snx5	0.20	1.00000000	mice
Prune1	-0.07	1.00000000	mice
Pdpn	0.29	1.00000000	mice
Scaf1	-0.06	1.00000000	mice
B9d2	-0.08	1.00000000	mice
Inpp5b	0.11	1.00000000	mice
Rex1bd	0.10	1.00000000	mice
Cdc123	0.11	1.00000000	mice
Fcgr3	-0.11	1.00000000	mice
Zkscan6	0.06	1.00000000	mice
Fam76a	-0.06	1.00000000	mice
Zfp944	-0.05	1.00000000	mice
Farsb	-0.07	1.00000000	mice
Cep250	-0.05	1.00000000	mice
Nr2f6	-0.04	1.00000000	mice
Lyrm2	0.04	1.00000000	mice
Tmem248	-0.07	1.00000000	mice
Ganab	-0.05	1.00000000	mice
Ulk1	-0.09	1.00000000	mice
Prdm10	-0.10	1.00000000	mice
Ufl1	-0.05	1.00000000	mice
Ppa2	-0.03	1.00000000	mice
Ankrd52	-0.06	1.00000000	mice
Ago1	-0.06	1.00000000	mice
Dcp1b	-0.05	1.00000000	mice
Stk11	-0.04	1.00000000	mice
Ap3m1	-0.04	1.00000000	mice
Pgm3	-0.05	1.00000000	mice
Neurl3	0.07	1.00000000	mice
Acot7	0.06	1.00000000	mice
Wdr41	-0.06	1.00000000	mice
Adnp2	0.04	1.00000000	mice
Arl4c	-0.30	1.00000000	mice
Uap1	-0.01	1.00000000	mice
Ndufs1	-0.04	1.00000000	mice
Orc2	-0.06	1.00000000	mice
Tcn2	-0.11	1.00000000	mice
Ppp1r37	-0.07	1.00000000	mice
Atic	-0.04	1.00000000	mice
Fam234a	-0.09	1.00000000	mice
Blnk	-0.08	1.00000000	mice
Klhdc3	-0.05	1.00000000	mice
Bcl2a1a	0.27	1.00000000	mice
Ptges3	-0.09	1.00000000	mice

Slc39a10	-0.06	1.00000000	mice
Ppp1r15b	-0.09	1.00000000	mice
Lactb	-0.04	1.00000000	mice
Pes1	-0.09	1.00000000	mice
Cacul1	-0.07	1.00000000	mice
Ptpa	-0.08	1.00000000	mice
Zfp1	-0.04	1.00000000	mice
Gdpd3	-0.06	1.00000000	mice
Rab5c	0.15	1.00000000	mice
Pcnx3	-0.06	1.00000000	mice
Socs7	-0.05	1.00000000	mice
Tada1	-0.03	1.00000000	mice
Pcf11	-0.05	1.00000000	mice
Phactr4	-0.05	1.00000000	mice
Skap2	0.16	1.00000000	mice
Sgpp1	-0.07	1.00000000	mice
Fam192a	0.10	1.00000000	mice
Zswim4	0.08	1.00000000	mice
Dgcr6	-0.02	1.00000000	mice
Cdkal1	-0.12	1.00000000	mice
Wdr75	-0.02	1.00000000	mice
Gipc1	0.07	1.00000000	mice
Abhd5	-0.05	1.00000000	mice
Wdr44	-0.13	1.00000000	mice
Dhx30	-0.07	1.00000000	mice
Dpp9	-0.08	1.00000000	mice
Psenen	0.12	1.00000000	mice
Bloc1s6	0.04	1.00000000	mice
Eps15	-0.07	1.00000000	mice
Atg2a	0.01	1.00000000	mice
Eldr	0.17	1.00000000	mice
Ldhb	-0.07	1.00000000	mice
Trpm4	0.06	1.00000000	mice
Akap10	-0.07	1.00000000	mice
Prpsap1	-0.05	1.00000000	mice
Isg20l2	-0.06	1.00000000	mice
Trmt10c	-0.04	1.00000000	mice
Iqsec2	0.12	1.00000000	mice
Abl2	-0.07	1.00000000	mice
L3mbtl3	-0.10	1.00000000	mice
Ap3s1	-0.04	1.00000000	mice
Atp5o	0.13	1.00000000	mice
Rabgap1l	-0.10	1.00000000	mice
Slc38a9	-0.08	1.00000000	mice
Zmat2	0.11	1.00000000	mice
Wdyhv1	0.05	1.00000000	mice

Dph6	-0.07	1.00000000	mice
Isy1	0.10	1.00000000	mice
Sf3b2	-0.04	1.00000000	mice
Pnkp	0.07	1.00000000	mice
Tex30	-0.09	1.00000000	mice
Ywhab	-0.09	1.00000000	mice
Chkb	-0.07	1.00000000	mice
Ube2j1	-0.07	1.00000000	mice
Etfdh	-0.06	1.00000000	mice
Utp15	-0.05	1.00000000	mice
Rfx3	-0.05	1.00000000	mice
Ldlr	0.10	1.00000000	mice
Ncf1	0.16	1.00000000	mice
Aggf1	-0.08	1.00000000	mice
Caprin1	-0.09	1.00000000	mice
Alkbh1	-0.02	1.00000000	mice
Trappc2l	0.12	1.00000000	mice
Trim35	-0.04	1.00000000	mice
Oxa1l	-0.03	1.00000000	mice
Heatr6	-0.05	1.00000000	mice
Fosb	-0.15	1.00000000	mice
Slc25a12	0.09	1.00000000	mice
Anapc5	-0.08	1.00000000	mice
Trabd	-0.03	1.00000000	mice
Cactin	-0.05	1.00000000	mice
Bach1	0.17	1.00000000	mice
Polr3b	-0.10	1.00000000	mice
Ranbp3	-0.05	1.00000000	mice
Fcf1	0.08	1.00000000	mice
Cct2	-0.07	1.00000000	mice
Ttyh3	-0.09	1.00000000	mice
Atl3	-0.02	1.00000000	mice
Ak6	0.06	1.00000000	mice
Smarcad1	-0.01	1.00000000	mice
Arrb2	0.19	1.00000000	mice
AU040320	-0.05	1.00000000	mice
Sco2	-0.03	1.00000000	mice
Vps28	0.05	1.00000000	mice
Irak3	-0.20	1.00000000	mice
Zfp53	0.07	1.00000000	mice
Incenp	-0.04	1.00000000	mice
Ube2j2	-0.09	1.00000000	mice
Cops3	-0.06	1.00000000	mice
Prkar1a	-0.08	1.00000000	mice
Bin3	0.10	1.00000000	mice
Tpp1	-0.06	1.00000000	mice

Rnpepl1	-0.06	1.00000000	mice
Stat6	-0.07	1.00000000	mice
Smad2	-0.07	1.00000000	mice
Osbpl9	-0.14	1.00000000	mice
Nfs1	-0.04	1.00000000	mice
Hlx	-0.10	1.00000000	mice
Kpna3	0.23	1.00000000	mice
Dennd4a	-0.01	1.00000000	mice
Gbe1	-0.10	1.00000000	mice
Cul4a	-0.04	1.00000000	mice
Psmd2	0.18	1.00000000	mice
Chchd4	-0.06	1.00000000	mice
Dut	0.00	1.00000000	mice
Ccdc12	0.12	1.00000000	mice
Dhrs7b	-0.06	1.00000000	mice
Arih2	0.17	1.00000000	mice
Nras	-0.08	1.00000000	mice
Ssr2	0.06	1.00000000	mice
Sharpin	0.06	1.00000000	mice
Rnf6	-0.07	1.00000000	mice
Ino80c	0.04	1.00000000	mice
Cry2	-0.03	1.00000000	mice
Cherp	-0.02	1.00000000	mice
Hcar2	0.42	1.00000000	mice
BC005624	-0.11	1.00000000	mice
Zc3h4	-0.07	1.00000000	mice
Coa3	0.15	1.00000000	mice
Ube2g2	-0.08	1.00000000	mice
C2cd2	-0.06	1.00000000	mice
Cops5	0.07	1.00000000	mice
Txnl4a	-0.06	1.00000000	mice
Atg9a	-0.07	1.00000000	mice
Ddhd1	0.00	1.00000000	mice
Ttl	-0.04	1.00000000	mice
Ahcyl1	-0.08	1.00000000	mice
Kat5	-0.01	1.00000000	mice
Fmc1	0.08	1.00000000	mice
Slc25a46	-0.06	1.00000000	mice
Utp11	0.08	1.00000000	mice
Gcn1	-0.03	1.00000000	mice
Kdm4a	-0.05	1.00000000	mice
Ppfia4	0.09	1.00000000	mice
Fbxo3	-0.05	1.00000000	mice
Urb2	-0.04	1.00000000	mice
Pcyt2	-0.02	1.00000000	mice
Tbc1d32	0.11	1.00000000	mice

Gatad1	-0.09	1.00000000	mice
Crebl2	-0.08	1.00000000	mice
2300009A05Rik	-0.04	1.00000000	mice
Pibf1	-0.09	1.00000000	mice
Aqr	-0.07	1.00000000	mice
Rsbn1	-0.01	1.00000000	mice
Ptma	0.15	1.00000000	mice
Asl	0.06	1.00000000	mice
Nub1	0.11	1.00000000	mice
Clns1a	-0.04	1.00000000	mice
Naga	-0.05	1.00000000	mice
Gca	0.13	1.00000000	mice
Ccdc162	-0.20	1.00000000	mice
Tmco3	-0.06	1.00000000	mice
Frs2	-0.08	1.00000000	mice
Cggbp1	0.12	1.00000000	mice
Plscr3	0.08	1.00000000	mice
AI662270	0.14	1.00000000	mice
Hilpda	0.40	1.00000000	mice
Emc10	-0.02	1.00000000	mice
Wfdc21	0.14	1.00000000	mice
Cyld	-0.05	1.00000000	mice
Lrba	-0.10	1.00000000	mice
Cox4i1	-0.08	1.00000000	mice
1810030O07Rik	-0.05	1.00000000	mice
Bri3bp	-0.07	1.00000000	mice
Exosc5	-0.06	1.00000000	mice
Rev1	-0.10	1.00000000	mice
Tbc1d20	-0.10	1.00000000	mice
Traf7	-0.02	1.00000000	mice
1810026B05Rik	-0.05	1.00000000	mice
Cops9	0.18	1.00000000	mice
Thoc1	-0.08	1.00000000	mice
Snrpb	-0.07	1.00000000	mice
Ilkap	-0.06	1.00000000	mice
Yipf1	0.03	1.00000000	mice
Plcb3	-0.02	1.00000000	mice
Cdk11b	-0.07	1.00000000	mice
Xlr	-0.04	1.00000000	mice
Capza1	-0.05	1.00000000	mice
Tmem256	0.12	1.00000000	mice
Casc3	-0.04	1.00000000	mice
Fam53a	-0.02	1.00000000	mice
Airn	0.15	1.00000000	mice
Aldh2	-0.10	1.00000000	mice
Derl2	0.08	1.00000000	mice

Pdcd7	-0.05	1.00000000	mice
Arf6	-0.05	1.00000000	mice
Aasdhppt	-0.07	1.00000000	mice
Ywhae	0.14	1.00000000	mice
Srm	-0.06	1.00000000	mice
Spop	0.09	1.00000000	mice
Ubr4	0.11	1.00000000	mice
Tmem184b	0.13	1.00000000	mice
Rnf144b	0.00	1.00000000	mice
Rab3d	-0.04	1.00000000	mice
Srsf2	0.15	1.00000000	mice
Cntln	-0.04	1.00000000	mice
Aldoa	-0.04	1.00000000	mice
Rabgef1	0.12	1.00000000	mice
Zc3h12c	0.30	1.00000000	mice
Pgrmc1	-0.03	1.00000000	mice
Naa30	-0.04	1.00000000	mice
Sugt1	0.11	1.00000000	mice
Rhoh	-0.06	1.00000000	mice
Nbas	-0.04	1.00000000	mice
Ppid	-0.05	1.00000000	mice
Rab12	0.14	1.00000000	mice
Tex14	0.36	1.00000000	mice
Cyth4	0.15	1.00000000	mice
Eif3c	0.12	1.00000000	mice
Rnps1	0.12	1.00000000	mice
Ift52	0.06	1.00000000	mice
Dis3l2	-0.06	1.00000000	mice
Ap1ar	-0.04	1.00000000	mice
Pacc1	0.07	1.00000000	mice
Top3a	-0.01	1.00000000	mice
Lims1	-0.18	1.00000000	mice
Tmem63a	-0.05	1.00000000	mice
Ddx54	-0.04	1.00000000	mice
Rbfa	0.08	1.00000000	mice
2610037D02Rik	0.08	1.00000000	mice
Ndufa1	0.13	1.00000000	mice
Erh	-0.07	1.00000000	mice
Zdhhc21	-0.06	1.00000000	mice
Rab18	-0.06	1.00000000	mice
Abcc1	0.00	1.00000000	mice
Spin1	-0.06	1.00000000	mice
Riok1	-0.05	1.00000000	mice
Opa1	-0.08	1.00000000	mice
Ptpn2	0.14	1.00000000	mice
Cdk12	-0.09	1.00000000	mice

Sh3bp1	0.09	1.00000000	mice
Lsm2	0.03	1.00000000	mice
Lrrk1	0.15	1.00000000	mice
Dcun1d1	-0.03	1.00000000	mice
Dnajc15	-0.03	1.00000000	mice
Kpna6	-0.06	1.00000000	mice
Fbxo42	-0.09	1.00000000	mice
Cenpb	-0.03	1.00000000	mice
2310033P09Rik	0.08	1.00000000	mice
Dtd1	-0.05	1.00000000	mice
Ncapd3	-0.05	1.00000000	mice
Supt20	-0.02	1.00000000	mice
Ctdnep1	-0.03	1.00000000	mice
Acp1	0.11	1.00000000	mice
Thada	-0.16	1.00000000	mice
Cox16	-0.05	1.00000000	mice
Stab1	0.06	1.00000000	mice
Sirpb1a	-0.05	1.00000000	mice
Cenpx	-0.04	1.00000000	mice
E2f3	0.09	1.00000000	mice
Rab22a	0.14	1.00000000	mice
Gpn2	-0.03	1.00000000	mice
P4ha1	0.13	1.00000000	mice
Uchl5	-0.07	1.00000000	mice
Uchl3	-0.06	1.00000000	mice
Ndufb3	0.13	1.00000000	mice
Rap1b	-0.10	1.00000000	mice
Atp7a	-0.04	1.00000000	mice
Fbxw2	-0.10	1.00000000	mice
Commd3	0.07	1.00000000	mice
Parp1	-0.06	1.00000000	mice
Ppp2cb	-0.06	1.00000000	mice
Snrpd2	0.15	1.00000000	mice
Smg1	-0.04	1.00000000	mice
Ikzf3	-0.14	1.00000000	mice
Nars	0.12	1.00000000	mice
Ppfibp2	-0.07	1.00000000	mice
Evl	0.22	1.00000000	mice
Dgat1	-0.03	1.00000000	mice
Ddx19a	-0.03	1.00000000	mice
Lyn	-0.09	1.00000000	mice
Zfp280c	-0.04	1.00000000	mice
Sclt1	-0.04	1.00000000	mice
Phykpl	0.05	1.00000000	mice
Pqbp1	-0.05	1.00000000	mice
Farsa	-0.10	1.00000000	mice

Rab9	0.11	1.00000000	mice
Nolc1	-0.04	1.00000000	mice
Rnf44	-0.05	1.00000000	mice
Stk19	0.09	1.00000000	mice
Frmd8	-0.06	1.00000000	mice
Ttc39b	-0.01	1.00000000	mice
Zfp143	0.04	1.00000000	mice
Ube4a	-0.10	1.00000000	mice
Ero1lb	-0.05	1.00000000	mice
Anks3	-0.05	1.00000000	mice
Snapin	-0.04	1.00000000	mice
Gabarapl2	-0.11	1.00000000	mice
Rrn3	-0.04	1.00000000	mice
Set	0.03	1.00000000	mice
Ckap4	0.06	1.00000000	mice
Fam98a	-0.04	1.00000000	mice
Hnrnpk	-0.05	1.00000000	mice
Fgfr1op	-0.06	1.00000000	mice
Arpc1a	-0.07	1.00000000	mice
Lsm1	-0.07	1.00000000	mice
Srcap	-0.07	1.00000000	mice
Eid3	0.08	1.00000000	mice
Abca7	-0.02	1.00000000	mice
H1f0	0.07	1.00000000	mice
Hnrnph2	-0.10	1.00000000	mice
Trmt61b	-0.07	1.00000000	mice
Polr3f	-0.03	1.00000000	mice
Rrp15	-0.02	1.00000000	mice
Carm1	-0.06	1.00000000	mice
Borcs6	-0.05	1.00000000	mice
Lrrc57	-0.03	1.00000000	mice
Ypel5	-0.08	1.00000000	mice
Dhps	-0.09	1.00000000	mice
Tpst2	0.12	1.00000000	mice
Psmd1	0.14	1.00000000	mice
Stard9	-0.11	1.00000000	mice
Ccdc50	-0.13	1.00000000	mice
Yrdc	-0.05	1.00000000	mice
Gramd1c	-0.06	1.00000000	mice
Fem1b	-0.02	1.00000000	mice
Zcchc7	-0.10	1.00000000	mice
Txnrd2	-0.05	1.00000000	mice
Gtf2a1	0.14	1.00000000	mice
Trim24	-0.05	1.00000000	mice
Atp6v0d1	-0.07	1.00000000	mice
Sf3a3	0.08	1.00000000	mice

Gosr2	0.11	1.00000000	mice
Crip2	0.08	1.00000000	mice
Sgsm3	-0.05	1.00000000	mice
Ak2	0.14	1.00000000	mice
Fip1l1	-0.07	1.00000000	mice
Dcun1d5	-0.04	1.00000000	mice
Dync1li1	-0.06	1.00000000	mice
Gpat4	-0.05	1.00000000	mice
Cish	0.11	1.00000000	mice
Tmem216	-0.02	1.00000000	mice
Kdm5c	-0.06	1.00000000	mice
Tlr13	0.06	1.00000000	mice
Arhgef3	-0.03	1.00000000	mice
Qtrt1	-0.03	1.00000000	mice
St13	-0.04	1.00000000	mice
Zfp318	-0.07	1.00000000	mice
Limk2	-0.06	1.00000000	mice
Rnf185	-0.12	1.00000000	mice
Ntng2	-0.06	1.00000000	mice
Rrm2b	-0.07	1.00000000	mice
Acsl1	-0.08	1.00000000	mice
Uqcr10	0.17	1.00000000	mice
Prpf8	-0.09	1.00000000	mice
Polg	-0.05	1.00000000	mice
Ilf3	0.15	1.00000000	mice
Natd1	-0.03	1.00000000	mice
Nubp2	0.07	1.00000000	mice
4930402H24Rik	-0.08	1.00000000	mice
Ifitm2	-0.07	1.00000000	mice
Ckap5	-0.10	1.00000000	mice
Pip5k1a	0.08	1.00000000	mice
Tmem33	-0.10	1.00000000	mice
Angel2	-0.07	1.00000000	mice
Aph1a	-0.08	1.00000000	mice
Abcf3	-0.02	1.00000000	mice
Kcmf1	0.14	1.00000000	mice
Gnl2	-0.03	1.00000000	mice
Fkbp8	-0.07	1.00000000	mice
Eif3b	-0.01	1.00000000	mice
Tns3	-0.02	1.00000000	mice
0610010K14Rik	0.08	1.00000000	mice
Pola2	0.09	1.00000000	mice
Dync1li2	-0.04	1.00000000	mice
Cyp4f18	-0.26	1.00000000	mice
Slc27a1	-0.02	1.00000000	mice
Ndufb10	-0.05	1.00000000	mice

Jaml	-0.33	1.00000000	mice
Inpp5f	-0.12	1.00000000	mice
Polr3gl	-0.02	1.00000000	mice
Lrrc63	0.10	1.00000000	mice
A630001O12Rik	0.03	1.00000000	mice
Srrt	0.10	1.00000000	mice
Wdtc1	0.09	1.00000000	mice
Tnip2	-0.07	1.00000000	mice
Slf1	-0.05	1.00000000	mice
Prmt3	-0.06	1.00000000	mice
Setd1a	-0.04	1.00000000	mice
Atl2	-0.04	1.00000000	mice
Rbm19	-0.05	1.00000000	mice
Exoc1	0.08	1.00000000	mice
Smc2	-0.03	1.00000000	mice
Commd9	0.06	1.00000000	mice
Ado	-0.02	1.00000000	mice
Kdelr2	-0.02	1.00000000	mice
Cgrrf1	-0.06	1.00000000	mice
Hcfc2	-0.05	1.00000000	mice
Adgre1	-0.05	1.00000000	mice
Ing4	-0.04	1.00000000	mice
Etfb	-0.05	1.00000000	mice
Afdn	-0.06	1.00000000	mice
Ube2d3	-0.06	1.00000000	mice
Borcs8	0.05	1.00000000	mice
Wasf2	-0.10	1.00000000	mice
Bola3	-0.02	1.00000000	mice
Pfkfb4	-0.07	1.00000000	mice
Nop2	-0.02	1.00000000	mice
Atad2b	-0.09	1.00000000	mice
Tspan17	0.05	1.00000000	mice
Hgs	-0.07	1.00000000	mice
Ate1	-0.06	1.00000000	mice
Acyp1	-0.04	1.00000000	mice
Nt5dc1	-0.09	1.00000000	mice
Ighm	-0.16	1.00000000	mice
Scly	0.04	1.00000000	mice
Slc36a1	-0.02	1.00000000	mice
2610001J05Rik	0.07	1.00000000	mice
Arpp19	0.12	1.00000000	mice
Secisbp2l	-0.07	1.00000000	mice
Lamp1	-0.03	1.00000000	mice
F13a1	-0.02	1.00000000	mice
Tmem241	-0.03	1.00000000	mice
AI504432	-0.07	1.00000000	mice

Psmd5	-0.06	1.00000000	mice
Ctsa	-0.07	1.00000000	mice
H13	-0.08	1.00000000	mice
Gorab	0.01	1.00000000	mice
Slc30a1	0.08	1.00000000	mice
Tjap1	0.06	1.00000000	mice
Zc3h18	-0.05	1.00000000	mice
Vamp4	-0.05	1.00000000	mice
Raet1e	0.06	1.00000000	mice
Zfp407	-0.11	1.00000000	mice
Zfp638	-0.07	1.00000000	mice
Ifngr2	0.10	1.00000000	mice
Snhg12	0.11	1.00000000	mice
Arid3b	-0.04	1.00000000	mice
Hadha	-0.07	1.00000000	mice
Slu7	0.07	1.00000000	mice
Slc15a4	0.01	1.00000000	mice
Arpc1b	0.06	1.00000000	mice
Cers2	-0.08	1.00000000	mice
Ube2q1	0.08	1.00000000	mice
Agtrap	0.09	1.00000000	mice
Arid4a	-0.06	1.00000000	mice
Dgcr2	-0.05	1.00000000	mice
Leo1	-0.03	1.00000000	mice
Slc35b2	0.08	1.00000000	mice
Alpk1	0.12	1.00000000	mice
Cdc37	0.12	1.00000000	mice
Ppia	-0.01	1.00000000	mice
Pcnp	0.09	1.00000000	mice
Rnf5	-0.04	1.00000000	mice
Ndufb8	0.10	1.00000000	mice
Ythdf1	-0.04	1.00000000	mice
Seh1l	-0.06	1.00000000	mice
Cyb5b	-0.05	1.00000000	mice
Asb3	-0.07	1.00000000	mice
Ei24	-0.03	1.00000000	mice
Pdrg1	0.04	1.00000000	mice
9130230L23Rik	-0.09	1.00000000	mice
S100a8	0.00	1.00000000	mice
Smurf1	-0.06	1.00000000	mice
Baz2a	-0.07	1.00000000	mice
Jak1	-0.10	1.00000000	mice
Irak4	0.07	1.00000000	mice
Ip6k1	-0.09	1.00000000	mice
Adh5	0.10	1.00000000	mice
Ptbp1	0.00	1.00000000	mice

Lrrc47	-0.03	1.00000000	mice
C330007P06Rik	-0.05	1.00000000	mice
D8Ertd738e	-0.06	1.00000000	mice
Cfap36	0.06	1.00000000	mice
Ablim1	-0.12	1.00000000	mice
Tbc1d17	0.00	1.00000000	mice
Trir	-0.02	1.00000000	mice
Erich1	-0.06	1.00000000	mice
Cdc5l	-0.14	1.00000000	mice
Tubb5	0.20	1.00000000	mice
Gon4l	-0.04	1.00000000	mice
Akr1b10	-0.01	1.00000000	mice
Gtpbp2	0.12	1.00000000	mice
Ankrd13a	-0.09	1.00000000	mice
Vmp1	0.07	1.00000000	mice
Cnppd1	-0.06	1.00000000	mice
Fkbp2	0.14	1.00000000	mice
Rtca	0.03	1.00000000	mice
Ndufaf8	0.14	1.00000000	mice
Glod4	-0.07	1.00000000	mice
Smarce1	0.10	1.00000000	mice
Crls1	-0.02	1.00000000	mice
Sf3b6	0.08	1.00000000	mice
Abhd13	-0.04	1.00000000	mice
Dennd6a	-0.06	1.00000000	mice
Cadm1	0.26	1.00000000	mice
Camk2d	0.08	1.00000000	mice
Ndufb2	0.13	1.00000000	mice
Nelfa	-0.06	1.00000000	mice
5031439G07Rik	-0.01	1.00000000	mice
Tsr1	-0.02	1.00000000	mice
Sqor	-0.08	1.00000000	mice
Glmp	0.06	1.00000000	mice
Prkaa1	-0.09	1.00000000	mice
Smap1	-0.08	1.00000000	mice
Elavl1	-0.05	1.00000000	mice
Vamp2	-0.06	1.00000000	mice
Slc2a1	0.06	1.00000000	mice
Gtf2h1	-0.07	1.00000000	mice
Prcc	-0.02	1.00000000	mice
Tial1	-0.05	1.00000000	mice
2310009B15Rik	0.05	1.00000000	mice
Hspbap1	-0.07	1.00000000	mice
Trim33	-0.07	1.00000000	mice
Tirap	-0.07	1.00000000	mice
Ss18l2	-0.01	1.00000000	mice

Tnrc18	-0.16	1.00000000	mice
Nae1	-0.02	1.00000000	mice
Bcr	0.00	1.00000000	mice
Dnaja1	0.13	1.00000000	mice
Fez2	0.06	1.00000000	mice
Sec22b	-0.05	1.00000000	mice
Ssh1	-0.06	1.00000000	mice
Uba6	-0.08	1.00000000	mice
Lsm7	0.07	1.00000000	mice
Adgre4	-0.17	1.00000000	mice
Emd	-0.10	1.00000000	mice
Fam204a	0.11	1.00000000	mice
Eml4	-0.10	1.00000000	mice
Btbd19	-0.06	1.00000000	mice
Inf2	-0.01	1.00000000	mice
Nol9	-0.03	1.00000000	mice
Ensa	0.09	1.00000000	mice
Cacfd1	-0.03	1.00000000	mice
Stoml2	0.07	1.00000000	mice
Dynll2	0.01	1.00000000	mice
Lsm12	0.12	1.00000000	mice
Atp5h	-0.06	1.00000000	mice
Rbm45	-0.04	1.00000000	mice
Gnl3l	-0.05	1.00000000	mice
Gabarapl1	-0.03	1.00000000	mice
2900097C17Rik	-0.04	1.00000000	mice
Trp53bp2	-0.09	1.00000000	mice
Krcc1	0.11	1.00000000	mice
Pigm	-0.04	1.00000000	mice
Pbx2	-0.04	1.00000000	mice
Ppil4	-0.04	1.00000000	mice
Fam49b	-0.05	1.00000000	mice
Snhg1	0.15	1.00000000	mice
Ammecr1l	-0.05	1.00000000	mice
Tatdn1	-0.05	1.00000000	mice
Naa38	0.05	1.00000000	mice
Rab5a	-0.04	1.00000000	mice
Luc7l3	0.13	1.00000000	mice
Pola1	-0.07	1.00000000	mice
Bicd2	-0.01	1.00000000	mice
Ncoa3	0.08	1.00000000	mice
Bop1	-0.05	1.00000000	mice
Slc7a6os	-0.04	1.00000000	mice
Ilk	-0.04	1.00000000	mice
Uckl1	0.05	1.00000000	mice
Cd300c2	0.17	1.00000000	mice

Lpgat1	-0.03	1.00000000	mice
Bmi1	-0.02	1.00000000	mice
Spryd3	-0.02	1.00000000	mice
Ap2m1	-0.02	1.00000000	mice
Fer	-0.02	1.00000000	mice
Skil	0.15	1.00000000	mice
Pus1	0.01	1.00000000	mice
Prpf40a	-0.06	1.00000000	mice
Spindoc	-0.04	1.00000000	mice
Htatip2	0.08	1.00000000	mice
Golga5	-0.07	1.00000000	mice
Aatf	-0.07	1.00000000	mice
2210016F16Rik	0.13	1.00000000	mice
Tnfrsf26	-0.03	1.00000000	mice
Btg1	-0.15	1.00000000	mice
Grhl1	-0.03	1.00000000	mice
Znrd1	0.06	1.00000000	mice
Slc25a39	-0.05	1.00000000	mice
Ubr7	-0.03	1.00000000	mice
Nhp2	-0.02	1.00000000	mice
Ndufs6	0.14	1.00000000	mice
Zfp217	-0.05	1.00000000	mice
Orai3	-0.04	1.00000000	mice
Ccdc47	-0.01	1.00000000	mice
Nsun2	-0.03	1.00000000	mice
Galnt6	-0.06	1.00000000	mice
Abi2	-0.04	1.00000000	mice
Nucks1	-0.04	1.00000000	mice
Ttc33	0.00	1.00000000	mice
Nmd3	-0.04	1.00000000	mice
Cul4b	0.00	1.00000000	mice
Emg1	-0.02	1.00000000	mice
Anapc10	0.07	1.00000000	mice
Gnpat	-0.02	1.00000000	mice
Galc	-0.02	1.00000000	mice
Ulk4	0.05	1.00000000	mice
Nsmce3	-0.04	1.00000000	mice
Ykt6	0.10	1.00000000	mice
Rgl2	-0.04	1.00000000	mice
Ktn1	0.02	1.00000000	mice
Tuba1a	0.22	1.00000000	mice
Ttc4	-0.04	1.00000000	mice
Capns1	-0.08	1.00000000	mice
Ahsa2	0.03	1.00000000	mice
Blvra	0.10	1.00000000	mice
Snx16	0.03	1.00000000	mice

Vti1b	-0.06	1.00000000	mice
Pex19	-0.06	1.00000000	mice
Cpne1	-0.04	1.00000000	mice
Srrd	0.05	1.00000000	mice
Nsrp1	0.06	1.00000000	mice
Csnk1g2	-0.01	1.00000000	mice
Renbp	-0.01	1.00000000	mice
Scrib	-0.03	1.00000000	mice
Synj1	0.12	1.00000000	mice
Trnau1ap	-0.04	1.00000000	mice
Slc35a2	-0.05	1.00000000	mice
Esrra	0.08	1.00000000	mice
Ndufa4	0.03	1.00000000	mice
Smg8	-0.04	1.00000000	mice
Tdg	0.07	1.00000000	mice
Atp1b3	-0.07	1.00000000	mice
Smim7	-0.02	1.00000000	mice
Bcat2	-0.02	1.00000000	mice
Dap	-0.05	1.00000000	mice
Armc1	-0.04	1.00000000	mice
Ip6k2	0.05	1.00000000	mice
Knop1	0.06	1.00000000	mice
Smg6	-0.08	1.00000000	mice
Epg5	-0.03	1.00000000	mice
Dhx40	-0.04	1.00000000	mice
Smarcc2	-0.04	1.00000000	mice
Nup107	-0.07	1.00000000	mice
Timm50	0.04	1.00000000	mice
Dnajc19	0.08	1.00000000	mice
Zmynd8	-0.05	1.00000000	mice
Trmt11	-0.02	1.00000000	mice
Pira2	0.24	1.00000000	mice
Utp18	-0.03	1.00000000	mice
Braf	0.11	1.00000000	mice
Cyp27a1	-0.06	1.00000000	mice
Chid1	0.02	1.00000000	mice
Atp6v0e	-0.06	1.00000000	mice
Srp14	-0.01	1.00000000	mice
Erap1	0.10	1.00000000	mice
Zfp445	-0.02	1.00000000	mice
Rwdd1	0.12	1.00000000	mice
Slc44a1	0.13	1.00000000	mice
Phkg2	-0.05	1.00000000	mice
Phf20l1	-0.07	1.00000000	mice
Clptm1l	-0.08	1.00000000	mice
Bzw1	-0.06	1.00000000	mice

Sra1	0.04	1.00000000	mice
Rufy1	-0.04	1.00000000	mice
Hif1an	-0.04	1.00000000	mice
Oxsr1	-0.03	1.00000000	mice
Wbp2	-0.05	1.00000000	mice
Fbxo7	0.08	1.00000000	mice
Atp8a1	0.18	1.00000000	mice
Tex2	-0.04	1.00000000	mice
Ndufa5	0.12	1.00000000	mice
Whamm	0.08	1.00000000	mice
Rngtt	-0.07	1.00000000	mice
Rbl1	0.10	1.00000000	mice
Lpcat3	-0.05	1.00000000	mice
Slc35b3	-0.05	1.00000000	mice
Xiap	0.14	1.00000000	mice
Cyb5r4	-0.06	1.00000000	mice
Als2	0.09	1.00000000	mice
B230219D22Rik	-0.03	1.00000000	mice
Klf11	-0.04	1.00000000	mice
Armc7	-0.08	1.00000000	mice
Jmjd6	-0.04	1.00000000	mice
Pdcd5	0.12	1.00000000	mice
Lman2	0.10	1.00000000	mice
Rce1	-0.03	1.00000000	mice
Urod	-0.03	1.00000000	mice
St3gal3	-0.04	1.00000000	mice
Nr2c1	-0.03	1.00000000	mice
Nup214	-0.07	1.00000000	mice
Wdr46	-0.05	1.00000000	mice
Fam114a2	-0.07	1.00000000	mice
Rraga	-0.02	1.00000000	mice
Vps26c	0.05	1.00000000	mice
Ppp2r5a	-0.06	1.00000000	mice
Usp50	0.04	1.00000000	mice
Pkm	0.11	1.00000000	mice
Atp13a2	-0.04	1.00000000	mice
Ppfia1	-0.08	1.00000000	mice
Spopl	-0.05	1.00000000	mice
Pik3r5	0.01	1.00000000	mice
Acsl5	-0.10	1.00000000	mice
LTO1	0.04	1.00000000	mice
Htt	-0.05	1.00000000	mice
Amz2	0.06	1.00000000	mice
Pex6	-0.04	1.00000000	mice
Tmed9	0.00	1.00000000	mice
Sh3glb2	-0.02	1.00000000	mice

Cwc15	-0.05	1.00000000	mice
Cemip2	0.05	1.00000000	mice
Entpd7	0.11	1.00000000	mice
Vapb	-0.05	1.00000000	mice
Lmbrd1	0.14	1.00000000	mice
Noc3l	-0.02	1.00000000	mice
Pafah1b2	-0.02	1.00000000	mice
Fuca2	-0.08	1.00000000	mice
Supt6	-0.06	1.00000000	mice
Ilvbl	-0.02	1.00000000	mice
Rtn4	0.10	1.00000000	mice
Ap1g1	-0.07	1.00000000	mice
Pdcd10	0.08	1.00000000	mice
Zfp131	0.14	1.00000000	mice
Nup98	0.14	1.00000000	mice
Psmb5	0.12	1.00000000	mice
Ndufb5	-0.02	1.00000000	mice
Nab1	0.04	1.00000000	mice
Tmem234	-0.03	1.00000000	mice
Sp4	-0.11	1.00000000	mice
Nsmce4a	-0.04	1.00000000	mice
Abhd17b	-0.08	1.00000000	mice
Ciapin1	0.06	1.00000000	mice
Nubp1	0.06	1.00000000	mice
Rab35	-0.03	1.00000000	mice
Ccdc80	0.03	1.00000000	mice
Gatd3a	-0.01	1.00000000	mice
Sdhaf2	0.02	1.00000000	mice
Ciao3	-0.05	1.00000000	mice
Gpatch11	0.06	1.00000000	mice
Il1r2	-0.53	1.00000000	mice
Zc3h13	-0.06	1.00000000	mice
Ubac2	-0.05	1.00000000	mice
Gars	-0.04	1.00000000	mice
Trappc11	-0.04	1.00000000	mice
Epb41	-0.02	1.00000000	mice
Ube2m	0.14	1.00000000	mice
Abt1	-0.03	1.00000000	mice
Abcf1	0.13	1.00000000	mice
Crot	-0.03	1.00000000	mice
Fam98c	-0.04	1.00000000	mice
Disc1	-0.05	1.00000000	mice
Pdk3	0.06	1.00000000	mice
Ppp1r7	0.06	1.00000000	mice
Pkp4	0.02	1.00000000	mice
Galnt11	-0.05	1.00000000	mice

Hacd3	-0.02	1.00000000	mice
Ppt1	0.07	1.00000000	mice
Clasp1	-0.05	1.00000000	mice
Fam172a	-0.03	1.00000000	mice
Khdc4	0.09	1.00000000	mice
Pdha1	-0.04	1.00000000	mice
Acadl	0.12	1.00000000	mice
Ap1b1	-0.01	1.00000000	mice
Scpep1	0.12	1.00000000	mice
Rbm10	-0.03	1.00000000	mice
Fchsd2	0.07	1.00000000	mice
Kdm7a	-0.19	1.00000000	mice
Slc8b1	0.11	1.00000000	mice
Gpatch8	-0.08	1.00000000	mice
Fam20c	-0.03	1.00000000	mice
Usp1	-0.03	1.00000000	mice
Zfp84	-0.01	1.00000000	mice
Ctbs	0.02	1.00000000	mice
Rnf181	0.08	1.00000000	mice
Ube2e1	-0.06	1.00000000	mice
Ago2	-0.06	1.00000000	mice
Dkc1	0.00	1.00000000	mice
Senp7	-0.03	1.00000000	mice
Toe1	-0.02	1.00000000	mice
Zfp26	-0.04	1.00000000	mice
Trp53inp1	-0.02	1.00000000	mice
Atg3	-0.10	1.00000000	mice
Taf15	0.15	1.00000000	mice
Pex2	-0.02	1.00000000	mice
Zfp386	-0.04	1.00000000	mice
Parp2	0.04	1.00000000	mice
Znrd2	0.04	1.00000000	mice
Dctn1	-0.05	1.00000000	mice
Npat	-0.03	1.00000000	mice
Fam114a1	-0.03	1.00000000	mice
Avl9	-0.06	1.00000000	mice
Ssbp2	-0.05	1.00000000	mice
Snap23	0.04	1.00000000	mice
Fxr1	0.10	1.00000000	mice
Tmem127	-0.04	1.00000000	mice
Rbm48	-0.02	1.00000000	mice
Gss	-0.03	1.00000000	mice
Zdhhc17	-0.01	1.00000000	mice
Rspry1	-0.05	1.00000000	mice
Vcl	-0.10	1.00000000	mice
Slc38a7	0.01	1.00000000	mice

Zfp729a	-0.03	1.00000000	mice
Zfp62	-0.02	1.00000000	mice
Kat6a	0.12	1.00000000	mice
Cog6	-0.05	1.00000000	mice
Usp16	-0.07	1.00000000	mice
Pnn	-0.03	1.00000000	mice
Isca1	-0.05	1.00000000	mice
Tinf2	0.03	1.00000000	mice
Atp10a	-0.18	1.00000000	mice
Orai2	0.05	1.00000000	mice
Hsf2	0.07	1.00000000	mice
Rbbp4	-0.03	1.00000000	mice
Prrc2a	-0.02	1.00000000	mice
Dhx36	-0.06	1.00000000	mice
Phf5a	-0.04	1.00000000	mice
Prps2	-0.02	1.00000000	mice
Ptcd2	0.04	1.00000000	mice
Fbxo4	0.09	1.00000000	mice
Psmg3	0.03	1.00000000	mice
Cipc	0.02	1.00000000	mice
Exoc6	-0.08	1.00000000	mice
Ints7	-0.05	1.00000000	mice
Dync1i2	0.13	1.00000000	mice
Ssr3	-0.08	1.00000000	mice
Acaa2	0.07	1.00000000	mice
Bcl2a1b	0.22	1.00000000	mice
Slc7a6	-0.03	1.00000000	mice
Raph1	0.14	1.00000000	mice
1110038B12Rik	0.09	1.00000000	mice
Adssl1	0.05	1.00000000	mice
Snhg3	-0.04	1.00000000	mice
Iws1	-0.05	1.00000000	mice
Cap1	-0.10	1.00000000	mice
Ing5	-0.05	1.00000000	mice
Ppp2r1b	0.00	1.00000000	mice
Cdc16	-0.03	1.00000000	mice
Pgk1	-0.05	1.00000000	mice
Plekhg2	0.02	1.00000000	mice
Golga1	-0.03	1.00000000	mice
Atf4	0.00	1.00000000	mice
Rnf187	-0.05	1.00000000	mice
Tecpr2	-0.05	1.00000000	mice
Zfp266	-0.03	1.00000000	mice
Riok2	-0.03	1.00000000	mice
Thoc6	0.06	1.00000000	mice
Sfmbt1	0.17	1.00000000	mice

Dda1	0.09	1.00000000	mice
Sbds	-0.06	1.00000000	mice
Ndufb4	0.13	1.00000000	mice
Arnt	-0.05	1.00000000	mice
Ap1s1	0.08	1.00000000	mice
Slc35d1	-0.06	1.00000000	mice
Snx8	0.06	1.00000000	mice
Sbno1	-0.07	1.00000000	mice
Arpc3	0.05	1.00000000	mice
Hmg20a	-0.05	1.00000000	mice
Phb	0.00	1.00000000	mice
Dgkz	-0.05	1.00000000	mice
Lrig2	-0.03	1.00000000	mice
Ptpn7	-0.04	1.00000000	mice
Camk1d	0.11	1.00000000	mice
Smim4	0.04	1.00000000	mice
1700123O20Rik	0.04	1.00000000	mice
Ccdc86	0.10	1.00000000	mice
Ubtd1	-0.03	1.00000000	mice
Tbpl1	0.13	1.00000000	mice
Krit1	-0.06	1.00000000	mice
Blzf1	-0.05	1.00000000	mice
Gpr108	0.08	1.00000000	mice
Birc3	0.06	1.00000000	mice
Tbc1d9b	0.00	1.00000000	mice
Psmd6	0.10	1.00000000	mice
Strap	-0.04	1.00000000	mice
Hnrnpu	-0.02	1.00000000	mice
Gsk3a	-0.05	1.00000000	mice
Hsd17b12	0.09	1.00000000	mice
Cmpk1	0.13	1.00000000	mice
Elk4	-0.11	1.00000000	mice
Rad17	-0.05	1.00000000	mice
Tiparp	-0.12	1.00000000	mice
Dtymk	-0.01	1.00000000	mice
Srp54a	-0.04	1.00000000	mice
Glce	-0.03	1.00000000	mice
Pole4	-0.04	1.00000000	mice
Tbc1d9	0.15	1.00000000	mice
Dpy19l1	-0.07	1.00000000	mice
Stard4	-0.02	1.00000000	mice
2310011J03Rik	0.07	1.00000000	mice
Ppp1r16b	-0.08	1.00000000	mice
Trmt13	-0.06	1.00000000	mice
Npm3	-0.04	1.00000000	mice
Trim26	0.08	1.00000000	mice

Itgam	-0.39	1.00000000	mice
Gtf3c3	-0.02	1.00000000	mice
Kdm2b	-0.05	1.00000000	mice
Igbp1	-0.06	1.00000000	mice
Srsf1	0.09	1.00000000	mice
Clk3	-0.04	1.00000000	mice
Frmd4a	-0.42	1.00000000	mice
Senp1	0.07	1.00000000	mice
Abcf2	0.01	1.00000000	mice
Atn1	-0.01	1.00000000	mice
Nostrin	-0.05	1.00000000	mice
Slc39a6	-0.02	1.00000000	mice
Sumf1	0.10	1.00000000	mice
Eif3j2	0.04	1.00000000	mice
B930036N10Rik	-0.02	1.00000000	mice
Fam50a	0.05	1.00000000	mice
Hnrnph3	0.07	1.00000000	mice
G3bp2	0.11	1.00000000	mice
Tbrg1	0.17	1.00000000	mice
Sdf4	0.06	1.00000000	mice
Extl3	0.05	1.00000000	mice
Sh3bgrl	-0.03	1.00000000	mice
Dctn6	-0.02	1.00000000	mice
Gga2	-0.01	1.00000000	mice
Lfng	-0.05	1.00000000	mice
Slc25a51	0.08	1.00000000	mice
Nfya	0.00	1.00000000	mice
Nudcd2	-0.01	1.00000000	mice
Trappc10	-0.08	1.00000000	mice
1500011B03Rik	-0.04	1.00000000	mice
Czib	0.03	1.00000000	mice
Disp1	-0.05	1.00000000	mice
Nrp1	0.15	1.00000000	mice
Bend6	0.03	1.00000000	mice
Triobp	0.08	1.00000000	mice
Cep350	-0.04	1.00000000	mice
Pip4k2b	-0.02	1.00000000	mice
Psmb1	0.10	1.00000000	mice
C1d	-0.04	1.00000000	mice
Ccdc58	0.04	1.00000000	mice
Tmem214	-0.02	1.00000000	mice
Tmem41b	-0.05	1.00000000	mice
Nrip1	0.09	1.00000000	mice
Slc26a2	0.08	1.00000000	mice
Twsg1	-0.01	1.00000000	mice
Fxr2	-0.02	1.00000000	mice

Txlng	-0.06	1.00000000	mice
Cisd2	-0.05	1.00000000	mice
Tmem14c	0.08	1.00000000	mice
Tmem179b	0.07	1.00000000	mice
Tomm70a	0.07	1.00000000	mice
Nrros	0.19	1.00000000	mice
Tor1a	0.05	1.00000000	mice
Soat1	-0.04	1.00000000	mice
Smim20	0.06	1.00000000	mice
Pex7	-0.05	1.00000000	mice
Snrnp40	0.07	1.00000000	mice
Chmp1b	-0.05	1.00000000	mice
Rap1gap2	0.04	1.00000000	mice
Ldha	0.15	1.00000000	mice
Vps35l	-0.05	1.00000000	mice
Gramd3	-0.03	1.00000000	mice
Eef1g	-0.03	1.00000000	mice
Dtx2	0.05	1.00000000	mice
Prkcb	0.05	1.00000000	mice
Chst12	-0.05	1.00000000	mice
Stk3	-0.04	1.00000000	mice
Edc4	-0.01	1.00000000	mice
Atg4d	0.03	1.00000000	mice
Itgb2	0.12	1.00000000	mice
Ppm1m	0.04	1.00000000	mice
Zeb1	-0.05	1.00000000	mice
Ahr	-0.05	1.00000000	mice
Immp1l	-0.02	1.00000000	mice
Wdr82	-0.03	1.00000000	mice
Tlk2	0.07	1.00000000	mice
Tm7sf3	0.09	1.00000000	mice
Nudt16l1	0.00	1.00000000	mice
Polb	0.04	1.00000000	mice
Ramac	-0.01	1.00000000	mice
Fyb	-0.03	1.00000000	mice
Asb8	-0.02	1.00000000	mice
Cfp	0.12	1.00000000	mice
Lrch4	0.03	1.00000000	mice
Smarca4	-0.03	1.00000000	mice
Rassf2	-0.12	1.00000000	mice
Ube2s	0.01	1.00000000	mice
BC003965	-0.05	1.00000000	mice
Bcl6	-0.11	1.00000000	mice
Srd5a3	0.03	1.00000000	mice
Isca2	0.05	1.00000000	mice
Spn	-0.03	1.00000000	mice

Tm2d3	-0.03	1.00000000	mice
Prpf4b	-0.01	1.00000000	mice
Echs1	-0.03	1.00000000	mice
Ythdf2	-0.04	1.00000000	mice
Usp53	-0.01	1.00000000	mice
Kdm1a	0.12	1.00000000	mice
Cnot2	-0.01	1.00000000	mice
1110065P20Rik	-0.03	1.00000000	mice
Naa35	-0.08	1.00000000	mice
Ift46	-0.02	1.00000000	mice
Nfxl1	0.03	1.00000000	mice
Golph3	-0.08	1.00000000	mice
Uba1	0.06	1.00000000	mice
Fermt3	0.12	1.00000000	mice
Ubxn4	-0.05	1.00000000	mice
Pign	-0.03	1.00000000	mice
Ptcd3	-0.03	1.00000000	mice
Ranbp10	-0.08	1.00000000	mice
Upf2	-0.05	1.00000000	mice
9530068E07Rik	0.09	1.00000000	mice
Chmp2b	-0.04	1.00000000	mice
Armh3	-0.08	1.00000000	mice
Ginm1	-0.04	1.00000000	mice
Lrmda	-0.09	1.00000000	mice
Ring1	0.02	1.00000000	mice
Aco2	-0.03	1.00000000	mice
Tia1	-0.04	1.00000000	mice
Copb2	0.06	1.00000000	mice
Tmem123	-0.03	1.00000000	mice
Usf1	-0.02	1.00000000	mice
Wdr12	-0.04	1.00000000	mice
Otud5	0.07	1.00000000	mice
Nfic	0.05	1.00000000	mice
Timm9	-0.03	1.00000000	mice
Ftsj3	-0.03	1.00000000	mice
Ldb1	0.03	1.00000000	mice
Cd300a	0.01	1.00000000	mice
Usp19	-0.05	1.00000000	mice
Coro1a	0.08	1.00000000	mice
Fam76b	-0.01	1.00000000	mice
Stimate	-0.04	1.00000000	mice
Ercc3	-0.02	1.00000000	mice
Sil1	-0.03	1.00000000	mice
Flt1	0.13	1.00000000	mice
Pcif1	-0.05	1.00000000	mice
Rexo4	0.02	1.00000000	mice

Chka	0.10	1.00000000	mice
Romo1	0.11	1.00000000	mice
Rgs19	0.06	1.00000000	mice
Dhx29	-0.01	1.00000000	mice
Eef1d	-0.02	1.00000000	mice
Pias1	-0.04	1.00000000	mice
C1galt1c1	0.06	1.00000000	mice
Zdhhc13	0.04	1.00000000	mice
Agpat3	-0.06	1.00000000	mice
Dennd2a	-0.02	1.00000000	mice
Fiz1	-0.03	1.00000000	mice
4930523C07Rik	-0.17	1.00000000	mice
Rabif	-0.01	1.00000000	mice
H3f3a	-0.10	1.00000000	mice
Vamp8	0.14	1.00000000	mice
Tut7	-0.07	1.00000000	mice
Phf23	-0.03	1.00000000	mice
Lsm5	0.09	1.00000000	mice
Usp45	-0.01	1.00000000	mice
Sf3a2	0.04	1.00000000	mice
Rab3il1	0.08	1.00000000	mice
Pigk	-0.03	1.00000000	mice
Papss2	0.04	1.00000000	mice
Prim2	-0.04	1.00000000	mice
Vps72	0.05	1.00000000	mice
Vezt	0.00	1.00000000	mice
Fbxl5	0.10	1.00000000	mice
Gtf3a	-0.02	1.00000000	mice
Gar1	-0.02	1.00000000	mice
Ctdsp1	-0.03	1.00000000	mice
Ascc2	-0.03	1.00000000	mice
Nvl	-0.04	1.00000000	mice
Abcb1b	0.07	1.00000000	mice
Tmem134	0.08	1.00000000	mice
Id1	0.11	1.00000000	mice
Rsrc1	-0.03	1.00000000	mice
Fth1	0.03	1.00000000	mice
Arhgef37	-0.14	1.00000000	mice
Acd	-0.01	1.00000000	mice
Nol10	-0.06	1.00000000	mice
Gprasp1	-0.04	1.00000000	mice
Zfc3h1	-0.02	1.00000000	mice
Tmem159	-0.02	1.00000000	mice
Tma16	0.11	1.00000000	mice
Erlin1	-0.05	1.00000000	mice
Srp68	-0.05	1.00000000	mice

Nt5c	0.10	1.00000000	mice
Chchd10	0.11	1.00000000	mice
Ndufa6	0.04	1.00000000	mice
Slc25a30	-0.05	1.00000000	mice
Polr2b	-0.04	1.00000000	mice
Nhlrc3	-0.01	1.00000000	mice
Lin7c	-0.02	1.00000000	mice
Tmem19	-0.04	1.00000000	mice
Ing2	0.04	1.00000000	mice
Ppme1	-0.06	1.00000000	mice
Ubap1	-0.04	1.00000000	mice
Vars	0.00	1.00000000	mice
Zbtb43	0.06	1.00000000	mice
Slc39a1	0.06	1.00000000	mice
Pfkp	-0.01	1.00000000	mice
Cib1	0.05	1.00000000	mice
Kat2b	-0.09	1.00000000	mice
Reps1	-0.01	1.00000000	mice
Crbn	-0.02	1.00000000	mice
Tspan13	-0.07	1.00000000	mice
Afg3l2	-0.02	1.00000000	mice
Tspan3	0.08	1.00000000	mice
Lclat1	-0.06	1.00000000	mice
Ccng2	-0.04	1.00000000	mice
Sap30l	-0.07	1.00000000	mice
Rab11fip1	0.15	1.00000000	mice
Prdx3	-0.05	1.00000000	mice
Dcaf13	0.07	1.00000000	mice
Bcl2	-0.13	1.00000000	mice
Fbxo30	-0.01	1.00000000	mice
Sp3os	0.04	1.00000000	mice
Sf3b3	-0.05	1.00000000	mice
Sema4a	-0.09	1.00000000	mice
Polr2j	0.06	1.00000000	mice
Gart	-0.02	1.00000000	mice
Ldlrad3	0.02	1.00000000	mice
Tbc1d2b	-0.10	1.00000000	mice
Zfp646	-0.02	1.00000000	mice
Rbm25	-0.02	1.00000000	mice
Ube2a	0.05	1.00000000	mice
Zfp91	-0.02	1.00000000	mice
Ppp2r3a	-0.04	1.00000000	mice
Phospho2	-0.03	1.00000000	mice
Eif4a1	0.09	1.00000000	mice
Ptprs	-0.05	1.00000000	mice
Eya3	0.04	1.00000000	mice

Cd200r4	0.01	1.00000000	mice
Dync2h1	-0.02	1.00000000	mice
Pole3	0.04	1.00000000	mice
Gpx1	-0.10	1.00000000	mice
Pik3r4	-0.04	1.00000000	mice
Lnpk	-0.05	1.00000000	mice
Clta	0.00	1.00000000	mice
Cdk5rap2	0.09	1.00000000	mice
Ptpra	-0.05	1.00000000	mice
Secisbp2	-0.04	1.00000000	mice
Ap4s1	-0.03	1.00000000	mice
Dars	-0.04	1.00000000	mice
Rsl24d1	-0.03	1.00000000	mice
Lysmd4	0.05	1.00000000	mice
Tex10	-0.03	1.00000000	mice
Hspa5	0.08	1.00000000	mice
Ncbp2	-0.02	1.00000000	mice
Nup62	-0.01	1.00000000	mice
Gripap1	0.07	1.00000000	mice
Ranbp2	0.15	1.00000000	mice
Tmem251	-0.07	1.00000000	mice
Iscu	-0.02	1.00000000	mice
Ldah	0.08	1.00000000	mice
Arhgef10l	-0.07	1.00000000	mice
Rfxap	-0.07	1.00000000	mice
Bet1l	-0.03	1.00000000	mice
Ccnh	0.03	1.00000000	mice
Ccl9	0.17	1.00000000	mice
Casp9	-0.03	1.00000000	mice
Tvp23b	0.00	1.00000000	mice
Zdhhc7	-0.03	1.00000000	mice
Emc7	-0.01	1.00000000	mice
Rab2b	0.01	1.00000000	mice
Ccng1	0.01	1.00000000	mice
Tspyl1	-0.07	1.00000000	mice
Tceanc2	0.02	1.00000000	mice
Tln1	0.04	1.00000000	mice
Spag9	0.08	1.00000000	mice
Gatd1	-0.01	1.00000000	mice
Cep57	-0.04	1.00000000	mice
Cybc1	-0.02	1.00000000	mice
Yif1a	0.06	1.00000000	mice
Lsm14a	-0.03	1.00000000	mice
Rab28	-0.08	1.00000000	mice
Rars	0.08	1.00000000	mice
Gatad2a	0.09	1.00000000	mice

Zfp236	-0.07	1.00000000	mice
Bbc3	-0.09	1.00000000	mice
Cyfip2	0.05	1.00000000	mice
Apoc2	0.08	1.00000000	mice
Slc30a7	0.09	1.00000000	mice
Enc1	0.09	1.00000000	mice
Rbm18	-0.05	1.00000000	mice
Rtraf	-0.02	1.00000000	mice
Tmem128	0.03	1.00000000	mice
Hint2	-0.01	1.00000000	mice
Arhgap27	-0.01	1.00000000	mice
Hmgcl	-0.05	1.00000000	mice
Pnpo	-0.04	1.00000000	mice
Guca1a	-0.02	1.00000000	mice
Cnot6	-0.05	1.00000000	mice
Oard1	0.00	1.00000000	mice
Chm	-0.07	1.00000000	mice
Tmem9b	-0.03	1.00000000	mice
Ufd1	0.05	1.00000000	mice
Ogfrl1	0.04	1.00000000	mice
Ube2i	0.10	1.00000000	mice
Cct4	-0.04	1.00000000	mice
Prkcsh	-0.05	1.00000000	mice
Tsga10	-0.06	1.00000000	mice
Tusc2	-0.01	1.00000000	mice
Lsm10	0.04	1.00000000	mice
Vps37c	0.04	1.00000000	mice
Wdr18	0.01	1.00000000	mice
Galns	0.04	1.00000000	mice
Lars	0.00	1.00000000	mice
Tmx2	0.03	1.00000000	mice
Sh3bgrl3	0.09	1.00000000	mice
Hhex	-0.05	1.00000000	mice
Slc12a4	0.03	1.00000000	mice
Dnajc10	-0.03	1.00000000	mice
Vma21	0.02	1.00000000	mice
Ncbp3	-0.05	1.00000000	mice
Nploc4	-0.02	1.00000000	mice
Eif5	0.01	1.00000000	mice
Ino80b	0.03	1.00000000	mice
Snhg4	-0.03	1.00000000	mice
Srrm2	-0.02	1.00000000	mice
Arpc4	0.08	1.00000000	mice
Txnip	-0.10	1.00000000	mice
Ssna1	-0.01	1.00000000	mice
Naa60	-0.03	1.00000000	mice

Acaa1a	0.08	1.00000000	mice
Trit1	0.00	1.00000000	mice
Cstf2t	-0.03	1.00000000	mice
Rnf138	-0.04	1.00000000	mice
Ppp5c	-0.03	1.00000000	mice
Grb2	0.08	1.00000000	mice
Usp5	-0.04	1.00000000	mice
Hnrnpc	-0.03	1.00000000	mice
Tmem38b	-0.04	1.00000000	mice
Slc10a7	-0.07	1.00000000	mice
1600020E01Rik	-0.03	1.00000000	mice
Stim2	-0.10	1.00000000	mice
Nuak2	0.09	1.00000000	mice
Ddx23	-0.04	1.00000000	mice
Ndst2	0.04	1.00000000	mice
Eif6	0.08	1.00000000	mice
Rell1	0.05	1.00000000	mice
Atad3a	-0.02	1.00000000	mice
Ccdc174	0.02	1.00000000	mice
Pqlc2	0.03	1.00000000	mice
Dnajb4	-0.09	1.00000000	mice
Surf1	0.03	1.00000000	mice
Sh3glb1	-0.03	1.00000000	mice
Unc45a	-0.03	1.00000000	mice
Sesn2	-0.03	1.00000000	mice
Anapc4	-0.05	1.00000000	mice
Fxyd5	-0.12	1.00000000	mice
Spata2	0.03	1.00000000	mice
Dgkh	-0.13	1.00000000	mice
Calr	0.19	1.00000000	mice
Srsf3	0.09	1.00000000	mice
Ntmt1	-0.04	1.00000000	mice
Emc2	-0.02	1.00000000	mice
Pym1	-0.02	1.00000000	mice
Clcn5	-0.03	1.00000000	mice
Stam	-0.07	1.00000000	mice
Snrpe	0.09	1.00000000	mice
Ppp1r21	-0.05	1.00000000	mice
Trip4	-0.03	1.00000000	mice
Itsn1	-0.05	1.00000000	mice
Ppih	0.05	1.00000000	mice
Limd1	0.03	1.00000000	mice
Zcrb1	0.07	1.00000000	mice
Itprip	-0.06	1.00000000	mice
Inppl1	0.04	1.00000000	mice
Trak1	-0.02	1.00000000	mice

Atad1	0.07	1.00000000	mice
Acsf2	0.00	1.00000000	mice
Haus8	0.01	1.00000000	mice
Rer1	0.07	1.00000000	mice
Pcid2	-0.01	1.00000000	mice
Ap2a1	-0.03	1.00000000	mice
Sf3a1	-0.01	1.00000000	mice
Zfp664	-0.06	1.00000000	mice
Zcchc9	0.08	1.00000000	mice
Zdhhc4	0.03	1.00000000	mice
Crtc2	-0.02	1.00000000	mice
Hfe	0.05	1.00000000	mice
Ndufb6	0.07	1.00000000	mice
Psen2	0.00	1.00000000	mice
Cog8	0.00	1.00000000	mice
Rbm3	-0.03	1.00000000	mice
Elp4	0.00	1.00000000	mice
Anapc7	0.00	1.00000000	mice
Tbcb	0.09	1.00000000	mice
Grpel1	-0.02	1.00000000	mice
Rmi1	0.00	1.00000000	mice
Nip7	-0.04	1.00000000	mice
Usp8	-0.07	1.00000000	mice
Lysmd3	-0.03	1.00000000	mice
Ofd1	-0.03	1.00000000	mice
Zkscan17	0.08	1.00000000	mice
Cbll1	-0.03	1.00000000	mice
Atp6v0a1	-0.01	1.00000000	mice
Arf2	-0.04	1.00000000	mice
Eif4e3	0.02	1.00000000	mice
Dpy30	0.03	1.00000000	mice
Gcc2	-0.02	1.00000000	mice
Nckap1l	-0.05	1.00000000	mice
Wrn	-0.05	1.00000000	mice
Sash1	-0.10	1.00000000	mice
Piezo1	0.05	1.00000000	mice
Atg10	-0.07	1.00000000	mice
Frg1	0.03	1.00000000	mice
Ppp6r1	0.00	1.00000000	mice
Sec23b	0.06	1.00000000	mice
Ccdc127	-0.04	1.00000000	mice
Tmem51	-0.05	1.00000000	mice
D230025D16Rik	-0.02	1.00000000	mice
Parl	-0.02	1.00000000	mice
Eif2ak1	-0.05	1.00000000	mice
6720427I07Rik	0.03	1.00000000	mice

Tmem63b	0.06	1.00000000	mice
Ascc1	-0.01	1.00000000	mice
Arl4a	-0.02	1.00000000	mice
Ngdn	0.05	1.00000000	mice
Tatdn2	-0.01	1.00000000	mice
Hp1bp3	-0.06	1.00000000	mice
B3gat3	-0.01	1.00000000	mice
Sf3b5	0.08	1.00000000	mice
Zbtb18	-0.02	1.00000000	mice
Actl6a	-0.03	1.00000000	mice
Prcp	0.11	1.00000000	mice
Ppp1r35	0.05	1.00000000	mice
Chd9	-0.04	1.00000000	mice
Vps11	0.00	1.00000000	mice
Rala	-0.02	1.00000000	mice
Dusp10	-0.16	1.00000000	mice
Arfgef2	0.00	1.00000000	mice
Fancc	-0.03	1.00000000	mice
Akt2	-0.08	1.00000000	mice
Washc5	-0.03	1.00000000	mice
Jagn1	-0.02	1.00000000	mice
Aim2	0.07	1.00000000	mice
Synj2bp	-0.05	1.00000000	mice
Adck1	-0.01	1.00000000	mice
Cnot8	0.03	1.00000000	mice
Rffl	0.26	1.00000000	mice
Traf6	-0.01	1.00000000	mice
Sfxn1	-0.02	1.00000000	mice
Jkamp	-0.01	1.00000000	mice
Atp6v1e1	0.10	1.00000000	mice
Zfp142	-0.02	1.00000000	mice
Pdia3	-0.01	1.00000000	mice
Laptm4a	0.07	1.00000000	mice
Rpp25l	0.05	1.00000000	mice
Plekhm2	0.14	1.00000000	mice
Osbpl8	0.04	1.00000000	mice
Chmp3	-0.05	1.00000000	mice
Sarnp	0.11	1.00000000	mice
Nmnat3	-0.01	1.00000000	mice
Dhx8	0.04	1.00000000	mice
Pmvk	0.05	1.00000000	mice
Rhbdf2	0.10	1.00000000	mice
Dnajc30	-0.03	1.00000000	mice
Nup50	0.07	1.00000000	mice
Erf	0.04	1.00000000	mice
Nop58	0.13	1.00000000	mice

Slc37a3	-0.05	1.00000000	mice
Ikzf5	-0.02	1.00000000	mice
Vdac1	-0.02	1.00000000	mice
2210408I21Rik	-0.02	1.00000000	mice
Borcs7	-0.06	1.00000000	mice
Fau	-0.06	1.00000000	mice
Camta1	-0.06	1.00000000	mice
Prelid1	0.08	1.00000000	mice
Cxxc1	0.04	1.00000000	mice
Lrch1	0.06	1.00000000	mice
Arhgap15	-0.09	1.00000000	mice
Ltv1	-0.02	1.00000000	mice
Ubap2l	0.06	1.00000000	mice
B4galt7	-0.01	1.00000000	mice
St3gal1	0.09	1.00000000	mice
Gabpa	-0.02	1.00000000	mice
Heatr5b	-0.02	1.00000000	mice
Slc16a3	-0.18	1.00000000	mice
4833439L19Rik	0.08	1.00000000	mice
Taf10	0.11	1.00000000	mice
Gps1	0.01	1.00000000	mice
Rad23a	0.03	1.00000000	mice
Ormdl1	-0.03	1.00000000	mice
Nemf	-0.03	1.00000000	mice
Dnttip2	-0.05	1.00000000	mice
Crebzf	-0.02	1.00000000	mice
Sf3b1	-0.03	1.00000000	mice
Tmem39a	-0.04	1.00000000	mice
Plin2	0.01	1.00000000	mice
2310009A05Rik	0.08	1.00000000	mice
Tmem167	0.08	1.00000000	mice
Agtpbp1	0.10	1.00000000	mice
Taok2	-0.02	1.00000000	mice
Kpna4	0.07	1.00000000	mice
Fis1	0.08	1.00000000	mice
Tnni2	-0.02	1.00000000	mice
Wdr45b	-0.04	1.00000000	mice
Larp7	0.04	1.00000000	mice
Ehd1	-0.07	1.00000000	mice
Slc12a2	-0.06	1.00000000	mice
Zfp182	-0.03	1.00000000	mice
Diaph2	-0.01	1.00000000	mice
Tnfrsf23	-0.08	1.00000000	mice
Faim	0.05	1.00000000	mice
Bod1l	-0.04	1.00000000	mice
Lta4h	-0.05	1.00000000	mice

Pwwp2a	-0.05	1.00000000	mice
Smim14	-0.04	1.00000000	mice
Nav1	-0.03	1.00000000	mice
Socs6	0.04	1.00000000	mice
Cebpd	-0.03	1.00000000	mice
Park7	0.06	1.00000000	mice
E030030I06Rik	-0.05	1.00000000	mice
Ap1s3	0.01	1.00000000	mice
Shb	-0.02	1.00000000	mice
Dohh	-0.06	1.00000000	mice
Hmgn5	0.01	1.00000000	mice
Csf2rb	0.12	1.00000000	mice
Prr14	-0.04	1.00000000	mice
Ezh1	-0.02	1.00000000	mice
Tra2a	0.09	1.00000000	mice
Bri3	-0.04	1.00000000	mice
Zfp984	-0.03	1.00000000	mice
Ethe1	0.05	1.00000000	mice
Vps41	-0.04	1.00000000	mice
Preb	-0.06	1.00000000	mice
Tpd52l2	-0.04	1.00000000	mice
Hivep3	0.27	1.00000000	mice
Fh1	0.00	1.00000000	mice
Slc39a9	0.00	1.00000000	mice
Gtf2f2	-0.04	1.00000000	mice
Eml2	0.00	1.00000000	mice
Trak2	-0.04	1.00000000	mice
Yif1b	0.05	1.00000000	mice
Stxbp1	0.01	1.00000000	mice
Scyl2	-0.03	1.00000000	mice
Pitrm1	-0.04	1.00000000	mice
Iqcb1	-0.03	1.00000000	mice
Slc39a2	0.15	1.00000000	mice
Nkapd1	-0.05	1.00000000	mice
Stx7	-0.01	1.00000000	mice
Slc37a2	-0.04	1.00000000	mice
Brix1	-0.04	1.00000000	mice
Gnptg	0.03	1.00000000	mice
Nkap	-0.01	1.00000000	mice
Zkscan3	0.00	1.00000000	mice
Sar1a	0.06	1.00000000	mice
Agpat4	-0.03	1.00000000	mice
Pum3	-0.03	1.00000000	mice
Erlec1	-0.02	1.00000000	mice
Rnf149	0.01	1.00000000	mice
Abhd17a	0.07	1.00000000	mice

Sft2d1	-0.04	1.00000000	mice
Dnajc13	0.09	1.00000000	mice
Fbxw11	0.10	1.00000000	mice
Cops6	0.06	1.00000000	mice
Igsf8	0.00	1.00000000	mice
Hspe1	-0.01	1.00000000	mice
Hdac2	-0.04	1.00000000	mice
Prmt7	-0.01	1.00000000	mice
Pwp1	0.01	1.00000000	mice
Exosc8	0.02	1.00000000	mice
Cpd	0.02	1.00000000	mice
Arf1	0.08	1.00000000	mice
Snapc5	0.03	1.00000000	mice
Cblb	0.10	1.00000000	mice
Gpr89	-0.01	1.00000000	mice
Atp6v1g1	0.00	1.00000000	mice
Ube2o	0.01	1.00000000	mice
Cyp4f16	-0.01	1.00000000	mice
Hnrnpa1	0.00	1.00000000	mice
Pigx	0.01	1.00000000	mice
Tmem115	-0.03	1.00000000	mice
Pam16	0.06	1.00000000	mice
Tigd2	-0.03	1.00000000	mice
Itgb1	-0.02	1.00000000	mice
Hps5	-0.03	1.00000000	mice
Cdipt	-0.03	1.00000000	mice
Trub2	-0.04	1.00000000	mice
Ddx18	0.00	1.00000000	mice
Ythdc2	-0.02	1.00000000	mice
Top3b	0.00	1.00000000	mice
Eif4ebp1	-0.08	1.00000000	mice
Srrm1	0.08	1.00000000	mice
Rnf2	0.05	1.00000000	mice
Bag1	0.09	1.00000000	mice
Akap8l	-0.02	1.00000000	mice
Bola2	0.12	1.00000000	mice
Klhl28	-0.04	1.00000000	mice
Kars	0.01	1.00000000	mice
Cdk19	0.01	1.00000000	mice
Cetn3	-0.03	1.00000000	mice
Sirt2	0.04	1.00000000	mice
Cbr3	0.05	1.00000000	mice
Cox18	0.03	1.00000000	mice
Leprotl1	-0.04	1.00000000	mice
Slc25a17	-0.01	1.00000000	mice
Atr	-0.05	1.00000000	mice

Csnk2a2	-0.01	1.00000000	mice
Vps33a	0.04	1.00000000	mice
Pik3c2a	0.09	1.00000000	mice
Strada	-0.02	1.00000000	mice
Cdip1	-0.04	1.00000000	mice
Zfpl1	0.05	1.00000000	mice
Cebpzos	0.03	1.00000000	mice
Stx5a	-0.01	1.00000000	mice
Tmem175	-0.03	1.00000000	mice
Gpn3	-0.04	1.00000000	mice
Rela	0.06	1.00000000	mice
Sike1	-0.06	1.00000000	mice
Khnyn	-0.03	1.00000000	mice
Hes1	0.02	1.00000000	mice
Larp1	-0.01	1.00000000	mice
Cs	0.00	1.00000000	mice
Katnbl1	-0.04	1.00000000	mice
Tyk2	0.00	1.00000000	mice
Tmem167b	0.03	1.00000000	mice
1110059E24Rik	-0.03	1.00000000	mice
Polr2c	0.08	1.00000000	mice
Clp1	0.04	1.00000000	mice
Cript	0.02	1.00000000	mice
Cfl2	0.06	1.00000000	mice
Bcap31	-0.01	1.00000000	mice
Nol12	0.04	1.00000000	mice
Psmd9	0.03	1.00000000	mice
Gnb1	-0.02	1.00000000	mice
Zc3h10	0.00	1.00000000	mice
Dcaf11	0.03	1.00000000	mice
Eci2	-0.05	1.00000000	mice
Tmx1	0.00	1.00000000	mice
Zfp991	0.04	1.00000000	mice
Cdc14a	0.03	1.00000000	mice
Ankhd1	0.09	1.00000000	mice
Nptn	-0.07	1.00000000	mice
Nans	-0.05	1.00000000	mice
Dbndd2	-0.03	1.00000000	mice
Gtf2a2	0.00	1.00000000	mice
Tceal8	0.00	1.00000000	mice
Fdx1	-0.03	1.00000000	mice
Rbck1	0.05	1.00000000	mice
Pogk	-0.02	1.00000000	mice
Rnaseh1	0.03	1.00000000	mice
Dnaja3	-0.02	1.00000000	mice
Uba2	-0.01	1.00000000	mice

Camkmt	-0.04	1.00000000	mice
Xpo1	0.00	1.00000000	mice
Pigf	0.00	1.00000000	mice
Dnajc8	-0.05	1.00000000	mice
Impad1	-0.03	1.00000000	mice
Rrp36	-0.03	1.00000000	mice
AI839979	-0.12	1.00000000	mice
Tmem176a	0.07	1.00000000	mice
Wdr20	0.02	1.00000000	mice
Dram2	-0.02	1.00000000	mice
Hmgn2	0.00	1.00000000	mice
Ep400	0.08	1.00000000	mice
Zfp809	-0.02	1.00000000	mice
Bms1	-0.04	1.00000000	mice
Clec1a	0.20	1.00000000	mice
Apex1	-0.01	1.00000000	mice
Zfp513	0.02	1.00000000	mice
Atp6v1a	-0.03	1.00000000	mice
Arglu1	0.09	1.00000000	mice
Rassf8	0.00	1.00000000	mice
Cd1d1	0.00	1.00000000	mice
Hps4	0.03	1.00000000	mice
Ddx52	0.02	1.00000000	mice
Frmd4b	0.08	1.00000000	mice
Psmg2	-0.02	1.00000000	mice
Polr2d	-0.04	1.00000000	mice
Pi4kb	-0.02	1.00000000	mice
Fancl	-0.05	1.00000000	mice
Pmpcb	0.06	1.00000000	mice
Dctn5	0.02	1.00000000	mice
Haus6	-0.06	1.00000000	mice
Ptp4a3	0.05	1.00000000	mice
Hikeshi	0.02	1.00000000	mice
Pcsk7	-0.02	1.00000000	mice
Slbp	0.06	1.00000000	mice
Ddx42	-0.02	1.00000000	mice
Zhx2	0.01	1.00000000	mice
Hibadh	0.08	1.00000000	mice
Abcd1	0.00	1.00000000	mice
Snrpa	0.05	1.00000000	mice
Phlda1	0.23	1.00000000	mice
Tmem161b	0.05	1.00000000	mice
Ten1	-0.07	1.00000000	mice
Slc25a32	-0.03	1.00000000	mice
Spg11	0.06	1.00000000	mice
Rab3gap2	-0.02	1.00000000	mice

Zmat5	0.05	1.00000000	mice
Ube2n	0.09	1.00000000	mice
Ndufaf7	-0.05	1.00000000	mice
Cbx3	-0.03	1.00000000	mice
Smpdl3b	-0.02	1.00000000	mice
Gpatch3	0.01	1.00000000	mice
Acp5	0.25	1.00000000	mice
Sgpl1	-0.04	1.00000000	mice
Tmem59	0.08	1.00000000	mice
Tle4	0.16	1.00000000	mice
Tox4	0.07	1.00000000	mice
Bcl2l1	0.10	1.00000000	mice
Rab29	-0.03	1.00000000	mice
Clptm1	0.04	1.00000000	mice
Sft2d2	0.00	1.00000000	mice
Fkbp15	-0.03	1.00000000	mice
Dgkg	-0.17	1.00000000	mice
Trip12	-0.01	1.00000000	mice
Srbd1	-0.02	1.00000000	mice
Nr6a1	-0.05	1.00000000	mice
Pom121	-0.03	1.00000000	mice
Itpa	0.03	1.00000000	mice
Esf1	-0.01	1.00000000	mice
Derl1	-0.04	1.00000000	mice
Ctif	-0.03	1.00000000	mice
Golga3	0.00	1.00000000	mice
Pitpnb	-0.04	1.00000000	mice
Son	0.13	1.00000000	mice
Tmem258	0.08	1.00000000	mice
Ccnt2	-0.09	1.00000000	mice
Eif4enif1	-0.02	1.00000000	mice
1700025G04Rik	-0.06	1.00000000	mice
Borcs5	-0.01	1.00000000	mice
Zfp207	0.00	1.00000000	mice
Nudc	0.09	1.00000000	mice
Gucd1	0.06	1.00000000	mice
Stxbp5	0.04	1.00000000	mice
P2ry6	-0.04	1.00000000	mice
Oaz2	-0.04	1.00000000	mice
Fam45a	-0.04	1.00000000	mice
Tpcn2	-0.05	1.00000000	mice
Senp3	-0.04	1.00000000	mice
Rnf217	-0.01	1.00000000	mice
Ankrd40	0.01	1.00000000	mice
Sap130	-0.02	1.00000000	mice
Snapc1	0.05	1.00000000	mice

Pcca	0.00	1.00000000	mice
Stat5a	0.09	1.00000000	mice
Tbkbp1	0.04	1.00000000	mice
Ppp4r3b	-0.01	1.00000000	mice
Dpm1	0.00	1.00000000	mice
Chpf2	-0.02	1.00000000	mice
Fubp3	0.00	1.00000000	mice
Bckdk	0.02	1.00000000	mice
Ints13	-0.03	1.00000000	mice
Cdc27	0.03	1.00000000	mice
Atf5	0.05	1.00000000	mice
Zrsr2	0.08	1.00000000	mice
Smndc1	0.06	1.00000000	mice
Syf2	0.05	1.00000000	mice
Ppp2r3c	0.08	1.00000000	mice
Il7r	0.15	1.00000000	mice
Stambp	0.03	1.00000000	mice
Atg4b	-0.05	1.00000000	mice
Chd4	-0.04	1.00000000	mice
Tmem209	0.02	1.00000000	mice
Cyp4v3	0.01	1.00000000	mice
Pdlim7	0.02	1.00000000	mice
Hacd4	-0.03	1.00000000	mice
Smu1	0.06	1.00000000	mice
Thoc2	0.00	1.00000000	mice
Tmem161a	-0.02	1.00000000	mice
Hsp90ab1	0.04	1.00000000	mice
Rab10	0.15	1.00000000	mice
Stambpl1	0.00	1.00000000	mice
Scfd1	0.02	1.00000000	mice
Ptpn23	0.07	1.00000000	mice
Supt4a	0.00	1.00000000	mice
Slc30a6	0.03	1.00000000	mice
Kif1bp	-0.05	1.00000000	mice
Ets2	0.05	1.00000000	mice
Lsm8	-0.02	1.00000000	mice
Bloc1s4	-0.04	1.00000000	mice
Ikbkg	0.03	1.00000000	mice
Get4	-0.02	1.00000000	mice
Actr1a	0.00	1.00000000	mice
Rnf25	0.03	1.00000000	mice
Pold3	0.03	1.00000000	mice
Ankrd28	0.00	1.00000000	mice
Ugp2	-0.05	1.00000000	mice
Fnip2	0.11	1.00000000	mice
Rbm4	-0.04	1.00000000	mice

Ccnk	0.00	1.00000000	mice
Cldnd1	0.04	1.00000000	mice
Pdcl3	0.07	1.00000000	mice
Slc4a7	0.09	1.00000000	mice
Sp3	-0.01	1.00000000	mice
Ewsr1	0.00	1.00000000	mice
Tlr4	0.11	1.00000000	mice
Eif3i	0.00	1.00000000	mice
Ruvbl1	0.04	1.00000000	mice
Ndufa12	0.15	1.00000000	mice
Slc25a33	-0.02	1.00000000	mice
Necap1	-0.03	1.00000000	mice
Zfyve27	-0.01	1.00000000	mice
Rnf121	0.02	1.00000000	mice
Zfp622	-0.02	1.00000000	mice
Spg21	-0.01	1.00000000	mice
Sdccag8	-0.01	1.00000000	mice
Fibp	-0.02	1.00000000	mice
Ccdc117	0.01	1.00000000	mice
Casp6	-0.02	1.00000000	mice
Lcmt1	0.03	1.00000000	mice
Sec62	0.00	1.00000000	mice
Trappc1	0.04	1.00000000	mice
Lamtor1	0.10	1.00000000	mice
Ndufs3	0.03	1.00000000	mice
C1galt1	-0.02	1.00000000	mice
Trappc6a	-0.01	1.00000000	mice
Ctr9	0.01	1.00000000	mice
Rif1	0.00	1.00000000	mice
Cep135	-0.01	1.00000000	mice
Arhgap12	0.00	1.00000000	mice
Car4	0.87	1.00000000	mice
Pabpn1	0.08	1.00000000	mice
Glcci1	-0.02	1.00000000	mice
Idh3a	0.00	1.00000000	mice
Sec23ip	-0.01	1.00000000	mice
Surf6	-0.01	1.00000000	mice
Slc44a2	0.11	1.00000000	mice
Cwf19l2	-0.04	1.00000000	mice
Alg1	0.02	1.00000000	mice
Acadsb	-0.02	1.00000000	mice
Tpgs1	0.03	1.00000000	mice
Hsd17b11	0.00	1.00000000	mice
Lactb2	0.05	1.00000000	mice
Crnkl1	0.04	1.00000000	mice
Ccdc107	0.00	1.00000000	mice

Dnajc25	-0.04	1.00000000	mice
Rbm17	-0.05	1.00000000	mice
Gcsh	-0.03	1.00000000	mice
Vhl	0.05	1.00000000	mice
Spsb2	0.05	1.00000000	mice
Tdp2	-0.04	1.00000000	mice
Dap3	0.00	1.00000000	mice
Gga1	0.06	1.00000000	mice
Ddx55	-0.01	1.00000000	mice
Abcc4	0.05	1.00000000	mice
Pias4	0.03	1.00000000	mice
Dvl3	-0.02	1.00000000	mice
Slc25a44	-0.03	1.00000000	mice
Ltb4r1	-0.06	1.00000000	mice
Phtf2	-0.03	1.00000000	mice
Pggt1b	0.05	1.00000000	mice
Zbtb7b	0.06	1.00000000	mice
Tor1b	0.00	1.00000000	mice
0610030E20Rik	0.01	1.00000000	mice
Txndc17	0.12	1.00000000	mice
Drap1	0.01	1.00000000	mice
Etf1	0.01	1.00000000	mice
Golga7	-0.03	1.00000000	mice
Ago3	0.02	1.00000000	mice
Ahsa1	-0.01	1.00000000	mice
Nop14	0.00	1.00000000	mice
Gfm2	0.03	1.00000000	mice
Shoc2	0.01	1.00000000	mice
Surf4	-0.03	1.00000000	mice
Lrch3	-0.03	1.00000000	mice
Slc35e1	0.01	1.00000000	mice
Ncstn	0.03	1.00000000	mice
Xab2	0.01	1.00000000	mice
Llgl1	0.02	1.00000000	mice
Cltb	-0.01	1.00000000	mice
Heg1	0.02	1.00000000	mice
Fundc2	0.02	1.00000000	mice
Azin1	0.09	1.00000000	mice
Baz1b	0.02	1.00000000	mice
Rab11fip2	-0.03	1.00000000	mice
Rras	-0.04	1.00000000	mice
Ctdspl2	-0.04	1.00000000	mice
Tmem205	0.06	1.00000000	mice
Brms1	0.04	1.00000000	mice
Llph	0.08	1.00000000	mice
Lias	-0.03	1.00000000	mice

Eif4a3	0.06	1.00000000	mice
Plpbp	0.02	1.00000000	mice
Cdk2ap1	-0.03	1.00000000	mice
Iah1	-0.03	1.00000000	mice
Ccar1	-0.01	1.00000000	mice
Yae1d1	0.13	1.00000000	mice
Pank2	0.04	1.00000000	mice
Samm50	0.09	1.00000000	mice
Asna1	-0.01	1.00000000	mice
Akap7	0.02	1.00000000	mice
Xrcc1	-0.02	1.00000000	mice
Tk2	0.01	1.00000000	mice
Eny2	-0.04	1.00000000	mice
Slx4ip	0.10	1.00000000	mice
Pycr2	0.00	1.00000000	mice
Siva1	-0.04	1.00000000	mice
Josd2	0.03	1.00000000	mice
Pno1	-0.04	1.00000000	mice
Slc25a1	-0.01	1.00000000	mice
Rxylt1	0.03	1.00000000	mice
Pnisr	-0.04	1.00000000	mice
Alkbh3	-0.04	1.00000000	mice
Slc35f6	0.04	1.00000000	mice
Idi1	0.10	1.00000000	mice
Kras	-0.09	1.00000000	mice
Ints12	-0.01	1.00000000	mice
Pak1ip1	-0.03	1.00000000	mice
Ppp2r2d	-0.02	1.00000000	mice
Desi1	0.05	1.00000000	mice
Ap5m1	-0.03	1.00000000	mice
Ndel1	-0.11	1.00000000	mice
Camkk2	-0.07	1.00000000	mice
Ston2	-0.01	1.00000000	mice
Ssu72	0.05	1.00000000	mice
Luzp1	0.09	1.00000000	mice
Rpp30	0.00	1.00000000	mice
Ccdc71l	0.04	1.00000000	mice
Tgoln1	0.04	1.00000000	mice
Bnip2	0.06	1.00000000	mice
Tbcc	-0.03	1.00000000	mice
Rab43	0.12	1.00000000	mice
Reep4	0.00	1.00000000	mice
Terf2	0.00	1.00000000	mice
Pcmt1	-0.07	1.00000000	mice
Utp3	0.07	1.00000000	mice
Sipa1l1	0.16	1.00000000	mice

Sirt1	-0.01	1.00000000	mice
Gskip	-0.05	1.00000000	mice
Caml	0.03	1.00000000	mice
Arhgap30	0.00	1.00000000	mice
Eif2b4	0.03	1.00000000	mice
Bub3	0.04	1.00000000	mice
Pigyl	0.00	1.00000000	mice
Cryzl1	-0.01	1.00000000	mice
2210016L21Rik	0.01	1.00000000	mice
Gan	-0.03	1.00000000	mice
Atp5f1	0.10	1.00000000	mice
Taz	-0.02	1.00000000	mice
Acot13	0.04	1.00000000	mice
Trappc13	0.00	1.00000000	mice
Slc20a2	0.01	1.00000000	mice
Pex13	0.01	1.00000000	mice
Nrbf2	0.03	1.00000000	mice
Usp39	-0.02	1.00000000	mice
Ube3b	0.03	1.00000000	mice
Fmr1	0.03	1.00000000	mice
Actr5	-0.01	1.00000000	mice
Cwc22	-0.07	1.00000000	mice
Stau1	0.06	1.00000000	mice
Oma1	0.05	1.00000000	mice
Rnase4	0.11	1.00000000	mice
Zbtb21	0.02	1.00000000	mice
Pex3	-0.03	1.00000000	mice
Cox19	-0.02	1.00000000	mice
Hal	-0.03	1.00000000	mice
Ddx47	0.02	1.00000000	mice
Parp8	0.11	1.00000000	mice
Pofut2	0.04	1.00000000	mice
Hjurp	-0.03	1.00000000	mice
Sympk	-0.04	1.00000000	mice
Taf1b	0.02	1.00000000	mice
Snx3	-0.01	1.00000000	mice
Lman2l	-0.02	1.00000000	mice
Rab1a	-0.02	1.00000000	mice
St3gal6	-0.24	1.00000000	mice
Tet3	0.04	1.00000000	mice
Ciao2a	0.08	1.00000000	mice
Tlr7	0.08	1.00000000	mice
Naip6	-0.04	1.00000000	mice
1700037H04Rik	-0.02	1.00000000	mice
Ube2d1	-0.04	1.00000000	mice
Sart3	-0.02	1.00000000	mice

Psmd13	0.04	1.00000000	mice
Pop7	0.05	1.00000000	mice
Card9	-0.03	1.00000000	mice
Timm8b	-0.02	1.00000000	mice
Chordc1	0.00	1.00000000	mice
Rubcnl	-0.03	1.00000000	mice
Rcan1	-0.03	1.00000000	mice
Gpsm3	-0.01	1.00000000	mice
Hspa14	-0.02	1.00000000	mice
Ptpn11	0.04	1.00000000	mice
Fbxw5	0.00	1.00000000	mice
Casp8ap2	-0.01	1.00000000	mice
Skiv2l	0.03	1.00000000	mice
Babam1	0.04	1.00000000	mice
Zfp598	0.01	1.00000000	mice
Rchy1	-0.07	1.00000000	mice
Commd10	-0.01	1.00000000	mice
Ggps1	0.06	1.00000000	mice
Ddx49	-0.01	1.00000000	mice
Cfh	-0.09	1.00000000	mice
Srebf2	-0.03	1.00000000	mice
Snx11	-0.03	1.00000000	mice
Eef1akmt4	-0.03	1.00000000	mice
Adam15	-0.03	1.00000000	mice
Bcl10	-0.03	1.00000000	mice
Rrm1	0.01	1.00000000	mice
Tppp3	-0.10	1.00000000	mice
Cpsf2	0.02	1.00000000	mice
Hcst	-0.07	1.00000000	mice
Prkab2	-0.03	1.00000000	mice
Ccnl2	-0.01	1.00000000	mice
Nop53	-0.01	1.00000000	mice
Ppp1r10	0.01	1.00000000	mice
Ldlrap1	0.08	1.00000000	mice
Rnasek	0.04	1.00000000	mice
Topors	0.01	1.00000000	mice
Lamtor3	0.04	1.00000000	mice
Rnf215	0.00	1.00000000	mice
Ppm1l	0.12	1.00000000	mice
Srsf9	0.06	1.00000000	mice
4930453N24Rik	-0.01	1.00000000	mice
Cln6	0.00	1.00000000	mice
Chchd2	-0.01	1.00000000	mice
Plekha2	-0.11	1.00000000	mice
Aspscr1	0.04	1.00000000	mice
Hint3	-0.01	1.00000000	mice

Csf2rb2	0.09	1.00000000	mice
Kcnn4	-0.02	1.00000000	mice
Cog4	-0.01	1.00000000	mice
Atp6v1f	0.08	1.00000000	mice
Nucb2	0.00	1.00000000	mice
Ech1	-0.01	1.00000000	mice
Tmem242	0.03	1.00000000	mice
Slc4a1ap	-0.02	1.00000000	mice
Spcs2	0.07	1.00000000	mice
Shkbp1	0.03	1.00000000	mice
Tmem199	0.00	1.00000000	mice
Cops4	0.04	1.00000000	mice
2610020C07Rik	-0.01	1.00000000	mice
Trap1	-0.01	1.00000000	mice
Pigu	0.06	1.00000000	mice
Gosr1	0.03	1.00000000	mice
Armc10	-0.03	1.00000000	mice
Zbtb8os	0.04	1.00000000	mice
Dcun1d3	0.00	1.00000000	mice
Cars	0.00	1.00000000	mice
Ublcp1	-0.03	1.00000000	mice
Prpsap2	-0.04	1.00000000	mice
Selenoh	-0.01	1.00000000	mice
Abraxas2	-0.03	1.00000000	mice
Pgam5	-0.01	1.00000000	mice
Fam104a	-0.03	1.00000000	mice
Eftud2	-0.01	1.00000000	mice
Stx17	-0.03	1.00000000	mice
Nsd2	0.09	1.00000000	mice
Nucb1	-0.04	1.00000000	mice
Rab3gap1	-0.03	1.00000000	mice
Zfp729b	-0.04	1.00000000	mice
Nxt1	-0.01	1.00000000	mice
Tgs1	0.09	1.00000000	mice
Ndufs2	0.00	1.00000000	mice
Gsn	0.06	1.00000000	mice
Pofut1	-0.01	1.00000000	mice
B3gnt2	0.06	1.00000000	mice
2410006H16Rik	0.04	1.00000000	mice
Pafah1b3	0.02	1.00000000	mice
Nifk	0.09	1.00000000	mice
2610507B11Rik	0.02	1.00000000	mice
2510009E07Rik	0.00	1.00000000	mice
Xpa	0.00	1.00000000	mice
Lage3	-0.04	1.00000000	mice
Sirt7	-0.03	1.00000000	mice

Zbtb24	-0.04	1.00000000	mice
Insig1	0.03	1.00000000	mice
Ik	0.01	1.00000000	mice
Arl1	0.03	1.00000000	mice
Calcrl	0.00	1.00000000	mice
Rprd1b	0.08	1.00000000	mice
Ndufab1	0.08	1.00000000	mice
Pstpip2	0.03	1.00000000	mice
Dusp6	-0.08	1.00000000	mice
Qars	-0.03	1.00000000	mice
Sipa1	-0.03	1.00000000	mice
Tmem181a	0.00	1.00000000	mice
Cnot4	-0.01	1.00000000	mice
Dnajc11	-0.04	1.00000000	mice
Zfp281	-0.07	1.00000000	mice
Smim12	-0.02	1.00000000	mice
Usp38	0.00	1.00000000	mice
Scpep1os	0.03	1.00000000	mice
Nfyb	0.02	1.00000000	mice
Tcerg1	-0.03	1.00000000	mice
Oser1	0.06	1.00000000	mice
Eefsec	-0.02	1.00000000	mice
Glt8d1	-0.02	1.00000000	mice
Ccndbp1	0.02	1.00000000	mice
Idh3b	0.07	1.00000000	mice
Ube2e3	0.06	1.00000000	mice
Trappc12	0.00	1.00000000	mice
Atp5l	0.04	1.00000000	mice
Cdk7	-0.02	1.00000000	mice
Yipf6	0.03	1.00000000	mice
Impdh2	-0.02	1.00000000	mice
Sdhaf4	0.03	1.00000000	mice
Sart1	0.05	1.00000000	mice
Zfp787	0.00	1.00000000	mice
a	0.02	1.00000000	mice
Setdb1	0.09	1.00000000	mice
Apmap	-0.03	1.00000000	mice
Cstf3	0.06	1.00000000	mice
Tkt	-0.01	1.00000000	mice
Elmod2	0.02	1.00000000	mice
Crkl	0.02	1.00000000	mice
Yipf5	-0.05	1.00000000	mice
Dennd1b	-0.07	1.00000000	mice
Ufsp2	-0.01	1.00000000	mice
Pim3	0.05	1.00000000	mice
Sept10	0.03	1.00000000	mice

Trappc6b	0.00	1.00000000	mice
Ttc17	-0.05	1.00000000	mice
Ppp1ca	0.04	1.00000000	mice
Tcf25	0.02	1.00000000	mice
Orc6	-0.04	1.00000000	mice
Eef1akmt1	0.02	1.00000000	mice
Rpn2	-0.01	1.00000000	mice
Dapk3	0.04	1.00000000	mice
Cab39l	0.05	1.00000000	mice
Smarcd1	0.01	1.00000000	mice
Snu13	0.00	1.00000000	mice
Bap1	0.00	1.00000000	mice
Rars2	0.01	1.00000000	mice
Snrpd3	0.06	1.00000000	mice
Wdr48	-0.06	1.00000000	mice
Nedd4l	0.01	1.00000000	mice
Gid8	0.04	1.00000000	mice
Smn1	0.04	1.00000000	mice
Pik3cb	-0.06	1.00000000	mice
Cd2ap	-0.02	1.00000000	mice
Tstd2	-0.02	1.00000000	mice
Washc4	0.02	1.00000000	mice
Anapc16	-0.03	1.00000000	mice
Sec61a1	-0.02	1.00000000	mice
Dhx38	0.04	1.00000000	mice
Armc8	0.01	1.00000000	mice
Smarcb1	0.05	1.00000000	mice
Kif3b	-0.02	1.00000000	mice
Sec11a	-0.03	1.00000000	mice
Nasp	0.11	1.00000000	mice
Wdr89	-0.04	1.00000000	mice
Unk	0.04	1.00000000	mice
Gadd45g	0.03	1.00000000	mice
Lgals3	0.10	1.00000000	mice
Ift20	0.00	1.00000000	mice
Adprh	0.00	1.00000000	mice
D1Ertd622e	0.02	1.00000000	mice
Clk2	0.00	1.00000000	mice
Lbh	-0.04	1.00000000	mice
Eipr1	0.03	1.00000000	mice
Eif3m	0.02	1.00000000	mice
Eif5a	0.09	1.00000000	mice
Ddx1	0.01	1.00000000	mice
Odr4	0.03	1.00000000	mice
Eri1	-0.04	1.00000000	mice
Slc35b1	-0.04	1.00000000	mice

Brwd3	0.00	1.00000000	mice
Brip1os	0.06	1.00000000	mice
Saysd1	-0.01	1.00000000	mice
Rap1a	0.15	1.00000000	mice
Got1	-0.03	1.00000000	mice
Lats2	-0.04	1.00000000	mice
Plod3	0.08	1.00000000	mice
Cct3	0.08	1.00000000	mice
Arrdc1	0.02	1.00000000	mice
A630001G21Rik	-0.04	1.00000000	mice
Bst1	0.12	1.00000000	mice
Ppp4c	-0.01	1.00000000	mice
Rad21	-0.04	1.00000000	mice
Anxa11	-0.03	1.00000000	mice
Exosc10	0.00	1.00000000	mice
Dguok	0.04	1.00000000	mice
Eloa	0.00	1.00000000	mice
Fubp1	0.02	1.00000000	mice
Apopt1	0.01	1.00000000	mice
Clcc1	-0.01	1.00000000	mice
Pdxk	0.04	1.00000000	mice
Rhoq	0.15	1.00000000	mice
Pdcd1lg2	0.03	1.00000000	mice
Rbm41	-0.02	1.00000000	mice
Sgta	0.06	1.00000000	mice
Snrnp200	-0.04	1.00000000	mice
Slc5a3	0.02	1.00000000	mice
Ciao1	-0.01	1.00000000	mice
Fbxo11	-0.05	1.00000000	mice
Rbbp6	0.03	1.00000000	mice
Hypk	0.04	1.00000000	mice
Kptn	0.01	1.00000000	mice
Tpm4	0.01	1.00000000	mice
Thumpd3	0.04	1.00000000	mice
Creb1	0.06	1.00000000	mice
Sap30bp	-0.01	1.00000000	mice
Zfp703	0.12	1.00000000	mice
Ndufa9	0.07	1.00000000	mice
Psmf1	0.02	1.00000000	mice
Dleu2	0.05	1.00000000	mice
Tomm5	0.03	1.00000000	mice
Peak1	-0.21	1.00000000	mice
Kif1c	0.00	1.00000000	mice
Ints6	0.06	1.00000000	mice
Sars	0.00	1.00000000	mice
Sec61b	-0.01	1.00000000	mice

Neat1	0.05	1.00000000	mice
Ing1	-0.03	1.00000000	mice
Hmgcs1	-0.01	1.00000000	mice
Naglu	0.00	1.00000000	mice
Pitpnm1	0.03	1.00000000	mice
Hnrnpab	0.09	1.00000000	mice
A430005L14Rik	0.03	1.00000000	mice
Chmp1a	0.03	1.00000000	mice
Churc1	0.03	1.00000000	mice
Polr1c	0.03	1.00000000	mice
Orc4	-0.01	1.00000000	mice
Polr2a	0.02	1.00000000	mice
Rnf139	-0.04	1.00000000	mice
Ctsl	0.26	1.00000000	mice
Snrpd1	0.10	1.00000000	mice
Strbp	-0.13	1.00000000	mice
Ormdl2	-0.04	1.00000000	mice
Rfc1	0.01	1.00000000	mice
Csf1r	0.00	1.00000000	mice
Chrac1	0.06	1.00000000	mice
Prpf38b	0.04	1.00000000	mice
Flot2	0.02	1.00000000	mice
Ube2v1	0.04	1.00000000	mice
Coq8b	-0.02	1.00000000	mice
Tbc1d13	0.00	1.00000000	mice
Anxa7	0.05	1.00000000	mice
Tuba4a	-0.05	1.00000000	mice
Cc2d1b	-0.02	1.00000000	mice
Nup93	-0.01	1.00000000	mice
Scamp3	0.02	1.00000000	mice
Pih1d1	0.03	1.00000000	mice
Pex5	-0.01	1.00000000	mice
Dpp3	0.04	1.00000000	mice
Chd3	0.01	1.00000000	mice
Pdcd2l	0.05	1.00000000	mice
Zbed4	-0.02	1.00000000	mice
Cenpa	-0.05	1.00000000	mice
Hras	0.06	1.00000000	mice
Lsm3	-0.02	1.00000000	mice
Rab3ip	-0.02	1.00000000	mice
Jpt2	0.00	1.00000000	mice
Gfm1	0.03	1.00000000	mice
Kctd10	-0.04	1.00000000	mice
Tut4	-0.11	1.00000000	mice
Atp13a1	0.00	1.00000000	mice
Ccdc146	-0.02	1.00000000	mice

Exosc3	-0.01	1.00000000	mice
Rnf41	0.00	1.00000000	mice
Cstf2	0.01	1.00000000	mice
Rabgap1	0.02	1.00000000	mice
Uimc1	0.01	1.00000000	mice
Samsn1	-0.13	1.00000000	mice
Lemd2	0.03	1.00000000	mice
Sept6	-0.02	1.00000000	mice
Dyrk2	0.08	1.00000000	mice
Polr3k	0.01	1.00000000	mice
Ufm1	0.04	1.00000000	mice
Dnase2a	0.07	1.00000000	mice
Ran	0.12	1.00000000	mice
Atxn7l1	-0.01	1.00000000	mice
Coro7	0.02	1.00000000	mice
Cep295	-0.01	1.00000000	mice
Fkbp3	0.01	1.00000000	mice
Rae1	0.02	1.00000000	mice
Dnajc4	0.02	1.00000000	mice
Ubxn2a	0.00	1.00000000	mice
Rtf1	0.03	1.00000000	mice
Gigyf2	0.03	1.00000000	mice
Treml4	0.19	1.00000000	mice
Tbk1	0.08	1.00000000	mice
Atp5g1	0.10	1.00000000	mice
Gnpda1	0.06	1.00000000	mice
Lmtk2	-0.01	1.00000000	mice
Nr4a2	-0.07	1.00000000	mice
Ino80e	0.00	1.00000000	mice
Btaf1	0.03	1.00000000	mice
Dhrs4	-0.02	1.00000000	mice
Uba3	0.00	1.00000000	mice
Nhsl2	0.01	1.00000000	mice
Trim25	-0.05	1.00000000	mice
Timm17a	0.07	1.00000000	mice
Cox6b1	0.08	1.00000000	mice
Elp5	0.03	1.00000000	mice
Prkra	-0.01	1.00000000	mice
Rbm15	0.00	1.00000000	mice
Sh2d3c	-0.15	1.00000000	mice
Dhrs1	-0.03	1.00000000	mice
Polr3c	0.04	1.00000000	mice
Ppp1r12c	0.00	1.00000000	mice
Golga2	0.00	1.00000000	mice
Ndufa8	0.08	1.00000000	mice
Ap3s2	-0.04	1.00000000	mice

Bud23	0.04	1.00000000	mice
Trmt11	0.02	1.00000000	mice
Atg14	-0.02	1.00000000	mice
D16Ertd472e	-0.05	1.00000000	mice
Ppp6c	-0.01	1.00000000	mice
Fzr1	0.02	1.00000000	mice
Plod1	0.00	1.00000000	mice
Rap2b	0.02	1.00000000	mice
Nsfl1c	0.05	1.00000000	mice
Csf2ra	0.04	1.00000000	mice
Depdc5	-0.06	1.00000000	mice
Trp53bp1	0.00	1.00000000	mice
Coq5	-0.01	1.00000000	mice
Clec4a2	0.04	1.00000000	mice
Tufm	0.02	1.00000000	mice
Zfp385a	0.03	1.00000000	mice
Commd7	0.05	1.00000000	mice
Dcakd	-0.01	1.00000000	mice
Prorsd1	0.04	1.00000000	mice
Gga3	-0.02	1.00000000	mice
Tcea1	0.06	1.00000000	mice
Smcr8	0.06	1.00000000	mice
Pcbd2	-0.03	1.00000000	mice
Anks1	0.02	1.00000000	mice
Selenot	0.03	1.00000000	mice
Aar2	0.01	1.00000000	mice
Tbc1d8	-0.05	1.00000000	mice
Rnf40	-0.01	1.00000000	mice
Fnbp4	0.10	1.00000000	mice
Ap1g2	-0.05	1.00000000	mice
Zfp330	-0.01	1.00000000	mice
Fam20b	-0.06	1.00000000	mice
Castor1	-0.01	1.00000000	mice
Clip2	0.00	1.00000000	mice
Pglyrp1	-0.20	1.00000000	mice
Ssr4	0.02	1.00000000	mice
Arhgef2	0.05	1.00000000	mice
Ndufv2	0.06	1.00000000	mice
Wtap	0.01	1.00000000	mice
Slc30a9	-0.02	1.00000000	mice
Drg1	-0.03	1.00000000	mice
Trem3	-0.19	1.00000000	mice
Txnl1	0.07	1.00000000	mice
Bud31	0.05	1.00000000	mice
Vrk3	0.02	1.00000000	mice
Urm1	0.02	1.00000000	mice

Cks2	0.11	1.00000000	mice
Gpatch2l	0.00	1.00000000	mice
Ehmt2	0.02	1.00000000	mice
Rnf168	-0.03	1.00000000	mice
Vps37a	0.01	1.00000000	mice
Adam8	-0.10	1.00000000	mice
Cep95	0.01	1.00000000	mice
C2cd3	-0.02	1.00000000	mice
Fbxo28	0.08	1.00000000	mice
Nelfe	0.06	1.00000000	mice
Ctse	-0.01	1.00000000	mice
Pafah1b1	-0.01	1.00000000	mice
Rnf14	-0.01	1.00000000	mice
Tomm40	0.01	1.00000000	mice
Snx1	0.07	1.00000000	mice
Rhbdd1	-0.02	1.00000000	mice
Cinp	0.01	1.00000000	mice
Nabp2	-0.01	1.00000000	mice
Trappc3	0.03	1.00000000	mice
Tfg	-0.04	1.00000000	mice
Rab11a	0.05	1.00000000	mice
Cog1	0.03	1.00000000	mice
Lpp	-0.03	1.00000000	mice
Fastk	0.01	1.00000000	mice
Thoc5	-0.03	1.00000000	mice
Havcr2	0.06	1.00000000	mice
Fam193a	0.04	1.00000000	mice
Zscan29	-0.03	1.00000000	mice
Clk1	0.03	1.00000000	mice
R3hdm2	0.03	1.00000000	mice
Cbx4	0.05	1.00000000	mice
Rnaset2a	0.01	1.00000000	mice
Stip1	0.08	1.00000000	mice
Inip	0.00	1.00000000	mice
Erlin2	0.04	1.00000000	mice
Pilrb1	-0.05	1.00000000	mice
Aurkaip1	0.04	1.00000000	mice
Lrrc8c	0.00	1.00000000	mice
Akna	-0.03	1.00000000	mice
Trmt6	0.00	1.00000000	mice
Cyhr1	-0.05	1.00000000	mice
Pfkl	-0.02	1.00000000	mice
Pdhx	0.03	1.00000000	mice
Commd8	0.00	1.00000000	mice
Acss2	-0.03	1.00000000	mice
Suz12	0.06	1.00000000	mice

Tmem50b	-0.02	1.00000000	mice
Inpp1	-0.03	1.00000000	mice
Ezh2	0.05	1.00000000	mice
Sigmar1	0.01	1.00000000	mice
Triap1	-0.01	1.00000000	mice
Depdc7	-0.05	1.00000000	mice
Bccip	0.02	1.00000000	mice
Coa7	0.01	1.00000000	mice
Tmem183a	0.04	1.00000000	mice
Zbtb17	0.03	1.00000000	mice
Smg9	0.01	1.00000000	mice
Zkscan1	0.02	1.00000000	mice
Atg4a	-0.01	1.00000000	mice
Nelfcd	0.03	1.00000000	mice
Il13ra1	0.00	1.00000000	mice
1110004F10Rik	0.04	1.00000000	mice
Tyrobp	-0.09	1.00000000	mice
Piwil2	0.00	1.00000000	mice
Timm13	0.04	1.00000000	mice
B3gnt5	-0.15	1.00000000	mice
Pip4p1	0.00	1.00000000	mice
Icosl	0.04	1.00000000	mice
Sub1	-0.02	1.00000000	mice
Cic	0.01	1.00000000	mice
Zcchc24	0.00	1.00000000	mice
Gtf2h2	0.01	1.00000000	mice
3110056K07Rik	-0.02	1.00000000	mice
Cmtm6	0.04	1.00000000	mice
Raly	0.07	1.00000000	mice
Pdcd11	-0.02	1.00000000	mice
Trappc5	0.01	1.00000000	mice
Ints9	0.00	1.00000000	mice
Arl6ip5	0.10	1.00000000	mice
Adamts6	0.02	1.00000000	mice
Ryr1	-0.02	1.00000000	mice
G6pdx	0.00	1.00000000	mice
Wdr74	-0.06	1.00000000	mice
Pias2	-0.03	1.00000000	mice
Cetn2	0.02	1.00000000	mice
Calm3	0.07	1.00000000	mice
Grk2	-0.02	1.00000000	mice
Fen1	0.05	1.00000000	mice
Sephs1	-0.01	1.00000000	mice
Naf1	-0.01	1.00000000	mice
Zfand2b	0.02	1.00000000	mice
Aifm1	0.00	1.00000000	mice

Ptms	0.12	1.00000000	mice
Ralgps1	-0.06	1.00000000	mice
Cux1	-0.02	1.00000000	mice
Btf3l4	0.00	1.00000000	mice
Ociad1	0.03	1.00000000	mice
Acsl3	-0.01	1.00000000	mice
Fam98b	0.03	1.00000000	mice
Ccdc9	0.02	1.00000000	mice
Cops2	0.04	1.00000000	mice
Gpkow	0.01	1.00000000	mice
Ap3d1	0.04	1.00000000	mice
Stard3nl	0.00	1.00000000	mice
Gcnt2	-0.15	1.00000000	mice
Dcbld2	0.09	1.00000000	mice
Ssbp4	0.08	1.00000000	mice
Grap2	0.24	1.00000000	mice
Tradd	0.00	1.00000000	mice
Sugp1	0.00	1.00000000	mice
Tmem192	0.06	1.00000000	mice
Ttc14	0.00	1.00000000	mice
Dock8	0.03	1.00000000	mice
Selplg	0.03	1.00000000	mice
Atp1a3	-0.06	1.00000000	mice
Nf1	-0.02	1.00000000	mice
Slc1a5	0.03	1.00000000	mice
Hnrnpdl	0.07	1.00000000	mice
Donson	0.00	1.00000000	mice
Arpc5l	0.07	1.00000000	mice
Rpain	0.01	1.00000000	mice
Galnt10	0.01	1.00000000	mice
Gpatch2	0.01	1.00000000	mice
Tmem126a	0.04	1.00000000	mice
Nlrc4	0.08	1.00000000	mice
Zdhhc5	0.02	1.00000000	mice
Nr1h2	0.02	1.00000000	mice
Aebp2	0.13	1.00000000	mice
Lcp1	0.02	1.00000000	mice
Prdm2	0.06	1.00000000	mice
Pde4b	-0.02	1.00000000	mice
Zwint	0.06	1.00000000	mice
Flvcr1	-0.05	1.00000000	mice
Snx18	-0.02	1.00000000	mice
Tfam	-0.02	1.00000000	mice
Fuom	-0.07	1.00000000	mice
Usp40	0.00	1.00000000	mice
Rbbp5	0.01	1.00000000	mice

Commd6	0.01	1.00000000	mice
Tfe3	0.01	1.00000000	mice
Ftl1-ps1	0.01	1.00000000	mice
Ccdc138	0.03	1.00000000	mice
Bend4	-0.04	1.00000000	mice
Dctn2	0.02	1.00000000	mice
Tm9sf4	0.00	1.00000000	mice
Lrif1	-0.01	1.00000000	mice
Krt80	-0.09	1.00000000	mice
Emc3	0.01	1.00000000	mice
Gtf2h5	-0.02	1.00000000	mice
Bscl2	0.01	1.00000000	mice
Ctso	0.03	1.00000000	mice
Plaa	-0.01	1.00000000	mice
Anxa5	0.03	1.00000000	mice
CAAA01118383.1	0.01	1.00000000	mice
Zfp36l1	0.11	1.00000000	mice
Ace	-0.10	1.00000000	mice
Usp42	0.01	1.00000000	mice
Akirin1	0.01	1.00000000	mice
Ndufv1	0.06	1.00000000	mice
Etv6	0.02	1.00000000	mice
Timm22	0.02	1.00000000	mice
Exosc9	-0.02	1.00000000	mice
Swt1	0.01	1.00000000	mice
Atxn1l	0.00	1.00000000	mice
Timm17b	-0.03	1.00000000	mice
Plxnc1	0.20	1.00000000	mice
Pgrmc2	-0.02	1.00000000	mice
Ostc	-0.01	1.00000000	mice
Rbm34	0.00	1.00000000	mice
Taf13	-0.02	1.00000000	mice
Apobr	0.01	1.00000000	mice
Spcs1	0.02	1.00000000	mice
Cyp51	-0.03	1.00000000	mice
Prelid3b	0.03	1.00000000	mice
Dnajb12	0.01	1.00000000	mice
Ncoa5	0.03	1.00000000	mice
Atf6b	0.00	1.00000000	mice
Dmtf1	0.00	1.00000000	mice
4933434E20Rik	0.02	1.00000000	mice
Afmid	0.03	1.00000000	mice
Rfk	-0.06	1.00000000	mice
Srp19	0.04	1.00000000	mice
Slc50a1	0.01	1.00000000	mice
Ppil2	0.03	1.00000000	mice

Cnot11	0.01	1.00000000	mice
Slc38a2	0.13	1.00000000	mice
Hdac3	-0.03	1.00000000	mice
Vps16	0.03	1.00000000	mice
Dgat2	0.00	1.00000000	mice
Sbf1	-0.03	1.00000000	mice
U2surp	0.04	1.00000000	mice
Wipi2	-0.01	1.00000000	mice
Bsg	0.05	1.00000000	mice
2310039H08Rik	0.03	1.00000000	mice
Ripor2	-0.37	1.00000000	mice
Ttpal	0.02	1.00000000	mice
Pura	0.04	1.00000000	mice
Rbmxl1	0.02	1.00000000	mice
Ccdc43	0.02	1.00000000	mice
Atp6v1h	0.07	1.00000000	mice
N6amt1	-0.05	1.00000000	mice
Nck2	0.03	1.00000000	mice
Otulin	0.05	1.00000000	mice
Anapc15	0.04	1.00000000	mice
Fosl2	0.05	1.00000000	mice
Bcl7b	0.01	1.00000000	mice
Sucla2	-0.02	1.00000000	mice
Sri	0.09	1.00000000	mice
Cass4	-0.13	1.00000000	mice
Zfr	0.08	1.00000000	mice
Sbno2	0.07	1.00000000	mice
Slc25a45	0.01	1.00000000	mice
Lig3	0.02	1.00000000	mice
Bcl2l13	0.00	1.00000000	mice
Etfa	-0.01	1.00000000	mice
Copg1	-0.01	1.00000000	mice
Irf3	0.02	1.00000000	mice
Ube2v2	0.01	1.00000000	mice
Timp2	0.12	1.00000000	mice
Alg5	0.06	1.00000000	mice
Cyth2	-0.04	1.00000000	mice
Lyl1	-0.02	1.00000000	mice
Vps36	0.01	1.00000000	mice
Ripk1	0.10	1.00000000	mice
2810004N23Rik	0.03	1.00000000	mice
Tmem68	0.04	1.00000000	mice
Cpsf3	-0.02	1.00000000	mice
Ddx56	0.02	1.00000000	mice
Arhgap4	0.03	1.00000000	mice
Elovl1	0.07	1.00000000	mice

Phf21a	-0.05	1.00000000	mice
Erp29	0.06	1.00000000	mice
Hmg20b	0.04	1.00000000	mice
Isoc1	-0.03	1.00000000	mice
Fabp4	0.27	1.00000000	mice
Vps26a	0.03	1.00000000	mice
Aldh16a1	0.02	1.00000000	mice
Tgfbrap1	-0.01	1.00000000	mice
Nop9	0.00	1.00000000	mice
Cpeb3	0.09	1.00000000	mice
Prmt1	0.05	1.00000000	mice
Lypla1	-0.03	1.00000000	mice
Zfp511	-0.01	1.00000000	mice
Ltbr	0.01	1.00000000	mice
Topbp1	-0.01	1.00000000	mice
Dr1	0.05	1.00000000	mice
Rai14	0.01	1.00000000	mice
Sec14l1	-0.01	1.00000000	mice
Pnrc2	-0.01	1.00000000	mice
Slc41a3	-0.01	1.00000000	mice
Bad	0.06	1.00000000	mice
Washc1	0.00	1.00000000	mice
Pinx1	-0.01	1.00000000	mice
Hacd2	0.04	1.00000000	mice
Tasor2	0.04	1.00000000	mice
Csrp2	0.02	1.00000000	mice
Orc3	-0.01	1.00000000	mice
Atp5d	0.06	1.00000000	mice
Babam2	-0.05	1.00000000	mice
Zcchc10	0.02	1.00000000	mice
Cdc34	0.00	1.00000000	mice
Smarcd2	-0.01	1.00000000	mice
Sfi1	0.05	1.00000000	mice
Flcn	0.02	1.00000000	mice
Pbdc1	0.05	1.00000000	mice
Plek	0.03	1.00000000	mice
Tmem230	0.03	1.00000000	mice
Rc3h2	-0.05	1.00000000	mice
Blcap	-0.01	1.00000000	mice
Nt5dc3	0.04	1.00000000	mice
Atxn7l3	0.04	1.00000000	mice
Birc2	0.06	1.00000000	mice
Fam160b2	-0.02	1.00000000	mice
Nipsnap3b	-0.04	1.00000000	mice
Jarid2	-0.15	1.00000000	mice
Sp2	-0.03	1.00000000	mice

Atp5c1	0.05	1.00000000	mice
Lxn	0.03	1.00000000	mice
Ubl4a	0.05	1.00000000	mice
Tiprl	-0.01	1.00000000	mice
Sdhc	-0.03	1.00000000	mice
Utp14a	0.00	1.00000000	mice
Atp2b4	0.07	1.00000000	mice
Ddx27	0.02	1.00000000	mice
Kxd1	-0.01	1.00000000	mice
Ssrp1	0.02	1.00000000	mice
Gabpb1	0.01	1.00000000	mice
Klf2	0.03	1.00000000	mice
Lzic	-0.03	1.00000000	mice
Nfu1	0.03	1.00000000	mice
Rbm27	-0.02	1.00000000	mice
Dync1h1	0.00	1.00000000	mice
Dnajb1	0.05	1.00000000	mice
Sec61a2	0.01	1.00000000	mice
Ppie	0.02	1.00000000	mice
Lrrc59	0.01	1.00000000	mice
Adpgk	0.00	1.00000000	mice
Stxbp2	0.01	1.00000000	mice
Dna2	-0.03	1.00000000	mice
Ccdc124	0.05	1.00000000	mice
Ust	-0.02	1.00000000	mice
5530601H04Rik	-0.03	1.00000000	mice
2410004B18Rik	0.02	1.00000000	mice
Cnep1r1	-0.01	1.00000000	mice
Grsf1	-0.01	1.00000000	mice
Poldip3	0.05	1.00000000	mice
Lsmem1	0.20	1.00000000	mice
Nfil3	0.08	1.00000000	mice
Cep192	0.01	1.00000000	mice
Ptger4	0.05	1.00000000	mice
Txndc15	0.01	1.00000000	mice
St8sia4	0.00	1.00000000	mice
Zfp868	-0.05	1.00000000	mice
Ciz1	0.05	1.00000000	mice
Ppip5k2	0.02	1.00000000	mice
Rgs2	0.01	1.00000000	mice
Polg2	0.01	1.00000000	mice
Rpa3	0.01	1.00000000	mice
Aig1	0.19	1.00000000	mice
Dpf2	0.06	1.00000000	mice
Snx17	0.00	1.00000000	mice
Ttc19	0.01	1.00000000	mice

Umad1	-0.02	1.00000000	mice
Ppdpf	0.01	1.00000000	mice
Glrx2	0.02	1.00000000	mice
Slc16a2	-0.02	1.00000000	mice
Yju2	-0.01	1.00000000	mice
Sec22a	-0.01	1.00000000	mice
Dlgap4	0.01	1.00000000	mice
Lrrc41	0.01	1.00000000	mice
Foxp4	0.04	1.00000000	mice
Srprb	-0.02	1.00000000	mice
Nudt13	0.02	1.00000000	mice
Pi4k2b	0.02	1.00000000	mice
Tax1bp1	0.04	1.00000000	mice
Prr3	0.02	1.00000000	mice
Zfp263	-0.01	1.00000000	mice
Skp1a	0.02	1.00000000	mice
Aga	0.01	1.00000000	mice
Rrp1	0.00	1.00000000	mice
Zc3h7a	0.01	1.00000000	mice
Ankra2	0.01	1.00000000	mice
Pwwp2b	0.03	1.00000000	mice
Slc39a14	0.02	1.00000000	mice
Gnb2	0.01	1.00000000	mice
Bfar	0.02	1.00000000	mice
Cuta	0.04	1.00000000	mice
Cbx1	0.03	1.00000000	mice
Btg3	-0.05	1.00000000	mice
Nup88	0.01	1.00000000	mice
Actr1b	0.05	1.00000000	mice
Ppp2r2a	0.04	1.00000000	mice
Pcna	0.08	1.00000000	mice
Pdss1	0.04	1.00000000	mice
Aagab	0.01	1.00000000	mice
Impact	0.09	1.00000000	mice
Virma	-0.05	1.00000000	mice
Polr3d	0.03	1.00000000	mice
Neu1	0.00	1.00000000	mice
S100a13	0.05	1.00000000	mice
Cd2bp2	0.02	1.00000000	mice
Hagh	-0.03	1.00000000	mice
Ahcyl2	-0.05	1.00000000	mice
Snip1	-0.02	1.00000000	mice
Nipsnap2	0.04	1.00000000	mice
Atmin	0.01	1.00000000	mice
Zfp106	0.02	1.00000000	mice
Zfp639	0.01	1.00000000	mice

Naa40	0.01	1.00000000	mice
Aen	0.04	1.00000000	mice
Umps	-0.02	1.00000000	mice
2210408F21Rik	0.06	1.00000000	mice
Zfyve16	0.03	1.00000000	mice
Pi16	-0.05	1.00000000	mice
Nudt18	0.02	1.00000000	mice
Eef1e1	0.01	1.00000000	mice
Il18	-0.14	1.00000000	mice
Becn1	0.03	1.00000000	mice
Uqcr11	0.10	1.00000000	mice
Khdrbs1	-0.01	1.00000000	mice
Elf1	0.03	1.00000000	mice
Cdk8	0.17	1.00000000	mice
Fam207a	0.00	1.00000000	mice
Asnsd1	0.01	1.00000000	mice
Cklf	0.03	1.00000000	mice
Tmbim6	0.00	1.00000000	mice
Hscb	0.05	1.00000000	mice
Vps4b	-0.02	1.00000000	mice
Commd2	0.03	1.00000000	mice
Banf1	0.02	1.00000000	mice
Eif1ad	0.04	1.00000000	mice
Ube2f	-0.02	1.00000000	mice
Sptlc1	0.00	1.00000000	mice
Egr2	0.07	1.00000000	mice
Spp1	0.30	1.00000000	mice
Ptrhd1	-0.02	1.00000000	mice
Naa10	0.02	1.00000000	mice
Hsd17b10	0.01	1.00000000	mice
Tars	0.01	1.00000000	mice
Wipf2	0.00	1.00000000	mice
Zfp715	0.00	1.00000000	mice
Dhrs11	0.02	1.00000000	mice
Slc9a8	0.03	1.00000000	mice
Vbp1	-0.01	1.00000000	mice
Creb3	-0.01	1.00000000	mice
Zfp410	0.02	1.00000000	mice
Cox6a1	0.01	1.00000000	mice
Utp23	0.02	1.00000000	mice
Epb41l1	0.04	1.00000000	mice
Pef1	0.01	1.00000000	mice
Upf3a	0.02	1.00000000	mice
Gramd1b	-0.08	1.00000000	mice
Hspa13	-0.02	1.00000000	mice
Vps51	0.00	1.00000000	mice

Abhd16a	0.00	1.00000000	mice
Gnai2	0.00	1.00000000	mice
Arl6ip4	-0.01	1.00000000	mice
Sh3gl1	-0.02	1.00000000	mice
Tceal9	0.04	1.00000000	mice
Efcab14	0.00	1.00000000	mice
Samd4b	0.06	1.00000000	mice
Tardbp	0.01	1.00000000	mice
Ccdc93	0.02	1.00000000	mice
Actn4	0.06	1.00000000	mice
Sdf2	0.03	1.00000000	mice
Fbxo38	0.04	1.00000000	mice
Naxd	0.01	1.00000000	mice
Ssb	0.02	1.00000000	mice
Exosc1	0.01	1.00000000	mice
Rgs10	0.05	1.00000000	mice
Arhgef7	0.04	1.00000000	mice
Ccr1	-0.05	1.00000000	mice
Epb41l2	0.03	1.00000000	mice
Ccdc90b	0.00	1.00000000	mice
Lyar	0.03	1.00000000	mice
Rad18	-0.05	1.00000000	mice
Ankrd49	0.01	1.00000000	mice
Uap1l1	0.06	1.00000000	mice
Hsbp1	0.05	1.00000000	mice
Dld	0.02	1.00000000	mice
Comtd1	0.01	1.00000000	mice
Tma7	0.04	1.00000000	mice
Supv3l1	-0.05	1.00000000	mice
4931406P16Rik	0.02	1.00000000	mice
Brox	0.01	1.00000000	mice
Clpx	0.02	1.00000000	mice
Txndc12	0.03	1.00000000	mice
Cant1	0.01	1.00000000	mice
Rbpj	-0.05	1.00000000	mice
Tug1	-0.02	1.00000000	mice
Pmf1	0.01	1.00000000	mice
Acot9	0.00	1.00000000	mice
Tbc1d22b	0.04	1.00000000	mice
Plagl2	0.06	1.00000000	mice
Zfand3	0.01	1.00000000	mice
Pdgfb	0.02	1.00000000	mice
Ncaph2	-0.03	1.00000000	mice
Napa	0.05	1.00000000	mice
Sf3b4	0.01	1.00000000	mice
Ntan1	0.06	1.00000000	mice

Epb41l4aos	0.00	1.00000000	mice
Vps53	-0.02	1.00000000	mice
Lsm4	0.00	1.00000000	mice
Prkab1	0.02	1.00000000	mice
Cops7a	0.03	1.00000000	mice
Ralgds	-0.02	1.00000000	mice
Eif5b	0.07	1.00000000	mice
Efr3a	0.03	1.00000000	mice
Csf1	-0.22	1.00000000	mice
Gyg	0.04	1.00000000	mice
Tnfaip8l2	0.03	1.00000000	mice
Plekhj1	0.00	1.00000000	mice
Hdgfl2	0.02	1.00000000	mice
Slc25a11	0.03	1.00000000	mice
Eif1ax	0.02	1.00000000	mice
Fgfr1op2	0.00	1.00000000	mice
Prpf4	-0.01	1.00000000	mice
Ubl7	-0.01	1.00000000	mice
Ufc1	0.06	1.00000000	mice
Sik1	-0.13	1.00000000	mice
Tnfrsf1a	0.07	1.00000000	mice
Spg7	0.00	1.00000000	mice
Rab1b	0.05	1.00000000	mice
Pts	0.02	1.00000000	mice
Phax	0.01	1.00000000	mice
Ccdc32	0.02	1.00000000	mice
Dazap1	0.01	1.00000000	mice
Dpm3	0.05	1.00000000	mice
Prkcd	0.02	1.00000000	mice
Papola	0.03	1.00000000	mice
Ccser2	0.01	1.00000000	mice
Plet1os	0.09	1.00000000	mice
Tifab	0.00	1.00000000	mice
Rbm4b	0.02	1.00000000	mice
Ggh	0.04	1.00000000	mice
Ubxn8	0.00	1.00000000	mice
Dnajc2	0.06	1.00000000	mice
Got2	-0.02	1.00000000	mice
Nudt5	0.03	1.00000000	mice
Thyn1	0.02	1.00000000	mice
Anxa6	0.03	1.00000000	mice
Snrnp25	0.01	1.00000000	mice
Brcc3	0.00	1.00000000	mice
Eif3g	0.04	1.00000000	mice
Gadd45a	-0.01	1.00000000	mice
Ndfip2	-0.03	1.00000000	mice

Bcl2l11	0.05	1.00000000	mice
Trp53inp2	0.02	1.00000000	mice
Prkaca	0.02	1.00000000	mice
Tmem185b	0.00	1.00000000	mice
Dnajc7	0.08	1.00000000	mice
Sgk3	0.02	1.00000000	mice
Pnkd	-0.01	1.00000000	mice
Dcp1a	0.02	1.00000000	mice
Tmem208	0.01	1.00000000	mice
Sesn3	-0.03	1.00000000	mice
Vdac2	0.04	1.00000000	mice
Cdk5	0.03	1.00000000	mice
Stn1	0.02	1.00000000	mice
Ccdc97	-0.01	1.00000000	mice
Leprot	0.03	1.00000000	mice
Il10ra	-0.01	1.00000000	mice
Ccnt1	0.03	1.00000000	mice
Timmdc1	0.01	1.00000000	mice
Hdhd2	0.04	1.00000000	mice
Hnrnpa3	0.02	1.00000000	mice
Zc3h15	0.03	1.00000000	mice
Trib1	-0.06	1.00000000	mice
Zfp367	-0.01	1.00000000	mice
Bex3	0.05	1.00000000	mice
R3hcc1l	0.01	1.00000000	mice
Adam17	0.00	1.00000000	mice
Dnlz	0.02	1.00000000	mice
Exosc7	-0.03	1.00000000	mice
Pot1b	-0.01	1.00000000	mice
Clpp	0.01	1.00000000	mice
D430042O09Rik	-0.01	1.00000000	mice
Cep164	-0.02	1.00000000	mice
Retreg3	-0.01	1.00000000	mice
Foxo3	-0.01	1.00000000	mice
Tmub2	0.02	1.00000000	mice
Plekha3	0.00	1.00000000	mice
Atp6v1c1	-0.01	1.00000000	mice
Supt5	0.02	1.00000000	mice
Slc7a5	-0.01	1.00000000	mice
Arrdc4	-0.07	1.00000000	mice
Cenpc1	0.04	1.00000000	mice
Zfp830	0.01	1.00000000	mice
Vamp7	0.01	1.00000000	mice
Lifr	-0.03	1.00000000	mice
Nsun6	0.01	1.00000000	mice
Cln5	-0.01	1.00000000	mice

Brd8	0.00	1.00000000	mice
Ccz1	0.04	1.00000000	mice
Ebag9	-0.01	1.00000000	mice
Stk16	0.01	1.00000000	mice
Tsfm	-0.02	1.00000000	mice
Cog2	-0.01	1.00000000	mice
Simc1	0.13	1.00000000	mice
Ndufb9	0.05	1.00000000	mice
Cdt1	-0.02	1.00000000	mice
Rbms2	0.04	1.00000000	mice
Utp6	0.01	1.00000000	mice
Uqcrc2	0.00	1.00000000	mice
Abcd3	0.04	1.00000000	mice
Cnpy3	-0.02	1.00000000	mice
Tmco1	0.08	1.00000000	mice
Furin	0.06	1.00000000	mice
Xrcc4	-0.02	1.00000000	mice
Nudt9	0.02	1.00000000	mice
Pik3ap1	0.00	1.00000000	mice
Taf1d	0.08	1.00000000	mice
Hsdl2	0.04	1.00000000	mice
Abhd2	-0.04	1.00000000	mice
Dmac1	0.00	1.00000000	mice
Fgd6	0.04	1.00000000	mice
Krtcap2	0.06	1.00000000	mice
Prpf6	0.00	1.00000000	mice
Hmgxb4	0.02	1.00000000	mice
Lman1	0.04	1.00000000	mice
Csnk1d	0.04	1.00000000	mice
5031425E22Rik	-0.04	1.00000000	mice
Stat4	-0.02	1.00000000	mice
Arfgap1	0.00	1.00000000	mice
Ppm1d	0.00	1.00000000	mice
Kctd5	0.01	1.00000000	mice
Ddost	0.02	1.00000000	mice
Pop5	0.05	1.00000000	mice
D10Wsu102e	-0.02	1.00000000	mice
Letm1	0.01	1.00000000	mice
Adap1	0.05	1.00000000	mice
Ebp	0.04	1.00000000	mice
Itgb1bp1	0.03	1.00000000	mice
Pik3cd	0.00	1.00000000	mice
Edem2	-0.01	1.00000000	mice
Wdr37	0.00	1.00000000	mice
Cpne3	0.03	1.00000000	mice
Baiap2	-0.01	1.00000000	mice

BC035044	-0.03	1.00000000	mice
Prkd3	-0.03	1.00000000	mice
Fbxw4	0.00	1.00000000	mice
Aarsd1	0.02	1.00000000	mice
Arfrp1	0.02	1.00000000	mice
Btbd7	0.05	1.00000000	mice
S100a4	-0.35	1.00000000	mice
Ralb	0.03	1.00000000	mice
Hps1	-0.01	1.00000000	mice
Zcchc17	-0.02	1.00000000	mice
U2af1	0.07	1.00000000	mice
Exoc7	0.01	1.00000000	mice
Lilra6	0.01	1.00000000	mice
Ints14	-0.11	1.00000000	mice
Rnf10	0.03	1.00000000	mice
Hdac9	0.04	1.00000000	mice
Ttf1	0.02	1.00000000	mice
Arpc5	0.01	1.00000000	mice
Pdia6	0.11	1.00000000	mice
Trappc4	0.00	1.00000000	mice
Dcps	0.01	1.00000000	mice
Dnajc24	-0.02	1.00000000	mice
Prpf3	0.01	1.00000000	mice
Ticam2	0.00	1.00000000	mice
Nufip1	0.00	1.00000000	mice
Ppwd1	0.02	1.00000000	mice
Nbr1	0.04	1.00000000	mice
Ric1	0.05	1.00000000	mice
Ncln	-0.01	1.00000000	mice
Nudt19	0.02	1.00000000	mice
1110059G10Rik	0.00	1.00000000	mice
Trnt1	0.00	1.00000000	mice
Socs4	-0.03	1.00000000	mice
Otulinl	0.12	1.00000000	mice
Ranbp1	0.12	1.00000000	mice
Lonp1	0.00	1.00000000	mice
Tnpo1	0.08	1.00000000	mice
Rbm22	-0.01	1.00000000	mice
Zdhhc6	0.00	1.00000000	mice
Snrpa1	0.00	1.00000000	mice
Tomm22	0.03	1.00000000	mice
Plcl1	0.02	1.00000000	mice
Thoc3	0.02	1.00000000	mice
Tlr6	-0.02	1.00000000	mice
Ugdh	0.02	1.00000000	mice
Vps52	0.01	1.00000000	mice

Tmem176b	-0.05	1.00000000	mice
Stx6	0.01	1.00000000	mice
Eno1	0.00	1.00000000	mice
Abracl	0.04	1.00000000	mice
Tra2b	0.05	1.00000000	mice
Dnase1l1	0.02	1.00000000	mice
Ccnl1	0.12	1.00000000	mice
Rdh11	0.00	1.00000000	mice
Nsa2	-0.03	1.00000000	mice
Cdk5rap3	0.03	1.00000000	mice
Chtop	0.02	1.00000000	mice
Dhx16	0.00	1.00000000	mice
Thoc7	0.02	1.00000000	mice
Gins4	0.02	1.00000000	mice
Wdr61	0.05	1.00000000	mice
Scyl1	0.05	1.00000000	mice
Tpcn1	-0.04	1.00000000	mice
Nudcd3	0.03	1.00000000	mice
Usp2	0.03	1.00000000	mice
Bet1	-0.04	1.00000000	mice
Ppp1r9b	0.03	1.00000000	mice
Acin1	0.04	1.00000000	mice
Dedd	0.03	1.00000000	mice
Lin52	0.01	1.00000000	mice
Vcan	-0.16	1.00000000	mice
Zyx	0.04	1.00000000	mice
Fbxl6	0.02	1.00000000	mice
Tubb6	0.04	1.00000000	mice
Ergic2	0.02	1.00000000	mice
Trp53	0.02	1.00000000	mice
Ppil3	0.00	1.00000000	mice
Rbm5	0.05	1.00000000	mice
Atg13	0.03	1.00000000	mice
Stx3	0.01	1.00000000	mice
Fam133b	0.04	1.00000000	mice
Ubap2	0.02	1.00000000	mice
Rab32	0.01	1.00000000	mice
Srsf11	0.04	1.00000000	mice
Rarg	-0.02	1.00000000	mice
Hnrnpa2b1	0.00	1.00000000	mice
Rnf126	0.01	1.00000000	mice
Enox2	0.01	1.00000000	mice
Chmp6	0.00	1.00000000	mice
Cnot3	0.03	1.00000000	mice
Nkiras2	0.00	1.00000000	mice
Susd3	-0.08	1.00000000	mice

Herc3	0.00	1.00000000	mice
Pknox1	-0.02	1.00000000	mice
Ccs	0.02	1.00000000	mice
Rab10os	-0.03	1.00000000	mice
Tango2	0.03	1.00000000	mice
Scaper	-0.01	1.00000000	mice
Bdp1	0.05	1.00000000	mice
Cct8	0.04	1.00000000	mice
Wsb1	0.08	1.00000000	mice
AI413582	0.01	1.00000000	mice
Nat9	0.01	1.00000000	mice
Cnot9	0.01	1.00000000	mice
Zswim7	0.00	1.00000000	mice
Arl3	0.03	1.00000000	mice
Slc9a3r1	-0.01	1.00000000	mice
Syap1	0.00	1.00000000	mice
E130307A14Rik	-0.01	1.00000000	mice
Gng2	0.09	1.00000000	mice
Serhl	0.03	1.00000000	mice
Dusp22	0.02	1.00000000	mice
Rheb	-0.01	1.00000000	mice
Traf5	-0.03	1.00000000	mice
Cox14	0.03	1.00000000	mice
Rpe	-0.01	1.00000000	mice
Gt(ROSA)26Sor	0.01	1.00000000	mice
Swi5	0.06	1.00000000	mice
Irf2bpl	-0.02	1.00000000	mice
Impa2	0.01	1.00000000	mice
Tubgcp2	0.00	1.00000000	mice
Ola1	0.01	1.00000000	mice
Cd200	0.09	1.00000000	mice
Cyb5r1	-0.02	1.00000000	mice
Nup188	0.02	1.00000000	mice
Dpp7	0.03	1.00000000	mice
Vezf1	0.01	1.00000000	mice
Plekhf2	0.05	1.00000000	mice
Acadvl	0.02	1.00000000	mice
Rinl	0.00	1.00000000	mice
Cnnm4	0.00	1.00000000	mice
Lars2	0.29	1.00000000	mice
Tsg101	0.03	1.00000000	mice
Zc3hc1	0.02	1.00000000	mice
Cdk4	-0.02	1.00000000	mice
Stk40	0.07	1.00000000	mice
Chuk	0.03	1.00000000	mice
Ttc9c	0.04	1.00000000	mice

Zfp710	-0.02	1.00000000	mice
Ccnyl1	0.03	1.00000000	mice
Fbrs	0.02	1.00000000	mice
Svip	0.01	1.00000000	mice
Ap2s1	0.00	1.00000000	mice
Serp1	0.05	1.00000000	mice
Gaa	0.01	1.00000000	mice
Rcsd1	0.00	1.00000000	mice
Pcgf3	0.00	1.00000000	mice
Hmgcr	0.02	1.00000000	mice
4632427E13Rik	-0.04	1.00000000	mice
Rp2	0.02	1.00000000	mice
Rrp7a	-0.01	1.00000000	mice
Eif4h	0.01	1.00000000	mice
Ndufv3	0.05	1.00000000	mice
Dnmt1	0.04	1.00000000	mice
Gadd45gip1	0.01	1.00000000	mice
Nagpa	0.01	1.00000000	mice
Usp12	0.02	1.00000000	mice
Upf1	0.01	1.00000000	mice
Ppp2r3d	0.02	1.00000000	mice
Tmed4	-0.01	1.00000000	mice
Use1	-0.02	1.00000000	mice
Cdc37l1	0.01	1.00000000	mice
Gabarap	0.01	1.00000000	mice
Flii	0.04	1.00000000	mice
Ctnnbl1	0.03	1.00000000	mice
Ppp1r13b	-0.02	1.00000000	mice
Ergic3	0.02	1.00000000	mice
Rcn2	0.00	1.00000000	mice
Nfkbil1	0.02	1.00000000	mice
Chchd1	0.06	1.00000000	mice
Fut8	-0.07	1.00000000	mice
Ano10	0.00	1.00000000	mice
Yipf4	0.04	1.00000000	mice
Ndufa10	0.04	1.00000000	mice
Ubxn1	0.05	1.00000000	mice
Dcxr	-0.01	1.00000000	mice
Snrnp48	0.02	1.00000000	mice
Btrc	0.00	1.00000000	mice
Anp32a	0.04	1.00000000	mice
Gpr107	0.01	1.00000000	mice
Stx8	0.05	1.00000000	mice
Rab7	-0.09	1.00000000	mice
Prpf19	0.03	1.00000000	mice
Tent2	0.01	1.00000000	mice

Lemd3	0.06	1.00000000	mice
Ahi1	-0.06	1.00000000	mice
Taf11	0.00	1.00000000	mice
Gtdc1	-0.15	1.00000000	mice
Diablo	0.00	1.00000000	mice
Rnaseh2a	0.00	1.00000000	mice
Kansl3	0.02	1.00000000	mice
Cops8	0.03	1.00000000	mice
Nxn	-0.06	1.00000000	mice
Plgrkt	0.03	1.00000000	mice
Sash3	0.00	1.00000000	mice
Cdca4	0.00	1.00000000	mice
Atp11a	0.02	1.00000000	mice
Nacc1	0.02	1.00000000	mice
Gtpbp1	0.03	1.00000000	mice
Eif1a	0.03	1.00000000	mice
Rfwd3	0.03	1.00000000	mice
Ptges	0.06	1.00000000	mice
Impa1	0.01	1.00000000	mice
Uxt	0.01	1.00000000	mice
Scyl3	-0.03	1.00000000	mice
Arhgdib	0.06	1.00000000	mice
Kmt2b	0.03	1.00000000	mice
Wbp11	0.04	1.00000000	mice
Cds1	0.03	1.00000000	mice
Ap1m1	0.01	1.00000000	mice
Fbxl2	0.02	1.00000000	mice
Rnf4	0.03	1.00000000	mice
Bloc1s2	0.04	1.00000000	mice
Il31ra	0.01	1.00000000	mice
Copz1	-0.03	1.00000000	mice
Zmiz2	-0.01	1.00000000	mice
Cfdp1	0.05	1.00000000	mice
Fabp5	0.38	1.00000000	mice
Erp44	0.03	1.00000000	mice
Vcp	0.06	1.00000000	mice
Polr2g	0.08	1.00000000	mice
Gnl1	0.02	1.00000000	mice
Scamp2	0.01	1.00000000	mice
Tmem70	0.01	1.00000000	mice
Sphk2	0.00	1.00000000	mice
Kri1	0.01	1.00000000	mice
A930007I19Rik	0.09	1.00000000	mice
Srfbp1	0.05	1.00000000	mice
Ccl25	0.00	1.00000000	mice
Smim26	0.02	1.00000000	mice

Terf1	-0.03	1.00000000	mice
Grcc10	0.02	1.00000000	mice
Tubgcp4	0.00	1.00000000	mice
Irak2	-0.04	1.00000000	mice
Axl	-0.09	1.00000000	mice
Fam214b	0.00	1.00000000	mice
Camta2	0.00	1.00000000	mice
Pmpca	0.02	1.00000000	mice
Ipcef1	-0.09	1.00000000	mice
Arl5a	0.02	1.00000000	mice
Cpsf4	0.01	1.00000000	mice
Ccnd2	-0.10	1.00000000	mice
Emilin2	-0.01	1.00000000	mice
Bag6	0.02	1.00000000	mice
Fam71f2	0.05	1.00000000	mice
As3mt	-0.01	1.00000000	mice
Rprd1a	0.02	1.00000000	mice
Alg13	0.01	1.00000000	mice
Odc1	0.01	1.00000000	mice
Card19	-0.15	1.00000000	mice
Hdac5	-0.01	1.00000000	mice
Insig2	-0.02	1.00000000	mice
Eps8	0.02	1.00000000	mice
Fam173a	0.02	1.00000000	mice
Timm10	-0.02	1.00000000	mice
Eif3j1	0.03	1.00000000	mice
Atpif1	0.07	1.00000000	mice
Kin	-0.01	1.00000000	mice
Fbh1	0.01	1.00000000	mice
Tm2d1	-0.01	1.00000000	mice
Caap1	-0.01	1.00000000	mice
AW549877	-0.01	1.00000000	mice
Smim15	0.02	1.00000000	mice
Phf20	0.02	1.00000000	mice
Gpalpp1	0.02	1.00000000	mice
Pold4	0.01	1.00000000	mice
Pxk	0.10	1.00000000	mice
Stx12	0.04	1.00000000	mice
Osbpl1a	0.00	1.00000000	mice
Ptgr2	0.02	1.00000000	mice
Tmem222	0.02	1.00000000	mice
Tle3	0.05	1.00000000	mice
Arid4b	-0.01	1.00000000	mice
Agpat1	0.04	1.00000000	mice
Slc39a13	0.03	1.00000000	mice
Helb	0.03	1.00000000	mice

Urgcp	-0.01	1.00000000	mice
Rab7b	0.13	1.00000000	mice
Pkn2	0.03	1.00000000	mice
Naaa	0.02	1.00000000	mice
Zfp992	0.02	1.00000000	mice
Nrbp1	0.02	1.00000000	mice
Cd47	0.02	1.00000000	mice
Hif1a	0.03	1.00000000	mice
Rwdd4a	0.01	1.00000000	mice
Selenof	0.01	1.00000000	mice
Suds3	0.02	1.00000000	mice
Spag7	0.01	1.00000000	mice
Ier3ip1	0.01	1.00000000	mice
Sc5d	-0.02	1.00000000	mice
Arfgap2	0.02	1.00000000	mice
Crcp	0.01	1.00000000	mice
Ndufaf2	-0.02	1.00000000	mice
Ubfd1	-0.01	1.00000000	mice
Fundc1	0.01	1.00000000	mice
Pgm2	-0.02	1.00000000	mice
P2ry14	0.07	1.00000000	mice
Utp4	-0.01	1.00000000	mice
Sdf2l1	0.07	1.00000000	mice
Was	-0.01	1.00000000	mice
Ostm1	0.02	1.00000000	mice
St3gal4	0.02	1.00000000	mice
Rab21	-0.02	1.00000000	mice
Saa3	5.85	0.00000000	pigs
Cfd	-2.84	0.00000000	pigs
Ftl1-ps1	2.03	0.00000000	pigs
Sod2	1.96	0.00000000	pigs
Pank3	1.51	0.00000000	pigs
Cavin3	-0.91	0.00000000	pigs
Cstb	1.54	0.00000000	pigs
Rnf19a	1.67	0.00000000	pigs
Arg1	2.45	0.00000000	pigs
Ctsc	1.26	0.00000000	pigs
Tesc	-0.88	0.00000000	pigs
Hpse	0.99	0.00000000	pigs
Abca1	1.52	0.00000000	pigs
Plin2	1.22	0.00000000	pigs
S100a6	-2.34	0.00000000	pigs
Tppp3	-1.73	0.00000000	pigs
Prdx1	1.10	0.00000000	pigs
Ctsb	1.38	0.00000000	pigs
Ucp2	-1.30	0.00000000	pigs

Npc2	1.44	0.00000000	pigs
Lrrk2	-1.48	0.00000000	pigs
Sdc2	1.49	0.00000000	pigs
Id3	1.04	0.00000000	pigs
Nrp2	1.11	0.00000000	pigs
Cpped1	0.86	0.00000000	pigs
Ido1	1.86	0.00000000	pigs
Gpnmb	1.84	0.00000000	pigs
Smpdl3a	1.16	0.00000000	pigs
Bst2	1.20	0.00000000	pigs
Spon2	1.03	0.00000000	pigs
Nupr1	2.18	0.00000000	pigs
Pdcd1lg2	0.97	0.00000000	pigs
Eng	0.64	0.00000000	pigs
Ifi27	-1.09	0.00000000	pigs
Dhrs9	-0.81	0.00000000	pigs
Ppif	1.19	0.00000000	pigs
Cytip	-1.04	0.00000000	pigs
Vav1	0.79	0.00000000	pigs
Npr3	-0.76	0.00000000	pigs
Atp6v1b2	0.96	0.00000000	pigs
Pkib	0.90	0.00000000	pigs
Lhfpl2	1.00	0.00000000	pigs
Epb41l3	1.17	0.00000000	pigs
Cst3	-1.51	0.00000000	pigs
Nek6	0.99	0.00000000	pigs
Cda	-1.49	0.00000000	pigs
Cyth1	0.98	0.00000000	pigs
Slc15a4	0.73	0.00000000	pigs
Cd163	1.23	0.00000000	pigs
Eepd1	1.14	0.00000000	pigs
Dram1	0.83	0.00000000	pigs
Litaf	0.90	0.00000000	pigs
Slamf8	0.54	0.00000000	pigs
Pdxk	0.91	0.00000000	pigs
Sgpl1	0.93	0.00000000	pigs
Thbs1	-2.26	0.00000000	pigs
Nebl	1.04	0.00000000	pigs
Plxna1	0.48	0.00000000	pigs
S100a4	-2.05	0.00000000	pigs
Itga4	-1.06	0.00000000	pigs
Slc16a7	0.94	0.00000000	pigs
Zhx2	1.11	0.00000000	pigs
Ago2	0.88	0.00000000	pigs
Ezr	-1.03	0.00000000	pigs
Lgmn	1.41	0.00000000	pigs

Tnfaip6	1.20	0.00000000	pigs
Cndp2	0.81	0.00000000	pigs
Ubl5	-0.78	0.00000000	pigs
Tsen2	0.69	0.00000000	pigs
F13a1	1.59	0.00000000	pigs
Scarb1	0.76	0.00000000	pigs
Vim	-0.96	0.00000000	pigs
Ggh	0.86	0.00000000	pigs
Slc2a5	0.63	0.00000000	pigs
Cdkn1a	0.70	0.00000000	pigs
Scpep1	0.87	0.00000000	pigs
Timp1	0.94	0.00000000	pigs
Ybey	0.47	0.00000000	pigs
Spsb1	0.69	0.00000000	pigs
Tuba1b	0.67	0.00000000	pigs
Wbp11	0.75	0.00000000	pigs
Socs1	0.69	0.00000000	pigs
Spp1	1.75	0.00000000	pigs
Eif4a1	0.67	0.00000000	pigs
Rgs18	-0.97	0.00000000	pigs
Ctsa	0.74	0.00000000	pigs
Lgals1	-0.98	0.00000000	pigs
Gns	0.77	0.00000000	pigs
Dmxl2	0.98	0.00000000	pigs
AA467197	1.41	0.00000000	pigs
Apoe	-0.03	0.00000000	pigs
Il7r	0.88	0.00000000	pigs
Tmem135	0.74	0.00000000	pigs
Irak3	0.88	0.00000000	pigs
Hsd3b7	0.55	0.00000000	pigs
Lrmda	1.40	0.00000000	pigs
Aco1	1.00	0.00000000	pigs
Vcam1	0.98	0.00000000	pigs
Rgl1	1.11	0.00000000	pigs
Bhlhe40	0.81	0.00000000	pigs
Agap3	0.54	0.00000000	pigs
Vegfa	0.78	0.00000000	pigs
Ptpn18	-0.71	0.00000000	pigs
Rala	0.78	0.00000000	pigs
Ifngr2	0.59	0.00000000	pigs
Por	0.59	0.00000000	pigs
Ubc	0.54	0.00000000	pigs
Rarres1	1.19	0.00000000	pigs
P4ha1	0.81	0.00000000	pigs
Fyn	0.93	0.00000000	pigs
Arsg	0.76	0.00000000	pigs

Acod1	0.89	0.00000000	pigs
Slc11a2	0.64	0.00000000	pigs
Tfrc	0.67	0.00000000	pigs
Gba	0.63	0.00000000	pigs
Arhgap15	-1.33	0.00000000	pigs
Serpinb10	-0.80	0.00000000	pigs
Tpi1	0.64	0.00000000	pigs
Sh3bp2	0.51	0.00000000	pigs
Dnase2a	0.62	0.00000000	pigs
Ap2m1	0.70	0.00000000	pigs
Cd82	0.54	0.00000000	pigs
Arpc1b	0.62	0.00000000	pigs
Lcor	0.71	0.00000000	pigs
Itgb8	0.99	0.00000000	pigs
Trpm2	0.39	0.00000000	pigs
Asap2	0.66	0.00000000	pigs
Cir1	0.48	0.00000000	pigs
Tmem163	-0.49	0.00000000	pigs
Ndrg1	0.75	0.00000000	pigs
Ifi30	0.82	0.00000000	pigs
Arhgap12	0.52	0.00000000	pigs
Trem2	0.47	0.00000000	pigs
Selenop	0.44	0.00000000	pigs
Slamf7	0.99	0.00000000	pigs
Igf1r	0.94	0.00000000	pigs
Tmem120b	0.78	0.00000000	pigs
Plgrkt	0.53	0.00000000	pigs
Ifit3	-1.08	0.00000000	pigs
Sh2b2	0.33	0.00000000	pigs
Samhd1	0.74	0.00000000	pigs
Fchsd2	1.07	0.00000000	pigs
Gnptab	0.59	0.00000000	pigs
Anxa1	-1.17	0.00000000	pigs
Fmnl3	0.60	0.00000000	pigs
Nyap2	0.87	0.00000000	pigs
Serping1	0.63	0.00000000	pigs
Ckb	0.38	0.00000000	pigs
Lrch1	1.03	0.00000000	pigs
Traf3	0.66	0.00000000	pigs
Ccr7	0.90	0.00000000	pigs
Slc23a2	0.66	0.00000000	pigs
Cyp3a57	0.58	0.00000000	pigs
Tspan4	0.35	0.00000000	pigs
Nfe2l2	0.75	0.00000000	pigs
Hsph1	0.89	0.00000000	pigs
Slc39a14	0.80	0.00000000	pigs

Nrp1	1.06	0.00000000	pigs
Gpcpd1	-0.90	0.00000000	pigs
Cyp1b1	1.24	0.00000000	pigs
Plekho1	0.65	0.00000000	pigs
Tubb5	0.58	0.00000000	pigs
Zranb1	0.40	0.00000000	pigs
Htra1	0.68	0.00000000	pigs
Sh3pxd2b	1.01	0.00000000	pigs
Dop1b	0.54	0.00000000	pigs
Atp13a3	0.67	0.00000000	pigs
Aldh2	0.67	0.00000000	pigs
Ninj1	0.75	0.00000000	pigs
Il1a	1.02	0.00000000	pigs
Kmo	0.67	0.00000000	pigs
Pip5k1a	0.46	0.00000000	pigs
Nr4a1	-0.98	0.00000000	pigs
Ctsh	0.84	0.00000000	pigs
Plxna2	0.43	0.00000000	pigs
Eif1	-0.43	0.00000000	pigs
Nedd4l	1.02	0.00000000	pigs
Ampd3	0.38	0.00000000	pigs
Arl8b	0.60	0.00000000	pigs
Wtap	0.66	0.00000000	pigs
H2-Ea	0.83	0.00000000	pigs
A2m	-0.37	0.00000000	pigs
Glmp	0.51	0.00000000	pigs
Gpr137b	0.75	0.00000000	pigs
Ttyh2	0.36	0.00000000	pigs
Kpnb1	0.55	0.00000000	pigs
Krt23	-0.25	0.00000000	pigs
Cers6	0.81	0.00000000	pigs
Xdh	0.64	0.00000000	pigs
Sypl	0.49	0.00000000	pigs
Ldlrad3	0.52	0.00000000	pigs
Gpr183	0.95	0.00000000	pigs
Epas1	0.26	0.00000000	pigs
Acap2	0.67	0.00000000	pigs
Ugcg	0.63	0.00000000	pigs
Glipr2	-0.81	0.00000000	pigs
Hectd4	0.68	0.00000000	pigs
Antxr2	0.88	0.00000000	pigs
Grn	0.58	0.00000000	pigs
Egln3	0.37	0.00000000	pigs
Batf3	0.66	0.00000000	pigs
Gla	0.46	0.00000000	pigs
Chka	0.61	0.00000000	pigs

Cmip	0.63	0.00000000	pigs
Dock10	0.93	0.00000000	pigs
Ddit4	0.66	0.00000000	pigs
Snrpd2	-0.65	0.00000000	pigs
Nsd2	0.32	0.00000000	pigs
Icmt	-0.66	0.00000000	pigs
Atp6v1a	0.55	0.00000000	pigs
Tnip3	0.77	0.00000000	pigs
Wfs1	0.30	0.00000000	pigs
Pdia3	0.51	0.00000000	pigs
Adam8	0.46	0.00000000	pigs
Igf2r	0.66	0.00000000	pigs
Slc2a6	0.47	0.00000000	pigs
Zfp292	0.49	0.00000000	pigs
Trio	0.86	0.00000000	pigs
Tnip1	0.62	0.00000000	pigs
Snx24	0.82	0.00000000	pigs
Cyp2j13	0.36	0.00000000	pigs
Pdhx	0.59	0.00000000	pigs
Dram2	0.50	0.00000000	pigs
Furin	0.52	0.00000000	pigs
Enpp4	0.44	0.00000000	pigs
Vmp1	0.74	0.00000000	pigs
Ccng2	0.42	0.00000000	pigs
Nod2	0.57	0.00000000	pigs
Ccdc170	-0.66	0.00000000	pigs
Pakap	0.57	0.00000000	pigs
Gpr35	0.24	0.00000000	pigs
Zfp804a	0.69	0.00000000	pigs
Calr	0.57	0.00000000	pigs
Comt	0.40	0.00000000	pigs
Rnf122	0.41	0.00000000	pigs
Ykt6	0.63	0.00000000	pigs
H2-Ab1	0.64	0.00000000	pigs
Bank1	-1.03	0.00000000	pigs
Il27	0.46	0.00000000	pigs
Tesk2	0.88	0.00000000	pigs
Ptpn2	0.56	0.00000000	pigs
Edem1	0.41	0.00000000	pigs
Bnip5	0.26	0.00000000	pigs
Csf3	0.90	0.00000000	pigs
Fscn1	0.48	0.00000000	pigs
Relt	0.27	0.00000000	pigs
Ostm1	0.46	0.00000000	pigs
Nfkb2	0.41	0.00000000	pigs
Aff1	0.75	0.00000000	pigs

Dennd1a	0.60	0.00000000	pigs
Arhgef10l	0.50	0.00000000	pigs
Egr1	-1.12	0.00000000	pigs
Npl	0.53	0.00000000	pigs
Asah1	0.61	0.00000000	pigs
Cdyl	0.55	0.00000000	pigs
Agps	0.30	0.00000000	pigs
Papss2	0.45	0.00000000	pigs
Epb41l2	0.66	0.00000000	pigs
B2m	0.34	0.00000000	pigs
Bnip3	0.70	0.00000000	pigs
Acp2	0.37	0.00000000	pigs
Dab2	0.37	0.00000000	pigs
Hsp90aa1	0.54	0.00000000	pigs
Capg	0.65	0.00000000	pigs
Shq1	0.39	0.00000000	pigs
Hspb1	0.56	0.00000000	pigs
Itgav	0.45	0.00000000	pigs
Socs3	0.63	0.00000000	pigs
Pdpn	0.89	0.00000000	pigs
Sik3	0.58	0.00000000	pigs
Nkg7	0.56	0.00000000	pigs
Fabp5	0.76	0.00000000	pigs
Snx6	0.62	0.00000000	pigs
N4bp1	0.65	0.00000000	pigs
Cxcr4	0.50	0.00000000	pigs
Ctsz	0.74	0.00000000	pigs
Higd1a	0.41	0.00000000	pigs
Ptges	0.61	0.00000000	pigs
Lipa	0.71	0.00000000	pigs
Abr	0.68	0.00000000	pigs
Slc22a3	0.61	0.00000000	pigs
Slc8b1	0.49	0.00000000	pigs
Cd9	-1.23	0.00000000	pigs
Ksr1	0.50	0.00000000	pigs
Cep192	0.41	0.00000000	pigs
Slc36a4	0.41	0.00000000	pigs
Slc45a4	0.34	0.00000000	pigs
Ppp1r13b	0.63	0.00000000	pigs
Dcun1d3	0.53	0.00000000	pigs
Cd300c	0.35	0.00000000	pigs
Atp6ap1	0.46	0.00000000	pigs
Ctnnb1	0.42	0.00000000	pigs
Gab2	0.66	0.00000000	pigs
Ugp2	0.51	0.00000000	pigs
Ctdsp1	-0.54	0.00000000	pigs

Phc2	0.29	0.00000000	pigs
Camsap2	0.39	0.00000000	pigs
Cyba	0.44	0.00000000	pigs
Gpr39	-0.61	0.00000000	pigs
Pacsin2	0.59	0.00000000	pigs
Slc43a2	0.50	0.00000000	pigs
Il21r	0.41	0.00000000	pigs
Lta4h	0.61	0.00000000	pigs
Slc30a5	0.46	0.00000000	pigs
Upp1	0.58	0.00000000	pigs
Slc15a3	0.41	0.00000000	pigs
Fut8	0.55	0.00000000	pigs
H2-Oa	0.39	0.00000000	pigs
Ccr5	0.27	0.00000000	pigs
Lsp1	-0.50	0.00000000	pigs
Arhgap10	0.82	0.00000000	pigs
Slc18a1	0.42	0.00000000	pigs
Ctss	0.47	0.00000000	pigs
Coq2	0.31	0.00000000	pigs
Hdac9	-0.75	0.00000000	pigs
Atp6v0b	0.47	0.00000000	pigs
Scarb2	0.73	0.00000000	pigs
Hmgb2	-0.86	0.00000000	pigs
Slc20a1	0.36	0.00000000	pigs
Rnf138	0.33	0.00000000	pigs
Cebpa	0.39	0.00000000	pigs
Prorp	0.51	0.00000000	pigs
Atp5k	-0.70	0.00000000	pigs
Snx2	0.52	0.00000000	pigs
Fam120a	0.54	0.00000000	pigs
Gnpda1	0.33	0.00000000	pigs
Rac2	-0.69	0.00000000	pigs
Laptm4a	0.39	0.00000000	pigs
Tpp1	0.31	0.00000000	pigs
Prelid2	-0.52	0.00000000	pigs
Stxbp1	0.48	0.00000000	pigs
Atp2b4	-0.85	0.00000000	pigs
Grb2	0.43	0.00000000	pigs
Rnf121	0.79	0.00000000	pigs
Il18	0.66	0.00000000	pigs
Wnk2	0.63	0.00000000	pigs
Rnd3	0.32	0.00000000	pigs
Dcbld2	0.67	0.00000000	pigs
Cry2	0.34	0.00000000	pigs
Il2ra	0.68	0.00000000	pigs
Ube2z	0.26	0.00000000	pigs

Aig1	0.32	0.00000000	pigs
Vps37c	0.23	0.00000000	pigs
Elob	-0.61	0.00000000	pigs
Snrpc	0.42	0.00000000	pigs
Rnf4	0.28	0.00000000	pigs
Arrdc3	0.44	0.00000000	pigs
Slc24a1	-0.56	0.00000000	pigs
Nfkbie	0.40	0.00000000	pigs
Tmem223	-0.70	0.00000000	pigs
Lcorl	0.40	0.00000000	pigs
Traf7	0.30	0.00000000	pigs
Galns	0.17	0.00000000	pigs
Adgre5	-0.98	0.00000000	pigs
Fam20a	0.38	0.00000000	pigs
Acvr1b	0.28	0.00000000	pigs
Sema3c	0.93	0.00000000	pigs
Clu	0.76	0.00000000	pigs
Ptprj	0.53	0.00000000	pigs
Agpat3	0.38	0.00000000	pigs
Vdr	0.37	0.00000000	pigs
P2ry6	0.20	0.00000000	pigs
Csnk1a1	0.45	0.00000000	pigs
Slc38a9	0.57	0.00000000	pigs
Avl9	0.38	0.00000000	pigs
Itgb5	0.28	0.00000000	pigs
Rybp	0.58	0.00000000	pigs
Slc3a2	0.32	0.00000000	pigs
Adap2	0.39	0.00000000	pigs
Frmd4b	0.53	0.00000000	pigs
Ywhaq	0.48	0.00000000	pigs
Syk	0.54	0.00000000	pigs
Emp3	-0.73	0.00000000	pigs
Zc3h12c	0.49	0.00000000	pigs
Tma7	-0.57	0.00000000	pigs
Actn1	0.34	0.00000000	pigs
Ctns	0.19	0.00000000	pigs
Dcaf5	0.33	0.00000000	pigs
Fmn1	0.39	0.00000000	pigs
Icam1	0.52	0.00000000	pigs
Flt1	-1.13	0.00000000	pigs
Txndc12	0.25	0.00000000	pigs
Atp1a1	0.42	0.00000000	pigs
Lman2	0.35	0.00000000	pigs
Clock	0.40	0.00000000	pigs
Neu1	0.27	0.00000000	pigs
Thyn1	0.21	0.00000000	pigs

Dnajb1	0.87	0.00000000	pigs
Pik3r1	0.69	0.00000000	pigs
Gpi1	0.44	0.00000000	pigs
Nceh1	0.31	0.00000000	pigs
Ndufb9	-0.63	0.00000000	pigs
Vwa5a	0.32	0.00000000	pigs
Zcchc14	0.45	0.00000000	pigs
Calu	0.26	0.00000000	pigs
S100a10	-0.91	0.00000000	pigs
Zmiz1	0.52	0.00000000	pigs
Lnx2	0.35	0.00000000	pigs
Cybrd1	0.43	0.00000000	pigs
B3glct	0.43	0.00000000	pigs
Pik3ap1	0.59	0.00000000	pigs
Pepd	0.25	0.00000000	pigs
Srgap1	-0.76	0.00000000	pigs
Etnk1	0.31	0.00000000	pigs
B4galt1	0.29	0.00000000	pigs
Stom	0.36	0.00000000	pigs
Tgm2	0.38	0.00000000	pigs
Kpna1	0.40	0.00000000	pigs
Tmtc2	0.51	0.00000000	pigs
Abl2	0.40	0.00000000	pigs
Rlf	0.37	0.00000000	pigs
Ppil6	-0.48	0.00000000	pigs
Ptbp2	0.31	0.00000000	pigs
Serpinb1a	-1.11	0.00000000	pigs
Cast	-0.58	0.00000000	pigs
Pxylp1	0.25	0.00000000	pigs
Fn1	-0.54	0.00000000	pigs
Zdhhc3	0.37	0.00000000	pigs
Arhgef11	0.39	0.00000000	pigs
Arrdc2	0.27	0.00000000	pigs
Tmf1	0.31	0.00000000	pigs
Ccpg1	-0.52	0.00000000	pigs
Stam	0.24	0.00000000	pigs
Tcf4	0.54	0.00000000	pigs
Rhoq	0.51	0.00000000	pigs
Aars	0.21	0.00000000	pigs
Sbno1	0.31	0.00000000	pigs
Uvrag	0.59	0.00000000	pigs
Rnf144b	-0.78	0.00000000	pigs
Zdhhc20	0.43	0.00000000	pigs
Prcp	0.47	0.00000000	pigs
Plek	-0.96	0.00000000	pigs
Tex10	0.30	0.00000000	pigs

Sh3bgrl3	-0.49	0.00000000	pigs
Kansl1	0.44	0.00000000	pigs
Tmem51	0.26	0.00000000	pigs
Psme1	-0.57	0.00000000	pigs
Ppp2r5e	0.40	0.00000000	pigs
Prpf39	0.28	0.00000000	pigs
Atp6v1c1	0.33	0.00000000	pigs
Tead3	0.25	0.00000000	pigs
Adora2a	0.27	0.00000000	pigs
Narf	0.19	0.00000000	pigs
Ulk2	0.22	0.00000000	pigs
Tcea3	0.14	0.00000000	pigs
Ltf	-1.80	0.00000000	pigs
Stim1	0.46	0.00000000	pigs
Hivep2	0.50	0.00000000	pigs
Cnot9	0.38	0.00000000	pigs
Slc7a7	0.43	0.00000000	pigs
Calhm6	0.32	0.00000000	pigs
Plod1	0.23	0.00000000	pigs
Nxt2	0.17	0.00000000	pigs
Atp11a	0.31	0.00000000	pigs
Cox7c	-0.43	0.00000000	pigs
Kdelr2	0.36	0.00000000	pigs
Arhgap18	0.47	0.00000000	pigs
Zdhhc18	0.42	0.00000000	pigs
Ncor2	0.37	0.00000000	pigs
Slc38a6	0.34	0.00000000	pigs
Ubtd2	0.35	0.00000000	pigs
Rac1	0.37	0.00000000	pigs
C4bp	-2.09	0.00000000	pigs
Smurf2	0.35	0.00000000	pigs
Vgll4	0.36	0.00000000	pigs
Rnase4	-0.77	0.00000000	pigs
Arl4c	0.40	0.00000000	pigs
Nasp	0.22	0.00000000	pigs
Smad7	0.34	0.00000000	pigs
Actb	-0.45	0.00000000	pigs
Cd81	0.37	0.00000000	pigs
Ereg	0.33	0.00000000	pigs
Lcp1	-0.70	0.00000000	pigs
F5	-0.42	0.00000000	pigs
Tcf12	0.49	0.00000000	pigs
Csf2rb2	0.42	0.00000000	pigs
Cltc	0.39	0.00000000	pigs
Pmepa1	0.27	0.00000000	pigs
Gatad2b	0.38	0.00000000	pigs

Nfkbia	0.64	0.00000000	pigs
Fam91a1	0.28	0.00000000	pigs
Pfkfb3	0.42	0.00000000	pigs
Rbm26	0.34	0.00000000	pigs
S1pr2	0.22	0.00000000	pigs
Xpo1	0.28	0.00000000	pigs
Fbxl20	0.44	0.00000000	pigs
Pde7a	0.54	0.00000000	pigs
Ppp6r3	0.33	0.00000000	pigs
Arl8a	0.30	0.00000000	pigs
Cnot2	0.35	0.00000000	pigs
Rora	0.21	0.00000000	pigs
Usp10	0.36	0.00000000	pigs
Tbc1d9	0.53	0.00000000	pigs
Creld1	0.18	0.00000000	pigs
Prkar1a	0.38	0.00000000	pigs
Ifit2	-0.76	0.00000000	pigs
Traf1	0.37	0.00000000	pigs
Slamf6	0.26	0.00000000	pigs
Cox17	-0.56	0.00000000	pigs
Smg1	0.35	0.00000000	pigs
Lrba	-0.47	0.00000000	pigs
Irf8	0.34	0.00000000	pigs
Itga2	-0.75	0.00000000	pigs
Retsat	0.20	0.00000000	pigs
Top3a	0.25	0.00000000	pigs
Tnfaip3	0.69	0.00000000	pigs
Blvrb	0.33	0.00000000	pigs
Zdhhc17	0.31	0.00000000	pigs
Vsir	-0.63	0.00000000	pigs
Canx	0.39	0.00000000	pigs
Lrch3	0.27	0.00000000	pigs
Hmgxb3	0.21	0.00000000	pigs
Trim24	0.44	0.00000000	pigs
Atp6ap2	0.36	0.00000000	pigs
Slc16a6	0.43	0.00000000	pigs
Lin54	0.27	0.00000000	pigs
Enpp2	0.36	0.00000000	pigs
Pter	0.20	0.00000000	pigs
Vps26c	0.25	0.00000000	pigs
Gapvd1	0.31	0.00000000	pigs
Hint3	0.19	0.00000000	pigs
Alas1	0.26	0.00000000	pigs
Galm	0.24	0.00000000	pigs
App	0.54	0.00000000	pigs
1810037I17Rik	0.37	0.00000000	pigs

Rnf19b	0.68	0.00000000	pigs
Il23r	0.22	0.00000000	pigs
Szrd1	0.29	0.00000000	pigs
Slc39a1	0.19	0.00000000	pigs
Bag3	0.47	0.00000000	pigs
Trp53bp2	0.20	0.00000000	pigs
Plxnd1	0.24	0.00000000	pigs
Piezo1	0.28	0.00000000	pigs
Car4	0.39	0.00000000	pigs
Abhd12	0.37	0.00000000	pigs
Ewsr1	0.30	0.00000000	pigs
Pik3r5	0.38	0.00000000	pigs
Runx3	0.34	0.00000000	pigs
Aff4	0.37	0.00000000	pigs
Rftn1	0.64	0.00000000	pigs
Siah1b	0.25	0.00000000	pigs
Il4i1	0.20	0.00000000	pigs
Prdx5	0.28	0.00000000	pigs
Cd274	0.32	0.00000000	pigs
Tfec	0.46	0.00000000	pigs
Tmem268	0.40	0.00000000	pigs
Wwox	0.46	0.00000000	pigs
Cyb561a3	0.21	0.00000000	pigs
Golga7	0.25	0.00000000	pigs
Kpna6	0.23	0.00000000	pigs
Cept1	0.25	0.00000000	pigs
Frmd4a	0.47	0.00000000	pigs
Zfyve9	-0.61	0.00000000	pigs
Bcat2	0.31	0.00000000	pigs
Slc35f6	0.16	0.00000000	pigs
2510039O18Rik	0.18	0.00000000	pigs
St3gal4	0.17	0.00000000	pigs
Vta1	0.29	0.00000000	pigs
P4hb	0.31	0.00000000	pigs
Pum2	0.37	0.00000000	pigs
Rab1a	0.30	0.00000000	pigs
Plagl1	0.37	0.00000000	pigs
Gna13	0.37	0.00000000	pigs
Ropn1l	-0.61	0.00000000	pigs
Pan3	0.38	0.00000000	pigs
Grk3	0.37	0.00000000	pigs
Golm1	0.22	0.00000000	pigs
Acaca	0.24	0.00000000	pigs
Stmn1	0.24	0.00000000	pigs
Reps1	0.36	0.00000000	pigs
Cubn	-0.38	0.00000000	pigs

Swap70	0.29	0.00000000	pigs
Zfp90	0.16	0.00000000	pigs
Gpr108	0.18	0.00000000	pigs
Hspa9	0.34	0.00000000	pigs
Bmp2k	0.45	0.00000000	pigs
Bri3	0.34	0.00000000	pigs
Noct	-0.86	0.00000000	pigs
Patl1	0.29	0.00000000	pigs
Zfp644	0.57	0.00000000	pigs
Adgrl2	0.69	0.00000000	pigs
Tmem263	0.28	0.00000000	pigs
Tab2	0.28	0.00000000	pigs
Slc4a7	0.17	0.00000000	pigs
Plcb1	-0.74	0.00000000	pigs
Stat5a	0.23	0.00000000	pigs
Jak1	0.35	0.00000000	pigs
Areg	0.92	0.00000000	pigs
Dusp2	-0.85	0.00000000	pigs
Washc5	0.37	0.00000000	pigs
Tcerg1	0.28	0.00000000	pigs
Prkn	-0.41	0.00000000	pigs
Tcirg1	0.15	0.00000000	pigs
Acer3	0.41	0.00000000	pigs
Pptc7	0.22	0.00000000	pigs
Usp3	0.35	0.00000000	pigs
Arf6	-0.55	0.00000000	pigs
Tmem129	0.23	0.00000000	pigs
Zfyve21	0.20	0.00000000	pigs
Prxl2a	0.00	0.00000000	pigs
Baz1b	0.30	0.00000000	pigs
Srgn	0.41	0.00000000	pigs
Rab5c	0.35	0.00000000	pigs
Tollip	0.23	0.00000000	pigs
Hps4	0.15	0.00000000	pigs
Vash1	0.23	0.00000000	pigs
Tyrobp	-0.61	0.00000000	pigs
Nr6a1	0.27	0.00000000	pigs
Galc	0.18	0.00000000	pigs
Nup98	0.19	0.00000000	pigs
Rhoh	0.40	0.00000000	pigs
Zfp263	0.19	0.00000000	pigs
Arid4b	0.45	0.00000000	pigs
Setd5	0.34	0.00000000	pigs
Tmem131l	0.51	0.00000000	pigs
Ipo9	0.24	0.00000000	pigs
Dusp16	0.67	0.00000000	pigs

Fnip1	0.41	0.00000000	pigs
Ano10	-0.49	0.00000000	pigs
Ppp1cc	0.30	0.00000000	pigs
Qser1	0.25	0.00000000	pigs
Rel	0.40	0.00000000	pigs
Stt3b	0.27	0.00000000	pigs
Tbc1d2b	0.45	0.00000000	pigs
Hif1a	0.42	0.00000000	pigs
Pdia4	0.41	0.00000000	pigs
Ppcs	0.27	0.00000000	pigs
Bcl2l1	0.18	0.00000000	pigs
Irf2bp2	0.29	0.00000000	pigs
Pcnx	0.28	0.00000000	pigs
Gpbp1	0.32	0.00000000	pigs
Crtc3	0.37	0.00000000	pigs
Stx3	0.28	0.00000000	pigs
Kdm2a	0.32	0.00000000	pigs
Tbc1d23	0.22	0.00000000	pigs
Foxk2	0.20	0.00000000	pigs
Spag9	0.38	0.00000000	pigs
Rbms1	0.52	0.00000000	pigs
Emsy	0.23	0.00000000	pigs
Serf2	-0.36	0.00000000	pigs
Alox5ap	-0.78	0.00000000	pigs
Slc19a2	0.34	0.00000000	pigs
Zzz3	0.29	0.00000000	pigs
Amfr	0.16	0.00000000	pigs
Tbc1d15	0.32	0.00000000	pigs
Plbd2	0.24	0.00000000	pigs
Smarca4	0.25	0.00000000	pigs
Il4ra	0.36	0.00000000	pigs
Sag	-0.37	0.00000000	pigs
Glrx3	0.28	0.00000000	pigs
Cfl1	0.40	0.00000000	pigs
Ap3d1	0.24	0.00000000	pigs
Ddx39a	0.17	0.00000000	pigs
Rab40b	0.21	0.00000000	pigs
Tpk1	0.22	0.00000000	pigs
Rab7b	0.33	0.00000000	pigs
Acsf2	0.22	0.00000000	pigs
Prrg4	0.20	0.00000000	pigs
Cecr2	0.20	0.00000000	pigs
Relch	0.35	0.00000000	pigs
Pdxdc1	0.19	0.00000000	pigs
Ghitm	0.25	0.00000000	pigs
Ddx31	0.41	0.00000000	pigs

Kdm3a	0.29	0.00000000	pigs
Rpp30	0.23	0.00000000	pigs
Slc30a7	0.27	0.00000000	pigs
Cep85l	0.17	0.00000000	pigs
Elk3	0.23	0.00000000	pigs
Ccdc167	0.21	0.00000000	pigs
Ero1a	0.33	0.00000000	pigs
Gpr85	0.22	0.00000000	pigs
Ube2k	0.30	0.00000000	pigs
Ncoa4	0.33	0.00000000	pigs
Tom1	0.21	0.00000000	pigs
Stat3	0.26	0.00000000	pigs
Efr3a	0.28	0.00000000	pigs
Rab9	0.28	0.00000000	pigs
Kpna3	0.25	0.00000000	pigs
Pxn	-0.51	0.00000000	pigs
Plekha5	-0.43	0.00000000	pigs
Trib1	-0.54	0.00000000	pigs
Dennd11	-0.78	0.00000000	pigs
Chordc1	0.29	0.00000000	pigs
Cbfa2t2	0.22	0.00000000	pigs
Fosl2	0.25	0.00000000	pigs
Gna12	0.24	0.00000000	pigs
Braf	0.40	0.00000000	pigs
Ramp2	-0.06	0.00000000	pigs
Snx12	0.19	0.00000000	pigs
Sdhd	0.30	0.00000000	pigs
Psmd4	0.38	0.00000000	pigs
Fam168b	0.26	0.00000000	pigs
Etv3	0.36	0.00000000	pigs
Vcp	0.28	0.00000000	pigs
Nampt	0.43	0.00000000	pigs
Ctdspl2	0.26	0.00000000	pigs
Atp6v0d1	0.24	0.00000000	pigs
Slc16a3	0.28	0.00000000	pigs
Snrnp48	0.17	0.00000000	pigs
Ptprc	-0.58	0.00000000	pigs
Pias2	0.24	0.00000000	pigs
Slco3a1	-0.48	0.00000000	pigs
Tsc1	0.09	0.00000000	pigs
Exosc7	0.15	0.00000000	pigs
Csnk1d	0.22	0.00000000	pigs
Renbp	0.13	0.00000000	pigs
Ubxn11	-0.33	0.00000000	pigs
Fuca1	0.19	0.00000000	pigs
Ogfrl1	0.31	0.00000000	pigs

Rad21	0.17	0.00000000	pigs
Zfp592	0.19	0.00000000	pigs
Sgk1	-0.86	0.00000000	pigs
Aco2	0.27	0.00000000	pigs
Pnrc2	0.20	0.00000000	pigs
Tsku	0.13	0.00000000	pigs
Il17c	0.08	0.00000000	pigs
Eed	0.21	0.00000000	pigs
Ube2d3	0.30	0.00000000	pigs
Slc66a2	0.34	0.00000000	pigs
Ptpn1	0.46	0.00000000	pigs
Lamp1	0.31	0.00000000	pigs
Commd4	0.31	0.00000000	pigs
Cdk8	0.18	0.00000000	pigs
Spen	0.34	0.00000000	pigs
Polb	0.31	0.00000000	pigs
Lsm14a	0.29	0.00000000	pigs
Abcg1	0.23	0.00000000	pigs
H1f3	0.46	0.00000000	pigs
Frs2	0.25	0.00000000	pigs
Rab22a	0.21	0.00000000	pigs
Trim33	0.22	0.00000000	pigs
Prdx3	0.27	0.00000000	pigs
Sestd1	0.33	0.00000000	pigs
Golt1b	0.18	0.00000000	pigs
Psmd3	0.21	0.00000000	pigs
Aldh3a2	0.20	0.00000000	pigs
Tmem184b	0.13	0.00000000	pigs
Ndel1	0.37	0.00000000	pigs
Sash1	0.20	0.00000000	pigs
Ada	0.17	0.00000000	pigs
Dmxl1	0.34	0.00000000	pigs
Slc36a1	0.18	0.00000000	pigs
Nt5dc2	0.18	0.00000000	pigs
Bud23	0.25	0.00000000	pigs
Polr2g	0.26	0.00000000	pigs
Fnip2	0.22	0.00000000	pigs
Dusp11	0.20	0.00000000	pigs
Ikbke	0.22	0.00000000	pigs
Nck2	0.39	0.00000000	pigs
Ammecr1	0.35	0.00000000	pigs
Ptpn9	0.36	0.00000000	pigs
Acadm	0.38	0.00000000	pigs
Slc25a36	0.26	0.00000000	pigs
Smarcc1	0.26	0.00000000	pigs
Cse1l	0.20	0.00000000	pigs

Tnpo3	0.31	0.00000000	pigs
Rnf145	0.20	0.00000000	pigs
Tmem144	0.13	0.00000000	pigs
Atp5j	-0.38	0.00000000	pigs
Calcrl	-0.58	0.00000000	pigs
Csf1r	0.36	0.00000000	pigs
Gpx1	-0.65	0.00000000	pigs
Jpt2	0.11	0.00000000	pigs
Tmem251	0.23	0.00000000	pigs
Flii	0.23	0.00000000	pigs
Lix1l	0.15	0.00000000	pigs
Jag1	0.25	0.00000000	pigs
Fubp1	0.22	0.00000000	pigs
Fbxo42	0.15	0.00000000	pigs
Psmc2	0.21	0.00000000	pigs
Gab3	0.14	0.00000000	pigs
Cracdl	0.47	0.00000000	pigs
Ctbp2	0.21	0.00000000	pigs
Stk17b	-0.56	0.00000000	pigs
Cert1	0.46	0.00000000	pigs
Tti2	0.26	0.00000000	pigs
Eif3h	-0.41	0.00000000	pigs
Inpp4b	0.39	0.00000000	pigs
Clcn7	0.20	0.00000000	pigs
Tcf20	0.18	0.00000000	pigs
Armc9	0.11	0.00000000	pigs
Grina	0.09	0.00000000	pigs
Ssbp3	0.39	0.00000000	pigs
Abtb2	0.77	0.00000000	pigs
Ehd1	0.25	0.00000000	pigs
Cdc27	0.21	0.00000000	pigs
Tnfaip2	0.29	0.00000000	pigs
Cxcl10	0.59	0.00000000	pigs
Ranbp2	0.31	0.00000000	pigs
Gfpt1	0.21	0.00000000	pigs
Slc39a6	0.18	0.00000000	pigs
Ifnar1	0.28	0.00000000	pigs
Larp4	0.24	0.00000000	pigs
Dync1li1	0.30	0.00000000	pigs
Rab3gap2	0.19	0.00000000	pigs
Arid3a	0.19	0.00000000	pigs
Arcn1	0.31	0.00000000	pigs
Trip10	0.15	0.00000000	pigs
Tnip2	0.19	0.00000000	pigs
Kif13a	-0.51	0.00000000	pigs
Ss18	0.29	0.00000000	pigs

Rnf169	0.42	0.00000000	pigs
Btg3	0.24	0.00000000	pigs
Ell	0.23	0.00000000	pigs
Pgm2	0.33	0.00000000	pigs
Rc3h2	0.18	0.00000000	pigs
Cacna1e	0.23	0.00000000	pigs
Lbr	-0.58	0.00000000	pigs
Ndufs1	0.20	0.00000000	pigs
Ncoa6	0.17	0.00000000	pigs
Sphk1	0.11	0.00000000	pigs
Sugp1	0.15	0.00000000	pigs
Gigyf2	0.26	0.00000000	pigs
Tmem19	0.18	0.00000000	pigs
Rhbdf2	0.30	0.00000000	pigs
Stard3nl	0.19	0.00000000	pigs
Nhlrc3	0.23	0.00000000	pigs
Ube2a	0.18	0.00000000	pigs
Zc3h14	0.21	0.00000000	pigs
Dock4	0.40	0.00000000	pigs
Fam193a	0.22	0.00000000	pigs
Brd4	0.30	0.00000000	pigs
Ebpl	0.19	0.00000000	pigs
Arl15	-0.91	0.00000000	pigs
Acbd3	0.24	0.00000000	pigs
Luzp1	0.24	0.00000000	pigs
Steap3	0.15	0.00000000	pigs
Ilrun	0.32	0.00000000	pigs
Vps37a	0.22	0.00000000	pigs
Sell	-0.84	0.00000000	pigs
Add3	-0.27	0.00000000	pigs
Cpsf6	1.39	0.00000000	pigs
Wdfy1	0.32	0.00000000	pigs
Stk3	0.36	0.00000000	pigs
Uba3	0.14	0.00000000	pigs
Utp6	0.23	0.00000000	pigs
Lrrc41	0.12	0.00000000	pigs
Peak1	0.28	0.00000000	pigs
Csf3r	0.32	0.00000000	pigs
Tnf	-0.53	0.00000000	pigs
Alkbh5	0.19	0.00000000	pigs
Apbb1ip	-0.54	0.00000000	pigs
Cox4i1	-0.28	0.00000000	pigs
Scaf8	0.18	0.00000000	pigs
Bcl2a1d	0.42	0.00000000	pigs
Xylt1	0.37	0.00000000	pigs
Klhl21	0.16	0.00000000	pigs

Pign	0.31	0.00000000	pigs
Usp12	0.19	0.00000000	pigs
Ankrd11	0.34	0.00000000	pigs
Bag2	0.13	0.00000000	pigs
Sema4a	0.20	0.00000000	pigs
Surf4	0.18	0.00000000	pigs
Il23a	0.83	0.00000000	pigs
Npc1	0.36	0.00000000	pigs
Ppfibp2	0.25	0.00000000	pigs
Ranbp3	0.14	0.00000000	pigs
Hyou1	0.19	0.00000000	pigs
Sptlc1	0.14	0.00000000	pigs
Topors	0.25	0.00000000	pigs
Atl2	0.17	0.00000000	pigs
Lrp8	0.14	0.00000000	pigs
Sec31a	0.35	0.00000000	pigs
Rab20	0.28	0.00000000	pigs
Xpo7	0.21	0.00000000	pigs
Appbp2	0.24	0.00000000	pigs
Ackr3	0.28	0.00000000	pigs
Ifitm6	-0.91	0.00000000	pigs
Gpr107	0.15	0.00000000	pigs
Orc4	0.23	0.00000000	pigs
Ppp2r5c	0.25	0.00000000	pigs
Trp53i11	-0.46	0.00000000	pigs
Trrap	0.17	0.00000000	pigs
Secisbp2l	0.16	0.00000000	pigs
Atp5md	-0.53	0.00000000	pigs
Bmx	-0.23	0.00000000	pigs
Retreg1	-0.37	0.00000000	pigs
Snx14	0.21	0.00000000	pigs
Ppp1cb	0.24	0.00000000	pigs
Zfp516	0.20	0.00000000	pigs
Arid2	0.39	0.00000000	pigs
Ppp3ca	-0.88	0.00000000	pigs
Tia1	0.16	0.00000000	pigs
Safb	0.18	0.00000000	pigs
Slc25a19	0.24	0.00000000	pigs
Pknox1	0.16	0.00000000	pigs
Ski	0.11	0.00000000	pigs
Dtx2	0.12	0.00000000	pigs
Abca3	0.15	0.00000000	pigs
Gosr1	0.21	0.00000000	pigs
Nemp2	0.13	0.00000000	pigs
Serinc3	0.14	0.00000000	pigs
Stx6	0.11	0.00000000	pigs

Psmg3	0.17	0.00000000	pigs
Cd24a	-1.21	0.00000000	pigs
Rbm18	0.26	0.00000000	pigs
Zfp148	0.25	0.00000000	pigs
Xab2	0.12	0.00000000	pigs
Poc1b	0.25	0.00000000	pigs
Sec62	-0.45	0.00000000	pigs
Hbb-bh2	-0.61	0.00000000	pigs
Hsd17b11	0.17	0.00000000	pigs
Ftsj1	0.14	0.00000000	pigs
Esd	-0.60	0.00000000	pigs
Dnpep	0.04	0.00000000	pigs
Tnpo1	0.20	0.00000000	pigs
Stx4a	0.17	0.00000000	pigs
Nfam1	0.27	0.00000000	pigs
Utrn	-0.52	0.00000000	pigs
Oaz1	-0.33	0.00000000	pigs
Gtf2h1	0.27	0.00000000	pigs
Ubash3b	0.22	0.00000000	pigs
Nck1	0.33	0.00000000	pigs
Tbc1d12	0.11	0.00000000	pigs
Arpc5	0.37	0.00000000	pigs
Pecr	0.12	0.00000000	pigs
Slc26a2	0.14	0.00000000	pigs
Cdk4	0.20	0.00000000	pigs
Pea15a	0.23	0.00000000	pigs
Dennd4c	0.27	0.00000000	pigs
Tnfrsf21	0.36	0.00000000	pigs
Ambra1	0.31	0.00000000	pigs
Vps11	0.14	0.00000000	pigs
Fpgs	-0.40	0.00000000	pigs
Cyth4	0.26	0.00000000	pigs
Tm6sf1	-0.35	0.00000000	pigs
Gusb	0.20	0.00000000	pigs
Adamts2	-0.37	0.00000000	pigs
Snai1	0.24	0.00000000	pigs
Yipf5	0.14	0.00000000	pigs
Ddhd1	0.18	0.00000000	pigs
Gga2	0.22	0.00000000	pigs
Akirin1	0.11	0.00000000	pigs
Commd8	0.20	0.00000000	pigs
Arhgap24	-0.66	0.00000000	pigs
Hacd3	0.13	0.00000000	pigs
Osbpl1a	0.26	0.00000000	pigs
Htr7	0.47	0.00000000	pigs
Ap1s1	0.18	0.00000000	pigs

Lmnb2	0.05	0.00000000	pigs
Slc43a3	0.15	0.00000000	pigs
Ip6k1	0.18	0.00000000	pigs
Nf1	0.32	0.00000000	pigs
Arid5a	0.19	0.00000000	pigs
Iqgap1	-0.48	0.00000000	pigs
Galnt7	0.34	0.00000000	pigs
Drg1	0.15	0.00000000	pigs
Ebi3	0.11	0.00000000	pigs
Prkx	0.13	0.00000000	pigs
Atp6v1h	0.15	0.00000000	pigs
Il15	-0.41	0.00000000	pigs
Phc3	0.19	0.00000000	pigs
Sec14l1	0.21	0.00000000	pigs
Cops4	0.17	0.00000000	pigs
Tmem33	0.18	0.00000000	pigs
Prkce	0.30	0.00000000	pigs
Pld3	0.23	0.00000000	pigs
Commd10	0.23	0.00000000	pigs
Tfip11	0.12	0.00000000	pigs
Dele1	0.16	0.00000000	pigs
Cnot4	0.30	0.00000000	pigs
A4galt	0.31	0.00000000	pigs
Usp37	0.27	0.00000000	pigs
Hdac4	0.30	0.00000000	pigs
Osbpl10	-0.47	0.00000000	pigs
Katnbl1	0.18	0.00000000	pigs
Rap1gap2	-0.52	0.00000000	pigs
9530068E07Rik	0.11	0.00000000	pigs
C1qa	0.05	0.00000000	pigs
Gch1	0.10	0.00000000	pigs
Cyfip2	-0.46	0.00000000	pigs
Stat4	0.32	0.00000000	pigs
Clptm1	0.15	0.00000000	pigs
Pcbd1	0.06	0.00000000	pigs
Ubr5	0.24	0.00000000	pigs
Nrf1	0.27	0.00000000	pigs
Pim3	0.14	0.00000000	pigs
Pfkl	0.12	0.00000000	pigs
Sumf1	0.15	0.00000000	pigs
Etl4	-0.79	0.00000000	pigs
Slamf1	0.19	0.00000000	pigs
Kdm5b	0.20	0.00000000	pigs
Ehbp1l1	0.22	0.00000000	pigs
Adora2b	0.21	0.00000000	pigs
Pnpla6	-0.15	0.00000000	pigs

Ngly1	0.25	0.00000000	pigs
Zfand6	0.31	0.00000000	pigs
Srebf1	0.14	0.00000000	pigs
Cpq	0.17	0.00000000	pigs
Hexb	0.21	0.00000000	pigs
Sppl3	0.26	0.00000000	pigs
Herc6	-0.73	0.00000000	pigs
Ptpn11	0.22	0.00000000	pigs
Elovl5	0.14	0.00000000	pigs
Gas2l3	0.18	0.00000000	pigs
Ankrd46	0.15	0.00000000	pigs
Cln3	0.14	0.00000000	pigs
Ecm1	0.33	0.00000000	pigs
Slc39a9	0.15	0.00000000	pigs
Ndst1	0.10	0.00000000	pigs
Zfp654	0.20	0.00000000	pigs
Copb1	0.19	0.00000000	pigs
Prex1	0.40	0.00000000	pigs
Ttc3	0.18	0.00000000	pigs
Jdp2	-0.60	0.00000000	pigs
Kbtbd2	0.14	0.00000000	pigs
Cul2	0.18	0.00000000	pigs
Cct3	0.27	0.00000000	pigs
Tomm7	-0.63	0.00000000	pigs
Pwwp2a	0.14	0.00000000	pigs
Herc4	0.29	0.00000000	pigs
Kat7	0.16	0.00000000	pigs
Zfyve26	0.16	0.00000000	pigs
1700025G04Rik	0.26	0.00000000	pigs
Adam28	0.14	0.00000000	pigs
Stk35	0.11	0.00000000	pigs
Dnajb9	0.25	0.00000000	pigs
Brwd3	0.18	0.00000000	pigs
Hnrnpa0	0.19	0.00000000	pigs
Wdr20	0.14	0.00000000	pigs
Eml4	0.30	0.00000000	pigs
Sos1	0.31	0.00000000	pigs
Slc38a7	0.10	0.00000000	pigs
Sf3b3	0.18	0.00000000	pigs
Tmem189	0.20	0.00000000	pigs
Fes	0.19	0.00000000	pigs
Sbno2	0.17	0.00000000	pigs
Etfa	0.21	0.00000000	pigs
Papolg	0.08	0.00000000	pigs
Ffar2	0.10	0.00000000	pigs
Hnrnpu	0.29	0.00000000	pigs

Hbp1	0.16	0.00000000	pigs
P2rx4	0.15	0.00000000	pigs
Tifa	0.14	0.00000000	pigs
Cct8	0.17	0.00000000	pigs
Acsl4	-0.89	0.00000000	pigs
Nup214	0.12	0.00000000	pigs
Tmed7	0.16	0.00000000	pigs
Zfp410	0.16	0.00000000	pigs
Dip2b	0.33	0.00000000	pigs
Cdkn1b	0.31	0.00000000	pigs
Tpcn1	0.11	0.00000000	pigs
H6pd	0.11	0.00000000	pigs
Tm2d3	0.16	0.00000000	pigs
Cybb	0.33	0.00000000	pigs
Ccs	0.19	0.00000000	pigs
Pum1	0.24	0.00000000	pigs
Panx1	0.12	0.00000000	pigs
Nme1	0.25	0.00000000	pigs
E2f4	0.19	0.00000000	pigs
Cacybp	0.29	0.00000000	pigs
Parp3	0.15	0.00000000	pigs
Iws1	0.18	0.00000000	pigs
Ppm1k	0.23	0.00000000	pigs
Bicd2	0.09	0.00000000	pigs
Atg16l1	0.11	0.00000000	pigs
Eps15	0.32	0.00000000	pigs
Smad4	0.22	0.00000000	pigs
Phip	0.16	0.00000000	pigs
Slc66a1	0.12	0.00000000	pigs
Yif1b	0.14	0.00000000	pigs
Srpk1	0.22	0.00000000	pigs
Srrt	0.15	0.00000000	pigs
Dock8	-0.48	0.00000000	pigs
Rhoc	0.18	0.00000000	pigs
Vps39	0.19	0.00000000	pigs
Smad5	0.12	0.00000000	pigs
Setd7	0.15	0.00000000	pigs
Smcr8	0.15	0.00000000	pigs
Pgs1	0.17	0.00000000	pigs
Psmb10	0.22	0.00000000	pigs
Scd2	0.19	0.00000000	pigs
Cd300lb	-0.20	0.00000000	pigs
Pim2	0.11	0.00000000	pigs
Plaat3	0.21	0.00000000	pigs
Rnf125	0.09	0.00000000	pigs
Deptor	0.21	0.00000000	pigs

Septin11	-0.48	0.00000000	pigs
Uso1	0.21	0.00000000	pigs
Lancl2	0.22	0.00000000	pigs
Anxa11	0.16	0.00000000	pigs
Cog6	0.17	0.00000000	pigs
Psmb7	0.19	0.00000000	pigs
Strn3	0.23	0.00000000	pigs
Gpatch3	0.14	0.00000000	pigs
Urm1	0.21	0.00000000	pigs
Smarcb1	0.04	0.00000000	pigs
Slc38a10	0.12	0.00000000	pigs
Vps35l	0.20	0.00000000	pigs
Tmem127	0.13	0.00000000	pigs
Dlg5	0.06	0.00000000	pigs
Itgb7	-0.32	0.00000000	pigs
Gucy2c	-1.55	0.00000000	pigs
Galk2	0.23	0.00000000	pigs
Grhpr	0.06	0.00000000	pigs
Arsa	0.09	0.00000000	pigs
Exoc5	0.16	0.00000000	pigs
Klhdc4	0.09	0.00000000	pigs
Tmem176a	0.21	0.00000000	pigs
Ttll1	0.03	0.00000000	pigs
Cep43	0.08	0.00000000	pigs
Laptm4b	0.11	0.00000000	pigs
Tmem181a	0.12	0.00000000	pigs
Axin1	0.15	0.00000000	pigs
Neto2	-0.50	0.00000000	pigs
Pspc1	0.29	0.00000000	pigs
Cd180	0.08	0.00000000	pigs
Rad51b	0.42	0.00000000	pigs
Trim21	0.12	0.00000000	pigs
Slc16a14	0.09	0.00000000	pigs
Eif4enif1	0.07	0.00000000	pigs
Il27ra	0.05	0.00000000	pigs
Nckipsd	0.12	0.00000000	pigs
Akap8l	0.12	0.00000000	pigs
Dyrk1a	0.25	0.00000000	pigs
Retreg2	0.08	0.00000000	pigs
Ap1b1	0.10	0.00000000	pigs
Rilpl2	0.30	0.00000000	pigs
Pla2g7	0.42	0.00000000	pigs
Ddb1	0.08	0.00000000	pigs
Tent2	0.19	0.00000000	pigs
Dync1h1	0.20	0.00000000	pigs
Ubr3	0.25	0.00000000	pigs

Pparg	0.12	0.00000000	pigs
Rsrp1	0.13	0.00000000	pigs
Arfgef2	0.17	0.00000000	pigs
Rgs14	-0.18	0.00000000	pigs
2410004B18Rik	0.18	0.00000000	pigs
Zswim8	0.11	0.00000000	pigs
Ccnd3	-0.52	0.00000000	pigs
Dcp1a	0.17	0.00000000	pigs
Lpp	0.29	0.00000000	pigs
Ap1ar	0.36	0.00000000	pigs
G2e3	0.16	0.00000000	pigs
B4galt5	0.47	0.00000000	pigs
Gnpda2	0.15	0.00000000	pigs
Npepps	0.18	0.00000000	pigs
Creb3	0.13	0.00000000	pigs
Kcnip1	0.27	0.00000000	pigs
Clec16a	0.13	0.00000000	pigs
Nemf	0.13	0.00000000	pigs
Qk	0.25	0.00000000	pigs
Lax1	-0.32	0.00000000	pigs
Per1	0.17	0.00000000	pigs
Stk24	0.26	0.00000000	pigs
Nt5dc1	-0.23	0.00000000	pigs
Fuca2	0.15	0.00000000	pigs
Ankhd1	0.31	0.00000000	pigs
Rfwd3	0.18	0.00000000	pigs
Nr2c2	0.19	0.00000000	pigs
Hk2	0.06	0.00000000	pigs
Sec23ip	0.12	0.00000000	pigs
Rprd2	0.18	0.00000000	pigs
Chst3	0.16	0.00000000	pigs
Gpm6b	0.30	0.00000000	pigs
Xpnpep1	0.14	0.00000000	pigs
Clcn5	0.30	0.00000000	pigs
Plod3	0.14	0.00000000	pigs
Ino80c	0.16	0.00000000	pigs
Usf1	0.16	0.00000000	pigs
Ddx54	0.11	0.00000000	pigs
Cebpg	0.08	0.00000000	pigs
Ric1	0.27	0.00000000	pigs
Ankib1	0.18	0.00000000	pigs
Ubqln1	0.18	0.00000000	pigs
Ywhah	0.10	0.00000000	pigs
Slc9a7	0.38	0.00000000	pigs
Kcnq1	0.14	0.00000000	pigs
Zc3h7a	0.16	0.00000000	pigs

Pafah1b2	0.15	0.00000000	pigs
Aplp2	0.15	0.00000000	pigs
Tnrc6b	-0.44	0.00000000	pigs
Zfr	0.18	0.00000000	pigs
Armcx3	0.11	0.00000000	pigs
Slc46a3	-0.28	0.00000000	pigs
Ppp1r37	0.07	0.00000000	pigs
Trnau1ap	0.10	0.00000000	pigs
Zeb2	0.39	0.00000000	pigs
Raf1	0.24	0.00000000	pigs
Tpt1	-0.29	0.00000000	pigs
Rtcb	0.16	0.00000000	pigs
Wdr36	0.15	0.00000000	pigs
Prpf40a	0.22	0.00000000	pigs
Smox	0.18	0.00000000	pigs
Runx1	0.42	0.00000000	pigs
Rpn2	0.16	0.00000000	pigs
Crebzf	0.09	0.00000000	pigs
Dcaf1	0.15	0.00000000	pigs
Tlk2	0.14	0.00000000	pigs
Kctd20	0.18	0.00000000	pigs
Sec13	0.19	0.00000000	pigs
Ppp2cb	0.07	0.00000000	pigs
Ankle2	0.09	0.00000000	pigs
Cd36	0.09	0.00000000	pigs
Otulinl	-0.50	0.00000000	pigs
Usp8	0.19	0.00000000	pigs
Orai2	0.09	0.00000000	pigs
Sae1	0.17	0.00000000	pigs
Lmbrd1	0.14	0.00000000	pigs
Rbm25	0.23	0.00000000	pigs
Slc31a2	0.17	0.00000000	pigs
Pkn2	0.25	0.00000000	pigs
Idua	0.10	0.00000000	pigs
Hspa8	0.33	0.00000000	pigs
Hmgxb4	0.14	0.00000000	pigs
Pdgfc	-0.56	0.00000000	pigs
Atxn2	0.22	0.00000000	pigs
Axl	0.19	0.00000000	pigs
Pdpk1	0.15	0.00000000	pigs
Ube2o	0.13	0.00000000	pigs
Entpd7	0.17	0.00000000	pigs
Cpt1c	-0.27	0.00000000	pigs
Pias1	0.28	0.00000000	pigs
Pou2f1	0.20	0.00000000	pigs
Osbpl3	0.62	0.00000000	pigs

Tm7sf3	0.08	0.00000000	pigs
Rab6a	0.18	0.00000000	pigs
Dvl3	0.13	0.00000000	pigs
Saraf	0.07	0.00000000	pigs
Acvr1	0.07	0.00000000	pigs
Cep95	0.14	0.00000000	pigs
Fam227b	-0.36	0.00000000	pigs
Snx3	0.27	0.00000000	pigs
Cemip2	-0.62	0.00000000	pigs
Ctdnep1	0.05	0.00000000	pigs
Stam2	0.18	0.00000000	pigs
Arl6ip5	0.07	0.00000000	pigs
Retreg3	0.10	0.00000000	pigs
Ccnh	0.11	0.00000000	pigs
Wipi2	0.09	0.00000000	pigs
Psmb4	0.27	0.00000000	pigs
Ubap2l	0.15	0.00000000	pigs
Smurf1	0.20	0.00000000	pigs
Plcg2	0.24	0.00000000	pigs
Cnot7	0.12	0.00000000	pigs
Hspd1	0.23	0.00000000	pigs
Iqgap2	0.37	0.00000000	pigs
Wdr74	0.20	0.00000000	pigs
Il10	0.27	0.00000000	pigs
Nop56	0.11	0.00000000	pigs
Fam53c	0.09	0.00000000	pigs
Acss1	0.17	0.00000000	pigs
Hmox1	0.16	0.00000000	pigs
Umps	0.14	0.00000000	pigs
Sap130	0.13	0.00000000	pigs
Ola1	0.24	0.00000000	pigs
Fnta	0.15	0.00000000	pigs
Gnb5	0.23	0.00000000	pigs
G0s2	0.00	0.00000000	pigs
Zfp236	0.20	0.00000000	pigs
Fubp3	0.13	0.00000000	pigs
Slc35a3	0.13	0.00000000	pigs
Kansl3	0.12	0.00000000	pigs
Soga1	0.09	0.00000000	pigs
Hspa5	0.32	0.00000000	pigs
Eif3l	0.20	0.00000000	pigs
Gnb1	0.20	0.00000000	pigs
Tmem35b	0.14	0.00000000	pigs
Cdc42ep2	0.15	0.00000000	pigs
Cnot1	0.25	0.00000000	pigs
Jade1	-0.82	0.00000000	pigs

Slc26a11	0.06	0.00000000	pigs
Chchd5	0.17	0.00000000	pigs
Ppp4c	0.20	0.00000000	pigs
Gsk3b	0.27	0.00000000	pigs
Psmc6	0.21	0.00000000	pigs
Pgm3	0.10	0.00000000	pigs
Tardbp	0.03	0.00000000	pigs
Phldb1	0.03	0.00000000	pigs
Spg20	0.13	0.00000000	pigs
Cox8a	-0.31	0.00000000	pigs
Oser1	0.12	0.00000000	pigs
Chuk	0.05	0.00000000	pigs
Ireb2	0.13	0.00000000	pigs
Wdr48	0.09	0.00000000	pigs
Bin1	0.14	0.00000000	pigs
Stx7	0.23	0.00000000	pigs
Arrdc4	0.36	0.00000000	pigs
Zfyve1	0.11	0.00000000	pigs
Zhx1	0.14	0.00000000	pigs
Fyttd1	0.12	0.00000000	pigs
Gle1	0.08	0.00000000	pigs
Trap1	0.10	0.00000000	pigs
Dars	0.22	0.00000000	pigs
Calm3	0.31	0.00000000	pigs
Sdcbp	0.24	0.00000000	pigs
Atxn7l1	0.24	0.00000000	pigs
Acsl3	0.10	0.00000000	pigs
Ncoa1	0.28	0.00000000	pigs
Ppm1h	0.36	0.00000000	pigs
Havcr2	0.18	0.00000000	pigs
Scamp1	0.12	0.00000000	pigs
Podn	0.38	0.00000000	pigs
Tbc1d4	0.24	0.00000000	pigs
Nptn	0.20	0.00000000	pigs
Rfc2	0.28	0.00000000	pigs
Creg1	0.11	0.00000000	pigs
Atp6v0a1	0.14	0.00000000	pigs
Fbp1	-0.43	0.00000000	pigs
Sec24d	0.37	0.00000000	pigs
Dazap2	0.12	0.00000000	pigs
Gatad2a	0.15	0.00000000	pigs
Btbd7	0.16	0.00000000	pigs
Cdk5rap3	0.09	0.00000000	pigs
Ciao2b	0.01	0.00000000	pigs
Ndufa13	-0.50	0.00000000	pigs
Sptan1	0.21	0.00000000	pigs

Sdad1	0.17	0.00000000	pigs
Ndufa4	-0.40	0.00000000	pigs
Eloa	0.14	0.00000000	pigs
Ahsa1	0.06	0.00000000	pigs
Arfgap3	0.14	0.00000000	pigs
Clk4	0.19	0.00000000	pigs
Niban2	0.16	0.00000000	pigs
Galnt10	0.21	0.00000000	pigs
Zbtb1	0.25	0.00000000	pigs
Aebp2	0.22	0.00000000	pigs
Clcn6	0.09	0.00000000	pigs
Ccar1	0.20	0.00000000	pigs
Rin2	0.17	0.00000000	pigs
Hpcal1	0.35	0.00000000	pigs
Ckap5	0.13	0.00000000	pigs
Prdm4	0.08	0.00000000	pigs
Trit1	0.11	0.00000000	pigs
Cep76	0.09	0.00000000	pigs
Nek9	0.17	0.00000000	pigs
Lpcat3	0.12	0.00000000	pigs
Hebp1	0.33	0.00000000	pigs
Ankrd50	0.10	0.00000000	pigs
Atf5	0.17	0.00000000	pigs
Anks3	0.07	0.00000000	pigs
Ube2e2	0.33	0.00000000	pigs
Pced1b	-0.33	0.00000000	pigs
Cmtm7	-0.43	0.00000000	pigs
Plagl2	0.09	0.00000000	pigs
Tgif2	0.14	0.00000000	pigs
Gnl2	0.16	0.00000000	pigs
Tada2a	0.13	0.00000000	pigs
Rpa2	0.15	0.00000000	pigs
Ddi2	0.11	0.00000000	pigs
Cdc42ep4	0.13	0.00000000	pigs
Wac	0.15	0.00000000	pigs
Ppfia1	0.15	0.00000000	pigs
Tor1aip2	0.11	0.00000000	pigs
Tmem132a	0.09	0.00000000	pigs
Cct4	0.11	0.00000000	pigs
Rcbtb1	0.12	0.00000000	pigs
Cux2	0.35	0.00000000	pigs
Rnase6	-0.68	0.00000000	pigs
Fnbp4	0.16	0.00000000	pigs
Kdm1a	0.19	0.00000000	pigs
Nsfl1c	0.08	0.00000000	pigs
Zfp326	0.13	0.00000000	pigs

Scyl1	0.09	0.00000000	pigs
Desi2	0.11	0.00000000	pigs
Hsp90b1	-0.02	0.00000000	pigs
Slc39a7	0.03	0.00000000	pigs
Kcnk13	0.08	0.00000000	pigs
Ahi1	0.37	0.00000000	pigs
C9orf72	0.13	0.00000000	pigs
Glis3	0.41	0.00000000	pigs
Ppip5k2	0.20	0.00000000	pigs
Rars2	0.10	0.00000000	pigs
Sccpdh	0.10	0.00000000	pigs
Tnik	0.38	0.00000000	pigs
Spg21	0.19	0.00000000	pigs
Zbtb34	0.13	0.00000000	pigs
Hat1	0.09	0.00000000	pigs
Tbl2	0.09	0.00000000	pigs
Rgs19	-0.38	0.00000000	pigs
Chd7	0.12	0.00000000	pigs
Fto	0.23	0.00000000	pigs
Ibtk	0.20	0.00000000	pigs
Bach1	-0.82	0.00000000	pigs
Tfg	0.15	0.00000000	pigs
Xpo5	0.13	0.00000000	pigs
Gsn	-0.69	0.00000000	pigs
Fem1b	0.06	0.00000000	pigs
Asxl1	0.19	0.00000000	pigs
Sin3a	0.13	0.00000000	pigs
Tlr6	0.18	0.00000000	pigs
Phlpp1	0.22	0.00000000	pigs
Kcmf1	0.19	0.00000000	pigs
8030462N17Rik	0.12	0.00000000	pigs
Tnfrsf17	-0.68	0.00000000	pigs
Rspry1	0.13	0.00000000	pigs
Tm9sf3	0.23	0.00000000	pigs
Ago1	0.19	0.00000000	pigs
Lrrc59	0.17	0.00000000	pigs
Sipa1l1	-0.63	0.00000000	pigs
Arhgdia	0.26	0.00000000	pigs
Vps35	0.22	0.00000000	pigs
Psmd2	0.25	0.00000000	pigs
Dyrk3	0.06	0.00000000	pigs
Cramp1	0.09	0.00000000	pigs
Otud4	0.10	0.00000000	pigs
Phb	0.20	0.00000000	pigs
Rabep1	0.19	0.00000000	pigs
Ap2a1	0.15	0.00000000	pigs

Slco4a1	0.15	0.00000000	pigs
Rbm6	0.17	0.00000000	pigs
4933434E20Rik	0.05	0.00000000	pigs
Araf	0.09	0.00000000	pigs
Acox1	0.17	0.00000000	pigs
Ppp1r9b	0.15	0.00000000	pigs
Actr1a	0.12	0.00000000	pigs
Rnf217	0.24	0.00000000	pigs
Ptp4a2	0.21	0.00000000	pigs
Cd40	0.23	0.00000000	pigs
Tmem65	-0.53	0.00000000	pigs
Ccdc93	0.21	0.00000000	pigs
Tmed3	0.07	0.00000000	pigs
Hdac3	0.09	0.00000000	pigs
Slc1a3	0.45	0.00000000	pigs
Lamtor1	0.23	0.00000000	pigs
Cxcl14	0.39	0.00000000	pigs
Rars	0.09	0.00000000	pigs
Ahcyl1	0.10	0.00000000	pigs
Ralgapb	0.14	0.00000000	pigs
Slc5a3	0.12	0.00000000	pigs
Hspe1-rs1	0.14	0.00000000	pigs
Egfl7	0.02	0.00000000	pigs
Tbc1d10a	0.08	0.00000000	pigs
Tcaim	0.11	0.00000000	pigs
Tcn2	0.03	0.00000000	pigs
Sec63	0.19	0.00000000	pigs
Pcmtd1	0.17	0.00000000	pigs
Copb2	0.18	0.00000000	pigs
Trip12	0.20	0.00000000	pigs
Rap2c	0.13	0.00000000	pigs
Birc6	0.25	0.00000000	pigs
Rrp1b	0.16	0.00000000	pigs
Fbxo28	0.10	0.00000000	pigs
Stx12	0.20	0.00000000	pigs
Ccdc22	0.05	0.00000000	pigs
Itga6	0.01	0.00000000	pigs
Acads	0.15	0.00000000	pigs
Sulf1	0.45	0.00000000	pigs
Fkbp4	0.11	0.00000000	pigs
Zbtb20	-0.57	0.00000000	pigs
Vat1	0.21	0.00000000	pigs
Nucks1	0.21	0.00000000	pigs
Prdm1	0.24	0.00000000	pigs
Cers5	0.11	0.00000000	pigs
Pltp	0.26	0.00000000	pigs

Scyl2	0.14	0.00000000	pigs
Klhl42	0.09	0.00000000	pigs
Rnmt	0.19	0.00000000	pigs
Uap1l1	0.06	0.00000000	pigs
St3gal1	0.19	0.00000000	pigs
Naa25	0.07	0.00000000	pigs
Cbfb	0.12	0.00000000	pigs
Arid5b	-0.36	0.00000000	pigs
Paxip1	0.08	0.00000000	pigs
Tbc1d9b	0.28	0.00000000	pigs
Edem3	0.07	0.00000000	pigs
Samd8	0.10	0.00000000	pigs
Jak2	0.23	0.00000000	pigs
Reep5	-0.35	0.00000000	pigs
Kdm6b	0.18	0.00000000	pigs
Fgl2	-0.57	0.00000000	pigs
Rictor	0.21	0.00000000	pigs
Tmem160	-0.45	0.00000000	pigs
Gtpbp1	0.08	0.00000000	pigs
Psmd11	0.18	0.00000000	pigs
Naa16	0.11	0.00000000	pigs
Tnfsf12	0.20	0.00000000	pigs
Nudt21	0.11	0.00000000	pigs
Sergef	0.17	0.00000000	pigs
Brd2	0.23	0.00000000	pigs
Luc7l	0.11	0.00000000	pigs
Cluh	0.14	0.00000000	pigs
Fbxo9	-0.71	0.00000000	pigs
Ccdc59	0.17	0.00000000	pigs
Polr2b	0.12	0.00000000	pigs
Capzb	0.23	0.00000000	pigs
Zfand5	0.23	0.00000000	pigs
Zhx3	0.15	0.00000000	pigs
Rin3	0.21	0.00000000	pigs
Gpx4	0.17	0.00000000	pigs
Tpst2	0.20	0.00000000	pigs
Otub1	0.16	0.00000000	pigs
Trim26	0.14	0.00000000	pigs
Caprin1	0.15	0.00000000	pigs
Prelid1	0.26	0.00000000	pigs
Eea1	0.19	0.00000000	pigs
Fnbp1	0.43	0.00000000	pigs
Ccnt2	0.13	0.00000000	pigs
Was	-0.45	0.00000000	pigs
Wipf2	0.10	0.00000000	pigs
Pacs2	0.06	0.00000000	pigs

Ube2v2	0.17	0.00000000	pigs
Hbegf	0.23	0.00000000	pigs
Tmem63b	0.06	0.00000000	pigs
Ywhab	-0.31	0.00000000	pigs
Tfe3	0.08	0.00000000	pigs
Scamp3	0.07	0.00000000	pigs
Dusp23	0.06	0.00000000	pigs
Fbxl19	0.06	0.00000000	pigs
Camk1	0.12	0.00000000	pigs
Prkcsh	0.07	0.00000000	pigs
Ube3a	0.19	0.00000000	pigs
Cinp	0.11	0.00000000	pigs
Serinc1	0.15	0.00000000	pigs
Gfod2	0.13	0.00000000	pigs
Atp11c	0.23	0.00000000	pigs
Smad3	0.30	0.00000000	pigs
Csk	0.21	0.00000000	pigs
Cdc42se1	0.17	0.00000000	pigs
Atf6b	0.10	0.00000000	pigs
Gatm	0.10	0.00000000	pigs
Ankrd13c	0.19	0.00000000	pigs
Camk2g	0.08	0.00000000	pigs
Wnk1	0.18	0.00000000	pigs
Rnf152	0.11	0.00000000	pigs
Kdm5c	0.08	0.00000000	pigs
Zmiz2	0.03	0.00000000	pigs
C1rl	0.09	0.00000000	pigs
Pcyox1	0.10	0.00000000	pigs
Dcxr	0.04	0.00000000	pigs
Efcab14	0.08	0.00000000	pigs
Rptor	0.16	0.00000000	pigs
Commd9	0.16	0.00000000	pigs
Rpia	-0.57	0.00000000	pigs
Xrcc6	0.09	0.00000000	pigs
Rbpj	0.28	0.00000000	pigs
Rai1	0.06	0.00000000	pigs
Atf2	0.14	0.00000000	pigs
Gucd1	0.08	0.00000000	pigs
Zbtb38	0.15	0.00000000	pigs
Eif2b5	0.14	0.00000000	pigs
Bloc1s2	0.12	0.00000000	pigs
Pigt	0.06	0.00000000	pigs
Ptrh1	0.11	0.00000000	pigs
Khdrbs1	0.10	0.00000000	pigs
Adam9	0.21	0.00000000	pigs
Cnot6	0.07	0.00000000	pigs

C1qb	-0.04	0.00000000	pigs
Pid1	0.20	0.00000000	pigs
Nup205	0.12	0.00000000	pigs
Epcam	-0.22	0.00000000	pigs
Aspscr1	0.13	0.00000000	pigs
Chml	0.09	0.00000000	pigs
Galnt2	0.11	0.00000000	pigs
Pds5a	0.16	0.00000000	pigs
Cnih1	0.14	0.00000000	pigs
Psme4	0.19	0.00000000	pigs
Agfg1	0.21	0.00000000	pigs
Dcaf10	0.05	0.00000000	pigs
Ranbp9	0.10	0.00000000	pigs
Abcc1	0.29	0.00000000	pigs
Brox	0.07	0.00000000	pigs
Oga	0.17	0.00000000	pigs
Tent4a	0.11	0.00000000	pigs
Sorl1	-0.80	0.00000000	pigs
Bcat1	0.24	0.00000000	pigs
Selenow	-0.53	0.00000000	pigs
Ptpre	-0.51	0.00000000	pigs
Tmem106a	0.10	0.00000000	pigs
Stau1	0.21	0.00000000	pigs
Glb1	0.14	0.00000000	pigs
Emg1	0.11	0.00000000	pigs
Lamtor3	0.23	0.00000000	pigs
Inhba	0.18	0.00000000	pigs
Baiap2	0.14	0.00000000	pigs
Tmem42	0.08	0.00000000	pigs
Pgd	-0.49	0.00000000	pigs
Ppp6r2	0.17	0.00000000	pigs
Ccdc174	0.08	0.00000000	pigs
Golga4	0.10	0.00000000	pigs
Retn	1.15	0.00000000	pigs
Cdc42bpb	0.08	0.00000000	pigs
Prpf31	0.11	0.00000000	pigs
Tmem259	0.09	0.00000000	pigs
Rrp15	0.12	0.00000000	pigs
Naa40	0.05	0.00000000	pigs
D16Ertd472e	-0.46	0.00000000	pigs
Tspan33	0.10	0.00000000	pigs
Yeats2	0.16	0.00000000	pigs
Rab5a	0.22	0.00000000	pigs
Lyrm1	0.10	0.00000000	pigs
Shc3	0.12	0.00000000	pigs
Aldh18a1	0.14	0.00000000	pigs

Sik2	0.28	0.00000000	pigs
Jmy	0.20	0.00000000	pigs
Itm2a	-0.26	0.00000000	pigs
Phactr4	0.08	0.00000000	pigs
Ero1b	0.10	0.00000000	pigs
Twf1	0.05	0.00000000	pigs
Ncoa2	-0.51	0.00000000	pigs
Pik3r4	0.10	0.00000000	pigs
Jkamp	0.02	0.00000000	pigs
Upf1	0.12	0.00000000	pigs
Pde8a	0.14	0.00000000	pigs
Dnajc18	0.07	0.00000000	pigs
Pnn	0.12	0.00000000	pigs
Socs7	0.07	0.00000000	pigs
Ppp4r3b	0.17	0.00000000	pigs
Rela	0.10	0.00000000	pigs
Ndufa9	0.12	0.00000000	pigs
Ttc1	0.08	0.00000000	pigs
St3gal5	0.05	0.00000000	pigs
Cand1	0.08	0.00000000	pigs
Tifab	0.14	0.00000000	pigs
Tmem11	0.13	0.00000000	pigs
Ptpn23	0.11	0.00000000	pigs
Sucla2	0.14	0.00000000	pigs
Tent5a	0.22	0.00000000	pigs
Ppard	0.12	0.00000000	pigs
Bend3	0.10	0.00000000	pigs
Ppp2r3c	0.12	0.00000000	pigs
Lmna	-0.65	0.00000000	pigs
Pgap2	0.04	0.00000000	pigs
Pnkp	0.09	0.00000000	pigs
Tspan3	0.08	0.00000000	pigs
Eya3	0.17	0.00000000	pigs
Srsf10	0.15	0.00000000	pigs
Cmtm3	0.15	0.00000000	pigs
Rprd1b	0.03	0.00000000	pigs
Hck	0.12	0.00000000	pigs
Gale	0.07	0.00000000	pigs
Ccnl1	0.22	0.00000000	pigs
Gnl1	0.10	0.00000000	pigs
Eef1b2	-0.32	0.00000000	pigs
Ccr1	0.15	0.00000000	pigs
Aldh7a1	0.10	0.00000000	pigs
Arel1	0.06	0.00000000	pigs
Gpaa1	0.09	0.00000000	pigs
Git2	0.11	0.00000000	pigs

Pip4p1	0.09	0.00000000	pigs
Ankrd28	-0.70	0.00000000	pigs
Txnrd1	0.09	0.00000000	pigs
Safb2	0.12	0.00000000	pigs
Srebf2	-0.47	0.00000000	pigs
Scfd1	0.15	0.00000000	pigs
Pmaip1	0.32	0.00000000	pigs
Pdk1	0.12	0.00000000	pigs
Ghrl	-0.83	0.00000000	pigs
Colgalt1	0.17	0.00000000	pigs
Tex9	-0.35	0.00000000	pigs
Tsn	0.11	0.00000000	pigs
Scamp2	0.07	0.00000000	pigs
Ctnnbl1	0.11	0.00000000	pigs
Cenpp	0.16	0.00000000	pigs
Wnt5b	-0.43	0.00000000	pigs
Get3	0.12	0.00000000	pigs
Sema7a	0.14	0.00000000	pigs
Tmem86a	0.09	0.00000000	pigs
Cdk12	0.18	0.00000000	pigs
Snrnp40	0.11	0.00000000	pigs
Ppp2r5d	0.07	0.00000000	pigs
Jak3	0.03	0.00000000	pigs
Wdr7	0.19	0.00000000	pigs
Guf1	0.10	0.00000000	pigs
Ranbp10	0.07	0.00000000	pigs
Zfp609	0.18	0.00000000	pigs
Dach1	-0.42	0.00000000	pigs
Ltn1	0.10	0.00000000	pigs
Atp6v1f	0.24	0.00000000	pigs
Inpp5d	0.20	0.00000000	pigs
Spred1	0.09	0.00000000	pigs
Kif27	0.10	0.00000000	pigs
Gtf3c4	0.08	0.00000000	pigs
Cd14	-0.09	0.00000000	pigs
Zfp143	0.14	0.00000000	pigs
St6galnac4	0.02	0.00000000	pigs
Plekha8	0.09	0.00000000	pigs
Ing2	0.05	0.00000000	pigs
Tmem140	0.10	0.00000000	pigs
Cybc1	0.11	0.00000000	pigs
Fam89a	-0.15	0.00000000	pigs
Irak1	0.06	0.00000000	pigs
Ptgr1	-0.68	0.00000000	pigs
Prpf3	0.10	0.00000000	pigs
Htra2	0.11	0.00000000	pigs

Hspa14	0.06	0.00000000	pigs
Sfswap	0.14	0.00000000	pigs
Cops3	0.13	0.00000000	pigs
Cyld	0.21	0.00000000	pigs
Sh2b3	0.11	0.00000000	pigs
Ddx23	0.05	0.00000000	pigs
Taf4	0.09	0.00000000	pigs
Pik3ca	0.15	0.00000000	pigs
Ccnc	0.15	0.00000000	pigs
Ankrd10	0.18	0.00000000	pigs
Baiap2l1	-0.05	0.00000000	pigs
Hus1	0.07	0.00000000	pigs
Traf6	0.11	0.00000000	pigs
Slc26a6	0.10	0.00000000	pigs
Yaf2	0.20	0.00000000	pigs
Hdac2	0.10	0.00000000	pigs
Il6st	0.13	0.00000000	pigs
Pdgfb	0.13	0.00000000	pigs
Ecpas	0.14	0.00000000	pigs
Dhrs11	0.09	0.00000000	pigs
Copg1	0.09	0.00000000	pigs
Eif4h	0.21	0.00000000	pigs
Serac1	0.16	0.00000000	pigs
Blzf1	0.11	0.00000000	pigs
Lgals3bp	0.24	0.00000000	pigs
Prkg1	0.38	0.00000000	pigs
Rras	0.22	0.00000000	pigs
Poldip3	0.09	0.00000000	pigs
Nifk	0.21	0.00000000	pigs
Nutf2	0.12	0.00000000	pigs
Dr1	0.12	0.00000000	pigs
Csf1	-0.48	0.00000000	pigs
Zdhhc9	0.08	0.00000000	pigs
Pot1b	0.07	0.00000000	pigs
Armc1	0.08	0.00000000	pigs
Ogfod1	0.10	0.00000000	pigs
Fjx1	0.18	0.00000000	pigs
AB124611	-0.34	0.00000000	pigs
Stip1	0.06	0.00000000	pigs
Tial1	0.17	0.00000000	pigs
Zbtb11	0.07	0.00000000	pigs
Arhgap22	0.13	0.00000000	pigs
Ubfd1	0.10	0.00000000	pigs
Fbxo38	0.10	0.00000000	pigs
Fads3	0.02	0.00000000	pigs
Pde3b	-0.44	0.00000000	pigs

Dennd2d	0.21	0.00000000	pigs
Bscl2	0.11	0.00000000	pigs
Ankfy1	0.20	0.00000000	pigs
Ly96	0.21	0.00000000	pigs
Dera	0.19	0.00000000	pigs
Pcgf5	0.13	0.00000000	pigs
Zfp622	0.13	0.00000000	pigs
Dpy19l4	0.07	0.00000000	pigs
Hsf1	0.10	0.00000000	pigs
Ncoa7	-0.34	0.00000000	pigs
Rc3h1	0.19	0.00000000	pigs
Nudt14	-0.27	0.00000000	pigs
Cog4	0.05	0.00000000	pigs
Wasl	0.09	0.00000000	pigs
Tgds	0.13	0.00000000	pigs
Foxj2	0.08	0.00000000	pigs
Lysmd3	0.11	0.00000000	pigs
Phyh	0.22	0.00000000	pigs
Septin2	0.11	0.00000000	pigs
Foxn3	0.29	0.00000000	pigs
Lin37	0.09	0.00000000	pigs
Eif5b	0.14	0.00000000	pigs
Aga	0.06	0.00000000	pigs
Zc3h18	0.07	0.00000000	pigs
Slc20a2	0.09	0.00000000	pigs
Tsr2	0.12	0.00000000	pigs
Limd1	0.10	0.00000000	pigs
Uxs1	0.07	0.00000000	pigs
Trim3	0.10	0.00000000	pigs
Trim28	0.12	0.00000000	pigs
Spint2	-0.44	0.00000000	pigs
Crybg1	0.23	0.00000000	pigs
Brap	0.10	0.00000000	pigs
Itga9	0.08	0.00000000	pigs
Pxk	0.14	0.00000000	pigs
Tmem87a	0.12	0.00000000	pigs
Prdm2	0.13	0.00000000	pigs
Rif1	0.08	0.00000000	pigs
Pdia5	0.08	0.00000000	pigs
Pnisr	0.11	0.00000000	pigs
Rpn1	0.13	0.00000000	pigs
Anapc13	0.11	0.00000000	pigs
Pih1d1	0.07	0.00000000	pigs
Psmd12	0.18	0.00000000	pigs
Ube2r2	0.21	0.00000000	pigs
Cops2	0.15	0.00000000	pigs

Ppp1r12a	0.30	0.00000000	pigs
Cop1	-0.48	0.00000000	pigs
Kdm6a	0.10	0.00000000	pigs
Rcan1	0.20	0.00000000	pigs
Ppp2r1a	0.04	0.00000000	pigs
Fam193b	0.06	0.00000000	pigs
Timm50	0.03	0.00000000	pigs
Ik	-0.31	0.00000000	pigs
Gatd1	0.03	0.00000000	pigs
Lpar1	0.15	0.00000000	pigs
Steap4	-0.53	0.00000000	pigs
Nvl	0.12	0.00000000	pigs
Slc17a5	0.11	0.00000000	pigs
Trappc11	0.08	0.00000000	pigs
Eif4g1	0.12	0.00000000	pigs
Dbr1	0.11	0.00000000	pigs
Tubg1	0.09	0.00000000	pigs
Gpbp1l1	0.09	0.00000000	pigs
Rbm41	0.08	0.00000000	pigs
Flvcr2	0.16	0.00000000	pigs
Arhgap17	0.19	0.00000000	pigs
Eri1	0.08	0.00000000	pigs
Emilin2	0.21	0.00000000	pigs
Lemd2	0.05	0.00000000	pigs
Echdc1	0.10	0.00000000	pigs
Ndufb1	-0.40	0.00000000	pigs
Bag6	0.03	0.00000000	pigs
Keap1	0.07	0.00000000	pigs
Coro1b	0.18	0.00000000	pigs
Bccip	0.11	0.00000001	pigs
Elmo2	0.10	0.00000001	pigs
Polr2m	0.07	0.00000001	pigs
Slc7a1	0.13	0.00000001	pigs
Zc3h12a	0.09	0.00000001	pigs
Taf1a	0.08	0.00000001	pigs
Sec24b	0.10	0.00000001	pigs
Coq5	0.05	0.00000001	pigs
Limk1	0.07	0.00000001	pigs
Ruvbl2	-0.01	0.00000001	pigs
Trmt1l	0.07	0.00000001	pigs
Elavl1	0.12	0.00000001	pigs
Trub2	0.10	0.00000001	pigs
Itpa	0.09	0.00000001	pigs
Cep120	0.16	0.00000001	pigs
Trp53bp1	0.10	0.00000001	pigs
Hsd17b4	0.23	0.00000001	pigs

Vcan	-0.43	0.00000001	pigs
Eif4e	0.11	0.00000001	pigs
Rnf2	0.22	0.00000001	pigs
Ercc3	0.06	0.00000001	pigs
Col3a1	0.12	0.00000001	pigs
Optn	0.18	0.00000001	pigs
Tmem205	0.02	0.00000001	pigs
Tmed9	0.11	0.00000001	pigs
Rbm15	0.10	0.00000001	pigs
Ap2a2	0.18	0.00000001	pigs
Gorasp2	0.12	0.00000001	pigs
Csrnp2	0.07	0.00000001	pigs
Ywhaz	-0.28	0.00000001	pigs
Atpsckmt	0.02	0.00000001	pigs
Nup54	0.16	0.00000001	pigs
Slc5a10	0.13	0.00000001	pigs
Krt10	-0.30	0.00000001	pigs
Khsrp	0.05	0.00000001	pigs
Oscar	0.02	0.00000001	pigs
Clpx	0.03	0.00000001	pigs
Elf4	0.03	0.00000001	pigs
Ssrp1	-0.02	0.00000001	pigs
Wee1	0.09	0.00000001	pigs
Pi4ka	0.08	0.00000001	pigs
Cpsf7	0.07	0.00000001	pigs
Txlng	0.12	0.00000001	pigs
Ppt1	0.18	0.00000001	pigs
Pafah1b1	0.18	0.00000001	pigs
Pkd1	0.06	0.00000001	pigs
Hvcn1	0.10	0.00000001	pigs
Ctdp1	0.08	0.00000001	pigs
Ccdc32	0.09	0.00000001	pigs
Dmac2	0.06	0.00000001	pigs
Psma5	0.21	0.00000001	pigs
Slc1a5	-0.41	0.00000001	pigs
Ddx55	0.04	0.00000001	pigs
Commd2	0.02	0.00000001	pigs
Ptdss1	0.12	0.00000001	pigs
Nol11	0.35	0.00000001	pigs
Rbm8a2	0.17	0.00000001	pigs
Zw10	0.08	0.00000001	pigs
Krr1	0.09	0.00000001	pigs
Alg3	0.08	0.00000001	pigs
Fabp3	-0.15	0.00000001	pigs
Zbtb7a	0.08	0.00000001	pigs
Slc35e1	0.06	0.00000001	pigs

Api5	0.12	0.00000002	pigs
Nr2c1	0.12	0.00000002	pigs
Ormdl1	0.06	0.00000002	pigs
Abcf3	0.12	0.00000002	pigs
Exog	0.04	0.00000002	pigs
Fam32a	0.07	0.00000002	pigs
Atp2c1	0.04	0.00000002	pigs
Taf8	0.07	0.00000002	pigs
Clta	0.18	0.00000002	pigs
Polr3e	0.07	0.00000002	pigs
Otulin	0.07	0.00000002	pigs
Fndc3a	0.22	0.00000002	pigs
Scarf1	0.05	0.00000002	pigs
Csde1	0.18	0.00000002	pigs
Dtx3l	-0.26	0.00000002	pigs
Vamp5	0.06	0.00000002	pigs
Wdr91	0.05	0.00000002	pigs
Sec23a	0.13	0.00000002	pigs
Ddost	0.30	0.00000002	pigs
Zmym2	0.09	0.00000002	pigs
Atxn10	0.04	0.00000002	pigs
Kctd3	0.03	0.00000002	pigs
Faf2	0.09	0.00000002	pigs
Spin1	0.06	0.00000002	pigs
Psmf1	0.09	0.00000002	pigs
Eif1b	-0.36	0.00000002	pigs
Smg7	0.13	0.00000002	pigs
Rfx5	0.08	0.00000002	pigs
Psme3ip1	0.14	0.00000002	pigs
Gpatch8	0.10	0.00000002	pigs
Rsf1	0.16	0.00000002	pigs
Opa1	0.07	0.00000002	pigs
Uox	-0.35	0.00000002	pigs
Pitpnb	0.14	0.00000002	pigs
BC004004	0.08	0.00000002	pigs
Cep83	0.07	0.00000002	pigs
Setd2	0.14	0.00000003	pigs
Srsf11	0.10	0.00000003	pigs
Zkscan1	0.09	0.00000003	pigs
Dnajc11	0.08	0.00000003	pigs
Cdc34	0.08	0.00000003	pigs
Erp29	0.12	0.00000003	pigs
Abhd13	0.13	0.00000003	pigs
Nmt1	0.17	0.00000003	pigs
Sart3	0.04	0.00000003	pigs
Alpk1	-0.26	0.00000003	pigs

Fam20c	-0.22	0.00000003	pigs
Necap2	0.17	0.00000003	pigs
Rsrc2	0.14	0.00000003	pigs
Wdr26	0.05	0.00000003	pigs
Ints1	0.04	0.00000003	pigs
Lrp6	0.15	0.00000003	pigs
Pmpca	0.05	0.00000003	pigs
Akr1b3	0.04	0.00000003	pigs
Sgsh	0.04	0.00000003	pigs
Rbm17	0.08	0.00000003	pigs
Arrb1	0.12	0.00000003	pigs
Tmx3	0.10	0.00000004	pigs
Atad2b	0.12	0.00000004	pigs
Polr3h	0.09	0.00000004	pigs
Dusp22	-0.35	0.00000004	pigs
Spns1	0.09	0.00000004	pigs
Smyd3	0.29	0.00000004	pigs
Dnajb12	0.09	0.00000004	pigs
Ehd4	0.19	0.00000004	pigs
Tgfbr1	0.10	0.00000004	pigs
Bag4	0.11	0.00000004	pigs
Cops6	0.10	0.00000004	pigs
Ints7	0.08	0.00000004	pigs
Dnm2	0.08	0.00000004	pigs
Bin2	-0.25	0.00000004	pigs
Klhl18	0.11	0.00000004	pigs
Tmem238	-0.48	0.00000004	pigs
Odf2	0.03	0.00000004	pigs
Naga	0.06	0.00000005	pigs
Rp2	0.12	0.00000005	pigs
Slc4a2	0.03	0.00000005	pigs
Pinx1	0.11	0.00000005	pigs
Arhgap31	0.21	0.00000005	pigs
Selenoi	0.16	0.00000005	pigs
Whamm	0.06	0.00000005	pigs
Hcfc2	0.08	0.00000005	pigs
Vapb	0.14	0.00000005	pigs
Eapp	-0.30	0.00000005	pigs
Ppil2	-0.01	0.00000005	pigs
Sh2d3c	-0.24	0.00000005	pigs
Sord	0.10	0.00000006	pigs
Usp16	0.09	0.00000006	pigs
Ganab	0.12	0.00000006	pigs
Fbxo6	0.15	0.00000006	pigs
Wdr37	0.09	0.00000006	pigs
Rasal2	-0.48	0.00000006	pigs

Klf4	0.23	0.00000006	pigs
Tmem39a	0.15	0.00000006	pigs
Smad2	0.09	0.00000006	pigs
Gemin4	0.11	0.00000006	pigs
Cd69	-0.47	0.00000006	pigs
Sec23b	0.05	0.00000006	pigs
Itgb1bp1	0.08	0.00000006	pigs
Hipk2	-0.63	0.00000006	pigs
Fam135a	-0.85	0.00000007	pigs
Pex14	0.09	0.00000007	pigs
Gorab	0.09	0.00000007	pigs
Senp6	0.15	0.00000007	pigs
Ivd	0.08	0.00000007	pigs
Hadhb	0.14	0.00000007	pigs
Zfp830	0.03	0.00000007	pigs
Larp1	0.02	0.00000007	pigs
Atp5pb	0.21	0.00000007	pigs
Cpeb3	-0.34	0.00000007	pigs
Yipf4	0.13	0.00000007	pigs
Riok1	0.06	0.00000008	pigs
Lrp10	0.09	0.00000008	pigs
Dpp3	0.05	0.00000008	pigs
G3bp1	0.12	0.00000008	pigs
Hnrnpr	0.07	0.00000008	pigs
Itch	0.11	0.00000008	pigs
Klhl24	0.07	0.00000008	pigs
Itm2c	0.05	0.00000009	pigs
Luc7l3	0.13	0.00000009	pigs
Fam102b	-0.10	0.00000009	pigs
Lmf1	0.02	0.00000009	pigs
Oas1e	-0.40	0.00000009	pigs
Camkk2	-0.25	0.00000009	pigs
Chpt1	-0.67	0.00000010	pigs
Leprot	0.01	0.00000010	pigs
Cdh23	-0.31	0.00000010	pigs
Znhit6	0.06	0.00000010	pigs
Topbp1	0.11	0.00000010	pigs
Sugt1	-0.02	0.00000011	pigs
Clk3	0.02	0.00000011	pigs
Uba6	0.10	0.00000011	pigs
Sec61g	-0.42	0.00000011	pigs
Kif21b	0.09	0.00000011	pigs
Nin	-0.45	0.00000011	pigs
Ccdc127	0.03	0.00000011	pigs
Septin9	0.10	0.00000011	pigs
Ypel2	0.03	0.00000011	pigs

Asb9	-0.25	0.00000011	pigs
Rapgef1	0.08	0.00000012	pigs
Suz12	0.03	0.00000012	pigs
Pfdn2	0.08	0.00000012	pigs
Dbndd2	0.13	0.00000012	pigs
Frat2	0.04	0.00000012	pigs
Epn1	0.06	0.00000012	pigs
Trim36	0.12	0.00000012	pigs
Dnajc13	0.18	0.00000012	pigs
Ap2b1	0.16	0.00000013	pigs
Sco1	0.06	0.00000013	pigs
Trpc4ap	0.10	0.00000013	pigs
Ric8b	0.07	0.00000013	pigs
Zfp36	-0.54	0.00000014	pigs
Huwe1	0.18	0.00000014	pigs
9930104L06Rik	0.08	0.00000014	pigs
Eipr1	0.09	0.00000014	pigs
Ccdc9	0.10	0.00000014	pigs
Aplf	0.06	0.00000014	pigs
Foxk1	0.07	0.00000014	pigs
Amh	0.09	0.00000014	pigs
Ccdc50	0.13	0.00000015	pigs
Wsb2	0.04	0.00000015	pigs
Atp2a2	0.15	0.00000015	pigs
Rwdd4a	-0.11	0.00000015	pigs
Ccl5	-0.55	0.00000015	pigs
Ypel3	-0.43	0.00000015	pigs
Usp39	0.02	0.00000015	pigs
Yy1	0.16	0.00000015	pigs
Dnttip2	0.12	0.00000015	pigs
Hps3	0.09	0.00000016	pigs
Enoph1	0.13	0.00000016	pigs
Ifngr1	0.09	0.00000016	pigs
Hmox2	0.06	0.00000016	pigs
Nup50	0.13	0.00000017	pigs
Vdac3	0.09	0.00000017	pigs
Traf5	0.11	0.00000017	pigs
Cab39	0.16	0.00000017	pigs
Ippk	0.07	0.00000017	pigs
Ap3m1	0.08	0.00000017	pigs
Casd1	0.04	0.00000017	pigs
Osgep	0.09	0.00000018	pigs
Klhdc10	0.08	0.00000018	pigs
Plaa	0.08	0.00000018	pigs
Selenof	0.15	0.00000018	pigs
Ccdc58	0.14	0.00000019	pigs

Oprm1	-0.27	0.00000019	pigs
Dhx58	0.13	0.00000019	pigs
Tmem64	0.05	0.00000020	pigs
Tgif1	0.07	0.00000020	pigs
Entpd4	0.05	0.00000020	pigs
Nus1	0.08	0.00000020	pigs
Siglec1	0.12	0.00000020	pigs
Ube2g2	0.10	0.00000020	pigs
Pgk1	0.17	0.00000021	pigs
Zmynd8	0.08	0.00000021	pigs
Zfp639	0.10	0.00000021	pigs
Zfp2	0.07	0.00000021	pigs
Nup133	0.05	0.00000021	pigs
Uhmk1	0.11	0.00000021	pigs
Psmd8	0.16	0.00000022	pigs
H1f4	-0.32	0.00000022	pigs
Sat1	0.16	0.00000022	pigs
Atg2b	0.09	0.00000022	pigs
Ppig	0.10	0.00000022	pigs
Tkfc	0.05	0.00000022	pigs
Arap1	0.07	0.00000023	pigs
Rangap1	0.02	0.00000023	pigs
Sft2d1	0.13	0.00000023	pigs
Lrrk1	0.16	0.00000024	pigs
Cln8	0.08	0.00000024	pigs
Ddx41	0.10	0.00000024	pigs
Usp38	0.09	0.00000024	pigs
Ints14	0.08	0.00000024	pigs
Plekha3	0.08	0.00000025	pigs
Ptp4a1	0.04	0.00000025	pigs
Bmf	0.04	0.00000025	pigs
Gsr	0.24	0.00000025	pigs
Emc4	0.02	0.00000027	pigs
Lyar	0.02	0.00000027	pigs
Zxdc	0.09	0.00000027	pigs
Steep1	0.09	0.00000028	pigs
Nup188	0.03	0.00000028	pigs
Eif3b	0.07	0.00000030	pigs
Atxn2l	0.05	0.00000030	pigs
Rad23b	0.16	0.00000030	pigs
Ndfip1	0.25	0.00000031	pigs
Rabl6	0.08	0.00000032	pigs
Zfp106	0.10	0.00000032	pigs
Galnt1	0.25	0.00000032	pigs
Ccny	0.18	0.00000034	pigs
Ifi35	0.15	0.00000034	pigs

Rnf216	0.05	0.00000034	pigs
Ppm1a	0.10	0.00000035	pigs
Itpr1	0.22	0.00000035	pigs
Usp14	0.07	0.00000035	pigs
Gabpb2	0.07	0.00000036	pigs
Eps15l1	0.08	0.00000036	pigs
Nagpa	0.04	0.00000036	pigs
Dohh	0.09	0.00000036	pigs
Kcnab3	0.11	0.00000037	pigs
Fbxo7	0.06	0.00000037	pigs
Ube2j1	0.08	0.00000037	pigs
Tubgcp2	0.05	0.00000038	pigs
Uqcrc1	0.19	0.00000038	pigs
Utp18	0.13	0.00000039	pigs
Cherp	0.08	0.00000039	pigs
Glrx5	0.07	0.00000039	pigs
Zfp451	0.00	0.00000039	pigs
Usb1	0.09	0.00000039	pigs
Dnajb4	0.08	0.00000040	pigs
Ncf4	0.20	0.00000040	pigs
Rabgap1	0.08	0.00000040	pigs
Tom1l2	0.10	0.00000041	pigs
Hk3	0.20	0.00000041	pigs
Tubgcp3	0.03	0.00000042	pigs
Pank2	0.09	0.00000044	pigs
Sun2	-0.38	0.00000044	pigs
Urod	0.05	0.00000045	pigs
Abhd2	-0.72	0.00000045	pigs
Irf4	-0.60	0.00000046	pigs
Trpm7	0.14	0.00000047	pigs
Gsto1	-0.05	0.00000048	pigs
Alkbh1	0.07	0.00000050	pigs
Abce1	0.15	0.00000050	pigs
Top2b	0.13	0.00000051	pigs
Tmem168	0.06	0.00000051	pigs
Irs2	0.29	0.00000051	pigs
Rab3gap1	0.10	0.00000051	pigs
Glce	0.16	0.00000051	pigs
Plin3	0.11	0.00000052	pigs
Cyb5a	-0.15	0.00000052	pigs
Iqsec1	0.03	0.00000053	pigs
Rcsd1	-0.38	0.00000054	pigs
Abcg3	-0.19	0.00000054	pigs
Rae1	0.05	0.00000056	pigs
Rubcn	0.08	0.00000057	pigs
Fkbp8	0.01	0.00000057	pigs

Rgs10	0.22	0.00000058	pigs
Tmem214	0.01	0.00000058	pigs
Tfdp2	-0.42	0.00000058	pigs
Ttc14	0.10	0.00000060	pigs
Ubqln4	0.04	0.00000061	pigs
Fbh1	0.10	0.00000061	pigs
Bud13	0.05	0.00000063	pigs
Arnt	0.22	0.00000063	pigs
Cep44	0.04	0.00000064	pigs
Psmc4	0.06	0.00000064	pigs
Naa15	0.12	0.00000065	pigs
Tnfsf13	0.07	0.00000066	pigs
Ctdsp2	0.07	0.00000068	pigs
Dync1i1	-0.69	0.00000069	pigs
Slc35d1	0.05	0.00000070	pigs
Lnpk	0.14	0.00000071	pigs
Phf23	0.13	0.00000071	pigs
Nars2	0.10	0.00000071	pigs
Acrbp	0.06	0.00000072	pigs
Cd86	-1.12	0.00000073	pigs
Isg15	-0.43	0.00000075	pigs
Ankrd33b	-0.52	0.00000075	pigs
Chfr	0.07	0.00000076	pigs
Prkab2	0.09	0.00000076	pigs
Sds	-0.03	0.00000077	pigs
Trpv2	0.08	0.00000079	pigs
Anapc15-ps	0.08	0.00000081	pigs
Relb	0.19	0.00000082	pigs
Dhx33	0.05	0.00000082	pigs
Ikbip	-0.01	0.00000083	pigs
Hdac1	0.05	0.00000084	pigs
B3gntl1	0.04	0.00000085	pigs
Chmp2b	0.11	0.00000086	pigs
Sirt1	0.07	0.00000086	pigs
Ankrd17	0.12	0.00000086	pigs
Tnrc6a	0.12	0.00000087	pigs
Glt8d1	0.03	0.00000087	pigs
Nudt22	0.05	0.00000089	pigs
Heatr6	0.12	0.00000094	pigs
Klhl15	0.29	0.00000095	pigs
Idh3b	0.05	0.00000098	pigs
Poglut3	0.04	0.00000100	pigs
Ei24	0.06	0.00000102	pigs
Slc40a1	-0.25	0.00000104	pigs
4930453N24Rik	0.15	0.00000104	pigs
Psmd10	0.06	0.00000104	pigs

Phrf1	0.07	0.00000104	pigs
Cerk	-0.28	0.00000105	pigs
E2f6	0.05	0.00000106	pigs
Slc27a1	0.08	0.00000107	pigs
Sesn2	0.09	0.00000108	pigs
Ddx18	0.08	0.00000108	pigs
Tamalin	0.14	0.00000110	pigs
Prkag1	0.05	0.00000111	pigs
Washc3	0.12	0.00000112	pigs
Zcchc8	0.05	0.00000112	pigs
Slc35b2	0.01	0.00000113	pigs
Gcn1	0.05	0.00000114	pigs
Tigar	-0.01	0.00000114	pigs
Ubr1	0.06	0.00000116	pigs
Ap5z1	0.08	0.00000116	pigs
Tsfm	0.04	0.00000117	pigs
Bin3	0.08	0.00000117	pigs
Pld2	0.08	0.00000117	pigs
Ddx17	0.15	0.00000117	pigs
Poldip2	0.12	0.00000117	pigs
Ogdh	0.11	0.00000118	pigs
Cadm1	0.01	0.00000119	pigs
Tns3	0.09	0.00000119	pigs
Fut11	0.07	0.00000120	pigs
Lsg1	0.04	0.00000123	pigs
Edf1	0.13	0.00000124	pigs
Oxsr1	0.09	0.00000124	pigs
Ehmt2	0.03	0.00000127	pigs
Pex13	0.04	0.00000130	pigs
Pck2	0.07	0.00000130	pigs
Vps16	0.05	0.00000133	pigs
Lonp1	0.03	0.00000135	pigs
Lmbrd2	0.06	0.00000136	pigs
Wrn	0.08	0.00000136	pigs
Ccm2	0.14	0.00000139	pigs
Commd1b	0.01	0.00000140	pigs
Slc35a4	0.06	0.00000141	pigs
Abraxas2	0.03	0.00000143	pigs
Erg28	0.07	0.00000147	pigs
Nxt1	0.06	0.00000152	pigs
Ankrd39	0.06	0.00000152	pigs
Dazap1	-0.36	0.00000155	pigs
Xpr1	0.14	0.00000156	pigs
Tbc1d10b	0.05	0.00000156	pigs
Sqle	-0.33	0.00000158	pigs
Acly	0.09	0.00000158	pigs

Prr14l	0.11	0.00000161	pigs
Utp15	0.07	0.00000164	pigs
Ttc22	-0.22	0.00000165	pigs
Nop58	0.08	0.00000168	pigs
Cox7a2	-0.27	0.00000169	pigs
Nit1	0.06	0.00000174	pigs
Accs	0.02	0.00000175	pigs
Rnf38	0.11	0.00000176	pigs
Sntb1	-0.33	0.00000176	pigs
Nufip1	0.08	0.00000178	pigs
Syvn1	0.08	0.00000178	pigs
Hnrnpul1	0.07	0.00000179	pigs
Ptcd3	0.11	0.00000180	pigs
Pram1	-0.27	0.00000180	pigs
Rnf111	0.10	0.00000184	pigs
Ecd	0.05	0.00000189	pigs
Aatf	0.10	0.00000190	pigs
Polr1c	0.11	0.00000191	pigs
Zbtb43	0.07	0.00000192	pigs
Rsbn1	0.02	0.00000194	pigs
Pitpnm1	0.06	0.00000195	pigs
Tgfbrap1	0.04	0.00000202	pigs
Gan	0.00	0.00000203	pigs
Tspo	-0.45	0.00000203	pigs
Ssr4	-0.30	0.00000207	pigs
Rbak	0.07	0.00000214	pigs
Ddx52	0.09	0.00000216	pigs
Atg2a	0.05	0.00000216	pigs
Nlk	0.11	0.00000218	pigs
Ergic1	0.06	0.00000222	pigs
Rbm42	0.09	0.00000223	pigs
Fgr	0.10	0.00000226	pigs
Pcm1	0.03	0.00000226	pigs
1810013L24Rik	0.13	0.00000228	pigs
Cpsf2	0.08	0.00000230	pigs
Alg8	0.04	0.00000233	pigs
Slc37a2	0.08	0.00000239	pigs
Psmd14	0.15	0.00000239	pigs
Dstn	-0.92	0.00000240	pigs
Ltbr	0.05	0.00000240	pigs
Bckdk	0.08	0.00000242	pigs
Ckap4	0.05	0.00000245	pigs
Osbpl8	0.28	0.00000245	pigs
Trim47	0.07	0.00000245	pigs
Zfp1	0.06	0.00000247	pigs
Fam114a1	0.08	0.00000253	pigs

Cpsf3	0.10	0.00000259	pigs
Psme3	0.09	0.00000266	pigs
Tiprl	0.06	0.00000266	pigs
Ctnna1	0.23	0.00000268	pigs
Ppbp	0.70	0.00000272	pigs
Chmp5	0.19	0.00000277	pigs
Pogz	0.04	0.00000286	pigs
Slc35b4	0.07	0.00000287	pigs
Fndc7	0.07	0.00000287	pigs
Plxnc1	0.26	0.00000288	pigs
Pi4kb	-0.01	0.00000290	pigs
Pkn1	-0.01	0.00000297	pigs
Smc4	0.11	0.00000297	pigs
Sulf2	-0.25	0.00000298	pigs
Ttc23l	0.05	0.00000300	pigs
Ndufs6	0.09	0.00000302	pigs
Psmc3	0.07	0.00000302	pigs
Arl1	0.05	0.00000302	pigs
Itsn2	0.13	0.00000303	pigs
Pigh	0.05	0.00000305	pigs
Nupl1	-0.06	0.00000308	pigs
Insig1	0.21	0.00000311	pigs
Dhx15	0.12	0.00000314	pigs
Romo1	-0.43	0.00000316	pigs
Tbc1d1	0.20	0.00000320	pigs
Capza2	0.25	0.00000323	pigs
Add1	0.10	0.00000324	pigs
Dcun1d4	0.02	0.00000327	pigs
Bmpr2	0.13	0.00000328	pigs
H2ac20	-0.26	0.00000329	pigs
Kidins220	0.07	0.00000332	pigs
Rabepk	0.05	0.00000341	pigs
Ripk2	0.05	0.00000345	pigs
Sumf2	0.02	0.00000351	pigs
Decr1	0.13	0.00000354	pigs
Phkg2	0.03	0.00000355	pigs
B3gnt5	0.11	0.00000363	pigs
Celf1	0.09	0.00000368	pigs
Gatb	0.06	0.00000370	pigs
Zmpste24	0.03	0.00000375	pigs
Acad9	0.06	0.00000378	pigs
Gdap2	0.06	0.00000380	pigs
Camkk1	0.00	0.00000387	pigs
Nadk2	0.04	0.00000390	pigs
Unk	0.06	0.00000392	pigs
Rab35	0.05	0.00000394	pigs

Atg4b	0.06	0.00000394	pigs
Ankrd22	-0.36	0.00000395	pigs
Ptar1	0.07	0.00000397	pigs
Vkorc1l1	0.09	0.00000397	pigs
Dgke	0.06	0.00000409	pigs
Dkc1	0.01	0.00000410	pigs
Ndufb7	-0.28	0.00000410	pigs
Suco	0.04	0.00000418	pigs
Ncstn	0.05	0.00000420	pigs
Sass6	0.06	0.00000421	pigs
Sap30bp	0.07	0.00000427	pigs
Frmpd4	-0.39	0.00000430	pigs
Qrsl1	0.06	0.00000433	pigs
Tuba4a	0.05	0.00000433	pigs
Dpp7	0.07	0.00000443	pigs
Srsf1	0.10	0.00000446	pigs
Stab1	-0.05	0.00000449	pigs
Ift52	0.06	0.00000465	pigs
Tob2	0.11	0.00000471	pigs
Hspa4	0.12	0.00000474	pigs
Slc1a2	-0.58	0.00000475	pigs
Atp10d	0.14	0.00000485	pigs
Piga	0.04	0.00000489	pigs
Ctsf	0.01	0.00000490	pigs
Yipf1	-0.40	0.00000495	pigs
Tasor2	0.04	0.00000502	pigs
Eif3a	0.15	0.00000514	pigs
Slc41a1	0.04	0.00000516	pigs
Ifrd2	0.08	0.00000518	pigs
Npat	0.06	0.00000523	pigs
Uevld	0.09	0.00000524	pigs
Got1	-0.02	0.00000528	pigs
Gpd2	0.05	0.00000535	pigs
Zfand2a	0.17	0.00000535	pigs
Plekhm2	0.18	0.00000540	pigs
Crat	0.05	0.00000545	pigs
Pcyt2	0.01	0.00000557	pigs
Hace1	0.11	0.00000574	pigs
Cfdp1	0.16	0.00000578	pigs
Elovl1	0.02	0.00000592	pigs
Cbll1	0.00	0.00000592	pigs
Naa50	0.11	0.00000604	pigs
Smim19	-0.08	0.00000608	pigs
Tmx1	0.04	0.00000612	pigs
Hltf	0.09	0.00000614	pigs
Aqr	0.03	0.00000617	pigs

Armc8	0.06	0.00000623	pigs
Bub3	0.04	0.00000646	pigs
Ssr2	-0.27	0.00000650	pigs
Erp44	0.12	0.00000657	pigs
Cnih4	0.01	0.00000659	pigs
Adpgk	0.04	0.00000659	pigs
Dpp4	0.08	0.00000681	pigs
Tcp11l1	0.05	0.00000684	pigs
Cmtr1	0.05	0.00000691	pigs
Lrpprc	0.05	0.00000697	pigs
Chp1	0.05	0.00000699	pigs
Hmbox1	0.12	0.00000706	pigs
Ilf3	0.08	0.00000710	pigs
Tmem101	0.08	0.00000721	pigs
Dnmt1	-0.04	0.00000731	pigs
Ntmt1	0.08	0.00000732	pigs
Hnrnpll	0.05	0.00000733	pigs
Cep128	0.06	0.00000742	pigs
Nadk	0.05	0.00000748	pigs
Ubr4	0.09	0.00000752	pigs
Dcp1b	0.06	0.00000759	pigs
Wdr47	0.03	0.00000769	pigs
Ralgapa1	0.19	0.00000787	pigs
Gemin8	0.04	0.00000794	pigs
Kdm4a	0.07	0.00000798	pigs
Apbb3	-0.03	0.00000800	pigs
Son	-0.24	0.00000805	pigs
Tnks2	0.00	0.00000807	pigs
Selenot	0.13	0.00000832	pigs
Snd1	0.20	0.00000835	pigs
Nktr	0.10	0.00000841	pigs
Ep400	0.07	0.00000847	pigs
Susd6	0.08	0.00000847	pigs
Ebag9	0.09	0.00000849	pigs
Emc1	0.04	0.00000855	pigs
Appl2	-0.58	0.00000857	pigs
Btf3	-0.22	0.00000859	pigs
Cdc37l1	0.08	0.00000879	pigs
Ifi44	-0.29	0.00000883	pigs
Ginm1	-0.48	0.00000886	pigs
Fmr1	0.10	0.00000887	pigs
Cd22	0.06	0.00000896	pigs
Grsf1	0.13	0.00000897	pigs
Slfn9	-0.15	0.00000909	pigs
Senp5	0.08	0.00000943	pigs
Itgal	-0.44	0.00000982	pigs

Osbpl2	-0.01	0.00000985	pigs
Tfeb	0.09	0.00001001	pigs
Nfyc	0.06	0.00001005	pigs
C1qc	0.23	0.00001005	pigs
Lamp2	0.12	0.00001014	pigs
Rcbtb2	0.08	0.00001020	pigs
Ak3	0.02	0.00001029	pigs
Tnni2	-0.25	0.00001038	pigs
Pgpep1	0.06	0.00001045	pigs
Strap	0.10	0.00001048	pigs
Rtca	0.05	0.00001056	pigs
Gramd1a	0.04	0.00001072	pigs
Sh3gl1	0.03	0.00001078	pigs
Smap2	0.10	0.00001086	pigs
Pikfyve	0.08	0.00001100	pigs
Reep4	0.06	0.00001118	pigs
Sufu	0.10	0.00001131	pigs
Lpcat1	0.02	0.00001143	pigs
Dmap1	0.07	0.00001177	pigs
Prep	0.11	0.00001185	pigs
Pias4	0.01	0.00001189	pigs
Llgl1	0.01	0.00001192	pigs
Ypel5	0.04	0.00001222	pigs
Coro1c	0.14	0.00001242	pigs
Nphp4	0.07	0.00001255	pigs
Crebbp	0.05	0.00001262	pigs
Tfam	0.03	0.00001266	pigs
Plau	-0.10	0.00001300	pigs
Fgd2	0.12	0.00001302	pigs
Pdcd11	0.03	0.00001307	pigs
Dscc1	0.05	0.00001324	pigs
Cops7a	0.10	0.00001329	pigs
Prrc1	0.04	0.00001339	pigs
Papola	0.15	0.00001348	pigs
Uba1	0.04	0.00001377	pigs
Nipa2	0.07	0.00001380	pigs
Rfxank	0.04	0.00001426	pigs
Rnf115	0.14	0.00001435	pigs
Katnal1	0.10	0.00001442	pigs
Txnl4a	0.02	0.00001443	pigs
Spata2	0.03	0.00001453	pigs
Eif5a2	0.07	0.00001456	pigs
Cdk9	0.05	0.00001461	pigs
Slc48a1	0.07	0.00001470	pigs
Vapa	0.13	0.00001475	pigs
Fh1	0.04	0.00001492	pigs

Cacul1	0.13	0.00001499	pigs
Ciao1	0.06	0.00001512	pigs
Qdpr	-0.27	0.00001520	pigs
Rgs1	-0.64	0.00001534	pigs
Dgkh	0.19	0.00001537	pigs
Rock2	0.04	0.00001572	pigs
H2bc14	0.05	0.00001589	pigs
Plcb4	-0.39	0.00001607	pigs
Fbxw11	0.17	0.00001648	pigs
Abhd5	0.15	0.00001666	pigs
Lrp12	0.05	0.00001698	pigs
Sf3b1	0.14	0.00001727	pigs
Vps45	0.08	0.00001732	pigs
Ncln	0.07	0.00001738	pigs
Ipo8	0.03	0.00001765	pigs
Rprd1a	0.10	0.00001772	pigs
Usp42	0.04	0.00001786	pigs
Bms1	0.08	0.00001786	pigs
Cog3	0.02	0.00001791	pigs
Aqp9	-0.23	0.00001800	pigs
Srp19	-0.28	0.00001840	pigs
Sar1a	0.05	0.00001864	pigs
Supt20	0.07	0.00001891	pigs
Exoc6	-0.39	0.00001917	pigs
Eprs	0.08	0.00001942	pigs
Sec61a1	0.05	0.00001959	pigs
Zfp598	0.08	0.00001976	pigs
Runx2	0.19	0.00002067	pigs
Fcer1g	-0.40	0.00002074	pigs
Tet2	0.13	0.00002104	pigs
Tmx4	-0.37	0.00002133	pigs
Thoc6	0.09	0.00002178	pigs
Helb	0.06	0.00002183	pigs
Cic	-0.02	0.00002220	pigs
Lemd3	0.03	0.00002226	pigs
Pds5b	0.05	0.00002256	pigs
Isg20	-0.24	0.00002259	pigs
Arhgap21	0.17	0.00002272	pigs
Tspan17	0.01	0.00002320	pigs
Brwd1	0.06	0.00002344	pigs
Taf15	0.08	0.00002377	pigs
Arfgef1	0.13	0.00002402	pigs
Sec22b	0.09	0.00002412	pigs
Golm2	0.03	0.00002413	pigs
Yipf6	0.07	0.00002511	pigs
Synj1	0.12	0.00002545	pigs

Usp20	0.04	0.00002576	pigs
Ythdc2	0.03	0.00002584	pigs
Hnrnph3	0.15	0.00002586	pigs
Bicra	0.08	0.00002590	pigs
Ppp1r12c	0.02	0.00002625	pigs
Cep295	0.07	0.00002678	pigs
Gga1	0.07	0.00002770	pigs
Glud1	-0.42	0.00002783	pigs
Capn10	0.09	0.00002844	pigs
Zpr1	0.05	0.00002880	pigs
Ppib	0.16	0.00002901	pigs
Farp1	0.03	0.00002950	pigs
Atg4c	-0.01	0.00002991	pigs
Dnajc14	0.04	0.00003033	pigs
Donson	0.05	0.00003053	pigs
Glul	-0.46	0.00003103	pigs
Dlst	0.07	0.00003164	pigs
Ly6d	-0.47	0.00003171	pigs
Prpf4	0.04	0.00003181	pigs
Dbt	0.08	0.00003232	pigs
Hnrnph1	0.09	0.00003263	pigs
Lztr1	0.05	0.00003304	pigs
Nol4l	0.10	0.00003362	pigs
Zfp207	0.08	0.00003451	pigs
Atp2b1	0.18	0.00003452	pigs
Trappc9	0.14	0.00003540	pigs
Rbm10	0.07	0.00003610	pigs
Xbp1	-0.01	0.00003612	pigs
Dnajc2	0.14	0.00003687	pigs
Cnnm4	0.09	0.00003763	pigs
0610030E20Rik	0.10	0.00003782	pigs
Pnkd	0.03	0.00003821	pigs
Pdcd7	0.05	0.00003977	pigs
Bhlhe41	-0.35	0.00003978	pigs
Cul3	0.10	0.00003994	pigs
Sdhb	0.14	0.00004130	pigs
Apba1	-0.23	0.00004148	pigs
Gadd45b	0.22	0.00004242	pigs
Cog1	0.05	0.00004242	pigs
Hira	0.03	0.00004257	pigs
Ccdc126	0.02	0.00004264	pigs
Rbmx	0.13	0.00004296	pigs
Fam168a	0.12	0.00004319	pigs
3110040N11Rik	-0.32	0.00004331	pigs
Plekhh1	-0.17	0.00004332	pigs
Prkca	0.15	0.00004344	pigs

Herpud1	-0.15	0.00004364	pigs
Sdhc	0.00	0.00004372	pigs
Kif2a	0.02	0.00004454	pigs
Cmklr1	-0.02	0.00004660	pigs
Ikzf3	-0.40	0.00004675	pigs
Septin6	0.08	0.00004697	pigs
Vps53	0.10	0.00004793	pigs
Polr2a	0.07	0.00004821	pigs
Adal	0.01	0.00004831	pigs
Snx18	0.08	0.00004855	pigs
Arhgef7	0.05	0.00004904	pigs
Cipc	0.07	0.00005005	pigs
Adss	0.10	0.00005020	pigs
Zyg11b	0.08	0.00005023	pigs
Actl6a	0.05	0.00005063	pigs
Cnpy3	0.06	0.00005066	pigs
Zfp335	0.05	0.00005182	pigs
Il1r1	0.17	0.00005244	pigs
Nsmce2	0.17	0.00005252	pigs
Atp13a2	0.04	0.00005283	pigs
Chchd6	-0.02	0.00005314	pigs
Prcc	0.05	0.00005329	pigs
C5ar1	-0.68	0.00005334	pigs
Vav3	-0.49	0.00005371	pigs
Frrs1	0.07	0.00005376	pigs
Slc7a11	-0.39	0.00005390	pigs
Rap1gds1	0.01	0.00005515	pigs
Trp53	0.08	0.00005617	pigs
Ttyh3	0.03	0.00005698	pigs
Gtdc1	0.14	0.00005699	pigs
Vti1b	0.04	0.00005711	pigs
Tmigd3	-0.01	0.00005730	pigs
Atmin	0.09	0.00005792	pigs
Ago4	0.04	0.00005818	pigs
Ubp1	0.07	0.00005824	pigs
Sav1	0.02	0.00005907	pigs
Crebrf	0.13	0.00005919	pigs
Rbm5	0.07	0.00005969	pigs
Dync1li2	0.03	0.00005973	pigs
Smg5	0.05	0.00005991	pigs
Eef1g	0.16	0.00006070	pigs
Pla2g2d	0.20	0.00006105	pigs
Fam222b	0.11	0.00006110	pigs
Ice2	0.06	0.00006111	pigs
Rexo5	0.05	0.00006211	pigs
Ppp6c	0.05	0.00006308	pigs

Arl6ip1	0.05	0.00006339	pigs
Prkci	0.07	0.00006369	pigs
Shisa5	0.06	0.00006386	pigs
Slc49a4	0.10	0.00006433	pigs
Fem1c	0.01	0.00006511	pigs
Stradb	0.08	0.00006547	pigs
Kdm5a	0.07	0.00006559	pigs
Cant1	0.01	0.00006601	pigs
Nras	0.02	0.00006609	pigs
Fbxo33	0.11	0.00006733	pigs
Fkbp15	0.09	0.00006743	pigs
Sult1a1	-0.37	0.00006761	pigs
Eps8	-0.31	0.00006911	pigs
Srp54a	0.09	0.00007063	pigs
Smndc1	0.07	0.00007120	pigs
Brd1	0.01	0.00007213	pigs
Nr4a2	-0.44	0.00007253	pigs
Fez2	0.05	0.00007361	pigs
Zfp638	0.01	0.00007512	pigs
Nrg1	-1.39	0.00007525	pigs
Ap4e1	0.05	0.00007554	pigs
Cgrrf1	0.05	0.00007565	pigs
Hars	-0.03	0.00007739	pigs
Prpf19	0.00	0.00007814	pigs
Flvcr1	0.00	0.00007866	pigs
Dennd4a	-0.47	0.00007909	pigs
Disp1	0.04	0.00007953	pigs
Pnpla2	-0.28	0.00008220	pigs
Ppp1r8	0.08	0.00008364	pigs
Taf3	0.09	0.00008378	pigs
Pphln1	0.06	0.00008533	pigs
Rassf2	0.29	0.00008827	pigs
Thap1	0.06	0.00008887	pigs
Wapl	0.14	0.00009002	pigs
Lonrf1	0.06	0.00009180	pigs
Usp31	0.05	0.00009196	pigs
Ulk3	0.04	0.00009259	pigs
Eif4ebp2	0.04	0.00009307	pigs
Gbf1	0.09	0.00009360	pigs
Pelp1	0.04	0.00009498	pigs
Ufd1	0.12	0.00009568	pigs
Zfp395	0.04	0.00009636	pigs
Tmem106b	0.06	0.00009669	pigs
Alcam	-0.70	0.00009720	pigs
Cep250	0.04	0.00009723	pigs
Pomp	0.11	0.00009863	pigs

Notch2	-0.83	0.00009944	pigs
Cdk13	0.09	0.00010120	pigs
Phf2	0.05	0.00010144	pigs
Spg7	0.08	0.00010215	pigs
Slc35a2	0.03	0.00010268	pigs
Klhl13	-0.12	0.00010345	pigs
Acot7	0.04	0.00010468	pigs
Letm1	0.02	0.00010563	pigs
Rnf166	0.06	0.00010874	pigs
Trim27	0.10	0.00010997	pigs
Abhd16a	0.00	0.00011050	pigs
Fermt3	0.15	0.00011375	pigs
Tubb4b	-0.62	0.00011607	pigs
Clasp1	0.04	0.00011880	pigs
Cdk16	0.02	0.00011944	pigs
Polr3d	0.06	0.00011949	pigs
Fbxo25	-0.23	0.00012256	pigs
Thoc2	0.12	0.00012296	pigs
Pnp	0.16	0.00012530	pigs
Lyn	0.12	0.00012632	pigs
Hs1bp3	0.05	0.00012797	pigs
Bpnt1	0.01	0.00012829	pigs
Rbm47	0.18	0.00012857	pigs
Tmem14a	-0.01	0.00012859	pigs
Cpox	0.04	0.00012885	pigs
Fbxo22	0.04	0.00012943	pigs
Chmp1a	0.04	0.00013097	pigs
Brd8	0.06	0.00013116	pigs
Tmcc1	-0.45	0.00013178	pigs
Zcchc17	0.08	0.00013336	pigs
Ints8	0.03	0.00013540	pigs
Spire1	0.05	0.00013653	pigs
Plekhm1	0.02	0.00013727	pigs
Nup88	0.05	0.00013805	pigs
Ilvbl	0.02	0.00013850	pigs
Nmi	0.15	0.00014282	pigs
Zfc3h1	0.06	0.00014388	pigs
Snx8	0.09	0.00014463	pigs
Vcpip1	0.04	0.00014614	pigs
Bmt2	0.09	0.00014662	pigs
Amn1	0.07	0.00014787	pigs
Slc29a1	0.04	0.00014856	pigs
Psd3	-0.11	0.00014908	pigs
Tsnax	0.07	0.00014912	pigs
Galntl5	-0.12	0.00015011	pigs
Polr1b	0.03	0.00015080	pigs

Kat14	0.00	0.00015373	pigs
Smarca5	0.09	0.00015780	pigs
Snx7	0.10	0.00015888	pigs
Drosha	0.01	0.00015918	pigs
Ano6	0.18	0.00016549	pigs
Fbxw4	0.09	0.00016586	pigs
Rpap3	0.06	0.00016667	pigs
Slmap	0.07	0.00016801	pigs
Usp9x	0.05	0.00016830	pigs
Abcd3	0.08	0.00016874	pigs
Ythdc1	0.08	0.00017092	pigs
Clk2	0.06	0.00017284	pigs
Tnks	-0.01	0.00017435	pigs
Hmces	-0.28	0.00017443	pigs
Exosc3	0.05	0.00017590	pigs
Bnip1	0.04	0.00017726	pigs
Ppp1r18	0.08	0.00017744	pigs
Rab33b	0.04	0.00017839	pigs
Tbc1d17	0.03	0.00017971	pigs
Gtf3c1	0.04	0.00018109	pigs
Cldnd1	0.00	0.00018284	pigs
D6Wsu163e	0.08	0.00018393	pigs
Fam98a	0.01	0.00018397	pigs
Srsf9	0.10	0.00018686	pigs
Slc25a3	-0.29	0.00019133	pigs
Unkl	0.06	0.00019397	pigs
Alkbh7	0.04	0.00019678	pigs
Cds2	-0.01	0.00020047	pigs
Trip4	0.09	0.00020505	pigs
Dhx38	0.07	0.00020567	pigs
Acvrl1	0.01	0.00020596	pigs
Ergic2	-0.05	0.00020598	pigs
Tpp2	0.02	0.00020693	pigs
Chm	0.08	0.00020892	pigs
Znhit3	0.03	0.00021096	pigs
Il17ra	-0.24	0.00021435	pigs
Aup1	0.03	0.00021919	pigs
Chd1l	-0.01	0.00022663	pigs
Zfand3	0.13	0.00022871	pigs
Dtwd2	0.11	0.00022910	pigs
Arf1	0.12	0.00023409	pigs
Lhpp	0.02	0.00023409	pigs
Tbxas1	-0.87	0.00023911	pigs
Slc11a1	-0.38	0.00024102	pigs
Tada3	0.06	0.00024115	pigs
Nfic	0.02	0.00024154	pigs

Aoah	0.04	0.00024253	pigs
Vps52	0.05	0.00024338	pigs
Gfod1	-0.48	0.00024588	pigs
Fam98b	0.09	0.00025071	pigs
Carm1	0.03	0.00025283	pigs
Scaf4	-0.02	0.00025767	pigs
C2	0.04	0.00026048	pigs
Ints6	0.06	0.00026103	pigs
Bcap31	0.08	0.00026635	pigs
Klhl28	0.00	0.00026649	pigs
Haus6	0.03	0.00026838	pigs
Usp15	-0.50	0.00027179	pigs
Wbp4	0.03	0.00028150	pigs
Cdc37	0.04	0.00028787	pigs
Nup37	0.10	0.00029022	pigs
Edc3	0.02	0.00029252	pigs
Rrp36	0.03	0.00029324	pigs
Yipf2	0.05	0.00029678	pigs
Grpel2	0.01	0.00029726	pigs
Gnpnat1	0.08	0.00030141	pigs
Fbxl3	0.05	0.00030179	pigs
5031439G07Rik	0.11	0.00030227	pigs
Spidr	0.07	0.00030436	pigs
Zfp384	0.05	0.00030887	pigs
Nrde2	0.08	0.00031102	pigs
Ets2	0.13	0.00031205	pigs
Tmem115	0.01	0.00031685	pigs
Gne	0.07	0.00031839	pigs
Bcl7c	0.01	0.00031935	pigs
Vps13a	0.11	0.00031989	pigs
Zdhhc7	0.02	0.00032416	pigs
Ppil1	0.02	0.00032625	pigs
Usp7	0.09	0.00034365	pigs
Trp53inp2	0.05	0.00035037	pigs
Rhob	0.08	0.00035274	pigs
Tmem104	0.02	0.00035345	pigs
Scnm1	0.01	0.00036032	pigs
Blmh	0.04	0.00036078	pigs
Pld4	-0.34	0.00036394	pigs
Spast	0.01	0.00036434	pigs
Tnk2	0.01	0.00037241	pigs
Nrbp1	0.11	0.00037356	pigs
Fam185a	0.07	0.00037961	pigs
Sdhaf2	0.13	0.00039007	pigs
Rfesd	0.06	0.00039218	pigs
Odc1	-0.54	0.00039636	pigs

Snap29	0.09	0.00040060	pigs
Wdfy3	0.18	0.00041244	pigs
Lrrc57	0.04	0.00041566	pigs
Igsf6	0.15	0.00041934	pigs
Cnnm3	0.03	0.00042038	pigs
Tagap1	-0.46	0.00042704	pigs
Park7	-0.26	0.00043629	pigs
Nfya	0.05	0.00043729	pigs
Supt3	-0.02	0.00043791	pigs
Prepl	0.06	0.00043967	pigs
Tceanc2	0.07	0.00044780	pigs
Paip1	0.04	0.00045136	pigs
Bcl3	0.07	0.00045629	pigs
Sirt7	0.09	0.00045656	pigs
Brpf3	0.01	0.00045719	pigs
Trak1	0.03	0.00045901	pigs
Lyst	-0.43	0.00046995	pigs
Taf5l	0.03	0.00047212	pigs
Txndc15	-0.08	0.00047935	pigs
Tgm1	0.04	0.00047949	pigs
Fbxo8	0.05	0.00048016	pigs
Rnf10	0.08	0.00048181	pigs
Bora	0.02	0.00048520	pigs
Naaa	0.14	0.00049948	pigs
Cul4b	0.05	0.00050867	pigs
Clec12b	-0.27	0.00051629	pigs
Ipo5	0.03	0.00052126	pigs
Abl1	0.07	0.00052341	pigs
Galk1	0.06	0.00053126	pigs
Derl2	0.08	0.00053722	pigs
Slc35g1	-0.15	0.00053968	pigs
Dclre1c	0.09	0.00055061	pigs
Arih2	-0.07	0.00055150	pigs
Agk	0.04	0.00055829	pigs
Hnrnpk	0.13	0.00056678	pigs
Pprc1	0.06	0.00056703	pigs
Virma	-0.01	0.00057396	pigs
Sptbn1	-0.22	0.00057676	pigs
Dock1	0.14	0.00057712	pigs
Pbxip1	-0.02	0.00058372	pigs
Grk5	-0.43	0.00058825	pigs
Ankrd37	-0.02	0.00058842	pigs
Arl13b	-0.02	0.00059256	pigs
Vps54	-0.01	0.00059892	pigs
Fbxo21	0.05	0.00059917	pigs
2310009B15Rik	-0.26	0.00060906	pigs

Dtnbp1	0.08	0.00061396	pigs
Slc25a24	-0.41	0.00061701	pigs
Gart	0.03	0.00061732	pigs
Prdx6	-0.40	0.00062182	pigs
Nme6	0.00	0.00062212	pigs
Ppp1r14bl	0.08	0.00062558	pigs
Src	0.02	0.00062615	pigs
Chsy1	0.09	0.00063038	pigs
Copg2	0.03	0.00063058	pigs
Zbtb40	0.06	0.00064378	pigs
Tcf7l2	0.09	0.00064617	pigs
Slc12a7	0.01	0.00065840	pigs
Tln1	-0.28	0.00065974	pigs
Atxn1	0.17	0.00066183	pigs
Slc6a6	-0.07	0.00066533	pigs
Nsa2	-0.35	0.00066567	pigs
Pnpo	0.08	0.00066943	pigs
Cd47	-0.32	0.00067875	pigs
Tlr2	0.06	0.00068127	pigs
Srsf5	-0.27	0.00069679	pigs
Asxl2	0.10	0.00069994	pigs
Prkcd	0.03	0.00070072	pigs
Sh3glb1	-0.20	0.00070403	pigs
Prkrip1	0.04	0.00070619	pigs
Rev3l	0.08	0.00070892	pigs
H2aj	0.04	0.00070999	pigs
Stxbp5	-0.51	0.00071684	pigs
Tspan14	0.09	0.00072938	pigs
Ppp2r5a	0.05	0.00073744	pigs
Nelfa	0.04	0.00073947	pigs
Xiap	0.10	0.00074383	pigs
C3	-0.13	0.00074705	pigs
Tax1bp3	0.08	0.00076897	pigs
Pdhb	0.07	0.00077120	pigs
Cyria	-0.73	0.00080066	pigs
Pcid2	0.01	0.00080713	pigs
Tmem154	-0.61	0.00081153	pigs
Srprb	0.08	0.00081309	pigs
Ndufs2	0.04	0.00082052	pigs
Arhgap45	-0.33	0.00082205	pigs
Eef1akmt3	0.06	0.00082839	pigs
Atg13	0.04	0.00085083	pigs
Irf2	-0.14	0.00085229	pigs
U2surp	0.10	0.00087445	pigs
Gspt2	-0.24	0.00087930	pigs
Rep15	0.07	0.00088930	pigs

Vps18	0.07	0.00092401	pigs
Amd2	0.08	0.00092846	pigs
Eif4e2	0.08	0.00093414	pigs
Nono	0.09	0.00093430	pigs
Lrrc42	-0.02	0.00094102	pigs
Rnf40	0.02	0.00098716	pigs
Dedd	0.10	0.00099606	pigs
Dnajb14	0.03	0.00099782	pigs
Fam98c	0.08	0.00101341	pigs
Fadd	0.04	0.00101520	pigs
Ubap2	0.12	0.00101524	pigs
Ier5	0.09	0.00101536	pigs
Prrc2c	0.16	0.00102603	pigs
Alg12	0.05	0.00102711	pigs
Ythdf3	0.08	0.00102859	pigs
Lrrc58	0.03	0.00103455	pigs
Adnp	0.05	0.00104188	pigs
Cwc22	0.00	0.00104993	pigs
Ccnq	-0.01	0.00107981	pigs
Cxcl3	0.15	0.00108621	pigs
Ubr2	0.10	0.00109916	pigs
Dnajc7	0.12	0.00111588	pigs
Idh3a	0.02	0.00111767	pigs
Ttc9c	0.03	0.00112081	pigs
Nipbl	0.12	0.00112809	pigs
Tax1bp1	0.03	0.00113661	pigs
Arf4	0.15	0.00115678	pigs
Pip5k1c	0.07	0.00116586	pigs
Ccdc142	0.01	0.00117052	pigs
Iqsec2	0.06	0.00118734	pigs
Utp4	0.05	0.00119002	pigs
Snx20	0.06	0.00119835	pigs
Fhod1	0.04	0.00121560	pigs
Ip6k2	-0.01	0.00125986	pigs
Clcc1	0.01	0.00127931	pigs
Lrrc8a	0.02	0.00128686	pigs
Tsr1	0.03	0.00129084	pigs
Clpb	0.02	0.00131209	pigs
Sinhcaf	0.07	0.00131308	pigs
Upf3b	-0.07	0.00131944	pigs
Gbp2b	0.00	0.00137476	pigs
Gabpb1	0.10	0.00139773	pigs
Bloc1s5	0.05	0.00140578	pigs
Vps25	0.02	0.00141577	pigs
Atf7ip2	0.05	0.00142840	pigs
Tmed5	0.10	0.00145450	pigs

Erbin	0.18	0.00145937	pigs
Flot1	0.08	0.00146120	pigs
Tbc1d22a	0.00	0.00146245	pigs
Pef1	0.08	0.00150130	pigs
Chd3	-0.03	0.00150611	pigs
Oxr1	-0.35	0.00152959	pigs
Rufy3	-0.01	0.00155272	pigs
Tbcd	0.04	0.00158102	pigs
Fbxw5	0.02	0.00159728	pigs
Acap1	-0.15	0.00162533	pigs
Epg5	0.10	0.00163998	pigs
Ddx47	0.03	0.00165318	pigs
Elf1	0.10	0.00165399	pigs
Twsg1	0.06	0.00165873	pigs
Piwil4	0.00	0.00168211	pigs
Dis3	-0.03	0.00169046	pigs
Phf21a	0.05	0.00169657	pigs
Zfhx3	0.01	0.00170064	pigs
Tpd52l2	0.04	0.00171714	pigs
Nisch	0.01	0.00174242	pigs
Wrap53	0.02	0.00177877	pigs
Wdr70	0.03	0.00179474	pigs
Dcaf13	0.04	0.00180381	pigs
Arhgef2	0.08	0.00181971	pigs
Telo2	0.02	0.00183393	pigs
Atp1b1	0.05	0.00184620	pigs
Ndufb8	0.11	0.00184663	pigs
Ggps1	0.01	0.00189163	pigs
Eftud2	0.10	0.00190333	pigs
Rcan3	-0.13	0.00190852	pigs
Bcl6	0.05	0.00192017	pigs
Zc3hc1	0.03	0.00192095	pigs
Dpyd	0.14	0.00192784	pigs
Baz2b	-0.36	0.00195391	pigs
Dcp2	0.07	0.00201075	pigs
Chd8	0.09	0.00203433	pigs
Srp72	0.06	0.00205021	pigs
Btbd9	0.13	0.00207855	pigs
Aldh16a1	0.10	0.00208025	pigs
Aspg	0.07	0.00209968	pigs
Prrc2a	0.03	0.00209986	pigs
Lcp2	0.17	0.00210935	pigs
Nagk	0.06	0.00211136	pigs
Bdp1	0.05	0.00211216	pigs
Nup93	0.04	0.00211447	pigs
Pcsk7	0.04	0.00212253	pigs

Trmt11	0.06	0.00224644	pigs
Bach2	0.19	0.00226168	pigs
Rbm14	0.03	0.00226754	pigs
Htatip2	-0.02	0.00227787	pigs
Lsm14b	0.02	0.00228365	pigs
Tmem147	-0.06	0.00228457	pigs
Tubb2b	0.00	0.00229120	pigs
Ttc13	-0.31	0.00233315	pigs
Aff2	0.27	0.00236449	pigs
Ctu2	0.05	0.00238408	pigs
Arhgap26	0.06	0.00238502	pigs
Ddx27	0.07	0.00241523	pigs
1700123O20Rik	0.02	0.00245094	pigs
Efl1	0.02	0.00245815	pigs
Tm9sf4	0.00	0.00245856	pigs
Ube3c	-0.01	0.00247041	pigs
Etfdh	0.06	0.00247855	pigs
Sart1	0.05	0.00249441	pigs
Ube2w	0.02	0.00250060	pigs
Flna	-0.35	0.00252110	pigs
Qrich1	0.06	0.00257500	pigs
Cish	0.16	0.00266435	pigs
Nadsyn1	0.03	0.00266483	pigs
Ubac1	0.04	0.00267875	pigs
Slc35a5	-0.01	0.00269457	pigs
Glyr1	0.04	0.00274170	pigs
Zfp697	0.00	0.00274174	pigs
Ccdc124	-0.02	0.00274807	pigs
Prkd3	0.08	0.00276951	pigs
Card19	0.06	0.00277488	pigs
Nufip2	0.07	0.00281274	pigs
Cux1	0.10	0.00286625	pigs
Ro60	0.01	0.00289115	pigs
Ccdc117	0.02	0.00294102	pigs
Nop10	0.17	0.00295129	pigs
Vezf1	0.02	0.00296824	pigs
Rnf103	-0.02	0.00299634	pigs
Ppp2r1b	-0.01	0.00299741	pigs
S100a8	-1.30	0.00300309	pigs
Tradd	0.12	0.00300584	pigs
Fbxo34	-0.01	0.00301648	pigs
Kdm4c	0.01	0.00304426	pigs
Zbtb24	0.05	0.00304957	pigs
Grcc10	-0.38	0.00308889	pigs
Polg	0.03	0.00317813	pigs
Sprtn	0.04	0.00321338	pigs

Smarce1	0.03	0.00322294	pigs
Ppp4r3a	0.08	0.00323453	pigs
Pdcd6	-0.38	0.00324161	pigs
Arl11	0.10	0.00335570	pigs
Atp2a3	0.04	0.00337133	pigs
Abhd17b	0.08	0.00338697	pigs
Trim23	0.08	0.00339572	pigs
Usp30	-0.02	0.00339813	pigs
Tmem192	0.04	0.00341916	pigs
Rnf170	0.03	0.00343616	pigs
Tmtc3	0.04	0.00353376	pigs
Ift46	-0.03	0.00355712	pigs
Dennd10	-0.01	0.00357265	pigs
Pdlim1	-0.37	0.00357749	pigs
Gpat4	-0.03	0.00358384	pigs
Zbtb41	0.02	0.00364500	pigs
Cdk6	0.11	0.00366393	pigs
Sar1b	0.13	0.00367139	pigs
Gss	-0.01	0.00371468	pigs
Cysltr2	-0.19	0.00372840	pigs
C2cd5	0.04	0.00376997	pigs
Ppp6r1	0.03	0.00382999	pigs
Atf1	0.03	0.00384978	pigs
Rpf2	0.06	0.00389975	pigs
Cdyl2	0.05	0.00391044	pigs
Tmem128	0.05	0.00391209	pigs
Rapgef2	0.18	0.00392469	pigs
Ugdh	-0.01	0.00399306	pigs
Cdc73	-0.01	0.00401147	pigs
Gltp	0.02	0.00409871	pigs
Inpp4a	0.02	0.00412687	pigs
Usf2	0.03	0.00413115	pigs
Rnf34	0.09	0.00414515	pigs
Rngtt	0.04	0.00418384	pigs
Polr3a	-0.01	0.00424900	pigs
Dock7	0.07	0.00434522	pigs
Paxbp1	0.03	0.00437677	pigs
C2cd3	-0.22	0.00438018	pigs
Acat3	-0.07	0.00441577	pigs
Pofut1	0.01	0.00441687	pigs
Fkbp5	0.11	0.00442782	pigs
Ergic3	-0.01	0.00444753	pigs
Rnf141	0.02	0.00445239	pigs
Lrrc75a	-0.12	0.00449634	pigs
Rfc3	0.21	0.00450760	pigs
Capn1	0.01	0.00452319	pigs

Inpp5b	-0.01	0.00454503	pigs
Arf5	0.01	0.00455689	pigs
Pus7	-0.05	0.00456232	pigs
Btbd2	0.02	0.00465076	pigs
Ktn1	0.08	0.00466994	pigs
Tysnd1	0.02	0.00474731	pigs
Zfp511	0.04	0.00481039	pigs
Adamtsl4	0.02	0.00484332	pigs
Plvap	-0.61	0.00487270	pigs
Psmb3	0.06	0.00487615	pigs
Cox11	0.06	0.00493167	pigs
Rab31	-0.43	0.00493781	pigs
Shmt2	0.08	0.00495615	pigs
Gaa	-0.01	0.00498827	pigs
Als2	0.05	0.00500249	pigs
Smarcd2	-0.01	0.00506296	pigs
Nhej1	0.07	0.00509977	pigs
Syne2	-0.37	0.00515606	pigs
Zcrb1	0.05	0.00517525	pigs
Gak	0.00	0.00517715	pigs
Dpagt1	0.06	0.00524119	pigs
Cops8	-0.05	0.00525659	pigs
Rbm19	0.08	0.00532318	pigs
Ddx56	0.04	0.00543765	pigs
Rida	0.10	0.00544804	pigs
Ezh1	-0.02	0.00547704	pigs
Tmem254a	0.01	0.00553101	pigs
Asph	0.04	0.00567496	pigs
Ap5m1	0.08	0.00567602	pigs
Rsl1d1	0.06	0.00568783	pigs
Dcaf11	0.01	0.00575366	pigs
Stx17	0.02	0.00579332	pigs
Dennd1c	0.04	0.00585903	pigs
Slain2	0.08	0.00593212	pigs
Fbxw7	0.13	0.00593436	pigs
Gys1	0.06	0.00595422	pigs
Nudcd3	0.05	0.00599144	pigs
Scoc	-0.03	0.00608856	pigs
Rcc2	0.01	0.00611141	pigs
Dync1i2	0.05	0.00612720	pigs
Abcd1	-0.01	0.00616867	pigs
Ntn4	0.07	0.00619243	pigs
Atxn7l3	0.04	0.00630316	pigs
Erbb4	-0.33	0.00634011	pigs
Sil1	-0.03	0.00634887	pigs
Cd44	0.11	0.00635928	pigs

Srp68	0.08	0.00638362	pigs
Rlim	0.07	0.00639245	pigs
Gpr34	0.03	0.00644169	pigs
Ilf2	0.04	0.00649376	pigs
Dhrs1	0.11	0.00653586	pigs
Rad17	0.03	0.00654946	pigs
Alg14	-0.01	0.00655247	pigs
Cyb561d2	-0.01	0.00655455	pigs
Aldh1l2	-0.01	0.00658344	pigs
Tdpoz8	0.10	0.00663063	pigs
Rnaseh2c	0.04	0.00665436	pigs
Zswim4	0.01	0.00666043	pigs
Txlna	0.07	0.00668224	pigs
Prmt5	0.04	0.00670189	pigs
Traf2	0.05	0.00673937	pigs
Dpf2	0.03	0.00680204	pigs
Bap1	-0.02	0.00681375	pigs
Rexo1	0.04	0.00696515	pigs
Uqcrc2	0.06	0.00700010	pigs
Usp48	0.02	0.00702163	pigs
Zfp800	0.04	0.00704746	pigs
Tango2	0.06	0.00709904	pigs
Paqr8	0.05	0.00711272	pigs
Pafah1b3	0.04	0.00719519	pigs
Apip	-0.01	0.00726982	pigs
Ddx6	0.11	0.00728049	pigs
Phf20l1	0.16	0.00734985	pigs
Clec7a	0.21	0.00735615	pigs
Tmem156	0.12	0.00737304	pigs
Gtf2b	0.08	0.00739659	pigs
Naa30	0.02	0.00745353	pigs
Crkl	0.00	0.00759746	pigs
Zfp280c	-0.05	0.00759827	pigs
Fam120b	0.03	0.00764647	pigs
Abhd18	0.04	0.00769998	pigs
Cds1	0.08	0.00772538	pigs
Klhl8	-0.08	0.00774555	pigs
Tlk1	0.08	0.00777308	pigs
Eif3j2	0.11	0.00785703	pigs
Nol10	0.03	0.00804285	pigs
Rcl1	0.06	0.00806356	pigs
Zfp407	0.08	0.00807371	pigs
Ash1l	0.09	0.00809217	pigs
Btbd6	0.06	0.00814741	pigs
Csgalnact2	0.15	0.00817661	pigs
Pigg	0.06	0.00831629	pigs

Lrrcc1	0.05	0.00845189	pigs
Rnaseh2b	-0.04	0.00845218	pigs
Acin1	0.02	0.00847370	pigs
Nhsl1	-0.19	0.00849179	pigs
Dap3	0.03	0.00849727	pigs
Srm	0.10	0.00854794	pigs
Htt	0.04	0.00863307	pigs
Nop2	0.04	0.00863590	pigs
Ythdf2	0.02	0.00864618	pigs
Gadd45a	0.52	0.00869780	pigs
Cwc15	0.06	0.00870265	pigs
Cflar	-0.13	0.00874657	pigs
Papss1	0.10	0.00879816	pigs
Hsf2	-0.02	0.00893643	pigs
Eif3m	0.09	0.00893916	pigs
Ftsj3	0.05	0.00897888	pigs
Bcl2l14	0.10	0.00908884	pigs
Gpr155	-0.02	0.00911681	pigs
Uchl3	0.06	0.00920748	pigs
Tcta	-0.01	0.00922205	pigs
Pcbp1	0.06	0.00931568	pigs
Akap11	0.04	0.00939481	pigs
Idi1	-0.42	0.00948860	pigs
Akr1a1	0.06	0.00960041	pigs
Ncbp3	0.02	0.00972075	pigs
Prim2	0.00	0.00976017	pigs
Kmt5b	0.04	0.00984580	pigs
Rabl3	0.01	0.00997850	pigs
Cilk1	-0.20	0.01006918	pigs
Haao	0.03	0.01012232	pigs
Kmt5a	0.03	0.01012628	pigs
Ankrd42	-0.09	0.01015738	pigs
Fam102a	0.00	0.01017954	pigs
Vbp1	0.13	0.01019635	pigs
Arl4a	-0.50	0.01032797	pigs
Hspa13	0.00	0.01035670	pigs
Tshz1	0.00	0.01043849	pigs
Jchain	-0.34	0.01058546	pigs
Snrnp70	0.01	0.01062254	pigs
Pccb	0.08	0.01066993	pigs
AW549877	-0.01	0.01082166	pigs
Abraxas1	-0.05	0.01083778	pigs
Emc10	-0.01	0.01087112	pigs
Psmd5	-0.03	0.01110770	pigs
Itpk1	0.08	0.01113609	pigs
AI837181	0.01	0.01157003	pigs

Snrpd1	-0.02	0.01177398	pigs
Stxbp3	0.06	0.01194307	pigs
Nkapd1	0.00	0.01204691	pigs
Ric8a	0.04	0.01220426	pigs
Ddb2	0.04	0.01229235	pigs
Scrn3	-0.03	0.01229270	pigs
Ndufaf5	0.04	0.01234440	pigs
Gnl3l	0.04	0.01256835	pigs
Uggt1	0.00	0.01260367	pigs
Plekhb2	0.04	0.01263378	pigs
Klhl36	0.05	0.01268981	pigs
Hscb	0.01	0.01270865	pigs
Ess2	0.06	0.01278573	pigs
Nfatc1	0.00	0.01280482	pigs
Scap	0.00	0.01291219	pigs
Colec12	-0.46	0.01337337	pigs
Ldb1	0.03	0.01343053	pigs
Dpp9	0.02	0.01358796	pigs
Abcc5	0.04	0.01368812	pigs
Polr2e	0.06	0.01386040	pigs
Tmppe	0.05	0.01394093	pigs
Abhd11	-0.36	0.01394828	pigs
Uri1	0.10	0.01406009	pigs
Ddx21	-0.34	0.01422687	pigs
Tirap	0.06	0.01433474	pigs
Cd79a	-0.32	0.01440444	pigs
Pja2	0.04	0.01448608	pigs
Esf1	0.07	0.01482088	pigs
Slc39a8	-0.44	0.01482393	pigs
Dld	-0.02	0.01505564	pigs
Slc25a22	0.00	0.01548066	pigs
Ankrd49	0.05	0.01560402	pigs
Ints3	0.03	0.01573784	pigs
Rmnd5b	0.02	0.01583496	pigs
Gtf2h3	-0.01	0.01593115	pigs
Snx13	0.05	0.01595380	pigs
B3gnt2	0.22	0.01607871	pigs
Fam162a	0.07	0.01622803	pigs
Pcgf3	0.05	0.01634422	pigs
Cox10	0.05	0.01638777	pigs
Stat1	-0.32	0.01641855	pigs
Lsm2	-0.01	0.01642089	pigs
Sephs1	0.02	0.01644331	pigs
Zranb2	0.07	0.01653188	pigs
Ift81	-0.23	0.01662286	pigs
Alg6	0.05	0.01678656	pigs

Spryd3	0.03	0.01731180	pigs
Sat2	0.04	0.01759435	pigs
Ptgs2	-0.34	0.01771061	pigs
Cep78	0.00	0.01771099	pigs
Plcd1	-0.19	0.01775093	pigs
Eif4e3	-0.03	0.01780727	pigs
Lasp1	0.03	0.01783198	pigs
Best1	-0.15	0.01818535	pigs
Prpf8	0.05	0.01831889	pigs
Tep1	0.03	0.01847715	pigs
Snx30	0.11	0.01852549	pigs
Samd4b	-0.02	0.01860750	pigs
Ascc2	0.03	0.01864864	pigs
Tesk1	0.06	0.01864951	pigs
Nbas	0.03	0.01874100	pigs
Tap1	-0.03	0.01878111	pigs
Cdk17	0.04	0.01884212	pigs
Ilkap	0.00	0.01894333	pigs
Dcps	-0.01	0.01910398	pigs
Lmnb1	-0.05	0.01915683	pigs
Orai3	0.04	0.01917965	pigs
Eif2ak2	-0.37	0.01918477	pigs
Tubgcp4	0.01	0.01919289	pigs
Polr2h	0.02	0.01932985	pigs
Supt4a	0.12	0.01960787	pigs
Ccni	0.01	0.01992252	pigs
Cfp	0.05	0.01998479	pigs
Abhd10	-0.13	0.01998651	pigs
Slc25a37	0.05	0.02026174	pigs
Banp	0.02	0.02058462	pigs
Slc50a1	-0.05	0.02068836	pigs
Abi1	0.04	0.02105613	pigs
Lrrc8d	0.01	0.02112640	pigs
Desi1	0.10	0.02120864	pigs
Nop53	0.03	0.02129873	pigs
Tbc1d8	-0.40	0.02173176	pigs
Ecsit	0.05	0.02174617	pigs
Snx9	0.07	0.02186743	pigs
Ccdc6	0.08	0.02187077	pigs
Tmbim6	0.08	0.02198194	pigs
Siah2	-0.32	0.02239597	pigs
Nt5c2	0.15	0.02245705	pigs
Rpf1	0.01	0.02314220	pigs
Ctr9	0.00	0.02327165	pigs
Chid1	0.02	0.02349485	pigs
Cpt1b	0.01	0.02393139	pigs

Nolc1	0.08	0.02397001	pigs
Hivep1	-0.44	0.02398518	pigs
Tmem183a	0.04	0.02423134	pigs
Secisbp2	-0.33	0.02451899	pigs
Gsap	-0.39	0.02494772	pigs
Snrpa1	-0.05	0.02500284	pigs
Sf3b6	0.04	0.02507544	pigs
Ccnt1	-0.03	0.02518761	pigs
Chd1	0.13	0.02526102	pigs
Ficd	-0.03	0.02528516	pigs
Adipor2	0.03	0.02534305	pigs
Dnajb2	-0.01	0.02547758	pigs
Togaram1	-0.01	0.02588277	pigs
Ppm1m	0.02	0.02595475	pigs
Ripk1	0.05	0.02601234	pigs
Tcof1	0.04	0.02609718	pigs
Tmem123	-0.23	0.02637158	pigs
Blm	-0.01	0.02652874	pigs
Rara	-0.38	0.02658273	pigs
Galnt6	0.02	0.02720200	pigs
Strn	0.06	0.02725723	pigs
Psmd1	0.07	0.02732712	pigs
Clp1	0.04	0.02737888	pigs
Rfc1	0.01	0.02741613	pigs
Cpm	-0.56	0.02752803	pigs
Cnep1r1	0.02	0.02774551	pigs
Pdia6	-0.16	0.02787445	pigs
Nr3c2	-0.15	0.02879930	pigs
Llph	0.11	0.02913667	pigs
Tnfrsf11a	-0.11	0.02917741	pigs
Txndc5	0.46	0.02983792	pigs
Cdc123	0.02	0.02995376	pigs
Slc31a1	0.02	0.02998111	pigs
Lpin1	0.05	0.03011125	pigs
Cdc42	0.13	0.03020505	pigs
Rbbp8	-0.03	0.03034962	pigs
Slc30a6	0.00	0.03046426	pigs
Senp2	0.01	0.03048230	pigs
Pdcl3	-0.04	0.03049917	pigs
Zbtb2	-0.01	0.03075068	pigs
Fxr2	0.02	0.03122878	pigs
Serpini1	-0.23	0.03136759	pigs
Etf1	0.06	0.03155400	pigs
Abhd3	0.05	0.03194498	pigs
Actr1b	0.00	0.03213931	pigs
Umad1	0.00	0.03246094	pigs

Iffo2	-0.02	0.03249965	pigs
Ccrl2	0.04	0.03294399	pigs
Exosc1	0.05	0.03305992	pigs
Tbc1d22b	0.05	0.03306167	pigs
Rev1	-0.01	0.03323789	pigs
Pfkfb4	0.00	0.03329665	pigs
Clptm1l	0.03	0.03336287	pigs
Spopl	0.02	0.03339077	pigs
Nenf	0.02	0.03363839	pigs
Lrrc47	0.01	0.03382961	pigs
Atp6v0a2	0.00	0.03389318	pigs
Spcs2	-0.09	0.03416873	pigs
Setbp1	-0.22	0.03432230	pigs
Crnkl1	0.03	0.03442843	pigs
Prkcq	0.10	0.03450147	pigs
Pde6d	-0.01	0.03457338	pigs
Serp1	-0.25	0.03475062	pigs
Cdc16	-0.03	0.03519287	pigs
Naa35	0.01	0.03529131	pigs
Stk11	-0.01	0.03536121	pigs
Yif1a	-0.01	0.03560162	pigs
Hipk3	-1.04	0.03600484	pigs
Eif4a3l1	0.05	0.03691069	pigs
Sike1	0.05	0.03800053	pigs
Nfe2l3	-0.19	0.03886140	pigs
Fbxo31	0.03	0.03904054	pigs
Ap1m1	-0.01	0.03950121	pigs
Chtop	0.05	0.03952268	pigs
Tmem248	0.05	0.04007210	pigs
H2az2	-0.29	0.04041475	pigs
Usp47	0.04	0.04058823	pigs
Ide	0.05	0.04082447	pigs
Terf2	-0.01	0.04084478	pigs
Lat2	0.04	0.04175735	pigs
Hnrnpa3	0.07	0.04194084	pigs
Os9	-0.02	0.04208684	pigs
Gnpat	0.01	0.04225950	pigs
Cdc40	0.03	0.04286865	pigs
Pop7	0.04	0.04363070	pigs
Psmd7	0.05	0.04473353	pigs
Rhbdd2	-0.02	0.04510349	pigs
Vps26a	0.00	0.04513874	pigs
Ybx3	0.12	0.04603395	pigs
Jarid2	0.04	0.04653773	pigs
Fam210b	-0.01	0.04660502	pigs
Txnl1	0.09	0.04675084	pigs

Ext1	0.12	0.04677363	pigs
Eif2ak3	0.10	0.04681372	pigs
Derl3	-0.72	0.04717322	pigs
Tomm70a	0.01	0.04718169	pigs
Ssh1	-0.05	0.04747855	pigs
Fastk	0.02	0.04775293	pigs
Supt16	0.02	0.04784214	pigs
Slc17a9	0.01	0.04813441	pigs
Puf60	0.02	0.04866259	pigs
Txk	-0.25	0.04884654	pigs
Slc8a1	0.15	0.04895468	pigs
Gfus	0.00	0.04948655	pigs
Sbds	0.01	0.05043727	pigs
Dot1l	-0.06	0.05060368	pigs
Ahctf1	-0.60	0.05064652	pigs
Trmt13	0.08	0.05083158	pigs
Stx18	0.03	0.05238958	pigs
Rasa3	-0.48	0.05256297	pigs
Sh3tc1	-0.15	0.05267293	pigs
Slc9a8	0.07	0.05283988	pigs
Trappc13	0.02	0.05327988	pigs
Xpo6	-0.68	0.05370812	pigs
Rubcnl	-0.51	0.05374483	pigs
Tor1a	0.00	0.05419780	pigs
AI413582	-0.01	0.05511123	pigs
Got2	-0.05	0.05551454	pigs
Hmgcr	-0.22	0.05561799	pigs
Lgals8	0.15	0.05602402	pigs
Ciita	0.04	0.05615033	pigs
Alms1	0.02	0.05699438	pigs
Sacm1l	0.01	0.05785129	pigs
Pgam5	0.02	0.05794791	pigs
Taf13	-0.05	0.05855151	pigs
Vopp1	0.03	0.05923135	pigs
Borcs5	0.12	0.05940797	pigs
Eif3f	-0.30	0.05953015	pigs
Erc1	-0.27	0.06032950	pigs
Nob1	0.02	0.06055592	pigs
Tmem60	0.07	0.06077331	pigs
Gripap1	0.00	0.06091808	pigs
Zmat5	-0.01	0.06243738	pigs
Sec22a	0.01	0.06244168	pigs
Golga3	0.02	0.06271847	pigs
Ufc1	-0.06	0.06282930	pigs
Cct7	0.07	0.06335496	pigs
Cttnbp2nl	-0.01	0.06370447	pigs

Ubxn6	-0.03	0.06382882	pigs
Gars	0.02	0.06488489	pigs
Smg9	0.02	0.06517974	pigs
Rrm2b	0.05	0.06521600	pigs
Fam126b	-0.04	0.06551620	pigs
Nipsnap3b	0.06	0.06588795	pigs
Rrp1	0.02	0.06684250	pigs
Vars	0.02	0.06717809	pigs
Bcl2l13	0.05	0.06835359	pigs
Prr7	-0.20	0.06938789	pigs
Lgals3	-0.13	0.07076513	pigs
Ccdc43	0.04	0.07116324	pigs
Tnfrsf1a	0.00	0.07255003	pigs
Rasgrp3	-0.14	0.07285794	pigs
Fhit	-0.24	0.07457306	pigs
Alkbh4	0.05	0.07526868	pigs
Atf6	0.04	0.07543002	pigs
Srpk2	0.05	0.07609510	pigs
Tlr4	0.08	0.07610440	pigs
Zfp341	0.02	0.07678985	pigs
Usp24	0.06	0.07756103	pigs
Aasdhppt	0.05	0.07770625	pigs
Cenpj	0.00	0.07778423	pigs
Hmg20a	0.02	0.07827711	pigs
Tram2	0.15	0.07909518	pigs
Ccdc85b	0.05	0.07918482	pigs
Gpatch1	0.00	0.07919395	pigs
Dph5	0.07	0.07945143	pigs
Ascc3	0.12	0.07959978	pigs
Fyb	0.02	0.07970379	pigs
Prkcb	0.06	0.07975023	pigs
Dctn4	-0.03	0.07986690	pigs
Zer1	0.01	0.08051130	pigs
Aar2	0.02	0.08098949	pigs
Abhd4	-0.15	0.08112076	pigs
Rnf13	0.06	0.08263273	pigs
Clns1a	0.04	0.08273613	pigs
Zfp146	-0.01	0.08333672	pigs
Trafd1	0.02	0.08365974	pigs
Ppp3cb	-0.02	0.08425227	pigs
Idh3g	0.06	0.08524681	pigs
St14	0.07	0.08542253	pigs
Hadh	0.09	0.08595521	pigs
Stag1	0.10	0.08596839	pigs
Lyplal1	0.05	0.08609393	pigs
Gpkow	0.05	0.08956625	pigs

Scyl3	0.00	0.09058805	pigs
Cul1	0.04	0.09288249	pigs
Fgd3	-0.04	0.09462788	pigs
Drap1	0.05	0.09476298	pigs
Pfdn1	-0.03	0.09483283	pigs
Taco1	0.01	0.09533819	pigs
Dnajc8	0.04	0.09538726	pigs
Auh	-0.56	0.09590109	pigs
Dek	-0.45	0.09693262	pigs
Taldo1	-0.22	0.09798611	pigs
Tmem175	0.03	0.09804251	pigs
Kcna3	-0.37	0.09826527	pigs
Fdft1	0.06	0.09868495	pigs
Gpr171	0.05	0.09979013	pigs
Irf3	-0.03	0.09989247	pigs
Rnf114	0.03	0.10096538	pigs
Cog2	0.01	0.10107423	pigs
LTO1	-0.02	0.10192413	pigs
Rrn3	0.01	0.10225553	pigs
Arhgap27	-0.11	0.10236581	pigs
Lactb2	-0.02	0.10291872	pigs
Itgb1	-0.36	0.10342098	pigs
Slc35b1	0.00	0.10483626	pigs
Arhgap5	-0.05	0.10763197	pigs
Arhgap35	0.06	0.10906781	pigs
Gnal	0.00	0.10909141	pigs
Plscr1	-0.35	0.10951541	pigs
Ly86	-0.01	0.10974334	pigs
Ubtd1	0.08	0.11065445	pigs
Ccnk	0.08	0.11098105	pigs
Tmem41b	-0.01	0.11120249	pigs
Atg4a-ps	0.01	0.11222095	pigs
Aamp	0.03	0.11334323	pigs
Fuom	-0.14	0.11351530	pigs
Ufl1	-0.04	0.11382848	pigs
Acsl5	0.02	0.11409858	pigs
Alox5	-0.18	0.11415665	pigs
Slc6a9	-0.04	0.11436219	pigs
Ndufa6	0.01	0.11446755	pigs
Zfp618	0.09	0.11577048	pigs
Csrnp1	0.05	0.11586837	pigs
P2ry12	-0.20	0.11651981	pigs
Ints11	0.02	0.11743249	pigs
Fbxo30	0.03	0.11752155	pigs
Atrx	0.08	0.11819963	pigs
Tmco3	-0.03	0.11868424	pigs

Cdkn2aip	-0.03	0.11965916	pigs
Pde4a	0.04	0.11998251	pigs
Trappc4	-0.33	0.12024845	pigs
Sf3a3	-0.04	0.12105007	pigs
Hnrnpm	0.08	0.12132385	pigs
Zfat	0.05	0.12250926	pigs
Cdk7	0.03	0.12280344	pigs
Simc1	0.00	0.12303931	pigs
Crybg3	-0.24	0.12347050	pigs
Prkar2a	0.00	0.12375760	pigs
Bod1l	0.00	0.12623421	pigs
Pmpcb	0.02	0.12702680	pigs
Vrk1	-0.21	0.12768930	pigs
Cops5	0.05	0.12994438	pigs
Rnf126	0.06	0.13047603	pigs
Nosip	-0.02	0.13073611	pigs
Phf6	0.02	0.13223080	pigs
Snapc1l	0.04	0.13225426	pigs
Sppl2a	0.10	0.13266822	pigs
Dctn6	0.05	0.13381013	pigs
Pold2	0.03	0.13508809	pigs
Rabgap1l	-0.34	0.13509247	pigs
Naf1	0.09	0.13534250	pigs
Wrnip1	-0.05	0.13639140	pigs
Foxo1	0.03	0.13927124	pigs
Immt	-0.03	0.14203076	pigs
Utp25	-0.02	0.14422893	pigs
Fam122b	0.03	0.14456416	pigs
Spring1	0.04	0.14649038	pigs
Sin3b	0.00	0.14820710	pigs
Slc25a28	0.05	0.14930093	pigs
Nudt5	0.06	0.15041226	pigs
Sars	-0.36	0.15263410	pigs
Tmco1	-0.28	0.15383724	pigs
Cars2	0.03	0.15411400	pigs
Uck2	0.07	0.15623765	pigs
Proser1	-0.01	0.15736313	pigs
Nedd9	-0.27	0.15751160	pigs
Rhbdd3	0.03	0.15772209	pigs
Pitrm1	-0.05	0.15883117	pigs
Hfe	0.00	0.15972123	pigs
Cyp51	-0.39	0.16276889	pigs
Tcea1	-0.26	0.16373174	pigs
Sec16a	0.06	0.16439455	pigs
Prpf4b	-0.33	0.16590144	pigs
Usp11	0.01	0.16595984	pigs

Cebpb	0.20	0.16654044	pigs
Cass4	-0.28	0.16757487	pigs
Dnajc16	0.05	0.16885378	pigs
Btbd10	-0.01	0.17094414	pigs
Itsn1	-0.10	0.17225379	pigs
Dnmt3a	0.00	0.17425882	pigs
Tmc1	-0.30	0.17433502	pigs
Brms1	0.03	0.17472791	pigs
Sertad3	0.05	0.17489091	pigs
Duoxa2	-0.30	0.17679365	pigs
Fcho1	0.06	0.17684210	pigs
Vezt	0.02	0.17727710	pigs
Gemin7	-0.16	0.18210196	pigs
Arap3	0.01	0.18210815	pigs
Ppp4r1	0.04	0.18316154	pigs
Tmem126b	0.03	0.18845051	pigs
Nubp2	-0.04	0.18935364	pigs
Ttc37	0.02	0.19244314	pigs
Dock2	-0.33	0.19364871	pigs
Unc119	-0.23	0.19376337	pigs
Fam53b	-0.05	0.19395650	pigs
Gde1	0.01	0.19395999	pigs
Cbx4	0.03	0.19401947	pigs
Sfmbt1	0.04	0.19980346	pigs
Zfp24	-0.02	0.20177895	pigs
Bsdc1	0.01	0.20269921	pigs
Rchy1	-0.01	0.20327781	pigs
Ndufaf8	-0.13	0.20440395	pigs
Exoc2	-0.04	0.20453592	pigs
Arid1a	-0.01	0.20471297	pigs
Dipk1a	0.03	0.20628680	pigs
Vamp4	0.02	0.20708031	pigs
Surf1	-0.21	0.20714159	pigs
Cisd3	-0.12	0.20980482	pigs
Btbd1	0.04	0.21104295	pigs
Vrk3	-0.02	0.21116158	pigs
1600012H06Rik	0.00	0.21399324	pigs
Elf2	0.04	0.21480333	pigs
Klhl2	-0.03	0.21728586	pigs
1110032A03Rik	0.01	0.21790039	pigs
Gcc2	-0.02	0.21839350	pigs
Tmem242	0.00	0.21910100	pigs
Ddx20	-0.01	0.21951815	pigs
Tufm	0.01	0.22162901	pigs
Ggcx	-0.06	0.22255615	pigs
Yars	-0.03	0.22354640	pigs

Rab3il1	-0.19	0.22456704	pigs
Copa	0.00	0.22742279	pigs
Pcca	-0.03	0.22838393	pigs
Thap11	0.00	0.23208871	pigs
Rabif	0.03	0.23485322	pigs
Adam10	-0.01	0.23890013	pigs
Gk5	0.23	0.24063486	pigs
Polr1a	-0.05	0.24108074	pigs
Gimap4	-0.22	0.24294947	pigs
Baz1a	0.15	0.24354732	pigs
Prkab1	-0.05	0.24867941	pigs
Prrc2b	0.00	0.24931170	pigs
Ciapin1	-0.07	0.24941526	pigs
Tmem92	-0.16	0.25035327	pigs
Hibadh	0.01	0.25079787	pigs
Zswim6	0.04	0.25258663	pigs
Slc35b3	0.05	0.25272991	pigs
Slc27a4	-0.02	0.25278723	pigs
Snrpf	0.00	0.25425791	pigs
Zfp330	-0.01	0.25433516	pigs
Irf5	-0.38	0.25532052	pigs
Ing1	0.03	0.25623606	pigs
Ppp1r35	-0.11	0.25700734	pigs
Exosc8	0.04	0.26049452	pigs
Parp1	-0.05	0.26267248	pigs
Pacc1	0.04	0.26284344	pigs
Rab8b	-0.35	0.26348099	pigs
Dcaf15	0.03	0.26714239	pigs
Polh	0.04	0.27263261	pigs
Rhot1	0.03	0.27368791	pigs
Exoc1	-0.02	0.27529476	pigs
Hnmt	0.15	0.28163047	pigs
Cry1	-0.03	0.28194273	pigs
Exosc10	0.02	0.28458765	pigs
Spag8	-0.49	0.29027907	pigs
9130409I23Rik	0.05	0.29188810	pigs
Vps50	0.02	0.29480824	pigs
Sfxn4	-0.02	0.29954792	pigs
Tsc22d2	-0.03	0.30005199	pigs
Synj2bp	0.08	0.30317068	pigs
Crtap	0.03	0.30393967	pigs
Lypla2	-0.04	0.30465028	pigs
Snrpa	0.00	0.30699763	pigs
Acadsb	-0.01	0.30732279	pigs
Bfar	0.05	0.31701350	pigs
Tmem106c	-0.04	0.32057617	pigs

Gtf3a	0.06	0.32458911	pigs
Abcf1	0.00	0.32692476	pigs
Lin7c	0.00	0.32704112	pigs
Dock11	-0.29	0.33155557	pigs
Nsmaf	0.02	0.33156727	pigs
Bud31	0.07	0.33250238	pigs
Rad23a	0.01	0.33796514	pigs
Creb3l2	0.04	0.34002767	pigs
9930021J03Rik	0.02	0.34298401	pigs
Zfp512b	-0.01	0.34308471	pigs
Orai1	0.02	0.34391116	pigs
Acbd5	-0.02	0.34432663	pigs
Necap1	0.01	0.35042980	pigs
Trp53rka	0.02	0.35055882	pigs
Rhbdd1	-0.04	0.35086608	pigs
Thop1	0.04	0.35283417	pigs
Snx10	0.22	0.35339977	pigs
Azin1	0.10	0.35613737	pigs
Plec	-0.27	0.35974872	pigs
Slc30a1	0.06	0.36245103	pigs
Pik3cd	0.00	0.36309648	pigs
Cd3eap	0.06	0.36770825	pigs
Idh2	0.20	0.37174168	pigs
Rbbp6	0.00	0.37189758	pigs
Erg	0.10	0.37483486	pigs
Glcci1	-0.34	0.38110185	pigs
Acad11	-0.09	0.38586836	pigs
Cul5	0.02	0.39107310	pigs
Kcnj13	-0.01	0.39140526	pigs
Stt3a	0.01	0.39253251	pigs
Tbc1d20	-0.09	0.39349978	pigs
Igfbp7	-0.12	0.39472825	pigs
Smc6	0.02	0.39550163	pigs
Ripor1	0.00	0.39572554	pigs
Becn1	-0.05	0.39725265	pigs
Fcho2	-0.30	0.39928382	pigs
Ankrd40	0.02	0.40114749	pigs
Nubp1	0.05	0.40429414	pigs
Ranbp1	0.02	0.40675134	pigs
Timm21	0.00	0.41679412	pigs
Hprt	-0.04	0.42307240	pigs
Arpc3	-0.16	0.43165785	pigs
Bptf	0.02	0.43311216	pigs
Tm9sf2	0.02	0.43452521	pigs
Kiz	0.02	0.43506144	pigs
Slc35c2	0.02	0.43949437	pigs

Tyw5	-0.01	0.43980174	pigs
Ccdc134	0.02	0.44175315	pigs
Uck1	-0.01	0.44292460	pigs
Iars2	-0.03	0.44650808	pigs
Rragc	-0.17	0.44713753	pigs
Gba2	0.00	0.44739186	pigs
Cutc	0.00	0.44770003	pigs
Tecpr1	-0.04	0.45226751	pigs
Sort1	-0.17	0.46686169	pigs
Ep300	0.04	0.46722524	pigs
Klf7	-0.02	0.46765634	pigs
Wdr11	0.03	0.47222748	pigs
Sdf4	0.07	0.47512330	pigs
Usp45	0.00	0.47785495	pigs
Gcfc2	0.03	0.47948843	pigs
Zfp672	0.03	0.48157591	pigs
Glmn	0.01	0.48226794	pigs
Jmjd6	0.02	0.48259643	pigs
Ifi44l	-0.31	0.48636046	pigs
Tanc2	0.03	0.48880687	pigs
Limd2	-0.19	0.48958688	pigs
Taf9b	0.00	0.49268400	pigs
Ints12	0.04	0.49464280	pigs
P2ry1	0.03	0.50321458	pigs
Vps37b	-0.03	0.50544293	pigs
Nr1h2	0.00	0.50726475	pigs
Pdss1	-0.01	0.50854124	pigs
Ppp1ca	-0.02	0.51745124	pigs
Otud6b	0.03	0.52209437	pigs
Dtx3	0.00	0.52969789	pigs
Igf2bp3	0.08	0.53364953	pigs
Dimt1	0.02	0.53804037	pigs
Usp6nl	0.12	0.53889217	pigs
Wbp2	0.00	0.54741575	pigs
Stk10	-0.28	0.55280865	pigs
Nme7	-0.25	0.55390532	pigs
Rmc1	0.06	0.55488365	pigs
Snx27	0.01	0.55617799	pigs
Zdhhc16	0.02	0.55746448	pigs
Hcls1	0.04	0.56026667	pigs
Xpa	0.06	0.56256127	pigs
Snrnp200	0.00	0.56366832	pigs
Osgin2	0.05	0.56487580	pigs
Serpinb9	0.06	0.56702678	pigs
Ezh2	0.02	0.57749836	pigs
Pdha1	0.04	0.57831146	pigs

Stap1	-0.01	0.58288505	pigs
Ier5l	0.05	0.58873049	pigs
Phf12	-0.07	0.59691986	pigs
AW554918	0.02	0.60441480	pigs
Kpna4	0.00	0.61551038	pigs
Syf2	0.03	0.61551769	pigs
Npm3	0.05	0.62018095	pigs
Fmc1	-0.32	0.62509163	pigs
Iffo1	0.01	0.62667281	pigs
Ints2	0.05	0.62755462	pigs
Ccl12	-1.15	0.62833901	pigs
Wars2	0.07	0.63109347	pigs
Numa1	-0.04	0.63449356	pigs
Pdzd11	-0.07	0.64644909	pigs
Sgf29	-0.05	0.65427713	pigs
Cdca4	0.00	0.65706659	pigs
Slc22a15	0.07	0.66718413	pigs
Rsad2	-0.18	0.67034247	pigs
Heatr1	0.01	0.67381265	pigs
Nudcd1	0.03	0.67891989	pigs
Fmnl1	0.00	0.68111412	pigs
Alkbh6	-0.02	0.68641541	pigs
Ndufa1	-0.35	0.68917616	pigs
Irf1	0.00	0.69133859	pigs
Gtf2i	-0.01	0.69423273	pigs
Dpy19l3	-0.17	0.69455240	pigs
Slc38a2	-0.06	0.69543774	pigs
Rxra	0.03	0.69860772	pigs
Osgepl1	0.00	0.70379634	pigs
Smc3	0.01	0.70697084	pigs
B4galt3	0.00	0.70776435	pigs
Atp5d	0.13	0.72111839	pigs
Pomgnt1	0.02	0.72129807	pigs
Cyb5r1	0.00	0.72329413	pigs
B3gnt3	0.01	0.72657597	pigs
Gid8	0.08	0.73274564	pigs
Trabd	-0.01	0.73693763	pigs
Wdr25	0.02	0.74177805	pigs
Heatr3	0.00	0.74475356	pigs
Wls	0.05	0.74983964	pigs
Tnfaip8l2	0.06	0.75254197	pigs
Pigv	-0.01	0.75608989	pigs
Klf12	-0.25	0.76542579	pigs
Wdr41	0.00	0.77080567	pigs
Slc25a38	-0.04	0.77676246	pigs
Polk	0.04	0.77793257	pigs

Sertad1	-0.04	0.78625558	pigs
Nfyb	0.00	0.78741631	pigs
Coq9	-0.01	0.79145654	pigs
Cd53	0.04	0.79207557	pigs
Chic2	0.04	0.79485713	pigs
Pink1	-0.19	0.79743078	pigs
Rbpms	0.03	0.79903580	pigs
Tbpl1	-0.01	0.80902035	pigs
Sh3kbp1	-0.32	0.80953786	pigs
Ppid	-0.06	0.81179415	pigs
Tspan31	-0.02	0.82229147	pigs
Apmap	0.05	0.82432078	pigs
Dynll2	-0.04	0.83051232	pigs
Slc25a26	0.04	0.83340557	pigs
Stoml2	-0.07	0.83579063	pigs
P3h1	-0.03	0.83626404	pigs
Armc10	-0.06	0.83833380	pigs
Chd6	-0.01	0.84516687	pigs
Bri3bp	0.00	0.84831558	pigs
Stub1	0.02	0.86699279	pigs
Wwp2	0.04	0.87209294	pigs
Usp5	0.02	0.88268775	pigs
Lrrc8c	-0.02	0.88613896	pigs
Scly	-0.07	0.89666636	pigs
Hook3	0.07	0.89666860	pigs
Nxf1	0.01	0.89878332	pigs
Serinc5	0.07	0.89893497	pigs
Rmnd5a	0.00	0.90197578	pigs
Tstd2	0.04	0.91389344	pigs
Dlg1	0.05	0.91468415	pigs
Ddx49	-0.01	0.91496172	pigs
Fam3a	-0.02	0.92129027	pigs
Smg6	-0.03	0.92500956	pigs
Acadvl	0.00	0.93027797	pigs
Pdpr	0.03	0.93286451	pigs
Atg101	0.00	0.94486724	pigs
Tsc22d4	0.01	0.94559108	pigs
Rab10	0.15	0.94763753	pigs
Hadha	0.03	0.95060285	pigs
Tex14	0.20	0.95195070	pigs
Ino80d	0.00	0.95285682	pigs
Pygm	0.01	0.95474841	pigs
Aida	-0.05	0.95587320	pigs
Gemin2	0.01	0.96034928	pigs
Ranbp17	-0.24	0.96537956	pigs
L3mbtl1	0.02	0.97087267	pigs

Pabpc4	0.00	0.98296901	pigs
Prr14	0.02	0.99153369	pigs
Amigo3	0.04	0.99371165	pigs
Eif4g3	-0.05	1.00000000	pigs
Chmp2a	0.04	1.00000000	pigs
Nhlrc2	0.02	1.00000000	pigs
Pdcd5	-0.37	1.00000000	pigs
Gnl3	0.00	1.00000000	pigs
Tfpt	0.02	1.00000000	pigs
Ccdc47	-0.04	1.00000000	pigs
Arfrp1	0.03	1.00000000	pigs
Rrs1	0.11	1.00000000	pigs
Smarcad1	0.00	1.00000000	pigs
Psmd13	-0.03	1.00000000	pigs
Plxdc2	-0.53	1.00000000	pigs
Hdgf	-0.23	1.00000000	pigs
Edrf1	-0.02	1.00000000	pigs
Acot8	0.03	1.00000000	pigs
Zbtb17	0.01	1.00000000	pigs
Atp5a1	-0.17	1.00000000	pigs
Rint1	0.01	1.00000000	pigs
Unc93b1	-0.04	1.00000000	pigs
Stk25	0.01	1.00000000	pigs
Cadps2	-0.28	1.00000000	pigs
Rb1cc1	-0.07	1.00000000	pigs
Odr4	0.00	1.00000000	pigs
Cenpu	-0.22	1.00000000	pigs
Aifm2	0.07	1.00000000	pigs
Atrn	0.07	1.00000000	pigs
Tlr8	-0.38	1.00000000	pigs
Gna15	-0.04	1.00000000	pigs
Cd38	-0.11	1.00000000	pigs
Heatr5b	0.01	1.00000000	pigs
Arsb	0.06	1.00000000	pigs
Agpat2	-0.29	1.00000000	pigs
Ptpra	-0.03	1.00000000	pigs
Ipo7	0.03	1.00000000	pigs
Zcchc7	0.11	1.00000000	pigs
Gpn1	0.04	1.00000000	pigs
Azi2	-0.01	1.00000000	pigs
Tut7	-0.10	1.00000000	pigs
Zzef1	0.09	1.00000000	pigs
Isyna1	0.02	1.00000000	pigs
Pigx	-0.14	1.00000000	pigs
Pqbp1	-0.07	1.00000000	pigs
Cox20	-0.22	1.00000000	pigs

Slc7a6os	-0.02	1.00000000	pigs
Parp12	-0.35	1.00000000	pigs
Zmym1	0.00	1.00000000	pigs
Aimp1	-0.26	1.00000000	pigs
Ube3b	-0.03	1.00000000	pigs
Usp28	0.01	1.00000000	pigs
Asb3	-0.03	1.00000000	pigs
Lrrc25	-0.03	1.00000000	pigs
Pcbp4	-0.03	1.00000000	pigs
Csnk2a2	-0.01	1.00000000	pigs
Ttc39c	0.09	1.00000000	pigs
Pick1	0.00	1.00000000	pigs
Cpt1a	-0.12	1.00000000	pigs
Dctn3	-0.03	1.00000000	pigs
Exoc6b	0.08	1.00000000	pigs
Snapc2	0.02	1.00000000	pigs
Aak1	-0.23	1.00000000	pigs
Rab43	0.01	1.00000000	pigs
Phax	-0.04	1.00000000	pigs
Scaf11	0.06	1.00000000	pigs
Lats1	-0.02	1.00000000	pigs
Trappc3	-0.02	1.00000000	pigs
Crtc2	0.02	1.00000000	pigs
Fkbp7	-0.04	1.00000000	pigs
Galnt11	0.07	1.00000000	pigs
Cactin	-0.01	1.00000000	pigs
Cebpz	0.03	1.00000000	pigs
Ccar2	0.03	1.00000000	pigs
Anapc1	-0.03	1.00000000	pigs
Samm50	0.05	1.00000000	pigs
Qpct	-0.22	1.00000000	pigs
Tmem161b	-0.01	1.00000000	pigs
Tcf3	-0.02	1.00000000	pigs
Hbs1l	0.07	1.00000000	pigs
Cnot10	-0.04	1.00000000	pigs
Fam174a	-0.04	1.00000000	pigs
Rex1bd	-0.35	1.00000000	pigs
St6gal1	-0.28	1.00000000	pigs
Hrh4	-0.03	1.00000000	pigs
Il6ra	-0.04	1.00000000	pigs
Kmt2e	0.14	1.00000000	pigs
Pcf11	0.04	1.00000000	pigs
Adm	0.03	1.00000000	pigs
Uchl5	-0.05	1.00000000	pigs
Gphn	0.01	1.00000000	pigs
Dus1l	-0.10	1.00000000	pigs

Akap8	0.01	1.00000000	pigs
Il1rap	-0.22	1.00000000	pigs
Fibp	0.02	1.00000000	pigs
4933400A11Rik	0.07	1.00000000	pigs
Tmub1	0.04	1.00000000	pigs
Prkcz	0.01	1.00000000	pigs
Smarcal1	0.01	1.00000000	pigs
Pgrmc1	-0.06	1.00000000	pigs
Apoo	0.00	1.00000000	pigs
Atg5	-0.06	1.00000000	pigs
Ptpa	0.01	1.00000000	pigs
Nf2	0.03	1.00000000	pigs
Edem2	-0.05	1.00000000	pigs
Pip4k2a	-0.50	1.00000000	pigs
Lxn	0.01	1.00000000	pigs
Ralb	0.02	1.00000000	pigs
Caml	-0.04	1.00000000	pigs
Dgkz	-0.07	1.00000000	pigs
Trnt1	-0.07	1.00000000	pigs
Ncf2	-0.34	1.00000000	pigs
Card11	0.30	1.00000000	pigs
Dnajc17	0.04	1.00000000	pigs
Yae1d1	-0.05	1.00000000	pigs
Kifbp	-0.03	1.00000000	pigs
B230219D22Rik	-0.06	1.00000000	pigs
Id2	0.05	1.00000000	pigs
Wdr61	-0.04	1.00000000	pigs
Slc35c1	0.01	1.00000000	pigs
Rhoa	0.10	1.00000000	pigs
Ift20	-0.28	1.00000000	pigs
Tfdp1	0.00	1.00000000	pigs
Plekhj1	-0.30	1.00000000	pigs
Ackr4	-0.34	1.00000000	pigs
Fmo4	0.05	1.00000000	pigs
Dolpp1	0.01	1.00000000	pigs
Gdi2	0.03	1.00000000	pigs
Bzw1	-0.11	1.00000000	pigs
Shc1	0.00	1.00000000	pigs
Phf8	0.02	1.00000000	pigs
Fndc3b	-0.04	1.00000000	pigs
Nap1l4	-0.06	1.00000000	pigs
Ppm1d	0.00	1.00000000	pigs
Bcl2l12	-0.01	1.00000000	pigs
Sharpin	0.01	1.00000000	pigs
Synrg	0.04	1.00000000	pigs
Nab1	-0.06	1.00000000	pigs

Isca2	0.01	1.00000000	pigs
Fbrs	-0.06	1.00000000	pigs
Xpo4	0.01	1.00000000	pigs
Eef2k	-0.01	1.00000000	pigs
Parp2	0.00	1.00000000	pigs
Supt6	-0.02	1.00000000	pigs
Dnajc5	0.03	1.00000000	pigs
Psmc1	-0.02	1.00000000	pigs
Shkbp1	0.02	1.00000000	pigs
Rrp8	-0.09	1.00000000	pigs
Ccndbp1	-0.01	1.00000000	pigs
Tcp1	0.07	1.00000000	pigs
Stat6	0.02	1.00000000	pigs
Aph1b	-0.23	1.00000000	pigs
Sqstm1	-0.10	1.00000000	pigs
Tmpo	0.01	1.00000000	pigs
Rnh1	-0.23	1.00000000	pigs
Galnt3	-0.17	1.00000000	pigs
Pycrl	0.06	1.00000000	pigs
Ammecr1l	0.00	1.00000000	pigs
Pak1ip1	-0.03	1.00000000	pigs
Eif2b3	-0.16	1.00000000	pigs
Elmod2	0.01	1.00000000	pigs
Carhsp1	0.03	1.00000000	pigs
Ptpn4	-0.07	1.00000000	pigs
Tbcb	0.02	1.00000000	pigs
Cmpk2	-0.22	1.00000000	pigs
Ublcp1	-0.02	1.00000000	pigs
Snrk	-0.05	1.00000000	pigs
Gspt1	0.03	1.00000000	pigs
Usp25	-0.03	1.00000000	pigs
Zc3h15	0.00	1.00000000	pigs
Prkdc	0.01	1.00000000	pigs
Ncf1	-0.08	1.00000000	pigs
Gps2	-0.25	1.00000000	pigs
Spred2	-0.05	1.00000000	pigs
Nup160	0.02	1.00000000	pigs
Wdr75	0.06	1.00000000	pigs
Laptm5	-0.18	1.00000000	pigs
Grk2	-0.01	1.00000000	pigs
Brk1	-0.27	1.00000000	pigs
Ptbp3	0.05	1.00000000	pigs
Gfm1	-0.30	1.00000000	pigs
Syne3	-0.01	1.00000000	pigs
U2af2	0.05	1.00000000	pigs
Pomt2	0.00	1.00000000	pigs

Cct5	0.04	1.00000000	pigs
Ostc	-0.02	1.00000000	pigs
Sp1	-0.06	1.00000000	pigs
Zbtb8os	0.03	1.00000000	pigs
Hsdl2	0.02	1.00000000	pigs
Trip11	-0.07	1.00000000	pigs
Ss18l2	0.04	1.00000000	pigs
Smim4	-0.20	1.00000000	pigs
Ifih1	-0.48	1.00000000	pigs
Uspl1	-0.02	1.00000000	pigs
Hspbap1	0.12	1.00000000	pigs
Pradc1	-0.03	1.00000000	pigs
Usf3	-0.10	1.00000000	pigs
Stim2	0.01	1.00000000	pigs
Abcb10	0.05	1.00000000	pigs
Pag1	-0.13	1.00000000	pigs
Slc30a9	-0.08	1.00000000	pigs
Zmym5	-0.02	1.00000000	pigs
Haus2	-0.05	1.00000000	pigs
4931406C07Rik	0.05	1.00000000	pigs
Adrm1	-0.02	1.00000000	pigs
Cdkal1	0.00	1.00000000	pigs
Anxa7	0.03	1.00000000	pigs
Rnf139	-0.02	1.00000000	pigs
Lig3	-0.01	1.00000000	pigs
Arv1	-0.20	1.00000000	pigs
Parp4	-0.21	1.00000000	pigs
Golga5	-0.03	1.00000000	pigs
Stau2	-0.02	1.00000000	pigs
Hsp90ab1	0.03	1.00000000	pigs
Sgta	-0.03	1.00000000	pigs
Skil	-0.10	1.00000000	pigs
Ylpm1	0.02	1.00000000	pigs
Avpi1	-0.11	1.00000000	pigs
Pgls	0.10	1.00000000	pigs
Nmral1	-0.04	1.00000000	pigs
Abcb7	0.01	1.00000000	pigs
Prkd2	-0.18	1.00000000	pigs
Cbl	0.05	1.00000000	pigs
Acaa1b	0.06	1.00000000	pigs
Parp8	-0.30	1.00000000	pigs
Ak6	-0.03	1.00000000	pigs
Atp8a1	-0.07	1.00000000	pigs
Actg1	0.06	1.00000000	pigs
Rest	-0.01	1.00000000	pigs
Wsb1	0.02	1.00000000	pigs

Pbrm1	-0.05	1.00000000	pigs
Egr2	-0.20	1.00000000	pigs
Pfkp	-0.06	1.00000000	pigs
Tmem30a	0.01	1.00000000	pigs
Lman1	0.00	1.00000000	pigs
Pfdn4	-0.02	1.00000000	pigs
Adsl	0.02	1.00000000	pigs
Gclm	-0.03	1.00000000	pigs
Entpd1	-0.09	1.00000000	pigs
Kctd10	0.04	1.00000000	pigs
Stpg4	-0.21	1.00000000	pigs
Rpa1	-0.01	1.00000000	pigs
Thoc1	-0.04	1.00000000	pigs
Tyk2	-0.11	1.00000000	pigs
Fbxl15	0.02	1.00000000	pigs
Snx25	0.04	1.00000000	pigs
Acaa2	-0.02	1.00000000	pigs
Thap7	-0.06	1.00000000	pigs
Prkacb	-0.02	1.00000000	pigs
Cdpf1	0.00	1.00000000	pigs
Rtn2	0.00	1.00000000	pigs
Cep162	-0.01	1.00000000	pigs
Zfp787	0.01	1.00000000	pigs
Wars	-0.03	1.00000000	pigs
4833439L19Rik	-0.08	1.00000000	pigs
Pik3c3	0.02	1.00000000	pigs
Rsrc1	0.01	1.00000000	pigs
Cul4a	-0.05	1.00000000	pigs
Cenpc1	0.00	1.00000000	pigs
Pdcd4	-0.27	1.00000000	pigs
Timm44	-0.01	1.00000000	pigs
Kctd12	0.02	1.00000000	pigs
Hacd4	0.01	1.00000000	pigs
Gls	0.04	1.00000000	pigs
Vps51	-0.05	1.00000000	pigs
Vti1a	0.02	1.00000000	pigs
Dcun1d5	0.00	1.00000000	pigs
Surf6	-0.01	1.00000000	pigs
Taf7	0.01	1.00000000	pigs
Dnajb6	-0.07	1.00000000	pigs
Nfrkb	0.01	1.00000000	pigs
Ttc27	-0.01	1.00000000	pigs
Lrrc45	-0.04	1.00000000	pigs
Dlat	0.00	1.00000000	pigs
Wdr77	0.05	1.00000000	pigs
Slc16a1	-0.25	1.00000000	pigs

Atf7ip	0.02	1.00000000	pigs
Kmt2c	0.00	1.00000000	pigs
Gps1	-0.06	1.00000000	pigs
Klf9	0.04	1.00000000	pigs
Fbrsl1	-0.07	1.00000000	pigs
Pou2af1	-0.08	1.00000000	pigs
Dhrs3	-0.03	1.00000000	pigs
Tmed10	0.00	1.00000000	pigs
Grb10	0.01	1.00000000	pigs
Washc1	0.00	1.00000000	pigs
Rbks	0.03	1.00000000	pigs
Pggt1b	-0.03	1.00000000	pigs
Eif3d	-0.03	1.00000000	pigs
Rrm1	0.02	1.00000000	pigs
Cox6a1	-0.21	1.00000000	pigs
Arglu1	-0.22	1.00000000	pigs
Ssbp2	-0.04	1.00000000	pigs
Exosc9	0.00	1.00000000	pigs
1700037H04Rik	-0.18	1.00000000	pigs
Tbrg4	-0.02	1.00000000	pigs
Slc39a13	0.02	1.00000000	pigs
Ssb	0.04	1.00000000	pigs
Fam3c	-0.14	1.00000000	pigs
Dido1	0.01	1.00000000	pigs
Rbm45	-0.02	1.00000000	pigs
Tra2a	-0.01	1.00000000	pigs
Wdr44	0.06	1.00000000	pigs
Hif1an	0.00	1.00000000	pigs
Ctbp1	-0.07	1.00000000	pigs
Cgas	-0.14	1.00000000	pigs
Gemin5	-0.01	1.00000000	pigs
Yju2	-0.04	1.00000000	pigs
Bahd1	0.00	1.00000000	pigs
Tpra1	0.02	1.00000000	pigs
Ufm1	0.06	1.00000000	pigs
Serpinb6b	-0.27	1.00000000	pigs
Syn1	-0.02	1.00000000	pigs
Ufsp2	0.04	1.00000000	pigs
Rcor1	-0.03	1.00000000	pigs
Nelfcd	-0.07	1.00000000	pigs
Ccdc115	-0.01	1.00000000	pigs
Fggy	-0.09	1.00000000	pigs
Gpatch11	0.04	1.00000000	pigs
Ctif	-0.01	1.00000000	pigs
Pdgfd	-0.10	1.00000000	pigs
1110065P20Rik	0.04	1.00000000	pigs

Alg5	0.03	1.00000000	pigs
Cnot8	-0.05	1.00000000	pigs
Nip7	0.05	1.00000000	pigs
Cdk19	-0.23	1.00000000	pigs
Arpc2	0.14	1.00000000	pigs
Isg20l2	-0.01	1.00000000	pigs
Plekhm3	-0.58	1.00000000	pigs
Nfkbid	-0.21	1.00000000	pigs
Ciz1	0.05	1.00000000	pigs
Gclc	-0.02	1.00000000	pigs
Nudc	-0.11	1.00000000	pigs
Ttll12	-0.23	1.00000000	pigs
Upf2	-0.02	1.00000000	pigs
Itgae	-0.02	1.00000000	pigs
Socs5	-0.03	1.00000000	pigs
Rpain	0.00	1.00000000	pigs
Rap1b	0.01	1.00000000	pigs
Slc9a6	0.00	1.00000000	pigs
Tssc4	0.01	1.00000000	pigs
Fbxl5	-0.23	1.00000000	pigs
Polr1f	0.01	1.00000000	pigs
Poc1a	0.02	1.00000000	pigs
Grk6	-0.24	1.00000000	pigs
Phf10	0.00	1.00000000	pigs
Dcaf8	0.00	1.00000000	pigs
Cers2	-0.07	1.00000000	pigs
Plekha1	-0.01	1.00000000	pigs
Gpr137	0.04	1.00000000	pigs
Dlgap4	-0.03	1.00000000	pigs
Actr2	0.07	1.00000000	pigs
Las1l	-0.01	1.00000000	pigs
Etv6	0.08	1.00000000	pigs
Snap47	-0.02	1.00000000	pigs
F11r	-0.05	1.00000000	pigs
Tlr7	-0.05	1.00000000	pigs
Pik3cb	0.00	1.00000000	pigs
Actr3	-0.16	1.00000000	pigs
Kdm2b	0.01	1.00000000	pigs
Cdc14a	0.00	1.00000000	pigs
Pgp	0.04	1.00000000	pigs
Dipk2a	0.03	1.00000000	pigs
Asns	-0.15	1.00000000	pigs
Col1a2	0.11	1.00000000	pigs
Coq4	-0.52	1.00000000	pigs
Slu7	0.01	1.00000000	pigs
Vars2	0.01	1.00000000	pigs

Tra2b	-0.24	1.00000000	pigs
Pskh1	-0.03	1.00000000	pigs
Nsf	-0.01	1.00000000	pigs
Numb	-0.53	1.00000000	pigs
Smpd4	-0.02	1.00000000	pigs
Cuta	-0.14	1.00000000	pigs
Tnfrsf13b	-0.15	1.00000000	pigs
Adnp2	0.06	1.00000000	pigs
Cwf19l1	-0.06	1.00000000	pigs
Otud3	-0.01	1.00000000	pigs
Ndfip2	0.00	1.00000000	pigs
Zc2hc1a	-0.25	1.00000000	pigs
Vps8	-0.02	1.00000000	pigs
Tfcp2	0.03	1.00000000	pigs
Plcl2	-0.27	1.00000000	pigs
Ralgapa2	-0.77	1.00000000	pigs
Arhgap30	0.00	1.00000000	pigs
Usp33	0.28	1.00000000	pigs
Zdhhc6	-0.05	1.00000000	pigs
Zfp36l1	-0.07	1.00000000	pigs
Adcy9	-0.01	1.00000000	pigs
Lsm3	-0.03	1.00000000	pigs
Elp2	0.02	1.00000000	pigs
Lin7a	-0.21	1.00000000	pigs
Usp19	0.02	1.00000000	pigs
Riok3	0.08	1.00000000	pigs
Pus7l	-0.12	1.00000000	pigs
Golgb1	-0.28	1.00000000	pigs
Phf1	0.00	1.00000000	pigs
Tlcd2	-0.04	1.00000000	pigs
Cetn3	-0.10	1.00000000	pigs
Sf3b2	-0.01	1.00000000	pigs
Akap10	-0.02	1.00000000	pigs
Cryl1	0.02	1.00000000	pigs
Eaf2	-0.25	1.00000000	pigs
Carmil1	-0.16	1.00000000	pigs
Pelo	-0.01	1.00000000	pigs
Ctps2	-0.26	1.00000000	pigs
Ccdc69	-0.36	1.00000000	pigs
Qtrt1	-0.10	1.00000000	pigs
St3gal6	0.03	1.00000000	pigs
Atg12	0.04	1.00000000	pigs
Exoc7	-0.01	1.00000000	pigs
Dad1	-0.38	1.00000000	pigs
Vps26b	-0.03	1.00000000	pigs
Eif4b	0.02	1.00000000	pigs

Tomm20	-0.09	1.00000000	pigs
Nbeal1	-0.01	1.00000000	pigs
Dguok	-0.07	1.00000000	pigs
Sgms1	-0.35	1.00000000	pigs
Hsd17b7	-0.01	1.00000000	pigs
Chchd4	-0.07	1.00000000	pigs
Bmpr1a	0.05	1.00000000	pigs
Ints9	0.04	1.00000000	pigs
Hypk	0.00	1.00000000	pigs
Senp1	-0.01	1.00000000	pigs
Usp46	-0.04	1.00000000	pigs
Lmtk2	-0.07	1.00000000	pigs
Inppl1	-0.03	1.00000000	pigs
Osbp	-0.02	1.00000000	pigs
Rbm28	0.00	1.00000000	pigs
Hlcs	-0.14	1.00000000	pigs
Cd37	-0.37	1.00000000	pigs
Arid4a	0.03	1.00000000	pigs
Ncbp1	0.01	1.00000000	pigs
Gramd1b	-0.12	1.00000000	pigs
Pla2g4a	-0.22	1.00000000	pigs
Arrb2	-0.24	1.00000000	pigs
Trp53inp1	-0.45	1.00000000	pigs
Diablo	-0.03	1.00000000	pigs
Tomm40	0.04	1.00000000	pigs
Sp3	0.02	1.00000000	pigs
Nsdhl	-0.02	1.00000000	pigs
Phactr1	-0.07	1.00000000	pigs
Sumo2	-0.17	1.00000000	pigs
Bloc1s6	0.06	1.00000000	pigs
Vps72	0.00	1.00000000	pigs
Nlrc5	-0.32	1.00000000	pigs
Gtf3c3	0.02	1.00000000	pigs
Chmp6	-0.02	1.00000000	pigs
Aven	-0.02	1.00000000	pigs
Abca5	-0.12	1.00000000	pigs
Eif2a	-0.22	1.00000000	pigs
Ap3b1	0.04	1.00000000	pigs
Dhx37	0.02	1.00000000	pigs
Vmo1	0.01	1.00000000	pigs
Tmem68	-0.04	1.00000000	pigs
Kdm4b	0.03	1.00000000	pigs
Kdm8	-0.15	1.00000000	pigs
Cstf3	-0.08	1.00000000	pigs
Kyat3	0.00	1.00000000	pigs
Znhit1	-0.07	1.00000000	pigs

Psmb9	0.09	1.00000000	pigs
Slc9a9	-0.05	1.00000000	pigs
Trim14	0.03	1.00000000	pigs
Tsga10	-0.08	1.00000000	pigs
G6pc3	-0.05	1.00000000	pigs
Faf1	0.00	1.00000000	pigs
Gar1	0.02	1.00000000	pigs
Exoc4	0.07	1.00000000	pigs
Tmem165	-0.07	1.00000000	pigs
B3gat3	-0.04	1.00000000	pigs
Setd3	-0.02	1.00000000	pigs
Sf3b4	-0.05	1.00000000	pigs
Fkbp3	-0.04	1.00000000	pigs
Krtcap2	-0.33	1.00000000	pigs
Psmb1	0.05	1.00000000	pigs
Arhgap25	0.01	1.00000000	pigs
Ipo4	-0.09	1.00000000	pigs
Ubap1	-0.45	1.00000000	pigs
Gnai3	-0.07	1.00000000	pigs
Bclaf1	0.04	1.00000000	pigs
Eif1ad	0.03	1.00000000	pigs
Sec11a	-0.05	1.00000000	pigs
Nol9	-0.04	1.00000000	pigs
Epb41	-0.18	1.00000000	pigs
Zfp131	-0.07	1.00000000	pigs
Gnb1l	0.01	1.00000000	pigs
Golim4	-0.04	1.00000000	pigs
Rab11fip2	0.04	1.00000000	pigs
Dpy30	-0.13	1.00000000	pigs
Tex264	-0.02	1.00000000	pigs
Invs	-0.01	1.00000000	pigs
U2af1l4	-0.09	1.00000000	pigs
Hectd1	0.00	1.00000000	pigs
Pik3c2a	0.02	1.00000000	pigs
Stk38l	-0.45	1.00000000	pigs
Slc24a4	-0.21	1.00000000	pigs
Bcar3	-0.03	1.00000000	pigs
Dcbld1	-0.02	1.00000000	pigs
Nup153	-0.04	1.00000000	pigs
Wdr82	0.00	1.00000000	pigs
Creld2	-0.04	1.00000000	pigs
Ccdc186	-0.08	1.00000000	pigs
Prmt7	-0.03	1.00000000	pigs
Zfpl1	-0.01	1.00000000	pigs
Vps4b	-0.17	1.00000000	pigs
Fam89b	-0.15	1.00000000	pigs

Disc1	-0.02	1.00000000	pigs
Ccdc97	0.03	1.00000000	pigs
Sigmar1	0.00	1.00000000	pigs
Rbm22	-0.05	1.00000000	pigs
4932411N23Rik	-0.01	1.00000000	pigs
Rgcc	0.08	1.00000000	pigs
Ppm1g	-0.24	1.00000000	pigs
Hs6st1	-0.01	1.00000000	pigs
Dnaaf2	-0.15	1.00000000	pigs
Stat2	0.01	1.00000000	pigs
Akap6	-0.13	1.00000000	pigs
Dctn2	-0.07	1.00000000	pigs
Uprt	-0.01	1.00000000	pigs
Ptpn13	-0.03	1.00000000	pigs
Rbm44	-0.28	1.00000000	pigs
Slc5a1	-0.02	1.00000000	pigs
Phlda2	-0.34	1.00000000	pigs
Esco1	0.02	1.00000000	pigs
Vps13d	-0.07	1.00000000	pigs
Trappc6b	-0.10	1.00000000	pigs
Rgs2	-0.15	1.00000000	pigs
Stk19	0.01	1.00000000	pigs
Osbpl11	-0.54	1.00000000	pigs
Tet3	0.02	1.00000000	pigs
Ptafr	-0.03	1.00000000	pigs
Hexim2	-0.03	1.00000000	pigs
Smim15	-0.01	1.00000000	pigs
Utp11	-0.04	1.00000000	pigs
Zmynd11	0.00	1.00000000	pigs
Lsm7	-0.15	1.00000000	pigs
Ciart	-0.27	1.00000000	pigs
Arfip1	-0.11	1.00000000	pigs
Dhx36	-0.01	1.00000000	pigs
Hook2	-0.11	1.00000000	pigs
Dynlrb1	-0.16	1.00000000	pigs
Ube2b	-0.16	1.00000000	pigs
Gnai2	0.05	1.00000000	pigs
Aurkaip1	-0.19	1.00000000	pigs
Nabp1	-0.18	1.00000000	pigs
Tmem59	-0.20	1.00000000	pigs
Ddx50	0.00	1.00000000	pigs
Dis3l	0.02	1.00000000	pigs
Trim25	-0.30	1.00000000	pigs
Ankrd12	-0.08	1.00000000	pigs
Slc41a2	-0.04	1.00000000	pigs
Rpap2	-0.22	1.00000000	pigs

Satb1	-0.17	1.00000000	pigs
Cib1	-0.25	1.00000000	pigs
Abcd4	-0.06	1.00000000	pigs
Tle5	-0.16	1.00000000	pigs
Rnf20	-0.03	1.00000000	pigs
Tmem107	-0.24	1.00000000	pigs
Stxbp2	0.06	1.00000000	pigs
Oxnad1	-0.05	1.00000000	pigs
Chd9	0.05	1.00000000	pigs
Ppil3	-0.04	1.00000000	pigs
Ebna1bp2	-0.03	1.00000000	pigs
Bola1	-0.22	1.00000000	pigs
Hlx	-0.09	1.00000000	pigs
Znfx1	-0.40	1.00000000	pigs
Nt5c3	-0.09	1.00000000	pigs
Tinf2	0.00	1.00000000	pigs
Kctd6	0.03	1.00000000	pigs
Ndufs3	0.06	1.00000000	pigs
Dhx40	-0.03	1.00000000	pigs
Tgfbr2	0.03	1.00000000	pigs
Junb	-0.12	1.00000000	pigs
Ankra2	-0.02	1.00000000	pigs
Rasa2	-0.07	1.00000000	pigs
Zbtb10	-0.07	1.00000000	pigs
Camta1	-0.08	1.00000000	pigs
Uhrf2	-0.16	1.00000000	pigs
Cep20	-0.04	1.00000000	pigs
Actr10	-0.11	1.00000000	pigs
Spats2l	0.00	1.00000000	pigs
Yrdc	-0.02	1.00000000	pigs
Prmt9	-0.03	1.00000000	pigs
Fam50a	-0.06	1.00000000	pigs
Taf4b	0.03	1.00000000	pigs
Utp3	-0.01	1.00000000	pigs
Rtf1	-0.07	1.00000000	pigs
Wbp1	-0.13	1.00000000	pigs
Sbf2	0.04	1.00000000	pigs
Nrd1	-0.01	1.00000000	pigs
Cebpd	-0.36	1.00000000	pigs
Gtpbp10	-0.01	1.00000000	pigs
St3gal3	-0.16	1.00000000	pigs
Rfng	0.00	1.00000000	pigs
Zeb1	-0.21	1.00000000	pigs
Nt5c3b	0.03	1.00000000	pigs
Shprh	-0.04	1.00000000	pigs
Enox2	-0.02	1.00000000	pigs

Eif2ak4	-0.03	1.00000000	pigs
Tmem109	-0.07	1.00000000	pigs
Tmub2	-0.03	1.00000000	pigs
Vps13c	-0.26	1.00000000	pigs
Zbtb44	-0.09	1.00000000	pigs
Nipal3	-0.01	1.00000000	pigs
Tor4a	-0.01	1.00000000	pigs
Timm8b	0.04	1.00000000	pigs
Tspan13	-0.21	1.00000000	pigs
Lrig2	-0.05	1.00000000	pigs
Fli1	-0.16	1.00000000	pigs
Acp1	-0.28	1.00000000	pigs
Ralbp1	-0.25	1.00000000	pigs
Atp9b	-0.02	1.00000000	pigs
Ccdc90b	-0.05	1.00000000	pigs
Pole4	-0.05	1.00000000	pigs
Pigu	-0.07	1.00000000	pigs
Hyal2	-0.18	1.00000000	pigs
Nol7	-0.21	1.00000000	pigs
Rnf146	-0.04	1.00000000	pigs
Abt1	0.05	1.00000000	pigs
Carnmt1	-0.06	1.00000000	pigs
Josd1	-0.02	1.00000000	pigs
Ppp2ca	-0.02	1.00000000	pigs
Diaph2	0.09	1.00000000	pigs
1810030O07Rik	-0.06	1.00000000	pigs
Cat	-0.18	1.00000000	pigs
Rgl2	-0.01	1.00000000	pigs
Terf1	-0.07	1.00000000	pigs
Fam214b	0.01	1.00000000	pigs
Pbx3	0.01	1.00000000	pigs
Capn7	-0.04	1.00000000	pigs
Emp1	-0.75	1.00000000	pigs
St3gal2	-0.06	1.00000000	pigs
Adcy7	-0.06	1.00000000	pigs
Kin	-0.17	1.00000000	pigs
Elp5	-0.03	1.00000000	pigs
Parm1	0.18	1.00000000	pigs
Ppp1r15b	-0.02	1.00000000	pigs
Gigyf1	-0.01	1.00000000	pigs
Jade3	0.04	1.00000000	pigs
Aif1	-0.09	1.00000000	pigs
Orc6	0.03	1.00000000	pigs
Pex7	-0.06	1.00000000	pigs
Strada	-0.05	1.00000000	pigs
Insyn2b	-0.15	1.00000000	pigs

Psmd6	-0.05	1.00000000	pigs
Bhlha15	-0.11	1.00000000	pigs
Bdh2	-0.01	1.00000000	pigs
Kdm1b	0.02	1.00000000	pigs
Ppan	0.04	1.00000000	pigs
Lrpap1	0.02	1.00000000	pigs
Kansl1l	0.06	1.00000000	pigs
Slc35f5	-0.01	1.00000000	pigs
Pi4k2b	-0.08	1.00000000	pigs
Eefsec	-0.01	1.00000000	pigs
Nup35	0.00	1.00000000	pigs
Rbm48	-0.04	1.00000000	pigs
Dusp6	-0.02	1.00000000	pigs
Tmed4	0.00	1.00000000	pigs
Pdlim5	-0.10	1.00000000	pigs
Atp5g3	-0.20	1.00000000	pigs
Exosc5	-0.02	1.00000000	pigs
Lpgat1	-0.47	1.00000000	pigs
Thoc7	-0.32	1.00000000	pigs
Xrn1	-0.01	1.00000000	pigs
Orc3	0.04	1.00000000	pigs
Rnpepl1	-0.10	1.00000000	pigs
Rnf149	-0.02	1.00000000	pigs
Emc7	-0.14	1.00000000	pigs
Parp16	-0.02	1.00000000	pigs
Rnf6	-0.06	1.00000000	pigs
Erap1	-0.17	1.00000000	pigs
Zyx	-0.32	1.00000000	pigs
Utp20	-0.02	1.00000000	pigs
Spata5	-0.28	1.00000000	pigs
Rhobtb3	-0.15	1.00000000	pigs
Cfap36	-0.23	1.00000000	pigs
Dag1	-0.01	1.00000000	pigs
Ccdc12	-0.09	1.00000000	pigs
Ddx5	-0.09	1.00000000	pigs
Trmt10c	0.02	1.00000000	pigs
Atp13a1	-0.01	1.00000000	pigs
Srbd1	-0.03	1.00000000	pigs
Txndc11	-0.21	1.00000000	pigs
Slc44a1	-0.06	1.00000000	pigs
Rad9a	-0.16	1.00000000	pigs
Nav1	-0.10	1.00000000	pigs
Kdm7a	-0.09	1.00000000	pigs
Nrros	-0.01	1.00000000	pigs
Dapk1	0.31	1.00000000	pigs
Cog5	-0.01	1.00000000	pigs

Noc3l	0.00	1.00000000	pigs
Nudt7	0.06	1.00000000	pigs
Kif1b	0.00	1.00000000	pigs
Slc4a1ap	-0.06	1.00000000	pigs
Kat5	-0.04	1.00000000	pigs
Rrp12	-0.02	1.00000000	pigs
Pde4b	-0.34	1.00000000	pigs
Akr7a5	-0.01	1.00000000	pigs
Larp1b	-0.01	1.00000000	pigs
Pstpip1	-0.23	1.00000000	pigs
Srsf7	0.06	1.00000000	pigs
Hddc2	-0.02	1.00000000	pigs
Fxr1	-0.08	1.00000000	pigs
Tcf25	-0.21	1.00000000	pigs
Fig4	-0.02	1.00000000	pigs
E2f3	0.00	1.00000000	pigs
Rfc4	-0.04	1.00000000	pigs
Rassf5	-0.04	1.00000000	pigs
Srsf3	0.11	1.00000000	pigs
Stk26	-0.03	1.00000000	pigs
Tbl3	-0.02	1.00000000	pigs
Wrap73	-0.02	1.00000000	pigs
Ghsr	-0.31	1.00000000	pigs
Snip1	-0.03	1.00000000	pigs
Traf3ip3	0.01	1.00000000	pigs
Uba5	0.01	1.00000000	pigs
Pcif1	-0.12	1.00000000	pigs
Agl	-0.05	1.00000000	pigs
Lrrfip2	0.04	1.00000000	pigs
Clip1	-0.29	1.00000000	pigs
Nlrp3	-0.82	1.00000000	pigs
Ppm1l	-0.32	1.00000000	pigs
Wdr3	0.02	1.00000000	pigs
Pno1	0.02	1.00000000	pigs
Ubac2	-0.10	1.00000000	pigs
Zdhhc13	-0.02	1.00000000	pigs
Hccs	-0.05	1.00000000	pigs
Rnf5	-0.02	1.00000000	pigs
Chchd3	0.01	1.00000000	pigs
Prpf38a	-0.06	1.00000000	pigs
C5ar2	-0.22	1.00000000	pigs
Surf2	0.02	1.00000000	pigs
Preb	-0.09	1.00000000	pigs
Hps1	-0.06	1.00000000	pigs
2610507B11Rik	-0.01	1.00000000	pigs
Ehmt1	-0.01	1.00000000	pigs

Herpud2	0.04	1.00000000	pigs
Smc2	0.00	1.00000000	pigs
Otud5	-0.03	1.00000000	pigs
Slirp	-0.15	1.00000000	pigs
Cyp7a1	-0.52	1.00000000	pigs
Churc1	-0.01	1.00000000	pigs
Crebl2	-0.08	1.00000000	pigs
Sting1	-0.22	1.00000000	pigs
Irak4	0.08	1.00000000	pigs
Tmem208	-0.02	1.00000000	pigs
9030025P20Rik	-0.04	1.00000000	pigs
Cyhr1	-0.01	1.00000000	pigs
Tg	-0.15	1.00000000	pigs
Entr1	-0.02	1.00000000	pigs
Cog7	0.01	1.00000000	pigs
Zfp827	-0.18	1.00000000	pigs
Dnttip1	-0.03	1.00000000	pigs
Smdt1	-0.26	1.00000000	pigs
Trmt10a	-0.02	1.00000000	pigs
Gtf3c6	0.02	1.00000000	pigs
Blvra	-0.01	1.00000000	pigs
Tex2	-0.01	1.00000000	pigs
Cdc42bpa	-0.11	1.00000000	pigs
Smchd1	-0.01	1.00000000	pigs
Cradd	-0.01	1.00000000	pigs
Fsd1l	-0.17	1.00000000	pigs
Clasp2	-0.27	1.00000000	pigs
Zfand1	-0.12	1.00000000	pigs
Ilk	-0.04	1.00000000	pigs
Cd80	0.04	1.00000000	pigs
Srek1ip1	-0.01	1.00000000	pigs
Spi1	-0.24	1.00000000	pigs
Tlnrd1	0.04	1.00000000	pigs
Tbca	-0.12	1.00000000	pigs
Chpf2	-0.01	1.00000000	pigs
Psme2	0.04	1.00000000	pigs
Olfm1	-0.17	1.00000000	pigs
Nat9	-0.03	1.00000000	pigs
Aspdh	-0.09	1.00000000	pigs
Yipf3	-0.12	1.00000000	pigs
Itih5	-0.07	1.00000000	pigs
Sfmbt2	-0.06	1.00000000	pigs
Pcdhga4	-0.06	1.00000000	pigs
Dennd1b	-0.44	1.00000000	pigs
Sdccag8	-0.24	1.00000000	pigs
Dennd2c	-0.07	1.00000000	pigs

Aldoart2	0.00	1.00000000	pigs
Rfx2	-0.04	1.00000000	pigs
Kcnab1	-0.19	1.00000000	pigs
Eif4g2	0.07	1.00000000	pigs
Cdc42ep3	-0.40	1.00000000	pigs
Coil	-0.03	1.00000000	pigs
Anp32e	-0.11	1.00000000	pigs
Bcl10	-0.06	1.00000000	pigs
Irf7	-0.04	1.00000000	pigs
Akip1	-0.05	1.00000000	pigs
Ndufa5	-0.29	1.00000000	pigs
Coasy	0.00	1.00000000	pigs
Fam120c	-0.02	1.00000000	pigs
Rxylt1	-0.05	1.00000000	pigs
Limk2	-0.25	1.00000000	pigs
Igsf8	0.04	1.00000000	pigs
Csnk1g3	0.00	1.00000000	pigs
Atp6v1d	-0.01	1.00000000	pigs
Eif2s1	0.05	1.00000000	pigs
Dgat2	0.06	1.00000000	pigs
Ndufs5	-0.19	1.00000000	pigs
Tapt1	0.03	1.00000000	pigs
Hax1	0.04	1.00000000	pigs
Bcas3	-0.24	1.00000000	pigs
Gemin6	-0.05	1.00000000	pigs
Nit2	-0.04	1.00000000	pigs
Herc2	-0.06	1.00000000	pigs
Zbtb22	-0.12	1.00000000	pigs
Smap1	-0.03	1.00000000	pigs
Thumpd2	0.04	1.00000000	pigs
Kansl2	-0.06	1.00000000	pigs
Ggnbp2	-0.03	1.00000000	pigs
Csnk1g2	-0.07	1.00000000	pigs
Uqcc1	0.01	1.00000000	pigs
Fundc1	-0.19	1.00000000	pigs
Eny2	-0.05	1.00000000	pigs
Adprs	-0.01	1.00000000	pigs
Trmt2a	-0.01	1.00000000	pigs
Brms1l	-0.06	1.00000000	pigs
Ap5b1	-0.02	1.00000000	pigs
Wdtc1	-0.04	1.00000000	pigs
Tmem203	-0.01	1.00000000	pigs
Clic5	-0.08	1.00000000	pigs
Rtraf	-0.31	1.00000000	pigs
Tbc1d16	0.01	1.00000000	pigs
Zadh2	-0.01	1.00000000	pigs

Emc2	-0.03	1.00000000	pigs
Cars	-0.06	1.00000000	pigs
Tmem38b	-0.32	1.00000000	pigs
Dctn5	0.00	1.00000000	pigs
Reep3	-0.10	1.00000000	pigs
Smyd2	0.01	1.00000000	pigs
Sel1l	-0.41	1.00000000	pigs
Arid1b	0.04	1.00000000	pigs
Nfxl1	0.03	1.00000000	pigs
Arhgap4	-0.09	1.00000000	pigs
Ap2s1	0.02	1.00000000	pigs
Dennd5b	-0.23	1.00000000	pigs
Apobec3	0.07	1.00000000	pigs
Dnaja3	-0.05	1.00000000	pigs
Ddx39b	-0.07	1.00000000	pigs
Pde12	-0.05	1.00000000	pigs
Klhdc3	-0.02	1.00000000	pigs
Akna	-0.02	1.00000000	pigs
Rbm39	-0.14	1.00000000	pigs
Srp9	-0.12	1.00000000	pigs
Uqcrfs1	0.03	1.00000000	pigs
Cdipt	-0.03	1.00000000	pigs
Slc35a1	-0.02	1.00000000	pigs
Spats2	-0.03	1.00000000	pigs
Rnft1	-0.38	1.00000000	pigs
Elp6	0.01	1.00000000	pigs
Sit1	-0.16	1.00000000	pigs
Dhx35	-0.02	1.00000000	pigs
Cd84	-0.06	1.00000000	pigs
Hsbp1	-0.34	1.00000000	pigs
Rfk	0.02	1.00000000	pigs
Pon2	0.02	1.00000000	pigs
Ndufa8	-0.01	1.00000000	pigs
Arhgef3	-0.17	1.00000000	pigs
Dhx8	-0.08	1.00000000	pigs
Sesn1	-0.04	1.00000000	pigs
Pik3r6	-0.14	1.00000000	pigs
Wdr5	-0.03	1.00000000	pigs
Rab7	-0.05	1.00000000	pigs
Hsd17b10	-0.13	1.00000000	pigs
Clk1	-0.02	1.00000000	pigs
Hdac5	-0.02	1.00000000	pigs
Sde2	0.00	1.00000000	pigs
Camta2	-0.25	1.00000000	pigs
Arfgap2	-0.01	1.00000000	pigs
Cnot6l	-0.17	1.00000000	pigs

Nr4a3	-0.46	1.00000000	pigs
Tnfsf8	0.00	1.00000000	pigs
Aqp3	-0.06	1.00000000	pigs
Vasp	-0.25	1.00000000	pigs
Tgfb1	0.03	1.00000000	pigs
Ern1	0.01	1.00000000	pigs
Dbi	-0.16	1.00000000	pigs
Pex1	-0.05	1.00000000	pigs
Sgms2	-0.20	1.00000000	pigs
Smpdl3b	-0.11	1.00000000	pigs
Zcchc9	0.02	1.00000000	pigs
Ubald1	-0.02	1.00000000	pigs
Tdrd3	0.00	1.00000000	pigs
L3mbtl3	0.01	1.00000000	pigs
Smim14	-0.07	1.00000000	pigs
Lpin2	-0.11	1.00000000	pigs
Wdr18	0.00	1.00000000	pigs
St13	0.00	1.00000000	pigs
Dnaja2	-0.16	1.00000000	pigs
Sphk2	-0.01	1.00000000	pigs
Fam114a2	-0.04	1.00000000	pigs
Xrcc5	-0.07	1.00000000	pigs
Ttc38	-0.05	1.00000000	pigs
Ccdc28b	-0.02	1.00000000	pigs
Ccser2	-0.23	1.00000000	pigs
Eif6	-0.02	1.00000000	pigs
Ap1s2	-0.25	1.00000000	pigs
Rassf8	0.01	1.00000000	pigs
Cd302	-0.07	1.00000000	pigs
Ndufa11	-0.06	1.00000000	pigs
Lclat1	-0.31	1.00000000	pigs
Vdac2	0.10	1.00000000	pigs
Gyg	-0.34	1.00000000	pigs
Anks1	-0.01	1.00000000	pigs
Casp1	-0.18	1.00000000	pigs
Herc1	-0.06	1.00000000	pigs
Dhx32	-0.08	1.00000000	pigs
Gtf2e2	-0.01	1.00000000	pigs
Slco2b1	0.04	1.00000000	pigs
Pigc	-0.15	1.00000000	pigs
Cnp	-0.02	1.00000000	pigs
Elk4	-0.01	1.00000000	pigs
Slc10a3	-0.16	1.00000000	pigs
Evl	0.07	1.00000000	pigs
Iars	-0.11	1.00000000	pigs
Rer1	-0.06	1.00000000	pigs

Tpr	-0.13	1.00000000	pigs
Klf13	-0.19	1.00000000	pigs
Atpaf2	-0.14	1.00000000	pigs
Cwc27	-0.06	1.00000000	pigs
Alad	0.00	1.00000000	pigs
Rnf44	-0.05	1.00000000	pigs
Nubpl	-0.05	1.00000000	pigs
Antkmt	-0.15	1.00000000	pigs
Tnfaip8	0.22	1.00000000	pigs
Taok2	-0.12	1.00000000	pigs
Il7	-0.24	1.00000000	pigs
Cuedc2	-0.11	1.00000000	pigs
Inpp5a	-0.09	1.00000000	pigs
Sipa1	-0.03	1.00000000	pigs
Pycard	-0.36	1.00000000	pigs
Deaf1	-0.10	1.00000000	pigs
Dnajc24	-0.03	1.00000000	pigs
Ptger4	0.01	1.00000000	pigs
Shfl	0.10	1.00000000	pigs
Paf1	-0.06	1.00000000	pigs
Gpsm3	-0.13	1.00000000	pigs
Rp9	-0.06	1.00000000	pigs
Nploc4	-0.07	1.00000000	pigs
Chchd1	-0.11	1.00000000	pigs
Gas2l1	0.05	1.00000000	pigs
Pet100	-0.20	1.00000000	pigs
Ccser1	0.13	1.00000000	pigs
Babam2	-0.06	1.00000000	pigs
Rab11a	-0.01	1.00000000	pigs
Ccr2	-0.18	1.00000000	pigs
Ube4b	-0.02	1.00000000	pigs
Ndufaf1	-0.10	1.00000000	pigs
Daam1	-0.08	1.00000000	pigs
Timm8a2	-0.01	1.00000000	pigs
Cyc1	-0.03	1.00000000	pigs
Ino80	0.00	1.00000000	pigs
Filip1l	-0.23	1.00000000	pigs
Ldlr	-0.06	1.00000000	pigs
Leng1	-0.10	1.00000000	pigs
Smc1a	0.03	1.00000000	pigs
Ttbk2	-0.08	1.00000000	pigs
Pin1	-0.05	1.00000000	pigs
Ppp1r21	0.02	1.00000000	pigs
Sesn3	-0.03	1.00000000	pigs
D8Ertd738e	-0.05	1.00000000	pigs
Epm2aip1	0.00	1.00000000	pigs

Gpr160	-0.10	1.00000000	pigs
Arxes2	-0.13	1.00000000	pigs
Psma7	-0.17	1.00000000	pigs
Shoc2	-0.22	1.00000000	pigs
Pms1	-0.04	1.00000000	pigs
Sidt2	-0.03	1.00000000	pigs
Casc3	-0.03	1.00000000	pigs
Nfia	-0.01	1.00000000	pigs
Rpa3	-0.34	1.00000000	pigs
Ell2	-0.22	1.00000000	pigs
Gtf2a1	-0.02	1.00000000	pigs
Dffa	-0.04	1.00000000	pigs
Nsrp1	0.00	1.00000000	pigs
Sh3glb2	-0.03	1.00000000	pigs
Parg	0.00	1.00000000	pigs
Eif3e	-0.22	1.00000000	pigs
Anxa5	-0.02	1.00000000	pigs
Slc10a7	-0.02	1.00000000	pigs
Dut	-0.09	1.00000000	pigs
Kif5b	-0.14	1.00000000	pigs
Ivns1abp	-0.14	1.00000000	pigs
Prmt2	-0.02	1.00000000	pigs
Slc9a3r1	-0.32	1.00000000	pigs
Wdr33	-0.01	1.00000000	pigs
Uros	-0.07	1.00000000	pigs
Acvr2a	-0.01	1.00000000	pigs
Dhx34	-0.40	1.00000000	pigs
Arl5b	-0.16	1.00000000	pigs
Wwc2	-0.01	1.00000000	pigs
Card9	-0.23	1.00000000	pigs
Prkaa1	-0.08	1.00000000	pigs
Atg7	-0.31	1.00000000	pigs
Usp1	-0.07	1.00000000	pigs
Pqlc3	-0.05	1.00000000	pigs
Bpnt2	-0.10	1.00000000	pigs
Fam111a	0.02	1.00000000	pigs
Gca	0.01	1.00000000	pigs
Smu1	-0.06	1.00000000	pigs
Pdlim7	-0.21	1.00000000	pigs
Nudt13	-0.12	1.00000000	pigs
Shld2	-0.16	1.00000000	pigs
Nelfb	0.01	1.00000000	pigs
Rcn2	-0.03	1.00000000	pigs
Abhd17c	-0.13	1.00000000	pigs
Tcp11	-0.08	1.00000000	pigs
Ndufv2	-0.02	1.00000000	pigs

Erh	-0.04	1.00000000	pigs
Rhof	-0.17	1.00000000	pigs
Psip1	-0.03	1.00000000	pigs
Pdcd10	0.02	1.00000000	pigs
Ninl	-0.02	1.00000000	pigs
Hdhd2	-0.02	1.00000000	pigs
Nars	-0.04	1.00000000	pigs
Cpne1	-0.04	1.00000000	pigs
Ikbkg	-0.07	1.00000000	pigs
Nfu1	-0.25	1.00000000	pigs
Arl5a	0.00	1.00000000	pigs
Cox18	-0.01	1.00000000	pigs
Tmed8	-0.02	1.00000000	pigs
Ube2j2	0.00	1.00000000	pigs
Ppp1r12b	0.00	1.00000000	pigs
Ankrd13a	-0.02	1.00000000	pigs
Eif2b4	-0.11	1.00000000	pigs
Il1r2	0.01	1.00000000	pigs
Spag1	-0.27	1.00000000	pigs
Naxd	-0.12	1.00000000	pigs
Tex30	-0.07	1.00000000	pigs
Polr2i	-0.09	1.00000000	pigs
Ubxn2a	-0.11	1.00000000	pigs
Slc7a5	0.06	1.00000000	pigs
Ptges3	-0.16	1.00000000	pigs
Yeats4	0.00	1.00000000	pigs
Rbx1	0.09	1.00000000	pigs
Suds3	-0.01	1.00000000	pigs
Ddx42	0.02	1.00000000	pigs
Arhgap6	-0.16	1.00000000	pigs
Nnt	-0.08	1.00000000	pigs
Kmt2b	-0.05	1.00000000	pigs
Crcp	-0.03	1.00000000	pigs
Hsd17b12	-0.20	1.00000000	pigs
Dnai3	-0.30	1.00000000	pigs
Tbc1d31	0.00	1.00000000	pigs
Ascc1	-0.07	1.00000000	pigs
Gpn3	-0.01	1.00000000	pigs
3110082I17Rik	-0.22	1.00000000	pigs
Lmbr1l	-0.05	1.00000000	pigs
Pygl	-0.24	1.00000000	pigs
Atad1	-0.11	1.00000000	pigs
Itpr2	-0.21	1.00000000	pigs
Slc25a33	-0.02	1.00000000	pigs
Dda1	-0.05	1.00000000	pigs
Skiv2l	-0.02	1.00000000	pigs

Phf3	-0.14	1.00000000	pigs
Tpmt	0.00	1.00000000	pigs
Brcc3	-0.01	1.00000000	pigs
Tmem131	-0.05	1.00000000	pigs
Adgrg6	-0.12	1.00000000	pigs
Tyw3	-0.02	1.00000000	pigs
Taok3	-0.25	1.00000000	pigs
Lpcat2	-0.20	1.00000000	pigs
Lactb	-0.02	1.00000000	pigs
Wipi1	-0.19	1.00000000	pigs
Tm2d2	-0.08	1.00000000	pigs
Ubl7	-0.11	1.00000000	pigs
Ap4s1	0.00	1.00000000	pigs
Fus	-0.10	1.00000000	pigs
Srfbp1	-0.05	1.00000000	pigs
Ctcf	-0.02	1.00000000	pigs
Ubxn4	0.01	1.00000000	pigs
Bicral	-0.05	1.00000000	pigs
Sec11c	-0.22	1.00000000	pigs
Arhgap9	-0.13	1.00000000	pigs
Obi1	0.00	1.00000000	pigs
Nedd4	-0.03	1.00000000	pigs
Tstd1	-0.41	1.00000000	pigs
Tm2d1	-0.07	1.00000000	pigs
Rfx7	-0.02	1.00000000	pigs
Acot13	-0.19	1.00000000	pigs
Ano8	-0.12	1.00000000	pigs
Dym	-0.04	1.00000000	pigs
Rassf4	-0.15	1.00000000	pigs
Nup107	-0.02	1.00000000	pigs
Smim7	-0.10	1.00000000	pigs
Prkaca	-0.06	1.00000000	pigs
Nupl2	-0.07	1.00000000	pigs
Tprgl	-0.10	1.00000000	pigs
Dennd5a	-0.17	1.00000000	pigs
Duox2	-0.03	1.00000000	pigs
Cmpk1	-0.03	1.00000000	pigs
B9d1	-0.34	1.00000000	pigs
Tnfrsf1b	0.02	1.00000000	pigs
Usp18	-0.01	1.00000000	pigs
Sri	-0.20	1.00000000	pigs
Nrip1	-0.22	1.00000000	pigs
Ahcyl2	-0.15	1.00000000	pigs
Srpr	-0.15	1.00000000	pigs
Bbx	-0.01	1.00000000	pigs
Dhdds	-0.10	1.00000000	pigs

Gdi1	-0.09	1.00000000	pigs
Psma3	-0.17	1.00000000	pigs
Ikzf5	-0.01	1.00000000	pigs
Rnpep	-0.09	1.00000000	pigs
Pbdc1	-0.04	1.00000000	pigs
Fam133b	-0.10	1.00000000	pigs
Zmym4	-0.02	1.00000000	pigs
Xrn2	-0.05	1.00000000	pigs
Ikbkb	0.03	1.00000000	pigs
Cep63	-0.09	1.00000000	pigs
Pcna	-0.13	1.00000000	pigs
Tgfbi	-0.30	1.00000000	pigs
Timmdc1	-0.06	1.00000000	pigs
Derpc	0.00	1.00000000	pigs
Apc	-0.01	1.00000000	pigs
Uqcrq	0.05	1.00000000	pigs
Cdc25b	-0.05	1.00000000	pigs
Capn15	-0.04	1.00000000	pigs
Cyb5b	-0.06	1.00000000	pigs
Ddx59	0.01	1.00000000	pigs
Pola1	-0.03	1.00000000	pigs
Trem1	-0.01	1.00000000	pigs
Ppa1	0.07	1.00000000	pigs
Afmid	-0.02	1.00000000	pigs
Bysl	-0.03	1.00000000	pigs
Rab28	-0.07	1.00000000	pigs
Elmod3	-0.01	1.00000000	pigs
Shld1	-0.16	1.00000000	pigs
Eef1d	-0.05	1.00000000	pigs
Ppp5c	-0.02	1.00000000	pigs
Gtpbp8	0.01	1.00000000	pigs
Ube2m	0.02	1.00000000	pigs
Trdmt1	-0.20	1.00000000	pigs
Setx	-0.02	1.00000000	pigs
Zmym6	-0.03	1.00000000	pigs
Prdm10	-0.03	1.00000000	pigs
Angel2	-0.04	1.00000000	pigs
Gck	-0.01	1.00000000	pigs
Tspan5	0.06	1.00000000	pigs
Polr2c	-0.01	1.00000000	pigs
Tnrc18	-0.07	1.00000000	pigs
Cpeb2	-0.31	1.00000000	pigs
Cmbl	0.00	1.00000000	pigs
Nudcd2	0.00	1.00000000	pigs
Aard	-0.03	1.00000000	pigs
Eloc	-0.09	1.00000000	pigs

Nsun3	-0.03	1.00000000	pigs
Hcfc1r1	-0.04	1.00000000	pigs
Zbtb33	0.01	1.00000000	pigs
Spsb2	-0.09	1.00000000	pigs
Fbxo39	0.10	1.00000000	pigs
Ulk1	-0.07	1.00000000	pigs
P2ry14	0.06	1.00000000	pigs
Agap1	-0.53	1.00000000	pigs
Tbck	-0.09	1.00000000	pigs
Kras	-0.03	1.00000000	pigs
Arhgap1	-0.05	1.00000000	pigs
Il10ra	-0.11	1.00000000	pigs
Pdk3	0.00	1.00000000	pigs
Sarm1	-0.03	1.00000000	pigs
Lrrc28	-0.06	1.00000000	pigs
Zfp422	-0.04	1.00000000	pigs
Pde4dip	0.02	1.00000000	pigs
Pdap1	-0.15	1.00000000	pigs
Ust	-0.21	1.00000000	pigs
Acsf3	-0.04	1.00000000	pigs
Htatsf1	-0.02	1.00000000	pigs
Atl3	-0.19	1.00000000	pigs
Snx29	-0.05	1.00000000	pigs
Kynu	-0.41	1.00000000	pigs
Crls1	-0.05	1.00000000	pigs
Alg2	-0.03	1.00000000	pigs
Tmem245	0.02	1.00000000	pigs
Zfp65	-0.06	1.00000000	pigs
Pcnx3	-0.03	1.00000000	pigs
Napepld	-0.03	1.00000000	pigs
Clpp	-0.07	1.00000000	pigs
Pam	-0.26	1.00000000	pigs
Rac3	-0.06	1.00000000	pigs
Dap	-0.06	1.00000000	pigs
R3hdm2	-0.08	1.00000000	pigs
Unc13d	-0.13	1.00000000	pigs
Bag5	-0.07	1.00000000	pigs
Ocel1	-0.05	1.00000000	pigs
Tmem222	-0.01	1.00000000	pigs
Adap1	-0.16	1.00000000	pigs
Ssr1	0.05	1.00000000	pigs
Evi2	-0.02	1.00000000	pigs
Itm2b	0.08	1.00000000	pigs
Saysd1	-0.02	1.00000000	pigs
Evi5	-0.23	1.00000000	pigs
Fos	-0.51	1.00000000	pigs

Rasgef1b	-0.09	1.00000000	pigs
Tst	-0.03	1.00000000	pigs
Tab3	-0.04	1.00000000	pigs
Ndufa10	-0.21	1.00000000	pigs
Arl14ep	-0.08	1.00000000	pigs
Ist1	-0.04	1.00000000	pigs
Psen1	-0.07	1.00000000	pigs
Pak2	-0.10	1.00000000	pigs
Ndufv1	-0.07	1.00000000	pigs
Lzic	-0.14	1.00000000	pigs
Amz2	-0.11	1.00000000	pigs
Kat2b	-0.04	1.00000000	pigs
Dpy19l1	-0.06	1.00000000	pigs
Nrep	-0.18	1.00000000	pigs
Usp22	-0.07	1.00000000	pigs
Fam117a	-0.12	1.00000000	pigs
Tle4	-0.45	1.00000000	pigs
Acot10	-0.14	1.00000000	pigs
Brd7	-0.04	1.00000000	pigs
Fam76b	0.00	1.00000000	pigs
B9d2	-0.14	1.00000000	pigs
Napa	-0.10	1.00000000	pigs
Tsen34	-0.18	1.00000000	pigs
Ttc33	0.01	1.00000000	pigs
Tbc1d2	-0.07	1.00000000	pigs
Crem	-0.15	1.00000000	pigs
Eif2b2	0.01	1.00000000	pigs
Plekhf2	-0.13	1.00000000	pigs
Ets1	-0.21	1.00000000	pigs
Rassf3	0.17	1.00000000	pigs
Ppp1r11	-0.03	1.00000000	pigs
Oard1	0.02	1.00000000	pigs
Rrbp1	-0.02	1.00000000	pigs
Taf11	-0.02	1.00000000	pigs
Taf1b	-0.14	1.00000000	pigs
Ankrd27	-0.09	1.00000000	pigs
Pde4d	-0.42	1.00000000	pigs
Nfkbiz	-0.24	1.00000000	pigs
Ring1	-0.09	1.00000000	pigs
Prss55	-0.24	1.00000000	pigs
Nsun2	-0.07	1.00000000	pigs
Srsf4	-0.06	1.00000000	pigs
Katna1	-0.07	1.00000000	pigs
Deup1	-0.04	1.00000000	pigs
Pes1	-0.10	1.00000000	pigs
Leng8	-0.10	1.00000000	pigs

Suclg2	-0.10	1.00000000	pigs
Ccng1	0.02	1.00000000	pigs
Lsm6	0.01	1.00000000	pigs
Oxa1l	-0.13	1.00000000	pigs
Aph1a	-0.12	1.00000000	pigs
Pnpt1	-0.10	1.00000000	pigs
Dbnl	-0.03	1.00000000	pigs
Plac9a	-0.10	1.00000000	pigs
Sgpp1	-0.19	1.00000000	pigs
Khk	-0.04	1.00000000	pigs
Arl6ip6	-0.06	1.00000000	pigs
Sap30l	-0.10	1.00000000	pigs
Slc5a6	-0.10	1.00000000	pigs
Tdp2	-0.05	1.00000000	pigs
Ift88	-0.13	1.00000000	pigs
Glipr1	-0.19	1.00000000	pigs
Nek4	-0.03	1.00000000	pigs
Rexo2	-0.07	1.00000000	pigs
Ppp1r10	-0.22	1.00000000	pigs
Nfe2l1	-0.07	1.00000000	pigs
Gramd3	0.06	1.00000000	pigs
9430038I01Rik	-0.01	1.00000000	pigs
Arntl	-0.12	1.00000000	pigs
Cpne3	-0.10	1.00000000	pigs
Suclg1	-0.17	1.00000000	pigs
Apaf1	-0.14	1.00000000	pigs
Casp8	0.02	1.00000000	pigs
Slc9a1	-0.02	1.00000000	pigs
Vps41	-0.11	1.00000000	pigs
Tmtc4	-0.03	1.00000000	pigs
Zdhhc4	-0.13	1.00000000	pigs
Rfc5	-0.05	1.00000000	pigs
Snap23	-0.02	1.00000000	pigs
Ipmk	-0.41	1.00000000	pigs
Ttc4	-0.04	1.00000000	pigs
Aifm1	-0.08	1.00000000	pigs
Dnajc10	-0.15	1.00000000	pigs
Paip2	-0.27	1.00000000	pigs
Derl1	-0.05	1.00000000	pigs
Ptpmt1	-0.15	1.00000000	pigs
Uhrf1bp1	-0.05	1.00000000	pigs
Tspan2	-0.11	1.00000000	pigs
Ubald2	-0.18	1.00000000	pigs
Cfap20	-0.17	1.00000000	pigs
Atxn7	-0.09	1.00000000	pigs
Tatdn1	-0.20	1.00000000	pigs

Tab1	0.00	1.00000000	pigs
Coq10b	-0.05	1.00000000	pigs
Cwf19l2	-0.09	1.00000000	pigs
Ralgps2	-0.06	1.00000000	pigs
Rfx1	-0.11	1.00000000	pigs
Nr2f6	0.01	1.00000000	pigs
Usp40	-0.05	1.00000000	pigs
Bend7	-0.01	1.00000000	pigs
Ccdc107	-0.13	1.00000000	pigs
Ube2h	-0.23	1.00000000	pigs
Fbxo3	-0.06	1.00000000	pigs
Klhl29	0.04	1.00000000	pigs
Ttf2	-0.08	1.00000000	pigs
Eif2ak1	-0.11	1.00000000	pigs
Slc46a2	0.01	1.00000000	pigs
Gas7	-0.21	1.00000000	pigs
Fah	-0.01	1.00000000	pigs
Socs6	-0.07	1.00000000	pigs
Ndufaf2	-0.10	1.00000000	pigs
Slc10a1	-0.17	1.00000000	pigs
Klhdc2	-0.17	1.00000000	pigs
Qsox2	-0.06	1.00000000	pigs
Fam149b	-0.09	1.00000000	pigs
Rbbp9	-0.01	1.00000000	pigs
Setdb2	-0.04	1.00000000	pigs
Nfx1	-0.08	1.00000000	pigs
Ndufab1	-0.20	1.00000000	pigs
Pou2f2	-0.01	1.00000000	pigs
Parp9	-0.03	1.00000000	pigs
Rad52	-0.11	1.00000000	pigs
Trim75	-0.17	1.00000000	pigs
Phf14	-0.11	1.00000000	pigs
Kat6b	-0.12	1.00000000	pigs
Ppwd1	0.00	1.00000000	pigs
Dhrs7b	-0.13	1.00000000	pigs
A430005L14Rik	0.00	1.00000000	pigs
Rbsn	-0.03	1.00000000	pigs
Fam214a	0.09	1.00000000	pigs
Hpf1	-0.01	1.00000000	pigs
Dhx30	-0.23	1.00000000	pigs
Uxt	-0.22	1.00000000	pigs
Frmd6	-0.02	1.00000000	pigs
Pnrc1	-0.11	1.00000000	pigs
Nde1	-0.08	1.00000000	pigs
Polr1d	-0.13	1.00000000	pigs
Dalrd3	-0.04	1.00000000	pigs

Btbd11	-0.07	1.00000000	pigs
Rpp21	-0.10	1.00000000	pigs
Thumpd3	-0.03	1.00000000	pigs
Psmb8	-0.04	1.00000000	pigs
Lpxn	-0.07	1.00000000	pigs
Slc37a1	-0.01	1.00000000	pigs
Ahr	-0.11	1.00000000	pigs
Klf2	-0.24	1.00000000	pigs
Calcoco2	-0.10	1.00000000	pigs
Cask	-0.22	1.00000000	pigs
Eif4ebp1	-0.18	1.00000000	pigs
Anapc7	-0.03	1.00000000	pigs
Fam189a1	-0.06	1.00000000	pigs
Tpgs1	-0.15	1.00000000	pigs
Ccdc25	-0.05	1.00000000	pigs
Dusp5	0.36	1.00000000	pigs
Nlrp12	0.01	1.00000000	pigs
Akap9	-0.09	1.00000000	pigs
Ccnb1ip1	-0.13	1.00000000	pigs
Dok1	-0.02	1.00000000	pigs
Accsl	-0.05	1.00000000	pigs
Crk	-0.10	1.00000000	pigs
Ube2q1	-0.10	1.00000000	pigs
Cltb	0.00	1.00000000	pigs
Naa20	-0.07	1.00000000	pigs
Specc1	-0.12	1.00000000	pigs
Rfx3	-0.19	1.00000000	pigs
Ehf	0.07	1.00000000	pigs
Ssna1	-0.19	1.00000000	pigs
Wdfy2	-0.02	1.00000000	pigs
Jmjd8	-0.16	1.00000000	pigs
Cep290	-0.10	1.00000000	pigs
Sap18	-0.15	1.00000000	pigs
Hmgcs1	-0.27	1.00000000	pigs
Wdr12	-0.04	1.00000000	pigs
Foxj3	-0.15	1.00000000	pigs
Gsdmd	-0.07	1.00000000	pigs
Cox7a2l	-0.18	1.00000000	pigs
Tmem50a	-0.17	1.00000000	pigs
Atr	-0.12	1.00000000	pigs
Hrh1	-0.05	1.00000000	pigs
Apex1	-0.03	1.00000000	pigs
Tpm4	0.04	1.00000000	pigs
Cox7a1	-0.38	1.00000000	pigs
Pitpnm2	-0.19	1.00000000	pigs
Akt2	-0.10	1.00000000	pigs

Vps28	-0.14	1.00000000	pigs
Trmt6	-0.17	1.00000000	pigs
Ptprk	-0.08	1.00000000	pigs
1110059G10Rik	-0.01	1.00000000	pigs
Hyi	-0.10	1.00000000	pigs
Eif2b1	-0.04	1.00000000	pigs
Naxe	-0.07	1.00000000	pigs
Rbmx2	0.00	1.00000000	pigs
Dicer1	-0.16	1.00000000	pigs
Eci3	0.00	1.00000000	pigs
Tgfa	0.17	1.00000000	pigs
Ubn2	-0.15	1.00000000	pigs
Arpc5l	-0.05	1.00000000	pigs
Spryd7	-0.07	1.00000000	pigs
Ubtf	-0.07	1.00000000	pigs
Vps36	-0.06	1.00000000	pigs
Asrgl1	-0.07	1.00000000	pigs
Fbl	-0.10	1.00000000	pigs
Xpot	-0.05	1.00000000	pigs
Nkap	-0.24	1.00000000	pigs
Vcpkmt	-0.05	1.00000000	pigs
Noa1	-0.08	1.00000000	pigs
Katnip	-0.07	1.00000000	pigs
Psmb2	0.01	1.00000000	pigs
Ncoa3	-0.26	1.00000000	pigs
Hras	-0.19	1.00000000	pigs
N4bp2	-0.04	1.00000000	pigs
Scand1	-0.19	1.00000000	pigs
Zfp664	-0.07	1.00000000	pigs
Ctnnd1	-0.09	1.00000000	pigs
Fyco1	-0.05	1.00000000	pigs
Cisd2	-0.07	1.00000000	pigs
Tnfsf9	-0.10	1.00000000	pigs
Prmt3	-0.05	1.00000000	pigs
Klc4	-0.06	1.00000000	pigs
Zfp251	-0.10	1.00000000	pigs
Cdin1	-0.06	1.00000000	pigs
Dcakd	-0.10	1.00000000	pigs
Cpd	-0.16	1.00000000	pigs
Dock5	-0.22	1.00000000	pigs
Ccdc180	-0.22	1.00000000	pigs
Ap1s3	0.01	1.00000000	pigs
Cdr2	-0.08	1.00000000	pigs
Tnrc6c	-0.12	1.00000000	pigs
Foxn2	-0.02	1.00000000	pigs
Slc25a13	-0.09	1.00000000	pigs

Afg1l	-0.05	1.00000000	pigs
5730455P16Rik	0.00	1.00000000	pigs
Ptk2b	-0.17	1.00000000	pigs
Ticam1	-0.17	1.00000000	pigs
Triobp	-0.11	1.00000000	pigs
Gin1	0.04	1.00000000	pigs
Rbm12b2	-0.10	1.00000000	pigs
Txnrd3	-0.07	1.00000000	pigs
Ints6l	-0.38	1.00000000	pigs
Tango6	-0.04	1.00000000	pigs
Dgkd	-0.02	1.00000000	pigs
Ubxn1	-0.12	1.00000000	pigs
Farsa	-0.04	1.00000000	pigs
Txnip	-0.24	1.00000000	pigs
Wdr45	-0.15	1.00000000	pigs
Lonp2	0.18	1.00000000	pigs
Sash3	-0.04	1.00000000	pigs
Eml3	-0.01	1.00000000	pigs
Peli1	-0.14	1.00000000	pigs
Cmah	-0.04	1.00000000	pigs
Ccdc66	-0.01	1.00000000	pigs
Fam13b	-0.18	1.00000000	pigs
Strbp	-0.06	1.00000000	pigs
Pibf1	-0.16	1.00000000	pigs
Recql	-0.12	1.00000000	pigs
Klhl20	-0.02	1.00000000	pigs
Tiparp	0.00	1.00000000	pigs
Plcb2	-0.08	1.00000000	pigs
Rbbp5	-0.20	1.00000000	pigs
Selenoo	-0.06	1.00000000	pigs
Nedd1	-0.05	1.00000000	pigs
Hnrnpd	0.01	1.00000000	pigs
Pam16	-0.05	1.00000000	pigs
Tmem184c	-0.05	1.00000000	pigs
Daglb	-0.16	1.00000000	pigs
Tk1	-0.05	1.00000000	pigs
Bclaf3	-0.04	1.00000000	pigs
Itgb2l	-0.05	1.00000000	pigs
Pop4	-0.06	1.00000000	pigs
Ndufb2	-0.02	1.00000000	pigs
Ccnl2	-0.06	1.00000000	pigs
Lima1	-0.12	1.00000000	pigs
Qars	-0.07	1.00000000	pigs
Fra10ac1	-0.02	1.00000000	pigs
Cap1	-0.05	1.00000000	pigs
Aagab	-0.06	1.00000000	pigs

Nav3	-0.22	1.00000000	pigs
Btf3l4	-0.21	1.00000000	pigs
Plaur	-0.09	1.00000000	pigs
Far1	-0.23	1.00000000	pigs
Samd4	-0.02	1.00000000	pigs
Aip	-0.07	1.00000000	pigs
Rnf14	-0.18	1.00000000	pigs
Tram1	-0.14	1.00000000	pigs
Camkmt	-0.07	1.00000000	pigs
Leo1	-0.06	1.00000000	pigs
Cmas	-0.14	1.00000000	pigs
Nfkb1	-0.26	1.00000000	pigs
Pwwp3a	-0.08	1.00000000	pigs
Nipsnap2	-0.11	1.00000000	pigs
Stx11	-0.30	1.00000000	pigs
Cers4	-0.08	1.00000000	pigs
Sap30	-0.16	1.00000000	pigs
Cox6a2	-0.05	1.00000000	pigs
Plpbp	0.00	1.00000000	pigs
Nelfe	-0.07	1.00000000	pigs
Acp4	-0.11	1.00000000	pigs
Gabarapl1	-0.12	1.00000000	pigs
Btg1b	-0.04	1.00000000	pigs
Tbrg1	-0.08	1.00000000	pigs
1110004F10Rik	0.00	1.00000000	pigs
Atpaf1	-0.05	1.00000000	pigs
Acbd6	-0.19	1.00000000	pigs
Ddrgk1	-0.20	1.00000000	pigs
Resf1	-0.23	1.00000000	pigs
Comtd1	-0.02	1.00000000	pigs
Adprh	-0.08	1.00000000	pigs
Rassf1	-0.24	1.00000000	pigs
Bag1	-0.07	1.00000000	pigs
Depdc1b	-0.05	1.00000000	pigs
Prkar2b	-0.19	1.00000000	pigs
Nsd1	-0.05	1.00000000	pigs
Cox5b-ps	0.02	1.00000000	pigs
Fam118b	0.00	1.00000000	pigs
Eif2s2	-0.08	1.00000000	pigs
BC003965	-0.05	1.00000000	pigs
Bid	-0.05	1.00000000	pigs
Kcnab2	-0.13	1.00000000	pigs
Snx17	-0.02	1.00000000	pigs
Rheb	-0.04	1.00000000	pigs
Kctd13	-0.05	1.00000000	pigs
Echs1	-0.24	1.00000000	pigs

Coq7	-0.23	1.00000000	pigs
Fam199x	-0.05	1.00000000	pigs
Insig2	-0.09	1.00000000	pigs
Drg2	-0.04	1.00000000	pigs
Jmjd1c	-0.07	1.00000000	pigs
Pald1	-0.06	1.00000000	pigs
Pex16	-0.04	1.00000000	pigs
Tdg	-0.06	1.00000000	pigs
Snrpb2	-0.23	1.00000000	pigs
Tmem126a	-0.03	1.00000000	pigs
Slc25a39	-0.14	1.00000000	pigs
Sco2	-0.04	1.00000000	pigs
Top1	0.08	1.00000000	pigs
Sertad2	-0.08	1.00000000	pigs
Cpeb4	-0.12	1.00000000	pigs
Prmt1	-0.07	1.00000000	pigs
Tnfsf13b	-0.09	1.00000000	pigs
Slc2a13	-0.13	1.00000000	pigs
D030056L22Rik	-0.01	1.00000000	pigs
Dynlt1a	-0.24	1.00000000	pigs
Nsmce1	-0.08	1.00000000	pigs
Acot4	-0.03	1.00000000	pigs
Ppp1r3b	-0.16	1.00000000	pigs
Arl6ip4	-0.11	1.00000000	pigs
Tor1aip1	-0.12	1.00000000	pigs
Arid3b	-0.10	1.00000000	pigs
Clic4	-0.29	1.00000000	pigs
Dynlt3	-0.07	1.00000000	pigs
Rwdd1	-0.14	1.00000000	pigs
Cbx1	-0.03	1.00000000	pigs
Ube2l6	-0.12	1.00000000	pigs
Olr1	-0.09	1.00000000	pigs
Rbbp7	-0.11	1.00000000	pigs
Clec5a	0.05	1.00000000	pigs
Bnip2	-0.09	1.00000000	pigs
S100a11	-0.20	1.00000000	pigs
Egfr	0.06	1.00000000	pigs
Dnajc3	-0.12	1.00000000	pigs
Ermp1	-0.03	1.00000000	pigs
4932438A13Rik	-0.09	1.00000000	pigs
Prnp	-0.31	1.00000000	pigs
Taf1d	-0.09	1.00000000	pigs
Nek1	-0.07	1.00000000	pigs
Timm10	-0.01	1.00000000	pigs
Dpp8	-0.09	1.00000000	pigs
Irak2	-0.39	1.00000000	pigs

Dennd6a	-0.05	1.00000000	pigs
Sfpq	-0.09	1.00000000	pigs
Nfil3	-0.25	1.00000000	pigs
Pim1	-0.09	1.00000000	pigs
Cstf1	-0.06	1.00000000	pigs
Cmc1	-0.13	1.00000000	pigs
Emb	-0.11	1.00000000	pigs
Kifap3	-0.09	1.00000000	pigs
Sclt1	-0.23	1.00000000	pigs
Hectd3	-0.06	1.00000000	pigs
Trex1	-0.08	1.00000000	pigs
Chmp7	-0.01	1.00000000	pigs
Creb5	-0.31	1.00000000	pigs
Fam172a	-0.17	1.00000000	pigs
Trim37	-0.08	1.00000000	pigs
Rap1a	-0.02	1.00000000	pigs
Anxa4	-0.06	1.00000000	pigs
Nfat5	-0.22	1.00000000	pigs
A830018L16Rik	-0.15	1.00000000	pigs
Gng10	-0.09	1.00000000	pigs
Oas2	-0.11	1.00000000	pigs
Immp1l	-0.20	1.00000000	pigs
AU040320	-0.02	1.00000000	pigs
Pgap1	0.08	1.00000000	pigs
Tipin	-0.04	1.00000000	pigs
Angptl4	-0.05	1.00000000	pigs
Ranbp6	-0.08	1.00000000	pigs
Clint1	-0.07	1.00000000	pigs
Arhgef18	-0.21	1.00000000	pigs
Xkr8	-0.13	1.00000000	pigs
Aftph	-0.09	1.00000000	pigs
Kcnq5	-0.01	1.00000000	pigs
Pithd1	-0.06	1.00000000	pigs
Gtf2f2	-0.09	1.00000000	pigs
Emc3	-0.16	1.00000000	pigs
Dnmbp	-0.23	1.00000000	pigs
Syap1	-0.29	1.00000000	pigs
Armh3	-0.15	1.00000000	pigs
Gadd45g	-0.01	1.00000000	pigs
Tbc1d5	-0.02	1.00000000	pigs
Ttc17	-0.20	1.00000000	pigs
Ercc8	-0.08	1.00000000	pigs
Ddx3x	-0.12	1.00000000	pigs
Vsig4	-0.24	1.00000000	pigs
Hnrnph2	-0.05	1.00000000	pigs
Gadd45gip1	-0.09	1.00000000	pigs

Rcor3	-0.01	1.00000000	pigs
Gtpbp4	-0.06	1.00000000	pigs
Srp14	-0.02	1.00000000	pigs
Gins2	-0.06	1.00000000	pigs
Agtpbp1	-0.09	1.00000000	pigs
Cabin1	-0.07	1.00000000	pigs
Itgam	-0.10	1.00000000	pigs
Sdf2	-0.10	1.00000000	pigs
Adcy4	0.01	1.00000000	pigs
Thumpd1	-0.07	1.00000000	pigs
Thap2	-0.07	1.00000000	pigs
Zfp276	-0.09	1.00000000	pigs
Pcmt1	-0.21	1.00000000	pigs
Septin1	-0.11	1.00000000	pigs
Gtf2f1	-0.02	1.00000000	pigs
Pml	-0.12	1.00000000	pigs
Rab2a	0.04	1.00000000	pigs
Coro1a	-0.18	1.00000000	pigs
Oxld1	-0.15	1.00000000	pigs
Lfng	-0.12	1.00000000	pigs
Stag2	-0.18	1.00000000	pigs
Tiam1	-0.18	1.00000000	pigs
Blnk	-0.22	1.00000000	pigs
Parn	-0.13	1.00000000	pigs
Cspp1	-0.13	1.00000000	pigs
Taf6	-0.06	1.00000000	pigs
Pafah2	-0.14	1.00000000	pigs
Dtx4	0.00	1.00000000	pigs
Ube2e3	-0.08	1.00000000	pigs
Isoc1	-0.19	1.00000000	pigs
Socs4	-0.04	1.00000000	pigs
Akt3	-0.24	1.00000000	pigs
Fam204a	-0.12	1.00000000	pigs
Cab39l	-0.06	1.00000000	pigs
Themis2	-0.10	1.00000000	pigs
Ncoa5	-0.07	1.00000000	pigs
Ssbp4	-0.21	1.00000000	pigs
Dennd4b	-0.08	1.00000000	pigs
Tsr3	-0.02	1.00000000	pigs
Btbd19	0.01	1.00000000	pigs
Klf5	-0.03	1.00000000	pigs
Trappc5	-0.20	1.00000000	pigs
Poglut1	-0.22	1.00000000	pigs
Parp14	-0.30	1.00000000	pigs
Nedd8	-0.07	1.00000000	pigs
Actr6	-0.12	1.00000000	pigs

Atp8b4	-0.07	1.00000000	pigs
Inip	0.01	1.00000000	pigs
Itprid2	-0.21	1.00000000	pigs
Chrac1	-0.21	1.00000000	pigs
Tmem47	-0.04	1.00000000	pigs
Bcap29	-0.08	1.00000000	pigs
Btaf1	-0.20	1.00000000	pigs
Rbm38	-0.06	1.00000000	pigs
Elp4	-0.09	1.00000000	pigs
Cwc25	-0.14	1.00000000	pigs
Txnl4b	-0.08	1.00000000	pigs
Swt1	-0.06	1.00000000	pigs
Fam177a	-0.11	1.00000000	pigs
Slc25a25	-0.08	1.00000000	pigs
Cks2	-0.26	1.00000000	pigs
Pts	-0.05	1.00000000	pigs
Tmem134	-0.16	1.00000000	pigs
Selenos	-0.35	1.00000000	pigs
Zc3h13	-0.04	1.00000000	pigs
Pld1	-0.23	1.00000000	pigs
Polm	-0.13	1.00000000	pigs
Rap2a	-0.12	1.00000000	pigs
Folr1	-0.17	1.00000000	pigs
Ssbp1	0.00	1.00000000	pigs
Ate1	-0.09	1.00000000	pigs
Hdac7	-0.11	1.00000000	pigs
Txndc16	0.03	1.00000000	pigs
Klhl7	-0.02	1.00000000	pigs
Atp5c1	0.03	1.00000000	pigs
Sugct	-0.10	1.00000000	pigs
Ska2	-0.07	1.00000000	pigs
Capn3	-0.12	1.00000000	pigs
Srrm2	-0.10	1.00000000	pigs
Ciao2a	-0.17	1.00000000	pigs
Snf8	-0.12	1.00000000	pigs
2810004N23Rik	-0.18	1.00000000	pigs
Gpt	-0.06	1.00000000	pigs
Nup155	-0.03	1.00000000	pigs
Lmo2	-0.06	1.00000000	pigs
Tmem63a	-0.06	1.00000000	pigs
Atm	-0.13	1.00000000	pigs
Rb1	-0.23	1.00000000	pigs
Nub1	-0.14	1.00000000	pigs
Snw1	-0.10	1.00000000	pigs
Parvb	-0.02	1.00000000	pigs
Rbck1	-0.08	1.00000000	pigs

Ckap2l	-0.04	1.00000000	pigs
Rbis	-0.08	1.00000000	pigs
Rsl24d1	-0.02	1.00000000	pigs
Stk16	-0.08	1.00000000	pigs
Prkag2	-0.19	1.00000000	pigs
Lamb1	0.12	1.00000000	pigs
Zdhhc5	-0.05	1.00000000	pigs
Plpp1	-0.26	1.00000000	pigs
Ttc39b	-0.15	1.00000000	pigs
Creb1	-0.17	1.00000000	pigs
Dcaf6	-0.12	1.00000000	pigs
Lmbr1	-0.10	1.00000000	pigs
Extl3	0.00	1.00000000	pigs
Capns1	-0.10	1.00000000	pigs
Commd3	-0.07	1.00000000	pigs
Wdr55	-0.08	1.00000000	pigs
Uap1	-0.02	1.00000000	pigs
Ndufb4	-0.30	1.00000000	pigs
Ptk2	-0.01	1.00000000	pigs
Gstk1	-0.05	1.00000000	pigs
Srxn1	-0.11	1.00000000	pigs
Smarca2	-0.04	1.00000000	pigs
Slc44a2	-0.02	1.00000000	pigs
Chmp4b	-0.01	1.00000000	pigs
Golga1	-0.08	1.00000000	pigs
Cideb	-0.06	1.00000000	pigs
Zfp322a	0.01	1.00000000	pigs
Dnase1l1	-0.20	1.00000000	pigs
Il13ra1	-0.17	1.00000000	pigs
Pnpla8	-0.34	1.00000000	pigs
Fundc2	-0.14	1.00000000	pigs
Ipo11	-0.10	1.00000000	pigs
Thap3	-0.02	1.00000000	pigs
Ndufb10	-0.10	1.00000000	pigs
Rpusd1	-0.03	1.00000000	pigs
Rsbn1l	-0.07	1.00000000	pigs
Snrpn	-0.14	1.00000000	pigs
Dop1a	-0.05	1.00000000	pigs
Slc25a17	-0.06	1.00000000	pigs
Zbtb25	-0.09	1.00000000	pigs
BC005624	-0.13	1.00000000	pigs
Fam8a1	-0.08	1.00000000	pigs
Slc25a12	-0.19	1.00000000	pigs
Trim38	0.08	1.00000000	pigs
Rnf220	-0.02	1.00000000	pigs
Anxa6	-0.22	1.00000000	pigs

Tnfsf4	-0.12	1.00000000	pigs
Taf12	-0.14	1.00000000	pigs
G3bp2	-0.10	1.00000000	pigs
Trim8	-0.06	1.00000000	pigs
Atp7a	-0.07	1.00000000	pigs
Ddx1	-0.07	1.00000000	pigs
Zcchc2	-0.02	1.00000000	pigs
Arap2	-0.03	1.00000000	pigs
Gxylt2	0.00	1.00000000	pigs
Zfp369	-0.10	1.00000000	pigs
Eogt	-0.11	1.00000000	pigs
Fgfr1op2	-0.19	1.00000000	pigs
Sdc3	-0.16	1.00000000	pigs
Itfg1	-0.24	1.00000000	pigs
Gcnt1	-0.10	1.00000000	pigs
C2cd2l	-0.05	1.00000000	pigs
Utp23	0.00	1.00000000	pigs
Iah1	-0.22	1.00000000	pigs
Plekha2	-0.08	1.00000000	pigs
Wdr13	-0.07	1.00000000	pigs
Tbc1d7	-0.14	1.00000000	pigs
Lap3	-0.07	1.00000000	pigs
Ttc28	-0.12	1.00000000	pigs
Slc25a20	-0.35	1.00000000	pigs
Tcp11l2	-0.08	1.00000000	pigs
Gpatch4	-0.03	1.00000000	pigs
Ldhc	-0.07	1.00000000	pigs
Leprotl1	-0.08	1.00000000	pigs
Dnlz	-0.17	1.00000000	pigs
Net1	-0.04	1.00000000	pigs
Cdkn2aipnl	-0.25	1.00000000	pigs
Nmd3	-0.04	1.00000000	pigs
Phtf2	-0.13	1.00000000	pigs
Lonrf3	-0.07	1.00000000	pigs
Cdadc1	-0.27	1.00000000	pigs
Timm22	-0.03	1.00000000	pigs
DPCD	-0.44	1.00000000	pigs
Lss	-0.05	1.00000000	pigs
Snupn	-0.02	1.00000000	pigs
Ccdc88c	-0.05	1.00000000	pigs
Exoc8	-0.05	1.00000000	pigs
Pin4	-0.09	1.00000000	pigs
Ncl	-0.11	1.00000000	pigs
Ankmy2	-0.07	1.00000000	pigs
Rtn4	-0.11	1.00000000	pigs
Adcy3	-0.05	1.00000000	pigs

Phf5a	-0.04	1.00000000	pigs
C030006K11Rik	-0.04	1.00000000	pigs
Oxct1	-0.25	1.00000000	pigs
4921524J17Rik	-0.05	1.00000000	pigs
Traf3ip2	-0.04	1.00000000	pigs
Tmem9b	-0.22	1.00000000	pigs
Sms	-0.12	1.00000000	pigs
Spg11	-0.07	1.00000000	pigs
Dhrs4	-0.15	1.00000000	pigs
Vps29	-0.05	1.00000000	pigs
Btrc	-0.13	1.00000000	pigs
Sec24a	-0.16	1.00000000	pigs
Il16	-0.11	1.00000000	pigs
Zdhhc14	0.03	1.00000000	pigs
Agpat1	-0.05	1.00000000	pigs
Clasrp	-0.02	1.00000000	pigs
Vrk2	-0.16	1.00000000	pigs
Tcfl5	-0.10	1.00000000	pigs
Eef2	-0.02	1.00000000	pigs
R3hdm1	-0.15	1.00000000	pigs
Dnajc1	-0.14	1.00000000	pigs
Tars	-0.14	1.00000000	pigs
Dhx57	-0.06	1.00000000	pigs
Vcl	-0.09	1.00000000	pigs
Lypla1	-0.14	1.00000000	pigs
Dtymk	-0.12	1.00000000	pigs
Psma4	-0.03	1.00000000	pigs
Crlf3	-0.08	1.00000000	pigs
Ppil4	-0.11	1.00000000	pigs
Dpysl2	-0.07	1.00000000	pigs
Zfand2b	-0.08	1.00000000	pigs
Czib	-0.17	1.00000000	pigs
Asap3	-0.17	1.00000000	pigs
Ift57	-0.18	1.00000000	pigs
Gpr82	0.08	1.00000000	pigs
Exoc3	-0.08	1.00000000	pigs
Fdx1	-0.17	1.00000000	pigs
Aasdh	-0.08	1.00000000	pigs
Endov	-0.22	1.00000000	pigs
Smpd2	-0.07	1.00000000	pigs
Cox5a	-0.03	1.00000000	pigs
Psap	-0.09	1.00000000	pigs
Flot2	-0.19	1.00000000	pigs
Raly	-0.16	1.00000000	pigs
Nsd3	-0.14	1.00000000	pigs
Dhx16	-0.05	1.00000000	pigs

H2-Ke6	-0.25	1.00000000	pigs
Fam20b	-0.03	1.00000000	pigs
Stk4	-0.06	1.00000000	pigs
Flad1	-0.06	1.00000000	pigs
Nek7	0.01	1.00000000	pigs
Hemgn	-0.08	1.00000000	pigs
Fosb	-0.36	1.00000000	pigs
Tfpi2	-0.05	1.00000000	pigs
Cfap97	-0.11	1.00000000	pigs
Tuba8	-0.03	1.00000000	pigs
Ccdc88a	-0.13	1.00000000	pigs
Nt5c	-0.09	1.00000000	pigs
Cnppd1	-0.03	1.00000000	pigs
Inpp5f	0.00	1.00000000	pigs
Ddx46	-0.05	1.00000000	pigs
Ubxn2b	-0.07	1.00000000	pigs
Snx16	-0.03	1.00000000	pigs
Zfp524	-0.07	1.00000000	pigs
Cggbp1	-0.14	1.00000000	pigs
Impdh1	-0.15	1.00000000	pigs
Atg16l2	-0.12	1.00000000	pigs
Chst15	-0.08	1.00000000	pigs
Cxxc1	-0.08	1.00000000	pigs
Plcl1	-0.54	1.00000000	pigs
Washc4	-0.09	1.00000000	pigs
Large1	-0.01	1.00000000	pigs
Pde7b	-0.12	1.00000000	pigs
Txndc9	-0.13	1.00000000	pigs
Hibch	-0.04	1.00000000	pigs
Ppme1	-0.16	1.00000000	pigs
Ptpdc1	-0.10	1.00000000	pigs
Col19a1	-0.17	1.00000000	pigs
Nfatc2	-0.11	1.00000000	pigs
Thrap3	-0.03	1.00000000	pigs
Spcs1	-0.41	1.00000000	pigs
Pir	-0.05	1.00000000	pigs
Soat1	-0.43	1.00000000	pigs
Ttc7	-0.10	1.00000000	pigs
Vps9d1	-0.02	1.00000000	pigs
Ruvbl1	-0.10	1.00000000	pigs
Hdgfl3	-0.06	1.00000000	pigs
Usp34	-0.07	1.00000000	pigs
Tmem97	-0.16	1.00000000	pigs
Grk4	-0.08	1.00000000	pigs
Dph1	-0.11	1.00000000	pigs
Anapc16	-0.13	1.00000000	pigs

Tmem170b	-0.07	1.00000000	pigs
Pex3	-0.06	1.00000000	pigs
Aopep	-0.13	1.00000000	pigs
Rdx	-0.34	1.00000000	pigs
Tasp1	-0.12	1.00000000	pigs
Guk1	-0.13	1.00000000	pigs
Trerf1	-0.09	1.00000000	pigs
Dcaf12	-0.11	1.00000000	pigs
Epc1	-0.08	1.00000000	pigs
Capn2	-0.13	1.00000000	pigs
Ppp1r15a	-0.30	1.00000000	pigs
A130010J15Rik	-0.10	1.00000000	pigs
Chst11	0.05	1.00000000	pigs
Slk	-0.13	1.00000000	pigs
R3hdm4	-0.27	1.00000000	pigs
Akap3	-0.08	1.00000000	pigs
Sltm	-0.11	1.00000000	pigs
Cpt2	-0.02	1.00000000	pigs
Sytl5	-0.08	1.00000000	pigs
Agpat4	-0.12	1.00000000	pigs
Kif3c	-0.21	1.00000000	pigs
Tk2	-0.05	1.00000000	pigs
Dhx29	-0.04	1.00000000	pigs
Col1a1	-0.03	1.00000000	pigs
Unc13b	-0.15	1.00000000	pigs
Gse1	-0.12	1.00000000	pigs
Enpp1	0.03	1.00000000	pigs
Kdelr3	-0.06	1.00000000	pigs
Pcmtd2	-0.06	1.00000000	pigs
Cdv3	-0.05	1.00000000	pigs
Ssr3	-0.17	1.00000000	pigs
Pum3	-0.13	1.00000000	pigs
Agpat5	-0.09	1.00000000	pigs
Orc2	-0.06	1.00000000	pigs
Pabpc1	0.03	1.00000000	pigs
Higd2a	-0.12	1.00000000	pigs
Uqcc3	-0.12	1.00000000	pigs
Scin	-0.02	1.00000000	pigs
Primpol	-0.27	1.00000000	pigs
Bzw2	-0.04	1.00000000	pigs
Rack1	-0.02	1.00000000	pigs
Hint1	-0.11	1.00000000	pigs
Focad	-0.12	1.00000000	pigs
Khnyn	-0.03	1.00000000	pigs
Trak2	-0.07	1.00000000	pigs
Trim44	-0.05	1.00000000	pigs

Notch1	-0.16	1.00000000	pigs
Tbl1xr1	-0.05	1.00000000	pigs
Tmem87b	-0.18	1.00000000	pigs
Slc39a11	-0.20	1.00000000	pigs
Tkt	-0.04	1.00000000	pigs
Olfml3	-0.14	1.00000000	pigs
Rufy1	-0.17	1.00000000	pigs
Abhd17a	-0.16	1.00000000	pigs
Serbp1	-0.09	1.00000000	pigs
Ifi204	-0.05	1.00000000	pigs
Eri3	-0.14	1.00000000	pigs
Pigk	-0.29	1.00000000	pigs
Ndufb11b	-0.16	1.00000000	pigs
Arhgef6	-0.15	1.00000000	pigs
Ulk4	-0.08	1.00000000	pigs
Znrf2	-0.02	1.00000000	pigs
Rsu1	-0.10	1.00000000	pigs
Snrnp35	-0.10	1.00000000	pigs
Camk1d	-0.11	1.00000000	pigs
Ubb	0.03	1.00000000	pigs
Idh1	-0.11	1.00000000	pigs
Csnk1g1	-0.01	1.00000000	pigs
Cmss1	-0.13	1.00000000	pigs
Oaz2	-0.18	1.00000000	pigs
Dmac2l	-0.08	1.00000000	pigs
Cep104	-0.16	1.00000000	pigs
Pik3ip1	-0.13	1.00000000	pigs
Dusp28	-0.16	1.00000000	pigs
Btk	-0.08	1.00000000	pigs
Rab18	-0.17	1.00000000	pigs
Shtn1	-0.18	1.00000000	pigs
Scfd2	0.08	1.00000000	pigs
Ethe1	-0.14	1.00000000	pigs
Nrbf2	-0.12	1.00000000	pigs
Ddx24	-0.14	1.00000000	pigs
Tmem241	-0.06	1.00000000	pigs
Dctpp1	-0.23	1.00000000	pigs
Cblb	-0.38	1.00000000	pigs
Immp2l	-0.07	1.00000000	pigs
Rnf214	-0.07	1.00000000	pigs
Prdx4	-0.11	1.00000000	pigs
Paxx	-0.26	1.00000000	pigs
Gnb4	-0.12	1.00000000	pigs
Gimap8	0.00	1.00000000	pigs
Ophn1	-0.13	1.00000000	pigs
Hgs	-0.14	1.00000000	pigs

Trib3	-0.05	1.00000000	pigs
Ubn1	-0.10	1.00000000	pigs
Anapc5	-0.05	1.00000000	pigs
Bnip3l	-0.11	1.00000000	pigs
Tor3a	-0.19	1.00000000	pigs
Tpst1	-0.25	1.00000000	pigs
Adar	-0.14	1.00000000	pigs
Rab11fip4	-0.10	1.00000000	pigs
Erlec1	-0.27	1.00000000	pigs
Rab4a	-0.09	1.00000000	pigs
Hmg20b	-0.18	1.00000000	pigs
Def8	-0.10	1.00000000	pigs
Pop5	-0.07	1.00000000	pigs
Prr5	-0.10	1.00000000	pigs
Cd164	-0.12	1.00000000	pigs
Tmem243	-0.11	1.00000000	pigs
Cnst	-0.12	1.00000000	pigs
Wwp1	-0.18	1.00000000	pigs
Ptpn6	-0.11	1.00000000	pigs
Asap1	-0.38	1.00000000	pigs
St7l	-0.10	1.00000000	pigs
Cd200r1	-0.05	1.00000000	pigs
Dpm2	-0.07	1.00000000	pigs
Zfp518a	-0.01	1.00000000	pigs
Tprkb	-0.19	1.00000000	pigs
Sh3bp5	-0.23	1.00000000	pigs
Nat10	-0.02	1.00000000	pigs
Tspan12	-0.10	1.00000000	pigs
Ogfr	-0.14	1.00000000	pigs
Dyrk1b	-0.11	1.00000000	pigs
Helz	-0.14	1.00000000	pigs
Ppa2	-0.14	1.00000000	pigs
Cd2bp2	-0.14	1.00000000	pigs
Ndufc2	-0.13	1.00000000	pigs
Tut4	-0.18	1.00000000	pigs
Rogdi	-0.15	1.00000000	pigs
Nup85	-0.01	1.00000000	pigs
Klf3	-0.27	1.00000000	pigs
Pacs1	-0.12	1.00000000	pigs
Dusp4	-0.09	1.00000000	pigs
Brd9	-0.06	1.00000000	pigs
Nfkbil1	-0.07	1.00000000	pigs
Fryl	-0.15	1.00000000	pigs
Hnrnpdl	-0.10	1.00000000	pigs
Rasal3	-0.06	1.00000000	pigs
Nbr1	-0.13	1.00000000	pigs

Cd2ap	-0.09	1.00000000	pigs
Epc2	-0.21	1.00000000	pigs
Trank1	-0.20	1.00000000	pigs
H13	-0.18	1.00000000	pigs
Ambn	-0.09	1.00000000	pigs
Terb1	-0.08	1.00000000	pigs
Snrpe	-0.18	1.00000000	pigs
Atf3	-0.32	1.00000000	pigs
Serpinb8	-0.07	1.00000000	pigs
Psmg4	-0.17	1.00000000	pigs
Ints13	-0.09	1.00000000	pigs
Pecam1	-0.23	1.00000000	pigs
Casp3	-0.07	1.00000000	pigs
C1qbp	-0.11	1.00000000	pigs
Rtf2	-0.06	1.00000000	pigs
Gnb2	0.01	1.00000000	pigs
Ubxn8	-0.13	1.00000000	pigs
Gcnt2	-0.08	1.00000000	pigs
Tmem164	-0.24	1.00000000	pigs
Timm17a	-0.05	1.00000000	pigs
Lsm8	-0.13	1.00000000	pigs
Pfdn6	-0.12	1.00000000	pigs
Wdfy4	-0.13	1.00000000	pigs
Eif3g	-0.09	1.00000000	pigs
Cnot11	-0.06	1.00000000	pigs
Atpif1	-0.08	1.00000000	pigs
Scimp	0.03	1.00000000	pigs
Krcc1	-0.12	1.00000000	pigs
Hip1	-0.09	1.00000000	pigs
Fcgr1	-0.23	1.00000000	pigs
Nr3c1	-0.08	1.00000000	pigs
Aimp2	-0.09	1.00000000	pigs
Skap2	-0.12	1.00000000	pigs
Fam126a	-0.26	1.00000000	pigs
Atg3	-0.05	1.00000000	pigs
Cyth3	-0.18	1.00000000	pigs
Acsl1	-0.54	1.00000000	pigs
Rell1	-0.05	1.00000000	pigs
Ift80	-0.19	1.00000000	pigs
Kat8	-0.12	1.00000000	pigs
Ptpro	-0.16	1.00000000	pigs
Denr	-0.08	1.00000000	pigs
Pdzd8	-0.20	1.00000000	pigs
Smim26	-0.06	1.00000000	pigs
Chst12	-0.10	1.00000000	pigs
Aprt	-0.14	1.00000000	pigs

Adk	-0.14	1.00000000	pigs
Trmt5	-0.05	1.00000000	pigs
Prr5l	-0.19	1.00000000	pigs
Dennd3	-0.25	1.00000000	pigs
Zc3hav1	-0.18	1.00000000	pigs
Ece1	-0.37	1.00000000	pigs
Akap7	-0.16	1.00000000	pigs
Esyt2	-0.21	1.00000000	pigs
Nacc2	-0.17	1.00000000	pigs
Nxpe3	-0.03	1.00000000	pigs
Enkur	-0.26	1.00000000	pigs
Slc25a4	-0.12	1.00000000	pigs
Dnajc19	-0.21	1.00000000	pigs
Arhgdib	-0.06	1.00000000	pigs
Cdk2ap2	-0.09	1.00000000	pigs
Ccnd2	-0.12	1.00000000	pigs
Abhd15	-0.02	1.00000000	pigs
Cd2	-0.10	1.00000000	pigs
Tmod3	-0.12	1.00000000	pigs
Tec	0.12	1.00000000	pigs
Fam13a	-0.19	1.00000000	pigs
Timm17b	-0.25	1.00000000	pigs
Zfp277	-0.08	1.00000000	pigs
Slc12a2	-0.05	1.00000000	pigs
Tle3	-0.16	1.00000000	pigs
Phactr2	-0.10	1.00000000	pigs
Cyb5r4	-0.16	1.00000000	pigs
Cfap410	-0.13	1.00000000	pigs
BC048507	-0.75	1.00000000	pigs
Klf6	-0.13	1.00000000	pigs
Vps13b	-0.02	1.00000000	pigs
Adgb	-0.06	1.00000000	pigs
Fbxl14	-0.08	1.00000000	pigs
Arhgap32	-0.20	1.00000000	pigs
Cibar1	-0.10	1.00000000	pigs
Crbn	-0.13	1.00000000	pigs
Adrb2	-0.06	1.00000000	pigs
Sptlc2	-0.17	1.00000000	pigs
Dusp1	-0.16	1.00000000	pigs
Ntn1	-0.18	1.00000000	pigs
Polr2f	-0.20	1.00000000	pigs
Rbl2	-0.17	1.00000000	pigs
Kdsr	-0.47	1.00000000	pigs
Wdr1	-0.18	1.00000000	pigs
Trmt112	-0.22	1.00000000	pigs
Lnpep	-0.10	1.00000000	pigs

Stambp	-0.14	1.00000000	pigs
Sema4d	-0.12	1.00000000	pigs
Nepro	-0.09	1.00000000	pigs
Glg1	-0.06	1.00000000	pigs
Scaper	-0.09	1.00000000	pigs
Dhx9	-0.12	1.00000000	pigs
Sp110	-0.16	1.00000000	pigs
Etfrf1	-0.14	1.00000000	pigs
G6pdx	-0.10	1.00000000	pigs
Pwwp2b	-0.11	1.00000000	pigs
Phf11a	-0.15	1.00000000	pigs
Tmem43	-0.10	1.00000000	pigs
Klhl9	-0.04	1.00000000	pigs
Adh5	-0.16	1.00000000	pigs
Zc3h6	-0.06	1.00000000	pigs
Nans	-0.10	1.00000000	pigs
Ndufb6	-0.12	1.00000000	pigs
Fam78a	-0.08	1.00000000	pigs
Nap1l1	-0.08	1.00000000	pigs
Csrp1	-0.13	1.00000000	pigs
Tet1	-0.08	1.00000000	pigs
Napg	-0.12	1.00000000	pigs
Epsti1	-0.08	1.00000000	pigs
Imp3	-0.13	1.00000000	pigs
Tma16	-0.08	1.00000000	pigs
Ttc7b	-0.20	1.00000000	pigs
Psmc5	-0.11	1.00000000	pigs
Pwp1	-0.13	1.00000000	pigs
Adi1	-0.28	1.00000000	pigs
Eif2d	-0.05	1.00000000	pigs
Ing3	-0.12	1.00000000	pigs
Emc9	-0.10	1.00000000	pigs
Dph6	-0.08	1.00000000	pigs
Appl1	-0.08	1.00000000	pigs
Coro2a	-0.05	1.00000000	pigs
Impa1	-0.11	1.00000000	pigs
Gbe1	-0.19	1.00000000	pigs
Taf10	-0.06	1.00000000	pigs
Smarcd1	-0.01	1.00000000	pigs
Thrb	-0.23	1.00000000	pigs
Septin7	-0.03	1.00000000	pigs
Tmem50b	-0.11	1.00000000	pigs
Xpc	-0.09	1.00000000	pigs
Traf4	-0.07	1.00000000	pigs
Rmdn1	-0.07	1.00000000	pigs
Csrp2	-0.06	1.00000000	pigs

Adam17	-0.24	1.00000000	pigs
Rock1	-0.11	1.00000000	pigs
Naa60	-0.13	1.00000000	pigs
Slc25a5	-0.09	1.00000000	pigs
Ppm1j	-0.04	1.00000000	pigs
Gna11	-0.10	1.00000000	pigs
Nfs1	-0.01	1.00000000	pigs
Zc3h7b	-0.15	1.00000000	pigs
Sntb2	-0.28	1.00000000	pigs
Tmem260	-0.13	1.00000000	pigs
Ptrh2	-0.15	1.00000000	pigs
Wasf2	-0.13	1.00000000	pigs
Tgs1	-0.13	1.00000000	pigs
Nudt1	-0.14	1.00000000	pigs
Klc2	-0.13	1.00000000	pigs
Fbxo11	-0.15	1.00000000	pigs
Rasa1	-0.12	1.00000000	pigs
Ndufs4	-0.19	1.00000000	pigs
Casp7	-0.05	1.00000000	pigs
Senp7	-0.11	1.00000000	pigs
Trps1	-0.42	1.00000000	pigs
Pigf	-0.12	1.00000000	pigs
Svopl	-0.09	1.00000000	pigs
Tcf7	-0.10	1.00000000	pigs
Tmem70	-0.09	1.00000000	pigs
Exosc4	-0.10	1.00000000	pigs
Uqcr10	-0.17	1.00000000	pigs
Ppox	-0.14	1.00000000	pigs
L3mbtl2	-0.04	1.00000000	pigs
Clcn3	-0.11	1.00000000	pigs
Skp1	-0.18	1.00000000	pigs
Siva1	-0.21	1.00000000	pigs
Ptpn12	-0.14	1.00000000	pigs
Gabpa	-0.08	1.00000000	pigs
Ccdc91	-0.18	1.00000000	pigs
Ndufa2	-0.03	1.00000000	pigs
Ccdc82	-0.10	1.00000000	pigs
Vwa8	-0.09	1.00000000	pigs
Ap1g1	-0.15	1.00000000	pigs
Smpd1	-0.17	1.00000000	pigs
Arrdc1	-0.11	1.00000000	pigs
Zc3h4	-0.08	1.00000000	pigs
Daxx	-0.06	1.00000000	pigs
Lancl1	-0.15	1.00000000	pigs
Ntan1	-0.28	1.00000000	pigs
Dnajc15	-0.21	1.00000000	pigs

Pitpna	-0.17	1.00000000	pigs
Bad	-0.10	1.00000000	pigs
Ikzf1	-0.09	1.00000000	pigs
Ppp2r2a	-0.14	1.00000000	pigs
Ogfod3	-0.18	1.00000000	pigs
Susd1	-0.09	1.00000000	pigs
Srrm1	-0.09	1.00000000	pigs
Acat1	-0.22	1.00000000	pigs
Stk40	-0.18	1.00000000	pigs
Pdcl	-0.09	1.00000000	pigs
Brd3	-0.09	1.00000000	pigs
Use1	-0.10	1.00000000	pigs
Ikzf2	-0.02	1.00000000	pigs
Erlin2	-0.04	1.00000000	pigs
Cnn2	-0.14	1.00000000	pigs
Heca	-0.13	1.00000000	pigs
Pip5k1b	-0.19	1.00000000	pigs
Phlpp2	-0.10	1.00000000	pigs
Terf2ip	-0.11	1.00000000	pigs
Pcyt1a	-0.36	1.00000000	pigs
Kars	-0.14	1.00000000	pigs
St8sia4	-0.15	1.00000000	pigs
Stk38	-0.25	1.00000000	pigs
Gpatch2	-0.10	1.00000000	pigs
Kif3b	-0.14	1.00000000	pigs
Niban1	-0.06	1.00000000	pigs
Arg2	-0.02	1.00000000	pigs
Sf3a1	-0.13	1.00000000	pigs
Calm1	-0.25	1.00000000	pigs
Polr2d	-0.07	1.00000000	pigs
Picalm	-0.19	1.00000000	pigs
Rasgrp4	-0.13	1.00000000	pigs
Urb2	-0.09	1.00000000	pigs
Acss3	-0.22	1.00000000	pigs
Zfp217	-0.04	1.00000000	pigs
Klhl5	-0.10	1.00000000	pigs
Diaph1	-0.10	1.00000000	pigs
Acadl	-0.03	1.00000000	pigs
Prpf6	-0.15	1.00000000	pigs
Ostf1	-0.07	1.00000000	pigs
Casp6	-0.11	1.00000000	pigs
Svil	-0.21	1.00000000	pigs
Hipk1	-0.18	1.00000000	pigs
Tasl	-0.06	1.00000000	pigs
Zbp1	-0.10	1.00000000	pigs
Ccdc15	-0.05	1.00000000	pigs

Ccdc28a	-0.12	1.00000000	pigs
2700062C07Rik	-0.16	1.00000000	pigs
Lamtor2	-0.14	1.00000000	pigs
Uhrf1bp1l	-0.17	1.00000000	pigs
Strn4	-0.07	1.00000000	pigs
Cdc26	-0.15	1.00000000	pigs
P2ry13	-0.04	1.00000000	pigs
Cep170	-0.22	1.00000000	pigs
Sun1	-0.14	1.00000000	pigs
Hacd2	-0.10	1.00000000	pigs
Plk3	-0.13	1.00000000	pigs
Imp4	-0.05	1.00000000	pigs
Snx4	-0.17	1.00000000	pigs
Eaf1	-0.18	1.00000000	pigs
Dock9	-0.12	1.00000000	pigs
Taok1	-0.21	1.00000000	pigs
Tasor	-0.13	1.00000000	pigs
Sod1	-0.26	1.00000000	pigs
Pus10	-0.12	1.00000000	pigs
Dis3l2	-0.17	1.00000000	pigs
Osbpl9	-0.11	1.00000000	pigs
Snapc4	-0.08	1.00000000	pigs
Unc50	-0.15	1.00000000	pigs
Atf4	-0.03	1.00000000	pigs
Klf10	-0.14	1.00000000	pigs
Twf2	-0.11	1.00000000	pigs
Fam76a	-0.10	1.00000000	pigs
Fbxl17	-0.11	1.00000000	pigs
Ankrd44	-0.10	1.00000000	pigs
Ier3	-0.17	1.00000000	pigs
Cyb561d1	-0.07	1.00000000	pigs
Cited2	-0.13	1.00000000	pigs
Lars	-0.19	1.00000000	pigs
Polr1h	-0.10	1.00000000	pigs
Snrnp25	-0.22	1.00000000	pigs
Cd151	-0.06	1.00000000	pigs
Pik3cg	-0.16	1.00000000	pigs
Commd5	-0.07	1.00000000	pigs
Ogt	-0.10	1.00000000	pigs
Acss2	-0.06	1.00000000	pigs
Ndufb3	-0.06	1.00000000	pigs
BC005537	-0.17	1.00000000	pigs
Hp1bp3	-0.17	1.00000000	pigs
Arpc4	-0.01	1.00000000	pigs
Txndc17	-0.09	1.00000000	pigs
Star	-0.10	1.00000000	pigs

Nfatc3	-0.07	1.00000000	pigs
Armt1	-0.08	1.00000000	pigs
Ndufaf3	-0.11	1.00000000	pigs
Tsc22d3	-0.17	1.00000000	pigs
Cd48	0.03	1.00000000	pigs
Slc12a6	-0.09	1.00000000	pigs
Stx8	-0.12	1.00000000	pigs
Gpank1	-0.02	1.00000000	pigs
Cdk14	-0.28	1.00000000	pigs
Slc2a9	-0.08	1.00000000	pigs
Prdx2	-0.14	1.00000000	pigs
Hk1	-0.09	1.00000000	pigs
Spsb3	-0.13	1.00000000	pigs
Slc39a10	-0.10	1.00000000	pigs
Nbn	-0.20	1.00000000	pigs
Zfp36l2	-0.18	1.00000000	pigs
Pus1	-0.03	1.00000000	pigs
Ddhd2	-0.09	1.00000000	pigs
Ehbp1	-0.08	1.00000000	pigs
Dapp1	-0.11	1.00000000	pigs
Phkb	-0.09	1.00000000	pigs
Ptprcap	-0.17	1.00000000	pigs
