# Supplementary material for: Heterogeneous viral-host response at single-cell resolution and viral adaptive mutational atlas in birds and mammals upon H5 influenza virus infection
Source: Natl Sci Rev. 2026 Jun 25;13(14):nwag380. doi: 10.1093/nsr/nwag380 (PMC13458601; doi:10.1093/nsr/nwag380)
Supplement: Supplementary file 1 [file nwag380_supplemental_files.zip › Supplementary Data 12.pdf]

plementary Data 12. Differentially expressed genes of NK across different species upon H5 HPAIV infect

gene	log2FoldChange	p value	host
Ly6e	2.05	0.00000000	chicken
Ifit2	1.79	0.00000000	chicken
Oasl2	1.74	0.00000000	chicken
Plppr5	1.96	0.00000000	chicken
Tox3	1.88	0.00000000	chicken
Stat1	1.60	0.00000000	chicken
Itga1	2.18	0.00000000	chicken
Smarcal1	-1.51	0.00000000	chicken
Trank1	1.77	0.00000000	chicken
Rack1	-1.66	0.00000000	chicken
Trim25	1.24	0.00000000	chicken
Chd7	1.61	0.00000000	chicken
Pde3b	1.06	0.00000000	chicken
Chsy1	1.51	0.00000000	chicken
Dennd1b	0.99	0.00000000	chicken
Tpt1	-1.41	0.00000000	chicken
Ppm1h	1.54	0.00000000	chicken
Rfx2	1.42	0.00000000	chicken
Rnf213	1.19	0.00000000	chicken
Pcsk2	1.24	0.00000000	chicken
Susd6	1.03	0.00000000	chicken
Cd44	0.87	0.00000000	chicken
Rsad2	1.60	0.00000000	chicken
Ccl9	1.69	0.00000000	chicken
Rbm39	-1.00	0.00000000	chicken
Plac8	1.45	0.00000000	chicken
Eef2	-1.17	0.00000000	chicken
Rbms1	1.32	0.00000000	chicken
Pabpc1	-0.94	0.00000000	chicken
Eef1b2	-1.35	0.00000000	chicken
Syt14	1.96	0.00000000	chicken
Glipr2	1.36	0.00000000	chicken
Ybx1	-1.23	0.00000000	chicken
Foxn3	1.32	0.00000000	chicken
Fryl	0.96	0.00000000	chicken
Cd247	1.09	0.00000000	chicken
Cblb	0.77	0.00000000	chicken
Samsn1	1.05	0.00000000	chicken
Prdx1	-1.30	0.00000000	chicken
Eef1d	-1.23	0.00000000	chicken
Rap1gds1	1.04	0.00000000	chicken

Dock10	0.97	0.00000000	chicken
Foxk1	1.02	0.00000000	chicken
Ahr	1.56	0.00000000	chicken
Aopep	1.31	0.00000000	chicken
Ddx60	1.02	0.00000000	chicken
Sema3c	1.56	0.00000000	chicken
Ifih1	1.33	0.00000000	chicken
Fasl	0.80	0.00000000	chicken
Epn2	1.18	0.00000000	chicken
Plcl2	1.05	0.00000000	chicken
Etv6	1.43	0.00000000	chicken
Tbl1xr1	1.11	0.00000000	chicken
Ptafr	0.70	0.00000000	chicken
Hivep1	1.21	0.00000000	chicken
Gnb1	0.92	0.00000000	chicken
Rasgef1a	1.28	0.00000000	chicken
Slbp	0.59	0.00000000	chicken
Pik3r1	0.94	0.00000000	chicken
Fbxl17	1.07	0.00000000	chicken
Gfod1	1.13	0.00000000	chicken
Nfatc1	1.09	0.00000000	chicken
Osbpl3	0.90	0.00000000	chicken
B3gnt4	0.88	0.00000000	chicken
Itpkb	1.01	0.00000000	chicken
Fbxo34	0.96	0.00000000	chicken
P2rx7	0.91	0.00000000	chicken
Ptprc	0.77	0.00000000	chicken
Rbm24	0.91	0.00000000	chicken
Ptpn2	0.83	0.00000000	chicken
Dusp16	1.05	0.00000000	chicken
Tgfbr2	0.98	0.00000000	chicken
Pik3cb	1.21	0.00000000	chicken
Slco3a1	1.12	0.00000000	chicken
Nme2	-1.08	0.00000000	chicken
Celf2	0.78	0.00000000	chicken
Ankrd6	1.32	0.00000000	chicken
Ptk2	1.15	0.00000000	chicken
Cnot2	0.88	0.00000000	chicken
Lrmda	1.14	0.00000000	chicken
Il2rb	0.88	0.00000000	chicken
Ets2	0.94	0.00000000	chicken
Rftn1	1.33	0.00000000	chicken
Glcci1	1.45	0.00000000	chicken
Disc1	1.07	0.00000000	chicken
Cmip	0.70	0.00000000	chicken
Atp2a2	0.96	0.00000000	chicken

Cep85l	0.84	0.00000000	chicken
Nadk	0.91	0.00000000	chicken
Rbm47	1.25	0.00000000	chicken
Setx	0.83	0.00000000	chicken
Ikzf2	1.06	0.00000000	chicken
Zbtb16	1.25	0.00000000	chicken
Rock2	0.98	0.00000000	chicken
Htr7	0.58	0.00000000	chicken
Fli1	1.00	0.00000000	chicken
Ppp6r2	0.76	0.00000000	chicken
Cmtr1	0.93	0.00000000	chicken
Ifng	0.67	0.00000000	chicken
Prr5	1.06	0.00000000	chicken
Gls	0.92	0.00000000	chicken
Irf8	0.60	0.00000000	chicken
Zfand6	0.82	0.00000000	chicken
Niban1	0.87	0.00000000	chicken
Rc3h1	0.89	0.00000000	chicken
Cbfb	1.14	0.00000000	chicken
Carmil1	1.11	0.00000000	chicken
Arid5b	0.96	0.00000000	chicken
Helz2	0.82	0.00000000	chicken
Eya3	0.82	0.00000000	chicken
Stat4	0.75	0.00000000	chicken
Hipk3	1.00	0.00000000	chicken
Jarid2	0.44	0.00000000	chicken
Scin	-1.31	0.00000000	chicken
Smchd1	0.88	0.00000000	chicken
Enah	0.76	0.00000000	chicken
Tbc1d5	0.99	0.00000000	chicken
Rnf214	-1.27	0.00000000	chicken
Dpyd	0.91	0.00000000	chicken
Vav3	0.62	0.00000000	chicken
Smad1	1.01	0.00000000	chicken
Fau	-0.87	0.00000000	chicken
Adsl	-1.22	0.00000000	chicken
Tiparp	0.74	0.00000000	chicken
Runx3	0.65	0.00000000	chicken
H3f3a	-0.80	0.00000000	chicken
Kif9	0.36	0.00000000	chicken
Dgkh	1.16	0.00000000	chicken
Ppp1r16b	0.99	0.00000000	chicken
Jak2	0.97	0.00000000	chicken
B2m	0.72	0.00000000	chicken
Rin3	1.00	0.00000000	chicken
Hivep2	0.68	0.00000000	chicken

Evi2a	0.64	0.00000000	chicken
Slc25a3	-0.93	0.00000000	chicken
Nck2	0.95	0.00000000	chicken
Eif1	-0.79	0.00000000	chicken
Samhd1	0.80	0.00000000	chicken
Ptpn22	0.67	0.00000000	chicken
Ndrg3	0.79	0.00000000	chicken
Dot1l	0.69	0.00000000	chicken
Fnbp1	0.66	0.00000000	chicken
Gnal	1.13	0.00000000	chicken
Hvcn1	0.81	0.00000000	chicken
Traf3	0.76	0.00000000	chicken
Adam19	0.50	0.00000000	chicken
Ncoa1	0.80	0.00000000	chicken
Ranbp9	0.63	0.00000000	chicken
Atp8a1	-1.29	0.00000000	chicken
Arhgap15	0.49	0.00000000	chicken
Nr3c2	1.46	0.00000000	chicken
Atp10a	0.82	0.00000000	chicken
Cbl	0.68	0.00000000	chicken
Vim	0.92	0.00000000	chicken
Nedd9	0.75	0.00000000	chicken
Parp8	1.23	0.00000000	chicken
Rnf19b	0.48	0.00000000	chicken
Dst	1.33	0.00000000	chicken
Rybp	0.74	0.00000000	chicken
Rras2	0.75	0.00000000	chicken
Prkag2	0.71	0.00000000	chicken
Arap2	0.87	0.00000000	chicken
Tmcc1	0.80	0.00000000	chicken
Dixdc1	0.32	0.00000000	chicken
Sbno2	0.74	0.00000000	chicken
Cpt1a	0.64	0.00000000	chicken
Wdfy2	0.72	0.00000000	chicken
Rubcnl	0.74	0.00000000	chicken
Eif3e	-0.97	0.00000000	chicken
Akt1	0.74	0.00000000	chicken
Suds3	0.72	0.00000000	chicken
Lrrc8d	0.66	0.00000000	chicken
Cass4	0.60	0.00000000	chicken
Slc16a6	0.46	0.00000000	chicken
Eif4a2	-0.60	0.00000000	chicken
Tle4	0.72	0.00000000	chicken
Itch	0.62	0.00000000	chicken
Nr1d2	-1.04	0.00000000	chicken
Igf2bp3	0.78	0.00000000	chicken

Pla2g4a	0.87	0.00000000	chicken
Cd74	1.01	0.00000000	chicken
Wbp11	0.58	0.00000000	chicken
Ehd3	0.85	0.00000000	chicken
Zc3hav1l	0.64	0.00000000	chicken
Traf2	0.61	0.00000000	chicken
Nap1l1	-0.71	0.00000000	chicken
Nr4a3	0.86	0.00000000	chicken
H3f3b	-0.66	0.00000000	chicken
Fyn	0.51	0.00000000	chicken
Skap2	0.96	0.00000000	chicken
Dpys	0.47	0.00000000	chicken
Smad3	1.03	0.00000000	chicken
Phactr2	0.62	0.00000000	chicken
Sdc4	0.65	0.00000000	chicken
Parp12	0.53	0.00000000	chicken
Rffl	0.63	0.00000000	chicken
Tut4	0.64	0.00000000	chicken
Ralgapa1	0.78	0.00000000	chicken
Cish	0.55	0.00000000	chicken
Crym	-1.38	0.00000000	chicken
Stat2	0.34	0.00000000	chicken
Ikzf1	0.54	0.00000000	chicken
Pde4b	0.63	0.00000000	chicken
Polk	0.52	0.00000000	chicken
Lrba	0.62	0.00000000	chicken
Ptpn12	0.73	0.00000000	chicken
Dusp1	0.53	0.00000000	chicken
Far1	0.59	0.00000000	chicken
Runx2	0.71	0.00000000	chicken
Xylt1	0.84	0.00000000	chicken
Gna13	0.62	0.00000000	chicken
Cert1	0.90	0.00000000	chicken
Rab2a	0.57	0.00000000	chicken
Picalm	1.10	0.00000000	chicken
Pnrc1	-0.50	0.00000000	chicken
Hnrnpa1	-0.88	0.00000000	chicken
Grap2	-1.13	0.00000000	chicken
Zbtb46	0.71	0.00000000	chicken
Oit3	0.51	0.00000000	chicken
Sh3kbp1	-0.70	0.00000000	chicken
Nav3	1.04	0.00000000	chicken
Gpx1	-0.90	0.00000000	chicken
Cd3g	-0.49	0.00000000	chicken
Tmem123	0.74	0.00000000	chicken
Obp2a	0.29	0.00000000	chicken

Borcs5	0.79	0.00000000	chicken
Exoc6	0.75	0.00000000	chicken
Zfp36l1	0.60	0.00000000	chicken
Eif3m	-0.83	0.00000000	chicken
Ubac2	0.93	0.00000000	chicken
Hipk1	0.55	0.00000000	chicken
Antxr2	0.90	0.00000000	chicken
Zfp593	-0.85	0.00000000	chicken
Cmtm7	0.71	0.00000000	chicken
Npas3	2.18	0.00000000	chicken
Trio	0.95	0.00000000	chicken
Arhgap17	0.63	0.00000000	chicken
Znfx1	0.51	0.00000000	chicken
Ptgs2	0.56	0.00000000	chicken
Etnk1	0.51	0.00000000	chicken
Arhgap10	0.90	0.00000000	chicken
Eif3l	-0.90	0.00000000	chicken
Acsbg2	0.56	0.00000000	chicken
Eif3f	-0.88	0.00000000	chicken
Eif3d	-0.87	0.00000000	chicken
Wwp1	0.88	0.00000000	chicken
Foxn2	0.59	0.00000000	chicken
Gramd4	0.59	0.00000000	chicken
Lasp1	0.57	0.00000000	chicken
Ikbke	0.42	0.00000000	chicken
Tox	-0.67	0.00000000	chicken
Eif4g2	-0.74	0.00000000	chicken
Pstpip1	0.59	0.00000000	chicken
Kmt2e	0.52	0.00000000	chicken
Itsn2	0.63	0.00000000	chicken
Ldlrad4	0.60	0.00000000	chicken
Cmpk2	0.33	0.00000000	chicken
Cdin1	0.44	0.00000000	chicken
Plpp1	0.61	0.00000000	chicken
Hipk2	0.80	0.00000000	chicken
Pik3cd	0.48	0.00000000	chicken
Phactr3	0.71	0.00000000	chicken
P4ha2	0.38	0.00000000	chicken
Bcl11b	0.84	0.00000000	chicken
Pou2f1	0.76	0.00000000	chicken
Cep164	-1.17	0.00000000	chicken
Exoc2	0.68	0.00000000	chicken
Cstf3	0.60	0.00000000	chicken
Lnpep	0.62	0.00000000	chicken
Arhgap6	-1.12	0.00000000	chicken
Limd1	0.46	0.00000000	chicken

Sntb1	0.70	0.00000000	chicken
Hnrnph2	-0.51	0.00000000	chicken
Cenpc1	0.38	0.00000000	chicken
Stxbp5	0.63	0.00000000	chicken
Ecpas	0.51	0.00000000	chicken
Eif4b	-0.80	0.00000000	chicken
Slc41a1	0.55	0.00000000	chicken
Gnai1	0.61	0.00000000	chicken
Atf4	-0.63	0.00000000	chicken
Setbp1	0.65	0.00000000	chicken
St8sia1	-1.63	0.00000000	chicken
Nup210	0.71	0.00000000	chicken
Stoml1	0.34	0.00000000	chicken
Zdhhc20	0.66	0.00000000	chicken
Gnas	0.64	0.00000000	chicken
Elmo1	-0.67	0.00000000	chicken
Prkd1	0.55	0.00000000	chicken
Ephx1	0.30	0.00000000	chicken
Setd5	0.49	0.00000000	chicken
Nsa2	-0.78	0.00000000	chicken
Spsb1	0.40	0.00000000	chicken
Pgs1	0.36	0.00000000	chicken
Trafd1	0.41	0.00000000	chicken
Fth1	-0.55	0.00000000	chicken
Erbin	0.63	0.00000000	chicken
Zeb2	-0.74	0.00000000	chicken
Dpf3	0.64	0.00000000	chicken
Rheb	0.46	0.00000000	chicken
Rnf130	-0.83	0.00000000	chicken
Sgms1	0.54	0.00000000	chicken
Zbtb38	0.55	0.00000000	chicken
Il1b	0.63	0.00000000	chicken
Srp9	0.39	0.00000000	chicken
Snx10	0.62	0.00000000	chicken
Cd83	0.59	0.00000000	chicken
Asxl3	1.16	0.00000000	chicken
Ergic1	0.57	0.00000000	chicken
Alox5	0.66	0.00000000	chicken
Tnfsf13b	0.54	0.00000000	chicken
Evl	0.64	0.00000000	chicken
Agpat4	0.72	0.00000000	chicken
Fbxl4	0.57	0.00000000	chicken
Nuak1	-1.14	0.00000000	chicken
N4bp1	-0.73	0.00000000	chicken
Clec16a	0.55	0.00000000	chicken
Hexim1	-0.72	0.00000000	chicken

Ppm1d	0.35	0.00000000	chicken
Trdmt1	0.41	0.00000000	chicken
Capn2	0.38	0.00000000	chicken
Cd7	0.74	0.00000000	chicken
Hao1	0.37	0.00000000	chicken
Wwox	0.72	0.00000000	chicken
Il27ra	-0.57	0.00000000	chicken
Rassf2	0.46	0.00000000	chicken
Rab28	0.53	0.00000000	chicken
Cnn3	0.32	0.00000000	chicken
Diaph2	0.52	0.00000000	chicken
Lpp	0.46	0.00000000	chicken
Hnrnpll	0.54	0.00000000	chicken
Phf2	0.65	0.00000000	chicken
Fer	0.66	0.00000000	chicken
Lao1	-0.71	0.00000000	chicken
Slc43a2	0.44	0.00000000	chicken
Herpud1	0.54	0.00000000	chicken
Eno1b	-0.63	0.00000000	chicken
Rtn4	0.57	0.00000000	chicken
Adipor2	0.49	0.00000000	chicken
Tomm20	-0.76	0.00000000	chicken
Pds5b	-1.01	0.00000000	chicken
Pgap1	0.47	0.00000000	chicken
Gramd1b	0.43	0.00000000	chicken
Basp1	0.59	0.00000000	chicken
Rpp30	0.62	0.00000000	chicken
Fndc3a	0.46	0.00000000	chicken
Ugt8a	0.34	0.00000000	chicken
Socs1	0.42	0.00000000	chicken
Bcas3	0.60	0.00000000	chicken
Fyttd1	0.41	0.00000000	chicken
Klf6	0.53	0.00000000	chicken
Dennd4a	0.51	0.00000000	chicken
Sgk1	0.46	0.00000000	chicken
Sptbn1	-0.76	0.00000000	chicken
Rnf216	0.39	0.00000000	chicken
Fbxo11	0.52	0.00000000	chicken
Rbpj	0.68	0.00000000	chicken
Lrrfip1	0.77	0.00000000	chicken
Irf1	0.54	0.00000000	chicken
Gpr34	0.31	0.00000000	chicken
Zmynd11	0.57	0.00000000	chicken
Cask	0.68	0.00000000	chicken
Hdac7	0.55	0.00000000	chicken
Rbmxl1	-0.77	0.00000000	chicken

Fnip1	0.59	0.00000000	chicken
Atf3	0.55	0.00000000	chicken
Cast	0.53	0.00000000	chicken
Tpk1	0.44	0.00000000	chicken
Cmss1	0.64	0.00000000	chicken
Smad5	0.43	0.00000000	chicken
Umad1	0.66	0.00000000	chicken
Ptprj	0.54	0.00000000	chicken
Phf12	0.49	0.00000000	chicken
Usp25	0.57	0.00000000	chicken
Saraf	0.38	0.00000000	chicken
Septin9	0.52	0.00000000	chicken
Clcn3	0.50	0.00000000	chicken
Disp1	0.55	0.00000000	chicken
Susd3	0.66	0.00000000	chicken
Prom1	0.54	0.00000000	chicken
Galnt1	0.55	0.00000001	chicken
Cox4i1	-0.67	0.00000001	chicken
Arsg	0.46	0.00000001	chicken
Sugt1	0.29	0.00000001	chicken
Dgkz	-0.69	0.00000001	chicken
Slc20a2	-0.91	0.00000001	chicken
Babam2	0.49	0.00000001	chicken
Taf3	0.54	0.00000001	chicken
Elavl1	0.43	0.00000001	chicken
Hpcal1	0.49	0.00000001	chicken
Hnrnph3	-0.63	0.00000001	chicken
Golga4	0.60	0.00000001	chicken
Tax1bp3	0.22	0.00000001	chicken
Anapc7	0.29	0.00000001	chicken
Nfatc2	0.60	0.00000001	chicken
Ndrg1	0.45	0.00000001	chicken
Vapb	0.46	0.00000001	chicken
Ndel1	0.38	0.00000001	chicken
Csnk1a1	0.48	0.00000001	chicken
Pim1	0.45	0.00000001	chicken
Itpr2	-0.92	0.00000002	chicken
Chd2	0.54	0.00000002	chicken
Nt5c3b	0.31	0.00000002	chicken
Deptor	0.32	0.00000002	chicken
Dedd	-0.69	0.00000002	chicken
Unc13d	-0.82	0.00000002	chicken
Smg1	0.43	0.00000002	chicken
Tia1	0.43	0.00000002	chicken
Auh	0.24	0.00000002	chicken
Ppp4r3a	0.42	0.00000002	chicken

Septin14	0.25	0.00000002	chicken
Vps13a	0.37	0.00000003	chicken
Tsc22d3	0.50	0.00000003	chicken
Farp1	0.55	0.00000003	chicken
Rbm33	0.49	0.00000003	chicken
Rala	0.49	0.00000003	chicken
Gsk3b	0.42	0.00000003	chicken
Far2	0.56	0.00000004	chicken
Pip4k2a	0.44	0.00000004	chicken
Ctnna1	0.52	0.00000005	chicken
Syngr2	0.23	0.00000006	chicken
Cd8a	0.41	0.00000006	chicken
Pxn	0.45	0.00000006	chicken
Slk	-0.45	0.00000006	chicken
Sema4d	0.55	0.00000007	chicken
Nbeal2	0.37	0.00000007	chicken
Ttc7	0.65	0.00000007	chicken
Per2	0.48	0.00000008	chicken
Lsm14a	-0.69	0.00000008	chicken
Rasa3	-0.77	0.00000008	chicken
Snrk	0.50	0.00000009	chicken
Ipmk	-0.70	0.00000010	chicken
Eml4	0.45	0.00000010	chicken
Pisd	0.26	0.00000010	chicken
Tasp1	0.45	0.00000011	chicken
Clock	0.36	0.00000011	chicken
Birc2	0.49	0.00000011	chicken
Cdyl2	0.38	0.00000012	chicken
Lsm12	0.29	0.00000012	chicken
Foxo1	0.59	0.00000012	chicken
Nmi	0.41	0.00000013	chicken
Cdkal1	0.48	0.00000013	chicken
Rock1	0.39	0.00000016	chicken
Capn10	0.23	0.00000016	chicken
Lrp12	0.55	0.00000016	chicken
Tmem229b	0.24	0.00000017	chicken
Ttc7b	0.48	0.00000018	chicken
Ctsc	0.30	0.00000018	chicken
Rfc1	0.38	0.00000020	chicken
Ifnar1	0.44	0.00000021	chicken
Arhgef3	0.43	0.00000022	chicken
Trim59	0.39	0.00000022	chicken
Tagap1	0.51	0.00000025	chicken
Zcchc14	0.37	0.00000025	chicken
Utp25	0.39	0.00000027	chicken
Rere	0.49	0.00000028	chicken

Pde6d	0.39	0.00000028	chicken
Dennd1a	0.37	0.00000028	chicken
Elf1	0.31	0.00000030	chicken
Heca	0.42	0.00000032	chicken
Pias1	0.43	0.00000032	chicken
Trip12	0.48	0.00000035	chicken
Rb1	0.51	0.00000036	chicken
Itgb1	0.51	0.00000037	chicken
Ppp3r1	0.34	0.00000041	chicken
Pacsin2	0.46	0.00000041	chicken
Fosb	0.32	0.00000043	chicken
Osbpl10	0.61	0.00000045	chicken
Rreb1	0.51	0.00000046	chicken
Xpr1	0.43	0.00000050	chicken
Gigyf2	0.40	0.00000051	chicken
Rab29	0.31	0.00000055	chicken
Ppp3ca	0.54	0.00000062	chicken
Tnks	0.43	0.00000065	chicken
Iqgap1	0.37	0.00000067	chicken
Ulbp1	-0.55	0.00000075	chicken
Phf6	0.35	0.00000078	chicken
Lrch1	0.57	0.00000078	chicken
Ndfip2	0.47	0.00000080	chicken
Suco	0.35	0.00000081	chicken
Bcl2	0.63	0.00000084	chicken
Zzz3	0.48	0.00000087	chicken
Exoc4	0.47	0.00000099	chicken
Arl15	0.47	0.00000103	chicken
Socs2	-0.69	0.00000109	chicken
Pudp	0.42	0.00000121	chicken
Il6st	0.40	0.00000140	chicken
Rhoa	-0.48	0.00000149	chicken
Nrip1	-0.53	0.00000152	chicken
Kctd17	0.21	0.00000162	chicken
Ginm1	0.30	0.00000172	chicken
BC005624	-0.79	0.00000183	chicken
Cabin1	0.36	0.00000189	chicken
Ybx3	-0.52	0.00000190	chicken
Sbno1	0.40	0.00000191	chicken
Heatr5a	0.47	0.00000192	chicken
Plcg2	0.54	0.00000197	chicken
Dnajc5	0.29	0.00000197	chicken
Nlrc5	0.46	0.00000197	chicken
Zbtb21	0.23	0.00000206	chicken
Fbxw11	0.35	0.00000209	chicken
Rab1a	0.33	0.00000235	chicken

Foxp1	0.33	0.00000236	chicken
Zbtb1	0.31	0.00000260	chicken
Calm1	-0.40	0.00000302	chicken
Acbd5	0.35	0.00000308	chicken
Rictor	0.43	0.00000314	chicken
Itpka	0.53	0.00000337	chicken
Sult4a1	0.37	0.00000365	chicken
Il20ra	-1.06	0.00000394	chicken
Urm1	-0.67	0.00000394	chicken
Chmp4b	0.39	0.00000419	chicken
Bicd2	0.52	0.00000429	chicken
Tmem135	0.45	0.00000440	chicken
Tec	0.34	0.00000454	chicken
Sos2	0.36	0.00000479	chicken
Hnrnpm	-0.52	0.00000491	chicken
Impa2	0.38	0.00000503	chicken
Eif5a2	-0.64	0.00000507	chicken
2210408I21Rik	0.41	0.00000510	chicken
Klf12	0.57	0.00000560	chicken
Wnk1	0.37	0.00000579	chicken
Arrdc1	0.20	0.00000673	chicken
Cdk17	0.42	0.00000680	chicken
Erlec1	0.25	0.00000682	chicken
Zfat	0.26	0.00000699	chicken
Fars2	0.45	0.00000734	chicken
Slmap	0.58	0.00000759	chicken
Fto	0.46	0.00000821	chicken
Fut8	0.37	0.00000847	chicken
Arpc3	0.43	0.00000864	chicken
Atp1b1	0.44	0.00000870	chicken
Prss45	0.40	0.00000872	chicken
Cyrib	0.33	0.00000897	chicken
Atic	-0.59	0.00000995	chicken
Sh3rf1	0.41	0.00001015	chicken
Lnpk	0.44	0.00001116	chicken
Ssu72	0.36	0.00001118	chicken
Akr1a1	0.21	0.00001158	chicken
Actg1	-0.46	0.00001169	chicken
Lin54	0.43	0.00001188	chicken
Rfx3	0.81	0.00001203	chicken
Arxes2	-0.50	0.00001356	chicken
Phf11a	0.34	0.00001398	chicken
D430041D05Rik	0.21	0.00001511	chicken
Slc29a4	-0.71	0.00001515	chicken
Ptger4	0.40	0.00001541	chicken
Calm2	-0.56	0.00001560	chicken

Rbm43	0.22	0.00001851	chicken
Vps13d	-0.70	0.00001864	chicken
Eml6	0.45	0.00001887	chicken
Sec14l1	0.45	0.00001996	chicken
Osbpl5	0.70	0.00002142	chicken
Card11	0.35	0.00002193	chicken
Nfe2l2	0.28	0.00002378	chicken
Nr2c2	0.34	0.00002434	chicken
Rab8b	0.33	0.00002468	chicken
Znhit6	0.24	0.00002633	chicken
Slc35a5	0.23	0.00002784	chicken
Pds5a	0.43	0.00003010	chicken
Lfng	0.23	0.00003040	chicken
Gbe1	0.45	0.00003084	chicken
Tnrc6c	0.40	0.00003178	chicken
Usp24	0.45	0.00003345	chicken
Pde4d	0.54	0.00003696	chicken
Llph	0.34	0.00003788	chicken
Apoa1	0.09	0.00003867	chicken
Xylb	0.23	0.00003871	chicken
Sclt1	-0.73	0.00003965	chicken
Spen	0.37	0.00003965	chicken
Rspry1	0.33	0.00004009	chicken
Ost4	0.27	0.00004117	chicken
Kmt5b	-0.65	0.00004272	chicken
Uxs1	0.61	0.00004700	chicken
Top1	0.33	0.00004861	chicken
Tub	0.31	0.00004904	chicken
Hnrnpr	-0.42	0.00004935	chicken
Tent5a	0.36	0.00005152	chicken
Rpgr	0.31	0.00005248	chicken
Vcl	0.52	0.00005327	chicken
Cytip	0.35	0.00005455	chicken
Cnot1	-0.41	0.00005623	chicken
Fbxo33	-0.61	0.00005957	chicken
Prelid3b	0.34	0.00006114	chicken
Csnk1d	0.33	0.00006185	chicken
Laptm5	0.39	0.00006852	chicken
Ttc39c	0.31	0.00007126	chicken
Kdm4c	0.34	0.00007133	chicken
Zeb1	0.41	0.00007136	chicken
Prkd3	0.40	0.00007457	chicken
Plekha1	0.37	0.00008126	chicken
Alox5ap	0.53	0.00008404	chicken
Nup153	0.31	0.00008890	chicken
Znrf3	0.45	0.00008931	chicken

Arl4c	0.22	0.00009041	chicken
Esrrg	0.40	0.00009195	chicken
Slc6a1	0.27	0.00009252	chicken
Nktr	0.31	0.00009292	chicken
Cds2	0.27	0.00009844	chicken
Prkcb	0.33	0.00010327	chicken
Arid4b	0.37	0.00010345	chicken
Crip1	0.57	0.00010555	chicken
Surf4	0.34	0.00010605	chicken
Gimap5	0.18	0.00010612	chicken
Cnot6	0.30	0.00010634	chicken
Tcf7	-0.73	0.00010715	chicken
Ccnl1	-0.38	0.00010806	chicken
Golgb1	-0.77	0.00011355	chicken
Fancc	0.42	0.00011394	chicken
Gga3	0.25	0.00011478	chicken
Rbm31y	-0.49	0.00012148	chicken
Cnpy3	0.29	0.00012303	chicken
Nfat5	0.30	0.00012648	chicken
Psmc3	-0.60	0.00013788	chicken
Ctbp1	0.35	0.00013839	chicken
Dlg1	0.32	0.00014111	chicken
Cd28	-0.90	0.00014270	chicken
Utrn	0.28	0.00014304	chicken
Clint1	0.34	0.00014399	chicken
Zc3h15	-0.59	0.00015029	chicken
Anxa6	0.35	0.00015194	chicken
Uba2	0.28	0.00015464	chicken
Sirt5	0.30	0.00015523	chicken
Qdpr	0.43	0.00015961	chicken
Cnot6l	0.36	0.00016796	chicken
Tcf12	0.33	0.00017998	chicken
Rel	0.42	0.00018307	chicken
Top2b	-0.61	0.00019257	chicken
Crem	0.44	0.00020562	chicken
Htr4	0.65	0.00020596	chicken
Pik3r6	0.39	0.00022764	chicken
Crtam	0.36	0.00024148	chicken
Orai1	0.15	0.00024806	chicken
Psme4	-0.53	0.00025249	chicken
Zfp423	0.51	0.00025782	chicken
Wdr1	0.33	0.00028700	chicken
Immp2l	0.78	0.00028875	chicken
Jak1	0.34	0.00029112	chicken
Sfpq	-0.47	0.00029466	chicken
Hnrnpa2b1	-0.38	0.00029765	chicken

Abcc1	0.49	0.00031400	chicken
D5Ertd579e	0.29	0.00032500	chicken
Aff2	0.30	0.00032566	chicken
Dync1li1	0.23	0.00033058	chicken
Tmem245	0.39	0.00033864	chicken
Shld2	0.39	0.00034009	chicken
Rabgap1	0.36	0.00034371	chicken
Stmn1	-0.57	0.00036375	chicken
Tm6sf1	-0.51	0.00036469	chicken
Cxcr4	0.46	0.00038374	chicken
Tomm7	-0.60	0.00039352	chicken
Gns	0.44	0.00040761	chicken
Tet2	0.49	0.00041399	chicken
Ppfibp1	0.36	0.00041405	chicken
Gfi1	0.27	0.00044051	chicken
Pgls	-0.54	0.00044254	chicken
Pdlim5	0.38	0.00045229	chicken
Zswim5	0.55	0.00045438	chicken
Zfp831	0.23	0.00047539	chicken
Sox13	-0.59	0.00048416	chicken
Acap2	0.31	0.00049890	chicken
Sla	-0.41	0.00051883	chicken
Fbrsl1	-0.59	0.00052344	chicken
Smg7	0.36	0.00053293	chicken
Tacc3	0.20	0.00054929	chicken
Fes	0.30	0.00054951	chicken
Hs6st1	0.55	0.00055698	chicken
Sec11c	0.21	0.00056058	chicken
Sbf2	0.67	0.00057677	chicken
Lcp1	0.42	0.00059601	chicken
Brd2	-0.47	0.00061669	chicken
Sft2d1	0.26	0.00062198	chicken
Prdm2	0.30	0.00065620	chicken
Dnajb6	0.31	0.00068550	chicken
Ern1	0.43	0.00070676	chicken
Pan3	0.33	0.00071022	chicken
Ssbp2	0.44	0.00078125	chicken
Nsmce2	0.31	0.00079125	chicken
Asxl2	0.43	0.00080472	chicken
Atf2	0.33	0.00084346	chicken
Errfi1	0.34	0.00085006	chicken
Hnrnpa3	-0.34	0.00086387	chicken
Gnai3	0.34	0.00087743	chicken
Cpne3	0.17	0.00092862	chicken
Ets1	0.31	0.00100206	chicken
Litaf	0.32	0.00100803	chicken

Car2	0.27	0.00103703	chicken
Sp4	-0.57	0.00106173	chicken
Ddx46	-0.57	0.00108526	chicken
Naa15	-0.66	0.00110433	chicken
Pcf11	-0.47	0.00116062	chicken
Rragd	0.37	0.00117850	chicken
Alcam	0.40	0.00119424	chicken
Herc2	0.35	0.00120947	chicken
Srsf3	-0.62	0.00127234	chicken
Fndc3b	0.34	0.00133042	chicken
Phtf2	0.39	0.00133411	chicken
Ywhah	0.35	0.00137550	chicken
Plcb4	-0.53	0.00138007	chicken
Zdhhc17	0.31	0.00138531	chicken
Zfp438	0.36	0.00139228	chicken
Klhl6	-0.56	0.00140383	chicken
Hectd4	0.36	0.00142397	chicken
Bhlhe40	0.49	0.00146302	chicken
Tcp11l2	0.39	0.00152170	chicken
Supt3	0.55	0.00156388	chicken
Rpap2	0.29	0.00159562	chicken
Eif3a	-0.41	0.00162849	chicken
Ube2q2	0.53	0.00164973	chicken
Qsox1	-0.60	0.00174936	chicken
Soat1	-0.57	0.00174995	chicken
Plin2	0.33	0.00178219	chicken
Ddx5	-0.25	0.00178875	chicken
Phf20	0.33	0.00179515	chicken
Cd47	0.34	0.00179814	chicken
AU040320	0.36	0.00184773	chicken
Arpc1b	-0.51	0.00185858	chicken
Ammecr1	0.38	0.00194269	chicken
Il16	-0.58	0.00213866	chicken
Akt3	0.46	0.00215431	chicken
Cul4b	-0.61	0.00216910	chicken
Tmtc4	0.31	0.00232544	chicken
Txndc11	0.22	0.00241520	chicken
Add1	0.43	0.00243396	chicken
Lmo2	0.29	0.00246783	chicken
Faf1	0.17	0.00248753	chicken
Lamp2	0.21	0.00248791	chicken
Skap1	0.52	0.00249466	chicken
Creb3l2	0.28	0.00260047	chicken
Ankrd39	-0.49	0.00270796	chicken
Asb5	-0.65	0.00279007	chicken
Chic2	-0.58	0.00280409	chicken

Kansl3	0.19	0.00283810	chicken
Gpatch1	0.27	0.00299210	chicken
Cox6a1	-0.49	0.00304977	chicken
L3mbtl3	0.44	0.00315025	chicken
Afap1l2	0.38	0.00321265	chicken
Ankrd10	0.25	0.00322687	chicken
Rab22a	0.37	0.00331791	chicken
Igfbp3	0.35	0.00334204	chicken
Arl4a	-0.61	0.00339782	chicken
Dusp10	0.34	0.00342382	chicken
Crygn	0.13	0.00348466	chicken
Rngtt	0.38	0.00363224	chicken
Fam107b	0.51	0.00374618	chicken
Rsu1	0.34	0.00376070	chicken
Impact	0.24	0.00378986	chicken
Arl3	0.22	0.00382392	chicken
Hspa5	0.37	0.00392978	chicken
Ube2e2	0.63	0.00399080	chicken
Tcf3	0.32	0.00405726	chicken
Acvr2a	0.46	0.00418196	chicken
Cep170	0.32	0.00424897	chicken
Cfap20	0.19	0.00433283	chicken
Azin1	0.28	0.00441170	chicken
Bclaf1	-0.40	0.00449614	chicken
Hbs1l	0.32	0.00451775	chicken
Glipr1l1	0.31	0.00461855	chicken
Taok3	0.36	0.00474468	chicken
Srsf1	-0.50	0.00487202	chicken
Irf4	0.39	0.00487509	chicken
Abca1	0.51	0.00488256	chicken
Eif2s3y	-0.56	0.00489953	chicken
Hsd17b7	-0.41	0.00490554	chicken
Dap	0.32	0.00498488	chicken
B3gnt2	0.28	0.00504239	chicken
Fam53a	0.51	0.00512382	chicken
Stau2	0.24	0.00518054	chicken
Glo1	-0.51	0.00524916	chicken
Pfkp	0.42	0.00528204	chicken
Bicral	0.19	0.00539472	chicken
Kif13a	0.49	0.00573895	chicken
Klf10	0.24	0.00591127	chicken
Ppip5k2	0.30	0.00592244	chicken
Cyth4	0.26	0.00594169	chicken
Cdc42se1	0.22	0.00600557	chicken
Slc49a4	0.46	0.00627168	chicken
Dhx36	-0.54	0.00640218	chicken

Adcy7	-0.63	0.00646225	chicken
Tdpoz8	0.28	0.00698700	chicken
Pcnt	0.21	0.00703986	chicken
Ranbp3	0.28	0.00711893	chicken
Ssbp1	-0.49	0.00729985	chicken
Cul2	0.31	0.00732678	chicken
Cdc14b	0.28	0.00734339	chicken
Elp4	0.18	0.00736243	chicken
Lrrc1	0.42	0.00756903	chicken
Lamp3	0.25	0.00766663	chicken
Stard8	-0.57	0.00770997	chicken
Rabl6	0.27	0.00787410	chicken
5031439G07Rik	0.29	0.00819372	chicken
Pkp4	0.31	0.00866096	chicken
Themis	0.20	0.00876909	chicken
Cdk14	-0.79	0.00886549	chicken
Efcab1	-0.52	0.00894537	chicken
Znrf2	0.39	0.01000625	chicken
Ly96	0.25	0.01003842	chicken
Slc38a1	0.22	0.01014831	chicken
Hook3	0.32	0.01026174	chicken
Cars	0.21	0.01082138	chicken
Timm44	-0.53	0.01092931	chicken
Eif2ak3	-0.51	0.01137305	chicken
Spg21	0.17	0.01158947	chicken
Cers5	0.15	0.01175385	chicken
Wasf2	0.25	0.01181292	chicken
Chd9	0.46	0.01195218	chicken
Rassf5	0.23	0.01199507	chicken
Sp3	-0.54	0.01232225	chicken
Kidins220	0.29	0.01296903	chicken
Slc2a3	-0.45	0.01297430	chicken
Trak2	0.21	0.01325612	chicken
Ubap1	0.30	0.01360949	chicken
Cdc42bpb	0.39	0.01388835	chicken
Grb2	0.24	0.01402392	chicken
Rasgrp1	0.30	0.01436255	chicken
Calcb	0.20	0.01455359	chicken
B3gntl1	-0.56	0.01517318	chicken
Vezt	0.28	0.01522561	chicken
Csrnp1	0.22	0.01529649	chicken
Rab20	0.34	0.01538936	chicken
Dnah12	-0.50	0.01576029	chicken
Prkce	-0.37	0.01632538	chicken
Wipf1	0.33	0.01640515	chicken
Atg2b	-0.59	0.01656861	chicken

Itpk1	0.18	0.01800148	chicken
Ccdc91	0.37	0.01837698	chicken
Hsp90ab1	-0.72	0.01855682	chicken
Abi1	0.35	0.01868916	chicken
Kif13b	0.27	0.01884018	chicken
Itga6	0.28	0.01886872	chicken
Slc24a3	-0.47	0.01935976	chicken
Usp38	-0.51	0.01972165	chicken
Zfp236	0.21	0.01985975	chicken
Tnfsf8	0.62	0.02020173	chicken
Cacybp	0.30	0.02089114	chicken
Orc1	0.30	0.02137759	chicken
Osbpl1a	0.31	0.02173853	chicken
Stard3nl	0.19	0.02211502	chicken
Ndufa4	-0.51	0.02215300	chicken
Atp6ap2	0.17	0.02264368	chicken
Phpt1	0.17	0.02340414	chicken
Lcor	0.39	0.02454519	chicken
Bin1	0.28	0.02483651	chicken
Gpd2	0.24	0.02485424	chicken
Rnf10	0.17	0.02510164	chicken
Ctdp1	0.58	0.02520346	chicken
Vps50	0.28	0.02521474	chicken
Sptlc2	0.44	0.02550024	chicken
Rcsd1	0.23	0.02585492	chicken
Tab2	0.24	0.02655729	chicken
Add3	-0.43	0.02683678	chicken
Poc1b	0.28	0.02684307	chicken
Klhl24	-0.65	0.02698291	chicken
Itm2a	-0.38	0.02703128	chicken
Lclat1	0.35	0.02769415	chicken
Upf2	0.23	0.02770768	chicken
Rhoh	0.25	0.02873821	chicken
Nfx1	0.33	0.02875203	chicken
Abcd3	0.19	0.02901958	chicken
Stx17	0.25	0.02942242	chicken
Slc9a9	-0.64	0.02944467	chicken
Arhgdib	-0.49	0.03084054	chicken
Dyrk1a	0.30	0.03085257	chicken
P2ry4	-0.48	0.03181252	chicken
Tubb4b	0.18	0.03184388	chicken
Stk4	0.19	0.03221635	chicken
Aak1	0.29	0.03326254	chicken
Eed	0.15	0.03469059	chicken
Arhgap26	0.23	0.03474490	chicken
Kpna1	0.22	0.03478470	chicken

Fxr1	0.19	0.03531373	chicken
Tmem50a	0.17	0.03564404	chicken
Osbp2	0.18	0.03605315	chicken
Sh2b3	0.17	0.03623522	chicken
Noc3l	0.22	0.03929100	chicken
Ppp6r3	0.26	0.04045469	chicken
Pkn2	-0.59	0.04204255	chicken
Fam102a	-0.52	0.04208311	chicken
Tent4b	0.16	0.04215305	chicken
Sun1	0.22	0.04240110	chicken
Nr3c1	0.30	0.04281527	chicken
Ccdc171	0.23	0.04368258	chicken
Adss	0.40	0.04423198	chicken
Vps37b	0.24	0.04440494	chicken
Aspg	0.29	0.04599947	chicken
Naca	-0.49	0.04816065	chicken
Sh3bp2	0.35	0.04937510	chicken
Arhgef18	0.14	0.05144294	chicken
Ssh2	0.27	0.05241083	chicken
Hdac1	0.17	0.05254814	chicken
Bend7	0.33	0.05288392	chicken
Zc3h12a	0.20	0.05412362	chicken
Lrrfip2	0.10	0.05506719	chicken
Ptdss1	0.27	0.05610783	chicken
Hopx	0.20	0.05622926	chicken
Oxr1	0.31	0.05755270	chicken
Ensa	0.12	0.06271323	chicken
Nf1	0.30	0.06321945	chicken
Rbsn	-0.54	0.06543285	chicken
Pcm1	0.28	0.06577937	chicken
Pnn	-0.52	0.06705002	chicken
Ciao2b	0.24	0.07001636	chicken
Il13ra1	-0.44	0.07052464	chicken
Nelfcd	0.27	0.07124177	chicken
Ascc3	0.20	0.07161104	chicken
Pde7b	0.66	0.07191453	chicken
Dis3	0.20	0.07205127	chicken
Fchsd2	0.16	0.07399794	chicken
Trappc9	0.18	0.07512543	chicken
Icos	-0.74	0.07606373	chicken
Ndufaf6	0.18	0.07736054	chicken
Plekhg5	0.25	0.07771423	chicken
Gpatch2	0.23	0.07787798	chicken
Trpv2	0.07	0.08007614	chicken
Cnnm2	0.37	0.08126331	chicken
Abraxas2	0.22	0.08150801	chicken

Hypk	0.13	0.08156432	chicken
Nol7	0.25	0.08324716	chicken
Tra2b	-0.37	0.08373640	chicken
Uchl3	0.16	0.08469370	chicken
Tex10	0.18	0.08546687	chicken
Ext1	-0.69	0.08574795	chicken
Rsbn1l	0.25	0.08846317	chicken
Rfx7	0.18	0.08913172	chicken
Garnl3	0.17	0.08948585	chicken
Htra2	-0.45	0.09038570	chicken
Ell	0.19	0.09055273	chicken
Anapc10	0.26	0.09075758	chicken
Dock2	-0.30	0.09451086	chicken
Aven	0.38	0.09469897	chicken
Pdhx	0.25	0.09654732	chicken
Ube2j1	0.26	0.09995066	chicken
Rab3gap2	0.25	0.10020619	chicken
Armh1	-0.42	0.10282168	chicken
Htra1	0.27	0.10398192	chicken
Gclc	-0.56	0.10508449	chicken
Zmpste24	0.20	0.10632818	chicken
R3hdm4	0.16	0.11027164	chicken
Ippk	0.17	0.11418550	chicken
Rab4a	0.23	0.11696555	chicken
Orc5	0.10	0.11906827	chicken
Vps8	0.20	0.11960489	chicken
Klhl5	0.26	0.11984091	chicken
Arih2	0.15	0.12040812	chicken
Casp3	0.22	0.12595239	chicken
Dock4	0.53	0.12626329	chicken
Sytl1	-0.45	0.12636597	chicken
Yap1	0.37	0.12642920	chicken
Rgs1	0.42	0.13258571	chicken
Jakmip1	0.20	0.13467526	chicken
Lsp1	0.23	0.13906055	chicken
Fam168a	0.29	0.13999664	chicken
Acp1	0.17	0.14332062	chicken
Atp2a3	-0.48	0.14774456	chicken
Rcor1	0.23	0.14794685	chicken
Eif2ak4	0.31	0.15091524	chicken
Trappc12	0.31	0.15572545	chicken
Larp4	0.23	0.15580755	chicken
Cbx3	-0.51	0.16486343	chicken
Upb1	-0.45	0.16518729	chicken
Pcmt1	0.22	0.16718681	chicken
Ube2g1	0.12	0.16849163	chicken

Bcr	-0.76	0.16884587	chicken
Adgrg1	-0.51	0.17025227	chicken
Hs2st1	0.24	0.17162485	chicken
Pfdn6	0.14	0.17168570	chicken
Sfxn1	0.20	0.17184707	chicken
Sbf1	0.17	0.17298847	chicken
Tln1	-0.46	0.17475536	chicken
Acot10	0.12	0.17641588	chicken
Erp44	0.24	0.17749646	chicken
Ccnd1	-0.54	0.17903455	chicken
Fbxo25	-0.48	0.18069484	chicken
Slc29a1	0.21	0.19456229	chicken
Dvl1	0.21	0.19693849	chicken
Nucks1	-0.35	0.19747363	chicken
Zfp148	0.23	0.19818159	chicken
Washc1	0.22	0.20783701	chicken
Stx8	0.20	0.20798142	chicken
Prr5l	-0.48	0.20949133	chicken
B4galt1	0.24	0.21003093	chicken
Rasa2	-0.27	0.21071374	chicken
Osbpl2	0.25	0.21477058	chicken
Atf7ip	0.26	0.22070633	chicken
4933400A11Rik	0.25	0.22519459	chicken
Atxn2	0.27	0.22603496	chicken
Sec16a	0.14	0.23191450	chicken
Tmed2	-0.50	0.23251624	chicken
Cst7	-0.67	0.23493769	chicken
Supt6	0.15	0.23887627	chicken
Tnrc6b	0.27	0.24279201	chicken
BC055324	0.13	0.24676905	chicken
Tmem258	0.17	0.24911934	chicken
Sri	0.15	0.24931472	chicken
Irf7	0.15	0.25212261	chicken
Ndufa9	-0.50	0.26002177	chicken
Gpd1l	0.17	0.26605192	chicken
Zfp511	0.20	0.26667126	chicken
Nek6	0.31	0.26690097	chicken
Nedd4	0.23	0.27319161	chicken
Usp44	0.09	0.28138376	chicken
Ccni	-0.40	0.28257213	chicken
Crim1	0.46	0.28418197	chicken
Nampt	0.28	0.28602389	chicken
Ifnar2	0.22	0.28688682	chicken
Ndufa13	-0.54	0.28698600	chicken
Bach1	0.33	0.29754968	chicken
Gpr68	0.33	0.29790595	chicken

Grap	0.25	0.29990636	chicken
Ddx3y	-0.31	0.30288852	chicken
Batf	0.17	0.30661420	chicken
Tbck	0.24	0.31453911	chicken
Aftph	0.23	0.32233042	chicken
Prex1	0.32	0.32997224	chicken
Sacm1l	0.21	0.33866608	chicken
Rc3h2	0.14	0.33898465	chicken
Fam122a	0.21	0.34365432	chicken
Pafah1b1	0.20	0.34452715	chicken
Uhrf1bp1l	0.27	0.34767922	chicken
Jmy	-0.59	0.35316845	chicken
Kdm1b	0.22	0.35399941	chicken
Hmgb1	-0.37	0.36983764	chicken
Septin8	-0.39	0.37349651	chicken
Phf21a	0.26	0.38242205	chicken
Tmem161b	0.22	0.38708635	chicken
Oxct1	0.37	0.39120899	chicken
Camk1d	-0.63	0.39180250	chicken
Eprs	0.16	0.39186160	chicken
Gpr183	0.23	0.39480126	chicken
Srgap1	0.10	0.40625814	chicken
Ppp1cc	0.21	0.41543638	chicken
Usp6nl	0.29	0.42393810	chicken
Stxbp1	0.19	0.43100004	chicken
Svbp	-0.42	0.43685826	chicken
Hmgcr	-0.46	0.44505851	chicken
Ncoa3	0.34	0.44990662	chicken
Tut7	0.18	0.45407912	chicken
Rnf4	0.13	0.45521609	chicken
Sting1	0.13	0.46483645	chicken
Szrd1	0.09	0.47029520	chicken
Cfdp1	0.16	0.47516008	chicken
Arfgap3	0.21	0.47731770	chicken
Trerf1	0.23	0.48377193	chicken
Bcl6	-0.64	0.48767720	chicken
Pdss1	0.12	0.49616130	chicken
Rsrc1	0.23	0.50681787	chicken
Wapl	-0.34	0.50714567	chicken
Kpna4	0.24	0.51564033	chicken
Lef1	-0.62	0.52774258	chicken
Arsb	0.20	0.52840523	chicken
Dhx15	-0.42	0.52895150	chicken
Nr4a2	-0.53	0.53533867	chicken
Eif3g	-0.44	0.53550095	chicken
Cat	-0.51	0.54885331	chicken

Pten	-0.21	0.55031284	chicken
Usp22	0.24	0.55670979	chicken
Cmtm3	-0.45	0.56460791	chicken
Lamp1	0.09	0.57429874	chicken
Psma7	0.20	0.57545178	chicken
Glb1	0.17	0.57651984	chicken
Tnrc6a	0.14	0.58334196	chicken
Phc3	0.17	0.58765751	chicken
Zfc3h1	0.22	0.58871012	chicken
Cbfa2t2	0.21	0.61363959	chicken
Foxi1	-0.44	0.61702922	chicken
Snx13	0.38	0.62720207	chicken
Spata5	0.32	0.63511252	chicken
Ubl3	0.34	0.64046522	chicken
Cnot4	0.24	0.65060552	chicken
Rab11fip2	0.07	0.65510628	chicken
Aagab	0.17	0.68483722	chicken
Nckap1	0.25	0.68503939	chicken
Cpne1	0.21	0.69068367	chicken
Fyco1	0.17	0.70052143	chicken
Satb1	0.46	0.71527482	chicken
Git2	-0.55	0.71831011	chicken
Zdhhc9	0.16	0.72105000	chicken
Ppp2r5c	0.33	0.72619211	chicken
Sesn3	0.26	0.72948490	chicken
Tulp1	0.16	0.75718300	chicken
Zfp652	0.18	0.77342547	chicken
Galnt2	0.23	0.78373361	chicken
Snx14	-0.59	0.78878242	chicken
Rnf13	0.19	0.79480727	chicken
Pnpt1	-0.40	0.79639612	chicken
Cx3cr1	0.21	0.81289253	chicken
Nub1	0.21	0.85033851	chicken
Gldc	-0.37	0.86062564	chicken
Dok6	0.31	0.87420332	chicken
Rsbn1	0.09	0.87969066	chicken
Btbd11	0.03	0.92486616	chicken
Grk3	0.18	0.93651744	chicken
Cltc	0.14	0.95827389	chicken
Dalrd3	0.15	0.96342598	chicken
Tsc22d2	0.23	0.98033155	chicken
Ythdc1	-0.25	0.98494338	chicken
Rb1cc1	-0.48	0.98797961	chicken
Arid2	0.37	0.98854634	chicken
Spidr	0.35	1.00000000	chicken
Tox2	0.18	1.00000000	chicken

Rcan1	0.22	1.00000000	chicken
Txn1	0.39	1.00000000	chicken
Ankhd1	0.29	1.00000000	chicken
Radx	0.18	1.00000000	chicken
Wsb1	0.18	1.00000000	chicken
Higd1a	0.14	1.00000000	chicken
Nek7	0.26	1.00000000	chicken
Siah2	0.14	1.00000000	chicken
Alg12	0.15	1.00000000	chicken
Otulin	-0.38	1.00000000	chicken
Jade1	0.21	1.00000000	chicken
Uqcrq	0.13	1.00000000	chicken
Ranbp10	0.23	1.00000000	chicken
Inpp4b	0.22	1.00000000	chicken
Csnk1e	0.11	1.00000000	chicken
Vti1a	0.30	1.00000000	chicken
Jpt2	0.09	1.00000000	chicken
Ggnbp2	0.11	1.00000000	chicken
Trrap	0.20	1.00000000	chicken
Pnrc2	0.18	1.00000000	chicken
Prkn	0.36	1.00000000	chicken
Atg4a-ps	0.12	1.00000000	chicken
Pin4	0.15	1.00000000	chicken
Iws1	-0.46	1.00000000	chicken
Gng2	-0.58	1.00000000	chicken
Zfp352	-0.44	1.00000000	chicken
Pinx1	0.17	1.00000000	chicken
Eea1	0.40	1.00000000	chicken
Optn	0.20	1.00000000	chicken
Epas1	0.34	1.00000000	chicken
Atp13a3	0.17	1.00000000	chicken
Fbxl5	0.22	1.00000000	chicken
Rhot1	0.09	1.00000000	chicken
Hivep3	0.19	1.00000000	chicken
Psmb1	-0.45	1.00000000	chicken
Wdr11	0.24	1.00000000	chicken
Ndufb6	0.16	1.00000000	chicken
Synj1	0.22	1.00000000	chicken
Bmt2	0.19	1.00000000	chicken
Upf3b	0.11	1.00000000	chicken
Efr3a	0.27	1.00000000	chicken
Atp5a1	-0.44	1.00000000	chicken
Gcnt4	0.18	1.00000000	chicken
Luc7l3	-0.47	1.00000000	chicken
Nasp	-0.54	1.00000000	chicken
Cbx4	0.15	1.00000000	chicken

Traf5	0.16	1.00000000	chicken
Fhod1	0.14	1.00000000	chicken
Ide	0.26	1.00000000	chicken
Dennd5a	0.22	1.00000000	chicken
Ibtk	0.20	1.00000000	chicken
Ttc28	0.14	1.00000000	chicken
Hras	-0.42	1.00000000	chicken
Fbxw5	0.12	1.00000000	chicken
Wdr59	0.17	1.00000000	chicken
Lpxn	0.06	1.00000000	chicken
2900026A02Rik	0.17	1.00000000	chicken
Dph6	0.10	1.00000000	chicken
Evi5	0.31	1.00000000	chicken
Lamc1	0.27	1.00000000	chicken
Large1	0.20	1.00000000	chicken
Dcbld2	0.14	1.00000000	chicken
Bivm	-0.45	1.00000000	chicken
Ssr2	-0.46	1.00000000	chicken
Gas7	-0.10	1.00000000	chicken
Sp1	0.12	1.00000000	chicken
Gnptab	0.24	1.00000000	chicken
Api5	-0.43	1.00000000	chicken
Bud13	0.06	1.00000000	chicken
Trp53bp2	0.15	1.00000000	chicken
Dusp7	-0.42	1.00000000	chicken
Trip11	-0.55	1.00000000	chicken
Ip6k1	0.13	1.00000000	chicken
8030462N17Rik	0.26	1.00000000	chicken
Zfp618	0.28	1.00000000	chicken
Qk	0.34	1.00000000	chicken
Slc25a13	0.28	1.00000000	chicken
Tef	-0.41	1.00000000	chicken
Vgll4	0.26	1.00000000	chicken
Plk2	-0.43	1.00000000	chicken
Garre1	0.18	1.00000000	chicken
Anp32e	-0.42	1.00000000	chicken
Aimp1	0.16	1.00000000	chicken
Tpcn1	-0.43	1.00000000	chicken
Fnip2	-0.49	1.00000000	chicken
P4hb	0.15	1.00000000	chicken
Prtg	0.19	1.00000000	chicken
Ino80c	0.19	1.00000000	chicken
Zfp451	0.22	1.00000000	chicken
Atxn7l1	0.20	1.00000000	chicken
Nvl	0.10	1.00000000	chicken
Birc6	-0.25	1.00000000	chicken

Ints10	0.11	1.00000000	chicken
Slc12a7	0.17	1.00000000	chicken
Rara	0.10	1.00000000	chicken
Pcgf3	0.20	1.00000000	chicken
Tax1bp1	0.48	1.00000000	chicken
Ncoa7	0.32	1.00000000	chicken
Polr1f	-0.43	1.00000000	chicken
Sem1	0.18	1.00000000	chicken
Notch2	0.26	1.00000000	chicken
Ufm1	0.11	1.00000000	chicken
Clns1a	0.12	1.00000000	chicken
Acsl3	-0.40	1.00000000	chicken
Lcp2	0.16	1.00000000	chicken
Uqcr11	0.13	1.00000000	chicken
Prdm11	0.16	1.00000000	chicken
Oxsr1	0.21	1.00000000	chicken
Usp37	0.12	1.00000000	chicken
Ahi1	0.28	1.00000000	chicken
Zdhhc14	0.30	1.00000000	chicken
Ndufa8	0.16	1.00000000	chicken
Dock11	-0.50	1.00000000	chicken
Slc35e3	0.14	1.00000000	chicken
Ube2f	0.13	1.00000000	chicken
Pggt1b	0.08	1.00000000	chicken
Supv3l1	0.16	1.00000000	chicken
Brms1l	0.19	1.00000000	chicken
Slc25a16	-0.50	1.00000000	chicken
Smap1	0.25	1.00000000	chicken
Itga4	0.24	1.00000000	chicken
Cd2ap	-0.34	1.00000000	chicken
Prpf4b	0.15	1.00000000	chicken
Tm7sf3	-0.32	1.00000000	chicken
Arrdc3	-0.30	1.00000000	chicken
Wac	0.27	1.00000000	chicken
Suclg2	0.06	1.00000000	chicken
Dynlrb1	0.10	1.00000000	chicken
Reep3	0.16	1.00000000	chicken
Osgin2	-0.32	1.00000000	chicken
Sec61a1	0.15	1.00000000	chicken
Ankrd46	0.12	1.00000000	chicken
Svil	0.30	1.00000000	chicken
Rgs9	-0.46	1.00000000	chicken
Txndc12	-0.33	1.00000000	chicken
Slc7a5	-0.49	1.00000000	chicken
Eif1b	-0.36	1.00000000	chicken
Eif5	-0.28	1.00000000	chicken

Zcchc2	0.19	1.00000000	chicken
Snx3	0.08	1.00000000	chicken
Nisch	0.14	1.00000000	chicken
Ube2v2	0.16	1.00000000	chicken
Ddx24	-0.51	1.00000000	chicken
Tpm1	-0.40	1.00000000	chicken
Btg2	0.21	1.00000000	chicken
Vps11	-0.41	1.00000000	chicken
Pdia3	-0.37	1.00000000	chicken
Clptm1l	-0.46	1.00000000	chicken
Tanc1	0.24	1.00000000	chicken
Ctss	0.14	1.00000000	chicken
Helb	0.09	1.00000000	chicken
Hsd17b4	0.18	1.00000000	chicken
Zfp207	-0.39	1.00000000	chicken
Ilrun	0.16	1.00000000	chicken
Surf6	0.17	1.00000000	chicken
Taok1	0.17	1.00000000	chicken
Scfd2	0.26	1.00000000	chicken
Tesk2	-0.57	1.00000000	chicken
Syne1	0.21	1.00000000	chicken
Tbcd	0.21	1.00000000	chicken
Smn1	0.14	1.00000000	chicken
Dicer1	0.19	1.00000000	chicken
Dnajb9	0.12	1.00000000	chicken
Pcca	0.21	1.00000000	chicken
Ccdc25	0.14	1.00000000	chicken
Trim33	0.07	1.00000000	chicken
Agps	0.12	1.00000000	chicken
Hdx	0.22	1.00000000	chicken
Bnip3	0.24	1.00000000	chicken
Rnf220	0.17	1.00000000	chicken
Ss18l2	-0.47	1.00000000	chicken
Ankrd13a	-0.35	1.00000000	chicken
Ahctf1	0.09	1.00000000	chicken
Slc12a2	-0.47	1.00000000	chicken
Cebpg	0.08	1.00000000	chicken
Cebpd	0.04	1.00000000	chicken
Fasn	-0.43	1.00000000	chicken
Bdp1	0.04	1.00000000	chicken
Commd1b	0.22	1.00000000	chicken
Pxk	0.18	1.00000000	chicken
Otud7a	0.43	1.00000000	chicken
Pcsk7	-0.34	1.00000000	chicken
Cdkn1b	0.21	1.00000000	chicken
Sap130	0.17	1.00000000	chicken

Eif4g3	0.21	1.00000000	chicken
Ankrd11	0.18	1.00000000	chicken
Gcc2	0.04	1.00000000	chicken
Smurf2	0.10	1.00000000	chicken
D630045J12Rik	0.24	1.00000000	chicken
Rab6a	0.27	1.00000000	chicken
Sms	0.12	1.00000000	chicken
Ywhag	0.13	1.00000000	chicken
Il10ra	0.22	1.00000000	chicken
Ric1	0.20	1.00000000	chicken
Usp8	0.12	1.00000000	chicken
Wasl	0.14	1.00000000	chicken
Arhgap30	-0.39	1.00000000	chicken
Prkci	0.17	1.00000000	chicken
Pphln1	0.20	1.00000000	chicken
Rlf	-0.43	1.00000000	chicken
Rnf34	0.08	1.00000000	chicken
Atp5g3	-0.37	1.00000000	chicken
Ccser2	0.29	1.00000000	chicken
Pbx1	0.10	1.00000000	chicken
Tnfrsf9	0.14	1.00000000	chicken
Slc25a36	0.19	1.00000000	chicken
Psma4	-0.40	1.00000000	chicken
Baz1b	0.12	1.00000000	chicken
Abhd17c	0.19	1.00000000	chicken
Ptprf	0.30	1.00000000	chicken
Cox10	0.13	1.00000000	chicken
Slc41a2	0.10	1.00000000	chicken
Pdpk1	-0.37	1.00000000	chicken
Fam133b	-0.54	1.00000000	chicken
1600014C10Rik	0.11	1.00000000	chicken
Armc8	0.16	1.00000000	chicken
Lsmem1	0.12	1.00000000	chicken
Gapvd1	0.17	1.00000000	chicken
Ndufc2	-0.30	1.00000000	chicken
Nos2	0.80	1.00000000	chicken
Ubxn7	0.06	1.00000000	chicken
Abhd2	0.17	1.00000000	chicken
Uri1	0.15	1.00000000	chicken
Kcnq1	-1.13	1.00000000	chicken
Eif3b	-0.38	1.00000000	chicken
Srpk2	-0.45	1.00000000	chicken
Triap1	-0.29	1.00000000	chicken
Arfip1	0.12	1.00000000	chicken
Polr2f	0.09	1.00000000	chicken
Prdm15	0.11	1.00000000	chicken

Abi2	0.21	1.00000000	chicken
Ccr7	0.17	1.00000000	chicken
Abhd5	0.07	1.00000000	chicken
Chchd3	0.09	1.00000000	chicken
Eefsec	0.22	1.00000000	chicken
Cd3e	-0.27	1.00000000	chicken
Nnt	0.06	1.00000000	chicken
B3gnt5	0.26	1.00000000	chicken
Abcc4	0.11	1.00000000	chicken
Qser1	-0.37	1.00000000	chicken
Lct	-0.32	1.00000000	chicken
Serpini1	0.23	1.00000000	chicken
Fhit	0.47	1.00000000	chicken
Foxk2	0.19	1.00000000	chicken
Dnaja1	0.13	1.00000000	chicken
Erc1	0.40	1.00000000	chicken
Kifbp	0.13	1.00000000	chicken
Hspa9	-0.44	1.00000000	chicken
Ube2h	0.21	1.00000000	chicken
Pfdn1	0.18	1.00000000	chicken
Ppp1r8	0.09	1.00000000	chicken
Zfand5	0.16	1.00000000	chicken
Rev3l	0.20	1.00000000	chicken
Jmjd1c	-0.33	1.00000000	chicken
Hsf1	-0.52	1.00000000	chicken
Prcc	0.04	1.00000000	chicken
Nans	0.15	1.00000000	chicken
Phf14	0.07	1.00000000	chicken
Phaf1	0.11	1.00000000	chicken
Secisbp2	0.17	1.00000000	chicken
Btbd7	0.09	1.00000000	chicken
Pitpna	0.18	1.00000000	chicken
Taf5l	0.10	1.00000000	chicken
Qrich1	0.16	1.00000000	chicken
Nol9	-0.40	1.00000000	chicken
Zcrb1	0.08	1.00000000	chicken
Tor1a	-0.32	1.00000000	chicken
Cog7	0.11	1.00000000	chicken
Stk17b	0.16	1.00000000	chicken
Banp	0.15	1.00000000	chicken
Arid1b	-0.30	1.00000000	chicken
Ttc1	0.05	1.00000000	chicken
Kat2b	0.12	1.00000000	chicken
Serbp1	-0.21	1.00000000	chicken
Raf1	0.15	1.00000000	chicken
Llgl2	0.08	1.00000000	chicken

Selenot	-0.44	1.00000000	chicken
Clk4	-0.43	1.00000000	chicken
Spcs1	0.07	1.00000000	chicken
Shoc2	0.14	1.00000000	chicken
Dlgap4	0.25	1.00000000	chicken
Adam10	0.20	1.00000000	chicken
Bnc2	-0.32	1.00000000	chicken
Sccpdh	-0.37	1.00000000	chicken
Reep2	-0.41	1.00000000	chicken
Ppp3cb	0.15	1.00000000	chicken
Zcchc10	0.08	1.00000000	chicken
Tcf20	0.23	1.00000000	chicken
Ubtd2	0.26	1.00000000	chicken
Efhd1	-0.45	1.00000000	chicken
Secisbp2l	0.09	1.00000000	chicken
Ttc17	0.14	1.00000000	chicken
Arl5a	-0.35	1.00000000	chicken
Sec61g	0.17	1.00000000	chicken
Fbxo30	0.13	1.00000000	chicken
Tob2	-0.44	1.00000000	chicken
Cdc25b	-0.44	1.00000000	chicken
Cry1	-0.38	1.00000000	chicken
Adat1	-0.36	1.00000000	chicken
Ero1b	0.20	1.00000000	chicken
Itm2b	0.10	1.00000000	chicken
Atg5	0.17	1.00000000	chicken
Zap70	0.20	1.00000000	chicken
Pals1	0.18	1.00000000	chicken
Kansl1	0.09	1.00000000	chicken
Bnip3l	-0.41	1.00000000	chicken
Setd3	0.20	1.00000000	chicken
Zfp704	0.28	1.00000000	chicken
Tada1	0.10	1.00000000	chicken
Gata3	0.10	1.00000000	chicken
Pde7a	0.34	1.00000000	chicken
Dip2b	0.09	1.00000000	chicken
Desi2	0.06	1.00000000	chicken
Il21r	0.13	1.00000000	chicken
Eepd1	0.13	1.00000000	chicken
Cnot7	-0.36	1.00000000	chicken
Fez2	0.22	1.00000000	chicken
Akap1	0.08	1.00000000	chicken
Tbc1d22a	0.15	1.00000000	chicken
Elovl1	0.09	1.00000000	chicken
Ube2w	0.17	1.00000000	chicken
Hexb	-0.45	1.00000000	chicken

Aff4	0.18	1.00000000	chicken
Jund	0.09	1.00000000	chicken
Ubfd1	0.04	1.00000000	chicken
Brf1	-0.37	1.00000000	chicken
Fam13b	0.08	1.00000000	chicken
Kcnk1	0.15	1.00000000	chicken
Ppp1r12a	0.14	1.00000000	chicken
Kif2a	0.16	1.00000000	chicken
Dusp5	0.18	1.00000000	chicken
Btbd9	0.14	1.00000000	chicken
Tapt1	-0.39	1.00000000	chicken
Srpr	0.16	1.00000000	chicken
Ankfy1	0.13	1.00000000	chicken
Etf1	-0.43	1.00000000	chicken
Rnf103	0.13	1.00000000	chicken
Kras	0.16	1.00000000	chicken
Clip1	0.13	1.00000000	chicken
Sap30l	0.13	1.00000000	chicken
Drosha	-0.41	1.00000000	chicken
Ncapd2	-0.38	1.00000000	chicken
Ldb1	0.02	1.00000000	chicken
Sod2	0.06	1.00000000	chicken
Rbx1	0.09	1.00000000	chicken
Ncoa2	0.14	1.00000000	chicken
Fubp3	0.06	1.00000000	chicken
Grk4	-0.39	1.00000000	chicken
Idua	0.18	1.00000000	chicken
Nudcd1	-0.39	1.00000000	chicken
Commd10	0.17	1.00000000	chicken
Il1rap	-0.50	1.00000000	chicken
Atad1	-0.41	1.00000000	chicken
Tbc1d1	0.26	1.00000000	chicken
Oga	-0.30	1.00000000	chicken
Sirt1	-0.47	1.00000000	chicken
Rora	-0.50	1.00000000	chicken
Plk3	0.19	1.00000000	chicken
Psmb4	0.09	1.00000000	chicken
Atp2b1	0.20	1.00000000	chicken
Ubox5	0.12	1.00000000	chicken
Rab3gap1	-0.45	1.00000000	chicken
Rassf1	-0.34	1.00000000	chicken
Cog3	0.12	1.00000000	chicken
Fbxo42	0.07	1.00000000	chicken
Tnpo1	0.12	1.00000000	chicken
Ezr	-0.29	1.00000000	chicken
Frs2	0.13	1.00000000	chicken

Ercc6	-0.45	1.00000000	chicken
Syf2	0.06	1.00000000	chicken
Cdv3	-0.34	1.00000000	chicken
Supt4a	0.14	1.00000000	chicken
Dstn	-0.17	1.00000000	chicken
Fbxo32	-0.40	1.00000000	chicken
Il18rap	0.10	1.00000000	chicken
Tom1l2	0.10	1.00000000	chicken
Zfand3	0.34	1.00000000	chicken
Stard4	-0.33	1.00000000	chicken
Cox5b-ps	0.08	1.00000000	chicken
Nrde2	0.05	1.00000000	chicken
Rbm6	0.11	1.00000000	chicken
Gtf2a2	-0.32	1.00000000	chicken
Ppm1a	0.17	1.00000000	chicken
Chd1	0.06	1.00000000	chicken
Arfgef2	0.07	1.00000000	chicken
Bsg	-0.35	1.00000000	chicken
Vps54	-0.03	1.00000000	chicken
Bmp2k	0.26	1.00000000	chicken
Fam172a	0.14	1.00000000	chicken
Stk24	0.14	1.00000000	chicken
Dnajc18	0.04	1.00000000	chicken
Smarca4	-0.38	1.00000000	chicken
Adrb2	-0.30	1.00000000	chicken
Ppp2r5d	0.11	1.00000000	chicken
Emp1	0.20	1.00000000	chicken
Cox5a	-0.42	1.00000000	chicken
Coro7	-0.37	1.00000000	chicken
Zfp318	-0.43	1.00000000	chicken
Uprt	0.10	1.00000000	chicken
Abhd18	-0.33	1.00000000	chicken
Ptpre	0.22	1.00000000	chicken
Ski	0.19	1.00000000	chicken
Pex1	-0.46	1.00000000	chicken
Slc25a14	0.11	1.00000000	chicken
Gne	0.10	1.00000000	chicken
Ppid	0.04	1.00000000	chicken
Cap1	0.11	1.00000000	chicken
Nptn	0.18	1.00000000	chicken
Lonp2	0.10	1.00000000	chicken
Hdgf	-0.33	1.00000000	chicken
Timm10	0.11	1.00000000	chicken
Ndufa10	-0.41	1.00000000	chicken
Pfdn5	0.14	1.00000000	chicken
AA467197	0.16	1.00000000	chicken

Prelid1	-0.33	1.00000000	chicken
Lmbrd2	0.12	1.00000000	chicken
St8sia4	0.44	1.00000000	chicken
Brox	0.09	1.00000000	chicken
Btf3	-0.41	1.00000000	chicken
Apoo	0.06	1.00000000	chicken
Bcar3	-0.44	1.00000000	chicken
Shisa5	0.03	1.00000000	chicken
Limk1	0.05	1.00000000	chicken
Sulf2	-0.38	1.00000000	chicken
Senp6	0.15	1.00000000	chicken
Eif4ebp1	-0.32	1.00000000	chicken
Xpo6	0.19	1.00000000	chicken
Cops4	0.07	1.00000000	chicken
Dazap1	0.11	1.00000000	chicken
Lyn	-0.44	1.00000000	chicken
Ubr5	0.21	1.00000000	chicken
Scyl2	-0.42	1.00000000	chicken
Abce1	-0.34	1.00000000	chicken
Atp6v1g1	0.05	1.00000000	chicken
Gxylt1	0.03	1.00000000	chicken
C3	0.09	1.00000000	chicken
Ctdspl	0.07	1.00000000	chicken
Sinhcaf	0.13	1.00000000	chicken
Itgb2l	-0.33	1.00000000	chicken
Crebrf	0.15	1.00000000	chicken
Ebna1bp2	0.05	1.00000000	chicken
Jup	0.08	1.00000000	chicken
Sh2d1a	0.05	1.00000000	chicken
Trmt11	0.19	1.00000000	chicken
Sh3bgrl3	0.20	1.00000000	chicken
Ctnnb1	0.04	1.00000000	chicken
Slx4	0.09	1.00000000	chicken
Rtraf	0.03	1.00000000	chicken
Smyd3	0.32	1.00000000	chicken
S100a10	0.19	1.00000000	chicken
Slc2a13	0.27	1.00000000	chicken
Arnt	0.11	1.00000000	chicken
Helz	0.16	1.00000000	chicken
Syne3	0.08	1.00000000	chicken
Hdac3	0.04	1.00000000	chicken
Unk	0.07	1.00000000	chicken
Hpf1	0.07	1.00000000	chicken
Stn1	-0.36	1.00000000	chicken
Stard5	0.09	1.00000000	chicken
Hif1a	0.31	1.00000000	chicken

Lyrm4	0.15	1.00000000	chicken
Srp14	0.05	1.00000000	chicken
Gtf3c1	0.06	1.00000000	chicken
Hacd3	0.07	1.00000000	chicken
4933427D14Rik	0.05	1.00000000	chicken
Dek	-0.42	1.00000000	chicken
Abracl	0.05	1.00000000	chicken
Rnaseh2b	0.16	1.00000000	chicken
Bmf	0.19	1.00000000	chicken
Ifi35	0.13	1.00000000	chicken
Zadh2	-0.29	1.00000000	chicken
Zranb2	0.11	1.00000000	chicken
Lzic	-0.24	1.00000000	chicken
Rnf144b	-0.41	1.00000000	chicken
Ubn1	-0.25	1.00000000	chicken
Dnajc1	-0.41	1.00000000	chicken
Ryk	0.09	1.00000000	chicken
Atg7	0.07	1.00000000	chicken
Aamp	0.08	1.00000000	chicken
Sub1	0.14	1.00000000	chicken
Trib2	0.15	1.00000000	chicken
Fam120a	0.08	1.00000000	chicken
Cdk9	0.08	1.00000000	chicken
Psmb7	-0.39	1.00000000	chicken
Pkm	0.14	1.00000000	chicken
Chka	-0.41	1.00000000	chicken
Sqle	-0.36	1.00000000	chicken
Pex13	0.04	1.00000000	chicken
Gyg	0.10	1.00000000	chicken
Slamf8	0.21	1.00000000	chicken
Hsd17b12	0.09	1.00000000	chicken
Tomm22	-0.31	1.00000000	chicken
Ythdc2	0.18	1.00000000	chicken
Arl6ip4	0.05	1.00000000	chicken
Psmg1	0.04	1.00000000	chicken
Rab35	0.06	1.00000000	chicken
Hmces	0.08	1.00000000	chicken
Ptpn7	0.09	1.00000000	chicken
Ptrh2	-0.34	1.00000000	chicken
Angel2	0.05	1.00000000	chicken
Lrrc8c	0.17	1.00000000	chicken
Specc1l	0.09	1.00000000	chicken
Ddrgk1	0.07	1.00000000	chicken
Trabd	0.11	1.00000000	chicken
Hnrnpab	-0.32	1.00000000	chicken
Fh1	-0.33	1.00000000	chicken

Pik3c2a	-0.38	1.00000000	chicken
Nfkb1	0.11	1.00000000	chicken
Ripor3	0.00	1.00000000	chicken
Fkbp1a	0.27	1.00000000	chicken
Fkbp5	0.09	1.00000000	chicken
Csgalnact2	0.08	1.00000000	chicken
Arhgap12	0.18	1.00000000	chicken
Oaz1	-0.34	1.00000000	chicken
Slc23a2	-0.46	1.00000000	chicken
Nkrf	-0.28	1.00000000	chicken
Ube3c	-0.31	1.00000000	chicken
Ndufa5	-0.32	1.00000000	chicken
Tnik	0.20	1.00000000	chicken
Pitpnb	0.10	1.00000000	chicken
Ngly1	-0.28	1.00000000	chicken
Fabp6	-0.28	1.00000000	chicken
Alg6	0.12	1.00000000	chicken
Luc7l2	-0.28	1.00000000	chicken
U2af2	0.05	1.00000000	chicken
Lemd3	0.15	1.00000000	chicken
Tmem168	0.15	1.00000000	chicken
Nob1	-0.25	1.00000000	chicken
Cotl1	-0.46	1.00000000	chicken
Kdm4b	0.08	1.00000000	chicken
Atad2b	0.20	1.00000000	chicken
Bcl2a1d	0.12	1.00000000	chicken
Pdhb	-0.32	1.00000000	chicken
Trps1	0.14	1.00000000	chicken
Lmbrd1	-0.30	1.00000000	chicken
Cox7b	-0.33	1.00000000	chicken
Paxbp1	-0.38	1.00000000	chicken
Pak2	0.15	1.00000000	chicken
Gnai2	-0.34	1.00000000	chicken
Sppl3	0.13	1.00000000	chicken
Atp1b3	-0.39	1.00000000	chicken
Pik3r4	0.13	1.00000000	chicken
Wdr37	0.09	1.00000000	chicken
Ino80d	-0.38	1.00000000	chicken
Sec62	0.11	1.00000000	chicken
Prrc2c	-0.18	1.00000000	chicken
Tmem131	0.10	1.00000000	chicken
Rpap1	0.04	1.00000000	chicken
Rabggtb	0.06	1.00000000	chicken
Eif3h	-0.16	1.00000000	chicken
Zfp609	0.09	1.00000000	chicken
Uqcrc2	-0.32	1.00000000	chicken

Tuba1b	0.09	1.00000000	chicken
Cntrl	0.01	1.00000000	chicken
Tmem101	0.09	1.00000000	chicken
Oxnad1	0.09	1.00000000	chicken
Ppia	-0.18	1.00000000	chicken
Xpo5	-0.43	1.00000000	chicken
Nup155	-0.28	1.00000000	chicken
Opa1	-0.32	1.00000000	chicken
Homer1	-0.43	1.00000000	chicken
Actn1	-0.28	1.00000000	chicken
Gpsm1	0.07	1.00000000	chicken
Tra2a	0.07	1.00000000	chicken
Fkbp11	0.08	1.00000000	chicken
Ankrd28	0.22	1.00000000	chicken
Nr4a1	0.00	1.00000000	chicken
Sf3a2	0.04	1.00000000	chicken
G3bp1	-0.36	1.00000000	chicken
Slc38a6	0.21	1.00000000	chicken
Tomm40l	-0.32	1.00000000	chicken
Dnm1l	0.08	1.00000000	chicken
Zdhhc8	0.03	1.00000000	chicken
Eftud2	-0.31	1.00000000	chicken
Strap	-0.37	1.00000000	chicken
Pfdn2	0.04	1.00000000	chicken
St7	0.24	1.00000000	chicken
Ankrd52	0.06	1.00000000	chicken
Xpa	0.11	1.00000000	chicken
Dido1	0.15	1.00000000	chicken
Slc39a9	-0.42	1.00000000	chicken
Edem2	0.09	1.00000000	chicken
Stk39	0.18	1.00000000	chicken
Cct7	-0.29	1.00000000	chicken
Sec24b	0.05	1.00000000	chicken
Cox19	0.03	1.00000000	chicken
Camk2d	0.18	1.00000000	chicken
Tle1	-0.28	1.00000000	chicken
Ndufb5	-0.36	1.00000000	chicken
Psmd12	-0.32	1.00000000	chicken
Sdha	0.11	1.00000000	chicken
Epc2	-0.42	1.00000000	chicken
Inpp5k	0.05	1.00000000	chicken
Gpalpp1	0.03	1.00000000	chicken
Gk5	-0.29	1.00000000	chicken
Nfatc3	0.16	1.00000000	chicken
Gpr107	-0.01	1.00000000	chicken
Ankib1	-0.43	1.00000000	chicken

Pfdn4	-0.25	1.00000000	chicken
Togaram1	0.13	1.00000000	chicken
Tnfaip8	0.07	1.00000000	chicken
Snu13	0.08	1.00000000	chicken
Brsk2	0.14	1.00000000	chicken
Pla2g7	0.08	1.00000000	chicken
Riok3	-0.45	1.00000000	chicken
Nkap	0.02	1.00000000	chicken
Sdf4	0.07	1.00000000	chicken
Btaf1	0.06	1.00000000	chicken
Rap1gap2	-0.35	1.00000000	chicken
Zfand4	-0.34	1.00000000	chicken
Acat1	0.04	1.00000000	chicken
Pfkfb3	0.03	1.00000000	chicken
Spast	0.09	1.00000000	chicken
Ppp1r13b	0.13	1.00000000	chicken
Tmem39a	-0.41	1.00000000	chicken
Ripk1	0.11	1.00000000	chicken
Skil	0.13	1.00000000	chicken
Ccnyl1	0.11	1.00000000	chicken
Csde1	-0.32	1.00000000	chicken
Anapc16	-0.29	1.00000000	chicken
Sart3	0.00	1.00000000	chicken
H2az1	0.18	1.00000000	chicken
Eif2s1	0.04	1.00000000	chicken
Ndufa2	0.01	1.00000000	chicken
Fam214a	-0.28	1.00000000	chicken
Napa	0.03	1.00000000	chicken
Ppil2	-0.38	1.00000000	chicken
Rprd1b	-0.39	1.00000000	chicken
Prep	0.13	1.00000000	chicken
Fbxw2	-0.32	1.00000000	chicken
Cc2d1b	0.02	1.00000000	chicken
Stk40	-0.39	1.00000000	chicken
Npepps	-0.01	1.00000000	chicken
Fgfr1op2	0.08	1.00000000	chicken
Ctdspl2	0.09	1.00000000	chicken
Ago3	-0.27	1.00000000	chicken
Igf1r	-0.39	1.00000000	chicken
Pdcd6	0.00	1.00000000	chicken
Ccdc6	0.09	1.00000000	chicken
Ap2b1	0.07	1.00000000	chicken
Pwwp2a	0.06	1.00000000	chicken
Acap3	0.11	1.00000000	chicken
Rpa1	0.10	1.00000000	chicken
Commd2	0.02	1.00000000	chicken

Ctr9	0.00	1.00000000	chicken
Batf3	0.16	1.00000000	chicken
Osbpl9	0.15	1.00000000	chicken
Sfmbt2	0.14	1.00000000	chicken
Commd8	0.08	1.00000000	chicken
Gse1	0.02	1.00000000	chicken
Psme3	-0.35	1.00000000	chicken
Fry	-0.20	1.00000000	chicken
Lamtor5	0.07	1.00000000	chicken
Psip1	0.12	1.00000000	chicken
Nupl1	0.07	1.00000000	chicken
Slc16a10	0.50	1.00000000	chicken
Aig1	0.13	1.00000000	chicken
BC048507	0.05	1.00000000	chicken
Acsl4	0.19	1.00000000	chicken
Extl3	-0.45	1.00000000	chicken
Nploc4	0.07	1.00000000	chicken
Usp48	-0.30	1.00000000	chicken
Erich1	0.19	1.00000000	chicken
Ttc37	0.11	1.00000000	chicken
Crkl	0.02	1.00000000	chicken
Zc3h6	0.11	1.00000000	chicken
Gnl3	-0.34	1.00000000	chicken
Csnk2a1	0.10	1.00000000	chicken
Armc1	0.05	1.00000000	chicken
Gosr2	-0.31	1.00000000	chicken
Vapa	-0.35	1.00000000	chicken
Scml2	-0.47	1.00000000	chicken
Vps29	0.04	1.00000000	chicken
Bcap29	0.11	1.00000000	chicken
Smarce1	-0.36	1.00000000	chicken
Gspt1	-0.31	1.00000000	chicken
Smc6	0.11	1.00000000	chicken
Peli1	0.17	1.00000000	chicken
Cib1	-0.14	1.00000000	chicken
Tmco1	0.08	1.00000000	chicken
Fbxo3	0.02	1.00000000	chicken
Spag9	0.20	1.00000000	chicken
Galnt7	-0.32	1.00000000	chicken
Cdc42	0.10	1.00000000	chicken
Eloc	0.08	1.00000000	chicken
Itpr1	-0.49	1.00000000	chicken
Zfp512	0.15	1.00000000	chicken
Htt	-0.39	1.00000000	chicken
Ap1g1	0.06	1.00000000	chicken
Rap1b	0.08	1.00000000	chicken

Ncbp1	0.09	1.00000000	chicken
Nr2c1	0.10	1.00000000	chicken
Dhx38	-0.30	1.00000000	chicken
Sfswap	-0.39	1.00000000	chicken
Aacs	-0.26	1.00000000	chicken
Xpo1	-0.30	1.00000000	chicken
Gpatch2l	0.15	1.00000000	chicken
Churc1	0.12	1.00000000	chicken
Ppib	-0.24	1.00000000	chicken
Kif5b	-0.32	1.00000000	chicken
Tfdp2	-0.27	1.00000000	chicken
Dock9	0.29	1.00000000	chicken
Cd226	0.11	1.00000000	chicken
Ppfia1	0.10	1.00000000	chicken
Tubgcp3	0.08	1.00000000	chicken
Cpeb2	-0.36	1.00000000	chicken
Ogfod3	0.01	1.00000000	chicken
Tmem167	0.14	1.00000000	chicken
Bub3	0.07	1.00000000	chicken
Phf5a	0.03	1.00000000	chicken
Ptprb	-0.04	1.00000000	chicken
Gemin4	0.08	1.00000000	chicken
Eipr1	-0.29	1.00000000	chicken
Rer1	-0.03	1.00000000	chicken
Fam8a1	0.06	1.00000000	chicken
Fas	0.27	1.00000000	chicken
Sync	0.10	1.00000000	chicken
Eef2k	0.06	1.00000000	chicken
Abtb1	-0.29	1.00000000	chicken
Rnf141	0.06	1.00000000	chicken
Snhg4	0.03	1.00000000	chicken
Vbp1	0.05	1.00000000	chicken
Wbp2nl	0.08	1.00000000	chicken
Chst10	0.07	1.00000000	chicken
Nlk	-0.02	1.00000000	chicken
Tbrg4	-0.31	1.00000000	chicken
Gadd45b	0.11	1.00000000	chicken
Tspan5	-0.16	1.00000000	chicken
Nup85	0.01	1.00000000	chicken
Eif2s2	0.06	1.00000000	chicken
Poll	0.09	1.00000000	chicken
Sptlc1	0.11	1.00000000	chicken
Cd164	0.11	1.00000000	chicken
Gosr1	0.13	1.00000000	chicken
Vps41	0.05	1.00000000	chicken
Rrp15	0.01	1.00000000	chicken

Rad23a	-0.32	1.00000000	chicken
Psmb6	0.01	1.00000000	chicken
Gpcpd1	0.04	1.00000000	chicken
Slc9a8	-0.02	1.00000000	chicken
Smap2	0.05	1.00000000	chicken
Parp1	-0.32	1.00000000	chicken
Prdx6	0.02	1.00000000	chicken
Usp10	0.05	1.00000000	chicken
Esd	-0.21	1.00000000	chicken
Sptan1	-0.37	1.00000000	chicken
Kansl1l	0.09	1.00000000	chicken
Krit1	0.01	1.00000000	chicken
Yeats2	-0.24	1.00000000	chicken
Hcfc2	0.04	1.00000000	chicken
Tspan4	0.17	1.00000000	chicken
Psma1	-0.01	1.00000000	chicken
Scap	0.02	1.00000000	chicken
Irs4	-0.29	1.00000000	chicken
Uvrag	-0.02	1.00000000	chicken
Cyth1	-0.28	1.00000000	chicken
Rac2	0.12	1.00000000	chicken
Tnip1	0.07	1.00000000	chicken
Dnpep	0.06	1.00000000	chicken
Rhof	0.04	1.00000000	chicken
Disp3	-0.38	1.00000000	chicken
Rangap1	0.05	1.00000000	chicken
Fos	0.22	1.00000000	chicken
Dip2c	-0.16	1.00000000	chicken
Prkar1a	0.10	1.00000000	chicken
Slc3a1	0.09	1.00000000	chicken
Ptpra	-0.31	1.00000000	chicken
Was	0.01	1.00000000	chicken
Txlna	0.05	1.00000000	chicken
Slc25a20	0.07	1.00000000	chicken
Sdhaf2	0.00	1.00000000	chicken
Rsl1d1	0.05	1.00000000	chicken
Ptp4a2	-0.36	1.00000000	chicken
Wdr12	0.14	1.00000000	chicken
Prkdc	-0.06	1.00000000	chicken
Tgs1	-0.33	1.00000000	chicken
Ndufb3	0.05	1.00000000	chicken
Fmnl1	-0.19	1.00000000	chicken
Arhgap24	-0.12	1.00000000	chicken
Edem3	0.09	1.00000000	chicken
Fbxo21	0.06	1.00000000	chicken
Amfr	0.11	1.00000000	chicken

Wdr4	-0.32	1.00000000	chicken
Ppard	0.06	1.00000000	chicken
Strbp	0.08	1.00000000	chicken
Tollip	0.05	1.00000000	chicken
Gsap	0.05	1.00000000	chicken
Prkar2a	-0.56	1.00000000	chicken
Setd2	-0.03	1.00000000	chicken
Sco1	0.03	1.00000000	chicken
Ndufs4	0.09	1.00000000	chicken
Bfar	-0.02	1.00000000	chicken
Cacul1	0.07	1.00000000	chicken
Epc1	-0.13	1.00000000	chicken
Ube2q1	-0.01	1.00000000	chicken
Cd81	-0.29	1.00000000	chicken
Ddx42	0.11	1.00000000	chicken
Atp1a1	-0.28	1.00000000	chicken
Napg	-0.05	1.00000000	chicken
Snrpd2	0.03	1.00000000	chicken
Uhrf2	0.10	1.00000000	chicken
Atp2c1	0.15	1.00000000	chicken
Gpatch8	0.02	1.00000000	chicken
Zc3h11a	0.11	1.00000000	chicken
Dgkd	0.07	1.00000000	chicken
Srsf10	0.08	1.00000000	chicken
Tnfrsf21	-0.24	1.00000000	chicken
Prkcd	-0.33	1.00000000	chicken
Exoc8	0.07	1.00000000	chicken
Nrp1	-0.25	1.00000000	chicken
Ift22	0.01	1.00000000	chicken
Hmbs	-0.27	1.00000000	chicken
Wbp1	-0.34	1.00000000	chicken
Atg16l1	0.06	1.00000000	chicken
Pxdc1	0.07	1.00000000	chicken
Taf15	0.04	1.00000000	chicken
Nipa2	0.05	1.00000000	chicken
Tmem183a	0.02	1.00000000	chicken
Runx1	0.10	1.00000000	chicken
Kdm5b	-0.04	1.00000000	chicken
Ctsz	0.06	1.00000000	chicken
Tmem186	-0.26	1.00000000	chicken
Psma2	0.08	1.00000000	chicken
Ankrd44	0.15	1.00000000	chicken
Dcaf5	0.05	1.00000000	chicken
Edc3	0.05	1.00000000	chicken
Gtf2e1	0.04	1.00000000	chicken
Vps35l	-0.33	1.00000000	chicken

Tfdp1	0.10	1.00000000	chicken
Nek9	0.13	1.00000000	chicken
Ptpn1	-0.35	1.00000000	chicken
Lmna	-0.42	1.00000000	chicken
Lgmn	0.15	1.00000000	chicken
Ttc27	-0.06	1.00000000	chicken
Nars	0.06	1.00000000	chicken
Xbp1	0.07	1.00000000	chicken
Ghdc	0.07	1.00000000	chicken
Zcchc8	-0.26	1.00000000	chicken
Arpc2	0.14	1.00000000	chicken
Klhl7	-0.21	1.00000000	chicken
Ndufb1	-0.26	1.00000000	chicken
Anxa11	-0.28	1.00000000	chicken
Pcnx	0.01	1.00000000	chicken
Ropn1l	-0.25	1.00000000	chicken
Cep112	0.21	1.00000000	chicken
Rbm12	0.00	1.00000000	chicken
Dapk1	0.19	1.00000000	chicken
Gpr18	-0.46	1.00000000	chicken
Cebpz	-0.32	1.00000000	chicken
Elf2	-0.40	1.00000000	chicken
Dcun1d1	-0.35	1.00000000	chicken
Ube2cbp	0.07	1.00000000	chicken
Dok1	0.00	1.00000000	chicken
Cyba	-0.31	1.00000000	chicken
Rad18	-0.42	1.00000000	chicken
Tshz2	0.19	1.00000000	chicken
Itm2c	0.12	1.00000000	chicken
Polr3a	0.09	1.00000000	chicken
Anapc15-ps	0.05	1.00000000	chicken
Ap3m2	-0.32	1.00000000	chicken
Dop1a	0.08	1.00000000	chicken
Exoc1	0.03	1.00000000	chicken
Fam177a	0.10	1.00000000	chicken
Slco4a1	0.07	1.00000000	chicken
Pno1	0.03	1.00000000	chicken
Rnmt	-0.25	1.00000000	chicken
Ppp1r12b	0.04	1.00000000	chicken
Rab18	-0.28	1.00000000	chicken
Cops3	0.05	1.00000000	chicken
Copz1	0.00	1.00000000	chicken
Polg2	-0.31	1.00000000	chicken
Katnbl1	0.08	1.00000000	chicken
Ptbp2	0.14	1.00000000	chicken
Arl8a	-0.03	1.00000000	chicken

Ncor2	-0.29	1.00000000	chicken
Atp6v0c	0.06	1.00000000	chicken
Pi4ka	-0.29	1.00000000	chicken
Atp11b	0.08	1.00000000	chicken
Vma21	-0.35	1.00000000	chicken
Agl	0.02	1.00000000	chicken
Ssr1	0.09	1.00000000	chicken
Snx24	-0.22	1.00000000	chicken
Lrch3	0.07	1.00000000	chicken
Snip1	-0.02	1.00000000	chicken
2210016F16Rik	0.04	1.00000000	chicken
Pik3c3	0.05	1.00000000	chicken
Vps18	0.05	1.00000000	chicken
Tlk1	0.09	1.00000000	chicken
Trappc8	0.12	1.00000000	chicken
Cdk6	0.06	1.00000000	chicken
Gtf2h5	-0.28	1.00000000	chicken
Glg1	0.06	1.00000000	chicken
Vmp1	0.08	1.00000000	chicken
Ncln	-0.03	1.00000000	chicken
Tcerg1	-0.27	1.00000000	chicken
Zc3h14	0.02	1.00000000	chicken
Psmd4	-0.02	1.00000000	chicken
Vcpip1	0.06	1.00000000	chicken
Rbm17	-0.04	1.00000000	chicken
Tars	0.07	1.00000000	chicken
Nsd3	0.07	1.00000000	chicken
Reep5	0.08	1.00000000	chicken
Vps13c	0.03	1.00000000	chicken
Cpeb4	0.11	1.00000000	chicken
Ccnd3	0.15	1.00000000	chicken
Rundc1	0.07	1.00000000	chicken
Ccnk	-0.03	1.00000000	chicken
Tor4a	0.05	1.00000000	chicken
Cwf19l2	0.07	1.00000000	chicken
Klhl42	-0.23	1.00000000	chicken
Serinc1	-0.26	1.00000000	chicken
Chchd10	-0.26	1.00000000	chicken
Qpctl	-0.21	1.00000000	chicken
Eny2	-0.19	1.00000000	chicken
Cpsf4l	0.05	1.00000000	chicken
Yipf5	-0.26	1.00000000	chicken
Arhgef12	-0.08	1.00000000	chicken
Stk26	-0.30	1.00000000	chicken
Ccdc59	-0.25	1.00000000	chicken
Rgs3	-0.41	1.00000000	chicken

Ccdc93	-0.30	1.00000000	chicken
Tram1l1	0.02	1.00000000	chicken
Rpap3	-0.01	1.00000000	chicken
Fadd	-0.03	1.00000000	chicken
Thyn1	-0.01	1.00000000	chicken
Pank3	-0.35	1.00000000	chicken
Blcap	0.05	1.00000000	chicken
Gfpt1	0.05	1.00000000	chicken
Ranbp1	0.05	1.00000000	chicken
Gpr155	-0.32	1.00000000	chicken
Ociad1	-0.23	1.00000000	chicken
Wdr77	-0.23	1.00000000	chicken
Ddb2	0.00	1.00000000	chicken
Apaf1	0.06	1.00000000	chicken
Slf2	0.04	1.00000000	chicken
Tcof1	-0.35	1.00000000	chicken
Bend3	-0.34	1.00000000	chicken
Tdrd3	0.12	1.00000000	chicken
St3gal2	-0.19	1.00000000	chicken
Rptor	0.00	1.00000000	chicken
Ppp2r5e	0.16	1.00000000	chicken
Hbp1	-0.05	1.00000000	chicken
Arpp19	-0.27	1.00000000	chicken
Ccndbp1	-0.24	1.00000000	chicken
Tasor	0.13	1.00000000	chicken
Avl9	-0.01	1.00000000	chicken
Bcor	0.06	1.00000000	chicken
Rtf2	0.06	1.00000000	chicken
Ncoa6	0.06	1.00000000	chicken
Pbrm1	0.03	1.00000000	chicken
Sytl3	0.00	1.00000000	chicken
Nudt14	0.09	1.00000000	chicken
Phax	-0.29	1.00000000	chicken
Ubxn2a	0.04	1.00000000	chicken
Hdgfl2	0.00	1.00000000	chicken
Ss18	0.01	1.00000000	chicken
Adar	0.02	1.00000000	chicken
Tes	-0.25	1.00000000	chicken
Sdhc	0.05	1.00000000	chicken
Lgals8	0.01	1.00000000	chicken
Cdc14a	0.16	1.00000000	chicken
Smc3	-0.27	1.00000000	chicken
Ndufaf8	-0.20	1.00000000	chicken
Flot1	-0.19	1.00000000	chicken
Luc7l	-0.31	1.00000000	chicken
Fkbp4	-0.39	1.00000000	chicken

Nckipsd	-0.02	1.00000000	chicken
Psmd1	0.07	1.00000000	chicken
Ttc33	0.06	1.00000000	chicken
Noc2l	-0.33	1.00000000	chicken
Slirp	0.00	1.00000000	chicken
Golim4	0.05	1.00000000	chicken
Hsp90b1	-0.25	1.00000000	chicken
Ahcyl1	0.00	1.00000000	chicken
Ralgapb	0.06	1.00000000	chicken
Rxra	0.11	1.00000000	chicken
Sec63	0.08	1.00000000	chicken
Strada	0.04	1.00000000	chicken
Adora2a	-0.29	1.00000000	chicken
Dcun1d4	0.10	1.00000000	chicken
Ubr2	-0.31	1.00000000	chicken
Elovl5	0.06	1.00000000	chicken
Rnf144a	-0.29	1.00000000	chicken
Ccz1	0.01	1.00000000	chicken
Slc1a4	-0.32	1.00000000	chicken
Cramp1	-0.30	1.00000000	chicken
Hlcs	0.13	1.00000000	chicken
Zdhhc21	0.06	1.00000000	chicken
Lamtor3	-0.02	1.00000000	chicken
Hcls1	0.02	1.00000000	chicken
Hadhb	-0.25	1.00000000	chicken
Bms1	-0.38	1.00000000	chicken
Sacs	-0.08	1.00000000	chicken
Sqstm1	0.07	1.00000000	chicken
9130409I23Rik	0.04	1.00000000	chicken
Rrp1b	0.03	1.00000000	chicken
Srf	-0.02	1.00000000	chicken
Ubash3a	-0.05	1.00000000	chicken
Dtx3l	0.06	1.00000000	chicken
Pwwp2b	0.05	1.00000000	chicken
Scaf11	0.04	1.00000000	chicken
Arid4a	0.18	1.00000000	chicken
Lap3	-0.31	1.00000000	chicken
Sec24a	0.04	1.00000000	chicken
Zfp800	0.07	1.00000000	chicken
Ddx19a	-0.29	1.00000000	chicken
Naa35	0.09	1.00000000	chicken
Ubtf	-0.29	1.00000000	chicken
Akap7	-0.32	1.00000000	chicken
Ube2j2	0.00	1.00000000	chicken
Cwc22	-0.24	1.00000000	chicken
Senp7	-0.31	1.00000000	chicken

Tsn	0.05	1.00000000	chicken
Tbc1d14	0.14	1.00000000	chicken
Dnmt3a	-0.23	1.00000000	chicken
Pomp	-0.01	1.00000000	chicken
Tmem223	0.00	1.00000000	chicken
Dbr1	0.01	1.00000000	chicken
Tgfb1	-0.26	1.00000000	chicken
Nrbp1	-0.05	1.00000000	chicken
Cdc42se2	0.02	1.00000000	chicken
Pmf1	-0.20	1.00000000	chicken
Ntng1	-0.32	1.00000000	chicken
Rgs18	0.01	1.00000000	chicken
Slc7a6os	0.08	1.00000000	chicken
Trmt1l	-0.25	1.00000000	chicken
Zc3h12d	0.07	1.00000000	chicken
Ppp2ca	0.10	1.00000000	chicken
H2az2	-0.32	1.00000000	chicken
Pdzd8	0.02	1.00000000	chicken
Psen1	-0.32	1.00000000	chicken
Yipf4	-0.05	1.00000000	chicken
Snap47	0.00	1.00000000	chicken
Srebf2	-0.31	1.00000000	chicken
Derl1	-0.30	1.00000000	chicken
Ralb	0.10	1.00000000	chicken
Ypel1	-0.27	1.00000000	chicken
Cirbp	-0.21	1.00000000	chicken
Pecam1	-0.32	1.00000000	chicken
Hprt	-0.01	1.00000000	chicken
Rbm19	0.03	1.00000000	chicken
Larp4b	-0.27	1.00000000	chicken
Tpd52l2	-0.01	1.00000000	chicken
Fdft1	-0.30	1.00000000	chicken
Ubl5b	-0.01	1.00000000	chicken
Usp34	0.09	1.00000000	chicken
Rab10	-0.22	1.00000000	chicken
Dapk2	0.02	1.00000000	chicken
Gtf3c6	-0.24	1.00000000	chicken
Ccdc88c	-0.08	1.00000000	chicken
Asns	-0.06	1.00000000	chicken
Vdac1	-0.01	1.00000000	chicken
S100a11	-0.08	1.00000000	chicken
Ndufa1	-0.05	1.00000000	chicken
Snx6	0.01	1.00000000	chicken
Dnajc19	0.02	1.00000000	chicken
Shprh	0.07	1.00000000	chicken
Brd7	-0.02	1.00000000	chicken

Atp5j2	0.08	1.00000000	chicken
Ppp1r21	0.00	1.00000000	chicken
Cst3	-0.26	1.00000000	chicken
Chm	-0.22	1.00000000	chicken
Atxn3	-0.30	1.00000000	chicken
Grcc10	-0.30	1.00000000	chicken
Tspan2	-0.29	1.00000000	chicken
App	-0.03	1.00000000	chicken
Ilf3	-0.37	1.00000000	chicken
Pdia6	0.05	1.00000000	chicken
Slc25a24	-0.25	1.00000000	chicken
Pdss2	-0.53	1.00000000	chicken
Ep300	0.05	1.00000000	chicken
Rbm26	0.13	1.00000000	chicken
Edem1	-0.22	1.00000000	chicken
4921524J17Rik	-0.34	1.00000000	chicken
Crb1	-0.24	1.00000000	chicken
Pxylp1	-0.31	1.00000000	chicken
Wdr61	-0.01	1.00000000	chicken
Cct5	0.06	1.00000000	chicken
Exoc3	-0.02	1.00000000	chicken
Ubr1	0.05	1.00000000	chicken
Rnf11	0.09	1.00000000	chicken
Tns3	0.12	1.00000000	chicken
Pcnp	-0.08	1.00000000	chicken
Sh3bp5	-0.03	1.00000000	chicken
Appl1	-0.06	1.00000000	chicken
Ipo9	-0.28	1.00000000	chicken
Slc30a4	-0.22	1.00000000	chicken
Akirin2	-0.19	1.00000000	chicken
Cul1	0.04	1.00000000	chicken
Ccny	-0.01	1.00000000	chicken
Ambra1	0.11	1.00000000	chicken
Ano5	0.08	1.00000000	chicken
Dock8	-0.23	1.00000000	chicken
Srsf6	-0.22	1.00000000	chicken
Pim3	0.03	1.00000000	chicken
Syncrip	-0.27	1.00000000	chicken
Denr	0.05	1.00000000	chicken
Btk	-0.29	1.00000000	chicken
Dusp23	-0.16	1.00000000	chicken
Zzef1	0.04	1.00000000	chicken
Dnajb12	-0.04	1.00000000	chicken
Lman2	-0.01	1.00000000	chicken
Slc6a6	-0.09	1.00000000	chicken
Rnf217	0.06	1.00000000	chicken

Tmx4	0.08	1.00000000	chicken
Snx9	0.02	1.00000000	chicken
Tmem120b	-0.04	1.00000000	chicken
Alkbh1	-0.27	1.00000000	chicken
Rsf1	0.07	1.00000000	chicken
Hp1bp3	-0.06	1.00000000	chicken
Dhdds	-0.04	1.00000000	chicken
Strn	-0.26	1.00000000	chicken
Rad23b	-0.30	1.00000000	chicken
Tctn3	-0.38	1.00000000	chicken
Sf3b3	-0.23	1.00000000	chicken
Dph3	-0.28	1.00000000	chicken
Armh3	-0.03	1.00000000	chicken
Cox7a2	-0.30	1.00000000	chicken
Tmem263	0.00	1.00000000	chicken
Abcb7	0.00	1.00000000	chicken
Snrpc	-0.23	1.00000000	chicken
Ypel2	-0.25	1.00000000	chicken
Plscr1	0.04	1.00000000	chicken
Nsf	-0.28	1.00000000	chicken
Smad2	0.00	1.00000000	chicken
Fam160b1	0.00	1.00000000	chicken
4932411N23Rik	0.01	1.00000000	chicken
Il18r1	-0.31	1.00000000	chicken
Sel1l	-0.31	1.00000000	chicken
Elp6	-0.20	1.00000000	chicken
Tbc1d12	0.08	1.00000000	chicken
Ccno	0.03	1.00000000	chicken
Pum3	-0.26	1.00000000	chicken
Sgk3	0.10	1.00000000	chicken
Ldah	-0.31	1.00000000	chicken
Ptpn14	-0.30	1.00000000	chicken
Cyria	0.08	1.00000000	chicken
Il12rb2	-0.03	1.00000000	chicken
Gbf1	0.16	1.00000000	chicken
Tmem19	-0.22	1.00000000	chicken
Vps37a	-0.08	1.00000000	chicken
Nfyc	-0.31	1.00000000	chicken
Rexo1	0.00	1.00000000	chicken
Uba3	0.02	1.00000000	chicken
Dennd4c	0.06	1.00000000	chicken
Gpbp1l1	0.00	1.00000000	chicken
Strn3	0.00	1.00000000	chicken
Rapgef2	0.07	1.00000000	chicken
Elk3	-0.10	1.00000000	chicken
Bin2	-0.01	1.00000000	chicken

Ciao1	0.00	1.00000000	chicken
Setdb2	-0.34	1.00000000	chicken
Fbxl3	-0.25	1.00000000	chicken
Lipa	-0.07	1.00000000	chicken
Chmp2b	-0.21	1.00000000	chicken
Kdm1a	0.02	1.00000000	chicken
Grk2	-0.05	1.00000000	chicken
Efl1	-0.01	1.00000000	chicken
LTO1	-0.05	1.00000000	chicken
Ireb2	-0.31	1.00000000	chicken
Ndufb8	-0.18	1.00000000	chicken
Ing3	-0.29	1.00000000	chicken
Atxn1	-0.27	1.00000000	chicken
Arf6	-0.22	1.00000000	chicken
Orc3	-0.01	1.00000000	chicken
Stk3	-0.15	1.00000000	chicken
Unc45a	-0.05	1.00000000	chicken
Lrrc34	-0.01	1.00000000	chicken
Ostc	-0.28	1.00000000	chicken
Slc9a6	0.03	1.00000000	chicken
Cyth3	0.07	1.00000000	chicken
Cog5	0.05	1.00000000	chicken
Wdr20	0.07	1.00000000	chicken
Ndufb7	0.04	1.00000000	chicken
Sbds	-0.04	1.00000000	chicken
Nmt2	-0.03	1.00000000	chicken
Ppa2	0.15	1.00000000	chicken
Rcc2	-0.19	1.00000000	chicken
Zfp827	-0.12	1.00000000	chicken
Kdm7a	0.15	1.00000000	chicken
Tnfsf11	-0.06	1.00000000	chicken
Eif1ad12	0.02	1.00000000	chicken
Bmpr2	-0.18	1.00000000	chicken
Gpatch4	-0.26	1.00000000	chicken
Ext2	0.00	1.00000000	chicken
Pcid2	-0.21	1.00000000	chicken
Bcat1	-0.43	1.00000000	chicken
Kdm5a	-0.25	1.00000000	chicken
Trmt13	-0.22	1.00000000	chicken
Naxd	0.02	1.00000000	chicken
Pdxdc1	-0.05	1.00000000	chicken
Tmx3	-0.07	1.00000000	chicken
Srsf11	-0.04	1.00000000	chicken
Utp20	-0.01	1.00000000	chicken
Gatad2a	-0.03	1.00000000	chicken
Ddhd1	0.00	1.00000000	chicken

Gtf2a1	-0.07	1.00000000	chicken
Ankle2	0.00	1.00000000	chicken
Tpd52	0.04	1.00000000	chicken
Csnk1g1	0.00	1.00000000	chicken
Naa40	-0.24	1.00000000	chicken
Sec13	-0.26	1.00000000	chicken
Tubg1	0.01	1.00000000	chicken
Slc36a4	-0.04	1.00000000	chicken
Slc22a3	0.11	1.00000000	chicken
Cdc123	0.06	1.00000000	chicken
Agfg1	0.06	1.00000000	chicken
Snrpf	-0.22	1.00000000	chicken
Prdx4	0.01	1.00000000	chicken
Ehbp1	0.02	1.00000000	chicken
Klhl2	0.05	1.00000000	chicken
Nipsnap2	0.06	1.00000000	chicken
Prmt9	-0.02	1.00000000	chicken
Nop58	-0.29	1.00000000	chicken
Pnpla8	-0.32	1.00000000	chicken
Tmsb4x	0.15	1.00000000	chicken
Tmem185a	-0.29	1.00000000	chicken
Chd5	-0.21	1.00000000	chicken
Cemip2	0.04	1.00000000	chicken
Smu1	-0.28	1.00000000	chicken
Appbp2	-0.33	1.00000000	chicken
Nfkbiz	-0.42	1.00000000	chicken
Dgkq	-0.27	1.00000000	chicken
Bri3	0.05	1.00000000	chicken
Isl2	0.00	1.00000000	chicken
Gars	-0.25	1.00000000	chicken
Jazf1	0.16	1.00000000	chicken
Creg1	-0.25	1.00000000	chicken
Hic1	0.08	1.00000000	chicken
Plcb1	0.31	1.00000000	chicken
Zfp654	0.03	1.00000000	chicken
Rpn1	-0.22	1.00000000	chicken
Taldo1	-0.21	1.00000000	chicken
Sik3	-0.04	1.00000000	chicken
Haus3	0.03	1.00000000	chicken
Vta1	-0.02	1.00000000	chicken
Rbm12b2	-0.01	1.00000000	chicken
Ncoa4	0.01	1.00000000	chicken
Cactin	0.01	1.00000000	chicken
Dnajc3	0.00	1.00000000	chicken
Agap1	0.15	1.00000000	chicken
Eomes	0.00	1.00000000	chicken

Ube2i	-0.05	1.00000000	chicken
Hdac11	0.03	1.00000000	chicken
Lbr	-0.23	1.00000000	chicken
Ints4	-0.04	1.00000000	chicken
Zfp638	0.00	1.00000000	chicken
Limd2	0.02	1.00000000	chicken
Tomm6	-0.24	1.00000000	chicken
Rtf1	-0.31	1.00000000	chicken
Ric8b	0.04	1.00000000	chicken
Ap3b1	0.11	1.00000000	chicken
Aktip	0.00	1.00000000	chicken
Larp1b	0.02	1.00000000	chicken
Gtpbp4	-0.28	1.00000000	chicken
Snrpd1	-0.28	1.00000000	chicken
Pik3ca	0.01	1.00000000	chicken
Tab3	-0.04	1.00000000	chicken
Kpna3	-0.23	1.00000000	chicken
Ints13	-0.29	1.00000000	chicken
Foxj2	-0.27	1.00000000	chicken
Fancm	-0.20	1.00000000	chicken
Tspan3	-0.20	1.00000000	chicken
Ttll5	-0.32	1.00000000	chicken
Enox2	0.04	1.00000000	chicken
Ebag9	-0.26	1.00000000	chicken
Atl2	0.04	1.00000000	chicken
Nono	-0.11	1.00000000	chicken
Afg3l2	-0.04	1.00000000	chicken
Capn7	0.07	1.00000000	chicken
Scaf4	-0.09	1.00000000	chicken
Rmnd1	-0.03	1.00000000	chicken
Tmed5	-0.31	1.00000000	chicken
Ubr7	-0.01	1.00000000	chicken
Trmt10c	-0.07	1.00000000	chicken
Dnajb14	-0.03	1.00000000	chicken
Hs6st2	0.06	1.00000000	chicken
Uhmk1	-0.29	1.00000000	chicken
Prpf40a	-0.26	1.00000000	chicken
Rlim	-0.29	1.00000000	chicken
Cradd	0.01	1.00000000	chicken
Atf6	-0.01	1.00000000	chicken
Polr2i	-0.01	1.00000000	chicken
St13	0.07	1.00000000	chicken
Psmd2	-0.01	1.00000000	chicken
Sppl2a	0.05	1.00000000	chicken
Bbx	0.06	1.00000000	chicken
Golm2	-0.30	1.00000000	chicken

Ctcf	-0.26	1.00000000	chicken
Arl8b	-0.04	1.00000000	chicken
Ncbp3	-0.05	1.00000000	chicken
Brd1	-0.20	1.00000000	chicken
Herc1	0.07	1.00000000	chicken
Clasp2	-0.20	1.00000000	chicken
Ap2a1	0.04	1.00000000	chicken
Tmpo	-0.05	1.00000000	chicken
Galc	-0.29	1.00000000	chicken
Lsg1	-0.23	1.00000000	chicken
Nhlrc2	-0.23	1.00000000	chicken
Psmd7	0.03	1.00000000	chicken
Bzw2	-0.09	1.00000000	chicken
Fam168b	-0.27	1.00000000	chicken
Btbd10	-0.27	1.00000000	chicken
Gphn	0.23	1.00000000	chicken
Ddx1	-0.33	1.00000000	chicken
Srsf2	-0.06	1.00000000	chicken
Kctd20	0.07	1.00000000	chicken
Hsf3	-0.25	1.00000000	chicken
Zbtb48	-0.19	1.00000000	chicken
Pgrmc2	0.04	1.00000000	chicken
Cct2	0.01	1.00000000	chicken
Tulp3	0.05	1.00000000	chicken
Ubn2	-0.10	1.00000000	chicken
Eif4e3	-0.07	1.00000000	chicken
Wdr3	-0.30	1.00000000	chicken
Commd5	0.04	1.00000000	chicken
Epb41	-0.25	1.00000000	chicken
Tmem259	-0.19	1.00000000	chicken
Stx2	-0.31	1.00000000	chicken
Fubp1	-0.23	1.00000000	chicken
Per3	0.12	1.00000000	chicken
Asf1a	-0.27	1.00000000	chicken
Sod1	-0.18	1.00000000	chicken
Nol8	0.09	1.00000000	chicken
Angel1	-0.05	1.00000000	chicken
Ncapg	-0.07	1.00000000	chicken
Trim24	-0.23	1.00000000	chicken
Sin3a	-0.01	1.00000000	chicken
Rint1	-0.03	1.00000000	chicken
Vamp4	-0.04	1.00000000	chicken
Snap29	-0.24	1.00000000	chicken
Akap8l	-0.19	1.00000000	chicken
Vps26a	-0.13	1.00000000	chicken
Azi2	0.00	1.00000000	chicken

Cox7a2l	-0.24	1.00000000	chicken
Gpr171	-0.17	1.00000000	chicken
Ubash3b	-0.08	1.00000000	chicken
Prkca	0.18	1.00000000	chicken
Cops5	0.05	1.00000000	chicken
Cdk8	0.14	1.00000000	chicken
Lpcat1	0.01	1.00000000	chicken
Tubgcp5	-0.21	1.00000000	chicken
Smarca2	-0.18	1.00000000	chicken
Trub1	-0.03	1.00000000	chicken
Ubqln4	0.01	1.00000000	chicken
Dmxl1	-0.01	1.00000000	chicken
Hk2	0.01	1.00000000	chicken
Wtap	0.07	1.00000000	chicken
Ik	-0.32	1.00000000	chicken
Vrk2	0.06	1.00000000	chicken
Dlst	-0.05	1.00000000	chicken
Nras	-0.24	1.00000000	chicken
Vdac2	-0.25	1.00000000	chicken
Get4	-0.04	1.00000000	chicken
Sde2	-0.25	1.00000000	chicken
Pms1	-0.22	1.00000000	chicken
Ccdc50	-0.06	1.00000000	chicken
Snx1	0.02	1.00000000	chicken
Polr2e	-0.02	1.00000000	chicken
Eif3j2	0.03	1.00000000	chicken
Casc3	-0.07	1.00000000	chicken
Ehmt1	-0.33	1.00000000	chicken
Sar1a	-0.25	1.00000000	chicken
D16Ertd472e	-0.24	1.00000000	chicken
Sec23ip	0.04	1.00000000	chicken
Akap9	0.05	1.00000000	chicken
Abcf2	-0.26	1.00000000	chicken
Poldip3	-0.06	1.00000000	chicken
Nus1	0.14	1.00000000	chicken
Tex2	-0.24	1.00000000	chicken
Als2	-0.01	1.00000000	chicken
Rpa3	0.00	1.00000000	chicken
Hnrnpk	-0.25	1.00000000	chicken
Prmt3	0.00	1.00000000	chicken
Pikfyve	-0.03	1.00000000	chicken
Nde1	-0.27	1.00000000	chicken
Rab7	-0.27	1.00000000	chicken
Rev1	0.00	1.00000000	chicken
Il2ra	-0.14	1.00000000	chicken
Ghitm	-0.09	1.00000000	chicken

Ulk2	-0.02	1.00000000	chicken
Chchd2	-0.25	1.00000000	chicken
Dkc1	0.05	1.00000000	chicken
Jpt1	-0.03	1.00000000	chicken
Emc6	-0.15	1.00000000	chicken
Uspl1	0.00	1.00000000	chicken
Wdsub1	-0.20	1.00000000	chicken
Nap1l4	-0.19	1.00000000	chicken
Zbtb41	-0.21	1.00000000	chicken
Polh	0.01	1.00000000	chicken
Rmnd5a	0.02	1.00000000	chicken
Gpi1	-0.22	1.00000000	chicken
Golga7	-0.20	1.00000000	chicken
Pag1	-0.12	1.00000000	chicken
Ywhaq	-0.15	1.00000000	chicken
Lpin1	0.07	1.00000000	chicken
Rap2c	0.01	1.00000000	chicken
Arpc5	-0.19	1.00000000	chicken
Lrmp	0.03	1.00000000	chicken
Pdcd5	-0.03	1.00000000	chicken
Sirt2	0.00	1.00000000	chicken
Rell1	-0.24	1.00000000	chicken
Ndufb9	0.03	1.00000000	chicken
Hmgcs1	0.01	1.00000000	chicken
Nipal3	-0.02	1.00000000	chicken
Rpp14	-0.28	1.00000000	chicken
Ndufb10	0.01	1.00000000	chicken
Rbis	-0.26	1.00000000	chicken
Wdr7	0.15	1.00000000	chicken
Tmtc3	-0.07	1.00000000	chicken
Ppp2r3c	-0.03	1.00000000	chicken
Usp47	0.01	1.00000000	chicken
Arf1	-0.25	1.00000000	chicken
Sh3glb1	-0.04	1.00000000	chicken
Sh3bgrl	0.07	1.00000000	chicken
Rab11fip1	-0.05	1.00000000	chicken
Sik1	-0.23	1.00000000	chicken
Smad6	0.05	1.00000000	chicken
Slc30a7	0.03	1.00000000	chicken
Arglu1	-0.12	1.00000000	chicken
Rnps1	-0.09	1.00000000	chicken
Klhdc4	-0.11	1.00000000	chicken
Dad1	0.04	1.00000000	chicken
Fam3c	-0.05	1.00000000	chicken
Ndufs7	-0.05	1.00000000	chicken
Clpx	-0.03	1.00000000	chicken

Psma3	-0.01	1.00000000	chicken
Rrbp1	-0.01	1.00000000	chicken
Stag2	0.03	1.00000000	chicken
Ppp2r2a	0.00	1.00000000	chicken
Pik3cg	-0.12	1.00000000	chicken
Uggt1	0.04	1.00000000	chicken
Rnf38	-0.03	1.00000000	chicken
Kpnb1	-0.25	1.00000000	chicken
Cnn2	-0.03	1.00000000	chicken
Ermn	-0.10	1.00000000	chicken
P4ha1	0.04	1.00000000	chicken
Lin7c	-0.21	1.00000000	chicken
Atp6v0a2	-0.07	1.00000000	chicken
Dnajc13	-0.02	1.00000000	chicken
Itgav	0.04	1.00000000	chicken
Ankrd13d	-0.19	1.00000000	chicken
Ssb	0.05	1.00000000	chicken
Cwc15	0.00	1.00000000	chicken
Txlng	-0.29	1.00000000	chicken
Ythdf3	-0.31	1.00000000	chicken
Snapin	-0.20	1.00000000	chicken
Park7	-0.25	1.00000000	chicken
Tmbim6	-0.04	1.00000000	chicken
Snapc4	-0.03	1.00000000	chicken
Carhsp1	0.09	1.00000000	chicken
Arfgap2	-0.04	1.00000000	chicken
Drg1	0.00	1.00000000	chicken
Laptm4a	-0.24	1.00000000	chicken
Nufip1	0.02	1.00000000	chicken
Esf1	-0.08	1.00000000	chicken
Dolpp1	-0.24	1.00000000	chicken
Abcg1	0.21	1.00000000	chicken
Hif1an	0.01	1.00000000	chicken
Tcea1	-0.14	1.00000000	chicken
Cul5	-0.26	1.00000000	chicken
Sgpl1	-0.26	1.00000000	chicken
Selenok	0.01	1.00000000	chicken
Wdr70	-0.35	1.00000000	chicken
Hmgb3	-0.20	1.00000000	chicken
Khdc4	-0.01	1.00000000	chicken
Kif21b	-0.06	1.00000000	chicken
Lpcat3	-0.16	1.00000000	chicken
Aldh18a1	-0.04	1.00000000	chicken
Fgd4	0.02	1.00000000	chicken
Ccdc126	-0.07	1.00000000	chicken
1110059G10Rik	-0.23	1.00000000	chicken

N4bp2l2	0.00	1.00000000	chicken
Bin3	-0.25	1.00000000	chicken
Fzd6	0.08	1.00000000	chicken
Ap1ar	-0.23	1.00000000	chicken
Wrn	-0.25	1.00000000	chicken
Atp5h	-0.01	1.00000000	chicken
Btrc	-0.02	1.00000000	chicken
Tmem30a	-0.20	1.00000000	chicken
Cab39	-0.07	1.00000000	chicken
Parg	-0.07	1.00000000	chicken
Tmem184b	-0.10	1.00000000	chicken
Rcor3	-0.20	1.00000000	chicken
Prpf8	-0.05	1.00000000	chicken
Naf1	-0.03	1.00000000	chicken
Atp6v1b2	0.04	1.00000000	chicken
Spry2	-0.07	1.00000000	chicken
Snx12	0.03	1.00000000	chicken
Emc2	-0.05	1.00000000	chicken
Impdh2	-0.01	1.00000000	chicken
Ube2o	-0.20	1.00000000	chicken
Usp45	0.01	1.00000000	chicken
Atg12	-0.17	1.00000000	chicken
Acap1	-0.23	1.00000000	chicken
Eif5b	-0.24	1.00000000	chicken
Inpp5f	-0.23	1.00000000	chicken
Tardbp	-0.06	1.00000000	chicken
Irak2	0.01	1.00000000	chicken
Srsf5	-0.02	1.00000000	chicken
Ago4	-0.01	1.00000000	chicken
Nelfe	0.00	1.00000000	chicken
Sf3a3	0.00	1.00000000	chicken
Pdha1	-0.05	1.00000000	chicken
Rab12	-0.19	1.00000000	chicken
Prdm10	0.02	1.00000000	chicken
Ndufv2	-0.19	1.00000000	chicken
Capzb	0.04	1.00000000	chicken
Smim19	-0.19	1.00000000	chicken
Aoah	-0.19	1.00000000	chicken
Rab9	0.02	1.00000000	chicken
Ncald	-0.20	1.00000000	chicken
Nop16	-0.17	1.00000000	chicken
Pgk1	-0.25	1.00000000	chicken
Rad54l2	0.02	1.00000000	chicken
Ppp6c	0.01	1.00000000	chicken
Apbb1ip	0.09	1.00000000	chicken
Cplane2	-0.03	1.00000000	chicken

Igf2bp2	-0.08	1.00000000	chicken
Ikzf3	0.02	1.00000000	chicken
Arhgap27	-0.34	1.00000000	chicken
Cope	0.01	1.00000000	chicken
Dnajb4	-0.19	1.00000000	chicken
Actr6	-0.04	1.00000000	chicken
Gabarapl1	-0.21	1.00000000	chicken
Ccpg1	0.00	1.00000000	chicken
Zfpm1	-0.03	1.00000000	chicken
Arfgef1	-0.20	1.00000000	chicken
Pum2	-0.01	1.00000000	chicken
Ikbkb	-0.26	1.00000000	chicken
Dhx37	-0.02	1.00000000	chicken
Nol11	-0.06	1.00000000	chicken
Btbd1	-0.24	1.00000000	chicken
Cd8b1	0.00	1.00000000	chicken
Dnaja4	-0.23	1.00000000	chicken
Capza2	0.08	1.00000000	chicken
Psmd6	-0.23	1.00000000	chicken
Eri3	-0.01	1.00000000	chicken
Armc2	-0.16	1.00000000	chicken
Tbp	0.00	1.00000000	chicken
Tm9sf2	-0.08	1.00000000	chicken
Dus3l	-0.17	1.00000000	chicken
1810013L24Rik	-0.26	1.00000000	chicken
Zfp292	-0.04	1.00000000	chicken
Fbxl20	-0.03	1.00000000	chicken
Ifrd1	0.08	1.00000000	chicken
Phf10	-0.30	1.00000000	chicken
Pcbp2	-0.23	1.00000000	chicken
Hps5	-0.12	1.00000000	chicken
Scfd1	0.03	1.00000000	chicken
Pde8a	0.26	1.00000000	chicken
4933434E20Rik	-0.19	1.00000000	chicken
Taf2	-0.05	1.00000000	chicken
Ppat	-0.03	1.00000000	chicken
Rabgap1l	-0.20	1.00000000	chicken
Ywhae	-0.05	1.00000000	chicken
Kat6a	0.03	1.00000000	chicken
Nop53	-0.09	1.00000000	chicken
Ercc8	-0.23	1.00000000	chicken
Uqcrb	0.02	1.00000000	chicken
Sumo3	-0.27	1.00000000	chicken
Tbk1	-0.25	1.00000000	chicken
Smyd2	0.05	1.00000000	chicken
Tfam	-0.02	1.00000000	chicken

Ppil3	-0.18	1.00000000	chicken
Rimbp2	0.07	1.00000000	chicken
Ipo5	-0.17	1.00000000	chicken
Ago1	-0.02	1.00000000	chicken
Ing4	-0.09	1.00000000	chicken
Ttyh3	0.05	1.00000000	chicken
Adnp	0.06	1.00000000	chicken
Fbxo22	0.00	1.00000000	chicken
Appl2	-0.18	1.00000000	chicken
Tnip2	-0.02	1.00000000	chicken
Trpc1	-0.09	1.00000000	chicken
Actr2	-0.17	1.00000000	chicken
Faf2	-0.08	1.00000000	chicken
Tmod3	-0.22	1.00000000	chicken
Yars	-0.07	1.00000000	chicken
Snx2	-0.17	1.00000000	chicken
Rbm5	-0.17	1.00000000	chicken
Ascc1	-0.19	1.00000000	chicken
Tspan14	-0.21	1.00000000	chicken
Zfp36l2	-0.18	1.00000000	chicken
Plagl1	-0.15	1.00000000	chicken
Rif1	-0.01	1.00000000	chicken
9030025P20Rik	-0.26	1.00000000	chicken
Lpin2	-0.18	1.00000000	chicken
Ttbk1	0.01	1.00000000	chicken
Usp3	0.02	1.00000000	chicken
Nsun2	-0.05	1.00000000	chicken
Coq8a	-0.01	1.00000000	chicken
Ndufab1	-0.16	1.00000000	chicken
Hspa4l	-0.12	1.00000000	chicken
Rrp9	-0.07	1.00000000	chicken
Lemd2	-0.06	1.00000000	chicken
Cbll1	-0.04	1.00000000	chicken
Pes1	-0.02	1.00000000	chicken
Gab3	-0.21	1.00000000	chicken
Gsdme	-0.20	1.00000000	chicken
Fbxw8	-0.07	1.00000000	chicken
Atp5j	0.02	1.00000000	chicken
Zyx	-0.15	1.00000000	chicken
Ltn1	-0.09	1.00000000	chicken
Slc9a7	0.04	1.00000000	chicken
Vrk1	-0.05	1.00000000	chicken
Cct8	-0.09	1.00000000	chicken
Ash1l	-0.03	1.00000000	chicken
Cerk	-0.22	1.00000000	chicken
Slc12a4	0.04	1.00000000	chicken

Rab11a	-0.06	1.00000000	chicken
Creld2	-0.23	1.00000000	chicken
Thoc5	-0.04	1.00000000	chicken
Adam17	-0.04	1.00000000	chicken
Polr2c	-0.07	1.00000000	chicken
Tmem106b	-0.05	1.00000000	chicken
Cse1l	-0.06	1.00000000	chicken
Slc35b1	-0.02	1.00000000	chicken
Siah1b	-0.23	1.00000000	chicken
Ip6k2	-0.02	1.00000000	chicken
Trpc4ap	-0.26	1.00000000	chicken
Tgif1	-0.25	1.00000000	chicken
Nsrp1	-0.04	1.00000000	chicken
Hsf2	-0.23	1.00000000	chicken
Prpf39	-0.25	1.00000000	chicken
Zmiz1	-0.31	1.00000000	chicken
Prkx	-0.17	1.00000000	chicken
Paf1	-0.04	1.00000000	chicken
Wdr26	-0.19	1.00000000	chicken
Bnip2	-0.03	1.00000000	chicken
Aco2	0.02	1.00000000	chicken
Pcnx2	-0.04	1.00000000	chicken
Dnaja2	-0.23	1.00000000	chicken
Ddx6	-0.20	1.00000000	chicken
Stam2	-0.29	1.00000000	chicken
Arl6ip5	0.03	1.00000000	chicken
Tmem33	-0.18	1.00000000	chicken
Yaf2	-0.29	1.00000000	chicken
Gtf3a	-0.07	1.00000000	chicken
Tnfaip3	-0.08	1.00000000	chicken
Txndc9	-0.02	1.00000000	chicken
Srsf7	-0.17	1.00000000	chicken
Klhl15	0.05	1.00000000	chicken
Usp7	0.02	1.00000000	chicken
Akap11	-0.05	1.00000000	chicken
Ccdc43	-0.27	1.00000000	chicken
Gorasp2	0.04	1.00000000	chicken
Dnlz	-0.08	1.00000000	chicken
Sertad2	-0.04	1.00000000	chicken
2700049A03Rik	-0.07	1.00000000	chicken
Psmc6	-0.03	1.00000000	chicken
Osbp	-0.21	1.00000000	chicken
Wdr44	-0.09	1.00000000	chicken
Cdc73	0.07	1.00000000	chicken
Rab14	-0.24	1.00000000	chicken
E2f6	-0.04	1.00000000	chicken

Sltm	-0.11	1.00000000	chicken
Arhgap11a	-0.03	1.00000000	chicken
Nedd8	-0.11	1.00000000	chicken
Rsrc2	0.00	1.00000000	chicken
Vamp3	-0.04	1.00000000	chicken
Snrnp25	-0.18	1.00000000	chicken
Igf2r	-0.25	1.00000000	chicken
Steep1	-0.01	1.00000000	chicken
Nelfa	-0.23	1.00000000	chicken
Xiap	-0.04	1.00000000	chicken
Ccnl2	-0.06	1.00000000	chicken
Tmem63a	-0.07	1.00000000	chicken
Ckap5	-0.06	1.00000000	chicken
Wdr41	-0.21	1.00000000	chicken
1110004F10Rik	0.05	1.00000000	chicken
Sav1	-0.02	1.00000000	chicken
Tfg	0.01	1.00000000	chicken
Rab11fip4	0.06	1.00000000	chicken
Yipf3	-0.04	1.00000000	chicken
Fbxl2	-0.47	1.00000000	chicken
Eif4a3l1	-0.23	1.00000000	chicken
Gtf2h1	0.00	1.00000000	chicken
Slc4a7	0.13	1.00000000	chicken
Cops8	-0.11	1.00000000	chicken
Thoc2	-0.01	1.00000000	chicken
Terf2	-0.22	1.00000000	chicken
Rab8a	-0.04	1.00000000	chicken
Taz	-0.09	1.00000000	chicken
Rad21	-0.26	1.00000000	chicken
Tomm70a	-0.14	1.00000000	chicken
Bmpr1a	-0.19	1.00000000	chicken
Inpp5d	0.03	1.00000000	chicken
Cnot10	-0.03	1.00000000	chicken
Uqcrfs1	-0.21	1.00000000	chicken
Tdg	-0.30	1.00000000	chicken
Slc39a11	-0.20	1.00000000	chicken
Coa5	-0.17	1.00000000	chicken
Tnks2	0.06	1.00000000	chicken
Phf3	-0.01	1.00000000	chicken
Tm9sf3	-0.10	1.00000000	chicken
Champ1	-0.15	1.00000000	chicken
Immt	-0.18	1.00000000	chicken
Idh2	-0.15	1.00000000	chicken
Psmd13	-0.09	1.00000000	chicken
Rnpc3	-0.24	1.00000000	chicken
Tbc1d15	-0.22	1.00000000	chicken

Uqcr10	0.01	1.00000000	chicken
Creb3	-0.16	1.00000000	chicken
Dip2a	-0.04	1.00000000	chicken
Esyt2	0.02	1.00000000	chicken
4833439L19Rik	-0.18	1.00000000	chicken
Tshz1	-0.16	1.00000000	chicken
Ythdf1	-0.25	1.00000000	chicken
Rbl1	-0.23	1.00000000	chicken
Pwp1	-0.26	1.00000000	chicken
Gstm4	-0.01	1.00000000	chicken
Ifi30	-0.19	1.00000000	chicken
Camk2g	0.07	1.00000000	chicken
Ercc3	-0.09	1.00000000	chicken
4932438A13Rik	-0.09	1.00000000	chicken
Dnajc2	-0.24	1.00000000	chicken
Actr3	0.04	1.00000000	chicken
Dhx30	-0.08	1.00000000	chicken
Efcab14	-0.14	1.00000000	chicken
Il7r	-0.13	1.00000000	chicken
Atp6v0e	-0.01	1.00000000	chicken
Eif2b3	-0.15	1.00000000	chicken
Zcchc17	-0.15	1.00000000	chicken
Pak1	0.10	1.00000000	chicken
Chuk	-0.24	1.00000000	chicken
Pdcd10	-0.24	1.00000000	chicken
Akap8	-0.08	1.00000000	chicken
Naa30	-0.21	1.00000000	chicken
Hnrnpu	-0.06	1.00000000	chicken
Hdac2	-0.12	1.00000000	chicken
Kdm3a	-0.21	1.00000000	chicken
Klhl13	0.02	1.00000000	chicken
Zfp711	-0.05	1.00000000	chicken
C2cd5	-0.17	1.00000000	chicken
Rnf114	-0.08	1.00000000	chicken
Gtf2f2	0.01	1.00000000	chicken
Hddc2	-0.14	1.00000000	chicken
Sf3a1	-0.20	1.00000000	chicken
Setd1b	-0.27	1.00000000	chicken
Zswim8	-0.15	1.00000000	chicken
Xrn2	-0.08	1.00000000	chicken
Ywhaz	-0.03	1.00000000	chicken
Whamm	-0.07	1.00000000	chicken
Cnp	-0.17	1.00000000	chicken
Stab1	-0.23	1.00000000	chicken
Acp2	-0.13	1.00000000	chicken
Bbs7	-0.04	1.00000000	chicken

Snrpb2	-0.13	1.00000000	chicken
Ssbp3	0.01	1.00000000	chicken
Rnf145	-0.05	1.00000000	chicken
Flnb	0.10	1.00000000	chicken
Usp20	-0.06	1.00000000	chicken
Slu7	-0.03	1.00000000	chicken
Wdr81	-0.17	1.00000000	chicken
Lims2	-0.08	1.00000000	chicken
Cltb	-0.03	1.00000000	chicken
Rab27a	-0.21	1.00000000	chicken
Prr14l	-0.28	1.00000000	chicken
Tmem104	-0.30	1.00000000	chicken
Rdx	-0.01	1.00000000	chicken
Brd8	-0.01	1.00000000	chicken
Eif2a	-0.19	1.00000000	chicken
Rasal2	0.01	1.00000000	chicken
Gtdc1	-0.33	1.00000000	chicken
Gpr137b	-0.08	1.00000000	chicken
Hspa4	-0.14	1.00000000	chicken
Sap30bp	0.00	1.00000000	chicken
Elk4	-0.09	1.00000000	chicken
Iqgap2	-0.19	1.00000000	chicken
Hdgfl3	-0.13	1.00000000	chicken
Kat6b	-0.05	1.00000000	chicken
Tsg101	-0.30	1.00000000	chicken
Ubqln1	-0.10	1.00000000	chicken
Zmynd8	-0.27	1.00000000	chicken
Erh	-0.04	1.00000000	chicken
Pdap1	0.00	1.00000000	chicken
Gorab	-0.08	1.00000000	chicken
Smndc1	-0.20	1.00000000	chicken
Arid3b	-0.18	1.00000000	chicken
Ptpn11	-0.07	1.00000000	chicken
Arhgap29	-0.11	1.00000000	chicken
Prpf3	-0.05	1.00000000	chicken
Nt5c3	-0.29	1.00000000	chicken
Ddx54	0.02	1.00000000	chicken
Nrd1	0.00	1.00000000	chicken
Pdia4	0.04	1.00000000	chicken
Ogt	0.04	1.00000000	chicken
Xpot	-0.23	1.00000000	chicken
Ccnd2	-0.17	1.00000000	chicken
Srebf1	-0.09	1.00000000	chicken
Dync1li2	-0.02	1.00000000	chicken
Nolc1	-0.17	1.00000000	chicken
Nup107	-0.14	1.00000000	chicken

Becn2	-0.16	1.00000000	chicken
Atp5pb	-0.05	1.00000000	chicken
Ap3s1	-0.15	1.00000000	chicken
Spg11	-0.15	1.00000000	chicken
Ppp2r2d	-0.09	1.00000000	chicken
Slc38a2	-0.09	1.00000000	chicken
Snapc5	-0.07	1.00000000	chicken
Syap1	-0.15	1.00000000	chicken
Arid3a	0.05	1.00000000	chicken
Etfa	-0.05	1.00000000	chicken
Txnip	-0.12	1.00000000	chicken
Naa25	-0.01	1.00000000	chicken
Otulinl	-0.09	1.00000000	chicken
Parvg	-0.30	1.00000000	chicken
Thada	-0.10	1.00000000	chicken
Utp11	0.03	1.00000000	chicken
BC048403	-0.20	1.00000000	chicken
Rprd1a	-0.05	1.00000000	chicken
Txnrd3	-0.22	1.00000000	chicken
Cox7c	-0.04	1.00000000	chicken
Slc66a2	-0.08	1.00000000	chicken
Cflar	0.06	1.00000000	chicken
Vps4b	-0.23	1.00000000	chicken
Bag1	-0.23	1.00000000	chicken
Rpf1	-0.17	1.00000000	chicken
Lmbr1	-0.09	1.00000000	chicken
Reck	-0.12	1.00000000	chicken
Atp5c1	-0.28	1.00000000	chicken
H2ac20	-0.01	1.00000000	chicken
Otud6b	-0.08	1.00000000	chicken
Ist1	-0.22	1.00000000	chicken
Thrap3	-0.05	1.00000000	chicken
Nucb2	-0.27	1.00000000	chicken
Calr	-0.04	1.00000000	chicken
Htatsf1	-0.10	1.00000000	chicken
AW554918	-0.02	1.00000000	chicken
Ints6l	-0.14	1.00000000	chicken
Pepd	-0.11	1.00000000	chicken
Adrm1	-0.22	1.00000000	chicken
Cyb5r4	-0.01	1.00000000	chicken
Zfp131	-0.21	1.00000000	chicken
Ssr3	-0.19	1.00000000	chicken
Ly75	-0.18	1.00000000	chicken
Sumo1	-0.21	1.00000000	chicken
Stt3b	0.00	1.00000000	chicken
Impa1	-0.13	1.00000000	chicken

Rars	-0.19	1.00000000	chicken
Ipo7	-0.02	1.00000000	chicken
Dym	-0.07	1.00000000	chicken
Crk	-0.17	1.00000000	chicken
Pak3	-0.13	1.00000000	chicken
Ppcs	-0.02	1.00000000	chicken
Usp14	-0.01	1.00000000	chicken
Carnmt1	-0.04	1.00000000	chicken
Edc4	-0.03	1.00000000	chicken
Sptssa	-0.06	1.00000000	chicken
Clcn5	-0.07	1.00000000	chicken
Slc2a8	-0.25	1.00000000	chicken
Acta2	0.05	1.00000000	chicken
Cnbp	-0.03	1.00000000	chicken
Lamtor2	-0.14	1.00000000	chicken
Rprd2	-0.27	1.00000000	chicken
Sipa1l1	-0.25	1.00000000	chicken
Krr1	-0.09	1.00000000	chicken
Ftsj3	-0.11	1.00000000	chicken
Cops7a	-0.17	1.00000000	chicken
Uso1	-0.02	1.00000000	chicken
Stk38l	-0.07	1.00000000	chicken
Aldoc	-0.15	1.00000000	chicken
Ppp1r7	0.02	1.00000000	chicken
Ankrd17	-0.08	1.00000000	chicken
Rmc1	-0.13	1.00000000	chicken
Psma5	-0.12	1.00000000	chicken
Zfyve21	-0.21	1.00000000	chicken
Rit1	-0.21	1.00000000	chicken
Cdc27	-0.08	1.00000000	chicken
Tacc1	-0.22	1.00000000	chicken
Zranb1	0.05	1.00000000	chicken
Lig3	-0.09	1.00000000	chicken
Copb1	-0.04	1.00000000	chicken
Nbn	-0.12	1.00000000	chicken
Hook1	-0.14	1.00000000	chicken
Ust	0.18	1.00000000	chicken
Srp72	-0.22	1.00000000	chicken
Pik3ap1	0.03	1.00000000	chicken
Aldh7a1	-0.11	1.00000000	chicken
Dars	-0.12	1.00000000	chicken
B230219D22Rik	-0.11	1.00000000	chicken
Ola1	0.03	1.00000000	chicken
Klf13	-0.14	1.00000000	chicken
Usp4	-0.22	1.00000000	chicken
Arl1	-0.12	1.00000000	chicken

Camsap2	-0.06	1.00000000	chicken
Asah1	-0.09	1.00000000	chicken
Hhex	-0.10	1.00000000	chicken
Zdhhc6	-0.06	1.00000000	chicken
Plekhm1	-0.09	1.00000000	chicken
Washc4	-0.27	1.00000000	chicken
Axin1	0.01	1.00000000	chicken
Ccdc174	-0.05	1.00000000	chicken
Vps33b	-0.15	1.00000000	chicken
Cdk19	-0.02	1.00000000	chicken
Sdcbp	-0.11	1.00000000	chicken
Tmem181a	-0.18	1.00000000	chicken
Rbm34	-0.17	1.00000000	chicken
Atp6v1e2	-0.08	1.00000000	chicken
Id2	-0.13	1.00000000	chicken
Kars	-0.11	1.00000000	chicken
Lonrf1	-0.10	1.00000000	chicken
Slc35e2	-0.07	1.00000000	chicken
Rilpl1	-0.20	1.00000000	chicken
Timm13	-0.07	1.00000000	chicken
Dock5	-0.12	1.00000000	chicken
Id3	-0.24	1.00000000	chicken
Gkap1	-0.11	1.00000000	chicken
Lonrf2	-0.14	1.00000000	chicken
Uchl5	-0.12	1.00000000	chicken
1110059E24Rik	-0.07	1.00000000	chicken
Abcc5	-0.23	1.00000000	chicken
Tpr	-0.01	1.00000000	chicken
3110082I17Rik	-0.17	1.00000000	chicken
Pcgf5	-0.09	1.00000000	chicken
Kmt2c	-0.03	1.00000000	chicken
Zfp644	-0.06	1.00000000	chicken
Psmc2	-0.19	1.00000000	chicken
Tpi1	-0.21	1.00000000	chicken
Tmem131l	-0.20	1.00000000	chicken
Arhgdia	-0.10	1.00000000	chicken
Dazap2	-0.08	1.00000000	chicken
Slc38a10	-0.09	1.00000000	chicken
Sat1	-0.01	1.00000000	chicken
Pik3r5	-0.12	1.00000000	chicken
Zfp326	-0.20	1.00000000	chicken
Sgsm2	-0.28	1.00000000	chicken
Atp6v0a1	-0.23	1.00000000	chicken
Plcg1	-0.19	1.00000000	chicken
Srp54a	-0.16	1.00000000	chicken
Papss1	-0.19	1.00000000	chicken

Snw1	-0.08	1.00000000	chicken
Hspe1-rs1	-0.04	1.00000000	chicken
Oser1	-0.14	1.00000000	chicken
Necap1	-0.06	1.00000000	chicken
Creb1	-0.08	1.00000000	chicken
Rbm14	-0.04	1.00000000	chicken
Dync1i2	-0.12	1.00000000	chicken
Ntan1	-0.28	1.00000000	chicken
Ptpn4	-0.13	1.00000000	chicken
Rcn2	0.05	1.00000000	chicken
Washc5	-0.11	1.00000000	chicken
R3hdm1	0.00	1.00000000	chicken
Larp1	-0.04	1.00000000	chicken
Ube2g2	-0.04	1.00000000	chicken
Fem1b	-0.16	1.00000000	chicken
Copg1	-0.04	1.00000000	chicken
Gsr	-0.16	1.00000000	chicken
Selenof	-0.04	1.00000000	chicken
Zrsr1	0.02	1.00000000	chicken
Ube2d2a	-0.08	1.00000000	chicken
Zc3h18	-0.18	1.00000000	chicken
Ostf1	-0.19	1.00000000	chicken
Nubpl	-0.19	1.00000000	chicken
Stat5b	-0.06	1.00000000	chicken
Ppp1cb	-0.13	1.00000000	chicken
C1qbp	-0.12	1.00000000	chicken
Ahrr	-0.01	1.00000000	chicken
Thoc7	-0.20	1.00000000	chicken
9530068E07Rik	-0.04	1.00000000	chicken
Tsnax	-0.10	1.00000000	chicken
Wdr91	-0.04	1.00000000	chicken
Eif2b5	-0.21	1.00000000	chicken
Lactb	-0.07	1.00000000	chicken
Pigk	0.07	1.00000000	chicken
Dld	-0.04	1.00000000	chicken
Atrx	0.02	1.00000000	chicken
Ice2	-0.11	1.00000000	chicken
Srsf4	-0.10	1.00000000	chicken
Tmem59	-0.04	1.00000000	chicken
Arhgef6	-0.10	1.00000000	chicken
Asap1	0.02	1.00000000	chicken
Herc3	-0.15	1.00000000	chicken
Cdc40	-0.15	1.00000000	chicken
Larp7	-0.06	1.00000000	chicken
Smim7	-0.05	1.00000000	chicken
Eif6	-0.11	1.00000000	chicken

Kdelr2	-0.25	1.00000000	chicken
Caprin1	-0.21	1.00000000	chicken
Atp5d	-0.09	1.00000000	chicken
Erlin2	-0.11	1.00000000	chicken
Insig1	-0.24	1.00000000	chicken
Eif4e	-0.05	1.00000000	chicken
Seh1l	-0.10	1.00000000	chicken
Dtnbp1	-0.10	1.00000000	chicken
Ewsr1	-0.13	1.00000000	chicken
Klf7	0.07	1.00000000	chicken
Ddx17	-0.16	1.00000000	chicken
Chmp5	-0.22	1.00000000	chicken
Parn	-0.11	1.00000000	chicken
AI597479	-0.16	1.00000000	chicken
Pibf1	-0.10	1.00000000	chicken
Chmp7	-0.16	1.00000000	chicken
Smarcd2	0.00	1.00000000	chicken
Atrn	-0.24	1.00000000	chicken
Rwdd1	-0.01	1.00000000	chicken
Ecd	-0.14	1.00000000	chicken
Anks1	-0.06	1.00000000	chicken
Sdhb	-0.02	1.00000000	chicken
Snrpb	-0.08	1.00000000	chicken
Irf2bp2	-0.24	1.00000000	chicken
Numa1	-0.06	1.00000000	chicken
Lpcat2b	-0.03	1.00000000	chicken
Ccnc	-0.05	1.00000000	chicken
Naa20	0.00	1.00000000	chicken
Crtc1	-0.19	1.00000000	chicken
Dync1h1	-0.04	1.00000000	chicken
Ube2e1	-0.05	1.00000000	chicken
Traf1	-0.18	1.00000000	chicken
Dgat2	-0.14	1.00000000	chicken
Washc2	-0.23	1.00000000	chicken
St6gal1	-0.04	1.00000000	chicken
Nbr1	-0.06	1.00000000	chicken
Ppp2cb	-0.16	1.00000000	chicken
Nr1h3	-0.11	1.00000000	chicken
Cd82	-0.14	1.00000000	chicken
Rad51b	0.03	1.00000000	chicken
Zc3h7a	-0.07	1.00000000	chicken
Ndufs2	-0.10	1.00000000	chicken
Tmem164	0.10	1.00000000	chicken
Smarca5	-0.19	1.00000000	chicken
Brpf3	-0.11	1.00000000	chicken
Cdk11b	-0.16	1.00000000	chicken

Gabpa	-0.17	1.00000000	chicken
Ppm1g	-0.10	1.00000000	chicken
Fggy	0.01	1.00000000	chicken
Utp6	-0.07	1.00000000	chicken
Gid8	-0.09	1.00000000	chicken
Ltf	-0.12	1.00000000	chicken
Brwd1	-0.03	1.00000000	chicken
Gnb4	-0.03	1.00000000	chicken
Fosl2	-0.22	1.00000000	chicken
Ccng2	-0.11	1.00000000	chicken
Stim2	-0.04	1.00000000	chicken
Khdrbs1	-0.06	1.00000000	chicken
Exosc10	-0.18	1.00000000	chicken
Ldhb	-0.03	1.00000000	chicken
Gstm5	-0.12	1.00000000	chicken
Snrpd3	-0.13	1.00000000	chicken
Pola1	-0.16	1.00000000	chicken
Klhdc1	-0.05	1.00000000	chicken
Eif2ak2	-0.15	1.00000000	chicken
Zfp384	-0.19	1.00000000	chicken
Paics	-0.21	1.00000000	chicken
Ezh2	-0.16	1.00000000	chicken
Ptp4a3	-0.07	1.00000000	chicken
Zfhx3	-0.19	1.00000000	chicken
Sufu	-0.04	1.00000000	chicken
Nudt16l1	-0.02	1.00000000	chicken
Riok1	-0.13	1.00000000	chicken
Zbed4	-0.18	1.00000000	chicken
Tmed7	-0.06	1.00000000	chicken
Rnf126	-0.07	1.00000000	chicken
Stk10	-0.01	1.00000000	chicken
Dbi	-0.16	1.00000000	chicken
Def6	-0.03	1.00000000	chicken
Ddx27	-0.15	1.00000000	chicken
Slc25a25	-0.18	1.00000000	chicken
Otud7b	-0.15	1.00000000	chicken
Aqr	-0.13	1.00000000	chicken
Nckap1l	-0.07	1.00000000	chicken
Kdm2a	-0.08	1.00000000	chicken
Pdk3	-0.03	1.00000000	chicken
Hectd1	-0.13	1.00000000	chicken
Ralbp1	-0.08	1.00000000	chicken
Amd2	-0.16	1.00000000	chicken
Ndufa12	-0.13	1.00000000	chicken
Tkt	-0.21	1.00000000	chicken
Rapgef1	-0.23	1.00000000	chicken

Erap1	-0.14	1.00000000	chicken
Esco1	-0.05	1.00000000	chicken
Tpp2	-0.15	1.00000000	chicken
Zfyve26	-0.09	1.00000000	chicken
Ugp2	-0.06	1.00000000	chicken
Arpc1a	-0.02	1.00000000	chicken
Jun	0.12	1.00000000	chicken
Cdc23	-0.11	1.00000000	chicken
Prkar1b	-0.14	1.00000000	chicken
Ndufb2	-0.11	1.00000000	chicken
Notch1	-0.12	1.00000000	chicken
Arfgap1	-0.09	1.00000000	chicken
Auts2	0.10	1.00000000	chicken
Cbx1	-0.07	1.00000000	chicken
Nup98	-0.16	1.00000000	chicken
Cep120	-0.10	1.00000000	chicken
Atp5b	-0.11	1.00000000	chicken
Prpsap2	-0.07	1.00000000	chicken
Gpr65	-0.12	1.00000000	chicken
Pcgf6	-0.09	1.00000000	chicken
Uqcrc1	-0.07	1.00000000	chicken
Crebbp	0.03	1.00000000	chicken
Ppif	-0.07	1.00000000	chicken
Ap3m1	-0.22	1.00000000	chicken
Hadh	-0.02	1.00000000	chicken
Wdr75	-0.17	1.00000000	chicken
Ldha	-0.02	1.00000000	chicken
Aebp2	-0.19	1.00000000	chicken
U2af1	-0.07	1.00000000	chicken
Suclg1	-0.06	1.00000000	chicken
Snrpe	-0.02	1.00000000	chicken
Hps3	-0.25	1.00000000	chicken
Sergef	-0.07	1.00000000	chicken
Ddx20	-0.18	1.00000000	chicken
Ank	-0.28	1.00000000	chicken
Copa	-0.07	1.00000000	chicken
Kif1b	-0.18	1.00000000	chicken
Ap2m1	-0.05	1.00000000	chicken
Fam126a	-0.03	1.00000000	chicken
Abraxas1	-0.07	1.00000000	chicken
Xpo7	-0.11	1.00000000	chicken
Dcaf1	-0.08	1.00000000	chicken
Phlpp2	-0.13	1.00000000	chicken
Rpf2	-0.03	1.00000000	chicken
Bcap31	-0.15	1.00000000	chicken
Fmr1	-0.20	1.00000000	chicken

Timm50	-0.18	1.00000000	chicken
Lat2	-0.06	1.00000000	chicken
Adarb1	-0.09	1.00000000	chicken
Pgam1	-0.16	1.00000000	chicken
Jade2	-0.09	1.00000000	chicken
Psmc1	-0.17	1.00000000	chicken
Ptch1	-0.26	1.00000000	chicken
Psma6	-0.18	1.00000000	chicken
Psme3ip1	-0.07	1.00000000	chicken
Pus7	0.03	1.00000000	chicken
Spty2d1	-0.05	1.00000000	chicken
Ilkap	-0.18	1.00000000	chicken
Tpp1	-0.10	1.00000000	chicken
Traf6	-0.22	1.00000000	chicken
Rilpl2	-0.08	1.00000000	chicken
Casp8ap2	-0.13	1.00000000	chicken
Rab5a	-0.07	1.00000000	chicken
Sec31a	-0.05	1.00000000	chicken
Rab43	-0.20	1.00000000	chicken
Phip	0.04	1.00000000	chicken
C2cd2l	-0.21	1.00000000	chicken
Phb2	-0.12	1.00000000	chicken
Ube2a	-0.12	1.00000000	chicken
Arhgap45	-0.18	1.00000000	chicken
Slc35b2	-0.21	1.00000000	chicken
Sik2	-0.04	1.00000000	chicken
Aldh5a1	-0.06	1.00000000	chicken
Heatr5b	-0.21	1.00000000	chicken
Sf3b6	-0.23	1.00000000	chicken
Pptc7	-0.16	1.00000000	chicken
Rnf111	-0.04	1.00000000	chicken
Ywhab	-0.05	1.00000000	chicken
Utp15	-0.10	1.00000000	chicken
Spz1	-0.11	1.00000000	chicken
Acadl	-0.17	1.00000000	chicken
Serpine2	-0.13	1.00000000	chicken
Srp68	-0.09	1.00000000	chicken
Rnf20	-0.12	1.00000000	chicken
Insrr	-0.19	1.00000000	chicken
Sec23b	-0.23	1.00000000	chicken
Ssrp1	-0.13	1.00000000	chicken
Hnrnpul1	-0.17	1.00000000	chicken
Gemin8	-0.09	1.00000000	chicken
Rsl24d1	-0.24	1.00000000	chicken
Irf2	-0.15	1.00000000	chicken
Pus10	-0.05	1.00000000	chicken

Sass6	-0.16	1.00000000	chicken
Ccdc186	-0.21	1.00000000	chicken
Gtf2b	-0.17	1.00000000	chicken
Rbbp4	-0.11	1.00000000	chicken
Spg7	-0.01	1.00000000	chicken
Aup1	-0.20	1.00000000	chicken
E2f5	-0.10	1.00000000	chicken
Fbxl22	-0.21	1.00000000	chicken
Hyou1	-0.15	1.00000000	chicken
Dis3l2	-0.18	1.00000000	chicken
Cbwd1	-0.17	1.00000000	chicken
Bach2	-0.19	1.00000000	chicken
Clk1	-0.22	1.00000000	chicken
Prmt7	-0.05	1.00000000	chicken
Elof1	-0.07	1.00000000	chicken
Ptbp1	-0.06	1.00000000	chicken
Hdac4	-0.43	1.00000000	chicken
Agpat5	-0.07	1.00000000	chicken
E2f4	-0.08	1.00000000	chicken
Cyb5b	-0.13	1.00000000	chicken
Isca1	-0.14	1.00000000	chicken
Srgap3	-0.01	1.00000000	chicken
Cwc25	-0.10	1.00000000	chicken
Fam204a	-0.19	1.00000000	chicken
Ogdh	-0.14	1.00000000	chicken
Csnk2a2	-0.08	1.00000000	chicken
Ube2k	-0.08	1.00000000	chicken
Trnt1	-0.07	1.00000000	chicken
Cyfip2	-0.15	1.00000000	chicken
Nup50	-0.11	1.00000000	chicken
Acbd3	-0.19	1.00000000	chicken
Dcaf13	-0.13	1.00000000	chicken
Ylpm1	-0.05	1.00000000	chicken
Fkbp3	-0.07	1.00000000	chicken
Clasp1	-0.13	1.00000000	chicken
Rbm45	-0.06	1.00000000	chicken
Paip1	-0.11	1.00000000	chicken
Relch	-0.02	1.00000000	chicken
Stk11	-0.22	1.00000000	chicken
Ube2b	-0.15	1.00000000	chicken
Erp29	-0.03	1.00000000	chicken
Bak1	-0.09	1.00000000	chicken
Pcna	-0.12	1.00000000	chicken
Fbxo45	-0.10	1.00000000	chicken
Prpf4	-0.07	1.00000000	chicken
Ndfip1	-0.14	1.00000000	chicken

Psmc5	-0.04	1.00000000	chicken
Smc4	-0.04	1.00000000	chicken
Nup214	-0.14	1.00000000	chicken
Tuba1a	-0.11	1.00000000	chicken
Ncbp2	-0.11	1.00000000	chicken
Phka2	-0.16	1.00000000	chicken
Srp19	-0.10	1.00000000	chicken
Pafah1b2	-0.25	1.00000000	chicken
Pccb	-0.13	1.00000000	chicken
Pmm2	-0.10	1.00000000	chicken
Ulk1	0.02	1.00000000	chicken
Nop14	-0.16	1.00000000	chicken
Phkb	-0.15	1.00000000	chicken
Copb2	-0.18	1.00000000	chicken
Plrg1	-0.19	1.00000000	chicken
Taf6	-0.16	1.00000000	chicken
Ube2l3	-0.26	1.00000000	chicken
Cdca4	-0.11	1.00000000	chicken
Atp5k	-0.20	1.00000000	chicken
Septin2	-0.09	1.00000000	chicken
Hspa8	-0.15	1.00000000	chicken
Ypel5	-0.07	1.00000000	chicken
Ppig	-0.14	1.00000000	chicken
Fus	-0.11	1.00000000	chicken
Arhgef7	0.02	1.00000000	chicken
Rsrp1	-0.17	1.00000000	chicken
Adk	-0.11	1.00000000	chicken
Rab39b	-0.15	1.00000000	chicken
Tm9sf5	-0.04	1.00000000	chicken
Wdr74	-0.09	1.00000000	chicken
Reps2	0.08	1.00000000	chicken
Trim8	-0.14	1.00000000	chicken
Nifk	-0.20	1.00000000	chicken
Yme1l1	-0.16	1.00000000	chicken
Tgfbr1	-0.17	1.00000000	chicken
Ktn1	-0.09	1.00000000	chicken
Hic2	0.10	1.00000000	chicken
Rab5if	-0.09	1.00000000	chicken
Sf3b5	-0.08	1.00000000	chicken
Ogfr	-0.11	1.00000000	chicken
Efhd2	-0.10	1.00000000	chicken
Eif2ak1	-0.07	1.00000000	chicken
Tex264	-0.10	1.00000000	chicken
Ate1	0.01	1.00000000	chicken
Kdm3b	-0.14	1.00000000	chicken
Actr1a	-0.16	1.00000000	chicken

Ilf2	-0.21	1.00000000	chicken
Nbas	-0.17	1.00000000	chicken
Wdr82	-0.19	1.00000000	chicken
Rnf166	0.09	1.00000000	chicken
Hells	-0.08	1.00000000	chicken
Inpp5a	0.10	1.00000000	chicken
Ppm1b	-0.10	1.00000000	chicken
Cop1	-0.17	1.00000000	chicken
9930021J03Rik	-0.18	1.00000000	chicken
Nol10	-0.01	1.00000000	chicken
Atp6v1a	-0.19	1.00000000	chicken
Paip2	-0.10	1.00000000	chicken
Dcp1b	-0.12	1.00000000	chicken
Spopl	-0.13	1.00000000	chicken
Twf2	-0.07	1.00000000	chicken
Odc1	-0.12	1.00000000	chicken
Slc9a3r1	-0.10	1.00000000	chicken
Pebp1	-0.06	1.00000000	chicken
Ivns1abp	-0.24	1.00000000	chicken
Usp19	-0.11	1.00000000	chicken
Wdr43	-0.19	1.00000000	chicken
Eif3i	-0.17	1.00000000	chicken
Inpp4a	0.07	1.00000000	chicken
Zbtb2	-0.14	1.00000000	chicken
U2surp	-0.07	1.00000000	chicken
Acaca	-0.10	1.00000000	chicken
Chchd1	-0.04	1.00000000	chicken
Hspd1	-0.10	1.00000000	chicken
Skp1	-0.16	1.00000000	chicken
Polr2b	-0.21	1.00000000	chicken
Eif4h	-0.09	1.00000000	chicken
Ttc13	-0.16	1.00000000	chicken
Zfp407	-0.01	1.00000000	chicken
Hsbp1	-0.06	1.00000000	chicken
Tlk2	-0.23	1.00000000	chicken
Nrbf2	-0.16	1.00000000	chicken
Hsp90aa1	-0.09	1.00000000	chicken
Ttc3	-0.02	1.00000000	chicken
Actb	0.51	1.00000000	chicken
Nxt2	-0.16	1.00000000	chicken
Prrc2b	-0.17	1.00000000	chicken
Eml3	-0.03	1.00000000	chicken
Pbxip1	-0.12	1.00000000	chicken
Bclaf3	-0.19	1.00000000	chicken
Nfkbib	-0.07	1.00000000	chicken
Atp9b	-0.16	1.00000000	chicken

Idh1	-0.07	1.00000000	chicken
Gzf1	-0.07	1.00000000	chicken
Gfpt2	-0.09	1.00000000	chicken
Nrf1	-0.09	1.00000000	chicken
Hmgn3	-0.03	1.00000000	chicken
Cnot8	-0.14	1.00000000	chicken
Rchy1	-0.16	1.00000000	chicken
C1galt1	-0.11	1.00000000	chicken
Rnf19a	-0.21	1.00000000	chicken
Btg1b	0.05	1.00000000	chicken
Kcnq5	-0.06	1.00000000	chicken
Ubp1	-0.16	1.00000000	chicken
Nop2	-0.11	1.00000000	chicken
Tmf1	-0.14	1.00000000	chicken
Crlf3	-0.07	1.00000000	chicken
Chmp2a	-0.16	1.00000000	chicken
Smg6	-0.11	1.00000000	chicken
Tfip11	-0.13	1.00000000	chicken
Slc10a7	-0.08	1.00000000	chicken
Kif5c	0.08	1.00000000	chicken
Arih1	-0.07	1.00000000	chicken
Dctn4	-0.10	1.00000000	chicken
Proser1	-0.11	1.00000000	chicken
Scaf8	-0.13	1.00000000	chicken
Zcchc7	-0.24	1.00000000	chicken
Spred2	-0.03	1.00000000	chicken
Arntl	-0.12	1.00000000	chicken
Pdcd4	-0.17	1.00000000	chicken
Wdr45b	-0.07	1.00000000	chicken
Ep400	-0.14	1.00000000	chicken
Rmdn3	-0.08	1.00000000	chicken
Naa50	-0.22	1.00000000	chicken
Slc39a10	-0.15	1.00000000	chicken
Slc25a5	-0.14	1.00000000	chicken
Ube4a	-0.14	1.00000000	chicken
Adck1	-0.13	1.00000000	chicken
Arhgap5	-0.15	1.00000000	chicken
Vcp	-0.13	1.00000000	chicken
Sarnp	-0.17	1.00000000	chicken
Ggps1	-0.13	1.00000000	chicken
Bap1	-0.13	1.00000000	chicken
Phf20l1	-0.16	1.00000000	chicken
Rbm22	-0.21	1.00000000	chicken
Rrs1	-0.18	1.00000000	chicken
Thumpd3	-0.11	1.00000000	chicken
Stat3	-0.04	1.00000000	chicken

Lancl2	-0.01	1.00000000	chicken
Snx20	-0.03	1.00000000	chicken
Cul3	0.00	1.00000000	chicken
Prpf19	-0.08	1.00000000	chicken
Pum1	0.02	1.00000000	chicken
Srrm1	-0.12	1.00000000	chicken
Zmat2	-0.16	1.00000000	chicken
Lbh	-0.08	1.00000000	chicken
Ndufb4	-0.08	1.00000000	chicken
Cd63	-0.19	1.00000000	chicken
Tbc1d2b	-0.09	1.00000000	chicken
Itfg1	-0.14	1.00000000	chicken
Tial1	-0.12	1.00000000	chicken
Slc30a9	-0.16	1.00000000	chicken
Stau1	-0.07	1.00000000	chicken
Zfp598	-0.08	1.00000000	chicken
Dcp1a	-0.12	1.00000000	chicken
Klf11	-0.11	1.00000000	chicken
Sdhd	-0.10	1.00000000	chicken
Rnf146	-0.14	1.00000000	chicken
Gnpat	-0.11	1.00000000	chicken
Pofut1	-0.14	1.00000000	chicken
Ube2r2	-0.16	1.00000000	chicken
Phlpp1	-0.02	1.00000000	chicken
Emc3	-0.13	1.00000000	chicken
Dnajc7	-0.15	1.00000000	chicken
Ube3a	-0.09	1.00000000	chicken
Bud31	-0.08	1.00000000	chicken
Blk	-0.03	1.00000000	chicken
Rab21	-0.17	1.00000000	chicken
Snx29	-0.14	1.00000000	chicken
Slc30a5	-0.02	1.00000000	chicken
Arcn1	-0.15	1.00000000	chicken
Fahd2a	-0.10	1.00000000	chicken
Fkbp8	-0.09	1.00000000	chicken
Herpud2	-0.08	1.00000000	chicken
Eif4e2	-0.18	1.00000000	chicken
Acvr1	-0.23	1.00000000	chicken
Acot7	-0.12	1.00000000	chicken
Exoc6b	-0.10	1.00000000	chicken
Nup205	-0.05	1.00000000	chicken
Ipo8	-0.04	1.00000000	chicken
Ppp1r10	-0.07	1.00000000	chicken
Iars	-0.12	1.00000000	chicken
Reps1	-0.06	1.00000000	chicken
Ino80	-0.16	1.00000000	chicken

Polr2l	-0.05	1.00000000	chicken
Cars2	-0.18	1.00000000	chicken
Nemf	-0.06	1.00000000	chicken
Gcn1	-0.20	1.00000000	chicken
Clk2	-0.15	1.00000000	chicken
Canx	-0.15	1.00000000	chicken
Pcmtd1	0.00	1.00000000	chicken
Cyp2r1	-0.04	1.00000000	chicken
Pnisr	-0.08	1.00000000	chicken
Timm17a	-0.08	1.00000000	chicken
Fam104a	-0.06	1.00000000	chicken
Ints2	-0.11	1.00000000	chicken
Sap18	-0.11	1.00000000	chicken
Peak1	-0.02	1.00000000	chicken
Upf1	-0.08	1.00000000	chicken
Lats1	-0.19	1.00000000	chicken
Uqcrh	-0.12	1.00000000	chicken
Letm1	-0.14	1.00000000	chicken
Nop56	-0.18	1.00000000	chicken
Bckdhb	-0.02	1.00000000	chicken
Srpk1	-0.15	1.00000000	chicken
Cnot9	-0.11	1.00000000	chicken
C1d	-0.20	1.00000000	chicken
Atxn7	0.07	1.00000000	chicken
Bop1	-0.07	1.00000000	chicken
Fbxo8	-0.12	1.00000000	chicken
Glrx5	-0.10	1.00000000	chicken
Ptp4a1	-0.18	1.00000000	chicken
Romo1	-0.14	1.00000000	chicken
Atxn10	-0.15	1.00000000	chicken
Eif4ebp2	-0.13	1.00000000	chicken
Zc3h13	-0.08	1.00000000	chicken
Brix1	-0.08	1.00000000	chicken
Scaper	-0.12	1.00000000	chicken
Tasor2	-0.09	1.00000000	chicken
Edf1	-0.12	1.00000000	chicken
Xpo4	-0.06	1.00000000	chicken
Tfrc	-0.17	1.00000000	chicken
Gdi2	-0.12	1.00000000	chicken
Fgd3	-0.06	1.00000000	chicken
Sel1l3	-0.13	1.00000000	chicken
Ints6	-0.14	1.00000000	chicken
Nabp1	-0.13	1.00000000	chicken
Eps15	-0.19	1.00000000	chicken
Smarcc1	-0.07	1.00000000	chicken
Usp16	-0.16	1.00000000	chicken

Prpf6	-0.11	1.00000000	chicken
Cog2	-0.10	1.00000000	chicken
Cul4a	-0.13	1.00000000	chicken
Sos1	-0.15	1.00000000	chicken
Psmd9	-0.14	1.00000000	chicken
Coro1c	0.07	1.00000000	chicken
Cyld	-0.09	1.00000000	chicken
Rbl2	-0.17	1.00000000	chicken
Gnl2	-0.19	1.00000000	chicken
Itpripl2	-0.13	1.00000000	chicken
Serp1	-0.04	1.00000000	chicken
Garem1	0.09	1.00000000	chicken
Ube2d1	-0.18	1.00000000	chicken
Polr3b	-0.07	1.00000000	chicken
Cdk12	-0.10	1.00000000	chicken
Cdc37	-0.10	1.00000000	chicken
Pogz	-0.08	1.00000000	chicken
Lars	-0.01	1.00000000	chicken
Ptpn6	-0.24	1.00000000	chicken
Ppil4	-0.12	1.00000000	chicken
Traf3ip3	-0.16	1.00000000	chicken
Ilk	-0.11	1.00000000	chicken
Scamp1	-0.13	1.00000000	chicken
Ufl1	-0.12	1.00000000	chicken
Nkiras2	-0.08	1.00000000	chicken
Cops2	-0.26	1.00000000	chicken
Zfp830	-0.18	1.00000000	chicken
Arfip2	-0.13	1.00000000	chicken
Zfp706	-0.12	1.00000000	chicken
Ndufs5	-0.10	1.00000000	chicken
Slain2	-0.09	1.00000000	chicken
Ift20	-0.09	1.00000000	chicken
Atf1	-0.12	1.00000000	chicken
Dcp2	-0.12	1.00000000	chicken
Wdr13	-0.08	1.00000000	chicken
Crnkl1	-0.12	1.00000000	chicken
Sdk1	-0.01	1.00000000	chicken
Lman1	-0.15	1.00000000	chicken
Wdr48	-0.05	1.00000000	chicken
Lars2	-0.15	1.00000000	chicken
Slc7a3	-0.14	1.00000000	chicken
Yy2	-0.21	1.00000000	chicken
Polr1c	-0.10	1.00000000	chicken
Papola	-0.09	1.00000000	chicken
Txnrd1	-0.03	1.00000000	chicken
Hnrnpd	-0.14	1.00000000	chicken

Pspc1	-0.05	1.00000000	chicken
Wipi2	-0.06	1.00000000	chicken
Tapbp	-0.06	1.00000000	chicken
Pdxk	-0.12	1.00000000	chicken
Thoc1	-0.14	1.00000000	chicken
Lrif1	-0.10	1.00000000	chicken
Ei24	-0.09	1.00000000	chicken
Kbtbd8	-0.12	1.00000000	chicken
Ppp1r2	-0.13	1.00000000	chicken
Eef1e1	-0.02	1.00000000	chicken
Cir1	-0.10	1.00000000	chicken
Cct6b	-0.09	1.00000000	chicken
Nab1	-0.13	1.00000000	chicken
Ifngr1	0.02	1.00000000	chicken
Sh3pxd2b	-0.12	1.00000000	chicken
Glyr1	-0.16	1.00000000	chicken
Psmb2	-0.13	1.00000000	chicken
Braf	-0.01	1.00000000	chicken
Tmem87a	-0.19	1.00000000	chicken
Rab5c	-0.12	1.00000000	chicken
Srfbp1	-0.13	1.00000000	chicken
Hk1	-0.09	1.00000000	chicken
Oat	-0.15	1.00000000	chicken
Trappc11	-0.11	1.00000000	chicken
Hint1	-0.08	1.00000000	chicken
Tmem165	-0.10	1.00000000	chicken
Rpn2	-0.12	1.00000000	chicken
Nsmaf	-0.08	1.00000000	chicken
Cep350	-0.10	1.00000000	chicken
H13	-0.13	1.00000000	chicken
Tmem115	-0.13	1.00000000	chicken
Snrpg	-0.10	1.00000000	chicken
Senp5	-0.17	1.00000000	chicken
Ppp2r5a	-0.14	1.00000000	chicken
1810058I24Rik	-0.04	1.00000000	chicken
Usf3	-0.15	1.00000000	chicken
Acad9	-0.12	1.00000000	chicken
Exoc5	-0.05	1.00000000	chicken
Smarca1	-0.10	1.00000000	chicken
Tgfbrap1	-0.18	1.00000000	chicken
Tcf25	-0.14	1.00000000	chicken
Dennd6a	-0.17	1.00000000	chicken
Tmed10	-0.04	1.00000000	chicken
Lrrc28	-0.08	1.00000000	chicken
Bzw1	-0.16	1.00000000	chicken
G3bp2	-0.09	1.00000000	chicken

Ctnnbl1	-0.12	1.00000000	chicken
Sesn1	0.01	1.00000000	chicken
Ptprs	-0.30	1.00000000	chicken
Abcd2	-0.06	1.00000000	chicken
Rac1	-0.11	1.00000000	chicken
Parp4	-0.18	1.00000000	chicken
Virma	-0.09	1.00000000	chicken
Rfxap	-0.15	1.00000000	chicken
Tprgl	-0.08	1.00000000	chicken
Glrx3	-0.18	1.00000000	chicken
Rufy3	-0.18	1.00000000	chicken
Fyb	-0.11	1.00000000	chicken
Dcun1d3	-0.13	1.00000000	chicken
Rab31	0.01	1.00000000	chicken
Letm2	-0.03	1.00000000	chicken
Ddx23	-0.12	1.00000000	chicken
Arpc4	-0.04	1.00000000	chicken
Paxip1	-0.07	1.00000000	chicken
Btf3l4	-0.05	1.00000000	chicken
Lag3	-0.12	1.00000000	chicken
Tcp1	-0.18	1.00000000	chicken
Osbpl8	-0.17	1.00000000	chicken
Otub1	-0.12	1.00000000	chicken
Arf4	-0.12	1.00000000	chicken
G2e3	-0.09	1.00000000	chicken
Polr2m	-0.10	1.00000000	chicken
Lrpprc	-0.08	1.00000000	chicken
Ing1	-0.07	1.00000000	chicken
Fam117a	-0.01	1.00000000	chicken
Stim1	-0.18	1.00000000	chicken
Ppp1ca	-0.13	1.00000000	chicken
BC005537	-0.14	1.00000000	chicken
Nqo2	-0.11	1.00000000	chicken
Ttf1	-0.15	1.00000000	chicken
Gzma	0.00	1.00000000	chicken
Wdr36	-0.11	1.00000000	chicken
Celf1	-0.08	1.00000000	chicken
Lta4h	-0.09	1.00000000	chicken
Utp3	-0.16	1.00000000	chicken
Lrch2	-0.15	1.00000000	chicken
Cnot11	-0.11	1.00000000	chicken
Plekho1	0.02	1.00000000	chicken
Psmd14	-0.12	1.00000000	chicken
Nanp	-0.15	1.00000000	chicken
Ubap2l	-0.08	1.00000000	chicken
Stx18	-0.06	1.00000000	chicken

Zfp143	-0.02	1.00000000	chicken
Ddost	-0.08	1.00000000	chicken
Acbd6	-0.11	1.00000000	chicken
Otud4	-0.21	1.00000000	chicken
Ppp4r1	-0.14	1.00000000	chicken
Clip4	-0.20	1.00000000	chicken
Ahsa2	-0.14	1.00000000	chicken
Stt3a	-0.10	1.00000000	chicken
Trpm7	-0.07	1.00000000	chicken
Kat7	-0.13	1.00000000	chicken
Golga1	-0.10	1.00000000	chicken
Ythdf2	-0.18	1.00000000	chicken
Usp33	-0.14	1.00000000	chicken
Sf3b1	-0.09	1.00000000	chicken
Usp15	-0.12	1.00000000	chicken
Tent2	-0.13	1.00000000	chicken
Rbbp6	-0.07	1.00000000	chicken
Taf4b	-0.02	1.00000000	chicken
Gpx4	-0.07	1.00000000	chicken
Xrn1	-0.19	1.00000000	chicken
Ran	-0.16	1.00000000	chicken
Ufd1	-0.08	1.00000000	chicken
Il18	-0.17	1.00000000	chicken
Slc35b3	-0.07	1.00000000	chicken
Dhx29	-0.06	1.00000000	chicken
Asb2	-0.18	1.00000000	chicken
Eif4g1	-0.12	1.00000000	chicken
Plac8	2.56	0.00000000	duck
Abtb2	2.61	0.00000000	duck
Snx10	2.80	0.00000000	duck
Fuca2	1.89	0.00000000	duck
S100a11	2.48	0.00000000	duck
Rac2	1.93	0.00000000	duck
Fabp3	1.95	0.00000000	duck
Hpgds	2.04	0.00000000	duck
Txn1	1.76	0.00000000	duck
Npc2	1.68	0.00000000	duck
Stat1	1.77	0.00000000	duck
F13a1	2.41	0.00000000	duck
Arhgdib	1.80	0.00000000	duck
Actb	1.65	0.00000000	duck
Fnip1	1.92	0.00000000	duck
Nfil3	1.95	0.00000000	duck
Anxa1	2.00	0.00000000	duck
Nfkbiz	1.50	0.00000000	duck
Crot	1.72	0.00000000	duck

Itsn1	2.11	0.00000000	duck
Ncf1	1.58	0.00000000	duck
Nfam1	1.85	0.00000000	duck
Aspg	2.09	0.00000000	duck
Sh3bgrl3	1.76	0.00000000	duck
Prok2	1.82	0.00000000	duck
Atp6v1b2	1.58	0.00000000	duck
Nampt	1.46	0.00000000	duck
Glrx	1.81	0.00000000	duck
Pgd	1.81	0.00000000	duck
Laptm5	1.30	0.00000000	duck
Srgn	1.15	0.00000000	duck
Lmnb1	1.72	0.00000000	duck
Arsb	1.85	0.00000000	duck
Angpt1	2.37	0.00000000	duck
Oat	1.51	0.00000000	duck
Sox6	-4.03	0.00000000	duck
Abcg1	1.83	0.00000000	duck
Smpd1	1.33	0.00000000	duck
Bri3	1.53	0.00000000	duck
G0s2	1.75	0.00000000	duck
Lrmda	2.29	0.00000000	duck
Gpx1	1.61	0.00000000	duck
Als2cl	1.64	0.00000000	duck
Ly96	1.55	0.00000000	duck
Trib1	1.36	0.00000000	duck
Ubl5b	1.27	0.00000000	duck
Pim3	1.37	0.00000000	duck
Cd9	1.43	0.00000000	duck
Ntng1	1.91	0.00000000	duck
Klf15	1.40	0.00000000	duck
Taldo1	1.46	0.00000000	duck
Ston2	1.71	0.00000000	duck
Vdac1	1.30	0.00000000	duck
Vsir	1.24	0.00000000	duck
Rhog	1.20	0.00000000	duck
Ptgs2	2.00	0.00000000	duck
Lamp1	1.10	0.00000000	duck
Arpc1b	1.33	0.00000000	duck
Ptafr	1.38	0.00000000	duck
Adora2b	1.51	0.00000000	duck
Cpb2	1.85	0.00000000	duck
Serpine2	1.26	0.00000000	duck
Chd7	1.38	0.00000000	duck
Klf13	-1.57	0.00000000	duck
Rxra	1.42	0.00000000	duck

Wdr37	1.41	0.00000000	duck
Ttc39b	1.25	0.00000000	duck
Numb	1.36	0.00000000	duck
Pid1	1.93	0.00000000	duck
Emb	1.30	0.00000000	duck
Wnt5b	1.22	0.00000000	duck
Tgds	1.15	0.00000000	duck
Ica1	1.57	0.00000000	duck
Trim24	-2.08	0.00000000	duck
Anxa11	1.18	0.00000000	duck
Dach2	1.37	0.00000000	duck
Sos1	-1.82	0.00000000	duck
Pnrc1	1.10	0.00000000	duck
Tpmt	1.17	0.00000000	duck
Lsp1	1.33	0.00000000	duck
Atp6v1h	1.20	0.00000000	duck
Samhd1	1.15	0.00000000	duck
Fzd6	1.51	0.00000000	duck
Amfr	1.03	0.00000000	duck
Dcaf6	1.34	0.00000000	duck
Riok3	1.00	0.00000000	duck
Atf4	1.06	0.00000000	duck
Nadk	1.25	0.00000000	duck
Traf3	0.88	0.00000000	duck
Inpp5d	-2.44	0.00000000	duck
Tax1bp1	1.19	0.00000000	duck
Csrp1	1.07	0.00000000	duck
Blnk	1.87	0.00000000	duck
Rundc1	1.15	0.00000000	duck
Coro1c	0.94	0.00000000	duck
Pkm	1.11	0.00000000	duck
Capn7	-3.47	0.00000000	duck
Tor4a	1.33	0.00000000	duck
Slc7a11	1.50	0.00000000	duck
Ypel5	1.18	0.00000000	duck
Chst12	1.03	0.00000000	duck
Atp6v1g3	1.12	0.00000000	duck
Bcas3	1.48	0.00000000	duck
Synm	-3.17	0.00000000	duck
Atf3	1.30	0.00000000	duck
Crem	1.03	0.00000000	duck
Dot1l	1.19	0.00000000	duck
Gch1	1.14	0.00000000	duck
Cask	1.70	0.00000000	duck
Actr2	0.99	0.00000000	duck
Klf6	1.17	0.00000000	duck

Diaph2	0.96	0.00000000	duck
Acss1	0.83	0.00000000	duck
Ipmk	-1.87	0.00000000	duck
Acsbg2	1.11	0.00000000	duck
Fry	1.15	0.00000000	duck
Optn	1.33	0.00000000	duck
Cenpu	1.19	0.00000000	duck
Atp6ap2	0.88	0.00000000	duck
Dop1b	1.28	0.00000000	duck
Sacm1l	1.18	0.00000000	duck
Panx2	0.96	0.00000000	duck
Tmem250-ps	1.01	0.00000000	duck
Kif21a	0.98	0.00000000	duck
Vav3	0.99	0.00000000	duck
Tnfaip3	0.86	0.00000000	duck
Cdk14	-2.08	0.00000000	duck
Epsti1	1.15	0.00000000	duck
Fem1c	0.98	0.00000000	duck
Rassf2	0.93	0.00000000	duck
Bambi	1.48	0.00000000	duck
Pam	1.28	0.00000000	duck
Pla2g4a	1.23	0.00000000	duck
Skap1	-1.74	0.00000000	duck
Dctn3	0.89	0.00000000	duck
Plekhb2	0.95	0.00000000	duck
Pigx	0.82	0.00000000	duck
Rnf19a	0.97	0.00000000	duck
Tst	1.07	0.00000000	duck
Ptprm	1.94	0.00000000	duck
Gng7	0.94	0.00000000	duck
Sat1	0.79	0.00000000	duck
Neurl1b	1.07	0.00000000	duck
Cebpb	0.64	0.00000000	duck
Gnpda1	0.80	0.00000000	duck
Tcirg1	0.98	0.00000000	duck
Rasa2	-1.70	0.00000000	duck
Kcnk5	0.95	0.00000000	duck
Ilk	0.95	0.00000000	duck
Acsl1	0.94	0.00000000	duck
Acod1	1.40	0.00000000	duck
Ctsc	1.00	0.00000000	duck
Arpc5	0.89	0.00000000	duck
Fli1	0.75	0.00000000	duck
Sh3kbp1	0.93	0.00000000	duck
Fth1	1.24	0.00000000	duck
Rnf19b	1.08	0.00000000	duck

Ero1a	0.95	0.00000000	duck
Zfp516	1.20	0.00000000	duck
Nabp1	0.96	0.00000000	duck
Ostm1	0.97	0.00000000	duck
Igsf9	1.04	0.00000000	duck
Plin2	0.93	0.00000000	duck
Polr2h	0.86	0.00000000	duck
Spi1	0.82	0.00000000	duck
Arpc3	0.92	0.00000000	duck
Cblb	-1.73	0.00000000	duck
Pef1	0.86	0.00000000	duck
Sesn1	-1.61	0.00000000	duck
Tbc1d2	0.85	0.00000000	duck
Nucb2	-1.43	0.00000000	duck
Plek	0.99	0.00000000	duck
Ccpg1	0.91	0.00000000	duck
Lpgat1	1.05	0.00000000	duck
Fam107b	0.77	0.00000000	duck
Atp6v1d	1.00	0.00000000	duck
Lasp1	1.01	0.00000000	duck
Yipf3	0.91	0.00000000	duck
Tubd1	0.98	0.00000000	duck
Foxo1	-2.03	0.00000000	duck
Prkd1	-1.98	0.00000000	duck
Itm2b	1.03	0.00000000	duck
Cebpa	1.09	0.00000000	duck
Gpr137b	1.15	0.00000000	duck
Trak1	1.19	0.00000000	duck
Klf10	0.92	0.00000000	duck
Asah1	1.01	0.00000000	duck
Ube3c	1.08	0.00000000	duck
Irak2	0.71	0.00000000	duck
Hibadh	1.28	0.00000000	duck
Crispld2	0.87	0.00000000	duck
Lrba	-1.78	0.00000000	duck
Psma7	0.88	0.00000000	duck
Lcp1	0.84	0.00000000	duck
Nudt12	0.98	0.00000000	duck
Zbtb44	0.88	0.00000000	duck
Tbc1d16	0.81	0.00000000	duck
Apobec1	0.74	0.00000000	duck
Slc39a13	0.71	0.00000000	duck
Atp6v0e	0.83	0.00000000	duck
Dtl	0.94	0.00000000	duck
Tulp1	0.76	0.00000000	duck
Hnrnpll	0.63	0.00000000	duck

Gabarapl2	1.06	0.00000000	duck
Il21r	-1.85	0.00000000	duck
Zc3h12a	0.94	0.00000000	duck
Zmynd11	-1.49	0.00000000	duck
Sh3bgrl	0.83	0.00000000	duck
Slc9a3r1	0.97	0.00000000	duck
Baz1b	-1.40	0.00000000	duck
Capg	1.00	0.00000000	duck
Chst13	0.76	0.00000000	duck
Plxnc1	0.83	0.00000000	duck
Dnajc3	0.82	0.00000000	duck
Ncald	-1.62	0.00000000	duck
Nrip1	0.60	0.00000000	duck
Pik3r1	-1.02	0.00000000	duck
Stom	0.96	0.00000000	duck
Itgav	1.02	0.00000000	duck
Ugt8a	0.83	0.00000000	duck
Cdc34	0.94	0.00000000	duck
Romo1	0.82	0.00000000	duck
Ninj1	0.76	0.00000000	duck
Epb41	-2.09	0.00000000	duck
Helq	0.89	0.00000000	duck
Gnai2	0.91	0.00000000	duck
Rab1a	0.81	0.00000000	duck
Capn10	-1.61	0.00000000	duck
Tet1	-1.70	0.00000000	duck
Limd2	0.86	0.00000000	duck
Adgrg3	0.81	0.00000000	duck
Arg2	0.89	0.00000000	duck
Tfrc	-1.40	0.00000000	duck
Sri	0.93	0.00000000	duck
Smarcd2	0.75	0.00000000	duck
Ptpre	0.68	0.00000000	duck
Oaz1	0.77	0.00000000	duck
Srp19	0.78	0.00000000	duck
Trim25	0.88	0.00000000	duck
Psmb2	0.91	0.00000000	duck
Pparg	0.44	0.00000000	duck
Lrrk2	0.79	0.00000000	duck
Phactr4	1.02	0.00000000	duck
Itk	-2.08	0.00000000	duck
Dazap2	0.61	0.00000000	duck
Tmed5	0.99	0.00000000	duck
Kif15	1.13	0.00000000	duck
Tspo	0.79	0.00000000	duck
Ehd4	0.76	0.00000000	duck

Trib2	-1.25	0.00000000	duck
Atp11c	0.98	0.00000000	duck
Serp1	0.79	0.00000000	duck
Dcun1d4	-1.34	0.00000000	duck
Tnrc6b	-2.57	0.00000000	duck
Rsu1	0.79	0.00000000	duck
Serinc5	-1.30	0.00000000	duck
Ikzf1	-1.41	0.00000000	duck
Nmnat2	0.81	0.00000000	duck
Irf1	-1.25	0.00000000	duck
Efhd2	0.88	0.00000000	duck
Frmd4b	1.07	0.00000000	duck
Cux1	0.74	0.00000000	duck
Arl6ip5	1.05	0.00000000	duck
Bud31	0.71	0.00000000	duck
Rnf166	0.71	0.00000000	duck
Diablo	0.73	0.00000000	duck
Acp2	0.81	0.00000000	duck
Psma3	0.86	0.00000000	duck
Garem1	0.91	0.00000000	duck
Arhgap15	0.85	0.00000000	duck
Abca3	0.80	0.00000000	duck
Jund	0.78	0.00000000	duck
Csf3r	0.79	0.00000000	duck
Rnf11	0.67	0.00000000	duck
Slc7a6	0.76	0.00000000	duck
Zc3h12d	-1.36	0.00000000	duck
Unc13d	0.87	0.00000000	duck
Pik3cb	0.77	0.00000000	duck
Nrp2	0.89	0.00000000	duck
Pank3	-1.14	0.00000000	duck
Itm2a	0.58	0.00000000	duck
Ankrd44	-1.44	0.00000000	duck
Pgm1	0.76	0.00000000	duck
Prelid3a	0.67	0.00000000	duck
Naga	0.68	0.00000000	duck
Suclg1	0.72	0.00000000	duck
Ap1s2	0.70	0.00000000	duck
Tox	-2.03	0.00000000	duck
Osbpl6	1.26	0.00000000	duck
Tcf7	-2.26	0.00000000	duck
Fyn	0.67	0.00000000	duck
Vmp1	0.79	0.00000000	duck
Afg3l2	0.87	0.00000000	duck
Hmox1	0.80	0.00000000	duck
Actr3	0.71	0.00000000	duck

Nceh1	0.63	0.00000000	duck
Runx3	-1.47	0.00000000	duck
Supt4a	0.74	0.00000000	duck
Rnf130	0.81	0.00000000	duck
Acap2	0.67	0.00000000	duck
Cnot6l	-1.36	0.00000000	duck
Tm6sf1	0.74	0.00000000	duck
Arhgef26	0.76	0.00000000	duck
Dusp7	0.84	0.00000000	duck
Lgmn	0.56	0.00000000	duck
Ifngr2	0.60	0.00000000	duck
Drd4	0.44	0.00000000	duck
Lta4h	0.71	0.00000000	duck
Hras	0.84	0.00000000	duck
Tnfaip8l1	0.81	0.00000000	duck
Ric1	0.79	0.00000000	duck
Jade1	-1.59	0.00000000	duck
5031439G07Rik	0.80	0.00000000	duck
H13	0.76	0.00000000	duck
Pik3c2a	-1.87	0.00000000	duck
Slc3a1	0.75	0.00000000	duck
Adm	0.76	0.00000000	duck
Tkt	0.87	0.00000000	duck
Cpt1a	0.65	0.00000000	duck
Btf3	0.87	0.00000000	duck
Slc1a2	1.33	0.00000000	duck
Jade2	-1.08	0.00000000	duck
Dstn	0.57	0.00000000	duck
Itpk1	-1.65	0.00000000	duck
Tspan18	-1.21	0.00000000	duck
Cyrib	0.65	0.00000000	duck
Nfatc1	-1.90	0.00000000	duck
Glipr2	0.58	0.00000000	duck
Heg1	-1.24	0.00000000	duck
Galnt1	0.54	0.00000000	duck
Ptpn22	-1.14	0.00000000	duck
Dicer1	-1.45	0.00000000	duck
Arid4b	-1.29	0.00000000	duck
Setbp1	-1.77	0.00000000	duck
Actn1	0.68	0.00000000	duck
Fosl2	0.59	0.00000000	duck
Tsc22d3	-1.23	0.00000000	duck
Larp1b	-1.77	0.00000000	duck
Stat4	-1.18	0.00000000	duck
Ngf	0.83	0.00000000	duck
Ralgapa2	-1.55	0.00000000	duck

Sema4a	0.72	0.00000000	duck
Cables1	1.02	0.00000000	duck
Rgs19	0.73	0.00000000	duck
Rfesd	-2.37	0.00000000	duck
Fam32a	0.74	0.00000000	duck
Hmgn3	-1.78	0.00000000	duck
Rgs3	-1.28	0.00000000	duck
Bst1	0.28	0.00000000	duck
Nudt21	0.86	0.00000000	duck
Dipk1b	0.57	0.00000000	duck
Sp4	-1.21	0.00000000	duck
Nos2	2.09	0.00000000	duck
Fkbp5	-1.38	0.00000000	duck
Exoc6b	-1.59	0.00000000	duck
Dusp1	0.79	0.00000000	duck
Dnajb9	0.73	0.00000000	duck
Hs6st1	-1.56	0.00000000	duck
Ppm1k	0.63	0.00000000	duck
Gsn	0.82	0.00000000	duck
Xpo6	-1.35	0.00000000	duck
Skap2	0.79	0.00000000	duck
Blmh	0.64	0.00000000	duck
Ephx1	0.58	0.00000000	duck
Atp6v1a	0.81	0.00000000	duck
Palld	-2.34	0.00000000	duck
Rai1	-1.31	0.00000000	duck
Cemip2	0.81	0.00000000	duck
Acadm	0.69	0.00000000	duck
Gsr	0.52	0.00000000	duck
Ginm1	0.58	0.00000000	duck
Sucnr1	0.67	0.00000000	duck
Slc9a9	-1.58	0.00000000	duck
Itgb1	0.61	0.00000000	duck
Pygl	0.66	0.00000000	duck
Akirin2	0.39	0.00000000	duck
Bach1	0.48	0.00000000	duck
Aplf	0.92	0.00000000	duck
Aplp2	0.47	0.00000000	duck
Cotl1	0.55	0.00000000	duck
Ctbs	0.69	0.00000000	duck
Wdr1	0.65	0.00000000	duck
Tmem258	0.75	0.00000000	duck
Jun	0.82	0.00000000	duck
Fzr1	0.72	0.00000000	duck
Dennd1b	-1.27	0.00000000	duck
Gnal	-1.78	0.00000000	duck

Abcc1	-1.80	0.00000000	duck
Rel	0.74	0.00000000	duck
Wdfy3	0.65	0.00000000	duck
Dgkz	-1.20	0.00000000	duck
Sstr4	0.59	0.00000000	duck
Erlec1	0.67	0.00000000	duck
Erbin	-1.40	0.00000000	duck
Eif2ak2	0.71	0.00000000	duck
Erp44	0.33	0.00000000	duck
Ndufb10	0.81	0.00000000	duck
Trps1	-0.84	0.00000000	duck
Abhd17a	0.63	0.00000000	duck
Pkn2	-1.50	0.00000000	duck
Slc12a4	-1.75	0.00000000	duck
Rgs1	-1.18	0.00000000	duck
Ralb	0.62	0.00000000	duck
Pik3ip1	-0.76	0.00000000	duck
Ptpn4	-1.77	0.00000000	duck
Ctss	0.88	0.00000000	duck
Wipi1	0.63	0.00000000	duck
Oser1	0.64	0.00000000	duck
Ago2	-1.46	0.00000000	duck
Zfp704	-1.08	0.00000000	duck
Lrpap1	0.70	0.00000000	duck
E2f2	0.60	0.00000000	duck
Traf5	-1.33	0.00000000	duck
Aopep	0.81	0.00000000	duck
Tnfrsf11a	0.67	0.00000000	duck
Anxa4	0.56	0.00000000	duck
Zfp706	0.69	0.00000000	duck
Itga4	-1.84	0.00000000	duck
Nt5m	0.46	0.00000000	duck
Samsn1	0.40	0.00000000	duck
Edem3	0.68	0.00000000	duck
Ctsb	0.70	0.00000000	duck
Phlpp2	0.55	0.00000000	duck
Rubcnl	0.59	0.00000000	duck
Efcab14	0.46	0.00000000	duck
Abca4	0.81	0.00000000	duck
Ilvbl	0.56	0.00000000	duck
Abca1	0.66	0.00000000	duck
Esd	0.59	0.00000000	duck
Sgpp1	0.44	0.00000000	duck
Tpm1	0.58	0.00000000	duck
Slc40a1	0.59	0.00000000	duck
Sh2b3	-0.92	0.00000000	duck

Rnf149	0.72	0.00000000	duck
Atp6v0c	0.75	0.00000000	duck
Chmp1a	0.58	0.00000000	duck
Slc15a4	0.57	0.00000001	duck
Jak2	-1.38	0.00000001	duck
Dctn6	0.64	0.00000001	duck
Arhgap10	-1.68	0.00000001	duck
Pard3	1.02	0.00000001	duck
Hpse	0.53	0.00000001	duck
Trappc3	0.65	0.00000001	duck
Adcy9	-1.84	0.00000001	duck
Ormdl2	0.74	0.00000001	duck
Sptan1	-1.58	0.00000001	duck
Glcci1	-1.96	0.00000001	duck
Baz1a	0.59	0.00000001	duck
Xxylt1	0.72	0.00000001	duck
Enpp4	0.47	0.00000001	duck
Pde7b	-1.58	0.00000001	duck
Bcl11b	-1.50	0.00000001	duck
Wdr89	0.58	0.00000001	duck
Slc7a3	0.84	0.00000001	duck
Syap1	0.75	0.00000001	duck
Eif5b	0.57	0.00000001	duck
Pdss1	0.62	0.00000001	duck
Palb2	0.65	0.00000001	duck
Sestd1	0.70	0.00000001	duck
Ephx4	0.42	0.00000001	duck
Zfand3	0.52	0.00000001	duck
Klhl7	-1.61	0.00000001	duck
Cass4	0.49	0.00000001	duck
Syngr2	0.54	0.00000002	duck
Bach2	-0.66	0.00000002	duck
Clip1	0.68	0.00000002	duck
As3mt	0.52	0.00000002	duck
Arih1	-1.17	0.00000002	duck
Atp5b	0.72	0.00000002	duck
Ssr2	0.66	0.00000002	duck
Ccdc88a	-1.63	0.00000002	duck
Agtpbp1	0.75	0.00000002	duck
Eif4ebp1	0.66	0.00000003	duck
Kctd10	0.67	0.00000003	duck
Ppp1ca	0.53	0.00000003	duck
Pak3	1.11	0.00000003	duck
Primpol	0.75	0.00000003	duck
Chmp4b	0.56	0.00000003	duck
Tpm3-rs7	0.59	0.00000003	duck

Fam102b	0.52	0.00000004	duck
Ripor3	-1.89	0.00000004	duck
Klf7	0.82	0.00000004	duck
Pold2	0.54	0.00000004	duck
Usp44	0.65	0.00000004	duck
Pacsin2	0.52	0.00000004	duck
Blk	-1.07	0.00000004	duck
Dtx2	0.61	0.00000004	duck
Eif3g	0.66	0.00000004	duck
Gabrr3	0.68	0.00000004	duck
Dcp1a	0.42	0.00000005	duck
Fem1a	0.50	0.00000005	duck
Osbpl1a	-1.28	0.00000005	duck
Echdc2	0.60	0.00000005	duck
Il2rb	-1.40	0.00000005	duck
Dusp16	0.70	0.00000005	duck
Rnf13	0.61	0.00000005	duck
Laptm4a	0.48	0.00000005	duck
Hbegf	0.27	0.00000005	duck
Eif1	0.51	0.00000005	duck
Pfdn1	0.57	0.00000006	duck
Ptpn2	-0.93	0.00000006	duck
Depdc1b	0.94	0.00000006	duck
Calm1	0.60	0.00000007	duck
Eps15	0.71	0.00000007	duck
Cebpd	0.50	0.00000007	duck
Abhd6	0.62	0.00000007	duck
Dtwd1	0.66	0.00000007	duck
Fhl3	0.69	0.00000008	duck
Abhd5	0.46	0.00000008	duck
Card11	-0.95	0.00000008	duck
Grb2	0.55	0.00000008	duck
Ube2j1	0.57	0.00000008	duck
Cxcr6	-1.04	0.00000009	duck
Chsy1	0.44	0.00000010	duck
Sp3	-0.94	0.00000010	duck
Ric8a	0.57	0.00000010	duck
Traf2	0.44	0.00000010	duck
Derl3	0.55	0.00000010	duck
Ski	0.47	0.00000010	duck
Sgms1	0.55	0.00000011	duck
Capzb	0.56	0.00000011	duck
Pou2f1	0.27	0.00000011	duck
Reep5	0.72	0.00000011	duck
Dynlt1a	0.67	0.00000011	duck
Lpxn	0.73	0.00000011	duck

Cd47	0.52	0.00000011	duck
Ensa	0.60	0.00000012	duck
Sppl3	0.63	0.00000012	duck
Upp1	0.62	0.00000013	duck
Dhrs3	0.52	0.00000015	duck
Cdc42se1	0.71	0.00000015	duck
Aff2	-1.73	0.00000016	duck
Nck2	0.40	0.00000018	duck
Zeb2	0.98	0.00000019	duck
Septin12	0.40	0.00000020	duck
Ssbp2	-2.00	0.00000020	duck
Golim4	0.67	0.00000022	duck
Scaf8	-1.13	0.00000022	duck
Tpi1	0.76	0.00000023	duck
Trmt1l	-0.86	0.00000024	duck
Pdcd10	0.56	0.00000024	duck
Btd	0.49	0.00000026	duck
Top1	0.27	0.00000027	duck
Arf6	0.63	0.00000028	duck
Pik3r5	0.37	0.00000029	duck
Rab27a	0.68	0.00000029	duck
Snrk	-1.15	0.00000031	duck
Ndufs4	0.58	0.00000034	duck
Nav3	-0.96	0.00000034	duck
Ldah	0.58	0.00000038	duck
Cdk5rap1	0.52	0.00000040	duck
Entpd2	0.37	0.00000045	duck
Znrf2	-1.30	0.00000045	duck
Pak2	0.43	0.00000049	duck
Nfkb2	0.68	0.00000049	duck
Ehd3	0.49	0.00000054	duck
Nsmce2	0.63	0.00000055	duck
Snx2	0.46	0.00000056	duck
Rab11fip2	0.64	0.00000060	duck
Cmtm7	-0.68	0.00000061	duck
Kcnab2	0.59	0.00000063	duck
Srgap1	-1.50	0.00000064	duck
Dars	-1.00	0.00000064	duck
Zdhhc15	0.67	0.00000066	duck
Blzf1	0.54	0.00000068	duck
Scube2	0.51	0.00000071	duck
Iqgap2	-0.99	0.00000071	duck
Sort1	0.45	0.00000072	duck
Hectd1	0.55	0.00000073	duck
Chfr	0.49	0.00000073	duck
Gramd3	0.61	0.00000074	duck

Itm2c	-1.30	0.00000075	duck
Ostf1	0.68	0.00000077	duck
Shprh	0.37	0.00000079	duck
Osbpl9	0.65	0.00000083	duck
Syn2	0.90	0.00000086	duck
Lrp8	0.65	0.00000087	duck
Lyn	0.33	0.00000091	duck
Nfkbie	0.82	0.00000094	duck
Dapk1	-1.03	0.00000095	duck
Ankrd28	0.50	0.00000095	duck
Atp23	0.48	0.00000096	duck
Ampd3	0.54	0.00000104	duck
Vps4b	0.64	0.00000106	duck
Polr2e	0.64	0.00000107	duck
Vps54	0.74	0.00000108	duck
Prdx1	0.54	0.00000108	duck
Timp4	0.64	0.00000109	duck
Itpka	0.56	0.00000111	duck
Rhoa	0.54	0.00000113	duck
Ssr1	0.58	0.00000116	duck
Poldip3	0.62	0.00000127	duck
Enpp3	0.72	0.00000130	duck
Znfx1	0.38	0.00000140	duck
Hsbp1	0.52	0.00000150	duck
Wdr81	0.52	0.00000156	duck
Sdk1	-0.89	0.00000163	duck
Nfe2l2	0.13	0.00000165	duck
Antxr2	0.15	0.00000169	duck
Snx17	0.59	0.00000176	duck
Serpini1	-1.16	0.00000177	duck
Ahsa1	0.49	0.00000188	duck
Gramd4	-1.15	0.00000191	duck
Ubr2	-1.27	0.00000195	duck
Dek	-0.72	0.00000220	duck
Herc4	0.41	0.00000226	duck
Itfg1	0.22	0.00000228	duck
Bhlhe41	0.45	0.00000233	duck
Fbxl4	0.31	0.00000242	duck
Gabarapl1	0.63	0.00000247	duck
Spopl	0.39	0.00000257	duck
Abcc4	-1.44	0.00000265	duck
Atp1b1	0.43	0.00000301	duck
Plcl2	-1.63	0.00000332	duck
Sh2d1a	-0.94	0.00000334	duck
Sdhb	0.50	0.00000340	duck
Kctd7	0.46	0.00000343	duck

Nuak1	0.58	0.00000345	duck
Ano10	0.94	0.00000362	duck
Smap2	0.43	0.00000363	duck
Lamtor5	0.63	0.00000370	duck
Arrdc4	0.39	0.00000376	duck
Dbnl	0.50	0.00000393	duck
Nup210l	0.48	0.00000401	duck
Rlim	0.50	0.00000406	duck
Sorbs2	-2.19	0.00000423	duck
Paqr7	0.47	0.00000424	duck
Tgfbr2	-0.85	0.00000436	duck
Tmem268	0.52	0.00000439	duck
Ythdc1	-0.88	0.00000443	duck
Hnrnpr	-0.80	0.00000448	duck
Ipo7	-0.82	0.00000456	duck
Asb3	0.70	0.00000495	duck
Il6ra	0.49	0.00000504	duck
Esrrg	-1.39	0.00000515	duck
Pdrg1	0.49	0.00000518	duck
Rasa1	-1.11	0.00000565	duck
Cept1	0.61	0.00000575	duck
Psma5	0.56	0.00000620	duck
Snx20	0.40	0.00000634	duck
Ndfip1	0.61	0.00000646	duck
Eif5a2	-1.03	0.00000646	duck
Sema3d	-1.60	0.00000663	duck
Efhd1	-1.13	0.00000664	duck
Ndufb6	0.56	0.00000691	duck
Bmp2k	0.63	0.00000716	duck
Txnrd1	1.04	0.00000722	duck
Dda1	0.48	0.00000733	duck
Ikzf2	0.66	0.00000739	duck
Psmb3	0.49	0.00000780	duck
Dele1	-1.16	0.00000809	duck
Cry1	-0.74	0.00000887	duck
Ubap1	0.45	0.00000899	duck
Arid4a	0.78	0.00000924	duck
Ccnh	0.49	0.00000936	duck
Cobll1	1.01	0.00000991	duck
Cntrl	-1.06	0.00000995	duck
Slc66a1	0.33	0.00001026	duck
Atp2b1	-1.20	0.00001218	duck
Rab7	0.62	0.00001219	duck
Cep170	-1.19	0.00001231	duck
Pds5b	-0.86	0.00001236	duck
Tmem35b	0.55	0.00001274	duck

Psmd2	0.58	0.00001392	duck
Wbp2nl	0.43	0.00001452	duck
Axin1	-1.20	0.00001480	duck
Fbxo8	0.51	0.00001482	duck
Cd82	-0.75	0.00001504	duck
Gpi1	0.57	0.00001614	duck
Ndufb3	0.48	0.00001635	duck
Plcb4	0.84	0.00001725	duck
Inpp5b	-0.62	0.00001744	duck
Furin	0.47	0.00001786	duck
Reps2	-0.01	0.00001808	duck
Coro1b	0.42	0.00001809	duck
Card9	0.36	0.00001859	duck
Slc25a16	-1.27	0.00001914	duck
Psma6	0.56	0.00001941	duck
Elovl5	0.45	0.00001996	duck
Adap2	0.46	0.00002101	duck
Rhoh	-0.61	0.00002121	duck
AU040320	0.51	0.00002148	duck
Plxna2	0.44	0.00002173	duck
Scarb1	0.34	0.00002208	duck
Ankrd11	0.42	0.00002212	duck
Adcy7	-1.98	0.00002242	duck
Eml4	0.69	0.00002271	duck
Tubb5	0.64	0.00002290	duck
Neto2	0.61	0.00002301	duck
Gata3	-0.43	0.00002385	duck
Rtf2	0.49	0.00002483	duck
Acox1	0.61	0.00002591	duck
Vapa	0.40	0.00002635	duck
Rpia	0.49	0.00002658	duck
Zc3h7a	-1.00	0.00002784	duck
Cers2	0.49	0.00002915	duck
Slc25a36	0.27	0.00002932	duck
Ost4	0.56	0.00002978	duck
Tbc1d2b	-1.06	0.00003037	duck
Gpr155	0.45	0.00003111	duck
Prkcq	-0.59	0.00003165	duck
Hipk3	0.60	0.00003245	duck
Lck	-0.50	0.00003332	duck
Zyx	0.38	0.00003354	duck
Calm2	0.42	0.00003429	duck
Gpr171	-1.00	0.00003462	duck
Ubash3a	-0.62	0.00003499	duck
Fam20c	0.48	0.00003503	duck
Taf12	0.60	0.00003556	duck

Dock9	-1.22	0.00003668	duck
Lpar4	-0.65	0.00003717	duck
Septin6	-0.77	0.00003740	duck
Ogfr	0.52	0.00003870	duck
Flii	0.57	0.00003910	duck
Auts2	-2.19	0.00004013	duck
Susd1	-0.30	0.00004269	duck
Fam193a	0.40	0.00004425	duck
Oxr1	-1.19	0.00004762	duck
Dapp1	0.18	0.00004944	duck
Fdx1	0.40	0.00005190	duck
Cnot9	0.47	0.00005383	duck
Syne3	-1.01	0.00005692	duck
Sh3pxd2a	0.55	0.00005699	duck
Endov	0.46	0.00005835	duck
Glod4	0.52	0.00005838	duck
Camk2g	0.43	0.00005979	duck
Relch	0.43	0.00006178	duck
Ddx5	-0.54	0.00006210	duck
Ppp2r2d	-0.86	0.00006434	duck
Disp1	-1.72	0.00006925	duck
Ankrd22	-0.35	0.00006937	duck
Ube2q2	-0.83	0.00006991	duck
Uhrf1bp1	-0.73	0.00007065	duck
Psmd6	0.34	0.00007101	duck
Btbd11	-0.12	0.00007115	duck
Atg7	0.45	0.00007157	duck
Ptpn6	0.62	0.00007350	duck
Rhoq	-0.53	0.00007380	duck
Rras2	-0.64	0.00007611	duck
Slc4a2	0.45	0.00007616	duck
Synrg	0.51	0.00007660	duck
Supt5	0.53	0.00008228	duck
Casp3	0.64	0.00008278	duck
Arntl2	0.25	0.00008490	duck
Ppara	0.79	0.00008534	duck
Nab1	0.63	0.00008908	duck
Pstpip1	-1.61	0.00009099	duck
Osbpl3	-1.03	0.00009220	duck
Zcchc7	0.29	0.00009242	duck
Zdhhc6	0.53	0.00009277	duck
Tgm4	0.87	0.00009289	duck
Commd4	0.58	0.00009386	duck
Ptp4a1	0.48	0.00009979	duck
Pamr1	0.42	0.00010118	duck
Tmem131	-1.04	0.00010170	duck

Lamtor2	0.49	0.00010310	duck
Spen	-1.10	0.00010459	duck
Stim2	-1.08	0.00010761	duck
Slc38a2	0.45	0.00010814	duck
Raver2	0.41	0.00010994	duck
Tmem42	0.39	0.00011440	duck
Ets1	-0.84	0.00011564	duck
Cdk13	-0.93	0.00012044	duck
Coro2a	0.50	0.00012344	duck
Wdr45b	0.46	0.00012703	duck
Caap1	0.41	0.00012768	duck
Cd3e	-0.64	0.00012844	duck
Bak1	-0.64	0.00013950	duck
Dcaf1	-0.87	0.00013957	duck
Adar	0.50	0.00013964	duck
Cd247	-1.09	0.00014096	duck
Sde2	0.28	0.00014447	duck
Coq5	0.41	0.00014792	duck
Tm9sf3	0.51	0.00015103	duck
Taar1	0.49	0.00015400	duck
Pgap2	0.31	0.00016527	duck
Paxip1	-1.17	0.00017059	duck
Scamp2	0.39	0.00017334	duck
Dcaf12	0.50	0.00017579	duck
Ncoa1	-0.58	0.00017635	duck
Rb1	-0.95	0.00017834	duck
Ppif	0.44	0.00017996	duck
Snx18	0.41	0.00018537	duck
Clcn7	0.49	0.00018736	duck
Agfg1	0.45	0.00018868	duck
B3gnt5	-0.65	0.00018916	duck
Vps18	0.41	0.00019079	duck
Tax1bp3	0.41	0.00019117	duck
Fam174a	0.44	0.00019257	duck
Grap2	-0.91	0.00020137	duck
Brsk2	-0.93	0.00020245	duck
Rasgef1b	0.49	0.00020538	duck
Ccdc71l	0.58	0.00020608	duck
Zfand2a	0.43	0.00022458	duck
Ash1l	-0.99	0.00022608	duck
Slc66a2	-0.63	0.00022961	duck
Galnt2	-0.75	0.00022999	duck
Herpud1	0.57	0.00023002	duck
Scml2	0.64	0.00023250	duck
Timp2	0.56	0.00024590	duck
Dnaja4	-1.01	0.00024613	duck

Rnf213	0.50	0.00024902	duck
Atp13a3	0.51	0.00025078	duck
Arhgap31	-0.58	0.00025728	duck
Atp10a	-0.59	0.00026077	duck
Cxcr4	-0.67	0.00026841	duck
Nt5e	0.41	0.00026860	duck
Ptx3	0.22	0.00028960	duck
Dennd5a	0.24	0.00028980	duck
Nudt3	0.18	0.00029276	duck
Yipf4	0.37	0.00029347	duck
Ppt1	0.37	0.00029401	duck
Ankrd27	-0.82	0.00029826	duck
Rasal2	-1.07	0.00030267	duck
Sub1	0.60	0.00030984	duck
Cops7a	0.36	0.00031629	duck
Ttc39a	0.32	0.00031839	duck
Samd4	0.67	0.00032531	duck
Nktr	-0.92	0.00033412	duck
Wdr91	0.37	0.00034002	duck
Ifnar1	-1.05	0.00035112	duck
Taok1	0.55	0.00035528	duck
Gab1	0.48	0.00036219	duck
Fam210b	0.33	0.00037319	duck
Cfd	0.59	0.00037449	duck
Amacr	0.55	0.00037627	duck
Tspan5	-0.78	0.00038144	duck
Naa16	-0.96	0.00038236	duck
Nsfl1c	0.44	0.00038756	duck
Ereg	0.33	0.00039327	duck
Fasn	-0.58	0.00039955	duck
Wdfy2	-0.92	0.00042819	duck
Pag1	-1.27	0.00043697	duck
Abraxas2	0.52	0.00044934	duck
Fam214a	0.60	0.00044990	duck
F3	0.79	0.00045568	duck
Slc26a4	0.39	0.00045804	duck
Dcp2	0.49	0.00046249	duck
Hk2	0.58	0.00046261	duck
Gpr27	0.30	0.00046744	duck
Prrc2b	-0.80	0.00046931	duck
Fam13a	0.60	0.00049472	duck
Pdlim5	0.72	0.00050808	duck
Urm1	0.57	0.00051002	duck
Ogt	-0.75	0.00054098	duck
Irf8	-0.87	0.00055328	duck
Ncf2	0.33	0.00056141	duck

Golph3l	0.39	0.00056586	duck
Slc17a5	0.27	0.00059123	duck
Rhbdf2	0.36	0.00059137	duck
Cep112	0.65	0.00063289	duck
Jmjd8	0.38	0.00066320	duck
Deptor	-0.48	0.00066678	duck
Psip1	-0.75	0.00067234	duck
Psmd7	0.53	0.00067336	duck
Aup1	0.52	0.00068068	duck
Nsmce1	-0.75	0.00070656	duck
Zfand5	0.64	0.00070769	duck
Acadl	0.36	0.00073762	duck
Kat6b	-0.78	0.00079309	duck
Dcun1d2	-1.34	0.00079412	duck
Slc4a7	-1.21	0.00079876	duck
Zmiz1	0.65	0.00080386	duck
Ints6	-0.71	0.00080621	duck
Irf2bpl	0.45	0.00081535	duck
Hic2	-1.05	0.00085167	duck
Prkx	-0.96	0.00085523	duck
Gxylt1	0.36	0.00087531	duck
Galnt17	0.50	0.00088930	duck
Sytl2	-0.59	0.00089099	duck
Bcap29	0.39	0.00090266	duck
Tiam1	-0.06	0.00090422	duck
Cdkn1a	0.35	0.00092929	duck
Ssbp3	-1.04	0.00094349	duck
Acvr1b	0.53	0.00094864	duck
Elovl6	-1.32	0.00095949	duck
Atp5g3	0.58	0.00096594	duck
Pdia5	0.32	0.00100350	duck
Gpatch2l	0.29	0.00101953	duck
Top3b	0.34	0.00102297	duck
Rbbp7	-0.61	0.00104047	duck
Lpar6	-1.09	0.00105200	duck
Per2	-0.95	0.00106741	duck
Parp10	0.28	0.00106828	duck
Ap2m1	0.40	0.00117838	duck
Jazf1	0.51	0.00119978	duck
Rnf144a	-1.22	0.00121451	duck
Crim1	-1.14	0.00127737	duck
Flnb	-1.28	0.00128018	duck
Echs1	0.37	0.00133331	duck
Hdac9	-0.87	0.00138227	duck
Far1	0.23	0.00143803	duck
Cog5	-1.26	0.00150876	duck

Klf12	-1.06	0.00152080	duck
Arhgef3	-0.93	0.00153449	duck
Gga2	-0.64	0.00155002	duck
Haus8	0.46	0.00159750	duck
Rbl1	-0.41	0.00160338	duck
1110059G10Rik	0.24	0.00161746	duck
Rap2a	0.33	0.00171247	duck
Herc1	-0.86	0.00174902	duck
Stk3	-1.37	0.00176226	duck
Sgce	-0.70	0.00188195	duck
Cryl1	0.41	0.00194390	duck
Evl	-0.88	0.00195779	duck
Lct	-0.50	0.00209352	duck
Ppp6c	0.45	0.00211812	duck
Abracl	0.46	0.00211936	duck
Prdm2	-0.70	0.00212684	duck
Cmtm6	0.54	0.00219242	duck
R3hdm1	-1.06	0.00230862	duck
Borcs5	0.54	0.00233685	duck
Rc3h1	-0.98	0.00235794	duck
Atp1a1	0.43	0.00244366	duck
Gusb	0.37	0.00246444	duck
Sf3b4	0.40	0.00248734	duck
Pgs1	0.49	0.00249212	duck
Sh3bp5	0.24	0.00250487	duck
Cwc15	0.43	0.00253411	duck
Chordc1	0.71	0.00255017	duck
Fcho2	0.19	0.00259857	duck
Kmt2e	-0.60	0.00262457	duck
Adrb1	-0.74	0.00267774	duck
Pgbd5	-0.67	0.00268428	duck
Traf7	0.39	0.00273428	duck
Inpp5a	-1.15	0.00277009	duck
Gak	-0.75	0.00279881	duck
Casd1	-1.66	0.00290043	duck
Pls3	-1.46	0.00290737	duck
Jade3	-0.81	0.00299671	duck
Hif1a	0.39	0.00303765	duck
Donson	0.34	0.00307497	duck
Prkag1	0.38	0.00309294	duck
Brd1	-0.94	0.00313706	duck
Naaladl2	-1.01	0.00314004	duck
Ndufs5	0.52	0.00324611	duck
Ammecr1	0.22	0.00337388	duck
Ank2	-0.85	0.00341411	duck
Ebag9	0.22	0.00342969	duck

Klhl12	0.23	0.00363585	duck
Sh3d19	0.44	0.00373716	duck
Cop1	0.03	0.00392231	duck
Zfp652	-0.73	0.00392998	duck
Adpgk	0.35	0.00405701	duck
Nkd1	0.24	0.00422895	duck
Zdhhc3	0.40	0.00426509	duck
Slc4a3	0.35	0.00432606	duck
Pdf	0.38	0.00449300	duck
Pstpip2	0.49	0.00450581	duck
Rpp38	0.30	0.00463358	duck
Kif5c	0.38	0.00465543	duck
Ciao2b	0.40	0.00475747	duck
Atp5a1	0.59	0.00485820	duck
Notch2	0.57	0.00490752	duck
Ubxn11	-0.41	0.00503504	duck
Smg5	0.35	0.00513570	duck
St3gal6	-0.83	0.00525908	duck
Rcn2	-0.89	0.00525980	duck
Zfhx3	0.50	0.00529876	duck
Rnf141	0.30	0.00537673	duck
Tmem251	0.39	0.00560339	duck
Cd74	-0.30	0.00575891	duck
Snx8	0.36	0.00579841	duck
2700049A03Rik	0.45	0.00600711	duck
Dcps	0.30	0.00602601	duck
Sec61g	0.59	0.00610153	duck
Usp25	0.57	0.00615551	duck
Cdk5rap2	-1.07	0.00628180	duck
Emc6	0.54	0.00634127	duck
Papln	0.43	0.00636038	duck
Unc119b	0.32	0.00636551	duck
Jmjd1c	-0.67	0.00652952	duck
Kansl1l	-0.85	0.00658826	duck
Rfc1	-0.64	0.00664548	duck
Fosb	1.21	0.00669332	duck
Usp48	-1.18	0.00671282	duck
Tdg	0.35	0.00676853	duck
Dennd6a	-0.65	0.00698458	duck
Stxbp1	0.43	0.00706750	duck
Tspan4	-1.01	0.00713121	duck
Zmat2	0.50	0.00725823	duck
Cluh	-0.75	0.00729925	duck
Rasa3	-1.19	0.00743258	duck
Arhgap29	0.36	0.00775740	duck
Galnt11	0.36	0.00792377	duck

Ube2h	-0.90	0.00804639	duck
Tmem165	0.41	0.00823413	duck
Nfatc2	-0.81	0.00847683	duck
P2ry13	0.52	0.00849213	duck
Sf3a2	0.43	0.00872307	duck
Eci3	0.34	0.00892499	duck
Rnf111	-0.91	0.00919840	duck
Fhod1	0.44	0.00929346	duck
Wsb1	0.28	0.00949478	duck
Klhdc1	0.55	0.00975928	duck
Etv3	0.37	0.00997043	duck
Appl2	0.58	0.00999940	duck
Trappc4	0.37	0.01010804	duck
Plod2	0.24	0.01012637	duck
Aff4	-0.68	0.01015997	duck
Otulinl	-0.66	0.01031254	duck
Nlrc3	-0.34	0.01066025	duck
Yaf2	0.57	0.01076727	duck
Rabl6	-0.57	0.01078346	duck
Rrbp1	0.56	0.01080319	duck
Ldlrad3	-0.05	0.01083379	duck
Prps2	-0.87	0.01106216	duck
Atp5j2	0.43	0.01148967	duck
Steep1	-0.45	0.01160164	duck
Pgm2	0.37	0.01170991	duck
Tmem183a	0.31	0.01182584	duck
Suz12	-0.80	0.01183955	duck
Leprot	0.41	0.01185985	duck
Ttc19	0.54	0.01247464	duck
Pik3ap1	0.12	0.01276036	duck
Gpr75	0.33	0.01278031	duck
Ipo5	-0.51	0.01300025	duck
Ppargc1b	0.47	0.01306560	duck
Rdh10	0.79	0.01323946	duck
Plagl1	-0.46	0.01362484	duck
Angptl2	-0.72	0.01366259	duck
Rpn1	0.38	0.01368996	duck
B2m	0.38	0.01375229	duck
Alg2	0.43	0.01375840	duck
Fam98a	-0.57	0.01379788	duck
Lims2	0.41	0.01380444	duck
Cds2	-0.54	0.01406344	duck
Nr3c1	0.23	0.01457994	duck
Hormad1	0.20	0.01482290	duck
Grpel1	0.38	0.01491254	duck
Pitpnm2	-0.30	0.01494059	duck

Tra2a	-0.71	0.01494405	duck
Prdm11	-0.47	0.01519690	duck
Pip4k2a	-0.68	0.01520732	duck
Tmco3	-1.01	0.01524413	duck
Il20ra	-0.73	0.01562873	duck
Slc25a24	0.11	0.01596814	duck
Fchsd2	0.65	0.01641229	duck
Eif2ak3	-0.58	0.01651966	duck
Fam126a	-1.16	0.01661626	duck
Pars2	0.38	0.01665684	duck
Swap70	0.40	0.01666646	duck
Hmgcr	0.49	0.01673497	duck
Hvcn1	0.45	0.01682392	duck
Adamtsl3	0.32	0.01797343	duck
Htra1	0.48	0.01810233	duck
B3gnt2	0.16	0.01810553	duck
Prkaa1	0.29	0.01818429	duck
Ywhah	-0.48	0.01831812	duck
Thap1	0.34	0.01908140	duck
Rspry1	-1.42	0.01954879	duck
Brip1	0.51	0.01963281	duck
Chic2	0.23	0.02027939	duck
Unc119	-0.58	0.02047383	duck
Park7	-0.52	0.02057770	duck
Plekha1	-0.58	0.02092168	duck
Rassf6	0.33	0.02106622	duck
Runx1	-0.74	0.02162640	duck
Luc7l3	-0.87	0.02212015	duck
Adgrg1	-0.53	0.02227608	duck
Arxes2	0.41	0.02247057	duck
Herc2	-1.33	0.02268996	duck
Sesn3	-0.46	0.02307562	duck
Phf20	-0.77	0.02384113	duck
Hsdl1	0.26	0.02416577	duck
Ubqln1	0.18	0.02428912	duck
Noct	0.45	0.02441553	duck
Dnajb6	0.22	0.02449521	duck
Ugp2	0.50	0.02455263	duck
Rnf128	0.47	0.02466202	duck
Svil	0.24	0.02493892	duck
Prr5	-1.01	0.02511421	duck
Cpne3	0.34	0.02558153	duck
Cnpy3	0.28	0.02573764	duck
Ggcx	0.29	0.02593131	duck
Odc1	0.59	0.02635652	duck
Setd1b	-0.87	0.02719372	duck

Srp68	0.47	0.02802217	duck
Rexo2	0.47	0.02847318	duck
Prrc1	0.30	0.02860454	duck
Stx7	0.32	0.02935431	duck
Rragc	0.34	0.02969335	duck
Abcc5	0.45	0.02976026	duck
Clec16a	-0.71	0.02979198	duck
Bod1l	-1.08	0.03015031	duck
Polr2d	0.38	0.03043206	duck
Bend5	0.39	0.03092634	duck
Nr3c2	-0.88	0.03096039	duck
Slc16a3	0.35	0.03175910	duck
Lipa	0.42	0.03193358	duck
Arf1	0.34	0.03284281	duck
Srf	0.43	0.03304009	duck
Cpd	0.18	0.03342009	duck
Pxk	0.12	0.03498344	duck
Chka	0.53	0.03530237	duck
Ier5	0.40	0.03559337	duck
Dntt	0.41	0.03560654	duck
Tspan8	0.43	0.03567579	duck
Rnf185	0.33	0.03620300	duck
Rmdn3	-0.66	0.03633869	duck
Arl8b	0.39	0.03646291	duck
Prkg2	0.50	0.03667426	duck
Nudt14	0.30	0.03732404	duck
Ctbp1	-0.74	0.03739824	duck
Grtp1	-0.93	0.03930403	duck
Tbc1d14	0.40	0.03950151	duck
Gtf2h5	0.39	0.03950816	duck
Capn15	0.06	0.03974064	duck
Esr1	0.47	0.04048717	duck
Spsb1	0.27	0.04060125	duck
Spata5	-0.89	0.04072233	duck
Acer2	-0.67	0.04073528	duck
Pcnt	-0.75	0.04139446	duck
Parvg	0.26	0.04242184	duck
Nf1	-0.82	0.04248977	duck
Cdk5r1	0.29	0.04440685	duck
Kif13a	0.17	0.04530945	duck
Clic4	-1.30	0.04590779	duck
Nsa2	0.54	0.04656748	duck
Erp29	0.42	0.04692896	duck
Sh3gl3	0.52	0.04754938	duck
Ccdc93	0.41	0.04778143	duck
Ripk1	0.49	0.04789944	duck

Rab11fip1	0.37	0.04797323	duck
Adam19	0.31	0.04829716	duck
Ubl7	0.33	0.04873976	duck
Itpr2	-0.75	0.04972705	duck
Srsf11	-0.60	0.04999091	duck
Hivep2	-0.46	0.05078175	duck
Hdx	-0.73	0.05177458	duck
Atp5j	0.39	0.05206743	duck
Hnrnpa3	-0.48	0.05225902	duck
Txnrd3	0.56	0.05306538	duck
Srgap3	0.18	0.05332371	duck
Sash1	-1.57	0.05386501	duck
Hdgfl3	-0.46	0.05390020	duck
Cdc42se2	0.34	0.05497697	duck
Zbtb38	-0.95	0.05541196	duck
Lbh	-0.73	0.05559705	duck
Rbm12b2	0.15	0.05607741	duck
Sccpdh	0.34	0.05644481	duck
Lpcat2b	0.31	0.05696812	duck
Lsm4	0.45	0.05761922	duck
Itprid2	0.26	0.05906015	duck
Txlng	-0.74	0.05981974	duck
Hpx	0.44	0.06000014	duck
Id2	0.64	0.06019757	duck
Rbm5	-0.63	0.06077026	duck
Klf3	-0.82	0.06214575	duck
Pxn	0.43	0.06232616	duck
Atrx	-1.00	0.06295309	duck
Lman2	0.40	0.06406651	duck
Csf1r	0.29	0.06491938	duck
Arhgap17	-0.71	0.06615965	duck
Arhgdia	0.45	0.06688951	duck
Desi2	-0.30	0.06693067	duck
Bmpr1a	-0.50	0.06707717	duck
Dlgap4	-0.63	0.06781851	duck
Cfap36	-0.50	0.06878517	duck
Nedd9	-0.82	0.06966706	duck
Tial1	-0.68	0.07028825	duck
Ube2q1	0.36	0.07180158	duck
Slc35a5	0.00	0.07285174	duck
Bicdl1	-0.56	0.07513691	duck
Tomm22	0.47	0.07532707	duck
Tecpr2	-0.98	0.07577384	duck
Nprl3	-0.86	0.07660885	duck
Ddx49	0.29	0.07763204	duck
A4galt	0.33	0.07823577	duck

Chp1	0.20	0.07842913	duck
Ppat	-0.70	0.08099344	duck
Pithd1	0.33	0.08236969	duck
Dcun1d5	0.32	0.08267739	duck
Rwdd4a	0.27	0.08285227	duck
Prkar2b	-0.71	0.08534255	duck
Reps1	-0.79	0.08945508	duck
Nme7	0.47	0.08969161	duck
Akt3	-0.64	0.09266512	duck
Sms	-0.67	0.09278582	duck
Slc35g1	0.21	0.09335062	duck
Bin2	0.43	0.09378253	duck
Zfp511	0.30	0.09439785	duck
Gadd45g	0.60	0.09644636	duck
Baiap2l1	0.56	0.09760010	duck
Ank1	-1.09	0.09828679	duck
Tomm7	0.58	0.09850068	duck
Nvl	-0.55	0.10053075	duck
Septin2	-0.44	0.10186944	duck
Hdac1	0.53	0.10308675	duck
Unc50	0.28	0.10595026	duck
Cry2	0.27	0.10616347	duck
Prr5l	-1.03	0.11037936	duck
Clasp1	-0.86	0.11156825	duck
Gdpd2	-0.47	0.11187034	duck
Fbrsl1	0.35	0.11191897	duck
Nr4a2	-0.62	0.11402287	duck
Stau1	-0.52	0.11412693	duck
S1pr4	0.34	0.11745006	duck
Ythdf2	0.37	0.11748930	duck
Gabra1	0.08	0.11799074	duck
Atxn7	-1.02	0.11838127	duck
Ccdc82	-0.46	0.11858459	duck
Impa1	0.43	0.11940341	duck
Nudt7	0.22	0.11990603	duck
Polr2f	0.37	0.12017616	duck
Zeb1	0.36	0.12208313	duck
Rnf20	0.23	0.12211023	duck
Slc9a4	0.13	0.12234088	duck
Tapt1	-1.01	0.12236983	duck
Ppm1d	0.29	0.12327354	duck
Rai2	-0.52	0.12542396	duck
Gramd1c	0.46	0.12562900	duck
Vcpip1	-0.71	0.12566990	duck
Setx	0.50	0.12907319	duck
Gda	0.42	0.12970601	duck

Psmf1	0.28	0.13111590	duck
Tusc2	0.35	0.14112806	duck
Sparc	0.25	0.14367523	duck
Ghitm	-0.67	0.14569984	duck
Ncln	0.30	0.14638443	duck
Hsf5	0.33	0.14652804	duck
Helz2	0.18	0.14674986	duck
Ppp1r12a	0.31	0.14725288	duck
Cisd2	0.27	0.14780085	duck
Apaf1	-0.72	0.14869391	duck
Kpna1	-0.70	0.15167608	duck
Cerk	-0.71	0.15230483	duck
Gramd2	0.25	0.15274946	duck
Birc6	0.17	0.15361720	duck
Nup43	-0.25	0.15641666	duck
Psmg3	0.32	0.15669956	duck
Psmb1	0.45	0.15692575	duck
Uri1	-0.97	0.15734378	duck
Ypel1	-0.87	0.15978345	duck
Golph3	0.41	0.16331390	duck
Ythdf1	-0.56	0.16481317	duck
Brca2	0.46	0.16488761	duck
Prkdc	-0.79	0.16500139	duck
Camsap2	-0.76	0.16740205	duck
Atxn7l1	-0.76	0.16780008	duck
Prpf4b	-0.58	0.17365028	duck
Ndufb4	0.38	0.17516371	duck
Elmo2	-0.47	0.17599332	duck
Dennd11	0.37	0.17814850	duck
Grid1	0.56	0.18104104	duck
Hypk	0.36	0.18488452	duck
Pnpla8	-0.03	0.18639603	duck
Ctdspl2	-0.49	0.18643393	duck
Ndufb2	0.42	0.18882288	duck
Pgpep1	0.33	0.18894388	duck
Sec13	0.25	0.18938276	duck
Ncapd2	0.28	0.19048035	duck
Xbp1	0.21	0.19092643	duck
Pgap4	0.31	0.19357018	duck
Tmem67	0.49	0.19419107	duck
Tlr4	0.24	0.19835075	duck
Cope	0.27	0.19880187	duck
Rnf41	0.30	0.20104596	duck
Usp6nl	-0.16	0.20268015	duck
Srsf4	0.41	0.20571185	duck
Prdx6	0.33	0.20947286	duck

Hsp90b1	-0.66	0.21212938	duck
Fhad1	0.33	0.21535217	duck
Tmem245	-0.39	0.21860747	duck
Psd	0.37	0.21932324	duck
Rpain	0.35	0.21958370	duck
Sucla2	0.26	0.22018248	duck
Rell1	0.06	0.22452280	duck
Chst10	-0.62	0.22463821	duck
Psmb4	0.38	0.22884332	duck
Armc2	-0.59	0.23057059	duck
Ppil2	-0.59	0.23064161	duck
Ngly1	-0.62	0.23475972	duck
Sirt6	0.32	0.23490092	duck
Trdmt1	0.36	0.23706418	duck
Rasgrp1	-0.37	0.23780669	duck
Rhobtb2	-0.27	0.24026066	duck
Zbtb7b	0.23	0.24050956	duck
Chmp2b	0.48	0.24578772	duck
Zfp407	-0.75	0.25020300	duck
Dipk2a	-0.93	0.25093151	duck
Septin8	-0.39	0.26174163	duck
Iqsec1	-1.70	0.26310372	duck
Pnpla7	-1.13	0.26502919	duck
Sox30	0.37	0.26665716	duck
Dgkh	0.80	0.26677162	duck
Bpnt2	0.25	0.27490702	duck
Pfn2	0.32	0.27758887	duck
Phc2	0.39	0.27953199	duck
Sirt5	0.26	0.28960173	duck
Nr4a1	0.63	0.29018990	duck
Adipor1	0.17	0.29341091	duck
Rnf115	0.26	0.29478970	duck
Kdm4c	-1.04	0.29605862	duck
Tes	0.34	0.29917708	duck
Eea1	-0.37	0.30734308	duck
Tasp1	0.35	0.30799442	duck
Trak2	-0.58	0.30825632	duck
Tmem230	0.22	0.30875914	duck
Sun1	-0.99	0.30884801	duck
Wbp4	-1.02	0.31049112	duck
Ndufaf3	0.42	0.31865609	duck
Vps13b	-0.98	0.31906937	duck
Ola1	-0.31	0.31928655	duck
Fig4	0.40	0.32141016	duck
DPCD	0.30	0.32214699	duck
Ppig	-0.62	0.32788316	duck

Carhsp1	-1.09	0.32863880	duck
Ttc39c	-0.87	0.33621338	duck
Otud4	-0.48	0.33970517	duck
Ccndbp1	-0.51	0.34493865	duck
Pole4	0.22	0.35301612	duck
Nptn	0.17	0.35678912	duck
Fam214b	0.20	0.36189948	duck
Skp1	0.08	0.36208434	duck
Ncor2	0.45	0.36408708	duck
Cmtm3	0.38	0.36784746	duck
Dpf3	-0.74	0.36945201	duck
Anp32e	-0.42	0.37347093	duck
Ppp6r2	-0.59	0.37414545	duck
Acss2	0.26	0.37602923	duck
Sult4a1	-0.48	0.37832645	duck
Ankrd12	-0.76	0.38038123	duck
Cdc73	-0.72	0.38086938	duck
P4ha1	-0.72	0.38252452	duck
Dmxl1	-1.00	0.39352803	duck
Tmem123	-0.63	0.39657040	duck
Khdrbs1	-0.46	0.39706117	duck
Dync1h1	-0.46	0.40216881	duck
Grk4	0.41	0.40794766	duck
Tmem231	0.31	0.41165775	duck
Hivep1	0.34	0.42201945	duck
Prmt3	-0.50	0.42984299	duck
Polr1e	0.31	0.43019814	duck
Prepl	0.29	0.43525230	duck
Rufy2	-1.15	0.43691470	duck
Lfng	0.41	0.45186190	duck
Atp6v0a2	-0.61	0.45976149	duck
Cep164	0.24	0.46653392	duck
Dennd6b	-0.38	0.46847449	duck
Gdi2	0.37	0.47045355	duck
Skil	-0.39	0.47256032	duck
Aqp3	-0.68	0.47397629	duck
Hnrnpa2b1	-0.49	0.47469697	duck
Guk1	0.32	0.47826340	duck
Ppp1r21	-0.69	0.47939195	duck
Atp6v0d1	0.40	0.48246571	duck
Klf4	0.52	0.48659906	duck
Azin1	0.27	0.49115607	duck
Tmem167	0.29	0.49860025	duck
Zc3h13	1.00	0.49888939	duck
Fam199x	-0.38	0.49993949	duck
Pcna	-0.38	0.50250443	duck

Runx2	-0.57	0.50594528	duck
Parp1	-0.70	0.51274107	duck
Esco1	-0.87	0.51334513	duck
Tbc1d9b	0.18	0.54567384	duck
Cert1	0.29	0.54807515	duck
Bin1	-0.46	0.55054830	duck
Nnt	-0.35	0.55893779	duck
Plekhh3	0.30	0.56701173	duck
Ppargc1a	0.53	0.57480765	duck
Hivep3	-0.70	0.57704433	duck
Stam2	-0.77	0.57742011	duck
Dnajb2	0.32	0.58160813	duck
Xpa	0.30	0.58820206	duck
Stk38l	-0.51	0.59004216	duck
St8sia1	0.20	0.60384899	duck
Pdp1	-0.82	0.60689728	duck
Tm2d2	0.40	0.61715066	duck
Lypla1	-0.44	0.61886735	duck
Tnni2	0.39	0.62100644	duck
Rangap1	0.31	0.62321481	duck
Iars	-0.56	0.63031561	duck
Cldn18	0.51	0.63558709	duck
Ppp1r13b	-0.53	0.66273154	duck
Rbm31y	0.43	0.66293161	duck
Zfyve28	0.32	0.66532169	duck
Tob1	-0.64	0.67352747	duck
Prdx3	0.42	0.68686894	duck
Ctdp1	-0.56	0.69152707	duck
Ywhaq	0.25	0.70392128	duck
Pcca	-0.45	0.70478582	duck
Tex264	0.32	0.70661743	duck
Dph5	0.04	0.70916743	duck
Phf6	-0.65	0.72884071	duck
Slc39a9	-0.76	0.72915932	duck
Fbxo22	-0.62	0.73210042	duck
Elf2	-0.39	0.73538857	duck
Tmbim6	0.39	0.74602721	duck
Nfkb1	0.21	0.74983300	duck
Zfp644	-0.81	0.75258892	duck
Tmco1	0.28	0.75644330	duck
Scd2	-0.88	0.77045259	duck
Rbfox2	0.35	0.77693604	duck
Btbd9	-1.22	0.78248773	duck
Lrrc8d	-0.59	0.78897594	duck
Kpna4	-0.08	0.79708747	duck
Bms1	-0.48	0.79810832	duck

Bfar	-0.40	0.80163070	duck
Chuk	-0.82	0.80267786	duck
Ctif	0.31	0.81566270	duck
Dna2	-0.93	0.83570610	duck
Kdm2a	0.54	0.85788519	duck
Rap2c	0.29	0.87118983	duck
Gnb1	0.20	0.88335288	duck
Bclaf1	-0.62	0.88655878	duck
Ubr3	-0.88	0.88876762	duck
Tfpi2	0.47	0.89636724	duck
Ncoa4	-0.66	0.92422088	duck
Arv1	-0.68	0.92738057	duck
Emsy	-0.48	0.93642915	duck
Ktn1	0.24	0.94848162	duck
Arhgap26	-0.08	0.96794732	duck
Stard4	0.28	0.97360739	duck
B4galt2	0.25	0.98717469	duck
Taok3	-0.73	0.99869155	duck
Rheb	0.29	1.00000000	duck
Trp53inp2	0.29	1.00000000	duck
Arid2	-0.97	1.00000000	duck
Slc25a13	-0.84	1.00000000	duck
Stx8	0.39	1.00000000	duck
Exoc5	0.29	1.00000000	duck
Fbxo11	-0.64	1.00000000	duck
Zfp711	-0.55	1.00000000	duck
Lrp6	0.17	1.00000000	duck
Ldb1	-0.41	1.00000000	duck
Tmem154	0.22	1.00000000	duck
Plekhf2	-0.57	1.00000000	duck
Ralbp1	0.03	1.00000000	duck
Abce1	-0.58	1.00000000	duck
Trmt10a	0.19	1.00000000	duck
Cdca7l	-0.33	1.00000000	duck
Smu1	0.22	1.00000000	duck
Prkar1b	0.15	1.00000000	duck
Zbtb21	0.45	1.00000000	duck
Parp11	0.15	1.00000000	duck
Fbxl20	0.38	1.00000000	duck
Adgrg2	0.48	1.00000000	duck
Tmem131l	-0.70	1.00000000	duck
Stk38	0.32	1.00000000	duck
Arhgap5	-0.34	1.00000000	duck
Zswim8	0.18	1.00000000	duck
Aco1	0.35	1.00000000	duck
Pik3ca	-0.90	1.00000000	duck

Rrm2b	0.35	1.00000000	duck
Slco3a1	-0.35	1.00000000	duck
Nek6	-0.11	1.00000000	duck
Tmem33	0.36	1.00000000	duck
Ano5	-0.51	1.00000000	duck
Cstf3	0.35	1.00000000	duck
Fbxo3	-1.65	1.00000000	duck
Fam120a	-0.74	1.00000000	duck
Ubap2	0.28	1.00000000	duck
Smad5	0.44	1.00000000	duck
Psmc3	0.29	1.00000000	duck
Helz	0.22	1.00000000	duck
Lrrcc1	-0.33	1.00000000	duck
Fxn	0.22	1.00000000	duck
Cyth4	-0.36	1.00000000	duck
Lonp2	-0.59	1.00000000	duck
Jmjd4	0.33	1.00000000	duck
Psmd12	0.26	1.00000000	duck
Prpf40a	0.28	1.00000000	duck
Syf2	0.31	1.00000000	duck
Inpp5k	-0.83	1.00000000	duck
Dlg1	-0.42	1.00000000	duck
Stat3	0.28	1.00000000	duck
Vps13d	-0.79	1.00000000	duck
Ip6k1	-0.35	1.00000000	duck
Rbx1	0.34	1.00000000	duck
Cib1	0.36	1.00000000	duck
P4hb	0.37	1.00000000	duck
Lmbr1	0.44	1.00000000	duck
Akap1	-0.31	1.00000000	duck
Cers5	0.27	1.00000000	duck
Zfp512	-0.52	1.00000000	duck
Tmx3	0.38	1.00000000	duck
Fbxo9	0.12	1.00000000	duck
Abi1	-0.07	1.00000000	duck
Tns3	-0.72	1.00000000	duck
Slc25a47	0.34	1.00000000	duck
Zswim6	0.17	1.00000000	duck
Usp31	-0.31	1.00000000	duck
Bclaf3	0.39	1.00000000	duck
Tmem199	0.31	1.00000000	duck
Tra2b	-0.47	1.00000000	duck
Serac1	0.33	1.00000000	duck
Dip2a	-0.88	1.00000000	duck
Nucks1	-0.46	1.00000000	duck
Ccdc134	0.30	1.00000000	duck

Fgd4	-1.14	1.00000000	duck
Psmb7	0.17	1.00000000	duck
Slc16a6	-0.57	1.00000000	duck
Ubtd2	-1.11	1.00000000	duck
Dnajc5	0.33	1.00000000	duck
Ndufv2	0.19	1.00000000	duck
Chd1	0.14	1.00000000	duck
Tbl1xr1	0.25	1.00000000	duck
Rnf146	-0.45	1.00000000	duck
Ino80	-0.70	1.00000000	duck
Sumo1	0.26	1.00000000	duck
Pspc1	-0.70	1.00000000	duck
Traf1	-0.34	1.00000000	duck
Arcn1	0.26	1.00000000	duck
Fndc3a	-0.74	1.00000000	duck
Dync1i2	0.26	1.00000000	duck
Lpcat3	0.49	1.00000000	duck
Bud23	-0.23	1.00000000	duck
Usp14	0.18	1.00000000	duck
Nhsl2	0.37	1.00000000	duck
Far2	-0.32	1.00000000	duck
Atp1b3	0.27	1.00000000	duck
Nus1	0.36	1.00000000	duck
Shc1	0.24	1.00000000	duck
Pin1	0.39	1.00000000	duck
Csde1	-0.67	1.00000000	duck
Ankrd13a	0.34	1.00000000	duck
Slc7a6os	0.28	1.00000000	duck
Eri1	0.34	1.00000000	duck
Gpalpp1	0.12	1.00000000	duck
Irf2	-0.49	1.00000000	duck
Swt1	-0.53	1.00000000	duck
Ppp6r3	-0.80	1.00000000	duck
Rsbn1l	0.08	1.00000000	duck
Iqgap1	0.10	1.00000000	duck
Hnrnpm	-0.47	1.00000000	duck
Vangl1	0.03	1.00000000	duck
Smad4	-0.58	1.00000000	duck
Ube2e2	-1.25	1.00000000	duck
Npc1	0.49	1.00000000	duck
Tcf3	-0.72	1.00000000	duck
Wdr43	-0.32	1.00000000	duck
Fam76a	0.34	1.00000000	duck
Rexo5	-0.25	1.00000000	duck
Abtb1	0.03	1.00000000	duck
Rabep1	-0.42	1.00000000	duck

Hecw2	-0.69	1.00000000	duck
Fam102a	-0.38	1.00000000	duck
Nol8	-0.33	1.00000000	duck
Exoc1	0.26	1.00000000	duck
Wrn	-0.93	1.00000000	duck
Atxn2	-0.78	1.00000000	duck
Wwp1	-0.98	1.00000000	duck
Tnrc6a	-0.62	1.00000000	duck
Clcn3	-0.65	1.00000000	duck
Rpa1	0.31	1.00000000	duck
Spata7	-0.43	1.00000000	duck
Babam2	0.30	1.00000000	duck
Puf60	0.26	1.00000000	duck
Slain2	-0.69	1.00000000	duck
Fxr1	-0.84	1.00000000	duck
Slc25a3	0.26	1.00000000	duck
Sla	-0.12	1.00000000	duck
Tpr	-0.66	1.00000000	duck
Prkcd	-0.08	1.00000000	duck
Slc9a1	0.35	1.00000000	duck
Rsl1d1	-0.32	1.00000000	duck
Creg1	-1.87	1.00000000	duck
Arglu1	-0.55	1.00000000	duck
Btf3l4	0.40	1.00000000	duck
Tbc1d5	-0.86	1.00000000	duck
Rnf214	0.34	1.00000000	duck
Usp10	0.20	1.00000000	duck
Smurf2	-0.65	1.00000000	duck
Fa2h	0.15	1.00000000	duck
Atp2a2	-0.18	1.00000000	duck
Creb3l2	0.25	1.00000000	duck
Asb7	0.10	1.00000000	duck
Sh3glb1	0.30	1.00000000	duck
Zbtb34	-0.70	1.00000000	duck
Psmc1	0.33	1.00000000	duck
Shld1	0.36	1.00000000	duck
Sbno1	0.11	1.00000000	duck
Usp1	-0.29	1.00000000	duck
Snap23	0.10	1.00000000	duck
Nek9	0.36	1.00000000	duck
Tmem241	0.28	1.00000000	duck
Eif4g2	0.24	1.00000000	duck
Smad7	0.31	1.00000000	duck
Shisa5	0.26	1.00000000	duck
Rpap3	-0.25	1.00000000	duck
Als2	-0.38	1.00000000	duck

Slc9a8	0.15	1.00000000	duck
Bmf	-0.39	1.00000000	duck
Tmtc3	0.19	1.00000000	duck
Phc1	0.14	1.00000000	duck
Atrn	0.53	1.00000000	duck
Nkiras2	0.23	1.00000000	duck
Ahctf1	-0.52	1.00000000	duck
Ppp3ca	0.11	1.00000000	duck
Klf9	-0.33	1.00000000	duck
B4galt1	0.37	1.00000000	duck
Ercc6	-0.70	1.00000000	duck
Setd5	0.02	1.00000000	duck
Pdcd6	0.12	1.00000000	duck
Bcl2	-0.28	1.00000000	duck
Sec62	0.18	1.00000000	duck
Adgrv1	-0.76	1.00000000	duck
Cdk6	-0.53	1.00000000	duck
Fgl2	0.36	1.00000000	duck
Ssr3	0.34	1.00000000	duck
Id3	-0.35	1.00000000	duck
Srsf10	-0.54	1.00000000	duck
Zfp654	0.07	1.00000000	duck
Imp3	0.31	1.00000000	duck
Tubgcp3	-0.34	1.00000000	duck
Idh1	0.33	1.00000000	duck
Dnm1l	-0.36	1.00000000	duck
Ccser2	-0.37	1.00000000	duck
Sel1l	-0.57	1.00000000	duck
Eif3i	0.37	1.00000000	duck
Serinc3	0.24	1.00000000	duck
Kdm3b	-0.61	1.00000000	duck
Psmd14	0.34	1.00000000	duck
Denr	0.26	1.00000000	duck
Ufm1	0.21	1.00000000	duck
Fkbp4	-0.28	1.00000000	duck
Lars2	-0.25	1.00000000	duck
Ralgps1	-0.63	1.00000000	duck
Cog2	-0.59	1.00000000	duck
Rnf145	0.07	1.00000000	duck
Rnf217	0.09	1.00000000	duck
Polr1c	0.13	1.00000000	duck
Llph	0.06	1.00000000	duck
Klhl5	-0.61	1.00000000	duck
Tmcc1	-0.11	1.00000000	duck
Lpcat1	-0.39	1.00000000	duck
Strip1	0.23	1.00000000	duck

Suclg2	-0.68	1.00000000	duck
Ddx28	-0.25	1.00000000	duck
Zdhhc17	0.08	1.00000000	duck
Ddx17	-0.86	1.00000000	duck
Ing3	-0.84	1.00000000	duck
Gfi1	-0.44	1.00000000	duck
Prkar2a	-0.62	1.00000000	duck
Spred2	0.24	1.00000000	duck
Pip5k1b	-0.08	1.00000000	duck
Cyth1	-0.27	1.00000000	duck
Scamp3	0.19	1.00000000	duck
Ddx23	0.32	1.00000000	duck
Sidt2	0.21	1.00000000	duck
Ndufa8	0.25	1.00000000	duck
Wdr4	-0.27	1.00000000	duck
Ipo11	-0.59	1.00000000	duck
Ube2b	0.06	1.00000000	duck
Tmx4	0.14	1.00000000	duck
Ecpas	-0.92	1.00000000	duck
Orai1	0.24	1.00000000	duck
Tacc3	-0.22	1.00000000	duck
Ss18	-0.45	1.00000000	duck
Zw10	-0.20	1.00000000	duck
Gnptab	0.16	1.00000000	duck
Elovl1	0.26	1.00000000	duck
Pgrmc2	-0.39	1.00000000	duck
Plod1	0.16	1.00000000	duck
Smarca2	-0.75	1.00000000	duck
Pm20d2	-0.35	1.00000000	duck
Cep120	0.19	1.00000000	duck
Polr2l	0.31	1.00000000	duck
Gtdc1	-0.59	1.00000000	duck
Dram1	0.22	1.00000000	duck
Rbm25	-0.39	1.00000000	duck
Zfp217	0.47	1.00000000	duck
Gtf2b	0.16	1.00000000	duck
Dhx15	-0.60	1.00000000	duck
Fnip2	-0.70	1.00000000	duck
Edem1	0.43	1.00000000	duck
Rad51b	-0.73	1.00000000	duck
Fubp1	-0.66	1.00000000	duck
Stmn1	-0.32	1.00000000	duck
Dgkq	-1.08	1.00000000	duck
Nufip1	-0.33	1.00000000	duck
Rae1	-0.60	1.00000000	duck
Parn	-0.60	1.00000000	duck

Gphn	-0.41	1.00000000	duck
Dock11	-0.66	1.00000000	duck
Lgals3	0.39	1.00000000	duck
Nlrc5	-0.76	1.00000000	duck
Polr2k	0.27	1.00000000	duck
Gpn1	-0.61	1.00000000	duck
Nrde2	-0.42	1.00000000	duck
Ell	0.14	1.00000000	duck
Bcl11a	0.20	1.00000000	duck
Lrig2	-0.63	1.00000000	duck
Yeats4	0.30	1.00000000	duck
Hnrnpu	-0.38	1.00000000	duck
Stag2	-0.64	1.00000000	duck
Gpr160	0.15	1.00000000	duck
Pex5	0.24	1.00000000	duck
Tfam	-0.37	1.00000000	duck
Slx4	0.24	1.00000000	duck
C2cd5	-0.68	1.00000000	duck
Dynlrb1	0.27	1.00000000	duck
Smdt1	0.29	1.00000000	duck
Snx9	-0.49	1.00000000	duck
Samm50	0.26	1.00000000	duck
Irak4	0.05	1.00000000	duck
Klhl21	0.22	1.00000000	duck
Reep2	0.19	1.00000000	duck
Fam3c	0.40	1.00000000	duck
Wsb2	0.27	1.00000000	duck
Zbtb2	-0.23	1.00000000	duck
Nbas	-0.69	1.00000000	duck
Kcnk1	-0.35	1.00000000	duck
Zranb2	-0.76	1.00000000	duck
Add1	-0.12	1.00000000	duck
Cnot6	-0.40	1.00000000	duck
Zdhhc12	0.19	1.00000000	duck
Isca2	0.18	1.00000000	duck
Cog1	0.21	1.00000000	duck
Pphln1	-0.62	1.00000000	duck
Pip5k1c	0.04	1.00000000	duck
Capns2	0.18	1.00000000	duck
Tars	0.26	1.00000000	duck
Tec	-0.08	1.00000000	duck
Ndufb1	0.42	1.00000000	duck
Itga1	-0.68	1.00000000	duck
Lrch3	0.17	1.00000000	duck
Gpr55	-0.27	1.00000000	duck
Tox2	-0.27	1.00000000	duck

Ubxn2b	0.37	1.00000000	duck
Atad2b	0.27	1.00000000	duck
Hmgb3	-0.23	1.00000000	duck
Ogdh	-0.21	1.00000000	duck
Hsd17b12	0.28	1.00000000	duck
Btg1b	-0.78	1.00000000	duck
Gna13	-0.38	1.00000000	duck
Ropn1l	0.21	1.00000000	duck
Emc3	0.25	1.00000000	duck
Gnat2	0.28	1.00000000	duck
Nrbf2	0.32	1.00000000	duck
Znrf3	-0.67	1.00000000	duck
Dync1li1	-0.41	1.00000000	duck
Smap1	-0.37	1.00000000	duck
Ubac2	-0.08	1.00000000	duck
Xrcc5	-0.31	1.00000000	duck
Ttf1	0.41	1.00000000	duck
Heatr5a	0.03	1.00000000	duck
Pde9a	0.29	1.00000000	duck
Lrmp	0.26	1.00000000	duck
Slc25a25	-0.24	1.00000000	duck
Ifitm10	0.26	1.00000000	duck
Dcn	0.11	1.00000000	duck
Thumpd3	0.27	1.00000000	duck
Thap12	-0.60	1.00000000	duck
Grk3	-0.61	1.00000000	duck
Cdc16	-0.35	1.00000000	duck
Slc39a11	0.25	1.00000000	duck
Pex13	0.31	1.00000000	duck
Cdyl2	-0.01	1.00000000	duck
Nfatc3	-0.68	1.00000000	duck
Nav1	0.19	1.00000000	duck
Anapc13	0.32	1.00000000	duck
St8sia4	0.38	1.00000000	duck
Serbp1	-0.38	1.00000000	duck
Lactb2	0.19	1.00000000	duck
Adnp2	-0.46	1.00000000	duck
Tmem248	0.22	1.00000000	duck
Btaf1	-0.83	1.00000000	duck
Etfrf1	-0.37	1.00000000	duck
Rbm19	-0.23	1.00000000	duck
Ciapin1	-0.45	1.00000000	duck
Rfwd3	0.18	1.00000000	duck
Tmem65	0.12	1.00000000	duck
Tmem104	0.16	1.00000000	duck
Rora	-0.24	1.00000000	duck

Slc9a5	0.32	1.00000000	duck
Erap1	0.26	1.00000000	duck
Rfc5	0.26	1.00000000	duck
Lcp2	0.25	1.00000000	duck
Ubox5	0.22	1.00000000	duck
Gpd2	-0.12	1.00000000	duck
Prpf4	-0.12	1.00000000	duck
Katnip	0.07	1.00000000	duck
Asap1	-0.77	1.00000000	duck
Lzic	0.10	1.00000000	duck
Plcb2	-0.06	1.00000000	duck
Ccdc12	0.30	1.00000000	duck
Nup93	-0.23	1.00000000	duck
Jak1	0.22	1.00000000	duck
Ptcd3	-0.22	1.00000000	duck
Plekha3	-0.25	1.00000000	duck
Agpat5	-0.42	1.00000000	duck
Abhd2	0.22	1.00000000	duck
Ppp1r3b	0.20	1.00000000	duck
Nfat5	-0.86	1.00000000	duck
Pdcd4	-0.27	1.00000000	duck
Txn2	0.11	1.00000000	duck
Ssbp1	0.45	1.00000000	duck
Gpatch8	-0.56	1.00000000	duck
Parp9	0.11	1.00000000	duck
Rad18	0.36	1.00000000	duck
Nono	0.40	1.00000000	duck
Lrrc8a	0.29	1.00000000	duck
Cnih1	0.21	1.00000000	duck
Hnrnpab	-0.59	1.00000000	duck
Kbtbd8	0.20	1.00000000	duck
Vps51	0.18	1.00000000	duck
Csnk1a1	0.16	1.00000000	duck
Dmxl2	-0.41	1.00000000	duck
Ano9	0.17	1.00000000	duck
Sipa1l1	-0.31	1.00000000	duck
Zbed4	-0.27	1.00000000	duck
Ncoa3	-0.48	1.00000000	duck
Pum2	-0.69	1.00000000	duck
Heatr1	-0.25	1.00000000	duck
Ppib	0.35	1.00000000	duck
Alpk1	0.16	1.00000000	duck
Hnrnph3	-0.42	1.00000000	duck
Epc1	-0.67	1.00000000	duck
Zfp207	-0.78	1.00000000	duck
Clta	0.35	1.00000000	duck

Tfdp1	-0.49	1.00000000	duck
Tiam2	-0.09	1.00000000	duck
Atp5c1	0.30	1.00000000	duck
Wdr82	0.19	1.00000000	duck
Sla2	-0.33	1.00000000	duck
Clp1	0.19	1.00000000	duck
Smc3	-0.40	1.00000000	duck
Rsrc1	0.19	1.00000000	duck
Nmt1	0.30	1.00000000	duck
Gpn3	-0.37	1.00000000	duck
Rock2	0.27	1.00000000	duck
Rab14	0.23	1.00000000	duck
Ubfd1	0.03	1.00000000	duck
B4galt5	-0.10	1.00000000	duck
Rbpj	0.12	1.00000000	duck
Ppil3	0.07	1.00000000	duck
Rufy1	0.17	1.00000000	duck
Secisbp2l	0.12	1.00000000	duck
Rapsn	0.16	1.00000000	duck
Vash1	-0.91	1.00000000	duck
Dazap1	-0.77	1.00000000	duck
Rab19	0.13	1.00000000	duck
Rab21	0.13	1.00000000	duck
Aptx	0.22	1.00000000	duck
Txk	-0.61	1.00000000	duck
Lsm14a	0.05	1.00000000	duck
Il17ra	0.29	1.00000000	duck
Entpd1	-0.47	1.00000000	duck
Pcmt1	-0.45	1.00000000	duck
Whamm	-0.51	1.00000000	duck
Picalm	-0.13	1.00000000	duck
Stt3b	-0.55	1.00000000	duck
Ablim1	-0.47	1.00000000	duck
Dvl1	-0.60	1.00000000	duck
Eif2d	-0.32	1.00000000	duck
Dcaf5	-0.09	1.00000000	duck
Wdr33	-0.55	1.00000000	duck
Gas7	0.52	1.00000000	duck
Nbeal2	-0.42	1.00000000	duck
Nlrx1	0.25	1.00000000	duck
Josd1	0.13	1.00000000	duck
Thoc1	-0.67	1.00000000	duck
Kbtbd4	0.16	1.00000000	duck
Rif1	-0.67	1.00000000	duck
Kremen1	-0.62	1.00000000	duck
Cmas	0.32	1.00000000	duck

Abca13	0.10	1.00000000	duck
Cap1	0.23	1.00000000	duck
Spats2	0.19	1.00000000	duck
Tmem222	0.24	1.00000000	duck
Kdelr2	0.27	1.00000000	duck
Pdxdc1	-0.50	1.00000000	duck
Fam98b	-0.30	1.00000000	duck
Fam185a	0.07	1.00000000	duck
Tmem50b	0.02	1.00000000	duck
Hspa4l	-0.45	1.00000000	duck
Eipr1	-0.96	1.00000000	duck
Lsm14b	-0.44	1.00000000	duck
Tm9sf2	0.19	1.00000000	duck
Esf1	-0.56	1.00000000	duck
Pbdc1	-0.23	1.00000000	duck
Cops2	-0.41	1.00000000	duck
Cadm1	-0.45	1.00000000	duck
Prpf19	0.27	1.00000000	duck
Shld2	0.34	1.00000000	duck
Srebf2	0.28	1.00000000	duck
Spast	-0.53	1.00000000	duck
Pde4b	-0.22	1.00000000	duck
Ttbk1	-0.24	1.00000000	duck
Zmynd19	-0.13	1.00000000	duck
Fam204a	0.06	1.00000000	duck
Slc37a1	-0.59	1.00000000	duck
Haus2	0.08	1.00000000	duck
Rapgef2	-0.62	1.00000000	duck
Arfgef2	0.05	1.00000000	duck
Pcgf5	-0.24	1.00000000	duck
Wdr77	-0.14	1.00000000	duck
Socs7	-0.35	1.00000000	duck
Ezh2	-0.38	1.00000000	duck
Trip12	-0.98	1.00000000	duck
Dnttip2	-0.42	1.00000000	duck
Kctd20	-0.70	1.00000000	duck
Natd1	0.27	1.00000000	duck
Ddx10	-0.39	1.00000000	duck
Pptc7	-0.52	1.00000000	duck
Fut11	0.18	1.00000000	duck
Ccdc126	0.09	1.00000000	duck
Sap30	0.22	1.00000000	duck
Gnl3	-0.20	1.00000000	duck
Cdc123	0.15	1.00000000	duck
Smc4	-0.49	1.00000000	duck
Dnajc11	-0.39	1.00000000	duck

Rsrp1	-0.66	1.00000000	duck
Tcp1	-0.29	1.00000000	duck
Heph	0.18	1.00000000	duck
Zbtb1	-0.21	1.00000000	duck
Lrrc45	-0.23	1.00000000	duck
Add3	0.00	1.00000000	duck
Srrm1	-0.38	1.00000000	duck
Thoc2	-0.57	1.00000000	duck
Snrpa1	-0.22	1.00000000	duck
Gpr107	0.16	1.00000000	duck
Ccng2	-0.28	1.00000000	duck
Poldip2	0.26	1.00000000	duck
Thada	-0.40	1.00000000	duck
Rrn3	-0.22	1.00000000	duck
Srpk2	0.07	1.00000000	duck
Zcchc10	0.24	1.00000000	duck
Cpne1	-0.59	1.00000000	duck
Sec24b	-0.68	1.00000000	duck
Orc4	0.21	1.00000000	duck
Arid1b	-0.47	1.00000000	duck
Raf1	-0.55	1.00000000	duck
Ccdc186	0.05	1.00000000	duck
Pcnx	-0.77	1.00000000	duck
Golga7	0.20	1.00000000	duck
Fut8	0.09	1.00000000	duck
Acaa2	-0.26	1.00000000	duck
Cdv3	0.19	1.00000000	duck
Dnaja2	0.15	1.00000000	duck
Senp6	-0.39	1.00000000	duck
Brd7	0.13	1.00000000	duck
Pdia4	0.26	1.00000000	duck
Slc4a9	0.07	1.00000000	duck
Dctn5	0.20	1.00000000	duck
Socs5	-0.38	1.00000000	duck
Tmem185a	-0.21	1.00000000	duck
Kat2b	-0.10	1.00000000	duck
Prrc2c	-0.38	1.00000000	duck
Arl15	-0.67	1.00000000	duck
Rbm22	0.30	1.00000000	duck
Fnta	-0.33	1.00000000	duck
Tmc7	-0.25	1.00000000	duck
Extl3	0.15	1.00000000	duck
Hook3	0.11	1.00000000	duck
Chmp5	0.10	1.00000000	duck
Tmem203	0.34	1.00000000	duck
Plekhj1	0.19	1.00000000	duck

Polr3e	-0.25	1.00000000	duck
Slc23a2	-0.17	1.00000000	duck
Ppm1f	0.18	1.00000000	duck
Sypl2	0.34	1.00000000	duck
Tmem138	0.14	1.00000000	duck
Ankle2	-0.31	1.00000000	duck
Gse1	0.24	1.00000000	duck
Scmh1	-0.22	1.00000000	duck
Bcat1	0.43	1.00000000	duck
Tnfsf8	-0.23	1.00000000	duck
Usp16	-0.32	1.00000000	duck
Sesn2	0.06	1.00000000	duck
Snorc	0.01	1.00000000	duck
Phkb	0.00	1.00000000	duck
Clk1	-0.63	1.00000000	duck
Kat6a	0.09	1.00000000	duck
Pla2g15	0.08	1.00000000	duck
Arsg	-0.49	1.00000000	duck
Trio	-0.29	1.00000000	duck
Ptges3	-0.59	1.00000000	duck
Bcl2l11	0.17	1.00000000	duck
Uhrf1	-0.21	1.00000000	duck
Nars	0.14	1.00000000	duck
Tbc1d8b	0.09	1.00000000	duck
Plekhm3	0.30	1.00000000	duck
Ulk1	-0.84	1.00000000	duck
Ppm1a	0.00	1.00000000	duck
Nsun2	-0.30	1.00000000	duck
Pld1	-0.70	1.00000000	duck
Haus3	-0.35	1.00000000	duck
Rb1cc1	0.04	1.00000000	duck
Nbr1	-0.73	1.00000000	duck
Tmem120a	0.21	1.00000000	duck
Snu13	0.31	1.00000000	duck
Selenof	0.15	1.00000000	duck
Dusp5	-0.44	1.00000000	duck
Fjx1	0.13	1.00000000	duck
Immp1l	-0.54	1.00000000	duck
Pwp1	0.35	1.00000000	duck
Noc2l	-0.30	1.00000000	duck
Vcpkmt	0.20	1.00000000	duck
Rpn2	0.30	1.00000000	duck
Ak2	-0.19	1.00000000	duck
Gpt2	-0.26	1.00000000	duck
Trmt44	-0.22	1.00000000	duck
Cacna1i	0.28	1.00000000	duck

Pdlim7	0.26	1.00000000	duck
Sf3b6	0.27	1.00000000	duck
Nelfcd	0.02	1.00000000	duck
Ptbp3	0.12	1.00000000	duck
Zcchc2	-0.43	1.00000000	duck
Rpap2	-0.41	1.00000000	duck
Psd3	0.46	1.00000000	duck
Azi2	-0.53	1.00000000	duck
Arhgef12	-1.30	1.00000000	duck
Naa15	-0.68	1.00000000	duck
Kansl2	-0.25	1.00000000	duck
Otud7a	-0.33	1.00000000	duck
Iqcb1	-0.28	1.00000000	duck
Afap1l2	-0.28	1.00000000	duck
Wipi2	0.15	1.00000000	duck
Bcor	-0.38	1.00000000	duck
Ube2i	0.06	1.00000000	duck
Lrch1	-0.76	1.00000000	duck
Arfip2	0.17	1.00000000	duck
Wdr26	0.12	1.00000000	duck
Nudc	0.27	1.00000000	duck
Nfe2l1	0.20	1.00000000	duck
Tmem50a	0.21	1.00000000	duck
Gpatch4	0.18	1.00000000	duck
Arfip1	0.14	1.00000000	duck
Senp5	0.27	1.00000000	duck
Snx1	0.16	1.00000000	duck
Arl6ip4	0.06	1.00000000	duck
Nolc1	-0.33	1.00000000	duck
Dcp1b	-0.16	1.00000000	duck
Susd6	-0.46	1.00000000	duck
Fam114a1	0.19	1.00000000	duck
Fam241a	-0.44	1.00000000	duck
Cul5	-0.61	1.00000000	duck
Eif4a2	0.25	1.00000000	duck
Hbp1	-0.63	1.00000000	duck
Sbno2	0.24	1.00000000	duck
Ube2e3	0.02	1.00000000	duck
Wdr44	-0.58	1.00000000	duck
Asf1a	-0.30	1.00000000	duck
Rprd1b	-0.48	1.00000000	duck
Gga3	0.23	1.00000000	duck
Eef1a1	0.03	1.00000000	duck
Ap3b1	0.15	1.00000000	duck
Slc30a1	0.24	1.00000000	duck
Atxn1l	0.20	1.00000000	duck

Cep83	0.22	1.00000000	duck
Polr1a	-0.23	1.00000000	duck
Gng2	-0.16	1.00000000	duck
Uchl3	0.12	1.00000000	duck
Zmpste24	0.07	1.00000000	duck
Zbtb16	-0.72	1.00000000	duck
Fam122b	-0.44	1.00000000	duck
Slc2a13	0.27	1.00000000	duck
Zmynd8	-0.33	1.00000000	duck
Sfmbt1	-0.42	1.00000000	duck
LTO1	-0.20	1.00000000	duck
Gosr1	0.10	1.00000000	duck
Fryl	-0.64	1.00000000	duck
Ctr9	-0.35	1.00000000	duck
Bmpr2	-0.53	1.00000000	duck
Chchd7	-0.28	1.00000000	duck
Setd7	-0.02	1.00000000	duck
Tnip1	0.21	1.00000000	duck
Hectd4	-0.30	1.00000000	duck
Tspan14	0.11	1.00000000	duck
Prkab1	0.24	1.00000000	duck
Chd9	-0.42	1.00000000	duck
Nrbp2	-0.17	1.00000000	duck
Plcl1	0.06	1.00000000	duck
Pdzd8	-0.14	1.00000000	duck
Cdkal1	-0.52	1.00000000	duck
Hipk1	-0.54	1.00000000	duck
Bzw1	0.11	1.00000000	duck
Ppp2r5d	-0.20	1.00000000	duck
Il16	-0.18	1.00000000	duck
Usp20	-0.05	1.00000000	duck
Nqo2	0.17	1.00000000	duck
Btrc	-0.54	1.00000000	duck
Bnc2	-0.52	1.00000000	duck
Eif5	-0.02	1.00000000	duck
Daglb	-0.41	1.00000000	duck
Etnk1	-0.55	1.00000000	duck
Pip5k1a	-0.34	1.00000000	duck
Htatsf1	-0.50	1.00000000	duck
Cilk1	-0.43	1.00000000	duck
Ptpa	0.01	1.00000000	duck
Kcnq5	-0.72	1.00000000	duck
Phf20l1	-0.51	1.00000000	duck
Kansl1	-0.41	1.00000000	duck
Pmepa1	0.26	1.00000000	duck
Cpeb3	-0.23	1.00000000	duck

Kdm5b	0.05	1.00000000	duck
Insr	0.14	1.00000000	duck
Sgsm3	0.28	1.00000000	duck
Acsl4	-0.11	1.00000000	duck
Ppcdc	0.22	1.00000000	duck
Acbd3	0.30	1.00000000	duck
Taf10	0.22	1.00000000	duck
Cnot4	0.07	1.00000000	duck
Ptpn12	0.01	1.00000000	duck
Hs6st3	-0.94	1.00000000	duck
Ccnl1	-0.34	1.00000000	duck
Lysmd2	0.19	1.00000000	duck
Pds5a	-0.42	1.00000000	duck
Yeats2	-0.18	1.00000000	duck
Tmem38a	-0.26	1.00000000	duck
Tsn	-0.36	1.00000000	duck
Prelid1	0.26	1.00000000	duck
Ythdc2	-0.31	1.00000000	duck
Lin54	-0.70	1.00000000	duck
Nop58	-0.27	1.00000000	duck
Alox5	-0.87	1.00000000	duck
Rdx	-0.54	1.00000000	duck
Atg2b	0.29	1.00000000	duck
Suox	-0.12	1.00000000	duck
Adgrg6	0.27	1.00000000	duck
Srfbp1	-0.41	1.00000000	duck
Garre1	0.24	1.00000000	duck
Ammecr1l	0.13	1.00000000	duck
Tnfsf11	0.43	1.00000000	duck
Akap11	-0.36	1.00000000	duck
Arhgap6	0.19	1.00000000	duck
Ciao2a	0.00	1.00000000	duck
Cables2	-0.26	1.00000000	duck
Ccdc6	-0.63	1.00000000	duck
Smarca5	0.02	1.00000000	duck
Saraf	0.19	1.00000000	duck
Rnf139	0.16	1.00000000	duck
Tpra1	-0.03	1.00000000	duck
Klhl36	0.19	1.00000000	duck
Hook1	-0.47	1.00000000	duck
Tlnrd1	0.21	1.00000000	duck
Tango2	-0.26	1.00000000	duck
C2cd2	-0.92	1.00000000	duck
Psmd13	0.13	1.00000000	duck
Cebpz	-0.35	1.00000000	duck
Adk	-0.19	1.00000000	duck

Crebrf	-0.71	1.00000000	duck
Ascc3	-0.90	1.00000000	duck
Ube2g2	0.05	1.00000000	duck
Pbx4	-0.76	1.00000000	duck
Sos2	-0.47	1.00000000	duck
Zc3h18	0.14	1.00000000	duck
Prkrip1	0.15	1.00000000	duck
Taf13	0.27	1.00000000	duck
Dpysl2	-0.04	1.00000000	duck
Tln2	-0.28	1.00000000	duck
Sirt7	0.18	1.00000000	duck
9130409I23Rik	0.20	1.00000000	duck
N4bp1	0.17	1.00000000	duck
Pias2	0.13	1.00000000	duck
Snrpc	0.16	1.00000000	duck
Clock	-0.58	1.00000000	duck
Ust	-0.13	1.00000000	duck
Slc35f5	0.12	1.00000000	duck
Sft2d1	0.19	1.00000000	duck
Zbtb7a	0.18	1.00000000	duck
Zfp513	0.20	1.00000000	duck
Atp11a	-0.51	1.00000000	duck
Cdc14a	-1.00	1.00000000	duck
Exoc2	0.02	1.00000000	duck
Gtf2e1	-0.68	1.00000000	duck
Atp9b	-0.40	1.00000000	duck
Fnbp1	0.31	1.00000000	duck
Cpsf2	-0.15	1.00000000	duck
Ric8b	-0.48	1.00000000	duck
Tnks2	-0.64	1.00000000	duck
Nek7	0.28	1.00000000	duck
Aoah	-0.02	1.00000000	duck
Gpr18	-0.26	1.00000000	duck
Arhgef6	0.23	1.00000000	duck
Bbx	0.32	1.00000000	duck
Sltm	-0.36	1.00000000	duck
Ppp1r16b	0.27	1.00000000	duck
Slc39a6	-0.07	1.00000000	duck
Rcc1l	0.21	1.00000000	duck
Sdf2l1	0.26	1.00000000	duck
Sox13	-0.12	1.00000000	duck
Uchl5	-0.27	1.00000000	duck
Fbxo30	-0.38	1.00000000	duck
Bcl7a	-0.29	1.00000000	duck
Ctdspl	-0.88	1.00000000	duck
Ei24	0.25	1.00000000	duck

Trpm7	0.21	1.00000000	duck
Golga4	-0.04	1.00000000	duck
Ptdss1	-0.34	1.00000000	duck
Vti1b	0.16	1.00000000	duck
Snrpd3	0.26	1.00000000	duck
Sec23a	-0.62	1.00000000	duck
Gper1	0.26	1.00000000	duck
Gpx3	0.27	1.00000000	duck
Spg21	0.08	1.00000000	duck
Hspa2	-0.95	1.00000000	duck
Tbc1d12	-0.64	1.00000000	duck
Ankrd17	-0.49	1.00000000	duck
Tcp11l2	-0.01	1.00000000	duck
Ehmt1	-0.41	1.00000000	duck
Ubash3b	-0.10	1.00000000	duck
Eif3e	-0.38	1.00000000	duck
Ccdc18	-0.60	1.00000000	duck
Gatad2a	-0.52	1.00000000	duck
Nipsnap1	0.10	1.00000000	duck
Rap1gap2	-0.27	1.00000000	duck
4932411N23Rik	-0.54	1.00000000	duck
Peak1	-0.85	1.00000000	duck
Ttc28	-0.35	1.00000000	duck
Fer	0.17	1.00000000	duck
Rnf220	0.01	1.00000000	duck
Dop1a	-0.36	1.00000000	duck
Crtam	-0.31	1.00000000	duck
Atf6	0.08	1.00000000	duck
Usb1	0.12	1.00000000	duck
Tet2	0.11	1.00000000	duck
Ppfia1	-0.49	1.00000000	duck
Zfat	-0.05	1.00000000	duck
Gucd1	0.07	1.00000000	duck
Lsm8	0.29	1.00000000	duck
Kat7	-0.14	1.00000000	duck
Zzef1	-0.84	1.00000000	duck
Zdhhc7	0.04	1.00000000	duck
Fam222b	-0.56	1.00000000	duck
Ptp4a2	0.18	1.00000000	duck
Ints13	-0.53	1.00000000	duck
Cdk17	-0.63	1.00000000	duck
Tyw3	-0.36	1.00000000	duck
Vamp4	0.21	1.00000000	duck
Lonp1	-0.18	1.00000000	duck
Sephs1	0.13	1.00000000	duck
Fndc3b	-0.19	1.00000000	duck

Ccdc127	0.13	1.00000000	duck
Gramd1b	-0.15	1.00000000	duck
Slc2a8	-0.19	1.00000000	duck
Vps53	0.18	1.00000000	duck
Csrnp1	-0.57	1.00000000	duck
Cog6	-0.33	1.00000000	duck
Ankrd10	0.21	1.00000000	duck
Chchd5	0.23	1.00000000	duck
Nol10	-0.31	1.00000000	duck
Zfp618	-0.45	1.00000000	duck
Igf2r	-0.32	1.00000000	duck
Hmces	0.16	1.00000000	duck
Pcm1	-0.38	1.00000000	duck
Traf6	-0.29	1.00000000	duck
Cdc37l1	-0.37	1.00000000	duck
Bub3	0.01	1.00000000	duck
Rpa2	-0.17	1.00000000	duck
Virma	-0.37	1.00000000	duck
Lonrf2	-0.22	1.00000000	duck
Tmem242	0.18	1.00000000	duck
Vps35l	-0.13	1.00000000	duck
Uba2	-0.47	1.00000000	duck
Snrpg	0.35	1.00000000	duck
Elk4	-0.20	1.00000000	duck
Eif4enif1	-0.36	1.00000000	duck
Jarid2	-0.27	1.00000000	duck
Hikeshi	-0.13	1.00000000	duck
Exosc3	0.22	1.00000000	duck
Tbc1d22a	-0.50	1.00000000	duck
Slk	-0.05	1.00000000	duck
Sertad2	-0.03	1.00000000	duck
Eif3m	0.23	1.00000000	duck
Itch	-0.69	1.00000000	duck
Susd3	0.16	1.00000000	duck
Atp2c1	-0.09	1.00000000	duck
Atp5pb	0.13	1.00000000	duck
Atg12	0.11	1.00000000	duck
Riok1	-0.22	1.00000000	duck
Snx13	0.06	1.00000000	duck
Tln1	0.15	1.00000000	duck
Dynlt3	0.16	1.00000000	duck
Lsm6	-0.12	1.00000000	duck
Sec11a	0.25	1.00000000	duck
Rassf5	-0.48	1.00000000	duck
Psmd1	-0.13	1.00000000	duck
Tesk2	-0.89	1.00000000	duck

Bdp1	-0.55	1.00000000	duck
Gsk3b	-0.26	1.00000000	duck
Ide	-0.69	1.00000000	duck
Trappc9	-0.61	1.00000000	duck
Galns	-0.07	1.00000000	duck
Uhrf2	-0.41	1.00000000	duck
Sec14l1	-0.65	1.00000000	duck
Alcam	0.44	1.00000000	duck
Pigf	-0.54	1.00000000	duck
Usp8	0.12	1.00000000	duck
Tbc1d1	0.17	1.00000000	duck
Snrnp200	0.29	1.00000000	duck
Triap1	0.15	1.00000000	duck
Qrich1	-0.24	1.00000000	duck
Cul2	-0.63	1.00000000	duck
Aida	-0.22	1.00000000	duck
Lars	-0.31	1.00000000	duck
Ints8	-0.63	1.00000000	duck
Tlk1	-0.58	1.00000000	duck
Tcf25	0.24	1.00000000	duck
Plekha7	0.31	1.00000000	duck
Fbxl14	0.16	1.00000000	duck
Lrp3	-0.58	1.00000000	duck
Evi5	-0.80	1.00000000	duck
Taf5	-0.34	1.00000000	duck
Slu7	-0.32	1.00000000	duck
Orai2	0.16	1.00000000	duck
Abhd13	0.13	1.00000000	duck
Slc35e1	0.10	1.00000000	duck
Lin7c	-0.19	1.00000000	duck
Nde1	0.13	1.00000000	duck
Arntl	-0.55	1.00000000	duck
Tle3	0.08	1.00000000	duck
Pbrm1	-0.52	1.00000000	duck
Ndufa10	-0.06	1.00000000	duck
Nae1	-0.38	1.00000000	duck
Atg16l1	-0.37	1.00000000	duck
Rnf4	0.00	1.00000000	duck
Yipf5	0.06	1.00000000	duck
Ybx1	-0.34	1.00000000	duck
Csgalnact2	0.21	1.00000000	duck
Ufd1	-0.34	1.00000000	duck
Tnip3	0.40	1.00000000	duck
Kin	0.17	1.00000000	duck
Zfp131	0.13	1.00000000	duck
Dnajb12	0.14	1.00000000	duck

Lmo2	-0.63	1.00000000	duck
Ercc3	-0.18	1.00000000	duck
Cse1l	-0.14	1.00000000	duck
Eif4a3l1	0.18	1.00000000	duck
Stard5	-0.33	1.00000000	duck
Arap2	-0.54	1.00000000	duck
Pcgf6	-0.36	1.00000000	duck
Invs	0.06	1.00000000	duck
Ahdc1	-0.06	1.00000000	duck
Tom1l2	-0.01	1.00000000	duck
Ehbp1	-0.39	1.00000000	duck
Ube2k	-0.03	1.00000000	duck
Oga	-0.33	1.00000000	duck
Pnisr	-0.57	1.00000000	duck
Desi1	0.06	1.00000000	duck
Rsrc2	-0.39	1.00000000	duck
Psme3	0.19	1.00000000	duck
Tspan12	-0.18	1.00000000	duck
Ubqln4	0.14	1.00000000	duck
Dtx3l	0.07	1.00000000	duck
Vamp3	0.09	1.00000000	duck
Peli1	0.18	1.00000000	duck
Cnnm2	-0.90	1.00000000	duck
Ufl1	-0.18	1.00000000	duck
Tut4	-0.13	1.00000000	duck
Ran	0.28	1.00000000	duck
Crk	0.02	1.00000000	duck
Hmgb2	-0.12	1.00000000	duck
Golga1	-0.48	1.00000000	duck
Rassf8	-0.26	1.00000000	duck
Pgam5	-0.16	1.00000000	duck
Sel1l3	-0.42	1.00000000	duck
Rragd	0.05	1.00000000	duck
Dcbld2	-0.20	1.00000000	duck
Zbtb43	-0.85	1.00000000	duck
Lonrf1	-0.54	1.00000000	duck
Asxl2	-0.19	1.00000000	duck
Ccdc47	-0.04	1.00000000	duck
Sgsm2	-0.63	1.00000000	duck
4933400A11Rik	0.13	1.00000000	duck
Dnajc2	-0.30	1.00000000	duck
Nipbl	-0.04	1.00000000	duck
Ptbp1	-0.40	1.00000000	duck
Pan3	-0.83	1.00000000	duck
Zfp326	-0.38	1.00000000	duck
Lhfpl2	0.29	1.00000000	duck

Anxa2	0.29	1.00000000	duck
Dpp9	0.08	1.00000000	duck
Ext2	-0.19	1.00000000	duck
Kdm5a	-0.20	1.00000000	duck
Ppm1l	-0.06	1.00000000	duck
Zdhhc9	0.26	1.00000000	duck
Sptlc1	-0.01	1.00000000	duck
Cbx3	-0.39	1.00000000	duck
Odf2	0.18	1.00000000	duck
Tnrc6c	-0.58	1.00000000	duck
Gtf2e2	-0.13	1.00000000	duck
Hdac4	-0.42	1.00000000	duck
Armh3	-0.89	1.00000000	duck
Rest	-0.32	1.00000000	duck
Gnas	-0.02	1.00000000	duck
Rab22a	0.00	1.00000000	duck
Zc3h3	0.15	1.00000000	duck
Scaf11	-0.49	1.00000000	duck
Dhrs7c	-0.32	1.00000000	duck
Rer1	0.15	1.00000000	duck
Anxa6	-0.35	1.00000000	duck
Eif4g3	-0.18	1.00000000	duck
Six4	0.22	1.00000000	duck
Hspa5	-0.25	1.00000000	duck
Tent4a	-0.40	1.00000000	duck
Naa25	-0.26	1.00000000	duck
Slc35b1	0.12	1.00000000	duck
Ssu72	-0.34	1.00000000	duck
Spry1	0.07	1.00000000	duck
Fgd5	-0.41	1.00000000	duck
Rhot1	0.05	1.00000000	duck
Pik3cd	0.13	1.00000000	duck
Psme3ip1	-0.27	1.00000000	duck
Trim23	0.05	1.00000000	duck
Letm1	0.18	1.00000000	duck
Cnot1	-0.33	1.00000000	duck
Slc51a	-0.10	1.00000000	duck
Bnip3l	-0.13	1.00000000	duck
Exosc7	-0.06	1.00000000	duck
Yy2	-0.09	1.00000000	duck
Atp13a1	0.09	1.00000000	duck
Dusp6	0.20	1.00000000	duck
Cab39	-0.44	1.00000000	duck
Uvrag	-0.21	1.00000000	duck
Abcd3	0.19	1.00000000	duck
Gtf2f2	-0.54	1.00000000	duck

Prune1	0.02	1.00000000	duck
Phf21a	0.31	1.00000000	duck
Papola	-0.60	1.00000000	duck
Il7r	0.03	1.00000000	duck
Strada	0.11	1.00000000	duck
Larp7	0.23	1.00000000	duck
Pafah1b2	0.13	1.00000000	duck
Bnip3	-0.15	1.00000000	duck
Atad1	-0.52	1.00000000	duck
Pdia6	0.27	1.00000000	duck
Gtf3c1	-0.18	1.00000000	duck
Npepps	-0.38	1.00000000	duck
Adam10	-0.09	1.00000000	duck
Srpk1	-0.25	1.00000000	duck
Ylpm1	-0.45	1.00000000	duck
Slc25a37	-0.73	1.00000000	duck
Homer1	-0.46	1.00000000	duck
Gabpb1	-0.01	1.00000000	duck
Creb1	-0.32	1.00000000	duck
Rnaseh2b	0.21	1.00000000	duck
Tubgcp5	-0.27	1.00000000	duck
Kdm1b	0.23	1.00000000	duck
Hes6	0.23	1.00000000	duck
Rabggtb	0.06	1.00000000	duck
Atxn10	-0.38	1.00000000	duck
Ap4e1	-0.36	1.00000000	duck
Cars	-0.49	1.00000000	duck
Eepd1	-0.48	1.00000000	duck
Sec11c	0.25	1.00000000	duck
Nup133	-0.41	1.00000000	duck
Cdk9	0.11	1.00000000	duck
Iws1	0.07	1.00000000	duck
Ppard	0.16	1.00000000	duck
Dusp11	-0.21	1.00000000	duck
Bcl2l1	-0.15	1.00000000	duck
Wnt4	0.20	1.00000000	duck
Slc35a3	0.15	1.00000000	duck
Anapc2	0.18	1.00000000	duck
Slc9a7	-0.22	1.00000000	duck
Rftn1	-0.44	1.00000000	duck
Eif3j2	0.12	1.00000000	duck
Eif3a	-0.56	1.00000000	duck
Tnip2	-0.51	1.00000000	duck
Hint1	-0.11	1.00000000	duck
Tnfrsf21	0.05	1.00000000	duck
Dnajc1	-0.45	1.00000000	duck

Osbpl2	0.04	1.00000000	duck
Pdcl3	0.19	1.00000000	duck
Chst7	-0.07	1.00000000	duck
Ppa2	-0.47	1.00000000	duck
Ssb	-0.02	1.00000000	duck
Cpeb2	-0.57	1.00000000	duck
Fbxw8	-0.36	1.00000000	duck
Noc3l	-0.31	1.00000000	duck
Tmem41b	-0.01	1.00000000	duck
Cdk7	-0.26	1.00000000	duck
Ncstn	0.12	1.00000000	duck
Nubp1	-0.22	1.00000000	duck
Snx12	0.26	1.00000000	duck
Eif3d	0.10	1.00000000	duck
Cbfb	-0.23	1.00000000	duck
Ppp2r5a	-0.09	1.00000000	duck
Epb41l2	-0.55	1.00000000	duck
Sec22b	0.10	1.00000000	duck
Vti1a	-0.61	1.00000000	duck
Csnk1g2	0.14	1.00000000	duck
Znhit6	-0.29	1.00000000	duck
Adap1	-0.46	1.00000000	duck
Ndufb9	0.27	1.00000000	duck
Trerf1	-0.74	1.00000000	duck
Stk17b	-0.52	1.00000000	duck
Slc43a2	-0.13	1.00000000	duck
Klhl15	-0.55	1.00000000	duck
Rfx2	-0.11	1.00000000	duck
Aak1	-0.13	1.00000000	duck
Arhgap1	0.11	1.00000000	duck
Vta1	0.19	1.00000000	duck
Tef	-0.50	1.00000000	duck
Nyx	0.00	1.00000000	duck
Insig1	0.28	1.00000000	duck
Cpeb4	-0.28	1.00000000	duck
Hp1bp3	-0.01	1.00000000	duck
Arf4	-0.44	1.00000000	duck
Tsc2	-0.28	1.00000000	duck
Adipor2	0.07	1.00000000	duck
Akap8	-0.46	1.00000000	duck
Camk1d	-0.72	1.00000000	duck
Rab5a	-0.03	1.00000000	duck
Pitpnm3	-0.09	1.00000000	duck
Trim59	-0.23	1.00000000	duck
Radx	-0.20	1.00000000	duck
Rab6a	0.00	1.00000000	duck

Uxs1	-0.64	1.00000000	duck
Jdp2	0.24	1.00000000	duck
Tomm20	-0.28	1.00000000	duck
Idh2	0.22	1.00000000	duck
Derl1	0.10	1.00000000	duck
Rab2a	0.18	1.00000000	duck
Gpc5	-0.07	1.00000000	duck
Slc30a7	0.13	1.00000000	duck
Vkorc1l1	-0.24	1.00000000	duck
Tent2	-0.37	1.00000000	duck
Pmm2	-0.27	1.00000000	duck
Fem1b	0.05	1.00000000	duck
Ptgfrn	-0.29	1.00000000	duck
Nip7	0.11	1.00000000	duck
Acvr1	-0.16	1.00000000	duck
Vma21	0.08	1.00000000	duck
Hsd17b4	-0.24	1.00000000	duck
Timm44	-0.17	1.00000000	duck
Cdc42bpb	0.08	1.00000000	duck
Hibch	0.15	1.00000000	duck
Ints11	-0.26	1.00000000	duck
Eprs	0.10	1.00000000	duck
Gpatch11	0.05	1.00000000	duck
Acot10	-0.08	1.00000000	duck
St13	-0.05	1.00000000	duck
Snx29	-0.41	1.00000000	duck
Stau2	-0.18	1.00000000	duck
Narf	-0.17	1.00000000	duck
Usp22	-0.59	1.00000000	duck
Sh3gl1	0.15	1.00000000	duck
Pdxk	0.20	1.00000000	duck
Ice2	-0.46	1.00000000	duck
Fbxw11	0.07	1.00000000	duck
Scrib	-0.31	1.00000000	duck
Smarca1	-0.17	1.00000000	duck
Fyttd1	0.18	1.00000000	duck
Kmt5b	0.03	1.00000000	duck
Thyn1	-0.04	1.00000000	duck
Kpnb1	-0.24	1.00000000	duck
Atm	-0.23	1.00000000	duck
Decr2	0.12	1.00000000	duck
Eaf1	0.05	1.00000000	duck
Trmt10c	0.13	1.00000000	duck
Paxbp1	-0.60	1.00000000	duck
Ampd2	0.06	1.00000000	duck
Lemd3	-0.34	1.00000000	duck

Dennd4a	-0.35	1.00000000	duck
St7l	-0.01	1.00000000	duck
Btbd7	-0.24	1.00000000	duck
Crnkl1	0.20	1.00000000	duck
Heatr5b	-0.43	1.00000000	duck
Zfp318	-0.24	1.00000000	duck
Bin3	0.12	1.00000000	duck
Rwdd1	0.20	1.00000000	duck
Leo1	-0.04	1.00000000	duck
Slc12a2	-0.51	1.00000000	duck
Hells	-0.15	1.00000000	duck
Ttc33	-0.01	1.00000000	duck
Gipc2	0.05	1.00000000	duck
Dgkd	-0.51	1.00000000	duck
Nupl1	-0.30	1.00000000	duck
Stag1	-0.26	1.00000000	duck
Srp54a	0.14	1.00000000	duck
Tpm4	-0.16	1.00000000	duck
Rnh1	0.08	1.00000000	duck
Foxj2	-0.11	1.00000000	duck
Ell2	-0.37	1.00000000	duck
Nup210	-0.38	1.00000000	duck
Prdm1	0.24	1.00000000	duck
Commd8	0.14	1.00000000	duck
Fam8a1	0.08	1.00000000	duck
Ext1	-0.22	1.00000000	duck
Cactin	0.11	1.00000000	duck
Pdpk1	-0.50	1.00000000	duck
Tmem11	-0.19	1.00000000	duck
Zcchc17	0.12	1.00000000	duck
Tk2	-0.08	1.00000000	duck
Rnf168	-0.34	1.00000000	duck
Abhd17c	-0.34	1.00000000	duck
Cog7	-0.17	1.00000000	duck
Dhdds	-0.08	1.00000000	duck
Taf5l	-0.32	1.00000000	duck
Gpatch1	-0.51	1.00000000	duck
Atmin	-0.03	1.00000000	duck
Dpm2	0.06	1.00000000	duck
Thoc7	0.11	1.00000000	duck
Efl1	-0.29	1.00000000	duck
Ube2g1	0.04	1.00000000	duck
Dapk2	-0.64	1.00000000	duck
G3bp2	0.03	1.00000000	duck
Pop4	-0.25	1.00000000	duck
Kdm4b	-0.37	1.00000000	duck

Prkd3	0.09	1.00000000	duck
Sash3	0.19	1.00000000	duck
Gnl2	0.15	1.00000000	duck
Krr1	0.10	1.00000000	duck
Ppm1g	-0.21	1.00000000	duck
Vps41	0.19	1.00000000	duck
Erich6	-0.11	1.00000000	duck
Usp4	0.11	1.00000000	duck
Gnptg	0.27	1.00000000	duck
Kifbp	-0.26	1.00000000	duck
Canx	0.05	1.00000000	duck
C1d	-0.14	1.00000000	duck
Epg5	-0.06	1.00000000	duck
Klhdc10	0.04	1.00000000	duck
Strn3	-0.49	1.00000000	duck
Bsdc1	0.05	1.00000000	duck
Ctnnb1	-0.03	1.00000000	duck
Fars2	-0.78	1.00000000	duck
Hsf1	-0.26	1.00000000	duck
Ggh	-0.05	1.00000000	duck
Pik3r6	-0.42	1.00000000	duck
Dnajb4	-0.66	1.00000000	duck
Lmtk2	-0.69	1.00000000	duck
Phf14	-0.23	1.00000000	duck
Dbi	-0.18	1.00000000	duck
Tmem39a	0.15	1.00000000	duck
Smad3	0.14	1.00000000	duck
Rbbp4	-0.19	1.00000000	duck
Tfdp2	0.23	1.00000000	duck
Zfr	-0.09	1.00000000	duck
Lats1	-0.24	1.00000000	duck
Ywhab	-0.29	1.00000000	duck
Slc45a1	-0.18	1.00000000	duck
Ap3m1	0.10	1.00000000	duck
Nifk	0.23	1.00000000	duck
Ankra2	-0.14	1.00000000	duck
Aig1	0.18	1.00000000	duck
Irf4	-0.15	1.00000000	duck
Rab11a	0.02	1.00000000	duck
Camta1	-0.26	1.00000000	duck
Ddx46	-0.23	1.00000000	duck
Txndc17	0.23	1.00000000	duck
Tmem9b	0.10	1.00000000	duck
Wnk1	-0.17	1.00000000	duck
Golgb1	-0.57	1.00000000	duck
Fbxo25	0.13	1.00000000	duck

Cep44	0.10	1.00000000	duck
Nap1l4	-0.35	1.00000000	duck
1700034I23Rik	0.18	1.00000000	duck
Tdrd3	-0.26	1.00000000	duck
Aprt	0.07	1.00000000	duck
Il6st	-0.03	1.00000000	duck
Phf5a	0.13	1.00000000	duck
Aebp2	0.10	1.00000000	duck
Dync1li2	-0.43	1.00000000	duck
Tank	0.14	1.00000000	duck
Bicral	-0.58	1.00000000	duck
Gga1	0.11	1.00000000	duck
Faf1	-0.24	1.00000000	duck
Ctnna1	0.24	1.00000000	duck
Rab10	-0.07	1.00000000	duck
Supv3l1	-0.28	1.00000000	duck
Kif5b	-0.11	1.00000000	duck
Washc4	-0.14	1.00000000	duck
Pisd	-0.20	1.00000000	duck
Eif3l	0.19	1.00000000	duck
Crtc1	-0.30	1.00000000	duck
Dpy30	0.07	1.00000000	duck
Areg	-0.26	1.00000000	duck
Ube2v2	-0.01	1.00000000	duck
Kctd2	0.15	1.00000000	duck
Adarb1	0.16	1.00000000	duck
Atf1	-0.12	1.00000000	duck
Dynll2	-0.08	1.00000000	duck
Dnajc7	-0.01	1.00000000	duck
Fmr1	0.01	1.00000000	duck
Slc30a5	0.14	1.00000000	duck
Wasf2	0.10	1.00000000	duck
Prkci	-0.26	1.00000000	duck
Wdr53	0.07	1.00000000	duck
Sin3b	-0.37	1.00000000	duck
Psmd10	0.10	1.00000000	duck
Aldoc	0.18	1.00000000	duck
Tnfaip1	-0.29	1.00000000	duck
2510039O18Rik	0.14	1.00000000	duck
Ncbp1	-0.25	1.00000000	duck
Sfxn1	0.15	1.00000000	duck
Smg1	-0.40	1.00000000	duck
Pdss2	-0.50	1.00000000	duck
Rtraf	0.09	1.00000000	duck
Cct8	0.11	1.00000000	duck
Stx11	0.17	1.00000000	duck

Rock1	0.09	1.00000000	duck
Nfyb	0.14	1.00000000	duck
Foxn2	-0.30	1.00000000	duck
Fbxw2	-0.09	1.00000000	duck
Cdk8	-0.62	1.00000000	duck
Upf2	-0.49	1.00000000	duck
Eri3	-0.35	1.00000000	duck
Spty2d1	-0.32	1.00000000	duck
Cdc26	0.27	1.00000000	duck
Ube2j2	0.08	1.00000000	duck
Tanc1	0.15	1.00000000	duck
Nras	0.03	1.00000000	duck
Fbxo46	0.14	1.00000000	duck
Adam17	-0.35	1.00000000	duck
Aff1	-0.44	1.00000000	duck
Psen1	0.03	1.00000000	duck
Kras	0.13	1.00000000	duck
Xpot	-0.34	1.00000000	duck
Pepd	0.14	1.00000000	duck
Ap1g1	0.01	1.00000000	duck
Jmy	0.00	1.00000000	duck
Zrsr1	0.23	1.00000000	duck
Lrp12	0.05	1.00000000	duck
Eya3	0.09	1.00000000	duck
Zcrb1	-0.02	1.00000000	duck
Sf3a1	0.06	1.00000000	duck
Plaa	0.12	1.00000000	duck
Litaf	-0.16	1.00000000	duck
Cdc42	0.00	1.00000000	duck
Top2b	-0.52	1.00000000	duck
Luzp1	-0.27	1.00000000	duck
Naa30	-0.54	1.00000000	duck
Wdr36	-0.04	1.00000000	duck
Tspan3	-0.40	1.00000000	duck
Rab12	-0.10	1.00000000	duck
Cdc27	-0.33	1.00000000	duck
Eloc	-0.07	1.00000000	duck
Kpna3	-0.20	1.00000000	duck
Ankmy2	-0.11	1.00000000	duck
Slf2	-0.33	1.00000000	duck
Pcgf3	-0.06	1.00000000	duck
Arsk	-0.48	1.00000000	duck
Naa60	0.27	1.00000000	duck
Pde8a	-0.18	1.00000000	duck
Ttc1	0.09	1.00000000	duck
Txnl1	-0.09	1.00000000	duck

Rnf126	0.13	1.00000000	duck
Cnot8	-0.06	1.00000000	duck
Ccnk	0.14	1.00000000	duck
Psmc2	0.10	1.00000000	duck
Fbxw5	-0.18	1.00000000	duck
Acad9	-0.29	1.00000000	duck
Dtymk	-0.10	1.00000000	duck
Bdh1	-0.16	1.00000000	duck
Tnfaip8	-0.32	1.00000000	duck
Trafd1	-0.02	1.00000000	duck
Rabepk	0.14	1.00000000	duck
Lrrfip2	0.25	1.00000000	duck
Rufy3	-0.01	1.00000000	duck
Ugcg	-0.50	1.00000000	duck
Tmed2	0.14	1.00000000	duck
Osbpl11	-0.39	1.00000000	duck
Lig3	-0.26	1.00000000	duck
Tle4	-0.09	1.00000000	duck
9930021J03Rik	0.00	1.00000000	duck
Impa2	-0.11	1.00000000	duck
Pwwp2a	0.12	1.00000000	duck
Smarce1	-0.03	1.00000000	duck
Selenoi	0.17	1.00000000	duck
Fkbp3	0.07	1.00000000	duck
Sufu	-0.64	1.00000000	duck
Rnf10	0.00	1.00000000	duck
Deaf1	0.00	1.00000000	duck
Veph1	-0.22	1.00000000	duck
Ttc17	-0.02	1.00000000	duck
Gnb4	-0.27	1.00000000	duck
Nfya	-0.06	1.00000000	duck
Sync	-0.17	1.00000000	duck
Rcsd1	-0.44	1.00000000	duck
Tti1	-0.31	1.00000000	duck
Pwwp2b	-0.26	1.00000000	duck
Yars	0.20	1.00000000	duck
Fasl	0.10	1.00000000	duck
Pknox1	-0.91	1.00000000	duck
Prpf6	-0.03	1.00000000	duck
Trim33	-0.31	1.00000000	duck
Dyrk1a	-0.47	1.00000000	duck
Gpbp1l1	-0.07	1.00000000	duck
Cnep1r1	-0.07	1.00000000	duck
Gna12	-0.38	1.00000000	duck
Arid3b	0.12	1.00000000	duck
Tbcd	-0.57	1.00000000	duck

Sart3	-0.31	1.00000000	duck
Zbtb41	-0.53	1.00000000	duck
Spag9	-0.16	1.00000000	duck
Prmt9	-0.17	1.00000000	duck
Acat1	-0.11	1.00000000	duck
Gabpa	-0.36	1.00000000	duck
Ube3b	-0.01	1.00000000	duck
Ctnnbl1	-0.50	1.00000000	duck
Ndst4	-0.19	1.00000000	duck
Rbm6	-0.13	1.00000000	duck
Rab3gap2	-0.38	1.00000000	duck
Edrf1	-0.24	1.00000000	duck
Atp2a3	-0.15	1.00000000	duck
Vdac3	0.09	1.00000000	duck
Atp11b	-0.40	1.00000000	duck
Kansl3	-0.14	1.00000000	duck
Esyt2	-0.13	1.00000000	duck
Ddx1	-0.43	1.00000000	duck
Gdpd5	0.23	1.00000000	duck
Vcl	-0.56	1.00000000	duck
Snx14	-0.47	1.00000000	duck
Vapb	0.03	1.00000000	duck
Tmem164	0.08	1.00000000	duck
Tsr3	0.13	1.00000000	duck
Ttc37	-0.40	1.00000000	duck
Clk2	0.08	1.00000000	duck
Nsmaf	-0.53	1.00000000	duck
Prkar1a	0.06	1.00000000	duck
Ambra1	-0.39	1.00000000	duck
Vps13a	-0.21	1.00000000	duck
Slc35b3	0.08	1.00000000	duck
Cd44	-0.22	1.00000000	duck
Angel2	-0.29	1.00000000	duck
Cnksr2	0.05	1.00000000	duck
Snrnp40	0.10	1.00000000	duck
Appbp2	-0.02	1.00000000	duck
Dcun1d3	-0.30	1.00000000	duck
Hip1	0.28	1.00000000	duck
Fubp3	0.10	1.00000000	duck
Anks1	-0.32	1.00000000	duck
Zbtb8os	0.24	1.00000000	duck
Fam76b	-0.13	1.00000000	duck
Arl5b	-0.27	1.00000000	duck
Tmem19	-0.20	1.00000000	duck
Zcchc8	-0.09	1.00000000	duck
Dtnbp1	-0.24	1.00000000	duck

Rac1	-0.26	1.00000000	duck
Sap130	0.23	1.00000000	duck
Ankrd52	-0.16	1.00000000	duck
Nr1h3	-0.06	1.00000000	duck
Surf4	0.24	1.00000000	duck
Smarcc1	-0.54	1.00000000	duck
Ccnyl1	-0.18	1.00000000	duck
Ncoa2	-0.05	1.00000000	duck
Hlf	0.10	1.00000000	duck
Ube2r2	-0.18	1.00000000	duck
Vezf1	-0.63	1.00000000	duck
Ipo9	0.03	1.00000000	duck
Rbm26	-0.33	1.00000000	duck
Anapc1	0.17	1.00000000	duck
Adat1	0.28	1.00000000	duck
Lman1	-0.04	1.00000000	duck
Ago3	-0.26	1.00000000	duck
Tada1	-0.40	1.00000000	duck
Ap2b1	0.05	1.00000000	duck
Ireb2	-0.39	1.00000000	duck
Ywhae	0.06	1.00000000	duck
Scaper	-0.34	1.00000000	duck
Hdac7	0.09	1.00000000	duck
Cfap20	0.07	1.00000000	duck
Foxp2	-1.06	1.00000000	duck
Clint1	0.03	1.00000000	duck
Pccb	-0.06	1.00000000	duck
Abat	-0.09	1.00000000	duck
Atp6v1c1	0.06	1.00000000	duck
Bloc1s4	0.02	1.00000000	duck
Nup214	-0.27	1.00000000	duck
Dennd1a	-0.56	1.00000000	duck
Ppp3cb	-0.34	1.00000000	duck
Pafah1b1	0.01	1.00000000	duck
Tbpl1	0.07	1.00000000	duck
Pogz	-0.27	1.00000000	duck
Clpx	0.16	1.00000000	duck
Arnt	-0.01	1.00000000	duck
Ptger4	0.31	1.00000000	duck
Zfp143	-0.17	1.00000000	duck
Scfd1	-0.30	1.00000000	duck
Ap1m1	0.08	1.00000000	duck
Actr6	0.00	1.00000000	duck
Bola3	0.06	1.00000000	duck
Ddx27	-0.22	1.00000000	duck
Dhx38	-0.01	1.00000000	duck

Srpr	-0.20	1.00000000	duck
Scaf4	-0.26	1.00000000	duck
Copg2	-0.15	1.00000000	duck
Acvr2a	0.03	1.00000000	duck
Ube4b	-0.21	1.00000000	duck
Wdr7	-0.55	1.00000000	duck
Slc7a10	-0.32	1.00000000	duck
Psmd4	0.06	1.00000000	duck
Dhx30	-0.24	1.00000000	duck
Fam167b	0.07	1.00000000	duck
Ubp1	-0.39	1.00000000	duck
Stt3a	0.13	1.00000000	duck
Erlin1	-0.03	1.00000000	duck
Phf3	-0.55	1.00000000	duck
Ate1	-0.15	1.00000000	duck
Phrf1	-0.26	1.00000000	duck
Auh	-0.17	1.00000000	duck
Pdcd5	-0.20	1.00000000	duck
Rp2	-0.19	1.00000000	duck
Asns	-0.13	1.00000000	duck
Ncoa5	-0.22	1.00000000	duck
Casp8ap2	-0.33	1.00000000	duck
Vdac2	0.10	1.00000000	duck
Foxk1	-0.51	1.00000000	duck
Usf3	0.09	1.00000000	duck
Vwa8	0.08	1.00000000	duck
Vezt	-0.15	1.00000000	duck
Dym	-0.34	1.00000000	duck
Ubn2	-0.21	1.00000000	duck
Slc25a44	0.03	1.00000000	duck
Katnbl1	-0.14	1.00000000	duck
Naa50	-0.39	1.00000000	duck
Rexo4	-0.11	1.00000000	duck
Fbxl17	-0.28	1.00000000	duck
Ing4	0.11	1.00000000	duck
Dram2	0.11	1.00000000	duck
Hspa8	-0.01	1.00000000	duck
Actr10	-0.02	1.00000000	duck
Zfp292	-0.28	1.00000000	duck
Hprt	-0.17	1.00000000	duck
Entpd4	-0.18	1.00000000	duck
Ing5	-0.32	1.00000000	duck
Akap8l	-0.07	1.00000000	duck
Bcl2l13	-0.37	1.00000000	duck
G3bp1	-0.27	1.00000000	duck
Zbtb37	-0.45	1.00000000	duck

Sec16a	-0.28	1.00000000	duck
Arfgap1	-0.19	1.00000000	duck
Rhof	-0.22	1.00000000	duck
Pten	-0.02	1.00000000	duck
Kdm2b	-0.04	1.00000000	duck
Nrbp1	-0.43	1.00000000	duck
Lgals8	-0.11	1.00000000	duck
Nfs1	-0.03	1.00000000	duck
Elk3	0.14	1.00000000	duck
Purb	0.21	1.00000000	duck
Uhmk1	0.06	1.00000000	duck
Rab39b	0.11	1.00000000	duck
Arhgap25	0.06	1.00000000	duck
Arpp19	0.06	1.00000000	duck
Nup50	-0.37	1.00000000	duck
Larp4b	-0.19	1.00000000	duck
Abcb7	-0.06	1.00000000	duck
Cep85l	-0.29	1.00000000	duck
Prpf3	0.17	1.00000000	duck
Nr2c2	-0.25	1.00000000	duck
Arid5b	0.02	1.00000000	duck
Nr2c1	-0.40	1.00000000	duck
Zfp148	-0.26	1.00000000	duck
Wapl	-0.39	1.00000000	duck
Pgrmc1	0.09	1.00000000	duck
Nr4a3	0.05	1.00000000	duck
Kdsr	-0.14	1.00000000	duck
Immt	-0.09	1.00000000	duck
Ptch1	-0.32	1.00000000	duck
Sptlc2	-0.30	1.00000000	duck
Ppp2ca	-0.29	1.00000000	duck
Slc30a4	-0.09	1.00000000	duck
Eef2	0.00	1.00000000	duck
Ppme1	-0.18	1.00000000	duck
Kctd5	-0.44	1.00000000	duck
Ubap2l	-0.32	1.00000000	duck
Fnbp1l	0.23	1.00000000	duck
Fbxw7	0.08	1.00000000	duck
Vdr	0.10	1.00000000	duck
Dok2	0.18	1.00000000	duck
Apoo	-0.13	1.00000000	duck
Slc25a5	0.00	1.00000000	duck
Ccnl2	-0.24	1.00000000	duck
Tshz2	-0.60	1.00000000	duck
Rexo1	-0.05	1.00000000	duck
Fos	0.26	1.00000000	duck

Appl1	0.04	1.00000000	duck
Pxylp1	-0.52	1.00000000	duck
Rab8b	-0.27	1.00000000	duck
Srp9	0.04	1.00000000	duck
4833439L19Rik	-0.22	1.00000000	duck
Slc38a10	0.07	1.00000000	duck
Btg2	-0.32	1.00000000	duck
Ppp4r3a	-0.51	1.00000000	duck
Eif4g1	-0.22	1.00000000	duck
Api5	0.09	1.00000000	duck
Erc1	-0.31	1.00000000	duck
Tsc1	-0.10	1.00000000	duck
Itpr1	-0.19	1.00000000	duck
Eefsec	-0.32	1.00000000	duck
Acadsb	-0.29	1.00000000	duck
Uhrf1bp1l	0.21	1.00000000	duck
Eif2b1	0.10	1.00000000	duck
Txlna	-0.33	1.00000000	duck
Ppp1cc	0.02	1.00000000	duck
Dhx8	0.11	1.00000000	duck
Zhx1	-0.09	1.00000000	duck
Akt1	0.15	1.00000000	duck
Srsf5	-0.25	1.00000000	duck
Snrpb	0.24	1.00000000	duck
Aftph	-0.12	1.00000000	duck
Zc3h15	0.09	1.00000000	duck
Tpp2	-0.31	1.00000000	duck
Dgke	-0.16	1.00000000	duck
Zfp638	-0.78	1.00000000	duck
Slc4a11	-0.28	1.00000000	duck
Tmem64	0.01	1.00000000	duck
Tmem30a	-0.13	1.00000000	duck
Fam168b	-0.13	1.00000000	duck
Yme1l1	0.11	1.00000000	duck
Btbd10	-0.43	1.00000000	duck
Avl9	0.01	1.00000000	duck
Get4	0.08	1.00000000	duck
Hspa4	-0.58	1.00000000	duck
Wwox	-0.67	1.00000000	duck
Nkrf	0.10	1.00000000	duck
Rnf169	-0.20	1.00000000	duck
Zfp592	0.31	1.00000000	duck
Pdk3	-0.76	1.00000000	duck
Arhgef7	-0.20	1.00000000	duck
Phactr2	-0.14	1.00000000	duck
Inpp4a	0.07	1.00000000	duck

Sema4d	-0.43	1.00000000	duck
Tut7	-0.13	1.00000000	duck
Siah1b	-0.19	1.00000000	duck
Snx4	-0.20	1.00000000	duck
Pi4kb	-0.04	1.00000000	duck
Armc1	-0.30	1.00000000	duck
Ap3s1	0.01	1.00000000	duck
Cisd1	-0.02	1.00000000	duck
Lonrf3	-0.37	1.00000000	duck
Plcg2	-0.27	1.00000000	duck
Paip1	0.09	1.00000000	duck
Copb2	0.01	1.00000000	duck
Upf1	-0.12	1.00000000	duck
Capza2	-0.04	1.00000000	duck
Cst7	0.11	1.00000000	duck
Cnot10	0.03	1.00000000	duck
Smchd1	-0.58	1.00000000	duck
Dctn4	0.07	1.00000000	duck
Cwf19l1	-0.05	1.00000000	duck
Nt5c3	-0.20	1.00000000	duck
Spg11	0.11	1.00000000	duck
Prelid3b	0.05	1.00000000	duck
Cnot2	-0.16	1.00000000	duck
Nedd4	-0.11	1.00000000	duck
Pitpnb	-0.24	1.00000000	duck
Pfkfb3	-0.58	1.00000000	duck
Rbis	-0.19	1.00000000	duck
Csnk2a2	-0.29	1.00000000	duck
Trabd	-0.03	1.00000000	duck
Bsg	-0.41	1.00000000	duck
Lclat1	-0.13	1.00000000	duck
Pik3c3	-0.26	1.00000000	duck
Cbx6	0.07	1.00000000	duck
Snrpf	0.28	1.00000000	duck
Ywhag	0.05	1.00000000	duck
Lmnb2	-0.17	1.00000000	duck
Cwc27	-0.62	1.00000000	duck
Ralgapa1	-0.55	1.00000000	duck
Rmc1	-0.17	1.00000000	duck
Rev1	-0.54	1.00000000	duck
Rab35	-0.40	1.00000000	duck
Usp47	-0.32	1.00000000	duck
Dsn1	0.05	1.00000000	duck
Ndufa6	0.15	1.00000000	duck
Emc2	0.13	1.00000000	duck
Hace1	0.17	1.00000000	duck

Pard3b	-0.60	1.00000000	duck
Arhgap45	-0.17	1.00000000	duck
Supt6	-0.01	1.00000000	duck
Sf3b1	-0.23	1.00000000	duck
Celf2	-0.08	1.00000000	duck
Grk2	0.17	1.00000000	duck
Zfyve26	0.06	1.00000000	duck
Agpat4	0.05	1.00000000	duck
Abraxas1	0.07	1.00000000	duck
Qk	-0.45	1.00000000	duck
Iffo2	0.07	1.00000000	duck
Hgs	-0.02	1.00000000	duck
Cacna1c	0.24	1.00000000	duck
Eif3b	-0.26	1.00000000	duck
Tent5a	0.17	1.00000000	duck
Ecd	-0.09	1.00000000	duck
Fes	0.05	1.00000000	duck
Lipc	0.08	1.00000000	duck
Cdk12	0.01	1.00000000	duck
Polk	-0.16	1.00000000	duck
Exosc10	-0.17	1.00000000	duck
Rnps1	0.08	1.00000000	duck
Nmt2	0.19	1.00000000	duck
Tmed10	-0.15	1.00000000	duck
Foxk2	-0.36	1.00000000	duck
Phtf2	-0.26	1.00000000	duck
Spg7	-0.01	1.00000000	duck
Upb1	0.05	1.00000000	duck
Bicd2	-0.08	1.00000000	duck
Gzf1	-0.08	1.00000000	duck
Nup98	-0.32	1.00000000	duck
Ranbp1	-0.13	1.00000000	duck
Foxo4	-0.07	1.00000000	duck
Smurf1	0.12	1.00000000	duck
Gapvd1	-0.51	1.00000000	duck
Git2	-0.07	1.00000000	duck
Golga5	0.03	1.00000000	duck
Cul3	-0.46	1.00000000	duck
Nedd4l	-0.05	1.00000000	duck
Cish	-0.64	1.00000000	duck
Wdr20	-0.53	1.00000000	duck
Exoc6	-0.10	1.00000000	duck
Ranbp10	-0.37	1.00000000	duck
Ppp4r1	0.06	1.00000000	duck
Ptpn11	-0.38	1.00000000	duck
Tmem184b	-0.36	1.00000000	duck

Lrif1	-0.08	1.00000000	duck
Braf	-0.32	1.00000000	duck
Hyou1	-0.06	1.00000000	duck
Bend7	0.08	1.00000000	duck
Lsm11	0.04	1.00000000	duck
Elf1	-0.26	1.00000000	duck
Tmem70	-0.05	1.00000000	duck
Tmf1	-0.03	1.00000000	duck
Ppp3cc	-0.17	1.00000000	duck
Galk2	-0.03	1.00000000	duck
Ranbp9	-0.53	1.00000000	duck
Septin7	-0.46	1.00000000	duck
Rictor	-0.20	1.00000000	duck
Osbpl8	-0.04	1.00000000	duck
Camkmt	-0.40	1.00000000	duck
Tmx1	-0.09	1.00000000	duck
Vps29	-0.01	1.00000000	duck
Faf2	-0.06	1.00000000	duck
Rere	-0.36	1.00000000	duck
Pdia3	0.05	1.00000000	duck
Luc7l	-0.01	1.00000000	duck
Epas1	-0.66	1.00000000	duck
Hif1an	-0.20	1.00000000	duck
AW554918	0.06	1.00000000	duck
Fam168a	-0.36	1.00000000	duck
Vps35	0.08	1.00000000	duck
Fgfr1op2	-0.37	1.00000000	duck
Gpr137c	-0.07	1.00000000	duck
Lrrc59	0.11	1.00000000	duck
Pex14	-0.14	1.00000000	duck
Cnst	-0.21	1.00000000	duck
Nsrp1	-0.30	1.00000000	duck
Ptbp2	-0.17	1.00000000	duck
Bcl6	0.14	1.00000000	duck
Stim1	-0.19	1.00000000	duck
Cwc25	0.00	1.00000000	duck
Ppp2r5e	-0.06	1.00000000	duck
Anapc5	0.06	1.00000000	duck
Copa	-0.04	1.00000000	duck
Alas1	-0.18	1.00000000	duck
Hbs1l	-0.59	1.00000000	duck
Selenok	0.02	1.00000000	duck
Drg1	-0.03	1.00000000	duck
Gnpda2	-0.21	1.00000000	duck
Sec63	-0.43	1.00000000	duck
Cops8	0.04	1.00000000	duck

Usp33	-0.36	1.00000000	duck
Ube3a	-0.24	1.00000000	duck
Commd2	0.09	1.00000000	duck
Oard1	-0.16	1.00000000	duck
Atf2	-0.40	1.00000000	duck
Xpnpep1	-0.24	1.00000000	duck
Pank4	-0.08	1.00000000	duck
Plekhm2	0.13	1.00000000	duck
Orc3	-0.14	1.00000000	duck
Papss1	0.20	1.00000000	duck
Sugt1	-0.43	1.00000000	duck
Ruvbl1	0.03	1.00000000	duck
Ints2	-0.03	1.00000000	duck
Larp1	-0.20	1.00000000	duck
Pdap1	-0.02	1.00000000	duck
Ubxn7	-0.15	1.00000000	duck
Rnpepl1	-0.27	1.00000000	duck
Sec31a	-0.09	1.00000000	duck
Ikbkb	-0.14	1.00000000	duck
Ybx3	-0.02	1.00000000	duck
Edc4	-0.14	1.00000000	duck
Tbc1d23	-0.16	1.00000000	duck
Igf2bp3	0.06	1.00000000	duck
Nfyc	-0.14	1.00000000	duck
Ep300	-0.27	1.00000000	duck
Scyl2	-0.21	1.00000000	duck
Etv6	-0.22	1.00000000	duck
Ube2d1	-0.36	1.00000000	duck
Tsc22d2	-0.36	1.00000000	duck
Cript	0.07	1.00000000	duck
Atic	0.07	1.00000000	duck
Arl4a	0.13	1.00000000	duck
Xpo4	-0.18	1.00000000	duck
Acbd5	-0.32	1.00000000	duck
Dusp10	-0.14	1.00000000	duck
Khdc4	-0.10	1.00000000	duck
Atg13	-0.16	1.00000000	duck
Copb1	-0.12	1.00000000	duck
Rngtt	0.04	1.00000000	duck
Nisch	-0.16	1.00000000	duck
Eif3f	-0.14	1.00000000	duck
Rsl24d1	-0.16	1.00000000	duck
Septin9	-0.34	1.00000000	duck
Adrm1	-0.27	1.00000000	duck
Pqlc3	-0.16	1.00000000	duck
Wbp11	-0.35	1.00000000	duck

Lemd2	0.08	1.00000000	duck
Clcc1	0.11	1.00000000	duck
Setdb1	-0.05	1.00000000	duck
Sppl2a	-0.15	1.00000000	duck
Abl1	-0.33	1.00000000	duck
Zcchc14	0.02	1.00000000	duck
Pcmtd1	-0.86	1.00000000	duck
Ndufa5	-0.01	1.00000000	duck
Cstf1	-0.32	1.00000000	duck
Ppil1	0.02	1.00000000	duck
Atxn1	-0.59	1.00000000	duck
Prkcb	-0.01	1.00000000	duck
Rbbp6	-0.18	1.00000000	duck
Chpf2	0.11	1.00000000	duck
Efr3a	-0.44	1.00000000	duck
Nol9	-0.04	1.00000000	duck
Plekha2	0.13	1.00000000	duck
Rab9	-0.19	1.00000000	duck
Rabif	-0.02	1.00000000	duck
Gtf2h1	-0.22	1.00000000	duck
Isca1	0.02	1.00000000	duck
Commd1b	0.04	1.00000000	duck
Cyld	-0.03	1.00000000	duck
Ccny	-0.27	1.00000000	duck
Ilrun	-0.56	1.00000000	duck
Ppp1r8	-0.20	1.00000000	duck
Rreb1	-0.71	1.00000000	duck
Ctsh	0.07	1.00000000	duck
Ythdf3	-0.31	1.00000000	duck
Ipo8	-0.27	1.00000000	duck
Golm2	-0.38	1.00000000	duck
Ap3d1	-0.23	1.00000000	duck
Sgpl1	-0.38	1.00000000	duck
Uggt1	-0.28	1.00000000	duck
Brwd3	-0.48	1.00000000	duck
Dip2b	0.03	1.00000000	duck
Aqr	-0.43	1.00000000	duck
Erh	-0.08	1.00000000	duck
Tcea1	-0.32	1.00000000	duck
Tm9sf4	0.03	1.00000000	duck
Gpr68	-0.31	1.00000000	duck
Rcor1	-0.26	1.00000000	duck
Rapgef1	-0.13	1.00000000	duck
Rnf216	0.06	1.00000000	duck
Ogfrl1	0.02	1.00000000	duck
Gnai3	-0.43	1.00000000	duck

Dnajc19	-0.02	1.00000000	duck
Rprd1a	-0.21	1.00000000	duck
Stk10	-0.08	1.00000000	duck
Vps37a	-0.10	1.00000000	duck
Nfrkb	-0.14	1.00000000	duck
Slc6a6	-0.31	1.00000000	duck
Edc3	-0.05	1.00000000	duck
Bcl7b	-0.01	1.00000000	duck
Prkce	-0.48	1.00000000	duck
Nipa2	0.04	1.00000000	duck
Pinx1	-0.33	1.00000000	duck
Lpin2	-0.70	1.00000000	duck
Pum1	-0.07	1.00000000	duck
N4bp2	-0.18	1.00000000	duck
Gns	-0.31	1.00000000	duck
Zfand6	-0.30	1.00000000	duck
Ppil4	-0.21	1.00000000	duck
Gigyf2	-0.32	1.00000000	duck
Crkl	-0.03	1.00000000	duck
Cdc40	0.02	1.00000000	duck
Ints1	-0.09	1.00000000	duck
Ube2a	-0.13	1.00000000	duck
Ralgapb	-0.43	1.00000000	duck
Cramp1	-0.35	1.00000000	duck
Acsf2	0.04	1.00000000	duck
Stx17	0.20	1.00000000	duck
Lrrc1	0.16	1.00000000	duck
Dph3	-0.15	1.00000000	duck
Sirt1	-0.34	1.00000000	duck
Acbd6	-0.05	1.00000000	duck
Ctcf	-0.15	1.00000000	duck
Rilpl2	0.12	1.00000000	duck
Eef1b2	-0.16	1.00000000	duck
Kif1b	-0.36	1.00000000	duck
Tob2	0.00	1.00000000	duck
Tmem59	-0.10	1.00000000	duck
Nop14	-0.05	1.00000000	duck
Ddost	0.07	1.00000000	duck
Surf6	-0.08	1.00000000	duck
Cwf19l2	-0.29	1.00000000	duck
Lmbrd2	0.04	1.00000000	duck
Tgs1	-0.05	1.00000000	duck
Celf1	-0.15	1.00000000	duck
Kif13b	-0.12	1.00000000	duck
Vps72	0.01	1.00000000	duck
Gcc2	-0.25	1.00000000	duck

Basp1	-0.33	1.00000000	duck
Smndc1	-0.02	1.00000000	duck
Trank1	-0.23	1.00000000	duck
Ndrg3	-0.58	1.00000000	duck
Gpatch2	-0.11	1.00000000	duck
Snap29	0.07	1.00000000	duck
Ndufs1	-0.23	1.00000000	duck
Rtf1	0.14	1.00000000	duck
Agl	-0.28	1.00000000	duck
Naa35	-0.03	1.00000000	duck
Sin3a	-0.20	1.00000000	duck
Ahcyl1	-0.03	1.00000000	duck
Hnrnpa0	0.08	1.00000000	duck
Tatdn1	-0.18	1.00000000	duck
Cc2d2a	-0.21	1.00000000	duck
Atg5	-0.64	1.00000000	duck
Rbm39	-0.04	1.00000000	duck
Sh3bp2	-0.17	1.00000000	duck
Taf3	-0.50	1.00000000	duck
Dcun1d1	-0.10	1.00000000	duck
Ndufs7	0.10	1.00000000	duck
Fam135a	-0.20	1.00000000	duck
Cyfip2	-0.27	1.00000000	duck
Aimp1	-0.10	1.00000000	duck
Nelfb	-0.07	1.00000000	duck
Adprh	0.02	1.00000000	duck
Eef1d	-0.03	1.00000000	duck
Eif2s2	-0.07	1.00000000	duck
Prkca	-0.27	1.00000000	duck
Nap1l1	-0.38	1.00000000	duck
Rad21	-0.03	1.00000000	duck
Trim8	0.12	1.00000000	duck
Eif4e	-0.26	1.00000000	duck
Trp53bp2	0.00	1.00000000	duck
Opa1	-0.08	1.00000000	duck
Ncbp3	-0.20	1.00000000	duck
Nfx1	-0.27	1.00000000	duck
Ranbp3	-0.41	1.00000000	duck
Hscb	-0.08	1.00000000	duck
Psma4	-0.24	1.00000000	duck
Zdhhc14	-0.50	1.00000000	duck
Suds3	-0.05	1.00000000	duck
Ubn1	-0.02	1.00000000	duck
Errfi1	-0.32	1.00000000	duck
Rrp1b	-0.27	1.00000000	duck
Eftud2	0.04	1.00000000	duck

Zfand4	-0.36	1.00000000	duck
Phf2	-0.16	1.00000000	duck
Ints10	0.01	1.00000000	duck
Batf	0.28	1.00000000	duck
Gfpt1	-0.51	1.00000000	duck
Zfp384	-0.12	1.00000000	duck
Pbx1	-0.09	1.00000000	duck
Fam172a	0.11	1.00000000	duck
Hmgxb4	-0.03	1.00000000	duck
Rsf1	-0.46	1.00000000	duck
Pitpna	-0.22	1.00000000	duck
Usp38	-0.27	1.00000000	duck
Xpr1	-0.34	1.00000000	duck
Sf3b3	-0.03	1.00000000	duck
Sar1a	-0.12	1.00000000	duck
Rit1	-0.31	1.00000000	duck
Eif4h	-0.06	1.00000000	duck
Stx2	-0.28	1.00000000	duck
Snx6	-0.21	1.00000000	duck
Specc1	-0.07	1.00000000	duck
Commd9	0.03	1.00000000	duck
Zmym2	-0.30	1.00000000	duck
Wac	-0.13	1.00000000	duck
Uba5	-0.14	1.00000000	duck
Rfng	-0.17	1.00000000	duck
Sppl2b	-0.01	1.00000000	duck
Smg7	0.02	1.00000000	duck
Htr4	-0.70	1.00000000	duck
St6gal1	-0.45	1.00000000	duck
Ino80d	0.00	1.00000000	duck
Disc1	0.21	1.00000000	duck
Ptpn1	-0.07	1.00000000	duck
Rbm17	-0.02	1.00000000	duck
Kbtbd2	-0.08	1.00000000	duck
Gpcpd1	-0.42	1.00000000	duck
Stk11	0.18	1.00000000	duck
Eif2b5	-0.01	1.00000000	duck
Hspa9	-0.07	1.00000000	duck
Adss	-0.12	1.00000000	duck
Stub1	-0.25	1.00000000	duck
Fam126b	-0.64	1.00000000	duck
Tcea2	-0.12	1.00000000	duck
Uprt	-0.18	1.00000000	duck
Xrn1	-0.09	1.00000000	duck
Cct5	-0.21	1.00000000	duck
Fbxl2	0.10	1.00000000	duck

Pnrc2	-0.15	1.00000000	duck
Spcs2	0.11	1.00000000	duck
Ppip5k2	-0.15	1.00000000	duck
Kidins220	-0.16	1.00000000	duck
Rgs9	-0.17	1.00000000	duck
Pex7	-0.07	1.00000000	duck
Cdk19	-0.48	1.00000000	duck
D5Ertd579e	-0.81	1.00000000	duck
Ino80c	0.06	1.00000000	duck
Emc7	-0.12	1.00000000	duck
Tbc1d15	-0.39	1.00000000	duck
Usp7	-0.33	1.00000000	duck
Aco2	-0.21	1.00000000	duck
Usp24	-0.21	1.00000000	duck
Zfp598	0.01	1.00000000	duck
Tlk2	-0.17	1.00000000	duck
Ccdc171	-0.08	1.00000000	duck
Pno1	0.13	1.00000000	duck
Smad2	-0.77	1.00000000	duck
Larp4	-0.05	1.00000000	duck
Sntb1	-0.03	1.00000000	duck
Ube4a	-0.13	1.00000000	duck
Snrpd1	0.23	1.00000000	duck
Vps36	0.00	1.00000000	duck
Tcerg1	-0.27	1.00000000	duck
Lactb	0.01	1.00000000	duck
Prdm10	-0.32	1.00000000	duck
Ssh2	-0.24	1.00000000	duck
Gtpbp1	-0.05	1.00000000	duck
Clasp2	-0.46	1.00000000	duck
Scarb2	-0.10	1.00000000	duck
Brms1l	-0.06	1.00000000	duck
Ist1	-0.01	1.00000000	duck
Fbxo34	-0.30	1.00000000	duck
Hadhb	-0.05	1.00000000	duck
Cnot11	-0.30	1.00000000	duck
Parp8	-0.44	1.00000000	duck
Sap30l	-0.21	1.00000000	duck
Dapk3	-0.03	1.00000000	duck
Scp2	-0.14	1.00000000	duck
Pnpla2	-0.02	1.00000000	duck
Ifih1	-0.12	1.00000000	duck
Cdkn1b	-0.50	1.00000000	duck
Slc38a1	0.14	1.00000000	duck
Ash2l	-0.05	1.00000000	duck
Ibtk	-0.13	1.00000000	duck

Rbm33	-0.11	1.00000000	duck
Slc41a3	-0.46	1.00000000	duck
Klhdc2	-0.23	1.00000000	duck
Dnaja1	-0.01	1.00000000	duck
Cep350	-0.23	1.00000000	duck
Casp9	-0.10	1.00000000	duck
Snw1	0.00	1.00000000	duck
Satb1	-0.37	1.00000000	duck
Proser1	-0.14	1.00000000	duck
Prpf39	-0.26	1.00000000	duck
Gars	-0.07	1.00000000	duck
Stxbp5	-0.56	1.00000000	duck
Etf1	-0.04	1.00000000	duck
Arih2	-0.11	1.00000000	duck
Prpf8	-0.01	1.00000000	duck
Ndufa4	0.00	1.00000000	duck
Slc25a12	-0.44	1.00000000	duck
Phb	0.09	1.00000000	duck
Nub1	-0.10	1.00000000	duck
Stk4	-0.09	1.00000000	duck
Zfp236	-0.51	1.00000000	duck
Prmt7	-0.14	1.00000000	duck
Stk40	-0.27	1.00000000	duck
Wars	-0.24	1.00000000	duck
Adora2a	-0.37	1.00000000	duck
Limk2	-0.24	1.00000000	duck
Eif2a	-0.13	1.00000000	duck
Tab2	-0.63	1.00000000	duck
Ggnbp2	-0.14	1.00000000	duck
Vgll4	-0.14	1.00000000	duck
Gtf2a1	-0.53	1.00000000	duck
Akap13	-0.17	1.00000000	duck
Cracr2a	-0.14	1.00000000	duck
Hadha	-0.11	1.00000000	duck
Cct2	0.14	1.00000000	duck
Acaca	-0.08	1.00000000	duck
Qser1	-0.11	1.00000000	duck
Cct3	-0.13	1.00000000	duck
Tmem87a	-0.23	1.00000000	duck
Ippk	-0.17	1.00000000	duck
Rad54l2	-0.12	1.00000000	duck
Fam13b	-0.19	1.00000000	duck
Smc6	-0.23	1.00000000	duck
Sdf4	0.00	1.00000000	duck
Zfp36l1	-0.17	1.00000000	duck
Taf15	0.06	1.00000000	duck

Rpf2	-0.02	1.00000000	duck
Cmtr1	-0.11	1.00000000	duck
Cgrrf1	-0.30	1.00000000	duck
Plrg1	-0.07	1.00000000	duck
Lamp2	-0.22	1.00000000	duck
Lcmt1	-0.16	1.00000000	duck
Wbp2	-0.17	1.00000000	duck
Ccdc174	-0.17	1.00000000	duck
Ap3m2	0.12	1.00000000	duck
Zranb1	-0.41	1.00000000	duck
Wdr70	-0.47	1.00000000	duck
Parg	-0.39	1.00000000	duck
Prpf38a	0.10	1.00000000	duck
Emc8	0.10	1.00000000	duck
Hipk2	-0.04	1.00000000	duck
Gfod1	-0.40	1.00000000	duck
Dhx36	-0.03	1.00000000	duck
Sec61a1	0.10	1.00000000	duck
Fbxo28	-0.04	1.00000000	duck
Elavl1	-0.32	1.00000000	duck
Atxn7l2	0.00	1.00000000	duck
Zfyve9	-0.43	1.00000000	duck
Rnmt	0.11	1.00000000	duck
Spag1	-0.13	1.00000000	duck
Sec23b	-0.02	1.00000000	duck
Scamp1	-0.18	1.00000000	duck
Tacc1	0.01	1.00000000	duck
Usp15	-0.11	1.00000000	duck
Niban1	0.00	1.00000000	duck
Dcaf13	-0.03	1.00000000	duck
Npepl1	-0.02	1.00000000	duck
Fam117a	0.09	1.00000000	duck
Rc3h2	-0.35	1.00000000	duck
Kars	0.10	1.00000000	duck
Polr2b	0.02	1.00000000	duck
Ubl3	0.22	1.00000000	duck
Rasgef1a	-0.32	1.00000000	duck
Lbr	-0.46	1.00000000	duck
Erlin2	-0.24	1.00000000	duck
Timp3	-0.20	1.00000000	duck
Prdm15	-0.01	1.00000000	duck
Il12rb2	0.12	1.00000000	duck
Pcyt2	-0.17	1.00000000	duck
Utp18	-0.13	1.00000000	duck
B3gnt4	0.05	1.00000000	duck
Eif4e2	0.10	1.00000000	duck

Gyg	0.00	1.00000000	duck
Fbxo33	0.01	1.00000000	duck
Lmbrd1	-0.19	1.00000000	duck
Crlf3	-0.08	1.00000000	duck
Ckap5	-0.11	1.00000000	duck
Cct7	0.18	1.00000000	duck
Rabgap1	-0.25	1.00000000	duck
Napg	-0.10	1.00000000	duck
Rbl2	-0.37	1.00000000	duck
Srp72	0.00	1.00000000	duck
Bzw2	-0.09	1.00000000	duck
Smim19	0.08	1.00000000	duck
Smyd2	0.11	1.00000000	duck
Csnk1g1	-0.25	1.00000000	duck
Slc10a7	-0.15	1.00000000	duck
Ilf2	-0.09	1.00000000	duck
Cyria	-0.32	1.00000000	duck
Wdr48	-0.33	1.00000000	duck
Stk26	-0.18	1.00000000	duck
Cnot7	-0.08	1.00000000	duck
Zfp438	-0.67	1.00000000	duck
Tent4b	-0.25	1.00000000	duck
Ip6k2	-0.14	1.00000000	duck
Ccdc117	-0.20	1.00000000	duck
Prkag2	-0.28	1.00000000	duck
Fgd3	-0.37	1.00000000	duck
Oit3	-0.46	1.00000000	duck
Nxt2	-0.47	1.00000000	duck
Btbd2	-0.31	1.00000000	duck
Plpp1	0.00	1.00000000	duck
Snrpb2	0.00	1.00000000	duck
Psmc6	-0.25	1.00000000	duck
Npl	-0.05	1.00000000	duck
Atl2	0.08	1.00000000	duck
Gclc	-0.31	1.00000000	duck
Leprotl1	-0.06	1.00000000	duck
Nat10	-0.11	1.00000000	duck
Arhgef18	-0.18	1.00000000	duck
Rab20	-0.24	1.00000000	duck
Itgb2l	-0.09	1.00000000	duck
Slc30a9	-0.42	1.00000000	duck
Slc7a5	0.01	1.00000000	duck
Klhl24	-0.15	1.00000000	duck
Ndufs6	0.10	1.00000000	duck
Rbm34	-0.23	1.00000000	duck
Carnmt1	-0.24	1.00000000	duck

Kif2a	0.02	1.00000000	duck
Ska2	-0.23	1.00000000	duck
Ttc7	-0.25	1.00000000	duck
Swi5	0.07	1.00000000	duck
Rap1b	-0.29	1.00000000	duck
Ddx55	-0.06	1.00000000	duck
Gab3	-0.43	1.00000000	duck
Ifngr1	-0.29	1.00000000	duck
Ergic1	-0.64	1.00000000	duck
Zbtb10	-0.01	1.00000000	duck
Clk4	-0.01	1.00000000	duck
Hexb	-0.14	1.00000000	duck
Abcd2	-0.23	1.00000000	duck
Tulp4	-0.25	1.00000000	duck
Bcas2	-0.17	1.00000000	duck
Zbtb20	0.04	1.00000000	duck
Phb2	-0.02	1.00000000	duck
Impact	0.16	1.00000000	duck
Rmnd1	-0.20	1.00000000	duck
Chaf1a	0.07	1.00000000	duck
Xpo7	-0.39	1.00000000	duck
Zzz3	-0.38	1.00000000	duck
Rap2b	-0.14	1.00000000	duck
Ccdc28a	-0.05	1.00000000	duck
Sf3a3	0.01	1.00000000	duck
Trappc11	-0.19	1.00000000	duck
Lancl2	-0.23	1.00000000	duck
Glyr1	-0.21	1.00000000	duck
Gorasp2	0.14	1.00000000	duck
Ccdc59	-0.16	1.00000000	duck
Pank2	0.00	1.00000000	duck
Tfg	-0.16	1.00000000	duck
Ern1	-0.18	1.00000000	duck
Omg	0.07	1.00000000	duck
Galc	0.08	1.00000000	duck
Srp14	-0.14	1.00000000	duck
Parp4	-0.13	1.00000000	duck
Nup205	-0.03	1.00000000	duck
Lcor	-0.04	1.00000000	duck
Abcc8	0.06	1.00000000	duck
Fyb	0.06	1.00000000	duck
Ulk2	-0.54	1.00000000	duck
Cir1	-0.23	1.00000000	duck
Otud7b	-0.18	1.00000000	duck
Hdac2	-0.13	1.00000000	duck
Pikfyve	-0.25	1.00000000	duck

Siah2	-0.08	1.00000000	duck
Slc45a4	-0.54	1.00000000	duck
Cbl	-0.09	1.00000000	duck
Zfp36l2	-0.05	1.00000000	duck
Rbms1	-0.02	1.00000000	duck
Bhlhe40	0.42	1.00000000	duck
Cd81	-0.42	1.00000000	duck
Zc3h12b	0.02	1.00000000	duck
Vps39	-0.09	1.00000000	duck
Cct4	0.04	1.00000000	duck
Senp7	-0.47	1.00000000	duck
Gpr141	-0.50	1.00000000	duck
Borcs6	-0.05	1.00000000	duck
Hnrnpd	-0.15	1.00000000	duck
Uap1l1	0.01	1.00000000	duck
Jpt1	-0.22	1.00000000	duck
Sod2	-0.15	1.00000000	duck
Dusp22	-0.59	1.00000000	duck
Hexim1	-0.07	1.00000000	duck
Tmem161b	-0.15	1.00000000	duck
Paip2	-0.06	1.00000000	duck
Gbe1	0.09	1.00000000	duck
Ddx60	-0.33	1.00000000	duck
Prex1	-0.16	1.00000000	duck
Kdm1a	-0.23	1.00000000	duck
Tubb4b	-0.07	1.00000000	duck
Caprin1	-0.07	1.00000000	duck
Cops5	-0.13	1.00000000	duck
Tpd52l2	-0.10	1.00000000	duck
Cflar	-0.52	1.00000000	duck
Trip11	-0.40	1.00000000	duck
Vps8	-0.22	1.00000000	duck
Fam78a	-0.07	1.00000000	duck
Ddb1	-0.23	1.00000000	duck
Rpf1	-0.06	1.00000000	duck
Usp34	-0.17	1.00000000	duck
Ncoa6	-0.34	1.00000000	duck
Naxd	-0.12	1.00000000	duck
Eps15l1	-0.16	1.00000000	duck
Rbsn	-0.31	1.00000000	duck
Gpbp1	-0.25	1.00000000	duck
Rnf34	-0.28	1.00000000	duck
Ndufs3	0.10	1.00000000	duck
Cacul1	-0.40	1.00000000	duck
Gtpbp10	-0.95	1.00000000	duck
Nsd1	-0.11	1.00000000	duck

Txndc9	-0.41	1.00000000	duck
Fam91a1	-0.21	1.00000000	duck
Lyrm7	-0.21	1.00000000	duck
Fbxl5	-0.49	1.00000000	duck
Arrdc3	-0.14	1.00000000	duck
Slc44a1	-0.02	1.00000000	duck
Ap1ar	-0.12	1.00000000	duck
Frs2	-0.47	1.00000000	duck
Nfia	-0.69	1.00000000	duck
Chchd3	-0.24	1.00000000	duck
Cul4b	-0.28	1.00000000	duck
Vps26a	-0.08	1.00000000	duck
Ywhaz	-0.21	1.00000000	duck
Ubxn2a	-0.10	1.00000000	duck
Nr1d2	-0.09	1.00000000	duck
Wiz	-0.15	1.00000000	duck
Tmem168	-0.15	1.00000000	duck
Plk2	-0.02	1.00000000	duck
Exoc7	-0.05	1.00000000	duck
Cops3	-0.17	1.00000000	duck
Fez2	-0.04	1.00000000	duck
Pola1	-0.07	1.00000000	duck
Rabgap1l	-0.22	1.00000000	duck
Arid1a	-0.32	1.00000000	duck
Eif2s1	-0.15	1.00000000	duck
Itpkb	-0.16	1.00000000	duck
Zpr1	-0.08	1.00000000	duck
Fbxo32	-0.36	1.00000000	duck
Smarcad1	-0.10	1.00000000	duck
Akap10	-0.17	1.00000000	duck
L3mbtl3	-0.08	1.00000000	duck
Herpud2	0.03	1.00000000	duck
Sod1	-0.20	1.00000000	duck
Tasor	-0.06	1.00000000	duck
Dennd4c	0.00	1.00000000	duck
Anapc7	-0.07	1.00000000	duck
Srek1ip1	-0.08	1.00000000	duck
Brd3	-0.42	1.00000000	duck
Xrn2	-0.32	1.00000000	duck
Idh3a	0.14	1.00000000	duck
Slc48a1	-0.07	1.00000000	duck
Fbxo38	-0.34	1.00000000	duck
Tgif1	0.10	1.00000000	duck
Nlk	-0.21	1.00000000	duck
Tnks	-0.43	1.00000000	duck
Ccni	-0.15	1.00000000	duck

Cops4	-0.06	1.00000000	duck
Tlcd3a	-0.15	1.00000000	duck
Selenoo	-0.39	1.00000000	duck
Rab8a	-0.14	1.00000000	duck
Dnajc16	-0.04	1.00000000	duck
Serinc1	-0.30	1.00000000	duck
Siva1	0.11	1.00000000	duck
Tnpo1	-0.48	1.00000000	duck
Fbxo45	-0.19	1.00000000	duck
Supt3	-0.27	1.00000000	duck
Pcif1	-0.08	1.00000000	duck
Gcn1	-0.03	1.00000000	duck
Tia1	-0.10	1.00000000	duck
Ergic2	-0.39	1.00000000	duck
Prdx4	-0.02	1.00000000	duck
Ddx6	-0.11	1.00000000	duck
Ppp2r2a	-0.25	1.00000000	duck
Strn	-0.19	1.00000000	duck
Ankrd13c	-0.40	1.00000000	duck
Ankfy1	-0.16	1.00000000	duck
Taf2	0.10	1.00000000	duck
Plec	-0.32	1.00000000	duck
Cdca4	0.03	1.00000000	duck
Gid8	-0.27	1.00000000	duck
Lsg1	0.04	1.00000000	duck
Srsf6	-0.02	1.00000000	duck
Gfod2	-0.18	1.00000000	duck
Psmg2	-0.07	1.00000000	duck
Fbxl22	-0.06	1.00000000	duck
Clptm1l	-0.13	1.00000000	duck
Rap1gds1	-0.12	1.00000000	duck
Dnajb14	-0.12	1.00000000	duck
Slc35a1	-0.02	1.00000000	duck
Slc27a1	0.05	1.00000000	duck
Gtf2a2	-0.13	1.00000000	duck
Bnip2	-0.21	1.00000000	duck
Ikbke	-0.15	1.00000000	duck
Epn2	-0.32	1.00000000	duck
Uso1	-0.29	1.00000000	duck
Usp45	-0.25	1.00000000	duck
Hat1	-0.09	1.00000000	duck
Glrx5	-0.36	1.00000000	duck
Reck	0.02	1.00000000	duck
Thrap3	-0.34	1.00000000	duck
Btbd1	-0.02	1.00000000	duck
Cltc	-0.16	1.00000000	duck

Hnrnpk	-0.07	1.00000000	duck
Rlf	-0.43	1.00000000	duck
Nup107	-0.08	1.00000000	duck
Pik3cg	-0.29	1.00000000	duck
Dlat	-0.16	1.00000000	duck
Ccnb2	0.04	1.00000000	duck
Ing1	-0.09	1.00000000	duck
Ero1b	-0.27	1.00000000	duck
Arl5a	-0.12	1.00000000	duck
Nelfa	-0.12	1.00000000	duck
Pi4ka	-0.30	1.00000000	duck
Rpa3	-0.22	1.00000000	duck
Traf3ip3	-0.38	1.00000000	duck
Stambpl1	-0.25	1.00000000	duck
Trappc8	-0.27	1.00000000	duck
Cbx1	-0.06	1.00000000	duck
Slc44a2	0.07	1.00000000	duck
Htt	-0.27	1.00000000	duck
Csl	0.03	1.00000000	duck
Scn8a	-0.11	1.00000000	duck
Shoc2	-0.15	1.00000000	duck
Bptf	-0.32	1.00000000	duck
Ago4	-0.09	1.00000000	duck
Hadh	0.01	1.00000000	duck
Gpr180	-0.06	1.00000000	duck
Fam53a	-0.26	1.00000000	duck
Phospho1	-0.15	1.00000000	duck
Ppp2r5c	-0.36	1.00000000	duck
Eif3h	-0.18	1.00000000	duck
Cmss1	-0.01	1.00000000	duck
Pgap1	-0.37	1.00000000	duck
Rab11fip4	-0.43	1.00000000	duck
Fmnl1	-0.01	1.00000000	duck
Igf1r	0.11	1.00000000	duck
Hmox2	-0.04	1.00000000	duck
Pdcl	-0.19	1.00000000	duck
Tab3	-0.12	1.00000000	duck
Dmap1	-0.08	1.00000000	duck
Unk	0.04	1.00000000	duck
Crybg3	-0.29	1.00000000	duck
Rtn4	-0.14	1.00000000	duck
Ociad1	-0.05	1.00000000	duck
Nhlrc2	-0.08	1.00000000	duck
Ube2o	-0.18	1.00000000	duck
Tspan2	-0.07	1.00000000	duck
Ppp2cb	-0.17	1.00000000	duck

Fam114a2	-0.15	1.00000000	duck
Cebpg	-0.10	1.00000000	duck
Dhx40	-0.23	1.00000000	duck
Suco	-0.18	1.00000000	duck
Epc2	-0.22	1.00000000	duck
Phf12	-0.26	1.00000000	duck
Cul1	-0.33	1.00000000	duck
Ccnt1	-0.11	1.00000000	duck
Nol11	-0.15	1.00000000	duck
Ubac1	-0.34	1.00000000	duck
Ndfip2	-0.30	1.00000000	duck
Agps	-0.10	1.00000000	duck
Pelo	-0.15	1.00000000	duck
Setd3	-0.23	1.00000000	duck
Ppp1cb	-0.25	1.00000000	duck
Gan	-0.16	1.00000000	duck
Hmbox1	-0.32	1.00000000	duck
Caml	0.03	1.00000000	duck
Xylt1	-0.60	1.00000000	duck
Kdm7a	-0.22	1.00000000	duck
Amd2	0.07	1.00000000	duck
Dock10	0.24	1.00000000	duck
Slc49a4	0.01	1.00000000	duck
Ascc2	-0.22	1.00000000	duck
Tpt1	5.66	0.00000000	pigeons
Cmip	5.02	0.00000000	pigeons
Rab33a	4.90	0.00000000	pigeons
Adss	3.53	0.00000000	pigeons
Cd3g	3.72	0.00000000	pigeons
Arpc2	3.25	0.00000000	pigeons
Atf7ip	2.93	0.00000000	pigeons
Dars	2.89	0.00000000	pigeons
Fyb	2.76	0.00000000	pigeons
Atp5b	2.36	0.00000000	pigeons
Igfbp7	-3.81	0.00000000	pigeons
Rgs1	1.94	0.00000000	pigeons
Cfd	-4.33	0.00000000	pigeons
Cnbp	2.22	0.00000000	pigeons
Sytl3	3.06	0.00000000	pigeons
Aqp1	-4.32	0.00000000	pigeons
Atp5g3	2.39	0.00000000	pigeons
Vim	2.72	0.00000000	pigeons
Ptprm	-4.41	0.00000000	pigeons
Niban1	3.03	0.00000000	pigeons
Dido1	2.34	0.00000000	pigeons
Epas1	-4.03	0.00000000	pigeons

Oga	2.33	0.00000000	pigeons
Wtap	2.23	0.00000000	pigeons
Phip	2.50	0.00000000	pigeons
Sap18	2.11	0.00000000	pigeons
Tiparp	2.67	0.00000000	pigeons
Pecam1	-3.60	0.00000000	pigeons
U2surp	2.23	0.00000000	pigeons
Ankhd1	2.26	0.00000000	pigeons
Pcf11	2.17	0.00000000	pigeons
Cyria	2.74	0.00000000	pigeons
Calcrl	-3.62	0.00000000	pigeons
Timp3	-3.62	0.00000000	pigeons
Rbms3	-3.75	0.00000000	pigeons
Trpc4ap	1.86	0.00000000	pigeons
Cxcr4	1.93	0.00000000	pigeons
Lpp	2.14	0.00000000	pigeons
Syncrip	1.91	0.00000000	pigeons
Tmtc1	-3.36	0.00000000	pigeons
Pfkp	2.29	0.00000000	pigeons
Txnip	1.97	0.00000000	pigeons
Anapc15-ps	1.72	0.00000000	pigeons
Ptprc	1.28	0.00000000	pigeons
Evl	1.31	0.00000000	pigeons
Adgrf5	-3.08	0.00000000	pigeons
Rgs2	3.35	0.00000000	pigeons
Sdcbp	1.83	0.00000000	pigeons
Zc3h12d	1.63	0.00000000	pigeons
Bcl11b	1.86	0.00000000	pigeons
Ldb2	-3.40	0.00000000	pigeons
Apbb1ip	1.97	0.00000000	pigeons
Adgrl4	-2.98	0.00000000	pigeons
Adgrl2	-3.29	0.00000000	pigeons
Tmem100	-3.07	0.00000000	pigeons
Lnpep	1.80	0.00000000	pigeons
Gsap	2.11	0.00000000	pigeons
Cd28	2.57	0.00000000	pigeons
Cklf	1.61	0.00000000	pigeons
Kars	1.61	0.00000000	pigeons
Cd247	1.30	0.00000000	pigeons
Sptbn1	-2.49	0.00000000	pigeons
Egr1	-2.82	0.00000000	pigeons
Syne2	-2.99	0.00000000	pigeons
Ptprb	-2.82	0.00000000	pigeons
Nova1	-3.09	0.00000000	pigeons
Tjp1	-3.03	0.00000000	pigeons
Adnp	1.61	0.00000000	pigeons

Arid5b	1.34	0.00000000	pigeons
Fos	-2.29	0.00000000	pigeons
Podxl	-2.54	0.00000000	pigeons
Cytip	1.53	0.00000000	pigeons
Akap12	-2.82	0.00000000	pigeons
Sparc	-2.67	0.00000000	pigeons
Rapgef5	-2.89	0.00000000	pigeons
Prep	1.79	0.00000000	pigeons
Rnf38	1.66	0.00000000	pigeons
Arhgef9	-2.70	0.00000000	pigeons
4932411N23Rik	-3.19	0.00000000	pigeons
Usp3	1.30	0.00000000	pigeons
Strap	1.43	0.00000000	pigeons
Plekhg1	-2.65	0.00000000	pigeons
Dll4	-2.46	0.00000000	pigeons
Phax	1.49	0.00000000	pigeons
Rasal2	-2.53	0.00000000	pigeons
Kifbp	1.45	0.00000000	pigeons
Atp5k	1.37	0.00000000	pigeons
Vcp	1.36	0.00000000	pigeons
Rgcc	-2.59	0.00000000	pigeons
Snrpd2	1.33	0.00000000	pigeons
Pomp	1.30	0.00000000	pigeons
Ndufa1	1.33	0.00000000	pigeons
Sox6	-2.52	0.00000000	pigeons
Arhgap31	-2.55	0.00000000	pigeons
Tgfbr1	1.48	0.00000000	pigeons
Rffl	1.48	0.00000000	pigeons
Cavin2	-2.56	0.00000000	pigeons
Fam189a1	-2.43	0.00000000	pigeons
Sdhc	1.25	0.00000000	pigeons
Gpr171	1.19	0.00000000	pigeons
Gpx3	-2.57	0.00000000	pigeons
Tnfaip8	1.13	0.00000000	pigeons
Ptger4	1.29	0.00000000	pigeons
Brd8	1.28	0.00000000	pigeons
Cdc37	1.24	0.00000000	pigeons
Sipa1l1	1.06	0.00000000	pigeons
Ccz1	1.27	0.00000000	pigeons
Sema6a	-2.48	0.00000000	pigeons
Gnai1	1.44	0.00000000	pigeons
Fermt2	-2.26	0.00000000	pigeons
App	1.67	0.00000000	pigeons
Rtf2	1.21	0.00000000	pigeons
Zfp352	-2.11	0.00000000	pigeons
Atp10a	1.25	0.00000000	pigeons

Sdc4	-1.71	0.00000000	pigeons
Rgs4	1.82	0.00000000	pigeons
Pcnp	1.20	0.00000000	pigeons
Csnk1d	1.30	0.00000000	pigeons
Eprs	1.33	0.00000000	pigeons
Arhgap15	0.93	0.00000000	pigeons
Afdn	-2.49	0.00000000	pigeons
Sdk1	-2.53	0.00000000	pigeons
Lpl	-2.25	0.00000000	pigeons
Dock2	1.18	0.00000000	pigeons
Zfp36l1	0.96	0.00000000	pigeons
Ankrd44	1.10	0.00000000	pigeons
Tdpoz8	1.30	0.00000000	pigeons
Ahsa2	1.46	0.00000000	pigeons
Nfia	-2.43	0.00000000	pigeons
Rad23b	1.34	0.00000000	pigeons
Eif4b	1.16	0.00000000	pigeons
Cdh5	-2.12	0.00000000	pigeons
Hecw2	-2.59	0.00000000	pigeons
Cacybp	1.21	0.00000000	pigeons
Stat1	1.17	0.00000000	pigeons
Fth1	-0.78	0.00000000	pigeons
Spcs1	1.15	0.00000000	pigeons
Kcne3	-2.10	0.00000000	pigeons
Tollip	1.20	0.00000000	pigeons
C1qbp	1.15	0.00000000	pigeons
Alox5ap	1.33	0.00000000	pigeons
Stat4	1.24	0.00000000	pigeons
Ccser2	1.15	0.00000000	pigeons
Sinhcaf	1.49	0.00000000	pigeons
Rhob	1.49	0.00000000	pigeons
Jcad	-2.07	0.00000000	pigeons
Pde4b	0.88	0.00000000	pigeons
Ankrd10	1.10	0.00000000	pigeons
Crim1	-2.33	0.00000000	pigeons
Pde10a	-2.41	0.00000000	pigeons
Corin	-2.37	0.00000000	pigeons
Sorbs1	-2.32	0.00000000	pigeons
Tmcc3	-2.22	0.00000000	pigeons
Trpv2	1.20	0.00000000	pigeons
Arap2	1.33	0.00000000	pigeons
Cd47	0.93	0.00000000	pigeons
Trrap	1.20	0.00000000	pigeons
Sp4	1.29	0.00000000	pigeons
Synm	-1.98	0.00000000	pigeons
Nop56	1.13	0.00000000	pigeons

Slirp	1.08	0.00000000	pigeons
Pmp22	-2.15	0.00000000	pigeons
Arvcf	-2.17	0.00000000	pigeons
Enox2	1.07	0.00000000	pigeons
Samsn1	0.81	0.00000000	pigeons
St8sia4	1.39	0.00000000	pigeons
Ccdc25	1.08	0.00000000	pigeons
Sfswap	1.08	0.00000000	pigeons
Plcg1	1.06	0.00000000	pigeons
Lcp2	1.03	0.00000000	pigeons
Col4a1	-2.13	0.00000000	pigeons
Wipf1	1.15	0.00000000	pigeons
Pim1	1.20	0.00000000	pigeons
Atp5c1	1.01	0.00000000	pigeons
Tcf7	2.60	0.00000000	pigeons
Gna13	0.88	0.00000000	pigeons
Zbtb20	-2.37	0.00000000	pigeons
Cav1	-1.93	0.00000000	pigeons
Ddit4	-1.71	0.00000000	pigeons
Abca1	1.11	0.00000000	pigeons
Cars	1.13	0.00000000	pigeons
Ezr	1.21	0.00000000	pigeons
Cnn3	-1.80	0.00000000	pigeons
Sdc2	-1.89	0.00000000	pigeons
Smad6	-2.07	0.00000000	pigeons
Prex2	-1.92	0.00000000	pigeons
Slmap	1.13	0.00000000	pigeons
Zdhhc20	1.18	0.00000000	pigeons
Plcb1	-1.68	0.00000000	pigeons
Npr3	-1.93	0.00000000	pigeons
Slc16a6	1.03	0.00000000	pigeons
Dock1	-1.98	0.00000000	pigeons
Apip	1.04	0.00000000	pigeons
Bmpr2	-2.14	0.00000000	pigeons
Slc9a3r2	-1.82	0.00000000	pigeons
Golm2	1.01	0.00000000	pigeons
Naa20	0.97	0.00000000	pigeons
Tut7	1.01	0.00000000	pigeons
Lrmp	1.11	0.00000000	pigeons
Runx1	0.95	0.00000000	pigeons
Arl4a	-2.25	0.00000000	pigeons
Tagap1	1.28	0.00000000	pigeons
Gadd45b	1.13	0.00000000	pigeons
Itga4	1.04	0.00000000	pigeons
Sec24d	1.10	0.00000000	pigeons
Polr1f	0.90	0.00000000	pigeons

Rybp	1.25	0.00000000	pigeons
Dock9	-2.25	0.00000000	pigeons
Tardbp	1.02	0.00000000	pigeons
Adcy7	0.86	0.00000000	pigeons
Tmem204	-1.80	0.00000000	pigeons
Fam107b	1.03	0.00000000	pigeons
Efnb2	-1.94	0.00000000	pigeons
Tox2	0.81	0.00000000	pigeons
Kcnc1	-1.84	0.00000000	pigeons
Itm2b	-1.99	0.00000000	pigeons
Prkce	-1.91	0.00000000	pigeons
Laptm4a	-1.89	0.00000000	pigeons
Snrk	0.78	0.00000000	pigeons
Zfp521	-1.66	0.00000000	pigeons
Rassf9	-1.68	0.00000000	pigeons
Dach1	-1.68	0.00000000	pigeons
Tbx3	-1.65	0.00000000	pigeons
Ptpn22	0.95	0.00000000	pigeons
Dock10	0.83	0.00000000	pigeons
Ndst4	1.53	0.00000000	pigeons
Tns1	-1.81	0.00000000	pigeons
Hpgd	-1.56	0.00000000	pigeons
S100a13	-1.63	0.00000000	pigeons
Sarnp	0.83	0.00000000	pigeons
Xpo6	0.80	0.00000000	pigeons
Ptpn14	-1.66	0.00000000	pigeons
Cdk14	1.23	0.00000000	pigeons
Trank1	1.06	0.00000000	pigeons
Ccdc85a	-1.68	0.00000000	pigeons
Bri3bp	0.85	0.00000000	pigeons
Dclk1	-1.90	0.00000000	pigeons
Zhx2	1.03	0.00000000	pigeons
Rasgef1a	1.31	0.00000000	pigeons
Il18rap	0.95	0.00000000	pigeons
Stard13	-1.84	0.00000000	pigeons
Pde3b	0.79	0.00000000	pigeons
Cd2ap	1.09	0.00000000	pigeons
Slc12a7	1.00	0.00000000	pigeons
Rars	0.86	0.00000000	pigeons
Fli1	-1.73	0.00000000	pigeons
Prr5	0.75	0.00000000	pigeons
Fgd5	-1.66	0.00000000	pigeons
Cdkn2aip	0.83	0.00000000	pigeons
Etnk1	0.79	0.00000000	pigeons
Cbx7	1.03	0.00000000	pigeons
Jun	-1.15	0.00000000	pigeons

Apcdd1	-1.65	0.00000000	pigeons
Tnik	0.81	0.00000000	pigeons
Rnf213	0.84	0.00000000	pigeons
Tead4	-1.70	0.00000000	pigeons
Rhoj	-1.50	0.00000000	pigeons
Inpp4b	1.54	0.00000000	pigeons
Sap30bp	0.81	0.00000000	pigeons
Il22ra2	1.03	0.00000000	pigeons
Fgl2	0.96	0.00000000	pigeons
Psme3ip1	0.77	0.00000000	pigeons
Tars	0.80	0.00000000	pigeons
Ptprn2	-1.75	0.00000000	pigeons
Acvrl1	-1.45	0.00000000	pigeons
Neo1	-1.63	0.00000000	pigeons
Hoxa3	-1.54	0.00000000	pigeons
Nars	0.82	0.00000000	pigeons
Gpd1l	1.03	0.00000000	pigeons
Galnt18	-1.52	0.00000000	pigeons
Ikzf1	0.96	0.00000000	pigeons
Celf2	0.74	0.00000000	pigeons
Pde7b	1.02	0.00000000	pigeons
Glrx3	0.72	0.00000000	pigeons
Spred2	-1.83	0.00000000	pigeons
Ablim1	-1.78	0.00000000	pigeons
Diaph2	0.49	0.00000000	pigeons
Tmbim1	0.97	0.00000000	pigeons
Fas	0.93	0.00000000	pigeons
Chfr	0.86	0.00000000	pigeons
Dock8	0.92	0.00000000	pigeons
Sec23ip	0.86	0.00000000	pigeons
Evi2a	0.88	0.00000000	pigeons
Tmem135	0.84	0.00000000	pigeons
Fgfr2	-1.57	0.00000000	pigeons
Creb5	-1.90	0.00000000	pigeons
Pggt1b	0.89	0.00000000	pigeons
Spred1	-1.49	0.00000000	pigeons
Stk38	0.79	0.00000000	pigeons
Kdm3a	0.84	0.00000000	pigeons
Lrch3	0.69	0.00000000	pigeons
Sik1	0.71	0.00000000	pigeons
Zbtb38	0.85	0.00000000	pigeons
Zfp366	-1.28	0.00000000	pigeons
Tgfbr3	-1.40	0.00000000	pigeons
Fbxl21	0.79	0.00000000	pigeons
Il1b	1.34	0.00000000	pigeons
Runx1t1	-1.56	0.00000000	pigeons

Antxr2	0.79	0.00000000	pigeons
Actb	-0.56	0.00000000	pigeons
Wdfy3	-1.55	0.00000000	pigeons
Phtf2	0.81	0.00000000	pigeons
Agap1	-1.58	0.00000000	pigeons
Bsg	-1.82	0.00000000	pigeons
Eif2ak3	0.85	0.00000000	pigeons
Cdk5r1	0.86	0.00000000	pigeons
Lpgat1	0.78	0.00000000	pigeons
Cnot1	0.66	0.00000000	pigeons
Plcg2	1.02	0.00000000	pigeons
Ramp2	-1.45	0.00000000	pigeons
Dlc1	-1.41	0.00000000	pigeons
Ski	-0.79	0.00000000	pigeons
Arhgap21	-1.93	0.00000000	pigeons
Cep170	0.71	0.00000000	pigeons
Tek	-1.27	0.00000000	pigeons
Chsy1	0.94	0.00000000	pigeons
Tent5a	1.07	0.00000000	pigeons
Sesn1	0.75	0.00000000	pigeons
Rpf2	0.57	0.00000000	pigeons
Ephb1	-1.44	0.00000000	pigeons
Rsbn1l	0.64	0.00000000	pigeons
Tmem63a	0.92	0.00000000	pigeons
Auts2	-1.58	0.00000000	pigeons
Rasgrf2	-1.32	0.00000000	pigeons
Sec62	0.65	0.00000000	pigeons
Osbpl10	-1.50	0.00000000	pigeons
Kdr	-1.33	0.00000000	pigeons
Tapbpl	0.68	0.00000000	pigeons
Klhl5	0.81	0.00000000	pigeons
Rai14	-1.23	0.00000000	pigeons
Colec10	-1.17	0.00000000	pigeons
Pusl1	0.79	0.00000000	pigeons
Ctbp1	0.68	0.00000000	pigeons
Ube3a	0.73	0.00000000	pigeons
Tmem123	0.69	0.00000000	pigeons
Foxn2	0.79	0.00000000	pigeons
Slc9a9	0.89	0.00000000	pigeons
Pik3cd	0.75	0.00000000	pigeons
Sla	0.78	0.00000000	pigeons
Pik3cg	0.62	0.00000000	pigeons
Rbpj	0.81	0.00000000	pigeons
Rbmx2	0.71	0.00000000	pigeons
Rc3h1	0.76	0.00000000	pigeons
Heg1	-1.06	0.00000000	pigeons

Pls3	-1.68	0.00000000	pigeons
Tnfaip3	0.76	0.00000000	pigeons
Atp5j2	0.66	0.00000000	pigeons
Stk17b	0.67	0.00000000	pigeons
Cobll1	-1.26	0.00000000	pigeons
Tjp2	-1.28	0.00000000	pigeons
Klf13	0.76	0.00000000	pigeons
Phf12	0.63	0.00000000	pigeons
Idh3b	0.60	0.00000000	pigeons
Abcg1	0.69	0.00000000	pigeons
Rock1	0.67	0.00000000	pigeons
Ddx28	0.73	0.00000000	pigeons
Atxn7l1	0.68	0.00000000	pigeons
Efemp1	-1.58	0.00000000	pigeons
Atp11b	0.61	0.00000000	pigeons
Tbp	0.65	0.00000000	pigeons
Rgs18	0.80	0.00000000	pigeons
Yars	0.65	0.00000000	pigeons
Gtpbp4	0.65	0.00000000	pigeons
Rel	0.65	0.00000000	pigeons
Cul2	0.58	0.00000000	pigeons
Sgms2	0.82	0.00000000	pigeons
Aars	0.60	0.00000000	pigeons
Rnf216	0.85	0.00000000	pigeons
Rassf3	-1.69	0.00000000	pigeons
Kcnj5	-1.68	0.00000000	pigeons
Tox	1.01	0.00000000	pigeons
Dock11	0.53	0.00000000	pigeons
Cgnl1	-1.14	0.00000000	pigeons
Prrg1	-1.20	0.00000000	pigeons
Cd44	0.60	0.00000000	pigeons
Trerf1	0.87	0.00000000	pigeons
Npas3	-1.78	0.00000000	pigeons
Irf4	0.91	0.00000000	pigeons
Ifngr1	0.74	0.00000000	pigeons
Cd74	-1.06	0.00000000	pigeons
Tfpi2	-1.61	0.00000000	pigeons
Wwc2	-1.13	0.00000000	pigeons
Dcn	-1.76	0.00000000	pigeons
Slc38a2	0.62	0.00000000	pigeons
Psip1	0.61	0.00000000	pigeons
Nckap1	-1.41	0.00000000	pigeons
Klhl15	0.70	0.00000000	pigeons
Nav3	0.91	0.00000000	pigeons
Banp	0.80	0.00000000	pigeons
Fbh1	0.63	0.00000000	pigeons

Bccip	0.58	0.00000000	pigeons
Arhgap30	0.63	0.00000000	pigeons
Khdc4	0.60	0.00000000	pigeons
Dgkq	0.96	0.00000000	pigeons
Cog3	0.58	0.00000000	pigeons
Blcap	0.62	0.00000000	pigeons
Rfc1	0.54	0.00000000	pigeons
Card11	0.88	0.00000000	pigeons
Ralgps2	-1.64	0.00000000	pigeons
Polr2i	0.60	0.00000000	pigeons
Dedd	0.57	0.00000000	pigeons
Spata5	0.68	0.00000000	pigeons
Ubtf	0.62	0.00000000	pigeons
Tent4a	0.69	0.00000000	pigeons
Gulp1	-1.20	0.00000000	pigeons
Sox18	-1.11	0.00000000	pigeons
Zfpm1	0.73	0.00000000	pigeons
Apba2	-1.08	0.00000000	pigeons
Ccdc88a	0.68	0.00000000	pigeons
Ddx17	0.41	0.00000000	pigeons
Lpar4	1.04	0.00000000	pigeons
Gpcpd1	0.78	0.00000000	pigeons
Nfatc3	0.59	0.00000000	pigeons
Vps13d	0.67	0.00000000	pigeons
Cbx6	0.83	0.00000000	pigeons
Tspan9	-1.54	0.00000000	pigeons
Wdr37	0.58	0.00000000	pigeons
Igf2bp3	0.83	0.00000000	pigeons
Tdrkh	0.86	0.00000000	pigeons
Nasp	0.67	0.00000000	pigeons
Dpyd	-1.53	0.00000000	pigeons
Pdgfb	-1.05	0.00000000	pigeons
Luc7l3	0.51	0.00000000	pigeons
Aoah	0.68	0.00000000	pigeons
Rnf19b	0.62	0.00000000	pigeons
Cttnbp2nl	-1.36	0.00000000	pigeons
Fam8a1	0.65	0.00000000	pigeons
Nanp	0.66	0.00000000	pigeons
Far2	-1.10	0.00000000	pigeons
Enah	-1.17	0.00000000	pigeons
Rfx7	0.88	0.00000000	pigeons
Terf2ip	0.55	0.00000000	pigeons
Kpnb1	0.67	0.00000000	pigeons
Tex2	-1.78	0.00000000	pigeons
Chd2	0.50	0.00000000	pigeons
Pim3	-1.70	0.00000000	pigeons

Lhpp	0.63	0.00000000	pigeons
Dennd2a	-1.34	0.00000000	pigeons
Kdelr3	0.53	0.00000000	pigeons
Siah2	0.77	0.00000000	pigeons
Eif2b4	0.55	0.00000000	pigeons
Ttl	0.50	0.00000000	pigeons
Adamts2	-1.41	0.00000000	pigeons
Cadps2	-1.28	0.00000000	pigeons
Ccng2	0.68	0.00000000	pigeons
Twsg1	-1.19	0.00000000	pigeons
Cep85l	0.66	0.00000000	pigeons
Btg1b	0.55	0.00000000	pigeons
Pstpip1	0.75	0.00000000	pigeons
Zfp777	0.50	0.00000000	pigeons
Lifr	-1.04	0.00000000	pigeons
Rbm26	0.51	0.00000000	pigeons
Dennd1b	0.53	0.00000000	pigeons
Strbp	0.54	0.00000000	pigeons
Psmc3ip	0.51	0.00000000	pigeons
Trim28	0.50	0.00000000	pigeons
Erg	-1.32	0.00000000	pigeons
Helz	0.54	0.00000000	pigeons
Sftpc	-3.29	0.00000000	pigeons
Syne1	0.54	0.00000000	pigeons
Kcnk5	0.96	0.00000000	pigeons
Il7r	0.70	0.00000000	pigeons
Dtd1	0.59	0.00000000	pigeons
Tspan18	-1.34	0.00000000	pigeons
Smc4	0.62	0.00000000	pigeons
Pdlim1	-1.09	0.00000000	pigeons
Ndufv3	0.48	0.00000000	pigeons
Ano5	0.70	0.00000000	pigeons
Ctdspl	-1.30	0.00000000	pigeons
Phldb2	-1.05	0.00000000	pigeons
Ubash3a	0.73	0.00000000	pigeons
Egfl7	-1.01	0.00000000	pigeons
Rxylt1	0.56	0.00000000	pigeons
Atp11a	-1.32	0.00000000	pigeons
Pnisr	0.45	0.00000000	pigeons
Caskin2	-1.09	0.00000000	pigeons
Enpp4	0.77	0.00000000	pigeons
Spidr	0.70	0.00000000	pigeons
Traf3ip3	0.60	0.00000000	pigeons
Thbs1	-1.37	0.00000000	pigeons
B2m	-0.23	0.00000000	pigeons
Col22a1	-1.23	0.00000000	pigeons

Lrba	0.55	0.00000000	pigeons
Dsp	0.60	0.00000000	pigeons
Arglu1	0.33	0.00000000	pigeons
Hook1	0.53	0.00000000	pigeons
Pikfyve	0.61	0.00000000	pigeons
Arrdc3	0.62	0.00000000	pigeons
S1pr1	-1.52	0.00000000	pigeons
Inpp5k	-0.64	0.00000000	pigeons
Serpind1	-1.32	0.00000000	pigeons
Ep400	0.46	0.00000000	pigeons
Sgms1	0.56	0.00000000	pigeons
Fnbp1	0.57	0.00000000	pigeons
Ttbk1	0.70	0.00000000	pigeons
Ets1	0.48	0.00000000	pigeons
Znfx1	0.42	0.00000000	pigeons
Dusp7	-1.72	0.00000000	pigeons
Ube2j1	0.52	0.00000000	pigeons
Thada	0.62	0.00000000	pigeons
Hbp1	0.25	0.00000000	pigeons
Cd4	0.85	0.00000000	pigeons
Ythdc2	0.67	0.00000000	pigeons
Slain2	0.48	0.00000000	pigeons
Runx3	0.55	0.00000000	pigeons
Braf	0.46	0.00000000	pigeons
Smchd1	0.35	0.00000000	pigeons
Amotl2	-0.89	0.00000000	pigeons
Ptdss1	0.61	0.00000000	pigeons
Ubac2	0.57	0.00000000	pigeons
Sh3kbp1	0.52	0.00000000	pigeons
Dpf3	0.88	0.00000000	pigeons
Agbl1	-1.12	0.00000000	pigeons
Fbxl5	0.54	0.00000000	pigeons
Ppp6r3	0.37	0.00000000	pigeons
Bmp6	-1.09	0.00000000	pigeons
Ncoa3	0.43	0.00000000	pigeons
Gcnt4	0.69	0.00000000	pigeons
Stox2	-1.32	0.00000000	pigeons
Stambp	0.48	0.00000000	pigeons
Cherp	0.44	0.00000000	pigeons
Ifnar1	0.58	0.00000000	pigeons
Cd3e	0.35	0.00000000	pigeons
Srpk1	0.47	0.00000000	pigeons
Crtc1	0.70	0.00000000	pigeons
Ergic1	0.43	0.00000001	pigeons
Rin2	0.58	0.00000001	pigeons
Kcnq5	0.78	0.00000001	pigeons

Ccdc6	0.55	0.00000001	pigeons
Ccm2	0.43	0.00000001	pigeons
Eapp	0.56	0.00000001	pigeons
Suv39h2	0.58	0.00000001	pigeons
Ehbp1	0.45	0.00000001	pigeons
Socs1	0.66	0.00000001	pigeons
Il2rb	0.82	0.00000001	pigeons
Eml6	-0.98	0.00000001	pigeons
Elf1	0.46	0.00000001	pigeons
Jak1	0.41	0.00000001	pigeons
Aff2	0.64	0.00000001	pigeons
Nrap	0.52	0.00000001	pigeons
Uhrf1bp1l	0.63	0.00000001	pigeons
Ptpn6	0.60	0.00000001	pigeons
Hax1	0.41	0.00000001	pigeons
Cmtm8	-1.02	0.00000002	pigeons
Satb1	0.74	0.00000002	pigeons
Baz2b	-1.31	0.00000002	pigeons
Pabpc1	0.38	0.00000002	pigeons
Tab2	0.42	0.00000002	pigeons
Acvr1	0.55	0.00000002	pigeons
Tbl1xr1	0.59	0.00000002	pigeons
Atg2b	0.50	0.00000003	pigeons
Shroom2	-0.88	0.00000003	pigeons
Dpysl2	-1.18	0.00000003	pigeons
Hdac3	0.41	0.00000003	pigeons
Aifm1	0.45	0.00000003	pigeons
Aip	0.48	0.00000003	pigeons
Jmjd1c	0.57	0.00000003	pigeons
Ripor1	-1.04	0.00000003	pigeons
Hnrnpll	0.65	0.00000004	pigeons
Zbtb1	0.55	0.00000004	pigeons
Cdc42se2	0.83	0.00000004	pigeons
Cdc42bpa	-1.38	0.00000004	pigeons
Adgrg1	-1.17	0.00000004	pigeons
Cyfip2	0.48	0.00000004	pigeons
Glcci1	0.73	0.00000005	pigeons
Cald1	-1.13	0.00000005	pigeons
Pik3r1	0.78	0.00000005	pigeons
Cab39	0.32	0.00000005	pigeons
Atad1	0.46	0.00000005	pigeons
Traf5	0.64	0.00000005	pigeons
Rictor	0.56	0.00000005	pigeons
Rspry1	0.61	0.00000005	pigeons
Blvra	0.74	0.00000005	pigeons
Kdm1b	0.56	0.00000005	pigeons

Slc4a7	0.60	0.00000006	pigeons
Tbk1	0.45	0.00000006	pigeons
Asxl1	0.68	0.00000006	pigeons
Dcp2	0.42	0.00000007	pigeons
Tcf7l2	-1.25	0.00000007	pigeons
Qk	-1.13	0.00000007	pigeons
Ppia	0.42	0.00000007	pigeons
Zmynd8	0.51	0.00000008	pigeons
Riok2	0.43	0.00000008	pigeons
LTO1	0.52	0.00000008	pigeons
Crip2	0.43	0.00000008	pigeons
Srsf11	0.39	0.00000008	pigeons
Fbxo33	0.37	0.00000009	pigeons
Lyn	-1.20	0.00000010	pigeons
Thyn1	0.46	0.00000011	pigeons
Usp7	0.28	0.00000011	pigeons
Traf6	0.50	0.00000011	pigeons
Gsk3b	-0.90	0.00000011	pigeons
Ranbp17	0.70	0.00000011	pigeons
Zeb2	-0.43	0.00000012	pigeons
Farp1	-0.91	0.00000013	pigeons
Sbno1	0.27	0.00000013	pigeons
Rbfox2	-1.45	0.00000013	pigeons
Rpa1	0.40	0.00000014	pigeons
Sacs	0.65	0.00000014	pigeons
Rbl2	0.42	0.00000014	pigeons
Nr4a3	0.61	0.00000016	pigeons
Zfp207	0.25	0.00000016	pigeons
Iqgap2	0.56	0.00000017	pigeons
Idua	0.49	0.00000018	pigeons
Il1rap	0.78	0.00000018	pigeons
Rftn1	0.69	0.00000018	pigeons
Smc6	0.56	0.00000019	pigeons
Laptm5	0.35	0.00000022	pigeons
Psme4	0.36	0.00000022	pigeons
Pdhx	0.47	0.00000023	pigeons
Capn7	0.52	0.00000025	pigeons
Zfp706	0.30	0.00000026	pigeons
Rasa2	0.54	0.00000026	pigeons
Cnot2	0.51	0.00000026	pigeons
Kansl1l	0.34	0.00000028	pigeons
Tnrc6c	0.54	0.00000034	pigeons
Pias1	0.35	0.00000035	pigeons
Numb	-1.35	0.00000036	pigeons
Fbxo32	0.58	0.00000037	pigeons
Pxdc1	-1.12	0.00000037	pigeons

Atp1b1	0.47	0.00000038	pigeons
Camk1d	1.06	0.00000039	pigeons
Utp25	0.44	0.00000039	pigeons
Polr1d	0.41	0.00000039	pigeons
Inip	0.40	0.00000039	pigeons
Nr4a1	0.53	0.00000039	pigeons
Oxr1	0.43	0.00000043	pigeons
Eif2ak2	0.43	0.00000045	pigeons
Ckb	-0.86	0.00000046	pigeons
Itprip	0.41	0.00000058	pigeons
Gars	0.42	0.00000058	pigeons
Prrc2c	0.32	0.00000058	pigeons
Jarid2	0.61	0.00000060	pigeons
Fam53a	0.52	0.00000069	pigeons
Slf2	0.45	0.00000069	pigeons
Atf3	-1.55	0.00000070	pigeons
Sfmbt1	0.77	0.00000077	pigeons
Atg16l1	0.51	0.00000077	pigeons
Gphn	-1.21	0.00000081	pigeons
Pcsk2	-0.95	0.00000083	pigeons
Rgl1	-0.94	0.00000083	pigeons
Pde1c	-1.30	0.00000083	pigeons
Pcid2	0.46	0.00000085	pigeons
Aktip	0.49	0.00000085	pigeons
Tcp1	0.40	0.00000089	pigeons
Ttc21b	0.51	0.00000093	pigeons
Reps2	-0.83	0.00000093	pigeons
Rbm33	0.23	0.00000101	pigeons
Surf4	0.47	0.00000102	pigeons
Prpf40a	0.49	0.00000111	pigeons
Hspa4	0.31	0.00000119	pigeons
Rasgrp1	0.51	0.00000120	pigeons
Nfatc2	0.59	0.00000134	pigeons
Zeb1	0.41	0.00000143	pigeons
Hsdl2	0.67	0.00000143	pigeons
Sytl1	0.51	0.00000158	pigeons
Ccnd3	0.54	0.00000159	pigeons
Itpr2	0.47	0.00000160	pigeons
Trmt11	0.40	0.00000163	pigeons
Jchain	0.45	0.00000165	pigeons
Vav3	0.61	0.00000175	pigeons
Kdm2b	0.50	0.00000185	pigeons
Cd8a	0.69	0.00000192	pigeons
Atp2b1	0.62	0.00000210	pigeons
Eif3b	0.29	0.00000217	pigeons
Runx2	0.81	0.00000218	pigeons

Pus10	0.53	0.00000232	pigeons
Fbxo11	0.37	0.00000234	pigeons
Slc25a43	0.48	0.00000245	pigeons
Fkbp5	0.48	0.00000247	pigeons
Rassf5	0.46	0.00000252	pigeons
Ice2	0.44	0.00000279	pigeons
Slc12a4	0.34	0.00000283	pigeons
Hif1a	0.45	0.00000296	pigeons
Aimp1	0.37	0.00000298	pigeons
Nfkbie	0.38	0.00000300	pigeons
Etv6	-0.80	0.00000308	pigeons
Nr4a2	0.58	0.00000310	pigeons
Dazap1	0.37	0.00000314	pigeons
Ssb	0.37	0.00000314	pigeons
Vps37b	0.37	0.00000320	pigeons
Utp20	0.40	0.00000327	pigeons
Cerk	0.60	0.00000331	pigeons
Cyld	0.40	0.00000341	pigeons
Clic4	-1.42	0.00000349	pigeons
Dock4	-1.13	0.00000351	pigeons
Sun2	0.53	0.00000366	pigeons
Gk5	-1.19	0.00000369	pigeons
Vta1	0.39	0.00000386	pigeons
Sav1	0.48	0.00000392	pigeons
Kdm3b	0.35	0.00000409	pigeons
Traf3	0.54	0.00000410	pigeons
Ctdp1	0.41	0.00000415	pigeons
Pacsin2	0.33	0.00000469	pigeons
Eif2b3	0.38	0.00000472	pigeons
Naa15	0.52	0.00000474	pigeons
Kansl1	0.27	0.00000516	pigeons
Qrich1	0.41	0.00000562	pigeons
Ikzf3	0.57	0.00000564	pigeons
Dnajc1	0.54	0.00000586	pigeons
Zbtb37	0.54	0.00000591	pigeons
Hipk1	0.27	0.00000597	pigeons
Vapb	0.31	0.00000649	pigeons
Elovl6	0.70	0.00000693	pigeons
Rcsd1	0.31	0.00000767	pigeons
Bbx	0.40	0.00000782	pigeons
Fgd6	-1.04	0.00000799	pigeons
Fnbp1l	-1.49	0.00000821	pigeons
Bcl2	0.72	0.00000828	pigeons
Ccdc82	0.44	0.00000837	pigeons
Tada1	0.42	0.00000850	pigeons
Supt3	0.61	0.00000919	pigeons

Slc6a15	0.66	0.00001008	pigeons
Ndufa13	0.37	0.00001080	pigeons
Cep44	0.40	0.00001095	pigeons
Bcl2l11	0.48	0.00001109	pigeons
Creg1	-1.29	0.00001140	pigeons
Celf1	0.32	0.00001142	pigeons
Ptpn2	0.36	0.00001143	pigeons
Kdm4b	0.44	0.00001249	pigeons
Pcmtd1	0.38	0.00001310	pigeons
Orai1	0.51	0.00001363	pigeons
Anxa5	-1.21	0.00001426	pigeons
Dok1	0.38	0.00001427	pigeons
Ppp4r3a	0.44	0.00001459	pigeons
Kcnq1	-1.01	0.00001463	pigeons
Far1	0.35	0.00001503	pigeons
Cep112	0.42	0.00001508	pigeons
Setbp1	0.67	0.00001645	pigeons
Tec	0.69	0.00001813	pigeons
Nbn	0.38	0.00001876	pigeons
Ogfr	0.45	0.00002101	pigeons
Pspc1	0.25	0.00002163	pigeons
Bcat1	0.48	0.00002174	pigeons
Btbd9	0.46	0.00002232	pigeons
Rfx2	0.45	0.00002273	pigeons
Ctla4	0.91	0.00002287	pigeons
Anapc1	0.48	0.00002335	pigeons
Ppm1k	0.37	0.00002336	pigeons
Stk3	0.40	0.00002342	pigeons
Akap9	0.43	0.00002402	pigeons
Gtf2a1	0.35	0.00002494	pigeons
Ibtk	0.46	0.00002561	pigeons
Hectd4	0.40	0.00002677	pigeons
Tspan2	0.46	0.00002832	pigeons
Ptges3	0.28	0.00002853	pigeons
Alox5	0.65	0.00002918	pigeons
Eif3m	0.35	0.00002960	pigeons
Fry	-0.91	0.00003211	pigeons
Rfx3	0.55	0.00003304	pigeons
Anxa2	0.44	0.00003417	pigeons
Lrrc31	0.40	0.00003427	pigeons
Atp2a3	0.43	0.00003431	pigeons
Hivep1	0.48	0.00003494	pigeons
Rbm12	0.44	0.00003539	pigeons
Kras	0.40	0.00003789	pigeons
Srsf10	0.41	0.00004038	pigeons
Galnt9	0.44	0.00004254	pigeons

Sat1	0.38	0.00004687	pigeons
Stom	0.26	0.00004882	pigeons
Csnk2a2	0.34	0.00004887	pigeons
Trio	-1.27	0.00004903	pigeons
L3mbtl3	0.36	0.00005031	pigeons
Nek6	0.46	0.00005414	pigeons
Frs2	0.54	0.00005446	pigeons
Efl1	0.39	0.00006108	pigeons
Thoc2	0.26	0.00006108	pigeons
Cmtm7	0.47	0.00007091	pigeons
Scaf4	0.35	0.00007213	pigeons
St3gal1	-1.08	0.00007329	pigeons
Snhg4	0.24	0.00007410	pigeons
Zranb2	0.45	0.00007412	pigeons
Ubn2	0.33	0.00007713	pigeons
Stx17	0.31	0.00007843	pigeons
Klhl7	0.36	0.00008250	pigeons
Atp1b3	-1.42	0.00008789	pigeons
Pla2r1	-0.86	0.00009074	pigeons
Psmg2	0.29	0.00009199	pigeons
Gab3	0.50	0.00009335	pigeons
Uxs1	0.39	0.00010036	pigeons
Svil	-0.63	0.00010184	pigeons
Ifnar2	0.33	0.00010322	pigeons
Exoc3	0.46	0.00010557	pigeons
Hnrnph3	0.17	0.00010577	pigeons
Atad5	0.55	0.00010699	pigeons
Xpo7	0.22	0.00010722	pigeons
Atxn2	0.25	0.00010805	pigeons
Thrap3	0.29	0.00011454	pigeons
Ust	0.73	0.00011597	pigeons
Zfc3h1	0.29	0.00011665	pigeons
Aspm	0.33	0.00012175	pigeons
Srpr	0.35	0.00012751	pigeons
Tra2a	0.15	0.00012831	pigeons
Ss18	0.56	0.00012897	pigeons
Ran	0.21	0.00013389	pigeons
Snrpe	0.32	0.00013939	pigeons
Dhx15	0.19	0.00014227	pigeons
Elavl1	0.28	0.00014606	pigeons
Xrn2	0.31	0.00015283	pigeons
Gtdc1	0.76	0.00015356	pigeons
Ep300	0.23	0.00015555	pigeons
Rad51b	0.67	0.00016408	pigeons
Sntb1	0.56	0.00016635	pigeons
Tnpo1	0.23	0.00017606	pigeons

Dennd2d	0.45	0.00017835	pigeons
Rpn1	0.35	0.00017929	pigeons
Rac2	0.27	0.00018037	pigeons
Scyl2	0.46	0.00019315	pigeons
Ing1	0.47	0.00020037	pigeons
Rap1gds1	0.46	0.00020219	pigeons
Spag6l	0.61	0.00020948	pigeons
Dennd4a	-1.19	0.00021978	pigeons
Dst	-0.34	0.00022176	pigeons
Hs6st1	-1.14	0.00026169	pigeons
Igf2r	0.24	0.00026408	pigeons
Ctso	0.42	0.00026628	pigeons
Srsf3	0.24	0.00026806	pigeons
Eml4	0.48	0.00026951	pigeons
Rpa2	0.31	0.00027661	pigeons
Ssrp1	0.30	0.00028500	pigeons
Cdca7l	-0.96	0.00029245	pigeons
Brd7	0.34	0.00029616	pigeons
Tbc1d14	0.45	0.00030374	pigeons
Stat2	0.41	0.00031575	pigeons
Ylpm1	0.29	0.00031814	pigeons
Pnldc1	0.28	0.00032986	pigeons
Bicd1	-1.07	0.00033757	pigeons
Yrdc	0.29	0.00033826	pigeons
Zmynd11	0.39	0.00034874	pigeons
Klf12	0.48	0.00035918	pigeons
Zfp384	0.42	0.00039024	pigeons
Gnb5	-0.93	0.00039785	pigeons
Rnasel	0.55	0.00040623	pigeons
Pxk	0.41	0.00044288	pigeons
Lpxn	0.46	0.00044324	pigeons
Btaf1	0.39	0.00044861	pigeons
Themis	0.48	0.00044938	pigeons
Snrnp35	0.42	0.00046140	pigeons
Pisd	0.29	0.00046987	pigeons
Tmem11	0.38	0.00047141	pigeons
Azi2	0.38	0.00048385	pigeons
Sash3	0.43	0.00049063	pigeons
Acap2	0.35	0.00049203	pigeons
Selenot	0.26	0.00050113	pigeons
Fmnl2	-1.06	0.00051035	pigeons
Rnf34	0.40	0.00051748	pigeons
Ctsc	0.37	0.00053011	pigeons
Agfg1	0.28	0.00056768	pigeons
Cttnbp2	-0.86	0.00056773	pigeons
Nap1l1	0.31	0.00056819	pigeons

Ik	0.22	0.00057195	pigeons
Bmp2k	0.25	0.00060682	pigeons
Kidins220	0.17	0.00060710	pigeons
Rrp1b	0.21	0.00060844	pigeons
Gnal	0.81	0.00061803	pigeons
Zfp711	0.27	0.00063794	pigeons
Eif3e	0.21	0.00064142	pigeons
Kat6a	0.20	0.00069946	pigeons
Csde1	0.25	0.00070183	pigeons
Tasp1	0.43	0.00071566	pigeons
Scarb2	-1.12	0.00071594	pigeons
Zc3h18	0.31	0.00074159	pigeons
Pkn2	0.20	0.00074342	pigeons
Tle1	0.66	0.00075351	pigeons
Nab1	0.42	0.00076154	pigeons
Rilpl2	0.36	0.00077546	pigeons
Sike1	0.24	0.00077576	pigeons
Slc39a6	0.39	0.00081989	pigeons
Tulp3	0.41	0.00085079	pigeons
Epn2	0.21	0.00090269	pigeons
Zcchc2	0.34	0.00090409	pigeons
Naxd	0.23	0.00094225	pigeons
Kpna1	0.14	0.00099100	pigeons
Uri1	0.40	0.00099503	pigeons
Nol9	0.27	0.00100634	pigeons
Arl6ip5	0.29	0.00116192	pigeons
Csnk1a1	0.28	0.00122657	pigeons
Chic2	0.34	0.00123106	pigeons
Dffb	0.37	0.00127195	pigeons
Txnrd1	-1.20	0.00128449	pigeons
Ptpn7	0.35	0.00129634	pigeons
Dusp10	0.31	0.00132076	pigeons
Tnks	0.29	0.00133864	pigeons
Cabin1	0.35	0.00134486	pigeons
Srsf1	0.18	0.00138400	pigeons
Gyg	0.19	0.00140923	pigeons
Grk3	0.16	0.00144504	pigeons
Acaca	0.34	0.00144519	pigeons
Rnf145	0.22	0.00144538	pigeons
Neurl1b	-1.12	0.00145022	pigeons
Adam19	0.38	0.00146776	pigeons
Letm1	0.30	0.00148684	pigeons
Irf1	0.35	0.00151305	pigeons
Gatad2a	0.29	0.00153383	pigeons
Tnfrsf18	0.87	0.00160277	pigeons
Osbpl11	0.39	0.00161414	pigeons

Xpo4	0.35	0.00163067	pigeons
Tnrc6a	0.15	0.00163688	pigeons
Cul1	0.22	0.00164413	pigeons
Usp16	0.40	0.00170841	pigeons
Cyfip1	-1.14	0.00171419	pigeons
Tspan4	-0.93	0.00173152	pigeons
Eif2s3y	0.21	0.00181630	pigeons
Cstf3	0.26	0.00182258	pigeons
Acp1	0.15	0.00182454	pigeons
Supt6	0.22	0.00186927	pigeons
Ndufb1	0.15	0.00188393	pigeons
Fyn	0.29	0.00190614	pigeons
Foxk1	0.33	0.00195965	pigeons
Actr6	0.36	0.00198783	pigeons
Susd6	0.28	0.00205900	pigeons
Snx20	0.25	0.00206247	pigeons
Dus2	0.35	0.00213490	pigeons
B3gntl1	0.43	0.00217373	pigeons
Ssbp2	-0.88	0.00218457	pigeons
Ogt	0.20	0.00222308	pigeons
Sanbr	0.48	0.00223466	pigeons
Crem	0.32	0.00226856	pigeons
Lmbrd1	0.29	0.00231556	pigeons
Rdx	0.26	0.00240039	pigeons
Smurf2	-0.96	0.00241984	pigeons
Ercc3	0.12	0.00245306	pigeons
Yap1	-0.96	0.00247793	pigeons
Snrpd3	0.30	0.00256284	pigeons
Prkd3	0.20	0.00258070	pigeons
Odc1	0.40	0.00258675	pigeons
Rapgef1	0.25	0.00262569	pigeons
Lsm14a	0.10	0.00262822	pigeons
Nup210	0.35	0.00264235	pigeons
Pknox1	0.31	0.00302537	pigeons
Irf7	0.33	0.00319770	pigeons
Sft2d1	0.05	0.00320933	pigeons
Nuak1	-0.86	0.00350861	pigeons
Bcl7a	0.36	0.00351640	pigeons
Rbfa	-0.89	0.00353839	pigeons
Ttll12	0.25	0.00354495	pigeons
Eif3g	0.23	0.00361108	pigeons
Capzb	0.24	0.00369256	pigeons
Ikbkb	0.34	0.00371871	pigeons
Ppil4	0.31	0.00372456	pigeons
Stx18	0.55	0.00376883	pigeons
Nfkb1	0.28	0.00378365	pigeons

Igf1r	0.62	0.00379263	pigeons
Erp29	0.20	0.00380661	pigeons
Fdx1	0.13	0.00384518	pigeons
Pten	0.24	0.00390063	pigeons
Tcof1	0.33	0.00392194	pigeons
Zbtb21	0.15	0.00394672	pigeons
Cbl	0.21	0.00406908	pigeons
Unc119b	0.26	0.00410500	pigeons
Tacc1	0.17	0.00416677	pigeons
2900026A02Rik	0.39	0.00416823	pigeons
Jade2	0.23	0.00419835	pigeons
Herc1	0.16	0.00424422	pigeons
Tmx1	0.33	0.00430868	pigeons
Jakmip2	0.43	0.00436265	pigeons
Gpr155	0.28	0.00472397	pigeons
Gramd1c	0.61	0.00494680	pigeons
Dram2	0.22	0.00508122	pigeons
Vcpip1	0.16	0.00509069	pigeons
Sccpdh	0.25	0.00517207	pigeons
Dnajc13	0.24	0.00524753	pigeons
Cnot6	0.07	0.00531465	pigeons
Baz1b	0.27	0.00550016	pigeons
Rcan1	-1.03	0.00557457	pigeons
Irak4	0.32	0.00561766	pigeons
Arhgap5	-1.24	0.00564213	pigeons
Sdf4	0.30	0.00565406	pigeons
Puf60	0.23	0.00584356	pigeons
Bzw1	0.26	0.00595700	pigeons
Snrpc	0.32	0.00604213	pigeons
Smg1	0.30	0.00611011	pigeons
Bin1	-0.75	0.00633466	pigeons
Arid4a	0.08	0.00641015	pigeons
Zcchc8	0.15	0.00645922	pigeons
Ppp1r9a	0.53	0.00654989	pigeons
Slc41a1	0.26	0.00656644	pigeons
Cwc15	0.16	0.00682879	pigeons
Gse1	0.30	0.00710139	pigeons
Nlk	0.20	0.00715377	pigeons
Gpd2	0.36	0.00727496	pigeons
Dnaja4	0.25	0.00733290	pigeons
Rbm34	0.20	0.00735449	pigeons
Ipo11	0.38	0.00735787	pigeons
Nebl	0.50	0.00742974	pigeons
4933400A11Rik	0.34	0.00747644	pigeons
Cul3	0.26	0.00748278	pigeons
Kif5c	0.68	0.00751652	pigeons

Prkx	0.42	0.00788430	pigeons
Gpatch8	0.33	0.00792816	pigeons
Kif21b	0.30	0.00827387	pigeons
Lrrfip2	0.22	0.00828889	pigeons
Pcif1	0.26	0.00871669	pigeons
Xpot	0.26	0.00878388	pigeons
Wac	0.14	0.00886082	pigeons
Ip6k2	0.13	0.00899543	pigeons
Skap1	-0.53	0.00900965	pigeons
Flnb	-1.11	0.00908510	pigeons
Il17ra	0.35	0.00932068	pigeons
Prdm1	0.50	0.00950843	pigeons
Asxl3	-1.08	0.00979929	pigeons
Hbs1l	0.12	0.00991942	pigeons
Exosc10	0.21	0.01020210	pigeons
Borcs5	0.32	0.01028194	pigeons
Slc25a16	0.41	0.01035612	pigeons
Ddx6	0.16	0.01046110	pigeons
Ncoa6	0.24	0.01048599	pigeons
Shq1	0.34	0.01090255	pigeons
Ldlrad3	0.04	0.01111086	pigeons
Inpp5a	-0.63	0.01217637	pigeons
Traf1	0.41	0.01231226	pigeons
Dicer1	0.24	0.01246351	pigeons
Copg1	0.29	0.01252906	pigeons
Ppp1r12a	0.29	0.01256707	pigeons
Rbm31y	-0.50	0.01271837	pigeons
Usp15	0.06	0.01277458	pigeons
Upf2	0.16	0.01280793	pigeons
Actg1	-0.30	0.01282691	pigeons
Ralgapb	0.22	0.01294337	pigeons
Lcp1	0.26	0.01296240	pigeons
Tcf12	-1.24	0.01302019	pigeons
Adgrg6	0.80	0.01321320	pigeons
Cript	0.24	0.01353027	pigeons
D630045J12Rik	0.53	0.01353166	pigeons
Tlk1	0.21	0.01370896	pigeons
Fndc3a	0.23	0.01371146	pigeons
Tial1	0.15	0.01378353	pigeons
Zw10	0.21	0.01383976	pigeons
Eif4h	0.21	0.01388495	pigeons
Sod2	0.33	0.01394834	pigeons
Zcchc10	0.27	0.01395223	pigeons
Prrc2b	0.29	0.01398568	pigeons
Lcor	0.27	0.01423163	pigeons
Hsd17b12	-1.14	0.01429841	pigeons

Kcnk1	0.41	0.01447997	pigeons
Rasa3	0.68	0.01448677	pigeons
Sart3	0.26	0.01455523	pigeons
Clk4	0.09	0.01461869	pigeons
Aagab	0.38	0.01472216	pigeons
C2cd5	0.26	0.01493772	pigeons
Luc7l	0.14	0.01552003	pigeons
Add1	0.16	0.01576834	pigeons
Lrig1	0.51	0.01659860	pigeons
Otud7a	0.71	0.01661736	pigeons
Wdr26	0.18	0.01677354	pigeons
Rbx1	0.37	0.01685269	pigeons
Ythdf3	0.27	0.01734566	pigeons
Lrmda	-0.91	0.01736928	pigeons
Ncoa2	0.48	0.01742670	pigeons
Setx	0.06	0.01758226	pigeons
Tfg	0.26	0.01758852	pigeons
Hcfc2	0.08	0.01764203	pigeons
Btrc	0.34	0.01766269	pigeons
Tacc3	0.22	0.01769722	pigeons
Rbm12b2	0.29	0.01775601	pigeons
N4bp1	0.25	0.01783717	pigeons
Bach2	0.59	0.01799045	pigeons
Skil	0.37	0.01810269	pigeons
Zrsr1	0.20	0.01843390	pigeons
Vrk1	0.35	0.01846727	pigeons
Ttc39c	0.35	0.01870442	pigeons
Wdr25	0.41	0.01870808	pigeons
Cks2	0.35	0.01913845	pigeons
Ube2v2	0.26	0.01919027	pigeons
Traf2	0.23	0.01929066	pigeons
Zfp326	0.17	0.01930393	pigeons
Arl3	0.23	0.01973320	pigeons
Spen	0.13	0.01974631	pigeons
Ndufa8	0.10	0.02008129	pigeons
Ahi1	0.46	0.02046508	pigeons
Slc16a10	0.51	0.02069194	pigeons
Ahr	0.43	0.02080122	pigeons
Stt3b	0.10	0.02089814	pigeons
Edem1	0.25	0.02112203	pigeons
Atp6v0e	0.24	0.02136708	pigeons
Gprin3	0.26	0.02142949	pigeons
Lrpprc	0.28	0.02145193	pigeons
Rpap2	0.25	0.02151777	pigeons
Akap11	0.21	0.02161733	pigeons
Rbbp4	0.19	0.02270229	pigeons

Notch2	0.31	0.02279983	pigeons
Gpr65	0.45	0.02285497	pigeons
Kctd20	0.30	0.02297847	pigeons
Zfp618	0.30	0.02343324	pigeons
Fbxw2	0.21	0.02389488	pigeons
Zbtb46	0.19	0.02451669	pigeons
Sh3gl1	-0.86	0.02456316	pigeons
Parn	0.15	0.02510278	pigeons
Nfil3	0.53	0.02525054	pigeons
Atp6v0c	-0.86	0.02556340	pigeons
Srsf5	0.09	0.02559549	pigeons
Qser1	0.16	0.02567544	pigeons
Spty2d1	-0.60	0.02611157	pigeons
Sesn3	0.28	0.02659052	pigeons
Itgb2l	0.31	0.02704526	pigeons
Pcnx	0.07	0.02758994	pigeons
Gpr18	0.38	0.02780337	pigeons
Get4	0.19	0.02812484	pigeons
Ddx46	0.18	0.02880804	pigeons
Oat	-0.55	0.02916323	pigeons
Osbpl8	0.25	0.02940620	pigeons
Ing3	0.27	0.02943363	pigeons
Ubr5	0.15	0.02957389	pigeons
Serinc5	0.48	0.02978074	pigeons
Atp10d	0.23	0.03013769	pigeons
Fam185a	0.30	0.03025052	pigeons
Def6	0.38	0.03037224	pigeons
Eif2a	0.50	0.03044773	pigeons
Cntrl	0.32	0.03072197	pigeons
Wdr44	0.26	0.03081163	pigeons
Acvr2a	0.19	0.03095552	pigeons
Tmcc1	0.18	0.03142218	pigeons
Ddx1	0.17	0.03171300	pigeons
Tmem222	0.23	0.03180950	pigeons
Zfp292	0.29	0.03216823	pigeons
Pik3c3	0.30	0.03244416	pigeons
Usp24	0.38	0.03258355	pigeons
Dhx36	0.18	0.03292454	pigeons
4932438A13Rik	0.13	0.03323103	pigeons
Arhgap25	0.32	0.03335375	pigeons
Atl2	0.24	0.03419989	pigeons
Apoa1	-1.73	0.03474483	pigeons
Rgs3	-0.91	0.03496997	pigeons
Denr	0.28	0.03511642	pigeons
Arhgef18	0.33	0.03532649	pigeons
Caprin1	0.13	0.03652101	pigeons

Lbr	0.12	0.03678943	pigeons
Zfat	0.33	0.03685638	pigeons
Wipi2	0.37	0.03725529	pigeons
Dcaf5	-0.08	0.03737886	pigeons
Kmt2e	0.18	0.03755456	pigeons
Gfod1	0.38	0.03769874	pigeons
Nfe2l1	0.08	0.03839925	pigeons
Lrrc47	0.33	0.03856526	pigeons
Ints13	0.21	0.03982047	pigeons
G3bp1	0.20	0.03989139	pigeons
Xpr1	0.34	0.04061395	pigeons
Prpf8	0.24	0.04129798	pigeons
Pum1	0.11	0.04147814	pigeons
Ric8b	0.16	0.04165611	pigeons
Crb1	-0.47	0.04460255	pigeons
Tfip11	0.32	0.04475137	pigeons
Lrch1	0.24	0.04526918	pigeons
Usp45	0.12	0.04561861	pigeons
Aco2	0.21	0.04594028	pigeons
Prkaa1	0.13	0.04634794	pigeons
Ankrd49	0.19	0.04698322	pigeons
Hpcal1	0.04	0.04722109	pigeons
Acsl5	0.47	0.04831080	pigeons
Elmod2	0.27	0.04884910	pigeons
Raf1	0.32	0.04930143	pigeons
Rhof	0.26	0.05027444	pigeons
Sash1	0.82	0.05088752	pigeons
Slc9a7	0.37	0.05170441	pigeons
Tcerg1	0.21	0.05210284	pigeons
Fyttd1	0.25	0.05247109	pigeons
Kif11	0.35	0.05265984	pigeons
1700034I23Rik	0.16	0.05293789	pigeons
Sptan1	-1.17	0.05351967	pigeons
Pigb	0.21	0.05387263	pigeons
Rlf	0.31	0.05399485	pigeons
Cpsf3	0.32	0.05443940	pigeons
Lamtor2	0.24	0.05618724	pigeons
Osbpl3	0.34	0.05660359	pigeons
Tnfrsf21	0.28	0.05822569	pigeons
Stag2	0.11	0.05863204	pigeons
Naa25	0.41	0.05891925	pigeons
Arpc5	0.13	0.05895370	pigeons
Bcr	0.21	0.05910499	pigeons
Gnl3	0.26	0.05923451	pigeons
Itpr1	0.30	0.06149373	pigeons
Ei24	0.09	0.06161937	pigeons

Arhgap26	-0.79	0.06259744	pigeons
Kdm5b	0.30	0.06378732	pigeons
Birc6	0.21	0.06393241	pigeons
Cotl1	0.30	0.06426470	pigeons
Dnajc9	-0.93	0.06487087	pigeons
Btf3	0.28	0.06552784	pigeons
Scoc	0.44	0.06582023	pigeons
Csgalnact2	0.40	0.06768471	pigeons
Iffo2	0.42	0.06832919	pigeons
Hnrnpu	0.14	0.06950451	pigeons
Cd81	-0.85	0.06959208	pigeons
Ascc1	0.15	0.07017289	pigeons
Aqr	0.26	0.07110050	pigeons
Efhd2	0.29	0.07231645	pigeons
Pes1	0.27	0.07347639	pigeons
Pdxk	0.32	0.07359497	pigeons
Pdpk1	0.17	0.07386557	pigeons
Ppp2r2a	0.06	0.07478907	pigeons
Csf1	0.41	0.07668822	pigeons
Sugt1	0.12	0.07876087	pigeons
Tnip3	0.40	0.08011515	pigeons
Prmt9	0.30	0.08064766	pigeons
Noct	0.37	0.08166813	pigeons
Ptpn9	0.24	0.08173129	pigeons
Tomm70a	0.23	0.08381552	pigeons
Gnpda2	0.15	0.08531536	pigeons
Limd2	0.23	0.08549038	pigeons
Deaf1	0.32	0.08627540	pigeons
Gpbp1l1	0.11	0.08810970	pigeons
Uchl5	0.18	0.08858239	pigeons
Trim24	0.22	0.08862940	pigeons
Sdf2l1	0.31	0.08890077	pigeons
Rpp30	0.31	0.09435616	pigeons
Lag3	0.21	0.09436555	pigeons
Nfatc1	0.52	0.09702639	pigeons
Soga1	0.32	0.09703782	pigeons
Ralgapa1	0.11	0.09788196	pigeons
Fam120a	0.16	0.09789273	pigeons
Khdrbs1	0.28	0.09810722	pigeons
Ttc17	0.11	0.09818930	pigeons
Osbpl9	0.04	0.09856882	pigeons
Sbds	0.34	0.09910189	pigeons
Parp6	0.12	0.10197496	pigeons
Limd1	0.36	0.10311600	pigeons
Nubpl	0.40	0.10377737	pigeons
Pms2	0.22	0.10384623	pigeons

Stk10	0.20	0.10406750	pigeons
Eif4a3l1	0.23	0.10430046	pigeons
Rab11fip4	0.27	0.10438973	pigeons
Gfi1	0.59	0.10461874	pigeons
Tbck	0.28	0.10854781	pigeons
Sgip1	0.15	0.11069521	pigeons
Nolc1	0.28	0.11165743	pigeons
Vmp1	0.13	0.11192860	pigeons
Esyt2	0.37	0.11429320	pigeons
Ilf3	0.17	0.11573663	pigeons
Lct	0.34	0.11634777	pigeons
Lhfpl5	0.34	0.11707447	pigeons
Dag1	0.38	0.11777507	pigeons
Gng2	0.34	0.11864030	pigeons
Hdac2	0.00	0.12519272	pigeons
Hook3	0.16	0.12616422	pigeons
Dnajb6	0.11	0.12619590	pigeons
Xrcc4	0.20	0.12684463	pigeons
Sf3b1	0.11	0.12925234	pigeons
Rab8b	0.13	0.13112174	pigeons
Ska2	0.15	0.13254287	pigeons
Nol11	0.24	0.13354006	pigeons
Ddx20	0.18	0.13610650	pigeons
Prrc1	0.35	0.13694556	pigeons
Fbxl3	0.25	0.14008603	pigeons
Fkbp1a	-1.08	0.14187447	pigeons
Twf1	-0.82	0.14194498	pigeons
Tmem19	0.32	0.14217377	pigeons
Agps	0.22	0.14288556	pigeons
Nmi	0.24	0.14664965	pigeons
Urb1	0.31	0.15005296	pigeons
Ipo7	0.13	0.15170380	pigeons
Dync1li1	0.20	0.15249591	pigeons
Tcea1	0.24	0.15314441	pigeons
Dapk1	0.28	0.15318124	pigeons
Nol8	0.15	0.15345126	pigeons
Fam3c	0.13	0.15382112	pigeons
Secisbp2	0.16	0.15499697	pigeons
Itpka	0.20	0.15672474	pigeons
Sulf2	0.89	0.15783094	pigeons
Usp10	0.22	0.15807689	pigeons
Klhdc2	0.26	0.15886543	pigeons
Heatr3	0.35	0.15936252	pigeons
Lonp1	0.25	0.15960159	pigeons
Arid5a	0.25	0.16197632	pigeons
Trmt10c	0.11	0.16363130	pigeons

Acer2	-0.82	0.16512726	pigeons
Pex13	0.29	0.16595740	pigeons
Scaper	0.19	0.16783250	pigeons
Atxn7	0.12	0.17064082	pigeons
Eif2s2	0.07	0.17150361	pigeons
Tmem185a	0.23	0.17337134	pigeons
Ubap1	0.28	0.17455397	pigeons
Sh3bgrl	0.20	0.17533030	pigeons
Arid2	0.21	0.17593670	pigeons
Sdha	0.28	0.17634442	pigeons
Dtwd2	0.33	0.17714109	pigeons
Cd40lg	0.46	0.17884240	pigeons
Exoc6	0.24	0.18255753	pigeons
Luzp1	-1.03	0.18334076	pigeons
Pcnx2	0.28	0.18414066	pigeons
Pdk1	0.18	0.18414949	pigeons
Cog6	0.29	0.18505221	pigeons
Sft2d2	0.27	0.18529752	pigeons
Ptk2	-0.97	0.18854039	pigeons
Wdr77	0.34	0.19135626	pigeons
Usp25	0.19	0.19218920	pigeons
Wdr1	0.05	0.19415134	pigeons
Usp47	0.11	0.19520110	pigeons
Dlgap4	0.15	0.19541393	pigeons
Pign	0.36	0.19580925	pigeons
Zbed4	0.13	0.19825784	pigeons
Tspan3	-1.01	0.19868962	pigeons
Bdp1	0.37	0.19870553	pigeons
Ubr1	0.16	0.20538106	pigeons
Dnaja3	0.24	0.20621409	pigeons
Parp9	0.26	0.20824833	pigeons
Sin3a	0.10	0.21337510	pigeons
Sec14l1	-0.91	0.21537728	pigeons
Fkbp4	0.20	0.21696359	pigeons
Aftph	0.05	0.21717293	pigeons
Dgkz	0.30	0.21938435	pigeons
Ppwd1	0.18	0.21980294	pigeons
Tnfaip8l1	0.35	0.22007732	pigeons
Cpt1a	0.17	0.22018631	pigeons
Stk26	0.04	0.22287499	pigeons
Pdcd7	0.20	0.22393031	pigeons
Pdhb	0.21	0.22436296	pigeons
Oxsr1	-0.97	0.22501225	pigeons
Bivm	0.28	0.22579414	pigeons
Dtx3l	0.22	0.23464630	pigeons
Itm2a	-0.58	0.23708137	pigeons

Iqgap1	0.11	0.24477835	pigeons
Dnajc17	0.20	0.24496145	pigeons
Usf3	0.37	0.25256171	pigeons
Lamc1	-1.00	0.25355315	pigeons
Tmem87a	0.09	0.25476303	pigeons
Nr2c2	0.22	0.25915971	pigeons
Pld1	0.22	0.25988604	pigeons
Snapc1l	0.31	0.26322622	pigeons
Dcaf13	0.23	0.26400168	pigeons
Ddx3y	0.23	0.26731344	pigeons
Tcf20	0.19	0.27058643	pigeons
Cdk17	-0.79	0.27497258	pigeons
Als2cl	-0.98	0.27628492	pigeons
Hacd2	0.08	0.28475299	pigeons
Arv1	0.26	0.28570960	pigeons
Ywhaz	0.08	0.29162208	pigeons
Zbtb43	0.26	0.29464883	pigeons
Vps26a	0.14	0.29730707	pigeons
Sf3a3	0.16	0.29859092	pigeons
Rpp40	0.24	0.30183266	pigeons
Patj	0.42	0.31406724	pigeons
Nup88	0.21	0.31408185	pigeons
Pofut1	0.14	0.31432442	pigeons
Sap130	0.16	0.31717205	pigeons
Cdk19	0.01	0.31725038	pigeons
Sema4d	0.38	0.32563789	pigeons
Tmem168	0.19	0.32836051	pigeons
Bclaf1	0.09	0.32921992	pigeons
Cct4	0.16	0.32977792	pigeons
Oxct1	0.26	0.33310970	pigeons
Axin1	0.24	0.33646647	pigeons
Gemin4	0.11	0.34023941	pigeons
Usp54	-0.95	0.34119865	pigeons
Gpn3	0.11	0.34365836	pigeons
Atg3	0.20	0.34377165	pigeons
Rnf139	0.26	0.34651093	pigeons
Lrp3	0.11	0.34849392	pigeons
Srsf2	0.13	0.35539342	pigeons
Dph6	0.19	0.35560083	pigeons
Jak2	0.26	0.35756040	pigeons
Ipo5	0.19	0.36097379	pigeons
Lrif1	0.35	0.37591525	pigeons
Slc4a11	0.26	0.37816512	pigeons
Phf3	0.13	0.38081264	pigeons
Xpa	0.03	0.38117718	pigeons
Aig1	0.26	0.38493507	pigeons

Inpp5d	0.29	0.38602734	pigeons
Ube2b	0.09	0.38706815	pigeons
Ifi35	0.03	0.39067102	pigeons
Gnb4	0.09	0.39503376	pigeons
Cep83	-0.99	0.40159145	pigeons
Eny2	0.22	0.40647562	pigeons
Nf1	0.14	0.40958862	pigeons
Sf3b3	0.14	0.41143869	pigeons
Cct2	0.14	0.41317521	pigeons
Galc	0.30	0.42595373	pigeons
Tbc1d15	0.08	0.42626956	pigeons
Abhd17c	0.24	0.42667775	pigeons
Sec61g	0.16	0.42679413	pigeons
Sec16a	0.16	0.42881008	pigeons
Ppp3cc	0.24	0.43015540	pigeons
Dhx38	0.10	0.44125872	pigeons
Dusp5	0.30	0.45065887	pigeons
Ywhae	0.01	0.45790844	pigeons
Ckap5	0.24	0.45980108	pigeons
Amd2	0.02	0.47074968	pigeons
Slc35a5	0.24	0.47409436	pigeons
Pard3b	-0.96	0.48051595	pigeons
Slc36a4	0.20	0.48410218	pigeons
Clk1	0.27	0.48613036	pigeons
Lrwd1	0.25	0.48855917	pigeons
Farp2	-0.61	0.49887973	pigeons
Anks1	-0.89	0.50171984	pigeons
Rit1	0.26	0.50218383	pigeons
Tnfsf11	0.92	0.50274233	pigeons
Eif4g1	0.21	0.50343114	pigeons
Pgam1	0.32	0.50351749	pigeons
Rhoa	-0.32	0.50721285	pigeons
Npc2	-1.09	0.51207832	pigeons
Parp1	0.20	0.51224587	pigeons
Efr3a	0.28	0.51397472	pigeons
Nop14	0.11	0.52257351	pigeons
Arfip1	0.16	0.52956602	pigeons
Zfp143	0.23	0.53266917	pigeons
Nhp2	0.15	0.54092626	pigeons
9930021J03Rik	0.16	0.55522895	pigeons
Commd7	0.25	0.55753632	pigeons
Bub3	0.10	0.56769021	pigeons
Stk39	0.28	0.57054493	pigeons
Uba2	0.24	0.57433059	pigeons
Zbtb49	0.29	0.57545260	pigeons
Rbms1	0.08	0.57879512	pigeons

Asxl2	0.19	0.58093553	pigeons
Usp44	0.26	0.58238746	pigeons
Upf1	0.06	0.59505844	pigeons
Kdm4c	0.20	0.59665363	pigeons
Fbxl22	0.36	0.59742771	pigeons
Zfp512b	0.29	0.60177382	pigeons
Capn15	0.18	0.60496099	pigeons
Polk	0.11	0.60644706	pigeons
Gpr68	0.45	0.60741613	pigeons
Ypel5	0.24	0.60902788	pigeons
Wasl	0.25	0.61045918	pigeons
Lrp12	0.23	0.61078802	pigeons
Yme1l1	0.16	0.61706850	pigeons
Smim14	-0.79	0.61854689	pigeons
Lrrc8d	0.20	0.62950518	pigeons
Kdm7a	0.38	0.63002986	pigeons
Ubp1	0.03	0.64722488	pigeons
Parp8	0.29	0.64853655	pigeons
Cysltr1	0.41	0.65635994	pigeons
Ndufs4	0.19	0.65669789	pigeons
Clk3	0.24	0.66682086	pigeons
Ap1g1	0.01	0.67226202	pigeons
Arhgap45	0.35	0.67559661	pigeons
Epc1	0.07	0.67696850	pigeons
Rsl1d1	0.22	0.67756398	pigeons
Tmem184b	0.13	0.68619916	pigeons
Asap1	0.22	0.68838381	pigeons
Nub1	0.20	0.70048450	pigeons
Prim2	0.15	0.70128220	pigeons
Anxa11	-0.04	0.70514956	pigeons
Nek7	0.18	0.70618501	pigeons
Ech1	0.24	0.70630403	pigeons
Orc3	0.24	0.70647180	pigeons
Ptbp2	0.12	0.70928614	pigeons
Ip6k1	0.17	0.70976062	pigeons
Nipa2	-0.02	0.71409110	pigeons
Pptc7	0.26	0.71531217	pigeons
Hnrnpr	0.17	0.71799724	pigeons
Ppp6r2	0.14	0.71980003	pigeons
Pias2	0.21	0.72707684	pigeons
Nusap1	-0.03	0.72839310	pigeons
Aldh7a1	0.25	0.73585554	pigeons
Herpud1	0.36	0.73849523	pigeons
Ube2w	0.13	0.74924846	pigeons
Gramd1b	0.45	0.75099156	pigeons
Atp9b	0.13	0.75832226	pigeons

Hace1	0.36	0.75892030	pigeons
Prkd1	0.29	0.77032556	pigeons
Ubash3b	0.42	0.77220770	pigeons
Golga5	0.22	0.77464251	pigeons
Ube2g2	0.23	0.78477748	pigeons
Sms	0.30	0.78685046	pigeons
Prpf38a	0.34	0.79585908	pigeons
Dusp15	0.19	0.79635474	pigeons
Setd5	0.03	0.80936275	pigeons
Smarcc1	0.16	0.81084660	pigeons
Tk2	0.11	0.85463261	pigeons
Carhsp1	0.16	0.85732380	pigeons
Reep5	0.02	0.85786543	pigeons
Spry1	0.31	0.86120991	pigeons
Slk	0.14	0.86771105	pigeons
Phb2	0.22	0.89162268	pigeons
Slc41a2	0.33	0.89801342	pigeons
Larp1	0.17	0.90227097	pigeons
Borcs7	0.26	0.90600726	pigeons
Dync1h1	0.06	0.90619826	pigeons
Phactr2	0.08	0.92273504	pigeons
Fam168a	-1.04	0.92813992	pigeons
Ybx1	-0.44	0.92860881	pigeons
Il10ra	0.36	0.93510317	pigeons
Sacm1l	0.08	0.93886559	pigeons
Ube2d2a	0.09	0.94468025	pigeons
Nap1l4	0.07	0.95791488	pigeons
Gabarapl2	0.01	0.95960192	pigeons
Sp3	0.16	0.97661297	pigeons
Ttc14	0.23	0.97711553	pigeons
Rrn3	0.18	0.97915487	pigeons
Scaf8	0.12	0.99653687	pigeons
Rsrc2	0.10	0.99859318	pigeons
Dis3	0.12	1.00000000	pigeons
Ppig	0.09	1.00000000	pigeons
Cdc42	0.10	1.00000000	pigeons
Kat7	0.12	1.00000000	pigeons
Naa50	0.12	1.00000000	pigeons
Smap2	0.17	1.00000000	pigeons
Stx8	0.23	1.00000000	pigeons
Zc3h7a	0.20	1.00000000	pigeons
Umad1	0.24	1.00000000	pigeons
Trafd1	0.24	1.00000000	pigeons
Ypel2	0.22	1.00000000	pigeons
Ndufa5	0.22	1.00000000	pigeons
Nfat5	-0.12	1.00000000	pigeons

Yeats4	0.26	1.00000000	pigeons
Zbtb11	-0.06	1.00000000	pigeons
Vapa	0.02	1.00000000	pigeons
Impdh2	0.25	1.00000000	pigeons
Top3b	0.18	1.00000000	pigeons
Eif2b1	0.14	1.00000000	pigeons
Nbeal2	0.35	1.00000000	pigeons
Pank3	0.06	1.00000000	pigeons
Fgfr1op2	0.16	1.00000000	pigeons
Utp23	0.38	1.00000000	pigeons
Tmem70	0.27	1.00000000	pigeons
Ncoa1	0.08	1.00000000	pigeons
Wdr82	0.08	1.00000000	pigeons
Ruvbl1	0.20	1.00000000	pigeons
Larp7	0.14	1.00000000	pigeons
Il21r	0.32	1.00000000	pigeons
Trdmt1	0.07	1.00000000	pigeons
Xpc	0.12	1.00000000	pigeons
Il27ra	0.28	1.00000000	pigeons
Eef1e1	-0.05	1.00000000	pigeons
Chd9	0.16	1.00000000	pigeons
Atm	0.25	1.00000000	pigeons
Psmd14	0.29	1.00000000	pigeons
Ankrd13a	0.20	1.00000000	pigeons
Zc3h15	0.12	1.00000000	pigeons
Xrn1	0.15	1.00000000	pigeons
Strada	0.29	1.00000000	pigeons
Pnpla8	-0.04	1.00000000	pigeons
Tex10	0.11	1.00000000	pigeons
Cdk6	0.26	1.00000000	pigeons
Rad51c	0.22	1.00000000	pigeons
Ythdf1	0.18	1.00000000	pigeons
Itga6	0.12	1.00000000	pigeons
Ints6	-1.02	1.00000000	pigeons
Ppid	0.12	1.00000000	pigeons
Atf6	0.05	1.00000000	pigeons
Utp3	0.15	1.00000000	pigeons
Park7	0.19	1.00000000	pigeons
Git2	0.22	1.00000000	pigeons
Tpr	0.13	1.00000000	pigeons
Nbas	0.14	1.00000000	pigeons
Ago2	0.06	1.00000000	pigeons
Dhx29	0.18	1.00000000	pigeons
Ndufaf2	0.28	1.00000000	pigeons
Prpsap2	0.14	1.00000000	pigeons
Tbc1d12	0.19	1.00000000	pigeons

Nsrp1	0.24	1.00000000	pigeons
Tab3	0.13	1.00000000	pigeons
Ngly1	0.12	1.00000000	pigeons
Atg12	0.25	1.00000000	pigeons
Ing4	0.14	1.00000000	pigeons
Nlrc3	0.24	1.00000000	pigeons
Slc25a28	0.25	1.00000000	pigeons
Pigt	0.24	1.00000000	pigeons
Nedd4	0.30	1.00000000	pigeons
Pyroxd1	0.34	1.00000000	pigeons
Nifk	0.15	1.00000000	pigeons
Copa	0.18	1.00000000	pigeons
Plxnc1	0.19	1.00000000	pigeons
Tm9sf3	-0.01	1.00000000	pigeons
Selenof	0.14	1.00000000	pigeons
Gid8	0.11	1.00000000	pigeons
Tmx3	0.14	1.00000000	pigeons
Ube2o	0.23	1.00000000	pigeons
Osbpl1a	-1.00	1.00000000	pigeons
Pigk	0.11	1.00000000	pigeons
Pfdn4	0.11	1.00000000	pigeons
Pno1	0.29	1.00000000	pigeons
Kmt2c	0.07	1.00000000	pigeons
Ssh2	0.08	1.00000000	pigeons
Atp13a1	0.26	1.00000000	pigeons
Ccdc50	-0.79	1.00000000	pigeons
Ncstn	0.27	1.00000000	pigeons
Ncoa5	0.15	1.00000000	pigeons
Wdr59	0.24	1.00000000	pigeons
Tmem131l	0.04	1.00000000	pigeons
Hps3	0.24	1.00000000	pigeons
Aurkaip1	0.22	1.00000000	pigeons
Rpap3	0.12	1.00000000	pigeons
Hexim1	0.14	1.00000000	pigeons
Cep104	0.09	1.00000000	pigeons
Plagl1	0.32	1.00000000	pigeons
Zcchc17	0.17	1.00000000	pigeons
Pik3c2a	-0.09	1.00000000	pigeons
Sf3a1	0.09	1.00000000	pigeons
Ssu72	0.09	1.00000000	pigeons
Lck	0.16	1.00000000	pigeons
Suds3	0.03	1.00000000	pigeons
Itch	0.12	1.00000000	pigeons
Wdr91	0.20	1.00000000	pigeons
Tprgl	0.22	1.00000000	pigeons
Setd2	0.03	1.00000000	pigeons

Rnf114	0.09	1.00000000	pigeons
Nfrkb	0.23	1.00000000	pigeons
Tmc7	0.09	1.00000000	pigeons
Nsmaf	0.26	1.00000000	pigeons
Atp6v0b	0.01	1.00000000	pigeons
Auh	0.18	1.00000000	pigeons
Rabggtb	0.25	1.00000000	pigeons
Nup54	0.30	1.00000000	pigeons
Fmr1	0.07	1.00000000	pigeons
Hivep2	0.25	1.00000000	pigeons
Trappc4	0.12	1.00000000	pigeons
4833439L19Rik	0.11	1.00000000	pigeons
Rcor3	0.15	1.00000000	pigeons
Erich1	0.07	1.00000000	pigeons
Hspa9	0.22	1.00000000	pigeons
Grk4	0.19	1.00000000	pigeons
Tshz2	0.69	1.00000000	pigeons
Cers5	0.15	1.00000000	pigeons
Jade1	0.02	1.00000000	pigeons
Txndc11	0.20	1.00000000	pigeons
Ttc33	0.25	1.00000000	pigeons
Unk	0.08	1.00000000	pigeons
Snrpd1	0.04	1.00000000	pigeons
Strn	0.22	1.00000000	pigeons
Gzf1	0.15	1.00000000	pigeons
Cdv3	0.01	1.00000000	pigeons
Ints10	0.24	1.00000000	pigeons
Cc2d2a	0.26	1.00000000	pigeons
Arntl2	-0.38	1.00000000	pigeons
Elk3	-0.85	1.00000000	pigeons
Plek	0.30	1.00000000	pigeons
Clns1a	0.16	1.00000000	pigeons
Eif4a2	0.17	1.00000000	pigeons
Aven	0.31	1.00000000	pigeons
Ankib1	0.20	1.00000000	pigeons
Fbxw7	0.28	1.00000000	pigeons
Ube2l3	0.08	1.00000000	pigeons
Tmem140	0.13	1.00000000	pigeons
Erbin	0.13	1.00000000	pigeons
Prkrip1	0.04	1.00000000	pigeons
Stx12	0.15	1.00000000	pigeons
Eif2b2	0.24	1.00000000	pigeons
Napepld	0.14	1.00000000	pigeons
F5	-1.02	1.00000000	pigeons
Arfgap1	0.05	1.00000000	pigeons
Rabgap1	0.05	1.00000000	pigeons

Prpf3	0.16	1.00000000	pigeons
Zfp318	0.28	1.00000000	pigeons
2510039O18Rik	0.15	1.00000000	pigeons
Cct6b	0.02	1.00000000	pigeons
Alkbh5	0.05	1.00000000	pigeons
Itgb1bp1	0.22	1.00000000	pigeons
Csrnp1	0.10	1.00000000	pigeons
Pcmt1	0.21	1.00000000	pigeons
Prelid3b	0.24	1.00000000	pigeons
Ppm1a	0.20	1.00000000	pigeons
Rexo2	0.10	1.00000000	pigeons
Aff4	0.00	1.00000000	pigeons
Arhgap10	0.17	1.00000000	pigeons
Sumo1	0.05	1.00000000	pigeons
Sbno2	0.16	1.00000000	pigeons
Tmem258	0.10	1.00000000	pigeons
Add3	0.33	1.00000000	pigeons
Rpa3	0.20	1.00000000	pigeons
Zbtb7a	0.22	1.00000000	pigeons
Ccny	0.24	1.00000000	pigeons
Vps41	0.20	1.00000000	pigeons
Gdpd5	0.81	1.00000000	pigeons
Pdcd10	0.06	1.00000000	pigeons
Lactb	0.10	1.00000000	pigeons
Vezt	0.10	1.00000000	pigeons
Lnpk	0.21	1.00000000	pigeons
Srp9	0.05	1.00000000	pigeons
Rhoh	0.10	1.00000000	pigeons
Rrbp1	0.20	1.00000000	pigeons
Tank	0.15	1.00000000	pigeons
Helz2	0.12	1.00000000	pigeons
Rangap1	0.08	1.00000000	pigeons
Clec16a	0.26	1.00000000	pigeons
Rpgr	0.19	1.00000000	pigeons
Paxip1	0.07	1.00000000	pigeons
Papss1	-0.01	1.00000000	pigeons
Nsd3	-0.08	1.00000000	pigeons
Fryl	-0.59	1.00000000	pigeons
Ttyh3	0.03	1.00000000	pigeons
Nubp1	0.09	1.00000000	pigeons
Chordc1	0.13	1.00000000	pigeons
Vps29	0.04	1.00000000	pigeons
Fggy	0.28	1.00000000	pigeons
Slc35b1	0.18	1.00000000	pigeons
Top1	0.04	1.00000000	pigeons
Eif3d	0.22	1.00000000	pigeons

Arf6	0.08	1.00000000	pigeons
Polg2	0.07	1.00000000	pigeons
Dcp1b	0.34	1.00000000	pigeons
Dcaf1	0.08	1.00000000	pigeons
Ipo8	0.10	1.00000000	pigeons
Aup1	0.21	1.00000000	pigeons
Ebf1	-0.80	1.00000000	pigeons
Ciapin1	0.13	1.00000000	pigeons
Srebf2	0.06	1.00000000	pigeons
Npepps	-0.08	1.00000000	pigeons
Exosc9	0.17	1.00000000	pigeons
Ostf1	0.04	1.00000000	pigeons
Acox1	0.18	1.00000000	pigeons
Aak1	0.15	1.00000000	pigeons
Rsrc1	0.13	1.00000000	pigeons
Slc39a9	0.18	1.00000000	pigeons
Cdc40	0.16	1.00000000	pigeons
Eif3l	0.16	1.00000000	pigeons
Rbm39	0.07	1.00000000	pigeons
Pbrm1	0.02	1.00000000	pigeons
St7l	0.19	1.00000000	pigeons
Tpi1	0.08	1.00000000	pigeons
Bnip2	0.06	1.00000000	pigeons
Pin4	0.18	1.00000000	pigeons
Tbcd	0.18	1.00000000	pigeons
Wnk1	0.15	1.00000000	pigeons
Smyd3	0.16	1.00000000	pigeons
Atmin	0.16	1.00000000	pigeons
Smarcb1	0.19	1.00000000	pigeons
Senp6	0.15	1.00000000	pigeons
Tbc1d4	0.07	1.00000000	pigeons
Nipsnap2	0.18	1.00000000	pigeons
Ccdc88c	0.23	1.00000000	pigeons
Psmd1	0.11	1.00000000	pigeons
Fam222b	0.33	1.00000000	pigeons
Emc2	0.14	1.00000000	pigeons
Rabl6	0.17	1.00000000	pigeons
Rapgef2	-0.84	1.00000000	pigeons
Zbtb2	0.02	1.00000000	pigeons
Tapt1	0.20	1.00000000	pigeons
Styk1	0.37	1.00000000	pigeons
Htt	0.04	1.00000000	pigeons
B4galt5	0.12	1.00000000	pigeons
Rab11a	0.02	1.00000000	pigeons
Rnmt	0.08	1.00000000	pigeons
Gsn	-1.11	1.00000000	pigeons

Ctcf	0.03	1.00000000	pigeons
Tgfbrap1	0.07	1.00000000	pigeons
Oma1	0.21	1.00000000	pigeons
Ccnd2	0.27	1.00000000	pigeons
Sltm	0.00	1.00000000	pigeons
Klhl36	0.19	1.00000000	pigeons
Hipk2	0.14	1.00000000	pigeons
Cbx3	0.01	1.00000000	pigeons
Tmem60	0.23	1.00000000	pigeons
Ttf1	0.14	1.00000000	pigeons
Golga1	0.16	1.00000000	pigeons
Krit1	0.08	1.00000000	pigeons
Asb2	0.21	1.00000000	pigeons
Cct8	0.10	1.00000000	pigeons
Slc43a2	0.31	1.00000000	pigeons
Kif13b	-0.07	1.00000000	pigeons
Galnt10	0.17	1.00000000	pigeons
Yaf2	0.16	1.00000000	pigeons
Dennd1a	0.05	1.00000000	pigeons
Gtf2f2	0.19	1.00000000	pigeons
Hic2	0.21	1.00000000	pigeons
Tubgcp3	0.14	1.00000000	pigeons
Ghdc	0.13	1.00000000	pigeons
Zbtb24	0.07	1.00000000	pigeons
Batf	0.27	1.00000000	pigeons
Rab3gap2	0.28	1.00000000	pigeons
Trmt6	0.18	1.00000000	pigeons
Uap1	0.26	1.00000000	pigeons
Topbp1	0.21	1.00000000	pigeons
Dnajc7	0.09	1.00000000	pigeons
Ncbp1	0.10	1.00000000	pigeons
Kmt5b	-0.12	1.00000000	pigeons
Dip2b	0.15	1.00000000	pigeons
Cltb	0.03	1.00000000	pigeons
Eif3i	-0.49	1.00000000	pigeons
Timm44	0.17	1.00000000	pigeons
Derl1	0.07	1.00000000	pigeons
Ube2a	0.03	1.00000000	pigeons
Zdhhc9	0.02	1.00000000	pigeons
Pfdn1	0.04	1.00000000	pigeons
Hddc2	0.06	1.00000000	pigeons
Wwc1	0.23	1.00000000	pigeons
Cpsf2	0.06	1.00000000	pigeons
Dck	0.11	1.00000000	pigeons
Nrip1	0.40	1.00000000	pigeons
Slc30a4	0.11	1.00000000	pigeons

Ndufs2	0.10	1.00000000	pigeons
Cops4	0.19	1.00000000	pigeons
Ddx23	0.17	1.00000000	pigeons
Prkca	0.15	1.00000000	pigeons
Btf3l4	0.10	1.00000000	pigeons
Fbxo42	0.20	1.00000000	pigeons
Kdm1a	0.22	1.00000000	pigeons
Coro1c	0.17	1.00000000	pigeons
Ints8	0.07	1.00000000	pigeons
Scfd1	0.15	1.00000000	pigeons
Nsl1	0.00	1.00000000	pigeons
Fam32a	-0.06	1.00000000	pigeons
Hspa13	0.21	1.00000000	pigeons
Lef1	0.69	1.00000000	pigeons
Cnot4	0.20	1.00000000	pigeons
Fchsd2	-0.07	1.00000000	pigeons
Ube2i	0.09	1.00000000	pigeons
Adnp2	-0.14	1.00000000	pigeons
Eif3f	0.02	1.00000000	pigeons
Vps35	0.19	1.00000000	pigeons
Klf3	-0.71	1.00000000	pigeons
Foxk2	0.07	1.00000000	pigeons
Rab22a	0.13	1.00000000	pigeons
Slc30a5	0.15	1.00000000	pigeons
Abracl	0.06	1.00000000	pigeons
Paxbp1	0.08	1.00000000	pigeons
Ifih1	0.14	1.00000000	pigeons
Samd8	0.13	1.00000000	pigeons
Aplp2	-0.93	1.00000000	pigeons
Wdr43	0.16	1.00000000	pigeons
Acsl4	0.07	1.00000000	pigeons
Eef2	0.15	1.00000000	pigeons
Dcun1d4	0.14	1.00000000	pigeons
Zfp830	0.20	1.00000000	pigeons
Ppa2	0.20	1.00000000	pigeons
Cish	0.20	1.00000000	pigeons
Anapc7	0.10	1.00000000	pigeons
Prr5l	0.13	1.00000000	pigeons
Sucla2	0.06	1.00000000	pigeons
Dnmt1	0.12	1.00000000	pigeons
Nup50	0.18	1.00000000	pigeons
Ppp2r5c	0.10	1.00000000	pigeons
Epb41l3	-0.16	1.00000000	pigeons
Gjd4	0.13	1.00000000	pigeons
Wdr20	0.01	1.00000000	pigeons
Psmd4	0.11	1.00000000	pigeons

Lpcat3	0.15	1.00000000	pigeons
Dok2	0.16	1.00000000	pigeons
Eif6	0.15	1.00000000	pigeons
Virma	0.21	1.00000000	pigeons
Atg13	0.26	1.00000000	pigeons
Rc3h2	0.15	1.00000000	pigeons
Uchl3	0.24	1.00000000	pigeons
Nktr	-0.06	1.00000000	pigeons
Psmd13	0.09	1.00000000	pigeons
Sirt1	0.15	1.00000000	pigeons
Pik3r5	-0.01	1.00000000	pigeons
Ints2	0.08	1.00000000	pigeons
Hmces	0.29	1.00000000	pigeons
Trappc3	0.24	1.00000000	pigeons
Copg2	0.09	1.00000000	pigeons
Pcm1	0.20	1.00000000	pigeons
Acp2	0.10	1.00000000	pigeons
Cog1	0.20	1.00000000	pigeons
Surf6	0.10	1.00000000	pigeons
Nedd9	0.15	1.00000000	pigeons
Fubp1	-0.02	1.00000000	pigeons
Arid1b	0.22	1.00000000	pigeons
Cnst	0.09	1.00000000	pigeons
Stard3nl	0.06	1.00000000	pigeons
Kpna6	0.11	1.00000000	pigeons
Zzz3	0.01	1.00000000	pigeons
Clpx	0.01	1.00000000	pigeons
Ankrd11	0.19	1.00000000	pigeons
Sos1	-0.05	1.00000000	pigeons
Pphln1	0.18	1.00000000	pigeons
Cmtr1	0.16	1.00000000	pigeons
Lmbr1	0.10	1.00000000	pigeons
Dhx40	0.12	1.00000000	pigeons
Nup93	0.18	1.00000000	pigeons
Gtf2h5	0.02	1.00000000	pigeons
Sin3b	0.07	1.00000000	pigeons
Ap2b1	-0.16	1.00000000	pigeons
Sfpq	0.05	1.00000000	pigeons
Heatr5b	0.12	1.00000000	pigeons
Sec23b	-0.03	1.00000000	pigeons
Ptpra	0.16	1.00000000	pigeons
Nemf	0.18	1.00000000	pigeons
Larp4	0.16	1.00000000	pigeons
Ankle2	0.12	1.00000000	pigeons
Plekhb2	0.18	1.00000000	pigeons
Naa30	0.05	1.00000000	pigeons

Ranbp10	0.20	1.00000000	pigeons
Snrpa1	0.12	1.00000000	pigeons
Psma6	-0.03	1.00000000	pigeons
Hs1bp3	0.03	1.00000000	pigeons
Nsmce1	0.16	1.00000000	pigeons
Ndufb9	0.10	1.00000000	pigeons
Gpr107	0.08	1.00000000	pigeons
Snw1	-0.05	1.00000000	pigeons
Bmt2	0.14	1.00000000	pigeons
Tmem41b	0.21	1.00000000	pigeons
Pip5k1a	-0.90	1.00000000	pigeons
Zfp644	0.08	1.00000000	pigeons
Itpkb	0.08	1.00000000	pigeons
Txlng	0.19	1.00000000	pigeons
Eif3h	0.09	1.00000000	pigeons
Bbs2	0.14	1.00000000	pigeons
Cflar	0.02	1.00000000	pigeons
Ywhab	0.08	1.00000000	pigeons
Ap1s2	0.15	1.00000000	pigeons
Chchd4	0.20	1.00000000	pigeons
Pla2g4a	0.19	1.00000000	pigeons
Fyco1	0.07	1.00000000	pigeons
Zfp148	0.09	1.00000000	pigeons
Cog5	-0.02	1.00000000	pigeons
Nfyb	0.08	1.00000000	pigeons
Edem3	0.01	1.00000000	pigeons
Comtd1	0.20	1.00000000	pigeons
Arhgef3	0.05	1.00000000	pigeons
Clp1	0.18	1.00000000	pigeons
Arid4b	0.09	1.00000000	pigeons
Polr2c	0.13	1.00000000	pigeons
Ubap2	0.03	1.00000000	pigeons
Fbxl4	0.07	1.00000000	pigeons
Pmm2	0.18	1.00000000	pigeons
Seh1l	0.13	1.00000000	pigeons
Samhd1	0.08	1.00000000	pigeons
Ppil3	0.04	1.00000000	pigeons
Usp33	0.04	1.00000000	pigeons
Fhl3	0.10	1.00000000	pigeons
Atp2a2	0.11	1.00000000	pigeons
Ctr9	0.08	1.00000000	pigeons
Adcy9	-0.17	1.00000000	pigeons
Dnm1l	0.09	1.00000000	pigeons
Ythdc1	0.02	1.00000000	pigeons
Nceh1	0.30	1.00000000	pigeons
Vcl	-0.73	1.00000000	pigeons

Api5	0.07	1.00000000	pigeons
Ccnk	-0.04	1.00000000	pigeons
Ttc1	-0.01	1.00000000	pigeons
Sun1	0.05	1.00000000	pigeons
Nek9	0.20	1.00000000	pigeons
Paip1	0.06	1.00000000	pigeons
Fem1a	0.12	1.00000000	pigeons
Pabpc4	0.11	1.00000000	pigeons
Plagl2	0.10	1.00000000	pigeons
Ppp2r2d	0.01	1.00000000	pigeons
Btbd7	0.14	1.00000000	pigeons
Bcas2	0.24	1.00000000	pigeons
Gps1	0.13	1.00000000	pigeons
Cul5	0.11	1.00000000	pigeons
Atf2	0.11	1.00000000	pigeons
Gramd4	0.25	1.00000000	pigeons
Fer	0.08	1.00000000	pigeons
Tmem35b	-0.12	1.00000000	pigeons
Pi4k2b	0.18	1.00000000	pigeons
Rsu1	0.02	1.00000000	pigeons
G3bp2	0.04	1.00000000	pigeons
Glg1	-0.10	1.00000000	pigeons
U2af1	-0.08	1.00000000	pigeons
Rab5a	-0.06	1.00000000	pigeons
Slc25a36	-0.02	1.00000000	pigeons
Kctd3	0.11	1.00000000	pigeons
Ehd3	0.00	1.00000000	pigeons
Bhlhe40	0.13	1.00000000	pigeons
Prpf4b	-0.05	1.00000000	pigeons
Emc3	-0.05	1.00000000	pigeons
Copb2	0.11	1.00000000	pigeons
Prkag2	0.15	1.00000000	pigeons
Ippk	-0.08	1.00000000	pigeons
Xrcc5	0.09	1.00000000	pigeons
Ezh2	0.14	1.00000000	pigeons
Ell	0.06	1.00000000	pigeons
Fkbp8	0.17	1.00000000	pigeons
Rpn2	0.04	1.00000000	pigeons
Tbc1d23	0.08	1.00000000	pigeons
Supt20	0.12	1.00000000	pigeons
Pnpt1	0.14	1.00000000	pigeons
Ppp1r27	0.19	1.00000000	pigeons
Gnai2	0.04	1.00000000	pigeons
Trps1	0.39	1.00000000	pigeons
Ralbp1	-0.84	1.00000000	pigeons
Stxbp6	0.30	1.00000000	pigeons

Hells	0.05	1.00000000	pigeons
Guk1	0.06	1.00000000	pigeons
Ppm1h	0.15	1.00000000	pigeons
Cstf1	0.16	1.00000000	pigeons
Dstn	-0.35	1.00000000	pigeons
Ube2v1	0.01	1.00000000	pigeons
Fuca2	0.16	1.00000000	pigeons
Nucb2	0.02	1.00000000	pigeons
Pwwp2a	0.22	1.00000000	pigeons
Cd99l2	0.09	1.00000000	pigeons
Nrf1	0.26	1.00000000	pigeons
Togaram1	0.05	1.00000000	pigeons
Itgb1	0.22	1.00000000	pigeons
Nudt21	0.12	1.00000000	pigeons
Zfp330	0.15	1.00000000	pigeons
Fcf1	0.16	1.00000000	pigeons
Naaa	0.08	1.00000000	pigeons
Tfdp2	0.25	1.00000000	pigeons
Tra2b	-0.04	1.00000000	pigeons
Phf20l1	-0.05	1.00000000	pigeons
Srp14	0.18	1.00000000	pigeons
Optn	0.05	1.00000000	pigeons
Ythdf2	0.04	1.00000000	pigeons
Snip1	-0.01	1.00000000	pigeons
Csnk1e	0.03	1.00000000	pigeons
Edc3	0.07	1.00000000	pigeons
Plekha1	0.06	1.00000000	pigeons
Ap3m1	0.13	1.00000000	pigeons
S100a10	-0.37	1.00000000	pigeons
Arhgef7	-0.84	1.00000000	pigeons
Lats1	0.00	1.00000000	pigeons
Wbp2nl	0.06	1.00000000	pigeons
Nfyc	0.18	1.00000000	pigeons
Srek1ip1	0.13	1.00000000	pigeons
Csnk2a1	0.17	1.00000000	pigeons
Casp7	0.11	1.00000000	pigeons
Bambi	-0.67	1.00000000	pigeons
Ppp1r7	0.09	1.00000000	pigeons
Commd9	0.04	1.00000000	pigeons
Cep350	0.04	1.00000000	pigeons
Phc3	0.08	1.00000000	pigeons
Nr1d2	-0.50	1.00000000	pigeons
Arih2	0.21	1.00000000	pigeons
Rnf20	0.05	1.00000000	pigeons
Psma1	0.12	1.00000000	pigeons
Glyr1	0.09	1.00000000	pigeons

Gins2	0.19	1.00000000	pigeons
Decr1	0.19	1.00000000	pigeons
Tomm20	0.01	1.00000000	pigeons
Ap3b1	0.16	1.00000000	pigeons
Uhrf2	0.03	1.00000000	pigeons
Psmd2	-0.01	1.00000000	pigeons
Ppp1r8	0.04	1.00000000	pigeons
Acadl	0.02	1.00000000	pigeons
Rab35	0.01	1.00000000	pigeons
Nlrc5	0.09	1.00000000	pigeons
Fam53b	0.14	1.00000000	pigeons
Psmc6	-0.01	1.00000000	pigeons
Ttc37	0.11	1.00000000	pigeons
Rlim	-0.10	1.00000000	pigeons
Klhl2	0.28	1.00000000	pigeons
Ilf2	0.01	1.00000000	pigeons
Nfx1	-0.11	1.00000000	pigeons
Ccdc59	0.10	1.00000000	pigeons
Tcf25	0.02	1.00000000	pigeons
Hsp90aa1	-0.03	1.00000000	pigeons
Acsl3	0.17	1.00000000	pigeons
Nfu1	0.11	1.00000000	pigeons
Bin3	-0.84	1.00000000	pigeons
Jakmip1	0.18	1.00000000	pigeons
Cdk7	0.04	1.00000000	pigeons
Nelfcd	0.09	1.00000000	pigeons
Nkrf	0.04	1.00000000	pigeons
Rassf2	0.16	1.00000000	pigeons
Sec24a	0.01	1.00000000	pigeons
Rmnd1	0.12	1.00000000	pigeons
Irf2	-0.13	1.00000000	pigeons
Tbc1d22a	-0.07	1.00000000	pigeons
Gfra3	0.16	1.00000000	pigeons
Pdcd4	0.13	1.00000000	pigeons
Nudt16l1	0.19	1.00000000	pigeons
Ttc28	-0.86	1.00000000	pigeons
Gnl2	0.06	1.00000000	pigeons
Vps13c	0.14	1.00000000	pigeons
Fam162a	0.05	1.00000000	pigeons
Pip5k1c	0.02	1.00000000	pigeons
Ptk2b	0.21	1.00000000	pigeons
Top2b	-0.14	1.00000000	pigeons
Tmed2	-0.04	1.00000000	pigeons
Ptges2	0.13	1.00000000	pigeons
Dnaja1	0.04	1.00000000	pigeons
Kifap3	0.10	1.00000000	pigeons

Kdelr2	-0.06	1.00000000	pigeons
Nipal3	0.10	1.00000000	pigeons
Hspa4l	0.19	1.00000000	pigeons
Itm2c	0.21	1.00000000	pigeons
Itfg1	0.13	1.00000000	pigeons
Sppl3	-0.09	1.00000000	pigeons
Ube2q1	-0.04	1.00000000	pigeons
Oaz1	0.10	1.00000000	pigeons
Snx17	0.00	1.00000000	pigeons
Pde8a	-0.39	1.00000000	pigeons
Dhx8	0.16	1.00000000	pigeons
Smarca5	-0.02	1.00000000	pigeons
Syf2	0.03	1.00000000	pigeons
Ndufa2	-0.06	1.00000000	pigeons
Hmgcs1	0.16	1.00000000	pigeons
Polr2k	0.01	1.00000000	pigeons
Cog7	0.15	1.00000000	pigeons
Sec24b	0.08	1.00000000	pigeons
Hlcs	0.13	1.00000000	pigeons
Cwf19l2	0.21	1.00000000	pigeons
Zfand1	-0.02	1.00000000	pigeons
Appbp2	0.04	1.00000000	pigeons
Bicral	0.02	1.00000000	pigeons
Zcrb1	0.09	1.00000000	pigeons
Usp4	-0.13	1.00000000	pigeons
Rcbtb2	0.02	1.00000000	pigeons
Primpol	0.20	1.00000000	pigeons
Cwc25	0.15	1.00000000	pigeons
Wwp1	0.00	1.00000000	pigeons
Noc2l	0.22	1.00000000	pigeons
Hmgcr	0.01	1.00000000	pigeons
Zdhhc3	0.05	1.00000000	pigeons
Spopl	0.00	1.00000000	pigeons
Psme3	0.15	1.00000000	pigeons
Strn3	-0.12	1.00000000	pigeons
Ddx42	0.03	1.00000000	pigeons
Fam177a	-0.89	1.00000000	pigeons
Rnpc3	-0.06	1.00000000	pigeons
Opa1	-0.03	1.00000000	pigeons
Tmed10	0.07	1.00000000	pigeons
Slu7	0.02	1.00000000	pigeons
Fkbp11	0.03	1.00000000	pigeons
Rev1	0.02	1.00000000	pigeons
Rer1	0.06	1.00000000	pigeons
Pds5a	-0.09	1.00000000	pigeons
Dgkh	-0.53	1.00000000	pigeons

Atrx	-0.06	1.00000000	pigeons
Cst7	0.19	1.00000000	pigeons
Fxr1	-0.21	1.00000000	pigeons
Noc3l	0.03	1.00000000	pigeons
Ap4b1	0.12	1.00000000	pigeons
Pum2	0.01	1.00000000	pigeons
Ndfip2	0.10	1.00000000	pigeons
Nutf2	0.00	1.00000000	pigeons
Dnajc10	-0.10	1.00000000	pigeons
Zfp654	0.00	1.00000000	pigeons
Heatr6	0.15	1.00000000	pigeons
Socs5	0.14	1.00000000	pigeons
Scaf11	0.01	1.00000000	pigeons
Tecpr2	-0.02	1.00000000	pigeons
Cct5	0.09	1.00000000	pigeons
Sptlc1	0.17	1.00000000	pigeons
Atp13a3	0.10	1.00000000	pigeons
Pik3cb	0.05	1.00000000	pigeons
Pals1	-0.56	1.00000000	pigeons
Tor1b	0.09	1.00000000	pigeons
Jagn1	0.16	1.00000000	pigeons
Nufip2	-0.12	1.00000000	pigeons
Alg12	0.10	1.00000000	pigeons
Haus3	0.26	1.00000000	pigeons
Lta4h	0.20	1.00000000	pigeons
Arcn1	0.03	1.00000000	pigeons
Zdhhc6	-0.03	1.00000000	pigeons
Rnaseh2b	-0.04	1.00000000	pigeons
Tfb2m	0.09	1.00000000	pigeons
Ist1	0.08	1.00000000	pigeons
Fbxw11	0.00	1.00000000	pigeons
Taldo1	-0.01	1.00000000	pigeons
Commd6	0.01	1.00000000	pigeons
Cdc23	0.21	1.00000000	pigeons
Trp73	0.18	1.00000000	pigeons
Ppp1cc	-0.03	1.00000000	pigeons
Acp6	0.18	1.00000000	pigeons
Hdx	-0.08	1.00000000	pigeons
Arhgdib	0.14	1.00000000	pigeons
Serbp1	0.14	1.00000000	pigeons
Aebp2	0.12	1.00000000	pigeons
Rap2c	0.01	1.00000000	pigeons
Nup214	0.22	1.00000000	pigeons
Dnajc2	0.05	1.00000000	pigeons
Acsbg2	0.16	1.00000000	pigeons
Ubqln4	0.05	1.00000000	pigeons

Ehd4	-0.84	1.00000000	pigeons
Grsf1	0.11	1.00000000	pigeons
Bcap29	0.01	1.00000000	pigeons
Atrn	-0.53	1.00000000	pigeons
Fbxl14	-0.01	1.00000000	pigeons
Ppm1b	-0.11	1.00000000	pigeons
Dync1li2	0.00	1.00000000	pigeons
Hif1an	0.17	1.00000000	pigeons
Rab3gap1	0.08	1.00000000	pigeons
Ankrd50	-0.76	1.00000000	pigeons
Atp6v0a2	-0.01	1.00000000	pigeons
Naa35	0.21	1.00000000	pigeons
Synj1	-0.06	1.00000000	pigeons
Zbtb48	0.10	1.00000000	pigeons
Brcc3	0.12	1.00000000	pigeons
Ccdc93	0.12	1.00000000	pigeons
Rere	-0.80	1.00000000	pigeons
Fbxl17	0.21	1.00000000	pigeons
Eif1ad12	-0.07	1.00000000	pigeons
Cct7	-0.01	1.00000000	pigeons
Cul4b	-0.16	1.00000000	pigeons
Arpc5l	0.11	1.00000000	pigeons
Dpm2	-0.03	1.00000000	pigeons
Dnajc5	0.05	1.00000000	pigeons
Rsbn1	0.11	1.00000000	pigeons
Nup85	0.07	1.00000000	pigeons
Nudcd1	0.04	1.00000000	pigeons
Abcc4	0.07	1.00000000	pigeons
Bfar	-0.01	1.00000000	pigeons
Cnnm2	-0.82	1.00000000	pigeons
Clcn3	0.12	1.00000000	pigeons
Klf7	-0.77	1.00000000	pigeons
Ehmt1	-0.10	1.00000000	pigeons
Spg21	0.12	1.00000000	pigeons
Polr2f	0.02	1.00000000	pigeons
Smg7	0.16	1.00000000	pigeons
Zdhhc17	0.02	1.00000000	pigeons
Ldb1	0.07	1.00000000	pigeons
Tet2	-0.52	1.00000000	pigeons
Snap23	-0.03	1.00000000	pigeons
Polr1e	0.09	1.00000000	pigeons
Tmpo	-0.12	1.00000000	pigeons
Ttc27	0.00	1.00000000	pigeons
Gtf2b	0.02	1.00000000	pigeons
Fosl2	0.09	1.00000000	pigeons
Ttc3	0.03	1.00000000	pigeons

Nr3c1	-0.01	1.00000000	pigeons
Polr2e	0.08	1.00000000	pigeons
Xpnpep1	0.15	1.00000000	pigeons
Syne3	0.11	1.00000000	pigeons
Nrbp1	0.08	1.00000000	pigeons
Eif4enif1	0.09	1.00000000	pigeons
Trappc8	0.17	1.00000000	pigeons
Supv3l1	0.03	1.00000000	pigeons
Ralgps1	0.06	1.00000000	pigeons
Shoc2	0.04	1.00000000	pigeons
Vps50	-0.44	1.00000000	pigeons
Hat1	0.06	1.00000000	pigeons
Smu1	-0.06	1.00000000	pigeons
Bms1	0.02	1.00000000	pigeons
Reps1	0.01	1.00000000	pigeons
Cds2	-0.02	1.00000000	pigeons
Pex14	0.00	1.00000000	pigeons
Brd3	0.03	1.00000000	pigeons
Eif2ak1	0.08	1.00000000	pigeons
Nsa2	0.02	1.00000000	pigeons
Zer1	0.06	1.00000000	pigeons
Angptl2	0.02	1.00000000	pigeons
Kpna4	-0.03	1.00000000	pigeons
Ppp1ca	0.02	1.00000000	pigeons
Scamp2	0.04	1.00000000	pigeons
Arsg	0.29	1.00000000	pigeons
Actr2	-0.01	1.00000000	pigeons
Sec61a2	0.07	1.00000000	pigeons
Snrpb2	-0.03	1.00000000	pigeons
Lrch2	0.11	1.00000000	pigeons
Wdr70	0.16	1.00000000	pigeons
Slc10a7	0.00	1.00000000	pigeons
Nus1	0.04	1.00000000	pigeons
Vps39	0.06	1.00000000	pigeons
Zswim8	-0.60	1.00000000	pigeons
Isca2	-0.02	1.00000000	pigeons
Gcc2	0.03	1.00000000	pigeons
Taf11	0.04	1.00000000	pigeons
Rundc3b	0.10	1.00000000	pigeons
Ankrd28	0.12	1.00000000	pigeons
Usb1	0.21	1.00000000	pigeons
Fam13b	-0.09	1.00000000	pigeons
Ppp1r2	0.12	1.00000000	pigeons
Gnpat	0.08	1.00000000	pigeons
Tnks2	-0.01	1.00000000	pigeons
Hipk3	0.16	1.00000000	pigeons

Itfg2	-0.13	1.00000000	pigeons
Helb	0.09	1.00000000	pigeons
Ola1	0.06	1.00000000	pigeons
Setdb1	0.04	1.00000000	pigeons
Tiprl	0.07	1.00000000	pigeons
Hsbp1	-0.15	1.00000000	pigeons
Glo1	-0.05	1.00000000	pigeons
R3hdm1	0.10	1.00000000	pigeons
Riok1	0.08	1.00000000	pigeons
Pebp1	0.00	1.00000000	pigeons
Rab21	-0.21	1.00000000	pigeons
Ankrd17	0.04	1.00000000	pigeons
Nadk	-0.02	1.00000000	pigeons
Tbc1d5	0.06	1.00000000	pigeons
Ddx51	0.12	1.00000000	pigeons
Cramp1	-0.08	1.00000000	pigeons
Tbc1d1	0.16	1.00000000	pigeons
Trappc2	0.07	1.00000000	pigeons
Blk	0.31	1.00000000	pigeons
Gosr1	0.06	1.00000000	pigeons
Hyou1	0.07	1.00000000	pigeons
Ranbp3	0.07	1.00000000	pigeons
Peak1	-0.03	1.00000000	pigeons
Usp12	-0.78	1.00000000	pigeons
Gosr2	0.10	1.00000000	pigeons
Tgfbr2	0.16	1.00000000	pigeons
Rnf6	-0.04	1.00000000	pigeons
Sf3a2	-0.03	1.00000000	pigeons
Stat3	0.14	1.00000000	pigeons
Yeats2	0.04	1.00000000	pigeons
Exoc4	-0.09	1.00000000	pigeons
Smpd2	0.11	1.00000000	pigeons
Abcc5	0.06	1.00000000	pigeons
Erh	0.05	1.00000000	pigeons
Txnrd3	0.13	1.00000000	pigeons
Ints11	0.06	1.00000000	pigeons
Sipa1l2	-0.27	1.00000000	pigeons
Tbrg4	0.12	1.00000000	pigeons
Phb	0.04	1.00000000	pigeons
Armc8	0.03	1.00000000	pigeons
Vps54	-0.47	1.00000000	pigeons
Bbs12	0.27	1.00000000	pigeons
Tmem242	0.07	1.00000000	pigeons
Ssbp3	0.06	1.00000000	pigeons
Fam76a	0.09	1.00000000	pigeons
Uba6	0.06	1.00000000	pigeons

Rbsn	0.13	1.00000000	pigeons
Dusp1	-0.29	1.00000000	pigeons
Pdss2	0.12	1.00000000	pigeons
Nr1h3	-0.20	1.00000000	pigeons
Utp6	0.16	1.00000000	pigeons
Klhl22	0.07	1.00000000	pigeons
Kif1b	-0.07	1.00000000	pigeons
Taf2	-0.14	1.00000000	pigeons
Polr2b	-0.01	1.00000000	pigeons
Cnn2	0.07	1.00000000	pigeons
Zdhhc21	0.12	1.00000000	pigeons
Rabepk	0.03	1.00000000	pigeons
Ubr2	-0.02	1.00000000	pigeons
Tma16	0.02	1.00000000	pigeons
Hdac4	-0.67	1.00000000	pigeons
Gbf1	-0.07	1.00000000	pigeons
Eif5a2	0.08	1.00000000	pigeons
Senp5	0.11	1.00000000	pigeons
Vezf1	-0.06	1.00000000	pigeons
Gns	-0.14	1.00000000	pigeons
Pi4ka	0.03	1.00000000	pigeons
Arhgdia	0.17	1.00000000	pigeons
Eif1b	0.02	1.00000000	pigeons
Wdr3	0.11	1.00000000	pigeons
Commd1b	0.01	1.00000000	pigeons
Llph	0.06	1.00000000	pigeons
Dctn3	-0.04	1.00000000	pigeons
Acvr1b	0.11	1.00000000	pigeons
Plcl2	0.26	1.00000000	pigeons
Usp34	-0.03	1.00000000	pigeons
Dda1	0.17	1.00000000	pigeons
Ube2g1	-0.15	1.00000000	pigeons
Ldlrad4	0.24	1.00000000	pigeons
Ctu2	0.16	1.00000000	pigeons
Gkap1	0.10	1.00000000	pigeons
Hdgfl3	0.01	1.00000000	pigeons
Ube2k	-0.04	1.00000000	pigeons
Odf2	0.07	1.00000000	pigeons
Ccdc117	0.00	1.00000000	pigeons
Tsr3	0.14	1.00000000	pigeons
Foxo1	0.11	1.00000000	pigeons
Phf20	-0.06	1.00000000	pigeons
Anp32e	-0.02	1.00000000	pigeons
Ppp1cb	-0.02	1.00000000	pigeons
Polr3a	0.03	1.00000000	pigeons
Ugcg	0.18	1.00000000	pigeons

Anxa6	0.05	1.00000000	pigeons
Tpp2	-0.09	1.00000000	pigeons
Rad21	0.01	1.00000000	pigeons
Psmd12	-0.02	1.00000000	pigeons
Lrrc28	0.02	1.00000000	pigeons
Fasl	0.36	1.00000000	pigeons
Fto	0.00	1.00000000	pigeons
Samm50	0.02	1.00000000	pigeons
Tpd52l2	-0.12	1.00000000	pigeons
Srpk2	-0.03	1.00000000	pigeons
Etl4	-0.54	1.00000000	pigeons
Atp23	0.07	1.00000000	pigeons
Nop58	0.02	1.00000000	pigeons
Nucks1	-0.07	1.00000000	pigeons
Ywhaq	-0.44	1.00000000	pigeons
Taf3	-0.10	1.00000000	pigeons
Fgd3	-0.59	1.00000000	pigeons
Ash2l	0.06	1.00000000	pigeons
Vrk2	0.13	1.00000000	pigeons
Whamm	0.12	1.00000000	pigeons
Gpi1	0.07	1.00000000	pigeons
Sync	0.17	1.00000000	pigeons
Eea1	-0.03	1.00000000	pigeons
Zbtb41	-0.01	1.00000000	pigeons
Tmem33	0.08	1.00000000	pigeons
Snx24	-0.38	1.00000000	pigeons
Tspan13	0.05	1.00000000	pigeons
Xxylt1	0.09	1.00000000	pigeons
Utp15	0.15	1.00000000	pigeons
Zbtb33	0.09	1.00000000	pigeons
Hmgb3	0.02	1.00000000	pigeons
Pak1ip1	0.18	1.00000000	pigeons
Rbm17	-0.01	1.00000000	pigeons
Fam98a	0.02	1.00000000	pigeons
Clasp2	-0.79	1.00000000	pigeons
Lemd3	-0.06	1.00000000	pigeons
Dmap1	-0.91	1.00000000	pigeons
Slf1	0.01	1.00000000	pigeons
Brox	0.02	1.00000000	pigeons
Rbm15	0.07	1.00000000	pigeons
Ammecr1	-0.02	1.00000000	pigeons
Heca	-0.26	1.00000000	pigeons
Ormdl1	0.17	1.00000000	pigeons
Thoc7	0.11	1.00000000	pigeons
Stk4	0.10	1.00000000	pigeons
Ergic2	0.09	1.00000000	pigeons

Taf5	-0.04	1.00000000	pigeons
Desi2	0.15	1.00000000	pigeons
Lcmt2	-0.45	1.00000000	pigeons
Hs6st2	0.04	1.00000000	pigeons
Snx12	0.07	1.00000000	pigeons
Atp6v1e2	-0.21	1.00000000	pigeons
Ctdspl2	-0.03	1.00000000	pigeons
Lrrc8b	-0.01	1.00000000	pigeons
Lpin2	-0.05	1.00000000	pigeons
Reep2	-0.05	1.00000000	pigeons
Rprd1b	-0.06	1.00000000	pigeons
Pdxdc1	0.03	1.00000000	pigeons
Arfgap2	0.02	1.00000000	pigeons
Uvrag	-0.08	1.00000000	pigeons
Crkl	0.03	1.00000000	pigeons
Psmc5	-0.11	1.00000000	pigeons
Glmn	0.16	1.00000000	pigeons
Zfp106	-0.02	1.00000000	pigeons
Supt4a	-0.10	1.00000000	pigeons
Pop4	0.07	1.00000000	pigeons
Fgd4	0.15	1.00000000	pigeons
Ubl3	-0.03	1.00000000	pigeons
Drg1	0.06	1.00000000	pigeons
Lrrfip1	0.14	1.00000000	pigeons
Cwc27	0.08	1.00000000	pigeons
Trim8	-0.18	1.00000000	pigeons
Stau1	0.10	1.00000000	pigeons
Ambra1	-0.08	1.00000000	pigeons
Ewsr1	0.09	1.00000000	pigeons
Slc35f5	0.13	1.00000000	pigeons
Pparg	-0.50	1.00000000	pigeons
Ccng1	-0.08	1.00000000	pigeons
Krr1	-0.06	1.00000000	pigeons
Acbd3	0.12	1.00000000	pigeons
Htra2	0.11	1.00000000	pigeons
Rab40c	0.00	1.00000000	pigeons
Pdap1	-0.03	1.00000000	pigeons
Arnt	0.01	1.00000000	pigeons
Ubap2l	0.06	1.00000000	pigeons
Phf21a	-0.02	1.00000000	pigeons
Cbll1	0.18	1.00000000	pigeons
Pitpnb	-0.04	1.00000000	pigeons
Pank4	0.10	1.00000000	pigeons
Rnf138	0.10	1.00000000	pigeons
Cebpz	0.10	1.00000000	pigeons
Gnb1	-0.15	1.00000000	pigeons

Esco1	-0.04	1.00000000	pigeons
Ppp1r13b	-0.71	1.00000000	pigeons
Xpo5	0.12	1.00000000	pigeons
Crlf3	0.01	1.00000000	pigeons
Tanc1	0.04	1.00000000	pigeons
Rwdd2b	0.07	1.00000000	pigeons
Taf15	0.04	1.00000000	pigeons
Ndufa10	0.04	1.00000000	pigeons
Atp6v0d1	-0.06	1.00000000	pigeons
Otud4	0.17	1.00000000	pigeons
Ap3s1	-0.43	1.00000000	pigeons
Eif4e2	0.10	1.00000000	pigeons
Gatad1	0.13	1.00000000	pigeons
Ghitm	-0.10	1.00000000	pigeons
Cog2	0.01	1.00000000	pigeons
Gga3	0.07	1.00000000	pigeons
Sde2	0.08	1.00000000	pigeons
Zbtb6	0.20	1.00000000	pigeons
Eftud2	0.05	1.00000000	pigeons
Plin2	-0.05	1.00000000	pigeons
Vps53	-0.05	1.00000000	pigeons
Bag1	-0.01	1.00000000	pigeons
Fam102a	-0.27	1.00000000	pigeons
Arpc3	0.08	1.00000000	pigeons
Cc2d1b	0.04	1.00000000	pigeons
Arpp19	-0.08	1.00000000	pigeons
Sppl2a	-0.03	1.00000000	pigeons
Copb1	-0.04	1.00000000	pigeons
Pnn	-0.05	1.00000000	pigeons
Tnip1	-0.10	1.00000000	pigeons
Lasp1	-0.03	1.00000000	pigeons
Camkmt	0.27	1.00000000	pigeons
Pdia6	0.02	1.00000000	pigeons
Scfd2	0.23	1.00000000	pigeons
Ptpn12	0.12	1.00000000	pigeons
Nhsl2	0.10	1.00000000	pigeons
Dis3l2	0.02	1.00000000	pigeons
Klhl24	0.05	1.00000000	pigeons
Swap70	0.06	1.00000000	pigeons
Cdc42bpb	-0.70	1.00000000	pigeons
Elp3	0.03	1.00000000	pigeons
Shisa5	-0.24	1.00000000	pigeons
Abr	0.04	1.00000000	pigeons
Zzef1	0.13	1.00000000	pigeons
Gcn1	-0.01	1.00000000	pigeons
Bsdc1	0.11	1.00000000	pigeons

Tmem131	-0.11	1.00000000	pigeons
Rsf1	-0.14	1.00000000	pigeons
Cast	-0.06	1.00000000	pigeons
Clasp1	-0.01	1.00000000	pigeons
Aimp2	0.04	1.00000000	pigeons
Gpalpp1	-0.11	1.00000000	pigeons
Dkc1	0.09	1.00000000	pigeons
Ddx24	0.12	1.00000000	pigeons
Psma7	-0.03	1.00000000	pigeons
Tgs1	0.14	1.00000000	pigeons
Polr2d	0.06	1.00000000	pigeons
Exoc2	0.07	1.00000000	pigeons
Atg5	-0.01	1.00000000	pigeons
Tor4a	0.13	1.00000000	pigeons
Dcp1a	0.03	1.00000000	pigeons
Dek	-0.03	1.00000000	pigeons
Spry2	-0.58	1.00000000	pigeons
Limk1	0.15	1.00000000	pigeons
Thumpd3	-0.01	1.00000000	pigeons
Etfa	-0.10	1.00000000	pigeons
C2cd2l	-0.04	1.00000000	pigeons
Zyx	-0.02	1.00000000	pigeons
Ndufa4	0.01	1.00000000	pigeons
Gtf2a2	-0.13	1.00000000	pigeons
C1galt1	0.11	1.00000000	pigeons
Esrrg	0.09	1.00000000	pigeons
Rab14	-0.05	1.00000000	pigeons
Rad54l2	-0.06	1.00000000	pigeons
Sec61a1	0.12	1.00000000	pigeons
Nabp1	-0.03	1.00000000	pigeons
Itgav	-0.01	1.00000000	pigeons
Dpy30	-0.07	1.00000000	pigeons
Vti1a	-0.04	1.00000000	pigeons
Znhit6	0.01	1.00000000	pigeons
Lmbrd2	-0.48	1.00000000	pigeons
Sdhaf2	0.10	1.00000000	pigeons
Rev3l	0.04	1.00000000	pigeons
Yipf3	-0.13	1.00000000	pigeons
Eif3j2	-0.03	1.00000000	pigeons
Immt	0.03	1.00000000	pigeons
Rabgap1l	0.03	1.00000000	pigeons
Skap2	-0.62	1.00000000	pigeons
Zfp319	0.05	1.00000000	pigeons
Ndufs3	0.06	1.00000000	pigeons
Rwdd4a	0.12	1.00000000	pigeons
Rnaseh1	0.00	1.00000000	pigeons

Trmt2a	0.08	1.00000000	pigeons
Oard1	-0.03	1.00000000	pigeons
Ptpn1	0.13	1.00000000	pigeons
Dynlrb1	-0.14	1.00000000	pigeons
Abce1	-0.08	1.00000000	pigeons
Ddx52	0.02	1.00000000	pigeons
Hadh	-0.10	1.00000000	pigeons
Telo2	-0.08	1.00000000	pigeons
Phf2	-0.01	1.00000000	pigeons
Sertad2	-0.04	1.00000000	pigeons
Ddx27	0.07	1.00000000	pigeons
Slc25a13	0.00	1.00000000	pigeons
Ide	-0.13	1.00000000	pigeons
Acap3	0.07	1.00000000	pigeons
St6gal1	0.11	1.00000000	pigeons
Taf12	0.07	1.00000000	pigeons
Ctnna1	-0.31	1.00000000	pigeons
Tpst2	0.09	1.00000000	pigeons
Rnf146	-0.06	1.00000000	pigeons
Adprm	0.02	1.00000000	pigeons
Alad	0.05	1.00000000	pigeons
Ggnbp2	-0.08	1.00000000	pigeons
Cfap97	0.02	1.00000000	pigeons
Ercc6	-0.06	1.00000000	pigeons
Flot2	0.06	1.00000000	pigeons
Tmem30a	-0.08	1.00000000	pigeons
Inpp4a	0.15	1.00000000	pigeons
Tia1	-0.02	1.00000000	pigeons
Nxt2	0.03	1.00000000	pigeons
Ccni	-0.09	1.00000000	pigeons
Ndufs1	0.02	1.00000000	pigeons
Htatsf1	-0.10	1.00000000	pigeons
Tmem230	-0.17	1.00000000	pigeons
Taok3	-0.08	1.00000000	pigeons
Pde7a	0.04	1.00000000	pigeons
Uso1	-0.18	1.00000000	pigeons
Katnbl1	0.02	1.00000000	pigeons
Ercc4	0.01	1.00000000	pigeons
Nsun2	0.01	1.00000000	pigeons
Capza2	-0.13	1.00000000	pigeons
Angel1	0.08	1.00000000	pigeons
Gnas	-0.14	1.00000000	pigeons
Uggt1	-0.14	1.00000000	pigeons
Slc35b3	0.09	1.00000000	pigeons
Ift20	0.04	1.00000000	pigeons
Crebrf	-0.15	1.00000000	pigeons

Cdc27	0.00	1.00000000	pigeons
Fars2	0.13	1.00000000	pigeons
Hmbs	-0.03	1.00000000	pigeons
Prmt3	0.15	1.00000000	pigeons
Scin	0.39	1.00000000	pigeons
Rae1	0.03	1.00000000	pigeons
Ppp3r1	-0.15	1.00000000	pigeons
Bcl2l13	-0.08	1.00000000	pigeons
Tsnax	0.02	1.00000000	pigeons
9130409I23Rik	-0.72	1.00000000	pigeons
Zfp831	0.18	1.00000000	pigeons
Stk11	0.01	1.00000000	pigeons
Srrm1	-0.05	1.00000000	pigeons
Nelfa	0.03	1.00000000	pigeons
Zfand3	-0.02	1.00000000	pigeons
Spg11	-0.11	1.00000000	pigeons
Tmem181a	0.07	1.00000000	pigeons
Zfp639	-0.03	1.00000000	pigeons
Swi5	-0.46	1.00000000	pigeons
Fbxo30	0.08	1.00000000	pigeons
Nup153	-0.09	1.00000000	pigeons
Homer1	-0.53	1.00000000	pigeons
Rnf11	-0.73	1.00000000	pigeons
Rbm6	-0.08	1.00000000	pigeons
Chgb	0.15	1.00000000	pigeons
Casp8ap2	-0.01	1.00000000	pigeons
Hspa8	-0.20	1.00000000	pigeons
Rragd	0.06	1.00000000	pigeons
Ccnyl1	-0.15	1.00000000	pigeons
Ndrg3	0.05	1.00000000	pigeons
Sub1	-0.06	1.00000000	pigeons
Rbm19	-0.07	1.00000000	pigeons
Tmem64	0.05	1.00000000	pigeons
Cdc16	0.03	1.00000000	pigeons
Ppm1f	-0.80	1.00000000	pigeons
Ccdc91	0.18	1.00000000	pigeons
Gnptab	0.02	1.00000000	pigeons
Eif4e	0.09	1.00000000	pigeons
Hdac11	0.18	1.00000000	pigeons
Tm9sf4	0.06	1.00000000	pigeons
Crnkl1	0.03	1.00000000	pigeons
Ppp1r16b	0.01	1.00000000	pigeons
Rexo1	0.00	1.00000000	pigeons
Gtf3c1	0.07	1.00000000	pigeons
Fam126a	0.09	1.00000000	pigeons
Acta2	0.20	1.00000000	pigeons

Psmc1	0.03	1.00000000	pigeons
Nop2	0.02	1.00000000	pigeons
Tecpr1	-0.04	1.00000000	pigeons
Pank2	0.08	1.00000000	pigeons
Pibf1	0.02	1.00000000	pigeons
Ero1b	-0.03	1.00000000	pigeons
Psmb3	0.05	1.00000000	pigeons
Cnot7	-0.08	1.00000000	pigeons
Zfp609	0.06	1.00000000	pigeons
Gata2	0.02	1.00000000	pigeons
Chid1	0.01	1.00000000	pigeons
Lysmd2	0.05	1.00000000	pigeons
Faf2	-0.04	1.00000000	pigeons
Fbxo38	-0.01	1.00000000	pigeons
Psma3	-0.07	1.00000000	pigeons
Prpf6	-0.14	1.00000000	pigeons
Polr2h	0.09	1.00000000	pigeons
Kif3b	0.03	1.00000000	pigeons
Timmdc1	0.08	1.00000000	pigeons
Nr2c1	-0.03	1.00000000	pigeons
Eefsec	0.04	1.00000000	pigeons
Idh3a	0.08	1.00000000	pigeons
Spcs2	0.07	1.00000000	pigeons
Snx9	-0.12	1.00000000	pigeons
Cipc	-0.05	1.00000000	pigeons
Tdrd3	-0.03	1.00000000	pigeons
Wdr61	0.03	1.00000000	pigeons
Tiam2	-0.07	1.00000000	pigeons
Aasdh	0.05	1.00000000	pigeons
Zfand6	-0.03	1.00000000	pigeons
Pdzd8	-0.03	1.00000000	pigeons
Vti1b	-0.01	1.00000000	pigeons
Nfs1	-0.06	1.00000000	pigeons
Gigyf2	0.00	1.00000000	pigeons
Diablo	0.11	1.00000000	pigeons
Slc35c1	0.11	1.00000000	pigeons
Etf1	-0.02	1.00000000	pigeons
Utp11	0.05	1.00000000	pigeons
Trip12	-0.08	1.00000000	pigeons
Rnf10	-0.05	1.00000000	pigeons
Tmem141	0.15	1.00000000	pigeons
Ppp3cb	-0.08	1.00000000	pigeons
Chka	-0.11	1.00000000	pigeons
Il18r1	0.20	1.00000000	pigeons
Arpc1b	-0.42	1.00000000	pigeons
Coq9	0.21	1.00000000	pigeons

Sbf1	0.05	1.00000000	pigeons
Invs	-0.09	1.00000000	pigeons
Ndufb6	-0.05	1.00000000	pigeons
Slc15a4	-0.12	1.00000000	pigeons
Eif2ak4	-0.06	1.00000000	pigeons
Fbrsl1	0.12	1.00000000	pigeons
Otulin	-0.01	1.00000000	pigeons
Ldah	-0.04	1.00000000	pigeons
Sec11a	-0.11	1.00000000	pigeons
Fam172a	0.01	1.00000000	pigeons
Nfkb2	0.07	1.00000000	pigeons
Prdm2	-0.11	1.00000000	pigeons
Exosc2	0.08	1.00000000	pigeons
Serpini1	-0.03	1.00000000	pigeons
Ccdc66	0.03	1.00000000	pigeons
Psmd3	0.04	1.00000000	pigeons
Ube2j2	-0.02	1.00000000	pigeons
Fam168b	0.01	1.00000000	pigeons
Smarca2	-0.05	1.00000000	pigeons
Vdac2	-0.16	1.00000000	pigeons
Psmb2	0.07	1.00000000	pigeons
Camk2d	-0.04	1.00000000	pigeons
Ndufb10	-0.12	1.00000000	pigeons
Galnt1	0.07	1.00000000	pigeons
Gpatch11	0.06	1.00000000	pigeons
Eri1	-0.10	1.00000000	pigeons
Tbpl1	-0.11	1.00000000	pigeons
Epcam	0.12	1.00000000	pigeons
Gpatch2l	0.01	1.00000000	pigeons
Srfbp1	0.00	1.00000000	pigeons
Fasn	-0.12	1.00000000	pigeons
Prdm15	-0.04	1.00000000	pigeons
Srp54a	-0.07	1.00000000	pigeons
Tns3	-0.70	1.00000000	pigeons
Tmem164	0.15	1.00000000	pigeons
Gne	0.13	1.00000000	pigeons
Hspa5	-0.36	1.00000000	pigeons
Pds5b	-0.04	1.00000000	pigeons
Nelfb	0.04	1.00000000	pigeons
Ppil1	-0.08	1.00000000	pigeons
Cdkn1b	-0.01	1.00000000	pigeons
Fam204a	-0.05	1.00000000	pigeons
Phf5a	0.02	1.00000000	pigeons
Nptn	-0.11	1.00000000	pigeons
Fbxw8	-0.15	1.00000000	pigeons
Ctsh	0.04	1.00000000	pigeons

Akt1	-0.17	1.00000000	pigeons
Hsph1	0.15	1.00000000	pigeons
Kat2b	-0.57	1.00000000	pigeons
Dnajc15	-0.04	1.00000000	pigeons
Susd3	0.03	1.00000000	pigeons
Ppp4r1	-0.20	1.00000000	pigeons
Poldip2	0.03	1.00000000	pigeons
Wapl	-0.01	1.00000000	pigeons
Dync1i2	-0.24	1.00000000	pigeons
Exoc7	-0.01	1.00000000	pigeons
Nmt2	-0.03	1.00000000	pigeons
Pafah1b1	-0.15	1.00000000	pigeons
Akap10	-0.03	1.00000000	pigeons
Ppm1g	-0.06	1.00000000	pigeons
Ppme1	0.00	1.00000000	pigeons
Hagh	0.06	1.00000000	pigeons
Tpk1	-0.48	1.00000000	pigeons
Snrnp25	-0.09	1.00000000	pigeons
Psmb7	-0.13	1.00000000	pigeons
Cdc14a	-0.20	1.00000000	pigeons
Kansl3	-0.07	1.00000000	pigeons
Exosc7	0.08	1.00000000	pigeons
Ufl1	-0.08	1.00000000	pigeons
Ndufb8	-0.07	1.00000000	pigeons
Fbxo9	-0.03	1.00000000	pigeons
Brf1	-0.13	1.00000000	pigeons
Pip4k2a	-0.11	1.00000000	pigeons
Irf2bpl	-0.11	1.00000000	pigeons
Fbxo48	0.08	1.00000000	pigeons
Immp2l	-0.27	1.00000000	pigeons
Stoml2	-0.08	1.00000000	pigeons
Prkcd	-0.06	1.00000000	pigeons
Dnajc3	-0.25	1.00000000	pigeons
Commd2	-0.02	1.00000000	pigeons
Tspan14	-0.18	1.00000000	pigeons
Cdkal1	-0.04	1.00000000	pigeons
Btbd10	-0.06	1.00000000	pigeons
Ccne1	0.05	1.00000000	pigeons
Slc2a13	-0.09	1.00000000	pigeons
Kbtbd2	-0.28	1.00000000	pigeons
Adam17	-0.12	1.00000000	pigeons
Smap1	-0.08	1.00000000	pigeons
Rheb	-0.21	1.00000000	pigeons
Wdr4	0.07	1.00000000	pigeons
Ovch2	0.27	1.00000000	pigeons
Flii	-0.01	1.00000000	pigeons

Samd4	0.06	1.00000000	pigeons
Tmem248	0.03	1.00000000	pigeons
Secisbp2l	-0.17	1.00000000	pigeons
Ak2	-0.56	1.00000000	pigeons
Lonp2	-0.08	1.00000000	pigeons
Timm13	-0.06	1.00000000	pigeons
Dnajb12	-0.16	1.00000000	pigeons
Rtcb	-0.01	1.00000000	pigeons
Bag5	-0.86	1.00000000	pigeons
Ecd	0.15	1.00000000	pigeons
Ppard	-0.13	1.00000000	pigeons
Slc25a24	-0.08	1.00000000	pigeons
Tes	-0.17	1.00000000	pigeons
Impa1	0.04	1.00000000	pigeons
Armt1	-0.01	1.00000000	pigeons
Gnptg	-0.09	1.00000000	pigeons
Trib2	-0.14	1.00000000	pigeons
Acat1	-0.06	1.00000000	pigeons
Ccndbp1	-0.15	1.00000000	pigeons
Rprd1a	-0.01	1.00000000	pigeons
Rac1	-0.59	1.00000000	pigeons
Rnps1	-0.05	1.00000000	pigeons
Riok3	-0.02	1.00000000	pigeons
Tm9sf2	-0.09	1.00000000	pigeons
Gtpbp1	0.01	1.00000000	pigeons
Usp37	-0.27	1.00000000	pigeons
Psmb1	-0.17	1.00000000	pigeons
Arl5a	-0.06	1.00000000	pigeons
Acsl1	-0.73	1.00000000	pigeons
Zfp512	0.10	1.00000000	pigeons
Dr1	-0.14	1.00000000	pigeons
Rmdn3	0.06	1.00000000	pigeons
Rsl24d1	-0.12	1.00000000	pigeons
Itpk1	-0.67	1.00000000	pigeons
Nop16	-0.04	1.00000000	pigeons
Clptm1l	-0.12	1.00000000	pigeons
Sil1	-0.40	1.00000000	pigeons
Nsfl1c	-0.03	1.00000000	pigeons
Rbm15b	-0.02	1.00000000	pigeons
Arhgap8	-0.02	1.00000000	pigeons
Bcar3	0.01	1.00000000	pigeons
Rcor1	-0.60	1.00000000	pigeons
Usp6nl	-0.22	1.00000000	pigeons
Gpatch1	-0.04	1.00000000	pigeons
Ap2a1	-0.11	1.00000000	pigeons
Psmf1	-0.01	1.00000000	pigeons

Rtf1	-0.18	1.00000000	pigeons
Lrpap1	-0.29	1.00000000	pigeons
Cacul1	-0.58	1.00000000	pigeons
Ino80c	-0.08	1.00000000	pigeons
Cnot6l	0.06	1.00000000	pigeons
Htr4	0.06	1.00000000	pigeons
Erlec1	0.00	1.00000000	pigeons
B3gnt2	0.05	1.00000000	pigeons
Rin3	-0.07	1.00000000	pigeons
Fubp3	-0.12	1.00000000	pigeons
Kdm2a	-0.04	1.00000000	pigeons
Sf3b5	-0.02	1.00000000	pigeons
Rab27a	-0.14	1.00000000	pigeons
Ppie	0.00	1.00000000	pigeons
Pnrc2	-0.11	1.00000000	pigeons
Fktn	-0.01	1.00000000	pigeons
Crk	-0.27	1.00000000	pigeons
Raly	0.08	1.00000000	pigeons
Rab2a	-0.16	1.00000000	pigeons
Pdia4	-0.02	1.00000000	pigeons
Ifngr2	-0.66	1.00000000	pigeons
Tnip2	-0.05	1.00000000	pigeons
Ssr2	-0.36	1.00000000	pigeons
Hnrnpa2b1	0.02	1.00000000	pigeons
Psmd9	-0.10	1.00000000	pigeons
Ebna1bp2	-0.18	1.00000000	pigeons
Angel2	-0.09	1.00000000	pigeons
Hp1bp3	-0.16	1.00000000	pigeons
Usp46	0.14	1.00000000	pigeons
Aatf	-0.08	1.00000000	pigeons
St8sia1	0.22	1.00000000	pigeons
Sri	-0.15	1.00000000	pigeons
Vgll4	-0.10	1.00000000	pigeons
Dalrd3	0.09	1.00000000	pigeons
Ctnnbl1	0.03	1.00000000	pigeons
Apaf1	-0.10	1.00000000	pigeons
Cask	0.10	1.00000000	pigeons
Rnls	0.04	1.00000000	pigeons
Atad2b	-0.04	1.00000000	pigeons
Narf	0.04	1.00000000	pigeons
Clcc1	-0.03	1.00000000	pigeons
Smad3	-0.24	1.00000000	pigeons
Nek1	0.04	1.00000000	pigeons
Hspd1	-0.05	1.00000000	pigeons
Ly75	-0.26	1.00000000	pigeons
Exoc1	-0.09	1.00000000	pigeons

Pdlim5	-0.12	1.00000000	pigeons
Hikeshi	0.04	1.00000000	pigeons
Parg	-0.14	1.00000000	pigeons
Ndufab1	0.00	1.00000000	pigeons
Hsp90b1	-0.37	1.00000000	pigeons
Uba3	-0.14	1.00000000	pigeons
Iars2	0.00	1.00000000	pigeons
Dyrk1a	-0.10	1.00000000	pigeons
Tmem208	0.02	1.00000000	pigeons
Abhd13	0.04	1.00000000	pigeons
Itsn2	-0.23	1.00000000	pigeons
Vps11	0.00	1.00000000	pigeons
Chd7	-0.52	1.00000000	pigeons
Gabpb1	0.04	1.00000000	pigeons
Brms1l	-0.12	1.00000000	pigeons
Nup107	-0.19	1.00000000	pigeons
Ccpg1	-0.21	1.00000000	pigeons
Ogdh	-0.03	1.00000000	pigeons
Lman2	-0.11	1.00000000	pigeons
Cnot8	0.04	1.00000000	pigeons
Ctnnbip1	0.02	1.00000000	pigeons
Ddb1	-0.06	1.00000000	pigeons
Pak2	-0.13	1.00000000	pigeons
Pcgf6	-0.13	1.00000000	pigeons
Dnajb14	0.01	1.00000000	pigeons
Ndufs8	-0.09	1.00000000	pigeons
Ccnl1	0.07	1.00000000	pigeons
Spz1	-0.17	1.00000000	pigeons
Isca1	-0.42	1.00000000	pigeons
Pdcd11	0.05	1.00000000	pigeons
Wipi1	0.00	1.00000000	pigeons
Sik3	0.00	1.00000000	pigeons
Rnh1	0.09	1.00000000	pigeons
Sec63	-0.17	1.00000000	pigeons
Tob1	-0.11	1.00000000	pigeons
Fam133b	-0.04	1.00000000	pigeons
Rbbp6	-0.12	1.00000000	pigeons
Ankmy2	-0.13	1.00000000	pigeons
Nol10	-0.09	1.00000000	pigeons
Farsb	0.02	1.00000000	pigeons
Psma4	-0.14	1.00000000	pigeons
Rnf13	-0.16	1.00000000	pigeons
Tmem167	-0.03	1.00000000	pigeons
Wbp11	-0.07	1.00000000	pigeons
Utp18	-0.09	1.00000000	pigeons
Pcmtd2	-0.04	1.00000000	pigeons

Appl2	-0.03	1.00000000	pigeons
Herpud2	-0.16	1.00000000	pigeons
Cdk12	-0.13	1.00000000	pigeons
Casp2	-0.14	1.00000000	pigeons
Ppp2r5a	-0.31	1.00000000	pigeons
Ascc3	-0.04	1.00000000	pigeons
Pik3ap1	0.05	1.00000000	pigeons
Rnf214	-0.18	1.00000000	pigeons
Tlk2	-0.21	1.00000000	pigeons
Atp7a	-0.07	1.00000000	pigeons
Psma5	-0.08	1.00000000	pigeons
Atp6v1d	-0.07	1.00000000	pigeons
Nrp1	-0.57	1.00000000	pigeons
Ets2	-0.40	1.00000000	pigeons
Zfp335	-0.20	1.00000000	pigeons
Ptger3	0.39	1.00000000	pigeons
Snx2	-0.31	1.00000000	pigeons
Tbc1d22b	0.04	1.00000000	pigeons
Pdcd5	-0.02	1.00000000	pigeons
Aacs	-0.77	1.00000000	pigeons
Ntan1	-0.05	1.00000000	pigeons
Cstf2	0.07	1.00000000	pigeons
Nupl1	-0.02	1.00000000	pigeons
Smarce1	-0.14	1.00000000	pigeons
Nup155	0.03	1.00000000	pigeons
Zfp518b	-0.12	1.00000000	pigeons
Cdc73	-0.28	1.00000000	pigeons
Sh3glb1	-0.56	1.00000000	pigeons
Utrn	-0.22	1.00000000	pigeons
Ube2d1	-0.08	1.00000000	pigeons
Akirin2	-0.14	1.00000000	pigeons
Ankrd13d	0.13	1.00000000	pigeons
Zranb1	-0.26	1.00000000	pigeons
Azin1	-0.25	1.00000000	pigeons
Nudc	-0.12	1.00000000	pigeons
Sdhaf4	0.03	1.00000000	pigeons
Washc5	0.02	1.00000000	pigeons
Hnrnpm	-0.24	1.00000000	pigeons
Serinc1	0.01	1.00000000	pigeons
Arpc1a	-0.13	1.00000000	pigeons
Cops7a	-0.03	1.00000000	pigeons
Chmp4b	-0.23	1.00000000	pigeons
Rora	0.02	1.00000000	pigeons
Ankfy1	-0.13	1.00000000	pigeons
Rab10	-0.10	1.00000000	pigeons
Pde12	0.13	1.00000000	pigeons

Zfhx3	-0.11	1.00000000	pigeons
Rab8a	-0.06	1.00000000	pigeons
Cdca4	-0.15	1.00000000	pigeons
Xbp1	-0.05	1.00000000	pigeons
Ube2f	0.00	1.00000000	pigeons
Ing2	-0.06	1.00000000	pigeons
Il6st	-0.07	1.00000000	pigeons
Lfng	-0.81	1.00000000	pigeons
Washc4	-0.05	1.00000000	pigeons
Rnf19a	-0.35	1.00000000	pigeons
Vps4b	-0.20	1.00000000	pigeons
Prdx3	-0.12	1.00000000	pigeons
Emc7	-0.47	1.00000000	pigeons
Taok1	-0.08	1.00000000	pigeons
Snx10	-0.78	1.00000000	pigeons
Ddx31	0.04	1.00000000	pigeons
Plrg1	-0.10	1.00000000	pigeons
Ftsj3	0.05	1.00000000	pigeons
Stxbp5	-0.27	1.00000000	pigeons
Appl1	-0.16	1.00000000	pigeons
Gclc	-0.02	1.00000000	pigeons
Brix1	0.01	1.00000000	pigeons
Fam104a	-0.02	1.00000000	pigeons
Pla2g7	-0.02	1.00000000	pigeons
Fancd2	-0.12	1.00000000	pigeons
Rprd2	-0.03	1.00000000	pigeons
Pou2f1	-0.11	1.00000000	pigeons
Chsy3	0.06	1.00000000	pigeons
Ikzf2	0.24	1.00000000	pigeons
Elmo1	0.09	1.00000000	pigeons
Suclg1	0.00	1.00000000	pigeons
Agtpbp1	0.00	1.00000000	pigeons
Cenpc1	-0.12	1.00000000	pigeons
Pfkfb3	-0.93	1.00000000	pigeons
R3hdm4	-0.01	1.00000000	pigeons
Cdyl2	-0.11	1.00000000	pigeons
Rufy1	-0.21	1.00000000	pigeons
Clock	-0.23	1.00000000	pigeons
Senp7	-0.16	1.00000000	pigeons
Trim33	0.00	1.00000000	pigeons
Ncbp3	0.05	1.00000000	pigeons
Bloc1s2	0.02	1.00000000	pigeons
Lclat1	-0.12	1.00000000	pigeons
Lonrf3	-0.55	1.00000000	pigeons
Eif1	0.04	1.00000000	pigeons
Creb3l2	-0.47	1.00000000	pigeons

Ncoa7	0.02	1.00000000	pigeons
Psma2	-0.04	1.00000000	pigeons
Ankrd39	-0.25	1.00000000	pigeons
Fam149b	0.05	1.00000000	pigeons
Epb41	-0.18	1.00000000	pigeons
Fbxo7	0.05	1.00000000	pigeons
Urm1	-0.15	1.00000000	pigeons
Zfp276	0.08	1.00000000	pigeons
Nfxl1	-0.03	1.00000000	pigeons
Pafah1b2	-0.06	1.00000000	pigeons
Vps36	-0.01	1.00000000	pigeons
Csrp1	-0.73	1.00000000	pigeons
Ern1	-0.08	1.00000000	pigeons
Dbr1	-0.08	1.00000000	pigeons
Adk	-0.59	1.00000000	pigeons
Ipmk	-0.11	1.00000000	pigeons
Tle3	-0.28	1.00000000	pigeons
Nans	-0.13	1.00000000	pigeons
Klhl6	0.01	1.00000000	pigeons
Lbh	-0.01	1.00000000	pigeons
Trak1	-0.67	1.00000000	pigeons
Apoo	-0.09	1.00000000	pigeons
Rcc1	-0.18	1.00000000	pigeons
Nup98	-0.12	1.00000000	pigeons
Upf3b	-0.09	1.00000000	pigeons
Pgs1	-0.08	1.00000000	pigeons
Clint1	-0.12	1.00000000	pigeons
Dpy19l4	-0.05	1.00000000	pigeons
Rock2	-0.55	1.00000000	pigeons
Atp2c1	-0.12	1.00000000	pigeons
Golga4	-0.59	1.00000000	pigeons
E2f6	-0.11	1.00000000	pigeons
Dus1l	-0.02	1.00000000	pigeons
Wdr47	-0.46	1.00000000	pigeons
Fmnl1	0.06	1.00000000	pigeons
Tob2	-0.27	1.00000000	pigeons
Esd	-0.21	1.00000000	pigeons
Nudcd3	0.03	1.00000000	pigeons
Dnaja2	-0.12	1.00000000	pigeons
Leo1	0.01	1.00000000	pigeons
Iws1	-0.13	1.00000000	pigeons
Rad17	-0.06	1.00000000	pigeons
Zfp131	0.01	1.00000000	pigeons
Elf2	-0.29	1.00000000	pigeons
Dip2a	0.03	1.00000000	pigeons
Fxyd6	-0.47	1.00000000	pigeons

Emc8	-0.13	1.00000000	pigeons
Rab18	-0.04	1.00000000	pigeons
Cmss1	-0.13	1.00000000	pigeons
Ctnnb1	-0.74	1.00000000	pigeons
Trim25	0.03	1.00000000	pigeons
Snx14	-0.55	1.00000000	pigeons
Ssbp1	-0.02	1.00000000	pigeons
Suclg2	-0.02	1.00000000	pigeons
Yipf5	-0.15	1.00000000	pigeons
Rabep1	0.02	1.00000000	pigeons
Ubfd1	-0.17	1.00000000	pigeons
Aspscr1	-0.06	1.00000000	pigeons
Ptp4a1	-0.22	1.00000000	pigeons
Drg2	0.03	1.00000000	pigeons
Wsb1	-0.11	1.00000000	pigeons
Rala	-0.08	1.00000000	pigeons
Xpo1	-0.16	1.00000000	pigeons
Agpat5	-0.37	1.00000000	pigeons
Ate1	-0.09	1.00000000	pigeons
Rpia	-0.06	1.00000000	pigeons
Erp44	-0.15	1.00000000	pigeons
Polr2l	-0.04	1.00000000	pigeons
Kin	-0.17	1.00000000	pigeons
Zfp236	-0.04	1.00000000	pigeons
Thoc1	-0.03	1.00000000	pigeons
Snap29	-0.08	1.00000000	pigeons
Zc3h13	-0.19	1.00000000	pigeons
Eci3	-0.03	1.00000000	pigeons
Syap1	-0.14	1.00000000	pigeons
Scp2	-0.12	1.00000000	pigeons
Actn1	-0.17	1.00000000	pigeons
Tcf3	-0.67	1.00000000	pigeons
Tmf1	-0.18	1.00000000	pigeons
Rnf111	-0.14	1.00000000	pigeons
Ireb2	-0.09	1.00000000	pigeons
Yipf4	-0.10	1.00000000	pigeons
Uspl1	0.00	1.00000000	pigeons
Dcbld2	-0.22	1.00000000	pigeons
Tmem65	0.04	1.00000000	pigeons
Esf1	-0.08	1.00000000	pigeons
Asap2	-0.10	1.00000000	pigeons
Phlpp2	-0.15	1.00000000	pigeons
Rnf220	-0.23	1.00000000	pigeons
Ext2	-0.11	1.00000000	pigeons
Itsn1	-0.21	1.00000000	pigeons
Ddx5	-0.12	1.00000000	pigeons

Commd4	-0.09	1.00000000	pigeons
Sh3bp5	-0.51	1.00000000	pigeons
Spg7	-0.02	1.00000000	pigeons
Jmy	-0.06	1.00000000	pigeons
Il12rb2	-0.29	1.00000000	pigeons
Tpmt	-0.02	1.00000000	pigeons
Usp14	0.03	1.00000000	pigeons
Pcgf5	-0.18	1.00000000	pigeons
Tpra1	0.06	1.00000000	pigeons
Eif4g2	-0.34	1.00000000	pigeons
Srp72	-0.16	1.00000000	pigeons
Eif5	-0.12	1.00000000	pigeons
Pdia3	-0.23	1.00000000	pigeons
Nufip1	-0.06	1.00000000	pigeons
Pik3ip1	-0.13	1.00000000	pigeons
Atf1	-0.05	1.00000000	pigeons
Capn11	-0.14	1.00000000	pigeons
Adam23	-0.02	1.00000000	pigeons
Specc1l	-0.50	1.00000000	pigeons
Extl3	-0.07	1.00000000	pigeons
Usp53	-0.47	1.00000000	pigeons
Phkb	0.02	1.00000000	pigeons
Ndufs7	-0.02	1.00000000	pigeons
Cyth3	-0.48	1.00000000	pigeons
Actr1a	-0.10	1.00000000	pigeons
Pank1	-0.10	1.00000000	pigeons
Taf5l	-0.67	1.00000000	pigeons
Prkcb	-0.03	1.00000000	pigeons
Trappc12	-0.11	1.00000000	pigeons
Dnmt3a	-0.04	1.00000000	pigeons
Zfand5	-0.10	1.00000000	pigeons
Ccnh	0.06	1.00000000	pigeons
Fbxo34	-0.04	1.00000000	pigeons
Rbm45	-0.05	1.00000000	pigeons
Armc1	-0.28	1.00000000	pigeons
Phf10	-0.11	1.00000000	pigeons
H13	-0.11	1.00000000	pigeons
Trip11	-0.48	1.00000000	pigeons
Phf6	-0.06	1.00000000	pigeons
Tmem266	-0.59	1.00000000	pigeons
Creld2	-0.12	1.00000000	pigeons
Kctd10	0.04	1.00000000	pigeons
Ash1l	-0.27	1.00000000	pigeons
Wsb2	0.09	1.00000000	pigeons
Tmem154	-0.12	1.00000000	pigeons
Pik3r4	-0.03	1.00000000	pigeons

Vps25	-0.13	1.00000000	pigeons
Ssr3	-0.37	1.00000000	pigeons
Sec23a	-0.16	1.00000000	pigeons
Ltf	-0.02	1.00000000	pigeons
Arhgef6	-0.26	1.00000000	pigeons
Exosc1	-0.21	1.00000000	pigeons
Dennd4c	-0.10	1.00000000	pigeons
Fut8	-0.58	1.00000000	pigeons
Ino80d	-0.10	1.00000000	pigeons
Lrig2	-0.03	1.00000000	pigeons
Dctn1	-0.32	1.00000000	pigeons
Psmd6	-0.16	1.00000000	pigeons
Snx29	-0.54	1.00000000	pigeons
Slc30a9	-0.18	1.00000000	pigeons
Ppp3ca	-0.18	1.00000000	pigeons
Nme7	-0.40	1.00000000	pigeons
Stx2	-0.16	1.00000000	pigeons
Rbl1	-0.21	1.00000000	pigeons
Serinc3	-0.72	1.00000000	pigeons
Snx25	-0.09	1.00000000	pigeons
Zc2hc1a	-0.15	1.00000000	pigeons
Tatdn1	-0.05	1.00000000	pigeons
Bzw2	-0.38	1.00000000	pigeons
Brwd1	-0.22	1.00000000	pigeons
Abcb7	-0.07	1.00000000	pigeons
Prdx1	-0.06	1.00000000	pigeons
G2e3	-0.21	1.00000000	pigeons
Abcc1	0.00	1.00000000	pigeons
Ltn1	-0.17	1.00000000	pigeons
Cisd1	-0.23	1.00000000	pigeons
Ndufv2	-0.01	1.00000000	pigeons
B3gnt5	-0.03	1.00000000	pigeons
Larp1b	-0.21	1.00000000	pigeons
Saraf	-0.22	1.00000000	pigeons
Ccdc186	-0.24	1.00000000	pigeons
Slc25a20	-0.01	1.00000000	pigeons
Rpap1	-0.08	1.00000000	pigeons
Snapc4	-0.03	1.00000000	pigeons
Dusp23	-0.06	1.00000000	pigeons
Tmem183a	0.00	1.00000000	pigeons
Zdhhc14	-0.24	1.00000000	pigeons
Gnai3	-0.04	1.00000000	pigeons
Ddx59	-0.06	1.00000000	pigeons
Prkar1a	-0.09	1.00000000	pigeons
Cops2	-0.08	1.00000000	pigeons
Picalm	-0.79	1.00000000	pigeons

Pinx1	-0.17	1.00000000	pigeons
Stau2	-0.19	1.00000000	pigeons
Cope	-0.16	1.00000000	pigeons
Actr8	-0.04	1.00000000	pigeons
Ino80	-0.44	1.00000000	pigeons
Wdfy1	-0.19	1.00000000	pigeons
Phkg1	-0.07	1.00000000	pigeons
Surf2	-0.05	1.00000000	pigeons
Ddrgk1	-0.27	1.00000000	pigeons
Dynlt3	-0.01	1.00000000	pigeons
Pam	-0.13	1.00000000	pigeons
Stam	0.02	1.00000000	pigeons
Nbr1	0.03	1.00000000	pigeons
Pdcd6	-0.16	1.00000000	pigeons
Ric1	-0.09	1.00000000	pigeons
Kpna3	-0.27	1.00000000	pigeons
St3gal6	-0.56	1.00000000	pigeons
Atxn1	-0.36	1.00000000	pigeons
Usp48	-0.26	1.00000000	pigeons
Cir1	-0.03	1.00000000	pigeons
Uhmk1	-0.15	1.00000000	pigeons
Lias	-0.06	1.00000000	pigeons
Afg3l2	-0.20	1.00000000	pigeons
Ebag9	-0.12	1.00000000	pigeons
Tmsb4x	-0.01	1.00000000	pigeons
Dusp6	-0.29	1.00000000	pigeons
Lsm8	-0.33	1.00000000	pigeons
Fhit	0.05	1.00000000	pigeons
Cenpt	-0.09	1.00000000	pigeons
Adipor1	-0.15	1.00000000	pigeons
Erc1	-0.48	1.00000000	pigeons
Zhx1	-0.26	1.00000000	pigeons
Tgif1	-0.16	1.00000000	pigeons
Rab6a	-0.20	1.00000000	pigeons
Ppm1d	-0.41	1.00000000	pigeons
Rtn4	-0.43	1.00000000	pigeons
Elovl5	-0.23	1.00000000	pigeons
Crip1	-0.25	1.00000000	pigeons
Arfgap3	-0.38	1.00000000	pigeons
Epc2	-0.30	1.00000000	pigeons
Hdac7	-0.42	1.00000000	pigeons
Adam10	-0.36	1.00000000	pigeons
Ncor2	-0.34	1.00000000	pigeons
Cops8	-0.23	1.00000000	pigeons
Sdhd	-0.25	1.00000000	pigeons
Ppil2	0.01	1.00000000	pigeons

Vamp3	-0.23	1.00000000	pigeons
Ccnl2	-0.27	1.00000000	pigeons
Slc35c2	-0.01	1.00000000	pigeons
Cfdp1	-0.11	1.00000000	pigeons
Jade3	-0.50	1.00000000	pigeons
Scpep1	0.02	1.00000000	pigeons
Atf4	-0.30	1.00000000	pigeons
Eif4ebp1	-0.48	1.00000000	pigeons
Cap1	-0.18	1.00000000	pigeons
Gabpa	-0.11	1.00000000	pigeons
Psmg3	-0.08	1.00000000	pigeons
Arf4	-0.15	1.00000000	pigeons
Btbd1	-0.11	1.00000000	pigeons
Syt11	-0.24	1.00000000	pigeons
Smg6	-0.23	1.00000000	pigeons
Polr3b	-0.07	1.00000000	pigeons
Proser1	-0.06	1.00000000	pigeons
Tdg	-0.16	1.00000000	pigeons
Tenm1	-0.22	1.00000000	pigeons
Gapvd1	-0.23	1.00000000	pigeons
Pola1	0.00	1.00000000	pigeons
Sh2d1a	-0.21	1.00000000	pigeons
Nhlrc2	-0.03	1.00000000	pigeons
Bcl6	-0.03	1.00000000	pigeons
Gpx1	-0.32	1.00000000	pigeons
Fkbp3	-0.12	1.00000000	pigeons
Psmd7	-0.17	1.00000000	pigeons
Notch1	-0.64	1.00000000	pigeons
Sap30l	-0.09	1.00000000	pigeons
Tfrc	-0.48	1.00000000	pigeons
Kif5b	-0.40	1.00000000	pigeons
Fam135a	-0.46	1.00000000	pigeons
Rap1b	-0.35	1.00000000	pigeons
Cd63	-0.27	1.00000000	pigeons
Nbeal1	-0.48	1.00000000	pigeons
Cnih1	-0.12	1.00000000	pigeons
Ptpn4	-0.15	1.00000000	pigeons
Cbfb	-0.05	1.00000000	pigeons
Ppfia1	-0.43	1.00000000	pigeons
Hsf2	-0.04	1.00000000	pigeons
Rgs9	-0.02	1.00000000	pigeons
Slc25a3	-0.24	1.00000000	pigeons
Blmh	-0.05	1.00000000	pigeons
Ppib	-0.02	1.00000000	pigeons
Nde1	-0.08	1.00000000	pigeons
Zfp622	-0.15	1.00000000	pigeons

Cpeb4	-0.77	1.00000000	pigeons
Eno1b	-0.17	1.00000000	pigeons
Lamp1	-0.72	1.00000000	pigeons
Btbd2	-0.01	1.00000000	pigeons
Ube4a	-0.06	1.00000000	pigeons
Lonrf1	-0.32	1.00000000	pigeons
Slc4a4	-0.48	1.00000000	pigeons
Rnf144a	-0.39	1.00000000	pigeons
Tnrc6b	-0.42	1.00000000	pigeons
Sf3b6	-0.15	1.00000000	pigeons
Chp1	-0.49	1.00000000	pigeons
Gpr137b	-0.14	1.00000000	pigeons
Dcun1d5	-0.32	1.00000000	pigeons
Abtb1	-0.21	1.00000000	pigeons
Chmp5	-0.33	1.00000000	pigeons
Zfand4	-0.05	1.00000000	pigeons
Nkiras2	-0.01	1.00000000	pigeons
Usp22	-0.63	1.00000000	pigeons
Slc23a2	-0.10	1.00000000	pigeons
Sel1l	-0.21	1.00000000	pigeons
Wdsub1	-0.02	1.00000000	pigeons
Eif5b	-0.62	1.00000000	pigeons
Hdac1	-0.08	1.00000000	pigeons
Hmgb1	-0.21	1.00000000	pigeons
Grb2	-0.15	1.00000000	pigeons
Ncald	-0.26	1.00000000	pigeons
Tef	-0.38	1.00000000	pigeons
Ndel1	-0.16	1.00000000	pigeons
Pin1	-0.07	1.00000000	pigeons
Dlg1	-0.33	1.00000000	pigeons
Cpsf4l	-0.15	1.00000000	pigeons
Prelid1	-0.12	1.00000000	pigeons
Bud31	-0.25	1.00000000	pigeons
Psmc3	-0.18	1.00000000	pigeons
Kif13a	-0.37	1.00000000	pigeons
Elp4	-0.07	1.00000000	pigeons
Rcbtb1	-0.71	1.00000000	pigeons
Rab7	-0.66	1.00000000	pigeons
Tdp2	-0.16	1.00000000	pigeons
Dgkd	-0.61	1.00000000	pigeons
Emp1	-0.21	1.00000000	pigeons
Heatr5a	-0.09	1.00000000	pigeons
Dlst	-0.10	1.00000000	pigeons
Stim2	-0.55	1.00000000	pigeons
Aplf	-0.10	1.00000000	pigeons
Ppt1	-0.46	1.00000000	pigeons

Txndc12	-0.32	1.00000000	pigeons
Raph1	-0.19	1.00000000	pigeons
Amot	-0.22	1.00000000	pigeons
Ndufs6	-0.37	1.00000000	pigeons
Naca	-0.15	1.00000000	pigeons
Adipor2	-0.18	1.00000000	pigeons
Gpatch2	-0.13	1.00000000	pigeons
Cd82	-0.20	1.00000000	pigeons
Nt5c3	-0.29	1.00000000	pigeons
Commd10	-0.19	1.00000000	pigeons
Slc25a25	-0.15	1.00000000	pigeons
Parvg	-0.42	1.00000000	pigeons
Amfr	-0.51	1.00000000	pigeons
Btg2	-0.63	1.00000000	pigeons
Rp2	-0.42	1.00000000	pigeons
Pmepa1	-0.48	1.00000000	pigeons
Smad5	-0.38	1.00000000	pigeons
Ptpn11	-0.13	1.00000000	pigeons
Ano10	-0.43	1.00000000	pigeons
Ahsa1	-0.19	1.00000000	pigeons
Ndufa6	-0.36	1.00000000	pigeons
Stk40	-0.09	1.00000000	pigeons
Disp1	-0.65	1.00000000	pigeons
Hecw1	-0.17	1.00000000	pigeons
Stx11	0.01	1.00000000	pigeons
Sprtn	-0.21	1.00000000	pigeons
Ostc	-0.24	1.00000000	pigeons
Setd3	-0.10	1.00000000	pigeons
Chmp2b	-0.40	1.00000000	pigeons
Lpcat1	-0.13	1.00000000	pigeons
Kif2a	-0.27	1.00000000	pigeons
Tkt	-0.20	1.00000000	pigeons
Atp1a1	-0.63	1.00000000	pigeons
Trpm7	-0.33	1.00000000	pigeons
Pwwp2b	-0.37	1.00000000	pigeons
Prdx4	-0.18	1.00000000	pigeons
Sec13	-0.09	1.00000000	pigeons
Poldip3	-0.13	1.00000000	pigeons
N4bp2l2	-0.34	1.00000000	pigeons
Anapc10	-0.19	1.00000000	pigeons
Tfdp1	-0.19	1.00000000	pigeons
Gcsh	0.02	1.00000000	pigeons
Rnaset2b	-0.42	1.00000000	pigeons
Nsmce2	-0.06	1.00000000	pigeons
Rcn2	-0.45	1.00000000	pigeons
Bach1	-0.31	1.00000000	pigeons

Cnpy3	-0.14	1.00000000	pigeons
Ptpre	-0.47	1.00000000	pigeons
Dmxl1	-0.26	1.00000000	pigeons
Sgpp1	-0.37	1.00000000	pigeons
Arhgef12	-0.46	1.00000000	pigeons
Psmb4	-0.41	1.00000000	pigeons
Cmtm3	-0.14	1.00000000	pigeons
Pitpna	-0.27	1.00000000	pigeons
Prkar2a	-0.23	1.00000000	pigeons
Abi1	-0.28	1.00000000	pigeons
Efcab14	-0.35	1.00000000	pigeons
Prr14l	-0.27	1.00000000	pigeons
Vwa8	-0.64	1.00000000	pigeons
Actr10	-0.11	1.00000000	pigeons
Clta	-0.13	1.00000000	pigeons
Wdr36	-0.14	1.00000000	pigeons
Stk24	-0.36	1.00000000	pigeons
Rptor	-0.05	1.00000000	pigeons
Nono	-0.13	1.00000000	pigeons
Acaa2	-0.11	1.00000000	pigeons
Prelid3a	-0.26	1.00000000	pigeons
Fnip1	-0.24	1.00000000	pigeons
Smc3	-0.18	1.00000000	pigeons
Slc25a5	0.01	1.00000000	pigeons
Eef1d	-0.10	1.00000000	pigeons
Tnfrsf8	-0.10	1.00000000	pigeons
Xylt1	-0.12	1.00000000	pigeons
Camsap2	-0.50	1.00000000	pigeons
Fbxo8	-0.12	1.00000000	pigeons
Abhd2	0.00	1.00000000	pigeons
Jazf1	-0.30	1.00000000	pigeons
Pan3	-0.40	1.00000000	pigeons
Dnajb4	-0.29	1.00000000	pigeons
Dctn6	-0.22	1.00000000	pigeons
Arl8b	-0.27	1.00000000	pigeons
Trib1	-0.24	1.00000000	pigeons
Klhdc1	-0.23	1.00000000	pigeons
Smyd2	-0.04	1.00000000	pigeons
Trip4	-0.08	1.00000000	pigeons
Tmem106b	-0.14	1.00000000	pigeons
Acad9	-0.08	1.00000000	pigeons
Il16	-0.38	1.00000000	pigeons
Ywhah	-0.20	1.00000000	pigeons
Timp2	-0.50	1.00000000	pigeons
Nrd1	-0.15	1.00000000	pigeons
Zdhhc7	-0.08	1.00000000	pigeons

Adrm1	-0.14	1.00000000	pigeons
Wbp4	-0.57	1.00000000	pigeons
Eva1c	0.05	1.00000000	pigeons
Klf6	-0.04	1.00000000	pigeons
Fez2	-0.30	1.00000000	pigeons
Shprh	-0.41	1.00000000	pigeons
Abcf2	-0.16	1.00000000	pigeons
Dtx2	-0.44	1.00000000	pigeons
Ube3c	-0.28	1.00000000	pigeons
Zfp598	-0.22	1.00000000	pigeons
Slc35a1	-0.33	1.00000000	pigeons
Irak2	-0.10	1.00000000	pigeons
Prmt7	-0.39	1.00000000	pigeons
2210408I21Rik	-0.19	1.00000000	pigeons
Rab12	-0.52	1.00000000	pigeons
Arih1	-0.29	1.00000000	pigeons
Cnot10	-0.11	1.00000000	pigeons
Rbm22	-0.21	1.00000000	pigeons
Zfp652	-0.26	1.00000000	pigeons
Dennd5a	-0.44	1.00000000	pigeons
Rnf130	0.05	1.00000000	pigeons
Tpm4	-0.26	1.00000000	pigeons
Nr3c2	-0.38	1.00000000	pigeons
Smndc1	-0.34	1.00000000	pigeons
Atp6v1c1	-0.43	1.00000000	pigeons
Usp38	-0.21	1.00000000	pigeons
Cops3	-0.31	1.00000000	pigeons
Tm2d1	-0.17	1.00000000	pigeons
Ppfibp1	-0.41	1.00000000	pigeons
Emc6	-0.10	1.00000000	pigeons
Ptp4a2	-0.26	1.00000000	pigeons
Fh1	-0.23	1.00000000	pigeons
Pard3	-0.48	1.00000000	pigeons
Psmc2	-0.12	1.00000000	pigeons
Sec11c	-0.24	1.00000000	pigeons
Plbd2	-0.28	1.00000000	pigeons
Sulf1	-0.08	1.00000000	pigeons
Arl1	-0.19	1.00000000	pigeons
Prkn	-0.08	1.00000000	pigeons
Rreb1	-0.30	1.00000000	pigeons
Fbxo25	-0.14	1.00000000	pigeons
Sema7a	-0.42	1.00000000	pigeons
Gpx4	-0.14	1.00000000	pigeons
Bnip1	-0.05	1.00000000	pigeons
Nvl	-0.51	1.00000000	pigeons
Cisd2	-0.14	1.00000000	pigeons

Snx13	-0.30	1.00000000	pigeons
Hmg20b	-0.24	1.00000000	pigeons
Naxe	-0.07	1.00000000	pigeons
Tnfrsf25	-0.42	1.00000000	pigeons
Edf1	-0.29	1.00000000	pigeons
Gbe1	-0.16	1.00000000	pigeons
Bcor	-0.58	1.00000000	pigeons
Rab5c	-0.38	1.00000000	pigeons
Gid4	-0.03	1.00000000	pigeons
Chmp6	-0.21	1.00000000	pigeons
Fzd6	-0.28	1.00000000	pigeons
Slc7a6os	-0.19	1.00000000	pigeons
Focad	-0.10	1.00000000	pigeons
Ddost	-0.28	1.00000000	pigeons
Arhgap12	-0.21	1.00000000	pigeons
Bloc1s6	-0.25	1.00000000	pigeons
Canx	-0.18	1.00000000	pigeons
Dcun1d1	-0.12	1.00000000	pigeons
Gpr39	-0.04	1.00000000	pigeons
Znrf1	-0.29	1.00000000	pigeons
Ube4b	-0.14	1.00000000	pigeons
Trappc11	-0.18	1.00000000	pigeons
Camk2g	-0.43	1.00000000	pigeons
Dym	-0.33	1.00000000	pigeons
Txndc17	-0.44	1.00000000	pigeons
Acbd5	-0.17	1.00000000	pigeons
Zfp516	-0.10	1.00000000	pigeons
Atp11c	-0.60	1.00000000	pigeons
Wdr7	-0.47	1.00000000	pigeons
Rif1	-0.18	1.00000000	pigeons
Cpne3	-0.38	1.00000000	pigeons
Tpm1	-0.30	1.00000000	pigeons
Taf13	-0.35	1.00000000	pigeons
Eef1b2	-0.08	1.00000000	pigeons
Faf1	-0.62	1.00000000	pigeons
Arxes2	-0.21	1.00000000	pigeons
Hnrnpab	-0.19	1.00000000	pigeons
Sh3bgrl3	-0.18	1.00000000	pigeons
Tmem9b	-0.12	1.00000000	pigeons
Exoc6b	-0.50	1.00000000	pigeons
Pef1	-0.22	1.00000000	pigeons
Ndufb5	-0.27	1.00000000	pigeons
Skp1	-0.33	1.00000000	pigeons
Szrd1	-0.13	1.00000000	pigeons
Larp4b	-0.29	1.00000000	pigeons
Tmem50a	-0.52	1.00000000	pigeons

Golt1b	-0.15	1.00000000	pigeons
Ppp2r5e	-0.32	1.00000000	pigeons
Ripk1	-0.11	1.00000000	pigeons
Immp1l	-0.30	1.00000000	pigeons
Atp6v1g1	-0.42	1.00000000	pigeons
Bicd2	-0.45	1.00000000	pigeons
Cdk8	-0.26	1.00000000	pigeons
Herc3	-0.26	1.00000000	pigeons
Pdk3	-0.43	1.00000000	pigeons
Gorasp2	-0.18	1.00000000	pigeons
Foxn3	-0.57	1.00000000	pigeons
Ccdc47	-0.15	1.00000000	pigeons
Txndc9	-0.27	1.00000000	pigeons
Sqstm1	-0.40	1.00000000	pigeons
Abcd2	-0.29	1.00000000	pigeons
Slc12a2	-0.36	1.00000000	pigeons
Tax1bp1	-0.36	1.00000000	pigeons
Atxn10	-0.15	1.00000000	pigeons
S100a11	-0.37	1.00000000	pigeons
Id2	-0.15	1.00000000	pigeons
Ap2m1	-0.32	1.00000000	pigeons
Ranbp1	-0.30	1.00000000	pigeons
Atp6v0a1	-0.53	1.00000000	pigeons
Lin54	-0.30	1.00000000	pigeons
Ing5	-0.20	1.00000000	pigeons
Cops5	-0.18	1.00000000	pigeons
Cry2	-0.16	1.00000000	pigeons
D5Ertd579e	-0.45	1.00000000	pigeons
Ndfip1	-0.34	1.00000000	pigeons
Lims2	-0.44	1.00000000	pigeons
Rell1	-0.39	1.00000000	pigeons
Slc38a10	-0.11	1.00000000	pigeons
Tmem165	-0.24	1.00000000	pigeons
Slc30a7	-0.21	1.00000000	pigeons
Lamp2	-0.48	1.00000000	pigeons
Atp6ap2	-0.32	1.00000000	pigeons
Dld	-0.23	1.00000000	pigeons
Dennd6a	-0.22	1.00000000	pigeons
Arhgap29	-0.42	1.00000000	pigeons
Tcn2	-0.21	1.00000000	pigeons
Hscb	-0.16	1.00000000	pigeons
Lyar	-0.09	1.00000000	pigeons
Pbx1	-0.53	1.00000000	pigeons
Lonrf2	-0.28	1.00000000	pigeons
Plekha3	-0.16	1.00000000	pigeons
Slc2a8	-0.17	1.00000000	pigeons

Zfp407	-0.09	1.00000000	pigeons
Pdcl3	-0.22	1.00000000	pigeons
Nip7	-0.02	1.00000000	pigeons
Ndufb3	-0.33	1.00000000	pigeons
Eif2s1	-0.35	1.00000000	pigeons
Ulk1	-0.54	1.00000000	pigeons
Nudt3	-0.13	1.00000000	pigeons
Grap2	-0.23	1.00000000	pigeons
Srp68	-0.20	1.00000000	pigeons
Grpel1	-0.05	1.00000000	pigeons
Disc1	-0.13	1.00000000	pigeons
Insr	-0.37	1.00000000	pigeons
Nqo2	-0.14	1.00000000	pigeons
Fundc1	-0.03	1.00000000	pigeons
Sod1	-0.35	1.00000000	pigeons
Kdm5a	-0.40	1.00000000	pigeons
Washc3	-0.20	1.00000000	pigeons
Hnrnpa3	-0.01	1.00000000	pigeons
Zfp592	-0.21	1.00000000	pigeons
Btbd11	0.02	1.00000000	pigeons
Spag9	-0.32	1.00000000	pigeons
Poc1b	-0.29	1.00000000	pigeons
Ube2h	-0.33	1.00000000	pigeons
Napg	-0.25	1.00000000	pigeons
Slc6a6	-0.49	1.00000000	pigeons
Edrf1	-0.28	1.00000000	pigeons
Ybx3	-0.21	1.00000000	pigeons
Ggps1	-0.37	1.00000000	pigeons
Trap1	-0.14	1.00000000	pigeons
Cebpg	-0.18	1.00000000	pigeons
Pla2g12a	-0.38	1.00000000	pigeons
Ufm1	-0.37	1.00000000	pigeons
Snx3	-0.41	1.00000000	pigeons
Fem1b	-0.19	1.00000000	pigeons
Zc3h12a	-0.53	1.00000000	pigeons
Sh3bp2	-0.43	1.00000000	pigeons
Akt3	-0.20	1.00000000	pigeons
Srp19	-0.22	1.00000000	pigeons
Tbc1d2b	-0.27	1.00000000	pigeons
Tmed5	-0.48	1.00000000	pigeons
Plk3	-0.23	1.00000000	pigeons
Erlin2	-0.05	1.00000000	pigeons
Lemd2	-0.15	1.00000000	pigeons
Necap2	-0.18	1.00000000	pigeons
Slx4	-0.27	1.00000000	pigeons
Hectd1	-0.23	1.00000000	pigeons

Dnajb2	-0.24	1.00000000	pigeons
Tmco1	-0.17	1.00000000	pigeons
Tln1	-0.28	1.00000000	pigeons
Psen1	-0.34	1.00000000	pigeons
Rwdd1	-0.27	1.00000000	pigeons
Nfkbiz	-0.57	1.00000000	pigeons
Ap1ar	-0.19	1.00000000	pigeons
Ndufaf7	-0.07	1.00000000	pigeons
Kat6b	-0.45	1.00000000	pigeons
Alg2	-0.19	1.00000000	pigeons
Clip1	-0.57	1.00000000	pigeons
Cpeb2	-0.10	1.00000000	pigeons
Sptlc2	-0.25	1.00000000	pigeons
Exoc5	-0.41	1.00000000	pigeons
Ctsb	-0.34	1.00000000	pigeons
Arntl	-0.25	1.00000000	pigeons
Oser1	-0.25	1.00000000	pigeons
Chmp1a	-0.28	1.00000000	pigeons
Asah1	-0.43	1.00000000	pigeons
Pcna	-0.33	1.00000000	pigeons
Gramd3	-0.08	1.00000000	pigeons
Tmbim6	-0.27	1.00000000	pigeons
Cactin	-0.37	1.00000000	pigeons
Sec31a	-0.45	1.00000000	pigeons
Zmat2	-0.15	1.00000000	pigeons
Golm1	-0.10	1.00000000	pigeons
Per3	-0.20	1.00000000	pigeons
P4ha1	-0.55	1.00000000	pigeons
Ttc7b	-0.33	1.00000000	pigeons
Nfe2l2	-0.47	1.00000000	pigeons
Arl6ip1	-0.36	1.00000000	pigeons
Setd7	-0.38	1.00000000	pigeons
Ciao1	-0.21	1.00000000	pigeons
Vps37a	-0.30	1.00000000	pigeons
Stam2	-0.56	1.00000000	pigeons
Smarcal1	-0.03	1.00000000	pigeons
Rnf149	-0.35	1.00000000	pigeons
Ralb	-0.53	1.00000000	pigeons
Cpne2	-0.20	1.00000000	pigeons
Bap1	-0.17	1.00000000	pigeons
Arl15	-0.33	1.00000000	pigeons
Ext1	-0.63	1.00000000	pigeons
Hsd17b4	-0.28	1.00000000	pigeons
Ypel1	-0.53	1.00000000	pigeons
Wasf2	-0.32	1.00000000	pigeons
Pdrg1	-0.25	1.00000000	pigeons

Pik3ca	-0.29	1.00000000	pigeons
Rb1	-0.33	1.00000000	pigeons
Eepd1	-0.34	1.00000000	pigeons
Atp8a1	-0.45	1.00000000	pigeons
Ube2q2	-0.43	1.00000000	pigeons
Bnip3l	-0.56	1.00000000	pigeons
Ppp2ca	-0.25	1.00000000	pigeons
Dot1l	-0.28	1.00000000	pigeons
Plcb4	-0.40	1.00000000	pigeons
Ifrd1	-0.24	1.00000000	pigeons
Ssr1	-0.23	1.00000000	pigeons
Cry1	-0.11	1.00000000	pigeons
Suco	-0.36	1.00000000	pigeons
Gtf2h1	-0.37	1.00000000	pigeons
Aff1	0.22	1.00000000	pigeons
Rb1cc1	-0.32	1.00000000	pigeons
Dbi	-0.30	1.00000000	pigeons
Oaz2	-0.27	1.00000000	pigeons
Nisch	-0.21	1.00000000	pigeons
Cltc	-0.49	1.00000000	pigeons
Tmem59	-0.26	1.00000000	pigeons
Ptbp1	-0.15	1.00000000	pigeons
Ndufa12	-0.19	1.00000000	pigeons
Ranbp9	-0.35	1.00000000	pigeons
Ppa1	-0.19	1.00000000	pigeons
Usp8	-0.37	1.00000000	pigeons
Cpne1	-0.45	1.00000000	pigeons
Rsrp1	-0.39	1.00000000	pigeons
Gata3	-0.20	1.00000000	pigeons
Srgap3	-0.16	1.00000000	pigeons
Sbf2	-0.48	1.00000000	pigeons
Eif4g3	-0.43	1.00000000	pigeons
Hhex	-0.35	1.00000000	pigeons
Prex1	-0.14	1.00000000	pigeons
Per2	-0.24	1.00000000	pigeons
Pald1	-0.43	1.00000000	pigeons
Ppp2r5d	-0.23	1.00000000	pigeons
Glipr2	-0.40	1.00000000	pigeons
Gsr	-0.24	1.00000000	pigeons
Cmtm4	-0.58	1.00000000	pigeons
Brd1	-0.45	1.00000000	pigeons
Pde4d	-0.08	1.00000000	pigeons
Sos2	-0.38	1.00000000	pigeons
Trim30a	1.56	0.00000000	mice
Bst2	1.57	0.00000000	mice
Zbp1	1.23	0.00000000	mice

Fam107b	-0.95	0.00000000	mice
Rnf213	1.44	0.00000000	mice
Isg15	1.53	0.00000000	mice
Stat1	1.31	0.00000000	mice
Ifi27l2a	2.52	0.00000000	mice
B2m	0.78	0.00000000	mice
Xaf1	0.94	0.00000000	mice
Ifit1	1.39	0.00000000	mice
Rtp4	1.00	0.00000000	mice
Ifi203	1.35	0.00000000	mice
Sp100	1.15	0.00000000	mice
Lgals3bp	0.87	0.00000000	mice
Parp14	1.27	0.00000000	mice
Herc6	1.49	0.00000000	mice
Samd9l	1.07	0.00000000	mice
Gbp7	1.06	0.00000000	mice
H2-K1	0.67	0.00000000	mice
Ifit3	0.93	0.00000000	mice
Nfkb1	1.15	0.00000000	mice
Parp9	1.09	0.00000000	mice
Irf7	0.96	0.00000000	mice
Slfn8	0.92	0.00000000	mice
Ifi209	0.93	0.00000000	mice
Oasl1	0.90	0.00000000	mice
Epsti1	1.09	0.00000000	mice
Ddx60	1.05	0.00000000	mice
Nfkbia	1.10	0.00000000	mice
Usp18	0.75	0.00000000	mice
Cd52	0.95	0.00000000	mice
Cxcl10	1.39	0.00000000	mice
Ifitm3	1.04	0.00000000	mice
Ifi44	0.93	0.00000000	mice
Ifi214	0.94	0.00000000	mice
Psme1	0.84	0.00000000	mice
4932438A13Rik	-0.74	0.00000000	mice
Ifih1	1.01	0.00000000	mice
Rsad2	1.22	0.00000000	mice
A930037H05Rik	0.72	0.00000000	mice
Tapbp	0.79	0.00000000	mice
Pml	0.87	0.00000000	mice
Phf11b	0.75	0.00000000	mice
Slfn2	0.89	0.00000000	mice
Gbp4	0.82	0.00000000	mice
Arhgap15	-0.76	0.00000000	mice
Ly6c2	1.49	0.00000000	mice
Tsc22d3	-0.78	0.00000000	mice

Ly6a	1.14	0.00000000	mice
H2-T23	0.81	0.00000000	mice
Dtx3l	0.78	0.00000000	mice
Dock10	-0.62	0.00000000	mice
Slfn5	1.09	0.00000000	mice
Gbp5	0.89	0.00000000	mice
Irgm1	0.61	0.00000000	mice
Eif1	-0.41	0.00000000	mice
Ifi47	0.84	0.00000000	mice
Icam1	1.04	0.00000000	mice
Eif2ak2	0.63	0.00000000	mice
Tnfrsf9	1.12	0.00000000	mice
Gbp6	0.54	0.00000000	mice
Psmb8	0.75	0.00000000	mice
Ifi206	0.50	0.00000000	mice
Pim1	0.95	0.00000000	mice
AY036118	0.81	0.00000000	mice
Ascc3	0.82	0.00000000	mice
Cxcl2	0.64	0.00000000	mice
Stat3	0.65	0.00000000	mice
Oas3	0.42	0.00000000	mice
Gadd45b	1.01	0.00000000	mice
Pdcd4	-0.65	0.00000000	mice
Ifi208	0.57	0.00000000	mice
A330040F15Rik	0.73	0.00000000	mice
Tap1	0.62	0.00000000	mice
AW112010	1.04	0.00000000	mice
Cd47	0.58	0.00000000	mice
Ccnd2	0.73	0.00000000	mice
Gbp2b	0.88	0.00000000	mice
Gbp3	0.50	0.00000000	mice
Scgb1a1	0.89	0.00000000	mice
Tmsb10	0.40	0.00000000	mice
Klf2	-0.63	0.00000000	mice
Stat2	0.64	0.00000000	mice
Gbp2	0.78	0.00000000	mice
Psme2	0.61	0.00000000	mice
Ddx58	0.70	0.00000000	mice
Ifit2	0.59	0.00000000	mice
Srsf5	-0.47	0.00000000	mice
Pnrc1	-0.50	0.00000000	mice
Gzmb	1.48	0.00000000	mice
Ccrl2	0.57	0.00000000	mice
Igtp	0.60	0.00000000	mice
Stx11	0.64	0.00000000	mice
Ly6c1	0.58	0.00000000	mice

Il7r	-0.94	0.00000000	mice
Heatr5a	-0.68	0.00000000	mice
Klhl6	-0.66	0.00000000	mice
Isg20	0.62	0.00000000	mice
Bach2	-0.46	0.00000000	mice
Sec61g	0.58	0.00000000	mice
Ddx24	0.59	0.00000000	mice
Casp4	0.58	0.00000000	mice
Relb	0.57	0.00000000	mice
Clec2d	0.43	0.00000000	mice
Tspo	0.64	0.00000000	mice
Bcl3	0.52	0.00000000	mice
Creb1	0.78	0.00000000	mice
Dip2b	-0.67	0.00000000	mice
Atp5e	0.58	0.00000000	mice
9930111J21Rik2	0.64	0.00000000	mice
Lgals1	0.94	0.00000000	mice
Cox6c	0.52	0.00000000	mice
H2-D1	0.34	0.00000000	mice
Ifi202b	0.41	0.00000000	mice
Tmsb4x	0.54	0.00000000	mice
Zfp36l2	-0.51	0.00000000	mice
Traf3	0.72	0.00000000	mice
Tcp11l2	-0.57	0.00000000	mice
Dhx58	0.30	0.00000000	mice
Psmb9	0.53	0.00000000	mice
Rnf145	-0.71	0.00000000	mice
Irf9	0.49	0.00000000	mice
Helz2	0.53	0.00000000	mice
Daxx	0.52	0.00000000	mice
Rel	0.73	0.00000000	mice
Traf1	0.62	0.00000000	mice
Etv6	0.73	0.00000000	mice
Irf1	0.65	0.00000000	mice
Plac8	0.98	0.00000000	mice
Ifi35	0.52	0.00000000	mice
Ifit3b	0.31	0.00000000	mice
Adar	0.46	0.00000000	mice
Epb41	-0.54	0.00000000	mice
Eif2ak3	-0.70	0.00000000	mice
Rasgrp2	-0.56	0.00000000	mice
Bzw2	-0.63	0.00000000	mice
Klf13	-0.45	0.00000000	mice
Samhd1	0.97	0.00000000	mice
Pde3b	-0.64	0.00000000	mice
Shisa5	0.51	0.00000000	mice

Pfn1	0.56	0.00000000	mice
Phf11c	0.40	0.00000000	mice
Actb	0.56	0.00000000	mice
Iigp1	0.69	0.00000000	mice
Tgfbr2	-0.58	0.00000000	mice
Ccr5	0.68	0.00000000	mice
Emb	-0.63	0.00000000	mice
Clic1	0.56	0.00000000	mice
Ctss	0.52	0.00000000	mice
Oas1a	0.28	0.00000000	mice
Ccl4	1.34	0.00000000	mice
Rras2	-1.01	0.00000000	mice
Ndufa13	0.49	0.00000000	mice
Lef1	-0.69	0.00000000	mice
Gbp9	0.42	0.00000000	mice
Ppa1	0.59	0.00000000	mice
Cxcl1	0.32	0.00000000	mice
Tor3a	0.45	0.00000000	mice
Zyg11b	-0.63	0.00000000	mice
Cox17	0.48	0.00000000	mice
Ptp4a2	0.44	0.00000000	mice
Jak1	-0.34	0.00000000	mice
Rnf157	0.68	0.00000000	mice
Itk	-0.60	0.00000000	mice
Gbp10	0.23	0.00000000	mice
Rassf3	-0.61	0.00000000	mice
Irf2	0.53	0.00000000	mice
4930444A19Rik	0.38	0.00000000	mice
Saa3	0.59	0.00000000	mice
Oas2	0.27	0.00000000	mice
Zbtb46	0.49	0.00000000	mice
Grap2	-0.66	0.00000000	mice
Nfkbiz	0.64	0.00000000	mice
Rab2a	-0.39	0.00000000	mice
Btbd9	-0.41	0.00000000	mice
Kdm6b	0.53	0.00000000	mice
Cyth1	-0.50	0.00000000	mice
Plaur	-0.56	0.00000000	mice
Ly6e	0.55	0.00000000	mice
Nfkbid	0.50	0.00000000	mice
Sp110	0.48	0.00000000	mice
Slc49a4	-0.62	0.00000000	mice
Cyba	0.55	0.00000000	mice
Atp5k	0.39	0.00000000	mice
Ifi204	0.36	0.00000000	mice
Klrk1	0.63	0.00000000	mice

Lncpint	-0.38	0.00000000	mice
Atp1b3	-0.44	0.00000000	mice
Hspa5	0.43	0.00000000	mice
Pde2a	-0.59	0.00000000	mice
Pde7a	-0.44	0.00000000	mice
Pag1	-0.57	0.00000000	mice
Ccnd3	-0.43	0.00000000	mice
Cd274	0.61	0.00000000	mice
Heca	-0.44	0.00000000	mice
Bhlhe40	0.63	0.00000000	mice
Ss18	-0.46	0.00000000	mice
Nampt	0.48	0.00000000	mice
Atp5mpl	0.42	0.00000000	mice
Btg1	0.34	0.00000000	mice
Irgm2	0.33	0.00000000	mice
Ccl3	0.98	0.00000000	mice
Xcl1	1.42	0.00000000	mice
Rab8b	0.49	0.00000000	mice
Gpr65	0.40	0.00000000	mice
Nmi	0.43	0.00000000	mice
Clic4	0.44	0.00000000	mice
Slfn1	0.79	0.00000000	mice
Samsn1	0.63	0.00000000	mice
Ifi211	0.25	0.00000000	mice
Lcp1	0.54	0.00000000	mice
Rgs16	0.54	0.00000000	mice
Celf2	-0.44	0.00000000	mice
Zup1	0.55	0.00000000	mice
Il2rg	0.41	0.00000000	mice
Znrf3	-0.40	0.00000000	mice
Rgs10	-0.76	0.00000000	mice
Trim12c	0.39	0.00000000	mice
Cirbp	-0.42	0.00000000	mice
Dbf4	-0.47	0.00000000	mice
Il1b	0.75	0.00000000	mice
Klhl24	-0.48	0.00000000	mice
Gem	0.68	0.00000000	mice
Plcxd2	-0.58	0.00000000	mice
Gbp8	0.42	0.00000000	mice
Ndufb1-ps	0.39	0.00000000	mice
Nlrc5	0.54	0.00000000	mice
Atxn1	0.82	0.00000000	mice
Parp12	0.24	0.00000000	mice
Ier5	0.40	0.00000000	mice
Trim56	0.35	0.00000000	mice
Txnip	-0.58	0.00000000	mice

Ttc39b	0.49	0.00000000	mice
Txn1	0.46	0.00000000	mice
Rnf125	-0.41	0.00000000	mice
B4galt5	0.52	0.00000000	mice
Ccm2	-0.52	0.00000000	mice
Insyn2b	0.46	0.00000000	mice
St8sia1	-0.63	0.00000000	mice
Sgms1	-0.66	0.00000000	mice
Arf4	0.37	0.00000000	mice
Slfn4	0.44	0.00000000	mice
Cd69	0.76	0.00000000	mice
Aim2	0.48	0.00000000	mice
H2-T24	0.20	0.00000000	mice
H2-T22	0.48	0.00000000	mice
Camk4	-0.69	0.00000000	mice
Ctsw	0.42	0.00000000	mice
Avl9	-0.47	0.00000000	mice
Sell	0.47	0.00000000	mice
Lyz2	0.45	0.00000000	mice
Osbpl3	0.56	0.00000000	mice
Jaml	0.33	0.00000000	mice
Lmbr1l	-0.41	0.00000000	mice
Ubn1	-0.39	0.00000000	mice
Ski	-0.41	0.00000000	mice
Brd1	-0.40	0.00000000	mice
Tmem256	0.24	0.00000000	mice
Atp5j	0.35	0.00000000	mice
Cpeb2	-0.63	0.00000000	mice
Arap2	-0.48	0.00000000	mice
Lpp	0.52	0.00000000	mice
Plaat3	0.38	0.00000000	mice
Ube2j1	-0.37	0.00000000	mice
Anxa2	0.54	0.00000000	mice
Crip1	0.58	0.00000000	mice
Chd4	0.47	0.00000000	mice
Thada	-0.57	0.00000000	mice
Dbi	0.34	0.00000000	mice
Psmb10	0.40	0.00000000	mice
Pet100	0.27	0.00000000	mice
Ndufa3	0.33	0.00000000	mice
Trim14	0.35	0.00000000	mice
S100a6	0.79	0.00000000	mice
Ube2b	-0.33	0.00000000	mice
Gpbp1	0.35	0.00000000	mice
Patj	-0.50	0.00000000	mice
Tsix	0.32	0.00000000	mice

Tmem173	0.19	0.00000000	mice
Atp5j2	0.39	0.00000000	mice
Ramp3	-0.43	0.00000000	mice
Usp12	-0.47	0.00000000	mice
Cst7	0.28	0.00000000	mice
Kdm6bos	0.23	0.00000000	mice
Rbm39	-0.21	0.00000000	mice
Abcg1	-0.43	0.00000000	mice
Nfkbie	0.34	0.00000000	mice
Hivep1	0.52	0.00000000	mice
Def6	-0.36	0.00000000	mice
Stk39	0.45	0.00000000	mice
Anxa6	0.41	0.00000001	mice
Pakap.1	-0.56	0.00000001	mice
Kdm3a	-0.42	0.00000001	mice
Rap1b	-0.25	0.00000001	mice
Tgif1	0.40	0.00000001	mice
Jmy	-0.35	0.00000001	mice
Uqcrb	0.31	0.00000001	mice
Prdx6	-0.38	0.00000001	mice
Tgtp2	0.18	0.00000001	mice
Ncr1	0.54	0.00000001	mice
Cox7c	0.33	0.00000001	mice
H2-Q6	0.26	0.00000001	mice
Tbc1d15	-0.37	0.00000001	mice
Usp48	-0.38	0.00000001	mice
Serbp1	0.30	0.00000001	mice
Tor1aip1	0.36	0.00000002	mice
Baz1a	0.39	0.00000002	mice
Cblb	-0.35	0.00000002	mice
Zeb2	0.62	0.00000002	mice
Lrrfip1	0.45	0.00000002	mice
Atp5l	0.32	0.00000002	mice
Acod1	0.55	0.00000003	mice
Oga	-0.36	0.00000003	mice
Lilr4b	0.31	0.00000003	mice
Ifngr1	-0.32	0.00000003	mice
Ypel3	-0.36	0.00000003	mice
Otulin	0.39	0.00000003	mice
Ttc39c	0.33	0.00000003	mice
Bcl2a1d	0.35	0.00000004	mice
Rmnd5a	-0.41	0.00000004	mice
4930455C13Rik	0.20	0.00000004	mice
Il10ra	0.32	0.00000004	mice
Pitpnc1	-0.39	0.00000004	mice
Tyrobp	0.58	0.00000004	mice

S100a13	0.38	0.00000004	mice
Adk	-0.80	0.00000005	mice
Jade1	-0.36	0.00000005	mice
Frat2	-0.29	0.00000005	mice
Cenpa	-0.45	0.00000006	mice
Il21r	-0.47	0.00000006	mice
Ankrd12	-0.39	0.00000006	mice
Irs2	-0.41	0.00000006	mice
Itm2b	0.31	0.00000007	mice
Picalm	0.49	0.00000007	mice
Eif2s2	0.36	0.00000007	mice
Serpinb9	0.51	0.00000008	mice
Smad7	-0.30	0.00000008	mice
Spp1	0.27	0.00000009	mice
Rab3ip	-0.55	0.00000010	mice
Ccr2	0.42	0.00000011	mice
Pgm2l1	-0.43	0.00000012	mice
Adora2a	0.35	0.00000012	mice
Tomm7	0.32	0.00000012	mice
Vps37b	-0.21	0.00000012	mice
Batf	0.32	0.00000013	mice
St3gal4	0.35	0.00000014	mice
Trim25	0.33	0.00000014	mice
Trafd1	0.30	0.00000015	mice
Tdrp	-0.34	0.00000015	mice
Tank	0.36	0.00000022	mice
Pomp	0.32	0.00000026	mice
H2-Q4	0.33	0.00000027	mice
Rflnb	-0.35	0.00000027	mice
Aldoa	0.31	0.00000028	mice
Peak1	0.43	0.00000029	mice
Runx2	0.39	0.00000032	mice
Bcl2a1b	0.40	0.00000032	mice
Rnf114	0.32	0.00000033	mice
H2-Q7	0.34	0.00000035	mice
Fam168b	-0.32	0.00000036	mice
Ifngr2	-0.39	0.00000038	mice
Olfr56	0.28	0.00000038	mice
Irf8	0.49	0.00000046	mice
Slc24a1	0.26	0.00000046	mice
Ncf4	0.27	0.00000054	mice
Gzma	0.61	0.00000055	mice
Elob	0.33	0.00000058	mice
Snrpf	0.33	0.00000060	mice
Bnip3l	-0.33	0.00000062	mice
Col3a1	0.17	0.00000065	mice

Ulbp1	0.26	0.00000066	mice
Ern1	0.53	0.00000069	mice
Tcof1	0.27	0.00000071	mice
Dusp10	-0.55	0.00000074	mice
Pnpt1	0.22	0.00000080	mice
Zcchc2	0.32	0.00000086	mice
Nabp1	0.38	0.00000088	mice
Ldlrap1	-0.26	0.00000094	mice
Ndufa2	0.27	0.00000094	mice
Cox5b	0.32	0.00000097	mice
Lyst	-0.51	0.00000098	mice
Vps54	0.47	0.00000109	mice
Arhgef18	-0.43	0.00000109	mice
Kcnn4	-0.53	0.00000112	mice
Pip4k2a	-0.44	0.00000117	mice
Lrrk1	0.46	0.00000122	mice
Ets1	-0.24	0.00000123	mice
Fam169b	-0.52	0.00000126	mice
Gvin1	0.19	0.00000132	mice
Tacc1	-0.38	0.00000136	mice
Pnpla8	-0.38	0.00000156	mice
Kcnk10	0.32	0.00000173	mice
Eif5a	0.38	0.00000184	mice
Ap1s3	0.37	0.00000188	mice
Aff4	-0.36	0.00000210	mice
Ctsb	0.28	0.00000217	mice
Birc3	0.42	0.00000219	mice
Rb1cc1	-0.34	0.00000242	mice
Nfkb2	0.27	0.00000245	mice
Zfp64	-0.41	0.00000275	mice
Ptpn6	0.24	0.00000309	mice
Lilrb4a	0.26	0.00000315	mice
Eef2	-0.25	0.00000322	mice
Atxn7	-0.29	0.00000324	mice
Atad2	-0.48	0.00000328	mice
Fcer1g	0.45	0.00000400	mice
Arl4c	-0.31	0.00000405	mice
Atp11b	-0.33	0.00000420	mice
1600014C10Rik	0.26	0.00000431	mice
4930595D18Rik	0.17	0.00000449	mice
Sidt1	-0.47	0.00000461	mice
Atp10a	0.44	0.00000513	mice
Tmem131l	-0.44	0.00000563	mice
1700084C06Rik	0.27	0.00000589	mice
Aff3	-0.52	0.00000593	mice
Ankrd50	-0.30	0.00000618	mice

Atp5md	0.28	0.00000668	mice
Rb1	-0.38	0.00000785	mice
Ptprc	0.25	0.00000795	mice
5330438D12Rik	0.32	0.00000804	mice
Ndufb7	0.32	0.00000832	mice
Nkg7	0.43	0.00000890	mice
Klhl8	0.16	0.00000905	mice
Selenok	-0.27	0.00000916	mice
Pik3ap1	0.42	0.00000935	mice
Abl1	-0.49	0.00001058	mice
Gba	0.13	0.00001069	mice
Hdac4	-0.46	0.00001083	mice
Trp53	0.29	0.00001106	mice
Atrn	-0.42	0.00001179	mice
Cd55	-0.35	0.00001181	mice
Cox7a2	0.32	0.00001238	mice
Tuba1a	-0.31	0.00001240	mice
Tbc1d1	0.47	0.00001284	mice
Setbp1	0.56	0.00001400	mice
Acp5	-0.27	0.00001643	mice
Phf21a	0.39	0.00001666	mice
Runx3	0.36	0.00001716	mice
Usp25	0.36	0.00001760	mice
Uqcc2	0.18	0.00001767	mice
Cdkn1b	-0.33	0.00001792	mice
Actn1	-0.38	0.00001882	mice
Vegfa	0.33	0.00001899	mice
Rhoa	-0.26	0.00002036	mice
Per1	-0.30	0.00002080	mice
Smarca2	-0.40	0.00002172	mice
AC149090.1	0.26	0.00002316	mice
Slc25a25	-0.37	0.00002365	mice
Sema4d	0.33	0.00002407	mice
Igf1r	-0.63	0.00002478	mice
Satb1	-0.30	0.00002546	mice
Cebpb	-0.46	0.00002658	mice
Zfand2a	-0.34	0.00002705	mice
Prr5l	0.20	0.00002764	mice
Ankrd11	-0.23	0.00002826	mice
Dennd4a	0.48	0.00002950	mice
Elovl6	0.39	0.00002978	mice
Osbpl9	-0.42	0.00003287	mice
2310001H17Rik	0.21	0.00003504	mice
Sumo2	0.26	0.00003611	mice
Ywhab	0.30	0.00003658	mice
Elmsan1	-0.32	0.00003876	mice

Rasa3	-0.31	0.00004031	mice
Dgka	-0.39	0.00004568	mice
2610035D17Rik	-0.29	0.00004580	mice
Capza2	0.30	0.00004875	mice
Dpm3	0.22	0.00005144	mice
Srp9	-0.29	0.00005491	mice
Uqcrq	0.28	0.00005499	mice
H3f3a	-0.21	0.00005566	mice
Cgas	0.19	0.00005745	mice
Selenow	0.32	0.00005798	mice
Sntb1	-0.61	0.00005860	mice
Rpa1	-0.35	0.00006046	mice
BC005537	-0.29	0.00006167	mice
Fbxo32	-0.32	0.00006275	mice
Coro1a	0.29	0.00006434	mice
Prex1	-0.44	0.00006461	mice
Lamp2	0.34	0.00006649	mice
Bicdl1	-0.41	0.00006857	mice
Oxct1	-0.40	0.00006998	mice
Itm2a	-0.40	0.00007041	mice
Ccl6	0.30	0.00007093	mice
Ogfr	0.27	0.00007288	mice
Tap2	0.30	0.00007840	mice
Tnf	0.26	0.00008001	mice
Sem1	0.26	0.00008312	mice
Kat6b	-0.39	0.00008385	mice
Tdrd7	0.21	0.00008540	mice
Ppp1ca	0.26	0.00008894	mice
Ssh2	-0.19	0.00009761	mice
B230307C23Rik	0.22	0.00010002	mice
Sftpc	-0.04	0.00010688	mice
Fas	0.33	0.00010799	mice
Lsm4	0.27	0.00011063	mice
Rbpj	0.39	0.00011609	mice
A930015D03Rik	0.29	0.00012372	mice
Tpt1	-0.20	0.00012542	mice
Scimp	0.19	0.00013036	mice
Tmem59	-0.30	0.00013131	mice
Slc35d1	-0.51	0.00013457	mice
Psma2	0.28	0.00015529	mice
Ndufc1	0.22	0.00015546	mice
Etnk1	0.34	0.00015822	mice
Ndufa1	0.27	0.00016518	mice
D16Ertd472e	0.28	0.00016525	mice
Ube4b	-0.37	0.00016926	mice
Crebrf	-0.27	0.00018987	mice

Sp140	0.25	0.00021587	mice
Tgfb1	0.33	0.00022046	mice
Serf2	0.24	0.00022121	mice
Esyt2	-0.24	0.00022923	mice
Cybb	0.30	0.00023565	mice
St6gal1	-0.32	0.00025214	mice
Sema4a	0.24	0.00025685	mice
Epb41l2	0.44	0.00025844	mice
Cox6b1	0.27	0.00027414	mice
S100a11	0.30	0.00029757	mice
Cox7b	0.28	0.00030490	mice
B3gnt2	0.34	0.00030597	mice
Krtcap2	0.27	0.00030844	mice
Hsd11b1	0.18	0.00030898	mice
Aoah	0.49	0.00031333	mice
Fam117b	-0.37	0.00032246	mice
Zzef1	-0.31	0.00032542	mice
Rundc3b	-0.31	0.00033097	mice
Reps1	-0.46	0.00033600	mice
Cflar	0.29	0.00033714	mice
Cdkn1a	0.35	0.00034012	mice
Gpr183	0.61	0.00036868	mice
Itga4	0.64	0.00038061	mice
Uqcr11	0.23	0.00038930	mice
Ndufa4	0.30	0.00040204	mice
Snrpd2	0.25	0.00041252	mice
Tecpr1	-0.38	0.00042654	mice
Dot1l	0.23	0.00043359	mice
Resf1	0.41	0.00043718	mice
Socs5	0.29	0.00044249	mice
Tiprl	-0.35	0.00045662	mice
Cyfip1	0.22	0.00047016	mice
Stim1	-0.34	0.00047982	mice
Klrb1f	0.18	0.00049047	mice
Eef1b2	-0.29	0.00051561	mice
Pnpla7	-0.32	0.00051812	mice
Eef1a1	-0.16	0.00055478	mice
Lrrc4	0.26	0.00056727	mice
Gfod1	0.38	0.00063730	mice
Kpna4	-0.27	0.00065145	mice
Parp10	0.17	0.00068520	mice
Slamf7	0.26	0.00069670	mice
Snrpb2	0.24	0.00070372	mice
Hif1a	0.51	0.00072995	mice
Jund	-0.29	0.00076483	mice
S1pr1	-0.27	0.00077144	mice

Ikzf2	0.71	0.00078641	mice
Pced1b	-0.28	0.00080306	mice
Bcl11b	-0.37	0.00082900	mice
Erap1	0.28	0.00084229	mice
Cmip	-0.27	0.00084591	mice
Optn	0.16	0.00089142	mice
Ppp1r18	0.21	0.00091771	mice
Jakmip1	-0.34	0.00094744	mice
Parp11	0.27	0.00098866	mice
Acbd5	-0.31	0.00101713	mice
Rtn4rl1	-0.42	0.00104290	mice
Rack1	-0.22	0.00105089	mice
Arhgap25	-0.26	0.00110086	mice
Klra3	0.45	0.00111649	mice
Romo1	0.20	0.00115977	mice
Klrg1	0.23	0.00116479	mice
Cd7	-0.42	0.00117370	mice
Ctdp1	-0.35	0.00121963	mice
AU020206	0.38	0.00122877	mice
Smurf1	-0.36	0.00126944	mice
Zbtb38	-0.31	0.00127312	mice
Dleu2	0.64	0.00136550	mice
Ndufv3	0.22	0.00140313	mice
Sytl2	-0.42	0.00148307	mice
Hs3st3b1	-0.40	0.00151497	mice
Zc3h12d	0.23	0.00157087	mice
Rhoq	0.32	0.00159926	mice
Klf6	-0.19	0.00160359	mice
Gria3	-0.20	0.00164179	mice
Fchsd2	0.30	0.00165296	mice
Jak2	0.51	0.00165443	mice
Actr3	0.27	0.00166304	mice
Tent5a	0.25	0.00177013	mice
Eif4ebp2	-0.30	0.00184122	mice
1810026B05Rik	-0.29	0.00188742	mice
Cd244a	0.18	0.00197658	mice
Kras	-0.22	0.00197956	mice
Nfe2l2	0.25	0.00201258	mice
Foxp1	-0.25	0.00212121	mice
Atp6v1a	0.22	0.00217748	mice
Usp9x	-0.21	0.00229628	mice
Slc9a7	0.23	0.00232721	mice
Pik3ip1	-0.23	0.00239249	mice
Acot7	0.24	0.00248510	mice
Fgd3	-0.30	0.00252045	mice
Sh2d1b1	0.16	0.00260350	mice

Dock8	0.37	0.00260602	mice
Smad3	0.33	0.00264881	mice
Tmlhe	-0.31	0.00265997	mice
Klk8	-0.25	0.00276589	mice
Ubald2	0.33	0.00276867	mice
Fndc3a	0.29	0.00278637	mice
Tnfaip2	0.16	0.00280917	mice
Edf1	0.24	0.00286166	mice
Sla	0.25	0.00298897	mice
Filip1l	0.28	0.00302875	mice
Gpr155	0.26	0.00306508	mice
Atp2b4	0.35	0.00309070	mice
Cfl1	0.24	0.00315553	mice
Cdadc1	-0.26	0.00328446	mice
Spred2	-0.29	0.00330584	mice
Hip1	0.33	0.00334853	mice
Rsbn1l	0.27	0.00340503	mice
Tom1l2	-0.32	0.00344144	mice
Fbrsl1	-0.29	0.00349263	mice
Cysltr2	0.24	0.00350334	mice
D5Ertd615e	0.25	0.00367034	mice
Cops9	0.20	0.00369465	mice
Anxa1	0.15	0.00384609	mice
Ankhd1	0.28	0.00387303	mice
1700016P03Rik	-0.23	0.00397121	mice
Irf5	0.14	0.00411363	mice
Tmem140	0.16	0.00447651	mice
Uba7	0.14	0.00479927	mice
Hcst	0.28	0.00497402	mice
Cd9	0.29	0.00510842	mice
Fam129a	0.33	0.00516316	mice
Hivep2	0.30	0.00524414	mice
S100a4	0.58	0.00532381	mice
Sipa1l2	-0.46	0.00534690	mice
Notch1	0.22	0.00535324	mice
Rgcc	-0.25	0.00535612	mice
Retreg1	-0.32	0.00563184	mice
Reep5	0.22	0.00585740	mice
Pde4d	-0.27	0.00608113	mice
Cmss1	0.53	0.00669669	mice
Gata3	0.24	0.00672239	mice
Dpm1	-0.25	0.00680850	mice
Wasf2	-0.27	0.00688333	mice
Wnt5b	-0.38	0.00700044	mice
St6galnac4	0.17	0.00708050	mice
Chmp2a	0.21	0.00713205	mice

Tax1bp1	-0.23	0.00715647	mice
Itgb1	0.25	0.00725464	mice
Arel1	0.20	0.00765254	mice
Dnajc19	0.21	0.00779939	mice
Kmt5b	-0.25	0.00821040	mice
Pacs1	-0.43	0.00828153	mice
Rrad	0.21	0.00831447	mice
Plscr1	0.16	0.00873040	mice
Add3	-0.20	0.00879842	mice
Gpr174	0.21	0.00884656	mice
Xiap	0.24	0.00898665	mice
Cdk11b	-0.16	0.00907447	mice
Tle4	-0.32	0.00961670	mice
Tada1	0.12	0.01001457	mice
Ndufa7	0.25	0.01019267	mice
Txnrd1	0.29	0.01033168	mice
Pcnt	-0.22	0.01040347	mice
Atg7	0.22	0.01051990	mice
Casp8	0.21	0.01056282	mice
Gsk3b	-0.24	0.01064764	mice
St8sia6	-0.32	0.01076819	mice
Nr4a1	0.40	0.01077175	mice
Pole3	0.11	0.01080960	mice
Atp5b	0.24	0.01115795	mice
Spty2d1	0.24	0.01145165	mice
Ciao2b	0.16	0.01148457	mice
Anp32b	0.22	0.01157120	mice
Klre1	0.25	0.01162838	mice
Adam17	0.32	0.01261277	mice
Ifnar2	0.19	0.01289824	mice
Rell1	-0.30	0.01301970	mice
Tsc22d4	0.26	0.01326217	mice
Sipa1l1	-0.28	0.01327154	mice
Cdk8	0.30	0.01340425	mice
Sptbn1	-0.27	0.01383872	mice
Gcnt2	-0.48	0.01412427	mice
Pycard	0.22	0.01420726	mice
Emp3	0.34	0.01437294	mice
Fam3c	-0.31	0.01454352	mice
Cish	0.17	0.01462049	mice
Klf3	-0.25	0.01463135	mice
Klra9	0.20	0.01582030	mice
Cytip	-0.19	0.01586869	mice
Rilpl2	0.21	0.01610061	mice
Nars2	-0.42	0.01664632	mice
Faah	-0.24	0.01677934	mice

Nckap5l	-0.13	0.01692884	mice
Smchd1	0.40	0.01743549	mice
Hspa4	-0.25	0.01783246	mice
Tnrc6b	-0.19	0.01872693	mice
Ptma	0.23	0.01880586	mice
Saraf	-0.22	0.01917558	mice
Gpr171	0.26	0.01931386	mice
Armc7	-0.31	0.01943183	mice
AI662270	0.23	0.01946009	mice
Pdap1	0.23	0.01957360	mice
Nedd8	0.25	0.01981725	mice
Lat2	0.15	0.02062354	mice
Ndufa11	0.24	0.02094023	mice
Ppp1r2	-0.28	0.02096272	mice
Arglu1	0.21	0.02141595	mice
Ttc7b	-0.28	0.02221424	mice
Zfr	0.25	0.02258548	mice
Ftl1	-0.16	0.02258578	mice
Gimap9	0.29	0.02372268	mice
Tprgl	-0.28	0.02466463	mice
Nr4a3	0.26	0.02535197	mice
Sec11a	-0.23	0.02840792	mice
Klhdc2	-0.32	0.02850965	mice
Cast	-0.29	0.02901294	mice
Ctps2	-0.23	0.02934935	mice
Brd2	0.28	0.03000380	mice
Arhgef3	0.35	0.03011239	mice
Themis	-0.39	0.03096806	mice
Set	0.23	0.03237916	mice
Zeb1	-0.37	0.03240017	mice
Riok3	-0.26	0.03288328	mice
Psma7	0.25	0.03398911	mice
Sik2	-0.30	0.03420283	mice
Clcn6	-0.25	0.03561731	mice
Lck	0.26	0.03583281	mice
Puf60	-0.25	0.03607632	mice
Lats2	-0.28	0.03609014	mice
Pan3	-0.18	0.03689575	mice
Narf	-0.23	0.03727343	mice
Atf3	0.21	0.03798840	mice
Slk	-0.27	0.03836652	mice
Cdkn2aip	-0.23	0.03845789	mice
Pcyt1a	0.31	0.03890423	mice
Galnt2	-0.36	0.04074685	mice
Cnot7	-0.24	0.04155024	mice
Clint1	-0.22	0.04158080	mice

Phlda1	0.27	0.04166543	mice
Fasl	0.30	0.04189449	mice
Ankrd55	-0.27	0.04260662	mice
Senp2	-0.24	0.04379249	mice
Arrb1	-0.29	0.04474825	mice
Smad4	-0.22	0.04486410	mice
Slc43a2	-0.25	0.04550353	mice
Unc93b1	0.13	0.04556986	mice
Smdt1	0.25	0.04576045	mice
Ptpn3	0.24	0.04604804	mice
Ccpg1	-0.26	0.04669460	mice
Capg	0.25	0.04701847	mice
Ifng	0.81	0.04830745	mice
Tmem258	0.24	0.04861457	mice
Ilrun	0.20	0.05084761	mice
Canx	0.23	0.05103147	mice
Chd2	-0.27	0.05233522	mice
Slc25a36	-0.23	0.05340555	mice
Slc15a3	0.15	0.05355098	mice
Ubl5	0.26	0.05397095	mice
Smurf2	-0.27	0.05435382	mice
Rabac1	-0.23	0.05443514	mice
Kansl1	-0.21	0.05937029	mice
Ssr2	0.17	0.05966641	mice
Polr2m	-0.25	0.06028215	mice
Anapc13	0.13	0.06041699	mice
Rlim	-0.24	0.06064099	mice
Ppard	-0.32	0.06238096	mice
Tsg101	-0.25	0.06291743	mice
Ccny	-0.25	0.06298871	mice
Tnrc6c	-0.22	0.06439882	mice
Hadhb	-0.22	0.06564932	mice
Chd6	-0.32	0.06630905	mice
Nfkbib	0.18	0.06650568	mice
Zfp644	-0.22	0.06771500	mice
Ech1	0.13	0.06804689	mice
Traj18	-0.14	0.07060337	mice
Uba52	0.24	0.07147869	mice
Tmco1	0.16	0.07238781	mice
Ccr7	-0.29	0.07321858	mice
Lrmda	0.30	0.07434050	mice
Atg2a	-0.23	0.07517796	mice
Ddx21	0.23	0.07540774	mice
Camk1d	-0.21	0.07758625	mice
Ptprcap	0.23	0.07772273	mice
Il12rb2	0.36	0.07774556	mice

Sik1	-0.26	0.08008749	mice
Psmb3	0.27	0.08302862	mice
Fundc2	0.19	0.08320904	mice
Unc119	0.13	0.08356067	mice
Trdc	0.20	0.08646737	mice
Ccdc50	0.15	0.08706873	mice
Vcpip1	0.24	0.08946169	mice
Cd82	0.20	0.09040712	mice
Junb	-0.22	0.09461146	mice
Son	0.20	0.09587802	mice
Inpp5a	-0.26	0.09903077	mice
Pim2	0.22	0.10007113	mice
Arhgap1	-0.26	0.10150316	mice
Ppp1r3b	0.25	0.10214984	mice
Crybg1	-0.22	0.10346992	mice
Ccnt1	-0.24	0.10614599	mice
Fes	0.14	0.10805999	mice
Itpkb	-0.24	0.10847926	mice
Hsh2d	0.14	0.10884733	mice
Calr	0.22	0.10973833	mice
Il4ra	-0.25	0.11229199	mice
Hnrnph1	-0.21	0.11288443	mice
Cplane1	-0.36	0.11315511	mice
Arhgef2	0.20	0.11441703	mice
C2cd3	0.16	0.11605047	mice
Fus	-0.19	0.11835695	mice
Psma4	0.22	0.12100431	mice
Ndufs5	0.16	0.12320842	mice
Clasp2	-0.28	0.12388865	mice
Fndc9	0.14	0.12410457	mice
Gimap1	0.22	0.12538869	mice
Cycs	0.23	0.12612067	mice
Ltb	0.28	0.12726697	mice
Psmc3	0.19	0.12808700	mice
Foxo1	-0.24	0.12817355	mice
Cdc37	0.22	0.13110527	mice
Rab4a	-0.24	0.13857222	mice
Zfp91	0.21	0.13871914	mice
Chsy1	0.23	0.13967839	mice
Zdhhc14	-0.23	0.14378527	mice
Gimap4	0.25	0.14527459	mice
Tshz1	-0.26	0.15334694	mice
Hnrnpa0	-0.18	0.15659026	mice
Ssbp3	-0.25	0.16126196	mice
Sec61b	0.22	0.16551581	mice
Ttc3	-0.32	0.16562995	mice

Il6st	-0.17	0.16646830	mice
Pdcd6	-0.27	0.16659107	mice
Arid5b	0.34	0.16738916	mice
Sidt2	-0.21	0.17179474	mice
Treml2	0.18	0.17561779	mice
Sun2	-0.24	0.18335091	mice
S100a9	0.04	0.18335995	mice
Tbrg1	0.21	0.18384841	mice
Adap1	0.11	0.18670583	mice
Nf1	0.23	0.20117471	mice
Etfb	0.14	0.20169054	mice
Tor1aip2	0.24	0.20312409	mice
Rabgap1l	0.31	0.20489623	mice
Glul	-0.17	0.21014046	mice
Sms	0.27	0.21442290	mice
Cmah	-0.17	0.23646808	mice
Acot2	-0.15	0.23652249	mice
Atg13	-0.23	0.24124036	mice
Frmd8	-0.21	0.24959385	mice
Tmem64	-0.19	0.25409648	mice
Il18rap	0.19	0.26559517	mice
Nab1	0.21	0.26622018	mice
Rsf1os1	0.18	0.27100356	mice
Xrn1	0.25	0.27139496	mice
Lysmd4	0.16	0.27158880	mice
Stt3b	-0.25	0.28361324	mice
Otud1	-0.14	0.28388048	mice
Ywhaq	-0.21	0.28414464	mice
Spcs2	0.19	0.28464037	mice
Dgkd	-0.25	0.29210657	mice
Klf9	-0.20	0.29218402	mice
Ndufs8	0.15	0.29269043	mice
Tmem70	0.11	0.29580943	mice
Numa1	-0.27	0.29585484	mice
Dpp4	-0.14	0.29588987	mice
Zc3h12a	0.15	0.29689989	mice
Cry1	-0.23	0.29850767	mice
Taf13	-0.19	0.29942768	mice
Vav1	0.13	0.30328295	mice
Insr	-0.19	0.30967525	mice
Bscl2	0.14	0.31658076	mice
Nae1	-0.18	0.32774621	mice
Gnai3	-0.21	0.33320114	mice
Cetn2	0.18	0.33742105	mice
Igkc	-0.32	0.33802356	mice
Gramd1b	0.39	0.34449579	mice

Csnk1g1	0.20	0.34559048	mice
Eif4e	0.19	0.34679728	mice
Klf7	-0.25	0.34902243	mice
Ext1	-0.24	0.35016244	mice
Calm1	0.18	0.35670584	mice
Nr3c1	-0.29	0.35854071	mice
Fkbp2	0.10	0.35897108	mice
Gtf2i	-0.30	0.36538747	mice
Cemip2	0.40	0.37147711	mice
Spata1	-0.27	0.37635566	mice
Dmrta1	-0.21	0.37690656	mice
U2af1	-0.21	0.38543014	mice
Emd	-0.22	0.38700092	mice
Eif1a	0.23	0.38745974	mice
Cmtm7	0.20	0.38893421	mice
Ube3a	0.19	0.38960420	mice
Lamtor2	0.17	0.39047283	mice
Klra7	0.39	0.39160872	mice
Fbxl3	-0.24	0.39923943	mice
Znrf1	-0.40	0.40513370	mice
Sec23b	0.19	0.41534093	mice
Slc12a7	-0.27	0.41708878	mice
Rnpc3	-0.22	0.41732190	mice
Brip1os	0.18	0.41772959	mice
Vcp	0.22	0.41911884	mice
Tubb2a	-0.31	0.41957695	mice
Ccdc12	0.13	0.42435981	mice
Lst1	0.14	0.42918655	mice
Pecam1	0.36	0.43750958	mice
Elf1	-0.18	0.43841262	mice
Ncoa2	-0.21	0.44230131	mice
Wdfy1	0.23	0.44251501	mice
Denr	0.18	0.44516557	mice
Zdhhc20	-0.21	0.44566162	mice
Rbl1	0.14	0.44725840	mice
Card6	-0.21	0.44728027	mice
Scaf8	-0.21	0.47996716	mice
Kdm5b	-0.25	0.48244058	mice
Pwp1	0.11	0.48525879	mice
Eprs	-0.22	0.48706593	mice
Pdxdc1	-0.22	0.48714354	mice
Tox	-0.35	0.49196280	mice
Galc	0.13	0.49336468	mice
Eif3c	0.19	0.49917402	mice
Csnk2a1	0.21	0.50014367	mice
Tfam	0.22	0.50802293	mice

Ccl5	0.27	0.52213521	mice
Anxa7	0.13	0.52479813	mice
Sh3bp5	-0.24	0.52859673	mice
Fam13b	-0.24	0.53542643	mice
Bcl2l1	0.20	0.56921389	mice
Prelid2	-0.20	0.57583814	mice
Spry2	0.19	0.58207239	mice
Nabp2	0.10	0.59155214	mice
Cmc1	0.15	0.59557795	mice
Calm2	-0.21	0.59821637	mice
Lmna	0.25	0.60860165	mice
Kbtbd2	-0.21	0.60948380	mice
Fis1	0.18	0.61987715	mice
Zc3h13	-0.22	0.62334174	mice
Pttg1	0.16	0.62571408	mice
Rhobtb2	-0.17	0.62711896	mice
Tmem160	0.20	0.63718354	mice
Cmtr1	0.14	0.64864439	mice
Kcmf1	0.16	0.65481720	mice
Lgals9	0.13	0.66701907	mice
Rnf31	0.16	0.68359848	mice
Rcan3	-0.15	0.69247131	mice
Zfp592	-0.27	0.70102876	mice
Hgsnat	0.29	0.72495106	mice
Cep57	-0.23	0.72872631	mice
Ssbp2	-0.29	0.73750680	mice
Cpne3	0.11	0.74642745	mice
Tmem134	0.16	0.74914827	mice
Rab5a	-0.21	0.76605612	mice
2510039O18Rik	-0.26	0.77063731	mice
Ndufs6	0.16	0.77623495	mice
Spag9	0.23	0.79342253	mice
Itgb2	0.25	0.79651557	mice
Scn1b	0.13	0.81981328	mice
Tnfsf10	0.12	0.82590375	mice
Cuta	0.16	0.83300070	mice
Atp11c	-0.22	0.84182175	mice
N4bp1	0.20	0.84279057	mice
Prdx2	0.11	0.84769550	mice
S1pr4	-0.18	0.85505516	mice
Pik3r5	0.26	0.88163856	mice
Snrpe	0.18	0.88564262	mice
Exoc5	-0.24	0.92931056	mice
AW011738	0.13	0.95491564	mice
P2ry14	0.18	0.95766802	mice
Uqcr10	0.18	0.96699273	mice

Tfdp2	-0.32	0.97854614	mice
Rnf103	-0.22	0.97885231	mice
Grina	0.17	0.99171971	mice
Rnh1	0.16	1.00000000	mice
Tmem163	0.20	1.00000000	mice
Plec	0.19	1.00000000	mice
Wbp4	0.11	1.00000000	mice
Serinc1	-0.21	1.00000000	mice
Trappc2l	0.11	1.00000000	mice
Tyk2	0.22	1.00000000	mice
Wwp2	-0.26	1.00000000	mice
Pear1	-0.28	1.00000000	mice
Evi2a	0.13	1.00000000	mice
Anapc2	-0.20	1.00000000	mice
Hist1h1c	-0.20	1.00000000	mice
Cd48	0.17	1.00000000	mice
Setd3	-0.22	1.00000000	mice
Phyh	0.15	1.00000000	mice
Chordc1	0.15	1.00000000	mice
Ttyh3	-0.17	1.00000000	mice
Fbxl12	-0.19	1.00000000	mice
Tgfbr3	-0.26	1.00000000	mice
Atp5o	0.17	1.00000000	mice
Ndufb9	0.15	1.00000000	mice
Hdac7	-0.25	1.00000000	mice
Ankfy1	0.23	1.00000000	mice
Gls	-0.13	1.00000000	mice
Sept11	0.26	1.00000000	mice
Rhob	-0.20	1.00000000	mice
Setdb2	0.24	1.00000000	mice
Adamts14	0.05	1.00000000	mice
Socs1	0.25	1.00000000	mice
Smim11	0.14	1.00000000	mice
Acpp	-0.27	1.00000000	mice
Atp5c1	0.19	1.00000000	mice
Rac2	0.18	1.00000000	mice
Ssr4	0.18	1.00000000	mice
Abca1	-0.21	1.00000000	mice
Ppm1h	-0.33	1.00000000	mice
Clnk	0.28	1.00000000	mice
Dnaja2	-0.22	1.00000000	mice
5031439G07Rik	-0.14	1.00000000	mice
Serinc3	-0.24	1.00000000	mice
Ddx41	0.12	1.00000000	mice
Smim14	-0.20	1.00000000	mice
Pacc1	0.18	1.00000000	mice

Rgs2	-0.18	1.00000000	mice
Casp3	0.14	1.00000000	mice
Kansl1l	0.22	1.00000000	mice
Ran	0.19	1.00000000	mice
Fbxo28	0.14	1.00000000	mice
Katna1	0.18	1.00000000	mice
Cdk2ap1	0.18	1.00000000	mice
Tram1	0.17	1.00000000	mice
Eif5b	0.21	1.00000000	mice
Gsap	0.24	1.00000000	mice
Stim2	0.24	1.00000000	mice
Rrbp1	0.14	1.00000000	mice
Tma7	0.16	1.00000000	mice
Usp45	0.14	1.00000000	mice
Rab19	0.13	1.00000000	mice
Gdi2	-0.21	1.00000000	mice
Psmg4	0.15	1.00000000	mice
Xpo1	0.23	1.00000000	mice
Orai1	0.22	1.00000000	mice
Naa20	0.22	1.00000000	mice
Ndufb3	0.14	1.00000000	mice
Cers6	0.21	1.00000000	mice
Ppp2r5c	-0.19	1.00000000	mice
Sqstm1	0.28	1.00000000	mice
Zfp369	-0.14	1.00000000	mice
Sh3pxd2a	-0.12	1.00000000	mice
Arl6ip5	0.20	1.00000000	mice
Lman1	0.12	1.00000000	mice
Pde4a	0.13	1.00000000	mice
Unkl	-0.20	1.00000000	mice
Plek	0.11	1.00000000	mice
Rnf38	-0.33	1.00000000	mice
Pias2	-0.29	1.00000000	mice
Rinl	0.21	1.00000000	mice
Dis3l2	0.22	1.00000000	mice
Rheb	0.12	1.00000000	mice
Pcbp2	-0.20	1.00000000	mice
Snrpd1	0.15	1.00000000	mice
Elmo2	0.12	1.00000000	mice
Bcl2	-0.21	1.00000000	mice
Cfdp1	0.13	1.00000000	mice
Crebzf	-0.21	1.00000000	mice
Ppm1a	-0.21	1.00000000	mice
Scfd2	-0.23	1.00000000	mice
Ptk2b	-0.18	1.00000000	mice
Sos2	-0.33	1.00000000	mice

Cul2	-0.28	1.00000000	mice
Sf1	-0.17	1.00000000	mice
Simc1	-0.14	1.00000000	mice
Nacc1	0.11	1.00000000	mice
Ankrd10	-0.21	1.00000000	mice
Cdip1	0.17	1.00000000	mice
Spata6	-0.17	1.00000000	mice
Arid1a	0.16	1.00000000	mice
Dnajb11	0.17	1.00000000	mice
Sytl3	0.26	1.00000000	mice
Utrn	-0.17	1.00000000	mice
Cnbp	-0.17	1.00000000	mice
Galnt6	-0.23	1.00000000	mice
Tmc6	-0.18	1.00000000	mice
Naa38	0.10	1.00000000	mice
Ccdc124	0.11	1.00000000	mice
Lgals8	0.10	1.00000000	mice
Usp22	-0.18	1.00000000	mice
Gadd45g	0.16	1.00000000	mice
Gramd3	-0.23	1.00000000	mice
Drap1	0.15	1.00000000	mice
Bend4	-0.23	1.00000000	mice
Rassf1	-0.29	1.00000000	mice
Acer2	-0.16	1.00000000	mice
Ube2l3	0.18	1.00000000	mice
St8sia4	-0.27	1.00000000	mice
Phf3	-0.20	1.00000000	mice
Tpi1	0.10	1.00000000	mice
Rasa2	-0.25	1.00000000	mice
Lnpk	-0.17	1.00000000	mice
Eif2s1	0.16	1.00000000	mice
Cnn2	0.15	1.00000000	mice
Smc4	-0.22	1.00000000	mice
D8Ertd738e	-0.20	1.00000000	mice
Top2b	-0.23	1.00000000	mice
Ubald1	-0.15	1.00000000	mice
Zfp101	-0.16	1.00000000	mice
Tex2	-0.16	1.00000000	mice
Capza1	0.16	1.00000000	mice
Rap1a	0.13	1.00000000	mice
Hexim1	-0.16	1.00000000	mice
Dennd5a	0.27	1.00000000	mice
Tut4	-0.12	1.00000000	mice
Psmc1	0.10	1.00000000	mice
Nptn	-0.17	1.00000000	mice
Plaa	-0.20	1.00000000	mice

Polk	0.13	1.00000000	mice
Il10rb	0.12	1.00000000	mice
Hnrnph2	-0.21	1.00000000	mice
Umad1	0.16	1.00000000	mice
Kif1b	-0.14	1.00000000	mice
Wdr45	-0.11	1.00000000	mice
H2afv	-0.18	1.00000000	mice
Slc41a2	-0.48	1.00000000	mice
Aurkaip1	0.11	1.00000000	mice
Gpr160	0.09	1.00000000	mice
Ddi2	-0.26	1.00000000	mice
Colgalt1	0.08	1.00000000	mice
Lonp2	-0.22	1.00000000	mice
Abcb1b	-0.27	1.00000000	mice
Atp6v0d1	-0.19	1.00000000	mice
Ttc14	-0.23	1.00000000	mice
Polr2l	0.08	1.00000000	mice
Cdc27	0.16	1.00000000	mice
Arid4a	-0.21	1.00000000	mice
Trpm7	-0.14	1.00000000	mice
Il18r1	0.14	1.00000000	mice
Itga6	-0.26	1.00000000	mice
Dazap2	-0.19	1.00000000	mice
Cd37	0.12	1.00000000	mice
Clk1	-0.11	1.00000000	mice
Lsm6	0.14	1.00000000	mice
Lcp2	0.19	1.00000000	mice
Rab33b	-0.16	1.00000000	mice
Stk4	-0.19	1.00000000	mice
Ubap2	-0.20	1.00000000	mice
Polr3b	-0.22	1.00000000	mice
Srpk2	-0.22	1.00000000	mice
Sf3b1	0.14	1.00000000	mice
Atg4b	-0.20	1.00000000	mice
H2-Aa	0.04	1.00000000	mice
Gimap3	-0.19	1.00000000	mice
Trmt1l	0.11	1.00000000	mice
Tpm3-rs7	0.16	1.00000000	mice
1810037I17Rik	0.20	1.00000000	mice
Usp53	-0.15	1.00000000	mice
Phf1	-0.15	1.00000000	mice
Lrp10	0.12	1.00000000	mice
Sacm1l	-0.32	1.00000000	mice
Fbxo11	0.29	1.00000000	mice
Elk3	0.12	1.00000000	mice
Plin2	0.13	1.00000000	mice

Ppp1cc	-0.18	1.00000000	mice
Bcor	0.17	1.00000000	mice
Dennd1b	0.22	1.00000000	mice
1110008P14Rik	0.12	1.00000000	mice
Tmprss6	0.09	1.00000000	mice
Dap3	-0.18	1.00000000	mice
Ndufb2	0.16	1.00000000	mice
Itgal	0.21	1.00000000	mice
Rhot1	-0.20	1.00000000	mice
Raet1e	0.21	1.00000000	mice
Yeats4	-0.14	1.00000000	mice
Srsf7	0.19	1.00000000	mice
Rabgef1	0.11	1.00000000	mice
Nktr	-0.19	1.00000000	mice
Rab37	0.09	1.00000000	mice
Vps13d	-0.23	1.00000000	mice
Ndrg1	-0.15	1.00000000	mice
Cd28	-0.17	1.00000000	mice
Atg4a	0.33	1.00000000	mice
Hpf1	0.13	1.00000000	mice
Sptan1	-0.16	1.00000000	mice
Rnf19a	-0.20	1.00000000	mice
S100a8	-0.04	1.00000000	mice
Setd5	0.19	1.00000000	mice
Tle1	-0.14	1.00000000	mice
Rnpep	0.14	1.00000000	mice
Arnt	-0.18	1.00000000	mice
Fto	-0.24	1.00000000	mice
Slc22a5	-0.12	1.00000000	mice
Ppp3cc	0.15	1.00000000	mice
Pik3cd	-0.28	1.00000000	mice
Syngr2	0.14	1.00000000	mice
Slc35b2	0.10	1.00000000	mice
Znhit1	0.09	1.00000000	mice
Abce1	0.13	1.00000000	mice
Fam53a	-0.15	1.00000000	mice
Prkcsh	0.08	1.00000000	mice
Prdx5	0.18	1.00000000	mice
Rlf	-0.18	1.00000000	mice
Aopep	0.22	1.00000000	mice
Cep120	-0.25	1.00000000	mice
Atp5d	0.18	1.00000000	mice
Hnrnpab	0.17	1.00000000	mice
Nucb1	0.16	1.00000000	mice
Ccdc85b	-0.12	1.00000000	mice
Wipi2	-0.18	1.00000000	mice

Smarca4	0.14	1.00000000	mice
Uhrf1bp1l	0.16	1.00000000	mice
Kat6a	0.16	1.00000000	mice
Rybp	-0.20	1.00000000	mice
Nfatc3	0.21	1.00000000	mice
Sorl1	-0.28	1.00000000	mice
Kmt2c	-0.18	1.00000000	mice
Rnf4	-0.22	1.00000000	mice
Tmed5	0.14	1.00000000	mice
Taf10	0.15	1.00000000	mice
B930036N10Rik	0.13	1.00000000	mice
Ndor1	-0.17	1.00000000	mice
Tm2d3	0.11	1.00000000	mice
Sesn3	-0.09	1.00000000	mice
Ssna1	-0.17	1.00000000	mice
Eif4g3	0.15	1.00000000	mice
Usp50	0.08	1.00000000	mice
Stat5b	-0.31	1.00000000	mice
Rapgef4	-0.15	1.00000000	mice
Polr2f	0.13	1.00000000	mice
Slirp	0.11	1.00000000	mice
Cnst	-0.20	1.00000000	mice
Dnajb4	0.11	1.00000000	mice
Dusp16	0.22	1.00000000	mice
Pou2f1	0.19	1.00000000	mice
Rab10	0.16	1.00000000	mice
Xylt1	-0.41	1.00000000	mice
Taldo1	0.11	1.00000000	mice
Zfp869	-0.19	1.00000000	mice
Gabpb2	-0.23	1.00000000	mice
Trio	0.17	1.00000000	mice
Acad9	-0.12	1.00000000	mice
Snrpg	0.15	1.00000000	mice
Krcc1	0.17	1.00000000	mice
Exoc3	-0.20	1.00000000	mice
Nipbl	-0.15	1.00000000	mice
Plekhm3	-0.17	1.00000000	mice
Serpinb6b	0.27	1.00000000	mice
Rngtt	-0.18	1.00000000	mice
Pnpla2	0.14	1.00000000	mice
Thoc1	-0.19	1.00000000	mice
Blm	0.12	1.00000000	mice
Lin54	0.29	1.00000000	mice
Dtl	-0.20	1.00000000	mice
Cd3d	-0.16	1.00000000	mice
Tpm3	0.17	1.00000000	mice

Dapk2	0.20	1.00000000	mice
Cks2	0.13	1.00000000	mice
Adprhl2	-0.14	1.00000000	mice
Klri2	0.20	1.00000000	mice
Pitpna	-0.23	1.00000000	mice
Ccsap	-0.11	1.00000000	mice
Ccng2	-0.17	1.00000000	mice
Tpk1	0.16	1.00000000	mice
Ctse	-0.21	1.00000000	mice
Pum1	0.16	1.00000000	mice
Gimap7	0.20	1.00000000	mice
Cog2	-0.15	1.00000000	mice
Usp49	-0.18	1.00000000	mice
Ier3ip1	0.10	1.00000000	mice
Psmd2	0.14	1.00000000	mice
Rnasek	0.17	1.00000000	mice
Pfdn1	0.09	1.00000000	mice
Fam49b	0.29	1.00000000	mice
Sh2d1a	0.16	1.00000000	mice
Atpif1	0.10	1.00000000	mice
Tbx21	0.13	1.00000000	mice
Ddost	0.16	1.00000000	mice
Tent4a	0.10	1.00000000	mice
Chmp5	0.12	1.00000000	mice
Ifnar1	0.18	1.00000000	mice
Litaf	0.14	1.00000000	mice
Calm3	0.13	1.00000000	mice
Psip1	-0.18	1.00000000	mice
Slc2a1	-0.21	1.00000000	mice
Grk5	0.19	1.00000000	mice
Dynll2	0.11	1.00000000	mice
Snx3	0.13	1.00000000	mice
Tjap1	0.14	1.00000000	mice
Numb	-0.20	1.00000000	mice
Phf6	0.22	1.00000000	mice
C2cd5	-0.23	1.00000000	mice
Gnl1	0.11	1.00000000	mice
Acadl	0.11	1.00000000	mice
Aak1	-0.24	1.00000000	mice
Aven	-0.23	1.00000000	mice
Hspa13	0.09	1.00000000	mice
Dhrs1	0.09	1.00000000	mice
Psen1	-0.20	1.00000000	mice
Id2	0.23	1.00000000	mice
Itgb1bp1	0.09	1.00000000	mice
Prmt1	0.12	1.00000000	mice

Slc3a2	0.13	1.00000000	mice
Braf	-0.14	1.00000000	mice
Dhx9	-0.17	1.00000000	mice
Ikzf1	-0.16	1.00000000	mice
Trat1	-0.15	1.00000000	mice
Eloc	0.13	1.00000000	mice
Hint1	0.17	1.00000000	mice
Txnl4a	0.11	1.00000000	mice
Tmx4	-0.25	1.00000000	mice
Ostc	0.15	1.00000000	mice
Itsn2	-0.17	1.00000000	mice
Tmbim4	0.13	1.00000000	mice
Gas7	0.21	1.00000000	mice
Lgals3	0.13	1.00000000	mice
Crlf3	-0.26	1.00000000	mice
Zfand3	0.16	1.00000000	mice
Sh3bgrl3	0.15	1.00000000	mice
Furin	0.28	1.00000000	mice
Rpp25l	0.08	1.00000000	mice
Cmas	-0.16	1.00000000	mice
Tdp2	-0.21	1.00000000	mice
Igf2r	0.11	1.00000000	mice
Ints7	-0.24	1.00000000	mice
Kpna6	0.08	1.00000000	mice
Jun	0.18	1.00000000	mice
Zbtb20	-0.18	1.00000000	mice
Xdh	0.18	1.00000000	mice
Pdia3	0.15	1.00000000	mice
Glcci1	0.29	1.00000000	mice
Cotl1	-0.18	1.00000000	mice
Eif1ax	0.10	1.00000000	mice
Eya1	0.33	1.00000000	mice
Cpd	0.11	1.00000000	mice
Tmem184b	0.13	1.00000000	mice
Parvg	0.08	1.00000000	mice
2410006H16Rik	0.16	1.00000000	mice
Rbm38	-0.15	1.00000000	mice
Chmp4b	0.23	1.00000000	mice
Cox7a2l	-0.18	1.00000000	mice
Dynlrb1	0.08	1.00000000	mice
Nmnat2	-0.23	1.00000000	mice
Pip5k1c	-0.17	1.00000000	mice
Plcg1	-0.12	1.00000000	mice
Srsf11	-0.12	1.00000000	mice
Cep68	0.12	1.00000000	mice
Rhoh	-0.21	1.00000000	mice

Rbm15	0.08	1.00000000	mice
Arih1	-0.10	1.00000000	mice
Rapgef2	-0.17	1.00000000	mice
Zfp52	0.17	1.00000000	mice
Slc25a39	0.08	1.00000000	mice
Xrcc5	-0.15	1.00000000	mice
Tspyl2	-0.10	1.00000000	mice
Arpc5	0.14	1.00000000	mice
Ppp2r5a	-0.18	1.00000000	mice
Snhg8	-0.15	1.00000000	mice
Timm13	0.09	1.00000000	mice
Rnf8	-0.18	1.00000000	mice
Afg1l	0.09	1.00000000	mice
Ppt1	0.10	1.00000000	mice
Tle5	-0.15	1.00000000	mice
Atp9b	-0.20	1.00000000	mice
Clta	-0.19	1.00000000	mice
Srsf9	0.12	1.00000000	mice
P2rx4	-0.14	1.00000000	mice
Tiparp	-0.23	1.00000000	mice
Slc25a12	0.13	1.00000000	mice
Arfgap1	-0.18	1.00000000	mice
Osbpl11	-0.23	1.00000000	mice
Swi5	0.10	1.00000000	mice
Fam241a	-0.07	1.00000000	mice
Tnks2	-0.17	1.00000000	mice
Elk4	-0.16	1.00000000	mice
Rpn2	0.15	1.00000000	mice
Prf1	0.25	1.00000000	mice
Wdr47	-0.19	1.00000000	mice
B4galnt1	-0.19	1.00000000	mice
Smim3	0.11	1.00000000	mice
Gdpd5	0.08	1.00000000	mice
Arhgap18	0.06	1.00000000	mice
Elovl5	0.21	1.00000000	mice
Ranbp10	-0.25	1.00000000	mice
Sfxn1	0.11	1.00000000	mice
Nrbf2	0.13	1.00000000	mice
Tmem167	0.14	1.00000000	mice
Sertad2	0.20	1.00000000	mice
Kdm7a	-0.22	1.00000000	mice
Prdx1	0.20	1.00000000	mice
Rac1	-0.15	1.00000000	mice
Eogt	-0.18	1.00000000	mice
Ano6	-0.25	1.00000000	mice
Lrrc58	-0.16	1.00000000	mice

Lipa	0.12	1.00000000	mice
Fbxo42	-0.22	1.00000000	mice
Zpr1	-0.15	1.00000000	mice
Fig4	-0.21	1.00000000	mice
Kdm5a	-0.19	1.00000000	mice
Stk40	0.15	1.00000000	mice
Ctsd	0.20	1.00000000	mice
Zdhhc15	0.03	1.00000000	mice
1810041H14Rik	-0.12	1.00000000	mice
Rttn	-0.23	1.00000000	mice
Prkag1	-0.19	1.00000000	mice
Fam76a	-0.14	1.00000000	mice
Pex2	0.10	1.00000000	mice
Sorbs1	-0.33	1.00000000	mice
Tfip11	0.12	1.00000000	mice
Zfp398	-0.18	1.00000000	mice
Cnot6l	-0.08	1.00000000	mice
Llph	0.15	1.00000000	mice
Ppid	0.12	1.00000000	mice
Pygl	-0.13	1.00000000	mice
Hps4	0.09	1.00000000	mice
Sept6	-0.19	1.00000000	mice
Nme1	0.23	1.00000000	mice
Gnai2	0.16	1.00000000	mice
Noct	0.11	1.00000000	mice
Rab5c	0.11	1.00000000	mice
Tle3	-0.13	1.00000000	mice
Srgap2	0.17	1.00000000	mice
Psmb5	0.11	1.00000000	mice
Klf16	-0.11	1.00000000	mice
Ifitm2	0.10	1.00000000	mice
Cma1	0.16	1.00000000	mice
Fam78b	0.12	1.00000000	mice
Fbxo46	0.05	1.00000000	mice
Trim35	-0.15	1.00000000	mice
Cnnm3	-0.15	1.00000000	mice
Ublcp1	0.07	1.00000000	mice
Ccdc125	-0.12	1.00000000	mice
Pin4	0.09	1.00000000	mice
Gse1	0.10	1.00000000	mice
Zranb2	0.11	1.00000000	mice
Elmo1	-0.13	1.00000000	mice
Bcas3	-0.26	1.00000000	mice
Setx	-0.17	1.00000000	mice
Gnb2	0.14	1.00000000	mice
Cmpk1	0.11	1.00000000	mice

Gnptg	0.09	1.00000000	mice
Cd14	0.02	1.00000000	mice
Pnp	0.12	1.00000000	mice
Gyg	0.07	1.00000000	mice
Pus1	0.11	1.00000000	mice
Ncoa3	0.15	1.00000000	mice
Nt5c2	-0.20	1.00000000	mice
Pam16	0.11	1.00000000	mice
Syne2	0.16	1.00000000	mice
Ptpn7	0.10	1.00000000	mice
Stx6	0.20	1.00000000	mice
Ralgps2	-0.14	1.00000000	mice
Dnah8	-0.20	1.00000000	mice
Tceal9	0.08	1.00000000	mice
Dync1h1	-0.19	1.00000000	mice
Eci1	0.05	1.00000000	mice
Syvn1	0.11	1.00000000	mice
Dlst	-0.18	1.00000000	mice
Sec23a	-0.18	1.00000000	mice
Eed	0.17	1.00000000	mice
Dcaf6	-0.22	1.00000000	mice
Coil	-0.15	1.00000000	mice
Atp2b1	-0.16	1.00000000	mice
Grcc10	0.19	1.00000000	mice
Ghitm	0.14	1.00000000	mice
Zfp110	-0.16	1.00000000	mice
Cog6	-0.19	1.00000000	mice
Smpdl3a	0.06	1.00000000	mice
Zfp827	0.10	1.00000000	mice
Cd3e	-0.12	1.00000000	mice
Ccdc25	0.14	1.00000000	mice
Bax	0.11	1.00000000	mice
Cbx4	-0.14	1.00000000	mice
Peli1	0.17	1.00000000	mice
Snhg6	0.09	1.00000000	mice
Lat	-0.13	1.00000000	mice
Arhgap45	-0.15	1.00000000	mice
Hnrnpa3	0.14	1.00000000	mice
Bola2	0.14	1.00000000	mice
Ctsz	0.11	1.00000000	mice
Sarnp	0.15	1.00000000	mice
Pvt1	0.17	1.00000000	mice
Nop53	-0.16	1.00000000	mice
Eif3j2	0.11	1.00000000	mice
Sh3bp1	0.06	1.00000000	mice
Abi1	-0.18	1.00000000	mice

Cdc14b	-0.22	1.00000000	mice
Ly9	0.07	1.00000000	mice
Pstpip1	0.09	1.00000000	mice
Igfbp4	-0.23	1.00000000	mice
Cry2	-0.17	1.00000000	mice
Fyco1	0.15	1.00000000	mice
Vasp	0.14	1.00000000	mice
Epc2	-0.15	1.00000000	mice
Commd4	0.09	1.00000000	mice
Stard3nl	0.07	1.00000000	mice
Snrpc	-0.14	1.00000000	mice
Znfx1	0.14	1.00000000	mice
Acadvl	0.07	1.00000000	mice
Ksr1	0.16	1.00000000	mice
Cpsf2	0.11	1.00000000	mice
Rhoc	0.07	1.00000000	mice
Arhgef11	-0.16	1.00000000	mice
Ehbp1l1	0.10	1.00000000	mice
Fxn	-0.14	1.00000000	mice
Grk4	-0.14	1.00000000	mice
Smad2	-0.19	1.00000000	mice
Camk2d	-0.21	1.00000000	mice
Rock1	0.13	1.00000000	mice
Herc4	-0.15	1.00000000	mice
Hps3	-0.14	1.00000000	mice
Actr2	0.14	1.00000000	mice
Stx4a	0.07	1.00000000	mice
Zmpste24	-0.19	1.00000000	mice
Lmnb1	0.22	1.00000000	mice
Cyth4	0.16	1.00000000	mice
Cxcr4	-0.14	1.00000000	mice
Pink1	-0.11	1.00000000	mice
Sec31a	-0.19	1.00000000	mice
Cdc25b	-0.12	1.00000000	mice
Gna13	-0.14	1.00000000	mice
Ppp2r5e	-0.16	1.00000000	mice
Inpp1	0.15	1.00000000	mice
Cnppd1	-0.15	1.00000000	mice
Chfr	-0.20	1.00000000	mice
Pfdn4	0.11	1.00000000	mice
Ndufb4	0.09	1.00000000	mice
Ash1l	0.12	1.00000000	mice
Ctsc	0.13	1.00000000	mice
Helz	-0.16	1.00000000	mice
Zer1	-0.13	1.00000000	mice
Sh3bgrl	0.14	1.00000000	mice

Ppm1k	0.06	1.00000000	mice
Lyn	0.11	1.00000000	mice
Ssr3	0.10	1.00000000	mice
Pi4ka	-0.16	1.00000000	mice
Zfp62	0.16	1.00000000	mice
Cuedc2	0.07	1.00000000	mice
Camsap2	-0.17	1.00000000	mice
Ldah	0.11	1.00000000	mice
Txndc11	-0.21	1.00000000	mice
Frg1	0.14	1.00000000	mice
Nlk	-0.17	1.00000000	mice
Cib1	0.10	1.00000000	mice
Usp3	-0.23	1.00000000	mice
Sept9	0.08	1.00000000	mice
Nipa2	0.13	1.00000000	mice
Pigm	-0.10	1.00000000	mice
Usp15	-0.15	1.00000000	mice
Wipf1	0.13	1.00000000	mice
Ocel1	-0.15	1.00000000	mice
Usp28	-0.16	1.00000000	mice
Zfand6	-0.21	1.00000000	mice
Bbs9	-0.14	1.00000000	mice
Snx10	0.05	1.00000000	mice
Kremen1	-0.12	1.00000000	mice
Sesn1	-0.24	1.00000000	mice
Rcbtb1	-0.10	1.00000000	mice
Tmcc1	-0.20	1.00000000	mice
Crtc3	-0.20	1.00000000	mice
Psenen	0.11	1.00000000	mice
Rnf220	-0.14	1.00000000	mice
Psmc5	0.09	1.00000000	mice
4833420G17Rik	-0.16	1.00000000	mice
Os9	0.11	1.00000000	mice
H2afy	0.11	1.00000000	mice
Senp7	0.10	1.00000000	mice
Axin1	-0.15	1.00000000	mice
Flnb	-0.18	1.00000000	mice
Pxk	-0.19	1.00000000	mice
H2afz	0.17	1.00000000	mice
Ssbp1	0.10	1.00000000	mice
Foxo3	-0.18	1.00000000	mice
Tmbim6	0.14	1.00000000	mice
Usp47	-0.15	1.00000000	mice
Nono	0.12	1.00000000	mice
S100a10	0.12	1.00000000	mice
Trib2	-0.08	1.00000000	mice

Cnp	0.14	1.00000000	mice
Casc4	-0.17	1.00000000	mice
Pgap2	0.11	1.00000000	mice
Dhrs11	-0.14	1.00000000	mice
Ddx18	0.13	1.00000000	mice
Cd164	0.09	1.00000000	mice
Nt5c3	0.11	1.00000000	mice
Asb13	0.10	1.00000000	mice
Irf2bpl	-0.22	1.00000000	mice
Rara	0.10	1.00000000	mice
Cdc42se1	-0.17	1.00000000	mice
Acox1	-0.15	1.00000000	mice
Eif3i	0.13	1.00000000	mice
Dek	0.14	1.00000000	mice
Zfas1	0.14	1.00000000	mice
Abhd13	-0.10	1.00000000	mice
Tapt1	-0.23	1.00000000	mice
Rcc2	-0.22	1.00000000	mice
Chka	-0.16	1.00000000	mice
Ctnna1	0.04	1.00000000	mice
Gab2	-0.39	1.00000000	mice
Slc12a6	-0.26	1.00000000	mice
Swap70	0.13	1.00000000	mice
Baz2a	0.10	1.00000000	mice
Ebna1bp2	0.09	1.00000000	mice
Tcf20	-0.18	1.00000000	mice
Cul3	-0.12	1.00000000	mice
B4galt7	-0.15	1.00000000	mice
Camta1	0.13	1.00000000	mice
Pcnx	0.19	1.00000000	mice
Adss	-0.15	1.00000000	mice
Ero1lb	-0.17	1.00000000	mice
Arpp19	0.13	1.00000000	mice
Tcf4	0.17	1.00000000	mice
Hsf1	-0.11	1.00000000	mice
Pafah1b1	0.12	1.00000000	mice
Fam91a1	-0.16	1.00000000	mice
Zfp950	0.06	1.00000000	mice
Slain1	-0.15	1.00000000	mice
Atox1	0.13	1.00000000	mice
Phip	0.16	1.00000000	mice
Zc3hav1	-0.06	1.00000000	mice
Inpp5b	0.08	1.00000000	mice
Ppia	-0.08	1.00000000	mice
Hmgn5	0.08	1.00000000	mice
0610010K14Rik	0.09	1.00000000	mice

Fth1	-0.10	1.00000000	mice
Ppp4r3b	0.12	1.00000000	mice
Crbn	-0.13	1.00000000	mice
Rnf19b	0.12	1.00000000	mice
Atp5f1	0.14	1.00000000	mice
Snx9	0.11	1.00000000	mice
Luc7l2	-0.11	1.00000000	mice
Stk38l	0.23	1.00000000	mice
Zfp655	-0.16	1.00000000	mice
Wtap	-0.14	1.00000000	mice
Pgap1	-0.10	1.00000000	mice
Sgip1	-0.14	1.00000000	mice
Iars	0.09	1.00000000	mice
Plxdc2	0.14	1.00000000	mice
2510002D24Rik	-0.10	1.00000000	mice
Rsrp1	0.14	1.00000000	mice
Lrrc47	-0.10	1.00000000	mice
Trac	-0.11	1.00000000	mice
Sh3kbp1	-0.14	1.00000000	mice
Rere	-0.20	1.00000000	mice
Nckap1l	-0.16	1.00000000	mice
Azi2	0.16	1.00000000	mice
Ahi1	0.13	1.00000000	mice
Lrrfip2	0.11	1.00000000	mice
Ptms	0.10	1.00000000	mice
Khdc4	-0.17	1.00000000	mice
Was	0.09	1.00000000	mice
Iqcb1	-0.11	1.00000000	mice
Far1os	0.08	1.00000000	mice
Ncapd3	-0.18	1.00000000	mice
Sec63	-0.13	1.00000000	mice
Tm2d2	-0.11	1.00000000	mice
Eif4a1	0.17	1.00000000	mice
Washc4	-0.19	1.00000000	mice
Ube2e3	0.15	1.00000000	mice
Glmp	0.08	1.00000000	mice
Atg16l1	-0.14	1.00000000	mice
Paip2	-0.15	1.00000000	mice
Arl4d	0.15	1.00000000	mice
Plcg2	0.11	1.00000000	mice
Fam118a	-0.08	1.00000000	mice
Rhof	0.13	1.00000000	mice
Cars	0.10	1.00000000	mice
Ncoa7	0.09	1.00000000	mice
Akna	0.10	1.00000000	mice
Cyc1	0.10	1.00000000	mice

Pon2	-0.21	1.00000000	mice
Plekha2	-0.25	1.00000000	mice
Cct8	0.14	1.00000000	mice
Nop58	0.16	1.00000000	mice
Etf1	-0.12	1.00000000	mice
Vrk1	-0.12	1.00000000	mice
Hcls1	0.10	1.00000000	mice
Ucp2	-0.17	1.00000000	mice
Rab3gap2	-0.10	1.00000000	mice
Hectd1	0.18	1.00000000	mice
Spsb1	-0.15	1.00000000	mice
Sub1	0.19	1.00000000	mice
Cbx5	0.07	1.00000000	mice
Sec11c	0.10	1.00000000	mice
Prkca	-0.13	1.00000000	mice
Ppp1r14b	0.15	1.00000000	mice
Brap	-0.15	1.00000000	mice
Pcnx3	-0.12	1.00000000	mice
Laptm4a	0.14	1.00000000	mice
Gng10	0.08	1.00000000	mice
Bola3	0.13	1.00000000	mice
Fnip2	0.07	1.00000000	mice
Fgfr2	0.15	1.00000000	mice
Elovl7	-0.17	1.00000000	mice
Zfp212	-0.12	1.00000000	mice
Oprm1	0.15	1.00000000	mice
Kmt2a	0.14	1.00000000	mice
Ap2s1	0.10	1.00000000	mice
Grk6	-0.19	1.00000000	mice
Sash3	0.13	1.00000000	mice
Angpt1	-0.31	1.00000000	mice
Fam162a	0.10	1.00000000	mice
Cdk6	0.11	1.00000000	mice
Zfp518a	-0.11	1.00000000	mice
Atp6ap2	-0.13	1.00000000	mice
Eif2ak1	-0.19	1.00000000	mice
Irf2bp2	0.11	1.00000000	mice
Klhdc4	0.08	1.00000000	mice
Wbp11	-0.16	1.00000000	mice
Sppl2a	0.15	1.00000000	mice
Gdpd3	-0.14	1.00000000	mice
Ddb2	-0.10	1.00000000	mice
Traf2	0.10	1.00000000	mice
Sdf2l1	0.05	1.00000000	mice
Ing3	-0.15	1.00000000	mice
Birc2	0.13	1.00000000	mice

Atp5a1	-0.15	1.00000000	mice
Lrpap1	0.08	1.00000000	mice
Pus7	-0.13	1.00000000	mice
Clca3a1	0.10	1.00000000	mice
Crot	-0.20	1.00000000	mice
Slc25a40	-0.12	1.00000000	mice
Uhrf2	-0.10	1.00000000	mice
Gabarap	0.14	1.00000000	mice
Tmed3	0.05	1.00000000	mice
St6galnac6	0.03	1.00000000	mice
Cdk5rap3	0.06	1.00000000	mice
Nin	-0.19	1.00000000	mice
Rnf138	-0.12	1.00000000	mice
Atp6v1d	0.09	1.00000000	mice
Ift80	-0.12	1.00000000	mice
Tnfaip8	0.14	1.00000000	mice
Lamp1	0.13	1.00000000	mice
Dpy19l1	0.06	1.00000000	mice
Apbb1ip	0.21	1.00000000	mice
Sfxn3	-0.13	1.00000000	mice
St3gal6	0.06	1.00000000	mice
Tatdn1	0.03	1.00000000	mice
Nedd9	-0.16	1.00000000	mice
Snw1	0.11	1.00000000	mice
Ogdh	-0.19	1.00000000	mice
Trim36	-0.17	1.00000000	mice
Zfp410	-0.20	1.00000000	mice
Arpc1a	-0.16	1.00000000	mice
Arid2	0.14	1.00000000	mice
Tasor	0.16	1.00000000	mice
Exoc6b	-0.12	1.00000000	mice
Rcsd1	0.09	1.00000000	mice
Cgrrf1	0.06	1.00000000	mice
Pde5a	0.15	1.00000000	mice
Wdr48	-0.14	1.00000000	mice
Snrpd3	0.11	1.00000000	mice
Ust	-0.31	1.00000000	mice
Snrpa1	0.11	1.00000000	mice
Adgre5	-0.12	1.00000000	mice
Tob1	-0.17	1.00000000	mice
Ssb	0.10	1.00000000	mice
Adipor1	-0.15	1.00000000	mice
Ttc19	-0.15	1.00000000	mice
Stard5	0.12	1.00000000	mice
Ggta1	-0.28	1.00000000	mice
Cdkal1	0.17	1.00000000	mice

Rbm6	-0.20	1.00000000	mice
Tgif2	0.12	1.00000000	mice
Cstb	0.14	1.00000000	mice
Coq5	-0.13	1.00000000	mice
BC004004	-0.15	1.00000000	mice
Gatad2a	0.13	1.00000000	mice
Polr3k	0.09	1.00000000	mice
Zfp36l1	0.25	1.00000000	mice
Pak1	0.21	1.00000000	mice
Wrnip1	0.07	1.00000000	mice
Kif21b	-0.15	1.00000000	mice
Hsp90aa1	0.17	1.00000000	mice
Appbp2	-0.16	1.00000000	mice
Tmpo	0.10	1.00000000	mice
Akap11	-0.14	1.00000000	mice
Apobec3	0.09	1.00000000	mice
Nfyc	-0.16	1.00000000	mice
Ass1	-0.13	1.00000000	mice
Cox4i1	0.11	1.00000000	mice
Tmed9	0.11	1.00000000	mice
Fmc1	0.06	1.00000000	mice
Tspyl1	-0.14	1.00000000	mice
Dnajc5	0.06	1.00000000	mice
Setd4	-0.11	1.00000000	mice
Ptpn18	0.18	1.00000000	mice
Ebp	0.09	1.00000000	mice
Gtf2b	0.14	1.00000000	mice
Aifm1	0.07	1.00000000	mice
Fbxw11	0.15	1.00000000	mice
Cep350	0.09	1.00000000	mice
Rrm1	-0.12	1.00000000	mice
Capns1	0.09	1.00000000	mice
Tra2a	-0.13	1.00000000	mice
Chpt1	0.12	1.00000000	mice
Ptpn2	0.11	1.00000000	mice
Arhgap27os3	-0.09	1.00000000	mice
Pabpc1	-0.10	1.00000000	mice
Rpa3	0.05	1.00000000	mice
Letm2	-0.08	1.00000000	mice
Sdhb	0.11	1.00000000	mice
Keap1	0.03	1.00000000	mice
Hsd17b12	-0.15	1.00000000	mice
Ece1	0.14	1.00000000	mice
Crim1	0.04	1.00000000	mice
Hbp1	-0.15	1.00000000	mice
Itpk1	-0.19	1.00000000	mice

Nsmce2	-0.15	1.00000000	mice
Efr3a	-0.14	1.00000000	mice
Dhrs7	0.09	1.00000000	mice
Tcf12	-0.18	1.00000000	mice
Nhlrc2	-0.19	1.00000000	mice
Ndufv2	0.13	1.00000000	mice
Gpatch2	0.10	1.00000000	mice
Ap1ar	-0.10	1.00000000	mice
Prdm11	-0.15	1.00000000	mice
H2-Q10	0.09	1.00000000	mice
Eya2	-0.30	1.00000000	mice
Cog5	-0.20	1.00000000	mice
Snx2	0.14	1.00000000	mice
Trim23	-0.11	1.00000000	mice
Cbx1	0.06	1.00000000	mice
Scml4	-0.14	1.00000000	mice
Ammecr1	0.14	1.00000000	mice
Tec	0.11	1.00000000	mice
Ndufa5	0.08	1.00000000	mice
Snrpa	0.08	1.00000000	mice
Itpr3	-0.18	1.00000000	mice
Klrb1a	0.06	1.00000000	mice
Cd226	0.14	1.00000000	mice
Tmem230	0.06	1.00000000	mice
Pdpr	-0.12	1.00000000	mice
Ankrd13c	-0.17	1.00000000	mice
Cops6	0.08	1.00000000	mice
Smc3	-0.13	1.00000000	mice
C87436	-0.10	1.00000000	mice
Gle1	-0.14	1.00000000	mice
Snrnp27	0.10	1.00000000	mice
Bcl9l	-0.17	1.00000000	mice
Wdr37	-0.17	1.00000000	mice
Smg1	-0.09	1.00000000	mice
Pprc1	-0.07	1.00000000	mice
Fermt3	0.10	1.00000000	mice
Cuedc1	0.06	1.00000000	mice
Prkd2	-0.20	1.00000000	mice
Npm1	-0.09	1.00000000	mice
Arhgap17	-0.22	1.00000000	mice
Aff1	0.15	1.00000000	mice
Dnaja1	0.16	1.00000000	mice
Ube2q1	0.09	1.00000000	mice
Trappc8	-0.15	1.00000000	mice
Atp5h	0.09	1.00000000	mice
Stx12	0.07	1.00000000	mice

Psmd4	0.13	1.00000000	mice
Ift140	-0.15	1.00000000	mice
Baz2b	-0.13	1.00000000	mice
Dhx32	-0.10	1.00000000	mice
Tomm34	-0.12	1.00000000	mice
Gprasp1	0.08	1.00000000	mice
Susd6	0.14	1.00000000	mice
Sun1	-0.10	1.00000000	mice
Brf1	-0.12	1.00000000	mice
Ago3	-0.14	1.00000000	mice
Dcaf17	-0.11	1.00000000	mice
Ndufc2	0.06	1.00000000	mice
Leng1	0.05	1.00000000	mice
Taf1	-0.16	1.00000000	mice
Itch	-0.15	1.00000000	mice
Slco3a1	0.33	1.00000000	mice
Rictor	-0.13	1.00000000	mice
Nr6a1	0.20	1.00000000	mice
Stx8	0.10	1.00000000	mice
Dennd4c	0.20	1.00000000	mice
Klhl12	-0.18	1.00000000	mice
Rab8a	0.13	1.00000000	mice
Dpm2	0.04	1.00000000	mice
Nol10	-0.17	1.00000000	mice
Irak4	0.08	1.00000000	mice
Rbpms	0.06	1.00000000	mice
Ube2n	0.13	1.00000000	mice
Hars	-0.12	1.00000000	mice
Cs	0.08	1.00000000	mice
Usf3	0.07	1.00000000	mice
Lrig2	-0.18	1.00000000	mice
Aacs	-0.12	1.00000000	mice
Immt	0.01	1.00000000	mice
Ppp1r11	0.09	1.00000000	mice
Tubgcp3	-0.11	1.00000000	mice
Josd2	0.06	1.00000000	mice
Tnfsf12	0.06	1.00000000	mice
Zfp942	-0.18	1.00000000	mice
C1galt1	0.11	1.00000000	mice
Tbcb	0.08	1.00000000	mice
Tapbpl	0.10	1.00000000	mice
Psmb4	0.10	1.00000000	mice
Fgf13	-0.20	1.00000000	mice
Ppp6r2	0.09	1.00000000	mice
Zrsr2	0.09	1.00000000	mice
Arid5a	-0.12	1.00000000	mice

Aida	0.11	1.00000000	mice
Nr2c2	-0.15	1.00000000	mice
Taf15	0.11	1.00000000	mice
Stmn1	-0.19	1.00000000	mice
Top2a	0.08	1.00000000	mice
Polr2a	-0.13	1.00000000	mice
Ddhd2	-0.17	1.00000000	mice
Csnk2b	0.11	1.00000000	mice
Hist1h1e	-0.21	1.00000000	mice
Cox18	0.06	1.00000000	mice
Acvr2a	-0.15	1.00000000	mice
Taf1b	-0.14	1.00000000	mice
Scmh1	-0.16	1.00000000	mice
Siah1a	-0.18	1.00000000	mice
Atxn7l1	-0.15	1.00000000	mice
Pitpnm2	-0.13	1.00000000	mice
Sav1	0.07	1.00000000	mice
Kctd9	0.07	1.00000000	mice
Dad1	0.15	1.00000000	mice
Smim15	0.07	1.00000000	mice
Casp7	0.07	1.00000000	mice
Cenpx	0.05	1.00000000	mice
Tatdn2	-0.13	1.00000000	mice
Gimap5	0.11	1.00000000	mice
Rfx3	-0.19	1.00000000	mice
Pop5	0.06	1.00000000	mice
Dguok	0.06	1.00000000	mice
Tnfaip8l2	0.07	1.00000000	mice
Guk1	-0.15	1.00000000	mice
9430038I01Rik	-0.14	1.00000000	mice
Rffl	0.10	1.00000000	mice
Arhgdib	-0.09	1.00000000	mice
Fndc3b	0.09	1.00000000	mice
Ndfip1	0.14	1.00000000	mice
Gtpbp1	-0.21	1.00000000	mice
Nsrp1	0.12	1.00000000	mice
Pglyrp1	0.07	1.00000000	mice
Egr1	0.18	1.00000000	mice
Vrk2	0.11	1.00000000	mice
Ergic3	0.06	1.00000000	mice
Ppp1r16b	0.08	1.00000000	mice
Trpv2	0.09	1.00000000	mice
Trbc2	-0.22	1.00000000	mice
Blcap	0.08	1.00000000	mice
Stx7	0.09	1.00000000	mice
Gpd1l	-0.09	1.00000000	mice

Higd1a	0.09	1.00000000	mice
Camkmt	0.26	1.00000000	mice
Cox14	-0.13	1.00000000	mice
Armcx3	0.07	1.00000000	mice
Cd2ap	-0.13	1.00000000	mice
Plekha5	-0.14	1.00000000	mice
Heatr3	-0.11	1.00000000	mice
Phf20	-0.16	1.00000000	mice
Ywhae	0.11	1.00000000	mice
Eif3e	-0.13	1.00000000	mice
Tbc1d10a	-0.09	1.00000000	mice
Hnrnpa2b1	0.11	1.00000000	mice
Vgll4	0.13	1.00000000	mice
Hsd17b10	0.05	1.00000000	mice
Klrc1	0.11	1.00000000	mice
Rasal3	-0.15	1.00000000	mice
Vps36	0.06	1.00000000	mice
Etfa	0.07	1.00000000	mice
Fam20b	0.02	1.00000000	mice
Gars	-0.13	1.00000000	mice
Tmem104	0.07	1.00000000	mice
Trerf1	0.03	1.00000000	mice
Erg28	0.08	1.00000000	mice
Skap2	0.11	1.00000000	mice
Washc5	-0.08	1.00000000	mice
Hibadh	-0.15	1.00000000	mice
Tinf2	0.08	1.00000000	mice
Pik3c2a	-0.09	1.00000000	mice
Arpc5l	0.07	1.00000000	mice
Aftph	0.16	1.00000000	mice
Polr3a	-0.09	1.00000000	mice
Rgs3	0.10	1.00000000	mice
Ints6	0.20	1.00000000	mice
Slc38a2	0.21	1.00000000	mice
Fam129b	-0.16	1.00000000	mice
Hmgb2	-0.14	1.00000000	mice
Abhd17a	0.09	1.00000000	mice
Zbtb1	0.17	1.00000000	mice
2300009A05Rik	-0.13	1.00000000	mice
Zswim8	0.09	1.00000000	mice
Adcy3	-0.12	1.00000000	mice
Tex264	-0.09	1.00000000	mice
Chkb	-0.17	1.00000000	mice
Abhd16a	0.06	1.00000000	mice
Rnf139	0.11	1.00000000	mice
Trak2	-0.16	1.00000000	mice

Fbxo33	-0.13	1.00000000	mice
H2afx	-0.12	1.00000000	mice
Cox20	0.07	1.00000000	mice
Kmt2e	-0.12	1.00000000	mice
Rap1gds1	0.23	1.00000000	mice
Neto2	-0.20	1.00000000	mice
N4bp2	-0.14	1.00000000	mice
Stx5a	-0.12	1.00000000	mice
Zfp207	0.07	1.00000000	mice
Stk25	-0.12	1.00000000	mice
Ube2h	-0.09	1.00000000	mice
Selplg	0.10	1.00000000	mice
Psma5	0.11	1.00000000	mice
Gng12	0.13	1.00000000	mice
1600020E01Rik	-0.10	1.00000000	mice
Gtf2ird2	-0.13	1.00000000	mice
Csnk1d	-0.14	1.00000000	mice
Hbs1l	0.17	1.00000000	mice
Zfp568	-0.16	1.00000000	mice
Gripap1	0.10	1.00000000	mice
Adprh	0.06	1.00000000	mice
Polr2k	0.06	1.00000000	mice
Elp4	-0.15	1.00000000	mice
Eomes	-0.01	1.00000000	mice
Rfc3	0.10	1.00000000	mice
Dtwd2	-0.08	1.00000000	mice
Zfp706	-0.15	1.00000000	mice
Caprin1	0.11	1.00000000	mice
Zfp868	-0.14	1.00000000	mice
Ugcg	0.15	1.00000000	mice
Cdk14	0.20	1.00000000	mice
Hnrnpll	0.10	1.00000000	mice
Tmem127	-0.12	1.00000000	mice
Psmd7	0.09	1.00000000	mice
Pdrg1	0.08	1.00000000	mice
Dhx29	-0.14	1.00000000	mice
Coa3	0.07	1.00000000	mice
Ssrp1	0.04	1.00000000	mice
Iws1	-0.18	1.00000000	mice
Huwe1	-0.09	1.00000000	mice
Dennd1c	0.06	1.00000000	mice
Pum3	0.08	1.00000000	mice
Cd200r4	0.02	1.00000000	mice
Fcho2	-0.13	1.00000000	mice
Arih2	0.17	1.00000000	mice
Tent4b	-0.17	1.00000000	mice

Banp	0.12	1.00000000	mice
Rrp1	-0.11	1.00000000	mice
Pde12	0.06	1.00000000	mice
Coa7	-0.08	1.00000000	mice
Ide	0.08	1.00000000	mice
Fam102a	-0.15	1.00000000	mice
Sfpq	-0.09	1.00000000	mice
Nup88	-0.13	1.00000000	mice
Spag7	0.04	1.00000000	mice
Ppp1r15b	0.06	1.00000000	mice
Emc2	0.07	1.00000000	mice
Ubn2	0.10	1.00000000	mice
Drg1	0.14	1.00000000	mice
Ubr1	-0.11	1.00000000	mice
Zfp664	-0.13	1.00000000	mice
Las1l	-0.16	1.00000000	mice
Ppp3cb	0.07	1.00000000	mice
Tspan13	-0.12	1.00000000	mice
Cyb5b	0.07	1.00000000	mice
Trappc13	0.07	1.00000000	mice
Fxr2	0.08	1.00000000	mice
Accs	-0.12	1.00000000	mice
Uap1	0.12	1.00000000	mice
Fancl	-0.18	1.00000000	mice
Gigyf2	0.10	1.00000000	mice
Acsf2	-0.15	1.00000000	mice
Pja1	-0.10	1.00000000	mice
Ddx19b	-0.12	1.00000000	mice
Trit1	-0.13	1.00000000	mice
Ppib	0.12	1.00000000	mice
Uqcrfs1	-0.11	1.00000000	mice
Vdac3	0.07	1.00000000	mice
Commd1	0.09	1.00000000	mice
Abcb1a	0.10	1.00000000	mice
Ctnnb1	0.07	1.00000000	mice
Zdhhc18	0.12	1.00000000	mice
Pbxip1	-0.11	1.00000000	mice
Add1	-0.14	1.00000000	mice
Htatsf1	-0.11	1.00000000	mice
Trpc4ap	-0.13	1.00000000	mice
Fam118b	0.06	1.00000000	mice
Pigk	0.04	1.00000000	mice
Ap1b1	-0.14	1.00000000	mice
Akap13	0.11	1.00000000	mice
Lman2	0.06	1.00000000	mice
Naa60	0.07	1.00000000	mice

Arntl	-0.11	1.00000000	mice
Riox1	-0.09	1.00000000	mice
Cfap20	-0.15	1.00000000	mice
Grb2	0.19	1.00000000	mice
Traf3ip3	-0.13	1.00000000	mice
Itga2	0.04	1.00000000	mice
Phykpl	0.06	1.00000000	mice
Asna1	0.05	1.00000000	mice
Ddhd1	-0.12	1.00000000	mice
Txk	-0.12	1.00000000	mice
Ptpn4	0.15	1.00000000	mice
Aggf1	-0.16	1.00000000	mice
Tbc1d4	-0.07	1.00000000	mice
Csnk1a1	-0.08	1.00000000	mice
Ppfia1	-0.16	1.00000000	mice
Clcn3	-0.22	1.00000000	mice
Gak	-0.12	1.00000000	mice
Ddx50	-0.13	1.00000000	mice
Psmc2	0.09	1.00000000	mice
Atp7a	-0.08	1.00000000	mice
Nfx1	-0.15	1.00000000	mice
Ankrd17	0.15	1.00000000	mice
Taf3	-0.20	1.00000000	mice
Zfhx2	0.07	1.00000000	mice
Psmd14	0.12	1.00000000	mice
Dop1a	-0.15	1.00000000	mice
Zfp622	0.12	1.00000000	mice
Pop4	-0.09	1.00000000	mice
Pdcd1lg2	0.12	1.00000000	mice
Tubb5	-0.11	1.00000000	mice
Dse	-0.10	1.00000000	mice
Api5	0.10	1.00000000	mice
Btbd11	-0.43	1.00000000	mice
Ube2m	0.09	1.00000000	mice
2210016F16Rik	0.05	1.00000000	mice
Nck1	0.06	1.00000000	mice
Ddx17	-0.15	1.00000000	mice
Ndufaf8	0.11	1.00000000	mice
Ahctf1	-0.14	1.00000000	mice
Icos	0.39	1.00000000	mice
Atg10	0.12	1.00000000	mice
Atp5g1	0.12	1.00000000	mice
Ahdc1	0.00	1.00000000	mice
Gtdc1	0.17	1.00000000	mice
Sulf2	0.08	1.00000000	mice
Tcf7l2	-0.11	1.00000000	mice

E330009J07Rik	0.07	1.00000000	mice
Rnf216	0.03	1.00000000	mice
Lactb	-0.13	1.00000000	mice
Nmt2	-0.13	1.00000000	mice
Tfe3	0.05	1.00000000	mice
Pdcd11	0.06	1.00000000	mice
Secisbp2	-0.18	1.00000000	mice
Pigx	0.04	1.00000000	mice
Slc16a10	-0.31	1.00000000	mice
Nxf1	-0.14	1.00000000	mice
Clcn5	0.08	1.00000000	mice
Ubl3	0.09	1.00000000	mice
Rora	-0.01	1.00000000	mice
Ddit3	0.09	1.00000000	mice
Lpin2	0.08	1.00000000	mice
Snx18	0.06	1.00000000	mice
Kdsr	-0.11	1.00000000	mice
Pfdn6	0.06	1.00000000	mice
Dclre1c	0.16	1.00000000	mice
Rars	0.06	1.00000000	mice
Bin2	-0.12	1.00000000	mice
Ptbp2	-0.13	1.00000000	mice
Ccdc117	0.08	1.00000000	mice
Gpsm1	-0.11	1.00000000	mice
Sec62	0.09	1.00000000	mice
Ezh2	0.09	1.00000000	mice
Vps29	0.10	1.00000000	mice
Xpo4	-0.11	1.00000000	mice
Stat6	0.05	1.00000000	mice
Ormdl3	0.05	1.00000000	mice
Psmc6	0.07	1.00000000	mice
Ehd1	-0.17	1.00000000	mice
Dipk2a	-0.09	1.00000000	mice
Zfp704	-0.21	1.00000000	mice
Eif1b	0.09	1.00000000	mice
Clasrp	-0.11	1.00000000	mice
Rnps1	0.08	1.00000000	mice
Kctd18	-0.11	1.00000000	mice
Hsdl1	-0.08	1.00000000	mice
Zfp407	0.09	1.00000000	mice
Slc38a10	-0.11	1.00000000	mice
Impdh2	-0.11	1.00000000	mice
Atp2a2	0.11	1.00000000	mice
Dhx40	0.01	1.00000000	mice
Cdc5l	0.05	1.00000000	mice
Kdm4b	-0.11	1.00000000	mice

Ncor1	0.08	1.00000000	mice
Ppargc1b	-0.11	1.00000000	mice
Wwp1	-0.12	1.00000000	mice
Ulk1	-0.14	1.00000000	mice
Egln1	-0.13	1.00000000	mice
Sord	-0.16	1.00000000	mice
Alcam	-0.06	1.00000000	mice
Zfp598	0.10	1.00000000	mice
Abracl	0.10	1.00000000	mice
Sc5d	0.03	1.00000000	mice
Slc9a6	-0.08	1.00000000	mice
Pde8a	-0.23	1.00000000	mice
Por	0.11	1.00000000	mice
Usp1	-0.11	1.00000000	mice
Exosc8	0.09	1.00000000	mice
D1Ertd622e	0.11	1.00000000	mice
Pde7b	0.19	1.00000000	mice
Plekhm2	-0.13	1.00000000	mice
Tbl1x	0.21	1.00000000	mice
Zdhhc5	-0.10	1.00000000	mice
Ccdc28a	-0.06	1.00000000	mice
Ing5	-0.12	1.00000000	mice
Gfm2	0.07	1.00000000	mice
Faf1	-0.15	1.00000000	mice
Agap3	-0.15	1.00000000	mice
Nap1l4	-0.12	1.00000000	mice
Zfp951	-0.09	1.00000000	mice
Abhd12	-0.20	1.00000000	mice
Copz1	0.05	1.00000000	mice
Utp23	0.08	1.00000000	mice
Nup155	-0.13	1.00000000	mice
Setd7	-0.09	1.00000000	mice
Atg4d	0.07	1.00000000	mice
Asxl1	-0.09	1.00000000	mice
Bcas2	-0.10	1.00000000	mice
Zfp638	0.12	1.00000000	mice
Hnrnpd	0.14	1.00000000	mice
Cldnd1	0.07	1.00000000	mice
Pnrc2	0.05	1.00000000	mice
Dlgap4	0.06	1.00000000	mice
Rab6a	-0.12	1.00000000	mice
Tbc1d2b	0.05	1.00000000	mice
Rab35	-0.15	1.00000000	mice
Coq2	0.02	1.00000000	mice
Uqcc1	-0.12	1.00000000	mice
Acbd3	-0.11	1.00000000	mice

Ppig	0.14	1.00000000	mice
Pxylp1	-0.13	1.00000000	mice
Rab5b	0.06	1.00000000	mice
Ube2i	0.10	1.00000000	mice
Shoc2	0.09	1.00000000	mice
Lsg1	-0.14	1.00000000	mice
Aip	-0.16	1.00000000	mice
Ell	-0.21	1.00000000	mice
Hnrnpdl	0.09	1.00000000	mice
Sema6a	0.26	1.00000000	mice
Taok1	0.11	1.00000000	mice
Gng5	0.07	1.00000000	mice
Atad1	0.08	1.00000000	mice
Ylpm1	-0.17	1.00000000	mice
Diaph3	-0.16	1.00000000	mice
Lancl1	-0.08	1.00000000	mice
Eipr1	-0.18	1.00000000	mice
Papss1	-0.12	1.00000000	mice
Kcnab2	-0.09	1.00000000	mice
Depdc5	-0.11	1.00000000	mice
Dstn	0.09	1.00000000	mice
Ormdl2	0.02	1.00000000	mice
Ythdc1	-0.11	1.00000000	mice
Psma3	0.09	1.00000000	mice
Rnf130	0.19	1.00000000	mice
Nt5dc1	-0.14	1.00000000	mice
Nr1d2	-0.13	1.00000000	mice
Tecr	0.08	1.00000000	mice
Ints11	0.05	1.00000000	mice
Vma21	0.06	1.00000000	mice
Tsc1	-0.15	1.00000000	mice
Vmp1	0.13	1.00000000	mice
Edem1	-0.13	1.00000000	mice
Dcaf5	0.13	1.00000000	mice
Trappc3	0.05	1.00000000	mice
E130307A14Rik	0.08	1.00000000	mice
Cyfip2	-0.09	1.00000000	mice
Trim8	-0.10	1.00000000	mice
Osbpl8	-0.11	1.00000000	mice
Gtf2a2	0.08	1.00000000	mice
Utp4	-0.16	1.00000000	mice
Cdk16	-0.07	1.00000000	mice
Spice1	-0.11	1.00000000	mice
Pcbp1	0.07	1.00000000	mice
Pacsin2	-0.26	1.00000000	mice
Atg2b	-0.13	1.00000000	mice

Lin52	-0.15	1.00000000	mice
Cdc14a	0.08	1.00000000	mice
Atf6	0.04	1.00000000	mice
Hira	-0.18	1.00000000	mice
Cwc15	0.07	1.00000000	mice
Zfp280c	-0.15	1.00000000	mice
Cfap36	0.02	1.00000000	mice
Chchd1	0.07	1.00000000	mice
Arl5c	0.12	1.00000000	mice
Racgap1	-0.10	1.00000000	mice
Triobp	0.06	1.00000000	mice
Chchd7	-0.08	1.00000000	mice
Rabep2	-0.12	1.00000000	mice
Ltn1	-0.11	1.00000000	mice
Dym	-0.12	1.00000000	mice
Haus2	-0.07	1.00000000	mice
Piga	-0.09	1.00000000	mice
Chchd3	0.12	1.00000000	mice
Cntrl	0.12	1.00000000	mice
Ints6l	-0.12	1.00000000	mice
Ube2v1	0.05	1.00000000	mice
Cap1	0.10	1.00000000	mice
Klrc2	0.03	1.00000000	mice
Rnf111	0.11	1.00000000	mice
Lsm14a	0.05	1.00000000	mice
Rabgap1	0.13	1.00000000	mice
Arf6	-0.14	1.00000000	mice
Polr3g	-0.07	1.00000000	mice
Gtf2f1	0.07	1.00000000	mice
Zfp335os	-0.10	1.00000000	mice
Taf1d	0.10	1.00000000	mice
Zfp472	-0.08	1.00000000	mice
Fbxo34	-0.12	1.00000000	mice
Tmem241	-0.01	1.00000000	mice
Cep63	-0.06	1.00000000	mice
Synj2bp	0.11	1.00000000	mice
Smim4	0.04	1.00000000	mice
Dynll1	0.12	1.00000000	mice
Utp14b	-0.09	1.00000000	mice
Rnf121	0.06	1.00000000	mice
Akt3	-0.14	1.00000000	mice
Cnnm2	-0.27	1.00000000	mice
Knop1	0.08	1.00000000	mice
Gnl3	0.05	1.00000000	mice
Naca	-0.10	1.00000000	mice
Ccnh	0.12	1.00000000	mice

Slc10a7	0.08	1.00000000	mice
A930005H10Rik	-0.08	1.00000000	mice
Dld	0.10	1.00000000	mice
Zfp263	-0.09	1.00000000	mice
Pabpn1	0.08	1.00000000	mice
Lmbrd1	-0.17	1.00000000	mice
Slc25a3	0.09	1.00000000	mice
Atp8b4	0.13	1.00000000	mice
Zfc3h1	-0.11	1.00000000	mice
Retreg3	-0.12	1.00000000	mice
Bcl7c	0.06	1.00000000	mice
Vamp8	0.07	1.00000000	mice
Sipa1l3	0.06	1.00000000	mice
Frmd6	-0.16	1.00000000	mice
Rad18	-0.14	1.00000000	mice
Clock	0.05	1.00000000	mice
Vcpkmt	0.05	1.00000000	mice
Arsb	-0.12	1.00000000	mice
Khsrp	0.04	1.00000000	mice
2610020C07Rik	0.04	1.00000000	mice
Rnf34	0.08	1.00000000	mice
Scaf1	-0.07	1.00000000	mice
Syf2	-0.11	1.00000000	mice
Cited2	-0.14	1.00000000	mice
Fos	0.17	1.00000000	mice
Bicral	0.07	1.00000000	mice
Eef2k	-0.13	1.00000000	mice
Ppp3ca	0.06	1.00000000	mice
Ntan1	-0.13	1.00000000	mice
Acsl3	-0.15	1.00000000	mice
Anxa11	0.10	1.00000000	mice
Srsf10	0.07	1.00000000	mice
Tspan14	-0.17	1.00000000	mice
Samm50	0.06	1.00000000	mice
Foxn3	-0.12	1.00000000	mice
Sec24d	-0.20	1.00000000	mice
Ralgps1	-0.09	1.00000000	mice
Lrrc28	-0.12	1.00000000	mice
Ctdsp2	-0.12	1.00000000	mice
Gng2	0.08	1.00000000	mice
Ppp4r1	-0.15	1.00000000	mice
4930469K13Rik	0.04	1.00000000	mice
Ttc13	-0.12	1.00000000	mice
Pgam5	-0.11	1.00000000	mice
Klf12	0.02	1.00000000	mice
Zswim7	-0.07	1.00000000	mice

Tubgcp4	0.06	1.00000000	mice
Ago2	-0.09	1.00000000	mice
Tmem14c	0.06	1.00000000	mice
Plagl2	0.06	1.00000000	mice
Emc7	0.09	1.00000000	mice
Zfp282	-0.10	1.00000000	mice
Pigu	0.02	1.00000000	mice
Psmd12	0.09	1.00000000	mice
Anapc5	-0.14	1.00000000	mice
2810013P06Rik	-0.08	1.00000000	mice
Plp2	0.02	1.00000000	mice
Fhit	-0.15	1.00000000	mice
Desi2	-0.13	1.00000000	mice
Hp1bp3	-0.12	1.00000000	mice
Commd8	0.02	1.00000000	mice
Tpst2	0.05	1.00000000	mice
Stag2	-0.09	1.00000000	mice
Hdlbp	-0.10	1.00000000	mice
Snapc5	-0.11	1.00000000	mice
Ube2d1	0.10	1.00000000	mice
Tmem234	-0.09	1.00000000	mice
Rer1	0.09	1.00000000	mice
Cradd	-0.21	1.00000000	mice
Lrrc8d	-0.19	1.00000000	mice
Banf1	0.09	1.00000000	mice
Coro1c	-0.17	1.00000000	mice
Atp5g3	0.13	1.00000000	mice
Lars2	0.20	1.00000000	mice
Ehd4	0.14	1.00000000	mice
Tango2	-0.11	1.00000000	mice
Sgsm2	-0.11	1.00000000	mice
Csrnp1	0.12	1.00000000	mice
Zfp142	0.07	1.00000000	mice
Btaf1	-0.12	1.00000000	mice
Ndufa8	0.05	1.00000000	mice
Ccdc88c	0.09	1.00000000	mice
H60b	0.10	1.00000000	mice
Gosr2	-0.09	1.00000000	mice
Pfkl	-0.10	1.00000000	mice
Sirt1	-0.06	1.00000000	mice
Xpo7	-0.14	1.00000000	mice
Svbp	0.06	1.00000000	mice
Sept1	0.11	1.00000000	mice
Psma1	0.10	1.00000000	mice
Tmem87a	-0.12	1.00000000	mice
Sumo1	0.09	1.00000000	mice

Nfil3	0.03	1.00000000	mice
Apc	-0.18	1.00000000	mice
Glis3	0.18	1.00000000	mice
Usp4	-0.13	1.00000000	mice
Pcm1	0.10	1.00000000	mice
Sla2	0.10	1.00000000	mice
Cst3	0.07	1.00000000	mice
Tshz3	-0.03	1.00000000	mice
Zfp277	0.04	1.00000000	mice
Arfgap2	-0.10	1.00000000	mice
Polg	-0.06	1.00000000	mice
Fopnl	-0.13	1.00000000	mice
Abt1	-0.09	1.00000000	mice
Dnajc15	0.04	1.00000000	mice
Selenoh	0.07	1.00000000	mice
Napa	-0.13	1.00000000	mice
BC005624	-0.15	1.00000000	mice
Hdac5	-0.08	1.00000000	mice
Fbxw8	0.05	1.00000000	mice
Lpxn	0.12	1.00000000	mice
Pcid2	-0.11	1.00000000	mice
Klf10	0.08	1.00000000	mice
Stambpl1	-0.17	1.00000000	mice
Skiv2l	-0.07	1.00000000	mice
Cyld	0.07	1.00000000	mice
Polr2i	0.05	1.00000000	mice
Rdh11	-0.07	1.00000000	mice
Eea1	0.01	1.00000000	mice
Ndufb10	0.05	1.00000000	mice
Rasa1	-0.09	1.00000000	mice
Tasor2	0.17	1.00000000	mice
Rbm3	0.09	1.00000000	mice
Ldha	0.10	1.00000000	mice
Haus3	-0.15	1.00000000	mice
Cers2	0.06	1.00000000	mice
Polr1c	0.07	1.00000000	mice
Cd3g	-0.02	1.00000000	mice
Cracr2a	-0.11	1.00000000	mice
Aplp2	-0.24	1.00000000	mice
Appl1	-0.17	1.00000000	mice
Tango6	-0.17	1.00000000	mice
Ipo9	-0.09	1.00000000	mice
Tbcd	-0.16	1.00000000	mice
Degs1	0.05	1.00000000	mice
Abcc5	-0.08	1.00000000	mice
Swt1	0.05	1.00000000	mice

Fam172a	0.16	1.00000000	mice
Pdzd8	-0.17	1.00000000	mice
Ttc37	0.08	1.00000000	mice
Ndufs7	0.03	1.00000000	mice
Oser1	-0.12	1.00000000	mice
Entr1	-0.08	1.00000000	mice
Dars	0.08	1.00000000	mice
Hs6st1	-0.06	1.00000000	mice
Rab12	0.09	1.00000000	mice
Epas1	-0.14	1.00000000	mice
Hilpda	0.14	1.00000000	mice
Taz	0.04	1.00000000	mice
Exosc3	0.06	1.00000000	mice
Tbc1d9b	0.08	1.00000000	mice
Ripk2	0.16	1.00000000	mice
Grk2	0.11	1.00000000	mice
Psmf1	-0.10	1.00000000	mice
Tnks	-0.12	1.00000000	mice
AW549877	-0.08	1.00000000	mice
Ddx3x	-0.11	1.00000000	mice
Smarcad1	0.04	1.00000000	mice
Phb2	0.09	1.00000000	mice
Cd74	-0.16	1.00000000	mice
Ddx46	-0.11	1.00000000	mice
Hnrnpk	0.09	1.00000000	mice
Ubc	-0.09	1.00000000	mice
Uqcrc2	0.08	1.00000000	mice
Golm1	-0.22	1.00000000	mice
Gpd2	-0.14	1.00000000	mice
Erlec1	0.04	1.00000000	mice
Arhgef6	0.06	1.00000000	mice
Pogk	0.07	1.00000000	mice
Ubac2	-0.15	1.00000000	mice
Cpne1	0.06	1.00000000	mice
Eef1e1	0.05	1.00000000	mice
Ints13	-0.05	1.00000000	mice
Usp7	-0.08	1.00000000	mice
Camkk2	0.08	1.00000000	mice
Ankmy2	-0.07	1.00000000	mice
Tk2	-0.08	1.00000000	mice
Ube3c	-0.18	1.00000000	mice
Caap1	0.03	1.00000000	mice
Pkn2	0.14	1.00000000	mice
Spr	-0.07	1.00000000	mice
Gpatch2l	0.00	1.00000000	mice
Rab28	0.06	1.00000000	mice

Tfg	-0.11	1.00000000	mice
Arhgap26	0.08	1.00000000	mice
Cc2d1b	-0.07	1.00000000	mice
Pdp1	-0.09	1.00000000	mice
Zmym1	-0.10	1.00000000	mice
Gtf3c6	0.02	1.00000000	mice
Bcl6	0.08	1.00000000	mice
Dpy30	-0.10	1.00000000	mice
Anks3	-0.04	1.00000000	mice
Snrnp70	-0.09	1.00000000	mice
Ppp2ca	0.09	1.00000000	mice
Parp8	0.14	1.00000000	mice
Iqgap2	0.11	1.00000000	mice
Rbms2	0.01	1.00000000	mice
Usf2	-0.11	1.00000000	mice
Agtrap	0.06	1.00000000	mice
Yju2	-0.08	1.00000000	mice
Ip6k1	-0.11	1.00000000	mice
Cdk19	-0.04	1.00000000	mice
Sfswap	-0.18	1.00000000	mice
Rwdd4a	0.04	1.00000000	mice
Erbin	0.15	1.00000000	mice
Pisd	-0.08	1.00000000	mice
Exosc5	0.05	1.00000000	mice
Nipal3	-0.09	1.00000000	mice
Zranb1	-0.06	1.00000000	mice
Ehd3	-0.07	1.00000000	mice
Lrch3	0.09	1.00000000	mice
Adcy7	-0.10	1.00000000	mice
Naa15	0.09	1.00000000	mice
Nsmce1	-0.12	1.00000000	mice
Gtf3c2	-0.10	1.00000000	mice
Tlk1	-0.16	1.00000000	mice
Gpr18	0.10	1.00000000	mice
Inpp4b	-0.35	1.00000000	mice
Asxl2	0.12	1.00000000	mice
Ubash3a	-0.03	1.00000000	mice
Cdk12	0.09	1.00000000	mice
Polh	-0.09	1.00000000	mice
Exd2	-0.08	1.00000000	mice
Coq10b	0.13	1.00000000	mice
Cables2	-0.03	1.00000000	mice
Plekha3	0.07	1.00000000	mice
Flcn	0.05	1.00000000	mice
C330007P06Rik	0.03	1.00000000	mice
Polr3gl	-0.08	1.00000000	mice

Nfia	-0.12	1.00000000	mice
Sdhaf2	0.04	1.00000000	mice
Gpx4	0.08	1.00000000	mice
BE692007	0.07	1.00000000	mice
Sfr1	-0.09	1.00000000	mice
Cnih4	0.06	1.00000000	mice
Prpf40a	0.14	1.00000000	mice
Atf2	-0.06	1.00000000	mice
Snrk	0.02	1.00000000	mice
Tpm4	-0.14	1.00000000	mice
Txlng	0.09	1.00000000	mice
Dcp2	0.03	1.00000000	mice
Selenoi	-0.11	1.00000000	mice
Tomm6	0.10	1.00000000	mice
Uba3	0.04	1.00000000	mice
Tpp1	0.03	1.00000000	mice
Arhgef7	-0.12	1.00000000	mice
Dkc1	0.05	1.00000000	mice
Rgl1	-0.16	1.00000000	mice
Asrgl1	0.03	1.00000000	mice
Arl8a	-0.14	1.00000000	mice
Ccdc59	0.08	1.00000000	mice
Tkt	0.05	1.00000000	mice
Pdlim1	-0.11	1.00000000	mice
Zfp692	-0.16	1.00000000	mice
Rbm45	-0.08	1.00000000	mice
Nxn	0.01	1.00000000	mice
Gypc	-0.07	1.00000000	mice
Adam9	0.06	1.00000000	mice
Vars	0.03	1.00000000	mice
Rfxap	-0.09	1.00000000	mice
Baiap2	-0.08	1.00000000	mice
Ola1	0.05	1.00000000	mice
Txndc16	-0.13	1.00000000	mice
Hmgb1	-0.09	1.00000000	mice
1110038B12Rik	0.05	1.00000000	mice
1810013L24Rik	-0.10	1.00000000	mice
Lrba	-0.09	1.00000000	mice
Osgin1	0.07	1.00000000	mice
Stard4	0.05	1.00000000	mice
St3gal1	-0.06	1.00000000	mice
Prr3	0.05	1.00000000	mice
Pitpnb	-0.14	1.00000000	mice
Ggnbp2	-0.07	1.00000000	mice
2700049A03Rik	-0.09	1.00000000	mice
Birc6	-0.07	1.00000000	mice

Ablim1	0.00	1.00000000	mice
Yipf3	-0.10	1.00000000	mice
Rex1bd	0.06	1.00000000	mice
Rad21	-0.15	1.00000000	mice
Pmpcb	0.05	1.00000000	mice
Car2	0.00	1.00000000	mice
Wdr36	-0.10	1.00000000	mice
Cpeb4	0.12	1.00000000	mice
Parp6	-0.09	1.00000000	mice
Nars	0.05	1.00000000	mice
Ntng2	0.02	1.00000000	mice
Faim	0.11	1.00000000	mice
Slc39a6	0.07	1.00000000	mice
Cdt1	-0.08	1.00000000	mice
Pdcd10	0.07	1.00000000	mice
Srsf1	0.06	1.00000000	mice
Sdcbp	-0.10	1.00000000	mice
Dmxl1	-0.17	1.00000000	mice
Pip4p1	-0.08	1.00000000	mice
Gab3	0.06	1.00000000	mice
Ccnk	-0.09	1.00000000	mice
Enpp1	-0.14	1.00000000	mice
Pttg1ip	-0.08	1.00000000	mice
Rpp21	0.06	1.00000000	mice
Tmem208	0.07	1.00000000	mice
Glrx3	-0.08	1.00000000	mice
Nbeal2	0.08	1.00000000	mice
Zfpm1	0.00	1.00000000	mice
Gps1	0.04	1.00000000	mice
Psmd10	0.03	1.00000000	mice
Arhgef1	-0.11	1.00000000	mice
Bop1	-0.12	1.00000000	mice
B3gat3	0.02	1.00000000	mice
Ethe1	-0.07	1.00000000	mice
Bex3	-0.13	1.00000000	mice
Clptm1l	0.05	1.00000000	mice
Gsn	-0.04	1.00000000	mice
Esd	0.08	1.00000000	mice
Cnep1r1	-0.09	1.00000000	mice
Emilin2	0.01	1.00000000	mice
Pcbp3	-0.13	1.00000000	mice
Nudt21	-0.13	1.00000000	mice
Zbtb4	-0.11	1.00000000	mice
Tagap	0.12	1.00000000	mice
Atg5	-0.10	1.00000000	mice
Cdk2ap2	-0.09	1.00000000	mice

Fbxl20	-0.11	1.00000000	mice
Eif3f	-0.10	1.00000000	mice
Prpf4b	0.13	1.00000000	mice
Hspbp1	-0.11	1.00000000	mice
Bnip2	0.06	1.00000000	mice
Sod2	0.07	1.00000000	mice
Ncoa4	0.07	1.00000000	mice
Snx4	-0.10	1.00000000	mice
Sh3glb1	-0.07	1.00000000	mice
Fkbp3	0.06	1.00000000	mice
Elf4	0.09	1.00000000	mice
Rnf167	-0.08	1.00000000	mice
Ptpre	0.08	1.00000000	mice
Isca1	-0.05	1.00000000	mice
Atp6v1h	-0.10	1.00000000	mice
Gsdmd	0.03	1.00000000	mice
Ndel1	-0.10	1.00000000	mice
Thyn1	0.04	1.00000000	mice
Crk	0.10	1.00000000	mice
Stk16	0.02	1.00000000	mice
Znrf2	0.04	1.00000000	mice
Panx1	0.04	1.00000000	mice
Lrig1	0.04	1.00000000	mice
Abcc1	0.06	1.00000000	mice
Smc6	-0.13	1.00000000	mice
Fam184a	-0.15	1.00000000	mice
Pik3cg	-0.20	1.00000000	mice
Rprd2	-0.07	1.00000000	mice
Agpat3	0.06	1.00000000	mice
Il6ra	-0.06	1.00000000	mice
Zswim4	0.12	1.00000000	mice
Tnip1	0.12	1.00000000	mice
Tnfrsf1b	0.03	1.00000000	mice
Vdac1	0.08	1.00000000	mice
Nup35	-0.10	1.00000000	mice
Dgcr2	0.06	1.00000000	mice
Tln1	0.06	1.00000000	mice
Zkscan3	0.10	1.00000000	mice
Pycr2	-0.05	1.00000000	mice
Rubcn	0.02	1.00000000	mice
Smim13	-0.05	1.00000000	mice
Shprh	-0.13	1.00000000	mice
Rp9	-0.07	1.00000000	mice
Sf3b6	-0.07	1.00000000	mice
Zbed4	-0.04	1.00000000	mice
Smc2	0.03	1.00000000	mice

Slc16a1	-0.07	1.00000000	mice
Ino80	-0.14	1.00000000	mice
Rnf187	0.07	1.00000000	mice
Cnot6	-0.10	1.00000000	mice
Topbp1	-0.18	1.00000000	mice
Ttc1	0.06	1.00000000	mice
Ubp1	-0.11	1.00000000	mice
Hmbox1	-0.15	1.00000000	mice
Efcab14	-0.14	1.00000000	mice
Nemf	0.09	1.00000000	mice
Rbis	0.04	1.00000000	mice
Stxbp3	-0.14	1.00000000	mice
Akap8	-0.11	1.00000000	mice
Polr1d	-0.08	1.00000000	mice
Cbfa2t2	-0.14	1.00000000	mice
Bfar	0.09	1.00000000	mice
Tent2	0.07	1.00000000	mice
Slc16a6	-0.12	1.00000000	mice
Nfatc1	-0.18	1.00000000	mice
Elp5	0.04	1.00000000	mice
Tbc1d8b	0.07	1.00000000	mice
Serpinb9b	-0.01	1.00000000	mice
Ptger4	0.08	1.00000000	mice
Pofut2	-0.11	1.00000000	mice
Vti1a	0.06	1.00000000	mice
C9orf72	-0.16	1.00000000	mice
Gtpbp2	0.05	1.00000000	mice
Pdlim5	0.11	1.00000000	mice
Ikzf5	0.04	1.00000000	mice
Farp1	0.07	1.00000000	mice
Ifitm10	0.02	1.00000000	mice
Dnajc13	0.10	1.00000000	mice
Ldb1	-0.12	1.00000000	mice
Atf7	-0.17	1.00000000	mice
Strbp	0.07	1.00000000	mice
Borcs8	0.04	1.00000000	mice
Polr2c	0.03	1.00000000	mice
Bri3	-0.07	1.00000000	mice
Atad2b	-0.11	1.00000000	mice
Atxn10	-0.14	1.00000000	mice
Uba2	0.08	1.00000000	mice
Stk38	-0.09	1.00000000	mice
Eps15	-0.13	1.00000000	mice
Rmnd5b	-0.10	1.00000000	mice
Tmem123	0.06	1.00000000	mice
Fam189b	-0.07	1.00000000	mice

Laptm5	0.06	1.00000000	mice
Tcf3	-0.10	1.00000000	mice
Gabarapl1	0.06	1.00000000	mice
Epg5	-0.13	1.00000000	mice
Lin9	-0.09	1.00000000	mice
Vsir	-0.08	1.00000000	mice
Nedd1	0.05	1.00000000	mice
Sh2d3c	-0.16	1.00000000	mice
Csrp1	-0.10	1.00000000	mice
Hcfc2	-0.10	1.00000000	mice
Got2	-0.10	1.00000000	mice
Sgpp1	-0.06	1.00000000	mice
Sec24c	-0.07	1.00000000	mice
Tmx3	0.05	1.00000000	mice
Zfp69	-0.07	1.00000000	mice
Glb1	0.06	1.00000000	mice
Arid4b	0.12	1.00000000	mice
Lin7c	0.08	1.00000000	mice
Trip12	0.07	1.00000000	mice
Eif5	-0.08	1.00000000	mice
Tmem108	-0.29	1.00000000	mice
Prpf38a	0.04	1.00000000	mice
Cdk13	-0.09	1.00000000	mice
B4galt1	-0.20	1.00000000	mice
Arl13b	0.09	1.00000000	mice
Gramd4	-0.13	1.00000000	mice
Zfp672	-0.14	1.00000000	mice
Nsd1	0.10	1.00000000	mice
Pou6f1	-0.06	1.00000000	mice
Trappc1	0.02	1.00000000	mice
Timm44	0.05	1.00000000	mice
Ube2k	-0.08	1.00000000	mice
Pik3c3	-0.09	1.00000000	mice
Arid3b	-0.07	1.00000000	mice
Wdtc1	0.05	1.00000000	mice
Cnot2	0.10	1.00000000	mice
Atr	0.06	1.00000000	mice
Ubr4	0.09	1.00000000	mice
Hmgn1	-0.09	1.00000000	mice
Ndufb6	0.05	1.00000000	mice
Ttc9c	0.04	1.00000000	mice
Stk11	-0.11	1.00000000	mice
Slc33a1	-0.10	1.00000000	mice
Sgta	0.06	1.00000000	mice
Ighm	-0.11	1.00000000	mice
Slc25a11	0.04	1.00000000	mice

Tspan5	0.06	1.00000000	mice
Samd8	-0.08	1.00000000	mice
Herpud1	0.08	1.00000000	mice
Tulp4	-0.16	1.00000000	mice
Hs2st1	-0.20	1.00000000	mice
Nsun4	-0.09	1.00000000	mice
Taf5	0.07	1.00000000	mice
Klhl7	-0.09	1.00000000	mice
Rassf2	-0.08	1.00000000	mice
Nt5dc3	-0.11	1.00000000	mice
Ppm1j	0.01	1.00000000	mice
Yipf1	0.02	1.00000000	mice
Phactr4	0.06	1.00000000	mice
Lsm12	0.08	1.00000000	mice
Bmt2	-0.08	1.00000000	mice
Phgdh	0.06	1.00000000	mice
Lypla2	0.06	1.00000000	mice
Pdcd6ip	-0.13	1.00000000	mice
Tmem147	0.04	1.00000000	mice
Tmem115	-0.10	1.00000000	mice
Rexo5	-0.09	1.00000000	mice
Cd8a	0.11	1.00000000	mice
Fnbp1	0.20	1.00000000	mice
Phf2	-0.11	1.00000000	mice
Brix1	-0.08	1.00000000	mice
Tmem37	0.05	1.00000000	mice
Ugp2	0.00	1.00000000	mice
Hhex	0.03	1.00000000	mice
Cep250	-0.11	1.00000000	mice
4930522L14Rik	-0.09	1.00000000	mice
Wars	0.03	1.00000000	mice
Supt5	0.05	1.00000000	mice
Sars	0.03	1.00000000	mice
Lig1	0.06	1.00000000	mice
Glg1	-0.08	1.00000000	mice
Plekhf2	0.09	1.00000000	mice
Csk	0.02	1.00000000	mice
Eya3	-0.17	1.00000000	mice
Dph5	-0.08	1.00000000	mice
Ube2s	-0.08	1.00000000	mice
Rab18	0.06	1.00000000	mice
Klrd1	0.13	1.00000000	mice
Ahsa2	0.03	1.00000000	mice
Srsf4	0.10	1.00000000	mice
Vezf1	0.07	1.00000000	mice
Zmynd8	0.07	1.00000000	mice

Cryzl1	0.05	1.00000000	mice
Rala	-0.14	1.00000000	mice
Armc8	0.04	1.00000000	mice
Gatad2b	-0.09	1.00000000	mice
Srp14	0.07	1.00000000	mice
Zfyve1	0.03	1.00000000	mice
Acsl5	0.02	1.00000000	mice
Cobll1	0.11	1.00000000	mice
Bcl7b	-0.05	1.00000000	mice
Wapl	-0.05	1.00000000	mice
Tnpo2	-0.08	1.00000000	mice
Snx1	0.04	1.00000000	mice
Bin3	0.04	1.00000000	mice
Terf2	-0.08	1.00000000	mice
Ak2	0.07	1.00000000	mice
Tomm70a	0.03	1.00000000	mice
Rbm15b	-0.08	1.00000000	mice
Vwa8	0.05	1.00000000	mice
Churc1	0.05	1.00000000	mice
Uba6	-0.13	1.00000000	mice
Atf7ip	0.09	1.00000000	mice
Nkap	0.06	1.00000000	mice
Zcchc10	0.07	1.00000000	mice
Hat1	0.07	1.00000000	mice
Thumpd3	0.05	1.00000000	mice
Tbpl1	0.05	1.00000000	mice
Ppp1r13b	-0.09	1.00000000	mice
Abhd17b	0.13	1.00000000	mice
Uchl5	0.05	1.00000000	mice
Atp2c1	-0.17	1.00000000	mice
Socs4	0.04	1.00000000	mice
Usp39	0.05	1.00000000	mice
Spsb3	-0.06	1.00000000	mice
Exoc2	0.08	1.00000000	mice
Slc37a2	0.05	1.00000000	mice
Slc25a51	0.04	1.00000000	mice
Cox11	-0.10	1.00000000	mice
Inpp4a	-0.09	1.00000000	mice
Itm2c	0.04	1.00000000	mice
Zmym5	-0.08	1.00000000	mice
Dedd	0.03	1.00000000	mice
Pom121	-0.08	1.00000000	mice
Tiam1	-0.05	1.00000000	mice
Rapgef6	0.13	1.00000000	mice
Psmd11	0.06	1.00000000	mice
Dnajc18	0.06	1.00000000	mice

Pmpca	0.01	1.00000000	mice
Ctr9	-0.08	1.00000000	mice
Cdv3	-0.12	1.00000000	mice
Ndufs4	0.05	1.00000000	mice
Pola1	0.10	1.00000000	mice
Srebf1	-0.06	1.00000000	mice
Gcc2	0.09	1.00000000	mice
2610037D02Rik	0.11	1.00000000	mice
Ap1m1	-0.08	1.00000000	mice
Ar	-0.31	1.00000000	mice
Dab2ip	0.02	1.00000000	mice
Ezr	-0.05	1.00000000	mice
Urm1	-0.08	1.00000000	mice
Ube2a	-0.11	1.00000000	mice
Ap2b1	0.08	1.00000000	mice
Sod1	0.02	1.00000000	mice
Got1	-0.09	1.00000000	mice
Sec22a	0.10	1.00000000	mice
Ncoa6	0.04	1.00000000	mice
Tm6sf1	-0.18	1.00000000	mice
Il27ra	0.06	1.00000000	mice
Eri1	0.08	1.00000000	mice
Abl2	-0.08	1.00000000	mice
Zfyve16	0.07	1.00000000	mice
Ulk2	-0.09	1.00000000	mice
Rbm4b	0.08	1.00000000	mice
Sf3b3	0.08	1.00000000	mice
Dnajc9	-0.11	1.00000000	mice
Cerk	-0.14	1.00000000	mice
Zbtb10	0.06	1.00000000	mice
3110056K07Rik	-0.09	1.00000000	mice
Herc2	0.07	1.00000000	mice
Pcsk7	-0.14	1.00000000	mice
Bsdc1	-0.08	1.00000000	mice
Ndufab1	0.03	1.00000000	mice
Slc20a1	-0.12	1.00000000	mice
Hinfp	-0.06	1.00000000	mice
AW554918	0.12	1.00000000	mice
Taf12	0.02	1.00000000	mice
Pgd	0.04	1.00000000	mice
Capn7	-0.10	1.00000000	mice
Stk19	0.07	1.00000000	mice
Zfp280d	-0.09	1.00000000	mice
Atp1b1	-0.11	1.00000000	mice
N4bp2l1	-0.07	1.00000000	mice
Erp44	0.08	1.00000000	mice

Dnttip1	-0.12	1.00000000	mice
Kcnq5	0.05	1.00000000	mice
Uvrag	0.03	1.00000000	mice
Caml	0.06	1.00000000	mice
Nol7	0.06	1.00000000	mice
Armh3	-0.13	1.00000000	mice
Pik3r1	0.09	1.00000000	mice
Inpp5k	-0.12	1.00000000	mice
Ankrd40	0.00	1.00000000	mice
Gstcd	0.06	1.00000000	mice
Csnk1g3	0.12	1.00000000	mice
Lemd3	-0.08	1.00000000	mice
Ttc5	-0.10	1.00000000	mice
Gbe1	0.05	1.00000000	mice
G3bp2	0.09	1.00000000	mice
Cwc27	-0.19	1.00000000	mice
Ppp1r37	-0.10	1.00000000	mice
Rnf13	0.07	1.00000000	mice
Gna11	-0.05	1.00000000	mice
Aldh9a1	-0.09	1.00000000	mice
Asb3	-0.12	1.00000000	mice
Tomm5	0.03	1.00000000	mice
Flvcr1	-0.04	1.00000000	mice
Prrc2a	-0.10	1.00000000	mice
Hspa8	0.15	1.00000000	mice
Il16	0.06	1.00000000	mice
Snhg4	-0.07	1.00000000	mice
Rasl11b	-0.11	1.00000000	mice
Styx	-0.12	1.00000000	mice
Ddx52	0.03	1.00000000	mice
Larp1	0.08	1.00000000	mice
Cr1l	-0.08	1.00000000	mice
Slc38a1	0.06	1.00000000	mice
Pkd1	-0.07	1.00000000	mice
Arpc3	-0.08	1.00000000	mice
Edem3	-0.13	1.00000000	mice
Psmd1	0.11	1.00000000	mice
Yipf6	0.04	1.00000000	mice
Arhgap21	0.04	1.00000000	mice
Gphn	0.15	1.00000000	mice
Cdca4	0.04	1.00000000	mice
Tuba4a	-0.08	1.00000000	mice
Abr	-0.03	1.00000000	mice
Pptc7	0.02	1.00000000	mice
Rbx1	0.11	1.00000000	mice
Rabif	-0.08	1.00000000	mice

Zbtb7a	0.03	1.00000000	mice
P2ry10	-0.08	1.00000000	mice
Zscan26	-0.08	1.00000000	mice
Evl	0.07	1.00000000	mice
Lbh	-0.13	1.00000000	mice
Fam78a	-0.07	1.00000000	mice
Rtraf	0.09	1.00000000	mice
Fbxo30	0.05	1.00000000	mice
Amd1	-0.10	1.00000000	mice
Ftx	-0.10	1.00000000	mice
Psmd6	0.06	1.00000000	mice
Sbds	-0.11	1.00000000	mice
Slc25a38	-0.07	1.00000000	mice
Zscan21	-0.08	1.00000000	mice
Ybx3	-0.03	1.00000000	mice
Slc5a3	0.03	1.00000000	mice
Wdr70	0.03	1.00000000	mice
Pi4k2b	-0.08	1.00000000	mice
Fam117a	-0.14	1.00000000	mice
F2r	-0.16	1.00000000	mice
Disc1	0.02	1.00000000	mice
Arfgef2	-0.05	1.00000000	mice
Rab1b	0.03	1.00000000	mice
Pip5k1a	0.10	1.00000000	mice
Abcb9	-0.13	1.00000000	mice
Dera	0.03	1.00000000	mice
Trmt6	-0.09	1.00000000	mice
Wdhd1	-0.06	1.00000000	mice
Sumo3	0.04	1.00000000	mice
Fancm	-0.10	1.00000000	mice
Ints14	-0.02	1.00000000	mice
Nup153	-0.10	1.00000000	mice
Terf2ip	-0.06	1.00000000	mice
Ahcyl2	-0.03	1.00000000	mice
Phf8	-0.08	1.00000000	mice
Cbx3	0.10	1.00000000	mice
Adh5	0.04	1.00000000	mice
Capzb	0.06	1.00000000	mice
Spindoc	-0.05	1.00000000	mice
Actl6a	-0.09	1.00000000	mice
Srsf3	-0.06	1.00000000	mice
Tbca	0.08	1.00000000	mice
Pim3	-0.11	1.00000000	mice
Sntb2	-0.16	1.00000000	mice
Hsd17b11	0.04	1.00000000	mice
Vamp3	-0.13	1.00000000	mice

Traf4	-0.11	1.00000000	mice
Apaf1	0.04	1.00000000	mice
Hif1an	-0.04	1.00000000	mice
Rufy1	-0.15	1.00000000	mice
Zmiz1	-0.19	1.00000000	mice
Bccip	0.05	1.00000000	mice
Ptprj	0.09	1.00000000	mice
Agpat4	-0.12	1.00000000	mice
Nip7	0.04	1.00000000	mice
Trappc10	-0.13	1.00000000	mice
Sec16a	-0.12	1.00000000	mice
Ramac	-0.06	1.00000000	mice
Ttll5	-0.09	1.00000000	mice
Asf1a	0.06	1.00000000	mice
Wdr83os	0.04	1.00000000	mice
Fam102b	-0.10	1.00000000	mice
Fem1b	0.03	1.00000000	mice
Sec13	0.04	1.00000000	mice
Fam122a	-0.05	1.00000000	mice
Prkab1	-0.07	1.00000000	mice
Ipcef1	-0.05	1.00000000	mice
Sacs	-0.08	1.00000000	mice
Ttc33	0.04	1.00000000	mice
Hagh	-0.03	1.00000000	mice
Nr4a2	-0.07	1.00000000	mice
Gla	0.07	1.00000000	mice
Utp3	0.03	1.00000000	mice
Uba1	0.05	1.00000000	mice
Pygb	-0.11	1.00000000	mice
Bsg	0.08	1.00000000	mice
Atf4	0.06	1.00000000	mice
Gga3	-0.06	1.00000000	mice
Tnfrsf1a	0.05	1.00000000	mice
Rab7	-0.11	1.00000000	mice
Fem1c	-0.11	1.00000000	mice
Tbc1d23	-0.12	1.00000000	mice
Ndufa6	-0.07	1.00000000	mice
Pbrm1	-0.13	1.00000000	mice
Dtx4	-0.05	1.00000000	mice
Lnx2	-0.04	1.00000000	mice
Bud31	0.07	1.00000000	mice
Grasp	-0.06	1.00000000	mice
Dag1	-0.10	1.00000000	mice
Opa3	0.04	1.00000000	mice
Ktn1	-0.09	1.00000000	mice
Nebl	-0.17	1.00000000	mice

Ganab	0.05	1.00000000	mice
Ccdc9	-0.07	1.00000000	mice
Fam222b	-0.14	1.00000000	mice
Srek1	-0.09	1.00000000	mice
Atic	-0.06	1.00000000	mice
Ciao2a	0.07	1.00000000	mice
Atp8b2	0.04	1.00000000	mice
Gnptab	0.08	1.00000000	mice
Eif3b	0.06	1.00000000	mice
Hmgxb4	0.01	1.00000000	mice
Kpnb1	-0.09	1.00000000	mice
Brk1	0.06	1.00000000	mice
Cdk4	0.03	1.00000000	mice
Galnt7	0.09	1.00000000	mice
Dock9	-0.10	1.00000000	mice
Gne	0.03	1.00000000	mice
Rnf7	-0.06	1.00000000	mice
Amfr	-0.10	1.00000000	mice
Elp2	-0.07	1.00000000	mice
Tnpo1	-0.12	1.00000000	mice
Tipin	0.04	1.00000000	mice
Nadk2	-0.05	1.00000000	mice
Imp4	0.04	1.00000000	mice
Hipk3	-0.13	1.00000000	mice
Fbxo21	-0.06	1.00000000	mice
Srpk1	-0.11	1.00000000	mice
Tmed10	0.09	1.00000000	mice
Ufl1	-0.07	1.00000000	mice
Extl3	0.01	1.00000000	mice
Lemd2	-0.07	1.00000000	mice
Atg3	0.04	1.00000000	mice
Vps13b	-0.08	1.00000000	mice
Vps16	0.06	1.00000000	mice
Abca7	0.05	1.00000000	mice
Bptf	-0.10	1.00000000	mice
Txndc17	0.05	1.00000000	mice
Fyb	-0.08	1.00000000	mice
Akirin1	0.07	1.00000000	mice
Utp25	-0.05	1.00000000	mice
Xpot	0.06	1.00000000	mice
Sap30bp	-0.11	1.00000000	mice
Ank	0.01	1.00000000	mice
Psph	0.03	1.00000000	mice
Hipk2	-0.14	1.00000000	mice
Srrd	0.03	1.00000000	mice
Elp1	-0.08	1.00000000	mice

Suz12	0.08	1.00000000	mice
Fam76b	0.08	1.00000000	mice
Cbll1	-0.13	1.00000000	mice
Rbm26	0.09	1.00000000	mice
Pmepa1	0.07	1.00000000	mice
Slc9a3r1	0.07	1.00000000	mice
Xlr4b	-0.07	1.00000000	mice
Tnrc6a	-0.10	1.00000000	mice
Tbk1	0.09	1.00000000	mice
Fryl	0.08	1.00000000	mice
Nfatc2	-0.10	1.00000000	mice
Slc9a8	0.07	1.00000000	mice
Ddx1	0.06	1.00000000	mice
Fau	-0.05	1.00000000	mice
Tagln2	0.09	1.00000000	mice
Zbtb11	-0.12	1.00000000	mice
Pik3ca	-0.05	1.00000000	mice
Fam133b	0.03	1.00000000	mice
Agpat5	-0.08	1.00000000	mice
1700017B05Rik	-0.11	1.00000000	mice
Fgl2	0.01	1.00000000	mice
Fnbp4	0.06	1.00000000	mice
Cox10	-0.09	1.00000000	mice
Twf1	0.03	1.00000000	mice
Senp1	0.01	1.00000000	mice
Esyt1	0.01	1.00000000	mice
Rwdd1	0.02	1.00000000	mice
Cstf3	0.05	1.00000000	mice
Agk	-0.12	1.00000000	mice
Ier2	-0.07	1.00000000	mice
Pdss2	-0.22	1.00000000	mice
Dync1li1	-0.11	1.00000000	mice
Npc2	0.10	1.00000000	mice
Vrk3	-0.07	1.00000000	mice
Hivep3	-0.11	1.00000000	mice
Heatr1	-0.09	1.00000000	mice
Pdia6	0.10	1.00000000	mice
Tigit	-0.07	1.00000000	mice
Slc22a15	0.05	1.00000000	mice
Ergic2	0.07	1.00000000	mice
Atp6v1g1	0.08	1.00000000	mice
Capn15	-0.13	1.00000000	mice
Sdha	-0.10	1.00000000	mice
Pam	0.04	1.00000000	mice
Isg20l2	-0.07	1.00000000	mice
Ankib1	-0.14	1.00000000	mice

Arpc1b	0.04	1.00000000	mice
Zdhhc17	0.02	1.00000000	mice
Vim	0.04	1.00000000	mice
Crlf2	0.03	1.00000000	mice
Bet1l	-0.11	1.00000000	mice
Kctd12	-0.09	1.00000000	mice
Runx1	-0.14	1.00000000	mice
Ube2f	0.02	1.00000000	mice
Tmx1	-0.08	1.00000000	mice
Zmym4	0.03	1.00000000	mice
Ddx39b	0.04	1.00000000	mice
Atraid	0.02	1.00000000	mice
Usp36	-0.11	1.00000000	mice
Calcoco1	-0.08	1.00000000	mice
Akt1	-0.02	1.00000000	mice
Sbno1	0.07	1.00000000	mice
Dcp1a	-0.12	1.00000000	mice
Atp6v1f	0.07	1.00000000	mice
Etv3	-0.06	1.00000000	mice
Ndufs2	0.06	1.00000000	mice
Snx25	-0.13	1.00000000	mice
Tmem251	-0.07	1.00000000	mice
Castor2	-0.04	1.00000000	mice
1110038F14Rik	0.01	1.00000000	mice
Elof1	0.04	1.00000000	mice
Borcs7	0.00	1.00000000	mice
Fam53b	-0.10	1.00000000	mice
Supt3	0.11	1.00000000	mice
Rcor1	0.04	1.00000000	mice
Vti1b	-0.08	1.00000000	mice
Ppip5k2	-0.09	1.00000000	mice
Srsf6	-0.05	1.00000000	mice
Svil	-0.15	1.00000000	mice
Cacybp	0.02	1.00000000	mice
Dtnb	-0.19	1.00000000	mice
Higd2a	-0.11	1.00000000	mice
Smarce1	0.07	1.00000000	mice
Rbm25	0.08	1.00000000	mice
Xpnpep1	0.03	1.00000000	mice
Siah2	-0.06	1.00000000	mice
Kif2a	-0.14	1.00000000	mice
Fibp	0.03	1.00000000	mice
Utp11	0.04	1.00000000	mice
Pms2	-0.09	1.00000000	mice
5530601H04Rik	-0.07	1.00000000	mice
Ehmt1	-0.11	1.00000000	mice

Fam173a	0.05	1.00000000	mice
Rab24	-0.06	1.00000000	mice
Sft2d1	-0.09	1.00000000	mice
Unk	-0.08	1.00000000	mice
Golga7	0.04	1.00000000	mice
Timm8b	0.03	1.00000000	mice
Cenpq	-0.08	1.00000000	mice
Dtymk	0.07	1.00000000	mice
Fuca2	-0.01	1.00000000	mice
Ppil3	0.03	1.00000000	mice
Ap4e1	-0.09	1.00000000	mice
Cep295	-0.08	1.00000000	mice
Jade2	-0.14	1.00000000	mice
Hnrnpl	-0.08	1.00000000	mice
Ubfd1	0.03	1.00000000	mice
Esf1	0.03	1.00000000	mice
Cops8	0.03	1.00000000	mice
Pcbd2	0.03	1.00000000	mice
Ddx23	-0.09	1.00000000	mice
Tyw5	-0.08	1.00000000	mice
Atg14	-0.04	1.00000000	mice
Odc1	-0.03	1.00000000	mice
Fam168a	-0.08	1.00000000	mice
Ddx54	0.07	1.00000000	mice
Wdr59	-0.09	1.00000000	mice
Dram2	0.06	1.00000000	mice
Tra2b	0.10	1.00000000	mice
Paxx	-0.06	1.00000000	mice
Entpd6	-0.07	1.00000000	mice
Gimap8	0.09	1.00000000	mice
Sp3	0.07	1.00000000	mice
Slc25a33	-0.09	1.00000000	mice
2210016L21Rik	0.05	1.00000000	mice
Ensa	-0.12	1.00000000	mice
Atp8a1	-0.13	1.00000000	mice
Kif23	0.07	1.00000000	mice
Nup107	-0.07	1.00000000	mice
Ngly1	-0.09	1.00000000	mice
Surf4	0.02	1.00000000	mice
Hmg20b	0.08	1.00000000	mice
Epm2aip1	0.03	1.00000000	mice
Dcaf13	-0.13	1.00000000	mice
Sephs1	0.03	1.00000000	mice
Btbd1	-0.13	1.00000000	mice
Tspan3	0.03	1.00000000	mice
Wdr74	0.06	1.00000000	mice

Chp1	-0.11	1.00000000	mice
Prpf8	-0.09	1.00000000	mice
Rock2	-0.05	1.00000000	mice
Tfdp1	0.06	1.00000000	mice
Upf3b	0.02	1.00000000	mice
Gnas	0.04	1.00000000	mice
Eid1	0.03	1.00000000	mice
Eif3h	-0.09	1.00000000	mice
Chtop	0.02	1.00000000	mice
Rsf1	0.04	1.00000000	mice
Pgs1	-0.09	1.00000000	mice
Gpatch8	-0.07	1.00000000	mice
Stam2	0.04	1.00000000	mice
Brox	-0.09	1.00000000	mice
Crem	0.09	1.00000000	mice
Psmb7	0.04	1.00000000	mice
Fkbp1a	0.07	1.00000000	mice
Bbip1	0.03	1.00000000	mice
Ube2v2	0.01	1.00000000	mice
Scarb2	-0.13	1.00000000	mice
Ints4	-0.09	1.00000000	mice
Klrb1c	0.00	1.00000000	mice
Hjurp	-0.06	1.00000000	mice
Tmem179b	0.04	1.00000000	mice
Vkorc1l1	-0.11	1.00000000	mice
Srgap3	0.08	1.00000000	mice
Psmc4	0.07	1.00000000	mice
Gpcpd1	0.03	1.00000000	mice
Hspa14	0.06	1.00000000	mice
Tbc1d22a	-0.11	1.00000000	mice
Ermp1	0.03	1.00000000	mice
Hdac1	0.06	1.00000000	mice
Zmat2	0.04	1.00000000	mice
Zfp68	-0.07	1.00000000	mice
Ptdss1	-0.12	1.00000000	mice
D430042O09Rik	0.03	1.00000000	mice
Efhd2	-0.01	1.00000000	mice
Pex13	0.10	1.00000000	mice
Zfp182	-0.10	1.00000000	mice
Cisd2	-0.06	1.00000000	mice
Sh2d2a	0.02	1.00000000	mice
Ctla4	0.24	1.00000000	mice
Dyrk2	-0.05	1.00000000	mice
Pogz	0.05	1.00000000	mice
Rfc2	0.04	1.00000000	mice
Emc10	0.04	1.00000000	mice

Ppp1r12c	-0.08	1.00000000	mice
Sugt1	0.02	1.00000000	mice
Nubp1	0.02	1.00000000	mice
Dexi	-0.07	1.00000000	mice
Znrd1	0.02	1.00000000	mice
Akr1b3	-0.08	1.00000000	mice
Snx29	-0.12	1.00000000	mice
Rap2b	-0.07	1.00000000	mice
Sbf1	-0.06	1.00000000	mice
Prdm1	0.05	1.00000000	mice
Bag4	-0.10	1.00000000	mice
Adnp	-0.10	1.00000000	mice
Rnf43	0.07	1.00000000	mice
Ercc5	-0.08	1.00000000	mice
Srrt	0.08	1.00000000	mice
Ostm1	-0.12	1.00000000	mice
Spast	0.05	1.00000000	mice
Cir1	-0.09	1.00000000	mice
Lasp1	-0.02	1.00000000	mice
Psmd8	0.07	1.00000000	mice
Prrc1	-0.11	1.00000000	mice
Unc50	0.06	1.00000000	mice
Naa50	0.06	1.00000000	mice
Prelid1	0.04	1.00000000	mice
Diablo	-0.06	1.00000000	mice
Gch1	-0.02	1.00000000	mice
Ndufaf4	0.05	1.00000000	mice
Glud1	-0.06	1.00000000	mice
Ciz1	0.03	1.00000000	mice
Arhgdia	-0.09	1.00000000	mice
Smg9	-0.11	1.00000000	mice
Dhx8	0.01	1.00000000	mice
Wsb1	-0.05	1.00000000	mice
Cop1	-0.06	1.00000000	mice
Lamtor4	0.03	1.00000000	mice
Slc25a26	-0.06	1.00000000	mice
Tmem259	0.05	1.00000000	mice
Pts	0.06	1.00000000	mice
Itprid2	-0.08	1.00000000	mice
Ptrhd1	0.06	1.00000000	mice
Eif4enif1	0.06	1.00000000	mice
Bzw1	-0.07	1.00000000	mice
Atxn1l	-0.07	1.00000000	mice
B9d2	0.01	1.00000000	mice
Tab2	0.10	1.00000000	mice
AI506816	-0.10	1.00000000	mice

Ddx39	0.02	1.00000000	mice
Akap8l	-0.09	1.00000000	mice
Slc20a2	0.05	1.00000000	mice
Cnot10	-0.08	1.00000000	mice
Carhsp1	0.04	1.00000000	mice
Larp7	-0.07	1.00000000	mice
Tmem131	-0.05	1.00000000	mice
Cfl2	0.05	1.00000000	mice
Supv3l1	0.04	1.00000000	mice
Rnf166	-0.07	1.00000000	mice
1810030O07Rik	0.08	1.00000000	mice
Impdh1	-0.05	1.00000000	mice
Stk26	-0.07	1.00000000	mice
Tpd52l2	0.08	1.00000000	mice
Phf20l1	-0.03	1.00000000	mice
Top1	0.08	1.00000000	mice
Tcf25	-0.08	1.00000000	mice
Ddrgk1	0.04	1.00000000	mice
Prkcb	-0.10	1.00000000	mice
Spen	0.05	1.00000000	mice
Ikbke	0.04	1.00000000	mice
Pigp	0.04	1.00000000	mice
Ash2l	-0.05	1.00000000	mice
Prkcq	0.16	1.00000000	mice
Esco1	-0.12	1.00000000	mice
Actr10	0.04	1.00000000	mice
Derl2	0.02	1.00000000	mice
Vps28	0.02	1.00000000	mice
Rnf149	0.09	1.00000000	mice
Klhl18	-0.10	1.00000000	mice
Nop9	0.06	1.00000000	mice
Ralbp1	-0.05	1.00000000	mice
Cox8a	0.04	1.00000000	mice
Nfrkb	-0.10	1.00000000	mice
Tfeb	0.05	1.00000000	mice
Nrf1	-0.09	1.00000000	mice
Tars	0.06	1.00000000	mice
Taf7	-0.04	1.00000000	mice
Wdr43	0.05	1.00000000	mice
Rfx7	-0.13	1.00000000	mice
Gnb4	0.07	1.00000000	mice
Fam149b	-0.08	1.00000000	mice
Papola	0.06	1.00000000	mice
Dcaf15	0.02	1.00000000	mice
Eif2b1	-0.05	1.00000000	mice
Dcp1b	-0.10	1.00000000	mice

Cyp51	-0.06	1.00000000	mice
Rhbdd1	0.01	1.00000000	mice
Naxd	-0.06	1.00000000	mice
Stag1	-0.07	1.00000000	mice
Ormdl1	0.02	1.00000000	mice
Pkn1	-0.13	1.00000000	mice
Wdr91	-0.05	1.00000000	mice
Dnajc2	0.04	1.00000000	mice
Aco2	0.01	1.00000000	mice
Smarca5	-0.08	1.00000000	mice
Jade3	-0.07	1.00000000	mice
Hopx	0.07	1.00000000	mice
Gatad1	0.02	1.00000000	mice
Cd53	0.06	1.00000000	mice
Dip2c	-0.13	1.00000000	mice
Zc3h18	-0.11	1.00000000	mice
Selenop	-0.10	1.00000000	mice
Pym1	-0.05	1.00000000	mice
Nfyb	0.05	1.00000000	mice
Prim2	-0.23	1.00000000	mice
Ccdc93	0.00	1.00000000	mice
Nek7	-0.01	1.00000000	mice
Thoc5	0.05	1.00000000	mice
Gsk3a	0.03	1.00000000	mice
Slf2	0.01	1.00000000	mice
Lcorl	0.07	1.00000000	mice
Plekhm1	-0.10	1.00000000	mice
Leng8	-0.06	1.00000000	mice
2610001J05Rik	0.01	1.00000000	mice
Tespa1	-0.04	1.00000000	mice
Wdr7	0.06	1.00000000	mice
Ninj1	0.07	1.00000000	mice
Kdm2b	0.05	1.00000000	mice
Rsl1d1	-0.08	1.00000000	mice
Fip1l1	-0.13	1.00000000	mice
Atxn2	0.03	1.00000000	mice
Hdac2	0.00	1.00000000	mice
Hypk	0.07	1.00000000	mice
Alyref	-0.08	1.00000000	mice
Rab43	-0.09	1.00000000	mice
Abcb7	0.03	1.00000000	mice
Srp19	0.03	1.00000000	mice
Ttf1	0.04	1.00000000	mice
Tom1	-0.12	1.00000000	mice
Rnf185	-0.09	1.00000000	mice
Tmed7	-0.10	1.00000000	mice

Card11	0.14	1.00000000	mice
Ssr1	0.08	1.00000000	mice
Dcaf11	0.06	1.00000000	mice
Khdrbs1	-0.02	1.00000000	mice
Golt1b	-0.08	1.00000000	mice
Far1	-0.09	1.00000000	mice
Sirt2	0.02	1.00000000	mice
Klhdc10	0.01	1.00000000	mice
Ak3	0.06	1.00000000	mice
Dctn3	0.05	1.00000000	mice
Dipk1a	-0.04	1.00000000	mice
Rprd1b	-0.08	1.00000000	mice
Tnrc18	0.01	1.00000000	mice
Herpud2	-0.08	1.00000000	mice
Chd1l	-0.09	1.00000000	mice
Crnkl1	0.03	1.00000000	mice
Cep128	0.01	1.00000000	mice
Zfp777	-0.08	1.00000000	mice
9530068E07Rik	0.04	1.00000000	mice
Bak1	0.01	1.00000000	mice
Zfp740	-0.07	1.00000000	mice
Rab11b	-0.11	1.00000000	mice
Arl6ip4	0.00	1.00000000	mice
Bcap29	-0.01	1.00000000	mice
Dusp2	-0.15	1.00000000	mice
Trp53inp2	-0.08	1.00000000	mice
Chmp1b	-0.07	1.00000000	mice
Atg16l2	0.07	1.00000000	mice
Pithd1	-0.05	1.00000000	mice
Clpx	-0.02	1.00000000	mice
Txnl1	0.04	1.00000000	mice
Hadha	-0.08	1.00000000	mice
Ptbp1	-0.08	1.00000000	mice
Scp2	-0.09	1.00000000	mice
Trnau1ap	0.02	1.00000000	mice
Clcf1	0.01	1.00000000	mice
Lactb2	-0.13	1.00000000	mice
Kif1bp	0.02	1.00000000	mice
Xpa	-0.10	1.00000000	mice
BC003965	-0.05	1.00000000	mice
Pex14	0.06	1.00000000	mice
Cope	0.00	1.00000000	mice
Crkl	0.04	1.00000000	mice
G6pdx	0.02	1.00000000	mice
Sugp2	-0.06	1.00000000	mice
Tmem183a	-0.11	1.00000000	mice

Bms1	-0.07	1.00000000	mice
Armcx5	0.03	1.00000000	mice
Lta4h	-0.10	1.00000000	mice
Alkbh5	-0.07	1.00000000	mice
Tsc22d1	-0.08	1.00000000	mice
Rptor	0.10	1.00000000	mice
Cog1	0.03	1.00000000	mice
Rnf113a2	0.04	1.00000000	mice
Unc45a	-0.08	1.00000000	mice
Nlrc3	-0.14	1.00000000	mice
Creld2	0.01	1.00000000	mice
Gabpa	0.00	1.00000000	mice
Tanc2	0.09	1.00000000	mice
Scaper	0.06	1.00000000	mice
Rrn3	-0.07	1.00000000	mice
Arhgap5	-0.07	1.00000000	mice
Tbcel	-0.09	1.00000000	mice
Vbp1	-0.08	1.00000000	mice
Chn2	-0.01	1.00000000	mice
Nup160	-0.10	1.00000000	mice
Zfp445	-0.08	1.00000000	mice
Chd1	-0.10	1.00000000	mice
Polg2	-0.01	1.00000000	mice
Abhd5	0.04	1.00000000	mice
Cetn3	-0.02	1.00000000	mice
Larp4	-0.05	1.00000000	mice
Olfm1	-0.17	1.00000000	mice
Erh	-0.10	1.00000000	mice
Emp1	-0.14	1.00000000	mice
Sypl	0.03	1.00000000	mice
Rab21	0.05	1.00000000	mice
Spata2	0.01	1.00000000	mice
Scfd1	0.00	1.00000000	mice
Kctd20	-0.03	1.00000000	mice
Nsfl1c	-0.08	1.00000000	mice
Pld3	0.04	1.00000000	mice
Zbtb17	0.05	1.00000000	mice
Ppp1r15a	-0.04	1.00000000	mice
Eif2b5	-0.08	1.00000000	mice
Hes6	-0.04	1.00000000	mice
Shld1	-0.04	1.00000000	mice
Leprot	-0.08	1.00000000	mice
Atn1	-0.08	1.00000000	mice
Plekhj1	0.04	1.00000000	mice
Ube2d2a	-0.08	1.00000000	mice
Akap9	-0.10	1.00000000	mice

Epc1	-0.11	1.00000000	mice
Stk24	-0.07	1.00000000	mice
Sympk	0.00	1.00000000	mice
Peli2	-0.13	1.00000000	mice
Hace1	-0.13	1.00000000	mice
Aph1c	0.09	1.00000000	mice
Anapc1	-0.09	1.00000000	mice
Wdr1	0.07	1.00000000	mice
Dnajb6	-0.05	1.00000000	mice
Rsl24d1	0.06	1.00000000	mice
Idh3g	0.02	1.00000000	mice
Psmb6	0.07	1.00000000	mice
Polr2d	-0.11	1.00000000	mice
Sec24b	-0.10	1.00000000	mice
Lpin1	-0.07	1.00000000	mice
Foxp4	-0.01	1.00000000	mice
Rbbp5	-0.09	1.00000000	mice
Zfp831	-0.10	1.00000000	mice
Tex261	0.05	1.00000000	mice
Kdm5c	-0.08	1.00000000	mice
Cpsf7	-0.06	1.00000000	mice
Ypel5	-0.06	1.00000000	mice
Alkbh3	-0.07	1.00000000	mice
Slc25a24	0.02	1.00000000	mice
Ankra2	0.01	1.00000000	mice
Parp4	0.06	1.00000000	mice
Aebp2	-0.03	1.00000000	mice
Cnot11	0.02	1.00000000	mice
2900026A02Rik	0.02	1.00000000	mice
Usp32	0.00	1.00000000	mice
Lfng	0.02	1.00000000	mice
Khnyn	-0.10	1.00000000	mice
CAAA01118383.1	-0.09	1.00000000	mice
Orc3	0.05	1.00000000	mice
Lias	-0.06	1.00000000	mice
Prxl2c	-0.05	1.00000000	mice
Atp6v0b	0.03	1.00000000	mice
Ubap2l	-0.01	1.00000000	mice
Dok2	-0.07	1.00000000	mice
Arid3a	-0.02	1.00000000	mice
Rsrc2	0.09	1.00000000	mice
Aldh3a2	-0.04	1.00000000	mice
Dhrs3	-0.01	1.00000000	mice
Patz1	-0.04	1.00000000	mice
Atp11a	-0.05	1.00000000	mice
Atp13a3	0.07	1.00000000	mice

Ibtk	-0.11	1.00000000	mice
Chd9	-0.10	1.00000000	mice
Sdad1	-0.04	1.00000000	mice
Ccnyl1	-0.05	1.00000000	mice
Basp1	-0.11	1.00000000	mice
Suv39h1	-0.04	1.00000000	mice
Abhd17c	-0.05	1.00000000	mice
Ube2d3	-0.05	1.00000000	mice
Suclg1	0.04	1.00000000	mice
Ppm1d	-0.08	1.00000000	mice
Zwint	-0.04	1.00000000	mice
Zfp326	-0.07	1.00000000	mice
Acap1	0.02	1.00000000	mice
Fry	0.04	1.00000000	mice
H2-Ab1	-0.05	1.00000000	mice
Nrip1	-0.01	1.00000000	mice
Ctsa	0.05	1.00000000	mice
Gpalpp1	-0.11	1.00000000	mice
Hscb	0.01	1.00000000	mice
Ankrd44	0.06	1.00000000	mice
Tmf1	0.04	1.00000000	mice
Ambra1	-0.04	1.00000000	mice
Rabep1	-0.07	1.00000000	mice
Als2	0.00	1.00000000	mice
Bcl2l11	-0.02	1.00000000	mice
Polr2h	-0.04	1.00000000	mice
Phtf2	-0.04	1.00000000	mice
Atl2	-0.09	1.00000000	mice
Abcf1	0.03	1.00000000	mice
Wasl	-0.08	1.00000000	mice
Jmjd6	-0.06	1.00000000	mice
Bcap31	0.04	1.00000000	mice
Antxr2	-0.01	1.00000000	mice
Trak1	-0.18	1.00000000	mice
Naa25	0.08	1.00000000	mice
Dap	0.04	1.00000000	mice
Brd8	-0.04	1.00000000	mice
Uimc1	0.06	1.00000000	mice
Scamp2	-0.10	1.00000000	mice
Dbr1	0.05	1.00000000	mice
Slc41a1	0.07	1.00000000	mice
Ankrd52	-0.06	1.00000000	mice
Nol8	-0.06	1.00000000	mice
Zfp367	-0.06	1.00000000	mice
Btf3	-0.04	1.00000000	mice
Phf12	0.09	1.00000000	mice

Kars	0.02	1.00000000	mice
Ccdc6	0.09	1.00000000	mice
Bet1	0.02	1.00000000	mice
Vps33b	0.02	1.00000000	mice
Ccdc71l	-0.09	1.00000000	mice
Ndfip2	-0.09	1.00000000	mice
Aagab	0.04	1.00000000	mice
Rassf5	-0.09	1.00000000	mice
Gadd45a	-0.10	1.00000000	mice
Lsm8	-0.04	1.00000000	mice
B3galnt2	0.04	1.00000000	mice
Zfp180	-0.07	1.00000000	mice
Fam89b	0.03	1.00000000	mice
Oip5os1	-0.11	1.00000000	mice
Zfp84	0.04	1.00000000	mice
Ifrd1	0.11	1.00000000	mice
Ube2g2	-0.06	1.00000000	mice
Smndc1	0.02	1.00000000	mice
Aasdhppt	0.03	1.00000000	mice
Ewsr1	0.06	1.00000000	mice
Asap2	-0.05	1.00000000	mice
H2afj	0.06	1.00000000	mice
Tbc1d22b	0.03	1.00000000	mice
Rbm48	-0.06	1.00000000	mice
Xpo6	-0.14	1.00000000	mice
Trim44	0.04	1.00000000	mice
Eif4b	-0.09	1.00000000	mice
Aamp	0.04	1.00000000	mice
Calu	0.03	1.00000000	mice
Frs2	-0.04	1.00000000	mice
Asap1	-0.06	1.00000000	mice
Pdk3	0.04	1.00000000	mice
Cdkn2aipnl	0.01	1.00000000	mice
Clk2	-0.06	1.00000000	mice
Cul4b	-0.08	1.00000000	mice
Ufm1	0.03	1.00000000	mice
Toe1	-0.05	1.00000000	mice
Bud23	-0.04	1.00000000	mice
Cog3	-0.10	1.00000000	mice
Zfp24	0.04	1.00000000	mice
Atp13a1	-0.06	1.00000000	mice
Ube2z	-0.08	1.00000000	mice
Slc39a9	0.03	1.00000000	mice
Lbr	0.06	1.00000000	mice
Pex16	-0.06	1.00000000	mice
Ptar1	-0.11	1.00000000	mice

Rev1	0.08	1.00000000	mice
Tpm1	-0.09	1.00000000	mice
Fgfr1op	0.05	1.00000000	mice
Plgrkt	0.04	1.00000000	mice
Sgk1	0.08	1.00000000	mice
Ecpas	0.08	1.00000000	mice
Pea15a	-0.12	1.00000000	mice
Ugdh	-0.08	1.00000000	mice
Dop1b	-0.08	1.00000000	mice
Dock11	-0.07	1.00000000	mice
Anks1	0.01	1.00000000	mice
Npm3	-0.05	1.00000000	mice
Arl4a	-0.08	1.00000000	mice
Psmb2	0.05	1.00000000	mice
Tfb2m	-0.08	1.00000000	mice
Rnf169	0.00	1.00000000	mice
Plcb4	-0.02	1.00000000	mice
Thrap3	-0.04	1.00000000	mice
Oxa1l	0.00	1.00000000	mice
Nup210	0.06	1.00000000	mice
1110059E24Rik	0.02	1.00000000	mice
Riok2	0.07	1.00000000	mice
Il17ra	-0.10	1.00000000	mice
Osbp	-0.10	1.00000000	mice
Zmat5	0.04	1.00000000	mice
Piezo1	0.04	1.00000000	mice
Eif4g1	0.03	1.00000000	mice
Prpf3	-0.07	1.00000000	mice
Zkscan14	-0.03	1.00000000	mice
Wac	0.08	1.00000000	mice
Lsm7	0.03	1.00000000	mice
Pnkp	0.03	1.00000000	mice
Dpf2	-0.09	1.00000000	mice
Exosc4	0.03	1.00000000	mice
Fam114a2	0.04	1.00000000	mice
Hras	0.01	1.00000000	mice
Vps37a	-0.10	1.00000000	mice
Yy1	-0.07	1.00000000	mice
Oaz2	0.03	1.00000000	mice
Bcl10	0.06	1.00000000	mice
Rmc1	-0.05	1.00000000	mice
Samd4b	0.05	1.00000000	mice
Nosip	-0.09	1.00000000	mice
Ncoa1	-0.09	1.00000000	mice
Irf3	0.04	1.00000000	mice
Nln	-0.16	1.00000000	mice

2410004B18Rik	-0.04	1.00000000	mice
Rab11fip2	-0.07	1.00000000	mice
Ube2j2	-0.08	1.00000000	mice
Nkapd1	0.02	1.00000000	mice
Smarcd1	-0.06	1.00000000	mice
Notch2	0.07	1.00000000	mice
Cebpz	-0.09	1.00000000	mice
Ramp1	-0.04	1.00000000	mice
Dcaf12	-0.07	1.00000000	mice
Hspd1	0.07	1.00000000	mice
Dennd6a	0.03	1.00000000	mice
Rab3gap1	-0.07	1.00000000	mice
Chd3	0.05	1.00000000	mice
Zbtb2	-0.08	1.00000000	mice
Farsb	-0.01	1.00000000	mice
Ppm1g	0.03	1.00000000	mice
Auh	-0.07	1.00000000	mice
Trf	-0.11	1.00000000	mice
Gxylt1	-0.15	1.00000000	mice
Cops4	0.02	1.00000000	mice
Tgfbr1	-0.07	1.00000000	mice
Ndufa12	0.02	1.00000000	mice
Lrrc8b	-0.10	1.00000000	mice
Srpr	0.04	1.00000000	mice
Trap1	-0.08	1.00000000	mice
Galk2	0.01	1.00000000	mice
Safb2	-0.11	1.00000000	mice
Snx27	0.04	1.00000000	mice
Slc9a1	-0.10	1.00000000	mice
Sf3b5	0.05	1.00000000	mice
Pno1	0.03	1.00000000	mice
Orai2	-0.07	1.00000000	mice
Nckap1	-0.13	1.00000000	mice
Smad5	0.05	1.00000000	mice
Rbm33	-0.08	1.00000000	mice
Ube2g1	-0.08	1.00000000	mice
Boll	0.02	1.00000000	mice
Rab9	0.04	1.00000000	mice
Taok3	0.02	1.00000000	mice
Ccnc	-0.11	1.00000000	mice
Nrbp1	-0.08	1.00000000	mice
Zfp87	0.02	1.00000000	mice
Dr1	-0.09	1.00000000	mice
Plekhb2	0.02	1.00000000	mice
Pnn	0.03	1.00000000	mice
Pspc1	-0.04	1.00000000	mice

Stt3a	0.00	1.00000000	mice
Ppm1b	-0.02	1.00000000	mice
Afg3l1	-0.09	1.00000000	mice
Tdg	-0.06	1.00000000	mice
Smarcb1	0.03	1.00000000	mice
Prep	-0.11	1.00000000	mice
Naa30	0.03	1.00000000	mice
Ttc4	-0.08	1.00000000	mice
Pdcl	-0.04	1.00000000	mice
Lsamp	0.14	1.00000000	mice
Prkcz	-0.05	1.00000000	mice
Kif13b	-0.06	1.00000000	mice
Wdr45b	-0.09	1.00000000	mice
Ubash3b	-0.14	1.00000000	mice
Trappc5	0.02	1.00000000	mice
Unc119b	0.01	1.00000000	mice
Dhx36	-0.07	1.00000000	mice
Dapp1	-0.15	1.00000000	mice
Tmed2	-0.06	1.00000000	mice
Aspscr1	-0.06	1.00000000	mice
Napsa	0.01	1.00000000	mice
Cwf19l2	-0.13	1.00000000	mice
Pfkfb3	0.09	1.00000000	mice
Rab2b	-0.07	1.00000000	mice
Srcap	-0.07	1.00000000	mice
Vamp2	-0.05	1.00000000	mice
Gtf3c3	-0.06	1.00000000	mice
Usp16	-0.10	1.00000000	mice
Insig2	0.03	1.00000000	mice
Fam98b	0.03	1.00000000	mice
Sf3a2	0.02	1.00000000	mice
Upf1	0.01	1.00000000	mice
Lrrc8a	-0.06	1.00000000	mice
Becn1	0.02	1.00000000	mice
Scyl3	-0.07	1.00000000	mice
5031425E22Rik	-0.09	1.00000000	mice
Hspa4l	-0.10	1.00000000	mice
Nfya	0.05	1.00000000	mice
Akr1a1	0.03	1.00000000	mice
Slc44a2	0.05	1.00000000	mice
Cnot3	0.02	1.00000000	mice
Sgf29	0.01	1.00000000	mice
Gabbr1	0.02	1.00000000	mice
Nsmaf	0.02	1.00000000	mice
Pfkp	-0.06	1.00000000	mice
Psd4	0.04	1.00000000	mice

Tmem175	-0.06	1.00000000	mice
Fam193b	-0.05	1.00000000	mice
Sri	0.02	1.00000000	mice
Fmnl1	0.00	1.00000000	mice
Brcc3	-0.09	1.00000000	mice
BC005561	0.02	1.00000000	mice
Wdyhv1	-0.06	1.00000000	mice
Cd5	0.25	1.00000000	mice
Bbx	0.00	1.00000000	mice
Pi4k2a	-0.07	1.00000000	mice
Nifk	0.06	1.00000000	mice
Spcs3	0.04	1.00000000	mice
Smg7	-0.10	1.00000000	mice
D130040H23Rik	0.01	1.00000000	mice
Tuba1c	0.03	1.00000000	mice
Ikzf3	0.05	1.00000000	mice
H2-Ke6	-0.04	1.00000000	mice
As3mt	-0.15	1.00000000	mice
E030030I06Rik	0.02	1.00000000	mice
Anapc11	-0.09	1.00000000	mice
Hmox2	0.06	1.00000000	mice
Ppp2r2d	0.04	1.00000000	mice
Rnasel	0.06	1.00000000	mice
Zfp36	0.05	1.00000000	mice
Rapgef1	0.09	1.00000000	mice
Fmr1	-0.06	1.00000000	mice
Pxn	0.02	1.00000000	mice
Desi1	-0.01	1.00000000	mice
Ubxn4	-0.08	1.00000000	mice
Tmem71	-0.02	1.00000000	mice
Ilvbl	-0.06	1.00000000	mice
Bambi	-0.08	1.00000000	mice
Dmtf1	0.05	1.00000000	mice
Ddt	0.05	1.00000000	mice
Pole4	-0.05	1.00000000	mice
Prps2	0.02	1.00000000	mice
Haus4	-0.08	1.00000000	mice
Col4a3bp	0.11	1.00000000	mice
Pinx1	-0.05	1.00000000	mice
Scap	-0.07	1.00000000	mice
Socs3	0.02	1.00000000	mice
Cabin1	-0.07	1.00000000	mice
Lcor	0.07	1.00000000	mice
Rbmxl1	0.05	1.00000000	mice
Eef1g	0.04	1.00000000	mice
Gtf2h1	-0.10	1.00000000	mice

Pcnp	0.03	1.00000000	mice
Slc4a2	0.01	1.00000000	mice
Tm9sf3	-0.09	1.00000000	mice
Emsy	0.03	1.00000000	mice
Ogt	-0.05	1.00000000	mice
Ipo8	-0.05	1.00000000	mice
Ccdc127	0.03	1.00000000	mice
Ppp6c	0.04	1.00000000	mice
Gnaq	0.09	1.00000000	mice
Nek1	-0.16	1.00000000	mice
Trmt10c	0.04	1.00000000	mice
Ubtf	-0.05	1.00000000	mice
Sugp1	-0.10	1.00000000	mice
Sike1	-0.12	1.00000000	mice
Trp53bp1	0.04	1.00000000	mice
Dnajc7	-0.08	1.00000000	mice
Cnot8	0.02	1.00000000	mice
Inpp5d	-0.17	1.00000000	mice
Rbm5	0.02	1.00000000	mice
Farsa	0.05	1.00000000	mice
2810004N23Rik	0.02	1.00000000	mice
Sdhc	0.03	1.00000000	mice
Pnisr	-0.07	1.00000000	mice
Pex19	-0.05	1.00000000	mice
Rai1	0.05	1.00000000	mice
Cltb	0.01	1.00000000	mice
Eif3m	-0.06	1.00000000	mice
Sec61a1	0.02	1.00000000	mice
Actr5	-0.05	1.00000000	mice
Focad	-0.06	1.00000000	mice
Ilf2	0.01	1.00000000	mice
R3hcc1l	-0.06	1.00000000	mice
Abcg2	0.04	1.00000000	mice
Fut8	-0.03	1.00000000	mice
Xbp1	-0.08	1.00000000	mice
Pja2	-0.08	1.00000000	mice
Ranbp2	-0.05	1.00000000	mice
Eif3k	-0.07	1.00000000	mice
Rrs1	-0.05	1.00000000	mice
Tmem11	-0.06	1.00000000	mice
Cox6a1	0.05	1.00000000	mice
Dlg1	-0.10	1.00000000	mice
Rc3h2	-0.07	1.00000000	mice
Rtf2	0.02	1.00000000	mice
Trip11	-0.04	1.00000000	mice
Disp1	-0.10	1.00000000	mice

Elovl1	0.01	1.00000000	mice
Tmem120a	-0.04	1.00000000	mice
4921524J17Rik	-0.05	1.00000000	mice
Rnf44	0.02	1.00000000	mice
Fam160b1	-0.05	1.00000000	mice
Sf3b2	-0.07	1.00000000	mice
Ap3m1	-0.04	1.00000000	mice
Impa1	0.01	1.00000000	mice
Pcf11	-0.02	1.00000000	mice
Eci2	0.00	1.00000000	mice
Zw10	0.02	1.00000000	mice
Ctdnep1	0.02	1.00000000	mice
Rab10os	-0.06	1.00000000	mice
Nadk	0.02	1.00000000	mice
Tjp2	-0.22	1.00000000	mice
5830408C22Rik	0.02	1.00000000	mice
Arfgef1	-0.07	1.00000000	mice
Sdccag8	0.11	1.00000000	mice
Tmem165	-0.07	1.00000000	mice
Pcmt1	-0.06	1.00000000	mice
Paxip1	-0.08	1.00000000	mice
Otud4	-0.09	1.00000000	mice
Nf2	-0.13	1.00000000	mice
Polr1a	-0.07	1.00000000	mice
Spg11	0.03	1.00000000	mice
Cd8b1	-0.02	1.00000000	mice
Zfp729b	-0.07	1.00000000	mice
Stard7	0.02	1.00000000	mice
Cep83	-0.03	1.00000000	mice
Dhx38	0.00	1.00000000	mice
Hexb	-0.05	1.00000000	mice
Phtf1	0.02	1.00000000	mice
Gnpda2	0.01	1.00000000	mice
Agps	-0.07	1.00000000	mice
Cables1	-0.23	1.00000000	mice
Aig1	0.08	1.00000000	mice
Dcun1d5	-0.09	1.00000000	mice
Ets2	0.07	1.00000000	mice
Acp1	0.01	1.00000000	mice
Pi4kb	0.02	1.00000000	mice
Alg5	0.02	1.00000000	mice
Rbbp7	-0.02	1.00000000	mice
Atxn7l3b	-0.07	1.00000000	mice
Chm	0.05	1.00000000	mice
Cenpc1	-0.09	1.00000000	mice
Tial1	-0.06	1.00000000	mice

Cdk9	-0.07	1.00000000	mice
Tpr	-0.10	1.00000000	mice
Trp53inp1	0.07	1.00000000	mice
Itgav	0.21	1.00000000	mice
Apool	0.01	1.00000000	mice
Tmem33	-0.02	1.00000000	mice
Tmed4	0.04	1.00000000	mice
Kpna1	-0.03	1.00000000	mice
Vps39	0.03	1.00000000	mice
Stau1	-0.07	1.00000000	mice
Pgrmc1	-0.05	1.00000000	mice
Leo1	-0.08	1.00000000	mice
Rnf168	0.00	1.00000000	mice
Eif3d	0.05	1.00000000	mice
Prkar1a	-0.05	1.00000000	mice
Afg3l2	-0.02	1.00000000	mice
Pdha1	-0.10	1.00000000	mice
Glrx	0.01	1.00000000	mice
Cdipt	0.03	1.00000000	mice
4632427E13Rik	0.02	1.00000000	mice
Dedd2	-0.06	1.00000000	mice
Wdr44	0.05	1.00000000	mice
Tmem222	-0.07	1.00000000	mice
Pdhb	-0.05	1.00000000	mice
Lpcat3	-0.09	1.00000000	mice
Ccnl1	-0.02	1.00000000	mice
Sf3a1	0.02	1.00000000	mice
Hnrnpul2	0.01	1.00000000	mice
Zbtb44	-0.12	1.00000000	mice
Pbdc1	0.05	1.00000000	mice
Pip4k2c	-0.06	1.00000000	mice
Ilf3	0.08	1.00000000	mice
Vps35l	0.01	1.00000000	mice
Ppp1cb	-0.12	1.00000000	mice
Klhl25	-0.01	1.00000000	mice
Hmg20a	0.03	1.00000000	mice
Pbx3	0.07	1.00000000	mice
Slc4a7	0.07	1.00000000	mice
Arl5a	0.05	1.00000000	mice
9430091E24Rik	-0.05	1.00000000	mice
Fbxo8	-0.12	1.00000000	mice
Ppp2r1a	-0.08	1.00000000	mice
Rabggtb	0.04	1.00000000	mice
Gpbp1l1	-0.06	1.00000000	mice
Golph3	0.02	1.00000000	mice
Uggt1	-0.09	1.00000000	mice

Dock4	-0.23	1.00000000	mice
Nr1h2	-0.08	1.00000000	mice
Smap1	0.05	1.00000000	mice
Usp14	-0.06	1.00000000	mice
Zfp512	0.03	1.00000000	mice
Otub1	0.03	1.00000000	mice
Rbm22	-0.07	1.00000000	mice
Fez2	0.04	1.00000000	mice
Otud5	0.02	1.00000000	mice
Ccdc174	-0.10	1.00000000	mice
Cyhr1	0.04	1.00000000	mice
Vamp7	0.02	1.00000000	mice
Tsn	-0.07	1.00000000	mice
Dicer1	0.05	1.00000000	mice
Nmd3	0.02	1.00000000	mice
Rpn1	0.03	1.00000000	mice
Eif6	-0.10	1.00000000	mice
Hsbp1	0.08	1.00000000	mice
Kbtbd11	0.02	1.00000000	mice
Vps26a	0.04	1.00000000	mice
Gpn3	-0.08	1.00000000	mice
Atp6v0c	0.06	1.00000000	mice
Haus6	-0.09	1.00000000	mice
Ada	0.01	1.00000000	mice
Rbm18	-0.09	1.00000000	mice
Commd3	0.00	1.00000000	mice
Pggt1b	-0.10	1.00000000	mice
Enox2	0.05	1.00000000	mice
Ero1l	0.02	1.00000000	mice
Neu1	0.01	1.00000000	mice
Scand1	0.05	1.00000000	mice
Sucla2	-0.01	1.00000000	mice
Ptpmt1	-0.05	1.00000000	mice
Tm2d1	-0.01	1.00000000	mice
Casp8ap2	0.02	1.00000000	mice
Sbno2	0.03	1.00000000	mice
Paip2b	-0.06	1.00000000	mice
Txn2	0.03	1.00000000	mice
Pdcl3	-0.02	1.00000000	mice
Btbd10	0.02	1.00000000	mice
Vps11	-0.05	1.00000000	mice
Bclaf1	-0.01	1.00000000	mice
Poc1b	0.00	1.00000000	mice
Ddb1	-0.08	1.00000000	mice
Cct2	0.03	1.00000000	mice
Thy1	0.08	1.00000000	mice

Poldip3	-0.09	1.00000000	mice
Htt	0.07	1.00000000	mice
Ahnak	-0.15	1.00000000	mice
Smyd3	-0.04	1.00000000	mice
Psma6	0.05	1.00000000	mice
Hpcal1	-0.12	1.00000000	mice
Qrfp	-0.11	1.00000000	mice
Spcs1	0.06	1.00000000	mice
Pola2	0.02	1.00000000	mice
Ubqln2	-0.04	1.00000000	mice
Snrnp40	0.02	1.00000000	mice
Brms1l	-0.09	1.00000000	mice
Utp18	-0.06	1.00000000	mice
Dbnl	0.01	1.00000000	mice
Tvp23b	-0.06	1.00000000	mice
Pgp	0.00	1.00000000	mice
Rab1a	-0.08	1.00000000	mice
Twsg1	-0.03	1.00000000	mice
Cep44	-0.06	1.00000000	mice
Prkaa1	0.00	1.00000000	mice
Heg1	-0.03	1.00000000	mice
Dync1i2	0.03	1.00000000	mice
Zfp800	-0.01	1.00000000	mice
Cnpy3	-0.07	1.00000000	mice
Zyx	0.05	1.00000000	mice
Oxsr1	0.02	1.00000000	mice
Insig1	-0.04	1.00000000	mice
Jarid2	0.08	1.00000000	mice
Limd2	-0.09	1.00000000	mice
Dnmt1	-0.07	1.00000000	mice
Pop7	0.00	1.00000000	mice
Lclat1	-0.06	1.00000000	mice
Tnfrsf18	0.09	1.00000000	mice
Trappc4	0.01	1.00000000	mice
Nos1ap	-0.09	1.00000000	mice
Emc3	0.02	1.00000000	mice
Supt6	0.02	1.00000000	mice
Cox5a	0.05	1.00000000	mice
Erich1	-0.06	1.00000000	mice
Siva1	-0.09	1.00000000	mice
Surf6	0.03	1.00000000	mice
Dcaf8	-0.11	1.00000000	mice
Fabp5	-0.03	1.00000000	mice
Naga	0.03	1.00000000	mice
Blmh	-0.11	1.00000000	mice
Sdhaf1	0.05	1.00000000	mice

Polr2j	0.02	1.00000000	mice
Pank2	0.00	1.00000000	mice
Jpt1	-0.05	1.00000000	mice
Rbm27	-0.01	1.00000000	mice
Slc25a5	0.06	1.00000000	mice
Kif16b	-0.10	1.00000000	mice
Vps18	-0.06	1.00000000	mice
Ahsa1	-0.02	1.00000000	mice
Rnf126	0.01	1.00000000	mice
Ppp2cb	-0.06	1.00000000	mice
Btbd7	0.00	1.00000000	mice
Brwd3	0.06	1.00000000	mice
Smox	0.09	1.00000000	mice
Pus10	0.04	1.00000000	mice
Upf2	0.07	1.00000000	mice
Rnf25	0.05	1.00000000	mice
Hdac3	-0.03	1.00000000	mice
Kansl3	-0.06	1.00000000	mice
Oaz1	0.04	1.00000000	mice
Agap2	-0.06	1.00000000	mice
Ctcf	0.02	1.00000000	mice
Sap30l	-0.08	1.00000000	mice
Tusc2	0.02	1.00000000	mice
Arpc4	0.04	1.00000000	mice
Bcr	-0.03	1.00000000	mice
Syne3	-0.02	1.00000000	mice
Cstf2	-0.06	1.00000000	mice
Tm9sf2	-0.01	1.00000000	mice
Ipo7	-0.09	1.00000000	mice
Arf3	-0.12	1.00000000	mice
Foxj3	-0.08	1.00000000	mice
Prdx3	0.01	1.00000000	mice
Eif4h	-0.06	1.00000000	mice
Fam98a	0.00	1.00000000	mice
Nudt4	-0.07	1.00000000	mice
Ppp1r21	0.02	1.00000000	mice
Hook3	-0.07	1.00000000	mice
Zc3h4	-0.04	1.00000000	mice
Chchd2	0.06	1.00000000	mice
Trim59	-0.02	1.00000000	mice
Camsap1	0.01	1.00000000	mice
Isy1	0.03	1.00000000	mice
Bicra	-0.01	1.00000000	mice
Ctsl	-0.01	1.00000000	mice
Rint1	-0.05	1.00000000	mice
Plekha1	-0.03	1.00000000	mice

Ebpl	0.00	1.00000000	mice
Dtnbp1	0.07	1.00000000	mice
Gtf2a1	0.04	1.00000000	mice
Arfgap3	0.01	1.00000000	mice
Fmnl3	-0.11	1.00000000	mice
Glyr1	-0.02	1.00000000	mice
Gpat4	-0.10	1.00000000	mice
Ncbp1	-0.10	1.00000000	mice
Taf4b	-0.04	1.00000000	mice
Pik3r4	0.04	1.00000000	mice
Cript	-0.06	1.00000000	mice
Tmem181a	0.02	1.00000000	mice
Arrb2	-0.02	1.00000000	mice
Copa	-0.04	1.00000000	mice
Cep95	0.00	1.00000000	mice
Dpysl2	-0.03	1.00000000	mice
Stam	-0.08	1.00000000	mice
Togaram1	0.03	1.00000000	mice
Ctbp1	-0.07	1.00000000	mice
Sepsecs	-0.04	1.00000000	mice
Katnbl1	-0.07	1.00000000	mice
Zc3hc1	-0.02	1.00000000	mice
Rabl6	-0.09	1.00000000	mice
Rnf123	-0.04	1.00000000	mice
Snrpb	0.02	1.00000000	mice
Rnaseh2c	0.01	1.00000000	mice
Dnajc25	0.02	1.00000000	mice
Idnk	0.02	1.00000000	mice
Nras	-0.07	1.00000000	mice
Ranbp1	0.06	1.00000000	mice
Faap100	-0.06	1.00000000	mice
Trrap	-0.08	1.00000000	mice
Tex14	0.08	1.00000000	mice
Hspe1	-0.03	1.00000000	mice
Acat2	0.03	1.00000000	mice
Prpf18	-0.07	1.00000000	mice
Rftn1	-0.07	1.00000000	mice
Ubxn7	-0.07	1.00000000	mice
Deaf1	-0.05	1.00000000	mice
Zfp346	0.00	1.00000000	mice
Dcps	0.01	1.00000000	mice
Sppl3	-0.06	1.00000000	mice
Ubxn1	0.04	1.00000000	mice
Napg	0.02	1.00000000	mice
Tollip	-0.02	1.00000000	mice
A930024E05Rik	0.02	1.00000000	mice

Tnik	-0.04	1.00000000	mice
Hk1	0.01	1.00000000	mice
Ssu72	0.03	1.00000000	mice
Acyp2	-0.05	1.00000000	mice
Traf6	-0.06	1.00000000	mice
Serp1	-0.07	1.00000000	mice
Arpc2	0.03	1.00000000	mice
Ikbkg	0.02	1.00000000	mice
Slc35e1	0.01	1.00000000	mice
Pdcd7	0.00	1.00000000	mice
Uspl1	0.03	1.00000000	mice
Ice1	-0.07	1.00000000	mice
Cul5	-0.08	1.00000000	mice
Trps1	-0.07	1.00000000	mice
Snx20	0.01	1.00000000	mice
Ofd1	-0.05	1.00000000	mice
Coa5	0.01	1.00000000	mice
Fam234a	-0.01	1.00000000	mice
Heatr6	-0.04	1.00000000	mice
Wdr20	-0.06	1.00000000	mice
Txndc15	0.00	1.00000000	mice
Ythdc2	-0.08	1.00000000	mice
Snd1	0.05	1.00000000	mice
Eml4	0.07	1.00000000	mice
Tmem65	-0.05	1.00000000	mice
Rxylt1	-0.04	1.00000000	mice
Gpc1	0.01	1.00000000	mice
Tmcc3	-0.16	1.00000000	mice
Hmgcr	0.02	1.00000000	mice
Zfp451	0.03	1.00000000	mice
Zcchc8	0.02	1.00000000	mice
Dlat	0.01	1.00000000	mice
Fars2	-0.02	1.00000000	mice
Ldlrad4	-0.03	1.00000000	mice
Sptlc2	0.01	1.00000000	mice
Atrnl1	-0.04	1.00000000	mice
Spryd3	-0.05	1.00000000	mice
Fxr1	0.03	1.00000000	mice
Kmt2b	0.03	1.00000000	mice
Rap2c	-0.01	1.00000000	mice
Vps37c	-0.04	1.00000000	mice
Gon4l	-0.06	1.00000000	mice
Ubl7	-0.04	1.00000000	mice
Rpf1	0.03	1.00000000	mice
Slmap	0.02	1.00000000	mice
Setd2	0.03	1.00000000	mice

Zfp362	-0.04	1.00000000	mice
Aar2	-0.06	1.00000000	mice
Ext2	0.02	1.00000000	mice
Nemp2	0.01	1.00000000	mice
Trappc12	-0.04	1.00000000	mice
Lztfl1	0.00	1.00000000	mice
Pebp1	-0.05	1.00000000	mice
Aldh2	0.00	1.00000000	mice
Fh1	0.00	1.00000000	mice
Ndrg3	0.05	1.00000000	mice
Lsm5	0.05	1.00000000	mice
Tbl1xr1	-0.09	1.00000000	mice
Nipsnap2	-0.09	1.00000000	mice
Ykt6	0.00	1.00000000	mice
Rab11a	-0.07	1.00000000	mice
D10Wsu102e	0.04	1.00000000	mice
Vcl	-0.15	1.00000000	mice
Hectd4	-0.05	1.00000000	mice
Terf1	0.04	1.00000000	mice
Rad54l2	0.01	1.00000000	mice
Rev3l	0.08	1.00000000	mice
Trp53bp2	0.03	1.00000000	mice
Uri1	-0.07	1.00000000	mice
Ncaph2	0.00	1.00000000	mice
Qsox1	-0.06	1.00000000	mice
4930417O13Rik	0.03	1.00000000	mice
Arhgap10	-0.03	1.00000000	mice
Ndufb8	0.04	1.00000000	mice
Dynlt3	0.02	1.00000000	mice
Brd7	-0.07	1.00000000	mice
Neurl3	-0.12	1.00000000	mice
Erp29	0.03	1.00000000	mice
Zmym2	-0.08	1.00000000	mice
Zfp710	0.02	1.00000000	mice
St7l	0.01	1.00000000	mice
Tmem41b	-0.04	1.00000000	mice
Fosl2	-0.04	1.00000000	mice
Slc7a5	0.03	1.00000000	mice
Cybc1	-0.03	1.00000000	mice
Vps41	0.07	1.00000000	mice
Sharpin	-0.06	1.00000000	mice
Setd1a	-0.03	1.00000000	mice
Slc25a16	-0.07	1.00000000	mice
Zbtb8os	-0.02	1.00000000	mice
Ap1g1	0.04	1.00000000	mice
Fam193a	0.02	1.00000000	mice

Psmd9	-0.05	1.00000000	mice
Ireb2	-0.06	1.00000000	mice
Zfp384	-0.06	1.00000000	mice
Scai	0.01	1.00000000	mice
2700097O09Rik	-0.01	1.00000000	mice
Skap1	-0.02	1.00000000	mice
Ric1	0.05	1.00000000	mice
Ptpn12	0.04	1.00000000	mice
Opa1	0.00	1.00000000	mice
Ube2r2	-0.08	1.00000000	mice
Nfs1	0.01	1.00000000	mice
Pikfyve	-0.02	1.00000000	mice
Parp2	0.03	1.00000000	mice
Ppp4c	-0.03	1.00000000	mice
Ndufs3	-0.10	1.00000000	mice
Psmb1	0.03	1.00000000	mice
Ddx43	0.02	1.00000000	mice
B230219D22Rik	-0.09	1.00000000	mice
Lysmd3	0.04	1.00000000	mice
Ei24	0.01	1.00000000	mice
2310011J03Rik	-0.07	1.00000000	mice
Topors	-0.05	1.00000000	mice
Vamp4	-0.05	1.00000000	mice
Homer1	0.07	1.00000000	mice
Ccdc47	0.04	1.00000000	mice
Zfat	0.04	1.00000000	mice
Eif4e2	0.05	1.00000000	mice
Klrc3	-0.03	1.00000000	mice
Zcchc17	-0.06	1.00000000	mice
Cwc22	-0.06	1.00000000	mice
Kctd10	0.01	1.00000000	mice
Pcna	0.02	1.00000000	mice
Cog4	0.03	1.00000000	mice
Diaph2	0.07	1.00000000	mice
Naa10	0.00	1.00000000	mice
Erf	-0.04	1.00000000	mice
Itpr1	-0.03	1.00000000	mice
Cat	0.01	1.00000000	mice
Ppdpf	0.04	1.00000000	mice
Virma	-0.05	1.00000000	mice
Lrif1	0.04	1.00000000	mice
Klhdc3	-0.06	1.00000000	mice
Ccni	-0.04	1.00000000	mice
Fbl	0.02	1.00000000	mice
Ube2q2	-0.04	1.00000000	mice
Tmem184c	-0.03	1.00000000	mice

Pex7	0.01	1.00000000	mice
Tmem50b	0.01	1.00000000	mice
Celf1	-0.05	1.00000000	mice
Galnt12	-0.05	1.00000000	mice
Nbas	-0.03	1.00000000	mice
Nfat5	0.09	1.00000000	mice
Galnt10	-0.07	1.00000000	mice
Chuk	0.01	1.00000000	mice
Prrc2c	0.00	1.00000000	mice
Acat1	-0.08	1.00000000	mice
Adpgk	-0.05	1.00000000	mice
Hsp90ab1	0.09	1.00000000	mice
Rsbn1	0.09	1.00000000	mice
Tpp2	-0.03	1.00000000	mice
Rexo4	-0.03	1.00000000	mice
Phactr2	-0.04	1.00000000	mice
Prr12	-0.06	1.00000000	mice
Sp4	-0.06	1.00000000	mice
Slc1a5	0.03	1.00000000	mice
Tor1b	0.04	1.00000000	mice
R3hdm4	-0.08	1.00000000	mice
5430405H02Rik	0.02	1.00000000	mice
Glrx2	-0.02	1.00000000	mice
Dock2	-0.03	1.00000000	mice
Errfi1	0.08	1.00000000	mice
Spg21	-0.08	1.00000000	mice
Ap3m2	-0.05	1.00000000	mice
Arl1	-0.05	1.00000000	mice
Cdc42se2	0.04	1.00000000	mice
Lrch1	0.03	1.00000000	mice
Rnf20	-0.05	1.00000000	mice
Rpap3	-0.05	1.00000000	mice
Lats1	-0.07	1.00000000	mice
Lsm2	0.04	1.00000000	mice
Clip1	-0.05	1.00000000	mice
Slc9a9	-0.08	1.00000000	mice
Zfp438	-0.08	1.00000000	mice
Prelid3b	-0.06	1.00000000	mice
Zmynd11	-0.06	1.00000000	mice
Timp2	0.07	1.00000000	mice
Gpsm3	0.01	1.00000000	mice
Ube4a	-0.11	1.00000000	mice
Dusp1	0.14	1.00000000	mice
Iah1	-0.05	1.00000000	mice
Nufip2	0.05	1.00000000	mice
Seh1l	-0.05	1.00000000	mice

Nop14	0.03	1.00000000	mice
Ythdf2	0.02	1.00000000	mice
Pcmtd2	-0.08	1.00000000	mice
Pfdn5	0.03	1.00000000	mice
Vapb	-0.06	1.00000000	mice
Iqgap1	-0.03	1.00000000	mice
Nol4l	-0.06	1.00000000	mice
Edrf1	-0.09	1.00000000	mice
Supt20	-0.02	1.00000000	mice
Anxa5	0.04	1.00000000	mice
Sirt7	-0.08	1.00000000	mice
Abraxas2	0.02	1.00000000	mice
8030462N17Rik	0.00	1.00000000	mice
Clpp	-0.05	1.00000000	mice
Angel2	-0.07	1.00000000	mice
Anapc16	0.01	1.00000000	mice
Gpatch11	0.01	1.00000000	mice
Dapk3	0.00	1.00000000	mice
Luc7l	0.04	1.00000000	mice
Nedd4	-0.01	1.00000000	mice
Nsa2	0.03	1.00000000	mice
Fbxo22	0.02	1.00000000	mice
Slc50a1	0.00	1.00000000	mice
Ankrd28	-0.05	1.00000000	mice
Vps4b	0.03	1.00000000	mice
Lmbrd2	-0.07	1.00000000	mice
Sin3b	-0.02	1.00000000	mice
Tubgcp5	-0.05	1.00000000	mice
Supt4a	0.05	1.00000000	mice
Cox16	0.01	1.00000000	mice
Fzd5	0.00	1.00000000	mice
Scpep1	0.01	1.00000000	mice
Fbxo38	0.03	1.00000000	mice
Hes1	-0.11	1.00000000	mice
Kif5b	-0.04	1.00000000	mice
Atp6v0e	0.02	1.00000000	mice
Tex10	-0.02	1.00000000	mice
Snx15	-0.04	1.00000000	mice
Hprt	0.01	1.00000000	mice
Rbm28	0.01	1.00000000	mice
Rin3	-0.18	1.00000000	mice
Zgpat	0.00	1.00000000	mice
Dnajc3	-0.07	1.00000000	mice
Rnaset2a	0.02	1.00000000	mice
Acaca	-0.07	1.00000000	mice
Gimap1os	-0.05	1.00000000	mice

Xrn2	0.01	1.00000000	mice
Uxt	-0.03	1.00000000	mice
Ddx6	-0.03	1.00000000	mice
Osbpl7	-0.04	1.00000000	mice
Trmt112	0.03	1.00000000	mice
Clec16a	-0.06	1.00000000	mice
Sap18	0.01	1.00000000	mice
Psme3	-0.04	1.00000000	mice
Fam53c	-0.06	1.00000000	mice
Iars2	-0.06	1.00000000	mice
Arid1b	0.06	1.00000000	mice
Ubxn2a	0.03	1.00000000	mice
Rpp30	-0.06	1.00000000	mice
Yes1	0.16	1.00000000	mice
Ranbp3	-0.06	1.00000000	mice
Baz1b	-0.03	1.00000000	mice
N4bp2l2	-0.10	1.00000000	mice
Trappc6a	0.00	1.00000000	mice
Actg1	0.06	1.00000000	mice
Ltv1	-0.06	1.00000000	mice
Ier5l	0.06	1.00000000	mice
Zdhhc9	-0.03	1.00000000	mice
Gapvd1	-0.01	1.00000000	mice
Snapc1	0.04	1.00000000	mice
Zbtb24	-0.03	1.00000000	mice
Uck2	0.02	1.00000000	mice
Ralgapa2	-0.08	1.00000000	mice
Nhp2	0.02	1.00000000	mice
Zfp160	0.03	1.00000000	mice
Tmem128	0.01	1.00000000	mice
Nsd3	-0.01	1.00000000	mice
Pxmp4	-0.06	1.00000000	mice
Selenos	0.03	1.00000000	mice
Dgat1	-0.09	1.00000000	mice
Arl15	-0.03	1.00000000	mice
Ap1s2	0.00	1.00000000	mice
Atp6v1c1	-0.06	1.00000000	mice
Slc25a20	0.00	1.00000000	mice
Zfp639	-0.04	1.00000000	mice
Tbc1d14	0.06	1.00000000	mice
Gskip	0.01	1.00000000	mice
Cnot4	0.05	1.00000000	mice
Ezh1	-0.06	1.00000000	mice
Rnaseh1	-0.02	1.00000000	mice
Nup188	-0.08	1.00000000	mice
Anp32a	0.04	1.00000000	mice

Snhg5	-0.01	1.00000000	mice
Idh3b	0.02	1.00000000	mice
Yrdc	-0.03	1.00000000	mice
Parg	0.01	1.00000000	mice
Arhgap30	0.04	1.00000000	mice
Slc45a4	-0.10	1.00000000	mice
Wls	-0.03	1.00000000	mice
Ndufaf2	-0.06	1.00000000	mice
Tomm40	0.01	1.00000000	mice
Atp1a1	-0.08	1.00000000	mice
Cdc34	0.02	1.00000000	mice
Kdelr2	0.03	1.00000000	mice
Strada	-0.08	1.00000000	mice
Senp3	0.01	1.00000000	mice
Rsu1	0.02	1.00000000	mice
Gdap2	-0.11	1.00000000	mice
Nxpe3	0.09	1.00000000	mice
Ahcyl1	0.00	1.00000000	mice
Phc2	-0.09	1.00000000	mice
Uba5	-0.06	1.00000000	mice
Arf5	-0.04	1.00000000	mice
Gins4	-0.01	1.00000000	mice
Ufd1	-0.05	1.00000000	mice
Sin3a	0.03	1.00000000	mice
Slc23a2	-0.09	1.00000000	mice
Zfp746	-0.04	1.00000000	mice
Spred1	-0.03	1.00000000	mice
Nup205	0.00	1.00000000	mice
Tbc1d12	-0.08	1.00000000	mice
Golph3l	0.01	1.00000000	mice
Ptpa	-0.09	1.00000000	mice
Capn2	-0.03	1.00000000	mice
Atxn2l	-0.10	1.00000000	mice
Taf2	0.02	1.00000000	mice
Nelfb	0.02	1.00000000	mice
Sept7	0.04	1.00000000	mice
Ano10	-0.06	1.00000000	mice
Gpr68	-0.04	1.00000000	mice
Rela	0.02	1.00000000	mice
Pds5a	-0.08	1.00000000	mice
Galnt11	-0.03	1.00000000	mice
Nol9	-0.05	1.00000000	mice
U2surp	0.05	1.00000000	mice
Zswim6	0.01	1.00000000	mice
Alkbh8	0.02	1.00000000	mice
Pold3	0.03	1.00000000	mice

Arhgap35	0.01	1.00000000	mice
Zfp507	-0.03	1.00000000	mice
Gltp	0.01	1.00000000	mice
Acap2	-0.06	1.00000000	mice
Elavl1	-0.04	1.00000000	mice
Nudt3	-0.03	1.00000000	mice
Sf3b4	-0.05	1.00000000	mice
Snap29	0.02	1.00000000	mice
Akirin2	-0.06	1.00000000	mice
Emc6	0.01	1.00000000	mice
Sec24a	-0.09	1.00000000	mice
Atp6ap1	-0.02	1.00000000	mice
Abcf3	-0.04	1.00000000	mice
Eif3j1	-0.04	1.00000000	mice
Cdkn2d	-0.05	1.00000000	mice
Rif1	0.03	1.00000000	mice
Snu13	-0.05	1.00000000	mice
Aimp1	0.07	1.00000000	mice
Riok1	-0.01	1.00000000	mice
Afdn	-0.02	1.00000000	mice
Tob2	0.07	1.00000000	mice
Commd7	0.00	1.00000000	mice
Srebf2	-0.06	1.00000000	mice
Bcl2l13	-0.03	1.00000000	mice
Tomm20	0.03	1.00000000	mice
Pigf	0.01	1.00000000	mice
H3f3b	0.02	1.00000000	mice
Babam2	0.01	1.00000000	mice
Park7	0.03	1.00000000	mice
Zfp26	-0.02	1.00000000	mice
Slf1	-0.08	1.00000000	mice
Sgpl1	-0.03	1.00000000	mice
Top3a	-0.07	1.00000000	mice
Acox3	0.01	1.00000000	mice
Hacd2	0.03	1.00000000	mice
Smg6	0.07	1.00000000	mice
St13	0.01	1.00000000	mice
Zhx1	0.00	1.00000000	mice
Adgrl4	-0.01	1.00000000	mice
Lims1	0.03	1.00000000	mice
Rab27a	0.01	1.00000000	mice
Taf11	0.02	1.00000000	mice
Kcnq1ot1	0.04	1.00000000	mice
Senp6	-0.01	1.00000000	mice
Rad23b	-0.07	1.00000000	mice
Ptpn1	0.00	1.00000000	mice

Cd2bp2	-0.04	1.00000000	mice
Zfp944	0.00	1.00000000	mice
Tmem50a	-0.06	1.00000000	mice
Pibf1	0.00	1.00000000	mice
Clip2	-0.01	1.00000000	mice
Larp4b	-0.01	1.00000000	mice
Zdhhc13	-0.05	1.00000000	mice
Txndc12	0.03	1.00000000	mice
Yeats2	-0.12	1.00000000	mice
Prpf6	-0.06	1.00000000	mice
Prr7	0.04	1.00000000	mice
Fkbp4	-0.04	1.00000000	mice
Rbm14	0.00	1.00000000	mice
Ddx49	-0.04	1.00000000	mice
Ccar1	0.02	1.00000000	mice
Btrc	0.06	1.00000000	mice
Rrp7a	0.03	1.00000000	mice
Snx30	-0.01	1.00000000	mice
Ubr5	0.04	1.00000000	mice
Slc2a3	0.02	1.00000000	mice
Frmd4b	-0.07	1.00000000	mice
Slc26a2	-0.04	1.00000000	mice
Zcrb1	0.00	1.00000000	mice
Zfyve27	-0.05	1.00000000	mice
Gimap6	-0.07	1.00000000	mice
Ttll3	-0.03	1.00000000	mice
Cops2	0.02	1.00000000	mice
Ddx27	-0.01	1.00000000	mice
Preb	0.01	1.00000000	mice
Ppp2r2a	0.06	1.00000000	mice
Rprd1a	-0.02	1.00000000	mice
Phf14	0.01	1.00000000	mice
Cct4	-0.04	1.00000000	mice
Il2rb	-0.02	1.00000000	mice
Hmgxb3	-0.01	1.00000000	mice
Nub1	-0.04	1.00000000	mice
Eps15l1	-0.12	1.00000000	mice
Fam126b	0.03	1.00000000	mice
Dnajc21	-0.03	1.00000000	mice
Clcn4	-0.01	1.00000000	mice
Sec22b	-0.07	1.00000000	mice
Luc7l3	0.01	1.00000000	mice
Tm9sf4	-0.03	1.00000000	mice
Psmd5	-0.03	1.00000000	mice
Cers5	-0.09	1.00000000	mice
Ankrd16	-0.01	1.00000000	mice

Cdyl	0.00	1.00000000	mice
Ccl25	-0.10	1.00000000	mice
Limk2	-0.08	1.00000000	mice
Rae1	-0.07	1.00000000	mice
2310033P09Rik	0.02	1.00000000	mice
1110004F10Rik	0.04	1.00000000	mice
Traf5	-0.03	1.00000000	mice
Ing1	-0.11	1.00000000	mice
Dusp5	0.04	1.00000000	mice
Sft2d2	-0.04	1.00000000	mice
Ckap5	-0.02	1.00000000	mice
Klhl15	-0.01	1.00000000	mice
Ppp3r1	0.02	1.00000000	mice
Sys1	-0.08	1.00000000	mice
Hacd3	0.01	1.00000000	mice
Glod4	0.00	1.00000000	mice
Slc25a46	-0.04	1.00000000	mice
Tbc1d5	0.04	1.00000000	mice
Fyn	0.08	1.00000000	mice
Gramd1a	-0.07	1.00000000	mice
Ric8b	0.02	1.00000000	mice
Prr13	-0.02	1.00000000	mice
Pank3	0.03	1.00000000	mice
Cdk5rap2	-0.07	1.00000000	mice
D330041H03Rik	-0.03	1.00000000	mice
Sra1	0.02	1.00000000	mice
Abcf2	-0.04	1.00000000	mice
Utp14a	0.03	1.00000000	mice
Lrpprc	-0.02	1.00000000	mice
Fbxl5	0.02	1.00000000	mice
Fbxo3	-0.06	1.00000000	mice
Nrros	0.02	1.00000000	mice
Pigt	-0.03	1.00000000	mice
Alkbh1	0.05	1.00000000	mice
Ccz1	0.04	1.00000000	mice
Chst12	-0.09	1.00000000	mice
Tnfaip3	0.04	1.00000000	mice
Thoc2	0.05	1.00000000	mice
Rbfa	-0.05	1.00000000	mice
Impad1	0.04	1.00000000	mice
Rcl1	0.00	1.00000000	mice
Jdp2	-0.03	1.00000000	mice
Fli1	0.08	1.00000000	mice
Use1	-0.04	1.00000000	mice
Phf5a	0.02	1.00000000	mice
Psmd13	-0.02	1.00000000	mice

Lamtor1	0.01	1.00000000	mice
P4ha1	0.08	1.00000000	mice
Sde2	0.02	1.00000000	mice
Helb	-0.04	1.00000000	mice
Il1rap	0.02	1.00000000	mice
Necap2	0.00	1.00000000	mice
Gabpb1	-0.03	1.00000000	mice
Timm22	0.00	1.00000000	mice
Prdm10	-0.10	1.00000000	mice
Tmem161b	0.04	1.00000000	mice
Tbc1d13	-0.05	1.00000000	mice
Xist	0.01	1.00000000	mice
Npepps	0.02	1.00000000	mice
Rbbp6	-0.04	1.00000000	mice
Cab39l	-0.04	1.00000000	mice
Sinhcaf	-0.01	1.00000000	mice
Slc30a5	-0.05	1.00000000	mice
Ybx1	0.03	1.00000000	mice
Azin1	0.01	1.00000000	mice
Ostf1	-0.05	1.00000000	mice
Osbpl2	-0.06	1.00000000	mice
Casd1	-0.02	1.00000000	mice
Relch	0.06	1.00000000	mice
Prpf19	0.01	1.00000000	mice
Ercc6	-0.03	1.00000000	mice
Itgb3	0.00	1.00000000	mice
Ube2o	0.00	1.00000000	mice
Exosc1	-0.03	1.00000000	mice
Kcnj8	-0.04	1.00000000	mice
Scaf4	0.03	1.00000000	mice
Vdac2	0.00	1.00000000	mice
Clpb	-0.07	1.00000000	mice
Pcyt2	-0.04	1.00000000	mice
Anp32e	0.02	1.00000000	mice
BC031181	-0.08	1.00000000	mice
Glce	0.03	1.00000000	mice
Tdrd3	-0.08	1.00000000	mice
Dda1	-0.06	1.00000000	mice
Snhg1	-0.04	1.00000000	mice
Tes	0.05	1.00000000	mice
Atp6v1b2	-0.04	1.00000000	mice
Xab2	-0.04	1.00000000	mice
Rbm4	0.01	1.00000000	mice
Adrm1	-0.02	1.00000000	mice
Prpsap1	-0.02	1.00000000	mice
Slc7a6	0.04	1.00000000	mice

Sephs2	-0.07	1.00000000	mice
Sdhd	-0.05	1.00000000	mice
Rnft1	0.00	1.00000000	mice
Synj1	0.02	1.00000000	mice
Eif4e3	0.02	1.00000000	mice
Ruvbl1	0.03	1.00000000	mice
Oat	-0.06	1.00000000	mice
Ap2a1	0.00	1.00000000	mice
Araf	0.00	1.00000000	mice
Smarcc2	-0.09	1.00000000	mice
Edc4	-0.06	1.00000000	mice
Nme2	0.06	1.00000000	mice
Hdac8	-0.11	1.00000000	mice
Fbxo7	-0.05	1.00000000	mice
Pigs	-0.01	1.00000000	mice
Abcc4	0.02	1.00000000	mice
Exoc1	-0.01	1.00000000	mice
Cwc25	-0.10	1.00000000	mice
Lax1	-0.04	1.00000000	mice
Trip4	-0.08	1.00000000	mice
Polr2e	-0.01	1.00000000	mice
Kif3b	-0.04	1.00000000	mice
Gan	-0.04	1.00000000	mice
Tmem164	-0.11	1.00000000	mice
Hgs	-0.04	1.00000000	mice
1110059G10Rik	-0.03	1.00000000	mice
Hdhd5	-0.02	1.00000000	mice
Rpe	-0.03	1.00000000	mice
Kdm3b	-0.04	1.00000000	mice
Cpsf3	0.03	1.00000000	mice
Paip1	0.00	1.00000000	mice
Cnksr3	-0.05	1.00000000	mice
Fam49a	-0.05	1.00000000	mice
Sart1	0.01	1.00000000	mice
Nsmce4a	-0.02	1.00000000	mice
Dido1	-0.05	1.00000000	mice
Pdss1	0.06	1.00000000	mice
Nol11	0.01	1.00000000	mice
Pde4b	-0.04	1.00000000	mice
Rnf10	0.02	1.00000000	mice
Ppfibp1	-0.04	1.00000000	mice
Zhx2	-0.03	1.00000000	mice
Lyar	0.03	1.00000000	mice
Ccser2	-0.06	1.00000000	mice
Nans	0.03	1.00000000	mice
Pitpnm1	-0.01	1.00000000	mice

Fam210a	-0.04	1.00000000	mice
Vapa	-0.02	1.00000000	mice
Tgfbrap1	-0.02	1.00000000	mice
Scd2	0.02	1.00000000	mice
Hmgcs1	0.04	1.00000000	mice
Rad23a	0.00	1.00000000	mice
Akt2	-0.04	1.00000000	mice
Bdp1	-0.01	1.00000000	mice
Dcaf1	-0.06	1.00000000	mice
Plekho2	0.02	1.00000000	mice
Slc35b3	-0.07	1.00000000	mice
Ndufb11	0.05	1.00000000	mice
Ptpn23	-0.03	1.00000000	mice
Parp1	0.03	1.00000000	mice
Nsun2	-0.01	1.00000000	mice
Inip	-0.03	1.00000000	mice
Jtb	-0.05	1.00000000	mice
Safb	0.00	1.00000000	mice
Ppp1r8	-0.02	1.00000000	mice
Trir	0.02	1.00000000	mice
Rexo1	-0.06	1.00000000	mice
Jmjd1c	0.05	1.00000000	mice
Coro7	-0.01	1.00000000	mice
Tsc22d2	0.01	1.00000000	mice
Prpf38b	0.02	1.00000000	mice
Stxbp5	0.04	1.00000000	mice
Acsl4	0.02	1.00000000	mice
Eloa	-0.01	1.00000000	mice
Chmp2b	0.02	1.00000000	mice
Nvl	-0.06	1.00000000	mice
Tardbp	0.02	1.00000000	mice
Arhgef10	-0.04	1.00000000	mice
Brd4	0.07	1.00000000	mice
Tmem87b	-0.07	1.00000000	mice
Hsdl2	0.05	1.00000000	mice
Klhl9	0.03	1.00000000	mice
Spint2	-0.05	1.00000000	mice
Grpel1	-0.08	1.00000000	mice
Slc38a6	-0.10	1.00000000	mice
Dbt	-0.05	1.00000000	mice
Actn4	0.04	1.00000000	mice
Atf1	-0.09	1.00000000	mice
Ftsj3	-0.01	1.00000000	mice
Emc8	-0.03	1.00000000	mice
Secisbp2l	0.01	1.00000000	mice
Ifi27	0.00	1.00000000	mice

Dcun1d4	-0.06	1.00000000	mice
Setd1b	-0.02	1.00000000	mice
Dnm1l	-0.03	1.00000000	mice
Rtn4	-0.02	1.00000000	mice
Tmem189	-0.12	1.00000000	mice
Tomm22	-0.03	1.00000000	mice
Bmpr2	-0.02	1.00000000	mice
Dock5	-0.21	1.00000000	mice
Cd96	0.01	1.00000000	mice
Rpia	-0.04	1.00000000	mice
Csnk1g2	0.02	1.00000000	mice
Tm9sf1	0.00	1.00000000	mice
Dhx30	-0.02	1.00000000	mice
Ift20	-0.09	1.00000000	mice
Acaa2	-0.01	1.00000000	mice
Dusp11	-0.06	1.00000000	mice
Kat7	0.01	1.00000000	mice
Zfp322a	-0.07	1.00000000	mice
Bloc1s6	-0.04	1.00000000	mice
Ttc17	-0.04	1.00000000	mice
Cdc40	-0.07	1.00000000	mice
Pdia4	-0.01	1.00000000	mice
Nck2	-0.09	1.00000000	mice
Nrarp	-0.07	1.00000000	mice
Dnajc8	-0.05	1.00000000	mice
Gpr132	-0.05	1.00000000	mice
Ist1	0.02	1.00000000	mice
Dpp8	-0.08	1.00000000	mice
Ilk	-0.07	1.00000000	mice
Ndufv1	-0.01	1.00000000	mice
P4hb	0.05	1.00000000	mice
Trim28	-0.06	1.00000000	mice
Copg2	-0.01	1.00000000	mice
R3hdm1	-0.02	1.00000000	mice
Fam174a	-0.04	1.00000000	mice
1700025G04Rik	-0.12	1.00000000	mice
Fam50a	-0.03	1.00000000	mice
Fancc	0.02	1.00000000	mice
Rad17	-0.01	1.00000000	mice
Timm10b	0.01	1.00000000	mice
Sel1l	-0.03	1.00000000	mice
Cask	-0.07	1.00000000	mice
Sae1	-0.08	1.00000000	mice
Prpf39	-0.02	1.00000000	mice
Hccs	-0.03	1.00000000	mice
Babam1	0.00	1.00000000	mice

Fbxo45	-0.04	1.00000000	mice
Rnf41	0.03	1.00000000	mice
Tmem245	-0.03	1.00000000	mice
Usp38	0.01	1.00000000	mice
Zbtb25	-0.01	1.00000000	mice
Upf3a	0.01	1.00000000	mice
Tmod3	-0.03	1.00000000	mice
I730030J21Rik	0.01	1.00000000	mice
Ppih	-0.01	1.00000000	mice
Ubac1	-0.01	1.00000000	mice
App	0.02	1.00000000	mice
Xndc1	-0.02	1.00000000	mice
Cacul1	-0.10	1.00000000	mice
Limk1	-0.03	1.00000000	mice
Plcl2	0.02	1.00000000	mice
Galnt1	-0.04	1.00000000	mice
Gadd45gip1	-0.03	1.00000000	mice
Rbm10	-0.07	1.00000000	mice
Etaa1	0.00	1.00000000	mice
Cnot9	0.00	1.00000000	mice
Syncrip	-0.01	1.00000000	mice
Entpd1	0.03	1.00000000	mice
Cct6a	0.00	1.00000000	mice
Itsn1	-0.05	1.00000000	mice
Lmo4	0.00	1.00000000	mice
Tmem62	-0.06	1.00000000	mice
Ankle2	-0.04	1.00000000	mice
Bysl	-0.01	1.00000000	mice
Syne1	-0.03	1.00000000	mice
Rtn3	0.01	1.00000000	mice
Irak1	-0.04	1.00000000	mice
A430005L14Rik	0.02	1.00000000	mice
Qtrt1	-0.03	1.00000000	mice
Ip6k2	-0.06	1.00000000	mice
Washc3	-0.03	1.00000000	mice
Phrf1	0.00	1.00000000	mice
Srsf2	0.01	1.00000000	mice
Endod1	-0.01	1.00000000	mice
Ubr7	-0.08	1.00000000	mice
Zfp53	0.05	1.00000000	mice
Lamtor3	-0.01	1.00000000	mice
Sbk1	-0.02	1.00000000	mice
Npc1	-0.03	1.00000000	mice
Ciapin1	-0.06	1.00000000	mice
Ap2a2	-0.01	1.00000000	mice
Snap23	-0.03	1.00000000	mice

Atp6v0a2	0.01	1.00000000	mice
Rc3h1	0.05	1.00000000	mice
Aph1a	0.00	1.00000000	mice
Hcfc1	0.00	1.00000000	mice
Ndufs1	0.01	1.00000000	mice
Tbck	-0.02	1.00000000	mice
Stip1	0.01	1.00000000	mice
Ate1	0.01	1.00000000	mice
Irak2	0.00	1.00000000	mice
Fam207a	-0.06	1.00000000	mice
Crebl2	-0.01	1.00000000	mice
Zzz3	-0.02	1.00000000	mice
Stub1	-0.04	1.00000000	mice
Nop10	0.01	1.00000000	mice
Tubb4b	-0.05	1.00000000	mice
4933406I18Rik	0.05	1.00000000	mice
Pcmtd1	-0.05	1.00000000	mice
Orc5	-0.02	1.00000000	mice
Zap70	0.00	1.00000000	mice
C1qbp	0.04	1.00000000	mice
Fbxo4	-0.01	1.00000000	mice
Thap12	-0.02	1.00000000	mice
Dyrk1a	0.00	1.00000000	mice
Reep3	0.01	1.00000000	mice
Tmem170b	-0.03	1.00000000	mice
Kri1	-0.02	1.00000000	mice
Crcp	-0.05	1.00000000	mice
Iffo2	0.00	1.00000000	mice
Suclg2	-0.02	1.00000000	mice
Apex2	-0.03	1.00000000	mice
Zc3h7a	-0.08	1.00000000	mice
Nasp	0.04	1.00000000	mice
Zadh2	-0.04	1.00000000	mice
Tmem120b	0.01	1.00000000	mice
Nolc1	0.03	1.00000000	mice
Fam32a	0.01	1.00000000	mice
Hax1	0.00	1.00000000	mice
Cep192	-0.09	1.00000000	mice
Cep97	0.01	1.00000000	mice
Tada3	-0.04	1.00000000	mice
Nom1	-0.01	1.00000000	mice
Strap	-0.04	1.00000000	mice
Acly	0.02	1.00000000	mice
Hsph1	-0.04	1.00000000	mice
Dph3	-0.05	1.00000000	mice
Ube2e1	-0.08	1.00000000	mice

Sap130	-0.02	1.00000000	mice
Trub2	-0.01	1.00000000	mice
Zdhhc3	-0.04	1.00000000	mice
Trmt11	0.01	1.00000000	mice
Arl2bp	-0.02	1.00000000	mice
Tmem9b	-0.04	1.00000000	mice
Abtb2	0.14	1.00000000	mice
Csnk1e	0.03	1.00000000	mice
0610030E20Rik	0.01	1.00000000	mice
Cd27	-0.03	1.00000000	mice
Nek9	0.01	1.00000000	mice
Utp20	-0.06	1.00000000	mice
Sar1a	-0.08	1.00000000	mice
Prkrip1	-0.09	1.00000000	mice
Trappc11	0.02	1.00000000	mice
Prkce	0.03	1.00000000	mice
Klri1	-0.05	1.00000000	mice
Dgkz	-0.07	1.00000000	mice
Ammecr1l	-0.04	1.00000000	mice
Tgs1	-0.01	1.00000000	mice
Sat1	0.06	1.00000000	mice
Zkscan1	-0.01	1.00000000	mice
Ivns1abp	-0.05	1.00000000	mice
Gpkow	0.07	1.00000000	mice
4930453N24Rik	-0.04	1.00000000	mice
Fubp1	0.03	1.00000000	mice
Pdpk1	0.00	1.00000000	mice
Polm	-0.03	1.00000000	mice
Brpf3	-0.05	1.00000000	mice
Hikeshi	-0.01	1.00000000	mice
Suds3	-0.03	1.00000000	mice
Yme1l1	-0.04	1.00000000	mice
Nod1	-0.12	1.00000000	mice
Trim33	0.04	1.00000000	mice
Ddx20	-0.03	1.00000000	mice
Fdxr	-0.03	1.00000000	mice
Zfp51	-0.02	1.00000000	mice
Ssbp4	-0.04	1.00000000	mice
Adam10	0.00	1.00000000	mice
Klhl28	0.00	1.00000000	mice
Ncstn	-0.01	1.00000000	mice
Wbp11	-0.01	1.00000000	mice
Nop56	-0.01	1.00000000	mice
Vps35	-0.02	1.00000000	mice
Usf1	0.00	1.00000000	mice
Slc25a4	-0.06	1.00000000	mice

Parn	-0.05	1.00000000	mice
Exosc9	0.01	1.00000000	mice
Prkci	-0.06	1.00000000	mice
Rab22a	-0.04	1.00000000	mice
Qk	-0.08	1.00000000	mice
Lzic	-0.03	1.00000000	mice
Ralgapb	-0.03	1.00000000	mice
Snx13	0.02	1.00000000	mice
Rfc1	0.00	1.00000000	mice
Raph1	-0.01	1.00000000	mice
Stxbp2	-0.01	1.00000000	mice
Kmt2d	0.00	1.00000000	mice
Josd1	-0.04	1.00000000	mice
Raly	-0.04	1.00000000	mice
Sfmbt1	0.05	1.00000000	mice
Proser1	-0.08	1.00000000	mice
Adipor2	-0.08	1.00000000	mice
Rnf40	0.01	1.00000000	mice
Rfk	-0.03	1.00000000	mice
Glrx5	-0.04	1.00000000	mice
Rnf14	0.01	1.00000000	mice
Rufy3	-0.07	1.00000000	mice
Capn1	0.00	1.00000000	mice
Ddx19a	-0.01	1.00000000	mice
Pds5b	-0.05	1.00000000	mice
Cramp1l	-0.05	1.00000000	mice
Cdk17	0.01	1.00000000	mice
Timm8a1	0.02	1.00000000	mice
Ncl	0.00	1.00000000	mice
Eif2b4	0.00	1.00000000	mice
Tanc1	-0.03	1.00000000	mice
Slu7	-0.02	1.00000000	mice
Zbtb16	-0.30	1.00000000	mice
Rreb1	-0.08	1.00000000	mice
Ralgapa1	0.03	1.00000000	mice
Oxr1	0.05	1.00000000	mice
Ankrd13a	0.01	1.00000000	mice
Rrnad1	-0.03	1.00000000	mice
Hipk1	0.01	1.00000000	mice
Ctla2b	-0.09	1.00000000	mice
Prkdc	-0.08	1.00000000	mice
Dnajc11	-0.01	1.00000000	mice
U2af2	0.00	1.00000000	mice
Sec23ip	0.00	1.00000000	mice
Ap3d1	-0.04	1.00000000	mice
Srprb	-0.01	1.00000000	mice

Rrm2b	0.00	1.00000000	mice
Rnpepl1	-0.01	1.00000000	mice
Ndufb5	0.05	1.00000000	mice
Ppp1r9b	-0.07	1.00000000	mice
Idh3a	-0.01	1.00000000	mice
Usp37	-0.05	1.00000000	mice
Zfp983	0.02	1.00000000	mice
Pdcd5	0.01	1.00000000	mice
Smu1	-0.05	1.00000000	mice
Pum2	-0.04	1.00000000	mice
Ilkap	-0.04	1.00000000	mice
Eif2ak4	-0.04	1.00000000	mice
Cherp	0.03	1.00000000	mice
Ppme1	-0.02	1.00000000	mice
Ufc1	-0.04	1.00000000	mice
Hnrnpc	0.06	1.00000000	mice
Zfp131	0.00	1.00000000	mice
Thap3	-0.05	1.00000000	mice
Tstd2	-0.02	1.00000000	mice
Bag1	-0.12	1.00000000	mice
Ppp1r10	0.03	1.00000000	mice
Dnajb1	0.02	1.00000000	mice
Ly75	-0.09	1.00000000	mice
Pign	-0.08	1.00000000	mice
Gns	-0.03	1.00000000	mice
Sertad1	0.02	1.00000000	mice
Smarcc1	0.04	1.00000000	mice
Prkd3	-0.01	1.00000000	mice
Git2	0.00	1.00000000	mice
Dennd3	-0.15	1.00000000	mice
Luzp1	-0.03	1.00000000	mice
Cbfb	-0.07	1.00000000	mice
Kxd1	0.00	1.00000000	mice
Fnta	-0.12	1.00000000	mice
Tbce	-0.07	1.00000000	mice
Klf4	0.03	1.00000000	mice
Vps50	-0.04	1.00000000	mice
Eny2	-0.03	1.00000000	mice
Ccdc88b	0.02	1.00000000	mice
Pik3cb	-0.14	1.00000000	mice
Gtf3c1	-0.05	1.00000000	mice
1500011B03Rik	0.02	1.00000000	mice
Rbm34	0.01	1.00000000	mice
Airn	-0.15	1.00000000	mice
Tmem126a	-0.08	1.00000000	mice
Bckdha	-0.04	1.00000000	mice

Cdk7	-0.05	1.00000000	mice
Sptssa	-0.09	1.00000000	mice
Tet2	-0.03	1.00000000	mice
Fam45a	-0.01	1.00000000	mice
Trmt2b	-0.02	1.00000000	mice
Smim10l1	-0.01	1.00000000	mice
Rnf24	-0.10	1.00000000	mice
Gid8	-0.07	1.00000000	mice
Hmgcl	0.01	1.00000000	mice
Lypla1	0.00	1.00000000	mice
Hint3	0.01	1.00000000	mice
Cdc26	0.01	1.00000000	mice
St7	-0.02	1.00000000	mice
Pvr	-0.05	1.00000000	mice
Pgls	-0.02	1.00000000	mice
Senp5	-0.01	1.00000000	mice
Dck	-0.03	1.00000000	mice
Sart3	-0.03	1.00000000	mice
Fam120a	-0.05	1.00000000	mice
Eif3g	0.00	1.00000000	mice
Dgkh	0.02	1.00000000	mice
Ppp6r1	0.00	1.00000000	mice
Rab14	-0.05	1.00000000	mice
Rnmt	-0.01	1.00000000	mice
Lrp6	-0.07	1.00000000	mice
Gigyf1	-0.07	1.00000000	mice
Dctn1	-0.05	1.00000000	mice
Vps13c	-0.02	1.00000000	mice
Zfp236	-0.02	1.00000000	mice
Psmd3	-0.03	1.00000000	mice
Lsm3	-0.05	1.00000000	mice
1810058I24Rik	-0.07	1.00000000	mice
Pmm2	-0.01	1.00000000	mice
Taf4	-0.08	1.00000000	mice
Rcn2	0.00	1.00000000	mice
Zcchc9	-0.03	1.00000000	mice
Zfp809	-0.04	1.00000000	mice
Calcrl	-0.07	1.00000000	mice
Faf2	0.04	1.00000000	mice
Triap1	-0.05	1.00000000	mice
Ipmk	-0.03	1.00000000	mice
Efl1	-0.10	1.00000000	mice
Tab3	-0.01	1.00000000	mice
Tmem135	0.03	1.00000000	mice
Phf10	-0.01	1.00000000	mice
Naa16	-0.03	1.00000000	mice

Zfp148	0.02	1.00000000	mice
Immp2l	-0.10	1.00000000	mice
Smc1a	0.01	1.00000000	mice
Brd9	-0.07	1.00000000	mice
Ctdspl2	0.03	1.00000000	mice
Cyb5r3	-0.02	1.00000000	mice
Gas5	-0.02	1.00000000	mice
Srp54a	-0.04	1.00000000	mice
Cbl	-0.07	1.00000000	mice
Rnf115	0.01	1.00000000	mice
Heatr5b	-0.02	1.00000000	mice
Dohh	-0.05	1.00000000	mice
Ino80b	-0.03	1.00000000	mice
Ncbp3	-0.05	1.00000000	mice
Tut7	-0.02	1.00000000	mice
Hmgn2	-0.01	1.00000000	mice
Derl1	0.00	1.00000000	mice
Zfp830	-0.01	1.00000000	mice
Ripk1	0.01	1.00000000	mice
Commd6	0.00	1.00000000	mice
Kat2b	0.02	1.00000000	mice
Rpa2	0.02	1.00000000	mice
Fdx2	-0.06	1.00000000	mice
Glo1	-0.04	1.00000000	mice
Rbm7	0.00	1.00000000	mice
Rad50	0.05	1.00000000	mice
Sf3a3	0.00	1.00000000	mice
Srrm1	0.02	1.00000000	mice
Shc1	-0.01	1.00000000	mice
Ccnt2	-0.02	1.00000000	mice
Tex30	-0.04	1.00000000	mice
Rnf181	0.01	1.00000000	mice
Otud7b	-0.14	1.00000000	mice
Usp24	-0.05	1.00000000	mice
Cdc123	-0.04	1.00000000	mice
Prkcd	0.04	1.00000000	mice
Gt(ROSA)26Sor	0.01	1.00000000	mice
Dnajb12	-0.05	1.00000000	mice
Dnajc14	-0.02	1.00000000	mice
Card19	-0.03	1.00000000	mice
Vps13a	0.01	1.00000000	mice
Atp2a3	-0.05	1.00000000	mice
Pde4dip	-0.05	1.00000000	mice
Copb2	-0.05	1.00000000	mice
Gspt1	0.06	1.00000000	mice
Nelfa	0.00	1.00000000	mice

Ubr2	0.03	1.00000000	mice
Umps	-0.02	1.00000000	mice
Cggbp1	0.00	1.00000000	mice
Naa40	-0.02	1.00000000	mice
Smarcd2	-0.01	1.00000000	mice
S100pbp	-0.06	1.00000000	mice
Selenon	0.00	1.00000000	mice
Soat1	0.00	1.00000000	mice
Epn1	-0.03	1.00000000	mice
Wdr89	-0.06	1.00000000	mice
Tox4	-0.06	1.00000000	mice
Snf8	0.03	1.00000000	mice
Strip1	-0.01	1.00000000	mice
Pdcd2l	-0.06	1.00000000	mice
Dctn4	-0.03	1.00000000	mice
Tmem168	0.00	1.00000000	mice
Ddx10	-0.02	1.00000000	mice
Ubxn8	-0.03	1.00000000	mice
Ywhaz	0.01	1.00000000	mice
Arrdc1	-0.03	1.00000000	mice
Nsf	-0.08	1.00000000	mice
Uhmk1	-0.02	1.00000000	mice
Polr2g	-0.01	1.00000000	mice
Smn1	-0.03	1.00000000	mice
Zfp787	-0.02	1.00000000	mice
Bag5	-0.02	1.00000000	mice
Ercc4	-0.05	1.00000000	mice
Fbxw2	-0.04	1.00000000	mice
Lars	0.03	1.00000000	mice
Rrp15	0.01	1.00000000	mice
Nob1	-0.03	1.00000000	mice
Abhd2	-0.02	1.00000000	mice
Pwwp2a	0.06	1.00000000	mice
Gna12	-0.06	1.00000000	mice
Paics	-0.03	1.00000000	mice
Rp2	0.00	1.00000000	mice
Cmtm6	-0.03	1.00000000	mice
Ap5m1	-0.03	1.00000000	mice
Dnmbp	-0.03	1.00000000	mice
Dst	-0.03	1.00000000	mice
Rit1	-0.03	1.00000000	mice
Wsb2	-0.02	1.00000000	mice
Prkx	-0.07	1.00000000	mice
Ercc3	0.02	1.00000000	mice
Prps1	-0.01	1.00000000	mice
Wdr5	-0.01	1.00000000	mice

Gid4	-0.03	1.00000000	mice
Kidins220	0.01	1.00000000	mice
Fasn	-0.05	1.00000000	mice
Osgep	-0.03	1.00000000	mice
Nsmce3	-0.05	1.00000000	mice
Phkg2	-0.08	1.00000000	mice
Pcgf3	-0.03	1.00000000	mice
Rbmx	-0.03	1.00000000	mice
Clasp1	0.04	1.00000000	mice
E2f3	-0.01	1.00000000	mice
Nol12	-0.02	1.00000000	mice
Golga2	-0.02	1.00000000	mice
Ddx55	-0.02	1.00000000	mice
Tnfrsf26	-0.01	1.00000000	mice
Fam192a	-0.01	1.00000000	mice
Lamtor5	-0.07	1.00000000	mice
Hnrnpul1	-0.02	1.00000000	mice
4921511C10Rik	0.03	1.00000000	mice
Ncoa5	-0.02	1.00000000	mice
Eapp	-0.02	1.00000000	mice
Ptpn22	-0.04	1.00000000	mice
Specc1l	-0.04	1.00000000	mice
Nrd1	0.03	1.00000000	mice
Pak1ip1	0.02	1.00000000	mice
Ripor2	-0.01	1.00000000	mice
Bloc1s4	-0.03	1.00000000	mice
Lsm1	-0.06	1.00000000	mice
Erc1	-0.01	1.00000000	mice
Tbc1d20	-0.05	1.00000000	mice
Lrrc8c	0.00	1.00000000	mice
Usp34	0.03	1.00000000	mice
Ascc2	-0.06	1.00000000	mice
Hspa9	-0.05	1.00000000	mice
Cnot1	-0.02	1.00000000	mice
Anapc10	-0.03	1.00000000	mice
Rnf2	0.01	1.00000000	mice
Casc3	-0.08	1.00000000	mice
Eif4a3	-0.08	1.00000000	mice
Nup214	-0.03	1.00000000	mice
Copb1	-0.06	1.00000000	mice
Stat4	0.07	1.00000000	mice
Zfp169	-0.05	1.00000000	mice
R3hdm2	-0.10	1.00000000	mice
Rexo2	-0.07	1.00000000	mice
Kdm4a	0.00	1.00000000	mice
Eif2s3x	0.01	1.00000000	mice

Serpinb6a	-0.01	1.00000000	mice
Pot1b	-0.01	1.00000000	mice
Purb	-0.01	1.00000000	mice
Sipa1	-0.02	1.00000000	mice
Tars2	-0.03	1.00000000	mice
Gtf2e2	-0.04	1.00000000	mice
Zfhx3	-0.04	1.00000000	mice
Rab11fip1	0.06	1.00000000	mice
Vps72	0.01	1.00000000	mice
Aqr	-0.05	1.00000000	mice
Bag6	-0.05	1.00000000	mice
Dctn6	-0.01	1.00000000	mice
Eif4g2	-0.04	1.00000000	mice
Slamf6	0.03	1.00000000	mice
Spin1	0.02	1.00000000	mice
Usp10	-0.02	1.00000000	mice
Rbl2	0.04	1.00000000	mice
Pepd	-0.03	1.00000000	mice
Hnrnpa1	-0.03	1.00000000	mice
Smim7	-0.04	1.00000000	mice
Setdb1	-0.01	1.00000000	mice
Prdm2	-0.01	1.00000000	mice
Gtf2f2	-0.10	1.00000000	mice
Gtpbp4	-0.03	1.00000000	mice
Dmac2	-0.04	1.00000000	mice
Ccndbp1	0.01	1.00000000	mice
Taf8	0.01	1.00000000	mice
Nbeal1	0.00	1.00000000	mice
Snip1	-0.01	1.00000000	mice
Taok2	-0.01	1.00000000	mice
4930402H24Rik	-0.05	1.00000000	mice
Cd247	0.02	1.00000000	mice
Fbxw4	-0.05	1.00000000	mice
Psme4	-0.01	1.00000000	mice
Tmem248	-0.04	1.00000000	mice
Bin1	-0.01	1.00000000	mice
Arhgap12	0.06	1.00000000	mice
0610012G03Rik	-0.04	1.00000000	mice
Skil	-0.03	1.00000000	mice
Pkp4	0.06	1.00000000	mice
Eml5	-0.04	1.00000000	mice
2610507B11Rik	0.02	1.00000000	mice
Nploc4	-0.04	1.00000000	mice
Dut	0.00	1.00000000	mice
Rspry1	-0.02	1.00000000	mice
Asnsd1	-0.03	1.00000000	mice

Cct5	0.01	1.00000000	mice
Paf1	-0.05	1.00000000	mice
Pdcd2	0.00	1.00000000	mice
Cds2	-0.06	1.00000000	mice
Bicd2	-0.02	1.00000000	mice
Pcgf5	0.07	1.00000000	mice
Nfe2l1	-0.01	1.00000000	mice
Cyth3	-0.03	1.00000000	mice
Trnt1	-0.01	1.00000000	mice
Zbtb43	0.00	1.00000000	mice
Igbp1	-0.05	1.00000000	mice
Edc3	-0.04	1.00000000	mice
Slc35a3	-0.05	1.00000000	mice
Arl5b	0.00	1.00000000	mice
Rbm17	0.00	1.00000000	mice
Pex3	-0.05	1.00000000	mice
Pdhx	-0.03	1.00000000	mice
Cenpb	-0.03	1.00000000	mice
Trim26	0.02	1.00000000	mice
Nsun6	-0.04	1.00000000	mice
Kpna3	-0.03	1.00000000	mice
Klc1	-0.03	1.00000000	mice
Gpx1	0.00	1.00000000	mice
Zxdc	0.00	1.00000000	mice
Cand1	-0.01	1.00000000	mice
Pex5	-0.03	1.00000000	mice
Wdr3	-0.03	1.00000000	mice
Wdr61	-0.01	1.00000000	mice
Atg101	-0.01	1.00000000	mice
Pak2	-0.03	1.00000000	mice
Rab4b	-0.07	1.00000000	mice
Arrdc3	-0.04	1.00000000	mice
Zfp292	-0.02	1.00000000	mice
Cic	0.01	1.00000000	mice
Emg1	-0.02	1.00000000	mice
Ncbp2	-0.02	1.00000000	mice
Xpr1	-0.03	1.00000000	mice
Rgs1	-0.24	1.00000000	mice
Fam120b	-0.04	1.00000000	mice
Fbxo9	-0.03	1.00000000	mice
Gfpt1	-0.04	1.00000000	mice
Sos1	-0.06	1.00000000	mice
Zfp729a	-0.01	1.00000000	mice
Qars	-0.03	1.00000000	mice
6720427I07Rik	0.03	1.00000000	mice
1700123O20Rik	0.00	1.00000000	mice

Prkacb	-0.05	1.00000000	mice
Spn	0.01	1.00000000	mice
Epb41l4aos	-0.01	1.00000000	mice
Dnajc10	-0.07	1.00000000	mice
Thoc7	-0.02	1.00000000	mice
Ubr3	-0.02	1.00000000	mice
Hdgf	0.02	1.00000000	mice
Arap1	0.02	1.00000000	mice
Dnajb9	0.01	1.00000000	mice
Flii	-0.03	1.00000000	mice
Foxn2	0.03	1.00000000	mice
Gpi1	-0.03	1.00000000	mice
Lmtk2	-0.06	1.00000000	mice
Cspp1	0.02	1.00000000	mice
Eif2a	0.03	1.00000000	mice
Utp15	0.01	1.00000000	mice
Pafah1b2	-0.06	1.00000000	mice
Prr14	-0.02	1.00000000	mice
Sp1	0.00	1.00000000	mice
Nudt18	-0.03	1.00000000	mice
Tmem30a	-0.01	1.00000000	mice
Btg2	0.07	1.00000000	mice
Utp6	0.00	1.00000000	mice
Vac14	-0.03	1.00000000	mice
Sh3bp5l	-0.03	1.00000000	mice
Rbks	0.01	1.00000000	mice
AI837181	0.01	1.00000000	mice
Naa35	-0.03	1.00000000	mice
Timm23	-0.06	1.00000000	mice
Ggct	-0.09	1.00000000	mice
Emc4	-0.01	1.00000000	mice
Brpf1	0.03	1.00000000	mice
Cyb5a	-0.04	1.00000000	mice
Fbxl14	-0.01	1.00000000	mice
Lrrc41	-0.04	1.00000000	mice
Rfwd3	0.02	1.00000000	mice
Bclaf3	0.07	1.00000000	mice
Ranbp9	-0.06	1.00000000	mice
Eif3l	-0.01	1.00000000	mice
Kin	-0.05	1.00000000	mice
Arcn1	0.02	1.00000000	mice
Wrn	-0.08	1.00000000	mice
Ptpn11	-0.04	1.00000000	mice
Snhg3	-0.04	1.00000000	mice
Ost4	-0.05	1.00000000	mice
Cndp2	0.00	1.00000000	mice

Ppil2	-0.02	1.00000000	mice
Slc38a9	0.03	1.00000000	mice
Lpcat4	-0.05	1.00000000	mice
Txlna	-0.02	1.00000000	mice
Tcea1	-0.03	1.00000000	mice
Fam214a	0.02	1.00000000	mice
Ube3b	-0.04	1.00000000	mice
Ppie	-0.02	1.00000000	mice
Acbd6	-0.07	1.00000000	mice
Uqcrc1	-0.02	1.00000000	mice
Glipr2	0.00	1.00000000	mice
Ppp1r7	0.00	1.00000000	mice
Ciao1	0.00	1.00000000	mice
Rrp1b	0.00	1.00000000	mice
Acin1	0.03	1.00000000	mice
Ptcd3	-0.03	1.00000000	mice
Psap	-0.05	1.00000000	mice
Trbc1	-0.01	1.00000000	mice
Galnt6os	0.00	1.00000000	mice
Zfp281	0.02	1.00000000	mice
Sdf4	0.00	1.00000000	mice
Usp42	-0.07	1.00000000	mice
Tgoln1	-0.03	1.00000000	mice
Acot8	0.00	1.00000000	mice
Spopl	-0.07	1.00000000	mice
Vps4a	-0.05	1.00000000	mice
Fcf1	-0.03	1.00000000	mice
Bcorl1	-0.04	1.00000000	mice
Arhgef12	0.05	1.00000000	mice
Kdm4c	-0.01	1.00000000	mice
Gpr108	0.00	1.00000000	mice
Chd7	0.04	1.00000000	mice
Vps26b	0.00	1.00000000	mice
Wars2	-0.06	1.00000000	mice
Timm9	0.00	1.00000000	mice
Snapc3	-0.02	1.00000000	mice
Dcaf10	0.04	1.00000000	mice
Otud6b	-0.01	1.00000000	mice
Ctdsp1	-0.02	1.00000000	mice
Ing2	-0.03	1.00000000	mice
Dgcr8	-0.02	1.00000000	mice
Fkbp15	0.01	1.00000000	mice
Clns1a	-0.03	1.00000000	mice
Foxj2	-0.07	1.00000000	mice
Dnajc24	-0.01	1.00000000	mice
Tpd52	-0.07	1.00000000	mice

Trmt1	0.01	1.00000000	mice
Zfp609	0.03	1.00000000	mice
Vav3	0.07	1.00000000	mice
Ddx31	-0.01	1.00000000	mice
Herc1	-0.04	1.00000000	mice
Ddx47	-0.05	1.00000000	mice
Prmt3	0.04	1.00000000	mice
Nmt1	-0.02	1.00000000	mice
Zfyve26	-0.05	1.00000000	mice
Ppp1r12b	0.01	1.00000000	mice
Itpr2	-0.02	1.00000000	mice
Snx6	-0.06	1.00000000	mice
Slc7a1	-0.02	1.00000000	mice
Papolg	0.03	1.00000000	mice
Slbp	0.03	1.00000000	mice
Wwox	0.00	1.00000000	mice
Twf2	-0.02	1.00000000	mice
Pgrmc2	-0.01	1.00000000	mice
Atm	-0.05	1.00000000	mice
Ppp2r3a	-0.03	1.00000000	mice
Ngdn	-0.05	1.00000000	mice
Arf1	-0.02	1.00000000	mice
Srp72	0.03	1.00000000	mice
Nufip1	-0.01	1.00000000	mice
Daglb	0.00	1.00000000	mice
Slc37a1	-0.01	1.00000000	mice
Hnrnpf	0.03	1.00000000	mice
Vezt	-0.01	1.00000000	mice
Cdc42	-0.01	1.00000000	mice
Slc25a17	-0.04	1.00000000	mice
G2e3	-0.04	1.00000000	mice
Pes1	0.00	1.00000000	mice
Akap10	-0.04	1.00000000	mice
Cd2	0.01	1.00000000	mice
Ythdf3	-0.02	1.00000000	mice
Eftud2	-0.03	1.00000000	mice
Syk	-0.15	1.00000000	mice
Kdm6a	0.04	1.00000000	mice
Brwd1	0.03	1.00000000	mice
Strn4	0.00	1.00000000	mice
Tmem192	-0.05	1.00000000	mice
Smim19	-0.02	1.00000000	mice
Gclm	-0.06	1.00000000	mice
Zfp260	0.04	1.00000000	mice
Cd44	-0.07	1.00000000	mice
Rap1gap2	-0.13	1.00000000	mice

Cxxc5	-0.06	1.00000000	mice
Wdr18	0.00	1.00000000	mice
Tbc1d10c	-0.05	1.00000000	mice
Pgk1	-0.01	1.00000000	mice
Cyth2	-0.03	1.00000000	mice
Dennd2d	-0.02	1.00000000	mice
Trmt61b	-0.01	1.00000000	mice
Rraga	-0.01	1.00000000	mice
Fgfr1op2	0.03	1.00000000	mice
Hmces	0.00	1.00000000	mice
Trim39	0.00	1.00000000	mice
Timm17a	-0.05	1.00000000	mice
Prpf31	-0.03	1.00000000	mice
Srgn	0.06	1.00000000	mice
Gclc	0.01	1.00000000	mice
Ikbkb	-0.05	1.00000000	mice
Exoc6	-0.01	1.00000000	mice
Slain2	-0.03	1.00000000	mice
Nup54	-0.02	1.00000000	mice
Sh3rf1	0.04	1.00000000	mice
Commd2	-0.03	1.00000000	mice
Brms1	-0.05	1.00000000	mice
Donson	0.04	1.00000000	mice
Agtpbp1	0.02	1.00000000	mice
Orc4	0.01	1.00000000	mice
Serinc5	-0.04	1.00000000	mice
Fxyd5	0.00	1.00000000	mice
Bmp2k	-0.10	1.00000000	mice
Atp5g2	-0.01	1.00000000	mice
Ptbp3	-0.01	1.00000000	mice
Limd1	0.00	1.00000000	mice
Casp2	-0.03	1.00000000	mice
Rnf219	-0.03	1.00000000	mice
Supt16	-0.06	1.00000000	mice
Neat1	-0.01	1.00000000	mice
Pias4	-0.01	1.00000000	mice
Patl1	-0.03	1.00000000	mice
Ociad1	-0.05	1.00000000	mice
Gna15	0.00	1.00000000	mice
Cebpg	0.01	1.00000000	mice
Scamp3	-0.03	1.00000000	mice
Rbm8a	0.00	1.00000000	mice
Trim24	0.00	1.00000000	mice
Snx17	0.00	1.00000000	mice
Large1	-0.06	1.00000000	mice
Imp3	-0.03	1.00000000	mice

Urgcp	-0.02	1.00000000	mice
Yars	-0.05	1.00000000	mice
Ctla2a	-0.07	1.00000000	mice
Polr3c	-0.05	1.00000000	mice
Fbxw7	0.02	1.00000000	mice
Shb	-0.01	1.00000000	mice
Pold4	-0.02	1.00000000	mice
Atrx	0.01	1.00000000	mice
Zfp81	0.01	1.00000000	mice
Cdc37l1	-0.04	1.00000000	mice
Zfp874b	-0.03	1.00000000	mice
Arhgap9	0.00	1.00000000	mice
Herc3	-0.05	1.00000000	mice
Hnrnpu	0.00	1.00000000	mice
Ctnnbl1	-0.06	1.00000000	mice
Dpp3	-0.01	1.00000000	mice
Rdx	-0.01	1.00000000	mice
Copg1	-0.03	1.00000000	mice
Plk3	0.10	1.00000000	mice
Anapc4	-0.07	1.00000000	mice
Zfp106	-0.02	1.00000000	mice
Larp1b	-0.13	1.00000000	mice
Trmt13	-0.05	1.00000000	mice
Ppp2r3c	-0.03	1.00000000	mice
Pten	-0.05	1.00000000	mice
Gart	-0.01	1.00000000	mice
Spidr	-0.08	1.00000000	mice
Szrd1	-0.03	1.00000000	mice
Dcun1d1	-0.02	1.00000000	mice
Slc7a6os	0.00	1.00000000	mice
Rnf11	-0.05	1.00000000	mice
Selenot	0.01	1.00000000	mice
Nucks1	-0.01	1.00000000	mice
Ccdc88a	-0.05	1.00000000	mice
Acd	-0.03	1.00000000	mice
Arl8b	-0.03	1.00000000	mice
Zfp654	-0.03	1.00000000	mice
Ttpal	0.00	1.00000000	mice
Ccng1	0.04	1.00000000	mice
Ing4	-0.03	1.00000000	mice
Dhx16	0.01	1.00000000	mice
Gosr1	-0.04	1.00000000	mice
Hps5	-0.01	1.00000000	mice
Fbxl17	-0.03	1.00000000	mice
Hnrnpm	0.03	1.00000000	mice
Clptm1	0.00	1.00000000	mice

Zfp511	-0.01	1.00000000	mice
Fnip1	0.05	1.00000000	mice
Gabarapl2	0.00	1.00000000	mice
Stk32c	-0.07	1.00000000	mice
Adprm	-0.03	1.00000000	mice
Gnl2	-0.01	1.00000000	mice
Cdc73	-0.07	1.00000000	mice
Phax	-0.04	1.00000000	mice
Rbms1	0.00	1.00000000	mice
Trabd	0.01	1.00000000	mice
Vps33a	-0.07	1.00000000	mice
Prpsap2	-0.06	1.00000000	mice
Pgam1	-0.06	1.00000000	mice
Serpini1	0.05	1.00000000	mice
Ehmt2	-0.02	1.00000000	mice
Ptpn9	-0.03	1.00000000	mice
Hsd17b4	-0.05	1.00000000	mice
Slc30a7	-0.02	1.00000000	mice
Atl3	0.05	1.00000000	mice
Npepl1	-0.03	1.00000000	mice
Exoc7	-0.03	1.00000000	mice
Golim4	-0.07	1.00000000	mice
Rmdn1	-0.02	1.00000000	mice
Zfp949	-0.02	1.00000000	mice
Eno1	-0.05	1.00000000	mice
6030458C11Rik	-0.02	1.00000000	mice
Itfg1	-0.05	1.00000000	mice
Prmt9	-0.07	1.00000000	mice
Dhx57	-0.05	1.00000000	mice
Echs1	-0.03	1.00000000	mice
Dcaf7	0.01	1.00000000	mice
4930523C07Rik	-0.03	1.00000000	mice
Txndc5	-0.02	1.00000000	mice
Tmem39a	-0.02	1.00000000	mice
Cklf	-0.04	1.00000000	mice
Hltf	0.05	1.00000000	mice
Phlpp2	-0.06	1.00000000	mice
Hsp90b1	-0.06	1.00000000	mice
Pkm	0.06	1.00000000	mice
Vps8	-0.04	1.00000000	mice
Golga1	-0.04	1.00000000	mice
Plrg1	0.01	1.00000000	mice
Nudcd3	-0.03	1.00000000	mice
Fuca1	-0.07	1.00000000	mice
Fam8a1	-0.03	1.00000000	mice
Slc35b1	-0.05	1.00000000	mice

Dcun1d3	-0.01	1.00000000	mice
Ipo5	-0.03	1.00000000	mice
A630001G21Rik	-0.05	1.00000000	mice
Snx14	0.00	1.00000000	mice
Thoc3	-0.03	1.00000000	mice
Snx5	-0.06	1.00000000	mice
a	-0.02	1.00000000	mice
Ep300	-0.02	1.00000000	mice
Tmco4	-0.07	1.00000000	mice
Rsrc1	0.02	1.00000000	mice
Amz2	-0.01	1.00000000	mice
Tmem209	-0.06	1.00000000	mice
Snrnp200	-0.02	1.00000000	mice
Agfg1	0.04	1.00000000	mice
Zc3h3	-0.01	1.00000000	mice
Zfp933	-0.06	1.00000000	mice
Zdhhc21	0.00	1.00000000	mice
Crebbp	0.03	1.00000000	mice
Gar1	0.00	1.00000000	mice
Ywhag	0.00	1.00000000	mice
Uqcc3	-0.03	1.00000000	mice
Lsp1	0.02	1.00000000	mice
Trim27	0.00	1.00000000	mice
Slc37a3	-0.07	1.00000000	mice
Fosb	0.33	1.00000000	mice
Rragc	-0.02	1.00000000	mice
Nceh1	0.02	1.00000000	mice
4933434E20Rik	-0.06	1.00000000	mice
Txndc9	0.00	1.00000000	mice
Daam1	-0.08	1.00000000	mice
Vps53	-0.03	1.00000000	mice
Ginm1	-0.01	1.00000000	mice
Rnf6	-0.05	1.00000000	mice
Ddx42	-0.01	1.00000000	mice
Ube2w	-0.01	1.00000000	mice
Chst11	-0.08	1.00000000	mice
Exosc10	-0.07	1.00000000	mice
Sptlc1	-0.04	1.00000000	mice
Csde1	-0.01	1.00000000	mice
Cers4	-0.05	1.00000000	mice
Cep135	0.00	1.00000000	mice
Ago1	-0.07	1.00000000	mice
Rab5if	-0.04	1.00000000	mice
Atp6v1e1	-0.03	1.00000000	mice
Yipf4	-0.03	1.00000000	mice
Fam126a	-0.02	1.00000000	mice

Dhx33	-0.02	1.00000000	mice
Ap4s1	-0.09	1.00000000	mice
Clk3	-0.03	1.00000000	mice
Blzf1	-0.02	1.00000000	mice
Eri3	-0.02	1.00000000	mice
Nup98	0.05	1.00000000	mice
Fam204a	0.01	1.00000000	mice
Ap3b1	0.00	1.00000000	mice
Atg12	-0.02	1.00000000	mice
Gtf2h5	-0.04	1.00000000	mice
Plxnc1	-0.26	1.00000000	mice
Krit1	-0.07	1.00000000	mice
Cactin	-0.03	1.00000000	mice
Taco1	-0.02	1.00000000	mice
Smad1	-0.01	1.00000000	mice
Gnpat	-0.02	1.00000000	mice
Ap2m1	-0.04	1.00000000	mice
Camk2g	-0.01	1.00000000	mice
Actr1b	-0.06	1.00000000	mice
Gpr146	0.03	1.00000000	mice
Ythdf1	-0.02	1.00000000	mice
Etfdh	0.03	1.00000000	mice
Ubxn6	-0.01	1.00000000	mice
Slc6a6	-0.02	1.00000000	mice
Tmem243	-0.02	1.00000000	mice
Cpq	-0.01	1.00000000	mice
Commd10	-0.07	1.00000000	mice
Dync1li2	-0.02	1.00000000	mice
Itgam	-0.16	1.00000000	mice
Nup93	-0.01	1.00000000	mice
Prcc	-0.03	1.00000000	mice
Stx16	-0.02	1.00000000	mice
Trim11	-0.02	1.00000000	mice
Ccdc43	-0.03	1.00000000	mice
AU040320	-0.02	1.00000000	mice
Zdhhc8	-0.04	1.00000000	mice
E2f4	-0.02	1.00000000	mice
Cant1	-0.03	1.00000000	mice
Ttc28	0.04	1.00000000	mice
Zfand5	0.00	1.00000000	mice
Nxt1	-0.01	1.00000000	mice
Nedd4l	0.01	1.00000000	mice
Eif1ad	-0.03	1.00000000	mice
Dtd1	0.03	1.00000000	mice
Actr1a	-0.05	1.00000000	mice
Fbxl6	0.00	1.00000000	mice

Pef1	-0.04	1.00000000	mice
Usp33	-0.03	1.00000000	mice
Nab2	-0.02	1.00000000	mice
Ergic1	-0.01	1.00000000	mice
Vhl	-0.03	1.00000000	mice
Otulinl	-0.07	1.00000000	mice
Acer3	0.00	1.00000000	mice
Nbr1	-0.03	1.00000000	mice
Nsd2	-0.04	1.00000000	mice
Rab31	-0.04	1.00000000	mice
Ints9	0.00	1.00000000	mice
Nisch	-0.01	1.00000000	mice
Yaf2	0.03	1.00000000	mice
Cyb5r4	-0.03	1.00000000	mice
Dhx15	-0.06	1.00000000	mice
Comt	-0.03	1.00000000	mice
Chd8	0.01	1.00000000	mice
Ptp4a3	-0.01	1.00000000	mice
D230025D16Rik	-0.03	1.00000000	mice
Prkch	0.00	1.00000000	mice
Ywhah	-0.03	1.00000000	mice
Lnpep	0.02	1.00000000	mice
Npat	0.01	1.00000000	mice
Cops5	-0.02	1.00000000	mice
Tnpo3	0.02	1.00000000	mice
Lrrc59	-0.05	1.00000000	mice
Uchl3	-0.02	1.00000000	mice
Sdf2	-0.02	1.00000000	mice
Slc25a28	-0.02	1.00000000	mice
Orc2	-0.03	1.00000000	mice
Srp68	-0.01	1.00000000	mice
Tnk2	-0.01	1.00000000	mice
Chrac1	-0.05	1.00000000	mice
Tcn2	0.00	1.00000000	mice
Wipf2	-0.02	1.00000000	mice
Leprotl1	-0.02	1.00000000	mice
Cul4a	-0.07	1.00000000	mice
Foxk2	-0.01	1.00000000	mice
Fam104a	-0.02	1.00000000	mice
D030056L22Rik	-0.03	1.00000000	mice
Dctn2	-0.06	1.00000000	mice
Rab27b	-0.11	1.00000000	mice
Ebag9	-0.03	1.00000000	mice
Phc3	0.08	1.00000000	mice
Aup1	0.01	1.00000000	mice
Dnajc1	0.00	1.00000000	mice

Adamts6	-0.15	1.00000000	mice
Adamts10	-0.06	1.00000000	mice
Hdgfl2	-0.02	1.00000000	mice
Wbp2	-0.02	1.00000000	mice
Spg7	-0.02	1.00000000	mice
Dtx2	-0.02	1.00000000	mice
Gprin3	0.00	1.00000000	mice
Gdi1	-0.02	1.00000000	mice
Pabpc4	0.01	1.00000000	mice
Tusc3	-0.07	1.00000000	mice
Nmrk1	-0.03	1.00000000	mice
Dnttip2	-0.01	1.00000000	mice
Ppp4r2	-0.05	1.00000000	mice
Zfp703	-0.02	1.00000000	mice
Skp1a	0.00	1.00000000	mice
H13	-0.01	1.00000000	mice
St6galnac3	-0.06	1.00000000	mice
Rhog	-0.01	1.00000000	mice
Zfp560	0.00	1.00000000	mice
E4f1	-0.03	1.00000000	mice
Diaph1	-0.02	1.00000000	mice
Foxk1	-0.05	1.00000000	mice
Kdm2a	-0.02	1.00000000	mice
Stk10	0.07	1.00000000	mice
Polr3f	-0.04	1.00000000	mice
Trappc9	0.02	1.00000000	mice
Clcc1	-0.02	1.00000000	mice
Ndufa9	-0.01	1.00000000	mice
Glipr1	-0.01	1.00000000	mice
Egln2	-0.03	1.00000000	mice
Bod1l	0.02	1.00000000	mice
Arhgap4	0.00	1.00000000	mice
Tcf7	0.01	1.00000000	mice
4931406P16Rik	-0.01	1.00000000	mice
Nt5c	-0.03	1.00000000	mice
Tceanc2	-0.04	1.00000000	mice
Golga5	-0.04	1.00000000	mice
Retreg2	-0.01	1.00000000	mice
Grap	0.02	1.00000000	mice
Acot9	0.02	1.00000000	mice
Ift52	0.00	1.00000000	mice
Tor1a	-0.02	1.00000000	mice
Isca2	-0.01	1.00000000	mice
Qrich1	0.00	1.00000000	mice
Uqcrh	0.00	1.00000000	mice
Ppm1l	-0.05	1.00000000	mice

Bach1	-0.05	1.00000000	mice
L3mbtl3	-0.01	1.00000000	mice
Eif4a2	-0.01	1.00000000	mice
Hnrnpr	0.00	1.00000000	mice
Zmiz2	-0.02	1.00000000	mice
Ecd	-0.04	1.00000000	mice
Sergef	-0.05	1.00000000	mice
Impact	0.02	1.00000000	mice
Pin1	-0.06	1.00000000	mice
Gnb1	-0.02	1.00000000	mice
Tet3	0.07	1.00000000	mice
Hip1r	-0.11	1.00000000	mice
Cct7	-0.03	1.00000000	mice
Zdhhc6	-0.02	1.00000000	mice
Kmt5a	0.00	1.00000000	mice
Rbm42	-0.07	1.00000000	mice
Ap3s1	-0.04	1.00000000	mice
Gopc	-0.07	1.00000000	mice
A930007I19Rik	-0.02	1.00000000	mice
Pcca	-0.05	1.00000000	mice
Cog8	-0.04	1.00000000	mice
Usp8	0.00	1.00000000	mice
Kansl2	0.00	1.00000000	mice
Stk3	-0.01	1.00000000	mice
Ggps1	-0.04	1.00000000	mice
Cbwd1	0.01	1.00000000	mice
Golga4	-0.02	1.00000000	mice
Sufu	-0.05	1.00000000	mice
Icam2	-0.03	1.00000000	mice
Tcp1	-0.02	1.00000000	mice
Necap1	-0.01	1.00000000	mice
Usp19	-0.04	1.00000000	mice
Plpbp	-0.04	1.00000000	mice
Ik	-0.03	1.00000000	mice
Hnrnph3	0.03	1.00000000	mice
Pura	0.00	1.00000000	mice
AB124611	-0.01	1.00000000	mice
Tlk2	-0.04	1.00000000	mice
Cnih1	-0.06	1.00000000	mice
Nfatc2ip	0.00	1.00000000	mice
Prpf4	-0.02	1.00000000	mice
Brd3	-0.04	1.00000000	mice
Eef1d	0.00	1.00000000	mice
Smim20	0.01	1.00000000	mice
Eif2b2	-0.03	1.00000000	mice
Slc25a30	-0.04	1.00000000	mice

Zfp330	0.00	1.00000000	mice
Aatf	-0.01	1.00000000	mice
Ipo11	-0.03	1.00000000	mice
Sh2b1	-0.06	1.00000000	mice
Sfi1	0.03	1.00000000	mice
Polb	0.01	1.00000000	mice
Fhl2	-0.04	1.00000000	mice
Golgb1	-0.05	1.00000000	mice
Slx4ip	0.00	1.00000000	mice
Ppp1r12a	0.02	1.00000000	mice
Uxs1	-0.05	1.00000000	mice
Nup210l	-0.01	1.00000000	mice
Cops3	-0.03	1.00000000	mice
Eif3a	0.00	1.00000000	mice
Ubqln1	0.04	1.00000000	mice
Spata13	-0.01	1.00000000	mice
Yipf5	0.00	1.00000000	mice
G3bp1	0.05	1.00000000	mice
Acadm	-0.03	1.00000000	mice
Cab39	-0.01	1.00000000	mice
Pqbp1	-0.02	1.00000000	mice
Timm17b	-0.03	1.00000000	mice
Kdelr1	-0.03	1.00000000	mice
Prkag2	0.00	1.00000000	mice
Hyou1	-0.03	1.00000000	mice
Paxbp1	-0.01	1.00000000	mice
Tug1	0.00	1.00000000	mice
Pfdn2	0.00	1.00000000	mice
Prkar2a	-0.06	1.00000000	mice
Sil1	-0.04	1.00000000	mice
Zfp1	-0.04	1.00000000	mice
Stx17	-0.04	1.00000000	mice
Psen2	-0.01	1.00000000	mice
Dstyk	0.01	1.00000000	mice
Golga3	0.00	1.00000000	mice
Ttc7	-0.08	1.00000000	mice
Gbf1	0.01	1.00000000	mice
Whamm	-0.08	1.00000000	mice
Creg1	0.00	1.00000000	mice
Sar1b	-0.06	1.00000000	mice
Anapc7	-0.01	1.00000000	mice
Snhg12	0.00	1.00000000	mice
Itgb7	-0.04	1.00000000	mice
Ndufa10	-0.04	1.00000000	mice
Cep85l	0.01	1.00000000	mice
Tubgcp2	-0.01	1.00000000	mice

Usp6nl	0.01	1.00000000	mice
Apopt1	-0.02	1.00000000	mice
Rnf146	-0.03	1.00000000	mice
Tasp1	0.02	1.00000000	mice
Slc31a1	-0.02	1.00000000	mice
Traf3ip2	0.01	1.00000000	mice
Trappc6b	0.01	1.00000000	mice
Slc30a9	-0.04	1.00000000	mice
Slc4a1ap	-0.01	1.00000000	mice
Tbc1d10b	-0.05	1.00000000	mice
Wdr82	0.00	1.00000000	mice
Rest	-0.03	1.00000000	mice
Zc3h15	-0.01	1.00000000	mice
Tcerg1	0.03	1.00000000	mice
Stn1	0.01	1.00000000	mice
Spata5	0.01	1.00000000	mice
Tigd2	-0.03	1.00000000	mice
Ppp6r3	-0.01	1.00000000	mice
Vta1	-0.04	1.00000000	mice
Usp31	0.02	1.00000000	mice
Rbbp4	-0.02	1.00000000	mice
Polr2b	-0.05	1.00000000	mice
Zcchc7	0.03	1.00000000	mice
Fbrs	-0.04	1.00000000	mice
Ccdc82	-0.03	1.00000000	mice
Zfp266	-0.04	1.00000000	mice
Zfp871	-0.02	1.00000000	mice
Pknox1	0.02	1.00000000	mice
Scyl2	0.04	1.00000000	mice
Sik3	0.10	1.00000000	mice
Cipc	0.03	1.00000000	mice
Snrnp48	-0.03	1.00000000	mice
Dpp9	-0.02	1.00000000	mice
Synrg	-0.02	1.00000000	mice
Xrcc4	-0.06	1.00000000	mice
Krr1	-0.04	1.00000000	mice
Cept1	-0.05	1.00000000	mice
Syap1	-0.05	1.00000000	mice
Fbxl2	-0.09	1.00000000	mice
Cltc	0.01	1.00000000	mice
Uso1	-0.02	1.00000000	mice
Gcn1	-0.01	1.00000000	mice
Kctd5	-0.04	1.00000000	mice
Aprt	0.00	1.00000000	mice
Ntmt1	-0.01	1.00000000	mice
Cpsf6	-0.02	1.00000000	mice

Lrmp	-0.09	1.00000000	mice
Nudc	-0.02	1.00000000	mice
Rbsn	-0.04	1.00000000	mice
Nipsnap3b	-0.04	1.00000000	mice
E130308A19Rik	0.00	1.00000000	mice
Urb2	0.03	1.00000000	mice
Parl	0.00	1.00000000	mice
Zc3h14	-0.05	1.00000000	mice
Iscu	-0.04	1.00000000	mice
Gsr	-0.04	1.00000000	mice
Btf3l4	-0.01	1.00000000	mice
Pphln1	-0.04	1.00000000	mice
Clk4	-0.01	1.00000000	mice
Eefsec	-0.03	1.00000000	mice
Nkiras1	-0.01	1.00000000	mice
Ptpra	-0.01	1.00000000	mice
Nup50	0.00	1.00000000	mice
Exosc7	-0.03	1.00000000	mice
Pias1	-0.02	1.00000000	mice
Klhl2	-0.11	1.00000000	mice
Odf2	-0.03	1.00000000	mice
Nus1	-0.04	1.00000000	mice
Strn3	0.01	1.00000000	mice
Aars	0.00	1.00000000	mice
Zfp397	-0.03	1.00000000	mice
Drosha	-0.06	1.00000000	mice
Sltm	0.03	1.00000000	mice
Ctso	0.01	1.00000000	mice
Phkb	-0.04	1.00000000	mice
Ubap1	-0.03	1.00000000	mice
Cux1	-0.04	1.00000000	mice
Clp1	-0.02	1.00000000	mice
Pa2g4	-0.05	1.00000000	mice
Rchy1	-0.01	1.00000000	mice
Letm1	-0.02	1.00000000	mice
Dennd1a	0.00	1.00000000	mice
Zfp646	-0.01	1.00000000	mice
Wdr26	0.00	1.00000000	mice
Gapdh	0.00	1.00000000	mice
Zfp652	-0.01	1.00000000	mice
Ccdc186	-0.02	1.00000000	mice
Fkbp8	-0.04	1.00000000	mice
Edem2	-0.06	1.00000000	mice
Dnm2	-0.01	1.00000000	mice
Vamp1	-0.01	1.00000000	mice
Nt5c3b	-0.01	1.00000000	mice

Cul1	-0.04	1.00000000	mice
Wdr75	-0.02	1.00000000	mice
Iqsec1	-0.09	1.00000000	mice
Sp2	0.00	1.00000000	mice
Arfrp1	-0.01	1.00000000	mice
Ell2	-0.03	1.00000000	mice
Btg3	-0.01	1.00000000	mice
Elf2	-0.01	1.00000000	mice
Prkaca	-0.01	1.00000000	mice
Cd6	0.21	1.00000000	mice
Bloc1s2	-0.03	1.00000000	mice
Ric8a	-0.02	1.00000000	mice
Gde1	-0.01	1.00000000	mice
Wnk1	0.08	1.00000000	mice
Tmem106b	-0.04	1.00000000	mice
Gnl3l	-0.01	1.00000000	mice
A330023F24Rik	-0.07	1.00000000	mice
Pcif1	-0.02	1.00000000	mice
Flna	-0.04	1.00000000	mice
4833439L19Rik	0.01	1.00000000	mice
Washc2	-0.07	1.00000000	mice
Armc1	-0.02	1.00000000	mice
Ccnl2	0.04	1.00000000	mice
Zfx	-0.02	1.00000000	mice
Rrp36	-0.04	1.00000000	mice
Cct3	-0.03	1.00000000	mice
Nap1l1	0.04	1.00000000	mice
Lamc1	0.05	1.00000000	mice
Cep170	-0.01	1.00000000	mice
Sh3gl1	-0.02	1.00000000	mice
Cnnm4	-0.02	1.00000000	mice
Eaf1	-0.04	1.00000000	mice
Ep400	0.03	1.00000000	mice
Pot1a	-0.01	1.00000000	mice
Cpsf4	0.01	1.00000000	mice
Slc39a7	-0.01	1.00000000	mice
Atxn7l3	0.02	1.00000000	mice
Sh2b3	-0.03	1.00000000	mice
Rbbp8	-0.01	1.00000000	mice
Sox5	-0.10	1.00000000	mice
Strn	-0.03	1.00000000	mice
Rbck1	-0.01	1.00000000	mice
Chic2	0.00	1.00000000	mice
Fyttd1	0.00	1.00000000	mice
Cdc42ep3	-0.06	1.00000000	mice
Prrc2b	0.06	1.00000000	mice

Prorsd1	-0.03	1.00000000	mice
Gps2	-0.01	1.00000000	mice
Sec61a2	-0.01	1.00000000	mice
Rtcb	-0.03	1.00000000	mice
Ddx5	0.01	1.00000000	mice
Ncor2	-0.01	1.00000000	mice
Smc5	0.01	1.00000000	mice
Arl6ip1	0.02	1.00000000	mice
Ino80e	-0.01	1.00000000	mice
Wdr33	-0.01	1.00000000	mice
Ppwd1	-0.01	1.00000000	mice
Noc2l	-0.02	1.00000000	mice
Gemin7	-0.04	1.00000000	mice
1700109H08Rik	0.03	1.00000000	mice
Dhps	-0.04	1.00000000	mice
Cdyl2	-0.06	1.00000000	mice
Cd84	-0.02	1.00000000	mice
Bbc3	-0.06	1.00000000	mice
Top3b	-0.04	1.00000000	mice
Stk17b	0.05	1.00000000	mice
Phlpp1	0.03	1.00000000	mice
Rbm41	-0.02	1.00000000	mice
Scaf11	0.00	1.00000000	mice
Twistnb	-0.02	1.00000000	mice
Arl6ip6	0.00	1.00000000	mice
Srfbp1	-0.06	1.00000000	mice
Kdm1a	-0.06	1.00000000	mice
Dhdds	-0.03	1.00000000	mice
Ubl4a	-0.05	1.00000000	mice
Raf1	-0.04	1.00000000	mice
Zfp955b	-0.06	1.00000000	mice
Dnajb2	-0.02	1.00000000	mice
Dnmt3a	-0.04	1.00000000	mice
Cxxc1	-0.01	1.00000000	mice
Zfp386	-0.03	1.00000000	mice
Ippk	-0.03	1.00000000	mice
Rab11fip3	-0.06	1.00000000	mice
Tfrc	0.01	1.00000000	mice
Tuba1b	-0.03	1.00000000	mice
9930021J03Rik	0.01	1.00000000	mice
D5Ertd579e	-0.04	1.00000000	mice
Smap2	-0.06	1.00000000	mice
Gga1	-0.02	1.00000000	mice
Cluap1	-0.03	1.00000000	mice
Snx12	-0.04	1.00000000	mice
Thumpd1	-0.03	1.00000000	mice

Cpt1a	-0.03	1.00000000	mice
Rangap1	0.00	1.00000000	mice
Spop	-0.03	1.00000000	mice
Ppp2r1b	-0.02	1.00000000	mice
Gorasp2	-0.05	1.00000000	mice
2410002F23Rik	0.03	1.00000000	mice
Chmp3	-0.01	1.00000000	mice
Fiz1	-0.01	1.00000000	mice
Socs7	-0.03	1.00000000	mice
Traf7	0.00	1.00000000	mice
Fkbp5	0.01	1.00000000	mice
Dazap1	-0.01	1.00000000	mice
Selenof	-0.02	1.00000000	mice
Zbtb41	-0.01	1.00000000	mice
Rap2a	-0.04	1.00000000	mice
Sult2b1	-0.10	1.00000000	mice
Bub3	0.01	1.00000000	mice
C1d	-0.02	1.00000000	mice
Slc35e2	0.04	1.00000000	mice
Samd3	-0.13	1.00000000	mice
Zdhhc7	0.00	1.00000000	mice
Atxn3	-0.02	1.00000000	mice
Gemin5	-0.02	1.00000000	mice
Tia1	0.06	1.00000000	mice
Suco	-0.02	1.00000000	mice
Ino80d	-0.01	1.00000000	mice
Cse1l	0.02	1.00000000	mice
Stx18	-0.05	1.00000000	mice
BC002059	-0.05	1.00000000	mice
Adsl	-0.02	1.00000000	mice
Ppil4	-0.01	1.00000000	mice
Ints8	0.00	1.00000000	mice
Ssh1	-0.09	1.00000000	mice
Ptges3	-0.02	1.00000000	mice
Ncald	-0.11	1.00000000	mice
Smg5	-0.04	1.00000000	mice
Tsnax	-0.02	1.00000000	mice
Lpgat1	-0.04	1.00000000	mice
Rasgrp1	0.02	1.00000000	mice
Srrm2	0.01	1.00000000	mice
Rtf1	0.00	1.00000000	mice
Ppp4r3a	0.02	1.00000000	mice
Immp1l	-0.03	1.00000000	mice
Ppat	-0.09	1.00000000	mice
Orc6	-0.02	1.00000000	mice
Taf5l	-0.04	1.00000000	mice

Csnk2a2	-0.04	1.00000000	mice
Ten1	-0.02	1.00000000	mice
Vopp1	-0.04	1.00000000	mice
Prdm4	-0.03	1.00000000	mice
Stat5a	-0.03	1.00000000	mice
Carnmt1	0.06	1.00000000	mice
Exoc4	0.01	1.00000000	mice
Socs6	-0.02	1.00000000	mice
Rab40c	-0.01	1.00000000	mice
Ttc27	-0.01	1.00000000	mice
Srbd1	-0.05	1.00000000	mice
Ubb	0.02	1.00000000	mice
0610010F05Rik	-0.07	1.00000000	mice
Gpr107	-0.02	1.00000000	mice
2900097C17Rik	-0.02	1.00000000	mice
Dync2h1	-0.06	1.00000000	mice
Prr14l	-0.02	1.00000000	mice
Ccs	-0.03	1.00000000	mice
Saa3	2.17	0.00000000	pigs
Timp1	1.75	0.00000000	pigs
Ago2	1.00	0.00000000	pigs
Ndfip1	0.93	0.00000000	pigs
Antxr2	1.17	0.00000000	pigs
Actb	-0.77	0.00000000	pigs
Dusp4	1.08	0.00000000	pigs
Runx2	0.91	0.00000000	pigs
Frmd4b	1.00	0.00000000	pigs
Aldh3a2	0.97	0.00000000	pigs
Cd2	-0.77	0.00000000	pigs
Pmaip1	0.90	0.00000000	pigs
Gpx1	-1.13	0.00000000	pigs
S100a6	-1.68	0.00000000	pigs
Cpm	-1.66	0.00000000	pigs
Prkca	1.09	0.00000000	pigs
Rbpj	0.97	0.00000000	pigs
Adgrg1	-1.03	0.00000000	pigs
Il7r	1.40	0.00000000	pigs
Ftl1-ps1	0.80	0.00000000	pigs
Cry2	0.77	0.00000000	pigs
Spock2	0.78	0.00000000	pigs
Nol11	0.74	0.00000000	pigs
Hsph1	0.98	0.00000000	pigs
Gna13	0.79	0.00000000	pigs
F2r	0.69	0.00000000	pigs
H2-Ob	0.67	0.00000000	pigs
Pspc1	0.76	0.00000000	pigs

Hif1a	0.89	0.00000000	pigs
Cxcr6	0.71	0.00000000	pigs
Grk3	0.85	0.00000000	pigs
Zswim6	0.74	0.00000000	pigs
Bhlhe40	0.86	0.00000000	pigs
Slc11a2	0.80	0.00000000	pigs
Iffo2	0.69	0.00000000	pigs
Niban1	1.22	0.00000000	pigs
Smpdl3a	0.46	0.00000000	pigs
Chst12	0.85	0.00000000	pigs
Smurf2	1.03	0.00000000	pigs
Rap1gap2	-1.02	0.00000000	pigs
Nck2	1.02	0.00000000	pigs
Hint3	0.49	0.00000000	pigs
1700025G04Rik	0.70	0.00000000	pigs
Atp2b4	0.74	0.00000000	pigs
Prkce	0.86	0.00000000	pigs
Rap1b	-0.64	0.00000000	pigs
Immp2l	-1.20	0.00000000	pigs
Lgmn	0.50	0.00000000	pigs
Snapc1l	0.87	0.00000000	pigs
Rfx2	1.03	0.00000000	pigs
Xylt1	0.83	0.00000000	pigs
Nme8	0.58	0.00000000	pigs
Dennd3	0.62	0.00000000	pigs
Tut4	-0.80	0.00000000	pigs
Rasa3	-0.96	0.00000000	pigs
Stat5a	0.76	0.00000000	pigs
Furin	1.11	0.00000000	pigs
Atp2a2	0.83	0.00000000	pigs
Cyth4	0.62	0.00000000	pigs
Dhrs3	0.71	0.00000000	pigs
Ppp1r12a	0.60	0.00000000	pigs
Lpin1	0.78	0.00000000	pigs
Tiparp	0.87	0.00000000	pigs
Smc4	0.70	0.00000000	pigs
Ppp2r5c	0.68	0.00000000	pigs
Anxa6	0.59	0.00000000	pigs
Anxa5	0.50	0.00000000	pigs
Poc1b	0.67	0.00000000	pigs
Zeb1	0.78	0.00000000	pigs
Rnf19a	0.87	0.00000000	pigs
Stat1	0.66	0.00000000	pigs
Cnot6l	0.56	0.00000000	pigs
Ccnl1	0.61	0.00000000	pigs
Hpse	0.44	0.00000000	pigs

Tsen2	0.69	0.00000000	pigs
Ogt	0.51	0.00000000	pigs
Fam102a	0.60	0.00000000	pigs
S100a4	-1.38	0.00000000	pigs
Abtb2	1.08	0.00000000	pigs
Rnf2	0.99	0.00000000	pigs
Ccdc50	0.67	0.00000000	pigs
Hspa8	0.53	0.00000000	pigs
Sik3	0.66	0.00000000	pigs
Ppp3ca	-0.70	0.00000000	pigs
Nfkb1	0.71	0.00000000	pigs
Fosb	0.59	0.00000000	pigs
Per1	0.59	0.00000000	pigs
Klf2	-0.96	0.00000000	pigs
Trpv2	0.47	0.00000000	pigs
Wnk1	0.62	0.00000000	pigs
Relb	0.60	0.00000000	pigs
Atp1a1	0.62	0.00000000	pigs
Irf8	0.58	0.00000000	pigs
Traf5	0.63	0.00000000	pigs
Ranbp9	0.62	0.00000000	pigs
Ahrr	0.54	0.00000000	pigs
Cdkn1a	0.49	0.00000000	pigs
Serf2	-0.43	0.00000000	pigs
Cfd	-0.91	0.00000000	pigs
Csnk1a1	0.45	0.00000000	pigs
Uvrag	0.62	0.00000000	pigs
AA467197	0.66	0.00000000	pigs
Pnp	0.69	0.00000000	pigs
Zcchc14	0.50	0.00000000	pigs
Slamf7	0.79	0.00000000	pigs
Fscn1	0.35	0.00000000	pigs
Arg1	0.58	0.00000000	pigs
Tbc1d4	0.90	0.00000000	pigs
Card11	0.72	0.00000000	pigs
Sptlc2	0.61	0.00000000	pigs
Ubl5	-0.69	0.00000000	pigs
Ctsb	0.45	0.00000000	pigs
Arid4b	0.46	0.00000000	pigs
Stambpl1	0.70	0.00000000	pigs
Top3a	0.42	0.00000000	pigs
Ndfip2	0.75	0.00000000	pigs
Lag3	0.83	0.00000000	pigs
Snx9	0.77	0.00000000	pigs
Zeb2	-0.65	0.00000000	pigs
Oas2	0.41	0.00000000	pigs

Tnip2	0.51	0.00000000	pigs
Atp11a	0.46	0.00000000	pigs
Sema4a	0.52	0.00000000	pigs
Dnajb1	0.55	0.00000000	pigs
Thsd7b	-0.91	0.00000000	pigs
Sun2	-0.73	0.00000000	pigs
Rpap2	0.49	0.00000000	pigs
Cx3cr1	-0.80	0.00000000	pigs
Efr3a	0.50	0.00000000	pigs
Itgal	-0.74	0.00000000	pigs
Ppp6r3	0.48	0.00000000	pigs
Inpp5d	0.44	0.00000000	pigs
Tmem131l	0.64	0.00000000	pigs
C1qa	0.44	0.00000000	pigs
Tuba1b	0.52	0.00000000	pigs
Pde7a	0.64	0.00000000	pigs
Ahi1	0.61	0.00000000	pigs
Ell2	0.51	0.00000000	pigs
Lpin2	0.65	0.00000000	pigs
Hsp90aa1	0.43	0.00000000	pigs
Tnip3	0.56	0.00000000	pigs
Chordc1	0.47	0.00000000	pigs
Piezo1	0.40	0.00000000	pigs
Ankrd11	0.44	0.00000000	pigs
Shisa5	0.37	0.00000000	pigs
Lcor	0.43	0.00000000	pigs
Rasgef1b	0.53	0.00000000	pigs
Phtf1	0.56	0.00000000	pigs
Chd1	0.42	0.00000000	pigs
Lbr	-0.68	0.00000000	pigs
Itk	0.47	0.00000000	pigs
Cd96	0.59	0.00000000	pigs
Vmp1	0.51	0.00000000	pigs
Sod1	0.44	0.00000000	pigs
Satb1	0.90	0.00000000	pigs
Sell	-0.87	0.00000000	pigs
Ahr	0.59	0.00000000	pigs
Atxn7l1	-0.70	0.00000000	pigs
Pik3ap1	0.58	0.00000000	pigs
Ppp4r3a	0.50	0.00000000	pigs
Klrg1	-0.75	0.00000000	pigs
Rasa2	0.42	0.00000000	pigs
Cd28	0.65	0.00000000	pigs
Bmp2k	0.57	0.00000000	pigs
Ccser2	0.53	0.00000000	pigs
Utrn	-0.52	0.00000000	pigs

Nr3c1	0.65	0.00000000	pigs
Flna	-0.77	0.00000000	pigs
Herpud1	0.42	0.00000000	pigs
Gfi1	0.53	0.00000000	pigs
Rp2	0.61	0.00000000	pigs
Il4ra	0.40	0.00000000	pigs
Sptan1	0.42	0.00000000	pigs
Tgfbr3	0.41	0.00000000	pigs
Gpr183	0.44	0.00000000	pigs
Prrc2b	0.44	0.00000000	pigs
Gramd3	0.81	0.00000000	pigs
Ranbp2	0.44	0.00000000	pigs
Ralgapa1	0.55	0.00000000	pigs
Il2ra	0.60	0.00000000	pigs
Traf1	0.66	0.00000000	pigs
Nedd4l	0.60	0.00000000	pigs
Usp18	0.43	0.00000000	pigs
Tma7	-0.55	0.00000000	pigs
Gcsam	0.49	0.00000000	pigs
Trat1	-0.51	0.00000000	pigs
Bag3	0.54	0.00000000	pigs
Nfkbiz	0.64	0.00000000	pigs
Scpep1	0.35	0.00000000	pigs
Tmem65	0.60	0.00000000	pigs
Ulk2	0.38	0.00000000	pigs
Edem3	0.52	0.00000000	pigs
Pmepa1	0.32	0.00000000	pigs
Cyp2j13	0.27	0.00000000	pigs
Selenos	0.39	0.00000000	pigs
Atp2b1	0.54	0.00000000	pigs
Spsb1	0.34	0.00000000	pigs
Acss1	0.30	0.00000000	pigs
Gls	0.40	0.00000000	pigs
Rev1	0.58	0.00000000	pigs
Fdft1	0.28	0.00000000	pigs
Nfat5	0.42	0.00000000	pigs
P4ha1	0.45	0.00000000	pigs
Isg20	0.40	0.00000000	pigs
Eif3a	0.38	0.00000000	pigs
Bmf	0.36	0.00000000	pigs
Cyth3	0.56	0.00000000	pigs
Rdx	-0.46	0.00000000	pigs
Ptprc	-0.24	0.00000000	pigs
Bcl6	0.34	0.00000000	pigs
Apbb1ip	-0.44	0.00000000	pigs
S1pr1	-0.71	0.00000000	pigs

Stat3	0.48	0.00000000	pigs
Dyrk1a	0.42	0.00000000	pigs
Srpk2	0.51	0.00000000	pigs
Eif4a1	0.43	0.00000000	pigs
Arhgap18	0.39	0.00000000	pigs
Kdm6a	0.43	0.00000000	pigs
Phactr2	-0.59	0.00000000	pigs
C4bp	-1.23	0.00000000	pigs
Plagl1	0.55	0.00000000	pigs
Itgae	0.51	0.00000000	pigs
Irf2bp2	0.39	0.00000000	pigs
Rsbn1	0.51	0.00000000	pigs
Ern1	0.46	0.00000000	pigs
Jmjd1c	0.43	0.00000000	pigs
Ier5	0.40	0.00000000	pigs
Itgb2l	-0.64	0.00000000	pigs
Ybey	0.33	0.00000000	pigs
Fam185a	0.51	0.00000000	pigs
Sik2	0.60	0.00000000	pigs
Arhgef18	-0.69	0.00000000	pigs
Susd1	-0.81	0.00000000	pigs
Ptprcap	-0.52	0.00000000	pigs
Apmap	0.35	0.00000000	pigs
Dennd5a	0.34	0.00000000	pigs
Odc1	0.40	0.00000000	pigs
Stk24	0.40	0.00000000	pigs
Ezh2	0.45	0.00000000	pigs
Cemip2	-0.56	0.00000000	pigs
Ankrd28	0.44	0.00000000	pigs
Cd69	0.53	0.00000000	pigs
Vps37b	0.44	0.00000000	pigs
Trp53i11	0.38	0.00000000	pigs
Ddx3x	0.32	0.00000000	pigs
Galnt3	0.34	0.00000000	pigs
Pdxk	0.35	0.00000000	pigs
Traf3	0.37	0.00000000	pigs
Rsrp1	0.41	0.00000000	pigs
Gpr34	0.31	0.00000000	pigs
Dctn4	0.43	0.00000000	pigs
Bst2	0.32	0.00000000	pigs
Cstb	0.42	0.00000000	pigs
Trim2	-0.70	0.00000000	pigs
Braf	0.44	0.00000000	pigs
Slc7a1	0.35	0.00000000	pigs
Galnt7	0.69	0.00000000	pigs
Cir1	0.31	0.00000000	pigs

Ccl5	-0.38	0.00000000	pigs
Klhl8	-0.90	0.00000000	pigs
Szrd1	0.41	0.00000000	pigs
Il21r	0.46	0.00000000	pigs
Gpbp1	0.36	0.00000000	pigs
Stx11	0.56	0.00000000	pigs
Ecpas	0.41	0.00000000	pigs
Cmip	0.39	0.00000000	pigs
Csrnp1	0.38	0.00000000	pigs
Emb	0.47	0.00000000	pigs
Arhgap24	0.84	0.00000000	pigs
Suco	0.48	0.00000000	pigs
Stat4	0.47	0.00000000	pigs
Traf3ip1	0.30	0.00000000	pigs
Ubqln1	0.38	0.00000001	pigs
Rab27a	0.41	0.00000001	pigs
Arid1b	0.40	0.00000001	pigs
Rel	0.56	0.00000001	pigs
Pitpnm3	0.37	0.00000001	pigs
Prpf39	0.35	0.00000001	pigs
Aplf	0.29	0.00000001	pigs
Pcgf5	0.29	0.00000001	pigs
Evl	0.42	0.00000001	pigs
C1qb	0.28	0.00000001	pigs
Setbp1	0.46	0.00000001	pigs
Peli1	0.43	0.00000001	pigs
Cd24a	-1.12	0.00000001	pigs
Fam102b	0.44	0.00000001	pigs
Tesc	-0.60	0.00000002	pigs
Rab1a	0.41	0.00000002	pigs
Ccdc6	0.49	0.00000002	pigs
Dennd4a	0.60	0.00000002	pigs
Zbtb10	0.42	0.00000002	pigs
Foxo1	0.57	0.00000002	pigs
Yars	0.40	0.00000002	pigs
Vav1	0.44	0.00000002	pigs
Ywhab	-0.46	0.00000002	pigs
Slc20a1	0.31	0.00000002	pigs
Cda	-1.33	0.00000003	pigs
Adgre5	-0.41	0.00000003	pigs
Cd8a	-0.59	0.00000003	pigs
Surf4	0.26	0.00000003	pigs
Rnf19b	0.47	0.00000003	pigs
Akirin1	0.40	0.00000003	pigs
A4galt	0.22	0.00000003	pigs
Cpeb2	0.41	0.00000004	pigs

Rhoh	0.50	0.00000005	pigs
Sh3bgrl3	-0.41	0.00000006	pigs
Icam1	0.41	0.00000006	pigs
Arl5b	0.36	0.00000006	pigs
Klf3	-0.67	0.00000006	pigs
Tfrc	0.33	0.00000007	pigs
Luc7l	0.35	0.00000008	pigs
Klf4	0.27	0.00000008	pigs
Inpp4b	0.49	0.00000009	pigs
Sit1	0.31	0.00000009	pigs
Zc3h12c	0.41	0.00000009	pigs
Hivep1	0.54	0.00000009	pigs
Slc23a2	0.44	0.00000011	pigs
Ap1ar	0.38	0.00000012	pigs
Itgb1bp1	0.34	0.00000013	pigs
Pde4b	0.34	0.00000014	pigs
Lrrk2	-1.47	0.00000015	pigs
Cstf3	0.32	0.00000015	pigs
Blk	0.68	0.00000016	pigs
Elob	-0.50	0.00000016	pigs
Zbtb2	0.37	0.00000017	pigs
Etnk1	0.33	0.00000017	pigs
Tent4a	0.30	0.00000018	pigs
Gpr171	0.34	0.00000018	pigs
Ucp2	-0.51	0.00000018	pigs
Nfkbia	0.56	0.00000021	pigs
4933434E20Rik	0.22	0.00000026	pigs
Gpbp1l1	0.33	0.00000027	pigs
Nfatc2	0.37	0.00000030	pigs
Rai1	0.22	0.00000030	pigs
Tm7sf3	0.25	0.00000031	pigs
Pawr	0.32	0.00000033	pigs
Scmh1	0.40	0.00000034	pigs
Fnbp1	0.39	0.00000039	pigs
Rgs10	0.22	0.00000040	pigs
Ptpn12	0.45	0.00000041	pigs
Chd7	0.24	0.00000042	pigs
Slc16a1	0.42	0.00000043	pigs
Qk	0.39	0.00000051	pigs
Dusp1	-0.78	0.00000058	pigs
Pum1	0.29	0.00000059	pigs
Gdi2	0.31	0.00000062	pigs
Nsd3	0.48	0.00000064	pigs
Hnrnph3	0.31	0.00000064	pigs
Fez2	0.46	0.00000065	pigs
Klrb1a	0.85	0.00000065	pigs

H2az2	-0.36	0.00000066	pigs
Ehd1	0.34	0.00000068	pigs
Dnah11	-0.65	0.00000069	pigs
Pank3	0.23	0.00000071	pigs
Gcnt1	0.37	0.00000071	pigs
Ifit2	-0.77	0.00000072	pigs
Rin3	0.46	0.00000078	pigs
Dusp11	0.28	0.00000086	pigs
Rab3gap2	0.34	0.00000090	pigs
Epsti1	0.40	0.00000096	pigs
Slc30a5	0.40	0.00000096	pigs
Ncoa7	0.40	0.00000100	pigs
Usp37	0.31	0.00000107	pigs
Ccnt2	0.27	0.00000109	pigs
Ets1	0.24	0.00000112	pigs
Ankrd27	0.20	0.00000117	pigs
Syne3	0.36	0.00000128	pigs
Avpi1	0.28	0.00000129	pigs
Zc3hav1	0.34	0.00000134	pigs
Rftn1	0.46	0.00000148	pigs
Cdh24	0.23	0.00000151	pigs
Ube2d3	0.28	0.00000157	pigs
Plek	-0.52	0.00000160	pigs
Baz1a	0.41	0.00000170	pigs
Eef2	0.30	0.00000183	pigs
Gpr35	0.16	0.00000185	pigs
Eed	0.33	0.00000188	pigs
St6gal1	0.50	0.00000188	pigs
Vim	0.38	0.00000192	pigs
Pik3cd	0.36	0.00000192	pigs
Cacna1e	0.27	0.00000224	pigs
Smurf1	0.45	0.00000224	pigs
Fndc3a	0.45	0.00000238	pigs
Zbtb16	0.41	0.00000261	pigs
Snx25	0.26	0.00000265	pigs
Phkg2	0.27	0.00000270	pigs
Adss	0.27	0.00000286	pigs
Sap30l	0.26	0.00000303	pigs
Nek9	0.28	0.00000348	pigs
Klf13	0.36	0.00000351	pigs
Phc3	0.35	0.00000373	pigs
Nop56	0.27	0.00000379	pigs
Cox7c	-0.44	0.00000417	pigs
Slc4a7	0.35	0.00000443	pigs
Fubp1	0.27	0.00000450	pigs
Pdpk1	0.37	0.00000476	pigs

Aco2	0.26	0.00000507	pigs
Jak2	0.40	0.00000511	pigs
Cytip	0.37	0.00000514	pigs
Ppm1k	0.33	0.00000517	pigs
Lpcat3	0.23	0.00000533	pigs
Hdac4	0.24	0.00000561	pigs
Pcnx4	0.28	0.00000570	pigs
Rapgef2	0.41	0.00000580	pigs
Tmem129	0.40	0.00000584	pigs
Sar1b	0.29	0.00000590	pigs
Dennd11	-0.93	0.00000614	pigs
Tln1	-0.44	0.00000624	pigs
Ubr4	0.30	0.00000664	pigs
Fam71e1	-0.38	0.00000678	pigs
Vcp	0.24	0.00000702	pigs
Siah1b	0.34	0.00000720	pigs
Usp12	0.29	0.00000743	pigs
Gadd45g	0.36	0.00000844	pigs
Brd4	0.30	0.00000878	pigs
S100a10	-0.46	0.00000886	pigs
Dnajc2	0.25	0.00000909	pigs
Stk17b	0.24	0.00000936	pigs
Tox2	0.39	0.00000988	pigs
Tet2	0.40	0.00001047	pigs
Coq2	0.31	0.00001058	pigs
Icos	0.50	0.00001061	pigs
Agfg1	0.27	0.00001086	pigs
Gigyf2	0.31	0.00001130	pigs
Echdc1	0.21	0.00001227	pigs
Zfp207	0.26	0.00001235	pigs
Nampt	0.32	0.00001265	pigs
Raf1	0.27	0.00001281	pigs
Pou2f1	0.37	0.00001307	pigs
Chst11	0.38	0.00001353	pigs
Aak1	-0.63	0.00001470	pigs
Rrp15	0.25	0.00001509	pigs
Cdv3	0.29	0.00001668	pigs
Pafah2	0.28	0.00001673	pigs
Slc36a4	0.29	0.00001927	pigs
Il27ra	0.29	0.00001931	pigs
Ube2z	0.23	0.00002122	pigs
Ypel3	-0.58	0.00002135	pigs
Rell1	0.24	0.00002169	pigs
Fabp5	0.21	0.00002204	pigs
Lgals3bp	0.27	0.00002210	pigs
Cnot1	0.21	0.00002278	pigs

Oxa1l	0.26	0.00002312	pigs
Ubash3a	0.36	0.00002441	pigs
Tmem135	0.39	0.00002448	pigs
Slain1	0.22	0.00002454	pigs
S1pr5	-0.43	0.00002591	pigs
Rab35	0.27	0.00002626	pigs
Sema6d	-0.59	0.00002657	pigs
Jmy	0.46	0.00002680	pigs
Ctnnb1	0.27	0.00002694	pigs
Ptprj	0.66	0.00002719	pigs
Abcg1	0.19	0.00002792	pigs
Pop7	0.21	0.00002811	pigs
Sec14l1	0.31	0.00002960	pigs
Trappc9	0.32	0.00003079	pigs
Nup50	0.30	0.00003088	pigs
Adgrg5	-0.60	0.00003154	pigs
Klhl42	0.22	0.00003253	pigs
Ube2e2	0.44	0.00003297	pigs
Rab9	0.30	0.00003325	pigs
Hsp90ab1	0.28	0.00003394	pigs
Lmtk2	0.28	0.00003892	pigs
Nfkbie	0.25	0.00003959	pigs
D2hgdh	0.35	0.00004239	pigs
Rbm26	0.33	0.00004434	pigs
Socs3	0.33	0.00004863	pigs
Crk	0.26	0.00004927	pigs
Ube2h	0.24	0.00004986	pigs
Crebbp	0.30	0.00005035	pigs
Dbi	-0.55	0.00005177	pigs
Gpr107	0.21	0.00005322	pigs
Zbtb43	0.31	0.00005601	pigs
Upf1	0.20	0.00005772	pigs
Gcnt2	0.57	0.00005977	pigs
Cxcr3	0.20	0.00005996	pigs
Xpr1	0.27	0.00006259	pigs
Clock	0.27	0.00006322	pigs
Pgs1	0.29	0.00006484	pigs
Tmed10	0.26	0.00006514	pigs
Ncoa3	0.29	0.00007138	pigs
Il6st	0.31	0.00007297	pigs
Ptbp2	0.31	0.00007573	pigs
Ppp2r1b	0.20	0.00007905	pigs
Psmg3	0.15	0.00008345	pigs
Kmt2e	0.18	0.00008457	pigs
D16Ertd472e	0.34	0.00008940	pigs
Lonrf1	0.32	0.00009189	pigs

Scml4	0.41	0.00009238	pigs
Adnp	0.27	0.00009719	pigs
L3mbtl1	0.23	0.00009839	pigs
Ikzf1	0.28	0.00009886	pigs
Gpr108	0.22	0.00009913	pigs
Zfand5	0.28	0.00009939	pigs
Safb2	0.30	0.00010290	pigs
Pi4k2b	0.22	0.00010654	pigs
Rgcc	0.37	0.00010955	pigs
Psme1	-0.53	0.00011079	pigs
Rgs19	-0.53	0.00011494	pigs
Rtn4	0.31	0.00011526	pigs
Cyth1	0.39	0.00011726	pigs
Wtap	0.26	0.00012027	pigs
Caprin1	0.32	0.00012535	pigs
Bnip3	0.28	0.00012912	pigs
Tab2	0.29	0.00013059	pigs
Ptger3	0.17	0.00013180	pigs
Luc7l3	0.30	0.00013359	pigs
Psmd11	0.20	0.00013766	pigs
Plgrkt	0.16	0.00013861	pigs
Cblb	0.30	0.00014156	pigs
Abca1	0.23	0.00014781	pigs
Gsk3b	0.27	0.00015297	pigs
Bend7	0.23	0.00015511	pigs
Git2	0.19	0.00015608	pigs
Derl1	0.26	0.00015653	pigs
Ndel1	0.37	0.00015706	pigs
Rnf139	0.30	0.00016145	pigs
Slc9a3r1	-0.53	0.00016209	pigs
Tnip1	0.28	0.00016362	pigs
Gzma	0.91	0.00017110	pigs
Fbxl5	0.25	0.00017299	pigs
Plcg2	0.59	0.00018509	pigs
Pafah1b1	0.26	0.00018520	pigs
Actr10	0.18	0.00018841	pigs
Narf	0.18	0.00018849	pigs
Sec23b	0.22	0.00018881	pigs
Pip4k2a	0.29	0.00019287	pigs
Slc6a6	0.23	0.00019454	pigs
Acot7	0.21	0.00019466	pigs
Rbm18	0.20	0.00020107	pigs
Usf1	0.21	0.00020188	pigs
Dip2b	0.28	0.00020284	pigs
Sec31a	0.26	0.00020539	pigs
Nfe2l2	0.30	0.00020816	pigs

Dkc1	0.18	0.00021777	pigs
Ksr1	0.25	0.00021881	pigs
Tamalin	0.23	0.00022639	pigs
Kdm6b	0.17	0.00023088	pigs
Cops2	0.18	0.00023557	pigs
Akap5	0.24	0.00023716	pigs
Gch1	0.34	0.00023780	pigs
Vapb	0.25	0.00025043	pigs
Cacybp	0.18	0.00026350	pigs
Klhdc10	0.62	0.00026453	pigs
Eif3m	0.26	0.00027119	pigs
Cnot9	0.41	0.00028220	pigs
Edem1	0.24	0.00028442	pigs
Clasp1	0.26	0.00028550	pigs
Fam135a	-0.74	0.00028650	pigs
Spen	0.34	0.00029729	pigs
Otulin	0.31	0.00030246	pigs
Nfkb2	0.22	0.00030459	pigs
Safb	0.22	0.00030729	pigs
St8sia4	0.38	0.00030866	pigs
N4bp1	0.29	0.00031034	pigs
Kdm4c	0.25	0.00031201	pigs
Psmd4	0.20	0.00031334	pigs
Tmem11	0.22	0.00031474	pigs
Snrnp48	0.26	0.00031477	pigs
C5ar1	-0.82	0.00032219	pigs
Llph	0.21	0.00034900	pigs
Slc25a36	0.17	0.00034943	pigs
Dync1h1	0.23	0.00035351	pigs
Gtpbp1	0.17	0.00035534	pigs
Syne2	0.26	0.00038311	pigs
Dgke	0.19	0.00039694	pigs
Adora2b	0.16	0.00039859	pigs
Snrpd2	-0.43	0.00041020	pigs
Aplp2	0.20	0.00042095	pigs
Pxk	0.34	0.00042411	pigs
Dcp2	0.29	0.00042911	pigs
Bzw1	-0.38	0.00043041	pigs
Gna12	0.19	0.00043488	pigs
Smndc1	0.29	0.00044033	pigs
Ldlrad3	-0.62	0.00044097	pigs
Lats1	0.19	0.00044343	pigs
Plekha1	0.38	0.00045202	pigs
Slc2a13	0.20	0.00045297	pigs
Aftph	0.28	0.00046495	pigs
Rassf5	0.29	0.00047482	pigs

Cand1	0.18	0.00048424	pigs
Bud23	0.28	0.00049957	pigs
Tcp11	0.19	0.00050406	pigs
Hspa9	0.20	0.00053952	pigs
Ikzf2	0.54	0.00054081	pigs
Lactb	0.26	0.00054356	pigs
Hcfc2	0.20	0.00055395	pigs
Serpinb9	0.28	0.00055456	pigs
Tspan12	0.26	0.00056120	pigs
Hnrnpll	0.31	0.00056124	pigs
Zfp90	0.20	0.00056280	pigs
Ppp1r9b	0.23	0.00056460	pigs
Top1	0.29	0.00057195	pigs
Zbtb11	0.21	0.00057839	pigs
Arhgap31	-0.55	0.00059186	pigs
Prpf40a	0.25	0.00061661	pigs
Prdm2	0.27	0.00061964	pigs
Eif3l	0.17	0.00062993	pigs
Cd38	-0.47	0.00063931	pigs
Zfp13	-0.49	0.00065698	pigs
Hbs1l	0.26	0.00068373	pigs
Prmt1	0.19	0.00069554	pigs
Pan3	0.31	0.00072773	pigs
Gan	0.35	0.00075467	pigs
Hbb-bh2	-0.89	0.00077489	pigs
Ythdf2	0.21	0.00078994	pigs
Zc3h7a	0.20	0.00086475	pigs
Srsf11	0.21	0.00087186	pigs
Atg16l1	0.19	0.00088679	pigs
Lnx2	0.21	0.00088907	pigs
Pygb	0.19	0.00091552	pigs
Rpia	0.25	0.00095910	pigs
Ptk2b	0.42	0.00105086	pigs
Ext1	0.34	0.00108516	pigs
Zcchc2	0.29	0.00109358	pigs
Urm1	0.15	0.00114261	pigs
Hnrnph1	0.22	0.00117860	pigs
Selenoi	0.25	0.00118063	pigs
Usp3	0.29	0.00119568	pigs
Ahsa1	0.18	0.00120902	pigs
Smg1	0.20	0.00130055	pigs
Psmd2	0.18	0.00138509	pigs
Wapl	0.20	0.00139858	pigs
Son	-0.31	0.00140493	pigs
Sod2	0.29	0.00142338	pigs
Ppp2ca	0.22	0.00143683	pigs

Saraf	0.21	0.00145097	pigs
Dock5	-0.49	0.00149624	pigs
Glrx3	0.19	0.00152751	pigs
Lmna	0.33	0.00155642	pigs
Uap1	0.27	0.00155670	pigs
Hspe1-rs1	0.25	0.00163815	pigs
Tpi1	0.28	0.00174497	pigs
Serpinb6b	-0.56	0.00176935	pigs
Tnfsf9	0.12	0.00178380	pigs
Hdac6	0.20	0.00178402	pigs
Steap4	-0.49	0.00180689	pigs
Zc3h13	0.20	0.00181840	pigs
Pim2	0.14	0.00183758	pigs
Pnisr	0.26	0.00185369	pigs
Clic5	-0.41	0.00187041	pigs
Ostf1	-0.43	0.00189601	pigs
Arap2	0.30	0.00194119	pigs
Lrch1	0.31	0.00194677	pigs
Ncapg	0.47	0.00195537	pigs
Gpnmb	0.17	0.00198399	pigs
Plekho1	0.23	0.00199606	pigs
Huwe1	0.26	0.00202195	pigs
Ddx5	0.21	0.00209222	pigs
Secisbp2l	0.15	0.00211829	pigs
Fus	0.24	0.00218507	pigs
Serinc5	0.33	0.00218892	pigs
Prex1	0.24	0.00224689	pigs
Csde1	0.27	0.00224756	pigs
Uqcrfs1	0.16	0.00228512	pigs
Esyt2	0.28	0.00240223	pigs
Iqgap1	0.23	0.00242263	pigs
Baz1b	0.20	0.00243202	pigs
Zbtb25	0.16	0.00245501	pigs
Sat1	0.23	0.00248062	pigs
Cpd	0.22	0.00249850	pigs
Dnajb2	0.15	0.00250285	pigs
Zfp704	0.37	0.00255983	pigs
Itpr1	0.30	0.00256011	pigs
Plaur	-0.85	0.00268536	pigs
Sms	0.27	0.00278498	pigs
Oxr1	0.17	0.00278964	pigs
Ap3d1	0.21	0.00282178	pigs
Ppp2r5d	0.14	0.00285660	pigs
Ncald	-0.42	0.00300316	pigs
Fam98b	0.15	0.00305903	pigs
Limk1	0.18	0.00314152	pigs

H1f3	0.22	0.00318795	pigs
Zmym2	0.23	0.00335447	pigs
St3gal5	0.30	0.00345154	pigs
Uxs1	0.32	0.00347420	pigs
Nemp2	0.22	0.00351985	pigs
Cd6	-0.55	0.00357350	pigs
Ero1b	0.26	0.00368734	pigs
Slc38a2	0.28	0.00379075	pigs
Tbc1d23	0.27	0.00379366	pigs
Zfp654	0.26	0.00388285	pigs
Vopp1	0.22	0.00393571	pigs
Ubr5	0.29	0.00393682	pigs
Slc25a13	0.17	0.00394699	pigs
Washc5	0.19	0.00394880	pigs
Amfr	0.20	0.00400224	pigs
Wac	0.22	0.00402554	pigs
Crebzf	0.19	0.00432928	pigs
Nrf1	0.25	0.00433834	pigs
Pcdhga4	0.11	0.00444991	pigs
Fosl2	0.24	0.00450094	pigs
Cox8a	-0.33	0.00472917	pigs
Ddb1	0.14	0.00473242	pigs
Fnta	0.10	0.00482854	pigs
Agpat4	0.23	0.00487972	pigs
Ablim3	0.22	0.00489882	pigs
Foxk1	0.24	0.00498280	pigs
Jak3	0.18	0.00508689	pigs
Ppp2r5e	0.30	0.00514929	pigs
Itpr2	0.28	0.00518217	pigs
Rheb	0.21	0.00519657	pigs
Zfp609	0.32	0.00532747	pigs
Rnf10	0.19	0.00541993	pigs
Tbca	0.17	0.00547280	pigs
Skap1	0.30	0.00547382	pigs
Sf3b3	0.22	0.00562401	pigs
Klf10	0.23	0.00565515	pigs
Smarca4	0.17	0.00572230	pigs
Snx27	0.28	0.00578845	pigs
Trim44	-0.55	0.00591087	pigs
Hpcal1	0.20	0.00594616	pigs
Anxa7	0.14	0.00612925	pigs
Arl8b	0.21	0.00626385	pigs
Dstn	0.22	0.00675420	pigs
Tmtc3	0.28	0.00688201	pigs
Nrg1	-0.94	0.00692928	pigs
Kif1b	0.24	0.00710107	pigs

Spred2	0.30	0.00718066	pigs
Pde8a	0.34	0.00719739	pigs
Ptpn1	0.28	0.00741704	pigs
Rela	0.21	0.00742843	pigs
Runx3	0.29	0.00751272	pigs
Pak1ip1	0.13	0.00772616	pigs
Ugp2	0.24	0.00773918	pigs
Serpinb1a	0.06	0.00779583	pigs
Frmd6	0.19	0.00783411	pigs
Fnbp4	0.25	0.00790533	pigs
Ptpn18	-0.43	0.00801762	pigs
Trim27	0.18	0.00810361	pigs
Cdc27	0.20	0.00840197	pigs
Rfk	0.19	0.00855115	pigs
Septin7	0.17	0.00873703	pigs
Fh1	0.13	0.00890831	pigs
Ywhah	0.23	0.00897411	pigs
Arl6ip5	0.17	0.00902335	pigs
Foxk2	0.22	0.00907531	pigs
Traf4	0.23	0.00916011	pigs
Plcg1	0.16	0.00918442	pigs
Rnf144b	-0.66	0.00929429	pigs
Ptdss1	0.20	0.00935297	pigs
Fam204a	0.16	0.00937781	pigs
Tomm70a	0.16	0.00968382	pigs
Rtraf	0.20	0.00986054	pigs
Arcn1	0.18	0.01051850	pigs
Arrdc2	0.15	0.01086188	pigs
Qrich1	0.19	0.01087055	pigs
Nek7	0.33	0.01094445	pigs
Strn3	0.26	0.01118622	pigs
Peak1	0.27	0.01130062	pigs
Rala	0.32	0.01149636	pigs
Stt3b	0.24	0.01163373	pigs
Rad21	0.16	0.01165603	pigs
Atp6v1b2	0.19	0.01169653	pigs
Ranbp3	0.19	0.01228958	pigs
Eif1	-0.25	0.01231984	pigs
Srsf7	0.13	0.01257832	pigs
Acly	0.13	0.01315165	pigs
Ly96	0.17	0.01336854	pigs
Ifi35	0.19	0.01344406	pigs
Dync1li2	0.09	0.01353878	pigs
Zranb1	0.24	0.01361791	pigs
Nup153	0.24	0.01369495	pigs
Nop2	0.14	0.01397459	pigs

Klf9	0.14	0.01424305	pigs
Etv6	0.25	0.01432933	pigs
Ppp1r15b	0.15	0.01516994	pigs
Dock2	-0.36	0.01539388	pigs
Ints6l	0.18	0.01561968	pigs
Stk26	0.29	0.01656247	pigs
Pdhx	0.18	0.01669414	pigs
Ubp1	0.20	0.01746250	pigs
Bach2	0.70	0.01762122	pigs
Slc25a3	0.18	0.01795350	pigs
Ctdspl2	0.16	0.01823142	pigs
Kdm2a	0.20	0.01844374	pigs
Actr1a	0.15	0.01846554	pigs
Cd8b1	-0.45	0.01849010	pigs
Trnau1ap	0.14	0.01904030	pigs
Wwox	0.30	0.01963610	pigs
Fbxo7	0.21	0.01963826	pigs
Ttc3	0.17	0.01980896	pigs
Yipf5	0.19	0.02019774	pigs
Tax1bp1	0.23	0.02102708	pigs
Thoc2	0.18	0.02135642	pigs
Atp5k	-0.44	0.02156057	pigs
Cbfb	0.20	0.02168295	pigs
Susd6	0.18	0.02173485	pigs
Cspp1	0.19	0.02200549	pigs
Tmem140	0.24	0.02215027	pigs
Plvap	-0.44	0.02234416	pigs
Tcerg1	0.18	0.02254281	pigs
Ghitm	0.21	0.02316473	pigs
Ewsr1	0.23	0.02324669	pigs
Supt3	0.29	0.02371446	pigs
Cdadc1	0.28	0.02375310	pigs
Trerf1	0.47	0.02390853	pigs
Pabpc4	0.12	0.02395759	pigs
Ptges3	0.21	0.02557606	pigs
Sds	-0.98	0.02595187	pigs
Rnf103	0.16	0.02663304	pigs
Zhx2	0.34	0.02669052	pigs
Tra2a	0.22	0.02681130	pigs
Smg7	0.21	0.02688575	pigs
Ncoa6	0.21	0.02690043	pigs
Tom1	0.19	0.02780420	pigs
Tubb4b	0.18	0.02784588	pigs
Arpc1b	0.15	0.02866875	pigs
Dcaf5	0.18	0.02886829	pigs
Pnkp	0.21	0.02894428	pigs

Grk6	-0.44	0.02921395	pigs
Hsbp1	0.22	0.02937846	pigs
Vcl	-0.46	0.03014622	pigs
Ppard	0.15	0.03034664	pigs
Ibtk	0.16	0.03053707	pigs
Esco1	0.25	0.03076896	pigs
Rc3h1	0.22	0.03077807	pigs
Abhd2	0.22	0.03119736	pigs
Fmnl3	0.15	0.03158280	pigs
Snx2	0.16	0.03192033	pigs
Zcchc8	0.16	0.03215627	pigs
Tmem123	0.23	0.03300857	pigs
Dmxl1	0.24	0.03304197	pigs
Fut8	0.35	0.03315692	pigs
Rhoq	-0.38	0.03372880	pigs
U2surp	0.22	0.03392425	pigs
Ugcg	0.23	0.03394398	pigs
Ilkap	0.16	0.03399848	pigs
Dbnl	-0.44	0.03425350	pigs
Atf4	0.23	0.03568012	pigs
Bach1	0.21	0.03574590	pigs
Ccr5	0.24	0.03636821	pigs
Hipk3	0.16	0.03637505	pigs
Eif4e	0.19	0.03679003	pigs
Selenot	0.16	0.03754335	pigs
Slc38a6	0.13	0.03768516	pigs
Qars	0.12	0.03865134	pigs
Cd80	0.25	0.03913829	pigs
Ppp4c	0.14	0.03945889	pigs
Cds2	0.20	0.04012150	pigs
Dap	0.25	0.04175415	pigs
Tiprl	0.13	0.04219780	pigs
Arhgap10	0.25	0.04263752	pigs
Snx13	0.20	0.04336903	pigs
Abhd13	0.22	0.04399314	pigs
Akt3	0.25	0.04501822	pigs
Stx6	0.15	0.04523410	pigs
Cyria	-0.35	0.04525947	pigs
Slc41a2	0.18	0.04539935	pigs
Setdb2	0.28	0.04709164	pigs
Sema4d	0.23	0.04874195	pigs
Crtam	0.44	0.04918089	pigs
Pfkp	0.19	0.05045872	pigs
Sdf4	0.26	0.05058724	pigs
Cert1	0.17	0.05089604	pigs
Rgs1	0.18	0.05110542	pigs

Cybb	0.20	0.05137368	pigs
Fbxw11	0.24	0.05189062	pigs
Naa15	0.14	0.05240741	pigs
Fndc7	0.13	0.05259049	pigs
Eif2ak3	0.27	0.05301726	pigs
Pip5k1a	0.06	0.05475549	pigs
Rb1cc1	0.13	0.05554943	pigs
Tmem71	-0.40	0.05585207	pigs
Hexim1	0.14	0.05638842	pigs
Ero1a	0.20	0.05665954	pigs
Ptpra	0.24	0.05834170	pigs
Apbb3	0.14	0.05903002	pigs
Hivep2	0.28	0.06245380	pigs
Tomm20	0.16	0.06252520	pigs
Nr4a3	0.28	0.06265225	pigs
Ptcd3	0.16	0.06344107	pigs
Orc4	0.18	0.06367724	pigs
Flvcr1	0.24	0.06369151	pigs
Skil	0.21	0.06374393	pigs
Rrp1	0.15	0.06480161	pigs
Nifk	0.19	0.06609790	pigs
Triobp	-0.40	0.06692583	pigs
Npc1	0.28	0.06808322	pigs
Cdyl	0.15	0.06842434	pigs
Hnrnpa0	0.22	0.07038957	pigs
Fryl	-0.34	0.07092103	pigs
Wdr47	0.17	0.07746379	pigs
Slc66a2	0.18	0.07813687	pigs
Xiap	0.26	0.07822020	pigs
Hectd4	0.18	0.07943447	pigs
Pacsin2	0.18	0.07953776	pigs
Ttc39c	0.26	0.08062929	pigs
Tmed7	0.15	0.08109856	pigs
Itga6	-0.49	0.08151679	pigs
Jarid2	0.19	0.08289660	pigs
Larp4	0.15	0.08400379	pigs
Socs1	0.16	0.08458474	pigs
Pgrmc1	0.15	0.08500576	pigs
Kdm1b	0.18	0.08524591	pigs
Pelo	0.14	0.08668615	pigs
Tia1	0.19	0.08748911	pigs
Rif1	0.18	0.08852005	pigs
Ddx54	0.12	0.08897348	pigs
Lsp1	-0.43	0.09148056	pigs
Fam222a	0.19	0.09194116	pigs
Tcf7	0.18	0.09265632	pigs

Far1	0.24	0.09312894	pigs
9930104L06Rik	0.11	0.09408076	pigs
Abce1	0.16	0.09474151	pigs
Hipk2	0.52	0.09547875	pigs
Isoc1	0.13	0.09560247	pigs
Bub3	0.14	0.09737834	pigs
Polr1d	0.22	0.09767656	pigs
Psmc6	0.14	0.09907991	pigs
Cnot2	0.30	0.10125054	pigs
Tnfaip3	0.26	0.10146986	pigs
Tfg	0.09	0.10162296	pigs
Atp10d	0.16	0.10162852	pigs
Rhobtb1	-0.41	0.10240205	pigs
Fam174a	0.12	0.10297798	pigs
Lix1l	0.12	0.10685477	pigs
Eif3d	0.15	0.10691974	pigs
Gpi1	0.12	0.10797976	pigs
Dhx15	0.17	0.11106005	pigs
Fcer1g	-0.56	0.11333939	pigs
Ctnna1	0.22	0.11354154	pigs
Axin1	0.18	0.11419517	pigs
Rac2	-0.21	0.11486117	pigs
Ipo7	0.26	0.11840975	pigs
Ankfy1	0.25	0.12290925	pigs
Glce	0.23	0.12314736	pigs
Ncoa4	0.13	0.12331399	pigs
Ccdc28b	0.13	0.12541295	pigs
Kctd3	0.18	0.12833201	pigs
Nipal1	-0.39	0.12889361	pigs
Zc3h12a	0.18	0.12894533	pigs
Ski	0.17	0.13014357	pigs
Abhd18	0.12	0.13055680	pigs
Akap8l	0.14	0.13109388	pigs
Cndp2	0.18	0.13143590	pigs
Plaat3	0.06	0.13414121	pigs
Tial1	0.19	0.13438924	pigs
Ccng1	0.13	0.13446598	pigs
Tgfb1	0.23	0.13668523	pigs
Daam1	0.18	0.13841331	pigs
Eif3j2	0.09	0.14197632	pigs
Otud4	0.15	0.14437935	pigs
Tnks	0.22	0.14669250	pigs
Cmas	0.19	0.14732487	pigs
Ube2g2	0.12	0.14804249	pigs
Capg	0.17	0.14881998	pigs
Dusp2	-0.49	0.15044669	pigs

Ubn1	0.20	0.15137363	pigs
Rab22a	0.26	0.15275477	pigs
Gemin5	0.16	0.15330929	pigs
Sorl1	-0.80	0.15404197	pigs
Cog6	0.10	0.15427347	pigs
Dram1	0.17	0.15469579	pigs
Ptpn3	0.14	0.15548483	pigs
Smad4	0.20	0.15610261	pigs
BC048507	0.14	0.15769145	pigs
Glul	0.19	0.15968225	pigs
Hspb1	0.16	0.16254447	pigs
Ino80c	0.12	0.16261345	pigs
Cct4	0.15	0.16323486	pigs
Agpat1	0.12	0.16548319	pigs
Ttc23l	0.18	0.16755453	pigs
Sh3bp5	-0.44	0.16855683	pigs
Uchl3	0.12	0.17158545	pigs
Wbp1l	0.18	0.17181055	pigs
Rab5a	0.15	0.18107724	pigs
Fbxo28	0.13	0.18114452	pigs
Tcp1	0.18	0.18237022	pigs
Brd2	0.19	0.18429945	pigs
Pptc7	0.11	0.18498758	pigs
Rybp	0.17	0.18522054	pigs
Krr1	0.12	0.18540007	pigs
Dtymk	0.08	0.18706740	pigs
Nptn	0.18	0.18924581	pigs
Hcls1	-0.38	0.18937592	pigs
Slc45a4	0.17	0.19255398	pigs
Greb1l	0.37	0.19658735	pigs
Dr1	0.14	0.20595503	pigs
Prorp	0.15	0.20696890	pigs
Heatr9	0.24	0.21041817	pigs
Btbd11	0.31	0.21104556	pigs
Csrnp2	0.13	0.21490678	pigs
Smarca2	0.24	0.21766420	pigs
Gla	0.18	0.22861950	pigs
Pml	0.23	0.22935694	pigs
Elavl1	0.11	0.23522804	pigs
Cilk1	0.31	0.23524997	pigs
Zfp36	0.31	0.23938907	pigs
Fam91a1	0.15	0.24041840	pigs
Rap1gds1	0.24	0.24163883	pigs
Sgpl1	0.14	0.24320717	pigs
Unc13d	0.07	0.24359113	pigs
Rae1	0.17	0.25024974	pigs

Tle4	0.30	0.25450853	pigs
Cox4i1	-0.25	0.25578267	pigs
Ddi2	0.14	0.25637219	pigs
Ciao2b	0.11	0.25704063	pigs
Cep85l	0.20	0.26108427	pigs
Eif4h	0.15	0.26607952	pigs
Rcor1	0.22	0.26643323	pigs
Dram2	0.09	0.26662415	pigs
Pex14	0.19	0.26669582	pigs
Cab39	0.25	0.26729165	pigs
Kansl1	0.17	0.26872483	pigs
Notch1	0.17	0.26993520	pigs
Setd5	0.19	0.27791885	pigs
Ddx20	0.11	0.27950014	pigs
Fbxo21	0.15	0.28451477	pigs
Ip6k1	0.18	0.28564882	pigs
Dcaf1	0.12	0.28643284	pigs
Cops4	0.13	0.29901595	pigs
Trmt11	0.09	0.30118865	pigs
Pcbd1	0.12	0.30122080	pigs
Vdac3	0.15	0.30657761	pigs
Ube2a	0.13	0.30663356	pigs
Slc38a10	0.16	0.30733655	pigs
Ccnd2	0.18	0.30893906	pigs
Rtf2	0.14	0.30931712	pigs
Cish	0.17	0.31816719	pigs
Wdfy3	-0.79	0.32167315	pigs
Ik	-0.44	0.32382002	pigs
Tmem19	0.15	0.33026068	pigs
Bclaf1	0.18	0.33336644	pigs
Acadl	0.15	0.33944421	pigs
Ube2k	0.23	0.33946373	pigs
Bcl9l	-0.27	0.34105570	pigs
Zfp236	0.08	0.34195801	pigs
Tmem181a	0.16	0.34320853	pigs
Rgs2	0.19	0.34585146	pigs
Stx7	0.10	0.34825186	pigs
S100a8	-1.15	0.35127612	pigs
Ndufb1	-0.42	0.35159994	pigs
Pi4kb	0.14	0.35258710	pigs
Hmgcr	0.08	0.35523255	pigs
Arpc4	-0.34	0.37506238	pigs
Tnfrsf9	0.22	0.37860799	pigs
Tasor2	0.15	0.39161090	pigs
Tec	0.21	0.39176831	pigs
Elf1	0.20	0.39212778	pigs

Pdlim5	0.18	0.39650114	pigs
Nop58	0.15	0.39857988	pigs
Ndrg1	0.23	0.39952358	pigs
Pth1r	-0.33	0.40167177	pigs
Egr1	-0.39	0.40532847	pigs
Smad3	0.28	0.40893569	pigs
Calm3	0.16	0.41291305	pigs
Zdhhc5	0.11	0.41293450	pigs
Ndufa4	-0.34	0.42071001	pigs
Plekha8	0.11	0.42077442	pigs
Grn	0.17	0.42177676	pigs
Trim75	0.15	0.42503610	pigs
Ywhaz	0.17	0.42882247	pigs
Zfp644	0.20	0.43115583	pigs
Unkl	0.08	0.43234448	pigs
Cd226	-0.48	0.43823721	pigs
Azin1	0.15	0.44896323	pigs
Tcf20	0.15	0.45546226	pigs
4930523C07Rik	0.17	0.45610751	pigs
Pja2	0.19	0.45624315	pigs
Rapgef1	0.13	0.45625020	pigs
Rock1	0.21	0.46315823	pigs
Pcca	0.23	0.46455709	pigs
Vamp5	-0.50	0.46491091	pigs
Ngly1	0.16	0.46675213	pigs
Gdpd5	0.14	0.47009266	pigs
Emsy	0.12	0.47283691	pigs
4932411N23Rik	0.14	0.47418469	pigs
Brwd3	0.25	0.47517283	pigs
Rc3h2	0.15	0.47600646	pigs
Zbtb17	0.18	0.47761694	pigs
Lysmd3	0.10	0.48257660	pigs
Eny2	0.12	0.48294152	pigs
Pcolce	-0.27	0.48617632	pigs
Elmo2	0.11	0.49344293	pigs
Prf1	0.21	0.49724243	pigs
Eif2s1	0.10	0.50106333	pigs
Sltm	0.15	0.50607603	pigs
Rbm39	0.15	0.51420978	pigs
Rap1a	0.19	0.51490292	pigs
4930453N24Rik	0.12	0.51528338	pigs
Polr1f	0.21	0.51588955	pigs
Cyld	0.16	0.51619320	pigs
Was	-0.38	0.52639438	pigs
Tgif2	0.15	0.53314231	pigs
Cdkn1b	-0.40	0.53586784	pigs

Cavin3	-0.47	0.54405972	pigs
Ccnk	0.15	0.54633692	pigs
Tspan31	0.18	0.54812082	pigs
Kcna3	0.12	0.55731967	pigs
Tcf12	0.31	0.55813957	pigs
Zfp36l1	0.14	0.56082044	pigs
Ptpn11	0.04	0.57151552	pigs
Rpn1	0.12	0.57281264	pigs
Cenpj	0.12	0.58159398	pigs
Atp23	0.23	0.58178206	pigs
Rnf145	0.17	0.58835389	pigs
Emc7	0.12	0.58850617	pigs
Nmral1	0.10	0.59479672	pigs
Laptm5	-0.16	0.59681423	pigs
Arl1	0.07	0.60099834	pigs
Inpp4a	0.14	0.60334720	pigs
Itm2a	0.17	0.60630467	pigs
Lsm14a	0.15	0.61020265	pigs
Tnfsf14	0.13	0.61652349	pigs
Sesn1	0.13	0.61947315	pigs
Dot1l	0.10	0.62141063	pigs
Zfp622	0.09	0.62650219	pigs
Rbl2	0.25	0.62882685	pigs
Rbm5	0.10	0.63241492	pigs
Ipo9	0.18	0.63657711	pigs
Lcp1	-0.18	0.64122067	pigs
Hes6	0.07	0.64152832	pigs
Smim14	-0.43	0.64770280	pigs
Cct3	0.11	0.65261056	pigs
Pfdn2	0.11	0.65463561	pigs
Rtca	0.09	0.65641314	pigs
Pnn	-0.33	0.65704478	pigs
Arhgap25	0.15	0.65881638	pigs
P2ry10	0.14	0.66075480	pigs
Armcx3	0.08	0.66185030	pigs
Dlg1	0.14	0.66554090	pigs
Zfp280c	0.18	0.66844933	pigs
Slc4a11	0.17	0.67111037	pigs
Ptpre	-0.34	0.69379118	pigs
Eef1g	0.18	0.69549969	pigs
Zfp1	0.12	0.70238391	pigs
Ip6k2	0.09	0.70384719	pigs
Birc6	0.19	0.70628845	pigs
Tpm4	0.17	0.70789678	pigs
Usp46	0.17	0.70916671	pigs
Stim1	0.20	0.71201545	pigs

Xpo7	0.14	0.71345686	pigs
Bloc1s5	0.12	0.72450094	pigs
Sdhb	0.12	0.72767670	pigs
Eif4b	0.15	0.73409155	pigs
Clk4	0.08	0.73543127	pigs
Prdx3	0.09	0.76055359	pigs
Tmem156	0.16	0.76298512	pigs
Themis2	-0.28	0.77395050	pigs
Uspl1	0.11	0.78655968	pigs
Ccdc186	0.12	0.79436508	pigs
Hsf1	0.13	0.79590249	pigs
Npc2	0.16	0.79626504	pigs
Socs7	0.09	0.80207455	pigs
Ruvbl2	0.10	0.80600508	pigs
Emp3	-0.42	0.81108132	pigs
Lman2	0.12	0.81403220	pigs
Synj1	0.14	0.82244963	pigs
Smap1	0.20	0.83473578	pigs
Lck	-0.29	0.83707240	pigs
Cdyl2	0.22	0.84213825	pigs
Umad1	0.11	0.85371261	pigs
Kpnb1	0.18	0.85407612	pigs
Ssb	0.12	0.85605711	pigs
Sfmbt2	0.17	0.86510347	pigs
Zfp326	0.11	0.87519225	pigs
Kpna6	0.10	0.88184132	pigs
Smarca5	0.20	0.89459575	pigs
Trip12	0.16	0.90184690	pigs
Npepps	0.12	0.90719181	pigs
Dusp10	0.13	0.90931020	pigs
Lat2	-0.49	0.90962315	pigs
Wbp2	0.17	0.91139102	pigs
Pfdn1	0.15	0.91458963	pigs
Ccdc59	0.14	0.92001784	pigs
Wdr11	0.08	0.94042861	pigs
Xab2	0.11	0.94340059	pigs
Dtx3l	-0.32	0.94473403	pigs
Ppil2	0.08	0.94577047	pigs
Hint1	0.14	0.95152744	pigs
Tbc1d15	0.16	0.96218817	pigs
Ube2j2	0.12	0.96760986	pigs
Anxa11	0.14	0.96787905	pigs
Psmd12	0.14	0.97708350	pigs
Ndufb2	0.10	0.98678641	pigs
Sec63	0.20	1.00000000	pigs
Tent5a	0.14	1.00000000	pigs

Taf1d	0.12	1.00000000	pigs
Nsmaf	0.11	1.00000000	pigs
Ect2	0.20	1.00000000	pigs
Psmd3	0.14	1.00000000	pigs
Vps29	0.11	1.00000000	pigs
Rpap3	0.14	1.00000000	pigs
Ndufa13	-0.37	1.00000000	pigs
Glud1	0.13	1.00000000	pigs
Pet100	-0.37	1.00000000	pigs
Idh2	0.08	1.00000000	pigs
Stxbp3	0.13	1.00000000	pigs
Atp5md	-0.43	1.00000000	pigs
Dapp1	-0.39	1.00000000	pigs
Zfp36l2	-0.20	1.00000000	pigs
Phf20l1	-0.32	1.00000000	pigs
Pias1	0.13	1.00000000	pigs
Kpna1	0.16	1.00000000	pigs
Dctpp1	-0.42	1.00000000	pigs
Pola1	0.32	1.00000000	pigs
Usp7	0.12	1.00000000	pigs
Rnf6	-0.39	1.00000000	pigs
Tlk2	0.14	1.00000000	pigs
Eogt	0.17	1.00000000	pigs
Aff4	0.14	1.00000000	pigs
Fbxo11	0.25	1.00000000	pigs
Wrnip1	0.13	1.00000000	pigs
Chic2	0.15	1.00000000	pigs
Zmiz1	0.20	1.00000000	pigs
Kcnab2	0.23	1.00000000	pigs
Lrrc41	0.08	1.00000000	pigs
Kif3a	0.14	1.00000000	pigs
Trmt10a	0.10	1.00000000	pigs
Hbegf	0.23	1.00000000	pigs
Kdm2b	0.20	1.00000000	pigs
Sh2d1a	-0.37	1.00000000	pigs
Patl1	0.07	1.00000000	pigs
Cct7	0.12	1.00000000	pigs
Bloc1s2	0.12	1.00000000	pigs
Ehd4	0.11	1.00000000	pigs
Csnk1d	0.10	1.00000000	pigs
Arrdc3	0.19	1.00000000	pigs
Naa50	0.20	1.00000000	pigs
Lypla1	0.03	1.00000000	pigs
Dnajc5	0.08	1.00000000	pigs
Nap1l4	0.08	1.00000000	pigs
Ripk1	0.09	1.00000000	pigs

Spag9	0.16	1.00000000	pigs
Arhgap26	-0.28	1.00000000	pigs
Adal	0.10	1.00000000	pigs
Cep68	0.05	1.00000000	pigs
Pdha1	0.09	1.00000000	pigs
Rab28	0.16	1.00000000	pigs
Slc12a2	0.11	1.00000000	pigs
Vti1b	0.05	1.00000000	pigs
Bcat2	0.08	1.00000000	pigs
Glipr1	0.13	1.00000000	pigs
Cyp51	0.08	1.00000000	pigs
Klrk1	0.44	1.00000000	pigs
Bzw2	0.08	1.00000000	pigs
Sin3a	0.11	1.00000000	pigs
Acap2	0.14	1.00000000	pigs
Cox7a2	-0.40	1.00000000	pigs
Eloc	0.08	1.00000000	pigs
Dbr1	0.09	1.00000000	pigs
Tmem87a	0.14	1.00000000	pigs
Herc6	-0.49	1.00000000	pigs
Vta1	0.11	1.00000000	pigs
Fgl2	-0.39	1.00000000	pigs
Rrp1b	0.16	1.00000000	pigs
Glmn	0.14	1.00000000	pigs
Sntb1	-0.37	1.00000000	pigs
Rab5c	0.13	1.00000000	pigs
Tmtc2	-0.50	1.00000000	pigs
Abl2	0.13	1.00000000	pigs
Usp10	0.11	1.00000000	pigs
Smc6	0.25	1.00000000	pigs
Rnft1	0.12	1.00000000	pigs
Ddx17	0.13	1.00000000	pigs
Ap2m1	0.13	1.00000000	pigs
Usp9x	0.16	1.00000000	pigs
Foxs1	0.12	1.00000000	pigs
Ndufb9	-0.43	1.00000000	pigs
Cd81	0.22	1.00000000	pigs
Usp6nl	0.14	1.00000000	pigs
Kynu	0.28	1.00000000	pigs
Cc2d1b	0.08	1.00000000	pigs
Usp22	0.13	1.00000000	pigs
Chmp2b	0.09	1.00000000	pigs
Idh3g	0.06	1.00000000	pigs
Septin6	-0.31	1.00000000	pigs
Gsap	0.23	1.00000000	pigs
Xkr6	0.21	1.00000000	pigs

Ifi30	0.08	1.00000000	pigs
Pik3ca	0.21	1.00000000	pigs
Erbin	0.17	1.00000000	pigs
Dnajb6	0.15	1.00000000	pigs
Naf1	0.26	1.00000000	pigs
Cct8	0.13	1.00000000	pigs
Nsun2	0.12	1.00000000	pigs
Psmd8	0.09	1.00000000	pigs
Ctdsp1	-0.31	1.00000000	pigs
Trim28	0.11	1.00000000	pigs
9130409I23Rik	0.00	1.00000000	pigs
4933400A11Rik	0.15	1.00000000	pigs
Gatad2b	0.13	1.00000000	pigs
Gas6	-0.32	1.00000000	pigs
Alkbh5	0.10	1.00000000	pigs
Rsrc2	0.17	1.00000000	pigs
Eif4g1	0.10	1.00000000	pigs
Lrrc8c	0.18	1.00000000	pigs
Wdr59	0.09	1.00000000	pigs
Carhsp1	0.10	1.00000000	pigs
Pomp	0.09	1.00000000	pigs
Hyi	0.13	1.00000000	pigs
Pqbp1	0.09	1.00000000	pigs
Net1	0.09	1.00000000	pigs
Tmem189	0.11	1.00000000	pigs
Tfe3	0.10	1.00000000	pigs
Ctsh	0.19	1.00000000	pigs
Ints6	0.28	1.00000000	pigs
Hdac2	0.11	1.00000000	pigs
Dmac2	0.14	1.00000000	pigs
Nasp	0.13	1.00000000	pigs
Hddc2	0.12	1.00000000	pigs
Arpc2	-0.20	1.00000000	pigs
Taf4b	0.17	1.00000000	pigs
Rac1	0.16	1.00000000	pigs
Serinc3	0.12	1.00000000	pigs
Tmem259	0.08	1.00000000	pigs
Cyb561a3	0.16	1.00000000	pigs
Csf1	0.32	1.00000000	pigs
Aff1	0.13	1.00000000	pigs
Xpo5	0.13	1.00000000	pigs
Ccny	0.17	1.00000000	pigs
Chchd5	0.13	1.00000000	pigs
Fcmr	0.10	1.00000000	pigs
Wdr37	0.11	1.00000000	pigs
Prkar2a	0.10	1.00000000	pigs

Rubcnl	-0.07	1.00000000	pigs
Taok1	0.15	1.00000000	pigs
Txnip	-0.44	1.00000000	pigs
4932438A13Rik	-0.47	1.00000000	pigs
Nos2	-0.29	1.00000000	pigs
Adh5	0.08	1.00000000	pigs
Eipr1	0.03	1.00000000	pigs
Parp9	0.11	1.00000000	pigs
Adsl	0.03	1.00000000	pigs
Cd37	0.14	1.00000000	pigs
Nudt21	0.11	1.00000000	pigs
Entpd4	0.10	1.00000000	pigs
Srsf1	0.05	1.00000000	pigs
Trio	0.13	1.00000000	pigs
Rhof	0.11	1.00000000	pigs
Nbn	-0.41	1.00000000	pigs
Nelfa	0.09	1.00000000	pigs
Gtf3c1	0.15	1.00000000	pigs
Cltc	0.18	1.00000000	pigs
Ubr2	0.21	1.00000000	pigs
Pard6b	0.22	1.00000000	pigs
Trem1	-0.41	1.00000000	pigs
Ptp4a2	0.14	1.00000000	pigs
Pus7	0.10	1.00000000	pigs
Kdm5c	0.15	1.00000000	pigs
Acat3	0.10	1.00000000	pigs
Cmtm7	-0.39	1.00000000	pigs
Galnt2	0.15	1.00000000	pigs
Drg1	0.12	1.00000000	pigs
Uggt1	0.06	1.00000000	pigs
Snx14	0.09	1.00000000	pigs
Api5	0.14	1.00000000	pigs
Slc37a1	0.16	1.00000000	pigs
Scarb2	0.08	1.00000000	pigs
Kdelr2	0.12	1.00000000	pigs
Fbxo30	0.09	1.00000000	pigs
Atg4b	0.08	1.00000000	pigs
Hnrnpu	0.16	1.00000000	pigs
Naa16	0.11	1.00000000	pigs
Exosc1	0.06	1.00000000	pigs
Bod1l	0.07	1.00000000	pigs
Hnrnph2	-0.38	1.00000000	pigs
Dock11	-0.41	1.00000000	pigs
Carm1	0.09	1.00000000	pigs
Tyrobp	0.13	1.00000000	pigs
Etf1	0.10	1.00000000	pigs

Higd1a	0.04	1.00000000	pigs
Gosr1	0.12	1.00000000	pigs
Anapc1	0.18	1.00000000	pigs
Plagl2	0.11	1.00000000	pigs
Gclc	0.15	1.00000000	pigs
Kif20b	0.12	1.00000000	pigs
Ist1	0.08	1.00000000	pigs
Rap2c	0.09	1.00000000	pigs
Fkbp5	0.25	1.00000000	pigs
Oser1	0.07	1.00000000	pigs
Hspa4	0.10	1.00000000	pigs
Sec61g	-0.31	1.00000000	pigs
P4hb	0.07	1.00000000	pigs
Tmem165	0.09	1.00000000	pigs
Pgap1	0.18	1.00000000	pigs
Nop53	0.15	1.00000000	pigs
Rabgap1l	0.21	1.00000000	pigs
Donson	0.17	1.00000000	pigs
Sec22b	0.03	1.00000000	pigs
Ptgr1	0.08	1.00000000	pigs
Adnp2	0.14	1.00000000	pigs
Ddx60	0.15	1.00000000	pigs
Sdcbp	0.13	1.00000000	pigs
Gipc2	-0.31	1.00000000	pigs
Gtf2f1	0.07	1.00000000	pigs
Ss18	0.23	1.00000000	pigs
Dnajb12	0.08	1.00000000	pigs
Crtc3	0.04	1.00000000	pigs
Sypl	0.09	1.00000000	pigs
Dnm2	0.11	1.00000000	pigs
Apoe	0.16	1.00000000	pigs
Usp25	0.17	1.00000000	pigs
Ttc7b	-0.31	1.00000000	pigs
Btf3	0.15	1.00000000	pigs
Fyco1	0.15	1.00000000	pigs
Nab1	0.13	1.00000000	pigs
Lxn	0.13	1.00000000	pigs
Ppa2	0.12	1.00000000	pigs
Oga	0.11	1.00000000	pigs
Dele1	0.08	1.00000000	pigs
Rad23a	0.09	1.00000000	pigs
Ing2	0.05	1.00000000	pigs
Tkt	0.09	1.00000000	pigs
Phc2	0.12	1.00000000	pigs
Trrap	0.12	1.00000000	pigs
Fam110a	0.11	1.00000000	pigs

Dlst	0.11	1.00000000	pigs
Kcmf1	0.13	1.00000000	pigs
Abcb7	0.02	1.00000000	pigs
Ahcyl2	-0.50	1.00000000	pigs
Dtx3	0.09	1.00000000	pigs
Comt	0.10	1.00000000	pigs
Nat10	0.10	1.00000000	pigs
Rpa3	0.15	1.00000000	pigs
Zc3h3	0.10	1.00000000	pigs
Dut	0.14	1.00000000	pigs
Aars	0.09	1.00000000	pigs
Ercc4	0.11	1.00000000	pigs
Tardbp	0.06	1.00000000	pigs
Snd1	0.15	1.00000000	pigs
Paqr3	0.09	1.00000000	pigs
Arl14ep	0.08	1.00000000	pigs
Cnnm4	0.13	1.00000000	pigs
Atg3	0.06	1.00000000	pigs
Tent2	0.11	1.00000000	pigs
Fbxl20	0.17	1.00000000	pigs
Hdgfl3	-0.43	1.00000000	pigs
Wdr20	0.16	1.00000000	pigs
Sqstm1	0.12	1.00000000	pigs
Nlrc3	0.20	1.00000000	pigs
Nol10	0.07	1.00000000	pigs
Nphp4	0.10	1.00000000	pigs
Tmf1	0.11	1.00000000	pigs
Utp6	0.12	1.00000000	pigs
Trps1	0.31	1.00000000	pigs
Slc22a15	0.07	1.00000000	pigs
Plpp1	0.21	1.00000000	pigs
Pias2	0.09	1.00000000	pigs
Llgl1	0.09	1.00000000	pigs
Vps16	0.09	1.00000000	pigs
Trim26	0.11	1.00000000	pigs
Arf1	0.11	1.00000000	pigs
Pycard	0.07	1.00000000	pigs
Arl6ip4	0.07	1.00000000	pigs
Ttc14	0.08	1.00000000	pigs
Tshz1	0.11	1.00000000	pigs
Iws1	0.13	1.00000000	pigs
1810013L24Rik	0.09	1.00000000	pigs
Tmem183a	0.07	1.00000000	pigs
G6pdx	0.10	1.00000000	pigs
Nisch	0.11	1.00000000	pigs
Cog1	0.04	1.00000000	pigs

Cnot4	0.18	1.00000000	pigs
Cog3	0.07	1.00000000	pigs
Ust	0.18	1.00000000	pigs
Klf12	-0.40	1.00000000	pigs
Txnl1	0.10	1.00000000	pigs
Cdk17	0.18	1.00000000	pigs
Cacul1	0.08	1.00000000	pigs
Ube2v2	0.07	1.00000000	pigs
Sugp1	0.07	1.00000000	pigs
Tmem106b	0.06	1.00000000	pigs
Pitpnb	0.11	1.00000000	pigs
Dicer1	0.07	1.00000000	pigs
Abcc1	0.15	1.00000000	pigs
Nsfl1c	0.08	1.00000000	pigs
Rasal2	-0.26	1.00000000	pigs
Lcorl	0.04	1.00000000	pigs
Cst7	-0.30	1.00000000	pigs
Picalm	0.18	1.00000000	pigs
Parp14	0.13	1.00000000	pigs
Dock10	-0.25	1.00000000	pigs
Ube3c	0.09	1.00000000	pigs
Calu	0.12	1.00000000	pigs
Nrp1	-0.31	1.00000000	pigs
B3gnt2	0.19	1.00000000	pigs
Reep3	0.11	1.00000000	pigs
Psmb10	0.10	1.00000000	pigs
Bms1	0.05	1.00000000	pigs
Rfwd3	0.11	1.00000000	pigs
Dennd2c	0.11	1.00000000	pigs
Bin2	-0.35	1.00000000	pigs
Trim25	0.09	1.00000000	pigs
Acox1	0.05	1.00000000	pigs
Akr1a1	0.08	1.00000000	pigs
Polr1b	0.08	1.00000000	pigs
Btbd1	0.14	1.00000000	pigs
Golm1	0.17	1.00000000	pigs
Lmbr1l	0.10	1.00000000	pigs
Pdxdc1	0.12	1.00000000	pigs
Vbp1	0.10	1.00000000	pigs
Coro1a	-0.23	1.00000000	pigs
Panx1	0.14	1.00000000	pigs
Hdac3	0.07	1.00000000	pigs
Ubc	0.12	1.00000000	pigs
Klhl15	0.18	1.00000000	pigs
Rlim	0.09	1.00000000	pigs
Ncf1	-0.49	1.00000000	pigs

Kdm3a	0.09	1.00000000	pigs
Dcps	0.05	1.00000000	pigs
Kansl2	0.06	1.00000000	pigs
Ndufs1	0.07	1.00000000	pigs
Cse1l	0.12	1.00000000	pigs
Hnrnpm	0.16	1.00000000	pigs
Tram1	0.11	1.00000000	pigs
Kansl3	0.10	1.00000000	pigs
Dnttip2	0.10	1.00000000	pigs
Psip1	0.09	1.00000000	pigs
Tmem168	0.04	1.00000000	pigs
Ccnq	0.07	1.00000000	pigs
Atg101	0.02	1.00000000	pigs
Dynlt1a	0.05	1.00000000	pigs
Rasgrp2	-0.39	1.00000000	pigs
Atp6v0b	0.11	1.00000000	pigs
Ints13	0.08	1.00000000	pigs
Fam168b	0.05	1.00000000	pigs
Pcbp4	0.06	1.00000000	pigs
Hmox2	0.12	1.00000000	pigs
Ccdc138	0.09	1.00000000	pigs
Dnajc3	0.11	1.00000000	pigs
Atp8a1	-0.15	1.00000000	pigs
Polr1c	0.10	1.00000000	pigs
Fam222b	0.11	1.00000000	pigs
Phf1	0.10	1.00000000	pigs
Pstpip1	0.10	1.00000000	pigs
Clic4	0.21	1.00000000	pigs
Acap1	0.06	1.00000000	pigs
Rrp36	0.03	1.00000000	pigs
Rnf114	0.10	1.00000000	pigs
Rpf2	0.08	1.00000000	pigs
Thyn1	0.07	1.00000000	pigs
Sec61a1	0.08	1.00000000	pigs
Trp53inp1	0.16	1.00000000	pigs
Nepro	0.05	1.00000000	pigs
Cinp	0.07	1.00000000	pigs
Katnbl1	0.08	1.00000000	pigs
Chtop	0.08	1.00000000	pigs
Eif1b	-0.37	1.00000000	pigs
Uhrf1bp1l	0.07	1.00000000	pigs
Scaf4	0.10	1.00000000	pigs
Chuk	0.13	1.00000000	pigs
Cpsf7	0.05	1.00000000	pigs
Atp6v0a2	0.08	1.00000000	pigs
Ifi44l	0.07	1.00000000	pigs

Tpst2	0.09	1.00000000	pigs
Cfap20	0.02	1.00000000	pigs
Golm2	0.06	1.00000000	pigs
Crem	0.11	1.00000000	pigs
Cuta	0.11	1.00000000	pigs
Got1	0.10	1.00000000	pigs
Dusp16	0.26	1.00000000	pigs
Zbtb8os	0.07	1.00000000	pigs
Ece1	-0.25	1.00000000	pigs
Tbpl1	0.14	1.00000000	pigs
Osbpl8	-0.26	1.00000000	pigs
Psap	0.11	1.00000000	pigs
Pafah1b3	0.07	1.00000000	pigs
Alcam	0.28	1.00000000	pigs
Gtf2h1	0.08	1.00000000	pigs
Nlk	0.08	1.00000000	pigs
Psmc2	0.02	1.00000000	pigs
Pggt1b	0.11	1.00000000	pigs
Arhgap15	-0.54	1.00000000	pigs
Fam210b	0.07	1.00000000	pigs
Tmem164	-0.36	1.00000000	pigs
Lrp10	0.09	1.00000000	pigs
Wipi2	0.08	1.00000000	pigs
Bmpr2	0.14	1.00000000	pigs
Phip	0.13	1.00000000	pigs
Rufy3	0.08	1.00000000	pigs
Emilin2	0.07	1.00000000	pigs
Zfp827	0.07	1.00000000	pigs
Synrg	0.08	1.00000000	pigs
Tsc22d2	0.20	1.00000000	pigs
Leprotl1	0.10	1.00000000	pigs
Rbm41	0.09	1.00000000	pigs
Sumo2	-0.25	1.00000000	pigs
Tcof1	0.09	1.00000000	pigs
Arl15	-0.41	1.00000000	pigs
Spi1	-0.31	1.00000000	pigs
2610507B11Rik	0.06	1.00000000	pigs
Zmym5	0.13	1.00000000	pigs
Gpr37	0.19	1.00000000	pigs
Camk4	0.30	1.00000000	pigs
Wasl	0.11	1.00000000	pigs
Abhd5	0.05	1.00000000	pigs
Card19	0.10	1.00000000	pigs
Cep95	0.05	1.00000000	pigs
Khdrbs1	0.09	1.00000000	pigs
Rock2	0.02	1.00000000	pigs

Pdia3	-0.23	1.00000000	pigs
Foxj2	0.08	1.00000000	pigs
Nupl1	0.08	1.00000000	pigs
Rab4a	-0.39	1.00000000	pigs
Ssr1	0.05	1.00000000	pigs
9430038I01Rik	0.05	1.00000000	pigs
Sclt1	0.10	1.00000000	pigs
Txndc17	0.11	1.00000000	pigs
Osbpl1a	0.07	1.00000000	pigs
Ankrd12	0.14	1.00000000	pigs
Gnl1	0.10	1.00000000	pigs
Hsd17b11	0.02	1.00000000	pigs
Bcl11b	0.19	1.00000000	pigs
Nr2c1	0.08	1.00000000	pigs
Rubcn	0.10	1.00000000	pigs
Zc3h18	0.12	1.00000000	pigs
Zfr	0.10	1.00000000	pigs
Uox	-0.26	1.00000000	pigs
Aspscr1	0.12	1.00000000	pigs
Kif3c	0.08	1.00000000	pigs
Ttf2	0.17	1.00000000	pigs
Apobec3	0.07	1.00000000	pigs
Plekha2	0.05	1.00000000	pigs
Pacc1	0.07	1.00000000	pigs
Phactr1	-0.80	1.00000000	pigs
Bud13	0.04	1.00000000	pigs
Tap1	0.08	1.00000000	pigs
Ttc13	-0.32	1.00000000	pigs
Prkcq	0.19	1.00000000	pigs
Slco3a1	0.18	1.00000000	pigs
Plekhm2	0.11	1.00000000	pigs
Katnal1	0.05	1.00000000	pigs
Ppp6r2	0.04	1.00000000	pigs
Taf3	0.13	1.00000000	pigs
Acsl3	0.10	1.00000000	pigs
Gfpt1	0.13	1.00000000	pigs
Psme4	0.07	1.00000000	pigs
Sap18	0.12	1.00000000	pigs
Numb	-0.61	1.00000000	pigs
Nkg7	-0.21	1.00000000	pigs
Srrt	0.08	1.00000000	pigs
Blmh	0.08	1.00000000	pigs
Ruvbl1	0.06	1.00000000	pigs
Prr5l	-0.34	1.00000000	pigs
Fli1	0.17	1.00000000	pigs
Phtf2	0.14	1.00000000	pigs

Rbx1	0.06	1.00000000	pigs
Dnajb9	0.09	1.00000000	pigs
Smyd3	0.15	1.00000000	pigs
Xrcc6	0.03	1.00000000	pigs
Cpne2	-0.32	1.00000000	pigs
Tle5	-0.36	1.00000000	pigs
C3	0.04	1.00000000	pigs
Nrbp1	0.06	1.00000000	pigs
Slc9a8	0.06	1.00000000	pigs
Csnk2a2	0.08	1.00000000	pigs
Rassf3	-0.47	1.00000000	pigs
Hmgxb3	0.07	1.00000000	pigs
Ufl1	0.06	1.00000000	pigs
Golga7	0.09	1.00000000	pigs
Naxd	0.08	1.00000000	pigs
Yeats2	0.04	1.00000000	pigs
Insig2	-0.27	1.00000000	pigs
Ccdc174	0.05	1.00000000	pigs
Zdhhc18	0.11	1.00000000	pigs
Klhl18	0.02	1.00000000	pigs
Fbh1	0.12	1.00000000	pigs
Wdr91	0.08	1.00000000	pigs
Bfar	0.11	1.00000000	pigs
Asb1	0.07	1.00000000	pigs
Thap1	0.03	1.00000000	pigs
Fbxo39	0.09	1.00000000	pigs
Hbp1	0.10	1.00000000	pigs
Zdhhc17	0.11	1.00000000	pigs
Hmgxb4	0.04	1.00000000	pigs
Ccnb1ip1	0.03	1.00000000	pigs
Rbm28	0.08	1.00000000	pigs
Poc1a	0.07	1.00000000	pigs
Coil	0.04	1.00000000	pigs
Dhx9	0.10	1.00000000	pigs
Phf11a	0.14	1.00000000	pigs
Plcl2	-0.27	1.00000000	pigs
Psmb1	0.08	1.00000000	pigs
Itsn2	0.08	1.00000000	pigs
Cst3	-0.44	1.00000000	pigs
Pcna	0.14	1.00000000	pigs
Atmin	0.10	1.00000000	pigs
Casp6	0.01	1.00000000	pigs
Rnf34	0.11	1.00000000	pigs
Twf2	0.10	1.00000000	pigs
Dennd10	0.11	1.00000000	pigs
Pdcd4	0.12	1.00000000	pigs

Ccs	0.04	1.00000000	pigs
Ppp1cc	0.06	1.00000000	pigs
Cherp	0.08	1.00000000	pigs
Wdr48	0.03	1.00000000	pigs
Coro1c	0.15	1.00000000	pigs
Osbpl9	0.11	1.00000000	pigs
Rrbp1	0.14	1.00000000	pigs
Sf3b1	0.13	1.00000000	pigs
Fbxo33	0.13	1.00000000	pigs
Dmd	-0.37	1.00000000	pigs
Eva1a	0.10	1.00000000	pigs
Sco1	0.07	1.00000000	pigs
Ftsj1	0.05	1.00000000	pigs
B4galt5	0.05	1.00000000	pigs
Csnk1g1	0.08	1.00000000	pigs
Dusp5	0.14	1.00000000	pigs
Dtna	-0.27	1.00000000	pigs
Tmem43	0.07	1.00000000	pigs
Arih2	0.10	1.00000000	pigs
Csnk1g2	0.09	1.00000000	pigs
Septin11	0.20	1.00000000	pigs
Brd1	0.10	1.00000000	pigs
Hace1	0.12	1.00000000	pigs
Tnfrsf1b	0.14	1.00000000	pigs
Wdr74	0.05	1.00000000	pigs
Fbxo4	0.07	1.00000000	pigs
Trak1	0.07	1.00000000	pigs
Kdm5b	0.10	1.00000000	pigs
Foxn3	-0.25	1.00000000	pigs
Plekhb2	0.07	1.00000000	pigs
Exoc5	0.07	1.00000000	pigs
Ilk	0.04	1.00000000	pigs
Tsc22d3	0.06	1.00000000	pigs
Rab40b	0.07	1.00000000	pigs
Ldlrad4	0.29	1.00000000	pigs
Nup93	0.08	1.00000000	pigs
Hells	0.03	1.00000000	pigs
Osgep	0.09	1.00000000	pigs
Ssr3	0.07	1.00000000	pigs
Strip1	0.08	1.00000000	pigs
Pcf11	0.06	1.00000000	pigs
Usp15	0.06	1.00000000	pigs
Otub1	0.01	1.00000000	pigs
Clip1	0.02	1.00000000	pigs
Ric8b	0.14	1.00000000	pigs
Lsm6	0.07	1.00000000	pigs

Nipsnap3b	0.03	1.00000000	pigs
Fam193a	0.03	1.00000000	pigs
Atxn1	0.25	1.00000000	pigs
Ikzf5	0.16	1.00000000	pigs
Scyl3	0.04	1.00000000	pigs
H13	0.12	1.00000000	pigs
Phax	0.03	1.00000000	pigs
Rnf122	0.15	1.00000000	pigs
Nucks1	-0.38	1.00000000	pigs
Cldnd1	0.09	1.00000000	pigs
Atf2	0.03	1.00000000	pigs
Vps54	0.15	1.00000000	pigs
Gng10	0.08	1.00000000	pigs
Tmem238	-0.02	1.00000000	pigs
Sbno2	0.13	1.00000000	pigs
S100a1	-0.48	1.00000000	pigs
Tubgcp3	0.08	1.00000000	pigs
Sec13	0.04	1.00000000	pigs
Zxdc	0.06	1.00000000	pigs
Ormdl1	0.03	1.00000000	pigs
Abcf1	0.05	1.00000000	pigs
Cul3	0.10	1.00000000	pigs
Lyst	-0.51	1.00000000	pigs
Fbrsl1	0.06	1.00000000	pigs
Idh3b	0.09	1.00000000	pigs
Rars2	0.15	1.00000000	pigs
S100a11	-0.51	1.00000000	pigs
Sel1l	0.08	1.00000000	pigs
Wdr44	0.20	1.00000000	pigs
Rnf126	0.04	1.00000000	pigs
Papolg	0.09	1.00000000	pigs
Cry1	0.12	1.00000000	pigs
Sar1a	0.05	1.00000000	pigs
Itga4	-0.26	1.00000000	pigs
Rictor	0.08	1.00000000	pigs
Cwf19l2	0.04	1.00000000	pigs
Sugt1	0.09	1.00000000	pigs
Arfgap3	0.13	1.00000000	pigs
Slmap	0.07	1.00000000	pigs
Poldip3	0.08	1.00000000	pigs
2210408I21Rik	-0.41	1.00000000	pigs
Wdr41	0.02	1.00000000	pigs
Limd2	-0.37	1.00000000	pigs
Fam162a	0.03	1.00000000	pigs
Agk	0.19	1.00000000	pigs
Clptm1l	0.07	1.00000000	pigs

Ttc7	0.09	1.00000000	pigs
Alas1	0.05	1.00000000	pigs
Lgals1	-1.05	1.00000000	pigs
Ggcx	0.06	1.00000000	pigs
Bnip3l	0.02	1.00000000	pigs
U2af1l4	0.05	1.00000000	pigs
Cd3e	-0.16	1.00000000	pigs
Eif3h	-0.27	1.00000000	pigs
Ralgapa2	0.10	1.00000000	pigs
Zbtb22	0.07	1.00000000	pigs
Traf7	0.08	1.00000000	pigs
Tsc22d4	0.06	1.00000000	pigs
Sinhcaf	0.12	1.00000000	pigs
Scg5	-0.36	1.00000000	pigs
Slc39a10	-0.39	1.00000000	pigs
Twf1	0.07	1.00000000	pigs
Abhd3	0.05	1.00000000	pigs
Nubpl	0.18	1.00000000	pigs
Naga	0.06	1.00000000	pigs
Polr2g	0.04	1.00000000	pigs
Dtwd2	0.13	1.00000000	pigs
Dnajc18	0.12	1.00000000	pigs
Sh3bp2	0.14	1.00000000	pigs
Diaph1	-0.25	1.00000000	pigs
Lsg1	0.01	1.00000000	pigs
Bptf	0.12	1.00000000	pigs
Isg20l2	0.04	1.00000000	pigs
Syt3	0.08	1.00000000	pigs
Sfswap	0.07	1.00000000	pigs
Ubap2l	0.06	1.00000000	pigs
Nufip2	0.08	1.00000000	pigs
Cnot8	0.05	1.00000000	pigs
Stk38	-0.32	1.00000000	pigs
Topors	0.08	1.00000000	pigs
Rnmt	0.12	1.00000000	pigs
Pip5k1b	-0.38	1.00000000	pigs
Tmub1	0.08	1.00000000	pigs
Polr3d	0.01	1.00000000	pigs
Pacs1	0.13	1.00000000	pigs
Stau1	0.07	1.00000000	pigs
Pik3c3	0.08	1.00000000	pigs
Arhgap27	-0.27	1.00000000	pigs
Atxn7	0.13	1.00000000	pigs
Cnih4	0.04	1.00000000	pigs
Cfdp1	-0.35	1.00000000	pigs
Usp16	0.08	1.00000000	pigs

Cflar	0.23	1.00000000	pigs
Pik3c2a	-0.39	1.00000000	pigs
Usp42	0.07	1.00000000	pigs
Rhbdd1	0.14	1.00000000	pigs
Chsy1	0.14	1.00000000	pigs
Pgls	0.04	1.00000000	pigs
Aatf	0.02	1.00000000	pigs
Dis3l2	0.08	1.00000000	pigs
Rrn3	0.07	1.00000000	pigs
Rtcb	0.05	1.00000000	pigs
Nans	0.08	1.00000000	pigs
Cdc25b	-0.28	1.00000000	pigs
Fgr	-0.36	1.00000000	pigs
Gemin7	-0.24	1.00000000	pigs
Riok1	0.05	1.00000000	pigs
Ndufs4	0.04	1.00000000	pigs
Gle1	0.08	1.00000000	pigs
Iqgap2	-0.24	1.00000000	pigs
Ccnc	0.07	1.00000000	pigs
Babam2	0.06	1.00000000	pigs
Brap	0.01	1.00000000	pigs
Slc5a10	0.07	1.00000000	pigs
Txndc5	0.04	1.00000000	pigs
Arid3b	0.03	1.00000000	pigs
Nus1	0.04	1.00000000	pigs
Ppt1	0.04	1.00000000	pigs
Zcrb1	0.06	1.00000000	pigs
Arhgef3	-0.31	1.00000000	pigs
Icmt	-0.16	1.00000000	pigs
Hs3st3b1	0.12	1.00000000	pigs
Spata2	0.07	1.00000000	pigs
Nars	0.03	1.00000000	pigs
Lmbrd2	0.08	1.00000000	pigs
Zfp639	0.01	1.00000000	pigs
Paxx	0.03	1.00000000	pigs
Zc3h14	0.02	1.00000000	pigs
Eif2ak2	0.07	1.00000000	pigs
Nadsyn1	0.08	1.00000000	pigs
Cops5	0.04	1.00000000	pigs
Paip1	0.02	1.00000000	pigs
Brwd1	0.05	1.00000000	pigs
Gapvd1	0.08	1.00000000	pigs
Snrpa1	0.05	1.00000000	pigs
Cdc73	0.03	1.00000000	pigs
Eya3	0.07	1.00000000	pigs
Usp48	0.07	1.00000000	pigs

G3bp2	0.04	1.00000000	pigs
Armh3	-0.43	1.00000000	pigs
Etv3	0.10	1.00000000	pigs
Plxnd1	-0.28	1.00000000	pigs
Slc25a25	0.11	1.00000000	pigs
Ptpn9	0.08	1.00000000	pigs
Tnpo3	0.06	1.00000000	pigs
Camta1	-0.01	1.00000000	pigs
Ddx47	0.05	1.00000000	pigs
Btbd7	0.07	1.00000000	pigs
Ccdc127	0.01	1.00000000	pigs
Rbm8a2	0.05	1.00000000	pigs
Pogz	0.04	1.00000000	pigs
Foxn2	0.14	1.00000000	pigs
Cenpp	0.20	1.00000000	pigs
Zfp39	0.12	1.00000000	pigs
Pik3cg	-0.33	1.00000000	pigs
Cops8	0.08	1.00000000	pigs
Nup85	0.08	1.00000000	pigs
Gpatch3	0.06	1.00000000	pigs
Ireb2	0.01	1.00000000	pigs
Dpy19l1	0.09	1.00000000	pigs
Usp38	0.07	1.00000000	pigs
Exoc6b	0.02	1.00000000	pigs
Rcbtb2	0.05	1.00000000	pigs
Crebrf	0.14	1.00000000	pigs
Elp1	0.06	1.00000000	pigs
Slc25a24	0.07	1.00000000	pigs
Cdk9	0.06	1.00000000	pigs
Tom1l2	0.08	1.00000000	pigs
Psmd1	0.05	1.00000000	pigs
Pfkl	0.02	1.00000000	pigs
Pbrm1	-0.37	1.00000000	pigs
Tmem260	-0.33	1.00000000	pigs
Tmed9	0.07	1.00000000	pigs
Ttc39b	0.08	1.00000000	pigs
Tfdp2	-0.32	1.00000000	pigs
Zfp335	0.03	1.00000000	pigs
Ifi44	0.09	1.00000000	pigs
Psmb4	0.04	1.00000000	pigs
Ergic2	-0.01	1.00000000	pigs
Anks1	0.16	1.00000000	pigs
Vav3	-0.28	1.00000000	pigs
Eloa	0.08	1.00000000	pigs
Spast	0.03	1.00000000	pigs
Isg15	0.05	1.00000000	pigs

Aco1	0.02	1.00000000	pigs
Chd1l	0.08	1.00000000	pigs
Bcap29	-0.32	1.00000000	pigs
Polr2i	0.02	1.00000000	pigs
Rnf40	0.06	1.00000000	pigs
2700049A03Rik	-0.23	1.00000000	pigs
Kdm5a	0.08	1.00000000	pigs
Papss1	0.07	1.00000000	pigs
Srsf9	0.00	1.00000000	pigs
Arhgef33	-0.27	1.00000000	pigs
Tbcd	0.05	1.00000000	pigs
Ptpn6	0.05	1.00000000	pigs
Senp1	0.03	1.00000000	pigs
Ctdsp2	0.04	1.00000000	pigs
Stradb	0.04	1.00000000	pigs
Krcc1	-0.28	1.00000000	pigs
Oxct1	-0.41	1.00000000	pigs
Zfp369	0.02	1.00000000	pigs
BC005537	0.07	1.00000000	pigs
Zfp146	0.06	1.00000000	pigs
Nup188	0.06	1.00000000	pigs
Ldlrap1	0.04	1.00000000	pigs
Sh2d3c	0.07	1.00000000	pigs
Ift80	0.05	1.00000000	pigs
Fam126b	0.02	1.00000000	pigs
Atxn2	0.06	1.00000000	pigs
Slc25a5	0.02	1.00000000	pigs
Phf6	0.09	1.00000000	pigs
Tmem101	0.07	1.00000000	pigs
Rbm12b2	0.04	1.00000000	pigs
Nol7	-0.32	1.00000000	pigs
Grb2	-0.27	1.00000000	pigs
Capzb	-0.26	1.00000000	pigs
Abt1	0.03	1.00000000	pigs
Sbds	0.06	1.00000000	pigs
Gfm1	0.07	1.00000000	pigs
Heatr6	0.07	1.00000000	pigs
Vps13a	0.10	1.00000000	pigs
Dcaf10	0.06	1.00000000	pigs
Tubb2b	-0.02	1.00000000	pigs
Eif5b	0.12	1.00000000	pigs
Zzz3	0.02	1.00000000	pigs
Acaca	0.00	1.00000000	pigs
Chrac1	-0.37	1.00000000	pigs
Serinc1	0.08	1.00000000	pigs
Gnb1	0.07	1.00000000	pigs

Ndufv1	0.01	1.00000000	pigs
Ccng2	-0.34	1.00000000	pigs
Nup133	0.08	1.00000000	pigs
Cdc37	0.03	1.00000000	pigs
Ap2a2	0.07	1.00000000	pigs
Tmem87b	0.06	1.00000000	pigs
Ppip5k2	0.08	1.00000000	pigs
Cdk8	0.31	1.00000000	pigs
Polk	0.02	1.00000000	pigs
Vapa	0.09	1.00000000	pigs
Kif5b	0.03	1.00000000	pigs
Rnaseh2c	0.04	1.00000000	pigs
Znfx1	-0.11	1.00000000	pigs
Btbd10	0.07	1.00000000	pigs
Ccdc107	-0.03	1.00000000	pigs
Numa1	0.04	1.00000000	pigs
Cbx4	0.06	1.00000000	pigs
Ppm1l	0.01	1.00000000	pigs
Otud3	0.06	1.00000000	pigs
Ss18l2	0.03	1.00000000	pigs
Etfrf1	-0.30	1.00000000	pigs
Txndc12	0.02	1.00000000	pigs
Hira	0.04	1.00000000	pigs
Helz	0.06	1.00000000	pigs
Lrch3	-0.04	1.00000000	pigs
Pdcl	-0.25	1.00000000	pigs
Dennd1c	0.06	1.00000000	pigs
Nkapd1	-0.02	1.00000000	pigs
Ccnt1	-0.34	1.00000000	pigs
Polh	0.01	1.00000000	pigs
Nol4l	-0.32	1.00000000	pigs
Tcf3	0.05	1.00000000	pigs
Cd86	0.05	1.00000000	pigs
Nmt1	0.05	1.00000000	pigs
Kat14	0.07	1.00000000	pigs
Rprd2	0.08	1.00000000	pigs
Ube2q1	-0.32	1.00000000	pigs
Arnt	0.05	1.00000000	pigs
Ankrd10	0.12	1.00000000	pigs
Rassf2	0.00	1.00000000	pigs
Cubn	-0.27	1.00000000	pigs
Zkscan1	0.05	1.00000000	pigs
Pex13	0.05	1.00000000	pigs
Eif3f	0.13	1.00000000	pigs
Gars	0.02	1.00000000	pigs
Tut7	-0.46	1.00000000	pigs

Rbm47	-0.65	1.00000000	pigs
Cept1	0.08	1.00000000	pigs
Esd	0.04	1.00000000	pigs
Lax1	0.17	1.00000000	pigs
Scnm1	0.04	1.00000000	pigs
Ube3b	0.02	1.00000000	pigs
Scfd2	0.10	1.00000000	pigs
Lrp11	0.11	1.00000000	pigs
Stxbp5	-0.45	1.00000000	pigs
Strn	0.10	1.00000000	pigs
Ppan	0.02	1.00000000	pigs
Endov	0.01	1.00000000	pigs
Cox6a1	-0.29	1.00000000	pigs
Hyal2	-0.01	1.00000000	pigs
Lemd3	0.01	1.00000000	pigs
Tsc1	0.05	1.00000000	pigs
Tgfbr1	0.05	1.00000000	pigs
Syap1	-0.31	1.00000000	pigs
Sec16a	0.07	1.00000000	pigs
Prcc	0.04	1.00000000	pigs
Sirt7	0.00	1.00000000	pigs
Ogfod1	0.08	1.00000000	pigs
H2aj	-0.04	1.00000000	pigs
Zfp664	0.04	1.00000000	pigs
Reps1	0.06	1.00000000	pigs
Rnf111	0.08	1.00000000	pigs
Gimap4	0.04	1.00000000	pigs
Tmem222	0.06	1.00000000	pigs
Add3	-0.35	1.00000000	pigs
Taf1a	0.07	1.00000000	pigs
Septin1	-0.27	1.00000000	pigs
Aida	0.04	1.00000000	pigs
Trim33	0.07	1.00000000	pigs
Gtf2h3	0.00	1.00000000	pigs
Cdc42se2	0.08	1.00000000	pigs
Arhgap17	0.08	1.00000000	pigs
Phf2	0.03	1.00000000	pigs
Nxt1	-0.01	1.00000000	pigs
Bpnt2	0.01	1.00000000	pigs
Uhrf2	0.07	1.00000000	pigs
Pgk1	0.08	1.00000000	pigs
Pycrl	0.04	1.00000000	pigs
Bysl	-0.22	1.00000000	pigs
Ccni	0.01	1.00000000	pigs
Rbm38	-0.32	1.00000000	pigs
Fam120a	0.04	1.00000000	pigs

Xxylt1	0.12	1.00000000	pigs
Ptafr	-0.14	1.00000000	pigs
Chm	0.03	1.00000000	pigs
Zfp277	0.05	1.00000000	pigs
Atf6	0.08	1.00000000	pigs
Atg5	0.08	1.00000000	pigs
Rasa1	0.04	1.00000000	pigs
St3gal2	0.08	1.00000000	pigs
Faxc	0.05	1.00000000	pigs
Tsn	0.05	1.00000000	pigs
Zdhhc13	0.09	1.00000000	pigs
Cfap36	0.03	1.00000000	pigs
Csf3r	-0.43	1.00000000	pigs
Arpc5	0.05	1.00000000	pigs
Xpo4	0.02	1.00000000	pigs
Capza2	0.07	1.00000000	pigs
Rbbp7	-0.36	1.00000000	pigs
Vps25	0.04	1.00000000	pigs
Usp8	0.07	1.00000000	pigs
Ric1	0.01	1.00000000	pigs
Ldlr	0.12	1.00000000	pigs
Ilf2	-0.01	1.00000000	pigs
Gtdc1	0.04	1.00000000	pigs
Ilf3	0.08	1.00000000	pigs
Ap2a1	0.04	1.00000000	pigs
Lsm14b	0.09	1.00000000	pigs
Stag1	0.14	1.00000000	pigs
Clpx	0.05	1.00000000	pigs
Wbp1	0.05	1.00000000	pigs
Sgpp1	0.08	1.00000000	pigs
Cmss1	0.06	1.00000000	pigs
Wipf2	0.03	1.00000000	pigs
Clcn5	0.12	1.00000000	pigs
Tm2d3	0.06	1.00000000	pigs
Abraxas2	0.08	1.00000000	pigs
Trp53	0.05	1.00000000	pigs
Npat	0.05	1.00000000	pigs
Bcl10	0.01	1.00000000	pigs
Ift46	0.06	1.00000000	pigs
Dapk2	0.00	1.00000000	pigs
Cdk12	0.06	1.00000000	pigs
Senp2	0.08	1.00000000	pigs
Inpp5f	0.12	1.00000000	pigs
Lipa	0.10	1.00000000	pigs
Ndufs2	-0.02	1.00000000	pigs
Flt1	0.14	1.00000000	pigs

Septin2	0.03	1.00000000	pigs
Flii	0.11	1.00000000	pigs
Faf2	0.07	1.00000000	pigs
Dnttip1	-0.01	1.00000000	pigs
Ammecr1	0.08	1.00000000	pigs
Gna15	0.14	1.00000000	pigs
Xrcc5	-0.26	1.00000000	pigs
Nenf	0.06	1.00000000	pigs
Polb	-0.01	1.00000000	pigs
Setd3	0.07	1.00000000	pigs
Tex2	0.09	1.00000000	pigs
Atp6v1f	-0.02	1.00000000	pigs
Flnb	0.11	1.00000000	pigs
Fam177a	0.01	1.00000000	pigs
Clasrp	0.07	1.00000000	pigs
Lhpp	0.00	1.00000000	pigs
Sec23ip	0.05	1.00000000	pigs
Rwdd4a	0.07	1.00000000	pigs
Atf6b	0.06	1.00000000	pigs
Tmem131	0.05	1.00000000	pigs
Fxr2	0.02	1.00000000	pigs
Cep152	0.07	1.00000000	pigs
Eif2ak1	0.05	1.00000000	pigs
Cct5	0.02	1.00000000	pigs
Nt5c2	0.08	1.00000000	pigs
Vamp4	0.08	1.00000000	pigs
Cbll1	0.07	1.00000000	pigs
Pdhb	0.03	1.00000000	pigs
Ppp2r2a	0.09	1.00000000	pigs
Ddx52	0.02	1.00000000	pigs
Supt20	0.02	1.00000000	pigs
Wdr26	0.00	1.00000000	pigs
Glyr1	0.05	1.00000000	pigs
Acsl4	-0.33	1.00000000	pigs
Cep135	0.07	1.00000000	pigs
Agap2	0.05	1.00000000	pigs
Skap2	-0.38	1.00000000	pigs
Nob1	0.04	1.00000000	pigs
C2cd5	0.07	1.00000000	pigs
Fam3c	0.06	1.00000000	pigs
Shld2	0.03	1.00000000	pigs
Nfatc1	0.05	1.00000000	pigs
Galc	0.03	1.00000000	pigs
Il10	-0.22	1.00000000	pigs
Dpf2	0.00	1.00000000	pigs
Srpr	0.03	1.00000000	pigs

Prdx5	0.05	1.00000000	pigs
Acsl5	0.09	1.00000000	pigs
Kpna4	0.08	1.00000000	pigs
Utp11	0.03	1.00000000	pigs
Nub1	0.11	1.00000000	pigs
Fxr1	0.06	1.00000000	pigs
Vsir	-0.34	1.00000000	pigs
Nktr	0.06	1.00000000	pigs
Bri3	0.04	1.00000000	pigs
Cd40lg	-0.38	1.00000000	pigs
Otud7b	0.00	1.00000000	pigs
G6pc3	0.09	1.00000000	pigs
Dcun1d4	0.03	1.00000000	pigs
Rab3gap1	0.05	1.00000000	pigs
Bola1	-0.01	1.00000000	pigs
Znhit6	-0.02	1.00000000	pigs
Slk	0.04	1.00000000	pigs
Fam32a	0.00	1.00000000	pigs
Tuba4a	-0.01	1.00000000	pigs
Ppp1cb	0.03	1.00000000	pigs
Arl4c	-0.36	1.00000000	pigs
Slc30a6	0.05	1.00000000	pigs
Serbp1	-0.19	1.00000000	pigs
Lrrc58	0.08	1.00000000	pigs
Cep83	0.05	1.00000000	pigs
Anapc15-ps	0.06	1.00000000	pigs
Cog5	0.05	1.00000000	pigs
Col19a1	0.03	1.00000000	pigs
Larp1b	0.07	1.00000000	pigs
Rnf115	0.07	1.00000000	pigs
Trit1	0.06	1.00000000	pigs
Gbe1	0.03	1.00000000	pigs
Gnl3	0.04	1.00000000	pigs
Stim2	0.05	1.00000000	pigs
Srp68	0.03	1.00000000	pigs
Spg7	0.05	1.00000000	pigs
Grcc10	-0.35	1.00000000	pigs
Prdm1	0.12	1.00000000	pigs
Top2b	0.09	1.00000000	pigs
Ttbk2	0.14	1.00000000	pigs
B4galt1	0.08	1.00000000	pigs
Vps52	0.04	1.00000000	pigs
Atl2	0.04	1.00000000	pigs
Ssrp1	0.04	1.00000000	pigs
Rab31	-0.44	1.00000000	pigs
Nono	0.04	1.00000000	pigs

Sbno1	0.10	1.00000000	pigs
Dctn6	0.01	1.00000000	pigs
Ykt6	0.05	1.00000000	pigs
Kmt2c	0.09	1.00000000	pigs
Naa25	0.00	1.00000000	pigs
Klhl28	-0.01	1.00000000	pigs
Ppp1r13b	0.11	1.00000000	pigs
Dpf3	0.18	1.00000000	pigs
Laptm4a	0.04	1.00000000	pigs
Cnot7	0.02	1.00000000	pigs
Sipa1	0.04	1.00000000	pigs
Trappc6b	0.00	1.00000000	pigs
Slc9a6	0.07	1.00000000	pigs
Rpn2	-0.02	1.00000000	pigs
Gclm	0.06	1.00000000	pigs
Psmc5	0.01	1.00000000	pigs
Zfyve1	0.04	1.00000000	pigs
Fnip1	0.07	1.00000000	pigs
Tbc1d5	-0.25	1.00000000	pigs
Psmc3	0.00	1.00000000	pigs
Cdkal1	0.05	1.00000000	pigs
Tmem243	0.04	1.00000000	pigs
Auh	0.01	1.00000000	pigs
Ipo5	0.01	1.00000000	pigs
S100pbp	0.03	1.00000000	pigs
Sf3a3	-0.02	1.00000000	pigs
Ap1s1	0.04	1.00000000	pigs
Tsfm	0.04	1.00000000	pigs
Pla2g4a	0.01	1.00000000	pigs
Ift20	0.09	1.00000000	pigs
Accsl	0.07	1.00000000	pigs
Rack1	0.10	1.00000000	pigs
Tbl3	0.06	1.00000000	pigs
Ablim1	-0.28	1.00000000	pigs
Senp6	0.08	1.00000000	pigs
Copa	0.02	1.00000000	pigs
Aga	0.01	1.00000000	pigs
Trmt2a	0.05	1.00000000	pigs
Lpcat2	-0.33	1.00000000	pigs
Gcn1	0.02	1.00000000	pigs
8030462N17Rik	0.06	1.00000000	pigs
Pim3	0.01	1.00000000	pigs
Slc35e3	-0.30	1.00000000	pigs
Eif4e2	0.03	1.00000000	pigs
Uba3	0.01	1.00000000	pigs
Adrm1	0.00	1.00000000	pigs

Rida	0.06	1.00000000	pigs
Zbtb1	0.07	1.00000000	pigs
Hlcs	-0.33	1.00000000	pigs
Ccar1	0.01	1.00000000	pigs
Taok3	-0.35	1.00000000	pigs
Pmpcb	0.02	1.00000000	pigs
Rnf125	-0.33	1.00000000	pigs
Btaf1	0.01	1.00000000	pigs
Lin54	0.05	1.00000000	pigs
Epn1	0.05	1.00000000	pigs
Psme3	-0.03	1.00000000	pigs
Prim2	-0.04	1.00000000	pigs
Eif3b	0.02	1.00000000	pigs
Prdx4	0.02	1.00000000	pigs
Lman1	0.02	1.00000000	pigs
Pdgfd	-0.35	1.00000000	pigs
Arfgef2	0.08	1.00000000	pigs
Zfc3h1	0.05	1.00000000	pigs
Ifnar1	-0.02	1.00000000	pigs
Hsdl2	0.02	1.00000000	pigs
Ccar2	0.01	1.00000000	pigs
Klf6	0.12	1.00000000	pigs
Prrc2a	0.02	1.00000000	pigs
Sidt2	0.06	1.00000000	pigs
Rlf	-0.02	1.00000000	pigs
Agpat5	0.04	1.00000000	pigs
Sdhd	0.00	1.00000000	pigs
Atm	-0.33	1.00000000	pigs
Stx17	0.08	1.00000000	pigs
Ermp1	0.04	1.00000000	pigs
Zfp830	0.00	1.00000000	pigs
Strap	-0.01	1.00000000	pigs
Nudt1	-0.29	1.00000000	pigs
Cdkn2c	0.08	1.00000000	pigs
Tlnrd1	-0.04	1.00000000	pigs
Dnajc13	-0.03	1.00000000	pigs
Pkn2	0.09	1.00000000	pigs
Srsf4	0.02	1.00000000	pigs
Snrpn	0.03	1.00000000	pigs
Secisbp2	-0.33	1.00000000	pigs
Dcxr	-0.01	1.00000000	pigs
Lancl1	0.03	1.00000000	pigs
Sgf29	0.06	1.00000000	pigs
Plk3	0.09	1.00000000	pigs
Hspa13	0.02	1.00000000	pigs
Bicd2	0.00	1.00000000	pigs

Atg2b	0.00	1.00000000	pigs
Zw10	0.03	1.00000000	pigs
Pds5a	0.05	1.00000000	pigs
Ifitm6	-0.41	1.00000000	pigs
Jchain	-0.31	1.00000000	pigs
Aqr	0.00	1.00000000	pigs
Slc25a4	-0.01	1.00000000	pigs
Olfml3	0.01	1.00000000	pigs
Nmi	0.04	1.00000000	pigs
Ints7	0.03	1.00000000	pigs
Snrpc	0.00	1.00000000	pigs
Dido1	0.04	1.00000000	pigs
Bcl3	0.05	1.00000000	pigs
Dgkd	0.13	1.00000000	pigs
Ldb1	0.07	1.00000000	pigs
Setd2	0.05	1.00000000	pigs
Larp1	0.05	1.00000000	pigs
Fam234a	0.08	1.00000000	pigs
Dctn3	-0.04	1.00000000	pigs
Gdap2	0.04	1.00000000	pigs
Ppp2r1a	0.02	1.00000000	pigs
Nr4a2	-0.28	1.00000000	pigs
Ankrd33b	-0.40	1.00000000	pigs
Orai1	0.02	1.00000000	pigs
Ndufb6	-0.01	1.00000000	pigs
Pabpc1	0.10	1.00000000	pigs
Usp39	-0.03	1.00000000	pigs
Ddost	0.02	1.00000000	pigs
Strbp	-0.22	1.00000000	pigs
Dctn5	0.00	1.00000000	pigs
Brd3	0.04	1.00000000	pigs
Trappc11	0.02	1.00000000	pigs
Desi1	0.07	1.00000000	pigs
Hrh4	-0.02	1.00000000	pigs
Ankrd46	0.02	1.00000000	pigs
P2ry14	-0.24	1.00000000	pigs
Tarbp1	0.03	1.00000000	pigs
Rprd1a	0.00	1.00000000	pigs
Rabep1	0.14	1.00000000	pigs
Heca	0.04	1.00000000	pigs
Phf10	0.03	1.00000000	pigs
Colgalt1	0.02	1.00000000	pigs
Gk5	-0.33	1.00000000	pigs
Vrk1	0.10	1.00000000	pigs
Gorasp2	0.03	1.00000000	pigs
Srgn	0.13	1.00000000	pigs

Fkbp3	-0.02	1.00000000	pigs
Dock8	-0.20	1.00000000	pigs
Appbp2	-0.04	1.00000000	pigs
Gzmk	0.30	1.00000000	pigs
Cetn3	0.03	1.00000000	pigs
Nipbl	0.08	1.00000000	pigs
Zfp143	-0.32	1.00000000	pigs
Cgas	-0.30	1.00000000	pigs
Samd3	-0.28	1.00000000	pigs
Ubfd1	0.01	1.00000000	pigs
Zfat	0.12	1.00000000	pigs
Kif2a	0.02	1.00000000	pigs
Ppid	0.04	1.00000000	pigs
Cd101	0.08	1.00000000	pigs
Sec24a	-0.04	1.00000000	pigs
Sf3b4	0.01	1.00000000	pigs
Copb1	-0.04	1.00000000	pigs
Dync1i2	0.06	1.00000000	pigs
Eps15	0.07	1.00000000	pigs
Akna	0.05	1.00000000	pigs
Cabin1	0.05	1.00000000	pigs
Sesn3	-0.34	1.00000000	pigs
Sde2	0.00	1.00000000	pigs
Kbtbd2	0.07	1.00000000	pigs
Ptar1	-0.02	1.00000000	pigs
Lrrc57	0.01	1.00000000	pigs
Ide	0.07	1.00000000	pigs
Emg1	0.04	1.00000000	pigs
Fbxo6	-0.29	1.00000000	pigs
Odr4	0.02	1.00000000	pigs
Itprid2	0.04	1.00000000	pigs
Sptlc1	0.00	1.00000000	pigs
Gnl2	0.00	1.00000000	pigs
Aamp	0.03	1.00000000	pigs
Golga4	0.06	1.00000000	pigs
Uqcrc1	0.05	1.00000000	pigs
Cyb5b	0.02	1.00000000	pigs
Ccdc115	0.02	1.00000000	pigs
LTO1	-0.01	1.00000000	pigs
Zdhhc20	0.14	1.00000000	pigs
Eif2b5	-0.03	1.00000000	pigs
Cdc123	0.02	1.00000000	pigs
Hmgcs1	0.00	1.00000000	pigs
Amh	-0.01	1.00000000	pigs
Sigirr	0.04	1.00000000	pigs
Epg5	-0.04	1.00000000	pigs

Cep44	0.01	1.00000000	pigs
Ikzf3	-0.14	1.00000000	pigs
Exosc5	0.00	1.00000000	pigs
Rfx7	0.13	1.00000000	pigs
Guf1	0.04	1.00000000	pigs
Prkcsh	-0.01	1.00000000	pigs
Nudcd3	0.02	1.00000000	pigs
Elp2	0.00	1.00000000	pigs
Cdc42ep3	-0.32	1.00000000	pigs
Dynlt3	-0.30	1.00000000	pigs
Ndufb10	-0.01	1.00000000	pigs
Phf23	-0.22	1.00000000	pigs
Eif2a	-0.04	1.00000000	pigs
Taf4	0.05	1.00000000	pigs
Akap10	0.03	1.00000000	pigs
Tmco3	0.05	1.00000000	pigs
Akap9	0.08	1.00000000	pigs
Krt10	-0.30	1.00000000	pigs
Zc3h8	0.06	1.00000000	pigs
Ptpn2	0.02	1.00000000	pigs
Ppig	0.01	1.00000000	pigs
Tmem106c	-0.02	1.00000000	pigs
Ap4e1	0.00	1.00000000	pigs
Hdac7	0.00	1.00000000	pigs
Fto	0.13	1.00000000	pigs
Rbm6	0.05	1.00000000	pigs
Nbas	0.06	1.00000000	pigs
Btf3l4	0.00	1.00000000	pigs
Agfg2	0.05	1.00000000	pigs
Dld	0.03	1.00000000	pigs
Ubap2	0.10	1.00000000	pigs
Odf2	-0.01	1.00000000	pigs
Ubb	0.07	1.00000000	pigs
B4galt4	0.03	1.00000000	pigs
Rbbp6	0.08	1.00000000	pigs
Kdm4b	0.02	1.00000000	pigs
Prkcb	-0.14	1.00000000	pigs
Atf7ip2	0.15	1.00000000	pigs
Ddx1	-0.05	1.00000000	pigs
Psmf1	-0.04	1.00000000	pigs
Scly	0.00	1.00000000	pigs
Sec23a	0.02	1.00000000	pigs
Ppcs	0.00	1.00000000	pigs
Tmem184c	0.03	1.00000000	pigs
Dnajb14	0.03	1.00000000	pigs
Rbm15	0.00	1.00000000	pigs

Commd1b	0.06	1.00000000	pigs
E2f3	0.06	1.00000000	pigs
Prepl	0.01	1.00000000	pigs
Abi1	0.06	1.00000000	pigs
Pi4ka	-0.01	1.00000000	pigs
Nat9	-0.01	1.00000000	pigs
Arhgap30	-0.27	1.00000000	pigs
Rer1	-0.31	1.00000000	pigs
Bccip	-0.04	1.00000000	pigs
Il1rap	-0.65	1.00000000	pigs
Nabp1	0.04	1.00000000	pigs
Ncl	-0.20	1.00000000	pigs
Orc6	0.01	1.00000000	pigs
Phkb	-0.32	1.00000000	pigs
Snrpd1	-0.01	1.00000000	pigs
Dock9	-0.36	1.00000000	pigs
Actr3	-0.13	1.00000000	pigs
Hemgn	-0.25	1.00000000	pigs
Adam9	0.07	1.00000000	pigs
Cuedc2	-0.03	1.00000000	pigs
Sec11c	0.01	1.00000000	pigs
Chmp1a	0.02	1.00000000	pigs
Gnptab	0.12	1.00000000	pigs
Lmnb1	-0.43	1.00000000	pigs
Ercc3	0.03	1.00000000	pigs
1110004F10Rik	0.04	1.00000000	pigs
Dop1a	0.01	1.00000000	pigs
Zpr1	0.05	1.00000000	pigs
Actr2	0.08	1.00000000	pigs
Nudt5	0.02	1.00000000	pigs
Pcmtd1	0.00	1.00000000	pigs
Pag1	0.15	1.00000000	pigs
Rara	0.02	1.00000000	pigs
Clptm1	0.03	1.00000000	pigs
Sf3b6	-0.02	1.00000000	pigs
Pop4	0.00	1.00000000	pigs
Zfp831	0.04	1.00000000	pigs
Pmpca	0.03	1.00000000	pigs
Dync1li1	-0.01	1.00000000	pigs
Cmc1	-0.29	1.00000000	pigs
Tmem268	-0.23	1.00000000	pigs
Gne	-0.01	1.00000000	pigs
Cd53	0.08	1.00000000	pigs
Hk1	0.01	1.00000000	pigs
Slc43a2	0.05	1.00000000	pigs
Gsn	-0.26	1.00000000	pigs

Pik3ip1	0.03	1.00000000	pigs
Zfp24	-0.01	1.00000000	pigs
Aasdhppt	0.01	1.00000000	pigs
Camk1d	-0.31	1.00000000	pigs
Sirt1	0.03	1.00000000	pigs
Sort1	0.08	1.00000000	pigs
Lrig2	0.00	1.00000000	pigs
Paxip1	0.02	1.00000000	pigs
Prkag2	0.04	1.00000000	pigs
Atp5pb	0.05	1.00000000	pigs
Polr2a	0.04	1.00000000	pigs
Prcp	0.04	1.00000000	pigs
Pafah1b2	0.04	1.00000000	pigs
Kiz	-0.01	1.00000000	pigs
Nbeal1	0.02	1.00000000	pigs
Gpn3	0.03	1.00000000	pigs
Inpp5a	0.00	1.00000000	pigs
Sergef	0.06	1.00000000	pigs
Ostm1	0.04	1.00000000	pigs
Hyou1	0.04	1.00000000	pigs
Atp5c1	0.02	1.00000000	pigs
Rsl1d1	0.05	1.00000000	pigs
Uros	0.06	1.00000000	pigs
Gbp2b	-0.07	1.00000000	pigs
Clnk	0.16	1.00000000	pigs
Ggps1	0.07	1.00000000	pigs
Uqcrc2	-0.01	1.00000000	pigs
Fos	-0.22	1.00000000	pigs
Klrd1	-0.22	1.00000000	pigs
Ctsc	0.16	1.00000000	pigs
Pecam1	-0.20	1.00000000	pigs
Irak1	0.00	1.00000000	pigs
Plekhf1	-0.21	1.00000000	pigs
Sdhaf2	0.01	1.00000000	pigs
Gata3	0.08	1.00000000	pigs
Pip5k1c	0.05	1.00000000	pigs
Necap1	-0.27	1.00000000	pigs
Azi2	0.01	1.00000000	pigs
Ankhd1	0.05	1.00000000	pigs
Trnt1	0.02	1.00000000	pigs
Rab10	0.07	1.00000000	pigs
Rbmx	-0.02	1.00000000	pigs
Gorab	0.03	1.00000000	pigs
Ppp4r3b	0.04	1.00000000	pigs
Dynlrb2	-0.25	1.00000000	pigs
Rnf38	0.05	1.00000000	pigs

Phb	0.00	1.00000000	pigs
Aebp2	0.03	1.00000000	pigs
Os9	-0.01	1.00000000	pigs
Atxn10	-0.02	1.00000000	pigs
Pqlc3	-0.04	1.00000000	pigs
Utp18	0.05	1.00000000	pigs
Hectd1	0.08	1.00000000	pigs
Aup1	0.00	1.00000000	pigs
Calm1	-0.16	1.00000000	pigs
R3hdm2	-0.31	1.00000000	pigs
Psmb3	0.07	1.00000000	pigs
St3gal4	0.02	1.00000000	pigs
Ptp4a1	-0.02	1.00000000	pigs
Adap1	-0.08	1.00000000	pigs
Akr1b3	0.06	1.00000000	pigs
AI413582	0.04	1.00000000	pigs
Sfpq	0.07	1.00000000	pigs
Rev3l	0.09	1.00000000	pigs
Cdk13	0.05	1.00000000	pigs
Cln3	0.01	1.00000000	pigs
Ccdc9	0.01	1.00000000	pigs
Uhmk1	-0.01	1.00000000	pigs
Cic	0.03	1.00000000	pigs
Ctcf	0.02	1.00000000	pigs
Rab18	0.01	1.00000000	pigs
Dnmt3a	0.03	1.00000000	pigs
Exosc10	0.02	1.00000000	pigs
Prpf4b	-0.26	1.00000000	pigs
Cep290	-0.02	1.00000000	pigs
Nipa2	0.02	1.00000000	pigs
Tox	0.24	1.00000000	pigs
Klhl36	0.04	1.00000000	pigs
Prkag1	0.01	1.00000000	pigs
Sp4	0.26	1.00000000	pigs
Galnt6	0.11	1.00000000	pigs
Fgd3	-0.35	1.00000000	pigs
Ddx46	-0.26	1.00000000	pigs
Ufd1	0.01	1.00000000	pigs
Gtf2b	-0.02	1.00000000	pigs
Slc10a1	-0.01	1.00000000	pigs
Snrnp25	-0.31	1.00000000	pigs
Brms1l	0.04	1.00000000	pigs
Phlpp2	0.04	1.00000000	pigs
Dazap1	-0.23	1.00000000	pigs
Yy1	0.04	1.00000000	pigs
Ddx21	0.01	1.00000000	pigs

Unc119	-0.28	1.00000000	pigs
Ciao1	0.01	1.00000000	pigs
Eef1d	0.04	1.00000000	pigs
Rnf157	0.03	1.00000000	pigs
Iars2	0.01	1.00000000	pigs
Ankrd17	0.07	1.00000000	pigs
Psmb7	-0.06	1.00000000	pigs
Fyb	-0.26	1.00000000	pigs
Frs2	0.00	1.00000000	pigs
Ncbp3	-0.02	1.00000000	pigs
Bdp1	0.00	1.00000000	pigs
Celf1	0.05	1.00000000	pigs
Smap2	-0.44	1.00000000	pigs
Kif3b	0.10	1.00000000	pigs
3110040N11Rik	-0.29	1.00000000	pigs
Slc1a5	0.05	1.00000000	pigs
Rasgef1a	-0.01	1.00000000	pigs
Slc39a11	-0.37	1.00000000	pigs
Nup214	0.01	1.00000000	pigs
Cep78	0.06	1.00000000	pigs
Txlna	-0.02	1.00000000	pigs
Tbrg4	0.00	1.00000000	pigs
Eif4ebp1	-0.07	1.00000000	pigs
Tgds	0.02	1.00000000	pigs
Dcaf13	-0.03	1.00000000	pigs
Gart	0.06	1.00000000	pigs
Ywhaq	-0.24	1.00000000	pigs
Il17ra	0.00	1.00000000	pigs
Rab43	0.02	1.00000000	pigs
Lrpap1	-0.02	1.00000000	pigs
Zdhhc14	0.13	1.00000000	pigs
Utp25	0.02	1.00000000	pigs
Kidins220	0.04	1.00000000	pigs
Nemf	-0.01	1.00000000	pigs
Arpc5l	-0.21	1.00000000	pigs
Elovl5	0.03	1.00000000	pigs
Clta	0.02	1.00000000	pigs
Yipf6	0.00	1.00000000	pigs
Rcc2	0.03	1.00000000	pigs
Scamp2	0.06	1.00000000	pigs
Ptbp3	0.11	1.00000000	pigs
Nup54	0.03	1.00000000	pigs
Arxes2	-0.02	1.00000000	pigs
Vasp	-0.29	1.00000000	pigs
Nuggc	-0.23	1.00000000	pigs
Arglu1	0.05	1.00000000	pigs

Zfyve26	-0.21	1.00000000	pigs
Ddx23	-0.01	1.00000000	pigs
Acadvl	-0.25	1.00000000	pigs
Haus6	-0.02	1.00000000	pigs
Togaram1	0.04	1.00000000	pigs
Dcp1a	0.05	1.00000000	pigs
Coq4	0.05	1.00000000	pigs
Cep162	-0.20	1.00000000	pigs
Ube2l6	-0.02	1.00000000	pigs
Lrrfip2	0.08	1.00000000	pigs
Acads	0.04	1.00000000	pigs
Lpp	-0.24	1.00000000	pigs
Eprs	-0.05	1.00000000	pigs
Sertad3	-0.35	1.00000000	pigs
Epc2	-0.29	1.00000000	pigs
Ncstn	0.01	1.00000000	pigs
Tnpo1	0.02	1.00000000	pigs
Gramd1a	0.01	1.00000000	pigs
Copg2	0.05	1.00000000	pigs
Ncoa2	0.12	1.00000000	pigs
Nsmce2	-0.27	1.00000000	pigs
Dpyd	0.02	1.00000000	pigs
Atp6v1h	-0.02	1.00000000	pigs
Thoc6	0.03	1.00000000	pigs
Znhit3	-0.02	1.00000000	pigs
Aip	0.02	1.00000000	pigs
Dhrs11	0.02	1.00000000	pigs
Prkdc	-0.03	1.00000000	pigs
Amph	0.11	1.00000000	pigs
2510039O18Rik	-0.06	1.00000000	pigs
Nosip	0.04	1.00000000	pigs
Rab7	0.04	1.00000000	pigs
Bloc1s6	0.03	1.00000000	pigs
Nrip1	0.02	1.00000000	pigs
Nmd3	-0.05	1.00000000	pigs
Ankrd13c	0.14	1.00000000	pigs
Spag8	-0.03	1.00000000	pigs
Slc3a2	0.05	1.00000000	pigs
Lgals8	0.04	1.00000000	pigs
Vps13b	-0.21	1.00000000	pigs
Taf9b	0.00	1.00000000	pigs
Pwp1	0.02	1.00000000	pigs
Ggnbp2	-0.03	1.00000000	pigs
Srp54a	0.03	1.00000000	pigs
Acsf2	-0.04	1.00000000	pigs
Phactr4	0.05	1.00000000	pigs

Btg1b	-0.13	1.00000000	pigs
Ppp2r5a	0.08	1.00000000	pigs
Plekhm3	-0.47	1.00000000	pigs
Fbxo8	-0.09	1.00000000	pigs
Khsrp	-0.01	1.00000000	pigs
Paxbp1	-0.02	1.00000000	pigs
Srp9	-0.02	1.00000000	pigs
R3hdm1	-0.27	1.00000000	pigs
Pdlim7	-0.28	1.00000000	pigs
Tcf25	-0.01	1.00000000	pigs
Snx18	-0.19	1.00000000	pigs
Taok2	0.01	1.00000000	pigs
Acbd6	-0.30	1.00000000	pigs
Capns1	-0.27	1.00000000	pigs
Fam53b	-0.11	1.00000000	pigs
Clec16a	0.00	1.00000000	pigs
Ola1	-0.06	1.00000000	pigs
Gpatch8	0.04	1.00000000	pigs
Snap29	-0.06	1.00000000	pigs
Dipk2a	0.05	1.00000000	pigs
Cebpd	0.09	1.00000000	pigs
Pcid2	0.00	1.00000000	pigs
Ufsp2	-0.01	1.00000000	pigs
Exoc2	0.06	1.00000000	pigs
Bnip2	0.00	1.00000000	pigs
Ppfia1	0.00	1.00000000	pigs
Dus1l	0.00	1.00000000	pigs
Ppil3	-0.34	1.00000000	pigs
Ell	0.07	1.00000000	pigs
Naa30	0.00	1.00000000	pigs
Stard4	0.07	1.00000000	pigs
Fam98a	-0.02	1.00000000	pigs
Crbn	-0.02	1.00000000	pigs
Plin2	0.02	1.00000000	pigs
Bud31	-0.03	1.00000000	pigs
Rngtt	0.01	1.00000000	pigs
Nup155	-0.19	1.00000000	pigs
Podn	-0.22	1.00000000	pigs
Psmc4	-0.05	1.00000000	pigs
Hacd4	0.03	1.00000000	pigs
Clint1	0.05	1.00000000	pigs
Aif1	0.07	1.00000000	pigs
Prdx2	-0.08	1.00000000	pigs
Ube2o	0.05	1.00000000	pigs
Psmd14	-0.02	1.00000000	pigs
Eml4	0.08	1.00000000	pigs

Uba6	0.04	1.00000000	pigs
Ddx49	0.02	1.00000000	pigs
Tma16	0.01	1.00000000	pigs
Tmem263	0.01	1.00000000	pigs
Acaa2	-0.02	1.00000000	pigs
Rbm22	0.02	1.00000000	pigs
Samhd1	-0.02	1.00000000	pigs
Rnf138	-0.01	1.00000000	pigs
Ndufv2	-0.01	1.00000000	pigs
Ddx39b	0.00	1.00000000	pigs
Dnajc11	0.01	1.00000000	pigs
Tet3	0.05	1.00000000	pigs
Ice2	-0.02	1.00000000	pigs
Arl8a	-0.01	1.00000000	pigs
Ssbp1	-0.03	1.00000000	pigs
Vkorc1l1	0.01	1.00000000	pigs
Cyb5a	-0.03	1.00000000	pigs
Esf1	0.07	1.00000000	pigs
Whrn	0.05	1.00000000	pigs
Plpbp	0.02	1.00000000	pigs
Hnrnpr	0.03	1.00000000	pigs
Rabl6	0.03	1.00000000	pigs
Prkd2	-0.30	1.00000000	pigs
Tmem30a	0.04	1.00000000	pigs
Slc35a1	-0.03	1.00000000	pigs
Wsb1	0.04	1.00000000	pigs
Pygm	0.07	1.00000000	pigs
Chd9	-0.21	1.00000000	pigs
Dcaf15	0.03	1.00000000	pigs
Golim4	0.03	1.00000000	pigs
Slc50a1	0.00	1.00000000	pigs
Nfu1	0.04	1.00000000	pigs
Pcnx	0.04	1.00000000	pigs
Eef2k	0.06	1.00000000	pigs
Gps2	-0.33	1.00000000	pigs
Tlcd2	-0.03	1.00000000	pigs
Rpf1	0.01	1.00000000	pigs
Vezf1	0.03	1.00000000	pigs
Sun1	0.04	1.00000000	pigs
Pigt	0.00	1.00000000	pigs
Psma5	-0.01	1.00000000	pigs
Rassf1	0.04	1.00000000	pigs
Recql	-0.28	1.00000000	pigs
Kat8	0.01	1.00000000	pigs
Tmem160	-0.05	1.00000000	pigs
Polr3e	-0.02	1.00000000	pigs

Ndufs6	0.01	1.00000000	pigs
Ash2l	0.02	1.00000000	pigs
Dennd2d	0.00	1.00000000	pigs
Iars	-0.02	1.00000000	pigs
St6galnac4	-0.31	1.00000000	pigs
Spry2	-0.05	1.00000000	pigs
Asxl1	0.07	1.00000000	pigs
Atp11c	-0.01	1.00000000	pigs
Tmem59	-0.01	1.00000000	pigs
Vezt	-0.20	1.00000000	pigs
Taf5l	0.00	1.00000000	pigs
Prkx	-0.27	1.00000000	pigs
Ctss	-0.03	1.00000000	pigs
Kat7	-0.05	1.00000000	pigs
Ppil4	0.00	1.00000000	pigs
Tpd52l2	-0.06	1.00000000	pigs
Bicdl1	0.04	1.00000000	pigs
Slc20a2	0.07	1.00000000	pigs
Pde4d	-0.21	1.00000000	pigs
Znrf2	0.08	1.00000000	pigs
Cdk6	0.17	1.00000000	pigs
Ptpn7	-0.03	1.00000000	pigs
Psmb2	0.00	1.00000000	pigs
Atrx	0.05	1.00000000	pigs
Trappc13	-0.03	1.00000000	pigs
Fundc1	0.01	1.00000000	pigs
Zfp131	-0.02	1.00000000	pigs
Klhl20	-0.29	1.00000000	pigs
Pdia6	-0.05	1.00000000	pigs
Acot10	-0.02	1.00000000	pigs
Grsf1	-0.03	1.00000000	pigs
Hprt	-0.22	1.00000000	pigs
Itpk1	-0.03	1.00000000	pigs
Smu1	0.03	1.00000000	pigs
Get3	-0.07	1.00000000	pigs
Rnf4	0.04	1.00000000	pigs
Stn1	0.01	1.00000000	pigs
Pde6d	-0.02	1.00000000	pigs
Rad51b	0.02	1.00000000	pigs
Btg3	0.02	1.00000000	pigs
Tsc22d1	0.00	1.00000000	pigs
Stoml2	-0.03	1.00000000	pigs
Parp2	0.01	1.00000000	pigs
Txlng	-0.05	1.00000000	pigs
Atp6ap1	0.03	1.00000000	pigs
Tmco1	-0.28	1.00000000	pigs

Ppp2r3c	0.04	1.00000000	pigs
Trmt112	0.01	1.00000000	pigs
Btbd9	-0.01	1.00000000	pigs
Arl5a	-0.03	1.00000000	pigs
Rcbtb1	-0.01	1.00000000	pigs
Ung	-0.29	1.00000000	pigs
Stat6	-0.02	1.00000000	pigs
Bcl2l13	0.01	1.00000000	pigs
Tnni2	-0.28	1.00000000	pigs
Fem1b	-0.02	1.00000000	pigs
Tmem120b	0.04	1.00000000	pigs
Hspd1	0.00	1.00000000	pigs
Bag4	-0.01	1.00000000	pigs
Derl2	-0.06	1.00000000	pigs
Crkl	0.02	1.00000000	pigs
Dis3	0.02	1.00000000	pigs
Pgm2	0.00	1.00000000	pigs
Suz12	0.04	1.00000000	pigs
Anapc7	0.01	1.00000000	pigs
Orc5	-0.28	1.00000000	pigs
Ubr3	0.00	1.00000000	pigs
Ankrd39	-0.03	1.00000000	pigs
Tmem97	-0.27	1.00000000	pigs
Pgam5	0.05	1.00000000	pigs
Ipo8	0.02	1.00000000	pigs
Ly9	-0.26	1.00000000	pigs
Dpm2	-0.03	1.00000000	pigs
Sfxn1	0.03	1.00000000	pigs
Cux1	-0.33	1.00000000	pigs
Cpsf3	0.03	1.00000000	pigs
Whamm	-0.03	1.00000000	pigs
Insyn2b	-0.34	1.00000000	pigs
Zfp512b	-0.01	1.00000000	pigs
Exoc7	-0.01	1.00000000	pigs
Eif3g	0.03	1.00000000	pigs
Copg1	-0.02	1.00000000	pigs
Commd8	0.01	1.00000000	pigs
Hdgf	-0.34	1.00000000	pigs
Nup107	-0.08	1.00000000	pigs
Prep	0.00	1.00000000	pigs
Arhgap4	-0.05	1.00000000	pigs
Ammecr1l	0.00	1.00000000	pigs
Xpnpep1	0.00	1.00000000	pigs
Tmem38b	-0.01	1.00000000	pigs
Lrpprc	-0.02	1.00000000	pigs
Cxxc5	-0.09	1.00000000	pigs

Clcn3	0.00	1.00000000	pigs
Zranb2	-0.01	1.00000000	pigs
Clk1	0.04	1.00000000	pigs
Tmem68	-0.02	1.00000000	pigs
Emp1	-0.09	1.00000000	pigs
Ndufa5	-0.01	1.00000000	pigs
Tcea1	-0.27	1.00000000	pigs
Rbm14	0.00	1.00000000	pigs
Dnajc7	0.03	1.00000000	pigs
Tbc1d20	-0.03	1.00000000	pigs
Igf2bp3	-0.27	1.00000000	pigs
Fyttd1	-0.05	1.00000000	pigs
Dhx16	0.02	1.00000000	pigs
Supt6	-0.07	1.00000000	pigs
Slc17a5	0.04	1.00000000	pigs
Caml	-0.05	1.00000000	pigs
Casd1	0.01	1.00000000	pigs
Lrmda	-0.38	1.00000000	pigs
Cnst	-0.03	1.00000000	pigs
Xpo6	-0.52	1.00000000	pigs
Ddhd1	0.01	1.00000000	pigs
Epm2aip1	0.04	1.00000000	pigs
Taf10	-0.29	1.00000000	pigs
Tab3	0.03	1.00000000	pigs
Ndufa8	-0.07	1.00000000	pigs
Fbxo38	0.03	1.00000000	pigs
Prr5	-0.24	1.00000000	pigs
Sgsm2	0.01	1.00000000	pigs
Clk3	0.03	1.00000000	pigs
Pofut1	-0.05	1.00000000	pigs
Uqcrq	-0.08	1.00000000	pigs
Atp13a3	0.05	1.00000000	pigs
Limk2	-0.50	1.00000000	pigs
Emc2	-0.03	1.00000000	pigs
Fmr1	-0.02	1.00000000	pigs
Sppl3	0.02	1.00000000	pigs
Irak2	0.21	1.00000000	pigs
Ccdc150	-0.01	1.00000000	pigs
Psma3	0.00	1.00000000	pigs
Rufy2	0.01	1.00000000	pigs
Ppp2cb	-0.06	1.00000000	pigs
Nup88	0.00	1.00000000	pigs
St7l	-0.06	1.00000000	pigs
Efl1	-0.01	1.00000000	pigs
5031439G07Rik	-0.17	1.00000000	pigs
Fermt3	0.01	1.00000000	pigs

Arhgap6	0.09	1.00000000	pigs
Retreg3	-0.01	1.00000000	pigs
Hat1	0.00	1.00000000	pigs
Hnrnpd	0.02	1.00000000	pigs
Ube3a	0.03	1.00000000	pigs
Rbbp8	0.04	1.00000000	pigs
Ap1g1	-0.07	1.00000000	pigs
Arf4	0.04	1.00000000	pigs
Ndufa9	-0.02	1.00000000	pigs
Ptpn4	0.19	1.00000000	pigs
Pknox1	0.01	1.00000000	pigs
Lrrc59	-0.03	1.00000000	pigs
Tubb5	0.06	1.00000000	pigs
Cep295	0.01	1.00000000	pigs
Fbxw4	0.00	1.00000000	pigs
Ccdc117	-0.07	1.00000000	pigs
Spint2	0.00	1.00000000	pigs
Thap7	-0.02	1.00000000	pigs
Arpc3	-0.18	1.00000000	pigs
Cep192	-0.01	1.00000000	pigs
Brcc3	0.00	1.00000000	pigs
Slc35b3	-0.02	1.00000000	pigs
Gtf2a1	-0.27	1.00000000	pigs
Sh3glb1	0.01	1.00000000	pigs
Cnih1	-0.04	1.00000000	pigs
Wdr82	0.05	1.00000000	pigs
Atxn2l	-0.02	1.00000000	pigs
Dpy19l3	-0.05	1.00000000	pigs
Rmc1	-0.05	1.00000000	pigs
Usp11	0.01	1.00000000	pigs
Gpat4	-0.07	1.00000000	pigs
Slc9a1	-0.04	1.00000000	pigs
Cebpg	0.00	1.00000000	pigs
Pfdn4	-0.01	1.00000000	pigs
Rab2a	0.02	1.00000000	pigs
Calr	0.04	1.00000000	pigs
Ikbkb	0.02	1.00000000	pigs
Zfp652	0.07	1.00000000	pigs
Prkab1	0.03	1.00000000	pigs
Focad	-0.34	1.00000000	pigs
Chchd3	-0.01	1.00000000	pigs
Smarcb1	-0.02	1.00000000	pigs
Eif4e3	-0.29	1.00000000	pigs
Tor1aip1	-0.05	1.00000000	pigs
Dennd1a	0.02	1.00000000	pigs
Hnrnpk	0.00	1.00000000	pigs

Dctn2	0.01	1.00000000	pigs
Lamtor2	-0.01	1.00000000	pigs
Tspan14	0.02	1.00000000	pigs
Ublcp1	-0.26	1.00000000	pigs
Dedd	0.01	1.00000000	pigs
Heatr3	0.04	1.00000000	pigs
Ints12	0.02	1.00000000	pigs
Exoc3	-0.02	1.00000000	pigs
Cmpk1	-0.05	1.00000000	pigs
Pbdc1	-0.06	1.00000000	pigs
Tmem63a	-0.04	1.00000000	pigs
Ccdc66	0.04	1.00000000	pigs
Txndc15	-0.03	1.00000000	pigs
Epc1	-0.21	1.00000000	pigs
Clk2	0.00	1.00000000	pigs
Rfc3	-0.26	1.00000000	pigs
Vps41	-0.32	1.00000000	pigs
Ep400	0.03	1.00000000	pigs
Txndc9	-0.05	1.00000000	pigs
Arid5a	0.04	1.00000000	pigs
Hdac9	-0.07	1.00000000	pigs
Fam117b	0.01	1.00000000	pigs
Cmah	-0.26	1.00000000	pigs
Dtnbp1	-0.04	1.00000000	pigs
Tbl1xr1	0.03	1.00000000	pigs
Pcyt1a	-0.25	1.00000000	pigs
Exog	0.03	1.00000000	pigs
Calcoco2	-0.02	1.00000000	pigs
Erh	-0.20	1.00000000	pigs
Rnf216	0.11	1.00000000	pigs
Ubap1	-0.07	1.00000000	pigs
Cat	0.00	1.00000000	pigs
Yaf2	0.02	1.00000000	pigs
Slc30a9	-0.02	1.00000000	pigs
Nmur1	-0.17	1.00000000	pigs
Denr	-0.09	1.00000000	pigs
Stub1	-0.05	1.00000000	pigs
Txnrd1	-0.02	1.00000000	pigs
Psen1	-0.10	1.00000000	pigs
Capn15	0.02	1.00000000	pigs
Lnpep	0.10	1.00000000	pigs
Agpat2	0.02	1.00000000	pigs
Fggy	0.07	1.00000000	pigs
Ltn1	-0.04	1.00000000	pigs
Ppa1	-0.02	1.00000000	pigs
Fam214a	0.04	1.00000000	pigs

Sec62	-0.30	1.00000000	pigs
Tmbim6	-0.16	1.00000000	pigs
Decr1	-0.01	1.00000000	pigs
Ranbp10	-0.01	1.00000000	pigs
Aldh16a1	0.02	1.00000000	pigs
Tbc1d22b	0.02	1.00000000	pigs
Tmem248	-0.05	1.00000000	pigs
Plekha3	-0.01	1.00000000	pigs
Ago1	0.02	1.00000000	pigs
Tnfsf12	-0.21	1.00000000	pigs
Nup210	0.00	1.00000000	pigs
Wsb2	-0.03	1.00000000	pigs
Diaph2	-0.19	1.00000000	pigs
Retreg1	0.15	1.00000000	pigs
Tlk1	-0.23	1.00000000	pigs
Foxj3	0.01	1.00000000	pigs
Ckap5	0.01	1.00000000	pigs
Cebpz	-0.02	1.00000000	pigs
Psmd7	-0.02	1.00000000	pigs
Taf5	0.02	1.00000000	pigs
Atp6ap2	0.00	1.00000000	pigs
Zfp422	-0.03	1.00000000	pigs
4833439L19Rik	-0.01	1.00000000	pigs
Fyn	0.10	1.00000000	pigs
Agps	-0.03	1.00000000	pigs
Leng8	0.01	1.00000000	pigs
Pwwp3a	-0.20	1.00000000	pigs
Dnajc8	-0.28	1.00000000	pigs
Sgta	-0.27	1.00000000	pigs
Xpc	-0.04	1.00000000	pigs
Tex14	0.02	1.00000000	pigs
Erp29	-0.02	1.00000000	pigs
Ythdc2	-0.01	1.00000000	pigs
Nfyc	-0.02	1.00000000	pigs
Sharpin	0.00	1.00000000	pigs
Acadm	-0.26	1.00000000	pigs
Scyl2	-0.02	1.00000000	pigs
Fdx1	-0.03	1.00000000	pigs
Rfc2	-0.02	1.00000000	pigs
Ptpn23	0.02	1.00000000	pigs
Cyba	0.02	1.00000000	pigs
Arhgap9	-0.02	1.00000000	pigs
Fam126a	-0.30	1.00000000	pigs
Adipor2	0.01	1.00000000	pigs
Ankra2	0.02	1.00000000	pigs
9530068E07Rik	-0.06	1.00000000	pigs

Prdm11	0.03	1.00000000	pigs
Dclre1c	-0.27	1.00000000	pigs
Bag6	-0.04	1.00000000	pigs
G3bp1	0.05	1.00000000	pigs
Hadha	-0.01	1.00000000	pigs
Papola	0.05	1.00000000	pigs
Leng1	-0.16	1.00000000	pigs
Tada1	-0.02	1.00000000	pigs
Prdx1	0.02	1.00000000	pigs
Dgkz	-0.25	1.00000000	pigs
Gfod1	0.13	1.00000000	pigs
Exosc9	-0.03	1.00000000	pigs
Trim24	0.01	1.00000000	pigs
Vps13c	-0.26	1.00000000	pigs
Anapc5	-0.04	1.00000000	pigs
Ippk	-0.01	1.00000000	pigs
Rad52	-0.25	1.00000000	pigs
Armc8	0.01	1.00000000	pigs
Utp23	-0.01	1.00000000	pigs
Trip4	-0.05	1.00000000	pigs
Ubxn11	0.01	1.00000000	pigs
Rbm10	-0.03	1.00000000	pigs
Cdk5rap2	0.01	1.00000000	pigs
Sp1	0.05	1.00000000	pigs
AU040320	-0.26	1.00000000	pigs
Pum2	0.05	1.00000000	pigs
Stam	0.01	1.00000000	pigs
Pot1b	0.04	1.00000000	pigs
Tmem128	-0.03	1.00000000	pigs
Ro60	-0.07	1.00000000	pigs
Puf60	-0.01	1.00000000	pigs
Rasgrp1	-0.12	1.00000000	pigs
Gbf1	0.03	1.00000000	pigs
Pms2	-0.16	1.00000000	pigs
Fbxo32	-0.05	1.00000000	pigs
Slc44a2	0.00	1.00000000	pigs
Dnase1l1	-0.19	1.00000000	pigs
Dis3l	-0.20	1.00000000	pigs
Appl1	-0.06	1.00000000	pigs
Keap1	-0.03	1.00000000	pigs
Nop10	-0.01	1.00000000	pigs
Tmem251	-0.05	1.00000000	pigs
Tmem245	-0.06	1.00000000	pigs
Cox6a2	-0.01	1.00000000	pigs
Abhd15	-0.21	1.00000000	pigs
Ap2s1	-0.02	1.00000000	pigs

Vps26a	-0.30	1.00000000	pigs
Arhgdib	-0.07	1.00000000	pigs
Eif4a3l1	-0.02	1.00000000	pigs
Ctdp1	-0.03	1.00000000	pigs
Glt8d1	0.03	1.00000000	pigs
Dnajc1	0.11	1.00000000	pigs
Ndufb8	-0.05	1.00000000	pigs
Nbr1	-0.08	1.00000000	pigs
Ssbp3	0.04	1.00000000	pigs
Polr1h	-0.14	1.00000000	pigs
Itm2b	-0.03	1.00000000	pigs
Ubxn4	-0.01	1.00000000	pigs
Lmbr1	-0.35	1.00000000	pigs
Ndufc2	-0.09	1.00000000	pigs
Galnt10	-0.15	1.00000000	pigs
Vps51	0.01	1.00000000	pigs
Arfgef1	0.00	1.00000000	pigs
Arid4a	0.02	1.00000000	pigs
Uprt	-0.03	1.00000000	pigs
Ddx24	-0.03	1.00000000	pigs
Cdk4	-0.08	1.00000000	pigs
Paip2	-0.18	1.00000000	pigs
Trap1	-0.02	1.00000000	pigs
Utp15	0.00	1.00000000	pigs
Eps15l1	-0.01	1.00000000	pigs
Nrbf2	-0.02	1.00000000	pigs
Cstf1	-0.02	1.00000000	pigs
Zdhhc7	0.03	1.00000000	pigs
Cpped1	-0.29	1.00000000	pigs
Folr1	-0.77	1.00000000	pigs
Bcas3	0.08	1.00000000	pigs
Iqsec1	-0.02	1.00000000	pigs
Bcap31	0.00	1.00000000	pigs
Gnal	-0.04	1.00000000	pigs
Nipal3	-0.02	1.00000000	pigs
Gadd45a	-0.27	1.00000000	pigs
Atg12	-0.03	1.00000000	pigs
Plekhg3	-0.17	1.00000000	pigs
Agpat3	0.00	1.00000000	pigs
Fbxo22	-0.03	1.00000000	pigs
Fasl	-0.03	1.00000000	pigs
Tbc1d9b	-0.02	1.00000000	pigs
Gnpnat1	-0.04	1.00000000	pigs
Snrpe	-0.25	1.00000000	pigs
Acer3	-0.02	1.00000000	pigs
Ascc3	0.02	1.00000000	pigs

Abraxas1	-0.16	1.00000000	pigs
Fkbp8	-0.02	1.00000000	pigs
Zbtb24	-0.03	1.00000000	pigs
Ncapd3	-0.15	1.00000000	pigs
Pygl	-0.28	1.00000000	pigs
Cul4a	-0.30	1.00000000	pigs
Unk	0.00	1.00000000	pigs
Plec	-0.05	1.00000000	pigs
Steep1	-0.04	1.00000000	pigs
Pdk3	0.00	1.00000000	pigs
Pcif1	0.02	1.00000000	pigs
Golt1b	-0.02	1.00000000	pigs
Slc4a1ap	-0.01	1.00000000	pigs
Gnai3	-0.04	1.00000000	pigs
Ktn1	0.00	1.00000000	pigs
Cnpy3	0.02	1.00000000	pigs
Tmem147	-0.07	1.00000000	pigs
Fbxo31	0.05	1.00000000	pigs
Snip1	-0.02	1.00000000	pigs
Tmem107	-0.28	1.00000000	pigs
Cd164	-0.01	1.00000000	pigs
Ccdc180	0.02	1.00000000	pigs
Rp9	-0.06	1.00000000	pigs
Cisd3	-0.32	1.00000000	pigs
Ogdh	-0.02	1.00000000	pigs
Crybg1	-0.26	1.00000000	pigs
Angel2	0.02	1.00000000	pigs
Dcaf6	0.01	1.00000000	pigs
Irf3	-0.02	1.00000000	pigs
Tspan3	-0.04	1.00000000	pigs
Telo2	0.02	1.00000000	pigs
Chmp6	-0.05	1.00000000	pigs
Il6ra	0.04	1.00000000	pigs
Hltf	-0.04	1.00000000	pigs
Scai	-0.03	1.00000000	pigs
Acin1	-0.03	1.00000000	pigs
Elp4	-0.01	1.00000000	pigs
Czib	-0.09	1.00000000	pigs
Inpp1	-0.02	1.00000000	pigs
Zfp217	0.00	1.00000000	pigs
Dnajc14	0.00	1.00000000	pigs
Srpk1	-0.02	1.00000000	pigs
Cited2	-0.04	1.00000000	pigs
Gcfc2	-0.07	1.00000000	pigs
Dek	-0.01	1.00000000	pigs
Tasp1	-0.33	1.00000000	pigs

Qser1	-0.01	1.00000000	pigs
Wars2	-0.05	1.00000000	pigs
Elp5	-0.01	1.00000000	pigs
Dhrs7b	0.03	1.00000000	pigs
Fkbp4	-0.02	1.00000000	pigs
Ipo4	-0.01	1.00000000	pigs
Stx4a	0.03	1.00000000	pigs
Tmem33	-0.06	1.00000000	pigs
Cd247	0.03	1.00000000	pigs
Snx6	-0.03	1.00000000	pigs
Rbms1	-0.29	1.00000000	pigs
Stx12	0.01	1.00000000	pigs
Disp1	-0.26	1.00000000	pigs
Creb1	0.12	1.00000000	pigs
Il12rb2	-0.12	1.00000000	pigs
Limd1	0.04	1.00000000	pigs
Gns	0.01	1.00000000	pigs
Prmt5	-0.03	1.00000000	pigs
Ubald1	0.00	1.00000000	pigs
Capn10	-0.03	1.00000000	pigs
Xk	-0.14	1.00000000	pigs
Ints8	0.00	1.00000000	pigs
Bbx	0.04	1.00000000	pigs
Cramp1	0.00	1.00000000	pigs
Hadhb	-0.04	1.00000000	pigs
Samm50	0.00	1.00000000	pigs
Actr6	-0.19	1.00000000	pigs
Ap3m1	-0.03	1.00000000	pigs
Zcchc24	-0.04	1.00000000	pigs
Resf1	-0.05	1.00000000	pigs
Kifbp	0.01	1.00000000	pigs
Saysd1	0.00	1.00000000	pigs
Kif7	-0.16	1.00000000	pigs
Chd6	-0.07	1.00000000	pigs
Naa20	-0.06	1.00000000	pigs
Ino80d	-0.08	1.00000000	pigs
Prr14l	-0.08	1.00000000	pigs
Smarcad1	0.01	1.00000000	pigs
Tada2a	0.00	1.00000000	pigs
Nup35	0.06	1.00000000	pigs
Rcan3	-0.02	1.00000000	pigs
Cdk1	-0.26	1.00000000	pigs
Golgb1	-0.11	1.00000000	pigs
Cxcr4	0.10	1.00000000	pigs
Lars	-0.06	1.00000000	pigs
Rfx1	-0.03	1.00000000	pigs

Zfp592	-0.06	1.00000000	pigs
Rnf13	-0.27	1.00000000	pigs
Atp6v1a	0.00	1.00000000	pigs
Tbc1d7	-0.04	1.00000000	pigs
Vps53	-0.03	1.00000000	pigs
Ubald2	0.05	1.00000000	pigs
Echs1	-0.05	1.00000000	pigs
Tpp2	0.02	1.00000000	pigs
Rexo2	-0.04	1.00000000	pigs
Qdpr	-0.26	1.00000000	pigs
Atp5g3	-0.22	1.00000000	pigs
Ube2j1	0.02	1.00000000	pigs
Usp40	-0.01	1.00000000	pigs
Tm2d2	-0.06	1.00000000	pigs
Pdap1	-0.28	1.00000000	pigs
Psmb8	0.06	1.00000000	pigs
Rora	0.20	1.00000000	pigs
Alkbh7	-0.06	1.00000000	pigs
Avl9	-0.03	1.00000000	pigs
Commd3	-0.02	1.00000000	pigs
Desi2	-0.05	1.00000000	pigs
Hdac1	-0.22	1.00000000	pigs
Arl6ip1	0.19	1.00000000	pigs
Chka	0.00	1.00000000	pigs
Ncr1	0.04	1.00000000	pigs
Pdcl3	-0.08	1.00000000	pigs
Acbd3	-0.08	1.00000000	pigs
Parn	0.01	1.00000000	pigs
Yipf3	-0.20	1.00000000	pigs
Prpf19	0.00	1.00000000	pigs
Tmem154	-0.39	1.00000000	pigs
Vcpip1	-0.05	1.00000000	pigs
Tm9sf2	0.02	1.00000000	pigs
Sertad1	-0.29	1.00000000	pigs
Acsl1	-0.90	1.00000000	pigs
Ap2b1	-0.02	1.00000000	pigs
Slc10a3	-0.02	1.00000000	pigs
Star	-0.15	1.00000000	pigs
Dnajc15	-0.07	1.00000000	pigs
Pdcd7	-0.04	1.00000000	pigs
Slc30a7	-0.02	1.00000000	pigs
Lasp1	0.01	1.00000000	pigs
Btrc	-0.29	1.00000000	pigs
Fbrs	-0.03	1.00000000	pigs
Usp14	-0.06	1.00000000	pigs
Elk3	-0.07	1.00000000	pigs

Cdc42	0.02	1.00000000	pigs
Eif4ebp2	-0.04	1.00000000	pigs
Cox18	0.08	1.00000000	pigs
Cyhr1	0.03	1.00000000	pigs
Adi1	-0.04	1.00000000	pigs
Vps28	-0.07	1.00000000	pigs
Slain2	0.01	1.00000000	pigs
Dhrs1	-0.05	1.00000000	pigs
H2-Ea	0.03	1.00000000	pigs
Spring1	-0.02	1.00000000	pigs
Atad2b	0.03	1.00000000	pigs
Inpp5b	-0.02	1.00000000	pigs
Osgin2	-0.03	1.00000000	pigs
Ndufa1	-0.29	1.00000000	pigs
Fam76a	-0.25	1.00000000	pigs
Arhgef6	-0.08	1.00000000	pigs
Kmt5b	0.03	1.00000000	pigs
Wdfy2	-0.01	1.00000000	pigs
Ifit3	-0.52	1.00000000	pigs
Banp	0.01	1.00000000	pigs
Atg7	-0.20	1.00000000	pigs
Tlr7	-0.05	1.00000000	pigs
Hypk	-0.07	1.00000000	pigs
Rbl1	0.00	1.00000000	pigs
Wwp2	-0.21	1.00000000	pigs
Ccdc82	-0.19	1.00000000	pigs
Sccpdh	-0.02	1.00000000	pigs
Zfp330	-0.06	1.00000000	pigs
Irak4	-0.23	1.00000000	pigs
Eomes	-0.14	1.00000000	pigs
Fbxl17	0.03	1.00000000	pigs
Stk4	-0.15	1.00000000	pigs
Wbp4	0.01	1.00000000	pigs
Tesk2	-0.08	1.00000000	pigs
Hus1	-0.04	1.00000000	pigs
Rab6a	-0.10	1.00000000	pigs
Serp1	-0.11	1.00000000	pigs
Polr2c	0.02	1.00000000	pigs
Rsl24d1	-0.07	1.00000000	pigs
2700062C07Rik	-0.26	1.00000000	pigs
Dpysl2	0.03	1.00000000	pigs
Armc10	-0.02	1.00000000	pigs
Nutf2	-0.04	1.00000000	pigs
St8sia1	-0.25	1.00000000	pigs
Snap23	-0.01	1.00000000	pigs
Usp45	-0.02	1.00000000	pigs

Ethe1	-0.06	1.00000000	pigs
Cd79a	-0.27	1.00000000	pigs
Rabgap1	-0.29	1.00000000	pigs
Gpsm3	-0.16	1.00000000	pigs
Cox5a	-0.05	1.00000000	pigs
Bicra	0.01	1.00000000	pigs
Zc3h15	-0.13	1.00000000	pigs
Cbx1	-0.14	1.00000000	pigs
Fbxo3	0.02	1.00000000	pigs
Kyat3	-0.25	1.00000000	pigs
Poldip2	0.00	1.00000000	pigs
Psmd6	-0.02	1.00000000	pigs
Pcmtd2	-0.04	1.00000000	pigs
Pdzd8	-0.02	1.00000000	pigs
Skp1	0.05	1.00000000	pigs
Acp5	-0.26	1.00000000	pigs
Ctsa	-0.04	1.00000000	pigs
Nsd1	-0.20	1.00000000	pigs
Atf3	0.05	1.00000000	pigs
Zfp800	-0.03	1.00000000	pigs
Fcho2	-0.28	1.00000000	pigs
Noct	-0.23	1.00000000	pigs
Creld1	0.00	1.00000000	pigs
Zap70	-0.01	1.00000000	pigs
Arhgef2	0.01	1.00000000	pigs
Eif2s2	-0.06	1.00000000	pigs
Sdad1	-0.04	1.00000000	pigs
Tbc1d31	-0.06	1.00000000	pigs
Ppp3cb	-0.01	1.00000000	pigs
Tmem203	-0.17	1.00000000	pigs
Apc	-0.29	1.00000000	pigs
Otud5	-0.01	1.00000000	pigs
Ipmk	-0.56	1.00000000	pigs
Rhot1	-0.01	1.00000000	pigs
Paqr8	0.01	1.00000000	pigs
Nedd4	-0.21	1.00000000	pigs
Nfkbil1	0.03	1.00000000	pigs
Aig1	0.10	1.00000000	pigs
Vti1a	-0.27	1.00000000	pigs
Usp47	-0.04	1.00000000	pigs
Tdrd3	0.00	1.00000000	pigs
Chmp2a	-0.03	1.00000000	pigs
Slc38a9	-0.32	1.00000000	pigs
Itch	-0.05	1.00000000	pigs
Nsmce1	-0.03	1.00000000	pigs
Ap5z1	0.00	1.00000000	pigs

Txnl4a	-0.05	1.00000000	pigs
Atp6v0d1	-0.07	1.00000000	pigs
Cracr2a	0.00	1.00000000	pigs
Ints14	-0.20	1.00000000	pigs
Rspry1	0.02	1.00000000	pigs
Cbl	-0.20	1.00000000	pigs
Ppp1r11	-0.16	1.00000000	pigs
Erc1	-0.19	1.00000000	pigs
Cbfa2t2	-0.25	1.00000000	pigs
Zfp341	0.01	1.00000000	pigs
Rrm1	-0.05	1.00000000	pigs
Nup98	-0.25	1.00000000	pigs
Notch2	-0.90	1.00000000	pigs
Amz2	-0.05	1.00000000	pigs
Ddit4	-0.16	1.00000000	pigs
Lemd2	-0.02	1.00000000	pigs
Taf11	-0.06	1.00000000	pigs
Zhx1	0.02	1.00000000	pigs
Shfl	-0.02	1.00000000	pigs
Ttc5	-0.01	1.00000000	pigs
Smc1a	-0.26	1.00000000	pigs
Fam120b	0.04	1.00000000	pigs
Tmem39a	0.01	1.00000000	pigs
Hsd17b12	0.00	1.00000000	pigs
Sec24b	-0.06	1.00000000	pigs
Tmem115	-0.03	1.00000000	pigs
Imp3	-0.15	1.00000000	pigs
Armc7	0.03	1.00000000	pigs
Tmx1	-0.11	1.00000000	pigs
Zdhhc3	-0.03	1.00000000	pigs
Nin	-0.33	1.00000000	pigs
Vps35l	-0.24	1.00000000	pigs
Ikbkg	-0.06	1.00000000	pigs
Aph1a	-0.20	1.00000000	pigs
G2e3	-0.05	1.00000000	pigs
Shkbp1	-0.03	1.00000000	pigs
Cnn2	-0.02	1.00000000	pigs
Cmtm3	-0.07	1.00000000	pigs
Rnpep	-0.25	1.00000000	pigs
Tm9sf3	0.00	1.00000000	pigs
Srsf10	-0.02	1.00000000	pigs
Ncbp1	-0.01	1.00000000	pigs
Cd44	0.01	1.00000000	pigs
Xpot	-0.03	1.00000000	pigs
Ugdh	0.00	1.00000000	pigs
Srp19	-0.08	1.00000000	pigs

Cul5	-0.08	1.00000000	pigs
Plekhj1	-0.07	1.00000000	pigs
Atp9b	-0.02	1.00000000	pigs
Smg6	-0.07	1.00000000	pigs
Nfe2l3	-0.03	1.00000000	pigs
Usp34	-0.20	1.00000000	pigs
Osbpl11	-0.08	1.00000000	pigs
Ehmt1	-0.09	1.00000000	pigs
Ephb6	-0.15	1.00000000	pigs
Cebpb	-0.36	1.00000000	pigs
Brox	-0.05	1.00000000	pigs
Nfil3	-0.09	1.00000000	pigs
Atp8b4	0.04	1.00000000	pigs
Fem1c	-0.08	1.00000000	pigs
Cant1	-0.03	1.00000000	pigs
Ranbp6	-0.23	1.00000000	pigs
Txndc11	-0.01	1.00000000	pigs
Tars	-0.02	1.00000000	pigs
Jazf1	-0.09	1.00000000	pigs
Nsf	0.01	1.00000000	pigs
Lancl2	-0.25	1.00000000	pigs
Cwc15	-0.06	1.00000000	pigs
Abhd12	-0.04	1.00000000	pigs
Wdr61	-0.11	1.00000000	pigs
Golga5	0.02	1.00000000	pigs
Zfpm1	-0.02	1.00000000	pigs
Stx18	-0.10	1.00000000	pigs
Tm2d1	-0.05	1.00000000	pigs
Bicral	-0.10	1.00000000	pigs
Ganab	-0.06	1.00000000	pigs
Entr1	-0.01	1.00000000	pigs
Oaz1	-0.14	1.00000000	pigs
Ankle2	-0.09	1.00000000	pigs
Nnt	-0.07	1.00000000	pigs
2410004B18Rik	-0.03	1.00000000	pigs
Gpaa1	-0.05	1.00000000	pigs
Tmem208	-0.18	1.00000000	pigs
Ankib1	-0.08	1.00000000	pigs
Irf2	-0.27	1.00000000	pigs
Trmt6	-0.06	1.00000000	pigs
Glcci1	-0.10	1.00000000	pigs
Smim15	-0.22	1.00000000	pigs
Etfa	-0.09	1.00000000	pigs
Eif3e	-0.02	1.00000000	pigs
Nt5c3	-0.03	1.00000000	pigs
Irak3	0.04	1.00000000	pigs

Gnpda2	-0.06	1.00000000	pigs
Commd10	-0.06	1.00000000	pigs
Gadd45gip1	-0.05	1.00000000	pigs
St3gal3	-0.20	1.00000000	pigs
Yipf2	-0.06	1.00000000	pigs
Cmtr1	-0.03	1.00000000	pigs
Fgfr1op2	-0.10	1.00000000	pigs
Araf	-0.03	1.00000000	pigs
Tnik	0.05	1.00000000	pigs
Phf8	-0.05	1.00000000	pigs
Lrrc47	-0.05	1.00000000	pigs
Pphln1	-0.03	1.00000000	pigs
Nras	-0.06	1.00000000	pigs
Arap3	-0.03	1.00000000	pigs
Ezr	-0.01	1.00000000	pigs
Tmx4	0.12	1.00000000	pigs
Bag5	-0.09	1.00000000	pigs
Cerk	-0.08	1.00000000	pigs
Hnrnpdl	0.02	1.00000000	pigs
Dhx37	-0.17	1.00000000	pigs
Traf3ip3	-0.18	1.00000000	pigs
Tnks2	-0.06	1.00000000	pigs
Timm17b	-0.08	1.00000000	pigs
Riok3	-0.10	1.00000000	pigs
B3gat3	-0.03	1.00000000	pigs
Fer	0.05	1.00000000	pigs
Dnajc10	-0.03	1.00000000	pigs
Wdfy1	-0.01	1.00000000	pigs
Orai3	0.00	1.00000000	pigs
Id2	0.01	1.00000000	pigs
Ddrgk1	-0.09	1.00000000	pigs
Socs4	-0.08	1.00000000	pigs
Cog2	0.01	1.00000000	pigs
Cwc27	-0.28	1.00000000	pigs
Upf2	-0.06	1.00000000	pigs
Tbc1d22a	-0.05	1.00000000	pigs
Ctps2	-0.12	1.00000000	pigs
Elf4	-0.07	1.00000000	pigs
Tanc2	-0.08	1.00000000	pigs
Ctsw	-0.10	1.00000000	pigs
Slc16a6	-0.21	1.00000000	pigs
Unc50	-0.04	1.00000000	pigs
Socs5	-0.25	1.00000000	pigs
Dip2a	-0.07	1.00000000	pigs
Spg11	-0.20	1.00000000	pigs
Cab39l	-0.08	1.00000000	pigs

Dad1	-0.27	1.00000000	pigs
Ctr9	-0.06	1.00000000	pigs
Srebf2	-0.03	1.00000000	pigs
Dnajc24	-0.04	1.00000000	pigs
Ep300	0.00	1.00000000	pigs
Elf2	-0.04	1.00000000	pigs
Cxcl3	-0.88	1.00000000	pigs
1700037H04Rik	-0.11	1.00000000	pigs
Lsm8	-0.06	1.00000000	pigs
Ndufa2	-0.26	1.00000000	pigs
Nufip1	-0.03	1.00000000	pigs
Snw1	-0.09	1.00000000	pigs
Vps36	-0.22	1.00000000	pigs
Taf13	-0.03	1.00000000	pigs
Kifap3	0.03	1.00000000	pigs
Htatip2	-0.19	1.00000000	pigs
Tollip	-0.05	1.00000000	pigs
Surf6	-0.03	1.00000000	pigs
Fbxl3	-0.05	1.00000000	pigs
Atp6v1c1	-0.07	1.00000000	pigs
Srrm2	-0.16	1.00000000	pigs
Edc3	-0.07	1.00000000	pigs
Sos1	-0.01	1.00000000	pigs
Lin7c	-0.02	1.00000000	pigs
Cul1	0.00	1.00000000	pigs
Xcl1	0.24	1.00000000	pigs
Pam	-0.11	1.00000000	pigs
Creb3	-0.05	1.00000000	pigs
Klhl2	-0.03	1.00000000	pigs
Ppp1r18	-0.24	1.00000000	pigs
Vps39	-0.07	1.00000000	pigs
Kdm7a	-0.28	1.00000000	pigs
Gps1	-0.08	1.00000000	pigs
Cdkn2aipnl	-0.12	1.00000000	pigs
Sap30	-0.06	1.00000000	pigs
Filip1l	-0.06	1.00000000	pigs
Ncf2	-0.06	1.00000000	pigs
Otud6b	-0.02	1.00000000	pigs
Dhx36	-0.20	1.00000000	pigs
Kctd10	0.00	1.00000000	pigs
Tmem127	0.01	1.00000000	pigs
Gab2	-0.61	1.00000000	pigs
Slc35c2	-0.20	1.00000000	pigs
Pbxip1	-0.06	1.00000000	pigs
Chchd1	-0.07	1.00000000	pigs
Ogfrl1	-0.03	1.00000000	pigs

Ppp6c	-0.12	1.00000000	pigs
Wdr33	-0.01	1.00000000	pigs
Bin1	-0.15	1.00000000	pigs
Pcm1	0.00	1.00000000	pigs
Uxt	-0.23	1.00000000	pigs
Kras	-0.22	1.00000000	pigs
Yju2	-0.03	1.00000000	pigs
Acp1	-0.09	1.00000000	pigs
Ybx3	0.00	1.00000000	pigs
Zfp407	-0.05	1.00000000	pigs
Usp24	-0.19	1.00000000	pigs
Terb1	-0.04	1.00000000	pigs
Ifngr1	-0.06	1.00000000	pigs
Cenpc1	-0.01	1.00000000	pigs
Akap7	-0.21	1.00000000	pigs
Hp1bp3	-0.23	1.00000000	pigs
Smchd1	-0.11	1.00000000	pigs
Sft2d1	-0.03	1.00000000	pigs
Ppp4r1	-0.02	1.00000000	pigs
Zfp106	-0.01	1.00000000	pigs
Sh2d2a	-0.20	1.00000000	pigs
Ssbp4	-0.21	1.00000000	pigs
Fancm	-0.13	1.00000000	pigs
Rbpms	-0.13	1.00000000	pigs
Ufm1	-0.26	1.00000000	pigs
Polr2e	-0.06	1.00000000	pigs
Klf8	-0.23	1.00000000	pigs
Syk	-0.16	1.00000000	pigs
Stpg4	-0.11	1.00000000	pigs
Sart3	-0.03	1.00000000	pigs
Nme1	-0.06	1.00000000	pigs
Aasdh	-0.13	1.00000000	pigs
Ubxn1	-0.06	1.00000000	pigs
Zbtb20	0.05	1.00000000	pigs
Chd8	-0.02	1.00000000	pigs
Ubd	0.09	1.00000000	pigs
Cfl1	-0.09	1.00000000	pigs
Cep128	0.00	1.00000000	pigs
Tmcc1	-0.35	1.00000000	pigs
Septin9	0.00	1.00000000	pigs
Atp2a3	-0.09	1.00000000	pigs
Nup160	0.02	1.00000000	pigs
Zfp148	-0.04	1.00000000	pigs
Slc35b1	-0.06	1.00000000	pigs
Spats2	-0.03	1.00000000	pigs
0610030E20Rik	-0.02	1.00000000	pigs

Gspt2	-0.21	1.00000000	pigs
Por	-0.15	1.00000000	pigs
Diablo	-0.20	1.00000000	pigs
Ubash3b	-0.12	1.00000000	pigs
Pnpt1	-0.03	1.00000000	pigs
Znhit1	-0.05	1.00000000	pigs
Ccne2	-0.06	1.00000000	pigs
Il16	-0.18	1.00000000	pigs
Cdc16	-0.18	1.00000000	pigs
Pank2	-0.05	1.00000000	pigs
Nhlrc2	-0.06	1.00000000	pigs
Tfdp1	-0.03	1.00000000	pigs
Gpr174	-0.06	1.00000000	pigs
Selenof	-0.02	1.00000000	pigs
Ahcyl1	-0.05	1.00000000	pigs
Tcaim	-0.03	1.00000000	pigs
Rcsd1	-0.24	1.00000000	pigs
Sec22a	-0.09	1.00000000	pigs
Casp8	0.00	1.00000000	pigs
Fbxw7	-0.14	1.00000000	pigs
Fbxo34	0.02	1.00000000	pigs
Dlgap4	-0.06	1.00000000	pigs
B3glct	-0.18	1.00000000	pigs
Atad1	-0.24	1.00000000	pigs
Timm17a	-0.03	1.00000000	pigs
Ptpn22	0.07	1.00000000	pigs
Itgb7	-0.23	1.00000000	pigs
Noa1	-0.16	1.00000000	pigs
Hipk1	0.05	1.00000000	pigs
Ash1l	0.00	1.00000000	pigs
Zfp451	-0.03	1.00000000	pigs
Prrc2c	0.01	1.00000000	pigs
Ccndbp1	-0.03	1.00000000	pigs
9030025P20Rik	-0.07	1.00000000	pigs
Bsdc1	0.00	1.00000000	pigs
Cage1	-0.18	1.00000000	pigs
Adpgk	-0.03	1.00000000	pigs
Igf2r	-0.01	1.00000000	pigs
Rcn2	-0.20	1.00000000	pigs
Tnrc6b	0.00	1.00000000	pigs
Smarcd1	-0.20	1.00000000	pigs
Srsf5	0.03	1.00000000	pigs
Smdt1	-0.16	1.00000000	pigs
Virma	-0.03	1.00000000	pigs
Irs2	-0.07	1.00000000	pigs
Drap1	-0.04	1.00000000	pigs

Pxn	-0.27	1.00000000	pigs
Trpm7	0.03	1.00000000	pigs
Cdkn2aip	-0.09	1.00000000	pigs
Aven	0.06	1.00000000	pigs
Ncor2	-0.07	1.00000000	pigs
Lamp2	-0.08	1.00000000	pigs
Tubgcp2	-0.05	1.00000000	pigs
Spg21	-0.10	1.00000000	pigs
Phf12	-0.10	1.00000000	pigs
Lrba	0.00	1.00000000	pigs
Polr2d	-0.09	1.00000000	pigs
Letm1	-0.04	1.00000000	pigs
Ppm1g	-0.06	1.00000000	pigs
Prpf3	-0.04	1.00000000	pigs
Cybrd1	-0.08	1.00000000	pigs
Rfc1	-0.06	1.00000000	pigs
Zfp410	-0.05	1.00000000	pigs
Casc3	-0.20	1.00000000	pigs
Ifi204	0.04	1.00000000	pigs
Serac1	0.00	1.00000000	pigs
Wdr5	-0.06	1.00000000	pigs
Plscr1	-0.01	1.00000000	pigs
Trp53bp1	-0.03	1.00000000	pigs
Ccdc69	-0.21	1.00000000	pigs
Selenow	-0.29	1.00000000	pigs
Prelid1	-0.04	1.00000000	pigs
Hnrnpa3	0.03	1.00000000	pigs
Gse1	-0.20	1.00000000	pigs
Chchd6	-0.06	1.00000000	pigs
Eif2b1	-0.06	1.00000000	pigs
Klf11	-0.23	1.00000000	pigs
Stk40	-0.20	1.00000000	pigs
Trafd1	-0.06	1.00000000	pigs
Gabpb1	0.00	1.00000000	pigs
Gramd1b	-0.22	1.00000000	pigs
Spcs2	-0.20	1.00000000	pigs
Kat2b	-0.20	1.00000000	pigs
Pin4	-0.05	1.00000000	pigs
Rprd1b	0.01	1.00000000	pigs
Fmo4	-0.14	1.00000000	pigs
Rttn	-0.02	1.00000000	pigs
Psmg4	-0.21	1.00000000	pigs
Bcl2l1	0.03	1.00000000	pigs
Tbc1d2b	0.03	1.00000000	pigs
1810037I17Rik	-0.03	1.00000000	pigs
Tob2	-0.04	1.00000000	pigs

Proser1	0.00	1.00000000	pigs
Strn4	-0.02	1.00000000	pigs
Ubtf	-0.21	1.00000000	pigs
Zmpste24	-0.01	1.00000000	pigs
Ulk4	-0.17	1.00000000	pigs
Sae1	-0.03	1.00000000	pigs
Rad17	-0.04	1.00000000	pigs
Cep104	-0.05	1.00000000	pigs
Usf2	-0.10	1.00000000	pigs
Tssc4	-0.05	1.00000000	pigs
Orc3	-0.18	1.00000000	pigs
Tsen34	-0.14	1.00000000	pigs
Snx10	-0.11	1.00000000	pigs
Hk3	-0.22	1.00000000	pigs
Crybg2	-0.28	1.00000000	pigs
Slc25a26	-0.04	1.00000000	pigs
Commd9	-0.03	1.00000000	pigs
Zfp292	0.00	1.00000000	pigs
Uso1	-0.10	1.00000000	pigs
Lsm7	-0.06	1.00000000	pigs
Upf3b	-0.06	1.00000000	pigs
Tk2	0.00	1.00000000	pigs
Oxsr1	-0.07	1.00000000	pigs
Vcan	-0.23	1.00000000	pigs
Stk19	-0.07	1.00000000	pigs
Rexo1	-0.03	1.00000000	pigs
Dhx8	-0.28	1.00000000	pigs
Ogfod3	-0.02	1.00000000	pigs
Ccdc91	0.01	1.00000000	pigs
Fbxo42	-0.05	1.00000000	pigs
Got2	0.01	1.00000000	pigs
Ttc17	-0.10	1.00000000	pigs
Pgd	-0.07	1.00000000	pigs
Thap11	-0.05	1.00000000	pigs
Pea15a	-0.06	1.00000000	pigs
Josd1	-0.06	1.00000000	pigs
H2-Ke6	-0.05	1.00000000	pigs
Gas7	0.15	1.00000000	pigs
Supt16	-0.09	1.00000000	pigs
Rufy1	-0.30	1.00000000	pigs
Ankrd37	-0.02	1.00000000	pigs
Tex10	-0.08	1.00000000	pigs
Cdip1	0.00	1.00000000	pigs
Dynll2	-0.19	1.00000000	pigs
Aurkaip1	-0.18	1.00000000	pigs
Ralgapb	-0.10	1.00000000	pigs

Sertad2	-0.01	1.00000000	pigs
Slc7a7	-0.06	1.00000000	pigs
Ddb2	-0.05	1.00000000	pigs
Nupl2	-0.14	1.00000000	pigs
Dhx30	-0.14	1.00000000	pigs
Cers2	-0.18	1.00000000	pigs
Nfatc3	-0.15	1.00000000	pigs
Dnajb4	-0.07	1.00000000	pigs
Chp1	-0.01	1.00000000	pigs
Psme3ip1	-0.08	1.00000000	pigs
Itm2c	-0.07	1.00000000	pigs
Cd84	-0.18	1.00000000	pigs
Asap1	-0.18	1.00000000	pigs
Eif2b4	-0.07	1.00000000	pigs
Antkmt	-0.13	1.00000000	pigs
Enpp4	0.07	1.00000000	pigs
Itga1	-0.05	1.00000000	pigs
B3gntl1	-0.04	1.00000000	pigs
Spidr	-0.03	1.00000000	pigs
Junb	-0.01	1.00000000	pigs
Zfp169	-0.13	1.00000000	pigs
Chst7	0.04	1.00000000	pigs
Dcakd	-0.16	1.00000000	pigs
Cass4	0.00	1.00000000	pigs
Srp72	-0.10	1.00000000	pigs
Nedd9	-0.09	1.00000000	pigs
Cdc37l1	0.02	1.00000000	pigs
Cops6	-0.03	1.00000000	pigs
Nelfe	-0.08	1.00000000	pigs
Serpini1	-0.09	1.00000000	pigs
Lamtor3	-0.10	1.00000000	pigs
Slc30a1	-0.02	1.00000000	pigs
Cnep1r1	-0.04	1.00000000	pigs
Ryk	0.02	1.00000000	pigs
Nagk	-0.08	1.00000000	pigs
Tbc1d1	0.01	1.00000000	pigs
Dera	-0.27	1.00000000	pigs
Tnfaip8	-0.09	1.00000000	pigs
Tle3	-0.24	1.00000000	pigs
Wrap73	-0.15	1.00000000	pigs
Csrp1	-0.05	1.00000000	pigs
Trp53rka	-0.07	1.00000000	pigs
Vwa8	-0.22	1.00000000	pigs
Nsa2	-0.07	1.00000000	pigs
Prkrip1	-0.05	1.00000000	pigs
Trim23	-0.02	1.00000000	pigs

Ubac2	-0.08	1.00000000	pigs
Rnf149	-0.12	1.00000000	pigs
Naa35	-0.08	1.00000000	pigs
Csnk1g3	0.00	1.00000000	pigs
Yrdc	-0.07	1.00000000	pigs
Abl1	-0.13	1.00000000	pigs
Pign	-0.09	1.00000000	pigs
Gspt1	-0.06	1.00000000	pigs
Sp3	-0.35	1.00000000	pigs
Slc35a3	-0.12	1.00000000	pigs
Ercc8	-0.04	1.00000000	pigs
Gemin2	-0.07	1.00000000	pigs
Arhgap21	0.03	1.00000000	pigs
AW554918	-0.02	1.00000000	pigs
Cyb561d2	-0.15	1.00000000	pigs
Trmt13	-0.01	1.00000000	pigs
Flot2	-0.12	1.00000000	pigs
Ranbp1	-0.03	1.00000000	pigs
Hdac5	0.01	1.00000000	pigs
Abhd16a	-0.03	1.00000000	pigs
Dennd1b	0.01	1.00000000	pigs
Ndufb11b	-0.17	1.00000000	pigs
Cenpw	-0.02	1.00000000	pigs
Atp5d	-0.10	1.00000000	pigs
Aifm1	-0.08	1.00000000	pigs
Katna1	-0.01	1.00000000	pigs
Uimc1	-0.06	1.00000000	pigs
Erap1	-0.20	1.00000000	pigs
Cwf19l1	-0.12	1.00000000	pigs
Extl3	-0.06	1.00000000	pigs
Thoc1	-0.06	1.00000000	pigs
Pnpla2	-0.11	1.00000000	pigs
Adam10	-0.11	1.00000000	pigs
Crtc1	-0.06	1.00000000	pigs
Ap1b1	-0.07	1.00000000	pigs
Rbm25	0.02	1.00000000	pigs
Psma4	-0.13	1.00000000	pigs
Snap47	-0.05	1.00000000	pigs
Prmt2	-0.06	1.00000000	pigs
Acbd5	-0.01	1.00000000	pigs
Pibf1	-0.21	1.00000000	pigs
Clpb	-0.12	1.00000000	pigs
Trmt1l	-0.09	1.00000000	pigs
Ccnh	-0.09	1.00000000	pigs
Ubl7	-0.01	1.00000000	pigs
Ripk2	0.02	1.00000000	pigs

Wrap53	-0.04	1.00000000	pigs
Gtpbp4	-0.09	1.00000000	pigs
Nup205	-0.01	1.00000000	pigs
Ciz1	-0.01	1.00000000	pigs
Atp13a2	-0.03	1.00000000	pigs
Ssh1	-0.01	1.00000000	pigs
Ddx42	-0.05	1.00000000	pigs
Sufu	-0.09	1.00000000	pigs
Dcaf8	-0.03	1.00000000	pigs
Adk	0.03	1.00000000	pigs
Rhoa	0.01	1.00000000	pigs
Zfp395	-0.17	1.00000000	pigs
Phf14	-0.15	1.00000000	pigs
Lef1	-0.12	1.00000000	pigs
Stmn1	-0.26	1.00000000	pigs
Efcab14	-0.15	1.00000000	pigs
Epb41	-0.12	1.00000000	pigs
Fam89b	-0.15	1.00000000	pigs
Tdg	-0.14	1.00000000	pigs
Cap1	-0.03	1.00000000	pigs
Tmem223	-0.26	1.00000000	pigs
Sqle	0.03	1.00000000	pigs
Bcl7c	-0.08	1.00000000	pigs
Xpo1	-0.05	1.00000000	pigs
9930021J03Rik	-0.04	1.00000000	pigs
Tango2	-0.08	1.00000000	pigs
Fam50a	-0.05	1.00000000	pigs
Pds5b	-0.07	1.00000000	pigs
Rad23b	-0.05	1.00000000	pigs
Ddx18	-0.09	1.00000000	pigs
Dpy30	-0.10	1.00000000	pigs
Ccpg1	-0.03	1.00000000	pigs
Slc35f5	-0.01	1.00000000	pigs
Ttc37	-0.10	1.00000000	pigs
Bpnt1	-0.08	1.00000000	pigs
Traf6	-0.05	1.00000000	pigs
Sdf2	-0.10	1.00000000	pigs
Commd4	-0.17	1.00000000	pigs
Smc3	-0.04	1.00000000	pigs
Uba5	-0.08	1.00000000	pigs
Taf15	-0.11	1.00000000	pigs
Crls1	-0.01	1.00000000	pigs
Ccdc15	-0.17	1.00000000	pigs
Ddhd2	-0.04	1.00000000	pigs
Tmem9b	-0.07	1.00000000	pigs
Acot13	-0.05	1.00000000	pigs

Arntl	0.07	1.00000000	pigs
Siva1	-0.11	1.00000000	pigs
Slc10a7	-0.09	1.00000000	pigs
Pnpla8	-0.15	1.00000000	pigs
Cdc40	-0.14	1.00000000	pigs
Ehbp1	-0.19	1.00000000	pigs
Ccnl2	-0.05	1.00000000	pigs
Ccdc22	-0.04	1.00000000	pigs
Rilpl2	-0.08	1.00000000	pigs
Cdin1	-0.15	1.00000000	pigs
Zc3h4	-0.16	1.00000000	pigs
Tmem41b	-0.03	1.00000000	pigs
Eif2ak4	-0.24	1.00000000	pigs
Bin3	-0.01	1.00000000	pigs
Ndufb7	-0.12	1.00000000	pigs
Glrx5	-0.05	1.00000000	pigs
Lcp2	-0.13	1.00000000	pigs
Smyd2	-0.04	1.00000000	pigs
Ak6	-0.07	1.00000000	pigs
Shq1	-0.07	1.00000000	pigs
Lnpk	-0.08	1.00000000	pigs
Oprm1	-0.20	1.00000000	pigs
Rsf1	0.03	1.00000000	pigs
Osbpl3	0.11	1.00000000	pigs
Terf1	-0.22	1.00000000	pigs
Akt2	-0.07	1.00000000	pigs
Plod3	-0.04	1.00000000	pigs
Kdsr	0.01	1.00000000	pigs
Rars	-0.01	1.00000000	pigs
Yipf1	-0.20	1.00000000	pigs
Arl6ip6	-0.06	1.00000000	pigs
Kdm8	-0.10	1.00000000	pigs
BC005624	-0.26	1.00000000	pigs
Sucla2	0.00	1.00000000	pigs
Kdm4a	-0.04	1.00000000	pigs
Ate1	-0.06	1.00000000	pigs
Nlrc5	-0.16	1.00000000	pigs
Cpne1	-0.17	1.00000000	pigs
Vrk2	-0.15	1.00000000	pigs
Kat6b	0.03	1.00000000	pigs
Lsm2	-0.24	1.00000000	pigs
Sdhc	-0.09	1.00000000	pigs
Gabpb2	-0.22	1.00000000	pigs
Nolc1	-0.07	1.00000000	pigs
Ypel5	-0.02	1.00000000	pigs
Ergic1	-0.14	1.00000000	pigs

Fnip2	0.01	1.00000000	pigs
Rnaseh2b	-0.03	1.00000000	pigs
Spata5	-0.25	1.00000000	pigs
Aldh18a1	-0.02	1.00000000	pigs
Clip4	-0.14	1.00000000	pigs
Rex1bd	-0.19	1.00000000	pigs
Dpp8	-0.25	1.00000000	pigs
Fibp	-0.09	1.00000000	pigs
Gga2	-0.17	1.00000000	pigs
Gak	-0.05	1.00000000	pigs
Spag16	0.00	1.00000000	pigs
Tipin	-0.19	1.00000000	pigs
Tfip11	-0.02	1.00000000	pigs
Ap1m1	-0.09	1.00000000	pigs
Zcchc9	-0.11	1.00000000	pigs
Rpa2	-0.03	1.00000000	pigs
Fbl	-0.07	1.00000000	pigs
Ppp1r35	-0.02	1.00000000	pigs
BC004004	-0.18	1.00000000	pigs
Kif21b	-0.02	1.00000000	pigs
Camk2g	-0.20	1.00000000	pigs
Evi2	-0.21	1.00000000	pigs
Apex1	-0.09	1.00000000	pigs
Herc4	0.07	1.00000000	pigs
B230219D22Rik	-0.12	1.00000000	pigs
Gtpbp8	-0.16	1.00000000	pigs
Psmb9	-0.13	1.00000000	pigs
Yipf4	-0.05	1.00000000	pigs
Slc9a7	0.00	1.00000000	pigs
Elovl6	0.00	1.00000000	pigs
Ubxn2a	-0.05	1.00000000	pigs
Hsd17b4	-0.03	1.00000000	pigs
Smpd1	-0.04	1.00000000	pigs
Plscr3	-0.23	1.00000000	pigs
Ap3b1	-0.17	1.00000000	pigs
Eea1	-0.15	1.00000000	pigs
E130308A19Rik	-0.09	1.00000000	pigs
Eif6	-0.21	1.00000000	pigs
Swt1	-0.15	1.00000000	pigs
Asxl2	-0.31	1.00000000	pigs
Herpud2	-0.08	1.00000000	pigs
Pepd	-0.14	1.00000000	pigs
Pole4	-0.15	1.00000000	pigs
Nfyb	-0.13	1.00000000	pigs
Nubp2	-0.08	1.00000000	pigs
Cyfip2	-0.01	1.00000000	pigs

Trappc5	-0.06	1.00000000	pigs
Ppp1r14bl	-0.18	1.00000000	pigs
Pbx3	-0.08	1.00000000	pigs
Entpd1	0.10	1.00000000	pigs
Smad7	-0.21	1.00000000	pigs
Srsf3	0.01	1.00000000	pigs
Wwp1	-0.07	1.00000000	pigs
Herc2	-0.17	1.00000000	pigs
Zc3h7b	-0.08	1.00000000	pigs
Itpa	-0.05	1.00000000	pigs
Fmnl1	-0.01	1.00000000	pigs
Deaf1	-0.11	1.00000000	pigs
Nfya	-0.06	1.00000000	pigs
Trank1	-0.34	1.00000000	pigs
Ddx55	-0.05	1.00000000	pigs
Camkmt	-0.09	1.00000000	pigs
Pnrc2	-0.20	1.00000000	pigs
Nfe2l1	-0.03	1.00000000	pigs
Senp7	-0.21	1.00000000	pigs
Sri	-0.05	1.00000000	pigs
Zbtb37	-0.04	1.00000000	pigs
Nkap	-0.04	1.00000000	pigs
Smim7	-0.20	1.00000000	pigs
Nr1h2	-0.09	1.00000000	pigs
Tprgl	-0.15	1.00000000	pigs
Casp3	-0.02	1.00000000	pigs
Tnfsf4	-0.13	1.00000000	pigs
Ccdc90b	-0.07	1.00000000	pigs
Cdc14a	-0.05	1.00000000	pigs
Rbm48	-0.01	1.00000000	pigs
Thrap3	0.00	1.00000000	pigs
Zbtb44	-0.07	1.00000000	pigs
Lfng	-0.10	1.00000000	pigs
Ppp1r10	-0.12	1.00000000	pigs
Tmod3	-0.01	1.00000000	pigs
Fam76b	0.00	1.00000000	pigs
Usf3	-0.07	1.00000000	pigs
Atp5a1	-0.17	1.00000000	pigs
Kcnab3	-0.12	1.00000000	pigs
Lyn	-0.18	1.00000000	pigs
Ccdc85b	-0.08	1.00000000	pigs
Cfp	-0.11	1.00000000	pigs
R3hdm4	-0.08	1.00000000	pigs
Ythdc1	-0.01	1.00000000	pigs
Prpf31	-0.07	1.00000000	pigs
Jade1	-0.09	1.00000000	pigs

Agl	-0.19	1.00000000	pigs
Htt	-0.07	1.00000000	pigs
Uck2	0.01	1.00000000	pigs
Cnot6	-0.15	1.00000000	pigs
Rsrc1	-0.17	1.00000000	pigs
Cks2	-0.25	1.00000000	pigs
Napg	-0.08	1.00000000	pigs
Anp32e	-0.22	1.00000000	pigs
Gadd45b	-0.08	1.00000000	pigs
Ctnnbl1	-0.19	1.00000000	pigs
Stau2	-0.06	1.00000000	pigs
Ankrd49	-0.20	1.00000000	pigs
Tgfbrap1	-0.06	1.00000000	pigs
Jak1	-0.01	1.00000000	pigs
Primpol	-0.13	1.00000000	pigs
Poglut1	-0.18	1.00000000	pigs
Coq9	-0.05	1.00000000	pigs
Polr2f	-0.20	1.00000000	pigs
Tmed5	-0.16	1.00000000	pigs
Sppl2a	-0.22	1.00000000	pigs
Snx17	-0.09	1.00000000	pigs
Akip1	-0.17	1.00000000	pigs
Katnip	-0.04	1.00000000	pigs
Park7	-0.16	1.00000000	pigs
Slc35e1	-0.07	1.00000000	pigs
Tfcp2	-0.11	1.00000000	pigs
Akap11	-0.09	1.00000000	pigs
Lrrc28	-0.05	1.00000000	pigs
Cox17	-0.16	1.00000000	pigs
Pfkfb3	-0.21	1.00000000	pigs
Hook3	-0.13	1.00000000	pigs
Atp2c1	-0.04	1.00000000	pigs
Oaz2	-0.02	1.00000000	pigs
Scamp1	-0.09	1.00000000	pigs
Rnf146	-0.12	1.00000000	pigs
Ankrd42	-0.10	1.00000000	pigs
Cyc1	-0.12	1.00000000	pigs
Wasf2	-0.04	1.00000000	pigs
Hnrnpul1	-0.02	1.00000000	pigs
Sars	-0.25	1.00000000	pigs
Phf3	-0.22	1.00000000	pigs
Far2	-0.16	1.00000000	pigs
Rb1	-0.13	1.00000000	pigs
Ythdf3	-0.07	1.00000000	pigs
Dhx29	-0.13	1.00000000	pigs
Thumpd1	-0.23	1.00000000	pigs

Thoc7	-0.21	1.00000000	pigs
Ufc1	-0.06	1.00000000	pigs
Cenph	-0.15	1.00000000	pigs
Ptger4	-0.02	1.00000000	pigs
Vgll4	0.00	1.00000000	pigs
Snrnp40	-0.07	1.00000000	pigs
Gnpda1	-0.11	1.00000000	pigs
Topbp1	-0.07	1.00000000	pigs
Bdh2	-0.09	1.00000000	pigs
Washc3	-0.30	1.00000000	pigs
Umps	-0.05	1.00000000	pigs
Litaf	0.09	1.00000000	pigs
Gabarapl1	-0.06	1.00000000	pigs
Rsad2	-0.09	1.00000000	pigs
Tomm7	-0.26	1.00000000	pigs
Emc4	-0.15	1.00000000	pigs
Akap8	-0.10	1.00000000	pigs
Tex264	-0.08	1.00000000	pigs
Clcn7	-0.03	1.00000000	pigs
Ginm1	-0.20	1.00000000	pigs
Slc39a6	-0.07	1.00000000	pigs
Gpcpd1	-0.39	1.00000000	pigs
Invs	-0.14	1.00000000	pigs
Fam172a	-0.07	1.00000000	pigs
Rep15	-0.04	1.00000000	pigs
Gltp	-0.05	1.00000000	pigs
Rfx3	-0.17	1.00000000	pigs
Vps26c	-0.06	1.00000000	pigs
Rfxank	-0.16	1.00000000	pigs
Taf12	-0.19	1.00000000	pigs
Stmn3	-0.20	1.00000000	pigs
Vps13d	-0.17	1.00000000	pigs
Acat1	-0.09	1.00000000	pigs
Nedd1	-0.07	1.00000000	pigs
Gpatch2	-0.20	1.00000000	pigs
Gab3	-0.09	1.00000000	pigs
Rnf14	-0.23	1.00000000	pigs
Pnrc1	-0.01	1.00000000	pigs
Zcchc7	-0.05	1.00000000	pigs
U2af2	-0.02	1.00000000	pigs
Ankrd13a	-0.05	1.00000000	pigs
Uqcr10	-0.15	1.00000000	pigs
C1qbp	-0.09	1.00000000	pigs
Ranbp17	-0.10	1.00000000	pigs
Hacd2	-0.10	1.00000000	pigs
Hmg20a	-0.19	1.00000000	pigs

Suclg1	-0.21	1.00000000	pigs
Eef1b2	0.01	1.00000000	pigs
Tmem60	-0.08	1.00000000	pigs
Galnt1	-0.14	1.00000000	pigs
Atp5j	-0.09	1.00000000	pigs
Sike1	-0.13	1.00000000	pigs
Fam117a	-0.24	1.00000000	pigs
Ing5	-0.08	1.00000000	pigs
Tm9sf4	-0.11	1.00000000	pigs
Cd48	-0.05	1.00000000	pigs
Bad	-0.08	1.00000000	pigs
Pld3	0.00	1.00000000	pigs
Optn	-0.07	1.00000000	pigs
Cyb5r4	-0.16	1.00000000	pigs
Hspa14	-0.08	1.00000000	pigs
Thumpd2	-0.13	1.00000000	pigs
Ivd	-0.16	1.00000000	pigs
Ubxn8	-0.18	1.00000000	pigs
Slc39a9	-0.12	1.00000000	pigs
Usp33	-0.14	1.00000000	pigs
Gpn1	-0.03	1.00000000	pigs
Rgs14	-0.11	1.00000000	pigs
Phf5a	-0.21	1.00000000	pigs
Rbks	-0.15	1.00000000	pigs
Tnrc6c	-0.11	1.00000000	pigs
Sgms1	-0.15	1.00000000	pigs
Rab11fip2	-0.12	1.00000000	pigs
Spire1	0.02	1.00000000	pigs
Bag1	-0.23	1.00000000	pigs
Cers4	-0.08	1.00000000	pigs
Ppp1r8	-0.08	1.00000000	pigs
Raly	-0.04	1.00000000	pigs
Capn2	-0.08	1.00000000	pigs
Samd8	-0.07	1.00000000	pigs
Snrpb2	-0.20	1.00000000	pigs
Blvrb	-0.10	1.00000000	pigs
Csgalnact2	-0.13	1.00000000	pigs
Nsd2	0.00	1.00000000	pigs
Kcnq5	-0.12	1.00000000	pigs
Sgk1	-0.15	1.00000000	pigs
Septin3	-0.10	1.00000000	pigs
Bmt2	-0.05	1.00000000	pigs
5730455P16Rik	-0.07	1.00000000	pigs
Clasp2	-0.12	1.00000000	pigs
Ubr1	-0.23	1.00000000	pigs
Becn1	-0.10	1.00000000	pigs

Rnf44	-0.08	1.00000000	pigs
Hsd17b10	-0.07	1.00000000	pigs
Immp1l	-0.07	1.00000000	pigs
1600012H06Rik	-0.11	1.00000000	pigs
Prpf8	-0.10	1.00000000	pigs
Snx29	-0.22	1.00000000	pigs
Timm22	-0.11	1.00000000	pigs
Pim1	-0.05	1.00000000	pigs
Pram1	-0.04	1.00000000	pigs
Ncoa5	-0.05	1.00000000	pigs
Dhx38	-0.05	1.00000000	pigs
Stk38l	-0.24	1.00000000	pigs
Btbd19	-0.06	1.00000000	pigs
Brms1	-0.08	1.00000000	pigs
Kars	-0.14	1.00000000	pigs
Gca	-0.06	1.00000000	pigs
Relch	-0.04	1.00000000	pigs
Rptor	-0.06	1.00000000	pigs
Brk1	-0.12	1.00000000	pigs
Slc25a39	-0.05	1.00000000	pigs
Adcy7	-0.09	1.00000000	pigs
Sf3a1	-0.06	1.00000000	pigs
Casp7	-0.14	1.00000000	pigs
Leo1	-0.16	1.00000000	pigs
Grk5	0.19	1.00000000	pigs
Trim37	-0.20	1.00000000	pigs
Ubxn6	-0.05	1.00000000	pigs
Galnt11	-0.22	1.00000000	pigs
Pde3b	0.08	1.00000000	pigs
Psma7	-0.06	1.00000000	pigs
Timm10	-0.09	1.00000000	pigs
Abhd17a	-0.10	1.00000000	pigs
Taf7	-0.11	1.00000000	pigs
Nek6	-0.05	1.00000000	pigs
Zfand6	-0.11	1.00000000	pigs
Phlda2	-0.21	1.00000000	pigs
Iah1	-0.15	1.00000000	pigs
Xbp1	-0.12	1.00000000	pigs
Zfp2	-0.07	1.00000000	pigs
Eftud2	-0.09	1.00000000	pigs
Ube2b	0.03	1.00000000	pigs
Alg5	-0.07	1.00000000	pigs
Cradd	-0.10	1.00000000	pigs
Tagap1	-0.16	1.00000000	pigs
Rbmx2	-0.08	1.00000000	pigs
Wipi1	-0.03	1.00000000	pigs

Rpp21	-0.06	1.00000000	pigs
Arf5	-0.10	1.00000000	pigs
Pwwp2a	-0.07	1.00000000	pigs
Slc25a12	-0.21	1.00000000	pigs
Ubn2	-0.14	1.00000000	pigs
Rfc4	-0.01	1.00000000	pigs
Hibch	-0.19	1.00000000	pigs
Ikbke	-0.13	1.00000000	pigs
Sec24d	-0.06	1.00000000	pigs
Zzef1	-0.05	1.00000000	pigs
Lin9	-0.17	1.00000000	pigs
Cd47	-0.14	1.00000000	pigs
Alms1	-0.15	1.00000000	pigs
Otulinl	-0.22	1.00000000	pigs
Sntb2	-0.14	1.00000000	pigs
Ghsr	-0.05	1.00000000	pigs
Ppme1	-0.07	1.00000000	pigs
Fkbp15	-0.23	1.00000000	pigs
Zbtb7a	-0.09	1.00000000	pigs
Sacm1l	-0.13	1.00000000	pigs
Capn1	-0.08	1.00000000	pigs
Syf2	-0.18	1.00000000	pigs
Slf1	-0.13	1.00000000	pigs
Smim19	-0.14	1.00000000	pigs
Ostc	-0.08	1.00000000	pigs
Runx1	0.00	1.00000000	pigs
Topaz1	0.02	1.00000000	pigs
Pop5	-0.11	1.00000000	pigs
Sh3kbp1	0.02	1.00000000	pigs
Cep164	-0.10	1.00000000	pigs
Wdr12	-0.08	1.00000000	pigs
Impa1	-0.10	1.00000000	pigs
Tigar	-0.08	1.00000000	pigs
Ap4s1	-0.09	1.00000000	pigs
Acadsb	-0.16	1.00000000	pigs
Srprb	-0.07	1.00000000	pigs
Dpy19l4	-0.02	1.00000000	pigs
St13	-0.15	1.00000000	pigs
Prkaa1	-0.12	1.00000000	pigs
Pkn1	-0.09	1.00000000	pigs
Dazap2	-0.10	1.00000000	pigs
Hacd3	-0.14	1.00000000	pigs
Ccdc12	-0.15	1.00000000	pigs
Dcun1d3	-0.10	1.00000000	pigs
Tmem161b	-0.11	1.00000000	pigs
Ndufb3	-0.26	1.00000000	pigs

Chmp5	-0.14	1.00000000	pigs
Ankrd44	-0.03	1.00000000	pigs
Tubgcp4	-0.08	1.00000000	pigs
Tg	-0.12	1.00000000	pigs
Btd	-0.14	1.00000000	pigs
Nr2c2	-0.21	1.00000000	pigs
Herc1	-0.03	1.00000000	pigs
Tet1	-0.02	1.00000000	pigs
Rab11a	-0.12	1.00000000	pigs
Klhl24	-0.10	1.00000000	pigs
Camta2	-0.11	1.00000000	pigs
Sap130	-0.19	1.00000000	pigs
Cers5	-0.08	1.00000000	pigs
Rcl1	-0.05	1.00000000	pigs
E2f4	-0.05	1.00000000	pigs
Nuak2	-0.10	1.00000000	pigs
Gnai2	-0.12	1.00000000	pigs
Aimp1	-0.13	1.00000000	pigs
Cactin	-0.09	1.00000000	pigs
Pus10	-0.10	1.00000000	pigs
Erlec1	-0.09	1.00000000	pigs
Zyx	-0.23	1.00000000	pigs
Nme6	-0.05	1.00000000	pigs
Cop1	-0.09	1.00000000	pigs
Ndufs5	-0.17	1.00000000	pigs
Dguok	-0.01	1.00000000	pigs
Nudcd2	-0.10	1.00000000	pigs
Nsl1	-0.14	1.00000000	pigs
Preb	-0.06	1.00000000	pigs
Ube4b	-0.04	1.00000000	pigs
Atpif1	-0.20	1.00000000	pigs
Dnlz	-0.14	1.00000000	pigs
Pigb	-0.12	1.00000000	pigs
Ift52	-0.14	1.00000000	pigs
Lmbrd1	-0.04	1.00000000	pigs
Pik3r4	-0.06	1.00000000	pigs
Ipo11	-0.16	1.00000000	pigs
Cep120	-0.05	1.00000000	pigs
Kin	-0.09	1.00000000	pigs
Add1	-0.12	1.00000000	pigs
Cwc22	-0.11	1.00000000	pigs
Eif4enif1	-0.06	1.00000000	pigs
Polr2m	-0.10	1.00000000	pigs
Eefsec	-0.05	1.00000000	pigs
Cpeb4	-0.13	1.00000000	pigs
Abr	-0.12	1.00000000	pigs

Yif1a	-0.05	1.00000000	pigs
Vps72	-0.07	1.00000000	pigs
Nvl	-0.26	1.00000000	pigs
Golga1	-0.09	1.00000000	pigs
Cox20	-0.14	1.00000000	pigs
Cul4b	-0.11	1.00000000	pigs
Lypla2	-0.09	1.00000000	pigs
Zcchc17	-0.09	1.00000000	pigs
Hsd17b7	-0.05	1.00000000	pigs
Dnmt1	-0.24	1.00000000	pigs
Gss	-0.01	1.00000000	pigs
Chd3	-0.14	1.00000000	pigs
Ndufab1	-0.13	1.00000000	pigs
Fam114a2	-0.07	1.00000000	pigs
Rhoc	-0.10	1.00000000	pigs
Anapc13	-0.15	1.00000000	pigs
Shoc2	-0.10	1.00000000	pigs
Crlf3	-0.12	1.00000000	pigs
Zbtb41	-0.06	1.00000000	pigs
Polm	-0.09	1.00000000	pigs
Atp6v1d	-0.10	1.00000000	pigs
Dph1	-0.06	1.00000000	pigs
Slamf6	-0.18	1.00000000	pigs
Dipk1a	-0.24	1.00000000	pigs
Ctdnep1	-0.07	1.00000000	pigs
Ccdc43	-0.10	1.00000000	pigs
Rnf5	-0.05	1.00000000	pigs
Pithd1	-0.15	1.00000000	pigs
Glipr2	-0.06	1.00000000	pigs
Coq10b	-0.16	1.00000000	pigs
Ccp110	-0.14	1.00000000	pigs
Naxe	-0.11	1.00000000	pigs
Zfp30	-0.07	1.00000000	pigs
Prpf38a	-0.20	1.00000000	pigs
Ivns1abp	-0.17	1.00000000	pigs
Dhx40	-0.02	1.00000000	pigs
Vdac2	-0.13	1.00000000	pigs
Naa40	-0.15	1.00000000	pigs
Slc39a14	-0.06	1.00000000	pigs
Ccdc126	-0.09	1.00000000	pigs
Ubtd2	-0.14	1.00000000	pigs
Ctbp1	-0.07	1.00000000	pigs
Emc9	-0.06	1.00000000	pigs
Rbm42	-0.18	1.00000000	pigs
Dffa	-0.05	1.00000000	pigs
Rmnd5a	-0.07	1.00000000	pigs

Ankmy2	-0.03	1.00000000	pigs
Kdm1a	-0.21	1.00000000	pigs
L3mbtl3	-0.12	1.00000000	pigs
Trabd	-0.09	1.00000000	pigs
Pex3	-0.10	1.00000000	pigs
Insig1	-0.09	1.00000000	pigs
Tspo	-0.18	1.00000000	pigs
Cmpk2	-0.11	1.00000000	pigs
Timm8b	-0.14	1.00000000	pigs
Ano6	-0.05	1.00000000	pigs
Uqcc3	-0.07	1.00000000	pigs
Taldo1	-0.08	1.00000000	pigs
Prkar1a	-0.14	1.00000000	pigs
Nt5c	-0.07	1.00000000	pigs
Gtf2e2	-0.09	1.00000000	pigs
Golga3	-0.17	1.00000000	pigs
Tbc1d10c	-0.16	1.00000000	pigs
Wdr1	-0.10	1.00000000	pigs
Zfp618	-0.07	1.00000000	pigs
Khnyn	-0.12	1.00000000	pigs
Dph6	-0.10	1.00000000	pigs
Jkamp	-0.10	1.00000000	pigs
H2ac20	-0.20	1.00000000	pigs
Ehbp1l1	-0.07	1.00000000	pigs
Churc1	-0.11	1.00000000	pigs
Cdc26	-0.10	1.00000000	pigs
Pex16	-0.07	1.00000000	pigs
Uri1	-0.10	1.00000000	pigs
Sytl3	-0.06	1.00000000	pigs
Agtpbp1	-0.11	1.00000000	pigs
Prkar2b	-0.05	1.00000000	pigs
Snrnp70	-0.11	1.00000000	pigs
Csk	-0.15	1.00000000	pigs
Alox5ap	-0.31	1.00000000	pigs
Eif4g2	-0.03	1.00000000	pigs
Tpr	-0.07	1.00000000	pigs
Slu7	-0.13	1.00000000	pigs
Dcaf11	-0.07	1.00000000	pigs
Stx8	-0.07	1.00000000	pigs
Fbxo9	-0.26	1.00000000	pigs
Scaf11	-0.12	1.00000000	pigs
Cep250	-0.13	1.00000000	pigs
Cybc1	-0.07	1.00000000	pigs
Ccdc124	-0.17	1.00000000	pigs
Pinx1	-0.04	1.00000000	pigs
Svil	-0.17	1.00000000	pigs

Ing1	-0.08	1.00000000	pigs
Rchy1	-0.06	1.00000000	pigs
Pitpna	-0.06	1.00000000	pigs
Brd8	-0.13	1.00000000	pigs
Psme2	-0.11	1.00000000	pigs
Cd244a	-0.17	1.00000000	pigs
Tpt1	0.00	1.00000000	pigs
Smg9	-0.06	1.00000000	pigs
Cnot10	-0.20	1.00000000	pigs
Ppm1d	-0.12	1.00000000	pigs
Gusb	-0.16	1.00000000	pigs
Crnkl1	-0.10	1.00000000	pigs
Hras	-0.11	1.00000000	pigs
Parp8	-0.09	1.00000000	pigs
Cast	-0.14	1.00000000	pigs
Arid5b	-0.01	1.00000000	pigs
Arhgap35	-0.14	1.00000000	pigs
Ndufs3	-0.10	1.00000000	pigs
Aprt	-0.12	1.00000000	pigs
Gtf3c6	-0.04	1.00000000	pigs
St3gal1	-0.10	1.00000000	pigs
Spns3	-0.20	1.00000000	pigs
Yeats4	-0.15	1.00000000	pigs
Ppm1a	-0.17	1.00000000	pigs
Tmed4	-0.21	1.00000000	pigs
Cdc34	-0.13	1.00000000	pigs
Xrn1	-0.09	1.00000000	pigs
Emc1	-0.07	1.00000000	pigs
Tspan5	0.06	1.00000000	pigs
Il10ra	-0.17	1.00000000	pigs
Cpne3	-0.10	1.00000000	pigs
Lap3	-0.09	1.00000000	pigs
Exoc1	-0.21	1.00000000	pigs
Gnb2	-0.16	1.00000000	pigs
Amd2	-0.09	1.00000000	pigs
Rbm19	-0.07	1.00000000	pigs
Vps37a	-0.15	1.00000000	pigs
Gabpa	-0.08	1.00000000	pigs
Senp5	-0.08	1.00000000	pigs
Phf21a	-0.21	1.00000000	pigs
Klc2	-0.08	1.00000000	pigs
Snx16	0.01	1.00000000	pigs
Emc3	-0.08	1.00000000	pigs
Rnf166	-0.06	1.00000000	pigs
Pcyox1	-0.11	1.00000000	pigs
Wdtc1	-0.13	1.00000000	pigs

Appl2	-0.06	1.00000000	pigs
Ulk1	-0.11	1.00000000	pigs
Arrdc1	-0.12	1.00000000	pigs
Cops3	-0.08	1.00000000	pigs
Aimp2	-0.18	1.00000000	pigs
Terf2	-0.08	1.00000000	pigs
Ttc1	-0.15	1.00000000	pigs
Ak3	-0.11	1.00000000	pigs
Pikfyve	-0.04	1.00000000	pigs
Cd3d	-0.04	1.00000000	pigs
Rgs3	-0.15	1.00000000	pigs
Cnot11	-0.10	1.00000000	pigs
Atf7ip	-0.05	1.00000000	pigs
Usp44	-0.10	1.00000000	pigs
Lrrc42	-0.06	1.00000000	pigs
Rtf1	-0.11	1.00000000	pigs
Klhdc4	-0.09	1.00000000	pigs
Aspdh	-0.06	1.00000000	pigs
Gigyf1	-0.06	1.00000000	pigs
Tdp2	-0.09	1.00000000	pigs
Stk39	-0.07	1.00000000	pigs
Jade2	-0.09	1.00000000	pigs
Fam189a1	-0.09	1.00000000	pigs
Nol9	-0.14	1.00000000	pigs
Trib2	-0.13	1.00000000	pigs
Cxxc1	-0.06	1.00000000	pigs
Uchl5	-0.16	1.00000000	pigs
Arfip1	-0.21	1.00000000	pigs
Nap1l1	-0.11	1.00000000	pigs
Lpgat1	-0.55	1.00000000	pigs
Krtcap2	-0.13	1.00000000	pigs
Dnaja2	-0.19	1.00000000	pigs
D8Ertd738e	-0.10	1.00000000	pigs
Plaa	-0.20	1.00000000	pigs
Lamp1	-0.09	1.00000000	pigs
Nedd8	-0.14	1.00000000	pigs
Dusp22	-0.17	1.00000000	pigs
Tfam	-0.13	1.00000000	pigs
Arf6	-0.12	1.00000000	pigs
Ahctf1	-0.26	1.00000000	pigs
C2cd3	-0.10	1.00000000	pigs
Ralb	-0.09	1.00000000	pigs
Gfus	-0.09	1.00000000	pigs
Cltb	-0.17	1.00000000	pigs
Fbxl14	-0.05	1.00000000	pigs
Pno1	-0.20	1.00000000	pigs

Stk11	-0.12	1.00000000	pigs
Nrd1	-0.07	1.00000000	pigs
Znrf3	-0.05	1.00000000	pigs
Reep5	-0.02	1.00000000	pigs
Rab8b	-0.19	1.00000000	pigs
Ebag9	-0.10	1.00000000	pigs
Prpf6	-0.17	1.00000000	pigs
Gabbr1	-0.16	1.00000000	pigs
Il12rb1	-0.04	1.00000000	pigs
Pgp	-0.01	1.00000000	pigs
Clcc1	-0.16	1.00000000	pigs
Xrn2	-0.13	1.00000000	pigs
Disc1	-0.17	1.00000000	pigs
Gtf3a	-0.11	1.00000000	pigs
Aopep	-0.12	1.00000000	pigs
Kmt2b	-0.10	1.00000000	pigs
Chfr	-0.17	1.00000000	pigs
Timmdc1	-0.11	1.00000000	pigs
Ddx6	-0.08	1.00000000	pigs
Hps3	-0.13	1.00000000	pigs
Zfand3	-0.09	1.00000000	pigs
Tnf	-0.14	1.00000000	pigs
Tgs1	-0.17	1.00000000	pigs
Cdr2	-0.21	1.00000000	pigs
Atg13	-0.19	1.00000000	pigs
Rest	-0.17	1.00000000	pigs
Nf1	-0.18	1.00000000	pigs
Polr2b	-0.11	1.00000000	pigs
Gpr18	-0.17	1.00000000	pigs
Arid1a	-0.18	1.00000000	pigs
Osgepl1	-0.15	1.00000000	pigs
Dalrd3	-0.06	1.00000000	pigs
Shld1	-0.06	1.00000000	pigs
Dusp28	-0.09	1.00000000	pigs
Sipa1l1	-0.11	1.00000000	pigs
Slc9a9	-0.01	1.00000000	pigs
Gripap1	-0.12	1.00000000	pigs
Gid8	-0.13	1.00000000	pigs
Ccsap	-0.14	1.00000000	pigs
Ppp1r12b	-0.07	1.00000000	pigs
Ifih1	-0.16	1.00000000	pigs
Ccdc93	-0.15	1.00000000	pigs
Pitpnm1	-0.05	1.00000000	pigs
Hmgb2	-0.18	1.00000000	pigs
Flad1	-0.06	1.00000000	pigs
Hgs	-0.11	1.00000000	pigs

Khk	-0.06	1.00000000	pigs
Cib1	-0.16	1.00000000	pigs
Snx8	-0.17	1.00000000	pigs
Smc2	-0.07	1.00000000	pigs
Higd2a	-0.17	1.00000000	pigs
Tsr2	-0.11	1.00000000	pigs
Sdccag8	-0.13	1.00000000	pigs
Snrnp200	-0.14	1.00000000	pigs
Itfg1	-0.16	1.00000000	pigs
Psmd13	-0.09	1.00000000	pigs
Snx3	-0.11	1.00000000	pigs
Canx	-0.10	1.00000000	pigs
Creld2	-0.08	1.00000000	pigs
Bid	-0.09	1.00000000	pigs
Nxf1	-0.14	1.00000000	pigs
Usp20	-0.21	1.00000000	pigs
Zdhhc15	-0.14	1.00000000	pigs
Vps4b	-0.17	1.00000000	pigs
Ghrl	-0.20	1.00000000	pigs
Polr2h	-0.09	1.00000000	pigs
Trdmt1	-0.16	1.00000000	pigs
Txk	0.08	1.00000000	pigs
Vps8	-0.18	1.00000000	pigs
Ppp1r15a	-0.06	1.00000000	pigs
Sting1	-0.10	1.00000000	pigs
Asb3	-0.19	1.00000000	pigs
Baz2b	-0.11	1.00000000	pigs
Wdr7	-0.17	1.00000000	pigs
Lsm3	-0.16	1.00000000	pigs
Dag1	-0.15	1.00000000	pigs
Pcgf3	-0.09	1.00000000	pigs
Trim8	-0.12	1.00000000	pigs
Igfbp7	-0.07	1.00000000	pigs
Ring1	-0.09	1.00000000	pigs
Ddx39a	-0.10	1.00000000	pigs
Pelp1	-0.08	1.00000000	pigs
Gcc2	-0.14	1.00000000	pigs
Stam2	-0.16	1.00000000	pigs
Rbis	-0.09	1.00000000	pigs
Ube2w	-0.18	1.00000000	pigs
Snrk	-0.11	1.00000000	pigs
Nrde2	-0.16	1.00000000	pigs
Pak2	-0.06	1.00000000	pigs
Cd5	-0.25	1.00000000	pigs
Cwc25	-0.12	1.00000000	pigs
Kctd13	-0.09	1.00000000	pigs

Ncoa1	-0.02	1.00000000	pigs
Slc7a6os	-0.07	1.00000000	pigs
Spcs1	-0.10	1.00000000	pigs
Etfdh	-0.09	1.00000000	pigs
Pnkd	-0.18	1.00000000	pigs
Rragc	-0.20	1.00000000	pigs
Zbtb38	-0.07	1.00000000	pigs
Cul2	-0.14	1.00000000	pigs
Slc25a37	-0.03	1.00000000	pigs
Pum3	-0.09	1.00000000	pigs
Scaper	-0.12	1.00000000	pigs
Cryl1	-0.12	1.00000000	pigs
Bckdhb	-0.16	1.00000000	pigs
Utp3	-0.12	1.00000000	pigs
Tra2b	-0.07	1.00000000	pigs
Arrb2	-0.11	1.00000000	pigs
Prkacb	-0.12	1.00000000	pigs
Ppp1ca	-0.20	1.00000000	pigs
Ppp1r16b	-0.12	1.00000000	pigs
Irf1	-0.12	1.00000000	pigs
Lonp2	-0.17	1.00000000	pigs
Nfx1	-0.16	1.00000000	pigs
Rap2a	-0.09	1.00000000	pigs
1700123O20Rik	-0.11	1.00000000	pigs
Smarce1	-0.10	1.00000000	pigs
Kctd20	-0.11	1.00000000	pigs
Fam168a	-0.06	1.00000000	pigs
Dcaf12	-0.13	1.00000000	pigs
Atf1	-0.18	1.00000000	pigs
Fundc2	-0.15	1.00000000	pigs
Rsu1	-0.21	1.00000000	pigs
Hook2	-0.05	1.00000000	pigs
Tmem50b	-0.10	1.00000000	pigs
Sass6	-0.07	1.00000000	pigs
Tsr3	-0.10	1.00000000	pigs
Brd9	-0.14	1.00000000	pigs
Ube2m	-0.11	1.00000000	pigs
Ube2e3	-0.13	1.00000000	pigs
Nek1	-0.11	1.00000000	pigs
Lclat1	-0.16	1.00000000	pigs
Atl3	-0.17	1.00000000	pigs
Ezh1	-0.14	1.00000000	pigs
Tmem50a	-0.16	1.00000000	pigs
Zbp1	-0.13	1.00000000	pigs
Htatsf1	-0.17	1.00000000	pigs
Prdx6	-0.12	1.00000000	pigs

Cep170	-0.11	1.00000000	pigs
Rnpepl1	-0.11	1.00000000	pigs
Nr4a1	-0.16	1.00000000	pigs
Pigu	-0.08	1.00000000	pigs
Immt	-0.12	1.00000000	pigs
Ccdc47	-0.14	1.00000000	pigs
Gtf2i	-0.20	1.00000000	pigs
Ssr4	-0.08	1.00000000	pigs
Fchsd2	-0.16	1.00000000	pigs
Itgam	-0.13	1.00000000	pigs
Ddx50	-0.17	1.00000000	pigs
B2m	0.00	1.00000000	pigs
Slc1a2	-0.30	1.00000000	pigs
Scfd1	-0.09	1.00000000	pigs
Dnmbp	-0.15	1.00000000	pigs
Prss55	-0.16	1.00000000	pigs
Parp1	-0.18	1.00000000	pigs
Rasal3	-0.16	1.00000000	pigs
Stard3nl	-0.09	1.00000000	pigs
Adar	-0.09	1.00000000	pigs
Lrrcc1	-0.08	1.00000000	pigs
Ebna1bp2	-0.08	1.00000000	pigs
3110082I17Rik	-0.17	1.00000000	pigs
Elk4	-0.13	1.00000000	pigs
Atrn	-0.12	1.00000000	pigs
Ubac1	-0.09	1.00000000	pigs
Pdgfa	-0.10	1.00000000	pigs
Plekhf2	-0.04	1.00000000	pigs
Actn1	-0.12	1.00000000	pigs
Sash3	-0.16	1.00000000	pigs
Ap5m1	-0.09	1.00000000	pigs
Ylpm1	-0.14	1.00000000	pigs
Dars	-0.15	1.00000000	pigs
Terf2ip	-0.15	1.00000000	pigs
Ccdc88c	-0.08	1.00000000	pigs
Cggbp1	-0.15	1.00000000	pigs
Nck1	-0.05	1.00000000	pigs
Bcl2a1d	-0.11	1.00000000	pigs
Spsb3	-0.09	1.00000000	pigs
Ninl	-0.12	1.00000000	pigs
Rsbn1l	-0.12	1.00000000	pigs
2300009A05Rik	-0.06	1.00000000	pigs
Pam16	-0.11	1.00000000	pigs
Dstyk	-0.12	1.00000000	pigs
Ptpdc1	-0.12	1.00000000	pigs
Ddx27	-0.14	1.00000000	pigs

Ccdc28a	-0.08	1.00000000	pigs
Tango6	-0.18	1.00000000	pigs
Grk2	-0.21	1.00000000	pigs
Itgb1	-0.09	1.00000000	pigs
Galk2	-0.09	1.00000000	pigs
Rbck1	-0.16	1.00000000	pigs
Srrm1	-0.03	1.00000000	pigs
Rwdd1	-0.21	1.00000000	pigs
Upp1	-0.18	1.00000000	pigs
Pitpnm2	-0.15	1.00000000	pigs
Slco5a1	-0.08	1.00000000	pigs
Pdia4	-0.12	1.00000000	pigs
Spns1	-0.11	1.00000000	pigs
Arid2	-0.12	1.00000000	pigs
Vps35	-0.17	1.00000000	pigs
Ntan1	-0.12	1.00000000	pigs
Nde1	-0.17	1.00000000	pigs
Lmf1	-0.10	1.00000000	pigs
Tinf2	-0.15	1.00000000	pigs
Parg	-0.14	1.00000000	pigs
Gsr	-0.01	1.00000000	pigs
Anxa1	-0.22	1.00000000	pigs
Ccnd3	-0.03	1.00000000	pigs
Tnrc18	-0.14	1.00000000	pigs
Hsp90b1	-0.12	1.00000000	pigs
Pdcd10	-0.16	1.00000000	pigs
Zmym1	-0.14	1.00000000	pigs
Pura	-0.11	1.00000000	pigs
Opa1	-0.18	1.00000000	pigs
Dennd6a	-0.16	1.00000000	pigs
Vps50	-0.15	1.00000000	pigs
Ndufaf2	-0.13	1.00000000	pigs
Stk10	-0.03	1.00000000	pigs
Flot1	-0.12	1.00000000	pigs
Dusp14	-0.11	1.00000000	pigs
Exoc6	-0.04	1.00000000	pigs
Rnf20	-0.08	1.00000000	pigs
Afg1l	-0.14	1.00000000	pigs
Srp14	-0.18	1.00000000	pigs
Slc35d1	-0.07	1.00000000	pigs
Pdcd1	-0.06	1.00000000	pigs
Thumpd3	-0.09	1.00000000	pigs
Pced1b	-0.02	1.00000000	pigs
Cdk7	-0.15	1.00000000	pigs
Prkaca	-0.12	1.00000000	pigs
Zfp438	-0.21	1.00000000	pigs

Eif2b3	-0.14	1.00000000	pigs
Gtf2f2	-0.14	1.00000000	pigs
Kpna3	-0.11	1.00000000	pigs
Ppib	-0.10	1.00000000	pigs
Imp4	-0.21	1.00000000	pigs
Uba1	-0.09	1.00000000	pigs
Aagab	-0.13	1.00000000	pigs
Trip11	-0.12	1.00000000	pigs
Cdipt	-0.07	1.00000000	pigs
Ndufa11	-0.17	1.00000000	pigs
Rnh1	-0.09	1.00000000	pigs
Zc3h6	-0.07	1.00000000	pigs
Tasor	-0.16	1.00000000	pigs
Snrpf	-0.19	1.00000000	pigs
Surf1	-0.12	1.00000000	pigs
Las1l	-0.09	1.00000000	pigs
Heatr5b	-0.14	1.00000000	pigs
Cdk2ap2	-0.22	1.00000000	pigs
Necap2	-0.08	1.00000000	pigs
Camkk2	-0.07	1.00000000	pigs
Dym	-0.21	1.00000000	pigs
Ube2r2	-0.08	1.00000000	pigs
Chml	-0.12	1.00000000	pigs
Emc10	-0.13	1.00000000	pigs
Abcd3	-0.08	1.00000000	pigs
Smad2	-0.16	1.00000000	pigs
Dapl1	0.00	1.00000000	pigs
Stag2	-0.09	1.00000000	pigs
Tcp11l2	-0.13	1.00000000	pigs
Scand1	-0.14	1.00000000	pigs
Srek1ip1	-0.10	1.00000000	pigs
Prmt9	-0.05	1.00000000	pigs
Sf3b2	-0.12	1.00000000	pigs
Chid1	-0.12	1.00000000	pigs
Lzic	-0.22	1.00000000	pigs
Tor4a	-0.07	1.00000000	pigs
Cdk19	-0.16	1.00000000	pigs
Cox5b-ps	-0.08	1.00000000	pigs
Brd7	-0.20	1.00000000	pigs
Sptbn1	-0.14	1.00000000	pigs
Faf1	-0.14	1.00000000	pigs
Cd2bp2	-0.13	1.00000000	pigs
Cnp	-0.13	1.00000000	pigs
Hmces	-0.11	1.00000000	pigs
Dcun1d5	-0.14	1.00000000	pigs
Gatad2a	-0.10	1.00000000	pigs

Rhob	-0.21	1.00000000	pigs
Cpt1a	-0.23	1.00000000	pigs
Psmc1	-0.11	1.00000000	pigs
Klhdc2	-0.15	1.00000000	pigs
Tes	-0.10	1.00000000	pigs
Smarcc1	-0.11	1.00000000	pigs
Arhgap45	-0.11	1.00000000	pigs
Sprtn	-0.10	1.00000000	pigs
Atr	-0.15	1.00000000	pigs
Arhgap12	-0.15	1.00000000	pigs
Zmynd11	-0.09	1.00000000	pigs
Rnf214	-0.12	1.00000000	pigs
Edf1	-0.13	1.00000000	pigs
Jakmip1	-0.15	1.00000000	pigs
Setx	-0.10	1.00000000	pigs
Ilrun	-0.10	1.00000000	pigs
Gpd2	-0.15	1.00000000	pigs
Lrrc8d	-0.05	1.00000000	pigs
Rcor3	-0.13	1.00000000	pigs
Guk1	-0.12	1.00000000	pigs
Pigc	-0.16	1.00000000	pigs
Ascc1	-0.16	1.00000000	pigs
Trak2	-0.16	1.00000000	pigs
Suclg2	-0.12	1.00000000	pigs
Dnaaf2	-0.06	1.00000000	pigs
Cstf2t	-0.08	1.00000000	pigs
Cdc42se1	-0.15	1.00000000	pigs
Actr1b	-0.10	1.00000000	pigs
Hmbox1	-0.16	1.00000000	pigs
Zmynd8	-0.19	1.00000000	pigs
Ndufb4	-0.18	1.00000000	pigs
Ccm2	-0.07	1.00000000	pigs
Ralbp1	-0.15	1.00000000	pigs
Sp110	-0.11	1.00000000	pigs
Tnrc6a	-0.13	1.00000000	pigs
Fam149b	-0.19	1.00000000	pigs
Klhl5	-0.14	1.00000000	pigs
Prmt7	-0.12	1.00000000	pigs
Abhd6	-0.13	1.00000000	pigs
Cox7a2l	-0.09	1.00000000	pigs
Tapt1	-0.11	1.00000000	pigs
Nfrkb	-0.10	1.00000000	pigs
Spopl	-0.07	1.00000000	pigs
Borcs5	-0.12	1.00000000	pigs
Rangap1	-0.14	1.00000000	pigs
Actg1	0.00	1.00000000	pigs

Abhd17b	-0.17	1.00000000	pigs
Romo1	-0.12	1.00000000	pigs
Usp1	-0.14	1.00000000	pigs
Taf8	-0.10	1.00000000	pigs
Idi1	-0.09	1.00000000	pigs
Fam133b	-0.14	1.00000000	pigs
Pex1	-0.12	1.00000000	pigs
Rnf217	-0.06	1.00000000	pigs
Supt4a	-0.10	1.00000000	pigs
Chmp4b	-0.12	1.00000000	pigs
Pik3r1	-0.10	1.00000000	pigs
Slc12a6	-0.19	1.00000000	pigs
Themis	0.07	1.00000000	pigs
Prr7	-0.13	1.00000000	pigs
Hpf1	-0.11	1.00000000	pigs
Exoc4	-0.05	1.00000000	pigs
Ccdc25	-0.17	1.00000000	pigs
Gyg	-0.15	1.00000000	pigs
Pvrig	-0.11	1.00000000	pigs
Pcmt1	-0.13	1.00000000	pigs
Cisd2	-0.21	1.00000000	pigs
Plxnc1	-0.19	1.00000000	pigs
Hspa5	-0.10	1.00000000	pigs
Washc4	-0.17	1.00000000	pigs
Snx4	-0.13	1.00000000	pigs
A130010J15Rik	-0.14	1.00000000	pigs
Arap1	-0.16	1.00000000	pigs
Zswim8	-0.04	1.00000000	pigs
Dennd4c	-0.15	1.00000000	pigs
Pdcd5	-0.23	1.00000000	pigs
Rab11fip3	-0.13	1.00000000	pigs
Edrf1	-0.16	1.00000000	pigs
Kat5	-0.09	1.00000000	pigs
Comtd1	-0.09	1.00000000	pigs
Prmt3	-0.12	1.00000000	pigs
Ing3	-0.16	1.00000000	pigs
Rbm17	-0.11	1.00000000	pigs
Rpa1	-0.14	1.00000000	pigs
Gemin4	-0.11	1.00000000	pigs
Tdpoz8	-0.09	1.00000000	pigs
Carnmt1	-0.10	1.00000000	pigs
Samd4b	-0.13	1.00000000	pigs
Gdi1	-0.07	1.00000000	pigs
Naa60	-0.11	1.00000000	pigs
Fig4	-0.17	1.00000000	pigs
Clstn1	-0.09	1.00000000	pigs

Pdcd6	-0.14	1.00000000	pigs
Sec11a	-0.13	1.00000000	pigs
Glg1	-0.16	1.00000000	pigs
Cep63	-0.14	1.00000000	pigs
Gpx4	-0.17	1.00000000	pigs
Copb2	-0.14	1.00000000	pigs
Erp44	-0.17	1.00000000	pigs
Dnajc19	-0.12	1.00000000	pigs
Tmx3	-0.19	1.00000000	pigs
Lamtor1	-0.16	1.00000000	pigs
Eapp	-0.16	1.00000000	pigs
Pcbp1	-0.08	1.00000000	pigs
H1f4	-0.16	1.00000000	pigs
Ubxn2b	-0.05	1.00000000	pigs
Zer1	-0.15	1.00000000	pigs
Sumf1	-0.10	1.00000000	pigs
Selenop	-0.09	1.00000000	pigs
N4bp2	-0.18	1.00000000	pigs
Dynlrb1	-0.15	1.00000000	pigs
Fmc1	-0.11	1.00000000	pigs
Nars2	-0.10	1.00000000	pigs
Zmym4	-0.17	1.00000000	pigs
Trappc4	-0.11	1.00000000	pigs
Tgfbr2	-0.01	1.00000000	pigs
Ttc33	-0.11	1.00000000	pigs
Fam13b	-0.14	1.00000000	pigs
Pfdn6	-0.15	1.00000000	pigs
Tbrg1	-0.17	1.00000000	pigs
Ppp1r12c	-0.10	1.00000000	pigs
Xpa	-0.16	1.00000000	pigs
Fndc3b	-0.57	1.00000000	pigs
Acot4	-0.11	1.00000000	pigs
Tgif1	-0.11	1.00000000	pigs
Fam78a	-0.12	1.00000000	pigs
Rab29	-0.13	1.00000000	pigs
Rnf169	-0.11	1.00000000	pigs
Ap1s2	-0.14	1.00000000	pigs
Ndufa10	-0.12	1.00000000	pigs
Zfp638	-0.17	1.00000000	pigs
Hars	-0.11	1.00000000	pigs
Ogfr	-0.09	1.00000000	pigs
Pef1	-0.06	1.00000000	pigs
Dyrk2	-0.11	1.00000000	pigs
Lgals3	0.07	1.00000000	pigs
Ssr2	-0.07	1.00000000	pigs
Arhgdia	-0.09	1.00000000	pigs

Nipsnap2	-0.09	1.00000000	pigs
Wdr45	-0.08	1.00000000	pigs
Scaf8	-0.14	1.00000000	pigs
Arel1	-0.07	1.00000000	pigs
Ctsf	-0.10	1.00000000	pigs
Trpc4ap	-0.14	1.00000000	pigs
2810004N23Rik	-0.19	1.00000000	pigs
Spryd7	-0.12	1.00000000	pigs
Wdr70	-0.10	1.00000000	pigs
Pik3r5	-0.04	1.00000000	pigs
Klhl7	-0.12	1.00000000	pigs
Siah2	-0.13	1.00000000	pigs
Trappc3	-0.11	1.00000000	pigs
Stambp	-0.16	1.00000000	pigs
P3h1	-0.07	1.00000000	pigs
Ttc38	-0.12	1.00000000	pigs
Tatdn1	-0.15	1.00000000	pigs
Dbt	-0.11	1.00000000	pigs
Adam17	-0.27	1.00000000	pigs
Tbck	-0.15	1.00000000	pigs
Tmem134	-0.20	1.00000000	pigs
Ambra1	-0.12	1.00000000	pigs
Snf8	-0.15	1.00000000	pigs
Nudc	-0.14	1.00000000	pigs
Ago4	-0.12	1.00000000	pigs
Trim38	-0.09	1.00000000	pigs
Ino80	-0.17	1.00000000	pigs
Stxbp2	-0.15	1.00000000	pigs
Nubp1	-0.10	1.00000000	pigs
Tbx21	-0.14	1.00000000	pigs
Vrk3	-0.13	1.00000000	pigs
H2-Ab1	0.00	1.00000000	pigs
Tradd	-0.07	1.00000000	pigs
Eif4g3	-0.09	1.00000000	pigs
Ndufaf3	-0.18	1.00000000	pigs
Hsf2	-0.16	1.00000000	pigs
Nsrp1	-0.11	1.00000000	pigs
Prkd3	-0.08	1.00000000	pigs
Hadh	-0.10	1.00000000	pigs
Ssna1	-0.15	1.00000000	pigs
Srbd1	-0.16	1.00000000	pigs
Lpxn	-0.08	1.00000000	pigs
Sap30bp	-0.05	1.00000000	pigs
Parp12	-0.21	1.00000000	pigs
Tstd2	-0.09	1.00000000	pigs
Pin1	-0.16	1.00000000	pigs

Apaf1	-0.04	1.00000000	pigs
