# Supplementary material for: Heterogeneous viral-host response at single-cell resolution and viral adaptive mutational atlas in birds and mammals upon H5 influenza virus infection
Source: Natl Sci Rev. 2026 Jun 25;13(14):nwag380. doi: 10.1093/nsr/nwag380 (PMC13458601; doi:10.1093/nsr/nwag380)
Supplement: Supplementary file 1 [file nwag380_supplemental_files.zip › Supplementary Data 13.pdf]

plementary Data 13. Differentially expressed genes of endothelial across different species upon H5 HPAIV infec

gene	log2FoldChange	p value	host
Sox6	-4.83	0.00000000	duck
Tnrc6b	-3.61	0.00000000	duck
Creg1	-3.62	0.00000000	duck
Vps13b	-3.55	0.00000000	duck
Rhag	-4.09	0.00000000	duck
Epb41	-3.70	0.00000000	duck
Itpk1	-4.17	0.00000000	duck
Ripor3	-2.79	0.00000000	duck
Atrx	-2.48	0.00000000	duck
Vash1	-3.26	0.00000000	duck
Narf	-3.84	0.00000000	duck
Ank1	-3.12	0.00000000	duck
Epas1	-2.42	0.00000000	duck
Sptb	-2.90	0.00000000	duck
Fos	2.17	0.00000000	duck
Synm	-2.15	0.00000000	duck
Fryl	-1.95	0.00000000	duck
Zmiz1	2.17	0.00000000	duck
Tfrc	-3.69	0.00000000	duck
Pmp22	2.05	0.00000000	duck
Sptan1	-2.90	0.00000000	duck
Jun	1.96	0.00000000	duck
Rfesd	-2.88	0.00000000	duck
Pls3	1.78	0.00000000	duck
Ipmk	-2.94	0.00000000	duck
Actb	1.70	0.00000000	duck
Herc2	-3.53	0.00000000	duck
D5Ertd579e	-3.24	0.00000000	duck
Zbtb16	2.16	0.00000000	duck
Anxa2	2.30	0.00000000	duck
Flnb	1.54	0.00000000	duck
Anxa5	1.78	0.00000000	duck
Igf1r	1.84	0.00000000	duck
Zbtb20	2.10	0.00000000	duck
Klf4	2.90	0.00000000	duck
Ifnar1	-2.76	0.00000000	duck
Pard3b	2.27	0.00000000	duck
Slc43a3	-2.40	0.00000000	duck
Herc1	-2.17	0.00000000	duck
Stxbp6	2.45	0.00000000	duck
Sema3d	-2.85	0.00000000	duck
Timp3	1.42	0.00000000	duck

Fxr1	-2.48	0.00000000	duck
Anxa1	3.20	0.00000000	duck
Fbrsl1	1.64	0.00000000	duck
Reep1	-2.79	0.00000000	duck
Dcun1d2	-2.77	0.00000000	duck
Cebpd	1.87	0.00000000	duck
Tmem108	3.12	0.00000000	duck
Pgrmc1	2.05	0.00000000	duck
Rundc1	1.52	0.00000000	duck
Slc25a37	-2.68	0.00000000	duck
Tmem64	1.81	0.00000000	duck
Nt5dc2	-2.25	0.00000000	duck
Foxp2	2.45	0.00000000	duck
Epb41l4a	2.22	0.00000000	duck
Carmil1	1.09	0.00000000	duck
Wwc2	1.96	0.00000000	duck
Ammecr1	-2.89	0.00000000	duck
Cldn18	2.74	0.00000000	duck
Ccny	1.44	0.00000000	duck
Fosb	2.01	0.00000000	duck
Nrp1	1.91	0.00000000	duck
Ptpn14	2.07	0.00000000	duck
Dusp1	2.27	0.00000000	duck
Stim2	1.67	0.00000000	duck
Arrdc3	1.42	0.00000000	duck
Ski	1.37	0.00000000	duck
Sh3bgrl	0.97	0.00000000	duck
Slc44a1	1.55	0.00000000	duck
Ptpn21	1.78	0.00000000	duck
Trak1	2.01	0.00000000	duck
Ncoa4	-2.67	0.00000000	duck
Pbx1	1.68	0.00000000	duck
Zswim6	1.90	0.00000000	duck
Dock5	1.81	0.00000000	duck
Jdp2	2.04	0.00000000	duck
Ppp3ca	1.99	0.00000000	duck
Lamc1	1.56	0.00000000	duck
Plxnb2	1.67	0.00000000	duck
Fgfr2	1.96	0.00000000	duck
Ndrg1	1.61	0.00000000	duck
Stat1	1.57	0.00000000	duck
Larp1b	-2.60	0.00000000	duck
Taok3	-2.34	0.00000000	duck
Rbms3	1.64	0.00000000	duck
Gata6	1.60	0.00000000	duck
Rassf9	1.55	0.00000000	duck

Ccser1	2.52	0.00000000	duck
Jund	1.59	0.00000000	duck
Tox2	1.85	0.00000000	duck
Lrrc1	1.82	0.00000000	duck
Rtn1	1.87	0.00000000	duck
Lmo7	2.61	0.00000000	duck
Emp1	1.83	0.00000000	duck
Nfil3	1.87	0.00000000	duck
Lrrn4	2.05	0.00000000	duck
Nr4a3	1.65	0.00000000	duck
Tpm2	1.39	0.00000000	duck
Ptprf	1.98	0.00000000	duck
Rap1b	1.28	0.00000000	duck
Azin1	1.28	0.00000000	duck
Arhgap21	1.19	0.00000000	duck
Rab20	1.68	0.00000000	duck
Wbp4	-2.36	0.00000000	duck
S100a11	1.94	0.00000000	duck
Actn1	1.26	0.00000000	duck
Slc6a6	-1.36	0.00000000	duck
Usp6nl	1.32	0.00000000	duck
Dctn3	1.37	0.00000000	duck
Ptprm	1.06	0.00000000	duck
Ctnna1	1.29	0.00000000	duck
Pard3	1.42	0.00000000	duck
Hipk3	1.67	0.00000000	duck
Ncor2	1.36	0.00000000	duck
Tmem35b	1.39	0.00000000	duck
Trip12	-2.44	0.00000000	duck
Zfp704	1.24	0.00000000	duck
Csrnp3	2.13	0.00000000	duck
Swap70	1.41	0.00000000	duck
Lmna	1.27	0.00000000	duck
Epcam	1.36	0.00000000	duck
Baiap2l1	1.77	0.00000000	duck
Calm1	1.18	0.00000000	duck
Zfhx3	1.24	0.00000000	duck
F3	1.55	0.00000000	duck
Rapgef2	1.94	0.00000000	duck
Spred1	1.63	0.00000000	duck
Hpx	2.30	0.00000000	duck
Stom	1.43	0.00000000	duck
Klf6	1.27	0.00000000	duck
Tcf7l2	1.39	0.00000000	duck
Atp12a	2.01	0.00000000	duck
Ddx17	-1.91	0.00000000	duck

Stard13	1.83	0.00000000	duck
Afap1	2.37	0.00000000	duck
Cux1	1.10	0.00000000	duck
Trio	1.51	0.00000000	duck
Smad6	1.25	0.00000000	duck
Dram1	1.24	0.00000000	duck
Nfkbiz	1.61	0.00000000	duck
Rufy3	1.50	0.00000000	duck
Plekha5	1.99	0.00000000	duck
Sema3c	2.05	0.00000000	duck
Calm2	1.49	0.00000000	duck
Frmd4b	1.28	0.00000000	duck
Snrpd1	1.09	0.00000000	duck
Gpcpd1	-2.01	0.00000000	duck
Hecw2	1.39	0.00000000	duck
Twsg1	1.34	0.00000000	duck
Csrp1	1.32	0.00000000	duck
Snrpg	1.22	0.00000000	duck
B3gat2	2.33	0.00000000	duck
Samhd1	1.53	0.00000000	duck
Sgpp1	1.23	0.00000000	duck
Setbp1	1.48	0.00000000	duck
Kras	1.18	0.00000000	duck
Snx30	1.25	0.00000000	duck
Rasal2	1.47	0.00000000	duck
Jak2	-0.98	0.00000000	duck
Abcc1	-2.83	0.00000000	duck
Slc46a2	1.65	0.00000000	duck
Jph1	-2.39	0.00000000	duck
Hipk2	1.10	0.00000000	duck
Itsn1	1.56	0.00000000	duck
Sec11c	1.19	0.00000000	duck
Bbx	1.10	0.00000000	duck
Ago2	-2.10	0.00000000	duck
Cdh1	1.33	0.00000000	duck
Nceh1	1.30	0.00000000	duck
Ndufa4	1.24	0.00000000	duck
Ccpg1	-1.75	0.00000000	duck
Cobl	1.79	0.00000000	duck
Egfr	1.60	0.00000000	duck
Sri	1.05	0.00000000	duck
Hlf	1.29	0.00000000	duck
Robo2	2.24	0.00000000	duck
Pacsin2	1.34	0.00000000	duck
Phf3	-2.07	0.00000000	duck
Nrg1	1.98	0.00000000	duck

Hspa8	1.94	0.00000000	duck
Yap1	1.26	0.00000000	duck
Unc5c	2.43	0.00000000	duck
Ano5	0.97	0.00000000	duck
Itpr2	1.16	0.00000000	duck
Nt5c3	-2.47	0.00000000	duck
Pard6b	1.40	0.00000000	duck
Wwtr1	1.47	0.00000000	duck
Asph	1.73	0.00000000	duck
Filip1	1.52	0.00000000	duck
Lrpap1	1.00	0.00000000	duck
Nhs	2.38	0.00000000	duck
Sh3bgrl3	1.32	0.00000000	duck
Eml4	1.31	0.00000000	duck
Dip2c	1.20	0.00000000	duck
Rai2	1.08	0.00000000	duck
Ndufb10	0.93	0.00000000	duck
Tjp1	1.13	0.00000000	duck
Arhgef3	1.32	0.00000000	duck
Zdhhc14	1.52	0.00000000	duck
Ost4	1.24	0.00000000	duck
Reep5	1.10	0.00000000	duck
Etv3	1.07	0.00000000	duck
Atp11a	2.11	0.00000000	duck
Gas7	1.14	0.00000000	duck
Smad7	1.27	0.00000000	duck
Cgnl1	1.29	0.00000000	duck
Cdc42bpa	1.19	0.00000000	duck
Ppfibp2	1.33	0.00000000	duck
Bcor	1.32	0.00000000	duck
St6gal1	-2.14	0.00000000	duck
Fam3c	1.26	0.00000000	duck
Ier5	1.26	0.00000000	duck
Cdo1	1.55	0.00000000	duck
Gpc4	1.38	0.00000000	duck
Rasef	1.49	0.00000000	duck
Fam13a	1.38	0.00000000	duck
Ube2h	-1.78	0.00000000	duck
Peak1	0.97	0.00000000	duck
Ssr1	1.11	0.00000000	duck
Dstn	0.95	0.00000000	duck
Csrnp1	1.27	0.00000000	duck
Epb41l5	1.23	0.00000000	duck
Cidea	1.26	0.00000000	duck
Pik3cb	1.35	0.00000000	duck
Rsu1	1.16	0.00000000	duck

Gpx3	1.43	0.00000000	duck
Tomm7	1.10	0.00000000	duck
Celsr1	1.32	0.00000000	duck
Trpm6	2.19	0.00000000	duck
Spred2	1.30	0.00000000	duck
Ccdc50	1.29	0.00000000	duck
Cpm	1.60	0.00000000	duck
Dnaja1	1.02	0.00000000	duck
Lrp5	1.21	0.00000000	duck
Zfp36l1	1.01	0.00000000	duck
Sdc2	1.47	0.00000000	duck
Pbx4	-2.32	0.00000000	duck
Fabp3	1.32	0.00000000	duck
Cd151	1.01	0.00000000	duck
Pde10a	1.43	0.00000000	duck
Rbm24	-1.96	0.00000000	duck
Klhl29	2.47	0.00000000	duck
Atf3	1.30	0.00000000	duck
Erp29	0.92	0.00000000	duck
Plac8	1.78	0.00000000	duck
Igsf9	0.96	0.00000000	duck
Ppib	1.44	0.00000000	duck
Prkce	1.14	0.00000000	duck
Gadd45g	1.44	0.00000000	duck
Atp5j	1.03	0.00000000	duck
Svil	1.12	0.00000000	duck
Arhgdia	0.96	0.00000000	duck
Slc4a4	2.17	0.00000000	duck
Ccn2	1.08	0.00000000	duck
Cd9	0.97	0.00000000	duck
Lasp1	1.08	0.00000000	duck
Fat1	1.39	0.00000000	duck
Naaa	1.49	0.00000000	duck
Irx3	1.16	0.00000000	duck
Fosl2	0.99	0.00000000	duck
Peli2	1.16	0.00000000	duck
Clta	1.07	0.00000000	duck
Cebpa	1.46	0.00000000	duck
Nebl	1.72	0.00000000	duck
Fnbp1l	1.05	0.00000000	duck
Plekha7	1.30	0.00000000	duck
Agap1	1.38	0.00000000	duck
Afdn	0.94	0.00000000	duck
Actn4	0.91	0.00000000	duck
Bmpr1a	1.29	0.00000000	duck
Irf2bpl	1.28	0.00000000	duck

Lhfp	1.50	0.00000000	duck
Osbpl5	1.73	0.00000000	duck
Smad3	0.98	0.00000000	duck
Sec61g	1.25	0.00000000	duck
Klf15	1.13	0.00000000	duck
Rpn1	0.90	0.00000000	duck
Trim8	1.24	0.00000000	duck
Cask	1.18	0.00000000	duck
Enpp1	1.21	0.00000000	duck
Als2cl	1.19	0.00000000	duck
Atp1a1	0.97	0.00000000	duck
Cpt1a	0.91	0.00000000	duck
Atp5b	1.10	0.00000000	duck
Itgb1	0.95	0.00000000	duck
Sema5a	1.70	0.00000000	duck
Tmcc1	-2.19	0.00000000	duck
Kdelr2	1.03	0.00000000	duck
Pmepa1	1.07	0.00000000	duck
Hivep3	1.93	0.00000000	duck
Ankrd12	-1.67	0.00000000	duck
Prex1	1.27	0.00000000	duck
Unc13b	1.42	0.00000000	duck
Id2	1.22	0.00000000	duck
Tle4	1.65	0.00000000	duck
Runx1t1	1.52	0.00000000	duck
Ppargc1a	1.28	0.00000000	duck
Bri3	0.94	0.00000000	duck
Srpk2	1.21	0.00000000	duck
Pdia4	1.17	0.00000000	duck
Stx19	1.11	0.00000000	duck
Tpm1	1.00	0.00000000	duck
Sh3gl3	1.11	0.00000000	duck
Slc35f3	1.73	0.00000000	duck
Npc1	1.35	0.00000000	duck
H13	1.20	0.00000000	duck
Wdfy3	1.00	0.00000000	duck
Baz2b	1.11	0.00000000	duck
Pkm	1.05	0.00000000	duck
Rnf223	1.42	0.00000000	duck
Rhpn2	0.87	0.00000000	duck
Dhrs3	1.25	0.00000000	duck
Veph1	1.25	0.00000000	duck
Tnrc18	0.94	0.00000000	duck
Tkt	0.82	0.00000000	duck
Atp5j2	0.96	0.00000000	duck
Alas1	1.15	0.00000000	duck

Dynll2	0.95	0.00000000	duck
Samd4	1.31	0.00000000	duck
Sema6a	1.46	0.00000000	duck
Slc30a10	-1.91	0.00000000	duck
Tnfsf10	1.23	0.00000000	duck
Pdlim5	1.27	0.00000000	duck
Arpc1b	1.34	0.00000000	duck
Echdc2	0.92	0.00000000	duck
Plpp7	-1.66	0.00000000	duck
Hk2	0.67	0.00000000	duck
Chka	1.75	0.00000000	duck
Dusp7	1.13	0.00000000	duck
Polr2f	0.99	0.00000000	duck
Irs2	1.26	0.00000000	duck
Bag5	-2.08	0.00000000	duck
Ccn1	1.37	0.00000000	duck
Tmem248	1.01	0.00000000	duck
Shroom2	1.11	0.00000000	duck
Arvcf	1.05	0.00000000	duck
Hsd17b12	0.96	0.00000000	duck
Sh3pxd2a	0.93	0.00000000	duck
Rbfox2	1.18	0.00000000	duck
Tmem258	0.82	0.00000000	duck
Tead1	1.10	0.00000000	duck
Nr3c2	1.31	0.00000000	duck
Ktn1	0.81	0.00000000	duck
Cdc14a	-2.57	0.00000000	duck
Dag1	0.81	0.00000000	duck
Efnb1	1.11	0.00000000	duck
Xdh	1.28	0.00000000	duck
Plcxd1	-2.38	0.00000000	duck
Clic4	1.03	0.00000000	duck
Romo1	0.69	0.00000000	duck
Adgrl2	0.84	0.00000000	duck
Dkk3	1.01	0.00000000	duck
Hmgb3	0.94	0.00000000	duck
Rel	1.04	0.00000000	duck
Canx	0.92	0.00000000	duck
Pdia6	0.88	0.00000000	duck
Ptpn3	1.14	0.00000000	duck
Prdm16	1.31	0.00000000	duck
Ctbs	0.82	0.00000000	duck
Akap13	1.21	0.00000000	duck
Ror1	1.39	0.00000000	duck
Aqp3	-2.48	0.00000000	duck
Ank3	1.63	0.00000000	duck

Slc22a23	1.12	0.00000000	duck
Sbno1	-1.65	0.00000000	duck
Sh3kbp1	1.04	0.00000000	duck
Fchsd2	1.19	0.00000000	duck
Sntb1	1.44	0.00000000	duck
Hif1a	0.86	0.00000000	duck
Itga9	1.43	0.00000000	duck
Rab11fip1	1.08	0.00000000	duck
Tmx3	0.84	0.00000000	duck
Cdkal1	0.99	0.00000000	duck
Naaladl2	1.42	0.00000000	duck
Cxadr	1.05	0.00000000	duck
Atp5g3	0.94	0.00000000	duck
Ssr3	0.89	0.00000000	duck
Cat	0.79	0.00000000	duck
Ctnnb1	0.70	0.00000000	duck
Smim20	0.90	0.00000000	duck
Fam13b	0.93	0.00000000	duck
Atp11b	0.76	0.00000000	duck
Thsd7a	1.23	0.00000000	duck
Atxn10	1.58	0.00000000	duck
Nek7	0.61	0.00000000	duck
Actr2	0.92	0.00000000	duck
Rnd3	1.31	0.00000000	duck
Ssr2	0.93	0.00000000	duck
Rbm47	1.09	0.00000000	duck
Ube2e2	1.63	0.00000000	duck
Lrmda	1.07	0.00000000	duck
Tiam1	1.18	0.00000000	duck
Ppl	0.97	0.00000000	duck
Ubr2	-2.16	0.00000000	duck
Fbxo3	-2.11	0.00000000	duck
Atp5a1	0.84	0.00000000	duck
Rhoa	0.95	0.00000000	duck
Tsc1	-2.03	0.00000000	duck
Osmr	1.28	0.00000000	duck
Creb5	1.79	0.00000000	duck
Pdk3	-2.04	0.00000000	duck
Arl6ip5	0.67	0.00000000	duck
Klf10	1.01	0.00000000	duck
Pag1	1.23	0.00000000	duck
Aptx	0.73	0.00000000	duck
Erh	0.66	0.00000000	duck
Ybx3	-1.86	0.00000000	duck
Nefl	1.31	0.00000000	duck
Naxd	0.83	0.00000000	duck

Atf4	0.98	0.00000000	duck
Atp1b3	0.89	0.00000000	duck
Parva	1.01	0.00000000	duck
Ssb	0.80	0.00000000	duck
Lifr	1.17	0.00000000	duck
Hmox1	1.91	0.00000000	duck
Dennd4c	1.08	0.00000000	duck
Rnf19b	1.08	0.00000000	duck
Adgrg6	1.44	0.00000000	duck
Tubb1	-1.74	0.00000000	duck
Ran	0.79	0.00000000	duck
Selenoo	-1.97	0.00000000	duck
Nsa2	0.64	0.00000000	duck
Ablim2	1.28	0.00000000	duck
Yaf2	0.83	0.00000000	duck
Fbxl17	1.09	0.00000000	duck
Syt16	1.10	0.00000000	duck
Plbd2	0.79	0.00000000	duck
Dock1	0.95	0.00000000	duck
Borcs6	0.75	0.00000000	duck
Rab12	0.93	0.00000000	duck
Arpc3	0.74	0.00000000	duck
Wnt5a	0.93	0.00000000	duck
Chd7	0.77	0.00000000	duck
Ndufa6	0.77	0.00000000	duck
Cpeb4	-1.91	0.00000000	duck
Septin11	1.30	0.00000000	duck
Id3	1.41	0.00000000	duck
Odc1	0.99	0.00000000	duck
Setd7	0.79	0.00000000	duck
Smpd1	0.82	0.00000000	duck
Ank	0.82	0.00000000	duck
Rbm31y	0.92	0.00000000	duck
Gxylt1	0.98	0.00000000	duck
Ext1	1.39	0.00000000	duck
Tln1	0.83	0.00000000	duck
Bhlhe40	1.16	0.00000000	duck
Nrp2	1.10	0.00000000	duck
Slc43a2	0.84	0.00000000	duck
Snx10	1.45	0.00000000	duck
Hnrnpk	0.65	0.00000000	duck
Tpm4	0.91	0.00000000	duck
Hsp90b1	1.11	0.00000000	duck
Cebpb	1.16	0.00000000	duck
Foxa2	0.62	0.00000000	duck
Lpar1	1.63	0.00000000	duck

Ranbp10	-2.63	0.00000000	duck
Acvr2a	1.03	0.00000000	duck
Npc2	0.93	0.00000000	duck
Adam10	-1.51	0.00000000	duck
Esrp2	1.08	0.00000000	duck
Prxl2c	0.97	0.00000000	duck
Bmpr1b	1.84	0.00000000	duck
Slc45a4	-1.93	0.00000000	duck
Pigx	0.57	0.00000000	duck
Snrpf	0.76	0.00000000	duck
Ica1	0.80	0.00000000	duck
Thoc2	-1.74	0.00000000	duck
Gpc5	1.33	0.00000000	duck
Pawr	1.00	0.00000000	duck
Hmgb2	1.04	0.00000000	duck
Pdzrn3	1.31	0.00000000	duck
Tent5a	1.26	0.00000000	duck
Zfp706	0.79	0.00000000	duck
Khdrbs3	1.47	0.00000000	duck
Trim25	0.97	0.00000000	duck
Elf3	1.33	0.00000000	duck
Slc35f5	0.85	0.00000000	duck
Aopep	1.53	0.00000000	duck
Phldb2	1.01	0.00000000	duck
Psd3	1.04	0.00000000	duck
Cd81	0.87	0.00000000	duck
Eif4ebp1	0.88	0.00000000	duck
Sipa1l1	0.92	0.00000000	duck
Urm1	0.79	0.00000000	duck
Pde5a	0.48	0.00000000	duck
Txn1	1.04	0.00000000	duck
Pfn2	0.58	0.00000000	duck
Arih1	-1.86	0.00000000	duck
Bicd2	0.76	0.00000000	duck
Sdc1	1.63	0.00000000	duck
Hadh	0.68	0.00000000	duck
Nucks1	0.71	0.00000000	duck
Serpinh1	0.96	0.00000000	duck
Nhsl1	1.18	0.00000000	duck
Lpin2	-2.01	0.00000000	duck
Gnai2	1.03	0.00000000	duck
Cmtm6	0.85	0.00000000	duck
Auh	0.57	0.00000000	duck
Ttc7	0.92	0.00000000	duck
Hint1	0.91	0.00000000	duck
Fam43a	0.62	0.00000000	duck

Ephx1	0.81	0.00000000	duck
Ndufb2	0.89	0.00000000	duck
Plcb4	1.12	0.00000000	duck
Reps2	0.93	0.00000000	duck
Creb3l2	0.72	0.00000000	duck
Ssbp2	-2.69	0.00000000	duck
Tmem131l	-1.55	0.00000000	duck
Spsb1	0.74	0.00000000	duck
Gna12	0.78	0.00000000	duck
Rere	0.81	0.00000000	duck
Cttnbp2nl	0.79	0.00000000	duck
Acvr1b	0.73	0.00000000	duck
Xpa	0.76	0.00000000	duck
Nupl1	0.79	0.00000000	duck
Slc26a9	0.79	0.00000000	duck
Rbms1	0.87	0.00000000	duck
Taf13	0.67	0.00000000	duck
Pdlim1	1.10	0.00000000	duck
Ppm1l	0.92	0.00000000	duck
Hacd4	2.10	0.00000000	duck
Fmn1	0.74	0.00000000	duck
Cnn3	0.64	0.00000000	duck
Fgd3	1.07	0.00000000	duck
Nprl3	-2.45	0.00000000	duck
Crybg1	0.77	0.00000000	duck
Sytl5	1.08	0.00000000	duck
Jag2	1.30	0.00000000	duck
Sh3rf1	1.13	0.00000000	duck
Rasgef1a	0.96	0.00000000	duck
Cdc42ep3	0.70	0.00000000	duck
Adamts2	1.00	0.00000000	duck
Ak2	-2.15	0.00000000	duck
Atrn	0.64	0.00000000	duck
Cyyr1	1.00	0.00000000	duck
Tmem98	0.80	0.00000000	duck
Ppp1ca	0.66	0.00000000	duck
Vav2	1.22	0.00000000	duck
Pdgfb	1.37	0.00000000	duck
Deptor	0.92	0.00000000	duck
Tob1	0.96	0.00000000	duck
Klf12	1.18	0.00000000	duck
Thsd4	1.64	0.00000000	duck
Park7	0.71	0.00000000	duck
Cfd	1.86	0.00000000	duck
Dpysl2	1.10	0.00000000	duck
Spry1	0.73	0.00000000	duck

Slc49a4	0.82	0.00000000	duck
Fgfr3	1.00	0.00000000	duck
Vmp1	0.57	0.00000000	duck
Wwox	1.43	0.00000000	duck
Fli1	1.33	0.00000000	duck
Sel1l3	0.84	0.00000000	duck
Shtn1	0.85	0.00000000	duck
Tanc2	0.95	0.00000000	duck
Agrn	0.94	0.00000000	duck
Nfkb1	0.74	0.00000000	duck
Igfbp3	1.18	0.00000000	duck
Ppp1r13b	0.76	0.00000000	duck
Plekhg4	0.71	0.00000000	duck
Sash1	0.86	0.00000000	duck
Sh3bp2	0.73	0.00000000	duck
Glod4	0.71	0.00000000	duck
Fth1	-1.04	0.00000000	duck
Nedd4l	0.73	0.00000000	duck
Togaram2	0.89	0.00000000	duck
Ddost	0.68	0.00000000	duck
Ackr3	-1.70	0.00000000	duck
Lims2	0.98	0.00000000	duck
Eif4a2	0.77	0.00000000	duck
Eea1	0.69	0.00000000	duck
Epsti1	0.71	0.00000000	duck
Lfng	0.98	0.00000000	duck
Epha5	1.15	0.00000000	duck
Elovl7	0.92	0.00000000	duck
Atp2a2	0.76	0.00000000	duck
Cert1	0.71	0.00000000	duck
Kalrn	1.74	0.00000000	duck
Oat	0.83	0.00000000	duck
Vps37b	0.83	0.00000000	duck
Rpn2	0.70	0.00000000	duck
Pdzd2	1.52	0.00000000	duck
Cobll1	0.59	0.00000000	duck
Serpine2	1.07	0.00000000	duck
Entpd5	-1.75	0.00000000	duck
Cep170b	0.74	0.00000000	duck
Notch2	0.70	0.00000000	duck
Ubac2	0.79	0.00000000	duck
Cavin1	0.87	0.00000000	duck
Zfand5	0.88	0.00000000	duck
Igf2bp3	1.00	0.00000000	duck
Ndfip1	0.77	0.00000000	duck
Hbegf	0.90	0.00000000	duck

Ncoa1	0.83	0.00000000	duck
Blvra	-1.59	0.00000000	duck
Rrbp1	0.64	0.00000000	duck
Pdia3	0.76	0.00000000	duck
Cited4	1.28	0.00000000	duck
Npas2	1.30	0.00000000	duck
Dbnl	0.89	0.00000000	duck
Bach2	1.28	0.00000000	duck
Wwc1	0.98	0.00000000	duck
Rhpn1	0.94	0.00000000	duck
Hsdl2	0.55	0.00000000	duck
Prdx4	0.64	0.00000000	duck
Fam177a	0.77	0.00000000	duck
Socs3	0.87	0.00000000	duck
Xxylt1	0.52	0.00000000	duck
S100a13	0.82	0.00000000	duck
Tubb4b	0.70	0.00000000	duck
Fnbp1	1.07	0.00000000	duck
Ppm1e	1.99	0.00000000	duck
Trappc9	0.81	0.00000000	duck
Ptprk	0.85	0.00000000	duck
Rnf145	0.69	0.00000000	duck
Sdad1	0.89	0.00000000	duck
Clip1	0.69	0.00000000	duck
Acer3	0.98	0.00000000	duck
Dctn6	0.64	0.00000000	duck
Osbpl1a	-1.86	0.00000000	duck
Prkcd	0.82	0.00000000	duck
Il6st	1.05	0.00000000	duck
Pdlim4	1.01	0.00000000	duck
Arf1	0.90	0.00000000	duck
Abca3	0.80	0.00000000	duck
Rock2	0.82	0.00000000	duck
Stt3a	0.62	0.00000000	duck
Sec62	0.65	0.00000000	duck
Fasn	1.02	0.00000001	duck
Pgm1	0.71	0.00000001	duck
Tacc3	-2.13	0.00000001	duck
Rassf8	0.64	0.00000001	duck
Bnip3l	-2.01	0.00000001	duck
Osbpl3	0.78	0.00000001	duck
Ncald	0.73	0.00000001	duck
Usp25	0.82	0.00000001	duck
Arhgef26	0.74	0.00000001	duck
Yes1	0.67	0.00000001	duck
Ppm1f	0.94	0.00000001	duck

Abtb2	1.40	0.00000001	duck
Dclk1	1.07	0.00000001	duck
Tlnrd1	1.33	0.00000001	duck
Atrnl1	1.33	0.00000001	duck
Ndufs7	0.59	0.00000001	duck
Vdac2	0.60	0.00000001	duck
Actr3	0.71	0.00000001	duck
Usp48	-1.68	0.00000001	duck
Prkca	1.05	0.00000001	duck
Ripor1	0.83	0.00000001	duck
Slc39a8	0.97	0.00000001	duck
Pim3	0.95	0.00000001	duck
Atp8b1	0.70	0.00000001	duck
Stk10	0.61	0.00000001	duck
Etl4	1.39	0.00000001	duck
Ttc28	0.75	0.00000001	duck
Tspan12	0.70	0.00000001	duck
Irx5	0.68	0.00000001	duck
Echs1	0.71	0.00000001	duck
1700034I23Rik	0.52	0.00000001	duck
Tubb5	0.72	0.00000001	duck
B3gnt7	0.92	0.00000001	duck
Inpp5a	1.13	0.00000001	duck
Slc9a7	0.71	0.00000001	duck
Rnf144a	1.29	0.00000001	duck
Ppic	0.58	0.00000001	duck
Lipa	0.63	0.00000001	duck
Stxbp1	0.86	0.00000001	duck
Irf7	0.92	0.00000001	duck
Pik3ip1	0.51	0.00000001	duck
Gsn	0.76	0.00000001	duck
Nab1	0.62	0.00000001	duck
Sorbs1	2.20	0.00000001	duck
Dusp10	0.98	0.00000001	duck
Ybx1	0.79	0.00000001	duck
Fndc5	0.81	0.00000001	duck
Plce1	0.99	0.00000001	duck
Bmpr2	0.50	0.00000001	duck
Arl9	0.59	0.00000001	duck
Rassf7	0.60	0.00000001	duck
Gpx2	-2.22	0.00000001	duck
Efna5	1.32	0.00000001	duck
Chsy3	1.42	0.00000001	duck
Hdac1	0.89	0.00000001	duck
Rhof	0.98	0.00000001	duck
Hmgn3	0.74	0.00000002	duck

Btg1b	0.66	0.00000002	duck
Ppp1cb	-1.47	0.00000002	duck
Spats2l	1.37	0.00000002	duck
Ndufa10	0.57	0.00000002	duck
Slc9a3r1	0.99	0.00000002	duck
Rnf130	1.13	0.00000002	duck
Efna1	0.58	0.00000002	duck
Rab11fip4	0.72	0.00000002	duck
Rac1	0.71	0.00000002	duck
Ttc39b	0.98	0.00000002	duck
Ap1ar	0.51	0.00000002	duck
Dhcr24	0.63	0.00000002	duck
Arfip2	0.63	0.00000002	duck
Pam	0.74	0.00000002	duck
Capn7	-1.90	0.00000002	duck
Eef2k	0.83	0.00000002	duck
Trp53bp2	0.78	0.00000002	duck
Atp1b1	0.78	0.00000002	duck
Cript	0.90	0.00000002	duck
Anapc2	1.23	0.00000003	duck
Sorbs3	0.63	0.00000003	duck
Tppp3	1.00	0.00000003	duck
Rab11fip2	0.79	0.00000003	duck
Tnfrsf19	0.82	0.00000003	duck
Fer	0.82	0.00000003	duck
Tmem164	0.59	0.00000003	duck
Ndufa8	0.58	0.00000003	duck
Prkd1	0.80	0.00000003	duck
Arhgap12	0.77	0.00000003	duck
Acer2	1.05	0.00000003	duck
Kctd20	-2.14	0.00000003	duck
Cttn	0.57	0.00000003	duck
Coro1c	0.93	0.00000003	duck
Tlcd1	0.68	0.00000003	duck
Lgals3	0.72	0.00000003	duck
Ilrun	-1.78	0.00000004	duck
Igfbp1	1.35	0.00000004	duck
Sestd1	0.49	0.00000004	duck
Tob2	0.84	0.00000004	duck
Ripk4	0.79	0.00000004	duck
Sgce	0.58	0.00000004	duck
Acvr1	0.72	0.00000004	duck
Tspan13	0.84	0.00000004	duck
Tor4a	1.12	0.00000004	duck
Tmem63b	0.83	0.00000004	duck
Fnip2	1.00	0.00000004	duck

Scnn1a	0.68	0.00000004	duck
Ndufb9	0.56	0.00000004	duck
Alcam	1.17	0.00000004	duck
Ndufs4	0.48	0.00000004	duck
Pik3ap1	0.65	0.00000004	duck
Tmem50a	0.64	0.00000005	duck
Nsmce2	0.77	0.00000005	duck
Foxa1	0.76	0.00000005	duck
Bcr	0.34	0.00000005	duck
Commd1b	0.95	0.00000005	duck
Cav2	0.83	0.00000005	duck
Fkbp4	0.69	0.00000005	duck
Upk1b	1.16	0.00000006	duck
Rcor1	0.80	0.00000006	duck
Rcn2	0.65	0.00000006	duck
Foxk1	0.52	0.00000006	duck
Tmem9b	0.49	0.00000006	duck
Tpmt	0.81	0.00000006	duck
Slc12a2	-2.37	0.00000006	duck
Atp2b1	0.59	0.00000006	duck
Gnptab	0.66	0.00000006	duck
Ciao2b	0.44	0.00000006	duck
Nudt21	0.87	0.00000006	duck
Cd44	1.56	0.00000007	duck
Hyi	0.44	0.00000007	duck
Acat1	0.55	0.00000007	duck
Col8a1	1.00	0.00000007	duck
Abcg1	0.80	0.00000007	duck
Guk1	0.42	0.00000008	duck
Rnf4	-1.71	0.00000008	duck
Gnptg	0.45	0.00000008	duck
Syngr2	0.63	0.00000008	duck
Spcs2	0.64	0.00000008	duck
Ergic1	0.39	0.00000009	duck
Amotl2	0.65	0.00000009	duck
Dbi	0.65	0.00000009	duck
Ogfrl1	0.75	0.00000010	duck
Gpr137b	0.70	0.00000010	duck
Baiap2	0.65	0.00000010	duck
Zc2hc1a	0.86	0.00000010	duck
Hspb8	0.60	0.00000010	duck
Syt8	0.78	0.00000010	duck
Cachd1	0.93	0.00000011	duck
Stx6	0.48	0.00000011	duck
Prelid1	0.65	0.00000011	duck
Kazn	0.79	0.00000011	duck

Hdac4	0.75	0.00000011	duck
Lman1	0.77	0.00000011	duck
Sdhb	0.62	0.00000012	duck
Pde8a	-1.05	0.00000012	duck
Tm2d2	0.53	0.00000012	duck
Aldoc	0.89	0.00000012	duck
Hunk	0.94	0.00000012	duck
Phlda2	1.09	0.00000012	duck
Insig1	1.18	0.00000013	duck
Psmb3	0.44	0.00000013	duck
Cables1	0.64	0.00000013	duck
Galnt1	0.58	0.00000013	duck
Rab7	0.71	0.00000014	duck
Tmem245	0.58	0.00000014	duck
Sdf2l1	0.44	0.00000014	duck
Spats2	0.65	0.00000014	duck
Npnt	0.45	0.00000014	duck
Lpcat3	0.52	0.00000015	duck
Ilf2	0.44	0.00000015	duck
Nifk	0.65	0.00000016	duck
Sox5	1.36	0.00000016	duck
Pcmtd1	-1.90	0.00000016	duck
Nap1l1	0.40	0.00000016	duck
Lratd2	0.76	0.00000016	duck
Ppp6r3	-2.06	0.00000017	duck
Gpi1	0.71	0.00000017	duck
AW554918	0.69	0.00000017	duck
Ywhab	0.54	0.00000017	duck
Ppara	0.62	0.00000018	duck
Psma3	0.70	0.00000018	duck
Carhsp1	1.04	0.00000018	duck
Lsm5	0.41	0.00000018	duck
Lzts3	0.67	0.00000019	duck
Kif21a	0.59	0.00000019	duck
Stat3	0.62	0.00000019	duck
Tns1	0.87	0.00000021	duck
Sft2d2	0.66	0.00000022	duck
Timm13	0.64	0.00000022	duck
Sgms2	0.83	0.00000022	duck
Arpc5	0.55	0.00000022	duck
Frrs1	-1.20	0.00000023	duck
Itm2a	-1.17	0.00000024	duck
Pnrc1	0.72	0.00000024	duck
Polr2d	0.52	0.00000024	duck
Fam172a	0.44	0.00000024	duck
Gsg1	0.81	0.00000025	duck

Cdc42bpb	0.49	0.00000025	duck
Tmod4	-0.99	0.00000025	duck
Tnfaip3	0.78	0.00000025	duck
Pcgf5	0.28	0.00000025	duck
Ctif	1.11	0.00000026	duck
Apbb2	1.25	0.00000027	duck
Rai14	1.72	0.00000027	duck
Txndc17	0.37	0.00000027	duck
Tgfbr3	0.93	0.00000027	duck
Cald1	1.10	0.00000028	duck
Rnf20	0.70	0.00000029	duck
Plagl1	0.61	0.00000030	duck
Lrba	0.78	0.00000032	duck
Ggcx	0.60	0.00000032	duck
Vill	0.76	0.00000032	duck
Psmb1	0.53	0.00000034	duck
Edem1	0.52	0.00000034	duck
Arxes2	0.72	0.00000035	duck
Purb	0.72	0.00000035	duck
Psma7	0.65	0.00000035	duck
Antkmt	0.58	0.00000036	duck
Rhou	0.61	0.00000036	duck
Iqgap1	0.41	0.00000037	duck
Herpud1	0.82	0.00000038	duck
Bicc1	0.83	0.00000038	duck
Itga1	0.49	0.00000039	duck
Arf6	0.63	0.00000040	duck
Fgfbp1	1.80	0.00000040	duck
Lurap1l	0.80	0.00000040	duck
Fermt2	0.82	0.00000041	duck
Pxylp1	0.70	0.00000041	duck
Slc20a2	0.53	0.00000041	duck
Ndufb1	0.62	0.00000042	duck
Ripk2	0.60	0.00000042	duck
Nav2	0.87	0.00000043	duck
Lcor	0.56	0.00000047	duck
Bnip3	0.62	0.00000047	duck
Cbfb	0.84	0.00000048	duck
Ptafr	1.02	0.00000049	duck
Zfp516	0.82	0.00000050	duck
Cav1	0.64	0.00000050	duck
Ei24	0.35	0.00000051	duck
Yipf3	0.49	0.00000051	duck
Emc2	0.60	0.00000052	duck
Cnih4	0.75	0.00000052	duck
Ostf1	0.65	0.00000053	duck

Sema5b	1.33	0.00000054	duck
Bin3	1.10	0.00000056	duck
Sccpdh	0.81	0.00000056	duck
Chst9	1.17	0.00000057	duck
Fat3	0.75	0.00000060	duck
Slc9a2	0.62	0.00000060	duck
B3gnt2	0.65	0.00000060	duck
Tab2	-1.92	0.00000061	duck
Pnisr	-1.57	0.00000065	duck
Arl13b	0.72	0.00000065	duck
Polr2l	0.53	0.00000066	duck
Ston2	0.59	0.00000066	duck
Septin8	0.65	0.00000067	duck
Kpna4	-1.65	0.00000067	duck
Kdm7a	-1.54	0.00000072	duck
Fyn	0.83	0.00000072	duck
Phb	0.44	0.00000072	duck
Tspan4	1.02	0.00000073	duck
Dennd11	0.70	0.00000076	duck
Gns	0.42	0.00000077	duck
Tmbim6	0.36	0.00000083	duck
Iqsec1	0.73	0.00000084	duck
Dut	0.76	0.00000087	duck
Taldo1	0.57	0.00000090	duck
Dyrk3	-1.89	0.00000091	duck
Anxa4	0.35	0.00000092	duck
Exph5	0.77	0.00000092	duck
Gdi2	0.44	0.00000093	duck
Wrn	-1.17	0.00000095	duck
Commd8	0.56	0.00000097	duck
Prdx6	0.50	0.00000098	duck
Acyp2	0.65	0.00000098	duck
Sec61a1	0.42	0.00000100	duck
Sptlc1	0.36	0.00000101	duck
Crot	0.59	0.00000105	duck
Psmb4	0.50	0.00000106	duck
Ccnyl1	0.53	0.00000109	duck
Nampt	0.68	0.00000116	duck
Ccnh	0.50	0.00000120	duck
Atp6v1d	0.67	0.00000120	duck
Lima1	0.71	0.00000122	duck
Gramd1c	0.60	0.00000124	duck
Ralbp1	0.89	0.00000126	duck
Arl8b	0.49	0.00000127	duck
Ncoa7	0.69	0.00000128	duck
Dazap2	0.57	0.00000129	duck

Znrf2	0.61	0.00000136	duck
Srp19	0.53	0.00000142	duck
Fmnl2	0.68	0.00000143	duck
Cmtm8	0.82	0.00000145	duck
Snapc5	0.50	0.00000146	duck
Rad51b	0.69	0.00000147	duck
Commd2	0.28	0.00000149	duck
Tfpi	1.72	0.00000153	duck
Arhgap28	0.99	0.00000158	duck
Polr2e	0.50	0.00000160	duck
Glcci1	0.36	0.00000160	duck
Cbx7	0.47	0.00000160	duck
Ptpn2	-1.24	0.00000162	duck
Sdhaf4	0.46	0.00000172	duck
Disc1	0.97	0.00000175	duck
Dgkh	0.88	0.00000180	duck
Fermt1	0.44	0.00000183	duck
C1s1	0.58	0.00000188	duck
Coro2a	0.59	0.00000188	duck
Slit3	1.12	0.00000192	duck
Otud6b	0.62	0.00000196	duck
Fzd8	0.59	0.00000197	duck
Arhgap42	0.84	0.00000200	duck
Tle3	0.47	0.00000202	duck
Kif1b	0.17	0.00000204	duck
Fam214a	0.30	0.00000207	duck
Arl1	0.35	0.00000212	duck
Commd10	0.55	0.00000213	duck
Gabpa	0.35	0.00000214	duck
Eif1	0.95	0.00000230	duck
Lmf1	0.67	0.00000230	duck
Acsf2	0.47	0.00000240	duck
Hspa12a	0.59	0.00000241	duck
Cnih1	0.61	0.00000241	duck
Capn2	0.54	0.00000242	duck
Tbc1d2	0.68	0.00000249	duck
Eif3f	0.45	0.00000252	duck
Snx17	0.49	0.00000252	duck
Kpna3	0.45	0.00000253	duck
Hddc2	0.40	0.00000258	duck
Fam174a	0.32	0.00000260	duck
Lmf2	0.48	0.00000264	duck
Asxl2	-1.17	0.00000269	duck
Fuca2	0.74	0.00000282	duck
Cct2	0.49	0.00000285	duck
Snrpb	0.48	0.00000293	duck

Imp3	0.57	0.00000294	duck
Arid5b	0.59	0.00000300	duck
Psma5	0.30	0.00000301	duck
Emc3	0.46	0.00000303	duck
Tgfbr2	1.02	0.00000312	duck
Snrpd3	0.29	0.00000330	duck
Tmed2	0.50	0.00000357	duck
Vav3	1.16	0.00000361	duck
Sort1	0.50	0.00000370	duck
Slc46a3	-1.57	0.00000387	duck
Smurf1	0.60	0.00000396	duck
Sema6d	1.36	0.00000412	duck
Tmem223	0.59	0.00000413	duck
Ctss	0.37	0.00000413	duck
Ilk	0.80	0.00000420	duck
Exoc6b	0.48	0.00000457	duck
Atp6ap2	0.50	0.00000459	duck
Itgav	0.53	0.00000467	duck
Zfyve27	0.45	0.00000468	duck
Arl15	0.56	0.00000476	duck
Anxa7	0.49	0.00000477	duck
Ddx54	0.49	0.00000478	duck
Tox3	0.95	0.00000487	duck
Pmm2	0.66	0.00000494	duck
Shisa5	0.68	0.00000502	duck
Arid1a	0.46	0.00000506	duck
Wdr1	0.45	0.00000510	duck
Nfia	0.78	0.00000515	duck
Pold2	0.38	0.00000515	duck
Fgd4	0.93	0.00000519	duck
Camta1	0.39	0.00000529	duck
Pola1	0.68	0.00000530	duck
Eif3g	0.47	0.00000533	duck
Wasl	0.38	0.00000550	duck
Asah1	0.41	0.00000561	duck
Saal1	0.64	0.00000562	duck
Scp2	0.21	0.00000563	duck
Ssrp1	0.39	0.00000571	duck
Ttc7b	0.71	0.00000573	duck
Itprid2	0.55	0.00000576	duck
Ntng1	0.95	0.00000611	duck
Dlgap4	0.52	0.00000611	duck
Txnrd1	-1.96	0.00000613	duck
Txn2	0.89	0.00000616	duck
Lrp4	0.78	0.00000635	duck
Emc6	0.67	0.00000637	duck

Dync1h1	0.34	0.00000643	duck
Vgll4	0.63	0.00000645	duck
Rcc2	0.61	0.00000648	duck
Col12a1	0.79	0.00000652	duck
Nr4a1	0.96	0.00000654	duck
Itgb6	0.79	0.00000656	duck
Grhl2	0.55	0.00000666	duck
Phlpp1	1.01	0.00000692	duck
Lsm8	0.59	0.00000694	duck
Slc44a3	0.73	0.00000705	duck
Cep89	0.52	0.00000713	duck
Lbr	-1.59	0.00000718	duck
Tnfaip8	0.54	0.00000726	duck
Suox	-1.70	0.00000730	duck
Lpcat1	0.68	0.00000736	duck
Raph1	0.56	0.00000745	duck
Fzd1	0.58	0.00000755	duck
Ssbp1	0.29	0.00000757	duck
Tmem243	0.89	0.00000761	duck
Tox	0.81	0.00000779	duck
Tmem199	0.62	0.00000784	duck
Limd2	0.91	0.00000785	duck
Fzd6	0.53	0.00000789	duck
P4hb	0.27	0.00000816	duck
Smim14	0.23	0.00000860	duck
Tbc1d5	0.73	0.00000884	duck
Ulk2	0.51	0.00000894	duck
Ptprs	0.81	0.00000899	duck
Zhx3	0.65	0.00000900	duck
Fgf2	0.83	0.00000905	duck
Lcp2	1.46	0.00000929	duck
Psma6	0.50	0.00000936	duck
Ipo5	0.49	0.00000937	duck
Nek6	0.55	0.00000982	duck
Septin9	0.97	0.00000988	duck
Taf15	0.37	0.00000989	duck
Ccni	0.21	0.00001024	duck
Esyt2	0.58	0.00001038	duck
Selenof	0.48	0.00001041	duck
Arhgdib	1.06	0.00001054	duck
Eml1	1.10	0.00001080	duck
Parp8	0.99	0.00001083	duck
Psmd7	0.24	0.00001089	duck
Tapt1	-1.73	0.00001100	duck
Zhx1	0.30	0.00001132	duck
Hip1	0.23	0.00001136	duck

Crybg3	-1.81	0.00001181	duck
Stmn1	0.58	0.00001198	duck
Sergef	0.79	0.00001257	duck
Hmgcr	0.58	0.00001263	duck
Hmgxb4	0.40	0.00001322	duck
Txndc12	0.51	0.00001329	duck
Glul	0.60	0.00001331	duck
Ephb3	0.69	0.00001344	duck
Senp5	0.28	0.00001361	duck
Smdt1	0.59	0.00001393	duck
Cope	0.56	0.00001446	duck
Cxxc4	1.30	0.00001456	duck
Pcca	0.67	0.00001478	duck
B3gnt5	0.38	0.00001490	duck
Tmem158	1.11	0.00001498	duck
Tspo	0.44	0.00001511	duck
Btf3l4	0.45	0.00001514	duck
Fam53b	0.95	0.00001523	duck
Casd1	-1.93	0.00001528	duck
Ccng2	0.35	0.00001575	duck
Lsm4	0.45	0.00001576	duck
Suclg2	0.45	0.00001604	duck
Cndp2	0.51	0.00001604	duck
Tpi1	0.57	0.00001617	duck
Dip2b	0.58	0.00001620	duck
Atp1b4	0.53	0.00001660	duck
Dusp5	0.63	0.00001690	duck
Esrrg	0.49	0.00001717	duck
Gli3	0.71	0.00001731	duck
Slc41a2	0.48	0.00001735	duck
Polr2k	0.53	0.00001823	duck
Stau2	0.64	0.00001866	duck
Rab31	0.59	0.00001898	duck
Nos2	0.99	0.00001919	duck
Ranbp1	0.27	0.00001947	duck
Decr2	0.45	0.00001993	duck
Sap30	0.44	0.00002003	duck
Ankrd28	-1.49	0.00002010	duck
Tm9sf3	0.42	0.00002022	duck
Pkp4	0.56	0.00002027	duck
Selenop	0.21	0.00002078	duck
Wdr37	0.63	0.00002099	duck
Il13ra1	0.59	0.00002108	duck
Eprs	0.36	0.00002174	duck
Retreg1	0.44	0.00002274	duck
Rdh10	0.40	0.00002277	duck

Angptl4	1.01	0.00002295	duck
Psmb2	0.33	0.00002313	duck
Zdhhc7	0.60	0.00002341	duck
Spryd7	0.47	0.00002362	duck
Tcirg1	0.48	0.00002390	duck
Commd4	0.51	0.00002390	duck
Srebf2	0.65	0.00002400	duck
Ttc33	0.28	0.00002421	duck
Spata5	0.68	0.00002499	duck
Thyn1	0.56	0.00002541	duck
Atp2b4	0.31	0.00002542	duck
Arg2	0.66	0.00002591	duck
Yeats2	0.69	0.00002592	duck
Bcl2	0.90	0.00002599	duck
Snx8	0.39	0.00002629	duck
Smap1	0.30	0.00002643	duck
Zyx	0.88	0.00002726	duck
Tle1	0.88	0.00002760	duck
Serinc5	0.49	0.00002787	duck
Srfbp1	0.38	0.00002834	duck
Phf21a	0.38	0.00002946	duck
Scoc	0.58	0.00002959	duck
Hdac7	0.73	0.00002999	duck
Akt3	0.97	0.00003004	duck
Kcnrg	0.47	0.00003036	duck
C3	0.54	0.00003041	duck
Cry1	0.53	0.00003085	duck
Acaca	0.68	0.00003095	duck
Agtpbp1	0.59	0.00003124	duck
Chpt1	0.55	0.00003129	duck
Gtf2a2	0.26	0.00003168	duck
Eif3i	0.32	0.00003169	duck
Ttf2	0.50	0.00003205	duck
Prss23	0.71	0.00003237	duck
Ftcd	0.86	0.00003255	duck
Ywhae	0.35	0.00003285	duck
Tns3	0.78	0.00003331	duck
Fam20c	0.73	0.00003335	duck
Vegfa	1.23	0.00003342	duck
Srgn	1.18	0.00003362	duck
Tc2n	0.70	0.00003407	duck
Adgrg2	0.68	0.00003488	duck
Hsbp1	0.30	0.00003499	duck
Large1	0.67	0.00003514	duck
Wee1	0.42	0.00003516	duck
Cnot9	0.50	0.00003518	duck

Gstk1	0.43	0.00003567	duck
Gpalpp1	0.50	0.00003579	duck
Ninj1	0.54	0.00003593	duck
Ippk	0.51	0.00003650	duck
Efnb2	1.09	0.00003665	duck
Net1	0.99	0.00003666	duck
Idh2	0.44	0.00003674	duck
Piezo2	1.31	0.00003747	duck
Atp13a3	0.21	0.00003780	duck
Art4	-0.96	0.00003784	duck
Garem1	0.47	0.00003787	duck
Pla2g4a	0.79	0.00003798	duck
Serinc2	0.44	0.00003887	duck
Cnpy3	0.61	0.00003917	duck
Igf2bp2	0.53	0.00003936	duck
Nup107	0.79	0.00004078	duck
Chp1	0.44	0.00004108	duck
Brix1	0.69	0.00004161	duck
Rexo2	0.39	0.00004223	duck
Nus1	0.43	0.00004249	duck
Tmem30a	0.36	0.00004306	duck
Lgals8	-1.45	0.00004343	duck
Tomm22	0.43	0.00004389	duck
Flot1	0.40	0.00004438	duck
Rxra	0.37	0.00004446	duck
Ano3	0.93	0.00004462	duck
Eif4ebp2	0.34	0.00004497	duck
Steap3	-1.64	0.00004530	duck
Slc15a4	0.25	0.00004561	duck
Ugp2	0.51	0.00004580	duck
Kitl	0.68	0.00004604	duck
Pip4p2	0.47	0.00004690	duck
Sub1	0.19	0.00004698	duck
Nubpl	0.59	0.00004811	duck
Acsbg2	0.83	0.00004841	duck
Ext2	0.61	0.00004847	duck
Rnf217	0.46	0.00004888	duck
Snrpc	0.40	0.00004965	duck
Rora	0.78	0.00005035	duck
Fam117b	0.58	0.00005225	duck
Hpgds	0.47	0.00005328	duck
Prkab1	0.35	0.00005413	duck
Sh3d19	0.70	0.00005495	duck
Zdhhc15	0.45	0.00005498	duck
Adm	0.75	0.00005515	duck
Bckdhb	0.55	0.00005516	duck

Rai1	0.70	0.00005813	duck
Pigt	0.53	0.00005865	duck
Osbp2	0.81	0.00005942	duck
Slc16a6	-1.40	0.00005980	duck
Zbtb10	0.60	0.00006017	duck
Ppip5k2	0.71	0.00006089	duck
Btbd11	0.91	0.00006168	duck
Optn	0.40	0.00006267	duck
Hs1bp3	0.46	0.00006298	duck
Zfp36l2	0.56	0.00006375	duck
Thbs1	1.19	0.00006440	duck
Hmgcs1	0.74	0.00006502	duck
Zc3h12a	0.70	0.00006588	duck
Lyn	0.59	0.00006671	duck
Igf2bp1	0.84	0.00006684	duck
Otud1	0.87	0.00006837	duck
Glis2	0.44	0.00006880	duck
Cped1	1.00	0.00007025	duck
Stk24	0.54	0.00007130	duck
Stam	0.53	0.00007132	duck
Supt5	0.58	0.00007147	duck
Pef1	0.43	0.00007265	duck
Ubl3	-0.08	0.00007398	duck
Grm7	0.68	0.00007599	duck
Isoc1	0.50	0.00007690	duck
Fam174b	0.44	0.00007798	duck
Slco2a1	0.75	0.00007887	duck
Pld1	0.56	0.00007891	duck
Ypel1	0.61	0.00007953	duck
Pak4	0.42	0.00008166	duck
Txnrd3	0.36	0.00008172	duck
Bzw2	0.37	0.00008281	duck
Bend7	0.50	0.00008283	duck
Pgap2	0.30	0.00008451	duck
Lamtor5	0.50	0.00008886	duck
Etv6	0.48	0.00008932	duck
Snx31	0.57	0.00009019	duck
Lpgat1	0.81	0.00009084	duck
Ndufb4	0.41	0.00009091	duck
Prdm11	0.77	0.00009145	duck
Fbln5	0.74	0.00009182	duck
Ppie	0.27	0.00009200	duck
Tgm2	0.69	0.00009422	duck
Nfkb2	0.34	0.00009639	duck
Lipg	0.53	0.00009649	duck
Pelo	0.51	0.00009653	duck

Cbx6	0.45	0.00009702	duck
Lrch1	0.35	0.00009739	duck
Cep85l	0.56	0.00009776	duck
Sh3yl1	0.45	0.00009815	duck
Tra2b	0.26	0.00010185	duck
Cadm1	0.71	0.00010342	duck
Hadhb	0.32	0.00010560	duck
Nfe2l1	0.31	0.00010613	duck
Tcp1	0.49	0.00010667	duck
L3mbtl3	0.61	0.00010674	duck
Nono	0.32	0.00010738	duck
Pdap1	0.24	0.00010940	duck
Chsy1	0.57	0.00011007	duck
Als2	0.67	0.00011045	duck
Appl2	0.37	0.00011140	duck
Lrp6	0.44	0.00011356	duck
Surf4	0.43	0.00011456	duck
Clint1	0.36	0.00011523	duck
Tst	0.43	0.00011607	duck
Dock7	0.66	0.00011735	duck
Rlf	-1.69	0.00012035	duck
Rab3il1	-1.56	0.00012144	duck
Stk26	-1.11	0.00012175	duck
Cisd1	0.41	0.00012538	duck
Hnrnpa0	0.12	0.00012631	duck
Lysmd2	0.38	0.00012674	duck
Atp5pb	0.37	0.00012926	duck
Tpk1	0.64	0.00012961	duck
Tnfsf15	0.93	0.00013220	duck
Lrrfip2	0.34	0.00013307	duck
Ghr	0.75	0.00013721	duck
Fam78a	-1.25	0.00013741	duck
Rac2	1.06	0.00013751	duck
St3gal1	0.28	0.00014045	duck
Agpat5	0.45	0.00014387	duck
Spock3	0.98	0.00014568	duck
Nipsnap2	0.30	0.00014649	duck
Nt5dc1	0.57	0.00014775	duck
Tenm2	0.89	0.00014815	duck
Lamtor2	0.29	0.00014971	duck
Rtf1	0.25	0.00014977	duck
Limch1	0.71	0.00015502	duck
Rbm19	0.50	0.00016009	duck
Eif2ak3	0.20	0.00016137	duck
Nabp1	0.53	0.00016192	duck
Ralgps2	0.51	0.00016212	duck

Ywhah	0.34	0.00016358	duck
Serinc3	0.22	0.00016517	duck
Immp2l	0.90	0.00016573	duck
Sgms1	-1.26	0.00016754	duck
Vwa8	0.42	0.00016758	duck
Ppp6c	0.49	0.00016767	duck
Ncoa2	0.13	0.00016781	duck
Limk2	0.45	0.00017055	duck
Btbd1	0.49	0.00017216	duck
Cdk5rap3	0.35	0.00017249	duck
Rnf19a	0.43	0.00017262	duck
Snx6	0.23	0.00017594	duck
Jazf1	0.59	0.00017689	duck
Snx2	0.14	0.00017875	duck
Bcl9	0.50	0.00018005	duck
Dlg1	0.63	0.00018626	duck
Sstr4	1.46	0.00018640	duck
Nedd4	0.44	0.00018707	duck
Kctd1	0.36	0.00018838	duck
Ptbp2	0.30	0.00018988	duck
Ankrd10	0.26	0.00019027	duck
Ano6	0.80	0.00019125	duck
Neo1	0.52	0.00019204	duck
Wdfy2	0.80	0.00019406	duck
Dynlrb1	0.38	0.00019512	duck
Dapk3	0.37	0.00019596	duck
Bcl9l	0.48	0.00020165	duck
Nsfl1c	0.43	0.00020254	duck
Rangap1	0.41	0.00020299	duck
Tuft1	0.40	0.00020311	duck
Tle5	0.37	0.00021064	duck
Siva1	0.45	0.00021144	duck
Nipa2	0.32	0.00021289	duck
Sema4d	0.74	0.00021530	duck
Pmvk	0.49	0.00021549	duck
Aldh7a1	0.48	0.00021637	duck
Dusp16	0.33	0.00021649	duck
Paqr7	0.57	0.00022072	duck
Sesn3	0.48	0.00022120	duck
Pid1	0.86	0.00022349	duck
Rgl1	0.85	0.00022805	duck
Ranbp17	0.83	0.00022930	duck
Suclg1	0.48	0.00023145	duck
Plekha1	0.44	0.00023248	duck
Ndufaf3	0.43	0.00023402	duck
Atic	0.52	0.00024105	duck

Acsl4	0.44	0.00024267	duck
Pinx1	0.42	0.00024296	duck
Cds1	0.41	0.00024613	duck
Cib1	0.53	0.00024633	duck
Abi2	0.66	0.00025085	duck
Ammecr1l	0.55	0.00025228	duck
Clmn	0.56	0.00025456	duck
Crls1	0.41	0.00025776	duck
Slc25a29	0.51	0.00026214	duck
Cemip2	1.40	0.00026658	duck
Rras2	0.35	0.00027335	duck
Dbt	0.68	0.00027841	duck
Tmem179b	0.44	0.00028331	duck
Clic3	0.48	0.00029028	duck
Kcnk1	0.42	0.00029699	duck
Pxn	0.48	0.00030199	duck
2210408I21Rik	0.65	0.00030597	duck
Tjp3	0.37	0.00030615	duck
Rnh1	0.51	0.00030957	duck
Larp4b	0.33	0.00031138	duck
Tmem242	0.44	0.00031538	duck
Ptpru	0.37	0.00032316	duck
Srp72	0.26	0.00032919	duck
Lamp2	0.56	0.00034299	duck
Ppa1	0.27	0.00034553	duck
Fam117a	-1.58	0.00034683	duck
Trappc3	0.32	0.00034864	duck
Polg2	0.76	0.00035301	duck
Add2	-1.02	0.00035451	duck
Rgs3	0.95	0.00035492	duck
Atg12	0.69	0.00036137	duck
Vps54	0.57	0.00036181	duck
Nvl	0.43	0.00036780	duck
Numb	0.47	0.00037401	duck
Pfdn4	0.55	0.00037443	duck
Rsl24d1	0.03	0.00037921	duck
Slc2a1	0.42	0.00038143	duck
Ophn1	0.75	0.00040410	duck
Sec11a	0.20	0.00041194	duck
Spry2	0.50	0.00041642	duck
Thada	0.61	0.00041799	duck
Tmco3	-1.71	0.00042577	duck
Ubn2	0.27	0.00042750	duck
Ccser2	0.53	0.00043142	duck
Dusp6	0.66	0.00044445	duck
Arfgef3	0.46	0.00044449	duck

Usp44	0.45	0.00044483	duck
Tom1l1	0.40	0.00044532	duck
Plpp2	0.31	0.00044596	duck
Adamts8	0.63	0.00044766	duck
Slc45a3	0.36	0.00046651	duck
Ccng1	0.42	0.00047171	duck
Traf3	0.41	0.00047343	duck
Plcg2	0.92	0.00047373	duck
Psmd2	0.08	0.00047597	duck
Bach1	-1.36	0.00048305	duck
Fam135a	0.78	0.00049216	duck
Smim13	0.44	0.00050028	duck
Stag2	-1.23	0.00051130	duck
Adcy9	-0.81	0.00051143	duck
Ulk1	-1.57	0.00051270	duck
Bfsp2	0.41	0.00051583	duck
Sik2	0.42	0.00052238	duck
Trim14	0.36	0.00052450	duck
Tbxas1	1.75	0.00053293	duck
Fndc3b	0.51	0.00053555	duck
Itpr1	0.65	0.00054123	duck
Ilvbl	0.42	0.00055782	duck
Soat1	0.45	0.00056345	duck
Lman2	0.58	0.00058041	duck
Slc44a2	0.40	0.00058108	duck
Fgd6	0.59	0.00058169	duck
Gnpat	0.34	0.00059096	duck
Itpripl2	0.49	0.00059348	duck
Hdgfl3	0.59	0.00060433	duck
Secisbp2	0.40	0.00060801	duck
Nck2	0.35	0.00061171	duck
Bud31	0.33	0.00061494	duck
Slit2	0.94	0.00062279	duck
Gas6	0.59	0.00062474	duck
Snrnp40	0.42	0.00063107	duck
Ebna1bp2	0.63	0.00063111	duck
Eif3d	0.20	0.00063431	duck
Arhgap29	0.78	0.00064224	duck
Fbxw7	0.42	0.00064258	duck
Zdhhc21	0.50	0.00064683	duck
Ostm1	0.63	0.00067311	duck
Tmco1	0.26	0.00067406	duck
Palld	-2.44	0.00068021	duck
Jmjd8	0.47	0.00068837	duck
Flvcr1	0.62	0.00069254	duck
Copa	0.33	0.00069917	duck

Plat	-1.51	0.00071037	duck
Rims1	0.76	0.00071133	duck
Ets1	0.58	0.00071517	duck
Pwp1	0.38	0.00072291	duck
Tax1bp3	0.39	0.00073414	duck
Oaz1	0.57	0.00075492	duck
Slc2a12	0.39	0.00075810	duck
Pwwp2a	0.34	0.00076042	duck
Abcb6	-1.03	0.00076382	duck
Afap1l2	0.48	0.00077769	duck
Klf7	0.34	0.00078216	duck
Zswim5	0.59	0.00080694	duck
Ccdc85c	0.58	0.00081917	duck
Ehbp1	0.55	0.00082018	duck
Ppid	0.32	0.00082032	duck
Cct5	0.26	0.00082363	duck
Rilpl2	0.34	0.00083079	duck
Zfp407	0.48	0.00084142	duck
Kif2a	0.49	0.00084256	duck
Tent5b	0.64	0.00084774	duck
Asap2	0.60	0.00085790	duck
Ell2	-1.09	0.00086414	duck
Arhgef6	0.37	0.00087460	duck
Endov	0.39	0.00088460	duck
Shroom3	0.50	0.00089653	duck
Vdac1	0.33	0.00089991	duck
Derl2	0.36	0.00090792	duck
Sparc	0.47	0.00092401	duck
Pfdn1	0.41	0.00093146	duck
Bud23	0.41	0.00093626	duck
Phf20l1	-1.82	0.00093731	duck
Rcbtb1	0.35	0.00093824	duck
Tmem59	0.29	0.00095714	duck
Klhl5	0.44	0.00096395	duck
Galnt3	0.42	0.00097324	duck
Lhfpl2	1.34	0.00098428	duck
Nfe2l2	-1.36	0.00098835	duck
Ivd	0.43	0.00100619	duck
Pgd	0.53	0.00100685	duck
Flii	0.55	0.00101685	duck
Rnf138	0.54	0.00102137	duck
Gipc2	0.12	0.00102256	duck
Cpeb2	0.57	0.00102473	duck
Smad1	0.70	0.00103185	duck
Cbx4	0.40	0.00104061	duck
Emp2	0.31	0.00104742	duck

Wnt7a	0.38	0.00105217	duck
Eif3m	0.09	0.00106258	duck
Pex10	0.23	0.00106506	duck
Cited2	0.53	0.00107319	duck
Errfi1	0.23	0.00107824	duck
Eif3b	0.26	0.00108063	duck
Akap12	0.88	0.00108751	duck
Psen1	0.29	0.00109872	duck
Tmem167	0.28	0.00110162	duck
Tor2a	0.43	0.00111035	duck
Arap2	0.37	0.00111332	duck
Tex14	0.48	0.00113854	duck
Fam3d	0.55	0.00114636	duck
Crlf1	0.38	0.00114636	duck
Skp1	-1.16	0.00118247	duck
Dnajc1	0.46	0.00118581	duck
Il17ra	0.43	0.00121964	duck
Arhgap24	0.39	0.00122513	duck
Castor2	0.24	0.00122513	duck
Epb41l1	0.38	0.00122607	duck
Aimp2	0.36	0.00123660	duck
Tubd1	0.70	0.00126736	duck
Plek2	0.65	0.00127350	duck
Id4	0.74	0.00129519	duck
Neto2	0.34	0.00129537	duck
Snx7	0.24	0.00129994	duck
Stc2	0.98	0.00131224	duck
Celf2	0.81	0.00132454	duck
Pros1	0.45	0.00132704	duck
Trim3	0.42	0.00133627	duck
Srbd1	0.44	0.00135540	duck
Bcl6	0.40	0.00136513	duck
Brd3	-1.60	0.00137401	duck
Prrc1	0.17	0.00138127	duck
Specc1	0.77	0.00141466	duck
Tanc1	0.30	0.00142153	duck
Pm20d2	0.49	0.00142374	duck
Rtraf	0.35	0.00142687	duck
Nop58	0.12	0.00147501	duck
Pdcd6	0.32	0.00150779	duck
Lsm3	0.40	0.00150868	duck
Emg1	0.40	0.00151058	duck
Tpcn1	0.97	0.00152911	duck
Laptm5	1.00	0.00154258	duck
Cnst	1.40	0.00156003	duck
Prrg3	0.28	0.00156603	duck

Fubp1	0.19	0.00157532	duck
Sun1	-1.67	0.00158580	duck
Cbx3	0.42	0.00159830	duck
B4galt1	0.56	0.00161737	duck
Grb10	0.84	0.00162363	duck
Grk3	0.84	0.00166081	duck
Slc38a1	0.21	0.00168840	duck
Yeats4	0.43	0.00169231	duck
Bpnt2	0.37	0.00171286	duck
Gorasp1	0.33	0.00171500	duck
Kctd14	0.40	0.00172880	duck
Srek1ip1	0.32	0.00174509	duck
Tmem203	0.50	0.00174637	duck
Chmp2b	0.45	0.00175308	duck
Rasa1	0.15	0.00177656	duck
Pepd	0.36	0.00179143	duck
Wnt16	0.41	0.00179344	duck
Dpyd	0.86	0.00179947	duck
Hikeshi	0.38	0.00182607	duck
Zdhhc6	0.37	0.00183816	duck
Tmem116	0.53	0.00183892	duck
Slc25a47	0.71	0.00186167	duck
Krr1	0.31	0.00188528	duck
Akt1	0.57	0.00191505	duck
Cdin1	0.50	0.00191513	duck
Acod1	0.80	0.00195935	duck
Cd83	0.57	0.00196964	duck
Bysl	0.42	0.00197769	duck
Tbc1d1	0.50	0.00201761	duck
Fem1c	0.25	0.00202515	duck
Golim4	0.42	0.00206823	duck
Pias2	0.38	0.00211862	duck
Ptprr	0.50	0.00215171	duck
Copb1	0.25	0.00215597	duck
Plbd1	0.50	0.00223709	duck
Vezf1	-1.16	0.00224380	duck
Chid1	0.28	0.00226021	duck
Prkrip1	0.29	0.00226634	duck
Rergl	0.46	0.00227613	duck
Ndufb6	0.24	0.00228717	duck
Rarres1	0.80	0.00235300	duck
Rab39b	0.48	0.00235553	duck
Shoc2	0.54	0.00236366	duck
G0s2	0.98	0.00241848	duck
Nppc	1.46	0.00243346	duck
Jade3	0.11	0.00255669	duck

Armh3	-1.66	0.00256935	duck
Itga6	0.64	0.00257586	duck
Smim19	0.44	0.00257664	duck
Rab27a	0.41	0.00261521	duck
Slc25a5	0.43	0.00262468	duck
Eif4a3l1	0.20	0.00263209	duck
Elp3	0.25	0.00264260	duck
Eps8	0.70	0.00264638	duck
Rbbp4	0.22	0.00268913	duck
Prox1	0.75	0.00273310	duck
Llgl2	0.32	0.00274295	duck
Pdcl3	0.25	0.00276091	duck
Nsd2	-0.07	0.00277988	duck
Sod3	0.53	0.00280215	duck
Lgi3	0.39	0.00280215	duck
Spsb4	0.44	0.00280933	duck
Snta1	0.46	0.00280933	duck
Nyx	0.59	0.00281575	duck
Ormdl2	0.60	0.00283909	duck
Ppard	0.23	0.00284579	duck
Ccdc28a	0.39	0.00285175	duck
Pcnt	0.29	0.00286679	duck
Abi1	0.05	0.00286783	duck
Dhx58	0.47	0.00287369	duck
Lad1	0.39	0.00293358	duck
Stag1	0.02	0.00297977	duck
Pgpep1	0.51	0.00300826	duck
Rsl1d1	0.42	0.00301001	duck
Amotl1	0.41	0.00308937	duck
Smu1	0.51	0.00312335	duck
Psip1	0.37	0.00313481	duck
Cstf2	0.24	0.00313720	duck
Esd	0.40	0.00314481	duck
Kpnb1	0.24	0.00314540	duck
Ubash3b	0.58	0.00319590	duck
Dusp22	-1.77	0.00321982	duck
Tmco4	-1.45	0.00322517	duck
Sf3a1	0.15	0.00322649	duck
Pkdcc	0.37	0.00325042	duck
Usp53	0.46	0.00326071	duck
Smarca2	0.59	0.00327351	duck
Cracdl	0.42	0.00329392	duck
Trib1	0.36	0.00330634	duck
Cyria	0.44	0.00333072	duck
Lypla1	0.40	0.00334035	duck
Psmc6	0.17	0.00334577	duck

Dnajb5	0.32	0.00340680	duck
Cgas	0.34	0.00342108	duck
Pfkfb3	-1.57	0.00342344	duck
Cap1	0.32	0.00342785	duck
Tmprss2	0.45	0.00343326	duck
Rassf2	0.32	0.00348415	duck
Smarcd2	0.27	0.00351852	duck
Tmed5	0.23	0.00353114	duck
Eif4e3	-1.44	0.00358343	duck
Utp20	0.28	0.00362175	duck
P3h2	0.53	0.00366529	duck
Osbpl6	0.59	0.00372056	duck
Arhgap5	0.24	0.00373859	duck
Rwdd1	0.33	0.00377015	duck
Ldb1	-1.39	0.00394355	duck
Stt3b	0.08	0.00399201	duck
Slc35a1	0.39	0.00399875	duck
Dach2	0.81	0.00411892	duck
Zfp395	0.41	0.00412485	duck
Rbm5	-1.15	0.00416032	duck
Taf2	0.31	0.00422197	duck
Ptbp3	0.11	0.00432658	duck
Wdr24	0.26	0.00437267	duck
Il6ra	0.43	0.00437267	duck
Lsm6	0.18	0.00438005	duck
Ptp4a2	0.24	0.00444273	duck
Cdc26	0.40	0.00450542	duck
Atp6v0c	0.32	0.00452843	duck
Cct8	0.22	0.00465466	duck
Cfap97	0.04	0.00472421	duck
Sc5d	0.38	0.00475562	duck
Ndufs5	0.35	0.00476784	duck
Aqp4	0.43	0.00480372	duck
Tmem106b	0.31	0.00486021	duck
Eef1a1	0.37	0.00495714	duck
Cldn1	0.45	0.00499941	duck
Arsb	0.45	0.00500706	duck
Abl1	0.27	0.00504635	duck
Fry	0.46	0.00506964	duck
Grk4	0.07	0.00511100	duck
Efl1	0.28	0.00511112	duck
Nuak2	0.38	0.00513697	duck
Pnrc2	0.20	0.00514701	duck
Hnrnpu	0.26	0.00523644	duck
Prdx1	-1.51	0.00527081	duck
Parp16	0.31	0.00531127	duck

Gabarapl2	0.20	0.00533091	duck
Ptk7	0.41	0.00534988	duck
Golph3l	0.48	0.00539633	duck
Pigc	0.41	0.00543398	duck
Pde6d	0.26	0.00546493	duck
Adora2b	0.83	0.00558207	duck
Tbc1d8	0.20	0.00561845	duck
Trerf1	0.40	0.00563783	duck
Atp23	0.26	0.00567698	duck
9130409I23Rik	0.30	0.00568433	duck
Clip2	0.49	0.00572080	duck
Gch1	0.63	0.00573406	duck
Dennd6a	-1.77	0.00575160	duck
Cpne8	0.47	0.00576363	duck
Erap1	0.23	0.00588350	duck
F2r	1.64	0.00592125	duck
Tead4	0.32	0.00592392	duck
Cnot6l	-1.41	0.00594135	duck
Nolc1	0.13	0.00597222	duck
Aldh6a1	0.32	0.00601643	duck
Aup1	0.19	0.00611943	duck
Nkx2-9	0.39	0.00624608	duck
Rfc5	0.42	0.00627568	duck
Slc16a7	0.62	0.00627839	duck
Tasor	0.36	0.00631695	duck
Golph3	0.27	0.00636276	duck
Alg2	0.60	0.00637309	duck
Acat3	0.38	0.00647482	duck
Dtx3l	0.27	0.00649190	duck
Nr4a2	0.58	0.00649575	duck
Tbc1d16	0.25	0.00652024	duck
Trim13	0.38	0.00664873	duck
Luzp1	0.25	0.00676137	duck
Slc5a8	0.29	0.00681499	duck
Rab25	0.32	0.00681499	duck
Rassf3	0.55	0.00686215	duck
Plxnc1	0.34	0.00691453	duck
Ssbp3	-1.41	0.00692899	duck
Ppil1	0.28	0.00694161	duck
Ap3m1	0.37	0.00704646	duck
Foxk2	-1.46	0.00708707	duck
Rbbp8	0.46	0.00710133	duck
Csnk1g2	0.17	0.00711482	duck
Pla2g12a	0.37	0.00714354	duck
Ginm1	0.16	0.00714850	duck
Slc2a13	0.50	0.00715281	duck

Dpy30	0.41	0.00721783	duck
Ahcyl1	0.20	0.00729572	duck
Ankdd1b	0.44	0.00734710	duck
Ptges3l	0.27	0.00735321	duck
Pgs1	0.40	0.00738065	duck
Fgf9	0.35	0.00742737	duck
Uspl1	0.46	0.00755077	duck
Lsg1	0.31	0.00759847	duck
Ugcg	0.34	0.00766481	duck
Rngtt	0.39	0.00770983	duck
Nrip1	0.53	0.00771064	duck
Cmss1	0.32	0.00781296	duck
Irf6	0.22	0.00782340	duck
Btf3	0.27	0.00788316	duck
Elovl1	0.31	0.00799162	duck
Enoph1	0.26	0.00799521	duck
Hspa2	0.58	0.00807071	duck
Tef	0.43	0.00819818	duck
Fibin	0.36	0.00822282	duck
Abcd3	0.21	0.00824226	duck
Lrrk2	0.40	0.00834808	duck
Lrrc8a	0.23	0.00835199	duck
Paip1	-0.05	0.00839644	duck
Chmp4b	0.40	0.00843242	duck
Eif2ak2	0.10	0.00845409	duck
Ehd3	0.28	0.00850320	duck
Samd8	0.32	0.00859107	duck
Clstn1	0.34	0.00863473	duck
Erc2	0.57	0.00863696	duck
Ago3	0.54	0.00869772	duck
Pgm2	0.50	0.00870886	duck
Adpgk	0.28	0.00883112	duck
Agps	0.35	0.00888575	duck
Ccdc12	0.28	0.00890453	duck
Nol7	0.51	0.00894443	duck
Htra1	0.67	0.00901426	duck
P2rx4	0.33	0.00902208	duck
Jmjd1c	0.17	0.00907287	duck
B4galnt3	0.51	0.00914931	duck
Gpx1	0.75	0.00926573	duck
Vps29	0.18	0.00929633	duck
Fam171a1	0.70	0.00938422	duck
Cry2	0.43	0.00941208	duck
Pds5b	0.38	0.00941309	duck
Rnf216	0.13	0.00942812	duck
Lin54	-1.54	0.00955602	duck

Uhrf1bp1l	0.39	0.00957725	duck
Wnt5b	0.39	0.00963346	duck
Nnt	0.55	0.00966958	duck
Rasa3	0.88	0.00974814	duck
Lancl1	0.31	0.00993447	duck
Lactb	0.45	0.01014431	duck
Bms1	0.35	0.01021741	duck
Ust	0.65	0.01026158	duck
Aida	0.27	0.01034842	duck
Trabd2b	0.84	0.01036747	duck
Kars	0.10	0.01044389	duck
Desi2	0.58	0.01060617	duck
Rsad2	0.60	0.01060863	duck
Slc34a2	0.75	0.01060863	duck
Zbtb7b	0.30	0.01060863	duck
Stard5	0.33	0.01062301	duck
Asap1	0.88	0.01065140	duck
Lonrf3	0.24	0.01078185	duck
Txndc9	-1.58	0.01080892	duck
Lmnb2	0.24	0.01083736	duck
Sumo1	0.23	0.01090349	duck
Cdv3	0.25	0.01090900	duck
Slc25a3	0.33	0.01098385	duck
Phactr4	0.44	0.01102011	duck
Vsnl1	0.53	0.01103358	duck
Synj2	0.69	0.01113731	duck
Stxbp5	0.42	0.01118711	duck
Prkar1b	0.59	0.01125018	duck
Vti1a	0.16	0.01137962	duck
Gpr161	0.38	0.01143635	duck
Smc3	0.41	0.01156020	duck
Ip6k2	0.35	0.01167505	duck
Tln2	-2.03	0.01170994	duck
Blnk	0.55	0.01174590	duck
Ccdc171	0.45	0.01190895	duck
Golgb1	-0.01	0.01193134	duck
Pak2	0.22	0.01193297	duck
Gtdc1	0.37	0.01195578	duck
B4galt5	0.26	0.01198404	duck
Ints6	0.34	0.01201457	duck
Rfc1	0.21	0.01202555	duck
Klf5	0.30	0.01216058	duck
Elk4	0.14	0.01224545	duck
Rab1a	0.23	0.01225778	duck
N4bp2	0.39	0.01227883	duck
Usp8	0.09	0.01234048	duck

Ogt	-1.55	0.01246765	duck
Abca2	0.33	0.01247223	duck
Blmh	0.14	0.01258186	duck
Gars	0.54	0.01268117	duck
Bace1	0.24	0.01286271	duck
Ptger4	0.41	0.01305323	duck
Galnt7	0.32	0.01305693	duck
Galnt2	0.45	0.01307498	duck
Psmg4	0.27	0.01314663	duck
Cxxc5	0.98	0.01314951	duck
Psmd6	0.26	0.01318908	duck
Pex7	0.30	0.01321304	duck
Sms	-0.09	0.01333996	duck
Abca1	0.53	0.01355627	duck
Ankrd50	0.48	0.01357513	duck
Stx17	0.78	0.01373179	duck
Abcb7	-1.70	0.01376392	duck
Ccna2	0.52	0.01404528	duck
Hells	0.60	0.01405973	duck
Borcs5	0.66	0.01418119	duck
Rpa2	0.37	0.01428355	duck
Dpp10	1.04	0.01450030	duck
Rap2b	0.48	0.01450725	duck
Coq7	0.34	0.01471441	duck
Cks1brt	0.44	0.01479637	duck
Sacs	1.11	0.01480316	duck
Flrt3	0.53	0.01509345	duck
Epn2	0.47	0.01520962	duck
Hyou1	0.12	0.01529968	duck
Rrm2b	0.37	0.01538820	duck
Get4	0.36	0.01546922	duck
Pars2	0.56	0.01566432	duck
Tmem19	0.18	0.01606518	duck
Rnmt	0.21	0.01615310	duck
Pdss2	0.26	0.01634966	duck
Ywhaq	0.33	0.01637549	duck
Tars	0.41	0.01648405	duck
Rab8b	0.15	0.01682539	duck
Gabarapl1	0.37	0.01728260	duck
Lrrc59	0.42	0.01747323	duck
Steap4	0.35	0.01748198	duck
Fam162a	0.14	0.01751031	duck
Arfip1	0.09	0.01766181	duck
Nop14	0.44	0.01780979	duck
Saraf	0.04	0.01787279	duck
Cdc42	0.35	0.01792548	duck

Wdr4	0.34	0.01819655	duck
Tmem184c	0.28	0.01828266	duck
Cacna1i	0.92	0.01834397	duck
Dnajb14	0.47	0.01839715	duck
Irag1	0.50	0.01846218	duck
Wdr43	0.38	0.01858803	duck
Vegfc	0.49	0.01872834	duck
Slc38a2	0.47	0.01885725	duck
Rab2a	0.34	0.01928565	duck
Ddx28	0.13	0.01939922	duck
D630045J12Rik	0.64	0.01944480	duck
Dpp7	-0.01	0.01949380	duck
Cdkn1a	0.28	0.01952152	duck
Xk	-1.04	0.02021648	duck
Tmed10	0.27	0.02036436	duck
Galnt10	0.45	0.02057421	duck
Hs6st2	0.48	0.02070309	duck
Hpse	1.30	0.02090381	duck
Icmt	0.22	0.02092569	duck
Timm8a2	0.30	0.02095708	duck
Ptgs2	0.66	0.02116518	duck
Galm	0.33	0.02121203	duck
A4galt	0.35	0.02139769	duck
Eif4h	-0.02	0.02186032	duck
2610507B11Rik	0.27	0.02239697	duck
Hexim1	0.31	0.02248322	duck
Uggt1	0.13	0.02273751	duck
Rab8a	0.20	0.02285816	duck
Cog7	0.47	0.02285976	duck
Pdgfrb	0.19	0.02299049	duck
Pole4	0.18	0.02308388	duck
Cpne3	0.12	0.02310745	duck
Synrg	0.14	0.02313752	duck
Irak2	0.27	0.02314590	duck
Tmem208	0.37	0.02316265	duck
Sgsh	0.31	0.02331803	duck
Itm2b	0.32	0.02335524	duck
Scarb2	0.38	0.02351364	duck
Orc3	-1.55	0.02352349	duck
Pcna	0.48	0.02389166	duck
Vopp1	-0.11	0.02419607	duck
Nip7	0.16	0.02419607	duck
Xrcc5	0.53	0.02427238	duck
Lmnb1	0.49	0.02432574	duck
Sytl2	0.92	0.02434811	duck
Sgpl1	0.42	0.02461582	duck

Gbe1	0.57	0.02468489	duck
As3mt	0.25	0.02540065	duck
Phf14	0.32	0.02540237	duck
Sowaha	0.32	0.02545649	duck
Sacm1l	0.10	0.02547268	duck
Hic2	-1.60	0.02615881	duck
Prkag1	0.41	0.02645421	duck
Dynlt1a	0.52	0.02650824	duck
Iqgap2	1.05	0.02791519	duck
Dusp8	0.40	0.02794677	duck
Cers2	0.18	0.02811916	duck
Chn1	0.41	0.02834356	duck
Gcnt4	0.43	0.02849529	duck
Pes1	0.30	0.02853316	duck
Slc25a46	0.22	0.02864375	duck
Sat1	0.43	0.02875016	duck
Cct4	0.24	0.02921696	duck
Aatf	0.22	0.02934147	duck
Wdr36	0.23	0.02941535	duck
Plin2	0.80	0.02950630	duck
Triap1	0.30	0.02958589	duck
Fbxl4	-2.05	0.02971142	duck
Pkp2	0.39	0.02992239	duck
Aco2	0.07	0.03032416	duck
Arhgap10	0.27	0.03036591	duck
Plekhb2	0.36	0.03044105	duck
Ythdc2	-1.65	0.03054723	duck
Emcn	0.87	0.03059722	duck
Nup205	0.30	0.03070827	duck
Gnpda1	0.19	0.03070827	duck
Lrrc47	0.27	0.03104653	duck
Ptpn12	0.33	0.03126016	duck
Ank2	0.38	0.03153247	duck
Slc31a1	0.24	0.03197653	duck
Usp1	0.41	0.03211761	duck
Zdhhc8	0.41	0.03253368	duck
9530077C05Rik	0.43	0.03283473	duck
Rhoj	0.74	0.03371455	duck
Slc30a1	0.09	0.03382976	duck
Unc119b	0.31	0.03391706	duck
Tmem251	0.38	0.03406668	duck
Gna11	0.17	0.03425604	duck
Lrrc8d	0.91	0.03437636	duck
Aldh18a1	0.23	0.03455354	duck
Ctsh	0.22	0.03488875	duck
Pik3ca	-1.69	0.03499084	duck

Uhmk1	-1.46	0.03512525	duck
Nucb2	0.19	0.03598304	duck
Slf1	0.35	0.03605475	duck
Snx4	0.13	0.03669274	duck
Dvl3	0.20	0.03679111	duck
Vps35	0.29	0.03730821	duck
Utp15	0.41	0.03736874	duck
Dpysl3	0.41	0.03743226	duck
Lox	0.76	0.03821660	duck
Dsel	0.25	0.03822076	duck
Pafah1b2	0.08	0.03840200	duck
Arhgap6	0.92	0.03915055	duck
Gda	0.49	0.03938628	duck
Hspa12b	0.46	0.03947272	duck
St14	0.30	0.03967258	duck
Psd	0.45	0.03977436	duck
Cdc42se1	0.34	0.03982161	duck
Ctsb	0.45	0.04056362	duck
Acp6	0.30	0.04072570	duck
Fdps	0.56	0.04086020	duck
Ehmt1	0.15	0.04088615	duck
Pdgfa	0.27	0.04102788	duck
Apba1	0.42	0.04126145	duck
Itgb8	0.32	0.04180524	duck
Sox9	0.24	0.04193912	duck
Slc1a2	0.06	0.04210809	duck
Dapk1	0.49	0.04253108	duck
Lemd3	-0.02	0.04257220	duck
Srsf6	0.15	0.04296942	duck
Ldah	0.40	0.04307319	duck
Lama5	0.31	0.04350899	duck
Ostc	0.20	0.04358061	duck
Slc40a1	0.06	0.04367483	duck
Xkr6	0.39	0.04404330	duck
Atp6v1b2	0.27	0.04409601	duck
Kif5b	0.08	0.04419470	duck
Dock9	0.45	0.04443562	duck
Syf2	0.06	0.04449118	duck
Pcyt2	0.25	0.04490409	duck
Ntpcr	0.25	0.04492971	duck
Slc25a44	0.34	0.04504767	duck
Dcbld1	0.32	0.04531811	duck
Pak3	0.27	0.04534892	duck
Capzb	0.14	0.04554462	duck
Kctd19	0.71	0.04609678	duck
Prelid3a	0.29	0.04621989	duck

Ano10	0.03	0.04633213	duck
Acadl	-0.08	0.04712023	duck
Rgs4	1.36	0.04727150	duck
Adamts3	0.43	0.04732732	duck
Tmed3	0.33	0.04783740	duck
Tspan6	0.24	0.04793048	duck
Uaca	0.18	0.04800152	duck
Xpo4	0.21	0.04814890	duck
Fut8	0.79	0.04821549	duck
Tmem41b	0.10	0.04843886	duck
Ing1	0.43	0.04864118	duck
Rnf13	0.13	0.04868843	duck
Dcaf5	-1.82	0.04937832	duck
Prkag2	0.17	0.04942978	duck
Hacd3	0.34	0.04994155	duck
Cbfa2t2	0.36	0.05078324	duck
Agr2	0.54	0.05114117	duck
Chmp4c	0.37	0.05158640	duck
Cacna1e	0.33	0.05180776	duck
Rc3h1	-1.27	0.05254714	duck
Psmd12	0.01	0.05293334	duck
Ankmy2	0.12	0.05345384	duck
Igfbp7	1.08	0.05351277	duck
Cyth3	0.64	0.05371275	duck
Tmem14a	0.24	0.05410231	duck
Anxa6	0.31	0.05463518	duck
Gng7	0.21	0.05546424	duck
Shcbp1	0.39	0.05647498	duck
Star	0.30	0.05682348	duck
Plek	0.33	0.05683876	duck
Pex16	0.29	0.05721784	duck
Ash2l	0.26	0.05858020	duck
Sinhcaf	0.58	0.05902117	duck
Cdk14	0.62	0.05914398	duck
Cd82	0.33	0.05934718	duck
Jade1	-1.48	0.05952161	duck
Ero1a	0.01	0.05958721	duck
Fst	0.39	0.05975796	duck
Bspry	0.10	0.06068072	duck
Bola3	0.11	0.06092713	duck
Polr1e	0.28	0.06105061	duck
Usp28	0.40	0.06208451	duck
Zmat2	0.10	0.06241546	duck
Rab3gap1	-0.03	0.06250840	duck
Pdcd10	0.13	0.06260977	duck
Lta4h	0.25	0.06329288	duck

Ccnb2	0.17	0.06343261	duck
Rab11fip3	0.33	0.06475853	duck
Tmem42	0.18	0.06484303	duck
Srpr	0.09	0.06509440	duck
Pan3	-1.24	0.06565505	duck
Pgam5	0.23	0.06595904	duck
Caap1	0.49	0.06597816	duck
Zw10	0.18	0.06597816	duck
Tfdp1	-1.39	0.06639016	duck
Surf6	0.28	0.06697092	duck
C1ra	0.19	0.06711031	duck
Extl3	0.11	0.06716399	duck
Nme7	-0.30	0.06745126	duck
Atad3a	0.17	0.06908262	duck
Supt4a	0.28	0.06960767	duck
Prok2	0.54	0.06993143	duck
Srf	0.22	0.07062492	duck
Sf3b4	0.04	0.07108754	duck
Tmem218	0.36	0.07145307	duck
Cwc27	-0.04	0.07187798	duck
Tdrd7	0.20	0.07213224	duck
DPCD	0.26	0.07250845	duck
Atp6v0e	0.14	0.07256320	duck
Pparg	0.53	0.07265537	duck
Ube2b	0.25	0.07273396	duck
Dtwd1	0.30	0.07291971	duck
Nme3	0.25	0.07291971	duck
Cse1l	0.25	0.07357486	duck
Ptch1	0.53	0.07490882	duck
Rsrp1	-1.36	0.07512644	duck
Wdr89	0.29	0.07540129	duck
Hes6	0.28	0.07540129	duck
Il1rapl2	0.63	0.07585068	duck
Rtca	0.34	0.07611353	duck
Rgma	0.32	0.07673261	duck
Tfam	0.26	0.07689765	duck
Iars	0.15	0.07692883	duck
Cks2	0.13	0.07713117	duck
Fubp3	-0.13	0.07784659	duck
Ccdc91	0.15	0.07795321	duck
Pank4	-1.39	0.07877973	duck
Plekhm3	0.38	0.08016438	duck
Phyhd1	0.52	0.08020882	duck
Htr1b	1.22	0.08041540	duck
Atxn2	-1.38	0.08068025	duck
Crispld2	0.42	0.08121244	duck

Zfyve28	0.47	0.08132889	duck
Pgap6	-1.04	0.08197731	duck
Sugct	0.71	0.08217148	duck
Dcp2	0.34	0.08259085	duck
Ptdss1	0.31	0.08334904	duck
Ttc32	0.59	0.08336475	duck
Vti1b	0.20	0.08433607	duck
Lamp1	0.55	0.08591109	duck
Vsir	0.42	0.08710747	duck
Ensa	0.23	0.08712289	duck
Cct7	0.15	0.08784635	duck
Psmg3	0.52	0.08909224	duck
Crb2	0.25	0.08912010	duck
Phc2	0.15	0.09016823	duck
Zfp521	1.37	0.09042405	duck
Tmem39a	0.25	0.09107882	duck
Edem3	-0.08	0.09108016	duck
Ap1s2	0.12	0.09251784	duck
Pigs	0.34	0.09418784	duck
Kctd10	0.15	0.09722954	duck
Rest	0.12	0.09883008	duck
Pop4	0.07	0.09924494	duck
Gale	0.26	0.09924720	duck
Kif13a	0.08	0.10075323	duck
Fez2	-0.16	0.10115324	duck
Rmdn1	0.32	0.10147790	duck
Pdcd5	0.16	0.10174110	duck
Pno1	0.29	0.10185565	duck
Gyg	0.36	0.10202426	duck
Lamc2	0.25	0.10213592	duck
Nrbp2	-1.40	0.10232254	duck
Fhod1	0.75	0.10279421	duck
Nsmce4a	0.47	0.10504915	duck
Rcbtb2	0.48	0.10541879	duck
Areg	0.24	0.10583556	duck
Got2	0.27	0.10685774	duck
Otulinl	0.77	0.10716860	duck
Atp6v0d1	0.15	0.10782910	duck
Far1	-0.02	0.10823825	duck
Vrk2	0.47	0.10846584	duck
Prrg1	0.30	0.10868591	duck
Tnip1	0.29	0.10886987	duck
Lrrc75a	0.71	0.11107063	duck
Eef1b2	0.24	0.11136130	duck
Ro60	0.29	0.11339538	duck
Slc39a13	0.15	0.11352463	duck

Robo1	0.54	0.11391106	duck
Sbno2	0.10	0.11508023	duck
Zmynd19	0.39	0.11544218	duck
Dync1li2	0.05	0.11576804	duck
Rrp7a	0.38	0.11642518	duck
Litaf	0.49	0.11684988	duck
Tmem97	0.27	0.11730401	duck
Sema3f	0.43	0.11730597	duck
Gchfr	1.23	0.11814309	duck
Pcyox1	0.16	0.11829976	duck
Vapa	0.13	0.11901729	duck
Tedc1	0.13	0.11988815	duck
Chst10	0.25	0.11990389	duck
Sephs1	0.32	0.12024994	duck
E2f6	0.15	0.12185797	duck
Nmt1	0.28	0.12564673	duck
Slc38a10	0.19	0.12620484	duck
Klhl21	-0.01	0.12628904	duck
Vat1	0.42	0.12654351	duck
5031439G07Rik	0.44	0.12725104	duck
Slc44a5	0.25	0.12800622	duck
Snx24	0.34	0.12812249	duck
Btbd9	-1.74	0.12985054	duck
Hsd17b4	0.06	0.13130827	duck
Bclaf3	0.37	0.13159112	duck
Idh3a	0.33	0.13193575	duck
Traf2	0.28	0.13284618	duck
Nectin1	0.23	0.13405176	duck
Nek9	0.17	0.13514129	duck
Ahsa1	0.24	0.13549720	duck
Lsmem1	0.35	0.13692799	duck
Ralgapa2	-1.75	0.13732954	duck
Gpatch2	0.23	0.13790213	duck
Cradd	0.33	0.13805574	duck
Vamp3	0.20	0.13871311	duck
Slc39a9	0.02	0.13905671	duck
Stk38l	0.16	0.13924046	duck
Ddx1	-0.05	0.13983405	duck
Pxdc1	0.37	0.14250032	duck
Tfg	0.36	0.14282077	duck
Ranbp9	-1.60	0.14294611	duck
Xrcc4	0.11	0.14320287	duck
Copg2	-1.57	0.14400184	duck
Pnpla7	-2.02	0.14573003	duck
Utp11	0.31	0.14691526	duck
Nsrp1	0.74	0.14772453	duck

Tmem165	0.37	0.14799899	duck
Sh2d4a	0.34	0.14887198	duck
Zbtb1	0.45	0.14906506	duck
Camk2g	-1.09	0.15193856	duck
Stx1a	0.22	0.15242827	duck
Pink1	0.25	0.15428304	duck
Rnf213	0.16	0.15666261	duck
Daglb	-0.03	0.15808080	duck
Ptbp1	0.27	0.15846443	duck
Zc3h18	-0.06	0.15909921	duck
Upf1	0.16	0.15931669	duck
Noc2l	0.19	0.16041531	duck
Dynlt3	-0.13	0.16081333	duck
Got1	0.35	0.16084875	duck
Dsg2	0.41	0.16243504	duck
Gorasp2	0.02	0.16304570	duck
Phtf2	0.60	0.16340610	duck
Tmtc2	0.56	0.16351229	duck
Eef1d	0.21	0.16406948	duck
Zdhhc13	0.15	0.16434348	duck
Wbp2nl	0.09	0.16568986	duck
Cldnd1	0.46	0.16641656	duck
Tbc1d4	0.73	0.16685574	duck
Noct	0.10	0.16879807	duck
Sec63	0.11	0.16941860	duck
Nup93	0.35	0.17125554	duck
Pdxk	0.12	0.17350222	duck
Rbl1	0.40	0.17427628	duck
Lsp1	0.57	0.17522214	duck
Prelid2	-1.25	0.17522214	duck
Plxdc2	1.00	0.17529943	duck
Fam171b	0.23	0.17592879	duck
Epb41l4b	0.03	0.17592879	duck
Plgrkt	0.49	0.18221356	duck
Ndufb3	0.05	0.18222728	duck
Fzd7	0.35	0.18338995	duck
Fbxl22	-1.00	0.18382332	duck
Slc9a6	0.25	0.18455131	duck
Phb2	0.18	0.18507551	duck
Ola1	-0.27	0.18805884	duck
Sdf2	0.24	0.19151717	duck
Tmem230	0.35	0.19202319	duck
Rnf166	0.23	0.19247089	duck
Slc2a9	0.44	0.19254418	duck
Nbeal2	0.40	0.19285708	duck
Ndufs3	0.07	0.19361369	duck

Ncstn	0.25	0.19490175	duck
Adk	0.68	0.19572166	duck
Papss1	0.26	0.19677010	duck
Psmd14	-0.04	0.19855410	duck
Rfc2	-0.02	0.19860673	duck
Chordc1	0.16	0.19873723	duck
Klhdc1	0.22	0.20265101	duck
Rbpms	0.25	0.20293740	duck
Pla2r1	0.42	0.20379358	duck
Golt1b	0.04	0.20665610	duck
Ptpn6	0.46	0.20782975	duck
Stk25	0.17	0.20881491	duck
Naa35	-0.14	0.21166534	duck
Pla2g15	0.20	0.21272725	duck
Flvcr2	0.51	0.21379561	duck
Amd2	0.24	0.21461973	duck
Pnpla2	0.11	0.21503940	duck
Tspan18	0.73	0.21608245	duck
Zfp462	0.37	0.21621864	duck
Galnt17	0.39	0.21684277	duck
Glrx5	-1.73	0.21869685	duck
Npl	-0.11	0.22016707	duck
Fars2	-0.13	0.22028398	duck
Nup155	0.44	0.22076542	duck
Psmf1	0.23	0.22079406	duck
Hat1	-0.06	0.22110879	duck
Crppa	0.29	0.22243007	duck
Scnn1b	0.24	0.22243007	duck
Snx1	0.17	0.22651981	duck
Laptm4a	0.64	0.22708582	duck
Parp9	0.02	0.22813949	duck
Fbxl14	0.31	0.22921754	duck
Ryk	0.30	0.23140002	duck
Ttbk2	0.12	0.23491089	duck
Zbtb7c	0.49	0.23564918	duck
Fhl4	0.25	0.23580241	duck
Hdac9	0.86	0.23583752	duck
Spg20	0.30	0.23586591	duck
Carnmt1	0.12	0.23595524	duck
Daam1	0.42	0.23755376	duck
Tatdn1	0.26	0.23864970	duck
Ttc39c	0.21	0.23864973	duck
Acaa1b	0.13	0.23992128	duck
Tlcd2	-0.84	0.24018743	duck
Phf6	-0.12	0.24219332	duck
Nol4l	0.26	0.24270523	duck

Gdf11	0.22	0.24644542	duck
Slc7a6	0.19	0.24760765	duck
Emc8	0.20	0.24862740	duck
Grpel1	0.01	0.24988726	duck
Polr2c	0.36	0.25004710	duck
Eif2a	0.03	0.25203734	duck
Slc16a10	-1.46	0.25284773	duck
Cep104	0.28	0.25437600	duck
Sde2	-1.12	0.25615340	duck
Pdia5	0.23	0.25622885	duck
Ube2j1	0.13	0.25631829	duck
Cnksr2	0.46	0.25771243	duck
Atg13	-0.09	0.26061661	duck
Tfpi2	0.88	0.26066482	duck
Sap130	0.18	0.26152057	duck
Timm17a	0.25	0.26245928	duck
Pdia2	0.25	0.26348160	duck
Ppif	0.29	0.26605924	duck
Tmem33	0.16	0.26794695	duck
Plekhm2	0.18	0.27046774	duck
Slc9a1	0.58	0.27124598	duck
Xpo5	0.41	0.27317556	duck
Ly96	0.31	0.27879139	duck
Hnrnph3	-0.06	0.27980187	duck
Hsph1	0.32	0.28100897	duck
Scd2	-0.10	0.28461448	duck
Gigyf2	-1.35	0.28593180	duck
Tgm4	0.60	0.28715593	duck
Bcar1	0.32	0.29028961	duck
Sypl2	0.18	0.29161748	duck
Slc35b2	0.16	0.29252185	duck
Fam234a	0.09	0.29740609	duck
Nmt2	0.24	0.30288389	duck
Traf7	0.13	0.30376150	duck
Nptn	-0.01	0.30398442	duck
Creld2	0.17	0.30426684	duck
Atp10d	0.48	0.30434541	duck
Ccdc125	0.44	0.30623095	duck
Dnajc7	-0.08	0.31193681	duck
Washc3	0.34	0.31407837	duck
Cstf3	0.03	0.31493206	duck
Coq5	0.34	0.31758113	duck
Trap1	0.15	0.31847785	duck
Klf13	0.02	0.31912504	duck
Rbm33	-1.39	0.32091435	duck
Nedd9	0.17	0.32213819	duck

Josd1	0.29	0.32704343	duck
Cmtm7	0.19	0.33082035	duck
Dync2li1	0.27	0.33105640	duck
Sh3bp4	0.55	0.33168979	duck
Fbxl7	0.84	0.33259503	duck
Cdc34	0.33	0.33413897	duck
Dnajc3	0.15	0.33418480	duck
Zbtb8os	0.22	0.33526528	duck
Ttc39a	0.34	0.34037846	duck
Zfp622	0.28	0.34226748	duck
Ntmt1	0.34	0.34314565	duck
Pemt	0.31	0.34609113	duck
Thumpd3	0.13	0.34967276	duck
Utp18	0.30	0.35515072	duck
Srsf4	0.25	0.36395034	duck
Atxn1l	0.06	0.37103032	duck
Hs6st1	0.30	0.37406043	duck
Bub3	0.05	0.37430999	duck
Wsb2	0.40	0.37461019	duck
Alg3	0.27	0.37528846	duck
Bmper	0.69	0.37729878	duck
Tes	0.34	0.37860065	duck
Gucd1	0.16	0.37925167	duck
Pfkl	0.21	0.38022719	duck
Kat6b	0.11	0.38094762	duck
Tnfrsf11a	0.14	0.38156271	duck
Dctn4	0.32	0.38392720	duck
Hspb11	0.42	0.38406918	duck
Cfap410	0.12	0.38924260	duck
Susd6	0.02	0.38995157	duck
Crym	0.74	0.39168711	duck
Hmbox1	0.36	0.39488938	duck
Nipa1	0.13	0.39689639	duck
Pde3a	1.75	0.40536869	duck
Basp1	0.14	0.41050548	duck
Smarce1	0.08	0.41073079	duck
Dennd2a	0.50	0.41172396	duck
Sec22b	0.30	0.41252822	duck
Polr2h	0.23	0.41370742	duck
Gpt2	0.35	0.41404080	duck
Chchd4	0.03	0.41419666	duck
Zcchc17	0.16	0.42092110	duck
Polr1c	0.30	0.42238425	duck
Colgalt2	0.33	0.42521527	duck
Efna2	0.33	0.42851608	duck
Ruvbl1	0.26	0.42883587	duck

Jmy	0.18	0.43011257	duck
Atp11c	0.28	0.43469896	duck
Tub	0.09	0.43922663	duck
Crtc1	0.12	0.44140237	duck
Dlg2	0.30	0.44272981	duck
Cab39l	0.75	0.44333540	duck
Fam32a	0.32	0.44353608	duck
Pik3r6	0.05	0.44871850	duck
Vps36	0.22	0.45213817	duck
Hnrnpll	0.00	0.45447474	duck
Slc12a4	-1.03	0.46170558	duck
Gulp1	0.48	0.46307233	duck
Tma16	0.26	0.46365869	duck
Ddx60	-1.94	0.46449754	duck
Cdkl5	0.34	0.46497244	duck
Rmi2	0.20	0.46728803	duck
Adipor1	0.06	0.46873555	duck
Prune2	0.56	0.47239489	duck
Ufm1	0.00	0.47246974	duck
Slc25a43	0.17	0.47782943	duck
C77080	0.21	0.47782943	duck
Ankra2	0.21	0.48009578	duck
Jpt2	0.21	0.48332337	duck
Nfam1	0.27	0.48648010	duck
Gnb1	0.02	0.48679529	duck
Ppm1h	0.35	0.49330267	duck
Sf3b6	0.18	0.49584706	duck
Zdhhc3	0.04	0.49647035	duck
Bak1	0.21	0.49742723	duck
Pcgf3	0.28	0.50345551	duck
Hivep1	0.21	0.50491296	duck
Nudc	0.16	0.50789561	duck
Orai2	0.19	0.50842506	duck
Sertad2	0.40	0.51041547	duck
Nudt1	0.28	0.51462978	duck
Lgmn	0.39	0.51681090	duck
Sltm	0.08	0.51744478	duck
Cep152	0.33	0.52052511	duck
Actr10	0.22	0.52406077	duck
Gfod1	0.78	0.52494656	duck
Nup85	0.33	0.52648460	duck
Clba1	0.41	0.52899061	duck
Agpat4	0.43	0.53280825	duck
Rab22a	0.02	0.53632894	duck
Sf3b3	0.36	0.53883441	duck
Purg	-1.28	0.53979431	duck

Cfap36	0.26	0.54240289	duck
Tmem183a	-1.05	0.54344364	duck
Wsb1	-0.11	0.54383865	duck
Oma1	0.12	0.54709636	duck
Vkorc1l1	0.25	0.55098756	duck
Crkl	0.15	0.56678637	duck
Klhl36	0.23	0.57144252	duck
Rmc1	-1.23	0.57152576	duck
Dhrs13	0.12	0.57641296	duck
Rlim	0.24	0.57665955	duck
Zbtb2	0.18	0.58105327	duck
Fam107b	0.25	0.58439603	duck
Slc35e1	0.05	0.59129190	duck
Trappc2l	0.06	0.59215386	duck
Parn	-0.31	0.59509543	duck
Hace1	0.27	0.59656881	duck
Pdrg1	0.01	0.60204199	duck
Stox2	0.65	0.60276569	duck
Aldh9a1	0.28	0.60611570	duck
Zcchc2	0.51	0.60822557	duck
Bcl2l11	0.28	0.61571559	duck
Bcas2	0.19	0.62034170	duck
Plcxd2	0.27	0.63411201	duck
Arf4	0.20	0.63507667	duck
Tacc2	0.26	0.63723932	duck
Bambi	0.55	0.63951651	duck
Tyw3	0.02	0.64006816	duck
4833439L19Rik	0.37	0.64312592	duck
Impdh2	0.13	0.64966825	duck
Fgfr1op2	0.17	0.65071294	duck
Batf3	0.15	0.65308343	duck
Daam2	0.31	0.65618489	duck
Ptchd1	0.32	0.66566897	duck
Prdm4	0.27	0.66749299	duck
Sema3a	1.51	0.66820250	duck
Hadha	0.12	0.67131952	duck
Copg1	0.09	0.67598234	duck
Coq2	0.81	0.67617766	duck
Atp10a	0.23	0.68675195	duck
Smad5	0.10	0.68926387	duck
Serp1	0.25	0.69075789	duck
Aox1	0.31	0.69143313	duck
Pitpnb	-0.10	0.69440042	duck
Galnt12	0.29	0.69460225	duck
Lsm7	0.33	0.69604754	duck
Lars2	0.19	0.69994802	duck

Slc35a3	0.29	0.70253766	duck
Kif13b	0.03	0.70300839	duck
Fuca1	0.21	0.70418772	duck
Nfatc2	0.27	0.70600748	duck
Srp68	0.19	0.70613993	duck
Adamtsl3	0.42	0.71055905	duck
Ikzf1	-1.29	0.71055927	duck
Nr1d2	0.07	0.71118919	duck
Chmp1a	0.20	0.71498558	duck
St6galnac4	0.27	0.72775414	duck
Rap2c	0.11	0.73318905	duck
Sh3glb1	0.16	0.73396146	duck
Pofut2	0.18	0.73781580	duck
Lrmp	0.73	0.73870976	duck
Anxa11	0.07	0.73904571	duck
Grb2	0.00	0.74193905	duck
Grid1	0.34	0.74582766	duck
Eif2b1	0.38	0.74774308	duck
Appl1	-0.09	0.75296151	duck
Hip1r	0.11	0.75626766	duck
Ccdc71l	0.14	0.75930028	duck
Senp7	-1.22	0.76101097	duck
Tyro3	0.30	0.76134796	duck
Adrm1	-0.04	0.76460057	duck
Trp53inp2	0.18	0.76685786	duck
Slc25a22	0.19	0.78233857	duck
Fam114a1	0.42	0.78418245	duck
Dcaf10	0.17	0.78437712	duck
Arl3	0.09	0.79146140	duck
Atp2a3	0.61	0.79202504	duck
Raver2	0.15	0.79337993	duck
Plekhf1	0.34	0.79349592	duck
Gpr63	0.33	0.79377251	duck
Tmem241	0.27	0.79851013	duck
Zc3h7b	0.10	0.80071529	duck
Rb1cc1	-1.41	0.80766388	duck
Rpa3	-0.06	0.80848507	duck
Ugdh	0.30	0.81758198	duck
Garre1	0.44	0.82490972	duck
Smim5	0.47	0.84237236	duck
Nckap1	-0.35	0.85067190	duck
Itga8	0.31	0.85749392	duck
Wdr82	0.19	0.85955777	duck
Cops7a	0.05	0.86694570	duck
Dnttip2	-0.14	0.87002724	duck
Slc35b1	0.00	0.87514758	duck

Poldip3	-0.08	0.87683290	duck
Arhgap25	0.92	0.87932645	duck
Gpatch4	0.17	0.88518307	duck
Tshz3	0.45	0.88998620	duck
Leprot	0.23	0.89017727	duck
Sgsm1	0.29	0.89522640	duck
Ctsc	0.08	0.90615657	duck
Taf12	0.22	0.90693166	duck
Ogfr	0.19	0.90769786	duck
Ppm1m	0.19	0.90944690	duck
Smarcb1	0.29	0.92430634	duck
Dek	0.09	0.92831391	duck
Poldip2	0.37	0.92930883	duck
Atoh8	0.60	0.93480187	duck
Foxc1	0.93	0.93678894	duck
Tfdp2	0.24	0.93866143	duck
Ttll7	0.37	0.94636787	duck
Rxylt1	0.32	0.94746169	duck
Efhd1	0.17	0.94999443	duck
Cyrib	0.27	0.95783762	duck
Scmh1	0.23	0.97098557	duck
Dock4	0.28	0.97470702	duck
Vps4b	0.04	0.97622450	duck
Rilpl1	0.27	0.97916637	duck
Cept1	-0.19	0.97920910	duck
Plec	-1.16	0.97998583	duck
Enah	0.01	0.98693925	duck
Plk2	0.69	0.98803692	duck
Lekr1	0.24	0.98807412	duck
Phf5a	0.10	0.99372384	duck
Slc41a3	-1.16	1.00000000	duck
Snf8	0.10	1.00000000	duck
Snx25	0.20	1.00000000	duck
Larp7	0.06	1.00000000	duck
Nup88	0.09	1.00000000	duck
Nfkbie	0.43	1.00000000	duck
Thoc7	0.13	1.00000000	duck
Podxl	0.73	1.00000000	duck
Pdcd11	0.06	1.00000000	duck
Denr	0.11	1.00000000	duck
Tusc2	0.10	1.00000000	duck
Eif5	-0.53	1.00000000	duck
Acox1	-0.04	1.00000000	duck
Htr2a	0.86	1.00000000	duck
Eif5a2	-1.56	1.00000000	duck
Rabggtb	-0.23	1.00000000	duck

Lpar6	0.64	1.00000000	duck
Amot	0.17	1.00000000	duck
Lpin1	0.50	1.00000000	duck
Slco3a1	0.69	1.00000000	duck
Shc1	0.21	1.00000000	duck
Pla2g7	0.44	1.00000000	duck
Dync1i2	-0.09	1.00000000	duck
Ttc9	0.38	1.00000000	duck
Sh3bp5	-1.27	1.00000000	duck
Pde12	0.17	1.00000000	duck
Tgs1	0.29	1.00000000	duck
Cbx8	0.20	1.00000000	duck
Gsr	0.10	1.00000000	duck
Taf10	0.14	1.00000000	duck
Heatr5a	-0.10	1.00000000	duck
Csgalnact2	0.28	1.00000000	duck
Evi5	-1.49	1.00000000	duck
Atg7	-0.35	1.00000000	duck
Coro1b	0.23	1.00000000	duck
Rapgef5	1.15	1.00000000	duck
Qk	0.41	1.00000000	duck
Ctbp2	0.69	1.00000000	duck
Filip1l	0.35	1.00000000	duck
Exoc3	-0.07	1.00000000	duck
Parp4	0.04	1.00000000	duck
Dnajb9	-1.57	1.00000000	duck
Ddx47	0.39	1.00000000	duck
Acad9	-0.03	1.00000000	duck
Poglut2	0.16	1.00000000	duck
Sec31a	-0.02	1.00000000	duck
Tnrc6c	0.41	1.00000000	duck
Pdik1l	-1.06	1.00000000	duck
Aven	-0.46	1.00000000	duck
Nae1	0.06	1.00000000	duck
Dcun1d5	0.18	1.00000000	duck
Polr2b	-0.13	1.00000000	duck
Fgd5	0.19	1.00000000	duck
Washc5	0.06	1.00000000	duck
Eefsec	-0.23	1.00000000	duck
Bche	0.29	1.00000000	duck
Tspan5	-1.40	1.00000000	duck
Tada2b	0.21	1.00000000	duck
Fndc3a	0.08	1.00000000	duck
Cep120	0.26	1.00000000	duck
Crmp1	0.29	1.00000000	duck
Arhgap1	0.02	1.00000000	duck

Rpf1	-0.01	1.00000000	duck
Wipi1	-1.21	1.00000000	duck
Selenoi	0.24	1.00000000	duck
Tmem70	0.02	1.00000000	duck
Dcun1d3	-1.64	1.00000000	duck
Hmg20b	0.22	1.00000000	duck
Abl2	0.45	1.00000000	duck
Lratd1	0.22	1.00000000	duck
Vps26a	0.30	1.00000000	duck
Atad2b	-1.19	1.00000000	duck
Steep1	-0.26	1.00000000	duck
Rusc2	0.42	1.00000000	duck
Cdk7	0.13	1.00000000	duck
Cmtm3	0.18	1.00000000	duck
Parp11	0.30	1.00000000	duck
Nde1	-1.55	1.00000000	duck
Zfp532	-0.29	1.00000000	duck
Atp2b2	0.60	1.00000000	duck
Psmb7	-0.14	1.00000000	duck
Rrp15	0.32	1.00000000	duck
Yars	0.09	1.00000000	duck
Gne	0.28	1.00000000	duck
Rala	-0.14	1.00000000	duck
Acp2	0.02	1.00000000	duck
Rer1	0.13	1.00000000	duck
Bcl7a	-0.18	1.00000000	duck
Zfp503	0.15	1.00000000	duck
Slc39a6	0.15	1.00000000	duck
Plppr5	0.32	1.00000000	duck
Snap47	0.27	1.00000000	duck
Ash1l	-0.89	1.00000000	duck
Hdac2	-0.24	1.00000000	duck
Rtf2	0.33	1.00000000	duck
Galnt18	0.15	1.00000000	duck
Bpnt1	0.30	1.00000000	duck
Pard6g	0.36	1.00000000	duck
Tm9sf2	-0.03	1.00000000	duck
Trib2	0.55	1.00000000	duck
Surf2	0.18	1.00000000	duck
Fam126a	0.07	1.00000000	duck
Tbc1d14	0.42	1.00000000	duck
Hormad1	0.35	1.00000000	duck
Psmd5	-0.01	1.00000000	duck
Acbd3	0.33	1.00000000	duck
Sema7a	0.20	1.00000000	duck
Ugt8a	0.34	1.00000000	duck

Hnrnpm	0.06	1.00000000	duck
Pex5	0.05	1.00000000	duck
Reep2	0.07	1.00000000	duck
Reln	1.08	1.00000000	duck
Cops7b	-0.01	1.00000000	duck
Kif15	0.46	1.00000000	duck
Tpm3-rs7	0.21	1.00000000	duck
Tmem231	0.25	1.00000000	duck
Sirt5	0.31	1.00000000	duck
Tmem38b	0.17	1.00000000	duck
Itgae	0.40	1.00000000	duck
Crnkl1	-0.09	1.00000000	duck
Kifap3	0.17	1.00000000	duck
Yae1d1	0.06	1.00000000	duck
Ift74	0.29	1.00000000	duck
Dhx36	0.10	1.00000000	duck
Ppm1k	0.09	1.00000000	duck
Dpm2	0.03	1.00000000	duck
Agap3	0.39	1.00000000	duck
Rad21	-0.04	1.00000000	duck
Tex264	0.16	1.00000000	duck
Vcl	0.02	1.00000000	duck
Tnpo1	0.10	1.00000000	duck
Hps3	0.26	1.00000000	duck
Aasdh	-0.91	1.00000000	duck
Wapl	-1.04	1.00000000	duck
Itgb4	0.12	1.00000000	duck
Tom1	0.17	1.00000000	duck
Slc25a26	0.05	1.00000000	duck
Rhbdf1	0.39	1.00000000	duck
AF366264	0.10	1.00000000	duck
Rasgef1b	0.82	1.00000000	duck
Exosc7	0.12	1.00000000	duck
Actl6a	0.21	1.00000000	duck
Irf2	-1.27	1.00000000	duck
Lyar	-0.06	1.00000000	duck
Akap1	0.22	1.00000000	duck
Sft2d1	0.03	1.00000000	duck
Ppt1	0.19	1.00000000	duck
Tmem43	0.11	1.00000000	duck
Fam189a2	0.30	1.00000000	duck
Caprin1	-0.28	1.00000000	duck
Vgll1	-1.03	1.00000000	duck
Snu13	0.02	1.00000000	duck
Cog1	0.19	1.00000000	duck
Runx2	-0.18	1.00000000	duck

Ino80c	0.11	1.00000000	duck
Sec13	-0.17	1.00000000	duck
Plxnd1	0.55	1.00000000	duck
Kdm2b	0.48	1.00000000	duck
Fam185a	0.08	1.00000000	duck
Exoc1	0.23	1.00000000	duck
Zmpste24	0.00	1.00000000	duck
B4galt2	0.19	1.00000000	duck
Arid2	-1.41	1.00000000	duck
Rbx1	0.03	1.00000000	duck
Pin1	0.13	1.00000000	duck
Dapp1	-0.03	1.00000000	duck
Vcpkmt	0.45	1.00000000	duck
Gorab	0.09	1.00000000	duck
Dcn	0.57	1.00000000	duck
Herpud2	0.07	1.00000000	duck
Psmd4	-0.08	1.00000000	duck
Btbd6	0.40	1.00000000	duck
Setx	0.04	1.00000000	duck
Ythdc1	-1.36	1.00000000	duck
Rab3b	0.20	1.00000000	duck
Eif2b2	0.26	1.00000000	duck
Gusb	0.35	1.00000000	duck
Smyd2	0.19	1.00000000	duck
Stim1	0.04	1.00000000	duck
Snrnp200	0.11	1.00000000	duck
Batf	0.16	1.00000000	duck
Ebag9	0.00	1.00000000	duck
Chchd1	0.26	1.00000000	duck
Lrig1	0.12	1.00000000	duck
Golga7	0.00	1.00000000	duck
Zc3h7a	-1.34	1.00000000	duck
Parvg	0.51	1.00000000	duck
Septin7	-0.12	1.00000000	duck
Camsap1	0.29	1.00000000	duck
Ppp1cc	0.11	1.00000000	duck
Adgra3	0.32	1.00000000	duck
Tiam2	0.77	1.00000000	duck
Heatr1	0.30	1.00000000	duck
Ptpre	0.00	1.00000000	duck
Rnf141	0.20	1.00000000	duck
Abhd17c	0.06	1.00000000	duck
Rreb1	-0.15	1.00000000	duck
Polr3k	-0.04	1.00000000	duck
Cwc25	0.15	1.00000000	duck
Golm1	0.48	1.00000000	duck

Diaph2	0.23	1.00000000	duck
Prdm10	-1.14	1.00000000	duck
Copb2	-0.23	1.00000000	duck
Slc45a1	0.25	1.00000000	duck
Gjc2	0.19	1.00000000	duck
Vps37c	-0.04	1.00000000	duck
Slc25a24	-1.43	1.00000000	duck
Cpsf2	0.08	1.00000000	duck
Fkbp3	-0.02	1.00000000	duck
Larp4	-0.23	1.00000000	duck
Slc35c2	0.05	1.00000000	duck
Arcn1	-0.06	1.00000000	duck
Elf2	0.08	1.00000000	duck
Slc2a8	0.09	1.00000000	duck
Rhobtb2	0.36	1.00000000	duck
Pdxdc1	0.23	1.00000000	duck
Usp5	0.23	1.00000000	duck
Scamp2	-0.06	1.00000000	duck
Parp10	0.11	1.00000000	duck
Prdx3	-0.09	1.00000000	duck
Ndufa5	-0.07	1.00000000	duck
Abracl	0.34	1.00000000	duck
Gpn1	-0.06	1.00000000	duck
Gab1	-1.12	1.00000000	duck
Scamp1	0.02	1.00000000	duck
Jpt1	-0.18	1.00000000	duck
Slc22a4	-0.79	1.00000000	duck
Pik3cd	0.38	1.00000000	duck
Arrdc4	0.19	1.00000000	duck
Brip1	0.08	1.00000000	duck
Brms1l	0.03	1.00000000	duck
Aoah	0.20	1.00000000	duck
Nol10	0.30	1.00000000	duck
Golga3	-0.27	1.00000000	duck
Lonp2	0.06	1.00000000	duck
Hmox2	0.14	1.00000000	duck
Lats1	-0.09	1.00000000	duck
Sppl3	-0.22	1.00000000	duck
Gtf3c1	-0.17	1.00000000	duck
Cog5	-0.23	1.00000000	duck
Kctd3	0.23	1.00000000	duck
Eif2ak1	0.17	1.00000000	duck
Socs7	0.22	1.00000000	duck
Rabl6	-0.04	1.00000000	duck
Otulin	0.32	1.00000000	duck
Dcun1d4	-0.23	1.00000000	duck

Wdr35	0.27	1.00000000	duck
Fgfrl1	1.08	1.00000000	duck
Capg	0.15	1.00000000	duck
Homer1	0.63	1.00000000	duck
Haus2	0.19	1.00000000	duck
Urb1	0.33	1.00000000	duck
Trappc6b	0.13	1.00000000	duck
Lbh	0.39	1.00000000	duck
Cactin	0.13	1.00000000	duck
Wfs1	0.18	1.00000000	duck
Slk	-0.01	1.00000000	duck
Chd9	-0.06	1.00000000	duck
Prdm2	-0.20	1.00000000	duck
Cmas	0.05	1.00000000	duck
Niban1	0.07	1.00000000	duck
Kctd9	0.30	1.00000000	duck
Acsl1	-0.03	1.00000000	duck
Eaf1	0.22	1.00000000	duck
Cep192	-0.10	1.00000000	duck
Dennd1b	0.02	1.00000000	duck
Ampd3	0.05	1.00000000	duck
Sgsm3	0.05	1.00000000	duck
Palb2	0.24	1.00000000	duck
Epha4	0.59	1.00000000	duck
Smim12	0.18	1.00000000	duck
Gpbp1	0.13	1.00000000	duck
Akap8l	-0.07	1.00000000	duck
Elp1	-0.13	1.00000000	duck
Dcbld2	-0.12	1.00000000	duck
Ndrg3	-0.16	1.00000000	duck
Hspa5	0.08	1.00000000	duck
Nutf2	-0.02	1.00000000	duck
Vcpip1	-1.42	1.00000000	duck
Trpm7	0.02	1.00000000	duck
Prkcz	-0.03	1.00000000	duck
Fzr1	0.06	1.00000000	duck
Cnot6	-0.18	1.00000000	duck
Fam102a	0.06	1.00000000	duck
Ttc27	0.10	1.00000000	duck
Erg28	0.07	1.00000000	duck
Chmp5	-0.10	1.00000000	duck
Psme3	0.09	1.00000000	duck
Qrich1	-0.05	1.00000000	duck
Pbdc1	-0.04	1.00000000	duck
Rarb	0.36	1.00000000	duck
Rnaseh2b	-0.24	1.00000000	duck

Tecpr1	0.29	1.00000000	duck
Farp2	0.08	1.00000000	duck
Caml	0.04	1.00000000	duck
Ppp4r1	0.31	1.00000000	duck
Tmem47	0.11	1.00000000	duck
Aco1	0.05	1.00000000	duck
Prmt3	0.05	1.00000000	duck
Dlg5	0.37	1.00000000	duck
Ppp1r3b	0.36	1.00000000	duck
Recql5	0.48	1.00000000	duck
Kifbp	0.05	1.00000000	duck
Plpbp	0.23	1.00000000	duck
Fbxo34	-1.52	1.00000000	duck
Stard4	0.30	1.00000000	duck
Ncf1	0.41	1.00000000	duck
Trp53rka	0.23	1.00000000	duck
Fam118b	0.15	1.00000000	duck
Sf3a3	0.37	1.00000000	duck
4933400A11Rik	0.26	1.00000000	duck
Rnf214	0.13	1.00000000	duck
Vdr	0.14	1.00000000	duck
Lmbrd1	0.28	1.00000000	duck
Ubl5b	0.35	1.00000000	duck
Slc4a2	0.02	1.00000000	duck
Ncoa3	-0.09	1.00000000	duck
Cotl1	0.19	1.00000000	duck
Hypk	-0.06	1.00000000	duck
Rnf121	0.12	1.00000000	duck
Pitpna	0.23	1.00000000	duck
Rbm22	0.02	1.00000000	duck
Slc39a11	0.05	1.00000000	duck
Syap1	0.30	1.00000000	duck
Zeb1	-1.53	1.00000000	duck
Nufip1	0.12	1.00000000	duck
Arhgap40	0.19	1.00000000	duck
Elmod2	0.18	1.00000000	duck
Nras	0.17	1.00000000	duck
Sirt6	0.09	1.00000000	duck
Tcf25	-0.02	1.00000000	duck
Dnajb2	0.06	1.00000000	duck
Prkx	-1.79	1.00000000	duck
Ywhaz	0.13	1.00000000	duck
Utp3	0.08	1.00000000	duck
Pdss1	0.13	1.00000000	duck
Foxn2	0.12	1.00000000	duck
Tm6sf1	0.35	1.00000000	duck

Dyrk2	0.15	1.00000000	duck
Heg1	1.02	1.00000000	duck
Tdp2	0.06	1.00000000	duck
Plaa	0.14	1.00000000	duck
Ddx18	0.26	1.00000000	duck
Zc3h12c	0.38	1.00000000	duck
Nop2	0.19	1.00000000	duck
Pdcl	-0.04	1.00000000	duck
Ece1	0.50	1.00000000	duck
Cnot11	-0.16	1.00000000	duck
Ttf1	-0.03	1.00000000	duck
Atp2c1	-0.18	1.00000000	duck
Tcf4	-0.16	1.00000000	duck
Picalm	-0.17	1.00000000	duck
Pxmp4	0.08	1.00000000	duck
Brox	0.03	1.00000000	duck
Chrac1	0.06	1.00000000	duck
Yme1l1	-0.24	1.00000000	duck
Arntl2	-0.03	1.00000000	duck
Auts2	0.39	1.00000000	duck
Srsf10	-0.21	1.00000000	duck
Rbm17	-0.14	1.00000000	duck
Coq9	0.20	1.00000000	duck
Tmem117	0.19	1.00000000	duck
Trmt1l	0.14	1.00000000	duck
Eps15	-0.11	1.00000000	duck
Bmp2	0.02	1.00000000	duck
Dhx29	0.16	1.00000000	duck
Ints1	0.11	1.00000000	duck
Smchd1	-1.50	1.00000000	duck
Itga2	0.51	1.00000000	duck
Tsr3	0.10	1.00000000	duck
Ssu72	-1.05	1.00000000	duck
Syt14	0.26	1.00000000	duck
Tacc1	0.22	1.00000000	duck
Tmem87a	-0.26	1.00000000	duck
Natd1	0.28	1.00000000	duck
Ndufv2	-0.16	1.00000000	duck
Ociad1	-0.13	1.00000000	duck
Arfgap2	0.12	1.00000000	duck
Ppp1r12a	0.13	1.00000000	duck
Pigk	-0.21	1.00000000	duck
Sass6	0.36	1.00000000	duck
C1qtnf12	0.31	1.00000000	duck
F13a1	0.13	1.00000000	duck
Tnip2	-0.24	1.00000000	duck

Calcrl	0.87	1.00000000	duck
Pacs2	0.31	1.00000000	duck
Slc7a3	0.25	1.00000000	duck
Ttpal	-0.68	1.00000000	duck
Osbpl2	-0.19	1.00000000	duck
Astn2	0.43	1.00000000	duck
Arhgap31	0.51	1.00000000	duck
Hook1	0.00	1.00000000	duck
Tgif1	0.04	1.00000000	duck
Tnks2	0.26	1.00000000	duck
Hivep2	-0.46	1.00000000	duck
Tcim	0.17	1.00000000	duck
Emb	0.10	1.00000000	duck
Lrrc39	0.76	1.00000000	duck
Sec24b	-1.20	1.00000000	duck
Lnpk	0.16	1.00000000	duck
Babam2	-0.47	1.00000000	duck
Rp2	0.04	1.00000000	duck
Ormdl1	0.07	1.00000000	duck
Dcaf13	0.09	1.00000000	duck
Raf1	0.06	1.00000000	duck
Sgcb	0.17	1.00000000	duck
Etnk1	0.32	1.00000000	duck
Gtf2b	0.07	1.00000000	duck
Zdhhc9	0.11	1.00000000	duck
Ube3c	-1.21	1.00000000	duck
Ankrd46	0.07	1.00000000	duck
Rcsd1	0.36	1.00000000	duck
Aspg	0.47	1.00000000	duck
Tspan3	-0.02	1.00000000	duck
Satb1	-0.01	1.00000000	duck
Tmem86a	0.21	1.00000000	duck
Gnal	0.68	1.00000000	duck
Rsf1	-1.51	1.00000000	duck
Cnot1	-0.18	1.00000000	duck
Nptx2	0.06	1.00000000	duck
Hnrnpab	-1.01	1.00000000	duck
Plcd1	0.19	1.00000000	duck
Zcchc14	-0.16	1.00000000	duck
Nectin3	0.22	1.00000000	duck
Zpr1	0.13	1.00000000	duck
Lamb2	0.22	1.00000000	duck
Pccb	0.02	1.00000000	duck
Tcerg1	-0.34	1.00000000	duck
Afg1l	-0.01	1.00000000	duck
Tmem185a	0.50	1.00000000	duck

Hnrnpa3	0.11	1.00000000	duck
Prkar1a	0.11	1.00000000	duck
Acadm	-0.07	1.00000000	duck
Asb7	-0.16	1.00000000	duck
Neurl1b	0.51	1.00000000	duck
Pdp1	-0.07	1.00000000	duck
Gnl3	-0.07	1.00000000	duck
Ddx27	-0.16	1.00000000	duck
Kif3a	-0.02	1.00000000	duck
Kdm5b	-0.04	1.00000000	duck
Wdr12	0.08	1.00000000	duck
Selenok	0.07	1.00000000	duck
Sh3bgrl2	0.10	1.00000000	duck
Vma21	0.09	1.00000000	duck
Srp9	-0.19	1.00000000	duck
Nob1	0.22	1.00000000	duck
Ythdf2	-0.12	1.00000000	duck
Nfyc	-0.06	1.00000000	duck
Wdsub1	0.00	1.00000000	duck
Prepl	0.31	1.00000000	duck
Npepps	0.07	1.00000000	duck
Slc7a5	0.46	1.00000000	duck
Smox	-1.32	1.00000000	duck
Rab10	-0.04	1.00000000	duck
Ift27	0.40	1.00000000	duck
Zfr	-0.11	1.00000000	duck
Fnta	0.11	1.00000000	duck
Zcrb1	-0.11	1.00000000	duck
Cep43	-1.01	1.00000000	duck
Otud7a	0.37	1.00000000	duck
Zfp131	0.04	1.00000000	duck
Rnps1	0.25	1.00000000	duck
Stk40	0.08	1.00000000	duck
Rnf128	-1.41	1.00000000	duck
Sod1	-0.01	1.00000000	duck
Fbn1	0.40	1.00000000	duck
St13	-0.07	1.00000000	duck
Klf11	0.24	1.00000000	duck
Derl3	0.27	1.00000000	duck
Fam98b	0.06	1.00000000	duck
Gtpbp1	0.07	1.00000000	duck
Tmf1	-0.23	1.00000000	duck
Habp4	0.09	1.00000000	duck
Atp5c1	0.01	1.00000000	duck
Pdgfc	0.46	1.00000000	duck
Nup98	-0.22	1.00000000	duck

Asf1a	0.02	1.00000000	duck
Scamp4	0.01	1.00000000	duck
F2rl3	0.96	1.00000000	duck
Fcho2	-1.33	1.00000000	duck
Nkiras2	0.19	1.00000000	duck
Ablim1	0.10	1.00000000	duck
Phrf1	0.03	1.00000000	duck
Dlat	0.06	1.00000000	duck
Adssl1	0.34	1.00000000	duck
Pcif1	0.03	1.00000000	duck
Ccdc18	-0.87	1.00000000	duck
Scamp3	0.18	1.00000000	duck
Ap2b1	0.07	1.00000000	duck
Ppig	-0.02	1.00000000	duck
Klhl18	0.25	1.00000000	duck
Stk38	0.15	1.00000000	duck
Eftud2	0.05	1.00000000	duck
Klhl22	0.17	1.00000000	duck
Zfpm1	0.43	1.00000000	duck
Cep112	0.15	1.00000000	duck
Lin52	-0.09	1.00000000	duck
Rab35	-0.04	1.00000000	duck
Paip2	0.09	1.00000000	duck
Fyb	0.62	1.00000000	duck
Pip5k1b	-0.26	1.00000000	duck
Rbis	-0.07	1.00000000	duck
Ppcs	0.19	1.00000000	duck
Zc3h15	-0.32	1.00000000	duck
Vps72	0.09	1.00000000	duck
Rmdn3	0.47	1.00000000	duck
Pstpip2	0.42	1.00000000	duck
Eif3j2	-0.23	1.00000000	duck
B9d1	0.39	1.00000000	duck
Timp2	0.20	1.00000000	duck
Tpd52l2	0.01	1.00000000	duck
Nod1	0.21	1.00000000	duck
Cpe	-0.02	1.00000000	duck
Alpk1	0.08	1.00000000	duck
Nfatc1	1.54	1.00000000	duck
Chpf2	0.07	1.00000000	duck
Strn	-0.08	1.00000000	duck
Yipf5	-0.26	1.00000000	duck
Gpr137c	0.07	1.00000000	duck
Cenpu	0.22	1.00000000	duck
Sec23a	-0.24	1.00000000	duck
Arglu1	-1.05	1.00000000	duck

Psmc3	-0.01	1.00000000	duck
Scfd2	0.13	1.00000000	duck
Tmtc1	0.55	1.00000000	duck
Pcnx	-1.45	1.00000000	duck
Timm44	-0.19	1.00000000	duck
Focad	0.36	1.00000000	duck
Ap2m1	-0.27	1.00000000	duck
Trmt10a	0.06	1.00000000	duck
Tcea1	0.04	1.00000000	duck
Phlda1	0.14	1.00000000	duck
Serpini1	0.05	1.00000000	duck
Aff1	-0.13	1.00000000	duck
Rflnb	0.15	1.00000000	duck
Nup210	0.03	1.00000000	duck
Psmc1	-0.17	1.00000000	duck
Slc30a5	0.08	1.00000000	duck
Unc50	0.14	1.00000000	duck
Gnl2	0.01	1.00000000	duck
Cep350	-1.21	1.00000000	duck
Syde2	0.20	1.00000000	duck
Cass4	0.56	1.00000000	duck
Ackr1	-0.72	1.00000000	duck
Zmat5	0.04	1.00000000	duck
Arl5b	-0.13	1.00000000	duck
Fam189a1	0.60	1.00000000	duck
Hectd1	-0.95	1.00000000	duck
Naga	0.04	1.00000000	duck
Ints11	0.04	1.00000000	duck
Trmt6	-0.11	1.00000000	duck
Pigl	0.07	1.00000000	duck
Pcmtd2	-0.16	1.00000000	duck
Cdc20	0.81	1.00000000	duck
Btg2	0.22	1.00000000	duck
Spire1	0.30	1.00000000	duck
Glrx	0.56	1.00000000	duck
Gcn1	-0.01	1.00000000	duck
Psmc2	-0.27	1.00000000	duck
Glb1	0.17	1.00000000	duck
Klhdc2	0.19	1.00000000	duck
Sucla2	-0.27	1.00000000	duck
Ciao3	-1.19	1.00000000	duck
Rnf41	0.07	1.00000000	duck
Creb1	0.01	1.00000000	duck
Lmo4	0.32	1.00000000	duck
Ube2q1	-0.04	1.00000000	duck
Acbd6	-0.20	1.00000000	duck

Rabgap1	-0.01	1.00000000	duck
Cntnap2	0.42	1.00000000	duck
Napg	-0.08	1.00000000	duck
Gemin5	0.03	1.00000000	duck
Sla	0.56	1.00000000	duck
Septin6	0.08	1.00000000	duck
Irx2	0.03	1.00000000	duck
Spag1	0.18	1.00000000	duck
Rab14	-0.26	1.00000000	duck
Fbxw11	-0.17	1.00000000	duck
Gramd1b	0.48	1.00000000	duck
Fmnl1	0.15	1.00000000	duck
Pigq	-0.94	1.00000000	duck
Susd3	0.30	1.00000000	duck
Pex2	0.02	1.00000000	duck
Ppp1r16b	0.33	1.00000000	duck
Cep83	-0.31	1.00000000	duck
Gnpda2	0.16	1.00000000	duck
Ankle2	0.06	1.00000000	duck
Suco	-0.22	1.00000000	duck
Rab3gap2	-0.32	1.00000000	duck
Rsbn1	0.07	1.00000000	duck
Abcc4	-1.13	1.00000000	duck
Samd11	0.17	1.00000000	duck
Commd9	0.32	1.00000000	duck
Pygl	-0.08	1.00000000	duck
Axin1	-1.22	1.00000000	duck
Bin1	0.31	1.00000000	duck
Wdr91	-0.25	1.00000000	duck
Rcan3	0.12	1.00000000	duck
Rap1gds1	0.04	1.00000000	duck
Aftph	0.13	1.00000000	duck
Nova1	0.96	1.00000000	duck
Riok1	0.07	1.00000000	duck
Fbxo32	-0.83	1.00000000	duck
Ubr3	-1.04	1.00000000	duck
Dmap1	-0.98	1.00000000	duck
Zfp512	-0.34	1.00000000	duck
Exoc2	-0.05	1.00000000	duck
Dtnbp1	-1.41	1.00000000	duck
Tpra1	-0.18	1.00000000	duck
Rbm34	-0.12	1.00000000	duck
Phtf1	-1.15	1.00000000	duck
Uba5	-0.07	1.00000000	duck
Klf9	0.24	1.00000000	duck
Ppp2r5c	-1.43	1.00000000	duck

Tbc1d23	-0.09	1.00000000	duck
Hexb	-0.07	1.00000000	duck
Fat4	0.75	1.00000000	duck
Ergic2	-0.36	1.00000000	duck
Ago4	-0.13	1.00000000	duck
Pank3	-0.08	1.00000000	duck
Hoxa5	0.31	1.00000000	duck
Nfyb	-0.09	1.00000000	duck
Dph3	0.04	1.00000000	duck
Exoc6	-0.12	1.00000000	duck
Gpatch2l	-1.37	1.00000000	duck
Kdsr	-0.35	1.00000000	duck
Tmem123	-0.08	1.00000000	duck
Slc6a4	0.36	1.00000000	duck
Wdr81	0.06	1.00000000	duck
Eif4g3	-1.02	1.00000000	duck
Slc35a5	-0.13	1.00000000	duck
Fbxo45	0.23	1.00000000	duck
Rnft1	-0.15	1.00000000	duck
Puf60	-0.12	1.00000000	duck
Idh1	0.06	1.00000000	duck
Fbxl2	0.02	1.00000000	duck
Ddx20	-0.02	1.00000000	duck
Reps1	0.00	1.00000000	duck
Trappc13	-0.01	1.00000000	duck
Dok2	0.40	1.00000000	duck
Pik3r1	0.22	1.00000000	duck
Pigf	-0.08	1.00000000	duck
Ptpn1	0.48	1.00000000	duck
St3gal3	0.00	1.00000000	duck
Kiz	0.29	1.00000000	duck
Slf2	0.15	1.00000000	duck
Arpp19	-0.10	1.00000000	duck
Eogt	0.52	1.00000000	duck
Acap2	-0.15	1.00000000	duck
Gclc	-0.74	1.00000000	duck
Crk	0.05	1.00000000	duck
Qser1	-0.41	1.00000000	duck
Slc30a9	-1.26	1.00000000	duck
Zc3h12b	0.02	1.00000000	duck
Smad2	-0.08	1.00000000	duck
Ska2	-0.20	1.00000000	duck
Nelfb	0.04	1.00000000	duck
Chchd7	0.01	1.00000000	duck
Tax1bp1	-0.70	1.00000000	duck
Scml2	-0.98	1.00000000	duck

Cisd2	-0.03	1.00000000	duck
Chst13	0.22	1.00000000	duck
Naa15	-0.36	1.00000000	duck
Dnajc21	0.09	1.00000000	duck
Slc35e3	0.09	1.00000000	duck
Zrsr1	-0.16	1.00000000	duck
Vta1	-0.07	1.00000000	duck
Snrnp35	0.38	1.00000000	duck
Usp47	-0.29	1.00000000	duck
Tbl1xr1	-1.12	1.00000000	duck
Fbf1	-0.12	1.00000000	duck
Wasf2	-0.13	1.00000000	duck
Zfp217	-0.18	1.00000000	duck
Isca2	-0.04	1.00000000	duck
Fam53a	-0.37	1.00000000	duck
Rad51ap1	-0.11	1.00000000	duck
Vps13d	-0.91	1.00000000	duck
AU040320	-0.17	1.00000000	duck
Nol8	0.05	1.00000000	duck
Rrn3	0.08	1.00000000	duck
Nipal3	-0.04	1.00000000	duck
Comt	-0.12	1.00000000	duck
Swi5	-0.34	1.00000000	duck
Katnip	0.03	1.00000000	duck
Angptl2	-0.01	1.00000000	duck
Add1	-1.19	1.00000000	duck
Jcad	0.30	1.00000000	duck
Oga	-0.01	1.00000000	duck
Cables2	-1.04	1.00000000	duck
Wars	-0.04	1.00000000	duck
Dsn1	0.12	1.00000000	duck
Bin2	0.14	1.00000000	duck
Dgkd	-0.12	1.00000000	duck
Scrib	0.02	1.00000000	duck
Tmcc3	0.76	1.00000000	duck
She	0.46	1.00000000	duck
Nol9	-0.06	1.00000000	duck
Ntn4	0.40	1.00000000	duck
Fgl2	1.01	1.00000000	duck
Plag1	-1.82	1.00000000	duck
Prpf19	0.04	1.00000000	duck
Zmym2	0.23	1.00000000	duck
Ndnf	0.34	1.00000000	duck
Fam114a2	0.05	1.00000000	duck
Aars	0.06	1.00000000	duck
Fbxo22	0.08	1.00000000	duck

Supt3	-0.25	1.00000000	duck
Cars	-0.05	1.00000000	duck
Rb1	-0.22	1.00000000	duck
Ttll1	-0.02	1.00000000	duck
Ccdc6	-0.30	1.00000000	duck
Ppp1r8	0.33	1.00000000	duck
Osbpl8	-0.02	1.00000000	duck
Tlk1	-0.06	1.00000000	duck
C2cd3	-0.91	1.00000000	duck
Fem1a	-0.25	1.00000000	duck
Rnf14	0.10	1.00000000	duck
Ciao2a	-0.17	1.00000000	duck
Bcl11a	-0.99	1.00000000	duck
Snx18	0.24	1.00000000	duck
Rell1	-0.05	1.00000000	duck
Rfx3	0.06	1.00000000	duck
Septin2	0.13	1.00000000	duck
Lix1	0.55	1.00000000	duck
Eny2	-0.10	1.00000000	duck
Sar1b	-0.04	1.00000000	duck
C1galt1	-0.12	1.00000000	duck
Wdr70	-0.26	1.00000000	duck
Tcp11l2	-1.16	1.00000000	duck
Acaa2	-0.27	1.00000000	duck
Glt8d1	0.12	1.00000000	duck
Gnai3	-0.33	1.00000000	duck
Czib	0.08	1.00000000	duck
Jmjd4	0.19	1.00000000	duck
Nek1	0.00	1.00000000	duck
Erp44	-0.22	1.00000000	duck
Vangl1	0.16	1.00000000	duck
Ddx10	0.08	1.00000000	duck
Aurkaip1	0.03	1.00000000	duck
Zcchc24	0.28	1.00000000	duck
Lclat1	-0.25	1.00000000	duck
Slc30a6	0.12	1.00000000	duck
Ereg	0.10	1.00000000	duck
Jarid2	-0.83	1.00000000	duck
Grk2	0.34	1.00000000	duck
Ildr2	0.27	1.00000000	duck
Snx12	-0.18	1.00000000	duck
Cebpz	-0.07	1.00000000	duck
Col5a2	0.29	1.00000000	duck
Smndc1	0.15	1.00000000	duck
Tank	0.02	1.00000000	duck
Rsrc1	-0.16	1.00000000	duck

Cish	-1.05	1.00000000	duck
Exosc3	-0.27	1.00000000	duck
Adnp2	-0.09	1.00000000	duck
Abhd17a	0.01	1.00000000	duck
Pank1	-0.06	1.00000000	duck
Chaf1a	-0.12	1.00000000	duck
Dctn5	0.26	1.00000000	duck
Lonp1	-0.10	1.00000000	duck
Fam199x	-0.07	1.00000000	duck
Casp7	0.00	1.00000000	duck
Anp32e	-0.30	1.00000000	duck
Cnppd1	0.05	1.00000000	duck
Spast	-0.16	1.00000000	duck
1110059G10Rik	-0.27	1.00000000	duck
Arfrp1	-0.20	1.00000000	duck
Sart3	0.02	1.00000000	duck
Il15	0.30	1.00000000	duck
Ap1m1	-0.12	1.00000000	duck
Strip1	0.10	1.00000000	duck
Tbc1d9b	0.00	1.00000000	duck
Nbas	-0.37	1.00000000	duck
Tor3a	0.20	1.00000000	duck
Vps11	0.23	1.00000000	duck
Hlcs	-0.40	1.00000000	duck
Lmbrd2	-0.36	1.00000000	duck
Syn2	-0.12	1.00000000	duck
Eif5b	-1.19	1.00000000	duck
Ublcp1	0.01	1.00000000	duck
Thoc5	0.03	1.00000000	duck
Trappc4	-0.16	1.00000000	duck
Ampd2	0.10	1.00000000	duck
Cdkn1b	0.12	1.00000000	duck
Nup35	0.09	1.00000000	duck
Usf3	-0.13	1.00000000	duck
Eipr1	-1.35	1.00000000	duck
Pip5k1a	-0.16	1.00000000	duck
Pdcd2	0.06	1.00000000	duck
Tmtc3	-0.49	1.00000000	duck
Baz1b	-0.20	1.00000000	duck
Trim33	-0.20	1.00000000	duck
Gna13	-0.35	1.00000000	duck
Rpf2	-0.31	1.00000000	duck
Prps2	0.09	1.00000000	duck
Dhx30	0.03	1.00000000	duck
Samm50	-0.32	1.00000000	duck
Ppp2cb	-0.22	1.00000000	duck

Psme3ip1	-0.17	1.00000000	duck
Ddx49	-0.09	1.00000000	duck
Ch25h	0.50	1.00000000	duck
Znfx1	0.00	1.00000000	duck
Nipsnap1	0.11	1.00000000	duck
Atp13a1	0.03	1.00000000	duck
Rab40c	0.09	1.00000000	duck
Ccdc186	0.02	1.00000000	duck
Rpa1	0.05	1.00000000	duck
Rab30	0.12	1.00000000	duck
Gatad1	0.20	1.00000000	duck
Taf1a	0.11	1.00000000	duck
Sdf4	-0.26	1.00000000	duck
Spi1	0.20	1.00000000	duck
Znhit6	-0.04	1.00000000	duck
Pdlim7	0.49	1.00000000	duck
Rbm26	-0.79	1.00000000	duck
Abraxas1	0.20	1.00000000	duck
Wdr7	-1.20	1.00000000	duck
Nuak1	0.05	1.00000000	duck
Ccp110	-0.27	1.00000000	duck
Tbc1d12	-0.02	1.00000000	duck
Dhx35	-0.01	1.00000000	duck
Ccdc66	-0.07	1.00000000	duck
Naa50	-0.21	1.00000000	duck
Chst11	-0.21	1.00000000	duck
Itch	-1.50	1.00000000	duck
Rnf10	-1.10	1.00000000	duck
Tex10	-0.06	1.00000000	duck
Ncbp3	-0.21	1.00000000	duck
Ubl7	0.16	1.00000000	duck
Eif4g2	0.03	1.00000000	duck
Cln6	-0.04	1.00000000	duck
Gpn3	-0.26	1.00000000	duck
Aimp1	-0.27	1.00000000	duck
Pknox1	-1.27	1.00000000	duck
Tnfaip1	0.01	1.00000000	duck
Hoxa3	0.32	1.00000000	duck
Rint1	-0.02	1.00000000	duck
Eepd1	0.43	1.00000000	duck
Fbxo28	-0.36	1.00000000	duck
Ccnl2	-0.07	1.00000000	duck
Uvrag	-0.24	1.00000000	duck
Drg1	0.14	1.00000000	duck
Zfyve9	-1.23	1.00000000	duck
Tmem104	-0.08	1.00000000	duck

Eya3	0.03	1.00000000	duck
Zfp507	0.08	1.00000000	duck
Phlpp2	-0.22	1.00000000	duck
Epb41l2	-0.06	1.00000000	duck
Hdx	-0.04	1.00000000	duck
Rab9	-0.32	1.00000000	duck
Cbx1	-0.21	1.00000000	duck
Gapvd1	-0.35	1.00000000	duck
Wbp11	-0.39	1.00000000	duck
Prpf4	0.03	1.00000000	duck
Trps1	0.00	1.00000000	duck
Tet2	-0.09	1.00000000	duck
Btbd7	-0.14	1.00000000	duck
Ascc3	-1.58	1.00000000	duck
Arhgap17	-0.16	1.00000000	duck
Snx14	-0.18	1.00000000	duck
Gng10	0.29	1.00000000	duck
Dbf4	0.00	1.00000000	duck
Qars	-0.09	1.00000000	duck
Cebpg	-0.09	1.00000000	duck
Rsbn1l	0.29	1.00000000	duck
Fam167b	0.21	1.00000000	duck
Clip4	-0.14	1.00000000	duck
Sin3a	-0.05	1.00000000	duck
Spg21	0.00	1.00000000	duck
Exoc7	-0.12	1.00000000	duck
Prr5	0.38	1.00000000	duck
Plod2	0.15	1.00000000	duck
Ankrd44	-0.15	1.00000000	duck
Prune1	0.16	1.00000000	duck
Elavl1	0.54	1.00000000	duck
Rab5a	-0.25	1.00000000	duck
Egfl7	0.57	1.00000000	duck
Sf3a2	-0.05	1.00000000	duck
Zbtb21	-0.04	1.00000000	duck
Zfp644	-1.44	1.00000000	duck
Tmcc2	0.39	1.00000000	duck
Azi2	-1.48	1.00000000	duck
Znrf3	-0.43	1.00000000	duck
Polr3d	0.04	1.00000000	duck
Npepl1	0.01	1.00000000	duck
Senp6	-0.52	1.00000000	duck
Gpr107	-0.19	1.00000000	duck
Golga4	-0.45	1.00000000	duck
Rexo4	0.11	1.00000000	duck
Syn3	0.11	1.00000000	duck

Desi1	-0.39	1.00000000	duck
Ddx5	0.30	1.00000000	duck
Kpna1	-0.10	1.00000000	duck
Pdzd8	-0.27	1.00000000	duck
Sfmbt1	-0.10	1.00000000	duck
Atxn1	-0.05	1.00000000	duck
Ttc13	-1.13	1.00000000	duck
Eri1	0.11	1.00000000	duck
Lrrk1	-0.20	1.00000000	duck
Zfp618	-0.14	1.00000000	duck
Tssc4	-0.03	1.00000000	duck
Tsr1	-0.11	1.00000000	duck
Rnf169	0.11	1.00000000	duck
Eif2b5	0.17	1.00000000	duck
Irf8	0.21	1.00000000	duck
Tubgcp3	-0.44	1.00000000	duck
Il16	-0.02	1.00000000	duck
Jag1	0.28	1.00000000	duck
Dym	-1.09	1.00000000	duck
Snx20	0.11	1.00000000	duck
Dnajb12	-0.22	1.00000000	duck
Lrrc58	-0.01	1.00000000	duck
Naa60	0.09	1.00000000	duck
Lsm14b	-0.15	1.00000000	duck
Dhx38	0.08	1.00000000	duck
Rspry1	-0.10	1.00000000	duck
Casp8ap2	-0.37	1.00000000	duck
Tango2	0.07	1.00000000	duck
Arl5a	-0.14	1.00000000	duck
Rhot1	-0.03	1.00000000	duck
Timm22	0.12	1.00000000	duck
Nars	-0.13	1.00000000	duck
Rbm25	-0.17	1.00000000	duck
Pacc1	0.46	1.00000000	duck
Sdk1	0.33	1.00000000	duck
Prkn	0.10	1.00000000	duck
Leo1	-0.35	1.00000000	duck
Atxn7l1	-1.26	1.00000000	duck
Bcl7b	-0.26	1.00000000	duck
2510039O18Rik	-0.08	1.00000000	duck
Drosha	-0.16	1.00000000	duck
Atp6v1h	-0.10	1.00000000	duck
Cct3	-0.37	1.00000000	duck
Cntrl	-0.20	1.00000000	duck
Ccnt1	0.09	1.00000000	duck
Lyrm2	0.05	1.00000000	duck

Rragc	0.02	1.00000000	duck
Reep3	-0.61	1.00000000	duck
Cops8	-0.31	1.00000000	duck
Brpf3	-0.80	1.00000000	duck
Arhgap26	-1.06	1.00000000	duck
Rmnd1	0.03	1.00000000	duck
Fbxw8	-0.18	1.00000000	duck
Zfp639	-0.18	1.00000000	duck
Cdc73	-1.01	1.00000000	duck
Dars	-0.26	1.00000000	duck
Armc9	-0.33	1.00000000	duck
Nfya	-0.32	1.00000000	duck
Ambra1	-0.31	1.00000000	duck
Zfp609	0.00	1.00000000	duck
Zcchc10	0.02	1.00000000	duck
Tsn	-0.42	1.00000000	duck
Tshz2	0.86	1.00000000	duck
Rabep1	-0.35	1.00000000	duck
Naa16	-0.56	1.00000000	duck
Zfp518b	-0.17	1.00000000	duck
Dph5	0.00	1.00000000	duck
Birc6	-0.94	1.00000000	duck
Cds2	-0.03	1.00000000	duck
Traf3ip3	-0.73	1.00000000	duck
Ppfia1	-0.33	1.00000000	duck
Tfcp2	-0.01	1.00000000	duck
Efemp1	0.74	1.00000000	duck
Cep20	-0.12	1.00000000	duck
Psmd10	0.14	1.00000000	duck
Unc13c	0.58	1.00000000	duck
Gtf2e2	0.12	1.00000000	duck
Ccdc127	0.01	1.00000000	duck
Srp54a	-0.23	1.00000000	duck
Scaf4	-0.14	1.00000000	duck
Arih2	-1.26	1.00000000	duck
Cdk12	-0.11	1.00000000	duck
Exosc10	-0.16	1.00000000	duck
Snx9	-1.29	1.00000000	duck
Vamp4	-0.03	1.00000000	duck
Ss18	-0.23	1.00000000	duck
Ppp2r5e	-0.44	1.00000000	duck
Cfap99	0.31	1.00000000	duck
Rars	0.14	1.00000000	duck
Tomm20	-0.33	1.00000000	duck
Zfp423	-1.90	1.00000000	duck
Ndufs6	-0.10	1.00000000	duck

Invs	0.53	1.00000000	duck
Aplf	0.30	1.00000000	duck
Pgrmc2	-0.28	1.00000000	duck
Erlin1	-0.36	1.00000000	duck
Rnf139	-0.22	1.00000000	duck
Sod2	-0.28	1.00000000	duck
Kbtbd2	-0.22	1.00000000	duck
Zfyve1	-1.00	1.00000000	duck
Slc25a36	-0.35	1.00000000	duck
Ccdc92	-0.03	1.00000000	duck
Sbspon	0.19	1.00000000	duck
Cuedc1	-1.21	1.00000000	duck
Pdzd11	0.06	1.00000000	duck
Pgap1	-0.21	1.00000000	duck
Crebrf	-1.01	1.00000000	duck
Ptx3	0.23	1.00000000	duck
Exoc8	-0.08	1.00000000	duck
Uxs1	0.12	1.00000000	duck
Kctd5	-0.38	1.00000000	duck
Llph	-0.10	1.00000000	duck
Hdac11	0.03	1.00000000	duck
Ccdc43	-0.03	1.00000000	duck
Ncbp1	-0.06	1.00000000	duck
Zfp143	-1.20	1.00000000	duck
Derl1	-0.25	1.00000000	duck
Camkmt	-0.32	1.00000000	duck
Sin3b	-0.28	1.00000000	duck
Usp10	-0.44	1.00000000	duck
Entpd1	0.20	1.00000000	duck
Ccdc32	0.07	1.00000000	duck
Tbc1d22a	-0.47	1.00000000	duck
Glipr2	0.05	1.00000000	duck
Slc24a2	0.21	1.00000000	duck
Prkcb	0.08	1.00000000	duck
Ppwd1	0.00	1.00000000	duck
Btaf1	-1.18	1.00000000	duck
Vdac3	-0.18	1.00000000	duck
Ell	-0.16	1.00000000	duck
Ireb2	-1.03	1.00000000	duck
Fstl1	0.29	1.00000000	duck
Antxr2	-0.33	1.00000000	duck
Pum2	-0.97	1.00000000	duck
Smg5	-0.12	1.00000000	duck
Prpf8	-0.15	1.00000000	duck
Wdr47	0.21	1.00000000	duck
Pogz	-0.26	1.00000000	duck

Kat6a	-0.32	1.00000000	duck
Zfp384	-0.03	1.00000000	duck
Erlec1	-0.32	1.00000000	duck
Rbpj	-0.26	1.00000000	duck
Diablo	-0.10	1.00000000	duck
Trafd1	-1.30	1.00000000	duck
Ddx23	0.08	1.00000000	duck
Oaz2	0.03	1.00000000	duck
Dop1b	-0.27	1.00000000	duck
Ankrd52	-0.30	1.00000000	duck
Enpp4	0.09	1.00000000	duck
Ppp1r1c	0.36	1.00000000	duck
Pum1	-0.24	1.00000000	duck
Fam126b	-1.20	1.00000000	duck
Usp24	-0.55	1.00000000	duck
Fam20b	-0.05	1.00000000	duck
Dstyk	-0.25	1.00000000	duck
Scai	-0.36	1.00000000	duck
Ephb1	0.13	1.00000000	duck
Prdm15	-0.09	1.00000000	duck
Trp53inp1	-0.21	1.00000000	duck
Trnt1	0.16	1.00000000	duck
Prex2	0.56	1.00000000	duck
Tiprl	0.14	1.00000000	duck
Sp3	-0.07	1.00000000	duck
Cwf19l1	-0.09	1.00000000	duck
Pex14	-0.10	1.00000000	duck
G3bp1	-0.29	1.00000000	duck
B4galt3	0.08	1.00000000	duck
Gabpb2	-0.77	1.00000000	duck
Adss	-0.28	1.00000000	duck
Wdr45b	-0.42	1.00000000	duck
Hbs1l	-1.26	1.00000000	duck
Stx11	0.35	1.00000000	duck
Snrpb2	-0.20	1.00000000	duck
Thap4	-0.68	1.00000000	duck
Snrk	-1.16	1.00000000	duck
Prkd3	-0.29	1.00000000	duck
Smc4	-0.01	1.00000000	duck
Scaper	-0.40	1.00000000	duck
Hcfc2	-0.04	1.00000000	duck
Rnf111	-1.28	1.00000000	duck
Setd5	-0.37	1.00000000	duck
Chic2	-1.42	1.00000000	duck
Dusp23	0.05	1.00000000	duck
Ahcyl2	-0.01	1.00000000	duck

Tmem138	-0.06	1.00000000	duck
Fam193a	-1.00	1.00000000	duck
Fam210a	0.11	1.00000000	duck
Fam76a	-0.10	1.00000000	duck
Unc13d	0.21	1.00000000	duck
Pithd1	-0.20	1.00000000	duck
Snap29	-0.27	1.00000000	duck
Vps41	-0.56	1.00000000	duck
Ccdc85a	0.16	1.00000000	duck
Cltc	-1.03	1.00000000	duck
Slc37a1	-1.20	1.00000000	duck
Dennd1a	-1.60	1.00000000	duck
Traf6	-0.01	1.00000000	duck
Tmc7	0.21	1.00000000	duck
Pde4b	-0.01	1.00000000	duck
Lcp1	0.64	1.00000000	duck
Xrcc2	0.42	1.00000000	duck
Pank2	-0.07	1.00000000	duck
Plekho1	0.39	1.00000000	duck
Abhd13	-0.18	1.00000000	duck
Acot10	-1.21	1.00000000	duck
Thoc3	0.21	1.00000000	duck
Snw1	-0.40	1.00000000	duck
Klhl12	-0.03	1.00000000	duck
Cdc37l1	-1.08	1.00000000	duck
Rnf146	0.04	1.00000000	duck
Pisd	0.30	1.00000000	duck
St8sia1	-0.05	1.00000000	duck
Tmem222	-0.09	1.00000000	duck
Chchd3	-0.52	1.00000000	duck
Rida	-0.09	1.00000000	duck
Plcl2	0.06	1.00000000	duck
Dnajc19	-0.33	1.00000000	duck
Agfg1	-1.25	1.00000000	duck
Rbbp7	-0.23	1.00000000	duck
Slc10a7	-0.55	1.00000000	duck
B2m	0.10	1.00000000	duck
Alg12	-0.25	1.00000000	duck
Fbxo25	-1.02	1.00000000	duck
Gnb4	-0.01	1.00000000	duck
Gramd4	0.03	1.00000000	duck
Vps13a	-1.02	1.00000000	duck
Nelfa	-0.31	1.00000000	duck
Uchl3	-0.26	1.00000000	duck
Ube2i	-0.09	1.00000000	duck
Zranb1	-0.46	1.00000000	duck

Adsl	-0.31	1.00000000	duck
Scrn3	-0.17	1.00000000	duck
Uso1	-0.31	1.00000000	duck
Slc1a4	-0.07	1.00000000	duck
Appbp2	-0.27	1.00000000	duck
Ripk1	-0.02	1.00000000	duck
Dnaja3	0.12	1.00000000	duck
Smurf2	-1.27	1.00000000	duck
Cyld	-0.02	1.00000000	duck
Nans	0.05	1.00000000	duck
Dtx2	0.13	1.00000000	duck
Ccnc	-0.10	1.00000000	duck
Aamdc	-0.79	1.00000000	duck
Clasp2	-1.06	1.00000000	duck
Pus1	0.04	1.00000000	duck
Armc1	-0.19	1.00000000	duck
Dus1l	-0.10	1.00000000	duck
Clasp1	-0.35	1.00000000	duck
Zcchc7	-0.57	1.00000000	duck
Fam122b	-0.30	1.00000000	duck
Noc3l	0.17	1.00000000	duck
Dnm3	0.03	1.00000000	duck
C2cd2	-0.71	1.00000000	duck
Nol11	-0.09	1.00000000	duck
Abhd17b	0.10	1.00000000	duck
Vwf	0.30	1.00000000	duck
Ide	-1.17	1.00000000	duck
Qsox2	-0.12	1.00000000	duck
Tpst1	-0.77	1.00000000	duck
Cdk6	-0.07	1.00000000	duck
Luc7l	-0.27	1.00000000	duck
Tmem184b	-1.01	1.00000000	duck
Ctcf	-0.32	1.00000000	duck
Tmem250-ps	-0.14	1.00000000	duck
Yipf6	-0.18	1.00000000	duck
Pds5a	-0.34	1.00000000	duck
Tm9sf4	-0.17	1.00000000	duck
Ints13	-0.52	1.00000000	duck
Agpat3	0.48	1.00000000	duck
Dync1li1	-0.29	1.00000000	duck
Abce1	-1.17	1.00000000	duck
Fam219a	-0.12	1.00000000	duck
Prpf38a	0.18	1.00000000	duck
LTO1	-0.22	1.00000000	duck
Gid8	-0.41	1.00000000	duck
Plekhj1	-0.09	1.00000000	duck

Sidt2	0.03	1.00000000	duck
Tut4	-1.06	1.00000000	duck
Abhd2	-0.22	1.00000000	duck
Carf	-0.06	1.00000000	duck
Pum3	-0.03	1.00000000	duck
Lonrf2	0.03	1.00000000	duck
Kmt5b	-0.51	1.00000000	duck
Syne3	-0.49	1.00000000	duck
Nhlrc2	-0.08	1.00000000	duck
Cacul1	-1.26	1.00000000	duck
Ipo8	-0.01	1.00000000	duck
Tmem50b	-0.14	1.00000000	duck
Plpp1	0.57	1.00000000	duck
Kctd2	-0.20	1.00000000	duck
Sec22a	-0.26	1.00000000	duck
Edc4	-0.02	1.00000000	duck
Bloc1s4	-0.36	1.00000000	duck
Pik3r5	-0.02	1.00000000	duck
Csf3r	0.18	1.00000000	duck
Ppp6r2	-0.85	1.00000000	duck
Eri3	-0.31	1.00000000	duck
Tmem161b	-0.18	1.00000000	duck
Adat1	-0.19	1.00000000	duck
G3bp2	-0.18	1.00000000	duck
Ube2e3	-0.49	1.00000000	duck
Lmbr1	-0.87	1.00000000	duck
Zfand2a	0.16	1.00000000	duck
Rock1	-0.25	1.00000000	duck
Ankrd22	-0.35	1.00000000	duck
Hsf2	-0.03	1.00000000	duck
Abcc5	-0.94	1.00000000	duck
Phf2	-0.60	1.00000000	duck
Ppp1r21	-1.10	1.00000000	duck
Cul5	-1.15	1.00000000	duck
Cxcr4	0.01	1.00000000	duck
Prpf4b	-0.23	1.00000000	duck
Adipor2	-0.25	1.00000000	duck
Inpp4a	-1.02	1.00000000	duck
Cdc123	-0.41	1.00000000	duck
Trip11	-1.14	1.00000000	duck
Uchl5	-0.12	1.00000000	duck
Ppp2r2a	-0.34	1.00000000	duck
Trim37	-0.16	1.00000000	duck
Etfrf1	-0.16	1.00000000	duck
Ube2o	0.19	1.00000000	duck
Slain2	-0.06	1.00000000	duck

Tut7	-0.35	1.00000000	duck
Stam2	-1.28	1.00000000	duck
Tbl2	-0.15	1.00000000	duck
Pgm2l1	-0.94	1.00000000	duck
Usp16	-0.22	1.00000000	duck
Emc7	-0.29	1.00000000	duck
Hscb	-0.18	1.00000000	duck
Eci3	-0.16	1.00000000	duck
Akap11	-0.33	1.00000000	duck
Bzw1	-0.28	1.00000000	duck
Zfp830	-0.16	1.00000000	duck
Vps18	-0.02	1.00000000	duck
Kdm1a	-1.14	1.00000000	duck
Fmr1	-0.41	1.00000000	duck
Stx7	-0.01	1.00000000	duck
Gnas	-0.08	1.00000000	duck
Ube2d1	-0.46	1.00000000	duck
Ino80	-0.44	1.00000000	duck
Osbpl9	-0.38	1.00000000	duck
Usp12	-0.19	1.00000000	duck
Washc4	-0.18	1.00000000	duck
Socs5	-0.97	1.00000000	duck
Uba2	-0.48	1.00000000	duck
Tnfaip8l1	0.07	1.00000000	duck
P4ha2	-0.29	1.00000000	duck
Lin7c	-0.54	1.00000000	duck
Kdm4c	-0.41	1.00000000	duck
Ncln	-0.20	1.00000000	duck
Snx13	0.05	1.00000000	duck
Camk1d	-0.11	1.00000000	duck
Cblb	-0.09	1.00000000	duck
Nup54	-0.05	1.00000000	duck
Clptm1l	-0.36	1.00000000	duck
Rabepk	-0.02	1.00000000	duck
Ldb2	0.71	1.00000000	duck
Arl4c	-0.10	1.00000000	duck
Actr6	0.01	1.00000000	duck
Pde7b	0.07	1.00000000	duck
Ccdc93	-0.24	1.00000000	duck
Gle1	-0.63	1.00000000	duck
Hmg20a	-0.29	1.00000000	duck
Peli1	-0.15	1.00000000	duck
Ube4a	-0.17	1.00000000	duck
Zfat	-1.27	1.00000000	duck
Gga1	-0.09	1.00000000	duck
Spag9	-0.01	1.00000000	duck

Sdr16c5	-0.21	1.00000000	duck
Ipo7	-0.18	1.00000000	duck
Usp7	-0.86	1.00000000	duck
Gtf2a1	-1.04	1.00000000	duck
Adar	-0.29	1.00000000	duck
Ap3s1	-0.46	1.00000000	duck
Plrg1	-0.26	1.00000000	duck
Ecd	-0.17	1.00000000	duck
Foxj2	-0.48	1.00000000	duck
Plekha3	-0.83	1.00000000	duck
Cpeb3	-1.37	1.00000000	duck
Bnc2	-0.68	1.00000000	duck
Taf5l	-1.04	1.00000000	duck
Letm1	-0.25	1.00000000	duck
Tti1	-0.14	1.00000000	duck
Adi1	-0.27	1.00000000	duck
Zdhhc12	-0.05	1.00000000	duck
Rnf11	-1.00	1.00000000	duck
Dmxl2	-0.41	1.00000000	duck
R3hdm1	-0.75	1.00000000	duck
Chfr	-0.24	1.00000000	duck
Xpnpep1	-0.12	1.00000000	duck
Phf12	-0.28	1.00000000	duck
Glyr1	-0.51	1.00000000	duck
Bcas3	-0.88	1.00000000	duck
Smc6	-0.34	1.00000000	duck
Plxna2	0.30	1.00000000	duck
Arhgap15	0.27	1.00000000	duck
Cul3	-1.45	1.00000000	duck
Pex13	-0.09	1.00000000	duck
Hagh	-0.88	1.00000000	duck
Uap1l1	-0.04	1.00000000	duck
Uba3	-0.86	1.00000000	duck
Nup50	-0.39	1.00000000	duck
Atp9b	-1.32	1.00000000	duck
Atg5	-1.59	1.00000000	duck
Cdk8	-1.22	1.00000000	duck
Tie1	0.10	1.00000000	duck
Plod1	-0.46	1.00000000	duck
Shld2	0.62	1.00000000	duck
Ubn1	-0.43	1.00000000	duck
Ctbp1	-0.92	1.00000000	duck
Rnls	-0.26	1.00000000	duck
Nrbf2	-0.25	1.00000000	duck
Crebl2	-0.35	1.00000000	duck
Disp1	-1.60	1.00000000	duck

Ankrd17	-0.24	1.00000000	duck
Pphln1	-0.39	1.00000000	duck
Kin	-0.13	1.00000000	duck
Aagab	-0.26	1.00000000	duck
Ric1	-0.42	1.00000000	duck
Eif3a	-0.22	1.00000000	duck
Pcgf6	-0.50	1.00000000	duck
Ubqln4	-0.32	1.00000000	duck
Dpp9	-0.31	1.00000000	duck
Pik3c3	-0.59	1.00000000	duck
Zzz3	-0.58	1.00000000	duck
Ctdp1	-0.88	1.00000000	duck
Wdr61	-0.11	1.00000000	duck
Ccnk	-0.36	1.00000000	duck
9930021J03Rik	-0.10	1.00000000	duck
Eif1b	0.16	1.00000000	duck
Rad51c	-0.05	1.00000000	duck
Uhrf2	-0.46	1.00000000	duck
Nr3c1	-1.05	1.00000000	duck
Ngly1	-1.11	1.00000000	duck
Ddx46	-0.34	1.00000000	duck
Ndfip2	-0.98	1.00000000	duck
Rfng	-0.21	1.00000000	duck
Psmd1	-0.69	1.00000000	duck
Pqlc3	0.08	1.00000000	duck
Smarca5	-0.27	1.00000000	duck
Ddb1	-1.07	1.00000000	duck
Adgrf5	0.43	1.00000000	duck
Ppm1g	-0.35	1.00000000	duck
Cep57	-0.74	1.00000000	duck
Capn15	-0.07	1.00000000	duck
Vps26b	-0.17	1.00000000	duck
Bst1	-0.15	1.00000000	duck
Prkci	-0.50	1.00000000	duck
Pak1ip1	-0.19	1.00000000	duck
Endod1	0.02	1.00000000	duck
Slc26a5	-0.81	1.00000000	duck
Tsc22d2	-0.20	1.00000000	duck
Ap3m2	-0.15	1.00000000	duck
Helz	-1.15	1.00000000	duck
Zfp638	-0.43	1.00000000	duck
Anapc1	-0.11	1.00000000	duck
Cryl1	-0.08	1.00000000	duck
Faf2	-0.38	1.00000000	duck
Wdr44	-0.16	1.00000000	duck
Pdpk1	-0.35	1.00000000	duck

Eif4g1	-0.17	1.00000000	duck
Clk2	-0.21	1.00000000	duck
Yy2	0.19	1.00000000	duck
Nsd1	-1.21	1.00000000	duck
Ap3b1	-1.05	1.00000000	duck
Pxk	-0.31	1.00000000	duck
Faap24	-0.40	1.00000000	duck
Ggh	-0.97	1.00000000	duck
Pofut1	-0.10	1.00000000	duck
Papola	-1.19	1.00000000	duck
Lcmt1	-0.07	1.00000000	duck
Cop1	-0.26	1.00000000	duck
Ccdc47	-0.43	1.00000000	duck
Ccnl1	-0.01	1.00000000	duck
Ppp2r5a	-0.57	1.00000000	duck
Rif1	-0.14	1.00000000	duck
Bod1l	-1.16	1.00000000	duck
Drd4	-0.21	1.00000000	duck
Etfdh	-0.10	1.00000000	duck
Tmem100	0.04	1.00000000	duck
Adcy7	-0.14	1.00000000	duck
Arhgap45	0.25	1.00000000	duck
Dazap1	-0.52	1.00000000	duck
Cdc40	-0.30	1.00000000	duck
Edc3	-0.28	1.00000000	duck
Fam204a	-0.47	1.00000000	duck
Htatsf1	-1.00	1.00000000	duck
Nbr1	-0.25	1.00000000	duck
Ric8b	-1.13	1.00000000	duck
Kdm3b	-0.59	1.00000000	duck
Gpr180	-0.39	1.00000000	duck
Fam168b	-1.17	1.00000000	duck
Ncoa6	-1.13	1.00000000	duck
Pou2f1	-1.03	1.00000000	duck
Aak1	-0.43	1.00000000	duck
Hibadh	-0.49	1.00000000	duck
Gpat4	-0.28	1.00000000	duck
Txlna	-0.20	1.00000000	duck
Lrrc8c	0.12	1.00000000	duck
Cfap20	-0.02	1.00000000	duck
Smarca1	-0.29	1.00000000	duck
Pdk4	-0.18	1.00000000	duck
Plcb1	-0.48	1.00000000	duck
Nsl1	-0.14	1.00000000	duck
Pptc7	-0.14	1.00000000	duck
Pcm1	-0.46	1.00000000	duck

Hectd4	-0.42	1.00000000	duck
Xrn1	-1.37	1.00000000	duck
P4ha1	-0.43	1.00000000	duck
Adgrl4	0.20	1.00000000	duck
Zfp513	-0.06	1.00000000	duck
Slc9a8	-0.22	1.00000000	duck
Sulf2	0.16	1.00000000	duck
Ccdc59	-0.42	1.00000000	duck
Pdhx	-0.24	1.00000000	duck
Tent4b	-0.75	1.00000000	duck
Rit1	-0.24	1.00000000	duck
Slc25a38	-0.60	1.00000000	duck
Tulp4	-0.45	1.00000000	duck
Rnf34	-0.12	1.00000000	duck
Igf2r	-1.19	1.00000000	duck
Kdm1b	0.08	1.00000000	duck
Atf2	-0.28	1.00000000	duck
Ube2a	-0.37	1.00000000	duck
Gatad2a	-0.20	1.00000000	duck
Zer1	-0.57	1.00000000	duck
Lrch3	-0.48	1.00000000	duck
Sppl2b	-0.09	1.00000000	duck
Pikfyve	-1.09	1.00000000	duck
Zzef1	-1.09	1.00000000	duck
Sirt7	-0.12	1.00000000	duck
Yod1	-0.18	1.00000000	duck
Acss1	-1.01	1.00000000	duck
Pik3c2a	-0.96	1.00000000	duck
Fam98a	-0.17	1.00000000	duck
Epb41l3	0.02	1.00000000	duck
Tent4a	-0.38	1.00000000	duck
Trdmt1	-1.05	1.00000000	duck
Csl	-0.11	1.00000000	duck
Cetn3	-0.28	1.00000000	duck
Git2	-0.22	1.00000000	duck
Suds3	-0.36	1.00000000	duck
C2cd5	-0.41	1.00000000	duck
Ibtk	-0.54	1.00000000	duck
Zfand4	-0.25	1.00000000	duck
Ipo9	-0.23	1.00000000	duck
Rpia	-0.29	1.00000000	duck
Thap1	-0.79	1.00000000	duck
Kat7	-0.16	1.00000000	duck
Ercc3	-0.07	1.00000000	duck
Ist1	-0.17	1.00000000	duck
Rchy1	0.04	1.00000000	duck

Zc3h13	-0.56	1.00000000	duck
Csnk1a1	-0.06	1.00000000	duck
Tcea2	0.09	1.00000000	duck
Rictor	-1.08	1.00000000	duck
Rabgap1l	-0.12	1.00000000	duck
Phkb	-0.60	1.00000000	duck
Lemd2	-0.32	1.00000000	duck
Tek	0.15	1.00000000	duck
Sh2b3	-0.93	1.00000000	duck
Dock11	-0.15	1.00000000	duck
Vezt	-0.94	1.00000000	duck
Klhl7	-0.16	1.00000000	duck
Tlk2	-0.30	1.00000000	duck
Skil	-0.06	1.00000000	duck
Rbm39	-0.37	1.00000000	duck
Skap2	-0.11	1.00000000	duck
Nsmaf	-0.28	1.00000000	duck
Clk4	-0.60	1.00000000	duck
Stub1	-0.45	1.00000000	duck
Ufl1	-0.36	1.00000000	duck
Nup214	-0.26	1.00000000	duck
Rtcb	-0.17	1.00000000	duck
Uimc1	-0.75	1.00000000	duck
Usp4	-0.32	1.00000000	duck
Arid3b	-0.25	1.00000000	duck
Ube2r2	-1.11	1.00000000	duck
Abcd2	-0.95	1.00000000	duck
Ecpas	-0.31	1.00000000	duck
Ipo11	-0.34	1.00000000	duck
Ywhag	-0.28	1.00000000	duck
Fbxo38	-0.93	1.00000000	duck
Cfl2	-0.76	1.00000000	duck
Arv1	-0.94	1.00000000	duck
Opa1	-1.35	1.00000000	duck
Ikbkb	-0.22	1.00000000	duck
Whamm	-0.09	1.00000000	duck
Ankrd13a	-0.19	1.00000000	duck
Rab21	-0.37	1.00000000	duck
Jmjd6	-0.21	1.00000000	duck
Rnf170	-0.87	1.00000000	duck
Ppa2	-0.57	1.00000000	duck
Srsf11	-0.46	1.00000000	duck
Ufd1	-0.35	1.00000000	duck
Dapk2	-0.38	1.00000000	duck
Abraxas2	-0.34	1.00000000	duck
Fbxl5	-0.94	1.00000000	duck

Lyrm1	-0.72	1.00000000	duck
Ptprb	0.44	1.00000000	duck
Tmem11	-0.36	1.00000000	duck
Hspa4l	-0.37	1.00000000	duck
Sos1	-0.92	1.00000000	duck
Fzd4	0.18	1.00000000	duck
Klhl2	-0.29	1.00000000	duck
Scfd1	-0.52	1.00000000	duck
Nubp1	-0.22	1.00000000	duck
Crem	0.34	1.00000000	duck
Aff2	0.18	1.00000000	duck
Eif2s1	-0.05	1.00000000	duck
Cerk	-0.98	1.00000000	duck
Eef2	-0.14	1.00000000	duck
Hspa14	-0.32	1.00000000	duck
Pigg	-0.48	1.00000000	duck
Cd47	0.05	1.00000000	duck
Prrc2c	-0.24	1.00000000	duck
Vps50	-0.24	1.00000000	duck
Slc35b3	-0.38	1.00000000	duck
Cops3	-0.96	1.00000000	duck
Vps13c	-1.00	1.00000000	duck
Aff4	-0.47	1.00000000	duck
Tesk2	-1.11	1.00000000	duck
Vps35l	-0.25	1.00000000	duck
Umad1	-1.40	1.00000000	duck
Zbtb37	-0.53	1.00000000	duck
Ctdspl2	-0.53	1.00000000	duck
Ero1b	-0.18	1.00000000	duck
Tec	-0.51	1.00000000	duck
Hp1bp3	-0.29	1.00000000	duck
Gabpb1	-0.87	1.00000000	duck
Ube4b	-1.18	1.00000000	duck
Six4	-0.22	1.00000000	duck
Gpatch1	-0.58	1.00000000	duck
Paxip1	-0.44	1.00000000	duck
Lrp3	-0.82	1.00000000	duck
Pnpla8	-0.96	1.00000000	duck
Bnip2	-0.33	1.00000000	duck
Kansl1	-0.86	1.00000000	duck
Gtf2f2	-0.97	1.00000000	duck
Nr6a1	-0.38	1.00000000	duck
Brd1	-0.62	1.00000000	duck
Prkar2a	-0.46	1.00000000	duck
Nap1l4	-0.14	1.00000000	duck
Atmin	-0.42	1.00000000	duck

Pitpnm2	-1.11	1.00000000	duck
Zfp711	-0.80	1.00000000	duck
Zbtb43	-0.95	1.00000000	duck
Crim1	-0.50	1.00000000	duck
Isca1	-0.24	1.00000000	duck
Wac	-0.94	1.00000000	duck
Psmd13	-0.24	1.00000000	duck
Usp38	-0.48	1.00000000	duck
Fbxo46	-0.14	1.00000000	duck
Zfand3	-0.68	1.00000000	duck
Wwp1	-0.28	1.00000000	duck
Bcar3	-0.14	1.00000000	duck
Chd1	-0.59	1.00000000	duck
Ube2g2	-0.08	1.00000000	duck
Dennd5a	-0.97	1.00000000	duck
Aplp2	0.32	1.00000000	duck
Ccdc126	-0.18	1.00000000	duck
Rpap3	-0.12	1.00000000	duck
Ythdf1	-0.39	1.00000000	duck
Bcl2l1	-0.52	1.00000000	duck
Cd74	0.86	1.00000000	duck
Btbd2	-0.89	1.00000000	duck
Abhd12	-0.04	1.00000000	duck
Sphk1	-0.68	1.00000000	duck
Kcnk5	-0.17	1.00000000	duck
Rnf220	-0.59	1.00000000	duck
Cops4	-0.26	1.00000000	duck
Nsun2	-0.14	1.00000000	duck
Ercc6	-1.11	1.00000000	duck
Dusp11	-0.09	1.00000000	duck
Ddx55	-0.05	1.00000000	duck
Phf20	-0.25	1.00000000	duck
Ezh2	-0.36	1.00000000	duck
Dcps	-0.22	1.00000000	duck
Prkaa1	-0.38	1.00000000	duck
Nsmce1	-0.59	1.00000000	duck
Osgin1	-0.87	1.00000000	duck
Atxn7	-0.44	1.00000000	duck
Kmt2e	-0.45	1.00000000	duck
Snx5	0.03	1.00000000	duck
Serbp1	-0.36	1.00000000	duck
Ikzf5	-0.26	1.00000000	duck
Slc7a6os	-0.08	1.00000000	duck
Rnf185	-0.16	1.00000000	duck
Lrif1	-0.58	1.00000000	duck
Npr3	0.07	1.00000000	duck

Fam168a	-0.20	1.00000000	duck
Insr	-1.17	1.00000000	duck
Riok3	-0.37	1.00000000	duck
Lrp12	-0.60	1.00000000	duck
Sav1	-0.19	1.00000000	duck
Vapb	-0.52	1.00000000	duck
Frs2	-0.85	1.00000000	duck
Ppil2	-0.55	1.00000000	duck
Eif3l	-0.40	1.00000000	duck
Tbc1d13	-0.12	1.00000000	duck
Kpna6	-0.87	1.00000000	duck
Srsf5	-0.51	1.00000000	duck
Dda1	-0.18	1.00000000	duck
Ints2	-0.41	1.00000000	duck
Braf	-0.16	1.00000000	duck
Etf1	-0.45	1.00000000	duck
Gak	-0.74	1.00000000	duck
Spg11	-0.38	1.00000000	duck
Foxo4	-0.44	1.00000000	duck
Osbpl11	-0.17	1.00000000	duck
Xrcc6	-0.34	1.00000000	duck
Eps15l1	-0.95	1.00000000	duck
Arid4a	-0.25	1.00000000	duck
Zfp236	-1.02	1.00000000	duck
Pi4kb	-0.19	1.00000000	duck
Leprotl1	-0.25	1.00000000	duck
Elovl5	-0.31	1.00000000	duck
Serac1	-0.57	1.00000000	duck
Fyttd1	-0.51	1.00000000	duck
Gpr155	-0.17	1.00000000	duck
Csnk2a2	-0.12	1.00000000	duck
Apoo	-0.39	1.00000000	duck
Ppil3	-0.19	1.00000000	duck
Rcc1l	-0.28	1.00000000	duck
Prpf3	-0.21	1.00000000	duck
Cog6	-0.21	1.00000000	duck
Ppme1	-0.75	1.00000000	duck
Prpf40a	-0.51	1.00000000	duck
Cpne1	-0.47	1.00000000	duck
Ppp2ca	-0.35	1.00000000	duck
Fbxo11	-0.27	1.00000000	duck
Cnot2	-1.13	1.00000000	duck
Ccnt2	-0.10	1.00000000	duck
Hacd2	-0.31	1.00000000	duck
Lrrc45	-0.14	1.00000000	duck
Arhgef18	-0.64	1.00000000	duck

Fig4	0.04	1.00000000	duck
Ogdh	-0.43	1.00000000	duck
Ttc17	-0.62	1.00000000	duck
Ift22	-0.16	1.00000000	duck
Btrc	-1.08	1.00000000	duck
Rpgr	-0.27	1.00000000	duck
Taf4	-0.67	1.00000000	duck
Kbtbd8	-0.72	1.00000000	duck
Bfar	-0.28	1.00000000	duck
Prkdc	-0.63	1.00000000	duck
Stk4	-0.50	1.00000000	duck
Atf1	-0.99	1.00000000	duck
Acbd5	-0.56	1.00000000	duck
Fam102b	-0.38	1.00000000	duck
Tet1	-0.59	1.00000000	duck
Bsdc1	-0.24	1.00000000	duck
Slc17a5	-0.89	1.00000000	duck
Pip5k1c	-0.27	1.00000000	duck
Nfrkb	-0.17	1.00000000	duck
Gkap1	-0.28	1.00000000	duck
Ifih1	-0.93	1.00000000	duck
Gata3	-0.36	1.00000000	duck
Ggnbp2	-0.49	1.00000000	duck
Tpp2	-0.41	1.00000000	duck
Rev1	-1.27	1.00000000	duck
Spopl	-0.47	1.00000000	duck
Usp14	-1.13	1.00000000	duck
Bcap29	-0.86	1.00000000	duck
Stk11	-0.33	1.00000000	duck
Zfp839	-0.07	1.00000000	duck
Rdx	-1.07	1.00000000	duck
Rragd	-0.42	1.00000000	duck
4932411N23Rik	-0.30	1.00000000	duck
Cnot8	-0.20	1.00000000	duck
Cers5	-0.31	1.00000000	duck
Khdrbs1	-0.35	1.00000000	duck
Dcaf12	-0.38	1.00000000	duck
Sil1	-0.44	1.00000000	duck
Ptpa	-0.30	1.00000000	duck
Arhgef7	-0.74	1.00000000	duck
Fkbp14	-0.25	1.00000000	duck
Dnajb4	-1.06	1.00000000	duck
Srp14	-0.43	1.00000000	duck
Esf1	-0.31	1.00000000	duck
Elk3	-0.09	1.00000000	duck
Sprtn	-0.17	1.00000000	duck

Nelfcd	-0.29	1.00000000	duck
Ubfd1	-0.46	1.00000000	duck
Chek2	-0.25	1.00000000	duck
Secisbp2l	-0.62	1.00000000	duck
Ifngr1	-0.17	1.00000000	duck
Ebf1	0.08	1.00000000	duck
Gab3	-0.79	1.00000000	duck
Itfg1	-0.51	1.00000000	duck
Cnot4	-0.62	1.00000000	duck
Cdpf1	-0.24	1.00000000	duck
Abca5	-0.14	1.00000000	duck
Ssh2	-0.61	1.00000000	duck
Smap2	-0.36	1.00000000	duck
Taf5	-0.27	1.00000000	duck
Brd7	-0.45	1.00000000	duck
Tnks	-0.48	1.00000000	duck
Slc25a25	-0.25	1.00000000	duck
Stau1	-0.30	1.00000000	duck
Zfp654	-0.33	1.00000000	duck
Pi4k2b	-0.42	1.00000000	duck
Rexo1	-0.66	1.00000000	duck
Polk	-0.25	1.00000000	duck
Katna1	-0.52	1.00000000	duck
Cdk19	-1.11	1.00000000	duck
Xpr1	-0.67	1.00000000	duck
Usp33	-0.71	1.00000000	duck
Dnajc2	-0.43	1.00000000	duck
Klhl15	-0.50	1.00000000	duck
Ap4e1	-0.79	1.00000000	duck
Arfgap1	-0.38	1.00000000	duck
Camsap2	-0.44	1.00000000	duck
Eif4enif1	-0.20	1.00000000	duck
Adamts9	-0.25	1.00000000	duck
Taf3	-0.94	1.00000000	duck
Cep170	-0.87	1.00000000	duck
Rrp1b	-1.18	1.00000000	duck
Heph	-0.26	1.00000000	duck
Rfx2	-0.20	1.00000000	duck
Cep290	-1.21	1.00000000	duck
Cgrrf1	-0.45	1.00000000	duck
Trak2	-0.29	1.00000000	duck
Psma4	-0.23	1.00000000	duck
Itpkb	0.02	1.00000000	duck
Trim24	-0.38	1.00000000	duck
Tsc2	-0.46	1.00000000	duck
Atg4b	-0.73	1.00000000	duck

Atp6v1c1	-0.44	1.00000000	duck
Kdm2a	-0.06	1.00000000	duck
Tra2a	-0.81	1.00000000	duck
Zfp318	-0.67	1.00000000	duck
Zfyve21	-0.09	1.00000000	duck
Sap30l	-0.77	1.00000000	duck
Rbl2	-0.19	1.00000000	duck
Dcaf17	-0.15	1.00000000	duck
Sesn1	-0.92	1.00000000	duck
Fbxl20	-0.21	1.00000000	duck
Nr2c2	-0.98	1.00000000	duck
Arl6ip4	-0.12	1.00000000	duck
Arhgap18	0.11	1.00000000	duck
Ing5	-0.81	1.00000000	duck
Sp4	-0.30	1.00000000	duck
Agl	-0.36	1.00000000	duck
Dyrk1a	-0.79	1.00000000	duck
Aprt	-0.73	1.00000000	duck
Nup133	-0.17	1.00000000	duck
Zfp326	-0.72	1.00000000	duck
Psmg2	-0.32	1.00000000	duck
Nfat5	-0.36	1.00000000	duck
Slc25a16	-0.67	1.00000000	duck
Ing4	-0.29	1.00000000	duck
Nudcd1	-0.17	1.00000000	duck
Qdpr	-0.46	1.00000000	duck
Cdh5	-0.16	1.00000000	duck
Ifitm10	-0.34	1.00000000	duck
Tasp1	-0.53	1.00000000	duck
Ifngr2	-0.47	1.00000000	duck
Kidins220	-0.66	1.00000000	duck
Txnl1	-0.61	1.00000000	duck
Gpd2	-1.22	1.00000000	duck
Srrm1	-0.44	1.00000000	duck
Lars	-0.41	1.00000000	duck
Brwd3	-0.96	1.00000000	duck
Lzic	-0.36	1.00000000	duck
Cbl	-0.44	1.00000000	duck
Nbn	-0.53	1.00000000	duck
Aasdhppt	-1.10	1.00000000	duck
Cmtr1	-0.24	1.00000000	duck
Gse1	-0.03	1.00000000	duck
Rapgef1	-0.35	1.00000000	duck
Iws1	-0.52	1.00000000	duck
Tbpl1	-0.16	1.00000000	duck
Pten	-0.36	1.00000000	duck

Fam8a1	-0.39	1.00000000	duck
Extl2	-0.65	1.00000000	duck
Top2b	-0.77	1.00000000	duck
Cflar	-0.63	1.00000000	duck
Ppp3cc	-0.35	1.00000000	duck
Zbed4	-0.27	1.00000000	duck
Trpc3	-0.03	1.00000000	duck
Zbtb38	-0.24	1.00000000	duck
Rab6a	-0.69	1.00000000	duck
Zfand6	-0.67	1.00000000	duck
Akap8	-0.62	1.00000000	duck
Scyl2	-0.54	1.00000000	duck
Setdb1	-0.88	1.00000000	duck
Asxl1	-0.46	1.00000000	duck
Ap3d1	-0.38	1.00000000	duck
Ttc37	-0.64	1.00000000	duck
Dnajc5	-0.42	1.00000000	duck
Dcaf6	-0.33	1.00000000	duck
Uhrf1bp1	-0.75	1.00000000	duck
Kdm4b	-1.01	1.00000000	duck
Nudt3	-1.02	1.00000000	duck
St3gal6	-0.39	1.00000000	duck
Prrc2b	-0.86	1.00000000	duck
Pdcd4	-0.42	1.00000000	duck
Nrbp1	-0.43	1.00000000	duck
Strn3	-0.67	1.00000000	duck
Gfpt1	-0.33	1.00000000	duck
Lyrm7	-0.73	1.00000000	duck
Ccndbp1	-0.88	1.00000000	duck
Stx2	-0.56	1.00000000	duck
Cpd	-0.18	1.00000000	duck
Dop1a	-0.97	1.00000000	duck
Hnrnpd	-0.33	1.00000000	duck
Lrig2	-0.37	1.00000000	duck
Pi4ka	-0.88	1.00000000	duck
Tia1	-0.57	1.00000000	duck
Rad54l2	-0.42	1.00000000	duck
Nf2	-0.23	1.00000000	duck
Anks1	-0.37	1.00000000	duck
Trim59	-0.42	1.00000000	duck
Scaf8	-1.07	1.00000000	duck
Dmxl1	-0.68	1.00000000	duck
Nisch	-0.98	1.00000000	duck
Slc4a7	-0.61	1.00000000	duck
Gosr1	-0.92	1.00000000	duck
Ythdf3	-0.85	1.00000000	duck

Naa25	-0.56	1.00000000	duck
C9orf72	-0.30	1.00000000	duck
Arsk	-1.01	1.00000000	duck
Immp1l	-0.78	1.00000000	duck
Fundc1	-0.51	1.00000000	duck
Adarb1	-0.71	1.00000000	duck
Xylt1	-0.39	1.00000000	duck
Slc25a21	-1.84	1.00000000	duck
Ankrd54	-0.59	1.00000000	duck
Scn8a	-0.59	1.00000000	duck
Wnk1	-0.35	1.00000000	duck
Tcf3	-0.70	1.00000000	duck
Upf2	-0.93	1.00000000	duck
Nkrf	-0.40	1.00000000	duck
Dagla	-0.55	1.00000000	duck
Prpf6	-0.31	1.00000000	duck
Tdg	0.01	1.00000000	duck
Lztr1	-0.62	1.00000000	duck
Per2	-0.52	1.00000000	duck
Ints10	-0.37	1.00000000	duck
G2e3	-0.22	1.00000000	duck
Rufy1	-0.23	1.00000000	duck
Cir1	-0.28	1.00000000	duck
Bend5	-0.55	1.00000000	duck
Clock	-1.01	1.00000000	duck
Btbd10	-0.64	1.00000000	duck
Lactb2	-0.59	1.00000000	duck
Arhgef12	-0.17	1.00000000	duck
Nr2c1	-0.45	1.00000000	duck
Zfp148	-0.94	1.00000000	duck
Kdm5a	-0.89	1.00000000	duck
N4bp2l1	-0.33	1.00000000	duck
Xpot	-0.55	1.00000000	duck
Ypel5	-0.40	1.00000000	duck
Samsn1	-0.26	1.00000000	duck
Stk3	-0.51	1.00000000	duck
Zfyve26	-0.59	1.00000000	duck
Suz12	-0.52	1.00000000	duck
Zbtb7a	-0.27	1.00000000	duck
Hook3	-0.52	1.00000000	duck
Api5	-0.56	1.00000000	duck
Tbc1d2b	-0.73	1.00000000	duck
N4bp1	-0.92	1.00000000	duck
Ints8	-0.75	1.00000000	duck
2700049A03Rik	-0.43	1.00000000	duck
Erbin	0.09	1.00000000	duck

Gdap2	-0.82	1.00000000	duck
Tbc1d8b	-0.91	1.00000000	duck
Spata2	-0.70	1.00000000	duck
Sec23b	-0.50	1.00000000	duck
Nfx1	-0.75	1.00000000	duck
Nfatc3	-0.82	1.00000000	duck
Insig2	-0.32	1.00000000	duck
Golga1	-0.62	1.00000000	duck
Zfp292	-0.84	1.00000000	duck
Gtf2h5	-0.37	1.00000000	duck
Clcn3	-0.61	1.00000000	duck
Bdh1	-0.54	1.00000000	duck
Fbxo9	-0.82	1.00000000	duck
Wdr33	-0.39	1.00000000	duck
Fxn	-0.70	1.00000000	duck
Cep250	-0.31	1.00000000	duck
Swt1	-0.21	1.00000000	duck
Adam17	-0.54	1.00000000	duck
Nf1	-1.02	1.00000000	duck
Arfgef2	-0.61	1.00000000	duck
Cdc42se2	-0.64	1.00000000	duck
Klhl24	-0.67	1.00000000	duck
Adora2a	-0.10	1.00000000	duck
Zmynd8	-1.01	1.00000000	duck
Gcc2	-0.50	1.00000000	duck
Fdx1	-0.38	1.00000000	duck
Fam76b	-0.27	1.00000000	duck
Bclaf1	-0.81	1.00000000	duck
Lancl2	-0.41	1.00000000	duck
Golga5	-0.95	1.00000000	duck
Ctr9	-0.40	1.00000000	duck
Zmat3	-0.22	1.00000000	duck
Smarcal1	-0.15	1.00000000	duck
Tab3	-0.84	1.00000000	duck
Gzf1	-0.30	1.00000000	duck
Setd1b	-0.74	1.00000000	duck
Prelid3b	-0.32	1.00000000	duck
Ubap1	-1.02	1.00000000	duck
Tbck	-0.53	1.00000000	duck
Ddx31	-0.77	1.00000000	duck
Rhbdd1	-0.40	1.00000000	duck
Trappc12	-0.22	1.00000000	duck
Cog2	-0.45	1.00000000	duck
Fbxo33	-0.40	1.00000000	duck
Slc23a2	-0.05	1.00000000	duck
Srgap3	-0.80	1.00000000	duck

Efcab14	-0.39	1.00000000	duck
Add3	-0.02	1.00000000	duck
Ddx6	-0.65	1.00000000	duck
Slc30a7	-0.66	1.00000000	duck
Hnrnpr	-0.39	1.00000000	duck
Ehd4	-0.52	1.00000000	duck
Dnaja2	-0.40	1.00000000	duck
Ormdl3	-0.47	1.00000000	duck
Tnrc6a	-0.59	1.00000000	duck
Heatr5b	-0.46	1.00000000	duck
Smad4	-0.73	1.00000000	duck
Ndufaf2	-0.70	1.00000000	duck
Phf10	-0.52	1.00000000	duck
Dcaf1	-0.74	1.00000000	duck
Dgkq	-0.57	1.00000000	duck
Tom1l2	-0.79	1.00000000	duck
Ptges3	-0.83	1.00000000	duck
Dock10	-0.57	1.00000000	duck
Stxbp3	-0.54	1.00000000	duck
Zbtb41	-0.88	1.00000000	duck
Usp45	-0.97	1.00000000	duck
Akap10	-0.38	1.00000000	duck
Zfp598	-0.63	1.00000000	duck
Ascc2	-0.47	1.00000000	duck
Avl9	-0.52	1.00000000	duck
Eif4e2	-0.48	1.00000000	duck
Hspa4	-0.67	1.00000000	duck
Immt	-0.42	1.00000000	duck
Clec16a	-0.76	1.00000000	duck
Ppp2r2d	-0.59	1.00000000	duck
Cwf19l2	-0.73	1.00000000	duck
Ttc1	-0.51	1.00000000	duck
Cdca4	-0.29	1.00000000	duck
Asb3	-0.54	1.00000000	duck
Ppm1a	-0.85	1.00000000	duck
Bcl2l13	-0.67	1.00000000	duck
Gga3	-0.74	1.00000000	duck
Rhbdf2	-0.25	1.00000000	duck
Spty2d1	-0.24	1.00000000	duck
Arid1b	-0.84	1.00000000	duck
Ube2v2	-1.05	1.00000000	duck
Wdr26	-0.56	1.00000000	duck
Cnot10	-0.41	1.00000000	duck
Shprh	-0.77	1.00000000	duck
Nlrc5	-0.60	1.00000000	duck
Zranb2	-0.76	1.00000000	duck

Tmem65	-0.75	1.00000000	duck
Pdk1	-0.76	1.00000000	duck
Smarcc1	-0.97	1.00000000	duck
Pcnx4	-0.88	1.00000000	duck
Fam222b	-0.97	1.00000000	duck
Spg7	-0.50	1.00000000	duck
Zcchc8	-0.43	1.00000000	duck
Ube2j2	-0.45	1.00000000	duck
Trmt10c	-0.18	1.00000000	duck
Supt6	-0.54	1.00000000	duck
Ldlrap1	-0.38	1.00000000	duck
Stx18	-0.84	1.00000000	duck
Spen	-0.65	1.00000000	duck
Tada1	0.05	1.00000000	duck
Pbrm1	-0.70	1.00000000	duck
Zmynd11	-1.01	1.00000000	duck
Ofd1	-0.54	1.00000000	duck
Cggbp1	-0.14	1.00000000	duck
Sult4a1	-0.53	1.00000000	duck
Cdc27	-0.81	1.00000000	duck
Larp1	-0.52	1.00000000	duck
Emsy	-0.38	1.00000000	duck
Sirt1	-0.61	1.00000000	duck
Dipk2a	-0.47	1.00000000	duck
Efr3a	-1.25	1.00000000	duck
Akirin2	-0.57	1.00000000	duck
Zeb2	-0.08	1.00000000	duck
Ern1	-0.44	1.00000000	duck
Eloc	-0.77	1.00000000	duck
Ankrd13c	-0.85	1.00000000	duck
Ubp1	-0.82	1.00000000	duck
Wipi2	-0.66	1.00000000	duck
Ing3	-0.42	1.00000000	duck
Lyve1	0.51	1.00000000	duck
Baz1a	-0.33	1.00000000	duck
Lrrc28	-0.69	1.00000000	duck
Tmx1	-0.39	1.00000000	duck
Trmt13	-0.44	1.00000000	duck
Ift80	-0.74	1.00000000	duck
Fnip1	-0.61	1.00000000	duck
Vps53	-0.49	1.00000000	duck
Anapc7	-0.27	1.00000000	duck
Rbbp5	-0.44	1.00000000	duck
Thoc1	-0.68	1.00000000	duck
Hprt	-0.84	1.00000000	duck
Siah1b	-0.61	1.00000000	duck

Tube1	-0.67	1.00000000	duck
Zswim8	-1.21	1.00000000	duck
Cops2	-0.53	1.00000000	duck
Zfp652	-0.67	1.00000000	duck
Atg2b	-0.84	1.00000000	duck
Trank1	-0.57	1.00000000	duck
Ylpm1	-0.88	1.00000000	duck
Alox5	-0.84	1.00000000	duck
Cops5	-0.44	1.00000000	duck
Abhd5	-0.87	1.00000000	duck
Ccdc174	-0.50	1.00000000	duck
Fto	-0.65	1.00000000	duck
Clk1	-0.86	1.00000000	duck
Cul1	-1.02	1.00000000	duck
Top1	-0.51	1.00000000	duck
Zc3h3	-0.58	1.00000000	duck
Zfp518a	-0.44	1.00000000	duck
Celf1	-0.79	1.00000000	duck
Cstf1	-0.17	1.00000000	duck
Ppp4r3a	-0.70	1.00000000	duck
Rpgrip1l	-0.22	1.00000000	duck
Esco1	-0.64	1.00000000	duck
Nrf1	-0.69	1.00000000	duck
Usp22	-0.81	1.00000000	duck
Ccdc88a	-0.23	1.00000000	duck
Tdrd3	-1.05	1.00000000	duck
Uri1	-0.85	1.00000000	duck
Fkbp5	-0.52	1.00000000	duck
Rheb	-0.55	1.00000000	duck
Fbxo30	-0.65	1.00000000	duck
Asns	-0.47	1.00000000	duck
Zfp207	-0.83	1.00000000	duck
Cdyl2	-0.52	1.00000000	duck
Ctps2	-0.64	1.00000000	duck
Ptpn11	-0.81	1.00000000	duck
Cnep1r1	-0.35	1.00000000	duck
Rae1	-0.80	1.00000000	duck
Dhx40	-0.59	1.00000000	duck
Akr1a1	-0.32	1.00000000	duck
Atg4c	-0.80	1.00000000	duck
Zfp821	-0.39	1.00000000	duck
Phactr2	-0.34	1.00000000	duck
Dnajc11	-0.31	1.00000000	duck
Snap23	-0.70	1.00000000	duck
Dusp19	-0.64	1.00000000	duck
Arid4b	-0.31	1.00000000	duck

Bmt2	-0.76	1.00000000	duck
Epc2	-0.70	1.00000000	duck
Wdr48	-0.74	1.00000000	duck
St3gal5	-0.17	1.00000000	duck
Tk2	-0.59	1.00000000	duck
Ice2	-0.71	1.00000000	duck
Smg1	-0.65	1.00000000	duck
Ube3a	-0.75	1.00000000	duck
Ralgps1	-0.33	1.00000000	duck
Wbp2	-0.45	1.00000000	duck
Ankrd11	-0.69	1.00000000	duck
Zfp592	-0.64	1.00000000	duck
Ahctf1	-0.73	1.00000000	duck
Dip2a	-0.49	1.00000000	duck
Rtn4	-0.33	1.00000000	duck
Fbxo8	-0.61	1.00000000	duck
Amfr	-0.57	1.00000000	duck
Dhdds	-0.31	1.00000000	duck
Tmem131	-0.71	1.00000000	duck
Gsk3b	-0.39	1.00000000	duck
Atp6v0a2	-0.60	1.00000000	duck
Hspa9	-1.06	1.00000000	duck
Dicer1	-1.01	1.00000000	duck
Usp20	-0.69	1.00000000	duck
Rmnd5a	-0.78	1.00000000	duck
Apool	-0.75	1.00000000	duck
Slc30a4	-0.70	1.00000000	duck
Impa1	-0.45	1.00000000	duck
Nub1	-0.60	1.00000000	duck
Pip4k2a	-0.54	1.00000000	duck
Fbxo7	-0.44	1.00000000	duck
Golm2	-0.64	1.00000000	duck
Elmo2	-0.67	1.00000000	duck
Tspan14	0.04	1.00000000	duck
Nxt2	-0.79	1.00000000	duck
Runx1	-0.44	1.00000000	duck
Rpap2	-0.52	1.00000000	duck
Slc25a15	-0.35	1.00000000	duck
Ubqln1	-0.81	1.00000000	duck
Rprd1a	-0.90	1.00000000	duck
Parg	-0.93	1.00000000	duck
Seh1l	-0.65	1.00000000	duck
Dcun1d1	-0.65	1.00000000	duck
Commd6	-0.27	1.00000000	duck
Nfs1	-0.36	1.00000000	duck
Cnnm2	-0.96	1.00000000	duck

Pafah1b1	-0.49	1.00000000	duck
Ubac1	-0.72	1.00000000	duck
Arl4a	-0.73	1.00000000	duck
Tmx4	-0.42	1.00000000	duck
Ctnnbl1	-0.65	1.00000000	duck
Inpp5d	-0.28	1.00000000	duck
Ralb	-0.43	1.00000000	duck
Sf3b1	-0.60	1.00000000	duck
Crbn	-0.58	1.00000000	duck
Usp15	-0.80	1.00000000	duck
Serinc1	-0.67	1.00000000	duck
Trappc8	-1.08	1.00000000	duck
Dcp1a	-0.61	1.00000000	duck
Zfp362	-0.38	1.00000000	duck
Sufu	-0.64	1.00000000	duck
Zbtb44	-0.61	1.00000000	duck
Paxbp1	-0.87	1.00000000	duck
Ptpn9	-0.21	1.00000000	duck
Tm2d1	-0.50	1.00000000	duck
Fam120a	-0.64	1.00000000	duck
Snx29	-0.85	1.00000000	duck
Sugt1	-0.79	1.00000000	duck
Reck	-0.61	1.00000000	duck
Abtb1	-0.51	1.00000000	duck
Rbm12b2	-0.67	1.00000000	duck
Iqcb1	-0.80	1.00000000	duck
Dhx15	-1.00	1.00000000	duck
Dnm1l	-0.29	1.00000000	duck
Ubxn7	-0.67	1.00000000	duck
Ackr4	-0.30	1.00000000	duck
Tbcd	-0.82	1.00000000	duck
Ep300	-0.38	1.00000000	duck
Fam91a1	-0.63	1.00000000	duck
Bmp2k	-0.67	1.00000000	duck
Smg7	-0.45	1.00000000	duck
Erlin2	-0.86	1.00000000	duck
Wdr3	-0.27	1.00000000	duck
Cul4b	-0.85	1.00000000	duck
Atad1	-0.91	1.00000000	duck
Jak1	-0.72	1.00000000	duck
Oxr1	-0.72	1.00000000	duck
Helq	-0.45	1.00000000	duck
Vps8	-0.47	1.00000000	duck
Rnf114	-0.89	1.00000000	duck
Tpr	-0.77	1.00000000	duck
Irf1	-0.36	1.00000000	duck

Ubap2	-0.85	1.00000000	duck
Rsrc2	-0.78	1.00000000	duck
Uggt2	-0.66	1.00000000	duck
Slc25a13	-1.22	1.00000000	duck
Wdtc1	-0.65	1.00000000	duck
Atp7a	-0.70	1.00000000	duck
Aqr	-0.71	1.00000000	duck
Arnt	-0.45	1.00000000	duck
Oser1	-0.25	1.00000000	duck
Arntl	-0.94	1.00000000	duck
Erc1	-0.74	1.00000000	duck
Aig1	-1.15	1.00000000	duck
Cluh	-0.83	1.00000000	duck
Gtf2h1	-0.75	1.00000000	duck
Pspc1	-0.89	1.00000000	duck
Lsm14a	-0.65	1.00000000	duck
Chuk	-1.00	1.00000000	duck
Cnot7	-0.81	1.00000000	duck
Lmtk2	-0.68	1.00000000	duck
Ctdspl	-0.29	1.00000000	duck
Snx16	-0.91	1.00000000	duck
Yipf4	-0.58	1.00000000	duck
Casp3	-0.41	1.00000000	duck
Rbks	-0.72	1.00000000	duck
Bdp1	-0.82	1.00000000	duck
Ints9	-0.87	1.00000000	duck
Tnfrsf21	-0.58	1.00000000	duck
Klf3	-0.78	1.00000000	duck
Ube2g1	-0.69	1.00000000	duck
Smarcad1	-0.54	1.00000000	duck
Usp34	-0.60	1.00000000	duck
Herc4	-0.64	1.00000000	duck
Susd1	-0.51	1.00000000	duck
Foxo1	-0.16	1.00000000	duck
Slc48a1	-0.32	1.00000000	duck
Ccdc117	-0.80	1.00000000	duck
Pex1	-0.68	1.00000000	duck
Cdk17	-0.50	1.00000000	duck
Hbp1	-0.68	1.00000000	duck
Nlk	-0.90	1.00000000	duck
Pkn2	-0.66	1.00000000	duck
Rbbp6	-0.58	1.00000000	duck
Lnx2	-0.57	1.00000000	duck
Cdk13	-0.57	1.00000000	duck
Ino80d	-0.92	1.00000000	duck
Tial1	-0.75	1.00000000	duck

Sec14l1	-0.33	1.00000000	duck
Aebp2	-0.66	1.00000000	duck
Sos2	-0.71	1.00000000	duck
Tent2	-0.36	1.00000000	duck
Rnf149	-0.58	1.00000000	duck
Parp1	-0.99	1.00000000	duck
Relch	-0.83	1.00000000	duck
Otud4	-0.47	1.00000000	duck
Dvl1	-1.03	1.00000000	duck
Thrap3	-0.54	1.00000000	duck
Atl2	-0.84	1.00000000	duck
Setd3	-1.03	1.00000000	duck
Slc9a3r2	-0.64	1.00000000	duck
Ckap4	-0.42	1.00000000	duck
Sppl2a	-0.79	1.00000000	duck
Tmem168	-0.55	1.00000000	duck
Sec16a	-0.67	1.00000000	duck
Virma	-0.60	1.00000000	duck
Edrf1	-0.64	1.00000000	duck
Helz2	-0.37	1.00000000	duck
Htt	-0.79	1.00000000	duck
C1d	-0.47	1.00000000	duck
Eif3e	-0.65	1.00000000	duck
Tbc1d15	-0.78	1.00000000	duck
Ppm1d	-0.65	1.00000000	duck
Atf6	-0.61	1.00000000	duck
Flot2	-0.48	1.00000000	duck
Hras	-0.49	1.00000000	duck
Stk17b	-0.60	1.00000000	duck
Tbk1	-0.72	1.00000000	duck
Xbp1	-0.41	1.00000000	duck
Lmo2	-0.27	1.00000000	duck
Bptf	-0.74	1.00000000	duck
Xpo7	-0.27	1.00000000	duck
Clcc1	-0.87	1.00000000	duck
Fem1b	-0.59	1.00000000	duck
Cramp1	-0.65	1.00000000	duck
Unk	-0.69	1.00000000	duck
Rbsn	-0.84	1.00000000	duck
Gpbp1l1	-0.71	1.00000000	duck
Faf1	-0.80	1.00000000	duck
Clpx	-0.79	1.00000000	duck
Bsg	-0.14	1.00000000	duck
Eaf2	-0.71	1.00000000	duck
Otud7b	-0.65	1.00000000	duck
Zbtb34	-0.80	1.00000000	duck

Ranbp3	-0.69	1.00000000	duck
Ate1	-0.58	1.00000000	duck
Slu7	-0.62	1.00000000	duck
Ube2k	-0.84	1.00000000	duck
Cdk5rap2	-0.71	1.00000000	duck
Chchd5	-0.45	1.00000000	duck
Inpp5k	-0.67	1.00000000	duck
Hgs	-0.39	1.00000000	duck
Slc25a12	-0.52	1.00000000	duck
Zfp438	-0.78	1.00000000	duck
Nipbl	-0.41	1.00000000	duck
Ip6k1	-0.59	1.00000000	duck
Hs6st3	-1.36	1.00000000	duck
Luc7l3	-0.84	1.00000000	duck
Proser2	-0.63	1.00000000	duck
Csde1	-0.53	1.00000000	duck
Trappc11	-0.44	1.00000000	duck
Sugp2	-0.44	1.00000000	duck
Cwc15	-0.55	1.00000000	duck
Nr1h3	-0.41	1.00000000	duck
Atp6v1a	-0.75	1.00000000	duck
Naa30	-0.75	1.00000000	duck
Ak3	-0.58	1.00000000	duck
Cul2	-0.53	1.00000000	duck
Ckap5	-0.47	1.00000000	duck
Txlng	-1.04	1.00000000	duck
Tarsl2	-0.75	1.00000000	duck
Odr4	-0.43	1.00000000	duck
Eif2s2	-0.60	1.00000000	duck
Nadk	-0.54	1.00000000	duck
Ciapin1	-0.63	1.00000000	duck
Ptpn4	-0.44	1.00000000	duck
Styk1	-0.36	1.00000000	duck
Sel1l	-0.77	1.00000000	duck
Hsf1	-0.54	1.00000000	duck
Epc1	-0.46	1.00000000	duck
Srpk1	-0.67	1.00000000	duck
Nrde2	-0.58	1.00000000	duck
Cdk5r1	-0.50	1.00000000	duck
Kansl1l	-0.76	1.00000000	duck
Per3	-0.77	1.00000000	duck
Hnrnpa2b1	-0.46	1.00000000	duck
Rc3h2	-0.85	1.00000000	duck
Dhx8	-0.54	1.00000000	duck
Gpatch8	-0.54	1.00000000	duck
Bicral	-0.44	1.00000000	duck

Acadsb	-0.84	1.00000000	duck
Vps37a	-0.67	1.00000000	duck
Ints4	-0.39	1.00000000	duck
Wdr20	-0.72	1.00000000	duck
Trim23	-0.65	1.00000000	duck
Elf1	-0.07	1.00000000	duck
Sptlc2	-0.63	1.00000000	duck
St7l	-0.48	1.00000000	duck
Ndufs1	-0.75	1.00000000	duck
Dcp1b	-1.05	1.00000000	duck
Cab39	-0.49	1.00000000	duck
Gphn	-1.05	1.00000000	duck
Eif4e	-0.72	1.00000000	duck
Nktr	-1.11	1.00000000	duck
Ankrd27	-0.83	1.00000000	duck
Atg16l1	-0.79	1.00000000	duck
Zdhhc17	-0.75	1.00000000	duck
Wiz	-0.88	1.00000000	duck
Dennd4a	-0.52	1.00000000	duck
Epg5	-0.67	1.00000000	duck
Xpo6	-0.83	1.00000000	duck
Gan	-0.73	1.00000000	duck
Ppp3cb	-0.64	1.00000000	duck
Ralgapb	-0.51	1.00000000	duck
Xrn2	-0.98	1.00000000	duck
Ubtd2	-0.49	1.00000000	duck
Ppargc1b	-0.53	1.00000000	duck
Hipk1	-0.87	1.00000000	duck
Afg3l2	-0.59	1.00000000	duck
Ubap2l	-0.55	1.00000000	duck
Txnrd2	-0.60	1.00000000	duck
Eif3h	-0.49	1.00000000	duck
Kat2b	-0.82	1.00000000	duck
Rbm6	-1.00	1.00000000	duck
Ankfy1	-0.51	1.00000000	duck
Dnaja4	-0.58	1.00000000	duck
Scaf11	-1.04	1.00000000	duck
Arsg	-0.44	1.00000000	duck
Dnajb6	-0.46	1.00000000	duck
Khdc4	-0.34	1.00000000	duck
Tsc22d3	-0.10	1.00000000	duck
Oard1	-0.46	1.00000000	duck
Prpf39	-0.72	1.00000000	duck
Apaf1	-0.60	1.00000000	duck
Supv3l1	-0.16	1.00000000	duck
Tecpr2	-0.36	1.00000000	duck

Capza2	-0.75	1.00000000	duck
Stx8	-1.20	1.00000000	duck
Pcmt1	-0.69	1.00000000	duck
Lonrf1	-0.70	1.00000000	duck
Rasa2	-0.56	1.00000000	duck
Vps39	-0.79	1.00000000	duck
Gtf2e1	-0.70	1.00000000	duck
Dot1l	-0.48	1.00000000	duck
Taok1	-0.91	1.00000000	duck
Jade2	-0.70	1.00000000	duck
Csnk1g1	-0.71	1.00000000	duck
Ralgapa1	-0.89	1.00000000	duck
Rprd1b	-0.99	1.00000000	duck
Cilk1	-0.66	1.00000000	duck
Itpka	-0.34	1.00000000	duck
Gpatch11	-0.72	1.00000000	duck
Ap1g1	-0.65	1.00000000	duck
Furin	-0.79	1.00000000	duck
Strada	-0.68	1.00000000	duck
Ube2q2	-0.70	1.00000000	duck
Ghitm	-0.60	1.00000000	duck
Rab11a	-0.40	1.00000000	duck
Pcf11	2.09	0.00000000	pigeons
App	3.40	0.00000000	pigeons
Tpt1	4.79	0.00000000	pigeons
Atf7ip	2.98	0.00000000	pigeons
Gnai1	1.89	0.00000000	pigeons
Sap18	1.83	0.00000000	pigeons
Acvr2b	2.34	0.00000000	pigeons
Vim	2.18	0.00000000	pigeons
Wtap	1.69	0.00000000	pigeons
Sgk1	3.61	0.00000000	pigeons
Phip	2.33	0.00000000	pigeons
Rab33a	5.00	0.00000000	pigeons
Nfib	2.40	0.00000000	pigeons
Rnf38	1.71	0.00000000	pigeons
Tmbim1	1.96	0.00000000	pigeons
Arpc2	1.96	0.00000000	pigeons
Gypc	1.71	0.00000000	pigeons
Oga	1.76	0.00000000	pigeons
Crip2	1.90	0.00000000	pigeons
Ivns1abp	2.59	0.00000000	pigeons
U2surp	2.00	0.00000000	pigeons
Lpp	1.97	0.00000000	pigeons
Tiparp	1.66	0.00000000	pigeons
Anpep	1.80	0.00000000	pigeons

Cnbp	1.89	0.00000000	pigeons
Pcdhgb4	1.73	0.00000000	pigeons
Adnp	1.71	0.00000000	pigeons
Dido1	2.13	0.00000000	pigeons
Bri3bp	2.63	0.00000000	pigeons
Hoxb7	2.70	0.00000000	pigeons
Jup	1.58	0.00000000	pigeons
Atp5b	1.99	0.00000000	pigeons
Txnip	4.21	0.00000000	pigeons
Vip	1.72	0.00000000	pigeons
Syncrip	1.58	0.00000000	pigeons
Slc12a7	1.75	0.00000000	pigeons
Tfpi2	3.17	0.00000000	pigeons
Ndufa1	1.41	0.00000000	pigeons
Atp5g3	1.45	0.00000000	pigeons
Ankhd1	1.42	0.00000000	pigeons
Pctp	1.43	0.00000000	pigeons
Cmip	1.68	0.00000000	pigeons
Tardbp	1.31	0.00000000	pigeons
Rhob	1.62	0.00000000	pigeons
Pomp	1.27	0.00000000	pigeons
Lnpep	1.35	0.00000000	pigeons
Cd2ap	1.47	0.00000000	pigeons
Sdcbp	1.18	0.00000000	pigeons
Anapc15-ps	1.12	0.00000000	pigeons
Gadd45b	1.48	0.00000000	pigeons
Cdc37	1.16	0.00000000	pigeons
Vcp	1.19	0.00000000	pigeons
Nfic	1.16	0.00000000	pigeons
Ccdc25	1.16	0.00000000	pigeons
Plscr1	1.25	0.00000000	pigeons
Nasp	1.33	0.00000000	pigeons
Slmap	1.25	0.00000000	pigeons
Enox2	1.11	0.00000000	pigeons
Trpc4ap	1.10	0.00000000	pigeons
Cklf	1.11	0.00000000	pigeons
Slirp	1.12	0.00000000	pigeons
Tollip	1.07	0.00000000	pigeons
Rad23b	1.21	0.00000000	pigeons
Strap	1.15	0.00000000	pigeons
Niban1	1.28	0.00000000	pigeons
Sdhc	0.93	0.00000000	pigeons
Zfp777	1.07	0.00000000	pigeons
Eprs	1.11	0.00000000	pigeons
Golm2	1.00	0.00000000	pigeons
Sarnp	1.00	0.00000000	pigeons

Inpp4b	1.85	0.00000000	pigeons
Pcnp	0.98	0.00000000	pigeons
Cacybp	1.01	0.00000000	pigeons
Kars	1.02	0.00000000	pigeons
Brd8	0.93	0.00000000	pigeons
Rnf103	1.06	0.00000000	pigeons
Vopp1	1.59	0.00000000	pigeons
Apip	0.99	0.00000000	pigeons
Atp5c1	0.93	0.00000000	pigeons
Tgfbr1	1.02	0.00000000	pigeons
Pcolce2	2.14	0.00000000	pigeons
Fbxl21	0.87	0.00000000	pigeons
Ubtf	0.87	0.00000000	pigeons
Il1b	2.08	0.00000000	pigeons
Tdpoz8	0.86	0.00000000	pigeons
Igf1r	1.95	0.00000000	pigeons
Phax	0.84	0.00000000	pigeons
Rgs1	2.40	0.00000000	pigeons
Sec23ip	0.91	0.00000000	pigeons
Cars	0.96	0.00000000	pigeons
Tapbpl	0.89	0.00000000	pigeons
Rin2	0.99	0.00000000	pigeons
Pfkfb1	0.96	0.00000000	pigeons
Ablim1	1.65	0.00000000	pigeons
Nars	0.81	0.00000000	pigeons
Cd74	1.20	0.00000000	pigeons
Ccz1	0.82	0.00000000	pigeons
Trrap	0.81	0.00000000	pigeons
Adss	0.98	0.00000000	pigeons
Sap30bp	0.73	0.00000000	pigeons
Sinhcaf	0.79	0.00000000	pigeons
Sec24d	0.83	0.00000000	pigeons
Kifbp	0.73	0.00000000	pigeons
Rtf2	0.82	0.00000000	pigeons
Csnk1d	0.76	0.00000000	pigeons
Zdhhc20	0.84	0.00000000	pigeons
Rybp	0.89	0.00000000	pigeons
Tbp	0.70	0.00000000	pigeons
Spcs1	0.76	0.00000000	pigeons
Cul4a	0.96	0.00000000	pigeons
Banp	0.84	0.00000000	pigeons
C1qbp	0.76	0.00000000	pigeons
Pip4p2	0.76	0.00000000	pigeons
Nanp	0.71	0.00000000	pigeons
Atp5k	0.73	0.00000000	pigeons
Fbh1	0.74	0.00000000	pigeons

Ahsa2	0.70	0.00000000	pigeons
Fam189a1	-2.51	0.00000000	pigeons
Rwdd2a	0.71	0.00000000	pigeons
Aktip	0.69	0.00000000	pigeons
Rxylt1	0.76	0.00000000	pigeons
Crtap	0.71	0.00000000	pigeons
Il1rap	0.99	0.00000000	pigeons
Nop56	0.65	0.00000000	pigeons
Cdkn2aip	0.62	0.00000000	pigeons
Ccn1	1.15	0.00000000	pigeons
Wtip	0.76	0.00000000	pigeons
Ikbip	0.66	0.00000000	pigeons
Blcap	0.56	0.00000000	pigeons
Tut7	0.79	0.00000000	pigeons
Prep	0.73	0.00000000	pigeons
Dpp4	0.78	0.00000000	pigeons
Dapl1	0.65	0.00000000	pigeons
Rars	0.59	0.00000000	pigeons
Lpl	1.24	0.00000000	pigeons
Tdrp	0.68	0.00000000	pigeons
Kcnj5	-1.96	0.00000000	pigeons
Rbmx2	0.59	0.00000000	pigeons
Psme3ip1	0.64	0.00000000	pigeons
Corin	-3.43	0.00000000	pigeons
Dctn2	0.66	0.00000000	pigeons
Snrpd2	0.60	0.00000000	pigeons
Tent5a	0.74	0.00000000	pigeons
Dst	-0.81	0.00000000	pigeons
Atp5j2	0.61	0.00000000	pigeons
Dars	0.69	0.00000000	pigeons
Tent4a	0.59	0.00000000	pigeons
Rgs2	1.09	0.00000000	pigeons
LTO1	0.62	0.00000000	pigeons
Trpv2	0.75	0.00000000	pigeons
Eif4b	0.55	0.00000000	pigeons
Polr2i	0.61	0.00000000	pigeons
Polr1d	0.62	0.00000000	pigeons
Calu	0.56	0.00000000	pigeons
Ralgps2	-2.43	0.00000000	pigeons
Eif2b4	0.57	0.00000000	pigeons
Kdm3a	0.58	0.00000000	pigeons
Sfswap	0.47	0.00000000	pigeons
Tars	0.53	0.00000000	pigeons
Syne2	-1.00	0.00000000	pigeons
Pfkp	0.72	0.00000000	pigeons
Dtd1	0.48	0.00000000	pigeons

Sfxn3	0.44	0.00000000	pigeons
Cyria	1.31	0.00000000	pigeons
Glrx3	0.51	0.00000000	pigeons
B2m	0.80	0.00000000	pigeons
Unc5c	1.69	0.00000000	pigeons
Cog3	0.47	0.00000000	pigeons
Fkbp5	1.23	0.00000000	pigeons
Naa20	0.44	0.00000000	pigeons
Tnpo3	0.45	0.00000000	pigeons
Gsk3b	-1.01	0.00000000	pigeons
Sars	0.52	0.00000000	pigeons
Igfbp7	-0.81	0.00000000	pigeons
Rgcc	-1.25	0.00000000	pigeons
Idh3b	0.40	0.00000000	pigeons
Gars	0.38	0.00000000	pigeons
Terf2ip	0.41	0.00000000	pigeons
Dedd	0.43	0.00000000	pigeons
Nova1	0.96	0.00000000	pigeons
Gcdh	0.42	0.00000000	pigeons
Aamp	0.39	0.00000000	pigeons
Eapp	0.47	0.00000000	pigeons
Inpp5a	-1.08	0.00000000	pigeons
Psmc3ip	0.38	0.00000000	pigeons
Bccip	0.41	0.00000000	pigeons
Dnmbp	0.37	0.00000000	pigeons
Tmem100	0.76	0.00000000	pigeons
Inpp5k	-0.81	0.00000000	pigeons
Insig1	1.04	0.00000000	pigeons
Dab2ip	0.38	0.00000000	pigeons
Plekhg1	-1.21	0.00000000	pigeons
F10	0.69	0.00000000	pigeons
Porcn	0.38	0.00000000	pigeons
Brap	0.40	0.00000000	pigeons
Dohh	0.35	0.00000000	pigeons
Ctnna3	1.41	0.00000000	pigeons
Dgkd	-1.25	0.00000000	pigeons
Utp25	0.38	0.00000000	pigeons
Pym1	0.35	0.00000000	pigeons
Cdca7l	-1.50	0.00000000	pigeons
Scube3	-1.42	0.00000000	pigeons
Zbtb20	-0.76	0.00000000	pigeons
Tex2	-1.33	0.00000000	pigeons
Epb41l3	-0.67	0.00000000	pigeons
Pls3	0.84	0.00000000	pigeons
Rasal2	-0.84	0.00000000	pigeons
Neurl1b	-1.83	0.00000000	pigeons

Sptlc2	0.99	0.00000000	pigeons
Vill	-1.16	0.00000000	pigeons
Sec31a	-1.27	0.00000000	pigeons
Cmtm8	-1.10	0.00000000	pigeons
Fkbp1a	-0.71	0.00000000	pigeons
Adamts2	-1.31	0.00000000	pigeons
Pla2g7	0.90	0.00000000	pigeons
Arhgap15	1.52	0.00000000	pigeons
Dclk1	-2.89	0.00000000	pigeons
Dnajc10	-1.50	0.00000000	pigeons
Esm1	1.03	0.00000000	pigeons
Lmo7	-1.01	0.00000000	pigeons
Dmap1	-1.37	0.00000000	pigeons
Rbfa	-1.56	0.00000000	pigeons
Atf3	1.88	0.00000000	pigeons
Fermt2	-0.83	0.00000000	pigeons
Ntrk2	0.99	0.00000000	pigeons
Hivep2	-0.98	0.00000000	pigeons
Timp3	0.55	0.00000000	pigeons
Arhgap31	-0.87	0.00000000	pigeons
Spty2d1	-0.76	0.00000000	pigeons
Efemp1	-0.94	0.00000000	pigeons
Calcrl	0.61	0.00000000	pigeons
Gpx3	-0.69	0.00000000	pigeons
Tcf15	0.42	0.00000000	pigeons
Usp28	-1.03	0.00000000	pigeons
Nfx1	0.92	0.00000000	pigeons
Retreg1	-1.33	0.00000000	pigeons
Nup107	-1.11	0.00000000	pigeons
Bmp6	0.94	0.00000000	pigeons
Hlf	-1.08	0.00000000	pigeons
Arhgef9	-0.60	0.00000000	pigeons
Stk24	-1.36	0.00000000	pigeons
Rnf19b	0.73	0.00000000	pigeons
Ets2	0.86	0.00000000	pigeons
Stom	0.81	0.00000000	pigeons
Elovl6	0.86	0.00000000	pigeons
Abcg1	0.49	0.00000000	pigeons
Tmtc1	-1.04	0.00000000	pigeons
Afdn	-0.78	0.00000000	pigeons
Srebf1	0.76	0.00000000	pigeons
Fli1	-0.59	0.00000000	pigeons
Npas3	-0.91	0.00000000	pigeons
Adgrl4	0.47	0.00000000	pigeons
2900026A02Rik	-1.06	0.00000000	pigeons
Dock2	0.64	0.00000000	pigeons

Sdk1	-0.67	0.00000000	pigeons
Cep83	-1.14	0.00000000	pigeons
Snap23	0.85	0.00000000	pigeons
Zc3h12d	1.24	0.00000000	pigeons
Pip4k2a	-0.46	0.00000000	pigeons
Spred2	-0.63	0.00000000	pigeons
Cd34	-0.79	0.00000000	pigeons
Sox6	-1.04	0.00000000	pigeons
Per2	-0.67	0.00000000	pigeons
Ly75	0.66	0.00000000	pigeons
Stk39	0.69	0.00000000	pigeons
Plcb1	-0.66	0.00000000	pigeons
Slc15a1	-0.92	0.00000000	pigeons
Itpkb	-0.63	0.00000000	pigeons
Stk17b	-1.02	0.00000000	pigeons
Fads2b	0.60	0.00000000	pigeons
Kcne3	-0.55	0.00000000	pigeons
Eml6	-1.23	0.00000000	pigeons
Rbfox2	-0.99	0.00000000	pigeons
Sla	0.73	0.00000000	pigeons
Tmem266	-1.12	0.00000000	pigeons
Kit	0.62	0.00000000	pigeons
Snx14	-1.02	0.00000000	pigeons
Agbl1	-1.25	0.00000000	pigeons
Tgfbr3	0.66	0.00000000	pigeons
Ski	-0.68	0.00000000	pigeons
Samsn1	1.08	0.00000000	pigeons
Pecam1	0.38	0.00000000	pigeons
Hdac4	-0.76	0.00000000	pigeons
Rhoa	-0.46	0.00000000	pigeons
Dach1	-0.89	0.00000000	pigeons
Samhd1	0.66	0.00000000	pigeons
Numb	-0.56	0.00000000	pigeons
Kdelr3	0.63	0.00000000	pigeons
Stox2	-0.79	0.00000000	pigeons
Tet2	-0.64	0.00000000	pigeons
Ptprm	-0.42	0.00000000	pigeons
Rere	-0.72	0.00000000	pigeons
Rassf3	-0.57	0.00000000	pigeons
Laptm5	0.47	0.00000000	pigeons
Cyth3	-0.84	0.00000000	pigeons
Auts2	-0.87	0.00000000	pigeons
Proc	-0.78	0.00000000	pigeons
Enah	-0.78	0.00000000	pigeons
Bin3	-1.34	0.00000000	pigeons
Rac2	0.36	0.00000000	pigeons

Khdrbs3	-0.94	0.00000000	pigeons
Adgra1	-1.05	0.00000000	pigeons
Nfatc1	-1.17	0.00000000	pigeons
Sytl4	0.49	0.00000000	pigeons
Disp1	0.80	0.00000000	pigeons
Atrn	-0.77	0.00000000	pigeons
Skap1	-0.49	0.00000000	pigeons
Kcnq1	-1.28	0.00000000	pigeons
Filip1	-0.80	0.00000000	pigeons
Pm20d2	-0.83	0.00000000	pigeons
Eif3i	-0.59	0.00000000	pigeons
Kcnc1	0.75	0.00000000	pigeons
Spsb4	-0.90	0.00000000	pigeons
Ccnl1	-0.43	0.00000000	pigeons
Hoxb5	0.72	0.00000000	pigeons
Ralbp1	-0.57	0.00000000	pigeons
Sqstm1	0.61	0.00000000	pigeons
4932411N23Rik	-0.44	0.00000000	pigeons
Arl4a	-0.40	0.00000000	pigeons
Ldb2	0.44	0.00000000	pigeons
Ano5	-0.81	0.00000000	pigeons
Slc5a1	-0.84	0.00000000	pigeons
Ints6	-0.84	0.00000000	pigeons
Arhgdib	0.52	0.00000000	pigeons
Gata3	0.72	0.00000000	pigeons
Txnrd2	-0.73	0.00000000	pigeons
Nr1d2	-0.58	0.00000000	pigeons
Dennd2a	-0.54	0.00000000	pigeons
Adgrl2	0.37	0.00000000	pigeons
Hs6st1	-0.68	0.00000000	pigeons
Rb1	-0.70	0.00000000	pigeons
Trio	-0.52	0.00000000	pigeons
Tspo	0.52	0.00000000	pigeons
Atp11a	-1.08	0.00000000	pigeons
Lcp1	0.63	0.00000000	pigeons
Crim1	-0.57	0.00000000	pigeons
Ssbp2	-0.83	0.00000000	pigeons
Atp9a	0.53	0.00000000	pigeons
Eef1b2	-0.40	0.00000000	pigeons
Rbms3	-0.41	0.00000000	pigeons
Pim3	-0.47	0.00000000	pigeons
Eif4g3	-0.75	0.00000000	pigeons
Cfap45	-0.75	0.00000000	pigeons
Polr3b	-0.93	0.00000000	pigeons
Tacc1	0.55	0.00000000	pigeons
Zfp352	-0.39	0.00000000	pigeons

Cbx7	0.49	0.00000000	pigeons
Usp22	-0.69	0.00000000	pigeons
Npr3	-0.85	0.00000000	pigeons
Srebf2	0.55	0.00000000	pigeons
Ippk	0.66	0.00000000	pigeons
Wdr35	-0.75	0.00000000	pigeons
Pgm3	0.57	0.00000000	pigeons
Sgip1	-0.89	0.00000000	pigeons
Cmpk2	0.47	0.00000000	pigeons
Cnnm2	-0.93	0.00000000	pigeons
Boc	-0.57	0.00000000	pigeons
Tmem204	-0.51	0.00000000	pigeons
Arhgap21	-0.61	0.00000000	pigeons
Sparc	-0.34	0.00000000	pigeons
S100a13	-0.45	0.00000000	pigeons
Arhgef7	-0.77	0.00000000	pigeons
Ehd4	-0.69	0.00000000	pigeons
Cobll1	-0.58	0.00000000	pigeons
Clcn1	-0.68	0.00000000	pigeons
Sema7a	-0.59	0.00000000	pigeons
Pias1	0.54	0.00000000	pigeons
Hecw2	-0.60	0.00000000	pigeons
Cask	-0.67	0.00000000	pigeons
Fam171a1	0.50	0.00000000	pigeons
Hgfac	-0.60	0.00000000	pigeons
Nr4a1	-0.55	0.00000000	pigeons
Vps54	-0.83	0.00000000	pigeons
Hspa5	-0.57	0.00000000	pigeons
Coro2b	0.55	0.00000000	pigeons
Sag	-0.56	0.00000000	pigeons
Jade3	-0.72	0.00000000	pigeons
Ppargc1a	-0.79	0.00000000	pigeons
Kif26a	-0.59	0.00000000	pigeons
Retsat	0.31	0.00000000	pigeons
Phf20	-0.65	0.00000000	pigeons
Phactr2	0.54	0.00000000	pigeons
Oxsr1	-0.66	0.00000000	pigeons
Bbs4	-0.51	0.00000000	pigeons
Lamp2	0.51	0.00000000	pigeons
Dock10	0.50	0.00000000	pigeons
Cald1	-0.59	0.00000000	pigeons
Lmo2	0.66	0.00000000	pigeons
Drd1	-0.44	0.00000000	pigeons
Rcbtb1	-0.64	0.00000000	pigeons
Acsbg2	0.46	0.00000000	pigeons
Ddx17	0.50	0.00000000	pigeons

Wdr7	-0.68	0.00000000	pigeons
Sfxn2	-0.53	0.00000000	pigeons
Klhl8	-0.54	0.00000000	pigeons
Scube2	-0.55	0.00000000	pigeons
Flnb	-0.51	0.00000000	pigeons
Lpin2	-0.68	0.00000000	pigeons
Erc1	-0.55	0.00000000	pigeons
Enpp6	-0.47	0.00000000	pigeons
Pkdcc	0.64	0.00000000	pigeons
Eif3k	-0.54	0.00000000	pigeons
Hs3st1	0.44	0.00000000	pigeons
Ino80	-0.75	0.00000000	pigeons
Exoc6b	-0.53	0.00000000	pigeons
Hpgd	0.53	0.00000000	pigeons
Gng10	-0.38	0.00000000	pigeons
Actg1	-0.25	0.00000000	pigeons
Foxn3	-0.48	0.00000000	pigeons
Ldlrad4	-0.64	0.00000000	pigeons
Rab11fip3	-0.61	0.00000000	pigeons
Sema6a	-0.53	0.00000000	pigeons
Aoah	-0.61	0.00000000	pigeons
Acaa1b	-0.55	0.00000000	pigeons
Epas1	0.36	0.00000000	pigeons
Lrrk2	0.50	0.00000000	pigeons
Cavin2	0.43	0.00000000	pigeons
Gigyf2	-0.59	0.00000000	pigeons
Ext1	-0.55	0.00000000	pigeons
Hdx	-0.53	0.00000000	pigeons
Tjp1	-0.36	0.00000000	pigeons
Fam13a	-0.52	0.00000000	pigeons
Ripor3	-0.49	0.00000000	pigeons
Ccny	-0.86	0.00000000	pigeons
Tspan9	-0.57	0.00000000	pigeons
Gk5	-0.48	0.00000000	pigeons
Sorbs1	-0.31	0.00000000	pigeons
Rbm31y	-0.36	0.00000000	pigeons
Klf7	-0.76	0.00000000	pigeons
Camk2g	-0.39	0.00000000	pigeons
Gnb5	-0.48	0.00000000	pigeons
Tfpi	0.59	0.00000000	pigeons
Hdac7	-0.63	0.00000000	pigeons
Oaz2	-0.47	0.00000000	pigeons
Ybx1	-0.43	0.00000000	pigeons
Cbfa2t3	0.44	0.00000000	pigeons
Zc3h12a	0.56	0.00000000	pigeons
Limd1	-0.64	0.00000000	pigeons

Arid1b	-0.50	0.00000000	pigeons
St6galnac3	-0.60	0.00000000	pigeons
Celf2	0.66	0.00000000	pigeons
Plcd1	-0.60	0.00000000	pigeons
Ankrd39	-0.41	0.00000000	pigeons
Agap1	-0.67	0.00000000	pigeons
Prickle1	-0.44	0.00000000	pigeons
Ttc28	-0.57	0.00000000	pigeons
Nmd3	0.31	0.00000000	pigeons
Hbp1	0.46	0.00000000	pigeons
Tub	-0.45	0.00000000	pigeons
Pard3b	-0.47	0.00000000	pigeons
Elmo1	-0.44	0.00000000	pigeons
Lifr	-0.44	0.00000000	pigeons
Hsd17b12	-0.41	0.00000000	pigeons
Chmp4c	-0.42	0.00000000	pigeons
Nyx	-0.43	0.00000000	pigeons
Elf1	-0.55	0.00000000	pigeons
Slf1	-0.52	0.00000000	pigeons
Efna2	-0.45	0.00000000	pigeons
Ptk2	-0.57	0.00000000	pigeons
Naca	-0.33	0.00000000	pigeons
Stat1	0.50	0.00000000	pigeons
Irf1	0.54	0.00000000	pigeons
Smarcal1	-0.26	0.00000000	pigeons
Spred1	-0.67	0.00000000	pigeons
Dot1l	-0.48	0.00000000	pigeons
Tcf12	-0.35	0.00000000	pigeons
Hsp90aa1	0.49	0.00000000	pigeons
Nav2	-0.67	0.00000000	pigeons
Tfrc	-0.19	0.00000000	pigeons
Ern1	-0.61	0.00000000	pigeons
Pnisr	0.51	0.00000000	pigeons
Ckap4	0.36	0.00000000	pigeons
Dll4	-0.37	0.00000000	pigeons
Luzp1	-0.44	0.00000000	pigeons
Slc16a6	0.37	0.00000000	pigeons
Ston1	0.42	0.00000000	pigeons
Sdc1	-0.46	0.00000000	pigeons
Slc6a6	0.05	0.00000000	pigeons
Disc1	0.44	0.00000000	pigeons
Tnip2	0.58	0.00000000	pigeons
Bin1	-0.50	0.00000000	pigeons
Fryl	-0.37	0.00000000	pigeons
Rnf144a	0.51	0.00000000	pigeons
Stard4	0.24	0.00000000	pigeons

Trim8	-0.48	0.00000000	pigeons
Drc7	-0.47	0.00000000	pigeons
Fbrsl1	-0.36	0.00000000	pigeons
Fmnl2	-0.42	0.00000000	pigeons
Ankrd44	0.53	0.00000000	pigeons
Sftpc	-0.48	0.00000000	pigeons
Apba2	-0.51	0.00000000	pigeons
Grip1	0.56	0.00000001	pigeons
F7	-0.42	0.00000001	pigeons
Colec10	-0.56	0.00000001	pigeons
Itm2a	0.27	0.00000001	pigeons
Pdgfb	0.45	0.00000001	pigeons
Trank1	0.55	0.00000001	pigeons
Aqp11	0.43	0.00000001	pigeons
Tspan6	0.37	0.00000001	pigeons
Cd9	0.53	0.00000001	pigeons
Tnfaip3	0.63	0.00000001	pigeons
Hic2	0.36	0.00000001	pigeons
F8	-0.49	0.00000001	pigeons
Tead4	-0.45	0.00000001	pigeons
Pwwp2b	-0.45	0.00000001	pigeons
Tnfrsf1a	0.37	0.00000001	pigeons
Adgrg1	0.41	0.00000001	pigeons
Slc35e3	-0.41	0.00000002	pigeons
Vav3	0.47	0.00000002	pigeons
Pde3b	-0.17	0.00000002	pigeons
Elovl5	0.38	0.00000002	pigeons
Raph1	-0.61	0.00000002	pigeons
Adcy8	0.52	0.00000003	pigeons
Tab3	0.46	0.00000003	pigeons
Ccdc50	0.33	0.00000003	pigeons
Wwtr1	-0.40	0.00000003	pigeons
Heg1	-0.32	0.00000003	pigeons
Far1	0.36	0.00000004	pigeons
Hoxb3	0.51	0.00000004	pigeons
Aldh1a2	-0.46	0.00000005	pigeons
Npc1	0.29	0.00000005	pigeons
Ptpn14	-0.39	0.00000005	pigeons
Sema3g	-0.54	0.00000005	pigeons
Limd2	0.31	0.00000005	pigeons
Snx10	0.62	0.00000006	pigeons
Hoxb6	0.41	0.00000006	pigeons
Upk1b	-0.37	0.00000006	pigeons
Ddit4	-0.19	0.00000006	pigeons
Ctso	0.27	0.00000006	pigeons
Cfd	-0.21	0.00000006	pigeons

Tsc2	-0.59	0.00000007	pigeons
Gpa33	0.38	0.00000010	pigeons
Ulk1	-0.58	0.00000010	pigeons
Crem	-0.36	0.00000010	pigeons
Zfp706	0.33	0.00000010	pigeons
Ppm1h	0.33	0.00000011	pigeons
Cacul1	-0.37	0.00000011	pigeons
Cry1	-0.46	0.00000011	pigeons
Bmp2	0.56	0.00000012	pigeons
Ubac2	0.35	0.00000013	pigeons
Acsl3	0.26	0.00000013	pigeons
Prelid3a	-0.48	0.00000013	pigeons
Capn7	0.29	0.00000013	pigeons
Itgb1	-0.34	0.00000014	pigeons
Slc46a2	-0.37	0.00000014	pigeons
Tfdp2	-0.59	0.00000014	pigeons
Tmem140	0.40	0.00000014	pigeons
Asxl3	0.35	0.00000016	pigeons
Klf13	-0.56	0.00000016	pigeons
Pus1	-0.37	0.00000017	pigeons
Rapgef2	-0.20	0.00000018	pigeons
Bet1	0.35	0.00000019	pigeons
Cpq	-0.57	0.00000021	pigeons
Stxbp5	-0.48	0.00000023	pigeons
Ptprc	0.69	0.00000026	pigeons
Syne1	0.35	0.00000037	pigeons
Pip5k1a	-0.44	0.00000040	pigeons
Aff1	-0.47	0.00000041	pigeons
Katnb1	-0.38	0.00000043	pigeons
Crip1	0.36	0.00000046	pigeons
Arvcf	-0.40	0.00000049	pigeons
Baz2b	-0.36	0.00000053	pigeons
Arpc5	0.37	0.00000054	pigeons
Wdsub1	-0.39	0.00000054	pigeons
Fgfr2	-0.32	0.00000054	pigeons
Podxl	-0.24	0.00000056	pigeons
Trib1	-0.21	0.00000062	pigeons
Usp12	0.41	0.00000065	pigeons
Klhl15	0.30	0.00000067	pigeons
Il11ra1	-0.25	0.00000068	pigeons
Ogfr	0.44	0.00000069	pigeons
Cit	-0.33	0.00000072	pigeons
Taf5l	-0.40	0.00000073	pigeons
Tmsb4x	0.29	0.00000075	pigeons
Hbs1l	0.38	0.00000077	pigeons
Caskin2	0.34	0.00000077	pigeons

Eva1c	0.34	0.00000078	pigeons
Glul	-0.33	0.00000094	pigeons
Skap2	0.49	0.00000110	pigeons
Khdrbs1	0.33	0.00000118	pigeons
Nubpl	0.41	0.00000118	pigeons
Rnf145	0.37	0.00000120	pigeons
Polk	0.40	0.00000124	pigeons
Plbd2	-0.41	0.00000131	pigeons
Tns1	-0.28	0.00000132	pigeons
Atg101	-0.36	0.00000170	pigeons
Evl	0.98	0.00000175	pigeons
Fbxl18	-0.61	0.00000187	pigeons
Bcl2l1	0.38	0.00000190	pigeons
Utrn	0.24	0.00000208	pigeons
Trak1	-0.56	0.00000246	pigeons
Fnbp1	-0.45	0.00000250	pigeons
Klf3	-0.33	0.00000304	pigeons
Rap1b	-0.33	0.00000306	pigeons
Ckb	0.35	0.00000368	pigeons
Daam2	-0.35	0.00000383	pigeons
Exoc5	0.40	0.00000390	pigeons
Hells	-0.39	0.00000413	pigeons
Srsf1	0.34	0.00000433	pigeons
Fat4	-0.31	0.00000443	pigeons
Plk3	-0.38	0.00000444	pigeons
Iqgap1	-0.37	0.00000463	pigeons
Serpind1	0.62	0.00000464	pigeons
Smc3	-0.43	0.00000479	pigeons
Clec16a	0.46	0.00000572	pigeons
Ptgs2	0.67	0.00000578	pigeons
Primpol	0.23	0.00000633	pigeons
Sox18	0.36	0.00000680	pigeons
Etl4	-0.42	0.00000684	pigeons
Itpk1	-0.36	0.00000762	pigeons
Ndufb9	0.26	0.00000804	pigeons
Nol11	-0.42	0.00000818	pigeons
Hs1bp3	0.36	0.00000851	pigeons
Vgll4	-0.37	0.00000928	pigeons
Epc1	-0.43	0.00000931	pigeons
Pbx1	-0.44	0.00000947	pigeons
Ttc21b	0.29	0.00000995	pigeons
Rbms1	0.28	0.00001032	pigeons
Dlgap4	-0.38	0.00001049	pigeons
Exoc4	-0.44	0.00001205	pigeons
Cldn12	0.31	0.00001207	pigeons
Plcg2	0.35	0.00001244	pigeons

Ndufa13	0.31	0.00001316	pigeons
Synj1	0.37	0.00001391	pigeons
Creb1	0.42	0.00001411	pigeons
Xpo6	0.40	0.00001428	pigeons
Pikfyve	-0.39	0.00001444	pigeons
Lamc1	-0.41	0.00001453	pigeons
Dytn	-0.40	0.00001547	pigeons
Sat1	0.52	0.00001663	pigeons
Ifngr1	0.40	0.00001665	pigeons
Smg6	-0.42	0.00001698	pigeons
Pxdc1	0.34	0.00001786	pigeons
Cdc42bpb	-0.43	0.00001837	pigeons
St3gal3	-0.40	0.00001881	pigeons
Foxn2	0.22	0.00001933	pigeons
Abcd3	0.28	0.00001980	pigeons
Il15	0.39	0.00001993	pigeons
Smad1	-0.49	0.00001997	pigeons
Ppp4r1	-0.46	0.00002174	pigeons
Ergic1	0.28	0.00002361	pigeons
Ift140	-0.39	0.00002453	pigeons
Fah	-0.29	0.00002461	pigeons
Ppp1r13b	-0.44	0.00002552	pigeons
Slc30a7	-0.40	0.00002771	pigeons
Fgf12	0.41	0.00002870	pigeons
Dicer1	0.31	0.00002937	pigeons
Nenf	-0.33	0.00003324	pigeons
Frzb	-0.30	0.00003598	pigeons
Per3	-0.38	0.00004015	pigeons
Cwc27	0.42	0.00004610	pigeons
Acadl	0.33	0.00004691	pigeons
Sec61g	0.37	0.00005071	pigeons
Tlnrd1	-0.40	0.00005129	pigeons
Fbxo7	0.32	0.00005184	pigeons
Sel1l	0.35	0.00005271	pigeons
Snrpa1	0.27	0.00005280	pigeons
Eif2ak2	0.36	0.00005538	pigeons
Arih1	-0.38	0.00005726	pigeons
Ddx28	0.26	0.00005804	pigeons
Sh3glb1	-0.38	0.00006071	pigeons
Lpcat3	0.27	0.00006489	pigeons
Fdx1	0.29	0.00006543	pigeons
Brwd1	-0.41	0.00007082	pigeons
Nmi	0.35	0.00007172	pigeons
Reep5	-0.36	0.00007811	pigeons
Wdr20	0.32	0.00007922	pigeons
Zdhhc9	0.22	0.00008400	pigeons

Vcl	-0.35	0.00008661	pigeons
Galc	0.30	0.00008675	pigeons
Ctdspl	0.19	0.00008689	pigeons
Txnrd1	-0.43	0.00008870	pigeons
Pde10a	-0.67	0.00009175	pigeons
Rusc2	-0.50	0.00009402	pigeons
Pald1	-0.37	0.00009798	pigeons
Slc4a4	-0.53	0.00010124	pigeons
Srp14	0.25	0.00010380	pigeons
Cebpg	0.27	0.00010444	pigeons
Rpn1	0.30	0.00010851	pigeons
Neo1	-0.28	0.00011366	pigeons
Cers5	0.22	0.00011482	pigeons
Xpo1	0.40	0.00012139	pigeons
Tmem181a	0.25	0.00012388	pigeons
Acvr2a	0.38	0.00012422	pigeons
Etv6	-0.37	0.00013104	pigeons
Fnip1	0.81	0.00014206	pigeons
Eif4g2	-0.25	0.00015057	pigeons
Anks1	-0.42	0.00015563	pigeons
Zfp106	0.38	0.00016592	pigeons
Prmt7	-0.54	0.00017611	pigeons
Slc9a3r2	-0.30	0.00018471	pigeons
Usp34	-0.48	0.00019684	pigeons
Zfhx4	-0.27	0.00019939	pigeons
Zfp469	-0.46	0.00024911	pigeons
Fndc3a	0.35	0.00025576	pigeons
Ncoa2	0.37	0.00025795	pigeons
Ube2q2	-0.30	0.00026301	pigeons
Naa15	0.30	0.00026466	pigeons
Polg2	-0.39	0.00027198	pigeons
Rundc3b	0.38	0.00027418	pigeons
Scoc	0.30	0.00028593	pigeons
Bloc1s6	-0.31	0.00031361	pigeons
Rnf11	-0.37	0.00032008	pigeons
Sec14l1	0.24	0.00032566	pigeons
Tbk1	0.38	0.00033784	pigeons
Arl6ip5	0.32	0.00035282	pigeons
L3mbtl3	0.27	0.00039200	pigeons
Tmem50b	0.26	0.00039209	pigeons
Tox2	0.34	0.00039322	pigeons
Tmbim6	-0.33	0.00040096	pigeons
Tcf7l2	-0.37	0.00041859	pigeons
Serac1	0.23	0.00042203	pigeons
Cpne1	-0.36	0.00043612	pigeons
Atf4	-0.20	0.00044810	pigeons

Insig2	0.14	0.00046476	pigeons
Prex1	-0.45	0.00046502	pigeons
Kat6b	-0.42	0.00047268	pigeons
Hsph1	0.59	0.00049681	pigeons
Pde4d	0.32	0.00056032	pigeons
Slc9a6	-0.42	0.00056139	pigeons
Nr4a2	-0.41	0.00059049	pigeons
Gpc5	0.39	0.00060052	pigeons
Arhgef6	-0.44	0.00060920	pigeons
Ppib	0.37	0.00065825	pigeons
Pole4	0.24	0.00068152	pigeons
Rfx3	-0.58	0.00068399	pigeons
Wsb1	-0.29	0.00068931	pigeons
Lrp6	0.32	0.00071037	pigeons
Arhgap28	0.44	0.00074496	pigeons
Acer2	0.34	0.00075710	pigeons
Rnf152	0.31	0.00076030	pigeons
Pla2g12b	-0.26	0.00079427	pigeons
Coq8a	-0.23	0.00082417	pigeons
Slc4a11	0.24	0.00084171	pigeons
Acer3	0.31	0.00085831	pigeons
Ifnar2	0.32	0.00086067	pigeons
Zfp407	-0.45	0.00087537	pigeons
Aacs	-0.42	0.00089233	pigeons
Itpr1	-0.58	0.00091206	pigeons
Atxn7	-0.38	0.00094955	pigeons
Amd2	0.30	0.00095292	pigeons
Znrf1	-0.39	0.00096129	pigeons
Gnb1l	-0.30	0.00097626	pigeons
Hhex	-0.34	0.00102149	pigeons
Cftr	0.31	0.00105572	pigeons
Gse1	0.33	0.00105889	pigeons
Abcf2	-0.35	0.00107977	pigeons
Fth1	0.19	0.00108406	pigeons
Bcl7a	0.26	0.00110845	pigeons
Nkd1	-0.31	0.00112181	pigeons
Synm	-0.24	0.00112308	pigeons
Hmox1	0.28	0.00114336	pigeons
Trp53i11	-0.30	0.00115874	pigeons
Dennd5a	-0.31	0.00124395	pigeons
Pcgf5	0.30	0.00126554	pigeons
Brd1	-0.36	0.00129652	pigeons
Cttnbp2	0.33	0.00131679	pigeons
Dstn	-0.21	0.00142628	pigeons
Sccpdh	0.22	0.00142664	pigeons
Grk3	-0.42	0.00143331	pigeons

Ppp1r12a	-0.35	0.00155874	pigeons
Ccni	-0.30	0.00156623	pigeons
Prr16	-0.33	0.00157029	pigeons
Tmem63a	0.25	0.00157419	pigeons
Cramp1	-0.39	0.00164344	pigeons
Hacd2	0.27	0.00183768	pigeons
Ccnl2	-0.37	0.00191671	pigeons
Pgam1	0.24	0.00194836	pigeons
Aprt	0.28	0.00196049	pigeons
Ube2w	0.30	0.00210421	pigeons
Snrk	-0.31	0.00215860	pigeons
Dnajb9	0.25	0.00220375	pigeons
Jade1	0.27	0.00223125	pigeons
Nvl	-0.38	0.00232445	pigeons
Ube2h	-0.30	0.00233140	pigeons
Jarid2	-0.35	0.00240717	pigeons
Tnks2	0.27	0.00241892	pigeons
Ccnd1	-0.37	0.00243833	pigeons
Suv39h2	0.18	0.00261600	pigeons
Psma6	0.31	0.00283984	pigeons
Dip2c	-0.40	0.00284370	pigeons
Eif5a2	-0.31	0.00288200	pigeons
E2f1	0.19	0.00295359	pigeons
Rnf13	0.29	0.00296950	pigeons
Adamtsl2	-0.34	0.00297457	pigeons
Exosc3	0.22	0.00314240	pigeons
Sh3bp2	0.33	0.00320698	pigeons
Hyal1	-0.25	0.00320955	pigeons
Ahr	-0.33	0.00321694	pigeons
C1galt1c1	0.25	0.00324662	pigeons
Glg1	-0.38	0.00337255	pigeons
Rsrp1	0.32	0.00338273	pigeons
Sh3pxd2a	-0.49	0.00343078	pigeons
Rnaset2b	0.32	0.00344310	pigeons
Lrch2	0.22	0.00358095	pigeons
Cyld	0.19	0.00364513	pigeons
Wbp2	0.20	0.00367879	pigeons
Ppid	0.26	0.00371682	pigeons
Ccser1	-0.57	0.00385699	pigeons
Ankrd50	-0.25	0.00386693	pigeons
Fxyd6	0.28	0.00390098	pigeons
Zfyve1	-0.39	0.00396401	pigeons
Cxcl12	-0.32	0.00432606	pigeons
Abhd17c	0.28	0.00439326	pigeons
Tmem59	0.28	0.00445295	pigeons
Slc15a4	0.24	0.00447197	pigeons

Usp10	0.28	0.00478137	pigeons
Kif5b	-0.28	0.00482029	pigeons
Hopx	-0.22	0.00483900	pigeons
Kpnb1	0.24	0.00499338	pigeons
Seh1l	0.25	0.00510641	pigeons
Osbpl3	-0.45	0.00533250	pigeons
Vwa1	0.36	0.00548959	pigeons
Ube2k	-0.42	0.00549423	pigeons
Usp6nl	-0.35	0.00551625	pigeons
Ccm2l	0.22	0.00575474	pigeons
Osbpl5	-0.20	0.00607356	pigeons
Slc25a16	0.24	0.00639002	pigeons
Dpf3	-0.37	0.00641758	pigeons
Efr3a	0.32	0.00694369	pigeons
Pard3	-0.38	0.00728664	pigeons
Rfc1	0.29	0.00738170	pigeons
Srpr	0.24	0.00758134	pigeons
Gnb4	-0.28	0.00759726	pigeons
Tpk1	-0.53	0.00763293	pigeons
Dock11	0.40	0.00800964	pigeons
Hif1a	0.33	0.00803942	pigeons
Hyi	-0.22	0.00824554	pigeons
Larp4b	-0.44	0.00833279	pigeons
Dusp15	0.22	0.00853685	pigeons
Ifnar1	0.16	0.00909834	pigeons
Tmem47	-0.29	0.00957504	pigeons
Lrrc8b	0.23	0.00996330	pigeons
Sbds	0.16	0.00997868	pigeons
Akap12	-0.19	0.01010838	pigeons
Pcna	-0.35	0.01028501	pigeons
Dpyd	-0.35	0.01073149	pigeons
Ankrd17	-0.34	0.01074143	pigeons
Snx25	-0.42	0.01075797	pigeons
Fuca1	-0.26	0.01111292	pigeons
Dock4	-0.52	0.01118152	pigeons
Sap130	-0.32	0.01145957	pigeons
Nfkb1	0.49	0.01148299	pigeons
Homer1	-0.53	0.01158534	pigeons
Sulf1	0.37	0.01218838	pigeons
Ano10	-0.30	0.01230070	pigeons
Pgs1	0.22	0.01276757	pigeons
Ntn4	0.33	0.01295380	pigeons
Nbn	0.16	0.01302292	pigeons
Lrrc8c	0.26	0.01328765	pigeons
Rxra	0.25	0.01366320	pigeons
Tmem150c	0.29	0.01402599	pigeons

Zfp516	-0.11	0.01447318	pigeons
Kifc3	-0.33	0.01493151	pigeons
Rdx	0.31	0.01527128	pigeons
Psip1	0.28	0.01544839	pigeons
Kitl	0.46	0.01561112	pigeons
Prss45	-0.34	0.01618098	pigeons
Ap2m1	0.27	0.01634365	pigeons
Ubr5	-0.39	0.01677338	pigeons
Csnk1a1	-0.26	0.01820007	pigeons
Zfp827	-0.34	0.01827938	pigeons
Eif3g	0.25	0.01868011	pigeons
Nhlrc2	0.17	0.01876154	pigeons
Edem1	0.28	0.01886237	pigeons
Tbc1d4	-0.34	0.01901484	pigeons
Kdm3b	-0.38	0.01910992	pigeons
Raf1	0.24	0.01928182	pigeons
Cstf3	0.20	0.01988145	pigeons
Chordc1	0.23	0.02016059	pigeons
Hoxa5	0.37	0.02059874	pigeons
Gemin4	0.18	0.02155125	pigeons
Ccnh	0.20	0.02169276	pigeons
Runx1t1	0.32	0.02202893	pigeons
Dtx3l	0.30	0.02292411	pigeons
Zfp644	0.25	0.02323968	pigeons
Arhgap26	-0.32	0.02411526	pigeons
Atp2b1	0.26	0.02479161	pigeons
Zfp423	-0.37	0.02487126	pigeons
Tatdn1	0.21	0.02527976	pigeons
Psen1	-0.31	0.02555409	pigeons
Ust	-0.41	0.02595481	pigeons
Sos1	-0.32	0.02666922	pigeons
Dnajc13	0.26	0.02765805	pigeons
Dyrk1a	-0.31	0.02789802	pigeons
Paqr7	0.30	0.02825173	pigeons
Rcc1l	-0.18	0.02832417	pigeons
Lyn	0.36	0.02997933	pigeons
Cpeb2	-0.14	0.03039264	pigeons
Smurf2	-0.22	0.03158159	pigeons
Dcbld1	0.31	0.03158761	pigeons
Dysf	-0.25	0.03169697	pigeons
Acsl1	0.44	0.03411854	pigeons
St8sia4	0.31	0.03646050	pigeons
Znfx1	0.32	0.03670178	pigeons
Pnrc2	0.29	0.03696550	pigeons
Kdm7a	-0.22	0.03752693	pigeons
Baz1b	0.29	0.03808705	pigeons

Zbtb11	-0.35	0.03904909	pigeons
Foxf1	0.27	0.04032784	pigeons
Sltm	-0.31	0.04039114	pigeons
Sec23b	0.17	0.04078560	pigeons
Zswim5	-0.21	0.04174098	pigeons
Polr3a	-0.45	0.04324633	pigeons
Itch	0.26	0.04431939	pigeons
Aplp2	0.24	0.04468781	pigeons
Rpn2	0.24	0.04479858	pigeons
Flot2	0.30	0.04490667	pigeons
Olfml2a	-0.21	0.04717913	pigeons
Flt4	-0.33	0.04971516	pigeons
Stx2	0.24	0.04978062	pigeons
Rbpj	0.29	0.05005480	pigeons
Trib2	-0.24	0.05158795	pigeons
Nfyb	0.24	0.05190648	pigeons
Samd11	-0.27	0.05388602	pigeons
Zdhhc3	0.26	0.05491868	pigeons
Timmdc1	0.12	0.05506007	pigeons
Ciapin1	0.22	0.05513795	pigeons
Tmem167	0.25	0.05522358	pigeons
Bicd2	0.17	0.05738922	pigeons
Elk3	-0.34	0.05887584	pigeons
Ryk	0.24	0.05903816	pigeons
Sucla2	0.21	0.05947663	pigeons
Jag2	0.45	0.05990547	pigeons
Fam3c	0.18	0.06007279	pigeons
Hivep1	-0.46	0.06095585	pigeons
Rictor	0.33	0.06167143	pigeons
C2cd5	0.26	0.06254731	pigeons
Naa25	0.20	0.06356791	pigeons
Slco2a1	0.29	0.06359252	pigeons
Agpat5	0.35	0.06410425	pigeons
Secisbp2l	0.26	0.06480959	pigeons
Bnip3	0.16	0.06553546	pigeons
Tnfrsf11a	-0.29	0.06612698	pigeons
Rbm26	0.27	0.06783300	pigeons
Plcb2	0.15	0.06904457	pigeons
Znhit6	0.30	0.06917370	pigeons
Nrbf2	0.19	0.06985933	pigeons
Flt1	-0.29	0.07043121	pigeons
Hpcal1	-0.20	0.07071970	pigeons
Clasp2	-0.28	0.07076234	pigeons
Arpc3	0.24	0.07169570	pigeons
Efnb2	-0.33	0.07425239	pigeons
Snx3	-0.27	0.07472493	pigeons

Dlg1	-0.47	0.07601386	pigeons
Gdpd1	0.25	0.07666326	pigeons
Aig1	0.25	0.07732607	pigeons
Ldah	0.24	0.07961564	pigeons
Ahi1	0.25	0.07985301	pigeons
Armc8	0.22	0.08027985	pigeons
Ccnyl1	0.27	0.08226452	pigeons
Jazf1	-0.27	0.08280524	pigeons
Sptbn1	0.15	0.08384497	pigeons
Tmcc3	-0.30	0.08401069	pigeons
Aplnr	-0.26	0.08699809	pigeons
Hddc2	0.25	0.08784707	pigeons
Ncoa7	0.44	0.08958769	pigeons
Ehd3	0.27	0.08998358	pigeons
Plekhb2	0.25	0.09329807	pigeons
Ttc4	0.25	0.09379202	pigeons
Dusp7	-0.12	0.09589097	pigeons
Abca1	0.27	0.09844292	pigeons
Srek1ip1	0.25	0.09934095	pigeons
Sesn1	0.24	0.10085677	pigeons
Wdr44	0.26	0.10214486	pigeons
Rmdn2	0.29	0.10239461	pigeons
Palmd	0.31	0.10302534	pigeons
Fyco1	0.29	0.10478054	pigeons
Bmp2k	0.29	0.10494540	pigeons
Sec61a2	0.17	0.10542120	pigeons
Ssh2	0.24	0.10714747	pigeons
Bcor	-0.46	0.10812272	pigeons
Zfp521	0.28	0.11009868	pigeons
Extl3	-0.23	0.11171265	pigeons
Taf15	0.21	0.11361262	pigeons
Hcn2	0.15	0.11891950	pigeons
Atp6v0c	-0.25	0.12079124	pigeons
Rnf114	0.26	0.12249054	pigeons
Rbm43	0.20	0.12708550	pigeons
Tinagl1	0.35	0.13019306	pigeons
Shq1	0.17	0.13265474	pigeons
Pik3c2a	-0.36	0.13378759	pigeons
Rnaseh2b	0.27	0.13489164	pigeons
Chmp1a	0.27	0.13802625	pigeons
Pik3ip1	0.25	0.13950591	pigeons
Vmp1	-0.33	0.14177146	pigeons
Tlk2	-0.38	0.14442348	pigeons
Nr2c2	0.24	0.14704452	pigeons
Rev1	-0.29	0.14835170	pigeons
Cltc	-0.31	0.15649185	pigeons

Ninj1	0.14	0.15661239	pigeons
Tmem168	0.18	0.15670858	pigeons
Col4a1	-0.20	0.16174345	pigeons
Phtf2	-0.31	0.16195111	pigeons
Ctss	0.20	0.16342010	pigeons
Dusp1	-0.06	0.16403819	pigeons
Dynll2	-0.19	0.16449393	pigeons
Cggbp1	0.23	0.16488591	pigeons
Ufl1	0.26	0.17102329	pigeons
Gtdc1	0.35	0.17524082	pigeons
Hsp90b1	-0.17	0.17615706	pigeons
Dennd4a	0.25	0.17746461	pigeons
Adamts3	-0.32	0.17881841	pigeons
Stag2	0.22	0.18172240	pigeons
Chd7	0.25	0.18427611	pigeons
Rnpc3	0.14	0.18573183	pigeons
Dok1	0.23	0.18653069	pigeons
Eef2	-0.22	0.18730461	pigeons
Nr3c1	-0.31	0.19300679	pigeons
Zcchc7	0.22	0.19428324	pigeons
Brf2	-0.18	0.20332613	pigeons
Il6st	0.25	0.21781902	pigeons
Kpna3	-0.37	0.21946213	pigeons
Rab8b	0.20	0.22532094	pigeons
Zfp800	0.18	0.22564786	pigeons
Tmem144	0.21	0.22597035	pigeons
Zfand6	-0.30	0.22732050	pigeons
Ccpg1	0.18	0.22820667	pigeons
Helz2	-0.30	0.23135641	pigeons
Lrpprc	0.24	0.23295161	pigeons
Hax1	0.22	0.23307430	pigeons
Eif5	0.28	0.23740838	pigeons
Fosl2	-0.26	0.23765882	pigeons
Pdxdc1	0.23	0.23769072	pigeons
Tnfrsf11b	-0.37	0.24194660	pigeons
Nlk	0.22	0.24734560	pigeons
Spata5	0.32	0.25356936	pigeons
Eng	0.20	0.25747118	pigeons
Atp9b	0.22	0.25872005	pigeons
Bend3	-0.28	0.25899707	pigeons
Ssr3	0.26	0.26043024	pigeons
Lipt2	0.15	0.26142437	pigeons
Adam10	-0.28	0.26303165	pigeons
Apcdd1	0.27	0.26938883	pigeons
Prelid3b	0.17	0.27117127	pigeons
Pccb	-0.26	0.27577251	pigeons

Trim59	-0.26	0.27670621	pigeons
Fut8	-0.38	0.28215502	pigeons
Ssrp1	0.25	0.28285512	pigeons
Zfp518a	0.23	0.28334636	pigeons
Elavl1	0.24	0.29097585	pigeons
Anapc1	0.21	0.29365772	pigeons
Tomm20	-0.28	0.29893180	pigeons
Ccng1	0.19	0.30028208	pigeons
Tns3	-0.28	0.30544211	pigeons
Use1	0.17	0.30661204	pigeons
Psmd3	0.16	0.32178305	pigeons
Tspan13	0.32	0.33033084	pigeons
Kdelr2	0.32	0.33932803	pigeons
Ptdss2	-0.26	0.34892108	pigeons
Nup133	-0.56	0.35077573	pigeons
Ap1g1	-0.38	0.35231731	pigeons
Tmtc3	0.26	0.35879947	pigeons
4933400A11Rik	0.11	0.36876954	pigeons
Pola1	-0.33	0.37352965	pigeons
Akap11	0.26	0.37628482	pigeons
Bzw2	-0.34	0.37675790	pigeons
Dtx2	0.32	0.38541010	pigeons
Rab37	-0.24	0.38846101	pigeons
Ltn1	0.20	0.39371026	pigeons
Dnajc15	0.27	0.40574390	pigeons
Zbtb1	0.18	0.40577525	pigeons
Smndc1	-0.24	0.40712593	pigeons
Ak2	-0.18	0.40883250	pigeons
Adgrf5	-0.16	0.41036917	pigeons
Wscd1	0.16	0.41283505	pigeons
Pam	0.28	0.41970109	pigeons
Ginm1	0.21	0.42153007	pigeons
Creb5	0.25	0.42264844	pigeons
Cdr2	0.28	0.42618573	pigeons
Sh2d3c	-0.24	0.43001720	pigeons
Upf1	-0.24	0.43139676	pigeons
Cnot2	0.25	0.43591621	pigeons
Bcap29	0.25	0.43640158	pigeons
Rasa2	0.22	0.43887522	pigeons
Ptpn21	0.25	0.43931539	pigeons
Hnrnpa3	-0.22	0.44123881	pigeons
Rnf213	0.27	0.44524744	pigeons
Casd1	0.20	0.44606059	pigeons
Prkd3	0.24	0.44624793	pigeons
Ghdc	0.20	0.44921115	pigeons
Dnajb4	0.47	0.47569826	pigeons

Frmd4a	0.27	0.47995995	pigeons
Ppp1r9a	-0.17	0.48894935	pigeons
Anxa5	-0.18	0.49478824	pigeons
Tnfrsf8	-0.23	0.50481322	pigeons
Scmh1	-0.21	0.50621551	pigeons
Tceanc2	0.16	0.50812924	pigeons
Atp6v0e	0.28	0.51088202	pigeons
Hdac2	0.17	0.51667488	pigeons
Wdr37	0.24	0.54137346	pigeons
Ostm1	0.17	0.54969535	pigeons
Samd4	-0.30	0.55023566	pigeons
Ppm1k	0.20	0.55173770	pigeons
Tpi1	0.15	0.56206349	pigeons
Trp53inp1	-0.28	0.56264726	pigeons
Rbm12b2	0.17	0.56769324	pigeons
Usp37	-0.48	0.56827285	pigeons
Nsmce2	0.41	0.56891516	pigeons
Nectin1	-0.27	0.57453039	pigeons
Ifrd1	-0.13	0.57826937	pigeons
Zbtb41	0.25	0.58401547	pigeons
Vta1	0.16	0.58921354	pigeons
Sppl2a	0.22	0.59325269	pigeons
Rsad2	0.27	0.59769006	pigeons
Xaf1	0.20	0.60157767	pigeons
Snn	0.24	0.63906280	pigeons
Gulp1	0.27	0.65443200	pigeons
Ccdc88c	-0.29	0.66353792	pigeons
Whamm	0.18	0.67977388	pigeons
Smad3	-0.26	0.68213480	pigeons
Lamtor2	0.25	0.68231398	pigeons
Atmin	0.15	0.68601719	pigeons
Gorasp2	0.20	0.69670277	pigeons
Ccser2	0.23	0.69886042	pigeons
9130409I23Rik	0.26	0.70240143	pigeons
Ctr9	0.17	0.72170456	pigeons
Fggy	0.31	0.75091067	pigeons
Trabd	0.17	0.75297657	pigeons
Tbc1d14	-0.27	0.77087619	pigeons
Tbc1d23	0.27	0.78263078	pigeons
Senp5	0.14	0.79855112	pigeons
Chmp4b	-0.26	0.81767791	pigeons
Kmt2c	-0.19	0.81791531	pigeons
Klhl7	0.26	0.84321206	pigeons
Rpa1	-0.34	0.84742186	pigeons
Cct2	-0.18	0.84938440	pigeons
Arfgap3	-0.27	0.85332104	pigeons

Reep1	-0.25	0.85611399	pigeons
Psmd2	0.12	0.86474732	pigeons
Rae1	0.15	0.86981547	pigeons
Ireb2	0.19	0.88372317	pigeons
Ttll12	0.23	0.89058075	pigeons
Egfl7	-0.19	0.89265253	pigeons
Pros1	0.19	0.91134990	pigeons
Erh	0.27	0.92942071	pigeons
Cep112	0.26	0.93245900	pigeons
Kdm4b	0.16	0.94609989	pigeons
Plat	-0.26	0.94764615	pigeons
Farp2	-0.23	0.94899651	pigeons
Exoc2	0.25	0.95580582	pigeons
Rasgrp3	-0.36	0.98003638	pigeons
Nmt2	0.11	1.00000000	pigeons
Nup153	-0.27	1.00000000	pigeons
Ssr1	-0.26	1.00000000	pigeons
Dkc1	0.16	1.00000000	pigeons
Dpysl2	-0.26	1.00000000	pigeons
Colgalt2	-0.27	1.00000000	pigeons
Gna13	0.31	1.00000000	pigeons
Ech1	0.17	1.00000000	pigeons
Comtd1	0.17	1.00000000	pigeons
Srpk2	-0.28	1.00000000	pigeons
Amotl1	0.19	1.00000000	pigeons
Wapl	-0.27	1.00000000	pigeons
Lyve1	0.18	1.00000000	pigeons
Ttll5	-0.32	1.00000000	pigeons
Ubn2	0.26	1.00000000	pigeons
Egr3	-0.23	1.00000000	pigeons
Rer1	0.22	1.00000000	pigeons
Zrsr1	0.26	1.00000000	pigeons
Uhrf1bp1	0.18	1.00000000	pigeons
Nab1	-0.28	1.00000000	pigeons
Snw1	0.22	1.00000000	pigeons
Sod1	0.23	1.00000000	pigeons
Stk38	0.15	1.00000000	pigeons
Uhmk1	0.13	1.00000000	pigeons
Borcs5	0.15	1.00000000	pigeons
Gramd3	0.26	1.00000000	pigeons
Cpsf4l	-0.33	1.00000000	pigeons
Trmt11	0.25	1.00000000	pigeons
Cdc42bpa	-0.19	1.00000000	pigeons
Phlpp2	-0.28	1.00000000	pigeons
Stmn1	-0.17	1.00000000	pigeons
Tsnax	0.19	1.00000000	pigeons

Fzd4	-0.26	1.00000000	pigeons
Stxbp6	0.38	1.00000000	pigeons
Fam222b	0.24	1.00000000	pigeons
Usf3	0.27	1.00000000	pigeons
Chpt1	0.17	1.00000000	pigeons
Ergic3	0.16	1.00000000	pigeons
Ctnna1	-0.27	1.00000000	pigeons
Capn11	0.21	1.00000000	pigeons
Arfrp1	0.23	1.00000000	pigeons
Chmp2b	-0.25	1.00000000	pigeons
Kansl1l	-0.23	1.00000000	pigeons
Zfp618	-0.23	1.00000000	pigeons
Rfx2	-0.27	1.00000000	pigeons
Trmt13	0.22	1.00000000	pigeons
Dock9	-0.13	1.00000000	pigeons
Qk	0.14	1.00000000	pigeons
Nifk	-0.17	1.00000000	pigeons
Serinc3	0.21	1.00000000	pigeons
Gna11	0.21	1.00000000	pigeons
Dgkz	0.17	1.00000000	pigeons
Atg12	0.19	1.00000000	pigeons
Rtcb	0.15	1.00000000	pigeons
Rdh10	-0.25	1.00000000	pigeons
Farp1	-0.32	1.00000000	pigeons
Sptan1	-0.17	1.00000000	pigeons
Tmem131l	-0.27	1.00000000	pigeons
Tank	0.25	1.00000000	pigeons
Parvg	0.19	1.00000000	pigeons
Vezt	0.18	1.00000000	pigeons
Nfatc2	-0.23	1.00000000	pigeons
Ube2l3	0.24	1.00000000	pigeons
Sorbs2	-0.19	1.00000000	pigeons
Vapb	0.29	1.00000000	pigeons
Eif2ak3	0.17	1.00000000	pigeons
Large1	-0.30	1.00000000	pigeons
Trp53bp2	-0.25	1.00000000	pigeons
Rbbp6	-0.20	1.00000000	pigeons
Lrrc1	0.26	1.00000000	pigeons
Sod2	0.18	1.00000000	pigeons
Pes1	0.12	1.00000000	pigeons
Cul1	0.18	1.00000000	pigeons
B4galt1	0.16	1.00000000	pigeons
Wipi1	0.18	1.00000000	pigeons
Unc50	0.16	1.00000000	pigeons
Pank3	0.30	1.00000000	pigeons
Lsmem1	-0.10	1.00000000	pigeons

Nrp2	-0.38	1.00000000	pigeons
Tmem106b	0.17	1.00000000	pigeons
Pmm2	0.18	1.00000000	pigeons
Aida	-0.24	1.00000000	pigeons
Gid4	0.22	1.00000000	pigeons
Ankle2	0.23	1.00000000	pigeons
Zranb2	0.23	1.00000000	pigeons
Zhx1	-0.26	1.00000000	pigeons
Wdr70	0.29	1.00000000	pigeons
Cisd2	-0.19	1.00000000	pigeons
Clip1	0.22	1.00000000	pigeons
Pi4k2b	0.22	1.00000000	pigeons
Prr14l	-0.29	1.00000000	pigeons
Lrpap1	0.21	1.00000000	pigeons
Lonp2	0.15	1.00000000	pigeons
Rap2c	0.25	1.00000000	pigeons
Lsm1	-0.20	1.00000000	pigeons
Cog5	0.35	1.00000000	pigeons
Zfand3	-0.28	1.00000000	pigeons
Tiam2	-0.31	1.00000000	pigeons
Snx16	0.23	1.00000000	pigeons
Gja4	-0.24	1.00000000	pigeons
Fgd3	-0.24	1.00000000	pigeons
Cflar	0.17	1.00000000	pigeons
Pik3cg	0.26	1.00000000	pigeons
Caap1	-0.25	1.00000000	pigeons
Eef1d	-0.16	1.00000000	pigeons
Lamtor3	0.11	1.00000000	pigeons
Ranbp3	0.10	1.00000000	pigeons
Arntl2	-0.17	1.00000000	pigeons
Slc39a13	0.19	1.00000000	pigeons
Fam177a	0.21	1.00000000	pigeons
Rfx7	0.25	1.00000000	pigeons
Ndufs6	-0.20	1.00000000	pigeons
Wbp1l	-0.25	1.00000000	pigeons
Ifi30	0.11	1.00000000	pigeons
Plekhf2	0.23	1.00000000	pigeons
Aldh9a1	-0.15	1.00000000	pigeons
Ptp4a1	0.21	1.00000000	pigeons
Pdpk1	0.19	1.00000000	pigeons
Chic1	0.18	1.00000000	pigeons
Cbx3	0.20	1.00000000	pigeons
Adcy9	-0.33	1.00000000	pigeons
Sh3gl1	-0.20	1.00000000	pigeons
Ptbp1	-0.18	1.00000000	pigeons
Tmem18	0.17	1.00000000	pigeons

Stam2	-0.24	1.00000000	pigeons
Vps13d	-0.25	1.00000000	pigeons
Pitpna	-0.30	1.00000000	pigeons
Gabpa	0.21	1.00000000	pigeons
Rin3	0.19	1.00000000	pigeons
Jag1	-0.17	1.00000000	pigeons
Alg12	-0.22	1.00000000	pigeons
Rap1gds1	0.27	1.00000000	pigeons
Ccnj	0.19	1.00000000	pigeons
Eml1	0.27	1.00000000	pigeons
Kdm1b	0.15	1.00000000	pigeons
Fam133b	-0.21	1.00000000	pigeons
Lyar	-0.28	1.00000000	pigeons
Usp8	0.24	1.00000000	pigeons
Bbx	0.28	1.00000000	pigeons
Atxn7l1	0.21	1.00000000	pigeons
Gpx1	0.06	1.00000000	pigeons
Erp29	0.28	1.00000000	pigeons
Ip6k1	-0.19	1.00000000	pigeons
Atp8a1	-0.26	1.00000000	pigeons
Nfu1	0.21	1.00000000	pigeons
Abcb10	-0.33	1.00000000	pigeons
Fbxl3	0.16	1.00000000	pigeons
Nol10	0.24	1.00000000	pigeons
Tmem33	0.12	1.00000000	pigeons
Decr1	0.19	1.00000000	pigeons
Rab3il1	-0.09	1.00000000	pigeons
Socs6	0.22	1.00000000	pigeons
Lamp1	0.18	1.00000000	pigeons
Zc2hc1a	-0.21	1.00000000	pigeons
Ap3s1	-0.24	1.00000000	pigeons
Pign	0.16	1.00000000	pigeons
Dbi	0.21	1.00000000	pigeons
Usp33	0.19	1.00000000	pigeons
Frmd4b	-0.20	1.00000000	pigeons
Sf3a3	0.16	1.00000000	pigeons
Elf2	-0.21	1.00000000	pigeons
Dennd4c	0.21	1.00000000	pigeons
Ibtk	0.27	1.00000000	pigeons
Ctdp1	0.22	1.00000000	pigeons
Rpap3	0.22	1.00000000	pigeons
Saal1	0.14	1.00000000	pigeons
Prkab1	0.12	1.00000000	pigeons
Smc6	0.20	1.00000000	pigeons
Lemd3	-0.34	1.00000000	pigeons
Selenof	0.22	1.00000000	pigeons

Plk2	0.37	1.00000000	pigeons
Ywhaq	-0.20	1.00000000	pigeons
Hnrnpll	0.24	1.00000000	pigeons
Rab11a	0.20	1.00000000	pigeons
Dnah12	-0.20	1.00000000	pigeons
Inpp4a	-0.23	1.00000000	pigeons
Arhgap12	-0.30	1.00000000	pigeons
Pef1	-0.18	1.00000000	pigeons
Gid8	0.14	1.00000000	pigeons
Nr3c2	0.17	1.00000000	pigeons
Atad1	0.15	1.00000000	pigeons
Tdg	-0.27	1.00000000	pigeons
Las1l	0.21	1.00000000	pigeons
Tmem117	0.16	1.00000000	pigeons
Tcp1	0.25	1.00000000	pigeons
Gnl3	0.21	1.00000000	pigeons
Tmed5	0.20	1.00000000	pigeons
Ddx3y	-0.08	1.00000000	pigeons
Cep44	0.19	1.00000000	pigeons
Rmnd1	0.18	1.00000000	pigeons
Htatsf1	0.16	1.00000000	pigeons
Cplx1	0.24	1.00000000	pigeons
Prkci	0.21	1.00000000	pigeons
Ptprb	-0.14	1.00000000	pigeons
Dram1	0.12	1.00000000	pigeons
Tmpo	-0.22	1.00000000	pigeons
Plcb4	-0.34	1.00000000	pigeons
Pld1	-0.27	1.00000000	pigeons
Comt	-0.05	1.00000000	pigeons
Slc39a9	0.15	1.00000000	pigeons
Pnpla8	0.17	1.00000000	pigeons
Tspan18	-0.29	1.00000000	pigeons
Bicd1	-0.17	1.00000000	pigeons
Isca2	0.19	1.00000000	pigeons
Pik3ca	-0.30	1.00000000	pigeons
Xrn2	0.15	1.00000000	pigeons
Epb41l1	-0.18	1.00000000	pigeons
Zc3h14	0.12	1.00000000	pigeons
Lias	0.16	1.00000000	pigeons
Osbpl8	0.19	1.00000000	pigeons
Fam53a	0.28	1.00000000	pigeons
Nudc	0.20	1.00000000	pigeons
Sema4c	0.14	1.00000000	pigeons
Nup214	0.16	1.00000000	pigeons
Zw10	0.16	1.00000000	pigeons
Nr2c1	0.15	1.00000000	pigeons

Rab9	0.10	1.00000000	pigeons
Pknox1	0.24	1.00000000	pigeons
Klf10	-0.21	1.00000000	pigeons
Arl1	0.17	1.00000000	pigeons
Cul4b	0.17	1.00000000	pigeons
Zfp507	0.18	1.00000000	pigeons
Lrp4	-0.22	1.00000000	pigeons
Dcun1d2	-0.23	1.00000000	pigeons
Zbtb18	0.19	1.00000000	pigeons
Rnf19a	0.22	1.00000000	pigeons
Slc35a5	0.20	1.00000000	pigeons
Eif2s2	0.21	1.00000000	pigeons
Phf3	-0.20	1.00000000	pigeons
Zdhhc14	-0.36	1.00000000	pigeons
Cpeb4	0.21	1.00000000	pigeons
Ednrb	-0.28	1.00000000	pigeons
Ubap2	-0.20	1.00000000	pigeons
Tapt1	-0.34	1.00000000	pigeons
Sike1	0.18	1.00000000	pigeons
Fkbp8	0.14	1.00000000	pigeons
Jcad	0.17	1.00000000	pigeons
Fam8a1	0.19	1.00000000	pigeons
Supt20	0.18	1.00000000	pigeons
Sf3a1	0.19	1.00000000	pigeons
Tmem43	0.12	1.00000000	pigeons
Gnal	-0.24	1.00000000	pigeons
Chac1	0.46	1.00000000	pigeons
Ccng2	0.22	1.00000000	pigeons
Rbm15b	-0.23	1.00000000	pigeons
Rab4a	0.12	1.00000000	pigeons
Atp11b	0.18	1.00000000	pigeons
Srpk1	0.19	1.00000000	pigeons
Atg3	0.14	1.00000000	pigeons
Adk	-0.30	1.00000000	pigeons
Rarres2	-0.21	1.00000000	pigeons
Id3	-0.14	1.00000000	pigeons
Ndufaf4	0.11	1.00000000	pigeons
Rpap2	0.17	1.00000000	pigeons
Sel1l3	0.17	1.00000000	pigeons
Fmr1	0.18	1.00000000	pigeons
Klf11	-0.17	1.00000000	pigeons
Vps25	-0.18	1.00000000	pigeons
Sash1	-0.17	1.00000000	pigeons
Afap1l2	-0.17	1.00000000	pigeons
Laptm4a	-0.08	1.00000000	pigeons
Ass1	0.22	1.00000000	pigeons

Pdcd7	0.20	1.00000000	pigeons
Ruvbl1	0.17	1.00000000	pigeons
Ezh1	0.15	1.00000000	pigeons
Dhx15	-0.26	1.00000000	pigeons
Rpap1	0.12	1.00000000	pigeons
Csrnp1	-0.20	1.00000000	pigeons
Rassf9	0.17	1.00000000	pigeons
Irak2	0.36	1.00000000	pigeons
Commd9	0.24	1.00000000	pigeons
Sbf2	-0.27	1.00000000	pigeons
Lrrfip1	0.57	1.00000000	pigeons
Cmtr1	0.18	1.00000000	pigeons
Cadps2	-0.27	1.00000000	pigeons
Adamts10	0.09	1.00000000	pigeons
Iqgap2	-0.18	1.00000000	pigeons
Tmem19	0.16	1.00000000	pigeons
E2f6	0.17	1.00000000	pigeons
Taf2	0.17	1.00000000	pigeons
Opa1	0.13	1.00000000	pigeons
Rai2	0.17	1.00000000	pigeons
Atp23	0.13	1.00000000	pigeons
Zcchc24	0.31	1.00000000	pigeons
Chsy1	0.28	1.00000000	pigeons
Fbxo25	0.15	1.00000000	pigeons
Hmgcs1	0.06	1.00000000	pigeons
Psmb4	-0.17	1.00000000	pigeons
Usp45	0.15	1.00000000	pigeons
Arhgef18	0.12	1.00000000	pigeons
Emc8	0.12	1.00000000	pigeons
Dda1	0.14	1.00000000	pigeons
Fas	0.14	1.00000000	pigeons
Bach1	0.22	1.00000000	pigeons
Armc1	0.18	1.00000000	pigeons
Bach2	0.18	1.00000000	pigeons
Ss18	0.18	1.00000000	pigeons
Helz	0.21	1.00000000	pigeons
Igf2r	0.18	1.00000000	pigeons
Parp9	0.17	1.00000000	pigeons
Tbc1d8	0.21	1.00000000	pigeons
Enox1	-0.31	1.00000000	pigeons
Smchd1	0.17	1.00000000	pigeons
Ska2	0.23	1.00000000	pigeons
Ptges2	0.10	1.00000000	pigeons
Slc40a1	-0.18	1.00000000	pigeons
Acat3	0.11	1.00000000	pigeons
Atp2c1	0.14	1.00000000	pigeons

Nxpe3	0.06	1.00000000	pigeons
Fbxo33	0.18	1.00000000	pigeons
Tcof1	0.25	1.00000000	pigeons
Caps2	0.21	1.00000000	pigeons
Pigb	0.20	1.00000000	pigeons
Lama4	0.39	1.00000000	pigeons
Fam168a	-0.32	1.00000000	pigeons
Kcnj15	0.18	1.00000000	pigeons
Trps1	0.18	1.00000000	pigeons
Anxa11	0.21	1.00000000	pigeons
Ddx1	0.20	1.00000000	pigeons
Rac1	-0.17	1.00000000	pigeons
Fgd4	-0.15	1.00000000	pigeons
Arhgef10	-0.18	1.00000000	pigeons
Prkar1a	-0.17	1.00000000	pigeons
Arhgef26	-0.17	1.00000000	pigeons
Tcn2	-0.20	1.00000000	pigeons
Cript	0.19	1.00000000	pigeons
Gbf1	-0.37	1.00000000	pigeons
Camk2d	-0.30	1.00000000	pigeons
Tef	-0.20	1.00000000	pigeons
Asf1a	0.10	1.00000000	pigeons
Rbx1	0.19	1.00000000	pigeons
Cdk19	0.19	1.00000000	pigeons
Ube2d1	0.18	1.00000000	pigeons
Cbx1	-0.10	1.00000000	pigeons
Atxn10	0.20	1.00000000	pigeons
Poc1a	0.15	1.00000000	pigeons
Slx4	-0.22	1.00000000	pigeons
Pim1	0.16	1.00000000	pigeons
Ttc17	0.17	1.00000000	pigeons
Ppp3ca	0.17	1.00000000	pigeons
Zbtb26	0.09	1.00000000	pigeons
Tiprl	0.08	1.00000000	pigeons
Abi1	-0.23	1.00000000	pigeons
Slc44a1	0.16	1.00000000	pigeons
Cdh5	-0.20	1.00000000	pigeons
Atp6v0b	0.23	1.00000000	pigeons
Tnks	-0.30	1.00000000	pigeons
Hipk3	0.09	1.00000000	pigeons
Zcchc17	0.13	1.00000000	pigeons
Ankrd49	0.19	1.00000000	pigeons
Zfat	-0.21	1.00000000	pigeons
Fbxl5	0.21	1.00000000	pigeons
Atp6ap2	0.13	1.00000000	pigeons
Rsl1d1	0.13	1.00000000	pigeons

Cdc14a	-0.20	1.00000000	pigeons
Rad21	0.16	1.00000000	pigeons
Ggps1	-0.29	1.00000000	pigeons
Fes	0.08	1.00000000	pigeons
Ube2d2a	0.15	1.00000000	pigeons
Atp6v1d	0.19	1.00000000	pigeons
Yrdc	0.16	1.00000000	pigeons
Cnn2	0.11	1.00000000	pigeons
Tmem11	0.19	1.00000000	pigeons
Immp1l	0.19	1.00000000	pigeons
Srsf3	0.15	1.00000000	pigeons
Ptges3	0.14	1.00000000	pigeons
Sh3d19	0.21	1.00000000	pigeons
Arhgap5	0.15	1.00000000	pigeons
Stx18	0.18	1.00000000	pigeons
Gne	0.25	1.00000000	pigeons
Stab1	-0.21	1.00000000	pigeons
Suclg1	0.16	1.00000000	pigeons
Rbbp4	0.16	1.00000000	pigeons
Ints13	0.18	1.00000000	pigeons
Rcan1	-0.23	1.00000000	pigeons
Actn1	0.35	1.00000000	pigeons
Tle3	-0.26	1.00000000	pigeons
Slc37a4	0.16	1.00000000	pigeons
Acyp2	0.09	1.00000000	pigeons
Nbeal1	0.22	1.00000000	pigeons
Slc22a23	0.18	1.00000000	pigeons
Rcor3	0.14	1.00000000	pigeons
Ttc14	0.14	1.00000000	pigeons
Osbpl2	0.18	1.00000000	pigeons
Brd3	0.18	1.00000000	pigeons
Hspa9	0.13	1.00000000	pigeons
Ypel5	0.17	1.00000000	pigeons
Helq	0.15	1.00000000	pigeons
Fubp3	-0.22	1.00000000	pigeons
Lrrc57	0.15	1.00000000	pigeons
Yipf5	0.12	1.00000000	pigeons
Cdkn1b	0.17	1.00000000	pigeons
Btbd6	0.14	1.00000000	pigeons
Rapgef1	0.11	1.00000000	pigeons
Wdfy1	0.11	1.00000000	pigeons
Polr2e	-0.21	1.00000000	pigeons
Aqp1	0.16	1.00000000	pigeons
Birc6	-0.19	1.00000000	pigeons
Cpsf3	-0.11	1.00000000	pigeons
Usp16	0.22	1.00000000	pigeons

Ppard	-0.13	1.00000000	pigeons
Ftsj3	0.09	1.00000000	pigeons
Pla2r1	-0.17	1.00000000	pigeons
Ext2	0.23	1.00000000	pigeons
Phf21a	-0.33	1.00000000	pigeons
Saraf	0.13	1.00000000	pigeons
Dach2	-0.20	1.00000000	pigeons
Washc5	0.12	1.00000000	pigeons
Stoml2	-0.13	1.00000000	pigeons
Edem3	0.17	1.00000000	pigeons
Gtf2h5	-0.19	1.00000000	pigeons
Faf1	-0.25	1.00000000	pigeons
Fam98a	0.14	1.00000000	pigeons
Bzw1	0.25	1.00000000	pigeons
Rab18	0.18	1.00000000	pigeons
Zcchc10	0.12	1.00000000	pigeons
Canx	0.14	1.00000000	pigeons
Lrp12	0.11	1.00000000	pigeons
Rftn2	-0.25	1.00000000	pigeons
Rcn2	-0.17	1.00000000	pigeons
Rb1cc1	-0.26	1.00000000	pigeons
Tmed2	0.13	1.00000000	pigeons
Phf10	0.11	1.00000000	pigeons
Usp14	-0.11	1.00000000	pigeons
Rock2	-0.21	1.00000000	pigeons
S1pr1	-0.09	1.00000000	pigeons
Ppm1g	-0.19	1.00000000	pigeons
Wasl	0.17	1.00000000	pigeons
Utp6	0.17	1.00000000	pigeons
Dffb	0.11	1.00000000	pigeons
Ppp1cb	-0.20	1.00000000	pigeons
Ube4a	-0.17	1.00000000	pigeons
Tm9sf2	0.09	1.00000000	pigeons
Kif5c	-0.17	1.00000000	pigeons
Erg	-0.17	1.00000000	pigeons
Rala	0.18	1.00000000	pigeons
Slc25a24	0.18	1.00000000	pigeons
Ppp1r27	0.19	1.00000000	pigeons
Diaph2	0.30	1.00000000	pigeons
Sdf2l1	0.17	1.00000000	pigeons
Rreb1	0.16	1.00000000	pigeons
Itpka	0.19	1.00000000	pigeons
Tada3	-0.15	1.00000000	pigeons
Ctnnbl1	0.05	1.00000000	pigeons
Ranbp10	-0.22	1.00000000	pigeons
Azi2	0.13	1.00000000	pigeons

Bcl6	0.17	1.00000000	pigeons
B4galt2	0.11	1.00000000	pigeons
Twsg1	-0.17	1.00000000	pigeons
Dapk2	-0.14	1.00000000	pigeons
Urb1	0.20	1.00000000	pigeons
Phldb2	-0.22	1.00000000	pigeons
Yeats2	-0.26	1.00000000	pigeons
Psmd12	0.16	1.00000000	pigeons
Poc1b	-0.24	1.00000000	pigeons
Ybx3	-0.25	1.00000000	pigeons
Vps50	0.19	1.00000000	pigeons
Ric1	-0.29	1.00000000	pigeons
Rnf141	0.08	1.00000000	pigeons
Gfra3	0.17	1.00000000	pigeons
Tmem87a	0.14	1.00000000	pigeons
Oxct1	0.17	1.00000000	pigeons
Pcmt1	0.07	1.00000000	pigeons
Cnot6	0.14	1.00000000	pigeons
Pitpnb	-0.21	1.00000000	pigeons
Atxn1l	0.19	1.00000000	pigeons
Lrif1	-0.16	1.00000000	pigeons
Uri1	-0.23	1.00000000	pigeons
Zfp512b	0.11	1.00000000	pigeons
Samd12	-0.25	1.00000000	pigeons
Polr2d	0.11	1.00000000	pigeons
Slc25a13	-0.24	1.00000000	pigeons
Dpy19l4	0.17	1.00000000	pigeons
Gpatch8	0.17	1.00000000	pigeons
Fam124b	0.17	1.00000000	pigeons
Pcnx2	0.12	1.00000000	pigeons
Galnt1	0.17	1.00000000	pigeons
Pinx1	0.18	1.00000000	pigeons
Ric8b	0.13	1.00000000	pigeons
Ccdc88a	0.20	1.00000000	pigeons
Sgpp1	0.15	1.00000000	pigeons
Rab6a	-0.17	1.00000000	pigeons
Nup35	0.04	1.00000000	pigeons
Ncoa1	-0.22	1.00000000	pigeons
Cnpy3	-0.16	1.00000000	pigeons
Stim2	0.13	1.00000000	pigeons
Faf2	-0.15	1.00000000	pigeons
Ppfibp1	-0.18	1.00000000	pigeons
Srrm1	-0.16	1.00000000	pigeons
Zc3h15	0.18	1.00000000	pigeons
Acap2	0.23	1.00000000	pigeons
Dennd1b	0.38	1.00000000	pigeons

Sdha	0.18	1.00000000	pigeons
Adipor2	0.09	1.00000000	pigeons
Itgb1bp1	0.13	1.00000000	pigeons
Hnrnpa2b1	0.13	1.00000000	pigeons
Ppara	-0.29	1.00000000	pigeons
Lypla1	0.16	1.00000000	pigeons
Dtwd1	0.21	1.00000000	pigeons
Fkbp3	0.18	1.00000000	pigeons
Pdap1	0.12	1.00000000	pigeons
Nbas	0.12	1.00000000	pigeons
Ripk1	0.22	1.00000000	pigeons
Rbm22	-0.17	1.00000000	pigeons
Eri3	-0.18	1.00000000	pigeons
Atxn1	-0.19	1.00000000	pigeons
Clasp1	-0.26	1.00000000	pigeons
Appl1	-0.18	1.00000000	pigeons
Ttc7b	-0.32	1.00000000	pigeons
Tecpr2	0.18	1.00000000	pigeons
Dll1	-0.23	1.00000000	pigeons
Rc3h2	0.12	1.00000000	pigeons
Klhdc2	0.07	1.00000000	pigeons
Edn1	0.02	1.00000000	pigeons
Rbm15	0.13	1.00000000	pigeons
Clptm1l	-0.22	1.00000000	pigeons
Fam172a	-0.30	1.00000000	pigeons
Tor1b	0.16	1.00000000	pigeons
Ttf1	0.19	1.00000000	pigeons
Vps4b	-0.17	1.00000000	pigeons
Clpx	0.17	1.00000000	pigeons
Wdr91	-0.11	1.00000000	pigeons
Gpbp1l1	0.13	1.00000000	pigeons
Tbx3	0.22	1.00000000	pigeons
Tjp2	-0.15	1.00000000	pigeons
Ddx6	0.10	1.00000000	pigeons
Acvr1b	0.19	1.00000000	pigeons
Washc4	0.14	1.00000000	pigeons
Iws1	-0.20	1.00000000	pigeons
Grtp1	0.20	1.00000000	pigeons
Copb2	0.18	1.00000000	pigeons
Zmynd8	0.18	1.00000000	pigeons
Emc7	0.19	1.00000000	pigeons
Crtc1	0.18	1.00000000	pigeons
Egr1	0.14	1.00000000	pigeons
Akap7	0.28	1.00000000	pigeons
Cnih1	0.10	1.00000000	pigeons
Vti1b	0.17	1.00000000	pigeons

Cops4	0.15	1.00000000	pigeons
Api5	0.13	1.00000000	pigeons
Ttc1	0.10	1.00000000	pigeons
Slc25a25	-0.15	1.00000000	pigeons
Smap2	0.22	1.00000000	pigeons
Fam219a	-0.18	1.00000000	pigeons
Stx17	0.10	1.00000000	pigeons
Adrb1	0.24	1.00000000	pigeons
Ccp110	-0.19	1.00000000	pigeons
Cnppd1	0.17	1.00000000	pigeons
Etnk1	0.19	1.00000000	pigeons
Exoc3	0.13	1.00000000	pigeons
Nub1	0.13	1.00000000	pigeons
Psmd9	0.16	1.00000000	pigeons
Fam32a	0.18	1.00000000	pigeons
Tial1	0.12	1.00000000	pigeons
Adgra2	0.17	1.00000000	pigeons
Tob2	-0.21	1.00000000	pigeons
Vcpip1	0.13	1.00000000	pigeons
Igf2bp3	0.22	1.00000000	pigeons
Stard8	-0.16	1.00000000	pigeons
Tab2	0.12	1.00000000	pigeons
Tmem30a	0.13	1.00000000	pigeons
Tmx3	0.09	1.00000000	pigeons
Klhdc1	0.10	1.00000000	pigeons
Lims2	0.19	1.00000000	pigeons
Arpp19	0.12	1.00000000	pigeons
Tmx1	0.12	1.00000000	pigeons
Dmxl1	0.25	1.00000000	pigeons
Creg1	-0.18	1.00000000	pigeons
Lmbr1	-0.21	1.00000000	pigeons
Emc3	0.23	1.00000000	pigeons
Btg2	0.38	1.00000000	pigeons
Cactin	-0.17	1.00000000	pigeons
Taldo1	0.09	1.00000000	pigeons
Pcmtd2	0.10	1.00000000	pigeons
Lrmda	-0.19	1.00000000	pigeons
Irf2bpl	-0.21	1.00000000	pigeons
Elovl1	-0.23	1.00000000	pigeons
Taf11	-0.12	1.00000000	pigeons
Taf4	0.16	1.00000000	pigeons
Nfe2l2	0.17	1.00000000	pigeons
Spry2	-0.23	1.00000000	pigeons
Wdr48	0.16	1.00000000	pigeons
Tra2b	-0.17	1.00000000	pigeons
Sfpq	-0.20	1.00000000	pigeons

Nr4a3	0.11	1.00000000	pigeons
Gatad2a	-0.22	1.00000000	pigeons
Tpd52l2	-0.22	1.00000000	pigeons
Arrdc3	0.22	1.00000000	pigeons
Plekha1	-0.20	1.00000000	pigeons
Arhgef12	-0.22	1.00000000	pigeons
Ddrgk1	0.16	1.00000000	pigeons
Sgce	-0.14	1.00000000	pigeons
Ncor2	-0.18	1.00000000	pigeons
Hipk2	-0.25	1.00000000	pigeons
Trip11	-0.18	1.00000000	pigeons
Tnfaip8	0.39	1.00000000	pigeons
Nabp1	-0.17	1.00000000	pigeons
Esf1	0.13	1.00000000	pigeons
Nfxl1	0.16	1.00000000	pigeons
Arpc1a	0.14	1.00000000	pigeons
H13	0.18	1.00000000	pigeons
Sec24a	0.17	1.00000000	pigeons
Pum2	0.17	1.00000000	pigeons
Ngly1	0.12	1.00000000	pigeons
Zdhhc8	-0.17	1.00000000	pigeons
Cttnbp2nl	-0.14	1.00000000	pigeons
Arfip1	0.18	1.00000000	pigeons
Cpd	-0.16	1.00000000	pigeons
Ppp2ca	-0.15	1.00000000	pigeons
Lca5	0.11	1.00000000	pigeons
Cipc	0.15	1.00000000	pigeons
Lin54	-0.22	1.00000000	pigeons
Tshz2	0.15	1.00000000	pigeons
Nupl1	0.14	1.00000000	pigeons
Nap1l1	-0.12	1.00000000	pigeons
Elovl7	0.17	1.00000000	pigeons
Spg7	0.12	1.00000000	pigeons
Bag3	0.36	1.00000000	pigeons
Insr	-0.21	1.00000000	pigeons
Setd3	0.08	1.00000000	pigeons
Rad51b	0.15	1.00000000	pigeons
Galnt18	-0.12	1.00000000	pigeons
Setx	-0.19	1.00000000	pigeons
Cep170	0.12	1.00000000	pigeons
Ptp4a2	0.14	1.00000000	pigeons
Sptlc1	0.16	1.00000000	pigeons
Prdx1	0.36	1.00000000	pigeons
Sh3bp5	0.15	1.00000000	pigeons
Ttc3	-0.21	1.00000000	pigeons
Psmd7	-0.16	1.00000000	pigeons

Osbpl1a	-0.20	1.00000000	pigeons
Aagab	0.15	1.00000000	pigeons
Amotl2	0.20	1.00000000	pigeons
Pbld2	-0.10	1.00000000	pigeons
Eif2ak4	-0.13	1.00000000	pigeons
Ube2j1	0.26	1.00000000	pigeons
Nuak1	0.29	1.00000000	pigeons
Ulk2	-0.16	1.00000000	pigeons
Ankmy2	0.10	1.00000000	pigeons
Acbd3	-0.20	1.00000000	pigeons
Rilpl1	-0.17	1.00000000	pigeons
Creb3l2	0.17	1.00000000	pigeons
Lman2	0.11	1.00000000	pigeons
Alkbh5	0.10	1.00000000	pigeons
Ciao1	-0.14	1.00000000	pigeons
Tek	0.12	1.00000000	pigeons
Asxl2	-0.11	1.00000000	pigeons
Tgfbrap1	0.13	1.00000000	pigeons
Cfap97	0.19	1.00000000	pigeons
Atf6	-0.19	1.00000000	pigeons
Snrpe	0.18	1.00000000	pigeons
Tmx4	0.11	1.00000000	pigeons
St3gal5	0.20	1.00000000	pigeons
Sun2	0.23	1.00000000	pigeons
Clk3	0.11	1.00000000	pigeons
Uba3	0.11	1.00000000	pigeons
Haus2	0.18	1.00000000	pigeons
Cd81	0.14	1.00000000	pigeons
Csrp1	-0.09	1.00000000	pigeons
Zmat2	0.18	1.00000000	pigeons
Usp46	0.04	1.00000000	pigeons
Smc4	0.25	1.00000000	pigeons
Ccdc85a	-0.20	1.00000000	pigeons
Heatr5a	0.20	1.00000000	pigeons
Ppil3	0.10	1.00000000	pigeons
Pkp4	-0.12	1.00000000	pigeons
Ube3a	0.13	1.00000000	pigeons
Pdcl	-0.12	1.00000000	pigeons
Bambi	-0.21	1.00000000	pigeons
Pigk	0.13	1.00000000	pigeons
Adar	0.09	1.00000000	pigeons
Eps8	0.20	1.00000000	pigeons
Vps26a	0.16	1.00000000	pigeons
Pdzd8	0.17	1.00000000	pigeons
Plag1	0.11	1.00000000	pigeons
Larp4	0.08	1.00000000	pigeons

Itga1	-0.14	1.00000000	pigeons
Emc2	0.16	1.00000000	pigeons
Rapgef5	0.07	1.00000000	pigeons
Heyl	-0.13	1.00000000	pigeons
Pex13	0.05	1.00000000	pigeons
Ubl3	0.03	1.00000000	pigeons
Htt	-0.18	1.00000000	pigeons
Focad	-0.16	1.00000000	pigeons
Rnf10	-0.24	1.00000000	pigeons
Aldh6a1	0.11	1.00000000	pigeons
Trappc2	0.13	1.00000000	pigeons
Fez2	-0.13	1.00000000	pigeons
Gas6	-0.15	1.00000000	pigeons
Slc39a10	0.20	1.00000000	pigeons
Ola1	0.12	1.00000000	pigeons
Pde8a	-0.20	1.00000000	pigeons
Ppp3cb	-0.19	1.00000000	pigeons
Pcsk2	-0.13	1.00000000	pigeons
Topbp1	0.16	1.00000000	pigeons
Cd247	0.38	1.00000000	pigeons
Entpd6	0.12	1.00000000	pigeons
Wac	-0.23	1.00000000	pigeons
Ramp2	0.19	1.00000000	pigeons
Zbtb37	-0.21	1.00000000	pigeons
Drosha	0.11	1.00000000	pigeons
Zfp384	0.10	1.00000000	pigeons
Ppig	-0.19	1.00000000	pigeons
Fgd6	0.08	1.00000000	pigeons
Soga1	0.15	1.00000000	pigeons
Ctnnb1	0.14	1.00000000	pigeons
Nop14	-0.14	1.00000000	pigeons
Ctcf	-0.19	1.00000000	pigeons
Ccdc174	0.08	1.00000000	pigeons
Klhl2	0.11	1.00000000	pigeons
Noct	-0.14	1.00000000	pigeons
Afg3l2	-0.15	1.00000000	pigeons
Epc2	-0.18	1.00000000	pigeons
Fgd5	-0.10	1.00000000	pigeons
Idh2	-0.15	1.00000000	pigeons
Psma2	0.08	1.00000000	pigeons
Tra2a	0.11	1.00000000	pigeons
Arf4	0.15	1.00000000	pigeons
Hmgb3	0.29	1.00000000	pigeons
Prkaa1	0.12	1.00000000	pigeons
Sec11c	0.19	1.00000000	pigeons
Psmd1	0.09	1.00000000	pigeons

Nox1	-0.10	1.00000000	pigeons
Rpp25l	-0.10	1.00000000	pigeons
Arhgap29	0.09	1.00000000	pigeons
Cnot6l	0.21	1.00000000	pigeons
Abr	0.16	1.00000000	pigeons
Txndc12	0.12	1.00000000	pigeons
Napepld	0.12	1.00000000	pigeons
Arpc1b	0.14	1.00000000	pigeons
Ptpn12	0.14	1.00000000	pigeons
Slc39a3	0.10	1.00000000	pigeons
Brox	-0.16	1.00000000	pigeons
Cradd	-0.16	1.00000000	pigeons
Phb2	0.17	1.00000000	pigeons
Prpf39	0.11	1.00000000	pigeons
Arih2	0.12	1.00000000	pigeons
Arhgap10	-0.06	1.00000000	pigeons
Serinc1	0.07	1.00000000	pigeons
Srsf11	0.10	1.00000000	pigeons
Pgpep1	0.15	1.00000000	pigeons
Tulp3	0.16	1.00000000	pigeons
Dpy19l3	0.06	1.00000000	pigeons
Ddb1	0.11	1.00000000	pigeons
Foxk2	0.17	1.00000000	pigeons
Ptpre	0.13	1.00000000	pigeons
Phldb1	-0.15	1.00000000	pigeons
Kazn	0.11	1.00000000	pigeons
Eif3m	0.16	1.00000000	pigeons
Wnk1	-0.22	1.00000000	pigeons
Agtr2	-0.11	1.00000000	pigeons
Parp1	-0.11	1.00000000	pigeons
Naxd	0.11	1.00000000	pigeons
Ash1l	-0.19	1.00000000	pigeons
Hs6st2	0.11	1.00000000	pigeons
Pofut1	0.08	1.00000000	pigeons
Pdrg1	0.14	1.00000000	pigeons
Stam	0.18	1.00000000	pigeons
Cldnd1	0.13	1.00000000	pigeons
Ogfrl1	-0.18	1.00000000	pigeons
Prkx	0.15	1.00000000	pigeons
Scarb2	0.15	1.00000000	pigeons
Nfrkb	0.09	1.00000000	pigeons
Tmem35b	0.16	1.00000000	pigeons
Smarcb1	0.12	1.00000000	pigeons
Ndufv3	0.16	1.00000000	pigeons
Stat2	0.10	1.00000000	pigeons
Alad	0.08	1.00000000	pigeons

Heatr5b	-0.16	1.00000000	pigeons
Bicc1	0.13	1.00000000	pigeons
Stk10	0.12	1.00000000	pigeons
Dctn1	-0.15	1.00000000	pigeons
Exoc8	0.07	1.00000000	pigeons
Nedd9	-0.16	1.00000000	pigeons
Pkn2	-0.14	1.00000000	pigeons
Osgepl1	0.13	1.00000000	pigeons
Gjd4	-0.24	1.00000000	pigeons
Eif2b1	0.10	1.00000000	pigeons
Tmem222	0.18	1.00000000	pigeons
Lasp1	-0.14	1.00000000	pigeons
Ndufa8	0.11	1.00000000	pigeons
Tgif1	-0.20	1.00000000	pigeons
Yipf1	0.11	1.00000000	pigeons
Sulf2	0.26	1.00000000	pigeons
Chst1	0.11	1.00000000	pigeons
Gtf2f2	0.22	1.00000000	pigeons
Cdc40	0.16	1.00000000	pigeons
Rab12	-0.25	1.00000000	pigeons
Dusp10	0.14	1.00000000	pigeons
Lsm5	0.09	1.00000000	pigeons
Stard3nl	0.17	1.00000000	pigeons
Nckap1	-0.23	1.00000000	pigeons
Otud7a	0.11	1.00000000	pigeons
Usp7	-0.13	1.00000000	pigeons
Arpc5l	0.16	1.00000000	pigeons
Phf12	0.15	1.00000000	pigeons
Inpp5f	-0.08	1.00000000	pigeons
Ankrd11	-0.10	1.00000000	pigeons
Xpot	0.07	1.00000000	pigeons
Rpf2	-0.25	1.00000000	pigeons
Tspan4	-0.11	1.00000000	pigeons
Sin3a	-0.21	1.00000000	pigeons
Nelfa	0.09	1.00000000	pigeons
Smg1	-0.11	1.00000000	pigeons
Gtpbp4	0.14	1.00000000	pigeons
Tmem184b	-0.17	1.00000000	pigeons
Parp8	0.09	1.00000000	pigeons
Spag9	-0.20	1.00000000	pigeons
Fry	-0.22	1.00000000	pigeons
Rell1	0.15	1.00000000	pigeons
Apold1	0.47	1.00000000	pigeons
Foxo1	-0.15	1.00000000	pigeons
Pde4b	0.12	1.00000000	pigeons
Lrrc59	0.18	1.00000000	pigeons

2210408I21Rik	-0.28	1.00000000	pigeons
Nelfb	0.09	1.00000000	pigeons
Drg2	0.11	1.00000000	pigeons
Abca4	-0.38	1.00000000	pigeons
Ripor1	-0.10	1.00000000	pigeons
Fem1a	0.10	1.00000000	pigeons
Wdr47	-0.13	1.00000000	pigeons
Asb5	-0.13	1.00000000	pigeons
Gclc	0.11	1.00000000	pigeons
Ctdspl2	0.12	1.00000000	pigeons
Abracl	0.18	1.00000000	pigeons
Sdc4	0.09	1.00000000	pigeons
Acvrl1	-0.14	1.00000000	pigeons
Idh1	0.11	1.00000000	pigeons
Hdgfl3	0.05	1.00000000	pigeons
Atp1b1	0.25	1.00000000	pigeons
Ncbp1	0.05	1.00000000	pigeons
Ccdc59	0.13	1.00000000	pigeons
Aak1	0.12	1.00000000	pigeons
Traf6	0.19	1.00000000	pigeons
S100a11	0.20	1.00000000	pigeons
Csnk2a2	0.14	1.00000000	pigeons
Plrg1	0.01	1.00000000	pigeons
Dock7	-0.10	1.00000000	pigeons
Gab2	-0.16	1.00000000	pigeons
Agfg1	0.11	1.00000000	pigeons
Srfbp1	0.16	1.00000000	pigeons
Dnajc28	0.12	1.00000000	pigeons
P4ha1	0.13	1.00000000	pigeons
Bmt2	0.08	1.00000000	pigeons
Psmd4	0.12	1.00000000	pigeons
Aebp2	0.14	1.00000000	pigeons
Tlk1	0.14	1.00000000	pigeons
Crkl	0.11	1.00000000	pigeons
Larp7	-0.15	1.00000000	pigeons
Arf6	-0.12	1.00000000	pigeons
Lxn	-0.09	1.00000000	pigeons
Btrc	0.01	1.00000000	pigeons
Trim35	0.16	1.00000000	pigeons
Ppp6r2	-0.16	1.00000000	pigeons
Cct8	0.12	1.00000000	pigeons
Cav1	0.13	1.00000000	pigeons
Cltb	-0.14	1.00000000	pigeons
Lmbrd1	0.16	1.00000000	pigeons
Ppp1cc	0.16	1.00000000	pigeons
Rflnb	0.15	1.00000000	pigeons

Arhgef28	0.11	1.00000000	pigeons
Trappc4	0.15	1.00000000	pigeons
Nol8	0.17	1.00000000	pigeons
Endou	-0.11	1.00000000	pigeons
Ankfy1	-0.16	1.00000000	pigeons
Arl8a	0.15	1.00000000	pigeons
Npepps	0.14	1.00000000	pigeons
Shroom2	0.21	1.00000000	pigeons
Bud31	0.13	1.00000000	pigeons
Gxylt1	-0.18	1.00000000	pigeons
Dcbld2	-0.11	1.00000000	pigeons
Hipk1	-0.17	1.00000000	pigeons
Ube2q1	-0.12	1.00000000	pigeons
F5	0.51	1.00000000	pigeons
Akap10	0.09	1.00000000	pigeons
Ccdc6	0.13	1.00000000	pigeons
Cdk12	-0.16	1.00000000	pigeons
Sertad2	-0.13	1.00000000	pigeons
Srp68	0.05	1.00000000	pigeons
Atp2a3	0.10	1.00000000	pigeons
Ggh	0.15	1.00000000	pigeons
Ppp6r3	0.10	1.00000000	pigeons
Thyn1	-0.14	1.00000000	pigeons
Zhx2	0.18	1.00000000	pigeons
Oxr1	0.20	1.00000000	pigeons
Ppp4r3a	0.12	1.00000000	pigeons
Vezf1	0.10	1.00000000	pigeons
Immp2l	-0.18	1.00000000	pigeons
Gabpb1	0.10	1.00000000	pigeons
Wbp4	-0.01	1.00000000	pigeons
Bub3	0.09	1.00000000	pigeons
Ywhaz	-0.17	1.00000000	pigeons
Hdac11	0.14	1.00000000	pigeons
Dennd1a	0.21	1.00000000	pigeons
Dync1li1	0.10	1.00000000	pigeons
Fam185a	0.09	1.00000000	pigeons
Adipor1	0.12	1.00000000	pigeons
Ctsb	0.13	1.00000000	pigeons
Rhot1	0.09	1.00000000	pigeons
Plin2	0.11	1.00000000	pigeons
Rmdn3	0.16	1.00000000	pigeons
Dhx37	-0.14	1.00000000	pigeons
Lfng	-0.12	1.00000000	pigeons
Tasp1	0.04	1.00000000	pigeons
Fam104a	0.11	1.00000000	pigeons
Zeb2	-0.19	1.00000000	pigeons

Ift80	-0.12	1.00000000	pigeons
Itgav	0.09	1.00000000	pigeons
Atp2b4	0.13	1.00000000	pigeons
Cep350	-0.15	1.00000000	pigeons
Srp9	0.15	1.00000000	pigeons
Esyt2	0.10	1.00000000	pigeons
Jade2	0.11	1.00000000	pigeons
Txndc15	0.09	1.00000000	pigeons
Amfr	-0.12	1.00000000	pigeons
Cbfb	-0.08	1.00000000	pigeons
Fgfr1op2	0.05	1.00000000	pigeons
Cog6	-0.14	1.00000000	pigeons
Edf1	0.10	1.00000000	pigeons
Hnrnpr	0.09	1.00000000	pigeons
R3hdm1	-0.16	1.00000000	pigeons
Sft2d1	0.20	1.00000000	pigeons
Ndufs7	-0.16	1.00000000	pigeons
Sec62	0.13	1.00000000	pigeons
Prrg1	-0.11	1.00000000	pigeons
Fem1b	0.09	1.00000000	pigeons
Arfgap2	0.07	1.00000000	pigeons
Akt3	-0.18	1.00000000	pigeons
Atp1a1	0.16	1.00000000	pigeons
Ntmt1	-0.08	1.00000000	pigeons
Exosc1	0.11	1.00000000	pigeons
Atp6v1h	0.12	1.00000000	pigeons
Ptprs	-0.15	1.00000000	pigeons
Hspb11	-0.05	1.00000000	pigeons
Rfc5	0.07	1.00000000	pigeons
Gnptab	0.14	1.00000000	pigeons
Nrf1	-0.17	1.00000000	pigeons
Fyttd1	-0.15	1.00000000	pigeons
Psmg2	0.10	1.00000000	pigeons
Elk4	0.15	1.00000000	pigeons
Bnip2	0.07	1.00000000	pigeons
Ppp2r5e	0.09	1.00000000	pigeons
Aspscr1	0.11	1.00000000	pigeons
Ehmt1	-0.13	1.00000000	pigeons
Deaf1	0.10	1.00000000	pigeons
Spg21	0.10	1.00000000	pigeons
Ppp3r1	-0.16	1.00000000	pigeons
Pus10	0.18	1.00000000	pigeons
Ndufb10	-0.10	1.00000000	pigeons
Spen	0.08	1.00000000	pigeons
Hikeshi	0.13	1.00000000	pigeons
Emp2	0.17	1.00000000	pigeons

Wbp2nl	0.04	1.00000000	pigeons
Ssbp1	0.17	1.00000000	pigeons
Psmb2	0.10	1.00000000	pigeons
Gkap1	0.13	1.00000000	pigeons
St8sia1	-0.12	1.00000000	pigeons
Commd6	0.12	1.00000000	pigeons
Lonrf1	0.18	1.00000000	pigeons
Nutf2	0.16	1.00000000	pigeons
Xpc	0.18	1.00000000	pigeons
Nfatc3	0.18	1.00000000	pigeons
Tal1	-0.13	1.00000000	pigeons
Fbxw2	0.09	1.00000000	pigeons
Usp47	-0.19	1.00000000	pigeons
Ttc37	-0.08	1.00000000	pigeons
Ppip5k2	0.04	1.00000000	pigeons
Golt1b	0.13	1.00000000	pigeons
Adarb1	-0.21	1.00000000	pigeons
Gata2	0.19	1.00000000	pigeons
Ppia	-0.10	1.00000000	pigeons
Wwc2	0.12	1.00000000	pigeons
Nsfl1c	0.10	1.00000000	pigeons
Dnaja1	0.10	1.00000000	pigeons
Smarca2	-0.18	1.00000000	pigeons
Clk4	0.17	1.00000000	pigeons
Baiap2	-0.09	1.00000000	pigeons
Ascc1	-0.17	1.00000000	pigeons
Fktn	-0.15	1.00000000	pigeons
Cpt2	-0.18	1.00000000	pigeons
Cct4	0.17	1.00000000	pigeons
Vamp4	0.09	1.00000000	pigeons
Telo2	-0.14	1.00000000	pigeons
Ipo13	-0.13	1.00000000	pigeons
Erp44	0.07	1.00000000	pigeons
Lin52	0.15	1.00000000	pigeons
Atg4c	0.09	1.00000000	pigeons
Trafd1	0.10	1.00000000	pigeons
Zfp276	-0.16	1.00000000	pigeons
Slc2a13	0.17	1.00000000	pigeons
Nt5dc1	-0.09	1.00000000	pigeons
Ipmk	0.13	1.00000000	pigeons
Ephb1	0.11	1.00000000	pigeons
Angptl2	0.07	1.00000000	pigeons
Commd10	-0.11	1.00000000	pigeons
Slc25a22	0.08	1.00000000	pigeons
Nfil3	0.13	1.00000000	pigeons
Clint1	0.12	1.00000000	pigeons

Tmem258	0.13	1.00000000	pigeons
Dhx29	0.02	1.00000000	pigeons
Parn	-0.16	1.00000000	pigeons
Thrap3	0.10	1.00000000	pigeons
Cdc14b	0.16	1.00000000	pigeons
Hspg2	-0.17	1.00000000	pigeons
Fundc1	-0.11	1.00000000	pigeons
Scamp1	0.09	1.00000000	pigeons
D630045J12Rik	-0.21	1.00000000	pigeons
Ltf	0.09	1.00000000	pigeons
Tmcc1	0.14	1.00000000	pigeons
Oser1	-0.10	1.00000000	pigeons
AU040320	0.05	1.00000000	pigeons
Ercc4	0.10	1.00000000	pigeons
Kdsr	0.10	1.00000000	pigeons
Ndufb1	0.15	1.00000000	pigeons
Rab3gap2	0.06	1.00000000	pigeons
Ttyh3	-0.12	1.00000000	pigeons
Acp1	0.17	1.00000000	pigeons
Prex2	-0.19	1.00000000	pigeons
Ndfip2	-0.11	1.00000000	pigeons
Tspan14	-0.14	1.00000000	pigeons
Nipal3	0.09	1.00000000	pigeons
Proser1	-0.14	1.00000000	pigeons
Gcc2	-0.14	1.00000000	pigeons
Dnajc3	0.13	1.00000000	pigeons
Zfp711	0.08	1.00000000	pigeons
Fer	0.14	1.00000000	pigeons
Ypel1	-0.12	1.00000000	pigeons
Nudt16l1	0.06	1.00000000	pigeons
Ttc27	0.07	1.00000000	pigeons
Fam13b	-0.13	1.00000000	pigeons
Nrbp1	0.10	1.00000000	pigeons
Capzb	0.06	1.00000000	pigeons
Thoc7	0.10	1.00000000	pigeons
Alcam	-0.15	1.00000000	pigeons
Desi1	0.08	1.00000000	pigeons
Nhp2	0.13	1.00000000	pigeons
Epn2	-0.11	1.00000000	pigeons
Rbm39	-0.14	1.00000000	pigeons
Peak1	0.11	1.00000000	pigeons
Cmtm4	-0.18	1.00000000	pigeons
Ddx5	-0.04	1.00000000	pigeons
Tmem245	0.16	1.00000000	pigeons
Golga5	0.07	1.00000000	pigeons
Tnip1	0.08	1.00000000	pigeons

Rnft1	0.09	1.00000000	pigeons
Slc35b3	0.07	1.00000000	pigeons
Kdm2b	0.15	1.00000000	pigeons
Eif4h	0.13	1.00000000	pigeons
Golga1	0.13	1.00000000	pigeons
Wdr3	-0.11	1.00000000	pigeons
Rnf215	0.03	1.00000000	pigeons
Oat	0.08	1.00000000	pigeons
Vegfc	0.48	1.00000000	pigeons
Eif3l	-0.12	1.00000000	pigeons
Zdhhc13	0.09	1.00000000	pigeons
Btf3l4	0.16	1.00000000	pigeons
Clta	-0.05	1.00000000	pigeons
Psmc6	0.16	1.00000000	pigeons
Smurf1	0.07	1.00000000	pigeons
Setdb1	-0.07	1.00000000	pigeons
Pik3r1	0.10	1.00000000	pigeons
Crebrf	0.15	1.00000000	pigeons
4833439L19Rik	0.14	1.00000000	pigeons
Sf3a2	-0.13	1.00000000	pigeons
Scaf11	0.07	1.00000000	pigeons
Rasgrf2	-0.10	1.00000000	pigeons
Arnt	0.09	1.00000000	pigeons
Rprd1b	-0.16	1.00000000	pigeons
Abce1	0.12	1.00000000	pigeons
Cpne2	-0.08	1.00000000	pigeons
Rrp1b	0.11	1.00000000	pigeons
Fbxl20	0.08	1.00000000	pigeons
Pik3cb	0.13	1.00000000	pigeons
Wls	-0.14	1.00000000	pigeons
Xpo5	0.12	1.00000000	pigeons
Actr8	0.07	1.00000000	pigeons
Trim24	0.11	1.00000000	pigeons
Ublcp1	0.10	1.00000000	pigeons
Tgfbr2	-0.13	1.00000000	pigeons
Dnaja4	0.19	1.00000000	pigeons
Trappc11	-0.07	1.00000000	pigeons
Ppp2r2b	0.14	1.00000000	pigeons
Lgmn	-0.14	1.00000000	pigeons
Ndufb8	0.20	1.00000000	pigeons
Pigt	0.04	1.00000000	pigeons
Rpa2	0.13	1.00000000	pigeons
Hoxb8	0.12	1.00000000	pigeons
Ddx52	0.10	1.00000000	pigeons
Polr2c	-0.09	1.00000000	pigeons
Slc43a2	-0.13	1.00000000	pigeons

Snx9	-0.11	1.00000000	pigeons
Dhx36	-0.16	1.00000000	pigeons
Ubqln4	-0.11	1.00000000	pigeons
Rab35	0.09	1.00000000	pigeons
Arid4b	0.12	1.00000000	pigeons
S100a10	-0.02	1.00000000	pigeons
Gcn1	-0.17	1.00000000	pigeons
Btg1b	0.16	1.00000000	pigeons
Dph6	0.15	1.00000000	pigeons
Cog7	0.12	1.00000000	pigeons
Braf	0.16	1.00000000	pigeons
Tbcd	0.06	1.00000000	pigeons
Txndc9	-0.15	1.00000000	pigeons
Ptpn2	0.16	1.00000000	pigeons
Eif2a	-0.10	1.00000000	pigeons
Ascc3	-0.12	1.00000000	pigeons
Cul5	0.06	1.00000000	pigeons
Ostf1	0.14	1.00000000	pigeons
Xylt1	-0.13	1.00000000	pigeons
Nfia	-0.12	1.00000000	pigeons
Tmem141	-0.14	1.00000000	pigeons
Hmgb2	0.14	1.00000000	pigeons
Xpr1	0.11	1.00000000	pigeons
Eed	0.16	1.00000000	pigeons
Foxp4	-0.08	1.00000000	pigeons
Rai14	0.20	1.00000000	pigeons
Naa50	0.07	1.00000000	pigeons
Lrp3	-0.19	1.00000000	pigeons
Zfp36l1	0.07	1.00000000	pigeons
Apoa1	-0.14	1.00000000	pigeons
Nipa2	-0.13	1.00000000	pigeons
Dip2b	0.07	1.00000000	pigeons
Scarf2	0.15	1.00000000	pigeons
Ei24	0.09	1.00000000	pigeons
Axin2	-0.14	1.00000000	pigeons
Rnf4	0.14	1.00000000	pigeons
Ap3m1	0.07	1.00000000	pigeons
Pusl1	0.09	1.00000000	pigeons
Ndufb3	-0.13	1.00000000	pigeons
Arcn1	0.08	1.00000000	pigeons
Ddhd1	0.02	1.00000000	pigeons
Sdf2	0.09	1.00000000	pigeons
Abi2	-0.11	1.00000000	pigeons
Uxs1	0.13	1.00000000	pigeons
Pdss2	0.10	1.00000000	pigeons
Spopl	0.08	1.00000000	pigeons

Prss23	0.18	1.00000000	pigeons
9930021J03Rik	0.13	1.00000000	pigeons
Ywhab	0.09	1.00000000	pigeons
Gbe1	-0.28	1.00000000	pigeons
Trappc8	0.08	1.00000000	pigeons
Eif3b	0.03	1.00000000	pigeons
Rcbtb2	0.09	1.00000000	pigeons
Smad5	-0.24	1.00000000	pigeons
Fkbp11	0.13	1.00000000	pigeons
Cttn	0.14	1.00000000	pigeons
Hoxa6	-0.12	1.00000000	pigeons
Fndc3b	0.19	1.00000000	pigeons
Wdr61	0.06	1.00000000	pigeons
Copa	0.13	1.00000000	pigeons
Gde1	-0.04	1.00000000	pigeons
Polr2l	0.08	1.00000000	pigeons
Vti1a	-0.14	1.00000000	pigeons
Efnb1	-0.17	1.00000000	pigeons
Selenot	0.10	1.00000000	pigeons
Nktr	-0.15	1.00000000	pigeons
Ncoa3	0.12	1.00000000	pigeons
Bhlhe40	-0.13	1.00000000	pigeons
Cnst	0.19	1.00000000	pigeons
Zc3h13	-0.10	1.00000000	pigeons
Zfp609	-0.12	1.00000000	pigeons
Zc3h7a	0.13	1.00000000	pigeons
Arglu1	0.06	1.00000000	pigeons
Urm1	-0.11	1.00000000	pigeons
Snx21	-0.08	1.00000000	pigeons
Atg16l1	-0.09	1.00000000	pigeons
Synrg	0.09	1.00000000	pigeons
Lonrf3	0.35	1.00000000	pigeons
Psmc2	0.08	1.00000000	pigeons
Snip1	-0.11	1.00000000	pigeons
Sf3b5	0.11	1.00000000	pigeons
Dip2a	-0.12	1.00000000	pigeons
Rsl24d1	0.12	1.00000000	pigeons
Ipo8	0.04	1.00000000	pigeons
Rad17	-0.13	1.00000000	pigeons
Zfp598	0.08	1.00000000	pigeons
Katnbl1	0.06	1.00000000	pigeons
Fam204a	-0.11	1.00000000	pigeons
Ccdc102a	-0.10	1.00000000	pigeons
Rbm18	-0.08	1.00000000	pigeons
Strn3	-0.16	1.00000000	pigeons
F2r	-0.12	1.00000000	pigeons

Ddhd2	0.08	1.00000000	pigeons
Kctd10	0.10	1.00000000	pigeons
Ets1	-0.08	1.00000000	pigeons
Pdcd5	0.06	1.00000000	pigeons
Gfod1	0.12	1.00000000	pigeons
Otud7b	-0.11	1.00000000	pigeons
Pcgf6	0.13	1.00000000	pigeons
Arhgap8	0.02	1.00000000	pigeons
Krr1	0.09	1.00000000	pigeons
Dus1l	-0.16	1.00000000	pigeons
Kat7	-0.17	1.00000000	pigeons
Kif2a	-0.10	1.00000000	pigeons
Enc1	-0.16	1.00000000	pigeons
Tpra1	0.12	1.00000000	pigeons
Hoxa3	-0.17	1.00000000	pigeons
Unk	0.10	1.00000000	pigeons
Tmem242	-0.12	1.00000000	pigeons
Hectd4	-0.02	1.00000000	pigeons
Dnm1l	0.04	1.00000000	pigeons
Zfand1	0.07	1.00000000	pigeons
Pik3c3	0.12	1.00000000	pigeons
Bbs2	0.06	1.00000000	pigeons
Tada1	0.10	1.00000000	pigeons
Rcc1	-0.03	1.00000000	pigeons
Slc38a2	0.26	1.00000000	pigeons
Luc7l3	0.16	1.00000000	pigeons
Snapc4	0.09	1.00000000	pigeons
Zfp518b	0.10	1.00000000	pigeons
Tspan3	0.09	1.00000000	pigeons
Gipc2	-0.07	1.00000000	pigeons
Skil	0.09	1.00000000	pigeons
St7l	0.06	1.00000000	pigeons
Sik1	0.19	1.00000000	pigeons
Rsbn1	0.03	1.00000000	pigeons
Vkorc1l1	0.09	1.00000000	pigeons
Stx8	0.13	1.00000000	pigeons
Rbm33	0.09	1.00000000	pigeons
Oma1	0.08	1.00000000	pigeons
Lsg1	0.13	1.00000000	pigeons
Rabgap1	0.17	1.00000000	pigeons
Jakmip3	-0.08	1.00000000	pigeons
Zc4h2	0.13	1.00000000	pigeons
Taf5	0.08	1.00000000	pigeons
Ppp2r5c	-0.14	1.00000000	pigeons
Agps	0.05	1.00000000	pigeons
Setd2	0.08	1.00000000	pigeons

Heca	-0.14	1.00000000	pigeons
Pebp1	0.06	1.00000000	pigeons
Nudt21	0.13	1.00000000	pigeons
Crk	0.13	1.00000000	pigeons
Hspa8	0.25	1.00000000	pigeons
Kif13b	0.07	1.00000000	pigeons
Tma16	-0.07	1.00000000	pigeons
Traf2	0.14	1.00000000	pigeons
Iars2	0.14	1.00000000	pigeons
Elmod2	0.08	1.00000000	pigeons
Sh3rf3	-0.11	1.00000000	pigeons
Ppme1	-0.06	1.00000000	pigeons
Zbtb2	-0.09	1.00000000	pigeons
Kmt2e	-0.09	1.00000000	pigeons
Etf1	0.09	1.00000000	pigeons
Zswim8	-0.17	1.00000000	pigeons
Usp24	0.11	1.00000000	pigeons
Zbtb38	0.17	1.00000000	pigeons
Tbc1d22a	0.13	1.00000000	pigeons
Ldb1	0.07	1.00000000	pigeons
Exoc1	0.08	1.00000000	pigeons
Gpx4	-0.10	1.00000000	pigeons
Vil1	0.03	1.00000000	pigeons
Exoc6	-0.09	1.00000000	pigeons
Odf2	-0.04	1.00000000	pigeons
Pbrm1	0.10	1.00000000	pigeons
Eefsec	0.05	1.00000000	pigeons
Zeb1	-0.08	1.00000000	pigeons
Chic2	0.09	1.00000000	pigeons
Sik3	-0.10	1.00000000	pigeons
Capza2	0.03	1.00000000	pigeons
Brf1	-0.19	1.00000000	pigeons
Sec24b	0.07	1.00000000	pigeons
Ankrd54	0.09	1.00000000	pigeons
Rev3l	-0.17	1.00000000	pigeons
Zmpste24	0.04	1.00000000	pigeons
Rab40b	0.10	1.00000000	pigeons
Rasa3	0.19	1.00000000	pigeons
Cpt1a	-0.07	1.00000000	pigeons
Ap1s2	0.09	1.00000000	pigeons
Ppp1r16b	-0.05	1.00000000	pigeons
Ubn1	0.10	1.00000000	pigeons
Zranb1	-0.24	1.00000000	pigeons
Gng12	-0.11	1.00000000	pigeons
Ctbp2	0.06	1.00000000	pigeons
Eif5b	-0.13	1.00000000	pigeons

Sirt6	0.12	1.00000000	pigeons
Fkbp4	-0.06	1.00000000	pigeons
Syt11	-0.13	1.00000000	pigeons
Atrx	-0.12	1.00000000	pigeons
Tbpl1	0.13	1.00000000	pigeons
Tpp2	-0.14	1.00000000	pigeons
Ttc19	0.08	1.00000000	pigeons
Tpr	0.12	1.00000000	pigeons
Scfd2	0.14	1.00000000	pigeons
Xpo4	0.02	1.00000000	pigeons
Ppil2	-0.13	1.00000000	pigeons
Cdca4	0.12	1.00000000	pigeons
Vps29	0.06	1.00000000	pigeons
Ilf2	-0.06	1.00000000	pigeons
Wdr26	0.06	1.00000000	pigeons
Creld2	-0.07	1.00000000	pigeons
Csde1	-0.12	1.00000000	pigeons
Abhd2	0.02	1.00000000	pigeons
Dync1h1	0.08	1.00000000	pigeons
Dnajc5	-0.09	1.00000000	pigeons
Eef1e1	0.10	1.00000000	pigeons
Ap2a1	0.07	1.00000000	pigeons
Cpsf2	0.07	1.00000000	pigeons
Becn2	0.11	1.00000000	pigeons
Thumpd3	-0.10	1.00000000	pigeons
Gyg	0.11	1.00000000	pigeons
Cish	-0.05	1.00000000	pigeons
Zmynd11	0.18	1.00000000	pigeons
Spz1	-0.09	1.00000000	pigeons
Hmbox1	-0.08	1.00000000	pigeons
Tanc2	-0.19	1.00000000	pigeons
Chd9	0.09	1.00000000	pigeons
Uvrag	-0.19	1.00000000	pigeons
Abl1	-0.09	1.00000000	pigeons
Bcr	0.06	1.00000000	pigeons
Dld	0.09	1.00000000	pigeons
Arel1	0.09	1.00000000	pigeons
Cyfip2	0.03	1.00000000	pigeons
Cpeb3	0.14	1.00000000	pigeons
Polr2k	0.12	1.00000000	pigeons
Hsf2	0.10	1.00000000	pigeons
Vps37a	-0.03	1.00000000	pigeons
Lats1	0.05	1.00000000	pigeons
Ifih1	0.02	1.00000000	pigeons
Arid5b	0.52	1.00000000	pigeons
Snx29	-0.14	1.00000000	pigeons

Ebag9	0.19	1.00000000	pigeons
Sin3b	0.04	1.00000000	pigeons
Eif3e	0.13	1.00000000	pigeons
Psmg1	-0.05	1.00000000	pigeons
Fnip2	0.09	1.00000000	pigeons
Fbxl7	-0.08	1.00000000	pigeons
Acyp1	-0.08	1.00000000	pigeons
Acot10	-0.10	1.00000000	pigeons
Zmat4	-0.07	1.00000000	pigeons
U2af1	-0.09	1.00000000	pigeons
Psmd13	-0.10	1.00000000	pigeons
Nrp1	-0.11	1.00000000	pigeons
Cep57	0.18	1.00000000	pigeons
4932438A13Rik	-0.18	1.00000000	pigeons
Ostc	0.11	1.00000000	pigeons
Xrcc4	0.17	1.00000000	pigeons
Aimp1	0.13	1.00000000	pigeons
Nnt	-0.11	1.00000000	pigeons
Txnrd3	-0.04	1.00000000	pigeons
Bfar	0.11	1.00000000	pigeons
Sbno2	0.06	1.00000000	pigeons
Taf3	0.11	1.00000000	pigeons
Utp18	-0.12	1.00000000	pigeons
Psmd6	0.10	1.00000000	pigeons
Rgma	-0.08	1.00000000	pigeons
Cebpz	0.08	1.00000000	pigeons
Usp4	-0.14	1.00000000	pigeons
Pla2g12a	0.14	1.00000000	pigeons
Nop16	0.05	1.00000000	pigeons
Gab1	0.12	1.00000000	pigeons
Tes	-0.21	1.00000000	pigeons
Tbc1d9b	-0.12	1.00000000	pigeons
Prkd1	0.06	1.00000000	pigeons
Snrnp25	-0.07	1.00000000	pigeons
Ssu72	0.06	1.00000000	pigeons
Ankrd13a	0.08	1.00000000	pigeons
Zfp639	-0.12	1.00000000	pigeons
Nr6a1	-0.07	1.00000000	pigeons
Ing5	0.04	1.00000000	pigeons
Adamts1	-0.12	1.00000000	pigeons
Dync1li2	-0.06	1.00000000	pigeons
Atf2	0.06	1.00000000	pigeons
Eif4ebp1	-0.08	1.00000000	pigeons
Gsr	-0.07	1.00000000	pigeons
Pwwp2a	-0.11	1.00000000	pigeons
Prkca	-0.11	1.00000000	pigeons

Vps36	0.11	1.00000000	pigeons
Snx17	-0.14	1.00000000	pigeons
Suds3	0.10	1.00000000	pigeons
Tomm70a	0.12	1.00000000	pigeons
Epb41l4a	-0.16	1.00000000	pigeons
Sub1	-0.13	1.00000000	pigeons
Ufsp2	-0.08	1.00000000	pigeons
Zzef1	-0.13	1.00000000	pigeons
Cep63	0.08	1.00000000	pigeons
Flii	0.08	1.00000000	pigeons
Tk2	0.16	1.00000000	pigeons
Naa30	0.11	1.00000000	pigeons
Cdv3	0.07	1.00000000	pigeons
Fto	0.16	1.00000000	pigeons
Ptgr2	0.11	1.00000000	pigeons
Tax1bp3	0.08	1.00000000	pigeons
Sacm1l	-0.20	1.00000000	pigeons
Gpatch1	0.09	1.00000000	pigeons
Kifap3	0.08	1.00000000	pigeons
Ndufs8	-0.07	1.00000000	pigeons
Ncbp3	-0.04	1.00000000	pigeons
Ip6k2	0.17	1.00000000	pigeons
Hmg20b	-0.09	1.00000000	pigeons
Lrba	0.05	1.00000000	pigeons
Slc2a8	0.06	1.00000000	pigeons
Noc2l	0.09	1.00000000	pigeons
Esco1	0.12	1.00000000	pigeons
Pan3	-0.20	1.00000000	pigeons
Ptpn4	-0.09	1.00000000	pigeons
Immt	0.05	1.00000000	pigeons
Ube2g2	0.11	1.00000000	pigeons
Btbd9	0.02	1.00000000	pigeons
Ero1b	0.10	1.00000000	pigeons
Rc3h1	-0.15	1.00000000	pigeons
Clstn2	0.10	1.00000000	pigeons
Suco	-0.10	1.00000000	pigeons
Btaf1	0.08	1.00000000	pigeons
Rilpl2	0.05	1.00000000	pigeons
Gtf2b	0.06	1.00000000	pigeons
Zbtb33	0.00	1.00000000	pigeons
Atp2a2	-0.19	1.00000000	pigeons
Prpf38a	0.08	1.00000000	pigeons
Casp8ap2	-0.10	1.00000000	pigeons
Elp4	0.25	1.00000000	pigeons
Stxbp1	0.04	1.00000000	pigeons
Lnpk	0.09	1.00000000	pigeons

Poldip3	0.08	1.00000000	pigeons
Fbxo9	0.05	1.00000000	pigeons
Sart3	0.08	1.00000000	pigeons
Tob1	-0.08	1.00000000	pigeons
Ndufs3	0.09	1.00000000	pigeons
Surf2	0.07	1.00000000	pigeons
Cops2	0.06	1.00000000	pigeons
Klhl3	-0.10	1.00000000	pigeons
Rab3gap1	0.07	1.00000000	pigeons
Ddx51	0.09	1.00000000	pigeons
Snx13	0.07	1.00000000	pigeons
Cant1	-0.02	1.00000000	pigeons
Phkg1	-0.10	1.00000000	pigeons
Fbxw8	0.04	1.00000000	pigeons
Snapc5	0.08	1.00000000	pigeons
Uspl1	0.04	1.00000000	pigeons
Cdc73	-0.16	1.00000000	pigeons
Ghitm	0.16	1.00000000	pigeons
Ccdc93	0.11	1.00000000	pigeons
Reck	0.12	1.00000000	pigeons
Ppm1f	-0.07	1.00000000	pigeons
Lrrk1	-0.04	1.00000000	pigeons
Rp2	0.08	1.00000000	pigeons
Trap1	0.03	1.00000000	pigeons
Hacd3	0.03	1.00000000	pigeons
Srsf5	-0.08	1.00000000	pigeons
Acads	0.05	1.00000000	pigeons
Gpatch2	0.14	1.00000000	pigeons
Wipi2	0.05	1.00000000	pigeons
Rab5c	-0.05	1.00000000	pigeons
Stk40	0.12	1.00000000	pigeons
Kif13a	0.13	1.00000000	pigeons
Lonp1	0.04	1.00000000	pigeons
Nemf	-0.05	1.00000000	pigeons
Eif2s1	0.09	1.00000000	pigeons
Nup98	-0.16	1.00000000	pigeons
Gtf2a1	0.04	1.00000000	pigeons
Cenpt	0.05	1.00000000	pigeons
Nop58	0.06	1.00000000	pigeons
Nup50	0.08	1.00000000	pigeons
Rsu1	0.11	1.00000000	pigeons
P3h2	-0.09	1.00000000	pigeons
Dnm1	-0.05	1.00000000	pigeons
Fbxw11	-0.14	1.00000000	pigeons
Brd7	0.07	1.00000000	pigeons
Tmed3	-0.07	1.00000000	pigeons

Psmc3	0.06	1.00000000	pigeons
Rab29	0.05	1.00000000	pigeons
Timm21	0.13	1.00000000	pigeons
Rnf130	-0.15	1.00000000	pigeons
Hps3	0.09	1.00000000	pigeons
Ahsa1	-0.13	1.00000000	pigeons
Usp44	-0.05	1.00000000	pigeons
Stau1	-0.07	1.00000000	pigeons
Anapc10	-0.10	1.00000000	pigeons
Pcdh1	-0.03	1.00000000	pigeons
Rnf216	0.06	1.00000000	pigeons
Psma3	-0.04	1.00000000	pigeons
Ide	0.10	1.00000000	pigeons
Tmem185a	0.00	1.00000000	pigeons
Scrn3	0.07	1.00000000	pigeons
Carhsp1	0.13	1.00000000	pigeons
Yes1	0.08	1.00000000	pigeons
Nolc1	0.08	1.00000000	pigeons
Sf3b1	-0.04	1.00000000	pigeons
Slc35f5	0.05	1.00000000	pigeons
Rab7	-0.08	1.00000000	pigeons
Sumo1	0.04	1.00000000	pigeons
Smim14	0.05	1.00000000	pigeons
Lpar4	0.10	1.00000000	pigeons
Fam120a	0.09	1.00000000	pigeons
Grb2	0.00	1.00000000	pigeons
Tmem70	-0.04	1.00000000	pigeons
Ccdc47	0.01	1.00000000	pigeons
Zfp148	0.08	1.00000000	pigeons
Dcun1d4	-0.12	1.00000000	pigeons
Exosc9	0.05	1.00000000	pigeons
Nrip1	0.00	1.00000000	pigeons
Stk26	0.10	1.00000000	pigeons
Ints11	-0.08	1.00000000	pigeons
Ube4b	0.04	1.00000000	pigeons
Zfp318	0.09	1.00000000	pigeons
Ptpra	0.05	1.00000000	pigeons
Nfyc	0.11	1.00000000	pigeons
Zfp821	-0.05	1.00000000	pigeons
Ralgapa1	0.07	1.00000000	pigeons
Vps35	-0.03	1.00000000	pigeons
Yod1	0.06	1.00000000	pigeons
Ralgapb	0.09	1.00000000	pigeons
Acaca	-0.10	1.00000000	pigeons
Ing2	-0.08	1.00000000	pigeons
Ube2g1	-0.05	1.00000000	pigeons

Erbin	0.10	1.00000000	pigeons
Dcaf1	-0.10	1.00000000	pigeons
Dync2h1	-0.13	1.00000000	pigeons
Ccdc117	0.09	1.00000000	pigeons
Wdfy3	0.05	1.00000000	pigeons
Fars2	-0.16	1.00000000	pigeons
Acsl5	0.05	1.00000000	pigeons
Gpi1	-0.11	1.00000000	pigeons
Skp1	0.14	1.00000000	pigeons
Fh1	0.12	1.00000000	pigeons
Lrrfip2	-0.11	1.00000000	pigeons
Pafah1b1	0.06	1.00000000	pigeons
Inpp5d	0.09	1.00000000	pigeons
Pcif1	0.07	1.00000000	pigeons
Pptc7	0.04	1.00000000	pigeons
Ctps2	0.12	1.00000000	pigeons
Dram2	0.11	1.00000000	pigeons
Ist1	0.07	1.00000000	pigeons
Snx19	-0.07	1.00000000	pigeons
Rlf	0.00	1.00000000	pigeons
Unc119b	-0.12	1.00000000	pigeons
Zfp366	-0.05	1.00000000	pigeons
Chid1	-0.15	1.00000000	pigeons
Zdhhc17	0.06	1.00000000	pigeons
Grk6	-0.08	1.00000000	pigeons
Pals1	-0.04	1.00000000	pigeons
Pgm2	-0.10	1.00000000	pigeons
Gpr107	-0.07	1.00000000	pigeons
Acly	-0.01	1.00000000	pigeons
Noc3l	0.11	1.00000000	pigeons
Arl3	0.04	1.00000000	pigeons
Sp4	0.06	1.00000000	pigeons
Aurkaip1	0.07	1.00000000	pigeons
Eif4a3l1	0.06	1.00000000	pigeons
Cnot10	-0.10	1.00000000	pigeons
Pacsin2	0.06	1.00000000	pigeons
Zfp292	0.15	1.00000000	pigeons
Tmem200b	-0.03	1.00000000	pigeons
Commd7	0.13	1.00000000	pigeons
Llph	0.11	1.00000000	pigeons
Usp48	-0.05	1.00000000	pigeons
Ccndbp1	0.13	1.00000000	pigeons
Lsm8	0.08	1.00000000	pigeons
Cmss1	-0.09	1.00000000	pigeons
Ell	0.02	1.00000000	pigeons
Gpatch2l	-0.08	1.00000000	pigeons

Prrc2c	0.05	1.00000000	pigeons
Rassf8	-0.09	1.00000000	pigeons
Aldh4a1	0.06	1.00000000	pigeons
Pabpc4	-0.03	1.00000000	pigeons
Togaram1	0.03	1.00000000	pigeons
Rock1	0.04	1.00000000	pigeons
Pten	0.05	1.00000000	pigeons
Prpsap2	0.07	1.00000000	pigeons
Scpep1	0.04	1.00000000	pigeons
Slc23a2	0.04	1.00000000	pigeons
Atp6v1c1	0.07	1.00000000	pigeons
Cwf19l2	0.09	1.00000000	pigeons
Sf3b6	-0.09	1.00000000	pigeons
Sec11a	0.06	1.00000000	pigeons
Eif4e	0.11	1.00000000	pigeons
Psmf1	-0.09	1.00000000	pigeons
Sufu	-0.07	1.00000000	pigeons
Hace1	0.09	1.00000000	pigeons
Optn	-0.10	1.00000000	pigeons
Dnajb14	-0.11	1.00000000	pigeons
Smpd2	-0.06	1.00000000	pigeons
Snx24	0.01	1.00000000	pigeons
Gnas	0.09	1.00000000	pigeons
Ralgps1	0.11	1.00000000	pigeons
Abhd18	0.05	1.00000000	pigeons
Clk2	-0.11	1.00000000	pigeons
Fbxl4	0.09	1.00000000	pigeons
Ndufa5	0.12	1.00000000	pigeons
Jagn1	0.02	1.00000000	pigeons
Eif1	0.13	1.00000000	pigeons
Bsdc1	-0.09	1.00000000	pigeons
Ppp2r2a	-0.13	1.00000000	pigeons
Arl6ip1	0.06	1.00000000	pigeons
Ndufv2	0.10	1.00000000	pigeons
Ubap1	-0.05	1.00000000	pigeons
Dynlrb1	0.10	1.00000000	pigeons
Sestd1	0.06	1.00000000	pigeons
Itga6	-0.11	1.00000000	pigeons
Tatdn3	-0.03	1.00000000	pigeons
Ilf3	-0.09	1.00000000	pigeons
Nr2f2	0.18	1.00000000	pigeons
Hlcs	0.06	1.00000000	pigeons
Hexim1	0.05	1.00000000	pigeons
Col22a1	0.08	1.00000000	pigeons
Sppl3	-0.14	1.00000000	pigeons
Tfdp1	-0.03	1.00000000	pigeons

Fbxo3	-0.11	1.00000000	pigeons
Fam53b	0.05	1.00000000	pigeons
Ece1	0.05	1.00000000	pigeons
Nr2f6	0.05	1.00000000	pigeons
Prpf40a	0.06	1.00000000	pigeons
Ifngr2	-0.10	1.00000000	pigeons
Arfgap1	0.06	1.00000000	pigeons
Cdc42se2	0.15	1.00000000	pigeons
Rpgrip1l	0.06	1.00000000	pigeons
Zfyve9	0.04	1.00000000	pigeons
Fut11	0.05	1.00000000	pigeons
Rnf111	0.05	1.00000000	pigeons
Chfr	0.02	1.00000000	pigeons
Nono	0.01	1.00000000	pigeons
Akirin2	-0.08	1.00000000	pigeons
Gosr1	-0.09	1.00000000	pigeons
Timp2	0.09	1.00000000	pigeons
Ube2cbp	-0.09	1.00000000	pigeons
Tssc4	0.09	1.00000000	pigeons
Kbtbd2	-0.04	1.00000000	pigeons
Tnpo1	-0.15	1.00000000	pigeons
Ap4s1	-0.03	1.00000000	pigeons
Ndfip1	-0.08	1.00000000	pigeons
Txndc17	-0.06	1.00000000	pigeons
Actr10	0.03	1.00000000	pigeons
Tspan15	0.12	1.00000000	pigeons
Ppil4	0.03	1.00000000	pigeons
Atp7a	0.03	1.00000000	pigeons
Apool	0.08	1.00000000	pigeons
Atp6v0a2	0.07	1.00000000	pigeons
Plpp3	-0.11	1.00000000	pigeons
Arntl	0.13	1.00000000	pigeons
Glmn	0.05	1.00000000	pigeons
Zdhhc7	0.10	1.00000000	pigeons
Prpf8	0.05	1.00000000	pigeons
Ppp1r7	0.06	1.00000000	pigeons
C2cd2l	-0.10	1.00000000	pigeons
Pdcd6	-0.03	1.00000000	pigeons
Ikbkb	0.07	1.00000000	pigeons
Gpatch11	-0.07	1.00000000	pigeons
Psma4	0.06	1.00000000	pigeons
Gnptg	-0.11	1.00000000	pigeons
Phf6	0.04	1.00000000	pigeons
Commd1b	-0.09	1.00000000	pigeons
Rab40c	0.11	1.00000000	pigeons
Copb1	0.05	1.00000000	pigeons

Ambra1	0.08	1.00000000	pigeons
Shoc2	-0.14	1.00000000	pigeons
Swt1	-0.12	1.00000000	pigeons
Pxk	0.12	1.00000000	pigeons
Trpm7	-0.09	1.00000000	pigeons
Sun1	-0.07	1.00000000	pigeons
Utp3	0.00	1.00000000	pigeons
Tnfrsf25	-0.11	1.00000000	pigeons
Pfdn4	0.11	1.00000000	pigeons
Wipf1	0.09	1.00000000	pigeons
Ikbke	0.09	1.00000000	pigeons
Cstf1	0.07	1.00000000	pigeons
Frs2	-0.06	1.00000000	pigeons
Pdlim5	0.12	1.00000000	pigeons
Anxa2	0.19	1.00000000	pigeons
Antxr2	0.26	1.00000000	pigeons
Zfp236	0.05	1.00000000	pigeons
Ddx59	0.02	1.00000000	pigeons
Oaz1	0.18	1.00000000	pigeons
Dusp6	-0.14	1.00000000	pigeons
Ankrd28	0.07	1.00000000	pigeons
Yeats4	-0.05	1.00000000	pigeons
Dcaf5	-0.15	1.00000000	pigeons
Cables2	-0.05	1.00000000	pigeons
Nfs1	0.08	1.00000000	pigeons
Scaf4	-0.08	1.00000000	pigeons
Ptdss1	0.07	1.00000000	pigeons
Exoc7	-0.06	1.00000000	pigeons
Adrm1	0.05	1.00000000	pigeons
Vwa8	0.02	1.00000000	pigeons
Cabin1	0.03	1.00000000	pigeons
Glce	0.11	1.00000000	pigeons
Rbm34	-0.09	1.00000000	pigeons
Dgkh	-0.08	1.00000000	pigeons
Ddx27	0.10	1.00000000	pigeons
Zcrb1	0.06	1.00000000	pigeons
Cdc27	-0.10	1.00000000	pigeons
Gzf1	0.03	1.00000000	pigeons
Pacs2	-0.06	1.00000000	pigeons
Slc44a3	0.06	1.00000000	pigeons
Uchl5	0.07	1.00000000	pigeons
Supt6	0.07	1.00000000	pigeons
Kin	0.08	1.00000000	pigeons
Hibadh	0.14	1.00000000	pigeons
Rimklb	0.04	1.00000000	pigeons
Fam234a	-0.10	1.00000000	pigeons

Ints10	0.09	1.00000000	pigeons
Ate1	-0.15	1.00000000	pigeons
Prdm2	0.05	1.00000000	pigeons
Ywhah	-0.06	1.00000000	pigeons
Sav1	-0.09	1.00000000	pigeons
Pmepa1	-0.12	1.00000000	pigeons
Eif2ak1	-0.06	1.00000000	pigeons
Cnot11	-0.06	1.00000000	pigeons
Prpf4b	0.08	1.00000000	pigeons
Stt3b	0.03	1.00000000	pigeons
Ebna1bp2	-0.03	1.00000000	pigeons
Ubtd1	0.10	1.00000000	pigeons
Ergic2	0.02	1.00000000	pigeons
Hspa4l	0.09	1.00000000	pigeons
Zfp410	0.11	1.00000000	pigeons
Cuedc1	-0.03	1.00000000	pigeons
Lpin1	0.08	1.00000000	pigeons
Ogt	-0.13	1.00000000	pigeons
Prr7	0.07	1.00000000	pigeons
Pcm1	0.12	1.00000000	pigeons
Bag1	-0.12	1.00000000	pigeons
Rnf24	-0.19	1.00000000	pigeons
Ndufab1	0.05	1.00000000	pigeons
Hsbp1	-0.07	1.00000000	pigeons
Nsd3	-0.04	1.00000000	pigeons
Chmp6	-0.09	1.00000000	pigeons
Dctn6	-0.09	1.00000000	pigeons
Tmem230	0.11	1.00000000	pigeons
Prpf6	0.02	1.00000000	pigeons
1700034I23Rik	0.02	1.00000000	pigeons
Abtb1	-0.07	1.00000000	pigeons
Smarcc1	-0.13	1.00000000	pigeons
Ezh2	-0.04	1.00000000	pigeons
Cops8	-0.10	1.00000000	pigeons
Hoxb4	0.01	1.00000000	pigeons
Exosc10	0.04	1.00000000	pigeons
Naa35	0.06	1.00000000	pigeons
Pdia6	0.14	1.00000000	pigeons
Zfp654	0.03	1.00000000	pigeons
Zfp143	-0.10	1.00000000	pigeons
Fam76a	0.01	1.00000000	pigeons
Trim33	-0.08	1.00000000	pigeons
Top2b	-0.04	1.00000000	pigeons
Zdhhc21	-0.11	1.00000000	pigeons
Tmem128	0.08	1.00000000	pigeons
Ypel2	0.01	1.00000000	pigeons

Dennd6a	0.11	1.00000000	pigeons
Fbxo8	0.07	1.00000000	pigeons
Atp1b3	0.08	1.00000000	pigeons
Kpna4	-0.06	1.00000000	pigeons
Zfhx3	-0.09	1.00000000	pigeons
Ing4	-0.01	1.00000000	pigeons
Abcd2	0.10	1.00000000	pigeons
Dnaja3	0.05	1.00000000	pigeons
Gtf2a2	0.09	1.00000000	pigeons
Actr6	0.02	1.00000000	pigeons
Eif2b3	0.08	1.00000000	pigeons
Nus1	0.05	1.00000000	pigeons
Zfp207	0.14	1.00000000	pigeons
Ndufb6	-0.08	1.00000000	pigeons
Gabarapl2	-0.09	1.00000000	pigeons
Dhx38	0.03	1.00000000	pigeons
Surf4	0.06	1.00000000	pigeons
Zcchc2	0.09	1.00000000	pigeons
Alas1	-0.11	1.00000000	pigeons
Scaf8	0.06	1.00000000	pigeons
Specc1l	-0.04	1.00000000	pigeons
S100a16	0.05	1.00000000	pigeons
Thoc1	0.09	1.00000000	pigeons
Scp2	0.03	1.00000000	pigeons
Paxip1	-0.07	1.00000000	pigeons
Bnip1	-0.07	1.00000000	pigeons
Ppa2	0.08	1.00000000	pigeons
Ylpm1	-0.06	1.00000000	pigeons
Gpn3	0.09	1.00000000	pigeons
Sh3bgrl3	-0.13	1.00000000	pigeons
Eif1b	0.05	1.00000000	pigeons
Shprh	-0.07	1.00000000	pigeons
Scn8a	0.05	1.00000000	pigeons
Prdx4	0.07	1.00000000	pigeons
Etfa	0.14	1.00000000	pigeons
Anxa6	0.06	1.00000000	pigeons
Abcb7	0.00	1.00000000	pigeons
Wdr89	-0.01	1.00000000	pigeons
Hnrnpab	-0.06	1.00000000	pigeons
Cpne3	-0.09	1.00000000	pigeons
Dlc1	-0.11	1.00000000	pigeons
Pak2	0.02	1.00000000	pigeons
Xpo7	0.08	1.00000000	pigeons
Uaca	-0.01	1.00000000	pigeons
Rpia	-0.02	1.00000000	pigeons
Cog2	0.05	1.00000000	pigeons

Tpx2	0.03	1.00000000	pigeons
Chmp7	-0.12	1.00000000	pigeons
Psme3	-0.03	1.00000000	pigeons
Blzf1	0.11	1.00000000	pigeons
Nup54	0.08	1.00000000	pigeons
Cap1	0.05	1.00000000	pigeons
Ptp4a3	0.01	1.00000000	pigeons
Tmem135	0.02	1.00000000	pigeons
Cdc42ep3	0.11	1.00000000	pigeons
Coro1c	0.09	1.00000000	pigeons
Nudcd1	0.06	1.00000000	pigeons
Pias2	0.04	1.00000000	pigeons
Cd99l2	0.04	1.00000000	pigeons
Tln1	-0.09	1.00000000	pigeons
Appbp2	0.07	1.00000000	pigeons
Tbc1d9	0.01	1.00000000	pigeons
Ildr2	0.09	1.00000000	pigeons
Park7	0.03	1.00000000	pigeons
Itsn2	0.08	1.00000000	pigeons
Dnm3	-0.05	1.00000000	pigeons
Rit1	0.08	1.00000000	pigeons
Tbc1d15	-0.10	1.00000000	pigeons
Rabep1	0.04	1.00000000	pigeons
Acat1	0.11	1.00000000	pigeons
Arv1	0.06	1.00000000	pigeons
Fhit	0.05	1.00000000	pigeons
Tex10	0.07	1.00000000	pigeons
Rtf1	0.02	1.00000000	pigeons
Susd6	0.14	1.00000000	pigeons
Slc35a1	0.06	1.00000000	pigeons
Lrch3	-0.08	1.00000000	pigeons
Tmem65	-0.06	1.00000000	pigeons
Pisd	-0.01	1.00000000	pigeons
Spidr	0.15	1.00000000	pigeons
Vav2	-0.08	1.00000000	pigeons
Wasf2	-0.07	1.00000000	pigeons
Strn	0.02	1.00000000	pigeons
Uckl1	-0.09	1.00000000	pigeons
N4bp2l2	-0.05	1.00000000	pigeons
Arhgef3	-0.11	1.00000000	pigeons
Hnrnph3	-0.08	1.00000000	pigeons
Btbd3	-0.07	1.00000000	pigeons
Lpar1	0.01	1.00000000	pigeons
Fam114a1	-0.09	1.00000000	pigeons
Nadk	0.14	1.00000000	pigeons
Nkiras2	0.05	1.00000000	pigeons

Srp19	-0.05	1.00000000	pigeons
Ndel1	-0.03	1.00000000	pigeons
Lsm14b	-0.01	1.00000000	pigeons
Uggt1	-0.06	1.00000000	pigeons
Celf1	-0.03	1.00000000	pigeons
Cul2	-0.03	1.00000000	pigeons
Cotl1	0.13	1.00000000	pigeons
Ndufs1	0.01	1.00000000	pigeons
Senp7	0.05	1.00000000	pigeons
Hsdl2	0.05	1.00000000	pigeons
Auh	-0.09	1.00000000	pigeons
Ppt1	-0.09	1.00000000	pigeons
Arxes2	-0.04	1.00000000	pigeons
Clns1a	0.08	1.00000000	pigeons
Slc38a6	-0.07	1.00000000	pigeons
Scyl2	0.05	1.00000000	pigeons
Uprt	-0.07	1.00000000	pigeons
Btf3	0.11	1.00000000	pigeons
Fam20b	0.02	1.00000000	pigeons
Snrpd1	-0.12	1.00000000	pigeons
Tubgcp3	-0.10	1.00000000	pigeons
Rbm17	-0.08	1.00000000	pigeons
Wdr82	0.06	1.00000000	pigeons
Jak2	-0.08	1.00000000	pigeons
Ripk2	-0.05	1.00000000	pigeons
Tpm1	-0.07	1.00000000	pigeons
Hmcn1	-0.10	1.00000000	pigeons
Xxylt1	0.11	1.00000000	pigeons
Caprin1	0.06	1.00000000	pigeons
Hat1	0.04	1.00000000	pigeons
Pias4	0.00	1.00000000	pigeons
Sergef	-0.08	1.00000000	pigeons
Il27ra	0.02	1.00000000	pigeons
Yme1l1	0.04	1.00000000	pigeons
Pabpc1	-0.03	1.00000000	pigeons
Trmt10c	0.08	1.00000000	pigeons
Jam2	0.05	1.00000000	pigeons
Commd4	-0.07	1.00000000	pigeons
Klf6	0.06	1.00000000	pigeons
Picalm	-0.03	1.00000000	pigeons
Cmas	-0.08	1.00000000	pigeons
Sirt1	0.04	1.00000000	pigeons
Nsrp1	-0.06	1.00000000	pigeons
Ppp2r5a	-0.09	1.00000000	pigeons
Vapa	-0.09	1.00000000	pigeons
Lmbrd2	0.00	1.00000000	pigeons

Cc2d1b	0.01	1.00000000	pigeons
Pgam5	0.06	1.00000000	pigeons
Gns	0.13	1.00000000	pigeons
Akap9	0.05	1.00000000	pigeons
Zfp335	0.00	1.00000000	pigeons
Dusp23	-0.04	1.00000000	pigeons
Eea1	-0.01	1.00000000	pigeons
Thoc2	0.06	1.00000000	pigeons
Usp53	0.02	1.00000000	pigeons
Tmem248	-0.02	1.00000000	pigeons
Snhg4	0.07	1.00000000	pigeons
Nol9	-0.06	1.00000000	pigeons
Prdx3	0.09	1.00000000	pigeons
Slc25a20	-0.06	1.00000000	pigeons
Ago2	-0.10	1.00000000	pigeons
P3h1	-0.03	1.00000000	pigeons
Dazap1	-0.14	1.00000000	pigeons
Cpsf4	0.01	1.00000000	pigeons
Gga3	0.09	1.00000000	pigeons
Sp3	0.04	1.00000000	pigeons
Pdia4	0.06	1.00000000	pigeons
Gja6	0.17	1.00000000	pigeons
Crnkl1	0.04	1.00000000	pigeons
Rspry1	-0.09	1.00000000	pigeons
Nap1l4	-0.05	1.00000000	pigeons
Ranbp17	0.11	1.00000000	pigeons
Slc25a36	0.06	1.00000000	pigeons
Dlg5	-0.10	1.00000000	pigeons
Ints12	-0.03	1.00000000	pigeons
Pds5a	0.04	1.00000000	pigeons
Ppp2r5d	0.02	1.00000000	pigeons
Itsn1	-0.07	1.00000000	pigeons
Tm9sf3	-0.09	1.00000000	pigeons
Zfp131	-0.09	1.00000000	pigeons
Ncald	-0.06	1.00000000	pigeons
Xpnpep1	0.04	1.00000000	pigeons
Shroom4	0.09	1.00000000	pigeons
Dym	0.01	1.00000000	pigeons
Sdf4	0.05	1.00000000	pigeons
Yap1	-0.06	1.00000000	pigeons
Chst14	-0.05	1.00000000	pigeons
Lmcd1	-0.08	1.00000000	pigeons
Nubp1	-0.05	1.00000000	pigeons
G3bp2	0.08	1.00000000	pigeons
Syt13	0.05	1.00000000	pigeons
Rwdd1	-0.07	1.00000000	pigeons

Supt3	0.04	1.00000000	pigeons
Aff2	-0.06	1.00000000	pigeons
Txndc11	0.07	1.00000000	pigeons
Agtpbp1	0.06	1.00000000	pigeons
Epha4	-0.13	1.00000000	pigeons
Ube2j2	-0.02	1.00000000	pigeons
Rprd2	-0.02	1.00000000	pigeons
Alg2	0.06	1.00000000	pigeons
Pex14	-0.05	1.00000000	pigeons
Vps45	-0.04	1.00000000	pigeons
Eif3h	-0.08	1.00000000	pigeons
Cry2	-0.01	1.00000000	pigeons
Tpm4	-0.07	1.00000000	pigeons
Yipf3	-0.03	1.00000000	pigeons
Vps11	0.06	1.00000000	pigeons
Slu7	-0.02	1.00000000	pigeons
Fam214a	-0.05	1.00000000	pigeons
Ift88	0.05	1.00000000	pigeons
Eny2	-0.03	1.00000000	pigeons
Eif4enif1	-0.12	1.00000000	pigeons
Fubp1	0.00	1.00000000	pigeons
Bcas2	0.09	1.00000000	pigeons
Gapvd1	-0.10	1.00000000	pigeons
Abcc4	0.00	1.00000000	pigeons
Tmed10	-0.07	1.00000000	pigeons
Ythdf1	-0.07	1.00000000	pigeons
Zcchc14	0.00	1.00000000	pigeons
Slc13a1	0.06	1.00000000	pigeons
N4bp1	0.07	1.00000000	pigeons
Ice2	0.01	1.00000000	pigeons
Sri	-0.04	1.00000000	pigeons
Fgr	-0.02	1.00000000	pigeons
Parg	-0.10	1.00000000	pigeons
Scaper	0.19	1.00000000	pigeons
Chsy3	-0.05	1.00000000	pigeons
Tubgcp5	0.11	1.00000000	pigeons
Ufm1	-0.11	1.00000000	pigeons
Phf20l1	-0.11	1.00000000	pigeons
Siah1b	0.05	1.00000000	pigeons
Bsg	-0.05	1.00000000	pigeons
Garnl3	-0.05	1.00000000	pigeons
Nudt3	0.04	1.00000000	pigeons
Podn	-0.02	1.00000000	pigeons
Esd	0.06	1.00000000	pigeons
Rgs3	-0.03	1.00000000	pigeons
Ik	0.06	1.00000000	pigeons

Gata6	0.05	1.00000000	pigeons
Acaa2	0.07	1.00000000	pigeons
Ankrd10	-0.03	1.00000000	pigeons
Nr1h3	0.05	1.00000000	pigeons
Pdia3	-0.07	1.00000000	pigeons
Adam23	0.04	1.00000000	pigeons
Ppm1a	0.04	1.00000000	pigeons
Uba6	0.04	1.00000000	pigeons
Fam168b	-0.10	1.00000000	pigeons
Psmb3	0.03	1.00000000	pigeons
Zfp330	0.11	1.00000000	pigeons
Tulp4	0.07	1.00000000	pigeons
Snx2	0.04	1.00000000	pigeons
Ranbp9	0.02	1.00000000	pigeons
Chp1	-0.01	1.00000000	pigeons
Nrd1	0.10	1.00000000	pigeons
Tmem9b	0.02	1.00000000	pigeons
Clstn1	0.03	1.00000000	pigeons
Hyou1	-0.05	1.00000000	pigeons
Eif1ad12	-0.05	1.00000000	pigeons
G3bp1	-0.01	1.00000000	pigeons
Tbc1d1	-0.06	1.00000000	pigeons
Hadhb	0.06	1.00000000	pigeons
Cir1	0.08	1.00000000	pigeons
Luc7l	0.07	1.00000000	pigeons
Prmt9	-0.04	1.00000000	pigeons
Golph3l	0.00	1.00000000	pigeons
Syf2	0.07	1.00000000	pigeons
Pin4	0.01	1.00000000	pigeons
Cul3	0.05	1.00000000	pigeons
Dag1	-0.03	1.00000000	pigeons
Zfp503	-0.04	1.00000000	pigeons
Commd2	-0.06	1.00000000	pigeons
Rsrc1	0.14	1.00000000	pigeons
Pdk1	0.05	1.00000000	pigeons
Crot	0.00	1.00000000	pigeons
Ankrd27	0.02	1.00000000	pigeons
Kat2b	-0.03	1.00000000	pigeons
Sec23a	-0.12	1.00000000	pigeons
Fam199x	-0.03	1.00000000	pigeons
Dnmt3a	0.01	1.00000000	pigeons
Gsn	0.03	1.00000000	pigeons
Plekho1	-0.09	1.00000000	pigeons
Apoo	-0.04	1.00000000	pigeons
Usp3	-0.06	1.00000000	pigeons
Serpinh1	-0.12	1.00000000	pigeons

Taf13	-0.05	1.00000000	pigeons
Ep400	0.00	1.00000000	pigeons
Tmem131	0.03	1.00000000	pigeons
Serinc5	-0.07	1.00000000	pigeons
Sdc2	-0.02	1.00000000	pigeons
Pou2f1	0.00	1.00000000	pigeons
Uhrf2	-0.08	1.00000000	pigeons
Cep250	-0.03	1.00000000	pigeons
Pcgf3	-0.04	1.00000000	pigeons
Rora	0.04	1.00000000	pigeons
Sipa1l1	0.01	1.00000000	pigeons
Pdk3	0.06	1.00000000	pigeons
Isca1	0.05	1.00000000	pigeons
Osbpl9	0.06	1.00000000	pigeons
Zfp622	0.01	1.00000000	pigeons
Hspa4	0.10	1.00000000	pigeons
Slc31a1	0.06	1.00000000	pigeons
Glyr1	-0.08	1.00000000	pigeons
Shisa5	0.03	1.00000000	pigeons
Cd151	0.09	1.00000000	pigeons
Napg	-0.11	1.00000000	pigeons
Ndufs4	0.03	1.00000000	pigeons
Slc35b1	0.01	1.00000000	pigeons
Ppm1d	0.03	1.00000000	pigeons
Ssb	0.09	1.00000000	pigeons
Hectd1	0.04	1.00000000	pigeons
Tbc1d5	-0.02	1.00000000	pigeons
Rab8a	0.01	1.00000000	pigeons
Zfc3h1	0.06	1.00000000	pigeons
Eogt	-0.03	1.00000000	pigeons
Gnpat	-0.06	1.00000000	pigeons
Cwc25	-0.04	1.00000000	pigeons
Casp2	-0.01	1.00000000	pigeons
Rffl	0.04	1.00000000	pigeons
Nans	0.04	1.00000000	pigeons
Rab14	0.02	1.00000000	pigeons
Pde6d	-0.02	1.00000000	pigeons
Nme7	-0.05	1.00000000	pigeons
Nusap1	-0.13	1.00000000	pigeons
Ndufa6	-0.07	1.00000000	pigeons
Tnrc6c	-0.14	1.00000000	pigeons
Ubr2	0.02	1.00000000	pigeons
Dlst	0.03	1.00000000	pigeons
Leo1	-0.07	1.00000000	pigeons
Surf6	0.04	1.00000000	pigeons
Zbed4	-0.04	1.00000000	pigeons

Pdlim1	0.05	1.00000000	pigeons
Nt5e	0.07	1.00000000	pigeons
Rsrc2	-0.09	1.00000000	pigeons
Enoph1	0.05	1.00000000	pigeons
Chst3	0.00	1.00000000	pigeons
Anks6	0.02	1.00000000	pigeons
Cerk	-0.11	1.00000000	pigeons
Tmem94	0.03	1.00000000	pigeons
Nup210	0.07	1.00000000	pigeons
Cdc42	0.05	1.00000000	pigeons
Ubac1	-0.08	1.00000000	pigeons
Dek	0.03	1.00000000	pigeons
Kdr	0.06	1.00000000	pigeons
Lrrc8a	-0.05	1.00000000	pigeons
Atp6v0a1	-0.08	1.00000000	pigeons
Kdm1a	0.02	1.00000000	pigeons
Virma	0.03	1.00000000	pigeons
Ehbp1	0.01	1.00000000	pigeons
Vps8	0.07	1.00000000	pigeons
Foxk1	-0.01	1.00000000	pigeons
Ddx23	0.05	1.00000000	pigeons
Elp3	-0.01	1.00000000	pigeons
Cgnl1	-0.04	1.00000000	pigeons
Sdhaf4	0.02	1.00000000	pigeons
Dock1	-0.08	1.00000000	pigeons
Dffa	0.02	1.00000000	pigeons
Ntan1	0.03	1.00000000	pigeons
Zbtb21	-0.12	1.00000000	pigeons
Carmil1	-0.15	1.00000000	pigeons
Dcp2	-0.12	1.00000000	pigeons
Xbp1	-0.04	1.00000000	pigeons
Dhx8	0.08	1.00000000	pigeons
Ssbp3	-0.07	1.00000000	pigeons
Ebf1	0.10	1.00000000	pigeons
Klhl6	0.08	1.00000000	pigeons
Dnajb2	0.02	1.00000000	pigeons
Ap2b1	0.08	1.00000000	pigeons
Psmb7	-0.05	1.00000000	pigeons
Cacna2d2	0.06	1.00000000	pigeons
Fbxo34	0.03	1.00000000	pigeons
Naaa	0.04	1.00000000	pigeons
Ptcd3	-0.03	1.00000000	pigeons
Dnajc7	0.12	1.00000000	pigeons
Fnbp1l	-0.01	1.00000000	pigeons
Git1	0.08	1.00000000	pigeons
Pde7a	0.01	1.00000000	pigeons

Rab5a	0.01	1.00000000	pigeons
Ramp3	0.20	1.00000000	pigeons
Asap1	0.18	1.00000000	pigeons
Denr	-0.05	1.00000000	pigeons
Ppp1r8	0.02	1.00000000	pigeons
Twf1	0.02	1.00000000	pigeons
Slk	-0.02	1.00000000	pigeons
Bbs9	-0.08	1.00000000	pigeons
Asb3	-0.02	1.00000000	pigeons
Serbp1	-0.01	1.00000000	pigeons
Nt5c3	0.06	1.00000000	pigeons
Zc3h12b	-0.06	1.00000000	pigeons
Tor2a	0.08	1.00000000	pigeons
Add3	-0.08	1.00000000	pigeons
Amdhd2	-0.06	1.00000000	pigeons
Ccnk	0.06	1.00000000	pigeons
Tm9sf4	0.03	1.00000000	pigeons
Lmo4	0.01	1.00000000	pigeons
Snx30	0.07	1.00000000	pigeons
Fxr1	0.07	1.00000000	pigeons
Arap3	-0.05	1.00000000	pigeons
Emc6	0.08	1.00000000	pigeons
Cds2	0.01	1.00000000	pigeons
Ogdh	-0.10	1.00000000	pigeons
Jmjd1c	-0.09	1.00000000	pigeons
Wdr36	-0.02	1.00000000	pigeons
Tarbp1	-0.10	1.00000000	pigeons
Csnk1e	-0.03	1.00000000	pigeons
Eif2s3y	0.02	1.00000000	pigeons
Fbxo28	-0.03	1.00000000	pigeons
Hp1bp3	-0.07	1.00000000	pigeons
Actr1a	-0.01	1.00000000	pigeons
Klhl36	0.08	1.00000000	pigeons
Blmh	-0.09	1.00000000	pigeons
Snx12	0.06	1.00000000	pigeons
Usp25	-0.10	1.00000000	pigeons
Timm13	-0.06	1.00000000	pigeons
Tbc1d12	-0.01	1.00000000	pigeons
Usp15	-0.08	1.00000000	pigeons
Bmpr2	-0.09	1.00000000	pigeons
Rapgef3	0.08	1.00000000	pigeons
Atp6v0d1	0.02	1.00000000	pigeons
Urb2	-0.02	1.00000000	pigeons
Btbd7	0.04	1.00000000	pigeons
Pfdn1	0.04	1.00000000	pigeons
Trappc12	-0.05	1.00000000	pigeons

Rnf214	-0.04	1.00000000	pigeons
Lbr	0.09	1.00000000	pigeons
Plpp2	0.05	1.00000000	pigeons
Sar1b	0.01	1.00000000	pigeons
Pmp22	0.10	1.00000000	pigeons
Golim4	0.06	1.00000000	pigeons
Nucks1	-0.05	1.00000000	pigeons
Uso1	-0.01	1.00000000	pigeons
Slc30a1	0.03	1.00000000	pigeons
Upf2	0.05	1.00000000	pigeons
Clk1	0.02	1.00000000	pigeons
Atl2	-0.11	1.00000000	pigeons
Gnai2	0.00	1.00000000	pigeons
Prkce	-0.05	1.00000000	pigeons
Necap1	0.05	1.00000000	pigeons
Pgm2l1	0.02	1.00000000	pigeons
Ran	0.07	1.00000000	pigeons
Rnf8	0.00	1.00000000	pigeons
Slain2	0.04	1.00000000	pigeons
Tlr3	0.12	1.00000000	pigeons
Golga4	-0.09	1.00000000	pigeons
Lta4h	0.01	1.00000000	pigeons
Lrig2	0.07	1.00000000	pigeons
Tmco1	0.12	1.00000000	pigeons
Lclat1	-0.02	1.00000000	pigeons
Cnot7	0.01	1.00000000	pigeons
Ckap5	-0.10	1.00000000	pigeons
Tanc1	0.06	1.00000000	pigeons
Ddost	-0.05	1.00000000	pigeons
Itfg1	-0.15	1.00000000	pigeons
Dnajc1	0.07	1.00000000	pigeons
Secisbp2	0.02	1.00000000	pigeons
Rheb	-0.07	1.00000000	pigeons
Reps1	0.00	1.00000000	pigeons
Hadh	0.06	1.00000000	pigeons
Rbl1	-0.10	1.00000000	pigeons
Scfd1	-0.01	1.00000000	pigeons
Psmb1	0.01	1.00000000	pigeons
Klhl24	-0.02	1.00000000	pigeons
Pspc1	0.01	1.00000000	pigeons
Srp72	0.09	1.00000000	pigeons
Pank4	0.05	1.00000000	pigeons
Eml4	0.01	1.00000000	pigeons
Eno1b	0.00	1.00000000	pigeons
Ap3b1	-0.08	1.00000000	pigeons
Zfand5	-0.05	1.00000000	pigeons

Fgf1	0.12	1.00000000	pigeons
Smtnl2	-0.01	1.00000000	pigeons
Srsf10	0.06	1.00000000	pigeons
Zfp652	-0.03	1.00000000	pigeons
Casp7	0.03	1.00000000	pigeons
Tubb6	-0.01	1.00000000	pigeons
Pla2g6	-0.05	1.00000000	pigeons
Ugp2	-0.01	1.00000000	pigeons
Trip4	0.03	1.00000000	pigeons
Leprot	-0.06	1.00000000	pigeons
Adnp2	-0.16	1.00000000	pigeons
Eepd1	0.07	1.00000000	pigeons
Nf1	0.02	1.00000000	pigeons
Smad6	0.02	1.00000000	pigeons
Atg5	0.02	1.00000000	pigeons
Smyd3	-0.11	1.00000000	pigeons
Zbtb7a	-0.04	1.00000000	pigeons
Vangl1	-0.12	1.00000000	pigeons
Ndufs2	0.04	1.00000000	pigeons
Rnf220	0.01	1.00000000	pigeons
Tbl1xr1	-0.19	1.00000000	pigeons
Hoxa7	0.06	1.00000000	pigeons
Pcnx	-0.03	1.00000000	pigeons
Prdm15	0.05	1.00000000	pigeons
Copg1	0.03	1.00000000	pigeons
Ankib1	0.05	1.00000000	pigeons
Invs	-0.07	1.00000000	pigeons
Bclaf1	-0.05	1.00000000	pigeons
Eci3	0.05	1.00000000	pigeons
Tbc1d2b	0.04	1.00000000	pigeons
Kmt5b	-0.02	1.00000000	pigeons
Psmc1	-0.09	1.00000000	pigeons
Yipf4	-0.02	1.00000000	pigeons
Setd7	-0.05	1.00000000	pigeons
Bcar1	0.09	1.00000000	pigeons
Oard1	-0.01	1.00000000	pigeons
Plekhm3	0.00	1.00000000	pigeons
Rtn4	-0.02	1.00000000	pigeons
Jak1	0.09	1.00000000	pigeons
Cgrrf1	-0.04	1.00000000	pigeons
Fos	0.25	1.00000000	pigeons
Rnf149	0.00	1.00000000	pigeons
Txlng	-0.12	1.00000000	pigeons
Nup85	-0.06	1.00000000	pigeons
Ndufa10	0.09	1.00000000	pigeons
Lgals8	0.00	1.00000000	pigeons

Ace3	-0.02	1.00000000	pigeons
Cdc23	-0.03	1.00000000	pigeons
F2rl2	0.00	1.00000000	pigeons
Lrch1	0.04	1.00000000	pigeons
Kansl1	-0.03	1.00000000	pigeons
Lysmd2	0.06	1.00000000	pigeons
Sh3kbp1	-0.10	1.00000000	pigeons
Ctu2	0.01	1.00000000	pigeons
Rbm12	0.07	1.00000000	pigeons
Vrk2	-0.07	1.00000000	pigeons
Trerf1	-0.10	1.00000000	pigeons
AW554918	0.02	1.00000000	pigeons
Csnk2a1	0.02	1.00000000	pigeons
Tia1	0.05	1.00000000	pigeons
Tmc7	0.01	1.00000000	pigeons
Akt1	0.01	1.00000000	pigeons
Washc3	-0.04	1.00000000	pigeons
Nhsl2	0.03	1.00000000	pigeons
Atad3a	-0.02	1.00000000	pigeons
Rbl2	0.02	1.00000000	pigeons
Rangap1	-0.04	1.00000000	pigeons
Efcab14	0.10	1.00000000	pigeons
Fbxo22	-0.04	1.00000000	pigeons
Rrn3	-0.04	1.00000000	pigeons
Pin1	-0.02	1.00000000	pigeons
Dync1i2	-0.08	1.00000000	pigeons
Herc1	0.12	1.00000000	pigeons
Rexo2	0.08	1.00000000	pigeons
Notch1	-0.05	1.00000000	pigeons
Eftud2	0.00	1.00000000	pigeons
Kdm4c	0.00	1.00000000	pigeons
Chd2	-0.11	1.00000000	pigeons
G2e3	-0.04	1.00000000	pigeons
Jund	0.10	1.00000000	pigeons
Phf2	-0.16	1.00000000	pigeons
Larp1b	0.04	1.00000000	pigeons
Dcun1d1	0.01	1.00000000	pigeons
Sh3bgrl	-0.03	1.00000000	pigeons
Sbno1	0.04	1.00000000	pigeons
Hmbs	-0.01	1.00000000	pigeons
Ubp1	-0.01	1.00000000	pigeons
Desi2	0.01	1.00000000	pigeons
Ptpn11	0.02	1.00000000	pigeons
Kif3a	-0.01	1.00000000	pigeons
Pepd	-0.08	1.00000000	pigeons
Ifi35	-0.01	1.00000000	pigeons

Psma5	-0.03	1.00000000	pigeons
Dtnbp1	0.13	1.00000000	pigeons
Zc3h18	-0.05	1.00000000	pigeons
Hspa12a	-0.02	1.00000000	pigeons
Esrrg	-0.16	1.00000000	pigeons
Cast	0.01	1.00000000	pigeons
Abcc5	-0.08	1.00000000	pigeons
Rab21	0.03	1.00000000	pigeons
Tprgl	-0.04	1.00000000	pigeons
Ubr1	0.09	1.00000000	pigeons
Tnrc6a	-0.02	1.00000000	pigeons
St3gal6	-0.15	1.00000000	pigeons
Plekha3	-0.02	1.00000000	pigeons
Nisch	0.05	1.00000000	pigeons
Cct5	0.02	1.00000000	pigeons
Pitrm1	0.02	1.00000000	pigeons
Iah1	-0.02	1.00000000	pigeons
Nipsnap2	-0.09	1.00000000	pigeons
Taok1	-0.02	1.00000000	pigeons
Sdhd	0.00	1.00000000	pigeons
Ywhae	-0.02	1.00000000	pigeons
Phb	0.09	1.00000000	pigeons
Polr2f	0.09	1.00000000	pigeons
Ndufb5	0.04	1.00000000	pigeons
Prkrip1	0.07	1.00000000	pigeons
Papss2	0.05	1.00000000	pigeons
Cd47	-0.01	1.00000000	pigeons
Gpr50	0.06	1.00000000	pigeons
Sil1	0.04	1.00000000	pigeons
Ncstn	0.02	1.00000000	pigeons
Fbxl17	0.09	1.00000000	pigeons
Pphln1	0.03	1.00000000	pigeons
Edc3	-0.02	1.00000000	pigeons
Cstf2	-0.08	1.00000000	pigeons
Rsf1	-0.04	1.00000000	pigeons
Smg7	-0.08	1.00000000	pigeons
Ncoa4	0.10	1.00000000	pigeons
Dnajc2	0.01	1.00000000	pigeons
Chmp5	-0.02	1.00000000	pigeons
Lemd2	0.03	1.00000000	pigeons
Stard13	0.07	1.00000000	pigeons
Ppp1r2	-0.02	1.00000000	pigeons
Scamp2	0.03	1.00000000	pigeons
Atp10d	0.02	1.00000000	pigeons
Trappc3	0.01	1.00000000	pigeons
Smarca5	-0.08	1.00000000	pigeons

Pibf1	0.09	1.00000000	pigeons
Copg2	0.00	1.00000000	pigeons
R3hdm4	0.00	1.00000000	pigeons
Jun	0.24	1.00000000	pigeons
Ube2e3	0.03	1.00000000	pigeons
Eif3f	0.02	1.00000000	pigeons
Homer2	-0.01	1.00000000	pigeons
Ctsh	-0.03	1.00000000	pigeons
Polr2b	-0.06	1.00000000	pigeons
Bnip3l	0.12	1.00000000	pigeons
Psen2	0.01	1.00000000	pigeons
Zcchc8	0.00	1.00000000	pigeons
Ddx55	0.02	1.00000000	pigeons
Bend7	-0.06	1.00000000	pigeons
Zfp830	0.06	1.00000000	pigeons
Krit1	0.10	1.00000000	pigeons
Paip1	-0.02	1.00000000	pigeons
Atp11c	-0.04	1.00000000	pigeons
Gnpda2	0.00	1.00000000	pigeons
Klhl22	-0.07	1.00000000	pigeons
Anp32e	0.01	1.00000000	pigeons
Asah1	0.00	1.00000000	pigeons
Acad9	-0.01	1.00000000	pigeons
Bag5	0.04	1.00000000	pigeons
Ak1	0.10	1.00000000	pigeons
Acsl4	0.03	1.00000000	pigeons
Ldlrad3	-0.12	1.00000000	pigeons
Dnmt1	0.01	1.00000000	pigeons
Ap1ar	-0.01	1.00000000	pigeons
Bicral	-0.15	1.00000000	pigeons
Kctd20	0.18	1.00000000	pigeons
Tmc5	0.05	1.00000000	pigeons
Lzic	0.05	1.00000000	pigeons
Arl15	0.04	1.00000000	pigeons
Nudcd3	0.11	1.00000000	pigeons
Hmgb1	0.00	1.00000000	pigeons
Rab27a	-0.08	1.00000000	pigeons
Snx33	0.05	1.00000000	pigeons
Tax1bp1	-0.02	1.00000000	pigeons
Smtn	0.01	1.00000000	pigeons
Slc12a2	0.01	1.00000000	pigeons
Nedd4	0.04	1.00000000	pigeons
Adgra3	0.03	1.00000000	pigeons
Lpcat1	0.07	1.00000000	pigeons
Rab10	-0.01	1.00000000	pigeons
Tpcn1	0.09	1.00000000	pigeons

Ube2a	-0.01	1.00000000	pigeons
Ccdc91	0.10	1.00000000	pigeons
Ube2v1	0.05	1.00000000	pigeons
Aftph	-0.12	1.00000000	pigeons
Pafah1b2	0.01	1.00000000	pigeons
Tmco3	0.01	1.00000000	pigeons
Fam162a	0.08	1.00000000	pigeons
Aamdc	0.04	1.00000000	pigeons
Epb41	0.05	1.00000000	pigeons
Apaf1	-0.10	1.00000000	pigeons
Bcl9	-0.05	1.00000000	pigeons
Derl1	-0.02	1.00000000	pigeons
Tcf25	-0.07	1.00000000	pigeons
Herc3	-0.05	1.00000000	pigeons
Papss1	-0.10	1.00000000	pigeons
Cfl2	0.03	1.00000000	pigeons
Zbtb48	0.05	1.00000000	pigeons
Cdk8	-0.06	1.00000000	pigeons
Rnf217	-0.01	1.00000000	pigeons
Pgd	-0.05	1.00000000	pigeons
St3gal1	-0.07	1.00000000	pigeons
Crebl2	-0.02	1.00000000	pigeons
B3galnt2	-0.01	1.00000000	pigeons
Pdcd10	-0.02	1.00000000	pigeons
Ube2f	0.02	1.00000000	pigeons
Pcdh19	0.07	1.00000000	pigeons
Ube2i	0.05	1.00000000	pigeons
Cisd1	0.01	1.00000000	pigeons
Cenpc1	-0.13	1.00000000	pigeons
Helb	0.05	1.00000000	pigeons
Rnf20	-0.01	1.00000000	pigeons
Ndufa12	-0.02	1.00000000	pigeons
Lcor	-0.11	1.00000000	pigeons
Smim4	0.03	1.00000000	pigeons
Fchsd2	0.01	1.00000000	pigeons
Tmem116	-0.12	1.00000000	pigeons
Taf12	0.03	1.00000000	pigeons
Gphn	-0.03	1.00000000	pigeons
Rims2	0.02	1.00000000	pigeons
Nectin3	0.04	1.00000000	pigeons
Gcnt4	-0.05	1.00000000	pigeons
Adam17	0.02	1.00000000	pigeons
Rnaseh1	0.00	1.00000000	pigeons
Tmem42	0.05	1.00000000	pigeons
Top1	-0.08	1.00000000	pigeons
Ptpn1	-0.04	1.00000000	pigeons

Aimp2	0.04	1.00000000	pigeons
Tnfsf10	-0.01	1.00000000	pigeons
Grk4	-0.04	1.00000000	pigeons
Tgs1	-0.01	1.00000000	pigeons
Atg2b	0.03	1.00000000	pigeons
2510039O18Rik	-0.01	1.00000000	pigeons
Prkn	0.04	1.00000000	pigeons
Yaf2	0.07	1.00000000	pigeons
Camsap2	-0.05	1.00000000	pigeons
Gtpbp1	0.01	1.00000000	pigeons
Abhd13	-0.03	1.00000000	pigeons
Slc10a7	-0.01	1.00000000	pigeons
Erich1	0.01	1.00000000	pigeons
Arl5a	-0.03	1.00000000	pigeons
Haus3	0.10	1.00000000	pigeons
Cxcr4	0.06	1.00000000	pigeons
Tmem208	-0.01	1.00000000	pigeons
Slc7a6os	0.06	1.00000000	pigeons
Heph	0.01	1.00000000	pigeons
Brms1l	0.10	1.00000000	pigeons
Zdhhc6	-0.05	1.00000000	pigeons
Rab31	0.03	1.00000000	pigeons
Ccdc126	-0.04	1.00000000	pigeons
Cdc16	0.04	1.00000000	pigeons
Ncoa6	0.03	1.00000000	pigeons
Cdc42ep1	0.01	1.00000000	pigeons
Gpcpd1	0.09	1.00000000	pigeons
Katnal1	0.00	1.00000000	pigeons
Kdm5b	-0.06	1.00000000	pigeons
Kbtbd11	0.02	1.00000000	pigeons
Gpr155	-0.04	1.00000000	pigeons
Vps41	0.08	1.00000000	pigeons
Pogz	-0.04	1.00000000	pigeons
Ppp2r2d	0.04	1.00000000	pigeons
Nacc2	-0.02	1.00000000	pigeons
Dhrs3	0.02	1.00000000	pigeons
Necap2	0.05	1.00000000	pigeons
Erlec1	0.03	1.00000000	pigeons
Amot	-0.02	1.00000000	pigeons
Wdfy2	-0.04	1.00000000	pigeons
Ddx42	-0.05	1.00000000	pigeons
Rnd3	-0.03	1.00000000	pigeons
Uba2	0.04	1.00000000	pigeons
Psme4	-0.02	1.00000000	pigeons
Nfkbiz	0.11	1.00000000	pigeons
Ppic	0.02	1.00000000	pigeons

Traf3	0.00	1.00000000	pigeons
Prkar2a	-0.06	1.00000000	pigeons
Nsl1	-0.06	1.00000000	pigeons
Pcdh12	-0.01	1.00000000	pigeons
Dis3	-0.01	1.00000000	pigeons
Epha2	-0.02	1.00000000	pigeons
Cfdp1	-0.01	1.00000000	pigeons
Spryd7	-0.02	1.00000000	pigeons
Ube2b	0.01	1.00000000	pigeons
Fzd6	-0.06	1.00000000	pigeons
Eif4e2	0.00	1.00000000	pigeons
Fblim1	-0.06	1.00000000	pigeons
Usp1	0.06	1.00000000	pigeons
Riok3	-0.04	1.00000000	pigeons
Lama5	0.00	1.00000000	pigeons
Srsf2	-0.01	1.00000000	pigeons
Tnrc6b	-0.16	1.00000000	pigeons
Ptprn2	0.04	1.00000000	pigeons
Ing1	0.07	1.00000000	pigeons
Dynlt3	0.07	1.00000000	pigeons
Cct6b	0.03	1.00000000	pigeons
Abcc1	0.01	1.00000000	pigeons
Clcn3	0.05	1.00000000	pigeons
Snap29	-0.05	1.00000000	pigeons
Kpna1	-0.12	1.00000000	pigeons
Sugt1	-0.03	1.00000000	pigeons
Lrrc58	-0.01	1.00000000	pigeons
Tfg	0.03	1.00000000	pigeons
Srf	-0.05	1.00000000	pigeons
Xrn1	-0.02	1.00000000	pigeons
Nsa2	-0.05	1.00000000	pigeons
Upf3b	0.01	1.00000000	pigeons
Lrrcc1	-0.04	1.00000000	pigeons
Fasn	-0.05	1.00000000	pigeons
Psma7	0.05	1.00000000	pigeons
Slc12a4	-0.03	1.00000000	pigeons
Nfya	0.02	1.00000000	pigeons
Ints8	0.00	1.00000000	pigeons
Asap2	-0.01	1.00000000	pigeons
Rbm6	-0.03	1.00000000	pigeons
Hmgcr	-0.03	1.00000000	pigeons
Efl1	-0.07	1.00000000	pigeons
Slc35c2	0.04	1.00000000	pigeons
Azin1	0.00	1.00000000	pigeons
Atad2b	-0.06	1.00000000	pigeons
Dr1	-0.07	1.00000000	pigeons

Acap3	0.02	1.00000000	pigeons
Cyfip1	0.00	1.00000000	pigeons
Reep2	-0.06	1.00000000	pigeons
Trpc1	-0.01	1.00000000	pigeons
Tmf1	0.04	1.00000000	pigeons
Ppil1	0.05	1.00000000	pigeons
Ghr	-0.04	1.00000000	pigeons
Aff4	0.02	1.00000000	pigeons
Rmnd5a	0.03	1.00000000	pigeons
Cachd1	-0.01	1.00000000	pigeons
Kdm5a	0.03	1.00000000	pigeons
Exog	-0.06	1.00000000	pigeons
Otulin	-0.01	1.00000000	pigeons
Cops5	-0.02	1.00000000	pigeons
Phkb	-0.01	1.00000000	pigeons
Zfp592	0.04	1.00000000	pigeons
Eif3d	-0.01	1.00000000	pigeons
Cnot4	-0.04	1.00000000	pigeons
Psma1	0.05	1.00000000	pigeons
Jmy	-0.06	1.00000000	pigeons
Lig3	-0.03	1.00000000	pigeons
Nptn	0.04	1.00000000	pigeons
Bms1	-0.05	1.00000000	pigeons
Ndufa4	0.03	1.00000000	pigeons
Ptk7	-0.02	1.00000000	pigeons
Ercc3	-0.02	1.00000000	pigeons
Ppm1b	0.09	1.00000000	pigeons
Lpgat1	0.08	1.00000000	pigeons
Srp54a	-0.02	1.00000000	pigeons
Rufy1	-0.04	1.00000000	pigeons
Zzz3	-0.02	1.00000000	pigeons
Appl2	0.03	1.00000000	pigeons
Btbd1	-0.05	1.00000000	pigeons
Rnf146	0.04	1.00000000	pigeons
Psmd14	0.01	1.00000000	pigeons
Tie1	-0.04	1.00000000	pigeons
Rabl6	-0.03	1.00000000	pigeons
Thsd1	0.05	1.00000000	pigeons
Tmem165	0.04	1.00000000	pigeons
Atxn2	0.06	1.00000000	pigeons
Pdcl3	0.03	1.00000000	pigeons
Gnai3	0.01	1.00000000	pigeons
Rnf34	-0.02	1.00000000	pigeons
Sap30l	0.02	1.00000000	pigeons
Ptbp2	0.02	1.00000000	pigeons
Ncoa5	-0.07	1.00000000	pigeons

Agpat3	0.01	1.00000000	pigeons
Nde1	0.05	1.00000000	pigeons
Ythdc1	0.01	1.00000000	pigeons
Cab39	-0.09	1.00000000	pigeons
Zfp523	0.04	1.00000000	pigeons
Sgms1	-0.04	1.00000000	pigeons
Borcs8	-0.02	1.00000000	pigeons
Setd5	-0.07	1.00000000	pigeons
Slc9a5	-0.02	1.00000000	pigeons
Cbl	0.04	1.00000000	pigeons
Pcmtd1	0.03	1.00000000	pigeons
Hnrnpu	-0.01	1.00000000	pigeons
Iqsec1	0.05	1.00000000	pigeons
Pmvk	0.01	1.00000000	pigeons
Btbd10	-0.07	1.00000000	pigeons
Ankrd46	-0.04	1.00000000	pigeons
Cnih4	0.00	1.00000000	pigeons
Pum1	-0.04	1.00000000	pigeons
Thada	-0.09	1.00000000	pigeons
Nsmce1	0.01	1.00000000	pigeons
Tcerg1	-0.02	1.00000000	pigeons
Irf2	-0.02	1.00000000	pigeons
Ppfia1	-0.06	1.00000000	pigeons
Rabepk	0.00	1.00000000	pigeons
Dpm2	-0.04	1.00000000	pigeons
Gtf2h1	-0.06	1.00000000	pigeons
Erlin2	0.06	1.00000000	pigeons
Rexo4	0.03	1.00000000	pigeons
Htra1	0.09	1.00000000	pigeons
Arl13b	-0.01	1.00000000	pigeons
Ercc6	-0.03	1.00000000	pigeons
Smarce1	0.01	1.00000000	pigeons
Spry1	0.01	1.00000000	pigeons
Nufip2	-0.05	1.00000000	pigeons
Pex7	-0.04	1.00000000	pigeons
Pds5b	-0.05	1.00000000	pigeons
Taok3	-0.04	1.00000000	pigeons
Osbpl10	-0.08	1.00000000	pigeons
Abca3	0.03	1.00000000	pigeons
Ppie	0.04	1.00000000	pigeons
Xpa	0.03	1.00000000	pigeons
Cops3	-0.03	1.00000000	pigeons
Clock	0.05	1.00000000	pigeons
Vps37b	-0.03	1.00000000	pigeons
Slc30a6	-0.01	1.00000000	pigeons
Bdp1	-0.02	1.00000000	pigeons

Syap1	-0.01	1.00000000	pigeons
Naxe	-0.04	1.00000000	pigeons
Sec63	-0.05	1.00000000	pigeons
Usp54	-0.16	1.00000000	pigeons
Camkmt	0.09	1.00000000	pigeons
Tcea1	-0.01	1.00000000	pigeons
Senp6	-0.02	1.00000000	pigeons
Rlim	-0.04	1.00000000	pigeons
Sec13	-0.04	1.00000000	pigeons
Stau2	-0.04	1.00000000	pigeons
Ammecr1	-0.01	1.00000000	pigeons
Bap1	-0.01	1.00000000	pigeons
Zfp704	0.06	1.00000000	pigeons
Castor1	-0.05	1.00000000	pigeons
Cntf	0.02	1.00000000	pigeons
Smu1	-0.01	1.00000000	pigeons
Kif1b	-0.02	1.00000000	pigeons
Tm2d1	0.03	1.00000000	pigeons
Gtpbp10	-0.01	1.00000000	pigeons
Dis3l2	0.00	1.00000000	pigeons
Ipo7	-0.06	1.00000000	pigeons
Wwox	0.06	1.00000000	pigeons
Ppa1	0.03	1.00000000	pigeons
Agl	0.08	1.00000000	pigeons
Nod1	0.02	1.00000000	pigeons
Fbxl14	0.01	1.00000000	pigeons
Hook1	-0.05	1.00000000	pigeons
Smap1	-0.02	1.00000000	pigeons
Dvl1	-0.03	1.00000000	pigeons
Puf60	-0.01	1.00000000	pigeons
Wdr1	-0.01	1.00000000	pigeons
Fkbp14	0.03	1.00000000	pigeons
Lcmt2	0.01	1.00000000	pigeons
Itfg2	-0.01	1.00000000	pigeons
Nucb2	0.02	1.00000000	pigeons
Ccdc85c	-0.02	1.00000000	pigeons
Atp6v1g1	-0.06	1.00000000	pigeons
Snrpb2	0.05	1.00000000	pigeons
Trip12	-0.05	1.00000000	pigeons
Atl1	-0.03	1.00000000	pigeons
Cd82	-0.04	1.00000000	pigeons
Stab2	-0.08	1.00000000	pigeons
Sms	0.07	1.00000000	pigeons
Ica1	-0.04	1.00000000	pigeons
Pdhx	0.08	1.00000000	pigeons
Kidins220	-0.05	1.00000000	pigeons

Ep300	-0.05	1.00000000	pigeons
Ino80c	0.01	1.00000000	pigeons
Dpy30	0.03	1.00000000	pigeons
Bend5	-0.14	1.00000000	pigeons
Sh3bp4	0.00	1.00000000	pigeons
Lonrf2	-0.06	1.00000000	pigeons
Chst10	-0.05	1.00000000	pigeons
Dnajc17	0.01	1.00000000	pigeons
Nek7	0.05	1.00000000	pigeons
Fbxo11	-0.09	1.00000000	pigeons
Smim3	0.02	1.00000000	pigeons
Rnmt	0.01	1.00000000	pigeons
Letm1	0.12	1.00000000	pigeons
Rabggtb	0.02	1.00000000	pigeons
Swap70	0.01	1.00000000	pigeons
Sf3b3	-0.07	1.00000000	pigeons
Clcc1	0.08	1.00000000	pigeons
Pi4ka	-0.02	1.00000000	pigeons
Ndrg1	-0.12	1.00000000	pigeons
Pde1c	0.13	1.00000000	pigeons
Ttc33	-0.06	1.00000000	pigeons
Nfe2l1	-0.01	1.00000000	pigeons
Cnn3	0.01	1.00000000	pigeons
Tcf3	-0.04	1.00000000	pigeons
Glo1	0.02	1.00000000	pigeons
Rif1	-0.03	1.00000000	pigeons
Arid2	-0.05	1.00000000	pigeons
Dcun1d5	0.02	1.00000000	pigeons
Sap30	0.01	1.00000000	pigeons
Snrpd3	0.01	1.00000000	pigeons
Svil	0.37	1.00000000	pigeons
Lrrc28	-0.05	1.00000000	pigeons
Dgke	-0.03	1.00000000	pigeons
Tdp2	-0.02	1.00000000	pigeons
Hspd1	-0.01	1.00000000	pigeons
Hid1	-0.02	1.00000000	pigeons
Vdac2	0.04	1.00000000	pigeons
Cwc15	0.02	1.00000000	pigeons
Hcfc2	0.01	1.00000000	pigeons
Zfyve21	0.00	1.00000000	pigeons
Atp6v1e2	-0.03	1.00000000	pigeons
Slc37a2	0.07	1.00000000	pigeons
Fancd2	-0.08	1.00000000	pigeons
Cbx6	-0.02	1.00000000	pigeons
B3glct	0.02	1.00000000	pigeons
Rhoj	-0.11	1.00000000	pigeons

Dclk2	0.08	1.00000000	pigeons
Timm44	0.02	1.00000000	pigeons
Cntrl	0.00	1.00000000	pigeons
Cer1	-0.01	1.00000000	pigeons
Lsm14a	0.01	1.00000000	pigeons
Pnn	0.02	1.00000000	pigeons
Tmem183a	0.05	1.00000000	pigeons
Hook3	-0.02	1.00000000	pigeons
Cbll1	-0.03	1.00000000	pigeons
Prkcd	-0.05	1.00000000	pigeons
Pdhb	-0.02	1.00000000	pigeons
Fam135a	-0.15	1.00000000	pigeons
Tecpr1	-0.03	1.00000000	pigeons
Rnf138	-0.06	1.00000000	pigeons
Rpgr	-0.04	1.00000000	pigeons
Cdkal1	-0.02	1.00000000	pigeons
Rel	0.10	1.00000000	pigeons
Rab2a	0.04	1.00000000	pigeons
Klf12	-0.03	1.00000000	pigeons
Rexo1	0.01	1.00000000	pigeons
Otud3	0.00	1.00000000	pigeons
Plxna1	-0.01	1.00000000	pigeons
Hsd17b4	0.03	1.00000000	pigeons
Ythdf2	-0.03	1.00000000	pigeons
Slc25a28	-0.03	1.00000000	pigeons
Rrbp1	-0.03	1.00000000	pigeons
Cdk17	-0.07	1.00000000	pigeons
Phlpp1	-0.02	1.00000000	pigeons
Zfpm1	0.03	1.00000000	pigeons
Aven	0.04	1.00000000	pigeons
Ctbp1	0.00	1.00000000	pigeons
Kat6a	-0.05	1.00000000	pigeons
Rbm19	0.06	1.00000000	pigeons
Vps39	-0.05	1.00000000	pigeons
Lsm6	-0.08	1.00000000	pigeons
Psmc5	0.03	1.00000000	pigeons
Vps53	-0.02	1.00000000	pigeons
Rabgap1l	-0.05	1.00000000	pigeons
Hnrnpm	-0.01	1.00000000	pigeons
Iffo1	-0.03	1.00000000	pigeons
Rbsn	-0.08	1.00000000	pigeons
Cdk6	0.06	1.00000000	pigeons
Nfat5	-0.04	1.00000000	pigeons
Usp38	-0.04	1.00000000	pigeons
Rfng	0.00	1.00000000	pigeons
Trp53bp1	-0.01	1.00000000	pigeons

Kctd3	0.04	1.00000000	pigeons
Cnot1	-0.04	1.00000000	pigeons
Hscb	0.01	1.00000000	pigeons
Bcl2l13	0.04	1.00000000	pigeons
Ddx46	-0.03	1.00000000	pigeons
Btbd2	-0.01	1.00000000	pigeons
P4hb	-0.03	1.00000000	pigeons
D5Ertd579e	-0.05	1.00000000	pigeons
Gpc1	-0.02	1.00000000	pigeons
Dnaja2	0.00	1.00000000	pigeons
Herpud2	0.00	1.00000000	pigeons
Cops7a	0.04	1.00000000	pigeons
Zfp319	-0.01	1.00000000	pigeons
Nbr1	0.02	1.00000000	pigeons
Fam110d	0.00	1.00000000	pigeons
Fbxw7	-0.02	1.00000000	pigeons
Glcci1	0.03	1.00000000	pigeons
Prrc2b	0.00	1.00000000	pigeons
Phf5a	0.03	1.00000000	pigeons
Rad54l2	-0.03	1.00000000	pigeons
Stk3	-0.05	1.00000000	pigeons
Tmem50a	0.02	1.00000000	pigeons
Clic4	0.00	1.00000000	pigeons
Lef1	-0.01	1.00000000	pigeons
Ralb	0.02	1.00000000	pigeons
Itm2b	0.00	1.00000000	pigeons
Eri1	-0.01	1.00000000	pigeons
Gnl2	0.02	1.00000000	pigeons
Rab22a	-0.02	1.00000000	pigeons
Plod1	-0.03	1.00000000	pigeons
Rwdd4a	0.02	1.00000000	pigeons
Slc37a3	-0.04	1.00000000	pigeons
Serpini1	0.01	1.00000000	pigeons
Klhl5	-0.02	1.00000000	pigeons
Slc48a1	0.03	1.00000000	pigeons
Ap1m1	-0.04	1.00000000	pigeons
Larp1	0.05	1.00000000	pigeons
Tshz3	0.03	1.00000000	pigeons
Ggnbp2	-0.02	1.00000000	pigeons
Aco2	-0.01	1.00000000	pigeons
Ythdf3	-0.04	1.00000000	pigeons
Acbd5	0.02	1.00000000	pigeons
Eif4a2	0.03	1.00000000	pigeons
Ipo5	-0.01	1.00000000	pigeons
Paxbp1	0.00	1.00000000	pigeons
Tdrd3	-0.03	1.00000000	pigeons

Ssr2	0.01	1.00000000	pigeons
Rptor	-0.02	1.00000000	pigeons
Dnajb12	0.00	1.00000000	pigeons
Dctn3	-0.05	1.00000000	pigeons
Ppp1r26	-0.02	1.00000000	pigeons
Tpmt	0.01	1.00000000	pigeons
Qser1	0.04	1.00000000	pigeons
Stx12	-0.03	1.00000000	pigeons
Hyal2	-0.03	1.00000000	pigeons
Rcsd1	0.05	1.00000000	pigeons
Lbh	-0.02	1.00000000	pigeons
Rbm45	0.00	1.00000000	pigeons
Slc41a1	0.05	1.00000000	pigeons
5031439G07Rik	0.00	1.00000000	pigeons
Zfp326	0.03	1.00000000	pigeons
Ndufa2	-0.03	1.00000000	pigeons
Prpf3	-0.04	1.00000000	pigeons
Angel2	-0.08	1.00000000	pigeons
Sipa1l2	0.08	1.00000000	pigeons
Commd8	0.00	1.00000000	pigeons
Add1	0.00	1.00000000	pigeons
Npc2	-0.04	1.00000000	pigeons
Psmg3	-0.01	1.00000000	pigeons
Fam76b	0.00	1.00000000	pigeons
Tfip11	-0.01	1.00000000	pigeons
Prelid1	0.06	1.00000000	pigeons
Tpm3-rs7	0.03	1.00000000	pigeons
Als2cl	0.12	1.00000000	pigeons
Cope	0.03	1.00000000	pigeons
Cct7	-0.01	1.00000000	pigeons
Kras	0.02	1.00000000	pigeons
Nelfcd	0.00	1.00000000	pigeons
Uchl3	0.04	1.00000000	pigeons
Dcxr	-0.01	1.00000000	pigeons
Cd63	0.05	1.00000000	pigeons
Cep85l	-0.11	1.00000000	pigeons
Gnb1	-0.03	1.00000000	pigeons
Rcor1	-0.04	1.00000000	pigeons
Kank3	-0.04	1.00000000	pigeons
Ube3c	-0.07	1.00000000	pigeons
Slc25a3	0.04	1.00000000	pigeons
Actb	0.08	1.00000000	pigeons
Lactb	0.02	1.00000000	pigeons
Gpalpp1	-0.07	1.00000000	pigeons
Nup93	-0.09	1.00000000	pigeons
Ppp1ca	-0.02	1.00000000	pigeons

Ubfd1	0.01	1.00000000	pigeons
Swi5	-0.02	1.00000000	pigeons
Slc20a2	-0.04	1.00000000	pigeons
Slc35c1	-0.02	1.00000000	pigeons
Ift20	-0.02	1.00000000	pigeons
Rgl1	-0.03	1.00000000	pigeons
Znrf3	-0.01	1.00000000	pigeons
Plcl2	0.03	1.00000000	pigeons
Id2	0.04	1.00000000	pigeons
Actr2	-0.01	1.00000000	pigeons
Nkrf	-0.03	1.00000000	pigeons
Ube2v2	-0.01	1.00000000	pigeons
Get4	-0.05	1.00000000	pigeons
Qrich1	0.10	1.00000000	pigeons
Reps2	0.01	1.00000000	pigeons
Aqr	0.03	1.00000000	pigeons
Zc3h7b	0.01	1.00000000	pigeons
Ino80d	-0.06	1.00000000	pigeons
Clp1	0.01	1.00000000	pigeons
Srgap1	-0.01	1.00000000	pigeons
Dennd3	-0.03	1.00000000	pigeons
Eif3j2	-0.02	1.00000000	pigeons
Sec16a	0.00	1.00000000	pigeons
B9d1	-0.05	1.00000000	pigeons
Qdpr	0.01	1.00000000	pigeons
Slc30a4	0.01	1.00000000	pigeons
Slc30a9	-0.01	1.00000000	pigeons
Ranbp1	-0.05	1.00000000	pigeons
Odc1	0.08	1.00000000	pigeons
Fyn	0.01	1.00000000	pigeons
Ccdc186	-0.01	1.00000000	pigeons
Arl8b	0.00	1.00000000	pigeons
Rsbn1l	-0.03	1.00000000	pigeons
Nek9	0.02	1.00000000	pigeons
Vps26b	0.00	1.00000000	pigeons
Arid4a	-0.03	1.00000000	pigeons
Sos2	-0.08	1.00000000	pigeons
Dnajb6	0.02	1.00000000	pigeons
Slf2	-0.06	1.00000000	pigeons
Sde2	-0.04	1.00000000	pigeons
Bcar3	0.04	1.00000000	pigeons
Zbtb46	0.00	1.00000000	pigeons
Arl5b	0.00	1.00000000	pigeons
Supt4a	-0.02	1.00000000	pigeons
Rhoc	0.01	1.00000000	pigeons
Tcf20	-0.07	1.00000000	pigeons

Ewsr1	0.01	1.00000000	pigeons
Zbtb16	-2.50	0.00000000	mice
B2m	1.52	0.00000000	mice
H2-K1	1.28	0.00000000	mice
Ifi44	2.21	0.00000000	mice
Ly6e	1.38	0.00000000	mice
Bst2	1.89	0.00000000	mice
Irf7	1.79	0.00000000	mice
Klf2	-1.57	0.00000000	mice
Ifit3	1.61	0.00000000	mice
Gbp7	1.73	0.00000000	mice
Tsc22d3	-1.36	0.00000000	mice
Stat1	1.60	0.00000000	mice
Gbp4	1.86	0.00000000	mice
Ifi27l2a	2.23	0.00000000	mice
Ifitm3	1.30	0.00000000	mice
Isg15	1.82	0.00000000	mice
AY036118	1.66	0.00000000	mice
Cdkn1a	-1.39	0.00000000	mice
Cyp26b1	-1.45	0.00000000	mice
Ifit3b	1.16	0.00000000	mice
Klf9	-1.28	0.00000000	mice
H2-D1	0.90	0.00000000	mice
Sgms1	-1.25	0.00000000	mice
Zbp1	1.12	0.00000000	mice
Tmem252	-1.82	0.00000000	mice
Rsad2	1.79	0.00000000	mice
Klf4	-1.27	0.00000000	mice
Xaf1	1.30	0.00000000	mice
Ifit1	1.55	0.00000000	mice
Eif1	-0.77	0.00000000	mice
Oasl2	1.27	0.00000000	mice
Ly6a	1.15	0.00000000	mice
Gbp6	1.28	0.00000000	mice
Ifit2	1.17	0.00000000	mice
Usp18	1.02	0.00000000	mice
Ddx60	1.11	0.00000000	mice
Trim30a	1.19	0.00000000	mice
Cd274	1.37	0.00000000	mice
Tnfsf10	1.24	0.00000000	mice
Cmss1	1.24	0.00000000	mice
Phf11d	1.06	0.00000000	mice
Lgals3bp	1.00	0.00000000	mice
Jund	-1.06	0.00000000	mice
Psmb8	1.12	0.00000000	mice
Herc6	1.10	0.00000000	mice

Ube2l6	0.75	0.00000000	mice
H2-T23	1.06	0.00000000	mice
Fam117b	-1.01	0.00000000	mice
Zfp608	1.25	0.00000000	mice
H2-T22	0.96	0.00000000	mice
Samd9l	0.91	0.00000000	mice
Slfn2	0.94	0.00000000	mice
Ccl4	1.30	0.00000000	mice
Tap1	0.93	0.00000000	mice
Npr3	-1.16	0.00000000	mice
Cxcl2	1.38	0.00000000	mice
Txnip	-0.75	0.00000000	mice
Psmb9	0.84	0.00000000	mice
Tapbp	0.83	0.00000000	mice
Cmpk2	0.84	0.00000000	mice
Znfx1	0.90	0.00000000	mice
Plcb4	-0.88	0.00000000	mice
Cebpd	-0.94	0.00000000	mice
Parp14	0.93	0.00000000	mice
Cd74	1.49	0.00000000	mice
Sh3pxd2a	-0.79	0.00000000	mice
Gbp3	0.89	0.00000000	mice
Snrk	-0.83	0.00000000	mice
Trafd1	0.75	0.00000000	mice
Fas	-0.87	0.00000000	mice
Sp100	0.77	0.00000000	mice
Rnf213	0.81	0.00000000	mice
Plaur	-0.84	0.00000000	mice
Il1b	1.16	0.00000000	mice
Samhd1	0.92	0.00000000	mice
Per1	-0.46	0.00000000	mice
Rtp4	0.78	0.00000000	mice
Btg2	-0.64	0.00000000	mice
Gbp2b	1.13	0.00000000	mice
Cxcl10	1.96	0.00000000	mice
Igtp	0.71	0.00000000	mice
Dnaja1	-0.62	0.00000000	mice
Pknox2	-1.04	0.00000000	mice
Sbno2	-1.05	0.00000000	mice
Hspa8	-0.58	0.00000000	mice
Ccny	-0.70	0.00000000	mice
Ifih1	0.72	0.00000000	mice
Gbp10	0.45	0.00000000	mice
Nup210l	-1.25	0.00000000	mice
S1pr1	-0.55	0.00000000	mice
Parp12	0.64	0.00000000	mice

Efnb1	-0.71	0.00000000	mice
Arl4a	-0.43	0.00000000	mice
Ifi203	1.01	0.00000000	mice
Cyp1a1	-0.73	0.00000000	mice
Camk1d	1.01	0.00000000	mice
Stat2	0.69	0.00000000	mice
H2-Q7	0.67	0.00000000	mice
Sik1	-0.54	0.00000000	mice
Iigp1	0.96	0.00000000	mice
Tpt1	-0.46	0.00000000	mice
H2-Aa	1.09	0.00000000	mice
Arl4d	-0.53	0.00000000	mice
Hecw2	-0.70	0.00000000	mice
Socs3	-0.83	0.00000000	mice
Wars	0.91	0.00000000	mice
Prkcg	-1.00	0.00000000	mice
Fosb	-0.86	0.00000000	mice
Ccnd3	-0.67	0.00000000	mice
9530026P05Rik	-0.88	0.00000000	mice
H2-Eb1	1.01	0.00000000	mice
H2-T24	0.69	0.00000000	mice
Acer2	-0.84	0.00000000	mice
Parp11	0.63	0.00000000	mice
Epas1	-0.43	0.00000000	mice
Phf11b	0.62	0.00000000	mice
Scgb1a1	1.34	0.00000000	mice
Tap2	0.75	0.00000000	mice
Ifi44l	0.42	0.00000000	mice
Atf3	-0.80	0.00000000	mice
Psme1	0.78	0.00000000	mice
Plxna2	-0.70	0.00000000	mice
H2-Q6	0.50	0.00000000	mice
Stmn1	-0.63	0.00000000	mice
Cttnbp2nl	-0.54	0.00000000	mice
Csrnp1	-0.64	0.00000000	mice
Cpne8	-0.86	0.00000000	mice
Dusp1	-0.59	0.00000000	mice
Cfb	0.59	0.00000000	mice
Psmb10	0.75	0.00000000	mice
Sorbs3	-0.39	0.00000000	mice
Ifi47	0.68	0.00000000	mice
Rin2	-0.82	0.00000000	mice
Bcl2l1	-0.62	0.00000000	mice
Calr	0.68	0.00000000	mice
Jdp2	-0.59	0.00000000	mice
Plac8	0.87	0.00000000	mice

Gpihbp1	-0.71	0.00000000	mice
Sult1a1	-0.37	0.00000000	mice
Ccl2	0.65	0.00000000	mice
Ctla2b	-0.48	0.00000000	mice
Oaz1	-0.44	0.00000000	mice
Daxx	0.67	0.00000000	mice
Klf7	-0.52	0.00000000	mice
Oas2	0.31	0.00000000	mice
H2-Q4	0.58	0.00000000	mice
Adamts9	-1.20	0.00000000	mice
Flt1	-0.56	0.00000000	mice
Spats2l	0.61	0.00000000	mice
Shisa5	0.64	0.00000000	mice
Klf13	-0.49	0.00000000	mice
Rhob	-0.58	0.00000000	mice
Ppp1r15a	-0.64	0.00000000	mice
Nnmt	-0.32	0.00000000	mice
Oas1a	0.37	0.00000000	mice
Foxf1	-0.46	0.00000000	mice
Ddit4	-0.36	0.00000000	mice
Il18bp	0.41	0.00000000	mice
Atp1a1	-0.60	0.00000000	mice
Ccnl1	-0.49	0.00000000	mice
Herpud1	-0.52	0.00000000	mice
Ifi202b	0.47	0.00000000	mice
Gbp5	0.72	0.00000000	mice
Ifi27	-0.34	0.00000000	mice
Cish	-0.65	0.00000000	mice
Aqp1	-0.53	0.00000000	mice
Lgals9	0.58	0.00000000	mice
Ifi204	0.49	0.00000000	mice
Rbm39	-0.43	0.00000000	mice
Uba7	0.43	0.00000000	mice
Dennd3	-0.62	0.00000000	mice
Tns1	-0.64	0.00000000	mice
Uqcrh	-0.42	0.00000000	mice
Slfn4	0.82	0.00000000	mice
Sec14l1	-0.42	0.00000001	mice
Ctss	0.83	0.00000001	mice
Dhx58	0.32	0.00000001	mice
Gbp9	0.68	0.00000001	mice
Ftl1	-0.36	0.00000001	mice
Agrn	0.46	0.00000001	mice
Aldh2	-0.55	0.00000002	mice
Ddx58	0.53	0.00000002	mice
Lyz2	0.78	0.00000002	mice

Bach2	-0.62	0.00000002	mice
Ier3	-0.76	0.00000003	mice
Ripk1	-0.68	0.00000004	mice
AW112010	0.85	0.00000004	mice
Glul	-0.44	0.00000005	mice
Dnajb1	-0.50	0.00000008	mice
Gbp2	0.76	0.00000009	mice
Cx3cr1	-0.45	0.00000009	mice
Fkbp5	-0.39	0.00000009	mice
Rin3	-0.50	0.00000010	mice
Cdk8	0.69	0.00000010	mice
Tubb2a	-0.49	0.00000012	mice
Gbp8	0.60	0.00000014	mice
Oasl1	0.65	0.00000015	mice
Serinc3	-0.36	0.00000016	mice
Cyyr1	-0.51	0.00000018	mice
Plpp1	-0.51	0.00000018	mice
Nf1	-0.54	0.00000026	mice
H2-Ab1	0.95	0.00000027	mice
Clic4	-0.35	0.00000028	mice
Adrb2	-0.37	0.00000030	mice
Lrp5	-0.41	0.00000031	mice
Nfkbia	-0.32	0.00000033	mice
Pnrc1	-0.42	0.00000034	mice
Pxdc1	-0.45	0.00000035	mice
Tcn2	-0.40	0.00000036	mice
Aff3	-0.58	0.00000037	mice
Fmo2	-0.25	0.00000040	mice
Baiap2	-0.36	0.00000045	mice
Igfbp4	-0.69	0.00000053	mice
Wsb1	-0.44	0.00000054	mice
Ddx3x	-0.45	0.00000057	mice
Atf4	-0.54	0.00000065	mice
Pde12	-0.31	0.00000076	mice
Igfbp7	0.85	0.00000096	mice
Dgkh	-0.49	0.00000103	mice
Eif2ak2	0.43	0.00000104	mice
Sftpc	0.06	0.00000107	mice
Dusp8	-0.33	0.00000107	mice
Nampt	0.66	0.00000109	mice
Rnf125	-0.41	0.00000118	mice
Cxcl1	0.78	0.00000119	mice
Sorbs1	-0.70	0.00000123	mice
Emp1	-0.72	0.00000129	mice
Adgrl3	-0.63	0.00000212	mice
Srgn	-0.32	0.00000212	mice

Hsp90b1	0.51	0.00000234	mice
Tiparp	-0.82	0.00000238	mice
Rbbp8	-0.54	0.00000283	mice
Isg20	0.53	0.00000290	mice
Cemip2	-0.65	0.00000308	mice
Ipmk	-0.51	0.00000328	mice
H13	-0.44	0.00000387	mice
Cerk	-0.30	0.00000396	mice
Slfn3	0.35	0.00000442	mice
Rapgef5	-0.49	0.00000489	mice
Sp110	0.49	0.00000492	mice
Ubn1	-0.43	0.00000498	mice
Psme2	0.54	0.00000513	mice
Spry1	-0.42	0.00000806	mice
Fermt2	-0.41	0.00000840	mice
Dad1	-0.40	0.00000965	mice
S100a16	-0.44	0.00000981	mice
Rassf1	-0.48	0.00000992	mice
Kdm6b	-0.59	0.00001030	mice
Prickle1	0.60	0.00001069	mice
Adamts6	0.42	0.00001162	mice
Shank3	-0.51	0.00001285	mice
Aak1	-0.48	0.00001376	mice
Arhgap29	0.46	0.00001540	mice
Fbrsl1	-0.36	0.00001566	mice
Epb41	-0.76	0.00001724	mice
Ppp1r2	-0.37	0.00001730	mice
Cmip	-0.47	0.00001832	mice
Pygm	-0.26	0.00002100	mice
Lims2	-0.36	0.00002303	mice
Dach1	0.80	0.00002325	mice
Klf6	-0.39	0.00002363	mice
Tsc22d1	-0.55	0.00002452	mice
Tox2	-0.42	0.00002755	mice
Ahr	0.51	0.00003333	mice
Rlim	-0.35	0.00003606	mice
Adamts1	-0.71	0.00003726	mice
Irf2bp2	-0.43	0.00004005	mice
Parvb	-0.43	0.00004716	mice
Ly6c1	0.53	0.00005251	mice
Lars2	0.67	0.00005640	mice
Rhoj	-0.54	0.00006679	mice
Btf3	-0.28	0.00007157	mice
Sertad1	-0.50	0.00007632	mice
Ost4	-0.32	0.00009786	mice
Klf3	-0.42	0.00009925	mice

Eif1a	-0.32	0.00010230	mice
Lpl	-0.65	0.00010436	mice
Dnajb9	-0.39	0.00012299	mice
Ube2b	-0.32	0.00013223	mice
Snu13	-0.35	0.00013283	mice
Trim12c	0.34	0.00013328	mice
Kras	-0.35	0.00013891	mice
Tm4sf1	-0.29	0.00013909	mice
Crybb3	-0.25	0.00013997	mice
Cdc42ep4	-0.36	0.00015110	mice
Ephx1	-0.33	0.00015679	mice
Stk4	0.58	0.00015746	mice
Cyth1	-0.39	0.00016017	mice
S100a1	-0.36	0.00016355	mice
Ly6c2	0.53	0.00016590	mice
Actg1	-0.34	0.00017794	mice
Gng2	-0.37	0.00020913	mice
H3f3a	-0.25	0.00021254	mice
Gbe1	-0.37	0.00022966	mice
Clk1	-0.38	0.00023261	mice
Tor3a	0.44	0.00024356	mice
Col4a1	0.45	0.00024928	mice
Prkch	-0.52	0.00026122	mice
Nop58	-0.35	0.00026233	mice
Slco1c1	-0.24	0.00026267	mice
Pon2	-0.33	0.00026384	mice
Cep85l	-0.39	0.00027316	mice
9930111J21Rik2	0.53	0.00028714	mice
Tpm3-rs7	-0.25	0.00030403	mice
Svil	-0.33	0.00031072	mice
Tlr3	0.27	0.00035554	mice
Cebpg	-0.33	0.00035998	mice
Thbd	-0.42	0.00036454	mice
Fkbp14	-0.23	0.00036964	mice
Chchd2	-0.29	0.00038935	mice
Dtx3l	0.35	0.00042671	mice
Etf1	-0.36	0.00044679	mice
Clic5	0.39	0.00046700	mice
Kdr	0.60	0.00048028	mice
Camta1	-0.45	0.00048992	mice
Junb	-0.51	0.00049139	mice
Efnb2	-0.49	0.00049611	mice
Bphl	-0.30	0.00049836	mice
Selenow	0.44	0.00058379	mice
Nmi	0.53	0.00061155	mice
Slfn5	0.35	0.00063945	mice

Ucp2	-0.31	0.00065715	mice
Raly	-0.34	0.00078137	mice
Clec2d	0.54	0.00086213	mice
Adar	0.31	0.00097303	mice
Elmsan1	-0.51	0.00104752	mice
Tspo	0.45	0.00105645	mice
Ier2	-0.53	0.00107214	mice
Errfi1	-0.35	0.00117388	mice
Arid5b	-0.47	0.00118561	mice
Nol10	-0.32	0.00128576	mice
Stat3	-0.42	0.00130053	mice
Esam	-0.31	0.00131137	mice
Ciita	0.32	0.00138015	mice
Qsox1	-0.37	0.00144209	mice
Ifi211	0.27	0.00160513	mice
Evl	-0.47	0.00163072	mice
Emcn	-0.29	0.00165344	mice
Irgm2	0.42	0.00166505	mice
Tgtp2	0.34	0.00174005	mice
Tbc1d8	-0.48	0.00189010	mice
Irf2bpl	-0.18	0.00196703	mice
Adgrl4	-0.54	0.00230008	mice
Entpd1	-0.53	0.00242749	mice
Shroom2	-0.52	0.00244569	mice
Sema5a	-0.73	0.00250003	mice
Il1rn	0.40	0.00272067	mice
Polr2m	-0.28	0.00283962	mice
Parvg	-0.17	0.00311358	mice
Ifi35	0.52	0.00311462	mice
Fos	-0.62	0.00318625	mice
Itk	-0.50	0.00327277	mice
Zup1	0.35	0.00357703	mice
Rap1a	-0.32	0.00372504	mice
Peak1	0.51	0.00387391	mice
Cmtr1	0.28	0.00408132	mice
S100a8	1.78	0.00418723	mice
a	-0.40	0.00458125	mice
Tmtc1	-0.58	0.00472051	mice
Sec61g	0.51	0.00500210	mice
Tspan7	-0.32	0.00503592	mice
Inpp5d	-0.39	0.00545369	mice
Sh2d3c	-0.31	0.00556194	mice
Gabarapl2	-0.25	0.00632502	mice
Hexim1	-0.33	0.00634667	mice
Tob1	-0.31	0.00650908	mice
1810013L24Rik	-0.30	0.00654258	mice

Polg2	-0.24	0.00672251	mice
Samd4b	-0.29	0.00697419	mice
Snhg1	-0.32	0.00722771	mice
Parp9	0.39	0.00734627	mice
Adgrl2	0.65	0.00754120	mice
Psma7	0.56	0.00762547	mice
Oser1	-0.27	0.00769796	mice
Cybb	0.60	0.00843708	mice
Heg1	-0.39	0.00859629	mice
Coq10b	-0.26	0.00862592	mice
Nop10	-0.19	0.00872197	mice
Lgalsl	-0.22	0.00913646	mice
Eef1a1	-0.20	0.00934251	mice
Bag3	-0.30	0.00983400	mice
Tead2	-0.25	0.00993840	mice
Tnfrsf10b	-0.25	0.01073880	mice
Creb5	-0.65	0.01111676	mice
St8sia4	0.56	0.01130237	mice
Psme2b	0.22	0.01220825	mice
Slc25a47	-0.17	0.01256601	mice
Purb	-0.21	0.01304884	mice
Odc1	-0.14	0.01400597	mice
Arid5a	-0.30	0.01444083	mice
Ppp2r2d	-0.23	0.01591153	mice
Ccnd1	0.69	0.01602096	mice
Dazap2	-0.30	0.01613717	mice
Gstm1	-0.25	0.01636775	mice
Zfp503	0.51	0.01646419	mice
Lyn	0.46	0.01649801	mice
Zfp36	-0.34	0.01681195	mice
Aff1	-0.39	0.01776222	mice
Slc35f5	-0.24	0.01834625	mice
Tmem223	-0.15	0.01835770	mice
Dennd5b	-0.40	0.02056768	mice
Pitpna	-0.28	0.02117401	mice
Nfu1	-0.18	0.02351129	mice
Serpina3g	0.63	0.02356118	mice
Ube2s	-0.26	0.02358794	mice
Taf4b	-0.42	0.02378519	mice
Pim3	-0.38	0.02508111	mice
Nrgn	0.02	0.02589839	mice
Acod1	0.51	0.02600122	mice
Bnip3l	-0.21	0.02705185	mice
Ptprb	-0.42	0.02836832	mice
Nr4a1	-0.20	0.02869708	mice
Canx	0.47	0.02888301	mice

Phf20	-0.27	0.02914149	mice
Rbbp6	-0.31	0.02921489	mice
Ywhah	-0.29	0.02972408	mice
Ccnd2	0.52	0.03000205	mice
Ptgs1	-0.26	0.03063226	mice
Ogfr	0.42	0.03067286	mice
Taf11	-0.22	0.03071113	mice
Eif4a2	-0.35	0.03072120	mice
Pqlc1	-0.31	0.03075782	mice
Adam19	0.24	0.03096633	mice
Arhgef7	-0.47	0.03100005	mice
Filip1l	0.47	0.03126981	mice
Pnp	-0.31	0.03292498	mice
Dock9	-0.36	0.03414835	mice
Jmjd6	-0.23	0.03416953	mice
Fam71a	-0.15	0.03506452	mice
Ppdpf	-0.18	0.03532142	mice
Oaz2	-0.39	0.03540285	mice
St5	0.52	0.03541917	mice
Cdr2	-0.21	0.03570739	mice
Chrm2	-0.34	0.03658058	mice
Bsdc1	-0.25	0.03729408	mice
Litaf	-0.44	0.03787612	mice
Tmsb4x	-0.17	0.03842752	mice
Dynll1	-0.16	0.03928095	mice
Pan3	-0.31	0.04083234	mice
Tbc1d22a	-0.29	0.04239835	mice
Higd2a	-0.24	0.04263438	mice
Rap1b	-0.26	0.04442616	mice
Pkp4	-0.41	0.04469161	mice
Zfp948	-0.31	0.04533001	mice
Gltp	-0.19	0.04794137	mice
Nup107	-0.39	0.04859593	mice
Aida	0.48	0.05050278	mice
Tacc1	-0.39	0.05134728	mice
Arih1	-0.42	0.05252257	mice
Slc49a4	-0.23	0.05406340	mice
Aard	-0.06	0.05521263	mice
Slc35e4	-0.16	0.05684956	mice
Epb41l2	-0.36	0.05688624	mice
Fzd4	-0.28	0.05727626	mice
Egr1	-0.19	0.05773281	mice
Slc25a3	-0.18	0.05975068	mice
Rbl1	0.20	0.06002053	mice
Tcp11l2	-0.31	0.06128197	mice
Nedd9	-0.30	0.06160220	mice

Snx21	-0.20	0.06207486	mice
Rapgef1	-0.40	0.06572953	mice
Bambi	-0.15	0.06804912	mice
Gpx4	-0.25	0.06843985	mice
Agfg1	0.51	0.07358155	mice
Ndufa2	0.25	0.07543688	mice
Spred1	0.40	0.07727566	mice
Arel1	0.29	0.07744866	mice
Rapgef4	-0.38	0.08119118	mice
Atp5c1	-0.22	0.08327347	mice
Impdh2	-0.30	0.08510262	mice
Cfl1	-0.16	0.08822180	mice
Ece1	-0.28	0.09159449	mice
Luzp1	-0.25	0.09188663	mice
Sp140	0.50	0.09418690	mice
Serping1	0.36	0.09508828	mice
Fbxo30	-0.24	0.09788151	mice
Nt5dc3	-0.39	0.09939371	mice
Pdcd4	-0.32	0.10315709	mice
Nlrc5	0.26	0.10362124	mice
H2afz	-0.19	0.10403359	mice
Sema6b	-0.20	0.10843524	mice
Ndufb1-ps	0.42	0.10969943	mice
Lef1	-0.17	0.11230780	mice
Tmem184b	-0.28	0.11491554	mice
BC028528	-0.26	0.11669530	mice
Spp1	0.34	0.11672963	mice
Ccdc85a	-0.40	0.12097223	mice
Ifi208	0.27	0.12175790	mice
Siah2	-0.17	0.12243347	mice
Ampd1	-0.19	0.12642290	mice
Cited2	-0.14	0.12891198	mice
Znrd1	-0.05	0.13388789	mice
Polr1d	-0.20	0.13512811	mice
Grap	-0.20	0.13537168	mice
Inpp5k	-0.26	0.13537760	mice
H1f0	0.47	0.13556723	mice
Lyve1	-0.38	0.13683778	mice
Prkg1	-0.45	0.13725495	mice
Jpt1	-0.29	0.14301790	mice
Ptprg	-0.33	0.14918789	mice
Banp	-0.17	0.14936706	mice
Zc3h18	-0.16	0.15116531	mice
Spag9	-0.34	0.15187534	mice
Rnf7	-0.18	0.15865616	mice
Spry4	-0.18	0.16104625	mice

Pabpc1	-0.20	0.17365112	mice
Ccrl2	0.51	0.17597581	mice
Fnip1	-0.36	0.17726636	mice
Tbc1d15	-0.21	0.18216953	mice
Ivns1abp	-0.20	0.18921439	mice
Slk	-0.32	0.20142167	mice
Ifrd1	-0.18	0.20143861	mice
Tmem50a	-0.20	0.20367325	mice
Nfkbiz	-0.32	0.20477400	mice
Rtn4	-0.30	0.21328961	mice
Notch1	-0.33	0.21658545	mice
Gnai3	-0.27	0.23537176	mice
Rabac1	-0.23	0.23578818	mice
Lrrc8c	-0.35	0.23925973	mice
Dysf	0.41	0.24150770	mice
Samd12	-0.51	0.24492334	mice
Rab12	-0.27	0.24975188	mice
Swap70	-0.32	0.25123827	mice
Pfn1	0.42	0.25451173	mice
Lsm1	-0.22	0.25863215	mice
Aff4	-0.32	0.26315297	mice
Grn	0.44	0.26655348	mice
Ttc19	-0.16	0.27817845	mice
Fmo1	-0.26	0.27921582	mice
Ube2k	-0.27	0.28159226	mice
Pgm2l1	-0.26	0.28568674	mice
Rock2	-0.28	0.28657860	mice
Tmem11	-0.22	0.29171760	mice
Pdlim5	-0.38	0.30040304	mice
Ttpal	-0.19	0.30689687	mice
Kcnq1	-0.29	0.30703741	mice
Acr	-0.12	0.31265504	mice
Safb2	-0.11	0.31432444	mice
1300002E11Rik	-0.11	0.31782892	mice
Atp1b3	-0.29	0.32263480	mice
Clk3	-0.21	0.32435622	mice
Ndufb5	-0.27	0.32884088	mice
Atp5k	0.28	0.33573611	mice
Atrnl1	-0.21	0.34108092	mice
Tnfaip2	0.43	0.34471180	mice
Ccnyl1	-0.22	0.34705758	mice
Vav3	-0.35	0.35328986	mice
Ston1	-0.22	0.35713502	mice
Trp53inp2	-0.11	0.36682020	mice
Atl2	-0.13	0.38478684	mice
Vgll4	-0.24	0.39144837	mice

Nfya	-0.28	0.39227544	mice
Cadm4	-0.23	0.39389351	mice
Il1a	0.49	0.39458903	mice
Armcx4	0.34	0.40241363	mice
Nrarp	-0.24	0.40593607	mice
Pf4	-0.25	0.40596472	mice
Casp12	0.23	0.40993333	mice
Fam107b	-0.30	0.41020140	mice
9630028H03Rik	-0.21	0.41137537	mice
Ptov1	-0.29	0.41138399	mice
Ppia	-0.17	0.42106206	mice
Alkbh6	-0.15	0.43524752	mice
Fxyd4	-0.13	0.44358266	mice
Sec24a	-0.32	0.44571137	mice
Ndufa3	0.20	0.46987367	mice
Srsf5	-0.27	0.47493399	mice
Isca1	-0.15	0.48561909	mice
Trak2	-0.17	0.49960992	mice
Esm1	-0.50	0.50336643	mice
Anapc16	-0.10	0.50979869	mice
Osmr	-0.40	0.51033621	mice
Tubb4b	-0.16	0.51075978	mice
Serpinb9	0.38	0.51245503	mice
Sox4	0.48	0.51864279	mice
Chic2	-0.21	0.52169933	mice
Nup98	-0.32	0.52722912	mice
Cebpb	0.00	0.53106067	mice
Stx3	-0.26	0.53211797	mice
Sfr1	-0.29	0.54172243	mice
Rack1	-0.20	0.54671300	mice
Glg1	-0.23	0.54693947	mice
Cdc42ep2	-0.27	0.55704205	mice
Zcchc2	0.24	0.55752966	mice
Adk	-0.33	0.56845534	mice
Shprh	0.16	0.57048603	mice
Cdca7l	-0.23	0.57358450	mice
Neat1	-0.35	0.60664326	mice
Paip2	-0.26	0.61243229	mice
Ubb	-0.13	0.61990337	mice
Hgf	0.28	0.62723727	mice
Sppl2a	0.41	0.63832290	mice
Nr3c2	0.50	0.64402959	mice
Tubb2b	-0.06	0.65187343	mice
Npm1	-0.18	0.65668118	mice
Cd151	-0.21	0.67317579	mice
Rph3al	-0.19	0.67599398	mice

Camk4	-0.64	0.69646497	mice
Ptpru	-0.21	0.69805047	mice
Smim11	-0.26	0.70850317	mice
Slc43a3	-0.16	0.73187034	mice
Ube2h	-0.35	0.73289264	mice
Srsf3	-0.29	0.74407365	mice
Eln	-0.62	0.75374596	mice
Sde2	-0.15	0.75488708	mice
Lmbrd2	-0.12	0.76410715	mice
Sptssa	-0.19	0.76544214	mice
Slc38a2	-0.19	0.76673352	mice
Eloc	-0.23	0.77587629	mice
B4galt4	-0.18	0.78161935	mice
Il2rg	0.38	0.80030891	mice
Fcer1g	0.71	0.81608229	mice
Edn1	-0.51	0.81684383	mice
Slc3a2	-0.27	0.82824511	mice
Ppp2r5b	-0.13	0.83037390	mice
Gtpbp4	-0.17	0.85200643	mice
Usp36	-0.21	0.86157365	mice
Styx	-0.19	0.87372736	mice
Adamts10	-0.19	0.89550405	mice
Lncpint	-0.40	0.91040479	mice
Ccnl2	-0.18	0.91620214	mice
Ccnt1	-0.16	0.93073824	mice
Kif13a	-0.36	0.96043264	mice
Galnt1	0.38	0.96260050	mice
Cracr2b	-0.19	0.97659497	mice
Cnot7	-0.18	0.98228437	mice
Ddx50	-0.22	0.99104169	mice
Baz1a	-0.31	0.99323118	mice
Tpm3	-0.33	1.00000000	mice
Trim21	0.20	1.00000000	mice
Bicral	-0.18	1.00000000	mice
Ddx5	-0.19	1.00000000	mice
Ccl5	0.42	1.00000000	mice
Scn7a	0.53	1.00000000	mice
Calcrl	0.31	1.00000000	mice
Psmg4	0.41	1.00000000	mice
Sipa1	-0.21	1.00000000	mice
Rora	-0.42	1.00000000	mice
Plk3	-0.16	1.00000000	mice
Timm23	-0.21	1.00000000	mice
Rilpl1	-0.18	1.00000000	mice
Alyref	-0.23	1.00000000	mice
Tra2a	-0.19	1.00000000	mice

Gja4	0.53	1.00000000	mice
Idh2	-0.23	1.00000000	mice
Nfe2l2	-0.23	1.00000000	mice
Cmas	-0.18	1.00000000	mice
Atp11c	-0.23	1.00000000	mice
Nkd2	-0.12	1.00000000	mice
Aopep	-0.28	1.00000000	mice
Ddrgk1	-0.16	1.00000000	mice
Nfia	0.40	1.00000000	mice
Dync1li2	-0.18	1.00000000	mice
Ceacam1	-0.20	1.00000000	mice
Ctnnb1	-0.21	1.00000000	mice
Slirp	0.29	1.00000000	mice
Pglyrp1	0.24	1.00000000	mice
Bach1	-0.29	1.00000000	mice
Hsp90aa1	-0.18	1.00000000	mice
Akap12	-0.28	1.00000000	mice
Snhg8	-0.27	1.00000000	mice
Pttg1ip	-0.24	1.00000000	mice
Tmod3	-0.24	1.00000000	mice
Prpf38a	-0.19	1.00000000	mice
Fhl1	-0.26	1.00000000	mice
Tnpo1	-0.26	1.00000000	mice
Arhgap28	-0.43	1.00000000	mice
Arl13b	-0.22	1.00000000	mice
Hipk3	-0.20	1.00000000	mice
Stk40	-0.18	1.00000000	mice
D8Ertd738e	-0.24	1.00000000	mice
Prpf4	-0.22	1.00000000	mice
Gcc1	-0.20	1.00000000	mice
Btg3	-0.22	1.00000000	mice
Csf1	0.25	1.00000000	mice
Skap1	-0.28	1.00000000	mice
Uimc1	-0.24	1.00000000	mice
Ets1	0.32	1.00000000	mice
Asap2	-0.33	1.00000000	mice
Paics	-0.10	1.00000000	mice
Cox7a2l	-0.23	1.00000000	mice
Tpst2	-0.26	1.00000000	mice
Nav1	-0.29	1.00000000	mice
Cldnd1	-0.24	1.00000000	mice
Dnttip2	-0.18	1.00000000	mice
Ern1	-0.27	1.00000000	mice
Selenok	-0.21	1.00000000	mice
Clip1	-0.26	1.00000000	mice
9530082P21Rik	0.19	1.00000000	mice

Ago2	-0.21	1.00000000	mice
Ski	-0.22	1.00000000	mice
Cyth3	0.38	1.00000000	mice
Fmnl2	-0.50	1.00000000	mice
Fbxw2	-0.23	1.00000000	mice
Bri3	-0.18	1.00000000	mice
Tle5	-0.28	1.00000000	mice
Pde4b	-0.21	1.00000000	mice
Tcirg1	0.30	1.00000000	mice
Dhrs3	-0.15	1.00000000	mice
Gna13	-0.22	1.00000000	mice
Ubn2	-0.25	1.00000000	mice
Chd1	-0.21	1.00000000	mice
Ppp2cb	-0.25	1.00000000	mice
1700123O20Rik	-0.14	1.00000000	mice
Naa15	-0.16	1.00000000	mice
Klhl24	-0.17	1.00000000	mice
Gfpt1	-0.18	1.00000000	mice
Pias1	-0.24	1.00000000	mice
Wipi2	-0.22	1.00000000	mice
Podxl	0.35	1.00000000	mice
Atg2a	-0.14	1.00000000	mice
Egln2	-0.25	1.00000000	mice
S100a9	1.65	1.00000000	mice
Ttll7	-0.11	1.00000000	mice
Ifi30	-0.17	1.00000000	mice
Gsn	-0.33	1.00000000	mice
Stx12	-0.23	1.00000000	mice
Rsrc1	0.24	1.00000000	mice
Armc1	-0.23	1.00000000	mice
Ssh2	-0.33	1.00000000	mice
Erh	-0.10	1.00000000	mice
Edf1	-0.08	1.00000000	mice
Rgs9	-0.12	1.00000000	mice
Acadsb	-0.24	1.00000000	mice
Huwe1	-0.32	1.00000000	mice
Atraid	-0.05	1.00000000	mice
Ndst1	-0.26	1.00000000	mice
Pnpla8	-0.27	1.00000000	mice
Cnot4	-0.25	1.00000000	mice
Pvr	-0.24	1.00000000	mice
Txndc11	-0.18	1.00000000	mice
Srsf6	-0.24	1.00000000	mice
Pkig	-0.08	1.00000000	mice
Gdpd1	-0.14	1.00000000	mice
Ramp3	-0.27	1.00000000	mice

Celf2	-0.21	1.00000000	mice
Acyp1	-0.14	1.00000000	mice
Ppard	-0.23	1.00000000	mice
Crip1	0.43	1.00000000	mice
Tax1bp1	-0.27	1.00000000	mice
Ift172	0.27	1.00000000	mice
Ncoa7	0.38	1.00000000	mice
Ddx24	0.32	1.00000000	mice
Zfp131	-0.17	1.00000000	mice
Gng10	-0.20	1.00000000	mice
Arhgap26	-0.28	1.00000000	mice
Traf3	0.26	1.00000000	mice
Rab11a	-0.13	1.00000000	mice
Crebrf	-0.28	1.00000000	mice
Lpin1	-0.29	1.00000000	mice
Atp8a1	-0.54	1.00000000	mice
Tle1	-0.12	1.00000000	mice
Gchfr	-0.16	1.00000000	mice
Tbx3	0.21	1.00000000	mice
Polr2c	-0.12	1.00000000	mice
Pgm2	-0.23	1.00000000	mice
Ap3s1	-0.11	1.00000000	mice
Cav2	-0.26	1.00000000	mice
Pde4d	-0.29	1.00000000	mice
Zfand5	-0.30	1.00000000	mice
Nt5c3	0.21	1.00000000	mice
Adam9	-0.17	1.00000000	mice
Ankrd13c	-0.19	1.00000000	mice
Klhl9	-0.22	1.00000000	mice
Nova2	-0.22	1.00000000	mice
Dbnl	0.32	1.00000000	mice
Tomm20	-0.09	1.00000000	mice
Plekhg1	0.43	1.00000000	mice
Prxl2c	-0.27	1.00000000	mice
B930036N10Rik	0.12	1.00000000	mice
Nop53	-0.11	1.00000000	mice
Irf1	-0.45	1.00000000	mice
Atp5j	-0.08	1.00000000	mice
Ptprs	-0.17	1.00000000	mice
Foxo3	-0.27	1.00000000	mice
Cbfa2t3	-0.23	1.00000000	mice
Setdb2	0.21	1.00000000	mice
Cox8a	-0.21	1.00000000	mice
Pum1	-0.24	1.00000000	mice
Tal1	-0.19	1.00000000	mice
Plvap	-0.23	1.00000000	mice

4930599N23Rik	0.19	1.00000000	mice
Cst3	-0.24	1.00000000	mice
Elp1	0.11	1.00000000	mice
Bex3	-0.10	1.00000000	mice
Iqgap1	-0.21	1.00000000	mice
Ace	-0.22	1.00000000	mice
Zcchc14	-0.22	1.00000000	mice
Cd93	-0.25	1.00000000	mice
Elmod3	-0.21	1.00000000	mice
Rab2a	-0.23	1.00000000	mice
Ldb2	-0.31	1.00000000	mice
Sf3b1	-0.22	1.00000000	mice
Rab4a	-0.15	1.00000000	mice
Jtb	0.04	1.00000000	mice
Ints13	-0.20	1.00000000	mice
Sash1	-0.24	1.00000000	mice
Fkbp4	-0.24	1.00000000	mice
Dock6	-0.20	1.00000000	mice
Hpgd	-0.16	1.00000000	mice
Gapdh	-0.18	1.00000000	mice
Hint2	-0.22	1.00000000	mice
Hnrnpl	-0.18	1.00000000	mice
Eif3h	-0.12	1.00000000	mice
Chka	-0.21	1.00000000	mice
Ascc3	0.45	1.00000000	mice
Pcf11	-0.19	1.00000000	mice
Rabgef1	-0.28	1.00000000	mice
Snx3	-0.09	1.00000000	mice
Jun	-0.33	1.00000000	mice
Rassf9	0.30	1.00000000	mice
Ythdc1	-0.21	1.00000000	mice
Slc44a2	-0.08	1.00000000	mice
Rab3a	-0.01	1.00000000	mice
Cables1	-0.16	1.00000000	mice
Bnip3	-0.09	1.00000000	mice
Actn1	0.25	1.00000000	mice
Traf7	-0.23	1.00000000	mice
Tm2d2	-0.13	1.00000000	mice
Ints6	-0.01	1.00000000	mice
Per2	-0.16	1.00000000	mice
Simc1	-0.07	1.00000000	mice
Farsb	0.18	1.00000000	mice
Ar	0.42	1.00000000	mice
Arhgap15	-0.66	1.00000000	mice
Parp10	0.23	1.00000000	mice
Elf4	-0.24	1.00000000	mice

Kpnb1	-0.24	1.00000000	mice
Lgals8	0.21	1.00000000	mice
Atp5mpl	0.41	1.00000000	mice
Fosl2	-0.18	1.00000000	mice
Trip10	-0.14	1.00000000	mice
Sdcbp2	-0.15	1.00000000	mice
A3galt2	-0.07	1.00000000	mice
Nek6	-0.15	1.00000000	mice
Net1	-0.12	1.00000000	mice
Naa20	-0.05	1.00000000	mice
Bud31	-0.11	1.00000000	mice
Hilpda	-0.42	1.00000000	mice
Ppp1r14b	-0.02	1.00000000	mice
Satb1	-0.46	1.00000000	mice
Tlr2	0.22	1.00000000	mice
Pde2a	-0.17	1.00000000	mice
Kptn	-0.12	1.00000000	mice
Sbf2	-0.26	1.00000000	mice
Stam	-0.25	1.00000000	mice
Sema6d	0.40	1.00000000	mice
Birc2	-0.17	1.00000000	mice
Rhebl1	-0.08	1.00000000	mice
Stc1	-0.21	1.00000000	mice
Prmt2	-0.10	1.00000000	mice
Crem	-0.09	1.00000000	mice
Rnf114	0.37	1.00000000	mice
Camk2n1	-0.26	1.00000000	mice
Erf	-0.14	1.00000000	mice
Arhgap25	0.01	1.00000000	mice
Dusp10	-0.29	1.00000000	mice
Kdm5c	-0.24	1.00000000	mice
Prkar1a	-0.17	1.00000000	mice
Phyhd1	-0.10	1.00000000	mice
Cpeb2	-0.21	1.00000000	mice
Wtip	-0.19	1.00000000	mice
Csde1	-0.18	1.00000000	mice
Col4a2	0.32	1.00000000	mice
Rictor	-0.25	1.00000000	mice
Rbm18	-0.17	1.00000000	mice
Csnk1e	-0.12	1.00000000	mice
Cdc42bpa	-0.29	1.00000000	mice
Zfp326	-0.26	1.00000000	mice
Irgm1	0.07	1.00000000	mice
Lrrc58	-0.13	1.00000000	mice
P4ha2	-0.11	1.00000000	mice
Taf7	-0.17	1.00000000	mice

Cox14	-0.07	1.00000000	mice
Ndufb11	-0.22	1.00000000	mice
Ccl6	0.54	1.00000000	mice
Kansl1l	-0.19	1.00000000	mice
Itsn2	-0.22	1.00000000	mice
Ciao2b	-0.02	1.00000000	mice
Notch4	-0.25	1.00000000	mice
Pdcd6	-0.09	1.00000000	mice
Prkce	-0.26	1.00000000	mice
Wfdc1	-0.20	1.00000000	mice
Sbds	-0.14	1.00000000	mice
Vat1	-0.16	1.00000000	mice
Eif4g2	-0.22	1.00000000	mice
Pmp22	-0.17	1.00000000	mice
Pde7b	0.42	1.00000000	mice
Spata1	-0.15	1.00000000	mice
Dcp2	0.31	1.00000000	mice
Fkbp8	-0.22	1.00000000	mice
Pde9a	-0.17	1.00000000	mice
Guk1	-0.14	1.00000000	mice
Nsd3	-0.27	1.00000000	mice
Tmem109	-0.20	1.00000000	mice
Ubxn4	-0.24	1.00000000	mice
Cbfa2t2	0.23	1.00000000	mice
Hdac4	-0.17	1.00000000	mice
Cuta	0.12	1.00000000	mice
Eef1b2	-0.07	1.00000000	mice
Tprgl	-0.18	1.00000000	mice
Abt1	-0.13	1.00000000	mice
H2afx	-0.04	1.00000000	mice
Ttc7b	-0.17	1.00000000	mice
Rgcc	-0.26	1.00000000	mice
Ndufc2	-0.08	1.00000000	mice
Irf9	0.30	1.00000000	mice
Lemd2	-0.09	1.00000000	mice
Wnk1	-0.15	1.00000000	mice
Ybx3	-0.18	1.00000000	mice
Cox17	0.41	1.00000000	mice
Nup155	-0.12	1.00000000	mice
Emg1	-0.06	1.00000000	mice
Crk	-0.18	1.00000000	mice
Irf2	0.28	1.00000000	mice
Trdmt1	0.16	1.00000000	mice
Ldb1	-0.18	1.00000000	mice
Kpna1	-0.16	1.00000000	mice
1700017B05Rik	-0.12	1.00000000	mice

Bclaf3	-0.20	1.00000000	mice
Pxk	0.31	1.00000000	mice
Fam111a	0.17	1.00000000	mice
Sfswap	-0.16	1.00000000	mice
Zfp710	0.34	1.00000000	mice
Zfp655	-0.10	1.00000000	mice
Cenpx	-0.08	1.00000000	mice
Gtf2h5	-0.11	1.00000000	mice
Ppp6r3	-0.20	1.00000000	mice
Ackr2	-0.14	1.00000000	mice
Sar1a	-0.14	1.00000000	mice
Cd1d1	-0.10	1.00000000	mice
Eif4a1	-0.20	1.00000000	mice
Sh2b3	-0.13	1.00000000	mice
Nfatc1	-0.33	1.00000000	mice
Egr3	-0.17	1.00000000	mice
Wbp1	-0.09	1.00000000	mice
Rb1cc1	-0.25	1.00000000	mice
Ing3	-0.20	1.00000000	mice
Cd52	0.38	1.00000000	mice
Anks1	-0.29	1.00000000	mice
Adipor1	-0.17	1.00000000	mice
Kdm3a	-0.21	1.00000000	mice
Sirt7	-0.07	1.00000000	mice
Trp53inp1	-0.15	1.00000000	mice
Pafah1b1	-0.18	1.00000000	mice
Polr3b	-0.11	1.00000000	mice
Spr	-0.10	1.00000000	mice
Atp5g2	-0.14	1.00000000	mice
Tead1	-0.41	1.00000000	mice
Lgals3	0.35	1.00000000	mice
Nars	0.25	1.00000000	mice
Gprin3	0.20	1.00000000	mice
Nbdy	-0.13	1.00000000	mice
Atp6v1f	-0.07	1.00000000	mice
Ykt6	0.24	1.00000000	mice
Sptan1	-0.24	1.00000000	mice
Txnrd1	-0.10	1.00000000	mice
Hpcal1	-0.27	1.00000000	mice
Selenof	-0.12	1.00000000	mice
Dusp3	-0.06	1.00000000	mice
Ubl3	-0.20	1.00000000	mice
Erbin	-0.27	1.00000000	mice
Ctdspl	-0.30	1.00000000	mice
Rnf38	-0.27	1.00000000	mice
Atf6	-0.21	1.00000000	mice

Akt2	-0.16	1.00000000	mice
Hap1	0.05	1.00000000	mice
Endou	-0.22	1.00000000	mice
F2r	0.17	1.00000000	mice
Cdk2ap1	-0.18	1.00000000	mice
Sun1	-0.20	1.00000000	mice
Tmem33	-0.20	1.00000000	mice
Ndufb10	-0.07	1.00000000	mice
Slc22a5	-0.14	1.00000000	mice
Yrdc	-0.11	1.00000000	mice
Fam174a	-0.06	1.00000000	mice
Lsmem1	-0.16	1.00000000	mice
Hspa4	-0.17	1.00000000	mice
Ccser2	-0.12	1.00000000	mice
Dnajb6	-0.17	1.00000000	mice
Cnot6l	-0.22	1.00000000	mice
Frat2	-0.14	1.00000000	mice
Fth1	0.01	1.00000000	mice
Retreg1	-0.16	1.00000000	mice
Il15	0.39	1.00000000	mice
Ackr3	-0.18	1.00000000	mice
Peg13	-0.11	1.00000000	mice
Ppp1r18	-0.20	1.00000000	mice
Rasgrp2	-0.06	1.00000000	mice
Tram2	0.29	1.00000000	mice
Hey1	-0.17	1.00000000	mice
Arnt	-0.16	1.00000000	mice
Lamtor2	-0.25	1.00000000	mice
Rpn1	0.23	1.00000000	mice
A330023F24Rik	-0.20	1.00000000	mice
Katna1	-0.15	1.00000000	mice
Ap3m1	0.28	1.00000000	mice
Vapa	-0.18	1.00000000	mice
Clasp2	-0.22	1.00000000	mice
Pdia3	0.26	1.00000000	mice
Tmem234	-0.03	1.00000000	mice
Fiz1	-0.08	1.00000000	mice
Akirin1	-0.09	1.00000000	mice
Nr1d1	0.19	1.00000000	mice
Sco2	-0.10	1.00000000	mice
Txn2	-0.14	1.00000000	mice
Sat1	-0.16	1.00000000	mice
Zfp521	0.47	1.00000000	mice
Stt3b	-0.33	1.00000000	mice
Taf1d	-0.17	1.00000000	mice
Gdi2	-0.14	1.00000000	mice

Stox2	0.27	1.00000000	mice
Sumo1	-0.11	1.00000000	mice
Kdelr1	-0.13	1.00000000	mice
Sik3	-0.26	1.00000000	mice
Slco2a1	-0.22	1.00000000	mice
Gvin1	0.14	1.00000000	mice
Tbc1d10a	-0.14	1.00000000	mice
Fis1	-0.17	1.00000000	mice
Gstk1	0.04	1.00000000	mice
Arl15	-0.10	1.00000000	mice
Dab2	0.37	1.00000000	mice
Nfat5	-0.03	1.00000000	mice
Castor1	-0.17	1.00000000	mice
Ptma	0.17	1.00000000	mice
Adipor2	-0.22	1.00000000	mice
Ppp1cc	-0.12	1.00000000	mice
Sgip1	-0.35	1.00000000	mice
Eif4a3	-0.16	1.00000000	mice
Dock4	0.44	1.00000000	mice
Elmo1	-0.33	1.00000000	mice
Pdlim1	-0.28	1.00000000	mice
Olfml2a	-0.06	1.00000000	mice
Slc25a25	-0.22	1.00000000	mice
Ercc1	-0.07	1.00000000	mice
Cpt1a	0.32	1.00000000	mice
Tom1	-0.12	1.00000000	mice
Nim1k	-0.13	1.00000000	mice
Zhx3	-0.13	1.00000000	mice
Bag6	-0.13	1.00000000	mice
Tjp2	0.28	1.00000000	mice
Cr1l	-0.23	1.00000000	mice
Card19	0.00	1.00000000	mice
Pik3cb	0.18	1.00000000	mice
Brwd3	-0.28	1.00000000	mice
Isy1	-0.01	1.00000000	mice
Pcif1	-0.16	1.00000000	mice
Ptpn9	0.31	1.00000000	mice
Hsbp1	-0.11	1.00000000	mice
Tmtc2	0.45	1.00000000	mice
Slc41a1	-0.04	1.00000000	mice
Vcp	0.31	1.00000000	mice
BC004004	-0.15	1.00000000	mice
AW011738	0.19	1.00000000	mice
Grina	0.25	1.00000000	mice
E2f3	0.10	1.00000000	mice
Atxn1	0.35	1.00000000	mice

Tsix	0.16	1.00000000	mice
Neurl1b	-0.05	1.00000000	mice
Smco4	-0.25	1.00000000	mice
Ankrd42	-0.05	1.00000000	mice
Rprd2	-0.09	1.00000000	mice
Dusp5	-0.14	1.00000000	mice
Arrdc4	0.42	1.00000000	mice
Qrich1	-0.10	1.00000000	mice
Jak1	-0.27	1.00000000	mice
Ift20	-0.06	1.00000000	mice
Kalrn	-0.48	1.00000000	mice
Top1	-0.14	1.00000000	mice
Dpm1	-0.17	1.00000000	mice
Ldha	-0.25	1.00000000	mice
Fli1	-0.19	1.00000000	mice
St7l	-0.12	1.00000000	mice
Bmpr2	-0.19	1.00000000	mice
Vps28	-0.07	1.00000000	mice
Ctsl	-0.03	1.00000000	mice
Hbegf	-0.13	1.00000000	mice
Dennd5a	-0.21	1.00000000	mice
Ranbp2	-0.10	1.00000000	mice
Scand1	0.37	1.00000000	mice
Tpp1	-0.18	1.00000000	mice
Keap1	0.28	1.00000000	mice
Slc39a9	-0.13	1.00000000	mice
Apobec3	0.18	1.00000000	mice
Sub1	-0.16	1.00000000	mice
Siva1	-0.15	1.00000000	mice
Brk1	-0.06	1.00000000	mice
Limk2	-0.26	1.00000000	mice
Rasa2	-0.24	1.00000000	mice
A930037H05Rik	0.21	1.00000000	mice
Pdhb	-0.07	1.00000000	mice
Trim56	0.28	1.00000000	mice
Ptms	-0.13	1.00000000	mice
Calm2	-0.14	1.00000000	mice
Sun2	-0.08	1.00000000	mice
Dusp6	0.34	1.00000000	mice
Hopx	-0.22	1.00000000	mice
Ing2	-0.22	1.00000000	mice
Wasf2	-0.15	1.00000000	mice
Arap3	-0.23	1.00000000	mice
Srp9	-0.15	1.00000000	mice
Arhgef1	-0.05	1.00000000	mice
Crybg3	0.25	1.00000000	mice

Ndel1	-0.16	1.00000000	mice
Gtf2h1	-0.16	1.00000000	mice
Piezo1	-0.12	1.00000000	mice
Ippk	-0.20	1.00000000	mice
Ube2g1	-0.18	1.00000000	mice
Tox	-0.32	1.00000000	mice
Bmp6	0.07	1.00000000	mice
Ubr5	-0.21	1.00000000	mice
Dcaf12	-0.10	1.00000000	mice
Piezo2	0.43	1.00000000	mice
Gnas	-0.12	1.00000000	mice
Aldoa	-0.09	1.00000000	mice
Arih2	-0.10	1.00000000	mice
Oip5os1	-0.10	1.00000000	mice
Tmem64	-0.24	1.00000000	mice
Sec63	-0.16	1.00000000	mice
Spata13	0.25	1.00000000	mice
Nes	0.04	1.00000000	mice
Ptpre	0.41	1.00000000	mice
Khdc4	-0.18	1.00000000	mice
Tcim	-0.11	1.00000000	mice
Pcsk7	-0.12	1.00000000	mice
Srsf7	-0.20	1.00000000	mice
Rfx7	-0.27	1.00000000	mice
Cd200	0.44	1.00000000	mice
Wdr12	-0.11	1.00000000	mice
Pald1	-0.22	1.00000000	mice
Dcaf6	-0.21	1.00000000	mice
Arf6	-0.09	1.00000000	mice
Flot2	-0.11	1.00000000	mice
Slc30a7	-0.23	1.00000000	mice
Npr1	-0.12	1.00000000	mice
Jcad	-0.17	1.00000000	mice
Exoc6b	-0.13	1.00000000	mice
Kctd10	-0.15	1.00000000	mice
Zfp423	0.20	1.00000000	mice
Nup188	-0.23	1.00000000	mice
Psmg1	-0.09	1.00000000	mice
Slco3a1	0.36	1.00000000	mice
Exoc6	0.22	1.00000000	mice
Ppfibp1	-0.23	1.00000000	mice
Itga2	0.35	1.00000000	mice
Gadd45b	-0.12	1.00000000	mice
Enho	-0.14	1.00000000	mice
Sesn1	-0.22	1.00000000	mice
Ppp2ca	-0.14	1.00000000	mice

Zswim6	-0.34	1.00000000	mice
Prickle2	-0.34	1.00000000	mice
Lmbr1l	-0.15	1.00000000	mice
Pigp	-0.22	1.00000000	mice
Tyrobp	0.32	1.00000000	mice
Casp4	0.28	1.00000000	mice
Hnrnpa0	-0.15	1.00000000	mice
Usp34	-0.14	1.00000000	mice
Hk1	-0.16	1.00000000	mice
Pitpnb	-0.09	1.00000000	mice
Glce	-0.15	1.00000000	mice
Cbx7	-0.20	1.00000000	mice
Ghr	0.39	1.00000000	mice
Lrrc4	0.14	1.00000000	mice
Tbl1xr1	0.19	1.00000000	mice
Ass1	0.10	1.00000000	mice
Zbtb46	0.31	1.00000000	mice
Ube2f	-0.25	1.00000000	mice
Rabggtb	0.02	1.00000000	mice
Eif3a	-0.23	1.00000000	mice
Uri1	-0.21	1.00000000	mice
Klf11	-0.13	1.00000000	mice
Rhbdf1	-0.14	1.00000000	mice
Bvht	-0.06	1.00000000	mice
App	0.24	1.00000000	mice
Rhpn2	-0.13	1.00000000	mice
Tgtp1	0.12	1.00000000	mice
Hes1	-0.06	1.00000000	mice
Casd1	-0.14	1.00000000	mice
Yy1	-0.13	1.00000000	mice
Trim41	-0.04	1.00000000	mice
Trim14	0.12	1.00000000	mice
Sacm1l	0.28	1.00000000	mice
Tinagl1	-0.31	1.00000000	mice
Ankrd33b	-0.29	1.00000000	mice
Nr4a2	-0.13	1.00000000	mice
Itm2b	-0.11	1.00000000	mice
Tspan18	-0.26	1.00000000	mice
Cbx6	-0.08	1.00000000	mice
Phf14	0.28	1.00000000	mice
Sdf2	-0.12	1.00000000	mice
Saysd1	-0.19	1.00000000	mice
Gclc	-0.23	1.00000000	mice
Yes1	-0.07	1.00000000	mice
Gab3	0.28	1.00000000	mice
Prps1	-0.05	1.00000000	mice

Chd3	-0.04	1.00000000	mice
Tmem14c	-0.10	1.00000000	mice
Zbtb11	-0.13	1.00000000	mice
Thoc1	-0.17	1.00000000	mice
Pgrmc1	-0.07	1.00000000	mice
Col3a1	0.18	1.00000000	mice
Sntb1	-0.22	1.00000000	mice
Ptpn2	-0.07	1.00000000	mice
Lap3	0.33	1.00000000	mice
Rbms3	-0.21	1.00000000	mice
Josd1	-0.07	1.00000000	mice
Colec12	-0.22	1.00000000	mice
Ergic1	-0.13	1.00000000	mice
3110082I17Rik	-0.12	1.00000000	mice
Rbm4b	-0.05	1.00000000	mice
Dll1	-0.13	1.00000000	mice
Pfkl	-0.02	1.00000000	mice
Kif2a	-0.17	1.00000000	mice
Hmgn1	-0.06	1.00000000	mice
Slc15a3	0.30	1.00000000	mice
Pxmp4	0.11	1.00000000	mice
Snip1	-0.14	1.00000000	mice
Cnbp	-0.13	1.00000000	mice
Btbd7	-0.21	1.00000000	mice
Frmd6	-0.23	1.00000000	mice
Rsu1	-0.11	1.00000000	mice
Rfx1	0.00	1.00000000	mice
Nprl3	0.09	1.00000000	mice
Elp5	-0.17	1.00000000	mice
Ttc32	-0.14	1.00000000	mice
Ubap2	-0.10	1.00000000	mice
Ccser1	-0.31	1.00000000	mice
Arl1	0.04	1.00000000	mice
Ahsa2	-0.21	1.00000000	mice
Tmem100	-0.14	1.00000000	mice
Ppp5c	-0.13	1.00000000	mice
Dennd4b	-0.06	1.00000000	mice
Tmbim6	0.17	1.00000000	mice
Tgfbr3	-0.20	1.00000000	mice
Dap	-0.11	1.00000000	mice
Rab10	-0.19	1.00000000	mice
Plec	0.25	1.00000000	mice
Fbxo7	0.17	1.00000000	mice
Plekhg2	-0.22	1.00000000	mice
Rassf3	-0.23	1.00000000	mice
Vwa5a	0.21	1.00000000	mice

Auts2	-0.49	1.00000000	mice
Prrc2a	-0.22	1.00000000	mice
Vkorc1	-0.09	1.00000000	mice
Lrch1	-0.17	1.00000000	mice
Cnot2	-0.10	1.00000000	mice
Il21r	-0.10	1.00000000	mice
Acot9	-0.15	1.00000000	mice
Abhd17b	-0.21	1.00000000	mice
Rnf217	-0.21	1.00000000	mice
Ripply3	-0.12	1.00000000	mice
Stx5a	-0.15	1.00000000	mice
Rnaseh2c	0.00	1.00000000	mice
Fyb	-0.19	1.00000000	mice
Cbx4	-0.11	1.00000000	mice
Ppp4r1	-0.21	1.00000000	mice
Pprc1	-0.04	1.00000000	mice
Arfgap3	-0.06	1.00000000	mice
Tox3	0.31	1.00000000	mice
Dgcr6	-0.12	1.00000000	mice
Ppip5k2	0.20	1.00000000	mice
Haus2	-0.15	1.00000000	mice
Jmjd1c	-0.23	1.00000000	mice
Trmt11	0.11	1.00000000	mice
Tcp1	-0.07	1.00000000	mice
Ankrd13a	-0.11	1.00000000	mice
Rusc2	-0.10	1.00000000	mice
Gid4	-0.10	1.00000000	mice
Bloc1s4	0.08	1.00000000	mice
Crip2	-0.14	1.00000000	mice
Arf5	-0.03	1.00000000	mice
S100a13	-0.14	1.00000000	mice
Tsg101	-0.12	1.00000000	mice
Dexi	-0.07	1.00000000	mice
U2af1	-0.13	1.00000000	mice
Arpc3	-0.04	1.00000000	mice
Ywhaq	-0.06	1.00000000	mice
Ostf1	-0.21	1.00000000	mice
Xpnpep1	-0.14	1.00000000	mice
Ptpn1	-0.19	1.00000000	mice
Atp5h	-0.11	1.00000000	mice
Pabpn1	-0.15	1.00000000	mice
Dhrs7b	-0.04	1.00000000	mice
Gabarap	-0.13	1.00000000	mice
Pdxdc1	-0.12	1.00000000	mice
Prdx2	-0.04	1.00000000	mice
Nova1	0.11	1.00000000	mice

Rrbp1	0.21	1.00000000	mice
Slc22a15	-0.08	1.00000000	mice
Cdk9	-0.09	1.00000000	mice
Cldn5	-0.08	1.00000000	mice
Fam162a	-0.11	1.00000000	mice
Shfl	0.17	1.00000000	mice
Eng	-0.16	1.00000000	mice
Arrdc2	-0.06	1.00000000	mice
Icam2	0.35	1.00000000	mice
Nrp1	0.36	1.00000000	mice
Casp8	0.27	1.00000000	mice
Ppp1r12c	-0.10	1.00000000	mice
Cenpj	0.20	1.00000000	mice
Pdgfb	0.30	1.00000000	mice
S100a11	0.42	1.00000000	mice
Nol11	-0.08	1.00000000	mice
Jade2	-0.15	1.00000000	mice
Tex261	0.01	1.00000000	mice
Capn15	-0.09	1.00000000	mice
Wdr59	-0.12	1.00000000	mice
Tmem59	-0.09	1.00000000	mice
Cog8	-0.14	1.00000000	mice
Ddx39	0.16	1.00000000	mice
Swi5	-0.01	1.00000000	mice
Anapc11	-0.09	1.00000000	mice
A930015D03Rik	0.18	1.00000000	mice
Tomm70a	0.28	1.00000000	mice
Csrp2	-0.15	1.00000000	mice
Fgfr3	-0.05	1.00000000	mice
Rnf216	-0.11	1.00000000	mice
Alpl	0.27	1.00000000	mice
Ttyh3	-0.11	1.00000000	mice
Atp2b1	-0.16	1.00000000	mice
Hmbox1	0.33	1.00000000	mice
Herc4	-0.19	1.00000000	mice
Sh3gl1	-0.13	1.00000000	mice
Pcna	-0.07	1.00000000	mice
Lrg1	0.47	1.00000000	mice
Kif3a	-0.12	1.00000000	mice
Gstm2	-0.13	1.00000000	mice
Emilin1	0.17	1.00000000	mice
Zfand2a	-0.12	1.00000000	mice
Prkx	-0.11	1.00000000	mice
Pym1	-0.07	1.00000000	mice
Ppm1a	-0.22	1.00000000	mice
Pex13	-0.04	1.00000000	mice

Tspan13	-0.14	1.00000000	mice
Dcbld1	0.40	1.00000000	mice
1810055G02Rik	-0.06	1.00000000	mice
Glcci1	0.25	1.00000000	mice
Sigirr	-0.11	1.00000000	mice
Plekha8	-0.15	1.00000000	mice
B4galt1	-0.24	1.00000000	mice
Ndrg1	-0.22	1.00000000	mice
Ogfod1	-0.05	1.00000000	mice
Smim13	-0.20	1.00000000	mice
Numb	-0.15	1.00000000	mice
Lmf2	0.19	1.00000000	mice
Helz	-0.10	1.00000000	mice
Pfkfb3	0.04	1.00000000	mice
D5Ertd579e	-0.17	1.00000000	mice
Tagln2	-0.14	1.00000000	mice
Smarcc1	-0.06	1.00000000	mice
Tnfsf13	-0.02	1.00000000	mice
Slc35e2	-0.10	1.00000000	mice
Cadm1	-0.01	1.00000000	mice
Dlgap4	-0.24	1.00000000	mice
Zfx	-0.07	1.00000000	mice
Srpr	-0.25	1.00000000	mice
Furin	-0.12	1.00000000	mice
Tmem256	0.31	1.00000000	mice
Ginm1	-0.20	1.00000000	mice
Zc3h12a	-0.10	1.00000000	mice
St6galnac3	-0.16	1.00000000	mice
Kazn	-0.16	1.00000000	mice
Ppp1cb	-0.11	1.00000000	mice
Tex30	-0.06	1.00000000	mice
Abracl	-0.14	1.00000000	mice
Cuedc2	-0.08	1.00000000	mice
Prdm10	-0.14	1.00000000	mice
Usp49	-0.05	1.00000000	mice
Gna14	0.40	1.00000000	mice
Gnl2	-0.08	1.00000000	mice
Vti1b	-0.12	1.00000000	mice
Ulk2	-0.11	1.00000000	mice
Tex264	-0.08	1.00000000	mice
Zfp36l1	-0.36	1.00000000	mice
Ifitm10	-0.03	1.00000000	mice
Nup54	-0.10	1.00000000	mice
A930005H10Rik	-0.03	1.00000000	mice
Iscu	-0.16	1.00000000	mice
Tmem161b	0.16	1.00000000	mice

Vegfc	-0.07	1.00000000	mice
Phb2	-0.01	1.00000000	mice
Xylt1	-0.08	1.00000000	mice
Rab5a	-0.11	1.00000000	mice
Tssc4	-0.07	1.00000000	mice
Pex11b	-0.06	1.00000000	mice
Rai14	-0.28	1.00000000	mice
Rnf19a	-0.11	1.00000000	mice
Lst1	0.31	1.00000000	mice
Plaat3	0.17	1.00000000	mice
Smpdl3a	-0.20	1.00000000	mice
Ubc	-0.24	1.00000000	mice
Cyld	0.22	1.00000000	mice
Smim10l1	-0.08	1.00000000	mice
Haus3	-0.08	1.00000000	mice
Enpp4	0.23	1.00000000	mice
Cry1	-0.15	1.00000000	mice
Rassf8	0.19	1.00000000	mice
Aco2	-0.13	1.00000000	mice
Dele1	-0.14	1.00000000	mice
Sf3b4	-0.05	1.00000000	mice
Dpy30	-0.11	1.00000000	mice
Cox7c	0.32	1.00000000	mice
Rnd3	-0.07	1.00000000	mice
Eif3k	-0.06	1.00000000	mice
Tmem147	-0.07	1.00000000	mice
Prelid2	-0.12	1.00000000	mice
Trappc6b	-0.19	1.00000000	mice
Rapgef6	-0.24	1.00000000	mice
Ppa1	0.31	1.00000000	mice
Ptpn12	-0.26	1.00000000	mice
Pola2	-0.09	1.00000000	mice
N4bp2	-0.11	1.00000000	mice
Usp25	0.26	1.00000000	mice
Herc1	-0.11	1.00000000	mice
Cyth2	-0.01	1.00000000	mice
Syne2	-0.04	1.00000000	mice
Zdhhc5	-0.16	1.00000000	mice
Irak1	-0.11	1.00000000	mice
Jarid2	0.28	1.00000000	mice
Ftsj3	-0.09	1.00000000	mice
Pir	-0.06	1.00000000	mice
Gabpb1	-0.11	1.00000000	mice
Itga6	-0.21	1.00000000	mice
Gls	-0.17	1.00000000	mice
Ddx10	-0.13	1.00000000	mice

Kat6a	0.17	1.00000000	mice
Smc6	-0.06	1.00000000	mice
Igkc	-0.19	1.00000000	mice
Pcdhgc4	-0.12	1.00000000	mice
Hypk	0.00	1.00000000	mice
Sec16a	-0.03	1.00000000	mice
Cul9	-0.06	1.00000000	mice
Zfp64	-0.08	1.00000000	mice
Bmyc	-0.10	1.00000000	mice
2010310C07Rik	-0.07	1.00000000	mice
Ktn1	0.25	1.00000000	mice
Glrx5	0.00	1.00000000	mice
Gemin7	-0.08	1.00000000	mice
Dusp11	-0.18	1.00000000	mice
Fads1	-0.16	1.00000000	mice
Dhx36	0.26	1.00000000	mice
Tle4	-0.05	1.00000000	mice
Shb	-0.19	1.00000000	mice
Preb	-0.11	1.00000000	mice
Ddah2	-0.23	1.00000000	mice
Desi2	-0.13	1.00000000	mice
Gramd3	-0.12	1.00000000	mice
Polr2a	-0.17	1.00000000	mice
Zbtb2	-0.16	1.00000000	mice
Cbl	0.25	1.00000000	mice
Cstf3	-0.14	1.00000000	mice
Lrrc49	-0.16	1.00000000	mice
Pdrg1	-0.02	1.00000000	mice
Asns	0.25	1.00000000	mice
B4galt5	-0.12	1.00000000	mice
Tsc22d2	-0.16	1.00000000	mice
2900026A02Rik	-0.13	1.00000000	mice
St6galnac6	-0.14	1.00000000	mice
Palmd	-0.13	1.00000000	mice
Pml	0.14	1.00000000	mice
Ppm1b	-0.15	1.00000000	mice
Tspan6	-0.03	1.00000000	mice
Strbp	-0.15	1.00000000	mice
Car2	-0.18	1.00000000	mice
Btg1	0.08	1.00000000	mice
Galnt18	-0.16	1.00000000	mice
Snhg12	-0.13	1.00000000	mice
Gpx1	-0.09	1.00000000	mice
Wwc2	-0.21	1.00000000	mice
Nedd4l	-0.20	1.00000000	mice
Pakap.1	-0.14	1.00000000	mice

Btbd3	-0.23	1.00000000	mice
Taf1	-0.15	1.00000000	mice
Rexo2	-0.02	1.00000000	mice
4932438A13Rik	-0.23	1.00000000	mice
Dcun1d1	-0.14	1.00000000	mice
Zmiz1	-0.21	1.00000000	mice
Hspa2	-0.06	1.00000000	mice
Bik	-0.02	1.00000000	mice
Ciao2a	-0.01	1.00000000	mice
Ubap1	-0.10	1.00000000	mice
Raph1	-0.22	1.00000000	mice
Senp7	0.25	1.00000000	mice
Polr2h	-0.06	1.00000000	mice
Tfb2m	-0.04	1.00000000	mice
Gpaa1	-0.10	1.00000000	mice
N4bp1	0.17	1.00000000	mice
Airn	-0.18	1.00000000	mice
Trnau1ap	-0.14	1.00000000	mice
Pigf	-0.10	1.00000000	mice
Agap1	-0.16	1.00000000	mice
Bhlhe40	-0.18	1.00000000	mice
Slc38a6	0.17	1.00000000	mice
Samd4	-0.15	1.00000000	mice
Prkca	-0.33	1.00000000	mice
Phyh	0.06	1.00000000	mice
Riok3	-0.16	1.00000000	mice
Lamb2	-0.15	1.00000000	mice
Tdrd3	0.16	1.00000000	mice
Ahsa1	-0.14	1.00000000	mice
Prdx4	-0.08	1.00000000	mice
Tuba1b	-0.17	1.00000000	mice
Exoc3l	0.13	1.00000000	mice
Selenop	-0.08	1.00000000	mice
Plekhh1	-0.12	1.00000000	mice
Pgam1	-0.01	1.00000000	mice
Pef1	-0.09	1.00000000	mice
Setdb1	-0.12	1.00000000	mice
Tcaf1	-0.04	1.00000000	mice
Tet3	-0.21	1.00000000	mice
Iqsec2	0.19	1.00000000	mice
Sparcl1	-0.14	1.00000000	mice
Cpne9	-0.11	1.00000000	mice
Gpi1	-0.01	1.00000000	mice
Tmed2	-0.08	1.00000000	mice
Ctla2a	-0.13	1.00000000	mice
Bcl2l13	-0.13	1.00000000	mice

Hspb11	-0.14	1.00000000	mice
Ctsz	0.45	1.00000000	mice
Phldb2	0.25	1.00000000	mice
Khnyn	-0.04	1.00000000	mice
Rnf181	-0.05	1.00000000	mice
Hint1	-0.06	1.00000000	mice
Rpf1	0.01	1.00000000	mice
Rttn	0.11	1.00000000	mice
Pde3a	0.01	1.00000000	mice
Sertad2	-0.27	1.00000000	mice
Atp5e	0.28	1.00000000	mice
Ccdc85b	0.47	1.00000000	mice
Snrpb	-0.05	1.00000000	mice
Limch1	-0.29	1.00000000	mice
Nbeal1	-0.25	1.00000000	mice
Eef1d	-0.07	1.00000000	mice
Nde1	-0.02	1.00000000	mice
Ube2v1	-0.06	1.00000000	mice
Atp6ap2	-0.06	1.00000000	mice
Prdx3	-0.05	1.00000000	mice
Fpgs	-0.09	1.00000000	mice
Topbp1	-0.05	1.00000000	mice
Cpped1	0.09	1.00000000	mice
Ier5	-0.13	1.00000000	mice
Pccb	0.12	1.00000000	mice
Gpatch2	0.16	1.00000000	mice
Chd2	-0.20	1.00000000	mice
Grk5	0.18	1.00000000	mice
Slc8a1	0.23	1.00000000	mice
Dnaja2	-0.06	1.00000000	mice
Wasl	-0.11	1.00000000	mice
Fam76a	-0.16	1.00000000	mice
Gtf2i	-0.22	1.00000000	mice
Cd9	-0.04	1.00000000	mice
Ubr3	0.19	1.00000000	mice
Lonp2	0.19	1.00000000	mice
Tppp3	-0.16	1.00000000	mice
Akap11	-0.16	1.00000000	mice
Nktr	-0.12	1.00000000	mice
Adss	0.01	1.00000000	mice
Psmb5	0.02	1.00000000	mice
Laptm4a	0.20	1.00000000	mice
Ctbp2	-0.09	1.00000000	mice
Pebp1	0.05	1.00000000	mice
Nenf	-0.03	1.00000000	mice
B230219D22Rik	-0.16	1.00000000	mice

Ncoa5	-0.15	1.00000000	mice
Rflnb	-0.11	1.00000000	mice
Ywhae	-0.11	1.00000000	mice
Rnf2	-0.19	1.00000000	mice
Naca	0.00	1.00000000	mice
Emc7	-0.12	1.00000000	mice
Cct5	-0.15	1.00000000	mice
Rfk	-0.16	1.00000000	mice
Kpna4	-0.14	1.00000000	mice
Scp2	-0.16	1.00000000	mice
Lcp1	0.38	1.00000000	mice
Fgfr2	-0.11	1.00000000	mice
Utp11	-0.17	1.00000000	mice
Trim8	-0.13	1.00000000	mice
Ypel3	-0.11	1.00000000	mice
Rab28	-0.11	1.00000000	mice
Zfp426	-0.14	1.00000000	mice
Nsg1	-0.02	1.00000000	mice
H2afv	0.01	1.00000000	mice
Rala	-0.23	1.00000000	mice
Sin3b	-0.07	1.00000000	mice
Ctnnbip1	-0.11	1.00000000	mice
Qars	-0.09	1.00000000	mice
Anp32a	-0.03	1.00000000	mice
Tm2d1	-0.11	1.00000000	mice
Commd3	0.02	1.00000000	mice
Rnf20	-0.06	1.00000000	mice
Pdia6	0.26	1.00000000	mice
Grpel1	0.10	1.00000000	mice
Usp19	-0.10	1.00000000	mice
Scarb1	-0.13	1.00000000	mice
Phtf2	-0.15	1.00000000	mice
Ubr2	-0.24	1.00000000	mice
Zfp358	-0.06	1.00000000	mice
Nhp2	-0.15	1.00000000	mice
Rpp21	0.21	1.00000000	mice
Grcc10	-0.06	1.00000000	mice
Tnik	-0.19	1.00000000	mice
Pink1	-0.13	1.00000000	mice
Gtf2b	-0.13	1.00000000	mice
Erich1	0.14	1.00000000	mice
Ppp2r2a	-0.17	1.00000000	mice
Puf60	-0.07	1.00000000	mice
Atxn3	-0.03	1.00000000	mice
Wdr26	-0.08	1.00000000	mice
Atg5	-0.07	1.00000000	mice

Pon3	-0.17	1.00000000	mice
Dgka	-0.10	1.00000000	mice
Shld1	-0.15	1.00000000	mice
Sh3rf1	0.23	1.00000000	mice
Ndfip2	-0.10	1.00000000	mice
1810037I17Rik	-0.08	1.00000000	mice
Itgb1bp1	-0.10	1.00000000	mice
Cavin2	-0.09	1.00000000	mice
Hid1	-0.13	1.00000000	mice
Cd63	0.24	1.00000000	mice
Psmd10	-0.08	1.00000000	mice
Rnf126	-0.10	1.00000000	mice
CAAA01118383.1	-0.03	1.00000000	mice
Ppp1r13b	-0.13	1.00000000	mice
Stxbp3	-0.06	1.00000000	mice
Zswim4	-0.08	1.00000000	mice
Slc16a2	-0.11	1.00000000	mice
Slc35b2	-0.05	1.00000000	mice
Hnrnpf	-0.13	1.00000000	mice
Cav1	-0.14	1.00000000	mice
Ctsb	0.30	1.00000000	mice
Tor1aip2	0.21	1.00000000	mice
Sema3g	-0.19	1.00000000	mice
Rnf168	0.19	1.00000000	mice
Serpini1	-0.09	1.00000000	mice
Tdp2	-0.11	1.00000000	mice
Cmtm3	-0.13	1.00000000	mice
Lsm8	-0.23	1.00000000	mice
Phf23	-0.06	1.00000000	mice
Trp53	0.20	1.00000000	mice
9230114K14Rik	-0.07	1.00000000	mice
B4galt7	0.08	1.00000000	mice
Nfil3	-0.07	1.00000000	mice
Gsg1	-0.10	1.00000000	mice
Aldh9a1	-0.12	1.00000000	mice
Iqsec1	-0.12	1.00000000	mice
Hhex	0.18	1.00000000	mice
Ccdc85c	0.23	1.00000000	mice
Cox6a1	0.00	1.00000000	mice
Ssrp1	-0.18	1.00000000	mice
Timm9	-0.07	1.00000000	mice
Ubxn6	-0.06	1.00000000	mice
Rab11b	-0.10	1.00000000	mice
Snrpc	-0.02	1.00000000	mice
Cog2	0.11	1.00000000	mice
Fat4	0.21	1.00000000	mice

Lgals1	0.33	1.00000000	mice
Tanc1	-0.16	1.00000000	mice
Trib1	-0.10	1.00000000	mice
Ssr3	0.28	1.00000000	mice
Chn2	-0.17	1.00000000	mice
Tbc1d12	-0.15	1.00000000	mice
Pmaip1	-0.07	1.00000000	mice
Brap	-0.14	1.00000000	mice
Ddx17	-0.08	1.00000000	mice
B230369F24Rik	-0.05	1.00000000	mice
Brpf1	-0.05	1.00000000	mice
BC005537	-0.06	1.00000000	mice
B3galnt2	0.02	1.00000000	mice
Tmem189	-0.10	1.00000000	mice
Uhmk1	-0.10	1.00000000	mice
Tusc2	-0.07	1.00000000	mice
Rdx	0.29	1.00000000	mice
Rdh11	-0.06	1.00000000	mice
Elk3	0.26	1.00000000	mice
Zcchc7	0.18	1.00000000	mice
Antxr2	-0.06	1.00000000	mice
Emb	-0.36	1.00000000	mice
Nrros	0.14	1.00000000	mice
Gjc1	-0.13	1.00000000	mice
Idi1	0.18	1.00000000	mice
Tle3	-0.03	1.00000000	mice
Plpp6	0.20	1.00000000	mice
Unc119b	-0.16	1.00000000	mice
Poc1b	-0.16	1.00000000	mice
Fam129b	-0.14	1.00000000	mice
Amz2	-0.09	1.00000000	mice
Pin4	0.19	1.00000000	mice
Pold4	-0.11	1.00000000	mice
Ppme1	-0.06	1.00000000	mice
Sos2	-0.12	1.00000000	mice
Sae1	-0.11	1.00000000	mice
Rasip1	-0.12	1.00000000	mice
Tgoln1	-0.09	1.00000000	mice
Clca3a1	0.10	1.00000000	mice
Nrp2	-0.21	1.00000000	mice
Abcc1	-0.07	1.00000000	mice
Tmem140	0.15	1.00000000	mice
Osgep	-0.04	1.00000000	mice
Snhg4	-0.08	1.00000000	mice
Arhgap45	-0.13	1.00000000	mice
Cstf2	0.12	1.00000000	mice

Gphn	0.30	1.00000000	mice
Glis2	0.14	1.00000000	mice
Ctsd	0.37	1.00000000	mice
Dennd4a	-0.19	1.00000000	mice
Prdx1	-0.05	1.00000000	mice
Foxp1	0.16	1.00000000	mice
Inpp4b	-0.23	1.00000000	mice
Elf2	-0.19	1.00000000	mice
Pi4ka	-0.21	1.00000000	mice
Cnpy4	-0.02	1.00000000	mice
2900097C17Rik	0.32	1.00000000	mice
Zfp511	-0.04	1.00000000	mice
Dapk1	-0.28	1.00000000	mice
Fyn	-0.20	1.00000000	mice
Itprip	-0.05	1.00000000	mice
Cnpy2	-0.19	1.00000000	mice
Ran	-0.06	1.00000000	mice
Tmc6	-0.07	1.00000000	mice
Zbtb38	-0.12	1.00000000	mice
Zfp518a	0.13	1.00000000	mice
Ammecr1l	-0.10	1.00000000	mice
Tubb6	-0.04	1.00000000	mice
Parp4	-0.18	1.00000000	mice
Degs1	-0.08	1.00000000	mice
Cops8	-0.11	1.00000000	mice
Opa1	-0.11	1.00000000	mice
Fkbp3	-0.10	1.00000000	mice
Ccdc107	-0.08	1.00000000	mice
Schip1	-0.34	1.00000000	mice
Ier5l	-0.03	1.00000000	mice
Dynlt3	-0.03	1.00000000	mice
Trak1	-0.15	1.00000000	mice
Arf1	-0.09	1.00000000	mice
Stard8	-0.18	1.00000000	mice
Pik3ca	-0.14	1.00000000	mice
Nod1	0.15	1.00000000	mice
Ctif	-0.05	1.00000000	mice
Gtf2a2	-0.11	1.00000000	mice
Trappc6a	-0.14	1.00000000	mice
Gpkow	-0.13	1.00000000	mice
Lamtor1	-0.10	1.00000000	mice
Grrp1	-0.01	1.00000000	mice
Whamm	0.20	1.00000000	mice
Atp5o	0.06	1.00000000	mice
Txndc17	-0.10	1.00000000	mice
Crtc3	-0.09	1.00000000	mice

Egfl7	0.17	1.00000000	mice
Npepps	-0.15	1.00000000	mice
Eif5	-0.12	1.00000000	mice
Trim16	-0.17	1.00000000	mice
Slc25a32	-0.06	1.00000000	mice
Hnrnpa1	-0.03	1.00000000	mice
Brd2	-0.06	1.00000000	mice
Slc7a1	-0.14	1.00000000	mice
Phpt1	0.01	1.00000000	mice
Ablim1	-0.08	1.00000000	mice
AC149090.1	0.16	1.00000000	mice
Anxa3	-0.12	1.00000000	mice
Rbm48	-0.11	1.00000000	mice
Fxr1	-0.13	1.00000000	mice
Eif3e	-0.06	1.00000000	mice
Arhgap17	-0.06	1.00000000	mice
Gle1	-0.10	1.00000000	mice
Plekhm3	-0.09	1.00000000	mice
Psma2	0.25	1.00000000	mice
Wdr70	-0.16	1.00000000	mice
Hspb1	-0.15	1.00000000	mice
Hmgb2	-0.12	1.00000000	mice
Hprt	-0.10	1.00000000	mice
Ankrd27	-0.12	1.00000000	mice
Ctps2	-0.08	1.00000000	mice
Sptlc2	-0.27	1.00000000	mice
P4ha1	-0.10	1.00000000	mice
Selplg	-0.06	1.00000000	mice
Trmt112	-0.08	1.00000000	mice
Pnkd	-0.15	1.00000000	mice
Ccdc12	0.18	1.00000000	mice
Dctn3	-0.04	1.00000000	mice
Kcnip4	0.34	1.00000000	mice
Pcyt1a	-0.13	1.00000000	mice
Pa2g4	-0.12	1.00000000	mice
Ppp1r9b	-0.05	1.00000000	mice
Exoc2	0.18	1.00000000	mice
Chmp6	-0.21	1.00000000	mice
Jam2	-0.06	1.00000000	mice
5031425E22Rik	-0.05	1.00000000	mice
Tns3	0.26	1.00000000	mice
Hotairm1	0.01	1.00000000	mice
Tsc22d4	0.19	1.00000000	mice
Dmtn	-0.06	1.00000000	mice
Exoc1	0.10	1.00000000	mice
Trim2	-0.17	1.00000000	mice

Tipin	0.10	1.00000000	mice
Sft2d3	-0.09	1.00000000	mice
Casp7	0.07	1.00000000	mice
Paip2b	-0.07	1.00000000	mice
Tnfaip3	-0.04	1.00000000	mice
Fzd6	0.14	1.00000000	mice
Smg1	-0.16	1.00000000	mice
Idh1	0.12	1.00000000	mice
Ephb4	-0.16	1.00000000	mice
Akr1a1	0.02	1.00000000	mice
Dag1	-0.16	1.00000000	mice
Cecr2	-0.11	1.00000000	mice
Fbxl18	-0.13	1.00000000	mice
Fndc3a	-0.20	1.00000000	mice
Dock2	-0.26	1.00000000	mice
Stab1	0.35	1.00000000	mice
Cmtm6	0.23	1.00000000	mice
Uchl5	-0.15	1.00000000	mice
Irs2	-0.04	1.00000000	mice
Ndufb2	-0.09	1.00000000	mice
Pcbp1	-0.15	1.00000000	mice
Tax1bp3	0.00	1.00000000	mice
Hspd1	-0.11	1.00000000	mice
Pcbp2	-0.18	1.00000000	mice
Tomm22	-0.13	1.00000000	mice
Ndufa12	-0.15	1.00000000	mice
Atg14	-0.07	1.00000000	mice
Txnl1	-0.09	1.00000000	mice
Cct2	-0.10	1.00000000	mice
Hoxb3	0.10	1.00000000	mice
4930523C07Rik	-0.21	1.00000000	mice
Josd2	-0.16	1.00000000	mice
Rab5if	0.03	1.00000000	mice
Ufm1	-0.06	1.00000000	mice
Cfap20	-0.11	1.00000000	mice
Faf2	0.03	1.00000000	mice
Togaram1	-0.04	1.00000000	mice
Amd1	-0.03	1.00000000	mice
Avpi1	-0.05	1.00000000	mice
Cmtm7	0.16	1.00000000	mice
Hist1h1e	0.09	1.00000000	mice
Spaca6	-0.08	1.00000000	mice
Vps50	0.08	1.00000000	mice
Strn3	-0.12	1.00000000	mice
Prdm11	-0.10	1.00000000	mice
Tmod2	0.25	1.00000000	mice

Plpp5	-0.02	1.00000000	mice
Cbr1	-0.07	1.00000000	mice
Nsfl1c	-0.12	1.00000000	mice
Tshz3	0.14	1.00000000	mice
Supt4a	-0.10	1.00000000	mice
Zfp335os	-0.11	1.00000000	mice
Kifc3	-0.15	1.00000000	mice
Pcmtd1	0.23	1.00000000	mice
Wdpcp	0.04	1.00000000	mice
Dync1i2	0.23	1.00000000	mice
Ppp6r2	0.23	1.00000000	mice
Fnta	-0.21	1.00000000	mice
Bod1	-0.16	1.00000000	mice
Anp32e	-0.03	1.00000000	mice
Prx	-0.17	1.00000000	mice
Grk6	-0.10	1.00000000	mice
Phf3	-0.20	1.00000000	mice
Naxd	-0.02	1.00000000	mice
Ndufb6	0.04	1.00000000	mice
Cnih1	-0.01	1.00000000	mice
Rabepk	-0.10	1.00000000	mice
Hsd17b11	-0.10	1.00000000	mice
Ccdc9	-0.08	1.00000000	mice
Cers4	-0.03	1.00000000	mice
Hus1	0.10	1.00000000	mice
Golga2	-0.11	1.00000000	mice
Gcc2	0.26	1.00000000	mice
Herpud2	-0.11	1.00000000	mice
Timm8b	0.08	1.00000000	mice
Polr2g	-0.07	1.00000000	mice
Smyd3	-0.18	1.00000000	mice
Ptpn14	-0.23	1.00000000	mice
Smagp	-0.15	1.00000000	mice
Creb3l2	-0.12	1.00000000	mice
Zdhhc6	-0.08	1.00000000	mice
Sec23a	-0.10	1.00000000	mice
Eogt	-0.16	1.00000000	mice
Tubgcp5	0.13	1.00000000	mice
Znrf1	-0.22	1.00000000	mice
Gsdmd	0.18	1.00000000	mice
N4bp3	-0.10	1.00000000	mice
Dok1	0.13	1.00000000	mice
Qdpr	-0.07	1.00000000	mice
Nadk	0.06	1.00000000	mice
Usp22	-0.06	1.00000000	mice
Ndufs2	-0.02	1.00000000	mice

Ndufb8	-0.05	1.00000000	mice
Parp8	0.15	1.00000000	mice
Tmed4	-0.06	1.00000000	mice
Ifnar1	0.12	1.00000000	mice
Hook3	-0.09	1.00000000	mice
Clock	-0.15	1.00000000	mice
Ssr2	-0.03	1.00000000	mice
Grap2	-0.15	1.00000000	mice
Tpm1	0.34	1.00000000	mice
Resf1	0.27	1.00000000	mice
Nfx1	0.01	1.00000000	mice
Dhrs1	-0.13	1.00000000	mice
Tln2	-0.19	1.00000000	mice
Birc3	-0.18	1.00000000	mice
Akt1	-0.16	1.00000000	mice
Tnfsf12	-0.22	1.00000000	mice
Ola1	0.09	1.00000000	mice
Spsb1	-0.11	1.00000000	mice
Crybg1	0.09	1.00000000	mice
Ccdc47	-0.13	1.00000000	mice
Kin	0.04	1.00000000	mice
Adcy4	-0.12	1.00000000	mice
Tom1l2	-0.20	1.00000000	mice
Tmem47	-0.12	1.00000000	mice
Spen	-0.10	1.00000000	mice
Stn1	-0.05	1.00000000	mice
Vrk2	0.12	1.00000000	mice
Azin1	-0.17	1.00000000	mice
Ublcp1	0.11	1.00000000	mice
Lta4h	0.20	1.00000000	mice
Rnf130	0.01	1.00000000	mice
Tspyl1	-0.16	1.00000000	mice
Tspan17	-0.05	1.00000000	mice
Atp5md	0.27	1.00000000	mice
Pdcl3	0.02	1.00000000	mice
Robo4	-0.13	1.00000000	mice
Zfp182	-0.06	1.00000000	mice
Senp2	-0.06	1.00000000	mice
Cyp4b1	-0.17	1.00000000	mice
Sdf2l1	0.10	1.00000000	mice
Spata2	-0.02	1.00000000	mice
Tmbim1	-0.14	1.00000000	mice
Tcf3	-0.25	1.00000000	mice
Fam92a	0.16	1.00000000	mice
Rapgef3	-0.09	1.00000000	mice
Eif4g1	-0.04	1.00000000	mice

2610307P16Rik	0.28	1.00000000	mice
Prrc2c	-0.11	1.00000000	mice
Gadd45g	-0.08	1.00000000	mice
Aplp2	-0.08	1.00000000	mice
Vmp1	-0.09	1.00000000	mice
Rela	-0.09	1.00000000	mice
Ogfrl1	0.26	1.00000000	mice
Timm17a	-0.04	1.00000000	mice
Hipk2	-0.22	1.00000000	mice
Abhd2	-0.11	1.00000000	mice
Naxe	0.04	1.00000000	mice
Ddit3	-0.16	1.00000000	mice
Bad	0.00	1.00000000	mice
Dcps	-0.03	1.00000000	mice
As3mt	-0.16	1.00000000	mice
Wwtr1	-0.19	1.00000000	mice
Ahcyl1	-0.15	1.00000000	mice
Slc8b1	0.02	1.00000000	mice
Nbas	0.23	1.00000000	mice
Cd47	0.12	1.00000000	mice
Hnrnpd	0.23	1.00000000	mice
Dhx40	-0.16	1.00000000	mice
Tmem248	-0.06	1.00000000	mice
Sox7	-0.17	1.00000000	mice
Phb	-0.11	1.00000000	mice
Eva1b	0.36	1.00000000	mice
Nos1ap	-0.17	1.00000000	mice
Golga7	0.02	1.00000000	mice
Osbpl11	-0.21	1.00000000	mice
Ahctf1	-0.14	1.00000000	mice
2210016L21Rik	0.20	1.00000000	mice
Adgrl1	-0.14	1.00000000	mice
Ppp1r16b	0.22	1.00000000	mice
Yap1	-0.12	1.00000000	mice
Scaf1	-0.04	1.00000000	mice
Tnfaip1	0.03	1.00000000	mice
Ppp1r15b	-0.13	1.00000000	mice
Eif2s2	0.25	1.00000000	mice
Cux1	-0.08	1.00000000	mice
Cct3	-0.10	1.00000000	mice
Nmd3	-0.10	1.00000000	mice
Serinc5	-0.19	1.00000000	mice
Sh3tc2	-0.16	1.00000000	mice
Gkap1	-0.11	1.00000000	mice
Zfp869	-0.10	1.00000000	mice
Prmt9	-0.04	1.00000000	mice

Riok1	-0.11	1.00000000	mice
Nucks1	0.24	1.00000000	mice
Phldb1	-0.12	1.00000000	mice
Cops9	-0.20	1.00000000	mice
Kank2	-0.10	1.00000000	mice
Secisbp2	0.09	1.00000000	mice
Rbm17	-0.04	1.00000000	mice
Bak1	0.18	1.00000000	mice
Psmc3	0.20	1.00000000	mice
Zfp787	-0.04	1.00000000	mice
Rgs10	-0.14	1.00000000	mice
Cep120	-0.06	1.00000000	mice
Tead4	-0.16	1.00000000	mice
Washc1	0.03	1.00000000	mice
Zfp641	0.17	1.00000000	mice
Tbx2	0.57	1.00000000	mice
Borcs7	0.00	1.00000000	mice
Tyw1	-0.06	1.00000000	mice
Pja1	-0.03	1.00000000	mice
Cog7	-0.03	1.00000000	mice
Slc9a3r2	-0.06	1.00000000	mice
Olfm1	0.16	1.00000000	mice
Tra2b	-0.13	1.00000000	mice
Selenot	0.22	1.00000000	mice
Ten1	-0.01	1.00000000	mice
Ryk	-0.07	1.00000000	mice
Foxo1	-0.03	1.00000000	mice
U2af2	-0.04	1.00000000	mice
Patz1	-0.04	1.00000000	mice
Zdhhc14	-0.18	1.00000000	mice
Creg1	-0.10	1.00000000	mice
Eml6	0.20	1.00000000	mice
Snx4	-0.15	1.00000000	mice
Chst15	-0.10	1.00000000	mice
Snx2	0.35	1.00000000	mice
Slc48a1	-0.19	1.00000000	mice
Trappc1	-0.10	1.00000000	mice
Psmb3	0.18	1.00000000	mice
Cox7a2	-0.15	1.00000000	mice
Cse1l	-0.03	1.00000000	mice
Spg7	0.16	1.00000000	mice
Coro2b	-0.15	1.00000000	mice
Nckap1	-0.22	1.00000000	mice
Enc1	0.18	1.00000000	mice
Sdha	-0.12	1.00000000	mice
Ctdspl2	-0.03	1.00000000	mice

Rc3h1	-0.14	1.00000000	mice
Ewsr1	-0.06	1.00000000	mice
Sugt1	-0.01	1.00000000	mice
Prss12	-0.08	1.00000000	mice
Lmna	-0.19	1.00000000	mice
Col4a3bp	-0.14	1.00000000	mice
Uaca	-0.14	1.00000000	mice
Zmym4	0.21	1.00000000	mice
Rab43	-0.05	1.00000000	mice
Nxf1	-0.12	1.00000000	mice
Vdac1	-0.22	1.00000000	mice
Dtymk	0.00	1.00000000	mice
Eif4h	-0.12	1.00000000	mice
Dlst	-0.11	1.00000000	mice
Gabpb2	-0.08	1.00000000	mice
Slc10a7	0.20	1.00000000	mice
Ppic	-0.09	1.00000000	mice
Ccnk	-0.07	1.00000000	mice
Ss18	-0.04	1.00000000	mice
Dennd4c	0.11	1.00000000	mice
Rnf138	-0.14	1.00000000	mice
Pdia4	0.22	1.00000000	mice
Pawr	-0.25	1.00000000	mice
Ttc27	-0.10	1.00000000	mice
Ammecr1	0.18	1.00000000	mice
Tmem126a	-0.11	1.00000000	mice
Rufy1	0.17	1.00000000	mice
Cacybp	0.02	1.00000000	mice
Uqcrfs1	-0.01	1.00000000	mice
Pura	-0.18	1.00000000	mice
Atp2a3	-0.09	1.00000000	mice
Lurap1l	0.21	1.00000000	mice
Cdk11b	-0.12	1.00000000	mice
Cdkn2d	-0.06	1.00000000	mice
Il1r1	0.12	1.00000000	mice
Kif13b	-0.13	1.00000000	mice
Gpx7	-0.03	1.00000000	mice
Apold1	-0.01	1.00000000	mice
Srsf2	-0.08	1.00000000	mice
Tgs1	-0.02	1.00000000	mice
2610020C07Rik	-0.08	1.00000000	mice
Kat6b	-0.03	1.00000000	mice
Tomm5	-0.06	1.00000000	mice
Hnrnpdl	0.20	1.00000000	mice
Lrrc8b	-0.14	1.00000000	mice
Arsj	0.15	1.00000000	mice

Ecpas	0.17	1.00000000	mice
Tor1aip1	0.22	1.00000000	mice
Laptm4b	0.13	1.00000000	mice
Agpat1	-0.04	1.00000000	mice
Prag1	-0.01	1.00000000	mice
Tgif2	-0.10	1.00000000	mice
Tnfrsf12a	-0.01	1.00000000	mice
4732471J01Rik	-0.07	1.00000000	mice
Cdkn2aip	-0.18	1.00000000	mice
Prelid1	-0.12	1.00000000	mice
Cops4	-0.17	1.00000000	mice
Ccnjl	-0.08	1.00000000	mice
Fbxl7	-0.16	1.00000000	mice
Asap1	-0.11	1.00000000	mice
Syngr2	-0.11	1.00000000	mice
Plcb1	0.11	1.00000000	mice
Dstn	-0.04	1.00000000	mice
Ptges3	-0.13	1.00000000	mice
Pdlim2	-0.02	1.00000000	mice
Glis3	-0.15	1.00000000	mice
Nrbp1	0.22	1.00000000	mice
Hspe1	0.08	1.00000000	mice
Tcf4	0.17	1.00000000	mice
Ubxn1	-0.10	1.00000000	mice
Cyp4f13	-0.05	1.00000000	mice
Cplane1	-0.13	1.00000000	mice
Dpp9	-0.07	1.00000000	mice
Acvr2a	0.19	1.00000000	mice
Carmil1	0.20	1.00000000	mice
Trim3	-0.06	1.00000000	mice
Slc25a5	-0.05	1.00000000	mice
Afg3l2	-0.11	1.00000000	mice
Edil3	-0.18	1.00000000	mice
Itsn1	0.17	1.00000000	mice
Blvra	0.23	1.00000000	mice
Tmem9b	-0.11	1.00000000	mice
Dab2ip	-0.03	1.00000000	mice
Prpf6	0.14	1.00000000	mice
Taf8	0.01	1.00000000	mice
Cyp2d22	-0.12	1.00000000	mice
Eif6	-0.02	1.00000000	mice
Tmcc3	-0.15	1.00000000	mice
Epha4	0.19	1.00000000	mice
Fancc	-0.06	1.00000000	mice
Sema4c	-0.09	1.00000000	mice
Lonrf3	-0.12	1.00000000	mice

Pim1	0.23	1.00000000	mice
9930021J03Rik	-0.07	1.00000000	mice
Trappc2l	0.06	1.00000000	mice
Scamp2	-0.09	1.00000000	mice
Snapc5	0.05	1.00000000	mice
Ythdf2	-0.10	1.00000000	mice
Ripor2	-0.07	1.00000000	mice
Atad2	-0.11	1.00000000	mice
Fbxo38	0.04	1.00000000	mice
Serpinb6a	-0.12	1.00000000	mice
Fbxl4	0.08	1.00000000	mice
Dchs1	-0.08	1.00000000	mice
Tmem127	0.15	1.00000000	mice
Tspan8	-0.03	1.00000000	mice
Ormdl2	-0.01	1.00000000	mice
Ergic2	-0.01	1.00000000	mice
Rdm1	-0.10	1.00000000	mice
Sdcbp	-0.18	1.00000000	mice
Cpd	-0.10	1.00000000	mice
Sfpq	-0.07	1.00000000	mice
1600020E01Rik	-0.10	1.00000000	mice
Smad6	-0.08	1.00000000	mice
Cdk16	-0.06	1.00000000	mice
Vasn	-0.04	1.00000000	mice
Ilf2	-0.05	1.00000000	mice
Tes	-0.05	1.00000000	mice
Xbp1	-0.11	1.00000000	mice
Add3	-0.22	1.00000000	mice
Luc7l	0.05	1.00000000	mice
Dph3	-0.15	1.00000000	mice
Arap2	0.22	1.00000000	mice
Disc1	-0.09	1.00000000	mice
Smim26	-0.04	1.00000000	mice
Prdm1	0.31	1.00000000	mice
Snx12	-0.13	1.00000000	mice
Edrf1	0.11	1.00000000	mice
Zdhhc9	-0.05	1.00000000	mice
Txlng	-0.18	1.00000000	mice
Fgd5	-0.07	1.00000000	mice
Lman2	-0.02	1.00000000	mice
Amn1	0.15	1.00000000	mice
Pde3b	-0.15	1.00000000	mice
Cox4i1	-0.05	1.00000000	mice
Zyx	-0.02	1.00000000	mice
Nxt1	-0.09	1.00000000	mice
Zcchc24	-0.12	1.00000000	mice

Gtf2h2	-0.11	1.00000000	mice
Capzb	-0.04	1.00000000	mice
Nectin3	-0.12	1.00000000	mice
Fam168b	-0.11	1.00000000	mice
Siae	-0.08	1.00000000	mice
Zfp622	-0.02	1.00000000	mice
Cx3cl1	-0.05	1.00000000	mice
Cep95	-0.14	1.00000000	mice
Sms	-0.20	1.00000000	mice
Cab39l	0.19	1.00000000	mice
Peli1	-0.15	1.00000000	mice
Sar1b	-0.02	1.00000000	mice
Riok2	-0.06	1.00000000	mice
Tbck	0.09	1.00000000	mice
Cenpc1	-0.20	1.00000000	mice
Desi1	-0.12	1.00000000	mice
Btbd2	-0.09	1.00000000	mice
Ormdl3	-0.03	1.00000000	mice
Prrg2	-0.05	1.00000000	mice
Uba1	-0.06	1.00000000	mice
Rnf187	-0.22	1.00000000	mice
Rabep1	-0.09	1.00000000	mice
Gab1	0.62	1.00000000	mice
Slc39a7	0.00	1.00000000	mice
Hyal2	-0.12	1.00000000	mice
Zfp871	-0.08	1.00000000	mice
Snx18	-0.12	1.00000000	mice
Rbm25	-0.09	1.00000000	mice
Immp1l	-0.10	1.00000000	mice
Gcn1	0.02	1.00000000	mice
Pycard	0.06	1.00000000	mice
Plxnd1	-0.14	1.00000000	mice
Casc4	0.08	1.00000000	mice
Oxr1	0.18	1.00000000	mice
Amotl2	0.26	1.00000000	mice
Cwf19l2	0.20	1.00000000	mice
Polr3gl	-0.05	1.00000000	mice
Cep63	-0.06	1.00000000	mice
2610507B11Rik	-0.04	1.00000000	mice
Ints6l	-0.08	1.00000000	mice
Lama4	0.27	1.00000000	mice
Ahdc1	-0.09	1.00000000	mice
Tnpo2	-0.07	1.00000000	mice
Rhbdf2	-0.16	1.00000000	mice
Srsf10	-0.20	1.00000000	mice
Gpatch1	0.16	1.00000000	mice

Wdfy3	-0.13	1.00000000	mice
Nipbl	-0.10	1.00000000	mice
Ikbkb	0.02	1.00000000	mice
Ppp3ca	0.22	1.00000000	mice
Srsf1	0.15	1.00000000	mice
Plcl1	0.00	1.00000000	mice
Arpc1a	0.12	1.00000000	mice
Senp1	-0.04	1.00000000	mice
Jmy	-0.13	1.00000000	mice
Pank3	0.18	1.00000000	mice
Snx10	0.10	1.00000000	mice
Wdr83os	-0.07	1.00000000	mice
Cep350	0.30	1.00000000	mice
Rxylt1	0.14	1.00000000	mice
Rab1b	0.17	1.00000000	mice
Naa60	0.09	1.00000000	mice
Supt3	-0.04	1.00000000	mice
Pitrm1	-0.05	1.00000000	mice
Prdx6	0.00	1.00000000	mice
Cox5a	-0.11	1.00000000	mice
Hnrnpr	-0.04	1.00000000	mice
4632427E13Rik	-0.13	1.00000000	mice
Zdhhc1	-0.05	1.00000000	mice
Pcdh12	0.08	1.00000000	mice
Epc1	-0.04	1.00000000	mice
Unc45a	-0.08	1.00000000	mice
Avil	-0.09	1.00000000	mice
Plekha1	0.23	1.00000000	mice
Atrx	-0.12	1.00000000	mice
Adgrg6	-0.18	1.00000000	mice
Fam89b	-0.11	1.00000000	mice
Ppp4r2	-0.13	1.00000000	mice
Ctsa	-0.04	1.00000000	mice
Rab33b	-0.01	1.00000000	mice
Top2b	-0.14	1.00000000	mice
Zfp397	-0.09	1.00000000	mice
Rbfox2	0.14	1.00000000	mice
Fendrr	-0.16	1.00000000	mice
Pcnx3	-0.14	1.00000000	mice
Aqr	0.07	1.00000000	mice
Npc1	0.03	1.00000000	mice
Odr4	-0.15	1.00000000	mice
Sucla2	0.02	1.00000000	mice
Rpp14	-0.06	1.00000000	mice
Slc9a1	0.00	1.00000000	mice
Ssh1	-0.14	1.00000000	mice

Fam104a	-0.06	1.00000000	mice
Bbip1	-0.08	1.00000000	mice
Ptpmt1	-0.07	1.00000000	mice
Hadha	-0.06	1.00000000	mice
Slc2a13	-0.14	1.00000000	mice
Dhx16	-0.17	1.00000000	mice
Sin3a	-0.08	1.00000000	mice
Glod4	-0.06	1.00000000	mice
Pdlim7	0.03	1.00000000	mice
Trappc10	-0.04	1.00000000	mice
Rfc2	0.18	1.00000000	mice
Kcmf1	-0.12	1.00000000	mice
Cic	-0.06	1.00000000	mice
Vsig10	0.11	1.00000000	mice
Napa	0.08	1.00000000	mice
Dnajc11	0.00	1.00000000	mice
Sik2	-0.13	1.00000000	mice
Nup210	-0.04	1.00000000	mice
Golt1b	-0.02	1.00000000	mice
Ebpl	0.02	1.00000000	mice
Chchd7	0.01	1.00000000	mice
Bcl7a	-0.13	1.00000000	mice
Polr2e	-0.03	1.00000000	mice
Phf1	-0.08	1.00000000	mice
Dip2c	0.22	1.00000000	mice
Ntn1	0.16	1.00000000	mice
Arl5b	-0.13	1.00000000	mice
Gar1	-0.05	1.00000000	mice
Gins4	0.16	1.00000000	mice
Otud5	-0.13	1.00000000	mice
Dvl1	-0.10	1.00000000	mice
Ube2d2a	-0.09	1.00000000	mice
Sap18	-0.08	1.00000000	mice
Slc25a30	-0.09	1.00000000	mice
Wbp2	-0.08	1.00000000	mice
Alkbh8	0.13	1.00000000	mice
Slc39a6	-0.15	1.00000000	mice
Abi3bp	-0.18	1.00000000	mice
Alg9	0.04	1.00000000	mice
Cyp20a1	-0.05	1.00000000	mice
Acbd6	0.20	1.00000000	mice
Dynll2	-0.01	1.00000000	mice
Arntl	-0.03	1.00000000	mice
Akt3	0.24	1.00000000	mice
Dhx29	0.11	1.00000000	mice
Fgd6	0.12	1.00000000	mice

Ubr1	-0.17	1.00000000	mice
Clp1	-0.10	1.00000000	mice
Kctd9	0.08	1.00000000	mice
Snhg3	-0.05	1.00000000	mice
1500011B03Rik	-0.10	1.00000000	mice
Gdpd3	-0.11	1.00000000	mice
Kdm1a	0.18	1.00000000	mice
Zc3hav1l	0.01	1.00000000	mice
Bcap31	-0.02	1.00000000	mice
0610010K14Rik	-0.06	1.00000000	mice
Zzef1	-0.14	1.00000000	mice
Efna1	0.25	1.00000000	mice
Aig1	0.29	1.00000000	mice
Zfp36l2	-0.10	1.00000000	mice
Sltm	-0.17	1.00000000	mice
Dnajb11	-0.02	1.00000000	mice
Jagn1	-0.08	1.00000000	mice
Acsl5	-0.12	1.00000000	mice
Sec22a	0.13	1.00000000	mice
Copz1	-0.05	1.00000000	mice
Slc30a9	-0.13	1.00000000	mice
Rnasek	-0.03	1.00000000	mice
Naf1	-0.16	1.00000000	mice
Rab7	0.02	1.00000000	mice
Crebl2	-0.08	1.00000000	mice
Gata6	-0.11	1.00000000	mice
Fgl2	0.29	1.00000000	mice
Chd9	-0.15	1.00000000	mice
Phc3	0.13	1.00000000	mice
Plekha3	-0.03	1.00000000	mice
Cycs	0.02	1.00000000	mice
Thoc6	-0.08	1.00000000	mice
Hsd17b10	-0.06	1.00000000	mice
Kdm3b	-0.05	1.00000000	mice
Gnl3	0.01	1.00000000	mice
Rnf144a	0.24	1.00000000	mice
Zfp110	-0.08	1.00000000	mice
Rbx1	-0.10	1.00000000	mice
Snrnp27	0.06	1.00000000	mice
Wdr45b	0.03	1.00000000	mice
Fmo5	-0.08	1.00000000	mice
Fam193a	-0.15	1.00000000	mice
4930486L24Rik	-0.09	1.00000000	mice
Gstp1	-0.10	1.00000000	mice
Ubl7	-0.04	1.00000000	mice
Lamp2	-0.14	1.00000000	mice

Denr	0.28	1.00000000	mice
Csnk1a1	-0.10	1.00000000	mice
Pigq	0.01	1.00000000	mice
Epc2	-0.17	1.00000000	mice
Stk10	-0.13	1.00000000	mice
Snrpd1	0.19	1.00000000	mice
Aip	-0.04	1.00000000	mice
Trim24	-0.19	1.00000000	mice
Zmat5	-0.07	1.00000000	mice
Pik3r1	0.21	1.00000000	mice
Lima1	0.05	1.00000000	mice
Plxdc2	-0.11	1.00000000	mice
Vps54	0.25	1.00000000	mice
Ralgps2	0.00	1.00000000	mice
Tmem106a	0.23	1.00000000	mice
Elob	0.24	1.00000000	mice
Plek	0.32	1.00000000	mice
Nr2f6	-0.16	1.00000000	mice
Cpsf2	0.21	1.00000000	mice
Elf1	-0.16	1.00000000	mice
Nasp	0.04	1.00000000	mice
Zcchc9	-0.14	1.00000000	mice
Sh3kbp1	-0.03	1.00000000	mice
Smap2	-0.07	1.00000000	mice
Colgalt1	-0.05	1.00000000	mice
E130102H24Rik	0.08	1.00000000	mice
Zfp704	-0.12	1.00000000	mice
Plin2	0.26	1.00000000	mice
Utp4	-0.04	1.00000000	mice
Pet100	0.08	1.00000000	mice
Ddx39b	-0.01	1.00000000	mice
Tmem165	-0.05	1.00000000	mice
Clptm1	-0.06	1.00000000	mice
Car4	0.17	1.00000000	mice
Ptdss1	-0.06	1.00000000	mice
Nos3	-0.12	1.00000000	mice
Prodh	-0.05	1.00000000	mice
Fam114a2	-0.09	1.00000000	mice
Garnl3	0.15	1.00000000	mice
Smcr8	-0.06	1.00000000	mice
Cetn2	-0.12	1.00000000	mice
Zbtb18	0.15	1.00000000	mice
Zfp180	-0.14	1.00000000	mice
Khdrbs1	-0.15	1.00000000	mice
St7	-0.11	1.00000000	mice
Nkd1	-0.07	1.00000000	mice

Ino80b	0.03	1.00000000	mice
Taco1	0.18	1.00000000	mice
Psme4	-0.11	1.00000000	mice
Pard3	-0.12	1.00000000	mice
Sp4	-0.13	1.00000000	mice
Hipk1	-0.02	1.00000000	mice
Noc2l	-0.05	1.00000000	mice
Atp6ap1	0.00	1.00000000	mice
Oat	-0.09	1.00000000	mice
Gsk3a	-0.08	1.00000000	mice
Zc3h7a	-0.13	1.00000000	mice
Ing4	-0.13	1.00000000	mice
Rab6a	-0.04	1.00000000	mice
Psmc1	0.07	1.00000000	mice
Lsg1	0.00	1.00000000	mice
Ltn1	0.15	1.00000000	mice
Klf10	0.19	1.00000000	mice
Tcf7l1	-0.17	1.00000000	mice
Hras	-0.02	1.00000000	mice
Tdrd7	0.14	1.00000000	mice
Hmgcs1	0.19	1.00000000	mice
Tshz2	0.19	1.00000000	mice
Fap	0.21	1.00000000	mice
Slc31a1	-0.12	1.00000000	mice
Bcar3	-0.16	1.00000000	mice
Grk2	0.09	1.00000000	mice
Tfdp2	-0.11	1.00000000	mice
Itpr2	-0.12	1.00000000	mice
Rasa1	-0.11	1.00000000	mice
Acot7	0.17	1.00000000	mice
Pbrm1	-0.09	1.00000000	mice
Btrc	0.19	1.00000000	mice
Ap2m1	-0.17	1.00000000	mice
Vps45	0.15	1.00000000	mice
Slc38a10	0.21	1.00000000	mice
Timp3	-0.20	1.00000000	mice
Pecam1	0.08	1.00000000	mice
Atp13a2	0.09	1.00000000	mice
Golph3l	-0.01	1.00000000	mice
Tcf20	0.14	1.00000000	mice
Fh1	-0.08	1.00000000	mice
Ddhd1	-0.15	1.00000000	mice
Ralgapa2	-0.15	1.00000000	mice
Trim69	-0.09	1.00000000	mice
Acot13	-0.09	1.00000000	mice
Atf7ip	0.21	1.00000000	mice

Inpp1	0.26	1.00000000	mice
Vwf	-0.45	1.00000000	mice
Tusc3	-0.12	1.00000000	mice
Zfp646	0.08	1.00000000	mice
Wipf2	-0.07	1.00000000	mice
Apol8	-0.01	1.00000000	mice
Akr7a5	-0.06	1.00000000	mice
Becn1	0.03	1.00000000	mice
Ntmt1	-0.01	1.00000000	mice
AU020206	0.38	1.00000000	mice
Tex10	0.03	1.00000000	mice
Pitpnc1	0.16	1.00000000	mice
Serf2	-0.12	1.00000000	mice
Kdm5a	-0.09	1.00000000	mice
Rtp3	0.11	1.00000000	mice
Cript	0.07	1.00000000	mice
BC029722	-0.10	1.00000000	mice
Thoc7	0.07	1.00000000	mice
Dgkd	-0.08	1.00000000	mice
Bag5	-0.23	1.00000000	mice
Gspt1	-0.04	1.00000000	mice
Pdcl	0.20	1.00000000	mice
Rmnd5a	0.02	1.00000000	mice
Lepr	-0.17	1.00000000	mice
Phf6	0.07	1.00000000	mice
Wdr13	-0.06	1.00000000	mice
Cds2	-0.07	1.00000000	mice
Ebna1bp2	-0.07	1.00000000	mice
Aurkaip1	-0.07	1.00000000	mice
Cd82	0.07	1.00000000	mice
Gimap4	0.25	1.00000000	mice
Nfrkb	0.08	1.00000000	mice
1110038B12Rik	-0.03	1.00000000	mice
Clptm1l	0.06	1.00000000	mice
Gpn3	-0.05	1.00000000	mice
Nek9	-0.09	1.00000000	mice
Arfgef1	-0.06	1.00000000	mice
Rragc	0.02	1.00000000	mice
Prkrip1	0.04	1.00000000	mice
Sirpa	0.26	1.00000000	mice
Inf2	-0.02	1.00000000	mice
Anxa4	0.20	1.00000000	mice
Rbm27	-0.02	1.00000000	mice
Orc3	0.12	1.00000000	mice
Tbcc	-0.02	1.00000000	mice
Pfdn5	0.02	1.00000000	mice

Dnajc18	-0.11	1.00000000	mice
Cyb5r1	-0.05	1.00000000	mice
Sirt1	-0.08	1.00000000	mice
E130307A14Rik	0.10	1.00000000	mice
Serp1	-0.08	1.00000000	mice
Gtf2f1	0.13	1.00000000	mice
Ranbp1	0.10	1.00000000	mice
Nfkb2	-0.05	1.00000000	mice
Adam12	0.22	1.00000000	mice
Cct4	0.10	1.00000000	mice
Anp32b	-0.01	1.00000000	mice
Dnajc8	0.25	1.00000000	mice
Usp54	-0.06	1.00000000	mice
Psmd2	0.08	1.00000000	mice
Zfr	-0.03	1.00000000	mice
Idh3b	-0.07	1.00000000	mice
Rrp15	-0.04	1.00000000	mice
Park7	-0.05	1.00000000	mice
Bcorl1	-0.12	1.00000000	mice
Tmem88	-0.16	1.00000000	mice
Cabp1	-0.05	1.00000000	mice
Ralbp1	-0.11	1.00000000	mice
Plpp3	-0.05	1.00000000	mice
Ensa	-0.02	1.00000000	mice
G3bp1	-0.05	1.00000000	mice
Wdr5	-0.01	1.00000000	mice
Rce1	-0.09	1.00000000	mice
Dcaf8	-0.03	1.00000000	mice
Taf13	0.04	1.00000000	mice
Ero1l	-0.10	1.00000000	mice
Fem1b	-0.11	1.00000000	mice
Rcor1	-0.08	1.00000000	mice
Zpr1	-0.09	1.00000000	mice
Rasal2	-0.17	1.00000000	mice
Uba5	-0.01	1.00000000	mice
Uap1	-0.08	1.00000000	mice
Erg	-0.01	1.00000000	mice
Rsrp1	-0.08	1.00000000	mice
Retreg2	-0.06	1.00000000	mice
5330438D12Rik	-0.07	1.00000000	mice
Wac	-0.14	1.00000000	mice
Ltv1	0.09	1.00000000	mice
Csad	-0.12	1.00000000	mice
Utp20	-0.08	1.00000000	mice
Srprb	0.03	1.00000000	mice
Tmem205	-0.04	1.00000000	mice

Phf20l1	-0.14	1.00000000	mice
Arpc2	-0.06	1.00000000	mice
Gse1	-0.11	1.00000000	mice
Psme3	-0.06	1.00000000	mice
Tbpl1	-0.07	1.00000000	mice
Sptlc1	0.05	1.00000000	mice
Ppa2	-0.06	1.00000000	mice
Lsm2	-0.15	1.00000000	mice
Hnrnpul1	0.00	1.00000000	mice
Spcs3	0.15	1.00000000	mice
Chtop	0.06	1.00000000	mice
Tfe3	-0.03	1.00000000	mice
Eif4e	-0.12	1.00000000	mice
Lamp1	0.00	1.00000000	mice
Parva	-0.18	1.00000000	mice
Ntan1	0.03	1.00000000	mice
Diablo	-0.02	1.00000000	mice
Pik3c3	-0.05	1.00000000	mice
Rras2	0.01	1.00000000	mice
Esd	0.03	1.00000000	mice
Dhps	0.11	1.00000000	mice
Pgpep1	0.00	1.00000000	mice
Drap1	-0.04	1.00000000	mice
Psma5	0.18	1.00000000	mice
Zfp592	-0.03	1.00000000	mice
Fam173a	-0.02	1.00000000	mice
Ppp1r37	-0.06	1.00000000	mice
Lsm10	-0.01	1.00000000	mice
Ehmt1	-0.06	1.00000000	mice
Hfe	-0.16	1.00000000	mice
Adprh	0.01	1.00000000	mice
Utp6	-0.07	1.00000000	mice
Nid1	0.22	1.00000000	mice
Chd6	0.18	1.00000000	mice
Nemf	0.18	1.00000000	mice
Nop14	-0.01	1.00000000	mice
Actr10	0.09	1.00000000	mice
Dpysl2	-0.06	1.00000000	mice
Dync1li1	-0.06	1.00000000	mice
Inppl1	-0.11	1.00000000	mice
Elp4	0.08	1.00000000	mice
Cndp2	-0.01	1.00000000	mice
Cmc2	-0.13	1.00000000	mice
Hmgcl	0.06	1.00000000	mice
Rxrb	-0.02	1.00000000	mice
BC031181	-0.10	1.00000000	mice

Bcl2l11	-0.02	1.00000000	mice
Nedd8	-0.11	1.00000000	mice
Tasor2	0.15	1.00000000	mice
Dido1	-0.02	1.00000000	mice
Plod2	-0.09	1.00000000	mice
Smim14	0.03	1.00000000	mice
Scn3b	0.24	1.00000000	mice
Eif4ebp2	-0.04	1.00000000	mice
Serpinb1a	0.18	1.00000000	mice
Ints8	0.09	1.00000000	mice
Stk35	-0.01	1.00000000	mice
Gsk3b	-0.09	1.00000000	mice
Ccn2	-0.15	1.00000000	mice
Slc35d1	-0.13	1.00000000	mice
Stard7	0.19	1.00000000	mice
Tnip2	-0.09	1.00000000	mice
Smad2	-0.13	1.00000000	mice
Wrnip1	-0.01	1.00000000	mice
Kti12	-0.02	1.00000000	mice
Cd99l2	-0.04	1.00000000	mice
Tcof1	0.15	1.00000000	mice
Nedd4	-0.10	1.00000000	mice
Eps15	0.18	1.00000000	mice
Dnah8	-0.10	1.00000000	mice
Cmtm8	0.05	1.00000000	mice
Capn2	-0.16	1.00000000	mice
Vcl	-0.17	1.00000000	mice
Evi5	-0.07	1.00000000	mice
Nras	-0.04	1.00000000	mice
Lin7c	0.04	1.00000000	mice
Zfp703	0.11	1.00000000	mice
Chmp4b	0.19	1.00000000	mice
Lix1l	-0.06	1.00000000	mice
Gdap2	-0.03	1.00000000	mice
Rftn1	-0.07	1.00000000	mice
Psmg2	-0.17	1.00000000	mice
Ccdc68	0.18	1.00000000	mice
Enpp2	-0.07	1.00000000	mice
Pak2	-0.11	1.00000000	mice
Smap1	-0.13	1.00000000	mice
Hibadh	-0.05	1.00000000	mice
Ppat	-0.03	1.00000000	mice
Fkbp2	0.10	1.00000000	mice
Edem2	0.23	1.00000000	mice
Nt5c	0.02	1.00000000	mice
Pcgf3	-0.11	1.00000000	mice

Rrp36	-0.16	1.00000000	mice
Syvn1	0.11	1.00000000	mice
Coro1c	0.01	1.00000000	mice
Znrf2	0.19	1.00000000	mice
Zfp462	0.02	1.00000000	mice
Ube2j1	-0.13	1.00000000	mice
BC005624	-0.10	1.00000000	mice
Bbc3	-0.10	1.00000000	mice
Zbed3	-0.07	1.00000000	mice
Gamt	-0.04	1.00000000	mice
Acsl1	0.07	1.00000000	mice
Runx1t1	0.10	1.00000000	mice
Agtpbp1	-0.02	1.00000000	mice
Klhdc4	0.11	1.00000000	mice
Pltp	0.01	1.00000000	mice
Ipo5	-0.06	1.00000000	mice
Adam15	0.12	1.00000000	mice
Dlg5	0.17	1.00000000	mice
Cd34	0.28	1.00000000	mice
Elk4	-0.02	1.00000000	mice
Tecr	-0.03	1.00000000	mice
Ing1	0.07	1.00000000	mice
Rit1	-0.01	1.00000000	mice
Fam98a	0.04	1.00000000	mice
Thap2	-0.09	1.00000000	mice
Tmeff1	-0.13	1.00000000	mice
Cfap69	0.08	1.00000000	mice
Stat5a	-0.09	1.00000000	mice
Foxn2	-0.05	1.00000000	mice
Peli2	-0.01	1.00000000	mice
Etnk1	0.21	1.00000000	mice
Ncl	-0.09	1.00000000	mice
1810030O07Rik	-0.02	1.00000000	mice
Thap12	0.08	1.00000000	mice
Abhd5	0.15	1.00000000	mice
Ugcg	0.24	1.00000000	mice
Anxa5	0.09	1.00000000	mice
Agpat3	0.02	1.00000000	mice
Gnai1	-0.05	1.00000000	mice
C2cd2l	-0.07	1.00000000	mice
Cd14	0.12	1.00000000	mice
Smim3	-0.05	1.00000000	mice
Sdhd	-0.17	1.00000000	mice
Phf5a	0.07	1.00000000	mice
Diaph1	-0.07	1.00000000	mice
Gde1	-0.02	1.00000000	mice

Hagh	0.06	1.00000000	mice
Zeb2	0.22	1.00000000	mice
Acaa2	-0.06	1.00000000	mice
Sept11	0.03	1.00000000	mice
Wdr48	-0.03	1.00000000	mice
Sdc4	-0.12	1.00000000	mice
Epn2	-0.14	1.00000000	mice
Unc93b1	0.16	1.00000000	mice
Jade1	-0.15	1.00000000	mice
Tceal8	-0.04	1.00000000	mice
Gata3	0.05	1.00000000	mice
Spop	-0.02	1.00000000	mice
Ep400	-0.04	1.00000000	mice
Lpcat1	0.00	1.00000000	mice
Spcs2	0.05	1.00000000	mice
Plscr2	0.25	1.00000000	mice
Appbp2	-0.07	1.00000000	mice
Rasa3	-0.19	1.00000000	mice
Rtraf	0.09	1.00000000	mice
Lpin2	-0.11	1.00000000	mice
Tbc1d17	0.06	1.00000000	mice
Igf1r	-0.04	1.00000000	mice
Tnrc6b	0.14	1.00000000	mice
Anapc1	0.13	1.00000000	mice
Aco1	0.01	1.00000000	mice
Csnk2a2	-0.02	1.00000000	mice
Dync1h1	0.21	1.00000000	mice
Hyou1	0.13	1.00000000	mice
Tram1	0.05	1.00000000	mice
Rab11fip2	0.07	1.00000000	mice
Ckb	0.12	1.00000000	mice
Cdc42	-0.02	1.00000000	mice
Sf3b6	0.13	1.00000000	mice
Vamp3	-0.03	1.00000000	mice
Akr1b3	-0.01	1.00000000	mice
Tcea1	0.16	1.00000000	mice
Gnb4	0.15	1.00000000	mice
Nosip	-0.02	1.00000000	mice
Synrg	0.25	1.00000000	mice
Tspan12	-0.08	1.00000000	mice
Spg11	-0.01	1.00000000	mice
Pde5a	0.03	1.00000000	mice
Got2	-0.09	1.00000000	mice
Snrnp70	-0.10	1.00000000	mice
L3mbtl3	-0.20	1.00000000	mice
Tmem229b	0.11	1.00000000	mice

Tspan2	-0.12	1.00000000	mice
Pmpca	-0.06	1.00000000	mice
Phactr1	0.24	1.00000000	mice
Zc2hc1a	0.11	1.00000000	mice
Dcaf1	-0.13	1.00000000	mice
Anapc4	0.14	1.00000000	mice
Cpne1	0.07	1.00000000	mice
Sgk3	0.16	1.00000000	mice
Eif1b	-0.03	1.00000000	mice
Bcam	-0.02	1.00000000	mice
Anxa11	-0.04	1.00000000	mice
Clta	0.02	1.00000000	mice
Sec24c	0.10	1.00000000	mice
Klhdc10	0.12	1.00000000	mice
Pced1b	0.12	1.00000000	mice
Ccni	0.00	1.00000000	mice
Tsen15	-0.09	1.00000000	mice
Vim	0.12	1.00000000	mice
Zfp598	-0.13	1.00000000	mice
Ifngr1	-0.15	1.00000000	mice
Sys1	-0.03	1.00000000	mice
Ptgfrn	-0.06	1.00000000	mice
Crls1	0.03	1.00000000	mice
Tvp23b	-0.03	1.00000000	mice
Pkd2	-0.21	1.00000000	mice
Arhgap10	-0.11	1.00000000	mice
Esyt1	-0.10	1.00000000	mice
Dusp7	0.18	1.00000000	mice
Rbm3	0.03	1.00000000	mice
Ssbp3	0.03	1.00000000	mice
Snrpb2	0.12	1.00000000	mice
Atf1	-0.07	1.00000000	mice
Grik2	-0.10	1.00000000	mice
Faf1	0.06	1.00000000	mice
Trir	0.01	1.00000000	mice
Atg16l1	-0.05	1.00000000	mice
Psmd14	0.19	1.00000000	mice
Higd1a	-0.07	1.00000000	mice
Pbxip1	-0.05	1.00000000	mice
Gfod2	-0.21	1.00000000	mice
Luc7l3	-0.01	1.00000000	mice
Il4ra	-0.09	1.00000000	mice
Nckap5l	-0.04	1.00000000	mice
Fyttd1	-0.07	1.00000000	mice
4833420G17Rik	0.04	1.00000000	mice
Trim28	-0.10	1.00000000	mice

Ankrd40	-0.08	1.00000000	mice
Cxcl16	0.24	1.00000000	mice
Rpain	-0.11	1.00000000	mice
Ccdc124	0.02	1.00000000	mice
Reps1	-0.08	1.00000000	mice
Vdac3	-0.07	1.00000000	mice
Zfand2b	-0.06	1.00000000	mice
Tmem222	-0.01	1.00000000	mice
Chpt1	-0.10	1.00000000	mice
Nudcd2	-0.14	1.00000000	mice
Trim44	0.12	1.00000000	mice
Pik3r3	-0.06	1.00000000	mice
Slc25a4	0.13	1.00000000	mice
Thoc5	-0.03	1.00000000	mice
Sec31a	-0.14	1.00000000	mice
Ell2	0.20	1.00000000	mice
Lrrc59	-0.04	1.00000000	mice
Bcl9l	0.16	1.00000000	mice
Supt6	-0.09	1.00000000	mice
Upf2	0.20	1.00000000	mice
Adnp	-0.03	1.00000000	mice
Ctdnep1	-0.20	1.00000000	mice
Fbxl6	-0.05	1.00000000	mice
Atp2a2	0.16	1.00000000	mice
Zmat2	0.19	1.00000000	mice
Uqcrq	0.23	1.00000000	mice
Cdk4	-0.01	1.00000000	mice
Pdha1	-0.10	1.00000000	mice
Paip1	-0.01	1.00000000	mice
Arhgef18	-0.07	1.00000000	mice
Ehd1	0.16	1.00000000	mice
Gadd45a	-0.11	1.00000000	mice
Sparc	-0.05	1.00000000	mice
Klhl15	-0.15	1.00000000	mice
Bloc1s6	0.11	1.00000000	mice
Nostrin	-0.03	1.00000000	mice
Zfp68	-0.03	1.00000000	mice
Actl6a	-0.05	1.00000000	mice
Ppp4c	-0.03	1.00000000	mice
St3gal6	0.17	1.00000000	mice
Abce1	0.13	1.00000000	mice
Abcf3	0.09	1.00000000	mice
Olfr56	0.19	1.00000000	mice
Acadm	0.04	1.00000000	mice
Tctex1d2	0.18	1.00000000	mice
Ankrd11	-0.08	1.00000000	mice

Slc22a23	0.17	1.00000000	mice
Etfb	-0.08	1.00000000	mice
Dnajc21	-0.01	1.00000000	mice
Trmt1	-0.02	1.00000000	mice
Camkmt	0.17	1.00000000	mice
Cntln	-0.05	1.00000000	mice
Gpr180	-0.06	1.00000000	mice
Clip2	-0.10	1.00000000	mice
Klk8	0.05	1.00000000	mice
Atg10	-0.11	1.00000000	mice
Ndufs7	0.00	1.00000000	mice
Cers2	-0.08	1.00000000	mice
Atp8b1	0.22	1.00000000	mice
Camsap2	-0.08	1.00000000	mice
Hdac1	-0.07	1.00000000	mice
Fkbp1a	0.21	1.00000000	mice
Srsf4	-0.05	1.00000000	mice
Ccdc25	0.17	1.00000000	mice
Gapvd1	-0.05	1.00000000	mice
Unkl	-0.05	1.00000000	mice
Layn	-0.02	1.00000000	mice
Rgs3	0.17	1.00000000	mice
Ubxn8	-0.05	1.00000000	mice
Gpat4	-0.10	1.00000000	mice
Vsir	0.12	1.00000000	mice
Plekhb2	-0.02	1.00000000	mice
4930444A19Rik	0.08	1.00000000	mice
Dohh	-0.02	1.00000000	mice
Nr2c1	0.05	1.00000000	mice
Fus	-0.04	1.00000000	mice
Hsp90ab1	-0.07	1.00000000	mice
Gprc5a	-0.22	1.00000000	mice
Eif3j1	-0.07	1.00000000	mice
Sp1	-0.10	1.00000000	mice
Prrg1	-0.22	1.00000000	mice
Ptprc	0.25	1.00000000	mice
Hnrnph2	0.12	1.00000000	mice
Itpkb	0.16	1.00000000	mice
Slbp	0.02	1.00000000	mice
Aamdc	-0.03	1.00000000	mice
Rap1gds1	-0.03	1.00000000	mice
Rnf146	-0.05	1.00000000	mice
Slc1a1	-0.04	1.00000000	mice
Cnppd1	-0.10	1.00000000	mice
Supt5	0.17	1.00000000	mice
Slc33a1	0.16	1.00000000	mice

Sox17	-0.01	1.00000000	mice
Bptf	-0.09	1.00000000	mice
Prkar2a	-0.02	1.00000000	mice
Pdia5	-0.02	1.00000000	mice
Brcc3	-0.13	1.00000000	mice
Fbh1	0.02	1.00000000	mice
Rbm4	-0.01	1.00000000	mice
Rnf122	-0.06	1.00000000	mice
Scube1	-0.02	1.00000000	mice
Csnk1d	-0.13	1.00000000	mice
Psmd11	0.02	1.00000000	mice
Sap130	-0.04	1.00000000	mice
Abi3	-0.03	1.00000000	mice
Ddx54	-0.01	1.00000000	mice
Dym	-0.17	1.00000000	mice
Fam133b	0.13	1.00000000	mice
Rock1	-0.11	1.00000000	mice
Rnf141	-0.12	1.00000000	mice
Zmym2	-0.04	1.00000000	mice
Phf21a	0.18	1.00000000	mice
Arf4	-0.10	1.00000000	mice
Nufip2	-0.04	1.00000000	mice
Stim1	-0.09	1.00000000	mice
Hexb	0.15	1.00000000	mice
Cdr2l	-0.06	1.00000000	mice
Upf1	-0.06	1.00000000	mice
Lnx2	0.15	1.00000000	mice
Dnttip1	0.02	1.00000000	mice
Eif2b4	0.16	1.00000000	mice
Tuba1a	-0.21	1.00000000	mice
Itpr1	-0.11	1.00000000	mice
Arhgef12	0.08	1.00000000	mice
Cytip	-0.06	1.00000000	mice
Rad23a	-0.03	1.00000000	mice
Tk2	0.09	1.00000000	mice
Zfp292	0.16	1.00000000	mice
Eif4b	0.09	1.00000000	mice
Zfp330	0.03	1.00000000	mice
Ripor1	-0.08	1.00000000	mice
Pld1	0.16	1.00000000	mice
Rnf121	0.15	1.00000000	mice
Ndufab1	0.04	1.00000000	mice
Scaf4	-0.02	1.00000000	mice
Cox10	0.14	1.00000000	mice
Pgk1	-0.03	1.00000000	mice
Stim2	-0.11	1.00000000	mice

Dcaf13	0.13	1.00000000	mice
Eprs	0.11	1.00000000	mice
Arap1	0.01	1.00000000	mice
Brwd1	-0.13	1.00000000	mice
Ppm1m	-0.07	1.00000000	mice
Acaa1a	0.05	1.00000000	mice
Unk	0.10	1.00000000	mice
St6galnac4	0.22	1.00000000	mice
Ube2z	-0.02	1.00000000	mice
Cdk5rap3	0.08	1.00000000	mice
Ptpn21	0.06	1.00000000	mice
Nsmce3	-0.11	1.00000000	mice
Slc4a2	0.08	1.00000000	mice
Lmbrd1	-0.06	1.00000000	mice
Fkrp	-0.07	1.00000000	mice
Dpm3	-0.04	1.00000000	mice
Dpy19l4	0.12	1.00000000	mice
Pfdn2	-0.03	1.00000000	mice
Tmem87b	-0.04	1.00000000	mice
Tsen34	-0.03	1.00000000	mice
Emc3	-0.01	1.00000000	mice
2300009A05Rik	-0.09	1.00000000	mice
Hax1	0.04	1.00000000	mice
Pwwp3a	-0.03	1.00000000	mice
Pnpla2	-0.07	1.00000000	mice
Tor2a	0.00	1.00000000	mice
Zfp212	-0.01	1.00000000	mice
Tpp2	-0.15	1.00000000	mice
Usf1	0.15	1.00000000	mice
Tpi1	-0.04	1.00000000	mice
Stag1	-0.04	1.00000000	mice
Tigd2	0.17	1.00000000	mice
Pgs1	-0.01	1.00000000	mice
Dnm3	0.10	1.00000000	mice
Cblb	-0.15	1.00000000	mice
Ankle2	0.08	1.00000000	mice
Gsap	0.10	1.00000000	mice
Foxn3	-0.10	1.00000000	mice
Prkaca	-0.07	1.00000000	mice
Axin1	-0.10	1.00000000	mice
Hmcn1	0.26	1.00000000	mice
Psen1	-0.06	1.00000000	mice
Rfx2	-0.03	1.00000000	mice
A4galt	-0.05	1.00000000	mice
Pak4	-0.12	1.00000000	mice
Tmem63b	-0.13	1.00000000	mice

Fundc1	0.02	1.00000000	mice
Llgl1	0.07	1.00000000	mice
Sfi1	0.18	1.00000000	mice
Reps2	0.10	1.00000000	mice
Stom	-0.07	1.00000000	mice
Ncapd3	-0.06	1.00000000	mice
Nhsl2	-0.04	1.00000000	mice
Cops7a	-0.07	1.00000000	mice
Dipk2b	0.06	1.00000000	mice
Stard3nl	-0.02	1.00000000	mice
Tex2	-0.03	1.00000000	mice
Atp5f1	0.03	1.00000000	mice
Gdi1	0.10	1.00000000	mice
Ptk2b	-0.08	1.00000000	mice
Exosc8	0.10	1.00000000	mice
Plscr4	-0.07	1.00000000	mice
Stau1	-0.14	1.00000000	mice
Rabgap1l	-0.02	1.00000000	mice
Anapc10	0.23	1.00000000	mice
Akap10	0.19	1.00000000	mice
Grasp	-0.12	1.00000000	mice
Fnip2	-0.03	1.00000000	mice
Ppp2r5c	-0.11	1.00000000	mice
Heatr5a	-0.15	1.00000000	mice
AW554918	0.05	1.00000000	mice
Cdip1	0.06	1.00000000	mice
Sec23ip	-0.08	1.00000000	mice
Etv3	0.01	1.00000000	mice
Gprasp1	-0.14	1.00000000	mice
Ppp2r1b	-0.01	1.00000000	mice
Rnf220	0.30	1.00000000	mice
Hmgn2	0.06	1.00000000	mice
Slc50a1	-0.12	1.00000000	mice
Fam214a	-0.10	1.00000000	mice
Srp19	-0.02	1.00000000	mice
Sept8	0.04	1.00000000	mice
Ppp1r12a	-0.10	1.00000000	mice
Hspa1b	0.65	1.00000000	mice
Stx16	0.07	1.00000000	mice
Smc4	0.20	1.00000000	mice
Bola3	-0.06	1.00000000	mice
Fuca1	-0.01	1.00000000	mice
Unc13b	-0.10	1.00000000	mice
Kdm4a	0.10	1.00000000	mice
Atrn	-0.19	1.00000000	mice
Thap3	0.00	1.00000000	mice

Ddah1	0.40	1.00000000	mice
Cnnm2	-0.09	1.00000000	mice
Pex19	-0.14	1.00000000	mice
Asna1	0.05	1.00000000	mice
Cnnm3	0.03	1.00000000	mice
Scaf8	-0.07	1.00000000	mice
Id2	-0.03	1.00000000	mice
Trap1	-0.12	1.00000000	mice
Bet1l	-0.04	1.00000000	mice
Tox4	0.03	1.00000000	mice
Borcs6	-0.07	1.00000000	mice
Eif5b	0.17	1.00000000	mice
Abhd17a	-0.07	1.00000000	mice
Dnajb12	0.03	1.00000000	mice
Nme2	0.22	1.00000000	mice
Ccnc	-0.01	1.00000000	mice
Spryd7	0.18	1.00000000	mice
Lactb	0.00	1.00000000	mice
Ptprj	-0.13	1.00000000	mice
Rbm7	-0.03	1.00000000	mice
Arid4a	-0.14	1.00000000	mice
Stk11	-0.06	1.00000000	mice
Casp8ap2	0.18	1.00000000	mice
Il10rb	-0.06	1.00000000	mice
Egln1	0.00	1.00000000	mice
Zfp644	-0.07	1.00000000	mice
Cxcl12	0.30	1.00000000	mice
Dtd1	-0.10	1.00000000	mice
Fam8a1	-0.03	1.00000000	mice
Smad5	-0.04	1.00000000	mice
Otud6b	-0.12	1.00000000	mice
Ttc37	0.10	1.00000000	mice
Nmt1	0.18	1.00000000	mice
Ccpg1	-0.09	1.00000000	mice
Ddx18	0.15	1.00000000	mice
Jazf1	0.14	1.00000000	mice
Dock10	-0.05	1.00000000	mice
Hoxb4	-0.03	1.00000000	mice
Dapk3	0.19	1.00000000	mice
Ubfd1	0.00	1.00000000	mice
Caap1	0.15	1.00000000	mice
Zfyve16	0.07	1.00000000	mice
Dedd2	-0.06	1.00000000	mice
Rchy1	-0.06	1.00000000	mice
Bmp1	-0.13	1.00000000	mice
Fbl	0.03	1.00000000	mice

Ebp	-0.08	1.00000000	mice
Ankrd12	-0.04	1.00000000	mice
Fam98b	0.04	1.00000000	mice
Ero1lb	0.04	1.00000000	mice
Tpk1	0.15	1.00000000	mice
Plscr3	0.15	1.00000000	mice
Hoxa5	0.12	1.00000000	mice
Hectd1	-0.11	1.00000000	mice
Kpna6	0.11	1.00000000	mice
Scarb2	-0.07	1.00000000	mice
Poglut3	-0.06	1.00000000	mice
Nipa2	-0.07	1.00000000	mice
Bag1	0.15	1.00000000	mice
Neurl3	-0.08	1.00000000	mice
Ptprk	-0.07	1.00000000	mice
Sf3b3	0.08	1.00000000	mice
Eif2a	-0.05	1.00000000	mice
Usf2	0.04	1.00000000	mice
2810004N23Rik	0.24	1.00000000	mice
Sema7a	0.02	1.00000000	mice
Txndc9	-0.03	1.00000000	mice
Aven	-0.13	1.00000000	mice
Bahd1	-0.03	1.00000000	mice
Extl3	0.11	1.00000000	mice
Srbd1	0.10	1.00000000	mice
Dclre1c	0.20	1.00000000	mice
Ezh2	-0.06	1.00000000	mice
Pex14	0.06	1.00000000	mice
Fgfr1op2	0.04	1.00000000	mice
Saraf	-0.12	1.00000000	mice
Zranb1	-0.10	1.00000000	mice
Plod1	-0.16	1.00000000	mice
Tspan9	-0.15	1.00000000	mice
Tcerg1	-0.07	1.00000000	mice
Rhoq	0.03	1.00000000	mice
Sept9	-0.01	1.00000000	mice
Sppl3	-0.09	1.00000000	mice
Nr2c2	-0.06	1.00000000	mice
Phkg2	0.02	1.00000000	mice
Eml1	-0.12	1.00000000	mice
Cep83	-0.07	1.00000000	mice
Xpo1	0.06	1.00000000	mice
Tulp4	0.01	1.00000000	mice
Cnot10	-0.11	1.00000000	mice
Zfp664	0.05	1.00000000	mice
Dph6	0.19	1.00000000	mice

Larp1	-0.01	1.00000000	mice
Aktip	0.06	1.00000000	mice
Cdk14	-0.03	1.00000000	mice
Zbtb21	-0.03	1.00000000	mice
Ap4s1	-0.02	1.00000000	mice
Tmem29	-0.06	1.00000000	mice
Rtn3	-0.04	1.00000000	mice
Dcp1a	0.02	1.00000000	mice
Rbl2	0.02	1.00000000	mice
Stard4	0.18	1.00000000	mice
Tmem87a	-0.06	1.00000000	mice
Fbxo8	0.12	1.00000000	mice
Cxcr4	0.23	1.00000000	mice
Klhl18	-0.11	1.00000000	mice
Pcmt1	-0.06	1.00000000	mice
Syf2	-0.05	1.00000000	mice
Clic1	0.20	1.00000000	mice
Get4	0.03	1.00000000	mice
Phlpp2	-0.04	1.00000000	mice
Usp37	-0.03	1.00000000	mice
Gipc1	0.01	1.00000000	mice
Pole4	0.12	1.00000000	mice
Rgs6	0.39	1.00000000	mice
Nup153	-0.03	1.00000000	mice
Tmem167	0.18	1.00000000	mice
Nudt21	-0.02	1.00000000	mice
Suclg1	-0.08	1.00000000	mice
Dip2b	-0.15	1.00000000	mice
Tmem159	-0.10	1.00000000	mice
Alkbh3	0.07	1.00000000	mice
Insr	0.10	1.00000000	mice
Rufy3	-0.07	1.00000000	mice
Agps	-0.07	1.00000000	mice
Fam43a	-0.02	1.00000000	mice
Irak2	0.05	1.00000000	mice
Isoc1	-0.07	1.00000000	mice
Dock1	0.16	1.00000000	mice
Pdap1	0.29	1.00000000	mice
Aldh3a2	-0.02	1.00000000	mice
Gatad1	-0.02	1.00000000	mice
Smim7	-0.04	1.00000000	mice
Otud7b	-0.12	1.00000000	mice
Tln1	0.24	1.00000000	mice
Atp6v1d	0.12	1.00000000	mice
Crtc2	-0.15	1.00000000	mice
Fam32a	-0.10	1.00000000	mice

Plekho1	-0.06	1.00000000	mice
Dcun1d5	-0.07	1.00000000	mice
Gpd2	-0.12	1.00000000	mice
Tmsb10	0.40	1.00000000	mice
Chuk	0.03	1.00000000	mice
Lamtor4	0.01	1.00000000	mice
Pllp	0.03	1.00000000	mice
Pmpcb	-0.08	1.00000000	mice
Nbn	0.04	1.00000000	mice
Sept7	-0.05	1.00000000	mice
Tada3	0.04	1.00000000	mice
Rnh1	0.13	1.00000000	mice
Vangl1	0.20	1.00000000	mice
Pdcd6ip	-0.08	1.00000000	mice
Arf3	0.24	1.00000000	mice
Hspa9	-0.10	1.00000000	mice
Imp3	0.05	1.00000000	mice
Setd7	-0.05	1.00000000	mice
Dlc1	0.19	1.00000000	mice
Sox13	-0.03	1.00000000	mice
Fam160a2	0.09	1.00000000	mice
Ssbp4	0.25	1.00000000	mice
Tsn	-0.04	1.00000000	mice
Gnb1	0.12	1.00000000	mice
Anapc7	0.01	1.00000000	mice
Pam16	0.03	1.00000000	mice
Csgalnact2	0.22	1.00000000	mice
Spns2	0.13	1.00000000	mice
Klhl28	-0.07	1.00000000	mice
Plscr1	-0.02	1.00000000	mice
Amotl1	-0.12	1.00000000	mice
Pank2	0.20	1.00000000	mice
Irf8	0.07	1.00000000	mice
Larp4	0.00	1.00000000	mice
Ilk	0.02	1.00000000	mice
Ago3	-0.07	1.00000000	mice
Ocln	0.03	1.00000000	mice
Smim20	0.06	1.00000000	mice
Atp5a1	-0.03	1.00000000	mice
Snapin	0.00	1.00000000	mice
Fam210a	-0.09	1.00000000	mice
Igbp1	-0.03	1.00000000	mice
Rrp7a	0.10	1.00000000	mice
Psmd4	0.28	1.00000000	mice
Edem1	-0.02	1.00000000	mice
Ncbp2	0.04	1.00000000	mice

Zfp830	-0.10	1.00000000	mice
Spty2d1	0.03	1.00000000	mice
Fbxo9	-0.07	1.00000000	mice
Cnih4	0.00	1.00000000	mice
Tmbim4	-0.01	1.00000000	mice
Slc35b3	0.08	1.00000000	mice
Inpp4a	-0.04	1.00000000	mice
Snrpa1	0.07	1.00000000	mice
Ankfy1	0.12	1.00000000	mice
Sox18	0.13	1.00000000	mice
Syn3	0.04	1.00000000	mice
Tgfbr2	0.07	1.00000000	mice
Smarca5	-0.01	1.00000000	mice
Ppp6c	-0.01	1.00000000	mice
Tbkbp1	0.05	1.00000000	mice
Nrep	0.03	1.00000000	mice
Faim	-0.06	1.00000000	mice
Ide	0.08	1.00000000	mice
Snx14	-0.02	1.00000000	mice
Hcfc1	0.09	1.00000000	mice
Exoc5	0.00	1.00000000	mice
Fam3c	0.12	1.00000000	mice
Ambra1	0.16	1.00000000	mice
Yipf3	0.04	1.00000000	mice
Heca	-0.04	1.00000000	mice
Qser1	-0.08	1.00000000	mice
Gabbr1	-0.03	1.00000000	mice
Zfp384	-0.02	1.00000000	mice
Slc1a5	0.00	1.00000000	mice
Tlr4	0.13	1.00000000	mice
Dmac1	0.11	1.00000000	mice
Fxr2	0.01	1.00000000	mice
Ganab	0.13	1.00000000	mice
Aarsd1	-0.06	1.00000000	mice
Bnip2	0.00	1.00000000	mice
Hmgn5	0.00	1.00000000	mice
Atp5b	0.07	1.00000000	mice
Fam13c	0.14	1.00000000	mice
Lclat1	0.07	1.00000000	mice
Tm9sf1	-0.05	1.00000000	mice
Abcc5	0.17	1.00000000	mice
Gpsm3	0.14	1.00000000	mice
Cdk2ap2	0.07	1.00000000	mice
Capza1	-0.09	1.00000000	mice
Slc20a2	0.01	1.00000000	mice
Dars	0.18	1.00000000	mice

Lrrk2	0.06	1.00000000	mice
Abcd3	0.12	1.00000000	mice
Stk24	-0.04	1.00000000	mice
Selenoh	0.09	1.00000000	mice
Kremen1	-0.02	1.00000000	mice
Nfix	0.19	1.00000000	mice
Tpra1	0.15	1.00000000	mice
Trf	0.04	1.00000000	mice
Hbp1	-0.10	1.00000000	mice
C2cd2	-0.01	1.00000000	mice
4921524J17Rik	0.14	1.00000000	mice
Lsamp	-0.06	1.00000000	mice
Pik3ap1	0.27	1.00000000	mice
Senp5	-0.09	1.00000000	mice
Prkaa2	0.13	1.00000000	mice
Hdac7	0.15	1.00000000	mice
Zhx2	-0.12	1.00000000	mice
Lin52	0.01	1.00000000	mice
AU022252	-0.03	1.00000000	mice
8030462N17Rik	0.14	1.00000000	mice
Tent2	0.16	1.00000000	mice
Pfdn1	0.31	1.00000000	mice
Acsl3	-0.05	1.00000000	mice
Zwint	-0.15	1.00000000	mice
Serpinb6b	0.31	1.00000000	mice
Pdcd10	-0.02	1.00000000	mice
Ehbp1l1	-0.05	1.00000000	mice
Nbea	-0.06	1.00000000	mice
Trmt61b	0.04	1.00000000	mice
Usp10	-0.04	1.00000000	mice
Plekho2	0.06	1.00000000	mice
Rpp30	0.04	1.00000000	mice
Sipa1l2	-0.10	1.00000000	mice
Ttc14	0.08	1.00000000	mice
Zc3h3	0.00	1.00000000	mice
Rasgef1b	0.11	1.00000000	mice
Ddx6	-0.02	1.00000000	mice
Rnf103	-0.04	1.00000000	mice
Xpot	-0.06	1.00000000	mice
Acbd3	-0.08	1.00000000	mice
Phrf1	-0.02	1.00000000	mice
U2surp	0.17	1.00000000	mice
Ndufaf2	-0.04	1.00000000	mice
Fam204a	-0.15	1.00000000	mice
Ssb	-0.07	1.00000000	mice
Avl9	-0.07	1.00000000	mice

Ltbp1	-0.10	1.00000000	mice
Hsdl2	-0.07	1.00000000	mice
Prkab1	-0.08	1.00000000	mice
Limd2	0.15	1.00000000	mice
Ssr1	-0.06	1.00000000	mice
Tmem134	0.08	1.00000000	mice
Orc2	0.07	1.00000000	mice
Slc35a4	0.01	1.00000000	mice
Gtf2a1	0.17	1.00000000	mice
Ap1s2	0.02	1.00000000	mice
Kdsr	-0.06	1.00000000	mice
Rab2b	-0.02	1.00000000	mice
Angel2	-0.08	1.00000000	mice
Araf	0.03	1.00000000	mice
Cinp	-0.06	1.00000000	mice
Atp6v0a2	0.10	1.00000000	mice
Nkapd1	-0.07	1.00000000	mice
Prr12	0.14	1.00000000	mice
Ksr1	-0.08	1.00000000	mice
Ttc1	-0.01	1.00000000	mice
Nudc	-0.02	1.00000000	mice
Kctd6	-0.06	1.00000000	mice
Rnf185	-0.10	1.00000000	mice
Tasor	-0.09	1.00000000	mice
Serpina3f	0.15	1.00000000	mice
Pdss1	-0.06	1.00000000	mice
Yeats4	0.00	1.00000000	mice
Eif2ak3	-0.10	1.00000000	mice
Uso1	-0.04	1.00000000	mice
Crlf3	-0.22	1.00000000	mice
Nacc1	0.09	1.00000000	mice
Frmd4b	0.12	1.00000000	mice
Zfp277	0.14	1.00000000	mice
Actn4	0.01	1.00000000	mice
Psmd13	-0.08	1.00000000	mice
Dnajc7	-0.05	1.00000000	mice
Dnm2	-0.06	1.00000000	mice
Zfpm1	-0.01	1.00000000	mice
Gnl3l	0.03	1.00000000	mice
Tardbp	-0.13	1.00000000	mice
Insig1	0.06	1.00000000	mice
Insig2	0.00	1.00000000	mice
Pea15a	-0.02	1.00000000	mice
Abl2	-0.10	1.00000000	mice
Snx25	0.09	1.00000000	mice
Prmt3	-0.08	1.00000000	mice

Yipf1	0.00	1.00000000	mice
Yaf2	-0.10	1.00000000	mice
Cd81	-0.04	1.00000000	mice
Nup93	0.12	1.00000000	mice
Ncoa6	0.06	1.00000000	mice
4921511C10Rik	-0.04	1.00000000	mice
Trappc5	-0.05	1.00000000	mice
Slc25a46	-0.05	1.00000000	mice
Ccdc138	-0.04	1.00000000	mice
Tubgcp4	0.06	1.00000000	mice
Chp1	-0.04	1.00000000	mice
Lrrc8d	0.08	1.00000000	mice
Iqcb1	-0.02	1.00000000	mice
Ndufv3	-0.02	1.00000000	mice
Pla2g12a	-0.03	1.00000000	mice
Wdr3	0.00	1.00000000	mice
Tmpo	-0.03	1.00000000	mice
Ncoa3	-0.09	1.00000000	mice
Iars2	-0.01	1.00000000	mice
Kdm7a	0.06	1.00000000	mice
Hnrnph1	-0.14	1.00000000	mice
Abhd16a	-0.02	1.00000000	mice
Ppp1r10	-0.20	1.00000000	mice
Bsg	-0.18	1.00000000	mice
Fmnl3	0.16	1.00000000	mice
Afdn	0.16	1.00000000	mice
Celsr2	0.04	1.00000000	mice
Xpr1	-0.14	1.00000000	mice
Kxd1	0.00	1.00000000	mice
Phlpp1	-0.06	1.00000000	mice
Renbp	0.05	1.00000000	mice
Ybx1	-0.07	1.00000000	mice
Acbd5	0.25	1.00000000	mice
Ppt1	0.03	1.00000000	mice
Nr2f2	0.14	1.00000000	mice
Rnf4	-0.12	1.00000000	mice
Creld1	-0.06	1.00000000	mice
Pspc1	-0.01	1.00000000	mice
R3hdm2	0.09	1.00000000	mice
Aamp	0.09	1.00000000	mice
Txnl4a	-0.04	1.00000000	mice
Slc9a8	-0.02	1.00000000	mice
Crkl	-0.03	1.00000000	mice
Pbx2	-0.03	1.00000000	mice
Raver2	0.20	1.00000000	mice
Pdzd8	-0.09	1.00000000	mice

Socs1	-0.14	1.00000000	mice
Scmh1	-0.08	1.00000000	mice
Zc3h4	-0.06	1.00000000	mice
Snx6	0.14	1.00000000	mice
Lpcat3	-0.10	1.00000000	mice
Dnajc30	-0.01	1.00000000	mice
Prpf8	0.05	1.00000000	mice
Chmp2b	0.09	1.00000000	mice
Prss23	-0.16	1.00000000	mice
Sbno1	-0.02	1.00000000	mice
Ppm1f	0.06	1.00000000	mice
Nudt13	0.11	1.00000000	mice
Kpna3	0.15	1.00000000	mice
Ntng2	0.01	1.00000000	mice
Poldip2	-0.15	1.00000000	mice
Farp1	-0.07	1.00000000	mice
Paqr7	-0.01	1.00000000	mice
Thap11	0.00	1.00000000	mice
Rcl1	-0.06	1.00000000	mice
Xpo6	-0.10	1.00000000	mice
Gtdc1	0.06	1.00000000	mice
Hnrnpab	-0.05	1.00000000	mice
Cdc42bpb	-0.10	1.00000000	mice
Ubtd2	-0.06	1.00000000	mice
Srsf9	0.09	1.00000000	mice
Klhdc2	0.01	1.00000000	mice
Vps26b	0.00	1.00000000	mice
Tpgs2	-0.12	1.00000000	mice
Capn7	-0.15	1.00000000	mice
Nt5dc1	0.10	1.00000000	mice
Emd	-0.02	1.00000000	mice
Stard13	-0.10	1.00000000	mice
Ccn1	0.21	1.00000000	mice
Asxl1	-0.13	1.00000000	mice
Smurf2	-0.14	1.00000000	mice
Bclaf1	-0.03	1.00000000	mice
Acyp2	-0.05	1.00000000	mice
Hadhb	-0.12	1.00000000	mice
Rnf139	0.21	1.00000000	mice
Rbmx	-0.06	1.00000000	mice
Abi1	-0.02	1.00000000	mice
Sephs1	0.07	1.00000000	mice
Twf1	-0.03	1.00000000	mice
Bcas3	0.20	1.00000000	mice
Dmxl2	0.10	1.00000000	mice
Rsl1d1	-0.01	1.00000000	mice

Fnbp1	-0.06	1.00000000	mice
Srgap1	0.16	1.00000000	mice
Ift46	0.04	1.00000000	mice
R3hdm4	-0.03	1.00000000	mice
Zfp91	0.16	1.00000000	mice
Ttc33	0.11	1.00000000	mice
Dusp23	0.12	1.00000000	mice
Ndufs8	-0.07	1.00000000	mice
Ahnak	0.03	1.00000000	mice
Por	-0.03	1.00000000	mice
Fstl1	-0.14	1.00000000	mice
Sacs	0.02	1.00000000	mice
Sumo3	-0.02	1.00000000	mice
Arhgef2	0.04	1.00000000	mice
Sft2d2	0.03	1.00000000	mice
Scyl2	-0.01	1.00000000	mice
Glyr1	-0.03	1.00000000	mice
Atp5g3	-0.10	1.00000000	mice
Dnlz	-0.01	1.00000000	mice
Numa1	-0.01	1.00000000	mice
Prcp	-0.02	1.00000000	mice
Zfp654	0.18	1.00000000	mice
Hdac8	0.09	1.00000000	mice
Tanc2	0.10	1.00000000	mice
Nudt3	-0.07	1.00000000	mice
Sec24d	-0.05	1.00000000	mice
Lbp	0.03	1.00000000	mice
Fez2	0.04	1.00000000	mice
Phlda1	0.12	1.00000000	mice
Leo1	0.05	1.00000000	mice
Enox2	0.13	1.00000000	mice
Fbxo11	-0.15	1.00000000	mice
Adamtsl5	-0.04	1.00000000	mice
Zfp467	-0.02	1.00000000	mice
Picalm	0.03	1.00000000	mice
Ppp2r3c	0.13	1.00000000	mice
Nt5c3b	-0.03	1.00000000	mice
Washc3	0.05	1.00000000	mice
Dgcr2	-0.05	1.00000000	mice
2410006H16Rik	0.04	1.00000000	mice
Wtap	0.06	1.00000000	mice
Btbd9	-0.08	1.00000000	mice
Uchl3	-0.05	1.00000000	mice
Gnpda2	-0.09	1.00000000	mice
Ppp1r8	-0.01	1.00000000	mice
Fdps	0.09	1.00000000	mice

Nin	0.09	1.00000000	mice
Ngdn	0.01	1.00000000	mice
Ppfia1	0.21	1.00000000	mice
Alg13	0.02	1.00000000	mice
Paxx	0.08	1.00000000	mice
Rpe	-0.01	1.00000000	mice
Znrf3	-0.03	1.00000000	mice
Rnf25	0.04	1.00000000	mice
Sp3	0.08	1.00000000	mice
Ell	-0.06	1.00000000	mice
Ccm2	-0.03	1.00000000	mice
Scamp3	0.02	1.00000000	mice
Ppil4	-0.10	1.00000000	mice
Clpx	-0.04	1.00000000	mice
Efl1	0.08	1.00000000	mice
Hdac2	-0.08	1.00000000	mice
Ctcf	0.00	1.00000000	mice
Nptn	-0.06	1.00000000	mice
Knop1	0.07	1.00000000	mice
Cdk12	-0.03	1.00000000	mice
Dusp2	0.19	1.00000000	mice
Sart1	0.02	1.00000000	mice
Zfp746	0.00	1.00000000	mice
Pcsk5	0.02	1.00000000	mice
Zfp62	-0.11	1.00000000	mice
Kars	0.17	1.00000000	mice
Naa16	-0.11	1.00000000	mice
Emc1	-0.01	1.00000000	mice
Trerf1	-0.11	1.00000000	mice
Wbp4	0.03	1.00000000	mice
Rbbp9	-0.04	1.00000000	mice
Scrib	0.01	1.00000000	mice
Hebp1	0.01	1.00000000	mice
Ptp4a2	-0.01	1.00000000	mice
Klhl20	-0.01	1.00000000	mice
Bcl9	0.07	1.00000000	mice
Phf2	0.07	1.00000000	mice
Cnn3	-0.07	1.00000000	mice
Timm10b	0.22	1.00000000	mice
Xab2	0.01	1.00000000	mice
Zfp560	0.03	1.00000000	mice
Ngly1	0.15	1.00000000	mice
Larp1b	-0.08	1.00000000	mice
Capza2	0.18	1.00000000	mice
Dop1a	0.15	1.00000000	mice
S1pr4	0.03	1.00000000	mice

Gpr107	0.14	1.00000000	mice
Psd3	0.01	1.00000000	mice
5530601H04Rik	0.11	1.00000000	mice
Slc30a1	0.17	1.00000000	mice
Scyl1	-0.05	1.00000000	mice
Narf	-0.04	1.00000000	mice
Dand5	-0.05	1.00000000	mice
Gipc3	0.08	1.00000000	mice
Snhg5	-0.02	1.00000000	mice
Chfr	0.01	1.00000000	mice
Kit	0.40	1.00000000	mice
Dnajc3	-0.06	1.00000000	mice
Nxpe3	0.19	1.00000000	mice
Bicra	-0.02	1.00000000	mice
Arl3	-0.01	1.00000000	mice
Lrig2	-0.06	1.00000000	mice
Zbtb1	-0.03	1.00000000	mice
Stx18	0.09	1.00000000	mice
Rtf2	0.17	1.00000000	mice
Rnf10	0.03	1.00000000	mice
Gdpd5	0.13	1.00000000	mice
Pigm	0.10	1.00000000	mice
Synm	-0.13	1.00000000	mice
Cks2	0.14	1.00000000	mice
Snrpd2	0.03	1.00000000	mice
Dnajc13	-0.02	1.00000000	mice
R3hcc1l	0.13	1.00000000	mice
Rcbtb2	-0.01	1.00000000	mice
Cttnbp2	-0.06	1.00000000	mice
Snap23	-0.08	1.00000000	mice
Nomo1	0.01	1.00000000	mice
Pgap2	0.02	1.00000000	mice
Acap2	-0.16	1.00000000	mice
Pros1	-0.12	1.00000000	mice
Nynrin	0.08	1.00000000	mice
Acot8	0.02	1.00000000	mice
Stk19	0.00	1.00000000	mice
Eif3f	0.04	1.00000000	mice
Sec62	0.18	1.00000000	mice
Uba52	0.24	1.00000000	mice
Chd8	0.13	1.00000000	mice
Casp3	0.05	1.00000000	mice
Rab21	0.25	1.00000000	mice
Ubac1	0.31	1.00000000	mice
Usp7	-0.13	1.00000000	mice
Stmn2	-0.03	1.00000000	mice

Kif5b	0.03	1.00000000	mice
Abcb1a	-0.10	1.00000000	mice
Uqcc1	0.07	1.00000000	mice
Wbp11	-0.09	1.00000000	mice
H3f3b	0.16	1.00000000	mice
Ddx55	0.09	1.00000000	mice
Fam126a	-0.04	1.00000000	mice
Aar2	0.04	1.00000000	mice
Creb1	-0.14	1.00000000	mice
Prkab2	-0.02	1.00000000	mice
Senp6	-0.12	1.00000000	mice
Tob2	-0.08	1.00000000	mice
Pop7	0.02	1.00000000	mice
Erp29	0.05	1.00000000	mice
Zdhhc17	0.11	1.00000000	mice
Tsc1	-0.05	1.00000000	mice
Dennd1b	0.20	1.00000000	mice
Lpp	0.17	1.00000000	mice
Alas1	0.01	1.00000000	mice
Aatf	0.10	1.00000000	mice
Ano10	-0.04	1.00000000	mice
Cdk5rap2	0.08	1.00000000	mice
Pdhx	-0.08	1.00000000	mice
Eef2	-0.03	1.00000000	mice
Runx1	-0.11	1.00000000	mice
Dpysl3	-0.10	1.00000000	mice
Zfyve9	-0.10	1.00000000	mice
Gramd1a	-0.11	1.00000000	mice
Nolc1	-0.01	1.00000000	mice
Pafah1b2	0.09	1.00000000	mice
Adrm1	0.14	1.00000000	mice
Eif4e2	0.08	1.00000000	mice
Clcn5	-0.04	1.00000000	mice
Plxna4	-0.05	1.00000000	mice
Smu1	0.02	1.00000000	mice
Eif4enif1	0.07	1.00000000	mice
Upf3a	0.02	1.00000000	mice
Slx4ip	0.08	1.00000000	mice
Fam13b	-0.02	1.00000000	mice
Cdk13	-0.06	1.00000000	mice
Arid1b	0.15	1.00000000	mice
Ccdc86	-0.06	1.00000000	mice
Fkbp7	-0.05	1.00000000	mice
Kdm4b	-0.07	1.00000000	mice
Leprot	-0.07	1.00000000	mice
Dnmt3a	0.04	1.00000000	mice

Asah1	-0.01	1.00000000	mice
Nfatc3	-0.05	1.00000000	mice
Romo1	0.20	1.00000000	mice
Lsm5	-0.01	1.00000000	mice
Copg2	0.18	1.00000000	mice
Stk25	0.02	1.00000000	mice
Rbsn	-0.11	1.00000000	mice
Lman1	-0.05	1.00000000	mice
Adrb1	0.13	1.00000000	mice
Sbf1	0.01	1.00000000	mice
Fibp	0.02	1.00000000	mice
Zfp715	0.02	1.00000000	mice
Nln	0.09	1.00000000	mice
Lage3	0.04	1.00000000	mice
Rcan1	0.01	1.00000000	mice
Lrrc57	-0.01	1.00000000	mice
Tmem250-ps	0.03	1.00000000	mice
Slc25a28	0.16	1.00000000	mice
Ppig	-0.04	1.00000000	mice
Eif4e3	0.06	1.00000000	mice
Lias	0.04	1.00000000	mice
Thrap3	0.02	1.00000000	mice
Psenen	0.08	1.00000000	mice
Ppid	0.06	1.00000000	mice
Pbdc1	-0.02	1.00000000	mice
Ppm1l	0.12	1.00000000	mice
Wipi1	0.00	1.00000000	mice
Tmem214	0.06	1.00000000	mice
Bcl2l2	0.02	1.00000000	mice
Sgpp1	-0.03	1.00000000	mice
Gnptg	0.00	1.00000000	mice
Timm17b	-0.03	1.00000000	mice
Kmt2e	-0.04	1.00000000	mice
Gak	0.04	1.00000000	mice
Smndc1	0.00	1.00000000	mice
Sec11c	-0.12	1.00000000	mice
Dpp8	0.18	1.00000000	mice
Dnajc14	0.03	1.00000000	mice
Ndrg3	-0.06	1.00000000	mice
Psip1	0.02	1.00000000	mice
Atp6v1e1	0.19	1.00000000	mice
Sdhb	-0.08	1.00000000	mice
Trpc4ap	-0.01	1.00000000	mice
Wdr61	0.11	1.00000000	mice
Son	0.10	1.00000000	mice
Zfp318	-0.02	1.00000000	mice

Ctnnd1	0.15	1.00000000	mice
Erap1	0.14	1.00000000	mice
Fam219b	-0.01	1.00000000	mice
Tmem71	-0.06	1.00000000	mice
Slc16a13	0.00	1.00000000	mice
Rmdn1	0.08	1.00000000	mice
Pcnx	-0.04	1.00000000	mice
Dctn6	0.03	1.00000000	mice
Pcm1	0.07	1.00000000	mice
Ruvbl1	-0.01	1.00000000	mice
Hspa5	-0.05	1.00000000	mice
Sqstm1	-0.01	1.00000000	mice
Dock11	-0.14	1.00000000	mice
Trpm4	-0.05	1.00000000	mice
Reep5	0.11	1.00000000	mice
Sufu	0.00	1.00000000	mice
Ywhab	0.20	1.00000000	mice
Vdac2	0.05	1.00000000	mice
Bace1	0.00	1.00000000	mice
Srp72	0.00	1.00000000	mice
Hs2st1	0.13	1.00000000	mice
Arhgef40	0.13	1.00000000	mice
Zfyve26	0.07	1.00000000	mice
Klc1	0.20	1.00000000	mice
Psmb1	0.19	1.00000000	mice
Htt	-0.08	1.00000000	mice
Hira	0.04	1.00000000	mice
Pithd1	-0.07	1.00000000	mice
Yipf4	0.08	1.00000000	mice
Prdx5	0.23	1.00000000	mice
Tkt	0.04	1.00000000	mice
Arhgap23	-0.08	1.00000000	mice
Ostc	0.09	1.00000000	mice
Gusb	0.00	1.00000000	mice
Cep128	-0.07	1.00000000	mice
Atr	0.07	1.00000000	mice
Churc1	-0.06	1.00000000	mice
Zfp516	-0.07	1.00000000	mice
Nsd2	-0.03	1.00000000	mice
Camkk2	0.03	1.00000000	mice
Vps37a	-0.02	1.00000000	mice
Dhx38	0.00	1.00000000	mice
Zadh2	-0.15	1.00000000	mice
Pag1	0.01	1.00000000	mice
Zfp217	0.05	1.00000000	mice
Tbc1d4	-0.08	1.00000000	mice

Bzw2	-0.03	1.00000000	mice
Hs6st1	0.10	1.00000000	mice
Tnfaip8	-0.10	1.00000000	mice
Cdc34	0.13	1.00000000	mice
Psma3	0.12	1.00000000	mice
Dst	0.11	1.00000000	mice
Zfp1	-0.06	1.00000000	mice
Ndufa4	-0.05	1.00000000	mice
Dcaf7	-0.01	1.00000000	mice
Ccar2	0.11	1.00000000	mice
Polr2d	0.05	1.00000000	mice
Ptk2	0.10	1.00000000	mice
Ino80	-0.06	1.00000000	mice
Itgav	0.27	1.00000000	mice
Hnrnpa3	-0.05	1.00000000	mice
Acacb	-0.02	1.00000000	mice
Hsd17b4	0.03	1.00000000	mice
Ankra2	0.04	1.00000000	mice
Cbx1	0.09	1.00000000	mice
Nr1h2	-0.04	1.00000000	mice
Dguok	0.05	1.00000000	mice
Taok3	-0.09	1.00000000	mice
Bckdhb	-0.02	1.00000000	mice
Zfp410	-0.06	1.00000000	mice
Trib2	0.04	1.00000000	mice
Tollip	0.05	1.00000000	mice
Ddx42	0.16	1.00000000	mice
Gclm	-0.01	1.00000000	mice
Commd2	-0.02	1.00000000	mice
Ramac	0.06	1.00000000	mice
Ociad1	0.03	1.00000000	mice
Gab2	0.01	1.00000000	mice
Git2	0.13	1.00000000	mice
Ccdc9b	0.07	1.00000000	mice
Gatd3a	-0.10	1.00000000	mice
Sema3f	0.18	1.00000000	mice
Lipe	-0.08	1.00000000	mice
Fance	-0.03	1.00000000	mice
Acox1	0.02	1.00000000	mice
Soat1	0.10	1.00000000	mice
Snx17	-0.05	1.00000000	mice
Pip4p2	0.06	1.00000000	mice
Cox6c	0.11	1.00000000	mice
Psmb6	0.04	1.00000000	mice
Klhl5	-0.02	1.00000000	mice
Tug1	-0.07	1.00000000	mice

Trp53bp2	-0.07	1.00000000	mice
Copa	0.21	1.00000000	mice
Purg	-0.06	1.00000000	mice
Sap30l	0.10	1.00000000	mice
Smarca2	-0.07	1.00000000	mice
Vapb	0.03	1.00000000	mice
Golga3	0.08	1.00000000	mice
Klf12	0.09	1.00000000	mice
Commd4	0.02	1.00000000	mice
Ube2d1	0.24	1.00000000	mice
Irf3	0.18	1.00000000	mice
Ecscr	0.16	1.00000000	mice
Afap1	0.05	1.00000000	mice
Slc6a6	0.10	1.00000000	mice
Atxn2l	-0.02	1.00000000	mice
Shc1	-0.04	1.00000000	mice
Samd8	0.06	1.00000000	mice
Zfp438	-0.10	1.00000000	mice
Cirbp	-0.04	1.00000000	mice
Pds5a	-0.09	1.00000000	mice
Cep104	0.01	1.00000000	mice
LTO1	0.07	1.00000000	mice
Capns1	0.07	1.00000000	mice
Cnot11	-0.06	1.00000000	mice
Id1	-0.05	1.00000000	mice
Gps2	0.14	1.00000000	mice
Arhgap5	-0.09	1.00000000	mice
Scfd1	0.10	1.00000000	mice
Api5	0.18	1.00000000	mice
Snx1	0.09	1.00000000	mice
Nsmf	-0.09	1.00000000	mice
Phactr4	0.10	1.00000000	mice
Cib1	0.03	1.00000000	mice
Atp6v0e	-0.03	1.00000000	mice
Drg1	0.07	1.00000000	mice
Ints4	0.11	1.00000000	mice
Babam1	0.15	1.00000000	mice
Nucb1	-0.03	1.00000000	mice
Stk17b	-0.04	1.00000000	mice
Usp8	0.10	1.00000000	mice
Bap1	0.01	1.00000000	mice
Taok1	-0.04	1.00000000	mice
Sidt2	0.08	1.00000000	mice
Cdc26	0.04	1.00000000	mice
Rtl8b	0.23	1.00000000	mice
Twsg1	0.08	1.00000000	mice

Cog3	0.06	1.00000000	mice
Iah1	-0.01	1.00000000	mice
Tbrg1	0.17	1.00000000	mice
Etv6	-0.04	1.00000000	mice
Ythdf3	0.00	1.00000000	mice
Tbc1d1	0.15	1.00000000	mice
Fam110a	-0.09	1.00000000	mice
Pdzd11	-0.04	1.00000000	mice
Ndufs5	0.26	1.00000000	mice
Phf10	0.02	1.00000000	mice
Scap	0.09	1.00000000	mice
Pts	0.07	1.00000000	mice
Usp16	-0.11	1.00000000	mice
Uqcrc1	0.25	1.00000000	mice
Ctbs	0.10	1.00000000	mice
Eps15l1	-0.03	1.00000000	mice
Ssna1	-0.01	1.00000000	mice
Smc1a	-0.03	1.00000000	mice
Arpc1b	0.08	1.00000000	mice
Ppp1r11	0.08	1.00000000	mice
Tyk2	-0.02	1.00000000	mice
Ccdc43	-0.01	1.00000000	mice
Sart3	-0.05	1.00000000	mice
Prcc	0.06	1.00000000	mice
Ccdc66	0.03	1.00000000	mice
Cdk7	-0.04	1.00000000	mice
Ubxn2a	-0.10	1.00000000	mice
Bmp2k	-0.05	1.00000000	mice
Timm13	0.04	1.00000000	mice
Pcgf5	0.11	1.00000000	mice
Sec11a	0.02	1.00000000	mice
Zfp106	-0.06	1.00000000	mice
Cant1	0.07	1.00000000	mice
Vsig2	-0.05	1.00000000	mice
Inpp5b	0.13	1.00000000	mice
BC003965	0.05	1.00000000	mice
Otulin	0.20	1.00000000	mice
Cyc1	-0.05	1.00000000	mice
Ptcd3	0.04	1.00000000	mice
Azi2	0.15	1.00000000	mice
Lztr1	0.04	1.00000000	mice
Exosc5	0.00	1.00000000	mice
Ctps	0.06	1.00000000	mice
Sec24b	-0.03	1.00000000	mice
Rnf167	-0.04	1.00000000	mice
2510009E07Rik	0.00	1.00000000	mice

Wls	-0.08	1.00000000	mice
Dhdds	-0.06	1.00000000	mice
Cat	0.25	1.00000000	mice
Papola	-0.01	1.00000000	mice
Wrn	0.12	1.00000000	mice
Srr	-0.07	1.00000000	mice
Bpnt1	-0.08	1.00000000	mice
Copb1	-0.07	1.00000000	mice
Ldah	-0.01	1.00000000	mice
Scly	0.04	1.00000000	mice
Vps13b	0.21	1.00000000	mice
Slc35a2	-0.01	1.00000000	mice
Sdc3	0.25	1.00000000	mice
Arhgap27	0.02	1.00000000	mice
Ptch1	0.14	1.00000000	mice
Tbc1d30	0.07	1.00000000	mice
Diaph2	0.37	1.00000000	mice
Csrp1	0.21	1.00000000	mice
Fgfr1	0.13	1.00000000	mice
Rnf215	-0.01	1.00000000	mice
Def6	0.06	1.00000000	mice
Tbc1d22b	-0.04	1.00000000	mice
Trim33	0.17	1.00000000	mice
Lamc1	0.03	1.00000000	mice
Svbp	0.03	1.00000000	mice
Plod3	0.17	1.00000000	mice
Pum3	0.05	1.00000000	mice
Hars	-0.03	1.00000000	mice
Ube2a	0.01	1.00000000	mice
Trip4	0.06	1.00000000	mice
Kmt2d	0.19	1.00000000	mice
Isca2	0.05	1.00000000	mice
Lrrc8a	-0.10	1.00000000	mice
Ralgapb	0.10	1.00000000	mice
Spin1	0.12	1.00000000	mice
Nr4a3	0.08	1.00000000	mice
Ech1	-0.10	1.00000000	mice
Dip2a	0.02	1.00000000	mice
Triobp	0.11	1.00000000	mice
Prkcd	0.23	1.00000000	mice
Rell1	-0.09	1.00000000	mice
Zzz3	0.00	1.00000000	mice
Nae1	0.07	1.00000000	mice
Odf2	-0.04	1.00000000	mice
Zc3h13	-0.16	1.00000000	mice
Ttc3	-0.03	1.00000000	mice

Dtnb	-0.02	1.00000000	mice
Xrn1	0.07	1.00000000	mice
Cbx3	0.04	1.00000000	mice
Rex1bd	0.22	1.00000000	mice
Zfp407	0.09	1.00000000	mice
Tmem204	0.25	1.00000000	mice
Lncppara	0.04	1.00000000	mice
Kansl2	-0.09	1.00000000	mice
Itpr3	-0.01	1.00000000	mice
Ppp3r1	0.16	1.00000000	mice
Smg9	0.01	1.00000000	mice
Cdc23	0.01	1.00000000	mice
Cdkal1	0.13	1.00000000	mice
Ndufc1	0.30	1.00000000	mice
Zfp597	-0.07	1.00000000	mice
Bbx	0.11	1.00000000	mice
Cpne3	0.07	1.00000000	mice
Ocrl	0.07	1.00000000	mice
Brix1	-0.10	1.00000000	mice
Eno1	0.08	1.00000000	mice
Rap2c	0.02	1.00000000	mice
Caprin1	0.10	1.00000000	mice
Neto2	0.00	1.00000000	mice
Taz	-0.12	1.00000000	mice
Baz2b	-0.09	1.00000000	mice
Nubp1	0.08	1.00000000	mice
Wdsub1	0.02	1.00000000	mice
Adh5	-0.02	1.00000000	mice
Sgpp2	0.07	1.00000000	mice
Cul3	-0.03	1.00000000	mice
Zdhhc20	-0.09	1.00000000	mice
Trpm7	-0.02	1.00000000	mice
Xrcc6	-0.02	1.00000000	mice
Ap4e1	-0.03	1.00000000	mice
Kansl3	-0.06	1.00000000	mice
Cand1	-0.05	1.00000000	mice
Stard9	-0.08	1.00000000	mice
Pdpr	-0.01	1.00000000	mice
Kmt5a	0.09	1.00000000	mice
Zfp445	0.02	1.00000000	mice
Rab4b	-0.10	1.00000000	mice
Tmx2	-0.08	1.00000000	mice
Ufl1	-0.02	1.00000000	mice
Hbs1l	-0.05	1.00000000	mice
Arhgap6	0.14	1.00000000	mice
Rbm5	0.17	1.00000000	mice

Cope	0.16	1.00000000	mice
Tmem230	0.13	1.00000000	mice
Zbtb8os	0.03	1.00000000	mice
Sharpin	0.05	1.00000000	mice
Slc24a4	0.01	1.00000000	mice
Arhgdib	-0.01	1.00000000	mice
Sos1	0.01	1.00000000	mice
Pcnx4	-0.02	1.00000000	mice
Armcx2	-0.03	1.00000000	mice
Tmem44	-0.04	1.00000000	mice
Txndc16	0.12	1.00000000	mice
Alg1	-0.05	1.00000000	mice
Nup50	0.00	1.00000000	mice
Ccdc82	0.11	1.00000000	mice
Fam78b	-0.08	1.00000000	mice
Fopnl	-0.01	1.00000000	mice
Ppp2r5a	-0.11	1.00000000	mice
Atf2	0.01	1.00000000	mice
Pik3ip1	0.07	1.00000000	mice
Upp1	0.20	1.00000000	mice
Carnmt1	-0.08	1.00000000	mice
Brf1	-0.06	1.00000000	mice
Slc4a1ap	-0.10	1.00000000	mice
Taldo1	0.04	1.00000000	mice
Atp8b2	0.03	1.00000000	mice
Plekhj1	0.06	1.00000000	mice
Pip5k1a	-0.04	1.00000000	mice
Bysl	-0.05	1.00000000	mice
Fam149b	0.15	1.00000000	mice
Nmt2	0.10	1.00000000	mice
Csnk2b	0.03	1.00000000	mice
Lztfl1	-0.02	1.00000000	mice
Tmem68	0.07	1.00000000	mice
Tbca	0.09	1.00000000	mice
Cmc4	-0.04	1.00000000	mice
Psmd8	0.07	1.00000000	mice
Zbtb44	-0.11	1.00000000	mice
Ehd4	0.11	1.00000000	mice
Casz1	0.16	1.00000000	mice
Gtpbp2	0.09	1.00000000	mice
Rgl1	0.11	1.00000000	mice
Kifap3	0.04	1.00000000	mice
Tpcn1	-0.04	1.00000000	mice
Ddx1	-0.05	1.00000000	mice
Ubtd1	-0.09	1.00000000	mice
Atg2b	0.03	1.00000000	mice

Pfdn6	0.00	1.00000000	mice
Ndufb4	0.00	1.00000000	mice
Naa25	0.05	1.00000000	mice
Atg101	-0.06	1.00000000	mice
Plekha5	-0.06	1.00000000	mice
Dnmt1	0.11	1.00000000	mice
Oxsr1	-0.04	1.00000000	mice
N4bp2l2	0.04	1.00000000	mice
Chsy1	0.07	1.00000000	mice
Sh3bp5	-0.06	1.00000000	mice
Vps53	-0.04	1.00000000	mice
1700109H08Rik	-0.01	1.00000000	mice
Virma	0.08	1.00000000	mice
Gstt2	0.15	1.00000000	mice
Abr	0.08	1.00000000	mice
Rnf31	0.10	1.00000000	mice
Cd36	-0.06	1.00000000	mice
Osbp	-0.05	1.00000000	mice
Hace1	-0.01	1.00000000	mice
Tnrc6c	-0.05	1.00000000	mice
Babam2	-0.06	1.00000000	mice
Ankrd28	-0.06	1.00000000	mice
Aplnr	0.01	1.00000000	mice
Hat1	0.01	1.00000000	mice
Eftud2	0.15	1.00000000	mice
Lbh	0.27	1.00000000	mice
Vezf1	0.06	1.00000000	mice
Trim35	0.00	1.00000000	mice
Tubgcp2	0.09	1.00000000	mice
Exosc1	-0.06	1.00000000	mice
Kat7	-0.05	1.00000000	mice
Tgfbr1	0.05	1.00000000	mice
Itch	0.01	1.00000000	mice
Ercc4	0.01	1.00000000	mice
Ndufa1	0.04	1.00000000	mice
Ubl5	0.11	1.00000000	mice
Actr1a	0.07	1.00000000	mice
Cdkn2aipnl	0.04	1.00000000	mice
Sgpl1	0.00	1.00000000	mice
Tesk1	0.00	1.00000000	mice
Pacs2	-0.04	1.00000000	mice
B4gat1	0.06	1.00000000	mice
Rnf34	0.00	1.00000000	mice
Kat2b	0.17	1.00000000	mice
Abcg2	-0.07	1.00000000	mice
Arpc5l	0.03	1.00000000	mice

Gtf2ird1	-0.12	1.00000000	mice
Dis3l2	0.08	1.00000000	mice
Rspry1	-0.07	1.00000000	mice
Lpar6	-0.08	1.00000000	mice
Tbc1d19	0.00	1.00000000	mice
Comt	-0.01	1.00000000	mice
Akt1s1	0.01	1.00000000	mice
Ick	-0.14	1.00000000	mice
D430042O09Rik	0.03	1.00000000	mice
Nup214	0.02	1.00000000	mice
Trps1	0.07	1.00000000	mice
Clk4	-0.08	1.00000000	mice
Tshz1	-0.05	1.00000000	mice
Zcrb1	0.05	1.00000000	mice
Lmo4	0.04	1.00000000	mice
Itga1	0.11	1.00000000	mice
Smc5	-0.11	1.00000000	mice
Idh3g	0.13	1.00000000	mice
Iffo2	0.10	1.00000000	mice
Vps41	0.02	1.00000000	mice
Atp2c1	0.05	1.00000000	mice
Golga4	0.12	1.00000000	mice
Ppm1h	0.12	1.00000000	mice
Smtn	0.01	1.00000000	mice
Scpep1	0.11	1.00000000	mice
Stt3a	0.15	1.00000000	mice
Serpinh1	0.14	1.00000000	mice
Rbm6	-0.02	1.00000000	mice
Zbtb4	-0.08	1.00000000	mice
Capn1	-0.02	1.00000000	mice
Zfp367	0.09	1.00000000	mice
Zfc3h1	-0.06	1.00000000	mice
Rnf14	0.19	1.00000000	mice
Gorasp2	-0.14	1.00000000	mice
Slc36a4	-0.01	1.00000000	mice
Bcas2	0.14	1.00000000	mice
Polr3c	-0.02	1.00000000	mice
Cul5	0.04	1.00000000	mice
Alkbh5	-0.05	1.00000000	mice
Trappc13	-0.04	1.00000000	mice
Hnrnpk	0.07	1.00000000	mice
Smarcd1	0.07	1.00000000	mice
Nrf1	0.24	1.00000000	mice
Rad23b	0.03	1.00000000	mice
Dtnbp1	-0.10	1.00000000	mice
Agtrap	-0.02	1.00000000	mice

Slc35a3	0.03	1.00000000	mice
Fam210b	-0.03	1.00000000	mice
Acadvl	0.00	1.00000000	mice
Acadl	0.13	1.00000000	mice
Rhog	0.07	1.00000000	mice
Hnrnph3	-0.06	1.00000000	mice
Ids	0.01	1.00000000	mice
Snx5	-0.03	1.00000000	mice
Atp6v0c	0.16	1.00000000	mice
Smim12	-0.04	1.00000000	mice
Usp40	0.03	1.00000000	mice
H2-Ke6	0.00	1.00000000	mice
Echs1	-0.07	1.00000000	mice
Shoc2	0.14	1.00000000	mice
Syde1	-0.08	1.00000000	mice
Kmt5b	-0.09	1.00000000	mice
Wwp1	0.11	1.00000000	mice
Sh2d4b	-0.05	1.00000000	mice
Cltb	0.18	1.00000000	mice
Ncaph2	0.01	1.00000000	mice
Pear1	0.09	1.00000000	mice
Arid3b	-0.08	1.00000000	mice
Tmlhe	0.13	1.00000000	mice
Cramp1l	-0.10	1.00000000	mice
Ireb2	0.13	1.00000000	mice
Ercc6	-0.01	1.00000000	mice
Tut7	0.02	1.00000000	mice
Gtf3c6	0.01	1.00000000	mice
Bzw1	0.04	1.00000000	mice
BC005561	0.11	1.00000000	mice
Zfp366	-0.17	1.00000000	mice
Nelfe	0.08	1.00000000	mice
Nxn	0.01	1.00000000	mice
Gskip	0.01	1.00000000	mice
Leng8	0.01	1.00000000	mice
Eif2s3x	0.11	1.00000000	mice
Actr1b	0.10	1.00000000	mice
Itm2c	-0.05	1.00000000	mice
Tgif1	0.11	1.00000000	mice
Brd1	-0.06	1.00000000	mice
Ubqln4	0.04	1.00000000	mice
Emc4	0.01	1.00000000	mice
Arhgap18	0.09	1.00000000	mice
Rnaseh1	0.07	1.00000000	mice
Gsr	0.20	1.00000000	mice
Rab8a	0.21	1.00000000	mice

Pkm	0.10	1.00000000	mice
Ptprd	0.10	1.00000000	mice
Pogz	-0.01	1.00000000	mice
Rnf111	0.13	1.00000000	mice
Nbl1	0.02	1.00000000	mice
Rab8b	0.10	1.00000000	mice
P2rx7	0.16	1.00000000	mice
Adgrf5	-0.06	1.00000000	mice
Prkaa1	0.02	1.00000000	mice
Cdadc1	-0.07	1.00000000	mice
Nelfb	-0.05	1.00000000	mice
Usp31	0.06	1.00000000	mice
Arl2	0.05	1.00000000	mice
Spats2	-0.10	1.00000000	mice
Plpbp	0.07	1.00000000	mice
Pard3b	0.01	1.00000000	mice
Rer1	-0.07	1.00000000	mice
Gng5	-0.05	1.00000000	mice
Arfgap2	0.02	1.00000000	mice
Chst1	0.18	1.00000000	mice
Edc3	0.00	1.00000000	mice
Eef1g	-0.02	1.00000000	mice
Hdgf	0.04	1.00000000	mice
Smim19	-0.03	1.00000000	mice
Rnf6	-0.01	1.00000000	mice
Smo	-0.04	1.00000000	mice
Ccdc174	0.09	1.00000000	mice
Pcdh7	0.24	1.00000000	mice
Ccng2	-0.11	1.00000000	mice
Atp5l	0.09	1.00000000	mice
Hmg20b	0.17	1.00000000	mice
Larp4b	0.01	1.00000000	mice
Scaper	0.12	1.00000000	mice
Ube2i	0.05	1.00000000	mice
Actr3	-0.05	1.00000000	mice
Ints9	0.05	1.00000000	mice
Ndufb9	0.10	1.00000000	mice
Sowahc	-0.10	1.00000000	mice
Jkamp	-0.06	1.00000000	mice
Ssu72	0.20	1.00000000	mice
Rrm2b	-0.03	1.00000000	mice
Tgfb1i1	0.03	1.00000000	mice
Cfap97	0.09	1.00000000	mice
Sept4	0.24	1.00000000	mice
Lfng	0.17	1.00000000	mice
Tmem106b	0.06	1.00000000	mice

Smarce1	0.02	1.00000000	mice
Il1rap	-0.04	1.00000000	mice
Trmt13	-0.07	1.00000000	mice
Ascc2	-0.04	1.00000000	mice
Bdp1	-0.09	1.00000000	mice
Cacul1	-0.03	1.00000000	mice
Smarca4	0.13	1.00000000	mice
Snrpf	0.19	1.00000000	mice
Acly	0.04	1.00000000	mice
Rgl2	0.12	1.00000000	mice
Siah1a	-0.07	1.00000000	mice
Selenon	0.04	1.00000000	mice
Polr3d	-0.02	1.00000000	mice
Usp38	-0.05	1.00000000	mice
Nfyb	0.01	1.00000000	mice
Cdyl2	-0.03	1.00000000	mice
Gnb2	0.11	1.00000000	mice
Nr6a1	-0.06	1.00000000	mice
Slc37a3	0.01	1.00000000	mice
Dpp3	0.00	1.00000000	mice
Ezh1	0.06	1.00000000	mice
Selenom	0.03	1.00000000	mice
Lnpk	0.07	1.00000000	mice
Dda1	0.06	1.00000000	mice
Nisch	0.12	1.00000000	mice
Abhd17c	0.09	1.00000000	mice
Bin3	0.06	1.00000000	mice
Fabp5	0.14	1.00000000	mice
Sdhaf2	0.17	1.00000000	mice
Taf3	-0.01	1.00000000	mice
Nap1l4	-0.06	1.00000000	mice
Slc17a5	0.10	1.00000000	mice
Gimap1	-0.08	1.00000000	mice
Tbc1d20	0.03	1.00000000	mice
Brd8	0.01	1.00000000	mice
Psmc5	0.04	1.00000000	mice
Pcdh1	0.09	1.00000000	mice
Ufc1	0.00	1.00000000	mice
3110056K07Rik	0.03	1.00000000	mice
Ttc28	-0.01	1.00000000	mice
Phc2	0.03	1.00000000	mice
Zfp369	-0.01	1.00000000	mice
Fgfr1op	0.09	1.00000000	mice
Aftph	0.10	1.00000000	mice
Rbm33	-0.02	1.00000000	mice
Exosc3	0.04	1.00000000	mice

Gas5	0.01	1.00000000	mice
Lmntd1	0.14	1.00000000	mice
Set	0.13	1.00000000	mice
Ndufv1	0.02	1.00000000	mice
Rasgrp3	0.13	1.00000000	mice
Immt	-0.03	1.00000000	mice
Vps4a	-0.06	1.00000000	mice
Ipo7	-0.16	1.00000000	mice
Caml	0.00	1.00000000	mice
Nkiras2	-0.01	1.00000000	mice
Ciao1	-0.02	1.00000000	mice
Dcaf11	0.15	1.00000000	mice
Rpap3	0.09	1.00000000	mice
Cntrl	-0.01	1.00000000	mice
Phip	-0.01	1.00000000	mice
Cog4	0.05	1.00000000	mice
Nrd1	0.12	1.00000000	mice
Unc50	0.05	1.00000000	mice
Arl8a	-0.10	1.00000000	mice
Trmt11	0.15	1.00000000	mice
Sarnp	-0.04	1.00000000	mice
Tor1b	0.06	1.00000000	mice
Creld2	0.16	1.00000000	mice
Tspan14	0.01	1.00000000	mice
Kbtbd2	0.06	1.00000000	mice
Hikeshi	-0.09	1.00000000	mice
Ppp4r3a	0.17	1.00000000	mice
Psmd1	0.10	1.00000000	mice
Bdkrb2	-0.08	1.00000000	mice
Tmub2	0.09	1.00000000	mice
Ino80c	0.07	1.00000000	mice
Pptc7	-0.05	1.00000000	mice
Lsp1	0.11	1.00000000	mice
Vps11	-0.10	1.00000000	mice
Zmynd11	-0.03	1.00000000	mice
Emc8	0.10	1.00000000	mice
Arhgap31	-0.10	1.00000000	mice
Zfp280c	0.05	1.00000000	mice
Zc3h7b	-0.08	1.00000000	mice
Icmt	0.11	1.00000000	mice
P2rx4	0.06	1.00000000	mice
Wdtc1	0.08	1.00000000	mice
Psen2	-0.10	1.00000000	mice
Tmem123	-0.02	1.00000000	mice
Btaf1	-0.01	1.00000000	mice
Ints7	-0.06	1.00000000	mice

Atp6v1c1	-0.06	1.00000000	mice
Gigyf2	0.08	1.00000000	mice
Arl2bp	0.12	1.00000000	mice
Ugp2	0.14	1.00000000	mice
Camsap1	0.03	1.00000000	mice
Glrx3	0.02	1.00000000	mice
Plgrkt	0.08	1.00000000	mice
Atp2b4	0.05	1.00000000	mice
Glmp	-0.02	1.00000000	mice
Pus1	0.06	1.00000000	mice
Selenos	0.22	1.00000000	mice
Sipa1l1	-0.01	1.00000000	mice
Rtca	0.11	1.00000000	mice
Lcor	-0.04	1.00000000	mice
Flii	0.00	1.00000000	mice
Nans	0.09	1.00000000	mice
Rb1	-0.02	1.00000000	mice
Prpf39	-0.02	1.00000000	mice
Camta2	0.02	1.00000000	mice
Stoml2	0.10	1.00000000	mice
Vps29	-0.02	1.00000000	mice
Dpf2	-0.04	1.00000000	mice
Tmeff2	0.20	1.00000000	mice
Tie1	0.10	1.00000000	mice
Ube2e2	0.18	1.00000000	mice
Cachd1	0.06	1.00000000	mice
Sem1	0.01	1.00000000	mice
Pikfyve	0.09	1.00000000	mice
Bod1l	-0.06	1.00000000	mice
Chrac1	0.05	1.00000000	mice
Timeless	0.02	1.00000000	mice
Tsc2	0.01	1.00000000	mice
Creb3	0.00	1.00000000	mice
Brms1	0.09	1.00000000	mice
Ndufs1	0.07	1.00000000	mice
Jak2	-0.05	1.00000000	mice
Tacc2	0.01	1.00000000	mice
Endod1	0.03	1.00000000	mice
Rab9	0.10	1.00000000	mice
Gla	0.03	1.00000000	mice
Trp53i11	0.04	1.00000000	mice
Nlk	0.00	1.00000000	mice
Pbx1	0.15	1.00000000	mice
Blmh	0.10	1.00000000	mice
Supt20	0.08	1.00000000	mice
Asf1a	-0.10	1.00000000	mice

Golm1	0.08	1.00000000	mice
B3gnt2	-0.04	1.00000000	mice
Slc25a51	0.04	1.00000000	mice
Paxip1	0.12	1.00000000	mice
Leprotl1	0.21	1.00000000	mice
Grb14	0.17	1.00000000	mice
Cwc27	0.10	1.00000000	mice
Frg1	0.16	1.00000000	mice
Spns1	0.04	1.00000000	mice
Ddx21	0.15	1.00000000	mice
Zfpl1	-0.10	1.00000000	mice
Appl1	-0.03	1.00000000	mice
Anxa6	0.03	1.00000000	mice
Cd38	-0.08	1.00000000	mice
Flywch1	0.06	1.00000000	mice
Iws1	0.00	1.00000000	mice
Ndufv2	0.17	1.00000000	mice
Cdc14a	0.05	1.00000000	mice
Pknox1	0.00	1.00000000	mice
Rac1	0.11	1.00000000	mice
Nlgn2	0.05	1.00000000	mice
Pggt1b	-0.04	1.00000000	mice
Tasp1	-0.06	1.00000000	mice
Dusp16	0.03	1.00000000	mice
Csnk2a1	-0.02	1.00000000	mice
Tapt1	-0.02	1.00000000	mice
Hdac3	-0.03	1.00000000	mice
Sympk	0.11	1.00000000	mice
Tmem181a	-0.01	1.00000000	mice
Scaf11	0.08	1.00000000	mice
S100a6	0.28	1.00000000	mice
Dop1b	0.13	1.00000000	mice
Dld	0.08	1.00000000	mice
Zfyve21	-0.06	1.00000000	mice
Arhgap35	0.12	1.00000000	mice
Il13ra1	0.03	1.00000000	mice
Rhoc	0.26	1.00000000	mice
Rad51ap1	0.07	1.00000000	mice
Gnao1	0.02	1.00000000	mice
Cox11	-0.03	1.00000000	mice
Dhrs7	-0.04	1.00000000	mice
Bloc1s2	-0.11	1.00000000	mice
E2f4	-0.07	1.00000000	mice
Srp14	0.01	1.00000000	mice
Sdhaf4	0.22	1.00000000	mice
Glrx2	-0.07	1.00000000	mice

Cep164	0.06	1.00000000	mice
Anapc13	0.15	1.00000000	mice
Ccm2l	0.15	1.00000000	mice
Dcaf5	0.09	1.00000000	mice
Fam114a1	-0.10	1.00000000	mice
Dcp1b	-0.01	1.00000000	mice
Fbxl17	0.07	1.00000000	mice
Nsmce1	0.05	1.00000000	mice
Dhcr24	0.10	1.00000000	mice
Tiprl	0.08	1.00000000	mice
Prpf18	-0.01	1.00000000	mice
Ttc9c	0.06	1.00000000	mice
Cstb	0.49	1.00000000	mice
Zcchc17	0.10	1.00000000	mice
Fbxw8	-0.11	1.00000000	mice
Ndufa13	0.20	1.00000000	mice
Bcor	0.01	1.00000000	mice
Rad50	0.05	1.00000000	mice
Tmem131	-0.03	1.00000000	mice
Rmc1	-0.03	1.00000000	mice
Dnajc5	0.01	1.00000000	mice
Acin1	-0.03	1.00000000	mice
Lrrc32	0.01	1.00000000	mice
Gxylt1	0.03	1.00000000	mice
Tomm6	0.11	1.00000000	mice
Cbfb	-0.02	1.00000000	mice
Dctn2	0.06	1.00000000	mice
Yif1b	0.10	1.00000000	mice
Ccdc34	-0.02	1.00000000	mice
Gatad2a	0.16	1.00000000	mice
Yars	-0.06	1.00000000	mice
Atp6v1g1	0.00	1.00000000	mice
Fam126b	0.09	1.00000000	mice
Zfp821	-0.06	1.00000000	mice
Eml4	-0.03	1.00000000	mice
Gan	-0.01	1.00000000	mice
Ostm1	0.05	1.00000000	mice
Slc4a7	-0.05	1.00000000	mice
Uqcrb	0.27	1.00000000	mice
Prmt1	-0.10	1.00000000	mice
Ipo8	0.06	1.00000000	mice
Dnajb14	-0.06	1.00000000	mice
Kdm5b	-0.05	1.00000000	mice
4931406P16Rik	-0.05	1.00000000	mice
Zfp236	0.08	1.00000000	mice
Vamp8	0.05	1.00000000	mice

Sars	0.08	1.00000000	mice
Yif1a	0.07	1.00000000	mice
Selenoi	0.03	1.00000000	mice
Pgrmc2	-0.03	1.00000000	mice
Xist	0.09	1.00000000	mice
Dctn5	0.09	1.00000000	mice
Lypla1	0.12	1.00000000	mice
Armc5	0.04	1.00000000	mice
Ik	0.11	1.00000000	mice
Psmc4	0.07	1.00000000	mice
Wdr47	0.02	1.00000000	mice
Plekhf2	-0.05	1.00000000	mice
Daam1	0.08	1.00000000	mice
Hsf1	0.01	1.00000000	mice
Snx16	-0.06	1.00000000	mice
Lhfp	0.09	1.00000000	mice
Socs5	0.09	1.00000000	mice
Hdx	-0.13	1.00000000	mice
Rnpep	0.07	1.00000000	mice
Nfkbil1	-0.03	1.00000000	mice
Pcid2	-0.07	1.00000000	mice
Cdh5	-0.04	1.00000000	mice
Ddx41	0.11	1.00000000	mice
Stk38	-0.08	1.00000000	mice
Rbbp7	0.08	1.00000000	mice
Ddx46	0.11	1.00000000	mice
Adpgk	0.00	1.00000000	mice
Tent4b	0.05	1.00000000	mice
Spag7	-0.08	1.00000000	mice
Gpcpd1	-0.02	1.00000000	mice
Chmp7	0.08	1.00000000	mice
Prr14	0.03	1.00000000	mice
Eci2	0.01	1.00000000	mice
Emp3	0.20	1.00000000	mice
Large1	0.09	1.00000000	mice
Sirt2	0.03	1.00000000	mice
Dmwd	-0.01	1.00000000	mice
Nagk	-0.07	1.00000000	mice
Xpo7	0.00	1.00000000	mice
Trim47	0.00	1.00000000	mice
Wdr37	-0.06	1.00000000	mice
Eif3g	0.17	1.00000000	mice
Commd10	0.13	1.00000000	mice
Timp2	0.18	1.00000000	mice
Vangl2	0.10	1.00000000	mice
Usp15	-0.05	1.00000000	mice

Dhx30	0.09	1.00000000	mice
Ak6	0.03	1.00000000	mice
Fam50a	0.10	1.00000000	mice
Fdx2	0.00	1.00000000	mice
Lims1	0.11	1.00000000	mice
Ppil2	0.04	1.00000000	mice
Tlk2	0.05	1.00000000	mice
Vps13d	0.13	1.00000000	mice
Ssbp2	0.05	1.00000000	mice
Tnip1	0.17	1.00000000	mice
Batf2	0.08	1.00000000	mice
Srsf11	0.02	1.00000000	mice
Nme1	0.13	1.00000000	mice
Nucb2	0.08	1.00000000	mice
Ncln	0.01	1.00000000	mice
Psmd3	0.19	1.00000000	mice
Bcap29	0.00	1.00000000	mice
Ap1g1	0.16	1.00000000	mice
Cmpk1	0.02	1.00000000	mice
Smn1	0.09	1.00000000	mice
Rnmt	0.01	1.00000000	mice
4933434E20Rik	-0.01	1.00000000	mice
Dgat1	-0.04	1.00000000	mice
G3bp2	0.21	1.00000000	mice
Nfs1	-0.06	1.00000000	mice
Sra1	0.10	1.00000000	mice
Spopl	0.08	1.00000000	mice
Nup160	-0.07	1.00000000	mice
Farp2	-0.05	1.00000000	mice
Txndc15	0.11	1.00000000	mice
Sh3d19	0.13	1.00000000	mice
Als2cl	-0.14	1.00000000	mice
St3gal4	0.01	1.00000000	mice
Bfar	-0.01	1.00000000	mice
Rxra	0.06	1.00000000	mice
Dusp12	0.01	1.00000000	mice
Ric8a	-0.08	1.00000000	mice
Clu	0.01	1.00000000	mice
Eefsec	0.06	1.00000000	mice
Ndufa11	0.11	1.00000000	mice
Spcs1	-0.06	1.00000000	mice
Tgfbrap1	0.01	1.00000000	mice
Hgsnat	0.06	1.00000000	mice
Rbm34	0.09	1.00000000	mice
Hnrnpul2	-0.06	1.00000000	mice
Gna12	0.00	1.00000000	mice

Rae1	0.03	1.00000000	mice
Tns2	-0.12	1.00000000	mice
2310033P09Rik	0.12	1.00000000	mice
Znhit1	0.16	1.00000000	mice
Galk2	0.09	1.00000000	mice
Fkbp15	0.06	1.00000000	mice
Cavin3	0.24	1.00000000	mice
Prex2	0.11	1.00000000	mice
Flnb	-0.12	1.00000000	mice
Hlx	0.05	1.00000000	mice
Sdhc	0.12	1.00000000	mice
Lamtor5	0.14	1.00000000	mice
Smarcd2	0.11	1.00000000	mice
Nifk	0.03	1.00000000	mice
Ngrn	0.01	1.00000000	mice
Krcc1	0.14	1.00000000	mice
Lama5	-0.01	1.00000000	mice
Atp5j2	0.12	1.00000000	mice
Actr2	0.21	1.00000000	mice
Aimp1	0.00	1.00000000	mice
Wwox	0.23	1.00000000	mice
Cdkn1b	0.01	1.00000000	mice
Cpsf7	-0.03	1.00000000	mice
Crnkl1	-0.02	1.00000000	mice
Kif26a	0.02	1.00000000	mice
Stat5b	-0.18	1.00000000	mice
Ercc5	0.04	1.00000000	mice
Dyrk1a	0.01	1.00000000	mice
Wdfy2	0.17	1.00000000	mice
Pi4k2a	-0.01	1.00000000	mice
Rbpms	-0.05	1.00000000	mice
Kctd12	0.14	1.00000000	mice
Armcx1	-0.01	1.00000000	mice
Rnf166	0.10	1.00000000	mice
Shroom3	-0.08	1.00000000	mice
Ube2j2	0.04	1.00000000	mice
Rcn1	-0.07	1.00000000	mice
Pip5k1c	-0.03	1.00000000	mice
Oxa1l	0.04	1.00000000	mice
Stxbp6	-0.02	1.00000000	mice
Foxk1	0.05	1.00000000	mice
Cdk2	-0.03	1.00000000	mice
Pigk	-0.07	1.00000000	mice
Pacc1	-0.02	1.00000000	mice
Ndufb7	0.12	1.00000000	mice
Zfp777	0.08	1.00000000	mice

Dync2h1	0.08	1.00000000	mice
D230025D16Rik	0.11	1.00000000	mice
Lysmd4	-0.05	1.00000000	mice
Ccnt2	0.03	1.00000000	mice
Sugp1	-0.05	1.00000000	mice
Ctbp1	-0.04	1.00000000	mice
Cdyl	0.04	1.00000000	mice
Zfp865	0.02	1.00000000	mice
Urgcp	0.00	1.00000000	mice
Rbms2	0.12	1.00000000	mice
Dhx9	0.01	1.00000000	mice
Pip5k1b	0.06	1.00000000	mice
Papss1	0.02	1.00000000	mice
Ophn1	0.16	1.00000000	mice
Cap1	0.02	1.00000000	mice
Ssbp1	0.06	1.00000000	mice
Ap3d1	0.11	1.00000000	mice
Commd1	0.22	1.00000000	mice
Ptbp1	0.12	1.00000000	mice
Lbr	0.14	1.00000000	mice
Carm1	0.10	1.00000000	mice
Ip6k1	-0.05	1.00000000	mice
Naa50	0.18	1.00000000	mice
Gata2	-0.08	1.00000000	mice
Sf3b2	0.13	1.00000000	mice
Ube2g2	0.01	1.00000000	mice
Derl2	0.01	1.00000000	mice
Rev3l	0.13	1.00000000	mice
Lactb2	0.07	1.00000000	mice
1110008P14Rik	0.08	1.00000000	mice
Eif4g3	0.12	1.00000000	mice
Phf8	0.05	1.00000000	mice
1110004F10Rik	0.16	1.00000000	mice
Apex2	0.08	1.00000000	mice
Tlnrd1	-0.05	1.00000000	mice
Ltbp4	-0.16	1.00000000	mice
Fto	-0.01	1.00000000	mice
Ccdc90b	-0.02	1.00000000	mice
Tmem208	0.04	1.00000000	mice
Ppp3cc	0.02	1.00000000	mice
Dhx57	0.12	1.00000000	mice
Dok4	-0.06	1.00000000	mice
Cul1	-0.01	1.00000000	mice
Skap2	-0.01	1.00000000	mice
Abhd13	0.04	1.00000000	mice
Atf5	0.07	1.00000000	mice

Ckap4	-0.03	1.00000000	mice
Fes	-0.02	1.00000000	mice
Sh3bgrl	0.11	1.00000000	mice
Arid4b	0.01	1.00000000	mice
Naa80	0.10	1.00000000	mice
Abcf1	0.19	1.00000000	mice
Ccdc186	0.00	1.00000000	mice
Atg9a	0.02	1.00000000	mice
Slc9a3r1	0.02	1.00000000	mice
Fry	-0.09	1.00000000	mice
Ocel1	0.21	1.00000000	mice
Vkorc1l1	0.08	1.00000000	mice
Cnot3	0.12	1.00000000	mice
Cdc37l1	-0.05	1.00000000	mice
Stimate	0.02	1.00000000	mice
Polr1c	0.06	1.00000000	mice
Chmp3	0.20	1.00000000	mice
Plekhm2	-0.04	1.00000000	mice
Eif2b5	0.10	1.00000000	mice
Cpsf4	0.08	1.00000000	mice
Tomm40	0.00	1.00000000	mice
Las1l	0.01	1.00000000	mice
Pcnp	0.04	1.00000000	mice
Soga1	0.00	1.00000000	mice
Add1	0.18	1.00000000	mice
Brd7	0.06	1.00000000	mice
Lxn	-0.03	1.00000000	mice
Orai2	0.04	1.00000000	mice
Vta1	-0.01	1.00000000	mice
9530068E07Rik	0.07	1.00000000	mice
Usp48	-0.02	1.00000000	mice
Ppp1r12b	0.00	1.00000000	mice
Atp13a1	0.12	1.00000000	mice
Phka1	-0.16	1.00000000	mice
Topors	0.02	1.00000000	mice
Cpq	0.07	1.00000000	mice
Rnf24	0.06	1.00000000	mice
Usp47	-0.03	1.00000000	mice
Smad4	-0.01	1.00000000	mice
Slc7a6os	-0.09	1.00000000	mice
Celf1	-0.05	1.00000000	mice
Hivep1	0.12	1.00000000	mice
Nt5c2	-0.02	1.00000000	mice
Rad17	-0.03	1.00000000	mice
Pip4p1	0.12	1.00000000	mice
Pgd	0.07	1.00000000	mice

Ift52	0.01	1.00000000	mice
Gars	0.09	1.00000000	mice
Rere	0.09	1.00000000	mice
Clec1a	-0.03	1.00000000	mice
Zranb2	0.04	1.00000000	mice
Cdc14b	0.05	1.00000000	mice
Rbmxl1	0.10	1.00000000	mice
Rai1	-0.01	1.00000000	mice
Gga1	0.11	1.00000000	mice
Snrnp200	-0.01	1.00000000	mice
Atg7	0.02	1.00000000	mice
Eif3i	0.22	1.00000000	mice
Orc5	-0.04	1.00000000	mice
Srp68	0.06	1.00000000	mice
Ercc3	0.01	1.00000000	mice
Rab27a	0.03	1.00000000	mice
Rcn2	0.16	1.00000000	mice
Prdm4	0.02	1.00000000	mice
Pum2	0.01	1.00000000	mice
Cfap36	0.15	1.00000000	mice
2610037D02Rik	0.13	1.00000000	mice
Klhl2	0.03	1.00000000	mice
Fer	0.13	1.00000000	mice
Rfc1	0.16	1.00000000	mice
Bmi1	-0.01	1.00000000	mice
Smad1	0.20	1.00000000	mice
Fam98c	-0.09	1.00000000	mice
Sdad1	-0.06	1.00000000	mice
Fcho2	0.02	1.00000000	mice
Tpd52l2	0.08	1.00000000	mice
Fam53c	0.01	1.00000000	mice
Rab14	0.02	1.00000000	mice
Rpp25l	0.08	1.00000000	mice
Sept6	0.06	1.00000000	mice
Fbxl5	0.10	1.00000000	mice
Glud1	-0.11	1.00000000	mice
Dlat	0.04	1.00000000	mice
Plaa	0.08	1.00000000	mice
Ifngr2	0.06	1.00000000	mice
Tm6sf1	0.06	1.00000000	mice
Atp6v1a	0.08	1.00000000	mice
Cox18	-0.04	1.00000000	mice
Fbxw11	0.09	1.00000000	mice
Specc1l	0.14	1.00000000	mice
Kctd2	0.08	1.00000000	mice
Ramp2	-0.03	1.00000000	mice

Sqor	0.02	1.00000000	mice
Dennd6a	0.13	1.00000000	mice
Uxt	-0.02	1.00000000	mice
Ptbp3	0.08	1.00000000	mice
Tet2	-0.05	1.00000000	mice
1810058I24Rik	-0.01	1.00000000	mice
Akap8l	0.01	1.00000000	mice
Derl1	0.14	1.00000000	mice
C1d	0.05	1.00000000	mice
Pacsin2	-0.05	1.00000000	mice
Pdpk1	0.04	1.00000000	mice
Prkdc	-0.07	1.00000000	mice
Nsmce2	0.11	1.00000000	mice
Aasdhppt	0.07	1.00000000	mice
Eipr1	0.04	1.00000000	mice
Pola1	0.10	1.00000000	mice
Ubtf	0.06	1.00000000	mice
Exosc10	0.05	1.00000000	mice
Rbm26	-0.01	1.00000000	mice
Fam53a	-0.07	1.00000000	mice
Emsy	0.08	1.00000000	mice
Atg13	0.00	1.00000000	mice
Gnl1	-0.01	1.00000000	mice
C87436	-0.01	1.00000000	mice
Prep	0.00	1.00000000	mice
Hist3h2a	0.13	1.00000000	mice
Pgm5	-0.02	1.00000000	mice
Rab22a	0.04	1.00000000	mice
Sept10	-0.01	1.00000000	mice
Notch2	0.02	1.00000000	mice
Gask1b	0.08	1.00000000	mice
Sh3bp4	0.00	1.00000000	mice
Kiz	-0.02	1.00000000	mice
Ttc23	0.09	1.00000000	mice
Hcfc1r1	-0.01	1.00000000	mice
Zc3hav1	-0.10	1.00000000	mice
Atg12	0.04	1.00000000	mice
Tmem241	-0.05	1.00000000	mice
Tfam	0.02	1.00000000	mice
Ankrd50	-0.06	1.00000000	mice
Prr14l	0.09	1.00000000	mice
Samm50	0.03	1.00000000	mice
C430049B03Rik	0.07	1.00000000	mice
Kdm4c	0.14	1.00000000	mice
Pes1	-0.03	1.00000000	mice
Pi4k2b	0.01	1.00000000	mice

Polk	0.05	1.00000000	mice
Nfkb1	0.14	1.00000000	mice
Wdr82	0.02	1.00000000	mice
Os9	0.02	1.00000000	mice
Stx2	0.09	1.00000000	mice
Pias2	0.05	1.00000000	mice
Cdkn2b	0.13	1.00000000	mice
Dock8	0.03	1.00000000	mice
Chkb	0.02	1.00000000	mice
Ankrd46	0.14	1.00000000	mice
Chmp5	0.20	1.00000000	mice
Upf3b	-0.12	1.00000000	mice
Atp6v1b2	0.04	1.00000000	mice
Zfp24	0.00	1.00000000	mice
Arhgef10	0.02	1.00000000	mice
Plin3	0.00	1.00000000	mice
F11r	0.09	1.00000000	mice
Utrn	0.16	1.00000000	mice
Cep295	0.09	1.00000000	mice
Crym	0.03	1.00000000	mice
Slc39a14	0.05	1.00000000	mice
Pnpt1	0.01	1.00000000	mice
Pycr2	0.07	1.00000000	mice
Zfp148	-0.04	1.00000000	mice
Snrpe	0.01	1.00000000	mice
Bcl6	0.03	1.00000000	mice
Syncrip	-0.02	1.00000000	mice
Ubash3b	-0.06	1.00000000	mice
Tmtc3	0.02	1.00000000	mice
Ankrd52	-0.04	1.00000000	mice
Zfp143	0.03	1.00000000	mice
Gch1	-0.10	1.00000000	mice
Acox3	0.05	1.00000000	mice
Crtap	0.00	1.00000000	mice
Limd1	0.03	1.00000000	mice
Trappc9	0.03	1.00000000	mice
Bcar1	0.02	1.00000000	mice
Fut11	0.05	1.00000000	mice
Trappc3	0.22	1.00000000	mice
Zfp740	0.05	1.00000000	mice
Atxn7l3	0.01	1.00000000	mice
Slc31a2	0.06	1.00000000	mice
Rnf8	0.02	1.00000000	mice
Zfp422	-0.01	1.00000000	mice
Hist1h2bc	-0.02	1.00000000	mice
Ube2d3	0.02	1.00000000	mice

Ebf1	0.32	1.00000000	mice
Yeats2	0.04	1.00000000	mice
Entr1	-0.11	1.00000000	mice
Ets2	-0.08	1.00000000	mice
Parg	0.05	1.00000000	mice
Hp1bp3	0.17	1.00000000	mice
Ttc39b	0.03	1.00000000	mice
Zrsr2	0.02	1.00000000	mice
Rab24	0.02	1.00000000	mice
Nvl	0.02	1.00000000	mice
Rab3gap1	-0.01	1.00000000	mice
Fam189a2	0.04	1.00000000	mice
Sft2d1	0.03	1.00000000	mice
Dmxl1	0.05	1.00000000	mice
Cwc22	0.07	1.00000000	mice
Tmem203	0.00	1.00000000	mice
Ckap5	0.17	1.00000000	mice
Lrrc47	-0.07	1.00000000	mice
Esyt2	0.05	1.00000000	mice
Eloa	-0.01	1.00000000	mice
Srrt	0.01	1.00000000	mice
Zfyve1	0.09	1.00000000	mice
Atp6v0d1	0.03	1.00000000	mice
Snhg6	0.20	1.00000000	mice
Prorsd1	0.01	1.00000000	mice
Tmed9	0.05	1.00000000	mice
Smad7	0.10	1.00000000	mice
Dot1l	0.12	1.00000000	mice
Ppm1g	-0.01	1.00000000	mice
Cox6b1	0.08	1.00000000	mice
Necap2	0.03	1.00000000	mice
Banf1	0.13	1.00000000	mice
Eea1	-0.02	1.00000000	mice
Slc16a9	0.05	1.00000000	mice
Ttll5	-0.05	1.00000000	mice
Fam76b	0.12	1.00000000	mice
Ext2	0.12	1.00000000	mice
Fam91a1	-0.11	1.00000000	mice
Sec61a1	0.04	1.00000000	mice
Ascc1	-0.02	1.00000000	mice
Llph	0.16	1.00000000	mice
Prrc2b	-0.05	1.00000000	mice
Smchd1	0.16	1.00000000	mice
2310001H17Rik	0.00	1.00000000	mice
Smurf1	0.04	1.00000000	mice
Akap9	0.12	1.00000000	mice

Ttc13	-0.02	1.00000000	mice
Tfg	-0.04	1.00000000	mice
Stx17	0.07	1.00000000	mice
Vps35l	0.12	1.00000000	mice
Pou6f1	-0.05	1.00000000	mice
Bckdha	0.19	1.00000000	mice
Polr2k	-0.02	1.00000000	mice
Nf2	-0.04	1.00000000	mice
Nfxl1	-0.10	1.00000000	mice
Akap13	-0.07	1.00000000	mice
Hsdl1	-0.02	1.00000000	mice
Grb2	0.05	1.00000000	mice
Far1	0.03	1.00000000	mice
Snd1	0.08	1.00000000	mice
Vopp1	-0.03	1.00000000	mice
Psmd7	0.04	1.00000000	mice
Gtf3a	-0.04	1.00000000	mice
Parp6	0.00	1.00000000	mice
Krr1	0.08	1.00000000	mice
Zdhhc4	0.10	1.00000000	mice
Frmd4a	0.12	1.00000000	mice
Sclt1	0.12	1.00000000	mice
Prkd2	0.16	1.00000000	mice
Ehd2	0.07	1.00000000	mice
Prex1	0.00	1.00000000	mice
Stx4a	0.06	1.00000000	mice
Abcc3	-0.02	1.00000000	mice
Shroom4	0.00	1.00000000	mice
Rrp1	0.00	1.00000000	mice
Rcc2	0.06	1.00000000	mice
Anks3	-0.02	1.00000000	mice
Pdcd7	0.05	1.00000000	mice
Skp1a	0.09	1.00000000	mice
Ddhd2	-0.10	1.00000000	mice
Slc25a11	0.03	1.00000000	mice
Ncbp3	-0.01	1.00000000	mice
Cnpy3	0.11	1.00000000	mice
Pgls	0.00	1.00000000	mice
Fars2	0.02	1.00000000	mice
Cfl2	0.02	1.00000000	mice
Adcy9	0.06	1.00000000	mice
2700081O15Rik	0.05	1.00000000	mice
Tmem41b	-0.02	1.00000000	mice
Tatdn2	0.02	1.00000000	mice
Cspp1	-0.04	1.00000000	mice
Carhsp1	0.06	1.00000000	mice

Tmem176b	0.14	1.00000000	mice
Cox7b	0.14	1.00000000	mice
2410004B18Rik	-0.04	1.00000000	mice
Golga1	-0.01	1.00000000	mice
Psmb2	0.10	1.00000000	mice
Wdr11	0.05	1.00000000	mice
Ppwd1	0.06	1.00000000	mice
Hgs	-0.01	1.00000000	mice
Erg28	0.03	1.00000000	mice
Tex14	0.02	1.00000000	mice
Setbp1	0.05	1.00000000	mice
Aifm1	0.09	1.00000000	mice
Cers5	-0.07	1.00000000	mice
Rnps1	0.09	1.00000000	mice
Tmem131l	-0.05	1.00000000	mice
Polr3k	0.10	1.00000000	mice
Cox20	0.06	1.00000000	mice
Twf2	0.10	1.00000000	mice
Cnot8	0.02	1.00000000	mice
Pde7a	-0.07	1.00000000	mice
Tbc1d16	0.06	1.00000000	mice
Brd9	0.05	1.00000000	mice
Ikzf5	0.01	1.00000000	mice
Uba2	-0.01	1.00000000	mice
Phkb	0.02	1.00000000	mice
Smarcb1	0.00	1.00000000	mice
Cul4a	-0.07	1.00000000	mice
Rgs12	-0.03	1.00000000	mice
Ash2l	0.08	1.00000000	mice
Timm44	0.17	1.00000000	mice
Lyst	0.12	1.00000000	mice
Naa38	-0.08	1.00000000	mice
Rreb1	0.10	1.00000000	mice
Ppp4r3b	0.02	1.00000000	mice
Usp24	-0.03	1.00000000	mice
Rab40c	-0.03	1.00000000	mice
Tbl1x	0.08	1.00000000	mice
Polg	-0.06	1.00000000	mice
Atox1	0.09	1.00000000	mice
Edc4	0.09	1.00000000	mice
S100pbp	0.03	1.00000000	mice
Foxk2	0.10	1.00000000	mice
Wdr43	0.17	1.00000000	mice
Kctd20	0.01	1.00000000	mice
Ier3ip1	-0.02	1.00000000	mice
Arhgap12	0.13	1.00000000	mice

Strn4	0.03	1.00000000	mice
Rint1	0.06	1.00000000	mice
Eapp	0.12	1.00000000	mice
Rabgap1	0.00	1.00000000	mice
Eif2ak4	-0.02	1.00000000	mice
Ankhd1	0.01	1.00000000	mice
Atp5g1	0.11	1.00000000	mice
Pdcd11	-0.01	1.00000000	mice
Ankib1	0.03	1.00000000	mice
Gtf3c3	0.06	1.00000000	mice
Ankrd17	-0.08	1.00000000	mice
Cluap1	0.00	1.00000000	mice
Fubp3	0.09	1.00000000	mice
Spred2	0.04	1.00000000	mice
Socs4	-0.06	1.00000000	mice
Slf2	-0.02	1.00000000	mice
Atp13a3	0.05	1.00000000	mice
Pex7	0.02	1.00000000	mice
Rab3ip	-0.06	1.00000000	mice
Rhoa	0.03	1.00000000	mice
Arf2	0.01	1.00000000	mice
Nat9	0.03	1.00000000	mice
Emc6	0.09	1.00000000	mice
Napg	0.06	1.00000000	mice
Ccz1	-0.05	1.00000000	mice
Cyb5a	0.05	1.00000000	mice
Tsfm	-0.10	1.00000000	mice
Htra2	0.00	1.00000000	mice
Anxa1	-0.01	1.00000000	mice
Rptor	0.13	1.00000000	mice
Ash1l	0.00	1.00000000	mice
Prpf40a	0.04	1.00000000	mice
Unc5b	-0.01	1.00000000	mice
Dnajc1	-0.04	1.00000000	mice
Smdt1	0.10	1.00000000	mice
Hltf	-0.01	1.00000000	mice
Xdh	-0.11	1.00000000	mice
Flt4	0.01	1.00000000	mice
Cxxc1	-0.05	1.00000000	mice
Safb	-0.02	1.00000000	mice
Cenpb	0.25	1.00000000	mice
Tceal9	-0.04	1.00000000	mice
Trrap	-0.04	1.00000000	mice
Exosc9	0.09	1.00000000	mice
Bcl2	0.04	1.00000000	mice
Stub1	0.01	1.00000000	mice

Eif2b2	0.01	1.00000000	mice
D030056L22Rik	0.09	1.00000000	mice
Bccip	0.03	1.00000000	mice
Psma4	0.12	1.00000000	mice
Fbxo42	0.08	1.00000000	mice
Arcn1	-0.01	1.00000000	mice
Tm9sf3	0.07	1.00000000	mice
Acat1	0.01	1.00000000	mice
Asxl2	0.04	1.00000000	mice
Rnd1	0.42	1.00000000	mice
Wdr45	0.02	1.00000000	mice
Cd164	0.11	1.00000000	mice
Abca7	0.00	1.00000000	mice
Vti1a	0.00	1.00000000	mice
Cdkl5	0.06	1.00000000	mice
Usp42	0.02	1.00000000	mice
Arhgef15	0.13	1.00000000	mice
Ttc5	0.06	1.00000000	mice
Hdgfl3	0.09	1.00000000	mice
Calm3	0.28	1.00000000	mice
Letm1	0.03	1.00000000	mice
Prelid3b	0.01	1.00000000	mice
Ube2n	0.09	1.00000000	mice
Rinl	0.05	1.00000000	mice
Hspa13	-0.03	1.00000000	mice
Rbm22	0.02	1.00000000	mice
Cryzl1	-0.01	1.00000000	mice
Arfgap1	-0.05	1.00000000	mice
Gtf2f2	0.11	1.00000000	mice
Chd7	0.14	1.00000000	mice
Luc7l2	-0.06	1.00000000	mice
Acad8	0.00	1.00000000	mice
Cyb5r3	0.08	1.00000000	mice
Sf3a1	-0.03	1.00000000	mice
Snrpa	0.05	1.00000000	mice
Bin1	0.01	1.00000000	mice
Homer1	0.04	1.00000000	mice
Copg1	0.05	1.00000000	mice
Rcor3	0.03	1.00000000	mice
Setd5	0.03	1.00000000	mice
Sec22b	0.20	1.00000000	mice
Ccng1	0.05	1.00000000	mice
Eri1	-0.02	1.00000000	mice
AU040320	0.05	1.00000000	mice
Afap1l1	-0.03	1.00000000	mice
Copb2	0.12	1.00000000	mice

Pdgfd	0.13	1.00000000	mice
Nufip1	0.11	1.00000000	mice
Nol8	-0.07	1.00000000	mice
Trp53bp1	0.06	1.00000000	mice
Arl6ip4	0.05	1.00000000	mice
Vamp7	0.03	1.00000000	mice
Parp1	0.04	1.00000000	mice
Rprml	0.23	1.00000000	mice
Il6st	-0.02	1.00000000	mice
Uqcr10	0.32	1.00000000	mice
Hmgxb3	0.05	1.00000000	mice
Ndufs3	-0.07	1.00000000	mice
Cdc42se2	0.08	1.00000000	mice
Actr8	0.11	1.00000000	mice
Ccndbp1	-0.05	1.00000000	mice
Necap1	-0.03	1.00000000	mice
Zbtb20	-0.02	1.00000000	mice
Rnf169	-0.07	1.00000000	mice
Slc23a2	-0.14	1.00000000	mice
Acd	0.11	1.00000000	mice
Arpc4	0.32	1.00000000	mice
Ctdsp2	0.09	1.00000000	mice
Atp9b	-0.06	1.00000000	mice
Fbxo45	0.02	1.00000000	mice
Bola2	0.21	1.00000000	mice
Itgb1	-0.04	1.00000000	mice
Eif1ad	0.01	1.00000000	mice
Srpk2	-0.01	1.00000000	mice
Endov	0.04	1.00000000	mice
Ilf3	0.02	1.00000000	mice
Ggps1	0.12	1.00000000	mice
Rab18	0.04	1.00000000	mice
Qsox2	-0.01	1.00000000	mice
Bcl10	-0.02	1.00000000	mice
Git1	0.19	1.00000000	mice
Zfp800	0.05	1.00000000	mice
Usp53	0.10	1.00000000	mice
Kmt2c	-0.01	1.00000000	mice
Arhgef3	-0.17	1.00000000	mice
Wbp11	-0.02	1.00000000	mice
Lipa	-0.01	1.00000000	mice
Lrrc28	0.00	1.00000000	mice
Zfp362	-0.08	1.00000000	mice
Chmp2a	0.04	1.00000000	mice
Bckdk	0.11	1.00000000	mice
Tial1	0.12	1.00000000	mice

Smpd1	0.06	1.00000000	mice
Prpsap2	0.02	1.00000000	mice
Hs3st1	0.02	1.00000000	mice
Wipf1	0.07	1.00000000	mice
Eed	0.02	1.00000000	mice
Csk	0.20	1.00000000	mice
Zbtb41	0.08	1.00000000	mice
Yipf6	-0.02	1.00000000	mice
Fam102a	-0.02	1.00000000	mice
Clk2	-0.01	1.00000000	mice
Brip1os	0.04	1.00000000	mice
Leng1	0.08	1.00000000	mice
Cep170	0.01	1.00000000	mice
Cd2ap	-0.01	1.00000000	mice
Stard3	-0.01	1.00000000	mice
Ccdc6	0.10	1.00000000	mice
Uxs1	0.07	1.00000000	mice
Prpsap1	0.04	1.00000000	mice
Pex3	0.06	1.00000000	mice
Ggnbp2	0.09	1.00000000	mice
Acer3	-0.06	1.00000000	mice
Polb	-0.04	1.00000000	mice
Pou2f1	0.12	1.00000000	mice
Naga	-0.06	1.00000000	mice
Bag4	-0.02	1.00000000	mice
Smim15	0.21	1.00000000	mice
Fbxo28	-0.06	1.00000000	mice
Alg5	-0.03	1.00000000	mice
Gfm1	0.07	1.00000000	mice
Reep1	-0.05	1.00000000	mice
Hif1an	0.04	1.00000000	mice
Eif1ax	0.06	1.00000000	mice
Rad54l2	0.09	1.00000000	mice
Kmt2a	0.00	1.00000000	mice
Hspa4l	0.11	1.00000000	mice
Dnajc25	-0.01	1.00000000	mice
Slc35e1	-0.03	1.00000000	mice
Parn	0.07	1.00000000	mice
Acsl4	0.03	1.00000000	mice
Clpb	-0.02	1.00000000	mice
S100a10	0.10	1.00000000	mice
Seh1l	0.00	1.00000000	mice
Kdm2a	0.03	1.00000000	mice
Nub1	0.07	1.00000000	mice
Fchsd2	-0.05	1.00000000	mice
Cetn3	-0.09	1.00000000	mice

Sh3tc1	0.03	1.00000000	mice
Slc39a10	0.05	1.00000000	mice
Ube3b	0.01	1.00000000	mice
Dcaf10	-0.07	1.00000000	mice
Fbxw9	-0.01	1.00000000	mice
Tfdp1	0.04	1.00000000	mice
Cir1	0.08	1.00000000	mice
Cep162	0.11	1.00000000	mice
Pphln1	-0.02	1.00000000	mice
Adnp2	0.03	1.00000000	mice
Usp32	-0.06	1.00000000	mice
Hectd4	-0.03	1.00000000	mice
Usp4	0.09	1.00000000	mice
Zfp609	0.16	1.00000000	mice
Elp2	0.03	1.00000000	mice
Tubgcp3	-0.06	1.00000000	mice
Tmx1	0.01	1.00000000	mice
Flot1	0.09	1.00000000	mice
Ei24	0.04	1.00000000	mice
Otud1	0.05	1.00000000	mice
Ndufaf8	0.04	1.00000000	mice
Dnm1l	-0.04	1.00000000	mice
Ift43	0.06	1.00000000	mice
Tgm2	0.22	1.00000000	mice
Eif2d	0.03	1.00000000	mice
Dek	0.24	1.00000000	mice
Prkd3	0.09	1.00000000	mice
Slc30a5	0.04	1.00000000	mice
Ppib	0.15	1.00000000	mice
Lmbr1	0.03	1.00000000	mice
Rnpc3	0.07	1.00000000	mice
Ypel5	0.05	1.00000000	mice
Ssr4	0.07	1.00000000	mice
Fastk	0.11	1.00000000	mice
Ak2	0.05	1.00000000	mice
Fbxw5	0.11	1.00000000	mice
Inpp5a	0.03	1.00000000	mice
Osbpl8	0.16	1.00000000	mice
Nudt9	0.05	1.00000000	mice
Trip12	0.04	1.00000000	mice
Lifr	0.05	1.00000000	mice
Srek1	-0.02	1.00000000	mice
Tspan15	-0.04	1.00000000	mice
Glt8d1	-0.03	1.00000000	mice
Htatsf1	-0.05	1.00000000	mice
Aspscr1	0.10	1.00000000	mice

Qtrt1	0.05	1.00000000	mice
Polr3f	0.02	1.00000000	mice
Serbp1	0.19	1.00000000	mice
Tdg	0.04	1.00000000	mice
Snx13	-0.05	1.00000000	mice
Ddi2	0.01	1.00000000	mice
Zfhx3	0.10	1.00000000	mice
Tmed3	0.22	1.00000000	mice
Dlg1	0.09	1.00000000	mice
Fryl	-0.02	1.00000000	mice
Pom121	0.04	1.00000000	mice
Zmym5	0.05	1.00000000	mice
Grsf1	0.00	1.00000000	mice
Trim26	0.09	1.00000000	mice
Polr2j	0.00	1.00000000	mice
Atxn1l	0.03	1.00000000	mice
Snx9	0.00	1.00000000	mice
Strada	-0.02	1.00000000	mice
Zfp280d	0.09	1.00000000	mice
Rpia	0.03	1.00000000	mice
Ppp2r5e	0.10	1.00000000	mice
Tbc1d9b	0.01	1.00000000	mice
Adgra2	0.08	1.00000000	mice
Rtn1	0.06	1.00000000	mice
Lemd3	0.02	1.00000000	mice
Pnisr	0.06	1.00000000	mice
Abcf2	-0.07	1.00000000	mice
Ccs	0.00	1.00000000	mice
Trim27	-0.04	1.00000000	mice
Ring1	0.04	1.00000000	mice
Cct6a	0.14	1.00000000	mice
Idh3a	0.06	1.00000000	mice
Usp33	0.05	1.00000000	mice
Tmem237	0.15	1.00000000	mice
Aph1a	0.14	1.00000000	mice
Ilkap	0.17	1.00000000	mice
Pdcd2l	0.07	1.00000000	mice
Clasp1	-0.02	1.00000000	mice
Ulk1	0.03	1.00000000	mice
Wdfy1	-0.02	1.00000000	mice
Tmem115	0.05	1.00000000	mice
Hacd3	-0.11	1.00000000	mice
Arl5a	0.07	1.00000000	mice
Riox1	0.00	1.00000000	mice
Abcg1	0.16	1.00000000	mice
Sdhaf1	-0.12	1.00000000	mice

Zfp282	-0.06	1.00000000	mice
Elovl1	0.10	1.00000000	mice
St13	-0.01	1.00000000	mice
Dazap1	0.28	1.00000000	mice
Spindoc	-0.05	1.00000000	mice
Lcmt1	-0.03	1.00000000	mice
Vps13a	-0.05	1.00000000	mice
Zfp706	0.02	1.00000000	mice
Tspan31	0.25	1.00000000	mice
Tbcel	0.13	1.00000000	mice
Psma1	0.17	1.00000000	mice
Hpf1	0.00	1.00000000	mice
Ston2	0.08	1.00000000	mice
Sike1	0.08	1.00000000	mice
Cald1	0.27	1.00000000	mice
Vamp4	0.09	1.00000000	mice
Wdr20	-0.07	1.00000000	mice
Slc25a17	0.01	1.00000000	mice
Poglut2	0.06	1.00000000	mice
Hmox2	0.12	1.00000000	mice
Ndufa7	0.03	1.00000000	mice
Zfp322a	0.06	1.00000000	mice
Lrpprc	-0.02	1.00000000	mice
Asb13	0.14	1.00000000	mice
Zfp281	-0.04	1.00000000	mice
Gtf3c1	-0.06	1.00000000	mice
Pkia	-0.08	1.00000000	mice
Uqcr11	0.13	1.00000000	mice
Zbtb10	0.00	1.00000000	mice
Cd24a	0.02	1.00000000	mice
Ube3c	0.11	1.00000000	mice
Sipa1l3	-0.03	1.00000000	mice
Cyhr1	0.09	1.00000000	mice
Eif3l	0.02	1.00000000	mice
Ddost	0.19	1.00000000	mice
Phlda3	-0.03	1.00000000	mice
Rheb	0.04	1.00000000	mice
Ipo11	0.09	1.00000000	mice
Rbm15	0.03	1.00000000	mice
Ric1	0.03	1.00000000	mice
Ndufa10	0.07	1.00000000	mice
Zfp386	0.08	1.00000000	mice
Tbc1d14	-0.06	1.00000000	mice
Chd4	0.13	1.00000000	mice
Wdr44	0.08	1.00000000	mice
U2af1l4	-0.03	1.00000000	mice

Crcp	0.01	1.00000000	mice
Reep3	0.03	1.00000000	mice
Cfdp1	0.13	1.00000000	mice
Gimap6	-0.02	1.00000000	mice
Dnajb4	0.13	1.00000000	mice
Psmc6	0.07	1.00000000	mice
Pkd1	0.12	1.00000000	mice
Armcx3	-0.07	1.00000000	mice
Nom1	0.06	1.00000000	mice
Tmcc2	0.23	1.00000000	mice
Eif2b3	0.01	1.00000000	mice
Gnaq	0.08	1.00000000	mice
1810026B05Rik	0.09	1.00000000	mice
Pinx1	0.01	1.00000000	mice
Rilpl2	0.06	1.00000000	mice
Igf2r	0.01	1.00000000	mice
Ergic3	0.09	1.00000000	mice
Nbr1	0.02	1.00000000	mice
Yod1	0.00	1.00000000	mice
1700025G04Rik	0.03	1.00000000	mice
Ranbp10	0.11	1.00000000	mice
Bcl7c	0.03	1.00000000	mice
Klhdc3	0.07	1.00000000	mice
Fam122a	0.02	1.00000000	mice
Arhgap1	0.17	1.00000000	mice
Prkag2	-0.03	1.00000000	mice
Colgalt2	0.09	1.00000000	mice
Gns	0.14	1.00000000	mice
Abca3	0.08	1.00000000	mice
Rab35	0.03	1.00000000	mice
Vrk3	0.06	1.00000000	mice
Immp2l	0.06	1.00000000	mice
Jag1	0.06	1.00000000	mice
Ptpn23	0.10	1.00000000	mice
Ndufa6	0.00	1.00000000	mice
Fibin	-0.01	1.00000000	mice
Fam118b	0.01	1.00000000	mice
Fam135a	0.01	1.00000000	mice
Ncbp1	0.00	1.00000000	mice
Tmcc1	0.03	1.00000000	mice
Arrb1	-0.07	1.00000000	mice
Lrp4	0.06	1.00000000	mice
Rab11fip3	0.06	1.00000000	mice
Rab31	0.08	1.00000000	mice
Tmem30a	0.02	1.00000000	mice
Ralb	0.03	1.00000000	mice

Pmepa1	0.02	1.00000000	mice
Cog5	0.00	1.00000000	mice
Pkn3	0.01	1.00000000	mice
C1galt1c1	-0.04	1.00000000	mice
Phtf1	0.01	1.00000000	mice
Tmem65	0.11	1.00000000	mice
Fbxo34	0.02	1.00000000	mice
Slc29a1	0.13	1.00000000	mice
Fndc1	-0.12	1.00000000	mice
Dcbld2	0.00	1.00000000	mice
Lsm4	0.07	1.00000000	mice
Hccs	0.00	1.00000000	mice
Cwc25	0.06	1.00000000	mice
Itprid2	0.06	1.00000000	mice
Strn	0.00	1.00000000	mice
Gcnt2	-0.03	1.00000000	mice
Tnpo3	-0.02	1.00000000	mice
Arrdc3	-0.01	1.00000000	mice
Ndufs4	0.11	1.00000000	mice
Tgfb1	-0.03	1.00000000	mice
Rab10os	0.01	1.00000000	mice
Srfbp1	0.05	1.00000000	mice
Rars	0.01	1.00000000	mice
Sh3glb2	0.15	1.00000000	mice
Ntn4	0.04	1.00000000	mice
Pin1	0.08	1.00000000	mice
Frs2	-0.01	1.00000000	mice
Dgkz	0.15	1.00000000	mice
Rab3gap2	-0.02	1.00000000	mice
Spata5	0.00	1.00000000	mice
Birc6	0.11	1.00000000	mice
Fmc1	-0.04	1.00000000	mice
2310011J03Rik	-0.02	1.00000000	mice
Epn1	0.08	1.00000000	mice
Stx6	0.01	1.00000000	mice
Imp4	-0.12	1.00000000	mice
Pan2	0.02	1.00000000	mice
AI837181	-0.01	1.00000000	mice
Kitl	0.08	1.00000000	mice
Ncoa1	-0.02	1.00000000	mice
2210408I21Rik	0.01	1.00000000	mice
Smg6	-0.03	1.00000000	mice
Cradd	0.05	1.00000000	mice
Secisbp2l	0.18	1.00000000	mice
Lamtor3	-0.06	1.00000000	mice
Ubr4	0.09	1.00000000	mice

Rasgef1a	0.02	1.00000000	mice
Rev1	0.08	1.00000000	mice
Gopc	0.02	1.00000000	mice
Kif1bp	0.04	1.00000000	mice
Xrcc4	0.04	1.00000000	mice
Itga4	0.01	1.00000000	mice
Taf10	0.09	1.00000000	mice
Rraga	0.01	1.00000000	mice
Ate1	-0.02	1.00000000	mice
Ranbp3	0.04	1.00000000	mice
Rnf145	0.03	1.00000000	mice
Suclg2	0.12	1.00000000	mice
Idnk	0.09	1.00000000	mice
Setd2	0.08	1.00000000	mice
Ndufaf4	0.02	1.00000000	mice
Scamp1	0.04	1.00000000	mice
Abhd4	0.00	1.00000000	mice
Galnt2	-0.01	1.00000000	mice
Vasp	0.10	1.00000000	mice
Gpr108	0.04	1.00000000	mice
Ghitm	0.09	1.00000000	mice
Suds3	0.11	1.00000000	mice
Nap1l1	0.07	1.00000000	mice
Washc4	-0.06	1.00000000	mice
Ppp6r1	0.19	1.00000000	mice
Crlf2	0.11	1.00000000	mice
Lzic	0.05	1.00000000	mice
Zcchc10	0.06	1.00000000	mice
Rbm15b	0.01	1.00000000	mice
Gem	0.18	1.00000000	mice
Gng12	0.07	1.00000000	mice
Efcab14	-0.05	1.00000000	mice
E130308A19Rik	0.04	1.00000000	mice
Trim25	-0.01	1.00000000	mice
Hnrnpll	0.09	1.00000000	mice
Usp46	0.04	1.00000000	mice
Ttc17	0.04	1.00000000	mice
Bend7	0.04	1.00000000	mice
Pde8a	-0.07	1.00000000	mice
Pcmtd2	0.04	1.00000000	mice
Cactin	0.01	1.00000000	mice
Ppt2	0.07	1.00000000	mice
Anapc2	-0.01	1.00000000	mice
Slu7	-0.03	1.00000000	mice
Abcc4	0.06	1.00000000	mice
Itgb5	0.12	1.00000000	mice

Ano6	-0.06	1.00000000	mice
Commd7	-0.03	1.00000000	mice
Ube2w	0.11	1.00000000	mice
Eaf1	0.06	1.00000000	mice
Clns1a	0.07	1.00000000	mice
Apln	0.12	1.00000000	mice
Ufsp2	0.04	1.00000000	mice
2610203C22Rik	0.06	1.00000000	mice
Sptbn1	-0.02	1.00000000	mice
Ppp1ca	0.10	1.00000000	mice
Hjurp	-0.04	1.00000000	mice
Tbcd	-0.03	1.00000000	mice
Stxbp1	0.03	1.00000000	mice
Lysmd2	0.18	1.00000000	mice
Rexo1	-0.04	1.00000000	mice
Txlna	0.03	1.00000000	mice
Slc25a39	0.16	1.00000000	mice
Kif26b	0.24	1.00000000	mice
Fau	0.01	1.00000000	mice
Bax	0.04	1.00000000	mice
Elmo2	0.03	1.00000000	mice
Samsn1	-0.13	1.00000000	mice
Erlec1	0.00	1.00000000	mice
Ndufa5	0.16	1.00000000	mice
Rwdd1	0.10	1.00000000	mice
Atp6v0e2	-0.09	1.00000000	mice
Arid1a	0.09	1.00000000	mice
Timm50	0.08	1.00000000	mice
Srrm2	-0.01	1.00000000	mice
Coro7	0.01	1.00000000	mice
Atp6v0b	0.17	1.00000000	mice
Susd6	0.07	1.00000000	mice
Zc3h12c	0.02	1.00000000	mice
Gpbp1l1	0.08	1.00000000	mice
Grb10	-0.06	1.00000000	mice
Slc12a7	0.07	1.00000000	mice
Lrrcc1	0.06	1.00000000	mice
Slc25a16	0.04	1.00000000	mice
Elavl1	0.00	1.00000000	mice
Coro1a	0.16	1.00000000	mice
Ubr7	0.01	1.00000000	mice
Eef1akmt1	0.07	1.00000000	mice
Ptpn18	0.11	1.00000000	mice
Ccdc115	0.00	1.00000000	mice
Spice1	0.06	1.00000000	mice
Crebbp	0.06	1.00000000	mice

Brpf3	-0.03	1.00000000	mice
Ralgapa1	0.04	1.00000000	mice
She	-0.02	1.00000000	mice
Opa3	-0.13	1.00000000	mice
Afg3l1	-0.02	1.00000000	mice
Vps13c	0.01	1.00000000	mice
Dhx8	0.09	1.00000000	mice
Plcg1	0.04	1.00000000	mice
Ube2q2	0.12	1.00000000	mice
Abi2	-0.04	1.00000000	mice
Adprhl2	-0.01	1.00000000	mice
Gosr1	0.08	1.00000000	mice
Smox	0.17	1.00000000	mice
Fam120b	0.00	1.00000000	mice
Prkacb	0.12	1.00000000	mice
Abhd12	0.03	1.00000000	mice
Zfp319	0.05	1.00000000	mice
Timmdc1	0.03	1.00000000	mice
Fem1c	-0.13	1.00000000	mice
Hmgcr	-0.02	1.00000000	mice
D1Ertd622e	0.02	1.00000000	mice
Tbcb	-0.04	1.00000000	mice
Ogt	0.05	1.00000000	mice
Fam120a	-0.02	1.00000000	mice
Sestd1	-0.03	1.00000000	mice
Exosc4	0.01	1.00000000	mice
Zkscan17	-0.05	1.00000000	mice
Socs6	0.03	1.00000000	mice
Eif3d	-0.01	1.00000000	mice
Stx11	0.24	1.00000000	mice
Hps3	0.04	1.00000000	mice
Aatk	0.02	1.00000000	mice
Pik3r4	-0.02	1.00000000	mice
Aars	0.02	1.00000000	mice
Irs1	-0.09	1.00000000	mice
Ciz1	-0.03	1.00000000	mice
Lsm6	-0.06	1.00000000	mice
Sf1	0.10	1.00000000	mice
Uqcc2	-0.06	1.00000000	mice
Ap3b1	0.08	1.00000000	mice
Rybp	0.09	1.00000000	mice
Rif1	-0.03	1.00000000	mice
Txn1	0.04	1.00000000	mice
Psmc2	0.19	1.00000000	mice
Tmem80	0.04	1.00000000	mice
Snx15	-0.03	1.00000000	mice

Tirap	0.08	1.00000000	mice
C130074G19Rik	0.11	1.00000000	mice
Firre	-0.04	1.00000000	mice
Gtf3c2	0.04	1.00000000	mice
Washc5	0.01	1.00000000	mice
Tmem63a	0.04	1.00000000	mice
Pcbp3	0.10	1.00000000	mice
Rbm8a	0.04	1.00000000	mice
Cyfip1	0.03	1.00000000	mice
Otub1	0.14	1.00000000	mice
Ctsc	0.28	1.00000000	mice
Synj2bp	0.05	1.00000000	mice
Tbk1	0.08	1.00000000	mice
Sil1	0.01	1.00000000	mice
Nsmce4a	0.06	1.00000000	mice
Dbndd2	0.05	1.00000000	mice
Pqbp1	-0.10	1.00000000	mice
Zc3h15	0.07	1.00000000	mice
Clcc1	0.06	1.00000000	mice
Pole3	0.08	1.00000000	mice
Armc8	0.07	1.00000000	mice
Ube2o	-0.06	1.00000000	mice
Arsa	0.04	1.00000000	mice
Zfp266	0.10	1.00000000	mice
Patl2	0.05	1.00000000	mice
Oga	0.01	1.00000000	mice
Cct7	-0.03	1.00000000	mice
Kif1c	0.17	1.00000000	mice
Snx29	0.02	1.00000000	mice
Sod1	0.17	1.00000000	mice
Zfp950	0.00	1.00000000	mice
Fam45a	0.05	1.00000000	mice
Ubqln2	0.02	1.00000000	mice
Tab3	-0.04	1.00000000	mice
Usp14	0.01	1.00000000	mice
Pip4k2a	-0.05	1.00000000	mice
Braf	0.08	1.00000000	mice
Psma6	0.03	1.00000000	mice
Timm21	0.00	1.00000000	mice
Senp3	0.04	1.00000000	mice
Hspa12b	0.07	1.00000000	mice
Nsa2	0.00	1.00000000	mice
Psmb7	0.18	1.00000000	mice
Ednrb	-0.13	1.00000000	mice
Dll4	0.01	1.00000000	mice
Thoc2	0.03	1.00000000	mice

Cyb5r4	0.11	1.00000000	mice
Smg5	-0.03	1.00000000	mice
Urm1	0.05	1.00000000	mice
1110059E24Rik	0.06	1.00000000	mice
Heatr6	0.02	1.00000000	mice
Vps26c	-0.03	1.00000000	mice
Ddx52	0.13	1.00000000	mice
Fam214b	-0.05	1.00000000	mice
Fam20b	-0.03	1.00000000	mice
Cdc42ep1	-0.04	1.00000000	mice
Adgre5	-0.01	1.00000000	mice
Rbbp4	0.11	1.00000000	mice
Ncald	0.12	1.00000000	mice
Fam3a	0.01	1.00000000	mice
Rgs2	0.04	1.00000000	mice
Zfp945	0.02	1.00000000	mice
Cacna1c	0.14	1.00000000	mice
Esco1	0.01	1.00000000	mice
Agpat5	0.12	1.00000000	mice
Vps37b	-0.08	1.00000000	mice
Cops2	0.04	1.00000000	mice
Antxr1	0.03	1.00000000	mice
Cacfd1	-0.11	1.00000000	mice
Gabpa	0.07	1.00000000	mice
Ldlrad3	0.07	1.00000000	mice
Fscn1	0.08	1.00000000	mice
Ric8b	0.08	1.00000000	mice
Plekha2	0.01	1.00000000	mice
Elp3	-0.05	1.00000000	mice
Zkscan1	0.05	1.00000000	mice
Wapl	-0.02	1.00000000	mice
Wscd1	-0.04	1.00000000	mice
Atxn10	0.00	1.00000000	mice
Id3	0.02	1.00000000	mice
Fgf1	0.02	1.00000000	mice
Rtf1	0.01	1.00000000	mice
Gpalpp1	0.04	1.00000000	mice
Zfp329	0.00	1.00000000	mice
Arl6ip6	-0.03	1.00000000	mice
Rprd1b	0.01	1.00000000	mice
Ube2e3	0.13	1.00000000	mice
Sp2	0.09	1.00000000	mice
Psap	0.14	1.00000000	mice
Dpagt1	0.14	1.00000000	mice
Setd1a	0.02	1.00000000	mice
Nup35	-0.06	1.00000000	mice

Thsd1	0.05	1.00000000	mice
Dock7	0.13	1.00000000	mice
Rmdn3	0.01	1.00000000	mice
Slc20a1	0.13	1.00000000	mice
Spata6	0.04	1.00000000	mice
Hip1	0.05	1.00000000	mice
Emc10	-0.04	1.00000000	mice
Tmem183a	0.02	1.00000000	mice
Ints3	0.07	1.00000000	mice
Ap3s2	-0.01	1.00000000	mice
Supt16	0.05	1.00000000	mice
Xpo4	0.02	1.00000000	mice
Rngtt	0.07	1.00000000	mice
Psmd12	0.07	1.00000000	mice
A430005L14Rik	-0.05	1.00000000	mice
Cdc5l	0.07	1.00000000	mice
Eri3	-0.10	1.00000000	mice
Atf6b	0.02	1.00000000	mice
Hsd17b12	0.24	1.00000000	mice
Ptp4a3	0.10	1.00000000	mice
2310009A05Rik	0.10	1.00000000	mice
Nsun6	-0.07	1.00000000	mice
Asah2	0.07	1.00000000	mice
Calm1	0.09	1.00000000	mice
Aebp2	0.11	1.00000000	mice
Clec14a	0.13	1.00000000	mice
Arhgap32	0.08	1.00000000	mice
Pitpnm2	-0.05	1.00000000	mice
Dleu2	-0.02	1.00000000	mice
Fam199x	0.02	1.00000000	mice
Col5a2	0.03	1.00000000	mice
Krtcap2	0.07	1.00000000	mice
Pik3c2a	-0.01	1.00000000	mice
Etfdh	0.07	1.00000000	mice
Cnksr3	0.05	1.00000000	mice
Zscan26	0.07	1.00000000	mice
Ciapin1	0.05	1.00000000	mice
Bud23	0.09	1.00000000	mice
Oxct1	0.04	1.00000000	mice
Ahcyl2	0.05	1.00000000	mice
Otud4	0.12	1.00000000	mice
Hadh	0.08	1.00000000	mice
Sec13	0.13	1.00000000	mice
Sgf29	0.07	1.00000000	mice
Cnep1r1	-0.04	1.00000000	mice
Slc25a36	0.15	1.00000000	mice

Tmem245	0.10	1.00000000	mice
Dicer1	0.06	1.00000000	mice
Cep192	0.06	1.00000000	mice
Fxyd5	0.18	1.00000000	mice
Vcpip1	0.04	1.00000000	mice
Snapc1	0.08	1.00000000	mice
Scd2	-0.05	1.00000000	mice
Orai3	0.03	1.00000000	mice
Kat14	0.03	1.00000000	mice
Cars	-0.02	1.00000000	mice
Hlcs	-0.03	1.00000000	mice
Paxbp1	0.03	1.00000000	mice
Apopt1	0.06	1.00000000	mice
Triap1	0.15	1.00000000	mice
Pak1ip1	0.05	1.00000000	mice
Ica1	-0.03	1.00000000	mice
Ushbp1	0.00	1.00000000	mice
6030458C11Rik	-0.01	1.00000000	mice
Nr3c1	0.08	1.00000000	mice
Csgalnact1	0.03	1.00000000	mice
Zbtb7a	0.07	1.00000000	mice
Pi4kb	-0.01	1.00000000	mice
Vipas39	0.03	1.00000000	mice
Srpk1	0.07	1.00000000	mice
Plxnc1	0.03	1.00000000	mice
Szrd1	-0.01	1.00000000	mice
Srebf2	0.13	1.00000000	mice
Ttc4	0.05	1.00000000	mice
Klhl12	0.02	1.00000000	mice
Homer3	0.03	1.00000000	mice
D10Wsu102e	-0.07	1.00000000	mice
Tmem161a	-0.09	1.00000000	mice
Hsd3b7	0.00	1.00000000	mice
Rest	0.08	1.00000000	mice
Pdss2	0.11	1.00000000	mice
Patl1	-0.02	1.00000000	mice
Tlk1	0.01	1.00000000	mice
Npc2	0.15	1.00000000	mice
Fundc2	0.09	1.00000000	mice
Wwp2	-0.06	1.00000000	mice
Atp6v0a1	0.02	1.00000000	mice
C1galt1	0.07	1.00000000	mice
Rsbn1	0.04	1.00000000	mice
Nol12	0.01	1.00000000	mice
Sgce	0.11	1.00000000	mice
Eif3c	0.10	1.00000000	mice

Hip1r	0.06	1.00000000	mice
Ralgds	0.02	1.00000000	mice
Ube2l3	0.11	1.00000000	mice
Gpatch8	0.06	1.00000000	mice
Lin54	0.01	1.00000000	mice
Tbx3os1	-0.14	1.00000000	mice
Sdccag8	0.07	1.00000000	mice
Sec23b	0.08	1.00000000	mice
Ist1	0.04	1.00000000	mice
Rbm38	0.06	1.00000000	mice
Lrrc41	0.13	1.00000000	mice
Nsmaf	-0.07	1.00000000	mice
Lrp10	0.12	1.00000000	mice
Dnajc2	0.19	1.00000000	mice
Ethe1	0.13	1.00000000	mice
Iars	0.07	1.00000000	mice
Zc3h14	0.08	1.00000000	mice
Syne1	0.05	1.00000000	mice
Slc25a12	0.05	1.00000000	mice
Etl4	0.07	1.00000000	mice
Hivep3	0.11	1.00000000	mice
Use1	0.22	1.00000000	mice
Ube3a	-0.01	1.00000000	mice
4833403J16Rik	0.03	1.00000000	mice
Ints14	-0.02	1.00000000	mice
Cttn	0.09	1.00000000	mice
Klhl7	0.05	1.00000000	mice
Cuedc1	0.07	1.00000000	mice
Nip7	0.02	1.00000000	mice
Cdc16	0.08	1.00000000	mice
Tpgs1	0.03	1.00000000	mice
Ddb1	0.00	1.00000000	mice
Gimap5	0.14	1.00000000	mice
Tecpr1	0.04	1.00000000	mice
Taok2	0.05	1.00000000	mice
Sntb2	-0.15	1.00000000	mice
Atn1	0.02	1.00000000	mice
Kcnh1	0.08	1.00000000	mice
Taf5l	0.09	1.00000000	mice
Ywhaz	0.02	1.00000000	mice
Nudcd3	0.12	1.00000000	mice
Ncstn	-0.04	1.00000000	mice
Clstn1	0.14	1.00000000	mice
Taf1b	0.00	1.00000000	mice
Sgk1	0.00	1.00000000	mice
Utp3	-0.02	1.00000000	mice

Vps72	0.14	1.00000000	mice
Tent4a	0.14	1.00000000	mice
Tmco1	0.07	1.00000000	mice
Parp2	0.03	1.00000000	mice
Cdk17	-0.01	1.00000000	mice
Npat	0.05	1.00000000	mice
Pih1d1	0.04	1.00000000	mice
Impa1	0.15	1.00000000	mice
Adam10	-0.04	1.00000000	mice
Fbxo3	0.05	1.00000000	mice
Ccdc59	0.08	1.00000000	mice
Nab1	0.21	1.00000000	mice
Cby1	-0.03	1.00000000	mice
Pofut2	0.12	1.00000000	mice
Parl	0.10	1.00000000	mice
Pxn	0.01	1.00000000	mice
Acvr1b	0.05	1.00000000	mice
Trim37	0.08	1.00000000	mice
Fam171a1	0.14	1.00000000	mice
Stam2	0.05	1.00000000	mice
Orc4	0.14	1.00000000	mice
Pacs1	-0.04	1.00000000	mice
Pcca	0.00	1.00000000	mice
Gpr146	0.21	1.00000000	mice
Rad21	0.01	1.00000000	mice
Grtp1	0.04	1.00000000	mice
St3gal2	0.06	1.00000000	mice
Ccdc50	0.04	1.00000000	mice
Usp3	0.00	1.00000000	mice
Kctd5	0.08	1.00000000	mice
Serpine1	0.22	1.00000000	mice
Wdr1	0.04	1.00000000	mice
Arhgap21	0.02	1.00000000	mice
Gtpbp1	0.02	1.00000000	mice
Crbn	0.11	1.00000000	mice
Sap30bp	0.01	1.00000000	mice
Cyb5b	0.07	1.00000000	mice
Zfand6	0.01	1.00000000	mice
Lrif1	0.07	1.00000000	mice
Tmem242	-0.10	1.00000000	mice
Efr3a	0.00	1.00000000	mice
Focad	-0.04	1.00000000	mice
Lcorl	-0.06	1.00000000	mice
Farsa	0.06	1.00000000	mice
Cisd1	0.01	1.00000000	mice
Uba6	0.07	1.00000000	mice

Krit1	0.08	1.00000000	mice
Syk	0.01	1.00000000	mice
0610012G03Rik	0.15	1.00000000	mice
Dcun1d4	0.07	1.00000000	mice
Zmpste24	0.04	1.00000000	mice
Vamp5	0.05	1.00000000	mice
Utp18	0.03	1.00000000	mice
Lsm14a	0.01	1.00000000	mice
Adgra3	-0.08	1.00000000	mice
Pigu	0.07	1.00000000	mice
Slc30a4	-0.01	1.00000000	mice
Arglu1	0.03	1.00000000	mice
Ubqln1	0.05	1.00000000	mice
Wars2	0.02	1.00000000	mice
Eif3m	-0.08	1.00000000	mice
Rcsd1	0.25	1.00000000	mice
Ep300	0.05	1.00000000	mice
Cmah	-0.01	1.00000000	mice
Zeb1	-0.09	1.00000000	mice
Ctnnbl1	-0.02	1.00000000	mice
Fip1l1	0.07	1.00000000	mice
Zfp868	0.02	1.00000000	mice
Il17rc	0.02	1.00000000	mice
Zfat	0.04	1.00000000	mice
Nol4l	0.08	1.00000000	mice
Apbb2	0.02	1.00000000	mice
Prkd1	-0.07	1.00000000	mice
Sumo2	0.15	1.00000000	mice
Ube2r2	0.15	1.00000000	mice
Rapgef2	0.07	1.00000000	mice
Cotl1	0.05	1.00000000	mice
P4hb	0.08	1.00000000	mice
Ppp1r21	-0.01	1.00000000	mice
Ccdc117	0.04	1.00000000	mice
Ythdc2	0.08	1.00000000	mice
Sec61a2	0.03	1.00000000	mice
Tcf7l2	0.07	1.00000000	mice
Traf5	-0.06	1.00000000	mice
Aggf1	0.05	1.00000000	mice
Tbc1d23	0.07	1.00000000	mice
Dnpep	0.02	1.00000000	mice
Rhbdd1	0.02	1.00000000	mice
4930402H24Rik	0.03	1.00000000	mice
Tank	0.10	1.00000000	mice
Card10	0.01	1.00000000	mice
Cherp	-0.05	1.00000000	mice

Casp6	0.02	1.00000000	mice
Ampd3	0.01	1.00000000	mice
Fyco1	0.07	1.00000000	mice
Tmem160	0.14	1.00000000	mice
Psmd9	0.05	1.00000000	mice
Pde8b	-0.16	1.00000000	mice
Pam	0.09	1.00000000	mice
Ehbp1	-0.08	1.00000000	mice
Tmco3	-0.03	1.00000000	mice
Xpc	-0.07	1.00000000	mice
Nop56	-0.03	1.00000000	mice
Ttl	0.14	1.00000000	mice
Ilrun	0.07	1.00000000	mice
6720427I07Rik	0.08	1.00000000	mice
Lyrm2	0.14	1.00000000	mice
Nipsnap2	0.02	1.00000000	mice
Ddx19a	0.10	1.00000000	mice
Lmtk2	0.02	1.00000000	mice
Tm9sf4	0.11	1.00000000	mice
Slc12a2	0.08	1.00000000	mice
Zdhhc8	-0.03	1.00000000	mice
Crppa	-0.03	1.00000000	mice
Zkscan3	0.02	1.00000000	mice
Dclk2	-0.01	1.00000000	mice
Slc39a11	0.04	1.00000000	mice
Pds5b	-0.02	1.00000000	mice
Gna11	0.14	1.00000000	mice
Smc3	0.01	1.00000000	mice
2510002D24Rik	0.05	1.00000000	mice
Ube2q1	0.06	1.00000000	mice
Terf2ip	-0.02	1.00000000	mice
Nek7	0.02	1.00000000	mice
Txndc5	0.00	1.00000000	mice
Wsb2	0.00	1.00000000	mice
Nabp1	0.10	1.00000000	mice
Bcr	-0.03	1.00000000	mice
Gbf1	0.07	1.00000000	mice
Acvr1	-0.03	1.00000000	mice
Akap7	-0.01	1.00000000	mice
Fam102b	-0.01	1.00000000	mice
Qk	-0.01	1.00000000	mice
Ndfip1	0.09	1.00000000	mice
Poldip3	0.12	1.00000000	mice
Taf4	0.01	1.00000000	mice
D16Ertd472e	0.01	1.00000000	mice
Smarcc2	-0.03	1.00000000	mice

Scoc	0.13	1.00000000	mice
Tomm7	0.09	1.00000000	mice
Rrp1b	0.04	1.00000000	mice
Tmem170b	-0.02	1.00000000	mice
Ppp2r1a	0.11	1.00000000	mice
Fbxw7	0.08	1.00000000	mice
Kansl1	0.00	1.00000000	mice
Pbx3	0.04	1.00000000	mice
Abl1	0.00	1.00000000	mice
Ctnna1	0.02	1.00000000	mice
Golim4	-0.03	1.00000000	mice
Rtl8a	0.22	1.00000000	mice
Spidr	-0.01	1.00000000	mice
Usp1	0.13	1.00000000	mice
Pus10	0.03	1.00000000	mice
Eif4ebp1	0.12	1.00000000	mice
Ubac2	0.00	1.00000000	mice
Pygb	-0.07	1.00000000	mice
Cpsf3	0.03	1.00000000	mice
Prpf3	0.09	1.00000000	mice
Coq2	0.03	1.00000000	mice
Nus1	0.03	1.00000000	mice
Tmx3	0.04	1.00000000	mice
Dr1	-0.07	1.00000000	mice
Deaf1	-0.01	1.00000000	mice
Terf1	0.02	1.00000000	mice
Usf3	0.04	1.00000000	mice
Rsf1	0.08	1.00000000	mice
Nploc4	0.07	1.00000000	mice
Ppie	0.15	1.00000000	mice
Atp11b	0.00	1.00000000	mice
Cwc15	0.10	1.00000000	mice
Sumf1	-0.04	1.00000000	mice
Nudt4	-0.14	1.00000000	mice
Ncor2	0.08	1.00000000	mice
Srp54a	0.08	1.00000000	mice
Kank3	0.07	1.00000000	mice
Rnf13	-0.06	1.00000000	mice
Golga5	-0.01	1.00000000	mice
Pde4dip	0.00	1.00000000	mice
Tut4	0.06	1.00000000	mice
Setd1b	0.04	1.00000000	mice
Apmap	0.07	1.00000000	mice
Polr2f	0.13	1.00000000	mice
Plekhm1	0.07	1.00000000	mice
Pno1	0.05	1.00000000	mice

Ubald2	0.16	1.00000000	mice
Alcam	-0.02	1.00000000	mice
Eid1	0.05	1.00000000	mice
Elof1	0.03	1.00000000	mice
Dnaja3	0.09	1.00000000	mice
Zfp512	0.04	1.00000000	mice
Rab13	0.12	1.00000000	mice
Ptpa	0.01	1.00000000	mice
Cdc73	-0.02	1.00000000	mice
Thada	-0.01	1.00000000	mice
Kdm6a	0.03	1.00000000	mice
Epb41l4aos	-0.06	1.00000000	mice
Ubp1	-0.08	1.00000000	mice
Zfp260	0.04	1.00000000	mice
Prpf38b	-0.03	1.00000000	mice
Fam49b	0.12	1.00000000	mice
Tmem167b	0.02	1.00000000	mice
Atp6v1h	0.04	1.00000000	mice
Rnft1	0.03	1.00000000	mice
Cisd2	0.02	1.00000000	mice
Nck1	0.03	1.00000000	mice
Baz2a	-0.07	1.00000000	mice
Actb	0.07	1.00000000	mice
Inafm2	0.01	1.00000000	mice
Cops6	0.05	1.00000000	mice
Trappc4	0.11	1.00000000	mice
Pfkp	0.03	1.00000000	mice
Ccdc88a	0.07	1.00000000	mice
Cd40	0.00	1.00000000	mice
Prkag1	-0.04	1.00000000	mice
Cnst	0.05	1.00000000	mice
Tjp1	0.04	1.00000000	mice
Exosc7	-0.02	1.00000000	mice
Rnf115	0.18	1.00000000	mice
Rbpj	0.20	1.00000000	mice
Atg16l2	0.02	1.00000000	mice
Gtf2e2	-0.02	1.00000000	mice
Zmiz2	0.02	1.00000000	mice
Fam168a	0.05	1.00000000	mice
Esf1	-0.10	1.00000000	mice
Yipf5	0.03	1.00000000	mice
Relb	0.17	1.00000000	mice
Snrnp40	0.15	1.00000000	mice
Impdh1	0.12	1.00000000	mice
Lrmda	-0.02	1.00000000	mice
Ranbp9	0.00	1.00000000	mice

Slain2	0.11	1.00000000	mice
Snx7	0.11	1.00000000	mice
Pxdn	0.00	1.00000000	mice
Zfp451	0.04	1.00000000	mice
Suz12	0.03	1.00000000	mice
Retreg3	-0.05	1.00000000	mice
Zfas1	0.14	1.00000000	mice
Cavin1	0.02	1.00000000	mice
3830406C13Rik	0.11	1.00000000	mice
Prrc1	0.15	1.00000000	mice
Bcl6b	0.06	1.00000000	mice
Csnk1g2	0.07	1.00000000	mice
Rara	-0.01	1.00000000	mice
Ppp2r3d	0.02	1.00000000	mice
Tmem243	-0.06	1.00000000	mice
Ctdp1	-0.05	1.00000000	mice
Vamp2	-0.06	1.00000000	mice
Usp45	-0.03	1.00000000	mice
Vps35	0.03	1.00000000	mice
Pip4k2b	0.03	1.00000000	mice
Srrm1	-0.02	1.00000000	mice
Lratd2	-0.04	1.00000000	mice
Tor1a	-0.02	1.00000000	mice
Rnaseh2a	0.09	1.00000000	mice
Eif3b	-0.07	1.00000000	mice
Pigx	0.00	1.00000000	mice
Cox5b	0.04	1.00000000	mice
Pdcd5	0.11	1.00000000	mice
Lypla2	0.02	1.00000000	mice
Klhl13	0.08	1.00000000	mice
Chst2	0.18	1.00000000	mice
Pkn2	0.10	1.00000000	mice
Etfa	0.13	1.00000000	mice
Uggt1	0.12	1.00000000	mice
Setx	0.00	1.00000000	mice
4930503L19Rik	0.00	1.00000000	mice
Dpp4	0.02	1.00000000	mice
Vps36	0.13	1.00000000	mice
Pofut1	0.00	1.00000000	mice
Gfm2	0.01	1.00000000	mice
Zfp207	0.00	1.00000000	mice
Scfd2	-0.02	1.00000000	mice
Vegfa	0.17	1.00000000	mice
Hmces	-0.01	1.00000000	mice
Exoc4	0.03	1.00000000	mice
Snx8	-0.12	1.00000000	mice

Phykpl	0.03	1.00000000	mice
Fbxl20	0.05	1.00000000	mice
Eny2	0.11	1.00000000	mice
Sec61b	0.15	1.00000000	mice
Ppp2r3a	0.02	1.00000000	mice
Cep57	0.01	1.00000000	mice
Czib	0.08	1.00000000	mice
Plcb3	0.08	1.00000000	mice
2510039O18Rik	0.11	1.00000000	mice
Tma7	0.04	1.00000000	mice
Ecd	0.01	1.00000000	mice
Hdac5	0.00	1.00000000	mice
Atpaf1	-0.01	1.00000000	mice
Arfrp1	-0.08	1.00000000	mice
Zfp568	0.04	1.00000000	mice
Pias4	0.09	1.00000000	mice
Kif1b	0.03	1.00000000	mice
Atxn7	-0.03	1.00000000	mice
Prpf40b	0.02	1.00000000	mice
Noct	0.18	1.00000000	mice
Xiap	-0.03	1.00000000	mice
Caskin2	-0.03	1.00000000	mice
Jup	0.11	1.00000000	mice
Atpif1	0.19	1.00000000	mice
Relch	-0.03	1.00000000	mice
4930453N24Rik	0.11	1.00000000	mice
Cab39	0.05	1.00000000	mice
Flvcr1	0.02	1.00000000	mice
Lmnb1	0.22	1.00000000	mice
Ppm1d	0.05	1.00000000	mice
Cast	0.13	1.00000000	mice
Rwdd4a	0.02	1.00000000	mice
Naa35	0.00	1.00000000	mice
Abca1	-0.02	1.00000000	mice
Itpripl2	0.12	1.00000000	mice
Rarg	0.18	1.00000000	mice
H2afy	0.06	1.00000000	mice
Surf4	0.08	1.00000000	mice
Cdk19	0.04	1.00000000	mice
Tmem259	-0.02	1.00000000	mice
Slc12a6	0.04	1.00000000	mice
Fbrs	0.03	1.00000000	mice
Ywhag	0.07	1.00000000	mice
Osbpl9	-0.05	1.00000000	mice
Uck2	0.07	1.00000000	mice
Sod2	0.19	1.00000000	mice

Ifitm2	0.07	1.00000000	mice
Uspl1	0.05	1.00000000	mice
Cdc27	0.02	1.00000000	mice
Lyar	0.16	1.00000000	mice
Sav1	0.17	1.00000000	mice
Ino80e	-0.01	1.00000000	mice
Ptar1	-0.03	1.00000000	mice
Tdrp	0.01	1.00000000	mice
Nfyc	0.06	1.00000000	mice
Hdhd2	0.01	1.00000000	mice
St3gal1	0.02	1.00000000	mice
Foxj3	-0.07	1.00000000	mice
Psmd6	0.05	1.00000000	mice
Ndufb3	0.05	1.00000000	mice
Ice1	0.02	1.00000000	mice
Filip1	0.00	1.00000000	mice
Ubl4a	0.05	1.00000000	mice
Fam219a	-0.03	1.00000000	mice
Slc39a1	-0.07	1.00000000	mice
Slc2a9	-0.07	1.00000000	mice
Tnrc18	0.10	1.00000000	mice
Tm2d3	0.10	1.00000000	mice
Pwwp2a	0.04	1.00000000	mice
Glo1	0.12	1.00000000	mice
Hmox1	0.16	1.00000000	mice
Nsf	0.12	1.00000000	mice
B3gat3	0.05	1.00000000	mice
Adam17	0.16	1.00000000	mice
Oard1	0.04	1.00000000	mice
Gas2l1	0.11	1.00000000	mice
Smarcad1	0.09	1.00000000	mice
Spry2	-0.08	1.00000000	mice
Atg4a	-0.06	1.00000000	mice
Rnaset2a	0.06	1.00000000	mice
Ptpn4	0.24	1.00000000	mice
Rel	0.11	1.00000000	mice
Ints10	0.00	1.00000000	mice
Tmem258	-0.01	1.00000000	mice
H2afj	0.18	1.00000000	mice
Erc1	0.03	1.00000000	mice
Csnk1g3	0.03	1.00000000	mice
1110059G10Rik	0.07	1.00000000	mice
Stk16	0.08	1.00000000	mice
Agk	0.07	1.00000000	mice
Pibf1	-0.02	1.00000000	mice
Slmap	0.04	1.00000000	mice

Cpsf6	0.01	1.00000000	mice
Tmem135	0.07	1.00000000	mice
Lrba	-0.01	1.00000000	mice
Fcf1	0.10	1.00000000	mice
Fndc3b	0.03	1.00000000	mice
Bms1	-0.07	1.00000000	mice
Stk3	0.01	1.00000000	mice
Top3b	0.07	1.00000000	mice
Trappc11	0.01	1.00000000	mice
Pdzd2	-0.10	1.00000000	mice
Calu	0.04	1.00000000	mice
Dhx15	0.05	1.00000000	mice
St3gal3	-0.07	1.00000000	mice
Sh3d21	0.00	1.00000000	mice
Lonp1	0.01	1.00000000	mice
Katnbl1	0.01	1.00000000	mice
Slc25a24	0.03	1.00000000	mice
Nkiras1	0.05	1.00000000	mice
Lsm12	0.07	1.00000000	mice
Gripap1	-0.02	1.00000000	mice
Ctso	-0.01	1.00000000	mice
Proser1	0.01	1.00000000	mice
Khsrp	0.07	1.00000000	mice
Ddx47	0.03	1.00000000	mice
Snapc3	0.06	1.00000000	mice
Polr2b	0.07	1.00000000	mice
Lzts2	0.01	1.00000000	mice
Atp7a	0.02	1.00000000	mice
Ap2a2	0.08	1.00000000	mice
Irak4	-0.01	1.00000000	mice
Dctn4	0.11	1.00000000	mice
Ccdc91	-0.13	1.00000000	mice
Zfp638	0.06	1.00000000	mice
Dnajc15	0.07	1.00000000	mice
Atm	0.05	1.00000000	mice
Snx27	0.08	1.00000000	mice
Amfr	0.07	1.00000000	mice
Ubald1	0.05	1.00000000	mice
Tmem39a	0.05	1.00000000	mice
Nudt5	-0.03	1.00000000	mice
Chm	0.00	1.00000000	mice
Ccdc127	0.05	1.00000000	mice
Dnmbp	-0.03	1.00000000	mice
Slc35a1	-0.02	1.00000000	mice
Dynlrb1	0.05	1.00000000	mice
Casc3	0.01	1.00000000	mice

Impad1	-0.06	1.00000000	mice
Brms1l	0.02	1.00000000	mice
Tspan5	0.03	1.00000000	mice
Hspa1a	0.28	1.00000000	mice
Atxn7l1	0.00	1.00000000	mice
Tfeb	-0.08	1.00000000	mice
Ino80d	0.06	1.00000000	mice
Xrn2	0.10	1.00000000	mice
Pepd	0.01	1.00000000	mice
Sh3bgrl3	0.22	1.00000000	mice
Pnrc2	0.08	1.00000000	mice
Trio	0.05	1.00000000	mice
Helz2	-0.07	1.00000000	mice
Cask	-0.04	1.00000000	mice
Epg5	0.03	1.00000000	mice
Spg21	0.05	1.00000000	mice
Syap1	0.08	1.00000000	mice
Zdhhc7	0.01	1.00000000	mice
Ube4a	0.03	1.00000000	mice
Tnfrsf1a	0.09	1.00000000	mice
Thra	0.02	1.00000000	mice
Thbs1	0.17	1.00000000	mice
Prkn	-0.05	1.00000000	mice
Gps1	0.11	1.00000000	mice
Arl6ip1	0.13	1.00000000	mice
Atg3	0.08	1.00000000	mice
Glb1	0.09	1.00000000	mice
Ggta1	-0.01	1.00000000	mice
Ube2m	0.12	1.00000000	mice
Plau	0.10	1.00000000	mice
Cd44	0.12	1.00000000	mice
Tmem179b	0.00	1.00000000	mice
Cdc40	-0.02	1.00000000	mice
Efhd2	-0.06	1.00000000	mice
Tril	0.08	1.00000000	mice
Tm9sf2	0.00	1.00000000	mice
Lrrfip2	0.09	1.00000000	mice
Ube4b	0.07	1.00000000	mice
Eif3j2	0.06	1.00000000	mice
Usp39	0.05	1.00000000	mice
Usp9x	0.06	1.00000000	mice
Rfx3	0.06	1.00000000	mice
Slc25a26	0.04	1.00000000	mice
Tmem176a	0.17	1.00000000	mice
Nabp2	0.10	1.00000000	mice
Prkcsh	0.05	1.00000000	mice

Bbs9	0.08	1.00000000	mice
Hnrnpa2b1	0.07	1.00000000	mice
Emc2	0.23	1.00000000	mice
Zswim8	0.02	1.00000000	mice
Kdm2b	0.05	1.00000000	mice
Acp1	0.04	1.00000000	mice
Npdc1	0.13	1.00000000	mice
Dkc1	0.03	1.00000000	mice
Ndufs6	0.07	1.00000000	mice
Srgap2	0.07	1.00000000	mice
Orc6	0.08	1.00000000	mice
Ankrd10	-0.08	1.00000000	mice
Dennd1a	-0.02	1.00000000	mice
Ltbr	-0.02	1.00000000	mice
Ap2s1	0.11	1.00000000	mice
Strap	0.02	1.00000000	mice
Ift140	-0.03	1.00000000	mice
Rbm10	-0.05	1.00000000	mice
Ipo9	0.00	1.00000000	mice
Spast	0.03	1.00000000	mice
Rnf41	0.03	1.00000000	mice
Commd6	0.05	1.00000000	mice
Slc44a1	0.01	1.00000000	mice
Alkbh1	0.06	1.00000000	mice
Plat	-0.19	1.00000000	mice
Pisd	0.01	1.00000000	mice
Asb3	0.05	1.00000000	mice
2700049A03Rik	0.01	1.00000000	mice
Gngt2	0.24	1.00000000	mice
Cabin1	0.06	1.00000000	mice
Wdr36	0.13	1.00000000	mice
Dnajc9	0.01	1.00000000	mice
Gt(ROSA)26Sor	-0.05	1.00000000	mice
Rgmb	0.05	1.00000000	mice
Eif2ak1	0.10	1.00000000	mice
Ikbip	-0.01	1.00000000	mice
Pmvk	0.00	1.00000000	mice
Cebpz	-0.03	1.00000000	mice
R3hdm1	0.06	1.00000000	mice
Arid2	0.01	1.00000000	mice
Nsrp1	0.14	1.00000000	mice
Vrk1	0.01	1.00000000	mice
Cdipt	0.04	1.00000000	mice
Ibtk	0.10	1.00000000	mice
Flna	0.19	1.00000000	mice
Pigyl	0.03	1.00000000	mice

Ndufaf3	0.12	1.00000000	mice
Atad1	0.08	1.00000000	mice
Ctdsp1	0.11	1.00000000	mice
Arhgef26	0.20	1.00000000	mice
0610010F05Rik	0.01	1.00000000	mice
Tmed10	0.09	1.00000000	mice
Ncoa2	0.01	1.00000000	mice
Uqcc3	0.03	1.00000000	mice
Dnajc24	0.04	1.00000000	mice
Ext1	0.08	1.00000000	mice
Clpp	0.01	1.00000000	mice
Cdc37	0.09	1.00000000	mice
Tpm4	0.16	1.00000000	mice
Sgta	0.08	1.00000000	mice
Nfe2l1	0.00	1.00000000	mice
Zfp532	0.10	1.00000000	mice
Glp1r	0.07	1.00000000	mice
Rmnd5b	-0.02	1.00000000	mice
Sypl	0.02	1.00000000	mice
Dimt1	-0.04	1.00000000	mice
Dgke	0.08	1.00000000	mice
Hsph1	0.03	1.00000000	mice
Epha2	0.00	1.00000000	mice
Ncor1	0.07	1.00000000	mice
Ppp1r7	0.08	1.00000000	mice
Stat6	-0.02	1.00000000	mice
Auh	0.10	1.00000000	mice
Sfxn3	0.00	1.00000000	mice
Nek1	0.08	1.00000000	mice
1110038F14Rik	0.17	1.00000000	mice
Il17rd	0.09	1.00000000	mice
Edem3	0.00	1.00000000	mice
Trappc2	0.04	1.00000000	mice
Fcgrt	0.09	1.00000000	mice
Atp5d	0.25	1.00000000	mice
Aga	0.06	1.00000000	mice
Vbp1	0.13	1.00000000	mice
Rcbtb1	0.04	1.00000000	mice
Lats1	0.03	1.00000000	mice
Ripk2	0.03	1.00000000	mice
Tsnax	0.01	1.00000000	mice
Snrpd3	0.18	1.00000000	mice
Atp11a	0.07	1.00000000	mice
Itpk1	0.06	1.00000000	mice
Clcn3	-0.01	1.00000000	mice
Pex2	0.08	1.00000000	mice

Ppp1r35	0.02	1.00000000	mice
Nuak2	0.01	1.00000000	mice
Prr13	0.02	1.00000000	mice
Tmed7	0.01	1.00000000	mice
Pnn	-0.03	1.00000000	mice
Slc25a37	0.09	1.00000000	mice
Hdgfl2	0.06	1.00000000	mice
Ppih	0.07	1.00000000	mice
Tmf1	-0.13	1.00000000	mice
Dedd	-0.03	1.00000000	mice
Tango2	-0.04	1.00000000	mice
Rffl	0.04	1.00000000	mice
Gpatch2l	0.03	1.00000000	mice
Cklf	0.07	1.00000000	mice
Cggbp1	-0.01	1.00000000	mice
Sf3b5	0.09	1.00000000	mice
Ubxn7	-0.02	1.00000000	mice
Stap2	-0.01	1.00000000	mice
Kidins220	0.02	1.00000000	mice
Abtb2	0.15	1.00000000	mice
Bet1	0.16	1.00000000	mice
Arhgef11	-0.04	1.00000000	mice
Ninj1	0.10	1.00000000	mice
Sf3a2	0.02	1.00000000	mice
Hmgxb4	0.07	1.00000000	mice
Rpn2	0.05	1.00000000	mice
Rbm28	0.16	1.00000000	mice
Nono	0.00	1.00000000	mice
Pwwp2b	0.08	1.00000000	mice
Rbm42	0.07	1.00000000	mice
Vezt	0.07	1.00000000	mice
Alg14	-0.02	1.00000000	mice
Rap2a	0.20	1.00000000	mice
Bicd2	0.16	1.00000000	mice
Cox19	0.03	1.00000000	mice
Cox16	0.17	1.00000000	mice
Cmc1	0.04	1.00000000	mice
Slc5a3	0.03	1.00000000	mice
2700054A10Rik	0.10	1.00000000	mice
Rbm41	0.02	1.00000000	mice
Fbxl3	0.02	1.00000000	mice
Rsbn1l	-0.05	1.00000000	mice
Tulp3	0.01	1.00000000	mice
Tek	0.18	1.00000000	mice
Gpatch11	0.24	1.00000000	mice
Hivep2	0.16	1.00000000	mice

Als2	0.04	1.00000000	mice
Tmem128	-0.04	1.00000000	mice
Rufy2	-0.11	1.00000000	mice
Cog6	0.03	1.00000000	mice
Gpr89	-0.03	1.00000000	mice
Herc2	0.09	1.00000000	mice
Naa10	0.07	1.00000000	mice
Borcs8	-0.04	1.00000000	mice
Rc3h2	0.03	1.00000000	mice
Snap29	0.04	1.00000000	mice
Yme1l1	0.09	1.00000000	mice
Pten	-0.02	1.00000000	mice
Baz1b	0.05	1.00000000	mice
Chordc1	0.00	1.00000000	mice
Tnxb	0.09	1.00000000	mice
Cyb561	0.07	1.00000000	mice
Oma1	-0.07	1.00000000	mice
Camk2d	0.06	1.00000000	mice
Ddx27	0.08	1.00000000	mice
Sema3c	0.27	1.00000000	mice
Tm7sf3	0.09	1.00000000	mice
Trabd	0.02	1.00000000	mice
Erp44	0.07	1.00000000	mice
Asb7	-0.02	1.00000000	mice
Zhx1	-0.01	1.00000000	mice
Jag2	-0.10	1.00000000	mice
Rnf40	-0.03	1.00000000	mice
Snw1	0.09	1.00000000	mice
Fam192a	-0.03	1.00000000	mice
Irf2bp1	-0.04	1.00000000	mice
Tcf12	0.06	1.00000000	mice
Ubap2l	0.06	1.00000000	mice
Arl6ip5	0.08	1.00000000	mice
Abraxas2	0.10	1.00000000	mice
Orai1	0.01	1.00000000	mice
Pold3	0.08	1.00000000	mice
Tfpi	0.08	1.00000000	mice
Zfp142	0.00	1.00000000	mice
Cep135	0.09	1.00000000	mice
Rrn3	0.08	1.00000000	mice
Cnot1	0.05	1.00000000	mice
Cul2	0.17	1.00000000	mice
Hspa14	0.11	1.00000000	mice
Ap5m1	-0.04	1.00000000	mice
0610030E20Rik	0.00	1.00000000	mice
Traf6	-0.04	1.00000000	mice

Cnp	0.11	1.00000000	mice
5031439G07Rik	-0.05	1.00000000	mice
Dbi	0.01	1.00000000	mice
Isg20l2	0.03	1.00000000	mice
Nkap	0.10	1.00000000	mice
Tpr	0.10	1.00000000	mice
Fbxw4	-0.05	1.00000000	mice
Btbd10	-0.01	1.00000000	mice
Pomp	-0.03	1.00000000	mice
Nhlrc2	0.08	1.00000000	mice
Camk2g	0.04	1.00000000	mice
Hnrnpc	0.05	1.00000000	mice
Nceh1	0.09	1.00000000	mice
Armh3	0.03	1.00000000	mice
Lmo7	0.01	1.00000000	mice
Psmd5	0.05	1.00000000	mice
Yae1d1	0.09	1.00000000	mice
Abcb7	0.01	1.00000000	mice
Phactr2	0.15	1.00000000	mice
Ints11	0.06	1.00000000	mice
Clint1	0.09	1.00000000	mice
G2e3	-0.05	1.00000000	mice
Tmem263	0.03	1.00000000	mice
Lrpap1	0.11	1.00000000	mice
Stx8	0.03	1.00000000	mice
Sort1	-0.03	1.00000000	mice
Aprt	0.16	1.00000000	mice
Nrbf2	0.12	1.00000000	mice
Gyg	0.09	1.00000000	mice
Lysmd3	0.05	1.00000000	mice
Blzf1	0.01	1.00000000	mice
8430419K02Rik	0.03	1.00000000	mice
Fhod1	0.03	1.00000000	mice
Zcchc8	0.00	1.00000000	mice
Ldlrad4	0.09	1.00000000	mice
Ecm1	0.03	1.00000000	mice
Hdlbp	0.07	1.00000000	mice
Pcbd2	0.08	1.00000000	mice
Specc1	0.03	1.00000000	mice
Twistnb	0.03	1.00000000	mice
Cbll1	-0.02	1.00000000	mice
Rnf19b	0.00	1.00000000	mice
Fig4	0.08	1.00000000	mice
Arpp19	0.13	1.00000000	mice
Cyba	0.13	1.00000000	mice
Pmm2	-0.02	1.00000000	mice

Agpat4	0.10	1.00000000	mice
Dnajc10	0.06	1.00000000	mice
Tbce	-0.03	1.00000000	mice
Tars	0.04	1.00000000	mice
Pls3	-0.02	1.00000000	mice
Gfod1	0.08	1.00000000	mice
Xxylt1	-0.06	1.00000000	mice
Trnt1	0.02	1.00000000	mice
Lnpep	0.17	1.00000000	mice
Vac14	0.07	1.00000000	mice
Zfp639	0.02	1.00000000	mice
Cnot6	0.10	1.00000000	mice
Plxna1	0.15	1.00000000	mice
Washc2	0.09	1.00000000	mice
Ifnar2	0.05	1.00000000	mice
Rbfa	0.05	1.00000000	mice
Plk2	0.05	1.00000000	mice
Tcf7	0.12	1.00000000	mice
Gimap8	0.00	1.00000000	mice
Il3ra	0.07	1.00000000	mice
Prpf19	0.10	1.00000000	mice
Serinc1	0.07	1.00000000	mice
C1qbp	-0.06	1.00000000	mice
Ube2v2	0.07	1.00000000	mice
Rnf214	0.08	1.00000000	mice
Cdc42se1	0.05	1.00000000	mice
Psmf1	0.05	1.00000000	mice
Setd3	0.02	1.00000000	mice
Anxa2	0.09	1.00000000	mice
Anapc5	0.04	1.00000000	mice
Ptgr2	0.01	1.00000000	mice
Nck2	-0.08	1.00000000	mice
Vps33a	0.07	1.00000000	mice
Ccdc71l	0.05	1.00000000	mice
Snf8	0.04	1.00000000	mice
Slit3	0.03	1.00000000	mice
Csnk1g1	0.01	1.00000000	mice
Stip1	-0.04	1.00000000	mice
Erlin2	0.09	1.00000000	mice
Zfp652	-0.01	1.00000000	mice
Depdc5	0.03	1.00000000	mice
Pard6g	0.10	1.00000000	mice
Ppp3cb	0.03	1.00000000	mice
Impact	0.01	1.00000000	mice
Suco	0.12	1.00000000	mice
Dram2	0.13	1.00000000	mice

St6galnac2	0.07	1.00000000	mice
Dmtf1	0.12	1.00000000	mice
Sertad3	0.05	1.00000000	mice
Tmem168	-0.10	1.00000000	mice
Zdhhc3	0.07	1.00000000	mice
Btbd1	0.09	1.00000000	mice
Uhrf1bp1l	0.00	1.00000000	mice
Nup205	0.02	1.00000000	mice
Cped1	0.07	1.00000000	mice
Fam193b	-0.03	1.00000000	mice
Pygo2	0.12	1.00000000	mice
Tab2	0.06	1.00000000	mice
Stag2	0.13	1.00000000	mice
Stx7	0.16	1.00000000	mice
Pja2	0.03	1.00000000	mice
Phf12	-0.02	1.00000000	mice
Rabep2	0.08	1.00000000	mice
Hmgb1	0.03	1.00000000	mice
Scarf1	0.13	1.00000000	mice
Golph3	-0.02	1.00000000	mice
Fnbp1l	-0.02	1.00000000	mice
C330007P06Rik	0.16	1.00000000	mice
Plbd2	0.03	1.00000000	mice
Rab5c	0.13	1.00000000	mice
Rangap1	0.02	1.00000000	mice
Rpa3	0.03	1.00000000	mice
Cops5	0.03	1.00000000	mice
Acaca	-0.03	1.00000000	mice
Sema6a	-0.02	1.00000000	mice
Ube2e1	0.03	1.00000000	mice
Dstyk	0.06	1.00000000	mice
Hmg20a	0.06	1.00000000	mice
Trip11	0.01	1.00000000	mice
Exoc3	0.06	1.00000000	mice
Ro60	0.00	1.00000000	mice
Crim1	0.04	1.00000000	mice
Taf15	0.02	1.00000000	mice
Hcfc2	0.04	1.00000000	mice
Rfxap	0.03	1.00000000	mice
Bub3	-0.09	1.00000000	mice
Rtcb	0.08	1.00000000	mice
Vma21	0.07	1.00000000	mice
Ddt	0.06	1.00000000	mice
Pfdn4	-0.05	1.00000000	mice
2310022B05Rik	0.02	1.00000000	mice
Exoc7	0.10	1.00000000	mice

Ccdc58	0.00	1.00000000	mice
Cltc	0.07	1.00000000	mice
Prkcq	0.05	1.00000000	mice
Gabarapl1	0.12	1.00000000	mice
Ap2b1	0.04	1.00000000	mice
Brd3	-0.04	1.00000000	mice
Xpa	0.11	1.00000000	mice
Fam172a	0.03	1.00000000	mice
Rnf11	-0.02	1.00000000	mice
Slc12a4	0.12	1.00000000	mice
Rhot1	0.01	1.00000000	mice
Lats2	0.01	1.00000000	mice
Nelfa	0.13	1.00000000	mice
Dcaf17	0.01	1.00000000	mice
Tmem219	0.00	1.00000000	mice
Prpf31	0.02	1.00000000	mice
Galnt11	0.01	1.00000000	mice
Coq5	0.03	1.00000000	mice
Paf1	0.04	1.00000000	mice
Strip1	0.01	1.00000000	mice
Coa3	0.15	1.00000000	mice
Snrnp48	0.06	1.00000000	mice
Ptpra	-0.01	1.00000000	mice
Galnt7	0.03	1.00000000	mice
Uqcrc2	0.11	1.00000000	mice
Ip6k2	0.05	1.00000000	mice
Chchd1	-0.09	1.00000000	mice
Slc25a20	0.10	1.00000000	mice
Ap1s1	0.04	1.00000000	mice
Aup1	0.14	1.00000000	mice
Lgmn	0.31	1.00000000	mice
Akirin2	-0.10	1.00000000	mice
Nars2	-0.01	1.00000000	mice
Tmem43	-0.01	1.00000000	mice
Stxbp5	0.06	1.00000000	mice
Dyrk2	0.09	1.00000000	mice
Ppp2r5d	0.04	1.00000000	mice
Cdk6	0.03	1.00000000	mice
Crebzf	-0.01	1.00000000	mice
Pop5	0.09	1.00000000	mice
Cs	0.14	1.00000000	mice
Gid8	0.00	1.00000000	mice
Pgp	-0.12	1.00000000	mice
Ptbp2	0.12	1.00000000	mice
Fdx1	-0.06	1.00000000	mice
Chchd3	0.04	1.00000000	mice

Ago1	-0.01	1.00000000	mice
Lrch3	0.08	1.00000000	mice
Hint3	0.02	1.00000000	mice
Dnajc19	0.12	1.00000000	mice
Hexa	0.17	1.00000000	mice
Trappc8	0.11	1.00000000	mice
Ap1m1	0.02	1.00000000	mice
Gulp1	0.03	1.00000000	mice
Skil	0.10	1.00000000	mice
Socs7	0.03	1.00000000	mice
Rnpepl1	0.18	1.00000000	mice
Tango6	0.05	1.00000000	mice
Kcnq1ot1	-0.01	1.00000000	mice
Laptm5	0.19	1.00000000	mice
Rab5b	0.03	1.00000000	mice
Rbm14	0.08	1.00000000	mice
Terf2	0.01	1.00000000	mice
Fnbp4	0.08	1.00000000	mice
Dcun1d3	-0.07	1.00000000	mice
Papolg	0.05	1.00000000	mice
Atxn7l3b	0.17	1.00000000	mice
Ptpn11	0.13	1.00000000	mice
Ezr	-0.07	1.00000000	mice
Sel1l	0.05	1.00000000	mice
Tapbpl	-0.05	1.00000000	mice
Cop1	0.02	1.00000000	mice
Ap1ar	0.06	1.00000000	mice
Rbis	0.12	1.00000000	mice
Itga3	0.01	1.00000000	mice
C2cd3	0.04	1.00000000	mice
Eif2s1	0.06	1.00000000	mice
Rabl6	0.08	1.00000000	mice
Fam222b	-0.02	1.00000000	mice
Sf3a3	0.11	1.00000000	mice
Ylpm1	-0.07	1.00000000	mice
Egflam	-0.05	1.00000000	mice
Raf1	0.07	1.00000000	mice
Scai	-0.06	1.00000000	mice
Txndc12	0.06	1.00000000	mice
Sri	0.08	1.00000000	mice
Clcn4	0.02	1.00000000	mice
Akap8	0.07	1.00000000	mice
Gimap9	0.02	1.00000000	mice
Ints2	-0.02	1.00000000	mice
4833439L19Rik	0.10	1.00000000	mice
Kif16b	-0.06	1.00000000	mice

Aldh6a1	0.00	1.00000000	mice
Ctr9	-0.04	1.00000000	mice
Sdf4	0.11	1.00000000	mice
Akip1	-0.01	1.00000000	mice
Dap3	0.03	1.00000000	mice
Zdhhc18	0.07	1.00000000	mice
Lsm3	0.04	1.00000000	mice
Hacd2	-0.02	1.00000000	mice
Cd53	0.27	1.00000000	mice
Chmp1b	-0.04	1.00000000	mice
Tpd52	0.12	1.00000000	mice
Nol7	0.11	1.00000000	mice
Arhgef5	0.08	1.00000000	mice
Anxa7	0.08	1.00000000	mice
Pkn1	0.12	1.00000000	mice
Lars	0.02	1.00000000	mice
Ccar1	0.13	1.00000000	mice
Ogdh	0.00	1.00000000	mice
Heatr5b	0.02	1.00000000	mice
Ptprm	0.09	1.00000000	mice
Apaf1	0.02	1.00000000	mice
2610001J05Rik	0.09	1.00000000	mice
Ugdh	0.05	1.00000000	mice
Lpgat1	0.03	1.00000000	mice
Emp2	0.12	1.00000000	mice
Zfand3	0.05	1.00000000	mice
Nipal3	0.17	1.00000000	mice
Utp14a	-0.01	1.00000000	mice
Tmx4	-0.04	1.00000000	mice
Bmt2	0.03	1.00000000	mice
Socs2	0.01	1.00000000	mice
Tcf25	0.02	1.00000000	mice
Fbxo22	0.00	1.00000000	mice
Srcap	0.00	1.00000000	mice
Hoxb5	0.12	1.00000000	mice
Snrpg	0.08	1.00000000	mice
Prkci	-0.04	1.00000000	mice
Slc35b1	0.03	1.00000000	mice
Itfg1	-0.01	1.00000000	mice
Slc38a9	-0.02	1.00000000	mice
Foxj2	0.07	1.00000000	mice
Npepl1	0.07	1.00000000	mice
Ndufa9	0.03	1.00000000	mice
Polr2i	0.07	1.00000000	mice
Pde6d	0.10	1.00000000	mice
Tjap1	0.03	1.00000000	mice

Tnks2	0.04	1.00000000	mice
Nipsnap3b	0.08	1.00000000	mice
Rprd1a	-0.05	1.00000000	mice
Ift57	-0.04	1.00000000	mice
Fubp1	0.12	1.00000000	mice
Cept1	0.00	1.00000000	mice
Optn	0.10	1.00000000	mice
Gpbp1	0.09	1.00000000	mice
Cgnl1	-0.11	1.00000000	mice
Vwa8	0.07	1.00000000	mice
Sfmbt1	0.03	1.00000000	mice
Rnf44	0.05	1.00000000	mice
Cflar	0.11	1.00000000	mice
Smim4	0.11	1.00000000	mice
Invs	-0.02	1.00000000	mice
D630045J12Rik	0.02	1.00000000	mice
Atxn2	0.02	1.00000000	mice
Atf7	0.03	1.00000000	mice
Cct8	0.13	1.00000000	mice
Ccdc32	0.08	1.00000000	mice
Afg1l	-0.05	1.00000000	mice
Rrm1	0.02	1.00000000	mice
Blvrb	0.09	1.00000000	mice
Pcdh17	0.09	1.00000000	mice
Swt1	0.09	1.00000000	mice
Camk1	0.16	1.00000000	mice
2810013P06Rik	-0.02	1.00000000	mice
Nfkbib	0.03	1.00000000	mice
Gosr2	0.07	1.00000000	mice
Cbwd1	0.00	1.00000000	mice
Clec16a	0.01	1.00000000	mice
Taf12	0.06	1.00000000	mice
Atg4b	0.00	1.00000000	mice
Pnpla6	0.06	1.00000000	mice
Fancl	0.05	1.00000000	mice
Ufd1	0.01	1.00000000	mice
Hist1h1c	0.08	1.00000000	mice
Cpeb3	0.02	1.00000000	mice
Vps26a	0.06	1.00000000	mice
Crot	0.08	1.00000000	mice
Cdc123	0.01	1.00000000	mice
Tnks1bp1	0.02	1.00000000	mice
Unc45b	0.17	1.00000000	mice
Arfgef2	-0.02	1.00000000	mice
Got1	0.05	1.00000000	mice
Tspan3	0.02	1.00000000	mice

Psmb4	0.17	1.00000000	mice
Nhsl1	0.05	1.00000000	mice
Cgrrf1	0.10	1.00000000	mice
Prkra	-0.08	1.00000000	mice
Fut8	0.00	1.00000000	mice
Smg7	0.05	1.00000000	mice
Rfwd3	0.12	1.00000000	mice
Ddx23	0.05	1.00000000	mice
Atad2b	0.10	1.00000000	mice
Dctn1	0.04	1.00000000	mice
Rlf	-0.01	1.00000000	mice
Ing5	-0.03	1.00000000	mice
Ncoa4	0.04	1.00000000	mice
Ttbk2	-0.03	1.00000000	mice
Arhgdia	0.09	1.00000000	mice
Ttf1	0.02	1.00000000	mice
Herc3	0.06	1.00000000	mice
Lsm7	0.12	1.00000000	mice
Tnrc6a	0.06	1.00000000	mice
Tmem50b	0.04	1.00000000	mice
Slf1	0.07	1.00000000	mice
Pigt	-0.07	1.00000000	mice
Larp7	0.10	1.00000000	mice
Rsrc2	0.07	1.00000000	mice
Cdv3	0.04	1.00000000	mice
Cpne2	0.01	1.00000000	mice
Apc	0.07	1.00000000	mice
Vps8	-0.03	1.00000000	mice
Fam53b	0.01	1.00000000	mice
Zxdc	0.06	1.00000000	mice
Ehmt2	-0.03	1.00000000	mice
Commd8	-0.03	1.00000000	mice
Eci1	0.07	1.00000000	mice
Uba3	-0.05	1.00000000	mice
Zyg11b	0.08	1.00000000	mice
Fmr1	-0.02	1.00000000	mice
Brd4	0.07	1.00000000	mice
Asb8	-0.01	1.00000000	mice
Cadps2	0.14	1.00000000	mice
Frmd8	0.05	1.00000000	mice
Lasp1	-0.03	1.00000000	mice
Zfp697	0.07	1.00000000	mice
Zfp942	-0.05	1.00000000	mice
Chst11	0.05	1.00000000	mice
Golgb1	0.06	1.00000000	mice
Gpr182	0.10	1.00000000	mice

Arl4c	-0.08	1.00000000	mice
Rsl24d1	0.05	1.00000000	mice
Slc26a2	0.04	1.00000000	mice
Lmo2	0.07	1.00000000	mice
Rp9	0.07	1.00000000	mice
Gas6	0.14	1.00000000	mice
Ggh	0.09	1.00000000	mice
Bcl7b	0.10	1.00000000	mice
Apool	0.05	1.00000000	mice
Ermp1	0.02	1.00000000	mice
Ccnh	-0.01	1.00000000	mice
Nckap5	0.21	1.00000000	mice
Tspan4	0.07	1.00000000	mice
Pnkp	-0.01	1.00000000	mice
Bcl3	0.08	1.00000000	mice
Cd2bp2	0.06	1.00000000	mice
Ap2a1	0.04	1.00000000	mice
Fbxl14	0.02	1.00000000	mice
Heatr3	0.02	1.00000000	mice
Rpa1	0.00	1.00000000	mice
Fbxo6	0.14	1.00000000	mice
Tia1	0.02	1.00000000	mice
Phax	0.11	1.00000000	mice
Krt80	0.10	1.00000000	mice
Rnf144b	0.01	1.00000000	mice
Rbms1	-0.02	1.00000000	mice
Icam1	0.04	1.00000000	mice
Nrip1	0.04	1.00000000	mice
Bgn	-0.05	1.00000000	mice
Synj1	0.08	1.00000000	mice
Eya3	0.07	1.00000000	mice
Nsd1	0.06	1.00000000	mice
Tbxa2r	-0.11	1.00000000	mice
Nup88	0.01	1.00000000	mice
Prpf4b	0.04	1.00000000	mice
Usp6nl	-0.01	1.00000000	mice
Kmt2b	0.00	1.00000000	mice
Tnks	-0.01	1.00000000	mice
Lrrfip1	0.11	1.00000000	mice
Prdm2	0.07	1.00000000	mice
Arpc5	0.13	1.00000000	mice
Rbck1	0.13	1.00000000	mice
Dpy19l1	0.07	1.00000000	mice
Inpp5f	0.04	1.00000000	mice
AW549877	0.04	1.00000000	mice
Fkbp9	0.07	1.00000000	mice

Zfp46	0.06	1.00000000	mice
Poglut1	-0.05	1.00000000	mice
Atl3	0.09	1.00000000	mice
Gng11	0.16	1.00000000	mice
Tmem164	0.20	1.00000000	mice
Nfic	0.13	1.00000000	mice
Lrp6	0.14	1.00000000	mice
Uvrag	0.02	1.00000000	mice
Ankrd44	0.12	1.00000000	mice
Nr1d2	0.10	1.00000000	mice
Elovl5	0.10	1.00000000	mice
Wdr7	0.02	1.00000000	mice
Thoc3	0.00	1.00000000	mice
Cops3	-0.01	1.00000000	mice
Ldlrap1	0.07	1.00000000	mice
Tbc1d5	0.00	1.00000000	mice
Hnrnpu	0.04	1.00000000	mice
Ndufa8	0.23	1.00000000	mice
Gnai2	0.07	1.00000000	mice
Surf1	0.03	1.00000000	mice
Itga5	0.05	1.00000000	mice
Atp9a	0.05	1.00000000	mice
Cfap298	0.02	1.00000000	mice
Fbxo33	0.06	1.00000000	mice
Brox	0.16	1.00000000	mice
Uhrf2	0.03	1.00000000	mice
Gon4l	0.08	1.00000000	mice
2210016F16Rik	0.04	1.00000000	mice
Lrrk1	0.09	1.00000000	mice
Osbpl2	0.05	1.00000000	mice
Gpr137b	0.07	1.00000000	mice
Rexo4	0.11	1.00000000	mice
Fam49a	0.01	1.00000000	mice
Dpm2	0.03	1.00000000	mice
9430038I01Rik	0.10	1.00000000	mice
Umad1	-0.12	1.00000000	mice
Taf2	0.05	1.00000000	mice
Tubb5	0.09	1.00000000	mice
Fbxl12	0.00	1.00000000	mice
Thumpd1	-0.06	1.00000000	mice
Usp12	0.14	1.00000000	mice
Hif1a	0.17	1.00000000	mice
Wiz	0.02	1.00000000	mice
Cbx5	-0.01	1.00000000	mice
Timm22	0.08	1.00000000	mice
Nsun2	0.10	1.00000000	mice

Ypel2	0.03	1.00000000	mice
Cep68	0.03	1.00000000	mice
Tomm34	0.12	1.00000000	mice
Trp53i13	0.06	1.00000000	mice
Sh3glb1	0.01	1.00000000	mice
Agap3	0.00	1.00000000	mice
Arl8b	0.11	1.00000000	mice
Dennd2c	0.06	1.00000000	mice
Pelo	0.00	1.00000000	mice
Sinhcaf	0.01	1.00000000	mice
Ythdf1	-0.01	1.00000000	mice
Gatad2b	-0.01	1.00000000	mice
Wdr33	0.02	1.00000000	mice
Rras	0.13	1.00000000	mice
Snn	0.16	1.00000000	mice
Kdelr2	0.12	1.00000000	mice
Cmtm4	0.02	1.00000000	mice
Naa30	0.06	1.00000000	mice
Drosha	0.04	1.00000000	mice
Cpeb4	0.12	1.00000000	mice
Apoe	-0.17	1.00000000	mice
Prim2	0.05	1.00000000	mice
Cul4b	0.02	1.00000000	mice
Pcnt	0.06	1.00000000	mice
Rab1a	0.04	1.00000000	mice
Hnrnpm	0.01	1.00000000	mice
Gigyf1	0.06	1.00000000	mice
Fam241a	0.04	1.00000000	mice
Fam160b1	-0.03	1.00000000	mice
Eif5a	0.18	1.00000000	mice
Vps4b	0.13	1.00000000	mice
Ak3	0.10	1.00000000	mice
Nfib	0.05	1.00000000	mice
Tmed5	0.12	1.00000000	mice
Bscl2	-0.08	1.00000000	mice
Zmynd8	0.04	1.00000000	mice
Cobll1	0.11	1.00000000	mice
Adgrg1	0.01	1.00000000	mice
Acvrl1	0.06	1.00000000	mice
Saa3	4.39	0.00000000	pigs
Pde5a	1.06	0.00000000	pigs
P4ha1	1.51	0.00000000	pigs
Cry2	0.70	0.00000000	pigs
Hsph1	1.23	0.00000000	pigs
Sod2	2.07	0.00000000	pigs
Bst2	1.00	0.00000000	pigs

Odc1	1.56	0.00000000	pigs
Calm1	-0.77	0.00000000	pigs
F13a1	2.04	0.00000001	pigs
Cav1	-1.14	0.00000001	pigs
Cryab	1.35	0.00000002	pigs
Rnf19b	1.34	0.00000002	pigs
Cd9	-1.01	0.00000002	pigs
Cavin2	-1.16	0.00000002	pigs
Sec62	-1.00	0.00000008	pigs
Klf10	0.80	0.00000017	pigs
Rcor1	0.88	0.00000032	pigs
Areg	1.45	0.00000040	pigs
Slc9a3r2	-0.99	0.00000047	pigs
Fkbp5	1.15	0.00000074	pigs
Lrmda	1.17	0.00000118	pigs
Pdxk	0.75	0.00000170	pigs
Ago2	0.84	0.00000220	pigs
S100a10	-1.17	0.00000266	pigs
Epsti1	0.57	0.00000321	pigs
Tamalin	1.20	0.00000621	pigs
Cd163	1.39	0.00001029	pigs
Ctsb	1.05	0.00001393	pigs
Arg1	1.39	0.00001727	pigs
Ccn3	-2.02	0.00001879	pigs
Nfkbia	0.94	0.00002070	pigs
Zfand5	1.02	0.00002179	pigs
Ctsc	1.29	0.00002581	pigs
Atp11a	0.77	0.00002741	pigs
Piezo1	0.70	0.00002922	pigs
Oas2	0.79	0.00003177	pigs
Il1a	1.12	0.00004559	pigs
Rgcc	1.37	0.00005120	pigs
Acap2	0.81	0.00005828	pigs
Slc20a1	0.74	0.00005905	pigs
Rsad2	0.99	0.00006094	pigs
Edn1	1.33	0.00008256	pigs
Socs1	0.91	0.00009071	pigs
Csrp2	0.96	0.00009370	pigs
Fbxo39	0.61	0.00009843	pigs
Ulk2	0.88	0.00010889	pigs
Cstb	0.90	0.00011147	pigs
Cyth1	0.86	0.00011547	pigs
Nampt	0.85	0.00013282	pigs
Dram1	0.64	0.00014980	pigs
Sptbn1	-0.62	0.00017362	pigs
Large1	0.62	0.00018494	pigs

H2-Ea	1.15	0.00021098	pigs
Jade3	0.63	0.00024176	pigs
Vav2	0.37	0.00027070	pigs
C1qa	0.60	0.00027694	pigs
Emcn	-1.03	0.00029551	pigs
Actb	-0.23	0.00033563	pigs
Lrrc8c	0.68	0.00033819	pigs
Plin3	0.64	0.00034897	pigs
Sec14l1	0.47	0.00036953	pigs
Arid4b	0.76	0.00037164	pigs
Hspa8	0.78	0.00042004	pigs
Hdac4	0.50	0.00053044	pigs
Abca1	1.17	0.00053613	pigs
Tiam1	0.50	0.00056982	pigs
Inpp5d	0.53	0.00060963	pigs
Ifi30	0.90	0.00066371	pigs
Tma7	-0.79	0.00067910	pigs
Sin3a	0.65	0.00068082	pigs
Pcdh7	-1.19	0.00069614	pigs
Ncor2	0.84	0.00072950	pigs
B4galt1	0.58	0.00080160	pigs
Hif1a	0.84	0.00087896	pigs
Capg	1.06	0.00089256	pigs
Noct	0.89	0.00091511	pigs
Nfkbiz	1.23	0.00096157	pigs
Per1	0.75	0.00098309	pigs
Gadd45g	1.27	0.00124720	pigs
Nckap5l	0.43	0.00132293	pigs
Pgm2	0.80	0.00139613	pigs
Gsdmd	0.35	0.00146242	pigs
Tnxb	-0.97	0.00151543	pigs
Slco2a1	0.61	0.00168842	pigs
Zeb2	0.98	0.00169332	pigs
Slc39a8	0.47	0.00170232	pigs
Lrrc32	0.57	0.00173149	pigs
Niban2	0.67	0.00194230	pigs
Ido1	0.93	0.00214405	pigs
Clic5	1.17	0.00234868	pigs
Rhoq	0.56	0.00252140	pigs
Cd24a	-1.48	0.00254481	pigs
Baiap2	1.48	0.00261665	pigs
Tcea2	-1.08	0.00262844	pigs
Gadd45a	1.04	0.00290897	pigs
Nav1	0.49	0.00300088	pigs
P3h2	-1.18	0.00307977	pigs
Cav2	-0.65	0.00312461	pigs

Cpsf6	1.55	0.00315420	pigs
Ski	0.38	0.00324413	pigs
Pdcd5	-0.96	0.00326412	pigs
Apoe	0.60	0.00339186	pigs
Tsc22d1	0.80	0.00350315	pigs
Bhlhe40	0.77	0.00419099	pigs
Cavin1	-0.68	0.00449315	pigs
Pecam1	-0.50	0.00461356	pigs
Lcor	0.56	0.00472078	pigs
Arrdc3	0.79	0.00481076	pigs
Icam1	1.18	0.00493008	pigs
Bcar1	0.51	0.00512722	pigs
Lrp11	0.62	0.00591875	pigs
Dusp5	0.82	0.00620021	pigs
Plxna4	0.51	0.00627341	pigs
Prkg1	1.14	0.00661521	pigs
Smad3	0.66	0.00667367	pigs
Ifngr2	0.82	0.00686514	pigs
Csf1	0.51	0.00729518	pigs
Cdk19	0.74	0.00747087	pigs
Rad51b	0.57	0.00764204	pigs
Psmg3	0.39	0.00806443	pigs
Smurf1	0.51	0.01009952	pigs
Hipk3	0.71	0.01011570	pigs
Usp18	0.61	0.01166610	pigs
Efnb1	0.53	0.01209604	pigs
Cyp2j13	0.18	0.01241879	pigs
Gpnmb	0.61	0.01340507	pigs
Gclc	0.99	0.01356332	pigs
Poc1b	0.38	0.01430097	pigs
Lrrc57	0.50	0.01458304	pigs
Spp1	0.51	0.01499629	pigs
Rab3gap2	0.35	0.01516977	pigs
Tead4	0.40	0.01577521	pigs
Ccnd1	-1.00	0.01590523	pigs
Arrdc4	0.68	0.01649822	pigs
Nod2	0.71	0.01679402	pigs
Hbp1	0.72	0.01702940	pigs
Eif2ak3	0.59	0.01724528	pigs
Ankrd11	0.63	0.01758975	pigs
Polr2f	-0.84	0.01766205	pigs
Ppif	0.62	0.01832368	pigs
Ppp6r3	0.57	0.01918877	pigs
Ifi44	0.32	0.02262926	pigs
Sox17	0.53	0.02589764	pigs
Ltbp4	-0.92	0.02721971	pigs

Spint2	0.73	0.02728027	pigs
Ugdh	0.61	0.02808140	pigs
Adrb2	0.42	0.02889113	pigs
Fmnl2	1.04	0.02971369	pigs
Azin1	0.90	0.03039494	pigs
Lgals3bp	0.50	0.03095855	pigs
Errfi1	0.73	0.03137451	pigs
Cir1	0.42	0.03202911	pigs
Dr1	0.52	0.03445691	pigs
Hmox1	0.70	0.03455267	pigs
Sbspon	-0.77	0.03485070	pigs
Stk24	0.63	0.03622728	pigs
Septin7	-0.58	0.03833670	pigs
Trip10	0.21	0.03952965	pigs
Snrpd2	-0.57	0.04275818	pigs
Frmd4a	0.80	0.04435206	pigs
Ell	0.50	0.04534096	pigs
Ccr7	0.92	0.04586014	pigs
Klhl21	1.56	0.04676374	pigs
Shisal1	0.18	0.04677048	pigs
Abtb2	0.78	0.04679328	pigs
Glyr1	0.58	0.04682078	pigs
Igf1r	0.87	0.04689837	pigs
Cd44	1.28	0.04914564	pigs
Ywhab	-0.52	0.05248213	pigs
Dot1l	0.35	0.05284252	pigs
Dusp6	0.58	0.05377291	pigs
Tcirg1	0.41	0.05405511	pigs
Cd14	0.94	0.05715358	pigs
Trp53bp2	0.24	0.05725422	pigs
Plekho1	0.23	0.05765589	pigs
Tgfb2	-1.13	0.05824019	pigs
Nuak2	0.30	0.05854769	pigs
Ndel1	0.71	0.05906618	pigs
Sparcl1	0.97	0.05930601	pigs
Tsku	0.30	0.05940201	pigs
AA467197	0.82	0.06120394	pigs
Fut8	0.78	0.06522995	pigs
Ptges	0.43	0.06562534	pigs
Zmym2	0.28	0.06562988	pigs
Bmp1	0.13	0.06579278	pigs
Ccnd3	0.70	0.06861821	pigs
Tlr2	0.82	0.07468148	pigs
Abi3bp	-0.83	0.07989270	pigs
Pde9a	0.13	0.08156327	pigs
Phldb2	-0.91	0.08348244	pigs

Ext1	0.66	0.08414396	pigs
Uvrag	0.96	0.08590614	pigs
Nup98	0.36	0.08699621	pigs
Clec3b	-0.82	0.09201077	pigs
Ube2l6	0.47	0.09237851	pigs
Synpr	-0.92	0.09389333	pigs
Cenpu	-0.39	0.09521611	pigs
Rap1b	0.66	0.09545901	pigs
Il4ra	0.64	0.09611959	pigs
Bud23	1.29	0.09670780	pigs
Igf2r	0.59	0.10205926	pigs
Ccl21d	-0.99	0.10276885	pigs
C1qb	0.71	0.10278471	pigs
Icmt	-0.59	0.10406437	pigs
Thrb	0.34	0.10413185	pigs
Csf1r	0.91	0.10618235	pigs
Kdr	-1.23	0.10925865	pigs
Ubqln1	0.50	0.11039755	pigs
Ubr4	0.44	0.11248822	pigs
Ndrg1	1.10	0.11476684	pigs
Foxs1	0.24	0.12236271	pigs
Simc1	0.37	0.12262975	pigs
Ahr	0.52	0.12368396	pigs
Cachd1	0.25	0.13252329	pigs
Sat1	0.57	0.13678405	pigs
Dpyd	0.53	0.13755461	pigs
Rnf19a	0.71	0.13843168	pigs
Traf3	0.55	0.14012296	pigs
Ccdc141	-0.88	0.14450596	pigs
Dyrk3	0.40	0.14477718	pigs
Cdh13	-0.71	0.15664550	pigs
Nfkbie	0.24	0.15707081	pigs
Irf8	1.03	0.15723350	pigs
Galk2	0.50	0.15765936	pigs
Rad1	0.27	0.16175239	pigs
5031439G07Rik	0.25	0.16738558	pigs
Pspc1	0.45	0.16742338	pigs
Btg1b	0.80	0.16980369	pigs
Thumpd2	0.46	0.17172526	pigs
Pgs1	0.31	0.17332032	pigs
Omd	-0.74	0.17383902	pigs
Sema6a	-0.64	0.17462099	pigs
Zc3h3	0.32	0.17673507	pigs
Slc6a6	0.59	0.17832169	pigs
Zswim6	0.72	0.18969183	pigs
Acer2	0.40	0.20292937	pigs

Slc23a2	0.56	0.20453684	pigs
Zranb1	0.53	0.20799789	pigs
Luzp1	0.71	0.21177390	pigs
Igsf6	0.30	0.21965865	pigs
Rhobtb1	0.36	0.22659049	pigs
Plec	-0.66	0.22839618	pigs
Ube2d3	0.53	0.23053755	pigs
Lpin2	0.60	0.23787924	pigs
Kctd12	0.49	0.23993850	pigs
Tnfrsf1b	0.60	0.24720412	pigs
Arid5a	0.65	0.24851691	pigs
Smad1	-0.78	0.25214076	pigs
Dab2	-0.60	0.25379910	pigs
Slc2a13	0.20	0.25455648	pigs
Nudt14	0.61	0.25577518	pigs
Slamf7	1.37	0.25874671	pigs
Fgl2	-0.98	0.26809374	pigs
Rhob	0.71	0.28040871	pigs
Dmxl1	0.58	0.28159135	pigs
Tmod3	-0.67	0.28380786	pigs
Gria3	-0.50	0.29513190	pigs
Gucd1	0.15	0.30056116	pigs
Slc31a1	0.52	0.32089655	pigs
Slc39a14	0.54	0.32432656	pigs
Ifi44l	0.45	0.32517444	pigs
Kank3	-0.60	0.32626110	pigs
Usp6nl	0.41	0.32763589	pigs
Etv6	0.51	0.32812393	pigs
Sgcd	-0.87	0.33854714	pigs
Galnt2	0.31	0.34260251	pigs
Lifr	0.47	0.34427397	pigs
Gata3	0.35	0.35192340	pigs
Herc1	0.40	0.35282527	pigs
Agrn	0.34	0.36179033	pigs
Isg15	0.04	0.37321369	pigs
Pml	0.47	0.37807789	pigs
Ets2	0.53	0.37823124	pigs
Cldnd1	-0.64	0.38347101	pigs
Tbc1d8	0.62	0.38462248	pigs
Fabp5	-0.84	0.38574701	pigs
Atxn1	0.50	0.38840474	pigs
Itgb5	0.32	0.39042167	pigs
Tmem44	0.11	0.39778914	pigs
Rab11a	-0.60	0.40746853	pigs
Sema3g	0.34	0.41067255	pigs
Strbp	0.26	0.41427569	pigs

Olfml2a	0.19	0.41689481	pigs
Rnf34	0.40	0.42347822	pigs
Pbx3	0.34	0.44308872	pigs
C1qc	0.58	0.44357436	pigs
Ehbp1l1	0.34	0.44429862	pigs
Ing2	0.23	0.44787523	pigs
Serf2	-0.45	0.44831650	pigs
Rab9	0.44	0.44982117	pigs
Agpat3	0.53	0.45085774	pigs
Tmtc1	-0.73	0.45550575	pigs
Adgb	0.45	0.45660904	pigs
Rgs3	0.30	0.46126468	pigs
Tmem245	-0.61	0.47192212	pigs
Pfkfb3	0.83	0.47725541	pigs
Ngly1	0.35	0.47994336	pigs
Ccdc175	0.24	0.49282112	pigs
Ddx31	0.27	0.52239016	pigs
Gna12	0.71	0.52281395	pigs
Hexb	0.24	0.53277558	pigs
Ero1a	0.34	0.53848298	pigs
Ctsz	0.86	0.54331515	pigs
Tfec	0.97	0.58599275	pigs
Lims2	0.30	0.59488658	pigs
Atad2b	0.46	0.65102266	pigs
Plxdc2	-1.00	0.65962212	pigs
Ptpn23	0.22	0.68253826	pigs
Phlpp1	0.53	0.68892764	pigs
Akap12	-0.85	0.70288782	pigs
Ubl5	-0.63	0.70297213	pigs
Dazap2	0.50	0.71670269	pigs
H2-Ab1	0.91	0.72770257	pigs
Gfus	0.34	0.73571689	pigs
Tmem184b	0.38	0.73860144	pigs
Itgb1	-0.46	0.74547773	pigs
Pard3	0.74	0.74975112	pigs
Klf2	0.93	0.75377375	pigs
Snx24	0.54	0.75980684	pigs
Ciart	0.44	0.76410329	pigs
Igf2bp3	0.84	0.76431667	pigs
Pdcd1lg2	0.43	0.78233180	pigs
Naalad2	-0.73	0.83356118	pigs
Plk3	0.59	0.83543995	pigs
Bzw2	0.40	0.84901221	pigs
Ezh2	0.45	0.85010016	pigs
Zfp654	0.47	0.86165610	pigs
Ttbk2	0.38	0.87311737	pigs

Eif4a1	0.70	0.88449977	pigs
Tbc1d22b	0.25	0.89203585	pigs
Cldn5	-0.67	0.89934747	pigs
Dennd5b	0.90	0.92320760	pigs
Tmem120b	0.25	0.93183675	pigs
Tk1	0.47	0.93183675	pigs
Cxcl10	0.45	0.93930717	pigs
Rgs2	1.54	0.94190470	pigs
Spag9	0.55	0.95079883	pigs
Snx25	0.43	0.95233050	pigs
Atp13a3	0.69	0.95520773	pigs
Chic1	0.24	0.96612377	pigs
Cebpb	0.57	0.96773847	pigs
Pbx1	0.40	0.97795866	pigs
Ubash3b	0.34	0.97898939	pigs
Cd93	0.46	1.00000000	pigs
Bmx	0.13	1.00000000	pigs
Btg3	0.66	1.00000000	pigs
Timp3	-0.53	1.00000000	pigs
Inpp4a	0.38	1.00000000	pigs
Snx10	0.89	1.00000000	pigs
Narf	0.56	1.00000000	pigs
Zfand2a	0.40	1.00000000	pigs
Clasp1	0.43	1.00000000	pigs
Usp46	0.40	1.00000000	pigs
Pdlim5	0.60	1.00000000	pigs
Sdcbp2	0.33	1.00000000	pigs
Tsc22d3	0.75	1.00000000	pigs
Phf21b	0.15	1.00000000	pigs
Proser1	0.40	1.00000000	pigs
Ddx60	0.31	1.00000000	pigs
Tmem14a	0.11	1.00000000	pigs
Kctd3	0.46	1.00000000	pigs
Cmpk2	0.27	1.00000000	pigs
Wwox	0.70	1.00000000	pigs
Evl	0.50	1.00000000	pigs
Slc15a3	0.33	1.00000000	pigs
Cybb	0.80	1.00000000	pigs
Ranbp10	0.09	1.00000000	pigs
Ppfibp1	-0.40	1.00000000	pigs
Prcp	0.41	1.00000000	pigs
Gpcpd1	-1.19	1.00000000	pigs
Chka	0.47	1.00000000	pigs
Arap3	0.27	1.00000000	pigs
Aldh3a2	0.43	1.00000000	pigs
Lama4	-0.63	1.00000000	pigs

Cmip	0.47	1.00000000	pigs
Tgfb1	0.67	1.00000000	pigs
Tm4sf1	-0.96	1.00000000	pigs
Atp2b4	-0.64	1.00000000	pigs
Ass1	0.36	1.00000000	pigs
Ranbp9	0.34	1.00000000	pigs
Herc4	0.57	1.00000000	pigs
Gprc5a	0.46	1.00000000	pigs
Ammecr1	0.63	1.00000000	pigs
Tspan12	0.51	1.00000000	pigs
Fam111a	0.36	1.00000000	pigs
Gch1	0.81	1.00000000	pigs
Psma5	0.26	1.00000000	pigs
Cnot9	0.41	1.00000000	pigs
Susd6	0.49	1.00000000	pigs
Tll1	-1.33	1.00000000	pigs
Lrrc8a	0.15	1.00000000	pigs
Stat3	0.49	1.00000000	pigs
Kit	0.44	1.00000000	pigs
Rnf138	0.21	1.00000000	pigs
Ccdc85c	0.27	1.00000000	pigs
Zbtb10	0.44	1.00000000	pigs
Spry4	0.19	1.00000000	pigs
Szrd1	0.41	1.00000000	pigs
Pdpn	-0.62	1.00000000	pigs
Csf2rb2	0.99	1.00000000	pigs
Grk5	0.53	1.00000000	pigs
Epb41l3	0.92	1.00000000	pigs
Ddah2	0.23	1.00000000	pigs
Tfcp2	0.05	1.00000000	pigs
Hif1an	0.15	1.00000000	pigs
Spindoc	0.37	1.00000000	pigs
Kmt2c	0.39	1.00000000	pigs
Adipor2	0.40	1.00000000	pigs
Lratd2	0.27	1.00000000	pigs
Nr6a1	0.45	1.00000000	pigs
Kdm6a	0.43	1.00000000	pigs
Pnp	0.66	1.00000000	pigs
Nr2f1	-0.81	1.00000000	pigs
Wscd1	0.29	1.00000000	pigs
Arsa	0.20	1.00000000	pigs
Snd1	0.30	1.00000000	pigs
Acin1	0.60	1.00000000	pigs
Tbc1d1	0.61	1.00000000	pigs
Cbl	0.18	1.00000000	pigs
Pou6f1	0.35	1.00000000	pigs

Ddhd1	0.16	1.00000000	pigs
Dcun1d4	0.11	1.00000000	pigs
Pdgfc	-0.76	1.00000000	pigs
Bag3	0.85	1.00000000	pigs
Rnd1	0.79	1.00000000	pigs
Chrm2	0.65	1.00000000	pigs
Ddx24	-0.54	1.00000000	pigs
Ndst1	0.18	1.00000000	pigs
Brd1	0.21	1.00000000	pigs
Relb	0.36	1.00000000	pigs
Angptl4	-1.17	1.00000000	pigs
Ackr3	-0.72	1.00000000	pigs
Grk3	0.34	1.00000000	pigs
Lmo2	-0.71	1.00000000	pigs
Psme4	0.20	1.00000000	pigs
Rasa2	0.40	1.00000000	pigs
Rarres2	0.28	1.00000000	pigs
Stk4	0.38	1.00000000	pigs
Ubp1	0.22	1.00000000	pigs
Cd302	0.22	1.00000000	pigs
Crip2	-0.73	1.00000000	pigs
S100a6	-1.07	1.00000000	pigs
Polr2g	0.38	1.00000000	pigs
Steap3	0.13	1.00000000	pigs
Orai2	0.17	1.00000000	pigs
Tnfrsf9	0.32	1.00000000	pigs
Socs3	0.49	1.00000000	pigs
Bcl2a1d	0.93	1.00000000	pigs
Tnfaip3	0.94	1.00000000	pigs
Pdzd8	0.43	1.00000000	pigs
Upp1	0.10	1.00000000	pigs
Ikbip	0.25	1.00000000	pigs
Il27ra	0.13	1.00000000	pigs
Nfe2l2	0.58	1.00000000	pigs
Dclk2	1.15	1.00000000	pigs
Pdzrn3	0.73	1.00000000	pigs
Eif2s2	-0.56	1.00000000	pigs
Rbbp6	0.46	1.00000000	pigs
Plod1	0.17	1.00000000	pigs
Cox7c	-0.52	1.00000000	pigs
Nemp2	0.19	1.00000000	pigs
Pgm5	-0.77	1.00000000	pigs
Cdk8	0.19	1.00000000	pigs
Tsc22d2	0.64	1.00000000	pigs
Dcaf6	0.61	1.00000000	pigs
Hdac9	-0.82	1.00000000	pigs

Irf7	0.30	1.00000000	pigs
Trim44	0.40	1.00000000	pigs
Nfkb2	0.06	1.00000000	pigs
Cabyr	1.25	1.00000000	pigs
Jak3	0.08	1.00000000	pigs
Baz1a	0.69	1.00000000	pigs
Eif2ak2	0.44	1.00000000	pigs
Scyl1	0.43	1.00000000	pigs
Tmem223	-0.66	1.00000000	pigs
Tpst2	0.45	1.00000000	pigs
Chp2	-0.62	1.00000000	pigs
Rptor	0.09	1.00000000	pigs
Prss55	0.43	1.00000000	pigs
Aak1	-0.65	1.00000000	pigs
Uox	-0.54	1.00000000	pigs
Tnfrsf11a	-0.60	1.00000000	pigs
Ahsa1	0.38	1.00000000	pigs
Tnfaip8l1	0.22	1.00000000	pigs
Tspo	-0.52	1.00000000	pigs
Galnt17	0.54	1.00000000	pigs
Ptp4a2	0.47	1.00000000	pigs
Fgd5	0.27	1.00000000	pigs
Ccdc88a	0.57	1.00000000	pigs
Nol4l	0.29	1.00000000	pigs
Pitpnb	0.45	1.00000000	pigs
Sumo2	-0.41	1.00000000	pigs
Fgr	0.11	1.00000000	pigs
Tmem63a	0.18	1.00000000	pigs
Ivns1abp	0.62	1.00000000	pigs
Ghitm	0.33	1.00000000	pigs
H2-Oa	0.09	1.00000000	pigs
Ppp6r2	0.27	1.00000000	pigs
Emp2	0.31	1.00000000	pigs
Prorp	0.43	1.00000000	pigs
Col4a2	0.64	1.00000000	pigs
Tinagl1	-0.82	1.00000000	pigs
Pcbd1	0.11	1.00000000	pigs
Prom1	0.24	1.00000000	pigs
Phf13	0.14	1.00000000	pigs
Hck	0.60	1.00000000	pigs
Aplnr	0.39	1.00000000	pigs
Atp6v1c1	0.23	1.00000000	pigs
Tsen2	0.57	1.00000000	pigs
Grb10	0.56	1.00000000	pigs
Tnip1	0.67	1.00000000	pigs
Sqle	-0.36	1.00000000	pigs

Osbpl2	0.15	1.00000000	pigs
Tbc1d10a	0.15	1.00000000	pigs
Il17d	0.10	1.00000000	pigs
Cnot6l	0.50	1.00000000	pigs
Elf4	0.14	1.00000000	pigs
Ssh1	0.53	1.00000000	pigs
Wdfy3	0.19	1.00000000	pigs
Ramp2	-0.39	1.00000000	pigs
Suco	0.47	1.00000000	pigs
Arrdc2	0.33	1.00000000	pigs
Sqstm1	0.57	1.00000000	pigs
Fzd5	0.33	1.00000000	pigs
Nadsyn1	0.13	1.00000000	pigs
Hdac1	0.42	1.00000000	pigs
Snx14	0.28	1.00000000	pigs
Limk1	0.10	1.00000000	pigs
Sdc2	0.34	1.00000000	pigs
Usp54	0.51	1.00000000	pigs
Stat1	0.53	1.00000000	pigs
Atp5k	-0.55	1.00000000	pigs
Neu1	0.09	1.00000000	pigs
Nedd9	0.43	1.00000000	pigs
Prkd1	0.25	1.00000000	pigs
Lipa	0.37	1.00000000	pigs
Cst3	-0.57	1.00000000	pigs
Pmepa1	0.27	1.00000000	pigs
Zmat3	0.10	1.00000000	pigs
Zfp583	0.10	1.00000000	pigs
Cuedc1	-0.52	1.00000000	pigs
Hbegf	0.42	1.00000000	pigs
Zfp423	0.18	1.00000000	pigs
Ccdc68	-0.89	1.00000000	pigs
Rgs1	1.37	1.00000000	pigs
Ap1ar	-0.63	1.00000000	pigs
Eno2	0.51	1.00000000	pigs
Becn1	0.15	1.00000000	pigs
Nat9	0.26	1.00000000	pigs
Gyg	0.35	1.00000000	pigs
Braf	0.47	1.00000000	pigs
Osbpl3	0.75	1.00000000	pigs
Ostm1	0.35	1.00000000	pigs
Adgre5	0.86	1.00000000	pigs
Ndufa4	-0.48	1.00000000	pigs
Rhbdf2	0.30	1.00000000	pigs
Prss23	-0.95	1.00000000	pigs
Pan3	0.40	1.00000000	pigs

Ets1	-0.37	1.00000000	pigs
E2f3	0.22	1.00000000	pigs
Akt3	0.54	1.00000000	pigs
Ubqln4	0.13	1.00000000	pigs
Abl2	0.28	1.00000000	pigs
Accsl	0.44	1.00000000	pigs
Tenm3	-0.92	1.00000000	pigs
Pqlc3	0.10	1.00000000	pigs
Usp12	0.14	1.00000000	pigs
Eea1	-0.60	1.00000000	pigs
Sh3bgrl3	-0.73	1.00000000	pigs
Idi1	-0.54	1.00000000	pigs
Pou2f1	0.51	1.00000000	pigs
Chst15	0.23	1.00000000	pigs
Lcorl	0.24	1.00000000	pigs
Hsp90aa1	0.54	1.00000000	pigs
Vegfa	1.22	1.00000000	pigs
Brwd3	0.50	1.00000000	pigs
Xpnpep1	0.20	1.00000000	pigs
Xylt1	0.94	1.00000000	pigs
Adam9	0.53	1.00000000	pigs
Ints6	0.61	1.00000000	pigs
Camsap2	0.54	1.00000000	pigs
Brd2	0.69	1.00000000	pigs
Swap70	0.44	1.00000000	pigs
Klhl32	-0.51	1.00000000	pigs
Ccser1	0.96	1.00000000	pigs
Tcf7l2	0.45	1.00000000	pigs
Gsto1	0.28	1.00000000	pigs
Cnn3	-0.45	1.00000000	pigs
Pcnx	0.52	1.00000000	pigs
Sgpp1	0.42	1.00000000	pigs
Dram2	0.29	1.00000000	pigs
Ablim3	0.14	1.00000000	pigs
Csnk1a1	0.42	1.00000000	pigs
B4galnt1	-0.40	1.00000000	pigs
Selenow	-0.49	1.00000000	pigs
Hipk2	0.57	1.00000000	pigs
Resf1	0.62	1.00000000	pigs
Rgs4	-0.37	1.00000000	pigs
Clpx	0.05	1.00000000	pigs
Rsrp1	0.59	1.00000000	pigs
Gna13	0.23	1.00000000	pigs
Safb	0.34	1.00000000	pigs
Rapgef1	0.15	1.00000000	pigs
Cstf3	0.26	1.00000000	pigs

Litaf	0.44	1.00000000	pigs
Gpr161	0.18	1.00000000	pigs
Cbfb	0.18	1.00000000	pigs
Pdlim4	-0.69	1.00000000	pigs
Ankdd1a	-0.32	1.00000000	pigs
Pias2	0.37	1.00000000	pigs
Shisa5	0.45	1.00000000	pigs
Sirt1	0.32	1.00000000	pigs
Slc43a2	0.30	1.00000000	pigs
Loxl2	0.12	1.00000000	pigs
Ldb2	-0.32	1.00000000	pigs
Rab28	0.27	1.00000000	pigs
Safb2	0.36	1.00000000	pigs
Arhgap27	0.27	1.00000000	pigs
Cadm1	0.06	1.00000000	pigs
Tiparp	0.59	1.00000000	pigs
Crtc1	0.09	1.00000000	pigs
Sfmbt1	0.12	1.00000000	pigs
Sgpl1	0.49	1.00000000	pigs
Acsf3	0.13	1.00000000	pigs
Kdm3a	0.30	1.00000000	pigs
Jak2	0.32	1.00000000	pigs
Ebpl	0.36	1.00000000	pigs
Bmper	0.24	1.00000000	pigs
Cyld	0.37	1.00000000	pigs
Heatr3	0.33	1.00000000	pigs
Arsg	0.58	1.00000000	pigs
Vldlr	0.26	1.00000000	pigs
Nit1	0.26	1.00000000	pigs
Kif1b	0.43	1.00000000	pigs
Nipal2	0.39	1.00000000	pigs
Ybey	0.17	1.00000000	pigs
Cdh18	-0.96	1.00000000	pigs
Cers6	-0.43	1.00000000	pigs
Adcy9	0.34	1.00000000	pigs
Efnb2	0.38	1.00000000	pigs
Zc3h12c	0.37	1.00000000	pigs
Ccdc115	0.27	1.00000000	pigs
Wdfy2	0.12	1.00000000	pigs
Ephb1	0.43	1.00000000	pigs
Tmem123	-0.69	1.00000000	pigs
Ppp1r9a	-0.78	1.00000000	pigs
Lap3	0.47	1.00000000	pigs
Il13ra1	0.35	1.00000000	pigs
Ntrk2	0.32	1.00000000	pigs
Donson	0.19	1.00000000	pigs

Sugct	0.30	1.00000000	pigs
Tubb5	-0.61	1.00000000	pigs
Zhx2	0.40	1.00000000	pigs
Cnnm4	0.19	1.00000000	pigs
Ccnl1	0.44	1.00000000	pigs
Exoc3	0.33	1.00000000	pigs
Furin	0.09	1.00000000	pigs
Parp12	0.08	1.00000000	pigs
Srpk2	0.44	1.00000000	pigs
Lrch3	0.28	1.00000000	pigs
Ppp1r13b	0.38	1.00000000	pigs
Nucks1	-0.44	1.00000000	pigs
Avil	0.20	1.00000000	pigs
Tm7sf3	0.18	1.00000000	pigs
Sox5	0.51	1.00000000	pigs
Htra1	0.21	1.00000000	pigs
Fbxl7	0.54	1.00000000	pigs
Zbtb16	0.53	1.00000000	pigs
Psap	0.42	1.00000000	pigs
Bcl3	0.17	1.00000000	pigs
Ciita	0.10	1.00000000	pigs
Tia1	0.21	1.00000000	pigs
Clip1	0.41	1.00000000	pigs
Rab40b	0.22	1.00000000	pigs
Cfap74	0.08	1.00000000	pigs
Arid3b	0.33	1.00000000	pigs
Zfp408	0.04	1.00000000	pigs
Agfg1	0.15	1.00000000	pigs
Epha4	-0.51	1.00000000	pigs
Slc6a9	0.23	1.00000000	pigs
Cbx4	0.43	1.00000000	pigs
Rbbp9	0.37	1.00000000	pigs
Spast	0.13	1.00000000	pigs
Sema3d	-0.53	1.00000000	pigs
Gtpbp1	0.27	1.00000000	pigs
Slc4a7	0.53	1.00000000	pigs
Sec16a	0.33	1.00000000	pigs
Tlr7	0.21	1.00000000	pigs
Acot8	0.05	1.00000000	pigs
Tmem165	0.31	1.00000000	pigs
Znfx1	0.47	1.00000000	pigs
Znrf3	0.16	1.00000000	pigs
Ptgfrn	-0.42	1.00000000	pigs
Znrf2	0.30	1.00000000	pigs
Spg11	0.21	1.00000000	pigs
Sdccag8	0.39	1.00000000	pigs

Tmem47	0.34	1.00000000	pigs
Guk1	-0.56	1.00000000	pigs
Nfkb1	0.48	1.00000000	pigs
Id2	0.53	1.00000000	pigs
Pik3ca	0.42	1.00000000	pigs
Sh3pxd2b	0.42	1.00000000	pigs
Tmem164	0.35	1.00000000	pigs
Pard6g	-0.56	1.00000000	pigs
Wdr25	0.14	1.00000000	pigs
Ctnna1	0.40	1.00000000	pigs
Ube2z	0.20	1.00000000	pigs
Cox6a1	-0.50	1.00000000	pigs
Zfand3	0.43	1.00000000	pigs
Emc4	0.30	1.00000000	pigs
Cish	0.24	1.00000000	pigs
Ifi27	0.60	1.00000000	pigs
Prdm2	0.43	1.00000000	pigs
Bfar	0.96	1.00000000	pigs
Acsl3	0.21	1.00000000	pigs
Tifa	0.31	1.00000000	pigs
Plin2	0.74	1.00000000	pigs
Zc3hav1	0.28	1.00000000	pigs
Hmgb2	-0.69	1.00000000	pigs
Adamts6	0.52	1.00000000	pigs
Slc50a1	0.17	1.00000000	pigs
Atp1a1	0.63	1.00000000	pigs
Arhgap23	0.31	1.00000000	pigs
Ptk2	0.29	1.00000000	pigs
Il17ra	0.03	1.00000000	pigs
Sema3f	0.03	1.00000000	pigs
Acvr1b	0.27	1.00000000	pigs
Abr	0.62	1.00000000	pigs
Rrbp1	0.28	1.00000000	pigs
Rgl1	0.55	1.00000000	pigs
Samm50	0.36	1.00000000	pigs
Kdm5c	0.14	1.00000000	pigs
Ssx2ip	-0.26	1.00000000	pigs
Trpm4	0.24	1.00000000	pigs
Wdr26	0.18	1.00000000	pigs
Pxdn	0.27	1.00000000	pigs
Traf1	0.54	1.00000000	pigs
Lman2l	0.17	1.00000000	pigs
Pam	-0.60	1.00000000	pigs
Rora	0.62	1.00000000	pigs
Nr2f2	-0.59	1.00000000	pigs
Wee1	0.18	1.00000000	pigs

Cfap20	0.88	1.00000000	pigs
Fermt3	0.25	1.00000000	pigs
Dbi	-0.17	1.00000000	pigs
Aig1	-0.65	1.00000000	pigs
Reep1	-0.65	1.00000000	pigs
Osbpl11	0.23	1.00000000	pigs
Fam167b	0.29	1.00000000	pigs
Tef	0.14	1.00000000	pigs
Pdk4	-0.62	1.00000000	pigs
Txndc5	0.14	1.00000000	pigs
Trak1	0.22	1.00000000	pigs
Cipc	0.05	1.00000000	pigs
Hpcal1	0.43	1.00000000	pigs
Inpp5f	0.41	1.00000000	pigs
Slit3	-0.23	1.00000000	pigs
Csrnp1	0.42	1.00000000	pigs
Rasd1	0.22	1.00000000	pigs
Ints6l	0.43	1.00000000	pigs
Ccdc9	0.29	1.00000000	pigs
Fam91a1	0.41	1.00000000	pigs
Rassf5	0.55	1.00000000	pigs
Plagl1	0.31	1.00000000	pigs
Arhgap17	0.25	1.00000000	pigs
Nup153	0.49	1.00000000	pigs
Galns	0.18	1.00000000	pigs
Stx17	0.32	1.00000000	pigs
Eif1	-0.43	1.00000000	pigs
Efna1	0.43	1.00000000	pigs
Dennd3	0.19	1.00000000	pigs
Nek4	0.29	1.00000000	pigs
Iqgap2	0.34	1.00000000	pigs
Coro2b	0.46	1.00000000	pigs
Lix1l	0.18	1.00000000	pigs
Smad7	0.20	1.00000000	pigs
Lxn	0.53	1.00000000	pigs
Efr3a	0.20	1.00000000	pigs
Atp6ap2	0.26	1.00000000	pigs
Ccnk	0.17	1.00000000	pigs
B3glct	0.13	1.00000000	pigs
Ywhaz	0.42	1.00000000	pigs
Slc35f5	0.17	1.00000000	pigs
Kansl1	0.38	1.00000000	pigs
Ptbp2	0.12	1.00000000	pigs
Gbp2b	0.10	1.00000000	pigs
Fnbp1l	0.26	1.00000000	pigs
Tfeb	0.15	1.00000000	pigs

Ubtd2	0.27	1.00000000	pigs
Oser1	0.14	1.00000000	pigs
Trim3	0.11	1.00000000	pigs
Borcs5	0.52	1.00000000	pigs
Ufsp2	0.22	1.00000000	pigs
Ddr1	0.06	1.00000000	pigs
Dnajb9	0.40	1.00000000	pigs
Kynu	0.44	1.00000000	pigs
Palld	0.09	1.00000000	pigs
Spsb1	0.46	1.00000000	pigs
Runx1	1.44	1.00000000	pigs
Gtf3c1	0.09	1.00000000	pigs
Ifi35	0.28	1.00000000	pigs
Nol11	0.64	1.00000000	pigs
Rsbn1	0.17	1.00000000	pigs
Pfkfb4	0.06	1.00000000	pigs
Jazf1	0.38	1.00000000	pigs
Cd2ap	0.47	1.00000000	pigs
Hdac6	0.17	1.00000000	pigs
Iqgap1	-0.22	1.00000000	pigs
Ppm1k	0.24	1.00000000	pigs
Palmd	-0.55	1.00000000	pigs
Ccdc6	0.11	1.00000000	pigs
Adamtsl4	-0.22	1.00000000	pigs
Ubr5	0.29	1.00000000	pigs
Snx12	0.27	1.00000000	pigs
Bst1	-0.62	1.00000000	pigs
Nufip2	0.42	1.00000000	pigs
Txnrd1	0.37	1.00000000	pigs
Axin1	-0.02	1.00000000	pigs
Nrp1	0.22	1.00000000	pigs
Galnt18	0.75	1.00000000	pigs
Nagk	0.35	1.00000000	pigs
Clec16a	0.15	1.00000000	pigs
Atxn2l	0.17	1.00000000	pigs
Sbno2	0.26	1.00000000	pigs
Ndufa13	-0.62	1.00000000	pigs
Ahcyl1	0.09	1.00000000	pigs
Thbs1	-0.63	1.00000000	pigs
Itpk1	0.34	1.00000000	pigs
Kdm4c	0.09	1.00000000	pigs
Ier5	0.67	1.00000000	pigs
Hectd1	-0.53	1.00000000	pigs
Ttc7b	0.18	1.00000000	pigs
Git2	0.36	1.00000000	pigs
Ankrd12	0.35	1.00000000	pigs

Rnf2	0.36	1.00000000	pigs
Acot10	0.12	1.00000000	pigs
Fmnl3	0.17	1.00000000	pigs
Bend7	0.33	1.00000000	pigs
Pik3ap1	0.83	1.00000000	pigs
1810037I17Rik	0.54	1.00000000	pigs
Stt3b	0.23	1.00000000	pigs
Pggt1b	0.32	1.00000000	pigs
Ogdh	0.26	1.00000000	pigs
Nek6	0.27	1.00000000	pigs
Cyyr1	-0.39	1.00000000	pigs
Dgkh	0.30	1.00000000	pigs
N4bp1	0.40	1.00000000	pigs
Wdr7	0.33	1.00000000	pigs
Churc1	0.13	1.00000000	pigs
Tob2	0.35	1.00000000	pigs
Nr2c2	0.43	1.00000000	pigs
Cdca7l	-0.44	1.00000000	pigs
Aco1	0.48	1.00000000	pigs
Slc45a4	0.19	1.00000000	pigs
Tgif2	0.12	1.00000000	pigs
Gramd3	0.62	1.00000000	pigs
Ap1g1	0.13	1.00000000	pigs
Tafa5	0.07	1.00000000	pigs
Gla	0.62	1.00000000	pigs
Amotl2	-0.51	1.00000000	pigs
Slc9a1	0.55	1.00000000	pigs
Zc3h12a	0.63	1.00000000	pigs
Thrap3	-0.47	1.00000000	pigs
Ncf4	0.44	1.00000000	pigs
Chp1	0.45	1.00000000	pigs
Kbtbd2	0.33	1.00000000	pigs
Ubfd1	0.20	1.00000000	pigs
Ccdc126	0.35	1.00000000	pigs
Rnf121	0.21	1.00000000	pigs
Trim75	0.35	1.00000000	pigs
Dlg5	0.17	1.00000000	pigs
Wdr37	0.40	1.00000000	pigs
Bcat2	0.33	1.00000000	pigs
Insig1	0.79	1.00000000	pigs
Chordc1	0.29	1.00000000	pigs
Qk	0.37	1.00000000	pigs
Prox1	-0.40	1.00000000	pigs
Higd1a	0.19	1.00000000	pigs
Bnip3	0.68	1.00000000	pigs
Ticam1	0.15	1.00000000	pigs

Cables1	0.48	1.00000000	pigs
Rarres1	0.38	1.00000000	pigs
Zfp280c	0.49	1.00000000	pigs
Zc2hc1a	-0.38	1.00000000	pigs
Ewsr1	0.41	1.00000000	pigs
Ebf1	-0.66	1.00000000	pigs
Rftn1	0.51	1.00000000	pigs
Prex2	0.53	1.00000000	pigs
Grn	0.39	1.00000000	pigs
Deptor	0.47	1.00000000	pigs
Cd40	0.28	1.00000000	pigs
Ddb1	0.17	1.00000000	pigs
Iffo2	0.17	1.00000000	pigs
Nck2	0.36	1.00000000	pigs
Pim3	0.47	1.00000000	pigs
B4galt4	0.03	1.00000000	pigs
Gcnt1	-0.43	1.00000000	pigs
Prkce	0.74	1.00000000	pigs
Psmd3	0.08	1.00000000	pigs
Zfp36l1	-0.37	1.00000000	pigs
Fubp3	0.40	1.00000000	pigs
1110032A03Rik	-0.01	1.00000000	pigs
Acsf2	0.16	1.00000000	pigs
Lin54	0.10	1.00000000	pigs
Foxk2	0.10	1.00000000	pigs
Tent2	0.06	1.00000000	pigs
Cox17	-0.23	1.00000000	pigs
Aplf	0.13	1.00000000	pigs
Fbxl17	0.31	1.00000000	pigs
Alkbh5	0.57	1.00000000	pigs
Pla2g6	0.04	1.00000000	pigs
Agpat4	0.28	1.00000000	pigs
Armc7	0.08	1.00000000	pigs
Ago4	0.36	1.00000000	pigs
Hsd3b7	-0.49	1.00000000	pigs
Ptprj	0.47	1.00000000	pigs
Ppic	0.22	1.00000000	pigs
Gbe1	0.43	1.00000000	pigs
Dusp16	0.34	1.00000000	pigs
Cast	-0.52	1.00000000	pigs
Chsy3	0.98	1.00000000	pigs
Samd4b	0.09	1.00000000	pigs
Ap2a1	0.18	1.00000000	pigs
Ldb1	0.35	1.00000000	pigs
Ptgs2	0.77	1.00000000	pigs
Sh3pxd2a	0.32	1.00000000	pigs

Crim1	-0.65	1.00000000	pigs
Ube2k	0.25	1.00000000	pigs
Rbm47	0.35	1.00000000	pigs
Bag5	0.21	1.00000000	pigs
Synj2	0.15	1.00000000	pigs
Eng	0.11	1.00000000	pigs
Uap1	0.53	1.00000000	pigs
Rcsd1	-0.85	1.00000000	pigs
Serpinf1	0.25	1.00000000	pigs
Col8a1	-0.83	1.00000000	pigs
Gripap1	0.22	1.00000000	pigs
Abcc3	0.10	1.00000000	pigs
Banp	0.15	1.00000000	pigs
Pank3	0.51	1.00000000	pigs
Apobec3	0.23	1.00000000	pigs
Fyn	0.45	1.00000000	pigs
Scaf4	0.23	1.00000000	pigs
Ppfia1	0.22	1.00000000	pigs
Fam117b	0.33	1.00000000	pigs
Yaf2	0.34	1.00000000	pigs
Cdyl	0.20	1.00000000	pigs
Hcfc2	0.06	1.00000000	pigs
Ostf1	-0.48	1.00000000	pigs
Acvr1	0.11	1.00000000	pigs
Shq1	0.29	1.00000000	pigs
Sipa1l1	0.27	1.00000000	pigs
Il6st	0.34	1.00000000	pigs
Pik3r1	0.50	1.00000000	pigs
Ctbp2	0.17	1.00000000	pigs
Unkl	0.38	1.00000000	pigs
Ripk2	0.30	1.00000000	pigs
Herc6	0.41	1.00000000	pigs
Endov	0.14	1.00000000	pigs
Ttc39c	0.07	1.00000000	pigs
Klhl4	-0.63	1.00000000	pigs
Sgta	0.06	1.00000000	pigs
Slc22a23	0.39	1.00000000	pigs
Plekha5	0.20	1.00000000	pigs
Smim26	-0.38	1.00000000	pigs
Camk1d	0.37	1.00000000	pigs
Grina	0.11	1.00000000	pigs
Ranbp2	0.40	1.00000000	pigs
Elf1	0.38	1.00000000	pigs
Csgalnact2	0.33	1.00000000	pigs
Cybrd1	0.35	1.00000000	pigs
Enah	0.27	1.00000000	pigs

Sec61g	-0.41	1.00000000	pigs
Rwdd4a	-0.02	1.00000000	pigs
Nexn	-0.37	1.00000000	pigs
Stxbp1	0.18	1.00000000	pigs
Adamtsl1	-0.19	1.00000000	pigs
Cd82	0.52	1.00000000	pigs
Ifit2	-0.64	1.00000000	pigs
Cd47	-0.46	1.00000000	pigs
Aurkaip1	-0.37	1.00000000	pigs
Parp3	0.15	1.00000000	pigs
Rufy1	-0.51	1.00000000	pigs
Tent4a	0.27	1.00000000	pigs
Ptpn1	0.32	1.00000000	pigs
Dbndd2	0.12	1.00000000	pigs
Copg1	0.09	1.00000000	pigs
Sdhaf2	0.03	1.00000000	pigs
Tmem86a	0.05	1.00000000	pigs
Ankrd37	0.35	1.00000000	pigs
Setd7	0.05	1.00000000	pigs
Hivep2	0.63	1.00000000	pigs
Parm1	0.12	1.00000000	pigs
Abhd12	0.52	1.00000000	pigs
Kdm4b	0.81	1.00000000	pigs
Srp54a	0.33	1.00000000	pigs
Tmem87a	0.13	1.00000000	pigs
Tmem88	0.25	1.00000000	pigs
Tcf12	0.24	1.00000000	pigs
Gse1	0.06	1.00000000	pigs
Ehd2	-0.42	1.00000000	pigs
Zfp804a	0.11	1.00000000	pigs
Fam193a	0.34	1.00000000	pigs
Xpr1	0.35	1.00000000	pigs
Tbkbp1	0.16	1.00000000	pigs
Ramp3	-0.46	1.00000000	pigs
Col15a1	0.37	1.00000000	pigs
Wbp11	0.10	1.00000000	pigs
Ube2o	0.10	1.00000000	pigs
Sft2d2	0.22	1.00000000	pigs
Gpbp1	0.30	1.00000000	pigs
Sspn	-0.52	1.00000000	pigs
Thoc7	-0.48	1.00000000	pigs
Plcg2	0.45	1.00000000	pigs
Vps37b	0.08	1.00000000	pigs
Tbx1	-0.58	1.00000000	pigs
Lpcat2	0.15	1.00000000	pigs
Dip2c	0.27	1.00000000	pigs

Atp9a	0.11	1.00000000	pigs
Gsr	0.10	1.00000000	pigs
Gadd45b	0.51	1.00000000	pigs
Atpif1	-0.44	1.00000000	pigs
Ubap2l	0.21	1.00000000	pigs
Gpr160	-0.59	1.00000000	pigs
Gjc1	0.13	1.00000000	pigs
Zfp830	0.19	1.00000000	pigs
Cox4i1	-0.35	1.00000000	pigs
Phf10	0.25	1.00000000	pigs
Ptpn2	0.33	1.00000000	pigs
Pnisr	0.41	1.00000000	pigs
Aif1	0.49	1.00000000	pigs
Esam	0.29	1.00000000	pigs
Kcmf1	0.29	1.00000000	pigs
Eif3l	0.22	1.00000000	pigs
Foxo1	0.25	1.00000000	pigs
Wwp2	0.21	1.00000000	pigs
Cdkn1a	0.24	1.00000000	pigs
Supt20	0.18	1.00000000	pigs
Dlc1	0.19	1.00000000	pigs
Setx	0.25	1.00000000	pigs
Itgb8	-0.52	1.00000000	pigs
Ndufb9	-0.55	1.00000000	pigs
Ddx41	0.18	1.00000000	pigs
Zdhhc23	0.01	1.00000000	pigs
Xxylt1	0.25	1.00000000	pigs
Fem1c	0.31	1.00000000	pigs
Abhd10	-0.35	1.00000000	pigs
Adamts12	0.58	1.00000000	pigs
Tspan13	0.34	1.00000000	pigs
Abhd18	0.31	1.00000000	pigs
Clcn7	0.20	1.00000000	pigs
Stk10	0.10	1.00000000	pigs
Slc16a1	0.71	1.00000000	pigs
Fjx1	0.06	1.00000000	pigs
Prickle3	0.01	1.00000000	pigs
Dnajc8	-0.45	1.00000000	pigs
Arhgef37	0.35	1.00000000	pigs
Lhfpl2	0.54	1.00000000	pigs
Arap1	0.31	1.00000000	pigs
Pde4b	0.29	1.00000000	pigs
Rap1gds1	0.17	1.00000000	pigs
Accs	0.18	1.00000000	pigs
Slc7a7	0.16	1.00000000	pigs
Dock10	0.38	1.00000000	pigs

Stk3	0.20	1.00000000	pigs
Ecm2	-0.35	1.00000000	pigs
Irf1	0.43	1.00000000	pigs
Elmo2	0.15	1.00000000	pigs
Ace	0.22	1.00000000	pigs
Phactr2	-0.41	1.00000000	pigs
Snx9	0.59	1.00000000	pigs
Vps52	0.13	1.00000000	pigs
Rnf122	0.32	1.00000000	pigs
Plcb4	-0.39	1.00000000	pigs
Cald1	-0.42	1.00000000	pigs
Plekhh3	0.15	1.00000000	pigs
Cnot8	0.17	1.00000000	pigs
Tesk2	0.35	1.00000000	pigs
Acbd5	0.25	1.00000000	pigs
Zbtb11	0.13	1.00000000	pigs
Herpud1	0.42	1.00000000	pigs
Fchsd2	0.49	1.00000000	pigs
Ston2	-0.59	1.00000000	pigs
Ugp2	0.66	1.00000000	pigs
Ppa2	0.28	1.00000000	pigs
Usp9x	0.30	1.00000000	pigs
Grk4	0.20	1.00000000	pigs
Trib1	0.25	1.00000000	pigs
Aldh1l2	0.04	1.00000000	pigs
Rrp15	0.14	1.00000000	pigs
Gpbp1l1	0.30	1.00000000	pigs
Ttc3	0.35	1.00000000	pigs
Emp1	-0.55	1.00000000	pigs
Arhgap15	-1.44	1.00000000	pigs
Dnpep	0.03	1.00000000	pigs
Srp14	-0.55	1.00000000	pigs
Als2	0.28	1.00000000	pigs
Ambra1	0.36	1.00000000	pigs
Nr2c1	0.02	1.00000000	pigs
Slc36a4	0.16	1.00000000	pigs
Brox	0.23	1.00000000	pigs
Ryk	0.10	1.00000000	pigs
Zmym1	-0.52	1.00000000	pigs
Plekha2	0.42	1.00000000	pigs
Plbd2	0.19	1.00000000	pigs
H1f3	0.79	1.00000000	pigs
Serpinh1	0.11	1.00000000	pigs
Lck	0.30	1.00000000	pigs
Bcl9	0.05	1.00000000	pigs
Sned1	0.30	1.00000000	pigs

Tigar	0.10	1.00000000	pigs
Rhbdl2	0.12	1.00000000	pigs
Hectd3	0.06	1.00000000	pigs
Sgip1	-0.54	1.00000000	pigs
Filip1	-0.76	1.00000000	pigs
Serpinb9	0.17	1.00000000	pigs
Zmynd8	0.26	1.00000000	pigs
Ripk1	0.22	1.00000000	pigs
Prpf39	0.39	1.00000000	pigs
B3gnt3	-0.02	1.00000000	pigs
Rab33b	0.17	1.00000000	pigs
Slc66a1	0.24	1.00000000	pigs
Rbm15b	0.24	1.00000000	pigs
Slc37a1	0.14	1.00000000	pigs
Pmp22	-0.61	1.00000000	pigs
Golga7	0.08	1.00000000	pigs
Picalm	0.25	1.00000000	pigs
Ralb	0.31	1.00000000	pigs
Lbr	-0.60	1.00000000	pigs
Taf5l	0.11	1.00000000	pigs
Cdc42bpb	0.13	1.00000000	pigs
Golga5	0.24	1.00000000	pigs
Ptpmt1	0.03	1.00000000	pigs
Rcan1	-0.96	1.00000000	pigs
Socs6	0.31	1.00000000	pigs
Dnmt3a	0.01	1.00000000	pigs
Cdc42ep5	-0.46	1.00000000	pigs
Creld1	0.05	1.00000000	pigs
Atg2a	0.14	1.00000000	pigs
Synj2bp	0.18	1.00000000	pigs
Ddx21	-0.45	1.00000000	pigs
Bicd2	0.03	1.00000000	pigs
Ube2r2	0.25	1.00000000	pigs
Anxa7	-0.40	1.00000000	pigs
Apbb2	0.51	1.00000000	pigs
Gtf2a1	1.15	1.00000000	pigs
Cyba	0.46	1.00000000	pigs
Stx12	0.34	1.00000000	pigs
Ubr2	0.32	1.00000000	pigs
Gtf2h1	0.01	1.00000000	pigs
Tex9	0.13	1.00000000	pigs
Tpm4	-0.31	1.00000000	pigs
Gpr183	0.97	1.00000000	pigs
Tspan9	0.32	1.00000000	pigs
Nf1	0.40	1.00000000	pigs
Acss2	0.25	1.00000000	pigs

St3gal2	0.09	1.00000000	pigs
Trmt112	-0.36	1.00000000	pigs
Top1	0.36	1.00000000	pigs
Serpinb1a	0.60	1.00000000	pigs
Ier3	0.88	1.00000000	pigs
Tmem185a	0.19	1.00000000	pigs
Dcps	0.27	1.00000000	pigs
Spidr	0.53	1.00000000	pigs
Slc39a11	0.25	1.00000000	pigs
Kat14	0.23	1.00000000	pigs
C4bp	-0.58	1.00000000	pigs
Dhtkd1	0.12	1.00000000	pigs
Ccn2	-0.53	1.00000000	pigs
Jmjd8	0.21	1.00000000	pigs
Kdm2a	0.31	1.00000000	pigs
Lonrf1	0.19	1.00000000	pigs
Dctd	0.07	1.00000000	pigs
Prkd2	0.07	1.00000000	pigs
Zhx1	-0.01	1.00000000	pigs
Ubn1	0.27	1.00000000	pigs
Oxct1	-0.40	1.00000000	pigs
Kdm4a	0.14	1.00000000	pigs
Dipk1b	-0.51	1.00000000	pigs
Jade2	0.11	1.00000000	pigs
Pdap1	-0.39	1.00000000	pigs
Calml4	-0.46	1.00000000	pigs
Tppp3	-0.93	1.00000000	pigs
Dpy19l1	0.34	1.00000000	pigs
Cog6	0.17	1.00000000	pigs
Rin2	0.61	1.00000000	pigs
Wipi2	0.22	1.00000000	pigs
Top3a	0.24	1.00000000	pigs
Gli2	-0.24	1.00000000	pigs
Pik3c3	0.27	1.00000000	pigs
Ogt	0.14	1.00000000	pigs
Psmb7	0.28	1.00000000	pigs
Elob	-0.34	1.00000000	pigs
Akip1	0.28	1.00000000	pigs
Calcoco2	-0.40	1.00000000	pigs
Tra2b	0.39	1.00000000	pigs
Il10ra	0.43	1.00000000	pigs
Slc25a36	0.22	1.00000000	pigs
Foxp2	-0.35	1.00000000	pigs
Arhgap12	0.09	1.00000000	pigs
Extl2	0.06	1.00000000	pigs
Epm2aip1	0.32	1.00000000	pigs

Psmg4	-0.36	1.00000000	pigs
Stim1	0.05	1.00000000	pigs
Lmtk2	0.03	1.00000000	pigs
Cmss1	-0.40	1.00000000	pigs
Bsdc1	0.00	1.00000000	pigs
Cradd	0.04	1.00000000	pigs
Cdc42ep2	0.15	1.00000000	pigs
Tsc1	0.14	1.00000000	pigs
Urm1	0.24	1.00000000	pigs
Wwtr1	0.43	1.00000000	pigs
Gnl2	0.22	1.00000000	pigs
Cndp2	0.22	1.00000000	pigs
Nedd4l	0.82	1.00000000	pigs
Scarb2	0.24	1.00000000	pigs
Ppp1r3d	0.11	1.00000000	pigs
Ubap1	0.12	1.00000000	pigs
Kank1	0.19	1.00000000	pigs
Rhobtb3	0.33	1.00000000	pigs
Gak	0.22	1.00000000	pigs
Lrrc8d	0.18	1.00000000	pigs
Adam12	0.35	1.00000000	pigs
Ptpn21	0.00	1.00000000	pigs
Cebpz	-0.40	1.00000000	pigs
Batf2	0.09	1.00000000	pigs
Dipk2b	0.20	1.00000000	pigs
Papss2	0.18	1.00000000	pigs
Kcnq1	0.27	1.00000000	pigs
Phactr1	0.49	1.00000000	pigs
Ppp4r3a	0.31	1.00000000	pigs
Arhgef7	0.08	1.00000000	pigs
Gipc2	-0.33	1.00000000	pigs
Ednrb	-1.43	1.00000000	pigs
Hnmt	0.60	1.00000000	pigs
Whamm	0.24	1.00000000	pigs
Slc38a2	0.40	1.00000000	pigs
Trps1	0.33	1.00000000	pigs
Ficd	0.18	1.00000000	pigs
Gdi2	0.42	1.00000000	pigs
Entr1	0.25	1.00000000	pigs
Acer3	0.29	1.00000000	pigs
Gab2	0.26	1.00000000	pigs
Arhgap32	0.12	1.00000000	pigs
Tmem263	0.11	1.00000000	pigs
Rep15	-0.42	1.00000000	pigs
Clec1a	0.12	1.00000000	pigs
Col1a2	0.51	1.00000000	pigs

Plac9a	-0.55	1.00000000	pigs
Scarb1	0.35	1.00000000	pigs
B3gnt5	-0.39	1.00000000	pigs
Ssbp3	-0.01	1.00000000	pigs
Ep300	0.39	1.00000000	pigs
Atrnl1	0.39	1.00000000	pigs
Itprip	0.15	1.00000000	pigs
Fam177a	0.14	1.00000000	pigs
Rai1	0.24	1.00000000	pigs
Pter	-0.01	1.00000000	pigs
Trim56	0.04	1.00000000	pigs
Lamc1	-0.67	1.00000000	pigs
Wac	0.16	1.00000000	pigs
Washc5	0.31	1.00000000	pigs
Hspb8	0.21	1.00000000	pigs
Tax1bp1	0.34	1.00000000	pigs
Arhgef2	0.03	1.00000000	pigs
Tnfrsf21	0.41	1.00000000	pigs
Eepd1	0.49	1.00000000	pigs
Tubb6	-0.34	1.00000000	pigs
Ninj1	0.21	1.00000000	pigs
Exoc4	0.27	1.00000000	pigs
Usp28	0.10	1.00000000	pigs
Pcbp1	-0.27	1.00000000	pigs
Edem3	0.25	1.00000000	pigs
Fuom	-0.31	1.00000000	pigs
Ggh	0.12	1.00000000	pigs
Slc35a2	0.06	1.00000000	pigs
Tjap1	0.13	1.00000000	pigs
Rictor	0.38	1.00000000	pigs
Eed	-0.03	1.00000000	pigs
Usp10	0.10	1.00000000	pigs
Eif3d	0.48	1.00000000	pigs
Klf9	0.16	1.00000000	pigs
Lats1	-0.04	1.00000000	pigs
Plce1	-0.50	1.00000000	pigs
Ss18l2	0.21	1.00000000	pigs
Tmem8b	0.21	1.00000000	pigs
Actr1a	0.08	1.00000000	pigs
Prdx5	0.26	1.00000000	pigs
Klhl2	0.20	1.00000000	pigs
Taco1	0.10	1.00000000	pigs
Kmt5a	0.02	1.00000000	pigs
Socs7	0.01	1.00000000	pigs
Pias1	0.22	1.00000000	pigs
Slc12a2	0.36	1.00000000	pigs

Lmbr1	0.14	1.00000000	pigs
Rlf	0.18	1.00000000	pigs
Cert1	0.23	1.00000000	pigs
Papolg	0.24	1.00000000	pigs
Fscn1	0.30	1.00000000	pigs
Tnfsf12	-0.31	1.00000000	pigs
Stk35	0.01	1.00000000	pigs
Selenof	-0.43	1.00000000	pigs
Katnip	0.21	1.00000000	pigs
Zfp618	0.41	1.00000000	pigs
Gas7	0.31	1.00000000	pigs
Agps	0.06	1.00000000	pigs
Nos3	-0.14	1.00000000	pigs
Itpr3	0.07	1.00000000	pigs
Retreg1	0.31	1.00000000	pigs
Slc9b1	-0.46	1.00000000	pigs
Itga3	-0.70	1.00000000	pigs
Rab11fip3	0.19	1.00000000	pigs
Phf8	0.07	1.00000000	pigs
Slc7a1	0.20	1.00000000	pigs
Cdr2	-0.04	1.00000000	pigs
Cacna1e	0.40	1.00000000	pigs
Lrch1	0.22	1.00000000	pigs
A830018L16Rik	0.17	1.00000000	pigs
Flt4	-0.29	1.00000000	pigs
Tmem150c	0.12	1.00000000	pigs
S1pr1	-0.41	1.00000000	pigs
Sugp2	0.08	1.00000000	pigs
Zfp90	0.24	1.00000000	pigs
Tcea1	-0.50	1.00000000	pigs
Egfl8	0.08	1.00000000	pigs
Prpf31	0.16	1.00000000	pigs
Ift52	0.06	1.00000000	pigs
Arv1	0.25	1.00000000	pigs
Fuca1	0.19	1.00000000	pigs
Tnfsf13b	0.26	1.00000000	pigs
Pick1	0.05	1.00000000	pigs
Srsf4	-0.70	1.00000000	pigs
Ndufa1	-0.51	1.00000000	pigs
Adora2a	0.25	1.00000000	pigs
Frmd3	-1.05	1.00000000	pigs
Fndc3b	0.30	1.00000000	pigs
Zfp532	0.12	1.00000000	pigs
Itgb2l	0.31	1.00000000	pigs
Lrrc45	0.25	1.00000000	pigs
Cd48	0.83	1.00000000	pigs

Trafd1	0.16	1.00000000	pigs
Dhrs7b	0.16	1.00000000	pigs
Nasp	0.65	1.00000000	pigs
Stab1	0.41	1.00000000	pigs
Exosc7	0.23	1.00000000	pigs
Ascc1	0.21	1.00000000	pigs
Col3a1	0.13	1.00000000	pigs
Appl1	-0.48	1.00000000	pigs
Cop1	0.31	1.00000000	pigs
Gabarapl1	0.22	1.00000000	pigs
Cgnl1	0.34	1.00000000	pigs
Birc6	0.25	1.00000000	pigs
Gng10	0.40	1.00000000	pigs
Cul4a	0.14	1.00000000	pigs
Dyrk1b	0.10	1.00000000	pigs
Pdpk1	0.09	1.00000000	pigs
Crkl	0.03	1.00000000	pigs
Gsk3b	0.38	1.00000000	pigs
Cic	0.13	1.00000000	pigs
Chst3	0.05	1.00000000	pigs
Gtf2b	0.52	1.00000000	pigs
Cep95	0.25	1.00000000	pigs
Dcaf5	0.17	1.00000000	pigs
Cep68	0.00	1.00000000	pigs
Dnajb1	0.97	1.00000000	pigs
Acp2	0.20	1.00000000	pigs
Atl2	0.18	1.00000000	pigs
Grk2	0.39	1.00000000	pigs
Arhgap6	0.40	1.00000000	pigs
Pld3	-0.32	1.00000000	pigs
Rasgef1b	0.78	1.00000000	pigs
Coq2	0.23	1.00000000	pigs
Hip1	0.10	1.00000000	pigs
Endod1	-0.51	1.00000000	pigs
Vps13a	0.24	1.00000000	pigs
Sos1	0.31	1.00000000	pigs
Epb41l4a	0.48	1.00000000	pigs
Eps15l1	0.19	1.00000000	pigs
Rcc2	0.04	1.00000000	pigs
Ubd	0.06	1.00000000	pigs
Yipf5	0.12	1.00000000	pigs
Kmt5b	0.22	1.00000000	pigs
Psmd11	0.27	1.00000000	pigs
Pmaip1	0.47	1.00000000	pigs
Fh1	0.08	1.00000000	pigs
Cdk6	0.53	1.00000000	pigs

Dennd5a	0.26	1.00000000	pigs
Cnot7	0.24	1.00000000	pigs
Agpat1	-0.03	1.00000000	pigs
Caskin2	-0.58	1.00000000	pigs
Traf2	0.10	1.00000000	pigs
Snx30	0.22	1.00000000	pigs
Laptm4b	0.28	1.00000000	pigs
Jakmip3	0.10	1.00000000	pigs
Csrnp2	0.43	1.00000000	pigs
Nufip1	-0.29	1.00000000	pigs
Qrfpr	-0.80	1.00000000	pigs
Cdc26	0.18	1.00000000	pigs
Thsd7a	-0.23	1.00000000	pigs
Gdi1	0.24	1.00000000	pigs
Gba	0.18	1.00000000	pigs
Dyrk1a	0.23	1.00000000	pigs
Pank2	0.04	1.00000000	pigs
Snx17	0.43	1.00000000	pigs
Fes	0.05	1.00000000	pigs
Psd3	0.00	1.00000000	pigs
Arpc5l	0.19	1.00000000	pigs
Ier5l	0.62	1.00000000	pigs
Uba3	0.21	1.00000000	pigs
Ehmt2	0.08	1.00000000	pigs
Trim27	-0.06	1.00000000	pigs
Far2	-0.62	1.00000000	pigs
Cep44	0.23	1.00000000	pigs
Tsc22d4	0.15	1.00000000	pigs
Anapc15-ps	0.19	1.00000000	pigs
Ddit4	0.52	1.00000000	pigs
Fzd6	-0.40	1.00000000	pigs
Sh2b3	0.21	1.00000000	pigs
Pepd	0.59	1.00000000	pigs
Stx8	0.29	1.00000000	pigs
Ccser2	0.05	1.00000000	pigs
Fnip1	0.33	1.00000000	pigs
Edem2	0.15	1.00000000	pigs
Ptprb	0.20	1.00000000	pigs
Hs6st1	0.31	1.00000000	pigs
Gramd1a	0.09	1.00000000	pigs
Tbc1d12	0.35	1.00000000	pigs
Cox5b-ps	-0.36	1.00000000	pigs
Hk1	0.21	1.00000000	pigs
Pik3r5	0.95	1.00000000	pigs
Wtap	0.44	1.00000000	pigs
Tspan11	0.15	1.00000000	pigs

Nupl1	0.16	1.00000000	pigs
Cds2	0.19	1.00000000	pigs
Hid1	0.12	1.00000000	pigs
Plekhg1	-0.41	1.00000000	pigs
Tarbp1	0.05	1.00000000	pigs
Capn2	-0.52	1.00000000	pigs
Rpap2	0.17	1.00000000	pigs
Nipbl	0.31	1.00000000	pigs
Cnot1	0.08	1.00000000	pigs
Casp8	0.11	1.00000000	pigs
Cep85l	-0.42	1.00000000	pigs
Pfkl	0.08	1.00000000	pigs
Pgd	0.24	1.00000000	pigs
Ggps1	-0.07	1.00000000	pigs
Doc2b	0.12	1.00000000	pigs
Rela	0.13	1.00000000	pigs
Zfr	0.28	1.00000000	pigs
Chd9	0.38	1.00000000	pigs
Prr14l	0.12	1.00000000	pigs
Tmem135	0.36	1.00000000	pigs
Laptm5	0.24	1.00000000	pigs
Zfy1	-0.57	1.00000000	pigs
Lman2	0.10	1.00000000	pigs
Emilin2	0.99	1.00000000	pigs
Atg7	-0.54	1.00000000	pigs
Gpihbp1	0.20	1.00000000	pigs
Shc1	-0.46	1.00000000	pigs
Cadps2	0.21	1.00000000	pigs
Rp2	0.43	1.00000000	pigs
Plpp1	-0.31	1.00000000	pigs
Fam204a	0.10	1.00000000	pigs
Brk1	-0.45	1.00000000	pigs
Unc13b	0.23	1.00000000	pigs
Numa1	0.05	1.00000000	pigs
Atp5md	-0.40	1.00000000	pigs
Nelfa	0.08	1.00000000	pigs
Sike1	0.06	1.00000000	pigs
Cramp1	0.16	1.00000000	pigs
Nde1	0.27	1.00000000	pigs
Lpcat3	0.09	1.00000000	pigs
A4galt	0.17	1.00000000	pigs
Atp2a2	0.20	1.00000000	pigs
Fam120b	-0.07	1.00000000	pigs
Tmed10	0.37	1.00000000	pigs
Rngtt	0.12	1.00000000	pigs
Dock8	-0.61	1.00000000	pigs

Skil	0.19	1.00000000	pigs
Card9	0.01	1.00000000	pigs
Vcpip1	0.08	1.00000000	pigs
Cyth3	0.22	1.00000000	pigs
Col4a5	-0.58	1.00000000	pigs
Galm	0.20	1.00000000	pigs
Rock1	0.24	1.00000000	pigs
Ywhaq	0.31	1.00000000	pigs
Fbxo4	0.04	1.00000000	pigs
Arfgef2	0.19	1.00000000	pigs
Cul3	0.28	1.00000000	pigs
Polr2i	-0.43	1.00000000	pigs
Kdm1a	0.14	1.00000000	pigs
Smim4	-0.30	1.00000000	pigs
Sugt1	0.08	1.00000000	pigs
Cox7a1	-0.46	1.00000000	pigs
Actr1b	0.06	1.00000000	pigs
Rhoh	0.30	1.00000000	pigs
Slc24a1	-0.36	1.00000000	pigs
Lamtor2	0.27	1.00000000	pigs
Prdm11	0.29	1.00000000	pigs
Stox2	-0.43	1.00000000	pigs
Stx11	0.42	1.00000000	pigs
Ccdc180	0.10	1.00000000	pigs
Lrif1	0.02	1.00000000	pigs
Tmub1	0.19	1.00000000	pigs
Ehmt1	0.03	1.00000000	pigs
Cyria	0.26	1.00000000	pigs
Ei24	0.30	1.00000000	pigs
Irak2	0.46	1.00000000	pigs
Fanci	-0.24	1.00000000	pigs
Npr1	0.00	1.00000000	pigs
Tmem65	0.31	1.00000000	pigs
Lrrfip2	0.00	1.00000000	pigs
Srsf11	0.27	1.00000000	pigs
Acad11	0.25	1.00000000	pigs
C3	0.77	1.00000000	pigs
Snx29	0.22	1.00000000	pigs
Scg3	0.41	1.00000000	pigs
Por	0.05	1.00000000	pigs
Lsm3	-0.35	1.00000000	pigs
Psmb10	0.16	1.00000000	pigs
Stat5a	0.13	1.00000000	pigs
Pde4a	0.17	1.00000000	pigs
Kremen1	0.02	1.00000000	pigs
Prdm1	0.01	1.00000000	pigs

Fancb	0.38	1.00000000	pigs
Fkbp15	0.11	1.00000000	pigs
Tanc1	-0.24	1.00000000	pigs
Eipr1	0.24	1.00000000	pigs
Trim2	0.11	1.00000000	pigs
Plxna2	0.06	1.00000000	pigs
Scyl2	0.08	1.00000000	pigs
Dusp23	0.15	1.00000000	pigs
Pdk1	0.03	1.00000000	pigs
Slc30a6	0.05	1.00000000	pigs
Ppp2r5c	0.30	1.00000000	pigs
Tiprl	0.11	1.00000000	pigs
Nt5c3b	0.23	1.00000000	pigs
Trim35	0.08	1.00000000	pigs
Api5	0.23	1.00000000	pigs
Carm1	0.12	1.00000000	pigs
Tmco1	-0.45	1.00000000	pigs
Gck	0.17	1.00000000	pigs
Galnt15	0.26	1.00000000	pigs
Retsat	0.04	1.00000000	pigs
Sh3kbp1	0.12	1.00000000	pigs
Runx1t1	-0.41	1.00000000	pigs
Dnttip1	0.23	1.00000000	pigs
Fbxo38	0.10	1.00000000	pigs
Prkag1	0.01	1.00000000	pigs
Cdyl2	0.07	1.00000000	pigs
Klhl15	0.27	1.00000000	pigs
Atf6b	-0.05	1.00000000	pigs
Tspan18	0.37	1.00000000	pigs
Ccdc22	-0.03	1.00000000	pigs
Nhlrc3	0.08	1.00000000	pigs
Thoc2	0.20	1.00000000	pigs
Ecm1	0.28	1.00000000	pigs
Sik2	0.24	1.00000000	pigs
Katnbl1	0.15	1.00000000	pigs
Synj1	0.27	1.00000000	pigs
Glt8d1	0.08	1.00000000	pigs
Htatsf1	0.02	1.00000000	pigs
Vmp1	0.34	1.00000000	pigs
Shld1	-0.78	1.00000000	pigs
Klc4	0.09	1.00000000	pigs
Daglb	0.17	1.00000000	pigs
Clk4	0.07	1.00000000	pigs
Lhfp	0.25	1.00000000	pigs
Adar	0.25	1.00000000	pigs
Rbm45	0.13	1.00000000	pigs

Nudt22	0.07	1.00000000	pigs
Adgra2	0.16	1.00000000	pigs
Filip1l	-0.38	1.00000000	pigs
Jag1	0.26	1.00000000	pigs
Snrpc	0.04	1.00000000	pigs
Erg28	0.13	1.00000000	pigs
Armc9	0.12	1.00000000	pigs
Limch1	-0.35	1.00000000	pigs
Fam168a	0.20	1.00000000	pigs
Dph1	-0.02	1.00000000	pigs
Ppme1	0.05	1.00000000	pigs
Shkbp1	0.11	1.00000000	pigs
Dip2a	0.16	1.00000000	pigs
Ctsa	0.20	1.00000000	pigs
Rlim	0.16	1.00000000	pigs
Itgam	0.18	1.00000000	pigs
Ddx1	-0.46	1.00000000	pigs
Rassf3	0.31	1.00000000	pigs
Rbpms	0.31	1.00000000	pigs
Clic4	0.80	1.00000000	pigs
Zfp236	-0.03	1.00000000	pigs
Abcb10	0.04	1.00000000	pigs
Eef2	0.27	1.00000000	pigs
Scly	0.11	1.00000000	pigs
Samd8	0.19	1.00000000	pigs
Fyttd1	0.04	1.00000000	pigs
Slc66a2	-0.03	1.00000000	pigs
Eya3	0.13	1.00000000	pigs
Pcmt1	-0.48	1.00000000	pigs
Fkbp4	-0.23	1.00000000	pigs
Cdc27	0.35	1.00000000	pigs
Ndfip1	0.50	1.00000000	pigs
Hpgd	0.63	1.00000000	pigs
Dst	-0.55	1.00000000	pigs
Krtcap2	-0.37	1.00000000	pigs
St3gal1	0.28	1.00000000	pigs
Snx8	0.19	1.00000000	pigs
Ulk1	0.03	1.00000000	pigs
Slc4a2	0.06	1.00000000	pigs
Nme6	0.00	1.00000000	pigs
Ifitm6	0.25	1.00000000	pigs
Ddb2	0.21	1.00000000	pigs
Ap1m1	0.05	1.00000000	pigs
Tra2a	0.11	1.00000000	pigs
Tnip2	0.34	1.00000000	pigs
Tgfbr2	-0.31	1.00000000	pigs

Gpx8	0.04	1.00000000	pigs
Prkar1b	0.29	1.00000000	pigs
Nova2	0.01	1.00000000	pigs
Cecr2	0.13	1.00000000	pigs
Nhsl2	0.24	1.00000000	pigs
Reps1	0.10	1.00000000	pigs
Per2	0.11	1.00000000	pigs
Fam102b	0.18	1.00000000	pigs
Igfbp5	-0.51	1.00000000	pigs
Sacs	0.08	1.00000000	pigs
Dop1b	0.04	1.00000000	pigs
Atp11c	0.35	1.00000000	pigs
Zfat	0.10	1.00000000	pigs
Slc49a4	-0.10	1.00000000	pigs
Sec22a	0.10	1.00000000	pigs
Ltbr	0.11	1.00000000	pigs
Nhej1	0.26	1.00000000	pigs
Ppm1e	0.26	1.00000000	pigs
Esco1	0.21	1.00000000	pigs
Pdhx	0.17	1.00000000	pigs
Abhd3	-0.02	1.00000000	pigs
Rnf115	0.09	1.00000000	pigs
Tmem97	-0.45	1.00000000	pigs
Plekha7	-0.01	1.00000000	pigs
Kidins220	0.04	1.00000000	pigs
Shroom4	0.09	1.00000000	pigs
Arhgap35	0.17	1.00000000	pigs
Ptk2b	0.31	1.00000000	pigs
Pea15a	-0.32	1.00000000	pigs
Recql	-0.34	1.00000000	pigs
Fam53c	0.05	1.00000000	pigs
Hmgxb3	0.12	1.00000000	pigs
Ash1l	0.31	1.00000000	pigs
Supt4a	0.11	1.00000000	pigs
2610507B11Rik	0.05	1.00000000	pigs
Rbl2	0.09	1.00000000	pigs
Xpo1	0.29	1.00000000	pigs
Abcg1	0.27	1.00000000	pigs
Stx3	0.17	1.00000000	pigs
Qars	0.27	1.00000000	pigs
Rab3gap1	-0.03	1.00000000	pigs
Gpr137	0.00	1.00000000	pigs
Eif4g1	0.03	1.00000000	pigs
Clcn5	0.04	1.00000000	pigs
Brd4	0.14	1.00000000	pigs
Hus1	0.04	1.00000000	pigs

Tle3	0.07	1.00000000	pigs
Irak3	0.30	1.00000000	pigs
Vcam1	0.01	1.00000000	pigs
Zfp326	-0.07	1.00000000	pigs
Stxbp2	0.21	1.00000000	pigs
2510039O18Rik	-0.04	1.00000000	pigs
Pdia4	0.17	1.00000000	pigs
Bcam	-0.44	1.00000000	pigs
Sec23b	0.30	1.00000000	pigs
Syk	0.30	1.00000000	pigs
B3gntl1	0.07	1.00000000	pigs
Rab4a	0.12	1.00000000	pigs
Tead2	0.06	1.00000000	pigs
C9orf72	0.31	1.00000000	pigs
Ppib	-0.22	1.00000000	pigs
Usp22	0.13	1.00000000	pigs
Ddx47	0.10	1.00000000	pigs
Ccl5	-0.33	1.00000000	pigs
Ksr1	0.18	1.00000000	pigs
Topors	0.03	1.00000000	pigs
Gab3	0.32	1.00000000	pigs
Zbtb40	0.12	1.00000000	pigs
Ascc3	0.13	1.00000000	pigs
Calcrl	0.57	1.00000000	pigs
Rex1bd	-0.43	1.00000000	pigs
Setd3	0.26	1.00000000	pigs
Cars	0.27	1.00000000	pigs
Tmem52	-0.57	1.00000000	pigs
Fam50a	0.20	1.00000000	pigs
Cda	-0.88	1.00000000	pigs
Peli2	-0.54	1.00000000	pigs
Rwdd1	-0.24	1.00000000	pigs
Psmb4	0.28	1.00000000	pigs
Klhl24	0.07	1.00000000	pigs
Olr1	0.16	1.00000000	pigs
Lactb	0.23	1.00000000	pigs
Lat2	0.15	1.00000000	pigs
Pmpca	0.17	1.00000000	pigs
Rsrc2	0.31	1.00000000	pigs
Zdhhc14	0.49	1.00000000	pigs
Zfp106	-0.34	1.00000000	pigs
Yipf2	-0.50	1.00000000	pigs
Sema3a	-0.14	1.00000000	pigs
Lta4h	0.09	1.00000000	pigs
Ptpn6	0.22	1.00000000	pigs
Gpi1	0.31	1.00000000	pigs

N4bp2	0.55	1.00000000	pigs
Tnpo1	0.06	1.00000000	pigs
Lmna	-0.40	1.00000000	pigs
Dtx3	0.04	1.00000000	pigs
Sparc	0.69	1.00000000	pigs
Casp8ap2	0.14	1.00000000	pigs
Bmp4	0.04	1.00000000	pigs
Ccdc186	0.04	1.00000000	pigs
Vstm4	0.12	1.00000000	pigs
Amotl1	0.11	1.00000000	pigs
Acsl4	-0.12	1.00000000	pigs
Pgrmc1	0.18	1.00000000	pigs
Dusp11	0.20	1.00000000	pigs
Pja2	0.15	1.00000000	pigs
Yju2	0.04	1.00000000	pigs
Sfmbt2	0.09	1.00000000	pigs
Rab3il1	0.03	1.00000000	pigs
Vwa8	0.12	1.00000000	pigs
Acss3	0.21	1.00000000	pigs
Gpat4	0.10	1.00000000	pigs
Akap9	-0.48	1.00000000	pigs
Taf4b	0.36	1.00000000	pigs
Nudt13	0.18	1.00000000	pigs
Nol7	-0.42	1.00000000	pigs
Creb5	0.32	1.00000000	pigs
Sgsm3	0.18	1.00000000	pigs
Eif4ebp2	-0.23	1.00000000	pigs
Crebbp	0.06	1.00000000	pigs
E2f6	0.23	1.00000000	pigs
Idh1	0.13	1.00000000	pigs
Eif4g3	0.27	1.00000000	pigs
Noc3l	-0.38	1.00000000	pigs
Ipo9	0.20	1.00000000	pigs
Abi1	0.06	1.00000000	pigs
Atp10d	0.38	1.00000000	pigs
Tln1	-0.20	1.00000000	pigs
Gucy2c	0.59	1.00000000	pigs
Tab2	0.04	1.00000000	pigs
Dbnl	0.10	1.00000000	pigs
Fuca2	0.05	1.00000000	pigs
Tbc1d2b	0.36	1.00000000	pigs
Csnk1g3	0.17	1.00000000	pigs
Kdm5a	0.22	1.00000000	pigs
Rbck1	0.00	1.00000000	pigs
Epg5	-0.07	1.00000000	pigs
Pls3	-0.25	1.00000000	pigs

Prkab1	0.04	1.00000000	pigs
Kdm1b	0.19	1.00000000	pigs
Aprt	-0.30	1.00000000	pigs
Pdlim1	-0.29	1.00000000	pigs
Lrba	0.18	1.00000000	pigs
4921524J17Rik	-0.42	1.00000000	pigs
Acss1	0.17	1.00000000	pigs
Zranb2	-0.52	1.00000000	pigs
Pwp1	-0.31	1.00000000	pigs
Ktn1	-0.33	1.00000000	pigs
Ntan1	-0.35	1.00000000	pigs
Vps37c	0.22	1.00000000	pigs
Ncoa4	-0.04	1.00000000	pigs
Rtf1	-0.42	1.00000000	pigs
Tspan5	-0.23	1.00000000	pigs
Ccdc93	0.24	1.00000000	pigs
Scarf1	0.00	1.00000000	pigs
Ctif	0.09	1.00000000	pigs
Timp1	0.62	1.00000000	pigs
Dstyk	0.00	1.00000000	pigs
Lrp6	0.04	1.00000000	pigs
Ddi2	-0.04	1.00000000	pigs
Ndufb7	-0.41	1.00000000	pigs
Tmem33	-0.07	1.00000000	pigs
Bptf	0.35	1.00000000	pigs
Acot4	0.00	1.00000000	pigs
Tbca	-0.27	1.00000000	pigs
Plau	0.03	1.00000000	pigs
C1qtnf2	-0.25	1.00000000	pigs
Dnase2a	0.23	1.00000000	pigs
Gprc5b	0.21	1.00000000	pigs
Plekhb2	0.25	1.00000000	pigs
Atad1	0.05	1.00000000	pigs
Adarb1	0.14	1.00000000	pigs
Erbin	0.31	1.00000000	pigs
Sertad1	0.33	1.00000000	pigs
Tlr4	0.18	1.00000000	pigs
Adamts9	-0.66	1.00000000	pigs
Plscr4	0.22	1.00000000	pigs
Ghsr	-0.29	1.00000000	pigs
Flrt2	-0.44	1.00000000	pigs
Cacul1	0.30	1.00000000	pigs
Slc44a1	0.26	1.00000000	pigs
Ddx3x	0.19	1.00000000	pigs
Sdhb	0.30	1.00000000	pigs
Fbln2	0.13	1.00000000	pigs

Taf13	-0.06	1.00000000	pigs
Antkmt	-0.28	1.00000000	pigs
Brd3	-0.49	1.00000000	pigs
Rassf8	-0.54	1.00000000	pigs
Parp14	0.03	1.00000000	pigs
Osbpl1a	0.06	1.00000000	pigs
Stx6	0.25	1.00000000	pigs
Zc3h18	0.10	1.00000000	pigs
Pfdn1	0.45	1.00000000	pigs
Gorasp1	0.15	1.00000000	pigs
Cox18	0.13	1.00000000	pigs
Ctdsp1	-0.29	1.00000000	pigs
Rph3al	-0.12	1.00000000	pigs
Ltbp1	0.59	1.00000000	pigs
Cinp	0.02	1.00000000	pigs
Gpr108	0.07	1.00000000	pigs
Cep83	-0.04	1.00000000	pigs
Commd4	0.18	1.00000000	pigs
Rnf144b	-0.02	1.00000000	pigs
Zmat5	0.05	1.00000000	pigs
Papola	0.31	1.00000000	pigs
Fbxo22	0.56	1.00000000	pigs
Tor3a	0.09	1.00000000	pigs
Pik3c2a	0.24	1.00000000	pigs
Ica1	0.21	1.00000000	pigs
Fam124a	0.14	1.00000000	pigs
Arl15	0.25	1.00000000	pigs
Smc4	0.29	1.00000000	pigs
Yars	-0.23	1.00000000	pigs
Ahctf1	-0.09	1.00000000	pigs
Ndufaf1	-0.20	1.00000000	pigs
Capn1	-0.06	1.00000000	pigs
Trmt5	-0.26	1.00000000	pigs
Ppcs	0.34	1.00000000	pigs
Inpp5a	0.17	1.00000000	pigs
Timm21	0.14	1.00000000	pigs
Atp8a1	-0.03	1.00000000	pigs
Kpna1	0.14	1.00000000	pigs
Rbm26	0.11	1.00000000	pigs
Kat2b	-0.41	1.00000000	pigs
Stxbp6	-0.27	1.00000000	pigs
Itga2	-0.69	1.00000000	pigs
Tmx4	0.66	1.00000000	pigs
Dcbld2	-0.47	1.00000000	pigs
Sec22c	0.04	1.00000000	pigs
Zmym5	0.13	1.00000000	pigs

Galnt1	-0.25	1.00000000	pigs
Adgrg1	0.11	1.00000000	pigs
Armh3	0.02	1.00000000	pigs
Ddx50	-0.42	1.00000000	pigs
Eri3	0.06	1.00000000	pigs
Dis3l2	0.16	1.00000000	pigs
Hs3st1	-0.51	1.00000000	pigs
Use1	-0.38	1.00000000	pigs
Tmod2	-0.49	1.00000000	pigs
Sorbs2	-0.62	1.00000000	pigs
Vwa5a	0.20	1.00000000	pigs
Rel	0.31	1.00000000	pigs
Naa15	0.17	1.00000000	pigs
Cdkn2c	0.11	1.00000000	pigs
Wdfy1	0.16	1.00000000	pigs
Gja1	-0.51	1.00000000	pigs
Dnaaf5	0.14	1.00000000	pigs
Sh3tc1	-0.11	1.00000000	pigs
Cox11	0.17	1.00000000	pigs
Zzef1	0.08	1.00000000	pigs
Pigu	0.04	1.00000000	pigs
Itgav	0.10	1.00000000	pigs
Fbxl20	0.19	1.00000000	pigs
Unk	0.18	1.00000000	pigs
Sec31a	0.06	1.00000000	pigs
Ppp1r9b	0.26	1.00000000	pigs
Park7	-0.25	1.00000000	pigs
Bcl2l13	0.15	1.00000000	pigs
Cdc123	0.25	1.00000000	pigs
Scmh1	0.02	1.00000000	pigs
Pias3	-0.03	1.00000000	pigs
N4bp3	0.08	1.00000000	pigs
Itga9	-0.19	1.00000000	pigs
Wsb1	-0.29	1.00000000	pigs
Flot2	-0.03	1.00000000	pigs
Egr1	-0.61	1.00000000	pigs
Fbn1	0.09	1.00000000	pigs
Rnf114	0.21	1.00000000	pigs
Crybg1	0.40	1.00000000	pigs
Sh3bp4	0.17	1.00000000	pigs
Arhgap10	0.31	1.00000000	pigs
Gaa	0.21	1.00000000	pigs
Thap3	0.25	1.00000000	pigs
Csnk2a2	0.13	1.00000000	pigs
Cwc22	0.11	1.00000000	pigs
Cul2	0.10	1.00000000	pigs

Cln3	0.03	1.00000000	pigs
Exoc2	0.17	1.00000000	pigs
Arntl	-0.15	1.00000000	pigs
Prrc2b	0.11	1.00000000	pigs
Irf2bp2	0.24	1.00000000	pigs
Dcaf15	0.19	1.00000000	pigs
Anp32e	-0.31	1.00000000	pigs
Abi2	-0.03	1.00000000	pigs
Afap1	0.17	1.00000000	pigs
Wrnip1	0.09	1.00000000	pigs
Rnf145	0.06	1.00000000	pigs
Gosr1	0.03	1.00000000	pigs
Osbpl8	0.26	1.00000000	pigs
Nek7	0.25	1.00000000	pigs
Ubxn6	0.07	1.00000000	pigs
Klf13	-0.04	1.00000000	pigs
Tmem131	0.18	1.00000000	pigs
Fhdc1	0.19	1.00000000	pigs
Spats2l	0.13	1.00000000	pigs
Poc1a	0.26	1.00000000	pigs
Pi4kb	0.10	1.00000000	pigs
Phip	0.26	1.00000000	pigs
Tfpi	-0.37	1.00000000	pigs
Slc3a2	0.31	1.00000000	pigs
Zfp2	0.04	1.00000000	pigs
Akr1b3	0.39	1.00000000	pigs
Adcy3	0.03	1.00000000	pigs
Smarcal1	0.32	1.00000000	pigs
Il1rapl1	0.17	1.00000000	pigs
Vat1l	0.19	1.00000000	pigs
Rpp38	0.03	1.00000000	pigs
Htt	0.09	1.00000000	pigs
Polr1d	-0.28	1.00000000	pigs
4833439L19Rik	0.23	1.00000000	pigs
Sipa1	0.14	1.00000000	pigs
Cited2	0.68	1.00000000	pigs
Clock	0.07	1.00000000	pigs
Repin1	0.07	1.00000000	pigs
Cdkl1	-0.43	1.00000000	pigs
Prex1	0.13	1.00000000	pigs
Rragc	0.11	1.00000000	pigs
9130409I23Rik	0.03	1.00000000	pigs
Dnase1l1	-0.19	1.00000000	pigs
Pllp	0.10	1.00000000	pigs
Fam98a	0.09	1.00000000	pigs
Larp4	0.21	1.00000000	pigs

Sbds	-0.36	1.00000000	pigs
St6galnac3	0.52	1.00000000	pigs
Atpaf2	-0.15	1.00000000	pigs
Ifrd2	-0.03	1.00000000	pigs
Sap30l	0.11	1.00000000	pigs
Gata2	0.18	1.00000000	pigs
Phf1	0.08	1.00000000	pigs
Asxl1	-0.01	1.00000000	pigs
Vps13d	0.04	1.00000000	pigs
Psmf1	0.15	1.00000000	pigs
Rbm6	0.20	1.00000000	pigs
Slc36a1	0.04	1.00000000	pigs
Purg	0.29	1.00000000	pigs
Wnk1	0.18	1.00000000	pigs
Wdr61	0.30	1.00000000	pigs
Nfic	-0.36	1.00000000	pigs
Dhrs11	0.03	1.00000000	pigs
Gnai3	0.30	1.00000000	pigs
Trim8	0.09	1.00000000	pigs
Tgfbr1	0.00	1.00000000	pigs
Lrp12	0.07	1.00000000	pigs
Csrp1	0.03	1.00000000	pigs
Prxl2a	0.20	1.00000000	pigs
Ppil2	0.07	1.00000000	pigs
Ykt6	0.29	1.00000000	pigs
Psmd10	0.13	1.00000000	pigs
Tyk2	0.03	1.00000000	pigs
Usp3	0.19	1.00000000	pigs
Sap18	-0.18	1.00000000	pigs
Hspa9	0.13	1.00000000	pigs
Gpatch3	-0.02	1.00000000	pigs
Hsd17b12	0.01	1.00000000	pigs
Ppa1	0.10	1.00000000	pigs
Pnpla2	-0.35	1.00000000	pigs
Hoxd8	-0.33	1.00000000	pigs
Relch	-0.03	1.00000000	pigs
Kank2	-0.37	1.00000000	pigs
Sirt7	0.15	1.00000000	pigs
Zfpm2	-0.33	1.00000000	pigs
Pdcd10	-0.26	1.00000000	pigs
Etv1	0.13	1.00000000	pigs
Srrm1	-0.29	1.00000000	pigs
Vopp1	0.09	1.00000000	pigs
Gpatch4	-0.26	1.00000000	pigs
Trim26	0.29	1.00000000	pigs
Trpm7	0.30	1.00000000	pigs

Nsa2	-0.19	1.00000000	pigs
Acly	0.04	1.00000000	pigs
Esyt2	0.19	1.00000000	pigs
Tmem39a	-0.02	1.00000000	pigs
Tns1	0.25	1.00000000	pigs
Dhx32	0.19	1.00000000	pigs
Dnajc13	0.15	1.00000000	pigs
Ralgapb	-0.08	1.00000000	pigs
Pcmtd1	0.25	1.00000000	pigs
Hyal2	0.36	1.00000000	pigs
Rab6a	0.01	1.00000000	pigs
Ppp6c	0.02	1.00000000	pigs
Pdss2	0.09	1.00000000	pigs
Sp4	-0.04	1.00000000	pigs
Zfp606	-0.06	1.00000000	pigs
Plekha8	0.03	1.00000000	pigs
Srp68	-0.02	1.00000000	pigs
Soga1	-0.15	1.00000000	pigs
Cyb561	0.23	1.00000000	pigs
Pin4	-0.40	1.00000000	pigs
Plekhm1	0.14	1.00000000	pigs
Arid4a	0.15	1.00000000	pigs
Ttll7	-0.17	1.00000000	pigs
Dgkd	-0.10	1.00000000	pigs
Sf3b6	-0.28	1.00000000	pigs
Canx	-0.25	1.00000000	pigs
Ptpn9	-0.05	1.00000000	pigs
Lrch2	0.06	1.00000000	pigs
Fam118b	-0.46	1.00000000	pigs
Hars	0.11	1.00000000	pigs
Odr4	-0.03	1.00000000	pigs
Ptprk	-0.37	1.00000000	pigs
Rac2	-0.25	1.00000000	pigs
Scpep1	0.34	1.00000000	pigs
Atp6v0b	0.31	1.00000000	pigs
Cers5	0.02	1.00000000	pigs
Smad2	0.16	1.00000000	pigs
Sec23a	0.14	1.00000000	pigs
Ptpn4	0.06	1.00000000	pigs
Smg7	0.11	1.00000000	pigs
Plcb1	0.23	1.00000000	pigs
Stxbp5	-0.13	1.00000000	pigs
Csk	0.25	1.00000000	pigs
Slc27a1	-0.06	1.00000000	pigs
Xpa	0.00	1.00000000	pigs
Iws1	0.31	1.00000000	pigs

Senp5	0.03	1.00000000	pigs
Rfwd3	0.03	1.00000000	pigs
Wfdc1	0.21	1.00000000	pigs
Ube2h	0.08	1.00000000	pigs
Prim2	0.15	1.00000000	pigs
Skap2	0.13	1.00000000	pigs
Leprot	-0.04	1.00000000	pigs
Arhgap25	0.16	1.00000000	pigs
Dad1	-0.47	1.00000000	pigs
Ssr2	-0.28	1.00000000	pigs
Fbh1	0.04	1.00000000	pigs
Ryr2	-0.61	1.00000000	pigs
Timmdc1	0.07	1.00000000	pigs
Ndufb8	0.38	1.00000000	pigs
Sav1	0.27	1.00000000	pigs
Zfp592	0.23	1.00000000	pigs
Angpt2	0.31	1.00000000	pigs
Egr2	0.10	1.00000000	pigs
1700025G04Rik	-0.44	1.00000000	pigs
Gtpbp2	0.18	1.00000000	pigs
Timm17a	-0.16	1.00000000	pigs
Retreg2	0.12	1.00000000	pigs
Agfg2	0.25	1.00000000	pigs
Pdgfd	-1.09	1.00000000	pigs
Abhd17b	0.05	1.00000000	pigs
Tlk2	-0.02	1.00000000	pigs
Antxr2	0.51	1.00000000	pigs
Rhoj	-0.04	1.00000000	pigs
Appbp2	-0.02	1.00000000	pigs
Phf21a	0.10	1.00000000	pigs
Akirin1	0.00	1.00000000	pigs
Cops8	0.21	1.00000000	pigs
Ncoa6	0.15	1.00000000	pigs
Nlk	-0.03	1.00000000	pigs
Cdc42se1	0.00	1.00000000	pigs
Sccpdh	0.18	1.00000000	pigs
Dynlt1a	-0.44	1.00000000	pigs
Vps26c	-0.14	1.00000000	pigs
Alms1	0.00	1.00000000	pigs
Zdhhc13	0.03	1.00000000	pigs
Nub1	0.76	1.00000000	pigs
Fam32a	0.15	1.00000000	pigs
Gtf3c6	0.04	1.00000000	pigs
Tnrc6c	-0.11	1.00000000	pigs
Tns3	0.31	1.00000000	pigs
Gan	0.08	1.00000000	pigs

C2cd2	-0.03	1.00000000	pigs
Vim	-0.60	1.00000000	pigs
Vps51	0.02	1.00000000	pigs
Gls	0.32	1.00000000	pigs
Trrap	0.00	1.00000000	pigs
Hnrnpk	-0.21	1.00000000	pigs
Ergic3	0.01	1.00000000	pigs
Lipc	-0.11	1.00000000	pigs
Dusp2	0.40	1.00000000	pigs
Arl8a	0.31	1.00000000	pigs
Garem1	0.26	1.00000000	pigs
Exosc10	-0.02	1.00000000	pigs
Prkca	0.25	1.00000000	pigs
Il7	-0.36	1.00000000	pigs
Zfp827	0.11	1.00000000	pigs
Fbxo7	0.05	1.00000000	pigs
Lrrc41	0.05	1.00000000	pigs
9030025P20Rik	-0.05	1.00000000	pigs
Azi2	0.12	1.00000000	pigs
Slc25a28	0.16	1.00000000	pigs
Nfatc3	0.18	1.00000000	pigs
Tcta	0.32	1.00000000	pigs
Zbtb17	0.16	1.00000000	pigs
Zcchc24	0.04	1.00000000	pigs
Emsy	0.02	1.00000000	pigs
Smyd3	0.19	1.00000000	pigs
Gphn	0.22	1.00000000	pigs
Vbp1	0.31	1.00000000	pigs
Txlng	0.06	1.00000000	pigs
Ctdspl2	0.12	1.00000000	pigs
Slc19a2	-0.21	1.00000000	pigs
Wdr70	0.13	1.00000000	pigs
Slk	-0.40	1.00000000	pigs
Erlec1	0.19	1.00000000	pigs
Serpine1	0.37	1.00000000	pigs
Nfat5	0.26	1.00000000	pigs
Atp2c1	-0.01	1.00000000	pigs
Trim38	0.15	1.00000000	pigs
Adsl	0.08	1.00000000	pigs
Peli1	0.11	1.00000000	pigs
H6pd	0.04	1.00000000	pigs
Tdpoz8	0.17	1.00000000	pigs
Denn2b	0.01	1.00000000	pigs
Bmerb1	0.05	1.00000000	pigs
Carmil1	0.11	1.00000000	pigs
Lrp2bp	-0.46	1.00000000	pigs

Ric1	0.14	1.00000000	pigs
Lrig2	-0.02	1.00000000	pigs
Setdb2	0.06	1.00000000	pigs
C130074G19Rik	-0.07	1.00000000	pigs
Zfp24	0.10	1.00000000	pigs
Tnfaip8	0.05	1.00000000	pigs
Fundc1	0.08	1.00000000	pigs
Ccar2	-0.07	1.00000000	pigs
Farp2	0.09	1.00000000	pigs
Stil	0.16	1.00000000	pigs
Tial1	0.15	1.00000000	pigs
Pip5k1a	-0.10	1.00000000	pigs
Tmem189	0.20	1.00000000	pigs
Ifit3	-0.06	1.00000000	pigs
Fbxw4	0.00	1.00000000	pigs
Tm2d3	0.07	1.00000000	pigs
Psme3ip1	0.77	1.00000000	pigs
Bmt2	0.16	1.00000000	pigs
Hacd1	-0.32	1.00000000	pigs
Echs1	0.08	1.00000000	pigs
Parp9	0.08	1.00000000	pigs
Slc30a7	0.03	1.00000000	pigs
Txndc11	0.47	1.00000000	pigs
Zfp507	0.27	1.00000000	pigs
Ctnnd1	-0.68	1.00000000	pigs
Ipo7	0.12	1.00000000	pigs
Ccnd2	0.19	1.00000000	pigs
Rmnd5a	0.21	1.00000000	pigs
Tle1	-0.49	1.00000000	pigs
Rapgef4	-0.30	1.00000000	pigs
Ntn1	0.02	1.00000000	pigs
Oga	0.08	1.00000000	pigs
Smc6	0.12	1.00000000	pigs
Ndufb1	-0.33	1.00000000	pigs
Fbxw7	0.06	1.00000000	pigs
Rbm48	-0.17	1.00000000	pigs
Lonrf3	0.44	1.00000000	pigs
Nup188	0.12	1.00000000	pigs
Tmem131l	0.20	1.00000000	pigs
Ttll1	-0.01	1.00000000	pigs
Fus	0.21	1.00000000	pigs
Ccdc117	-0.02	1.00000000	pigs
Fosl2	0.12	1.00000000	pigs
Cog1	0.10	1.00000000	pigs
Ifih1	0.16	1.00000000	pigs
Ppp4c	0.24	1.00000000	pigs

P4ha2	0.09	1.00000000	pigs
Tmc7	-0.02	1.00000000	pigs
Bloc1s2	0.13	1.00000000	pigs
Vkorc1l1	0.10	1.00000000	pigs
Ift46	-0.06	1.00000000	pigs
Ostc	0.21	1.00000000	pigs
Tmem259	-0.04	1.00000000	pigs
Prpsap1	0.08	1.00000000	pigs
Nid1	-0.03	1.00000000	pigs
Pi4ka	-0.03	1.00000000	pigs
Lmcd1	0.15	1.00000000	pigs
Bclaf3	-0.07	1.00000000	pigs
Ankrd40	0.19	1.00000000	pigs
Pcdh19	0.03	1.00000000	pigs
Tmem154	-0.35	1.00000000	pigs
Prmt5	0.28	1.00000000	pigs
Kmt2e	0.20	1.00000000	pigs
Polr2e	-0.34	1.00000000	pigs
Tmem238	-0.17	1.00000000	pigs
Svil	0.26	1.00000000	pigs
Trim33	0.09	1.00000000	pigs
Cybc1	0.09	1.00000000	pigs
Ndufa5	-0.36	1.00000000	pigs
Gnptab	0.13	1.00000000	pigs
Smg6	0.06	1.00000000	pigs
Tnfaip8l3	-0.34	1.00000000	pigs
Pcid2	-0.01	1.00000000	pigs
Erh	-0.18	1.00000000	pigs
Zswim8	0.12	1.00000000	pigs
Ldlrad4	0.07	1.00000000	pigs
Cox8a	-0.35	1.00000000	pigs
Etfdh	0.29	1.00000000	pigs
Taf4	0.00	1.00000000	pigs
Septin8	0.05	1.00000000	pigs
Tbc1d8b	0.14	1.00000000	pigs
Tex10	-0.02	1.00000000	pigs
Nfil3	0.40	1.00000000	pigs
Dlgap4	0.17	1.00000000	pigs
Brf2	-0.20	1.00000000	pigs
Ubxn8	0.10	1.00000000	pigs
Ppp2ca	0.24	1.00000000	pigs
Cct4	0.20	1.00000000	pigs
Hmces	0.11	1.00000000	pigs
Psip1	0.01	1.00000000	pigs
Suclg2	0.24	1.00000000	pigs
B3galnt2	0.13	1.00000000	pigs

Ddx5	0.26	1.00000000	pigs
Zfp362	-0.02	1.00000000	pigs
Id3	-0.17	1.00000000	pigs
Eloa	0.09	1.00000000	pigs
Nupr1	0.04	1.00000000	pigs
Gnb1l	-0.04	1.00000000	pigs
Camkk2	-0.02	1.00000000	pigs
Atp6ap1	0.13	1.00000000	pigs
Hexim2	0.07	1.00000000	pigs
Csnk1g1	0.10	1.00000000	pigs
Ppp2r1a	0.15	1.00000000	pigs
Gprc5c	0.10	1.00000000	pigs
Ptx3	-0.62	1.00000000	pigs
Immp1l	-0.28	1.00000000	pigs
Kctd9	-0.48	1.00000000	pigs
Ankrd28	0.02	1.00000000	pigs
Ninl	0.05	1.00000000	pigs
Smc3	-0.28	1.00000000	pigs
Wdr44	0.10	1.00000000	pigs
Tpp2	-0.07	1.00000000	pigs
Cbfa2t2	0.06	1.00000000	pigs
Gsn	-0.67	1.00000000	pigs
Wbp2	0.04	1.00000000	pigs
Zfp277	0.07	1.00000000	pigs
Thap1	0.04	1.00000000	pigs
Zdhhc3	0.07	1.00000000	pigs
Stat6	0.10	1.00000000	pigs
Tex14	-0.13	1.00000000	pigs
Ppp2r5e	0.18	1.00000000	pigs
Phlpp2	0.29	1.00000000	pigs
Grpel2	0.11	1.00000000	pigs
Thbs3	-0.07	1.00000000	pigs
Acat3	0.10	1.00000000	pigs
Ocrl	-0.02	1.00000000	pigs
Ehd4	0.13	1.00000000	pigs
Rnf5	-0.17	1.00000000	pigs
Desi2	0.03	1.00000000	pigs
Haus3	0.07	1.00000000	pigs
Klf4	0.44	1.00000000	pigs
Fggy	0.01	1.00000000	pigs
Sik3	0.28	1.00000000	pigs
Polb	0.29	1.00000000	pigs
Dicer1	0.10	1.00000000	pigs
Cd151	-0.41	1.00000000	pigs
Slc38a10	0.31	1.00000000	pigs
BC005537	0.20	1.00000000	pigs

Lemd3	0.10	1.00000000	pigs
Hspa13	0.02	1.00000000	pigs
Twf1	0.03	1.00000000	pigs
Tspan6	0.10	1.00000000	pigs
Coq4	0.13	1.00000000	pigs
Coq9	0.09	1.00000000	pigs
Nfs1	-0.01	1.00000000	pigs
Chchd3	0.02	1.00000000	pigs
Ube2e2	0.20	1.00000000	pigs
Tmem107	0.33	1.00000000	pigs
Dennd4a	0.24	1.00000000	pigs
Ccnt2	0.14	1.00000000	pigs
Arhgap28	-0.41	1.00000000	pigs
Arfip1	0.20	1.00000000	pigs
Kpna3	0.17	1.00000000	pigs
Capn7	0.20	1.00000000	pigs
Timm10	-0.33	1.00000000	pigs
Rars	0.10	1.00000000	pigs
Sorbs1	0.23	1.00000000	pigs
Nup214	0.00	1.00000000	pigs
Scamp2	-0.06	1.00000000	pigs
Glmp	0.03	1.00000000	pigs
Usp11	0.02	1.00000000	pigs
Bckdhb	-0.44	1.00000000	pigs
Ank	0.19	1.00000000	pigs
Sel1l	-0.13	1.00000000	pigs
Eif1b	-0.37	1.00000000	pigs
Ppp1r12a	0.07	1.00000000	pigs
Nup88	-0.11	1.00000000	pigs
Tcp11	0.00	1.00000000	pigs
Sdc3	-0.08	1.00000000	pigs
C5ar2	-0.44	1.00000000	pigs
Tbc1d7	0.11	1.00000000	pigs
Cbx1	-0.31	1.00000000	pigs
Sugp1	-0.01	1.00000000	pigs
Jam2	-0.10	1.00000000	pigs
Abcd4	0.24	1.00000000	pigs
Fryl	0.20	1.00000000	pigs
Selenom	-0.36	1.00000000	pigs
Cct7	-0.36	1.00000000	pigs
Gars	-0.34	1.00000000	pigs
Oxsr1	-0.01	1.00000000	pigs
Rap1a	0.23	1.00000000	pigs
Slc29a1	-0.01	1.00000000	pigs
Kdm2b	0.09	1.00000000	pigs
Scfd1	0.11	1.00000000	pigs

Plaat3	-0.13	1.00000000	pigs
Etnk1	0.33	1.00000000	pigs
Adal	0.10	1.00000000	pigs
Zbtb37	0.06	1.00000000	pigs
Cab39l	0.23	1.00000000	pigs
Nhlrc2	0.06	1.00000000	pigs
Vgll4	0.12	1.00000000	pigs
Bcorl1	0.07	1.00000000	pigs
Fbxl4	-0.07	1.00000000	pigs
Nutf2	-0.31	1.00000000	pigs
Cytip	-0.24	1.00000000	pigs
Ncbp1	0.13	1.00000000	pigs
Hivep1	0.42	1.00000000	pigs
Pacs1	0.11	1.00000000	pigs
Ubc	0.23	1.00000000	pigs
Casp3	0.12	1.00000000	pigs
Glul	0.12	1.00000000	pigs
Helz	0.19	1.00000000	pigs
Dyrk2	0.14	1.00000000	pigs
Ankib1	0.02	1.00000000	pigs
Dhrs3	-0.43	1.00000000	pigs
Slc44a5	-0.24	1.00000000	pigs
Sbno1	0.21	1.00000000	pigs
Pde8a	0.01	1.00000000	pigs
Golga7b	0.12	1.00000000	pigs
Alg14	0.16	1.00000000	pigs
Ift81	-0.23	1.00000000	pigs
Rev3l	0.32	1.00000000	pigs
Akap10	0.20	1.00000000	pigs
Septin11	0.45	1.00000000	pigs
Rbmx2	-0.12	1.00000000	pigs
Fbxo42	-0.05	1.00000000	pigs
Zfp335	0.06	1.00000000	pigs
Cops6	0.10	1.00000000	pigs
Nt5e	-0.33	1.00000000	pigs
Lekr1	0.10	1.00000000	pigs
Eif3a	0.14	1.00000000	pigs
Pafah1b1	0.22	1.00000000	pigs
Taok2	0.09	1.00000000	pigs
Ttc9	-0.13	1.00000000	pigs
Gtf2i	0.10	1.00000000	pigs
Cd99l2	-0.03	1.00000000	pigs
Rragb	-0.21	1.00000000	pigs
Serinc5	-0.28	1.00000000	pigs
Tfrc	-0.04	1.00000000	pigs
Popdc3	0.19	1.00000000	pigs

Ucp2	-0.35	1.00000000	pigs
Wdr91	-0.07	1.00000000	pigs
Taf7	0.15	1.00000000	pigs
Dhx57	-0.27	1.00000000	pigs
Wdtc1	0.05	1.00000000	pigs
Usp24	0.02	1.00000000	pigs
Ptpra	0.20	1.00000000	pigs
Nup155	-0.32	1.00000000	pigs
Diaph1	0.10	1.00000000	pigs
Son	-0.10	1.00000000	pigs
Slc25a21	-0.43	1.00000000	pigs
Zdhhc20	0.36	1.00000000	pigs
Zbtb41	0.01	1.00000000	pigs
Kmt2b	0.02	1.00000000	pigs
Tecpr1	0.16	1.00000000	pigs
Slc35b3	-0.03	1.00000000	pigs
Zranb3	0.05	1.00000000	pigs
Chsy1	0.10	1.00000000	pigs
Pcyt1a	0.21	1.00000000	pigs
Asb13	-0.19	1.00000000	pigs
Lgals1	0.14	1.00000000	pigs
St8sia4	-0.04	1.00000000	pigs
Ogfrl1	0.37	1.00000000	pigs
Fermt2	-0.41	1.00000000	pigs
Snw1	-0.29	1.00000000	pigs
Ccpg1	-0.32	1.00000000	pigs
Fam76b	0.06	1.00000000	pigs
Crat	0.03	1.00000000	pigs
Pld5	-0.15	1.00000000	pigs
Ppp2r5d	-0.08	1.00000000	pigs
Snx6	0.38	1.00000000	pigs
Taf8	0.16	1.00000000	pigs
Phf6	0.19	1.00000000	pigs
Prmt1	0.18	1.00000000	pigs
Stag2	-0.28	1.00000000	pigs
Spire1	-0.12	1.00000000	pigs
Gatad2b	0.13	1.00000000	pigs
Wdr41	0.07	1.00000000	pigs
Dgke	-0.20	1.00000000	pigs
Smarca2	0.15	1.00000000	pigs
Sec22b	0.08	1.00000000	pigs
Dab2ip	0.02	1.00000000	pigs
Fnip2	0.07	1.00000000	pigs
Plcd3	-0.04	1.00000000	pigs
Slco2b1	0.09	1.00000000	pigs
Trp53bp1	0.07	1.00000000	pigs

Bach1	0.37	1.00000000	pigs
Fam214a	0.09	1.00000000	pigs
Pon2	0.09	1.00000000	pigs
Dcbld1	0.10	1.00000000	pigs
Strada	0.05	1.00000000	pigs
Ints12	-0.42	1.00000000	pigs
Pdrg1	0.10	1.00000000	pigs
Tspan17	0.29	1.00000000	pigs
Optn	-0.06	1.00000000	pigs
Snx2	-0.17	1.00000000	pigs
Dis3	-0.13	1.00000000	pigs
Wsb2	-0.05	1.00000000	pigs
Paqr8	0.01	1.00000000	pigs
Denr	0.25	1.00000000	pigs
Crppa	0.09	1.00000000	pigs
Cdkal1	0.28	1.00000000	pigs
Dcp2	0.26	1.00000000	pigs
Cpeb3	0.11	1.00000000	pigs
Kdm5b	-0.07	1.00000000	pigs
Nsd2	0.07	1.00000000	pigs
Kcnn3	0.25	1.00000000	pigs
Dnttip2	-0.40	1.00000000	pigs
Daam1	0.17	1.00000000	pigs
Kctd6	0.00	1.00000000	pigs
Ppt1	-0.03	1.00000000	pigs
Lgmn	0.41	1.00000000	pigs
Trappc9	-0.07	1.00000000	pigs
Cactin	0.42	1.00000000	pigs
Arhgap21	-0.03	1.00000000	pigs
Pigk	-0.28	1.00000000	pigs
Oas1e	-0.06	1.00000000	pigs
Wipf2	-0.07	1.00000000	pigs
Ube2w	0.04	1.00000000	pigs
Tcp11l2	0.10	1.00000000	pigs
Rbm5	0.12	1.00000000	pigs
Slc11a2	0.27	1.00000000	pigs
Fam13a	0.20	1.00000000	pigs
Trim24	0.03	1.00000000	pigs
Ippk	0.03	1.00000000	pigs
Stx2	0.09	1.00000000	pigs
Plekha1	-0.01	1.00000000	pigs
Ankrd55	-0.52	1.00000000	pigs
Cdc42ep4	0.27	1.00000000	pigs
Tbc1d16	0.11	1.00000000	pigs
Psmd14	-0.03	1.00000000	pigs
Ifnar1	0.19	1.00000000	pigs

Cpsf7	-0.11	1.00000000	pigs
Isyna1	-0.20	1.00000000	pigs
Nrbp2	0.09	1.00000000	pigs
Ankrd17	0.23	1.00000000	pigs
Dhx37	-0.08	1.00000000	pigs
Tollip	0.12	1.00000000	pigs
Wdhd1	-0.21	1.00000000	pigs
Rapgef5	0.18	1.00000000	pigs
Hmgxb4	-0.25	1.00000000	pigs
Cmklr1	0.03	1.00000000	pigs
Ccdc3	-0.17	1.00000000	pigs
Fam126b	0.13	1.00000000	pigs
Fundc2	0.03	1.00000000	pigs
Ophn1	0.09	1.00000000	pigs
Ormdl1	-0.03	1.00000000	pigs
Fbxo33	0.04	1.00000000	pigs
Cpt2	-0.09	1.00000000	pigs
Rtn4	0.38	1.00000000	pigs
Tbc1d5	0.17	1.00000000	pigs
Ing3	0.11	1.00000000	pigs
Bok	0.23	1.00000000	pigs
Erap1	-0.18	1.00000000	pigs
Nfya	0.21	1.00000000	pigs
Rras	-0.28	1.00000000	pigs
9930012K11Rik	0.02	1.00000000	pigs
Tal1	-0.18	1.00000000	pigs
Siah1b	-0.20	1.00000000	pigs
Asxl2	0.08	1.00000000	pigs
Lrrc59	0.03	1.00000000	pigs
Fosb	0.10	1.00000000	pigs
Ndfip2	0.02	1.00000000	pigs
Telo2	0.08	1.00000000	pigs
Dlg1	-0.39	1.00000000	pigs
Dcun1d3	0.29	1.00000000	pigs
Cox7a2	-0.27	1.00000000	pigs
Nsd1	-0.02	1.00000000	pigs
Ubl7	-0.01	1.00000000	pigs
Ube3a	0.18	1.00000000	pigs
Lmf1	0.07	1.00000000	pigs
Dusp28	0.01	1.00000000	pigs
Nup93	-0.04	1.00000000	pigs
Ndufb4	-0.15	1.00000000	pigs
Aida	-0.06	1.00000000	pigs
Il1rap	0.17	1.00000000	pigs
Snrnp48	-0.03	1.00000000	pigs
Hyou1	0.19	1.00000000	pigs

Ubtd1	-0.02	1.00000000	pigs
Rab22a	0.11	1.00000000	pigs
Itpr2	0.21	1.00000000	pigs
Scn3a	-0.15	1.00000000	pigs
Wdr47	0.03	1.00000000	pigs
Elp3	0.14	1.00000000	pigs
Wdsub1	-0.41	1.00000000	pigs
Ppp4r1	-0.08	1.00000000	pigs
Gnpnat1	-0.29	1.00000000	pigs
Usp15	0.22	1.00000000	pigs
Rbm18	0.11	1.00000000	pigs
Ap1s1	0.30	1.00000000	pigs
Prep	0.18	1.00000000	pigs
Ccn1	-0.69	1.00000000	pigs
Senp6	0.08	1.00000000	pigs
Ndufa10	-0.18	1.00000000	pigs
Fndc7	-0.07	1.00000000	pigs
Rpain	0.00	1.00000000	pigs
Agpat5	0.20	1.00000000	pigs
Abcc1	0.06	1.00000000	pigs
Sash1	0.47	1.00000000	pigs
Bzw1	-0.21	1.00000000	pigs
Iqsec2	-0.05	1.00000000	pigs
Sfxn3	-0.06	1.00000000	pigs
Sphk1	0.16	1.00000000	pigs
Tmem183a	0.01	1.00000000	pigs
Taf10	0.24	1.00000000	pigs
Gucy1a1	-0.60	1.00000000	pigs
Cxcr4	0.52	1.00000000	pigs
Dpy19l3	0.33	1.00000000	pigs
Uros	0.09	1.00000000	pigs
Ankhd1	0.18	1.00000000	pigs
Arhgef18	0.02	1.00000000	pigs
Rnf216	-0.10	1.00000000	pigs
Xpc	-0.03	1.00000000	pigs
Pgm3	0.09	1.00000000	pigs
Acsl5	0.14	1.00000000	pigs
Rcn2	0.95	1.00000000	pigs
Brd7	0.06	1.00000000	pigs
Dcun1d5	0.04	1.00000000	pigs
Pip4k2a	0.21	1.00000000	pigs
Naa50	0.04	1.00000000	pigs
Ppp2r2a	0.11	1.00000000	pigs
Phc2	0.11	1.00000000	pigs
Pabpc1	0.30	1.00000000	pigs
Dlat	-0.13	1.00000000	pigs

Reln	-0.13	1.00000000	pigs
Ctr9	0.17	1.00000000	pigs
Trio	0.24	1.00000000	pigs
Insig2	-0.01	1.00000000	pigs
Zhx3	0.10	1.00000000	pigs
Scaf1	0.09	1.00000000	pigs
Nipsnap3b	-0.25	1.00000000	pigs
Fam168b	0.16	1.00000000	pigs
Chst11	0.25	1.00000000	pigs
Ppp1r21	0.18	1.00000000	pigs
Taf1d	0.29	1.00000000	pigs
Zfpl1	0.08	1.00000000	pigs
Plod2	0.13	1.00000000	pigs
Lemd2	-0.06	1.00000000	pigs
Gnb1	0.20	1.00000000	pigs
Etl4	-0.23	1.00000000	pigs
Ube2v2	0.22	1.00000000	pigs
Ccdc191	0.14	1.00000000	pigs
Nrd1	0.19	1.00000000	pigs
Kdm6b	0.09	1.00000000	pigs
Slc6a2	-0.06	1.00000000	pigs
Fnbp1	-0.47	1.00000000	pigs
Tst	0.16	1.00000000	pigs
Xpot	-0.12	1.00000000	pigs
Ing1	0.62	1.00000000	pigs
Arl6ip6	0.09	1.00000000	pigs
Smdt1	0.21	1.00000000	pigs
Lrrk1	-0.52	1.00000000	pigs
Ankmy2	-0.05	1.00000000	pigs
Rnf141	0.03	1.00000000	pigs
Snx27	0.00	1.00000000	pigs
Ftl1-ps1	0.62	1.00000000	pigs
Ptprs	-0.29	1.00000000	pigs
Hspb1	-0.12	1.00000000	pigs
Col1a1	0.26	1.00000000	pigs
Tbccd1	-0.01	1.00000000	pigs
Asns	0.10	1.00000000	pigs
Rhoa	0.17	1.00000000	pigs
Tubgcp2	0.05	1.00000000	pigs
Exosc5	0.24	1.00000000	pigs
Gpr4	0.02	1.00000000	pigs
Cep290	0.22	1.00000000	pigs
Ccm2	0.08	1.00000000	pigs
Idh3b	-0.01	1.00000000	pigs
Ap5m1	0.00	1.00000000	pigs
Tet2	0.34	1.00000000	pigs

H1f4	0.77	1.00000000	pigs
Ttc14	-0.01	1.00000000	pigs
Ino80	0.04	1.00000000	pigs
Smarcc1	0.02	1.00000000	pigs
Fam133b	-0.33	1.00000000	pigs
Nudcd1	0.11	1.00000000	pigs
Trim25	0.16	1.00000000	pigs
3110082I17Rik	0.10	1.00000000	pigs
Osbpl6	-0.41	1.00000000	pigs
Srgn	0.78	1.00000000	pigs
Txnl4b	-0.02	1.00000000	pigs
Odf2	0.04	1.00000000	pigs
Kif27	0.10	1.00000000	pigs
Dip2b	0.20	1.00000000	pigs
Tsc2	0.09	1.00000000	pigs
B4galt5	0.19	1.00000000	pigs
Atp5pb	0.23	1.00000000	pigs
Tspan3	-0.27	1.00000000	pigs
Rnf152	0.04	1.00000000	pigs
Hspa14	0.39	1.00000000	pigs
Anapc7	-0.35	1.00000000	pigs
Nceh1	0.02	1.00000000	pigs
Fbxo6	0.01	1.00000000	pigs
Cwf19l1	-0.45	1.00000000	pigs
Cmtm7	-0.20	1.00000000	pigs
Pex1	-0.26	1.00000000	pigs
Rae1	0.02	1.00000000	pigs
Acox1	0.14	1.00000000	pigs
Abhd11	-0.19	1.00000000	pigs
Yap1	0.04	1.00000000	pigs
Cog5	0.04	1.00000000	pigs
Hk3	0.25	1.00000000	pigs
Cyp51	-0.37	1.00000000	pigs
Commd8	0.01	1.00000000	pigs
Btf3l4	0.09	1.00000000	pigs
Sft2d1	-0.05	1.00000000	pigs
Tfpi2	-0.13	1.00000000	pigs
Hcfc1r1	-0.12	1.00000000	pigs
Hoxd9	-0.21	1.00000000	pigs
Eml3	-0.02	1.00000000	pigs
Dedd	0.00	1.00000000	pigs
Nr4a1	-0.31	1.00000000	pigs
Pnrc1	0.28	1.00000000	pigs
Add3	-0.22	1.00000000	pigs
Clcc1	0.04	1.00000000	pigs
Il33	-0.17	1.00000000	pigs

Rhbdd1	-0.30	1.00000000	pigs
8030462N17Rik	0.08	1.00000000	pigs
Pias4	-0.02	1.00000000	pigs
Blzf1	0.08	1.00000000	pigs
Nmral1	0.13	1.00000000	pigs
Zfyve1	-0.02	1.00000000	pigs
Btbd19	0.14	1.00000000	pigs
Kif5b	0.16	1.00000000	pigs
Apold1	-0.16	1.00000000	pigs
Uqcrc1	0.02	1.00000000	pigs
Zfp148	-0.41	1.00000000	pigs
Aldh16a1	0.07	1.00000000	pigs
Rrp12	0.03	1.00000000	pigs
B2m	0.18	1.00000000	pigs
Zfp131	0.16	1.00000000	pigs
Senp8	0.15	1.00000000	pigs
Elovl1	-0.30	1.00000000	pigs
Pkn3	-0.15	1.00000000	pigs
Polk	0.07	1.00000000	pigs
Col5a2	-0.44	1.00000000	pigs
Dvl3	0.12	1.00000000	pigs
Lars2	0.03	1.00000000	pigs
Prmt9	-0.31	1.00000000	pigs
Commd9	0.05	1.00000000	pigs
Wdr59	0.22	1.00000000	pigs
Zbtb1	0.17	1.00000000	pigs
Taldo1	0.12	1.00000000	pigs
Gabbr1	0.02	1.00000000	pigs
Zfp369	-0.04	1.00000000	pigs
Tmem41b	0.08	1.00000000	pigs
Tmem30a	0.18	1.00000000	pigs
H2aj	0.08	1.00000000	pigs
Romo1	-0.22	1.00000000	pigs
Cd34	-0.09	1.00000000	pigs
Amd2	0.60	1.00000000	pigs
Selenop	0.09	1.00000000	pigs
Tuba1b	-0.17	1.00000000	pigs
Guf1	0.13	1.00000000	pigs
Atp6v1f	0.42	1.00000000	pigs
Pcf11	0.30	1.00000000	pigs
Sgms1	0.22	1.00000000	pigs
Nr4a2	-0.28	1.00000000	pigs
Psme2	0.35	1.00000000	pigs
Klf12	-0.28	1.00000000	pigs
Ubr3	0.17	1.00000000	pigs
Wars2	-0.04	1.00000000	pigs

Slc25a13	0.16	1.00000000	pigs
Rgs12	-0.26	1.00000000	pigs
Twsg1	-0.65	1.00000000	pigs
Ypel5	0.02	1.00000000	pigs
Pycrl	-0.14	1.00000000	pigs
Cnot2	0.24	1.00000000	pigs
Gfod2	0.28	1.00000000	pigs
Adam17	0.13	1.00000000	pigs
Selenot	0.30	1.00000000	pigs
Chd1l	-0.04	1.00000000	pigs
Dennd6a	-0.01	1.00000000	pigs
Lhpp	0.12	1.00000000	pigs
Exoc6b	0.18	1.00000000	pigs
Ppp1cb	0.16	1.00000000	pigs
Otud6b	-0.43	1.00000000	pigs
Slc10a1	-0.08	1.00000000	pigs
Tmem106a	0.20	1.00000000	pigs
Prkrip1	0.03	1.00000000	pigs
Nudcd3	-0.03	1.00000000	pigs
Atg3	0.14	1.00000000	pigs
Cbll1	-0.04	1.00000000	pigs
Ube2b	-0.20	1.00000000	pigs
Ccdc25	0.27	1.00000000	pigs
Fmo4	-0.06	1.00000000	pigs
Nono	0.06	1.00000000	pigs
Vcp	0.08	1.00000000	pigs
Rb1cc1	0.19	1.00000000	pigs
Slc39a7	-0.02	1.00000000	pigs
Cfl2	-0.09	1.00000000	pigs
Vrk3	0.09	1.00000000	pigs
Usp37	0.03	1.00000000	pigs
Pcdh12	-0.05	1.00000000	pigs
Tada2a	0.00	1.00000000	pigs
Itga1	-0.39	1.00000000	pigs
Crebrf	0.01	1.00000000	pigs
Rbm22	-0.12	1.00000000	pigs
Ppp1r18	-0.04	1.00000000	pigs
Copb1	-0.40	1.00000000	pigs
Bag6	-0.07	1.00000000	pigs
9930104L06Rik	0.02	1.00000000	pigs
2700049A03Rik	-0.24	1.00000000	pigs
Gemin4	0.03	1.00000000	pigs
Spns2	0.01	1.00000000	pigs
Anxa6	-0.09	1.00000000	pigs
Ubap2	0.18	1.00000000	pigs
Tango6	-0.09	1.00000000	pigs

Epn2	0.09	1.00000000	pigs
Ubb	0.24	1.00000000	pigs
Tinf2	0.08	1.00000000	pigs
Unc93b1	-0.06	1.00000000	pigs
Tpt1	-0.01	1.00000000	pigs
Ppp2r1b	0.01	1.00000000	pigs
Klhl3	0.00	1.00000000	pigs
Fkbp3	-0.29	1.00000000	pigs
Palm	-0.27	1.00000000	pigs
1700123O20Rik	0.21	1.00000000	pigs
Tuba8	0.05	1.00000000	pigs
Ybx3	0.16	1.00000000	pigs
Zcchc2	0.04	1.00000000	pigs
Kif3a	0.03	1.00000000	pigs
Zfand6	0.18	1.00000000	pigs
Hexim1	0.65	1.00000000	pigs
Hat1	0.06	1.00000000	pigs
Slc41a1	-0.16	1.00000000	pigs
Itgb1bp1	-0.01	1.00000000	pigs
Serp1	0.09	1.00000000	pigs
Atp5g3	-0.20	1.00000000	pigs
Cnot11	-0.03	1.00000000	pigs
Naa25	0.13	1.00000000	pigs
Setd2	0.20	1.00000000	pigs
Usp42	0.00	1.00000000	pigs
Hdac3	-0.06	1.00000000	pigs
Spc24	-0.33	1.00000000	pigs
Eapp	-0.43	1.00000000	pigs
Rasa3	0.34	1.00000000	pigs
Tmem205	0.07	1.00000000	pigs
Sdhc	0.02	1.00000000	pigs
Smurf2	0.01	1.00000000	pigs
Snrpe	-0.22	1.00000000	pigs
Trim23	0.06	1.00000000	pigs
Ilrun	0.06	1.00000000	pigs
Plxnc1	0.27	1.00000000	pigs
Fam185a	0.11	1.00000000	pigs
Snip1	-0.07	1.00000000	pigs
Tm2d2	-0.07	1.00000000	pigs
Yes1	0.37	1.00000000	pigs
Pptc7	-0.08	1.00000000	pigs
Top2b	0.11	1.00000000	pigs
Rad52	-0.04	1.00000000	pigs
Tab3	-0.05	1.00000000	pigs
Runx2	0.45	1.00000000	pigs
Lancl1	-0.25	1.00000000	pigs

Tmem50a	-0.31	1.00000000	pigs
Nup160	-0.18	1.00000000	pigs
Nr4a3	0.19	1.00000000	pigs
Nfatc1	0.34	1.00000000	pigs
Wdr77	0.03	1.00000000	pigs
Ik	-0.28	1.00000000	pigs
Garre1	-0.04	1.00000000	pigs
Utp15	0.07	1.00000000	pigs
Nid2	-0.22	1.00000000	pigs
Lrrk2	-0.85	1.00000000	pigs
Enoph1	0.01	1.00000000	pigs
Scin	-0.32	1.00000000	pigs
Snx18	0.09	1.00000000	pigs
Npc2	0.23	1.00000000	pigs
Zfp248	0.19	1.00000000	pigs
Ncl	-0.16	1.00000000	pigs
Irf3	-0.03	1.00000000	pigs
Paxbp1	0.09	1.00000000	pigs
Hgs	0.14	1.00000000	pigs
Rgl2	0.16	1.00000000	pigs
Creg1	0.03	1.00000000	pigs
Atm	-0.37	1.00000000	pigs
Ust	0.02	1.00000000	pigs
Ccdc14	-0.06	1.00000000	pigs
Epas1	-0.19	1.00000000	pigs
Stap1	-0.59	1.00000000	pigs
Cobll1	0.03	1.00000000	pigs
Tsen34	0.05	1.00000000	pigs
Gatad2a	0.08	1.00000000	pigs
Timm17b	0.03	1.00000000	pigs
Tmem60	0.19	1.00000000	pigs
Gna11	0.01	1.00000000	pigs
Vapb	0.04	1.00000000	pigs
Fcer1g	0.02	1.00000000	pigs
Baz1b	0.05	1.00000000	pigs
Edc3	0.09	1.00000000	pigs
Ssr3	-0.19	1.00000000	pigs
Frs2	0.12	1.00000000	pigs
Creb1	0.15	1.00000000	pigs
Slc25a38	0.41	1.00000000	pigs
Fbrs	0.16	1.00000000	pigs
Ccdc91	-0.14	1.00000000	pigs
Rasgrf2	0.11	1.00000000	pigs
Hsp90ab1	0.16	1.00000000	pigs
Ipmk	-0.22	1.00000000	pigs
Slc5a1	0.00	1.00000000	pigs

Slc35f6	0.06	1.00000000	pigs
Ctnnbl1	0.08	1.00000000	pigs
Lgals3	-0.15	1.00000000	pigs
C1qbp	-0.36	1.00000000	pigs
Tor1aip2	-0.06	1.00000000	pigs
Zdhhc16	-0.09	1.00000000	pigs
Cep162	-0.33	1.00000000	pigs
Tbck	0.07	1.00000000	pigs
Ube3c	0.08	1.00000000	pigs
Slc25a5	0.27	1.00000000	pigs
Gtpbp8	0.06	1.00000000	pigs
Decr1	-0.09	1.00000000	pigs
Dctn2	-0.35	1.00000000	pigs
Spen	0.06	1.00000000	pigs
Enho	-0.55	1.00000000	pigs
Atp6v1h	0.08	1.00000000	pigs
Tnrc6b	-0.22	1.00000000	pigs
Kif2a	0.09	1.00000000	pigs
Vps41	0.16	1.00000000	pigs
AI837181	0.01	1.00000000	pigs
Add1	-0.25	1.00000000	pigs
Nqo1	-0.15	1.00000000	pigs
Cdc42bpa	0.38	1.00000000	pigs
Ttc23l	0.25	1.00000000	pigs
Pdxdc1	-0.11	1.00000000	pigs
Usp40	0.05	1.00000000	pigs
Phlda2	-0.18	1.00000000	pigs
Casp7	-0.03	1.00000000	pigs
St3gal6	-0.16	1.00000000	pigs
Ncf1	0.32	1.00000000	pigs
Slc25a17	0.00	1.00000000	pigs
Ralgps2	-0.27	1.00000000	pigs
Tmem100	-0.13	1.00000000	pigs
Amfr	-0.13	1.00000000	pigs
Atr	0.00	1.00000000	pigs
Anks3	0.23	1.00000000	pigs
Bmp2	-0.04	1.00000000	pigs
Stau1	0.09	1.00000000	pigs
Ccdc61	0.13	1.00000000	pigs
Nelfe	-0.04	1.00000000	pigs
Blvrb	0.01	1.00000000	pigs
Sigirr	-0.05	1.00000000	pigs
Reps2	-0.02	1.00000000	pigs
Dnaja2	-0.28	1.00000000	pigs
Dnaja3	-0.26	1.00000000	pigs
Tmtc4	0.12	1.00000000	pigs

Rgs19	-0.30	1.00000000	pigs
Blvra	0.01	1.00000000	pigs
Igfbp7	0.42	1.00000000	pigs
Reep3	-0.23	1.00000000	pigs
Blmh	0.22	1.00000000	pigs
Unc5b	-0.03	1.00000000	pigs
Rap2c	-0.01	1.00000000	pigs
Cops4	0.31	1.00000000	pigs
Tcerg1	0.17	1.00000000	pigs
Zbtb18	0.02	1.00000000	pigs
Arl8b	0.20	1.00000000	pigs
Ep400	-0.05	1.00000000	pigs
Tmem251	0.27	1.00000000	pigs
Tbpl1	-0.02	1.00000000	pigs
Rnf40	0.08	1.00000000	pigs
Ccdc32	0.22	1.00000000	pigs
Rnft1	-0.03	1.00000000	pigs
Tut4	-0.35	1.00000000	pigs
Atp6v1a	0.02	1.00000000	pigs
Tnrc6a	0.01	1.00000000	pigs
Cdk9	0.07	1.00000000	pigs
Nrip1	-0.15	1.00000000	pigs
Fbxw5	-0.06	1.00000000	pigs
Csnk1d	-0.07	1.00000000	pigs
Ruvbl2	0.02	1.00000000	pigs
Atp8b1	0.25	1.00000000	pigs
Cap1	0.19	1.00000000	pigs
Dnajc19	-0.39	1.00000000	pigs
Ccdc137	-0.05	1.00000000	pigs
Sertad3	0.09	1.00000000	pigs
Pnn	-0.36	1.00000000	pigs
Zfp652	-0.24	1.00000000	pigs
Hsd17b4	0.07	1.00000000	pigs
Hipk1	0.09	1.00000000	pigs
Tradd	0.20	1.00000000	pigs
Fam227b	0.12	1.00000000	pigs
Lypla1	0.30	1.00000000	pigs
Trit1	0.13	1.00000000	pigs
Emc9	0.13	1.00000000	pigs
Rrp1	-0.16	1.00000000	pigs
Cdipt	0.19	1.00000000	pigs
Kcnab3	-0.15	1.00000000	pigs
Slmap	-0.01	1.00000000	pigs
Smarcd1	0.14	1.00000000	pigs
Arhgap26	0.06	1.00000000	pigs
Rpa2	0.03	1.00000000	pigs

Arfgef1	0.04	1.00000000	pigs
Pde4dip	0.16	1.00000000	pigs
Arl6ip4	0.04	1.00000000	pigs
Pafah2	-0.07	1.00000000	pigs
Poldip2	-0.06	1.00000000	pigs
Cltb	-0.27	1.00000000	pigs
Yeats4	0.28	1.00000000	pigs
H2az2	-0.23	1.00000000	pigs
Tbcd	0.26	1.00000000	pigs
Fhod1	0.02	1.00000000	pigs
Eif4h	-0.21	1.00000000	pigs
Dnm3	-0.14	1.00000000	pigs
Exoc5	0.02	1.00000000	pigs
Asap2	-0.10	1.00000000	pigs
Akap8l	-0.03	1.00000000	pigs
Cdc73	0.12	1.00000000	pigs
Rbbp8	0.03	1.00000000	pigs
Wrap73	-0.03	1.00000000	pigs
Lsm14b	0.04	1.00000000	pigs
Tcf3	0.07	1.00000000	pigs
Tnks	0.09	1.00000000	pigs
Fam193b	0.00	1.00000000	pigs
Ankrd27	0.20	1.00000000	pigs
B230219D22Rik	0.05	1.00000000	pigs
Chtop	0.14	1.00000000	pigs
Nckap1	0.08	1.00000000	pigs
Aff4	0.22	1.00000000	pigs
Tmem128	-0.08	1.00000000	pigs
Rbm17	0.15	1.00000000	pigs
Znhit6	0.03	1.00000000	pigs
Gapvd1	0.02	1.00000000	pigs
Vps13b	0.12	1.00000000	pigs
Ergic1	-0.09	1.00000000	pigs
Lsm8	-0.54	1.00000000	pigs
Kras	0.14	1.00000000	pigs
Irak4	-0.11	1.00000000	pigs
Sec24a	0.23	1.00000000	pigs
Rpa3	0.16	1.00000000	pigs
Nop2	0.24	1.00000000	pigs
B9d1	-0.08	1.00000000	pigs
Uqcr10	-0.24	1.00000000	pigs
Pknox1	-0.13	1.00000000	pigs
Elf2	0.08	1.00000000	pigs
Asb3	-0.02	1.00000000	pigs
Phc3	-0.11	1.00000000	pigs
Peak1	0.34	1.00000000	pigs

Rhoc	0.22	1.00000000	pigs
Bub3	0.06	1.00000000	pigs
Gigyf2	-0.05	1.00000000	pigs
Dap	-0.02	1.00000000	pigs
Tcaf1	-0.11	1.00000000	pigs
Bbs9	-0.03	1.00000000	pigs
Pef1	-0.37	1.00000000	pigs
Cdk17	0.17	1.00000000	pigs
Pop4	0.03	1.00000000	pigs
Riox2	-0.26	1.00000000	pigs
Asah1	0.20	1.00000000	pigs
Kat7	-0.09	1.00000000	pigs
Evi5	0.05	1.00000000	pigs
Thsd4	0.32	1.00000000	pigs
Dmac2	-0.31	1.00000000	pigs
Babam2	0.10	1.00000000	pigs
Larp1b	0.11	1.00000000	pigs
Dchs1	-0.10	1.00000000	pigs
Hira	0.12	1.00000000	pigs
Tmem140	0.05	1.00000000	pigs
Ncbp3	-0.03	1.00000000	pigs
Gas6	0.12	1.00000000	pigs
Pprc1	-0.07	1.00000000	pigs
Rapgef3	-0.13	1.00000000	pigs
Prdx4	0.02	1.00000000	pigs
Tmem243	-0.28	1.00000000	pigs
Zfp521	-0.37	1.00000000	pigs
Raf1	0.07	1.00000000	pigs
Scfd2	0.15	1.00000000	pigs
Sptan1	0.36	1.00000000	pigs
Zmiz2	0.21	1.00000000	pigs
Ypel2	0.01	1.00000000	pigs
Ints8	0.01	1.00000000	pigs
Prkag2	0.37	1.00000000	pigs
Frmd4b	0.38	1.00000000	pigs
Chmp4b	0.10	1.00000000	pigs
Orc4	-0.05	1.00000000	pigs
Gxylt1	0.04	1.00000000	pigs
Sltm	0.25	1.00000000	pigs
Arid2	0.07	1.00000000	pigs
Lnpk	-0.02	1.00000000	pigs
Rap2a	-0.33	1.00000000	pigs
Phactr4	0.23	1.00000000	pigs
Atp6v0a1	-0.47	1.00000000	pigs
Wasf3	-0.37	1.00000000	pigs
Xbp1	0.09	1.00000000	pigs

Itsn2	0.12	1.00000000	pigs
Dnm2	0.24	1.00000000	pigs
Slc39a9	0.06	1.00000000	pigs
Tfam	0.17	1.00000000	pigs
Ogfod3	-0.06	1.00000000	pigs
Tmx1	0.00	1.00000000	pigs
Commd1b	0.04	1.00000000	pigs
Rbmx	0.27	1.00000000	pigs
Tlr3	0.06	1.00000000	pigs
Syne3	-0.03	1.00000000	pigs
Armcx2	0.06	1.00000000	pigs
Rassf2	0.06	1.00000000	pigs
Tdp2	-0.09	1.00000000	pigs
Plod3	-0.09	1.00000000	pigs
Sipa1l2	-0.47	1.00000000	pigs
Trabd	0.22	1.00000000	pigs
Fblim1	0.12	1.00000000	pigs
Dpp9	0.03	1.00000000	pigs
Acbd3	0.05	1.00000000	pigs
Cnep1r1	0.01	1.00000000	pigs
Cyhr1	-0.07	1.00000000	pigs
Rsu1	0.28	1.00000000	pigs
Tnrc18	-0.14	1.00000000	pigs
Tap1	0.08	1.00000000	pigs
Fxr1	-0.27	1.00000000	pigs
Dtnbp1	0.11	1.00000000	pigs
Sdcbp	0.20	1.00000000	pigs
Disc1	0.22	1.00000000	pigs
Dock1	0.14	1.00000000	pigs
Ptov1	-0.05	1.00000000	pigs
Rbm12b2	0.01	1.00000000	pigs
Pcdhga4	0.10	1.00000000	pigs
Dock2	0.29	1.00000000	pigs
Tada1	0.09	1.00000000	pigs
Gpaa1	0.00	1.00000000	pigs
Arl6	-0.03	1.00000000	pigs
Luc7l3	0.08	1.00000000	pigs
Cxxc1	-0.15	1.00000000	pigs
Camta1	0.05	1.00000000	pigs
Rnpep	-0.20	1.00000000	pigs
Fyco1	-0.32	1.00000000	pigs
Psmd2	0.03	1.00000000	pigs
Spg7	0.09	1.00000000	pigs
Snapc1l	0.14	1.00000000	pigs
Klhl42	0.06	1.00000000	pigs
Eif3g	-0.22	1.00000000	pigs

Rnd3	0.30	1.00000000	pigs
Ptbp3	0.20	1.00000000	pigs
Atxn7l1	-0.04	1.00000000	pigs
Usp48	-0.05	1.00000000	pigs
Zfp30	0.10	1.00000000	pigs
Ndufab1	-0.28	1.00000000	pigs
Efcab11	0.10	1.00000000	pigs
Gpd2	0.11	1.00000000	pigs
Anxa1	-0.09	1.00000000	pigs
D6Wsu163e	0.16	1.00000000	pigs
Slc8a1	0.20	1.00000000	pigs
Chd1	0.14	1.00000000	pigs
Ncoa3	0.10	1.00000000	pigs
Chchd5	0.29	1.00000000	pigs
Zdhhc15	-0.32	1.00000000	pigs
Atf2	-0.02	1.00000000	pigs
Ptcd3	-0.06	1.00000000	pigs
Scaf11	0.08	1.00000000	pigs
Pum1	0.10	1.00000000	pigs
Cdkn2aip	0.21	1.00000000	pigs
Chst12	-0.29	1.00000000	pigs
Gcnt3	-0.27	1.00000000	pigs
Ankrd10	0.01	1.00000000	pigs
Phkb	0.01	1.00000000	pigs
Ndufc2	-0.32	1.00000000	pigs
Rprd1a	0.14	1.00000000	pigs
Itpa	0.08	1.00000000	pigs
Atg2b	0.03	1.00000000	pigs
Ssbp2	-0.26	1.00000000	pigs
Atxn10	-0.07	1.00000000	pigs
Brms1l	0.06	1.00000000	pigs
Araf	0.07	1.00000000	pigs
Hibch	-0.45	1.00000000	pigs
Ptpn18	-0.01	1.00000000	pigs
Coro1c	0.10	1.00000000	pigs
Aatf	-0.05	1.00000000	pigs
Ppp2r3a	-0.47	1.00000000	pigs
Plagl2	0.11	1.00000000	pigs
Tcp11l1	-0.03	1.00000000	pigs
Tspan14	-0.07	1.00000000	pigs
Strn	0.14	1.00000000	pigs
Arpc1b	0.29	1.00000000	pigs
Copb2	-0.35	1.00000000	pigs
Vdac2	0.12	1.00000000	pigs
Fbxo3	-0.04	1.00000000	pigs
Eif4e	0.07	1.00000000	pigs

Ccndbp1	-0.13	1.00000000	pigs
Galnt7	-0.26	1.00000000	pigs
Dync1i2	-0.35	1.00000000	pigs
Dynll2	-0.09	1.00000000	pigs
Smtn	-0.23	1.00000000	pigs
Aff1	0.23	1.00000000	pigs
Rexo2	-0.24	1.00000000	pigs
Fbxw11	0.15	1.00000000	pigs
2310022B05Rik	-0.30	1.00000000	pigs
Snrpa	-0.02	1.00000000	pigs
Bcl6	0.01	1.00000000	pigs
Pir	0.09	1.00000000	pigs
Ggcx	0.10	1.00000000	pigs
Cyc1	-0.24	1.00000000	pigs
Alcam	-0.18	1.00000000	pigs
Procr	0.18	1.00000000	pigs
Ttf2	-0.04	1.00000000	pigs
Chic2	-0.06	1.00000000	pigs
Pi4k2b	0.03	1.00000000	pigs
Copa	-0.10	1.00000000	pigs
Ccdc85a	-0.12	1.00000000	pigs
Ccny	-0.01	1.00000000	pigs
Sclt1	-0.28	1.00000000	pigs
Obscn	-0.65	1.00000000	pigs
Lamtor1	0.18	1.00000000	pigs
Dstn	-0.41	1.00000000	pigs
Usp16	-0.12	1.00000000	pigs
Ebag9	-0.06	1.00000000	pigs
Cry1	0.17	1.00000000	pigs
Itm2a	-0.33	1.00000000	pigs
Klhl29	0.16	1.00000000	pigs
Wdr12	0.01	1.00000000	pigs
Serbp1	-0.13	1.00000000	pigs
Prmt3	-0.29	1.00000000	pigs
Colgalt1	-0.02	1.00000000	pigs
Glcci1	0.07	1.00000000	pigs
Rp9	-0.09	1.00000000	pigs
Gid8	0.10	1.00000000	pigs
Tmem268	0.21	1.00000000	pigs
Atg13	0.09	1.00000000	pigs
Tgfbr3	-0.07	1.00000000	pigs
Uqcc1	0.06	1.00000000	pigs
Pdgfa	0.26	1.00000000	pigs
Trnau1ap	0.07	1.00000000	pigs
Opa1	0.05	1.00000000	pigs
Cyfip2	-0.16	1.00000000	pigs

Taok1	0.05	1.00000000	pigs
Ubac1	-0.23	1.00000000	pigs
Tmem101	-0.02	1.00000000	pigs
Pet100	-0.33	1.00000000	pigs
Colgalt2	-0.20	1.00000000	pigs
Abl1	0.17	1.00000000	pigs
Tmem106b	0.10	1.00000000	pigs
Hspa12b	-0.18	1.00000000	pigs
Rubcn	0.19	1.00000000	pigs
Klf16	0.08	1.00000000	pigs
Pdia3	0.17	1.00000000	pigs
Sumf2	0.10	1.00000000	pigs
Zfp9	0.24	1.00000000	pigs
Fam117a	0.64	1.00000000	pigs
Arhgap5	-0.08	1.00000000	pigs
Fads3	0.18	1.00000000	pigs
Ndufs1	-0.01	1.00000000	pigs
Arl4a	0.09	1.00000000	pigs
Hnrnpd	-0.14	1.00000000	pigs
Galnt11	-0.27	1.00000000	pigs
Apbb1ip	-0.17	1.00000000	pigs
Etfa	-0.20	1.00000000	pigs
1600012H06Rik	-0.20	1.00000000	pigs
Ncstn	0.21	1.00000000	pigs
Kif1c	0.06	1.00000000	pigs
Ilf3	-0.03	1.00000000	pigs
Pcnx4	-0.10	1.00000000	pigs
Slc25a4	-0.30	1.00000000	pigs
Serinc1	0.20	1.00000000	pigs
Pgp	0.05	1.00000000	pigs
Rftn2	0.03	1.00000000	pigs
Wars	0.09	1.00000000	pigs
Pdp1	0.14	1.00000000	pigs
Zfp65	-0.09	1.00000000	pigs
F11r	0.02	1.00000000	pigs
Atp6v0a2	-0.01	1.00000000	pigs
Cdkn1b	-0.42	1.00000000	pigs
Plekhg5	0.02	1.00000000	pigs
Epc1	-0.19	1.00000000	pigs
Fnbp4	0.23	1.00000000	pigs
Fbrsl1	0.37	1.00000000	pigs
Itih5	-0.21	1.00000000	pigs
Tspan31	0.05	1.00000000	pigs
Washc4	0.02	1.00000000	pigs
Actg1	-0.09	1.00000000	pigs
Slc12a7	-0.06	1.00000000	pigs

Entpd4	-0.04	1.00000000	pigs
Tomm7	-0.04	1.00000000	pigs
Ipo4	-0.25	1.00000000	pigs
Itm2b	-0.17	1.00000000	pigs
Abhd5	0.08	1.00000000	pigs
Dtymk	-0.23	1.00000000	pigs
Syne2	0.17	1.00000000	pigs
Dek	-0.11	1.00000000	pigs
Eva1c	0.19	1.00000000	pigs
Gpx3	0.02	1.00000000	pigs
Elp6	0.18	1.00000000	pigs
Amot	-0.10	1.00000000	pigs
Uchl5	-0.39	1.00000000	pigs
Cep112	0.18	1.00000000	pigs
Tbc1d15	0.04	1.00000000	pigs
Czib	-0.36	1.00000000	pigs
Ptp4a1	-0.11	1.00000000	pigs
Pcna	0.33	1.00000000	pigs
Polr1h	-0.23	1.00000000	pigs
Ly96	-0.04	1.00000000	pigs
Snx13	0.10	1.00000000	pigs
Rfx1	-0.07	1.00000000	pigs
Calu	0.08	1.00000000	pigs
Traf3ip2	-0.04	1.00000000	pigs
Cxxc5	0.02	1.00000000	pigs
Pot1b	-0.35	1.00000000	pigs
Pgap2	0.04	1.00000000	pigs
Tssc4	0.02	1.00000000	pigs
Rdx	0.33	1.00000000	pigs
Wrn	-0.10	1.00000000	pigs
Zbp1	0.13	1.00000000	pigs
Lzic	0.01	1.00000000	pigs
Paqr3	0.07	1.00000000	pigs
D8Ertd738e	0.13	1.00000000	pigs
Cavin3	0.39	1.00000000	pigs
Ndufb3	-0.11	1.00000000	pigs
Cant1	0.05	1.00000000	pigs
Chuk	0.02	1.00000000	pigs
Ugcg	0.06	1.00000000	pigs
Rnf166	-0.01	1.00000000	pigs
Nectin3	-0.05	1.00000000	pigs
Trp53i11	0.00	1.00000000	pigs
Gnl1	-0.02	1.00000000	pigs
Atp9b	0.15	1.00000000	pigs
Atp6v1b2	0.30	1.00000000	pigs
Elmod3	-0.03	1.00000000	pigs

Acp1	-0.21	1.00000000	pigs
Osbpl5	0.06	1.00000000	pigs
Dnajc1	0.23	1.00000000	pigs
Zxdc	-0.03	1.00000000	pigs
Dcaf8	0.05	1.00000000	pigs
Cilk1	-0.42	1.00000000	pigs
Tmed5	-0.17	1.00000000	pigs
Ocel1	0.16	1.00000000	pigs
Prkn	-0.03	1.00000000	pigs
Bcl10	-0.34	1.00000000	pigs
Ndufb11b	-0.26	1.00000000	pigs
Ptpn3	-0.22	1.00000000	pigs
Zdhhc17	-0.04	1.00000000	pigs
Nrg1	-0.31	1.00000000	pigs
Ifngr1	-0.28	1.00000000	pigs
Exog	0.03	1.00000000	pigs
Smg1	-0.02	1.00000000	pigs
Prkcb	-0.18	1.00000000	pigs
Faf2	0.09	1.00000000	pigs
Sfxn1	0.01	1.00000000	pigs
Parva	-0.04	1.00000000	pigs
Fbxl3	0.16	1.00000000	pigs
S100a4	-0.61	1.00000000	pigs
Nme7	-0.14	1.00000000	pigs
Rmc1	-0.05	1.00000000	pigs
Tmem9b	-0.42	1.00000000	pigs
Slc35a3	0.05	1.00000000	pigs
Slc25a29	0.01	1.00000000	pigs
Tasor	0.01	1.00000000	pigs
Nars2	-0.05	1.00000000	pigs
2510009E07Rik	-0.33	1.00000000	pigs
Phrf1	-0.05	1.00000000	pigs
Nr3c2	0.14	1.00000000	pigs
Yipf6	-0.02	1.00000000	pigs
Pop5	-0.23	1.00000000	pigs
Pde7a	0.01	1.00000000	pigs
Pag1	-0.28	1.00000000	pigs
Pfdn2	-0.43	1.00000000	pigs
Ankrd49	0.08	1.00000000	pigs
Cnnm3	0.07	1.00000000	pigs
Crem	-0.29	1.00000000	pigs
Cpd	0.18	1.00000000	pigs
Stradb	0.10	1.00000000	pigs
Smpdl3b	-0.27	1.00000000	pigs
Cdc37l1	0.18	1.00000000	pigs
Fam102a	0.15	1.00000000	pigs

Nup35	0.05	1.00000000	pigs
Cd53	0.20	1.00000000	pigs
Cdc25b	0.03	1.00000000	pigs
Vta1	-0.12	1.00000000	pigs
Cyp3a57	-0.27	1.00000000	pigs
Dock5	-0.21	1.00000000	pigs
Nudc	-0.35	1.00000000	pigs
Anxa11	0.12	1.00000000	pigs
Txndc12	0.10	1.00000000	pigs
Thoc6	-0.07	1.00000000	pigs
Glmn	0.03	1.00000000	pigs
Fgd6	0.09	1.00000000	pigs
Arhgef6	-0.09	1.00000000	pigs
Got2	0.64	1.00000000	pigs
Ppm1d	-0.04	1.00000000	pigs
Tgif1	-0.30	1.00000000	pigs
Eif2b2	0.00	1.00000000	pigs
Rnf146	-0.11	1.00000000	pigs
Ythdf2	0.18	1.00000000	pigs
Phf11a	0.05	1.00000000	pigs
Spryd7	0.09	1.00000000	pigs
Ablim2	0.08	1.00000000	pigs
BC005624	-0.39	1.00000000	pigs
Slc4a1ap	0.01	1.00000000	pigs
Hspe1-rs1	0.15	1.00000000	pigs
Prkcz	0.22	1.00000000	pigs
Khk	-0.03	1.00000000	pigs
4933434E20Rik	-0.07	1.00000000	pigs
Clvs1	0.17	1.00000000	pigs
Ppm1h	0.06	1.00000000	pigs
Rbms1	-0.26	1.00000000	pigs
Rufy3	0.05	1.00000000	pigs
Iars2	-0.21	1.00000000	pigs
Ehbp1	-0.40	1.00000000	pigs
Nup50	-0.07	1.00000000	pigs
Zswim4	0.07	1.00000000	pigs
Slc35e1	0.02	1.00000000	pigs
Pdcd4	0.22	1.00000000	pigs
Zfp609	0.27	1.00000000	pigs
Rpa1	-0.13	1.00000000	pigs
Tfip11	0.30	1.00000000	pigs
1810013L24Rik	0.18	1.00000000	pigs
Vps28	-0.49	1.00000000	pigs
Hhat	-0.03	1.00000000	pigs
Ice2	-0.07	1.00000000	pigs
Tmem70	-0.03	1.00000000	pigs

Gabpb1	0.18	1.00000000	pigs
Pds5a	0.01	1.00000000	pigs
Smarca1	0.24	1.00000000	pigs
Ip6k2	0.21	1.00000000	pigs
Atg5	0.04	1.00000000	pigs
Zfc3h1	0.36	1.00000000	pigs
Ndufa6	-0.03	1.00000000	pigs
Enpp6	0.22	1.00000000	pigs
Appl2	-0.02	1.00000000	pigs
Col18a1	0.35	1.00000000	pigs
Sf3a3	-0.29	1.00000000	pigs
Rab7	0.02	1.00000000	pigs
Cops3	0.10	1.00000000	pigs
Lima1	-0.39	1.00000000	pigs
Flnc	-0.16	1.00000000	pigs
Slc40a1	-0.10	1.00000000	pigs
Comt	-0.01	1.00000000	pigs
Ptprc	-0.18	1.00000000	pigs
Slc25a12	-0.33	1.00000000	pigs
Ttc1	-0.19	1.00000000	pigs
Gbf1	0.12	1.00000000	pigs
Tlcd2	0.03	1.00000000	pigs
Tom1l2	-0.16	1.00000000	pigs
Dnajc7	-0.05	1.00000000	pigs
Nek1	0.02	1.00000000	pigs
Orc3	-0.22	1.00000000	pigs
Bicd1	-0.06	1.00000000	pigs
Supt16	-0.25	1.00000000	pigs
Wbp1	-0.09	1.00000000	pigs
Tmem134	0.03	1.00000000	pigs
Plekhm2	0.11	1.00000000	pigs
Hspa5	0.20	1.00000000	pigs
Pdk3	0.16	1.00000000	pigs
Clptm1l	0.08	1.00000000	pigs
Eif4e2	0.03	1.00000000	pigs
Hibadh	0.22	1.00000000	pigs
Zfp13	-0.19	1.00000000	pigs
Tex2	0.02	1.00000000	pigs
Zdhhc9	-0.03	1.00000000	pigs
Slc7a6os	0.05	1.00000000	pigs
Zfp444	0.09	1.00000000	pigs
Entpd1	-0.38	1.00000000	pigs
Naa35	-0.06	1.00000000	pigs
Arhgap31	0.07	1.00000000	pigs
Rpn2	-0.24	1.00000000	pigs
Galc	0.08	1.00000000	pigs

Sh3rf1	-0.25	1.00000000	pigs
Nfia	-0.21	1.00000000	pigs
Pkdcc	0.01	1.00000000	pigs
Arsb	0.06	1.00000000	pigs
Ubn2	-0.02	1.00000000	pigs
Tmco3	0.12	1.00000000	pigs
Arid1b	0.10	1.00000000	pigs
Zfp407	0.17	1.00000000	pigs
Egfl7	-0.39	1.00000000	pigs
Rab1a	-0.07	1.00000000	pigs
Wdr74	0.42	1.00000000	pigs
Bmp6	0.35	1.00000000	pigs
Wwp1	-0.06	1.00000000	pigs
Ptpn14	0.05	1.00000000	pigs
Klf6	0.19	1.00000000	pigs
Tada3	-0.08	1.00000000	pigs
Tnpo3	-0.02	1.00000000	pigs
Agl	-0.04	1.00000000	pigs
Chd6	-0.04	1.00000000	pigs
Notch1	-0.06	1.00000000	pigs
Cacna2d1	0.16	1.00000000	pigs
Snf8	-0.22	1.00000000	pigs
Bcl7a	0.01	1.00000000	pigs
S100pbp	-0.02	1.00000000	pigs
Nipsnap2	-0.24	1.00000000	pigs
Ciz1	-0.14	1.00000000	pigs
Slc20a2	-0.12	1.00000000	pigs
Rab11fip2	-0.08	1.00000000	pigs
Apip	-0.18	1.00000000	pigs
Syn3	-0.04	1.00000000	pigs
Ssna1	-0.16	1.00000000	pigs
Pcgf2	0.03	1.00000000	pigs
Lypla2	0.15	1.00000000	pigs
Pigc	-0.03	1.00000000	pigs
Ciao2a	-0.17	1.00000000	pigs
Kat8	-0.07	1.00000000	pigs
AU040320	0.08	1.00000000	pigs
Ap2a2	0.11	1.00000000	pigs
H1f0	-0.42	1.00000000	pigs
Wdr48	-0.11	1.00000000	pigs
Kctd10	0.08	1.00000000	pigs
Fam3a	-0.27	1.00000000	pigs
Rpusd1	-0.01	1.00000000	pigs
Pum3	-0.24	1.00000000	pigs
Prdx3	0.09	1.00000000	pigs
Pde7b	0.14	1.00000000	pigs

Ell2	0.06	1.00000000	pigs
Clk1	0.00	1.00000000	pigs
Gnl3l	-0.20	1.00000000	pigs
Trp53	0.03	1.00000000	pigs
Ctnnb1	0.08	1.00000000	pigs
Kdsr	-0.30	1.00000000	pigs
Pikfyve	-0.01	1.00000000	pigs
Sp1	-0.07	1.00000000	pigs
Rbm15	0.12	1.00000000	pigs
Aftph	0.16	1.00000000	pigs
Fadd	0.10	1.00000000	pigs
Il15	-0.09	1.00000000	pigs
Katnal1	-0.12	1.00000000	pigs
Stt3a	-0.06	1.00000000	pigs
Ppm1l	0.09	1.00000000	pigs
Crebzf	-0.06	1.00000000	pigs
Phldb1	-0.13	1.00000000	pigs
Ncoa1	0.07	1.00000000	pigs
Galt	0.06	1.00000000	pigs
Oard1	-0.11	1.00000000	pigs
B3gnt2	0.19	1.00000000	pigs
Sppl3	0.09	1.00000000	pigs
Cnot4	0.04	1.00000000	pigs
Sec23ip	-0.05	1.00000000	pigs
Nav2	-0.05	1.00000000	pigs
Patl1	0.09	1.00000000	pigs
Etv3	-0.08	1.00000000	pigs
Ilkap	-0.09	1.00000000	pigs
Spag1	0.24	1.00000000	pigs
Kpna4	0.16	1.00000000	pigs
Terf1	-0.18	1.00000000	pigs
Sucla2	-0.18	1.00000000	pigs
Tmem109	-0.22	1.00000000	pigs
Wdr82	0.14	1.00000000	pigs
Slc39a1	0.04	1.00000000	pigs
Ecpas	0.10	1.00000000	pigs
Zfyve26	0.27	1.00000000	pigs
Ano6	0.09	1.00000000	pigs
Ube2a	0.02	1.00000000	pigs
Nlrc5	-0.11	1.00000000	pigs
Lrsam1	-0.03	1.00000000	pigs
Dmxl2	0.05	1.00000000	pigs
Anks1	-0.03	1.00000000	pigs
Zfp438	-0.07	1.00000000	pigs
Btbd2	0.21	1.00000000	pigs
Ric8b	0.07	1.00000000	pigs

Ccng1	0.16	1.00000000	pigs
Dcaf11	0.08	1.00000000	pigs
Oaz1	0.25	1.00000000	pigs
Atp2b1	0.12	1.00000000	pigs
Dusp22	0.01	1.00000000	pigs
L3hypdh	-0.11	1.00000000	pigs
Nrbp1	0.09	1.00000000	pigs
Cmtm8	-0.27	1.00000000	pigs
Itsn1	-0.02	1.00000000	pigs
Taf12	0.18	1.00000000	pigs
Tmem161b	-0.28	1.00000000	pigs
Bri3	-0.10	1.00000000	pigs
Elovl5	0.01	1.00000000	pigs
Gnal	0.01	1.00000000	pigs
Sgsm2	0.08	1.00000000	pigs
Utp25	-0.07	1.00000000	pigs
Bahd1	-0.03	1.00000000	pigs
Pnpo	0.04	1.00000000	pigs
Sh3bp5	-0.06	1.00000000	pigs
Ywhah	-0.20	1.00000000	pigs
Srprb	-0.05	1.00000000	pigs
Aldh18a1	-0.23	1.00000000	pigs
Lrpap1	0.17	1.00000000	pigs
Pkp4	-0.22	1.00000000	pigs
Jmjd1c	0.33	1.00000000	pigs
Pitpnm2	-0.68	1.00000000	pigs
Wdr5	0.23	1.00000000	pigs
Pmpcb	-0.03	1.00000000	pigs
Sap30bp	0.12	1.00000000	pigs
Pdia6	-0.09	1.00000000	pigs
Nrp2	-0.20	1.00000000	pigs
Thyn1	0.07	1.00000000	pigs
Lamp1	0.20	1.00000000	pigs
Hes1	0.12	1.00000000	pigs
Sec13	0.03	1.00000000	pigs
Coro1b	-0.15	1.00000000	pigs
Eloc	-0.20	1.00000000	pigs
Prkcg	-0.04	1.00000000	pigs
Prdx1	0.48	1.00000000	pigs
Ppil4	-0.24	1.00000000	pigs
Ppl	-0.19	1.00000000	pigs
Amn1	-0.34	1.00000000	pigs
Oxnad1	0.06	1.00000000	pigs
Taf6	0.06	1.00000000	pigs
Stim2	-0.12	1.00000000	pigs
Samd4	-0.50	1.00000000	pigs

Brf1	-0.07	1.00000000	pigs
Ddost	0.09	1.00000000	pigs
Usp19	-0.09	1.00000000	pigs
Nus1	0.02	1.00000000	pigs
Nr2f6	-0.15	1.00000000	pigs
Gcnt2	-0.15	1.00000000	pigs
Ppp5c	-0.06	1.00000000	pigs
Tatdn1	-0.19	1.00000000	pigs
Rpf2	-0.20	1.00000000	pigs
Tmcc3	-0.01	1.00000000	pigs
Parn	0.13	1.00000000	pigs
Psme1	-0.25	1.00000000	pigs
Cemip2	-0.18	1.00000000	pigs
Clasrp	0.16	1.00000000	pigs
Cfdp1	-0.24	1.00000000	pigs
Fli1	0.01	1.00000000	pigs
Arf5	0.16	1.00000000	pigs
Inip	-0.05	1.00000000	pigs
Ndrg2	-0.33	1.00000000	pigs
Crls1	-0.01	1.00000000	pigs
Smarca4	0.05	1.00000000	pigs
Zfp622	0.19	1.00000000	pigs
Klhl9	-0.16	1.00000000	pigs
Tcf20	0.04	1.00000000	pigs
Slc38a9	-0.07	1.00000000	pigs
Lpgat1	-0.40	1.00000000	pigs
Pakap	-0.19	1.00000000	pigs
Togaram1	-0.05	1.00000000	pigs
Slc25a37	-0.05	1.00000000	pigs
Strap	0.02	1.00000000	pigs
Ankrd42	0.25	1.00000000	pigs
Exoc6	-0.24	1.00000000	pigs
Cntln	0.10	1.00000000	pigs
Nrbf2	-0.32	1.00000000	pigs
Nt5c2	0.18	1.00000000	pigs
Keap1	0.17	1.00000000	pigs
Thap2	-0.03	1.00000000	pigs
Nuak1	0.08	1.00000000	pigs
Ppp2cb	0.09	1.00000000	pigs
Trib2	-0.39	1.00000000	pigs
Ddx56	0.17	1.00000000	pigs
Ankra2	0.13	1.00000000	pigs
Eif6	-0.24	1.00000000	pigs
Abhd17c	-0.18	1.00000000	pigs
Chst7	-0.22	1.00000000	pigs
St3gal4	-0.07	1.00000000	pigs

Cdkn1c	1.01	1.00000000	pigs
Washc3	-0.15	1.00000000	pigs
Rnf10	0.08	1.00000000	pigs
Ino80d	0.07	1.00000000	pigs
C77080	0.04	1.00000000	pigs
Acat1	-0.15	1.00000000	pigs
Srsf7	0.47	1.00000000	pigs
Stx18	-0.21	1.00000000	pigs
Tyrobp	0.56	1.00000000	pigs
Zkscan1	-0.11	1.00000000	pigs
Idh2	0.14	1.00000000	pigs
Ergic2	-0.20	1.00000000	pigs
Ppp1r3b	-0.14	1.00000000	pigs
Rhbdd2	-0.04	1.00000000	pigs
Itga4	-0.88	1.00000000	pigs
Tspan15	-0.04	1.00000000	pigs
Inppl1	0.18	1.00000000	pigs
Pik3c2b	0.05	1.00000000	pigs
Rasal2	0.37	1.00000000	pigs
Ufc1	0.00	1.00000000	pigs
Hnrnph1	0.24	1.00000000	pigs
Depp1	-0.19	1.00000000	pigs
Brms1	0.00	1.00000000	pigs
Actr2	-0.11	1.00000000	pigs
Angel2	-0.01	1.00000000	pigs
Arnt	-0.35	1.00000000	pigs
Rassf9	-0.18	1.00000000	pigs
Adnp	-0.09	1.00000000	pigs
Ghrl	0.03	1.00000000	pigs
Chd7	-0.06	1.00000000	pigs
Fam98b	0.04	1.00000000	pigs
Cmc1	-0.16	1.00000000	pigs
Lancl2	0.03	1.00000000	pigs
Akt1s1	0.03	1.00000000	pigs
Kansl3	0.07	1.00000000	pigs
Smo	0.07	1.00000000	pigs
Sufu	0.04	1.00000000	pigs
Gnpda2	0.12	1.00000000	pigs
Lasp1	-0.38	1.00000000	pigs
Timm50	0.02	1.00000000	pigs
Ptdss1	-0.07	1.00000000	pigs
Gart	0.02	1.00000000	pigs
Drosha	-0.01	1.00000000	pigs
Rybp	0.12	1.00000000	pigs
Hras	-0.18	1.00000000	pigs
Loxl1	0.22	1.00000000	pigs

Sdf2	-0.13	1.00000000	pigs
Arfgef3	-0.29	1.00000000	pigs
Dcakd	0.35	1.00000000	pigs
Siah2	-0.22	1.00000000	pigs
Eny2	-0.22	1.00000000	pigs
Foxk1	0.12	1.00000000	pigs
Zcwpw1	0.09	1.00000000	pigs
Akap11	-0.12	1.00000000	pigs
Trim16	-0.02	1.00000000	pigs
Arf4	0.20	1.00000000	pigs
Clstn1	-0.08	1.00000000	pigs
Hook3	0.22	1.00000000	pigs
Senp2	-0.05	1.00000000	pigs
Atxn2	0.18	1.00000000	pigs
Naga	0.01	1.00000000	pigs
Ncald	-0.67	1.00000000	pigs
Sox18	0.21	1.00000000	pigs
Lrrc42	0.07	1.00000000	pigs
Chd8	-0.01	1.00000000	pigs
Zc3h4	-0.01	1.00000000	pigs
Rb1	-0.06	1.00000000	pigs
Pitpna	0.05	1.00000000	pigs
Inpp1	0.05	1.00000000	pigs
Zmpste24	-0.04	1.00000000	pigs
G2e3	0.03	1.00000000	pigs
Prkar2a	-0.08	1.00000000	pigs
Cpne8	-0.11	1.00000000	pigs
Itpr1	0.01	1.00000000	pigs
9930021J03Rik	0.05	1.00000000	pigs
Ndn	-0.02	1.00000000	pigs
Nubpl	-0.05	1.00000000	pigs
Bicral	-0.01	1.00000000	pigs
Invs	0.09	1.00000000	pigs
Slc10a7	0.02	1.00000000	pigs
Eif4e3	0.03	1.00000000	pigs
Cd164	0.17	1.00000000	pigs
Yeats2	0.08	1.00000000	pigs
Cbx6	-0.26	1.00000000	pigs
Fam8a1	0.12	1.00000000	pigs
Idh3a	-0.30	1.00000000	pigs
Prkaca	0.04	1.00000000	pigs
Dis3l	-0.07	1.00000000	pigs
Nudt21	-0.10	1.00000000	pigs
Cep192	0.18	1.00000000	pigs
Jup	-0.05	1.00000000	pigs
Tti2	-0.03	1.00000000	pigs

Hmg20a	-0.03	1.00000000	pigs
Rcbtb2	-0.09	1.00000000	pigs
Puf60	-0.02	1.00000000	pigs
Pyroxd1	-0.12	1.00000000	pigs
Dennd6b	0.04	1.00000000	pigs
Igf1	-0.23	1.00000000	pigs
Pogz	0.07	1.00000000	pigs
Dhx9	0.26	1.00000000	pigs
Rtn4rl2	-0.10	1.00000000	pigs
Rexo5	-0.09	1.00000000	pigs
Nsd3	-0.01	1.00000000	pigs
Zfp36l2	-0.08	1.00000000	pigs
Arsk	0.03	1.00000000	pigs
Prcc	0.00	1.00000000	pigs
Zfp664	0.12	1.00000000	pigs
Lyplal1	0.13	1.00000000	pigs
Srfbp1	-0.26	1.00000000	pigs
Smyd2	-0.02	1.00000000	pigs
Ckap5	-0.04	1.00000000	pigs
Sec61a1	-0.29	1.00000000	pigs
Ddx54	-0.04	1.00000000	pigs
Enpep	-0.36	1.00000000	pigs
Btd	-0.23	1.00000000	pigs
Chpt1	0.01	1.00000000	pigs
Gemin5	-0.15	1.00000000	pigs
Ilk	-0.26	1.00000000	pigs
Idua	-0.13	1.00000000	pigs
Dhx34	0.17	1.00000000	pigs
Trmt13	-0.10	1.00000000	pigs
Ubald2	0.07	1.00000000	pigs
Efcab14	0.01	1.00000000	pigs
Rbm19	0.17	1.00000000	pigs
D630045J12Rik	-0.03	1.00000000	pigs
Cox6a2	-0.01	1.00000000	pigs
Tab1	-0.07	1.00000000	pigs
Tasor2	-0.01	1.00000000	pigs
Pwwp2a	-0.04	1.00000000	pigs
Derl1	-0.29	1.00000000	pigs
Dop1a	-0.27	1.00000000	pigs
Lyve1	0.21	1.00000000	pigs
Selenoo	0.01	1.00000000	pigs
Yy1	-0.02	1.00000000	pigs
Nsmf	-0.07	1.00000000	pigs
Dlst	-0.31	1.00000000	pigs
Tmem19	0.01	1.00000000	pigs
Imp3	0.00	1.00000000	pigs

Srrt	0.08	1.00000000	pigs
Ahcyl2	0.08	1.00000000	pigs
Slc1a2	0.19	1.00000000	pigs
Fn1	0.31	1.00000000	pigs
Eftud2	0.08	1.00000000	pigs
Camta2	0.15	1.00000000	pigs
Ppp1cc	0.26	1.00000000	pigs
Samd12	0.33	1.00000000	pigs
Psmd13	0.01	1.00000000	pigs
Nabp1	0.19	1.00000000	pigs
Tle4	-0.37	1.00000000	pigs
Rtca	-0.04	1.00000000	pigs
Asph	-0.34	1.00000000	pigs
Cdk14	-0.62	1.00000000	pigs
Zmiz1	0.06	1.00000000	pigs
Pde4d	0.05	1.00000000	pigs
Rbm28	-0.22	1.00000000	pigs
Prmt2	0.18	1.00000000	pigs
Hspg2	-0.32	1.00000000	pigs
Pxk	0.02	1.00000000	pigs
Cfap36	-0.24	1.00000000	pigs
Ccdc85b	0.02	1.00000000	pigs
Hnrnpa3	-0.01	1.00000000	pigs
Rack1	0.29	1.00000000	pigs
Tmem248	-0.09	1.00000000	pigs
Bcas3	-0.36	1.00000000	pigs
Gclm	0.19	1.00000000	pigs
Cherp	-0.04	1.00000000	pigs
Nol10	-0.24	1.00000000	pigs
Syf2	-0.24	1.00000000	pigs
Nolc1	-0.24	1.00000000	pigs
Usf1	0.04	1.00000000	pigs
Casc3	-0.28	1.00000000	pigs
Ilf2	0.04	1.00000000	pigs
Dpysl2	-0.06	1.00000000	pigs
Vasp	-0.26	1.00000000	pigs
Aldh7a1	-0.06	1.00000000	pigs
Ranbp1	-0.20	1.00000000	pigs
Drg2	0.02	1.00000000	pigs
Vsig10	0.01	1.00000000	pigs
Fam189a2	-0.04	1.00000000	pigs
Slc25a26	0.10	1.00000000	pigs
Abraxas1	-0.20	1.00000000	pigs
Dennd1a	0.00	1.00000000	pigs
Swt1	-0.14	1.00000000	pigs
Hprt	-0.08	1.00000000	pigs

Eef1d	-0.16	1.00000000	pigs
Kif13a	-0.25	1.00000000	pigs
Sf3a1	-0.02	1.00000000	pigs
Npepps	0.09	1.00000000	pigs
Pygl	0.17	1.00000000	pigs
Lyrm1	-0.06	1.00000000	pigs
Slc25a25	-0.03	1.00000000	pigs
Plin5	-0.10	1.00000000	pigs
Gpr182	-0.24	1.00000000	pigs
Chd3	0.17	1.00000000	pigs
Trim72	-0.06	1.00000000	pigs
Apaf1	-0.06	1.00000000	pigs
Sell	-0.46	1.00000000	pigs
Hgf	0.00	1.00000000	pigs
Fkbp9	-0.03	1.00000000	pigs
Ctsh	0.26	1.00000000	pigs
Vps45	-0.13	1.00000000	pigs
Eif5b	-0.12	1.00000000	pigs
Cmpk1	0.03	1.00000000	pigs
Arhgdia	-0.14	1.00000000	pigs
Rusc2	-0.12	1.00000000	pigs
Dpy30	-0.25	1.00000000	pigs
Pram1	-0.02	1.00000000	pigs
Eml1	-0.36	1.00000000	pigs
Nup85	-0.14	1.00000000	pigs
Smap2	0.05	1.00000000	pigs
Fam124b	-0.03	1.00000000	pigs
Arhgef15	-0.12	1.00000000	pigs
Arl14ep	-0.32	1.00000000	pigs
Ric8a	-0.03	1.00000000	pigs
Ddx20	0.00	1.00000000	pigs
Ppm1f	-0.44	1.00000000	pigs
Pde3b	0.39	1.00000000	pigs
Tgm2	0.07	1.00000000	pigs
Plekhg3	-0.08	1.00000000	pigs
Prrc2a	-0.08	1.00000000	pigs
Usp20	-0.02	1.00000000	pigs
Rnf217	0.12	1.00000000	pigs
Dpf2	-0.15	1.00000000	pigs
Nsf	-0.11	1.00000000	pigs
Erc1	0.03	1.00000000	pigs
Pbdc1	0.13	1.00000000	pigs
Dennd10	-0.22	1.00000000	pigs
Dipk2a	0.03	1.00000000	pigs
Zcchc8	-0.16	1.00000000	pigs
Nudt7	-0.03	1.00000000	pigs

Yipf3	-0.36	1.00000000	pigs
Cep295	-0.07	1.00000000	pigs
Rtn2	0.13	1.00000000	pigs
Sh3bp2	-0.06	1.00000000	pigs
Kars	-0.12	1.00000000	pigs
Ocln	-0.19	1.00000000	pigs
Tmbim6	-0.16	1.00000000	pigs
Npc1	0.14	1.00000000	pigs
Slc16a9	-0.57	1.00000000	pigs
Acsl1	0.01	1.00000000	pigs
Tpi1	0.35	1.00000000	pigs
Brcc3	-0.06	1.00000000	pigs
Faxc	-0.32	1.00000000	pigs
Fer	-0.06	1.00000000	pigs
Atg101	-0.01	1.00000000	pigs
Ndufs2	0.02	1.00000000	pigs
Cwc27	-0.22	1.00000000	pigs
Slc1a5	-0.06	1.00000000	pigs
Psmd4	0.01	1.00000000	pigs
Serinc3	-0.04	1.00000000	pigs
Vps26a	-0.24	1.00000000	pigs
Glrx3	0.09	1.00000000	pigs
Tpp1	0.09	1.00000000	pigs
R3hdm4	0.00	1.00000000	pigs
Akt2	-0.11	1.00000000	pigs
Phf5a	-0.10	1.00000000	pigs
Tipin	-0.23	1.00000000	pigs
Tmx3	0.00	1.00000000	pigs
Arxes2	-0.32	1.00000000	pigs
Adk	0.02	1.00000000	pigs
Nedd4	-0.48	1.00000000	pigs
BC048507	-0.18	1.00000000	pigs
Rnf214	-0.06	1.00000000	pigs
Nme1	-0.15	1.00000000	pigs
Cog3	-0.05	1.00000000	pigs
Tcfl5	-0.05	1.00000000	pigs
Card19	-0.11	1.00000000	pigs
Atp5d	-0.16	1.00000000	pigs
Zfyve21	0.30	1.00000000	pigs
Ap3b1	-0.28	1.00000000	pigs
Pole4	-0.33	1.00000000	pigs
Pcbp4	0.09	1.00000000	pigs
Tagap1	0.59	1.00000000	pigs
Cd69	-0.24	1.00000000	pigs
Tlnrd1	0.06	1.00000000	pigs
Lmo7	-0.36	1.00000000	pigs

Luc7l	0.02	1.00000000	pigs
Nxpe3	-0.16	1.00000000	pigs
Polr2c	-0.21	1.00000000	pigs
Kctd20	-0.24	1.00000000	pigs
Rock2	-0.10	1.00000000	pigs
Tctn3	-0.20	1.00000000	pigs
Dnajb6	0.14	1.00000000	pigs
Ireb2	-0.14	1.00000000	pigs
Actr6	-0.07	1.00000000	pigs
Pkn2	0.02	1.00000000	pigs
Rmnd5b	0.15	1.00000000	pigs
Dnajb4	-0.26	1.00000000	pigs
Pus7	-0.34	1.00000000	pigs
Wdr75	-0.05	1.00000000	pigs
Tor1aip1	0.03	1.00000000	pigs
Sorbs3	-0.20	1.00000000	pigs
Cept1	-0.03	1.00000000	pigs
Etf1	0.08	1.00000000	pigs
Zfp217	0.02	1.00000000	pigs
Sms	-0.03	1.00000000	pigs
Aagab	0.20	1.00000000	pigs
Zc3h7a	-0.04	1.00000000	pigs
Nemf	-0.46	1.00000000	pigs
Clptm1	-0.10	1.00000000	pigs
Vav3	0.04	1.00000000	pigs
Vsir	-0.04	1.00000000	pigs
H2ac20	0.01	1.00000000	pigs
Arid5b	-0.27	1.00000000	pigs
Abcd3	-0.06	1.00000000	pigs
Nob1	-0.09	1.00000000	pigs
Ammecr1l	0.03	1.00000000	pigs
Tec	-0.03	1.00000000	pigs
Cyb561a3	-0.07	1.00000000	pigs
Auh	-0.09	1.00000000	pigs
Shprh	-0.20	1.00000000	pigs
Prr5	-0.22	1.00000000	pigs
Acp5	-0.13	1.00000000	pigs
Eps15	0.10	1.00000000	pigs
Arl6ip1	0.11	1.00000000	pigs
Snap23	-0.02	1.00000000	pigs
Vapa	-0.12	1.00000000	pigs
Trip6	-0.24	1.00000000	pigs
Cux1	0.11	1.00000000	pigs
Atp6v1d	-0.01	1.00000000	pigs
Ghr	0.46	1.00000000	pigs
Nfatc2	-0.13	1.00000000	pigs

Alas1	-0.07	1.00000000	pigs
Tmem39b	-0.12	1.00000000	pigs
Gtpbp4	-0.27	1.00000000	pigs
Lrp5	0.04	1.00000000	pigs
Rab10	0.13	1.00000000	pigs
Polm	-0.04	1.00000000	pigs
Arhgap18	0.11	1.00000000	pigs
Surf4	-0.10	1.00000000	pigs
Pacs2	-0.05	1.00000000	pigs
Impa1	-0.29	1.00000000	pigs
Fam110d	-0.17	1.00000000	pigs
Psmd8	-0.15	1.00000000	pigs
Zfp524	-0.24	1.00000000	pigs
Ssb	-0.16	1.00000000	pigs
Cfp	-0.04	1.00000000	pigs
Iah1	-0.21	1.00000000	pigs
Lsg1	0.11	1.00000000	pigs
Ppp1r8	0.06	1.00000000	pigs
Tmem168	-0.21	1.00000000	pigs
Adss	0.00	1.00000000	pigs
Rai14	-0.04	1.00000000	pigs
Plekhf2	-0.03	1.00000000	pigs
Kansl2	-0.22	1.00000000	pigs
Ube3b	-0.13	1.00000000	pigs
Prkar1a	0.12	1.00000000	pigs
Twf2	0.17	1.00000000	pigs
Prkab2	-0.02	1.00000000	pigs
Stk25	0.09	1.00000000	pigs
Sdhd	0.10	1.00000000	pigs
Pabpc4	0.06	1.00000000	pigs
Crtap	0.09	1.00000000	pigs
Faim	-0.23	1.00000000	pigs
Junb	0.74	1.00000000	pigs
Nipal3	-0.07	1.00000000	pigs
Pus10	-0.06	1.00000000	pigs
Caprin1	0.18	1.00000000	pigs
Sae1	0.05	1.00000000	pigs
Bdp1	-0.28	1.00000000	pigs
Efl1	-0.41	1.00000000	pigs
Nbr1	0.00	1.00000000	pigs
Rpf1	-0.21	1.00000000	pigs
Ptrh2	0.13	1.00000000	pigs
Orc2	0.07	1.00000000	pigs
Bysl	0.06	1.00000000	pigs
Lmbr1l	-0.03	1.00000000	pigs
Cacybp	-0.23	1.00000000	pigs

Nop53	0.11	1.00000000	pigs
Osbpl9	-0.04	1.00000000	pigs
Spr	-0.18	1.00000000	pigs
Zdhhc18	0.18	1.00000000	pigs
Irf2	0.04	1.00000000	pigs
Clpb	0.04	1.00000000	pigs
Aspdh	-0.06	1.00000000	pigs
Zc3h6	-0.20	1.00000000	pigs
Ttc27	-0.05	1.00000000	pigs
Hspb11	-0.16	1.00000000	pigs
Rcor3	0.03	1.00000000	pigs
Pink1	-0.10	1.00000000	pigs
Nktr	0.03	1.00000000	pigs
Trpm6	-0.02	1.00000000	pigs
Itprid2	0.09	1.00000000	pigs
Immp2l	0.12	1.00000000	pigs
Ldlrad3	-0.08	1.00000000	pigs
Topaz1	-0.04	1.00000000	pigs
Angptl2	0.03	1.00000000	pigs
Upf3b	-0.14	1.00000000	pigs
Trmt11	0.05	1.00000000	pigs
Klhdc4	-0.02	1.00000000	pigs
Fmr1	0.07	1.00000000	pigs
Fam120a	-0.09	1.00000000	pigs
Las1l	-0.08	1.00000000	pigs
Klf11	-0.03	1.00000000	pigs
Dpp8	-0.03	1.00000000	pigs
Gtdc1	0.00	1.00000000	pigs
Unc50	-0.07	1.00000000	pigs
Hoxa5	-0.01	1.00000000	pigs
S100a11	0.24	1.00000000	pigs
Pex14	0.08	1.00000000	pigs
Ndufs3	-0.18	1.00000000	pigs
Tbrg1	-0.12	1.00000000	pigs
Ccdc28b	-0.27	1.00000000	pigs
Stk39	-0.06	1.00000000	pigs
Cul4b	-0.12	1.00000000	pigs
Tubg1	0.17	1.00000000	pigs
Fbxo9	-0.26	1.00000000	pigs
Dysf	-0.01	1.00000000	pigs
Emp3	0.22	1.00000000	pigs
Ccdc174	-0.27	1.00000000	pigs
Echdc1	0.28	1.00000000	pigs
Cct5	0.10	1.00000000	pigs
Desi1	0.07	1.00000000	pigs
Eef1g	-0.01	1.00000000	pigs

Ssbp1	-0.24	1.00000000	pigs
Ints9	-0.18	1.00000000	pigs
Bag1	-0.06	1.00000000	pigs
Styk1	-0.07	1.00000000	pigs
Ncln	-0.14	1.00000000	pigs
Cwc15	-0.26	1.00000000	pigs
Fam89b	-0.06	1.00000000	pigs
Pcgf5	-0.10	1.00000000	pigs
Pald1	-0.10	1.00000000	pigs
Tmem62	-0.07	1.00000000	pigs
Usp31	-0.09	1.00000000	pigs
Nedd1	-0.03	1.00000000	pigs
Xpo5	0.08	1.00000000	pigs
Trap1	-0.06	1.00000000	pigs
Zer1	-0.40	1.00000000	pigs
Ankrd39	0.35	1.00000000	pigs
Trmt10a	-0.11	1.00000000	pigs
Jmjd6	0.01	1.00000000	pigs
Edem1	-0.14	1.00000000	pigs
Gk5	-0.17	1.00000000	pigs
Bccip	-0.09	1.00000000	pigs
Elovl6	-0.04	1.00000000	pigs
Riok1	0.12	1.00000000	pigs
Slc12a4	-0.06	1.00000000	pigs
Sf3b1	0.14	1.00000000	pigs
Nit2	0.08	1.00000000	pigs
Rfx5	0.01	1.00000000	pigs
2310009B15Rik	-0.13	1.00000000	pigs
Cttnbp2nl	-0.04	1.00000000	pigs
Phkg2	-0.01	1.00000000	pigs
Cggbp1	-0.29	1.00000000	pigs
Uso1	-0.02	1.00000000	pigs
Lsmem1	0.08	1.00000000	pigs
Dhx40	0.05	1.00000000	pigs
Tmed3	-0.09	1.00000000	pigs
Polr3k	0.10	1.00000000	pigs
Actl6a	0.14	1.00000000	pigs
Agpat2	0.53	1.00000000	pigs
Rbm10	-0.05	1.00000000	pigs
Zdhhc5	-0.13	1.00000000	pigs
Fam149b	-0.21	1.00000000	pigs
Lrrc58	-0.15	1.00000000	pigs
Grcc10	-0.22	1.00000000	pigs
Fez2	-0.14	1.00000000	pigs
Jchain	-0.02	1.00000000	pigs
Golga4	0.10	1.00000000	pigs

Osmr	0.19	1.00000000	pigs
Rtl6	-0.01	1.00000000	pigs
Lrrc47	-0.21	1.00000000	pigs
Hsbp1	-0.15	1.00000000	pigs
Naxe	0.01	1.00000000	pigs
Wdr3	0.12	1.00000000	pigs
Alkbh7	-0.06	1.00000000	pigs
Nkap	-0.09	1.00000000	pigs
Copg2	0.02	1.00000000	pigs
Ufl1	-0.06	1.00000000	pigs
Pecr	0.03	1.00000000	pigs
Phf3	0.20	1.00000000	pigs
Rfx7	0.05	1.00000000	pigs
Btbd3	0.03	1.00000000	pigs
Anxa4	0.13	1.00000000	pigs
Trim47	0.06	1.00000000	pigs
Drap1	-0.25	1.00000000	pigs
Usf3	-0.20	1.00000000	pigs
Ctdnep1	-0.17	1.00000000	pigs
Uqcc3	-0.15	1.00000000	pigs
Actr10	-0.14	1.00000000	pigs
Tapt1	0.04	1.00000000	pigs
Ltbp3	-0.15	1.00000000	pigs
Fbxo34	-0.18	1.00000000	pigs
Rspry1	-0.09	1.00000000	pigs
Tead1	-0.04	1.00000000	pigs
Parp4	0.09	1.00000000	pigs
Tram2	-0.04	1.00000000	pigs
Sulf2	0.00	1.00000000	pigs
Pip5k1c	-0.07	1.00000000	pigs
Klhl20	-0.07	1.00000000	pigs
Zc3h7b	-0.38	1.00000000	pigs
Rab43	-0.04	1.00000000	pigs
Uqcrfs1	0.06	1.00000000	pigs
Lss	-0.24	1.00000000	pigs
Gpr155	-0.12	1.00000000	pigs
Dnmbp	-0.34	1.00000000	pigs
Carhsp1	-0.31	1.00000000	pigs
Acbd6	0.08	1.00000000	pigs
Usp53	0.05	1.00000000	pigs
Slf1	-0.14	1.00000000	pigs
Xiap	-0.19	1.00000000	pigs
Golt1b	0.16	1.00000000	pigs
Cep97	-0.17	1.00000000	pigs
Ptpn11	0.12	1.00000000	pigs
Hmbox1	-0.25	1.00000000	pigs

Zbtb7a	0.01	1.00000000	pigs
Golga3	-0.35	1.00000000	pigs
Kpna6	0.00	1.00000000	pigs
Pno1	-0.20	1.00000000	pigs
Dync1li2	-0.13	1.00000000	pigs
Atf4	-0.11	1.00000000	pigs
Fgfr1op2	-0.39	1.00000000	pigs
Cfap97	-0.25	1.00000000	pigs
Thoc1	0.02	1.00000000	pigs
Ccdc82	-0.02	1.00000000	pigs
Fibp	0.01	1.00000000	pigs
Vps54	0.01	1.00000000	pigs
Bcl9l	0.17	1.00000000	pigs
Cox20	-0.18	1.00000000	pigs
Pop7	0.16	1.00000000	pigs
Nfix	-0.41	1.00000000	pigs
Tasp1	0.01	1.00000000	pigs
Pfkp	-0.18	1.00000000	pigs
Hbb-bh2	-0.30	1.00000000	pigs
Trmt2a	-0.02	1.00000000	pigs
Ppp1r16b	0.09	1.00000000	pigs
Snapc5	-0.10	1.00000000	pigs
Auts2	-0.10	1.00000000	pigs
Srrm2	0.12	1.00000000	pigs
Sh3gl1	0.03	1.00000000	pigs
Caml	-0.21	1.00000000	pigs
Anxa5	0.02	1.00000000	pigs
Xpo6	-0.25	1.00000000	pigs
Slc44a2	-0.08	1.00000000	pigs
Prx	-0.05	1.00000000	pigs
Pgap1	0.08	1.00000000	pigs
Ublcp1	0.13	1.00000000	pigs
Polr3d	0.13	1.00000000	pigs
Rnf20	0.16	1.00000000	pigs
Pdlim3	-0.30	1.00000000	pigs
Avl9	-0.06	1.00000000	pigs
Cnot6	-0.14	1.00000000	pigs
Pnkp	0.14	1.00000000	pigs
Plcb3	-0.08	1.00000000	pigs
Nap1l1	0.15	1.00000000	pigs
Ddx39a	0.00	1.00000000	pigs
Gdap2	0.00	1.00000000	pigs
Kirrel	0.10	1.00000000	pigs
Trip4	-0.10	1.00000000	pigs
Gga2	0.02	1.00000000	pigs
Psma3	-0.09	1.00000000	pigs

Ptpdc1	0.00	1.00000000	pigs
Enpp3	-0.19	1.00000000	pigs
Dym	0.06	1.00000000	pigs
Rnf169	-0.14	1.00000000	pigs
Sirt5	-0.14	1.00000000	pigs
Smpd1	-0.04	1.00000000	pigs
Gsap	0.05	1.00000000	pigs
Tbrg4	-0.34	1.00000000	pigs
Paxip1	0.10	1.00000000	pigs
Ipo8	-0.12	1.00000000	pigs
Dnajc14	-0.12	1.00000000	pigs
P3h1	-0.01	1.00000000	pigs
Adpgk	-0.03	1.00000000	pigs
Armc10	0.17	1.00000000	pigs
Zbtb8os	-0.14	1.00000000	pigs
Ttc21b	-0.35	1.00000000	pigs
Acaa1b	-0.08	1.00000000	pigs
Ccp110	0.01	1.00000000	pigs
Wdr18	-0.05	1.00000000	pigs
Gnai2	0.13	1.00000000	pigs
Dync2h1	-0.15	1.00000000	pigs
Acox2	-0.16	1.00000000	pigs
Klhdc1	-0.11	1.00000000	pigs
Atf1	0.05	1.00000000	pigs
Ap3d1	0.12	1.00000000	pigs
Rfxank	-0.12	1.00000000	pigs
Cers2	-0.15	1.00000000	pigs
Nfx1	-0.17	1.00000000	pigs
Pdcd6	-0.29	1.00000000	pigs
Itgb4	-0.17	1.00000000	pigs
Psmd1	0.02	1.00000000	pigs
Sgf29	0.09	1.00000000	pigs
Psmc1	-0.03	1.00000000	pigs
Fem1b	-0.02	1.00000000	pigs
Triobp	-0.27	1.00000000	pigs
Gba2	-0.02	1.00000000	pigs
Tmco4	-0.07	1.00000000	pigs
Ss18	-0.09	1.00000000	pigs
Satb1	-0.36	1.00000000	pigs
Snrpd1	0.15	1.00000000	pigs
Tbc1d9	0.12	1.00000000	pigs
Asf1a	-0.03	1.00000000	pigs
Wdr20	-0.14	1.00000000	pigs
Dtx3l	-0.34	1.00000000	pigs
Hsd17b10	-0.25	1.00000000	pigs
Spg20	-0.03	1.00000000	pigs

Sh3glb1	-0.08	1.00000000	pigs
Lrrcc1	-0.15	1.00000000	pigs
Ctss	0.64	1.00000000	pigs
Oxr1	-0.34	1.00000000	pigs
Cdk7	-0.15	1.00000000	pigs
Scaper	-0.04	1.00000000	pigs
Rfx2	0.02	1.00000000	pigs
Eif2b1	0.07	1.00000000	pigs
Hectd4	0.14	1.00000000	pigs
Cog7	0.05	1.00000000	pigs
Cep89	0.15	1.00000000	pigs
Sec11a	-0.19	1.00000000	pigs
Arpc2	-0.07	1.00000000	pigs
Dock4	0.03	1.00000000	pigs
Cpeb2	0.07	1.00000000	pigs
Serpini1	-0.13	1.00000000	pigs
Atp5j	-0.02	1.00000000	pigs
Ciao1	0.14	1.00000000	pigs
Retreg3	-0.20	1.00000000	pigs
Trnt1	-0.07	1.00000000	pigs
Fam89a	0.15	1.00000000	pigs
Ring1	0.06	1.00000000	pigs
Flt1	-0.21	1.00000000	pigs
Sephs1	0.17	1.00000000	pigs
Camk2g	0.04	1.00000000	pigs
Nmnat2	-0.53	1.00000000	pigs
Fam222b	-0.15	1.00000000	pigs
Hrh1	-0.24	1.00000000	pigs
Leng8	-0.17	1.00000000	pigs
Ap4s1	-0.07	1.00000000	pigs
Zeb1	-0.17	1.00000000	pigs
Txndc16	-0.16	1.00000000	pigs
Apmap	0.06	1.00000000	pigs
Foxn2	0.13	1.00000000	pigs
Fbxo31	0.07	1.00000000	pigs
Rufy2	-0.28	1.00000000	pigs
Cops7a	0.10	1.00000000	pigs
Ikzf5	0.04	1.00000000	pigs
Arhgef11	-0.03	1.00000000	pigs
Tmem260	0.15	1.00000000	pigs
Lpcat4	-0.06	1.00000000	pigs
Srp9	0.02	1.00000000	pigs
4930453N24Rik	0.09	1.00000000	pigs
Tmem203	-0.23	1.00000000	pigs
Sun1	-0.15	1.00000000	pigs
Aamp	-0.03	1.00000000	pigs

Lsm14a	-0.09	1.00000000	pigs
Rfesd	-0.01	1.00000000	pigs
Inpp5b	-0.06	1.00000000	pigs
Ogfr	-0.15	1.00000000	pigs
Golim4	-0.21	1.00000000	pigs
Atpaf1	-0.29	1.00000000	pigs
Txnl4a	-0.04	1.00000000	pigs
Vcan	0.08	1.00000000	pigs
Dtwd1	0.05	1.00000000	pigs
Rc3h1	-0.28	1.00000000	pigs
Cask	0.10	1.00000000	pigs
Acads	0.01	1.00000000	pigs
Slc38a6	0.31	1.00000000	pigs
Tmem126a	-0.19	1.00000000	pigs
Fam174b	-0.12	1.00000000	pigs
Lclat1	-0.15	1.00000000	pigs
Ccdc12	-0.04	1.00000000	pigs
Coasy	-0.06	1.00000000	pigs
Dmac2l	-0.21	1.00000000	pigs
Slu7	-0.13	1.00000000	pigs
Hnrnph2	-0.09	1.00000000	pigs
Rprd1b	0.04	1.00000000	pigs
Ltn1	0.04	1.00000000	pigs
Pex7	-0.37	1.00000000	pigs
Glis3	0.16	1.00000000	pigs
Ago1	0.15	1.00000000	pigs
Ccdc124	-0.31	1.00000000	pigs
Nup54	0.00	1.00000000	pigs
Agap3	-0.01	1.00000000	pigs
Ccdc88c	0.09	1.00000000	pigs
Qsox1	-0.04	1.00000000	pigs
Cgas	0.13	1.00000000	pigs
Prrc1	-0.15	1.00000000	pigs
Uqcrc2	-0.20	1.00000000	pigs
Cog2	0.06	1.00000000	pigs
Reck	-0.07	1.00000000	pigs
Eif2b4	0.07	1.00000000	pigs
Idh3g	-0.08	1.00000000	pigs
Fbxl14	-0.03	1.00000000	pigs
Ccnc	0.04	1.00000000	pigs
Pparg	-0.38	1.00000000	pigs
Adamts3	-0.46	1.00000000	pigs
Tbc1d9b	-0.20	1.00000000	pigs
Wipi1	-0.23	1.00000000	pigs
Sec24b	-0.23	1.00000000	pigs
Tbc1d22a	-0.03	1.00000000	pigs

G6pdx	0.06	1.00000000	pigs
Arhgef3	0.34	1.00000000	pigs
Wls	-0.22	1.00000000	pigs
Taok3	-0.11	1.00000000	pigs
Prdx2	0.06	1.00000000	pigs
Aip	-0.25	1.00000000	pigs
Heatr5b	-0.38	1.00000000	pigs
Ssr4	-0.25	1.00000000	pigs
Ncoa7	0.20	1.00000000	pigs
Adgrl4	-0.73	1.00000000	pigs
Wdr33	-0.27	1.00000000	pigs
Sfrp5	-0.01	1.00000000	pigs
Aspscr1	0.06	1.00000000	pigs
Tmem204	-0.42	1.00000000	pigs
Btbd1	-0.24	1.00000000	pigs
Itgb3	-0.11	1.00000000	pigs
Prpf6	-0.29	1.00000000	pigs
Arpc4	-0.11	1.00000000	pigs
Lars	-0.21	1.00000000	pigs
Zbtb22	-0.18	1.00000000	pigs
Lcp1	-0.42	1.00000000	pigs
Dennd2a	-0.33	1.00000000	pigs
Tbc1d32	-0.16	1.00000000	pigs
Gusb	-0.08	1.00000000	pigs
Lrrc2	-0.02	1.00000000	pigs
Tpra1	0.06	1.00000000	pigs
Rnf103	-0.29	1.00000000	pigs
Naa60	0.11	1.00000000	pigs
Pinx1	-0.13	1.00000000	pigs
Chm	-0.25	1.00000000	pigs
Chmp5	0.03	1.00000000	pigs
Tmem38b	-0.14	1.00000000	pigs
Chrac1	-0.24	1.00000000	pigs
Calr	0.25	1.00000000	pigs
Tmem87b	-0.34	1.00000000	pigs
Pms1	-0.36	1.00000000	pigs
Srp19	-0.25	1.00000000	pigs
Thumpd1	-0.30	1.00000000	pigs
Dnajc5	-0.11	1.00000000	pigs
Brwd1	0.04	1.00000000	pigs
Cyp2r1	-0.18	1.00000000	pigs
P2rx7	-0.10	1.00000000	pigs
Pygm	-0.01	1.00000000	pigs
Snrpa1	-0.22	1.00000000	pigs
Rgmb	-0.02	1.00000000	pigs
Supt6	0.04	1.00000000	pigs

Ccnq	0.01	1.00000000	pigs
Bloc1s6	-0.03	1.00000000	pigs
Leo1	-0.34	1.00000000	pigs
Aven	-0.08	1.00000000	pigs
Nmt1	-0.42	1.00000000	pigs
Nebl	0.20	1.00000000	pigs
Crtc2	0.02	1.00000000	pigs
Gorab	-0.17	1.00000000	pigs
Rtcb	0.20	1.00000000	pigs
Tprgl	-0.31	1.00000000	pigs
Ezr	0.08	1.00000000	pigs
Ppid	0.27	1.00000000	pigs
Pex13	-0.02	1.00000000	pigs
Sap130	-0.02	1.00000000	pigs
Ddx6	-0.20	1.00000000	pigs
Rpap3	-0.10	1.00000000	pigs
Snrpb2	-0.35	1.00000000	pigs
Nop56	-0.15	1.00000000	pigs
Vars	-0.24	1.00000000	pigs
Polr1c	-0.09	1.00000000	pigs
Ddx55	-0.11	1.00000000	pigs
Atrx	-0.02	1.00000000	pigs
Slc35a5	-0.30	1.00000000	pigs
Dhrs1	0.12	1.00000000	pigs
Acp6	-0.07	1.00000000	pigs
Cand1	-0.14	1.00000000	pigs
Jarid2	-0.01	1.00000000	pigs
Yrdc	-0.03	1.00000000	pigs
AI597479	-0.06	1.00000000	pigs
Zfand1	-0.11	1.00000000	pigs
Erlin2	-0.09	1.00000000	pigs
Ldlr	-0.19	1.00000000	pigs
Col27a1	-0.25	1.00000000	pigs
Hypk	-0.18	1.00000000	pigs
Afdn	-0.07	1.00000000	pigs
Uqcrq	-0.28	1.00000000	pigs
Folr1	0.01	1.00000000	pigs
Lrrc3b	-0.48	1.00000000	pigs
Imp4	-0.05	1.00000000	pigs
P4hb	0.12	1.00000000	pigs
Kdm7a	-0.20	1.00000000	pigs
Derl2	-0.08	1.00000000	pigs
Tns2	-0.09	1.00000000	pigs
Arhgef12	-0.08	1.00000000	pigs
Tsr3	-0.16	1.00000000	pigs
Zbtb20	-0.06	1.00000000	pigs

Klhdc3	-0.02	1.00000000	pigs
Rabgap1l	-0.51	1.00000000	pigs
Rab31	-0.11	1.00000000	pigs
Abraxas2	-0.06	1.00000000	pigs
Tsnax	-0.17	1.00000000	pigs
Slc35a1	-0.17	1.00000000	pigs
Scnm1	-0.04	1.00000000	pigs
Sf3b3	0.01	1.00000000	pigs
Klhl5	-0.09	1.00000000	pigs
Btbd6	-0.07	1.00000000	pigs
Polr3f	-0.08	1.00000000	pigs
Cdk16	-0.02	1.00000000	pigs
Ctdspl	0.15	1.00000000	pigs
Bicra	0.10	1.00000000	pigs
Tfdp2	-0.06	1.00000000	pigs
Tshz3	0.03	1.00000000	pigs
Atf3	-0.27	1.00000000	pigs
Dpy19l4	-0.14	1.00000000	pigs
Abhd13	-0.03	1.00000000	pigs
Rabl3	-0.15	1.00000000	pigs
Ypel3	0.05	1.00000000	pigs
Fkbp14	-0.07	1.00000000	pigs
Kin	-0.29	1.00000000	pigs
Rabl6	0.03	1.00000000	pigs
Fubp1	0.06	1.00000000	pigs
Grb2	0.57	1.00000000	pigs
Tmem160	-0.14	1.00000000	pigs
Pdcl	-0.17	1.00000000	pigs
Tshz1	-0.08	1.00000000	pigs
Sumf1	-0.22	1.00000000	pigs
Eda	0.03	1.00000000	pigs
Tstd2	-0.11	1.00000000	pigs
Thumpd3	-0.03	1.00000000	pigs
Tmcc1	-0.31	1.00000000	pigs
Terf2	-0.11	1.00000000	pigs
Cnp	0.14	1.00000000	pigs
Btbd7	-0.25	1.00000000	pigs
Suz12	-0.14	1.00000000	pigs
Txndc15	0.15	1.00000000	pigs
Fhit	-0.30	1.00000000	pigs
Setbp1	0.07	1.00000000	pigs
Txnip	-0.06	1.00000000	pigs
Tprkb	0.13	1.00000000	pigs
Spcs1	-0.31	1.00000000	pigs
Tmed4	-0.07	1.00000000	pigs
Hrh4	-0.08	1.00000000	pigs

Papss1	-0.20	1.00000000	pigs
Wasl	-0.02	1.00000000	pigs
Atmin	0.03	1.00000000	pigs
Prpf8	0.09	1.00000000	pigs
Gpr107	-0.19	1.00000000	pigs
Fbxl5	-0.33	1.00000000	pigs
L3mbtl3	-0.19	1.00000000	pigs
Cenpc1	-0.07	1.00000000	pigs
Nisch	-0.13	1.00000000	pigs
Ndufa8	-0.13	1.00000000	pigs
Golga1	-0.29	1.00000000	pigs
Arfgap3	-0.10	1.00000000	pigs
Ppm1a	-0.16	1.00000000	pigs
Edf1	0.03	1.00000000	pigs
Zdhhc7	-0.03	1.00000000	pigs
Kifap3	-0.14	1.00000000	pigs
Impdh1	-0.10	1.00000000	pigs
Zfand2b	-0.22	1.00000000	pigs
Rcl1	0.02	1.00000000	pigs
Topbp1	-0.02	1.00000000	pigs
Dctn4	-0.05	1.00000000	pigs
Trmt6	-0.09	1.00000000	pigs
Tsen15	0.05	1.00000000	pigs
Usp45	-0.10	1.00000000	pigs
Terf2ip	-0.07	1.00000000	pigs
Vdac3	0.08	1.00000000	pigs
Slc25a39	-0.27	1.00000000	pigs
Rprd2	-0.20	1.00000000	pigs
Arpc3	0.09	1.00000000	pigs
Trak2	-0.13	1.00000000	pigs
Cpne3	-0.21	1.00000000	pigs
Gask1b	-0.24	1.00000000	pigs
Gulp1	-0.54	1.00000000	pigs
Utp11	0.13	1.00000000	pigs
Commd5	-0.13	1.00000000	pigs
Vps13c	-0.05	1.00000000	pigs
Fan1	-0.03	1.00000000	pigs
Bpnt1	0.03	1.00000000	pigs
E2f4	0.09	1.00000000	pigs
Uhrf1bp1l	0.06	1.00000000	pigs
Il18	-0.19	1.00000000	pigs
Klhl7	-0.19	1.00000000	pigs
Foxj2	-0.05	1.00000000	pigs
Ercc8	-0.29	1.00000000	pigs
Ptpre	-0.26	1.00000000	pigs
Tbl3	-0.18	1.00000000	pigs

Sgms2	0.02	1.00000000	pigs
Akr7a5	-0.01	1.00000000	pigs
Vrk2	-0.18	1.00000000	pigs
Cep135	-0.19	1.00000000	pigs
Zfp451	-0.08	1.00000000	pigs
Rcbtb1	-0.09	1.00000000	pigs
Nfkbid	-0.01	1.00000000	pigs
Trappc4	-0.12	1.00000000	pigs
Ift80	-0.18	1.00000000	pigs
Ddx52	-0.01	1.00000000	pigs
Trpc1	-0.01	1.00000000	pigs
Gpatch8	0.00	1.00000000	pigs
Cpne2	-0.03	1.00000000	pigs
Ints3	0.02	1.00000000	pigs
Hltf	-0.22	1.00000000	pigs
Rab12	0.16	1.00000000	pigs
Nans	-0.10	1.00000000	pigs
Tomm20	-0.06	1.00000000	pigs
Ptges3	-0.07	1.00000000	pigs
Insr	0.03	1.00000000	pigs
Zfp143	-0.14	1.00000000	pigs
Dipk1a	0.07	1.00000000	pigs
Ccnt1	-0.31	1.00000000	pigs
Zfp207	-0.02	1.00000000	pigs
Prickle1	-0.08	1.00000000	pigs
Nsmce2	0.05	1.00000000	pigs
Ndst3	-0.02	1.00000000	pigs
Uevld	-0.08	1.00000000	pigs
Srebf1	-0.16	1.00000000	pigs
Zfp518a	-0.09	1.00000000	pigs
Pik3r4	0.01	1.00000000	pigs
Pds5b	0.03	1.00000000	pigs
Creb3l2	-0.30	1.00000000	pigs
Pros1	-0.07	1.00000000	pigs
Prpf3	0.16	1.00000000	pigs
Snrnp25	-0.25	1.00000000	pigs
Ak6	-0.06	1.00000000	pigs
Smap1	0.05	1.00000000	pigs
Saraf	-0.18	1.00000000	pigs
Rars2	-0.06	1.00000000	pigs
Rnf139	-0.09	1.00000000	pigs
Tmem63b	-0.32	1.00000000	pigs
Ap1s2	-0.21	1.00000000	pigs
Stxbp4	-0.05	1.00000000	pigs
Sting1	-0.01	1.00000000	pigs
Pdha1	-0.05	1.00000000	pigs

Fam174a	-0.15	1.00000000	pigs
Fdx1	-0.21	1.00000000	pigs
Aimp1	-0.26	1.00000000	pigs
Pcm1	-0.09	1.00000000	pigs
Skp1	0.16	1.00000000	pigs
Traf6	-0.12	1.00000000	pigs
Phb	0.00	1.00000000	pigs
C2cd3	-0.02	1.00000000	pigs
Aurkb	-0.24	1.00000000	pigs
Cmtm3	0.03	1.00000000	pigs
Ythdc1	-0.07	1.00000000	pigs
Snrk	-0.12	1.00000000	pigs
Zfp292	-0.14	1.00000000	pigs
Dennd1b	0.12	1.00000000	pigs
Rbm8a2	-0.13	1.00000000	pigs
Cd200	-0.04	1.00000000	pigs
Stk40	-0.02	1.00000000	pigs
Pacsin2	0.12	1.00000000	pigs
Cpq	0.02	1.00000000	pigs
Nploc4	0.01	1.00000000	pigs
Ganab	-0.26	1.00000000	pigs
Cltc	0.10	1.00000000	pigs
Srgap1	-0.07	1.00000000	pigs
Dguok	-0.13	1.00000000	pigs
Pip4k2b	-0.08	1.00000000	pigs
Dctn6	-0.26	1.00000000	pigs
Tln2	-0.33	1.00000000	pigs
Tbl2	0.21	1.00000000	pigs
Dock9	0.21	1.00000000	pigs
Srsf3	0.40	1.00000000	pigs
Pibf1	-0.24	1.00000000	pigs
Cdk2ap2	-0.04	1.00000000	pigs
Stard8	-0.14	1.00000000	pigs
Eml4	-0.02	1.00000000	pigs
C2cd5	-0.02	1.00000000	pigs
Llph	-0.18	1.00000000	pigs
Uspl1	-0.12	1.00000000	pigs
Pbxip1	0.06	1.00000000	pigs
Crcp	0.09	1.00000000	pigs
Ccdc97	-0.08	1.00000000	pigs
Creb3	0.06	1.00000000	pigs
Fam199x	0.01	1.00000000	pigs
Otulinl	-0.14	1.00000000	pigs
Kitl	-0.49	1.00000000	pigs
Spon2	-0.38	1.00000000	pigs
Znhit3	-0.02	1.00000000	pigs

Lsm2	-0.11	1.00000000	pigs
Commd10	-0.07	1.00000000	pigs
Uba6	-0.01	1.00000000	pigs
Tbc1d23	-0.07	1.00000000	pigs
Ddx39b	0.09	1.00000000	pigs
2210408I21Rik	-0.10	1.00000000	pigs
Pes1	-0.09	1.00000000	pigs
1110004F10Rik	0.04	1.00000000	pigs
Cmbl	-0.22	1.00000000	pigs
Sytl4	0.01	1.00000000	pigs
Bin3	-0.01	1.00000000	pigs
Zfp366	-0.13	1.00000000	pigs
Klhdc10	-0.06	1.00000000	pigs
Nmd3	-0.11	1.00000000	pigs
Htatip2	0.02	1.00000000	pigs
Sgce	-0.10	1.00000000	pigs
Tubgcp3	-0.06	1.00000000	pigs
Psmc6	0.00	1.00000000	pigs
Rif1	-0.53	1.00000000	pigs
Ythdf3	-0.21	1.00000000	pigs
Wapl	-0.06	1.00000000	pigs
Rac1	0.03	1.00000000	pigs
Slc39a6	-0.18	1.00000000	pigs
Cabin1	-0.05	1.00000000	pigs
Txnl1	-0.25	1.00000000	pigs
Cxcl3	-0.18	1.00000000	pigs
Fbxo30	-0.08	1.00000000	pigs
Traf7	0.07	1.00000000	pigs
Qsox2	-0.19	1.00000000	pigs
Pafah1b2	-0.22	1.00000000	pigs
Vps53	-0.19	1.00000000	pigs
Sh2d3c	-0.10	1.00000000	pigs
Jdp2	0.26	1.00000000	pigs
Snx33	-0.07	1.00000000	pigs
Klf7	-0.02	1.00000000	pigs
Nudcd2	0.08	1.00000000	pigs
Rbm25	-0.07	1.00000000	pigs
Sds	-0.35	1.00000000	pigs
Ttc33	-0.05	1.00000000	pigs
Katna1	-0.06	1.00000000	pigs
Fam3c	-0.16	1.00000000	pigs
Rabep1	-0.06	1.00000000	pigs
Sema4d	-0.07	1.00000000	pigs
Tsn	-0.19	1.00000000	pigs
Flot1	-0.10	1.00000000	pigs
Strn3	0.06	1.00000000	pigs

Psmb1	0.09	1.00000000	pigs
Zbtb44	0.05	1.00000000	pigs
Exosc8	-0.11	1.00000000	pigs
Abhd15	-0.22	1.00000000	pigs
Net1	-0.35	1.00000000	pigs
Dut	-0.15	1.00000000	pigs
Scn1b	-0.17	1.00000000	pigs
Arhgap1	-0.05	1.00000000	pigs
B4galt2	0.04	1.00000000	pigs
Fam110b	0.03	1.00000000	pigs
Psmd5	0.03	1.00000000	pigs
Trmt1l	-0.01	1.00000000	pigs
Hace1	0.00	1.00000000	pigs
Crot	-0.25	1.00000000	pigs
Rhbdf1	-0.06	1.00000000	pigs
Spry1	-0.03	1.00000000	pigs
Srek1ip1	-0.32	1.00000000	pigs
Slain2	-0.16	1.00000000	pigs
Ankrd13a	-0.05	1.00000000	pigs
Gramd1b	-0.06	1.00000000	pigs
Bcl6b	-0.23	1.00000000	pigs
Zfp12	-0.06	1.00000000	pigs
Zadh2	-0.22	1.00000000	pigs
S100a8	-0.56	1.00000000	pigs
Aplp2	0.10	1.00000000	pigs
Zfp36	-0.10	1.00000000	pigs
Dennd2c	0.01	1.00000000	pigs
Utp23	-0.31	1.00000000	pigs
Poldip3	-0.12	1.00000000	pigs
Ist1	-0.14	1.00000000	pigs
Golgb1	-0.18	1.00000000	pigs
Sh3glb2	-0.09	1.00000000	pigs
U2surp	-0.02	1.00000000	pigs
Ppp1r15b	0.06	1.00000000	pigs
Dctpp1	0.36	1.00000000	pigs
Xrn2	-0.15	1.00000000	pigs
Ripor1	-0.20	1.00000000	pigs
Bbs7	-0.05	1.00000000	pigs
Tfb1m	-0.20	1.00000000	pigs
Hfm1	-0.18	1.00000000	pigs
Prdx6	-0.15	1.00000000	pigs
Hmox2	-0.02	1.00000000	pigs
Nfu1	0.07	1.00000000	pigs
Umps	-0.18	1.00000000	pigs
4933400A11Rik	0.00	1.00000000	pigs
Alg5	-0.21	1.00000000	pigs

Sde2	0.01	1.00000000	pigs
Jak1	0.16	1.00000000	pigs
Slc39a13	-0.06	1.00000000	pigs
Csf3r	-0.01	1.00000000	pigs
Upf1	-0.11	1.00000000	pigs
Vps29	-0.14	1.00000000	pigs
Pld1	0.08	1.00000000	pigs
Khdrbs1	0.02	1.00000000	pigs
Uhrf2	-0.02	1.00000000	pigs
Snap29	-0.27	1.00000000	pigs
Echdc2	-0.14	1.00000000	pigs
Tsga10	-0.22	1.00000000	pigs
Stub1	-0.01	1.00000000	pigs
Gtf3c3	-0.20	1.00000000	pigs
Ptger2	-0.28	1.00000000	pigs
Vti1a	-0.02	1.00000000	pigs
Uxt	0.03	1.00000000	pigs
Rnf125	-0.09	1.00000000	pigs
Etfrf1	0.04	1.00000000	pigs
Dock6	-0.33	1.00000000	pigs
Tgs1	-0.09	1.00000000	pigs
Adcy4	-0.15	1.00000000	pigs
Pdgfb	-0.26	1.00000000	pigs
Arhgef26	0.31	1.00000000	pigs
Nek9	-0.16	1.00000000	pigs
Siae	-0.10	1.00000000	pigs
Zcchc17	0.08	1.00000000	pigs
Naa16	-0.20	1.00000000	pigs
Josd1	-0.06	1.00000000	pigs
Psmc2	-0.06	1.00000000	pigs
Epb41l2	0.11	1.00000000	pigs
Ephb4	-0.06	1.00000000	pigs
Rell1	0.20	1.00000000	pigs
Scyl3	0.07	1.00000000	pigs
Psmb3	-0.22	1.00000000	pigs
Rtraf	0.06	1.00000000	pigs
Rnf14	-0.23	1.00000000	pigs
Ciao2b	-0.20	1.00000000	pigs
Zfp146	-0.24	1.00000000	pigs
Heatr1	-0.13	1.00000000	pigs
Far1	-0.22	1.00000000	pigs
Zmym6	-0.12	1.00000000	pigs
Get3	-0.16	1.00000000	pigs
Lmbrd1	-0.01	1.00000000	pigs
Rc3h2	-0.35	1.00000000	pigs
Ldah	0.08	1.00000000	pigs

Ppp1r14bl	0.01	1.00000000	pigs
Taf15	-0.23	1.00000000	pigs
Exosc9	0.00	1.00000000	pigs
Apbb3	-0.02	1.00000000	pigs
Epb41	-0.43	1.00000000	pigs
Hadhb	-0.07	1.00000000	pigs
Anapc16	-0.05	1.00000000	pigs
Aga	0.08	1.00000000	pigs
Ndufa9	-0.11	1.00000000	pigs
Gpn3	-0.18	1.00000000	pigs
Uprt	0.01	1.00000000	pigs
Letm1	-0.22	1.00000000	pigs
Fndc3a	0.10	1.00000000	pigs
Zfp28	-0.13	1.00000000	pigs
Clk3	-0.14	1.00000000	pigs
Rad23b	-0.03	1.00000000	pigs
Aimp2	-0.07	1.00000000	pigs
Nxf1	0.01	1.00000000	pigs
Smim19	-0.27	1.00000000	pigs
Dach1	-0.58	1.00000000	pigs
Naa30	-0.09	1.00000000	pigs
Celsr1	0.23	1.00000000	pigs
Pin1	0.04	1.00000000	pigs
Ttc28	0.12	1.00000000	pigs
Cnst	-0.23	1.00000000	pigs
Spats2	0.11	1.00000000	pigs
Epb41l5	0.00	1.00000000	pigs
Naf1	-0.13	1.00000000	pigs
Hsf1	0.42	1.00000000	pigs
Polr2d	-0.29	1.00000000	pigs
Dnajb12	0.01	1.00000000	pigs
Cspp1	-0.06	1.00000000	pigs
Stau2	0.01	1.00000000	pigs
Ttc13	-0.25	1.00000000	pigs
Rheb	0.01	1.00000000	pigs
Plaur	-0.13	1.00000000	pigs
Rasgrp3	-0.33	1.00000000	pigs
Setd5	-0.08	1.00000000	pigs
Sult1a1	-0.09	1.00000000	pigs
Bmpr2	0.08	1.00000000	pigs
Ctdp1	0.09	1.00000000	pigs
Usp30	0.01	1.00000000	pigs
Qser1	-0.10	1.00000000	pigs
Plxnd1	0.00	1.00000000	pigs
Rnf111	-0.04	1.00000000	pigs
Tmem254a	-0.26	1.00000000	pigs

Nbn	-0.12	1.00000000	pigs
Pdia5	-0.19	1.00000000	pigs
Aifm1	-0.09	1.00000000	pigs
Arpc5	0.04	1.00000000	pigs
Dcaf12	-0.22	1.00000000	pigs
Uchl3	-0.18	1.00000000	pigs
Rnf150	-0.03	1.00000000	pigs
Snrnp70	0.00	1.00000000	pigs
Hmg20b	0.08	1.00000000	pigs
Zfp260	-0.03	1.00000000	pigs
Nrep	-0.10	1.00000000	pigs
Abhd2	-0.38	1.00000000	pigs
Emg1	0.10	1.00000000	pigs
Vezf1	-0.20	1.00000000	pigs
Srpr	-0.18	1.00000000	pigs
Flad1	-0.01	1.00000000	pigs
Rnf220	-0.47	1.00000000	pigs
Arl1	-0.22	1.00000000	pigs
Trip11	-0.18	1.00000000	pigs
Hspa4	-0.24	1.00000000	pigs
Fstl1	-0.06	1.00000000	pigs
Thap7	-0.14	1.00000000	pigs
Xab2	-0.12	1.00000000	pigs
Uba1	-0.08	1.00000000	pigs
Ddx59	0.02	1.00000000	pigs
Plek	-0.04	1.00000000	pigs
Eif4b	0.19	1.00000000	pigs
Sar1b	-0.11	1.00000000	pigs
Uhmk1	-0.29	1.00000000	pigs
Trappc6b	0.10	1.00000000	pigs
Card10	0.02	1.00000000	pigs
Rbm14	-0.09	1.00000000	pigs
Dync1h1	-0.08	1.00000000	pigs
Lmnb1	-0.07	1.00000000	pigs
Dusp19	0.02	1.00000000	pigs
Rac3	-0.05	1.00000000	pigs
G3bp1	0.01	1.00000000	pigs
Snrnp200	-0.03	1.00000000	pigs
Abcb7	-0.06	1.00000000	pigs
Eif3e	0.08	1.00000000	pigs
R3hdm2	-0.05	1.00000000	pigs
Ranbp6	-0.07	1.00000000	pigs
Orai1	-0.01	1.00000000	pigs
Tmem147	-0.15	1.00000000	pigs
Krt10	-0.27	1.00000000	pigs
Tm2d1	-0.25	1.00000000	pigs

Npdc1	-0.22	1.00000000	pigs
Tmed7	-0.21	1.00000000	pigs
Ndufv2	-0.22	1.00000000	pigs
Nfyb	-0.02	1.00000000	pigs
Hpf1	0.06	1.00000000	pigs
Efna5	-0.13	1.00000000	pigs
R3hdm1	0.07	1.00000000	pigs
Ptar1	-0.11	1.00000000	pigs
Pkib	-0.24	1.00000000	pigs
Pnpt1	-0.18	1.00000000	pigs
Larp1	0.08	1.00000000	pigs
Npr3	0.09	1.00000000	pigs
Rfx3	-0.25	1.00000000	pigs
Xrcc6	0.07	1.00000000	pigs
L2hgdh	-0.18	1.00000000	pigs
Chchd4	0.03	1.00000000	pigs
Arl6ip5	-0.09	1.00000000	pigs
Zfp395	-0.08	1.00000000	pigs
Inpp4b	-0.26	1.00000000	pigs
Scap	-0.17	1.00000000	pigs
Chmp6	-0.14	1.00000000	pigs
Cfl1	0.12	1.00000000	pigs
Sorl1	-0.45	1.00000000	pigs
Lsm6	0.23	1.00000000	pigs
Jade1	-0.16	1.00000000	pigs
Ube2e3	-0.25	1.00000000	pigs
Emc7	-0.06	1.00000000	pigs
Niban1	0.06	1.00000000	pigs
Pus1	0.02	1.00000000	pigs
Socs4	-0.10	1.00000000	pigs
Nnt	-0.03	1.00000000	pigs
Pak2	-0.09	1.00000000	pigs
Utrn	-0.05	1.00000000	pigs
LTO1	0.11	1.00000000	pigs
Adam10	-0.04	1.00000000	pigs
Emc2	-0.05	1.00000000	pigs
Sesn3	-0.36	1.00000000	pigs
Frmd6	-0.17	1.00000000	pigs
Rtn1	-0.09	1.00000000	pigs
Kif3b	-0.13	1.00000000	pigs
Ccdc173	-0.01	1.00000000	pigs
Trp53inp1	0.16	1.00000000	pigs
Robo1	-0.08	1.00000000	pigs
Nras	-0.02	1.00000000	pigs
Cdh23	-0.06	1.00000000	pigs
Btbd9	-0.16	1.00000000	pigs

Txndc17	-0.12	1.00000000	pigs
Ccdc167	-0.11	1.00000000	pigs
Nup133	-0.22	1.00000000	pigs
Cmas	-0.09	1.00000000	pigs
Vps35l	-0.17	1.00000000	pigs
Tnks2	0.02	1.00000000	pigs
Txndc9	-0.07	1.00000000	pigs
Smc1a	-0.09	1.00000000	pigs
Wasf2	-0.19	1.00000000	pigs
Anapc13	0.11	1.00000000	pigs
Pelp1	-0.07	1.00000000	pigs
Paf1	-0.16	1.00000000	pigs
Pafah1b3	-0.02	1.00000000	pigs
Rnf126	0.00	1.00000000	pigs
Ethe1	-0.18	1.00000000	pigs
Gcn1	-0.13	1.00000000	pigs
Prxl2c	-0.09	1.00000000	pigs
1700037H04Rik	0.03	1.00000000	pigs
Pcca	-0.13	1.00000000	pigs
Oxa1l	-0.28	1.00000000	pigs
Tpbg	-0.11	1.00000000	pigs
Adgra3	-0.40	1.00000000	pigs
Uri1	-0.10	1.00000000	pigs
Zpr1	0.06	1.00000000	pigs
Trappc13	-0.06	1.00000000	pigs
Vps50	-0.03	1.00000000	pigs
Gns	0.12	1.00000000	pigs
Itga6	-0.21	1.00000000	pigs
Srsf1	-0.13	1.00000000	pigs
Sestd1	-0.06	1.00000000	pigs
Snrpn	-0.17	1.00000000	pigs
Prkdc	-0.02	1.00000000	pigs
Khnyn	0.02	1.00000000	pigs
Siva1	-0.03	1.00000000	pigs
Shoc2	-0.02	1.00000000	pigs
Nbas	-0.02	1.00000000	pigs
Arl3	-0.07	1.00000000	pigs
Pcgf3	-0.05	1.00000000	pigs
Acaa2	-0.02	1.00000000	pigs
Ctcf	-0.06	1.00000000	pigs
Kdelr3	0.04	1.00000000	pigs
Herpud2	0.11	1.00000000	pigs
Wdr13	-0.11	1.00000000	pigs
Fbl	-0.07	1.00000000	pigs
Pwwp3a	-0.08	1.00000000	pigs
Lrrc66	0.02	1.00000000	pigs

Pard3b	-0.28	1.00000000	pigs
Utp18	-0.10	1.00000000	pigs
Numb	-0.05	1.00000000	pigs
Aopep	-0.01	1.00000000	pigs
Npat	-0.25	1.00000000	pigs
Tma16	-0.17	1.00000000	pigs
Zcchc14	0.01	1.00000000	pigs
Ino80c	-0.10	1.00000000	pigs
Pde1c	-0.05	1.00000000	pigs
Cd81	0.11	1.00000000	pigs
Arl13b	-0.25	1.00000000	pigs
Dynlt3	-0.15	1.00000000	pigs
Rest	-0.10	1.00000000	pigs
Ccng2	-0.20	1.00000000	pigs
Alg6	-0.29	1.00000000	pigs
Wdr45	-0.06	1.00000000	pigs
B3gat3	-0.25	1.00000000	pigs
Itm2c	-0.09	1.00000000	pigs
Secisbp2l	-0.28	1.00000000	pigs
Armc8	-0.03	1.00000000	pigs
Pkig	-0.04	1.00000000	pigs
Syap1	0.03	1.00000000	pigs
Tdg	0.20	1.00000000	pigs
Rfk	-0.24	1.00000000	pigs
Capn5	-0.14	1.00000000	pigs
Dnlz	-0.16	1.00000000	pigs
Sh3bgrl2	0.03	1.00000000	pigs
Sergef	-0.13	1.00000000	pigs
Antxr1	-0.13	1.00000000	pigs
Fto	0.00	1.00000000	pigs
Selenoi	-0.09	1.00000000	pigs
Suclg1	-0.03	1.00000000	pigs
Srebf2	-0.16	1.00000000	pigs
Cd3eap	0.04	1.00000000	pigs
Stk19	-0.19	1.00000000	pigs
Clec14a	-0.03	1.00000000	pigs
Pawr	-0.05	1.00000000	pigs
Dcxr	0.03	1.00000000	pigs
Slc38a4	-0.14	1.00000000	pigs
Dag1	-0.13	1.00000000	pigs
Lyn	-0.12	1.00000000	pigs
Rbbp5	-0.16	1.00000000	pigs
Ccar1	-0.21	1.00000000	pigs
Sod1	-0.03	1.00000000	pigs
Tek	-0.14	1.00000000	pigs
Vps25	-0.06	1.00000000	pigs

Tmem242	-0.12	1.00000000	pigs
Dpp4	-0.12	1.00000000	pigs
Tmem214	-0.07	1.00000000	pigs
Ift57	0.08	1.00000000	pigs
Vat1	-0.46	1.00000000	pigs
Cep63	-0.16	1.00000000	pigs
Ppp1r10	0.58	1.00000000	pigs
Arid1a	0.02	1.00000000	pigs
Cat	-0.22	1.00000000	pigs
Tnik	0.07	1.00000000	pigs
Tgfb1i1	-0.11	1.00000000	pigs
Rtn4r	-0.03	1.00000000	pigs
Vash1	-0.08	1.00000000	pigs
Bad	0.04	1.00000000	pigs
Vps37a	-0.13	1.00000000	pigs
Fgfr1	0.00	1.00000000	pigs
Eif3h	-0.12	1.00000000	pigs
Syngr1	-0.32	1.00000000	pigs
Prepl	-0.05	1.00000000	pigs
Lrpprc	-0.19	1.00000000	pigs
Bap1	-0.20	1.00000000	pigs
Gnai1	-0.11	1.00000000	pigs
Nkapd1	-0.16	1.00000000	pigs
Sun2	-0.06	1.00000000	pigs
Cpt1a	-0.02	1.00000000	pigs
Ak5	-0.21	1.00000000	pigs
Tmed9	-0.13	1.00000000	pigs
Otud7b	-0.31	1.00000000	pigs
Stk16	-0.02	1.00000000	pigs
Rapgef2	0.10	1.00000000	pigs
Sgk1	0.15	1.00000000	pigs
Cnot10	-0.12	1.00000000	pigs
Tmem208	-0.23	1.00000000	pigs
Abhd4	0.26	1.00000000	pigs
Zfp516	-0.08	1.00000000	pigs
Robo4	-0.12	1.00000000	pigs
Oaz2	-0.21	1.00000000	pigs
Otulin	-0.15	1.00000000	pigs
Ddhd2	0.05	1.00000000	pigs
Ccdc59	-0.23	1.00000000	pigs
Lgals8	-0.09	1.00000000	pigs
Chst9	-0.42	1.00000000	pigs
Ppp1r35	0.00	1.00000000	pigs
Cdk13	-0.12	1.00000000	pigs
Shank3	-0.28	1.00000000	pigs
AW554918	-0.05	1.00000000	pigs

Tle5	-0.19	1.00000000	pigs
Adm	-0.10	1.00000000	pigs
Supt3	-0.22	1.00000000	pigs
Nab1	-0.17	1.00000000	pigs
Gnb2	-0.02	1.00000000	pigs
Pkia	-0.10	1.00000000	pigs
Nars	-0.15	1.00000000	pigs
Camkmt	-0.02	1.00000000	pigs
Ppp1r12c	-0.01	1.00000000	pigs
Cisd2	-0.25	1.00000000	pigs
Ube2q1	-0.02	1.00000000	pigs
Edrf1	-0.06	1.00000000	pigs
Cep104	0.03	1.00000000	pigs
Iars	-0.36	1.00000000	pigs
Urgcp	-0.15	1.00000000	pigs
Vps72	-0.31	1.00000000	pigs
Rrp1b	-0.12	1.00000000	pigs
Golm2	0.02	1.00000000	pigs
Ip6k1	-0.03	1.00000000	pigs
Phf23	-0.07	1.00000000	pigs
Vti1b	0.26	1.00000000	pigs
Agtpbp1	-0.18	1.00000000	pigs
Smad5	-0.14	1.00000000	pigs
Tbc1d17	0.07	1.00000000	pigs
Nmt2	-0.38	1.00000000	pigs
Eaf1	-0.02	1.00000000	pigs
Srm	0.14	1.00000000	pigs
Trappc5	-0.21	1.00000000	pigs
Eif4ebp1	-0.06	1.00000000	pigs
Slc22a17	-0.13	1.00000000	pigs
Sema6b	-0.08	1.00000000	pigs
Nr1h2	-0.08	1.00000000	pigs
Rsrc1	0.12	1.00000000	pigs
Ddx18	-0.29	1.00000000	pigs
Dph6	-0.06	1.00000000	pigs
Creld2	0.05	1.00000000	pigs
Btrc	-0.30	1.00000000	pigs
Sfswap	-0.12	1.00000000	pigs
Uxs1	-0.23	1.00000000	pigs
Spata6	-0.09	1.00000000	pigs
Prmt7	-0.02	1.00000000	pigs
Tom1	0.06	1.00000000	pigs
Lyar	-0.25	1.00000000	pigs
Sharpin	0.06	1.00000000	pigs
Pim1	-0.06	1.00000000	pigs
Gli3	-0.03	1.00000000	pigs

Fam234b	-0.16	1.00000000	pigs
Ankrd6	-0.05	1.00000000	pigs
Srpk1	-0.07	1.00000000	pigs
Tor1a	-0.03	1.00000000	pigs
Exoc1	0.00	1.00000000	pigs
Utp3	-0.06	1.00000000	pigs
Spcs2	-0.13	1.00000000	pigs
Ahi1	0.28	1.00000000	pigs
Acaca	-0.37	1.00000000	pigs
Acadsb	0.07	1.00000000	pigs
Rxra	-0.04	1.00000000	pigs
Ppp3ca	-0.15	1.00000000	pigs
Fam114a2	-0.25	1.00000000	pigs
Dync2i2	-0.08	1.00000000	pigs
Ankrd46	0.00	1.00000000	pigs
Slc15a4	-0.03	1.00000000	pigs
Akr1a1	-0.03	1.00000000	pigs
Elp5	-0.03	1.00000000	pigs
Scaf8	-0.09	1.00000000	pigs
Slc5a10	-0.06	1.00000000	pigs
Dnajc18	-0.21	1.00000000	pigs
Zfp879	0.10	1.00000000	pigs
Ikbkb	-0.14	1.00000000	pigs
Stoml2	-0.05	1.00000000	pigs
Bag4	-0.02	1.00000000	pigs
Osbpl10	-0.34	1.00000000	pigs
1810030O07Rik	-0.07	1.00000000	pigs
Dph5	0.01	1.00000000	pigs
Nfkbil1	-0.21	1.00000000	pigs
Nmi	-0.07	1.00000000	pigs
Rfc4	-0.15	1.00000000	pigs
Smim14	0.21	1.00000000	pigs
Crlf3	0.06	1.00000000	pigs
Col4a6	-0.16	1.00000000	pigs
Klhl36	0.04	1.00000000	pigs
Cep126	-0.20	1.00000000	pigs
Cuta	-0.15	1.00000000	pigs
Ccnl2	0.00	1.00000000	pigs
Ube2j1	0.02	1.00000000	pigs
Tmem129	0.13	1.00000000	pigs
Hdac5	-0.11	1.00000000	pigs
Plscr1	-0.02	1.00000000	pigs
Bcap31	-0.12	1.00000000	pigs
Eif4enif1	-0.20	1.00000000	pigs
Glg1	-0.16	1.00000000	pigs
Dhx15	-0.06	1.00000000	pigs

Dnajc15	-0.19	1.00000000	pigs
Tor4a	-0.13	1.00000000	pigs
Otud5	0.13	1.00000000	pigs
Smim15	-0.12	1.00000000	pigs
Ablim1	0.21	1.00000000	pigs
Raly	0.02	1.00000000	pigs
Lin37	0.28	1.00000000	pigs
Kifbp	-0.28	1.00000000	pigs
Pcsk6	0.01	1.00000000	pigs
Rrm1	-0.17	1.00000000	pigs
Wtip	-0.12	1.00000000	pigs
Copz2	0.02	1.00000000	pigs
Ap2s1	0.18	1.00000000	pigs
Atf7ip	0.06	1.00000000	pigs
Ankrd13c	-0.16	1.00000000	pigs
Scamp1	-0.08	1.00000000	pigs
Fsd1l	-0.11	1.00000000	pigs
Cox5a	-0.11	1.00000000	pigs
Uck2	-0.10	1.00000000	pigs
Tpst1	-0.04	1.00000000	pigs
Cdca4	-0.08	1.00000000	pigs
Comtd1	-0.28	1.00000000	pigs
Timm22	-0.28	1.00000000	pigs
Faf1	0.00	1.00000000	pigs
Ap2m1	-0.06	1.00000000	pigs
Nubp2	-0.20	1.00000000	pigs
Tars	-0.19	1.00000000	pigs
Rab27a	-0.08	1.00000000	pigs
Anapc5	-0.17	1.00000000	pigs
Nsrp1	-0.27	1.00000000	pigs
Rab18	-0.08	1.00000000	pigs
Cdk12	0.00	1.00000000	pigs
Pacc1	-0.32	1.00000000	pigs
Tbc1d31	-0.10	1.00000000	pigs
Lysmd2	-0.18	1.00000000	pigs
Atg12	0.04	1.00000000	pigs
Bcap29	-0.12	1.00000000	pigs
Plekhj1	0.28	1.00000000	pigs
Slc10a3	-0.10	1.00000000	pigs
Pcmtd2	0.08	1.00000000	pigs
Khsrp	-0.21	1.00000000	pigs
Enpp4	0.09	1.00000000	pigs
Pex16	-0.13	1.00000000	pigs
Cerk	-0.09	1.00000000	pigs
Tomm40	-0.06	1.00000000	pigs
Ntn4	-0.16	1.00000000	pigs

Higd2a	-0.09	1.00000000	pigs
Ctbp1	-0.30	1.00000000	pigs
Gkap1	0.00	1.00000000	pigs
Sri	0.23	1.00000000	pigs
Ppard	-0.18	1.00000000	pigs
Spring1	0.06	1.00000000	pigs
Chfr	-0.23	1.00000000	pigs
Cflar	-0.01	1.00000000	pigs
Calm3	-0.05	1.00000000	pigs
Atp5a1	0.03	1.00000000	pigs
Hnrnpdl	0.00	1.00000000	pigs
Cnrip1	0.09	1.00000000	pigs
Tlk1	0.03	1.00000000	pigs
Sdad1	-0.21	1.00000000	pigs
Bbx	0.00	1.00000000	pigs
Ddx17	0.07	1.00000000	pigs
Crk	0.03	1.00000000	pigs
Trerf1	-0.10	1.00000000	pigs
Tgfbrap1	-0.13	1.00000000	pigs
Acadvl	-0.08	1.00000000	pigs
Hyi	-0.09	1.00000000	pigs
Rnf44	-0.05	1.00000000	pigs
Ap2b1	-0.26	1.00000000	pigs
Tmem115	-0.11	1.00000000	pigs
Prpf40a	0.07	1.00000000	pigs
Scoc	-0.05	1.00000000	pigs
Gfod1	-0.17	1.00000000	pigs
Tmem59	0.09	1.00000000	pigs
Grk6	-0.04	1.00000000	pigs
Ftsj1	-0.08	1.00000000	pigs
Ankfy1	0.00	1.00000000	pigs
Clns1a	-0.20	1.00000000	pigs
Phf14	-0.33	1.00000000	pigs
Rfc1	-0.04	1.00000000	pigs
Gstk1	-0.08	1.00000000	pigs
Lnpep	0.02	1.00000000	pigs
Calhm2	-0.07	1.00000000	pigs
Brd8	-0.09	1.00000000	pigs
Notch2	-1.00	1.00000000	pigs
Strn4	-0.07	1.00000000	pigs
Rnf4	-0.19	1.00000000	pigs
Capzb	-0.05	1.00000000	pigs
Nadk2	-0.11	1.00000000	pigs
Cttn	-0.19	1.00000000	pigs
Casp6	-0.14	1.00000000	pigs
Poglut1	-0.06	1.00000000	pigs

Ap1b1	-0.22	1.00000000	pigs
Elk3	-0.14	1.00000000	pigs
Yif1b	0.12	1.00000000	pigs
Prrc2c	0.18	1.00000000	pigs
Ccdc47	-0.03	1.00000000	pigs
Nin	-0.08	1.00000000	pigs
Cct8	-0.30	1.00000000	pigs
Klf3	0.05	1.00000000	pigs
Ogfod1	-0.18	1.00000000	pigs
Hexdc	-0.06	1.00000000	pigs
Cisd3	-0.03	1.00000000	pigs
Usp14	-0.11	1.00000000	pigs
Fut10	-0.07	1.00000000	pigs
Abhd17a	-0.16	1.00000000	pigs
Spata5	0.06	1.00000000	pigs
Pfdn6	-0.10	1.00000000	pigs
Usp33	-0.24	1.00000000	pigs
Zfp787	-0.15	1.00000000	pigs
Stk11	-0.05	1.00000000	pigs
Dzip3	0.00	1.00000000	pigs
Pelo	0.04	1.00000000	pigs
Ints7	-0.14	1.00000000	pigs
Tpm1	0.24	1.00000000	pigs
Dnajc2	0.08	1.00000000	pigs
Hmcn1	-0.23	1.00000000	pigs
Spopl	-0.20	1.00000000	pigs
Cdkl5	-0.09	1.00000000	pigs
Commd2	-0.37	1.00000000	pigs
Polr2b	0.04	1.00000000	pigs
Castor1	-0.24	1.00000000	pigs
Nop10	0.05	1.00000000	pigs
U2af2	-0.16	1.00000000	pigs
Cd2bp2	-0.10	1.00000000	pigs
Ndufs4	-0.10	1.00000000	pigs
Itch	0.02	1.00000000	pigs
Hadha	-0.26	1.00000000	pigs
Rpp21	-0.08	1.00000000	pigs
Ralgapa2	0.14	1.00000000	pigs
Smad4	-0.18	1.00000000	pigs
Rad17	-0.05	1.00000000	pigs
Tkt	-0.14	1.00000000	pigs
Rbis	-0.22	1.00000000	pigs
Olfml3	-0.29	1.00000000	pigs
Dhrs4	-0.08	1.00000000	pigs
Usp7	-0.17	1.00000000	pigs
Gpm6a	-0.14	1.00000000	pigs

Arrb1	-0.11	1.00000000	pigs
Zbtb38	-0.11	1.00000000	pigs
Ipo5	-0.07	1.00000000	pigs
Vwf	-0.07	1.00000000	pigs
Clcn3	-0.06	1.00000000	pigs
Tmem68	-0.22	1.00000000	pigs
Ubxn1	0.02	1.00000000	pigs
Usp1	-0.05	1.00000000	pigs
Gpx4	0.03	1.00000000	pigs
Cep128	0.05	1.00000000	pigs
Agk	-0.18	1.00000000	pigs
Trip12	-0.08	1.00000000	pigs
Ift88	-0.04	1.00000000	pigs
Spi1	0.02	1.00000000	pigs
Gamt	-0.12	1.00000000	pigs
Polr1f	-0.21	1.00000000	pigs
Lnx2	-0.13	1.00000000	pigs
Znhit1	0.29	1.00000000	pigs
Sco1	-0.07	1.00000000	pigs
Rbfox2	-0.18	1.00000000	pigs
Pkd1	-0.19	1.00000000	pigs
Ylpm1	0.08	1.00000000	pigs
Ccs	-0.15	1.00000000	pigs
Stat2	-0.04	1.00000000	pigs
Sap30	-0.02	1.00000000	pigs
Xpo4	-0.17	1.00000000	pigs
Phax	-0.03	1.00000000	pigs
Rara	0.08	1.00000000	pigs
Ctdsp2	-0.23	1.00000000	pigs
Radil	-0.01	1.00000000	pigs
Id1	-0.08	1.00000000	pigs
Abhd16a	-0.15	1.00000000	pigs
Nenf	-0.21	1.00000000	pigs
Senp7	-0.43	1.00000000	pigs
Ppp4r3b	-0.23	1.00000000	pigs
Xkr6	0.06	1.00000000	pigs
Ppp1r15a	-0.28	1.00000000	pigs
Rnf6	-0.07	1.00000000	pigs
Polr2h	-0.04	1.00000000	pigs
Ufd1	-0.09	1.00000000	pigs
Bclaf1	-0.05	1.00000000	pigs
BC004004	-0.17	1.00000000	pigs
Ankrd50	-0.33	1.00000000	pigs
Cpped1	0.40	1.00000000	pigs
Rabgap1	-0.40	1.00000000	pigs
Prkci	-0.02	1.00000000	pigs

Otub1	-0.18	1.00000000	pigs
Ccdc127	-0.14	1.00000000	pigs
Atl3	0.11	1.00000000	pigs
Erp29	-0.14	1.00000000	pigs
Cdkn2aipnl	0.00	1.00000000	pigs
Serac1	-0.15	1.00000000	pigs
Fam76a	-0.19	1.00000000	pigs
Il1r1	-0.01	1.00000000	pigs
Psma7	-0.05	1.00000000	pigs
Exoc7	0.22	1.00000000	pigs
Synpo	-0.19	1.00000000	pigs
Fam13b	-0.33	1.00000000	pigs
Dctn5	-0.09	1.00000000	pigs
Uggt1	-0.19	1.00000000	pigs
Ushbp1	-0.25	1.00000000	pigs
Adi1	-0.13	1.00000000	pigs
Enkur	-0.17	1.00000000	pigs
Bola1	-0.04	1.00000000	pigs
Clasp2	-0.10	1.00000000	pigs
Nup107	0.01	1.00000000	pigs
Rarg	0.04	1.00000000	pigs
Bdh2	0.04	1.00000000	pigs
Klhl8	0.07	1.00000000	pigs
Srgap3	-0.07	1.00000000	pigs
Tsr2	-0.14	1.00000000	pigs
L3mbtl1	0.04	1.00000000	pigs
Stambp	0.16	1.00000000	pigs
Cryl1	-0.14	1.00000000	pigs
Vps39	0.00	1.00000000	pigs
Erg	-0.20	1.00000000	pigs
Sox13	-0.03	1.00000000	pigs
Cnih4	-0.20	1.00000000	pigs
Ube2m	-0.05	1.00000000	pigs
Farp1	0.11	1.00000000	pigs
Vamp5	-0.10	1.00000000	pigs
Iqsec1	-0.18	1.00000000	pigs
Vps26b	-0.09	1.00000000	pigs
Chmp2a	0.24	1.00000000	pigs
Dvl1	-0.10	1.00000000	pigs
Pced1b	0.05	1.00000000	pigs
Hnrnpm	0.04	1.00000000	pigs
Upf2	-0.14	1.00000000	pigs
Pgk1	-0.18	1.00000000	pigs
Gfpt1	-0.09	1.00000000	pigs
Afap1l1	-0.20	1.00000000	pigs
Nadk	-0.10	1.00000000	pigs

Adnp2	-0.04	1.00000000	pigs
Pola1	-0.06	1.00000000	pigs
Tpk1	-0.25	1.00000000	pigs
Spag8	-0.18	1.00000000	pigs
Napg	-0.01	1.00000000	pigs
Zfp512b	-0.09	1.00000000	pigs
Ap3m1	-0.10	1.00000000	pigs
Plvap	0.09	1.00000000	pigs
Cep120	-0.14	1.00000000	pigs
Ankle2	-0.29	1.00000000	pigs
Snrpf	0.09	1.00000000	pigs
Pcdh1	-0.47	1.00000000	pigs
Coq10b	0.01	1.00000000	pigs
Laptm4a	-0.01	1.00000000	pigs
Dynlrb1	-0.01	1.00000000	pigs
Ssr1	0.00	1.00000000	pigs
Pde6d	-0.07	1.00000000	pigs
Smarca5	-0.07	1.00000000	pigs
Stam	-0.05	1.00000000	pigs
Nosip	-0.08	1.00000000	pigs
Kif26b	-0.40	1.00000000	pigs
Ddah1	-0.08	1.00000000	pigs
Slc4a4	0.21	1.00000000	pigs
Stmn1	-0.14	1.00000000	pigs
Prnp	0.01	1.00000000	pigs
Spg21	-0.08	1.00000000	pigs
Hacd2	-0.14	1.00000000	pigs
Igsf3	0.07	1.00000000	pigs
Rab11fip5	-0.06	1.00000000	pigs
Tk2	-0.10	1.00000000	pigs
Cibar1	-0.19	1.00000000	pigs
Ulk4	-0.27	1.00000000	pigs
Ubac2	-0.35	1.00000000	pigs
Hps3	-0.02	1.00000000	pigs
Septin2	-0.05	1.00000000	pigs
Psmd12	-0.11	1.00000000	pigs
Dhx29	-0.32	1.00000000	pigs
Tango2	-0.14	1.00000000	pigs
Triqk	-0.10	1.00000000	pigs
Ddx27	-0.10	1.00000000	pigs
Gtf2e2	-0.27	1.00000000	pigs
Dnajc3	-0.23	1.00000000	pigs
Rfc2	0.16	1.00000000	pigs
Ccdc66	-0.02	1.00000000	pigs
Rnasel	-0.14	1.00000000	pigs
Dhx30	-0.21	1.00000000	pigs

Eefsec	0.10	1.00000000	pigs
Ptpn12	-0.08	1.00000000	pigs
Iba57	-0.14	1.00000000	pigs
Chmp2b	-0.26	1.00000000	pigs
Snx4	-0.35	1.00000000	pigs
Obi1	-0.22	1.00000000	pigs
Eif2s1	-0.03	1.00000000	pigs
Bod1l	0.21	1.00000000	pigs
Kdelr2	-0.27	1.00000000	pigs
Ufm1	-0.11	1.00000000	pigs
Cpne1	-0.05	1.00000000	pigs
Nostrin	-0.48	1.00000000	pigs
Timm8b	-0.15	1.00000000	pigs
Trim28	-0.07	1.00000000	pigs
Tnfaip1	-0.19	1.00000000	pigs
Nedd8	-0.06	1.00000000	pigs
Sntb2	-0.15	1.00000000	pigs
Hcls1	0.10	1.00000000	pigs
Kiz	-0.13	1.00000000	pigs
Ppp2r3c	0.09	1.00000000	pigs
Pxn	0.17	1.00000000	pigs
Slc35e2	-0.08	1.00000000	pigs
Fdft1	0.08	1.00000000	pigs
Pdlim7	0.18	1.00000000	pigs
Rab5a	-0.03	1.00000000	pigs
Slc12a6	-0.09	1.00000000	pigs
Psmb9	0.14	1.00000000	pigs
Layn	-0.03	1.00000000	pigs
Snx3	-0.12	1.00000000	pigs
Jmy	0.04	1.00000000	pigs
Cdc42se2	-0.15	1.00000000	pigs
Dcaf13	-0.02	1.00000000	pigs
Rhot1	-0.12	1.00000000	pigs
Clint1	-0.09	1.00000000	pigs
Adamts1	-0.12	1.00000000	pigs
Atf6	-0.01	1.00000000	pigs
Synrg	-0.27	1.00000000	pigs
Ncoa5	0.00	1.00000000	pigs
Prpf38a	-0.17	1.00000000	pigs
Usp34	0.09	1.00000000	pigs
G3bp2	-0.03	1.00000000	pigs
Psmc4	0.21	1.00000000	pigs
Arhgap42	-0.16	1.00000000	pigs
Lin7c	-0.10	1.00000000	pigs
Homer1	0.37	1.00000000	pigs
Dennd11	-0.38	1.00000000	pigs

Efcc1	-0.10	1.00000000	pigs
Poglut3	-0.13	1.00000000	pigs
Eif2b3	-0.16	1.00000000	pigs
Sacm1l	-0.14	1.00000000	pigs
Fnta	-0.14	1.00000000	pigs
Prkaa1	-0.06	1.00000000	pigs
Armcx3	-0.23	1.00000000	pigs
Acvr2a	-0.20	1.00000000	pigs
Eif3f	0.00	1.00000000	pigs
Slc22a3	0.23	1.00000000	pigs
Pus7l	-0.20	1.00000000	pigs
Brd9	-0.09	1.00000000	pigs
Amh	0.14	1.00000000	pigs
Sulf1	-0.27	1.00000000	pigs
Cdc42	0.00	1.00000000	pigs
Rnmt	-0.19	1.00000000	pigs
Pcif1	-0.11	1.00000000	pigs
Pomp	-0.22	1.00000000	pigs
Eif2ak4	-0.20	1.00000000	pigs
Trank1	-0.25	1.00000000	pigs
Cc2d2a	-0.08	1.00000000	pigs
Smc2	-0.19	1.00000000	pigs
Slco3a1	-0.40	1.00000000	pigs
Cks2	-0.31	1.00000000	pigs
Elp2	-0.16	1.00000000	pigs
Arl5b	0.09	1.00000000	pigs
Osbp	0.01	1.00000000	pigs
Pak1ip1	-0.01	1.00000000	pigs
Rad21	-0.10	1.00000000	pigs
Igf2bp2	0.02	1.00000000	pigs
Slc30a9	-0.11	1.00000000	pigs
Nptn	-0.06	1.00000000	pigs
Pkn1	-0.09	1.00000000	pigs
Trpc6	-0.27	1.00000000	pigs
Crbn	-0.17	1.00000000	pigs
Pdhb	-0.11	1.00000000	pigs
Hdgf	-0.07	1.00000000	pigs
Smarcad1	-0.14	1.00000000	pigs
St13	0.03	1.00000000	pigs
Rbbp7	-0.05	1.00000000	pigs
Qdpr	-0.01	1.00000000	pigs
Bnip3l	-0.10	1.00000000	pigs
Dpp7	-0.10	1.00000000	pigs
Tbc1d19	-0.19	1.00000000	pigs
Sp110	0.09	1.00000000	pigs
Ncoa2	0.05	1.00000000	pigs

Erp44	-0.07	1.00000000	pigs
Thsd1	-0.25	1.00000000	pigs
Atp7a	-0.10	1.00000000	pigs
Sh3d19	-0.30	1.00000000	pigs
Igfbp4	-0.07	1.00000000	pigs
Actn1	-0.04	1.00000000	pigs
Nfib	0.02	1.00000000	pigs
Zfp639	-0.15	1.00000000	pigs
Hdac7	-0.24	1.00000000	pigs
Kazn	-0.05	1.00000000	pigs
Bcar3	-0.15	1.00000000	pigs
Bmp2k	0.05	1.00000000	pigs
Haus6	-0.23	1.00000000	pigs
Chchd1	-0.09	1.00000000	pigs
B9d2	-0.10	1.00000000	pigs
Ccdc58	-0.01	1.00000000	pigs
Eprs	-0.19	1.00000000	pigs
Necap2	0.00	1.00000000	pigs
Tubb4b	0.10	1.00000000	pigs
Ccdc18	-0.02	1.00000000	pigs
Podn	0.05	1.00000000	pigs
Smad6	-0.31	1.00000000	pigs
Arhgap29	0.00	1.00000000	pigs
Tbc1d4	0.07	1.00000000	pigs
Ndufb10	0.13	1.00000000	pigs
Zyg11b	-0.14	1.00000000	pigs
Herc2	-0.25	1.00000000	pigs
Osgin2	-0.19	1.00000000	pigs
Bid	-0.19	1.00000000	pigs
Snx16	-0.03	1.00000000	pigs
Emc10	-0.05	1.00000000	pigs
Zzz3	0.00	1.00000000	pigs
Rbsn	-0.13	1.00000000	pigs
Ackr2	-0.31	1.00000000	pigs
Smchd1	-0.07	1.00000000	pigs
Lysmd3	-0.25	1.00000000	pigs
Arih2	-0.24	1.00000000	pigs
Immt	-0.32	1.00000000	pigs
Crebl2	0.07	1.00000000	pigs
Zfp704	-0.08	1.00000000	pigs
Cass4	-0.18	1.00000000	pigs
2410004B18Rik	-0.29	1.00000000	pigs
Usp38	-0.02	1.00000000	pigs
Cops2	-0.18	1.00000000	pigs
Lonp2	-0.15	1.00000000	pigs
Tsfm	-0.01	1.00000000	pigs

Dmtn	-0.21	1.00000000	pigs
Ccni	0.11	1.00000000	pigs
Ppp1r12b	-0.23	1.00000000	pigs
Fmo1	-1.11	1.00000000	pigs
Gspt1	-0.01	1.00000000	pigs
Rilpl2	0.30	1.00000000	pigs
Trdmt1	0.01	1.00000000	pigs
Hebp1	-0.17	1.00000000	pigs
Zbtb25	-0.04	1.00000000	pigs
Rtf2	-0.15	1.00000000	pigs
Ano10	0.02	1.00000000	pigs
Rer1	-0.10	1.00000000	pigs
Gab1	-0.15	1.00000000	pigs
Leprotl1	-0.19	1.00000000	pigs
Cuedc2	-0.03	1.00000000	pigs
5730455P16Rik	-0.16	1.00000000	pigs
Ibtk	-0.08	1.00000000	pigs
Spred1	-0.24	1.00000000	pigs
Dnmt1	-0.21	1.00000000	pigs
Pik3cb	-0.12	1.00000000	pigs
Ppef1	-0.16	1.00000000	pigs
Ubtf	-0.08	1.00000000	pigs
Psme3	-0.10	1.00000000	pigs
Stk17b	-0.21	1.00000000	pigs
Hmgcr	-0.25	1.00000000	pigs
Sesn1	-0.09	1.00000000	pigs
Rfc3	0.06	1.00000000	pigs
Glce	-0.13	1.00000000	pigs
Car4	-0.91	1.00000000	pigs
Stx7	0.04	1.00000000	pigs
9430038I01Rik	-0.06	1.00000000	pigs
Rcan3	-0.04	1.00000000	pigs
Socs5	-0.18	1.00000000	pigs
Polr2m	0.14	1.00000000	pigs
St7l	-0.32	1.00000000	pigs
Aph1a	-0.30	1.00000000	pigs
Rpp30	-0.17	1.00000000	pigs
Hsp90b1	0.11	1.00000000	pigs
Chmp1a	-0.20	1.00000000	pigs
Krcc1	-0.32	1.00000000	pigs
Rbm42	0.00	1.00000000	pigs
Dtwd2	-0.12	1.00000000	pigs
Gpr137c	-0.20	1.00000000	pigs
Lsm7	-0.15	1.00000000	pigs
Fam53b	-0.08	1.00000000	pigs
Asrgl1	-0.15	1.00000000	pigs

Cep164	-0.12	1.00000000	pigs
Smarcb1	-0.23	1.00000000	pigs
Ube4b	-0.13	1.00000000	pigs
Pnrc2	0.06	1.00000000	pigs
Gps1	-0.07	1.00000000	pigs
Pbrm1	0.03	1.00000000	pigs
Ift20	-0.11	1.00000000	pigs
Tmem127	-0.02	1.00000000	pigs
Nol9	-0.08	1.00000000	pigs
H2-Ke6	-0.12	1.00000000	pigs
Ndufs6	0.02	1.00000000	pigs
Focad	-0.03	1.00000000	pigs
Primpol	-0.05	1.00000000	pigs
Ccdc62	-0.21	1.00000000	pigs
Il6ra	-0.40	1.00000000	pigs
Cct3	0.03	1.00000000	pigs
Gigyf1	0.03	1.00000000	pigs
Ddx23	-0.12	1.00000000	pigs
Bud31	-0.11	1.00000000	pigs
Wwc2	-0.27	1.00000000	pigs
Tbx3	-0.34	1.00000000	pigs
Phf2	-0.15	1.00000000	pigs
Nudt5	-0.08	1.00000000	pigs
Sbf2	0.01	1.00000000	pigs
Rnf13	-0.12	1.00000000	pigs
Elavl1	-0.07	1.00000000	pigs
Pik3ip1	-0.05	1.00000000	pigs
She	-0.31	1.00000000	pigs
Nifk	0.00	1.00000000	pigs
Tjp1	-0.05	1.00000000	pigs
Ccm2l	-0.23	1.00000000	pigs
Rnpepl1	-0.09	1.00000000	pigs
Tdrd3	-0.14	1.00000000	pigs
Arl4d	-0.15	1.00000000	pigs
Ralbp1	-0.14	1.00000000	pigs
Rubcnl	-0.32	1.00000000	pigs
Bcl2l1	0.04	1.00000000	pigs
Rchy1	0.02	1.00000000	pigs
Smim7	-0.09	1.00000000	pigs
Actr3	0.09	1.00000000	pigs
D16Ertd472e	-0.15	1.00000000	pigs
Rev1	0.36	1.00000000	pigs
Carnmt1	-0.02	1.00000000	pigs
Phyh	-0.03	1.00000000	pigs
Crybg3	-0.18	1.00000000	pigs
Dusp1	0.19	1.00000000	pigs

Pnpla8	-0.43	1.00000000	pigs
Gpsm2	-0.16	1.00000000	pigs
Zfp644	-0.04	1.00000000	pigs
Tmem184c	-0.19	1.00000000	pigs
Reep5	-0.11	1.00000000	pigs
Rnase4	0.05	1.00000000	pigs
Sf3b4	0.09	1.00000000	pigs
Cdc42ep1	-0.14	1.00000000	pigs
Pla2g4a	-0.32	1.00000000	pigs
Rida	0.11	1.00000000	pigs
Crtc3	-0.31	1.00000000	pigs
Anapc1	-0.04	1.00000000	pigs
Zfp384	0.02	1.00000000	pigs
Cep20	-0.09	1.00000000	pigs
Eif4g2	0.10	1.00000000	pigs
Cgrrf1	0.04	1.00000000	pigs
Tnfsf4	0.14	1.00000000	pigs
Slc9a8	0.13	1.00000000	pigs
Arf6	0.05	1.00000000	pigs
Dcaf1	-0.26	1.00000000	pigs
A130010J15Rik	0.03	1.00000000	pigs
Pura	-0.07	1.00000000	pigs
DPCD	-0.11	1.00000000	pigs
Pdcl3	-0.22	1.00000000	pigs
Nrf1	-0.06	1.00000000	pigs
Brap	0.08	1.00000000	pigs
Cul1	-0.18	1.00000000	pigs
Fam151b	-0.17	1.00000000	pigs
Golph3l	-0.13	1.00000000	pigs
Ankrd44	-0.48	1.00000000	pigs
Fam210b	0.16	1.00000000	pigs
Nfyc	-0.17	1.00000000	pigs
Itfg1	-0.09	1.00000000	pigs
Sp3	-0.32	1.00000000	pigs
Prpf4b	-0.03	1.00000000	pigs
Tm9sf4	-0.07	1.00000000	pigs
Arglu1	-0.07	1.00000000	pigs
Wdr1	-0.24	1.00000000	pigs
Plekhm3	-0.42	1.00000000	pigs
Ikbkg	-0.12	1.00000000	pigs
Bckdk	-0.14	1.00000000	pigs
Jcad	-0.32	1.00000000	pigs
Klhl28	-0.04	1.00000000	pigs
Fam120c	0.03	1.00000000	pigs
Zcrb1	-0.03	1.00000000	pigs
Cd109	-0.02	1.00000000	pigs

Elk4	-0.19	1.00000000	pigs
Pde12	-0.10	1.00000000	pigs
Cnih1	-0.23	1.00000000	pigs
Aco2	0.04	1.00000000	pigs
Cplane1	0.04	1.00000000	pigs
Thap11	-0.24	1.00000000	pigs
Xrcc5	-0.05	1.00000000	pigs
Fut11	-0.12	1.00000000	pigs
Dapk3	-0.13	1.00000000	pigs
Dgkz	-0.29	1.00000000	pigs
Ubxn2a	0.06	1.00000000	pigs
Akap8	-0.23	1.00000000	pigs
Slc48a1	-0.15	1.00000000	pigs
Taf1a	-0.07	1.00000000	pigs
Scand1	-0.02	1.00000000	pigs
Glrx5	-0.05	1.00000000	pigs
Atp6v0d1	-0.09	1.00000000	pigs
Dync1li1	-0.12	1.00000000	pigs
Arl5a	-0.07	1.00000000	pigs
Ppip5k2	0.04	1.00000000	pigs
Tufm	0.04	1.00000000	pigs
Cdh5	-0.02	1.00000000	pigs
Htr4	-0.11	1.00000000	pigs
Hscb	-0.11	1.00000000	pigs
Nxn	-0.02	1.00000000	pigs
Csde1	-0.04	1.00000000	pigs
Ubr1	0.09	1.00000000	pigs
Klc2	0.09	1.00000000	pigs
Tfe3	-0.11	1.00000000	pigs
Lactb2	-0.14	1.00000000	pigs
Ttc17	-0.17	1.00000000	pigs
Vps4b	0.01	1.00000000	pigs
Cul5	-0.26	1.00000000	pigs
Cnppd1	0.13	1.00000000	pigs
Tmf1	-0.15	1.00000000	pigs
Pear1	0.01	1.00000000	pigs
Cdk4	-0.14	1.00000000	pigs
Ndufaf2	-0.10	1.00000000	pigs
Bpnt2	-0.17	1.00000000	pigs
Cenpp	-0.03	1.00000000	pigs
Spsb3	0.04	1.00000000	pigs
Zmynd11	-0.17	1.00000000	pigs
Zfp341	-0.12	1.00000000	pigs
Flii	-0.03	1.00000000	pigs
Ppm1g	-0.23	1.00000000	pigs
Atp1b1	0.05	1.00000000	pigs

Nbea	-0.26	1.00000000	pigs
Rimklb	-0.22	1.00000000	pigs
Gpr176	-0.16	1.00000000	pigs
Ddx42	-0.17	1.00000000	pigs
Arrdc1	-0.06	1.00000000	pigs
Rin3	-0.08	1.00000000	pigs
Ptgr1	0.19	1.00000000	pigs
Eogt	-0.01	1.00000000	pigs
Psmd6	-0.25	1.00000000	pigs
Snrnp40	-0.16	1.00000000	pigs
Cdv3	0.03	1.00000000	pigs
Sar1a	-0.13	1.00000000	pigs
Eef1b2	0.17	1.00000000	pigs
Dock7	-0.23	1.00000000	pigs
Epn1	-0.18	1.00000000	pigs
St3gal5	0.04	1.00000000	pigs
Glb1	-0.04	1.00000000	pigs
Tcf4	-0.13	1.00000000	pigs
Ndufb6	-0.04	1.00000000	pigs
Fcho2	-0.06	1.00000000	pigs
Ezh1	0.08	1.00000000	pigs
Plaa	-0.18	1.00000000	pigs
Cebpd	-0.06	1.00000000	pigs
Utp6	0.09	1.00000000	pigs
Ro60	0.00	1.00000000	pigs
Flna	0.12	1.00000000	pigs
Zfp503	-0.08	1.00000000	pigs
Psmc5	-0.21	1.00000000	pigs
Dld	-0.06	1.00000000	pigs
Eef2k	-0.03	1.00000000	pigs
St6gal1	-0.13	1.00000000	pigs
Psmb8	-0.02	1.00000000	pigs
Septin6	-0.15	1.00000000	pigs
Arhgdib	-0.20	1.00000000	pigs
Nfrkb	-0.11	1.00000000	pigs
Trpc4ap	-0.03	1.00000000	pigs
Ppp2r5a	-0.06	1.00000000	pigs
Fam172a	-0.18	1.00000000	pigs
Rint1	-0.04	1.00000000	pigs
Dars	-0.01	1.00000000	pigs
Tspan4	-0.08	1.00000000	pigs
Exoc8	-0.07	1.00000000	pigs
Nf2	-0.17	1.00000000	pigs
Srbd1	-0.20	1.00000000	pigs
Zcchc7	-0.10	1.00000000	pigs
Gemin2	-0.03	1.00000000	pigs

Ndufb2	-0.02	1.00000000	pigs
Hadh	-0.17	1.00000000	pigs
Tnfrsf1a	-0.24	1.00000000	pigs
Fos	-0.24	1.00000000	pigs
Usp47	-0.15	1.00000000	pigs
Acvrl1	-0.03	1.00000000	pigs
Zfp410	-0.19	1.00000000	pigs
Hsd17b7	-0.14	1.00000000	pigs
Lamtor3	0.08	1.00000000	pigs
Pithd1	-0.16	1.00000000	pigs
Gas2l1	-0.03	1.00000000	pigs
Yif1a	-0.05	1.00000000	pigs
Txlna	-0.13	1.00000000	pigs
Lamb1	-0.19	1.00000000	pigs
Golm1	-0.21	1.00000000	pigs
Vps8	-0.37	1.00000000	pigs
Otud4	-0.28	1.00000000	pigs
2810004N23Rik	0.28	1.00000000	pigs
Stc1	-0.05	1.00000000	pigs
Tm6sf1	-0.18	1.00000000	pigs
Sertad2	-0.20	1.00000000	pigs
Taf3	-0.37	1.00000000	pigs
Rnf157	-0.32	1.00000000	pigs
Fam171a1	-0.17	1.00000000	pigs
Scamp3	-0.18	1.00000000	pigs
Nbeal1	-0.23	1.00000000	pigs
Septin10	-0.05	1.00000000	pigs
Patj	-0.04	1.00000000	pigs
Tfg	0.00	1.00000000	pigs
Nsfl1c	-0.01	1.00000000	pigs
Pkd2	-0.03	1.00000000	pigs
Ing5	-0.03	1.00000000	pigs
Adgrf5	-0.31	1.00000000	pigs
Sfpq	0.21	1.00000000	pigs
Pqbp1	0.03	1.00000000	pigs
Galnt10	-0.05	1.00000000	pigs
Aldh2	0.03	1.00000000	pigs
Nr3c1	-0.02	1.00000000	pigs
Rab29	-0.05	1.00000000	pigs
Ttc39b	0.06	1.00000000	pigs
Hnrnpul1	-0.07	1.00000000	pigs
Nsun2	0.03	1.00000000	pigs
Polh	-0.14	1.00000000	pigs
Tc2n	0.00	1.00000000	pigs
Rfng	-0.08	1.00000000	pigs
Dffa	-0.05	1.00000000	pigs

Sypl	-0.14	1.00000000	pigs
1110059G10Rik	0.03	1.00000000	pigs
Plscr3	-0.16	1.00000000	pigs
Usf2	-0.10	1.00000000	pigs
Prkcsh	-0.12	1.00000000	pigs
Tspyl2	0.09	1.00000000	pigs
Epc2	-0.19	1.00000000	pigs
Krr1	-0.25	1.00000000	pigs
Dapk1	0.32	1.00000000	pigs
Ola1	-0.06	1.00000000	pigs
Srsf10	-0.04	1.00000000	pigs
Thra	-0.25	1.00000000	pigs
Slc9a9	-0.22	1.00000000	pigs
Rab5c	0.01	1.00000000	pigs
Col19a1	-0.17	1.00000000	pigs
Hnrnpu	0.04	1.00000000	pigs
Naa20	-0.10	1.00000000	pigs
Zfp511	-0.14	1.00000000	pigs
Prpf19	-0.02	1.00000000	pigs
Prkx	-0.15	1.00000000	pigs
Phtf1	-0.13	1.00000000	pigs
Cnpy3	-0.08	1.00000000	pigs
Foxn3	0.02	1.00000000	pigs
Glipr2	0.17	1.00000000	pigs
Rbx1	0.16	1.00000000	pigs
Ppp1r11	-0.01	1.00000000	pigs
Rab2a	-0.10	1.00000000	pigs
Samhd1	0.02	1.00000000	pigs
Tmtc3	-0.30	1.00000000	pigs
Tcf7	-0.23	1.00000000	pigs
Eif3b	-0.15	1.00000000	pigs
Ppp3cb	-0.07	1.00000000	pigs
Tut7	0.02	1.00000000	pigs
Gpatch11	-0.10	1.00000000	pigs
Tbcb	-0.16	1.00000000	pigs
Ranbp17	-0.17	1.00000000	pigs
Srsf9	-0.08	1.00000000	pigs
Cse1l	0.26	1.00000000	pigs
Slc35b2	-0.10	1.00000000	pigs
Tcp1	0.16	1.00000000	pigs
Vcl	0.00	1.00000000	pigs
Ccdc15	-0.14	1.00000000	pigs
Parg	-0.18	1.00000000	pigs
Gfm1	-0.06	1.00000000	pigs
Limk2	-0.33	1.00000000	pigs
Cwc25	-0.14	1.00000000	pigs

Epha2	-0.27	1.00000000	pigs
Afg1l	-0.06	1.00000000	pigs
Slc35d1	-0.10	1.00000000	pigs
Paip1	-0.24	1.00000000	pigs
Ide	0.04	1.00000000	pigs
Aldh1a2	0.03	1.00000000	pigs
Csnk1g2	-0.17	1.00000000	pigs
Coro1a	-0.02	1.00000000	pigs
Gabpa	-0.24	1.00000000	pigs
AW549877	0.10	1.00000000	pigs
Pofut1	-0.27	1.00000000	pigs
Cyb5r4	-0.05	1.00000000	pigs
4932411N23Rik	-0.27	1.00000000	pigs
Ssbp4	-0.05	1.00000000	pigs
Arf1	-0.05	1.00000000	pigs
Cmtr1	-0.09	1.00000000	pigs
Zfhx3	-0.33	1.00000000	pigs
Slc39a10	-0.26	1.00000000	pigs
Psmb2	0.00	1.00000000	pigs
Wnt9a	0.53	1.00000000	pigs
Parp1	0.03	1.00000000	pigs
Cnn2	-0.13	1.00000000	pigs
Psma4	-0.07	1.00000000	pigs
Tiam2	0.01	1.00000000	pigs
H13	-0.17	1.00000000	pigs
Rnh1	0.00	1.00000000	pigs
Abce1	0.00	1.00000000	pigs
Dhx36	-0.20	1.00000000	pigs
Eif2d	-0.01	1.00000000	pigs
Clybl	-0.06	1.00000000	pigs
Tbc1d20	-0.16	1.00000000	pigs
Kyat3	-0.10	1.00000000	pigs
Snupn	-0.05	1.00000000	pigs
Pum2	-0.22	1.00000000	pigs
Zdhhc6	-0.14	1.00000000	pigs
F8	-0.20	1.00000000	pigs
Snx19	-0.10	1.00000000	pigs
Pam16	-0.24	1.00000000	pigs
Rbms3	0.09	1.00000000	pigs
Plat	0.18	1.00000000	pigs
Nos2	0.00	1.00000000	pigs
Lrp10	-0.14	1.00000000	pigs
Fam126a	-0.17	1.00000000	pigs
Eif2a	0.01	1.00000000	pigs
Stk38	-0.20	1.00000000	pigs
Shld2	-0.19	1.00000000	pigs

Crnkl1	-0.17	1.00000000	pigs
Ccdc69	-0.16	1.00000000	pigs
Cpeb4	0.03	1.00000000	pigs
Avpi1	-0.15	1.00000000	pigs
Taf11	0.03	1.00000000	pigs
Aup1	-0.16	1.00000000	pigs
Fah	-0.03	1.00000000	pigs
Cyb5b	-0.12	1.00000000	pigs
Asap1	-0.02	1.00000000	pigs
Stk38l	-0.11	1.00000000	pigs
Eif2b5	-0.09	1.00000000	pigs
Rbm41	-0.17	1.00000000	pigs
Plgrkt	0.04	1.00000000	pigs
Heatr6	-0.27	1.00000000	pigs
Cdc37	-0.01	1.00000000	pigs
Commd3	-0.05	1.00000000	pigs
Otud3	-0.03	1.00000000	pigs
Diablo	-0.02	1.00000000	pigs
Rala	-0.11	1.00000000	pigs
Fkbp7	-0.11	1.00000000	pigs
Tcn2	-0.17	1.00000000	pigs
Smu1	-0.20	1.00000000	pigs
Rab35	-0.18	1.00000000	pigs
Sec24d	-0.18	1.00000000	pigs
Slc25a3	0.17	1.00000000	pigs
Cd79a	-0.11	1.00000000	pigs
Apoa1	-0.15	1.00000000	pigs
Tubb2b	0.14	1.00000000	pigs
Snai1	0.28	1.00000000	pigs
Ndufaf3	-0.09	1.00000000	pigs
Bcr	-0.14	1.00000000	pigs
Akna	0.04	1.00000000	pigs
Sars	-0.29	1.00000000	pigs
Piezo2	-0.18	1.00000000	pigs
Bnip2	-0.19	1.00000000	pigs
Sart3	-0.11	1.00000000	pigs
Cntn3	-0.26	1.00000000	pigs
Phf12	0.15	1.00000000	pigs
Arap2	-0.54	1.00000000	pigs
Gtf2h3	-0.01	1.00000000	pigs
Ginm1	-0.17	1.00000000	pigs
Surf1	0.10	1.00000000	pigs
Dnajc11	-0.13	1.00000000	pigs
Serpinb6b	0.01	1.00000000	pigs
Stom	-0.02	1.00000000	pigs
Cblb	-0.51	1.00000000	pigs

Tm9sf3	-0.12	1.00000000	pigs
Rpn1	-0.05	1.00000000	pigs
Rbpj	0.38	1.00000000	pigs
Dazap1	-0.01	1.00000000	pigs
Sptlc1	-0.23	1.00000000	pigs
Ate1	-0.06	1.00000000	pigs
Diaph2	0.04	1.00000000	pigs
Eps8	-0.33	1.00000000	pigs
Lman1	-0.40	1.00000000	pigs
Adrm1	-0.07	1.00000000	pigs
Apex1	-0.07	1.00000000	pigs
Pitrm1	0.08	1.00000000	pigs
Emilin1	-0.16	1.00000000	pigs
Yipf4	-0.16	1.00000000	pigs
Pm20d2	-0.03	1.00000000	pigs
Prelid1	0.16	1.00000000	pigs
Virma	-0.29	1.00000000	pigs
Zfp282	-0.11	1.00000000	pigs
Adam15	-0.12	1.00000000	pigs
Rbks	-0.10	1.00000000	pigs
Ttll12	-0.04	1.00000000	pigs
Slc16a13	-0.15	1.00000000	pigs
Hacd3	-0.14	1.00000000	pigs
Ttc37	-0.23	1.00000000	pigs
Deaf1	-0.21	1.00000000	pigs
Foxf1	-0.39	1.00000000	pigs
Ece1	-0.15	1.00000000	pigs
Pigt	-0.19	1.00000000	pigs
G6pc3	-0.09	1.00000000	pigs
Pigf	-0.10	1.00000000	pigs
Disp1	-0.59	1.00000000	pigs
Ggnbp2	-0.01	1.00000000	pigs
Tmem106c	-0.14	1.00000000	pigs
Rrn3	-0.12	1.00000000	pigs
Tie1	-0.12	1.00000000	pigs
Cfd	0.32	1.00000000	pigs
Adgrl2	-0.62	1.00000000	pigs
Lrrc28	-0.18	1.00000000	pigs
Ptprm	-0.30	1.00000000	pigs
Hspd1	-0.14	1.00000000	pigs
Cebpg	-0.09	1.00000000	pigs
Rnf38	-0.18	1.00000000	pigs
Ctsf	-0.22	1.00000000	pigs
Capns1	-0.06	1.00000000	pigs
Lpp	-0.01	1.00000000	pigs
Ccdc90b	-0.20	1.00000000	pigs

Nt5c3	-0.17	1.00000000	pigs
Pgam5	-0.26	1.00000000	pigs
Kansl1l	-0.15	1.00000000	pigs
Drg1	-0.02	1.00000000	pigs
Aasdh	-0.13	1.00000000	pigs
Dcp1a	-0.29	1.00000000	pigs
Vezt	-0.04	1.00000000	pigs
Shfl	-0.17	1.00000000	pigs
Stam2	-0.18	1.00000000	pigs
Btaf1	-0.23	1.00000000	pigs
Rgs16	0.71	1.00000000	pigs
Amz2	-0.13	1.00000000	pigs
Grsf1	-0.28	1.00000000	pigs
Rsl24d1	-0.22	1.00000000	pigs
Pphln1	-0.22	1.00000000	pigs
Sdf4	-0.07	1.00000000	pigs
Huwe1	-0.04	1.00000000	pigs
Rilpl1	-0.18	1.00000000	pigs
Surf6	0.01	1.00000000	pigs
Srsf5	0.11	1.00000000	pigs
Akap7	-0.23	1.00000000	pigs
Dmd	-0.23	1.00000000	pigs
Tm9sf2	-0.04	1.00000000	pigs
Lfng	-0.19	1.00000000	pigs
Nudt1	-0.13	1.00000000	pigs
Washc1	-0.26	1.00000000	pigs
Smarcd2	0.09	1.00000000	pigs
Ube2j2	-0.31	1.00000000	pigs
Sppl2a	-0.15	1.00000000	pigs
Tes	0.11	1.00000000	pigs
Cwf19l2	-0.12	1.00000000	pigs
Prkacb	-0.08	1.00000000	pigs
Fig4	-0.32	1.00000000	pigs
Cdadc1	-0.27	1.00000000	pigs
Eif3j2	-0.07	1.00000000	pigs
Atrn	-0.30	1.00000000	pigs
Ero1b	-0.17	1.00000000	pigs
Fkbp8	-0.18	1.00000000	pigs
Baz2b	-0.08	1.00000000	pigs
Alox15	-0.11	1.00000000	pigs
Pgls	0.03	1.00000000	pigs
Gltp	-0.18	1.00000000	pigs
Eva1b	0.01	1.00000000	pigs
Tubgcp4	-0.13	1.00000000	pigs
Snap47	-0.06	1.00000000	pigs
Rbm39	0.09	1.00000000	pigs

Nvl	-0.10	1.00000000	pigs
Tmem222	0.04	1.00000000	pigs
Galk1	0.01	1.00000000	pigs
Gatd1	-0.15	1.00000000	pigs
Jkamp	0.01	1.00000000	pigs
Cpsf2	-0.02	1.00000000	pigs
Casd1	-0.12	1.00000000	pigs
Eif4a3l1	-0.14	1.00000000	pigs
Nsmaf	-0.16	1.00000000	pigs
Zyx	0.13	1.00000000	pigs
Sf3b2	-0.07	1.00000000	pigs
Gps2	-0.26	1.00000000	pigs
Ndufv1	0.01	1.00000000	pigs
Bcl7c	-0.09	1.00000000	pigs
Zc3h15	-0.08	1.00000000	pigs
Carf	-0.15	1.00000000	pigs
Psmc3	-0.15	1.00000000	pigs
Ddx46	-0.21	1.00000000	pigs
Ccdc142	-0.23	1.00000000	pigs
Pex3	-0.13	1.00000000	pigs
Emc3	-0.03	1.00000000	pigs
Hook2	0.09	1.00000000	pigs
Esd	-0.09	1.00000000	pigs
Tardbp	0.03	1.00000000	pigs
Os9	-0.18	1.00000000	pigs
Plekha3	-0.08	1.00000000	pigs
Cdc14a	-0.08	1.00000000	pigs
Acot13	0.06	1.00000000	pigs
Adh5	-0.19	1.00000000	pigs
Xpo7	-0.09	1.00000000	pigs
Dnajb14	-0.20	1.00000000	pigs
Ralgapa1	0.02	1.00000000	pigs
Bms1	-0.10	1.00000000	pigs
Ptpa	-0.19	1.00000000	pigs
Clta	0.00	1.00000000	pigs
Rnf149	-0.06	1.00000000	pigs
Xrn1	-0.10	1.00000000	pigs
Wdr36	-0.01	1.00000000	pigs
Ppil3	-0.14	1.00000000	pigs
Aasdhppt	-0.21	1.00000000	pigs
Ccdc50	0.02	1.00000000	pigs
Aebp2	-0.10	1.00000000	pigs
Foxj3	0.05	1.00000000	pigs
4932438A13Rik	-0.05	1.00000000	pigs
Cetn3	-0.18	1.00000000	pigs
Wbp4	-0.30	1.00000000	pigs

Ubald1	0.03	1.00000000	pigs
3110040N11Rik	0.07	1.00000000	pigs
Depdc1b	-0.14	1.00000000	pigs
Cops5	0.03	1.00000000	pigs
Bud13	-0.09	1.00000000	pigs
U2af1l4	-0.15	1.00000000	pigs
Sec63	-0.09	1.00000000	pigs
Ssrp1	-0.13	1.00000000	pigs
Septin9	-0.04	1.00000000	pigs
Slc9a6	-0.07	1.00000000	pigs
Atg4c	-0.13	1.00000000	pigs
Srp72	0.03	1.00000000	pigs
Sil1	-0.08	1.00000000	pigs
Cox7a2l	-0.09	1.00000000	pigs
Ipo11	0.24	1.00000000	pigs
Gabpb2	0.01	1.00000000	pigs
Hnrnpa0	-0.06	1.00000000	pigs
Ubxn4	-0.23	1.00000000	pigs
Stxbp3	-0.17	1.00000000	pigs
Lyst	-0.25	1.00000000	pigs
Paip2	-0.14	1.00000000	pigs
Spin1	-0.23	1.00000000	pigs
Fbxo28	-0.19	1.00000000	pigs
Dus1l	0.07	1.00000000	pigs
Ccdc43	-0.04	1.00000000	pigs
Kpnb1	-0.03	1.00000000	pigs
Terb1	0.02	1.00000000	pigs
Eif3m	0.06	1.00000000	pigs
Hnrnpr	0.08	1.00000000	pigs
Phf20l1	-0.03	1.00000000	pigs
Btf3	-0.09	1.00000000	pigs
Btbd10	-0.19	1.00000000	pigs
Slpi	-1.22	1.00000000	pigs
Cfap20dc	-0.01	1.00000000	pigs
Smarce1	-0.20	1.00000000	pigs
Nap1l4	-0.14	1.00000000	pigs
Plk2	0.33	1.00000000	pigs
Fam135a	-0.29	1.00000000	pigs
Lrrc1	0.00	1.00000000	pigs
Snx7	-0.26	1.00000000	pigs
Armc1	-0.06	1.00000000	pigs
Pfdn4	-0.16	1.00000000	pigs
Flnb	-0.06	1.00000000	pigs
Dcaf10	-0.16	1.00000000	pigs
Gnb4	-0.13	1.00000000	pigs
Bach2	-0.66	1.00000000	pigs

Uba5	0.05	1.00000000	pigs
Vps35	0.03	1.00000000	pigs
Dalrd3	0.08	1.00000000	pigs
Zfp64	-0.15	1.00000000	pigs
Dkc1	-0.11	1.00000000	pigs
Glud1	-0.15	1.00000000	pigs
Serping1	0.04	1.00000000	pigs
Tet1	0.04	1.00000000	pigs
Lzts2	-0.09	1.00000000	pigs
Cyb5a	-0.10	1.00000000	pigs
Alox5ap	-0.09	1.00000000	pigs
Brpf3	-0.01	1.00000000	pigs
Chid1	-0.08	1.00000000	pigs
Tfpt	-0.08	1.00000000	pigs
Pcdh17	-0.14	1.00000000	pigs
Gtf2f1	0.01	1.00000000	pigs
Ppig	-0.15	1.00000000	pigs
Cep170	-0.19	1.00000000	pigs
Fbxo21	-0.08	1.00000000	pigs
Spred2	-0.34	1.00000000	pigs
Extl3	-0.51	1.00000000	pigs
Hbs1l	-0.29	1.00000000	pigs
Lmbrd2	-0.10	1.00000000	pigs
Ebna1bp2	0.08	1.00000000	pigs
Nfe2l1	-0.09	1.00000000	pigs
Utp4	-0.09	1.00000000	pigs
Kat6b	-0.23	1.00000000	pigs
Spryd3	0.13	1.00000000	pigs
Pdpr	-0.12	1.00000000	pigs
Qrich1	0.01	1.00000000	pigs
Rdh10	-0.03	1.00000000	pigs
Cdin1	-0.19	1.00000000	pigs
Zfyve9	-0.20	1.00000000	pigs
Rasa1	-0.20	1.00000000	pigs
Rab30	-0.06	1.00000000	pigs
Atxn7	-0.10	1.00000000	pigs
Capza2	0.05	1.00000000	pigs
Psmd7	-0.25	1.00000000	pigs
Apc	-0.01	1.00000000	pigs
Zc3h14	-0.09	1.00000000	pigs
Zfp422	-0.25	1.00000000	pigs
Heca	-0.02	1.00000000	pigs
Atp5c1	0.09	1.00000000	pigs
Ddx49	-0.10	1.00000000	pigs
2700062C07Rik	0.11	1.00000000	pigs
Eif2ak1	-0.13	1.00000000	pigs

Fam20b	0.02	1.00000000	pigs
Sptlc2	0.09	1.00000000	pigs
Dhx8	-0.13	1.00000000	pigs
Gtf3a	-0.13	1.00000000	pigs
Necap1	-0.11	1.00000000	pigs
Dusp4	0.14	1.00000000	pigs
Rsbn1l	-0.25	1.00000000	pigs
Tmem11	-0.07	1.00000000	pigs
Alg11	0.00	1.00000000	pigs
Gnpda1	-0.03	1.00000000	pigs
Ska2	-0.15	1.00000000	pigs
Tbl1xr1	-0.17	1.00000000	pigs
Pnkd	-0.02	1.00000000	pigs
Nxt1	-0.14	1.00000000	pigs
Zfp358	-0.16	1.00000000	pigs
Nox4	-0.33	1.00000000	pigs
Nsmce1	-0.10	1.00000000	pigs
Rnaseh2c	-0.03	1.00000000	pigs
Adamts10	-0.40	1.00000000	pigs
Nop58	-0.09	1.00000000	pigs
Rexo1	-0.14	1.00000000	pigs
Zfp800	-0.08	1.00000000	pigs
Pdzd11	-0.27	1.00000000	pigs
Rab8b	0.00	1.00000000	pigs
Pdcd7	0.11	1.00000000	pigs
Ints13	-0.13	1.00000000	pigs
Limd1	-0.03	1.00000000	pigs
Tanc2	0.07	1.00000000	pigs
Pcyox1	-0.17	1.00000000	pigs
Esf1	-0.25	1.00000000	pigs
Trappc11	-0.28	1.00000000	pigs
Zmym4	-0.03	1.00000000	pigs
Nelfb	-0.25	1.00000000	pigs
Ecd	-0.04	1.00000000	pigs
Rad23a	-0.26	1.00000000	pigs
Ctps2	-0.05	1.00000000	pigs
Hp1bp3	0.04	1.00000000	pigs
Zw10	-0.16	1.00000000	pigs
Slc25a24	-0.06	1.00000000	pigs
Trim37	-0.15	1.00000000	pigs
Fbxo11	0.07	1.00000000	pigs
Enpp1	-0.20	1.00000000	pigs
Plcg1	-0.18	1.00000000	pigs
9530068E07Rik	-0.07	1.00000000	pigs
BC003965	-0.08	1.00000000	pigs
Hsd17b11	-0.26	1.00000000	pigs

Gcc2	-0.19	1.00000000	pigs
Cab39	-0.10	1.00000000	pigs
Pip5k1b	-0.15	1.00000000	pigs
Gpx1	0.04	1.00000000	pigs
Stx4a	-0.03	1.00000000	pigs
Hsf2	-0.14	1.00000000	pigs
Abcf1	-0.15	1.00000000	pigs
Tex264	-0.17	1.00000000	pigs
Sinhcaf	-0.14	1.00000000	pigs
Elp4	-0.16	1.00000000	pigs
Dennd4c	-0.26	1.00000000	pigs
Secisbp2	-0.15	1.00000000	pigs
Rpia	0.07	1.00000000	pigs
Ranbp3	-0.17	1.00000000	pigs
Ythdc2	-0.27	1.00000000	pigs
Zcchc9	-0.13	1.00000000	pigs
Tax1bp3	-0.09	1.00000000	pigs
Hint1	0.23	1.00000000	pigs
Lamp2	0.00	1.00000000	pigs
Usp8	-0.12	1.00000000	pigs
Agap1	0.10	1.00000000	pigs
Arcn1	-0.06	1.00000000	pigs
Gpr180	-0.19	1.00000000	pigs
Nrde2	-0.09	1.00000000	pigs
Zfp94	-0.19	1.00000000	pigs
Ndufa2	-0.06	1.00000000	pigs
Tmtc2	0.29	1.00000000	pigs
St6galnac4	-0.10	1.00000000	pigs
Stpg4	0.03	1.00000000	pigs
Coil	-0.24	1.00000000	pigs
Prkd3	-0.38	1.00000000	pigs
Tpr	-0.04	1.00000000	pigs
Cib1	-0.22	1.00000000	pigs
Klhdc2	-0.18	1.00000000	pigs
Trappc3	-0.19	1.00000000	pigs
Hdgfl3	0.04	1.00000000	pigs
Ccdc125	-0.19	1.00000000	pigs
Dpysl3	-0.07	1.00000000	pigs
Ap4e1	-0.07	1.00000000	pigs
Phtf2	-0.07	1.00000000	pigs
Leng1	0.10	1.00000000	pigs
Tpd52l2	-0.24	1.00000000	pigs
Hsdl2	-0.03	1.00000000	pigs
Ppp1ca	-0.08	1.00000000	pigs
Tomm70a	-0.08	1.00000000	pigs
Nubp1	0.19	1.00000000	pigs

Rassf1	-0.23	1.00000000	pigs
Ern1	-0.16	1.00000000	pigs
Fam114a1	-0.26	1.00000000	pigs
Parp8	-0.12	1.00000000	pigs
Ccnb1ip1	-0.16	1.00000000	pigs
Vamp4	-0.08	1.00000000	pigs
Nck1	-0.25	1.00000000	pigs
Zbtb46	-0.35	1.00000000	pigs
Gar1	-0.10	1.00000000	pigs
Prickle2	-0.73	1.00000000	pigs
Gja4	-0.31	1.00000000	pigs
Pign	-0.19	1.00000000	pigs
Acadm	0.10	1.00000000	pigs
Cdc40	-0.17	1.00000000	pigs
Zfp330	-0.06	1.00000000	pigs
Git1	-0.24	1.00000000	pigs
Rsl1d1	-0.15	1.00000000	pigs
Pgm2l1	-0.17	1.00000000	pigs
Atg14	-0.08	1.00000000	pigs
Nhsl1	0.04	1.00000000	pigs
Tmem126b	-0.17	1.00000000	pigs
Orc5	-0.36	1.00000000	pigs
App	0.05	1.00000000	pigs
Grik4	-0.07	1.00000000	pigs
Tram1	-0.22	1.00000000	pigs
Ndufs5	-0.19	1.00000000	pigs
Zc3h13	-0.07	1.00000000	pigs
Ehd1	-0.06	1.00000000	pigs
Bin1	-0.05	1.00000000	pigs
Slc35b1	-0.15	1.00000000	pigs
Uaca	-0.34	1.00000000	pigs
Slc30a5	-0.26	1.00000000	pigs
Fam189a1	-0.15	1.00000000	pigs
Ccnh	-0.13	1.00000000	pigs
Hlcs	-0.34	1.00000000	pigs
Rsf1	-0.02	1.00000000	pigs
Klhl18	-0.23	1.00000000	pigs
Naxd	-0.08	1.00000000	pigs
Zfp638	-0.31	1.00000000	pigs
Dtx1	-0.01	1.00000000	pigs
Plcd1	-0.02	1.00000000	pigs
Osgepl1	-0.20	1.00000000	pigs
Plcl2	-0.08	1.00000000	pigs
Gorasp2	-0.16	1.00000000	pigs
Dll4	0.03	1.00000000	pigs
Gnl3	0.19	1.00000000	pigs

Utp20	-0.09	1.00000000	pigs
Ddrgk1	-0.08	1.00000000	pigs
Gcfc2	-0.28	1.00000000	pigs
Ctnnbip1	-0.02	1.00000000	pigs
Ak3	-0.13	1.00000000	pigs
Hmgcs1	0.61	1.00000000	pigs
Ruvbl1	0.05	1.00000000	pigs
Fam71e1	-0.06	1.00000000	pigs
Nipa2	0.01	1.00000000	pigs
Daxx	-0.13	1.00000000	pigs
Rrp36	-0.14	1.00000000	pigs
Sec11c	-0.20	1.00000000	pigs
Uck1	-0.14	1.00000000	pigs
Zc3h8	-0.27	1.00000000	pigs
Abcg3	-0.06	1.00000000	pigs
Pih1d1	-0.09	1.00000000	pigs
Dnajc10	-0.05	1.00000000	pigs
Gpn1	-0.05	1.00000000	pigs
Dera	-0.18	1.00000000	pigs
Riok3	-0.06	1.00000000	pigs
Cers4	-0.21	1.00000000	pigs
Yipf1	-0.23	1.00000000	pigs
Vps36	-0.09	1.00000000	pigs
Tmem50b	-0.11	1.00000000	pigs
Fmc1	0.02	1.00000000	pigs
Gpatch2	-0.32	1.00000000	pigs
Aqr	-0.10	1.00000000	pigs
Smndc1	-0.18	1.00000000	pigs
Tet3	-0.40	1.00000000	pigs
Selenos	-0.15	1.00000000	pigs
Dync2li1	-0.16	1.00000000	pigs
Cd274	0.01	1.00000000	pigs
Tcf25	-0.22	1.00000000	pigs
Paxx	-0.11	1.00000000	pigs
Bscl2	0.00	1.00000000	pigs
Fam162a	0.10	1.00000000	pigs
Tmem43	-0.25	1.00000000	pigs
Usp25	-0.16	1.00000000	pigs
Aars	0.00	1.00000000	pigs
Steep1	0.08	1.00000000	pigs
Exosc4	-0.01	1.00000000	pigs
Zfp462	-0.24	1.00000000	pigs
Hdac2	-0.05	1.00000000	pigs
Cmah	-0.02	1.00000000	pigs
Dctn3	0.02	1.00000000	pigs
Sart1	-0.19	1.00000000	pigs

Fbxo8	-0.10	1.00000000	pigs
Cyp2s1	-0.24	1.00000000	pigs
Fbxo25	0.05	1.00000000	pigs
Ndufa11	0.02	1.00000000	pigs
Tmpo	-0.16	1.00000000	pigs
Hnrnph3	0.02	1.00000000	pigs
Gtf2f2	0.02	1.00000000	pigs
St3gal3	-0.03	1.00000000	pigs
Gadd45gip1	-0.03	1.00000000	pigs
Hnrnpll	-0.23	1.00000000	pigs
Cdc34	0.05	1.00000000	pigs
Psen1	-0.12	1.00000000	pigs
Stag1	-0.06	1.00000000	pigs
Dscc1	-0.08	1.00000000	pigs
Stard3nl	-0.02	1.00000000	pigs
Neto2	-0.24	1.00000000	pigs
