# Supplementary material for: Heterogeneous viral-host response at single-cell resolution and viral adaptive mutational atlas in birds and mammals upon H5 influenza virus infection
Source: Natl Sci Rev. 2026 Jun 25;13(14):nwag380. doi: 10.1093/nsr/nwag380 (PMC13458601; doi:10.1093/nsr/nwag380)
Supplement: Supplementary file 1 [file nwag380_supplemental_files.zip › Supplementary Data 14.pdf]

Supplementary Data 14. Differentially expressed genes of neutrophils across different species upon H5 HPAIV infection.

gene	log2FoldChange	p value	host
Ifit2	5.09	0.00000000	chicken
Sgk1	3.74	0.00000000	chicken
Smarcal1	-2.92	0.00000000	chicken
Tpt1	-3.03	0.00000000	chicken
Ccl9	2.76	0.00000000	chicken
Nos2	5.13	0.00000000	chicken
Plod2	3.69	0.00000000	chicken
Cd3g	-3.28	0.00000000	chicken
Tfec	3.77	0.00000000	chicken
Spi1	2.17	0.00000000	chicken
Tasl	3.06	0.00000000	chicken
Acod1	2.72	0.00000000	chicken
H3f3a	-2.54	0.00000000	chicken
Il1b	3.16	0.00000000	chicken
Itga4	-2.95	0.00000000	chicken
Tcf7	-3.65	0.00000000	chicken
Il6	4.53	0.00000000	chicken
Ptprj	2.88	0.00000000	chicken
Grb10	3.03	0.00000000	chicken
Snx10	2.48	0.00000000	chicken
Birc2	2.44	0.00000000	chicken
Hpgd	3.00	0.00000000	chicken
Pparg	2.70	0.00000000	chicken
Alcam	3.53	0.00000000	chicken
Plin2	2.38	0.00000000	chicken
Prdm1	3.49	0.00000000	chicken
Vmp1	2.66	0.00000000	chicken
Tax1bp1	2.57	0.00000000	chicken
Oasl2	2.74	0.00000000	chicken
Btg1b	-2.30	0.00000000	chicken
Eef1b2	-2.76	0.00000000	chicken
Rasgef1a	3.13	0.00000000	chicken
Eef1d	-2.63	0.00000000	chicken
Rack1	-2.76	0.00000000	chicken
Cd3e	-2.29	0.00000000	chicken
Kcnq5	-2.95	0.00000000	chicken
Trak1	3.08	0.00000000	chicken
Tgm4	3.83	0.00000000	chicken
Dgkh	2.46	0.00000000	chicken
Osbpl5	3.04	0.00000000	chicken
Cd83	2.95	0.00000000	chicken

Jarid2	-1.61	0.00000000	chicken
Bst1	2.17	0.00000000	chicken
Ets1	-2.62	0.00000000	chicken
Fap	3.73	0.00000000	chicken
Tnfaip3	1.97	0.00000000	chicken
Eef2	-2.34	0.00000000	chicken
Nme2	-2.34	0.00000000	chicken
F3	3.53	0.00000000	chicken
Aff2	-1.96	0.00000000	chicken
Rnaset2b	2.38	0.00000000	chicken
Slc44a3	3.28	0.00000000	chicken
Igdcc4	2.89	0.00000000	chicken
Rab20	1.56	0.00000000	chicken
Ifih1	4.27	0.00000000	chicken
Dusp1	1.98	0.00000000	chicken
Fnbp1	-2.15	0.00000000	chicken
Cd247	-2.43	0.00000000	chicken
Dennd4a	1.52	0.00000000	chicken
Socs3	1.99	0.00000000	chicken
Zfp593	-1.88	0.00000000	chicken
Pde3b	-1.70	0.00000000	chicken
Bcl11b	-2.12	0.00000000	chicken
Ckap2l	3.24	0.00000000	chicken
Rabgap1l	-2.09	0.00000000	chicken
Serbp1	-1.89	0.00000000	chicken
Fau	-2.05	0.00000000	chicken
Nus1	1.92	0.00000000	chicken
Spidr	2.73	0.00000000	chicken
Hivep1	1.81	0.00000000	chicken
Tnip2	1.54	0.00000000	chicken
Creb5	2.85	0.00000000	chicken
Pla2r1	2.04	0.00000000	chicken
Pid1	2.35	0.00000000	chicken
Cblb	-2.49	0.00000000	chicken
Baz2b	2.15	0.00000000	chicken
Zc3h12d	-2.79	0.00000000	chicken
Ybx1	-1.97	0.00000000	chicken
Stat4	-2.33	0.00000000	chicken
Flvcr2	2.37	0.00000000	chicken
Atf3	1.88	0.00000000	chicken
Casp1	2.64	0.00000000	chicken
Pla2g4a	2.31	0.00000000	chicken
Trio	2.58	0.00000000	chicken
Actg1	-1.20	0.00000000	chicken
Sdc4	3.14	0.00000000	chicken
St3gal6	2.17	0.00000000	chicken

Trank1	2.56	0.00000000	chicken
Brsk2	-3.16	0.00000000	chicken
Rubcnl	1.62	0.00000000	chicken
Bzw2	1.62	0.00000000	chicken
Zeb2	1.33	0.00000000	chicken
BC016579	2.35	0.00000000	chicken
Ly75	2.22	0.00000000	chicken
Dram1	1.95	0.00000000	chicken
Anxa2	1.65	0.00000000	chicken
Rgs1	2.09	0.00000000	chicken
Klf6	1.36	0.00000000	chicken
Ppa2	2.09	0.00000000	chicken
Hpcal1	-2.06	0.00000000	chicken
Lpgat1	2.10	0.00000000	chicken
Zfp516	1.77	0.00000000	chicken
Nsmce2	1.75	0.00000000	chicken
F13a1	2.33	0.00000000	chicken
Pabpc1	-1.37	0.00000000	chicken
Angpt1	1.72	0.00000000	chicken
Lrba	-1.99	0.00000000	chicken
Sbf2	2.29	0.00000000	chicken
Bach1	1.27	0.00000000	chicken
Foxo1	-2.34	0.00000000	chicken
Xdh	1.95	0.00000000	chicken
Bach2	-2.22	0.00000000	chicken
Fos	1.66	0.00000000	chicken
Sptbn1	-1.50	0.00000000	chicken
Antxr2	1.48	0.00000000	chicken
Fry	1.72	0.00000000	chicken
Helz2	3.17	0.00000000	chicken
Chd7	1.40	0.00000000	chicken
Haus6	1.76	0.00000000	chicken
Eif1	-1.67	0.00000000	chicken
Susd1	2.44	0.00000000	chicken
Il10ra	1.71	0.00000000	chicken
Ddx5	-1.29	0.00000000	chicken
Arxes2	-1.81	0.00000000	chicken
Ncf4	1.74	0.00000000	chicken
Kcnma1	2.38	0.00000000	chicken
Lipa	1.83	0.00000000	chicken
Ptgs2	2.77	0.00000000	chicken
Tmsb4x	-0.94	0.00000000	chicken
Scin	-1.91	0.00000000	chicken
Hnrnpa3	-1.43	0.00000000	chicken
Pdlim5	1.20	0.00000000	chicken
Ddx3y	-1.28	0.00000000	chicken

Slc39a13	1.79	0.00000000	chicken
Calcb	-1.98	0.00000000	chicken
Zpld1	2.64	0.00000000	chicken
Nfe2l2	1.24	0.00000000	chicken
Acta2	2.45	0.00000000	chicken
Zdhhc20	1.95	0.00000000	chicken
Ints14	1.30	0.00000000	chicken
Syne1	-1.64	0.00000000	chicken
Gcg	2.65	0.00000000	chicken
Ankrd44	-1.97	0.00000000	chicken
Dpp4	3.62	0.00000000	chicken
Lgmn	1.57	0.00000000	chicken
Itm2a	-1.44	0.00000000	chicken
Elmo1	-2.08	0.00000000	chicken
Tln2	3.59	0.00000000	chicken
Net1	1.53	0.00000000	chicken
Socs1	1.83	0.00000000	chicken
Tet2	1.48	0.00000000	chicken
Nfkbiz	1.25	0.00000000	chicken
B2m	-1.11	0.00000000	chicken
Etv6	1.52	0.00000000	chicken
Cflar	2.23	0.00000000	chicken
Aplp2	1.29	0.00000000	chicken
Hif1a	1.29	0.00000000	chicken
Il27ra	-1.89	0.00000000	chicken
Septin10	1.67	0.00000000	chicken
Arid1b	-1.85	0.00000000	chicken
Rnf19b	1.64	0.00000000	chicken
Rasgef1b	1.93	0.00000000	chicken
Uvrag	1.44	0.00000000	chicken
Ccnl1	-1.49	0.00000000	chicken
Dusp16	1.73	0.00000000	chicken
Ninj1	1.21	0.00000000	chicken
Il20ra	-2.31	0.00000000	chicken
Dpp8	1.76	0.00000000	chicken
Thsd4	1.77	0.00000000	chicken
Dst	3.08	0.00000000	chicken
Dock7	1.59	0.00000000	chicken
Nap1l1	-1.85	0.00000000	chicken
Plxna1	1.71	0.00000000	chicken
Lao1	1.84	0.00000000	chicken
Slk	-1.83	0.00000000	chicken
Zfp704	-1.94	0.00000000	chicken
Spred1	2.27	0.00000000	chicken
Csrnp1	1.47	0.00000000	chicken
Sema3c	2.50	0.00000000	chicken

Laptm5	-1.83	0.00000000	chicken
Fyco1	2.74	0.00000000	chicken
Ptafr	0.93	0.00000000	chicken
Erc1	2.37	0.00000000	chicken
Insr	1.19	0.00000000	chicken
Traf3	1.32	0.00000000	chicken
Pag1	-1.93	0.00000000	chicken
Rngtt	1.49	0.00000000	chicken
Tnfsf8	-1.79	0.00000000	chicken
Dock2	-2.10	0.00000000	chicken
Phactr2	-1.98	0.00000000	chicken
Icos	-2.08	0.00000000	chicken
Susd6	-1.69	0.00000000	chicken
Grap2	-1.87	0.00000000	chicken
Atp6v0d2	1.23	0.00000000	chicken
Prex1	-1.29	0.00000000	chicken
Pdgfa	2.04	0.00000000	chicken
Abcc5	1.58	0.00000000	chicken
Ear1	1.15	0.00000000	chicken
Pcmtd1	-1.83	0.00000000	chicken
Als2cl	1.20	0.00000000	chicken
Lef1	-1.75	0.00000000	chicken
Tox	-1.91	0.00000000	chicken
Wnk1	1.95	0.00000000	chicken
Wdfy3	1.60	0.00000000	chicken
Ptprc	-0.99	0.00000000	chicken
Ppard	1.39	0.00000000	chicken
Prkce	-1.92	0.00000000	chicken
Ralb	1.77	0.00000000	chicken
Plscr1	0.94	0.00000000	chicken
Syde2	1.18	0.00000000	chicken
Tasor2	1.24	0.00000000	chicken
Pfkfb3	1.37	0.00000000	chicken
5031439G07Rik	1.69	0.00000000	chicken
Htra1	1.41	0.00000000	chicken
Pde7b	-2.15	0.00000000	chicken
Zdhhc14	1.78	0.00000000	chicken
Tmem123	1.50	0.00000000	chicken
Rnf213	1.34	0.00000000	chicken
Zbtb46	2.02	0.00000000	chicken
Eif3l	-1.46	0.00000000	chicken
Nr4a3	1.10	0.00000000	chicken
Hnrnph2	-1.35	0.00000000	chicken
Pik3ap1	1.15	0.00000000	chicken
Sntb1	1.46	0.00000000	chicken
Irf4	-1.91	0.00000000	chicken

Ptpn2	1.25	0.00000000	chicken
Fermt1	1.48	0.00000000	chicken
Rras2	-1.77	0.00000000	chicken
Hbegf	1.43	0.00000000	chicken
Bcl6	1.48	0.00000000	chicken
Abcc1	1.62	0.00000000	chicken
Lyn	0.98	0.00000000	chicken
Ccdc88a	1.69	0.00000000	chicken
Rbm39	-1.23	0.00000000	chicken
Nmi	1.26	0.00000000	chicken
Nadk	1.13	0.00000000	chicken
Abcc4	1.54	0.00000000	chicken
Cd200	1.52	0.00000000	chicken
Sipa1l2	1.27	0.00000000	chicken
Snap47	1.41	0.00000000	chicken
Rsad2	2.34	0.00000000	chicken
Atxn7l1	1.33	0.00000000	chicken
Dnajb9	1.29	0.00000000	chicken
Zfp352	1.89	0.00000000	chicken
Syt16	2.04	0.00000000	chicken
Ch25h	1.40	0.00000000	chicken
Irf1	1.64	0.00000000	chicken
Lsmem1	1.21	0.00000000	chicken
Txnrd1	2.37	0.00000000	chicken
Runx3	-2.01	0.00000000	chicken
H3f3b	-1.03	0.00000000	chicken
Cpne8	1.84	0.00000000	chicken
Arhgap11a	1.72	0.00000000	chicken
Ikzf1	-1.88	0.00000000	chicken
Dclk3	1.88	0.00000000	chicken
Ly96	1.43	0.00000000	chicken
Tcf7l2	1.96	0.00000000	chicken
Dock10	-1.21	0.00000000	chicken
Fabp3	1.98	0.00000000	chicken
Ikzf3	-1.60	0.00000000	chicken
Sh3bp2	1.44	0.00000000	chicken
Ubtd2	1.25	0.00000000	chicken
Polk	2.11	0.00000000	chicken
Parvb	1.38	0.00000000	chicken
Ybx3	-1.68	0.00000000	chicken
Pacsin2	1.06	0.00000000	chicken
Lbh	-1.95	0.00000000	chicken
Il1r2	0.96	0.00000000	chicken
Tia1	1.45	0.00000000	chicken
Jun	1.18	0.00000000	chicken
Arhgap12	1.34	0.00000000	chicken

Tnfrsf9	-1.81	0.00000000	chicken
Fam102a	-1.62	0.00000000	chicken
Eif3e	-1.56	0.00000000	chicken
Bmpr1b	2.33	0.00000000	chicken
Cd81	0.99	0.00000000	chicken
Btg2	1.38	0.00000000	chicken
Runx1	-1.33	0.00000000	chicken
Ctps2	2.47	0.00000000	chicken
Npas2	1.68	0.00000000	chicken
Vgll4	1.56	0.00000000	chicken
Prkar2a	-1.91	0.00000000	chicken
Il18rap	1.86	0.00000000	chicken
Dek	-1.56	0.00000000	chicken
Rcor1	1.14	0.00000000	chicken
Rgs18	1.35	0.00000000	chicken
Gab1	1.54	0.00000000	chicken
Eepd1	1.57	0.00000000	chicken
Chst13	1.10	0.00000000	chicken
Adamtsl2	1.15	0.00000000	chicken
Klf12	-1.71	0.00000000	chicken
Tomm20	-1.42	0.00000000	chicken
Klf13	-1.48	0.00000000	chicken
Coro1c	-0.99	0.00000000	chicken
Cd28	-1.79	0.00000000	chicken
Dennd1b	-1.12	0.00000000	chicken
Slc17a6	1.33	0.00000000	chicken
G0s2	2.84	0.00000000	chicken
Bcl2a1d	1.29	0.00000000	chicken
Apbb1ip	-1.38	0.00000000	chicken
Ppm1d	1.40	0.00000000	chicken
Rdh10	1.67	0.00000000	chicken
Sat1	1.22	0.00000000	chicken
Atp10d	1.42	0.00000000	chicken
Ctsc	0.85	0.00000000	chicken
Svbp	-1.21	0.00000000	chicken
2900026A02Rik	-2.36	0.00000000	chicken
S100a6	1.04	0.00000001	chicken
Acsl3	-1.70	0.00000001	chicken
Tenm3	2.23	0.00000001	chicken
Osbpl3	-1.82	0.00000001	chicken
Slc43a2	-1.53	0.00000001	chicken
Tsc22d3	1.23	0.00000001	chicken
Eif4a2	-0.97	0.00000001	chicken
Ccl20	3.59	0.00000001	chicken
H2az2	-1.28	0.00000001	chicken
Ptpn14	-1.66	0.00000001	chicken

Ssu72	1.30	0.00000001	chicken
Eya3	1.25	0.00000001	chicken
Cd82	-1.55	0.00000001	chicken
Dio2	2.69	0.00000001	chicken
Ywhag	1.10	0.00000001	chicken
Phactr3	1.32	0.00000001	chicken
Ets2	1.23	0.00000001	chicken
Hnrnpa2b1	-1.20	0.00000001	chicken
Dusp5	1.26	0.00000001	chicken
Sh3kbp1	-1.57	0.00000001	chicken
S100a10	0.93	0.00000001	chicken
Spire1	2.13	0.00000002	chicken
Tgif1	0.88	0.00000002	chicken
Spata5	1.93	0.00000002	chicken
Acot10	0.56	0.00000002	chicken
Csnk2a2	-1.30	0.00000002	chicken
Grk3	-1.41	0.00000002	chicken
P2ry13	1.03	0.00000002	chicken
Slc29a4	-1.39	0.00000002	chicken
Commd1b	1.36	0.00000002	chicken
Hipk2	1.64	0.00000002	chicken
Fnip1	0.84	0.00000002	chicken
Swap70	1.00	0.00000003	chicken
Fam13a	1.26	0.00000003	chicken
Ythdc1	-1.07	0.00000003	chicken
Emp1	1.79	0.00000003	chicken
Pard3	1.59	0.00000003	chicken
Baiap2l1	1.11	0.00000003	chicken
Dll1	2.47	0.00000003	chicken
Ipmk	-1.66	0.00000003	chicken
Hnrnpa1	-1.29	0.00000003	chicken
Lrp6	1.44	0.00000004	chicken
Adgrd1	1.47	0.00000004	chicken
Ago3	-1.72	0.00000004	chicken
Brip1	2.00	0.00000004	chicken
Dnajc1	-1.63	0.00000004	chicken
Pan3	-1.42	0.00000005	chicken
Tlr4	0.91	0.00000006	chicken
Slc2a3	-1.25	0.00000006	chicken
Dennd1a	1.83	0.00000007	chicken
Prnp	0.97	0.00000007	chicken
Pgs1	0.79	0.00000007	chicken
Dennd4c	1.38	0.00000007	chicken
Jak1	-1.00	0.00000008	chicken
Stk40	0.69	0.00000008	chicken
Pim3	1.31	0.00000008	chicken

Fgd4	1.54	0.00000008	chicken
Hectd2	1.37	0.00000009	chicken
Spag16	-1.84	0.00000009	chicken
Capn3	1.40	0.00000009	chicken
Kcnb1	1.56	0.00000009	chicken
Aco1	1.01	0.00000010	chicken
Calm2	-1.38	0.00000011	chicken
Cst3	-2.37	0.00000011	chicken
Cxadr	1.37	0.00000011	chicken
Fkbp5	-1.15	0.00000011	chicken
Gnas	1.21	0.00000012	chicken
Scg5	1.14	0.00000013	chicken
Itpr2	-1.67	0.00000013	chicken
Ifngr1	-1.55	0.00000013	chicken
Tnip3	1.47	0.00000014	chicken
Numb	1.11	0.00000015	chicken
Cdc73	-1.76	0.00000015	chicken
Ptpn22	-1.34	0.00000018	chicken
Btbd11	0.93	0.00000020	chicken
Glrx	1.07	0.00000020	chicken
Kat6b	-1.63	0.00000021	chicken
Ifnar1	0.58	0.00000021	chicken
Fbxl2	-1.75	0.00000021	chicken
Slc25a3	-1.48	0.00000022	chicken
Slc22a3	-1.79	0.00000022	chicken
Atg7	2.39	0.00000023	chicken
Nptx2	1.40	0.00000024	chicken
Wwp1	1.21	0.00000025	chicken
Ttc39a	0.88	0.00000025	chicken
Rbm6	0.91	0.00000027	chicken
Ube3c	1.19	0.00000029	chicken
Gab2	1.30	0.00000033	chicken
Csf3	1.35	0.00000035	chicken
Gbp8	1.35	0.00000041	chicken
Inpp5d	-1.37	0.00000045	chicken
Ppfibp1	-1.40	0.00000048	chicken
Atp1b3	1.03	0.00000056	chicken
Inhbb	1.10	0.00000058	chicken
Eif3f	-1.11	0.00000060	chicken
Slc8a1	1.24	0.00000062	chicken
Cyrib	-1.31	0.00000066	chicken
Arhgap31	1.05	0.00000069	chicken
Cytip	-1.22	0.00000069	chicken
Slc38a6	-1.38	0.00000071	chicken
Csta1	1.99	0.00000074	chicken
B3gnt5	1.24	0.00000080	chicken

B3gnt2	-1.45	0.00000083	chicken
G3bp1	-1.14	0.00000089	chicken
Neurl1b	1.88	0.00000089	chicken
Ptpre	1.62	0.00000095	chicken
Cdk14	-1.48	0.00000102	chicken
Smap1	-1.42	0.00000108	chicken
Csf2ra	0.78	0.00000118	chicken
Arhgap15	-1.11	0.00000119	chicken
Srsf10	-1.16	0.00000123	chicken
Sh3bp5	1.11	0.00000131	chicken
Pacc1	1.20	0.00000139	chicken
Pou2f1	1.17	0.00000142	chicken
Adap2	0.88	0.00000143	chicken
Pdss2	-1.49	0.00000149	chicken
Zap70	-1.10	0.00000151	chicken
Igdcc3	1.47	0.00000155	chicken
Obp2a	2.18	0.00000161	chicken
Inpp5a	-1.13	0.00000165	chicken
Ndnf	0.96	0.00000170	chicken
Gns	1.25	0.00000175	chicken
Tbc1d7	0.94	0.00000175	chicken
Cox6a1	-1.20	0.00000180	chicken
Nek10	1.64	0.00000198	chicken
Basp1	0.80	0.00000207	chicken
Sumo3	-0.98	0.00000225	chicken
Fhod1	1.15	0.00000232	chicken
Bend6	1.44	0.00000234	chicken
Cyfip2	-1.40	0.00000240	chicken
Wnt5b	1.42	0.00000252	chicken
Qk	1.07	0.00000259	chicken
Lbr	-1.16	0.00000276	chicken
Rhoa	-0.84	0.00000281	chicken
Ankrd42	0.77	0.00000288	chicken
Nfatc2	-1.19	0.00000292	chicken
Prdx6	0.99	0.00000296	chicken
Arsg	1.07	0.00000350	chicken
Htr7	0.97	0.00000371	chicken
Tssc4	0.69	0.00000375	chicken
Itpr1	-1.96	0.00000401	chicken
Fryl	-1.26	0.00000420	chicken
Eif2s3y	-1.10	0.00000433	chicken
Crtc1	-1.27	0.00000442	chicken
Rab30	1.10	0.00000446	chicken
Gpr68	0.90	0.00000468	chicken
Urm1	-1.17	0.00000516	chicken
Birc6	-1.31	0.00000537	chicken

Atp11c	0.95	0.00000553	chicken
Egr1	1.07	0.00000573	chicken
Hk2	0.93	0.00000589	chicken
Cubn	1.34	0.00000599	chicken
Gpat3	1.01	0.00000649	chicken
Sbk2	0.67	0.00000659	chicken
Arf6	-1.34	0.00000662	chicken
Rftn2	1.19	0.00000771	chicken
Il15	0.82	0.00000798	chicken
Hectd1	1.09	0.00000847	chicken
Ifngr2	0.79	0.00000983	chicken
Hnrnpr	-1.38	0.00001000	chicken
Cry1	-1.66	0.00001013	chicken
Pfdn1	1.03	0.00001019	chicken
Ssh2	-1.52	0.00001029	chicken
Blk	-1.24	0.00001031	chicken
Ppil4	-1.68	0.00001035	chicken
Slc35e2	1.00	0.00001037	chicken
Sorbs1	1.06	0.00001054	chicken
Pde8a	1.59	0.00001064	chicken
Gnai1	-1.23	0.00001118	chicken
Zc3hav1l	1.05	0.00001120	chicken
Sp3	-1.50	0.00001132	chicken
Gata3	-0.88	0.00001289	chicken
Cpt1a	1.05	0.00001293	chicken
Acsl1	0.44	0.00001303	chicken
Nupl1	1.67	0.00001310	chicken
Snapc1l	1.03	0.00001320	chicken
Tmem106b	-1.28	0.00001328	chicken
Ssbp3	-1.18	0.00001383	chicken
Ttll5	1.92	0.00001394	chicken
Zfp521	-1.60	0.00001413	chicken
Arhgef3	-1.40	0.00001466	chicken
Kdm4b	-1.57	0.00001556	chicken
Daw1	2.36	0.00001606	chicken
Satb1	-1.56	0.00001621	chicken
Xpo6	-1.44	0.00001666	chicken
Rapgef2	-0.61	0.00001730	chicken
Anxa6	-0.92	0.00001758	chicken
Pard3b	2.09	0.00001784	chicken
Arid4b	-1.39	0.00001830	chicken
Babam2	1.03	0.00001943	chicken
Atp11a	0.75	0.00001968	chicken
Atp1b1	0.85	0.00002007	chicken
Sash1	1.50	0.00002100	chicken
Lamp3	1.14	0.00002158	chicken

Thoc2	-1.14	0.00002194	chicken
Fbln5	0.93	0.00002215	chicken
Aldh1a3	1.50	0.00002228	chicken
Cd200r1	1.02	0.00002373	chicken
Psme4	-1.19	0.00002408	chicken
Add3	-0.85	0.00002548	chicken
Iqcb1	0.78	0.00002566	chicken
Hsp90ab1	-1.99	0.00002574	chicken
Rassf3	1.01	0.00002640	chicken
Sestd1	0.88	0.00002659	chicken
Tmed10	-1.27	0.00002682	chicken
Uxs1	-1.12	0.00002683	chicken
Klhl18	0.88	0.00002730	chicken
Insc	1.72	0.00002745	chicken
Gadd45b	0.83	0.00002777	chicken
Fgf2	0.75	0.00002828	chicken
Lrmda	1.42	0.00002843	chicken
Tbc1d1	1.09	0.00002847	chicken
Bhlhe40	1.00	0.00002870	chicken
Hnrnpll	-1.36	0.00002893	chicken
Glipr2	0.82	0.00002968	chicken
Sergef	1.88	0.00002983	chicken
Arih2	1.01	0.00003081	chicken
Slc2a9	1.04	0.00003227	chicken
Larp4	0.97	0.00003587	chicken
Rp2	0.73	0.00003627	chicken
Tspan13	0.69	0.00003690	chicken
Emb	-2.21	0.00003747	chicken
C9orf72	0.92	0.00003922	chicken
Cirbp	-1.23	0.00003937	chicken
BC051665	1.04	0.00004017	chicken
Tll2	1.37	0.00004103	chicken
Grk4	-1.12	0.00004113	chicken
Grap	-0.92	0.00004257	chicken
C1qa	0.73	0.00004272	chicken
Tgfbr2	-0.94	0.00004297	chicken
Cacnb2	2.31	0.00004340	chicken
Ulbp1	-1.30	0.00004468	chicken
Inhba	1.89	0.00004845	chicken
Wdsub1	0.91	0.00005659	chicken
Lhfpl2	0.68	0.00005689	chicken
Zmynd11	-1.24	0.00005766	chicken
Sh3bgrl3	-1.41	0.00005797	chicken
Eif3m	-1.05	0.00005919	chicken
Prpsap1	0.80	0.00005921	chicken
Tmem266	0.91	0.00005938	chicken

Calm1	-1.05	0.00005946	chicken
Tmem268	0.89	0.00005998	chicken
Cep192	0.78	0.00006216	chicken
Rab38	0.62	0.00006341	chicken
Tox2	-1.09	0.00006528	chicken
Pdzd8	-1.22	0.00006729	chicken
Ccser1	0.81	0.00006924	chicken
Hmgb1	-1.10	0.00007095	chicken
Taf3	-1.33	0.00007120	chicken
Cstf3	0.98	0.00007258	chicken
Runx2	-1.87	0.00007329	chicken
Csde1	-1.15	0.00007500	chicken
Bnip3l	-1.23	0.00008108	chicken
Tbxas1	0.96	0.00008191	chicken
Slc2a6	0.84	0.00008687	chicken
Stk17b	-1.26	0.00008982	chicken
Bin1	1.14	0.00009326	chicken
Wwox	-2.17	0.00009601	chicken
Hibadh	0.34	0.00009620	chicken
Chac1	0.72	0.00009620	chicken
Dstn	-0.74	0.00009661	chicken
Grid1	1.36	0.00010195	chicken
Hsf2bp	0.75	0.00010375	chicken
Litaf	0.61	0.00011983	chicken
Rasa3	-1.25	0.00013129	chicken
Rpn1	-0.94	0.00014020	chicken
Cybb	0.88	0.00014166	chicken
Tbc1d15	0.84	0.00014370	chicken
Il13ra1	1.11	0.00014658	chicken
Sqstm1	1.29	0.00014989	chicken
Hacd4	1.43	0.00015021	chicken
Slc4a10	0.98	0.00015497	chicken
Ly6e	1.23	0.00016771	chicken
Cndp2	0.74	0.00016993	chicken
Ifng	1.83	0.00017893	chicken
Hsp90b1	-1.23	0.00018437	chicken
Klf15	0.70	0.00018800	chicken
Cyria	-1.05	0.00018882	chicken
Ss18l1	0.88	0.00019224	chicken
Add1	1.52	0.00019304	chicken
Galnt1	-0.99	0.00019364	chicken
Inpp4b	-1.50	0.00019463	chicken
Rab2a	-1.11	0.00020797	chicken
Ttll1	0.70	0.00021133	chicken
Nacc2	0.73	0.00021296	chicken
Tbk1	-1.18	0.00022411	chicken

Chsy1	0.76	0.00022820	chicken
Tnrc6c	-1.23	0.00023204	chicken
Rbm47	0.92	0.00023633	chicken
Eps15	0.75	0.00023940	chicken
Nucks1	-1.20	0.00024833	chicken
Garem1	1.03	0.00025303	chicken
Scfd1	-0.86	0.00026460	chicken
Necab2	1.45	0.00026783	chicken
Atp11b	-1.41	0.00027038	chicken
Tspan18	0.72	0.00027712	chicken
Rbm17	0.94	0.00028619	chicken
Cep170	1.46	0.00029701	chicken
Nol9	-1.29	0.00029844	chicken
Eloa	0.73	0.00030038	chicken
Rsph3b	0.87	0.00030725	chicken
Slc15a4	1.11	0.00031237	chicken
Scn8a	0.59	0.00031360	chicken
Sugt1	0.87	0.00034118	chicken
Fbxo34	-1.18	0.00034266	chicken
Rragd	0.57	0.00036270	chicken
Ddx60	0.46	0.00037799	chicken
Golim4	1.09	0.00040457	chicken
Nsa2	-1.11	0.00040768	chicken
Rbm31y	-1.24	0.00041532	chicken
Tubb1	0.85	0.00041596	chicken
Ammecr1	0.84	0.00041725	chicken
Lars2	0.73	0.00042211	chicken
4932438A13Rik	0.59	0.00042394	chicken
Tmem50a	-0.80	0.00045855	chicken
Traf5	-0.97	0.00046357	chicken
Hivep3	0.81	0.00047089	chicken
Sft2d1	1.07	0.00049528	chicken
Reps1	-1.15	0.00049879	chicken
Steap4	1.31	0.00050236	chicken
Cxcr4	-1.39	0.00051518	chicken
Rassf5	0.58	0.00052084	chicken
Tmprss7	0.84	0.00052197	chicken
Primpol	0.75	0.00052197	chicken
Dlx1	1.30	0.00052197	chicken
Kazn	0.71	0.00052197	chicken
Ankrd17	-1.25	0.00052608	chicken
Arpc1b	-1.16	0.00052940	chicken
Dnm1	0.59	0.00055257	chicken
Jdp2	0.82	0.00055315	chicken
Golga5	0.96	0.00056498	chicken
Slc10a7	0.50	0.00057413	chicken

Cox7b	-1.08	0.00057565	chicken
Rad51b	0.97	0.00058952	chicken
Cd2ap	0.99	0.00060500	chicken
Disc1	0.48	0.00064669	chicken
Tnrc6b	-1.03	0.00064957	chicken
Rhoq	0.86	0.00065371	chicken
Phlda2	1.28	0.00066706	chicken
Itga2	1.11	0.00066722	chicken
Ppp1cc	-1.12	0.00067590	chicken
Il2rb	-1.40	0.00067935	chicken
Iqsec1	1.24	0.00070976	chicken
Lsm14a	-1.19	0.00074142	chicken
Trib1	0.16	0.00074492	chicken
Comt	0.68	0.00079700	chicken
Ube2e1	0.67	0.00081423	chicken
Pi4ka	-1.21	0.00085280	chicken
Irf7	0.95	0.00085916	chicken
Vegfa	0.87	0.00089122	chicken
Cited4	0.83	0.00091534	chicken
Naca	-1.10	0.00093454	chicken
Usp25	0.82	0.00094377	chicken
Hopx	1.14	0.00104482	chicken
Plcg1	-0.79	0.00104697	chicken
Rassf2	0.80	0.00108440	chicken
Tpd52l2	0.58	0.00108762	chicken
Fbxo11	1.14	0.00110687	chicken
Cemip2	0.83	0.00112308	chicken
Cdc42ep4	0.67	0.00114517	chicken
Crip1	-1.49	0.00115017	chicken
Arpc2	-0.79	0.00115825	chicken
Ndufa4	-0.90	0.00116585	chicken
Ppia	-1.04	0.00116592	chicken
Tpr	-1.00	0.00117797	chicken
Cnot1	-1.00	0.00118766	chicken
Pdia3	-1.28	0.00124939	chicken
Slc46a2	0.68	0.00127937	chicken
Trpm7	1.90	0.00132378	chicken
Plekhm3	0.68	0.00132759	chicken
Lysmd2	0.77	0.00136939	chicken
Itga8	1.96	0.00137350	chicken
Spty2d1	-0.97	0.00140940	chicken
Klf10	0.82	0.00141785	chicken
Tspan9	0.66	0.00143663	chicken
Plcb2	0.74	0.00144569	chicken
Ank	0.74	0.00150152	chicken
Lrrk1	1.01	0.00151338	chicken

Rbpj	1.04	0.00154085	chicken
Fam171b	0.59	0.00159832	chicken
Epb41	-1.12	0.00160937	chicken
Rcc1l	0.56	0.00161497	chicken
Arfgef3	1.81	0.00167026	chicken
Cpeb2	1.19	0.00169662	chicken
Wdfy2	0.50	0.00169781	chicken
Arhgap24	1.71	0.00172746	chicken
Stat3	-1.20	0.00180001	chicken
Nr3c1	0.63	0.00182168	chicken
Rasa2	-0.93	0.00183493	chicken
Klf7	0.77	0.00185094	chicken
Faf1	0.58	0.00191971	chicken
Bcl2l1	0.48	0.00198369	chicken
Vsig4	0.93	0.00203084	chicken
Ckb	0.79	0.00208181	chicken
Chn1	0.73	0.00211752	chicken
Arhgef12	-0.97	0.00215092	chicken
Prkg2	0.91	0.00217740	chicken
Eml4	-0.97	0.00221076	chicken
Rad54b	0.68	0.00227957	chicken
Fsbp	0.65	0.00232395	chicken
Atp2a3	-1.05	0.00233303	chicken
Atp6v1h	0.32	0.00234311	chicken
Tomm7	-1.04	0.00234328	chicken
Neurl1a	0.87	0.00237581	chicken
Ankrd22	0.99	0.00237581	chicken
Enpp6	0.79	0.00244748	chicken
Hmgn3	-1.00	0.00247021	chicken
Bend5	0.66	0.00254461	chicken
Upf1	-1.06	0.00256313	chicken
Rgs13	0.88	0.00258502	chicken
Ccni	-0.84	0.00267774	chicken
Timp4	0.91	0.00270377	chicken
Hhex	1.08	0.00279032	chicken
Spsb1	0.51	0.00279382	chicken
Abce1	-1.05	0.00280143	chicken
Ampd3	0.61	0.00282843	chicken
Rab8b	-1.01	0.00291120	chicken
Supt3	-1.14	0.00291763	chicken
Arhgap45	-1.12	0.00292379	chicken
Ncf1	0.50	0.00297171	chicken
Zc3h12a	0.61	0.00299637	chicken
Utp6	0.68	0.00305370	chicken
Septin9	-1.04	0.00312150	chicken
Il18r1	0.87	0.00321044	chicken

Stat1	0.63	0.00321633	chicken
Arhgef18	0.79	0.00327163	chicken
Azin1	0.59	0.00328210	chicken
Prkn	-1.58	0.00334366	chicken
Nampt	0.70	0.00337681	chicken
Clptm1l	-1.00	0.00339343	chicken
Gsta1	0.70	0.00348192	chicken
Pkn2	-1.17	0.00351493	chicken
Sec24b	0.87	0.00355020	chicken
Slc9a3r1	-0.89	0.00358434	chicken
Ddhd1	0.93	0.00366021	chicken
Ddrgk1	-0.61	0.00382409	chicken
Pip4k2a	-1.16	0.00383835	chicken
Saxo1	0.61	0.00392081	chicken
Eif3d	-1.07	0.00392223	chicken
Nfatc3	-1.31	0.00404541	chicken
Ankdd1b	0.74	0.00415938	chicken
Vps50	0.96	0.00419094	chicken
Stk26	0.72	0.00425327	chicken
Rgs2	1.03	0.00434504	chicken
Ppp1r12a	-0.81	0.00442712	chicken
C1qc	0.71	0.00449579	chicken
Ubxn2a	0.67	0.00451951	chicken
Golga4	1.05	0.00451969	chicken
Hs6st2	-0.83	0.00483036	chicken
Tcirg1	1.08	0.00485299	chicken
Cdc25b	-0.93	0.00489746	chicken
Bbs7	-0.91	0.00492162	chicken
Ndrg1	0.98	0.00512316	chicken
Abtb1	1.16	0.00514787	chicken
Plk3	0.62	0.00516465	chicken
Dock4	0.91	0.00519139	chicken
Ppib	-1.07	0.00519394	chicken
Secisbp2l	0.68	0.00536032	chicken
Jak2	0.78	0.00537680	chicken
Ubr2	-1.00	0.00545685	chicken
Cdc42se2	-1.36	0.00549476	chicken
Vav3	0.76	0.00559066	chicken
Ube2j1	0.77	0.00567738	chicken
Ncf2	0.55	0.00575132	chicken
Phtf2	-0.97	0.00585673	chicken
Atp6v1c2	0.58	0.00617179	chicken
Trat1	-0.72	0.00617733	chicken
Chst11	1.74	0.00622310	chicken
Ctbp1	-1.09	0.00630385	chicken
Vcan	0.94	0.00632423	chicken

Calml3	0.46	0.00645741	chicken
Slc39a12	0.83	0.00645741	chicken
Prdm5	1.02	0.00645741	chicken
Sytl1	-0.70	0.00655432	chicken
Atp5j2	-1.08	0.00660728	chicken
Tcf3	-0.97	0.00663119	chicken
Tmed5	-0.83	0.00723388	chicken
Atp6v1a	0.64	0.00726948	chicken
Sap18	-0.69	0.00740776	chicken
Sec63	-1.23	0.00779415	chicken
Osbpl6	0.98	0.00780680	chicken
Dlgap1	0.60	0.00787083	chicken
Psma7	1.15	0.00805038	chicken
Samd4	0.48	0.00827182	chicken
Drd4	-0.77	0.00836195	chicken
Eif2ak3	-1.20	0.00864336	chicken
Rhob	0.76	0.00866283	chicken
Ikzf2	-1.58	0.00868988	chicken
Hmgcr	-1.34	0.00876174	chicken
Arl2bp	0.78	0.00883745	chicken
Slc22a23	1.04	0.00898098	chicken
Cox7a2	-0.86	0.00898783	chicken
Nabp1	0.72	0.00910635	chicken
Rc3h1	-1.01	0.00916330	chicken
Eps8	1.00	0.00918738	chicken
Fmnl1	-0.60	0.00945586	chicken
Rab11fip1	-0.66	0.00947378	chicken
Rims2	0.76	0.00997504	chicken
Plcd1	0.82	0.01006831	chicken
Fggy	0.49	0.01019547	chicken
Osbpl1a	-0.81	0.01023201	chicken
Lta4h	-1.07	0.01031026	chicken
Cd86	0.81	0.01061398	chicken
Cdh13	0.75	0.01061398	chicken
Cd8a	-0.75	0.01068786	chicken
Cyp2r1	0.80	0.01093434	chicken
Cat	-0.80	0.01117861	chicken
Rnf149	-0.96	0.01129483	chicken
Dhx36	-0.98	0.01149149	chicken
Irak2	0.50	0.01161799	chicken
Bank1	0.23	0.01172668	chicken
Acsbg2	0.76	0.01226729	chicken
Plcxd1	0.43	0.01242296	chicken
Atp6v1b2	0.43	0.01258485	chicken
Rock1	-0.29	0.01276849	chicken
Themis2	0.55	0.01320708	chicken

Arl4c	0.68	0.01339836	chicken
Fosl2	0.88	0.01370916	chicken
Dnajb4	0.63	0.01436539	chicken
Rusc2	0.80	0.01440869	chicken
Ugp2	0.66	0.01466224	chicken
Cep44	0.97	0.01474747	chicken
Snx24	0.58	0.01478293	chicken
Hmgcs1	0.85	0.01498728	chicken
Api5	-1.02	0.01535168	chicken
Anp32e	-0.85	0.01538512	chicken
Eif5a2	-0.94	0.01548547	chicken
Reps2	-1.32	0.01550774	chicken
Sanbr	0.65	0.01551361	chicken
Fn1	0.06	0.01576939	chicken
Slc66a1	0.56	0.01580269	chicken
Gabrr2	0.61	0.01592183	chicken
Cnnm2	0.86	0.01596925	chicken
Zfand3	0.85	0.01663967	chicken
Fmnl2	0.73	0.01707755	chicken
Kank1	0.83	0.01711012	chicken
Dpp10	1.27	0.01723900	chicken
Kctd17	0.60	0.01741231	chicken
Pea15a	0.34	0.01741231	chicken
Tmem120b	-0.86	0.01771619	chicken
Rnf128	0.71	0.01773201	chicken
Bend3	-1.09	0.01787886	chicken
1700034I23Rik	0.46	0.01821840	chicken
Eif3h	-0.92	0.01828748	chicken
Foxs1	0.52	0.01841098	chicken
Psmd7	0.56	0.01846026	chicken
Glcci1	-1.23	0.01893573	chicken
Stradb	0.65	0.01902953	chicken
Wdr35	0.57	0.01914415	chicken
Casp8	0.91	0.01927851	chicken
Nfatc1	-0.90	0.01934717	chicken
Nme7	1.35	0.01946587	chicken
Samsn1	0.64	0.01970399	chicken
Snrnp25	-0.78	0.02009440	chicken
Saraf	0.55	0.02009861	chicken
Btf3	-0.65	0.02034400	chicken
Ccser2	-1.02	0.02047728	chicken
Arg2	0.77	0.02173514	chicken
Scap	-1.08	0.02261083	chicken
Wtap	-0.99	0.02284286	chicken
Fam126a	-1.19	0.02303212	chicken
Ankrd28	0.95	0.02360093	chicken

Ankrd10	-0.97	0.02428208	chicken
Parp1	-0.83	0.02527140	chicken
Ngf	0.76	0.02538957	chicken
Tctn3	-0.72	0.02558605	chicken
Alpk1	0.74	0.02738908	chicken
Tifa	0.48	0.02851119	chicken
Ccn1	1.32	0.02873521	chicken
Dbr1	-0.70	0.02960832	chicken
Cln5	0.54	0.03024801	chicken
Sesn1	-1.49	0.03094445	chicken
Arpc4	-0.88	0.03147848	chicken
Pstpip2	0.50	0.03148203	chicken
Cpb2	0.66	0.03172811	chicken
Cap2	0.62	0.03288546	chicken
Cd36	1.39	0.03335529	chicken
Prkag2	0.75	0.03337195	chicken
Pacrg	1.22	0.03367098	chicken
Rhot1	0.60	0.03393921	chicken
Chuk	-1.24	0.03434029	chicken
Actr2	-0.93	0.03472663	chicken
Eif5	0.67	0.03497530	chicken
Inpp5k	0.60	0.03505339	chicken
Zbtb1	-0.74	0.03595900	chicken
Rreb1	0.75	0.03617732	chicken
Tshz2	-1.09	0.03642725	chicken
Smarca2	-1.11	0.03751364	chicken
Kif1b	-1.11	0.03761926	chicken
Brca2	0.67	0.03804528	chicken
Pdk4	0.68	0.04059605	chicken
Tubd1	0.39	0.04072900	chicken
Gcnt4	-1.04	0.04101190	chicken
Aldh18a1	-0.77	0.04172020	chicken
Dync2i1	0.84	0.04177639	chicken
Ap1s2	0.44	0.04198236	chicken
Ap4e1	0.52	0.04316834	chicken
Ift122	0.55	0.04328839	chicken
Glo1	-0.67	0.04333629	chicken
Ccdc122	0.46	0.04336512	chicken
Inpp4a	-0.83	0.04351732	chicken
Cyba	-1.50	0.04655641	chicken
Csgalnact1	1.04	0.04659934	chicken
Arhgef38	0.36	0.04659934	chicken
Pik3cg	-1.03	0.04711609	chicken
Tns1	0.40	0.04725718	chicken
Rab14	-0.97	0.04933019	chicken
Icosl	0.44	0.04973368	chicken

Rbm43	0.51	0.04991862	chicken
BC004004	0.49	0.05014509	chicken
Zdhhc7	0.54	0.05019049	chicken
Fth1	0.20	0.05100793	chicken
Hsf3	-1.07	0.05230562	chicken
Prelid3b	0.50	0.05291101	chicken
Klf5	0.40	0.05298809	chicken
Camkmt	0.58	0.05318764	chicken
Hnrnpu	-1.10	0.05331031	chicken
Ptpra	-0.90	0.05561743	chicken
Psma6	0.53	0.05596678	chicken
Xbp1	-0.96	0.05644416	chicken
Narf	0.91	0.06057303	chicken
Cyfip1	0.77	0.06199247	chicken
Hvcn1	-1.37	0.06380427	chicken
Pacs2	0.22	0.06664948	chicken
Ddx19a	-0.88	0.06712278	chicken
N4bp1	-1.09	0.06713149	chicken
Eno1b	-0.66	0.06750107	chicken
Ckap5	0.67	0.06929626	chicken
Hivep2	-0.81	0.06954236	chicken
Psmd14	0.70	0.07201950	chicken
Atrx	-0.72	0.07228518	chicken
Ndufv2	-0.64	0.07340620	chicken
Calcrl	1.30	0.07360696	chicken
Zmiz1	-1.08	0.07417223	chicken
Bbx	-1.27	0.07588903	chicken
Wtip	0.45	0.07602847	chicken
Fgd3	-1.01	0.08040826	chicken
Frmd3	0.84	0.08046743	chicken
Pdss1	0.52	0.08390596	chicken
Ndel1	0.75	0.08595301	chicken
Slc13a1	0.51	0.08604997	chicken
Nrip1	-1.28	0.08662995	chicken
Rab5if	-0.63	0.08742789	chicken
Txnip	0.68	0.08935829	chicken
Rfxap	-0.72	0.08990207	chicken
Lpar5	-0.60	0.09004391	chicken
Tmbim6	-0.65	0.09200026	chicken
Clint1	-0.95	0.09221567	chicken
Eaf2	0.37	0.09333894	chicken
Rimbp2	0.74	0.09504345	chicken
Sirt1	-0.96	0.09793092	chicken
Slc9a9	-0.94	0.09868047	chicken
Rap1gap2	-0.78	0.09886272	chicken
Plekho1	0.87	0.10224492	chicken

E2f3	0.32	0.10623548	chicken
Gpr137b	0.76	0.10692573	chicken
Fosb	0.60	0.10848686	chicken
Tiam1	0.41	0.10857652	chicken
Atl2	-1.08	0.10932664	chicken
Atad3a	0.41	0.10968952	chicken
Arhgap29	-0.97	0.10994996	chicken
Cep76	0.46	0.11017061	chicken
Gnal	-1.16	0.11054714	chicken
Tm4sf19	0.91	0.11060191	chicken
Il17rd	0.68	0.11060191	chicken
Ppp2r5e	1.07	0.11110195	chicken
F7	0.41	0.11126240	chicken
Fkbp3	-0.52	0.11229345	chicken
Slc2a5	0.57	0.11391194	chicken
Cox4i1	-0.99	0.11636085	chicken
St6gal1	-1.10	0.11704756	chicken
Zup1	0.38	0.11959568	chicken
Itgb2l	-1.13	0.11976623	chicken
Sinhcaf	0.67	0.12014143	chicken
Arhgef28	0.37	0.12087738	chicken
Atad5	1.02	0.12161756	chicken
Plekha7	0.43	0.12383224	chicken
Kcnh5	0.80	0.12383224	chicken
Tk2	0.45	0.12383224	chicken
Il19	0.30	0.12383224	chicken
Tmcc1	0.60	0.12542421	chicken
Alas1	0.75	0.12806545	chicken
Cish	-1.04	0.12833050	chicken
Disp3	-0.79	0.12977893	chicken
Atp13a3	0.71	0.12990897	chicken
Vps33b	-0.80	0.13098191	chicken
Ggps1	-1.07	0.13149925	chicken
Pds5b	-0.95	0.13262538	chicken
Gpr65	-1.08	0.13316062	chicken
Rfx2	0.63	0.13356083	chicken
Dglucy	0.67	0.13544093	chicken
Evl	-1.16	0.13673713	chicken
Agbl1	1.14	0.13852582	chicken
Csf1	0.07	0.13854924	chicken
Eif3a	-1.03	0.14125234	chicken
Sdk1	-1.25	0.14222258	chicken
Pik3r4	0.51	0.14262105	chicken
Aoah	0.50	0.14277386	chicken
Rbmxl1	-0.92	0.14327963	chicken
Tcerg1	-0.94	0.14361152	chicken

Fbrsl1	-0.80	0.14366249	chicken
Irf2bpl	0.33	0.14537646	chicken
Stt3b	-0.96	0.15461980	chicken
Auts2	-0.96	0.15476368	chicken
Lama3	1.70	0.15490294	chicken
Nras	-0.72	0.15494446	chicken
Eif4b	-1.04	0.15524480	chicken
Ttc28	0.44	0.15787237	chicken
Il16	-0.67	0.15808169	chicken
Scaf4	0.64	0.15823965	chicken
Casc3	0.55	0.15842044	chicken
Ctdp1	-0.99	0.15849774	chicken
Fyb	0.99	0.16248085	chicken
Dnmt3a	-1.15	0.16301644	chicken
Rbks	0.31	0.16362731	chicken
Itsn1	0.67	0.17035356	chicken
Plcl2	-1.13	0.17108013	chicken
Bcl2l15	0.51	0.17132230	chicken
Psap	0.42	0.17146855	chicken
Batf3	-0.33	0.17179991	chicken
Agap1	1.29	0.17289470	chicken
Aebp2	0.74	0.17381068	chicken
Tnfsf15	0.69	0.17930085	chicken
Pgls	-0.94	0.18456969	chicken
Esyt2	-1.11	0.18491466	chicken
Ankrd39	-0.42	0.18764653	chicken
Spats2l	0.74	0.18770540	chicken
Jazf1	0.50	0.18894425	chicken
Cenpu	0.94	0.19011575	chicken
Hdgfl3	-0.62	0.19543294	chicken
Hnrnpk	-1.04	0.19643936	chicken
Wapl	-0.72	0.19653255	chicken
Zdhhc8	0.52	0.19699586	chicken
Lcp1	0.70	0.19730245	chicken
Clpx	0.31	0.19762253	chicken
Prkd1	-0.92	0.19837790	chicken
Bag3	0.62	0.20136516	chicken
Ttll9	0.35	0.20136516	chicken
Dcun1d4	-0.96	0.20218337	chicken
Lpcat2b	-0.91	0.20521977	chicken
Actn1	-0.61	0.20992939	chicken
Capn10	0.72	0.21109413	chicken
Rab27a	-0.77	0.21605541	chicken
Iws1	-0.98	0.21943426	chicken
Cacnb4	0.23	0.21946486	chicken
Flnb	0.95	0.22003682	chicken

Cox5a	-0.51	0.22149605	chicken
Sf3b1	-0.94	0.22254637	chicken
Slamf8	-0.90	0.22443390	chicken
Stat2	0.25	0.22520673	chicken
Atad2b	1.30	0.22716875	chicken
C1qtnf12	0.39	0.22759968	chicken
Lpp	0.46	0.22769177	chicken
Wdr45b	0.62	0.22772150	chicken
Txnrd2	0.38	0.23138300	chicken
Ptger4	0.35	0.23486513	chicken
Itgb3	0.65	0.23520666	chicken
Hnrnpab	-0.87	0.24947629	chicken
Rab3il1	0.40	0.25217785	chicken
Zfc3h1	0.45	0.25306750	chicken
Hesx1	0.36	0.25513325	chicken
Psma4	-1.03	0.25588029	chicken
Nucb2	-1.04	0.25599162	chicken
Epcam	0.60	0.25933686	chicken
Zfp438	0.52	0.25939846	chicken
Rin2	0.25	0.26047847	chicken
Ugcg	0.56	0.26075763	chicken
Tph2	0.55	0.26112468	chicken
Papss1	-0.97	0.26765075	chicken
Igsf3	0.73	0.26816791	chicken
Dars	-0.91	0.27610188	chicken
Vac14	0.47	0.28539056	chicken
Ccnd1	-1.39	0.28987274	chicken
Adnp	-0.97	0.29139700	chicken
Kars	-0.74	0.29818117	chicken
Idh2	-0.46	0.30365526	chicken
Laptm4a	0.60	0.30573031	chicken
Gpr1	0.82	0.30786388	chicken
Ogt	-1.05	0.30821369	chicken
Smarce1	-0.81	0.31158710	chicken
Wdr25	0.76	0.31265465	chicken
Tulp4	0.67	0.31275894	chicken
Abca2	-0.88	0.31915311	chicken
Samd8	0.50	0.32046187	chicken
Lrmp	0.68	0.32190636	chicken
Slc15a2	0.42	0.32693650	chicken
Dner	0.61	0.32693650	chicken
Ube2l3	0.68	0.32694119	chicken
Itm2b	-1.06	0.32867046	chicken
Colec11	0.26	0.32922634	chicken
Klhl7	-0.73	0.33042081	chicken
Ccdc141	0.50	0.33274952	chicken

Ddx46	-1.16	0.34161284	chicken
Ska3	0.33	0.34256653	chicken
Hpgds	0.64	0.34256653	chicken
Ripk2	0.33	0.34293670	chicken
Cntrl	-0.98	0.34580486	chicken
Ywhaq	-0.57	0.34868981	chicken
Irf5	0.47	0.35487698	chicken
Pde4b	0.44	0.35685903	chicken
Rnf214	-1.19	0.35776284	chicken
Rarb	0.42	0.35954392	chicken
Eif3k	0.40	0.35954392	chicken
Fitm2	0.30	0.35954392	chicken
C3	0.69	0.36085528	chicken
Tbl1xr1	0.66	0.36402873	chicken
Dusp23	-0.56	0.36507006	chicken
Apoo	0.55	0.36561377	chicken
Lyg2	0.82	0.36655671	chicken
Zc3h7a	-0.88	0.36835440	chicken
Otud7b	-0.81	0.37158132	chicken
Acadsb	0.69	0.37230088	chicken
Sec23b	-0.91	0.37637683	chicken
Rilpl2	-0.54	0.38119002	chicken
Agfg1	0.59	0.38257615	chicken
Itgav	0.66	0.39694371	chicken
Setbp1	-0.91	0.40288851	chicken
Hlx	0.32	0.40511993	chicken
Rhof	-0.93	0.40938611	chicken
Atf7ip	0.70	0.41292047	chicken
Pdpk1	-0.84	0.43056769	chicken
Ubr1	-1.14	0.43170859	chicken
Tal1	0.59	0.43754712	chicken
Azin2	0.41	0.44214993	chicken
Cnn3	0.46	0.44618027	chicken
Senp5	0.31	0.44623542	chicken
Polr1f	-0.68	0.44900852	chicken
Itga1	-1.20	0.45214489	chicken
Tmem120a	0.67	0.45590720	chicken
Ppp4r1	-1.08	0.45783834	chicken
Parp12	0.38	0.46570483	chicken
Dnajc3	-0.90	0.46754245	chicken
Car2	-0.35	0.47608496	chicken
Galc	-1.03	0.47771161	chicken
Clcn7	0.45	0.48103992	chicken
Srpk2	-1.08	0.48174187	chicken
Itgb1bp1	0.24	0.48192266	chicken
Dnah12	0.73	0.48374979	chicken

Bclaf1	-0.92	0.48623311	chicken
Pax5	0.98	0.48787254	chicken
Rpa3	-0.98	0.49888958	chicken
Rev3l	0.58	0.50197693	chicken
Chp1	0.56	0.50545332	chicken
Sh3gl1	0.66	0.50882682	chicken
Tmem131	-0.96	0.50966740	chicken
Alox5	0.62	0.51225948	chicken
Slc37a1	0.63	0.52699968	chicken
Romo1	-0.85	0.53048441	chicken
Oxct1	-0.61	0.53265119	chicken
St8sia4	-1.21	0.53901452	chicken
Rad18	-0.87	0.54325078	chicken
Cpq	0.61	0.56372155	chicken
Agl	-0.51	0.57051464	chicken
Stim2	-1.07	0.57139320	chicken
Rabl6	-0.73	0.57867597	chicken
Pde10a	0.40	0.58100753	chicken
Orc1	0.52	0.58108730	chicken
Dapp1	0.54	0.58162268	chicken
Trak2	0.27	0.59887327	chicken
Zfp462	-0.55	0.60752586	chicken
Naa15	-0.89	0.61439538	chicken
Tmem164	0.37	0.61507052	chicken
Snx9	0.32	0.62135781	chicken
Fam168b	-1.04	0.62238879	chicken
Zc3h6	0.39	0.63219239	chicken
Ubash3a	-0.97	0.63260168	chicken
Prkar2b	0.61	0.65098277	chicken
Piga	0.65	0.65512118	chicken
Luc7l2	-0.91	0.65682098	chicken
Trpm5	0.37	0.67129223	chicken
Chrna5	0.31	0.67129223	chicken
Nbeal2	-1.05	0.67258704	chicken
Frmd4a	0.45	0.67695440	chicken
Irf8	0.45	0.68122570	chicken
Slc7a6os	-0.62	0.69218869	chicken
Oat	-0.66	0.71036859	chicken
Adsl	-0.68	0.73135762	chicken
Smc4	-0.71	0.73346511	chicken
Rnf141	0.96	0.73426168	chicken
Sppl2a	0.93	0.73626100	chicken
Rasgrp1	0.67	0.74706894	chicken
Tmtc2	0.63	0.75484777	chicken
Lcp2	-0.78	0.76377639	chicken
Rcor3	-0.55	0.76784613	chicken

Srgap3	-1.06	0.77191793	chicken
Aldh1a2	0.43	0.77197399	chicken
Timp2	0.33	0.79027350	chicken
Ackr2	0.24	0.79468887	chicken
Pm20d2	0.64	0.79570089	chicken
Cndp1	0.49	0.79599422	chicken
Emc3	-0.73	0.79793425	chicken
Cmtm4	0.55	0.79987077	chicken
Kpna4	-0.84	0.80023293	chicken
Clcn3	-1.00	0.81623971	chicken
Ctss	0.48	0.82000866	chicken
Gypc	0.43	0.83396166	chicken
Trps1	-0.50	0.83963973	chicken
Tomm6	-0.75	0.85577484	chicken
Por	0.36	0.86782146	chicken
Gnai2	-0.76	0.86788271	chicken
Stk3	-0.92	0.87991312	chicken
Xrn2	-0.92	0.88138978	chicken
Jmy	-0.76	0.89400042	chicken
Tjp2	0.38	0.89403768	chicken
Rbm14	-0.77	0.90009683	chicken
Ilf2	-0.71	0.91379658	chicken
Ppp1r10	0.57	0.91437146	chicken
Kif3b	0.37	0.91809413	chicken
Ackr4	0.20	0.91823533	chicken
Acox1	0.46	0.93003185	chicken
Atg4b	0.17	0.93471049	chicken
Plbd1	0.89	0.97149756	chicken
Arhgap30	-0.82	0.97285370	chicken
Klhl6	-0.90	0.98106016	chicken
Tagap1	0.79	0.98814008	chicken
Tnpo1	0.79	1.00000000	chicken
Use1	0.32	1.00000000	chicken
Phf12	-0.94	1.00000000	chicken
Pex13	0.36	1.00000000	chicken
Tedc1	0.32	1.00000000	chicken
Akr7a5	0.48	1.00000000	chicken
Eif3b	-0.81	1.00000000	chicken
Adgrg2	0.57	1.00000000	chicken
Seh1l	0.18	1.00000000	chicken
Ndufa5	-0.72	1.00000000	chicken
Ccnd2	-0.71	1.00000000	chicken
Slc4a2	0.08	1.00000000	chicken
Pepd	0.46	1.00000000	chicken
Ano5	0.55	1.00000000	chicken
Rora	-0.83	1.00000000	chicken

Pak2	-1.02	1.00000000	chicken
Cnot6	0.23	1.00000000	chicken
Cebpb	0.18	1.00000000	chicken
Carf	0.51	1.00000000	chicken
Kcnj2	0.50	1.00000000	chicken
Derl1	-0.75	1.00000000	chicken
Igf1r	0.43	1.00000000	chicken
Nptn	0.54	1.00000000	chicken
Rpp30	0.40	1.00000000	chicken
Arglu1	-0.91	1.00000000	chicken
Ercc3	0.47	1.00000000	chicken
Asap1	-0.44	1.00000000	chicken
Rufy3	-1.04	1.00000000	chicken
Wasl	-0.80	1.00000000	chicken
Gpx1	-0.19	1.00000000	chicken
Dpf3	-1.05	1.00000000	chicken
Atp5j	-0.73	1.00000000	chicken
Dgkd	-0.79	1.00000000	chicken
Amn	0.26	1.00000000	chicken
Hecw2	0.46	1.00000000	chicken
Gpc5	0.44	1.00000000	chicken
Ints2	1.12	1.00000000	chicken
Sgk3	0.32	1.00000000	chicken
Vps4b	-0.70	1.00000000	chicken
Dennd3	0.73	1.00000000	chicken
Trafd1	0.47	1.00000000	chicken
Fbxw2	-0.73	1.00000000	chicken
Sec62	-0.78	1.00000000	chicken
Rprd1a	-0.70	1.00000000	chicken
Inpp5f	-0.90	1.00000000	chicken
Galnt2	-0.71	1.00000000	chicken
Itgb6	0.39	1.00000000	chicken
Il1r1	0.60	1.00000000	chicken
Kmt2c	-0.61	1.00000000	chicken
Plxnd1	0.59	1.00000000	chicken
Npepps	0.60	1.00000000	chicken
C2cd2	0.25	1.00000000	chicken
1110004F10Rik	-0.72	1.00000000	chicken
Fndc3a	0.71	1.00000000	chicken
Tmpo	-0.73	1.00000000	chicken
Scube2	0.30	1.00000000	chicken
Dnah8	0.74	1.00000000	chicken
Snap91	0.71	1.00000000	chicken
Tspan2	-0.52	1.00000000	chicken
Vwa8	0.46	1.00000000	chicken
App	0.65	1.00000000	chicken

Hlcs	-1.02	1.00000000	chicken
Ndufa13	-0.71	1.00000000	chicken
Unc50	0.36	1.00000000	chicken
Lpin2	-0.81	1.00000000	chicken
Paxbp1	-0.94	1.00000000	chicken
Ahi1	-0.86	1.00000000	chicken
Arpp19	-0.72	1.00000000	chicken
Atp5d	-0.89	1.00000000	chicken
Tspan5	-1.21	1.00000000	chicken
Rin3	0.51	1.00000000	chicken
Cdk6	0.43	1.00000000	chicken
Crlf2	0.07	1.00000000	chicken
Arhgef7	-1.06	1.00000000	chicken
Caprin1	-0.81	1.00000000	chicken
Cdk19	-1.08	1.00000000	chicken
Rnd3	0.77	1.00000000	chicken
Rnf144a	1.43	1.00000000	chicken
Unc13d	-0.78	1.00000000	chicken
Slc49a4	0.53	1.00000000	chicken
Rapgef5	0.87	1.00000000	chicken
Niban1	-0.92	1.00000000	chicken
Zranb1	0.25	1.00000000	chicken
Gas7	0.58	1.00000000	chicken
Galns	0.46	1.00000000	chicken
Trip11	0.51	1.00000000	chicken
Crym	-0.66	1.00000000	chicken
Sppl3	0.48	1.00000000	chicken
Arap2	0.73	1.00000000	chicken
Sri	0.48	1.00000000	chicken
C1qb	0.35	1.00000000	chicken
Csnk1d	-0.83	1.00000000	chicken
Dnajc13	0.43	1.00000000	chicken
Akt1	0.45	1.00000000	chicken
Extl3	-1.23	1.00000000	chicken
Dazap1	-0.88	1.00000000	chicken
Zc3h12c	0.27	1.00000000	chicken
Park7	-0.83	1.00000000	chicken
Ypel2	-0.49	1.00000000	chicken
Cd7	-0.66	1.00000000	chicken
F5	0.72	1.00000000	chicken
Itpkb	-1.01	1.00000000	chicken
Epc1	-0.67	1.00000000	chicken
Pgap2	0.21	1.00000000	chicken
Rasal2	0.60	1.00000000	chicken
Tmed2	-0.90	1.00000000	chicken
Gzma	-1.51	1.00000000	chicken

Ero1b	-0.53	1.00000000	chicken
P4htm	0.28	1.00000000	chicken
Eloc	0.39	1.00000000	chicken
Arfgap3	0.48	1.00000000	chicken
Slco3a1	-0.73	1.00000000	chicken
Impa1	-0.65	1.00000000	chicken
Khdc4	0.37	1.00000000	chicken
Dab2	0.49	1.00000000	chicken
Arhgap17	-1.06	1.00000000	chicken
Nop58	-0.87	1.00000000	chicken
Esd	-0.36	1.00000000	chicken
Usp36	0.37	1.00000000	chicken
Chka	0.72	1.00000000	chicken
Sytl3	-1.08	1.00000000	chicken
Uck2	0.25	1.00000000	chicken
Ass1	0.19	1.00000000	chicken
Nav3	1.13	1.00000000	chicken
Galnt9	0.43	1.00000000	chicken
Osgin2	-0.62	1.00000000	chicken
Tuba1b	-1.09	1.00000000	chicken
Zfand5	0.36	1.00000000	chicken
Gramd4	-0.70	1.00000000	chicken
Ik	-0.57	1.00000000	chicken
Nfyc	-0.81	1.00000000	chicken
Dgkz	-0.87	1.00000000	chicken
Klhl13	0.50	1.00000000	chicken
Rel	0.59	1.00000000	chicken
Snrpe	-0.74	1.00000000	chicken
Rab7b	0.49	1.00000000	chicken
Dpep2	0.32	1.00000000	chicken
Scpep1	0.35	1.00000000	chicken
Crlf3	-0.47	1.00000000	chicken
Usp14	0.56	1.00000000	chicken
Cacul1	0.39	1.00000000	chicken
Sdc1	0.76	1.00000000	chicken
Adamts3	0.43	1.00000000	chicken
Cttnbp2nl	0.39	1.00000000	chicken
9930021J03Rik	-1.02	1.00000000	chicken
Pias1	-0.83	1.00000000	chicken
Eif3g	-0.77	1.00000000	chicken
Itga9	0.47	1.00000000	chicken
Luzp1	0.22	1.00000000	chicken
Cdc42	-0.81	1.00000000	chicken
Acap3	0.32	1.00000000	chicken
Osbpl8	0.16	1.00000000	chicken
Ndufb4	-0.52	1.00000000	chicken

Cib1	-0.84	1.00000000	chicken
Ctdspl	-0.81	1.00000000	chicken
Tshz1	-0.55	1.00000000	chicken
Stard3nl	0.42	1.00000000	chicken
Supv3l1	0.46	1.00000000	chicken
Ptrh2	-0.69	1.00000000	chicken
Tpp2	-0.72	1.00000000	chicken
Pum1	-0.98	1.00000000	chicken
Cd38	0.49	1.00000000	chicken
Fkbp1b	0.70	1.00000000	chicken
Plekhm2	0.32	1.00000000	chicken
Blm	0.32	1.00000000	chicken
Rab11fip4	0.54	1.00000000	chicken
Atp5a1	-0.53	1.00000000	chicken
Ralbp1	-0.87	1.00000000	chicken
Sav1	-0.38	1.00000000	chicken
Faf2	0.32	1.00000000	chicken
Dmxl1	-1.03	1.00000000	chicken
Gstcd	0.32	1.00000000	chicken
Pnpla6	0.18	1.00000000	chicken
Slc40a1	1.44	1.00000000	chicken
Tmem131l	-0.82	1.00000000	chicken
Timm17a	-0.55	1.00000000	chicken
Corin	0.48	1.00000000	chicken
Helz	0.45	1.00000000	chicken
Csnk1a1	-0.79	1.00000000	chicken
Sting1	0.37	1.00000000	chicken
Eaf1	0.30	1.00000000	chicken
Abraxas2	0.15	1.00000000	chicken
Arl8b	0.59	1.00000000	chicken
Fbxo22	0.54	1.00000000	chicken
Nek6	0.40	1.00000000	chicken
Etnk1	-0.83	1.00000000	chicken
Atp6v1g1	0.58	1.00000000	chicken
Edf1	-0.90	1.00000000	chicken
Stmn1	-0.75	1.00000000	chicken
Rybp	0.45	1.00000000	chicken
Ndufa10	-0.84	1.00000000	chicken
Cops8	-0.48	1.00000000	chicken
Ube2q2	0.50	1.00000000	chicken
Cert1	0.86	1.00000000	chicken
Necap2	0.30	1.00000000	chicken
Slc9a7	-0.98	1.00000000	chicken
Kif21a	0.39	1.00000000	chicken
Dhx15	-0.78	1.00000000	chicken
Sms	-0.73	1.00000000	chicken

Lnpep	-0.37	1.00000000	chicken
Pfdn2	-0.68	1.00000000	chicken
Fbxo32	-0.60	1.00000000	chicken
Atp6v1g3	0.38	1.00000000	chicken
Uhrf2	-0.46	1.00000000	chicken
Cstb	0.39	1.00000000	chicken
Aopep	0.57	1.00000000	chicken
Zfand2a	0.55	1.00000000	chicken
U2surp	-0.75	1.00000000	chicken
Wbp1	-0.63	1.00000000	chicken
Gch1	0.52	1.00000000	chicken
Irf2	0.66	1.00000000	chicken
Bmp2k	0.63	1.00000000	chicken
Ston2	0.32	1.00000000	chicken
Qdpr	-0.48	1.00000000	chicken
Il13ra2	0.50	1.00000000	chicken
Slc35a5	0.29	1.00000000	chicken
Ndfip1	-0.75	1.00000000	chicken
Actb	-0.26	1.00000000	chicken
Pde4d	-0.58	1.00000000	chicken
Fmn1	0.66	1.00000000	chicken
Sass6	-0.50	1.00000000	chicken
Capza2	-0.93	1.00000000	chicken
Apoa1	0.58	1.00000000	chicken
Frmd4b	0.27	1.00000000	chicken
Eif4e	-0.94	1.00000000	chicken
Pdgfb	0.37	1.00000000	chicken
Btrc	-0.91	1.00000000	chicken
Rcn2	-0.70	1.00000000	chicken
Cisd2	0.24	1.00000000	chicken
Oser1	0.38	1.00000000	chicken
Akap6	0.65	1.00000000	chicken
Eif2ak1	0.55	1.00000000	chicken
Pcsk7	-0.87	1.00000000	chicken
Emilin2	0.47	1.00000000	chicken
Rmnd1	-0.53	1.00000000	chicken
Nap1l4	-0.95	1.00000000	chicken
Dlec1	0.18	1.00000000	chicken
Ppp1r9a	0.42	1.00000000	chicken
Sfpq	-0.84	1.00000000	chicken
Tirap	0.46	1.00000000	chicken
Nub1	0.48	1.00000000	chicken
Tmem154	-0.92	1.00000000	chicken
Nup205	-0.57	1.00000000	chicken
Prom1	0.23	1.00000000	chicken
Rnf19a	0.44	1.00000000	chicken

Sf3b3	-0.89	1.00000000	chicken
Sgpl1	0.30	1.00000000	chicken
Grcc10	-0.58	1.00000000	chicken
St14	0.56	1.00000000	chicken
Slbp	0.24	1.00000000	chicken
Ppid	0.43	1.00000000	chicken
Hkdc1	0.41	1.00000000	chicken
Htatsf1	-0.62	1.00000000	chicken
Rap1gds1	-1.10	1.00000000	chicken
Tada2b	0.12	1.00000000	chicken
Trabd	0.43	1.00000000	chicken
Atp5c1	-0.56	1.00000000	chicken
Nin	0.32	1.00000000	chicken
Btbd1	-0.69	1.00000000	chicken
Dipk2a	-0.02	1.00000000	chicken
Ust	-1.07	1.00000000	chicken
Acap1	-0.72	1.00000000	chicken
Polq	0.66	1.00000000	chicken
Brd1	-0.78	1.00000000	chicken
Lpcat1	-0.86	1.00000000	chicken
Siah1b	0.31	1.00000000	chicken
Exoc5	0.51	1.00000000	chicken
Abca4	0.85	1.00000000	chicken
Zswim8	0.52	1.00000000	chicken
Srgap1	0.78	1.00000000	chicken
Kif5b	-0.55	1.00000000	chicken
Bcl2	-0.78	1.00000000	chicken
Syne3	0.32	1.00000000	chicken
Dhrs3	0.49	1.00000000	chicken
Cdk17	-0.51	1.00000000	chicken
Ankmy2	0.59	1.00000000	chicken
Lztr1	0.27	1.00000000	chicken
Rdx	-0.94	1.00000000	chicken
Smc3	1.43	1.00000000	chicken
Lrch3	-0.64	1.00000000	chicken
Champ1	-0.69	1.00000000	chicken
Peli2	0.73	1.00000000	chicken
Slc25a13	0.30	1.00000000	chicken
Rgs10	0.35	1.00000000	chicken
Arhgap25	0.35	1.00000000	chicken
Dld	-0.38	1.00000000	chicken
Lman2	-0.55	1.00000000	chicken
Iqck	0.28	1.00000000	chicken
Rasd1	0.55	1.00000000	chicken
Smad2	-0.50	1.00000000	chicken
Anapc16	-0.63	1.00000000	chicken

Dip2b	0.43	1.00000000	chicken
Rbms3	1.50	1.00000000	chicken
Fbxl5	0.35	1.00000000	chicken
Nelfcd	0.34	1.00000000	chicken
Tfpi2	0.67	1.00000000	chicken
Rnls	0.56	1.00000000	chicken
Rffl	0.70	1.00000000	chicken
Id3	0.50	1.00000000	chicken
Hmox1	0.79	1.00000000	chicken
Psmb7	-0.75	1.00000000	chicken
Gatm	-0.19	1.00000000	chicken
Lpxn	0.47	1.00000000	chicken
Peli1	-0.53	1.00000000	chicken
Abhd12	0.27	1.00000000	chicken
Fes	0.48	1.00000000	chicken
Ttc7b	0.33	1.00000000	chicken
Sdc2	0.58	1.00000000	chicken
Znrf2	-0.98	1.00000000	chicken
C87436	0.28	1.00000000	chicken
Pwwp2b	0.16	1.00000000	chicken
Virma	-0.56	1.00000000	chicken
Herc2	-0.90	1.00000000	chicken
Fez2	0.57	1.00000000	chicken
Trappc12	-0.66	1.00000000	chicken
Atp6v1d	0.55	1.00000000	chicken
Ptbp1	-0.58	1.00000000	chicken
Hspa9	-0.89	1.00000000	chicken
Hyou1	-0.76	1.00000000	chicken
Stk10	-0.74	1.00000000	chicken
Gipc2	0.26	1.00000000	chicken
Slc7a5	-1.07	1.00000000	chicken
Rnf217	-1.00	1.00000000	chicken
Dnajc2	-0.59	1.00000000	chicken
Slc35f1	1.27	1.00000000	chicken
Ikbkb	0.17	1.00000000	chicken
Rnpepl1	0.39	1.00000000	chicken
Ppil2	-1.03	1.00000000	chicken
Zfyve9	0.34	1.00000000	chicken
Zfp423	0.44	1.00000000	chicken
Atic	-0.58	1.00000000	chicken
Ubr5	-0.89	1.00000000	chicken
Csf1r	0.02	1.00000000	chicken
Rtf1	-0.89	1.00000000	chicken
Ski	0.55	1.00000000	chicken
Cops7a	-0.43	1.00000000	chicken
Lonrf3	0.63	1.00000000	chicken

Ssr2	-0.62	1.00000000	chicken
B230219D22Rik	-0.59	1.00000000	chicken
Tln1	-0.52	1.00000000	chicken
Hmgb3	-0.55	1.00000000	chicken
Srsf11	-1.00	1.00000000	chicken
Rbis	-0.57	1.00000000	chicken
Fam120b	0.35	1.00000000	chicken
Lrrc75a	0.43	1.00000000	chicken
Usp22	0.48	1.00000000	chicken
Lrp1	0.37	1.00000000	chicken
Fgl2	0.43	1.00000000	chicken
Sfswap	-0.86	1.00000000	chicken
Dicer1	-0.65	1.00000000	chicken
Skil	-0.81	1.00000000	chicken
Gnl3	-0.81	1.00000000	chicken
Abat	-0.49	1.00000000	chicken
Tut4	0.39	1.00000000	chicken
Smim14	0.13	1.00000000	chicken
Arhgap10	-0.84	1.00000000	chicken
Cdk5rap1	0.06	1.00000000	chicken
Sdhd	-0.53	1.00000000	chicken
Ctsb	0.28	1.00000000	chicken
Wbp1l	-0.55	1.00000000	chicken
Aimp1	-0.78	1.00000000	chicken
Zswim5	0.84	1.00000000	chicken
Zfp292	-0.69	1.00000000	chicken
Xpa	0.36	1.00000000	chicken
Nfil3	-0.15	1.00000000	chicken
Uspl1	-0.49	1.00000000	chicken
Eif4ebp1	-0.69	1.00000000	chicken
Taok1	0.12	1.00000000	chicken
Sbf1	-0.62	1.00000000	chicken
U2af1	-0.85	1.00000000	chicken
Dcp2	-0.63	1.00000000	chicken
Kcnq3	-1.33	1.00000000	chicken
Gclc	-0.80	1.00000000	chicken
Ifrd1	0.64	1.00000000	chicken
Ostm1	0.10	1.00000000	chicken
Nudt3	0.27	1.00000000	chicken
Eps15l1	0.35	1.00000000	chicken
Rhoh	-0.77	1.00000000	chicken
Smox	0.46	1.00000000	chicken
Dock11	-0.61	1.00000000	chicken
Notch2	-0.76	1.00000000	chicken
Fyn	0.66	1.00000000	chicken
Plac8	1.50	1.00000000	chicken

9130409I23Rik	-0.74	1.00000000	chicken
Chic2	-0.77	1.00000000	chicken
Zc3h15	-0.80	1.00000000	chicken
Arid4a	-0.83	1.00000000	chicken
Tmem170b	0.22	1.00000000	chicken
Hexb	-0.62	1.00000000	chicken
Prdx1	-0.85	1.00000000	chicken
Gapvd1	0.49	1.00000000	chicken
Klhl20	0.54	1.00000000	chicken
Tmem185a	-0.76	1.00000000	chicken
Was	-0.57	1.00000000	chicken
D430041D05Rik	0.57	1.00000000	chicken
Rnf13	0.48	1.00000000	chicken
Kdm3b	-0.70	1.00000000	chicken
Smarca1	-0.89	1.00000000	chicken
St3gal2	-0.52	1.00000000	chicken
Cdkn1b	-0.82	1.00000000	chicken
Ppm1a	-0.83	1.00000000	chicken
Zfp207	0.28	1.00000000	chicken
Pbrm1	-0.78	1.00000000	chicken
Ostf1	-0.57	1.00000000	chicken
Scaf8	-0.95	1.00000000	chicken
Tmem59	-0.61	1.00000000	chicken
Nt5e	0.48	1.00000000	chicken
C2cd5	-0.87	1.00000000	chicken
Tsen2	0.32	1.00000000	chicken
Cpne2	0.32	1.00000000	chicken
Dr1	-0.52	1.00000000	chicken
Ncoa2	-0.87	1.00000000	chicken
Thra	0.21	1.00000000	chicken
Rpap1	-0.51	1.00000000	chicken
Vti1b	0.16	1.00000000	chicken
Dis3l2	-0.79	1.00000000	chicken
Ttbk1	-0.63	1.00000000	chicken
Gkap1	0.45	1.00000000	chicken
Zbtb2	0.03	1.00000000	chicken
Sem1	0.47	1.00000000	chicken
Abcd2	0.85	1.00000000	chicken
Atf4	-0.49	1.00000000	chicken
Snap23	0.33	1.00000000	chicken
Exoc4	-0.84	1.00000000	chicken
Hras	0.29	1.00000000	chicken
Nqo2	0.08	1.00000000	chicken
Casp3	0.48	1.00000000	chicken
Uqcrc2	-0.64	1.00000000	chicken
Stx17	-0.02	1.00000000	chicken

Cfap45	0.22	1.00000000	chicken
Dnajb12	0.25	1.00000000	chicken
Ranbp10	0.42	1.00000000	chicken
Clip4	-0.82	1.00000000	chicken
Rap2c	-0.01	1.00000000	chicken
Canx	-0.65	1.00000000	chicken
Slc38a2	-0.59	1.00000000	chicken
Git2	-0.93	1.00000000	chicken
Rnf114	-0.63	1.00000000	chicken
Smg1	0.56	1.00000000	chicken
Dera	0.15	1.00000000	chicken
Ube2v2	-0.74	1.00000000	chicken
Dnah10	0.22	1.00000000	chicken
Vti1a	0.23	1.00000000	chicken
Pik3r5	0.46	1.00000000	chicken
Fancd2	0.41	1.00000000	chicken
Psmd3	0.38	1.00000000	chicken
Glis1	0.37	1.00000000	chicken
Desi2	-0.57	1.00000000	chicken
Sorcs2	0.56	1.00000000	chicken
Tiparp	-0.56	1.00000000	chicken
Pik3ca	-0.78	1.00000000	chicken
Bmpr2	-1.03	1.00000000	chicken
Hsph1	0.86	1.00000000	chicken
Nrros	0.44	1.00000000	chicken
Trerf1	-0.78	1.00000000	chicken
Col22a1	0.11	1.00000000	chicken
Ywhab	-0.57	1.00000000	chicken
Ldlrad4	0.44	1.00000000	chicken
Sos2	-0.81	1.00000000	chicken
Ndufs7	-0.56	1.00000000	chicken
F10	0.45	1.00000000	chicken
Prelid1	-0.62	1.00000000	chicken
Ip6k2	0.42	1.00000000	chicken
Ankrd46	0.33	1.00000000	chicken
Zbtb49	0.30	1.00000000	chicken
Ovch2	0.29	1.00000000	chicken
Appl1	0.36	1.00000000	chicken
Skap1	-0.71	1.00000000	chicken
Zfp131	-0.73	1.00000000	chicken
Tmem165	-0.36	1.00000000	chicken
Pdia4	-0.72	1.00000000	chicken
Wdr48	-0.62	1.00000000	chicken
Psmg1	0.20	1.00000000	chicken
Rnf150	0.25	1.00000000	chicken
Ilrun	0.15	1.00000000	chicken

Sf3a1	-0.60	1.00000000	chicken
Ghitm	-0.68	1.00000000	chicken
Lclat1	0.82	1.00000000	chicken
Rac2	-0.01	1.00000000	chicken
P2rx5	0.37	1.00000000	chicken
Zmat2	-0.48	1.00000000	chicken
Spred2	0.10	1.00000000	chicken
Rbm24	0.02	1.00000000	chicken
Il2ra	-1.62	1.00000000	chicken
Ndfip2	0.21	1.00000000	chicken
Hephl1	0.46	1.00000000	chicken
Aldoc	0.27	1.00000000	chicken
Vps26a	0.40	1.00000000	chicken
Fkbp1a	-0.72	1.00000000	chicken
Adrb2	-0.62	1.00000000	chicken
Tnfrsf21	0.34	1.00000000	chicken
Phka2	-0.48	1.00000000	chicken
Dnajc5	0.23	1.00000000	chicken
Slc16a10	0.23	1.00000000	chicken
Ralgps2	0.57	1.00000000	chicken
Kdm5b	-0.78	1.00000000	chicken
Cpeb4	0.32	1.00000000	chicken
Rell1	-0.74	1.00000000	chicken
Cmpk2	0.32	1.00000000	chicken
Ogfrl1	0.43	1.00000000	chicken
Atp6v0a1	0.32	1.00000000	chicken
Tomm22	-0.48	1.00000000	chicken
Vldlr	0.40	1.00000000	chicken
Glrx3	-0.51	1.00000000	chicken
Xylt1	0.56	1.00000000	chicken
Skap2	0.23	1.00000000	chicken
Ctsz	0.11	1.00000000	chicken
Gdap2	0.28	1.00000000	chicken
Yeats2	-0.82	1.00000000	chicken
Asb5	-0.71	1.00000000	chicken
C1galt1	-0.58	1.00000000	chicken
Kif13b	0.24	1.00000000	chicken
Tbc1d9b	0.26	1.00000000	chicken
Ift20	-0.70	1.00000000	chicken
Stxbp5	-1.11	1.00000000	chicken
Pfdn4	-0.81	1.00000000	chicken
Usp38	-0.77	1.00000000	chicken
Nr3c2	-0.72	1.00000000	chicken
Shld1	0.34	1.00000000	chicken
Ndufb1	-0.31	1.00000000	chicken
Pibf1	0.43	1.00000000	chicken

Pxn	0.36	1.00000000	chicken
Cdc14b	0.49	1.00000000	chicken
Rmrp	0.13	1.00000000	chicken
Csnk1g1	0.20	1.00000000	chicken
Dynlt1a	0.19	1.00000000	chicken
Gpatch1	0.13	1.00000000	chicken
Sdf4	-0.82	1.00000000	chicken
Rbm26	-0.68	1.00000000	chicken
Ing4	-0.69	1.00000000	chicken
Nlrc5	0.29	1.00000000	chicken
Rbl1	-0.72	1.00000000	chicken
Tnfrsf1a	0.46	1.00000000	chicken
Foxk1	0.53	1.00000000	chicken
Axin1	0.15	1.00000000	chicken
Bdh2	0.37	1.00000000	chicken
Cd300c	0.22	1.00000000	chicken
Zfp644	-0.76	1.00000000	chicken
Prdm15	-0.05	1.00000000	chicken
Pecam1	0.24	1.00000000	chicken
Foxp1	-0.68	1.00000000	chicken
Tent5a	-0.73	1.00000000	chicken
Adk	-0.59	1.00000000	chicken
Snhg4	-0.73	1.00000000	chicken
Pik3r1	-0.67	1.00000000	chicken
Nelfa	-0.81	1.00000000	chicken
Acot7	-0.52	1.00000000	chicken
Lgr4	0.24	1.00000000	chicken
Def6	-0.68	1.00000000	chicken
Tcp1	-0.66	1.00000000	chicken
Wipi2	0.37	1.00000000	chicken
Sclt1	-0.80	1.00000000	chicken
Fndc3b	0.38	1.00000000	chicken
Ppp1ca	-0.57	1.00000000	chicken
Coro7	-0.39	1.00000000	chicken
Cyb5r2	0.53	1.00000000	chicken
Ostc	-0.71	1.00000000	chicken
Fkbp8	-0.51	1.00000000	chicken
Lamp1	0.07	1.00000000	chicken
Rbm5	-0.73	1.00000000	chicken
Hnrnpd	-0.70	1.00000000	chicken
Ntan1	-0.64	1.00000000	chicken
Htra2	-0.57	1.00000000	chicken
Tra2a	-0.92	1.00000000	chicken
Erh	-0.50	1.00000000	chicken
Itpk1	-1.07	1.00000000	chicken
Fgf18	0.33	1.00000000	chicken

Unc119b	-0.42	1.00000000	chicken
Cwc15	-0.44	1.00000000	chicken
Clip1	0.32	1.00000000	chicken
Trmt10c	-0.43	1.00000000	chicken
Gtdc1	0.49	1.00000000	chicken
Ccrl2	0.25	1.00000000	chicken
Pms2	0.41	1.00000000	chicken
Rab11a	0.35	1.00000000	chicken
Vdr	0.45	1.00000000	chicken
Spic	0.42	1.00000000	chicken
Hspa13	0.10	1.00000000	chicken
Ikbke	0.13	1.00000000	chicken
Itm2c	0.30	1.00000000	chicken
Cep350	-0.45	1.00000000	chicken
Rprd1b	-0.61	1.00000000	chicken
Nup93	0.51	1.00000000	chicken
Spz1	-0.90	1.00000000	chicken
Isl2	0.55	1.00000000	chicken
Ddost	-0.55	1.00000000	chicken
Ddx24	-0.70	1.00000000	chicken
Ep300	-0.83	1.00000000	chicken
Xpo1	-0.77	1.00000000	chicken
Tubb6	0.32	1.00000000	chicken
Qser1	-0.58	1.00000000	chicken
Cyb5a	0.10	1.00000000	chicken
Card9	-0.25	1.00000000	chicken
Atp6v0d1	0.43	1.00000000	chicken
Rnf144b	0.46	1.00000000	chicken
Ddx31	0.49	1.00000000	chicken
Uqcc2	0.25	1.00000000	chicken
Ranbp3	0.04	1.00000000	chicken
Cpne3	0.48	1.00000000	chicken
Stard4	-0.38	1.00000000	chicken
Pcnx	-0.58	1.00000000	chicken
Aagab	-0.75	1.00000000	chicken
Trub1	0.23	1.00000000	chicken
AU040320	0.17	1.00000000	chicken
Hnrnpul1	0.30	1.00000000	chicken
Nup214	-0.62	1.00000000	chicken
4930453N24Rik	0.33	1.00000000	chicken
Tmem223	-0.42	1.00000000	chicken
Arid5a	-0.03	1.00000000	chicken
Nop14	0.14	1.00000000	chicken
Paip1	-0.45	1.00000000	chicken
BC005537	0.49	1.00000000	chicken
Rbl2	-0.59	1.00000000	chicken

Kctd20	0.24	1.00000000	chicken
Riok3	0.10	1.00000000	chicken
Bcas3	1.25	1.00000000	chicken
Traf6	-0.60	1.00000000	chicken
Snu13	-0.63	1.00000000	chicken
Dusp7	0.32	1.00000000	chicken
Crebbp	-0.74	1.00000000	chicken
Arhgap26	0.29	1.00000000	chicken
Oxsr1	0.45	1.00000000	chicken
Rcc2	-0.56	1.00000000	chicken
Spast	-0.60	1.00000000	chicken
Wdr77	-0.41	1.00000000	chicken
Pik3cb	0.49	1.00000000	chicken
Hmg20a	-0.57	1.00000000	chicken
Bnip2	-0.61	1.00000000	chicken
Tada1	0.43	1.00000000	chicken
Xiap	0.05	1.00000000	chicken
Plcg2	0.17	1.00000000	chicken
Senp7	-0.94	1.00000000	chicken
Tcf20	0.50	1.00000000	chicken
Lrrc8a	0.30	1.00000000	chicken
Lpin1	-0.39	1.00000000	chicken
G3bp2	-0.68	1.00000000	chicken
Akap9	-0.96	1.00000000	chicken
Kmt2e	-0.74	1.00000000	chicken
Abcd3	0.29	1.00000000	chicken
Vamp4	0.17	1.00000000	chicken
Tspo	0.10	1.00000000	chicken
Pspc1	-0.43	1.00000000	chicken
Atp5pb	-0.67	1.00000000	chicken
Tmem19	0.06	1.00000000	chicken
Pisd	0.19	1.00000000	chicken
Tmx3	-0.42	1.00000000	chicken
Slc12a4	0.15	1.00000000	chicken
Snrk	-0.52	1.00000000	chicken
Aspg	-0.94	1.00000000	chicken
Anxa11	-0.38	1.00000000	chicken
Cpsf2	0.43	1.00000000	chicken
Tcof1	-0.77	1.00000000	chicken
Zfp148	0.38	1.00000000	chicken
Zfp827	1.13	1.00000000	chicken
Fubp3	-0.77	1.00000000	chicken
Slc25a26	0.24	1.00000000	chicken
Gltp	0.60	1.00000000	chicken
Plxnb2	0.75	1.00000000	chicken
Golm2	-0.53	1.00000000	chicken

Ube2k	0.14	1.00000000	chicken
Vcl	-0.62	1.00000000	chicken
Ndufb9	-0.50	1.00000000	chicken
Rev1	-0.76	1.00000000	chicken
Cmtm3	-0.54	1.00000000	chicken
Dpysl2	0.27	1.00000000	chicken
Ireb2	-0.38	1.00000000	chicken
Bdp1	-0.47	1.00000000	chicken
Nmrk1	0.31	1.00000000	chicken
Sin3a	-0.39	1.00000000	chicken
Arpc5	-0.54	1.00000000	chicken
Gpatch3	0.37	1.00000000	chicken
Prrc2c	-0.46	1.00000000	chicken
Rab3gap2	0.22	1.00000000	chicken
Lmnb1	0.44	1.00000000	chicken
Znrf3	-0.68	1.00000000	chicken
Gzf1	-0.45	1.00000000	chicken
Rad23b	-0.67	1.00000000	chicken
Smim12	-0.31	1.00000000	chicken
Dsn1	0.29	1.00000000	chicken
Rad54l2	0.65	1.00000000	chicken
Zfand6	0.19	1.00000000	chicken
Itprid2	1.23	1.00000000	chicken
Rspry1	0.68	1.00000000	chicken
Vcp	0.33	1.00000000	chicken
Hbp1	-0.07	1.00000000	chicken
Bcap31	-0.46	1.00000000	chicken
Slc24a4	1.03	1.00000000	chicken
Adnp2	0.10	1.00000000	chicken
Ttc27	0.24	1.00000000	chicken
Plch2	0.04	1.00000000	chicken
Snrpd3	-0.62	1.00000000	chicken
Syap1	0.28	1.00000000	chicken
Ogdh	0.45	1.00000000	chicken
Arhgdia	-0.47	1.00000000	chicken
Lrp3	0.18	1.00000000	chicken
Tmem255a	0.11	1.00000000	chicken
Hs6st1	-1.58	1.00000000	chicken
Gtf2h5	-0.63	1.00000000	chicken
Golgb1	-0.63	1.00000000	chicken
Ndufb8	-0.36	1.00000000	chicken
Ssbp1	-0.70	1.00000000	chicken
Ewsr1	-0.72	1.00000000	chicken
Golga7	-0.55	1.00000000	chicken
Vps13c	0.33	1.00000000	chicken
Stab1	0.22	1.00000000	chicken

Pstpip1	-0.73	1.00000000	chicken
Zfp407	-0.37	1.00000000	chicken
Tnks2	0.42	1.00000000	chicken
Gigyf2	0.26	1.00000000	chicken
Cast	0.29	1.00000000	chicken
Arntl	0.31	1.00000000	chicken
A1bg	0.32	1.00000000	chicken
Prmt9	0.01	1.00000000	chicken
Taf5	-0.47	1.00000000	chicken
Ankib1	-0.69	1.00000000	chicken
Tubb4b	0.19	1.00000000	chicken
Phax	-0.44	1.00000000	chicken
Gphn	-0.95	1.00000000	chicken
Crot	0.05	1.00000000	chicken
Scp2	-0.50	1.00000000	chicken
Trim24	-0.15	1.00000000	chicken
Washc5	0.22	1.00000000	chicken
2410004B18Rik	0.29	1.00000000	chicken
Abhd6	0.32	1.00000000	chicken
Rtkn2	0.26	1.00000000	chicken
Itgb1	0.32	1.00000000	chicken
Uqcrc1	-0.35	1.00000000	chicken
Timm13	-0.52	1.00000000	chicken
Hsbp1	-0.52	1.00000000	chicken
Ube2h	-0.61	1.00000000	chicken
Fam204a	-0.31	1.00000000	chicken
Sec61g	-0.49	1.00000000	chicken
Slx4ip	0.61	1.00000000	chicken
Id2	-0.72	1.00000000	chicken
Siva1	-0.56	1.00000000	chicken
Exoc2	-0.76	1.00000000	chicken
Stau1	-0.38	1.00000000	chicken
Psmb2	-0.62	1.00000000	chicken
Cers5	0.25	1.00000000	chicken
Brd2	-0.64	1.00000000	chicken
Gxylt1	-0.53	1.00000000	chicken
Nr4a1	-0.49	1.00000000	chicken
Sumo1	-0.52	1.00000000	chicken
Chmp4b	-0.60	1.00000000	chicken
Rbbp6	0.14	1.00000000	chicken
Arl6ip5	0.38	1.00000000	chicken
Grn	-0.11	1.00000000	chicken
Camsap2	-0.70	1.00000000	chicken
Efhd2	-1.20	1.00000000	chicken
Rmc1	0.06	1.00000000	chicken
Limd2	-0.62	1.00000000	chicken

Hic1	0.24	1.00000000	chicken
Terf2	-0.42	1.00000000	chicken
Cdk1	0.21	1.00000000	chicken
Ndufb10	-0.53	1.00000000	chicken
Tra2b	-0.64	1.00000000	chicken
Usp20	0.31	1.00000000	chicken
Arfgef2	-0.47	1.00000000	chicken
Qsox1	-0.61	1.00000000	chicken
Tfdp2	-0.81	1.00000000	chicken
Sesn2	0.05	1.00000000	chicken
Prpsap2	-0.62	1.00000000	chicken
Ezr	-0.64	1.00000000	chicken
Cyth4	0.27	1.00000000	chicken
Galnt7	-0.44	1.00000000	chicken
Gne	-0.55	1.00000000	chicken
Stt3a	-0.59	1.00000000	chicken
Nat10	-0.46	1.00000000	chicken
Kdm5a	-0.65	1.00000000	chicken
Bnip3	-1.00	1.00000000	chicken
Foxn3	0.38	1.00000000	chicken
Ndufb5	-0.49	1.00000000	chicken
Aspm	0.12	1.00000000	chicken
Nmt1	0.20	1.00000000	chicken
Parp4	-0.52	1.00000000	chicken
Il6ra	0.01	1.00000000	chicken
Harbi1	0.14	1.00000000	chicken
Epn2	0.24	1.00000000	chicken
Il18	0.37	1.00000000	chicken
Ascc1	0.18	1.00000000	chicken
Stk38l	-0.58	1.00000000	chicken
Fahd2a	-0.44	1.00000000	chicken
Lamtor2	-0.43	1.00000000	chicken
Hemk1	-0.58	1.00000000	chicken
S100a11	-0.43	1.00000000	chicken
Kmt5b	-0.76	1.00000000	chicken
Ndufa9	-0.75	1.00000000	chicken
Stam2	0.21	1.00000000	chicken
Aup1	-0.30	1.00000000	chicken
Stau2	0.35	1.00000000	chicken
Igsf6	0.37	1.00000000	chicken
Rnh1	0.06	1.00000000	chicken
Septin8	0.16	1.00000000	chicken
Yy2	0.18	1.00000000	chicken
Cnot7	-0.47	1.00000000	chicken
Wsb1	0.18	1.00000000	chicken
Wwp2	0.31	1.00000000	chicken

Nfe2l1	0.14	1.00000000	chicken
Gcc2	-0.66	1.00000000	chicken
Csf3r	0.67	1.00000000	chicken
Cyth3	0.22	1.00000000	chicken
Ubr7	-0.45	1.00000000	chicken
Capzb	-0.45	1.00000000	chicken
Ttc17	0.25	1.00000000	chicken
Suds3	-0.79	1.00000000	chicken
Zbtb38	-0.50	1.00000000	chicken
Txlng	-0.55	1.00000000	chicken
Timp3	0.73	1.00000000	chicken
Fhit	-1.48	1.00000000	chicken
Snx2	0.13	1.00000000	chicken
Kif5c	-0.54	1.00000000	chicken
Twf2	0.29	1.00000000	chicken
Chchd10	-0.70	1.00000000	chicken
Cdk11b	0.32	1.00000000	chicken
Uqcrb	-0.45	1.00000000	chicken
Snrpd1	-0.52	1.00000000	chicken
Ssr3	-0.44	1.00000000	chicken
Paip2	-0.43	1.00000000	chicken
Dnaaf9	0.28	1.00000000	chicken
Pank3	0.08	1.00000000	chicken
Slc7a3	0.39	1.00000000	chicken
Nfic	0.44	1.00000000	chicken
Rock2	-0.78	1.00000000	chicken
Hipk1	-0.75	1.00000000	chicken
Capn11	-0.20	1.00000000	chicken
Camk1d	-1.13	1.00000000	chicken
Ppp3cb	-0.92	1.00000000	chicken
Ywhah	-0.54	1.00000000	chicken
Cab39	-0.72	1.00000000	chicken
Elovl5	-0.76	1.00000000	chicken
Cbx3	-0.83	1.00000000	chicken
Ndufb6	-0.35	1.00000000	chicken
Saal1	0.26	1.00000000	chicken
Nt5c3	0.62	1.00000000	chicken
Vps13d	-0.63	1.00000000	chicken
Phip	-0.88	1.00000000	chicken
Crebl2	0.28	1.00000000	chicken
Clasp2	0.61	1.00000000	chicken
Ndufc2	-0.59	1.00000000	chicken
Arrdc2	0.36	1.00000000	chicken
Dctn2	-0.41	1.00000000	chicken
Lsm4	0.21	1.00000000	chicken
Dennd5a	-0.07	1.00000000	chicken

Kdm1a	0.12	1.00000000	chicken
Tm9sf5	-0.62	1.00000000	chicken
Anxa1	0.14	1.00000000	chicken
Psmc5	-0.38	1.00000000	chicken
Commd8	0.28	1.00000000	chicken
Atp7a	0.09	1.00000000	chicken
Atp2b1	-0.55	1.00000000	chicken
Naxd	0.18	1.00000000	chicken
Tcf25	-0.52	1.00000000	chicken
Creld2	-0.46	1.00000000	chicken
Gsk3b	-0.30	1.00000000	chicken
Raf1	-0.63	1.00000000	chicken
Atp6v0c	-0.07	1.00000000	chicken
Slc39a8	0.30	1.00000000	chicken
Trim59	0.14	1.00000000	chicken
Nr4a2	-0.70	1.00000000	chicken
Tacc1	0.25	1.00000000	chicken
Rabep1	0.21	1.00000000	chicken
Ext1	-0.96	1.00000000	chicken
Gfod1	0.45	1.00000000	chicken
Slc2a8	-0.39	1.00000000	chicken
Cstf1	-0.51	1.00000000	chicken
Upf3b	-0.51	1.00000000	chicken
Trim25	0.19	1.00000000	chicken
Baz1b	-0.96	1.00000000	chicken
Taok3	-0.89	1.00000000	chicken
Otud7a	-0.89	1.00000000	chicken
Chd1	0.28	1.00000000	chicken
Ost4	0.29	1.00000000	chicken
Prpf19	-0.45	1.00000000	chicken
Zfp654	0.86	1.00000000	chicken
Atp5g3	-0.73	1.00000000	chicken
Dkc1	-0.64	1.00000000	chicken
Dnajc19	-0.40	1.00000000	chicken
Crnkl1	-0.58	1.00000000	chicken
Endov	0.20	1.00000000	chicken
Rsbn1l	-0.43	1.00000000	chicken
Slain2	-0.52	1.00000000	chicken
Psip1	-0.49	1.00000000	chicken
Usp47	-0.69	1.00000000	chicken
Nhlrc2	-0.67	1.00000000	chicken
Zzz3	0.43	1.00000000	chicken
Taf4	0.05	1.00000000	chicken
Rsrp1	-0.89	1.00000000	chicken
Tmem63a	-0.54	1.00000000	chicken
Gtf2f2	-0.76	1.00000000	chicken

Tgfbrap1	0.09	1.00000000	chicken
Tmco1	0.13	1.00000000	chicken
Rnpc3	-0.69	1.00000000	chicken
Ambra1	0.50	1.00000000	chicken
Uhmk1	-0.40	1.00000000	chicken
Pik3cd	0.05	1.00000000	chicken
Slc12a7	-0.69	1.00000000	chicken
Dnajc15	-0.32	1.00000000	chicken
Abhd17c	0.09	1.00000000	chicken
Sh3pxd2b	-0.15	1.00000000	chicken
Farp1	0.01	1.00000000	chicken
Otud4	-0.67	1.00000000	chicken
Osbp2	-0.53	1.00000000	chicken
Ticam1	0.02	1.00000000	chicken
Uba3	0.12	1.00000000	chicken
Pum2	-0.69	1.00000000	chicken
Acap2	0.35	1.00000000	chicken
Rfx7	-0.43	1.00000000	chicken
Snx1	-0.26	1.00000000	chicken
Ncald	-0.47	1.00000000	chicken
Borcs6	0.43	1.00000000	chicken
Nudc	-0.04	1.00000000	chicken
Adarb1	-0.65	1.00000000	chicken
Top1	-0.78	1.00000000	chicken
Fbxo8	0.23	1.00000000	chicken
Trpv2	0.03	1.00000000	chicken
Sec13	-0.55	1.00000000	chicken
Ap3m2	0.04	1.00000000	chicken
Tsn	-0.37	1.00000000	chicken
Ehd3	0.25	1.00000000	chicken
Ddx1	-0.61	1.00000000	chicken
Ermn	-0.22	1.00000000	chicken
Vdac1	0.23	1.00000000	chicken
Tmed7	-0.49	1.00000000	chicken
Hgs	0.06	1.00000000	chicken
Rab10	-0.46	1.00000000	chicken
Selenot	-0.52	1.00000000	chicken
Ube2e2	0.31	1.00000000	chicken
Arl6ip1	-0.74	1.00000000	chicken
Nusap1	0.18	1.00000000	chicken
Atp5k	-0.94	1.00000000	chicken
Ube3a	-0.58	1.00000000	chicken
Lfng	0.11	1.00000000	chicken
Cep128	0.32	1.00000000	chicken
Chm	-0.65	1.00000000	chicken
Erap1	-0.43	1.00000000	chicken

Slc35e3	0.11	1.00000000	chicken
Cyld	-0.47	1.00000000	chicken
Taf2	-0.40	1.00000000	chicken
Zfp706	-0.62	1.00000000	chicken
Srf	-0.42	1.00000000	chicken
Tial1	-0.71	1.00000000	chicken
Elof1	0.45	1.00000000	chicken
Tacc3	0.17	1.00000000	chicken
Pxk	0.37	1.00000000	chicken
Gpbp1l1	-0.40	1.00000000	chicken
Sox5	0.30	1.00000000	chicken
Tmem33	-0.33	1.00000000	chicken
Xpo7	-0.51	1.00000000	chicken
Gpd2	-0.36	1.00000000	chicken
Chd2	0.40	1.00000000	chicken
Zfp36l2	-0.96	1.00000000	chicken
Cct2	-0.68	1.00000000	chicken
Azi2	-0.37	1.00000000	chicken
Dalrd3	0.59	1.00000000	chicken
Dis3	0.07	1.00000000	chicken
Stx8	-0.08	1.00000000	chicken
Clk4	-0.51	1.00000000	chicken
Arhgap21	0.57	1.00000000	chicken
Lamtor1	0.13	1.00000000	chicken
Nfkb1	0.26	1.00000000	chicken
Rgs14	0.25	1.00000000	chicken
Thoc1	-0.68	1.00000000	chicken
Nifk	-0.59	1.00000000	chicken
Braf	-0.48	1.00000000	chicken
Ess2	0.11	1.00000000	chicken
Tdpoz8	0.28	1.00000000	chicken
Prrc2b	-0.60	1.00000000	chicken
Srsf7	-0.42	1.00000000	chicken
Rnf185	0.20	1.00000000	chicken
Rap2b	0.12	1.00000000	chicken
Ctr9	-0.55	1.00000000	chicken
Prep	-0.44	1.00000000	chicken
Prkrip1	0.06	1.00000000	chicken
Rere	-0.77	1.00000000	chicken
Arf4	0.53	1.00000000	chicken
Bcar3	-0.59	1.00000000	chicken
Rchy1	-0.52	1.00000000	chicken
E2f5	0.01	1.00000000	chicken
Sp1	0.06	1.00000000	chicken
Cars	0.03	1.00000000	chicken
Strada	0.11	1.00000000	chicken

Afg3l2	0.17	1.00000000	chicken
Cramp1	-0.42	1.00000000	chicken
Tubgcp5	-0.62	1.00000000	chicken
Rars2	0.18	1.00000000	chicken
Pxylp1	0.27	1.00000000	chicken
Tmem53	0.29	1.00000000	chicken
Pogz	-0.49	1.00000000	chicken
Gpatch4	0.31	1.00000000	chicken
Eml3	-0.29	1.00000000	chicken
Ssb	-0.75	1.00000000	chicken
Fgr	0.15	1.00000000	chicken
Ints13	-0.60	1.00000000	chicken
Hnrnph3	-0.52	1.00000000	chicken
Cdc40	-0.62	1.00000000	chicken
Crim1	-0.87	1.00000000	chicken
Srpr	-0.51	1.00000000	chicken
Vrk2	0.23	1.00000000	chicken
Jund	0.16	1.00000000	chicken
Ercc6	-0.75	1.00000000	chicken
Plekha1	-0.58	1.00000000	chicken
Ubp1	0.15	1.00000000	chicken
Eif4g3	-0.61	1.00000000	chicken
Kat2b	0.11	1.00000000	chicken
Ccdc126	-0.53	1.00000000	chicken
Plekhm1	0.03	1.00000000	chicken
Yaf2	0.16	1.00000000	chicken
Brd8	0.20	1.00000000	chicken
Hipk3	-0.50	1.00000000	chicken
Dock8	-0.71	1.00000000	chicken
Ngly1	0.13	1.00000000	chicken
H13	-0.47	1.00000000	chicken
Ube2w	0.24	1.00000000	chicken
Tef	-0.66	1.00000000	chicken
Radx	0.26	1.00000000	chicken
Znfx1	0.43	1.00000000	chicken
Xpr1	0.21	1.00000000	chicken
Cops4	0.29	1.00000000	chicken
Fam120a	-0.71	1.00000000	chicken
Vnn3	0.39	1.00000000	chicken
Washc1	-0.50	1.00000000	chicken
Ahctf1	0.31	1.00000000	chicken
Tlk2	0.15	1.00000000	chicken
Tspan4	-0.63	1.00000000	chicken
Clcn5	0.32	1.00000000	chicken
Lin54	0.23	1.00000000	chicken
Gabpa	-0.46	1.00000000	chicken

Ddx6	-0.64	1.00000000	chicken
Rab11fip2	0.35	1.00000000	chicken
Pcf11	0.15	1.00000000	chicken
Dip2a	-0.09	1.00000000	chicken
Dnm1l	0.28	1.00000000	chicken
Lemd3	0.27	1.00000000	chicken
Stx12	0.13	1.00000000	chicken
Tesk2	-0.03	1.00000000	chicken
Prpf39	-0.65	1.00000000	chicken
Hook1	0.08	1.00000000	chicken
Atxn7	0.16	1.00000000	chicken
Bin2	-0.48	1.00000000	chicken
Adss	0.13	1.00000000	chicken
Pten	-0.31	1.00000000	chicken
Suco	0.48	1.00000000	chicken
Rnf103	-0.04	1.00000000	chicken
1110059E24Rik	0.20	1.00000000	chicken
Akirin2	0.33	1.00000000	chicken
Strn	-0.76	1.00000000	chicken
Fermt2	0.26	1.00000000	chicken
Tex2	0.14	1.00000000	chicken
Srrm1	-0.43	1.00000000	chicken
Nktr	-0.32	1.00000000	chicken
Zfpm1	0.28	1.00000000	chicken
Creg1	-0.93	1.00000000	chicken
Tkt	-0.10	1.00000000	chicken
Sde2	-0.67	1.00000000	chicken
Lgals3	0.11	1.00000000	chicken
Sec14l1	-0.11	1.00000000	chicken
Pdcd6	0.06	1.00000000	chicken
Ctcf	-0.37	1.00000000	chicken
Cdc37	0.43	1.00000000	chicken
Nedd8	-0.59	1.00000000	chicken
Spen	0.26	1.00000000	chicken
Smad1	0.25	1.00000000	chicken
Xrn1	-0.26	1.00000000	chicken
Svil	-0.14	1.00000000	chicken
Golga3	-0.03	1.00000000	chicken
Dync1h1	-0.41	1.00000000	chicken
Fmr1	-0.40	1.00000000	chicken
Ppp4r3a	0.19	1.00000000	chicken
Tfrc	-0.57	1.00000000	chicken
Usp48	-0.61	1.00000000	chicken
Pptc7	-0.05	1.00000000	chicken
Dcaf13	-0.56	1.00000000	chicken
Vps13a	-0.50	1.00000000	chicken

Ube2o	-0.47	1.00000000	chicken
Invs	-0.25	1.00000000	chicken
Bcat1	-0.72	1.00000000	chicken
Reck	0.04	1.00000000	chicken
Cd63	-0.74	1.00000000	chicken
Tspan3	-0.57	1.00000000	chicken
Wdr11	-0.55	1.00000000	chicken
Cmip	-0.35	1.00000000	chicken
Gpr107	0.06	1.00000000	chicken
Actr3	-0.36	1.00000000	chicken
Creb1	0.22	1.00000000	chicken
Sel1l	-0.70	1.00000000	chicken
Chmp2a	0.03	1.00000000	chicken
Acbd3	-0.58	1.00000000	chicken
Cmklr1	0.16	1.00000000	chicken
Ube2a	-0.57	1.00000000	chicken
Kctd6	0.09	1.00000000	chicken
Gabpb1	0.09	1.00000000	chicken
Fdft1	-0.09	1.00000000	chicken
Adipor2	0.05	1.00000000	chicken
Tbc1d14	-0.94	1.00000000	chicken
Ctnnbl1	-0.47	1.00000000	chicken
Dot1l	0.14	1.00000000	chicken
Get4	-0.52	1.00000000	chicken
Strap	-0.51	1.00000000	chicken
Smu1	-0.50	1.00000000	chicken
Ei24	-0.34	1.00000000	chicken
Ahsa1	-0.40	1.00000000	chicken
Bag1	-0.61	1.00000000	chicken
Nasp	-0.64	1.00000000	chicken
Phf10	-0.55	1.00000000	chicken
Srsf5	-0.55	1.00000000	chicken
Sgms1	0.40	1.00000000	chicken
H2az1	-0.31	1.00000000	chicken
Eri3	0.06	1.00000000	chicken
Gpatch2l	0.04	1.00000000	chicken
Rab5a	-0.56	1.00000000	chicken
Tspan14	-1.17	1.00000000	chicken
Tubb5	-0.48	1.00000000	chicken
Tnfrsf11a	0.04	1.00000000	chicken
Ccdc82	-0.47	1.00000000	chicken
Limd1	-0.01	1.00000000	chicken
Adcy7	-0.82	1.00000000	chicken
Etfa	0.32	1.00000000	chicken
Sec11c	-0.37	1.00000000	chicken
Nup107	0.20	1.00000000	chicken

Vps54	0.10	1.00000000	chicken
Senp6	-0.69	1.00000000	chicken
Rnf166	-0.62	1.00000000	chicken
Phaf1	0.21	1.00000000	chicken
Emc1	0.02	1.00000000	chicken
Cops3	0.35	1.00000000	chicken
5730455P16Rik	-0.40	1.00000000	chicken
Pnisr	-0.66	1.00000000	chicken
Cracr2b	-0.27	1.00000000	chicken
Psma1	0.13	1.00000000	chicken
Crygn	-0.04	1.00000000	chicken
Tst	-0.46	1.00000000	chicken
Rftn1	-0.38	1.00000000	chicken
Ppp2r2d	-0.54	1.00000000	chicken
Tnfrsf26	0.11	1.00000000	chicken
Plek	0.44	1.00000000	chicken
Tbc1d8b	0.03	1.00000000	chicken
Ap3b1	-0.77	1.00000000	chicken
Ndufab1	-0.36	1.00000000	chicken
Lrp8	-0.17	1.00000000	chicken
Wdr37	0.12	1.00000000	chicken
Arfgef1	-0.19	1.00000000	chicken
Clns1a	-0.40	1.00000000	chicken
Yipf3	-0.49	1.00000000	chicken
Adck1	-0.42	1.00000000	chicken
Atp2a2	0.25	1.00000000	chicken
Sdhaf2	-0.44	1.00000000	chicken
Zfp236	-0.09	1.00000000	chicken
Ppif	0.24	1.00000000	chicken
Lmbrd2	-0.49	1.00000000	chicken
Nup98	-0.67	1.00000000	chicken
Pam	1.20	1.00000000	chicken
Dad1	-0.38	1.00000000	chicken
Luc7l	-0.73	1.00000000	chicken
Htt	-0.86	1.00000000	chicken
Sos1	-0.98	1.00000000	chicken
Socs6	0.00	1.00000000	chicken
Gbf1	-0.70	1.00000000	chicken
Crebrf	-0.05	1.00000000	chicken
Ugt8a	0.42	1.00000000	chicken
D16Ertd472e	-0.48	1.00000000	chicken
Cog7	-0.57	1.00000000	chicken
Arhgef6	-0.68	1.00000000	chicken
Llph	0.24	1.00000000	chicken
Lin7c	0.20	1.00000000	chicken
Ripk1	0.24	1.00000000	chicken

Eml6	0.64	1.00000000	chicken
Zfp830	-0.40	1.00000000	chicken
Klhl5	0.23	1.00000000	chicken
Pmepa1	0.39	1.00000000	chicken
Rab1a	0.11	1.00000000	chicken
Tnfaip8	-0.74	1.00000000	chicken
Zfp384	-0.46	1.00000000	chicken
Washc2	-0.85	1.00000000	chicken
Wdr36	-0.37	1.00000000	chicken
Eef1e1	-0.65	1.00000000	chicken
Amdhd2	0.05	1.00000000	chicken
Dock9	-0.86	1.00000000	chicken
Elk3	-0.65	1.00000000	chicken
Psmb1	-0.69	1.00000000	chicken
Hdac7	-0.54	1.00000000	chicken
Chfr	0.22	1.00000000	chicken
Cwc22	-0.40	1.00000000	chicken
Ssbp2	-0.33	1.00000000	chicken
Slc45a4	-0.25	1.00000000	chicken
Adssl1	-0.25	1.00000000	chicken
Slmap	-1.08	1.00000000	chicken
Hdgf	-0.40	1.00000000	chicken
Atp6v0e	-0.63	1.00000000	chicken
Rbbp5	0.07	1.00000000	chicken
Pnrc1	-0.05	1.00000000	chicken
Ptpn6	-0.65	1.00000000	chicken
Rtn4	-0.36	1.00000000	chicken
Iqgap1	-0.78	1.00000000	chicken
Chmp2b	-0.39	1.00000000	chicken
Atp6v1e2	-0.15	1.00000000	chicken
Wdr43	-0.55	1.00000000	chicken
Zfp451	0.31	1.00000000	chicken
Acaa1b	0.21	1.00000000	chicken
Exoc6	0.16	1.00000000	chicken
Stk11	-0.65	1.00000000	chicken
Eprs	-0.41	1.00000000	chicken
Tab3	0.21	1.00000000	chicken
Sdcbp	0.17	1.00000000	chicken
Eif4a3l1	-0.58	1.00000000	chicken
AI597479	0.07	1.00000000	chicken
Dtx2	0.88	1.00000000	chicken
Cask	-0.79	1.00000000	chicken
Nubpl	-0.22	1.00000000	chicken
D630045J12Rik	0.45	1.00000000	chicken
Snx20	-0.21	1.00000000	chicken
Grk2	-0.02	1.00000000	chicken

Thoc7	-0.33	1.00000000	chicken
Rb1	-0.58	1.00000000	chicken
Uba2	0.15	1.00000000	chicken
Naa40	0.20	1.00000000	chicken
Tsc22d2	0.06	1.00000000	chicken
Tdrd3	0.11	1.00000000	chicken
Cplane2	-0.62	1.00000000	chicken
Lyrm1	0.22	1.00000000	chicken
Erp44	-0.14	1.00000000	chicken
Sarnp	-0.40	1.00000000	chicken
Ptpn12	0.04	1.00000000	chicken
Sh2d1a	-0.53	1.00000000	chicken
Hnrnpm	-0.46	1.00000000	chicken
Csgalnact2	-0.10	1.00000000	chicken
Camk2d	0.18	1.00000000	chicken
Pikfyve	0.17	1.00000000	chicken
Gid8	-0.45	1.00000000	chicken
Kbtbd8	-0.03	1.00000000	chicken
Ldah	-0.74	1.00000000	chicken
Rab18	0.20	1.00000000	chicken
Pdap1	-0.40	1.00000000	chicken
Vps37b	-0.55	1.00000000	chicken
Sacs	0.32	1.00000000	chicken
Pwp1	0.11	1.00000000	chicken
Prkca	-0.78	1.00000000	chicken
Rnf11	-1.14	1.00000000	chicken
Cdc27	-0.54	1.00000000	chicken
Auh	0.37	1.00000000	chicken
Pex1	0.26	1.00000000	chicken
Hectd4	-0.57	1.00000000	chicken
Strn3	0.41	1.00000000	chicken
Slc1a4	-0.43	1.00000000	chicken
Usf3	-0.07	1.00000000	chicken
Immp2l	-0.95	1.00000000	chicken
Sh3glb1	0.04	1.00000000	chicken
Eif3i	-0.16	1.00000000	chicken
Lrch2	0.56	1.00000000	chicken
Gstm4	-0.36	1.00000000	chicken
Vapa	-0.62	1.00000000	chicken
Pik3c2a	-0.82	1.00000000	chicken
Rpa2	0.08	1.00000000	chicken
Celf1	-0.37	1.00000000	chicken
Arid3a	-0.55	1.00000000	chicken
Naa25	-0.53	1.00000000	chicken
Ide	-0.51	1.00000000	chicken
St13	-0.30	1.00000000	chicken

Kras	-0.46	1.00000000	chicken
Atg12	-0.40	1.00000000	chicken
Pnn	-0.41	1.00000000	chicken
Sacm1l	0.03	1.00000000	chicken
Jmjd1c	0.51	1.00000000	chicken
Trappc9	-0.18	1.00000000	chicken
Carhsp1	-0.70	1.00000000	chicken
Bri3	0.02	1.00000000	chicken
Atp5h	-0.35	1.00000000	chicken
Cul3	-0.40	1.00000000	chicken
Tmem183a	-0.40	1.00000000	chicken
Snap29	0.20	1.00000000	chicken
Psme3	-0.47	1.00000000	chicken
Anapc10	-0.10	1.00000000	chicken
Nop56	-0.44	1.00000000	chicken
Parp8	-0.88	1.00000000	chicken
Polr2l	-0.55	1.00000000	chicken
Cox7a2l	-0.32	1.00000000	chicken
Tomm70a	-0.65	1.00000000	chicken
Glyr1	-0.43	1.00000000	chicken
Fam13b	-0.66	1.00000000	chicken
Uqcrh	-0.39	1.00000000	chicken
Cd47	0.35	1.00000000	chicken
Zbtb21	0.09	1.00000000	chicken
Nedd4	-0.43	1.00000000	chicken
Zfp592	0.35	1.00000000	chicken
Cog5	-0.66	1.00000000	chicken
Setd3	-0.39	1.00000000	chicken
Glg1	-0.44	1.00000000	chicken
Atp1a1	0.14	1.00000000	chicken
Fam172a	-0.71	1.00000000	chicken
Osbpl9	0.05	1.00000000	chicken
Sipa1l1	-0.77	1.00000000	chicken
Tlk1	-0.73	1.00000000	chicken
Taz	-0.38	1.00000000	chicken
Srsf1	-0.65	1.00000000	chicken
Sla	0.33	1.00000000	chicken
Ubap2l	0.14	1.00000000	chicken
Ppat	-0.37	1.00000000	chicken
Cpsf4l	-0.73	1.00000000	chicken
Bms1	-0.35	1.00000000	chicken
Fkbp4	-0.34	1.00000000	chicken
Denr	-0.15	1.00000000	chicken
Ptp4a2	-0.50	1.00000000	chicken
Chchd1	-0.12	1.00000000	chicken
Ep400	-0.59	1.00000000	chicken

Arih1	0.36	1.00000000	chicken
Whamm	-0.10	1.00000000	chicken
Tnnt3	0.37	1.00000000	chicken
4921524J17Rik	0.10	1.00000000	chicken
Dph3	-0.50	1.00000000	chicken
Tfg	-0.18	1.00000000	chicken
Rab39b	-0.43	1.00000000	chicken
Numa1	-0.11	1.00000000	chicken
Prpf40a	-0.60	1.00000000	chicken
Kif2a	-0.63	1.00000000	chicken
Psma3	-0.57	1.00000000	chicken
Ash1l	-0.68	1.00000000	chicken
Slc3a1	-0.12	1.00000000	chicken
Rnf145	-0.70	1.00000000	chicken
Ubl3	-0.62	1.00000000	chicken
Pdcd5	-0.39	1.00000000	chicken
Brf1	-0.21	1.00000000	chicken
Gpr183	0.41	1.00000000	chicken
Ergic1	0.08	1.00000000	chicken
Klhl24	-0.52	1.00000000	chicken
Npc2	-0.02	1.00000000	chicken
Pak3	-0.12	1.00000000	chicken
Kifbp	-1.31	1.00000000	chicken
Tpm1	-0.17	1.00000000	chicken
Ehbp1	0.45	1.00000000	chicken
Katnbl1	-0.43	1.00000000	chicken
Notch1	-0.55	1.00000000	chicken
Zfyve1	0.07	1.00000000	chicken
Rbbp4	-0.24	1.00000000	chicken
Dop1b	0.34	1.00000000	chicken
Soat1	0.12	1.00000000	chicken
Ago4	-0.27	1.00000000	chicken
Tpcn1	-0.55	1.00000000	chicken
Rara	-0.63	1.00000000	chicken
Gnb4	-0.82	1.00000000	chicken
Dync1li2	0.09	1.00000000	chicken
Gpalpp1	-0.54	1.00000000	chicken
Kat6a	-0.56	1.00000000	chicken
Fli1	0.60	1.00000000	chicken
Smap2	0.07	1.00000000	chicken
Vps39	-0.07	1.00000000	chicken
Adar	-0.42	1.00000000	chicken
Dyrk2	0.16	1.00000000	chicken
Tbc1d22a	-0.22	1.00000000	chicken
Ilf3	-0.52	1.00000000	chicken
Nxpe3	0.00	1.00000000	chicken

Herc3	0.03	1.00000000	chicken
Trpc4ap	-0.48	1.00000000	chicken
Rilp	0.09	1.00000000	chicken
Eipr1	-0.58	1.00000000	chicken
Tmem241	-0.13	1.00000000	chicken
Zbtb8os	-0.34	1.00000000	chicken
Slc25a24	-0.34	1.00000000	chicken
Gabarapl1	-0.62	1.00000000	chicken
Decr1	0.05	1.00000000	chicken
Vapb	-0.37	1.00000000	chicken
Slc25a5	-0.54	1.00000000	chicken
Gstm5	-0.26	1.00000000	chicken
Naa50	-0.02	1.00000000	chicken
Tm9sf2	-0.14	1.00000000	chicken
Tasor	-0.62	1.00000000	chicken
Nkrf	-0.33	1.00000000	chicken
Ltn1	-0.39	1.00000000	chicken
Hspe1-rs1	-0.51	1.00000000	chicken
Pnrc2	0.03	1.00000000	chicken
Otulinl	-0.35	1.00000000	chicken
Ndufaf8	-0.52	1.00000000	chicken
Aatf	0.10	1.00000000	chicken
Nrp2	0.15	1.00000000	chicken
Adam17	-0.26	1.00000000	chicken
Zeb1	-0.50	1.00000000	chicken
Tnik	-0.22	1.00000000	chicken
Umad1	-0.72	1.00000000	chicken
Lrrc8d	-0.80	1.00000000	chicken
Bsg	-0.69	1.00000000	chicken
D5Ertd579e	0.02	1.00000000	chicken
Cfdp1	0.04	1.00000000	chicken
Zdhhc6	-0.47	1.00000000	chicken
Fus	-0.48	1.00000000	chicken
Lmbrd1	-0.62	1.00000000	chicken
Setd2	-0.43	1.00000000	chicken
Zfp711	-0.48	1.00000000	chicken
Snrpf	-0.40	1.00000000	chicken
Appl2	0.02	1.00000000	chicken
Pdia6	-0.34	1.00000000	chicken
Ccdc91	-0.26	1.00000000	chicken
Idh1	-0.27	1.00000000	chicken
Nudt16l1	-0.37	1.00000000	chicken
Pinx1	-0.10	1.00000000	chicken
Cd164	-0.47	1.00000000	chicken
Setd1b	-0.40	1.00000000	chicken
Ppp6r3	0.07	1.00000000	chicken

Sdhb	-0.43	1.00000000	chicken
Cerk	-0.50	1.00000000	chicken
Ptdss1	-0.41	1.00000000	chicken
Polr1c	-0.30	1.00000000	chicken
Rae1	-0.22	1.00000000	chicken
Wipf1	-0.41	1.00000000	chicken
Slc45a3	-0.14	1.00000000	chicken
Adam19	-0.65	1.00000000	chicken
Aig1	-0.47	1.00000000	chicken
Pcca	-0.08	1.00000000	chicken
Churc1	-0.31	1.00000000	chicken
Hk1	-0.15	1.00000000	chicken
Larp7	-0.53	1.00000000	chicken
Snx8	-0.57	1.00000000	chicken
Pdxdc1	0.15	1.00000000	chicken
Prkx	0.26	1.00000000	chicken
Enox2	-0.37	1.00000000	chicken
Smad6	-0.35	1.00000000	chicken
Sar1a	-0.38	1.00000000	chicken
Prdx4	-0.33	1.00000000	chicken
Lcor	-0.10	1.00000000	chicken
Serp1	-0.25	1.00000000	chicken
Fbxo45	-0.18	1.00000000	chicken
Ttc19	0.39	1.00000000	chicken
Itch	0.05	1.00000000	chicken
Ppm1h	0.00	1.00000000	chicken
Foxk2	-0.21	1.00000000	chicken
Prpf6	-0.38	1.00000000	chicken
Lats1	-0.34	1.00000000	chicken
Tcea1	-0.25	1.00000000	chicken
Ranbp9	0.11	1.00000000	chicken
Zyx	-0.35	1.00000000	chicken
Pde7a	-0.40	1.00000000	chicken
Flot2	-0.32	1.00000000	chicken
Lnpk	-0.16	1.00000000	chicken
Snx4	0.07	1.00000000	chicken
Tmem181a	0.05	1.00000000	chicken
Fh1	-0.47	1.00000000	chicken
Fbxl22	-0.03	1.00000000	chicken
Supt6	0.02	1.00000000	chicken
Adam23	-0.03	1.00000000	chicken
Zbtb8b	0.13	1.00000000	chicken
Rab31	-0.58	1.00000000	chicken
B4galt5	0.14	1.00000000	chicken
Kdm3a	-0.42	1.00000000	chicken
Sp4	-0.34	1.00000000	chicken

Srsf3	-0.40	1.00000000	chicken
Cactin	-0.05	1.00000000	chicken
2700049A03Rik	0.15	1.00000000	chicken
Gk5	-0.04	1.00000000	chicken
Gng2	-0.59	1.00000000	chicken
Bud31	-0.40	1.00000000	chicken
Nckipsd	0.17	1.00000000	chicken
Asb8	0.08	1.00000000	chicken
Hook3	0.10	1.00000000	chicken
Pomp	-0.37	1.00000000	chicken
Cop1	-1.09	1.00000000	chicken
Nr1h3	-0.86	1.00000000	chicken
Sf3a3	-0.43	1.00000000	chicken
Hsp90aa1	-0.80	1.00000000	chicken
Sptlc1	0.12	1.00000000	chicken
Polr2b	-0.28	1.00000000	chicken
Tbcd	-0.06	1.00000000	chicken
Ranbp1	-0.23	1.00000000	chicken
Cdyl2	0.05	1.00000000	chicken
Snapc4	0.13	1.00000000	chicken
Tob2	-0.62	1.00000000	chicken
Dnaja1	-0.20	1.00000000	chicken
Efr3a	0.03	1.00000000	chicken
Zfp326	-0.65	1.00000000	chicken
Nmt2	-0.60	1.00000000	chicken
Eif4e2	0.23	1.00000000	chicken
Klf11	0.02	1.00000000	chicken
Optn	-0.16	1.00000000	chicken
C1d	-0.55	1.00000000	chicken
Sf3b5	-0.35	1.00000000	chicken
Tpst1	0.11	1.00000000	chicken
Prr5l	-0.73	1.00000000	chicken
Txndc9	-0.46	1.00000000	chicken
Tgfb1	-0.63	1.00000000	chicken
Lrrfip1	-0.22	1.00000000	chicken
Plekhd1	0.05	1.00000000	chicken
Tubg1	-0.42	1.00000000	chicken
Pcnp	-0.35	1.00000000	chicken
Dnase2b	0.23	1.00000000	chicken
Glt1d1	0.03	1.00000000	chicken
R3hdm1	-0.50	1.00000000	chicken
Pls3	0.07	1.00000000	chicken
Plagl1	-0.21	1.00000000	chicken
Btf3l4	-0.51	1.00000000	chicken
Btbd9	-0.73	1.00000000	chicken
Zfp143	0.12	1.00000000	chicken

Gnb1	0.26	1.00000000	chicken
Atg101	-0.01	1.00000000	chicken
Zfyve26	-0.57	1.00000000	chicken
Ncbp2	0.12	1.00000000	chicken
Cnn2	-0.55	1.00000000	chicken
Bak1	-0.29	1.00000000	chicken
Aftph	-0.61	1.00000000	chicken
Acaca	0.08	1.00000000	chicken
Gdi2	-0.51	1.00000000	chicken
Ube2i	-0.04	1.00000000	chicken
Ubqln1	-0.06	1.00000000	chicken
Serpine2	0.07	1.00000000	chicken
Itfg1	-0.54	1.00000000	chicken
Aff4	-0.33	1.00000000	chicken
Lrrfip2	0.10	1.00000000	chicken
Abhd2	-0.21	1.00000000	chicken
Sec11a	-0.46	1.00000000	chicken
3110082I17Rik	-0.41	1.00000000	chicken
Arhgef9	0.20	1.00000000	chicken
Dlg1	0.14	1.00000000	chicken
Cul4a	0.05	1.00000000	chicken
Eif2ak2	0.10	1.00000000	chicken
Agpat4	0.22	1.00000000	chicken
Ubash3b	0.04	1.00000000	chicken
Rpp14	-0.48	1.00000000	chicken
Hdac2	-0.42	1.00000000	chicken
Srp72	-0.36	1.00000000	chicken
Tmem115	-0.38	1.00000000	chicken
Vbp1	-0.40	1.00000000	chicken
Gtf2a2	-0.28	1.00000000	chicken
Sod2	-0.44	1.00000000	chicken
Blcap	-0.42	1.00000000	chicken
Akap1	0.32	1.00000000	chicken
Kpna3	0.03	1.00000000	chicken
Armh1	-0.04	1.00000000	chicken
Nf1	1.14	1.00000000	chicken
S100a4	-0.05	1.00000000	chicken
Eif2b3	-0.18	1.00000000	chicken
Ibtk	-0.83	1.00000000	chicken
Cherp	-0.10	1.00000000	chicken
Shoc2	0.40	1.00000000	chicken
Ccdc6	-0.47	1.00000000	chicken
Phf20l1	-0.72	1.00000000	chicken
Grb2	-0.51	1.00000000	chicken
Acp2	0.05	1.00000000	chicken
Parvg	0.04	1.00000000	chicken

Sae1	0.09	1.00000000	chicken
Smarcc1	-0.20	1.00000000	chicken
Zmynd8	-0.64	1.00000000	chicken
Qrich1	-0.35	1.00000000	chicken
Tnfsf13b	0.39	1.00000000	chicken
Tapt1	-0.32	1.00000000	chicken
Ubxn7	-0.16	1.00000000	chicken
Nolc1	0.06	1.00000000	chicken
Hps5	-0.50	1.00000000	chicken
Slc7a11	0.38	1.00000000	chicken
Pik3c3	-0.38	1.00000000	chicken
Rbm45	-0.45	1.00000000	chicken
Rer1	-0.28	1.00000000	chicken
Pcnt	-0.15	1.00000000	chicken
Sh3bgrl	-0.27	1.00000000	chicken
Atp9b	-0.65	1.00000000	chicken
Ncoa6	-0.49	1.00000000	chicken
Fam53a	-0.08	1.00000000	chicken
Taf6	-0.38	1.00000000	chicken
Arpc1a	-0.22	1.00000000	chicken
Traf1	-0.65	1.00000000	chicken
Rab35	-0.57	1.00000000	chicken
Asns	-0.64	1.00000000	chicken
Per3	0.15	1.00000000	chicken
Tapbp	-0.33	1.00000000	chicken
Creb3	0.32	1.00000000	chicken
Phf3	-0.53	1.00000000	chicken
Dnaja2	-0.52	1.00000000	chicken
Ppp1r7	-0.34	1.00000000	chicken
Eif4h	-0.43	1.00000000	chicken
Nob1	0.16	1.00000000	chicken
Akap11	-0.23	1.00000000	chicken
Ankhd1	-0.48	1.00000000	chicken
Dtx3l	-0.13	1.00000000	chicken
P4ha1	0.45	1.00000000	chicken
Gfpt1	0.25	1.00000000	chicken
Prmt7	0.09	1.00000000	chicken
Ube2f	0.61	1.00000000	chicken
Polr2f	-0.41	1.00000000	chicken
Timm44	-0.50	1.00000000	chicken
Oxr1	-0.13	1.00000000	chicken
Slc37a3	-0.08	1.00000000	chicken
Tut7	0.04	1.00000000	chicken
Phf14	-0.76	1.00000000	chicken
Rnaseh2b	-0.27	1.00000000	chicken
Ing3	-0.55	1.00000000	chicken

L3mbtl3	-0.03	1.00000000	chicken
Copb1	-0.08	1.00000000	chicken
Npc1	-0.07	1.00000000	chicken
Cct6b	-0.01	1.00000000	chicken
Pds5a	-0.08	1.00000000	chicken
Copb2	-0.46	1.00000000	chicken
BC048507	0.15	1.00000000	chicken
Xrcc4	-0.43	1.00000000	chicken
Ttyh3	-0.59	1.00000000	chicken
Btaf1	-0.46	1.00000000	chicken
Camk2g	-0.80	1.00000000	chicken
Smchd1	0.20	1.00000000	chicken
Tasp1	0.14	1.00000000	chicken
Fbxo25	-0.62	1.00000000	chicken
Ythdc2	-0.41	1.00000000	chicken
Herpud1	0.15	1.00000000	chicken
F2r	0.23	1.00000000	chicken
Erp29	-0.11	1.00000000	chicken
Ubl5b	-0.43	1.00000000	chicken
C4b	0.04	1.00000000	chicken
Gtf2b	-0.60	1.00000000	chicken
Fer	-0.61	1.00000000	chicken
E2f6	-0.32	1.00000000	chicken
Cdv3	-0.25	1.00000000	chicken
Nbn	0.42	1.00000000	chicken
Ankrd13a	-1.00	1.00000000	chicken
Rsrc1	-0.37	1.00000000	chicken
Prr5	0.27	1.00000000	chicken
Elf2	-0.54	1.00000000	chicken
Scyl2	-0.44	1.00000000	chicken
Kansl3	-0.28	1.00000000	chicken
Psmd1	-0.02	1.00000000	chicken
Aven	-0.05	1.00000000	chicken
Foxj2	-0.62	1.00000000	chicken
Prpf8	-0.40	1.00000000	chicken
Ndufa1	-0.27	1.00000000	chicken
Rbsn	-0.55	1.00000000	chicken
Htr2a	0.05	1.00000000	chicken
Ppp2cb	-0.45	1.00000000	chicken
Ldb2	-0.97	1.00000000	chicken
Ap2a1	-0.35	1.00000000	chicken
Socs5	0.26	1.00000000	chicken
Cdc42se1	-0.56	1.00000000	chicken
Nrf1	-0.50	1.00000000	chicken
Trp53bp2	0.32	1.00000000	chicken
Ino80	-0.54	1.00000000	chicken

Dap	-0.25	1.00000000	chicken
Fxr1	-0.93	1.00000000	chicken
Slc2a13	0.01	1.00000000	chicken
Appbp2	0.05	1.00000000	chicken
Brd7	-0.17	1.00000000	chicken
Ankrd11	-0.24	1.00000000	chicken
Esf1	-0.09	1.00000000	chicken
Dtnbp1	-0.44	1.00000000	chicken
Gab3	-1.27	1.00000000	chicken
Cradd	-0.38	1.00000000	chicken
Kpna6	-0.01	1.00000000	chicken
Ube2g2	-0.46	1.00000000	chicken
Rangap1	-0.42	1.00000000	chicken
Taf4b	-0.51	1.00000000	chicken
Il1rap	-0.19	1.00000000	chicken
Dnajb6	0.03	1.00000000	chicken
Atg3	0.19	1.00000000	chicken
Cnot8	-0.47	1.00000000	chicken
Zc3h18	-0.48	1.00000000	chicken
Srsf2	-0.45	1.00000000	chicken
Nbas	-0.74	1.00000000	chicken
Ints6l	-0.13	1.00000000	chicken
Psmd13	-0.33	1.00000000	chicken
Ubtf	-0.01	1.00000000	chicken
Atp6v1c1	-0.18	1.00000000	chicken
Kcnq1	-0.36	1.00000000	chicken
Phf21a	-0.25	1.00000000	chicken
Nudcd3	-0.05	1.00000000	chicken
Pin4	0.01	1.00000000	chicken
Poc1b	-0.45	1.00000000	chicken
Dbi	-0.39	1.00000000	chicken
Anxa5	0.21	1.00000000	chicken
Fars2	-0.48	1.00000000	chicken
Nfkb2	-0.60	1.00000000	chicken
Crispld2	-0.05	1.00000000	chicken
Uqcrfs1	-0.27	1.00000000	chicken
Nrd1	0.08	1.00000000	chicken
Ino80d	-0.15	1.00000000	chicken
Cfap20	-0.27	1.00000000	chicken
Cox10	-0.53	1.00000000	chicken
Ube2e3	0.28	1.00000000	chicken
Fnip2	0.32	1.00000000	chicken
Dym	-0.06	1.00000000	chicken
Zfp512	-0.32	1.00000000	chicken
Gtf2a1	0.11	1.00000000	chicken
Ywhae	-0.53	1.00000000	chicken

Lsp1	-0.34	1.00000000	chicken
Setdb2	-0.06	1.00000000	chicken
Nr2c1	-0.36	1.00000000	chicken
Nufip1	-0.38	1.00000000	chicken
Zfp609	-0.04	1.00000000	chicken
Srpk1	-0.47	1.00000000	chicken
Ndufb2	-0.38	1.00000000	chicken
Utrn	0.11	1.00000000	chicken
Atp6v0a2	-0.21	1.00000000	chicken
Cacybp	0.10	1.00000000	chicken
Ythdf2	-0.11	1.00000000	chicken
Phf11a	0.03	1.00000000	chicken
Cds2	-0.07	1.00000000	chicken
Usp33	-0.03	1.00000000	chicken
Nploc4	0.17	1.00000000	chicken
Pla2g15	-0.63	1.00000000	chicken
Zcchc10	-0.13	1.00000000	chicken
Atp5b	-0.25	1.00000000	chicken
Prpf4b	-0.56	1.00000000	chicken
Cnpy3	0.10	1.00000000	chicken
Tanc1	-0.15	1.00000000	chicken
Abhd5	0.05	1.00000000	chicken
Syf2	0.06	1.00000000	chicken
Epc2	-0.53	1.00000000	chicken
Lyrm4	-0.29	1.00000000	chicken
Dpyd	-0.87	1.00000000	chicken
Zfat	0.05	1.00000000	chicken
Noc2l	-0.27	1.00000000	chicken
Smg6	-0.18	1.00000000	chicken
Snx29	-0.18	1.00000000	chicken
Ube2cbp	-0.29	1.00000000	chicken
Zfhx3	-0.35	1.00000000	chicken
Cst7	-0.42	1.00000000	chicken
Trrap	-0.27	1.00000000	chicken
Dvl1	0.17	1.00000000	chicken
1110059G10Rik	0.27	1.00000000	chicken
Akr1a1	0.26	1.00000000	chicken
Thoc5	-0.51	1.00000000	chicken
Bcas2	-0.15	1.00000000	chicken
Ncbp3	-0.02	1.00000000	chicken
Cd44	0.35	1.00000000	chicken
Psmc2	-0.33	1.00000000	chicken
Nr1d2	-0.46	1.00000000	chicken
Rsrc2	-0.65	1.00000000	chicken
Ccz1	-0.73	1.00000000	chicken
Zcchc2	-0.45	1.00000000	chicken

Sptlc2	-0.50	1.00000000	chicken
Ywhaz	-0.38	1.00000000	chicken
Rab33b	0.21	1.00000000	chicken
Polr2e	-0.07	1.00000000	chicken
Syncrip	-0.36	1.00000000	chicken
Bend7	0.16	1.00000000	chicken
Slc66a2	-0.37	1.00000000	chicken
Rab12	-0.50	1.00000000	chicken
Sbno2	0.08	1.00000000	chicken
Cyb5r4	-0.05	1.00000000	chicken
Fgfr1op2	-0.42	1.00000000	chicken
Pbx1	-0.05	1.00000000	chicken
Snrpb	-0.37	1.00000000	chicken
Rsu1	0.27	1.00000000	chicken
Ell	-0.37	1.00000000	chicken
Ptp4a1	-0.84	1.00000000	chicken
Hint1	-0.41	1.00000000	chicken
Ndufs5	-0.48	1.00000000	chicken
Rtf2	-0.60	1.00000000	chicken
Nfkbib	-0.35	1.00000000	chicken
Zc3h11a	-0.48	1.00000000	chicken
Necap1	-0.40	1.00000000	chicken
Ggnbp2	-0.09	1.00000000	chicken
Zfp598	-0.07	1.00000000	chicken
Pafah1b2	-0.27	1.00000000	chicken
Smndc1	-0.42	1.00000000	chicken
Slc38a1	-0.27	1.00000000	chicken
Slco2b1	-0.04	1.00000000	chicken
Card11	-0.33	1.00000000	chicken
Gadd45a	0.03	1.00000000	chicken
Brap	-0.34	1.00000000	chicken
Pus10	-0.04	1.00000000	chicken
Rit1	-0.04	1.00000000	chicken
Eefsec	0.18	1.00000000	chicken
Phlpp2	-0.35	1.00000000	chicken
Slc16a3	-0.94	1.00000000	chicken
Tex10	-0.15	1.00000000	chicken
Rnmt	-0.39	1.00000000	chicken
Plxnc1	-0.10	1.00000000	chicken
Tmem230	0.25	1.00000000	chicken
Arfip1	-0.45	1.00000000	chicken
Impa2	-0.18	1.00000000	chicken
Nipsnap2	-0.25	1.00000000	chicken
Kdm4c	-0.76	1.00000000	chicken
Prdm2	-0.46	1.00000000	chicken
Vrk1	-0.43	1.00000000	chicken

Arpc3	0.20	1.00000000	chicken
Rapgef1	-0.33	1.00000000	chicken
Cebpg	-0.25	1.00000000	chicken
Reep2	-0.25	1.00000000	chicken
Ndufs4	-0.35	1.00000000	chicken
Rnf20	-0.47	1.00000000	chicken
Nkiras2	-0.30	1.00000000	chicken
Psmc6	-0.01	1.00000000	chicken
Zcchc7	-0.44	1.00000000	chicken
Dach1	-0.11	1.00000000	chicken
Ppp6r2	-0.48	1.00000000	chicken
Fubp1	1.11	1.00000000	chicken
Slc4a7	0.23	1.00000000	chicken
Esrrg	-0.01	1.00000000	chicken
Rab7	0.03	1.00000000	chicken
Rbm19	-0.54	1.00000000	chicken
Far1	-0.08	1.00000000	chicken
Txndc11	-0.41	1.00000000	chicken
Aif1l	-1.40	1.00000000	chicken
Cdc42bpb	-0.08	1.00000000	chicken
Gnai3	-0.19	1.00000000	chicken
Ndufs2	-0.38	1.00000000	chicken
Commd4	-0.24	1.00000000	chicken
Ube2q1	-0.26	1.00000000	chicken
Pdcd4	-0.13	1.00000000	chicken
Cyth1	-0.44	1.00000000	chicken
Ifi30	-0.63	1.00000000	chicken
Washc4	0.09	1.00000000	chicken
Arhgdib	-0.43	1.00000000	chicken
Ric8b	-0.58	1.00000000	chicken
Yeats4	0.09	1.00000000	chicken
Tab2	-0.07	1.00000000	chicken
Cox5b-ps	-0.23	1.00000000	chicken
Septin14	-0.05	1.00000000	chicken
Borcs5	-0.11	1.00000000	chicken
Brwd1	-0.48	1.00000000	chicken
Sf3b6	-0.47	1.00000000	chicken
Tpk1	-0.35	1.00000000	chicken
Tec	-0.35	1.00000000	chicken
Kif4	-0.14	1.00000000	chicken
Tdg	-0.48	1.00000000	chicken
Dnaja4	-0.63	1.00000000	chicken
Phf20	-0.05	1.00000000	chicken
Nono	-0.37	1.00000000	chicken
Ube2r2	-0.55	1.00000000	chicken
Gtf2h1	-0.35	1.00000000	chicken

Stn1	-0.06	1.00000000	chicken
Ube4a	0.06	1.00000000	chicken
Lpcat3	-0.37	1.00000000	chicken
Ncoa1	-0.56	1.00000000	chicken
Arl1	-0.45	1.00000000	chicken
Thap4	-0.10	1.00000000	chicken
Pxdc1	0.06	1.00000000	chicken
Unk	-0.09	1.00000000	chicken
Elf1	0.21	1.00000000	chicken
Gbe1	-0.29	1.00000000	chicken
Ssr1	-0.19	1.00000000	chicken
Usp16	0.27	1.00000000	chicken
Rxra	-0.08	1.00000000	chicken
Txlna	-0.28	1.00000000	chicken
Acsl4	-0.64	1.00000000	chicken
Arcn1	-0.26	1.00000000	chicken
Atad1	-0.27	1.00000000	chicken
Rcbtb1	0.08	1.00000000	chicken
Vamp3	-0.19	1.00000000	chicken
Trib2	-0.35	1.00000000	chicken
Arl15	-0.60	1.00000000	chicken
Anapc15-ps	-0.32	1.00000000	chicken
Spopl	-0.19	1.00000000	chicken
Ecd	-0.27	1.00000000	chicken
Ebag9	-0.42	1.00000000	chicken
Gars	-0.10	1.00000000	chicken
Stk4	-0.67	1.00000000	chicken
Ift22	-0.05	1.00000000	chicken
Rars	-0.30	1.00000000	chicken
Cdk9	-0.06	1.00000000	chicken
Aco2	-0.35	1.00000000	chicken
Gpr155	-0.06	1.00000000	chicken
Tuba1a	-0.33	1.00000000	chicken
Picalm	-0.31	1.00000000	chicken
Tmem41b	-0.01	1.00000000	chicken
Bub3	0.16	1.00000000	chicken
Kpna1	-0.32	1.00000000	chicken
Thumpd3	-0.14	1.00000000	chicken
Ntmt1	0.16	1.00000000	chicken
Chmp7	0.07	1.00000000	chicken
Cul4b	-0.56	1.00000000	chicken
Rptor	-0.31	1.00000000	chicken
Phf2	-0.18	1.00000000	chicken
Dido1	-0.56	1.00000000	chicken
Ptch1	1.00	1.00000000	chicken
Zcchc17	-0.06	1.00000000	chicken

Ppfia1	-0.33	1.00000000	chicken
Ghdc	-0.45	1.00000000	chicken
Tcp11l2	-0.41	1.00000000	chicken
Usp34	-0.16	1.00000000	chicken
Btbd7	0.12	1.00000000	chicken
Atp13a2	0.11	1.00000000	chicken
Ehmt1	-0.44	1.00000000	chicken
Ndufb7	0.23	1.00000000	chicken
Acad9	0.19	1.00000000	chicken
Ttf1	-0.30	1.00000000	chicken
Rnf4	-0.10	1.00000000	chicken
Insig1	-0.01	1.00000000	chicken
9530068E07Rik	0.00	1.00000000	chicken
Ubox5	0.05	1.00000000	chicken
Ccndbp1	-0.45	1.00000000	chicken
Tom1	-0.17	1.00000000	chicken
Ahr	-0.45	1.00000000	chicken
Reep3	-0.01	1.00000000	chicken
Vezf1	0.01	1.00000000	chicken
Prkcb	-0.25	1.00000000	chicken
P2rx7	-0.52	1.00000000	chicken
Tardbp	-0.11	1.00000000	chicken
Wrn	-0.70	1.00000000	chicken
Cybc1	-0.41	1.00000000	chicken
Hsd17b12	0.11	1.00000000	chicken
Hexim1	-0.30	1.00000000	chicken
Ttc3	0.21	1.00000000	chicken
Surf6	0.01	1.00000000	chicken
Bcr	-0.75	1.00000000	chicken
Ccdc93	0.01	1.00000000	chicken
Ss18l2	-0.46	1.00000000	chicken
Sertad2	0.05	1.00000000	chicken
Srp68	-0.33	1.00000000	chicken
Ccdc88c	0.25	1.00000000	chicken
Psmb4	-0.21	1.00000000	chicken
Cars2	-0.19	1.00000000	chicken
Zcrb1	0.09	1.00000000	chicken
Ly86	-0.30	1.00000000	chicken
Akap8	-0.39	1.00000000	chicken
Rab29	-0.19	1.00000000	chicken
Srebf2	-0.54	1.00000000	chicken
Sap130	-0.22	1.00000000	chicken
Ing1	-0.37	1.00000000	chicken
Vps41	-1.06	1.00000000	chicken
Csl	0.03	1.00000000	chicken
Ilkap	-0.33	1.00000000	chicken

Cep112	-0.08	1.00000000	chicken
Rab8a	-0.19	1.00000000	chicken
Oaz1	0.14	1.00000000	chicken
Phlpp1	-0.30	1.00000000	chicken
Snrpg	-0.25	1.00000000	chicken
Igf2r	-0.50	1.00000000	chicken
Ppp3r1	-0.04	1.00000000	chicken
Arhgap6	-0.55	1.00000000	chicken
Rrp1b	-0.42	1.00000000	chicken
Isca1	-0.36	1.00000000	chicken
Slu7	-0.27	1.00000000	chicken
Asxl2	0.09	1.00000000	chicken
Cox7c	-0.34	1.00000000	chicken
Nr2c2	-0.11	1.00000000	chicken
Ufd1	-0.37	1.00000000	chicken
Oga	-0.44	1.00000000	chicken
Chid1	0.12	1.00000000	chicken
Cltb	-0.06	1.00000000	chicken
Tiam2	-0.81	1.00000000	chicken
Cep85l	-0.16	1.00000000	chicken
Sgsm2	-0.55	1.00000000	chicken
Lig3	-0.14	1.00000000	chicken
4932411N23Rik	-0.30	1.00000000	chicken
Prcc	-0.28	1.00000000	chicken
Psmc3	-0.42	1.00000000	chicken
Xpot	-0.30	1.00000000	chicken
Lrch1	-0.57	1.00000000	chicken
Nol11	-0.42	1.00000000	chicken
Sec24a	0.02	1.00000000	chicken
Dynlrb1	-0.16	1.00000000	chicken
Tomm40l	-0.30	1.00000000	chicken
Srp54a	0.08	1.00000000	chicken
Dedd	-0.38	1.00000000	chicken
Nab1	-0.08	1.00000000	chicken
Hmbs	-0.23	1.00000000	chicken
Ccdc186	-0.46	1.00000000	chicken
Ftsj3	-0.10	1.00000000	chicken
Rb1cc1	0.17	1.00000000	chicken
Abcf2	-0.21	1.00000000	chicken
Hspd1	0.07	1.00000000	chicken
Ccnd3	-0.09	1.00000000	chicken
Rcsd1	0.11	1.00000000	chicken
Evi5	-0.41	1.00000000	chicken
Lsm1	-0.02	1.00000000	chicken
C1qbp	-0.34	1.00000000	chicken
Heatr5a	-0.70	1.00000000	chicken

Gpcpd1	-0.09	1.00000000	chicken
Yipf5	0.12	1.00000000	chicken
Ints6	0.15	1.00000000	chicken
Usp4	0.06	1.00000000	chicken
Scaf11	-0.57	1.00000000	chicken
Ubqln4	-0.18	1.00000000	chicken
Rab21	-0.04	1.00000000	chicken
Tgfbr1	0.08	1.00000000	chicken
Ccpg1	-0.85	1.00000000	chicken
Pfkp	-0.59	1.00000000	chicken
Dcaf1	-0.28	1.00000000	chicken
Ktn1	-0.12	1.00000000	chicken
Samhd1	0.05	1.00000000	chicken
Elovl1	-0.49	1.00000000	chicken
Ddx27	-0.46	1.00000000	chicken
Gorasp2	0.03	1.00000000	chicken
Cc2d1b	0.08	1.00000000	chicken
Asah1	-0.51	1.00000000	chicken
Eif4g1	-0.44	1.00000000	chicken
Zdhhc17	-0.07	1.00000000	chicken
Cwc25	0.00	1.00000000	chicken
Ldhb	0.11	1.00000000	chicken
Ap3s1	-0.28	1.00000000	chicken
Nfkbie	0.02	1.00000000	chicken
Tpp1	-0.13	1.00000000	chicken
Tm9sf3	0.23	1.00000000	chicken
Fbxw11	-0.36	1.00000000	chicken
Atp8a1	-0.61	1.00000000	chicken
Esco1	-0.01	1.00000000	chicken
Dlgap4	-0.09	1.00000000	chicken
4833439L19Rik	-0.28	1.00000000	chicken
Calr	-0.39	1.00000000	chicken
Aurkaip1	-0.18	1.00000000	chicken
Parg	-0.41	1.00000000	chicken
Zcchc8	-0.19	1.00000000	chicken
Actr6	-0.29	1.00000000	chicken
Ube2g1	-0.08	1.00000000	chicken
Hcls1	0.00	1.00000000	chicken
Rnf220	0.03	1.00000000	chicken
Csrp1	0.13	1.00000000	chicken
Disp1	-0.07	1.00000000	chicken
Cbfb	-0.26	1.00000000	chicken
Serinc1	-0.37	1.00000000	chicken
Hps3	-0.51	1.00000000	chicken
Ttc13	0.07	1.00000000	chicken
Lrp12	-0.25	1.00000000	chicken

Slc20a2	-0.36	1.00000000	chicken
Ddx17	-0.41	1.00000000	chicken
Erc2	-0.14	1.00000000	chicken
Fam214a	0.08	1.00000000	chicken
Rad21	-0.27	1.00000000	chicken
Prkcd	-0.44	1.00000000	chicken
Cnot6l	-0.53	1.00000000	chicken
Chchd5	0.04	1.00000000	chicken
Wdr26	0.03	1.00000000	chicken
Tpd52	-0.43	1.00000000	chicken
Sptan1	-0.72	1.00000000	chicken
Ivns1abp	-0.42	1.00000000	chicken
Cmtr1	-0.57	1.00000000	chicken
Actr1a	-0.12	1.00000000	chicken
Xpo4	-0.27	1.00000000	chicken
Tmem245	-0.34	1.00000000	chicken
Rprd2	-0.24	1.00000000	chicken
Uqcr10	-0.07	1.00000000	chicken
Gsdme	-0.29	1.00000000	chicken
Tram1l1	-0.18	1.00000000	chicken
Lars	-0.22	1.00000000	chicken
Srp19	-0.08	1.00000000	chicken
Ice2	-0.27	1.00000000	chicken
Gls	0.00	1.00000000	chicken
Rad23a	-0.40	1.00000000	chicken
Ndufb3	0.02	1.00000000	chicken
Ahdc1	-0.22	1.00000000	chicken
Smim7	-0.27	1.00000000	chicken
Atxn1	-0.36	1.00000000	chicken
Slc39a9	-0.43	1.00000000	chicken
Eif2b5	-0.02	1.00000000	chicken
Cnbp	-0.19	1.00000000	chicken
Irs4	0.02	1.00000000	chicken
Orai2	0.00	1.00000000	chicken
Sub1	-0.57	1.00000000	chicken
Nvl	-0.03	1.00000000	chicken
Tnip1	0.17	1.00000000	chicken
Diaph2	-0.04	1.00000000	chicken
Ifnar2	-0.21	1.00000000	chicken
Gspt1	-0.31	1.00000000	chicken
Sbno1	-0.26	1.00000000	chicken
Gna13	-0.08	1.00000000	chicken
Steep1	-0.03	1.00000000	chicken
Ubn2	-0.16	1.00000000	chicken
Unc45a	-0.20	1.00000000	chicken
B4galt1	-0.20	1.00000000	chicken

Gpi1	-0.37	1.00000000	chicken
Zfyve21	-0.29	1.00000000	chicken
Susd3	-0.25	1.00000000	chicken
Rapsn	-0.16	1.00000000	chicken
Vps11	-0.46	1.00000000	chicken
Tnrc6a	-0.39	1.00000000	chicken
Nsd3	-0.03	1.00000000	chicken
Idh3a	0.05	1.00000000	chicken
Jpt1	-0.42	1.00000000	chicken
Ptpn11	-0.12	1.00000000	chicken
Larp4b	-0.34	1.00000000	chicken
E2f4	-0.38	1.00000000	chicken
Vma21	-0.17	1.00000000	chicken
Clec16a	-0.06	1.00000000	chicken
Ap1g1	-0.40	1.00000000	chicken
Klhdc4	-0.11	1.00000000	chicken
Atrn	0.09	1.00000000	chicken
Ppp2ca	-0.39	1.00000000	chicken
Pfn2	0.15	1.00000000	chicken
Ryk	-0.32	1.00000000	chicken
Usp3	-0.19	1.00000000	chicken
Commd10	-0.06	1.00000000	chicken
Aqr	-0.01	1.00000000	chicken
Eapp	-0.01	1.00000000	chicken
Ubn1	-0.41	1.00000000	chicken
Hspa4l	-0.38	1.00000000	chicken
Ctsh	0.09	1.00000000	chicken
Wasf2	-0.06	1.00000000	chicken
Pcbp2	-0.41	1.00000000	chicken
Hbs1l	-0.37	1.00000000	chicken
Cpne1	0.11	1.00000000	chicken
Herc1	-0.36	1.00000000	chicken
Itsn2	-0.24	1.00000000	chicken
Atp10a	-0.42	1.00000000	chicken
Cap1	-0.55	1.00000000	chicken
Dnajc7	-0.11	1.00000000	chicken
Relch	-0.48	1.00000000	chicken
Eif6	0.09	1.00000000	chicken
Wdr70	-0.16	1.00000000	chicken
Ncor2	0.21	1.00000000	chicken
Lonrf2	0.02	1.00000000	chicken
Smurf2	0.18	1.00000000	chicken
Rnf146	-0.46	1.00000000	chicken
Pfdn6	0.21	1.00000000	chicken
Uggt1	-0.33	1.00000000	chicken
Acadl	-0.13	1.00000000	chicken

Pim1	-0.59	1.00000000	chicken
Zfp318	-0.69	1.00000000	chicken
Gyg	-0.37	1.00000000	chicken
Itpka	-0.46	1.00000000	chicken
Pcna	-0.22	1.00000000	chicken
Txn1	-0.45	1.00000000	chicken
Per2	-0.14	1.00000000	chicken
Serpinb8	0.44	1.00000000	chicken
Dcaf5	-0.33	1.00000000	chicken
Efl1	0.02	1.00000000	chicken
Crem	-0.17	1.00000000	chicken
Tob1	-0.46	1.00000000	chicken
Erbin	-0.27	1.00000000	chicken
Glrx5	-0.32	1.00000000	chicken
Erlec1	0.04	1.00000000	chicken
BC048403	-0.46	1.00000000	chicken
Vim	-0.19	1.00000000	chicken
Pmf1	-0.08	1.00000000	chicken
Psmd9	-0.27	1.00000000	chicken
Ilk	0.07	1.00000000	chicken
Gatad2a	-0.27	1.00000000	chicken
Rictor	-0.42	1.00000000	chicken
Vps37a	-0.01	1.00000000	chicken
Efhd1	-0.32	1.00000000	chicken
Smyd3	0.41	1.00000000	chicken
Chchd2	-0.54	1.00000000	chicken
Traf3ip3	-0.15	1.00000000	chicken
Srsf6	-0.24	1.00000000	chicken
Pitpnb	-0.42	1.00000000	chicken
Slc39a10	-0.45	1.00000000	chicken
Ndufs8	0.13	1.00000000	chicken
Nrbp1	-0.18	1.00000000	chicken
Tle4	-0.01	1.00000000	chicken
Ccnk	-0.16	1.00000000	chicken
Lasp1	-0.34	1.00000000	chicken
Synj1	0.52	1.00000000	chicken
Cct7	-0.19	1.00000000	chicken
Pcm1	-0.57	1.00000000	chicken
Rheb	-0.34	1.00000000	chicken
Armc8	-0.34	1.00000000	chicken
Fas	-0.30	1.00000000	chicken
Osbpl2	-0.26	1.00000000	chicken
Tcf12	-0.23	1.00000000	chicken
Cotl1	-0.56	1.00000000	chicken
Irf2bp2	-0.01	1.00000000	chicken
Rab6a	-0.13	1.00000000	chicken

Pcgf5	-0.31	1.00000000	chicken
Tmf1	-0.18	1.00000000	chicken
Ythdf3	-0.19	1.00000000	chicken
Tmem87a	-0.47	1.00000000	chicken
Dapk1	-0.41	1.00000000	chicken
Utp3	-0.33	1.00000000	chicken
Cnot10	-0.25	1.00000000	chicken
Casp8ap2	-0.27	1.00000000	chicken
Lmo2	-0.19	1.00000000	chicken
Higd1a	-0.36	1.00000000	chicken
Stag2	-0.41	1.00000000	chicken
Usp10	-0.02	1.00000000	chicken
Ypel1	-0.35	1.00000000	chicken
Srp9	-0.08	1.00000000	chicken
Napa	-0.18	1.00000000	chicken
Tmem9b	-0.16	1.00000000	chicken
Psen1	-0.43	1.00000000	chicken
Ntng1	-0.25	1.00000000	chicken
Ulk2	-0.07	1.00000000	chicken
Qars	-0.17	1.00000000	chicken
Pdk3	-0.06	1.00000000	chicken
Pola1	-0.20	1.00000000	chicken
Lman1	-0.22	1.00000000	chicken
Surf4	0.09	1.00000000	chicken
Pcdhgb4	0.09	1.00000000	chicken
Bclaf3	-0.35	1.00000000	chicken
Wdr81	-0.20	1.00000000	chicken
Rac1	-0.23	1.00000000	chicken
Alox5ap	-0.25	1.00000000	chicken
Csnk1e	0.11	1.00000000	chicken
Aktip	-0.12	1.00000000	chicken
Rsl24d1	-0.32	1.00000000	chicken
Tmx4	-0.44	1.00000000	chicken
Ptpn1	-0.39	1.00000000	chicken
Il6st	0.06	1.00000000	chicken
Taldo1	-0.39	1.00000000	chicken
Copa	-0.02	1.00000000	chicken
Sec31a	-0.11	1.00000000	chicken
Cnih1	-0.04	1.00000000	chicken
Slc1a2	-0.42	1.00000000	chicken
Kansl1l	-0.10	1.00000000	chicken
Trim33	-0.24	1.00000000	chicken
Kdelr2	-0.23	1.00000000	chicken
Wdr7	-0.19	1.00000000	chicken
Gnptab	-0.38	1.00000000	chicken
Ric1	-0.20	1.00000000	chicken

Gpx4	-0.12	1.00000000	chicken
Plekhb2	-0.26	1.00000000	chicken
Rnf216	-0.16	1.00000000	chicken
Upf2	-0.12	1.00000000	chicken
Smg7	-0.07	1.00000000	chicken
Ahrr	-0.34	1.00000000	chicken
Hadh	0.06	1.00000000	chicken
Ern1	-0.42	1.00000000	chicken
Ola1	-0.11	1.00000000	chicken
Top2b	0.13	1.00000000	chicken
Usp37	-0.05	1.00000000	chicken
Psmd4	0.12	1.00000000	chicken
Arnt	-0.09	1.00000000	chicken
Commd2	-0.11	1.00000000	chicken
Tmem39a	-0.12	1.00000000	chicken
Pgd	-0.39	1.00000000	chicken
Tmem104	-0.39	1.00000000	chicken
Nfat5	-0.62	1.00000000	chicken
Rassf1	-0.10	1.00000000	chicken
Tollip	-0.37	1.00000000	chicken
Tent4b	-0.24	1.00000000	chicken
Ss18	-0.23	1.00000000	chicken
Cbfa2t3	0.23	1.00000000	chicken
Zfp36l1	-0.07	1.00000000	chicken
Cebpd	-0.11	1.00000000	chicken
Pex14	-0.15	1.00000000	chicken
Wdr3	-0.25	1.00000000	chicken
Nars	0.04	1.00000000	chicken
Rnf130	0.08	1.00000000	chicken
Pik3r6	-0.30	1.00000000	chicken
Hdc	-2.90	1.00000000	chicken
Ensa	0.13	1.00000000	chicken
Nanp	-0.08	1.00000000	chicken
Slc44a1	-0.48	1.00000000	chicken
Cdca4	-0.30	1.00000000	chicken
Banp	-0.20	1.00000000	chicken
Slco4a1	-0.14	1.00000000	chicken
Ran	-0.23	1.00000000	chicken
Rexo1	-0.20	1.00000000	chicken
Arid2	0.12	1.00000000	chicken
Arl4a	0.01	1.00000000	chicken
Ppig	-0.41	1.00000000	chicken
Nek9	0.05	1.00000000	chicken
Ahsa2	-0.17	1.00000000	chicken
4933400A11Rik	0.03	1.00000000	chicken
Ptk2	0.01	1.00000000	chicken

Snx6	-0.35	1.00000000	chicken
Usp7	-0.41	1.00000000	chicken
Ypel5	-0.29	1.00000000	chicken
Rab5c	-0.20	1.00000000	chicken
Lamtor3	-0.27	1.00000000	chicken
Ubac2	-0.58	1.00000000	chicken
Rbms1	-0.29	1.00000000	chicken
Gosr1	-0.37	1.00000000	chicken
Tle1	0.41	1.00000000	chicken
Cnot9	-0.01	1.00000000	chicken
Ndufa2	0.04	1.00000000	chicken
Leo1	0.05	1.00000000	chicken
Rnf111	-0.21	1.00000000	chicken
Sptssa	-0.35	1.00000000	chicken
Clk1	-0.45	1.00000000	chicken
Hypk	-0.12	1.00000000	chicken
Hspa8	-0.18	1.00000000	chicken
Lactb	-0.34	1.00000000	chicken
Naa30	-0.44	1.00000000	chicken
Usp49	-0.21	1.00000000	chicken
Egr2	-0.23	1.00000000	chicken
Ppp2r5c	-0.34	1.00000000	chicken
Atf1	-0.26	1.00000000	chicken
Rgs19	-0.03	1.00000000	chicken
Ppt1	-0.08	1.00000000	chicken
Cebpz	-0.15	1.00000000	chicken
Prkd3	0.03	1.00000000	chicken
Sec61a1	-0.22	1.00000000	chicken
Psmd2	-0.03	1.00000000	chicken
Abracl	-0.12	1.00000000	chicken
Ttc7	0.05	1.00000000	chicken
Sap30bp	-0.17	1.00000000	chicken
Trim8	-0.30	1.00000000	chicken
Larp1	-0.28	1.00000000	chicken
Ppp2r2a	-0.13	1.00000000	chicken
C2cd2l	-0.19	1.00000000	chicken
Plrg1	-0.29	1.00000000	chicken
Smpd1	-0.01	1.00000000	chicken
Cul5	-0.20	1.00000000	chicken
Ap2m1	-0.36	1.00000000	chicken
Trip12	-0.02	1.00000000	chicken
Agps	-0.16	1.00000000	chicken
Amfr	-0.21	1.00000000	chicken
Akap8l	-0.19	1.00000000	chicken
Rabgap1	-0.45	1.00000000	chicken
Tsg101	-0.31	1.00000000	chicken

Ccny	0.41	1.00000000	chicken
Rsf1	-0.27	1.00000000	chicken
Tubb2a	-0.26	1.00000000	chicken
Elk4	-0.14	1.00000000	chicken
Spg7	-0.20	1.00000000	chicken
Opa1	-0.40	1.00000000	chicken
Usp6nl	-0.49	1.00000000	chicken
Nxt2	-0.31	1.00000000	chicken
Ddx23	-0.14	1.00000000	chicken
Atp6ap2	-0.29	1.00000000	chicken
Gpatch8	-0.13	1.00000000	chicken
Slc30a9	-0.12	1.00000000	chicken
Fyttd1	-0.27	1.00000000	chicken
Adam10	-0.40	1.00000000	chicken
Acss2	-0.04	1.00000000	chicken
Gpatch2	-0.44	1.00000000	chicken
Fasn	-0.34	1.00000000	chicken
Tmod3	-0.24	1.00000000	chicken
Nckap1l	-0.18	1.00000000	chicken
Hcfc2	-0.17	1.00000000	chicken
Ctnnb1	0.04	1.00000000	chicken
Rfc1	-0.32	1.00000000	chicken
Celf2	0.04	1.00000000	chicken
Ppp1r2	-0.06	1.00000000	chicken
Atf6	-0.25	1.00000000	chicken
Abcg1	-0.10	1.00000000	chicken
Fam3c	0.03	1.00000000	chicken
P4hb	-0.28	1.00000000	chicken
Eif1ad12	-0.29	1.00000000	chicken
Nipa2	-0.09	1.00000000	chicken
Psma2	-0.11	1.00000000	chicken
Setx	-0.18	1.00000000	chicken
Yme1l1	-0.20	1.00000000	chicken
Srebf1	-0.11	1.00000000	chicken
Syt14	-0.39	1.00000000	chicken
Lamtor5	-0.12	1.00000000	chicken
Lmna	-0.30	1.00000000	chicken
Kdm7a	0.03	1.00000000	chicken
Ppp1r13b	-0.31	1.00000000	chicken
Ahcyl1	-0.27	1.00000000	chicken
1600014C10Rik	-0.15	1.00000000	chicken
Exoc3	-0.75	1.00000000	chicken
Pfdn5	-0.53	1.00000000	chicken
Fam107b	-0.14	1.00000000	chicken
Golga1	-0.53	1.00000000	chicken
Slc2a1	-0.16	1.00000000	chicken

Uso1	-0.34	1.00000000	chicken
Etf1	-0.25	1.00000000	chicken
Thrap3	-0.15	1.00000000	chicken
Cul2	-0.20	1.00000000	chicken
Dyrk1a	-0.70	1.00000000	chicken
Kif13a	-0.80	1.00000000	chicken
Slc6a6	0.34	1.00000000	chicken
Traf2	-0.19	1.00000000	chicken
Ipo5	-0.17	1.00000000	chicken
Hdac4	-0.29	1.00000000	chicken
Dazap2	-0.15	1.00000000	chicken
Edem1	-0.15	1.00000000	chicken
Rfk	-0.10	1.00000000	chicken
Ncoa3	-0.09	1.00000000	chicken
Krit1	-0.21	1.00000000	chicken
Arf1	-0.13	1.00000000	chicken
Aacs	-0.31	1.00000000	chicken
Zbed4	-0.25	1.00000000	chicken
Acvr1	-0.15	1.00000000	chicken
Chchd3	-0.31	1.00000000	chicken
Spcs1	-0.10	1.00000000	chicken
Dus3l	-0.08	1.00000000	chicken
Bicd2	0.02	1.00000000	chicken
Lims2	-0.20	1.00000000	chicken
Wac	0.03	1.00000000	chicken
Rab3a	-0.30	1.00000000	chicken
Abi1	-0.27	1.00000000	chicken
Cnot4	-0.51	1.00000000	chicken
Dock5	-0.27	1.00000000	chicken
Polg2	-0.16	1.00000000	chicken
Rpf2	-0.06	1.00000000	chicken
Nup50	0.12	1.00000000	chicken
Capn15	-0.34	1.00000000	chicken
Stk24	-0.25	1.00000000	chicken
Sik1	-0.33	1.00000000	chicken
Il17ra	-0.34	1.00000000	chicken
Bcap29	-0.21	1.00000000	chicken
Foxn2	-0.43	1.00000000	chicken
Tor1a	-0.16	1.00000000	chicken
Rab3gap1	-0.26	1.00000000	chicken
Gcn1	-0.09	1.00000000	chicken
Cbwd1	-0.03	1.00000000	chicken
Uri1	-0.64	1.00000000	chicken
Ppp2r5a	-0.40	1.00000000	chicken
Ptprs	-0.55	1.00000000	chicken
Bcor	-0.34	1.00000000	chicken

Rgs9	-0.45	1.00000000	chicken
Rrs1	-0.25	1.00000000	chicken
Fbxo33	-0.37	1.00000000	chicken
Plekhf2	-0.02	1.00000000	chicken
Dcp1b	-0.31	1.00000000	chicken
Skp1	-0.16	1.00000000	chicken
AA467197	-0.34	1.00000000	chicken
Snx13	-0.29	1.00000000	chicken
Ttc39b	-0.30	1.00000000	chicken
Pdcd10	0.07	1.00000000	chicken
Tfdp1	-0.29	1.00000000	chicken
Rlim	-0.18	1.00000000	chicken
Tnks	-0.17	1.00000000	chicken
Ppp3ca	-0.01	1.00000000	chicken
Btk	-0.16	1.00000000	chicken
Ncoa4	-0.06	1.00000000	chicken
Evi2a	-0.26	1.00000000	chicken
Slc25a16	-0.32	1.00000000	chicken
Eif2s2	-0.07	1.00000000	chicken
Slc25a25	0.09	1.00000000	chicken
Atf2	-0.36	1.00000000	chicken
Smarca4	-0.17	1.00000000	chicken
Zzef1	0.05	1.00000000	chicken
Ifi35	-0.13	1.00000000	chicken
Kat7	-0.33	1.00000000	chicken
Psmd12	-0.40	1.00000000	chicken
Wdr20	-0.08	1.00000000	chicken
Kidins220	-0.36	1.00000000	chicken
Uqcrq	-0.06	1.00000000	chicken
Aak1	-0.10	1.00000000	chicken
Usp24	-0.49	1.00000000	chicken
Wdr82	-0.35	1.00000000	chicken
Nedd9	-0.13	1.00000000	chicken
Rpn2	-0.20	1.00000000	chicken
Emc2	-0.21	1.00000000	chicken
Ubap2	-0.13	1.00000000	chicken
Sun1	-0.16	1.00000000	chicken
Dip2c	-0.08	1.00000000	chicken
Eif5b	0.31	1.00000000	chicken
Kpnb1	-0.12	1.00000000	chicken
Zcchc14	-0.20	1.00000000	chicken
Atp6v0b	-0.01	1.00000000	chicken
Rlf	-0.68	1.00000000	chicken
Psma5	-0.25	1.00000000	chicken
Cog2	-0.18	1.00000000	chicken
Psmd6	-0.10	1.00000000	chicken

Bud13	-0.48	1.00000000	chicken
Tmem184b	-0.23	1.00000000	chicken
Acvr2a	-0.13	1.00000000	chicken
Lonrf1	-0.23	1.00000000	chicken
Usp44	-0.11	1.00000000	chicken
Snw1	-0.27	1.00000000	chicken
Dcun1d3	-0.60	1.00000000	chicken
Uchl5	0.04	1.00000000	chicken
Stim1	-0.27	1.00000000	chicken
Uchl3	-0.18	1.00000000	chicken
Snrpc	-0.23	1.00000000	chicken
Jade2	-0.36	1.00000000	chicken
Ccnl2	-0.27	1.00000000	chicken
Cnot2	-0.07	1.00000000	chicken
Vdac2	-0.38	1.00000000	chicken
Sdha	-0.19	1.00000000	chicken
Stard8	-0.23	1.00000000	chicken
Pla2g7	-0.15	1.00000000	chicken
Abca1	0.48	1.00000000	chicken
Trappc8	-0.56	1.00000000	chicken
Sec16a	-0.13	1.00000000	chicken
Arl6ip4	-0.17	1.00000000	chicken
Arhgap5	-0.33	1.00000000	chicken
Edc4	-0.19	1.00000000	chicken
Gorab	-0.17	1.00000000	chicken
Rap1b	-0.17	1.00000000	chicken
Gnl2	-0.03	1.00000000	chicken
Gosr2	-0.12	1.00000000	chicken
Itpripl2	-0.09	1.00000000	chicken
Nsrp1	-0.14	1.00000000	chicken
Rab9	-0.31	1.00000000	chicken
Rabggtb	-0.34	1.00000000	chicken
Fadd	-0.12	1.00000000	chicken
Ccng2	-0.02	1.00000000	chicken
Arhgap27	-0.43	1.00000000	chicken
Copz1	-0.14	1.00000000	chicken
Cab39l	-0.09	1.00000000	chicken
Sod1	-0.09	1.00000000	chicken
Pgk1	-0.25	1.00000000	chicken
Pgam1	0.19	1.00000000	chicken
Iars	-0.36	1.00000000	chicken
Tvp23b	-0.07	1.00000000	chicken
Wdr1	-0.04	1.00000000	chicken
Cdkal1	-0.04	1.00000000	chicken
Snx14	-0.03	1.00000000	chicken
Homer1	-0.33	1.00000000	chicken

Odc1	-0.21	1.00000000	chicken
Ap2b1	-0.03	1.00000000	chicken
Igf2bp3	-0.34	1.00000000	chicken
Sqle	-0.23	1.00000000	chicken
Ttl	-0.10	1.00000000	chicken
Otulin	-0.29	1.00000000	chicken
Rab43	-0.22	1.00000000	chicken
Slc9a8	-0.01	1.00000000	chicken
Nde1	-0.39	1.00000000	chicken
Ubl7	-0.20	1.00000000	chicken
Iqgap2	-0.22	1.00000000	chicken
Heca	-0.16	1.00000000	chicken
Rtraf	-0.11	1.00000000	chicken
Slc25a20	-0.10	1.00000000	chicken
Eea1	-0.05	1.00000000	chicken
Papola	-0.17	1.00000000	chicken
Tent2	-0.28	1.00000000	chicken
Heatr5b	-0.32	1.00000000	chicken
Ezh2	-0.05	1.00000000	chicken
Ak2	-0.32	1.00000000	chicken
Rbm33	-0.14	1.00000000	chicken
Rbm22	-0.19	1.00000000	chicken
Pitpna	0.06	1.00000000	chicken
Uhrf1bp1l	-0.34	1.00000000	chicken
Armh3	-0.35	1.00000000	chicken
Tnfsf11	-0.38	1.00000000	chicken
Pkm	-0.03	1.00000000	chicken
Suclg2	-0.18	1.00000000	chicken
Ppp1cb	-0.11	1.00000000	chicken
Eif1b	0.01	1.00000000	chicken
Chd9	-0.34	1.00000000	chicken
Taf15	-0.07	1.00000000	chicken
Pnpla8	-0.24	1.00000000	chicken
Luc7l3	-0.52	1.00000000	chicken
Limk1	-0.11	1.00000000	chicken
Ppp1r16b	-0.61	1.00000000	chicken
Ptpn4	-0.34	1.00000000	chicken
Tes	-0.28	1.00000000	chicken
Pafah1b1	-0.20	1.00000000	chicken
Dcun1d1	0.11	1.00000000	chicken
Pnpla2	-0.50	1.00000000	chicken
Vps35l	-0.42	1.00000000	chicken
Lrpprc	-0.65	1.00000000	chicken
Alkbh5	-0.42	1.00000000	chicken
Carnmt1	0.03	1.00000000	chicken
Sik3	-0.34	1.00000000	chicken

Amd2	-0.12	1.00000000	chicken
4933434E20Rik	-0.14	1.00000000	chicken
Usp8	-0.24	1.00000000	chicken
Sirt2	-0.04	1.00000000	chicken
Ube2d2a	-0.13	1.00000000	chicken
Sars	-0.15	1.00000000	chicken
Szrd1	-0.22	1.00000000	chicken
Sltm	-0.12	1.00000000	chicken
Nuak1	-0.54	1.00000000	chicken
Dync1li1	-0.14	1.00000000	chicken
Ncoa7	-0.28	1.00000000	chicken
Ube2j2	-0.17	1.00000000	chicken
Slc25a36	-0.17	1.00000000	chicken
Gsr	0.06	1.00000000	chicken
Tor4a	-0.21	1.00000000	chicken
1810013L24Rik	-0.02	1.00000000	chicken
Psmb6	0.15	1.00000000	chicken
St7	-0.70	1.00000000	chicken
Atp2c1	-0.23	1.00000000	chicken
Ipo7	-0.30	1.00000000	chicken
Dnlz	-0.04	1.00000000	chicken
Eif4g2	-0.04	1.00000000	chicken
Dhx30	-0.26	1.00000000	chicken
Ylpm1	-0.26	1.00000000	chicken
Clasp1	0.02	1.00000000	chicken
Errfi1	-0.08	1.00000000	chicken
Ythdf1	-0.25	1.00000000	chicken
Tbc1d5	-0.36	1.00000000	chicken
Crk	-0.13	1.00000000	chicken
Jade1	-0.29	1.00000000	chicken
Ralgapa1	-0.42	1.00000000	chicken
Nop53	-0.02	1.00000000	chicken
Traf7	-0.28	1.00000000	chicken
Chmp5	-0.12	1.00000000	chicken
Cltc	-0.21	1.00000000	chicken
Cops2	-0.07	1.00000000	chicken
Ssrp1	-0.14	1.00000000	chicken
Nkap	-0.21	1.00000000	chicken
Adrm1	-0.26	1.00000000	chicken
Batf	-0.52	1.00000000	chicken
Hsf1	-0.56	1.00000000	chicken
Slc23a2	-0.21	1.00000000	chicken
Pdxk	0.13	1.00000000	chicken
Paxip1	-0.33	1.00000000	chicken
Smarca5	-0.29	1.00000000	chicken
Arid5b	-0.04	1.00000000	chicken

Dusp10	-0.02	1.00000000	chicken
Usp15	-0.13	1.00000000	chicken
Cct5	-0.21	1.00000000	chicken
St8sia1	-0.01	1.00000000	chicken
Stx2	-0.54	1.00000000	chicken
Tpi1	0.05	1.00000000	chicken
Prkar1a	-0.15	1.00000000	chicken
Thada	-0.41	1.00000000	chicken
Gtpbp4	-0.28	1.00000000	chicken
Lamp2	-0.13	1.00000000	chicken
Otub1	-0.08	1.00000000	chicken
Fam168a	-0.10	1.00000000	chicken
Ube2d1	-0.19	1.00000000	chicken
Timm50	-0.21	1.00000000	chicken
Spag9	-0.32	1.00000000	chicken
Chd5	-0.48	1.00000000	chicken
Exoc6b	-0.04	1.00000000	chicken
B3gntl1	-0.82	1.00000000	chicken
Ldha	0.58	1.00000000	chicken
Rbx1	-0.19	1.00000000	chicken
Zfp638	-0.27	1.00000000	chicken
Ankrd52	-0.04	1.00000000	chicken
Usp45	-0.15	1.00000000	chicken
Cul1	-0.37	1.00000000	chicken
Edc3	0.01	1.00000000	chicken
Lemd2	-0.50	1.00000000	chicken
Hspa5	0.20	1.00000000	chicken
Scaper	-0.43	1.00000000	chicken
Ginm1	0.01	1.00000000	chicken
Poldip3	-0.20	1.00000000	chicken
Fam133b	-0.11	1.00000000	chicken
Zmpste24	-0.23	1.00000000	chicken
Vps29	-0.09	1.00000000	chicken
Dnajb14	0.07	1.00000000	chicken
Slc30a5	-0.22	1.00000000	chicken
Bzw1	-0.32	1.00000000	chicken
Yipf4	-0.36	1.00000000	chicken
Tmem168	-0.25	1.00000000	chicken
Exoc1	-0.18	1.00000000	chicken
Rnf34	-0.34	1.00000000	chicken
Ist1	-0.25	1.00000000	chicken
Atxn3	-0.26	1.00000000	chicken
Ascc3	-0.69	1.00000000	chicken
Abcb7	-0.09	1.00000000	chicken
Vezt	-0.11	1.00000000	chicken
Acp1	-0.22	1.00000000	chicken

Dhx38	-0.41	1.00000000	chicken
Rrbp1	-0.18	1.00000000	chicken
Nup153	-0.15	1.00000000	chicken
Arrdc3	-0.17	1.00000000	chicken
Atg2b	-0.23	1.00000000	chicken
Rnf126	-0.05	1.00000000	chicken
Atg5	-0.27	1.00000000	chicken
Csnk2a1	-0.12	1.00000000	chicken
Zc3h13	0.15	1.00000000	chicken
Slc35b2	-0.21	1.00000000	chicken
Gga3	-0.07	1.00000000	chicken
Dcp1a	-0.23	1.00000000	chicken
Ctnna1	-0.31	1.00000000	chicken
Cnp	-0.16	1.00000000	chicken
Ciapin1	-0.13	1.00000000	chicken
Pofut1	-0.14	1.00000000	chicken
Psme3ip1	-0.27	1.00000000	chicken
Slirp	-0.17	1.00000000	chicken
Snapin	-0.11	1.00000000	chicken
Ccdc43	-0.23	1.00000000	chicken
Setd5	-0.34	1.00000000	chicken
Ptbp2	-0.30	1.00000000	chicken
Nck2	-0.14	1.00000000	chicken
Prpf3	-0.13	1.00000000	chicken
Ppp1r21	-0.23	1.00000000	chicken
Prr14l	-0.34	1.00000000	chicken
Eif3j2	-0.35	1.00000000	chicken
Smn1	-0.06	1.00000000	chicken
Cd74	-1.87	1.00000000	chicken
Armc1	-0.22	1.00000000	chicken
Ube2b	-0.32	1.00000000	chicken
Rnps1	-0.10	1.00000000	chicken
Nisch	-0.41	1.00000000	chicken
Rnf10	-0.13	1.00000000	chicken
Prdm10	-0.47	1.00000000	chicken
Ppm1b	-0.34	1.00000000	chicken
Kansl1	-0.18	1.00000000	chicken
Ogfr	-0.10	1.00000000	chicken
Fam177a	-0.11	1.00000000	chicken
Tmem135	-0.16	1.00000000	chicken
Reep5	-0.10	1.00000000	chicken
Snip1	-0.17	1.00000000	chicken
Txnrd3	-0.10	1.00000000	chicken
Psmc1	-0.15	1.00000000	chicken
Fbxl17	-0.35	1.00000000	chicken
Tgfbi	-0.53	1.00000000	chicken

Sdhc	-0.12	1.00000000	chicken
Stxbp1	-0.18	1.00000000	chicken
Nop16	-0.18	1.00000000	chicken
Elavl1	-0.38	1.00000000	chicken
Atxn2	-0.13	1.00000000	chicken
Trmt1l	-0.31	1.00000000	chicken
Clk2	-0.27	1.00000000	chicken
Wbp2nl	-0.24	1.00000000	chicken
BC005624	-0.25	1.00000000	chicken
Lsm12	-0.21	1.00000000	chicken
Igf2bp2	-0.11	1.00000000	chicken
Smad5	-0.20	1.00000000	chicken
Pphln1	-0.15	1.00000000	chicken
Cmtm7	0.06	1.00000000	chicken
Cir1	-0.34	1.00000000	chicken
Pdhb	-0.17	1.00000000	chicken
Sik2	-0.14	1.00000000	chicken
Rac2	1.95	0.00000000	duck
Snx10	3.13	0.00000000	duck
F13a1	2.41	0.00000000	duck
Fyn	1.88	0.00000000	duck
Plac8	2.84	0.00000000	duck
Sipa1l1	-2.20	0.00000000	duck
Susd6	-1.58	0.00000000	duck
Npc2	1.69	0.00000000	duck
Stat1	1.73	0.00000000	duck
Stk17b	-1.49	0.00000000	duck
Zeb2	2.15	0.00000000	duck
Alox5	-2.36	0.00000000	duck
Arl6ip5	1.71	0.00000000	duck
Bri3	1.85	0.00000000	duck
Epn2	-1.58	0.00000000	duck
Xpo6	-2.25	0.00000000	duck
Samhd1	2.11	0.00000000	duck
Nos2	4.15	0.00000000	duck
Rundc1	1.86	0.00000000	duck
Gnai3	-1.51	0.00000000	duck
Akap13	2.27	0.00000000	duck
Sh3bgrl3	1.40	0.00000000	duck
Txn1	1.51	0.00000000	duck
Nampt	1.41	0.00000000	duck
Il16	-1.52	0.00000000	duck
Cdk17	-1.65	0.00000000	duck
Dop1b	2.03	0.00000000	duck
Ncor2	1.75	0.00000000	duck
Sos1	-1.98	0.00000000	duck

Ppp1cb	-1.35	0.00000000	duck
Nxt2	-1.69	0.00000000	duck
Abcg1	1.73	0.00000000	duck
Ly96	1.57	0.00000000	duck
Eef1a1	1.09	0.00000000	duck
Fabp3	1.83	0.00000000	duck
Rnf19b	1.53	0.00000000	duck
Fbrsl1	1.55	0.00000000	duck
Optn	1.87	0.00000000	duck
Samsn1	-0.75	0.00000000	duck
Antxr2	-1.27	0.00000000	duck
Paxip1	-2.11	0.00000000	duck
Tnip2	-1.79	0.00000000	duck
Abtb2	1.49	0.00000000	duck
Laptm5	1.00	0.00000000	duck
Fzd6	2.01	0.00000000	duck
Dot1l	1.29	0.00000000	duck
Aspg	2.31	0.00000000	duck
Dennd4a	-1.50	0.00000000	duck
Eef1b2	1.21	0.00000000	duck
Ctsb	1.42	0.00000000	duck
Oat	1.36	0.00000000	duck
S100a11	1.45	0.00000000	duck
Sox6	-2.94	0.00000000	duck
Fuca2	1.42	0.00000000	duck
Itgb1	1.51	0.00000000	duck
Klf15	1.74	0.00000000	duck
Zfand2a	2.11	0.00000000	duck
Ccng2	-1.51	0.00000000	duck
Psmb2	1.23	0.00000000	duck
Acss1	1.28	0.00000000	duck
Fbxl5	-1.16	0.00000000	duck
Ero1a	1.35	0.00000000	duck
Ankrd22	-1.63	0.00000000	duck
Prok2	1.76	0.00000000	duck
Top1	-0.93	0.00000000	duck
Smpd1	1.26	0.00000000	duck
Itga4	-1.82	0.00000000	duck
Blnk	1.70	0.00000000	duck
Ogfr	1.25	0.00000000	duck
Anxa11	1.11	0.00000000	duck
Atf4	1.26	0.00000000	duck
Lrmda	2.13	0.00000000	duck
Ostm1	1.29	0.00000000	duck
Acsl4	-1.05	0.00000000	duck
Ptgs2	1.90	0.00000000	duck

Lyn	-0.91	0.00000000	duck
Ppp2ca	-1.40	0.00000000	duck
Fry	1.42	0.00000000	duck
Txnrd1	1.68	0.00000000	duck
Eepd1	-2.01	0.00000000	duck
Atp6v1h	1.31	0.00000000	duck
Epsti1	1.49	0.00000000	duck
Wdr37	1.41	0.00000000	duck
Crot	1.24	0.00000000	duck
Csrp1	1.19	0.00000000	duck
Zfp706	1.17	0.00000000	duck
Cish	-1.79	0.00000000	duck
Limd2	1.17	0.00000000	duck
Cd44	-1.06	0.00000000	duck
Hbegf	-1.21	0.00000000	duck
Ergic1	-1.72	0.00000000	duck
Cdc42se2	1.20	0.00000000	duck
Celf2	1.77	0.00000000	duck
Ankrd28	1.45	0.00000000	duck
Aopep	1.49	0.00000000	duck
Angpt1	1.44	0.00000000	duck
Anxa1	1.03	0.00000000	duck
Dctn3	1.10	0.00000000	duck
Ogdh	-1.31	0.00000000	duck
Hpgds	1.12	0.00000000	duck
Gpr137b	1.22	0.00000000	duck
Ccpg1	0.93	0.00000000	duck
Ppp1r12a	1.03	0.00000000	duck
Lamp1	0.88	0.00000000	duck
Ktn1	1.26	0.00000000	duck
Acod1	2.56	0.00000000	duck
Vsir	1.05	0.00000000	duck
Wnk1	-1.29	0.00000000	duck
Arid4a	1.07	0.00000000	duck
Synm	-2.33	0.00000000	duck
Herc4	1.09	0.00000000	duck
Btf3	1.14	0.00000000	duck
Pgd	1.09	0.00000000	duck
Taldo1	1.11	0.00000000	duck
Zdhhc15	1.08	0.00000000	duck
Trim25	0.99	0.00000000	duck
Arhgdib	0.86	0.00000000	duck
Syap1	1.09	0.00000000	duck
Lims2	1.26	0.00000000	duck
Atp6v1g3	1.26	0.00000000	duck
Elovl6	-2.50	0.00000000	duck

Csnk2a2	-1.14	0.00000000	duck
Gch1	1.10	0.00000000	duck
Rnf130	0.87	0.00000000	duck
Eif5b	1.15	0.00000000	duck
Reep5	1.14	0.00000000	duck
Tgds	1.21	0.00000000	duck
Sec61g	1.04	0.00000000	duck
Atp5b	1.04	0.00000000	duck
Ncf1	1.16	0.00000000	duck
Tec	-1.11	0.00000000	duck
Bcap29	0.94	0.00000000	duck
Eif2ak2	0.97	0.00000000	duck
Trank1	0.67	0.00000000	duck
Rhog	1.03	0.00000000	duck
Rnf213	1.00	0.00000000	duck
Itsn1	0.97	0.00000000	duck
Apobec1	1.31	0.00000000	duck
Atp11a	-1.75	0.00000000	duck
Nfam1	0.90	0.00000000	duck
Cnot9	1.00	0.00000000	duck
Sacm1l	0.94	0.00000000	duck
Ntng1	1.39	0.00000000	duck
Lmnb1	0.88	0.00000000	duck
Usp10	0.92	0.00000000	duck
Rnf19a	1.01	0.00000000	duck
Abi1	-1.31	0.00000000	duck
Acsbg2	0.98	0.00000000	duck
Rsu1	1.03	0.00000000	duck
Calm1	1.02	0.00000000	duck
Eef1d	0.96	0.00000000	duck
Fchsd2	1.22	0.00000000	duck
Bst1	-0.97	0.00000000	duck
Cd81	-1.64	0.00000000	duck
Nap1l1	1.09	0.00000000	duck
Ypel5	0.96	0.00000000	duck
Aff1	0.95	0.00000000	duck
Atp6v1b2	0.89	0.00000000	duck
Plcg2	1.19	0.00000000	duck
Gabarapl2	0.93	0.00000000	duck
Cpb2	1.32	0.00000000	duck
Ugcg	-1.43	0.00000000	duck
Aff4	-1.15	0.00000000	duck
Suclg1	0.85	0.00000000	duck
Atp11c	0.88	0.00000000	duck
Acadm	0.92	0.00000000	duck
Eef2	0.86	0.00000000	duck

Ston2	1.33	0.00000000	duck
Cask	1.04	0.00000000	duck
Eif3h	0.81	0.00000000	duck
Sec63	-1.67	0.00000000	duck
Cebpb	-0.74	0.00000000	duck
Prdx1	0.83	0.00000000	duck
Rnf166	0.90	0.00000000	duck
Phactr4	0.98	0.00000000	duck
Pde4b	-0.94	0.00000000	duck
Dgkh	1.30	0.00000000	duck
Atp5g3	0.96	0.00000000	duck
Dync1i2	0.90	0.00000000	duck
Tspan14	0.82	0.00000000	duck
Tiam1	-1.32	0.00000000	duck
Dach2	0.75	0.00000000	duck
Zfand5	1.03	0.00000000	duck
Bud31	0.86	0.00000000	duck
Canx	0.87	0.00000000	duck
Jmjd1c	-1.18	0.00000000	duck
Trappc3	0.90	0.00000000	duck
Syngr2	0.88	0.00000000	duck
Pgm1	0.82	0.00000000	duck
Tmem258	0.86	0.00000000	duck
Btg2	-0.98	0.00000000	duck
Pla2g15	0.94	0.00000000	duck
Sestd1	1.16	0.00000000	duck
Erp29	0.85	0.00000000	duck
Cop1	-1.23	0.00000000	duck
Yipf3	0.84	0.00000000	duck
Eif3g	0.87	0.00000000	duck
Eprs	0.88	0.00000000	duck
Tubb5	0.91	0.00000000	duck
Peli1	0.96	0.00000000	duck
Atp6v1a	0.91	0.00000000	duck
Dda1	0.83	0.00000000	duck
Fam3c	0.95	0.00000000	duck
Zdhhc17	0.94	0.00000000	duck
Ica1	0.91	0.00000000	duck
Ctsc	0.89	0.00000000	duck
Nr4a3	1.25	0.00000000	duck
Zfp217	1.01	0.00000000	duck
Btf3l4	0.81	0.00000000	duck
Atf3	0.76	0.00000000	duck
Vdac1	0.81	0.00000000	duck
Diablo	0.80	0.00000000	duck
Sumo1	0.72	0.00000000	duck

Ppp3ca	0.85	0.00000000	duck
Lamtor5	0.81	0.00000000	duck
Klf13	-1.35	0.00000000	duck
Riok3	0.76	0.00000000	duck
Hibadh	0.69	0.00000000	duck
Tcirg1	0.86	0.00000000	duck
Znfx1	0.73	0.00000000	duck
Lrpap1	0.89	0.00000000	duck
Asxl2	0.71	0.00000000	duck
Atp6v0e	0.78	0.00000000	duck
Polr2h	0.79	0.00000000	duck
Psma7	0.82	0.00000000	duck
Spats2	0.80	0.00000000	duck
Eif3i	0.80	0.00000000	duck
Atp5j	0.78	0.00000000	duck
Slc39a9	-1.44	0.00000000	duck
Ccdc71l	0.88	0.00000000	duck
Urm1	0.78	0.00000000	duck
Kif1b	0.79	0.00000000	duck
Ccdc93	0.85	0.00000000	duck
Usp6nl	-1.31	0.00000000	duck
Dnajc3	0.76	0.00000000	duck
Snrpg	0.79	0.00000000	duck
Fam214a	0.93	0.00000000	duck
Hsbp1	0.75	0.00000000	duck
Dtwd1	0.89	0.00000000	duck
Sla	-0.94	0.00000000	duck
Grk4	0.84	0.00000000	duck
Tmem250-ps	0.79	0.00000000	duck
Sub1	0.76	0.00000000	duck
Pik3r5	-0.63	0.00000000	duck
Pdrg1	0.75	0.00000000	duck
Bcor	-1.54	0.00000000	duck
Lcp2	0.74	0.00000000	duck
Rbx1	0.76	0.00000000	duck
Chmp4b	0.71	0.00000000	duck
Zfp326	0.88	0.00000000	duck
Akirin2	-0.71	0.00000000	duck
Kcnk5	0.80	0.00000000	duck
Dnajb9	0.95	0.00000000	duck
Larp7	0.73	0.00000000	duck
Frmd4b	1.15	0.00000000	duck
Ndufb6	0.76	0.00000000	duck
Nus1	0.94	0.00000000	duck
Rab20	1.08	0.00000000	duck
Fnip1	0.52	0.00000000	duck

Fosb	1.38	0.00000000	duck
Ralb	0.79	0.00000000	duck
Ubap2	0.83	0.00000000	duck
Osbpl9	0.72	0.00000000	duck
Lrp3	-1.12	0.00000000	duck
Ilk	0.79	0.00000000	duck
Jarid2	1.18	0.00000000	duck
Lipa	0.84	0.00000000	duck
Hspa8	1.22	0.00000000	duck
Nsa2	0.75	0.00000000	duck
Lpcat3	0.75	0.00000000	duck
Ndufs4	0.71	0.00000000	duck
Ulk2	0.77	0.00000000	duck
Chmp2b	0.89	0.00000000	duck
Atrn	1.08	0.00000000	duck
Slc23a2	0.79	0.00000000	duck
Arsb	0.86	0.00000000	duck
Ndfip1	0.76	0.00000000	duck
Supt4a	0.77	0.00000000	duck
Rubcnl	0.98	0.00000000	duck
Cib1	0.73	0.00000000	duck
Nfil3	0.76	0.00000000	duck
Bambi	1.24	0.00000000	duck
Glrx	0.69	0.00000000	duck
Nadk	0.65	0.00000000	duck
Tst	0.79	0.00000000	duck
Gas7	1.01	0.00000000	duck
Edem1	0.81	0.00000000	duck
Pef1	0.73	0.00000000	duck
Appl2	0.96	0.00000000	duck
Fzr1	0.77	0.00000000	duck
Gab1	1.07	0.00000000	duck
Polr2e	0.75	0.00000000	duck
Sde2	0.64	0.00000000	duck
Gpi1	0.70	0.00000000	duck
Hnrnpll	-0.80	0.00000000	duck
Ndufs5	0.70	0.00000000	duck
Edem3	0.67	0.00000000	duck
Klhdc1	0.82	0.00000000	duck
Cdc42	-0.71	0.00000000	duck
Lasp1	0.70	0.00000000	duck
Pid1	1.04	0.00000000	duck
Nr4a1	0.90	0.00000000	duck
Srp19	0.72	0.00000000	duck
Pik3ap1	-0.69	0.00000000	duck
Tmem35b	0.73	0.00000000	duck

Ythdf2	0.79	0.00000000	duck
Vmp1	0.66	0.00000000	duck
Xylt1	-1.24	0.00000000	duck
Pnrc1	0.63	0.00000000	duck
Slc16a6	-1.35	0.00000000	duck
Fbxw11	0.68	0.00000000	duck
Erap1	0.66	0.00000000	duck
Rab10	0.65	0.00000000	duck
Slc6a6	-1.03	0.00000000	duck
Arg2	0.80	0.00000000	duck
Smarcd2	0.71	0.00000000	duck
Cert1	0.86	0.00000000	duck
Agtpbp1	0.82	0.00000000	duck
Erlec1	0.77	0.00000000	duck
Setx	0.73	0.00000000	duck
Psmd2	0.71	0.00000000	duck
Dctn6	0.69	0.00000000	duck
Htr4	-1.56	0.00000000	duck
Ptprm	1.38	0.00000000	duck
Grpel1	0.82	0.00000000	duck
Rell1	-1.05	0.00000000	duck
Foxk1	0.83	0.00000000	duck
Chordc1	0.94	0.00000000	duck
Trak1	0.82	0.00000000	duck
Ndufb10	0.69	0.00000000	duck
Zmiz1	1.05	0.00000000	duck
Oit3	-1.42	0.00000000	duck
Nfkbiz	0.60	0.00000000	duck
Arpc3	0.66	0.00000000	duck
Wdfy3	1.62	0.00000000	duck
Atp5a1	0.66	0.00000000	duck
Gnpda1	0.71	0.00000000	duck
Hivep2	0.77	0.00000000	duck
Kctd10	0.69	0.00000000	duck
Cdk5rap1	0.68	0.00000000	duck
Tmem183a	0.61	0.00000000	duck
Ormdl2	0.68	0.00000000	duck
Psma3	0.71	0.00000000	duck
Ost4	0.67	0.00000000	duck
Tnrc6c	0.68	0.00000000	duck
Naga	0.69	0.00000000	duck
Atf6	0.72	0.00000000	duck
Sdhb	0.67	0.00000000	duck
Romo1	0.71	0.00000000	duck
Tmx3	0.64	0.00000000	duck
Scarb2	-1.44	0.00000000	duck

Rab1a	0.65	0.00000000	duck
Atp6ap2	0.69	0.00000000	duck
Fhl3	0.64	0.00000000	duck
Abca3	0.77	0.00000000	duck
Prdm1	0.87	0.00000000	duck
Snrk	-1.17	0.00000000	duck
Pla2g4a	0.64	0.00000000	duck
Nceh1	0.76	0.00000000	duck
Tmem230	0.67	0.00000000	duck
Zswim6	0.67	0.00000000	duck
Gpd2	0.52	0.00000000	duck
Xxylt1	0.70	0.00000000	duck
Pum2	-1.28	0.00000000	duck
Ap2m1	0.65	0.00000000	duck
Dtx3l	0.59	0.00000000	duck
Diaph2	0.59	0.00000000	duck
Arhgef26	0.85	0.00000000	duck
Ap3d1	0.65	0.00000000	duck
Ube3c	0.71	0.00000000	duck
Stard5	-1.08	0.00000000	duck
Kdm5a	-1.07	0.00000000	duck
Dbnl	0.69	0.00000000	duck
Rb1cc1	0.54	0.00000000	duck
Erp44	-0.94	0.00000000	duck
Ak2	0.49	0.00000000	duck
Sash3	0.69	0.00000000	duck
Sf3a2	0.66	0.00000000	duck
Adss	0.64	0.00000000	duck
Tfdp2	0.88	0.00000000	duck
Ndufb2	0.66	0.00000000	duck
Sh3kbp1	0.60	0.00000000	duck
Zdhhc6	0.65	0.00000000	duck
Atp5j2	0.65	0.00000000	duck
Endov	0.69	0.00000000	duck
Hmox1	0.89	0.00000000	duck
Eif4ebp1	0.66	0.00000000	duck
Phactr2	1.43	0.00000000	duck
Fkbp5	-1.42	0.00000000	duck
Pik3r6	0.81	0.00000000	duck
Rtn4	0.62	0.00000000	duck
Nrp2	0.86	0.00000000	duck
Atp1b3	0.60	0.00000000	duck
Palld	-2.03	0.00000000	duck
Slc7a11	0.89	0.00000000	duck
Atp1a1	0.80	0.00000000	duck
Cwc15	0.59	0.00000000	duck

Lyrm7	0.67	0.00000000	duck
Depdc1b	0.67	0.00000000	duck
Ndufa8	0.60	0.00000000	duck
Nck2	-0.84	0.00000000	duck
Gadd45g	0.86	0.00000000	duck
Actb	0.50	0.00000000	duck
Acp2	0.63	0.00000000	duck
Rab27a	0.71	0.00000000	duck
Kif21a	0.59	0.00000000	duck
Eps15	0.59	0.00000000	duck
Prex1	0.47	0.00000000	duck
B2m	0.48	0.00000000	duck
Cst7	0.74	0.00000000	duck
Ndufa4	0.68	0.00000000	duck
Bach1	0.49	0.00000000	duck
Pfkfb3	-1.07	0.00000000	duck
Atp6v0c	0.65	0.00000000	duck
Lsm8	0.52	0.00000000	duck
Snx17	0.59	0.00000000	duck
Esd	0.59	0.00000000	duck
Rrbp1	0.64	0.00000000	duck
Caprin1	0.50	0.00000000	duck
Eif3d	0.58	0.00000000	duck
Cbfb	0.72	0.00000000	duck
Ei24	0.56	0.00000000	duck
Dhx36	0.59	0.00000000	duck
Ppib	0.65	0.00000000	duck
Iqgap1	-0.95	0.00000000	duck
Psmd7	0.61	0.00000000	duck
Parvg	-0.70	0.00000000	duck
Eif3a	0.52	0.00000000	duck
Gng7	0.61	0.00000000	duck
Ptpn1	0.57	0.00000000	duck
Itm2a	0.46	0.00000000	duck
Dcaf6	0.51	0.00000000	duck
Camk2g	0.56	0.00000000	duck
Susd3	0.70	0.00000000	duck
Prdx6	0.59	0.00000000	duck
Snrpd3	0.57	0.00000000	duck
Tm2d2	0.60	0.00000000	duck
Tmem241	0.62	0.00000000	duck
Lrrc8d	0.90	0.00000000	duck
Paqr7	0.56	0.00000000	duck
Zbtb44	0.60	0.00000000	duck
Gsr	0.60	0.00000000	duck
Derl3	0.58	0.00000000	duck

Rapgef1	0.72	0.00000000	duck
Abcc4	-1.67	0.00000000	duck
Fam174a	0.61	0.00000000	duck
Septin7	0.44	0.00000000	duck
Drd4	-0.94	0.00000000	duck
Kif5b	0.52	0.00000000	duck
Cisd2	0.58	0.00000000	duck
Lgmn	0.37	0.00000000	duck
Ski	0.48	0.00000000	duck
Psmb4	0.56	0.00000000	duck
Ppt1	0.55	0.00000000	duck
Gper1	0.64	0.00000000	duck
Slc9a3r1	0.60	0.00000000	duck
Itm2b	0.65	0.00000000	duck
Cd86	0.70	0.00000000	duck
Taf3	0.46	0.00000000	duck
Ahsa1	0.60	0.00000000	duck
Nab1	0.58	0.00000000	duck
Pou2f1	0.52	0.00000000	duck
Ttc39b	0.47	0.00000000	duck
Tax1bp1	0.40	0.00000000	duck
Oser1	0.56	0.00000000	duck
Gpx1	0.52	0.00000000	duck
Abcc5	0.82	0.00000000	duck
N4bp1	0.54	0.00000000	duck
Pdcd10	0.59	0.00000000	duck
Wbp2nl	0.59	0.00000000	duck
Atp13a3	0.67	0.00000000	duck
Ubl5b	0.52	0.00000000	duck
Adap2	0.61	0.00000000	duck
Rpn2	0.53	0.00000000	duck
Errfi1	0.67	0.00000000	duck
Tmem65	0.62	0.00000000	duck
Hsd17b12	0.61	0.00000000	duck
Cebpa	0.67	0.00000000	duck
Ube2q1	0.59	0.00000000	duck
Dock10	0.90	0.00000000	duck
Zzz3	0.56	0.00000000	duck
Pde8a	-1.32	0.00000000	duck
Thoc7	0.50	0.00000000	duck
Bcas3	0.71	0.00000000	duck
Vps54	0.59	0.00000000	duck
Septin12	0.55	0.00000000	duck
Psma5	0.55	0.00000000	duck
Bptf	0.44	0.00000000	duck
Prkcd	0.55	0.00000000	duck

Nt5m	0.55	0.00000000	duck
Kars	0.53	0.00000000	duck
Clta	0.60	0.00000000	duck
Ldah	0.57	0.00000000	duck
Eif3m	0.50	0.00000000	duck
Ssr2	0.60	0.00000000	duck
Ppp1ca	0.59	0.00000000	duck
Rnf34	-1.18	0.00000000	duck
Prpf39	0.61	0.00000000	duck
Dram1	0.67	0.00000000	duck
Nmnat2	0.61	0.00000000	duck
Psmb1	0.59	0.00000000	duck
Nucb2	-0.89	0.00000000	duck
Gtf2b	0.58	0.00000000	duck
Trdmt1	0.58	0.00000000	duck
Eif4a2	0.54	0.00000000	duck
Wdr81	0.59	0.00000000	duck
Lsp1	0.56	0.00000000	duck
Lemd2	0.54	0.00000000	duck
Rfesd	-1.75	0.00000000	duck
Lamtor2	0.57	0.00000000	duck
Sntb1	1.03	0.00000000	duck
Zc3h13	0.82	0.00000000	duck
Nono	0.66	0.00000000	duck
Wdr82	0.57	0.00000000	duck
Znrf2	0.56	0.00000000	duck
Aimp1	0.52	0.00000000	duck
Enpp4	-0.95	0.00000000	duck
Ndufb3	0.57	0.00000000	duck
Aplf	0.76	0.00000000	duck
Serinc1	0.37	0.00000000	duck
Ccdc12	0.53	0.00000000	duck
Pold2	0.56	0.00000000	duck
Lgals3	0.55	0.00000000	duck
Glod4	0.55	0.00000000	duck
Ttf1	0.65	0.00000000	duck
Tmem33	0.54	0.00000000	duck
Rad51b	-1.05	0.00000000	duck
Aebp2	0.45	0.00000000	duck
Lsg1	0.75	0.00000000	duck
Arf1	0.55	0.00000000	duck
Tpmt	0.62	0.00000000	duck
Lbr	0.43	0.00000000	duck
Pum1	0.49	0.00000000	duck
Acsf2	0.55	0.00000000	duck
Rbms1	0.65	0.00000000	duck

Fgl2	0.54	0.00000000	duck
Eif2s2	0.54	0.00000000	duck
Alcam	1.37	0.00000000	duck
Rnf11	0.53	0.00000000	duck
Itpka	0.53	0.00000000	duck
Arl8b	0.52	0.00000000	duck
Oaz1	0.50	0.00000000	duck
Atp23	0.53	0.00000000	duck
Rc3h1	0.40	0.00000000	duck
Ndufb9	0.50	0.00000000	duck
Sema4a	0.55	0.00000000	duck
Bmp2k	0.48	0.00000000	duck
Sft2d1	0.51	0.00000000	duck
Usp47	0.39	0.00000000	duck
Abhd17a	0.53	0.00000000	duck
Golim4	0.49	0.00000000	duck
Vgll4	0.56	0.00000000	duck
Slc22a3	0.69	0.00000000	duck
Basp1	-1.43	0.00000000	duck
Srp68	0.55	0.00000000	duck
Ppara	0.72	0.00000000	duck
Supt5	0.58	0.00000000	duck
Rbbp7	-1.14	0.00000000	duck
Gpbp1l1	0.40	0.00000000	duck
Psmd6	0.58	0.00000000	duck
Hdac1	0.52	0.00000000	duck
Chst12	0.53	0.00000000	duck
Afg3l2	0.46	0.00000000	duck
Ctss	0.79	0.00000000	duck
Pard3	0.94	0.00000000	duck
Rexo2	0.53	0.00000000	duck
Tspan8	0.57	0.00000000	duck
Satb1	0.64	0.00000000	duck
Tulp1	0.56	0.00000000	duck
Numb	0.57	0.00000000	duck
Chka	0.76	0.00000000	duck
Nudt12	0.58	0.00000000	duck
Lamp2	0.46	0.00000000	duck
Zmat2	0.46	0.00000000	duck
Synrg	0.55	0.00000000	duck
Psme3	0.51	0.00000000	duck
Dazap2	0.54	0.00000000	duck
Aoah	0.57	0.00000000	duck
Adar	0.46	0.00000000	duck
Avl9	0.53	0.00000000	duck
Fam13a	0.83	0.00000000	duck

Ereg	-1.00	0.00000000	duck
Atp6v0d1	0.53	0.00000000	duck
Pdia4	0.52	0.00000000	duck
Acox1	0.49	0.00000000	duck
Arxes2	0.52	0.00000000	duck
Samm50	0.43	0.00000000	duck
Cnot4	0.42	0.00000000	duck
Zbtb21	0.56	0.00000000	duck
Hif1a	-0.80	0.00000000	duck
Rcsd1	0.48	0.00000000	duck
Sp3	-1.10	0.00000000	duck
Puf60	0.47	0.00000000	duck
Rpa1	0.60	0.00000000	duck
Hvcn1	0.58	0.00000000	duck
Tspo	0.51	0.00000000	duck
Gab3	0.61	0.00000000	duck
Cemip2	0.52	0.00000000	duck
Smdt1	0.54	0.00000000	duck
Ppp2r2d	-1.10	0.00000000	duck
Xbp1	0.54	0.00000000	duck
Lmbr1	0.94	0.00000000	duck
Dynlrb1	0.47	0.00000000	duck
Stom	0.47	0.00000000	duck
Cdc42bpb	0.51	0.00000000	duck
Cops7a	0.50	0.00000000	duck
Ptpre	-0.67	0.00000000	duck
Foxj2	0.43	0.00000000	duck
Stt3a	0.44	0.00000000	duck
Fem1c	0.52	0.00000000	duck
Rictor	0.41	0.00000000	duck
Spsb1	0.54	0.00000000	duck
Id2	0.73	0.00000000	duck
Slc39a11	0.58	0.00000000	duck
Rpn1	0.49	0.00000000	duck
Bhlhe41	0.54	0.00000000	duck
Gtf2h5	0.50	0.00000000	duck
Dcp1b	-1.50	0.00000000	duck
Lpxn	0.55	0.00000000	duck
Psmd12	0.48	0.00000000	duck
Zdhhc14	0.51	0.00000000	duck
Palb2	0.54	0.00000000	duck
Panx2	0.56	0.00000000	duck
Rsbn1l	0.44	0.00000000	duck
Cpa6	0.63	0.00000000	duck
Cep83	0.59	0.00000000	duck
Gpatch2l	0.46	0.00000000	duck

Gpr27	0.47	0.00000000	duck
Ssbp1	0.44	0.00000000	duck
Cpne3	0.44	0.00000000	duck
Ccnk	0.42	0.00000000	duck
Susd1	0.19	0.00000000	duck
Psd3	0.55	0.00000000	duck
Stambpl1	-1.12	0.00000000	duck
Tbc1d16	0.45	0.00000000	duck
Rpain	0.51	0.00000000	duck
Cep112	0.63	0.00000000	duck
Rnf20	0.47	0.00000000	duck
Fa2h	-0.96	0.00000000	duck
5031439G07Rik	0.44	0.00000000	duck
Spred2	0.39	0.00000000	duck
Tgm4	1.79	0.00000000	duck
St8sia4	0.62	0.00000000	duck
Ankrd13a	0.49	0.00000000	duck
Ndufb4	0.46	0.00000000	duck
Bbx	0.53	0.00000000	duck
Ube2b	-0.94	0.00000000	duck
Plekhd1	-0.75	0.00000000	duck
Sod1	0.46	0.00000000	duck
As3mt	0.44	0.00000000	duck
Psen1	0.43	0.00000000	duck
Lmbr1l	-0.66	0.00000000	duck
Scamp2	0.47	0.00000000	duck
H13	0.56	0.00000000	duck
2700049A03Rik	0.72	0.00000000	duck
Snap29	0.41	0.00000000	duck
Ckap5	0.45	0.00000000	duck
Natd1	0.45	0.00000000	duck
Gdi2	0.48	0.00000000	duck
Arhgdia	0.48	0.00000000	duck
Atg2b	0.47	0.00000000	duck
Parp9	0.41	0.00000000	duck
Ptbp3	0.41	0.00000000	duck
Capn15	0.47	0.00000000	duck
Ubqln1	0.43	0.00000000	duck
Yaf2	0.46	0.00000000	duck
Polr2d	0.44	0.00000000	duck
Pdia3	0.47	0.00000000	duck
Tsc22d3	-1.23	0.00000000	duck
P4hb	0.48	0.00000000	duck
Rasa2	-1.30	0.00000000	duck
Htra1	0.69	0.00000000	duck
Pigx	0.49	0.00000000	duck

Baiap2l1	0.56	0.00000000	duck
Appl1	0.49	0.00000000	duck
Slc10a7	0.62	0.00000000	duck
Nup98	0.40	0.00000000	duck
Tomm22	0.45	0.00000000	duck
Gsn	0.55	0.00000000	duck
Cdc42se1	0.51	0.00000000	duck
Nifk	0.49	0.00000000	duck
Gnas	0.35	0.00000000	duck
Rrp1b	0.47	0.00000000	duck
Prdx3	0.40	0.00000000	duck
Nsfl1c	0.46	0.00000000	duck
Zc3h3	0.67	0.00000000	duck
Pak3	0.80	0.00000000	duck
Ran	0.44	0.00000000	duck
Sstr4	0.49	0.00000000	duck
Abca1	0.41	0.00000000	duck
Serp1	0.47	0.00000000	duck
Tbc1d1	0.58	0.00000000	duck
Jmjd8	0.45	0.00000000	duck
Ppp4r3a	0.33	0.00000000	duck
Luc7l	0.51	0.00000000	duck
Gns	0.41	0.00000000	duck
Helz	0.35	0.00000000	duck
Ndufv2	0.36	0.00000000	duck
Gna13	-0.88	0.00000000	duck
Ehd4	0.41	0.00000000	duck
Llph	0.41	0.00000000	duck
Rtf2	0.42	0.00000000	duck
Bcl2l1	-1.02	0.00000000	duck
Rad21	0.38	0.00000000	duck
Smad7	0.64	0.00000000	duck
Tbc1d15	0.35	0.00000000	duck
Wnt5b	0.34	0.00000000	duck
Jund	0.57	0.00000000	duck
Ngf	0.44	0.00000000	duck
Gpr155	0.33	0.00000000	duck
Pdxk	0.65	0.00000000	duck
Cnot2	0.35	0.00000000	duck
Ifngr2	-0.77	0.00000000	duck
Ppif	0.54	0.00000000	duck
Prkab1	0.44	0.00000000	duck
Hypk	0.46	0.00000000	duck
Ndufaf3	0.43	0.00000000	duck
Hmgcr	0.56	0.00000000	duck
Commd1b	0.48	0.00000000	duck

Polr2l	0.47	0.00000000	duck
Phf21a	0.49	0.00000000	duck
Gtpbp10	-2.53	0.00000000	duck
Nipbl	0.37	0.00000000	duck
Nek6	-1.17	0.00000000	duck
Nipa2	0.43	0.00000000	duck
Bclaf3	0.53	0.00000000	duck
Alpk1	0.51	0.00000000	duck
Il6ra	0.46	0.00000000	duck
Cmss1	0.62	0.00000000	duck
Soat1	0.54	0.00000000	duck
Crnkl1	0.46	0.00000000	duck
Psmb3	0.44	0.00000000	duck
Wdr89	0.43	0.00000000	duck
Tmx4	0.40	0.00000000	duck
Snx2	0.36	0.00000000	duck
Dgkd	0.56	0.00000000	duck
Snrpc	0.43	0.00000000	duck
Pkm	0.41	0.00000000	duck
Gga2	0.49	0.00000000	duck
Ubn2	0.48	0.00000000	duck
Sh3bgrl	0.37	0.00000000	duck
Igf2r	0.42	0.00000000	duck
Tdg	0.45	0.00000000	duck
Rock2	0.49	0.00000000	duck
Zcrb1	0.41	0.00000000	duck
Ccnh	0.41	0.00000000	duck
Il18	0.51	0.00000000	duck
Eif3f	0.41	0.00000000	duck
Pgap4	0.48	0.00000000	duck
Kdm7a	-0.72	0.00000000	duck
Tars	0.39	0.00000000	duck
Cdv3	0.43	0.00000000	duck
Plekhb2	0.42	0.00000000	duck
Rbbp6	0.37	0.00000000	duck
Hook3	0.38	0.00000000	duck
Fam204a	0.37	0.00000000	duck
Usp7	0.35	0.00000000	duck
Phb2	0.42	0.00000000	duck
Psmd13	0.43	0.00000000	duck
Ctsh	0.44	0.00000000	duck
Ncapd2	0.45	0.00000000	duck
Kctd2	0.42	0.00000000	duck
Selenof	0.47	0.00000000	duck
Arhgap15	0.47	0.00000000	duck
Tut4	0.39	0.00000000	duck

Flii	0.42	0.00000000	duck
Arhgap25	0.42	0.00000000	duck
Usp8	0.33	0.00000000	duck
Atic	0.46	0.00000000	duck
Sucnr1	0.53	0.00000000	duck
Helq	0.45	0.00000000	duck
Bzw2	0.45	0.00000000	duck
Rbm31y	0.50	0.00000000	duck
Coro1b	0.41	0.00000000	duck
Rxra	0.49	0.00000000	duck
Eci3	0.39	0.00000000	duck
Rasgef1a	0.44	0.00000000	duck
Fam76a	0.45	0.00000000	duck
Slc66a1	0.42	0.00000000	duck
Wipi1	0.35	0.00000000	duck
Scaf11	0.35	0.00000000	duck
Uprt	0.38	0.00000000	duck
Zmpste24	0.41	0.00000000	duck
Pam	0.36	0.00000000	duck
Gfi1	-0.79	0.00000000	duck
Bnip3	-1.03	0.00000000	duck
Ppargc1a	0.61	0.00000000	duck
Chmp5	0.40	0.00000000	duck
Zc3h18	0.35	0.00000000	duck
Ap3b1	0.34	0.00000000	duck
Tubd1	0.40	0.00000000	duck
Dusp16	0.47	0.00000000	duck
Cul1	0.34	0.00000000	duck
Ap1s2	0.39	0.00000000	duck
Psmd4	0.41	0.00000000	duck
Cdk5r1	-0.87	0.00000000	duck
Psmd14	0.37	0.00000000	duck
Hsdl1	0.45	0.00000000	duck
Bub3	0.37	0.00000000	duck
Smim5	0.50	0.00000000	duck
Commd4	0.41	0.00000000	duck
Ankrd10	0.42	0.00000000	duck
Dsn1	0.39	0.00000000	duck
Stx8	0.35	0.00000000	duck
Fbxo25	0.35	0.00000000	duck
Esr1	0.46	0.00000000	duck
Pamr1	0.51	0.00000000	duck
Vdac2	0.45	0.00000000	duck
Vps35l	0.38	0.00000000	duck
Fem1a	0.41	0.00000000	duck
Cwf19l2	0.42	0.00000000	duck

Secisbp2l	0.30	0.00000000	duck
Birc6	-0.64	0.00000000	duck
Snrpb	0.44	0.00000000	duck
Ilvbl	0.42	0.00000000	duck
Nudt21	0.44	0.00000000	duck
Ciao2b	0.44	0.00000000	duck
Rragc	0.41	0.00000000	duck
Echs1	0.41	0.00000000	duck
Wdr26	0.41	0.00000000	duck
Sf3b6	0.41	0.00000000	duck
Emc3	0.39	0.00000000	duck
Atp2a2	0.35	0.00000000	duck
Rp2	-1.00	0.00000000	duck
Ndufs3	0.39	0.00000000	duck
Rab7	0.48	0.00000000	duck
Nudt14	0.38	0.00000000	duck
Fkbp3	0.37	0.00000000	duck
Tmem203	0.40	0.00000000	duck
Rtf1	0.33	0.00000000	duck
Actr10	0.38	0.00000000	duck
Dipk1b	0.43	0.00000000	duck
Plcl1	0.59	0.00000000	duck
Papss1	0.44	0.00000000	duck
Sh3glb1	0.42	0.00000000	duck
Unc13d	0.41	0.00000000	duck
Sec11a	0.41	0.00000000	duck
St13	0.35	0.00000000	duck
Cct8	0.41	0.00000000	duck
Hivep1	0.33	0.00000000	duck
Slc35a5	-0.90	0.00000000	duck
Slc7a10	-0.89	0.00000000	duck
Ostf1	0.47	0.00000000	duck
Sh3pxd2a	0.45	0.00000000	duck
Zc3h15	0.36	0.00000000	duck
Epc2	0.29	0.00000000	duck
Rassf5	0.42	0.00000000	duck
Ndufb1	0.39	0.00000000	duck
Sec62	0.45	0.00000000	duck
Acvr2a	0.38	0.00000000	duck
Pgap2	0.52	0.00000000	duck
Abracl	0.39	0.00000000	duck
Acot7	0.41	0.00000000	duck
Rilpl2	0.39	0.00000000	duck
Eri1	0.35	0.00000000	duck
Sppl3	0.40	0.00000000	duck
Sec14l1	0.37	0.00000000	duck

Usp33	0.28	0.00000000	duck
Kif13a	0.43	0.00000000	duck
Prkaa1	0.43	0.00000000	duck
Aup1	0.40	0.00000000	duck
G3bp1	0.31	0.00000000	duck
Sbno1	0.34	0.00000000	duck
Ier5	0.46	0.00000000	duck
Clip1	0.44	0.00000000	duck
Plekha2	0.43	0.00000000	duck
Tcp11l2	0.38	0.00000000	duck
Ndufa5	0.37	0.00000000	duck
Fbxo45	0.38	0.00000000	duck
Acap2	-0.70	0.00000000	duck
Areg	-1.13	0.00000000	duck
Slc30a9	0.39	0.00000000	duck
Vav3	-0.46	0.00000000	duck
Clcn7	0.44	0.00000000	duck
Xpo4	0.47	0.00000000	duck
Ctbs	0.46	0.00000000	duck
Adgrg3	0.41	0.00000000	duck
Dctn4	0.36	0.00000000	duck
Parp10	0.41	0.00000000	duck
Nudc	0.38	0.00000000	duck
Dynll2	0.37	0.00000000	duck
Fam185a	0.31	0.00000000	duck
Rheb	0.35	0.00000000	duck
Cmtm6	0.43	0.00000000	duck
Tnni2	0.47	0.00000000	duck
Usp14	0.35	0.00000000	duck
Csf1r	0.43	0.00000000	duck
Ube2i	0.29	0.00000000	duck
Sema3d	-1.36	0.00000000	duck
Gpalpp1	0.37	0.00000000	duck
Gda	0.39	0.00000000	duck
Txnrd3	0.34	0.00000000	duck
Saraf	0.40	0.00000000	duck
Brca2	0.54	0.00000000	duck
Pdss1	0.39	0.00000000	duck
Larp4b	0.33	0.00000000	duck
Osbpl6	0.76	0.00000000	duck
Ppat	-0.93	0.00000000	duck
Gusb	0.38	0.00000000	duck
Fmr1	0.28	0.00000000	duck
Als2cl	0.36	0.00000000	duck
Lman2	0.38	0.00000000	duck
Iws1	0.36	0.00000000	duck

Ric8a	0.39	0.00000000	duck
Arhgef6	0.39	0.00000000	duck
Fcho2	0.25	0.00000000	duck
Slc25a36	0.31	0.00000000	duck
Entpd2	0.34	0.00000000	duck
Galnt11	0.40	0.00000000	duck
Srpk2	0.36	0.00000000	duck
Tgif1	0.41	0.00000000	duck
Ephx4	0.38	0.00000000	duck
Pdia5	0.45	0.00000000	duck
Fig4	0.31	0.00000000	duck
Coq5	0.36	0.00000000	duck
Vps41	0.41	0.00000000	duck
Arfgef2	0.37	0.00000000	duck
Lrrc59	0.34	0.00000000	duck
Polr2b	0.36	0.00000000	duck
Krr1	0.42	0.00000000	duck
Neurl1b	0.19	0.00000000	duck
Slc1a2	0.60	0.00000000	duck
Psmc1	0.33	0.00000000	duck
Tpi1	0.38	0.00000000	duck
Pparg	-0.88	0.00000000	duck
Dapp1	-1.03	0.00000000	duck
Pithd1	0.35	0.00000000	duck
Rufy1	0.42	0.00000000	duck
Prelid1	0.39	0.00000000	duck
Unc119b	0.33	0.00000000	duck
Anxa2	0.76	0.00000000	duck
Vma21	0.33	0.00000000	duck
Zdhhc12	0.35	0.00000000	duck
Tlcd3a	-0.87	0.00000000	duck
Nsd1	0.32	0.00000000	duck
Rnf216	0.38	0.00000000	duck
Ugp2	0.37	0.00000000	duck
Eif3l	0.36	0.00000000	duck
Ube4b	0.29	0.00000000	duck
Cmas	0.39	0.00000000	duck
Fdx1	0.34	0.00000000	duck
Tcp1	0.35	0.00000000	duck
Ubl7	0.36	0.00000000	duck
Wsb2	0.35	0.00000000	duck
Kdelr2	0.34	0.00000000	duck
St8sia1	0.53	0.00000000	duck
Helz2	0.30	0.00000000	duck
Atp6v1c1	0.35	0.00000000	duck
Srp72	0.34	0.00000000	duck

Pxylp1	0.52	0.00000000	duck
Shc1	0.34	0.00000000	duck
Ppargc1b	0.40	0.00000000	duck
F3	0.94	0.00000000	duck
Naxd	0.47	0.00000000	duck
Golga7	0.33	0.00000000	duck
Cobll1	0.72	0.00000000	duck
Imp3	0.34	0.00000000	duck
Wdfy2	0.57	0.00000000	duck
Ube2g1	0.30	0.00000000	duck
Cers5	0.35	0.00000000	duck
Prpf40a	0.38	0.00000000	duck
Tcerg1	0.32	0.00000000	duck
Ankmy2	0.35	0.00000000	duck
Pwp1	0.46	0.00000000	duck
Idh3a	0.43	0.00000000	duck
Psmc3	0.35	0.00000000	duck
Taf12	0.38	0.00000000	duck
Sgsm3	0.36	0.00000000	duck
Tspan3	0.26	0.00000000	duck
Gla	0.36	0.00000000	duck
Exosc3	0.39	0.00000000	duck
Atxn1	0.23	0.00000000	duck
Aak1	0.36	0.00000000	duck
Dph5	0.33	0.00000000	duck
Larp1	0.36	0.00000000	duck
Lta4h	0.37	0.00000000	duck
Picalm	0.35	0.00000000	duck
Acsl1	-0.55	0.00000000	duck
Plekha1	0.42	0.00000000	duck
Slc25a5	0.34	0.00000000	duck
Rangap1	0.37	0.00000000	duck
Ranbp1	0.36	0.00000000	duck
Kif2a	0.39	0.00000000	duck
Tcf25	0.35	0.00000000	duck
Heatr5a	0.20	0.00000000	duck
Psmd1	0.30	0.00000000	duck
Slc9a5	0.38	0.00000000	duck
Dcp2	0.28	0.00000000	duck
Csnk1a1	0.33	0.00000000	duck
Spg21	0.36	0.00000000	duck
Nmt1	0.34	0.00000000	duck
Eif4a3l1	0.31	0.00000000	duck
Pdia6	0.35	0.00000000	duck
Tmtc3	0.31	0.00000000	duck
Tmf1	0.32	0.00000000	duck

Psmc6	0.24	0.00000000	duck
Ptger4	0.37	0.00000000	duck
Ubap1	0.27	0.00000000	duck
Gnai2	0.40	0.00000000	duck
Cope	0.35	0.00000000	duck
Bend5	0.41	0.00000000	duck
Scarb1	0.32	0.00000000	duck
Klf10	0.42	0.00000000	duck
Htt	0.30	0.00000000	duck
Rnf220	0.35	0.00000000	duck
Slc49a4	0.47	0.00000000	duck
Bcl6	0.52	0.00000000	duck
Rsl24d1	0.33	0.00000000	duck
Wdr48	0.28	0.00000000	duck
Dcp1a	-0.66	0.00000000	duck
Zfp654	0.31	0.00000000	duck
Caap1	0.34	0.00000000	duck
Lemd3	0.40	0.00000000	duck
Rer1	0.34	0.00000000	duck
Tmed5	0.35	0.00000000	duck
Osbp2	0.47	0.00000000	duck
Sirt6	0.32	0.00000000	duck
Donson	0.43	0.00000000	duck
Arhgap1	0.35	0.00000000	duck
Prpf6	0.25	0.00000000	duck
Gabrr3	0.61	0.00000000	duck
Cables1	0.40	0.00000000	duck
Eif3j2	0.26	0.00000000	duck
Ano10	0.22	0.00000000	duck
Smarca2	0.31	0.00000000	duck
Dcaf12	0.29	0.00000000	duck
Top3a	0.34	0.00000000	duck
Aptx	0.31	0.00000000	duck
Dhx58	0.33	0.00000000	duck
Aco1	0.34	0.00000000	duck
Casp3	0.45	0.00000000	duck
Ptbp2	0.30	0.00000000	duck
Btd	0.39	0.00000000	duck
Sbno2	0.35	0.00000000	duck
Ralbp1	0.23	0.00000000	duck
Papolg	0.35	0.00000000	duck
Tmed10	-0.51	0.00000000	duck
Raver2	0.42	0.00000000	duck
Ociad1	0.32	0.00000000	duck
Slc37a1	0.28	0.00000000	duck
Tnfaip8	0.36	0.00000000	duck

Rab9	0.32	0.00000000	duck
Taf13	0.36	0.00000000	duck
Odc1	0.34	0.00000000	duck
Plekhm2	0.35	0.00000000	duck
Tmem199	0.34	0.00000000	duck
Pitpnb	0.29	0.00000000	duck
Golph3	0.33	0.00000000	duck
Prelid3a	0.32	0.00000000	duck
Nhsl2	0.35	0.00000000	duck
Rbm33	0.15	0.00000000	duck
Elovl1	0.36	0.00000000	duck
Serinc3	0.31	0.00000000	duck
Rnf144a	0.33	0.00000000	duck
Cog7	0.40	0.00000000	duck
Tmem154	-0.96	0.00000000	duck
Gnb4	0.38	0.00000000	duck
Tmco1	0.35	0.00000000	duck
Slc26a4	0.39	0.00000000	duck
Nek9	0.38	0.00000000	duck
Gramd3	0.33	0.00000000	duck
Irf2bpl	0.37	0.00000000	duck
Eml4	0.50	0.00000000	duck
Fbxl4	0.29	0.00000000	duck
Snx8	0.35	0.00000000	duck
Adgrg2	0.60	0.00000000	duck
Jpt1	0.29	0.00000000	duck
Bak1	0.35	0.00000000	duck
Stx7	0.30	0.00000000	duck
Cc2d2a	-1.11	0.00000000	duck
Txn2	0.29	0.00000000	duck
Bnc2	-0.64	0.00000000	duck
Tm6sf1	0.35	0.00000000	duck
Chst13	-0.46	0.00000000	duck
Napg	0.28	0.00000000	duck
Rab22a	0.29	0.00000000	duck
Wdr24	0.33	0.00000000	duck
Acbd3	0.31	0.00000000	duck
Tmem167	0.30	0.00000000	duck
Tmem9b	0.35	0.00000000	duck
Sf3a1	0.31	0.00000000	duck
Rpp38	0.38	0.00000000	duck
Btbd11	-1.00	0.00000000	duck
Rlim	0.35	0.00000000	duck
N4bp2	0.43	0.00000000	duck
Rab14	0.32	0.00000000	duck
Nbeal2	-0.81	0.00000000	duck

G3bp2	0.26	0.00000000	duck
Ube2h	0.16	0.00000000	duck
Rab39b	0.33	0.00000000	duck
Ccdc59	0.35	0.00000000	duck
Copa	0.34	0.00000000	duck
Bzw1	0.26	0.00000000	duck
Nfatc3	0.27	0.00000000	duck
Dcaf13	0.33	0.00000000	duck
Utp18	0.33	0.00000000	duck
Sigmar1	0.30	0.00000000	duck
Arcn1	0.31	0.00000000	duck
Adpgk	0.30	0.00000000	duck
Zdhhc9	0.34	0.00000000	duck
Opa1	0.18	0.00000000	duck
Slc4a2	0.36	0.00000000	duck
Trmt10a	0.35	0.00000000	duck
Shoc2	0.37	0.00000000	duck
Atg7	0.33	0.00000000	duck
Nptn	0.29	0.00000000	duck
Leprot	0.34	0.00000000	duck
Lysmd2	0.29	0.00000000	duck
Aff2	0.72	0.00000000	duck
Thap1	0.31	0.00000000	duck
Pbx1	0.28	0.00000000	duck
Trim23	0.25	0.00000000	duck
Pgs1	0.35	0.00000000	duck
Rcc1l	0.36	0.00000000	duck
Tex264	0.29	0.00000000	duck
Cnih1	0.33	0.00000000	duck
Lpin2	0.21	0.00000000	duck
DPCD	0.35	0.00000000	duck
Cct4	0.30	0.00000000	duck
Atp6v1d	0.35	0.00000000	duck
Nfkb2	0.38	0.00000000	duck
Erlin2	0.28	0.00000000	duck
Slc35b1	0.31	0.00000000	duck
Arpc5	0.35	0.00000000	duck
Slc44a2	0.36	0.00000000	duck
Eif3b	0.34	0.00000000	duck
Cdc123	0.35	0.00000000	duck
Txnl1	0.21	0.00000000	duck
Chmp1a	0.36	0.00000000	duck
Kidins220	0.35	0.00000000	duck
Ap3m2	0.36	0.00000000	duck
Tbl1xr1	0.30	0.00000000	duck
Efhd2	0.38	0.00000000	duck

Rpf2	0.23	0.00000000	duck
Klhl21	0.32	0.00000000	duck
Hebp1	0.26	0.00000000	duck
Tbc1d2	0.34	0.00000000	duck
Rbm17	0.27	0.00000000	duck
Nkrf	0.31	0.00000000	duck
Extl3	0.37	0.00000000	duck
Ncln	0.34	0.00000000	duck
Lrp8	0.08	0.00000000	duck
Stxbp1	0.34	0.00000000	duck
Kpna4	0.26	0.00000000	duck
Ubn1	0.27	0.00000000	duck
Rab2a	0.35	0.00000000	duck
Tdrd3	0.18	0.00000000	duck
Prkar1b	0.38	0.00000000	duck
Klhl24	0.24	0.00000000	duck
Celf1	0.20	0.00000000	duck
Cpeb4	0.17	0.00000000	duck
Bhlhe40	0.50	0.00000000	duck
Psmf1	0.28	0.00000000	duck
Vta1	0.28	0.00000000	duck
Ddx46	0.22	0.00000000	duck
Sptan1	-1.23	0.00000000	duck
Slc7a5	0.40	0.00000000	duck
Cryl1	0.27	0.00000000	duck
Ufm1	0.25	0.00000000	duck
Fam8a1	0.22	0.00000000	duck
Exoc5	0.32	0.00000000	duck
Wdr91	0.35	0.00000000	duck
Ripk1	0.31	0.00000000	duck
Rab11fip1	0.26	0.00000000	duck
Capg	0.38	0.00000000	duck
Fam32a	0.32	0.00000000	duck
Hmces	0.30	0.00000000	duck
Tut7	0.31	0.00000000	duck
Slc7a6	0.13	0.00000000	duck
Uri1	0.22	0.00000000	duck
Suclg2	0.32	0.00000000	duck
Yeats4	0.34	0.00000000	duck
Rnf115	0.34	0.00000000	duck
Faf2	0.31	0.00000000	duck
Ube2a	0.28	0.00000000	duck
Hecw2	-1.08	0.00000000	duck
Tmx1	0.28	0.00000000	duck
Aftph	0.27	0.00000000	duck
Gzf1	0.33	0.00000000	duck

Chd9	0.30	0.00000000	duck
Txndc17	0.31	0.00000000	duck
Guk1	0.29	0.00000000	duck
Snrpd1	0.31	0.00000000	duck
Kcnab2	0.33	0.00000000	duck
Srsf4	0.28	0.00000000	duck
Nip7	0.29	0.00000000	duck
Git2	0.29	0.00000000	duck
Cdkn1a	0.43	0.00000000	duck
Fam20c	0.39	0.00000000	duck
Gpatch4	0.30	0.00000000	duck
Polr2f	0.34	0.00000000	duck
Pcgf5	0.40	0.00000000	duck
Hnrnpk	0.35	0.00000000	duck
Cubn	0.31	0.00000000	duck
Papln	0.33	0.00000000	duck
Tbc1d9b	0.26	0.00000000	duck
Bmpr2	-1.09	0.00000000	duck
Azin1	0.36	0.00000000	duck
Laptm4a	-0.35	0.00000000	duck
Pik3cb	0.31	0.00000000	duck
Gosr1	0.27	0.00000000	duck
Lbh	0.39	0.00000000	duck
Snw1	0.27	0.00000000	duck
Cenpu	0.35	0.00000000	duck
Asap1	0.39	0.00000000	duck
Zfand6	0.09	0.00000000	duck
Serac1	0.42	0.00000000	duck
Tmem248	0.27	0.00000000	duck
Abcb7	0.26	0.00000000	duck
Acadl	0.27	0.00000000	duck
Trmu	0.33	0.00000000	duck
Cnot1	-0.79	0.00000000	duck
Tacc3	-0.01	0.00000000	duck
Dnajc5	0.28	0.00000000	duck
Tbc1d22a	0.25	0.00000000	duck
Eftud2	0.29	0.00000000	duck
Naa50	0.26	0.00000000	duck
Rspry1	0.26	0.00000000	duck
Ptpn6	0.32	0.00000000	duck
Fbxl20	0.29	0.00000000	duck
Dram2	0.30	0.00000000	duck
Bcat1	0.39	0.00000000	duck
Rnps1	0.29	0.00000000	duck
1110059G10Rik	-0.80	0.00000000	duck
Disc1	0.48	0.00000000	duck

Rrm2b	0.29	0.00000000	duck
Zfyve28	-0.05	0.00000000	duck
Asb3	0.37	0.00000000	duck
Babam2	0.24	0.00000000	duck
Smyd2	0.34	0.00000000	duck
Cd47	0.25	0.00000000	duck
Ccdc77	-0.72	0.00000000	duck
Psmc2	0.25	0.00000000	duck
Upf1	0.26	0.00000000	duck
Bin2	0.33	0.00000000	duck
Ifitm10	0.38	0.00000000	duck
Dntt	0.43	0.00000000	duck
Reps2	-0.99	0.00000000	duck
Smc6	0.33	0.00000000	duck
Tmem50a	0.34	0.00000000	duck
Fbxo8	0.24	0.00000000	duck
Slc38a2	-0.44	0.00000000	duck
Usp34	0.19	0.00000000	duck
Plek	0.22	0.00000000	duck
Rnf13	0.30	0.00000000	duck
Ywhab	0.23	0.00000000	duck
Hspa9	0.32	0.00000000	duck
Sf3b4	0.32	0.00000000	duck
Cnpy3	0.27	0.00000000	duck
Pafah1b1	0.25	0.00000000	duck
Rhot1	0.38	0.00000000	duck
Ppp1r14c	0.45	0.00000000	duck
Ttc1	0.22	0.00000000	duck
Slc43a2	0.32	0.00000000	duck
Pygl	0.33	0.00000000	duck
Ddx60	0.10	0.00000000	duck
Desi1	0.21	0.00000000	duck
Plcb2	-1.23	0.00000000	duck
Nlk	0.22	0.00000000	duck
Smcr8	0.25	0.00000000	duck
Vapa	0.30	0.00000000	duck
Ikbke	0.31	0.00000000	duck
Swi5	0.26	0.00000000	duck
Eif3e	0.18	0.00000000	duck
Galns	0.22	0.00000000	duck
Kat6a	0.29	0.00000000	duck
Derl1	0.26	0.00000000	duck
Pank4	0.27	0.00000000	duck
Ppp6c	0.30	0.00000000	duck
Xpr1	0.20	0.00000000	duck
Ywhae	0.25	0.00000000	duck

Tbc1d14	0.30	0.00000000	duck
Decr2	0.27	0.00000000	duck
Actr3	0.31	0.00000000	duck
Ndufa6	0.27	0.00000000	duck
Atp6v1c2	0.37	0.00000000	duck
Lonrf3	-0.83	0.00000000	duck
Tkt	0.24	0.00000000	duck
Lpcat2b	-0.78	0.00000000	duck
B4galt2	0.29	0.00000000	duck
Sidt2	0.31	0.00000000	duck
Gcc2	0.26	0.00000000	duck
Ostc	0.28	0.00000000	duck
Zfp36l1	-0.48	0.00000000	duck
Srf	0.32	0.00000000	duck
Arpc1b	0.35	0.00000000	duck
Creb3l2	0.26	0.00000000	duck
Arntl2	0.25	0.00000000	duck
Ssr1	0.34	0.00000000	duck
Phc2	0.31	0.00000000	duck
Prelid3b	-0.69	0.00000000	duck
Sod2	0.28	0.00000000	duck
Josd1	0.27	0.00000000	duck
Trim33	0.20	0.00000000	duck
Bsg	-0.69	0.00000000	duck
Vamp4	0.31	0.00000000	duck
Ube2r2	0.19	0.00000000	duck
Brip1	0.33	0.00000000	duck
Hipk3	0.22	0.00000000	duck
Tmem67	0.36	0.00000000	duck
Slc25a3	0.36	0.00000000	duck
Larp1b	-1.26	0.00000000	duck
Lrp12	0.21	0.00000000	duck
Foxn2	0.28	0.00000000	duck
Haus8	0.31	0.00000000	duck
Polr1e	0.23	0.00000000	duck
Ntmt1	0.29	0.00000000	duck
Hes6	0.36	0.00000000	duck
Cry2	0.28	0.00000000	duck
Brd7	0.23	0.00000000	duck
Slc35f5	0.29	0.00000000	duck
Zfp131	0.26	0.00000000	duck
Cacna1i	0.39	0.00000000	duck
Usp4	0.26	0.00000000	duck
Slc38a1	0.23	0.00000000	duck
Tusc2	0.29	0.00000000	duck
Zcchc7	0.14	0.00000000	duck

Pin1	0.28	0.00000000	duck
Smad5	0.28	0.00000000	duck
Snu13	0.29	0.00000000	duck
Exoc2	0.18	0.00000000	duck
Grid1	0.34	0.00000000	duck
Pank2	0.29	0.00000000	duck
Fbxo32	0.31	0.00000000	duck
Usp25	0.19	0.00000000	duck
Ndrg3	-1.09	0.00000000	duck
Epb41	-1.80	0.00000000	duck
Rsrc1	0.36	0.00000000	duck
Ttc33	0.19	0.00000000	duck
Itfg1	-0.86	0.00000000	duck
Sec61a1	0.26	0.00000000	duck
Crkl	0.25	0.00000000	duck
Smad3	0.29	0.00000000	duck
Surf4	0.30	0.00000000	duck
Eea1	0.32	0.00000000	duck
Cactin	0.29	0.00000000	duck
Senp5	0.26	0.00000000	duck
Lsm4	0.28	0.00000000	duck
Sap30	0.27	0.00000000	duck
Dnajc19	0.25	0.00000000	duck
Ppm1k	0.26	0.00000000	duck
Fth1	0.30	0.00000000	duck
Trip11	0.05	0.00000000	duck
Rwdd1	0.31	0.00000000	duck
Tom1	0.30	0.00000000	duck
Eif4e	0.15	0.00000000	duck
Plcb4	0.40	0.00000000	duck
Lrmp	0.29	0.00000000	duck
Dcaf1	-0.80	0.00000000	duck
Itpkb	0.29	0.00000000	duck
Commd9	0.27	0.00000000	duck
Stk11	0.27	0.00000000	duck
Rnf149	0.23	0.00000000	duck
Zbtb7b	0.29	0.00000000	duck
Tnpo1	0.19	0.00000000	duck
Sgms1	0.13	0.00000000	duck
Tank	0.24	0.00000000	duck
Nipsnap1	0.25	0.00000000	duck
Glipr2	-0.02	0.00000000	duck
Cidea	0.35	0.00000000	duck
Veph1	-1.00	0.00000000	duck
Tob2	0.24	0.00000000	duck
Slc2a13	0.71	0.00000000	duck

Zcchc14	0.35	0.00000000	duck
Ube2g2	0.23	0.00000000	duck
Nop14	0.33	0.00000000	duck
Srrm1	0.24	0.00000000	duck
Lclat1	0.15	0.00000000	duck
Wdr45b	0.25	0.00000000	duck
AW554918	0.38	0.00000000	duck
Gars	0.24	0.00000000	duck
Arnt	0.23	0.00000000	duck
Camta1	0.25	0.00000000	duck
Idh1	0.19	0.00000000	duck
Casp8ap2	0.32	0.00000000	duck
Aplp2	0.27	0.00000000	duck
Npc1	0.36	0.00000000	duck
Fyb	0.26	0.00000000	duck
Ip6k2	0.22	0.00000000	duck
Itpr1	0.30	0.00000000	duck
Tor4a	0.17	0.00000000	duck
Pdcl3	0.24	0.00000000	duck
Anapc13	0.27	0.00000000	duck
Smap1	0.28	0.00000000	duck
Rnf128	0.36	0.00000000	duck
Pfn2	0.31	0.00000000	duck
Sema4d	-1.52	0.00000000	duck
Phf5a	0.26	0.00000000	duck
Anxa4	0.23	0.00000000	duck
Stag1	0.16	0.00000000	duck
Nisch	0.15	0.00000000	duck
Coro1c	0.29	0.00000000	duck
Ppp1cc	0.24	0.00000000	duck
Psmg3	0.29	0.00000000	duck
Amd2	0.32	0.00000000	duck
Nsrp1	0.22	0.00000000	duck
Rnf141	0.26	0.00000000	duck
Prrc2c	0.15	0.00000000	duck
Atf2	0.13	0.00000000	duck
Nudt7	0.28	0.00000000	duck
Rab21	0.27	0.00000000	duck
Wsb1	0.22	0.00000000	duck
Vps37c	0.22	0.00000000	duck
Sat1	-0.34	0.00000000	duck
Tmem242	0.25	0.00000000	duck
Smim20	0.26	0.00000000	duck
Cyria	0.10	0.00000000	duck
Ssr3	0.34	0.00000000	duck
Purb	0.31	0.00000000	duck

Relch	0.06	0.00000000	duck
Cog1	0.25	0.00000000	duck
Dennd4c	0.30	0.00000000	duck
Jazf1	0.39	0.00000000	duck
Dnaja1	0.31	0.00000000	duck
Ubfd1	0.25	0.00000000	duck
Hadha	0.24	0.00000000	duck
Skap2	0.20	0.00000000	duck
Scml2	0.32	0.00000000	duck
Cript	0.27	0.00000000	duck
Nyx	-0.78	0.00000000	duck
Trappc4	0.26	0.00000000	duck
Zfat	0.29	0.00000000	duck
Ccni	-0.68	0.00000000	duck
Hscb	0.24	0.00000000	duck
Hnrnpr	0.06	0.00000000	duck
Sap130	0.28	0.00000000	duck
Cdc34	0.29	0.00000000	duck
Kbtbd8	0.21	0.00000000	duck
Kif5c	0.34	0.00000000	duck
Tnrc6b	-1.78	0.00000000	duck
Irak2	-0.49	0.00000000	duck
Hmox2	0.23	0.00000000	duck
Clp1	0.29	0.00000000	duck
Etnk1	0.17	0.00000000	duck
Chchd5	0.22	0.00000000	duck
Gramd1c	0.29	0.00000000	duck
Insig1	0.40	0.00000000	duck
Abraxas1	0.30	0.00000000	duck
Zfp292	0.07	0.00000000	duck
C3	0.25	0.00000000	duck
Xrn2	0.10	0.00000000	duck
Slc7a3	0.32	0.00000000	duck
Kdm1b	0.21	0.00000000	duck
Ppm1d	0.23	0.00000000	duck
Sgpl1	-0.77	0.00000000	duck
Slc25a47	0.30	0.00000000	duck
Paxbp1	0.15	0.00000000	duck
Ctnna1	0.33	0.00000000	duck
Yy2	0.15	0.00000000	duck
Arl1	0.25	0.00000000	duck
Thrap3	0.12	0.00000000	duck
Ambra1	0.17	0.00000000	duck
Letm1	0.21	0.00000000	duck
Pars2	0.24	0.00000000	duck
Grb2	0.28	0.00000000	duck

Ncoa2	0.11	0.00000000	duck
Crem	0.24	0.00000000	duck
Scamp3	0.27	0.00000000	duck
Sirt1	0.11	0.00000000	duck
Chp1	-0.73	0.00000000	duck
Crispld2	0.29	0.00000000	duck
Lgals8	0.16	0.00000000	duck
Pip5k1b	-0.89	0.00000000	duck
Clic4	0.31	0.00000000	duck
Ifih1	0.10	0.00000000	duck
Acsl3	0.31	0.00000000	duck
Trio	0.45	0.00000000	duck
Pigt	0.26	0.00000000	duck
Tln2	0.20	0.00000000	duck
Cdyl2	0.11	0.00000000	duck
Isca2	0.25	0.00000000	duck
Tnfsf11	0.33	0.00000000	duck
Hbs1l	0.06	0.00000000	duck
Tmem123	-1.04	0.00000000	duck
Aig1	0.26	0.00000000	duck
Stim1	0.22	0.00000000	duck
9130409I23Rik	0.25	0.00000000	duck
Prkd3	0.27	0.00000000	duck
Tnfrsf11a	-0.99	0.00000000	duck
Rbl2	0.17	0.00000000	duck
Ddx1	0.14	0.00000000	duck
Chd1	0.19	0.00000000	duck
Ern1	0.31	0.00000000	duck
Ybx1	0.22	0.00000000	duck
Golm2	0.12	0.00000000	duck
Ubqln4	0.23	0.00000000	duck
Sucla2	0.20	0.00000000	duck
Asah1	0.25	0.00000000	duck
Rbm6	-0.79	0.00000000	duck
Smarce1	0.16	0.00000000	duck
Cnot11	0.20	0.00000000	duck
Itgav	0.20	0.00000000	duck
Klhl36	0.29	0.00000000	duck
Gtf2a1	0.10	0.00000000	duck
Ipmk	-1.20	0.00000000	duck
Abhd2	0.21	0.00000000	duck
Emc8	0.26	0.00000000	duck
Hspa4	0.21	0.00000000	duck
Frs2	0.24	0.00000000	duck
Dnajb2	0.24	0.00000000	duck
Rabggtb	0.19	0.00000000	duck

Smarca5	0.20	0.00000000	duck
Phb	0.21	0.00000000	duck
Ldlrad3	-1.18	0.00000000	duck
1700034I23Rik	0.29	0.00000000	duck
Nmt2	0.27	0.00000000	duck
Ctif	0.27	0.00000000	duck
Fer	0.30	0.00000000	duck
Insr	0.21	0.00000000	duck
Hlcs	0.23	0.00000000	duck
Nelfcd	0.24	0.00000000	duck
Arl6ip4	0.24	0.00000000	duck
Gatad2a	0.22	0.00000000	duck
Ctcf	0.16	0.00000000	duck
Fez2	0.20	0.00000000	duck
Tes	0.20	0.00000000	duck
Pgm2	0.26	0.00000000	duck
Wars	0.25	0.00000000	duck
Tcea1	0.22	0.00000000	duck
Zbtb7a	0.20	0.00000000	duck
Cep44	0.21	0.00000000	duck
Gclc	0.24	0.00000000	duck
Dip2b	0.26	0.00000000	duck
Tmem42	0.24	0.00000000	duck
Tomm7	0.29	0.00000000	duck
Dennd5a	-0.60	0.00000000	duck
Kdsr	0.26	0.00000000	duck
Irak4	0.21	0.00000000	duck
Tspan4	0.34	0.00000000	duck
Pld1	0.32	0.00000000	duck
Brms1l	0.17	0.00000000	duck
Ammecr1l	0.25	0.00000000	duck
Adcy9	-1.07	0.00000000	duck
Cct2	0.27	0.00000000	duck
Uchl3	0.23	0.00000000	duck
Dapk1	0.52	0.00000000	duck
Cops4	0.19	0.00000000	duck
Snrnp200	0.20	0.00000000	duck
Pnpla2	0.19	0.00000000	duck
Ddx49	0.23	0.00000000	duck
Paip1	0.17	0.00000000	duck
Prkag1	0.29	0.00000000	duck
Cdkal1	0.25	0.00000000	duck
Disp1	-0.91	0.00000000	duck
Tor2a	0.21	0.00000000	duck
Ikzf2	0.22	0.00000000	duck
Slc35b3	0.25	0.00000000	duck

Tfg	0.22	0.00000000	duck
Adk	0.21	0.00000000	duck
Vps13d	-1.11	0.00000000	duck
Nfyc	0.21	0.00000000	duck
Atmin	0.21	0.00000000	duck
Nkiras2	0.23	0.00000000	duck
Vps35	0.23	0.00000000	duck
Atf1	0.08	0.00000000	duck
Vapb	0.11	0.00000000	duck
Tsc22d2	0.21	0.00000000	duck
Slc25a37	-0.88	0.00000000	duck
Rbis	0.22	0.00000000	duck
Txndc12	0.21	0.00000000	duck
Aco2	0.20	0.00000000	duck
Ap3m1	0.23	0.00000000	duck
Agpat4	0.27	0.00000000	duck
Ppfia1	0.15	0.00000000	duck
Abl1	0.18	0.00000000	duck
Ube2k	0.07	0.00000000	duck
Kdm2a	0.23	0.00000000	duck
Ddost	0.25	0.00000000	duck
Uhmk1	0.20	0.00000000	duck
Psmb7	0.12	0.00000000	duck
Fbxo33	0.20	0.00000000	duck
Kif13b	0.32	0.00000000	duck
Hdac2	0.17	0.00000000	duck
Kdm1a	0.14	0.00000000	duck
Nup54	-0.61	0.00000000	duck
Lrrc8a	0.31	0.00000000	duck
Ate1	0.06	0.00000000	duck
Ncoa6	0.17	0.00000000	duck
Tifa	0.27	0.00000000	duck
Kpnb1	0.19	0.00000000	duck
Igf2bp3	0.25	0.00000000	duck
Ndufs7	0.24	0.00000000	duck
Sypl2	0.24	0.00000000	duck
Spag9	0.23	0.00000000	duck
Tacc1	0.25	0.00000000	duck
Snrpf	0.23	0.00000000	duck
Prpf19	0.22	0.00000000	duck
Angptl2	-0.93	0.00000000	duck
Hpse	-0.62	0.00000000	duck
Ints13	0.19	0.00000000	duck
Kbtbd4	0.24	0.00000000	duck
Arglu1	0.03	0.00000000	duck
Cpsf2	0.19	0.00000000	duck

Ino80d	0.17	0.00000000	duck
Phlpp2	0.14	0.00000000	duck
Stim2	0.42	0.00000000	duck
Lzic	0.18	0.00000000	duck
Ddx23	0.21	0.00000000	duck
Unk	0.29	0.00000000	duck
Sec23b	0.21	0.00000000	duck
Rnf139	0.22	0.00000000	duck
Nufip1	0.23	0.00000000	duck
Tubb4b	0.20	0.00000000	duck
Cramp1	0.16	0.00000000	duck
Rab11fip4	0.24	0.00000000	duck
Slc4a9	-0.82	0.00000000	duck
Tet1	-1.17	0.00000000	duck
Rap2a	0.23	0.00000000	duck
Tlnrd1	0.28	0.00000000	duck
Psma6	0.27	0.00000000	duck
Syne3	0.35	0.00000000	duck
9930021J03Rik	0.11	0.00000000	duck
Scaf4	0.11	0.00000000	duck
Fam114a2	0.22	0.00000000	duck
Slc35g1	0.28	0.00000000	duck
Nap1l4	0.16	0.00000000	duck
Srpk1	0.11	0.00000000	duck
Trappc2l	0.20	0.00000000	duck
Ywhag	0.24	0.00000000	duck
Pepd	0.44	0.00000000	duck
Nlrc5	0.09	0.00000000	duck
Tmem138	0.21	0.00000000	duck
Pdcd6	0.24	0.00000000	duck
Arrdc3	-0.82	0.00000000	duck
Bola3	0.22	0.00000000	duck
Ppp2r5a	0.10	0.00000000	duck
B3gnt4	0.26	0.00000000	duck
Cdk12	0.22	0.00000000	duck
Zpr1	0.19	0.00000000	duck
Jade2	-0.80	0.00000000	duck
Whamm	0.22	0.00000000	duck
Flnb	0.24	0.00000000	duck
Gramd2	0.23	0.00000000	duck
Cops8	0.18	0.00000000	duck
Pcif1	0.18	0.00000000	duck
Srebf2	0.24	0.00000000	duck
Dcaf5	0.12	0.00000000	duck
Abca4	0.29	0.00000000	duck
Hnrnpab	0.07	0.00000000	duck

Adora2b	0.31	0.00000000	duck
Btbd1	0.21	0.00000000	duck
Tanc1	0.23	0.00000000	duck
Adrm1	0.16	0.00000000	duck
Vps29	0.20	0.00000000	duck
Impa1	0.20	0.00000000	duck
Snrnp40	0.21	0.00000000	duck
Vps51	0.23	0.00000000	duck
Nr4a2	0.37	0.00000000	duck
Ptpn11	0.15	0.00000000	duck
Exoc6	0.23	0.00000000	duck
Zcchc17	0.24	0.00000000	duck
Ptx3	-1.06	0.00000000	duck
Wipi2	0.18	0.00000000	duck
Gyg	0.20	0.00000000	duck
Ubl3	0.23	0.00000000	duck
Gpatch11	0.18	0.00000000	duck
Isca1	0.15	0.00000000	duck
Pmvk	0.18	0.00000000	duck
Septin9	0.22	0.00000000	duck
Tmem70	0.21	0.00000000	duck
Prune1	0.20	0.00000000	duck
Cdc40	0.09	0.00000000	duck
Clint1	0.08	0.00000000	duck
Sptlc1	0.22	0.00000000	duck
Sirt5	0.21	0.00000000	duck
Uba3	0.19	0.00000000	duck
Naa30	0.09	0.00000000	duck
Ranbp3	0.07	0.00000000	duck
Pafah1b2	0.21	0.00000000	duck
Phc1	0.25	0.00000000	duck
Lman1	0.21	0.00000000	duck
Gpbp1	0.16	0.00000000	duck
Sh3gl3	0.30	0.00000000	duck
Tmbim6	0.25	0.00000000	duck
Galk1	0.22	0.00000000	duck
Aldoc	0.27	0.00000000	duck
Fhod1	0.24	0.00000000	duck
Nr1h3	0.16	0.00000000	duck
Kpna3	0.20	0.00000000	duck
Tnks2	0.13	0.00000000	duck
Rpia	0.17	0.00000000	duck
Pdap1	0.16	0.00000000	duck
Hipk2	0.33	0.00000000	duck
Anapc2	0.24	0.00000000	duck
Edc3	0.17	0.00000000	duck

Prkrip1	0.20	0.00000000	duck
Pcna	0.22	0.00000000	duck
Rel	0.28	0.00000000	duck
Clcc1	0.19	0.00000000	duck
Rtraf	0.16	0.00000000	duck
Zrsr1	0.18	0.00000000	duck
Ptpa	0.22	0.00000000	duck
Sel1l	0.03	0.00000000	duck
4933400A11Rik	0.22	0.00000000	duck
Ankrd13c	0.16	0.00000000	duck
Rnf10	0.14	0.00000000	duck
Kdm4b	0.15	0.00000000	duck
Cnot7	0.13	0.00000000	duck
Rnf41	0.20	0.00000000	duck
St7l	0.16	0.00000000	duck
Armc1	0.01	0.00000000	duck
Phf3	-0.05	0.00000000	duck
Tle3	0.25	0.00000000	duck
Oga	0.08	0.00000000	duck
Taok1	0.11	0.00000000	duck
Tia1	0.19	0.00000000	duck
Nr3c1	0.03	0.00000000	duck
Cwc25	0.18	0.00000000	duck
Cldn12	-0.51	0.00000000	duck
Cep85l	-1.01	0.00000000	duck
Smarcc1	0.16	0.00000000	duck
Neto2	0.32	0.00000000	duck
Cnot6	0.14	0.00000000	duck
Rhbdf2	0.16	0.00000000	duck
Bcl2l11	0.21	0.00000000	duck
Ccdc88a	0.05	0.00000000	duck
Eif2s1	0.16	0.00000000	duck
Smg1	0.03	0.00000000	duck
Copb1	0.13	0.00000000	duck
Garre1	0.25	0.00000000	duck
Tet2	0.19	0.00000000	duck
Ywhaz	0.18	0.00000000	duck
Kdm3b	-0.01	0.00000000	duck
Azi2	-0.11	0.00000000	duck
Ccdc134	0.21	0.00000000	duck
Nrip1	-0.62	0.00000000	duck
Tmem39a	0.19	0.00000000	duck
Kras	0.19	0.00000000	duck
Atp9b	0.17	0.00000000	duck
Slc38a10	0.19	0.00000000	duck
Themis2	0.19	0.00000000	duck

Hadh	0.17	0.00000000	duck
Arfgap2	0.19	0.00000000	duck
Atp2a3	0.22	0.00000000	duck
Rdx	0.06	0.00000000	duck
Rit1	0.11	0.00000000	duck
Nde1	0.16	0.00000000	duck
Rbbp4	0.20	0.00000000	duck
Tm9sf2	0.12	0.00000000	duck
Rbks	0.19	0.00000000	duck
Itpr2	0.23	0.00000000	duck
Taf15	0.20	0.00000000	duck
Usp20	0.19	0.00000000	duck
Cops3	0.11	0.00000000	duck
Ogfrl1	-0.84	0.00000000	duck
Sec11c	0.20	0.00000000	duck
Rdm1	-0.46	0.00000000	duck
Taf10	0.18	0.00000000	duck
Ccny	-0.27	0.00000000	duck
Taar1	0.29	0.00000000	duck
Acad9	0.16	0.00000000	duck
Rev1	0.06	0.00000000	duck
Nfyb	0.17	0.00000000	duck
Jade1	-0.75	0.00000000	duck
Odf2	0.21	0.00000000	duck
Atp13a1	0.17	0.00000000	duck
Tk2	0.17	0.00000000	duck
Siah1b	0.03	0.00000000	duck
Nt5dc2	-0.59	0.00000000	duck
Kmt5b	0.13	0.00000000	duck
Pde6d	0.15	0.00000000	duck
Dhx38	0.17	0.00000000	duck
Vps37a	0.13	0.00000000	duck
Srpr	0.16	0.00000000	duck
Larp4	0.12	0.00000000	duck
Polr2c	0.17	0.00000000	duck
Atad2b	0.16	0.00000000	duck
Creb5	0.47	0.00000000	duck
Dlat	0.17	0.00000000	duck
Pi4ka	0.14	0.00000000	duck
Ppil3	0.14	0.00000000	duck
Ptp4a2	0.17	0.00000000	duck
Gpr75	0.24	0.00000000	duck
Trp53inp2	0.20	0.00000000	duck
Actr6	0.18	0.00000000	duck
Rbm34	0.13	0.00000000	duck
Zfyve26	0.19	0.00000000	duck

Hint1	0.17	0.00000000	duck
Ggps1	0.19	0.00000000	duck
Dbi	0.20	0.00000000	duck
Heph	0.13	0.00000000	duck
Adm	-0.02	0.00000000	duck
Gnl2	0.16	0.00000000	duck
Rngtt	0.28	0.00000000	duck
Atg13	0.18	0.00000000	duck
Cept1	0.17	0.00000000	duck
Wbp1l	0.15	0.00000000	duck
Srgn	0.21	0.00000000	duck
Emc2	0.12	0.00000000	duck
Spcs2	0.18	0.00000000	duck
Copb2	0.18	0.00000000	duck
Ccnyl1	0.19	0.00000000	duck
Dcun1d1	0.16	0.00000000	duck
Nol9	0.15	0.00000000	duck
Pwwp2a	0.20	0.00000000	duck
Hnrnpm	0.14	0.00000000	duck
Trappc8	0.07	0.00000000	duck
Ssbp2	-1.05	0.00000000	duck
Ap3s1	0.14	0.00000000	duck
Ilf2	0.13	0.00000000	duck
Emb	0.21	0.00000000	duck
D5Ertd579e	-0.99	0.00000000	duck
Smg7	0.12	0.00000000	duck
Ino80	0.15	0.00000000	duck
Yipf4	0.06	0.00000000	duck
Arpp19	0.11	0.00000000	duck
Atp5c1	0.20	0.00000000	duck
Timp2	0.17	0.00000000	duck
Lactb	0.15	0.00000000	duck
Cluh	0.14	0.00000000	duck
Zbtb8os	0.22	0.00000000	duck
Capn7	-0.88	0.00000000	duck
A4galt	0.23	0.00000000	duck
Cars	0.19	0.00000000	duck
Slc30a5	0.17	0.00000000	duck
Ncstn	0.19	0.00000000	duck
Hdac7	0.25	0.00000000	duck
Dnajc1	0.09	0.00000000	duck
Fam193a	0.12	0.00000000	duck
Ppp1r8	0.14	0.00000000	duck
Lrif1	0.14	0.00000000	duck
Ank1	-0.79	0.00000000	duck
Zfyve9	0.23	0.00000000	duck

Exoc1	0.19	0.00000000	duck
Dynlt3	0.13	0.00000000	duck
Parp11	0.22	0.00000000	duck
Ptafr	0.23	0.00000000	duck
Klf11	0.20	0.00000000	duck
Ube3b	0.18	0.00000000	duck
Supt7l	0.16	0.00000000	duck
Fnbp1l	0.24	0.00000000	duck
Sar1a	-0.55	0.00000000	duck
Trim8	0.24	0.00000000	duck
Anapc1	0.14	0.00000000	duck
Amacr	0.18	0.00000000	duck
Ist1	0.19	0.00000000	duck
Klf6	0.22	0.00000000	duck
Tbc1d5	0.15	0.00000000	duck
Sla2	-0.53	0.00000000	duck
Thoc2	0.07	0.00000000	duck
Wdr53	0.17	0.00000000	duck
Lmbrd1	0.22	0.00000000	duck
Dvl1	0.22	0.00000000	duck
Etv3	0.15	0.00000000	duck
Zfp598	0.15	0.00000000	duck
Uba2	0.07	0.00000000	duck
Sdf4	0.19	0.00000000	duck
E2f2	0.18	0.00000000	duck
Tasor	0.09	0.00000000	duck
Brox	0.15	0.00000000	duck
Ampd3	0.11	0.00000000	duck
Fn1	-0.60	0.00000000	duck
Pik3cg	0.15	0.00000000	duck
Thyn1	0.20	0.00000000	duck
Ubxn2a	0.16	0.00000000	duck
AU040320	0.17	0.00000000	duck
Get4	0.14	0.00000000	duck
Cul4b	0.10	0.00000000	duck
Suds3	0.11	0.00000000	duck
Cab39	0.16	0.00000000	duck
Bcl7b	0.13	0.00000000	duck
Dtnbp1	0.07	0.00000000	duck
Chpf2	0.20	0.00000000	duck
Washc5	0.19	0.00000000	duck
Thoc5	0.17	0.00000000	duck
Ss18	0.08	0.00000000	duck
Shisa5	0.12	0.00000000	duck
Tfpi2	0.29	0.00000000	duck
Vti1b	0.13	0.00000000	duck

Zfp36l2	0.23	0.00000000	duck
Ndufs6	0.18	0.00000000	duck
Fut8	0.10	0.00000000	duck
Rsf1	-0.04	0.00000000	duck
Tmem231	0.18	0.00000000	duck
Tax1bp3	0.15	0.00000000	duck
Usf3	0.15	0.00000000	duck
Hp1bp3	0.15	0.00000000	duck
Bcl2	0.30	0.00000000	duck
Golga4	-0.13	0.00000000	duck
Herpud2	0.12	0.00000000	duck
Hdac4	0.18	0.00000000	duck
Acbd6	0.10	0.00000000	duck
Nfya	0.15	0.00000000	duck
Zfp148	0.01	0.00000000	duck
Tshz2	0.23	0.00000000	duck
Adipor1	0.16	0.00000000	duck
Fam78a	0.14	0.00000000	duck
Ccdc47	0.13	0.00000000	duck
Tmem41b	0.09	0.00000000	duck
Nqo2	0.21	0.00000000	duck
Akap10	0.08	0.00000000	duck
Rnaseh2b	0.14	0.00000000	duck
Ints2	0.16	0.00000000	duck
Spg7	0.11	0.00000000	duck
Yars	0.21	0.00000000	duck
Tmem222	0.20	0.00000000	duck
Igsf9	0.26	0.00000000	duck
Irf2	0.03	0.00000000	duck
Fam13b	0.16	0.00000000	duck
Tmem11	0.15	0.00000000	duck
Slu7	0.06	0.00000000	duck
Ahcyl2	0.18	0.00000000	duck
Aqr	0.05	0.00000000	duck
Senp7	0.02	0.00000000	duck
Chic2	0.07	0.00000000	duck
Ubap2l	0.13	0.00000000	duck
Ddx17	-0.61	0.00000000	duck
Ric1	0.12	0.00000000	duck
Ing3	0.05	0.00000000	duck
Tpp2	0.15	0.00000000	duck
Ccnt1	0.25	0.00000000	duck
Hsd17b4	0.09	0.00000000	duck
Fyttd1	0.10	0.00000000	duck
Camk1d	-1.21	0.00000000	duck
Ugt8a	-0.71	0.00000000	duck

Dcps	0.16	0.00000000	duck
Srsf11	0.08	0.00000000	duck
Enpp3	0.16	0.00000000	duck
Dapk2	0.03	0.00000000	duck
Tm9sf4	0.13	0.00000000	duck
Ifnar1	-0.86	0.00000000	duck
Ppp1r3c	0.20	0.00000000	duck
Ivd	0.14	0.00000000	duck
Srp14	0.12	0.00000000	duck
Pi4kb	0.12	0.00000000	duck
Arf4	0.16	0.00000000	duck
Taf2	0.15	0.00000000	duck
Derl2	0.18	0.00000000	duck
Reep2	0.19	0.00000000	duck
Atl2	0.04	0.00000000	duck
Arid1a	0.12	0.00000000	duck
Slc9a1	0.22	0.00000000	duck
Spg11	0.16	0.00000000	duck
Tsr3	0.12	0.00000000	duck
Impact	0.22	0.00000000	duck
Snx13	0.13	0.00000000	duck
Tom1l2	0.06	0.00000000	duck
Rgs9	-0.74	0.00000000	duck
Adap1	0.13	0.00000000	duck
Cdkn1b	-0.83	0.00000000	duck
Zranb1	0.06	0.00000000	duck
Caml	0.18	0.00000000	duck
Rgs19	0.20	0.00000000	duck
Sertad2	-0.72	0.00000000	duck
Pola1	0.33	0.00000000	duck
Tent5a	0.28	0.00000000	duck
Pmm2	0.14	0.00000000	duck
Smu1	0.21	0.00000000	duck
Pspc1	-0.04	0.00000000	duck
Limk2	-0.65	0.00000000	duck
Dstn	-0.24	0.00000000	duck
R3hdm1	0.04	0.00000000	duck
Zfr	0.03	0.00000000	duck
Ndfip2	0.02	0.00000000	duck
Nras	0.11	0.00000000	duck
Orai1	0.15	0.00000000	duck
Specc1	0.20	0.00000000	duck
Sort1	0.16	0.00000000	duck
Ep300	0.07	0.00000000	duck
Rpf1	0.10	0.00000000	duck
Api5	0.08	0.00000000	duck

Stard4	0.19	0.00000000	duck
Cstf3	-0.02	0.00000000	duck
Itch	-0.10	0.00000000	duck
Esco1	-0.06	0.00000000	duck
Bsdc1	0.14	0.00000000	duck
Scp2	0.11	0.00000000	duck
Tmem165	0.15	0.00000000	duck
Ankrd11	0.16	0.00000000	duck
Nek7	0.04	0.00000000	duck
Snx1	0.12	0.00000000	duck
Strn3	0.05	0.00000000	duck
Sf3a3	0.12	0.00000000	duck
Prpf8	0.14	0.00000000	duck
Ythdf1	0.09	0.00000000	duck
Hsp90b1	0.11	0.00000000	duck
Glrx5	0.02	0.00000000	duck
Swap70	0.09	0.00000000	duck
Arhgap10	-0.77	0.00000000	duck
Pex13	0.11	0.00000000	duck
Usp24	0.05	0.00000000	duck
Cmtm3	0.10	0.00000000	duck
2510039O18Rik	0.11	0.00000000	duck
Ppp3cb	0.00	0.00000000	duck
Gtf2h1	0.02	0.00000000	duck
Pqlc3	0.14	0.00000000	duck
Slc26a5	0.20	0.00000000	duck
Nup50	0.07	0.00000000	duck
Ppp2r5e	-0.12	0.00000000	duck
Fbxw7	0.15	0.00000000	duck
Ap1m1	0.10	0.00000000	duck
Rmdn3	0.14	0.00000000	duck
Tomm20	0.09	0.00000000	duck
Akt1	0.15	0.00000000	duck
Rprd1a	0.04	0.00000000	duck
Emc6	0.13	0.00000000	duck
Traf6	0.14	0.00000000	duck
Furin	0.12	0.00000000	duck
Gpatch2	0.31	0.00000000	duck
Hlf	0.27	0.00000000	duck
Crebl2	0.08	0.00000000	duck
Pdcl	0.04	0.00000000	duck
Slc4a3	0.19	0.00000000	duck
Echdc2	0.16	0.00000000	duck
Nup214	0.15	0.00000000	duck
Lcor	0.18	0.00000000	duck
Nub1	0.01	0.00000000	duck

Sri	0.18	0.00000000	duck
Faf1	0.03	0.00000000	duck
Cmtr1	0.04	0.00000000	duck
Zhx1	0.09	0.00000000	duck
Rala	0.10	0.00000000	duck
Xpa	0.10	0.00000000	duck
Ginm1	-0.43	0.00000000	duck
Commd2	0.09	0.00000000	duck
Shld1	0.14	0.00000000	duck
Smndc1	0.06	0.00000000	duck
Psme3ip1	0.15	0.00000000	duck
Abhd13	0.11	0.00000000	duck
Nelfa	0.03	0.00000000	duck
Suox	0.07	0.00000000	duck
Csf3r	-0.33	0.00000000	duck
Rac1	0.11	0.00000000	duck
Aprt	0.16	0.00000000	duck
Clk4	0.04	0.00000000	duck
Dph3	0.16	0.00000000	duck
Gnptg	0.13	0.00000000	duck
Dtx4	0.15	0.00000000	duck
Ikbkb	0.17	0.00000000	duck
Npepl1	0.11	0.00000000	duck
Vps72	0.13	0.00000000	duck
Nfe2l1	0.13	0.00000000	duck
Ube4a	0.08	0.00000000	duck
Hormad1	-0.61	0.00000000	duck
Kansl1	0.00	0.00000000	duck
Ssh2	-0.48	0.00000000	duck
Usp38	0.06	0.00000000	duck
Cct3	0.10	0.00000000	duck
Copg2	0.04	0.00000000	duck
Cacna1c	-0.87	0.00000000	duck
Cdk9	0.15	0.00000000	duck
Uhrf1bp1l	0.17	0.00000000	duck
Trappc9	0.22	0.00000000	duck
Xpot	0.05	0.00000000	duck
Rbm26	-0.12	0.00000000	duck
Cotl1	-0.51	0.00000000	duck
Hexb	0.15	0.00000000	duck
Nat10	0.20	0.00000000	duck
Pard3b	-0.81	0.00000000	duck
Hbp1	0.05	0.00000000	duck
Nfx1	0.13	0.00000000	duck
Nsmce2	0.04	0.00000000	duck
Fubp1	0.09	0.00000000	duck

Atxn7	0.02	0.00000000	duck
Svil	-0.68	0.00000000	duck
Fgd3	0.08	0.00000000	duck
Sec31a	0.15	0.00000000	duck
Abhd6	0.19	0.00000000	duck
Pcyt2	0.08	0.00000000	duck
Tpd52l2	0.12	0.00000000	duck
Chchd3	-0.08	0.00000000	duck
Nfkb1	-0.30	0.00000000	duck
Fam210b	0.19	0.00000000	duck
Rnmt	0.08	0.00000000	duck
Kbtbd2	0.08	0.00000000	duck
Gxylt1	0.11	0.00000000	duck
Add3	-0.72	0.00000000	duck
Slc39a13	-0.60	0.00000000	duck
Herpud1	0.18	0.00000000	duck
Atxn1l	0.14	0.00000000	duck
Oard1	0.00	0.00000000	duck
Slc44a1	0.18	0.00000000	duck
Fem1b	0.10	0.00000000	duck
Pinx1	0.04	0.00000000	duck
Zcchc2	-0.04	0.00000000	duck
Entpd1	0.13	0.00000000	duck
Strip1	0.15	0.00000000	duck
Braf	-0.02	0.00000000	duck
Psd	0.18	0.00000000	duck
Ska2	0.07	0.00000000	duck
Slc22a23	0.13	0.00000000	duck
Golph3l	0.19	0.00000000	duck
Drg1	0.13	0.00000000	duck
Spast	-0.03	0.00000000	duck
B4galt5	-0.62	0.00000000	duck
Fbxo22	0.09	0.00000000	duck
Plcl2	0.00	0.00000000	duck
Hras	0.19	0.00000000	duck
Prdm15	0.14	0.00000000	duck
Zfyve27	0.14	0.00000000	duck
Gabpa	0.07	0.00000000	duck
B4galnt3	-0.50	0.00000000	duck
Kif15	0.26	0.00000000	duck
Smc3	0.10	0.00000000	duck
Sesn1	0.11	0.00000000	duck
Gpr160	0.13	0.00000000	duck
Timp4	0.20	0.00000000	duck
Tsc1	-0.16	0.00000000	duck
Plekhm3	0.16	0.00000000	duck

Sar1b	0.11	0.00000000	duck
Ahcyl1	0.10	0.00000000	duck
Pelo	0.13	0.00000000	duck
Tspan2	-0.66	0.00000000	duck
Cblb	-0.54	0.00000000	duck
Itprid2	0.15	0.00000000	duck
C1d	0.10	0.00000000	duck
Vps36	0.06	0.00000000	duck
Bnip2	0.04	0.00000000	duck
Yipf5	0.10	0.00000000	duck
Pnisr	-0.08	0.00000000	duck
Plxna2	0.12	0.00000000	duck
Samd4	0.12	0.00000000	duck
Cwc27	-0.04	0.00000000	duck
Sec22b	0.13	0.00000000	duck
Bcl11a	0.21	0.00000000	duck
Trerf1	0.06	0.00000000	duck
Ube2e3	-0.06	0.00000000	duck
Fbxo9	0.06	0.00000000	duck
Klhdc2	0.11	0.00000000	duck
Cfap20	0.10	0.00000000	duck
Per2	-0.06	0.00000000	duck
Ppp2r2a	-0.14	0.00000000	duck
Fbxl17	0.16	0.00000000	duck
Hnrnph3	0.04	0.00000000	duck
Pds5a	0.05	0.00000000	duck
Pop4	0.13	0.00000000	duck
Gtf2a2	0.07	0.00000000	duck
Dars	0.01	0.00000000	duck
Lactb2	0.10	0.00000000	duck
Ikzf1	-1.12	0.00000000	duck
Pdlim7	0.18	0.00000000	duck
Phrf1	0.04	0.00000000	duck
Ibtk	-0.02	0.00000000	duck
Vps4b	0.15	0.00000000	duck
Kat2b	-0.01	0.00000000	duck
Slc25a44	0.13	0.00000000	duck
Ppp1r3d	0.13	0.00000000	duck
Cxcr4	0.59	0.00000000	duck
Hnrnpa3	-0.48	0.00000000	duck
Lin7c	0.07	0.00000000	duck
Ikzf5	0.07	0.00000000	duck
Csrnp1	-0.64	0.00000000	duck
Ralgapa1	-0.06	0.00000000	duck
Pag1	0.05	0.00000000	duck
Naa15	0.05	0.00000000	duck

Gabarapl1	0.08	0.00000000	duck
Dnaja2	0.05	0.00000000	duck
Wdr44	0.02	0.00000000	duck
Adipor2	-0.44	0.00000000	duck
Dhx30	0.15	0.00000000	duck
Cul3	-0.10	0.00000000	duck
Nrros	0.12	0.00000000	duck
Rsrc2	-0.02	0.00000000	duck
Txlna	0.05	0.00000000	duck
Dmap1	0.11	0.00000000	duck
Dcun1d3	-0.08	0.00000000	duck
Tmem30a	0.12	0.00000000	duck
Fbxo28	0.03	0.00000000	duck
Surf6	0.09	0.00000000	duck
Rcor1	0.11	0.00000000	duck
Phf20	-0.18	0.00000000	duck
Notch2	0.15	0.00000000	duck
Rab35	0.05	0.00000000	duck
Klhdc10	0.12	0.00000000	duck
Slc25a25	0.10	0.00000000	duck
Ascc2	0.11	0.00000000	duck
Btg1b	-0.02	0.00000000	duck
Emc7	0.10	0.00000000	duck
Vps13b	-1.07	0.00000000	duck
Ggnbp2	0.05	0.00000000	duck
Wdr33	-0.09	0.00000000	duck
Calm2	-0.54	0.00000000	duck
Septin6	0.08	0.00000000	duck
Ubp1	0.00	0.00000000	duck
Zfp513	0.10	0.00000000	duck
Alg2	0.18	0.00000000	duck
Trmt10c	0.07	0.00000000	duck
Pxn	0.08	0.00000000	duck
Zfp512	0.13	0.00000000	duck
Btrc	-0.07	0.00000000	duck
Btbd10	-0.05	0.00000000	duck
Atxn7l2	-0.50	0.00000000	duck
Exoc7	0.06	0.00000000	duck
Cdk19	-0.10	0.00000000	duck
Fjx1	0.12	0.00000000	duck
Blmh	0.10	0.00000000	duck
Traf3	-0.31	0.00000000	duck
Vdac3	0.04	0.00000000	duck
Xrn1	-0.08	0.00000000	duck
Fmnl1	0.09	0.00000000	duck
Rock1	-0.41	0.00000000	duck

Vcl	0.13	0.00000000	duck
P4ha1	-0.74	0.00000000	duck
Scrib	-0.05	0.00000000	duck
Ttc17	-0.02	0.00000000	duck
Clpx	-0.01	0.00000000	duck
Ets1	0.05	0.00000000	duck
Tmed2	0.15	0.00000000	duck
Nedd9	-0.67	0.00000000	duck
Vezt	0.08	0.00000000	duck
Lin52	0.06	0.00000000	duck
Prpf38a	0.07	0.00000000	duck
Cdca4	0.06	0.00000000	duck
Zfp511	0.11	0.00000000	duck
Sppl2a	0.10	0.00000000	duck
Nsl1	0.12	0.00000000	duck
Cnot8	0.07	0.00000000	duck
Smim19	0.11	0.00000000	duck
Spopl	-0.40	0.00000000	duck
Rbm22	0.10	0.00000000	duck
Pogz	0.06	0.00000000	duck
Sirt7	0.09	0.00000000	duck
Galnt17	0.18	0.00000000	duck
Six4	0.12	0.00000000	duck
Parp4	0.04	0.00000000	duck
Tent4b	-0.03	0.00000000	duck
Rnpepl1	0.14	0.00000000	duck
Dennd11	0.15	0.00000000	duck
Lmnb2	0.08	0.00000000	duck
Itga1	0.06	0.00000000	duck
Golga5	0.05	0.00000000	duck
Dazap1	-0.04	0.00000000	duck
Garem1	-0.08	0.00000000	duck
Elovl5	0.06	0.00000000	duck
Lcp1	-0.16	0.00000000	duck
Fxn	-0.01	0.00000000	duck
Ccdc117	0.00	0.00000000	duck
Otulinl	0.15	0.00000000	duck
Fbxl14	0.13	0.00000000	duck
Rbm25	-0.01	0.00000000	duck
Tab3	0.00	0.00000000	duck
Pole4	0.12	0.00000000	duck
Erlin1	0.01	0.00000000	duck
Noct	0.07	0.00000000	duck
Il17ra	0.04	0.00000000	duck
Pten	-0.03	0.00000000	duck
Ppm1f	0.06	0.00000000	duck

Selenok	0.09	0.00000000	duck
Glyr1	0.03	0.00000000	duck
Adam19	0.08	0.00000000	duck
Gorasp2	0.13	0.00000000	duck
Snx12	0.08	0.00000000	duck
Gcn1	0.08	0.00000000	duck
Zcchc8	0.02	0.00000000	duck
Cux1	0.14	0.00000000	duck
Ap1g1	-0.02	0.00000000	duck
Orc4	0.08	0.00000000	duck
Acot11	0.12	0.00000000	duck
Orc3	-0.67	0.00000000	duck
Hexim1	-0.04	0.00000000	duck
Timp3	0.35	0.00000001	duck
Sh3gl1	0.11	0.00000001	duck
Jak2	-0.88	0.00000001	duck
Ctnnbl1	0.06	0.00000001	duck
Emsy	0.05	0.00000001	duck
Rnh1	0.10	0.00000001	duck
G0s2	0.09	0.00000001	duck
Tmem164	-0.06	0.00000001	duck
Atxn7l1	-0.91	0.00000001	duck
Plaa	0.05	0.00000001	duck
Ap1ar	0.08	0.00000001	duck
Rbpj	-0.02	0.00000001	duck
Tnrc6a	-0.16	0.00000001	duck
Slc25a16	-0.79	0.00000001	duck
Smarcad1	0.15	0.00000001	duck
Eif4e2	0.03	0.00000001	duck
Plrg1	0.06	0.00000001	duck
Osbpl2	0.06	0.00000001	duck
Inpp5d	0.06	0.00000001	duck
Nabp1	0.09	0.00000001	duck
Vps18	-0.01	0.00000001	duck
Vcpkmt	0.11	0.00000002	duck
Orai2	0.05	0.00000002	duck
Rsrp1	-0.59	0.00000002	duck
Acss2	0.10	0.00000002	duck
Nbr1	-0.01	0.00000002	duck
Lmtk2	0.00	0.00000002	duck
Ccdc127	0.08	0.00000002	duck
Rest	-0.05	0.00000002	duck
Smap2	-0.40	0.00000002	duck
Tmem161b	0.05	0.00000002	duck
Efcab14	-0.46	0.00000002	duck
Zfp618	-0.09	0.00000002	duck

Srp9	0.02	0.00000002	duck
Btaf1	-0.08	0.00000003	duck
Tbpl1	0.03	0.00000003	duck
Sh2b2	0.11	0.00000003	duck
Tlk2	0.03	0.00000003	duck
Bin3	0.05	0.00000003	duck
Nkd1	-0.01	0.00000003	duck
Ap2b1	0.04	0.00000003	duck
Nlrx1	0.11	0.00000003	duck
Srsf6	0.05	0.00000003	duck
Nubp1	-0.03	0.00000003	duck
Npl	0.00	0.00000003	duck
Tmc3	-0.55	0.00000004	duck
Ide	-0.02	0.00000004	duck
Wdr20	-0.19	0.00000004	duck
Qrich1	0.06	0.00000004	duck
Ppp4r1	0.08	0.00000004	duck
Stt3b	0.01	0.00000004	duck
Dusp5	0.19	0.00000005	duck
Ddx27	0.04	0.00000005	duck
Nr2c2	-0.15	0.00000005	duck
Scfd1	0.01	0.00000006	duck
Slx4	0.10	0.00000006	duck
Wasf2	0.07	0.00000006	duck
Plekhj1	0.07	0.00000007	duck
Nol11	0.03	0.00000007	duck
Prpf4b	0.04	0.00000007	duck
Cd9	0.16	0.00000007	duck
Csgalnact2	0.01	0.00000008	duck
Hace1	0.11	0.00000008	duck
Klhl5	0.03	0.00000008	duck
Usp22	-0.05	0.00000008	duck
Strada	0.03	0.00000008	duck
Cyfip2	-0.02	0.00000008	duck
Atrx	-0.30	0.00000010	duck
Psmg2	0.08	0.00000011	duck
Ubr2	-0.79	0.00000011	duck
Amfr	0.06	0.00000012	duck
Tpm3-rs7	0.14	0.00000013	duck
Invs	-0.93	0.00000013	duck
Phf6	0.03	0.00000014	duck
Tnfaip3	-0.25	0.00000014	duck
Tmem185a	0.10	0.00000014	duck
Srsf5	-0.04	0.00000015	duck
Rabgap1l	0.04	0.00000016	duck
Foxk2	-0.08	0.00000018	duck

Cul2	-0.05	0.00000018	duck
Clasp2	-0.07	0.00000018	duck
Stx2	0.01	0.00000019	duck
Dctn5	0.08	0.00000021	duck
Cnep1r1	0.03	0.00000021	duck
Cnst	0.14	0.00000022	duck
Bloc1s4	0.03	0.00000022	duck
Ilrun	-0.20	0.00000022	duck
Csnk1g2	0.02	0.00000023	duck
Card9	0.07	0.00000027	duck
Nup210	0.01	0.00000027	duck
Gpr141	-0.84	0.00000029	duck
Fryl	-0.24	0.00000029	duck
Hif1an	0.04	0.00000030	duck
Nr1d2	0.01	0.00000032	duck
Rad54l2	0.06	0.00000032	duck
Slc35a3	0.07	0.00000034	duck
Uso1	-0.06	0.00000034	duck
Ube2q2	-0.55	0.00000034	duck
Stk4	0.02	0.00000036	duck
Hsf5	0.08	0.00000039	duck
Carnmt1	0.05	0.00000041	duck
Haus2	0.04	0.00000050	duck
Cir1	-0.06	0.00000051	duck
Hspa5	0.00	0.00000053	duck
Efl1	0.00	0.00000056	duck
Serbp1	0.00	0.00000058	duck
Rhoa	0.15	0.00000059	duck
Sccpdh	-0.61	0.00000059	duck
Srgap3	-0.62	0.00000065	duck
Btbd7	0.01	0.00000070	duck
Snorc	0.06	0.00000072	duck
Psma4	0.01	0.00000073	duck
Gpn3	0.03	0.00000076	duck
Lsm14a	0.00	0.00000078	duck
Ric8b	0.06	0.00000078	duck
Dpm2	0.09	0.00000082	duck
Rprd1b	-0.03	0.00000083	duck
Tpr	-0.07	0.00000086	duck
Qser1	-0.02	0.00000087	duck
Rap1gds1	0.15	0.00000091	duck
Appbp2	0.01	0.00000091	duck
Usp45	-0.16	0.00000094	duck
Rnf126	0.06	0.00000098	duck
Pdp1	-0.01	0.00000099	duck
Dgkq	0.13	0.00000099	duck

Asb7	0.10	0.00000104	duck
Sdf2l1	0.00	0.00000111	duck
Dennd1b	0.20	0.00000119	duck
Sppl2b	0.04	0.00000121	duck
Dtl	-0.22	0.00000122	duck
Rnd3	0.12	0.00000124	duck
Vangl1	0.07	0.00000131	duck
Gfpt1	-1.04	0.00000132	duck
Btbd2	-0.02	0.00000136	duck
Spty2d1	0.04	0.00000138	duck
Noc2l	0.07	0.00000157	duck
Dpy30	-0.02	0.00000157	duck
Arid5b	-0.30	0.00000163	duck
Tm2d1	0.04	0.00000171	duck
Fgfr1op2	0.00	0.00000173	duck
Ttc19	0.12	0.00000176	duck
Zdhhc7	-0.06	0.00000186	duck
Vps13a	-0.10	0.00000227	duck
Hmgb2	0.06	0.00000260	duck
Akap8l	0.06	0.00000280	duck
Fam114a1	0.03	0.00000294	duck
Eif5	0.07	0.00000312	duck
Ago4	-0.05	0.00000312	duck
Ensa	0.09	0.00000335	duck
Ube2o	-0.03	0.00000335	duck
Uggt1	-0.02	0.00000340	duck
Zswim8	-0.08	0.00000342	duck
Abcc1	-0.97	0.00000348	duck
Galnt1	-0.45	0.00000360	duck
Suco	0.02	0.00000370	duck
Lcmt1	0.08	0.00000396	duck
Dnajb6	-0.31	0.00000398	duck
Pkn2	-0.13	0.00000409	duck
Arih2	0.03	0.00000418	duck
Osbpl11	0.00	0.00000427	duck
Dek	0.05	0.00000460	duck
Bend7	0.09	0.00000462	duck
Gga1	0.05	0.00000476	duck
Gga3	0.02	0.00000478	duck
Blzf1	-0.01	0.00000478	duck
Tnfaip8l1	0.07	0.00000480	duck
Fam120a	-0.17	0.00000489	duck
Acaca	0.06	0.00000526	duck
Hipk1	-0.11	0.00000526	duck
Nuak1	-0.70	0.00000550	duck
Kin	-0.03	0.00000619	duck

Ttc37	0.02	0.00000637	duck
Ankfy1	0.00	0.00000641	duck
Syn2	-0.10	0.00000665	duck
Slf2	0.02	0.00000678	duck
Rai1	0.08	0.00000702	duck
Pcmtd1	-0.34	0.00000776	duck
Plod1	-0.02	0.00000782	duck
Fasn	0.07	0.00000833	duck
Eif2a	-0.01	0.00000860	duck
Dtx2	-0.08	0.00000895	duck
Lrp6	-0.12	0.00000902	duck
Sap30l	-0.01	0.00001000	duck
Ankrd12	-0.59	0.00001021	duck
Rabif	-0.37	0.00001038	duck
Rmc1	-0.05	0.00001043	duck
Tmem184b	-0.66	0.00001103	duck
Itpk1	-0.31	0.00001200	duck
Ppme1	0.03	0.00001208	duck
Cdc27	-0.03	0.00001263	duck
Txlng	-0.07	0.00001303	duck
Ubac2	0.04	0.00001358	duck
Ppil4	0.03	0.00001396	duck
Entpd4	-0.48	0.00001410	duck
Fam107b	-0.16	0.00001453	duck
Eif2ak3	0.03	0.00001577	duck
Cyrib	-0.06	0.00001723	duck
Ube2j2	0.05	0.00001856	duck
Dync1li1	0.02	0.00001924	duck
Gnat2	0.16	0.00001927	duck
Gpr180	0.03	0.00001998	duck
Pcm1	-0.04	0.00002002	duck
Dhx8	0.03	0.00002027	duck
Pxk	-0.68	0.00002521	duck
Gbe1	0.06	0.00002624	duck
B4galt1	-0.03	0.00002864	duck
Prrc1	-0.06	0.00003046	duck
Smchd1	-0.13	0.00003089	duck
Dync1h1	-0.07	0.00003144	duck
Trp53bp2	0.01	0.00003171	duck
Nupl1	0.06	0.00003190	duck
Phf20l1	-0.20	0.00003257	duck
Arid1b	0.03	0.00003277	duck
Slc7a6os	-0.01	0.00003696	duck
Klhl12	-0.57	0.00003976	duck
Srsf10	-0.08	0.00004084	duck
Dcun1d4	-0.05	0.00004150	duck

Stx11	0.04	0.00004206	duck
Tmem251	0.08	0.00004223	duck
Hnrnpu	-0.02	0.00004336	duck
Xpo7	-0.06	0.00004349	duck
Ncoa4	-0.61	0.00004395	duck
Zbtb37	-0.65	0.00004721	duck
Top2b	-0.20	0.00005049	duck
Chrdl1	-0.45	0.00005133	duck
Spen	-0.22	0.00005461	duck
Vps39	-0.01	0.00006307	duck
Skp1	0.03	0.00006507	duck
Plekha7	0.05	0.00006925	duck
Smurf1	0.01	0.00007541	duck
Usp44	-0.46	0.00007593	duck
Unc50	0.01	0.00007748	duck
Tubb1	0.00	0.00008542	duck
Osbpl3	-0.75	0.00009364	duck
Atp2c1	-0.19	0.00009553	duck
Rab11a	-0.52	0.00009765	duck
Tlr4	-0.56	0.00009788	duck
Denr	0.00	0.00009981	duck
Usp15	-0.07	0.00010072	duck
Raf1	-0.07	0.00010428	duck
Atxn2	-0.17	0.00010446	duck
Rdh10	-0.43	0.00010625	duck
Slc41a3	0.00	0.00011085	duck
Atad1	-0.06	0.00011158	duck
Supt6	-0.04	0.00011673	duck
Prmt7	-0.01	0.00011941	duck
Omg	0.04	0.00012230	duck
Nars	-0.02	0.00014116	duck
Ap4s1	0.03	0.00014136	duck
Bnip3l	-0.53	0.00014557	duck
Rap2b	0.02	0.00015545	duck
Plekhh3	0.11	0.00016138	duck
Acot10	-0.10	0.00016761	duck
Vps8	-0.02	0.00017839	duck
Trip12	-0.85	0.00018586	duck
Lrch3	-0.21	0.00018965	duck
Smad4	-0.02	0.00019226	duck
Arid3b	0.01	0.00019344	duck
Ankra2	0.06	0.00019796	duck
Acvr1	0.04	0.00020411	duck
Pdzd8	-0.04	0.00020471	duck
Reep3	0.00	0.00021701	duck
Slc17a5	-0.06	0.00021921	duck

Ciao2a	-0.40	0.00022094	duck
Cpt1a	0.06	0.00022264	duck
Cers2	0.04	0.00022499	duck
Ctnnb1	-0.20	0.00022879	duck
Etf1	0.04	0.00022911	duck
Arhgap29	-0.13	0.00023032	duck
Pde7b	0.09	0.00023405	duck
Leprotl1	0.01	0.00023531	duck
Trafd1	0.00	0.00024778	duck
Suz12	0.02	0.00024905	duck
Ubash3b	0.04	0.00028639	duck
Ranbp9	-0.29	0.00028916	duck
Hmbox1	-0.20	0.00029963	duck
Zbtb41	-0.06	0.00030785	duck
Setd7	-0.06	0.00031004	duck
Crebrf	-0.18	0.00032462	duck
Khdrbs1	-0.05	0.00032489	duck
Bclaf1	-0.12	0.00034327	duck
Vamp3	0.08	0.00035234	duck
Tmem131l	-0.14	0.00037419	duck
Rad52	-0.50	0.00037555	duck
Snx6	-0.02	0.00039311	duck
Ccdc186	-0.17	0.00039818	duck
Cdc73	-0.17	0.00041561	duck
Vash1	-0.68	0.00047249	duck
Arl15	0.00	0.00049610	duck
Cnot10	-0.07	0.00051012	duck
Adcy7	-0.58	0.00051442	duck
Tcea2	0.09	0.00055133	duck
Fli1	-0.08	0.00055330	duck
Rcn2	0.01	0.00056182	duck
Slc4a11	-0.37	0.00056275	duck
Stx17	-0.05	0.00058240	duck
Eloc	-0.02	0.00058697	duck
Arhgef7	0.11	0.00061127	duck
Dync1li2	-0.07	0.00061508	duck
Nucks1	-0.04	0.00061836	duck
Impa2	-0.01	0.00063925	duck
Poldip2	0.03	0.00064390	duck
Cstf1	0.02	0.00064825	duck
Cntrl	-0.01	0.00065023	duck
Chuk	-0.20	0.00068582	duck
Spi1	-0.36	0.00070354	duck
Tesk2	-0.10	0.00070619	duck
Pcnx	-0.09	0.00075785	duck
Parg	-0.13	0.00076656	duck

Pdpk1	-0.12	0.00080234	duck
Ccdc174	0.04	0.00081158	duck
Thumpd3	-0.01	0.00085310	duck
Thada	0.01	0.00086516	duck
Efr3a	-0.19	0.00090759	duck
Nsmce1	-0.06	0.00092103	duck
Ulk1	-0.69	0.00092458	duck
Siva1	-0.37	0.00095808	duck
Ranbp10	-0.05	0.00097979	duck
Stk10	-0.49	0.00098906	duck
Slc9a4	-0.10	0.00104589	duck
Ppig	-0.08	0.00108500	duck
Crlf3	-0.02	0.00123739	duck
Rabgap1	-0.04	0.00131425	duck
Ccnl2	-0.01	0.00138816	duck
Ice2	-0.04	0.00138919	duck
Galk2	-0.08	0.00139137	duck
Nup210l	0.07	0.00148865	duck
Leo1	-0.56	0.00154146	duck
Ipo7	-0.48	0.00159803	duck
Nrbf2	-0.05	0.00162299	duck
Epas1	-0.73	0.00168710	duck
Arfip1	-0.10	0.00170740	duck
Nme7	-0.81	0.00175137	duck
Abce1	-0.04	0.00180851	duck
Gnb1	-0.37	0.00183844	duck
Itgb2l	-0.35	0.00186941	duck
Cul5	-0.14	0.00196380	duck
Tmem268	0.02	0.00198894	duck
Arl4a	-0.02	0.00211921	duck
Clasp1	-0.11	0.00226728	duck
Rab8a	-0.03	0.00236197	duck
Jmjd4	0.01	0.00236704	duck
Clcn3	-0.16	0.00251105	duck
Zfand4	-0.05	0.00263554	duck
Nolc1	0.04	0.00266123	duck
Slc35e1	-0.07	0.00268128	duck
Golga1	-0.09	0.00275418	duck
Apoo	-0.61	0.00287666	duck
Polk	-0.15	0.00288342	duck
Pip4k2a	-0.22	0.00289684	duck
Wwox	0.17	0.00345629	duck
Cd247	0.07	0.00355757	duck
Ccdc126	-0.51	0.00360571	duck
Abcd2	-0.07	0.00363489	duck
Foxo4	-0.07	0.00394241	duck

Rnf185	-0.05	0.00426486	duck
Ptpn4	-0.78	0.00450580	duck
Inpp5k	-0.01	0.00454318	duck
Etv6	-0.15	0.00474172	duck
Carhsp1	-0.03	0.00482115	duck
Fam117a	-0.03	0.00482123	duck
Sltm	0.01	0.00482752	duck
Rasa3	0.01	0.00562297	duck
Cgrrf1	-0.05	0.00585593	duck
Fnbp1	0.06	0.00593049	duck
Shprh	-0.44	0.00598385	duck
Arhgef18	-0.50	0.00645381	duck
Bcl2l13	-0.14	0.00651060	duck
Tmem120a	-0.45	0.00676941	duck
Atp1b1	0.04	0.00694979	duck
Setd3	-0.13	0.00710146	duck
Dhx40	-0.05	0.00716716	duck
Arntl	-0.03	0.00724731	duck
Cdk7	-0.02	0.00732241	duck
Inpp5a	0.06	0.00761383	duck
Crtc1	-0.14	0.00761959	duck
Adnp2	0.00	0.00781315	duck
Nrbp2	-0.14	0.00821939	duck
Cbl	-0.53	0.00858793	duck
Ythdf3	-0.15	0.00894658	duck
Ninj1	-0.39	0.00917057	duck
Nfat5	-0.16	0.00937693	duck
Elf1	-0.04	0.00955033	duck
Kctd20	-0.14	0.00966755	duck
Ctdspl	-0.37	0.01024171	duck
Gtpbp1	0.00	0.01161018	duck
Snx20	-0.54	0.01231106	duck
Pstpip1	-0.35	0.01232038	duck
Cfl2	-0.03	0.01234793	duck
Niban1	-0.31	0.01271422	duck
Ints10	-0.01	0.01289459	duck
Nt5e	-0.06	0.01322488	duck
Tmem59	-0.02	0.01322524	duck
Fam102b	-0.61	0.01388231	duck
Gapvd1	-0.09	0.01501533	duck
Tsc2	-0.03	0.01510208	duck
Lrrk2	0.01	0.01543237	duck
Fam168b	-0.16	0.01602776	duck
Bicral	-0.56	0.01764979	duck
Bicd2	-0.07	0.01790408	duck
Ropn1l	-0.01	0.01861358	duck

Ogt	-0.51	0.01874696	duck
Erh	-0.59	0.01937683	duck
Cerk	-0.09	0.02002419	duck
Gan	-0.01	0.02126358	duck
Rap1b	-0.50	0.02278200	duck
Slc9a8	-0.09	0.02352431	duck
Zbtb38	-0.03	0.02410935	duck
Kpna1	-0.25	0.02521242	duck
Cebpd	-0.21	0.02553621	duck
Ppp6r3	-0.27	0.02566635	duck
Pptc7	-0.06	0.02595101	duck
Tm9sf3	0.08	0.02623000	duck
Nfrkb	-0.04	0.02725103	duck
Ccdc28a	0.00	0.02739833	duck
Dcun1d5	-0.06	0.02839012	duck
Abcd3	-0.10	0.02893405	duck
Pgrmc1	0.02	0.03016537	duck
Srek1ip1	0.04	0.03039578	duck
Sart3	-0.02	0.03128086	duck
Clock	-0.11	0.03191694	duck
Prpf3	-0.03	0.03664881	duck
Abhd5	-0.44	0.03734160	duck
Osbpl1a	-0.77	0.03867731	duck
Scaper	-0.01	0.03893207	duck
Ing4	-0.01	0.03947676	duck
Luc7l3	-0.07	0.04078669	duck
Snx14	-0.12	0.04290122	duck
Naa60	-0.13	0.04545804	duck
Traf7	0.01	0.04578337	duck
Rfx2	-0.70	0.04699723	duck
B3gnt2	-0.13	0.04714746	duck
Ndufs1	-0.03	0.04720783	duck
Ippk	-0.55	0.04790474	duck
Nipal4	-0.33	0.04918217	duck
Hnrnpd	-0.02	0.05105842	duck
Rlf	-0.12	0.05216572	duck
Vezf1	-0.25	0.05641885	duck
Zfhx3	0.03	0.06395954	duck
Kctd7	-0.11	0.06826823	duck
Pim3	0.03	0.06897566	duck
Exosc7	-0.10	0.07424519	duck
Zzef1	-0.17	0.07862041	duck
Ythdc1	-0.15	0.08220831	duck
Cdk8	-0.16	0.08410062	duck
Dyrk1a	-0.52	0.09262338	duck
Pdlim5	0.09	0.09470362	duck

Cap1	-0.28	0.10192856	duck
Fbxo3	-0.79	0.10228511	duck
Trim24	-0.57	0.10272907	duck
Prrc2b	-0.09	0.10675301	duck
Stub1	-0.07	0.10685102	duck
Cyth1	-0.36	0.10731705	duck
Slc30a7	-0.15	0.10906778	duck
Ube3a	-0.20	0.11012729	duck
Rabepk	0.01	0.11217572	duck
Tent2	-0.13	0.11443545	duck
Ubxn2b	-0.13	0.12068942	duck
Tnfsf8	-0.08	0.12079813	duck
Gpatch1	-0.05	0.12253099	duck
Cracr2a	-0.63	0.13074594	duck
Apaf1	-0.07	0.13079583	duck
Ppp3cc	-0.06	0.13567574	duck
Far1	-0.25	0.13710174	duck
Txndc9	-0.13	0.14698643	duck
Chsy1	-0.53	0.16164274	duck
Tmem131	-0.16	0.16217070	duck
Zfp384	-0.16	0.16547393	duck
Dnajb14	-0.04	0.16742830	duck
Adam17	-0.07	0.16932341	duck
Lars	-0.09	0.17038632	duck
Cwf19l1	-0.02	0.17903045	duck
Kat6b	-0.14	0.18382644	duck
Dennd1a	-0.30	0.18825743	duck
Vps26a	-0.06	0.19154489	duck
Rnf146	-0.05	0.19304674	duck
St6gal1	-0.51	0.19366565	duck
Syf2	-0.07	0.19655772	duck
Cops5	-0.13	0.19817096	duck
Bdp1	-0.30	0.19943031	duck
Paip2	-0.04	0.21280031	duck
Eaf1	-0.46	0.22221225	duck
Traf2	0.00	0.22912825	duck
Zfand3	-0.27	0.24090957	duck
Arhgap26	-0.56	0.26533344	duck
Tecpr2	-0.14	0.27315672	duck
Hk2	-0.06	0.27739572	duck
Rassf8	-0.43	0.27946451	duck
Sgpp1	-0.46	0.28902669	duck
Pik3c2a	-0.14	0.29357053	duck
Fbxo34	-0.08	0.30080426	duck
Kdm4c	-0.10	0.31208866	duck
Arhgap17	-0.07	0.34293885	duck

Adamtsl3	-0.02	0.35119738	duck
Zfp207	-0.22	0.37067317	duck
Uxs1	-0.01	0.37730343	duck
Eif4g3	-0.44	0.39509891	duck
Slc4a7	-0.30	0.41171242	duck
Ptges3	-0.21	0.43070368	duck
Gipc2	-0.10	0.43433844	duck
Jak1	0.07	0.45725893	duck
Tmcc1	-0.33	0.47354932	duck
Sugt1	-0.13	0.47485590	duck
Dpp9	-0.05	0.47636607	duck
Crim1	-0.90	0.50392993	duck
Zdhhc3	-0.23	0.51740502	duck
Scd2	-0.15	0.52512054	duck
Ppp6r2	-0.06	0.57118890	duck
Atp5pb	0.00	0.59644999	duck
Ralgapa2	-0.62	0.59997541	duck
Wwp1	-0.18	0.60203577	duck
Pan3	-0.21	0.63038102	duck
Crybg3	-0.32	0.64560964	duck
Rab3il1	0.08	0.64628716	duck
Atp2b1	-0.08	0.65396131	duck
Tab2	-0.27	0.67050693	duck
Ampd2	-0.39	0.72546088	duck
Sin3a	-0.09	0.76911054	duck
Kmt2e	-0.10	0.78081196	duck
Tef	-0.22	0.85823273	duck
Zfp318	-0.13	0.93550385	duck
Ell	-0.31	0.96966141	duck
Lrch1	-0.12	0.98734504	duck
Plod2	-0.12	1.00000000	duck
Jade3	-0.23	1.00000000	duck
Kifbp	-0.07	1.00000000	duck
Hat1	-0.10	1.00000000	duck
Tmem87a	-0.15	1.00000000	duck
Ehmt1	-0.09	1.00000000	duck
Trappc11	-0.16	1.00000000	duck
Dnajc7	-0.12	1.00000000	duck
Bod1l	-0.38	1.00000000	duck
Gna12	-0.18	1.00000000	duck
Fes	0.00	1.00000000	duck
Dnajb4	-0.23	1.00000000	duck
Exd1	-0.34	1.00000000	duck
Supv3l1	0.03	1.00000000	duck
Camkmt	-0.20	1.00000000	duck
Txk	-0.47	1.00000000	duck

Uap1l1	-0.04	1.00000000	duck
Ccnl1	-0.24	1.00000000	duck
Zc3h7b	-0.05	1.00000000	duck
Fndc3a	-0.15	1.00000000	duck
Pnrc2	-0.07	1.00000000	duck
Nvl	-0.35	1.00000000	duck
Dusp1	0.00	1.00000000	duck
Ankrd44	-0.69	1.00000000	duck
Fam53a	-0.28	1.00000000	duck
Arhgap45	-0.34	1.00000000	duck
Zfp644	-0.36	1.00000000	duck
Rreb1	-0.08	1.00000000	duck
Sptlc2	-0.12	1.00000000	duck
Nav1	-0.10	1.00000000	duck
Prkag2	-0.65	1.00000000	duck
Tpra1	-0.12	1.00000000	duck
Fam214b	-0.41	1.00000000	duck
Ttc7	-0.60	1.00000000	duck
Prkar1a	0.03	1.00000000	duck
Reps1	-0.56	1.00000000	duck
Ifngr1	-0.65	1.00000000	duck
Zcchc10	-0.10	1.00000000	duck
Capza2	-0.35	1.00000000	duck
Clptm1l	-0.09	1.00000000	duck
Abraxas2	-0.14	1.00000000	duck
Nf1	-0.16	1.00000000	duck
Evi5	-0.73	1.00000000	duck
Selenoo	-0.54	1.00000000	duck
Pknox1	-0.24	1.00000000	duck
Gak	-0.39	1.00000000	duck
Ctdp1	-0.13	1.00000000	duck
Grk3	-0.16	1.00000000	duck
Rpa3	-0.09	1.00000000	duck
Hspa4l	-0.08	1.00000000	duck
Atg16l1	-0.11	1.00000000	duck
Cep350	-0.46	1.00000000	duck
Gucd1	-0.16	1.00000000	duck
Ggh	-0.15	1.00000000	duck
Ash1l	-0.28	1.00000000	duck
Upp1	-0.53	1.00000000	duck
Eps15l1	-0.10	1.00000000	duck
Arap2	-0.13	1.00000000	duck
Cables2	-0.14	1.00000000	duck
Cdk13	-0.27	1.00000000	duck
Zfp592	-0.12	1.00000000	duck
Fxr1	-0.72	1.00000000	duck

Rab5a	-0.09	1.00000000	duck
Rfwd3	-0.32	1.00000000	duck
Vdr	-0.28	1.00000000	duck
Grk2	-0.05	1.00000000	duck
Slc3a1	-0.09	1.00000000	duck
Atp11b	-0.22	1.00000000	duck
Eif4h	-0.11	1.00000000	duck
Fbxl22	-0.06	1.00000000	duck
Nt5c3	-0.20	1.00000000	duck
Rb1	-0.18	1.00000000	duck
Hspa2	-0.12	1.00000000	duck
Lats1	-0.09	1.00000000	duck
Tmem50b	-0.41	1.00000000	duck
Wac	-0.30	1.00000000	duck
Uhrf2	-0.22	1.00000000	duck
Stam2	-0.19	1.00000000	duck
Rbm5	-0.19	1.00000000	duck
Pcmt1	-0.27	1.00000000	duck
Arsg	-0.47	1.00000000	duck
Plpp1	-0.37	1.00000000	duck
Dnajc16	-0.07	1.00000000	duck
Ptpn2	-0.37	1.00000000	duck
Pak2	-0.22	1.00000000	duck
Stk40	-0.07	1.00000000	duck
Ubac1	-0.11	1.00000000	duck
Ankrd46	-0.37	1.00000000	duck
Phf2	-0.32	1.00000000	duck
Ireb2	-0.18	1.00000000	duck
Sync	-0.35	1.00000000	duck
Nsmaf	-0.19	1.00000000	duck
Pgpep1	-0.11	1.00000000	duck
Tasor2	-0.07	1.00000000	duck
Prepl	-0.09	1.00000000	duck
Arid4b	-0.30	1.00000000	duck
Wiz	-0.16	1.00000000	duck
Phf12	-0.20	1.00000000	duck
Etfrf1	-0.36	1.00000000	duck
Gpr107	-0.12	1.00000000	duck
Ano9	-0.40	1.00000000	duck
Zmym2	-0.20	1.00000000	duck
Osbpl8	-0.20	1.00000000	duck
Rfng	-0.10	1.00000000	duck
Zfp638	-0.28	1.00000000	duck
Gigyf2	-0.16	1.00000000	duck
Clk2	-0.09	1.00000000	duck
Ing1	-0.13	1.00000000	duck

Klf7	-0.09	1.00000000	duck
Kansl1l	-0.35	1.00000000	duck
Chd7	-0.27	1.00000000	duck
Fam241a	-0.05	1.00000000	duck
Gpcpd1	-0.70	1.00000000	duck
Lrba	-0.44	1.00000000	duck
Fam126a	-0.36	1.00000000	duck
Ubox5	-0.06	1.00000000	duck
Agl	-0.23	1.00000000	duck
Borcs5	-0.28	1.00000000	duck
Rnf217	-0.61	1.00000000	duck
Rhof	-0.42	1.00000000	duck
Kdm5b	-0.32	1.00000000	duck
Fst	-0.03	1.00000000	duck
Rc3h2	-0.24	1.00000000	duck
Dok2	-0.09	1.00000000	duck
Epc1	-0.26	1.00000000	duck
Rap2c	-0.23	1.00000000	duck
Parp8	-0.64	1.00000000	duck
Smg5	-0.16	1.00000000	duck
Ppp2r5c	-0.39	1.00000000	duck
Trib1	0.02	1.00000000	duck
Sun1	-0.38	1.00000000	duck
Cpeb2	-0.27	1.00000000	duck
Ipo9	-0.09	1.00000000	duck
Ralgps1	-0.36	1.00000000	duck
Pstpip2	-0.36	1.00000000	duck
Slc45a4	-0.31	1.00000000	duck
Fbxo11	-0.37	1.00000000	duck
Slc40a1	-0.08	1.00000000	duck
Ecpas	-0.17	1.00000000	duck
Primpol	-0.23	1.00000000	duck
Ctbp1	-0.63	1.00000000	duck
Rnf4	-0.17	1.00000000	duck
Adprh	-0.12	1.00000000	duck
Ptpn12	-0.48	1.00000000	duck
Herc2	-0.84	1.00000000	duck
Tapt1	-0.65	1.00000000	duck
Ttc39c	-0.13	1.00000000	duck
Spag1	-0.38	1.00000000	duck
Prdm10	-0.23	1.00000000	duck
P2ry13	-0.26	1.00000000	duck
Zfp516	-0.10	1.00000000	duck
Ago2	-0.66	1.00000000	duck
Capzb	-0.06	1.00000000	duck
Lrrfip2	-0.27	1.00000000	duck

Pacsin2	-0.13	1.00000000	duck
Nrbp1	-0.23	1.00000000	duck
Ube2v2	-0.46	1.00000000	duck
Tbcd	-0.33	1.00000000	duck
Golgb1	-0.20	1.00000000	duck
Anapc7	-0.08	1.00000000	duck
Dock11	-0.54	1.00000000	duck
Tle4	-0.12	1.00000000	duck
Upb1	-0.22	1.00000000	duck
Sec16a	-0.19	1.00000000	duck
Steep1	-0.13	1.00000000	duck
Kctd5	-0.34	1.00000000	duck
Ripor3	-0.85	1.00000000	duck
Ptbp1	-0.17	1.00000000	duck
Eya3	-0.28	1.00000000	duck
Clec16a	-0.17	1.00000000	duck
Cd74	-0.18	1.00000000	duck
Casp9	-0.08	1.00000000	duck
Slk	-0.43	1.00000000	duck
Edrf1	-0.26	1.00000000	duck
Rassf2	-0.19	1.00000000	duck
Gsk3b	-0.35	1.00000000	duck
Elavl1	-0.23	1.00000000	duck
Cass4	-0.17	1.00000000	duck
Ddb1	-0.22	1.00000000	duck
Pank3	-0.68	1.00000000	duck
Sephs1	-0.06	1.00000000	duck
Actn1	-0.01	1.00000000	duck
Plxnc1	-0.26	1.00000000	duck
Pitpna	-0.34	1.00000000	duck
Gtf2f2	-0.22	1.00000000	duck
Ppa2	-0.50	1.00000000	duck
Actr2	0.03	1.00000000	duck
Sgsm2	-0.42	1.00000000	duck
Srp54a	-0.18	1.00000000	duck
Anp32e	-0.19	1.00000000	duck
Narf	-0.26	1.00000000	duck
Zfp438	-0.36	1.00000000	duck
Slc35a1	-0.10	1.00000000	duck
Zfp407	-0.09	1.00000000	duck
Kdm2b	-0.45	1.00000000	duck
Ncf2	-0.42	1.00000000	duck
Pik3r1	-0.26	1.00000000	duck
Arl5b	-0.20	1.00000000	duck
Scube2	-0.29	1.00000000	duck
Ssbp3	-0.47	1.00000000	duck

Arrdc4	-0.42	1.00000000	duck
Pitpnm3	-0.53	1.00000000	duck
Erc1	-0.42	1.00000000	duck
L3mbtl3	-0.18	1.00000000	duck
Serpine2	-0.04	1.00000000	duck
Zbtb20	-0.59	1.00000000	duck
Pikfyve	-0.25	1.00000000	duck
Strn	-0.49	1.00000000	duck
Fam135a	-0.30	1.00000000	duck
Tpm1	-0.31	1.00000000	duck
Usp48	-0.67	1.00000000	duck
Cnot6l	-0.52	1.00000000	duck
Herc1	-0.44	1.00000000	duck
Dusp10	-0.60	1.00000000	duck
Ino80c	-0.20	1.00000000	duck
Ncbp3	-0.14	1.00000000	duck
Ehd3	-0.14	1.00000000	duck
Prkcb	-0.32	1.00000000	duck
Tasp1	-0.37	1.00000000	duck
Phkb	-0.48	1.00000000	duck
Acaa2	-0.50	1.00000000	duck
Ralgapb	-0.26	1.00000000	duck
Dhdds	-0.21	1.00000000	duck
Erbin	-0.20	1.00000000	duck
Prkg2	-0.17	1.00000000	duck
Snap23	-0.34	1.00000000	duck
Nudt3	-0.18	1.00000000	duck
Pnpla8	-0.56	1.00000000	duck
Slc25a24	-0.53	1.00000000	duck
Cebpg	-0.16	1.00000000	duck
Atg5	-0.41	1.00000000	duck
Fbxo38	-0.21	1.00000000	duck
Dhrs3	-0.23	1.00000000	duck
Cct5	-0.15	1.00000000	duck
Rasgrp1	-0.30	1.00000000	duck
Zfp711	-0.29	1.00000000	duck
Snx4	-0.32	1.00000000	duck
Ube2j1	-0.17	1.00000000	duck
Nhlrc2	-0.10	1.00000000	duck
Nfe2l2	-0.20	1.00000000	duck
Pip5k1c	-0.41	1.00000000	duck
Trpm7	-0.33	1.00000000	duck
Acvr1b	-0.13	1.00000000	duck
Rbm12b2	-0.26	1.00000000	duck
Cpeb3	-0.44	1.00000000	duck
Anapc5	-0.24	1.00000000	duck

Poldip3	-0.07	1.00000000	duck
Pdss2	-0.38	1.00000000	duck
Rnf111	-0.32	1.00000000	duck
Adam10	-0.20	1.00000000	duck
Rab19	-0.30	1.00000000	duck
Vcpip1	-0.28	1.00000000	duck
Tra2a	-0.37	1.00000000	duck
Stk3	-0.53	1.00000000	duck
Ebag9	-0.40	1.00000000	duck
Sf3b1	-0.17	1.00000000	duck
Inpp4a	-0.21	1.00000000	duck
Hectd1	-0.03	1.00000000	duck
Ephx1	-0.27	1.00000000	duck
Elk3	-0.40	1.00000000	duck
Capn10	-0.33	1.00000000	duck
Upf2	-0.25	1.00000000	duck
Gid8	-0.21	1.00000000	duck
Dnaja4	-0.23	1.00000000	duck
Stat3	-0.18	1.00000000	duck
Ago3	-0.18	1.00000000	duck
Slc12a4	-0.54	1.00000000	duck
Vti1a	-0.53	1.00000000	duck
Rab11fip2	-0.25	1.00000000	duck
Fosl2	-0.04	1.00000000	duck
Brd3	-0.21	1.00000000	duck
Tra2b	-0.23	1.00000000	duck
Sesn2	-0.13	1.00000000	duck
Ddx5	-0.17	1.00000000	duck
Zfp652	-0.28	1.00000000	duck
Ptp4a1	-0.12	1.00000000	duck
Dym	-0.40	1.00000000	duck
Add1	-0.44	1.00000000	duck
Cbx3	-0.28	1.00000000	duck
Rexo1	-0.32	1.00000000	duck
Rab8b	-0.35	1.00000000	duck
Zbtb34	-0.39	1.00000000	duck
Armh3	-0.34	1.00000000	duck
Eif1	-0.06	1.00000000	duck
Bcl7a	-0.13	1.00000000	duck
Sh3bp2	-0.33	1.00000000	duck
Jun	-0.02	1.00000000	duck
Eif4g2	-0.01	1.00000000	duck
Zeb1	-0.39	1.00000000	duck
Ywhaq	-0.02	1.00000000	duck
Prr5l	-0.35	1.00000000	duck
Creg1	-0.68	1.00000000	duck

Bpnt2	-0.35	1.00000000	duck
Batf	-0.10	1.00000000	duck
Stag2	-0.35	1.00000000	duck
Tob1	-0.36	1.00000000	duck
Jdp2	-0.31	1.00000000	duck
Baz1a	-0.17	1.00000000	duck
Esyt2	-0.44	1.00000000	duck
Vps53	-0.24	1.00000000	duck
Epg5	-0.34	1.00000000	duck
Borcs6	-0.38	1.00000000	duck
Ccdc6	-0.37	1.00000000	duck
Papola	-0.34	1.00000000	duck
Tial1	-0.34	1.00000000	duck
Fam91a1	-0.27	1.00000000	duck
Prkce	-0.40	1.00000000	duck
Yme1l1	-0.38	1.00000000	duck
Scyl2	-0.42	1.00000000	duck
Ero1b	-0.39	1.00000000	duck
Sec13	-0.25	1.00000000	duck
Cacul1	-0.63	1.00000000	duck
Tfdp1	-0.20	1.00000000	duck
Rragd	-0.37	1.00000000	duck
Baz1b	-0.40	1.00000000	duck
Brwd3	-0.50	1.00000000	duck
Rasgef1b	-0.34	1.00000000	duck
Sos2	-0.31	1.00000000	duck
Cltc	-0.21	1.00000000	duck
Rnf145	-0.37	1.00000000	duck
Ehbp1	-0.34	1.00000000	duck
Pfdn1	-0.30	1.00000000	duck
Cdk5rap2	-0.35	1.00000000	duck
Arid2	-0.50	1.00000000	duck
Ubtd2	-0.50	1.00000000	duck
Cops2	-0.30	1.00000000	duck
Ghitm	-0.29	1.00000000	duck
Tiam2	-0.43	1.00000000	duck
Nup107	-0.19	1.00000000	duck
Deaf1	-0.17	1.00000000	duck
Agps	-0.28	1.00000000	duck
Dusp7	-0.09	1.00000000	duck
Phospho1	-0.19	1.00000000	duck
Pik3ca	-0.47	1.00000000	duck
S1pr4	-0.22	1.00000000	duck
Tmem104	-0.36	1.00000000	duck
Qk	-0.37	1.00000000	duck
Anxa6	-0.16	1.00000000	duck

Dnajb12	-0.29	1.00000000	duck
Gnal	-0.42	1.00000000	duck
Wdr1	-0.15	1.00000000	duck
Slc15a4	-0.31	1.00000000	duck
Tln1	-0.12	1.00000000	duck
Slc27a1	-0.16	1.00000000	duck
Ubr3	-0.45	1.00000000	duck
Lpgat1	-0.32	1.00000000	duck
Khdc4	-0.23	1.00000000	duck
Akap8	-0.24	1.00000000	duck
Fam126b	-0.46	1.00000000	duck
St3gal6	-0.43	1.00000000	duck
Dynlt1a	-0.14	1.00000000	duck
Nfia	-0.43	1.00000000	duck
Sh3bp5	-0.38	1.00000000	duck
Arf6	-0.22	1.00000000	duck
Lipc	-0.18	1.00000000	duck
Lfng	-0.34	1.00000000	duck
Stk38	-0.24	1.00000000	duck
Nfkbie	-0.22	1.00000000	duck
Ppp2cb	-0.31	1.00000000	duck
Setd5	-0.33	1.00000000	duck
Setd1b	-0.33	1.00000000	duck
Clk1	-0.40	1.00000000	duck
Ssu72	-0.19	1.00000000	duck
Stk26	-0.20	1.00000000	duck
Wapl	-0.38	1.00000000	duck
Kcnq5	-0.57	1.00000000	duck
Snx18	-0.34	1.00000000	duck
Fbxo46	-0.15	1.00000000	duck
Chfr	-0.34	1.00000000	duck
Slain2	-0.26	1.00000000	duck
Ngly1	-0.31	1.00000000	duck
Cdc14a	-0.72	1.00000000	duck
Tnfrsf21	-0.27	1.00000000	duck
Ankrd17	-0.36	1.00000000	duck
Rere	-0.27	1.00000000	duck
Plec	-0.36	1.00000000	duck
Agfg1	-0.14	1.00000000	duck
Ppm1a	-0.27	1.00000000	duck
Btbd9	-0.54	1.00000000	duck
Arih1	-0.59	1.00000000	duck
Cpne1	-0.45	1.00000000	duck
Ctr9	-0.28	1.00000000	duck
Zyx	-0.24	1.00000000	duck
Tnks	-0.28	1.00000000	duck

Ufd1	-0.26	1.00000000	duck
Ybx3	-0.26	1.00000000	duck
Rab6a	-0.28	1.00000000	duck
Zfp236	-0.41	1.00000000	duck
Gpatch8	-0.42	1.00000000	duck
Pbrm1	-0.41	1.00000000	duck
Tnip1	-0.23	1.00000000	duck
Bcas2	-0.25	1.00000000	duck
Crk	-0.26	1.00000000	duck
Hnrnpa2b1	-0.28	1.00000000	duck
Csde1	-0.28	1.00000000	duck
Fos	0.11	1.00000000	duck
Wbp2	-0.27	1.00000000	duck
Gabpb1	-0.35	1.00000000	duck
Lonrf1	-0.41	1.00000000	duck
Gabra1	-0.40	1.00000000	duck
Slc30a4	-0.29	1.00000000	duck
Cflar	-0.53	1.00000000	duck
Dusp11	-0.25	1.00000000	duck
Zc3h12a	-0.12	1.00000000	duck
Taok3	-0.46	1.00000000	duck
Lrrc1	-0.42	1.00000000	duck
Hs6st1	-0.14	1.00000000	duck
Ptpn9	-0.16	1.00000000	duck
Abtb1	-0.35	1.00000000	duck
Cdc37l1	-0.22	1.00000000	duck
Ahctf1	-0.26	1.00000000	duck
Runx1	-0.23	1.00000000	duck
Eif5a2	-0.35	1.00000000	duck
Auh	-0.45	1.00000000	duck
Ppard	-0.23	1.00000000	duck
Stxbp5	-0.56	1.00000000	duck
Smurf2	-0.50	1.00000000	duck
Rbm39	-0.07	1.00000000	duck
Ppp1r3b	-0.23	1.00000000	duck
Adora2a	-0.41	1.00000000	duck
Abca13	-0.47	1.00000000	duck
Brd1	-0.54	1.00000000	duck
Pdk3	-0.46	1.00000000	duck
Usb1	-0.23	1.00000000	duck
Cpd	-0.29	1.00000000	duck
Dhx15	-0.35	1.00000000	duck
Cyld	-0.32	1.00000000	duck
Plin2	0.02	1.00000000	duck
Zbtb43	-0.32	1.00000000	duck
Ddx6	-0.13	1.00000000	duck

Fgd4	-0.42	1.00000000	duck
Hip1	-0.12	1.00000000	duck
Traf3ip3	-0.33	1.00000000	duck
Nedd4l	-0.22	1.00000000	duck
Psip1	-0.26	1.00000000	duck
Setdb1	-0.32	1.00000000	duck
Slc16a3	-0.18	1.00000000	duck
Rassf6	-0.41	1.00000000	duck
Smad2	-0.38	1.00000000	duck
Scaf8	-0.44	1.00000000	duck
Ammecr1	-0.49	1.00000000	duck
Gfod2	-0.27	1.00000000	duck
Klhl15	-0.25	1.00000000	duck
F10	4.07	0.00000000	pigeons
Tpt1	4.50	0.00000000	pigeons
Vim	4.39	0.00000000	pigeons
Cyria	5.67	0.00000000	pigeons
Rab33a	6.60	0.00000000	pigeons
Arpc2	3.23	0.00000000	pigeons
Il1rap	4.91	0.00000000	pigeons
Il1b	6.84	0.00000000	pigeons
Rgs2	3.96	0.00000000	pigeons
Cd2ap	3.93	0.00000000	pigeons
App	4.40	0.00000000	pigeons
Pcf11	3.02	0.00000000	pigeons
Vsig4	3.04	0.00000000	pigeons
Plscr1	2.97	0.00000000	pigeons
Gpat3	3.06	0.00000000	pigeons
Lnpep	2.85	0.00000000	pigeons
Lpp	3.03	0.00000000	pigeons
Sdcbp	2.51	0.00000000	pigeons
Txnip	2.91	0.00000000	pigeons
Cfd	-5.00	0.00000000	pigeons
Dapp1	2.32	0.00000000	pigeons
Fos	-3.68	0.00000000	pigeons
Dido1	2.50	0.00000000	pigeons
Trib1	2.33	0.00000000	pigeons
Steap4	2.66	0.00000000	pigeons
Aqp1	-4.80	0.00000000	pigeons
Tollip	2.46	0.00000000	pigeons
Cklf	2.30	0.00000000	pigeons
Apbb1ip	2.38	0.00000000	pigeons
Atf7ip	3.01	0.00000000	pigeons
Niban1	2.79	0.00000000	pigeons
Ptprm	-4.83	0.00000000	pigeons
Tgfbr1	2.70	0.00000000	pigeons

Wtap	2.20	0.00000000	pigeons
Fyb	2.33	0.00000000	pigeons
Igfbp7	-4.21	0.00000000	pigeons
Adss	2.12	0.00000000	pigeons
Tmbim1	2.23	0.00000000	pigeons
Ankhd1	2.22	0.00000000	pigeons
Timp3	-4.12	0.00000000	pigeons
Epas1	-4.01	0.00000000	pigeons
Scaper	2.95	0.00000000	pigeons
Far1	1.96	0.00000000	pigeons
Pparg	1.81	0.00000000	pigeons
Gadd45b	2.59	0.00000000	pigeons
Fgd4	1.85	0.00000000	pigeons
Cmip	2.74	0.00000000	pigeons
Fnip1	1.66	0.00000000	pigeons
Rin3	1.83	0.00000000	pigeons
Adora2b	1.77	0.00000000	pigeons
Ppm1b	1.86	0.00000000	pigeons
Calcrl	-4.03	0.00000000	pigeons
Hbegf	2.01	0.00000000	pigeons
Rbms3	-4.16	0.00000000	pigeons
Atp5b	1.74	0.00000000	pigeons
Sap18	1.73	0.00000000	pigeons
Pecam1	-3.08	0.00000000	pigeons
Htr1f	2.45	0.00000000	pigeons
Pfkfb1	2.16	0.00000000	pigeons
Lpgat1	1.67	0.00000000	pigeons
U2surp	1.84	0.00000000	pigeons
Lrrfip1	1.82	0.00000000	pigeons
Ldb2	-3.98	0.00000000	pigeons
Rasal2	-3.92	0.00000000	pigeons
Cd9	1.57	0.00000000	pigeons
Rapgef5	-3.55	0.00000000	pigeons
Pknox1	1.86	0.00000000	pigeons
Ovch2	2.03	0.00000000	pigeons
Prokr2	1.68	0.00000000	pigeons
Elovl7	1.83	0.00000000	pigeons
Cars	1.76	0.00000000	pigeons
Arap2	1.83	0.00000000	pigeons
Strap	1.69	0.00000000	pigeons
Rmdn2	1.81	0.00000000	pigeons
Pla2g4a	1.78	0.00000000	pigeons
B4galt5	1.73	0.00000000	pigeons
Tmem268	1.77	0.00000000	pigeons
Slc12a7	1.92	0.00000000	pigeons
Ddit4	-3.16	0.00000000	pigeons

Nsmce2	1.86	0.00000000	pigeons
Cbfb	1.52	0.00000000	pigeons
Dot1l	1.32	0.00000000	pigeons
Btg2	1.59	0.00000000	pigeons
Atp1b1	1.54	0.00000000	pigeons
Angpt1	2.22	0.00000000	pigeons
Spi1	1.52	0.00000000	pigeons
Tjp1	-3.63	0.00000000	pigeons
Ptgs2	2.44	0.00000000	pigeons
Adgrl2	-3.62	0.00000000	pigeons
Stom	1.31	0.00000000	pigeons
Crim1	-3.30	0.00000000	pigeons
Phip	1.84	0.00000000	pigeons
Asap1	1.47	0.00000000	pigeons
Sun2	1.47	0.00000000	pigeons
Adnp	1.66	0.00000000	pigeons
Cnbp	1.53	0.00000000	pigeons
Sat1	1.21	0.00000000	pigeons
Tmtc1	-3.79	0.00000000	pigeons
Rasa2	1.55	0.00000000	pigeons
Adgrl4	-3.53	0.00000000	pigeons
Oxr1	1.94	0.00000000	pigeons
Sparc	-3.30	0.00000000	pigeons
Grb2	1.23	0.00000000	pigeons
Ets2	1.52	0.00000000	pigeons
Ptpre	1.65	0.00000000	pigeons
Svil	1.62	0.00000000	pigeons
Nars	1.52	0.00000000	pigeons
Greb1	1.64	0.00000000	pigeons
Hsp90aa1	-2.01	0.00000000	pigeons
Disc1	1.83	0.00000000	pigeons
Fa2h	1.36	0.00000000	pigeons
Tmem100	-3.38	0.00000000	pigeons
Alox5ap	1.41	0.00000000	pigeons
Antxr2	1.43	0.00000000	pigeons
Hecw2	-3.48	0.00000000	pigeons
Snx20	1.37	0.00000000	pigeons
Ptpn6	1.32	0.00000000	pigeons
Ube2w	1.30	0.00000000	pigeons
Nedd4	1.56	0.00000000	pigeons
Sdk1	-3.45	0.00000000	pigeons
Zdhhc20	1.53	0.00000000	pigeons
Tpd52	1.38	0.00000000	pigeons
Dram1	1.46	0.00000000	pigeons
Oga	1.49	0.00000000	pigeons
Pank3	1.53	0.00000000	pigeons

Plin2	1.65	0.00000000	pigeons
4932411N23Rik	-2.87	0.00000000	pigeons
Slc6a6	1.28	0.00000000	pigeons
Vcp	1.35	0.00000000	pigeons
Als2cl	1.29	0.00000000	pigeons
Rbm6	1.35	0.00000000	pigeons
Ube2j1	1.31	0.00000000	pigeons
Adgrf5	-3.24	0.00000000	pigeons
Pstpip1	1.66	0.00000000	pigeons
Ptafr	1.29	0.00000000	pigeons
Plcb1	-3.22	0.00000000	pigeons
Nova1	-3.32	0.00000000	pigeons
Syne2	-3.24	0.00000000	pigeons
Slc38a2	1.31	0.00000000	pigeons
Zc3h12a	1.24	0.00000000	pigeons
Trpc4ap	1.30	0.00000000	pigeons
Syncrip	1.43	0.00000000	pigeons
Sptbn1	-2.27	0.00000000	pigeons
Pomp	1.32	0.00000000	pigeons
Sec23ip	1.34	0.00000000	pigeons
Pik3r5	1.12	0.00000000	pigeons
Arid5b	1.43	0.00000000	pigeons
Itgb2l	1.14	0.00000000	pigeons
Tnip2	1.37	0.00000000	pigeons
Slc2a6	1.36	0.00000000	pigeons
Rab11fip4	1.21	0.00000000	pigeons
Ptprb	-3.17	0.00000000	pigeons
Capn3	1.65	0.00000000	pigeons
Fbh1	1.75	0.00000000	pigeons
Slc16a3	1.33	0.00000000	pigeons
Dock2	1.31	0.00000000	pigeons
Atf3	1.52	0.00000000	pigeons
Zfp352	-2.62	0.00000000	pigeons
Rnf19b	1.23	0.00000000	pigeons
Lonrf3	1.36	0.00000000	pigeons
Arhgdib	1.15	0.00000000	pigeons
Akap12	-3.19	0.00000000	pigeons
Serpinb1a	1.23	0.00000000	pigeons
Susd1	1.28	0.00000000	pigeons
Pfdn1	1.20	0.00000000	pigeons
Bcl7a	1.29	0.00000000	pigeons
Wipf1	1.41	0.00000000	pigeons
Tiam1	1.34	0.00000000	pigeons
Kdm7a	1.18	0.00000000	pigeons
Stard13	-3.20	0.00000000	pigeons
Samsn1	1.04	0.00000000	pigeons

Sdhc	1.19	0.00000000	pigeons
Nadk	1.08	0.00000000	pigeons
Clec16a	1.13	0.00000000	pigeons
Nfia	-2.97	0.00000000	pigeons
Fabp3	1.20	0.00000000	pigeons
Fgl2	1.24	0.00000000	pigeons
Prep	1.37	0.00000000	pigeons
Sfswap	1.31	0.00000000	pigeons
Rxra	1.15	0.00000000	pigeons
Rp2	1.09	0.00000000	pigeons
Sox6	-2.88	0.00000000	pigeons
Rgcc	-3.00	0.00000000	pigeons
Snx10	1.26	0.00000000	pigeons
Pfkp	1.45	0.00000000	pigeons
Cd74	-2.57	0.00000000	pigeons
Afdn	-3.01	0.00000000	pigeons
Acsbg2	1.05	0.00000000	pigeons
Arhgap15	1.18	0.00000000	pigeons
Rap2c	1.11	0.00000000	pigeons
Rtf2	1.12	0.00000000	pigeons
Cdk5r1	1.21	0.00000000	pigeons
Gpx3	-2.88	0.00000000	pigeons
Trpm1	1.46	0.00000000	pigeons
Rfwd3	1.27	0.00000000	pigeons
Zfp516	1.28	0.00000000	pigeons
Pik3cg	1.16	0.00000000	pigeons
Actn1	1.14	0.00000000	pigeons
Gpx1	1.07	0.00000000	pigeons
Ergic1	1.11	0.00000000	pigeons
Syne1	1.11	0.00000000	pigeons
Itga4	1.21	0.00000000	pigeons
Plcg2	1.06	0.00000000	pigeons
Snx4	1.21	0.00000000	pigeons
Polr1d	1.26	0.00000000	pigeons
Dennd1b	1.19	0.00000000	pigeons
Il1rl1	1.53	0.00000000	pigeons
Cavin2	-2.87	0.00000000	pigeons
Prok2	1.39	0.00000000	pigeons
Pim3	1.26	0.00000000	pigeons
Tagap1	1.59	0.00000000	pigeons
Syk	1.28	0.00000000	pigeons
Nfkbiz	1.17	0.00000000	pigeons
Raf1	1.13	0.00000000	pigeons
Rnaset2b	0.96	0.00000000	pigeons
Atp5g3	1.11	0.00000000	pigeons
Dock9	-2.74	0.00000000	pigeons

Eepd1	1.22	0.00000000	pigeons
Tiparp	1.26	0.00000000	pigeons
Zdhhc2	1.17	0.00000000	pigeons
Rac2	1.10	0.00000000	pigeons
Arl6ip5	1.03	0.00000000	pigeons
Braf	1.05	0.00000000	pigeons
Cers5	0.99	0.00000000	pigeons
Tut7	1.17	0.00000000	pigeons
Pcnp	1.06	0.00000000	pigeons
Ndufa1	1.07	0.00000000	pigeons
Snap23	0.98	0.00000000	pigeons
Zfp36l1	1.25	0.00000000	pigeons
Trpm8	1.25	0.00000000	pigeons
Cnn2	1.04	0.00000000	pigeons
Nek6	1.08	0.00000000	pigeons
Csrnp1	1.07	0.00000000	pigeons
Cacybp	1.02	0.00000000	pigeons
Rasgef1a	2.09	0.00000000	pigeons
Tnfaip8l1	1.04	0.00000000	pigeons
Atxn7l1	1.23	0.00000000	pigeons
Il16	0.90	0.00000000	pigeons
Cbl	1.04	0.00000000	pigeons
Enox2	1.08	0.00000000	pigeons
Ugp2	0.96	0.00000000	pigeons
Lrrk2	1.05	0.00000000	pigeons
Dtx2	0.92	0.00000000	pigeons
Ccdc88a	0.96	0.00000000	pigeons
Arhgap31	-2.81	0.00000000	pigeons
Pgs1	0.91	0.00000000	pigeons
Unc13d	0.99	0.00000000	pigeons
Sorbs1	-2.78	0.00000000	pigeons
Tmem71	1.12	0.00000000	pigeons
Rlim	0.88	0.00000000	pigeons
C3	1.02	0.00000000	pigeons
Tnks	1.13	0.00000000	pigeons
Dll4	-2.76	0.00000000	pigeons
Podxl	-2.75	0.00000000	pigeons
Col4a1	-2.73	0.00000000	pigeons
Coro1c	0.93	0.00000000	pigeons
Tnfaip3	0.84	0.00000000	pigeons
Heg1	-2.66	0.00000000	pigeons
Rab29	1.03	0.00000000	pigeons
Rasgrp1	1.10	0.00000000	pigeons
Chst13	0.85	0.00000000	pigeons
Rgs18	1.05	0.00000000	pigeons
Fam189a1	-2.84	0.00000000	pigeons

Lrch3	0.98	0.00000000	pigeons
Nucb2	0.90	0.00000000	pigeons
Fbxl21	1.07	0.00000000	pigeons
Dap	1.23	0.00000000	pigeons
Dars	1.13	0.00000000	pigeons
Rassf5	0.96	0.00000000	pigeons
Hacd2	0.94	0.00000000	pigeons
Kctd12	1.24	0.00000000	pigeons
Itpr1	1.07	0.00000000	pigeons
Kifbp	0.95	0.00000000	pigeons
Nrbf2	0.97	0.00000000	pigeons
Stx11	1.02	0.00000000	pigeons
Pgd	0.90	0.00000000	pigeons
Stat1	0.93	0.00000000	pigeons
Lyn	0.85	0.00000000	pigeons
Sema6a	-2.70	0.00000000	pigeons
Abtb1	0.86	0.00000000	pigeons
Laptm5	0.83	0.00000000	pigeons
Add3	0.90	0.00000000	pigeons
Slc9a9	1.27	0.00000000	pigeons
Notch2	0.93	0.00000000	pigeons
Dock11	0.81	0.00000000	pigeons
Brd8	0.94	0.00000000	pigeons
Adcy7	0.97	0.00000000	pigeons
Prelid3b	1.02	0.00000000	pigeons
Cndp2	1.04	0.00000000	pigeons
Tardbp	0.97	0.00000000	pigeons
Gabrr2	1.10	0.00000000	pigeons
Lcp1	0.77	0.00000000	pigeons
Acsl1	1.08	0.00000000	pigeons
Ptprc	0.82	0.00000000	pigeons
Pcgf5	1.12	0.00000000	pigeons
Pmp22	-2.61	0.00000000	pigeons
Eif2ak3	0.95	0.00000000	pigeons
Kcne3	-2.58	0.00000000	pigeons
Kdm3a	1.04	0.00000000	pigeons
Sucnr1	1.06	0.00000000	pigeons
Skap1	-2.56	0.00000000	pigeons
Evi2a	0.99	0.00000000	pigeons
Noct	0.96	0.00000000	pigeons
Gpr27	0.93	0.00000000	pigeons
Dusp10	1.10	0.00000000	pigeons
Iqgap1	0.89	0.00000000	pigeons
Slc45a4	1.12	0.00000000	pigeons
Wapl	0.94	0.00000000	pigeons
Vmp1	0.91	0.00000000	pigeons

Parn	0.87	0.00000000	pigeons
Csf1r	0.91	0.00000000	pigeons
Arhgap30	0.88	0.00000000	pigeons
Ppard	0.84	0.00000000	pigeons
Chsy1	1.32	0.00000000	pigeons
Tm6sf1	0.98	0.00000000	pigeons
Lpl	-2.49	0.00000000	pigeons
Yrdc	0.84	0.00000000	pigeons
Tial1	0.76	0.00000000	pigeons
Rbm33	0.89	0.00000000	pigeons
Vav3	0.91	0.00000000	pigeons
Ccnyl1	0.87	0.00000000	pigeons
Rsu1	1.04	0.00000000	pigeons
Tcf12	-2.00	0.00000000	pigeons
Sla	1.11	0.00000000	pigeons
Itch	0.82	0.00000000	pigeons
Atp5k	0.85	0.00000000	pigeons
Ezr	0.95	0.00000000	pigeons
Rnf38	1.24	0.00000000	pigeons
Rlf	0.84	0.00000000	pigeons
Ppa2	0.98	0.00000000	pigeons
Cyld	0.91	0.00000000	pigeons
Pik3cb	0.88	0.00000000	pigeons
Itprip	0.89	0.00000000	pigeons
Cpne8	0.79	0.00000000	pigeons
Grk4	0.90	0.00000000	pigeons
Cdh5	-2.40	0.00000000	pigeons
Rassf2	0.90	0.00000000	pigeons
Limd2	0.81	0.00000000	pigeons
Pip5k1b	0.93	0.00000000	pigeons
Shisa5	0.76	0.00000000	pigeons
B3gnt2	0.90	0.00000000	pigeons
Atp11b	0.81	0.00000000	pigeons
Npr3	-2.51	0.00000000	pigeons
Ints7	0.98	0.00000000	pigeons
Akirin2	0.83	0.00000000	pigeons
Ccm2	0.82	0.00000000	pigeons
Ccz1	0.83	0.00000000	pigeons
Trim36	1.01	0.00000000	pigeons
Atp6v1h	1.01	0.00000000	pigeons
Nop58	0.72	0.00000000	pigeons
Crip2	0.80	0.00000000	pigeons
Ccng2	1.05	0.00000000	pigeons
Pgam1	0.81	0.00000000	pigeons
Plekhb2	0.66	0.00000000	pigeons
Chmp4b	0.79	0.00000000	pigeons

Atp6v0e	0.71	0.00000000	pigeons
Ptpra	0.85	0.00000000	pigeons
Sri	0.73	0.00000000	pigeons
Gnb1	0.80	0.00000000	pigeons
Egr1	-1.80	0.00000000	pigeons
Pard3b	-2.45	0.00000000	pigeons
Acox1	0.87	0.00000000	pigeons
Corin	-2.70	0.00000000	pigeons
Cst7	1.00	0.00000000	pigeons
Ivns1abp	0.90	0.00000000	pigeons
Arpc5	0.69	0.00000000	pigeons
Ap1s2	0.76	0.00000000	pigeons
Slmap	0.89	0.00000000	pigeons
Plek	0.66	0.00000000	pigeons
Kmt2c	0.86	0.00000000	pigeons
Arhgef9	-2.47	0.00000000	pigeons
Efnb2	-2.50	0.00000000	pigeons
Lfng	0.95	0.00000000	pigeons
Pik3ap1	0.82	0.00000000	pigeons
Apip	0.89	0.00000000	pigeons
Trrap	0.82	0.00000000	pigeons
Fermt2	-2.48	0.00000000	pigeons
Arpc1b	0.75	0.00000000	pigeons
Dock8	0.82	0.00000000	pigeons
Pias1	0.85	0.00000000	pigeons
Slc40a1	0.88	0.00000000	pigeons
Dok2	0.70	0.00000000	pigeons
Hps5	0.90	0.00000000	pigeons
Anxa2	0.84	0.00000000	pigeons
Skap2	0.91	0.00000000	pigeons
Arvcf	-2.41	0.00000000	pigeons
Borcs5	1.15	0.00000000	pigeons
Kars	0.82	0.00000000	pigeons
Ube3a	0.83	0.00000000	pigeons
Cebpb	0.77	0.00000000	pigeons
S1pr1	-2.35	0.00000000	pigeons
Pak2	0.77	0.00000000	pigeons
Celf2	1.32	0.00000000	pigeons
Ssh2	0.73	0.00000000	pigeons
Vps37b	0.77	0.00000000	pigeons
Dock1	-2.36	0.00000000	pigeons
Stk17b	0.86	0.00000000	pigeons
Synm	-2.31	0.00000000	pigeons
Plekhf2	0.78	0.00000000	pigeons
Blcap	0.83	0.00000000	pigeons
Tars	0.85	0.00000000	pigeons

Irak2	0.87	0.00000000	pigeons
Gorasp1	0.82	0.00000000	pigeons
Rapgef2	-2.32	0.00000000	pigeons
Zfand3	0.89	0.00000000	pigeons
Evl	1.01	0.00000000	pigeons
Atp6v0d1	0.71	0.00000000	pigeons
Gbf1	0.93	0.00000000	pigeons
Ube2v1	0.68	0.00000000	pigeons
Ppm1a	0.86	0.00000000	pigeons
Icosl	1.09	0.00000000	pigeons
Pmepa1	0.77	0.00000000	pigeons
Anxa11	0.82	0.00000000	pigeons
Sap30bp	0.80	0.00000000	pigeons
Scap	0.98	0.00000000	pigeons
Plekhg1	-2.43	0.00000000	pigeons
Noc3l	0.85	0.00000000	pigeons
Nde1	0.74	0.00000000	pigeons
Atp13a3	0.91	0.00000000	pigeons
Foxn2	0.70	0.00000000	pigeons
Arl14ep	0.75	0.00000000	pigeons
Phax	0.80	0.00000000	pigeons
Ly96	0.76	0.00000000	pigeons
Lpcat2b	0.77	0.00000000	pigeons
Pde8a	1.63	0.00000000	pigeons
Bmp2k	0.80	0.00000000	pigeons
Nfe2l1	0.65	0.00000000	pigeons
Rock1	0.85	0.00000000	pigeons
Wdr1	0.73	0.00000000	pigeons
Tmem204	-2.28	0.00000000	pigeons
Ndufaf1	0.86	0.00000000	pigeons
Anxa6	0.70	0.00000000	pigeons
Eaf1	0.80	0.00000000	pigeons
Slirp	0.75	0.00000000	pigeons
Cd44	0.80	0.00000000	pigeons
Ssbp2	-2.30	0.00000000	pigeons
Anapc15-ps	0.72	0.00000000	pigeons
Phf20l1	0.72	0.00000000	pigeons
Setd2	0.67	0.00000000	pigeons
Apmap	0.81	0.00000000	pigeons
Snrpd2	0.69	0.00000000	pigeons
Actb	0.65	0.00000000	pigeons
Zyx	0.66	0.00000000	pigeons
Edem1	0.60	0.00000000	pigeons
Xpo6	0.67	0.00000000	pigeons
Ehbp1	0.86	0.00000000	pigeons
Hspa8	-2.04	0.00000000	pigeons

Capza2	0.65	0.00000000	pigeons
Ptprj	0.67	0.00000000	pigeons
Tmem154	0.60	0.00000000	pigeons
Eno1b	0.68	0.00000000	pigeons
Jchain	0.76	0.00000000	pigeons
Nsd3	0.76	0.00000000	pigeons
Banp	1.03	0.00000000	pigeons
Ankrd22	0.82	0.00000000	pigeons
Cipc	0.76	0.00000000	pigeons
Gyg	0.75	0.00000000	pigeons
Jcad	-2.21	0.00000000	pigeons
Cav1	-2.18	0.00000000	pigeons
Vcl	0.97	0.00000000	pigeons
Tmem63a	0.61	0.00000000	pigeons
Csf3r	0.68	0.00000000	pigeons
Arhgef18	0.73	0.00000000	pigeons
Smap2	0.64	0.00000000	pigeons
Eprs	0.89	0.00000000	pigeons
Dennd4c	0.98	0.00000000	pigeons
Serpine2	0.76	0.00000000	pigeons
Agps	0.54	0.00000000	pigeons
Cstf3	0.69	0.00000000	pigeons
Atxn1	0.75	0.00000000	pigeons
Pla2g15	0.87	0.00000000	pigeons
Cog3	0.78	0.00000000	pigeons
Nfkb2	0.60	0.00000000	pigeons
Cntrl	0.72	0.00000000	pigeons
Igf2r	0.81	0.00000000	pigeons
Cnn3	-2.09	0.00000000	pigeons
Cdc37	0.64	0.00000000	pigeons
Frs2	0.94	0.00000000	pigeons
Washc5	0.80	0.00000000	pigeons
Rassf3	-2.19	0.00000000	pigeons
Ywhaz	0.73	0.00000000	pigeons
Rbfox2	-2.19	0.00000000	pigeons
Arid4a	0.78	0.00000000	pigeons
Btbd11	0.67	0.00000000	pigeons
Sdc2	-2.13	0.00000000	pigeons
Ino80c	0.80	0.00000000	pigeons
Ncoa2	0.82	0.00000000	pigeons
Kbtbd8	0.80	0.00000000	pigeons
Atp6v1a	0.81	0.00000000	pigeons
Atp6ap2	0.63	0.00000000	pigeons
Pde4b	0.73	0.00000000	pigeons
Gna13	0.63	0.00000000	pigeons
Lrmp	0.70	0.00000000	pigeons

Capzb	0.67	0.00000000	pigeons
Arl4a	-1.92	0.00000000	pigeons
Ctdspl	-1.82	0.00000000	pigeons
Ugt8a	0.62	0.00000000	pigeons
Zbtb20	-1.54	0.00000000	pigeons
Plcl2	0.93	0.00000000	pigeons
Foxk2	0.80	0.00000000	pigeons
Acod1	1.00	0.00000000	pigeons
Tapbpl	0.71	0.00000000	pigeons
Trps1	-2.07	0.00000000	pigeons
Lamtor3	0.76	0.00000000	pigeons
Sec63	0.64	0.00000000	pigeons
Tgfbr3	-2.16	0.00000000	pigeons
Bicd1	-2.16	0.00000000	pigeons
Galc	0.73	0.00000000	pigeons
Dgat2	0.72	0.00000000	pigeons
Tec	0.78	0.00000000	pigeons
Emc3	0.51	0.00000000	pigeons
Plekha8	0.68	0.00000000	pigeons
Actg1	0.72	0.00000000	pigeons
Naa20	0.70	0.00000000	pigeons
Fnbp1l	-2.19	0.00000000	pigeons
Ostm1	0.60	0.00000000	pigeons
Slc9a3r2	-2.11	0.00000000	pigeons
Clic4	-2.03	0.00000000	pigeons
Trio	-1.66	0.00000000	pigeons
Kctd10	0.64	0.00000000	pigeons
Prex2	-2.18	0.00000000	pigeons
Tns1	-2.10	0.00000000	pigeons
Abi1	0.76	0.00000000	pigeons
Ints13	0.59	0.00000000	pigeons
Klhl22	0.64	0.00000000	pigeons
Pitpna	0.51	0.00000000	pigeons
Atp6v1c1	0.70	0.00000000	pigeons
Ankrd44	0.66	0.00000000	pigeons
Clpx	0.62	0.00000000	pigeons
Il17ra	0.73	0.00000000	pigeons
Wdfy3	1.02	0.00000000	pigeons
Neo1	-2.08	0.00000000	pigeons
Epn2	0.65	0.00000000	pigeons
Chgb	0.65	0.00000000	pigeons
Rad23b	0.67	0.00000000	pigeons
Adipor2	0.60	0.00000000	pigeons
Smtnl2	0.82	0.00000000	pigeons
Emc2	0.61	0.00000000	pigeons
Fam107b	0.88	0.00000000	pigeons

Ski	-1.30	0.00000000	pigeons
Tnfaip8	0.78	0.00000000	pigeons
Ppp1cb	0.53	0.00000000	pigeons
Sh3bp5	0.80	0.00000000	pigeons
Ncstn	0.67	0.00000000	pigeons
Osbpl1a	-2.05	0.00000000	pigeons
Trdmt1	0.83	0.00000000	pigeons
Psme4	0.73	0.00000000	pigeons
Il27ra	0.54	0.00000000	pigeons
Secisbp2l	0.53	0.00000000	pigeons
Ptk2b	0.76	0.00000000	pigeons
Actr2	0.59	0.00000000	pigeons
Tbx3	-1.96	0.00000000	pigeons
Gsn	0.56	0.00000000	pigeons
Ttl	0.63	0.00000000	pigeons
Rhof	0.58	0.00000000	pigeons
Ttc21b	0.60	0.00000000	pigeons
Stk26	0.50	0.00000000	pigeons
Agtpbp1	0.86	0.00000000	pigeons
Ptprn2	-2.11	0.00000000	pigeons
Spred2	-2.06	0.00000000	pigeons
Slc15a4	0.63	0.00000000	pigeons
Ube2l3	0.46	0.00000000	pigeons
Efemp1	-2.06	0.00000000	pigeons
Atp6v0b	0.50	0.00000000	pigeons
Trpv2	0.71	0.00000000	pigeons
Tdpoz8	0.68	0.00000000	pigeons
Rheb	0.50	0.00000000	pigeons
Kcnc1	-2.01	0.00000000	pigeons
Sgms1	0.62	0.00000000	pigeons
Fdx1	0.65	0.00000000	pigeons
Runx1	0.66	0.00000000	pigeons
Pdlim5	0.73	0.00000000	pigeons
Bcat1	0.56	0.00000000	pigeons
Ptpn14	-1.95	0.00000000	pigeons
Npas3	-2.24	0.00000000	pigeons
Fgd5	-2.04	0.00000000	pigeons
Irak4	0.68	0.00000000	pigeons
Sinhcaf	0.87	0.00000000	pigeons
Pstpip2	0.54	0.00000000	pigeons
Pdzd8	0.66	0.00000000	pigeons
Ganc	0.71	0.00000000	pigeons
Spg21	0.61	0.00000000	pigeons
Pld4	0.62	0.00000000	pigeons
Cep128	1.01	0.00000000	pigeons
Pou2f1	0.80	0.00000000	pigeons

Dph5	0.62	0.00000000	pigeons
Spcs2	0.60	0.00000000	pigeons
Rassf9	-1.96	0.00000000	pigeons
Galnt18	-2.06	0.00000000	pigeons
Hnrnpll	0.47	0.00000000	pigeons
Optn	0.54	0.00000000	pigeons
Fryl	-1.66	0.00000000	pigeons
Sarnp	0.60	0.00000000	pigeons
Stk40	0.63	0.00000000	pigeons
Net1	0.98	0.00000000	pigeons
Itm2b	-1.38	0.00000000	pigeons
Srpr	0.56	0.00000000	pigeons
Socs7	0.63	0.00000000	pigeons
Golm2	0.64	0.00000000	pigeons
Nhsl2	0.54	0.00000000	pigeons
Agpat2	0.65	0.00000000	pigeons
Glrx3	0.55	0.00000000	pigeons
Cdc42bpa	-1.94	0.00000000	pigeons
Arpc3	0.51	0.00000000	pigeons
Ehd3	0.59	0.00000000	pigeons
Dedd	0.57	0.00000000	pigeons
Tmem164	0.62	0.00000000	pigeons
Gpd2	0.71	0.00000000	pigeons
Trappc3	0.50	0.00000000	pigeons
Rab11a	0.46	0.00000000	pigeons
Ptp4a1	0.49	0.00000000	pigeons
Klf15	0.83	0.00000000	pigeons
Sytl1	0.65	0.00000000	pigeons
Ubr5	0.53	0.00000000	pigeons
Pcnx	0.55	0.00000000	pigeons
Cdc42	0.52	0.00000000	pigeons
Nup210	0.49	0.00000000	pigeons
Wbp2	0.55	0.00000000	pigeons
Apcdd1	-1.89	0.00000000	pigeons
Cd47	0.58	0.00000000	pigeons
Herpud2	0.58	0.00000000	pigeons
Ccdc25	0.65	0.00000000	pigeons
Oaz1	0.56	0.00000000	pigeons
Rnf13	0.49	0.00000000	pigeons
Cast	0.57	0.00000000	pigeons
Il10	0.61	0.00000000	pigeons
Gse1	0.55	0.00000000	pigeons
Efl1	0.67	0.00000000	pigeons
Dapk1	0.64	0.00000000	pigeons
Dlc1	-1.93	0.00000000	pigeons
Xpnpep1	0.50	0.00000000	pigeons

Naa15	0.52	0.00000000	pigeons
Stambp	0.63	0.00000000	pigeons
Papss1	0.71	0.00000000	pigeons
Kidins220	0.60	0.00000000	pigeons
Ubtd2	0.66	0.00000000	pigeons
Appl2	0.45	0.00000000	pigeons
Ankrd11	0.53	0.00000000	pigeons
Abcc4	0.77	0.00000000	pigeons
Tead4	-2.07	0.00000000	pigeons
Osbpl10	-1.96	0.00000000	pigeons
Chd9	0.60	0.00000000	pigeons
Tfec	0.89	0.00000000	pigeons
Zfp366	-1.79	0.00000000	pigeons
Wdr44	0.48	0.00000000	pigeons
Rab25	0.60	0.00000000	pigeons
Dusp6	0.54	0.00000000	pigeons
Lpxn	0.65	0.00000000	pigeons
Plxnc1	0.47	0.00000000	pigeons
Bst1	0.52	0.00000000	pigeons
Zc3h18	0.37	0.00000000	pigeons
Fhl3	0.59	0.00000000	pigeons
G0s2	0.75	0.00000000	pigeons
Rnf213	0.54	0.00000000	pigeons
Ccnd2	0.57	0.00000000	pigeons
Pole4	0.52	0.00000000	pigeons
Runx1t1	-1.93	0.00000000	pigeons
Tkt	0.51	0.00000000	pigeons
Wipi1	0.46	0.00000000	pigeons
Ap1g1	0.32	0.00000000	pigeons
Rab10	0.36	0.00000000	pigeons
Tmem106b	0.67	0.00000000	pigeons
Stx7	0.60	0.00000000	pigeons
Limk1	0.58	0.00000000	pigeons
Tcirg1	0.57	0.00000000	pigeons
Dclk1	-2.08	0.00000000	pigeons
Fam104a	0.55	0.00000000	pigeons
Spcs1	0.59	0.00000000	pigeons
Acaca	0.59	0.00000000	pigeons
Hpgd	-1.88	0.00000000	pigeons
Helq	0.61	0.00000000	pigeons
Arhgap5	-1.82	0.00000000	pigeons
Golim4	0.48	0.00000000	pigeons
Angptl2	0.91	0.00000000	pigeons
Atp11c	0.47	0.00000000	pigeons
Nipa2	0.43	0.00000000	pigeons
Acvr1b	0.58	0.00000000	pigeons

Iqgap2	0.54	0.00000000	pigeons
Vps53	0.57	0.00000000	pigeons
Atp5c1	0.51	0.00000000	pigeons
Akt3	-1.99	0.00000000	pigeons
Il22ra2	0.57	0.00000000	pigeons
Ginm1	0.43	0.00000000	pigeons
Sbno2	0.46	0.00000000	pigeons
Unc93b1	0.62	0.00000000	pigeons
Idh1	0.48	0.00000000	pigeons
Ccdc85a	-1.95	0.00000000	pigeons
Aff4	0.40	0.00000000	pigeons
Hck	0.59	0.00000000	pigeons
Tek	-1.74	0.00000000	pigeons
Dnajc13	0.46	0.00000000	pigeons
Helz2	0.48	0.00000000	pigeons
Gpr65	0.72	0.00000000	pigeons
Pggt1b	0.67	0.00000000	pigeons
Rars	0.57	0.00000000	pigeons
Ndel1	0.41	0.00000000	pigeons
Ifngr1	0.56	0.00000000	pigeons
Ube2k	0.62	0.00000000	pigeons
Spopl	0.45	0.00000000	pigeons
Ttc39b	0.69	0.00000000	pigeons
Crkl	0.53	0.00000000	pigeons
Nin	0.55	0.00000000	pigeons
Hs6st1	-1.92	0.00000000	pigeons
Csnk1d	0.53	0.00000000	pigeons
Dcn	-2.19	0.00000000	pigeons
Znfx1	0.48	0.00000000	pigeons
Odf2	0.47	0.00000000	pigeons
Igsf6	0.49	0.00000000	pigeons
Hectd1	0.65	0.00000000	pigeons
Cep112	0.43	0.00000000	pigeons
Zfp148	0.49	0.00000000	pigeons
Tank	0.51	0.00000000	pigeons
Ccni	0.51	0.00000000	pigeons
Tmem30a	0.42	0.00000000	pigeons
Plcb2	0.60	0.00000000	pigeons
Get4	0.40	0.00000000	pigeons
Fgfr2	-1.86	0.00000000	pigeons
Hivep1	0.70	0.00000000	pigeons
Smcr8	0.52	0.00000000	pigeons
Tbp	0.53	0.00000000	pigeons
Asb3	0.81	0.00000000	pigeons
Vps4b	0.40	0.00000000	pigeons
Zfp706	0.37	0.00000000	pigeons

Etf1	0.39	0.00000000	pigeons
Rev3l	0.80	0.00000000	pigeons
Ube2d2a	0.36	0.00000000	pigeons
Agap1	-1.74	0.00000000	pigeons
Ap3m1	0.41	0.00000000	pigeons
Adamtsl2	0.68	0.00000000	pigeons
Arhgef38	0.68	0.00000000	pigeons
Smarcal1	-1.37	0.00000000	pigeons
Igf1r	0.59	0.00000000	pigeons
Cxadr	0.82	0.00000000	pigeons
Bsg	-1.60	0.00000000	pigeons
Ankle2	0.46	0.00000000	pigeons
Lcp2	0.40	0.00000000	pigeons
Yap1	-1.87	0.00000000	pigeons
Ppp1cc	0.38	0.00000000	pigeons
Fkbp1a	-2.02	0.00000000	pigeons
5031439G07Rik	0.43	0.00000000	pigeons
Rab5a	0.37	0.00000000	pigeons
Slc25a17	0.54	0.00000000	pigeons
Nfe2l2	0.37	0.00000000	pigeons
Zfp521	-1.86	0.00000000	pigeons
Zbtb21	0.44	0.00000000	pigeons
Nudt3	0.43	0.00000000	pigeons
Sftpc	-3.90	0.00000000	pigeons
Etl4	-1.85	0.00000000	pigeons
Parp6	0.46	0.00000000	pigeons
Trabd	0.41	0.00000000	pigeons
Fyn	0.45	0.00000000	pigeons
Osbpl9	0.49	0.00000000	pigeons
Fam53a	0.76	0.00000000	pigeons
Trib2	-1.86	0.00000000	pigeons
Trafd1	0.45	0.00000000	pigeons
Ephb1	-1.68	0.00000000	pigeons
Ass1	0.53	0.00000000	pigeons
Jmjd1c	0.58	0.00000000	pigeons
Arntl	0.74	0.00000000	pigeons
Dgkd	-2.02	0.00000000	pigeons
Sec16a	0.43	0.00000000	pigeons
Usp4	0.41	0.00000000	pigeons
Thbs1	-2.17	0.00000000	pigeons
Stau2	0.66	0.00000000	pigeons
Auts2	-1.78	0.00000000	pigeons
Ascc2	0.47	0.00000000	pigeons
Ubap1	0.46	0.00000000	pigeons
Klhl15	0.40	0.00000000	pigeons
Rhoj	-1.74	0.00000000	pigeons

Ptdss1	0.60	0.00000000	pigeons
D5Ertd579e	0.51	0.00000000	pigeons
Gtf2h1	0.44	0.00000000	pigeons
Pde10a	-1.80	0.00000000	pigeons
Sgk1	0.67	0.00000000	pigeons
Hp1bp3	0.35	0.00000000	pigeons
Glyr1	0.37	0.00000000	pigeons
Nckap1	-1.77	0.00000000	pigeons
Btbd9	0.71	0.00000000	pigeons
Ogfr	0.37	0.00000000	pigeons
Zc3h7a	0.43	0.00000000	pigeons
Sucla2	0.41	0.00000000	pigeons
Btaf1	0.45	0.00000000	pigeons
Ewsr1	0.36	0.00000000	pigeons
Tbc1d24	0.51	0.00000000	pigeons
Csgalnact2	0.58	0.00000000	pigeons
Tent5a	0.71	0.00000000	pigeons
Exoc3	0.53	0.00000000	pigeons
Acvrl1	-1.65	0.00000000	pigeons
Rpf2	0.52	0.00000000	pigeons
Il31ra	0.53	0.00000000	pigeons
Itpr2	0.50	0.00000000	pigeons
Cul2	0.43	0.00000000	pigeons
Dpyd	-1.72	0.00000000	pigeons
Nup214	0.35	0.00000000	pigeons
Hif1a	0.44	0.00000000	pigeons
Qk	-1.14	0.00000000	pigeons
Rybp	0.74	0.00000000	pigeons
Aamp	0.55	0.00000000	pigeons
Eif4b	0.47	0.00000000	pigeons
Zfp207	0.36	0.00000000	pigeons
Oat	0.35	0.00000000	pigeons
Ostf1	0.39	0.00000000	pigeons
Arid5a	0.53	0.00000000	pigeons
Gpatch8	0.51	0.00000000	pigeons
Hs1bp3	0.42	0.00000000	pigeons
Klhdc10	0.47	0.00000000	pigeons
Sppl2a	0.38	0.00000000	pigeons
Nus1	0.36	0.00000000	pigeons
Stc1	0.54	0.00000000	pigeons
Traf3	0.64	0.00000000	pigeons
Tfpi2	-2.03	0.00000000	pigeons
Nr3c2	-1.80	0.00000000	pigeons
Ippk	0.48	0.00000000	pigeons
Nfatc3	0.52	0.00000000	pigeons
Fes	0.49	0.00000000	pigeons

Acap2	0.34	0.00000000	pigeons
Id2	0.52	0.00000000	pigeons
Eya3	0.51	0.00000000	pigeons
Arl3	0.34	0.00000000	pigeons
Ssu72	0.28	0.00000000	pigeons
Cobll1	-1.67	0.00000000	pigeons
Pspc1	0.41	0.00000000	pigeons
Tab3	0.42	0.00000000	pigeons
Wdr37	0.47	0.00000000	pigeons
Impa2	0.59	0.00000000	pigeons
Taldo1	0.30	0.00000000	pigeons
Nrap	0.45	0.00000000	pigeons
Gsr	0.34	0.00000000	pigeons
Wnt5b	0.50	0.00000000	pigeons
Yipf4	0.36	0.00000000	pigeons
Ninj1	0.51	0.00000000	pigeons
Sync	0.49	0.00000000	pigeons
Arfip1	0.45	0.00000000	pigeons
Helz	0.38	0.00000000	pigeons
Cacnb4	0.53	0.00000000	pigeons
Herc1	0.32	0.00000000	pigeons
Inip	0.46	0.00000000	pigeons
Ifnar1	0.35	0.00000000	pigeons
Erbin	0.36	0.00000000	pigeons
Rnf111	0.44	0.00000000	pigeons
Pten	0.23	0.00000000	pigeons
Basp1	0.47	0.00000000	pigeons
E2f3	0.37	0.00000000	pigeons
Rasgrf2	-1.59	0.00000000	pigeons
Agtrap	0.47	0.00000000	pigeons
Tbc1d1	0.52	0.00000000	pigeons
Spidr	0.75	0.00000000	pigeons
Cul1	0.37	0.00000000	pigeons
Ldlrad4	0.88	0.00000000	pigeons
Ntan1	0.22	0.00000000	pigeons
Kif1b	0.52	0.00000000	pigeons
Elovl5	0.23	0.00000000	pigeons
Klhl5	0.51	0.00000000	pigeons
Smad3	0.76	0.00000000	pigeons
Ddhd1	0.39	0.00000000	pigeons
Rai14	-1.63	0.00000000	pigeons
Rpa1	0.38	0.00000000	pigeons
Gnpda2	0.48	0.00000000	pigeons
Arhgef3	-1.79	0.00000000	pigeons
Cotl1	0.39	0.00000000	pigeons
Azi2	0.46	0.00000000	pigeons

Tmem181a	0.58	0.00000000	pigeons
Ogdh	0.39	0.00000000	pigeons
Rab20	0.43	0.00000000	pigeons
Stam2	0.32	0.00000000	pigeons
Zfp777	0.47	0.00000000	pigeons
Cd63	0.27	0.00000000	pigeons
Spns3	0.48	0.00000000	pigeons
Psma3	0.22	0.00000000	pigeons
Gpr176	0.50	0.00000000	pigeons
Xrn2	0.23	0.00000000	pigeons
Ap2b1	0.37	0.00000000	pigeons
Lamc1	-1.75	0.00000000	pigeons
Cdkn2aip	0.46	0.00000000	pigeons
Zfand5	0.33	0.00000000	pigeons
Scamp2	0.34	0.00000000	pigeons
Tax1bp1	0.55	0.00000000	pigeons
Vps26a	0.52	0.00000000	pigeons
Gsk3b	-0.90	0.00000000	pigeons
Arhgap12	0.44	0.00000000	pigeons
Fmnl2	-1.55	0.00000000	pigeons
Ablim1	-1.75	0.00000000	pigeons
Arhgap45	0.47	0.00000000	pigeons
Smarce1	0.38	0.00000000	pigeons
Rad54l2	0.41	0.00000000	pigeons
Rab11fip2	0.44	0.00000000	pigeons
Slc4a7	0.82	0.00000000	pigeons
Spred1	-1.62	0.00000000	pigeons
Sfxn1	0.49	0.00000000	pigeons
Swap70	0.37	0.00000000	pigeons
Atp2b1	0.48	0.00000000	pigeons
Hook3	0.24	0.00000000	pigeons
Paqr3	0.45	0.00000000	pigeons
Sos2	0.33	0.00000000	pigeons
Txndc11	0.51	0.00000000	pigeons
Serpind1	-1.50	0.00000000	pigeons
Tjp2	-1.55	0.00000000	pigeons
Pdap1	0.26	0.00000000	pigeons
Erlin1	0.39	0.00000000	pigeons
Gdap2	0.45	0.00000000	pigeons
Pnpla8	0.37	0.00000000	pigeons
Lcor	0.38	0.00000000	pigeons
Senp1	0.42	0.00000000	pigeons
Sin3a	0.35	0.00000000	pigeons
Kynu	0.53	0.00000000	pigeons
Zdhhc9	0.41	0.00000000	pigeons
Lifr	-1.54	0.00000000	pigeons

Psmg2	0.37	0.00000000	pigeons
Stox2	-1.60	0.00000000	pigeons
Atf2	0.33	0.00000000	pigeons
Exoc2	0.51	0.00000000	pigeons
Rcsd1	0.25	0.00000000	pigeons
Sec24a	0.36	0.00000000	pigeons
Thoc2	0.33	0.00000000	pigeons
Lnpk	0.42	0.00000000	pigeons
Sh3glb1	0.28	0.00000000	pigeons
Nmi	0.39	0.00000000	pigeons
Serpini1	0.39	0.00000000	pigeons
Ap2a1	0.27	0.00000000	pigeons
Ube2v2	0.29	0.00000000	pigeons
Fmnl1	0.33	0.00000000	pigeons
Cttnbp2nl	-1.69	0.00000000	pigeons
Rabgap1	0.38	0.00000000	pigeons
Tmed10	0.23	0.00000000	pigeons
Fh1	0.36	0.00000000	pigeons
Ddx17	0.19	0.00000000	pigeons
Cald1	-1.64	0.00000000	pigeons
Psph	0.46	0.00000000	pigeons
Upf3b	0.25	0.00000000	pigeons
Arhgap21	-1.72	0.00000000	pigeons
Kdr	-1.54	0.00000000	pigeons
Lonp1	0.35	0.00000000	pigeons
Dynlrb1	0.18	0.00000000	pigeons
Ythdf3	0.33	0.00000000	pigeons
Kif2a	0.53	0.00000000	pigeons
Pdpk1	0.29	0.00000000	pigeons
Psme3ip1	0.43	0.00000000	pigeons
Fli1	-1.06	0.00000000	pigeons
Ppp6r2	0.33	0.00000000	pigeons
Appbp2	0.35	0.00000000	pigeons
Slc25a16	0.45	0.00000000	pigeons
Sdha	0.35	0.00000000	pigeons
Yeats4	0.37	0.00000000	pigeons
Ctbs	0.40	0.00000000	pigeons
Wdr26	0.35	0.00000000	pigeons
Celf1	0.35	0.00000000	pigeons
Usp44	0.34	0.00000000	pigeons
Chfr	0.43	0.00000000	pigeons
Pias2	0.34	0.00000000	pigeons
Ctsb	0.39	0.00000000	pigeons
Hsp90b1	-1.69	0.00000000	pigeons
Dok1	0.32	0.00000000	pigeons
Col22a1	-1.44	0.00000000	pigeons

Rnf216	0.54	0.00000000	pigeons
Ptpn9	0.46	0.00000000	pigeons
Ugcg	0.47	0.00000000	pigeons
Ralb	0.30	0.00000000	pigeons
Srp9	0.16	0.00000000	pigeons
Ttc13	0.44	0.00000000	pigeons
Nbeal2	0.41	0.00000000	pigeons
Cnot6l	0.30	0.00000000	pigeons
Dach1	-1.54	0.00000000	pigeons
Cgnl1	-1.56	0.00000000	pigeons
Tmem87a	0.19	0.00000000	pigeons
Vat1l	0.54	0.00000000	pigeons
Vps35	0.35	0.00000000	pigeons
Rbm12	0.35	0.00000000	pigeons
Atp2a3	0.29	0.00000000	pigeons
Nrp1	-1.48	0.00000000	pigeons
Ddrgk1	0.23	0.00000000	pigeons
Foxf1	-1.34	0.00000000	pigeons
Neurl1b	-1.40	0.00000000	pigeons
Agbl1	-1.48	0.00000000	pigeons
Pard3	0.78	0.00000000	pigeons
Gab1	0.94	0.00000000	pigeons
Rer1	0.22	0.00000000	pigeons
Ltn1	0.33	0.00000000	pigeons
Wdr20	0.35	0.00000000	pigeons
Atp11a	0.37	0.00000000	pigeons
Rbbp8nl	0.49	0.00000000	pigeons
Usp33	0.32	0.00000000	pigeons
Hbs1l	0.22	0.00000000	pigeons
Acp1	0.24	0.00000000	pigeons
Rbm34	0.18	0.00000000	pigeons
Slc25a24	0.23	0.00000000	pigeons
Tle3	0.26	0.00000000	pigeons
Rnf149	0.36	0.00000000	pigeons
Nfil3	0.41	0.00000000	pigeons
Scarb2	-1.48	0.00000000	pigeons
Syf2	0.18	0.00000000	pigeons
Ncf2	0.27	0.00000000	pigeons
Osbpl8	0.23	0.00000000	pigeons
Gnai3	0.31	0.00000000	pigeons
Ankfy1	0.34	0.00000000	pigeons
Dennd2a	-1.60	0.00000000	pigeons
Bach1	0.25	0.00000000	pigeons
Kansl1	0.43	0.00000000	pigeons
Vdac2	0.18	0.00000000	pigeons
Rfx2	0.43	0.00000000	pigeons

Fgr	0.24	0.00000000	pigeons
Ndfip2	0.34	0.00000000	pigeons
Jak1	0.34	0.00000000	pigeons
Ralgps2	-1.76	0.00000000	pigeons
Sppl3	0.21	0.00000000	pigeons
Gpbp1l1	0.20	0.00000000	pigeons
Stk38	0.34	0.00000000	pigeons
Usp7	0.14	0.00000000	pigeons
Wdr82	0.18	0.00000000	pigeons
Prpf8	0.20	0.00000000	pigeons
Tecpr2	0.36	0.00000000	pigeons
Tab2	0.30	0.00000000	pigeons
Grb10	0.54	0.00000000	pigeons
Vwa8	0.32	0.00000000	pigeons
Edem3	0.33	0.00000000	pigeons
Hexim1	0.44	0.00000000	pigeons
Kdm5a	0.29	0.00000000	pigeons
Chst15	0.61	0.00000000	pigeons
Srp68	0.31	0.00000000	pigeons
Bud31	0.20	0.00000000	pigeons
Lrrc56	0.46	0.00000000	pigeons
Gpcpd1	0.39	0.00000000	pigeons
Ptpn1	0.38	0.00000000	pigeons
Zfp654	0.23	0.00000000	pigeons
Tsnax	0.42	0.00000000	pigeons
Fam168b	0.30	0.00000000	pigeons
P2rx1	0.38	0.00000000	pigeons
Cab39	0.20	0.00000000	pigeons
Klhl6	0.26	0.00000000	pigeons
Cadps2	-1.41	0.00000000	pigeons
Btbd1	0.37	0.00000000	pigeons
Zdhhc13	0.33	0.00000000	pigeons
Tmem184b	0.20	0.00000000	pigeons
Egfl7	-1.37	0.00000000	pigeons
Cnot6	0.21	0.00000000	pigeons
Asns	0.42	0.00000000	pigeons
Tnrc6b	-1.63	0.00000000	pigeons
St3gal1	-1.45	0.00000000	pigeons
Ide	0.24	0.00000000	pigeons
Capn15	0.33	0.00000000	pigeons
Stxbp1	0.36	0.00000000	pigeons
9130409I23Rik	0.39	0.00000000	pigeons
Ripor3	-1.52	0.00000000	pigeons
Plrg1	0.26	0.00000000	pigeons
Srebf2	0.35	0.00000000	pigeons
Gulp1	-1.54	0.00000000	pigeons

Sh3bgrl3	0.18	0.00000000	pigeons
Fbxl5	0.26	0.00000000	pigeons
Rab21	0.18	0.00000000	pigeons
Akt1	0.25	0.00000000	pigeons
Uba3	0.30	0.00000000	pigeons
Cnot2	0.49	0.00000000	pigeons
Phf12	0.24	0.00000000	pigeons
Nrbp2	0.47	0.00000000	pigeons
Rilpl2	0.20	0.00000000	pigeons
Lrrc8b	0.41	0.00000000	pigeons
Gata3	-1.45	0.00000000	pigeons
Pdcd6	0.22	0.00000000	pigeons
Fndc3a	0.38	0.00000000	pigeons
Sgpp1	0.27	0.00000000	pigeons
Stx3	0.42	0.00000000	pigeons
Zfp512b	0.38	0.00000000	pigeons
Rint1	0.36	0.00000000	pigeons
Psmc2	0.29	0.00000000	pigeons
Clk4	0.11	0.00000000	pigeons
Ccdc93	0.31	0.00000000	pigeons
Apaf1	0.36	0.00000000	pigeons
Fth1	0.36	0.00000000	pigeons
Ramp3	-1.37	0.00000000	pigeons
Narf	0.25	0.00000000	pigeons
Pdgfb	-1.30	0.00000000	pigeons
Atxn2	0.19	0.00000000	pigeons
Zfat	0.17	0.00000000	pigeons
Nr4a3	0.76	0.00000000	pigeons
Ncoa6	0.29	0.00000000	pigeons
Rapgef1	0.35	0.00000000	pigeons
Ncam1	0.53	0.00000000	pigeons
Cebpg	0.32	0.00000000	pigeons
Ube2h	0.29	0.00000000	pigeons
Eef1e1	0.14	0.00000000	pigeons
U2af1	0.22	0.00000000	pigeons
Gas7	0.32	0.00000000	pigeons
Tmem42	0.33	0.00000000	pigeons
S100a11	0.38	0.00000000	pigeons
Pigf	0.52	0.00000000	pigeons
Aak1	0.24	0.00000000	pigeons
Dynlt3	0.26	0.00000000	pigeons
Atp6v1e2	0.20	0.00000000	pigeons
Npepps	0.28	0.00000000	pigeons
Kdm4c	0.43	0.00000000	pigeons
Sptlc2	0.17	0.00000000	pigeons
Rab22a	0.36	0.00000000	pigeons

Thumpd3	0.22	0.00000000	pigeons
Zhx1	0.23	0.00000000	pigeons
Tax1bp3	0.24	0.00000000	pigeons
Ano5	0.47	0.00000000	pigeons
Efcab14	0.16	0.00000000	pigeons
Atg2b	0.28	0.00000000	pigeons
Ppp1r12a	0.43	0.00000000	pigeons
Ankrd13a	0.32	0.00000000	pigeons
Vta1	0.27	0.00000000	pigeons
Becn2	0.29	0.00000000	pigeons
Adam17	0.20	0.00000000	pigeons
Arid3b	0.44	0.00000000	pigeons
Xylt1	0.35	0.00000000	pigeons
Gcc2	0.23	0.00000000	pigeons
Anxa5	-1.61	0.00000000	pigeons
Sash1	-1.35	0.00000000	pigeons
Colec10	-1.28	0.00000000	pigeons
Lrba	-1.52	0.00000000	pigeons
Pam	0.48	0.00000000	pigeons
Hagh	0.21	0.00000000	pigeons
Atad1	0.16	0.00000000	pigeons
Sash3	0.36	0.00000000	pigeons
Srf	0.37	0.00000000	pigeons
Serinc5	0.33	0.00000000	pigeons
Nr2c2	0.23	0.00000000	pigeons
Tdg	0.19	0.00000000	pigeons
Ric8b	0.41	0.00000000	pigeons
Kpnb1	0.21	0.00000000	pigeons
Per2	0.37	0.00000000	pigeons
Exoc6b	-1.47	0.00000000	pigeons
Aplf	0.33	0.00000000	pigeons
Tm9sf2	0.11	0.00000000	pigeons
Kat2b	0.32	0.00000000	pigeons
Bmp6	-1.35	0.00000000	pigeons
Tgfbr2	-1.63	0.00000000	pigeons
Rbm38	0.34	0.00000000	pigeons
Rbx1	0.22	0.00000000	pigeons
Asxl3	-1.32	0.00000000	pigeons
Atp6v1b2	0.41	0.00000000	pigeons
Zfhx3	0.45	0.00000000	pigeons
Atp1b3	-1.51	0.00000000	pigeons
Fgd3	0.30	0.00000000	pigeons
Enah	-1.24	0.00000000	pigeons
Caskin2	-1.22	0.00000000	pigeons
Rnf6	0.38	0.00000000	pigeons
Fbxw7	0.52	0.00000000	pigeons

Tsen2	0.34	0.00000000	pigeons
Rbm26	0.19	0.00000000	pigeons
Rc3h2	0.32	0.00000000	pigeons
Ppp2r2d	0.16	0.00000000	pigeons
Elovl1	0.22	0.00000000	pigeons
Chpt1	0.33	0.00000000	pigeons
Abhd2	0.23	0.00000000	pigeons
S100a13	-1.72	0.00000000	pigeons
Gtf2a1	0.14	0.00000000	pigeons
Eya2	0.44	0.00000000	pigeons
Ubqln1	0.32	0.00000000	pigeons
Nedd9	0.39	0.00000000	pigeons
Tmem123	0.28	0.00000000	pigeons
Fat4	-1.24	0.00000000	pigeons
Pdlim1	-1.34	0.00000000	pigeons
Gatad2a	0.28	0.00000000	pigeons
Elf1	0.35	0.00000000	pigeons
Fyttd1	0.28	0.00000000	pigeons
Prrg1	-1.28	0.00000000	pigeons
Prkce	-0.98	0.00000000	pigeons
Dnase2b	0.39	0.00000000	pigeons
Pdcd10	0.16	0.00000000	pigeons
Dda1	0.26	0.00000000	pigeons
Pnisr	0.18	0.00000000	pigeons
Phldb2	-1.37	0.00000000	pigeons
Cap1	0.22	0.00000000	pigeons
Ccnk	0.17	0.00000000	pigeons
Kdm2a	0.29	0.00000000	pigeons
Nf1	0.20	0.00000000	pigeons
Zfand1	0.31	0.00000000	pigeons
Hpse	0.32	0.00000000	pigeons
Traf6	0.25	0.00000000	pigeons
Psmb7	0.09	0.00000000	pigeons
Zfp644	0.27	0.00000000	pigeons
Aftph	0.23	0.00000000	pigeons
Rad21	0.29	0.00000000	pigeons
Zbtb49	0.46	0.00000000	pigeons
Atp6v0c	0.26	0.00000000	pigeons
Prkcz	0.55	0.00000000	pigeons
Nceh1	0.35	0.00000000	pigeons
4933400A11Rik	0.25	0.00000000	pigeons
Tes	0.31	0.00000000	pigeons
Cnot1	0.13	0.00000000	pigeons
Sf3a3	0.14	0.00000000	pigeons
Rell1	0.31	0.00000000	pigeons
Fxyd6	-1.39	0.00000000	pigeons

Dennd5a	0.28	0.00000000	pigeons
Twsg1	-1.36	0.00000000	pigeons
Abcc5	0.41	0.00000000	pigeons
Bcl2l1	0.22	0.00000000	pigeons
Emp2	-1.26	0.00000000	pigeons
Gapvd1	0.28	0.00000000	pigeons
Bckdhb	0.55	0.00000000	pigeons
Klhl25	0.42	0.00000000	pigeons
Pde4d	0.59	0.00000000	pigeons
Reep1	-1.19	0.00000000	pigeons
Dnajc3	0.09	0.00000000	pigeons
Atp7a	0.20	0.00000000	pigeons
Sec22a	0.37	0.00000000	pigeons
Gns	0.34	0.00000000	pigeons
Cct6b	0.14	0.00000000	pigeons
Atp6v1g1	0.22	0.00000000	pigeons
Pcsk2	-1.28	0.00000000	pigeons
Hace1	0.44	0.00000000	pigeons
Sf3a2	0.22	0.00000000	pigeons
Rhbdf2	0.31	0.00000000	pigeons
Dnajb14	0.40	0.00000000	pigeons
Ccnl2	0.16	0.00000000	pigeons
Slc3a1	0.41	0.00000000	pigeons
Unc13b	0.44	0.00000000	pigeons
Galnt1	0.28	0.00000000	pigeons
St3gal6	0.12	0.00000000	pigeons
Tspan18	-1.43	0.00000000	pigeons
Cdca7l	-1.29	0.00000000	pigeons
Rab8b	0.19	0.00000000	pigeons
Otulin	0.24	0.00000000	pigeons
Arih2	0.25	0.00000000	pigeons
Prrc2c	0.20	0.00000000	pigeons
Amotl2	-1.22	0.00000000	pigeons
Ogfrl1	0.27	0.00000000	pigeons
Pafah1b1	0.03	0.00000000	pigeons
Vps37a	0.30	0.00000000	pigeons
Kin	0.20	0.00000000	pigeons
Lbr	0.12	0.00000000	pigeons
Gpn3	0.15	0.00000000	pigeons
Pik3c2a	0.15	0.00000000	pigeons
Baiap2l1	0.42	0.00000000	pigeons
Tmem120a	0.34	0.00000000	pigeons
Cep135	0.41	0.00000000	pigeons
Rangap1	0.19	0.00000000	pigeons
F5	0.40	0.00000000	pigeons
Klhl24	0.35	0.00000000	pigeons

Wwc2	-1.24	0.00000000	pigeons
Utp3	0.18	0.00000000	pigeons
Ech1	0.24	0.00000000	pigeons
Ctss	0.28	0.00000000	pigeons
Cabin1	0.18	0.00000000	pigeons
Dstn	0.37	0.00000000	pigeons
Ogt	0.18	0.00000000	pigeons
Clk1	0.11	0.00000000	pigeons
Cdc14a	0.30	0.00000000	pigeons
Rexo1	0.18	0.00000000	pigeons
Rgs6	0.88	0.00000000	pigeons
Pik3cd	0.23	0.00000000	pigeons
Hlf	-1.37	0.00000000	pigeons
Sema7a	-1.40	0.00000000	pigeons
Pip4k2a	0.39	0.00000000	pigeons
Tmem248	0.17	0.00000000	pigeons
Bag3	0.60	0.00000000	pigeons
Psmd1	0.07	0.00000000	pigeons
Ube2a	0.11	0.00000000	pigeons
Ep400	0.15	0.00000000	pigeons
Setd5	0.17	0.00000000	pigeons
Srsf5	0.06	0.00000000	pigeons
Cops7a	0.19	0.00000000	pigeons
Usp54	-1.50	0.00000000	pigeons
Rap1gds1	0.33	0.00000000	pigeons
Sec61a2	0.30	0.00000000	pigeons
Ppp3r1	0.10	0.00000000	pigeons
Gpr21	0.45	0.00000000	pigeons
Sox18	-1.15	0.00000000	pigeons
Lrif1	0.23	0.00000000	pigeons
Cpeb4	0.14	0.00000000	pigeons
Pde1c	-1.43	0.00000000	pigeons
Fosl2	0.22	0.00000000	pigeons
Phf20	0.21	0.00000000	pigeons
Pou1f1	0.50	0.00000000	pigeons
Lims2	-1.45	0.00000000	pigeons
Rab8a	0.17	0.00000000	pigeons
Atp2a2	0.20	0.00000000	pigeons
Nxpe3	0.30	0.00000000	pigeons
Zbtb43	0.35	0.00000000	pigeons
Zfp518a	0.29	0.00000000	pigeons
Tspan32	0.39	0.00000000	pigeons
Psmb1	0.18	0.00000000	pigeons
Snip1	0.19	0.00000000	pigeons
Ash1l	0.11	0.00000000	pigeons
Akr1a1	0.32	0.00000000	pigeons

Rsbn1l	0.15	0.00000000	pigeons
Zcrb1	0.15	0.00000000	pigeons
Card9	0.35	0.00000000	pigeons
Npepl1	0.27	0.00000000	pigeons
Tmtc4	0.32	0.00000000	pigeons
Tbc1d23	0.10	0.00000000	pigeons
Stk3	0.30	0.00000000	pigeons
Phospho1	0.29	0.00000000	pigeons
Sntb1	0.49	0.00000000	pigeons
Ttc17	0.15	0.00000000	pigeons
Klf7	0.25	0.00000000	pigeons
Dazap1	0.08	0.00000000	pigeons
Ncln	0.35	0.00000000	pigeons
Usp15	0.22	0.00000000	pigeons
Dock10	0.35	0.00000000	pigeons
Smad6	-1.28	0.00000000	pigeons
Twf2	0.24	0.00000000	pigeons
Ythdf2	0.16	0.00000000	pigeons
Csde1	-0.03	0.00000000	pigeons
Farp1	-1.26	0.00000000	pigeons
Smad1	-1.15	0.00000000	pigeons
Tcerg1	0.11	0.00000000	pigeons
Slc35a5	0.33	0.00000000	pigeons
Sirt1	0.12	0.00000000	pigeons
Dcun1d5	0.07	0.00000000	pigeons
Prmt9	0.19	0.00000000	pigeons
Rcor1	0.33	0.00000000	pigeons
Pak3	0.57	0.00000000	pigeons
Prpf40a	0.08	0.00000000	pigeons
Ppp3ca	0.37	0.00000000	pigeons
Zdhhc3	0.20	0.00000000	pigeons
Ccdc40	0.40	0.00000000	pigeons
Raph1	-1.23	0.00000000	pigeons
F2r	-1.20	0.00000000	pigeons
Csnk2a1	0.31	0.00000000	pigeons
Pus7l	0.42	0.00000000	pigeons
Adipor1	0.28	0.00000000	pigeons
Sbno1	0.06	0.00000000	pigeons
Arrdc3	-1.07	0.00000000	pigeons
Atl2	0.07	0.00000000	pigeons
Flii	0.12	0.00000000	pigeons
Etv6	-0.91	0.00000000	pigeons
Prkag2	0.33	0.00000000	pigeons
Rspry1	0.32	0.00000000	pigeons
Nr2f2	-1.16	0.00000000	pigeons
Cltc	0.00	0.00000000	pigeons

Alkbh5	0.31	0.00000000	pigeons
Ntn4	-1.17	0.00000000	pigeons
Tmem33	0.18	0.00000000	pigeons
Sdad1	0.28	0.00000000	pigeons
Ikzf2	0.35	0.00000000	pigeons
Tmcc3	-1.33	0.00000000	pigeons
Tmem167	0.15	0.00000000	pigeons
R3hdm4	0.24	0.00000000	pigeons
Arsb	0.34	0.00000000	pigeons
Dock4	-1.41	0.00000000	pigeons
Lrrfip2	0.24	0.00000000	pigeons
Ppp4r3a	0.13	0.00000000	pigeons
Xrcc4	0.61	0.00000000	pigeons
Crk	0.12	0.00000000	pigeons
Traf2	0.22	0.00000000	pigeons
Blmh	0.16	0.00000000	pigeons
Thoc5	0.28	0.00000000	pigeons
Sugt1	0.14	0.00000000	pigeons
Riok3	0.13	0.00000000	pigeons
Etfa	0.10	0.00000000	pigeons
Nfkb1	0.19	0.00000000	pigeons
Dr1	0.01	0.00000000	pigeons
Vapa	0.11	0.00000000	pigeons
L3mbtl3	0.18	0.00000000	pigeons
Bzw2	0.27	0.00000000	pigeons
Scaf11	0.17	0.00000000	pigeons
Atg13	0.24	0.00000000	pigeons
Yme1l1	0.16	0.00000000	pigeons
Fry	0.21	0.00000000	pigeons
Acadl	0.13	0.00000000	pigeons
Ifrd1	0.28	0.00000000	pigeons
Ly75	-0.03	0.00000000	pigeons
Dcaf1	0.07	0.00000000	pigeons
Ncf4	0.33	0.00000000	pigeons
Sfmbt1	0.43	0.00000000	pigeons
Eng	-1.16	0.00000000	pigeons
Ep300	0.11	0.00000000	pigeons
Usp28	-1.13	0.00000000	pigeons
Rnaseh1	0.18	0.00000000	pigeons
Arrdc4	0.29	0.00000000	pigeons
Sec11a	0.09	0.00000000	pigeons
Aagab	0.29	0.00000000	pigeons
Omg	0.36	0.00000000	pigeons
Rbfa	-1.18	0.00000000	pigeons
Ik	0.00	0.00000000	pigeons
AU040320	0.24	0.00000000	pigeons

Eif2s3y	0.08	0.00000000	pigeons
Dynlt1a	0.30	0.00000000	pigeons
Rab18	0.24	0.00000000	pigeons
Taok3	0.01	0.00000000	pigeons
Bnip2	0.01	0.00000000	pigeons
Nfatc1	-1.50	0.00000000	pigeons
Anxa7	0.30	0.00000000	pigeons
Gnb5	-1.16	0.00000000	pigeons
Ext1	-1.24	0.00000000	pigeons
Trp53i11	-1.14	0.00000000	pigeons
Wnk1	0.33	0.00000000	pigeons
Slc35f5	0.19	0.00000000	pigeons
Gigyf2	0.21	0.00000000	pigeons
Spen	0.23	0.00000000	pigeons
Plpp3	-1.08	0.00000000	pigeons
Actr8	0.17	0.00000000	pigeons
Elmo1	0.44	0.00000000	pigeons
Ndufs1	0.22	0.00000000	pigeons
Ssbp1	0.14	0.00000000	pigeons
Gnat2	0.41	0.00000000	pigeons
Enpp4	0.31	0.00000000	pigeons
Csnk2a2	0.02	0.00000000	pigeons
Tacc1	0.13	0.00000000	pigeons
4833439L19Rik	0.21	0.00000000	pigeons
Ibtk	0.15	0.00000000	pigeons
Copg1	0.16	0.00000000	pigeons
Sec62	0.10	0.00000000	pigeons
Unc5c	-1.24	0.00000000	pigeons
Sfpq	-0.02	0.00000000	pigeons
Anapc1	0.17	0.00000000	pigeons
N4bp1	0.15	0.00000000	pigeons
Gcnt4	0.27	0.00000000	pigeons
Slc9a3r1	0.28	0.00000000	pigeons
Jade3	0.51	0.00000000	pigeons
Tmem59	0.00	0.00000000	pigeons
Rnmt	0.12	0.00000000	pigeons
Eml4	0.68	0.00000000	pigeons
Tcf7l2	-1.40	0.00000000	pigeons
Strn	0.11	0.00000000	pigeons
Pag1	0.35	0.00000000	pigeons
Trank1	0.26	0.00000000	pigeons
Jun	-0.39	0.00000000	pigeons
Orc3	0.38	0.00000000	pigeons
Slc35b3	0.24	0.00000000	pigeons
Smg7	0.22	0.00000000	pigeons
Epb41l3	0.70	0.00000000	pigeons

Wasf2	-0.06	0.00000000	pigeons
Lin54	0.27	0.00000000	pigeons
Pkn2	0.10	0.00000000	pigeons
Dnajb12	0.06	0.00000000	pigeons
Apoo	0.17	0.00000000	pigeons
Itfg1	0.13	0.00000000	pigeons
Camk2g	0.18	0.00000000	pigeons
Cmtm8	-1.06	0.00000000	pigeons
Kcnj5	-1.60	0.00000000	pigeons
Ubxn6	0.28	0.00000000	pigeons
Nt5c3	0.08	0.00000000	pigeons
Gnl2	0.25	0.00000000	pigeons
Sbf2	0.29	0.00000000	pigeons
Emc6	0.15	0.00000000	pigeons
Ywhae	-0.07	0.00000000	pigeons
Zfp335	0.10	0.00000000	pigeons
Rnf166	0.30	0.00000000	pigeons
Kcnq5	0.37	0.00000000	pigeons
Lmbrd1	0.30	0.00000000	pigeons
Smg1	0.13	0.00000000	pigeons
Acsl3	0.26	0.00000000	pigeons
Cnot7	0.13	0.00000000	pigeons
Kdm5b	0.18	0.00000000	pigeons
Gzf1	0.15	0.00000000	pigeons
Ncoa5	0.18	0.00000000	pigeons
Vrk1	0.19	0.00000000	pigeons
Atp9a	0.28	0.00000000	pigeons
Snw1	0.01	0.00000000	pigeons
Ptk2	-1.23	0.00000000	pigeons
Pabpc1	0.33	0.00000000	pigeons
Qsox1	0.32	0.00000000	pigeons
Dnajc7	0.06	0.00000000	pigeons
Opa1	0.17	0.00000000	pigeons
Erlec1	0.15	0.00000000	pigeons
Ralgapa1	0.13	0.00000000	pigeons
Gnai2	0.23	0.00000000	pigeons
Irf2bpl	0.14	0.00000000	pigeons
Acp2	0.24	0.00000000	pigeons
Slc9a8	0.33	0.00000000	pigeons
Arg2	0.37	0.00000000	pigeons
Lrrc1	0.23	0.00000000	pigeons
Afg3l2	0.09	0.00000000	pigeons
Bcr	0.13	0.00000000	pigeons
Nipal3	0.19	0.00000000	pigeons
Osbpl2	0.22	0.00000000	pigeons
Bbx	0.23	0.00000000	pigeons

Stx12	0.20	0.00000000	pigeons
Apba2	-1.24	0.00000000	pigeons
Chmp5	-0.05	0.00000000	pigeons
Naa35	0.19	0.00000000	pigeons
Crot	0.30	0.00000000	pigeons
Rpn2	0.04	0.00000000	pigeons
Prkcb	0.18	0.00000000	pigeons
Stat3	0.16	0.00000000	pigeons
Rab14	0.21	0.00000000	pigeons
Bend5	0.09	0.00000000	pigeons
Usp20	0.20	0.00000000	pigeons
Senp5	0.19	0.00000000	pigeons
Nsrp1	0.11	0.00000000	pigeons
Add1	0.06	0.00000000	pigeons
Thada	0.41	0.00000000	pigeons
Parp9	0.14	0.00000000	pigeons
Nrd1	0.12	0.00000000	pigeons
Snx2	0.16	0.00000000	pigeons
Stx8	0.13	0.00000000	pigeons
Npc1	0.28	0.00000000	pigeons
Ppp1ca	0.13	0.00000000	pigeons
Copb2	0.01	0.00000000	pigeons
Psma2	0.11	0.00000000	pigeons
Rasgrp3	-1.18	0.00000000	pigeons
Ranbp3	0.19	0.00000000	pigeons
Fuca2	0.29	0.00000000	pigeons
Sike1	0.07	0.00000000	pigeons
Surf4	0.11	0.00000000	pigeons
Lrp1	0.30	0.00000000	pigeons
4932438A13Rik	0.03	0.00000000	pigeons
Kdm1a	0.07	0.00000000	pigeons
Reep5	0.03	0.00000000	pigeons
Sdf2l1	0.08	0.00000000	pigeons
Exosc7	0.26	0.00000000	pigeons
Fgd6	-1.05	0.00000000	pigeons
Ubr2	0.10	0.00000000	pigeons
Rexo2	0.03	0.00000000	pigeons
Fam76a	0.19	0.00000000	pigeons
Qrich1	0.06	0.00000000	pigeons
Stat2	0.24	0.00000000	pigeons
Rpp30	0.23	0.00000000	pigeons
Psmd9	0.02	0.00000000	pigeons
Tmed2	0.05	0.00000000	pigeons
Lta4h	0.22	0.00000000	pigeons
Adgrg1	-1.11	0.00000000	pigeons
Pdrg1	0.21	0.00000000	pigeons

Fbxo28	0.24	0.00000000	pigeons
Rab7	0.02	0.00000000	pigeons
Lama4	-1.11	0.00000000	pigeons
Arl5a	0.09	0.00000000	pigeons
Cdk6	-1.31	0.00000000	pigeons
Cpsf3	0.10	0.00000000	pigeons
Pum1	0.05	0.00000000	pigeons
Vdr	0.32	0.00000000	pigeons
Pde3b	-1.05	0.00000000	pigeons
Cdc42bpb	-1.50	0.00000000	pigeons
Hipk2	-1.18	0.00000000	pigeons
Dusp16	0.52	0.00000000	pigeons
Lpcat3	0.18	0.00000000	pigeons
Ppp3cb	0.08	0.00000000	pigeons
Capn7	0.16	0.00000000	pigeons
Rbm39	0.17	0.00000000	pigeons
Ankrd13d	0.18	0.00000000	pigeons
Wdfy1	0.08	0.00000000	pigeons
Tmem222	0.09	0.00000000	pigeons
Osbpl11	0.21	0.00000000	pigeons
Tor1b	0.13	0.00000000	pigeons
Stx17	0.10	0.00000000	pigeons
Csnk1e	0.06	0.00000000	pigeons
Snx18	0.30	0.00000000	pigeons
Smchd1	0.06	0.00000000	pigeons
Larp7	0.08	0.00000000	pigeons
Rnf114	0.20	0.00000000	pigeons
Smurf2	-1.26	0.00000000	pigeons
Pacsin2	0.22	0.00000000	pigeons
Ube2q1	0.04	0.00000000	pigeons
Fgfr1op2	0.12	0.00000000	pigeons
Nelfcd	0.11	0.00000000	pigeons
Crbn	0.15	0.00000000	pigeons
Dennd2d	0.25	0.00000000	pigeons
Stk4	0.14	0.00000000	pigeons
Cdk17	-1.35	0.00000000	pigeons
Ap3b1	0.23	0.00000000	pigeons
Rnasel	0.31	0.00000000	pigeons
Umad1	0.43	0.00000000	pigeons
Ripor1	-1.11	0.00000000	pigeons
Sccpdh	0.12	0.00000000	pigeons
Ppp2r2a	-0.06	0.00000000	pigeons
Cep350	0.06	0.00000000	pigeons
Uhrf1bp1l	0.17	0.00000000	pigeons
Unc45a	0.30	0.00000000	pigeons
Togaram1	0.09	0.00000000	pigeons

Cct4	0.00	0.00000000	pigeons
Bub3	0.15	0.00000000	pigeons
Il18	0.31	0.00000000	pigeons
Eif4h	-0.09	0.00000000	pigeons
D630045J12Rik	-1.13	0.00000000	pigeons
Jarid2	-0.75	0.00000000	pigeons
Tmx1	0.08	0.00000000	pigeons
Eif1	0.22	0.00000000	pigeons
Hsbp1	-0.06	0.00000000	pigeons
Strada	0.24	0.00000000	pigeons
Ythdf1	0.10	0.00000000	pigeons
Pgm2	0.25	0.00000000	pigeons
Golph3l	0.24	0.00000000	pigeons
Tmem41b	0.16	0.00000000	pigeons
Scaf4	0.08	0.00000000	pigeons
Zfc3h1	0.18	0.00000000	pigeons
Rpn1	0.08	0.00000000	pigeons
Dnm1l	0.26	0.00000000	pigeons
Agfg1	0.04	0.00000000	pigeons
Git2	0.23	0.00000000	pigeons
Bpnt1	0.20	0.00000000	pigeons
Ireb2	0.09	0.00000000	pigeons
Htra1	-1.16	0.00000000	pigeons
Atg5	0.18	0.00000000	pigeons
Chordc1	0.01	0.00000000	pigeons
Cul3	0.09	0.00000000	pigeons
Armc1	0.13	0.00000000	pigeons
Dennd4a	0.06	0.00000000	pigeons
Luzp1	-1.31	0.00000000	pigeons
Arcn1	0.04	0.00000000	pigeons
Atg12	0.17	0.00000000	pigeons
Adgrg6	0.18	0.00000000	pigeons
Cul5	0.17	0.00000000	pigeons
Ptbp1	0.21	0.00000000	pigeons
Axin2	-1.09	0.00000000	pigeons
Ttc3	0.13	0.00000000	pigeons
Syt11	0.00	0.00000000	pigeons
Phf2	0.14	0.00000000	pigeons
Smarca5	0.10	0.00000000	pigeons
Btf3l4	-0.10	0.00000000	pigeons
Pdhx	0.15	0.00000000	pigeons
Clip1	0.11	0.00000000	pigeons
Psmc3	0.04	0.00000000	pigeons
Necap1	0.24	0.00000000	pigeons
Champ1	0.22	0.00000000	pigeons
Lamp2	0.00	0.00000000	pigeons

Blzf1	0.16	0.00000000	pigeons
Rgs3	-1.03	0.00000000	pigeons
Tpm1	0.00	0.00000000	pigeons
Prpf3	0.19	0.00000000	pigeons
Arpc1a	0.06	0.00000000	pigeons
Tbxas1	0.27	0.00000000	pigeons
Ypel5	0.00	0.00000000	pigeons
Yipf3	0.14	0.00000000	pigeons
Bcap29	0.02	0.00000000	pigeons
Ercc6	0.20	0.00000000	pigeons
Vav2	-1.07	0.00000000	pigeons
Tasp1	0.29	0.00000000	pigeons
Il20ra	0.30	0.00000000	pigeons
Rhobtb1	0.46	0.00000000	pigeons
Stk10	0.04	0.00000000	pigeons
Vps18	0.22	0.00000000	pigeons
Pnrc2	0.10	0.00000000	pigeons
Rbbp6	0.12	0.00000000	pigeons
Eif3j2	0.01	0.00000000	pigeons
R3hdm1	0.12	0.00000000	pigeons
Rel	0.13	0.00000000	pigeons
Klf10	0.14	0.00000000	pigeons
Ndrg1	0.10	0.00000000	pigeons
Plekhm2	0.18	0.00000000	pigeons
Ppm1f	-1.43	0.00000000	pigeons
Cdc27	0.19	0.00000000	pigeons
Comt	0.57	0.00000000	pigeons
Pts	0.26	0.00000000	pigeons
Ypel1	-1.27	0.00000000	pigeons
Brox	0.08	0.00000000	pigeons
Nfu1	0.13	0.00000000	pigeons
Ncor2	0.04	0.00000000	pigeons
Psmc1	0.04	0.00000000	pigeons
Phf3	0.24	0.00000000	pigeons
Ankrd10	-1.08	0.00000000	pigeons
Spg11	0.14	0.00000000	pigeons
Ndufs4	0.21	0.00000000	pigeons
Wbp2nl	0.14	0.00000000	pigeons
Slc22a23	-1.14	0.00000000	pigeons
Sik3	0.10	0.00000000	pigeons
Haao	0.37	0.00000000	pigeons
Psmc5	0.07	0.00000000	pigeons
Nlk	0.15	0.00000000	pigeons
Zbtb38	0.02	0.00000000	pigeons
Ilf3	0.12	0.00000000	pigeons
Atp13a1	0.13	0.00000000	pigeons

Aqr	0.06	0.00000000	pigeons
Slc25a34	0.23	0.00000000	pigeons
Prdm2	0.05	0.00000000	pigeons
Sgsm3	0.27	0.00000000	pigeons
Usp14	0.14	0.00000000	pigeons
Slk	0.20	0.00000000	pigeons
Eif3g	0.09	0.00000000	pigeons
Copa	0.06	0.00000000	pigeons
Apoa1	-2.05	0.00000000	pigeons
Alox5	0.11	0.00000000	pigeons
Kdm1b	0.19	0.00000000	pigeons
Ell	0.03	0.00000000	pigeons
Rgs19	0.27	0.00000000	pigeons
Rabggtb	0.13	0.00000000	pigeons
Creb1	0.20	0.00000000	pigeons
Riok1	0.16	0.00000000	pigeons
Larp4b	-0.09	0.00000000	pigeons
Nsl1	0.01	0.00000000	pigeons
Eif2b3	0.05	0.00000000	pigeons
Tlk2	0.04	0.00000000	pigeons
Tmem230	0.01	0.00000000	pigeons
Kdm4b	0.05	0.00000000	pigeons
Zbtb7a	0.09	0.00000000	pigeons
Caprin1	-0.05	0.00000000	pigeons
Rnf138	0.13	0.00000000	pigeons
Zdhhc7	0.19	0.00000000	pigeons
Ube4b	0.15	0.00000000	pigeons
Pcsk7	0.17	0.00000000	pigeons
Sf3b1	-0.03	0.00000000	pigeons
Adnp2	0.05	0.00000000	pigeons
Pls3	-1.48	0.00000000	pigeons
Klhl18	0.22	0.00000000	pigeons
Ppp3cc	0.15	0.00000000	pigeons
Setd3	0.08	0.00000000	pigeons
Polr1e	0.15	0.00000000	pigeons
Pign	0.13	0.00000000	pigeons
Esyt2	0.01	0.00000000	pigeons
Gpr137b	0.09	0.00000000	pigeons
Slc39a9	0.24	0.00000000	pigeons
Lsm14a	-0.16	0.00000000	pigeons
Creg1	-1.54	0.00000000	pigeons
Fbxw11	0.06	0.00000000	pigeons
Tmem258	-0.05	0.00000000	pigeons
Ptbp2	0.12	0.00000000	pigeons
Gabpa	0.07	0.00000000	pigeons
Thoc7	0.07	0.00000000	pigeons

Ppil3	0.13	0.00000000	pigeons
Ggnbp2	0.01	0.00000000	pigeons
Tnip1	0.03	0.00000000	pigeons
Ccng1	0.15	0.00000000	pigeons
Abracl	0.00	0.00000000	pigeons
Ndufv3	0.00	0.00000000	pigeons
Ripk1	0.03	0.00000000	pigeons
Zeb1	0.34	0.00000000	pigeons
Samd8	0.21	0.00000000	pigeons
Rbm15	0.23	0.00000000	pigeons
Srp14	0.15	0.00000000	pigeons
Srsf1	-0.17	0.00000000	pigeons
Acer2	-1.06	0.00000000	pigeons
Tm9sf3	0.08	0.00000000	pigeons
Slc12a2	-1.22	0.00000000	pigeons
Aup1	0.26	0.00000000	pigeons
Psma6	-0.06	0.00000000	pigeons
Pin4	0.07	0.00000000	pigeons
Stx18	0.13	0.00000000	pigeons
Cdc42se2	0.17	0.00000000	pigeons
Carhsp1	0.04	0.00000000	pigeons
Ssrp1	0.13	0.00000000	pigeons
Ifi35	-0.08	0.00000000	pigeons
Atl1	0.15	0.00000000	pigeons
Kif11	0.22	0.00000000	pigeons
Sf3b6	-0.03	0.00000000	pigeons
Herpud1	0.16	0.00000000	pigeons
Vezf1	0.06	0.00000000	pigeons
Fam3c	-0.05	0.00000000	pigeons
Zfp318	0.22	0.00000000	pigeons
Smarcc2	0.18	0.00000000	pigeons
Ing4	0.11	0.00000000	pigeons
Ostc	0.04	0.00000000	pigeons
Psmd14	0.15	0.00000000	pigeons
Gps1	0.13	0.00000000	pigeons
Prkar1a	-0.07	0.00000000	pigeons
Cdk12	0.08	0.00000000	pigeons
Ciapin1	0.16	0.00000000	pigeons
Sod2	0.05	0.00000000	pigeons
Pmm2	0.19	0.00000000	pigeons
Eif4g1	0.16	0.00000000	pigeons
Cd82	-1.23	0.00000000	pigeons
Gtf2h5	-0.05	0.00000000	pigeons
Ncbp1	0.15	0.00000000	pigeons
Lnx2	0.32	0.00000000	pigeons
Saal1	0.35	0.00000000	pigeons

Haus2	0.22	0.00000000	pigeons
Ndufb1	-0.09	0.00000000	pigeons
Spdl1	0.21	0.00000000	pigeons
Synj1	-0.06	0.00000000	pigeons
Negr1	0.55	0.00000000	pigeons
Adora2a	0.22	0.00000000	pigeons
Setd7	0.01	0.00000000	pigeons
Supt6	0.06	0.00000000	pigeons
Fem1c	0.26	0.00000000	pigeons
Tpk1	-1.17	0.00000000	pigeons
Taf2	0.00	0.00000000	pigeons
Scyl2	0.11	0.00000000	pigeons
Rab3gap2	0.11	0.00000000	pigeons
Bcl2l13	0.10	0.00000000	pigeons
Phka2	0.15	0.00000000	pigeons
Clcn7	0.19	0.00000000	pigeons
Edn1	-1.11	0.00000000	pigeons
Cnnm2	-1.01	0.00000000	pigeons
Dhx40	0.20	0.00000000	pigeons
Slc37a3	0.18	0.00000000	pigeons
Tubb6	-0.97	0.00000000	pigeons
Slc25a5	0.13	0.00000000	pigeons
Lrmda	-1.13	0.00000000	pigeons
Ppil4	0.02	0.00000000	pigeons
Dlst	0.13	0.00000000	pigeons
Ccndbp1	-0.01	0.00000000	pigeons
Dnajc5	0.11	0.00000000	pigeons
Ica1	-1.04	0.00000000	pigeons
Ddx6	-0.06	0.00000000	pigeons
Srp54a	0.00	0.00000000	pigeons
Ifnar2	-0.13	0.00000000	pigeons
Esrp2	0.06	0.00000000	pigeons
Ubp1	0.00	0.00000000	pigeons
Xpa	-0.06	0.00000000	pigeons
4933427D14Rik	0.11	0.00000000	pigeons
Ints6	-1.32	0.00000000	pigeons
Nhlrc2	0.09	0.00000000	pigeons
Usp30	0.15	0.00000000	pigeons
Adam22	0.36	0.00000000	pigeons
Rbm15b	0.08	0.00000000	pigeons
Abr	0.12	0.00000000	pigeons
Chmp2b	0.10	0.00000000	pigeons
Impa1	0.18	0.00000000	pigeons
Fam168a	-1.24	0.00000000	pigeons
Fam114a2	0.10	0.00000000	pigeons
Tbc1d5	0.25	0.00000000	pigeons

Snx17	-0.03	0.00000000	pigeons
Fancm	0.28	0.00000000	pigeons
Copb1	0.02	0.00000000	pigeons
Cul4b	-0.11	0.00000000	pigeons
Esd	0.02	0.00000000	pigeons
Gk5	-1.38	0.00000000	pigeons
Nusap1	-0.14	0.00000000	pigeons
Dync1li1	-0.03	0.00000000	pigeons
Cript	0.03	0.00000000	pigeons
Plagl1	0.33	0.00000000	pigeons
Ddb1	0.06	0.00000000	pigeons
Rab27a	-0.05	0.00000000	pigeons
Nsun2	0.10	0.00000000	pigeons
Tbpl1	0.05	0.00000000	pigeons
Psma7	-0.20	0.00000000	pigeons
B3gntl1	0.29	0.00000001	pigeons
Alcam	-1.01	0.00000001	pigeons
Dicer1	0.11	0.00000001	pigeons
Rnpc3	0.23	0.00000001	pigeons
Nr3c1	0.20	0.00000001	pigeons
Dis3l2	0.09	0.00000001	pigeons
Uba2	0.00	0.00000001	pigeons
Pigt	0.17	0.00000001	pigeons
Hmox1	0.25	0.00000001	pigeons
Poldip3	0.02	0.00000001	pigeons
Tbc1d9b	0.09	0.00000001	pigeons
Fbxo42	0.13	0.00000001	pigeons
Sipa1l2	0.30	0.00000001	pigeons
Dnajc10	0.18	0.00000001	pigeons
Ddx11	0.17	0.00000001	pigeons
Irf7	0.16	0.00000001	pigeons
Ric1	0.01	0.00000001	pigeons
Hdac2	0.10	0.00000001	pigeons
Wipi2	0.09	0.00000001	pigeons
Ybx1	-1.40	0.00000001	pigeons
Wdr25	0.25	0.00000001	pigeons
Hnrnpm	-0.07	0.00000001	pigeons
Tspan13	-1.06	0.00000001	pigeons
Arfrp1	0.15	0.00000001	pigeons
Wdr81	0.18	0.00000001	pigeons
Ankmy2	0.10	0.00000001	pigeons
Ranbp17	-0.96	0.00000001	pigeons
Dcun1d2	-1.00	0.00000001	pigeons
Ppp1r2	-0.03	0.00000001	pigeons
Zfp469	0.27	0.00000001	pigeons
Esco1	0.11	0.00000001	pigeons

Hip1	0.22	0.00000001	pigeons
Exoc6	-0.01	0.00000001	pigeons
Fam32a	-0.22	0.00000001	pigeons
Trappc2	0.08	0.00000001	pigeons
Nop14	-0.08	0.00000001	pigeons
Nr4a1	0.13	0.00000001	pigeons
Ublcp1	0.07	0.00000001	pigeons
Seh1l	0.06	0.00000001	pigeons
Ankrd50	-1.05	0.00000001	pigeons
Nf2	0.17	0.00000001	pigeons
Gnb4	0.13	0.00000001	pigeons
Ywhaq	0.03	0.00000001	pigeons
Gid8	0.02	0.00000001	pigeons
Glul	-0.96	0.00000001	pigeons
Nap1l1	-1.31	0.00000001	pigeons
Cass4	0.10	0.00000001	pigeons
Entpd4	0.15	0.00000001	pigeons
Foxo1	-1.20	0.00000001	pigeons
Ptp4a2	-0.24	0.00000002	pigeons
Rai2	0.03	0.00000002	pigeons
Pdcl3	0.05	0.00000002	pigeons
Rnf10	0.03	0.00000002	pigeons
Sft2d1	0.00	0.00000002	pigeons
Zbtb26	0.08	0.00000002	pigeons
Nek7	0.09	0.00000002	pigeons
Atad2b	-0.07	0.00000002	pigeons
Aff2	-0.99	0.00000002	pigeons
Suds3	-0.07	0.00000002	pigeons
Ppfibp1	-1.00	0.00000002	pigeons
Spag9	-0.11	0.00000002	pigeons
Zfp384	0.13	0.00000002	pigeons
Tia1	0.11	0.00000002	pigeons
Rictor	-0.02	0.00000002	pigeons
Usp48	-0.10	0.00000002	pigeons
Phb2	-0.06	0.00000002	pigeons
Denr	0.02	0.00000002	pigeons
Bcl6	0.27	0.00000002	pigeons
Cwc15	-0.11	0.00000002	pigeons
Immp2l	-1.09	0.00000002	pigeons
Dlg1	-1.06	0.00000003	pigeons
Lamtor2	-0.06	0.00000003	pigeons
Nfyb	0.19	0.00000003	pigeons
Gabpb1	0.08	0.00000003	pigeons
Tk2	0.02	0.00000003	pigeons
Rubcnl	-1.37	0.00000003	pigeons
Ran	-0.20	0.00000003	pigeons

Fas	0.09	0.00000003	pigeons
Lrpap1	-0.12	0.00000003	pigeons
Fam172a	0.05	0.00000003	pigeons
Ufm1	-0.03	0.00000003	pigeons
Gucd1	0.13	0.00000003	pigeons
Luc7l	-0.11	0.00000003	pigeons
Rad17	0.17	0.00000003	pigeons
Gnptab	0.04	0.00000003	pigeons
Reep2	0.20	0.00000003	pigeons
Hlx	0.22	0.00000003	pigeons
Ulk1	-0.11	0.00000003	pigeons
Exoc1	0.06	0.00000004	pigeons
Gtf2f2	0.09	0.00000004	pigeons
Tbc1d15	-0.04	0.00000004	pigeons
Kpna6	0.11	0.00000004	pigeons
Ppp2r5d	0.04	0.00000004	pigeons
Chp1	-0.12	0.00000004	pigeons
Selenof	-0.17	0.00000004	pigeons
Tfg	0.07	0.00000005	pigeons
Cds2	0.02	0.00000005	pigeons
Brwd1	-0.07	0.00000005	pigeons
Rc3h1	-0.03	0.00000005	pigeons
Arl8a	0.07	0.00000005	pigeons
Elavl1	-0.03	0.00000005	pigeons
Vil1	0.13	0.00000005	pigeons
Cltb	-0.16	0.00000005	pigeons
Wsb1	0.03	0.00000006	pigeons
Gpalpp1	-0.12	0.00000006	pigeons
Dip2b	0.10	0.00000006	pigeons
Itfg2	-0.03	0.00000006	pigeons
Rnf146	-0.12	0.00000006	pigeons
Aff1	-0.98	0.00000006	pigeons
Tmem135	-1.18	0.00000007	pigeons
Pwwp2a	0.05	0.00000007	pigeons
Sec14l1	-1.39	0.00000007	pigeons
Chaf1a	0.14	0.00000007	pigeons
Stag2	0.23	0.00000007	pigeons
Ppp6r3	0.04	0.00000007	pigeons
Tspan4	-1.13	0.00000007	pigeons
Hsf2	0.14	0.00000007	pigeons
Snx27	0.10	0.00000008	pigeons
Nup50	0.00	0.00000008	pigeons
Ints4	0.14	0.00000008	pigeons
Rbm12b2	0.12	0.00000008	pigeons
Trerf1	0.12	0.00000008	pigeons
Gstcd	0.28	0.00000009	pigeons

Txlna	0.09	0.00000009	pigeons
Hat1	0.11	0.00000009	pigeons
Gpr107	0.02	0.00000010	pigeons
Invs	0.16	0.00000010	pigeons
Itgav	0.01	0.00000011	pigeons
Pfkfb3	0.21	0.00000011	pigeons
Cenpf	0.34	0.00000011	pigeons
Zbtb11	0.10	0.00000011	pigeons
Zcchc17	0.02	0.00000011	pigeons
Ppara	0.27	0.00000012	pigeons
Kpna4	-0.15	0.00000012	pigeons
Tbc1d16	0.22	0.00000012	pigeons
Atp2c1	-0.08	0.00000012	pigeons
Alas1	0.13	0.00000012	pigeons
Srsf11	-0.16	0.00000013	pigeons
St7l	0.10	0.00000014	pigeons
Sdf4	-0.14	0.00000014	pigeons
Prdm15	0.12	0.00000014	pigeons
Stxbp5	0.09	0.00000014	pigeons
Ddx23	0.06	0.00000015	pigeons
Taok1	0.01	0.00000015	pigeons
Prex1	0.11	0.00000016	pigeons
Dusp1	0.62	0.00000017	pigeons
Ppp2r5c	0.08	0.00000017	pigeons
Slc10a7	0.15	0.00000017	pigeons
Phf21a	0.07	0.00000019	pigeons
Fkbp8	0.13	0.00000019	pigeons
Zfand6	0.09	0.00000020	pigeons
Chd7	0.47	0.00000021	pigeons
Fam91a1	0.10	0.00000021	pigeons
Bcl11a	0.33	0.00000022	pigeons
Nr4a2	0.45	0.00000022	pigeons
Setdb1	0.00	0.00000022	pigeons
Fancd2	-0.11	0.00000024	pigeons
Trim33	0.03	0.00000024	pigeons
Ngly1	-0.07	0.00000024	pigeons
Zswim7	0.18	0.00000024	pigeons
Synrg	0.17	0.00000025	pigeons
Fmr1	-0.05	0.00000025	pigeons
Sulf2	0.08	0.00000026	pigeons
Sos1	-0.09	0.00000026	pigeons
Bzw1	-0.12	0.00000026	pigeons
Camk2d	-1.23	0.00000026	pigeons
Fez2	0.01	0.00000027	pigeons
Sh3kbp1	0.17	0.00000027	pigeons
Sar1b	0.08	0.00000027	pigeons

Lasp1	0.03	0.00000027	pigeons
Kcnq1	-1.04	0.00000028	pigeons
Ikbke	0.01	0.00000029	pigeons
Eef1b2	-1.24	0.00000034	pigeons
Lig4	0.17	0.00000036	pigeons
Ppm1e	0.24	0.00000037	pigeons
Tbk1	0.09	0.00000037	pigeons
Upf2	-0.15	0.00000039	pigeons
Fem1a	0.00	0.00000040	pigeons
Luc7l3	0.00	0.00000040	pigeons
Bicral	-0.12	0.00000041	pigeons
Atp6v1d	0.02	0.00000041	pigeons
Stard3nl	0.01	0.00000041	pigeons
Pgpep1	0.10	0.00000043	pigeons
Nrbp1	0.02	0.00000044	pigeons
Nr1h3	0.00	0.00000045	pigeons
Pyroxd1	0.17	0.00000046	pigeons
Itgb3	0.00	0.00000048	pigeons
Cish	0.12	0.00000051	pigeons
Secisbp2	-0.07	0.00000053	pigeons
Nap1l4	-0.10	0.00000054	pigeons
Psmd2	0.16	0.00000055	pigeons
Slc35a1	0.19	0.00000055	pigeons
Lrrc57	0.12	0.00000055	pigeons
Ckb	-1.08	0.00000057	pigeons
Psmd12	-0.14	0.00000057	pigeons
Birc6	0.11	0.00000058	pigeons
Zfp410	0.15	0.00000062	pigeons
Skil	0.23	0.00000065	pigeons
Psen1	-0.13	0.00000069	pigeons
Ankrd17	0.06	0.00000069	pigeons
Rnf20	-0.03	0.00000077	pigeons
Zcchc8	-0.12	0.00000079	pigeons
Slco4c1	0.26	0.00000079	pigeons
Erp44	-0.02	0.00000084	pigeons
Ube4a	0.06	0.00000084	pigeons
Nufip2	-0.16	0.00000086	pigeons
Ankib1	0.08	0.00000089	pigeons
Bmt2	0.16	0.00000089	pigeons
Zfp592	-0.08	0.00000091	pigeons
Rragc	0.17	0.00000094	pigeons
Hcfc2	0.06	0.00000098	pigeons
Gphn	-1.02	0.00000102	pigeons
Lrp12	-0.04	0.00000106	pigeons
Cdk7	0.06	0.00000106	pigeons
Ctsa	0.07	0.00000108	pigeons

Ncoa7	0.11	0.00000108	pigeons
Pcmtd2	-0.11	0.00000109	pigeons
Ap2m1	-0.19	0.00000111	pigeons
Fggy	0.11	0.00000113	pigeons
Usp8	-0.18	0.00000115	pigeons
Atg3	-0.03	0.00000117	pigeons
Vcpip1	-0.03	0.00000120	pigeons
Cask	-0.89	0.00000123	pigeons
Vps29	-0.07	0.00000125	pigeons
Stxbp6	-1.07	0.00000128	pigeons
Rragd	0.05	0.00000130	pigeons
Puf60	0.07	0.00000132	pigeons
Aurkaip1	0.00	0.00000135	pigeons
Fbxw2	0.00	0.00000136	pigeons
Adcy9	-1.14	0.00000137	pigeons
Ambra1	-0.07	0.00000137	pigeons
Trp53bp2	-0.96	0.00000138	pigeons
Zbtb2	-0.10	0.00000139	pigeons
Eif3m	-0.11	0.00000140	pigeons
Wdtc1	0.11	0.00000141	pigeons
Slc30a5	0.14	0.00000148	pigeons
Tmcc1	0.03	0.00000152	pigeons
Ywhab	-0.12	0.00000152	pigeons
Eif2ak1	0.02	0.00000154	pigeons
Ctu2	0.15	0.00000155	pigeons
Grpel1	0.11	0.00000157	pigeons
Usp24	-0.04	0.00000159	pigeons
Fasn	0.09	0.00000161	pigeons
Nipsnap2	0.13	0.00000165	pigeons
Iws1	-0.08	0.00000165	pigeons
Rab5c	-0.07	0.00000168	pigeons
Pdia6	-0.07	0.00000170	pigeons
Soga1	0.12	0.00000172	pigeons
Ypel2	0.12	0.00000176	pigeons
Rap1b	-0.07	0.00000181	pigeons
Zfp711	-0.10	0.00000182	pigeons
Cbll1	0.11	0.00000184	pigeons
Ranbp1	-0.22	0.00000184	pigeons
Ndufs2	-0.03	0.00000191	pigeons
Eif3d	-0.08	0.00000194	pigeons
Prkar2a	0.03	0.00000197	pigeons
Rab6a	-0.09	0.00000199	pigeons
Zfp292	0.10	0.00000201	pigeons
Avl9	0.13	0.00000202	pigeons
Zfp622	0.00	0.00000205	pigeons
Stx16	0.06	0.00000206	pigeons

Immt	0.01	0.00000207	pigeons
Foxn3	-1.00	0.00000209	pigeons
Cyfip2	0.03	0.00000210	pigeons
Dync1li2	-0.18	0.00000212	pigeons
Ube2j2	-0.07	0.00000212	pigeons
Zranb2	-0.05	0.00000215	pigeons
Slc25a3	-0.19	0.00000215	pigeons
Crlf3	0.04	0.00000218	pigeons
Llph	-0.08	0.00000222	pigeons
Osbpl3	0.04	0.00000224	pigeons
Lactb	-0.03	0.00000229	pigeons
Rprd2	-0.02	0.00000234	pigeons
Sacm1l	-0.07	0.00000237	pigeons
Ube3c	-0.13	0.00000238	pigeons
Cep85l	-0.13	0.00000243	pigeons
Rcbtb1	0.01	0.00000243	pigeons
Ctns	0.14	0.00000246	pigeons
Kifap3	0.04	0.00000256	pigeons
Ppp2ca	-0.07	0.00000263	pigeons
Ubap2	-0.04	0.00000266	pigeons
Laptm4a	-0.97	0.00000278	pigeons
Kpna1	-0.06	0.00000279	pigeons
Zrsr1	-0.11	0.00000283	pigeons
Asah1	-0.19	0.00000284	pigeons
Uggt1	0.07	0.00000290	pigeons
Derl1	-0.02	0.00000290	pigeons
Snx12	0.06	0.00000293	pigeons
Uri1	-0.05	0.00000308	pigeons
Ammecr1	0.04	0.00000311	pigeons
Arhgap25	0.04	0.00000320	pigeons
Sde2	-0.16	0.00000322	pigeons
Zfp831	0.12	0.00000323	pigeons
Azin1	0.08	0.00000347	pigeons
Vwf	0.34	0.00000352	pigeons
Krit1	0.09	0.00000354	pigeons
Pdhb	0.01	0.00000361	pigeons
D2hgdh	0.09	0.00000368	pigeons
Hspa5	-1.30	0.00000383	pigeons
Sec61g	-0.24	0.00000393	pigeons
Hipk3	0.02	0.00000394	pigeons
Dnaja2	-0.23	0.00000404	pigeons
Prpf6	-0.05	0.00000407	pigeons
Clint1	-0.10	0.00000412	pigeons
Tln1	-0.17	0.00000416	pigeons
Lrrc8d	-0.01	0.00000432	pigeons
Necap2	-0.07	0.00000433	pigeons

Gfra3	0.06	0.00000444	pigeons
Usf3	0.10	0.00000450	pigeons
Btbd10	0.10	0.00000453	pigeons
Smndc1	-0.16	0.00000456	pigeons
Hoxa5	-1.11	0.00000457	pigeons
Rab2a	-0.06	0.00000459	pigeons
Eif2s2	-0.12	0.00000466	pigeons
Zzz3	-0.10	0.00000471	pigeons
Itgb1	-1.18	0.00000493	pigeons
Ndufb9	-0.07	0.00000495	pigeons
Daglb	0.16	0.00000534	pigeons
Smpd2	0.09	0.00000539	pigeons
Ttc1	0.04	0.00000547	pigeons
Rab9	-0.01	0.00000563	pigeons
Nbas	-0.01	0.00000589	pigeons
Klf3	-1.23	0.00000613	pigeons
Rpa2	0.00	0.00000619	pigeons
Tmem104	0.19	0.00000639	pigeons
Gosr1	-0.01	0.00000644	pigeons
Dync1h1	0.14	0.00000651	pigeons
Shoc2	-0.08	0.00000652	pigeons
Iqsec1	0.05	0.00000660	pigeons
Trip12	-0.01	0.00000661	pigeons
Rae1	0.09	0.00000663	pigeons
Lonp2	-0.08	0.00000676	pigeons
Atrn	-1.19	0.00000760	pigeons
Gpr155	-0.02	0.00000765	pigeons
Fkbp3	-0.09	0.00000773	pigeons
Cdk8	0.16	0.00000781	pigeons
Wdr45b	0.12	0.00000810	pigeons
Rbm22	-0.07	0.00000816	pigeons
Cpsf4l	-0.01	0.00000874	pigeons
Ramp2	-1.21	0.00000879	pigeons
G3bp2	-0.03	0.00000910	pigeons
Ube2q2	-0.18	0.00000969	pigeons
Fyco1	0.14	0.00000982	pigeons
Tshz2	-1.06	0.00001043	pigeons
Nisch	-0.08	0.00001071	pigeons
Slc25a22	0.14	0.00001168	pigeons
Prelid3a	-0.11	0.00001181	pigeons
Actr1a	-0.14	0.00001204	pigeons
Tmem9b	-0.13	0.00001212	pigeons
Lats1	-0.13	0.00001242	pigeons
Abce1	-0.18	0.00001256	pigeons
Nlrc5	0.22	0.00001390	pigeons
AW554918	0.30	0.00001421	pigeons

Cmas	0.24	0.00001457	pigeons
Hadh	-0.17	0.00001459	pigeons
Pnpla2	0.11	0.00001478	pigeons
Fkbp15	0.11	0.00001519	pigeons
Pank2	0.08	0.00001597	pigeons
Ubqln4	0.01	0.00001606	pigeons
Sf3a1	-0.12	0.00001610	pigeons
Dpp9	0.10	0.00001613	pigeons
Arhgap29	-0.01	0.00001663	pigeons
Slc23a2	0.12	0.00001799	pigeons
Rbm18	0.04	0.00001813	pigeons
Pisd	-0.07	0.00001839	pigeons
Cdc40	0.06	0.00001861	pigeons
Lrpprc	0.08	0.00002003	pigeons
Amot	-0.09	0.00002014	pigeons
Wac	-0.09	0.00002072	pigeons
Ipo7	-0.17	0.00002091	pigeons
Rtf1	-0.13	0.00002129	pigeons
Numa1	0.12	0.00002239	pigeons
Larp4	0.04	0.00002256	pigeons
Lrig2	0.00	0.00002295	pigeons
Larp1	-0.03	0.00002334	pigeons
Commd7	-0.02	0.00002382	pigeons
Khdrbs1	0.01	0.00002392	pigeons
Cmtm3	0.07	0.00002445	pigeons
Lbh	-1.01	0.00002461	pigeons
Ascc3	0.18	0.00002462	pigeons
Upf1	-0.19	0.00002525	pigeons
Cpt1a	0.03	0.00002568	pigeons
Ndufb8	-0.17	0.00002572	pigeons
Btbd7	0.03	0.00002645	pigeons
S100a10	0.22	0.00002684	pigeons
Paxbp1	-0.16	0.00002702	pigeons
Txnrd1	-0.76	0.00002743	pigeons
Smu1	-0.06	0.00002823	pigeons
Virma	-0.03	0.00002836	pigeons
Cmtr1	0.07	0.00002858	pigeons
Ext2	-0.05	0.00003072	pigeons
Inpp5a	0.22	0.00003192	pigeons
Tnrc6a	-0.12	0.00003193	pigeons
Nfyc	0.00	0.00003297	pigeons
Limk2	0.14	0.00003498	pigeons
Hectd4	-0.08	0.00003557	pigeons
Smurf1	0.10	0.00003734	pigeons
Ei24	0.04	0.00003878	pigeons
Nhp2	-0.03	0.00003935	pigeons

Adar	0.03	0.00003950	pigeons
Pogz	0.11	0.00003952	pigeons
Slc9a6	-0.04	0.00004110	pigeons
Anks1	-1.13	0.00004114	pigeons
Lmo2	-0.97	0.00004214	pigeons
Vps13d	-0.07	0.00004231	pigeons
Sub1	-0.13	0.00004251	pigeons
Hax1	0.02	0.00004306	pigeons
Psme3	-0.09	0.00004659	pigeons
Ptges3	-0.26	0.00004674	pigeons
Rhoh	-0.01	0.00004738	pigeons
Rnf34	-0.09	0.00004924	pigeons
2510039O18Rik	-0.01	0.00005149	pigeons
Atxn1l	0.05	0.00005481	pigeons
Setx	-0.14	0.00005495	pigeons
Cd81	-1.07	0.00005660	pigeons
Xbp1	-0.01	0.00005890	pigeons
Dnajb2	-0.07	0.00005942	pigeons
Cops8	-0.17	0.00006151	pigeons
Cdh23	0.13	0.00006254	pigeons
Taf13	-0.10	0.00006319	pigeons
Fam214a	-0.04	0.00006466	pigeons
Dtnbp1	0.15	0.00006688	pigeons
Epc1	-0.09	0.00006753	pigeons
Pin1	-0.02	0.00007248	pigeons
Smarcc1	-0.11	0.00007296	pigeons
Csf1	0.26	0.00007329	pigeons
Vti1b	0.03	0.00007443	pigeons
Tpd52l2	-0.11	0.00007477	pigeons
Nsfl1c	0.04	0.00007570	pigeons
Paip1	0.02	0.00008198	pigeons
Tpr	-0.17	0.00008215	pigeons
Fam185a	0.10	0.00008399	pigeons
Rprd1a	0.09	0.00008472	pigeons
Pigl	0.24	0.00008780	pigeons
Cope	-0.10	0.00008794	pigeons
Ndufb6	-0.26	0.00008964	pigeons
Hmg20b	0.00	0.00009086	pigeons
Jakmip1	0.02	0.00009179	pigeons
Inpp5d	-1.15	0.00009364	pigeons
Fam120a	-0.19	0.00009742	pigeons
Pafah1b2	-0.09	0.00011072	pigeons
Clcn3	0.00	0.00011149	pigeons
Sf3b3	-0.15	0.00011450	pigeons
Stim2	-1.15	0.00011857	pigeons
Kpna3	-0.08	0.00011940	pigeons

Dgkz	0.03	0.00012836	pigeons
Rab35	-0.17	0.00013203	pigeons
Ipo8	0.05	0.00013369	pigeons
Nfkbie	-0.13	0.00013678	pigeons
Sh2d1a	-1.05	0.00014277	pigeons
Ebag9	0.01	0.00014602	pigeons
Acot10	0.04	0.00014976	pigeons
Rfc1	-0.06	0.00015348	pigeons
Zc3h15	-0.15	0.00015667	pigeons
Rwdd4a	-0.01	0.00016305	pigeons
H13	-0.14	0.00016327	pigeons
Gpi1	-0.03	0.00016358	pigeons
Ate1	-0.04	0.00016435	pigeons
Vamp4	0.07	0.00017197	pigeons
Supt4a	-0.27	0.00017277	pigeons
Tcp1	-0.20	0.00017327	pigeons
Npnt	0.09	0.00017729	pigeons
Hvcn1	0.06	0.00018795	pigeons
Hyou1	0.01	0.00018992	pigeons
Ube2g1	-0.19	0.00019033	pigeons
Tmem131	-0.04	0.00019247	pigeons
Taf3	-0.16	0.00019398	pigeons
Uhmk1	-0.01	0.00019531	pigeons
Smc6	0.05	0.00019911	pigeons
Snrpd3	-0.18	0.00020334	pigeons
Nub1	-0.11	0.00020644	pigeons
Nfya	0.07	0.00021472	pigeons
Vgll4	0.01	0.00021957	pigeons
Cdc73	-0.16	0.00022124	pigeons
Atp6v0a2	0.00	0.00022249	pigeons
Sin3b	-0.01	0.00022859	pigeons
Kdelr2	-0.30	0.00023144	pigeons
Ehd4	-0.28	0.00023974	pigeons
Kras	-0.18	0.00024577	pigeons
Lonrf1	-0.11	0.00024834	pigeons
Upp1	0.08	0.00025539	pigeons
Hdx	-1.14	0.00025779	pigeons
Rbl2	-0.07	0.00025826	pigeons
Arid2	0.05	0.00026229	pigeons
Ero1a	0.09	0.00027129	pigeons
Tet2	0.13	0.00027741	pigeons
Ifi30	-0.28	0.00028053	pigeons
Tspan14	-0.05	0.00028822	pigeons
Tmx3	-0.23	0.00029404	pigeons
Ube2i	-0.20	0.00029567	pigeons
Pik3r4	0.08	0.00029920	pigeons

Ctnna1	-0.14	0.00031355	pigeons
Gtf2a2	-0.13	0.00031447	pigeons
Gfod1	-0.84	0.00031751	pigeons
Tmem11	-0.02	0.00031902	pigeons
Cir1	-0.15	0.00034171	pigeons
Tbc1d12	-0.01	0.00034174	pigeons
Gna11	-0.11	0.00034458	pigeons
Trappc11	-0.05	0.00034602	pigeons
Ghdc	0.03	0.00035058	pigeons
Actr10	-0.07	0.00035088	pigeons
Pcif1	0.00	0.00035964	pigeons
Satb1	-0.07	0.00036370	pigeons
Ldah	0.03	0.00038468	pigeons
Eef1d	-1.27	0.00038970	pigeons
Ints11	0.04	0.00039459	pigeons
Tnpo1	-0.20	0.00039770	pigeons
Hddc2	-0.06	0.00040481	pigeons
Dclk2	0.15	0.00040522	pigeons
Fndc3b	0.15	0.00040594	pigeons
Usp10	-0.12	0.00041036	pigeons
Spty2d1	-0.59	0.00041301	pigeons
Zranb1	-0.25	0.00042562	pigeons
Arf4	-0.27	0.00042595	pigeons
Eef2	-0.23	0.00045108	pigeons
Bclaf1	-0.17	0.00045206	pigeons
Pphln1	0.05	0.00046250	pigeons
Snhg4	-0.25	0.00046669	pigeons
Nkrf	-0.08	0.00046991	pigeons
Top1	-0.18	0.00047137	pigeons
Dync1i2	-0.13	0.00047286	pigeons
Pex13	0.04	0.00047443	pigeons
Rnf145	0.03	0.00047887	pigeons
Api5	-0.17	0.00048162	pigeons
Clp1	-0.04	0.00048468	pigeons
Exoc4	-0.10	0.00049215	pigeons
G3bp1	-0.16	0.00049416	pigeons
Tex2	0.16	0.00049819	pigeons
Srp72	-0.16	0.00053716	pigeons
Dus1l	-0.07	0.00053905	pigeons
Sh3bgrl	-0.13	0.00053918	pigeons
Aco2	-0.15	0.00054241	pigeons
Psma1	-0.20	0.00055194	pigeons
Rsl24d1	-0.27	0.00056564	pigeons
Ssr2	-0.12	0.00057986	pigeons
Fam135a	-0.20	0.00059214	pigeons
Nudc	-0.01	0.00061047	pigeons

Nup98	-0.25	0.00062004	pigeons
Rsrc2	-0.23	0.00062967	pigeons
Ercc3	-0.11	0.00065277	pigeons
Cct2	-0.26	0.00065515	pigeons
Lrrc59	0.03	0.00067673	pigeons
Xpo7	-0.08	0.00067838	pigeons
Txndc17	-0.26	0.00068302	pigeons
Rufy1	-0.17	0.00071622	pigeons
Cflar	-0.25	0.00072148	pigeons
Dhx36	-0.17	0.00072735	pigeons
Ptpn2	-0.18	0.00072843	pigeons
Washc4	0.04	0.00073827	pigeons
Auh	-0.06	0.00075463	pigeons
Aimp2	-0.03	0.00078894	pigeons
Eif4a3l1	0.04	0.00080431	pigeons
Dnajb9	-0.04	0.00080764	pigeons
Tmf1	-0.22	0.00081240	pigeons
Utp23	-0.01	0.00083188	pigeons
Txndc12	-0.12	0.00083790	pigeons
Tcea1	-0.17	0.00085025	pigeons
Dnmt3a	-0.01	0.00087878	pigeons
Zfp507	0.04	0.00092515	pigeons
Polr2c	-0.03	0.00096905	pigeons
Ppp1r13b	-1.25	0.00098643	pigeons
Lrrc8a	0.01	0.00098828	pigeons
Tmem208	-0.04	0.00098909	pigeons
Arhgap26	-0.20	0.00099086	pigeons
Zfp276	-0.01	0.00103224	pigeons
Ctr9	-0.08	0.00104635	pigeons
Prpf4b	-0.21	0.00108271	pigeons
Aimp1	-0.31	0.00115805	pigeons
Ino80	-0.22	0.00116926	pigeons
Ube2b	-0.22	0.00117883	pigeons
Apool	0.01	0.00119742	pigeons
Sdc4	-0.50	0.00120446	pigeons
Usp38	-0.12	0.00120567	pigeons
Rac1	-0.29	0.00121177	pigeons
Kbtbd2	-0.10	0.00122611	pigeons
Sap30	0.00	0.00123592	pigeons
Dmap1	-1.08	0.00126646	pigeons
Sav1	-0.02	0.00129013	pigeons
Kdelr3	-0.11	0.00132678	pigeons
Gab3	0.01	0.00135079	pigeons
Cln5	-0.04	0.00135852	pigeons
Erg	-1.05	0.00141569	pigeons
Slc25a36	-0.06	0.00142842	pigeons

Gtdc1	0.03	0.00143568	pigeons
Eny2	-0.11	0.00147192	pigeons
Tmem185a	-0.04	0.00147945	pigeons
Acss2	0.10	0.00153107	pigeons
Exoc7	-0.10	0.00153113	pigeons
Tmem245	0.08	0.00155255	pigeons
Kdm3b	-0.27	0.00155515	pigeons
Ebf1	-1.04	0.00156517	pigeons
Adrm1	-0.16	0.00173240	pigeons
Nr2c1	-0.06	0.00190794	pigeons
Cpne3	-0.21	0.00192570	pigeons
Tapt1	-0.19	0.00194235	pigeons
Tmem183a	-0.06	0.00196012	pigeons
Eif3e	-0.22	0.00197306	pigeons
Ss18	-0.15	0.00201834	pigeons
Ano10	-0.91	0.00205289	pigeons
Cfdp1	-0.14	0.00214011	pigeons
Adamts2	-1.06	0.00215418	pigeons
Ppil1	-0.07	0.00223441	pigeons
Natd1	0.12	0.00227316	pigeons
Washc3	0.01	0.00230917	pigeons
Tmsb4x	-0.23	0.00233268	pigeons
Atrx	-0.18	0.00235878	pigeons
Itgb1bp1	0.01	0.00239062	pigeons
Yipf5	0.02	0.00239700	pigeons
Yipf1	-0.04	0.00239782	pigeons
Ttc37	-0.03	0.00259314	pigeons
Wbp4	-1.15	0.00263449	pigeons
Suco	-0.05	0.00264052	pigeons
Rnf4	-0.16	0.00267665	pigeons
Tspan2	0.04	0.00277101	pigeons
Abca3	-0.17	0.00277765	pigeons
Arl8b	-0.14	0.00278169	pigeons
Rpia	-0.13	0.00281308	pigeons
Arf6	-0.27	0.00281757	pigeons
Etnk1	-0.19	0.00284744	pigeons
Tob1	-0.18	0.00297305	pigeons
Eif3b	-0.20	0.00302572	pigeons
Iars2	-0.08	0.00306672	pigeons
Pinx1	-0.05	0.00307206	pigeons
Eif3f	-0.26	0.00327451	pigeons
Eif4g2	0.15	0.00331224	pigeons
Il6st	-0.81	0.00343206	pigeons
Yod1	-0.02	0.00361389	pigeons
Phc3	0.01	0.00363672	pigeons
Suclg1	-0.03	0.00368059	pigeons

Eif2ak2	-0.07	0.00383067	pigeons
Tada1	-0.12	0.00390532	pigeons
Rabl6	-0.18	0.00395198	pigeons
Nptn	-0.18	0.00398122	pigeons
Psmb2	-0.21	0.00404326	pigeons
Edf1	-0.15	0.00429955	pigeons
Hipk1	-0.27	0.00438551	pigeons
Ranbp9	-0.13	0.00442579	pigeons
Tox	-0.82	0.00445727	pigeons
Uchl5	0.01	0.00449552	pigeons
Ubn2	-0.18	0.00453715	pigeons
Slc2a13	0.13	0.00494530	pigeons
Cbx6	-0.16	0.00510966	pigeons
Cep83	-1.11	0.00515612	pigeons
Ufsp2	0.06	0.00521542	pigeons
Tns3	-0.94	0.00527857	pigeons
Arih1	-0.29	0.00542314	pigeons
Eif1ad12	-0.18	0.00546992	pigeons
Pbrm1	-0.18	0.00574862	pigeons
Copg2	-0.10	0.00592963	pigeons
Abcc1	-1.04	0.00596399	pigeons
Dlgap4	-0.09	0.00599074	pigeons
Ciao1	0.00	0.00620251	pigeons
Zc3h12b	0.15	0.00636443	pigeons
Rit1	0.00	0.00637482	pigeons
Scpep1	-0.06	0.00647969	pigeons
Cenpu	0.28	0.00660877	pigeons
Ralgapb	-0.12	0.00667616	pigeons
Golga5	-0.03	0.00677751	pigeons
Bcas2	-0.07	0.00679718	pigeons
Gpatch1	-0.18	0.00689299	pigeons
Cops3	-0.23	0.00713316	pigeons
Krr1	-0.11	0.00720911	pigeons
Ankrd28	0.08	0.00722701	pigeons
Phlpp2	-0.19	0.00729687	pigeons
Arnt	-0.20	0.00738957	pigeons
Polr2d	-0.05	0.00739046	pigeons
Hmgb2	-0.05	0.00767814	pigeons
Fam13b	-0.31	0.00805135	pigeons
Golga4	-1.05	0.00822026	pigeons
Tcf25	-0.10	0.00826559	pigeons
Rmnd1	-0.04	0.00841583	pigeons
Ttc39a	0.04	0.00857120	pigeons
Sptlc1	-0.03	0.00870616	pigeons
Pds5b	-0.99	0.00908701	pigeons
Tpi1	-0.03	0.00926121	pigeons

Dcbld2	-0.16	0.00942613	pigeons
Leo1	-0.13	0.00946734	pigeons
Pcmt1	-0.19	0.00980319	pigeons
Rbpj	-0.13	0.01014774	pigeons
Unc119b	-0.12	0.01045242	pigeons
Ndufb10	-0.33	0.01108161	pigeons
Rfx3	-0.81	0.01125623	pigeons
Lcmt2	-0.87	0.01127942	pigeons
Cpsf2	-0.07	0.01178711	pigeons
Sf3b5	-0.16	0.01185457	pigeons
Ckap5	-0.23	0.01255336	pigeons
Gnas	-0.31	0.01255663	pigeons
Nrf1	-0.06	0.01274562	pigeons
Rsf1	-0.20	0.01280338	pigeons
Ing5	-0.17	0.01288285	pigeons
Cxcr4	0.16	0.01315073	pigeons
Rprd1b	-0.10	0.01383155	pigeons
Nans	-0.11	0.01410671	pigeons
Ggps1	-0.19	0.01416045	pigeons
Lmo3	-1.09	0.01477336	pigeons
Ctsh	-0.10	0.01515443	pigeons
Crebl2	-0.13	0.01556580	pigeons
Uap1	-0.04	0.01561115	pigeons
Tmem168	-0.10	0.01619089	pigeons
Cdv3	-0.35	0.01694040	pigeons
Serinc3	-0.37	0.01697772	pigeons
Dhx38	-0.12	0.01701784	pigeons
Drc7	0.11	0.01713404	pigeons
Zc3h12d	0.16	0.01781738	pigeons
Rbm45	-0.08	0.01858641	pigeons
Fbxo9	-0.13	0.01873483	pigeons
Psmd6	-0.07	0.01913146	pigeons
Tlk1	-0.19	0.01926050	pigeons
Zfp830	-0.07	0.02065663	pigeons
Slc45a3	-0.24	0.02066992	pigeons
Clk3	0.05	0.02122098	pigeons
Ap3s1	-0.80	0.02192305	pigeons
Alpk1	0.12	0.02233696	pigeons
Ripk2	0.00	0.02290371	pigeons
Cyth3	-0.95	0.02294042	pigeons
Irf2	-0.32	0.02447770	pigeons
Ino80d	-0.15	0.02499024	pigeons
Abcg1	0.04	0.02546471	pigeons
Phactr2	-0.10	0.02558115	pigeons
Rb1	0.00	0.02577720	pigeons
Slain2	-0.21	0.02595960	pigeons

Naxd	-0.30	0.02596610	pigeons
Baz1b	-0.30	0.02640979	pigeons
Eif4e	-0.12	0.02702478	pigeons
Atf6	-0.19	0.02702590	pigeons
Usp6nl	-0.26	0.02747263	pigeons
Zbtb33	-0.05	0.02772011	pigeons
Trak1	-1.01	0.02856705	pigeons
Sp4	-0.13	0.02955507	pigeons
Prkd3	-0.19	0.02955521	pigeons
Csnk1a1	-0.05	0.03034489	pigeons
Usp45	-0.17	0.03175415	pigeons
Polr2f	-0.20	0.03189168	pigeons
Pola1	-0.94	0.03476298	pigeons
Akap10	-0.17	0.03547142	pigeons
Ndrg3	-0.02	0.03639224	pigeons
Chic2	-0.10	0.03695211	pigeons
Rgs9	-0.24	0.03766916	pigeons
Farp2	0.02	0.03776901	pigeons
Tcn2	-0.11	0.04109440	pigeons
Tmco1	-0.21	0.04112263	pigeons
Znhit6	-0.10	0.04492704	pigeons
Btbd2	-0.03	0.04499929	pigeons
Ern1	-0.17	0.04551884	pigeons
Brf1	-0.21	0.04563377	pigeons
Wdr36	-0.07	0.04565338	pigeons
Selenot	-0.32	0.04658131	pigeons
Pds5a	-0.16	0.04740556	pigeons
Rnaseh2b	-0.24	0.04860584	pigeons
Ndufb3	-0.32	0.04937980	pigeons
Vti1a	0.16	0.04947019	pigeons
Ndufs3	-0.11	0.04996031	pigeons
Kif5b	-0.18	0.05018841	pigeons
Cpne1	-0.23	0.05095394	pigeons
Hhex	-0.20	0.05263078	pigeons
Oard1	-0.23	0.05294944	pigeons
Sec11c	-0.30	0.05302131	pigeons
Abcd2	-0.20	0.05422646	pigeons
Ube2g2	-0.11	0.05432674	pigeons
Rpap2	-0.03	0.05489865	pigeons
Srrm1	-0.30	0.05581060	pigeons
Plekha1	-0.32	0.05647108	pigeons
Ipo5	-0.07	0.05930213	pigeons
Taf5l	-1.09	0.06079658	pigeons
Sart3	-0.10	0.06218882	pigeons
Cdc16	-0.15	0.06481489	pigeons
Skp1	-0.42	0.06748529	pigeons

Fchsd2	-0.30	0.06829490	pigeons
Xpot	-0.13	0.06930634	pigeons
Ola1	-0.15	0.07006683	pigeons
Dym	-0.21	0.07029261	pigeons
Pum2	-0.24	0.07385137	pigeons
Smc3	-0.21	0.07579719	pigeons
Usp12	-0.96	0.07584546	pigeons
Ikzf1	-0.23	0.07657587	pigeons
Ndufa13	-0.14	0.07762728	pigeons
Agl	-0.15	0.07995096	pigeons
Slc16a10	0.37	0.08299750	pigeons
Ddx5	0.13	0.08326849	pigeons
Rnf19a	-0.13	0.08503371	pigeons
Lin52	0.04	0.08654378	pigeons
Pitrm1	-0.06	0.08818899	pigeons
Akap9	-0.98	0.08922601	pigeons
Tbc1d2b	-0.18	0.09025407	pigeons
Chid1	-0.05	0.09156374	pigeons
Klhl36	-0.01	0.09228041	pigeons
Stx2	-0.18	0.09737896	pigeons
B4galt1	-0.03	0.09909599	pigeons
Klhdc1	-0.05	0.10420029	pigeons
Fam8a1	-0.15	0.10540164	pigeons
Cebpz	-0.10	0.10696456	pigeons
Psmc6	-0.37	0.10816646	pigeons
Hsd17b4	-0.11	0.11352447	pigeons
Arl6ip1	-0.32	0.11541788	pigeons
Cyfip1	0.09	0.11705514	pigeons
Rsrc1	-0.12	0.11725209	pigeons
Supt20	-0.11	0.11999669	pigeons
Dcaf5	-0.29	0.12691987	pigeons
Atg101	-0.13	0.13753326	pigeons
Zfp438	-0.01	0.13923630	pigeons
Snap29	-0.24	0.13937331	pigeons
Asxl2	-0.15	0.14270027	pigeons
Faf2	-0.17	0.14663873	pigeons
Glg1	-0.22	0.14836920	pigeons
Naa30	-0.21	0.15016473	pigeons
Rnf130	-0.34	0.15312346	pigeons
Tcf3	-0.11	0.16094350	pigeons
Srp19	-0.22	0.16096199	pigeons
Tomm70a	-0.15	0.16247118	pigeons
Adgrl3	0.13	0.16678343	pigeons
Phb	-0.15	0.16774749	pigeons
Itpkb	-0.51	0.16846080	pigeons
Dhx15	-0.36	0.17102024	pigeons

Aplp2	-1.13	0.17555517	pigeons
Pabpc4	-0.05	0.19259486	pigeons
Lrp3	-0.32	0.19475751	pigeons
Gabarapl2	-0.39	0.19597258	pigeons
Tnks2	-0.27	0.19706425	pigeons
Ttc7b	-0.18	0.20260802	pigeons
Scfd1	-0.17	0.20516569	pigeons
Hbp1	-0.28	0.20524146	pigeons
Edc3	-0.10	0.20905682	pigeons
Nudcd3	0.04	0.21359128	pigeons
Gpatch2l	-0.10	0.21644498	pigeons
Larp1b	-0.26	0.22037054	pigeons
Snx19	-0.07	0.22062945	pigeons
Zzef1	-0.12	0.22268948	pigeons
Adam10	-0.28	0.22566964	pigeons
Ubl3	-0.24	0.22976074	pigeons
Abca1	-0.21	0.23261040	pigeons
Ccdc88c	0.10	0.23767851	pigeons
Clptm1l	-0.27	0.24221824	pigeons
Bhlhe40	-0.13	0.24313778	pigeons
Rmdn3	-0.11	0.24404743	pigeons
Ndufa2	-0.30	0.25626329	pigeons
Eif5b	-0.29	0.26044358	pigeons
Htatsf1	-0.22	0.26178339	pigeons
Heatr5a	-0.21	0.26451150	pigeons
Smarca2	-0.18	0.26674240	pigeons
Usp16	-0.22	0.26870923	pigeons
Trappc8	-0.25	0.26923321	pigeons
Cops4	-0.18	0.27532844	pigeons
Sdhd	-0.39	0.28299913	pigeons
Emc7	-0.21	0.28987546	pigeons
Lemd2	-0.08	0.29160802	pigeons
Letm1	-0.21	0.29747359	pigeons
Zfp618	-0.15	0.30102677	pigeons
Pik3ca	-1.15	0.30170977	pigeons
Dusp7	-0.86	0.31155518	pigeons
Smad5	-0.88	0.31577902	pigeons
Cc2d1b	-0.05	0.31672050	pigeons
Rnf214	-0.30	0.32468704	pigeons
Hnrnph3	-0.35	0.33141130	pigeons
Kat6b	-0.87	0.33582233	pigeons
Camsap2	-1.03	0.33593216	pigeons
Rusc2	-0.25	0.33861966	pigeons
Ybx3	-1.04	0.34243366	pigeons
Zhx2	0.11	0.34746458	pigeons
Ap1ar	-0.10	0.36387177	pigeons

Klf13	-0.13	0.38431973	pigeons
Psma4	-0.36	0.38484443	pigeons
Phkb	-0.14	0.39756995	pigeons
Per3	-0.06	0.40333713	pigeons
Clk2	-0.15	0.41796736	pigeons
Disp1	-0.76	0.42792304	pigeons
Prkn	0.22	0.43192346	pigeons
Ufl1	-0.16	0.43485471	pigeons
Srsf3	-0.45	0.43562435	pigeons
Slc25a20	-0.06	0.45557005	pigeons
Ghitm	-0.39	0.46052461	pigeons
Atp9b	-0.23	0.47046577	pigeons
Nelfb	-0.10	0.47257828	pigeons
Psmb3	-0.12	0.49350159	pigeons
Fubp3	-0.18	0.52276607	pigeons
Eif2ak4	-0.24	0.52701193	pigeons
Prrc2b	-0.37	0.54524445	pigeons
Cramp1	-0.24	0.55568329	pigeons
Rpap3	-0.15	0.56222680	pigeons
Naaa	-0.10	0.59049885	pigeons
Tomm20	-0.23	0.59387545	pigeons
Epb41	-0.19	0.60124026	pigeons
Kif13b	-0.30	0.60994229	pigeons
Cct8	-0.29	0.61777844	pigeons
Ddx27	-0.24	0.62754013	pigeons
Blvra	-0.03	0.63301319	pigeons
Rsl1d1	-0.11	0.64357112	pigeons
Poc1b	-0.13	0.64753287	pigeons
Sumo1	-0.37	0.66022604	pigeons
Dcp2	-0.17	0.68789350	pigeons
Nifk	-0.30	0.69264675	pigeons
Cdkal1	0.04	0.69476639	pigeons
Ralbp1	-0.38	0.70529741	pigeons
Nsa2	-0.23	0.73294509	pigeons
Sel1l	-0.42	0.73514161	pigeons
Pfdn4	-0.16	0.74584471	pigeons
Flot2	-0.09	0.75284244	pigeons
Park7	-0.13	0.75973388	pigeons
Nono	-0.19	0.77799854	pigeons
Ppme1	-0.09	0.79727479	pigeons
Cpd	-0.11	0.80461447	pigeons
Gpx4	-0.34	0.82553359	pigeons
Trpm7	-0.24	0.85093187	pigeons
Ssr1	-0.09	0.91180252	pigeons
Esf1	-0.10	0.96015089	pigeons
Ndufs8	-0.39	0.96661926	pigeons

Amd2	-0.23	0.97394346	pigeons
Ing3	-0.12	1.00000000	pigeons
Zfp143	-0.24	1.00000000	pigeons
Edrf1	-0.15	1.00000000	pigeons
Cnot10	-0.31	1.00000000	pigeons
E2f6	-0.17	1.00000000	pigeons
Chmp6	-0.18	1.00000000	pigeons
Prelid1	-0.41	1.00000000	pigeons
Gjd4	-0.30	1.00000000	pigeons
Rbms1	-0.29	1.00000000	pigeons
Sec13	-0.14	1.00000000	pigeons
Rab37	-0.21	1.00000000	pigeons
C2cd2l	-0.11	1.00000000	pigeons
Zdhhc17	-0.26	1.00000000	pigeons
Sh3bp2	-1.15	1.00000000	pigeons
Pla2g7	-0.23	1.00000000	pigeons
Prkcd	-0.13	1.00000000	pigeons
Pim1	-0.23	1.00000000	pigeons
Ndufa4	-0.45	1.00000000	pigeons
Pxk	-0.16	1.00000000	pigeons
Xrn1	-0.27	1.00000000	pigeons
Thrap3	-0.25	1.00000000	pigeons
Srpk1	-0.22	1.00000000	pigeons
Angel2	-0.12	1.00000000	pigeons
Itsn1	-0.82	1.00000000	pigeons
Hmbs	-0.18	1.00000000	pigeons
Amdhd2	-0.15	1.00000000	pigeons
Rock2	-0.93	1.00000000	pigeons
Sqstm1	-0.31	1.00000000	pigeons
Hoxa3	-0.98	1.00000000	pigeons
Rabepk	-0.36	1.00000000	pigeons
Sap130	-0.23	1.00000000	pigeons
Prkrip1	-0.26	1.00000000	pigeons
Capn11	-0.22	1.00000000	pigeons
Parg	-0.30	1.00000000	pigeons
Tlnrd1	-0.93	1.00000000	pigeons
Ctcf	-0.43	1.00000000	pigeons
Dpysl2	-0.98	1.00000000	pigeons
Bag1	-0.11	1.00000000	pigeons
Armc8	-0.25	1.00000000	pigeons
Zmynd8	-0.21	1.00000000	pigeons
Ssr3	-0.36	1.00000000	pigeons
Btf3	-0.27	1.00000000	pigeons
Zfp236	-0.07	1.00000000	pigeons
Fbxo33	-0.22	1.00000000	pigeons
Tmbim6	-0.38	1.00000000	pigeons

Serbp1	-0.27	1.00000000	pigeons
Rbm17	-0.18	1.00000000	pigeons
Snrpe	-0.45	1.00000000	pigeons
Cstf2	-0.17	1.00000000	pigeons
Vapb	-0.28	1.00000000	pigeons
Ranbp10	-0.17	1.00000000	pigeons
Inpp4a	-0.09	1.00000000	pigeons
Lamp1	-0.32	1.00000000	pigeons
Uso1	-0.18	1.00000000	pigeons
Kat6a	-0.35	1.00000000	pigeons
Vps41	-0.21	1.00000000	pigeons
Fam204a	-0.35	1.00000000	pigeons
Crebrf	-0.28	1.00000000	pigeons
Hmbox1	-0.13	1.00000000	pigeons
Cnot4	-0.16	1.00000000	pigeons
Tmpo	-0.91	1.00000000	pigeons
Ncoa4	-0.22	1.00000000	pigeons
Srsf2	-0.19	1.00000000	pigeons
Vps25	-0.17	1.00000000	pigeons
Fam199x	-0.07	1.00000000	pigeons
Fam126a	0.04	1.00000000	pigeons
Bdp1	-0.25	1.00000000	pigeons
Ulk2	-0.26	1.00000000	pigeons
Erp29	-0.42	1.00000000	pigeons
Nup107	-1.02	1.00000000	pigeons
Crem	0.07	1.00000000	pigeons
Ift20	-0.19	1.00000000	pigeons
Dyrk1a	-0.17	1.00000000	pigeons
Polk	-0.30	1.00000000	pigeons
Surf6	-0.18	1.00000000	pigeons
Stam	-0.21	1.00000000	pigeons
Senp7	-0.34	1.00000000	pigeons
Bnip3l	-0.42	1.00000000	pigeons
Dctn1	-0.27	1.00000000	pigeons
Pitpnb	-0.34	1.00000000	pigeons
Usp47	-0.36	1.00000000	pigeons
Zbed4	-0.14	1.00000000	pigeons
Elmo2	-0.30	1.00000000	pigeons
Trip11	-0.27	1.00000000	pigeons
Commd1b	-0.36	1.00000000	pigeons
Lonrf2	-0.27	1.00000000	pigeons
Ly86	-1.01	1.00000000	pigeons
Alg12	-0.13	1.00000000	pigeons
Stt3b	-0.29	1.00000000	pigeons
Rasa3	-0.23	1.00000000	pigeons
Sec31a	-1.05	1.00000000	pigeons

Saraf	-0.25	1.00000000	pigeons
Camkmt	0.24	1.00000000	pigeons
Polr2l	-0.25	1.00000000	pigeons
Urm1	-0.43	1.00000000	pigeons
Ccser1	-0.76	1.00000000	pigeons
Shroom2	-0.74	1.00000000	pigeons
Nubp1	-0.18	1.00000000	pigeons
Rgl1	-0.68	1.00000000	pigeons
Uxs1	-0.82	1.00000000	pigeons
Arhgef6	-0.35	1.00000000	pigeons
Dcp1a	-0.13	1.00000000	pigeons
Phlpp1	-0.11	1.00000000	pigeons
Tbc1d22a	-0.37	1.00000000	pigeons
Spz1	-0.39	1.00000000	pigeons
Tdrd3	-0.24	1.00000000	pigeons
Ctnnb1	-0.99	1.00000000	pigeons
Kansl1l	-0.23	1.00000000	pigeons
Dytn	0.01	1.00000000	pigeons
Tnfsf15	0.00	1.00000000	pigeons
Cep44	-0.26	1.00000000	pigeons
Fbxl14	-0.26	1.00000000	pigeons
Hoxa7	-0.66	1.00000000	pigeons
Ddx1	-0.37	1.00000000	pigeons
Rev1	-0.39	1.00000000	pigeons
Pikfyve	-0.31	1.00000000	pigeons
Usp25	-0.30	1.00000000	pigeons
Erc1	-0.76	1.00000000	pigeons
Abcb7	-0.29	1.00000000	pigeons
Bend7	-0.18	1.00000000	pigeons
Ubac2	-0.21	1.00000000	pigeons
Spata5	-0.09	1.00000000	pigeons
Nktr	-0.87	1.00000000	pigeons
Atp8a1	-0.09	1.00000000	pigeons
Smap1	-0.20	1.00000000	pigeons
Snrpb2	-0.36	1.00000000	pigeons
Irf1	0.04	1.00000000	pigeons
Eif4g3	-0.13	1.00000000	pigeons
Fam177a	-0.89	1.00000000	pigeons
Qser1	-0.44	1.00000000	pigeons
Exoc5	-0.27	1.00000000	pigeons
Zfp131	-0.13	1.00000000	pigeons
Hdac4	-0.89	1.00000000	pigeons
Sh3pxd2a	-0.16	1.00000000	pigeons
Cpeb3	0.04	1.00000000	pigeons
Sertad2	0.12	1.00000000	pigeons
Ccser2	-0.67	1.00000000	pigeons

Acad9	-0.24	1.00000000	pigeons
Nab1	-0.23	1.00000000	pigeons
Oaz2	-0.74	1.00000000	pigeons
Sipa1l1	-0.67	1.00000000	pigeons
Oxct1	-0.36	1.00000000	pigeons
Nucks1	-0.95	1.00000000	pigeons
Rhoa	0.03	1.00000000	pigeons
Polr2e	-0.34	1.00000000	pigeons
Ccny	-0.92	1.00000000	pigeons
Crlf2	0.03	1.00000000	pigeons
Arhgef7	-0.86	1.00000000	pigeons
Erh	-0.43	1.00000000	pigeons
Arhgap8	-0.02	1.00000000	pigeons
Sptan1	-0.22	1.00000000	pigeons
Brd7	-0.33	1.00000000	pigeons
Slc41a1	-0.16	1.00000000	pigeons
Hnrnpa3	-0.06	1.00000000	pigeons
Zc3h13	-0.36	1.00000000	pigeons
Dbi	-0.55	1.00000000	pigeons
Slc35c2	-0.42	1.00000000	pigeons
Eif5	-0.31	1.00000000	pigeons
Slc25a25	-0.32	1.00000000	pigeons
Hsd17b12	-0.72	1.00000000	pigeons
Ccpg1	-0.39	1.00000000	pigeons
Cry1	-0.97	1.00000000	pigeons
Tmed5	-0.44	1.00000000	pigeons
Phf10	-0.21	1.00000000	pigeons
Usp34	-0.41	1.00000000	pigeons
Cacul1	-0.48	1.00000000	pigeons
Tmem35b	-0.25	1.00000000	pigeons
Diaph2	0.13	1.00000000	pigeons
Pi4ka	-0.35	1.00000000	pigeons
Tmem50a	-0.43	1.00000000	pigeons
Rbm31y	-0.90	1.00000000	pigeons
Cmtm4	-0.51	1.00000000	pigeons
Ddx46	-0.31	1.00000000	pigeons
Smim14	-0.83	1.00000000	pigeons
Nek9	-0.16	1.00000000	pigeons
Hdac7	-1.05	1.00000000	pigeons
Tmem242	-0.33	1.00000000	pigeons
Ube2f	-0.29	1.00000000	pigeons
Bfar	-0.26	1.00000000	pigeons
Nol10	-0.18	1.00000000	pigeons
Csrp1	-0.98	1.00000000	pigeons
Slc25a37	-0.20	1.00000000	pigeons
Ubr1	-0.34	1.00000000	pigeons

Ndufa10	-0.26	1.00000000	pigeons
Tob2	-0.36	1.00000000	pigeons
Ccdc126	-0.24	1.00000000	pigeons
Alg2	-0.12	1.00000000	pigeons
Oxsr1	-0.39	1.00000000	pigeons
Pcm1	-0.28	1.00000000	pigeons
Cct5	-0.45	1.00000000	pigeons
Sms	-0.18	1.00000000	pigeons
Dgkh	-0.86	1.00000000	pigeons
Hnrnpa2b1	-0.57	1.00000000	pigeons
Xpr1	-0.18	1.00000000	pigeons
Cog5	-0.22	1.00000000	pigeons
Esrrg	-0.01	1.00000000	pigeons
Gtf2b	-0.37	1.00000000	pigeons
Grk3	-0.36	1.00000000	pigeons
Rgs1	-0.02	1.00000000	pigeons
Rffl	-0.21	1.00000000	pigeons
Unk	-0.82	1.00000000	pigeons
Zbtb41	-0.19	1.00000000	pigeons
Zswim8	-0.28	1.00000000	pigeons
Fnbp1	-0.50	1.00000000	pigeons
Chmp1a	-0.28	1.00000000	pigeons
Arhgap10	-0.75	1.00000000	pigeons
Fbxo32	0.04	1.00000000	pigeons
Pcna	-0.87	1.00000000	pigeons
Txndc9	-0.42	1.00000000	pigeons
Tnrc6c	-0.32	1.00000000	pigeons
Cd247	-0.62	1.00000000	pigeons
Dst	0.18	1.00000000	pigeons
Prr14l	-0.23	1.00000000	pigeons
Rcn2	-0.85	1.00000000	pigeons
Eif5a2	-0.58	1.00000000	pigeons
Kdm2b	-0.19	1.00000000	pigeons
Ldlrad3	-0.40	1.00000000	pigeons
Acsl4	-0.79	1.00000000	pigeons
Cbx3	-0.44	1.00000000	pigeons
Ppp4r1	-0.45	1.00000000	pigeons
Samhd1	-0.42	1.00000000	pigeons
Hnrnpr	-0.46	1.00000000	pigeons
Ttc28	-0.59	1.00000000	pigeons
Ddx3y	0.03	1.00000000	pigeons
Fbxl3	-0.25	1.00000000	pigeons
Tmem165	-0.27	1.00000000	pigeons
Rabgap1l	-0.20	1.00000000	pigeons
Zdhhc14	-0.74	1.00000000	pigeons
Golga1	-0.15	1.00000000	pigeons

Uvrag	-0.32	1.00000000	pigeons
Cops2	-0.28	1.00000000	pigeons
Aebp2	-0.11	1.00000000	pigeons
Pwwp2b	-0.38	1.00000000	pigeons
Lmbr1	-0.31	1.00000000	pigeons
Glipr2	-1.00	1.00000000	pigeons
Eif2s1	-0.30	1.00000000	pigeons
Pef1	-0.33	1.00000000	pigeons
Trim8	-0.59	1.00000000	pigeons
Ndufa6	-0.33	1.00000000	pigeons
Snrk	-0.80	1.00000000	pigeons
Psip1	-0.45	1.00000000	pigeons
Ddx42	-0.37	1.00000000	pigeons
Ppia	-0.32	1.00000000	pigeons
Slc30a9	-0.45	1.00000000	pigeons
Scoc	-0.86	1.00000000	pigeons
Pank1	-0.13	1.00000000	pigeons
Parvg	-0.37	1.00000000	pigeons
Acvr2a	-0.20	1.00000000	pigeons
Numb	-0.32	1.00000000	pigeons
Ccdc6	-0.29	1.00000000	pigeons
Cog2	-0.25	1.00000000	pigeons
Foxk1	-0.23	1.00000000	pigeons
Plekha3	-0.22	1.00000000	pigeons
Zcchc2	-0.35	1.00000000	pigeons
Fkbp11	-0.32	1.00000000	pigeons
Arl15	-0.54	1.00000000	pigeons
Picalm	-0.32	1.00000000	pigeons
Tspan3	-1.04	1.00000000	pigeons
Slx4	-0.25	1.00000000	pigeons
Plcb4	-0.24	1.00000000	pigeons
Syap1	-0.33	1.00000000	pigeons
Pde7b	-0.46	1.00000000	pigeons
Ccnl1	-0.57	1.00000000	pigeons
Tgfbrap1	-0.26	1.00000000	pigeons
Ndufa12	-0.43	1.00000000	pigeons
Reps1	-0.45	1.00000000	pigeons
Cct7	-0.30	1.00000000	pigeons
Fubp1	-0.51	1.00000000	pigeons
Ppib	-0.36	1.00000000	pigeons
Uspl1	-0.28	1.00000000	pigeons
Tfrc	-0.86	1.00000000	pigeons
Brms1l	-0.32	1.00000000	pigeons
Commd10	-0.19	1.00000000	pigeons
Rcbtb2	-0.29	1.00000000	pigeons
Ppp2r5e	-0.73	1.00000000	pigeons

Ascc1	-0.30	1.00000000	pigeons
Eif3h	-0.40	1.00000000	pigeons
Prdx1	-0.90	1.00000000	pigeons
Hmgb1	-0.68	1.00000000	pigeons
Fzd6	-0.36	1.00000000	pigeons
Kmt2e	-0.17	1.00000000	pigeons
Snx3	-0.40	1.00000000	pigeons
Ago2	-0.25	1.00000000	pigeons
Smc4	-0.33	1.00000000	pigeons
Dtx3l	-0.39	1.00000000	pigeons
Utrn	-0.24	1.00000000	pigeons
Cops5	-0.32	1.00000000	pigeons
Hmgcr	-0.24	1.00000000	pigeons
Tiam2	-0.80	1.00000000	pigeons
Tspan9	-0.28	1.00000000	pigeons
Arglu1	-0.55	1.00000000	pigeons
Fbxl17	0.00	1.00000000	pigeons
Cdca4	-0.79	1.00000000	pigeons
Xpo1	-0.86	1.00000000	pigeons
Sap30l	-0.23	1.00000000	pigeons
Cwc27	-0.35	1.00000000	pigeons
Atf4	-0.82	1.00000000	pigeons
Pdia3	-0.52	1.00000000	pigeons
Gpatch2	-0.25	1.00000000	pigeons
Rora	-0.15	1.00000000	pigeons
Itsn2	-0.42	1.00000000	pigeons
Agpat5	-0.78	1.00000000	pigeons
Slc12a4	-0.93	1.00000000	pigeons
Chd2	-0.27	1.00000000	pigeons
Fbxo11	-0.42	1.00000000	pigeons
Lman2	-0.54	1.00000000	pigeons
Zmynd11	-0.78	1.00000000	pigeons
Tulp3	-0.29	1.00000000	pigeons
Sik1	-0.72	1.00000000	pigeons
Zcchc24	-0.07	1.00000000	pigeons
Zdhhc6	-0.59	1.00000000	pigeons
Hspa9	-0.44	1.00000000	pigeons
Ifngr2	-0.49	1.00000000	pigeons
Bag5	-0.58	1.00000000	pigeons
Eea1	-0.25	1.00000000	pigeons
Pnn	-0.80	1.00000000	pigeons
Dmxl1	-0.82	1.00000000	pigeons
Slu7	-0.41	1.00000000	pigeons
Casp7	-0.31	1.00000000	pigeons
Atxn7	-0.24	1.00000000	pigeons
Snx29	-0.47	1.00000000	pigeons

Inpp5k	0.03	1.00000000	pigeons
Pxdc1	-0.37	1.00000000	pigeons
Aoah	-0.33	1.00000000	pigeons
Rtn4	-0.63	1.00000000	pigeons
Psmd7	-0.42	1.00000000	pigeons
Scaf8	-0.39	1.00000000	pigeons
Glo1	-0.34	1.00000000	pigeons
Psmb4	-0.52	1.00000000	pigeons
Klf6	-0.33	1.00000000	pigeons
Crip1	-0.38	1.00000000	pigeons
Cnpy3	-0.40	1.00000000	pigeons
Sgpl1	-0.25	1.00000000	pigeons
Nbeal1	-0.44	1.00000000	pigeons
Gbe1	-0.12	1.00000000	pigeons
Heca	-0.77	1.00000000	pigeons
Bmpr2	-0.42	1.00000000	pigeons
Klhl8	-0.30	1.00000000	pigeons
Srgap3	-0.34	1.00000000	pigeons
Herc3	-0.32	1.00000000	pigeons
Zfp652	-0.70	1.00000000	pigeons
Amfr	-0.45	1.00000000	pigeons
Reps2	-0.69	1.00000000	pigeons
Cerk	-0.60	1.00000000	pigeons
Pla2r1	-0.49	1.00000000	pigeons
Usp22	-0.73	1.00000000	pigeons
Zcchc14	-0.16	1.00000000	pigeons
Hpcal1	-0.85	1.00000000	pigeons
Atp1a1	-0.51	1.00000000	pigeons
Notch1	-0.52	1.00000000	pigeons
Lrch1	-0.42	1.00000000	pigeons
Pbx1	-0.81	1.00000000	pigeons
Snx14	-0.84	1.00000000	pigeons
Ctsz	-0.23	1.00000000	pigeons
Sec23a	-0.40	1.00000000	pigeons
Fxr1	-0.54	1.00000000	pigeons
Pdia4	-0.45	1.00000000	pigeons
Fut8	-0.33	1.00000000	pigeons
Zbtb1	-0.35	1.00000000	pigeons
Rdx	-0.88	1.00000000	pigeons
Cstf1	-0.32	1.00000000	pigeons
Hspa4	-0.44	1.00000000	pigeons
Camk1d	-0.31	1.00000000	pigeons
Lysmd2	-0.27	1.00000000	pigeons
Sh3gl1	-0.63	1.00000000	pigeons
Epc2	-0.44	1.00000000	pigeons
Nfat5	-0.40	1.00000000	pigeons

Rsrp1	-0.82	1.00000000	pigeons
Ccdc50	-0.83	1.00000000	pigeons
Creb5	-0.35	1.00000000	pigeons
Aven	-0.20	1.00000000	pigeons
Nol9	-0.48	1.00000000	pigeons
Nme7	-0.44	1.00000000	pigeons
Btrc	-0.50	1.00000000	pigeons
Cdkn1b	-0.85	1.00000000	pigeons
Ncoa3	-0.40	1.00000000	pigeons
Lgmn	-0.37	1.00000000	pigeons
Tbl1xr1	-0.38	1.00000000	pigeons
Oser1	-0.34	1.00000000	pigeons
Abca4	-0.33	1.00000000	pigeons
Wdr7	-0.67	1.00000000	pigeons
Ndfip1	-0.59	1.00000000	pigeons
Fer	-0.33	1.00000000	pigeons
Abcf2	-0.39	1.00000000	pigeons
Isca1	-0.51	1.00000000	pigeons
Arid1b	-0.07	1.00000000	pigeons
Slc13a1	-0.12	1.00000000	pigeons
Zfp326	-0.51	1.00000000	pigeons
Jade1	-0.68	1.00000000	pigeons
Tmem266	-0.33	1.00000000	pigeons
P4ha1	-0.71	1.00000000	pigeons
Napg	-0.46	1.00000000	pigeons
Tra2a	-0.61	1.00000000	pigeons
Eif4ebp1	-0.70	1.00000000	pigeons
Nvl	-0.42	1.00000000	pigeons
Gclc	-0.41	1.00000000	pigeons
Strn3	-0.63	1.00000000	pigeons
Pan3	-0.85	1.00000000	pigeons
Psma5	-0.33	1.00000000	pigeons
Slc16a6	-0.28	1.00000000	pigeons
Ndufa5	-0.43	1.00000000	pigeons
Usp3	-0.48	1.00000000	pigeons
Itm2a	-0.26	1.00000000	pigeons
Canx	-0.59	1.00000000	pigeons
Chmp7	-0.33	1.00000000	pigeons
Slf2	-0.49	1.00000000	pigeons
Cep170	-0.48	1.00000000	pigeons
Dnajb4	-0.83	1.00000000	pigeons
B2m	0.06	1.00000000	pigeons
Anp32e	-0.82	1.00000000	pigeons
Zfp407	-0.11	1.00000000	pigeons
Tra2b	-0.65	1.00000000	pigeons
Clock	-0.53	1.00000000	pigeons

Slc4a4	-0.49	1.00000000	pigeons
Insr	-0.66	1.00000000	pigeons
Tmem131l	-0.35	1.00000000	pigeons
Rnf144a	-0.76	1.00000000	pigeons
Ythdc1	-0.53	1.00000000	pigeons
Homer1	-0.26	1.00000000	pigeons
Htt	-0.47	1.00000000	pigeons
Tbc1d14	-0.52	1.00000000	pigeons
C2cd5	-0.30	1.00000000	pigeons
Top2b	-0.74	1.00000000	pigeons
Slc4a11	-0.72	1.00000000	pigeons
Naca	-0.75	1.00000000	pigeons
Dnajb6	-0.87	1.00000000	pigeons
Dek	-0.62	1.00000000	pigeons
Fbrsl1	-0.11	1.00000000	pigeons
Jade2	-0.72	1.00000000	pigeons
Shprh	-0.75	1.00000000	pigeons
Timp2	-0.55	1.00000000	pigeons
Slc39a8	-0.29	1.00000000	pigeons
Rreb1	-0.45	1.00000000	pigeons
Rcan1	-0.83	1.00000000	pigeons
Akap11	-0.52	1.00000000	pigeons
Snx13	-0.51	1.00000000	pigeons
Hnrnpu	-0.57	1.00000000	pigeons
Ipmk	-0.44	1.00000000	pigeons
Rab12	-0.58	1.00000000	pigeons
Tcf20	-0.28	1.00000000	pigeons
Plk3	-0.60	1.00000000	pigeons
Srsf10	-0.45	1.00000000	pigeons
Dnaja4	-0.41	1.00000000	pigeons
Ttyh3	-0.73	1.00000000	pigeons
Rere	-0.42	1.00000000	pigeons
Gramd4	-0.22	1.00000000	pigeons
Lpin2	-0.73	1.00000000	pigeons
Fam133b	-0.70	1.00000000	pigeons
Clasp1	-0.64	1.00000000	pigeons
Flnb	-0.88	1.00000000	pigeons
Ppig	-0.56	1.00000000	pigeons
Ctdp1	-0.48	1.00000000	pigeons
Vps54	-0.62	1.00000000	pigeons
Rnf220	-0.82	1.00000000	pigeons
Baz2b	-0.75	1.00000000	pigeons
Fkbp4	-0.81	1.00000000	pigeons
Tpm4	-0.86	1.00000000	pigeons
Gnal	-0.25	1.00000000	pigeons
Slc30a1	-0.48	1.00000000	pigeons

Zeb2	-0.01	1.00000000	pigeons
Rftn2	-0.30	1.00000000	pigeons
Sod1	-0.76	1.00000000	pigeons
Cpeb2	-0.35	1.00000000	pigeons
Lclat1	-0.51	1.00000000	pigeons
Ppt1	-0.52	1.00000000	pigeons
Zbtb46	-0.50	1.00000000	pigeons
Adk	-0.18	1.00000000	pigeons
2900026A02Rik	-0.52	1.00000000	pigeons
Arfgap3	-0.48	1.00000000	pigeons
Bin1	-0.71	1.00000000	pigeons
Ctbp1	-0.29	1.00000000	pigeons
Rb1cc1	-0.46	1.00000000	pigeons
Kmt5b	-0.62	1.00000000	pigeons
Ptpn12	-0.69	1.00000000	pigeons
Btg1b	-0.08	1.00000000	pigeons
Atp6v0a1	-0.71	1.00000000	pigeons
Pip5k1a	-0.70	1.00000000	pigeons
Dnaja1	-0.51	1.00000000	pigeons
Bcor	-0.63	1.00000000	pigeons
Atg16l1	-0.68	1.00000000	pigeons
Hivep2	-0.07	1.00000000	pigeons
Ssb	-0.52	1.00000000	pigeons
Yaf2	-0.56	1.00000000	pigeons
Pdk3	-0.48	1.00000000	pigeons
Ip6k2	-0.77	1.00000000	pigeons
Lemd3	-0.47	1.00000000	pigeons
Kif13a	-0.43	1.00000000	pigeons
Snrpd1	-0.66	1.00000000	pigeons
Nrp2	-0.48	1.00000000	pigeons
Bicd2	-0.58	1.00000000	pigeons
Elf2	-0.72	1.00000000	pigeons
Cdk19	-0.48	1.00000000	pigeons
Ptpn4	-0.58	1.00000000	pigeons
Sp3	-0.59	1.00000000	pigeons
Nr1d2	-0.60	1.00000000	pigeons
Pik3r1	-0.31	1.00000000	pigeons
Ppm1d	-0.49	1.00000000	pigeons
Ahsa1	-0.50	1.00000000	pigeons
Clasp2	-0.38	1.00000000	pigeons
Jak2	-0.58	1.00000000	pigeons
Arhgef12	-0.83	1.00000000	pigeons
Rassf6	-0.63	1.00000000	pigeons
Pcmtd1	-0.47	1.00000000	pigeons
Nfx1	-0.65	1.00000000	pigeons
Bloc1s6	-0.59	1.00000000	pigeons

Nrip1	-0.41	1.00000000	pigeons
Bin3	-0.71	1.00000000	pigeons
Arxes2	-0.58	1.00000000	pigeons
Nup153	-0.61	1.00000000	pigeons
Smg6	-0.70	1.00000000	pigeons
Faf1	-0.65	1.00000000	pigeons
Rnf11	-0.56	1.00000000	pigeons
Dus2	-0.47	1.00000000	pigeons
Eif3i	-0.85	1.00000000	pigeons
Brd1	-0.54	1.00000000	pigeons
Susd6	-0.61	1.00000000	pigeons
Sltm	-0.66	1.00000000	pigeons
Npc2	-0.86	1.00000000	pigeons
Slc7a11	0.24	1.00000000	pigeons
Hnrnpab	-0.81	1.00000000	pigeons
Ppp2r5a	-0.75	1.00000000	pigeons
Arid4b	-0.61	1.00000000	pigeons
Tfdp1	-0.53	1.00000000	pigeons
Eif4a2	-0.29	1.00000000	pigeons
Sun1	-0.68	1.00000000	pigeons
Itpk1	-0.70	1.00000000	pigeons
Tpp2	-0.51	1.00000000	pigeons
Srpk2	-0.53	1.00000000	pigeons
Adam23	-0.42	1.00000000	pigeons
Ets1	-0.52	1.00000000	pigeons
Rsad2	5.07	0.00000000	mice
Isg15	2.55	0.00000000	mice
Herc6	3.46	0.00000000	mice
Trim30a	2.58	0.00000000	mice
Ifitm3	2.74	0.00000000	mice
Slfn5	3.14	0.00000000	mice
Slfn4	3.25	0.00000000	mice
Ifi27l2a	3.26	0.00000000	mice
Parp14	2.83	0.00000000	mice
Ifit3	3.60	0.00000000	mice
Ifit1	4.37	0.00000000	mice
Ifit2	3.80	0.00000000	mice
Irf7	2.53	0.00000000	mice
Ifit3b	2.92	0.00000000	mice
Rnf213	3.04	0.00000000	mice
Isg20	2.70	0.00000000	mice
Ddx60	2.75	0.00000000	mice
Oasl1	2.35	0.00000000	mice
B2m	1.52	0.00000000	mice
Ifih1	2.45	0.00000000	mice
Gbp2b	2.79	0.00000000	mice

Usp18	2.61	0.00000000	mice
Zbp1	2.49	0.00000000	mice
Oasl2	2.70	0.00000000	mice
Slfn8	2.15	0.00000000	mice
Ly6e	2.04	0.00000000	mice
Irgm1	2.36	0.00000000	mice
Cmpk2	2.62	0.00000000	mice
Rtp4	1.75	0.00000000	mice
Trafd1	2.01	0.00000000	mice
Plac8	2.46	0.00000000	mice
Trim30c	2.94	0.00000000	mice
Slfn1	1.76	0.00000000	mice
Ifi47	2.45	0.00000000	mice
Ddx58	1.88	0.00000000	mice
Gbp2	2.22	0.00000000	mice
Gbp7	2.11	0.00000000	mice
Il1b	1.75	0.00000000	mice
Cd274	2.26	0.00000000	mice
Oas3	1.59	0.00000000	mice
1600014C10Rik	1.93	0.00000000	mice
Samd9l	1.60	0.00000000	mice
Xaf1	1.84	0.00000000	mice
Ascc3	1.55	0.00000000	mice
Cxcl10	4.61	0.00000000	mice
Parp9	1.70	0.00000000	mice
Ctss	1.65	0.00000000	mice
H2-K1	1.20	0.00000000	mice
Nampt	1.70	0.00000000	mice
A930037H05Rik	2.09	0.00000000	mice
Gbp5	2.60	0.00000000	mice
Nlrc5	1.75	0.00000000	mice
Ccrl2	1.71	0.00000000	mice
Csrnp1	1.39	0.00000000	mice
Irf1	2.01	0.00000000	mice
Tor1aip1	1.37	0.00000000	mice
Stat2	1.57	0.00000000	mice
Slfn2	1.17	0.00000000	mice
Sppl2a	1.63	0.00000000	mice
Epsti1	1.83	0.00000000	mice
Acod1	1.01	0.00000000	mice
Clec2d	1.51	0.00000000	mice
Sp100	1.02	0.00000000	mice
Zup1	1.69	0.00000000	mice
Gbp3	1.56	0.00000000	mice
Cd44	-0.83	0.00000000	mice
Casp4	1.53	0.00000000	mice

Smox	0.98	0.00000000	mice
Ifit1bl2	1.35	0.00000000	mice
Ifitm6	1.46	0.00000000	mice
Tor3a	1.46	0.00000000	mice
Oas2	1.26	0.00000000	mice
Alcam	-1.21	0.00000000	mice
Tspo	1.32	0.00000000	mice
Wfdc17	2.44	0.00000000	mice
Bst2	1.48	0.00000000	mice
Hcar2	1.60	0.00000000	mice
9930111J21Rik2	1.39	0.00000000	mice
Stat1	1.31	0.00000000	mice
Btg2	1.03	0.00000000	mice
Tap1	1.23	0.00000000	mice
Ier5	1.05	0.00000000	mice
Pim1	0.98	0.00000000	mice
Eif2ak2	1.31	0.00000000	mice
Fap	1.93	0.00000000	mice
Nmi	1.28	0.00000000	mice
Dtx3l	1.18	0.00000000	mice
Irgm2	1.17	0.00000000	mice
Junb	1.06	0.00000000	mice
G0s2	1.54	0.00000000	mice
Ccl4	3.63	0.00000000	mice
Zc3hav1	1.13	0.00000000	mice
H2-D1	0.64	0.00000000	mice
Ly6c2	1.97	0.00000000	mice
Samhd1	1.12	0.00000000	mice
Ccl6	-1.04	0.00000000	mice
Oas1a	1.02	0.00000000	mice
Atf3	1.42	0.00000000	mice
Iqgap1	-0.83	0.00000000	mice
Fam107b	-1.23	0.00000000	mice
Igtp	1.06	0.00000000	mice
Helz2	1.01	0.00000000	mice
4930599N23Rik	1.20	0.00000000	mice
Clic4	1.45	0.00000000	mice
Parp12	1.08	0.00000000	mice
Chmp4b	0.92	0.00000000	mice
Inpp5d	-0.82	0.00000000	mice
Gadd45b	1.43	0.00000000	mice
Daxx	1.13	0.00000000	mice
Zfp608	-1.33	0.00000000	mice
Znfx1	1.20	0.00000000	mice
Ier3	1.33	0.00000000	mice
Egr1	1.31	0.00000000	mice

Il1a	1.96	0.00000000	mice
Actb	-0.52	0.00000000	mice
Cdk17	-1.09	0.00000000	mice
Sorl1	-0.79	0.00000000	mice
Ncf2	-0.85	0.00000000	mice
Usp25	1.07	0.00000000	mice
Ifi202b	1.34	0.00000000	mice
Olfr56	1.28	0.00000000	mice
Tor1aip2	1.00	0.00000000	mice
Fos	1.23	0.00000000	mice
Tdrd7	0.95	0.00000000	mice
Tmem140	1.03	0.00000000	mice
Lilr4b	0.71	0.00000000	mice
Ptgs2	1.41	0.00000000	mice
Tmsb4x	-0.41	0.00000000	mice
Fcgr1	1.09	0.00000000	mice
Tiparp	1.00	0.00000000	mice
Gbp9	0.85	0.00000000	mice
Xylt1	-0.77	0.00000000	mice
Jmjd1c	-1.08	0.00000000	mice
Lcp1	-0.68	0.00000000	mice
Wbp1l	-1.19	0.00000000	mice
Itgal	-0.96	0.00000000	mice
Ifit1bl1	1.09	0.00000000	mice
Nt5c3	1.03	0.00000000	mice
Itgam	-1.00	0.00000000	mice
Actg1	-0.55	0.00000000	mice
St3gal5	-1.13	0.00000000	mice
Ttc39c	0.94	0.00000000	mice
Ifi35	0.86	0.00000000	mice
Slc15a3	0.71	0.00000000	mice
Ptprc	-0.56	0.00000000	mice
Apbb1ip	-0.91	0.00000000	mice
Nfkbia	1.01	0.00000000	mice
Cmip	-0.66	0.00000000	mice
D530033B14Rik	0.90	0.00000000	mice
Ucp2	-0.90	0.00000000	mice
Entpd1	-0.65	0.00000000	mice
Adgre5	-0.82	0.00000000	mice
Stk17b	-0.73	0.00000000	mice
Nlrp3	0.90	0.00000000	mice
Socs1	1.12	0.00000000	mice
D8Ertd738e	-0.97	0.00000000	mice
Tyrobp	-0.49	0.00000000	mice
Ssh2	-1.03	0.00000000	mice
Ccnd3	-1.18	0.00000000	mice

Cdkn1a	1.14	0.00000000	mice
Rnf114	0.78	0.00000000	mice
Il1f9	1.05	0.00000000	mice
Ogfr	0.73	0.00000000	mice
Eif1	-0.46	0.00000000	mice
Casp8	0.81	0.00000000	mice
Ankfy1	0.79	0.00000000	mice
Tgfbr1	-1.10	0.00000000	mice
Sipa1l2	-1.02	0.00000000	mice
Ube2l6	0.75	0.00000000	mice
Cd9	-0.53	0.00000000	mice
Fcgr4	1.04	0.00000000	mice
Eepd1	-1.17	0.00000000	mice
Arf4	0.88	0.00000000	mice
Zfp36l2	-1.01	0.00000000	mice
Elmsan1	-1.03	0.00000000	mice
Serf2	-0.68	0.00000000	mice
Il1rn	0.97	0.00000000	mice
Smchd1	0.83	0.00000000	mice
Slc12a6	-1.14	0.00000000	mice
Pitpna	-0.97	0.00000000	mice
Shisa5	0.79	0.00000000	mice
Ripor2	-0.94	0.00000000	mice
Casp1	0.76	0.00000000	mice
Irf9	0.73	0.00000000	mice
Ccl3	2.53	0.00000000	mice
H2-T23	0.75	0.00000000	mice
Nars2	-1.21	0.00000000	mice
Cotl1	-0.81	0.00000000	mice
Scgb1a1	1.01	0.00000000	mice
Srsf5	-0.71	0.00000000	mice
Pnp	0.96	0.00000000	mice
Parp10	0.71	0.00000000	mice
Ets2	0.56	0.00000000	mice
Peli1	1.00	0.00000000	mice
Vps37b	0.73	0.00000000	mice
Tent5a	0.93	0.00000000	mice
Dock5	-1.14	0.00000000	mice
Tnfaip3	1.05	0.00000000	mice
Tapbp	0.75	0.00000000	mice
Ifi204	1.07	0.00000000	mice
Npc2	1.03	0.00000000	mice
Lgals9	0.74	0.00000000	mice
Psmb8	0.79	0.00000000	mice
Ehd4	0.81	0.00000000	mice
Diaph1	-0.76	0.00000000	mice

Slfn3	0.95	0.00000000	mice
Actr3	-0.61	0.00000000	mice
Upp1	0.91	0.00000000	mice
Tbrg1	0.70	0.00000000	mice
Hdac4	-0.92	0.00000000	mice
Trim12c	0.74	0.00000000	mice
Clec4a3	0.74	0.00000000	mice
Ifi207	1.33	0.00000000	mice
Plk3	-0.73	0.00000000	mice
5430427O19Rik	0.81	0.00000000	mice
Ankrd44	-0.86	0.00000000	mice
Pten	-0.87	0.00000000	mice
Wnk1	-0.75	0.00000000	mice
Retreg1	-0.66	0.00000000	mice
Tlr4	0.87	0.00000000	mice
Tagap	0.99	0.00000000	mice
Actn1	-0.95	0.00000000	mice
Trim56	0.69	0.00000000	mice
Coro1c	-0.99	0.00000000	mice
Dgkd	-1.00	0.00000000	mice
Rasa2	-1.14	0.00000000	mice
Hectd1	-0.68	0.00000000	mice
Pdlim5	-0.94	0.00000000	mice
Itpkb	-0.89	0.00000000	mice
Runx2	-1.07	0.00000000	mice
H2-Q7	0.71	0.00000000	mice
Hipk1	-0.90	0.00000000	mice
Dhx58	0.56	0.00000000	mice
Antxr2	-0.63	0.00000000	mice
H2-T22	0.70	0.00000000	mice
Tsc22d3	-0.90	0.00000000	mice
Tgif1	0.77	0.00000000	mice
Themis2	0.64	0.00000000	mice
Prkce	0.85	0.00000000	mice
Rapgef1	-1.05	0.00000000	mice
AY036118	0.73	0.00000000	mice
Elmo1	-0.74	0.00000000	mice
Rap2c	0.71	0.00000000	mice
4930430E12Rik	0.78	0.00000000	mice
Nudt4	-0.68	0.00000000	mice
Ifi211	0.64	0.00000000	mice
Acsl1	-1.02	0.00000000	mice
Jund	-0.61	0.00000000	mice
Ppp1r15a	0.79	0.00000000	mice
Il18bp	0.63	0.00000000	mice
Nt5c2	-0.85	0.00000000	mice

Fgd3	-0.69	0.00000000	mice
Ctsb	0.81	0.00000000	mice
Gbp8	0.66	0.00000000	mice
Capzb	-0.69	0.00000000	mice
Ifi209	0.85	0.00000000	mice
Ly6c1	0.72	0.00000000	mice
Lyst	-0.69	0.00000000	mice
Atp6v0e	-0.72	0.00000000	mice
Adar	0.61	0.00000000	mice
Il1r2	-0.59	0.00000000	mice
Pcgf5	0.81	0.00000000	mice
Smim3	-0.82	0.00000000	mice
H2-T24	0.57	0.00000000	mice
Pitpnc1	-1.05	0.00000000	mice
Ezr	-0.73	0.00000000	mice
Treml2	0.60	0.00000000	mice
Osm	0.98	0.00000000	mice
Cab39	-0.86	0.00000000	mice
Trim30d	0.56	0.00000000	mice
Reep3	-0.92	0.00000000	mice
Peak1	-1.09	0.00000000	mice
Pml	0.58	0.00000000	mice
Cox8a	-0.60	0.00000000	mice
Nedd9	-0.78	0.00000000	mice
Itgax	-0.82	0.00000000	mice
Nsd3	0.75	0.00000000	mice
Irf2	0.70	0.00000000	mice
Ist1	0.57	0.00000000	mice
Trim30b	0.70	0.00000000	mice
Lamp2	0.57	0.00000000	mice
Ifi203	1.02	0.00000000	mice
Lims1	-0.91	0.00000000	mice
Cd52	0.55	0.00000000	mice
Laptm5	-0.61	0.00000000	mice
Hspa4l	-1.04	0.00000000	mice
Dock2	-0.54	0.00000000	mice
Dennd4a	-0.48	0.00000000	mice
Celf2	-0.82	0.00000000	mice
Tbc1d23	-0.90	0.00000000	mice
Alox5ap	-0.53	0.00000000	mice
Endov	0.64	0.00000000	mice
Chst11	-0.51	0.00000000	mice
Zcchc2	0.72	0.00000000	mice
Gpcpd1	-0.70	0.00000000	mice
Kansl1	-0.89	0.00000000	mice
H2-Q4	0.56	0.00000000	mice

Lrg1	0.72	0.00000000	mice
Cytip	-0.65	0.00000000	mice
Lipg	0.60	0.00000000	mice
F3	0.76	0.00000000	mice
Cybb	0.99	0.00000000	mice
Rab2a	-0.67	0.00000000	mice
Ifi44	0.87	0.00000000	mice
Cpne2	-0.92	0.00000000	mice
Sgms1	-0.93	0.00000000	mice
Lilrb4a	0.48	0.00000000	mice
Schip1	0.90	0.00000000	mice
Ly6a	0.69	0.00000000	mice
Cd300lb	-0.75	0.00000000	mice
Arpc2	-0.46	0.00000000	mice
Rassf3	-0.80	0.00000000	mice
Etnk1	0.72	0.00000000	mice
Pygl	-0.74	0.00000000	mice
Zfp36	0.96	0.00000000	mice
Psmb9	0.63	0.00000000	mice
Oaz1	-0.58	0.00000000	mice
Pttg1	0.62	0.00000000	mice
Fxyd5	-0.45	0.00000000	mice
Psme1	0.67	0.00000000	mice
Rap1b	-0.47	0.00000000	mice
Itga4	-0.77	0.00000000	mice
Taldo1	-0.43	0.00000000	mice
Il15ra	0.52	0.00000000	mice
Sla	-0.79	0.00000000	mice
Gab2	-0.63	0.00000000	mice
Socs3	0.76	0.00000000	mice
Gbp4	0.53	0.00000000	mice
Gsk3b	-0.74	0.00000000	mice
Slfn9	0.51	0.00000000	mice
Tm6sf1	-0.80	0.00000000	mice
Selenok	-0.63	0.00000000	mice
Gvin1	0.40	0.00000000	mice
Sept7	-0.74	0.00000000	mice
Sp110	0.58	0.00000000	mice
1600002D24Rik	0.72	0.00000000	mice
Sirpb1a	-0.83	0.00000000	mice
Lpp	0.81	0.00000000	mice
Iqgap2	0.74	0.00000000	mice
Sh3bp5	-0.90	0.00000000	mice
Nr4a1	0.82	0.00000000	mice
Ifrd1	0.69	0.00000000	mice
Azi2	0.59	0.00000000	mice

Txnip	-0.67	0.00000000	mice
Steap4	0.80	0.00000000	mice
Fau	-0.34	0.00000000	mice
Ikzf1	-0.94	0.00000000	mice
Vim	-0.77	0.00000000	mice
Laptm4a	0.55	0.00000000	mice
1600020E01Rik	0.62	0.00000000	mice
Nfkbiz	0.53	0.00000000	mice
Prr5l	0.51	0.00000000	mice
Gtf2e2	-0.74	0.00000000	mice
Nadk	-0.67	0.00000000	mice
Dclre1c	0.63	0.00000000	mice
Chd7	-0.68	0.00000000	mice
Arpc5	-0.51	0.00000000	mice
Nfatc1	-0.80	0.00000000	mice
Nod2	0.53	0.00000000	mice
Cxcl1	1.24	0.00000000	mice
Synj1	-0.64	0.00000000	mice
Tnf	1.09	0.00000000	mice
Pfn1	-0.43	0.00000000	mice
Ubc	0.61	0.00000000	mice
Pias1	-0.70	0.00000000	mice
Kcnab1	0.71	0.00000000	mice
C5ar1	-0.53	0.00000000	mice
Gng5	-0.49	0.00000000	mice
Ccnd2	0.56	0.00000000	mice
Cd80	-0.70	0.00000000	mice
Cd300a	-0.61	0.00000000	mice
Calm2	-0.57	0.00000000	mice
Ergic1	-0.75	0.00000000	mice
Ubash3b	-0.97	0.00000000	mice
Arhgdib	-0.53	0.00000000	mice
Taok1	-0.75	0.00000000	mice
Grn	0.57	0.00000000	mice
Lsmem1	0.96	0.00000000	mice
Saa3	0.68	0.00000000	mice
Sdcbp	0.45	0.00000000	mice
Igf1r	-0.54	0.00000000	mice
Btbd9	-0.47	0.00000000	mice
Grk5	-0.96	0.00000000	mice
Ptbp3	-0.56	0.00000000	mice
Snrk	-0.78	0.00000000	mice
Sin3b	-0.75	0.00000000	mice
AU019990	-0.79	0.00000000	mice
Prkcb	-0.70	0.00000000	mice
Gfod1	-0.72	0.00000000	mice

Il15	0.77	0.00000000	mice
Abr	-0.55	0.00000000	mice
Luc7l2	-0.73	0.00000000	mice
Dok3	-0.72	0.00000000	mice
Tcn2	-0.64	0.00000000	mice
Arhgap15	-0.56	0.00000000	mice
Atp2b1	-0.43	0.00000000	mice
Pglyrp1	-0.59	0.00000000	mice
Btg1	-0.30	0.00000000	mice
Sipa1l1	0.70	0.00000000	mice
Uba7	0.38	0.00000000	mice
Cmss1	0.64	0.00000000	mice
Cyth1	-0.54	0.00000000	mice
Zeb2	0.50	0.00000000	mice
Pfdn5	-0.57	0.00000000	mice
Pmvk	-0.63	0.00000000	mice
Tpm4	-0.55	0.00000000	mice
Icam1	0.71	0.00000000	mice
Dusp6	-0.68	0.00000000	mice
Fndc3a	0.79	0.00000000	mice
Lyn	-0.37	0.00000000	mice
Lgals8	0.49	0.00000000	mice
Tent4a	0.39	0.00000000	mice
Ilrun	0.58	0.00000000	mice
Pak2	-0.58	0.00000000	mice
Zeb2os	0.44	0.00000000	mice
Adam8	-0.60	0.00000000	mice
Ski	-0.76	0.00000000	mice
Vav3	-0.78	0.00000000	mice
Sat1	0.53	0.00000000	mice
Rab20	0.48	0.00000000	mice
Fbxl5	0.78	0.00000000	mice
Setdb2	0.41	0.00000000	mice
Rlf	-0.66	0.00000000	mice
Nfkb1	0.46	0.00000000	mice
AW112010	0.55	0.00000000	mice
Csf3r	-0.37	0.00000000	mice
Cass4	-0.66	0.00000000	mice
Il17ra	-0.66	0.00000000	mice
Ctsz	0.59	0.00000000	mice
Cx3cr1	0.60	0.00000000	mice
Klf3	-0.68	0.00000000	mice
Cgas	0.38	0.00000000	mice
Per2	-0.58	0.00000000	mice
Zswim6	-0.57	0.00000000	mice
Enthd1	0.66	0.00000000	mice

Fbxo11	-0.54	0.00000000	mice
Baz2b	-0.40	0.00000000	mice
Prkag1	-0.68	0.00000000	mice
Ptpn22	-0.70	0.00000000	mice
Bcl2l1	0.61	0.00000000	mice
Fabp5	0.65	0.00000000	mice
Fkbp5	-0.57	0.00000000	mice
Wdr26	-0.60	0.00000000	mice
Rdh12	-0.66	0.00000000	mice
Anxa11	-0.57	0.00000000	mice
Ralgps1	-0.55	0.00000000	mice
Tuba1c	-0.65	0.00000000	mice
Clec5a	-0.76	0.00000000	mice
Ago2	-0.47	0.00000000	mice
Syk	-0.42	0.00000000	mice
Coq10b	0.48	0.00000000	mice
Alox5	-0.70	0.00000000	mice
Lpin2	-0.61	0.00000000	mice
Asap1	-0.67	0.00000000	mice
Emb	-0.62	0.00000000	mice
Whamm	0.39	0.00000000	mice
Dhrs7	-0.63	0.00000000	mice
Raf1	-0.59	0.00000000	mice
Stard3	0.39	0.00000000	mice
Irf2bp2	-0.60	0.00000000	mice
Taf7	0.50	0.00000000	mice
Ddit3	0.51	0.00000000	mice
Gcnt2	-0.51	0.00000000	mice
Art3	0.44	0.00000000	mice
Jarid2	-0.75	0.00000000	mice
4930469K13Rik	0.44	0.00000000	mice
Ostf1	-0.50	0.00000000	mice
Cyfip2	-0.70	0.00000000	mice
Vcpip1	0.48	0.00000000	mice
Cers6	0.77	0.00000000	mice
Apobec1	0.46	0.00000000	mice
A930015D03Rik	0.46	0.00000000	mice
Arid5a	0.53	0.00000000	mice
Gpi1	-0.63	0.00000000	mice
Pgam1	-0.67	0.00000000	mice
Inhba	0.63	0.00000000	mice
Sap30	0.38	0.00000000	mice
Ubxn4	-0.61	0.00000000	mice
Tpd52	-0.35	0.00000000	mice
Plin2	0.51	0.00000000	mice
Ppp2ca	-0.56	0.00000000	mice

Vps13d	-0.78	0.00000000	mice
Il18rap	-0.66	0.00000000	mice
Pdcd6	-0.61	0.00000000	mice
Pfkfb3	-0.74	0.00000000	mice
Tagln2	-0.85	0.00000000	mice
Trim25	0.49	0.00000000	mice
Gnaq	-0.57	0.00000000	mice
Zfas1	0.37	0.00000000	mice
Cnnm2	-0.75	0.00000000	mice
Sell	0.44	0.00000000	mice
Atp9b	-0.81	0.00000000	mice
Traf1	0.65	0.00000000	mice
Nptn	-0.66	0.00000000	mice
Iscu	-0.61	0.00000000	mice
Tmsb10	0.77	0.00000000	mice
Dennd5a	-0.58	0.00000000	mice
Usb1	0.35	0.00000000	mice
Rhoa	-0.45	0.00000000	mice
Cdc42	-0.39	0.00000000	mice
Rock1	-0.52	0.00000000	mice
Rrbp1	0.57	0.00000000	mice
Arid5b	0.70	0.00000000	mice
Wipf1	-0.58	0.00000000	mice
Coro1a	-0.44	0.00000000	mice
Nod1	0.51	0.00000000	mice
Lrrfip1	-0.51	0.00000000	mice
Trim26	0.36	0.00000000	mice
Pan3	-0.73	0.00000000	mice
Washc2	-0.61	0.00000000	mice
4932438A13Rik	-0.60	0.00000000	mice
Ubb	0.37	0.00000000	mice
Ost4	-0.54	0.00000000	mice
Nt5e	-0.63	0.00000000	mice
S100a11	-0.29	0.00000000	mice
Pip4k2a	-0.68	0.00000000	mice
Hk2	0.37	0.00000000	mice
Il6ra	-0.59	0.00000000	mice
Dhrs9	-0.50	0.00000000	mice
Coq2	0.37	0.00000000	mice
Fcho2	-0.65	0.00000000	mice
Dazap2	-0.49	0.00000000	mice
Rbm25	-0.61	0.00000000	mice
Tnfaip2	0.52	0.00000000	mice
Slc9a3r1	-0.63	0.00000000	mice
Ndfip2	-0.54	0.00000000	mice
Eif4g3	-0.64	0.00000000	mice

Tax1bp1	-0.46	0.00000000	mice
Zyx	-0.54	0.00000000	mice
Cln3	-0.59	0.00000000	mice
Gla	0.42	0.00000000	mice
Jdp2	-0.47	0.00000000	mice
Rhoh	0.43	0.00000000	mice
Irf5	0.43	0.00000000	mice
Nfam1	-0.62	0.00000000	mice
Prkcq	0.45	0.00000000	mice
N4bp2l2	-0.64	0.00000000	mice
Uhrf1bp1l	-0.66	0.00000000	mice
Dip2b	-0.74	0.00000000	mice
Trappc9	-0.68	0.00000000	mice
Ranbp2	0.60	0.00000000	mice
Ap2b1	-0.65	0.00000000	mice
Filip1l	0.35	0.00000000	mice
Gcnt1	-0.65	0.00000000	mice
Nfkbie	0.48	0.00000000	mice
Zmiz1	-0.69	0.00000000	mice
Bcl3	0.32	0.00000000	mice
Anxa3	-0.58	0.00000000	mice
Sgk3	0.39	0.00000000	mice
Cd69	0.70	0.00000000	mice
Arpc1b	-0.41	0.00000000	mice
St3gal6	-0.53	0.00000000	mice
Cd33	-0.54	0.00000000	mice
Alas1	-0.62	0.00000000	mice
Adipor2	-0.59	0.00000000	mice
Acap2	-0.67	0.00000000	mice
Brd4	-0.56	0.00000000	mice
Clint1	-0.57	0.00000000	mice
Zmynd11	-0.66	0.00000000	mice
Gsap	0.42	0.00000000	mice
Iqsec1	-0.58	0.00000000	mice
Sh3kbp1	-0.67	0.00000000	mice
Neurl3	-0.53	0.00000000	mice
Klhl2	-0.63	0.00000000	mice
Nipbl	-0.69	0.00000000	mice
Zfand3	-0.54	0.00000000	mice
Pbxip1	-0.59	0.00000000	mice
Cyp4f18	-0.49	0.00000000	mice
Xrn2	0.40	0.00000000	mice
Fyb	-0.55	0.00000000	mice
Kmt2e	-0.52	0.00000000	mice
Rnf149	-0.33	0.00000000	mice
Psme2	0.36	0.00000000	mice

Ctnnb1	-0.39	0.00000000	mice
Nabp1	0.36	0.00000000	mice
Pacsin2	-0.69	0.00000000	mice
Cd300c2	-0.56	0.00000000	mice
Chrm3	-0.48	0.00000000	mice
Birc3	0.39	0.00000000	mice
Slc28a2	0.38	0.00000000	mice
Fgr	-0.56	0.00000000	mice
A530032D15Rik	0.38	0.00000000	mice
Tmcc1	-0.67	0.00000000	mice
Tln1	-0.50	0.00000000	mice
Rac2	-0.39	0.00000000	mice
Ifitm2	-0.39	0.00000000	mice
Lpcat2	-0.56	0.00000000	mice
Tgfbr2	-0.57	0.00000000	mice
Scaf8	-0.61	0.00000000	mice
Col4a3bp	-0.71	0.00000000	mice
Spata13	-0.66	0.00000000	mice
Yeats4	-0.46	0.00000000	mice
Cyth4	-0.55	0.00000000	mice
Clta	-0.55	0.00000000	mice
Lcp2	0.38	0.00000000	mice
Gak	-0.53	0.00000000	mice
Dleu2	-0.58	0.00000000	mice
Cdkn2d	0.44	0.00000000	mice
Sod2	0.43	0.00000000	mice
Csprs	0.31	0.00000000	mice
Tspan14	-0.68	0.00000000	mice
Klf13	-0.60	0.00000000	mice
Eea1	-0.57	0.00000000	mice
Tgoln1	0.36	0.00000000	mice
Cflar	0.51	0.00000000	mice
Xbp1	0.37	0.00000000	mice
Stk4	-0.67	0.00000000	mice
Cd101	-0.57	0.00000000	mice
Ttc7	-0.57	0.00000000	mice
Cdh23	-0.44	0.00000000	mice
Asph	0.36	0.00000000	mice
Heca	-0.57	0.00000000	mice
Acsl4	-0.68	0.00000000	mice
D1Ertd622e	0.37	0.00000000	mice
Fpr3	0.55	0.00000000	mice
Ptprj	-0.45	0.00000000	mice
Psap	-0.35	0.00000000	mice
Cd3d	-0.43	0.00000000	mice
Wapl	-0.50	0.00000000	mice

Cop1	-0.64	0.00000000	mice
Sik3	-0.34	0.00000000	mice
Ndel1	-0.37	0.00000000	mice
Adamtsl4	0.29	0.00000000	mice
Gabarap	-0.31	0.00000000	mice
Homer1	0.58	0.00000000	mice
Ppp6r3	-0.55	0.00000000	mice
Ypel3	-0.44	0.00000000	mice
Prkar1a	-0.59	0.00000000	mice
Rab6a	-0.58	0.00000000	mice
Nrg1	0.81	0.00000000	mice
Ankrd22	-0.52	0.00000000	mice
Sh3glb1	-0.43	0.00000000	mice
Prdx6	-0.57	0.00000000	mice
Il18	0.36	0.00000000	mice
Crispld2	-0.38	0.00000000	mice
Fcer1g	0.26	0.00000000	mice
Smpdl3a	0.33	0.00000000	mice
Kmt5b	-0.51	0.00000000	mice
Adssl1	-0.48	0.00000000	mice
Abi1	-0.51	0.00000000	mice
Ctsc	0.44	0.00000000	mice
Kras	-0.43	0.00000000	mice
Rnpep	0.30	0.00000000	mice
Sik2	-0.55	0.00000000	mice
Ubald2	0.31	0.00000000	mice
Cdk13	-0.58	0.00000000	mice
Zfhx3	-0.62	0.00000000	mice
Cycs	0.53	0.00000000	mice
Fam241a	0.34	0.00000000	mice
Vegfa	0.44	0.00000000	mice
Gyg	0.42	0.00000000	mice
Vamp5	-0.51	0.00000000	mice
Nr3c1	-0.54	0.00000000	mice
Fam49b	-0.50	0.00000000	mice
Foxn2	-0.58	0.00000000	mice
Slc27a4	-0.51	0.00000000	mice
Ccnl1	0.38	0.00000000	mice
Retnlg	-0.64	0.00000000	mice
Parp4	-0.61	0.00000000	mice
Ppp4r1	-0.46	0.00000000	mice
Arpc4	-0.47	0.00000000	mice
Arih1	-0.52	0.00000000	mice
Sos2	-0.54	0.00000000	mice
Elk3	-0.65	0.00000000	mice
Gsn	-0.52	0.00000000	mice

Agap1	-0.58	0.00000000	mice
Rgs2	-0.46	0.00000000	mice
Arpc3	-0.36	0.00000000	mice
Ncf4	-0.42	0.00000000	mice
Bnip3l	-0.49	0.00000000	mice
Rnf34	0.35	0.00000000	mice
Fuca1	-0.49	0.00000000	mice
Snx20	0.31	0.00000000	mice
Ripk1	0.44	0.00000000	mice
St8sia4	-0.69	0.00000000	mice
Arl11	-0.49	0.00000000	mice
Otud5	0.30	0.00000000	mice
Rheb	-0.55	0.00000000	mice
Cyba	-0.37	0.00000000	mice
Cast	-0.61	0.00000000	mice
Kdm5b	-0.52	0.00000000	mice
Zc3h12c	0.69	0.00000000	mice
A330023F24Rik	-0.56	0.00000000	mice
Trem3	-0.40	0.00000000	mice
Fbxo31	-0.36	0.00000000	mice
Nckap1l	-0.53	0.00000000	mice
Snx14	-0.45	0.00000000	mice
Atp6ap2	-0.53	0.00000000	mice
Nceh1	0.44	0.00000000	mice
Tnrc18	-0.45	0.00000000	mice
Tmem184b	0.29	0.00000000	mice
Spi1	-0.39	0.00000000	mice
Impact	0.29	0.00000000	mice
Arhgap18	-0.59	0.00000000	mice
Pla2g7	-0.35	0.00000000	mice
Cdk14	0.48	0.00000000	mice
Drap1	-0.52	0.00000000	mice
Fmnl1	-0.44	0.00000000	mice
Tet2	-0.51	0.00000000	mice
Plk2	0.45	0.00000000	mice
Nfkbid	0.45	0.00000000	mice
Itgb2	-0.44	0.00000000	mice
Strbp	-0.59	0.00000000	mice
Eif5	-0.39	0.00000000	mice
Prr14l	-0.55	0.00000000	mice
C130050O18Rik	-0.45	0.00000000	mice
Tmem154	-0.53	0.00000000	mice
Rasa3	-0.59	0.00000000	mice
Atg7	-0.70	0.00000001	mice
Resf1	0.29	0.00000001	mice
Dtd1	-0.38	0.00000001	mice

Aqp9	-0.52	0.00000001	mice
Dgkh	-0.66	0.00000001	mice
Psmb10	0.27	0.00000001	mice
Def6	-0.43	0.00000001	mice
5430425K12Rik	-0.46	0.00000001	mice
Zfc3h1	-0.55	0.00000001	mice
Lasp1	-0.49	0.00000001	mice
Usp32	-0.52	0.00000001	mice
BC005624	-0.46	0.00000001	mice
Dync1li1	-0.57	0.00000001	mice
Rhof	-0.43	0.00000001	mice
Arhgap25	-0.61	0.00000001	mice
Dgkz	-0.48	0.00000001	mice
Cd164	0.34	0.00000001	mice
Got2	-0.46	0.00000001	mice
Svbp	0.23	0.00000001	mice
Klhl6	-0.57	0.00000001	mice
Cap1	-0.33	0.00000001	mice
Btbd1	-0.51	0.00000001	mice
Ube4a	-0.49	0.00000001	mice
Leprotl1	-0.47	0.00000001	mice
Kpna4	0.30	0.00000001	mice
AB124611	-0.49	0.00000001	mice
Strn	-0.51	0.00000002	mice
Tmem131l	-0.68	0.00000002	mice
Hgsnat	-0.43	0.00000002	mice
Rabac1	-0.44	0.00000002	mice
Pde1a	0.53	0.00000002	mice
Arel1	0.34	0.00000002	mice
Snap29	0.26	0.00000002	mice
Slc22a15	-0.48	0.00000002	mice
Plekha2	-0.50	0.00000002	mice
Arl6ip1	0.40	0.00000002	mice
Vapa	-0.49	0.00000002	mice
Ccl5	0.40	0.00000003	mice
Zfp638	-0.52	0.00000003	mice
Clasp1	-0.75	0.00000003	mice
Cfap54	-0.52	0.00000003	mice
Pde2a	-0.52	0.00000003	mice
Rcsd1	-0.47	0.00000004	mice
Prkar2b	-0.44	0.00000004	mice
Chd2	-0.46	0.00000004	mice
Trappc8	-0.50	0.00000005	mice
Fosl2	0.35	0.00000005	mice
Gnai3	-0.48	0.00000005	mice
Tra2a	0.41	0.00000006	mice

B230208H11Rik	-0.40	0.00000006	mice
Ptk2b	-0.19	0.00000006	mice
Otud7b	-0.46	0.00000006	mice
Chil1	0.42	0.00000006	mice
Zdhhc14	-0.63	0.00000007	mice
Ghitm	0.24	0.00000008	mice
Itm2b	0.25	0.00000008	mice
Fgd4	-0.66	0.00000008	mice
Lamb3	0.34	0.00000009	mice
Rap1gap2	-0.79	0.00000012	mice
Chmp5	0.27	0.00000013	mice
Ifitm1	0.65	0.00000014	mice
Ankrd33b	0.28	0.00000014	mice
Slf2	-0.52	0.00000014	mice
Tmem43	-0.42	0.00000015	mice
Hsd11b1	0.37	0.00000015	mice
Dido1	-0.40	0.00000015	mice
Nktr	-0.44	0.00000015	mice
Itch	-0.59	0.00000015	mice
Ogt	-0.46	0.00000016	mice
Ifngr2	-0.49	0.00000016	mice
Arhgdia	-0.48	0.00000018	mice
Arhgap26	-0.52	0.00000018	mice
Ncor1	-0.44	0.00000018	mice
Sfswap	-0.51	0.00000019	mice
Pdlim7	-0.47	0.00000020	mice
Ppp2r5a	-0.40	0.00000022	mice
Trpc4ap	-0.50	0.00000022	mice
Txnrd1	0.30	0.00000022	mice
Ppp1r13b	-0.49	0.00000024	mice
Dmxl2	-0.61	0.00000024	mice
Nfe2l2	0.27	0.00000026	mice
Cstdc4	0.63	0.00000027	mice
Xrn1	0.30	0.00000027	mice
Notch1	-0.46	0.00000030	mice
Arf1	-0.39	0.00000030	mice
Plekhm3	-0.58	0.00000031	mice
Pi16	-0.43	0.00000031	mice
Numb	-0.57	0.00000032	mice
Tecr	-0.42	0.00000034	mice
Hmox2	0.25	0.00000034	mice
Tmem164	-0.66	0.00000035	mice
Fcgr3	0.35	0.00000036	mice
Per1	-0.41	0.00000039	mice
Sirpa	-0.40	0.00000040	mice
Ppp1r12a	-0.39	0.00000042	mice

Sel1l	-0.47	0.00000043	mice
Wdr33	-0.55	0.00000044	mice
Tprgl	-0.38	0.00000045	mice
Tbc1d2	-0.45	0.00000046	mice
Hnrnpf	-0.48	0.00000048	mice
Picalm	-0.30	0.00000050	mice
Stx11	0.35	0.00000051	mice
Pdcd6ip	-0.52	0.00000052	mice
Svil	-0.51	0.00000053	mice
Sirpb1b	-0.48	0.00000054	mice
Grb2	-0.39	0.00000057	mice
Crebbp	-0.45	0.00000066	mice
Gsdme	-0.48	0.00000067	mice
Nfil3	0.35	0.00000071	mice
Sorbs1	-0.31	0.00000073	mice
Vps13b	-0.54	0.00000074	mice
Dph3	-0.46	0.00000074	mice
Rbm39	-0.32	0.00000076	mice
Capza1	-0.36	0.00000088	mice
Larp1	0.26	0.00000092	mice
Arl5b	0.25	0.00000097	mice
Atg3	-0.45	0.00000098	mice
Gpx4	-0.40	0.00000100	mice
Flna	-0.43	0.00000107	mice
Ythdf3	-0.42	0.00000111	mice
Cfap43	-0.43	0.00000115	mice
Abtb2	0.36	0.00000122	mice
Rab14	-0.47	0.00000132	mice
Vamp8	-0.48	0.00000137	mice
Tacc1	-0.47	0.00000138	mice
Tex14	0.25	0.00000149	mice
Pdzd8	-0.47	0.00000154	mice
Csnk1a1	-0.39	0.00000155	mice
Ptpn1	-0.34	0.00000167	mice
Cebpz	-0.43	0.00000167	mice
4933432I03Rik	0.26	0.00000168	mice
Ankrd12	-0.46	0.00000170	mice
Fmr1	0.30	0.00000175	mice
F630028O10Rik	-0.57	0.00000182	mice
Fam13b	-0.53	0.00000201	mice
Tmbim6	0.28	0.00000214	mice
Notch2	-0.35	0.00000216	mice
Xpr1	-0.56	0.00000224	mice
Ralgds	0.37	0.00000237	mice
Crip1	0.19	0.00000240	mice
Rab8b	0.20	0.00000240	mice

Stk40	-0.43	0.00000243	mice
Aldoa	-0.48	0.00000245	mice
Cpd	-0.47	0.00000258	mice
Slc25a3	-0.40	0.00000263	mice
Dyrk1a	-0.42	0.00000273	mice
Havcr2	-0.50	0.00000311	mice
Cxcl2	0.80	0.00000330	mice
B430306N03Rik	0.29	0.00000331	mice
Ythdf1	0.20	0.00000336	mice
Vsir	-0.45	0.00000344	mice
Cmtr1	0.21	0.00000345	mice
Lrch1	-0.63	0.00000363	mice
Hsh2d	0.23	0.00000372	mice
Fpr2	0.25	0.00000393	mice
Sycp2	0.33	0.00000409	mice
Cfl1	-0.31	0.00000421	mice
Cul3	-0.45	0.00000445	mice
Ankhd1	-0.50	0.00000465	mice
Timm17b	-0.32	0.00000494	mice
Carhsp1	0.27	0.00000518	mice
Pdlim2	0.22	0.00000535	mice
Acsl5	-0.46	0.00000551	mice
Yaf2	-0.42	0.00000563	mice
Rnf24	0.21	0.00000568	mice
Usp34	-0.48	0.00000582	mice
Hmces	-0.38	0.00000586	mice
Lmnb1	0.20	0.00000596	mice
Oga	-0.41	0.00000634	mice
Tmed10	-0.38	0.00000663	mice
Ppp1r2	-0.35	0.00000682	mice
Rnf130	-0.45	0.00000701	mice
Btf3	-0.36	0.00000780	mice
Rnf141	-0.49	0.00000790	mice
Litaf	0.22	0.00000812	mice
Irak3	-0.50	0.00000831	mice
Lfng	-0.44	0.00000907	mice
Kdm6b	0.21	0.00000931	mice
Elk4	-0.44	0.00000962	mice
Ntng2	0.52	0.00000976	mice
Dusp5	0.31	0.00001000	mice
Smim14	-0.41	0.00001053	mice
Tlr2	0.24	0.00001055	mice
Anks1	-0.45	0.00001075	mice
Serinc3	-0.36	0.00001114	mice
Trpm2	-0.42	0.00001145	mice
Clcn7	0.28	0.00001149	mice

Arl4c	-0.40	0.00001168	mice
Ggnbp2	-0.46	0.00001243	mice
Hnrnpk	-0.47	0.00001271	mice
Rab31	-0.58	0.00001338	mice
Olr1	-0.55	0.00001410	mice
Slc9a9	-0.49	0.00001429	mice
Kdm3a	-0.40	0.00001499	mice
Senp2	-0.46	0.00001515	mice
Atp5l	-0.38	0.00001563	mice
Selenow	0.32	0.00001570	mice
Il10	0.24	0.00001594	mice
Pum2	-0.43	0.00001637	mice
Rffl	0.43	0.00001746	mice
Rel	0.25	0.00001836	mice
Cbl	-0.54	0.00001866	mice
Plxnc1	-0.57	0.00001884	mice
Inpp5k	-0.37	0.00001900	mice
Zfand4	-0.35	0.00001903	mice
Fnip1	-0.30	0.00001955	mice
Selplg	-0.37	0.00002103	mice
Ppp1cb	-0.45	0.00002254	mice
Dym	-0.45	0.00002270	mice
Id1	0.38	0.00002429	mice
Rnf111	-0.47	0.00002442	mice
Rreb1	-0.51	0.00002551	mice
Denr	0.30	0.00002586	mice
Tbce	-0.40	0.00002689	mice
Rasip1	0.31	0.00002697	mice
Gna13	0.32	0.00002703	mice
Papola	-0.42	0.00002736	mice
Lmo2	0.20	0.00002941	mice
Srsf11	-0.42	0.00003004	mice
Dennd1b	-0.44	0.00003065	mice
Ptpre	-0.51	0.00003073	mice
Pde7b	0.42	0.00003143	mice
Pira2	-0.39	0.00003183	mice
Trem1	0.25	0.00003239	mice
Son	-0.33	0.00003382	mice
Bbx	0.48	0.00003565	mice
Rab37	0.29	0.00003596	mice
Trim8	-0.39	0.00003958	mice
Sf3b1	-0.34	0.00003976	mice
Dnajc5	-0.47	0.00004039	mice
Kctd12	-0.40	0.00004058	mice
Crybg1	0.17	0.00004306	mice
Eif2ak3	-0.37	0.00004369	mice

Bst1	0.23	0.00004405	mice
Ppm1a	-0.41	0.00004414	mice
Vps54	0.41	0.00004603	mice
Snx13	-0.56	0.00004652	mice
Washc4	0.30	0.00004872	mice
BC005537	-0.39	0.00004893	mice
Emd	-0.43	0.00005063	mice
Optn	0.25	0.00005393	mice
Ndufb1-ps	0.25	0.00005470	mice
Fbxw7	-0.46	0.00005622	mice
F5	-0.51	0.00005675	mice
Tbk1	0.23	0.00006034	mice
Arfgef1	-0.50	0.00006238	mice
Akt1	-0.39	0.00006360	mice
Id2	-0.65	0.00006436	mice
Rnf19a	-0.54	0.00006600	mice
Top1	-0.41	0.00007170	mice
Ppp1r3b	0.27	0.00007813	mice
Rab3gap2	-0.44	0.00008046	mice
Gnai2	-0.23	0.00008342	mice
Slc43a2	-0.50	0.00008360	mice
Pik3cb	-0.49	0.00008530	mice
Ndst1	-0.29	0.00008570	mice
Csde1	-0.36	0.00009437	mice
Slc7a11	-0.21	0.00009642	mice
Atxn7l1	-0.53	0.00009796	mice
Ddx50	-0.44	0.00010092	mice
R3hdm4	-0.41	0.00010193	mice
Exoc5	-0.44	0.00010360	mice
Tbc1d10c	-0.24	0.00010618	mice
Rprd2	0.28	0.00010625	mice
Amfr	-0.34	0.00011769	mice
St3gal1	-0.62	0.00011893	mice
Rtn3	-0.41	0.00012286	mice
Creg1	-0.41	0.00012603	mice
Snx18	-0.31	0.00012858	mice
Tnfrsf21	-0.48	0.00013766	mice
1810058I24Rik	-0.35	0.00015108	mice
Rybp	0.33	0.00015126	mice
Kif23	-0.41	0.00015745	mice
Ccpg1	-0.35	0.00015921	mice
Elmod3	0.15	0.00016936	mice
Usp3	-0.45	0.00017262	mice
Cul1	-0.45	0.00018171	mice
Vps35	-0.37	0.00018795	mice
I830077J02Rik	0.20	0.00020313	mice

Ankrd11	-0.34	0.00020438	mice
Plgrkt	0.24	0.00020702	mice
Pnpla7	-0.44	0.00020955	mice
Hexim1	-0.40	0.00021139	mice
Plekho2	0.26	0.00021997	mice
Wdfy4	-0.41	0.00022890	mice
Arap3	-0.36	0.00023387	mice
Rubcnl	-0.44	0.00023706	mice
A530064D06Rik	0.19	0.00027246	mice
Sh3bgrl3	-0.36	0.00027436	mice
Hcls1	-0.38	0.00027857	mice
Unc93b1	0.21	0.00028493	mice
Glud1	-0.52	0.00031592	mice
Rictor	-0.40	0.00031646	mice
Dusp2	0.39	0.00033731	mice
Tmem156	-0.43	0.00034770	mice
Kdm2a	-0.45	0.00038913	mice
Lonp2	-0.45	0.00039181	mice
Ndufaf7	-0.34	0.00039278	mice
Herpud1	-0.46	0.00039534	mice
Ly6g	0.18	0.00039536	mice
1810037I17Rik	0.23	0.00040337	mice
Yme1l1	-0.37	0.00040484	mice
Tomm6	-0.42	0.00040874	mice
Nek6	-0.41	0.00041451	mice
Ssu72	-0.31	0.00041541	mice
Lypd6b	-0.53	0.00042132	mice
Pak1	0.39	0.00042277	mice
Kat6a	0.24	0.00042746	mice
Plcl2	-0.43	0.00043618	mice
Il1rap	-0.40	0.00047029	mice
Hip1	-0.51	0.00047853	mice
Elf4	-0.49	0.00048339	mice
Pde3b	-0.49	0.00050759	mice
Ywhaz	-0.31	0.00051402	mice
Tnfaip6	0.29	0.00052652	mice
Sqstm1	0.31	0.00053999	mice
Ttc7b	-0.36	0.00054104	mice
Glipr2	-0.44	0.00054690	mice
Ddx3x	-0.34	0.00056289	mice
Anxa1	-0.41	0.00056343	mice
Spen	-0.34	0.00056705	mice
Arrb2	-0.37	0.00058147	mice
Pstpip2	-0.55	0.00060442	mice
Ppt1	-0.39	0.00061697	mice
Cnbp	-0.40	0.00063300	mice

Fli1	-0.51	0.00063711	mice
Tnrc6b	-0.37	0.00064664	mice
Daglb	-0.37	0.00065089	mice
Srgap2	0.25	0.00067787	mice
Gpd2	-0.47	0.00068804	mice
Hp1bp3	-0.29	0.00069926	mice
Eif4a1	-0.38	0.00073909	mice
Tpt1	-0.20	0.00076290	mice
Rbm3	-0.38	0.00078482	mice
Fes	-0.36	0.00078812	mice
Cd63	-0.45	0.00078849	mice
Opa3	0.21	0.00079857	mice
Cnr2	-0.38	0.00084195	mice
Dusp1	0.38	0.00084632	mice
Brk1	-0.43	0.00088722	mice
Atf1	-0.47	0.00089081	mice
Ppp2r5c	-0.44	0.00089619	mice
Wdr1	-0.42	0.00093219	mice
Braf	-0.30	0.00096558	mice
Nono	0.17	0.00098987	mice
Stambpl1	-0.41	0.00108208	mice
Hpcal1	-0.41	0.00109319	mice
Cept1	0.18	0.00109804	mice
Sftpc	-0.64	0.00112712	mice
Rbpj	0.21	0.00120498	mice
Klf7	-0.46	0.00122457	mice
Prkaa1	-0.34	0.00126136	mice
Purb	-0.37	0.00126582	mice
Gpr35	-0.43	0.00130154	mice
Atrnl1	0.26	0.00136277	mice
Adam17	-0.29	0.00137167	mice
Gpx1	-0.37	0.00140671	mice
Smurf2	-0.40	0.00143260	mice
Psen1	-0.34	0.00143437	mice
Phc2	0.10	0.00144241	mice
Ubn1	-0.38	0.00149979	mice
Tle5	-0.33	0.00152804	mice
C3	0.16	0.00153597	mice
Rnf216	-0.47	0.00153891	mice
Sypl	-0.41	0.00155286	mice
Kdm5c	-0.39	0.00159955	mice
Erc1	-0.41	0.00177646	mice
Kmt2c	-0.39	0.00183993	mice
Cdyl	-0.46	0.00187189	mice
Acer3	0.31	0.00188239	mice
Rnf11	-0.33	0.00188658	mice

Lgmn	0.23	0.00190457	mice
Pip5k1c	-0.34	0.00191576	mice
Plekhg3	-0.47	0.00196549	mice
Sephs2	-0.36	0.00204266	mice
Herc4	-0.44	0.00204434	mice
Cdk6	-0.57	0.00209529	mice
Zzef1	-0.35	0.00209903	mice
Txnl1	-0.41	0.00210879	mice
Gramd1a	-0.33	0.00216362	mice
Sp4	-0.45	0.00219320	mice
Cask	-0.44	0.00220420	mice
Supt20	-0.34	0.00224921	mice
Kif2a	-0.33	0.00229657	mice
Susd1	-0.43	0.00255137	mice
Gca	0.15	0.00261410	mice
1700017B05Rik	-0.40	0.00261674	mice
Gng2	-0.37	0.00275571	mice
Prpf6	-0.35	0.00282664	mice
Rgcc	-0.54	0.00282868	mice
Prr13	-0.34	0.00289341	mice
Ddx5	-0.19	0.00294291	mice
Lmbrd1	-0.41	0.00310945	mice
Rock2	-0.53	0.00312898	mice
Egr3	0.21	0.00322020	mice
Rnase6	0.26	0.00328302	mice
Cxcr2	0.28	0.00338572	mice
Il13ra1	0.15	0.00342943	mice
Satb1	-0.35	0.00352634	mice
Sgms2	-0.20	0.00363058	mice
Lrmp	-0.28	0.00367278	mice
Il1bos	0.19	0.00367404	mice
Foxp1	-0.48	0.00374460	mice
Csf1	-0.22	0.00383885	mice
Pafah1b1	-0.31	0.00403923	mice
Leng8	-0.31	0.00410356	mice
Fbxl3	-0.36	0.00431242	mice
Cxcr5	0.25	0.00438191	mice
Aak1	-0.51	0.00440634	mice
Prdx1	0.22	0.00455143	mice
Arrdc4	-0.37	0.00475164	mice
Tap2	0.17	0.00477112	mice
Srpk2	-0.33	0.00480237	mice
Wwp2	-0.39	0.00510575	mice
Slc25a33	-0.38	0.00514019	mice
Foxn3	-0.59	0.00561549	mice
Hilpda	0.62	0.00584016	mice

Fam32a	-0.36	0.00603998	mice
Kcmf1	-0.46	0.00622023	mice
Lcor	-0.44	0.00634765	mice
Fkbp15	-0.47	0.00640492	mice
Vasp	-0.25	0.00646026	mice
Snx2	0.21	0.00655994	mice
Psma5	0.12	0.00686700	mice
Fth1	-0.18	0.00687458	mice
S100a6	-0.21	0.00691117	mice
Trf	-0.51	0.00691295	mice
Ipcef1	-0.51	0.00702936	mice
Inpp5a	-0.35	0.00744028	mice
Wac	-0.41	0.00756787	mice
Bzw1	-0.35	0.00786436	mice
Wdfy3	-0.47	0.00835421	mice
Pik3r1	-0.34	0.00847729	mice
Cdc42ep2	0.17	0.00865389	mice
Gatad2b	-0.41	0.00868564	mice
Ddx17	-0.39	0.00874719	mice
Arsg	-0.37	0.00907649	mice
Tm9sf3	-0.40	0.00913208	mice
Ahcyl1	-0.33	0.00931613	mice
Atp7a	-0.41	0.00953500	mice
Atf2	-0.43	0.00955832	mice
Sema4a	-0.42	0.00961502	mice
Ralgapa1	-0.25	0.00985057	mice
N4bp1	0.07	0.01063971	mice
Elf2	-0.43	0.01076074	mice
Oser1	0.16	0.01180050	mice
Armc7	-0.27	0.01198207	mice
Adgrg3	-0.32	0.01202902	mice
Adipor1	-0.27	0.01217506	mice
Arrdc3	-0.41	0.01223342	mice
Pnisr	-0.37	0.01226171	mice
Bcl2a1a	0.34	0.01266449	mice
Fam110a	-0.37	0.01268555	mice
Strn3	-0.46	0.01308101	mice
Tmem33	0.32	0.01365887	mice
Asxl1	-0.33	0.01403259	mice
Nsrp1	-0.36	0.01420230	mice
Kdm7a	-0.46	0.01425522	mice
Swt1	-0.36	0.01428280	mice
Kif5b	-0.42	0.01531450	mice
Cep120	-0.27	0.01547895	mice
Clec4n	-0.36	0.01591053	mice
Sec11c	-0.32	0.01637750	mice

Fyco1	0.15	0.01640143	mice
Epc1	-0.37	0.01642415	mice
C130026I21Rik	0.16	0.01658147	mice
Pecam1	0.22	0.01670293	mice
Rc3h2	-0.41	0.01685768	mice
Srsf7	0.18	0.01704255	mice
Dck	0.16	0.01729300	mice
Skil	0.25	0.01739964	mice
Ube2k	-0.44	0.01753242	mice
Ybx1	-0.26	0.01789739	mice
App	-0.47	0.01806957	mice
Tle4	0.22	0.01863307	mice
Lrch3	-0.39	0.01945610	mice
Skap2	-0.34	0.01968604	mice
Pfkp	-0.35	0.02013995	mice
Usp2	-0.38	0.02019818	mice
Rbm26	-0.43	0.02044973	mice
Birc2	0.19	0.02082024	mice
Rasgrp2	-0.31	0.02167398	mice
Calm3	-0.33	0.02187598	mice
Nisch	-0.36	0.02188428	mice
Eloc	0.29	0.02243141	mice
Chp1	-0.37	0.02270664	mice
Sesn2	-0.35	0.02291607	mice
Insyn2b	0.16	0.02294621	mice
Arf3	-0.37	0.02313999	mice
Phf3	-0.38	0.02418780	mice
Apoe	0.26	0.02470559	mice
Sun2	-0.33	0.02513880	mice
Setd2	-0.39	0.02562537	mice
Rapgef6	-0.35	0.02567094	mice
BC031181	-0.35	0.02639452	mice
Epc2	-0.42	0.02754902	mice
Slain2	-0.32	0.02823639	mice
Casp3	-0.41	0.02836934	mice
Pnpla8	-0.37	0.02879593	mice
Riok3	-0.38	0.02884152	mice
Cst3	-0.12	0.02926453	mice
Pcbp2	-0.33	0.03119640	mice
Uvrag	-0.30	0.03231636	mice
Rbm5	-0.35	0.03270399	mice
Tbc1d15	-0.30	0.03407661	mice
Rap1a	-0.38	0.03547695	mice
Plekhn1	-0.34	0.03584006	mice
Grina	0.18	0.03620928	mice
Pnpla2	-0.39	0.03675342	mice

Pcmtd1	-0.36	0.03820173	mice
Cox4i1	-0.33	0.03826971	mice
Arhgef1	-0.31	0.03878414	mice
Plbd1	-0.39	0.03880495	mice
Sra1	-0.38	0.03901754	mice
Snx24	-0.48	0.03941507	mice
Stag2	-0.42	0.03957065	mice
Klf2	-0.28	0.03962884	mice
Abl2	0.14	0.04015960	mice
Rpe	0.13	0.04059905	mice
Hk3	0.22	0.04150995	mice
Dlg1	-0.46	0.04281871	mice
Tmbim4	-0.31	0.04286439	mice
Ets1	-0.44	0.04299359	mice
Wdr37	-0.30	0.04535654	mice
Cd47	0.20	0.04552180	mice
Abracl	-0.29	0.04562119	mice
Ip6k1	-0.33	0.04645825	mice
Nrf1	-0.36	0.04846136	mice
Fas	0.13	0.04878479	mice
Ngly1	-0.36	0.05011017	mice
Fbrs	-0.36	0.05136680	mice
Zfp236	-0.34	0.05253901	mice
Ecpas	-0.47	0.05636341	mice
Wasf2	-0.45	0.05645414	mice
Cstdc5	0.55	0.05648493	mice
Fus	-0.30	0.05719484	mice
Cox6b1	-0.35	0.05784656	mice
Ccdc12	-0.36	0.06195372	mice
Prdm2	-0.39	0.06227557	mice
Cd37	-0.39	0.06275315	mice
Lsp1	-0.22	0.06512057	mice
Tapt1	-0.31	0.06713135	mice
Lrrfip2	-0.35	0.06721369	mice
Pdcd4	-0.41	0.06888907	mice
Nsmce2	-0.43	0.06973791	mice
Dtnbp1	-0.39	0.06987824	mice
Glyr1	-0.33	0.07007338	mice
Ndufa7	-0.32	0.07045341	mice
Gpbp1	0.17	0.07142834	mice
Smad7	-0.44	0.07151908	mice
Jak2	-0.40	0.07425123	mice
Insr	-0.33	0.07441909	mice
Adprh	-0.38	0.07632274	mice
Tirap	-0.33	0.07643330	mice
Vav1	0.27	0.07697433	mice

Ap1g1	-0.40	0.08143058	mice
Gns	-0.37	0.08213728	mice
Nub1	0.18	0.08302191	mice
Cd86	0.36	0.08370733	mice
Ifngr1	-0.33	0.08388301	mice
Rnf19b	0.18	0.08506150	mice
Pkn1	-0.32	0.08794036	mice
Usp4	-0.28	0.08898474	mice
St3gal4	0.09	0.09104038	mice
Eif4g2	-0.37	0.09205878	mice
Dennd3	-0.31	0.09534951	mice
Tent2	-0.41	0.09650229	mice
Zswim4	-0.29	0.09862026	mice
Preb	-0.32	0.10661488	mice
Tbl1xr1	-0.38	0.10955082	mice
Dock11	-0.50	0.10985124	mice
Hp	-0.25	0.11379560	mice
5031439G07Rik	-0.38	0.11429306	mice
Gpatch2l	-0.36	0.11998420	mice
Rlim	-0.30	0.12182179	mice
Pet100	0.19	0.12585112	mice
Aftph	-0.41	0.12738957	mice
Vrk1	-0.32	0.12755017	mice
Nsd1	-0.32	0.13227592	mice
Itsn2	-0.35	0.13274059	mice
Rab11b	-0.31	0.13593208	mice
Agtpbp1	-0.40	0.13961459	mice
Smap2	-0.41	0.13986900	mice
Foxo3	-0.44	0.14300015	mice
Zc3h7a	0.18	0.14796004	mice
Atrn	-0.30	0.15070346	mice
Unc119	-0.30	0.15253629	mice
Gatad2a	0.16	0.15344479	mice
Grk2	-0.30	0.15441905	mice
Ambra1	-0.40	0.15695305	mice
Ing2	0.16	0.15744732	mice
Anxa5	0.10	0.15969023	mice
Sppl3	-0.40	0.15997043	mice
Hat1	0.20	0.16189018	mice
Anxa2	-0.22	0.16250235	mice
Anp32a	-0.31	0.16363808	mice
Ppp1r16b	-0.31	0.16603070	mice
Dgkg	-0.50	0.17015179	mice
Zfand5	0.21	0.17565192	mice
Epb41	-0.32	0.17575277	mice
Cdk2ap2	-0.48	0.17942542	mice

Ndfip1	-0.33	0.18082092	mice
Ppp1r18	-0.31	0.18152054	mice
Calm1	-0.16	0.18154148	mice
Cox7c	0.15	0.18173747	mice
Usp15	-0.38	0.18448214	mice
Abtb1	-0.25	0.18637123	mice
Far1	-0.34	0.19057016	mice
Esyt2	-0.45	0.19406262	mice
Hnrnpu	-0.33	0.19962880	mice
Dnm2	-0.35	0.20688578	mice
Hook3	-0.38	0.20770569	mice
Rasgef1b	0.34	0.21494958	mice
Hist1h1c	-0.31	0.22167724	mice
Scarb2	-0.41	0.22299339	mice
Prrc2c	-0.35	0.22890044	mice
Krit1	-0.39	0.23021419	mice
Sec14l1	-0.39	0.23107137	mice
Kctd20	-0.33	0.23198188	mice
Ggct	0.18	0.24438751	mice
Eldr	0.21	0.25315423	mice
Naa35	-0.28	0.25350395	mice
Zfp868	-0.28	0.25378789	mice
Fndc3b	-0.17	0.25574773	mice
Ptp4a2	-0.31	0.26246793	mice
Gramd1b	-0.28	0.26964575	mice
Slc44a1	-0.41	0.27274864	mice
Cdc73	-0.39	0.28315376	mice
Eif4ebp2	-0.37	0.28814719	mice
Plaur	-0.17	0.29412000	mice
Cep83	-0.51	0.30497818	mice
5031425E22Rik	-0.37	0.30864368	mice
Ftl1	-0.07	0.31116482	mice
Tecpr1	0.19	0.31642760	mice
Slc2a6	0.09	0.31994925	mice
Sntb2	-0.34	0.33015965	mice
Rap1gds1	-0.30	0.33363355	mice
Jak1	-0.20	0.34529469	mice
Sh3bgrl	0.04	0.34705573	mice
Zscan29	-0.26	0.34888325	mice
Sfi1	0.19	0.34901459	mice
Batf	0.13	0.35215897	mice
Senp6	-0.33	0.36232556	mice
Nedd4l	-0.36	0.36611427	mice
Rc3h1	-0.39	0.39020995	mice
Rnasel	0.20	0.39514917	mice
Wbp11	-0.27	0.39865511	mice

Rnf169	-0.36	0.40069912	mice
Sec24a	0.25	0.40273314	mice
Fam102a	-0.36	0.40396132	mice
Atp8b4	0.27	0.40838333	mice
Safb	-0.32	0.41084122	mice
Zfp871	-0.25	0.42023457	mice
Basp1	0.23	0.42518383	mice
Gse1	-0.27	0.42913562	mice
Nfatc3	-0.43	0.43155922	mice
Slc25a5	-0.31	0.43428388	mice
Tmem219	0.09	0.43776671	mice
Orai1	-0.36	0.44032882	mice
Raet1e	-0.30	0.45789449	mice
Rmnd5a	-0.28	0.45857015	mice
Ythdc1	-0.35	0.46194306	mice
Sertad2	0.16	0.46342887	mice
Atp6ap1	-0.31	0.46569522	mice
Zfp703	0.15	0.46828180	mice
Itgb1	0.13	0.47331912	mice
Sirpb1c	-0.32	0.47354740	mice
S100a10	0.20	0.48077103	mice
Avl9	-0.38	0.48080912	mice
Ulk4	0.19	0.48191500	mice
Relch	-0.32	0.48939964	mice
Dmtf1	-0.24	0.48976644	mice
Vgll4	-0.32	0.49452251	mice
Atp2c1	-0.31	0.52762755	mice
Dnajc13	0.08	0.53794734	mice
Fam120b	-0.29	0.55457710	mice
Cd300lf	0.18	0.55501269	mice
D16Ertd472e	0.15	0.55933703	mice
Akt3	0.16	0.56454883	mice
Gripap1	0.13	0.56795712	mice
Ikbkb	-0.30	0.58415713	mice
Tcf25	-0.33	0.58600287	mice
Camk2d	0.19	0.60045335	mice
Gnb1	-0.34	0.61237879	mice
Gtdc1	-0.30	0.62094854	mice
Csf1r	-0.37	0.62409799	mice
Vcl	-0.40	0.62509007	mice
Vamp3	-0.26	0.66818390	mice
Stk10	-0.37	0.67770926	mice
Bptf	-0.32	0.68412764	mice
Nsf	-0.40	0.68594222	mice
Srp14	-0.29	0.68600358	mice
Hivep2	0.23	0.70785254	mice

Ube2b	-0.19	0.70965521	mice
Ube2j2	-0.27	0.71339623	mice
AW011738	0.15	0.74310142	mice
Cdk11b	-0.31	0.75696274	mice
Adam10	-0.34	0.76281399	mice
Kidins220	-0.26	0.81510181	mice
Slc20a1	0.19	0.81674513	mice
Katna1	0.14	0.82688413	mice
Ankrd13a	-0.33	0.84227980	mice
Glg1	-0.44	0.84965922	mice
Etf1	-0.20	0.85449686	mice
Taf10	-0.24	0.87046233	mice
Fem1c	-0.23	0.87181583	mice
Sub1	-0.22	0.87633038	mice
Grcc10	-0.32	0.88359639	mice
Add1	-0.28	0.88621850	mice
Usf2	-0.29	0.89263976	mice
Zfx	-0.33	0.89844923	mice
Kif1b	-0.33	0.92038296	mice
Rnf38	-0.33	0.94153489	mice
Chmp2a	0.11	0.94310369	mice
Spag9	-0.10	0.94364487	mice
Nfkb2	0.10	0.94872405	mice
Wdfy2	-0.35	0.96468414	mice
Dock10	0.08	0.97435480	mice
Chchd2	-0.27	0.97656000	mice
Ncoa2	-0.33	0.98141001	mice
Tm9sf2	-0.34	0.98836273	mice
Rac1	-0.29	0.99975974	mice
Ehd1	-0.23	1.00000000	mice
Tnfsf13b	-0.33	1.00000000	mice
Flot1	0.12	1.00000000	mice
Dpm1	-0.31	1.00000000	mice
H2-Eb1	0.26	1.00000000	mice
Ttc39b	0.17	1.00000000	mice
Crk	-0.39	1.00000000	mice
Clk1	-0.25	1.00000000	mice
Wipf2	-0.27	1.00000000	mice
Sec61g	0.20	1.00000000	mice
Gpr141	-0.33	1.00000000	mice
Sidt2	-0.31	1.00000000	mice
Lrrk2	0.20	1.00000000	mice
Ndufb9	-0.34	1.00000000	mice
Tet3	0.12	1.00000000	mice
Prpf40a	-0.39	1.00000000	mice
Reps1	-0.34	1.00000000	mice

Oxr1	-0.33	1.00000000	mice
Qk	-0.27	1.00000000	mice
Pgap2	0.12	1.00000000	mice
Klhl24	-0.29	1.00000000	mice
Appbp2	-0.34	1.00000000	mice
Ube2d2a	-0.24	1.00000000	mice
Ppp4c	-0.29	1.00000000	mice
Glul	-0.30	1.00000000	mice
Fam168b	-0.30	1.00000000	mice
Ppp1r3d	0.14	1.00000000	mice
B3gnt2	0.18	1.00000000	mice
Tnfaip8	-0.31	1.00000000	mice
Slc16a6	-0.29	1.00000000	mice
Ccdc146	0.15	1.00000000	mice
Rassf5	-0.30	1.00000000	mice
Sugt1	-0.25	1.00000000	mice
S100a8	0.44	1.00000000	mice
Xdh	0.13	1.00000000	mice
Cdc37l1	-0.25	1.00000000	mice
Ubp1	-0.28	1.00000000	mice
Gpsm3	-0.25	1.00000000	mice
Trpv2	-0.33	1.00000000	mice
Gsr	-0.28	1.00000000	mice
Safb2	-0.28	1.00000000	mice
Nlrp12	-0.26	1.00000000	mice
Edem1	-0.30	1.00000000	mice
Efhd2	0.09	1.00000000	mice
Aldh2	-0.28	1.00000000	mice
Vps18	-0.31	1.00000000	mice
Arl15	-0.61	1.00000000	mice
Nap1l1	-0.38	1.00000000	mice
4732465J04Rik	-0.31	1.00000000	mice
Snx3	-0.35	1.00000000	mice
Nr4a3	0.15	1.00000000	mice
Bin2	0.12	1.00000000	mice
H2afj	-0.25	1.00000000	mice
U2af1	-0.24	1.00000000	mice
Ppp4r3b	0.12	1.00000000	mice
Fbxo38	-0.32	1.00000000	mice
Smyd3	-0.44	1.00000000	mice
P2ry2	-0.25	1.00000000	mice
Ralb	-0.28	1.00000000	mice
Zfr	-0.34	1.00000000	mice
Phf1	-0.24	1.00000000	mice
Psma7	0.15	1.00000000	mice
Igf2r	-0.26	1.00000000	mice

Zfp961	0.12	1.00000000	mice
Slc31a2	0.21	1.00000000	mice
Klf10	-0.28	1.00000000	mice
Gadd45g	0.33	1.00000000	mice
Rab5if	0.11	1.00000000	mice
Fnip2	0.04	1.00000000	mice
Arhgap30	0.18	1.00000000	mice
Crem	0.16	1.00000000	mice
Atg16l2	-0.21	1.00000000	mice
Cd24a	-0.16	1.00000000	mice
Runx1	-0.32	1.00000000	mice
Gng10	-0.28	1.00000000	mice
Pde4b	0.08	1.00000000	mice
Pkm	-0.24	1.00000000	mice
Lmtk2	-0.27	1.00000000	mice
Sptlc2	-0.36	1.00000000	mice
Ssr3	0.09	1.00000000	mice
Sh2b2	0.11	1.00000000	mice
Smarca2	-0.33	1.00000000	mice
Limd2	-0.31	1.00000000	mice
Aim2	-0.38	1.00000000	mice
Capg	0.19	1.00000000	mice
Ube2q1	0.12	1.00000000	mice
Rbbp6	-0.29	1.00000000	mice
Rasa1	-0.32	1.00000000	mice
Rb1cc1	-0.29	1.00000000	mice
Inpp4a	-0.35	1.00000000	mice
Clec4e	0.25	1.00000000	mice
Abca1	0.15	1.00000000	mice
Tbc1d14	-0.37	1.00000000	mice
B4galt1	-0.35	1.00000000	mice
Smad4	-0.31	1.00000000	mice
Ap1s2	-0.29	1.00000000	mice
Cdkn1b	-0.24	1.00000000	mice
Rhog	-0.26	1.00000000	mice
Tcf4	0.14	1.00000000	mice
Aff4	-0.26	1.00000000	mice
Ankrd13c	-0.34	1.00000000	mice
Eml4	-0.43	1.00000000	mice
Rp9	-0.28	1.00000000	mice
Tns3	-0.45	1.00000000	mice
4930581F22Rik	-0.24	1.00000000	mice
Zfp148	-0.30	1.00000000	mice
Dek	0.11	1.00000000	mice
Rabgap1	-0.25	1.00000000	mice
Fis1	-0.27	1.00000000	mice

Gapdh	-0.27	1.00000000	mice
Sf3b5	-0.23	1.00000000	mice
Xpo6	-0.32	1.00000000	mice
Rell1	-0.31	1.00000000	mice
Pip5k1b	-0.39	1.00000000	mice
Lyz2	0.22	1.00000000	mice
R3hdm2	-0.35	1.00000000	mice
Hopx	-0.30	1.00000000	mice
Ipo7	-0.33	1.00000000	mice
Cirbp	-0.26	1.00000000	mice
Pxk	-0.38	1.00000000	mice
Prpf39	-0.28	1.00000000	mice
Sf3b2	-0.30	1.00000000	mice
Arid1b	-0.41	1.00000000	mice
Sde2	0.17	1.00000000	mice
Nfkbib	0.08	1.00000000	mice
H2-Aa	0.14	1.00000000	mice
Cd82	-0.29	1.00000000	mice
Zfp652	-0.28	1.00000000	mice
Cnot1	-0.33	1.00000000	mice
Cript	-0.34	1.00000000	mice
Rab11a	-0.25	1.00000000	mice
Hck	0.16	1.00000000	mice
Srxn1	-0.33	1.00000000	mice
Add3	-0.24	1.00000000	mice
Fgl2	-0.18	1.00000000	mice
H3f3b	-0.10	1.00000000	mice
Tnks	-0.37	1.00000000	mice
Ppm1b	-0.27	1.00000000	mice
Fuca2	-0.30	1.00000000	mice
Nrd1	-0.29	1.00000000	mice
N4bp2l1	-0.34	1.00000000	mice
Arpp19	-0.32	1.00000000	mice
Psmd4	0.12	1.00000000	mice
Bri3	0.22	1.00000000	mice
Pbx1	-0.25	1.00000000	mice
Hnrnpa0	-0.28	1.00000000	mice
Csf2ra	-0.24	1.00000000	mice
Gch1	0.13	1.00000000	mice
Pcna	0.07	1.00000000	mice
Paip2	-0.26	1.00000000	mice
Tspan5	-0.33	1.00000000	mice
Usp8	-0.30	1.00000000	mice
Btbd19	-0.25	1.00000000	mice
Prkch	-0.49	1.00000000	mice
Ttyh3	-0.25	1.00000000	mice

Cdk8	0.18	1.00000000	mice
Csf2rb	0.15	1.00000000	mice
Pxn	-0.25	1.00000000	mice
Pilrb1	0.07	1.00000000	mice
Nme2	0.13	1.00000000	mice
Tlr6	0.09	1.00000000	mice
Crebl2	-0.21	1.00000000	mice
Slc25a37	0.11	1.00000000	mice
Phrf1	-0.23	1.00000000	mice
Hdlbp	-0.25	1.00000000	mice
Zfp292	-0.28	1.00000000	mice
Ep300	-0.35	1.00000000	mice
Cstf3	-0.27	1.00000000	mice
Gadd45a	0.11	1.00000000	mice
Pcf11	-0.31	1.00000000	mice
Hnrnpul2	-0.24	1.00000000	mice
Grk6	-0.20	1.00000000	mice
Lnpep	-0.26	1.00000000	mice
Arid4a	0.07	1.00000000	mice
Ppp6c	-0.27	1.00000000	mice
Runx3	0.12	1.00000000	mice
Smarca5	-0.26	1.00000000	mice
Nfat5	-0.32	1.00000000	mice
Plcg2	-0.33	1.00000000	mice
Atxn7l3b	-0.33	1.00000000	mice
Haus8	-0.31	1.00000000	mice
Jpt1	0.12	1.00000000	mice
Spty2d1	0.11	1.00000000	mice
Stk38	-0.29	1.00000000	mice
Hspa8	0.17	1.00000000	mice
Smg7	0.07	1.00000000	mice
Akirin1	-0.31	1.00000000	mice
Snx10	0.05	1.00000000	mice
Taf1	-0.30	1.00000000	mice
Akr1a1	0.08	1.00000000	mice
Tspan13	0.17	1.00000000	mice
Lrrc8d	-0.30	1.00000000	mice
Capza2	-0.19	1.00000000	mice
Tle3	-0.32	1.00000000	mice
AA467197	0.46	1.00000000	mice
Celf1	-0.39	1.00000000	mice
Tnfrsf1b	-0.18	1.00000000	mice
Nipsnap3b	0.08	1.00000000	mice
Zfand6	-0.38	1.00000000	mice
Clasp2	-0.28	1.00000000	mice
Tmed2	-0.27	1.00000000	mice

Kdm5a	-0.30	1.00000000	mice
Pilrb2	-0.26	1.00000000	mice
Syncrip	-0.25	1.00000000	mice
Tbc1d1	0.10	1.00000000	mice
Fbxl12	-0.25	1.00000000	mice
Huwe1	-0.36	1.00000000	mice
Cdk12	-0.32	1.00000000	mice
Supt6	-0.29	1.00000000	mice
Timm8b	-0.26	1.00000000	mice
Pank2	0.07	1.00000000	mice
2310001H17Rik	0.13	1.00000000	mice
Fmnl3	-0.22	1.00000000	mice
Vti1a	-0.31	1.00000000	mice
Trub2	-0.23	1.00000000	mice
Zfp407	-0.42	1.00000000	mice
Sgpl1	-0.31	1.00000000	mice
Card19	-0.24	1.00000000	mice
Bcl2a1d	0.13	1.00000000	mice
Wsb1	0.19	1.00000000	mice
Copa	-0.31	1.00000000	mice
Hist1h2be	0.06	1.00000000	mice
Fkbp8	-0.24	1.00000000	mice
Arhgap24	0.08	1.00000000	mice
Shoc2	-0.32	1.00000000	mice
Tes	-0.32	1.00000000	mice
Slc3a2	0.02	1.00000000	mice
Rhov	0.13	1.00000000	mice
Rnf125	-0.21	1.00000000	mice
Spop	-0.26	1.00000000	mice
Ndrg1	-0.29	1.00000000	mice
Cpeb2	-0.21	1.00000000	mice
Vamp4	-0.24	1.00000000	mice
Gpkow	-0.26	1.00000000	mice
Herpud2	-0.24	1.00000000	mice
Cd14	-0.24	1.00000000	mice
Pcbp1	-0.31	1.00000000	mice
Cir1	-0.19	1.00000000	mice
Lamtor4	-0.26	1.00000000	mice
Tarm1	0.14	1.00000000	mice
S100a9	0.23	1.00000000	mice
Dmxl1	-0.24	1.00000000	mice
Glipr1	-0.26	1.00000000	mice
Rngtt	-0.39	1.00000000	mice
Entpd3	0.12	1.00000000	mice
Cdc5l	-0.25	1.00000000	mice
Plp2	-0.25	1.00000000	mice

Plekhj1	-0.18	1.00000000	mice
Brd1	-0.25	1.00000000	mice
Plscr1	0.05	1.00000000	mice
Lst1	0.13	1.00000000	mice
Ppp1r12c	-0.22	1.00000000	mice
Sp3	-0.29	1.00000000	mice
Gng12	-0.33	1.00000000	mice
Rev3l	-0.34	1.00000000	mice
Rsu1	-0.29	1.00000000	mice
Ptbp2	-0.23	1.00000000	mice
Sec61b	-0.21	1.00000000	mice
Psmd8	0.09	1.00000000	mice
Api5	-0.25	1.00000000	mice
Ptpn6	-0.27	1.00000000	mice
Ogfrl1	0.09	1.00000000	mice
Arpc1a	-0.26	1.00000000	mice
Cstb	-0.37	1.00000000	mice
Ccm2	-0.29	1.00000000	mice
Cnn2	-0.24	1.00000000	mice
Ttc17	-0.22	1.00000000	mice
Arap1	-0.21	1.00000000	mice
Prkca	-0.31	1.00000000	mice
Nin	-0.22	1.00000000	mice
Tomm22	-0.22	1.00000000	mice
Sbds	-0.19	1.00000000	mice
Jun	0.28	1.00000000	mice
Hnrnpc	-0.25	1.00000000	mice
Rcbtb2	-0.29	1.00000000	mice
Prex1	-0.31	1.00000000	mice
Zdhhc20	-0.27	1.00000000	mice
Ptpn12	-0.21	1.00000000	mice
Tpr	-0.26	1.00000000	mice
Lcorl	-0.40	1.00000000	mice
Kdm6a	-0.25	1.00000000	mice
Tmod3	-0.35	1.00000000	mice
Pram1	-0.21	1.00000000	mice
Il2rg	0.01	1.00000000	mice
Pcnx	-0.28	1.00000000	mice
Gbe1	-0.30	1.00000000	mice
Calr	0.11	1.00000000	mice
Pqlc1	-0.18	1.00000000	mice
Ilf3	0.06	1.00000000	mice
Bmt2	-0.31	1.00000000	mice
Eif3a	-0.30	1.00000000	mice
B4galt5	0.04	1.00000000	mice
Rb1	-0.35	1.00000000	mice

Hnrnpl	-0.28	1.00000000	mice
Stam2	-0.30	1.00000000	mice
Evi2	0.02	1.00000000	mice
Phf20l1	-0.28	1.00000000	mice
Stat6	-0.26	1.00000000	mice
Sp2	-0.19	1.00000000	mice
Gngt2	-0.23	1.00000000	mice
Igsf6	-0.27	1.00000000	mice
Scaf4	-0.34	1.00000000	mice
Sema4d	-0.21	1.00000000	mice
Stfa2l1	0.29	1.00000000	mice
Tnpo3	-0.29	1.00000000	mice
Ddx46	-0.26	1.00000000	mice
Hmox1	0.13	1.00000000	mice
Pcnp	-0.23	1.00000000	mice
Sri	0.04	1.00000000	mice
Fryl	-0.34	1.00000000	mice
Psmd14	-0.39	1.00000000	mice
Chmp3	-0.25	1.00000000	mice
Atf6	-0.34	1.00000000	mice
Rbms1	-0.20	1.00000000	mice
Treml4	-0.32	1.00000000	mice
Ubr4	0.13	1.00000000	mice
Dot1l	0.09	1.00000000	mice
Rab7	0.14	1.00000000	mice
Tmem234	-0.24	1.00000000	mice
Rabgef1	0.12	1.00000000	mice
Tnfrsf1a	0.13	1.00000000	mice
Nrbp1	-0.21	1.00000000	mice
Lncpint	-0.28	1.00000000	mice
Rin3	-0.25	1.00000000	mice
Noct	-0.30	1.00000000	mice
Pfdn2	-0.21	1.00000000	mice
Rbm33	-0.22	1.00000000	mice
Cr1l	-0.24	1.00000000	mice
Hcst	-0.21	1.00000000	mice
Psma3	0.06	1.00000000	mice
Tbc1d2b	-0.28	1.00000000	mice
Atp11b	-0.24	1.00000000	mice
Tug1	-0.28	1.00000000	mice
Hspbap1	0.09	1.00000000	mice
Spatc1	0.09	1.00000000	mice
Ube2m	0.08	1.00000000	mice
Traf3	-0.37	1.00000000	mice
Cks2	0.08	1.00000000	mice
Ahcyl2	0.11	1.00000000	mice

Etv3	0.14	1.00000000	mice
Soat1	0.04	1.00000000	mice
Hnrnpm	-0.27	1.00000000	mice
Usp47	-0.28	1.00000000	mice
Tmem41b	-0.28	1.00000000	mice
Rab5c	0.07	1.00000000	mice
Cux1	0.00	1.00000000	mice
Atp5e	-0.18	1.00000000	mice
Slk	-0.26	1.00000000	mice
Pde8a	-0.24	1.00000000	mice
Selenot	0.00	1.00000000	mice
Hbp1	-0.26	1.00000000	mice
Twf2	-0.22	1.00000000	mice
Gpr65	-0.24	1.00000000	mice
Ubr5	-0.35	1.00000000	mice
Rsf1	-0.26	1.00000000	mice
Rab9	0.10	1.00000000	mice
Birc6	-0.25	1.00000000	mice
Atp6v1f	-0.24	1.00000000	mice
Phyh	-0.26	1.00000000	mice
Ubl5	-0.22	1.00000000	mice
Ssbp2	0.15	1.00000000	mice
Tcf20	-0.31	1.00000000	mice
Vps36	-0.20	1.00000000	mice
Thbs1	-0.42	1.00000000	mice
Sh3bp1	-0.26	1.00000000	mice
Hexa	-0.24	1.00000000	mice
Sfr1	-0.24	1.00000000	mice
Rflnb	-0.20	1.00000000	mice
Eps15	-0.28	1.00000000	mice
Eef1b2	-0.24	1.00000000	mice
Slc38a2	-0.30	1.00000000	mice
Siglece	-0.23	1.00000000	mice
Tnrc6a	-0.25	1.00000000	mice
Inpp4b	0.24	1.00000000	mice
Atp6v0d1	-0.22	1.00000000	mice
Tmx4	-0.25	1.00000000	mice
Nrbf2	-0.17	1.00000000	mice
Tmem59	-0.24	1.00000000	mice
Tcf7l2	0.08	1.00000000	mice
Ptma	0.01	1.00000000	mice
Uba52	0.05	1.00000000	mice
Csnk1g1	-0.32	1.00000000	mice
Tmem243	0.09	1.00000000	mice
Ube2j1	-0.20	1.00000000	mice
Wsb2	-0.28	1.00000000	mice

Spp1	0.33	1.00000000	mice
Dedd2	0.12	1.00000000	mice
Sik1	-0.25	1.00000000	mice
Btbd7	-0.35	1.00000000	mice
Vps4b	-0.16	1.00000000	mice
Tab2	-0.27	1.00000000	mice
Cmah	-0.28	1.00000000	mice
Hsd17b11	-0.25	1.00000000	mice
Tpst2	-0.17	1.00000000	mice
St13	-0.18	1.00000000	mice
Clip1	-0.27	1.00000000	mice
Tmem127	-0.22	1.00000000	mice
Cpsf6	-0.19	1.00000000	mice
Gtf2a2	-0.20	1.00000000	mice
Sf1	-0.26	1.00000000	mice
Ubap1	0.08	1.00000000	mice
Gdi1	-0.19	1.00000000	mice
Fmnl2	-0.41	1.00000000	mice
Ugp2	-0.28	1.00000000	mice
Hist1h2bc	-0.24	1.00000000	mice
Pspc1	-0.23	1.00000000	mice
Ceacam1	-0.24	1.00000000	mice
Ccdc174	-0.21	1.00000000	mice
Lamtor2	-0.23	1.00000000	mice
Higd1a	-0.26	1.00000000	mice
Atp11a	0.02	1.00000000	mice
Ddx24	0.06	1.00000000	mice
Samd8	-0.29	1.00000000	mice
Ube2i	-0.26	1.00000000	mice
Atp6v1e1	-0.21	1.00000000	mice
Stxbp3	0.13	1.00000000	mice
Rnf13	-0.27	1.00000000	mice
Cln5	-0.20	1.00000000	mice
Pabpc1	-0.21	1.00000000	mice
Camk1d	-0.17	1.00000000	mice
Arl8b	-0.31	1.00000000	mice
Actr2	0.10	1.00000000	mice
Fam76a	-0.25	1.00000000	mice
Rnf167	-0.23	1.00000000	mice
Srrd	0.07	1.00000000	mice
Ptafr	0.13	1.00000000	mice
Clec4d	0.09	1.00000000	mice
Ano6	-0.37	1.00000000	mice
Ranbp9	-0.37	1.00000000	mice
Znrf2	0.04	1.00000000	mice
Dnajc3	-0.25	1.00000000	mice

Sft2d2	0.03	1.00000000	mice
Arg2	0.07	1.00000000	mice
Taok3	-0.21	1.00000000	mice
Lipa	-0.22	1.00000000	mice
Eif4h	-0.23	1.00000000	mice
A530013C23Rik	0.08	1.00000000	mice
Slpi	0.16	1.00000000	mice
Cnih4	-0.21	1.00000000	mice
Git2	-0.24	1.00000000	mice
Faf1	-0.25	1.00000000	mice
Tmem71	0.07	1.00000000	mice
Crebrf	0.03	1.00000000	mice
Haus2	-0.28	1.00000000	mice
Tfeb	-0.26	1.00000000	mice
Elavl1	-0.23	1.00000000	mice
Zc3h15	-0.24	1.00000000	mice
Zcchc8	-0.20	1.00000000	mice
Atp5j2	0.07	1.00000000	mice
Zfp106	-0.29	1.00000000	mice
Cdc40	-0.23	1.00000000	mice
Ifnar2	-0.25	1.00000000	mice
Ing3	0.10	1.00000000	mice
Ccnl2	-0.29	1.00000000	mice
Prkcd	-0.28	1.00000000	mice
Btk	-0.23	1.00000000	mice
Scamp2	-0.24	1.00000000	mice
Zc3h13	-0.25	1.00000000	mice
Wfdc21	0.24	1.00000000	mice
Gphn	0.01	1.00000000	mice
Saraf	-0.21	1.00000000	mice
Sertad1	0.04	1.00000000	mice
Edf1	-0.20	1.00000000	mice
Phf21a	0.04	1.00000000	mice
Pbx2	-0.23	1.00000000	mice
2410006H16Rik	0.06	1.00000000	mice
Hspa5	0.10	1.00000000	mice
Nsa2	-0.29	1.00000000	mice
Atg2a	0.13	1.00000000	mice
Sp140	0.06	1.00000000	mice
BC004004	-0.19	1.00000000	mice
Ptges3	-0.22	1.00000000	mice
Pla1a	0.01	1.00000000	mice
Dennd1c	-0.18	1.00000000	mice
Esco1	-0.24	1.00000000	mice
Clec1a	-0.05	1.00000000	mice
Uqcrh	-0.23	1.00000000	mice

Tm9sf4	-0.23	1.00000000	mice
Ndufb11	-0.19	1.00000000	mice
Cd53	0.10	1.00000000	mice
Pgs1	0.03	1.00000000	mice
Sfpq	-0.14	1.00000000	mice
Tpm3	-0.19	1.00000000	mice
Atp13a3	-0.29	1.00000000	mice
Usp7	-0.22	1.00000000	mice
Atxn7	-0.23	1.00000000	mice
Neat1	0.14	1.00000000	mice
Srrm1	-0.22	1.00000000	mice
Zhx2	0.05	1.00000000	mice
Cyfip1	0.03	1.00000000	mice
Tmem38b	-0.27	1.00000000	mice
Atp1a3	-0.26	1.00000000	mice
Rbm47	-0.32	1.00000000	mice
Trip11	-0.26	1.00000000	mice
Pik3cg	-0.20	1.00000000	mice
Baz2a	-0.26	1.00000000	mice
Atp11c	-0.34	1.00000000	mice
Bach2	-0.29	1.00000000	mice
Pde4d	-0.29	1.00000000	mice
Rab1a	0.07	1.00000000	mice
Nrros	0.06	1.00000000	mice
Tomm20	-0.23	1.00000000	mice
Selenon	-0.22	1.00000000	mice
H2afy	0.15	1.00000000	mice
Slc44a2	-0.24	1.00000000	mice
Suds3	-0.21	1.00000000	mice
Aplp2	-0.30	1.00000000	mice
Vps28	-0.24	1.00000000	mice
Tnip1	-0.31	1.00000000	mice
Cyld	0.07	1.00000000	mice
Stx7	-0.05	1.00000000	mice
Dnttip1	-0.17	1.00000000	mice
Tmem167	-0.02	1.00000000	mice
Bmp2k	-0.31	1.00000000	mice
A630001O12Rik	0.02	1.00000000	mice
Cox6c	0.14	1.00000000	mice
Phf8	-0.19	1.00000000	mice
Pnkp	-0.17	1.00000000	mice
Snx27	-0.26	1.00000000	mice
Dennd1a	0.21	1.00000000	mice
Relb	-0.02	1.00000000	mice
Rnasek	-0.19	1.00000000	mice
Bcl2a1b	-0.17	1.00000000	mice

Lcn2	-0.05	1.00000000	mice
Aurkaip1	-0.15	1.00000000	mice
Samd4b	0.05	1.00000000	mice
Gdi2	-0.31	1.00000000	mice
Thrap3	-0.26	1.00000000	mice
Abcg1	-0.33	1.00000000	mice
Slc25a20	0.02	1.00000000	mice
Rtn4	-0.27	1.00000000	mice
Khdrbs1	-0.25	1.00000000	mice
Rabgap1l	-0.25	1.00000000	mice
Ubac2	-0.26	1.00000000	mice
Lemd3	-0.26	1.00000000	mice
Rnf115	-0.25	1.00000000	mice
Cd74	0.09	1.00000000	mice
Copb1	-0.22	1.00000000	mice
Ap3s1	-0.23	1.00000000	mice
Arf6	-0.23	1.00000000	mice
Cds2	-0.20	1.00000000	mice
Atp1a1	-0.23	1.00000000	mice
Cebpd	0.15	1.00000000	mice
Gabpb1	-0.24	1.00000000	mice
Cped1	0.18	1.00000000	mice
Zfyve26	-0.23	1.00000000	mice
Slc2a3	0.06	1.00000000	mice
Cmpk1	0.00	1.00000000	mice
Yy1	-0.26	1.00000000	mice
Hip1r	-0.26	1.00000000	mice
Lonrf1	-0.18	1.00000000	mice
Cmtm7	-0.14	1.00000000	mice
Vta1	-0.25	1.00000000	mice
Ube2r2	-0.27	1.00000000	mice
Ipmk	-0.25	1.00000000	mice
Zmynd8	-0.20	1.00000000	mice
Arcn1	-0.22	1.00000000	mice
Gpbp1l1	-0.18	1.00000000	mice
Stt3b	-0.24	1.00000000	mice
Ss18	-0.18	1.00000000	mice
H2-Ab1	0.11	1.00000000	mice
Chm	-0.27	1.00000000	mice
Vti1b	-0.24	1.00000000	mice
Ppib	0.05	1.00000000	mice
Canx	-0.22	1.00000000	mice
Rab11fip1	0.10	1.00000000	mice
Ninj1	-0.13	1.00000000	mice
Cd300ld	-0.18	1.00000000	mice
Gtf2f1	0.03	1.00000000	mice

Arf5	-0.23	1.00000000	mice
Tmem30a	-0.23	1.00000000	mice
Bmpr2	-0.35	1.00000000	mice
Klf6	0.11	1.00000000	mice
Phactr2	-0.27	1.00000000	mice
Txn1	0.08	1.00000000	mice
Eif4e	-0.25	1.00000000	mice
Uso1	-0.20	1.00000000	mice
Rbm7	0.03	1.00000000	mice
Tmem50a	-0.27	1.00000000	mice
Trir	-0.22	1.00000000	mice
Naca	-0.30	1.00000000	mice
Usp16	-0.26	1.00000000	mice
Ly96	0.05	1.00000000	mice
Ier2	0.09	1.00000000	mice
Hipk2	-0.33	1.00000000	mice
Nup153	-0.02	1.00000000	mice
Rnf145	-0.24	1.00000000	mice
Ppig	-0.22	1.00000000	mice
Eif4e2	-0.22	1.00000000	mice
Ppp1ca	-0.18	1.00000000	mice
Kdm2b	0.11	1.00000000	mice
Slc11a1	-0.13	1.00000000	mice
Nudt18	-0.16	1.00000000	mice
Lrrc25	0.06	1.00000000	mice
Thoc2	-0.28	1.00000000	mice
Tom1	-0.25	1.00000000	mice
Cd200r1	0.02	1.00000000	mice
Ran	0.02	1.00000000	mice
Psmb3	-0.02	1.00000000	mice
Osbpl8	-0.27	1.00000000	mice
Syf2	-0.19	1.00000000	mice
Snrnp70	-0.20	1.00000000	mice
Sdf4	-0.23	1.00000000	mice
Dusp11	-0.19	1.00000000	mice
Tsc22d2	-0.20	1.00000000	mice
Fam193a	-0.21	1.00000000	mice
Sem1	0.09	1.00000000	mice
Hif1a	-0.14	1.00000000	mice
Srrm2	-0.15	1.00000000	mice
Evi2a	0.04	1.00000000	mice
Pcif1	-0.17	1.00000000	mice
Pip5k1a	-0.22	1.00000000	mice
Srpr	-0.01	1.00000000	mice
Use1	-0.01	1.00000000	mice
Hsp90ab1	0.07	1.00000000	mice

Ppp1cc	-0.20	1.00000000	mice
Rbm27	-0.15	1.00000000	mice
Rprd1b	0.03	1.00000000	mice
Brwd1	-0.18	1.00000000	mice
Ubap2l	-0.22	1.00000000	mice
Pacs1	-0.16	1.00000000	mice
Arid1a	-0.01	1.00000000	mice
Pstpip1	-0.26	1.00000000	mice
Ncl	-0.21	1.00000000	mice
Dap	0.01	1.00000000	mice
Pilra	-0.18	1.00000000	mice
Scaper	-0.15	1.00000000	mice
Lhfpl2	-0.37	1.00000000	mice
Zfp91	0.02	1.00000000	mice
Cdc42se2	-0.25	1.00000000	mice
Ern1	0.00	1.00000000	mice
Ubl3	-0.24	1.00000000	mice
Amn1	0.08	1.00000000	mice
Csnk1g3	-0.26	1.00000000	mice
Set	-0.02	1.00000000	mice
Ero1lb	-0.24	1.00000000	mice
Atp6v0c	0.21	1.00000000	mice
Adnp	-0.15	1.00000000	mice
Smg1	-0.21	1.00000000	mice
Ywhae	-0.18	1.00000000	mice
Scaf11	-0.26	1.00000000	mice
Syap1	-0.18	1.00000000	mice
Clk4	-0.22	1.00000000	mice
Lamtor1	-0.20	1.00000000	mice
Kansl3	-0.21	1.00000000	mice
Vipas39	0.05	1.00000000	mice
Csnk1e	0.03	1.00000000	mice
Ifnar1	0.04	1.00000000	mice
Smap1	-0.23	1.00000000	mice
Zfp281	-0.22	1.00000000	mice
Slc38a1	0.02	1.00000000	mice
Atox1	-0.16	1.00000000	mice
Rtf2	-0.25	1.00000000	mice
Pik3r5	0.09	1.00000000	mice
Lars2	0.10	1.00000000	mice
Slc23a2	0.10	1.00000000	mice
Bcl10	-0.21	1.00000000	mice
Fgfr2	-0.18	1.00000000	mice
Capn1	0.02	1.00000000	mice
Ggps1	0.03	1.00000000	mice
Eif3f	0.02	1.00000000	mice

Itpr1	-0.06	1.00000000	mice
Zfp622	-0.19	1.00000000	mice
Pgd	-0.19	1.00000000	mice
Slu7	-0.21	1.00000000	mice
Sec63	-0.19	1.00000000	mice
Slc6a6	-0.17	1.00000000	mice
Creb5	0.28	1.00000000	mice
Zfp207	-0.22	1.00000000	mice
Dtx4	-0.01	1.00000000	mice
Kpna1	-0.18	1.00000000	mice
Stag1	-0.30	1.00000000	mice
Lsm14a	-0.06	1.00000000	mice
Trps1	-0.01	1.00000000	mice
Rapgef2	0.09	1.00000000	mice
Dapp1	-0.02	1.00000000	mice
Ralbp1	-0.32	1.00000000	mice
Fxr1	0.01	1.00000000	mice
Slc16a3	-0.03	1.00000000	mice
Hnrnpd	0.07	1.00000000	mice
Zmat2	-0.23	1.00000000	mice
Strap	-0.02	1.00000000	mice
Bcor	-0.18	1.00000000	mice
Tmem189	-0.20	1.00000000	mice
Blcap	-0.17	1.00000000	mice
Stk11	-0.18	1.00000000	mice
Cox5a	-0.20	1.00000000	mice
Eif1a	0.00	1.00000000	mice
Stim2	-0.23	1.00000000	mice
Herc1	-0.22	1.00000000	mice
Tob2	0.01	1.00000000	mice
Rassf2	-0.10	1.00000000	mice
Psmb4	0.00	1.00000000	mice
Zcchc7	-0.23	1.00000000	mice
Neu1	-0.22	1.00000000	mice
Rnf138	-0.21	1.00000000	mice
Adgrv1	-0.14	1.00000000	mice
Snx29	-0.18	1.00000000	mice
Tiam1	-0.28	1.00000000	mice
Ppm1h	-0.40	1.00000000	mice
Rbm42	-0.17	1.00000000	mice
Dhx40	0.25	1.00000000	mice
Hnrnpul1	-0.18	1.00000000	mice
Rasgrp4	-0.13	1.00000000	mice
Hmgn2	-0.17	1.00000000	mice
Lysmd3	-0.14	1.00000000	mice
Ewsr1	-0.18	1.00000000	mice

Gtf2b	-0.16	1.00000000	mice
Actr10	-0.23	1.00000000	mice
Borcs5	0.03	1.00000000	mice
Ddi2	-0.18	1.00000000	mice
Bag1	0.02	1.00000000	mice
Orai2	0.01	1.00000000	mice
Trip12	-0.21	1.00000000	mice
Tmem165	-0.19	1.00000000	mice
Bclaf1	-0.15	1.00000000	mice
Snx4	-0.18	1.00000000	mice
G3bp2	-0.03	1.00000000	mice
Kdm3b	-0.21	1.00000000	mice
Stk24	-0.05	1.00000000	mice
Gabarapl2	-0.20	1.00000000	mice
Tmem131	0.22	1.00000000	mice
Arhgap45	-0.22	1.00000000	mice
Polr1d	-0.18	1.00000000	mice
Csnk1d	0.06	1.00000000	mice
Ston2	-0.24	1.00000000	mice
Atp6v0b	-0.20	1.00000000	mice
Lrmda	0.04	1.00000000	mice
Ell	-0.17	1.00000000	mice
Hmgcr	-0.16	1.00000000	mice
Pdpn	0.01	1.00000000	mice
Hacd4	-0.14	1.00000000	mice
Xiap	-0.01	1.00000000	mice
Nck1	-0.20	1.00000000	mice
Lilra6	-0.18	1.00000000	mice
Emp3	0.04	1.00000000	mice
Rest	-0.18	1.00000000	mice
Bbip1	-0.22	1.00000000	mice
Snf8	-0.16	1.00000000	mice
Top2b	-0.19	1.00000000	mice
Slc9a1	0.03	1.00000000	mice
Hal	-0.18	1.00000000	mice
Ppp1r15b	-0.08	1.00000000	mice
Emilin2	0.10	1.00000000	mice
Stat5b	-0.26	1.00000000	mice
Ndufa3	-0.03	1.00000000	mice
Csk	-0.23	1.00000000	mice
Rab10os	0.00	1.00000000	mice
Fbxo42	-0.29	1.00000000	mice
Ensa	-0.17	1.00000000	mice
Tut4	0.09	1.00000000	mice
Atrx	-0.12	1.00000000	mice
Crtc3	-0.24	1.00000000	mice

Dgat1	-0.18	1.00000000	mice
Ankrd17	-0.01	1.00000000	mice
Dynll1	-0.22	1.00000000	mice
Eapp	-0.20	1.00000000	mice
Skp1a	-0.02	1.00000000	mice
Tut7	-0.08	1.00000000	mice
Atp5f1	-0.21	1.00000000	mice
Hmgb1	-0.20	1.00000000	mice
Sar1a	-0.20	1.00000000	mice
Trib1	0.06	1.00000000	mice
Cers2	-0.17	1.00000000	mice
Uqcr10	-0.13	1.00000000	mice
Bcap31	-0.19	1.00000000	mice
Tcp11l2	-0.16	1.00000000	mice
Higd2a	-0.15	1.00000000	mice
Trp53inp1	-0.16	1.00000000	mice
Inpp1	-0.02	1.00000000	mice
Cdv3	-0.23	1.00000000	mice
Eef1a1	-0.24	1.00000000	mice
Cxcr4	0.03	1.00000000	mice
Ncoa1	-0.30	1.00000000	mice
Ppp1r11	-0.22	1.00000000	mice
Rab10	-0.23	1.00000000	mice
Qrich1	-0.19	1.00000000	mice
Cacul1	-0.21	1.00000000	mice
Ino80d	-0.21	1.00000000	mice
H2afz	-0.15	1.00000000	mice
Atg16l1	-0.15	1.00000000	mice
Dock8	0.00	1.00000000	mice
Efcab14	-0.24	1.00000000	mice
Tmem258	-0.14	1.00000000	mice
Dnajb9	0.01	1.00000000	mice
Ik	-0.20	1.00000000	mice
Dnaja1	-0.18	1.00000000	mice
Smg6	-0.21	1.00000000	mice
H3f3a	-0.05	1.00000000	mice
Os9	-0.16	1.00000000	mice
Ext1	0.03	1.00000000	mice
Cnppd1	0.02	1.00000000	mice
Tnrc6c	-0.20	1.00000000	mice
Reps2	-0.40	1.00000000	mice
Ostc	-0.14	1.00000000	mice
Erbin	-0.13	1.00000000	mice
Psma2	0.02	1.00000000	mice
Usp9x	-0.25	1.00000000	mice
Prelid1	0.00	1.00000000	mice

P4hb	-0.19	1.00000000	mice
Dnajc1	-0.31	1.00000000	mice
Cox7a2	-0.20	1.00000000	mice
Srsf2	0.00	1.00000000	mice
Dnajb6	0.06	1.00000000	mice
Agps	-0.19	1.00000000	mice
Vps26a	0.01	1.00000000	mice
Tatdn2	0.01	1.00000000	mice
Ldha	-0.23	1.00000000	mice
Rtf1	-0.01	1.00000000	mice
Poldip3	-0.21	1.00000000	mice
Pik3ca	-0.16	1.00000000	mice
Ccndbp1	-0.18	1.00000000	mice
Cnot6l	-0.22	1.00000000	mice
Lrif1	-0.03	1.00000000	mice
Ptpn18	-0.19	1.00000000	mice
Cwc15	-0.13	1.00000000	mice
Akna	-0.14	1.00000000	mice
Cox14	-0.05	1.00000000	mice
Sltm	-0.20	1.00000000	mice
Ltb	0.11	1.00000000	mice
Gpr132	-0.09	1.00000000	mice
Pdpk1	-0.10	1.00000000	mice
Agpat4	-0.29	1.00000000	mice
Serbp1	-0.03	1.00000000	mice
Rnf10	-0.14	1.00000000	mice
Tnpo1	-0.26	1.00000000	mice
Nfia	-0.10	1.00000000	mice
Tpp2	-0.17	1.00000000	mice
Scp2	-0.24	1.00000000	mice
Npepps	0.08	1.00000000	mice
Atad2b	-0.19	1.00000000	mice
Tgm2	-0.15	1.00000000	mice
Ywhah	-0.19	1.00000000	mice
Snrpb	-0.22	1.00000000	mice
Tma7	-0.04	1.00000000	mice
Rfx3	-0.10	1.00000000	mice
Ndufa4	0.01	1.00000000	mice
Srp9	-0.15	1.00000000	mice
Klra2	0.04	1.00000000	mice
Ptgs2os2	-0.18	1.00000000	mice
Supt4a	-0.17	1.00000000	mice
Sec62	-0.22	1.00000000	mice
Hnrnph2	-0.18	1.00000000	mice
Ube2d3	-0.11	1.00000000	mice
Gigyf2	-0.23	1.00000000	mice

Ube2h	-0.17	1.00000000	mice
Akt2	-0.17	1.00000000	mice
Ptms	-0.15	1.00000000	mice
Sipa1l3	-0.17	1.00000000	mice
Ctdp1	-0.11	1.00000000	mice
Eif3k	-0.13	1.00000000	mice
Il4ra	-0.05	1.00000000	mice
Tkt	-0.17	1.00000000	mice
Gk	-0.11	1.00000000	mice
Acbd3	-0.13	1.00000000	mice
Rbm38	0.01	1.00000000	mice
Ccng2	-0.02	1.00000000	mice
Cox5b	-0.12	1.00000000	mice
Hnrnph1	-0.26	1.00000000	mice
Larp1b	-0.13	1.00000000	mice
Pmaip1	0.00	1.00000000	mice
Nf1	0.00	1.00000000	mice
Crlf2	-0.21	1.00000000	mice
Sumo2	-0.20	1.00000000	mice
Fnbp1	-0.26	1.00000000	mice
Lpgat1	-0.17	1.00000000	mice
Sys1	-0.18	1.00000000	mice
Khdc4	0.01	1.00000000	mice
Rnh1	0.00	1.00000000	mice
Plekhf2	-0.02	1.00000000	mice
Dusp16	0.08	1.00000000	mice
Psma1	-0.17	1.00000000	mice
Timp2	0.02	1.00000000	mice
Tmem123	-0.19	1.00000000	mice
Ube2a	-0.12	1.00000000	mice
Tec	-0.12	1.00000000	mice
4833407H14Rik	0.01	1.00000000	mice
Spin1	-0.01	1.00000000	mice
Rsrp1	0.05	1.00000000	mice
Zbtb11	-0.14	1.00000000	mice
Nuak2	-0.13	1.00000000	mice
Setd5	-0.24	1.00000000	mice
Usp38	-0.19	1.00000000	mice
Rbbp4	-0.13	1.00000000	mice
Cnot4	-0.28	1.00000000	mice
Acin1	-0.19	1.00000000	mice
Cdc42ep3	-0.08	1.00000000	mice
Ocel1	-0.15	1.00000000	mice
Lamp1	0.04	1.00000000	mice
Ndufb8	0.00	1.00000000	mice
Tmpo	-0.16	1.00000000	mice

Sec11a	-0.22	1.00000000	mice
Tmed5	-0.17	1.00000000	mice
Tnfrsf26	-0.05	1.00000000	mice
Cpne1	-0.18	1.00000000	mice
Simc1	0.06	1.00000000	mice
Ccni	-0.15	1.00000000	mice
Rbbp8	-0.07	1.00000000	mice
Jaml	0.00	1.00000000	mice
Psd4	-0.09	1.00000000	mice
Oat	-0.14	1.00000000	mice
Rilpl2	-0.13	1.00000000	mice
Crlf3	-0.07	1.00000000	mice
Tgfb1	0.01	1.00000000	mice
Arhgef3	-0.10	1.00000000	mice
Amd1	-0.17	1.00000000	mice
Cxcl3	0.10	1.00000000	mice
Ywhaq	-0.04	1.00000000	mice
Pfkfb4	-0.04	1.00000000	mice
Stx6	-0.05	1.00000000	mice
Gsk3a	0.01	1.00000000	mice
Akap13	-0.24	1.00000000	mice
Nup98	0.10	1.00000000	mice
Zranb1	-0.17	1.00000000	mice
Cd244a	-0.23	1.00000000	mice
Fam172a	-0.02	1.00000000	mice
Fbxw2	-0.19	1.00000000	mice
Slc9a8	-0.11	1.00000000	mice
Kdm4c	-0.19	1.00000000	mice
Sumo1	-0.20	1.00000000	mice
Rer1	-0.14	1.00000000	mice
Cpt1a	-0.08	1.00000000	mice
Abhd17b	-0.15	1.00000000	mice
Rab32	-0.16	1.00000000	mice
Sirt2	-0.16	1.00000000	mice
Gnas	-0.17	1.00000000	mice
Il10rb	-0.01	1.00000000	mice
Ppard	-0.14	1.00000000	mice
Tlr13	-0.18	1.00000000	mice
Pabpn1	-0.16	1.00000000	mice
Srgn	0.05	1.00000000	mice
1700016P03Rik	-0.01	1.00000000	mice
Bnip2	-0.17	1.00000000	mice
Isy1	-0.15	1.00000000	mice
Rbm6	-0.15	1.00000000	mice
Golgb1	-0.16	1.00000000	mice
Nufip2	-0.17	1.00000000	mice

Rchy1	0.00	1.00000000	mice
Tnks2	-0.16	1.00000000	mice
Hsp90b1	-0.08	1.00000000	mice
Sec24b	-0.15	1.00000000	mice
Rrp1	-0.14	1.00000000	mice
Atp5mpl	-0.03	1.00000000	mice
Fgfr1op2	-0.04	1.00000000	mice
Tbc1d8	0.03	1.00000000	mice
Hivep1	-0.20	1.00000000	mice
Txndc17	-0.02	1.00000000	mice
A730081D07Rik	0.02	1.00000000	mice
Taf15	-0.06	1.00000000	mice
Flvcr1	-0.12	1.00000000	mice
Syne1	-0.21	1.00000000	mice
Csnk2b	-0.02	1.00000000	mice
Cox7a2l	-0.18	1.00000000	mice
Dhx15	-0.11	1.00000000	mice
Edem2	-0.17	1.00000000	mice
Snrpg	-0.01	1.00000000	mice
Actn4	0.00	1.00000000	mice
Tmem251	-0.18	1.00000000	mice
Tmed7	-0.14	1.00000000	mice
Sh2d3c	-0.07	1.00000000	mice
Ndufs4	-0.09	1.00000000	mice
Zeb1	-0.03	1.00000000	mice
Parp8	0.06	1.00000000	mice
Upf2	-0.04	1.00000000	mice
Khnyn	-0.16	1.00000000	mice
Srsf3	-0.04	1.00000000	mice
Ube2f	-0.22	1.00000000	mice
Nab1	-0.10	1.00000000	mice
Hk1	0.10	1.00000000	mice
Ccar1	-0.16	1.00000000	mice
Dnajc8	-0.19	1.00000000	mice
Acbd5	-0.16	1.00000000	mice
Arhgap17	-0.24	1.00000000	mice
Atp5d	-0.06	1.00000000	mice
Raly	-0.02	1.00000000	mice
Pum1	-0.19	1.00000000	mice
Bach1	-0.15	1.00000000	mice
Galnt11	0.02	1.00000000	mice
Ube2g1	-0.16	1.00000000	mice
Arnt	-0.20	1.00000000	mice
Hnrnpa2b1	-0.04	1.00000000	mice
Eno1	-0.14	1.00000000	mice
Pcyt1a	-0.06	1.00000000	mice

Dynlrb1	-0.14	1.00000000	mice
Atp6v0a1	-0.19	1.00000000	mice
Ptpn23	-0.02	1.00000000	mice
Spidr	-0.15	1.00000000	mice
Degs1	-0.15	1.00000000	mice
Lgals3	-0.10	1.00000000	mice
Smc6	-0.28	1.00000000	mice
Zbtb20	-0.06	1.00000000	mice
Slco3a1	-0.14	1.00000000	mice
Ubtd1	0.00	1.00000000	mice
Rhob	0.10	1.00000000	mice
Gda	-0.19	1.00000000	mice
Tbc1d5	-0.18	1.00000000	mice
Tnfrsf23	-0.28	1.00000000	mice
Timm10b	-0.06	1.00000000	mice
Cited2	0.02	1.00000000	mice
Gtf2a1	-0.10	1.00000000	mice
Cd68	0.01	1.00000000	mice
Rab21	-0.15	1.00000000	mice
Cpeb4	-0.17	1.00000000	mice
Wtap	-0.16	1.00000000	mice
Sf3b6	-0.17	1.00000000	mice
Lbr	-0.15	1.00000000	mice
Rhbdf2	-0.06	1.00000000	mice
Pttg1ip	-0.17	1.00000000	mice
Atf4	-0.07	1.00000000	mice
Smndc1	-0.13	1.00000000	mice
Prpf38b	-0.11	1.00000000	mice
Eif4ebp1	-0.13	1.00000000	mice
Zdhhc18	-0.08	1.00000000	mice
Ints6	0.05	1.00000000	mice
Fnbp4	-0.02	1.00000000	mice
Tspyl1	-0.13	1.00000000	mice
C9orf72	-0.02	1.00000000	mice
Klra17	-0.15	1.00000000	mice
Tox4	-0.20	1.00000000	mice
Il16	-0.06	1.00000000	mice
Arfgef2	-0.01	1.00000000	mice
Hmgb2	-0.02	1.00000000	mice
Gnb2	-0.10	1.00000000	mice
Stap1	-0.06	1.00000000	mice
Cep350	-0.05	1.00000000	mice
Ppp2r2a	-0.20	1.00000000	mice
9830107B12Rik	-0.16	1.00000000	mice
Fbxo30	-0.05	1.00000000	mice
Psmd11	-0.15	1.00000000	mice

Ctsh	-0.02	1.00000000	mice
Hsp90aa1	-0.14	1.00000000	mice
Polr2a	-0.11	1.00000000	mice
Ctse	0.03	1.00000000	mice
Atg101	-0.14	1.00000000	mice
Cblb	-0.21	1.00000000	mice
Zfp36l1	-0.06	1.00000000	mice
Ric1	-0.14	1.00000000	mice
Phlda1	-0.16	1.00000000	mice
Sptan1	-0.14	1.00000000	mice
Was	-0.15	1.00000000	mice
Rela	-0.21	1.00000000	mice
Rnaset2a	-0.14	1.00000000	mice
Pik3c2a	-0.14	1.00000000	mice
Hnrnpab	-0.13	1.00000000	mice
Etv6	0.12	1.00000000	mice
Arid4b	-0.07	1.00000000	mice
Vmp1	-0.06	1.00000000	mice
Plekhm2	-0.04	1.00000000	mice
Rsrc2	-0.13	1.00000000	mice
Sgk1	-0.10	1.00000000	mice
Dnaja2	-0.13	1.00000000	mice
Epb41l2	-0.09	1.00000000	mice
Chic2	-0.17	1.00000000	mice
Dop1b	-0.02	1.00000000	mice
Clic1	-0.05	1.00000000	mice
Vdac2	-0.04	1.00000000	mice
Hdc	-0.06	1.00000000	mice
Stat4	0.05	1.00000000	mice
Ndufa2	-0.03	1.00000000	mice
Osgin1	0.01	1.00000000	mice
Rnf144a	0.06	1.00000000	mice
Arhgap9	-0.10	1.00000000	mice
Rara	-0.05	1.00000000	mice
Eif3h	-0.17	1.00000000	mice
Sarnp	-0.07	1.00000000	mice
Rala	-0.15	1.00000000	mice
Phip	-0.22	1.00000000	mice
Eya3	-0.29	1.00000000	mice
Cc2d2b	-0.41	1.00000000	mice
Ppia	-0.17	1.00000000	mice
Brd2	-0.15	1.00000000	mice
Slbp	-0.08	1.00000000	mice
Cdc27	0.06	1.00000000	mice
Susd6	-0.03	1.00000000	mice
Phlpp1	-0.01	1.00000000	mice

Acss2	-0.09	1.00000000	mice
Chka	-0.08	1.00000000	mice
Ube2n	-0.14	1.00000000	mice
Cisd2	-0.04	1.00000000	mice
Spcs2	-0.07	1.00000000	mice
Ubxn1	-0.13	1.00000000	mice
Smad3	-0.14	1.00000000	mice
Dhx38	-0.16	1.00000000	mice
Dstn	-0.21	1.00000000	mice
Ubn2	-0.11	1.00000000	mice
Serinc1	-0.15	1.00000000	mice
Ahi1	-0.02	1.00000000	mice
Elf1	-0.11	1.00000000	mice
Becn1	-0.14	1.00000000	mice
Nars	-0.17	1.00000000	mice
Snap23	0.00	1.00000000	mice
Olfr372	0.00	1.00000000	mice
Tmem134	-0.17	1.00000000	mice
Ifitm10	0.00	1.00000000	mice
Abcc1	-0.08	1.00000000	mice
Uqcrq	-0.12	1.00000000	mice
Irak2	0.01	1.00000000	mice
Fam111a	-0.23	1.00000000	mice
Golph3	-0.14	1.00000000	mice
Tcea1	-0.08	1.00000000	mice
Fpr1	-0.12	1.00000000	mice
Flcn	-0.11	1.00000000	mice
Rnf185	-0.16	1.00000000	mice
Larp4b	-0.17	1.00000000	mice
Cdc42bpg	-0.15	1.00000000	mice
Krcc1	-0.17	1.00000000	mice
Aff1	-0.02	1.00000000	mice
Furin	-0.06	1.00000000	mice
Acta2	-0.15	1.00000000	mice
Tomm7	-0.07	1.00000000	mice
Zfand2a	-0.16	1.00000000	mice
Kat2b	-0.11	1.00000000	mice
Dync1h1	-0.12	1.00000000	mice
Ppp3ca	-0.11	1.00000000	mice
Fbxw11	-0.19	1.00000000	mice
Atp5b	-0.04	1.00000000	mice
Nedd8	-0.15	1.00000000	mice
Ywhag	-0.05	1.00000000	mice
Jmy	-0.15	1.00000000	mice
N4bp2	-0.07	1.00000000	mice
Eef1g	-0.05	1.00000000	mice

Sbno1	0.03	1.00000000	mice
Pts	-0.01	1.00000000	mice
Airn	-0.08	1.00000000	mice
Hspa4	-0.18	1.00000000	mice
Tinf2	-0.05	1.00000000	mice
Znrf1	-0.18	1.00000000	mice
Ctsa	-0.05	1.00000000	mice
Bcl2l11	0.03	1.00000000	mice
Snu13	-0.07	1.00000000	mice
Bcl6	-0.04	1.00000000	mice
Nr4a2	0.03	1.00000000	mice
Zfp263	-0.16	1.00000000	mice
Rbks	-0.14	1.00000000	mice
Reep5	-0.11	1.00000000	mice
Hlx	-0.09	1.00000000	mice
Ergic2	-0.09	1.00000000	mice
Ssb	-0.08	1.00000000	mice
Ap3b1	-0.10	1.00000000	mice
Atf7ip	0.01	1.00000000	mice
Ash1l	-0.21	1.00000000	mice
Baz1a	-0.07	1.00000000	mice
Bend4	-0.13	1.00000000	mice
Dnajb1	-0.05	1.00000000	mice
Rbx1	-0.02	1.00000000	mice
Osbpl9	-0.03	1.00000000	mice
Atp6v1b2	-0.14	1.00000000	mice
Abhd5	-0.03	1.00000000	mice
Psmb1	-0.06	1.00000000	mice
Ywhab	-0.23	1.00000000	mice
Tra2b	0.01	1.00000000	mice
Asprv1	0.07	1.00000000	mice
Hsbp1	-0.08	1.00000000	mice
Pdcd10	-0.01	1.00000000	mice
Atxn1	-0.16	1.00000000	mice
Cfp	-0.04	1.00000000	mice
Diaph2	-0.27	1.00000000	mice
Rsbn1l	-0.12	1.00000000	mice
Cebpb	-0.02	1.00000000	mice
Bin3	-0.08	1.00000000	mice
Dync1i2	-0.13	1.00000000	mice
Pag1	0.03	1.00000000	mice
Tgfbi	0.00	1.00000000	mice
Bdp1	-0.15	1.00000000	mice
Eif4a2	-0.12	1.00000000	mice
Tob1	-0.10	1.00000000	mice
Eef2	-0.10	1.00000000	mice

Sap18	-0.08	1.00000000	mice
Psmb7	-0.18	1.00000000	mice
Cmtm6	-0.03	1.00000000	mice
Rad21	-0.15	1.00000000	mice
Ccdc88b	-0.03	1.00000000	mice
Atp5md	-0.01	1.00000000	mice
Rab24	-0.04	1.00000000	mice
Pik3cd	-0.16	1.00000000	mice
Cox6a1	-0.13	1.00000000	mice
Tubb4b	-0.05	1.00000000	mice
Psip1	-0.14	1.00000000	mice
Cd93	-0.15	1.00000000	mice
Arih2	0.01	1.00000000	mice
Depdc5	-0.03	1.00000000	mice
Slc8b1	-0.07	1.00000000	mice
Dbi	-0.08	1.00000000	mice
Bcas3	-0.19	1.00000000	mice
Ell2	0.00	1.00000000	mice
Itpr2	-0.03	1.00000000	mice
Rab4b	-0.08	1.00000000	mice
Cltc	-0.08	1.00000000	mice
Ube2l3	-0.12	1.00000000	mice
Cdk9	-0.08	1.00000000	mice
Tpm3-rs7	-0.05	1.00000000	mice
Scand1	-0.07	1.00000000	mice
Itprip	-0.14	1.00000000	mice
Trpm7	-0.08	1.00000000	mice
Atp6v1h	-0.05	1.00000000	mice
Fry	-0.12	1.00000000	mice
Napsa	-0.10	1.00000000	mice
Apobr	-0.05	1.00000000	mice
Eml1	-0.04	1.00000000	mice
Lmo4	-0.11	1.00000000	mice
Rabep1	-0.08	1.00000000	mice
Fbxl17	-0.08	1.00000000	mice
Ppp2r1a	-0.11	1.00000000	mice
E030030I06Rik	-0.12	1.00000000	mice
Pik3ap1	-0.09	1.00000000	mice
Erp29	-0.07	1.00000000	mice
Nop53	-0.14	1.00000000	mice
Psme4	-0.03	1.00000000	mice
Fam174a	-0.11	1.00000000	mice
Ctsl	-0.19	1.00000000	mice
Rere	-0.12	1.00000000	mice
Vps29	-0.11	1.00000000	mice
Lats2	-0.13	1.00000000	mice

Stat3	-0.02	1.00000000	mice
Slc49a4	-0.16	1.00000000	mice
Bsg	-0.11	1.00000000	mice
Chd4	-0.02	1.00000000	mice
H13	-0.05	1.00000000	mice
Ldlrad4	-0.14	1.00000000	mice
Atp5g2	-0.06	1.00000000	mice
Derl1	-0.15	1.00000000	mice
Ptpn2	-0.03	1.00000000	mice
Agfg1	-0.07	1.00000000	mice
Sfxn5	-0.04	1.00000000	mice
Atp6v1c1	-0.04	1.00000000	mice
Pnrc1	0.00	1.00000000	mice
Cwc25	-0.10	1.00000000	mice
Itgav	-0.08	1.00000000	mice
Ube2s	-0.14	1.00000000	mice
Tsc22d4	-0.08	1.00000000	mice
Fam129a	-0.17	1.00000000	mice
Elob	-0.02	1.00000000	mice
Irs2	-0.10	1.00000000	mice
Ndufa13	-0.10	1.00000000	mice
Ublcp1	-0.14	1.00000000	mice
Naip2	-0.13	1.00000000	mice
Frrs1	-0.14	1.00000000	mice
E230032D23Rik	-0.05	1.00000000	mice
Sbno2	-0.05	1.00000000	mice
Ndufa6	-0.06	1.00000000	mice
Plek	-0.02	1.00000000	mice
Vezf1	-0.17	1.00000000	mice
Spg21	-0.05	1.00000000	mice
Rragc	-0.14	1.00000000	mice
Tacstd2	-0.06	1.00000000	mice
Tlk2	-0.09	1.00000000	mice
Dctn3	-0.05	1.00000000	mice
Il10ra	-0.10	1.00000000	mice
Rab43	-0.08	1.00000000	mice
Cd84	-0.04	1.00000000	mice
Fyn	-0.17	1.00000000	mice
Ypel5	-0.15	1.00000000	mice
Fosb	0.01	1.00000000	mice
Taf13	-0.10	1.00000000	mice
Atxn10	-0.06	1.00000000	mice
Cox17	0.01	1.00000000	mice
Dgat2	-0.05	1.00000000	mice
Zcrb1	-0.13	1.00000000	mice
Npm1	-0.19	1.00000000	mice

Heatr1	-0.06	1.00000000	mice
Psenen	-0.06	1.00000000	mice
Rab44	-0.01	1.00000000	mice
Arl8a	-0.10	1.00000000	mice
Wdr7	-0.14	1.00000000	mice
Zfp869	-0.10	1.00000000	mice
Uba1	-0.11	1.00000000	mice
Gas5	-0.10	1.00000000	mice
Arglu1	-0.08	1.00000000	mice
Cggbp1	-0.11	1.00000000	mice
Pirb	-0.11	1.00000000	mice
Gpat3	-0.11	1.00000000	mice
Atp5j	-0.12	1.00000000	mice
Sh3bp2	-0.16	1.00000000	mice
Atp5c1	-0.01	1.00000000	mice
Por	-0.02	1.00000000	mice
Stim1	-0.16	1.00000000	mice
Flii	-0.08	1.00000000	mice
Atp5h	-0.08	1.00000000	mice
Tank	-0.08	1.00000000	mice
Rab22a	-0.13	1.00000000	mice
4933434E20Rik	-0.14	1.00000000	mice
Bhlhe40	-0.02	1.00000000	mice
Ifi27	-0.08	1.00000000	mice
Tfip11	-0.09	1.00000000	mice
Atp8a1	-0.11	1.00000000	mice
Numa1	-0.08	1.00000000	mice
Ppp4r2	-0.10	1.00000000	mice
Znrd1	-0.07	1.00000000	mice
Wdtc1	-0.10	1.00000000	mice
Stx12	-0.07	1.00000000	mice
Fam133b	-0.13	1.00000000	mice
Rab8a	-0.09	1.00000000	mice
Wbp2	-0.09	1.00000000	mice
Prkx	-0.12	1.00000000	mice
Napa	-0.10	1.00000000	mice
Pgk1	-0.15	1.00000000	mice
Atp6v1g1	-0.06	1.00000000	mice
Dock4	-0.13	1.00000000	mice
Snrpc	-0.12	1.00000000	mice
Dcaf12	-0.06	1.00000000	mice
Cdc42se1	-0.07	1.00000000	mice
Eif5a	-0.05	1.00000000	mice
Utrn	-0.11	1.00000000	mice
Kansl1l	-0.06	1.00000000	mice
Tnfsf14	-0.09	1.00000000	mice

Itprid2	-0.08	1.00000000	mice
1810026B05Rik	-0.09	1.00000000	mice
Slc31a1	-0.12	1.00000000	mice
Tctex1d2	-0.15	1.00000000	mice
Wdr45b	-0.13	1.00000000	mice
Cdip1	-0.14	1.00000000	mice
Slc40a1	-0.11	1.00000000	mice
Dlgap4	-0.07	1.00000000	mice
Ncoa3	-0.12	1.00000000	mice
Tmem248	-0.10	1.00000000	mice
Rab27a	-0.09	1.00000000	mice
Babam2	-0.07	1.00000000	mice
Ggta1	-0.13	1.00000000	mice
G3bp1	-0.08	1.00000000	mice
Chd1	-0.19	1.00000000	mice
Patl1	-0.04	1.00000000	mice
Poc1b	-0.03	1.00000000	mice
Atp5a1	-0.13	1.00000000	mice
Tbc1d20	-0.07	1.00000000	mice
Ep400	-0.08	1.00000000	mice
Atp6v1a	-0.11	1.00000000	mice
Eif2s2	-0.09	1.00000000	mice
1810013L24Rik	-0.09	1.00000000	mice
Psmb2	-0.11	1.00000000	mice
Hnrnpa3	-0.06	1.00000000	mice
Fbxo33	-0.10	1.00000000	mice
Zfp706	-0.08	1.00000000	mice
Serp1	-0.05	1.00000000	mice
Tmem14c	-0.09	1.00000000	mice
Gls	-0.10	1.00000000	mice
Suco	-0.03	1.00000000	mice
Ciapin1	-0.01	1.00000000	mice
Lrp10	-0.06	1.00000000	mice
Hnrnpll	-0.11	1.00000000	mice
Ugcg	-0.12	1.00000000	mice
Eed	-0.17	1.00000000	mice
Fermt3	-0.12	1.00000000	mice
Pomp	-0.09	1.00000000	mice
Xist	-0.13	1.00000000	mice
Arl6ip5	-0.12	1.00000000	mice
Pkn2	-0.09	1.00000000	mice
Ppp1r10	-0.10	1.00000000	mice
Atp6v1d	-0.10	1.00000000	mice
Ncf1	-0.05	1.00000000	mice
Arhgef2	-0.06	1.00000000	mice
Prkd3	-0.12	1.00000000	mice

Prdx5	0.04	1.00000000	mice
Smdt1	-0.04	1.00000000	mice
Ebi3	-0.13	1.00000000	mice
Pds5a	-0.09	1.00000000	mice
Atpif1	-0.07	1.00000000	mice
Cpne3	-0.09	1.00000000	mice
Pdia3	-0.10	1.00000000	mice
Slc2a1	-0.14	1.00000000	mice
Ctsd	0.02	1.00000000	mice
Ccnt1	-0.15	1.00000000	mice
Qsox1	-0.11	1.00000000	mice
Clec4a2	-0.16	1.00000000	mice
Zfp131	-0.05	1.00000000	mice
Esd	-0.08	1.00000000	mice
Frg1	-0.10	1.00000000	mice
Atp1b3	-0.11	1.00000000	mice
Lman2	-0.11	1.00000000	mice
Ccr1	-0.01	1.00000000	mice
Ndufb7	-0.08	1.00000000	mice
Glrx	-0.08	1.00000000	mice
Gpr137b	-0.06	1.00000000	mice
Aph1c	-0.10	1.00000000	mice
Selenof	-0.11	1.00000000	mice
Zc3h12a	-0.08	1.00000000	mice
Btaf1	-0.11	1.00000000	mice
Yipf4	-0.08	1.00000000	mice
Ier3ip1	-0.07	1.00000000	mice
Fam104a	-0.09	1.00000000	mice
Ddx6	0.03	1.00000000	mice
CAAA01118383.1	-0.14	1.00000000	mice
Creb1	-0.13	1.00000000	mice
Hnrnpdl	-0.09	1.00000000	mice
Hmgcl	-0.08	1.00000000	mice
Dach1	-0.04	1.00000000	mice
Azin1	-0.09	1.00000000	mice
Samsn1	0.00	1.00000000	mice
Ubr1	-0.04	1.00000000	mice
Caprin1	-0.11	1.00000000	mice
B9d2	-0.09	1.00000000	mice
Rab5a	-0.12	1.00000000	mice
Rack1	-0.15	1.00000000	mice
Plec	-0.17	1.00000000	mice
Dennd11	-1.57	0.00000000	pigs
Uvrag	3.27	0.00000000	pigs
Ube2h	-1.21	0.00000000	pigs
Lcp1	-1.51	0.00000000	pigs

Cstb	2.41	0.00000000	pigs
Lrrk2	-2.10	0.00000000	pigs
Lpgat1	-1.90	0.00000000	pigs
Fam135a	-2.10	0.00000000	pigs
Ppp3ca	-1.49	0.00000000	pigs
Hmgb2	-1.17	0.00000000	pigs
Spp1	2.45	0.00000000	pigs
Arl15	-2.50	0.00000000	pigs
Dock8	-1.53	0.00000000	pigs
Ostf1	-1.15	0.00000000	pigs
Pam	-2.23	0.00000000	pigs
Itga2	-2.08	0.00000000	pigs
Nin	-1.32	0.00000000	pigs
Pde3b	-1.96	0.00000000	pigs
Arhgap15	-1.08	0.00000000	pigs
Ipmk	-1.56	0.00000000	pigs
Ppif	3.26	0.00000000	pigs
Ptprc	-0.96	0.00000000	pigs
Notch2	-1.00	0.00000000	pigs
Arrdc4	2.22	0.00000000	pigs
B2m	0.73	0.00000000	pigs
Xpo6	-1.32	0.00000000	pigs
Cda	-1.08	0.00000000	pigs
Dock5	-1.47	0.00000000	pigs
C4bp	-2.56	0.00000000	pigs
Slamf7	4.11	0.00000000	pigs
Saa3	3.87	0.00000000	pigs
Ucp2	-1.91	0.00000000	pigs
Folr1	-1.34	0.00000000	pigs
Icam1	3.50	0.00000000	pigs
Cd24a	-1.49	0.00000000	pigs
Ftl1-ps1	3.56	0.00000000	pigs
Abca1	1.57	0.00000000	pigs
Plxnc1	-1.29	0.00000000	pigs
Auh	-1.36	0.00000000	pigs
Tspo	-1.39	0.00000000	pigs
Nod2	2.29	0.00000000	pigs
Cyria	-1.32	0.00000000	pigs
Nfkbia	1.25	0.00000000	pigs
Parp8	-2.06	0.00000000	pigs
Bhlhe40	2.17	0.00000000	pigs
Cblb	-1.95	0.00000000	pigs
Tlr2	1.95	0.00000000	pigs
Ifit2	-1.22	0.00000000	pigs
Sell	-1.03	0.00000000	pigs
Serpinb1a	-1.46	0.00000000	pigs

Tnfaip3	1.79	0.00000000	pigs
St6gal1	-1.38	0.00000000	pigs
Rap1gap2	-1.46	0.00000000	pigs
Stk17b	-1.15	0.00000000	pigs
Zc3h12c	3.17	0.00000000	pigs
Rnf144b	-1.57	0.00000000	pigs
Picalm	-1.00	0.00000000	pigs
Alox5ap	-0.99	0.00000000	pigs
Actb	-0.88	0.00000000	pigs
Tgfa	-1.38	0.00000000	pigs
Pnrc1	1.26	0.00000000	pigs
Lyst	-1.21	0.00000000	pigs
Cop1	-1.23	0.00000000	pigs
Nfe2l2	2.33	0.00000000	pigs
Pnpla8	-1.17	0.00000000	pigs
Gadd45b	2.31	0.00000000	pigs
Tbc1d15	1.94	0.00000000	pigs
Cyld	1.88	0.00000000	pigs
Fbxo9	-1.38	0.00000000	pigs
Cytip	-0.95	0.00000000	pigs
Tle4	-1.62	0.00000000	pigs
Herc6	-1.12	0.00000000	pigs
C5ar1	-0.87	0.00000000	pigs
Atxn1	-1.30	0.00000000	pigs
Crispld2	-1.37	0.00000000	pigs
Tlr4	1.87	0.00000000	pigs
Tnfaip2	1.97	0.00000000	pigs
Exoc6	-1.55	0.00000000	pigs
Hcls1	-1.12	0.00000000	pigs
Batf3	2.11	0.00000000	pigs
Rnf19a	2.22	0.00000000	pigs
Cdk12	2.00	0.00000000	pigs
Prelid2	-2.02	0.00000000	pigs
Erg	-1.46	0.00000000	pigs
Ppard	-1.21	0.00000000	pigs
Dhx34	-1.36	0.00000000	pigs
Tnrc6b	-1.27	0.00000000	pigs
Wdfy3	-0.90	0.00000000	pigs
Gpcpd1	-1.02	0.00000000	pigs
Dram1	1.90	0.00000000	pigs
Patl1	1.83	0.00000000	pigs
Steap4	-1.15	0.00000000	pigs
Il1a	1.93	0.00000000	pigs
Iqgap1	-0.97	0.00000000	pigs
Fgd3	-1.15	0.00000000	pigs
Numb	-0.77	0.00000000	pigs

Rac2	-0.93	0.00000000	pigs
Adgre5	-0.87	0.00000000	pigs
Il4ra	1.42	0.00000000	pigs
Dock2	-1.13	0.00000000	pigs
Fnbp1	-1.34	0.00000000	pigs
Cyth1	1.25	0.00000000	pigs
Pip5k1b	-1.63	0.00000000	pigs
Plekhm3	-0.87	0.00000000	pigs
Vav1	1.87	0.00000000	pigs
Rnf216	-1.27	0.00000000	pigs
Ncf1	-0.94	0.00000000	pigs
Igf2r	2.52	0.00000000	pigs
Acsl4	-0.73	0.00000000	pigs
Zfp292	1.89	0.00000000	pigs
Pik3ap1	1.51	0.00000000	pigs
Stat1	-1.12	0.00000000	pigs
Trpm7	2.16	0.00000000	pigs
Phf12	-1.21	0.00000000	pigs
Sqstm1	1.55	0.00000000	pigs
Tmem19	1.64	0.00000000	pigs
B3gnt2	2.13	0.00000000	pigs
Ncoa3	-1.34	0.00000000	pigs
Rragc	1.81	0.00000000	pigs
Inpp5d	-1.50	0.00000000	pigs
Sh3kbp1	-1.23	0.00000000	pigs
Ier3	1.81	0.00000000	pigs
Tnfsf13b	-1.12	0.00000000	pigs
Plcb1	-1.40	0.00000000	pigs
Ttc13	-1.31	0.00000000	pigs
Akr1c13	1.39	0.00000000	pigs
Cpd	-1.37	0.00000000	pigs
Washc5	1.81	0.00000000	pigs
Sorl1	-0.70	0.00000000	pigs
Pdxk	1.55	0.00000000	pigs
Bcl2a1d	1.32	0.00000000	pigs
Wtap	1.61	0.00000000	pigs
Csf1	2.24	0.00000000	pigs
Plcl2	-1.40	0.00000000	pigs
Pag1	-1.01	0.00000000	pigs
Itga4	-1.86	0.00000000	pigs
Lbr	-1.08	0.00000000	pigs
Slc18a1	1.66	0.00000000	pigs
Pacs1	-1.32	0.00000000	pigs
Rab1a	1.30	0.00000000	pigs
Ugcg	-1.32	0.00000000	pigs
Laptm4a	1.20	0.00000000	pigs

Tmcc1	-1.07	0.00000000	pigs
Ctsa	1.54	0.00000000	pigs
Limk2	-0.76	0.00000000	pigs
Coro1a	-0.86	0.00000000	pigs
Ifit3	-0.71	0.00000000	pigs
Cebpd	-1.06	0.00000000	pigs
Cfd	-0.75	0.00000000	pigs
Ano10	-1.17	0.00000000	pigs
Ptpn1	0.91	0.00000000	pigs
Sclt1	-1.62	0.00000000	pigs
Rac1	1.10	0.00000000	pigs
Cyfip2	-1.19	0.00000000	pigs
Clip1	-0.99	0.00000000	pigs
Rab7	0.91	0.00000000	pigs
Rab31	-0.78	0.00000000	pigs
Abhd2	-0.86	0.00000000	pigs
Ell	0.93	0.00000000	pigs
Traf1	1.69	0.00000000	pigs
Ifnar1	-0.91	0.00000000	pigs
S100a11	-1.22	0.00000000	pigs
Stx11	1.31	0.00000000	pigs
H2-Ea	1.67	0.00000000	pigs
Retn	2.20	0.00000000	pigs
Iqgap2	2.65	0.00000000	pigs
Btaf1	-1.24	0.00000000	pigs
Kdm7a	-0.89	0.00000000	pigs
Itgb7	-1.06	0.00000000	pigs
Ubr2	-0.88	0.00000000	pigs
Slc23a2	1.65	0.00000000	pigs
Pxn	-0.78	0.00000000	pigs
Pik3cg	-1.24	0.00000000	pigs
Fli1	-1.04	0.00000000	pigs
Lmnb1	-0.76	0.00000000	pigs
Dach1	-1.28	0.00000000	pigs
Jak1	1.19	0.00000000	pigs
Ankhd1	1.06	0.00000000	pigs
Stxbp5	-1.17	0.00000000	pigs
Snx10	1.14	0.00000000	pigs
Rnf122	1.56	0.00000000	pigs
Pafah2	-1.01	0.00000000	pigs
Cux1	-1.12	0.00000000	pigs
Rab10	2.12	0.00000000	pigs
4932438A13Rik	-1.15	0.00000000	pigs
Rnf169	-1.21	0.00000000	pigs
Gpat4	-0.65	0.00000000	pigs
Lpcat2	-1.11	0.00000000	pigs

Srpk2	-0.96	0.00000000	pigs
Tra2b	-0.89	0.00000000	pigs
Ctss	1.56	0.00000000	pigs
Ido1	2.56	0.00000000	pigs
Emb	-0.88	0.00000000	pigs
Secisbp2	-0.98	0.00000000	pigs
Cd82	1.63	0.00000000	pigs
Gbe1	-1.04	0.00000000	pigs
Cnot6l	-0.99	0.00000000	pigs
Fam111a	-1.16	0.00000000	pigs
Cast	-0.88	0.00000000	pigs
Dgkd	-0.93	0.00000000	pigs
Sat1	-0.57	0.00000000	pigs
Rara	-0.74	0.00000000	pigs
Was	-0.86	0.00000000	pigs
Rgs18	-0.78	0.00000000	pigs
Arg1	2.04	0.00000000	pigs
Timp1	1.97	0.00000000	pigs
Ago2	1.69	0.00000000	pigs
Nrp1	2.03	0.00000000	pigs
S100a8	-1.54	0.00000000	pigs
Arhgap26	-0.78	0.00000000	pigs
Sh3bgrl3	-0.88	0.00000000	pigs
Ncoa2	-0.90	0.00000000	pigs
Zdhhc18	1.09	0.00000000	pigs
Stk38l	-0.84	0.00000000	pigs
Asap1	-0.89	0.00000000	pigs
Znfx1	-0.76	0.00000000	pigs
Nlrp3	1.79	0.00000000	pigs
Plaur	-0.67	0.00000000	pigs
Ccnd3	-0.66	0.00000000	pigs
Daglb	-1.01	0.00000000	pigs
Ier5	1.48	0.00000000	pigs
Ksr1	1.33	0.00000000	pigs
Ccnl1	1.21	0.00000000	pigs
Cep170	-1.39	0.00000000	pigs
Gse1	-0.90	0.00000000	pigs
Crebrf	1.19	0.00000000	pigs
Sp3	-0.53	0.00000000	pigs
Ankrd44	-0.96	0.00000000	pigs
Tgfbi	-0.89	0.00000000	pigs
Ecm1	-0.78	0.00000000	pigs
Arhgdib	-0.71	0.00000000	pigs
Srgn	0.45	0.00000000	pigs
Tbc1d2b	1.75	0.00000000	pigs
Baz1a	1.23	0.00000000	pigs

Zhx2	1.72	0.00000000	pigs
Nol11	-1.10	0.00000000	pigs
R3hdm4	-0.70	0.00000000	pigs
Gyg	-0.96	0.00000000	pigs
Atp6ap1	1.71	0.00000000	pigs
Crk	1.38	0.00000000	pigs
Slc25a36	1.48	0.00000000	pigs
Ndrg1	1.50	0.00000000	pigs
Cap1	-0.73	0.00000000	pigs
Apbb1ip	-0.72	0.00000000	pigs
Vav3	-0.78	0.00000000	pigs
Pik3cb	-0.96	0.00000000	pigs
Osbpl2	-0.83	0.00000000	pigs
Ubb	0.73	0.00000000	pigs
Actr3	-0.57	0.00000000	pigs
Tmem131l	1.98	0.00000000	pigs
Rnase6	-0.92	0.00000000	pigs
Pde4b	-0.48	0.00000000	pigs
Osbpl9	-0.80	0.00000000	pigs
Diaph1	-0.66	0.00000000	pigs
Dctpp1	-0.80	0.00000000	pigs
Lrrc8d	-1.01	0.00000000	pigs
Asap3	-0.79	0.00000000	pigs
Baz2b	-1.18	0.00000000	pigs
Sult1a1	-0.76	0.00000000	pigs
Ccdc69	-0.79	0.00000000	pigs
Rapgef1	-0.86	0.00000000	pigs
Herpud1	1.32	0.00000000	pigs
P2ry14	1.59	0.00000000	pigs
Nt5c3	-0.75	0.00000000	pigs
Slmap	-0.96	0.00000000	pigs
Rnf2	1.84	0.00000000	pigs
Tpm4	-1.03	0.00000000	pigs
Nlrc5	-0.69	0.00000000	pigs
Zdhhc7	-0.90	0.00000000	pigs
Tmem131	-0.95	0.00000000	pigs
Carhsp1	-0.76	0.00000000	pigs
Prkd3	-0.96	0.00000000	pigs
Il18	-1.14	0.00000000	pigs
Gpbp1	0.83	0.00000000	pigs
Pspc1	1.43	0.00000000	pigs
Atp6v0b	1.47	0.00000000	pigs
Tti2	1.55	0.00000000	pigs
Ccpg1	-0.73	0.00000000	pigs
Ivns1abp	-0.62	0.00000000	pigs
H2-Ab1	1.46	0.00000000	pigs

Fgl2	-0.85	0.00000000	pigs
Lgals3	-0.70	0.00000000	pigs
Cd48	1.04	0.00000000	pigs
Cdc123	-0.79	0.00000000	pigs
Ap1ar	1.85	0.00000000	pigs
Oaz1	-0.66	0.00000000	pigs
Poglut1	-0.86	0.00000000	pigs
Slc3a2	1.18	0.00000000	pigs
Ell2	-0.62	0.00000000	pigs
Rock2	-0.87	0.00000000	pigs
Neto2	-1.86	0.00000000	pigs
Slc40a1	-0.80	0.00000000	pigs
Nfkbiz	1.35	0.00000000	pigs
F5	-0.83	0.00000000	pigs
AA467197	1.59	0.00000000	pigs
Phactr2	-0.96	0.00000000	pigs
Atad1	-0.89	0.00000000	pigs
Igsf6	1.36	0.00000000	pigs
Rbm47	-0.54	0.00000000	pigs
Arid4b	0.95	0.00000000	pigs
Csrnp1	1.29	0.00000000	pigs
Susd1	-0.93	0.00000000	pigs
Tnni2	-0.79	0.00000000	pigs
Noct	-0.68	0.00000000	pigs
Wdfy1	0.92	0.00000000	pigs
Vps54	1.50	0.00000000	pigs
Tnip1	0.81	0.00000000	pigs
Fnip2	1.52	0.00000000	pigs
Ikbkb	1.17	0.00000000	pigs
Ubald2	-0.69	0.00000000	pigs
Pced1b	-0.76	0.00000000	pigs
Nhlrc3	1.39	0.00000000	pigs
Coro1c	-0.78	0.00000000	pigs
Nrp2	1.44	0.00000000	pigs
Ets2	0.86	0.00000000	pigs
Trip11	-1.02	0.00000000	pigs
Dmxl2	2.35	0.00000000	pigs
Eif4g3	-0.69	0.00000000	pigs
Rabgap1l	-0.81	0.00000000	pigs
Clcn7	1.14	0.00000000	pigs
Galnt17	-1.29	0.00000000	pigs
Acot10	-0.84	0.00000000	pigs
Galnt1	1.77	0.00000000	pigs
Fggy	-0.88	0.00000000	pigs
Lat2	1.15	0.00000000	pigs
Dennd5a	-0.45	0.00000000	pigs

Il6ra	-0.55	0.00000000	pigs
Arl8b	1.40	0.00000000	pigs
Isg15	-0.50	0.00000000	pigs
Socs3	1.50	0.00000000	pigs
Ppp1r3c	-0.91	0.00000000	pigs
Tln1	-0.67	0.00000000	pigs
Wfs1	1.13	0.00000000	pigs
Pip4k2a	-1.06	0.00000000	pigs
Gnai3	-0.74	0.00000000	pigs
Pde4d	-0.86	0.00000000	pigs
Egr1	-0.60	0.00000000	pigs
Trank1	-0.72	0.00000000	pigs
Irak3	1.27	0.00000000	pigs
Scarb1	-1.21	0.00000000	pigs
Smap2	-0.45	0.00000000	pigs
Zc3hav1	-0.71	0.00000000	pigs
Dusp16	1.30	0.00000000	pigs
Snx3	-0.61	0.00000000	pigs
Ifi204	1.26	0.00000000	pigs
Tmx4	-0.93	0.00000000	pigs
Zeb2	1.64	0.00000000	pigs
Irs2	-0.79	0.00000000	pigs
Ints6l	-1.15	0.00000000	pigs
Padi4	-0.74	0.00000000	pigs
Adgrg3	-0.72	0.00000000	pigs
Stk38	-0.63	0.00000000	pigs
Aph1b	-0.73	0.00000000	pigs
Ppp2r5a	-0.81	0.00000000	pigs
Prss55	-1.02	0.00000000	pigs
Bcas3	-0.90	0.00000000	pigs
Cks2	-0.93	0.00000000	pigs
Ptbp3	-0.71	0.00000000	pigs
Rnf149	-0.47	0.00000000	pigs
Arhgap30	-0.68	0.00000000	pigs
Rassf5	-0.67	0.00000000	pigs
Tsc22d3	-0.46	0.00000000	pigs
Tmem154	-0.49	0.00000000	pigs
Arhgap45	-0.65	0.00000000	pigs
Irak2	1.80	0.00000000	pigs
Fos	-0.65	0.00000000	pigs
Phex	1.95	0.00000000	pigs
Rgs19	-0.54	0.00000000	pigs
Anxa1	-0.97	0.00000000	pigs
Exoc1	-0.74	0.00000000	pigs
Rel	1.02	0.00000000	pigs
Taldo1	-0.59	0.00000000	pigs

Eif4a1	1.15	0.00000000	pigs
Sod2	1.14	0.00000000	pigs
Itgal	-0.74	0.00000000	pigs
Slc9a3r1	-0.64	0.00000000	pigs
Fbxl5	-0.70	0.00000000	pigs
Gucy2c	-0.97	0.00000000	pigs
Glipr2	-0.66	0.00000000	pigs
Os9	-0.67	0.00000000	pigs
Bmx	-0.79	0.00000000	pigs
Opa1	-0.74	0.00000000	pigs
Ncoa1	-0.78	0.00000000	pigs
Cnga4	-0.93	0.00000000	pigs
Dock11	-0.95	0.00000000	pigs
Cyb5r4	-0.74	0.00000000	pigs
Pgd	-0.62	0.00000000	pigs
S100a6	-0.97	0.00000000	pigs
Gns	1.09	0.00000000	pigs
Tnfaip8	-0.85	0.00000000	pigs
Spin1	-0.71	0.00000000	pigs
Zbtb25	1.05	0.00000000	pigs
Zfp36	1.51	0.00000000	pigs
Pik3r5	0.85	0.00000000	pigs
Ankrd42	-0.95	0.00000000	pigs
Sdccag8	-1.05	0.00000000	pigs
Dtx3l	-0.69	0.00000000	pigs
Oscar	-0.56	0.00000000	pigs
Arhgap32	-0.95	0.00000000	pigs
Ckap2l	1.46	0.00000000	pigs
Plbd2	-0.63	0.00000000	pigs
Lgmn	1.03	0.00000000	pigs
Sntb1	-1.04	0.00000000	pigs
Erbin	1.86	0.00000000	pigs
Atp8b4	-0.65	0.00000000	pigs
Fyb	-0.73	0.00000000	pigs
Heca	-0.71	0.00000000	pigs
Rnase4	-0.62	0.00000000	pigs
Zyx	-0.51	0.00000000	pigs
Spi1	-0.49	0.00000000	pigs
Rb1	-1.01	0.00000000	pigs
Hipk2	-0.75	0.00000000	pigs
Cmip	-0.48	0.00000000	pigs
Plcb4	-1.42	0.00000000	pigs
Nedd9	-0.70	0.00000000	pigs
Cd69	-0.78	0.00000000	pigs
Cbl	-0.60	0.00000000	pigs
Clnk	-1.01	0.00000000	pigs

Psmb8	-0.59	0.00000000	pigs
Lasp1	-0.51	0.00000000	pigs
Fam102b	-1.60	0.00000000	pigs
Stxbp6	-0.98	0.00000000	pigs
Antxr2	-0.52	0.00000000	pigs
Gadd45a	-0.62	0.00000000	pigs
Nfkb1	0.58	0.00000000	pigs
Ogdh	-0.59	0.00000000	pigs
Bst2	0.94	0.00000000	pigs
Sri	-0.59	0.00000000	pigs
Klhl2	-0.74	0.00000000	pigs
C5ar2	-0.54	0.00000000	pigs
Pld1	-0.81	0.00000000	pigs
Cep43	-0.66	0.00000000	pigs
Hivep2	1.66	0.00000000	pigs
Aff4	0.93	0.00000000	pigs
Gtf2f2	-1.00	0.00000000	pigs
Tagap1	1.21	0.00000000	pigs
Arhgef6	-0.81	0.00000000	pigs
Eaf1	-0.63	0.00000000	pigs
Hnrnpll	-0.75	0.00000000	pigs
Rock1	-0.54	0.00000000	pigs
Cdh23	-0.61	0.00000000	pigs
Thbs1	-1.90	0.00000000	pigs
Slc30a5	-1.10	0.00000000	pigs
Hnrnph1	1.09	0.00000000	pigs
Scaf11	-0.61	0.00000000	pigs
Alox5	-0.65	0.00000000	pigs
Spock2	-0.77	0.00000000	pigs
Atf4	1.21	0.00000000	pigs
Irf2	-0.57	0.00000000	pigs
Scarb2	1.69	0.00000000	pigs
Grk6	-0.65	0.00000000	pigs
Nfkb2	1.12	0.00000000	pigs
Stk40	-0.55	0.00000000	pigs
Atxn7	-0.73	0.00000000	pigs
Fnip1	0.80	0.00000000	pigs
Tmem41b	-0.59	0.00000000	pigs
Tet2	-0.56	0.00000000	pigs
Tmc1	-0.79	0.00000000	pigs
Cd274	1.35	0.00000000	pigs
B4galt5	0.79	0.00000000	pigs
Tmem71	-0.72	0.00000000	pigs
Ifih1	-0.54	0.00000000	pigs
Ticam1	1.19	0.00000000	pigs
Eif3a	0.93	0.00000000	pigs

Usp15	-0.51	0.00000000	pigs
Atp6v1b2	1.24	0.00000000	pigs
Vsir	-0.39	0.00000000	pigs
Ubn2	-0.69	0.00000000	pigs
Parp12	-0.42	0.00000000	pigs
Rcsd1	-0.64	0.00000000	pigs
Sgk1	-0.55	0.00000000	pigs
Trp53i11	-0.56	0.00000000	pigs
Wdr70	-0.80	0.00000000	pigs
Taok3	-0.86	0.00000000	pigs
Akap7	-0.78	0.00000000	pigs
Helz	-0.50	0.00000000	pigs
Tec	-0.48	0.00000000	pigs
Pla2g4a	-0.82	0.00000000	pigs
Kat5	-0.58	0.00000000	pigs
Spg11	-0.68	0.00000000	pigs
Peli1	0.51	0.00000000	pigs
Cpt1c	-0.74	0.00000000	pigs
Ptprj	-0.54	0.00000000	pigs
Stat3	0.99	0.00000000	pigs
Slc12a6	-0.77	0.00000000	pigs
Entpd1	-0.85	0.00000000	pigs
Smcr8	0.86	0.00000000	pigs
Rab8b	-0.40	0.00000000	pigs
Itm2b	-0.50	0.00000000	pigs
Ctps2	-0.64	0.00000000	pigs
Il22ra2	-0.77	0.00000000	pigs
Plcg2	-0.41	0.00000000	pigs
Inpp5a	-0.56	0.00000000	pigs
Snx2	1.20	0.00000000	pigs
Dusp1	-0.43	0.00000000	pigs
Atp6v1c1	1.14	0.00000000	pigs
Jmjd1c	-0.39	0.00000000	pigs
Gimap4	-0.72	0.00000000	pigs
Ccdc88c	-0.91	0.00000000	pigs
Dhrs9	-0.45	0.00000000	pigs
Lpin2	-0.60	0.00000000	pigs
Hip1	-0.63	0.00000000	pigs
Hsp90aa1	1.73	0.00000000	pigs
Fndc3b	-0.51	0.00000000	pigs
Trim75	-0.44	0.00000000	pigs
Zbtb1	1.10	0.00000000	pigs
Smarcc1	1.04	0.00000000	pigs
Exoc2	-0.64	0.00000000	pigs
Npc2	0.81	0.00000000	pigs
Ccdc47	-0.61	0.00000000	pigs

Fcho2	-0.58	0.00000000	pigs
Phf21a	-0.67	0.00000000	pigs
Arhgap12	1.25	0.00000000	pigs
Runx2	-0.73	0.00000000	pigs
Sema4a	1.06	0.00000000	pigs
Vnn1	-0.46	0.00000000	pigs
Fchsd2	0.80	0.00000000	pigs
Ncoa7	-0.71	0.00000000	pigs
Srebf2	-0.59	0.00000000	pigs
Pmaip1	0.90	0.00000000	pigs
Foxn3	1.11	0.00000000	pigs
Lrch1	-0.42	0.00000000	pigs
Chmp4b	-0.55	0.00000000	pigs
Cdkn1b	1.13	0.00000000	pigs
G0s2	0.60	0.00000000	pigs
Rgs2	-0.30	0.00000000	pigs
Rasa2	-0.53	0.00000000	pigs
Aplp2	1.20	0.00000000	pigs
Insig1	1.10	0.00000000	pigs
Gbp2b	-0.53	0.00000000	pigs
Bri3	1.15	0.00000000	pigs
Ubxn11	-0.57	0.00000000	pigs
Tfec	0.94	0.00000000	pigs
Ahctf1	-0.39	0.00000000	pigs
Nsmce2	1.34	0.00000000	pigs
Cd300lb	-0.55	0.00000000	pigs
Lonp2	-0.77	0.00000000	pigs
Syap1	-0.49	0.00000000	pigs
Gla	1.06	0.00000000	pigs
Pgk1	-0.51	0.00000000	pigs
Cubn	-0.95	0.00000000	pigs
Sap30	-0.56	0.00000000	pigs
Ccl5	2.01	0.00000000	pigs
Tyrobp	-0.37	0.00000000	pigs
Ralgapa2	-0.84	0.00000000	pigs
Hecw1	-0.64	0.00000000	pigs
Id2	-0.51	0.00000000	pigs
Zfp467	-0.58	0.00000000	pigs
Slfn9	-0.56	0.00000000	pigs
Il1r2	1.24	0.00000000	pigs
AB124611	-0.56	0.00000000	pigs
Ifitm6	-0.34	0.00000000	pigs
Tbc1d7	-0.62	0.00000000	pigs
St13	0.99	0.00000000	pigs
Fnbp4	1.25	0.00000000	pigs
Sptlc2	-0.64	0.00000000	pigs

Wdr26	-0.53	0.00000000	pigs
Tfdp2	-0.63	0.00000000	pigs
Vim	-0.64	0.00000000	pigs
Hdac4	1.35	0.00000000	pigs
Ap1g1	-0.60	0.00000000	pigs
Upp1	-0.39	0.00000000	pigs
Ywhaq	-0.75	0.00000000	pigs
Flot2	-0.49	0.00000000	pigs
Ankrd22	-0.57	0.00000000	pigs
Tnpo3	-0.55	0.00000000	pigs
Arid4a	-0.54	0.00000000	pigs
Spred2	-0.74	0.00000000	pigs
Slc1a2	-0.64	0.00000000	pigs
Svil	0.73	0.00000000	pigs
Dip2b	0.98	0.00000000	pigs
Nr4a2	-0.61	0.00000000	pigs
Pdk3	-0.69	0.00000000	pigs
Fam168a	-0.79	0.00000000	pigs
Lamp1	0.88	0.00000000	pigs
Cd47	-0.48	0.00000000	pigs
Senp6	-0.64	0.00000000	pigs
Eif2ak2	-0.34	0.00000000	pigs
Tut7	-0.33	0.00000000	pigs
Gnb4	-0.52	0.00000000	pigs
Samd4b	-0.48	0.00000000	pigs
Klhl28	-0.86	0.00000000	pigs
Gnai2	-0.42	0.00000000	pigs
Ifngr2	0.82	0.00000000	pigs
Gcnt2	-0.83	0.00000000	pigs
C1qa	0.85	0.00000000	pigs
Gramd1a	-0.49	0.00000000	pigs
Htt	-0.59	0.00000000	pigs
Llph	0.95	0.00000000	pigs
Arf6	-0.49	0.00000000	pigs
Brd4	0.83	0.00000000	pigs
B4galt1	-0.48	0.00000000	pigs
Gtf2h1	0.86	0.00000000	pigs
Ubap2l	1.02	0.00000000	pigs
St8sia4	-0.50	0.00000000	pigs
Il13ra1	-0.39	0.00000000	pigs
Klhl24	1.13	0.00000000	pigs
Cep120	-0.56	0.00000000	pigs
Ccm2l	-0.48	0.00000000	pigs
Atp2b1	-0.55	0.00000000	pigs
Pdlim7	-0.37	0.00000000	pigs
Klhl5	-0.73	0.00000000	pigs

Nfatc3	-0.49	0.00000000	pigs
Orai1	-0.52	0.00000000	pigs
Tgif2	0.98	0.00000000	pigs
Cebpb	0.34	0.00000000	pigs
Snx18	-0.47	0.00000000	pigs
Btbd9	0.75	0.00000000	pigs
Rassf3	-0.56	0.00000000	pigs
Tmco1	-0.47	0.00000000	pigs
Phactr1	-0.28	0.00000000	pigs
Tm9sf3	1.31	0.00000000	pigs
Txk	-0.66	0.00000000	pigs
Wdfy4	-0.50	0.00000000	pigs
Tkt	-0.42	0.00000000	pigs
Eif4e3	-0.56	0.00000000	pigs
Ergic1	-0.48	0.00000000	pigs
Cyp4f18	-0.47	0.00000000	pigs
Nt5c2	-0.58	0.00000000	pigs
Usp9x	-0.45	0.00000000	pigs
Wasf2	-0.46	0.00000000	pigs
Uhrf1bp1l	-0.57	0.00000000	pigs
Add3	-0.47	0.00000000	pigs
Emilin2	1.16	0.00000000	pigs
Tnip3	1.09	0.00000000	pigs
Rnf19b	0.74	0.00000000	pigs
Qk	0.72	0.00000000	pigs
Cxcr4	1.10	0.00000000	pigs
Rcl1	-0.47	0.00000000	pigs
Zranb2	1.06	0.00000000	pigs
Etv6	-0.39	0.00000000	pigs
Tmem268	1.11	0.00000000	pigs
Gaa	-0.44	0.00000000	pigs
Ptpre	-0.46	0.00000000	pigs
Adgb	1.08	0.00000000	pigs
Erp44	1.05	0.00000000	pigs
Bag3	1.41	0.00000000	pigs
Fbxo33	-0.59	0.00000000	pigs
Cds1	-0.86	0.00000000	pigs
Sipa1	-0.42	0.00000000	pigs
Ppp1r3b	-0.32	0.00000000	pigs
Arhgap9	-0.40	0.00000000	pigs
Btbd11	-0.76	0.00000000	pigs
Zfp407	-0.64	0.00000000	pigs
Glyr1	-0.45	0.00000000	pigs
Slc25a28	-0.37	0.00000000	pigs
Atp13a3	0.96	0.00000000	pigs
Slc16a6	-0.54	0.00000000	pigs

Sh3bp2	0.82	0.00000000	pigs
Atp8a1	-0.96	0.00000000	pigs
Hipk1	-0.37	0.00000000	pigs
Pram1	-0.39	0.00000000	pigs
Pik3cd	-0.43	0.00000000	pigs
Rnft1	-0.52	0.00000000	pigs
Nectin1	-0.44	0.00000000	pigs
Slco4c1	-0.52	0.00000000	pigs
Rap1a	-0.40	0.00000000	pigs
Card9	-0.49	0.00000000	pigs
Adar	-0.38	0.00000000	pigs
Nek7	-0.53	0.00000000	pigs
Ifi44	-0.35	0.00000000	pigs
Etf1	0.84	0.00000000	pigs
Agtpbp1	-0.62	0.00000000	pigs
Atp1a1	1.15	0.00000000	pigs
Dcun1d4	-0.53	0.00000000	pigs
Arhgap6	-0.74	0.00000000	pigs
Jak2	-0.41	0.00000000	pigs
Atg3	1.01	0.00000000	pigs
Slc43a2	1.14	0.00000000	pigs
Sh2d3c	-0.45	0.00000000	pigs
Ptafr	-0.32	0.00000000	pigs
Trappc4	-0.42	0.00000000	pigs
Khnyn	-0.38	0.00000000	pigs
Skap2	-0.36	0.00000000	pigs
Kdm4b	-0.42	0.00000000	pigs
Chp1	-0.45	0.00000000	pigs
Rasa3	-0.40	0.00000000	pigs
Bnip3l	-0.32	0.00000000	pigs
Ctsc	0.78	0.00000000	pigs
Dennd2c	-0.87	0.00000000	pigs
Exoc4	-0.66	0.00000000	pigs
Epb41	-0.53	0.00000000	pigs
Sds	-0.27	0.00000000	pigs
Apaf1	-0.55	0.00000000	pigs
Trip12	-0.34	0.00000000	pigs
Rubcnl	-0.44	0.00000000	pigs
Gpat3	-0.49	0.00000000	pigs
Arpc3	-0.35	0.00000000	pigs
Klf10	1.02	0.00000000	pigs
Esd	-0.47	0.00000000	pigs
Plin5	-0.41	0.00000000	pigs
Gigyf2	1.11	0.00000000	pigs
Itgb2l	-0.28	0.00000000	pigs
Nagk	0.83	0.00000000	pigs

Plekhg3	-0.40	0.00000000	pigs
Crlf3	-0.40	0.00000000	pigs
Dhrs3	0.79	0.00000000	pigs
Cds2	-0.36	0.00000000	pigs
Ube2d3	0.52	0.00000000	pigs
Dgat2	-0.23	0.00000000	pigs
Cdk2ap2	-0.47	0.00000000	pigs
Resf1	-0.49	0.00000000	pigs
Psme4	-0.61	0.00000000	pigs
Actg1	-0.52	0.00000000	pigs
Ninj1	1.16	0.00000000	pigs
Fbxw11	0.96	0.00000000	pigs
St3gal2	-0.52	0.00000000	pigs
Cpeb3	-0.97	0.00000000	pigs
Ptpn12	-0.39	0.00000000	pigs
Ppp2ca	-0.35	0.00000000	pigs
Rnf44	-0.36	0.00000000	pigs
Cmpk2	-0.35	0.00000000	pigs
Ctdsp1	-0.40	0.00000000	pigs
Clasp1	-0.41	0.00000000	pigs
Sppl2a	0.98	0.00000000	pigs
Arpc5	-0.33	0.00000000	pigs
Herc1	-0.36	0.00000000	pigs
Apmap	-0.38	0.00000000	pigs
Htr7	-0.81	0.00000000	pigs
Gsk3b	-0.44	0.00000000	pigs
Vdr	-0.33	0.00000000	pigs
Ssh1	-0.49	0.00000000	pigs
Tiam1	-0.50	0.00000000	pigs
Ppfibp2	-0.55	0.00000000	pigs
Arl6ip1	-0.37	0.00000000	pigs
Clta	0.84	0.00000000	pigs
Shld1	-0.72	0.00000000	pigs
Parp14	-0.40	0.00000000	pigs
Prmt9	-0.54	0.00000000	pigs
Stim1	-0.59	0.00000000	pigs
Stat2	-0.40	0.00000000	pigs
G6pdx	-0.35	0.00000000	pigs
Cflar	0.77	0.00000000	pigs
Casp1	0.81	0.00000000	pigs
Cd9	-0.29	0.00000000	pigs
Coq10b	-0.34	0.00000000	pigs
Prdx1	0.74	0.00000000	pigs
Katnip	-0.50	0.00000000	pigs
Kmt2c	-0.33	0.00000000	pigs
Ddx46	-0.59	0.00000000	pigs

Tmem164	-0.44	0.00000000	pigs
Braf	0.54	0.00000000	pigs
Adam10	-0.46	0.00000000	pigs
Tbc1d10c	-0.40	0.00000000	pigs
Rprd1b	-0.48	0.00000000	pigs
Dock10	0.99	0.00000000	pigs
Rell1	-0.47	0.00000000	pigs
Snrk	-0.39	0.00000000	pigs
Nrg1	-0.43	0.00000000	pigs
Glipr1	-0.41	0.00000000	pigs
Olfml3	-0.34	0.00000000	pigs
Tsen2	0.87	0.00000000	pigs
Prex1	-0.14	0.00000000	pigs
Septin12	-0.42	0.00000000	pigs
Tra2a	0.92	0.00000000	pigs
Tle3	-0.30	0.00000000	pigs
Psma3	-0.37	0.00000000	pigs
Rab11fip4	-0.43	0.00000000	pigs
Nr3c1	-0.43	0.00000000	pigs
Ilrun	0.84	0.00000000	pigs
Klf3	-0.43	0.00000000	pigs
Prdm1	0.91	0.00000000	pigs
Slc11a1	-0.43	0.00000000	pigs
Pias1	0.50	0.00000000	pigs
Ifngr1	-0.32	0.00000000	pigs
Tsen34	-0.36	0.00000000	pigs
Pik3c2b	-0.41	0.00000000	pigs
Ptk2b	0.74	0.00000000	pigs
Vcpip1	-0.44	0.00000000	pigs
Ubl5	-0.34	0.00000000	pigs
Pip5k1a	0.90	0.00000000	pigs
Tmem135	1.07	0.00000000	pigs
Gnb1	0.68	0.00000000	pigs
Slc36a4	-0.45	0.00000000	pigs
Hbb-bh2	-0.57	0.00000000	pigs
Slc16a3	-0.37	0.00000000	pigs
Ptpn6	-0.30	0.00000000	pigs
Odc1	0.45	0.00000000	pigs
Smndc1	-0.33	0.00000000	pigs
Il2ra	0.92	0.00000000	pigs
Csnk1g1	-0.68	0.00000000	pigs
Sec62	-0.38	0.00000000	pigs
Ndufb2	-0.39	0.00000000	pigs
N4bp1	1.10	0.00000000	pigs
Afg1l	-0.65	0.00000000	pigs
Bin3	-0.50	0.00000000	pigs

Trp53inp1	-0.43	0.00000000	pigs
Ero1b	-0.44	0.00000000	pigs
Gabpb1	0.86	0.00000000	pigs
Nabp1	-0.29	0.00000000	pigs
Hnrnpu	0.87	0.00000000	pigs
Tst	-0.35	0.00000000	pigs
Haus8	-0.43	0.00000000	pigs
Olr1	0.62	0.00000000	pigs
Cers4	0.61	0.00000000	pigs
Aph1a	-0.32	0.00000000	pigs
Ypel3	-0.25	0.00000000	pigs
Casp3	-0.38	0.00000000	pigs
Ssbp3	-0.59	0.00000000	pigs
Ppfia1	-0.41	0.00000000	pigs
Ubash3a	-0.33	0.00000000	pigs
Tmem248	-0.51	0.00000000	pigs
Saraf	-0.37	0.00000000	pigs
Cab39	-0.37	0.00000000	pigs
Pik3ip1	-0.42	0.00000000	pigs
Kpna1	-0.46	0.00000000	pigs
Tspan14	-0.33	0.00000000	pigs
Ern1	-0.36	0.00000000	pigs
Limd2	-0.31	0.00000000	pigs
Kdm1a	-0.40	0.00000000	pigs
Trafd1	-0.29	0.00000000	pigs
Arap3	-0.29	0.00000000	pigs
Nfia	-0.69	0.00000000	pigs
Ints6	1.15	0.00000000	pigs
Arpc1b	-0.25	0.00000000	pigs
Yaf2	0.97	0.00000000	pigs
Rgcc	0.77	0.00000000	pigs
Fryl	-0.42	0.00000000	pigs
Pim1	0.79	0.00000000	pigs
Lrp10	-0.32	0.00000000	pigs
Pdzd8	-0.51	0.00000000	pigs
Tbxas1	-0.45	0.00000000	pigs
Actn1	-0.27	0.00000000	pigs
Ifi44l	-0.23	0.00000000	pigs
Flna	-0.31	0.00000000	pigs
Pdcd1lg2	1.36	0.00000000	pigs
Cemip2	-0.62	0.00000000	pigs
Anxa11	-0.29	0.00000000	pigs
Dtnbp1	-0.37	0.00000000	pigs
Rap2a	-0.39	0.00000000	pigs
Eif1	0.47	0.00000000	pigs
Chit1	-0.35	0.00000000	pigs

Ikzf1	-0.40	0.00000000	pigs
Elf2	-0.37	0.00000000	pigs
Psme1	-0.35	0.00000000	pigs
Ndufb9	-0.34	0.00000000	pigs
Ccnt1	-0.34	0.00000000	pigs
Spidr	-0.47	0.00000000	pigs
Dnajc13	0.90	0.00000000	pigs
Csta1	-0.56	0.00000000	pigs
Sec63	0.77	0.00000000	pigs
Bend7	-0.35	0.00000000	pigs
Agpat2	-0.33	0.00000000	pigs
Hnrnpa3	0.76	0.00000000	pigs
Gnptab	1.20	0.00000000	pigs
Tbc1d8b	-0.40	0.00000000	pigs
Npepps	-0.34	0.00000000	pigs
Tspan2	-0.36	0.00000000	pigs
Stk10	-0.24	0.00000000	pigs
Dapp1	-0.22	0.00000000	pigs
Dhrs11	-0.28	0.00000000	pigs
Elob	-0.28	0.00000000	pigs
Klf13	-0.25	0.00000000	pigs
Lgals8	-0.21	0.00000000	pigs
Cxcl3	-0.30	0.00000000	pigs
Itm2c	-0.24	0.00000000	pigs
Asxl2	-0.47	0.00000000	pigs
Lsp1	-0.30	0.00000000	pigs
Akna	-0.28	0.00000000	pigs
Wipi1	-0.32	0.00000000	pigs
Ap3d1	0.73	0.00000000	pigs
Acsl1	-0.43	0.00000000	pigs
Cd14	-0.24	0.00000000	pigs
Rnf145	-0.32	0.00000000	pigs
Gatad2b	-0.43	0.00000000	pigs
Crybg1	-0.36	0.00000000	pigs
Arhgap27	-0.35	0.00000000	pigs
Nup155	-0.38	0.00000000	pigs
Rgs14	-0.28	0.00000000	pigs
Cdv3	-0.25	0.00000000	pigs
Ubc	0.71	0.00000000	pigs
Pkn1	-0.36	0.00000000	pigs
Arpc2	-0.26	0.00000000	pigs
Ppp2r1b	-0.33	0.00000000	pigs
Stxbp2	-0.25	0.00000000	pigs
Rbms1	-0.38	0.00000000	pigs
Triobp	-0.23	0.00000000	pigs
Glg1	-0.51	0.00000000	pigs

Luc7l	-0.24	0.00000000	pigs
Lamb3	0.72	0.00000000	pigs
Diaph2	0.67	0.00000000	pigs
Rbpj	0.86	0.00000000	pigs
Sipa1l1	-0.14	0.00000000	pigs
Capzb	-0.24	0.00000000	pigs
Slc45a4	-0.35	0.00000000	pigs
Ankrd13a	-0.23	0.00000000	pigs
9930021J03Rik	-0.38	0.00000000	pigs
Foxn2	-0.31	0.00000000	pigs
Irf3	-0.27	0.00000000	pigs
Nrd1	-0.43	0.00000000	pigs
Nck2	-0.32	0.00000000	pigs
Tnfaip6	0.65	0.00000000	pigs
Reep5	-0.31	0.00000000	pigs
Arrb2	-0.20	0.00000000	pigs
Pdss1	-0.38	0.00000000	pigs
Nxpe3	-0.32	0.00000000	pigs
Eps15	-0.34	0.00000000	pigs
Phf20l1	-0.33	0.00000000	pigs
Skil	0.88	0.00000000	pigs
Fosb	-0.31	0.00000000	pigs
Dynlt1a	-0.30	0.00000000	pigs
Tap1	-0.18	0.00000000	pigs
Itprid2	-0.37	0.00000000	pigs
Erap1	-0.31	0.00000000	pigs
Vapa	0.84	0.00000000	pigs
Gtpbp1	-0.24	0.00000000	pigs
Ankrd11	0.90	0.00000000	pigs
Orc4	0.90	0.00000000	pigs
Tmem50b	-0.33	0.00000000	pigs
Sin3a	0.83	0.00000000	pigs
Rfx1	-0.28	0.00000000	pigs
Acap1	-0.31	0.00000000	pigs
Evi2	-0.16	0.00000000	pigs
Sec24a	-0.35	0.00000000	pigs
Klf7	-0.39	0.00000000	pigs
Pak2	-0.29	0.00000000	pigs
Tm6sf1	-0.29	0.00000000	pigs
Pecam1	-0.43	0.00000000	pigs
Dhdds	-0.25	0.00000000	pigs
Tgfbr1	-0.31	0.00000000	pigs
Tet3	1.00	0.00000000	pigs
Ptpn18	-0.33	0.00000000	pigs
Stat5a	0.89	0.00000000	pigs
Txnl4b	-0.34	0.00000000	pigs

Fundc1	-0.26	0.00000000	pigs
Sash3	-0.21	0.00000000	pigs
Zfp36l1	-0.38	0.00000000	pigs
Per1	0.80	0.00000000	pigs
Dusp2	0.49	0.00000000	pigs
Gng10	-0.22	0.00000000	pigs
Srsf10	-0.36	0.00000000	pigs
Zfand5	-0.26	0.00000000	pigs
Fem1c	-0.28	0.00000000	pigs
Calr	0.72	0.00000000	pigs
Ccni	-0.25	0.00000000	pigs
Camkk1	-0.29	0.00000000	pigs
Bmpr2	-0.45	0.00000000	pigs
Septin9	-0.20	0.00000000	pigs
Lonrf1	0.80	0.00000000	pigs
Gpx1	0.89	0.00000000	pigs
Zfp330	-0.31	0.00000000	pigs
Ldhc	-0.37	0.00000000	pigs
Safb2	0.81	0.00000000	pigs
Arv1	-0.25	0.00000000	pigs
Epc2	-0.31	0.00000000	pigs
Lclat1	-0.33	0.00000000	pigs
Kin	-0.28	0.00000000	pigs
Ppt1	-0.26	0.00000000	pigs
Phip	-0.36	0.00000000	pigs
Gas7	-0.15	0.00000000	pigs
Pcmtd1	0.97	0.00000000	pigs
Eif3e	-0.26	0.00000000	pigs
Ncf2	-0.22	0.00000000	pigs
Lfng	-0.21	0.00000000	pigs
Abi1	-0.24	0.00000000	pigs
Nfat5	0.62	0.00000000	pigs
Clk1	-0.21	0.00000000	pigs
Cul4a	-0.25	0.00000000	pigs
Kcnj15	-0.35	0.00000000	pigs
Plk3	0.74	0.00000000	pigs
Gsn	-0.23	0.00000000	pigs
Eif4h	0.83	0.00000000	pigs
B3galnt1	-0.32	0.00000000	pigs
Tgfbr2	-0.27	0.00000000	pigs
Oxr1	-0.31	0.00000000	pigs
Senp2	-0.30	0.00000000	pigs
Cep104	-0.25	0.00000000	pigs
Otud5	-0.23	0.00000000	pigs
Snai1	-0.20	0.00000000	pigs
Slc15a3	0.75	0.00000000	pigs

Itgam	-0.24	0.00000000	pigs
Chsy1	-0.44	0.00000000	pigs
Fermt3	-0.25	0.00000000	pigs
Xiap	0.78	0.00000000	pigs
Snd1	-0.25	0.00000000	pigs
Plekhb2	0.51	0.00000000	pigs
Tmem59	-0.14	0.00000000	pigs
Sppl3	0.83	0.00000000	pigs
Cdk17	-0.71	0.00000000	pigs
Eef2	0.70	0.00000000	pigs
Ncl	-0.19	0.00000000	pigs
Csnk1g2	-0.21	0.00000000	pigs
Nptn	0.81	0.00000000	pigs
Oxsr1	-0.36	0.00000000	pigs
Ampd2	-0.24	0.00000000	pigs
Rtn2	-0.43	0.00000000	pigs
Aftph	-0.26	0.00000000	pigs
Dcun1d3	-0.27	0.00000000	pigs
Nufip2	0.72	0.00000000	pigs
Zfp148	-0.26	0.00000000	pigs
Hspa8	1.00	0.00000000	pigs
Capn1	-0.21	0.00000000	pigs
Ralbp1	-0.25	0.00000000	pigs
Ankrd33b	-0.08	0.00000000	pigs
Arid3b	-0.27	0.00000000	pigs
Cggbp1	-0.20	0.00000000	pigs
Nadk	-0.16	0.00000000	pigs
Ankra2	-0.21	0.00000000	pigs
Fmnl1	-0.13	0.00000000	pigs
Ubxn8	-0.24	0.00000000	pigs
Wdr1	-0.17	0.00000000	pigs
Hacd4	-0.18	0.00000000	pigs
Nbn	-0.41	0.00000000	pigs
Zbp1	-0.14	0.00000000	pigs
Tnik	-0.85	0.00000000	pigs
Xylt1	-0.17	0.00000000	pigs
Pgm2	-0.23	0.00000000	pigs
Tfip11	0.81	0.00000000	pigs
Rab11a	-0.18	0.00000000	pigs
Dnm2	-0.19	0.00000000	pigs
Rngtt	-0.33	0.00000000	pigs
Timm8b	-0.20	0.00000000	pigs
Bnip3	-0.17	0.00000000	pigs
Pfkfb3	-0.25	0.00000000	pigs
Def8	-0.23	0.00000000	pigs
Edn1	1.06	0.00000000	pigs

Nup153	-0.26	0.00000000	pigs
Pstpip1	-0.11	0.00000000	pigs
Rilpl2	0.88	0.00000000	pigs
Serac1	-0.31	0.00000000	pigs
Ank1	0.90	0.00000000	pigs
Wnk1	0.78	0.00000000	pigs
Ptp4a1	-0.26	0.00000000	pigs
Itpr2	-0.36	0.00000000	pigs
Lonrf3	0.89	0.00000000	pigs
Riok3	-0.22	0.00000000	pigs
Kat6b	-0.39	0.00000000	pigs
Eif4b	0.70	0.00000000	pigs
Lyn	-0.19	0.00000000	pigs
Nbr1	-0.24	0.00000000	pigs
Nipbl	-0.20	0.00000000	pigs
Ece1	-0.21	0.00000000	pigs
Eapp	-0.20	0.00000000	pigs
Actr2	-0.22	0.00000000	pigs
Ethe1	-0.18	0.00000000	pigs
Ubap1	-0.21	0.00000000	pigs
Apoe	0.67	0.00000000	pigs
Fam13b	-0.26	0.00000000	pigs
Rabgap1	0.85	0.00000000	pigs
Lemd2	-0.21	0.00000000	pigs
Relt	-0.20	0.00000000	pigs
Brwd1	-0.18	0.00000000	pigs
Ginm1	-0.22	0.00000000	pigs
G3bp2	-0.17	0.00000000	pigs
Txnip	0.20	0.00000000	pigs
Hnrnpul1	-0.17	0.00000000	pigs
Vps13d	-0.36	0.00000000	pigs
Gk5	-0.27	0.00000000	pigs
Camta2	-0.22	0.00000000	pigs
Otub2	0.76	0.00000000	pigs
Pds5b	-0.32	0.00000000	pigs
Trak1	-0.30	0.00000000	pigs
Hdac1	-0.26	0.00000000	pigs
Cd44	-0.15	0.00000000	pigs
Lamtor3	0.67	0.00000000	pigs
Scaf8	-0.26	0.00000000	pigs
Fbxo34	-0.20	0.00000000	pigs
Ghsr	-0.17	0.00000000	pigs
Vps8	-0.34	0.00000000	pigs
P4ha1	0.96	0.00000000	pigs
Wdr74	0.67	0.00000000	pigs
Vcan	-0.45	0.00000000	pigs

Sdhd	-0.21	0.00000000	pigs
Cog3	0.83	0.00000000	pigs
Ranbp2	0.79	0.00000000	pigs
Traf3	0.58	0.00000000	pigs
Stk4	-0.14	0.00000000	pigs
Gab2	-0.18	0.00000000	pigs
Ddx5	0.52	0.00000000	pigs
Tmod3	-0.21	0.00000000	pigs
Tnfrsf21	-0.31	0.00000000	pigs
Phf23	0.45	0.00000000	pigs
Emc3	-0.23	0.00000000	pigs
Nmral1	-0.18	0.00000000	pigs
H2az2	-0.21	0.00000000	pigs
Entpd4	-0.17	0.00000000	pigs
Zfp207	0.76	0.00000000	pigs
Esyt2	-0.27	0.00000000	pigs
Idh2	-0.20	0.00000000	pigs
Cpt1a	-0.17	0.00000000	pigs
Bach1	-0.11	0.00000000	pigs
Vps26b	-0.22	0.00000000	pigs
Gnb2	-0.15	0.00000000	pigs
Gsap	0.58	0.00000000	pigs
Uap1	-0.23	0.00000000	pigs
Prkd2	-0.19	0.00000000	pigs
Dennd3	-0.14	0.00000000	pigs
Wnt5b	-0.10	0.00000000	pigs
Dhx8	-0.14	0.00000000	pigs
Sec23b	-0.18	0.00000000	pigs
Itch	-0.20	0.00000000	pigs
Arglu1	-0.19	0.00000000	pigs
Usp37	-0.64	0.00000000	pigs
Ralb	-0.13	0.00000000	pigs
Asrgl1	-0.21	0.00000000	pigs
Calm3	-0.08	0.00000000	pigs
Lnpep	-0.23	0.00000000	pigs
Hp1bp3	-0.16	0.00000000	pigs
Sp1	-0.30	0.00000000	pigs
Tmigd3	-0.12	0.00000000	pigs
Fbxw7	-0.28	0.00000000	pigs
Tmem127	-0.14	0.00000000	pigs
Prrc2c	0.58	0.00000000	pigs
Cstf3	0.65	0.00000000	pigs
Cldnd1	-0.18	0.00000000	pigs
Osbpl11	-0.26	0.00000000	pigs
Nsmce1	-0.20	0.00000000	pigs
Babam2	-0.15	0.00000000	pigs

Rlf	0.74	0.00000000	pigs
Lmo2	-0.20	0.00000000	pigs
Tpst1	-0.33	0.00000000	pigs
Oga	0.69	0.00000000	pigs
Gpsm3	-0.14	0.00000000	pigs
Adam17	-0.15	0.00000000	pigs
Stom	0.77	0.00000000	pigs
Rsrc2	0.69	0.00000000	pigs
Wbp2	0.63	0.00000000	pigs
Oas1e	-0.10	0.00000000	pigs
Akap10	-0.19	0.00000000	pigs
Golgb1	-0.19	0.00000000	pigs
Ppp1r18	-0.11	0.00000000	pigs
Ulk1	-0.15	0.00000000	pigs
Psap	0.73	0.00000000	pigs
Ppp2r5c	-0.08	0.00000000	pigs
Relb	0.52	0.00000000	pigs
Thrap3	0.73	0.00000000	pigs
Txlng	-0.34	0.00000000	pigs
Ube2l6	-0.08	0.00000000	pigs
Cyth4	-0.11	0.00000000	pigs
Il18rap	-0.15	0.00000000	pigs
Cox8a	-0.10	0.00000000	pigs
Fcer1g	-0.16	0.00000000	pigs
Vti1a	-0.24	0.00000000	pigs
Adora2a	0.65	0.00000000	pigs
Usp18	-0.13	0.00000000	pigs
Dcp2	-0.18	0.00000000	pigs
Sugt1	-0.46	0.00000000	pigs
1810013L24Rik	-0.12	0.00000000	pigs
Slc25a3	-0.15	0.00000000	pigs
Tmem140	-0.10	0.00000000	pigs
Rogdi	-0.07	0.00000000	pigs
Cdc16	-0.22	0.00000000	pigs
Ezr	-0.20	0.00000000	pigs
Trmt1l	-0.16	0.00000000	pigs
Setd5	0.75	0.00000000	pigs
Slc37a1	-0.07	0.00000000	pigs
Cgas	-0.18	0.00000000	pigs
Bid	-0.19	0.00000000	pigs
Sec24b	-0.14	0.00000000	pigs
Themis2	-0.04	0.00000000	pigs
Adam8	-0.05	0.00000000	pigs
Arg2	0.61	0.00000000	pigs
Zfp609	-0.46	0.00000000	pigs
Gripap1	-0.16	0.00000000	pigs

Pfkfb4	-0.11	0.00000000	pigs
Atf3	0.48	0.00000000	pigs
Hnrnph3	0.58	0.00000000	pigs
Il17ra	-0.12	0.00000000	pigs
Cd84	0.00	0.00000000	pigs
Luc7l3	0.63	0.00000000	pigs
Ccr1	-0.02	0.00000000	pigs
Csk	-0.14	0.00000000	pigs
Nr1h2	-0.11	0.00000000	pigs
Shoc2	-0.13	0.00000000	pigs
Tcp11l2	-0.10	0.00000000	pigs
Slc6a9	-0.09	0.00000000	pigs
Cpeb2	-0.29	0.00000000	pigs
Acin1	-0.07	0.00000000	pigs
Rnf38	-0.16	0.00000000	pigs
Tgm1	0.02	0.00000000	pigs
Rybp	0.75	0.00000000	pigs
Swt1	-0.15	0.00000000	pigs
Rhoc	-0.08	0.00000000	pigs
Cmtr1	-0.20	0.00000000	pigs
Cfl1	-0.13	0.00000000	pigs
Git2	-0.15	0.00000000	pigs
Scyl2	-0.18	0.00000000	pigs
Kcnj2	0.63	0.00000000	pigs
P2ry12	-0.18	0.00000000	pigs
Hdac5	-0.15	0.00000000	pigs
Pank3	0.63	0.00000000	pigs
Pcnx	-0.15	0.00000000	pigs
Ankrd17	0.58	0.00000000	pigs
Usp25	-0.23	0.00000000	pigs
Vps4b	-0.13	0.00000000	pigs
Numa1	-0.11	0.00000000	pigs
Ube2r2	-0.11	0.00000000	pigs
Oas2	-0.10	0.00000000	pigs
Kbtbd7	-0.23	0.00000000	pigs
Ppp6r3	0.70	0.00000000	pigs
Ubn1	-0.16	0.00000000	pigs
Ash1l	0.66	0.00000000	pigs
Safb	0.67	0.00000000	pigs
Ago1	0.62	0.00000000	pigs
Kif21b	-0.10	0.00000000	pigs
Ccdc180	-0.16	0.00000000	pigs
Hook3	-0.15	0.00000000	pigs
Hook2	-0.18	0.00000000	pigs
Rnf13	-0.14	0.00000000	pigs
Chst15	-0.15	0.00000000	pigs

Kansl1	-0.21	0.00000000	pigs
Arfip1	-0.13	0.00000000	pigs
Wsb1	0.61	0.00000000	pigs
Arid1a	-0.13	0.00000000	pigs
Cox7a2l	-0.10	0.00000000	pigs
Arf1	0.59	0.00000000	pigs
Faf2	-0.17	0.00000000	pigs
Rfwd3	0.69	0.00000000	pigs
Zc3h12a	0.64	0.00000000	pigs
G3bp1	-0.15	0.00000000	pigs
Hivep1	0.74	0.00000000	pigs
Flii	-0.12	0.00000000	pigs
Prkab1	-0.07	0.00000000	pigs
Rbks	-0.20	0.00000000	pigs
Nop10	-0.03	0.00000000	pigs
Psd3	-0.31	0.00000000	pigs
Parp9	-0.12	0.00000000	pigs
Il1rap	0.23	0.00000000	pigs
Gnl2	0.64	0.00000000	pigs
Anxa7	-0.11	0.00000000	pigs
Anks1	-0.20	0.00000000	pigs
Vrk3	-0.13	0.00000000	pigs
Wdr45	-0.11	0.00000000	pigs
Gna13	0.64	0.00000000	pigs
Kdm2a	-0.09	0.00000000	pigs
Nktr	-0.11	0.00000000	pigs
Ep300	-0.07	0.00000000	pigs
Abhd5	-0.14	0.00000000	pigs
Bmp2k	-0.17	0.00000000	pigs
Rgs10	0.62	0.00000000	pigs
P3h1	-0.09	0.00000000	pigs
Ppp4r1	-0.17	0.00000000	pigs
Tsc22d4	-0.10	0.00000000	pigs
Zfand6	-0.09	0.00000000	pigs
Kctd20	-0.11	0.00000000	pigs
Pxk	-0.01	0.00000000	pigs
Rhbdd1	-0.13	0.00000000	pigs
Cnot4	0.65	0.00000000	pigs
Tpt1	0.21	0.00000000	pigs
Zfp369	0.63	0.00000000	pigs
Rc3h1	0.69	0.00000000	pigs
Hnrnpd	0.52	0.00000000	pigs
Sfpq	0.61	0.00000000	pigs
Pabpc1	-0.14	0.00000000	pigs
Wdr33	0.52	0.00000000	pigs
Rnf111	-0.14	0.00000000	pigs

Ric1	1.04	0.00000000	pigs
Ptpn9	-0.22	0.00000000	pigs
Laptm5	-0.08	0.00000001	pigs
Niban1	0.04	0.00000001	pigs
Nr4a3	-0.26	0.00000001	pigs
Ppp1r13b	-0.18	0.00000001	pigs
4933400A11Rik	-0.15	0.00000001	pigs
Hk3	-0.07	0.00000001	pigs
Dlg1	-0.23	0.00000001	pigs
Gapt	-0.10	0.00000001	pigs
Hmox2	-0.04	0.00000001	pigs
Brpf3	-0.12	0.00000002	pigs
Sp110	-0.07	0.00000002	pigs
Gls	0.63	0.00000002	pigs
Tex2	-0.04	0.00000002	pigs
Ostm1	0.60	0.00000002	pigs
Stau1	-0.18	0.00000002	pigs
Zfp36l2	-0.09	0.00000002	pigs
Sos1	-0.21	0.00000003	pigs
Lmbr1l	-0.11	0.00000003	pigs
Tpi1	0.54	0.00000003	pigs
Epg5	0.05	0.00000003	pigs
Itsn2	0.61	0.00000003	pigs
Ripk1	-0.04	0.00000003	pigs
Ppp1cb	0.59	0.00000004	pigs
Lcp2	0.45	0.00000005	pigs
Esco1	0.61	0.00000006	pigs
Dctn4	-0.13	0.00000006	pigs
Slc7a11	0.77	0.00000006	pigs
Cert1	0.91	0.00000006	pigs
Srsf5	0.54	0.00000007	pigs
Sar1b	0.61	0.00000007	pigs
Fbxo38	-0.08	0.00000007	pigs
Cndp1	-0.13	0.00000008	pigs
Hpcal1	0.55	0.00000008	pigs
Tbc1d4	0.69	0.00000009	pigs
Bcl6	-0.12	0.00000010	pigs
Kdm4c	0.68	0.00000011	pigs
Raly	0.55	0.00000012	pigs
Nsd1	-0.19	0.00000012	pigs
Cdc42se1	0.02	0.00000014	pigs
Capn3	-0.06	0.00000015	pigs
St7l	-0.19	0.00000017	pigs
Txnrd1	0.18	0.00000019	pigs
Tmed10	-0.14	0.00000021	pigs
Tnfrsf1a	-0.06	0.00000022	pigs

Chuk	-0.13	0.00000022	pigs
Setx	-0.25	0.00000022	pigs
Tram1	-0.09	0.00000022	pigs
Cd37	-0.06	0.00000023	pigs
Fam76a	-0.06	0.00000025	pigs
Cpne1	-0.06	0.00000025	pigs
Sbno1	0.53	0.00000026	pigs
Pitpna	-0.01	0.00000026	pigs
Pan3	-0.05	0.00000028	pigs
Fam120a	-0.11	0.00000030	pigs
Ap3b1	-0.19	0.00000035	pigs
Eif2ak3	-0.22	0.00000035	pigs
Psmb3	-0.06	0.00000042	pigs
Grk2	-0.02	0.00000048	pigs
Dync1li1	0.55	0.00000049	pigs
Atg2a	-0.02	0.00000053	pigs
Kras	-0.07	0.00000057	pigs
Uox	0.59	0.00000061	pigs
Ubxn2b	-0.06	0.00000068	pigs
Gtf2a1	-0.07	0.00000070	pigs
Spen	0.71	0.00000071	pigs
Fam168b	0.56	0.00000076	pigs
Prkar1a	-0.08	0.00000080	pigs
Pef1	-0.06	0.00000086	pigs
Rab2a	0.59	0.00000086	pigs
Eloc	-0.09	0.00000092	pigs
Cdc42	-0.09	0.00000092	pigs
Grn	0.73	0.00000104	pigs
Dynlrb1	-0.11	0.00000141	pigs
Atp5a1	0.09	0.00000167	pigs
Crebbp	-0.04	0.00000172	pigs
Dym	-0.30	0.00000173	pigs
Ywhaz	-0.06	0.00000180	pigs
Smarca5	-0.07	0.00000183	pigs
Pm20d2	0.51	0.00000191	pigs
Grb2	0.00	0.00000193	pigs
Tmbim6	-0.06	0.00000213	pigs
Pdia3	0.53	0.00000215	pigs
Stk26	-0.08	0.00000227	pigs
Chst11	0.42	0.00000240	pigs
Ubr3	-0.17	0.00000268	pigs
Kmt5b	-0.08	0.00000278	pigs
Cpeb4	-0.15	0.00000309	pigs
Tfeb	-0.03	0.00000343	pigs
Kdm6b	0.56	0.00000366	pigs
4930453N24Rik	-0.11	0.00000390	pigs

Pml	-0.01	0.00000397	pigs
Sumo2	0.51	0.00000477	pigs
Ffar2	-0.02	0.00000479	pigs
Dap	-0.21	0.00000503	pigs
Nfil3	-0.09	0.00000512	pigs
Ppa1	-0.42	0.00000524	pigs
Nmi	0.00	0.00000561	pigs
Elf1	-0.24	0.00000599	pigs
Cnot8	-0.10	0.00000635	pigs
Calcoco2	-0.06	0.00000656	pigs
Zc3h13	-0.03	0.00000734	pigs
Fkbp5	-0.24	0.00000752	pigs
Ik	-0.07	0.00000762	pigs
Snf8	-0.06	0.00000762	pigs
Ccrl2	0.00	0.00000821	pigs
Isg20	-0.03	0.00000841	pigs
Alpk1	-0.09	0.00000897	pigs
Hip1r	0.62	0.00000946	pigs
Rab9	0.53	0.00000947	pigs
Cd164	0.52	0.00000975	pigs
Snapc1l	-0.12	0.00001016	pigs
Srek1ip1	-0.09	0.00001017	pigs
Hsp90b1	0.45	0.00001024	pigs
Usp34	-0.04	0.00001033	pigs
Ube3c	-0.03	0.00001071	pigs
Irf2bp2	0.64	0.00001085	pigs
Cst3	0.23	0.00001091	pigs
Chd7	-0.04	0.00001130	pigs
Ubr1	-0.05	0.00001197	pigs
Ppp1r10	-0.02	0.00001221	pigs
Fbxo11	-0.10	0.00001230	pigs
Pja2	-0.08	0.00001382	pigs
Csnk1a1	0.51	0.00001386	pigs
Dazap1	0.00	0.00001430	pigs
Atp6ap2	0.58	0.00001438	pigs
Rsad2	0.03	0.00001451	pigs
Srsf3	0.58	0.00001530	pigs
Kdm5b	-0.16	0.00001647	pigs
Nub1	0.10	0.00001795	pigs
Rhob	0.64	0.00001811	pigs
9130409I23Rik	-0.09	0.00001858	pigs
Dennd4a	-0.09	0.00002020	pigs
Arih2	-0.14	0.00002112	pigs
Rcor1	0.01	0.00002113	pigs
Pygm	0.01	0.00002272	pigs
Zfand3	-0.05	0.00002487	pigs

Nt5dc2	0.01	0.00002604	pigs
Pim3	0.44	0.00002795	pigs
Foxj2	-0.03	0.00003581	pigs
Chmp5	-0.03	0.00003746	pigs
Zfp326	0.57	0.00003816	pigs
Taf10	-0.03	0.00004022	pigs
Atg16l2	0.00	0.00004891	pigs
Psmf1	0.50	0.00005293	pigs
Tpst2	0.72	0.00005396	pigs
Plscr1	0.02	0.00005860	pigs
Sf3b2	-0.03	0.00006583	pigs
Top2b	0.02	0.00007444	pigs
Gca	-0.11	0.00010053	pigs
Suco	-0.06	0.00010250	pigs
Rbck1	-0.03	0.00010903	pigs
Phf11a	-0.15	0.00011331	pigs
Ankrd12	0.68	0.00012921	pigs
Ap2b1	-0.04	0.00014946	pigs
Atrx	-0.11	0.00019348	pigs
Zfp638	-0.06	0.00020367	pigs
Rab3gap2	-0.04	0.00021302	pigs
Cwc25	-0.03	0.00021492	pigs
Arid1b	-0.08	0.00024145	pigs
Tapt1	-0.01	0.00029570	pigs
Ubr5	0.00	0.00043018	pigs
Epc1	0.08	0.00048625	pigs
Rlim	0.02	0.00054620	pigs
Rnf146	0.43	0.00055343	pigs
Lactb	-0.03	0.00057145	pigs
Eif3h	-0.03	0.00063551	pigs
Hck	0.04	0.00063815	pigs
Sh3glb1	0.06	0.00065695	pigs
Wapl	0.54	0.00069065	pigs
Rrp1b	0.47	0.00077285	pigs
Tmem87a	0.06	0.00081836	pigs
Ifi35	-0.03	0.00083869	pigs
Zranb1	0.08	0.00088641	pigs
Lcor	0.52	0.00096405	pigs
Fxr1	0.52	0.00108250	pigs
Tnrc18	0.07	0.00110812	pigs
Tnfrsf1b	0.23	0.00116322	pigs
Fam172a	-0.15	0.00116338	pigs
Psme3ip1	0.01	0.00118137	pigs
Atf7ip	0.58	0.00126464	pigs
Btf3	0.57	0.00130164	pigs
Sik3	-0.06	0.00130259	pigs

Cox7a2	-0.03	0.00134023	pigs
Septin7	0.04	0.00140542	pigs
Btg1b	0.17	0.00144658	pigs
Ube2w	-0.01	0.00154061	pigs
Hnrnph2	0.01	0.00157372	pigs
Psmb1	0.00	0.00174346	pigs
Rassf1	0.43	0.00184777	pigs
Cdadc1	-0.16	0.00194000	pigs
B3gnt5	0.58	0.00237032	pigs
Csgalnact2	-0.11	0.00257575	pigs
Serinc1	0.04	0.00444668	pigs
Nampt	0.55	0.00454812	pigs
Furin	0.60	0.00487296	pigs
Sf3a1	-0.01	0.00489915	pigs
BC005537	0.01	0.00491125	pigs
Ubp1	0.00	0.00519113	pigs
Ist1	0.06	0.00524484	pigs
Cyba	0.57	0.00532436	pigs
Rev1	0.30	0.00540312	pigs
Ddx41	0.03	0.00601108	pigs
Sap18	0.46	0.00636782	pigs
Bin2	0.46	0.00640112	pigs
Tnks	0.05	0.00647923	pigs
Gtf2b	0.53	0.00657943	pigs
Slc6a6	0.68	0.00682705	pigs
Dnajb9	0.60	0.00715269	pigs
Stx17	0.04	0.00754545	pigs
Arid3a	0.51	0.00812980	pigs
Smu1	-0.01	0.00954632	pigs
Tsc22d2	-0.20	0.01091812	pigs
Bptf	0.01	0.01207216	pigs
Lamp2	0.67	0.01252343	pigs
Cry2	-0.10	0.01311245	pigs
Spag9	0.58	0.01353267	pigs
Cndp2	0.45	0.01394574	pigs
Bclaf1	0.54	0.01537106	pigs
Son	-0.01	0.01621049	pigs
Washc3	0.45	0.01646841	pigs
Rap2c	0.49	0.01828099	pigs
Wdr41	-0.01	0.01859811	pigs
Rp2	0.05	0.01875328	pigs
Snx13	0.00	0.01911292	pigs
Clcn5	0.52	0.01935384	pigs
Pum2	0.51	0.01948100	pigs
Pcf11	0.50	0.02300345	pigs
Zbtb20	-0.20	0.02325679	pigs

Supt6	0.02	0.02331885	pigs
Raf1	0.47	0.02416025	pigs
Lamtor1	0.54	0.02498483	pigs
Rsf1	0.00	0.02569407	pigs
Pdcd10	0.00	0.02749999	pigs
Clec7a	-0.15	0.02827127	pigs
Ppp2r3c	-0.01	0.02958202	pigs
Ppp2r2a	0.42	0.03102387	pigs
Susd6	0.36	0.03202879	pigs
Plekhf2	0.50	0.03268793	pigs
Gramd3	-0.11	0.03396495	pigs
Cybrd1	0.54	0.03463906	pigs
Plekho1	0.50	0.03535249	pigs
Ascc1	0.56	0.03562846	pigs
Hacd2	0.62	0.03679357	pigs
Szrd1	0.44	0.03926456	pigs
Galnt10	0.09	0.04055263	pigs
Rbbp6	0.43	0.04314286	pigs
Rin3	0.08	0.04587924	pigs
Nfya	0.01	0.04724188	pigs
Rhoh	0.57	0.04756167	pigs
Psen1	0.25	0.04787183	pigs
Bbx	-0.17	0.05096589	pigs
Arpc4	0.08	0.05144186	pigs
Pomp	-0.02	0.05218740	pigs
Xrn1	0.06	0.05544968	pigs
Cass4	0.66	0.05602145	pigs
Rfc2	0.49	0.05652111	pigs
Crem	-0.06	0.05767132	pigs
Spast	-0.03	0.06018445	pigs
Glis3	0.84	0.06275943	pigs
Pnrc2	-0.07	0.06462399	pigs
Csf3r	0.28	0.06738303	pigs
Gdi1	0.03	0.06909886	pigs
Usp10	-0.04	0.07178968	pigs
Serinc3	0.49	0.07582308	pigs
Stag2	-0.05	0.07609253	pigs
Entpd7	0.03	0.08020658	pigs
Xcr1	0.04	0.08033260	pigs
Dicer1	0.49	0.08091029	pigs
Ppp6r2	0.06	0.08320098	pigs
Ndfip2	-0.05	0.08954675	pigs
Tg	0.00	0.09377444	pigs
Spopl	0.02	0.09381284	pigs
Gatd1	0.44	0.09568859	pigs
Tbc1d1	-0.07	0.09727100	pigs

Tinagl1	0.07	0.09873845	pigs
Rxra	0.52	0.10506886	pigs
St3gal4	0.01	0.10666828	pigs
Khdrbs1	0.46	0.10708391	pigs
Ddx6	0.07	0.11003668	pigs
Ralgapa1	0.36	0.11207165	pigs
Chic2	0.55	0.14212194	pigs
Kif1b	0.03	0.15702380	pigs
Eif4ebp1	-0.02	0.15952185	pigs
Rhoa	0.47	0.16615072	pigs
Stx7	0.00	0.18705205	pigs
Josd1	0.07	0.18832187	pigs
Ankfy1	0.60	0.20666064	pigs
Icmt	0.44	0.22005871	pigs
Rtf2	0.07	0.23369651	pigs
Zcchc2	-0.03	0.24084662	pigs
Rnf10	0.11	0.24330055	pigs
Stag1	0.07	0.25413898	pigs
Creb1	0.42	0.28014879	pigs
Nup98	-0.03	0.28168177	pigs
Rassf2	0.39	0.33799964	pigs
Ywhab	0.02	0.34180490	pigs
Irak4	0.03	0.34860370	pigs
Plekhm1	0.04	0.35450257	pigs
Tax1bp1	0.40	0.35899197	pigs
Pgs1	0.08	0.37856063	pigs
Upf1	0.11	0.40789314	pigs
Brwd3	0.00	0.45293705	pigs
Hnrnpm	0.46	0.45564070	pigs
Agfg1	0.51	0.46679984	pigs
Ski	0.04	0.49033612	pigs
Tma7	0.01	0.50940673	pigs
Smurf2	0.07	0.52115481	pigs
Psmb9	0.07	0.53056844	pigs
Gtdc1	0.49	0.53714223	pigs
Plau	0.10	0.58726408	pigs
Pet100	0.09	0.59497370	pigs
Fgr	0.48	0.60066512	pigs
Fosl2	0.10	0.62756097	pigs
Cnot1	0.47	0.64187455	pigs
Ppp1r12a	0.39	0.73034568	pigs
Eml4	0.35	0.74273650	pigs
Csde1	0.46	0.78469486	pigs
Dpyd	0.00	1.00000000	pigs
Rbm39	0.41	1.00000000	pigs
Endov	0.11	1.00000000	pigs

Bag1	0.06	1.00000000	pigs
Cnr2	0.12	1.00000000	pigs
Plagl2	0.43	1.00000000	pigs
Dhx36	-0.03	1.00000000	pigs
Dcaf11	0.48	1.00000000	pigs
Dyrk1a	0.41	1.00000000	pigs
Prdx5	0.19	1.00000000	pigs
Csnk1d	0.05	1.00000000	pigs
Rasgef1b	0.60	1.00000000	pigs
Washc4	0.07	1.00000000	pigs
Nf1	0.11	1.00000000	pigs
Vasp	0.02	1.00000000	pigs
Ddx3x	0.18	1.00000000	pigs
Ranbp9	0.13	1.00000000	pigs
Zbtb22	0.18	1.00000000	pigs
Cd81	0.45	1.00000000	pigs
Utrn	-0.01	1.00000000	pigs
Ube2q1	0.07	1.00000000	pigs
BC005624	0.05	1.00000000	pigs
Huwe1	0.46	1.00000000	pigs
Fam32a	0.05	1.00000000	pigs
Fus	0.40	1.00000000	pigs
Nsd3	0.01	1.00000000	pigs
Fundc2	0.04	1.00000000	pigs
Plek	0.25	1.00000000	pigs
Arrdc3	0.42	1.00000000	pigs
Snrpc	0.37	1.00000000	pigs
Wdr82	0.45	1.00000000	pigs
Ahi1	0.29	1.00000000	pigs
Rad21	0.13	1.00000000	pigs
Necap1	0.10	1.00000000	pigs
Gdi2	0.41	1.00000000	pigs
Ptpn2	0.41	1.00000000	pigs
Dcaf12	0.07	1.00000000	pigs
Por	0.09	1.00000000	pigs
Ncf4	0.13	1.00000000	pigs
Tab2	0.11	1.00000000	pigs
Ube3a	0.03	1.00000000	pigs
Ambra1	0.09	1.00000000	pigs
Psme2	0.07	1.00000000	pigs
Eepd1	0.79	1.00000000	pigs
Acap2	0.30	1.00000000	pigs
Rictor	0.12	1.00000000	pigs
Samd8	0.40	1.00000000	pigs
Rbx1	0.11	1.00000000	pigs
Pnisr	0.39	1.00000000	pigs

Fam126a	0.10	1.00000000	pigs
Ssr1	0.38	1.00000000	pigs
Dhx58	0.08	1.00000000	pigs
Rab18	0.07	1.00000000	pigs
Gatad2a	0.21	1.00000000	pigs
Atp5pb	0.07	1.00000000	pigs
Arsb	0.47	1.00000000	pigs
Smurf1	0.01	1.00000000	pigs
P2ry13	0.13	1.00000000	pigs
Dcaf5	0.13	1.00000000	pigs
Hif1a	0.01	1.00000000	pigs
Chmp2a	0.18	1.00000000	pigs
Ythdc1	0.40	1.00000000	pigs
Sf3b6	0.08	1.00000000	pigs
Irf1	0.17	1.00000000	pigs
4833439L19Rik	0.15	1.00000000	pigs
Rbm14	0.38	1.00000000	pigs
Dek	0.06	1.00000000	pigs
Nfkbie	0.41	1.00000000	pigs
Acsl5	0.27	1.00000000	pigs
Atp6v1f	0.17	1.00000000	pigs
Dusp5	0.39	1.00000000	pigs
Herpud2	0.02	1.00000000	pigs
Clpx	0.06	1.00000000	pigs
Ccng2	0.11	1.00000000	pigs
Sbds	-0.21	1.00000000	pigs
Stpg4	0.35	1.00000000	pigs
Zswim6	-0.01	1.00000000	pigs
Supt4a	0.41	1.00000000	pigs
Klf6	0.32	1.00000000	pigs
Ndfip1	0.86	1.00000000	pigs
Chd3	0.38	1.00000000	pigs
Snap23	0.09	1.00000000	pigs
Tor1aip1	0.04	1.00000000	pigs
Hbp1	0.16	1.00000000	pigs
Wwp2	0.14	1.00000000	pigs
Trps1	-0.27	1.00000000	pigs
Ctnnb1	0.45	1.00000000	pigs
Arhgap25	0.28	1.00000000	pigs
Atp6v0a1	0.17	1.00000000	pigs
Dnajb6	0.18	1.00000000	pigs
Ndel1	0.29	1.00000000	pigs
Nfkbid	0.47	1.00000000	pigs
Tnfsf4	0.42	1.00000000	pigs
Ip6k1	0.15	1.00000000	pigs
Csf2rb2	0.29	1.00000000	pigs

Pacsin2	0.20	1.00000000	pigs
E2f3	0.12	1.00000000	pigs
Asprv1	0.35	1.00000000	pigs
Atp6v0d1	0.42	1.00000000	pigs
Dot1l	0.17	1.00000000	pigs
Ubr4	0.33	1.00000000	pigs
Syk	0.15	1.00000000	pigs
Plin3	0.46	1.00000000	pigs
Tbpl1	0.07	1.00000000	pigs
Paip2	0.16	1.00000000	pigs
Ccnt2	0.43	1.00000000	pigs
Nrf1	0.09	1.00000000	pigs
Flot1	0.53	1.00000000	pigs
Traf7	0.45	1.00000000	pigs
Top1	0.18	1.00000000	pigs
Ly9	0.14	1.00000000	pigs
Tuba1b	0.48	1.00000000	pigs
Shisa5	0.18	1.00000000	pigs
Tbc1d23	0.42	1.00000000	pigs
Herc4	0.10	1.00000000	pigs
Hmgxb4	0.34	1.00000000	pigs
Cisd2	0.13	1.00000000	pigs
Itpr1	0.07	1.00000000	pigs
1700037H04Rik	0.17	1.00000000	pigs
Hspb1	0.19	1.00000000	pigs
Klhl15	-0.20	1.00000000	pigs
Usp3	0.34	1.00000000	pigs
Papola	0.10	1.00000000	pigs
Arap1	0.27	1.00000000	pigs
Slc38a2	0.10	1.00000000	pigs
Rbm25	0.39	1.00000000	pigs
Ruvbl2	0.30	1.00000000	pigs
Prdm2	0.14	1.00000000	pigs
Vps37b	0.27	1.00000000	pigs
Chd1	0.32	1.00000000	pigs
Ppp4c	0.20	1.00000000	pigs
Zfp644	0.06	1.00000000	pigs
Vps13b	0.47	1.00000000	pigs
Hmox1	0.53	1.00000000	pigs
Edem1	0.42	1.00000000	pigs
Snw1	0.09	1.00000000	pigs
Arf4	0.12	1.00000000	pigs
Sdcbp	0.23	1.00000000	pigs
Pum1	0.41	1.00000000	pigs
Clk4	0.34	1.00000000	pigs
Zfc3h1	0.10	1.00000000	pigs

Crkl	0.10	1.00000000	pigs
Smad4	0.40	1.00000000	pigs
Tbl1xr1	0.19	1.00000000	pigs
Nek9	0.06	1.00000000	pigs
Vezf1	0.35	1.00000000	pigs
Kpna4	0.34	1.00000000	pigs
Borcs5	0.06	1.00000000	pigs
Zdhhc5	0.11	1.00000000	pigs
Parp16	0.15	1.00000000	pigs
Fndc3a	0.09	1.00000000	pigs
Akr1b3	0.32	1.00000000	pigs
Taok1	0.35	1.00000000	pigs
Zeb1	0.25	1.00000000	pigs
Azin1	-0.03	1.00000000	pigs
Rhbdf2	0.40	1.00000000	pigs
Arid2	0.53	1.00000000	pigs
Irf7	0.38	1.00000000	pigs
Cnn2	0.23	1.00000000	pigs
Ube2b	0.08	1.00000000	pigs
Parn	0.09	1.00000000	pigs
Srrm2	0.38	1.00000000	pigs
Klf2	0.25	1.00000000	pigs
Tmem92	0.38	1.00000000	pigs
Cdkn1a	0.17	1.00000000	pigs
Stk3	0.38	1.00000000	pigs
Ppbp	0.36	1.00000000	pigs
Leng8	0.17	1.00000000	pigs
Trib1	0.09	1.00000000	pigs
Atf1	0.33	1.00000000	pigs
Efr3a	0.32	1.00000000	pigs
Upf2	0.47	1.00000000	pigs
Litaf	0.17	1.00000000	pigs
Prpf40a	0.39	1.00000000	pigs
Gclc	0.34	1.00000000	pigs
Avl9	0.57	1.00000000	pigs
Dazap2	0.32	1.00000000	pigs
Slc9a8	0.22	1.00000000	pigs
Inpp4a	0.92	1.00000000	pigs
Caml	0.26	1.00000000	pigs
Kif5b	0.36	1.00000000	pigs
Bcl3	0.44	1.00000000	pigs
Btbd19	0.38	1.00000000	pigs
Cul1	0.19	1.00000000	pigs
Skp1	0.15	1.00000000	pigs
Abcc1	0.44	1.00000000	pigs
D16Ertd472e	0.16	1.00000000	pigs

Galc	0.19	1.00000000	pigs
Dhx9	0.17	1.00000000	pigs
Pcbp1	0.35	1.00000000	pigs
Cd101	0.14	1.00000000	pigs
Ptp4a2	0.37	1.00000000	pigs
Twf2	0.26	1.00000000	pigs
Slu7	0.17	1.00000000	pigs
Kdm6a	0.16	1.00000000	pigs
Ppm1m	0.16	1.00000000	pigs
Tyk2	0.18	1.00000000	pigs
Ccdc186	0.35	1.00000000	pigs
Capg	0.16	1.00000000	pigs
Vmp1	0.18	1.00000000	pigs
Ppp1r15b	0.21	1.00000000	pigs
Aif1	0.09	1.00000000	pigs
Rhoq	0.14	1.00000000	pigs
Arl5b	0.04	1.00000000	pigs
Snip1	0.15	1.00000000	pigs
Rela	0.33	1.00000000	pigs
Bzw1	0.23	1.00000000	pigs
Gfod1	0.25	1.00000000	pigs
Hint1	0.16	1.00000000	pigs
Zdhhc20	0.21	1.00000000	pigs
Lsm14a	0.33	1.00000000	pigs
Tfe3	0.18	1.00000000	pigs
Kdm5c	0.19	1.00000000	pigs
Ypel5	0.22	1.00000000	pigs
Nr2c2	0.16	1.00000000	pigs
Jarid2	0.47	1.00000000	pigs
Nab1	0.32	1.00000000	pigs
Cep85l	0.06	1.00000000	pigs
Fam214a	0.53	1.00000000	pigs
Trim26	0.22	1.00000000	pigs
Sh3bp5	0.13	1.00000000	pigs
Serf2	0.17	1.00000000	pigs
Adnp	0.21	1.00000000	pigs
Runx1	0.18	1.00000000	pigs
Tspan31	0.28	1.00000000	pigs
Kdm5a	0.31	1.00000000	pigs
Eif4g2	0.33	1.00000000	pigs
Sgms1	0.13	1.00000000	pigs
Ogt	0.33	1.00000000	pigs
Erc1	0.19	1.00000000	pigs
Il10ra	0.17	1.00000000	pigs
Fbxl3	0.25	1.00000000	pigs
Cox6a1	0.31	1.00000000	pigs

Setd2	0.35	1.00000000	pigs
Usp8	0.20	1.00000000	pigs
Treml2	0.14	1.00000000	pigs
Arid5b	0.17	1.00000000	pigs
Hnrnpdl	0.33	1.00000000	pigs
Ahr	0.09	1.00000000	pigs
Gbf1	0.18	1.00000000	pigs
Rap1b	0.24	1.00000000	pigs
Ppm1h	0.74	1.00000000	pigs
Bicral	0.21	1.00000000	pigs
Gabarapl1	0.20	1.00000000	pigs
Klhl8	0.04	1.00000000	pigs
Cdk13	0.27	1.00000000	pigs
Iqsec1	0.15	1.00000000	pigs
Sbno2	0.32	1.00000000	pigs
Brd2	0.38	1.00000000	pigs
Rnf114	0.22	1.00000000	pigs
Cd53	0.00	1.00000000	pigs
Rack1	0.41	1.00000000	pigs
Hnrnpk	0.31	1.00000000	pigs
Tmem50a	0.28	1.00000000	pigs
Psmb4	0.24	1.00000000	pigs
Wwp1	0.09	1.00000000	pigs
Stxbp3	0.32	1.00000000	pigs
Serping1	0.27	1.00000000	pigs
Tbc1d9b	0.41	1.00000000	pigs
Ythdf3	0.22	1.00000000	pigs
Prkcb	0.30	1.00000000	pigs
Hipk3	0.31	1.00000000	pigs
Yy1	0.35	1.00000000	pigs
Slc7a7	0.33	1.00000000	pigs
Ppp4r3b	0.27	1.00000000	pigs
Sema7a	0.21	1.00000000	pigs
Ppp1r15a	0.07	1.00000000	pigs
Ly96	0.20	1.00000000	pigs
Fbxl20	0.30	1.00000000	pigs
Lpp	0.40	1.00000000	pigs
Rb1cc1	0.31	1.00000000	pigs
Tcf20	0.21	1.00000000	pigs
Ptger4	0.25	1.00000000	pigs
Ccdc9	0.26	1.00000000	pigs
Calm1	0.21	1.00000000	pigs
Cltc	0.16	1.00000000	pigs
Pnp	0.17	1.00000000	pigs
Aff1	0.48	1.00000000	pigs
Kdm1b	0.25	1.00000000	pigs

Ggnbp2	0.33	1.00000000	pigs
Rab5c	0.28	1.00000000	pigs
Slc5a10	0.26	1.00000000	pigs
Eef1b2	0.27	1.00000000	pigs
Ddit4	0.11	1.00000000	pigs
Nol7	0.21	1.00000000	pigs
Cox4i1	0.29	1.00000000	pigs
Plekha2	0.25	1.00000000	pigs
Wac	0.26	1.00000000	pigs
Nfam1	0.26	1.00000000	pigs
Cul3	0.27	1.00000000	pigs
Add1	0.08	1.00000000	pigs
Lrpap1	0.25	1.00000000	pigs
Hbs1l	0.27	1.00000000	pigs
Tmem156	0.13	1.00000000	pigs
Pafah1b1	0.21	1.00000000	pigs
Capza2	0.30	1.00000000	pigs
Sf3b1	0.22	1.00000000	pigs
Arfgef1	0.33	1.00000000	pigs
Slc22a15	0.31	1.00000000	pigs
Tom1	0.09	1.00000000	pigs
Sec61g	0.24	1.00000000	pigs
Syf2	0.29	1.00000000	pigs
C9orf72	0.22	1.00000000	pigs
Fam193a	0.30	1.00000000	pigs
Atp11c	0.29	1.00000000	pigs
Abcg1	0.29	1.00000000	pigs
Rab5a	0.21	1.00000000	pigs
Arhgef2	0.23	1.00000000	pigs
Tm9sf2	0.29	1.00000000	pigs
S100a10	0.26	1.00000000	pigs
Tiparp	0.22	1.00000000	pigs
Sema4d	0.26	1.00000000	pigs
Pcmt1	0.24	1.00000000	pigs
Pou2f1	0.23	1.00000000	pigs
Stk24	0.27	1.00000000	pigs
Kcmf1	0.27	1.00000000	pigs
Nup50	0.26	1.00000000	pigs
Prorp	0.11	1.00000000	pigs
Atad2b	0.29	1.00000000	pigs
Ewsr1	0.23	1.00000000	pigs
Ndufa2	0.27	1.00000000	pigs
Pcgf5	0.24	1.00000000	pigs
Trem1	0.11	1.00000000	pigs
Jchain	0.29	1.00000000	pigs
Vps26a	0.15	1.00000000	pigs

Kmt2e	0.06	1.00000000	pigs
Epsti1	0.29	1.00000000	pigs
Strn3	0.31	1.00000000	pigs
Edem3	0.22	1.00000000	pigs
Ddx17	0.28	1.00000000	pigs
Atf6	0.28	1.00000000	pigs
Arnt	0.25	1.00000000	pigs
Hectd1	0.25	1.00000000	pigs
