# Supplementary material for: Heterogeneous viral-host response at single-cell resolution and viral adaptive mutational atlas in birds and mammals upon H5 influenza virus infection
Source: Natl Sci Rev. 2026 Jun 25;13(14):nwag380. doi: 10.1093/nsr/nwag380 (PMC13458601; doi:10.1093/nsr/nwag380)
Supplement: Supplementary file 1 [file nwag380_supplemental_files.zip › Supplementary Data 15.pdf]

upplementary Data 15. Differentially expressed genes of B cells across different species upon H5 HPAIV infectio

gene	log2FoldChange	p value	host
Prokr2	2.38	0.00000000	chicken
Stat2	1.31	0.00000000	chicken
Ifit2	1.46	0.00000015	chicken
Ube2h	1.20	0.00000219	chicken
Oasl2	1.02	0.00000297	chicken
Stat1	1.79	0.00001020	chicken
Ebf1	1.20	0.00003391	chicken
Ust	2.20	0.00004135	chicken
Rftn1	1.29	0.00013261	chicken
Exoc6	1.26	0.00013770	chicken
Gpr183	1.75	0.00018244	chicken
Tgfbr2	1.73	0.00038909	chicken
Ptk2	1.61	0.00048687	chicken
Trank1	1.07	0.00088591	chicken
Rfx3	2.57	0.00094380	chicken
Spi1	1.50	0.00150653	chicken
Elf1	1.02	0.00193034	chicken
Bcl11a	1.06	0.00250828	chicken
Prdm2	1.48	0.00301696	chicken
Dgkd	1.04	0.00401196	chicken
Oit3	1.15	0.00592564	chicken
Abi1	1.29	0.01394751	chicken
Pde4d	1.12	0.01410921	chicken
Tasl	1.12	0.01483411	chicken
Bmpr2	1.55	0.01548057	chicken
Tedc1	1.64	0.01610946	chicken
Vav3	1.07	0.02309000	chicken
Sbf2	1.22	0.03484146	chicken
Ssu72	1.20	0.04770182	chicken
Dock9	1.01	0.06670252	chicken
Cstf3	1.04	0.08787561	chicken
Ncoa3	1.65	0.08878832	chicken
Foxo1	1.00	0.08883257	chicken
Foxn3	1.18	0.09925763	chicken
Rgs1	1.28	0.09937926	chicken
Trim25	0.69	0.02279989	chicken
Asb12	0.56	0.02530912	chicken
Birc6	0.75	0.08928929	chicken
Tbc1d5	1.24	0.10216555	chicken
Stk38l	1.63	0.11040640	chicken
Ikzf1	1.25	0.12299666	chicken
Hivep1	1.19	0.12585109	chicken

Nap1l1	-1.56	0.15312538	chicken
Actb	-1.57	0.15511382	chicken
Kcnk1	1.23	0.18231165	chicken
Ccl9	0.62	0.19116278	chicken
Socs6	0.87	0.19145736	chicken
Angpt1	0.71	0.21075813	chicken
Znrf2	1.07	0.22580547	chicken
Prdx1	-1.30	0.26481848	chicken
Tmpo	1.09	0.27178857	chicken
Nr3c2	1.88	0.28357431	chicken
Hopx	0.69	0.37062965	chicken
Utrn	1.15	0.40404699	chicken
Sesn1	1.24	0.49407306	chicken
Fam107b	0.99	0.51587087	chicken
Urm1	-1.45	0.52358866	chicken
Nr2c2	0.90	0.53744104	chicken
Cxcr4	0.95	0.55528958	chicken
Arid4b	0.96	0.58130195	chicken
Eif3f	-1.42	0.70644454	chicken
Dennd1b	0.94	0.70691612	chicken
Etv6	1.18	0.71928705	chicken
Cotl1	-1.40	0.81191527	chicken
Atad2b	1.25	0.90656277	chicken
Pik3r1	0.97	0.98692994	chicken
Rimbp2	1.01	0.98993892	chicken
Sh3kbp1	1.07	1.00000000	chicken
Pds5a	1.09	1.00000000	chicken
Nub1	1.07	1.00000000	chicken
Pex12	0.41	1.00000000	chicken
Pan3	0.68	1.00000000	chicken
Eno1b	-1.11	1.00000000	chicken
Gigyf2	0.75	1.00000000	chicken
Ctdp1	1.04	1.00000000	chicken
Eif3e	-1.19	1.00000000	chicken
Eif3m	-1.09	1.00000000	chicken
Gnb1	0.93	1.00000000	chicken
Glcci1	1.28	1.00000000	chicken
H2ac20	0.78	1.00000000	chicken
Calcb	0.94	1.00000000	chicken
Arpc1b	-1.23	1.00000000	chicken
Ptprj	1.11	1.00000000	chicken
Ankrd44	0.86	1.00000000	chicken
Foxp1	0.65	1.00000000	chicken
Jak2	1.59	1.00000000	chicken
Shoc2	0.72	1.00000000	chicken
Coro7	-1.06	1.00000000	chicken

Tfap4	0.69	1.00000000	chicken
Snx13	1.24	1.00000000	chicken
Slc9a3r1	-1.08	1.00000000	chicken
Znfx1	1.15	1.00000000	chicken
Prom1	0.93	1.00000000	chicken
Rhoh	0.96	1.00000000	chicken
Hspe1-rs1	-1.15	1.00000000	chicken
Cox7b	-1.24	1.00000000	chicken
Emp1	1.40	1.00000000	chicken
Wbp1l	0.64	1.00000000	chicken
Zcchc7	0.82	1.00000000	chicken
Sun2	0.55	1.00000000	chicken
Eif3h	-0.74	1.00000000	chicken
Ergic1	0.83	1.00000000	chicken
Smchd1	1.15	1.00000000	chicken
Dpysl4	0.77	1.00000000	chicken
Rc3h1	0.85	1.00000000	chicken
Zeb1	0.66	1.00000000	chicken
Rap1gds1	0.98	1.00000000	chicken
Btrc	0.80	1.00000000	chicken
Tdrd1	0.46	1.00000000	chicken
Ide	1.27	1.00000000	chicken
Irf1	0.96	1.00000000	chicken
Hnrnpa1	-1.15	1.00000000	chicken
Esyt2	0.99	1.00000000	chicken
Pias1	0.90	1.00000000	chicken
Itga6	1.38	1.00000000	chicken
Fbxw11	0.77	1.00000000	chicken
Disp1	1.18	1.00000000	chicken
Eif3d	-1.00	1.00000000	chicken
Gfpt2	1.00	1.00000000	chicken
Prex1	0.96	1.00000000	chicken
Rapsn	0.63	1.00000000	chicken
Nfyc	0.79	1.00000000	chicken
Apbb1ip	0.83	1.00000000	chicken
Sh3bgrl	-0.99	1.00000000	chicken
Ttyh3	-1.04	1.00000000	chicken
Pde7b	1.71	1.00000000	chicken
Alcam	0.79	1.00000000	chicken
Zc3h12c	0.88	1.00000000	chicken
Rhoa	-0.71	1.00000000	chicken
Uba2	0.72	1.00000000	chicken
Tbc1d12	0.88	1.00000000	chicken
Usp25	0.70	1.00000000	chicken
Bach2	0.67	1.00000000	chicken
Galnt1	-0.86	1.00000000	chicken

Fli1	1.49	1.00000000	chicken
Gab3	0.89	1.00000000	chicken
Cpt1a	0.67	1.00000000	chicken
D5Ertd579e	1.01	1.00000000	chicken
Ankrd33b	0.79	1.00000000	chicken
Thra	0.68	1.00000000	chicken
Cep85l	0.81	1.00000000	chicken
Cds2	0.54	1.00000000	chicken
Ppp1r12a	0.51	1.00000000	chicken
Chd7	0.93	1.00000000	chicken
Rsbn1l	0.88	1.00000000	chicken
Fam102a	-1.20	1.00000000	chicken
Irak1bp1	0.28	1.00000000	chicken
Cox4i1	-0.91	1.00000000	chicken
Cox5a	-1.09	1.00000000	chicken
Baz2b	1.07	1.00000000	chicken
Oat	-0.98	1.00000000	chicken
Rnf141	1.06	1.00000000	chicken
Stx2	0.77	1.00000000	chicken
Arap2	0.72	1.00000000	chicken
Calm1	-0.85	1.00000000	chicken
Tomm6	-0.94	1.00000000	chicken
Serbp1	-0.69	1.00000000	chicken
Trps1	1.08	1.00000000	chicken
Epn2	0.75	1.00000000	chicken
Ppib	-0.94	1.00000000	chicken
Per2	0.62	1.00000000	chicken
Sgpl1	-1.02	1.00000000	chicken
Slc4a7	0.84	1.00000000	chicken
Sh2b3	0.53	1.00000000	chicken
Inpp5d	0.93	1.00000000	chicken
Obp2a	0.42	1.00000000	chicken
Dcp1b	0.94	1.00000000	chicken
Tspan3	0.76	1.00000000	chicken
Gclc	-1.27	1.00000000	chicken
Nedd9	0.93	1.00000000	chicken
Dennd5b	0.76	1.00000000	chicken
Eif3g	-0.98	1.00000000	chicken
Arhgdia	-0.84	1.00000000	chicken
Ppp6r3	0.61	1.00000000	chicken
Itga4	-0.73	1.00000000	chicken
Dennd5a	0.79	1.00000000	chicken
Ndel1	0.65	1.00000000	chicken
Psma7	-0.92	1.00000000	chicken
Erbin	0.81	1.00000000	chicken
Sap18	-0.94	1.00000000	chicken

Ebag9	0.69	1.00000000	chicken
Tsc22d3	0.59	1.00000000	chicken
Gnb4	0.79	1.00000000	chicken
Fam120a	0.74	1.00000000	chicken
Slc30a6	0.52	1.00000000	chicken
Snx2	-1.01	1.00000000	chicken
Zfp516	0.76	1.00000000	chicken
Nfkbiz	0.82	1.00000000	chicken
Rac2	-0.89	1.00000000	chicken
Ppfibp1	-0.99	1.00000000	chicken
Synj1	0.99	1.00000000	chicken
Hbp1	0.59	1.00000000	chicken
Ifih1	0.52	1.00000000	chicken
App	1.16	1.00000000	chicken
Dynlt1a	0.58	1.00000000	chicken
Bmt2	0.88	1.00000000	chicken
Cyba	-0.88	1.00000000	chicken
Osbpl3	0.69	1.00000000	chicken
Slc35a5	0.60	1.00000000	chicken
Scaf11	0.85	1.00000000	chicken
Glo1	-0.90	1.00000000	chicken
Atic	-0.93	1.00000000	chicken
Dock7	1.10	1.00000000	chicken
Add3	-0.69	1.00000000	chicken
Pwwp2b	0.83	1.00000000	chicken
Inpp4a	1.14	1.00000000	chicken
Dido1	0.69	1.00000000	chicken
Tcf7	-2.24	1.00000000	chicken
Dalrd3	0.79	1.00000000	chicken
Gnai2	-0.96	1.00000000	chicken
Gnpnat1	0.70	1.00000000	chicken
Elavl1	0.81	1.00000000	chicken
Tcp11l2	0.62	1.00000000	chicken
Ppp1r10	0.74	1.00000000	chicken
Kdm4b	0.72	1.00000000	chicken
Smarcd1	0.47	1.00000000	chicken
Gpd2	0.31	1.00000000	chicken
Ppm1l	0.80	1.00000000	chicken
Phtf2	0.70	1.00000000	chicken
Sptbn1	-0.92	1.00000000	chicken
Dapk1	0.73	1.00000000	chicken
Ssbp1	-0.69	1.00000000	chicken
Eif3l	-0.70	1.00000000	chicken
Itm2a	-0.79	1.00000000	chicken
Dynlt3	0.50	1.00000000	chicken
Zbtb2	0.70	1.00000000	chicken

Erp29	-0.73	1.00000000	chicken
Tm9sf3	-0.87	1.00000000	chicken
Rfx2	0.74	1.00000000	chicken
Kras	0.81	1.00000000	chicken
Ttc37	0.82	1.00000000	chicken
Il1b	0.44	1.00000000	chicken
Prdx4	-0.71	1.00000000	chicken
Ttf1	-0.87	1.00000000	chicken
Cd44	0.79	1.00000000	chicken
Nf1	0.74	1.00000000	chicken
Gphn	1.34	1.00000000	chicken
Plk3	0.53	1.00000000	chicken
Fbxl22	0.65	1.00000000	chicken
Chd5	0.39	1.00000000	chicken
Pank2	0.51	1.00000000	chicken
Rarg	0.31	1.00000000	chicken
Slc38a1	0.95	1.00000000	chicken
Zmynd11	1.02	1.00000000	chicken
Smarca5	-0.77	1.00000000	chicken
Dars	0.84	1.00000000	chicken
Trmt11	0.56	1.00000000	chicken
Ddx52	0.53	1.00000000	chicken
Ap1m1	0.40	1.00000000	chicken
Pcm1	0.60	1.00000000	chicken
Atp2b4	0.85	1.00000000	chicken
Phlpp1	1.15	1.00000000	chicken
Arpc4	-0.90	1.00000000	chicken
Gas8	0.42	1.00000000	chicken
Bank1	0.93	1.00000000	chicken
Arid5b	0.57	1.00000000	chicken
Zzz3	0.77	1.00000000	chicken
Lcp1	-0.81	1.00000000	chicken
Atf3	0.44	1.00000000	chicken
Zfp644	0.98	1.00000000	chicken
Dysf	0.55	1.00000000	chicken
Dennd4a	0.70	1.00000000	chicken
Zfp593	-0.71	1.00000000	chicken
Atxn7l1	0.85	1.00000000	chicken
Ube2w	0.68	1.00000000	chicken
Bcl2	0.80	1.00000000	chicken
Ccdc127	0.20	1.00000000	chicken
Slc25a5	-0.71	1.00000000	chicken
Dusp1	0.50	1.00000000	chicken
Evi2a	0.58	1.00000000	chicken
Ahctf1	0.95	1.00000000	chicken
Slc5a1	1.02	1.00000000	chicken

Csrnp1	0.58	1.00000000	chicken
Pde8a	1.71	1.00000000	chicken
Zdhhc17	0.74	1.00000000	chicken
Tomm20	-0.64	1.00000000	chicken
Nuak1	0.56	1.00000000	chicken
Ric8b	1.09	1.00000000	chicken
Nlrc5	0.45	1.00000000	chicken
Btf3	-0.70	1.00000000	chicken
Cdk14	0.74	1.00000000	chicken
H2az2	-1.20	1.00000000	chicken
Cpe	0.91	1.00000000	chicken
Fbxl5	0.48	1.00000000	chicken
Hipk3	0.90	1.00000000	chicken
Rfx7	0.57	1.00000000	chicken
Ppp2r5c	0.73	1.00000000	chicken
Slc12a4	0.87	1.00000000	chicken
Snx10	0.88	1.00000000	chicken
Sult4a1	0.64	1.00000000	chicken
Cd47	-0.65	1.00000000	chicken
Thoc2	0.55	1.00000000	chicken
Tubg1	0.79	1.00000000	chicken
Entpd5	0.56	1.00000000	chicken
Denr	-0.70	1.00000000	chicken
Htr4	1.09	1.00000000	chicken
Tle4	0.67	1.00000000	chicken
Bcr	0.87	1.00000000	chicken
Hsp90ab1	-1.03	1.00000000	chicken
Cdkal1	0.43	1.00000000	chicken
Vav2	0.70	1.00000000	chicken
Heatr6	0.29	1.00000000	chicken
Irf5	0.52	1.00000000	chicken
Runx1	-0.59	1.00000000	chicken
Dglucy	0.83	1.00000000	chicken
Crem	-1.12	1.00000000	chicken
Slbp	0.88	1.00000000	chicken
Sdhd	-0.69	1.00000000	chicken
Rbbp5	0.53	1.00000000	chicken
Rit1	0.55	1.00000000	chicken
Fyn	-0.88	1.00000000	chicken
Ccdc85c	0.69	1.00000000	chicken
Tpd52l2	0.50	1.00000000	chicken
Eif3a	-0.61	1.00000000	chicken
Dyrk1a	0.94	1.00000000	chicken
Pde3b	0.61	1.00000000	chicken
Cmtr1	0.95	1.00000000	chicken
Ash1l	0.77	1.00000000	chicken

Cnppd1	0.34	1.00000000	chicken
Ahrr	-1.09	1.00000000	chicken
Aopep	0.27	1.00000000	chicken
Tubgcp3	0.68	1.00000000	chicken
Ago2	0.61	1.00000000	chicken
Vps51	0.25	1.00000000	chicken
Tlr6	0.55	1.00000000	chicken
Serp1	-0.84	1.00000000	chicken
Zfp352	0.70	1.00000000	chicken
Hnrnpab	-0.39	1.00000000	chicken
Yy2	0.70	1.00000000	chicken
Ccni	-0.76	1.00000000	chicken
Qrich1	0.56	1.00000000	chicken
Tapbp	-0.77	1.00000000	chicken
Pdcd7	0.45	1.00000000	chicken
Pcgf1	0.42	1.00000000	chicken
Add1	0.91	1.00000000	chicken
Ppia	-0.91	1.00000000	chicken
Taf15	0.81	1.00000000	chicken
Tut7	0.75	1.00000000	chicken
Gucy1b1	0.40	1.00000000	chicken
Crlf3	-0.81	1.00000000	chicken
Nelfa	-0.72	1.00000000	chicken
Dock2	-0.66	1.00000000	chicken
Slc25a3	-0.67	1.00000000	chicken
Dync2i1	0.22	1.00000000	chicken
Prelid1	-0.57	1.00000000	chicken
Cdk19	0.63	1.00000000	chicken
Grap	-0.83	1.00000000	chicken
Phf20	0.56	1.00000000	chicken
Arpc2	-0.58	1.00000000	chicken
Hif1a	0.62	1.00000000	chicken
Nktr	0.84	1.00000000	chicken
Hivep2	0.58	1.00000000	chicken
Slc9a9	-1.49	1.00000000	chicken
Jund	0.25	1.00000000	chicken
Hat1	0.40	1.00000000	chicken
Tent4b	0.63	1.00000000	chicken
Csnk2a2	-0.90	1.00000000	chicken
N4bp2l2	0.48	1.00000000	chicken
Dicer1	0.70	1.00000000	chicken
Gtf2h1	0.69	1.00000000	chicken
Cd81	-0.80	1.00000000	chicken
Nrp2	-0.82	1.00000000	chicken
Ncf4	0.65	1.00000000	chicken
Snx9	0.80	1.00000000	chicken

Ndufaf6	0.54	1.00000000	chicken
Stx8	0.90	1.00000000	chicken
Ecpas	0.40	1.00000000	chicken
Slc16a6	0.36	1.00000000	chicken
Tax1bp3	0.62	1.00000000	chicken
Hcls1	-0.70	1.00000000	chicken
Phf2	0.67	1.00000000	chicken
Ralgapa1	0.98	1.00000000	chicken
Eif2s3y	-0.73	1.00000000	chicken
Baz1b	0.47	1.00000000	chicken
Rnf19b	0.44	1.00000000	chicken
Prr14l	0.69	1.00000000	chicken
Foxk2	0.64	1.00000000	chicken
Kcnq1	-1.16	1.00000000	chicken
Anp32e	-0.82	1.00000000	chicken
Tomm40l	-0.66	1.00000000	chicken
Rasgef1a	1.68	1.00000000	chicken
Zfyve26	-0.85	1.00000000	chicken
Ybx3	-0.78	1.00000000	chicken
Gna13	0.47	1.00000000	chicken
Epb41	-0.73	1.00000000	chicken
Ipmk	-0.68	1.00000000	chicken
Ssr3	-0.52	1.00000000	chicken
Hectd4	-0.69	1.00000000	chicken
Exoc2	0.88	1.00000000	chicken
Cox7c	-0.40	1.00000000	chicken
Fus	0.43	1.00000000	chicken
Psen1	0.65	1.00000000	chicken
Baiap2	0.24	1.00000000	chicken
Tagap1	0.53	1.00000000	chicken
Btg2	0.67	1.00000000	chicken
Eif3b	-0.58	1.00000000	chicken
Hdgf	-0.84	1.00000000	chicken
Itpr1	0.83	1.00000000	chicken
Fam32a	0.46	1.00000000	chicken
Rbm33	0.74	1.00000000	chicken
Tmem214	0.18	1.00000000	chicken
Chordc1	-0.57	1.00000000	chicken
Sdcbp	-0.64	1.00000000	chicken
Elf2	0.49	1.00000000	chicken
Kidins220	-0.41	1.00000000	chicken
Lnx2	0.86	1.00000000	chicken
Rsrc2	0.52	1.00000000	chicken
Prkar2a	-1.00	1.00000000	chicken
Ercc6	-0.89	1.00000000	chicken
Rras2	0.83	1.00000000	chicken

Yeats2	-0.74	1.00000000	chicken
Cytip	0.42	1.00000000	chicken
Cd247	-0.77	1.00000000	chicken
Sec24b	0.51	1.00000000	chicken
Zfp608	1.13	1.00000000	chicken
Pin4	-0.60	1.00000000	chicken
P4htm	0.28	1.00000000	chicken
Cc2d1b	-0.66	1.00000000	chicken
Ilk	-0.57	1.00000000	chicken
Arhgap29	-0.75	1.00000000	chicken
Uba3	0.74	1.00000000	chicken
Lrba	0.53	1.00000000	chicken
Sytl1	-0.73	1.00000000	chicken
Ptbp2	0.76	1.00000000	chicken
Pgls	-0.74	1.00000000	chicken
Ifngr1	-0.61	1.00000000	chicken
Zfand3	0.57	1.00000000	chicken
Zdhhc20	0.70	1.00000000	chicken
Itgb2l	-0.67	1.00000000	chicken
Pou2f1	1.09	1.00000000	chicken
Pfdn4	-0.76	1.00000000	chicken
Palb2	0.35	1.00000000	chicken
Rab8b	-0.70	1.00000000	chicken
Prcp	-0.81	1.00000000	chicken
Fbxl20	0.64	1.00000000	chicken
Stn1	-0.85	1.00000000	chicken
Drosha	-0.79	1.00000000	chicken
Uqcrb	-0.63	1.00000000	chicken
Dis3l2	-0.91	1.00000000	chicken
Hbs1l	0.81	1.00000000	chicken
Atrn	-0.97	1.00000000	chicken
Lonrf1	0.58	1.00000000	chicken
Adssl1	0.61	1.00000000	chicken
Stim1	-0.68	1.00000000	chicken
Rbmx2	0.55	1.00000000	chicken
Spty2d1	-0.76	1.00000000	chicken
Arxes2	-0.56	1.00000000	chicken
Prkrip1	-0.59	1.00000000	chicken
Ctnnbl1	-0.57	1.00000000	chicken
Rasa3	0.74	1.00000000	chicken
Iqgap2	1.21	1.00000000	chicken
Snrpd3	-0.68	1.00000000	chicken
Rassf2	0.67	1.00000000	chicken
Tab3	0.54	1.00000000	chicken
Atp5b	-0.75	1.00000000	chicken
Slc9a7	0.98	1.00000000	chicken

Rev3l	0.88	1.00000000	chicken
Eif2ak3	0.69	1.00000000	chicken
Rasal1	-0.74	1.00000000	chicken
Pes1	-0.71	1.00000000	chicken
Gbf1	1.05	1.00000000	chicken
Ddx19a	-0.50	1.00000000	chicken
Ate1	0.84	1.00000000	chicken
Btbd19	0.43	1.00000000	chicken
Sh3pxd2b	-1.08	1.00000000	chicken
Slc9a8	0.52	1.00000000	chicken
Lpgat1	1.03	1.00000000	chicken
Zbtb21	0.64	1.00000000	chicken
Pdia3	-0.71	1.00000000	chicken
Tuba1a	0.46	1.00000000	chicken
Sec61g	-0.50	1.00000000	chicken
Pgd	-0.58	1.00000000	chicken
Calm2	-0.81	1.00000000	chicken
Rpgr	1.03	1.00000000	chicken
Cox7a2	-0.68	1.00000000	chicken
Rgs14	0.30	1.00000000	chicken
Uqcrh	-0.75	1.00000000	chicken
Ndufaf1	0.83	1.00000000	chicken
Srp72	-0.61	1.00000000	chicken
Arpc1a	-0.57	1.00000000	chicken
Ccnd2	0.41	1.00000000	chicken
Ythdf3	0.38	1.00000000	chicken
Elk3	0.26	1.00000000	chicken
Rabggtb	0.62	1.00000000	chicken
Ndufa1	-0.40	1.00000000	chicken
Rell1	-0.58	1.00000000	chicken
Lyrm4	1.02	1.00000000	chicken
Ulk2	0.79	1.00000000	chicken
Kcnq5	0.47	1.00000000	chicken
Elovl5	0.88	1.00000000	chicken
Lrig2	0.27	1.00000000	chicken
Nol7	-0.52	1.00000000	chicken
Ppfia1	0.82	1.00000000	chicken
Ubl5b	-0.51	1.00000000	chicken
Aldh8a1	0.36	1.00000000	chicken
Sgms2	0.30	1.00000000	chicken
Cog6	0.59	1.00000000	chicken
Slc25a36	0.86	1.00000000	chicken
Traf3	0.61	1.00000000	chicken
Smurf2	0.28	1.00000000	chicken
Arfip1	0.45	1.00000000	chicken
Abcc1	0.66	1.00000000	chicken

Sar1a	-0.59	1.00000000	chicken
Fam124b	0.18	1.00000000	chicken
Tmem131l	0.70	1.00000000	chicken
Dnajc16	0.40	1.00000000	chicken
Cd3e	-0.80	1.00000000	chicken
Atxn3	-0.68	1.00000000	chicken
Pcnx	0.88	1.00000000	chicken
Rpp38	-0.51	1.00000000	chicken
Phb	-0.45	1.00000000	chicken
Ncoa7	0.54	1.00000000	chicken
Asap1	-0.68	1.00000000	chicken
Cradd	0.94	1.00000000	chicken
Bzw1	-0.62	1.00000000	chicken
Chchd10	0.68	1.00000000	chicken
Ndufb2	-0.19	1.00000000	chicken
Nip7	-0.58	1.00000000	chicken
Rilpl2	-0.49	1.00000000	chicken
Oxnad1	0.40	1.00000000	chicken
Syne1	-0.61	1.00000000	chicken
Gsk3b	0.62	1.00000000	chicken
Arhgap24	0.36	1.00000000	chicken
Nfatc3	0.44	1.00000000	chicken
Wwox	1.20	1.00000000	chicken
Nbl1	-0.76	1.00000000	chicken
Nde1	0.38	1.00000000	chicken
Prep	-0.69	1.00000000	chicken
Rab10	-0.59	1.00000000	chicken
Usp7	0.69	1.00000000	chicken
Dennd1a	0.99	1.00000000	chicken
Clec16a	0.50	1.00000000	chicken
Il2rb	-0.62	1.00000000	chicken
Sptssa	-0.49	1.00000000	chicken
Cdc14a	0.87	1.00000000	chicken
Ccnt1	0.19	1.00000000	chicken
Hadh	0.34	1.00000000	chicken
Rab18	0.50	1.00000000	chicken
Lpcat3	0.62	1.00000000	chicken
Stmn1	-1.03	1.00000000	chicken
Aco2	-0.65	1.00000000	chicken
Ift22	0.54	1.00000000	chicken
Spen	0.45	1.00000000	chicken
Ncor2	-0.38	1.00000000	chicken
Dnajc19	-0.52	1.00000000	chicken
Dvl1	0.67	1.00000000	chicken
Nucks1	-0.55	1.00000000	chicken
Ndufa4	-0.63	1.00000000	chicken

Kansl1l	0.69	1.00000000	chicken
Arfgef2	0.58	1.00000000	chicken
Orai1	0.15	1.00000000	chicken
Cflar	0.55	1.00000000	chicken
Dhx36	-0.60	1.00000000	chicken
Nfatc1	0.79	1.00000000	chicken
Cdca7l	-0.60	1.00000000	chicken
Laptm5	-0.39	1.00000000	chicken
Sbno2	0.40	1.00000000	chicken
Evi5	0.71	1.00000000	chicken
Hsd17b12	-0.82	1.00000000	chicken
Eif2s1	-0.56	1.00000000	chicken
Tbc1d4	-0.78	1.00000000	chicken
Gstm4	-0.61	1.00000000	chicken
Akirin2	-0.47	1.00000000	chicken
Lsp1	-0.66	1.00000000	chicken
Atxn1	-0.89	1.00000000	chicken
Jun	0.31	1.00000000	chicken
Kif21b	-0.52	1.00000000	chicken
Pgam1	-0.64	1.00000000	chicken
Txnrd1	-0.75	1.00000000	chicken
Tasp1	0.88	1.00000000	chicken
Cnot11	-0.51	1.00000000	chicken
Slc22a3	-0.82	1.00000000	chicken
Rnf8	-0.52	1.00000000	chicken
Pigyl	-0.44	1.00000000	chicken
Insig2	-0.46	1.00000000	chicken
Dusp7	-0.53	1.00000000	chicken
Nup153	0.45	1.00000000	chicken
P2rx7	0.48	1.00000000	chicken
Wrn	-0.75	1.00000000	chicken
Hspa4l	-0.73	1.00000000	chicken
Rnf111	0.46	1.00000000	chicken
Gtdc1	-0.82	1.00000000	chicken
Stag2	0.88	1.00000000	chicken
Psma4	-0.66	1.00000000	chicken
Gne	0.57	1.00000000	chicken
Tnks	0.45	1.00000000	chicken
Aldoc	-0.54	1.00000000	chicken
Abce1	-0.57	1.00000000	chicken
Ndfip1	-0.55	1.00000000	chicken
Snrnp35	0.54	1.00000000	chicken
Edf1	-0.53	1.00000000	chicken
Sod1	-0.44	1.00000000	chicken
Smg7	0.75	1.00000000	chicken
Pfdn6	-0.53	1.00000000	chicken

Fadd	0.29	1.00000000	chicken
Atp6v1e2	-0.54	1.00000000	chicken
Ndufv3	-0.44	1.00000000	chicken
Abca2	-0.62	1.00000000	chicken
Prpf4	-0.51	1.00000000	chicken
Eps15	0.43	1.00000000	chicken
Atp5g3	-0.56	1.00000000	chicken
Yars	0.38	1.00000000	chicken
Pmepa1	0.38	1.00000000	chicken
Fbxo33	-0.78	1.00000000	chicken
Zfp407	0.69	1.00000000	chicken
Ifi30	-0.49	1.00000000	chicken
Pigf	0.76	1.00000000	chicken
Pgs1	0.27	1.00000000	chicken
Pkn2	0.59	1.00000000	chicken
Secisbp2	0.32	1.00000000	chicken
Top2b	-0.32	1.00000000	chicken
Gtf2f2	0.88	1.00000000	chicken
Azin2	0.41	1.00000000	chicken
Vbp1	0.52	1.00000000	chicken
Atl2	0.39	1.00000000	chicken
Lsg1	-0.64	1.00000000	chicken
Rad54l2	0.27	1.00000000	chicken
Amdhd2	0.23	1.00000000	chicken
Ankrd39	-0.55	1.00000000	chicken
Pgap1	0.54	1.00000000	chicken
Kmt2e	0.63	1.00000000	chicken
Hpcal1	-0.65	1.00000000	chicken
Kpna1	0.68	1.00000000	chicken
Zc3h13	0.40	1.00000000	chicken
Ywhah	0.62	1.00000000	chicken
Dip2b	0.70	1.00000000	chicken
U2af2	-0.41	1.00000000	chicken
Stt3a	-0.56	1.00000000	chicken
Svbp	-0.51	1.00000000	chicken
Alg2	0.42	1.00000000	chicken
Plxnc1	-0.90	1.00000000	chicken
Smc4	1.12	1.00000000	chicken
Rere	0.37	1.00000000	chicken
Rdx	-0.61	1.00000000	chicken
Trip12	0.63	1.00000000	chicken
Cdc37	-0.61	1.00000000	chicken
Nol11	0.42	1.00000000	chicken
Abcb8	-0.45	1.00000000	chicken
Ddrgk1	-0.49	1.00000000	chicken
Tmem138	-0.44	1.00000000	chicken

Chst13	-0.51	1.00000000	chicken
Ppp2r2a	0.29	1.00000000	chicken
Romo1	-0.29	1.00000000	chicken
Brd1	0.41	1.00000000	chicken
Selenot	-0.65	1.00000000	chicken
Tomm22	-0.52	1.00000000	chicken
Lrmp	0.62	1.00000000	chicken
Pknox1	0.31	1.00000000	chicken
Ppp1r16b	0.45	1.00000000	chicken
Uhmk1	-0.43	1.00000000	chicken
Hao1	0.09	1.00000000	chicken
Cd83	0.56	1.00000000	chicken
Atp5h	-0.33	1.00000000	chicken
Akr1e1	-0.64	1.00000000	chicken
Garre1	0.78	1.00000000	chicken
Plcl2	0.97	1.00000000	chicken
Tia1	0.51	1.00000000	chicken
Cyp2r1	0.47	1.00000000	chicken
Bcl11b	-0.91	1.00000000	chicken
Esd	-0.49	1.00000000	chicken
Pax5	0.43	1.00000000	chicken
Cnot6	0.64	1.00000000	chicken
Kars	-0.33	1.00000000	chicken
Kifbp	0.46	1.00000000	chicken
Prrc2b	0.30	1.00000000	chicken
Tmem140	0.32	1.00000000	chicken
Tec	-0.67	1.00000000	chicken
Kansl1	0.39	1.00000000	chicken
Dgkz	-0.74	1.00000000	chicken
Luc7l3	-0.65	1.00000000	chicken
Gns	-0.59	1.00000000	chicken
Pla2r1	0.35	1.00000000	chicken
Nf2	0.34	1.00000000	chicken
Prpsap2	-0.41	1.00000000	chicken
B4galt6	0.36	1.00000000	chicken
Wwp1	-0.57	1.00000000	chicken
Qars	-0.40	1.00000000	chicken
Xbp1	-0.57	1.00000000	chicken
Sdhaf4	0.31	1.00000000	chicken
Setx	0.64	1.00000000	chicken
Fhod1	0.43	1.00000000	chicken
Ap2a1	0.77	1.00000000	chicken
Ctnnb1	0.49	1.00000000	chicken
Igf2r	-0.78	1.00000000	chicken
Ndufs4	-0.50	1.00000000	chicken
Derl1	-0.60	1.00000000	chicken

Rbm39	-0.26	1.00000000	chicken
Vrk2	0.76	1.00000000	chicken
Galc	-0.78	1.00000000	chicken
Rbbp7	-0.43	1.00000000	chicken
Elp4	0.65	1.00000000	chicken
Ulk1	-0.47	1.00000000	chicken
Psmb4	-0.53	1.00000000	chicken
Arhgdib	-0.55	1.00000000	chicken
Rab5if	-0.36	1.00000000	chicken
Dld	0.55	1.00000000	chicken
Traf6	0.36	1.00000000	chicken
Cntrl	0.58	1.00000000	chicken
Pik3c3	0.74	1.00000000	chicken
Cnnm2	-0.57	1.00000000	chicken
Plac8	0.51	1.00000000	chicken
Hspa4	-0.64	1.00000000	chicken
Gfod1	-0.78	1.00000000	chicken
Nrbp1	0.24	1.00000000	chicken
Cisd2	0.32	1.00000000	chicken
Nolc1	-0.60	1.00000000	chicken
Bsg	-0.51	1.00000000	chicken
Ensa	0.34	1.00000000	chicken
Togaram1	0.38	1.00000000	chicken
Adh5	-0.45	1.00000000	chicken
Dph5	0.21	1.00000000	chicken
Pitrm1	0.51	1.00000000	chicken
Pacc1	0.29	1.00000000	chicken
Ccnd1	-0.57	1.00000000	chicken
Wapl	0.42	1.00000000	chicken
Orc5	0.99	1.00000000	chicken
Grap2	-0.63	1.00000000	chicken
Abhd14b	-0.33	1.00000000	chicken
Ube2g1	0.53	1.00000000	chicken
Tbk1	0.56	1.00000000	chicken
Slc39a10	-0.25	1.00000000	chicken
Syngr2	0.37	1.00000000	chicken
C3	0.40	1.00000000	chicken
Hmces	-0.90	1.00000000	chicken
Slf2	0.70	1.00000000	chicken
Nlk	0.44	1.00000000	chicken
Tnrc6c	0.44	1.00000000	chicken
Klf13	-0.61	1.00000000	chicken
Tnik	-0.44	1.00000000	chicken
Ndufb1	-0.63	1.00000000	chicken
Cnot9	-0.40	1.00000000	chicken
Osbpl9	0.58	1.00000000	chicken

Utp20	-0.62	1.00000000	chicken
Ccdc61	0.24	1.00000000	chicken
Socs1	0.19	1.00000000	chicken
Il20ra	-1.05	1.00000000	chicken
Nck2	0.69	1.00000000	chicken
Sra1	0.34	1.00000000	chicken
Gadd45b	-0.68	1.00000000	chicken
Uqcrfs1	-0.45	1.00000000	chicken
Zfp148	0.60	1.00000000	chicken
Hmgb1	-0.59	1.00000000	chicken
Ank	-0.48	1.00000000	chicken
Ddx20	0.41	1.00000000	chicken
Tlk1	0.67	1.00000000	chicken
Zmiz1	-0.85	1.00000000	chicken
Arhgap39	-0.63	1.00000000	chicken
Tmem70	-0.39	1.00000000	chicken
Asah1	-0.52	1.00000000	chicken
Txndc16	-0.49	1.00000000	chicken
Cd28	-0.87	1.00000000	chicken
2900026A02Rik	-0.53	1.00000000	chicken
B3gntl1	-0.69	1.00000000	chicken
Ppp3r1	0.41	1.00000000	chicken
Zc3h12d	0.51	1.00000000	chicken
Tnpo1	0.81	1.00000000	chicken
Rab2a	-0.63	1.00000000	chicken
Slc36a4	0.81	1.00000000	chicken
Pde7a	0.50	1.00000000	chicken
4930453N24Rik	-0.49	1.00000000	chicken
Scg5	-0.49	1.00000000	chicken
Rfxap	-0.57	1.00000000	chicken
Skp1	-0.60	1.00000000	chicken
Ndufb5	-0.46	1.00000000	chicken
Gapvd1	0.48	1.00000000	chicken
Cnot2	0.48	1.00000000	chicken
Ranbp3	0.79	1.00000000	chicken
Cdc27	0.37	1.00000000	chicken
Pmpcb	-0.46	1.00000000	chicken
Fchsd2	-0.68	1.00000000	chicken
Tob2	-0.63	1.00000000	chicken
Tnrc6a	0.36	1.00000000	chicken
Cnot4	0.98	1.00000000	chicken
Lef1	-0.68	1.00000000	chicken
Scyl3	-0.37	1.00000000	chicken
Uaca	-0.64	1.00000000	chicken
Rabepk	-0.30	1.00000000	chicken
Chka	0.26	1.00000000	chicken

Ndufb7	-0.50	1.00000000	chicken
Ndufab1	-0.39	1.00000000	chicken
Ift80	0.20	1.00000000	chicken
Atp5j	-0.78	1.00000000	chicken
Cnih1	-0.42	1.00000000	chicken
Synrg	-0.45	1.00000000	chicken
Utp4	0.29	1.00000000	chicken
Cyrib	0.57	1.00000000	chicken
Ahsa1	-0.07	1.00000000	chicken
Kpnb1	-0.49	1.00000000	chicken
Ago1	0.50	1.00000000	chicken
Ndufc2	-0.38	1.00000000	chicken
Tcf12	0.67	1.00000000	chicken
Blnk	0.71	1.00000000	chicken
Zfp652	0.35	1.00000000	chicken
Vac14	-0.48	1.00000000	chicken
Impdh2	-0.53	1.00000000	chicken
Ublcp1	0.23	1.00000000	chicken
Adcy7	-0.49	1.00000000	chicken
Fasn	-0.66	1.00000000	chicken
Oga	-0.58	1.00000000	chicken
Runx2	-0.62	1.00000000	chicken
Itfg2	-0.42	1.00000000	chicken
Epcam	-0.44	1.00000000	chicken
Tyk2	-0.34	1.00000000	chicken
Wdr81	-0.56	1.00000000	chicken
Ralgps2	-0.82	1.00000000	chicken
Tfrc	-0.54	1.00000000	chicken
Exoc6b	0.31	1.00000000	chicken
Plagl1	0.53	1.00000000	chicken
Zfp740	0.29	1.00000000	chicken
Sec13	-0.62	1.00000000	chicken
Arid1b	0.78	1.00000000	chicken
Zfp451	0.51	1.00000000	chicken
Cpne1	0.54	1.00000000	chicken
Scaf8	0.22	1.00000000	chicken
Emc3	-0.46	1.00000000	chicken
Nans	-0.27	1.00000000	chicken
Wasf2	0.69	1.00000000	chicken
Ddx1	0.55	1.00000000	chicken
Vopp1	0.65	1.00000000	chicken
2210016F16Rik	0.59	1.00000000	chicken
Eif4g2	-0.50	1.00000000	chicken
Nemf	-0.53	1.00000000	chicken
Pald1	-0.45	1.00000000	chicken
Ddx24	-0.49	1.00000000	chicken

Angel2	0.42	1.00000000	chicken
Snap23	0.19	1.00000000	chicken
Sdhc	-0.41	1.00000000	chicken
Rpn1	-0.58	1.00000000	chicken
Gsr	-0.39	1.00000000	chicken
Arrdc3	-0.57	1.00000000	chicken
Gga3	0.26	1.00000000	chicken
Dcbld2	0.76	1.00000000	chicken
Ptpn7	-0.55	1.00000000	chicken
Tnip1	-0.49	1.00000000	chicken
Itpka	0.54	1.00000000	chicken
Cdk8	0.81	1.00000000	chicken
Rufy3	0.54	1.00000000	chicken
Fmnl1	-0.51	1.00000000	chicken
Ubash3b	-0.59	1.00000000	chicken
Vcl	0.24	1.00000000	chicken
Cdkn2aip	-0.43	1.00000000	chicken
Ankrd10	0.56	1.00000000	chicken
Eif4b	-0.55	1.00000000	chicken
Qsox1	-0.55	1.00000000	chicken
Itgav	-0.49	1.00000000	chicken
Adsl	-0.56	1.00000000	chicken
Prkaa1	0.31	1.00000000	chicken
Rad21	-0.61	1.00000000	chicken
Zfp598	0.35	1.00000000	chicken
Dstn	-0.45	1.00000000	chicken
Ak2	-0.39	1.00000000	chicken
Slc43a2	0.67	1.00000000	chicken
Arsg	0.10	1.00000000	chicken
Nol4l	0.75	1.00000000	chicken
Ubr5	0.57	1.00000000	chicken
Rabgap1l	-0.47	1.00000000	chicken
Rasgrp1	-0.59	1.00000000	chicken
Slamf1	0.28	1.00000000	chicken
Sh3rf3	0.47	1.00000000	chicken
Insr	0.18	1.00000000	chicken
Bnip2	-0.39	1.00000000	chicken
Tmf1	0.47	1.00000000	chicken
Syf2	-0.37	1.00000000	chicken
Fbxo22	0.61	1.00000000	chicken
Btaf1	0.73	1.00000000	chicken
Pik3cb	0.65	1.00000000	chicken
Arhgef9	0.72	1.00000000	chicken
Ssrp1	-0.44	1.00000000	chicken
Slc16a10	0.94	1.00000000	chicken
Ermp1	0.40	1.00000000	chicken

Ppp1r37	0.42	1.00000000	chicken
Szrd1	0.35	1.00000000	chicken
Naca	-0.41	1.00000000	chicken
Psmb7	-0.30	1.00000000	chicken
Osbpl8	-0.70	1.00000000	chicken
Timm17a	-0.47	1.00000000	chicken
Arglu1	0.45	1.00000000	chicken
Exosc2	-0.40	1.00000000	chicken
Rock2	0.56	1.00000000	chicken
Ubap2	-0.52	1.00000000	chicken
Wac	0.52	1.00000000	chicken
Orc3	0.51	1.00000000	chicken
Tfeb	0.23	1.00000000	chicken
Srp14	-0.62	1.00000000	chicken
Akt1	0.42	1.00000000	chicken
Eipr1	-0.24	1.00000000	chicken
Arl1	0.40	1.00000000	chicken
Pdia6	-0.42	1.00000000	chicken
Asxl2	0.46	1.00000000	chicken
Ptpn6	-0.32	1.00000000	chicken
Ahi1	0.85	1.00000000	chicken
Rnf13	0.50	1.00000000	chicken
Prpf8	-0.48	1.00000000	chicken
Ino80	0.35	1.00000000	chicken
N4bp1	-0.41	1.00000000	chicken
Sdk1	-0.54	1.00000000	chicken
Gng2	-0.52	1.00000000	chicken
Gan	-0.46	1.00000000	chicken
Bap1	0.19	1.00000000	chicken
Elmo1	-0.42	1.00000000	chicken
Scube2	0.40	1.00000000	chicken
Hvcn1	-0.39	1.00000000	chicken
Tcof1	-0.65	1.00000000	chicken
Prr5	0.71	1.00000000	chicken
Glrx5	-0.55	1.00000000	chicken
Pcnt	-0.47	1.00000000	chicken
Gnai3	-0.47	1.00000000	chicken
Polr1d	-0.39	1.00000000	chicken
Ampd3	0.24	1.00000000	chicken
Cse1l	-0.54	1.00000000	chicken
Tut4	0.47	1.00000000	chicken
Bcap29	-0.57	1.00000000	chicken
Hs6st2	-0.42	1.00000000	chicken
Stk3	0.83	1.00000000	chicken
Fbxo8	-0.65	1.00000000	chicken
Nutf2	-0.36	1.00000000	chicken

Etv5	0.27	1.00000000	chicken
Ip6k2	0.37	1.00000000	chicken
Pcid2	-0.44	1.00000000	chicken
Rars	-0.29	1.00000000	chicken
Lars	-0.44	1.00000000	chicken
Dock8	-0.56	1.00000000	chicken
Cib1	-0.46	1.00000000	chicken
Tnrc6b	0.37	1.00000000	chicken
Cyb5a	-0.29	1.00000000	chicken
Aoah	-0.44	1.00000000	chicken
Ola1	-0.61	1.00000000	chicken
Brsk2	-0.56	1.00000000	chicken
Tmtc4	0.39	1.00000000	chicken
Terf2	0.29	1.00000000	chicken
Txnip	0.48	1.00000000	chicken
Rbl1	-0.38	1.00000000	chicken
Pfn2	-0.34	1.00000000	chicken
Tmem223	-0.41	1.00000000	chicken
Nras	-0.36	1.00000000	chicken
Soat1	-0.51	1.00000000	chicken
Dtnbp1	0.58	1.00000000	chicken
Wdr70	-0.37	1.00000000	chicken
Tkt	-0.66	1.00000000	chicken
Pi4ka	0.95	1.00000000	chicken
Akr1a1	-0.42	1.00000000	chicken
Tmem186	-0.38	1.00000000	chicken
Rin3	-0.27	1.00000000	chicken
Clstn3	0.40	1.00000000	chicken
Trim8	0.47	1.00000000	chicken
Tpm1	-0.32	1.00000000	chicken
Tapt1	0.46	1.00000000	chicken
Rlf	-0.53	1.00000000	chicken
Ogfr	-0.36	1.00000000	chicken
Bclaf1	0.40	1.00000000	chicken
Rab39b	0.40	1.00000000	chicken
Vps13a	0.19	1.00000000	chicken
Fkbp4	-0.59	1.00000000	chicken
Rbpj	0.49	1.00000000	chicken
Wnk1	0.68	1.00000000	chicken
Ostc	-0.47	1.00000000	chicken
Nampt	0.33	1.00000000	chicken
Zer1	0.40	1.00000000	chicken
Nucb2	-0.44	1.00000000	chicken
Bnip3l	-0.67	1.00000000	chicken
Prdx6	-0.52	1.00000000	chicken
Ankrd13a	-0.55	1.00000000	chicken

Fam133b	-0.54	1.00000000	chicken
Ywhaq	-0.35	1.00000000	chicken
Ndufs2	0.46	1.00000000	chicken
Sap30l	-0.46	1.00000000	chicken
Gpn2	0.21	1.00000000	chicken
Cmtm3	-0.48	1.00000000	chicken
Ttc13	-0.58	1.00000000	chicken
Tmem65	-0.50	1.00000000	chicken
Ppp2r5e	0.51	1.00000000	chicken
Dyrk2	0.62	1.00000000	chicken
Rnf139	-0.32	1.00000000	chicken
Foxi1	-0.40	1.00000000	chicken
Nop16	0.22	1.00000000	chicken
Itch	0.38	1.00000000	chicken
Snx4	0.40	1.00000000	chicken
Snhg4	0.47	1.00000000	chicken
Parp1	-0.48	1.00000000	chicken
Helz	0.16	1.00000000	chicken
Grb2	0.30	1.00000000	chicken
Smarca4	-0.51	1.00000000	chicken
Ctns	0.48	1.00000000	chicken
Camsap2	0.64	1.00000000	chicken
Spcs1	-0.55	1.00000000	chicken
Tctn3	-0.24	1.00000000	chicken
Pard3	-0.87	1.00000000	chicken
Cdc42se2	0.88	1.00000000	chicken
Thyn1	-0.89	1.00000000	chicken
Gpi1	-0.39	1.00000000	chicken
Ppif	-0.50	1.00000000	chicken
Slc49a4	0.47	1.00000000	chicken
Arl13b	0.49	1.00000000	chicken
Nipa2	0.60	1.00000000	chicken
Cers5	0.36	1.00000000	chicken
Slf1	0.14	1.00000000	chicken
Ndufb4	-0.42	1.00000000	chicken
Sp4	0.38	1.00000000	chicken
Fahd2a	-0.41	1.00000000	chicken
Tardbp	0.44	1.00000000	chicken
Hnrnpd	0.28	1.00000000	chicken
Clock	0.62	1.00000000	chicken
Bckdhb	1.15	1.00000000	chicken
Swap70	0.46	1.00000000	chicken
Bpnt1	-0.43	1.00000000	chicken
Sccpdh	-0.36	1.00000000	chicken
Lmo2	0.63	1.00000000	chicken
Htra2	-0.46	1.00000000	chicken

Traf1	-0.47	1.00000000	chicken
Etnk1	0.37	1.00000000	chicken
Fbxw7	-0.55	1.00000000	chicken
Ift57	0.35	1.00000000	chicken
Rab12	0.53	1.00000000	chicken
Nedd4	0.75	1.00000000	chicken
Foxk1	0.58	1.00000000	chicken
P2ry6	-0.49	1.00000000	chicken
Sppl3	0.34	1.00000000	chicken
Cdv3	-0.47	1.00000000	chicken
Blk	0.74	1.00000000	chicken
Il6st	0.44	1.00000000	chicken
Naa50	-0.71	1.00000000	chicken
Clpx	-0.39	1.00000000	chicken
Ythdf1	-0.66	1.00000000	chicken
Dcaf13	-0.55	1.00000000	chicken
Ywhag	0.27	1.00000000	chicken
Cd164	-0.45	1.00000000	chicken
Ints6	0.44	1.00000000	chicken
Plekho1	-0.40	1.00000000	chicken
Fkbp8	0.52	1.00000000	chicken
Gimap5	0.35	1.00000000	chicken
Glipr2	0.29	1.00000000	chicken
Kdm4c	0.49	1.00000000	chicken
Smad2	-0.55	1.00000000	chicken
Bicral	0.49	1.00000000	chicken
Satb1	-0.91	1.00000000	chicken
Adrm1	0.01	1.00000000	chicken
Zfp36l2	-0.37	1.00000000	chicken
Rundc1	0.31	1.00000000	chicken
Ubox5	0.21	1.00000000	chicken
Ppp2ca	0.28	1.00000000	chicken
Eny2	-0.34	1.00000000	chicken
Trerf1	0.30	1.00000000	chicken
Kif3a	0.39	1.00000000	chicken
Dph6	-0.79	1.00000000	chicken
Ubr2	0.33	1.00000000	chicken
Arhgap12	0.40	1.00000000	chicken
Ntan1	0.35	1.00000000	chicken
Syne3	0.45	1.00000000	chicken
Btbd11	-0.65	1.00000000	chicken
Sfswap	0.35	1.00000000	chicken
Pacsin2	0.44	1.00000000	chicken
Dot1l	0.43	1.00000000	chicken
Rwdd1	-0.44	1.00000000	chicken
Wbp4	-0.43	1.00000000	chicken

Actr3	-0.51	1.00000000	chicken
Nhlrc2	0.53	1.00000000	chicken
Lonrf2	0.67	1.00000000	chicken
Bcar3	-0.59	1.00000000	chicken
Hint1	-0.21	1.00000000	chicken
Eif5	-0.59	1.00000000	chicken
Tmem243	0.42	1.00000000	chicken
Psmd6	-0.42	1.00000000	chicken
Cdc25b	-0.49	1.00000000	chicken
Senp7	0.34	1.00000000	chicken
S100a11	-0.28	1.00000000	chicken
Gpatch11	-0.15	1.00000000	chicken
Txn1	-0.42	1.00000000	chicken
Jmjd1c	0.20	1.00000000	chicken
Gpbp1l1	-0.30	1.00000000	chicken
Uqcc1	-0.15	1.00000000	chicken
Endov	0.15	1.00000000	chicken
Sdha	0.32	1.00000000	chicken
Ppp3cb	0.70	1.00000000	chicken
Napg	0.35	1.00000000	chicken
Abcf2	-0.47	1.00000000	chicken
Ubac2	0.39	1.00000000	chicken
Nr4a3	0.73	1.00000000	chicken
Crb1	0.46	1.00000000	chicken
Slc25a13	0.56	1.00000000	chicken
Ssr2	-0.59	1.00000000	chicken
Rnf214	-0.49	1.00000000	chicken
Trib2	-0.41	1.00000000	chicken
Als2	-0.46	1.00000000	chicken
Snrpe	-0.51	1.00000000	chicken
Psma2	-0.36	1.00000000	chicken
Ube2o	0.30	1.00000000	chicken
Sptan1	-0.44	1.00000000	chicken
Znhit6	0.66	1.00000000	chicken
Aldh5a1	-0.34	1.00000000	chicken
Ap3m2	-0.42	1.00000000	chicken
Bmf	0.32	1.00000000	chicken
Gabarapl2	-0.32	1.00000000	chicken
Ldhb	-0.60	1.00000000	chicken
Ebna1bp2	-0.16	1.00000000	chicken
Cd82	-0.60	1.00000000	chicken
Pef1	-0.33	1.00000000	chicken
Cnp	-0.38	1.00000000	chicken
Kyat3	0.40	1.00000000	chicken
Immt	-0.30	1.00000000	chicken
Fndc3a	0.53	1.00000000	chicken

Zbtb46	0.66	1.00000000	chicken
Nr4a2	0.37	1.00000000	chicken
Akap1	0.28	1.00000000	chicken
Radx	-0.43	1.00000000	chicken
Chd2	0.39	1.00000000	chicken
Tfdp1	-0.33	1.00000000	chicken
Dusp5	0.18	1.00000000	chicken
Blcap	-0.40	1.00000000	chicken
Adrb2	0.53	1.00000000	chicken
Bcl9l	0.56	1.00000000	chicken
Nars	-0.43	1.00000000	chicken
Ndufa5	-0.47	1.00000000	chicken
Csnk1g1	-0.42	1.00000000	chicken
Suco	-0.37	1.00000000	chicken
Ssb	-0.13	1.00000000	chicken
Prpf6	0.37	1.00000000	chicken
Pum3	-0.45	1.00000000	chicken
Parn	-0.34	1.00000000	chicken
Npc1	-0.44	1.00000000	chicken
Xpo1	-0.62	1.00000000	chicken
Ermn	0.39	1.00000000	chicken
Tctn1	0.51	1.00000000	chicken
Tcea2	0.15	1.00000000	chicken
Ldb1	-0.45	1.00000000	chicken
Pik3r4	0.25	1.00000000	chicken
Sdf4	0.80	1.00000000	chicken
Slc2a3	-0.42	1.00000000	chicken
Ubr7	-0.47	1.00000000	chicken
Trp53bp2	0.13	1.00000000	chicken
Vma21	-0.53	1.00000000	chicken
Rnpc3	-0.36	1.00000000	chicken
Aftph	0.84	1.00000000	chicken
Golgb1	-0.43	1.00000000	chicken
Nudt16l1	-0.40	1.00000000	chicken
Trmt10c	0.39	1.00000000	chicken
Stau1	-0.45	1.00000000	chicken
Tmco3	0.45	1.00000000	chicken
Gatad2b	0.30	1.00000000	chicken
Abcd2	-0.48	1.00000000	chicken
Pebp1	-0.46	1.00000000	chicken
Atxn2	0.31	1.00000000	chicken
Ctnna1	0.61	1.00000000	chicken
Pfdn2	-0.56	1.00000000	chicken
Pcf11	0.23	1.00000000	chicken
Ypel5	0.65	1.00000000	chicken
B3gnt4	-0.39	1.00000000	chicken

Slc39a6	-0.51	1.00000000	chicken
Taok1	0.61	1.00000000	chicken
Tnfsf13b	-0.66	1.00000000	chicken
Rnf10	0.16	1.00000000	chicken
Avl9	0.58	1.00000000	chicken
Nr4a1	0.15	1.00000000	chicken
Atp11a	0.14	1.00000000	chicken
Traf3ip1	0.32	1.00000000	chicken
Bcdin3d	0.12	1.00000000	chicken
Csnk1d	-0.06	1.00000000	chicken
Cox6a1	-0.57	1.00000000	chicken
Tanc1	0.28	1.00000000	chicken
Smarce1	-0.32	1.00000000	chicken
Ero1b	0.43	1.00000000	chicken
Arhgap15	0.18	1.00000000	chicken
Ostf1	-0.53	1.00000000	chicken
Klhl6	0.42	1.00000000	chicken
Ankrd11	0.27	1.00000000	chicken
Tomm70a	0.39	1.00000000	chicken
4933400A11Rik	-0.40	1.00000000	chicken
Timm13	-0.38	1.00000000	chicken
Qk	0.46	1.00000000	chicken
Sap30	-0.34	1.00000000	chicken
Atg13	0.21	1.00000000	chicken
St8sia4	0.53	1.00000000	chicken
Plrg1	0.22	1.00000000	chicken
Slc39a11	-0.46	1.00000000	chicken
Ttll5	-0.42	1.00000000	chicken
Cnn2	-0.46	1.00000000	chicken
Brca2	0.01	1.00000000	chicken
U2af1	0.29	1.00000000	chicken
Clns1a	-0.43	1.00000000	chicken
Llph	0.64	1.00000000	chicken
Snrpc	-0.38	1.00000000	chicken
Arpc5	-0.42	1.00000000	chicken
Was	-0.31	1.00000000	chicken
Clip1	0.38	1.00000000	chicken
Fam104a	0.36	1.00000000	chicken
Atxn7	0.68	1.00000000	chicken
Rngtt	-0.34	1.00000000	chicken
Osgin2	-0.45	1.00000000	chicken
Apoa1	0.23	1.00000000	chicken
Brip1	-0.44	1.00000000	chicken
Chst15	0.60	1.00000000	chicken
Pcsk7	-0.46	1.00000000	chicken
Psmd13	-0.17	1.00000000	chicken

Smad5	0.61	1.00000000	chicken
Pik3ap1	0.38	1.00000000	chicken
Rprd1b	-0.39	1.00000000	chicken
Rbm12	0.42	1.00000000	chicken
Nfatc2	-0.53	1.00000000	chicken
Cxcl13	0.04	1.00000000	chicken
Herpud1	0.27	1.00000000	chicken
Psmg4	-0.24	1.00000000	chicken
Stt3b	-0.36	1.00000000	chicken
Pdlim5	-0.66	1.00000000	chicken
Ralbp1	-0.09	1.00000000	chicken
Hspa8	-0.66	1.00000000	chicken
Tmed10	-0.45	1.00000000	chicken
Ascc2	-0.34	1.00000000	chicken
Tnfrsf9	-0.43	1.00000000	chicken
Rab7	0.06	1.00000000	chicken
Stk10	-0.42	1.00000000	chicken
Dapp1	0.30	1.00000000	chicken
Ubl3	0.37	1.00000000	chicken
Slx4	0.35	1.00000000	chicken
Pnrc2	0.31	1.00000000	chicken
Psmc2	-0.36	1.00000000	chicken
4932438A13Rik	0.46	1.00000000	chicken
Lats1	0.68	1.00000000	chicken
Aldh7a1	0.21	1.00000000	chicken
Svil	-0.43	1.00000000	chicken
Lsm12	0.44	1.00000000	chicken
Tpd52	-0.37	1.00000000	chicken
Otulin	-0.38	1.00000000	chicken
Derl2	-0.30	1.00000000	chicken
Usp24	0.51	1.00000000	chicken
Rbm6	0.66	1.00000000	chicken
Nup214	-0.43	1.00000000	chicken
Zfp706	-0.45	1.00000000	chicken
Ap1ar	-0.32	1.00000000	chicken
Ppip5k2	0.40	1.00000000	chicken
Bclaf3	0.62	1.00000000	chicken
Twnk	-0.39	1.00000000	chicken
Cct8	-0.25	1.00000000	chicken
Sbf1	-0.40	1.00000000	chicken
Abcb11	-0.50	1.00000000	chicken
Lyrm9	0.48	1.00000000	chicken
Rnf213	0.62	1.00000000	chicken
Psme3	-0.44	1.00000000	chicken
Decr1	-0.35	1.00000000	chicken
Tulp3	0.60	1.00000000	chicken

Rnf144a	0.03	1.00000000	chicken
Crebrf	0.52	1.00000000	chicken
Rft1	-0.44	1.00000000	chicken
Utp18	-0.29	1.00000000	chicken
Rexo2	-0.06	1.00000000	chicken
Spc25	-0.46	1.00000000	chicken
Hyou1	-0.52	1.00000000	chicken
Egr3	0.27	1.00000000	chicken
Slc15a4	0.46	1.00000000	chicken
Herc2	0.55	1.00000000	chicken
Tmed2	-0.41	1.00000000	chicken
Arl6ip5	-0.41	1.00000000	chicken
Akap8l	-0.41	1.00000000	chicken
Trio	0.80	1.00000000	chicken
Dap	0.16	1.00000000	chicken
Sbno1	0.19	1.00000000	chicken
Katnbl1	0.34	1.00000000	chicken
Ccdc186	0.42	1.00000000	chicken
Zmat5	0.02	1.00000000	chicken
5730455P16Rik	-0.35	1.00000000	chicken
Dazap2	-0.41	1.00000000	chicken
Ntsr1	-0.33	1.00000000	chicken
Scfd2	0.28	1.00000000	chicken
Selenof	0.42	1.00000000	chicken
Snrpf	-0.40	1.00000000	chicken
Stxbp1	-0.33	1.00000000	chicken
Scaper	0.19	1.00000000	chicken
Parvg	-0.01	1.00000000	chicken
Cept1	0.37	1.00000000	chicken
Hecw2	-0.57	1.00000000	chicken
Timp2	-0.34	1.00000000	chicken
Kbtbd2	0.28	1.00000000	chicken
Ippk	0.55	1.00000000	chicken
Csnk1a1	0.31	1.00000000	chicken
Ttbk1	0.39	1.00000000	chicken
Arl5a	0.37	1.00000000	chicken
Pim1	-0.39	1.00000000	chicken
Arl3	0.51	1.00000000	chicken
Snrpb	-0.30	1.00000000	chicken
Slc45a3	0.11	1.00000000	chicken
Arhgef3	0.36	1.00000000	chicken
Slc20a2	-0.12	1.00000000	chicken
Rb1cc1	0.58	1.00000000	chicken
Prpf39	-0.42	1.00000000	chicken
Clasp2	-0.41	1.00000000	chicken
Ubqln4	-0.30	1.00000000	chicken

Lpcat1	0.53	1.00000000	chicken
Tmem164	0.56	1.00000000	chicken
Rbms2	0.19	1.00000000	chicken
Fyttd1	0.39	1.00000000	chicken
Atp6v1b2	-0.52	1.00000000	chicken
Ube2e3	0.47	1.00000000	chicken
Ptcd3	0.21	1.00000000	chicken
Gemin5	-0.37	1.00000000	chicken
Uqcr11	-0.26	1.00000000	chicken
Commd8	-0.33	1.00000000	chicken
Zfp318	0.16	1.00000000	chicken
Psmd2	-0.33	1.00000000	chicken
Scaf4	0.22	1.00000000	chicken
Zzef1	-0.46	1.00000000	chicken
Ivns1abp	-0.26	1.00000000	chicken
Fbxl4	0.84	1.00000000	chicken
Ap1g1	0.53	1.00000000	chicken
Cyb5r4	-0.63	1.00000000	chicken
Cst3	-0.59	1.00000000	chicken
Fer	0.24	1.00000000	chicken
Ints8	-0.44	1.00000000	chicken
Sec11a	-0.45	1.00000000	chicken
Tnfrsf26	-0.36	1.00000000	chicken
Pola1	0.40	1.00000000	chicken
Acot10	-0.39	1.00000000	chicken
Uvrag	0.42	1.00000000	chicken
Rbm34	-0.28	1.00000000	chicken
Wipf1	0.37	1.00000000	chicken
Rprd2	-0.24	1.00000000	chicken
Spred2	-0.73	1.00000000	chicken
Eif4g3	0.48	1.00000000	chicken
Lpxn	0.42	1.00000000	chicken
Sik2	0.43	1.00000000	chicken
C4b	-0.27	1.00000000	chicken
Ndufb8	-0.38	1.00000000	chicken
Pals1	0.56	1.00000000	chicken
Fam241a	-0.41	1.00000000	chicken
Ireb2	0.29	1.00000000	chicken
Ubap1	0.32	1.00000000	chicken
Ncbp3	-0.37	1.00000000	chicken
Ptp4a1	-0.32	1.00000000	chicken
Taf4b	-0.11	1.00000000	chicken
Copz1	-0.30	1.00000000	chicken
Pbxip1	-0.36	1.00000000	chicken
Rheb	0.42	1.00000000	chicken
Rexo1	0.40	1.00000000	chicken

Slc23a2	0.39	1.00000000	chicken
Cd79b	0.20	1.00000000	chicken
Uri1	0.23	1.00000000	chicken
Zfp326	0.33	1.00000000	chicken
Tmem161b	0.32	1.00000000	chicken
Tmem87a	-0.32	1.00000000	chicken
Rhof	-0.37	1.00000000	chicken
Lcor	0.55	1.00000000	chicken
Immp1l	0.38	1.00000000	chicken
Arhgef11	0.26	1.00000000	chicken
Ik	-0.36	1.00000000	chicken
Thrap3	0.47	1.00000000	chicken
Exosc7	0.38	1.00000000	chicken
Zbtb34	0.51	1.00000000	chicken
Vezt	0.23	1.00000000	chicken
Itpripl2	0.16	1.00000000	chicken
Sf3b1	0.25	1.00000000	chicken
Stk17b	-0.54	1.00000000	chicken
Uqcrc1	-0.32	1.00000000	chicken
Rnps1	-0.18	1.00000000	chicken
Nkiras2	0.51	1.00000000	chicken
Zdhhc7	0.28	1.00000000	chicken
Lonp2	0.26	1.00000000	chicken
Cyfip2	-0.42	1.00000000	chicken
Bcl2l13	-0.41	1.00000000	chicken
Ess2	0.18	1.00000000	chicken
Ccdc47	-0.27	1.00000000	chicken
Tmcc3	0.20	1.00000000	chicken
Dip2a	-0.56	1.00000000	chicken
Khdc4	0.22	1.00000000	chicken
Whamm	0.30	1.00000000	chicken
Psmd1	-0.20	1.00000000	chicken
Gpx4	-0.20	1.00000000	chicken
Fam3c	-0.34	1.00000000	chicken
Tln1	-0.35	1.00000000	chicken
Polg2	0.43	1.00000000	chicken
Extl3	-0.13	1.00000000	chicken
Isca1	-0.30	1.00000000	chicken
Arnt	0.11	1.00000000	chicken
Nbas	-0.46	1.00000000	chicken
Edem1	0.00	1.00000000	chicken
Qpctl	-0.38	1.00000000	chicken
Naxd	-0.43	1.00000000	chicken
Pmm2	-0.35	1.00000000	chicken
Dtx2	0.33	1.00000000	chicken
Ncoa2	0.34	1.00000000	chicken

Pptc7	0.47	1.00000000	chicken
Strada	-0.24	1.00000000	chicken
Eif4a2	-0.20	1.00000000	chicken
Nat10	-0.14	1.00000000	chicken
Tmem192	-0.02	1.00000000	chicken
Rc3h2	0.40	1.00000000	chicken
Panx1	0.24	1.00000000	chicken
Abcb7	0.61	1.00000000	chicken
Grk3	0.36	1.00000000	chicken
Stau2	-0.39	1.00000000	chicken
Ift140	0.41	1.00000000	chicken
Dus1l	-0.30	1.00000000	chicken
Dph3	0.23	1.00000000	chicken
Skap2	0.56	1.00000000	chicken
Dnajc10	-0.44	1.00000000	chicken
Ccnl2	0.32	1.00000000	chicken
Edrf1	0.21	1.00000000	chicken
Ibtk	-0.53	1.00000000	chicken
Fry	0.41	1.00000000	chicken
Ciao2b	-0.27	1.00000000	chicken
Ndufa12	0.30	1.00000000	chicken
Psme4	-0.44	1.00000000	chicken
Zc3h6	0.28	1.00000000	chicken
Prkce	-0.77	1.00000000	chicken
Fkbp5	-0.22	1.00000000	chicken
Trp53rka	-0.20	1.00000000	chicken
Fam126a	-0.46	1.00000000	chicken
Tcf25	-0.43	1.00000000	chicken
Canx	-0.45	1.00000000	chicken
Zfand2b	-0.22	1.00000000	chicken
Sars	-0.19	1.00000000	chicken
Wdr48	-0.25	1.00000000	chicken
Plek	-0.38	1.00000000	chicken
Cd300c	-0.29	1.00000000	chicken
Akt2	-0.23	1.00000000	chicken
Polr2m	-0.24	1.00000000	chicken
Rcsd1	-0.37	1.00000000	chicken
Gramd1b	-0.33	1.00000000	chicken
Sec31a	-0.21	1.00000000	chicken
Igfbp7	-0.21	1.00000000	chicken
Slain2	-0.50	1.00000000	chicken
Bin2	-0.40	1.00000000	chicken
Cars	-0.37	1.00000000	chicken
Ttc27	-0.39	1.00000000	chicken
Npepl1	-0.27	1.00000000	chicken
Iqcb1	0.09	1.00000000	chicken

Otud4	0.23	1.00000000	chicken
Agap1	0.16	1.00000000	chicken
Chst2	0.03	1.00000000	chicken
Pphln1	1.03	1.00000000	chicken
Cox19	0.45	1.00000000	chicken
Pdap1	-0.33	1.00000000	chicken
Foxn2	0.76	1.00000000	chicken
Tbc1d23	0.54	1.00000000	chicken
Nploc4	-0.44	1.00000000	chicken
Stk38	0.36	1.00000000	chicken
Rfk	0.96	1.00000000	chicken
Tm9sf5	-0.37	1.00000000	chicken
Ube2q1	0.69	1.00000000	chicken
Commd2	-0.32	1.00000000	chicken
Ncstn	0.29	1.00000000	chicken
Cpsf3	-0.35	1.00000000	chicken
Cactin	-0.39	1.00000000	chicken
Hsf1	-0.41	1.00000000	chicken
Senp5	-0.18	1.00000000	chicken
Parp12	-0.26	1.00000000	chicken
Wdfy1	-0.28	1.00000000	chicken
Cybb	-0.26	1.00000000	chicken
Antxr2	0.17	1.00000000	chicken
Ppm1d	0.29	1.00000000	chicken
Coa5	-0.36	1.00000000	chicken
Tpcn1	-0.26	1.00000000	chicken
Txndc5	-0.40	1.00000000	chicken
Arpc3	-0.19	1.00000000	chicken
Xxylt1	0.34	1.00000000	chicken
Plekhm1	0.24	1.00000000	chicken
Ilrun	0.21	1.00000000	chicken
3110082I17Rik	-0.16	1.00000000	chicken
Zdhhc8	-0.54	1.00000000	chicken
Rbm5	0.35	1.00000000	chicken
Ascc3	0.50	1.00000000	chicken
Nudcd1	-0.29	1.00000000	chicken
Igf2bp3	0.51	1.00000000	chicken
Strap	0.00	1.00000000	chicken
Cramp1	0.32	1.00000000	chicken
Kdm2b	0.26	1.00000000	chicken
Dctn1	-0.17	1.00000000	chicken
Slc35b2	0.32	1.00000000	chicken
Lrp12	0.57	1.00000000	chicken
Pdxk	-0.35	1.00000000	chicken
Cry1	-0.38	1.00000000	chicken
Picalm	0.35	1.00000000	chicken

Gpatch2	0.48	1.00000000	chicken
Saraf	0.15	1.00000000	chicken
Haus2	-0.25	1.00000000	chicken
Rabl6	-0.06	1.00000000	chicken
BC048403	-0.33	1.00000000	chicken
Pepd	-0.57	1.00000000	chicken
Noc2l	-0.55	1.00000000	chicken
Atp1b3	-0.33	1.00000000	chicken
Ppcs	-0.33	1.00000000	chicken
Klf6	0.54	1.00000000	chicken
Smg6	-0.36	1.00000000	chicken
Srbd1	-0.20	1.00000000	chicken
Fzr1	0.14	1.00000000	chicken
Arfgef1	0.35	1.00000000	chicken
Lsm8	-0.24	1.00000000	chicken
Tmem135	0.78	1.00000000	chicken
Ezr	0.63	1.00000000	chicken
Arf4	0.30	1.00000000	chicken
Specc1l	0.24	1.00000000	chicken
Opa1	0.69	1.00000000	chicken
Zcchc14	0.44	1.00000000	chicken
Tpp2	-0.39	1.00000000	chicken
Crebbp	0.37	1.00000000	chicken
Bud13	0.23	1.00000000	chicken
Pstpip1	0.57	1.00000000	chicken
Rexo5	-0.30	1.00000000	chicken
Zdhhc6	-0.38	1.00000000	chicken
Rassf3	-0.32	1.00000000	chicken
Eef1e1	-0.43	1.00000000	chicken
Ddx18	0.37	1.00000000	chicken
Rfc1	-0.56	1.00000000	chicken
Grk4	0.48	1.00000000	chicken
Wdr47	0.24	1.00000000	chicken
Eml4	-0.32	1.00000000	chicken
Zcchc17	-0.32	1.00000000	chicken
Sdhaf2	-0.22	1.00000000	chicken
Atf6	0.18	1.00000000	chicken
Arpp19	-0.54	1.00000000	chicken
Eif3j2	-0.30	1.00000000	chicken
Ldha	-0.28	1.00000000	chicken
Wtap	0.28	1.00000000	chicken
Supt3	0.24	1.00000000	chicken
Acat1	-0.28	1.00000000	chicken
Cyp51	-0.31	1.00000000	chicken
Ptpre	-0.41	1.00000000	chicken
Pskh1	0.30	1.00000000	chicken

Atf1	0.26	1.00000000	chicken
Dbi	-0.26	1.00000000	chicken
Usp20	0.24	1.00000000	chicken
Fkbp1a	-0.32	1.00000000	chicken
Nbeal1	0.63	1.00000000	chicken
Siah1b	-0.09	1.00000000	chicken
Pak3	0.44	1.00000000	chicken
Sik1	-0.57	1.00000000	chicken
Lzic	-0.35	1.00000000	chicken
Rnf34	0.19	1.00000000	chicken
Slc16a3	-0.22	1.00000000	chicken
Rubcnl	0.24	1.00000000	chicken
Tfdp2	-0.48	1.00000000	chicken
St3gal2	0.17	1.00000000	chicken
Lcp2	-0.13	1.00000000	chicken
Atp5a1	-0.51	1.00000000	chicken
Slc12a7	-0.46	1.00000000	chicken
Rprd1a	0.50	1.00000000	chicken
Nrd1	-0.22	1.00000000	chicken
Ddx5	-0.26	1.00000000	chicken
Cog3	-0.31	1.00000000	chicken
Armh1	-0.39	1.00000000	chicken
Borcs5	0.60	1.00000000	chicken
P2ry4	-0.29	1.00000000	chicken
Pfkfb3	0.70	1.00000000	chicken
Irf8	0.28	1.00000000	chicken
Prkci	-0.29	1.00000000	chicken
Toe1	0.77	1.00000000	chicken
Fez2	0.19	1.00000000	chicken
Tvp23b	-0.35	1.00000000	chicken
Smn1	-0.33	1.00000000	chicken
Ndufaf2	0.62	1.00000000	chicken
Sos2	0.44	1.00000000	chicken
Tmem258	-0.26	1.00000000	chicken
Acp2	-0.21	1.00000000	chicken
Coro1c	-0.33	1.00000000	chicken
Sgms1	0.30	1.00000000	chicken
Lbh	-0.16	1.00000000	chicken
Arid2	0.28	1.00000000	chicken
Wwp2	-0.25	1.00000000	chicken
Vgll4	0.35	1.00000000	chicken
Eif2ak1	-0.43	1.00000000	chicken
Tbc1d25	0.16	1.00000000	chicken
Zfhx3	-0.44	1.00000000	chicken
Rpp14	-0.38	1.00000000	chicken
Ipo9	-0.19	1.00000000	chicken

Atm	-0.50	1.00000000	chicken
Atf2	0.43	1.00000000	chicken
Srpr	-0.23	1.00000000	chicken
Kctd20	0.41	1.00000000	chicken
Zfp609	0.00	1.00000000	chicken
Acot7	-0.33	1.00000000	chicken
Cct7	-0.31	1.00000000	chicken
Usp1	-0.26	1.00000000	chicken
Lamp2	-0.37	1.00000000	chicken
Trip11	-0.28	1.00000000	chicken
Bbln	0.27	1.00000000	chicken
BC005624	-0.26	1.00000000	chicken
Rfc5	-0.31	1.00000000	chicken
Sqstm1	0.47	1.00000000	chicken
Aifm1	-0.21	1.00000000	chicken
Emc1	-0.19	1.00000000	chicken
Eri1	0.10	1.00000000	chicken
Setd6	0.19	1.00000000	chicken
Cops7a	-0.19	1.00000000	chicken
Trappc8	0.74	1.00000000	chicken
Tcea1	-0.18	1.00000000	chicken
Atp1a1	-0.30	1.00000000	chicken
Cacul1	0.38	1.00000000	chicken
Cxcr5	0.22	1.00000000	chicken
Rara	-0.28	1.00000000	chicken
Hdx	0.10	1.00000000	chicken
Ankrd17	0.49	1.00000000	chicken
Rab22a	-0.15	1.00000000	chicken
Nhp2	-0.26	1.00000000	chicken
Setd1b	0.23	1.00000000	chicken
Park7	-0.30	1.00000000	chicken
Noc3l	0.24	1.00000000	chicken
Vps11	-0.39	1.00000000	chicken
Ndufb9	-0.26	1.00000000	chicken
Slc30a4	-0.14	1.00000000	chicken
Rsl24d1	-0.40	1.00000000	chicken
Rbm12b2	-0.05	1.00000000	chicken
Aip	0.20	1.00000000	chicken
Tax1bp1	0.22	1.00000000	chicken
Ctbp1	0.21	1.00000000	chicken
Dlgap4	0.37	1.00000000	chicken
Rtraf	-0.04	1.00000000	chicken
Trub1	0.36	1.00000000	chicken
Ufd1	-0.28	1.00000000	chicken
Pank3	-0.44	1.00000000	chicken
Zhx2	0.52	1.00000000	chicken

Kif2a	-0.47	1.00000000	chicken
Ankhd1	0.27	1.00000000	chicken
Syngr3	0.39	1.00000000	chicken
Celf1	0.49	1.00000000	chicken
Trim59	0.38	1.00000000	chicken
Erp44	0.27	1.00000000	chicken
Stab1	-0.50	1.00000000	chicken
Herc1	0.58	1.00000000	chicken
Tmem64	-0.44	1.00000000	chicken
Ubxn2a	0.20	1.00000000	chicken
Dync1li2	0.56	1.00000000	chicken
Ptpn2	0.25	1.00000000	chicken
Dync1li1	-0.35	1.00000000	chicken
Eif2ak2	0.35	1.00000000	chicken
Samsn1	0.78	1.00000000	chicken
Dis3l	0.30	1.00000000	chicken
Zbtb41	0.35	1.00000000	chicken
Sass6	0.61	1.00000000	chicken
Lao1	-0.47	1.00000000	chicken
Pggt1b	0.04	1.00000000	chicken
Taldo1	-0.22	1.00000000	chicken
Thop1	0.25	1.00000000	chicken
Oxsr1	-0.33	1.00000000	chicken
Srpk2	-0.36	1.00000000	chicken
Btf3l4	-0.29	1.00000000	chicken
2510002D24Rik	0.27	1.00000000	chicken
Rnf113a1	0.17	1.00000000	chicken
Abcc5	-0.26	1.00000000	chicken
Omg	0.30	1.00000000	chicken
Spsb1	0.15	1.00000000	chicken
Chchd1	-0.38	1.00000000	chicken
Ptprs	0.41	1.00000000	chicken
Dhx15	-0.44	1.00000000	chicken
Hlcs	-0.36	1.00000000	chicken
Atp6v1g1	-0.26	1.00000000	chicken
Id3	0.43	1.00000000	chicken
Rbm22	-0.29	1.00000000	chicken
Fh1	-0.33	1.00000000	chicken
Galnt2	0.26	1.00000000	chicken
1110059G10Rik	-0.36	1.00000000	chicken
Ndufaf4	0.51	1.00000000	chicken
Nupl1	0.17	1.00000000	chicken
Ubr1	0.50	1.00000000	chicken
Rnf11	-0.48	1.00000000	chicken
Fubp1	0.47	1.00000000	chicken
Efhd1	-0.42	1.00000000	chicken

Ndufb3	-0.39	1.00000000	chicken
Notch1	-0.38	1.00000000	chicken
Ascc1	-0.26	1.00000000	chicken
Irf7	0.35	1.00000000	chicken
Ckap5	0.35	1.00000000	chicken
Jade3	-0.47	1.00000000	chicken
Gxylt1	0.11	1.00000000	chicken
Amfr	0.19	1.00000000	chicken
Dek	-0.41	1.00000000	chicken
Tlk2	-0.36	1.00000000	chicken
BC048507	-0.28	1.00000000	chicken
Bcat1	0.49	1.00000000	chicken
Capza2	-0.37	1.00000000	chicken
Ranbp10	0.74	1.00000000	chicken
Smndc1	-0.29	1.00000000	chicken
Abhd17c	0.61	1.00000000	chicken
Copb1	0.39	1.00000000	chicken
Ddx59	0.20	1.00000000	chicken
Lta4h	0.13	1.00000000	chicken
Nkrf	-0.37	1.00000000	chicken
Ppp2r2d	0.28	1.00000000	chicken
Cct2	-0.03	1.00000000	chicken
Gpr65	-0.66	1.00000000	chicken
Gen1	0.32	1.00000000	chicken
Ly6e	0.42	1.00000000	chicken
Uprt	-0.50	1.00000000	chicken
Cnot6l	0.40	1.00000000	chicken
Cat	-0.35	1.00000000	chicken
Siah2	-0.35	1.00000000	chicken
Camsap1	0.17	1.00000000	chicken
Hcfc2	0.40	1.00000000	chicken
Cerk	-0.58	1.00000000	chicken
Rnf217	-0.36	1.00000000	chicken
Ifrd1	-0.17	1.00000000	chicken
Lpar5	0.29	1.00000000	chicken
Ppp4r1	-0.07	1.00000000	chicken
Ep300	0.23	1.00000000	chicken
Nxpe3	0.18	1.00000000	chicken
Lemd2	0.10	1.00000000	chicken
Actr2	-0.35	1.00000000	chicken
Tmem33	-0.33	1.00000000	chicken
Nfya	0.40	1.00000000	chicken
Fgd3	-0.25	1.00000000	chicken
Rbms1	-0.39	1.00000000	chicken
Rock1	-0.02	1.00000000	chicken
Erh	-0.31	1.00000000	chicken

Ktn1	-0.20	1.00000000	chicken
Cblb	0.25	1.00000000	chicken
Dcp2	0.39	1.00000000	chicken
Irf2bp2	0.11	1.00000000	chicken
Prmt7	0.48	1.00000000	chicken
Rhot1	0.33	1.00000000	chicken
Rnf103	0.37	1.00000000	chicken
Ythdf2	0.35	1.00000000	chicken
Pik3cd	-0.44	1.00000000	chicken
Yeats4	0.16	1.00000000	chicken
Ciao1	0.00	1.00000000	chicken
Odr4	0.27	1.00000000	chicken
Ubtf	0.29	1.00000000	chicken
Gtf2b	0.37	1.00000000	chicken
Abcc4	-0.51	1.00000000	chicken
Cyb561a3	-0.44	1.00000000	chicken
Slc24a4	-0.24	1.00000000	chicken
Gins3	0.51	1.00000000	chicken
Pnisr	0.27	1.00000000	chicken
Basp1	-0.20	1.00000000	chicken
Cpeb2	-0.67	1.00000000	chicken
Phc3	0.32	1.00000000	chicken
Anapc16	0.38	1.00000000	chicken
Zfp207	0.41	1.00000000	chicken
Wdr11	0.15	1.00000000	chicken
Tmem9b	-0.15	1.00000000	chicken
Ranbp9	0.19	1.00000000	chicken
Vps33b	-0.30	1.00000000	chicken
Hsp90b1	-0.17	1.00000000	chicken
Chn1	0.37	1.00000000	chicken
Erlin2	-0.11	1.00000000	chicken
Rbm17	-0.62	1.00000000	chicken
Fbxo5	0.23	1.00000000	chicken
Ints12	0.08	1.00000000	chicken
Snip1	0.21	1.00000000	chicken
Qdpr	-0.34	1.00000000	chicken
Taf2	0.51	1.00000000	chicken
Dnajc15	0.52	1.00000000	chicken
Hells	-0.39	1.00000000	chicken
Dip2c	0.62	1.00000000	chicken
Atp11b	0.25	1.00000000	chicken
Slc41a1	0.44	1.00000000	chicken
Nrbf2	0.36	1.00000000	chicken
D6Wsu163e	0.36	1.00000000	chicken
Stim2	0.25	1.00000000	chicken
Ctsb	-0.23	1.00000000	chicken

Sel1l	0.42	1.00000000	chicken
Rab27a	-0.33	1.00000000	chicken
Il2ra	0.37	1.00000000	chicken
Tmem184c	0.23	1.00000000	chicken
Slu7	0.13	1.00000000	chicken
Arhgap45	-0.31	1.00000000	chicken
Cd2ap	-0.24	1.00000000	chicken
Nudc	-0.21	1.00000000	chicken
Lrif1	-0.20	1.00000000	chicken
Rnf4	0.37	1.00000000	chicken
Stk11	0.40	1.00000000	chicken
Ddx6	-0.08	1.00000000	chicken
Chic2	0.30	1.00000000	chicken
Nmi	0.11	1.00000000	chicken
Xrn2	0.06	1.00000000	chicken
Pdcd5	-0.28	1.00000000	chicken
Usp15	-0.15	1.00000000	chicken
Zc3hav1l	0.52	1.00000000	chicken
Ccdc126	-0.37	1.00000000	chicken
Zranb1	0.23	1.00000000	chicken
Bin1	-0.10	1.00000000	chicken
Glipr1l1	0.17	1.00000000	chicken
Parp4	0.33	1.00000000	chicken
Xpo5	-0.19	1.00000000	chicken
Fam53a	0.21	1.00000000	chicken
Tmem259	0.12	1.00000000	chicken
Luc7l2	0.15	1.00000000	chicken
Ywhab	-0.42	1.00000000	chicken
Rapgef2	0.28	1.00000000	chicken
Eya3	0.31	1.00000000	chicken
Naf1	-0.25	1.00000000	chicken
Nup93	-0.04	1.00000000	chicken
Atp5k	-0.10	1.00000000	chicken
Pkm	-0.03	1.00000000	chicken
Zdhhc14	0.69	1.00000000	chicken
Dcun1d4	-0.50	1.00000000	chicken
Ctss	-0.29	1.00000000	chicken
Plekha1	-0.37	1.00000000	chicken
Gosr2	0.33	1.00000000	chicken
Ptprc	0.21	1.00000000	chicken
Pik3r6	0.16	1.00000000	chicken
Ncoa4	-0.35	1.00000000	chicken
Atp13a1	0.16	1.00000000	chicken
Pten	-0.19	1.00000000	chicken
Kif13a	0.73	1.00000000	chicken
Vps25	0.45	1.00000000	chicken

Sec63	0.47	1.00000000	chicken
St6gal1	0.28	1.00000000	chicken
Dipk1a	-0.33	1.00000000	chicken
Nfkb1	0.43	1.00000000	chicken
Ppp3ca	-0.14	1.00000000	chicken
Phf21a	0.50	1.00000000	chicken
Ptger4	0.43	1.00000000	chicken
Eif4ebp1	-0.33	1.00000000	chicken
Zcchc4	0.11	1.00000000	chicken
Kdm2a	0.41	1.00000000	chicken
Xiap	0.43	1.00000000	chicken
Pxk	0.35	1.00000000	chicken
Fos	-0.48	1.00000000	chicken
Paip1	0.54	1.00000000	chicken
Ppp1ca	-0.23	1.00000000	chicken
Dram1	0.11	1.00000000	chicken
Surf6	-0.23	1.00000000	chicken
Ruvbl1	-0.23	1.00000000	chicken
Rab35	-0.14	1.00000000	chicken
Ccm2	0.42	1.00000000	chicken
Tspan5	0.47	1.00000000	chicken
Nol9	-0.37	1.00000000	chicken
Ap2m1	-0.38	1.00000000	chicken
Atmin	0.07	1.00000000	chicken
Srebf2	-0.25	1.00000000	chicken
Vps35l	-0.10	1.00000000	chicken
Txn2	0.10	1.00000000	chicken
Upf3b	-0.28	1.00000000	chicken
St3gal6	-0.22	1.00000000	chicken
Psmc3	0.29	1.00000000	chicken
Casp7	0.12	1.00000000	chicken
Rnf146	0.11	1.00000000	chicken
Dnajb12	0.18	1.00000000	chicken
Lgals8	0.63	1.00000000	chicken
Csrp1	0.25	1.00000000	chicken
Setd3	0.31	1.00000000	chicken
Ppat	0.88	1.00000000	chicken
Stat4	0.45	1.00000000	chicken
Anapc1	0.40	1.00000000	chicken
Dpyd	0.65	1.00000000	chicken
Hsbp1	-0.41	1.00000000	chicken
C2cd5	-0.16	1.00000000	chicken
Eapp	0.19	1.00000000	chicken
Tdg	0.47	1.00000000	chicken
Cdkn1b	0.51	1.00000000	chicken
Fam98a	0.14	1.00000000	chicken

Casp2	0.43	1.00000000	chicken
Ppwd1	0.25	1.00000000	chicken
Kdsr	-0.37	1.00000000	chicken
Tollip	-0.14	1.00000000	chicken
Cdc42bpb	0.05	1.00000000	chicken
Eif1	-0.08	1.00000000	chicken
Atp2a2	0.33	1.00000000	chicken
Fbxw5	0.16	1.00000000	chicken
Pdhx	0.31	1.00000000	chicken
Pik3r5	-0.41	1.00000000	chicken
Nln	0.00	1.00000000	chicken
Elof1	0.21	1.00000000	chicken
Gosr1	0.44	1.00000000	chicken
Ccnl1	-0.25	1.00000000	chicken
Dbr1	0.17	1.00000000	chicken
Gpat4	-0.36	1.00000000	chicken
Capzb	0.41	1.00000000	chicken
Emg1	0.11	1.00000000	chicken
Golga3	0.05	1.00000000	chicken
Reep2	0.32	1.00000000	chicken
Sumo3	-0.36	1.00000000	chicken
Xpa	0.19	1.00000000	chicken
Klhl15	0.31	1.00000000	chicken
Lnpk	0.14	1.00000000	chicken
Dnajc18	-0.03	1.00000000	chicken
Hddc2	-0.25	1.00000000	chicken
Clcn7	-0.32	1.00000000	chicken
Cubn	0.29	1.00000000	chicken
Chst10	0.39	1.00000000	chicken
Sat1	-0.27	1.00000000	chicken
Taf8	0.22	1.00000000	chicken
Fam214a	-0.11	1.00000000	chicken
Slc30a9	-0.25	1.00000000	chicken
Yipf3	-0.36	1.00000000	chicken
Pdhb	-0.44	1.00000000	chicken
Tbc1d9b	0.29	1.00000000	chicken
Glt1d1	0.25	1.00000000	chicken
Ttc7	0.55	1.00000000	chicken
Uspl1	0.35	1.00000000	chicken
Ndufb10	0.06	1.00000000	chicken
Ccdc137	-0.26	1.00000000	chicken
Fbxl3	0.15	1.00000000	chicken
Rbm31y	0.32	1.00000000	chicken
Taok3	0.34	1.00000000	chicken
Rad23b	-0.49	1.00000000	chicken
Gfm1	-0.27	1.00000000	chicken

Arid5a	0.17	1.00000000	chicken
Sh3bp2	1.20	1.00000000	chicken
Samd11	-0.44	1.00000000	chicken
Rab4a	0.48	1.00000000	chicken
Nop2	-0.35	1.00000000	chicken
Wdr13	-0.14	1.00000000	chicken
Gnptab	0.57	1.00000000	chicken
Ist1	-0.38	1.00000000	chicken
Krr1	0.13	1.00000000	chicken
Champ1	-0.33	1.00000000	chicken
Ipo5	-0.15	1.00000000	chicken
Osbp2	0.17	1.00000000	chicken
Enpp4	-0.25	1.00000000	chicken
Nup85	-0.21	1.00000000	chicken
Srsf6	0.03	1.00000000	chicken
Ehmt1	0.61	1.00000000	chicken
Golga7	-0.30	1.00000000	chicken
Snap47	0.43	1.00000000	chicken
Tst	0.16	1.00000000	chicken
Vta1	0.23	1.00000000	chicken
Ccser2	-0.34	1.00000000	chicken
Papola	0.30	1.00000000	chicken
Ecd	-0.20	1.00000000	chicken
Cars2	0.19	1.00000000	chicken
Rnmt	-0.33	1.00000000	chicken
Rsu1	0.62	1.00000000	chicken
Szt2	0.10	1.00000000	chicken
Sfpq	-0.40	1.00000000	chicken
Aplp2	-0.11	1.00000000	chicken
Vps45	-0.02	1.00000000	chicken
Ilf2	-0.31	1.00000000	chicken
Fam120b	0.35	1.00000000	chicken
Tmed7	-0.22	1.00000000	chicken
Sbds	0.42	1.00000000	chicken
Tecpr2	0.63	1.00000000	chicken
Gnb1l	0.42	1.00000000	chicken
Lrp8	0.84	1.00000000	chicken
Lfng	0.14	1.00000000	chicken
Klhl7	-0.26	1.00000000	chicken
Cab39	-0.40	1.00000000	chicken
Snrpb2	-0.14	1.00000000	chicken
Slc6a6	-0.37	1.00000000	chicken
Hdac11	0.52	1.00000000	chicken
Strn	0.01	1.00000000	chicken
Naif1	-0.12	1.00000000	chicken
Lipa	-0.19	1.00000000	chicken

Fbxw8	0.50	1.00000000	chicken
Rab43	-0.10	1.00000000	chicken
Scap	-0.15	1.00000000	chicken
Stard3nl	-0.20	1.00000000	chicken
Golim4	-0.02	1.00000000	chicken
Ghitm	0.18	1.00000000	chicken
Washc4	0.35	1.00000000	chicken
Poll	0.05	1.00000000	chicken
Zbtb38	0.36	1.00000000	chicken
Spg7	-0.09	1.00000000	chicken
Bahcc1	-0.39	1.00000000	chicken
Coq9	-0.19	1.00000000	chicken
Nfrkb	0.28	1.00000000	chicken
Sgf29	0.06	1.00000000	chicken
Fbxo48	0.13	1.00000000	chicken
P4hb	-0.20	1.00000000	chicken
Polr2l	-0.35	1.00000000	chicken
Fmr1	0.45	1.00000000	chicken
Srsf2	-0.37	1.00000000	chicken
Arf1	-0.35	1.00000000	chicken
Pnpt1	-0.36	1.00000000	chicken
Trdmt1	0.27	1.00000000	chicken
Nckap1l	-0.30	1.00000000	chicken
Xrcc5	0.44	1.00000000	chicken
Sf3a1	-0.32	1.00000000	chicken
Cops4	0.21	1.00000000	chicken
Atp5d	-0.11	1.00000000	chicken
Stam2	-0.39	1.00000000	chicken
Pdcd11	-0.49	1.00000000	chicken
Virma	-0.18	1.00000000	chicken
Ube2i	-0.20	1.00000000	chicken
Rasgrp3	-0.41	1.00000000	chicken
Casp8ap2	0.62	1.00000000	chicken
Tab2	-0.02	1.00000000	chicken
Snx14	-0.34	1.00000000	chicken
Adss	-0.28	1.00000000	chicken
Zcchc8	-0.30	1.00000000	chicken
Ppp4r3a	0.24	1.00000000	chicken
Bop1	-0.25	1.00000000	chicken
Dedd	-0.44	1.00000000	chicken
Rab6a	-0.25	1.00000000	chicken
Klhdc1	0.82	1.00000000	chicken
Ddx51	0.23	1.00000000	chicken
Braf	0.47	1.00000000	chicken
Ddx23	0.39	1.00000000	chicken
Ikzf3	0.16	1.00000000	chicken

Dnmt3a	-0.28	1.00000000	chicken
Lrig1	-0.06	1.00000000	chicken
Snrpg	-0.40	1.00000000	chicken
Sil1	-0.22	1.00000000	chicken
Papss1	-0.33	1.00000000	chicken
Fnbp1	-0.01	1.00000000	chicken
Prdm10	0.21	1.00000000	chicken
Nvl	0.24	1.00000000	chicken
Psmb1	-0.20	1.00000000	chicken
Raf1	0.22	1.00000000	chicken
Acaca	0.26	1.00000000	chicken
Klhl2	0.40	1.00000000	chicken
Aatf	-0.33	1.00000000	chicken
Psmd12	0.27	1.00000000	chicken
Fbxw2	-0.16	1.00000000	chicken
Vps8	-0.19	1.00000000	chicken
Ctr9	-0.18	1.00000000	chicken
Lamc1	0.44	1.00000000	chicken
Ccnc	-0.34	1.00000000	chicken
Syk	0.46	1.00000000	chicken
Vps37c	0.42	1.00000000	chicken
Rab11fip2	0.25	1.00000000	chicken
Supt6	0.33	1.00000000	chicken
Nup107	-0.31	1.00000000	chicken
Ifnar2	0.29	1.00000000	chicken
Tmx3	0.02	1.00000000	chicken
Atp6v0d1	-0.33	1.00000000	chicken
Ccdc88c	-0.34	1.00000000	chicken
Uxs1	0.37	1.00000000	chicken
Taf5	-0.23	1.00000000	chicken
Ctdspl	0.36	1.00000000	chicken
Atg7	0.19	1.00000000	chicken
Creg1	-0.46	1.00000000	chicken
Tmx4	0.60	1.00000000	chicken
Dohh	0.12	1.00000000	chicken
Pign	0.13	1.00000000	chicken
Elp2	0.18	1.00000000	chicken
Foxj2	-0.19	1.00000000	chicken
Atp6v0b	-0.13	1.00000000	chicken
Jarid2	0.11	1.00000000	chicken
Hnrnph3	0.28	1.00000000	chicken
Stk24	0.36	1.00000000	chicken
Ift52	0.15	1.00000000	chicken
E2f4	-0.32	1.00000000	chicken
Cul1	0.62	1.00000000	chicken
Camk1d	0.00	1.00000000	chicken

Tram1l1	0.23	1.00000000	chicken
Prkdc	0.06	1.00000000	chicken
Cbx3	-0.10	1.00000000	chicken
Ern1	-0.43	1.00000000	chicken
Tcf20	0.19	1.00000000	chicken
Polr2d	0.26	1.00000000	chicken
Ly75	0.28	1.00000000	chicken
Nabp1	-0.37	1.00000000	chicken
Nfe2l2	-0.44	1.00000000	chicken
Dcun1d5	0.14	1.00000000	chicken
Slc7a11	-0.19	1.00000000	chicken
Slc38a6	-0.21	1.00000000	chicken
Cog7	-0.28	1.00000000	chicken
Icosl	-0.21	1.00000000	chicken
Dph1	-0.26	1.00000000	chicken
Trabd	0.34	1.00000000	chicken
Zfp831	0.22	1.00000000	chicken
Sf3a2	-0.24	1.00000000	chicken
Cass4	0.06	1.00000000	chicken
Ipo11	0.05	1.00000000	chicken
Gnl3	-0.30	1.00000000	chicken
Tgfbrap1	-0.12	1.00000000	chicken
Sgsm2	-0.19	1.00000000	chicken
Tent5a	-0.34	1.00000000	chicken
9530068E07Rik	0.51	1.00000000	chicken
Smap1	0.09	1.00000000	chicken
Nelfe	-0.25	1.00000000	chicken
Fam184a	0.52	1.00000000	chicken
Hnrnpa3	-0.20	1.00000000	chicken
Wdr12	0.09	1.00000000	chicken
Dhx37	-0.36	1.00000000	chicken
Klf11	-0.26	1.00000000	chicken
Rnf166	-0.38	1.00000000	chicken
Zeb2	-0.02	1.00000000	chicken
Aldh18a1	0.78	1.00000000	chicken
Cir1	-0.51	1.00000000	chicken
Cdk7	-0.11	1.00000000	chicken
Atf7ip	0.27	1.00000000	chicken
Arhgap27	0.06	1.00000000	chicken
Ppp1r7	0.32	1.00000000	chicken
Rmnd1	0.15	1.00000000	chicken
Fbxo38	-0.29	1.00000000	chicken
Ptdss1	0.44	1.00000000	chicken
Churc1	0.24	1.00000000	chicken
Pxylp1	0.29	1.00000000	chicken
Tbl3	-0.19	1.00000000	chicken

Esf1	-0.34	1.00000000	chicken
Cmss1	0.12	1.00000000	chicken
Acbd3	0.21	1.00000000	chicken
Creb3	-0.24	1.00000000	chicken
Diaph2	0.01	1.00000000	chicken
Gatad2a	0.51	1.00000000	chicken
Irf2	0.09	1.00000000	chicken
Psmd14	0.21	1.00000000	chicken
Cpne3	-0.24	1.00000000	chicken
Itprid2	-0.32	1.00000000	chicken
Dennd4c	0.23	1.00000000	chicken
Uqcrq	-0.24	1.00000000	chicken
Bicd2	0.35	1.00000000	chicken
Tnks2	-0.35	1.00000000	chicken
Efl1	0.37	1.00000000	chicken
Tpk1	-0.37	1.00000000	chicken
Gcn1	0.39	1.00000000	chicken
Bdh1	-0.18	1.00000000	chicken
Dnaja1	0.40	1.00000000	chicken
Sag	-0.03	1.00000000	chicken
Cyth3	0.18	1.00000000	chicken
Hspa9	-0.27	1.00000000	chicken
Fut8	0.12	1.00000000	chicken
Plekhd1	0.04	1.00000000	chicken
Sik3	0.05	1.00000000	chicken
Rel	0.61	1.00000000	chicken
Ndufs8	-0.30	1.00000000	chicken
Supv3l1	-0.33	1.00000000	chicken
Usp19	-0.15	1.00000000	chicken
Nup88	0.41	1.00000000	chicken
Camk2d	0.02	1.00000000	chicken
Rnf144b	0.19	1.00000000	chicken
Rrp9	0.24	1.00000000	chicken
Nkap	0.29	1.00000000	chicken
Eed	-0.19	1.00000000	chicken
Pds5b	-0.14	1.00000000	chicken
Anapc13	0.17	1.00000000	chicken
Rnf220	0.27	1.00000000	chicken
Nup50	0.16	1.00000000	chicken
Gpr180	-0.14	1.00000000	chicken
Prcc	-0.20	1.00000000	chicken
Adprh	0.24	1.00000000	chicken
Zfp236	0.13	1.00000000	chicken
Hk1	-0.32	1.00000000	chicken
Stard4	0.06	1.00000000	chicken
Txnrd3	0.01	1.00000000	chicken

Ttc3	-0.14	1.00000000	chicken
Hprt	-0.13	1.00000000	chicken
Pdxdc1	0.34	1.00000000	chicken
Adam23	0.03	1.00000000	chicken
Snrpd1	-0.32	1.00000000	chicken
Fam13b	0.12	1.00000000	chicken
Sirt7	0.11	1.00000000	chicken
Dnaja4	-0.48	1.00000000	chicken
Ei24	-0.32	1.00000000	chicken
Stx7	0.31	1.00000000	chicken
Timm50	0.15	1.00000000	chicken
Tes	-0.13	1.00000000	chicken
Pdk3	0.40	1.00000000	chicken
Sec31b	0.10	1.00000000	chicken
Nup210	-0.01	1.00000000	chicken
Limd1	0.24	1.00000000	chicken
Klhl36	-0.12	1.00000000	chicken
Atp6ap2	-0.33	1.00000000	chicken
Sdhb	-0.21	1.00000000	chicken
Nol8	-0.34	1.00000000	chicken
Itgb1	-0.23	1.00000000	chicken
Tmbim6	-0.36	1.00000000	chicken
Khdrbs1	0.13	1.00000000	chicken
Upf1	-0.27	1.00000000	chicken
Eprs	0.49	1.00000000	chicken
Vcp	-0.30	1.00000000	chicken
Helb	0.06	1.00000000	chicken
Larp1b	0.24	1.00000000	chicken
Atp6v1d	-0.36	1.00000000	chicken
Smap2	-0.13	1.00000000	chicken
Nphp1	0.46	1.00000000	chicken
Cul3	-0.27	1.00000000	chicken
Plekha3	-0.19	1.00000000	chicken
Rpap2	-0.32	1.00000000	chicken
Dctn2	-0.31	1.00000000	chicken
Wdr75	-0.36	1.00000000	chicken
Api5	-0.23	1.00000000	chicken
Anapc15-ps	0.04	1.00000000	chicken
Actr10	-0.27	1.00000000	chicken
Hscb	-0.12	1.00000000	chicken
Phf12	0.05	1.00000000	chicken
Fbxo32	0.51	1.00000000	chicken
Ehd3	0.60	1.00000000	chicken
Arhgap26	0.53	1.00000000	chicken
Cap1	-0.27	1.00000000	chicken
Bms1	-0.13	1.00000000	chicken

Slc25a26	0.01	1.00000000	chicken
Tbrg4	-0.20	1.00000000	chicken
Anxa2	0.25	1.00000000	chicken
Rtf2	0.02	1.00000000	chicken
Scpep1	-0.28	1.00000000	chicken
Ralgapb	0.29	1.00000000	chicken
Lmbrd2	-0.40	1.00000000	chicken
Tmem266	-0.07	1.00000000	chicken
Fkbp15	0.67	1.00000000	chicken
Gstm5	-0.25	1.00000000	chicken
Bag1	0.13	1.00000000	chicken
Tomm7	-0.22	1.00000000	chicken
Ythdc1	0.23	1.00000000	chicken
Lmbrd1	0.44	1.00000000	chicken
Xylt1	0.25	1.00000000	chicken
Adar	0.45	1.00000000	chicken
Bcas2	-0.10	1.00000000	chicken
Vim	0.33	1.00000000	chicken
C1qbp	-0.07	1.00000000	chicken
Vamp4	-0.20	1.00000000	chicken
Rnf126	-0.16	1.00000000	chicken
Trim24	-0.39	1.00000000	chicken
Atad5	-0.26	1.00000000	chicken
Jak1	-0.23	1.00000000	chicken
Prmt9	0.00	1.00000000	chicken
Cbx4	0.14	1.00000000	chicken
Sec61a1	-0.30	1.00000000	chicken
Fbxo11	0.24	1.00000000	chicken
Dr1	-0.12	1.00000000	chicken
Lin7c	-0.33	1.00000000	chicken
Tgs1	-0.39	1.00000000	chicken
Ube2f	0.12	1.00000000	chicken
Wbp1	-0.20	1.00000000	chicken
Washc2	-0.31	1.00000000	chicken
Sun1	0.46	1.00000000	chicken
Gramd4	-0.08	1.00000000	chicken
Fyco1	0.14	1.00000000	chicken
Cdc42	0.08	1.00000000	chicken
Ttc7b	-0.46	1.00000000	chicken
Adam10	0.19	1.00000000	chicken
Gorasp2	-0.28	1.00000000	chicken
Ankrd52	0.06	1.00000000	chicken
Adnp2	-0.21	1.00000000	chicken
2310033P09Rik	0.08	1.00000000	chicken
Rtn4	-0.28	1.00000000	chicken
Ncapd2	-0.13	1.00000000	chicken

Pms2	0.07	1.00000000	chicken
Gtpbp4	-0.31	1.00000000	chicken
Dlgap1	-0.11	1.00000000	chicken
Ube2l3	0.24	1.00000000	chicken
Pitpnm3	0.43	1.00000000	chicken
Zbed4	-0.17	1.00000000	chicken
Arl4a	0.29	1.00000000	chicken
Tnfsf10	-0.18	1.00000000	chicken
Trappc12	0.37	1.00000000	chicken
Hspd1	-0.43	1.00000000	chicken
Urb2	0.16	1.00000000	chicken
Slc25a24	0.28	1.00000000	chicken
Ppie	-0.09	1.00000000	chicken
Tmem168	0.25	1.00000000	chicken
Pnrc1	-0.14	1.00000000	chicken
Carhsp1	-0.23	1.00000000	chicken
Traf3ip3	0.22	1.00000000	chicken
Ttc17	-0.14	1.00000000	chicken
Trmt1l	-0.18	1.00000000	chicken
Arih1	0.20	1.00000000	chicken
Ccr7	0.38	1.00000000	chicken
Smarcd2	-0.14	1.00000000	chicken
Ufl1	0.53	1.00000000	chicken
Fam204a	-0.19	1.00000000	chicken
Lamp1	-0.28	1.00000000	chicken
Trappc13	0.13	1.00000000	chicken
Phax	-0.24	1.00000000	chicken
Ypel1	0.31	1.00000000	chicken
Vps13c	-0.41	1.00000000	chicken
Hlf	-0.33	1.00000000	chicken
Ppp1r13b	0.42	1.00000000	chicken
Rnf168	-0.06	1.00000000	chicken
Rasal2	-0.37	1.00000000	chicken
Sde2	-0.25	1.00000000	chicken
Nbeal2	-0.10	1.00000000	chicken
Prkca	-0.15	1.00000000	chicken
Fmnl2	0.36	1.00000000	chicken
Dcaf5	0.41	1.00000000	chicken
Naa20	-0.18	1.00000000	chicken
Wsb1	-0.12	1.00000000	chicken
Gnl2	-0.17	1.00000000	chicken
Celf2	-0.22	1.00000000	chicken
Ino80d	0.26	1.00000000	chicken
Lasp1	-0.30	1.00000000	chicken
Pfkp	0.62	1.00000000	chicken
Tm6sf1	-0.20	1.00000000	chicken

Actn1	0.04	1.00000000	chicken
Hook1	-0.25	1.00000000	chicken
Kpna3	-0.13	1.00000000	chicken
Ppp1cc	-0.18	1.00000000	chicken
Eif4g1	-0.12	1.00000000	chicken
Tdpoz8	0.38	1.00000000	chicken
Slc30a5	-0.08	1.00000000	chicken
Chpt1	0.00	1.00000000	chicken
Nek7	0.36	1.00000000	chicken
Jade1	0.12	1.00000000	chicken
Arhgap17	0.51	1.00000000	chicken
Srp54a	0.17	1.00000000	chicken
Abcg1	0.00	1.00000000	chicken
Zfp800	0.28	1.00000000	chicken
Ercc8	0.30	1.00000000	chicken
Rbsn	-0.12	1.00000000	chicken
Psmc5	-0.16	1.00000000	chicken
Nfkbib	0.31	1.00000000	chicken
Chsy1	-0.13	1.00000000	chicken
Rsrp1	0.14	1.00000000	chicken
Ghdc	-0.09	1.00000000	chicken
Tmem106b	-0.04	1.00000000	chicken
Cwc27	0.60	1.00000000	chicken
Qser1	0.04	1.00000000	chicken
Cyb5b	0.18	1.00000000	chicken
Glyr1	0.34	1.00000000	chicken
Zfp704	0.34	1.00000000	chicken
Sub1	-0.08	1.00000000	chicken
Dusp10	0.45	1.00000000	chicken
Ndufa10	-0.21	1.00000000	chicken
Vps50	0.31	1.00000000	chicken
Cop1	0.36	1.00000000	chicken
Camkmt	-0.02	1.00000000	chicken
Phb2	0.15	1.00000000	chicken
Unc119b	-0.16	1.00000000	chicken
Rab3gap2	-0.32	1.00000000	chicken
Dusp23	-0.21	1.00000000	chicken
Vps13d	0.63	1.00000000	chicken
Lbr	0.33	1.00000000	chicken
Setd5	0.24	1.00000000	chicken
Slc35e2	-0.21	1.00000000	chicken
Sap30bp	0.40	1.00000000	chicken
Hsf3	0.12	1.00000000	chicken
Rnf19a	0.47	1.00000000	chicken
Hira	0.40	1.00000000	chicken
Laptm4a	-0.19	1.00000000	chicken

Spata5	0.16	1.00000000	chicken
Cox10	0.49	1.00000000	chicken
Ddx27	0.34	1.00000000	chicken
Dcun1d3	-0.14	1.00000000	chicken
Aktip	0.86	1.00000000	chicken
Usp22	0.13	1.00000000	chicken
Polr2e	-0.23	1.00000000	chicken
Far2	0.01	1.00000000	chicken
Tvp23a	0.20	1.00000000	chicken
Nup155	-0.19	1.00000000	chicken
Notch2	0.22	1.00000000	chicken
Prelid3b	0.39	1.00000000	chicken
Vpreb3	-0.59	1.00000000	chicken
Pag1	0.31	1.00000000	chicken
Atp10a	0.02	1.00000000	chicken
Ogfod3	0.30	1.00000000	chicken
Exoc4	0.38	1.00000000	chicken
BC005537	0.07	1.00000000	chicken
Cdk12	0.17	1.00000000	chicken
Tfg	-0.31	1.00000000	chicken
Pcmtd1	0.14	1.00000000	chicken
Utp23	-0.20	1.00000000	chicken
Sel1l3	-0.13	1.00000000	chicken
Gyg	-0.08	1.00000000	chicken
Gtf2e1	0.34	1.00000000	chicken
Lonrf3	0.28	1.00000000	chicken
Crip1	0.26	1.00000000	chicken
Bri3	-0.28	1.00000000	chicken
Ankrd28	-0.18	1.00000000	chicken
Zfand6	0.24	1.00000000	chicken
Psmb2	-0.28	1.00000000	chicken
Rrp7a	-0.01	1.00000000	chicken
Acsbg2	-0.27	1.00000000	chicken
Polr3e	-0.06	1.00000000	chicken
Tmem120b	0.65	1.00000000	chicken
Hipk1	-0.07	1.00000000	chicken
Prim2	0.05	1.00000000	chicken
Slirp	0.21	1.00000000	chicken
Zfyve9	0.28	1.00000000	chicken
Pcmt1	0.12	1.00000000	chicken
Srf	-0.14	1.00000000	chicken
Srm	-0.36	1.00000000	chicken
Nup205	0.31	1.00000000	chicken
Alkbh5	0.18	1.00000000	chicken
Cbll1	-0.27	1.00000000	chicken
Wdr91	0.22	1.00000000	chicken

Ikbkb	-0.21	1.00000000	chicken
Gpn3	0.35	1.00000000	chicken
Pias2	0.28	1.00000000	chicken
Atp8a1	0.39	1.00000000	chicken
Lap3	0.31	1.00000000	chicken
Vps29	-0.29	1.00000000	chicken
Aplf	-0.05	1.00000000	chicken
Vapb	0.26	1.00000000	chicken
Traf2	-0.21	1.00000000	chicken
Psma1	-0.04	1.00000000	chicken
Prkd1	0.61	1.00000000	chicken
Cox16	0.35	1.00000000	chicken
P4ha1	-0.07	1.00000000	chicken
2210408I21Rik	0.68	1.00000000	chicken
Acsl3	-0.39	1.00000000	chicken
Slc7a6os	-0.30	1.00000000	chicken
Fancm	-0.19	1.00000000	chicken
Fxr1	0.14	1.00000000	chicken
Slc16a1	0.05	1.00000000	chicken
Rnf149	-0.23	1.00000000	chicken
Golga4	0.93	1.00000000	chicken
Dnajc3	-0.05	1.00000000	chicken
Rnf114	0.00	1.00000000	chicken
Abhd2	0.11	1.00000000	chicken
Zfp821	0.03	1.00000000	chicken
Smc3	0.26	1.00000000	chicken
AW554918	0.04	1.00000000	chicken
Tbcd	-0.17	1.00000000	chicken
Rbmxl1	0.12	1.00000000	chicken
Ccdc59	-0.27	1.00000000	chicken
Glb1	0.29	1.00000000	chicken
Pex14	0.30	1.00000000	chicken
Tnfrsf21	-0.30	1.00000000	chicken
Pdcd6	0.50	1.00000000	chicken
Ube2r2	-0.36	1.00000000	chicken
Pdia4	-0.18	1.00000000	chicken
Sacs	-0.23	1.00000000	chicken
Fam168b	0.11	1.00000000	chicken
Gp1bb	-0.22	1.00000000	chicken
Slmap	0.10	1.00000000	chicken
Runx3	-0.18	1.00000000	chicken
Arl15	0.70	1.00000000	chicken
Aspm	0.39	1.00000000	chicken
Tiparp	-0.28	1.00000000	chicken
Gspt1	0.21	1.00000000	chicken
Pik3cg	-0.16	1.00000000	chicken

Adarb1	0.22	1.00000000	chicken
Zfp654	0.29	1.00000000	chicken
Thoc1	-0.33	1.00000000	chicken
Ss18	0.11	1.00000000	chicken
Lrrc8d	0.06	1.00000000	chicken
Hmgcr	0.18	1.00000000	chicken
Ddost	-0.20	1.00000000	chicken
Zfp639	0.19	1.00000000	chicken
Ubxn7	-0.17	1.00000000	chicken
Nasp	-0.25	1.00000000	chicken
Lrch2	0.51	1.00000000	chicken
Acaa2	0.35	1.00000000	chicken
Ppil1	0.04	1.00000000	chicken
Gtf3c6	0.26	1.00000000	chicken
Rpap3	0.55	1.00000000	chicken
Asf1a	-0.41	1.00000000	chicken
Atrx	0.19	1.00000000	chicken
Dtwd2	0.26	1.00000000	chicken
Nfe2l1	0.44	1.00000000	chicken
Bcor	0.37	1.00000000	chicken
Sh3glb1	0.16	1.00000000	chicken
Cops8	0.09	1.00000000	chicken
Ptch1	-0.27	1.00000000	chicken
Dhx30	0.05	1.00000000	chicken
Rffl	0.16	1.00000000	chicken
Atf4	-0.29	1.00000000	chicken
Nsrp1	-0.01	1.00000000	chicken
Ctsc	-0.08	1.00000000	chicken
Wdr20	0.55	1.00000000	chicken
Bmp2k	0.49	1.00000000	chicken
Slc35e3	0.19	1.00000000	chicken
Prpf19	-0.15	1.00000000	chicken
Eloc	0.04	1.00000000	chicken
Clcn5	-0.46	1.00000000	chicken
Llgl2	-0.44	1.00000000	chicken
Ice2	0.17	1.00000000	chicken
Bcl6	-0.07	1.00000000	chicken
Appl2	-0.21	1.00000000	chicken
Napa	-0.28	1.00000000	chicken
Srp9	-0.33	1.00000000	chicken
Senp6	0.48	1.00000000	chicken
Kat2b	0.16	1.00000000	chicken
Kif5c	0.37	1.00000000	chicken
Rae1	-0.15	1.00000000	chicken
Tuba1b	0.08	1.00000000	chicken
Upf2	0.00	1.00000000	chicken

Pdpk1	0.07	1.00000000	chicken
Smarca2	0.02	1.00000000	chicken
Rab8a	-0.24	1.00000000	chicken
Irs4	0.01	1.00000000	chicken
Otud6b	-0.18	1.00000000	chicken
Ifi35	-0.12	1.00000000	chicken
Cep89	0.17	1.00000000	chicken
Pomp	0.37	1.00000000	chicken
Atp2a3	-0.12	1.00000000	chicken
Srsf7	-0.36	1.00000000	chicken
Spag9	0.05	1.00000000	chicken
Pex16	-0.08	1.00000000	chicken
Ndufs1	-0.10	1.00000000	chicken
Paics	-0.10	1.00000000	chicken
Gnpat	0.16	1.00000000	chicken
Lrrc28	-0.15	1.00000000	chicken
Ncoa6	-0.31	1.00000000	chicken
Arl6ip4	0.22	1.00000000	chicken
Sp3	0.15	1.00000000	chicken
Acbd6	0.03	1.00000000	chicken
Fbxo9	0.29	1.00000000	chicken
Stk26	0.23	1.00000000	chicken
Rad18	-0.20	1.00000000	chicken
Ppa2	0.11	1.00000000	chicken
Aqr	-0.12	1.00000000	chicken
Phkb	-0.30	1.00000000	chicken
Sirt1	0.03	1.00000000	chicken
Spryd7	-0.11	1.00000000	chicken
Cog2	0.00	1.00000000	chicken
Nap1l4	0.14	1.00000000	chicken
Ap1s2	0.00	1.00000000	chicken
Nop56	0.20	1.00000000	chicken
Ncbp1	-0.03	1.00000000	chicken
Ski	-0.04	1.00000000	chicken
Tatdn3	-0.24	1.00000000	chicken
Ddhd1	-0.31	1.00000000	chicken
Niban1	0.16	1.00000000	chicken
Tmcc1	0.43	1.00000000	chicken
Ctcf	0.31	1.00000000	chicken
Vps37b	-0.12	1.00000000	chicken
Tmco1	0.02	1.00000000	chicken
Rgs9	-0.32	1.00000000	chicken
Supt4a	0.33	1.00000000	chicken
Stxbp3	-0.30	1.00000000	chicken
Pak1ip1	-0.12	1.00000000	chicken
Bcap31	-0.21	1.00000000	chicken

Hdac7	0.16	1.00000000	chicken
Ndufa6	0.00	1.00000000	chicken
Trpc4ap	0.31	1.00000000	chicken
Nsmce2	0.26	1.00000000	chicken
Vps41	0.67	1.00000000	chicken
Btbd7	0.35	1.00000000	chicken
Aff2	0.08	1.00000000	chicken
Tprgl	-0.25	1.00000000	chicken
Kmt5b	0.06	1.00000000	chicken
Rab1a	-0.07	1.00000000	chicken
Brd2	-0.01	1.00000000	chicken
Slk	-0.11	1.00000000	chicken
Cyfip1	-0.30	1.00000000	chicken
Dnajb14	0.44	1.00000000	chicken
Snx8	-0.32	1.00000000	chicken
Polr1f	0.12	1.00000000	chicken
Adk	0.00	1.00000000	chicken
Arid4a	0.14	1.00000000	chicken
Tpi1	-0.26	1.00000000	chicken
Zfp365	0.29	1.00000000	chicken
Xpr1	-0.21	1.00000000	chicken
Babam2	0.45	1.00000000	chicken
Eif3i	-0.26	1.00000000	chicken
Nudt7	-0.12	1.00000000	chicken
Hras	0.04	1.00000000	chicken
Ccndbp1	0.30	1.00000000	chicken
Atrip	-0.21	1.00000000	chicken
Pdpr	0.24	1.00000000	chicken
Tiam2	0.49	1.00000000	chicken
Bbs7	-0.13	1.00000000	chicken
Pigk	-0.05	1.00000000	chicken
Gpr137b	0.27	1.00000000	chicken
Ncoa5	-0.16	1.00000000	chicken
Dcun1d1	-0.23	1.00000000	chicken
Chuk	-0.31	1.00000000	chicken
Rmnd5a	-0.19	1.00000000	chicken
Bivm	-0.30	1.00000000	chicken
Anapc10	0.28	1.00000000	chicken
Arl8a	-0.07	1.00000000	chicken
Rcan1	0.16	1.00000000	chicken
Chchd2	-0.23	1.00000000	chicken
Casc3	0.14	1.00000000	chicken
Ranbp1	-0.34	1.00000000	chicken
Exoc5	-0.29	1.00000000	chicken
Ip6k1	0.02	1.00000000	chicken
Igf1r	-0.24	1.00000000	chicken

Klhl42	-0.31	1.00000000	chicken
Emc2	-0.10	1.00000000	chicken
Wdr59	0.13	1.00000000	chicken
Ociad1	-0.15	1.00000000	chicken
Steep1	-0.10	1.00000000	chicken
Pdcl3	-0.05	1.00000000	chicken
Nop14	-0.11	1.00000000	chicken
Zfp438	-0.04	1.00000000	chicken
Stxbp5	0.76	1.00000000	chicken
Sarnp	0.07	1.00000000	chicken
Hsp90aa1	-1.86	1.00000000	chicken
Erich1	-0.28	1.00000000	chicken
Pspc1	0.15	1.00000000	chicken
Pum1	0.05	1.00000000	chicken
Eif2b5	0.24	1.00000000	chicken
Cdk11b	0.03	1.00000000	chicken
Ilf3	0.02	1.00000000	chicken
Usp6nl	0.65	1.00000000	chicken
Lrrc8b	0.22	1.00000000	chicken
Ltn1	-0.34	1.00000000	chicken
Brd7	-0.20	1.00000000	chicken
Commd10	0.24	1.00000000	chicken
Nxt2	0.12	1.00000000	chicken
Serinc1	0.01	1.00000000	chicken
Zfp830	-0.34	1.00000000	chicken
G3bp2	-0.34	1.00000000	chicken
Sharpin	0.07	1.00000000	chicken
Rad51b	-0.10	1.00000000	chicken
Cbr1	-0.12	1.00000000	chicken
Aff4	0.47	1.00000000	chicken
Tfip11	0.48	1.00000000	chicken
Cct5	0.34	1.00000000	chicken
Rnf145	-0.39	1.00000000	chicken
Tbck	0.27	1.00000000	chicken
Ruvbl2	0.20	1.00000000	chicken
Alkbh8	0.11	1.00000000	chicken
Yaf2	-0.12	1.00000000	chicken
Nckipsd	0.40	1.00000000	chicken
Tmem50b	0.07	1.00000000	chicken
Zfand5	-0.18	1.00000000	chicken
Zswim8	-0.39	1.00000000	chicken
Inpp5k	-0.07	1.00000000	chicken
Riok1	-0.22	1.00000000	chicken
Lamb1	0.10	1.00000000	chicken
Lrpprc	0.40	1.00000000	chicken
Dmxl1	-0.15	1.00000000	chicken

Iws1	0.26	1.00000000	chicken
Dnajc13	0.24	1.00000000	chicken
Pde4b	0.26	1.00000000	chicken
Osbpl5	-0.12	1.00000000	chicken
Smim14	0.04	1.00000000	chicken
Rbm38	-0.45	1.00000000	chicken
Cebpz	-0.05	1.00000000	chicken
Rnls	0.23	1.00000000	chicken
Tssc4	-0.10	1.00000000	chicken
Gzf1	-0.13	1.00000000	chicken
Phf14	0.29	1.00000000	chicken
Riok3	0.08	1.00000000	chicken
Ints13	0.41	1.00000000	chicken
Cnot10	0.14	1.00000000	chicken
Ube2d1	-0.12	1.00000000	chicken
Emc6	-0.01	1.00000000	chicken
Pigv	-0.13	1.00000000	chicken
Ctps	-0.09	1.00000000	chicken
Btk	-0.19	1.00000000	chicken
Napepld	0.23	1.00000000	chicken
Ngly1	-0.01	1.00000000	chicken
Ttc33	0.13	1.00000000	chicken
Unc45a	0.00	1.00000000	chicken
Rnf38	0.53	1.00000000	chicken
Klf12	-0.10	1.00000000	chicken
Wdr4	-0.01	1.00000000	chicken
Agpat5	0.06	1.00000000	chicken
Wdr7	0.16	1.00000000	chicken
Suclg1	-0.11	1.00000000	chicken
Tom1l2	0.09	1.00000000	chicken
Sar1b	0.03	1.00000000	chicken
Eefsec	-0.18	1.00000000	chicken
Appl1	-0.32	1.00000000	chicken
Ppig	-0.16	1.00000000	chicken
Gdpd5	0.43	1.00000000	chicken
Smdt1	0.27	1.00000000	chicken
Lamtor2	-0.12	1.00000000	chicken
Sos1	0.18	1.00000000	chicken
Zranb2	-0.15	1.00000000	chicken
Tlr7	0.17	1.00000000	chicken
Prrc2c	-0.19	1.00000000	chicken
Lpin2	-0.10	1.00000000	chicken
Trp73	-0.24	1.00000000	chicken
Lemd3	-0.14	1.00000000	chicken
Pum2	0.09	1.00000000	chicken
Gpr171	0.15	1.00000000	chicken

Appbp2	0.16	1.00000000	chicken
Tmem30a	-0.17	1.00000000	chicken
Zc3h15	-0.16	1.00000000	chicken
Stk4	-0.16	1.00000000	chicken
Pak2	0.24	1.00000000	chicken
Prpf4b	0.30	1.00000000	chicken
Scyl2	-0.23	1.00000000	chicken
Gls	0.30	1.00000000	chicken
Zrsr1	0.42	1.00000000	chicken
Slc38a2	0.10	1.00000000	chicken
Fdft1	0.05	1.00000000	chicken
Ckap4	-0.03	1.00000000	chicken
Rnf130	0.30	1.00000000	chicken
Ppid	0.38	1.00000000	chicken
Rbbp6	0.17	1.00000000	chicken
Prkcb	0.39	1.00000000	chicken
Ino80c	-0.02	1.00000000	chicken
Fra10ac1	0.35	1.00000000	chicken
Utp15	-0.24	1.00000000	chicken
Dusp14	0.22	1.00000000	chicken
Atp1b1	-0.26	1.00000000	chicken
Pms1	-0.08	1.00000000	chicken
Rufy1	-0.39	1.00000000	chicken
Vps53	0.03	1.00000000	chicken
Aacs	0.12	1.00000000	chicken
Brms1l	0.37	1.00000000	chicken
Vti1a	-0.22	1.00000000	chicken
Clint1	-0.09	1.00000000	chicken
Abtb1	-0.13	1.00000000	chicken
Klhl14	-0.01	1.00000000	chicken
Dlst	-0.18	1.00000000	chicken
Ubn1	-0.21	1.00000000	chicken
Rab5b	0.20	1.00000000	chicken
Pcbp2	-0.18	1.00000000	chicken
Dgkh	0.60	1.00000000	chicken
Kdm5a	0.08	1.00000000	chicken
Wsb2	-0.21	1.00000000	chicken
Arl8b	0.47	1.00000000	chicken
Txndc11	-0.18	1.00000000	chicken
Stx17	0.07	1.00000000	chicken
Nab1	-0.01	1.00000000	chicken
Ppil2	0.03	1.00000000	chicken
Smarcc2	-0.05	1.00000000	chicken
Eif4h	-0.31	1.00000000	chicken
Pafah1b2	-0.26	1.00000000	chicken
Srsf1	-0.21	1.00000000	chicken

Adck1	-0.15	1.00000000	chicken
Fgfr1op2	0.19	1.00000000	chicken
Ubac1	0.16	1.00000000	chicken
Larp4b	-0.04	1.00000000	chicken
Nmt2	-0.23	1.00000000	chicken
Tiprl	0.27	1.00000000	chicken
Usp38	-0.24	1.00000000	chicken
Slc25a16	0.16	1.00000000	chicken
Pfdn5	-0.11	1.00000000	chicken
Ncoa1	0.14	1.00000000	chicken
F5	0.06	1.00000000	chicken
Ccpg1	-0.13	1.00000000	chicken
Cep350	-0.06	1.00000000	chicken
Hexb	-0.03	1.00000000	chicken
Ap2b1	0.00	1.00000000	chicken
Odc1	-0.39	1.00000000	chicken
Ndufa13	-0.19	1.00000000	chicken
Impa1	0.31	1.00000000	chicken
Copa	-0.18	1.00000000	chicken
Rap2a	-0.14	1.00000000	chicken
Srebf1	-0.15	1.00000000	chicken
Tsnax	-0.25	1.00000000	chicken
Hadha	-0.01	1.00000000	chicken
Nr1h3	-0.06	1.00000000	chicken
Cnot7	-0.06	1.00000000	chicken
Cbfb	-0.02	1.00000000	chicken
Itsn2	0.12	1.00000000	chicken
Pld4	0.06	1.00000000	chicken
Pus10	0.33	1.00000000	chicken
Nono	0.15	1.00000000	chicken
Guk1	-0.05	1.00000000	chicken
Pm20d2	0.46	1.00000000	chicken
Ccnyl1	0.27	1.00000000	chicken
U2surp	0.27	1.00000000	chicken
Ahcyl1	0.06	1.00000000	chicken
Rbm26	0.17	1.00000000	chicken
Hectd1	-0.11	1.00000000	chicken
Traf5	-0.08	1.00000000	chicken
Alg6	-0.06	1.00000000	chicken
Bud23	-0.01	1.00000000	chicken
Uqcr10	-0.24	1.00000000	chicken
Hsf2	-0.15	1.00000000	chicken
Vps26c	0.27	1.00000000	chicken
Ext2	0.52	1.00000000	chicken
Tm9sf2	0.04	1.00000000	chicken
Bpnt2	0.18	1.00000000	chicken

Nptn	0.31	1.00000000	chicken
Pus7	0.14	1.00000000	chicken
Rap1b	0.10	1.00000000	chicken
Sqle	-0.18	1.00000000	chicken
Rpf2	-0.18	1.00000000	chicken
Xrn1	0.20	1.00000000	chicken
Ccnd3	0.14	1.00000000	chicken
Azi2	-0.27	1.00000000	chicken
Rmdn3	0.08	1.00000000	chicken
Ctsz	-0.04	1.00000000	chicken
Srsf11	-0.15	1.00000000	chicken
Pwp1	-0.36	1.00000000	chicken
Ccdc43	-0.18	1.00000000	chicken
Nubp2	0.24	1.00000000	chicken
Vrk1	0.39	1.00000000	chicken
Evl	-0.05	1.00000000	chicken
Phf5a	0.10	1.00000000	chicken
Sufu	0.21	1.00000000	chicken
Erlec1	0.33	1.00000000	chicken
Ddx3y	-0.09	1.00000000	chicken
Akap9	0.30	1.00000000	chicken
Dctn4	0.47	1.00000000	chicken
Faf1	0.11	1.00000000	chicken
Ankrd27	-0.06	1.00000000	chicken
Tmed5	-0.28	1.00000000	chicken
Vstm4	-0.29	1.00000000	chicken
Ndufaf8	-0.06	1.00000000	chicken
Uba6	-0.26	1.00000000	chicken
Borcs6	0.01	1.00000000	chicken
Slc2a8	-0.19	1.00000000	chicken
Cyld	-0.18	1.00000000	chicken
Smu1	-0.34	1.00000000	chicken
Siva1	0.24	1.00000000	chicken
Lgmn	-0.10	1.00000000	chicken
Ube2e2	0.76	1.00000000	chicken
Btbd9	0.10	1.00000000	chicken
Wbp2	0.08	1.00000000	chicken
Galnt6	0.10	1.00000000	chicken
Washc1	-0.14	1.00000000	chicken
Clptm1l	-0.21	1.00000000	chicken
Nelfcd	-0.18	1.00000000	chicken
Fbxl17	0.01	1.00000000	chicken
Ppp2cb	-0.05	1.00000000	chicken
Calr	-0.17	1.00000000	chicken
Rilpl1	0.14	1.00000000	chicken
Aimp1	-0.12	1.00000000	chicken

Reps1	0.08	1.00000000	chicken
Snapc4	0.01	1.00000000	chicken
Insig1	-0.24	1.00000000	chicken
Sf3b6	-0.20	1.00000000	chicken
Cltb	-0.05	1.00000000	chicken
Ep400	0.07	1.00000000	chicken
Rp2	0.28	1.00000000	chicken
G3bp1	0.14	1.00000000	chicken
Dazap1	0.07	1.00000000	chicken
Ube2d2a	-0.10	1.00000000	chicken
Faf2	-0.04	1.00000000	chicken
Phaf1	0.01	1.00000000	chicken
Dpysl2	0.28	1.00000000	chicken
Jmy	0.03	1.00000000	chicken
Tmem183a	-0.17	1.00000000	chicken
Cops2	0.22	1.00000000	chicken
Ptpn22	-0.07	1.00000000	chicken
Srsf3	0.13	1.00000000	chicken
Nnt	-0.16	1.00000000	chicken
Rpn2	-0.12	1.00000000	chicken
Ing3	0.04	1.00000000	chicken
Trafd1	0.24	1.00000000	chicken
Syap1	-0.11	1.00000000	chicken
Btg1b	0.08	1.00000000	chicken
Oaz2	0.14	1.00000000	chicken
Eif5b	-0.22	1.00000000	chicken
Cep112	0.67	1.00000000	chicken
Ylpm1	-0.33	1.00000000	chicken
Erc1	0.04	1.00000000	chicken
Cwf19l2	-0.35	1.00000000	chicken
Hypk	0.05	1.00000000	chicken
Smarca1	-0.20	1.00000000	chicken
Abracl	0.08	1.00000000	chicken
Chchd3	0.37	1.00000000	chicken
Srrm1	0.04	1.00000000	chicken
2700049A03Rik	-0.40	1.00000000	chicken
Pitpnb	-0.19	1.00000000	chicken
Snx29	0.26	1.00000000	chicken
Gpcpd1	-0.15	1.00000000	chicken
Abhd18	0.21	1.00000000	chicken
Scamp1	-0.19	1.00000000	chicken
Hdac4	-0.18	1.00000000	chicken
Abi2	0.31	1.00000000	chicken
Iqgap1	0.11	1.00000000	chicken
Fbxo34	-0.32	1.00000000	chicken
Dym	-0.05	1.00000000	chicken

Hdgfl2	-0.08	1.00000000	chicken
Osbp	-0.14	1.00000000	chicken
Chid1	0.85	1.00000000	chicken
Nop53	-0.05	1.00000000	chicken
Ggps1	-0.14	1.00000000	chicken
Ints2	0.21	1.00000000	chicken
Sft2d1	-0.24	1.00000000	chicken
Cast	0.31	1.00000000	chicken
Sh3bgrl3	-0.22	1.00000000	chicken
Trpm7	0.25	1.00000000	chicken
Bend7	0.22	1.00000000	chicken
Nisch	0.24	1.00000000	chicken
Prkcd	-0.05	1.00000000	chicken
Wdr82	0.13	1.00000000	chicken
Phlpp2	0.10	1.00000000	chicken
Wdr36	0.30	1.00000000	chicken
Fosl2	-0.29	1.00000000	chicken
Dcn	-0.38	1.00000000	chicken
Ndufv2	-0.08	1.00000000	chicken
Ube2e1	0.30	1.00000000	chicken
Cstf2	0.04	1.00000000	chicken
Nifk	-0.12	1.00000000	chicken
Acad9	0.08	1.00000000	chicken
Reep5	0.34	1.00000000	chicken
Hdgfl3	0.11	1.00000000	chicken
Uqcc2	-0.08	1.00000000	chicken
Ddx46	-0.20	1.00000000	chicken
Lct	-0.05	1.00000000	chicken
Lnpep	-0.18	1.00000000	chicken
Cdk6	-0.24	1.00000000	chicken
Gatd3a	0.43	1.00000000	chicken
Vps26a	-0.21	1.00000000	chicken
Cox7a2l	-0.32	1.00000000	chicken
Exosc10	0.21	1.00000000	chicken
Slc10a7	0.13	1.00000000	chicken
Strbp	0.17	1.00000000	chicken
Spopl	-0.02	1.00000000	chicken
Slc35b3	-0.04	1.00000000	chicken
Dus3l	0.11	1.00000000	chicken
Tubgcp5	0.03	1.00000000	chicken
Atp6v1a	0.10	1.00000000	chicken
Ddx17	0.16	1.00000000	chicken
Cmip	-0.25	1.00000000	chicken
Cnot8	0.17	1.00000000	chicken
Usf3	0.45	1.00000000	chicken
Cct4	-0.19	1.00000000	chicken

Lrrfip2	0.65	1.00000000	chicken
Chmp5	-0.31	1.00000000	chicken
Pik3c2a	-0.24	1.00000000	chicken
Zfc3h1	-0.17	1.00000000	chicken
4921524J17Rik	-0.12	1.00000000	chicken
Acap1	0.19	1.00000000	chicken
Golga1	-0.09	1.00000000	chicken
Slc38a10	0.21	1.00000000	chicken
Nus1	0.10	1.00000000	chicken
Becn2	-0.13	1.00000000	chicken
Usp34	0.09	1.00000000	chicken
Aurkaip1	0.04	1.00000000	chicken
Ldah	-0.48	1.00000000	chicken
Dad1	0.07	1.00000000	chicken
Uchl5	0.04	1.00000000	chicken
Bdp1	0.50	1.00000000	chicken
Slc25a14	-0.21	1.00000000	chicken
Chp1	-0.14	1.00000000	chicken
Osbpl2	-0.03	1.00000000	chicken
Pik3ca	0.08	1.00000000	chicken
Cdc73	0.00	1.00000000	chicken
Prkx	0.60	1.00000000	chicken
Ran	-0.18	1.00000000	chicken
Tsc22d2	0.35	1.00000000	chicken
Pdss2	-0.36	1.00000000	chicken
Paip2	-0.27	1.00000000	chicken
Znrf3	0.03	1.00000000	chicken
Jcad	-0.03	1.00000000	chicken
Seh1l	-0.10	1.00000000	chicken
Spg11	-0.14	1.00000000	chicken
8030462N17Rik	-0.20	1.00000000	chicken
Utp11	-0.13	1.00000000	chicken
Kansl3	0.04	1.00000000	chicken
Kdm7a	-0.15	1.00000000	chicken
Ddx54	-0.01	1.00000000	chicken
Hsph1	-0.10	1.00000000	chicken
Dhdds	0.06	1.00000000	chicken
Actr6	0.16	1.00000000	chicken
Hhex	-0.14	1.00000000	chicken
Naa35	0.70	1.00000000	chicken
Taf13	-0.08	1.00000000	chicken
Trnt1	-0.15	1.00000000	chicken
Sap130	0.74	1.00000000	chicken
Snapin	-0.13	1.00000000	chicken
Ago4	0.13	1.00000000	chicken
Psap	0.09	1.00000000	chicken

Nsun2	-0.29	1.00000000	chicken
Sfxn1	0.22	1.00000000	chicken
Anapc7	-0.07	1.00000000	chicken
Trrap	0.15	1.00000000	chicken
Aebp2	-0.05	1.00000000	chicken
Atg2b	-0.10	1.00000000	chicken
Smim19	0.03	1.00000000	chicken
Ext1	0.14	1.00000000	chicken
Fbxl2	-0.39	1.00000000	chicken
Atg5	-0.25	1.00000000	chicken
Vapa	-0.24	1.00000000	chicken
Phka2	0.07	1.00000000	chicken
Ryk	0.05	1.00000000	chicken
S100a10	-0.17	1.00000000	chicken
Ciapin1	0.06	1.00000000	chicken
Trmt13	-0.01	1.00000000	chicken
Rcor3	0.32	1.00000000	chicken
Ap3b1	0.20	1.00000000	chicken
Alkbh1	0.22	1.00000000	chicken
Bach1	-0.05	1.00000000	chicken
Ogdh	-0.16	1.00000000	chicken
Bcl7a	0.46	1.00000000	chicken
Fem1b	0.00	1.00000000	chicken
Zfp512	0.00	1.00000000	chicken
Ints7	-0.18	1.00000000	chicken
Jpt2	0.41	1.00000000	chicken
Lpp	-0.04	1.00000000	chicken
Grk2	-0.24	1.00000000	chicken
Rbl2	-0.08	1.00000000	chicken
Nrf1	-0.04	1.00000000	chicken
Snx25	0.33	1.00000000	chicken
Arhgef18	-0.04	1.00000000	chicken
Sec24a	0.03	1.00000000	chicken
Sephs1	0.16	1.00000000	chicken
Oxr1	-0.20	1.00000000	chicken
Nek10	1.01	1.00000000	chicken
Samm50	-0.19	1.00000000	chicken
Aars	0.20	1.00000000	chicken
Polr3b	0.11	1.00000000	chicken
Ube2b	0.03	1.00000000	chicken
Ehbp1	-0.74	1.00000000	chicken
Tex264	-0.13	1.00000000	chicken
Rab3il1	0.15	1.00000000	chicken
Cct6b	-0.03	1.00000000	chicken
Nfat5	0.14	1.00000000	chicken
Tmem131	0.43	1.00000000	chicken

Nbr1	0.01	1.00000000	chicken
Rab31	-0.15	1.00000000	chicken
Dok3	-0.07	1.00000000	chicken
Ppp1r2	0.40	1.00000000	chicken
Sri	-0.03	1.00000000	chicken
Zcchc2	0.15	1.00000000	chicken
Zfyve21	0.24	1.00000000	chicken
Fam76b	0.42	1.00000000	chicken
Tle5	0.07	1.00000000	chicken
Heatr5b	0.12	1.00000000	chicken
Usp47	0.18	1.00000000	chicken
Plekha8	0.41	1.00000000	chicken
Strn3	0.10	1.00000000	chicken
Flot2	0.32	1.00000000	chicken
Bhlhe40	-0.02	1.00000000	chicken
Ndufa2	0.18	1.00000000	chicken
Ntng1	0.07	1.00000000	chicken
Dtd1	0.18	1.00000000	chicken
Ddx41	0.10	1.00000000	chicken
Ndfip2	0.15	1.00000000	chicken
Polr2i	-0.13	1.00000000	chicken
Ccdc174	-0.03	1.00000000	chicken
Gtf2a1	0.47	1.00000000	chicken
Rcl1	-0.18	1.00000000	chicken
Gabpb1	0.09	1.00000000	chicken
Thg1l	0.05	1.00000000	chicken
Limk1	0.03	1.00000000	chicken
Tgfbr1	-0.05	1.00000000	chicken
Kif1b	0.20	1.00000000	chicken
Leo1	0.14	1.00000000	chicken
Nemp2	-0.16	1.00000000	chicken
Poldip3	-0.05	1.00000000	chicken
Oser1	-0.09	1.00000000	chicken
Il18	-0.20	1.00000000	chicken
Ss18l2	0.16	1.00000000	chicken
Gid8	0.02	1.00000000	chicken
Snx12	-0.12	1.00000000	chicken
Pdzd8	0.30	1.00000000	chicken
Dlg1	0.62	1.00000000	chicken
Ywhaz	0.22	1.00000000	chicken
Psmf1	0.10	1.00000000	chicken
Pprc1	0.25	1.00000000	chicken
Csnk2a1	-0.06	1.00000000	chicken
Dtx3l	-0.04	1.00000000	chicken
Tfam	0.20	1.00000000	chicken
Ube2v2	0.13	1.00000000	chicken

Cul4a	-0.30	1.00000000	chicken
Ssr1	-0.26	1.00000000	chicken
Cep170	-0.16	1.00000000	chicken
Thoc7	0.13	1.00000000	chicken
Gtf3a	0.11	1.00000000	chicken
Polr2f	0.33	1.00000000	chicken
Kdm5b	-0.01	1.00000000	chicken
Lamp3	0.01	1.00000000	chicken
Rreb1	0.64	1.00000000	chicken
Tcerg1	-0.20	1.00000000	chicken
Armc2	0.11	1.00000000	chicken
Ano5	-0.06	1.00000000	chicken
Tpr	0.23	1.00000000	chicken
Oaz1	-0.19	1.00000000	chicken
Taf5l	-0.16	1.00000000	chicken
Rangap1	0.42	1.00000000	chicken
Sugt1	-0.25	1.00000000	chicken
Spz1	-0.15	1.00000000	chicken
Ccdc50	-0.05	1.00000000	chicken
Ube2cbp	-0.16	1.00000000	chicken
Tmem115	0.00	1.00000000	chicken
Csde1	-0.06	1.00000000	chicken
B3gnt3	-0.01	1.00000000	chicken
Cherp	0.02	1.00000000	chicken
Rgs19	0.10	1.00000000	chicken
Hp1bp3	0.38	1.00000000	chicken
Gabarapl1	0.03	1.00000000	chicken
Hk2	0.09	1.00000000	chicken
Dpf3	0.05	1.00000000	chicken
Rad17	0.21	1.00000000	chicken
Kpna4	0.08	1.00000000	chicken
Pdlim7	-0.30	1.00000000	chicken
Afg3l2	-0.03	1.00000000	chicken
Rer1	-0.22	1.00000000	chicken
Phf11a	-0.08	1.00000000	chicken
Sergef	-0.32	1.00000000	chicken
Pafah1b1	0.02	1.00000000	chicken
Wbp2nl	0.25	1.00000000	chicken
Hmbs	0.08	1.00000000	chicken
Pitpna	-0.39	1.00000000	chicken
Rnaseh1	-0.14	1.00000000	chicken
Ptrh2	-0.23	1.00000000	chicken
Inpp5a	-0.12	1.00000000	chicken
Txlng	-0.18	1.00000000	chicken
Sirt2	-0.23	1.00000000	chicken
Gpatch4	0.31	1.00000000	chicken

Slc4a1ap	0.32	1.00000000	chicken
Chd9	0.16	1.00000000	chicken
Wdr37	0.15	1.00000000	chicken
Dcp1a	0.09	1.00000000	chicken
Cbfa2t3	0.14	1.00000000	chicken
Lhpp	0.11	1.00000000	chicken
Ogt	-0.07	1.00000000	chicken
Prkd3	0.02	1.00000000	chicken
Paxip1	-0.02	1.00000000	chicken
Atp13a3	0.17	1.00000000	chicken
Vdac2	-0.03	1.00000000	chicken
Eif1b	-0.07	1.00000000	chicken
Scfd1	0.00	1.00000000	chicken
Tmem104	0.59	1.00000000	chicken
Ptpra	-0.13	1.00000000	chicken
Esrrg	-0.11	1.00000000	chicken
Limd2	0.01	1.00000000	chicken
Drg1	0.05	1.00000000	chicken
Thoc5	0.04	1.00000000	chicken
Pudp	0.31	1.00000000	chicken
Uso1	0.34	1.00000000	chicken
Acsl4	-0.06	1.00000000	chicken
Dnajc2	-0.16	1.00000000	chicken
Rapgef1	0.00	1.00000000	chicken
Adipor2	-0.22	1.00000000	chicken
Fan1	-0.01	1.00000000	chicken
Txlna	0.15	1.00000000	chicken
Atp6v0e	-0.11	1.00000000	chicken
Fryl	0.12	1.00000000	chicken
Wdr77	0.18	1.00000000	chicken
Gcc2	-0.19	1.00000000	chicken
Dok1	0.04	1.00000000	chicken
Eif2ak4	-0.05	1.00000000	chicken
Slc39a9	0.03	1.00000000	chicken
Pip4k2a	0.12	1.00000000	chicken
Sltm	-0.19	1.00000000	chicken
Ube2j1	0.01	1.00000000	chicken
AU040320	-0.18	1.00000000	chicken
Hspa5	-0.05	1.00000000	chicken
Tbc1d14	-0.11	1.00000000	chicken
Apoo	0.13	1.00000000	chicken
Acap2	0.46	1.00000000	chicken
Chd1	0.11	1.00000000	chicken
Ifnar1	0.26	1.00000000	chicken
Plin2	0.12	1.00000000	chicken
Smyd3	0.18	1.00000000	chicken

Ndufs3	-0.07	1.00000000	chicken
Zfp827	-0.06	1.00000000	chicken
Ipo7	0.04	1.00000000	chicken
Sf3a3	0.28	1.00000000	chicken
Ing4	0.06	1.00000000	chicken
Usp14	-0.30	1.00000000	chicken
Csk	0.18	1.00000000	chicken
Spats2l	0.16	1.00000000	chicken
Zbtb26	0.19	1.00000000	chicken
St13	0.18	1.00000000	chicken
Gorab	0.24	1.00000000	chicken
Otulinl	0.30	1.00000000	chicken
Cope	-0.13	1.00000000	chicken
Bok	-0.03	1.00000000	chicken
Pnn	-0.07	1.00000000	chicken
Pgrmc2	0.12	1.00000000	chicken
Tnnt3	0.13	1.00000000	chicken
Zfp143	0.01	1.00000000	chicken
Fcf1	0.03	1.00000000	chicken
Eif4a3l1	0.17	1.00000000	chicken
Ptpn12	0.03	1.00000000	chicken
Srsf10	-0.07	1.00000000	chicken
Timp3	0.10	1.00000000	chicken
Rab29	0.07	1.00000000	chicken
Cdk17	0.07	1.00000000	chicken
Tmem167	-0.18	1.00000000	chicken
Ccdc25	0.35	1.00000000	chicken
Tspan14	0.25	1.00000000	chicken
Usp16	0.24	1.00000000	chicken
Cyth1	0.27	1.00000000	chicken
Wdr43	-0.11	1.00000000	chicken
Arhgef12	0.13	1.00000000	chicken
Lyn	-0.16	1.00000000	chicken
Psmd4	-0.10	1.00000000	chicken
Ets1	0.20	1.00000000	chicken
Krit1	0.12	1.00000000	chicken
Rtf1	0.13	1.00000000	chicken
Tex10	0.07	1.00000000	chicken
Tent2	-0.27	1.00000000	chicken
Cldnd1	-0.03	1.00000000	chicken
Ubtd2	0.33	1.00000000	chicken
Gnai1	0.15	1.00000000	chicken
Tgif1	0.00	1.00000000	chicken
Glg1	0.07	1.00000000	chicken
Galnt7	-0.13	1.00000000	chicken
Rab33b	-0.18	1.00000000	chicken

Clk4	0.02	1.00000000	chicken
Prmt3	0.16	1.00000000	chicken
Sptlc1	0.09	1.00000000	chicken
Id2	-0.16	1.00000000	chicken
Uggt1	-0.05	1.00000000	chicken
Timm8a2	0.14	1.00000000	chicken
Kctd2	0.34	1.00000000	chicken
Fkbp3	0.28	1.00000000	chicken
Numa1	-0.11	1.00000000	chicken
Ubp1	-0.03	1.00000000	chicken
Snrnp25	-0.23	1.00000000	chicken
Ap4e1	-0.23	1.00000000	chicken
Ahsa2	-0.01	1.00000000	chicken
Pmf1	-0.11	1.00000000	chicken
Ptpn4	0.06	1.00000000	chicken
Thada	-0.14	1.00000000	chicken
Nt5c3	0.32	1.00000000	chicken
Hnrnpa2b1	-0.01	1.00000000	chicken
Rsbn1	-0.04	1.00000000	chicken
Rev1	0.04	1.00000000	chicken
Parg	0.20	1.00000000	chicken
Rad23a	-0.21	1.00000000	chicken
Kdm1a	0.28	1.00000000	chicken
Iba57	0.07	1.00000000	chicken
Abraxas2	0.21	1.00000000	chicken
Epc2	0.35	1.00000000	chicken
Rab14	-0.01	1.00000000	chicken
Usp37	-0.17	1.00000000	chicken
Ndufb6	0.18	1.00000000	chicken
Aven	0.39	1.00000000	chicken
Usp45	0.24	1.00000000	chicken
Ldlrad4	0.01	1.00000000	chicken
Tfb1m	0.39	1.00000000	chicken
Ggnbp2	0.12	1.00000000	chicken
Clcn3	0.12	1.00000000	chicken
Dnajc7	-0.05	1.00000000	chicken
Xpo6	-0.03	1.00000000	chicken
Garnl3	0.03	1.00000000	chicken
Epc1	-0.09	1.00000000	chicken
Akap7	0.71	1.00000000	chicken
Lman2	0.07	1.00000000	chicken
Dbf4	0.05	1.00000000	chicken
Phf10	0.10	1.00000000	chicken
D16Ertd472e	-0.11	1.00000000	chicken
Rab5c	-0.04	1.00000000	chicken
Ubap2l	0.14	1.00000000	chicken

Pinx1	0.04	1.00000000	chicken
Pfdn1	-0.16	1.00000000	chicken
Rcor1	0.16	1.00000000	chicken
Stk40	-0.08	1.00000000	chicken
Gpr18	0.32	1.00000000	chicken
Grn	-0.21	1.00000000	chicken
Atp2b1	-0.43	1.00000000	chicken
Ap3s1	0.23	1.00000000	chicken
Zbtb1	-0.05	1.00000000	chicken
Lpin1	0.06	1.00000000	chicken
Plscr1	0.08	1.00000000	chicken
Atp5pb	-0.30	1.00000000	chicken
Prpf3	-0.25	1.00000000	chicken
Egr2	0.09	1.00000000	chicken
Nqo2	0.14	1.00000000	chicken
Slc25a25	-0.26	1.00000000	chicken
Peli1	-0.18	1.00000000	chicken
Bysl	0.24	1.00000000	chicken
Pogz	-0.06	1.00000000	chicken
Gfpt1	0.20	1.00000000	chicken
Nubp1	-0.15	1.00000000	chicken
Dnm1l	0.15	1.00000000	chicken
Lars2	0.19	1.00000000	chicken
Ubl7	0.06	1.00000000	chicken
Fbxo25	-0.17	1.00000000	chicken
Atxn10	0.46	1.00000000	chicken
Smg1	0.31	1.00000000	chicken
1110004F10Rik	0.13	1.00000000	chicken
Cd72	-0.13	1.00000000	chicken
Ccdc93	-0.08	1.00000000	chicken
Hif1an	0.12	1.00000000	chicken
Sdad1	-0.16	1.00000000	chicken
Poc1a	-0.18	1.00000000	chicken
Tacc1	-0.11	1.00000000	chicken
Fundc1	0.03	1.00000000	chicken
Nsf	0.16	1.00000000	chicken
Snw1	0.27	1.00000000	chicken
Banp	0.18	1.00000000	chicken
Agps	0.14	1.00000000	chicken
Nup98	-0.19	1.00000000	chicken
Ube2k	0.25	1.00000000	chicken
Exoc3	-0.12	1.00000000	chicken
Aagab	0.35	1.00000000	chicken
Brd8	0.58	1.00000000	chicken
Iars	0.25	1.00000000	chicken
Serpinb8	0.15	1.00000000	chicken

Bcas3	0.51	1.00000000	chicken
Sucla2	0.25	1.00000000	chicken
Skil	0.03	1.00000000	chicken
Sec23b	-0.02	1.00000000	chicken
Flot1	-0.19	1.00000000	chicken
Pno1	0.01	1.00000000	chicken
Tdrd3	0.16	1.00000000	chicken
Ssh2	0.12	1.00000000	chicken
Numb	0.16	1.00000000	chicken
Acvr1	0.17	1.00000000	chicken
Atp10d	-0.15	1.00000000	chicken
Zc3h14	0.21	1.00000000	chicken
Emc8	-0.09	1.00000000	chicken
Ccnk	-0.02	1.00000000	chicken
Smim12	0.21	1.00000000	chicken
Ptp4a2	-0.26	1.00000000	chicken
Csl	-0.06	1.00000000	chicken
Nelfb	-0.04	1.00000000	chicken
Pank4	-0.06	1.00000000	chicken
Tfec	-1.02	1.00000000	chicken
Atp6v0c	0.09	1.00000000	chicken
Crk	-0.04	1.00000000	chicken
Poc1b	0.07	1.00000000	chicken
Pdcd10	-0.16	1.00000000	chicken
Eif2a	-0.15	1.00000000	chicken
Utp3	0.00	1.00000000	chicken
Cd63	-0.15	1.00000000	chicken
Septin2	0.19	1.00000000	chicken
Actr1a	-0.04	1.00000000	chicken
Shld2	0.00	1.00000000	chicken
Commd1b	0.00	1.00000000	chicken
Glrx3	-0.17	1.00000000	chicken
Etfrf1	0.08	1.00000000	chicken
Tmem263	0.05	1.00000000	chicken
Eif4e	-0.26	1.00000000	chicken
9930021J03Rik	0.13	1.00000000	chicken
Pcna	-0.27	1.00000000	chicken
Commd5	0.04	1.00000000	chicken
Hdac2	-0.01	1.00000000	chicken
Ftsj3	-0.06	1.00000000	chicken
Ambra1	0.00	1.00000000	chicken
Akap11	0.44	1.00000000	chicken
B4galt5	-0.02	1.00000000	chicken
Elmo2	-0.14	1.00000000	chicken
Zc3h18	0.16	1.00000000	chicken
AI597479	-0.05	1.00000000	chicken

C2cd2l	0.68	1.00000000	chicken
Arhgef6	0.10	1.00000000	chicken
Psma3	0.19	1.00000000	chicken
Smim20	-0.17	1.00000000	chicken
Neil1	-0.15	1.00000000	chicken
Gbe1	0.00	1.00000000	chicken
Sec62	0.15	1.00000000	chicken
Pnpla8	-0.19	1.00000000	chicken
Ubn2	0.44	1.00000000	chicken
Cltc	-0.04	1.00000000	chicken
Cul5	0.00	1.00000000	chicken
Susd6	0.21	1.00000000	chicken
Rab3gap1	-0.24	1.00000000	chicken
Tmem184b	-0.05	1.00000000	chicken
Optn	0.02	1.00000000	chicken
Dhx38	-0.08	1.00000000	chicken
Tbl1xr1	-0.01	1.00000000	chicken
Hccs	-0.15	1.00000000	chicken
Atg16l1	-0.20	1.00000000	chicken
Tcf3	-0.22	1.00000000	chicken
Ahr	0.34	1.00000000	chicken
Dnajc1	-0.02	1.00000000	chicken
P2rx5	0.02	1.00000000	chicken
Lrrfip1	-0.04	1.00000000	chicken
Xpo4	-0.17	1.00000000	chicken
Ube3a	-0.39	1.00000000	chicken
Tef	-0.21	1.00000000	chicken
Sp1	0.01	1.00000000	chicken
4933434E20Rik	0.33	1.00000000	chicken
Srpk1	-0.17	1.00000000	chicken
Rictor	-0.31	1.00000000	chicken
Lin54	0.04	1.00000000	chicken
Phf20l1	-0.18	1.00000000	chicken
Agfg1	-0.18	1.00000000	chicken
Cfdp1	0.09	1.00000000	chicken
Thap4	-0.16	1.00000000	chicken
Elp3	0.22	1.00000000	chicken
Suds3	-0.04	1.00000000	chicken
Paxbp1	-0.07	1.00000000	chicken
Lrch3	-0.03	1.00000000	chicken
Btbd10	-0.20	1.00000000	chicken
1810058I24Rik	-0.07	1.00000000	chicken
Nfyb	0.03	1.00000000	chicken
Lamtor3	0.06	1.00000000	chicken
Tcp1	-0.10	1.00000000	chicken
Osbpl1a	0.33	1.00000000	chicken

Hexim1	-0.06	1.00000000	chicken
Tbp	0.02	1.00000000	chicken
Nsmaf	0.08	1.00000000	chicken
Uchl3	0.29	1.00000000	chicken
Gpr155	0.07	1.00000000	chicken
Kdm3a	-0.09	1.00000000	chicken
Smc6	0.03	1.00000000	chicken
Srsf5	0.17	1.00000000	chicken
Edc4	-0.04	1.00000000	chicken
Tnfaip3	-0.14	1.00000000	chicken
Sf3b3	-0.22	1.00000000	chicken
Akap8	-0.05	1.00000000	chicken
Psmd7	0.22	1.00000000	chicken
Prpf38a	-0.15	1.00000000	chicken
Wipi2	0.16	1.00000000	chicken
Ergic2	-0.05	1.00000000	chicken
Gtf2a2	0.35	1.00000000	chicken
Pdcd4	0.21	1.00000000	chicken
Vps35	0.16	1.00000000	chicken
Cdca4	-0.18	1.00000000	chicken
Prkag2	-0.05	1.00000000	chicken
Hikeshi	-0.08	1.00000000	chicken
Chfr	0.18	1.00000000	chicken
Ttc39b	0.14	1.00000000	chicken
Pacs2	-0.36	1.00000000	chicken
Usp3	-0.01	1.00000000	chicken
Setbp1	-0.17	1.00000000	chicken
Proser1	-0.14	1.00000000	chicken
Nipsnap2	0.15	1.00000000	chicken
Golm2	-0.09	1.00000000	chicken
Eif4ebp2	0.11	1.00000000	chicken
Kif5b	0.19	1.00000000	chicken
Ttc1	0.24	1.00000000	chicken
Pikfyve	0.03	1.00000000	chicken
Gpatch8	-0.02	1.00000000	chicken
Letm2	-0.15	1.00000000	chicken
Tatdn1	-0.03	1.00000000	chicken
Rdm1	0.25	1.00000000	chicken
Pbrm1	0.39	1.00000000	chicken
Heca	0.00	1.00000000	chicken
Cpsf4l	0.08	1.00000000	chicken
Invs	-0.28	1.00000000	chicken
Slc12a2	-0.40	1.00000000	chicken
Sin3a	-0.09	1.00000000	chicken
Irf4	0.02	1.00000000	chicken
Zfp638	0.07	1.00000000	chicken

Orc1	0.05	1.00000000	chicken
Tgfb1	0.08	1.00000000	chicken
Cstf1	-0.11	1.00000000	chicken
Brf1	-0.01	1.00000000	chicken
Armc8	0.09	1.00000000	chicken
Hnrnpul1	0.20	1.00000000	chicken
Bud31	-0.12	1.00000000	chicken
Cnot1	-0.22	1.00000000	chicken
Ewsr1	0.26	1.00000000	chicken
Nufip1	0.06	1.00000000	chicken
Cox5b-ps	0.16	1.00000000	chicken
Acot8	0.03	1.00000000	chicken
Aak1	-0.25	1.00000000	chicken
Arfip2	-0.13	1.00000000	chicken
Ube2q2	0.12	1.00000000	chicken
B4galt2	0.10	1.00000000	chicken
Sec16a	0.14	1.00000000	chicken
Lmna	-0.10	1.00000000	chicken
Chic1	0.19	1.00000000	chicken
Susd3	0.18	1.00000000	chicken
Senp1	0.04	1.00000000	chicken
Kbtbd8	0.04	1.00000000	chicken
Asns	-0.07	1.00000000	chicken
Sms	0.11	1.00000000	chicken
Bub3	-0.03	1.00000000	chicken
Ttc39a	-0.20	1.00000000	chicken
Itpk1	0.30	1.00000000	chicken
Rbbp4	0.03	1.00000000	chicken
Tasor	0.01	1.00000000	chicken
Ipo8	0.23	1.00000000	chicken
Ccdc91	-0.03	1.00000000	chicken
Itga2	-0.17	1.00000000	chicken
Nfs1	0.05	1.00000000	chicken
Kmt2c	-0.04	1.00000000	chicken
Tmem181a	0.16	1.00000000	chicken
Bbx	0.22	1.00000000	chicken
Creb1	0.16	1.00000000	chicken
Psmd9	-0.01	1.00000000	chicken
Cdyl2	-0.08	1.00000000	chicken
Setd2	0.00	1.00000000	chicken
H3f3b	0.01	1.00000000	chicken
Zfp292	-0.11	1.00000000	chicken
Ifngr2	-0.22	1.00000000	chicken
Agl	0.04	1.00000000	chicken
Slc9a6	0.03	1.00000000	chicken
Copb2	-0.22	1.00000000	chicken

Uhrf1bp1l	0.29	1.00000000	chicken
Crtc1	-0.22	1.00000000	chicken
Nek9	0.03	1.00000000	chicken
Otub1	0.23	1.00000000	chicken
Bhlhe41	0.05	1.00000000	chicken
Wdr3	-0.18	1.00000000	chicken
Tnfsf11	0.01	1.00000000	chicken
Rybp	-0.09	1.00000000	chicken
Spast	0.00	1.00000000	chicken
Ppil4	0.30	1.00000000	chicken
Cdc14b	0.21	1.00000000	chicken
Ezh2	0.46	1.00000000	chicken
Eif4e2	0.05	1.00000000	chicken
4933427D14Rik	0.06	1.00000000	chicken
Adamtsl2	-0.28	1.00000000	chicken
R3hdm1	0.44	1.00000000	chicken
Rnf20	0.24	1.00000000	chicken
Camk2g	0.27	1.00000000	chicken
B3gnt2	0.35	1.00000000	chicken
Ppm1b	0.01	1.00000000	chicken
Nol10	0.25	1.00000000	chicken
Cd38	0.40	1.00000000	chicken
Htatsf1	-0.11	1.00000000	chicken
Azin1	-0.20	1.00000000	chicken
Htt	0.25	1.00000000	chicken
Gemin8	-0.05	1.00000000	chicken
Anxa6	-0.04	1.00000000	chicken
Umad1	0.19	1.00000000	chicken
Carnmt1	-0.15	1.00000000	chicken
Ppm1g	-0.26	1.00000000	chicken
Nrde2	-0.08	1.00000000	chicken
Hadhb	0.06	1.00000000	chicken
Eml3	0.16	1.00000000	chicken
Dnpep	-0.03	1.00000000	chicken
Lman1	-0.18	1.00000000	chicken
Fhit	0.18	1.00000000	chicken
Septin9	0.06	1.00000000	chicken
Abca1	-0.01	1.00000000	chicken
Otud7a	0.27	1.00000000	chicken
Sec14l1	0.23	1.00000000	chicken
Nob1	-0.21	1.00000000	chicken
Arid3a	0.00	1.00000000	chicken
Fam168a	0.25	1.00000000	chicken
Cops5	0.10	1.00000000	chicken
Triap1	-0.07	1.00000000	chicken
Wdr44	-0.26	1.00000000	chicken

Rbis	0.23	1.00000000	chicken
Snu13	0.14	1.00000000	chicken
Ccdc88a	0.21	1.00000000	chicken
Tet2	0.03	1.00000000	chicken
Rb1	0.56	1.00000000	chicken
Irak2	0.28	1.00000000	chicken
Nr1d2	-0.02	1.00000000	chicken
Atp6v1c1	-0.17	1.00000000	chicken
Nr3c1	0.17	1.00000000	chicken
Sart3	0.08	1.00000000	chicken
Blmh	0.06	1.00000000	chicken
Tubb5	0.05	1.00000000	chicken
Srfbp1	0.03	1.00000000	chicken
Zfat	0.38	1.00000000	chicken
Bin3	-0.18	1.00000000	chicken
Hdac3	-0.09	1.00000000	chicken
Senp8	-0.11	1.00000000	chicken
Polr1c	-0.16	1.00000000	chicken
Rrp1b	0.04	1.00000000	chicken
Ubqln1	-0.09	1.00000000	chicken
Pcgf3	0.34	1.00000000	chicken
Terf2ip	0.04	1.00000000	chicken
Sema4d	0.54	1.00000000	chicken
Fggy	-0.26	1.00000000	chicken
Tmtc3	0.65	1.00000000	chicken
Dcaf1	0.24	1.00000000	chicken
Cdc40	-0.16	1.00000000	chicken
Syncrip	0.05	1.00000000	chicken
Ankib1	0.27	1.00000000	chicken
Pcnp	-0.22	1.00000000	chicken
Rcc2	0.14	1.00000000	chicken
Vamp3	-0.10	1.00000000	chicken
Kat6b	-0.20	1.00000000	chicken
Wdr26	0.05	1.00000000	chicken
Rnaseh2b	-0.02	1.00000000	chicken
Itfg1	-0.04	1.00000000	chicken
Tnip2	-0.03	1.00000000	chicken
Pccb	0.01	1.00000000	chicken
Zfp711	0.06	1.00000000	chicken
Dop1a	0.15	1.00000000	chicken
Zcrb1	0.13	1.00000000	chicken
Relch	0.15	1.00000000	chicken
Tspan4	0.21	1.00000000	chicken
Ltf	-0.11	1.00000000	chicken
Hivep3	-0.07	1.00000000	chicken
Fubp3	-0.14	1.00000000	chicken

Polr2c	0.32	1.00000000	chicken
Yme1l1	0.13	1.00000000	chicken
Ints11	-0.04	1.00000000	chicken
Suclg2	-0.21	1.00000000	chicken
Anxa11	0.52	1.00000000	chicken
Ing1	-0.05	1.00000000	chicken
Ppard	-0.16	1.00000000	chicken
Zfyve1	-0.15	1.00000000	chicken
Rac1	-0.14	1.00000000	chicken
Srgap3	0.10	1.00000000	chicken
Gnas	-0.13	1.00000000	chicken
Naa30	-0.03	1.00000000	chicken
Paf1	0.06	1.00000000	chicken
Zc3h7a	-0.07	1.00000000	chicken
Sppl2a	-0.34	1.00000000	chicken
C1d	0.07	1.00000000	chicken
Acvr2a	0.13	1.00000000	chicken
Atg4a-ps	0.07	1.00000000	chicken
Dhx8	-0.06	1.00000000	chicken
Bak1	-0.09	1.00000000	chicken
Cwc25	-0.08	1.00000000	chicken
Edc3	0.14	1.00000000	chicken
Pdss1	0.02	1.00000000	chicken
Upb1	-0.24	1.00000000	chicken
Dnajb6	-0.39	1.00000000	chicken
Eea1	-0.31	1.00000000	chicken
Arf6	0.08	1.00000000	chicken
Cops3	0.09	1.00000000	chicken
Psma6	0.15	1.00000000	chicken
Zfp131	-0.09	1.00000000	chicken
Prdx3	0.01	1.00000000	chicken
Immp2l	1.13	1.00000000	chicken
Washc5	0.01	1.00000000	chicken
Cemip2	-0.06	1.00000000	chicken
Nfkb2	-0.03	1.00000000	chicken
Prpf40a	-0.03	1.00000000	chicken
Amd2	0.04	1.00000000	chicken
Eif2b3	0.28	1.00000000	chicken
Taf3	0.13	1.00000000	chicken
Il17ra	0.24	1.00000000	chicken
Smarcc1	-0.07	1.00000000	chicken
Tmem123	0.55	1.00000000	chicken
Gtf2h5	0.08	1.00000000	chicken
Phactr2	-0.34	1.00000000	chicken
Cog5	0.13	1.00000000	chicken
Gabpa	-0.08	1.00000000	chicken

Hps5	-0.02	1.00000000	chicken
Clk2	-0.03	1.00000000	chicken
Deaf1	-0.02	1.00000000	chicken
Pgk1	0.02	1.00000000	chicken
Clasp1	-0.05	1.00000000	chicken
Trappc11	0.22	1.00000000	chicken
Ppp6r2	0.08	1.00000000	chicken
Btbd1	0.16	1.00000000	chicken
Zdhhc18	-0.12	1.00000000	chicken
Bend3	0.19	1.00000000	chicken
Ctdspl2	-0.23	1.00000000	chicken
Ctsa	-0.19	1.00000000	chicken
Hnrnpk	0.22	1.00000000	chicken
Ost4	0.07	1.00000000	chicken
Tra2b	0.22	1.00000000	chicken
Zfp36l1	-0.09	1.00000000	chicken
Snx6	-0.09	1.00000000	chicken
E2f6	-0.22	1.00000000	chicken
Crnkl1	0.26	1.00000000	chicken
Nudt14	0.32	1.00000000	chicken
Dsn1	0.04	1.00000000	chicken
Tnfaip8	0.00	1.00000000	chicken
Usp33	-0.05	1.00000000	chicken
Zbtb48	-0.13	1.00000000	chicken
Trappc9	0.09	1.00000000	chicken
Guf1	0.14	1.00000000	chicken
Nedd8	0.04	1.00000000	chicken
Hook3	-0.01	1.00000000	chicken
Polr2b	0.28	1.00000000	chicken
Impa2	-0.13	1.00000000	chicken
Pcgf5	-0.03	1.00000000	chicken
Lancl2	-0.12	1.00000000	chicken
Hnrnpm	-0.07	1.00000000	chicken
Tsg101	0.32	1.00000000	chicken
Plcg2	0.25	1.00000000	chicken
Phip	0.25	1.00000000	chicken
Grpel1	-0.14	1.00000000	chicken
Naa40	-0.02	1.00000000	chicken
Ska2	-0.11	1.00000000	chicken
Gdi2	0.11	1.00000000	chicken
Hmgcs1	0.06	1.00000000	chicken
Nek6	-0.04	1.00000000	chicken
Taf6	-0.10	1.00000000	chicken
Smim7	-0.04	1.00000000	chicken
Rab21	0.09	1.00000000	chicken
Ddx42	0.15	1.00000000	chicken

Eftud2	0.60	1.00000000	chicken
Fam122b	0.17	1.00000000	chicken
Arid3b	0.10	1.00000000	chicken
Gemin4	0.03	1.00000000	chicken
Uhrf2	0.04	1.00000000	chicken
Klhl24	-0.18	1.00000000	chicken
Cask	-0.19	1.00000000	chicken
Ndufa9	0.03	1.00000000	chicken
Jpt1	-0.11	1.00000000	chicken
Cdk9	-0.06	1.00000000	chicken
Klf10	0.01	1.00000000	chicken
Snap91	0.41	1.00000000	chicken
Rabep1	0.28	1.00000000	chicken
Idh3a	0.13	1.00000000	chicken
Brix1	0.04	1.00000000	chicken
Ufm1	0.11	1.00000000	chicken
Ift20	-0.03	1.00000000	chicken
Rsrc1	0.46	1.00000000	chicken
Psmb6	0.37	1.00000000	chicken
Wdr1	0.06	1.00000000	chicken
Hmgb2	-0.10	1.00000000	chicken
Samhd1	0.34	1.00000000	chicken
Rpf1	-0.18	1.00000000	chicken
Dnlz	-0.03	1.00000000	chicken
Golt1b	0.18	1.00000000	chicken
Cplane2	-0.07	1.00000000	chicken
Chm	0.34	1.00000000	chicken
Hnrnpu	0.20	1.00000000	chicken
Stat3	-0.16	1.00000000	chicken
Lsmem1	-0.02	1.00000000	chicken
Tra2a	-0.05	1.00000000	chicken
Tasor2	0.18	1.00000000	chicken
Cybc1	0.05	1.00000000	chicken
Atad1	-0.03	1.00000000	chicken
Smarcad1	0.59	1.00000000	chicken
Naa15	-0.03	1.00000000	chicken
Syt11	0.16	1.00000000	chicken
Il16	-0.16	1.00000000	chicken
Xpo7	-0.12	1.00000000	chicken
Cnbp	0.25	1.00000000	chicken
Yipf4	0.15	1.00000000	chicken
Per3	-0.14	1.00000000	chicken
Fmc1	-0.09	1.00000000	chicken
Rptor	0.13	1.00000000	chicken
Ripk1	-0.13	1.00000000	chicken
Lamtor1	0.21	1.00000000	chicken

Ptpn11	0.53	1.00000000	chicken
Atp5j2	0.05	1.00000000	chicken
Zmat2	-0.05	1.00000000	chicken
Rnaset2b	-0.46	1.00000000	chicken
Ell	-0.05	1.00000000	chicken
Tpp1	0.05	1.00000000	chicken
Cert1	-0.05	1.00000000	chicken
1810013L24Rik	0.07	1.00000000	chicken
Xpot	-0.06	1.00000000	chicken
Slc7a5	-0.05	1.00000000	chicken
Reck	0.13	1.00000000	chicken
Kdm3b	0.18	1.00000000	chicken
Utp25	0.09	1.00000000	chicken
B230219D22Rik	0.25	1.00000000	chicken
Supt20	-0.08	1.00000000	chicken
9030025P20Rik	0.05	1.00000000	chicken
Drg2	0.18	1.00000000	chicken
Atp6v0a2	-0.09	1.00000000	chicken
Klhl18	-0.15	1.00000000	chicken
Usp49	-0.17	1.00000000	chicken
Sh3bp5	0.34	1.00000000	chicken
Hnrnpr	0.11	1.00000000	chicken
Ppp1cb	0.15	1.00000000	chicken
Armc1	-0.02	1.00000000	chicken
Cdr2	-0.14	1.00000000	chicken
Nop58	-0.11	1.00000000	chicken
Cdc37l1	0.29	1.00000000	chicken
Spg21	0.12	1.00000000	chicken
Ube2j2	-0.04	1.00000000	chicken
Ywhae	-0.16	1.00000000	chicken
Arhgef7	0.01	1.00000000	chicken
Septin14	-0.01	1.00000000	chicken
Sf3b5	0.00	1.00000000	chicken
Ube3c	0.17	1.00000000	chicken
Git2	-0.18	1.00000000	chicken
Gsdme	0.06	1.00000000	chicken
Ythdc2	-0.14	1.00000000	chicken
Klf7	0.44	1.00000000	chicken
Reep3	0.09	1.00000000	chicken
Ccser1	-0.11	1.00000000	chicken
Srp19	-0.04	1.00000000	chicken
Hps3	0.14	1.00000000	chicken
AA467197	-0.14	1.00000000	chicken
Ilkap	-0.10	1.00000000	chicken
Eri3	-0.03	1.00000000	chicken
Pak1	0.13	1.00000000	chicken

Rbm14	-0.10	1.00000000	chicken
Ptbp1	0.08	1.00000000	chicken
Ssbp3	-0.12	1.00000000	chicken
Arhgap6	1.59	1.00000000	chicken
Aig1	-0.03	1.00000000	chicken
Ncbp2	0.57	1.00000000	chicken
Prkar1a	0.10	1.00000000	chicken
Fars2	0.54	1.00000000	chicken
Desi2	0.11	1.00000000	chicken
Birc2	-0.09	1.00000000	chicken
Itpkb	0.06	1.00000000	chicken
Atp2c1	-0.06	1.00000000	chicken
Gpalpp1	-0.04	1.00000000	chicken
Trim33	0.06	1.00000000	chicken
Eps15l1	0.06	1.00000000	chicken
Tmem39a	0.19	1.00000000	chicken
Slc25a20	0.13	1.00000000	chicken
Armh3	-0.25	1.00000000	chicken
Rbm24	0.11	1.00000000	chicken
Eif6	0.00	1.00000000	chicken
Ube4a	0.11	1.00000000	chicken
Slc7a3	0.00	1.00000000	chicken
Dennd6a	0.10	1.00000000	chicken
Dock11	0.05	1.00000000	chicken
Pofut1	0.07	1.00000000	chicken
Polr3a	-0.27	1.00000000	chicken
Bnip3	0.12	1.00000000	chicken
Psme3ip1	-0.06	1.00000000	chicken
Itga1	0.32	1.00000000	chicken
Pecam1	-0.16	1.00000000	chicken
Rbx1	0.06	1.00000000	chicken
Ppp2r5a	0.33	1.00000000	chicken
Zc3h11a	0.04	1.00000000	chicken
Cwc15	0.17	1.00000000	chicken
Tmem59	-0.03	1.00000000	chicken
Eif2s2	0.25	1.00000000	chicken
Adam17	0.33	1.00000000	chicken
H2az1	0.02	1.00000000	chicken
Gpc5	-0.44	1.00000000	chicken
Tox	0.37	1.00000000	chicken
Ccng2	-0.14	1.00000000	chicken
Dkc1	0.13	1.00000000	chicken
Casp3	-0.08	1.00000000	chicken
Zfp423	0.09	1.00000000	chicken
Tsn	0.57	1.00000000	chicken
Taz	-0.08	1.00000000	chicken

Cwc22	-0.06	1.00000000	chicken
Selenok	0.13	1.00000000	chicken
Ppm1a	0.16	1.00000000	chicken
Atp6v0a1	-0.13	1.00000000	chicken
Dock10	0.36	1.00000000	chicken
Pdha1	0.45	1.00000000	chicken
Cirbp	0.03	1.00000000	chicken
Slc26a5	-0.06	1.00000000	chicken
Rif1	0.18	1.00000000	chicken
Zmpste24	0.17	1.00000000	chicken
Timm44	0.10	1.00000000	chicken
Psip1	0.12	1.00000000	chicken
Nfxl1	-0.19	1.00000000	chicken
Zyx	-0.10	1.00000000	chicken
Hibch	0.06	1.00000000	chicken
Xpnpep1	0.04	1.00000000	chicken
Adora2a	-0.09	1.00000000	chicken
Hmgb3	0.05	1.00000000	chicken
Axin1	0.42	1.00000000	chicken
Jade2	-0.08	1.00000000	chicken
Nrip1	-0.33	1.00000000	chicken
Adnp	-0.17	1.00000000	chicken
Atp9b	0.33	1.00000000	chicken
Lsm14a	0.04	1.00000000	chicken
Rlim	0.03	1.00000000	chicken
Brwd1	0.49	1.00000000	chicken
Rbm19	-0.14	1.00000000	chicken
Psma5	-0.08	1.00000000	chicken
Luc7l	0.24	1.00000000	chicken
Ccdc6	0.09	1.00000000	chicken
Dhx29	-0.03	1.00000000	chicken
Higd1a	-0.10	1.00000000	chicken
Kat6a	0.02	1.00000000	chicken
Cdc42se1	0.24	1.00000000	chicken
Nmt1	0.43	1.00000000	chicken
Hnrnph2	0.01	1.00000000	chicken
Far1	0.32	1.00000000	chicken
Ccny	-0.08	1.00000000	chicken
Clk1	0.15	1.00000000	chicken
Cul2	-0.18	1.00000000	chicken
Sumo1	-0.10	1.00000000	chicken
Psmc1	0.18	1.00000000	chicken
Rsf1	-0.03	1.00000000	chicken
Dnaja2	-0.12	1.00000000	chicken
Wdsub1	0.05	1.00000000	chicken
Sla	-0.23	1.00000000	chicken

Klhl13	-0.05	1.00000000	chicken
Nr2c1	0.14	1.00000000	chicken
Arcn1	-0.10	1.00000000	chicken
Lamtor5	0.79	1.00000000	chicken
Edem3	0.10	1.00000000	chicken
Polr2h	0.00	1.00000000	chicken
Cep164	-0.09	1.00000000	chicken
Vps54	0.30	1.00000000	chicken
Sfmbt2	-0.15	1.00000000	chicken
Ap3m1	0.17	1.00000000	chicken
Ptpn1	-0.15	1.00000000	chicken
Lrch1	0.23	1.00000000	chicken
4932411N23Rik	0.01	1.00000000	chicken
Gars	0.06	1.00000000	chicken
Erap1	-0.04	1.00000000	chicken
Oxct1	0.20	1.00000000	chicken
Cul4b	0.07	1.00000000	chicken
Larp7	0.43	1.00000000	chicken
Eif1ad12	0.04	1.00000000	chicken
Zpld1	0.70	1.00000000	chicken
Tmod3	0.03	1.00000000	chicken
Pou2af1	0.07	1.00000000	chicken
Tex9	-0.07	1.00000000	chicken
D430041D05Rik	-0.03	1.00000000	chicken
Zfp384	0.00	1.00000000	chicken
Gtf3c1	0.15	1.00000000	chicken
Sinhcaf	-0.03	1.00000000	chicken
Rab9	-0.09	1.00000000	chicken
Gpatch1	0.28	1.00000000	chicken
Spidr	0.28	1.00000000	chicken
Atp5c1	0.18	1.00000000	chicken
Ago3	0.02	1.00000000	chicken
Lmbr1	-0.01	1.00000000	chicken
Rabgap1	0.19	1.00000000	chicken
Wdr74	0.02	1.00000000	chicken
Rbm45	-0.07	1.00000000	chicken
Thumpd3	0.10	1.00000000	chicken
Atg12	0.05	1.00000000	chicken
Tmem165	0.22	1.00000000	chicken
Csgalnact2	0.04	1.00000000	chicken
Chmp7	0.05	1.00000000	chicken
Fnip1	-0.16	1.00000000	chicken
Efr3a	0.19	1.00000000	chicken
Kat7	-0.24	1.00000000	chicken
Tial1	0.12	1.00000000	chicken
Ndufs5	0.04	1.00000000	chicken

Uqcrc2	0.33	1.00000000	chicken
Unk	0.06	1.00000000	chicken
Ube2a	0.21	1.00000000	chicken
Abat	0.12	1.00000000	chicken
Ppp1r21	-0.07	1.00000000	chicken
St7	0.46	1.00000000	chicken
Acp1	0.09	1.00000000	chicken
Vmp1	-0.10	1.00000000	chicken
Brox	0.34	1.00000000	chicken
Top1	0.17	1.00000000	chicken
Dnajb4	0.01	1.00000000	chicken
Chmp4b	0.07	1.00000000	chicken
Rrp15	0.47	1.00000000	chicken
Adat1	-0.32	1.00000000	chicken
Vps18	-0.08	1.00000000	chicken
Rchy1	0.07	1.00000000	chicken
Def6	0.13	1.00000000	chicken
Lactb	0.03	1.00000000	chicken
Rpap1	-0.11	1.00000000	chicken
Lrrk1	-0.10	1.00000000	chicken
Card11	-0.08	1.00000000	chicken
Tbc1d22a	0.22	1.00000000	chicken
Fam172a	0.12	1.00000000	chicken
Vps4b	-0.10	1.00000000	chicken
Twf2	-0.20	1.00000000	chicken
Rimklb	0.22	1.00000000	chicken
Cnpy3	-0.03	1.00000000	chicken
Vps39	0.20	1.00000000	chicken
Sav1	-0.05	1.00000000	chicken
Rsl1d1	0.17	1.00000000	chicken
Psmd3	0.33	1.00000000	chicken
Gpr107	-0.07	1.00000000	chicken
Asb5	-0.04	1.00000000	chicken
Utp6	0.58	1.00000000	chicken
Get4	0.04	1.00000000	chicken
Letm1	0.42	1.00000000	chicken
Bzw2	0.16	1.00000000	chicken
R3hdm4	-0.02	1.00000000	chicken
Txnrd2	0.11	1.00000000	chicken
Cbwd1	0.55	1.00000000	chicken
Tmem245	-0.02	1.00000000	chicken
Dync1h1	-0.13	1.00000000	chicken
Parp8	-0.27	1.00000000	chicken
Ammecr1	0.35	1.00000000	chicken
Fbrsl1	0.14	1.00000000	chicken
Grcc10	-0.02	1.00000000	chicken

Naa25	-0.28	1.00000000	chicken
Itpr2	-0.01	1.00000000	chicken
Usp10	-0.01	1.00000000	chicken
Sipa1l1	0.21	1.00000000	chicken
Cacybp	0.46	1.00000000	chicken
Sem1	0.06	1.00000000	chicken
Pcca	-0.28	1.00000000	chicken
Ndufs7	0.02	1.00000000	chicken
Glod5	-0.11	1.00000000	chicken
Nsd3	0.01	1.00000000	chicken
Npepps	0.27	1.00000000	chicken
Psmb3	-0.07	1.00000000	chicken
Ankrd13d	-0.04	1.00000000	chicken
Stx18	0.28	1.00000000	chicken
Ccdc82	-0.10	1.00000000	chicken
Chmp2a	-0.07	1.00000000	chicken
Surf4	0.01	1.00000000	chicken
Nadk	0.19	1.00000000	chicken
Arhgap30	-0.01	1.00000000	chicken
Phf3	0.22	1.00000000	chicken
Kdelr2	0.12	1.00000000	chicken
Obi1	-0.08	1.00000000	chicken
Zfp592	0.08	1.00000000	chicken
Cyria	0.10	1.00000000	chicken
Klhdc4	0.07	1.00000000	chicken
Larp4	-0.16	1.00000000	chicken
Etfa	0.01	1.00000000	chicken
Flnb	0.04	1.00000000	chicken
Lclat1	0.30	1.00000000	chicken
Usp48	-0.01	1.00000000	chicken
Timm10	0.04	1.00000000	chicken
Etf1	0.06	1.00000000	chicken
Itgb7	-0.22	1.00000000	chicken
Dis3	0.26	1.00000000	chicken
Lig3	-0.07	1.00000000	chicken
Adgra3	-0.14	1.00000000	chicken
LTO1	0.16	1.00000000	chicken
Tbc1d15	0.08	1.00000000	chicken
Ric1	0.08	1.00000000	chicken
Dync1i2	0.20	1.00000000	chicken
Usp4	-0.07	1.00000000	chicken
Necap1	0.14	1.00000000	chicken
Rab11a	-0.21	1.00000000	chicken
Rab5a	-0.22	1.00000000	chicken
Angel1	-0.06	1.00000000	chicken
Sptlc2	0.31	1.00000000	chicken

Rrs1	-0.05	1.00000000	chicken
Arfgap3	-0.02	1.00000000	chicken
D630045J12Rik	-0.05	1.00000000	chicken
Ttc4	0.05	1.00000000	chicken
Rasa2	-0.18	1.00000000	chicken
Arntl2	0.29	1.00000000	chicken
Srp68	0.05	1.00000000	chicken
Exoc1	0.12	1.00000000	chicken
Slamf8	-0.08	1.00000000	chicken
H13	0.08	1.00000000	chicken
Caprin1	-0.04	1.00000000	chicken
Larp1	-0.18	1.00000000	chicken
Plekha2	-0.09	1.00000000	chicken
Ints6l	-0.03	1.00000000	chicken
Abraxas1	-0.07	1.00000000	chicken
Copg1	0.13	1.00000000	chicken
Necap2	0.15	1.00000000	chicken
Hacd3	-0.08	1.00000000	chicken
Aup1	-0.01	1.00000000	chicken
Sertad2	-0.10	1.00000000	chicken
Ercc3	-0.12	1.00000000	chicken
Nfx1	-0.06	1.00000000	chicken
Slc25a32	0.37	1.00000000	chicken
Slc30a7	0.29	1.00000000	chicken
Snap29	-0.36	1.00000000	chicken
Ly96	0.16	1.00000000	chicken
Tmem185a	-0.16	1.00000000	chicken
Acbd5	-0.01	1.00000000	chicken
Fam122a	0.07	1.00000000	chicken
Wdr61	-0.06	1.00000000	chicken
Herpud2	0.34	1.00000000	chicken
Ccz1	0.04	1.00000000	chicken
Atp13a2	0.01	1.00000000	chicken
Rcn2	-0.06	1.00000000	chicken
Zmynd8	0.19	1.00000000	chicken
Fam160b1	-0.05	1.00000000	chicken
Secisbp2l	-0.08	1.00000000	chicken
Ube2g2	0.26	1.00000000	chicken
Lyar	0.15	1.00000000	chicken
Psmc6	0.04	1.00000000	chicken
Pcgf6	-0.06	1.00000000	chicken
Zcchc10	-0.17	1.00000000	chicken
Pwwp2a	0.22	1.00000000	chicken
Ntmt1	0.03	1.00000000	chicken
Smarcal1	-2.38	0.00000000	chicken
Tpt1	-2.30	0.00000000	chicken

Rack1	-2.51	0.00000001	chicken
Eef1b2	-2.24	0.00000002	chicken
Ybx1	-2.38	0.00000010	chicken
Eef2	-1.60	0.00000728	chicken
Actg1	-1.75	0.00004918	chicken
Fth1	-1.26	0.00005915	chicken
B2m	-1.30	0.00006824	chicken
Eef1d	-1.83	0.00007284	chicken
Pabpc1	-1.28	0.00014166	chicken
Nme2	-1.77	0.00016858	chicken
Cd3g	-2.15	0.00061588	chicken
Tmsb4x	-1.11	0.00383009	chicken
Eepd1	-2.24	0.00460858	chicken
H3f3a	-1.13	0.00589525	chicken
Ulbp1	-1.45	0.00665107	chicken
Fau	-1.55	0.00917355	chicken
Il27ra	-1.62	0.00919195	chicken
Cd74	-1.20	0.01056211	chicken
Nsa2	-1.59	0.04294885	chicken
Eif5a2	-1.46	0.06542408	chicken
Gpx1	-1.58	0.06546027	chicken
Anxa1	3.32	0.00000000	duck
Plac8	2.13	0.00000000	duck
Fabp3	1.97	0.00000000	duck
Als2cl	3.55	0.00000002	duck
Wnt5b	1.53	0.00000002	duck
Nos2	3.36	0.00000002	duck
Abtb2	4.34	0.00000014	duck
Crispld2	1.73	0.00000037	duck
Jun	2.44	0.00000214	duck
Nfam1	2.50	0.00000272	duck
Angpt1	2.49	0.00000336	duck
Rxra	2.42	0.00000464	duck
Glrx	2.85	0.00000607	duck
Prok2	1.49	0.00000627	duck
Laptm5	2.95	0.00000751	duck
Arg2	1.49	0.00000856	duck
Pim3	2.41	0.00001755	duck
Kcnk5	1.91	0.00002118	duck
Slc38a2	1.83	0.00003742	duck
Tnfaip3	2.34	0.00003872	duck
Cpb2	1.95	0.00006388	duck
Crot	2.18	0.00007071	duck
Lmnb1	2.07	0.00009540	duck
Cebpb	2.25	0.00010434	duck
Fosl2	2.33	0.00015805	duck

Jund	2.00	0.00023424	duck
Pik3r5	2.14	0.00023987	duck
Ptafr	2.36	0.00026605	duck
Atp6v1b2	2.02	0.00051511	duck
Wdfy3	2.30	0.00058036	duck
Serpine2	1.94	0.00072329	duck
Itsn1	2.69	0.00072507	duck
Zc3h12a	2.56	0.00076834	duck
Hnrnpll	2.80	0.00077887	duck
Fgfbp1	2.43	0.00106044	duck
Acsl1	2.60	0.00119583	duck
Cd9	2.36	0.00135587	duck
Dusp1	2.50	0.00136238	duck
Cebpd	2.17	0.00142696	duck
F13a1	1.29	0.00148312	duck
Ankrd22	1.56	0.00235380	duck
Fth1	2.25	0.00264756	duck
Optn	1.41	0.00276933	duck
Ica1	1.47	0.00278679	duck
Abca1	2.04	0.00278783	duck
Atf3	2.25	0.00311547	duck
Tbc1d2	1.14	0.00356225	duck
Pla2g4a	1.96	0.00401415	duck
Tax1bp1	2.71	0.00434787	duck
Actb	2.34	0.00449529	duck
Samsn1	2.70	0.00454638	duck
Adora2b	1.71	0.00477130	duck
Cass4	1.38	0.00500551	duck
Kif21a	1.20	0.00623136	duck
Drd4	2.04	0.00672350	duck
Tm6sf1	1.09	0.00700112	duck
Lcp1	1.86	0.00724666	duck
S100a11	3.09	0.00740838	duck
Chd7	2.35	0.00765869	duck
Hpgds	1.61	0.00776117	duck
Chst13	2.19	0.00790387	duck
Nfkbiz	1.92	0.01009823	duck
Upp1	1.30	0.01071870	duck
Fam102b	1.94	0.01330989	duck
Rbms3	2.61	0.01379267	duck
Runx1t1	1.73	0.01448338	duck
Lrpap1	1.11	0.01448338	duck
Lpar1	1.48	0.01448338	duck
Ephx1	1.19	0.01451198	duck
Bri3	1.60	0.01492228	duck
Pam	2.07	0.01759916	duck

Klf15	1.02	0.01761597	duck
Pgd	1.78	0.01806364	duck
Adamtsl3	1.16	0.01873820	duck
Npc2	1.76	0.01931327	duck
Sh3gl3	1.02	0.01992908	duck
Pparg	2.64	0.02305543	duck
Osbpl6	2.10	0.03754672	duck
Rab20	1.61	0.03908170	duck
Scube2	1.53	0.03976886	duck
Chsy1	1.86	0.04019853	duck
Stat1	1.60	0.04250694	duck
Bach1	1.60	0.04636539	duck
Trib1	2.42	0.04870947	duck
Lamp1	2.07	0.05067791	duck
Arhgap29	1.68	0.05204819	duck
Ikzf2	1.32	0.08303399	duck
Slc40a1	1.54	0.09710105	duck
Nt5e	0.66	0.01873820	duck
Cebpa	1.00	0.01873820	duck
P2ry13	0.96	0.09616476	duck
Emb	2.15	0.10342533	duck
Aspg	2.36	0.10901962	duck
Septin9	-1.37	0.10917474	duck
Ercc6	-1.23	0.11244337	duck
Lrmda	2.29	0.12593626	duck
Rnf144b	-1.88	0.13685184	duck
Pde4b	1.41	0.14743556	duck
Bach2	-1.11	0.15292909	duck
Sucnr1	1.67	0.15341946	duck
Tgm4	2.83	0.16177961	duck
Vdac1	0.83	0.16469499	duck
Sdc2	1.61	0.17057398	duck
Slc39a6	-1.10	0.17354751	duck
Rgs1	-1.26	0.19160395	duck
Tmem87a	-1.40	0.19444802	duck
Rap1gds1	-1.70	0.19465950	duck
Plxnb2	1.20	0.19539771	duck
Nhs	1.03	0.19539771	duck
Ptprk	1.29	0.19539771	duck
Dipk1b	0.67	0.19539771	duck
Tulp1	0.50	0.19539771	duck
C3	0.54	0.19539771	duck
Naa16	-1.63	0.20093945	duck
Rab12	-1.10	0.20155074	duck
Tmem131	-1.27	0.20423162	duck
Rnf19b	1.95	0.20713341	duck

Atxn7	-1.54	0.20931273	duck
Tbc1d5	-1.65	0.21243517	duck
Hbegf	1.03	0.22992339	duck
Egfr	1.52	0.23234259	duck
Cldn18	2.02	0.23234259	duck
Zc3h12d	-0.76	0.23911010	duck
Fuca2	1.71	0.23987176	duck
Prkd1	-1.90	0.25019954	duck
Acod1	0.84	0.26179357	duck
Col12a1	1.19	0.26179357	duck
Foxo1	-1.02	0.27632010	duck
Fzd6	1.58	0.28922086	duck
Dek	-1.42	0.29359498	duck
Fubp1	-1.27	0.31819808	duck
Pcgf5	-1.47	0.34085141	duck
Sccpdh	1.66	0.35631593	duck
Traf5	-1.61	0.36125828	duck
Ssbp3	-1.55	0.36199076	duck
N4bp2	-0.68	0.36443210	duck
Trabd	-1.24	0.36502060	duck
Atp1b1	1.93	0.37481996	duck
Csf3r	1.36	0.38592342	duck
Snx10	2.06	0.39182882	duck
Kif15	1.24	0.40252442	duck
Scaf11	-1.35	0.40947837	duck
Ilk	1.08	0.40978478	duck
Hormad1	0.98	0.42974357	duck
Scaf8	-1.26	0.49094977	duck
Prelid3b	1.41	0.51844989	duck
L3mbtl3	-1.45	0.53352959	duck
Oat	1.59	0.53693338	duck
Pan3	-1.17	0.55108212	duck
Pid1	1.66	0.55499626	duck
Bmf	-1.25	0.55690132	duck
Kremen1	-1.46	0.55935516	duck
Reps2	1.96	0.61682347	duck
Irak2	1.75	0.61737736	duck
Ppargc1a	1.54	0.62036203	duck
Ugt8a	0.82	0.63314158	duck
Hvcn1	-1.24	0.64728840	duck
Ranbp10	-1.30	0.67643704	duck
Nrip1	1.06	0.70106328	duck
Casp3	1.50	0.70226379	duck
Anxa11	1.60	0.72323432	duck
Abcg1	1.97	0.72449812	duck
Efhd2	0.89	0.74634177	duck

Fos	2.12	0.75991060	duck
Numb	1.98	0.77082444	duck
Usp22	-1.29	0.77758672	duck
Lrp12	-1.43	0.78566951	duck
Exoc6	-1.45	0.78654082	duck
Ubp1	-1.45	0.83897499	duck
Pik3ip1	-1.13	0.85593851	duck
Fbxw7	-1.65	0.87776007	duck
Zbtb16	2.19	0.88360133	duck
Ada	-1.01	0.89766257	duck
Rabl6	-1.39	0.89996439	duck
Zfp516	2.56	0.90533593	duck
Park7	-1.34	0.95929384	duck
Antxr2	1.41	0.96745970	duck
Klhl5	-1.60	0.98191941	duck
Lig3	-1.47	0.98481768	duck
Rdx	-1.20	0.98598701	duck
Pls3	1.26	0.99379912	duck
Herpud1	1.53	0.99623270	duck
Zmynd11	-1.57	1.00000000	duck
Gpr137b	-0.88	1.00000000	duck
Prelid3a	1.17	1.00000000	duck
Ptprm	1.41	1.00000000	duck
Cd83	-1.34	1.00000000	duck
Trim24	-1.60	1.00000000	duck
Ereg	2.09	1.00000000	duck
Smarca2	-0.97	1.00000000	duck
Atf4	1.67	1.00000000	duck
Hmgn3	-1.10	1.00000000	duck
Actn1	1.86	1.00000000	duck
Nadk	1.53	1.00000000	duck
Klf6	1.94	1.00000000	duck
Ppp2r5e	-1.28	1.00000000	duck
Cpt1a	1.72	1.00000000	duck
Ythdf1	-0.91	1.00000000	duck
Zbtb20	2.48	1.00000000	duck
Ubtd2	-1.02	1.00000000	duck
Cluh	-1.23	1.00000000	duck
Hivep2	-0.68	1.00000000	duck
Prdm15	-0.74	1.00000000	duck
Apoo	-1.11	1.00000000	duck
Ctdp1	-1.20	1.00000000	duck
Lcp2	1.75	1.00000000	duck
Anxa4	0.64	1.00000000	duck
Slc35b3	-1.42	1.00000000	duck
Rbm5	-0.86	1.00000000	duck

Ythdc1	-0.96	1.00000000	duck
Trps1	-1.30	1.00000000	duck
Ppp4r3a	-1.14	1.00000000	duck
Clock	-1.18	1.00000000	duck
Hnrnpr	-0.79	1.00000000	duck
Adgrg3	0.57	1.00000000	duck
Acsbg2	1.37	1.00000000	duck
Snx29	-1.91	1.00000000	duck
Sh3bgrl	1.40	1.00000000	duck
Usp1	-1.00	1.00000000	duck
Tspan18	-1.42	1.00000000	duck
Pxylp1	-1.10	1.00000000	duck
Ide	-1.25	1.00000000	duck
Ostm1	1.18	1.00000000	duck
Dicer1	-1.17	1.00000000	duck
Txlng	-1.12	1.00000000	duck
Fem1c	1.37	1.00000000	duck
Ccdc6	-1.09	1.00000000	duck
Slc9a2	0.75	1.00000000	duck
Fgf23	0.75	1.00000000	duck
Cpne8	0.27	1.00000000	duck
Zfpm2	0.40	1.00000000	duck
Khdrbs3	0.49	1.00000000	duck
Sash1	0.42	1.00000000	duck
Plcb1	0.47	1.00000000	duck
Pkdcc	0.85	1.00000000	duck
Slc35f3	0.63	1.00000000	duck
Fgl1	0.65	1.00000000	duck
Fgf10	1.39	1.00000000	duck
Npas3	1.09	1.00000000	duck
Syde2	0.56	1.00000000	duck
Smad3	1.41	1.00000000	duck
Fgf7	0.70	1.00000000	duck
Tox3	0.41	1.00000000	duck
Pdgfrb	0.92	1.00000000	duck
Slc35e4	0.29	1.00000000	duck
Plbd2	0.86	1.00000000	duck
Kcnj2	1.04	1.00000000	duck
Atp12a	0.85	1.00000000	duck
Lgi3	0.31	1.00000000	duck
Adm2	0.40	1.00000000	duck
Apobec1	0.47	1.00000000	duck
Syn2	0.80	1.00000000	duck
Plekhh3	0.45	1.00000000	duck
Zfp318	-1.14	1.00000000	duck
Parp1	-0.86	1.00000000	duck

Rasa2	-0.79	1.00000000	duck
Abcb7	-1.35	1.00000000	duck
Cflar	-1.30	1.00000000	duck
Spi1	1.56	1.00000000	duck
Vegfc	1.20	1.00000000	duck
Dnajb5	0.83	1.00000000	duck
Add1	-1.19	1.00000000	duck
Ttc39b	1.87	1.00000000	duck
Hmox1	1.14	1.00000000	duck
Tmem245	-1.28	1.00000000	duck
Tmem67	0.66	1.00000000	duck
Ppm1k	0.41	1.00000000	duck
Dhx58	0.47	1.00000000	duck
Larp4	-1.10	1.00000000	duck
Ikzf1	-1.29	1.00000000	duck
Ctbp1	-0.82	1.00000000	duck
Ankrd10	-1.01	1.00000000	duck
Kdm4c	-1.50	1.00000000	duck
Acss1	0.83	1.00000000	duck
Arhgef3	-0.20	1.00000000	duck
Grid1	1.22	1.00000000	duck
Depdc1b	1.60	1.00000000	duck
Unc13d	0.86	1.00000000	duck
Ptgs2	1.31	1.00000000	duck
Plin2	1.60	1.00000000	duck
Trmt11	-0.96	1.00000000	duck
Esco1	-1.12	1.00000000	duck
Tfpi2	0.85	1.00000000	duck
Ncoa7	-1.36	1.00000000	duck
Acap2	1.85	1.00000000	duck
Lrrc47	-1.17	1.00000000	duck
Tnfrsf11a	1.32	1.00000000	duck
Tor4a	0.96	1.00000000	duck
Ppp1cc	-0.89	1.00000000	duck
Sat1	1.15	1.00000000	duck
Ywhah	-0.80	1.00000000	duck
Epsti1	0.98	1.00000000	duck
Robo2	1.25	1.00000000	duck
Fam78a	-0.71	1.00000000	duck
Rbm33	-0.91	1.00000000	duck
Cd47	1.36	1.00000000	duck
Neurl1b	1.20	1.00000000	duck
Fyn	1.82	1.00000000	duck
Cdk6	-1.93	1.00000000	duck
Dach2	1.33	1.00000000	duck
Rmdn3	-0.44	1.00000000	duck

Ptpre	1.18	1.00000000	duck
Rexo1	-1.21	1.00000000	duck
Ttbk1	-0.62	1.00000000	duck
Atp6ap2	1.14	1.00000000	duck
Smim14	-1.05	1.00000000	duck
Sema4a	1.08	1.00000000	duck
Trak1	1.84	1.00000000	duck
Tbpl1	-0.97	1.00000000	duck
Znrf3	-1.66	1.00000000	duck
Vav2	-1.01	1.00000000	duck
Srgn	2.12	1.00000000	duck
Ctsb	1.61	1.00000000	duck
G0s2	2.10	1.00000000	duck
Pld4	-0.85	1.00000000	duck
Vps13b	-1.68	1.00000000	duck
Nfkb1	1.19	1.00000000	duck
Rhoh	-0.33	1.00000000	duck
Akap1	-0.88	1.00000000	duck
Rras2	-0.95	1.00000000	duck
Hdac2	-1.13	1.00000000	duck
Ago2	-1.56	1.00000000	duck
Usp53	-1.70	1.00000000	duck
Zc3h13	-0.24	1.00000000	duck
Arhgef12	-0.52	1.00000000	duck
Tbk1	-0.88	1.00000000	duck
Gadd45g	1.54	1.00000000	duck
Luc7l3	-0.97	1.00000000	duck
Tra2a	-0.83	1.00000000	duck
Dars	-0.61	1.00000000	duck
Srp14	-0.93	1.00000000	duck
Wnk1	1.55	1.00000000	duck
Dnttip2	-1.37	1.00000000	duck
Elovl5	0.85	1.00000000	duck
Arid4b	-0.72	1.00000000	duck
Bcas3	1.89	1.00000000	duck
Phactr4	1.19	1.00000000	duck
Zcchc2	-0.96	1.00000000	duck
Nedd4	-1.63	1.00000000	duck
Nfatc2	-1.28	1.00000000	duck
Zfp512	-1.07	1.00000000	duck
Casp6	-0.78	1.00000000	duck
Ipo7	-1.04	1.00000000	duck
Ppp6r2	-0.80	1.00000000	duck
Amfr	1.23	1.00000000	duck
Lyrm7	-1.04	1.00000000	duck
Nsd1	-0.74	1.00000000	duck

Phf20l1	-1.34	1.00000000	duck
Serinc5	-1.28	1.00000000	duck
Baz1b	-0.32	1.00000000	duck
Fgd3	-1.06	1.00000000	duck
Eif3e	-0.82	1.00000000	duck
Sec23b	-0.78	1.00000000	duck
Tcp1	-0.89	1.00000000	duck
Zfyve28	2.39	1.00000000	duck
Tmem131l	-0.94	1.00000000	duck
Yap1	1.31	1.00000000	duck
Panx2	0.93	1.00000000	duck
Cxcr5	-1.00	1.00000000	duck
Btbd9	-1.64	1.00000000	duck
Hk2	1.35	1.00000000	duck
Rif1	-1.04	1.00000000	duck
Lonrf1	-1.21	1.00000000	duck
Cep170	-0.94	1.00000000	duck
Cxcr4	-0.52	1.00000000	duck
St3gal5	-1.20	1.00000000	duck
Laptm4a	0.98	1.00000000	duck
Rufy2	-1.03	1.00000000	duck
Gpx1	1.87	1.00000000	duck
Hsp90b1	-0.21	1.00000000	duck
Aff1	1.39	1.00000000	duck
Bcl2	-1.53	1.00000000	duck
Enpp4	1.13	1.00000000	duck
Srsf10	-0.70	1.00000000	duck
Cep85l	-0.98	1.00000000	duck
Cwc27	-1.37	1.00000000	duck
Zeb2	1.65	1.00000000	duck
Ntng1	1.96	1.00000000	duck
Arhgdib	1.87	1.00000000	duck
Zswim6	0.97	1.00000000	duck
Hibadh	1.86	1.00000000	duck
Susd6	-0.82	1.00000000	duck
Ifitm10	0.56	1.00000000	duck
Fnip1	1.63	1.00000000	duck
Zc3h12c	-1.38	1.00000000	duck
Thada	-1.36	1.00000000	duck
Acp2	1.10	1.00000000	duck
Hras	0.98	1.00000000	duck
Lims2	1.39	1.00000000	duck
Fut8	-1.29	1.00000000	duck
Pisd	-0.92	1.00000000	duck
Ubac2	-1.02	1.00000000	duck
Swt1	-1.06	1.00000000	duck

Rad51b	-1.11	1.00000000	duck
Btbd7	-0.83	1.00000000	duck
Pnrc1	0.95	1.00000000	duck
Slc25a24	0.78	1.00000000	duck
Clec16a	-0.79	1.00000000	duck
Tnrc6b	-1.94	1.00000000	duck
Prkcq	-1.03	1.00000000	duck
Adnp2	-0.97	1.00000000	duck
Dot1l	1.94	1.00000000	duck
Atp6v0e	1.09	1.00000000	duck
Ell	1.38	1.00000000	duck
Rasgrp3	-0.92	1.00000000	duck
Iqgap2	-1.09	1.00000000	duck
Kat6b	-0.67	1.00000000	duck
Dmxl1	-1.21	1.00000000	duck
Zmym2	-0.98	1.00000000	duck
Rsl1d1	-0.84	1.00000000	duck
Ralgapa1	-1.11	1.00000000	duck
Whamm	-0.81	1.00000000	duck
Dip2a	-0.93	1.00000000	duck
Galnt7	-1.33	1.00000000	duck
Zfyve26	-1.12	1.00000000	duck
Jarid2	-0.94	1.00000000	duck
Sms	-1.08	1.00000000	duck
Acmsd	-1.41	1.00000000	duck
Stt3b	-0.65	1.00000000	duck
Bicd2	-0.53	1.00000000	duck
Fbxo33	-0.81	1.00000000	duck
Slc9a7	-0.70	1.00000000	duck
Gsk3b	0.72	1.00000000	duck
Dusp10	-0.58	1.00000000	duck
Cdk19	-1.33	1.00000000	duck
Atp6v1a	1.47	1.00000000	duck
Lypla1	-0.89	1.00000000	duck
Sos1	-0.92	1.00000000	duck
Dele1	-0.99	1.00000000	duck
Rprd1b	-0.85	1.00000000	duck
Actr3	1.41	1.00000000	duck
Lbh	-0.79	1.00000000	duck
Pxn	0.73	1.00000000	duck
Rab14	-0.89	1.00000000	duck
Wbp11	-1.01	1.00000000	duck
Dapk1	-0.77	1.00000000	duck
Dgkd	-0.58	1.00000000	duck
Arl6ip5	1.10	1.00000000	duck
Traf3ip3	-0.63	1.00000000	duck

Scarb2	0.39	1.00000000	duck
Ppp1r3c	0.49	1.00000000	duck
Gnai3	1.24	1.00000000	duck
Ifngr2	1.43	1.00000000	duck
Cilk1	-0.94	1.00000000	duck
Ptbp2	-0.42	1.00000000	duck
Scaf4	-0.94	1.00000000	duck
Cog6	-0.96	1.00000000	duck
Tle5	-0.66	1.00000000	duck
Hectd1	1.39	1.00000000	duck
Slc4a2	0.35	1.00000000	duck
Zfp236	-1.14	1.00000000	duck
Pkm	1.27	1.00000000	duck
Dnah17	-0.72	1.00000000	duck
Dusp7	1.75	1.00000000	duck
Vps13d	-0.71	1.00000000	duck
Dnajb9	0.87	1.00000000	duck
Kalrn	1.15	1.00000000	duck
Cfd	0.95	1.00000000	duck
Abca13	1.43	1.00000000	duck
Prdm10	-0.76	1.00000000	duck
Hmgxb4	-0.70	1.00000000	duck
Pnisr	-0.63	1.00000000	duck
Actr2	1.30	1.00000000	duck
Foxk2	-1.06	1.00000000	duck
Eif5a2	-0.85	1.00000000	duck
Ss18	-0.86	1.00000000	duck
Dock11	-0.91	1.00000000	duck
Dennd2d	-0.71	1.00000000	duck
Dmxl2	0.46	1.00000000	duck
Slc16a3	1.00	1.00000000	duck
Xpo4	-0.43	1.00000000	duck
Znrf2	-0.85	1.00000000	duck
Gsg1l	-1.55	1.00000000	duck
Armh3	-1.20	1.00000000	duck
Irag1	0.70	1.00000000	duck
Nucks1	-0.63	1.00000000	duck
Erbin	-0.82	1.00000000	duck
Gusb	0.87	1.00000000	duck
Zdhhc8	-0.91	1.00000000	duck
Hspa8	1.45	1.00000000	duck
Fndc3a	-0.98	1.00000000	duck
Garem1	2.04	1.00000000	duck
Susd1	0.50	1.00000000	duck
Siah2	-0.16	1.00000000	duck
Ppp6r3	-0.64	1.00000000	duck

Sp3	-0.55	1.00000000	duck
Elf1	-0.78	1.00000000	duck
Rabep1	-1.02	1.00000000	duck
Taldo1	0.73	1.00000000	duck
Zfp652	-0.92	1.00000000	duck
Gnpda1	0.58	1.00000000	duck
Rasgef1b	0.85	1.00000000	duck
Tasp1	-0.42	1.00000000	duck
Ywhab	-0.69	1.00000000	duck
Capn7	-1.21	1.00000000	duck
Rbm34	-0.90	1.00000000	duck
Basp1	-0.91	1.00000000	duck
Kctd5	-0.36	1.00000000	duck
Pank3	-0.83	1.00000000	duck
Ap1s2	1.20	1.00000000	duck
Baiap2l1	0.71	1.00000000	duck
Dipk1a	-1.00	1.00000000	duck
Cdc123	1.11	1.00000000	duck
Foxi1	-0.73	1.00000000	duck
Creb3l2	0.89	1.00000000	duck
Zfand2a	1.33	1.00000000	duck
Abcc4	-1.30	1.00000000	duck
Ubac1	-0.74	1.00000000	duck
Prpf4b	-0.67	1.00000000	duck
Sh3pxd2a	1.07	1.00000000	duck
Slc7a11	1.31	1.00000000	duck
Cttn	-0.67	1.00000000	duck
Sh3bgrl3	1.79	1.00000000	duck
Cep170b	0.92	1.00000000	duck
Gab1	2.15	1.00000000	duck
Prkdc	-1.07	1.00000000	duck
Caprin1	-0.79	1.00000000	duck
Xpo7	-0.85	1.00000000	duck
Prkar2b	-1.29	1.00000000	duck
Phf20	-0.71	1.00000000	duck
Slc25a16	-0.58	1.00000000	duck
Nop58	-0.71	1.00000000	duck
Fam126a	-0.87	1.00000000	duck
Jade2	-0.76	1.00000000	duck
Tia1	-0.53	1.00000000	duck
Ccndbp1	-0.93	1.00000000	duck
Acsl4	1.13	1.00000000	duck
Naa15	-1.20	1.00000000	duck
Lrmp	-0.85	1.00000000	duck
Dhrs7c	-0.94	1.00000000	duck
Usp45	-0.50	1.00000000	duck

Ipo5	-0.58	1.00000000	duck
Dcaf6	1.77	1.00000000	duck
Noc2l	-0.72	1.00000000	duck
Ap1m1	-0.77	1.00000000	duck
Polr3e	-0.84	1.00000000	duck
Cdc73	-1.26	1.00000000	duck
Ctss	1.47	1.00000000	duck
Lin7c	-0.61	1.00000000	duck
Adam17	-0.89	1.00000000	duck
Ulk2	-0.78	1.00000000	duck
Wbp4	-0.99	1.00000000	duck
Cct2	-0.81	1.00000000	duck
4932411N23Rik	-0.85	1.00000000	duck
Ddx24	-0.82	1.00000000	duck
Itm2a	1.19	1.00000000	duck
Srsf11	-0.67	1.00000000	duck
Tbc1d22a	-1.12	1.00000000	duck
Pgs1	-0.74	1.00000000	duck
Hbp1	-0.90	1.00000000	duck
Atic	-0.80	1.00000000	duck
Eps8	0.72	1.00000000	duck
Tmem243	0.96	1.00000000	duck
Sh3rf1	1.15	1.00000000	duck
Slit2	0.92	1.00000000	duck
Cdo1	1.37	1.00000000	duck
Lifr	0.37	1.00000000	duck
Ak7	0.87	1.00000000	duck
Cachd1	1.27	1.00000000	duck
Tmem120a	0.35	1.00000000	duck
Slc9a4	0.98	1.00000000	duck
Capns2	0.68	1.00000000	duck
Usp6nl	1.28	1.00000000	duck
Dhrs3	0.35	1.00000000	duck
Serbp1	-0.88	1.00000000	duck
Card11	-0.93	1.00000000	duck
Traf3	1.62	1.00000000	duck
Rgs3	-0.51	1.00000000	duck
Pdlim5	1.12	1.00000000	duck
Ackr4	0.93	1.00000000	duck
Vegfa	0.52	1.00000000	duck
Kcnj5	0.38	1.00000000	duck
Synm	-2.77	1.00000000	duck
Nprl3	-1.02	1.00000000	duck
Emc2	-0.83	1.00000000	duck
Tpk1	-1.25	1.00000000	duck
Wdr77	-0.59	1.00000000	duck

Txnrd1	1.94	1.00000000	duck
Trnt1	-0.67	1.00000000	duck
Slc5a1	-0.91	1.00000000	duck
Uhrf2	-1.27	1.00000000	duck
Gab2	0.24	1.00000000	duck
Pdia5	0.28	1.00000000	duck
Rnd3	0.43	1.00000000	duck
Sox6	-3.45	1.00000000	duck
Tmem164	-0.57	1.00000000	duck
Kdm3b	-0.65	1.00000000	duck
Rpap2	-1.09	1.00000000	duck
Yipf4	0.76	1.00000000	duck
Vash1	-1.02	1.00000000	duck
Tbc1d1	-1.05	1.00000000	duck
Castor2	0.15	1.00000000	duck
Ngf	0.32	1.00000000	duck
Proser2	0.64	1.00000000	duck
Pamr1	0.36	1.00000000	duck
Ppil4	-0.32	1.00000000	duck
Hnrnpd	-0.73	1.00000000	duck
Senp6	-0.75	1.00000000	duck
Nup133	-0.80	1.00000000	duck
Hnrnpa3	-0.58	1.00000000	duck
Atp6v0a2	-0.96	1.00000000	duck
Rnf166	1.01	1.00000000	duck
Parp8	-0.94	1.00000000	duck
Gfod1	-1.12	1.00000000	duck
Ubap1	-0.79	1.00000000	duck
Polr1a	-0.96	1.00000000	duck
Gpbp1l1	-0.82	1.00000000	duck
Ncf1	1.71	1.00000000	duck
Sun1	-0.49	1.00000000	duck
Nup50	-0.52	1.00000000	duck
Pdcd4	-0.92	1.00000000	duck
Gfod2	0.80	1.00000000	duck
Uhrf1bp1	-0.73	1.00000000	duck
Bfar	-0.57	1.00000000	duck
Dhx37	-0.52	1.00000000	duck
Gapvd1	-0.90	1.00000000	duck
Zbtb38	-1.10	1.00000000	duck
Supv3l1	-0.78	1.00000000	duck
Ing3	-0.63	1.00000000	duck
Suz12	-0.72	1.00000000	duck
Psmg3	-0.67	1.00000000	duck
Scrib	-0.44	1.00000000	duck
Smu1	1.37	1.00000000	duck

Pde10a	1.93	1.00000000	duck
Slc25a47	1.36	1.00000000	duck
Nfil3	1.60	1.00000000	duck
Prdm2	-0.80	1.00000000	duck
Cbfa2t3	-1.04	1.00000000	duck
Eif3a	-0.40	1.00000000	duck
Eea1	-0.47	1.00000000	duck
Gpatch1	-0.78	1.00000000	duck
Dennd5a	0.89	1.00000000	duck
Paxbp1	-0.83	1.00000000	duck
Efcab14	1.17	1.00000000	duck
Wdr43	-0.80	1.00000000	duck
Bcas2	-0.32	1.00000000	duck
Slc35a5	0.69	1.00000000	duck
Fhod1	0.76	1.00000000	duck
Nol8	-0.71	1.00000000	duck
Gpbp1	-0.62	1.00000000	duck
Stom	1.31	1.00000000	duck
Gpr18	-0.64	1.00000000	duck
Phf2	-0.69	1.00000000	duck
Pcgf6	-0.97	1.00000000	duck
Rtf1	-0.51	1.00000000	duck
Pptc7	-0.69	1.00000000	duck
Ryk	-0.80	1.00000000	duck
Stag2	-0.62	1.00000000	duck
Veph1	0.63	1.00000000	duck
Rfc1	-0.78	1.00000000	duck
Septin11	-0.96	1.00000000	duck
Tab2	-0.93	1.00000000	duck
Hat1	-0.60	1.00000000	duck
Ino80	-0.72	1.00000000	duck
Crem	1.23	1.00000000	duck
Rcsd1	-0.73	1.00000000	duck
Pus10	-0.78	1.00000000	duck
4930523C07Rik	-0.53	1.00000000	duck
Lpcat2b	0.58	1.00000000	duck
Immp1l	-0.90	1.00000000	duck
Uvrag	-0.54	1.00000000	duck
Dop1b	1.63	1.00000000	duck
Gch1	0.86	1.00000000	duck
Cables1	1.42	1.00000000	duck
Rfxank	0.50	1.00000000	duck
Tm9sf3	1.20	1.00000000	duck
Fam172a	-1.12	1.00000000	duck
Arhgap17	-0.64	1.00000000	duck
Zfand3	1.07	1.00000000	duck

Eif1	1.30	1.00000000	duck
Phrf1	-0.74	1.00000000	duck
Zbed4	-0.82	1.00000000	duck
Pinx1	-1.10	1.00000000	duck
Dennd5b	-1.27	1.00000000	duck
Kat2b	0.48	1.00000000	duck
Arid5b	1.15	1.00000000	duck
Zfp148	-0.66	1.00000000	duck
Tapt1	-1.23	1.00000000	duck
Blk	0.18	1.00000000	duck
Rab3gap2	-0.62	1.00000000	duck
Tbc1d15	-0.88	1.00000000	duck
St6gal1	-0.81	1.00000000	duck
Fundc1	-0.53	1.00000000	duck
Usp12	-0.75	1.00000000	duck
Nutf2	-0.72	1.00000000	duck
Cep290	-1.34	1.00000000	duck
Atp2b1	-0.67	1.00000000	duck
Plekha7	1.60	1.00000000	duck
Cnot6l	-0.83	1.00000000	duck
Urm1	-0.51	1.00000000	duck
Fam91a1	-0.52	1.00000000	duck
Ccdc134	-0.67	1.00000000	duck
Anp32e	-0.32	1.00000000	duck
Auh	-0.96	1.00000000	duck
Dusp6	0.80	1.00000000	duck
Pikfyve	-0.95	1.00000000	duck
Uri1	-0.72	1.00000000	duck
Eif4e	-0.42	1.00000000	duck
Clcn7	0.79	1.00000000	duck
Uba2	-0.60	1.00000000	duck
Tpp2	-1.04	1.00000000	duck
Slc7a6	1.12	1.00000000	duck
Fam241a	-0.95	1.00000000	duck
Gtf3c1	-1.08	1.00000000	duck
Rilpl2	-0.41	1.00000000	duck
Cnot1	-0.29	1.00000000	duck
Atp11b	-0.33	1.00000000	duck
Ints7	-0.61	1.00000000	duck
Supt6	-0.59	1.00000000	duck
Tcf3	-0.92	1.00000000	duck
Frmd4b	0.42	1.00000000	duck
Tlcd1	0.86	1.00000000	duck
Erich6	-0.61	1.00000000	duck
Wsb1	-0.91	1.00000000	duck
Rrp1b	-0.84	1.00000000	duck

Lpcat3	1.20	1.00000000	duck
Sntb1	1.21	1.00000000	duck
Slc22a23	0.58	1.00000000	duck
Fgf2	0.76	1.00000000	duck
Pds5b	-0.28	1.00000000	duck
Dip2b	-0.21	1.00000000	duck
Ankrd27	-0.60	1.00000000	duck
Hbs1l	-0.89	1.00000000	duck
Ap3b1	-0.54	1.00000000	duck
Sdf4	-0.70	1.00000000	duck
Naa25	-0.94	1.00000000	duck
Noc3l	-0.63	1.00000000	duck
Tasor2	-0.64	1.00000000	duck
Focad	-0.93	1.00000000	duck
Fa2h	0.45	1.00000000	duck
Pstpip2	0.86	1.00000000	duck
Gdi2	-0.41	1.00000000	duck
Tbl1xr1	-0.52	1.00000000	duck
Atf6	-0.59	1.00000000	duck
Zc3h7a	-0.43	1.00000000	duck
Vmp1	1.67	1.00000000	duck
Esr1	0.67	1.00000000	duck
Ndst4	0.72	1.00000000	duck
Sirt1	-0.63	1.00000000	duck
Setdb1	-0.76	1.00000000	duck
Gramd1c	0.44	1.00000000	duck
Golm2	-0.74	1.00000000	duck
Srgap3	1.50	1.00000000	duck
Psip1	0.09	1.00000000	duck
Alox5	1.63	1.00000000	duck
Rbm26	-0.39	1.00000000	duck
Dtl	0.58	1.00000000	duck
Emc6	1.08	1.00000000	duck
Xpr1	-0.74	1.00000000	duck
Tut4	-0.62	1.00000000	duck
Gas7	1.53	1.00000000	duck
Btaf1	-0.83	1.00000000	duck
Usp48	-0.73	1.00000000	duck
Tsn	-0.67	1.00000000	duck
Rasgrp1	-0.72	1.00000000	duck
Usp24	-0.47	1.00000000	duck
Rnf146	-0.70	1.00000000	duck
9130409I23Rik	1.20	1.00000000	duck
Dtx2	1.12	1.00000000	duck
Pitpnb	-0.30	1.00000000	duck
Chst10	-0.63	1.00000000	duck

Nxpe3	-0.72	1.00000000	duck
Nup205	-0.56	1.00000000	duck
Il21r	-0.79	1.00000000	duck
Trim66	-0.89	1.00000000	duck
Nufip1	-0.36	1.00000000	duck
Nck2	1.55	1.00000000	duck
Vps41	-0.50	1.00000000	duck
Nsrp1	-1.09	1.00000000	duck
Aimp1	-0.95	1.00000000	duck
Tet2	-0.48	1.00000000	duck
Ankrd52	-0.52	1.00000000	duck
Dazap1	-0.64	1.00000000	duck
Nktr	-0.69	1.00000000	duck
Abce1	-0.60	1.00000000	duck
Heatr5b	-0.84	1.00000000	duck
Runx1	-1.09	1.00000000	duck
Nupl1	-0.47	1.00000000	duck
Pdk3	-1.02	1.00000000	duck
Ptcd3	-0.60	1.00000000	duck
Plpp7	-0.73	1.00000000	duck
Arntl	-1.04	1.00000000	duck
Usp16	-0.71	1.00000000	duck
Prmt3	-0.78	1.00000000	duck
Gphn	-1.21	1.00000000	duck
Irf2	-0.62	1.00000000	duck
Ascc3	-0.82	1.00000000	duck
Ctnnb1	1.19	1.00000000	duck
Smyd2	-0.58	1.00000000	duck
Irf1	-0.48	1.00000000	duck
Ubr2	-1.04	1.00000000	duck
Hspa4	-0.98	1.00000000	duck
Taok3	-0.69	1.00000000	duck
Plek	1.68	1.00000000	duck
Appl2	-0.81	1.00000000	duck
Iars	-0.89	1.00000000	duck
Stxbp5	1.06	1.00000000	duck
Hivep3	-1.37	1.00000000	duck
Trp53bp1	-0.59	1.00000000	duck
Cdv3	-0.71	1.00000000	duck
Polr2l	-0.59	1.00000000	duck
Hectd4	-0.63	1.00000000	duck
Btg2	1.23	1.00000000	duck
Reps1	-1.10	1.00000000	duck
Rcbtb2	-0.65	1.00000000	duck
Otud4	-0.70	1.00000000	duck
Thap1	-0.69	1.00000000	duck

Smc4	-0.69	1.00000000	duck
Eif5b	0.95	1.00000000	duck
Pitpnm2	-0.27	1.00000000	duck
Svil	0.74	1.00000000	duck
Sipa1l1	1.28	1.00000000	duck
Eepd1	1.19	1.00000000	duck
Oser1	0.41	1.00000000	duck
Ets1	-0.29	1.00000000	duck
Tmco4	-0.69	1.00000000	duck
Nbas	-0.62	1.00000000	duck
Cdkal1	-0.74	1.00000000	duck
Kidins220	-0.45	1.00000000	duck
Bzw2	-0.52	1.00000000	duck
Ndufa10	-0.58	1.00000000	duck
Rab27a	1.31	1.00000000	duck
Sltm	-0.31	1.00000000	duck
Vdr	1.56	1.00000000	duck
Herc2	-1.07	1.00000000	duck
Ago3	-0.85	1.00000000	duck
Arhgef6	-0.62	1.00000000	duck
Arsb	1.36	1.00000000	duck
Pbdc1	-0.64	1.00000000	duck
Derl3	0.63	1.00000000	duck
Chek2	-0.91	1.00000000	duck
Rnaseh2b	-0.71	1.00000000	duck
Dtx1	-0.38	1.00000000	duck
Nampt	1.27	1.00000000	duck
Plekhb2	1.49	1.00000000	duck
Stmn1	-0.56	1.00000000	duck
Kifbp	-0.83	1.00000000	duck
Opa1	-0.46	1.00000000	duck
Pgm2	1.07	1.00000000	duck
Klhl15	-0.63	1.00000000	duck
Pgm1	0.90	1.00000000	duck
Nnt	-0.61	1.00000000	duck
Arl5a	-0.44	1.00000000	duck
Brd1	0.37	1.00000000	duck
Ola1	-1.00	1.00000000	duck
Fbxw8	-0.93	1.00000000	duck
Eps15l1	-0.76	1.00000000	duck
Sf3a1	-0.44	1.00000000	duck
Tasor	-0.25	1.00000000	duck
Zfp644	-0.61	1.00000000	duck
Fam120a	-0.51	1.00000000	duck
Cyth1	-0.61	1.00000000	duck
Adm	0.98	1.00000000	duck

Jdp2	0.81	1.00000000	duck
Utp18	-0.62	1.00000000	duck
Ppp2r5a	-0.74	1.00000000	duck
Igf2r	1.07	1.00000000	duck
Exoc2	-0.12	1.00000000	duck
Acsf2	-0.60	1.00000000	duck
Kcnk1	-0.89	1.00000000	duck
Tns3	0.83	1.00000000	duck
Nfatc1	-0.69	1.00000000	duck
Snrk	-0.77	1.00000000	duck
Fkbp4	-0.69	1.00000000	duck
Bcl6	1.07	1.00000000	duck
Obi1	-0.53	1.00000000	duck
Smarcad1	-0.54	1.00000000	duck
Pkn2	-0.68	1.00000000	duck
Ireb2	-0.89	1.00000000	duck
Wrn	-1.20	1.00000000	duck
Ncoa4	-0.66	1.00000000	duck
Tpm4	-0.74	1.00000000	duck
Cntrl	-0.44	1.00000000	duck
Gtf2e1	-0.56	1.00000000	duck
Tgfbr2	-0.52	1.00000000	duck
Azi2	-0.45	1.00000000	duck
Kdm2b	-0.60	1.00000000	duck
Bms1	-0.55	1.00000000	duck
Acox1	1.30	1.00000000	duck
Atm	-0.74	1.00000000	duck
Emsy	-0.58	1.00000000	duck
Nudt3	1.07	1.00000000	duck
Kat7	-0.49	1.00000000	duck
Eaf1	-0.54	1.00000000	duck
Rrp15	-0.46	1.00000000	duck
Amot	-0.55	1.00000000	duck
Gng2	-0.38	1.00000000	duck
Rpap1	-0.53	1.00000000	duck
Csnk1g1	-0.74	1.00000000	duck
Prkar2a	-1.02	1.00000000	duck
Arhgap26	-0.93	1.00000000	duck
Pex7	-0.53	1.00000000	duck
Hnrnpm	-0.21	1.00000000	duck
Psmc1	-0.40	1.00000000	duck
Uxs1	-0.68	1.00000000	duck
Rngtt	-0.72	1.00000000	duck
Rab11fip4	0.76	1.00000000	duck
Pik3ca	-0.65	1.00000000	duck
Tmem161b	-0.91	1.00000000	duck

Btrc	-1.08	1.00000000	duck
Brsk2	-1.15	1.00000000	duck
Rbpj	-0.68	1.00000000	duck
Fam122b	-0.68	1.00000000	duck
Asah1	1.81	1.00000000	duck
Srpk2	-0.70	1.00000000	duck
Vdac3	-0.41	1.00000000	duck
Pgam5	-0.71	1.00000000	duck
Riok3	1.42	1.00000000	duck
Aplp2	1.26	1.00000000	duck
Sla	1.04	1.00000000	duck
Elf2	-0.73	1.00000000	duck
Dstn	0.74	1.00000000	duck
Zhx1	-0.61	1.00000000	duck
Rab7	0.92	1.00000000	duck
Snap29	-0.61	1.00000000	duck
Tgds	0.76	1.00000000	duck
Tasl	-0.61	1.00000000	duck
Ripk2	0.62	1.00000000	duck
Filip1	1.45	1.00000000	duck
Nmnat2	0.45	1.00000000	duck
Alg3	0.25	1.00000000	duck
Fam20c	0.45	1.00000000	duck
Zfp143	-0.84	1.00000000	duck
St8sia4	-0.02	1.00000000	duck
Fosb	1.30	1.00000000	duck
Pigx	1.01	1.00000000	duck
Atxn1	-1.02	1.00000000	duck
Cdca7l	-0.56	1.00000000	duck
Hsf2	-0.62	1.00000000	duck
Dna2	-0.74	1.00000000	duck
Rhbdf1	-0.64	1.00000000	duck
Cacna1i	-0.47	1.00000000	duck
Alg13	-0.38	1.00000000	duck
Wdr7	-0.76	1.00000000	duck
Igf2bp2	0.64	1.00000000	duck
Naaladl2	1.00	1.00000000	duck
Rasgef1a	-0.86	1.00000000	duck
Supt5	0.95	1.00000000	duck
Dtwd1	1.12	1.00000000	duck
Ncbp3	-0.64	1.00000000	duck
Cul3	-0.17	1.00000000	duck
Tlcd3a	0.50	1.00000000	duck
Bcl7a	-0.42	1.00000000	duck
Ipo8	-0.58	1.00000000	duck
Etfdh	-0.68	1.00000000	duck

Atp6v1h	1.72	1.00000000	duck
Akap8	-0.42	1.00000000	duck
Ube2h	-0.72	1.00000000	duck
Wdr26	-0.62	1.00000000	duck
Tubgcp5	-0.47	1.00000000	duck
Fam214b	0.65	1.00000000	duck
Fam126b	0.35	1.00000000	duck
Pspc1	-0.33	1.00000000	duck
Nqo2	0.39	1.00000000	duck
Spred1	0.51	1.00000000	duck
Glyr1	-0.47	1.00000000	duck
Bdh1	-0.70	1.00000000	duck
Ski	1.16	1.00000000	duck
Cotl1	-0.63	1.00000000	duck
Ccnl1	-0.57	1.00000000	duck
Wapl	-0.68	1.00000000	duck
R3hdm1	-0.49	1.00000000	duck
Zc3h12b	-0.57	1.00000000	duck
Zfp598	-0.44	1.00000000	duck
Pigf	-0.71	1.00000000	duck
Acot10	-0.45	1.00000000	duck
Pik3cg	-0.23	1.00000000	duck
Wdr24	0.57	1.00000000	duck
Slit3	1.15	1.00000000	duck
B4galt7	0.20	1.00000000	duck
Hnf4a	0.58	1.00000000	duck
Tnfsf15	0.80	1.00000000	duck
Sdad1	-0.58	1.00000000	duck
Eef1b2	-0.78	1.00000000	duck
Areg	0.96	1.00000000	duck
Unc50	0.74	1.00000000	duck
Fam98a	-0.63	1.00000000	duck
Chp1	1.18	1.00000000	duck
Rbsn	-0.66	1.00000000	duck
C2cd3	-0.42	1.00000000	duck
Stx2	-0.95	1.00000000	duck
Vps36	-0.69	1.00000000	duck
Ybx1	-0.75	1.00000000	duck
Rab11fip2	0.89	1.00000000	duck
Cct8	0.86	1.00000000	duck
Anapc1	-0.73	1.00000000	duck
Top1	1.23	1.00000000	duck
Gpatch2	-0.90	1.00000000	duck
Farsb	-0.62	1.00000000	duck
Agpat3	-0.89	1.00000000	duck
Rrm2b	-0.47	1.00000000	duck

Rcl1	-0.51	1.00000000	duck
Ro60	-0.50	1.00000000	duck
Bud23	-0.53	1.00000000	duck
Kars	-0.20	1.00000000	duck
Atp2a3	-0.57	1.00000000	duck
Zswim5	0.85	1.00000000	duck
Sh2b3	-0.66	1.00000000	duck
Setd3	-0.52	1.00000000	duck
Brd7	-0.71	1.00000000	duck
Tmx3	-0.17	1.00000000	duck
Klf3	-0.93	1.00000000	duck
Trappc3	1.08	1.00000000	duck
Ptger4	0.28	1.00000000	duck
Smg1	-0.41	1.00000000	duck
Scyl2	-0.72	1.00000000	duck
Psd3	1.15	1.00000000	duck
Xpot	-0.90	1.00000000	duck
Pcnx	-0.77	1.00000000	duck
Aopep	-1.06	1.00000000	duck
Uspl1	-0.59	1.00000000	duck
Unk	-0.70	1.00000000	duck
Rbbp4	-0.16	1.00000000	duck
2210408I21Rik	0.17	1.00000000	duck
Etv4	0.24	1.00000000	duck
Eef1d	-0.71	1.00000000	duck
Tax1bp3	0.38	1.00000000	duck
Dync1li1	-0.61	1.00000000	duck
Adam10	-0.43	1.00000000	duck
Sinhcaf	-0.55	1.00000000	duck
B3gnt2	1.20	1.00000000	duck
Psmd14	-0.28	1.00000000	duck
Pex1	-0.93	1.00000000	duck
Tmem123	-0.56	1.00000000	duck
Ddx1	-0.46	1.00000000	duck
Parg	-0.68	1.00000000	duck
Hdac1	0.74	1.00000000	duck
Pphln1	-0.95	1.00000000	duck
Ccr7	-0.41	1.00000000	duck
Ehbp1	-0.79	1.00000000	duck
Pgap1	-0.75	1.00000000	duck
Scaper	-1.07	1.00000000	duck
Atp6v1d	1.19	1.00000000	duck
Eipr1	-0.65	1.00000000	duck
Pik3r4	-0.46	1.00000000	duck
Senp5	-0.65	1.00000000	duck
Nup93	-0.77	1.00000000	duck

Itfg1	0.95	1.00000000	duck
Rad54l2	-0.61	1.00000000	duck
Angptl2	-0.72	1.00000000	duck
Khdrbs1	-0.73	1.00000000	duck
Ahctf1	-0.47	1.00000000	duck
Arf1	1.38	1.00000000	duck
Usp33	-0.18	1.00000000	duck
Plaa	-0.49	1.00000000	duck
Ppip5k1	-0.38	1.00000000	duck
Dym	-0.65	1.00000000	duck
Pacsin2	0.81	1.00000000	duck
Oxnad1	-0.52	1.00000000	duck
Herc1	-0.39	1.00000000	duck
Atad2b	0.41	1.00000000	duck
Atp6v0c	0.92	1.00000000	duck
Psma4	-0.68	1.00000000	duck
Zfp608	-0.05	1.00000000	duck
Pla2g6	-0.40	1.00000000	duck
Xrcc2	-0.50	1.00000000	duck
Hace1	-0.68	1.00000000	duck
Wdsub1	-0.70	1.00000000	duck
Gfm1	-0.36	1.00000000	duck
Arid3b	-0.65	1.00000000	duck
Otud7a	-0.92	1.00000000	duck
Lcmt1	-0.46	1.00000000	duck
Anxa6	-0.19	1.00000000	duck
Arap2	-0.85	1.00000000	duck
Hdac9	-0.81	1.00000000	duck
Septin7	-0.43	1.00000000	duck
Styk1	0.64	1.00000000	duck
Eif4e3	0.60	1.00000000	duck
Stambpl1	-0.68	1.00000000	duck
Slc1a4	1.21	1.00000000	duck
Lrrc45	-0.61	1.00000000	duck
Ndufaf6	-0.58	1.00000000	duck
Itm2b	1.08	1.00000000	duck
Pola1	-1.00	1.00000000	duck
Hprt	0.61	1.00000000	duck
Rad17	-0.56	1.00000000	duck
Cbx6	-0.77	1.00000000	duck
Tmem65	0.73	1.00000000	duck
Heatr1	-0.41	1.00000000	duck
Rdh10	1.25	1.00000000	duck
Ptpn1	0.88	1.00000000	duck
Nckipsd	-0.60	1.00000000	duck
Vwa8	-0.73	1.00000000	duck

Lrp6	0.10	1.00000000	duck
Ndfip2	-0.46	1.00000000	duck
Slc44a2	-0.57	1.00000000	duck
Agpat5	-0.73	1.00000000	duck
Ddx46	-0.94	1.00000000	duck
Ube4a	-0.44	1.00000000	duck
Sap30	0.61	1.00000000	duck
Fam98b	-0.29	1.00000000	duck
Zfp831	-0.79	1.00000000	duck
Pafah1b1	-0.67	1.00000000	duck
Nlk	-0.68	1.00000000	duck
Relch	0.80	1.00000000	duck
Smchd1	-0.66	1.00000000	duck
Sesn1	-0.36	1.00000000	duck
Tnks2	-0.51	1.00000000	duck
Ctdspl2	0.10	1.00000000	duck
Naf1	-0.48	1.00000000	duck
Sri	1.03	1.00000000	duck
Golga5	-0.65	1.00000000	duck
Slc25a13	-0.60	1.00000000	duck
Ern1	-0.78	1.00000000	duck
Prkag2	-0.46	1.00000000	duck
Cdc16	0.17	1.00000000	duck
Bcr	0.30	1.00000000	duck
Hnrnpa2b1	-0.07	1.00000000	duck
Eri3	-0.46	1.00000000	duck
Nedd9	-0.34	1.00000000	duck
Zfp704	0.14	1.00000000	duck
Rsf1	-0.71	1.00000000	duck
Prkx	-0.51	1.00000000	duck
Prpf4	-0.45	1.00000000	duck
Creb1	-0.65	1.00000000	duck
Cep112	-0.66	1.00000000	duck
Commd6	-0.44	1.00000000	duck
Tarbp1	-0.57	1.00000000	duck
Uba6	-0.44	1.00000000	duck
Cep95	-0.56	1.00000000	duck
Nf1	-0.39	1.00000000	duck
Cramp1	-0.11	1.00000000	duck
LTO1	-0.75	1.00000000	duck
Exoc6b	-0.90	1.00000000	duck
Dph5	-0.51	1.00000000	duck
Sos2	-0.70	1.00000000	duck
Itpr2	-0.41	1.00000000	duck
Tst	0.82	1.00000000	duck
Ccdc66	-0.64	1.00000000	duck

Snx25	-0.25	1.00000000	duck
Ncoa5	-0.57	1.00000000	duck
Frs2	-0.65	1.00000000	duck
Smad5	0.83	1.00000000	duck
Gnl3	-0.44	1.00000000	duck
Lbr	-0.66	1.00000000	duck
Glipr2	1.04	1.00000000	duck
Hdgfl3	-0.48	1.00000000	duck
Chst7	-0.58	1.00000000	duck
Nsun2	-0.55	1.00000000	duck
Suclg2	-0.78	1.00000000	duck
Eif2a	-0.72	1.00000000	duck
Palld	-2.25	1.00000000	duck
Eps15	-0.63	1.00000000	duck
Ppil1	-0.33	1.00000000	duck
Nr2c1	-0.38	1.00000000	duck
Smg7	-0.08	1.00000000	duck
Nsmce1	-0.48	1.00000000	duck
Lrrcc1	-0.57	1.00000000	duck
Kansl2	-0.32	1.00000000	duck
Pdzd8	-1.00	1.00000000	duck
Sidt2	0.61	1.00000000	duck
Snrnp40	-0.33	1.00000000	duck
Qk	-0.89	1.00000000	duck
Tbc1d14	-0.35	1.00000000	duck
Dop1a	-0.58	1.00000000	duck
Prkci	-0.43	1.00000000	duck
Tbck	-0.98	1.00000000	duck
Dclre1a	-0.28	1.00000000	duck
Setd1b	-0.18	1.00000000	duck
Rlf	-0.46	1.00000000	duck
Crlf3	-0.32	1.00000000	duck
G3bp1	-0.64	1.00000000	duck
Csnk1g3	-0.49	1.00000000	duck
Cdk8	-0.69	1.00000000	duck
Xrn2	-0.66	1.00000000	duck
Cbx3	-0.04	1.00000000	duck
Lmbrd1	0.05	1.00000000	duck
Slc66a2	-0.74	1.00000000	duck
Vps8	-0.72	1.00000000	duck
Fbxo21	-0.66	1.00000000	duck
Stx11	1.24	1.00000000	duck
Phf14	-1.05	1.00000000	duck
Usp25	-0.33	1.00000000	duck
Pm20d2	-0.63	1.00000000	duck
Stradb	-0.53	1.00000000	duck

Edc4	-0.48	1.00000000	duck
Caml	-0.56	1.00000000	duck
Rexo5	-0.62	1.00000000	duck
Rnf149	1.05	1.00000000	duck
Smc3	-0.44	1.00000000	duck
Ppp3ca	-0.66	1.00000000	duck
Znhit6	-0.42	1.00000000	duck
Slc12a4	-0.81	1.00000000	duck
Sel1l	-0.71	1.00000000	duck
Krr1	-0.55	1.00000000	duck
Plekhf2	0.07	1.00000000	duck
Galnt1	-0.72	1.00000000	duck
Srf	0.67	1.00000000	duck
Tfrc	-1.16	1.00000000	duck
Ggnbp2	-0.48	1.00000000	duck
Dcp1a	0.81	1.00000000	duck
Rbm25	-0.48	1.00000000	duck
Nolc1	-0.65	1.00000000	duck
Calm2	0.69	1.00000000	duck
Tlk1	-0.55	1.00000000	duck
Ubxn7	-0.70	1.00000000	duck
Lrp5	-0.79	1.00000000	duck
Hikeshi	-0.38	1.00000000	duck
Ranbp1	-0.03	1.00000000	duck
Itpka	0.90	1.00000000	duck
Riok1	-0.77	1.00000000	duck
Atad1	-0.73	1.00000000	duck
Pcnt	-0.76	1.00000000	duck
Khdc4	-0.26	1.00000000	duck
Phf21a	-0.48	1.00000000	duck
Dcun1d2	-1.31	1.00000000	duck
Ldb1	-0.60	1.00000000	duck
Dctn3	0.97	1.00000000	duck
Sergef	-0.84	1.00000000	duck
Vps35l	-0.58	1.00000000	duck
Ccser1	0.93	1.00000000	duck
Ston2	0.63	1.00000000	duck
Tex10	-0.67	1.00000000	duck
Gnpda2	-0.45	1.00000000	duck
Ssb	-0.23	1.00000000	duck
Chic2	-0.33	1.00000000	duck
Tfb1m	-0.59	1.00000000	duck
Btbd10	-0.65	1.00000000	duck
Ppt1	0.44	1.00000000	duck
Uba5	-0.45	1.00000000	duck
Kdm7a	1.00	1.00000000	duck

Rab8a	-0.33	1.00000000	duck
Ppig	-0.46	1.00000000	duck
Aida	-0.60	1.00000000	duck
Phlpp2	1.42	1.00000000	duck
Snap23	-0.55	1.00000000	duck
Exoc3	-0.46	1.00000000	duck
Ints9	-0.44	1.00000000	duck
Supt3	-0.91	1.00000000	duck
Slc35a1	-0.40	1.00000000	duck
Tlr2	-0.45	1.00000000	duck
Oma1	-0.46	1.00000000	duck
Sass6	-0.54	1.00000000	duck
Actl6a	-0.45	1.00000000	duck
Zfp821	-0.37	1.00000000	duck
Nlrc3	-0.32	1.00000000	duck
Golgb1	-0.61	1.00000000	duck
Irs2	0.94	1.00000000	duck
Ralgps1	-0.68	1.00000000	duck
Wdr44	-0.63	1.00000000	duck
Ppme1	-0.71	1.00000000	duck
Stau1	-0.62	1.00000000	duck
Klhl7	-0.51	1.00000000	duck
Rictor	-0.68	1.00000000	duck
Slc25a12	-0.66	1.00000000	duck
Nle1	-0.56	1.00000000	duck
Ulk1	-0.61	1.00000000	duck
Zfp217	-0.24	1.00000000	duck
Slc35b1	0.54	1.00000000	duck
Ipo11	0.06	1.00000000	duck
Alg2	0.58	1.00000000	duck
Nabp1	1.05	1.00000000	duck
Socs7	0.09	1.00000000	duck
Pik3c3	-0.43	1.00000000	duck
Bclaf1	-0.49	1.00000000	duck
Stk10	-0.52	1.00000000	duck
Gtdc1	-0.38	1.00000000	duck
Cog5	-0.06	1.00000000	duck
Gnat2	0.53	1.00000000	duck
Gnb4	-0.46	1.00000000	duck
Helz2	0.55	1.00000000	duck
Pard3b	1.60	1.00000000	duck
Sin3a	-0.64	1.00000000	duck
Edar	-0.67	1.00000000	duck
Trp53inp2	-0.47	1.00000000	duck
Cop1	0.58	1.00000000	duck
Acbd6	-0.47	1.00000000	duck

Hddc2	-0.45	1.00000000	duck
Ptpn3	0.97	1.00000000	duck
Sec13	-0.32	1.00000000	duck
Abraxas1	-0.35	1.00000000	duck
Gps1	-0.34	1.00000000	duck
Kdm5b	-0.31	1.00000000	duck
Zfp407	-1.14	1.00000000	duck
Spry2	-0.57	1.00000000	duck
Ginm1	1.39	1.00000000	duck
Strn3	-0.48	1.00000000	duck
Cul5	-0.47	1.00000000	duck
Dnajc3	1.03	1.00000000	duck
Abhd17c	-0.90	1.00000000	duck
Sf3b1	-0.46	1.00000000	duck
Rsrc1	-0.81	1.00000000	duck
Pin1	-0.42	1.00000000	duck
Akap10	-0.60	1.00000000	duck
Tlr7	-0.25	1.00000000	duck
Abat	-0.31	1.00000000	duck
Eogt	-0.53	1.00000000	duck
Plekha3	-0.62	1.00000000	duck
Nrp2	1.32	1.00000000	duck
Srpk1	-0.11	1.00000000	duck
Hnrnpa0	-0.33	1.00000000	duck
Lta4h	0.37	1.00000000	duck
Pla2g15	0.87	1.00000000	duck
Ddx10	-0.36	1.00000000	duck
Parn	-0.82	1.00000000	duck
Rnf115	0.45	1.00000000	duck
Apaf1	-0.77	1.00000000	duck
Pak3	1.38	1.00000000	duck
Phf6	-0.65	1.00000000	duck
Chst2	-0.44	1.00000000	duck
Sacs	-0.53	1.00000000	duck
Pak1ip1	-0.45	1.00000000	duck
Stx18	-0.62	1.00000000	duck
F2rl1	-0.43	1.00000000	duck
Atg4b	-0.55	1.00000000	duck
Terf2	-0.32	1.00000000	duck
Oxr1	-0.60	1.00000000	duck
Trdmt1	-0.83	1.00000000	duck
Wdr36	-0.48	1.00000000	duck
Agps	-0.68	1.00000000	duck
Daglb	-0.58	1.00000000	duck
Lgals3	1.03	1.00000000	duck
Cactin	-0.56	1.00000000	duck

Slu7	-0.44	1.00000000	duck
Ints8	-0.63	1.00000000	duck
Zbtb41	-0.42	1.00000000	duck
Tcea1	-0.61	1.00000000	duck
Paxip1	-0.56	1.00000000	duck
Vangl1	0.51	1.00000000	duck
Chfr	0.67	1.00000000	duck
Chmp2b	1.11	1.00000000	duck
Pik3cd	-0.12	1.00000000	duck
Stub1	-0.38	1.00000000	duck
Edc3	-0.25	1.00000000	duck
Stk3	-0.70	1.00000000	duck
Zbtb26	-0.51	1.00000000	duck
Sdf2l1	1.06	1.00000000	duck
Hmbox1	-0.52	1.00000000	duck
Spast	-0.66	1.00000000	duck
Rbm19	-0.54	1.00000000	duck
Tatdn1	-0.39	1.00000000	duck
Fam204a	-0.56	1.00000000	duck
Robo1	0.48	1.00000000	duck
Pcyt1b	0.67	1.00000000	duck
Stx17	-0.46	1.00000000	duck
Eif4a2	0.67	1.00000000	duck
Ano5	-0.19	1.00000000	duck
Acadl	-0.31	1.00000000	duck
Ammecr1l	-0.27	1.00000000	duck
Chd9	-0.29	1.00000000	duck
Septin2	-0.48	1.00000000	duck
Zfp622	-0.19	1.00000000	duck
Zbtb10	-0.10	1.00000000	duck
Nup107	0.23	1.00000000	duck
Swi5	-0.40	1.00000000	duck
Slc6a6	-0.67	1.00000000	duck
Axin1	-0.67	1.00000000	duck
Ttc4	-0.12	1.00000000	duck
Bcl2l11	-0.61	1.00000000	duck
Npas2	0.77	1.00000000	duck
Col8a1	0.78	1.00000000	duck
Epb41l4a	0.82	1.00000000	duck
Abcc8	0.57	1.00000000	duck
Aldh9a1	1.21	1.00000000	duck
Rfwd3	0.95	1.00000000	duck
Clip2	0.26	1.00000000	duck
Asf1a	-0.50	1.00000000	duck
Tkt	1.18	1.00000000	duck
Pdss2	-0.61	1.00000000	duck

Ptpn12	-1.13	1.00000000	duck
Tgs1	0.21	1.00000000	duck
Snw1	0.14	1.00000000	duck
Kank4	-0.38	1.00000000	duck
Washc3	-0.43	1.00000000	duck
Uhrf1bp1l	-0.56	1.00000000	duck
Pcare	-0.56	1.00000000	duck
Guf1	-0.41	1.00000000	duck
Alpk2	-0.39	1.00000000	duck
Impdh2	-0.48	1.00000000	duck
Crbn	-0.58	1.00000000	duck
Gse1	-0.37	1.00000000	duck
Rbl1	-0.39	1.00000000	duck
Timm17a	-0.27	1.00000000	duck
Tnfsf13b	0.60	1.00000000	duck
Rarb	0.73	1.00000000	duck
Gcg	0.47	1.00000000	duck
Top2b	-0.30	1.00000000	duck
Crtc1	-0.34	1.00000000	duck
Sbno2	-0.25	1.00000000	duck
Faf1	-0.68	1.00000000	duck
Tln1	-0.46	1.00000000	duck
Csde1	-0.58	1.00000000	duck
Gtf2f2	-0.66	1.00000000	duck
Tubgcp3	-0.75	1.00000000	duck
Atg4c	-0.62	1.00000000	duck
Ppp2r2a	-0.35	1.00000000	duck
Dhdds	-0.28	1.00000000	duck
Prkce	-0.57	1.00000000	duck
Polr2c	0.85	1.00000000	duck
Jade1	-0.83	1.00000000	duck
Nrp1	0.31	1.00000000	duck
Slc16a1	-0.54	1.00000000	duck
Taf15	-0.42	1.00000000	duck
Sap30l	-0.28	1.00000000	duck
Naxd	-0.52	1.00000000	duck
Agpat4	0.22	1.00000000	duck
Epcam	0.51	1.00000000	duck
Zfyve21	0.24	1.00000000	duck
Tnrc18	0.74	1.00000000	duck
Arv1	-0.26	1.00000000	duck
Rac1	-0.56	1.00000000	duck
Calm1	0.68	1.00000000	duck
Rb1cc1	-0.59	1.00000000	duck
Snrpb2	-0.42	1.00000000	duck
Atg16l1	-0.49	1.00000000	duck

Disp1	-0.70	1.00000000	duck
Adam19	-0.53	1.00000000	duck
Tmx1	-0.58	1.00000000	duck
Fbxo11	-0.29	1.00000000	duck
Zfp207	-0.40	1.00000000	duck
Sgms1	0.74	1.00000000	duck
Afdn	0.40	1.00000000	duck
Akap12	0.32	1.00000000	duck
Uaca	0.66	1.00000000	duck
Hsdl1	0.23	1.00000000	duck
Nfat5	-0.48	1.00000000	duck
Hes6	-0.61	1.00000000	duck
Nap1l1	-0.49	1.00000000	duck
Pum2	-0.54	1.00000000	duck
Fbxl2	-0.20	1.00000000	duck
Zdhhc17	-0.76	1.00000000	duck
Jmjd6	0.83	1.00000000	duck
Cep250	-0.38	1.00000000	duck
Ctif	0.25	1.00000000	duck
Taf2	-0.35	1.00000000	duck
Gatad2a	-0.18	1.00000000	duck
Htatsf1	-0.43	1.00000000	duck
Ddx20	-0.21	1.00000000	duck
Erp29	-0.36	1.00000000	duck
Phf10	-0.58	1.00000000	duck
Ppat	-0.18	1.00000000	duck
Gpr141	0.21	1.00000000	duck
Aven	-0.86	1.00000000	duck
Tmem104	0.24	1.00000000	duck
Tmem242	-0.60	1.00000000	duck
Cog2	-0.43	1.00000000	duck
Ggps1	-0.76	1.00000000	duck
Rev1	-0.51	1.00000000	duck
Wdfy2	-0.51	1.00000000	duck
Ccr4	-0.35	1.00000000	duck
Wrnip1	-0.40	1.00000000	duck
Trmt6	-0.40	1.00000000	duck
Prmt9	-0.28	1.00000000	duck
Wee1	-0.31	1.00000000	duck
Zc3h14	-0.26	1.00000000	duck
Shld2	-0.61	1.00000000	duck
Rnpc3	-0.56	1.00000000	duck
Idh2	-0.27	1.00000000	duck
Zbtb6	-0.38	1.00000000	duck
Srm	-0.30	1.00000000	duck
Pelo	-0.35	1.00000000	duck

Cd151	-0.42	1.00000000	duck
Taf3	-0.58	1.00000000	duck
Gdpd5	-1.17	1.00000000	duck
Phtf1	-0.67	1.00000000	duck
Dhx36	-0.49	1.00000000	duck
Syap1	0.91	1.00000000	duck
Pwwp2b	-0.57	1.00000000	duck
Esf1	-0.82	1.00000000	duck
Vps39	-0.60	1.00000000	duck
Pcca	-0.61	1.00000000	duck
Hivep1	-0.35	1.00000000	duck
Zcchc7	-0.01	1.00000000	duck
Rassf3	-0.62	1.00000000	duck
Slc30a1	0.30	1.00000000	duck
Spata2	-0.46	1.00000000	duck
Tars	-0.38	1.00000000	duck
Trim37	-0.54	1.00000000	duck
Iars2	-0.35	1.00000000	duck
Epb41l5	-0.27	1.00000000	duck
Thap12	-0.39	1.00000000	duck
Xpo5	-0.32	1.00000000	duck
Pdpk1	-0.48	1.00000000	duck
Ogt	-0.25	1.00000000	duck
Proser1	-0.44	1.00000000	duck
Ttf1	-0.59	1.00000000	duck
Eef2k	-0.78	1.00000000	duck
Copg2	-0.64	1.00000000	duck
Wdr70	-0.91	1.00000000	duck
Fndc3b	-0.09	1.00000000	duck
Strn	-0.33	1.00000000	duck
Zfp592	-0.48	1.00000000	duck
Gpr34	-0.35	1.00000000	duck
Ndrg3	0.98	1.00000000	duck
Trank1	-0.70	1.00000000	duck
Ncoa3	-0.12	1.00000000	duck
Crk	-0.33	1.00000000	duck
Ckap5	-0.44	1.00000000	duck
Sptlc1	-0.54	1.00000000	duck
Hp1bp3	-0.49	1.00000000	duck
Kcnq5	1.35	1.00000000	duck
Lct	-0.07	1.00000000	duck
Ptpn22	-0.34	1.00000000	duck
Btbd2	-0.60	1.00000000	duck
Fubp3	-0.51	1.00000000	duck
Rel	-0.48	1.00000000	duck
Bnip3	-0.40	1.00000000	duck

Slc25a3	0.79	1.00000000	duck
Jak2	-0.91	1.00000000	duck
Ube2e3	-0.78	1.00000000	duck
Arih1	-0.37	1.00000000	duck
Pcmtd1	-0.51	1.00000000	duck
Zc3h3	1.11	1.00000000	duck
Pde7b	-1.55	1.00000000	duck
Prdm1	0.77	1.00000000	duck
Tmem185a	-0.55	1.00000000	duck
Ccdc93	-0.19	1.00000000	duck
Epc2	-0.43	1.00000000	duck
Casp8ap2	0.91	1.00000000	duck
Rnf111	-0.54	1.00000000	duck
Ddx17	-0.46	1.00000000	duck
Ube2e2	-1.85	1.00000000	duck
Eif4g3	-0.60	1.00000000	duck
Psen1	-0.67	1.00000000	duck
Naa30	-0.37	1.00000000	duck
Tnfaip1	-0.42	1.00000000	duck
Alk	-0.54	1.00000000	duck
Sec22b	-0.27	1.00000000	duck
Fan1	-0.35	1.00000000	duck
Adal	-0.34	1.00000000	duck
Ptpdc1	-0.48	1.00000000	duck
Pskh1	-0.41	1.00000000	duck
Fbxw5	-0.32	1.00000000	duck
Prkcz	-0.43	1.00000000	duck
Tube1	-0.48	1.00000000	duck
B4galnt4	-0.43	1.00000000	duck
Nedd4l	-0.33	1.00000000	duck
Zc3h15	-0.66	1.00000000	duck
Nrbf2	-0.62	1.00000000	duck
Pdhx	-0.53	1.00000000	duck
Tti1	-0.45	1.00000000	duck
Nol10	-0.57	1.00000000	duck
Nfyc	-0.30	1.00000000	duck
Naaa	0.08	1.00000000	duck
Tbxas1	1.00	1.00000000	duck
Abhd17b	-0.48	1.00000000	duck
Tent4a	-0.63	1.00000000	duck
Npr3	-0.54	1.00000000	duck
Otud7b	-0.41	1.00000000	duck
Grb2	-0.36	1.00000000	duck
Nkrf	-0.42	1.00000000	duck
Rnf168	-0.37	1.00000000	duck
Lmbrd2	-0.54	1.00000000	duck

Rc3h2	-0.55	1.00000000	duck
Rere	0.01	1.00000000	duck
Eif2s2	-0.39	1.00000000	duck
Il17ra	0.82	1.00000000	duck
Ppm1l	-0.49	1.00000000	duck
Zfp609	-0.36	1.00000000	duck
Akirin2	1.01	1.00000000	duck
Phf3	-0.42	1.00000000	duck
Slc25a20	0.49	1.00000000	duck
B4galt4	-0.42	1.00000000	duck
Gpn1	-0.47	1.00000000	duck
Raver2	0.91	1.00000000	duck
Nlrc5	-0.36	1.00000000	duck
Cwf19l1	0.16	1.00000000	duck
Cenpm	-0.32	1.00000000	duck
Ssh2	-0.38	1.00000000	duck
Pmm2	-0.38	1.00000000	duck
Gtf2h1	-0.49	1.00000000	duck
Stk38	-0.55	1.00000000	duck
Clasp2	-0.34	1.00000000	duck
Cwf19l2	0.00	1.00000000	duck
Ttc19	1.23	1.00000000	duck
Rpa2	-0.26	1.00000000	duck
Arf6	1.14	1.00000000	duck
Dynll2	0.31	1.00000000	duck
Gtf2e2	-0.25	1.00000000	duck
Gprin3	-0.63	1.00000000	duck
Dbf4	-0.48	1.00000000	duck
Adora2a	0.74	1.00000000	duck
Tmx4	0.09	1.00000000	duck
Hells	-0.46	1.00000000	duck
Ptx3	1.03	1.00000000	duck
Aplf	1.58	1.00000000	duck
Ppm1g	-0.44	1.00000000	duck
Btg1b	-0.29	1.00000000	duck
Ampd2	0.48	1.00000000	duck
Rbis	-0.02	1.00000000	duck
Dync1li2	-0.44	1.00000000	duck
Ap2m1	0.74	1.00000000	duck
Ripk1	0.37	1.00000000	duck
Arhgap6	-1.23	1.00000000	duck
Fam168b	-0.49	1.00000000	duck
Cdc42se2	-0.36	1.00000000	duck
Fam20b	-0.55	1.00000000	duck
Adss	-0.30	1.00000000	duck
Zfhx3	1.14	1.00000000	duck

Dpp7	-0.35	1.00000000	duck
Pank4	-0.43	1.00000000	duck
Napg	-0.57	1.00000000	duck
Ap3s1	0.01	1.00000000	duck
Crebrf	-0.64	1.00000000	duck
Ddx27	-0.51	1.00000000	duck
Naa50	-0.04	1.00000000	duck
Exog	-0.35	1.00000000	duck
Smurf1	-0.54	1.00000000	duck
Adprh	-0.24	1.00000000	duck
Nbn	-0.33	1.00000000	duck
Chrac1	-0.23	1.00000000	duck
Erich1	-0.58	1.00000000	duck
Aprt	-0.35	1.00000000	duck
Pip4k2b	-0.25	1.00000000	duck
Evi5l	-0.30	1.00000000	duck
Pitpnm1	-0.36	1.00000000	duck
Ccdc34	-0.41	1.00000000	duck
Cd44	0.70	1.00000000	duck
Uchl3	-0.08	1.00000000	duck
Letm1	-0.46	1.00000000	duck
Tfam	-0.60	1.00000000	duck
Agtpbp1	0.42	1.00000000	duck
Trib2	-0.28	1.00000000	duck
Tec	0.95	1.00000000	duck
Pot1b	-0.49	1.00000000	duck
Otud6b	-0.33	1.00000000	duck
Babam2	-0.46	1.00000000	duck
Acox3	-0.59	1.00000000	duck
Efl1	-0.01	1.00000000	duck
Nae1	-0.35	1.00000000	duck
Tmem108	0.21	1.00000000	duck
Pdcl3	-0.57	1.00000000	duck
Fam114a2	-0.25	1.00000000	duck
Dnajc5	0.96	1.00000000	duck
Cope	1.01	1.00000000	duck
Zrsr1	-0.48	1.00000000	duck
Smarcc1	0.06	1.00000000	duck
Ppp3cb	-0.79	1.00000000	duck
Chaf1a	-0.20	1.00000000	duck
Akap11	-0.44	1.00000000	duck
Ctr9	-0.41	1.00000000	duck
Saraf	0.47	1.00000000	duck
Ptpn9	0.35	1.00000000	duck
Srebf2	-0.05	1.00000000	duck
Ccny	-0.66	1.00000000	duck

Commd2	-0.52	1.00000000	duck
Scrn3	-0.23	1.00000000	duck
Rtraf	-0.17	1.00000000	duck
Nol9	-0.03	1.00000000	duck
Ankle2	-0.59	1.00000000	duck
Tfdp1	-0.68	1.00000000	duck
Lmo2	-0.80	1.00000000	duck
Slc9a3r1	0.98	1.00000000	duck
Clk1	-0.32	1.00000000	duck
Kctd2	0.30	1.00000000	duck
Elp2	-0.25	1.00000000	duck
Rnf139	-0.19	1.00000000	duck
Katnip	-0.27	1.00000000	duck
Insr	-0.16	1.00000000	duck
Phf12	-0.46	1.00000000	duck
Nek9	1.12	1.00000000	duck
Ice2	-0.21	1.00000000	duck
Commd10	-0.97	1.00000000	duck
Cpeb4	0.08	1.00000000	duck
Cenpu	1.34	1.00000000	duck
Srbd1	0.01	1.00000000	duck
Nrde2	-0.04	1.00000000	duck
Wiz	-0.57	1.00000000	duck
Pef1	0.45	1.00000000	duck
Zmynd19	-0.41	1.00000000	duck
Ncf2	0.67	1.00000000	duck
Edem3	1.48	1.00000000	duck
Lsm14a	-0.44	1.00000000	duck
Abhd17a	0.26	1.00000000	duck
Ankra2	-0.34	1.00000000	duck
Ankrd11	0.39	1.00000000	duck
Uchl5	0.19	1.00000000	duck
Pds5a	-0.20	1.00000000	duck
Kdm2a	1.13	1.00000000	duck
Dusp22	-0.63	1.00000000	duck
Gcn1	-0.08	1.00000000	duck
Iws1	-0.51	1.00000000	duck
Cisd2	-0.18	1.00000000	duck
Elk3	-0.53	1.00000000	duck
Adcy9	-1.28	1.00000000	duck
Tpd52l2	-0.58	1.00000000	duck
Psmb2	0.92	1.00000000	duck
Txn1	1.20	1.00000000	duck
Spen	0.10	1.00000000	duck
Arl4a	0.34	1.00000000	duck
Vopp1	-0.48	1.00000000	duck

Stim2	-0.13	1.00000000	duck
Zfp36l1	0.80	1.00000000	duck
Elmo2	-0.09	1.00000000	duck
Nelfa	-0.38	1.00000000	duck
Cebpz	-0.50	1.00000000	duck
Ube2o	-0.20	1.00000000	duck
Phc3	-0.27	1.00000000	duck
Urb1	-0.39	1.00000000	duck
Sema3d	-2.08	1.00000000	duck
Rpp40	-0.22	1.00000000	duck
Etaa1	-0.31	1.00000000	duck
Dck	-0.32	1.00000000	duck
Dock7	-0.33	1.00000000	duck
Gpr55	-0.38	1.00000000	duck
Shq1	-0.37	1.00000000	duck
Sfmbt1	-0.48	1.00000000	duck
Ciapin1	-0.31	1.00000000	duck
Aatf	-0.52	1.00000000	duck
Atp13a2	-0.28	1.00000000	duck
Bsdc1	-0.28	1.00000000	duck
Polrmt	-0.32	1.00000000	duck
Eif3l	-0.27	1.00000000	duck
Ensa	0.92	1.00000000	duck
Ddx18	-0.35	1.00000000	duck
Rap1gap2	-0.47	1.00000000	duck
Syne3	-0.58	1.00000000	duck
Runx2	-0.33	1.00000000	duck
Fkbp15	-0.53	1.00000000	duck
Kpna1	-0.28	1.00000000	duck
Eif3b	-0.50	1.00000000	duck
Tmem250-ps	0.65	1.00000000	duck
Nop2	-0.37	1.00000000	duck
Immt	-0.25	1.00000000	duck
Rnf185	0.53	1.00000000	duck
Kdm1b	-0.67	1.00000000	duck
Tada1	-0.22	1.00000000	duck
Eif3m	-0.13	1.00000000	duck
Prdx3	-0.33	1.00000000	duck
Zw10	-0.39	1.00000000	duck
Wdr88	-0.28	1.00000000	duck
Tfg	-0.29	1.00000000	duck
Ccdc174	-0.36	1.00000000	duck
Parp4	-0.25	1.00000000	duck
Zcchc8	-0.36	1.00000000	duck
Zbtb1	-0.56	1.00000000	duck
Cyrib	-0.30	1.00000000	duck

AF366264	-0.38	1.00000000	duck
Eftud2	-0.71	1.00000000	duck
B3gntl1	-0.45	1.00000000	duck
Rhog	1.09	1.00000000	duck
Prpf6	-0.36	1.00000000	duck
Afg3l2	0.92	1.00000000	duck
Slc45a1	-0.41	1.00000000	duck
Phka2	-0.46	1.00000000	duck
Ifnar1	-0.46	1.00000000	duck
Sde2	0.78	1.00000000	duck
Ebna1bp2	-0.28	1.00000000	duck
Rsrc2	-0.10	1.00000000	duck
Ankrd28	0.85	1.00000000	duck
Arhgap5	-0.54	1.00000000	duck
Dok3	-0.13	1.00000000	duck
Tfip11	-0.49	1.00000000	duck
Fbxo46	-0.31	1.00000000	duck
Slc48a1	-0.22	1.00000000	duck
Retreg1	-0.16	1.00000000	duck
Rfc5	-0.32	1.00000000	duck
Gpc4	0.34	1.00000000	duck
Pmepa1	-0.66	1.00000000	duck
Dnmt1	-0.26	1.00000000	duck
Surf6	-0.46	1.00000000	duck
Pi4k2b	-0.53	1.00000000	duck
Exosc2	-0.34	1.00000000	duck
Trim8	-0.12	1.00000000	duck
Tmem42	0.23	1.00000000	duck
Tmem63b	1.80	1.00000000	duck
Trabd2b	0.77	1.00000000	duck
Bcl9l	0.45	1.00000000	duck
Dpp9	0.89	1.00000000	duck
Ctla4	0.43	1.00000000	duck
Pars2	-0.22	1.00000000	duck
Actr10	-0.25	1.00000000	duck
Fbxl22	-0.40	1.00000000	duck
Usp20	-0.62	1.00000000	duck
Lars	-0.49	1.00000000	duck
Fbxw2	-0.34	1.00000000	duck
Txnl1	-0.03	1.00000000	duck
Blnk	0.05	1.00000000	duck
Ttc7	0.13	1.00000000	duck
Dpysl2	-0.20	1.00000000	duck
Sart3	-0.21	1.00000000	duck
Ufl1	-0.37	1.00000000	duck
Gpalpp1	0.72	1.00000000	duck

Zfp638	-0.39	1.00000000	duck
Trp53i11	-0.03	1.00000000	duck
Fut7	0.31	1.00000000	duck
Il6ra	0.55	1.00000000	duck
Txndc5	-0.48	1.00000000	duck
Gnptab	-0.30	1.00000000	duck
Atxn10	-0.60	1.00000000	duck
Cops2	0.48	1.00000000	duck
Atp6v0d1	0.72	1.00000000	duck
Smg5	0.74	1.00000000	duck
Slc30a7	-0.33	1.00000000	duck
Purb	-0.29	1.00000000	duck
Jazf1	0.76	1.00000000	duck
Anxa5	0.83	1.00000000	duck
Bysl	0.46	1.00000000	duck
Tmed5	0.77	1.00000000	duck
Ankfy1	-0.38	1.00000000	duck
Pex2	-0.40	1.00000000	duck
Rnf4	-0.57	1.00000000	duck
Eya3	-0.27	1.00000000	duck
Sin3b	-0.50	1.00000000	duck
Fbxl4	0.83	1.00000000	duck
Sfmbt2	-0.67	1.00000000	duck
Napepld	-0.33	1.00000000	duck
Zbtb14	-0.21	1.00000000	duck
Neurod6	-0.81	1.00000000	duck
Aldh5a1	-0.27	1.00000000	duck
Pnpt1	-0.27	1.00000000	duck
Desi2	-0.26	1.00000000	duck
Elmod2	-0.38	1.00000000	duck
Zfp330	-0.34	1.00000000	duck
Nup54	-0.22	1.00000000	duck
Lix1	-0.32	1.00000000	duck
Clba1	-0.32	1.00000000	duck
Lnpk	-0.30	1.00000000	duck
Dipk2a	-0.76	1.00000000	duck
Gpr171	-0.40	1.00000000	duck
Ccne1	-0.30	1.00000000	duck
Sfxn1	-0.19	1.00000000	duck
Tmem186	-0.24	1.00000000	duck
Gnb1l	-0.38	1.00000000	duck
Triap1	-0.22	1.00000000	duck
Cep131	-0.22	1.00000000	duck
Vps37d	-0.20	1.00000000	duck
Nt5c3b	-0.20	1.00000000	duck
Smarcd1	-0.25	1.00000000	duck

Per3	-0.43	1.00000000	duck
Pik3c2b	-0.21	1.00000000	duck
Scamp4	-0.21	1.00000000	duck
Fkbp7	-0.36	1.00000000	duck
Otoa	-0.41	1.00000000	duck
Ttc13	-0.58	1.00000000	duck
Zer1	-0.60	1.00000000	duck
Lsp1	1.17	1.00000000	duck
Ube3c	0.90	1.00000000	duck
Klf7	1.11	1.00000000	duck
Atmin	-0.43	1.00000000	duck
Hspa2	1.24	1.00000000	duck
Ube2a	-0.20	1.00000000	duck
Rfng	-0.11	1.00000000	duck
Tnfsf8	-0.28	1.00000000	duck
Top3a	0.44	1.00000000	duck
Tubd1	1.70	1.00000000	duck
Dnajc2	-0.70	1.00000000	duck
Tmem184b	-0.67	1.00000000	duck
Abca5	-0.56	1.00000000	duck
Pcm1	-0.49	1.00000000	duck
Cwc25	0.96	1.00000000	duck
Ints6	-0.38	1.00000000	duck
Slc23a2	1.07	1.00000000	duck
Hmces	0.45	1.00000000	duck
Tmem86a	1.06	1.00000000	duck
Osbp2	0.78	1.00000000	duck
Srpr	-0.49	1.00000000	duck
Tafa5	0.79	1.00000000	duck
Pdzrn3	0.20	1.00000000	duck
Arid1a	-0.41	1.00000000	duck
Tnip2	1.08	1.00000000	duck
Skil	-0.30	1.00000000	duck
Nfs1	-0.25	1.00000000	duck
Rheb	-0.32	1.00000000	duck
Ptpn4	-0.79	1.00000000	duck
Spg7	-0.44	1.00000000	duck
Zfand6	-0.27	1.00000000	duck
Isca1	-0.45	1.00000000	duck
Nat10	-0.54	1.00000000	duck
Tle4	-0.61	1.00000000	duck
Fancc	-0.76	1.00000000	duck
Psmd12	-0.47	1.00000000	duck
4933400A11Rik	-0.25	1.00000000	duck
Tnks	-0.59	1.00000000	duck
Sesn3	-0.18	1.00000000	duck

Hif1an	-0.25	1.00000000	duck
Arhgef18	1.04	1.00000000	duck
Cdk5rap1	0.54	1.00000000	duck
Slc39a13	1.04	1.00000000	duck
Klf12	-0.63	1.00000000	duck
Aig1	-0.74	1.00000000	duck
Tpm3-rs7	0.63	1.00000000	duck
Psmg2	-0.14	1.00000000	duck
Gatad1	-0.06	1.00000000	duck
Commd8	0.32	1.00000000	duck
Fancm	-0.14	1.00000000	duck
Slc31a1	-0.06	1.00000000	duck
Fmnl1	0.77	1.00000000	duck
Rfc3	-0.34	1.00000000	duck
Fnbp1l	-0.52	1.00000000	duck
Fer	-0.45	1.00000000	duck
Hgs	-0.21	1.00000000	duck
Tpmt	0.94	1.00000000	duck
Timp3	1.30	1.00000000	duck
Susd3	-0.39	1.00000000	duck
Leo1	0.93	1.00000000	duck
Lemd2	-0.24	1.00000000	duck
Vezt	-0.76	1.00000000	duck
Prpf3	-0.12	1.00000000	duck
Efr3a	-0.74	1.00000000	duck
Ric8b	-0.74	1.00000000	duck
Yipf3	0.87	1.00000000	duck
Ercc3	-0.45	1.00000000	duck
Acads	0.70	1.00000000	duck
Bicral	-0.10	1.00000000	duck
Gan	1.03	1.00000000	duck
Plekha5	0.87	1.00000000	duck
Gpsm1	0.21	1.00000000	duck
Nhlrc2	-0.21	1.00000000	duck
Braf	-0.32	1.00000000	duck
Rbm39	0.45	1.00000000	duck
Cubn	0.13	1.00000000	duck
Suclg1	-0.54	1.00000000	duck
Smim19	0.35	1.00000000	duck
Tmed10	-0.19	1.00000000	duck
Trappc12	-0.32	1.00000000	duck
Spred2	1.20	1.00000000	duck
Stk26	-0.25	1.00000000	duck
Wdr1	1.13	1.00000000	duck
2700049A03Rik	-0.42	1.00000000	duck
Eif2ak2	0.63	1.00000000	duck

Elavl1	-0.40	1.00000000	duck
Clasp1	-0.13	1.00000000	duck
Gabarapl1	0.65	1.00000000	duck
Ssr1	1.18	1.00000000	duck
Pcgf3	-0.60	1.00000000	duck
Ppp1r21	-0.70	1.00000000	duck
Cars	1.00	1.00000000	duck
Psmd7	0.20	1.00000000	duck
Blzf1	0.81	1.00000000	duck
Insyn2b	-0.35	1.00000000	duck
Slc16a10	0.13	1.00000000	duck
Zbtb24	0.13	1.00000000	duck
Smpd1	0.63	1.00000000	duck
Atf2	-0.01	1.00000000	duck
Ccnk	-0.48	1.00000000	duck
Zfp800	-0.37	1.00000000	duck
Gpr180	-0.54	1.00000000	duck
Tmcc3	-0.61	1.00000000	duck
Snx16	-0.73	1.00000000	duck
Drosha	-0.43	1.00000000	duck
Utp23	-0.43	1.00000000	duck
Lyplal1	-0.49	1.00000000	duck
Orc3	-0.34	1.00000000	duck
Prim2	-0.56	1.00000000	duck
Cfap97	-0.52	1.00000000	duck
Rapsn	-0.26	1.00000000	duck
Ighmbp2	-0.36	1.00000000	duck
Bag5	-0.49	1.00000000	duck
Snapc1l	-0.31	1.00000000	duck
Carf	-0.39	1.00000000	duck
Fastkd2	-0.20	1.00000000	duck
Spata6	-0.35	1.00000000	duck
Trpc1	-0.20	1.00000000	duck
Rfc4	-0.23	1.00000000	duck
Fanci	-0.31	1.00000000	duck
Gins3	-0.64	1.00000000	duck
Tradd	-0.29	1.00000000	duck
Stk32a	-0.28	1.00000000	duck
Cdc23	-0.30	1.00000000	duck
Necab3	-0.18	1.00000000	duck
Gtf3c4	-0.21	1.00000000	duck
Pmpca	-0.19	1.00000000	duck
Dnajc16	-0.17	1.00000000	duck
Pabpc4	-0.21	1.00000000	duck
Cd79b	-0.42	1.00000000	duck
Atxn2	0.01	1.00000000	duck

Thoc1	-0.62	1.00000000	duck
Nelfb	-0.34	1.00000000	duck
Tmem167	-0.44	1.00000000	duck
Rab1a	0.94	1.00000000	duck
Rab35	-0.50	1.00000000	duck
Tlk2	-0.06	1.00000000	duck
Gna12	-0.04	1.00000000	duck
Ttll1	-0.45	1.00000000	duck
Arf4	-0.21	1.00000000	duck
Ash1l	-0.25	1.00000000	duck
Atp5j2	0.83	1.00000000	duck
Cdk7	-0.27	1.00000000	duck
Got2	-0.26	1.00000000	duck
Lpar6	-0.37	1.00000000	duck
Celf2	-0.09	1.00000000	duck
Hadhb	0.26	1.00000000	duck
Tcf7l2	0.32	1.00000000	duck
Adamts2	0.34	1.00000000	duck
Hmgb2	-0.10	1.00000000	duck
Aoah	0.40	1.00000000	duck
Dazap2	0.79	1.00000000	duck
Ero1a	1.11	1.00000000	duck
Npepl1	-0.37	1.00000000	duck
Stk32c	-0.34	1.00000000	duck
Rabepk	-0.21	1.00000000	duck
Sugp2	-0.29	1.00000000	duck
Scfd1	-0.43	1.00000000	duck
Ssbp1	-0.35	1.00000000	duck
Smurf2	-0.30	1.00000000	duck
Nub1	-0.18	1.00000000	duck
Zbtb2	-0.15	1.00000000	duck
Romo1	0.93	1.00000000	duck
Zzz3	-0.17	1.00000000	duck
Ppm1d	0.08	1.00000000	duck
Adgrg2	-0.66	1.00000000	duck
Noct	0.57	1.00000000	duck
Hook1	-0.32	1.00000000	duck
Pwwp2a	-0.41	1.00000000	duck
Unc119b	0.75	1.00000000	duck
Arsk	-0.57	1.00000000	duck
Ap1ar	-0.49	1.00000000	duck
Shoc2	-0.43	1.00000000	duck
Ldah	0.69	1.00000000	duck
Agk	0.32	1.00000000	duck
D2hgdh	-0.12	1.00000000	duck
Rubcn	0.02	1.00000000	duck

Ing5	-0.45	1.00000000	duck
Cdca4	-0.02	1.00000000	duck
Ptbp3	0.78	1.00000000	duck
Golga4	-0.03	1.00000000	duck
Nab1	-0.32	1.00000000	duck
Nek1	-0.64	1.00000000	duck
Pdrg1	0.44	1.00000000	duck
Heatr3	-0.34	1.00000000	duck
Prkaa1	-0.45	1.00000000	duck
Sft2d2	-0.26	1.00000000	duck
Rchy1	-0.24	1.00000000	duck
Oaz1	0.70	1.00000000	duck
Bod1l	-0.03	1.00000000	duck
Cep152	-0.40	1.00000000	duck
Uba3	-0.36	1.00000000	duck
Clk2	0.24	1.00000000	duck
Stk25	-0.43	1.00000000	duck
Amd2	-0.05	1.00000000	duck
Rnf220	-0.45	1.00000000	duck
Ccdc127	-0.36	1.00000000	duck
Cdc27	-0.23	1.00000000	duck
Pias2	0.00	1.00000000	duck
Wdr45b	0.68	1.00000000	duck
Rfesd	-1.30	1.00000000	duck
Phb2	-0.12	1.00000000	duck
Irf4	-0.12	1.00000000	duck
Pex13	-0.19	1.00000000	duck
Rmnd5a	-0.66	1.00000000	duck
Itpkb	-0.49	1.00000000	duck
Acvr1	0.12	1.00000000	duck
Nars2	-0.45	1.00000000	duck
Cdkn1b	0.30	1.00000000	duck
Trerf1	0.06	1.00000000	duck
Elp3	-0.38	1.00000000	duck
Stau2	-0.32	1.00000000	duck
Ambra1	-0.33	1.00000000	duck
Fhl3	0.16	1.00000000	duck
Htt	0.04	1.00000000	duck
Grk4	-0.29	1.00000000	duck
Tiam1	1.01	1.00000000	duck
Lsm14b	-0.35	1.00000000	duck
Sppl2b	-0.38	1.00000000	duck
Prpf19	0.01	1.00000000	duck
Niban1	0.54	1.00000000	duck
Dennd4a	0.32	1.00000000	duck
Cnot10	-0.09	1.00000000	duck

Ncald	-0.22	1.00000000	duck
Ninj1	1.06	1.00000000	duck
Cse1l	-0.26	1.00000000	duck
Rabgap1l	-0.35	1.00000000	duck
Clcc1	-0.38	1.00000000	duck
Tomm7	-0.11	1.00000000	duck
Virma	-0.37	1.00000000	duck
Tbc1d12	-0.75	1.00000000	duck
Larp1b	-1.05	1.00000000	duck
Tulp4	0.00	1.00000000	duck
Tk2	-0.15	1.00000000	duck
Washc4	-0.58	1.00000000	duck
Lonrf2	-0.06	1.00000000	duck
Tmem168	-0.21	1.00000000	duck
Zfyve1	-0.17	1.00000000	duck
Nbeal2	0.83	1.00000000	duck
Polk	-0.35	1.00000000	duck
Haus2	-0.45	1.00000000	duck
Sgsm2	-0.53	1.00000000	duck
Ilf2	-0.19	1.00000000	duck
Paip2	-0.25	1.00000000	duck
B2m	0.65	1.00000000	duck
Errfi1	-0.20	1.00000000	duck
Itm2c	-0.43	1.00000000	duck
Rnf13	0.75	1.00000000	duck
Pigk	-0.55	1.00000000	duck
Gper1	-0.05	1.00000000	duck
Vav3	0.71	1.00000000	duck
Recql	-0.11	1.00000000	duck
Snrpg	-0.27	1.00000000	duck
Eif4h	-0.32	1.00000000	duck
Ppp1r12a	0.44	1.00000000	duck
Arhgap15	0.42	1.00000000	duck
Cers5	-0.23	1.00000000	duck
Ezh2	-0.17	1.00000000	duck
Ankrd13a	-0.14	1.00000000	duck
Gab3	-0.69	1.00000000	duck
Smap1	-0.27	1.00000000	duck
Zc3h18	-0.27	1.00000000	duck
Dcaf1	-0.34	1.00000000	duck
Ehd4	0.82	1.00000000	duck
Lsm5	-0.20	1.00000000	duck
Plekho1	-0.18	1.00000000	duck
Heatr5a	1.62	1.00000000	duck
Rabgap1	-0.49	1.00000000	duck
Dbnl	0.81	1.00000000	duck

Xrcc4	-0.55	1.00000000	duck
Siva1	-0.23	1.00000000	duck
Pofut1	-0.31	1.00000000	duck
Copb1	-0.44	1.00000000	duck
Eif3j2	0.06	1.00000000	duck
Galk2	-0.52	1.00000000	duck
Nup43	-0.11	1.00000000	duck
Dennd6a	-0.47	1.00000000	duck
Fam193a	0.66	1.00000000	duck
Rap2b	0.01	1.00000000	duck
Atp23	1.23	1.00000000	duck
Dvl1	0.00	1.00000000	duck
1700034I23Rik	-0.40	1.00000000	duck
St8sia1	-0.47	1.00000000	duck
Zzef1	-0.28	1.00000000	duck
Smarce1	-0.01	1.00000000	duck
Cul2	-0.36	1.00000000	duck
Alg12	-0.41	1.00000000	duck
Adprm	-0.22	1.00000000	duck
Klhl24	-0.28	1.00000000	duck
Rnf216	-0.13	1.00000000	duck
Ubn1	-0.11	1.00000000	duck
Rmc1	0.28	1.00000000	duck
Oga	-0.44	1.00000000	duck
Wdr91	-0.39	1.00000000	duck
Camkmt	-0.60	1.00000000	duck
Ppp1ca	0.84	1.00000000	duck
Plec	-0.09	1.00000000	duck
Ssu72	-0.25	1.00000000	duck
Snrpd3	-0.22	1.00000000	duck
Lyn	-0.31	1.00000000	duck
Rab2a	0.69	1.00000000	duck
Eloc	-0.29	1.00000000	duck
Tspan12	-0.22	1.00000000	duck
Entpd5	-0.16	1.00000000	duck
Taf5	-0.23	1.00000000	duck
Ralgapb	-0.13	1.00000000	duck
Tspo	0.52	1.00000000	duck
Ppp2r5d	0.31	1.00000000	duck
Klf10	0.75	1.00000000	duck
Kif2a	-0.31	1.00000000	duck
Tsc1	0.10	1.00000000	duck
Slc4a11	-0.04	1.00000000	duck
Ppa1	-0.14	1.00000000	duck
Usp31	-0.22	1.00000000	duck
Snx1	0.40	1.00000000	duck

Abhd2	-0.20	1.00000000	duck
Pigg	0.13	1.00000000	duck
Tsc2	-0.39	1.00000000	duck
Tssc4	-0.37	1.00000000	duck
Prmt7	-0.53	1.00000000	duck
Fbxl20	-0.05	1.00000000	duck
Tmem154	-0.51	1.00000000	duck
Ccnc	-0.27	1.00000000	duck
Nfxl1	-0.60	1.00000000	duck
Arfip1	-0.18	1.00000000	duck
Ndufa4	-0.17	1.00000000	duck
Chrna9	0.51	1.00000000	duck
Hipk1	-0.42	1.00000000	duck
Ptrhd1	-0.04	1.00000000	duck
Ddb1	-0.12	1.00000000	duck
Tank	-0.01	1.00000000	duck
Tnr	-0.03	1.00000000	duck
Dpy19l3	-0.06	1.00000000	duck
Trmt2a	-0.14	1.00000000	duck
Nudcd1	-0.31	1.00000000	duck
Naa60	1.31	1.00000000	duck
Txlna	-0.11	1.00000000	duck
Clcn3	-0.07	1.00000000	duck
Gorasp2	-0.26	1.00000000	duck
Zfp277	-0.35	1.00000000	duck
Zfp507	-0.60	1.00000000	duck
Cnot11	-0.19	1.00000000	duck
9930021J03Rik	-0.65	1.00000000	duck
Arhgap45	-0.18	1.00000000	duck
Fam32a	0.82	1.00000000	duck
Eefsec	-0.31	1.00000000	duck
Ric1	1.00	1.00000000	duck
Itpa	0.44	1.00000000	duck
Camta1	-0.17	1.00000000	duck
Rac2	0.82	1.00000000	duck
Ibtk	-0.36	1.00000000	duck
Fars2	-0.72	1.00000000	duck
Tusc2	0.56	1.00000000	duck
Cct3	-0.18	1.00000000	duck
Abhd13	0.93	1.00000000	duck
Tnpo1	-0.47	1.00000000	duck
Tesk2	-0.78	1.00000000	duck
Bak1	-0.33	1.00000000	duck
Dhx30	-0.37	1.00000000	duck
Gnas	-0.63	1.00000000	duck
Ptp4a1	0.45	1.00000000	duck

Arhgef26	0.30	1.00000000	duck
Serac1	0.07	1.00000000	duck
Wdr4	-0.22	1.00000000	duck
Slc10a7	1.27	1.00000000	duck
Phb	-0.21	1.00000000	duck
Tnfaip8	-0.44	1.00000000	duck
Diablo	0.84	1.00000000	duck
Rnmt	-0.06	1.00000000	duck
Snu13	0.56	1.00000000	duck
Epg5	-0.05	1.00000000	duck
Haus3	-0.19	1.00000000	duck
Bdp1	-0.50	1.00000000	duck
Xrcc5	-0.29	1.00000000	duck
Psmd10	-0.13	1.00000000	duck
Ptdss2	-0.23	1.00000000	duck
Exosc9	-0.19	1.00000000	duck
Borcs6	0.05	1.00000000	duck
Iqcb1	-0.03	1.00000000	duck
Ints10	-0.04	1.00000000	duck
Ints12	-0.31	1.00000000	duck
Tmem231	-0.29	1.00000000	duck
Ago4	-0.66	1.00000000	duck
Ogdh	0.87	1.00000000	duck
Sft2d1	-0.34	1.00000000	duck
Psd	0.73	1.00000000	duck
Fam13a	0.48	1.00000000	duck
P4ha2	1.03	1.00000000	duck
Srrm1	-0.39	1.00000000	duck
Bcl2l13	-0.43	1.00000000	duck
Trmt11	-0.33	1.00000000	duck
Pla2g4f	-0.42	1.00000000	duck
Ubap2	-0.37	1.00000000	duck
Atp5g3	-0.01	1.00000000	duck
Stxbp1	0.92	1.00000000	duck
Fbxo38	0.11	1.00000000	duck
Tmem35b	0.76	1.00000000	duck
Traf1	0.10	1.00000000	duck
Vapa	-0.45	1.00000000	duck
Zfyve9	-0.41	1.00000000	duck
Thoc7	-0.11	1.00000000	duck
Acvr1b	0.88	1.00000000	duck
Ppil2	-0.47	1.00000000	duck
Ero1b	-0.34	1.00000000	duck
Eif4ebp1	0.60	1.00000000	duck
Dynlt1a	0.08	1.00000000	duck
Klhdc3	0.37	1.00000000	duck

Aco1	0.94	1.00000000	duck
Galns	0.49	1.00000000	duck
Usp47	0.04	1.00000000	duck
Prdx1	-0.35	1.00000000	duck
Nipsnap1	-0.15	1.00000000	duck
Arhgap42	-0.19	1.00000000	duck
Tmem183a	0.52	1.00000000	duck
Rpap3	-0.24	1.00000000	duck
Lrrk1	-0.31	1.00000000	duck
Smim20	-0.18	1.00000000	duck
Ccdc50	-0.32	1.00000000	duck
Txndc15	0.37	1.00000000	duck
Itgav	0.73	1.00000000	duck
Acbd3	-0.19	1.00000000	duck
Lpxn	0.08	1.00000000	duck
Commd9	-0.32	1.00000000	duck
Zfyve27	-0.30	1.00000000	duck
Wac	-0.25	1.00000000	duck
Rae1	-0.43	1.00000000	duck
Sugt1	-0.40	1.00000000	duck
Ppp1r3b	0.48	1.00000000	duck
Gtpbp1	-0.30	1.00000000	duck
Tspan14	0.61	1.00000000	duck
Ccdc117	-0.28	1.00000000	duck
Cemip2	-0.45	1.00000000	duck
Ankrd54	-0.07	1.00000000	duck
Scfd2	-0.24	1.00000000	duck
Pcmtd2	-0.05	1.00000000	duck
Cog1	0.41	1.00000000	duck
Rsbn1	-0.11	1.00000000	duck
Pik3ap1	0.33	1.00000000	duck
Dcun1d4	0.14	1.00000000	duck
Dlgap4	-0.17	1.00000000	duck
Hmg20a	-0.15	1.00000000	duck
Arhgap12	-0.47	1.00000000	duck
Pafah1b2	-0.25	1.00000000	duck
Tnfrsf21	-0.28	1.00000000	duck
B4galt5	0.41	1.00000000	duck
Tcf7	-1.05	1.00000000	duck
Glrx5	-0.47	1.00000000	duck
Nbr1	-0.08	1.00000000	duck
Osbpl1a	-0.92	1.00000000	duck
Lamp2	0.75	1.00000000	duck
Fgfr1op2	0.03	1.00000000	duck
Wbp2nl	0.74	1.00000000	duck
Ccnt1	0.76	1.00000000	duck

Notch2	0.11	1.00000000	duck
Ano9	0.36	1.00000000	duck
Hdac4	-0.45	1.00000000	duck
Dntt	-0.36	1.00000000	duck
Yipf5	-0.44	1.00000000	duck
Gars	-0.12	1.00000000	duck
Ash2l	0.34	1.00000000	duck
Polr3b	-0.35	1.00000000	duck
Capzb	0.03	1.00000000	duck
Gpr107	-0.37	1.00000000	duck
Klhdc1	-0.03	1.00000000	duck
Rap2a	0.13	1.00000000	duck
Lmna	0.49	1.00000000	duck
Pou2f1	1.71	1.00000000	duck
Psmb7	-0.38	1.00000000	duck
Atp13a3	1.40	1.00000000	duck
Spg11	0.86	1.00000000	duck
Extl3	-0.04	1.00000000	duck
Pqlc3	-0.29	1.00000000	duck
Vps13a	-0.26	1.00000000	duck
Ripor3	-0.93	1.00000000	duck
Eif4a3l1	-0.25	1.00000000	duck
Tubb5	0.75	1.00000000	duck
Emc8	-0.40	1.00000000	duck
Pcnx2	-0.38	1.00000000	duck
Secisbp2l	-0.15	1.00000000	duck
Nr2c2	-0.39	1.00000000	duck
Sepsecs	-0.41	1.00000000	duck
C2cd5	-0.51	1.00000000	duck
Tbc1d31	-0.35	1.00000000	duck
Cpsf4	-0.21	1.00000000	duck
Fto	-0.75	1.00000000	duck
Eif3d	-0.16	1.00000000	duck
Osbpl3	-0.08	1.00000000	duck
Taf13	0.49	1.00000000	duck
Sema4d	-0.60	1.00000000	duck
Emc7	-0.45	1.00000000	duck
Prpf38a	-0.30	1.00000000	duck
Hps3	-0.37	1.00000000	duck
Lamc1	0.15	1.00000000	duck
Reep2	0.82	1.00000000	duck
Usf3	0.34	1.00000000	duck
Akap13	-0.28	1.00000000	duck
Bmt2	-0.38	1.00000000	duck
Rgs9	0.06	1.00000000	duck
Psmb3	0.63	1.00000000	duck

Llph	-0.36	1.00000000	duck
Ywhae	-0.17	1.00000000	duck
Esyt2	-0.28	1.00000000	duck
Sdk1	-0.34	1.00000000	duck
Dus1l	-0.27	1.00000000	duck
Pdcd2	-0.20	1.00000000	duck
Sppl3	1.09	1.00000000	duck
Nr4a3	0.93	1.00000000	duck
Tomm20	-0.13	1.00000000	duck
Sod2	-0.19	1.00000000	duck
Pex14	-0.67	1.00000000	duck
Trmt44	-0.21	1.00000000	duck
Samd8	-0.35	1.00000000	duck
Gnai2	0.45	1.00000000	duck
Zfp410	-0.25	1.00000000	duck
Asxl1	-0.14	1.00000000	duck
Arl8b	0.64	1.00000000	duck
Prdx6	0.56	1.00000000	duck
Exosc7	-0.06	1.00000000	duck
Samhd1	0.83	1.00000000	duck
Taf4b	-0.40	1.00000000	duck
Tmem94	-0.25	1.00000000	duck
Oard1	0.03	1.00000000	duck
Rufy3	-0.07	1.00000000	duck
Fancf	-0.15	1.00000000	duck
Snorc	-0.20	1.00000000	duck
Yae1d1	-0.14	1.00000000	duck
Ruvbl1	0.25	1.00000000	duck
Pum1	-0.35	1.00000000	duck
Cul1	-0.16	1.00000000	duck
Bnip1	-0.39	1.00000000	duck
Cgrrf1	0.36	1.00000000	duck
Dcaf10	-0.35	1.00000000	duck
Spout1	-0.31	1.00000000	duck
Rad51ap1	-0.42	1.00000000	duck
Fchsd2	0.63	1.00000000	duck
Ubl5b	0.97	1.00000000	duck
Snrpf	0.24	1.00000000	duck
Ttk	-0.10	1.00000000	duck
Atg12	-0.12	1.00000000	duck
Tfcp2	0.43	1.00000000	duck
Tbc1d2b	0.04	1.00000000	duck
Narf	-0.50	1.00000000	duck
Papolg	0.06	1.00000000	duck
Fam199x	0.36	1.00000000	duck
Fxr1	-0.57	1.00000000	duck

Dmap1	-0.49	1.00000000	duck
Trappc13	-0.27	1.00000000	duck
Wdr61	-0.17	1.00000000	duck
Arfgef2	-0.22	1.00000000	duck
Vezf1	0.16	1.00000000	duck
Fh1	-0.18	1.00000000	duck
Tmem258	0.74	1.00000000	duck
Sypl2	-0.20	1.00000000	duck
Cc2d2a	-0.25	1.00000000	duck
Tmco1	0.88	1.00000000	duck
Ttc37	-0.08	1.00000000	duck
Slc9a8	-0.31	1.00000000	duck
Slc25a37	-0.75	1.00000000	duck
Lin54	-0.14	1.00000000	duck
Atp13a1	0.19	1.00000000	duck
Cdk5rap2	-0.24	1.00000000	duck
Qser1	-0.48	1.00000000	duck
Dpm2	-0.16	1.00000000	duck
Fgl2	0.96	1.00000000	duck
Zbtb49	-0.43	1.00000000	duck
Papss1	-0.42	1.00000000	duck
Angel2	-0.38	1.00000000	duck
Ralbp1	-0.34	1.00000000	duck
Csnk2a2	-0.32	1.00000000	duck
Trmu	-0.35	1.00000000	duck
Wdr12	-0.21	1.00000000	duck
Poli	-0.23	1.00000000	duck
Suds3	-0.31	1.00000000	duck
Ttc33	-0.47	1.00000000	duck
Socs5	0.03	1.00000000	duck
Hook3	0.87	1.00000000	duck
Sec11a	-0.13	1.00000000	duck
Rnf144a	-0.48	1.00000000	duck
Cep57	-0.31	1.00000000	duck
Sav1	-0.56	1.00000000	duck
Smim13	-0.31	1.00000000	duck
Alpk1	0.55	1.00000000	duck
Srp72	0.47	1.00000000	duck
Zfat	0.08	1.00000000	duck
Atg5	-0.43	1.00000000	duck
Vps37c	0.30	1.00000000	duck
Rxylt1	-0.18	1.00000000	duck
Vrk1	-0.26	1.00000000	duck
Eif2d	-0.29	1.00000000	duck
Prkd3	-0.03	1.00000000	duck
Clp1	-0.21	1.00000000	duck

Nthl1	-0.05	1.00000000	duck
Gnl2	-0.19	1.00000000	duck
Copa	-0.21	1.00000000	duck
Cul4b	-0.02	1.00000000	duck
Rbm17	-0.12	1.00000000	duck
Fam222b	-0.30	1.00000000	duck
Gosr1	-0.33	1.00000000	duck
Rexo4	0.09	1.00000000	duck
Arrdc4	0.31	1.00000000	duck
Pik3r6	1.16	1.00000000	duck
Endov	0.55	1.00000000	duck
Sort1	0.43	1.00000000	duck
Kmt2e	-0.26	1.00000000	duck
Ap3d1	-0.10	1.00000000	duck
Klhl2	-0.46	1.00000000	duck
Ptdss1	-0.14	1.00000000	duck
Btbd1	-0.30	1.00000000	duck
Rasal2	-0.07	1.00000000	duck
Pfkfb3	0.68	1.00000000	duck
Cnot2	-0.06	1.00000000	duck
Arglu1	-0.09	1.00000000	duck
Rmnd1	-0.09	1.00000000	duck
Dynlrb1	-0.39	1.00000000	duck
Slc41a2	-0.06	1.00000000	duck
Gtf3c6	-0.23	1.00000000	duck
Tiam2	-0.57	1.00000000	duck
Pigh	-0.30	1.00000000	duck
Grsf1	-0.08	1.00000000	duck
Cep192	-0.45	1.00000000	duck
Hic2	-0.48	1.00000000	duck
Foxo4	-0.33	1.00000000	duck
Alcam	-0.32	1.00000000	duck
Reck	-0.23	1.00000000	duck
Kpna3	-0.04	1.00000000	duck
Slc26a5	-0.21	1.00000000	duck
Iah1	-0.13	1.00000000	duck
Arl9	-0.13	1.00000000	duck
Esrrg	-1.02	1.00000000	duck
Pla2g12a	0.00	1.00000000	duck
Snx12	0.40	1.00000000	duck
Rfx2	0.88	1.00000000	duck
Ephx4	-0.30	1.00000000	duck
Ing1	-0.39	1.00000000	duck
Pogz	-0.01	1.00000000	duck
N6amt1	-0.13	1.00000000	duck
Ttc7b	0.06	1.00000000	duck

Fam3c	-0.26	1.00000000	duck
Polg2	-0.08	1.00000000	duck
Slc2a8	-0.11	1.00000000	duck
Rad54b	-0.16	1.00000000	duck
Il20ra	-0.34	1.00000000	duck
Cbx1	-0.48	1.00000000	duck
Ap3m1	-0.17	1.00000000	duck
Scp2	-0.10	1.00000000	duck
Ogfr	-0.30	1.00000000	duck
Pum3	-0.05	1.00000000	duck
Shisa5	0.73	1.00000000	duck
Snrnp35	-0.12	1.00000000	duck
Prkab1	-0.05	1.00000000	duck
Stk16	0.41	1.00000000	duck
Gxylt1	0.80	1.00000000	duck
St3gal3	-0.33	1.00000000	duck
Tox	-0.32	1.00000000	duck
Rars	-0.02	1.00000000	duck
Nras	0.40	1.00000000	duck
Reep3	-0.68	1.00000000	duck
Cdpf1	0.17	1.00000000	duck
Fbxo22	-0.02	1.00000000	duck
Katnbl1	-0.35	1.00000000	duck
Yars	0.55	1.00000000	duck
Lrif1	-0.31	1.00000000	duck
Snx4	0.24	1.00000000	duck
Tenm4	1.75	1.00000000	duck
Phospho1	0.42	1.00000000	duck
Dlg2	0.61	1.00000000	duck
Psma3	0.79	1.00000000	duck
Dnajc19	-0.11	1.00000000	duck
Ndufb6	-0.02	1.00000000	duck
Rhoq	-0.24	1.00000000	duck
Parp9	0.31	1.00000000	duck
Nipal3	-0.23	1.00000000	duck
Cmtm7	-0.17	1.00000000	duck
Crkl	-0.32	1.00000000	duck
Eif3i	-0.15	1.00000000	duck
Dnajb2	0.35	1.00000000	duck
Rc3h1	-0.01	1.00000000	duck
Tom1l2	0.09	1.00000000	duck
Cnot4	0.49	1.00000000	duck
Ccdc126	-0.39	1.00000000	duck
Cct7	-0.19	1.00000000	duck
Slain2	-0.11	1.00000000	duck
Trappc9	-0.20	1.00000000	duck

Dock9	-0.10	1.00000000	duck
Umad1	-0.07	1.00000000	duck
Rbbp5	-0.27	1.00000000	duck
Pfpl	-0.21	1.00000000	duck
Lsm7	-0.13	1.00000000	duck
Ndufb4	-0.38	1.00000000	duck
Spry1	0.69	1.00000000	duck
Aak1	-0.47	1.00000000	duck
Casp2	-0.31	1.00000000	duck
Lrrc42	-0.29	1.00000000	duck
Aagab	-0.26	1.00000000	duck
Surf4	0.28	1.00000000	duck
Rcor1	-0.16	1.00000000	duck
Zfp36l2	0.48	1.00000000	duck
Arpc1b	0.85	1.00000000	duck
Rp2	0.82	1.00000000	duck
Rangap1	0.70	1.00000000	duck
Sgpp1	0.99	1.00000000	duck
Ap4e1	0.55	1.00000000	duck
Acaca	-0.11	1.00000000	duck
Fgd6	-0.29	1.00000000	duck
Thap4	-0.20	1.00000000	duck
Lrrc8d	0.09	1.00000000	duck
Ncoa6	-0.12	1.00000000	duck
Wdr81	0.45	1.00000000	duck
Ppif	1.00	1.00000000	duck
Rad21	-0.17	1.00000000	duck
Smap2	-0.16	1.00000000	duck
Ciao3	-0.36	1.00000000	duck
Lrig2	0.19	1.00000000	duck
Snx14	-0.11	1.00000000	duck
Ptbp1	0.10	1.00000000	duck
Stard4	0.48	1.00000000	duck
Gla	0.31	1.00000000	duck
Orc4	0.49	1.00000000	duck
Sardh	0.62	1.00000000	duck
Cdkn1a	0.33	1.00000000	duck
Pcyt2	0.17	1.00000000	duck
Ddx6	-0.24	1.00000000	duck
Hlcs	-0.35	1.00000000	duck
Spire1	-0.36	1.00000000	duck
Utp25	-0.26	1.00000000	duck
Cisd1	-0.35	1.00000000	duck
Clint1	-0.16	1.00000000	duck
Gfpt1	0.11	1.00000000	duck
Lsm3	-0.18	1.00000000	duck

Gtf3c3	0.19	1.00000000	duck
Elk4	-0.30	1.00000000	duck
Snx8	0.88	1.00000000	duck
Gpatch8	-0.24	1.00000000	duck
Xpo6	-0.40	1.00000000	duck
Cmtm6	1.02	1.00000000	duck
Ppil3	-0.28	1.00000000	duck
P2rx7	-0.17	1.00000000	duck
Prpf40a	0.44	1.00000000	duck
Arfip2	0.33	1.00000000	duck
Eny2	0.02	1.00000000	duck
Dnajb4	-0.53	1.00000000	duck
Gas8	0.29	1.00000000	duck
Rhbdf2	0.33	1.00000000	duck
Ybx3	-0.09	1.00000000	duck
Fam76b	-0.36	1.00000000	duck
Ccdc186	-0.56	1.00000000	duck
Eci3	0.69	1.00000000	duck
Abcd2	-0.34	1.00000000	duck
Nop14	-0.40	1.00000000	duck
Ly96	0.84	1.00000000	duck
Xylt1	-0.49	1.00000000	duck
Zfp830	-0.28	1.00000000	duck
Pus1	-0.12	1.00000000	duck
Ccdc71l	0.35	1.00000000	duck
Stk11	-0.09	1.00000000	duck
Foxn2	-0.30	1.00000000	duck
Ylpm1	-0.61	1.00000000	duck
Pip5k1a	-0.09	1.00000000	duck
Ggcx	0.46	1.00000000	duck
Osgin2	-0.05	1.00000000	duck
Psmd1	-0.54	1.00000000	duck
Cops4	0.17	1.00000000	duck
Asph	0.69	1.00000000	duck
Appl1	0.00	1.00000000	duck
Pygl	1.02	1.00000000	duck
Chst12	0.26	1.00000000	duck
Tlr4	0.26	1.00000000	duck
Filip1l	0.40	1.00000000	duck
Pld1	-0.07	1.00000000	duck
Rmi2	-0.05	1.00000000	duck
2610507B11Rik	0.02	1.00000000	duck
Exosc6	0.03	1.00000000	duck
Dis3	0.18	1.00000000	duck
Hmgb3	-0.14	1.00000000	duck
Srsf5	-0.08	1.00000000	duck

Ints13	-0.23	1.00000000	duck
Copg1	-0.17	1.00000000	duck
Ube2v2	0.27	1.00000000	duck
Smarca1	-0.12	1.00000000	duck
Ppib	1.79	1.00000000	duck
St3gal6	-0.68	1.00000000	duck
Itprid2	0.18	1.00000000	duck
Nvl	-0.17	1.00000000	duck
Nipsnap2	-0.18	1.00000000	duck
Lrch1	-0.40	1.00000000	duck
Zfp618	-0.54	1.00000000	duck
Gbe1	-0.36	1.00000000	duck
Ssbp2	-1.16	1.00000000	duck
Lman1	-0.16	1.00000000	duck
Cstf3	-0.11	1.00000000	duck
Tpi1	0.79	1.00000000	duck
Rreb1	0.70	1.00000000	duck
2510039O18Rik	0.18	1.00000000	duck
Nudt5	0.26	1.00000000	duck
Gorab	0.11	1.00000000	duck
Camsap2	-0.21	1.00000000	duck
Tfdp2	-0.06	1.00000000	duck
Rab22a	0.55	1.00000000	duck
Alg5	-0.25	1.00000000	duck
Radx	-0.23	1.00000000	duck
Sec22a	-0.19	1.00000000	duck
Rasa1	0.13	1.00000000	duck
Iqub	0.13	1.00000000	duck
Xkr8	0.08	1.00000000	duck
Thoc3	-0.15	1.00000000	duck
A4galt	0.10	1.00000000	duck
Blmh	-0.15	1.00000000	duck
Vps35	-0.31	1.00000000	duck
Clptm1l	-0.02	1.00000000	duck
Pcna	0.00	1.00000000	duck
Ric8a	0.59	1.00000000	duck
Tob2	0.30	1.00000000	duck
Tacc3	-0.82	1.00000000	duck
Plekha8	-0.38	1.00000000	duck
Tbl3	-0.20	1.00000000	duck
Etv3	0.19	1.00000000	duck
Fbln5	0.02	1.00000000	duck
Kcnip1	0.35	1.00000000	duck
Arxes2	-0.28	1.00000000	duck
Rpp38	0.36	1.00000000	duck
Naga	0.35	1.00000000	duck

Ldlrap1	-0.17	1.00000000	duck
Tiprl	-0.24	1.00000000	duck
Traf7	0.57	1.00000000	duck
Fam210a	-0.54	1.00000000	duck
Pbx4	-0.50	1.00000000	duck
Chchd7	-0.19	1.00000000	duck
Abca3	0.53	1.00000000	duck
Snrpd1	-0.04	1.00000000	duck
Zfr	-0.14	1.00000000	duck
Rapgef1	-0.37	1.00000000	duck
Spata5	-0.71	1.00000000	duck
Stam2	-0.33	1.00000000	duck
Ngly1	0.06	1.00000000	duck
Dcbld2	-0.27	1.00000000	duck
Tub	-0.27	1.00000000	duck
Tmem106b	0.04	1.00000000	duck
Tbc1d4	-0.33	1.00000000	duck
Usp34	-0.27	1.00000000	duck
Rragd	0.16	1.00000000	duck
Ccnh	-0.24	1.00000000	duck
Ube4b	-0.13	1.00000000	duck
Pdcd5	-0.29	1.00000000	duck
Cnksr2	1.20	1.00000000	duck
Insig2	-0.23	1.00000000	duck
Gfm2	-0.03	1.00000000	duck
Wsb2	-0.14	1.00000000	duck
Cdc34	0.82	1.00000000	duck
Pdap1	-0.60	1.00000000	duck
Etnk1	-0.12	1.00000000	duck
Foxj2	-0.22	1.00000000	duck
Actr6	-0.44	1.00000000	duck
Cpsf2	0.40	1.00000000	duck
Gtf3c5	-0.16	1.00000000	duck
Lats1	-0.09	1.00000000	duck
Srfbp1	-0.11	1.00000000	duck
Esd	0.88	1.00000000	duck
Tmem128	-0.12	1.00000000	duck
Top3b	-0.11	1.00000000	duck
Kif21b	-0.11	1.00000000	duck
Ercc8	0.21	1.00000000	duck
Cops8	-0.35	1.00000000	duck
N4bp2l1	-0.22	1.00000000	duck
Yeats2	-0.23	1.00000000	duck
Sugp1	-0.01	1.00000000	duck
Spg21	-0.13	1.00000000	duck
Setbp1	-0.34	1.00000000	duck

Ndfip1	0.61	1.00000000	duck
Wdr76	-0.22	1.00000000	duck
Hdac11	-0.27	1.00000000	duck
Anks1	-0.49	1.00000000	duck
Anxa2	1.14	1.00000000	duck
Taf5l	-0.17	1.00000000	duck
Nup214	0.03	1.00000000	duck
Slc25a15	-0.02	1.00000000	duck
Atg14	-0.04	1.00000000	duck
Zfp639	-0.08	1.00000000	duck
Slc27a1	-0.20	1.00000000	duck
Trappc8	0.06	1.00000000	duck
Zfp711	-0.10	1.00000000	duck
Thumpd2	-0.19	1.00000000	duck
Polr3k	-0.23	1.00000000	duck
Dapp1	0.53	1.00000000	duck
Plekhm2	-0.24	1.00000000	duck
Stk4	0.15	1.00000000	duck
Kpnb1	-0.10	1.00000000	duck
Bloc1s4	-0.10	1.00000000	duck
Aldh18a1	-0.19	1.00000000	duck
Acot11	-0.19	1.00000000	duck
Gar1	-0.03	1.00000000	duck
Utp3	-0.05	1.00000000	duck
Srek1ip1	-0.08	1.00000000	duck
Tedc1	0.26	1.00000000	duck
Eif2b1	-0.01	1.00000000	duck
Nup160	0.18	1.00000000	duck
Gtf2b	0.83	1.00000000	duck
Nudc	-0.29	1.00000000	duck
Txnrd2	1.14	1.00000000	duck
Zbtb44	0.48	1.00000000	duck
Ctcf	-0.33	1.00000000	duck
Thoc2	-0.23	1.00000000	duck
Vps26a	0.02	1.00000000	duck
Lancl2	-0.03	1.00000000	duck
Jmjd4	0.40	1.00000000	duck
Phc1	-0.04	1.00000000	duck
Cfl2	-0.29	1.00000000	duck
Rpf1	-0.15	1.00000000	duck
Tamm41	-0.17	1.00000000	duck
Dlat	0.04	1.00000000	duck
Ldlrad3	-0.13	1.00000000	duck
Lars2	0.12	1.00000000	duck
Ssrp1	-0.12	1.00000000	duck
Zdhhc6	-0.22	1.00000000	duck

Ugcg	0.49	1.00000000	duck
Zdhhc15	0.75	1.00000000	duck
Ankmy2	-0.07	1.00000000	duck
Rai2	0.27	1.00000000	duck
Drg1	-0.20	1.00000000	duck
Orai2	0.08	1.00000000	duck
Ip6k2	0.18	1.00000000	duck
Nmd3	-0.02	1.00000000	duck
Cldnd1	0.46	1.00000000	duck
Akt1	-0.30	1.00000000	duck
Tada2a	-0.22	1.00000000	duck
Slc66a1	-0.18	1.00000000	duck
Dnaja1	0.07	1.00000000	duck
Ska2	-0.37	1.00000000	duck
Nyx	0.59	1.00000000	duck
Bpnt2	0.09	1.00000000	duck
Ndufs1	-0.41	1.00000000	duck
Rgl1	-0.26	1.00000000	duck
Cd81	0.06	1.00000000	duck
Ppp2r5c	-0.25	1.00000000	duck
Nars	1.09	1.00000000	duck
Yy2	0.47	1.00000000	duck
5031439G07Rik	1.34	1.00000000	duck
Nudt1	-0.16	1.00000000	duck
Clstn1	-0.45	1.00000000	duck
Ank1	-0.71	1.00000000	duck
Abtb1	0.58	1.00000000	duck
Dtnbp1	-0.42	1.00000000	duck
Hsf1	-0.07	1.00000000	duck
Reep5	0.82	1.00000000	duck
Tsr3	-0.02	1.00000000	duck
Cyria	-0.38	1.00000000	duck
Bud31	0.50	1.00000000	duck
Sf3a3	-0.07	1.00000000	duck
Glod4	0.44	1.00000000	duck
Cdin1	0.07	1.00000000	duck
Vapb	-0.10	1.00000000	duck
Nfkbie	0.26	1.00000000	duck
Sh3bp5	-0.28	1.00000000	duck
Dip2c	-0.23	1.00000000	duck
Ublcp1	-0.17	1.00000000	duck
Eif4enif1	-0.27	1.00000000	duck
Slc17a5	0.33	1.00000000	duck
Ufm1	0.30	1.00000000	duck
Btbd11	-0.73	1.00000000	duck
Traf2	0.57	1.00000000	duck

Sec22c	-0.21	1.00000000	duck
Fbh1	-0.20	1.00000000	duck
Cpsf7	-0.36	1.00000000	duck
Slc25a36	-0.12	1.00000000	duck
Fmr1	0.12	1.00000000	duck
Cib2	0.29	1.00000000	duck
Vps4b	0.46	1.00000000	duck
Rnf41	0.25	1.00000000	duck
Bhlhe41	-0.13	1.00000000	duck
Acbd5	0.02	1.00000000	duck
Prpf39	1.29	1.00000000	duck
Ckap4	-0.20	1.00000000	duck
Slc25a26	-0.41	1.00000000	duck
Pus3	-0.23	1.00000000	duck
Atp1b3	0.15	1.00000000	duck
Slc4a4	-0.44	1.00000000	duck
Stard5	-0.23	1.00000000	duck
Lyrm1	-0.32	1.00000000	duck
Spsb3	-0.24	1.00000000	duck
Gpn3	-0.18	1.00000000	duck
Arl3	0.61	1.00000000	duck
Lgals8	0.27	1.00000000	duck
Adipor2	-0.20	1.00000000	duck
Fbxo30	-0.44	1.00000000	duck
Golim4	0.47	1.00000000	duck
Lrrc59	-0.10	1.00000000	duck
Zfp292	-0.29	1.00000000	duck
Peli1	-0.30	1.00000000	duck
Dennd4c	-0.33	1.00000000	duck
Ndc1	-0.22	1.00000000	duck
Thap11	-0.28	1.00000000	duck
Fnta	-0.22	1.00000000	duck
Ddx55	-0.29	1.00000000	duck
Cir1	0.15	1.00000000	duck
Rubcnl	0.76	1.00000000	duck
Rab11fip1	-0.13	1.00000000	duck
Dcaf5	-0.25	1.00000000	duck
Lhfpl4	0.76	1.00000000	duck
Luzp1	0.46	1.00000000	duck
Rassf5	0.03	1.00000000	duck
Snapc4	-0.22	1.00000000	duck
Dstyk	-0.25	1.00000000	duck
Tob1	0.39	1.00000000	duck
Phc2	0.45	1.00000000	duck
Zfp532	0.13	1.00000000	duck
Igf2bp3	-0.17	1.00000000	duck

Zbtb37	-0.34	1.00000000	duck
Chic1	0.49	1.00000000	duck
Serpind1	-0.22	1.00000000	duck
Sec11c	0.79	1.00000000	duck
Slf2	-0.16	1.00000000	duck
Evi5	-0.85	1.00000000	duck
Capn15	0.50	1.00000000	duck
Fem1a	0.22	1.00000000	duck
Slc35e3	-0.11	1.00000000	duck
Exd2	-0.16	1.00000000	duck
Dcaf17	-0.05	1.00000000	duck
Fut11	-0.07	1.00000000	duck
Cep89	-0.03	1.00000000	duck
Nudt7	-0.12	1.00000000	duck
Rnft1	-0.07	1.00000000	duck
Tab3	-0.08	1.00000000	duck
Ndufs4	0.71	1.00000000	duck
Lyar	0.71	1.00000000	duck
Stk17b	0.37	1.00000000	duck
Ube2j1	0.91	1.00000000	duck
Ascc2	-0.09	1.00000000	duck
Rcbtb1	0.50	1.00000000	duck
Etf1	0.12	1.00000000	duck
Chgb	0.45	1.00000000	duck
Arsg	-0.24	1.00000000	duck
Paip1	0.74	1.00000000	duck
Pde12	-0.15	1.00000000	duck
Ppp3cc	0.19	1.00000000	duck
Kat14	-0.15	1.00000000	duck
Sema4g	-0.18	1.00000000	duck
Leprot	-0.18	1.00000000	duck
Yeats4	-0.05	1.00000000	duck
Chpf2	-0.05	1.00000000	duck
Arel1	0.26	1.00000000	duck
Hyi	-0.01	1.00000000	duck
Rtca	0.06	1.00000000	duck
Clpx	0.02	1.00000000	duck
Setd7	0.52	1.00000000	duck
Dda1	0.67	1.00000000	duck
Zmat2	-0.27	1.00000000	duck
Ccdc82	0.63	1.00000000	duck
Cyld	0.27	1.00000000	duck
Ppcs	-0.13	1.00000000	duck
Chid1	0.60	1.00000000	duck
Slc1a2	0.26	1.00000000	duck
Palb2	0.27	1.00000000	duck

Fam167b	-0.03	1.00000000	duck
Aptx	0.68	1.00000000	duck
Akap8l	-0.04	1.00000000	duck
Agfg1	0.73	1.00000000	duck
Ist1	-0.11	1.00000000	duck
Zfp438	0.37	1.00000000	duck
Srp9	0.40	1.00000000	duck
Dgkq	-0.24	1.00000000	duck
Adk	0.15	1.00000000	duck
Sumo1	0.28	1.00000000	duck
Brwd3	-0.49	1.00000000	duck
Id2	-0.03	1.00000000	duck
Cbx8	-0.18	1.00000000	duck
Wfs1	0.26	1.00000000	duck
Tbc1d23	0.16	1.00000000	duck
Pias4	-0.31	1.00000000	duck
Ap2b1	-0.15	1.00000000	duck
Erlec1	1.01	1.00000000	duck
Aqr	0.18	1.00000000	duck
Rab5a	0.53	1.00000000	duck
Rnf213	-0.26	1.00000000	duck
Lclat1	0.35	1.00000000	duck
Wdr33	0.07	1.00000000	duck
Rhot1	0.14	1.00000000	duck
Lrp8	0.66	1.00000000	duck
Wdr59	-0.18	1.00000000	duck
Rtf2	-0.07	1.00000000	duck
Sertad2	0.43	1.00000000	duck
Pard3	0.50	1.00000000	duck
Ppip5k2	-0.21	1.00000000	duck
Abraxas2	-0.30	1.00000000	duck
Copb2	0.12	1.00000000	duck
Ube3a	0.26	1.00000000	duck
Rbbp7	0.22	1.00000000	duck
Psmd2	0.67	1.00000000	duck
Prdm4	0.16	1.00000000	duck
Dhx35	0.31	1.00000000	duck
Prkca	0.69	1.00000000	duck
Fam53a	-0.39	1.00000000	duck
Rock1	0.42	1.00000000	duck
Fam135a	0.07	1.00000000	duck
Tial1	0.08	1.00000000	duck
Exoc7	-0.48	1.00000000	duck
Atl2	-0.01	1.00000000	duck
Dag1	-0.08	1.00000000	duck
Npepps	-0.20	1.00000000	duck

Sh3bp2	-0.68	1.00000000	duck
Coro1c	0.51	1.00000000	duck
Rnf19a	-0.29	1.00000000	duck
Atg7	-0.67	1.00000000	duck
Clta	0.76	1.00000000	duck
Pnpla8	-0.54	1.00000000	duck
Fbxl17	0.05	1.00000000	duck
Gcfc2	-0.71	1.00000000	duck
Cbx7	0.57	1.00000000	duck
Bank1	0.84	1.00000000	duck
Slc16a14	0.25	1.00000000	duck
Erlin2	-0.36	1.00000000	duck
Tyw3	-0.29	1.00000000	duck
Ubfd1	-0.34	1.00000000	duck
Eprs	0.01	1.00000000	duck
Nr4a1	0.55	1.00000000	duck
Scai	-0.43	1.00000000	duck
Zfp362	0.07	1.00000000	duck
Rrn3	-0.22	1.00000000	duck
Borcs5	-0.09	1.00000000	duck
Atrn	-0.09	1.00000000	duck
Txk	-0.14	1.00000000	duck
Anapc5	-0.23	1.00000000	duck
Seh1l	-0.31	1.00000000	duck
Gpr160	0.24	1.00000000	duck
Parvg	-0.30	1.00000000	duck
Nsd2	0.62	1.00000000	duck
Jpt1	-0.17	1.00000000	duck
Igfbp7	-0.11	1.00000000	duck
Habp4	-0.18	1.00000000	duck
Trmt13	-0.19	1.00000000	duck
Selenok	-0.12	1.00000000	duck
Nup98	0.25	1.00000000	duck
Ttc39a	-0.10	1.00000000	duck
Pi4kb	-0.08	1.00000000	duck
Api5	0.77	1.00000000	duck
Plekha2	-0.02	1.00000000	duck
Eef2	-0.27	1.00000000	duck
Sirt5	-0.30	1.00000000	duck
Atp5pb	0.21	1.00000000	duck
Zranb1	-0.48	1.00000000	duck
Rundc1	0.50	1.00000000	duck
Pyroxd1	-0.13	1.00000000	duck
Jam2	-0.11	1.00000000	duck
Cnih4	-0.11	1.00000000	duck
Gle1	-0.20	1.00000000	duck

Vcpkmt	-0.15	1.00000000	duck
Odc1	0.48	1.00000000	duck
Traf6	0.06	1.00000000	duck
Vcl	0.57	1.00000000	duck
Smarca5	0.62	1.00000000	duck
Vps18	0.20	1.00000000	duck
Vcpip1	-0.43	1.00000000	duck
Ptpn11	-0.47	1.00000000	duck
Osbpl8	0.36	1.00000000	duck
Larp7	-0.27	1.00000000	duck
Kdm1a	0.11	1.00000000	duck
Taf4	-0.15	1.00000000	duck
Dcaf12	0.28	1.00000000	duck
Tmem60	-0.16	1.00000000	duck
Guk1	-0.18	1.00000000	duck
Insig1	-0.11	1.00000000	duck
Plag1	0.28	1.00000000	duck
Dclk2	-0.17	1.00000000	duck
Cbln4	-0.05	1.00000000	duck
Klhl11	-0.11	1.00000000	duck
Wdtc1	-0.07	1.00000000	duck
Rnf126	-0.10	1.00000000	duck
Xpc	0.21	1.00000000	duck
Gramd1b	-0.16	1.00000000	duck
Dnajc7	-0.33	1.00000000	duck
Caap1	0.75	1.00000000	duck
Sacm1l	1.02	1.00000000	duck
Zfp423	-0.37	1.00000000	duck
Plxnc1	0.64	1.00000000	duck
Smndc1	0.17	1.00000000	duck
Tma16	-0.06	1.00000000	duck
Lnx2	0.46	1.00000000	duck
Wdr3	-0.06	1.00000000	duck
Ogfrl1	0.04	1.00000000	duck
Cog7	-0.04	1.00000000	duck
Ppie	0.13	1.00000000	duck
Rbms1	-0.09	1.00000000	duck
Eif4g1	-0.17	1.00000000	duck
Deaf1	-0.16	1.00000000	duck
Ndufa8	0.18	1.00000000	duck
Gga2	0.30	1.00000000	duck
Usp14	0.31	1.00000000	duck
Tmem141	0.12	1.00000000	duck
Gpatch11	-0.34	1.00000000	duck
Idh3a	0.00	1.00000000	duck
Cux1	0.28	1.00000000	duck

Acvr2a	-0.43	1.00000000	duck
Bola3	0.07	1.00000000	duck
Itch	0.79	1.00000000	duck
Abhd12	0.01	1.00000000	duck
Itpk1	-0.07	1.00000000	duck
Cpm	-0.31	1.00000000	duck
Smad4	-0.25	1.00000000	duck
Slc25a38	-0.21	1.00000000	duck
Diaph2	0.61	1.00000000	duck
Pfn2	0.20	1.00000000	duck
Slc16a6	0.08	1.00000000	duck
Plrg1	0.32	1.00000000	duck
Adgrv1	0.53	1.00000000	duck
Npl	0.62	1.00000000	duck
Gna11	-0.13	1.00000000	duck
Tent2	-0.29	1.00000000	duck
Gipc2	-0.05	1.00000000	duck
Atg13	0.97	1.00000000	duck
Camk1d	1.10	1.00000000	duck
Emg1	0.56	1.00000000	duck
Ctbs	0.24	1.00000000	duck
Trim25	0.36	1.00000000	duck
Cep350	-0.31	1.00000000	duck
Ppfia1	-0.06	1.00000000	duck
Senp7	0.22	1.00000000	duck
Rnf130	0.50	1.00000000	duck
Aco2	0.10	1.00000000	duck
Cdc26	0.14	1.00000000	duck
Rpa3	-0.28	1.00000000	duck
Cdk12	0.29	1.00000000	duck
Kpna4	0.02	1.00000000	duck
Cep120	0.16	1.00000000	duck
Derl1	-0.12	1.00000000	duck
Kin	0.11	1.00000000	duck
Chmp1a	0.41	1.00000000	duck
Camk2g	-0.01	1.00000000	duck
Bptf	0.03	1.00000000	duck
Acsl3	0.25	1.00000000	duck
Cmss1	-0.19	1.00000000	duck
Epn2	0.56	1.00000000	duck
Pithd1	-0.04	1.00000000	duck
Kctd20	-0.43	1.00000000	duck
Bbx	0.08	1.00000000	duck
Tcerg1	-0.13	1.00000000	duck
Tecpr2	0.73	1.00000000	duck
Itgb2l	0.59	1.00000000	duck

Dnajc11	0.19	1.00000000	duck
Qars	0.03	1.00000000	duck
Wdr11	-0.25	1.00000000	duck
Scamp5	-0.22	1.00000000	duck
Gda	-0.51	1.00000000	duck
Snrpa1	-0.13	1.00000000	duck
Btf3	0.58	1.00000000	duck
Cltc	0.09	1.00000000	duck
Cdc42bpb	0.17	1.00000000	duck
Hyou1	-0.11	1.00000000	duck
Hadha	-0.18	1.00000000	duck
Scpep1	0.40	1.00000000	duck
Tmcc1	-0.12	1.00000000	duck
Rit1	0.03	1.00000000	duck
Rbm12b2	-0.33	1.00000000	duck
Arid2	0.04	1.00000000	duck
Ddx49	0.28	1.00000000	duck
Plcb2	0.26	1.00000000	duck
Tlnrd1	-0.02	1.00000000	duck
Nek7	-0.04	1.00000000	duck
Pten	-0.13	1.00000000	duck
Nudt12	0.87	1.00000000	duck
Luc7l	0.25	1.00000000	duck
Pgm2l1	-0.42	1.00000000	duck
Bnip3l	0.61	1.00000000	duck
Arpc3	0.68	1.00000000	duck
Sec61g	0.11	1.00000000	duck
Czib	-0.25	1.00000000	duck
Ube2g2	-0.19	1.00000000	duck
Lpgat1	-0.12	1.00000000	duck
Rer1	0.20	1.00000000	duck
Tgfbr3	1.05	1.00000000	duck
Bzw1	0.33	1.00000000	duck
Pnpla7	0.28	1.00000000	duck
Baz1a	0.39	1.00000000	duck
Abracl	0.35	1.00000000	duck
Lgmn	0.83	1.00000000	duck
Vamp3	0.49	1.00000000	duck
Irak4	0.16	1.00000000	duck
Josd1	0.37	1.00000000	duck
Casp9	0.26	1.00000000	duck
Tmem9b	-0.10	1.00000000	duck
Psmb1	0.75	1.00000000	duck
Hpse	-0.30	1.00000000	duck
Tmem203	0.39	1.00000000	duck
Glb1	-0.24	1.00000000	duck

Arl5b	-0.02	1.00000000	duck
Tcirg1	0.45	1.00000000	duck
Als2	-0.05	1.00000000	duck
Osbpl2	0.09	1.00000000	duck
Ammecr1	0.09	1.00000000	duck
Lmbr1	0.91	1.00000000	duck
Echdc2	0.30	1.00000000	duck
Uhmk1	0.41	1.00000000	duck
Ntmt1	0.29	1.00000000	duck
Dgkz	-0.16	1.00000000	duck
Lonp2	0.10	1.00000000	duck
Atrx	-0.34	1.00000000	duck
Hint1	-0.16	1.00000000	duck
Lpin2	-0.29	1.00000000	duck
Pold2	0.21	1.00000000	duck
Serp1	0.62	1.00000000	duck
Tent4b	-0.01	1.00000000	duck
Gpc5	0.35	1.00000000	duck
Ccnl2	0.21	1.00000000	duck
Lonp1	0.15	1.00000000	duck
Adgrg6	0.94	1.00000000	duck
Ormdl2	-0.24	1.00000000	duck
Pdcd11	-0.26	1.00000000	duck
Gpt2	0.04	1.00000000	duck
Tdg	0.34	1.00000000	duck
Cers2	-0.07	1.00000000	duck
Rai1	0.39	1.00000000	duck
Exosc3	0.35	1.00000000	duck
Cnot7	0.00	1.00000000	duck
Ccpg1	1.02	1.00000000	duck
Hipk3	0.32	1.00000000	duck
Wwox	0.29	1.00000000	duck
Rnls	0.09	1.00000000	duck
Dusp11	-0.01	1.00000000	duck
Katnb1	0.03	1.00000000	duck
Ncstn	0.04	1.00000000	duck
Arnt	-0.01	1.00000000	duck
Rcn2	-0.05	1.00000000	duck
Poldip2	0.11	1.00000000	duck
Panx1	0.32	1.00000000	duck
Sirt7	0.29	1.00000000	duck
Ccdc171	0.06	1.00000000	duck
Ube2i	-0.27	1.00000000	duck
Atp6v1c1	0.24	1.00000000	duck
Ccdc47	0.00	1.00000000	duck
Zyx	0.88	1.00000000	duck

Ube2q1	0.61	1.00000000	duck
Ino80c	-0.15	1.00000000	duck
Trappc4	-0.14	1.00000000	duck
Pradc1	0.29	1.00000000	duck
Tex14	0.50	1.00000000	duck
Cdk17	0.33	1.00000000	duck
Psmd13	0.63	1.00000000	duck
Nup155	-0.35	1.00000000	duck
Tgif1	-0.03	1.00000000	duck
Tpr	0.70	1.00000000	duck
Slc38a10	0.86	1.00000000	duck
Srsf6	-0.12	1.00000000	duck
Utp20	-0.01	1.00000000	duck
Nuak1	0.73	1.00000000	duck
Ecd	0.22	1.00000000	duck
Lztr1	0.96	1.00000000	duck
Rwdd1	0.09	1.00000000	duck
Ak2	-0.70	1.00000000	duck
Wdr37	1.22	1.00000000	duck
Kctd7	0.93	1.00000000	duck
Sf3b6	0.13	1.00000000	duck
Puf60	0.62	1.00000000	duck
Rock2	0.04	1.00000000	duck
Polr2f	0.06	1.00000000	duck
Nup210	-0.13	1.00000000	duck
Steep1	-0.04	1.00000000	duck
Pmvk	0.34	1.00000000	duck
Bsg	-0.18	1.00000000	duck
Spats2	0.39	1.00000000	duck
Pcnx4	-0.32	1.00000000	duck
Kansl1	-0.01	1.00000000	duck
C2cd2	-0.18	1.00000000	duck
Zbtb34	0.11	1.00000000	duck
Fnip2	-0.24	1.00000000	duck
Plekhj1	0.60	1.00000000	duck
Jmjd8	0.01	1.00000000	duck
Nudt14	0.49	1.00000000	duck
Il16	-0.16	1.00000000	duck
Trappc11	0.34	1.00000000	duck
Aebp2	0.01	1.00000000	duck
Cpne1	0.14	1.00000000	duck
Ikbkb	0.03	1.00000000	duck
Ank3	-0.37	1.00000000	duck
Carnmt1	0.17	1.00000000	duck
Vps51	-0.16	1.00000000	duck
Lrrc28	-0.51	1.00000000	duck

Dnajb12	-0.04	1.00000000	duck
Ofd1	-0.36	1.00000000	duck
Rbm6	-0.15	1.00000000	duck
Ranbp3	0.15	1.00000000	duck
Fam107b	0.37	1.00000000	duck
Lmf2	0.15	1.00000000	duck
Gpatch3	0.23	1.00000000	duck
Cpeb3	-0.69	1.00000000	duck
Rabl3	-0.19	1.00000000	duck
Pank2	0.02	1.00000000	duck
Dnajb6	0.60	1.00000000	duck
Skp1	-0.45	1.00000000	duck
Usp4	0.09	1.00000000	duck
Sdhaf4	-0.23	1.00000000	duck
Ddx5	-0.23	1.00000000	duck
Ccdc88a	0.25	1.00000000	duck
Nrf1	0.24	1.00000000	duck
Ythdf3	-0.02	1.00000000	duck
Papola	-0.06	1.00000000	duck
Rint1	0.20	1.00000000	duck
Rbks	0.24	1.00000000	duck
Dlg5	0.55	1.00000000	duck
Rnf141	0.19	1.00000000	duck
Eml4	0.96	1.00000000	duck
Ppp4r1	-0.31	1.00000000	duck
Exd1	-0.14	1.00000000	duck
Celf1	0.11	1.00000000	duck
Vps26b	-0.13	1.00000000	duck
Ift74	0.80	1.00000000	duck
Sestd1	0.65	1.00000000	duck
Dnaja4	-0.23	1.00000000	duck
Ccng2	0.70	1.00000000	duck
Camsap1	0.03	1.00000000	duck
Zfp518a	0.03	1.00000000	duck
Tbl2	-0.37	1.00000000	duck
Slc30a4	-0.31	1.00000000	duck
Galc	0.37	1.00000000	duck
Pdxdc1	-0.15	1.00000000	duck
Psmc2	-0.25	1.00000000	duck
Fuca1	0.06	1.00000000	duck
Sh3glb1	0.43	1.00000000	duck
Odr4	-0.11	1.00000000	duck
Ttc28	0.12	1.00000000	duck
Tbcd	0.44	1.00000000	duck
Ppp1r8	0.28	1.00000000	duck
Slc30a5	-0.34	1.00000000	duck

Brd3	0.15	1.00000000	duck
G2e3	-0.37	1.00000000	duck
Leprotl1	-0.35	1.00000000	duck
Utp15	-0.12	1.00000000	duck
Il6st	1.17	1.00000000	duck
Adipor1	0.34	1.00000000	duck
Sucla2	0.54	1.00000000	duck
Ikbke	0.08	1.00000000	duck
Rrbp1	0.47	1.00000000	duck
Spag9	0.23	1.00000000	duck
Rpusd2	-0.04	1.00000000	duck
Peak1	-0.16	1.00000000	duck
Rnps1	0.67	1.00000000	duck
Dut	0.44	1.00000000	duck
Fasn	1.49	1.00000000	duck
Ttc14	0.93	1.00000000	duck
Smc6	-1.00	1.00000000	duck
Rhoa	0.33	1.00000000	duck
Cep128	-0.16	1.00000000	duck
Nsmce2	0.88	1.00000000	duck
Fam76a	0.24	1.00000000	duck
Iffo2	0.61	1.00000000	duck
Igsf9	0.95	1.00000000	duck
Cish	-0.39	1.00000000	duck
Dok2	-0.05	1.00000000	duck
Polr3h	0.15	1.00000000	duck
Tmem251	0.52	1.00000000	duck
Tmem30a	0.12	1.00000000	duck
Atg2b	0.19	1.00000000	duck
Kat6a	-0.04	1.00000000	duck
Slc39a9	0.51	1.00000000	duck
Capza2	0.83	1.00000000	duck
Vps37b	-0.22	1.00000000	duck
Ppp1r13b	-0.41	1.00000000	duck
Gpr137c	0.32	1.00000000	duck
Dgke	-0.27	1.00000000	duck
Snx6	0.09	1.00000000	duck
Pip4k2a	0.39	1.00000000	duck
AW554918	0.30	1.00000000	duck
Nt5c3	-0.37	1.00000000	duck
Pdss1	0.35	1.00000000	duck
Hibch	-0.12	1.00000000	duck
Fry	-0.31	1.00000000	duck
Cyfip2	1.05	1.00000000	duck
Txndc9	-0.11	1.00000000	duck
Cdc14a	-0.43	1.00000000	duck

Tnfsf11	0.67	1.00000000	duck
Ddx23	0.03	1.00000000	duck
Asns	0.65	1.00000000	duck
Tert	0.17	1.00000000	duck
Ergic1	0.24	1.00000000	duck
Abi1	0.33	1.00000000	duck
Hmgcr	0.24	1.00000000	duck
Nubp1	-0.01	1.00000000	duck
Usp10	-0.02	1.00000000	duck
Nucb2	-0.08	1.00000000	duck
Pstpip1	-0.05	1.00000000	duck
Gabpb1	0.09	1.00000000	duck
Inpp4a	0.61	1.00000000	duck
Stat3	0.83	1.00000000	duck
Nsl1	-0.39	1.00000000	duck
Zfp827	-0.39	1.00000000	duck
Pak2	0.36	1.00000000	duck
Cnep1r1	0.26	1.00000000	duck
Cab39	0.01	1.00000000	duck
Igf1r	-0.01	1.00000000	duck
Pno1	0.19	1.00000000	duck
Arntl2	-0.18	1.00000000	duck
Pgrmc2	0.00	1.00000000	duck
Cops7b	-0.09	1.00000000	duck
Prr5l	0.68	1.00000000	duck
Xxylt1	0.57	1.00000000	duck
Tatdn3	-0.18	1.00000000	duck
Gcc2	-0.32	1.00000000	duck
Arhgef7	0.53	1.00000000	duck
Cmas	0.22	1.00000000	duck
Ppp2cb	0.33	1.00000000	duck
Sufu	-0.33	1.00000000	duck
Wdr89	0.15	1.00000000	duck
Rab11a	0.56	1.00000000	duck
Ppp6c	0.10	1.00000000	duck
Cdc42se1	-0.13	1.00000000	duck
Upf2	0.16	1.00000000	duck
Ube2q2	-0.13	1.00000000	duck
Srp68	0.41	1.00000000	duck
Acat1	0.02	1.00000000	duck
E2f6	0.08	1.00000000	duck
Lrrc1	0.06	1.00000000	duck
Npc1	0.56	1.00000000	duck
Xbp1	0.33	1.00000000	duck
Gnb1	0.33	1.00000000	duck
Slc3a1	-0.01	1.00000000	duck

Snf8	-0.02	1.00000000	duck
Uggt2	-0.01	1.00000000	duck
Ythdf2	-0.08	1.00000000	duck
Ran	-0.10	1.00000000	duck
Cep83	0.09	1.00000000	duck
Rcc2	0.40	1.00000000	duck
Zfp706	0.01	1.00000000	duck
Tomm22	0.01	1.00000000	duck
Thumpd3	0.60	1.00000000	duck
Ilrun	0.27	1.00000000	duck
Ap3m2	0.50	1.00000000	duck
Ktn1	0.12	1.00000000	duck
Cdk9	0.24	1.00000000	duck
Ubl7	-0.05	1.00000000	duck
Rsrp1	-0.39	1.00000000	duck
Pepd	0.23	1.00000000	duck
Sik2	-0.11	1.00000000	duck
Hecw2	-0.28	1.00000000	duck
Hacd2	-0.17	1.00000000	duck
Ccng1	-0.10	1.00000000	duck
Dcp1b	0.30	1.00000000	duck
Sf3a2	0.53	1.00000000	duck
Scn8a	0.11	1.00000000	duck
Sec24b	0.27	1.00000000	duck
Chuk	0.02	1.00000000	duck
Ube2b	1.08	1.00000000	duck
Slc37a1	0.22	1.00000000	duck
Tmbim6	-0.11	1.00000000	duck
St13	0.22	1.00000000	duck
Nfkb2	0.46	1.00000000	duck
Cask	1.13	1.00000000	duck
Timp2	-0.04	1.00000000	duck
Ptgfrn	0.32	1.00000000	duck
Wdr82	0.74	1.00000000	duck
Cct5	0.06	1.00000000	duck
Sugct	-0.62	1.00000000	duck
Ppid	0.40	1.00000000	duck
Gpr155	0.53	1.00000000	duck
Supt7l	0.01	1.00000000	duck
Ppp2ca	0.32	1.00000000	duck
Rlim	0.59	1.00000000	duck
Creg1	-0.25	1.00000000	duck
Wdr20	0.20	1.00000000	duck
Far2	-0.31	1.00000000	duck
Rnf10	0.38	1.00000000	duck
Slc35e1	0.56	1.00000000	duck

Slc41a3	1.05	1.00000000	duck
Dhx38	0.08	1.00000000	duck
Zswim8	0.28	1.00000000	duck
Avl9	-0.11	1.00000000	duck
Btf3l4	0.48	1.00000000	duck
Heatr6	0.11	1.00000000	duck
Slc2a13	0.73	1.00000000	duck
Nmt1	0.00	1.00000000	duck
Poll	-0.24	1.00000000	duck
Cpne3	0.79	1.00000000	duck
Nfe2l2	-0.03	1.00000000	duck
Ubqln1	-0.14	1.00000000	duck
Cstf2	0.44	1.00000000	duck
Csrnp1	0.34	1.00000000	duck
Lrch3	-0.04	1.00000000	duck
Lcor	-0.39	1.00000000	duck
Nifk	-0.01	1.00000000	duck
Irf7	0.52	1.00000000	duck
Lpcat1	0.31	1.00000000	duck
Ints4	-0.13	1.00000000	duck
Rad51c	-0.34	1.00000000	duck
Lrp3	0.28	1.00000000	duck
4933427D14Rik	0.25	1.00000000	duck
Coq2	-0.07	1.00000000	duck
Elp1	0.03	1.00000000	duck
Parp11	-0.03	1.00000000	duck
Bin2	-0.16	1.00000000	duck
Syf2	0.50	1.00000000	duck
Golga7	0.17	1.00000000	duck
Ino80d	0.19	1.00000000	duck
Cap1	0.24	1.00000000	duck
Pes1	0.18	1.00000000	duck
Rnf214	0.24	1.00000000	duck
Sptlc2	-0.02	1.00000000	duck
Fkbp3	0.00	1.00000000	duck
Bloc1s6	-0.15	1.00000000	duck
Fam8a1	-0.34	1.00000000	duck
Plcl1	0.23	1.00000000	duck
Socs3	0.32	1.00000000	duck
Lysmd2	0.15	1.00000000	duck
Fbxo34	0.34	1.00000000	duck
Fam114a1	0.18	1.00000000	duck
Cacna1c	0.26	1.00000000	duck
Trappc6b	0.30	1.00000000	duck
Nup85	0.58	1.00000000	duck
Nr3c1	0.46	1.00000000	duck

Hsd17b12	0.06	1.00000000	duck
Bmpr2	0.26	1.00000000	duck
Adap1	0.32	1.00000000	duck
Rsbn1l	0.27	1.00000000	duck
Edem1	0.19	1.00000000	duck
Tango2	0.01	1.00000000	duck
Hipk2	-0.20	1.00000000	duck
Kif13b	-0.31	1.00000000	duck
Faf2	0.00	1.00000000	duck
Rad52	0.16	1.00000000	duck
Eef1a1	-0.20	1.00000000	duck
Eif3f	0.18	1.00000000	duck
Plekhm3	-0.05	1.00000000	duck
Rad18	0.07	1.00000000	duck
Setd5	0.86	1.00000000	duck
Adpgk	0.16	1.00000000	duck
Pknox1	-0.12	1.00000000	duck
Ust	-0.01	1.00000000	duck
Brip1	-0.14	1.00000000	duck
Fam117a	0.38	1.00000000	duck
Timm13	0.99	1.00000000	duck
Trp53bp2	0.61	1.00000000	duck
Lonrf3	0.92	1.00000000	duck
Prrc2c	-0.01	1.00000000	duck
Adsl	-0.24	1.00000000	duck
Zfp513	0.36	1.00000000	duck
Ift22	-0.34	1.00000000	duck
Limd2	-0.07	1.00000000	duck
Eri1	0.12	1.00000000	duck
Zcrb1	-0.20	1.00000000	duck
Csnk1a1	0.70	1.00000000	duck
Tmem11	-0.15	1.00000000	duck
Atf1	-0.33	1.00000000	duck
Tmem230	0.05	1.00000000	duck
Raph1	0.55	1.00000000	duck
Dpagt1	0.05	1.00000000	duck
Csrp1	0.54	1.00000000	duck
Tmc7	-0.12	1.00000000	duck
Cnnm2	-0.05	1.00000000	duck
Clip1	0.55	1.00000000	duck
Pi4ka	-0.14	1.00000000	duck
Sdhb	-0.13	1.00000000	duck
Tnrc6a	0.30	1.00000000	duck
Erap1	0.87	1.00000000	duck
Cfap20	-0.03	1.00000000	duck
Bhlhe40	0.38	1.00000000	duck

Erlin1	-0.22	1.00000000	duck
Txndc17	-0.06	1.00000000	duck
Helq	0.27	1.00000000	duck
Trio	-0.21	1.00000000	duck
Ubr3	-0.09	1.00000000	duck
Spopl	0.03	1.00000000	duck
Ctps	0.40	1.00000000	duck
Sgce	0.14	1.00000000	duck
Plod2	0.48	1.00000000	duck
Fem1b	-0.11	1.00000000	duck
Iqsec1	-0.24	1.00000000	duck
Sirt6	0.32	1.00000000	duck
Ctnnbl1	0.00	1.00000000	duck
Gclc	-0.07	1.00000000	duck
Tmem59	-0.15	1.00000000	duck
Helz	-0.29	1.00000000	duck
AU040320	0.38	1.00000000	duck
Rnf114	0.25	1.00000000	duck
Nt5dc1	0.12	1.00000000	duck
Cacul1	-0.30	1.00000000	duck
D5Ertd579e	0.39	1.00000000	duck
Nom1	0.17	1.00000000	duck
C1d	-0.26	1.00000000	duck
Spcs2	-0.04	1.00000000	duck
Pbrm1	-0.02	1.00000000	duck
Ndufb3	0.02	1.00000000	duck
Smcr8	0.05	1.00000000	duck
Spty2d1	0.33	1.00000000	duck
Dcp2	-0.06	1.00000000	duck
Cebpg	-0.09	1.00000000	duck
Zcchc14	-0.28	1.00000000	duck
Fez2	-0.26	1.00000000	duck
Ankrd46	0.10	1.00000000	duck
Denr	0.03	1.00000000	duck
Tmem138	0.14	1.00000000	duck
Tes	0.15	1.00000000	duck
Pomt2	-0.02	1.00000000	duck
Dync1i2	0.27	1.00000000	duck
Dcun1d1	0.42	1.00000000	duck
Gpcpd1	-0.09	1.00000000	duck
Klhdc2	-0.19	1.00000000	duck
Slc43a2	0.26	1.00000000	duck
Lrrc8a	0.26	1.00000000	duck
Cnih1	-0.19	1.00000000	duck
Lrig1	-0.32	1.00000000	duck
Ptp4a2	0.57	1.00000000	duck

Pip5k1c	-0.24	1.00000000	duck
Ube2g1	0.01	1.00000000	duck
Dtymk	0.18	1.00000000	duck
Fdx1	0.22	1.00000000	duck
Vdac2	0.47	1.00000000	duck
N4bp1	-0.08	1.00000000	duck
Nemp1	0.41	1.00000000	duck
Vgll4	-0.17	1.00000000	duck
Wasf2	0.31	1.00000000	duck
Gzf1	0.13	1.00000000	duck
Xpa	0.08	1.00000000	duck
Zfp326	0.41	1.00000000	duck
Pigt	0.41	1.00000000	duck
Kdm5a	0.62	1.00000000	duck
Bend3	0.61	1.00000000	duck
Kbtbd2	0.05	1.00000000	duck
Kif13a	0.69	1.00000000	duck
Ccne2	0.07	1.00000000	duck
Pccb	0.32	1.00000000	duck
Qdpr	-0.12	1.00000000	duck
Trpm6	0.89	1.00000000	duck
Lemd3	0.17	1.00000000	duck
Nf2	-0.22	1.00000000	duck
Lrrfip2	0.54	1.00000000	duck
Hnrnpk	0.31	1.00000000	duck
Slc49a4	0.56	1.00000000	duck
Bcap29	0.49	1.00000000	duck
Ctps2	-0.11	1.00000000	duck
Slc30a6	0.00	1.00000000	duck
Kifap3	0.35	1.00000000	duck
Alas1	0.69	1.00000000	duck
Plpbp	-0.13	1.00000000	duck
Jade3	-0.23	1.00000000	duck
Sox5	-0.64	1.00000000	duck
Tpra1	0.27	1.00000000	duck
Strada	0.05	1.00000000	duck
Immp2l	-0.88	1.00000000	duck
Pnpla2	-0.11	1.00000000	duck
Cep164	0.02	1.00000000	duck
Ctsc	-0.16	1.00000000	duck
Shprh	0.29	1.00000000	duck
Ncln	0.04	1.00000000	duck
Gga1	0.47	1.00000000	duck
Ciao2b	0.16	1.00000000	duck
Echs1	0.23	1.00000000	duck
Amacr	-0.16	1.00000000	duck

Ugp2	-0.09	1.00000000	duck
Hadh	0.45	1.00000000	duck
Tmem50a	0.32	1.00000000	duck
Otulinl	0.24	1.00000000	duck
Saal1	0.52	1.00000000	duck
Atad3a	0.70	1.00000000	duck
Rnf169	0.06	1.00000000	duck
Ankrd17	-0.03	1.00000000	duck
Rab6a	-0.13	1.00000000	duck
Tacc1	0.40	1.00000000	duck
Ankrd13c	0.08	1.00000000	duck
Rragc	0.36	1.00000000	duck
Ccni	0.19	1.00000000	duck
Capg	0.49	1.00000000	duck
Nsmaf	-0.02	1.00000000	duck
Tmem165	0.85	1.00000000	duck
Scamp1	-0.29	1.00000000	duck
Rspry1	-0.33	1.00000000	duck
Impa2	-0.02	1.00000000	duck
Pdcl	-0.09	1.00000000	duck
Lmnb2	-0.10	1.00000000	duck
Hypk	-0.18	1.00000000	duck
Rab9	0.48	1.00000000	duck
Rnf138	0.13	1.00000000	duck
Prkcd	-0.28	1.00000000	duck
Slc7a5	-0.15	1.00000000	duck
Ecpas	0.16	1.00000000	duck
Ddx31	-0.27	1.00000000	duck
Vamp4	0.08	1.00000000	duck
Ate1	-0.17	1.00000000	duck
Prpf8	0.10	1.00000000	duck
Gyg	0.09	1.00000000	duck
Ube2d1	0.19	1.00000000	duck
Cfap36	1.10	1.00000000	duck
Asb3	0.39	1.00000000	duck
Hcfc2	0.53	1.00000000	duck
Ints2	0.30	1.00000000	duck
Lsm4	0.29	1.00000000	duck
Tmem241	0.03	1.00000000	duck
Tln2	0.04	1.00000000	duck
Vps50	-0.32	1.00000000	duck
Septin8	-0.09	1.00000000	duck
Ghitm	0.04	1.00000000	duck
Imp3	0.33	1.00000000	duck
Ccdc12	0.08	1.00000000	duck
Rpn2	0.52	1.00000000	duck

Abitram	0.05	1.00000000	duck
Tmem222	0.04	1.00000000	duck
Pex5	0.19	1.00000000	duck
Clic4	0.29	1.00000000	duck
Scamp3	0.14	1.00000000	duck
Scd2	0.48	1.00000000	duck
Lyrm2	-0.08	1.00000000	duck
Dagla	-0.04	1.00000000	duck
Pex10	-0.01	1.00000000	duck
Ing4	-0.02	1.00000000	duck
Tcf25	0.02	1.00000000	duck
Cds2	-0.01	1.00000000	duck
Ano10	-0.07	1.00000000	duck
Gsr	0.08	1.00000000	duck
Lrwd1	0.12	1.00000000	duck
Get4	0.55	1.00000000	duck
Arpp19	0.93	1.00000000	duck
Rnf6	0.17	1.00000000	duck
Creld2	0.25	1.00000000	duck
Csl	-0.02	1.00000000	duck
Nfx1	0.04	1.00000000	duck
Hspa5	0.40	1.00000000	duck
Rpa1	0.05	1.00000000	duck
Zdhhc3	0.52	1.00000000	duck
Slx4	-0.07	1.00000000	duck
Rpn1	0.35	1.00000000	duck
Pag1	-0.04	1.00000000	duck
Poc1a	-0.23	1.00000000	duck
Uggt1	0.95	1.00000000	duck
Crym	0.59	1.00000000	duck
Slc35c2	0.65	1.00000000	duck
Fzr1	0.13	1.00000000	duck
Hdx	-0.09	1.00000000	duck
Psma5	0.14	1.00000000	duck
Pfdn1	0.71	1.00000000	duck
Ralgps2	0.09	1.00000000	duck
Prelid1	0.37	1.00000000	duck
Coq5	0.39	1.00000000	duck
Ergic2	-0.18	1.00000000	duck
Cert1	1.07	1.00000000	duck
Bin3	0.18	1.00000000	duck
Lsm6	0.60	1.00000000	duck
Ttc1	0.11	1.00000000	duck
Phtf2	0.31	1.00000000	duck
S1pr4	-0.14	1.00000000	duck
Pbx1	0.64	1.00000000	duck

Arl6ip4	-0.17	1.00000000	duck
P4ha1	-0.12	1.00000000	duck
Pwp1	0.11	1.00000000	duck
Rell1	0.61	1.00000000	duck
Dcun1d3	0.84	1.00000000	duck
Helb	-0.06	1.00000000	duck
Wdr48	0.22	1.00000000	duck
Prdx4	-0.13	1.00000000	duck
Gnal	0.15	1.00000000	duck
Stx8	0.46	1.00000000	duck
Zfp131	0.18	1.00000000	duck
Taf10	-0.08	1.00000000	duck
Lzic	-0.19	1.00000000	duck
G3bp2	-0.08	1.00000000	duck
Zranb2	0.75	1.00000000	duck
Osbpl9	0.16	1.00000000	duck
Slc9a6	0.41	1.00000000	duck
Ppp2r3c	0.29	1.00000000	duck
Sars	0.08	1.00000000	duck
Foxk1	0.77	1.00000000	duck
Sh3gl1	-0.14	1.00000000	duck
Lin52	-0.14	1.00000000	duck
Arpc5	0.58	1.00000000	duck
Armc1	0.21	1.00000000	duck
Nr1d2	0.79	1.00000000	duck
Ppard	0.31	1.00000000	duck
Flnb	0.69	1.00000000	duck
Tcea2	-0.03	1.00000000	duck
Ehd3	-0.20	1.00000000	duck
Nr1h3	0.30	1.00000000	duck
Ikzf5	-0.07	1.00000000	duck
Ift122	0.04	1.00000000	duck
Raf1	-0.17	1.00000000	duck
Eif4g2	0.30	1.00000000	duck
Fbxl5	-0.10	1.00000000	duck
Slc12a2	0.81	1.00000000	duck
Rabggtb	-0.04	1.00000000	duck
Gemin5	0.04	1.00000000	duck
Bcl2l1	-0.29	1.00000000	duck
Eif2s1	-0.05	1.00000000	duck
Trmt10c	0.32	1.00000000	duck
Zc3h7b	0.22	1.00000000	duck
Lmtk2	0.07	1.00000000	duck
Aldh7a1	0.38	1.00000000	duck
Sec63	0.68	1.00000000	duck
Lipa	0.29	1.00000000	duck

Samd11	0.24	1.00000000	duck
Dnajc1	-0.44	1.00000000	duck
Cmtr1	-0.09	1.00000000	duck
Ccdc59	0.45	1.00000000	duck
Bcor	0.66	1.00000000	duck
Arfgap2	0.00	1.00000000	duck
Dnaja3	-0.11	1.00000000	duck
Cmtm3	0.23	1.00000000	duck
Ip6k1	0.24	1.00000000	duck
Gak	0.17	1.00000000	duck
Zc3hc1	0.53	1.00000000	duck
Snrpb	0.33	1.00000000	duck
Eif4e2	0.08	1.00000000	duck
Aasdh	0.20	1.00000000	duck
Telo2	-0.03	1.00000000	duck
Ccnt2	0.07	1.00000000	duck
Qrich1	0.32	1.00000000	duck
Gpatch2l	-0.22	1.00000000	duck
Ankrd12	-0.22	1.00000000	duck
Timm44	0.23	1.00000000	duck
Cript	-0.01	1.00000000	duck
Id3	0.42	1.00000000	duck
Atp2a2	-0.20	1.00000000	duck
Grpel1	0.29	1.00000000	duck
Samd4	-0.13	1.00000000	duck
Fam162a	-0.14	1.00000000	duck
Lamtor2	0.53	1.00000000	duck
Impa1	0.10	1.00000000	duck
Acadsb	0.27	1.00000000	duck
Nde1	0.17	1.00000000	duck
Ccser2	0.38	1.00000000	duck
Lactb	0.75	1.00000000	duck
Pigl	0.34	1.00000000	duck
Sprtn	-0.24	1.00000000	duck
Itgb1	0.00	1.00000000	duck
Dpy30	0.45	1.00000000	duck
Hsbp1	0.31	1.00000000	duck
Utp11	-0.06	1.00000000	duck
Tm9sf4	0.12	1.00000000	duck
Cstf1	-0.08	1.00000000	duck
Ddost	0.53	1.00000000	duck
Klhl12	-0.13	1.00000000	duck
Tspan8	0.23	1.00000000	duck
Pgap2	1.02	1.00000000	duck
Pm20d1	0.72	1.00000000	duck
Kbtbd8	0.16	1.00000000	duck

Cpsf3	0.17	1.00000000	duck
Lman2	0.02	1.00000000	duck
Per2	0.19	1.00000000	duck
Thrap3	0.14	1.00000000	duck
Limk2	0.05	1.00000000	duck
Bmp2k	0.08	1.00000000	duck
Gns	-0.07	1.00000000	duck
Arhgdia	0.03	1.00000000	duck
Gtpbp10	0.17	1.00000000	duck
Acsf3	-0.19	1.00000000	duck
Papln	0.63	1.00000000	duck
Dynlt3	0.65	1.00000000	duck
Itga1	0.18	1.00000000	duck
Zfp654	0.54	1.00000000	duck
Crnkl1	0.23	1.00000000	duck
Rbm31y	0.33	1.00000000	duck
Ppa2	-0.16	1.00000000	duck
Hint3	-0.25	1.00000000	duck
Furin	-0.06	1.00000000	duck
Prrc1	-0.05	1.00000000	duck
Tmem223	0.07	1.00000000	duck
Ep300	0.30	1.00000000	duck
Csgalnact2	0.04	1.00000000	duck
Derl2	0.20	1.00000000	duck
Pigs	0.29	1.00000000	duck
Rgs19	0.22	1.00000000	duck
Hmg20b	-0.05	1.00000000	duck
Nr3c2	0.87	1.00000000	duck
Rap2c	0.78	1.00000000	duck
Selenop	0.41	1.00000000	duck
Ccdc18	-0.19	1.00000000	duck
Nelfcd	-0.25	1.00000000	duck
Ralb	0.48	1.00000000	duck
Chd5	0.30	1.00000000	duck
Anapc7	-0.07	1.00000000	duck
Pxk	0.45	1.00000000	duck
Adcy7	-0.06	1.00000000	duck
Tmem38a	-0.06	1.00000000	duck
Acaa2	-0.14	1.00000000	duck
Eif3h	-0.01	1.00000000	duck
Tsc22d2	0.73	1.00000000	duck
Sf3b4	0.49	1.00000000	duck
Rpp14	0.30	1.00000000	duck
Taf1a	-0.06	1.00000000	duck
Atp1a1	0.15	1.00000000	duck
Dlg1	-0.09	1.00000000	duck

Ostf1	0.36	1.00000000	duck
Rala	0.62	1.00000000	duck
Dram1	0.25	1.00000000	duck
Usp7	-0.34	1.00000000	duck
Pdk1	0.37	1.00000000	duck
Wbp2	0.39	1.00000000	duck
Uso1	0.27	1.00000000	duck
Ttc17	0.65	1.00000000	duck
Tent5a	0.07	1.00000000	duck
Odf2	0.01	1.00000000	duck
Gsn	0.28	1.00000000	duck
Sh3kbp1	-0.05	1.00000000	duck
Hscb	0.18	1.00000000	duck
Decr2	0.21	1.00000000	duck
Snx20	0.66	1.00000000	duck
Vps29	0.08	1.00000000	duck
Chmp5	0.33	1.00000000	duck
Nol7	0.05	1.00000000	duck
Rnh1	0.13	1.00000000	duck
Hs6st1	0.06	1.00000000	duck
Desi1	0.40	1.00000000	duck
Rbx1	0.29	1.00000000	duck
Fbxo25	0.26	1.00000000	duck
Snx24	-0.23	1.00000000	duck
Prkar1b	0.42	1.00000000	duck
Tyms	0.05	1.00000000	duck
Cib1	0.48	1.00000000	duck
Yod1	-0.04	1.00000000	duck
Ndufs6	0.28	1.00000000	duck
Tm2d1	0.18	1.00000000	duck
Arhgap25	0.11	1.00000000	duck
Suco	0.11	1.00000000	duck
Rnf34	0.15	1.00000000	duck
Trp53inp1	0.19	1.00000000	duck
Ugdh	0.18	1.00000000	duck
Fam118b	0.20	1.00000000	duck
Thyn1	0.44	1.00000000	duck
Tut7	0.31	1.00000000	duck
Scamp2	0.05	1.00000000	duck
Rb1	0.71	1.00000000	duck
Wdr75	-0.01	1.00000000	duck
Txnrd3	0.10	1.00000000	duck
Atp5b	-0.02	1.00000000	duck
Dnm1l	0.12	1.00000000	duck
Dnaja2	-0.12	1.00000000	duck
Acadm	0.54	1.00000000	duck

Slc17a9	0.77	1.00000000	duck
Rest	-0.26	1.00000000	duck
Cradd	0.24	1.00000000	duck
Hnrnph3	0.22	1.00000000	duck
Adrm1	0.77	1.00000000	duck
Atp2c1	-0.03	1.00000000	duck
Gpatch4	0.12	1.00000000	duck
Rassf8	-0.52	1.00000000	duck
Ube2k	0.43	1.00000000	duck
Arcn1	0.29	1.00000000	duck
Vti1b	0.34	1.00000000	duck
Sash3	0.50	1.00000000	duck
Arrdc3	0.54	1.00000000	duck
Bloc1s2	0.03	1.00000000	duck
Appbp2	0.41	1.00000000	duck
Wars	0.09	1.00000000	duck
Serinc3	0.62	1.00000000	duck
Zbtb18	0.10	1.00000000	duck
Nkiras2	0.04	1.00000000	duck
Larp4b	0.22	1.00000000	duck
Pdxk	0.63	1.00000000	duck
Fnbp1	-0.18	1.00000000	duck
Polr2b	-0.21	1.00000000	duck
Vps54	0.24	1.00000000	duck
Sf3b3	0.26	1.00000000	duck
Dennd11	0.08	1.00000000	duck
Fam177a	0.17	1.00000000	duck
Cutc	0.20	1.00000000	duck
Pdia4	0.62	1.00000000	duck
Chmp4b	0.26	1.00000000	duck
Ints1	-0.03	1.00000000	duck
Smdt1	0.04	1.00000000	duck
Cdc42	0.15	1.00000000	duck
Dsn1	0.25	1.00000000	duck
Vkorc1l1	-0.05	1.00000000	duck
Slc25a5	0.57	1.00000000	duck
Abi2	-0.24	1.00000000	duck
Tnip1	0.27	1.00000000	duck
Smarcd2	0.08	1.00000000	duck
Birc6	0.07	1.00000000	duck
Tmem268	0.30	1.00000000	duck
Ier5	0.62	1.00000000	duck
Ssr3	0.53	1.00000000	duck
Prkag1	-0.05	1.00000000	duck
Cbfb	1.01	1.00000000	duck
Zmym3	0.08	1.00000000	duck

Gtf2a2	0.23	1.00000000	duck
Rfx3	0.01	1.00000000	duck
Neo1	0.25	1.00000000	duck
Uap1l1	0.07	1.00000000	duck
Hnrnpab	-0.02	1.00000000	duck
Dock10	0.47	1.00000000	duck
Suox	0.44	1.00000000	duck
Gna13	0.33	1.00000000	duck
Trim23	-0.14	1.00000000	duck
Ippk	0.03	1.00000000	duck
Satb1	0.41	1.00000000	duck
Ncoa1	0.52	1.00000000	duck
Trpm7	0.37	1.00000000	duck
Gpd2	-0.17	1.00000000	duck
Slc7a3	0.06	1.00000000	duck
Tspan3	0.39	1.00000000	duck
Xpnpep1	-0.11	1.00000000	duck
Gng7	0.11	1.00000000	duck
Vps72	0.03	1.00000000	duck
Gabarapl2	0.20	1.00000000	duck
Supt4a	0.06	1.00000000	duck
Pign	0.75	1.00000000	duck
Slc25a44	0.19	1.00000000	duck
Fryl	-0.37	1.00000000	duck
Tnfaip8l1	0.29	1.00000000	duck
Kcnab2	0.45	1.00000000	duck
Smim12	0.22	1.00000000	duck
Ptpn2	-0.11	1.00000000	duck
Pitpna	0.76	1.00000000	duck
Eif5	0.05	1.00000000	duck
Ap4s1	0.30	1.00000000	duck
Bcl7b	0.26	1.00000000	duck
Rassf2	0.50	1.00000000	duck
Washc5	0.63	1.00000000	duck
Taf12	0.46	1.00000000	duck
Kdelr2	0.07	1.00000000	duck
Tmem41b	0.69	1.00000000	duck
Fxn	0.25	1.00000000	duck
Gigyf2	-0.08	1.00000000	duck
Prkar1a	0.28	1.00000000	duck
Dhx29	-0.15	1.00000000	duck
Ndufb9	0.08	1.00000000	duck
Dusp5	0.36	1.00000000	duck
Atp11c	0.50	1.00000000	duck
Hlf	0.36	1.00000000	duck
Add3	0.18	1.00000000	duck

Fli1	0.08	1.00000000	duck
Fbxo3	-0.63	1.00000000	duck
Wipi2	0.54	1.00000000	duck
Ctnna1	0.94	1.00000000	duck
Batf	0.22	1.00000000	duck
Klhdc4	0.33	1.00000000	duck
Syngr2	0.15	1.00000000	duck
Zbtb7a	0.10	1.00000000	duck
Rab21	0.00	1.00000000	duck
Nol11	-0.23	1.00000000	duck
Ttc39c	0.40	1.00000000	duck
Ciao2a	0.74	1.00000000	duck
H13	0.75	1.00000000	duck
Zdhhc9	0.10	1.00000000	duck
Nsfl1c	0.24	1.00000000	duck
P4hb	0.55	1.00000000	duck
Hexim1	0.03	1.00000000	duck
Ywhaz	0.35	1.00000000	duck
Tubb4b	0.19	1.00000000	duck
Dtx3l	0.35	1.00000000	duck
Pde6d	-0.15	1.00000000	duck
Scmh1	-0.27	1.00000000	duck
Pdp1	0.38	1.00000000	duck
Cdyl2	0.47	1.00000000	duck
Fbxo28	0.36	1.00000000	duck
Fam174a	0.63	1.00000000	duck
Natd1	0.21	1.00000000	duck
Vps37a	-0.01	1.00000000	duck
Ncor2	0.53	1.00000000	duck
Sec62	0.34	1.00000000	duck
Bub3	0.28	1.00000000	duck
Sgpl1	0.22	1.00000000	duck
Kdsr	0.21	1.00000000	duck
Pik3c2a	0.35	1.00000000	duck
Uimc1	0.32	1.00000000	duck
Taok1	0.19	1.00000000	duck
Ubqln4	0.26	1.00000000	duck
Vma21	0.13	1.00000000	duck
Nus1	0.25	1.00000000	duck
Atp5a1	0.61	1.00000000	duck
Ormdl3	0.87	1.00000000	duck
Rnf145	0.26	1.00000000	duck
Gk5	-0.04	1.00000000	duck
Cops5	-0.32	1.00000000	duck
Secisbp2	-0.26	1.00000000	duck
Gnptg	0.18	1.00000000	duck

Nsa2	0.25	1.00000000	duck
Smyd3	0.08	1.00000000	duck
Kansl1l	0.23	1.00000000	duck
Atxn1l	0.19	1.00000000	duck
Tor2a	0.35	1.00000000	duck
Adat1	0.21	1.00000000	duck
Zbtb43	0.06	1.00000000	duck
Sec31a	0.21	1.00000000	duck
Cep44	-0.05	1.00000000	duck
Exoc1	0.11	1.00000000	duck
Ttc27	0.39	1.00000000	duck
Ndufb2	0.30	1.00000000	duck
Hnrnpu	0.11	1.00000000	duck
Zdhhc7	0.27	1.00000000	duck
Ext2	0.49	1.00000000	duck
Cnot9	0.24	1.00000000	duck
Polr1e	0.12	1.00000000	duck
Sec23a	0.03	1.00000000	duck
St7l	0.15	1.00000000	duck
Grk2	0.46	1.00000000	duck
Ext1	0.61	1.00000000	duck
Smpd4	0.12	1.00000000	duck
Naa35	-0.07	1.00000000	duck
Selenoo	-0.22	1.00000000	duck
Psmc6	0.19	1.00000000	duck
Klhdc10	0.26	1.00000000	duck
Qsox2	0.12	1.00000000	duck
Yipf6	0.74	1.00000000	duck
Fbrsl1	0.12	1.00000000	duck
Aff4	0.10	1.00000000	duck
Psmd6	0.27	1.00000000	duck
Upf1	0.38	1.00000000	duck
Vsir	0.57	1.00000000	duck
Ube2j2	0.31	1.00000000	duck
Brms1l	0.01	1.00000000	duck
Slc7a6os	0.77	1.00000000	duck
Trip11	0.05	1.00000000	duck
Rbbp6	0.36	1.00000000	duck
Ccnyl1	-0.06	1.00000000	duck
Epb41l2	0.67	1.00000000	duck
Tm9sf2	0.35	1.00000000	duck
Fam214a	0.58	1.00000000	duck
Tmem50b	0.01	1.00000000	duck
Znfx1	0.05	1.00000000	duck
Erp44	0.37	1.00000000	duck
Golph3	0.26	1.00000000	duck

Pdcd10	0.26	1.00000000	duck
Tspan2	0.04	1.00000000	duck
Prepl	0.04	1.00000000	duck
Capn10	0.26	1.00000000	duck
Pcif1	0.31	1.00000000	duck
Inpp5k	0.15	1.00000000	duck
Polr1c	0.38	1.00000000	duck
Dbi	0.69	1.00000000	duck
Ccdc28a	0.20	1.00000000	duck
Cpd	0.52	1.00000000	duck
Fam13b	0.35	1.00000000	duck
Rab40c	0.20	1.00000000	duck
Psmb4	0.06	1.00000000	duck
Tm2d2	0.27	1.00000000	duck
Zcchc17	-0.12	1.00000000	duck
Cnot8	0.10	1.00000000	duck
Oit3	0.58	1.00000000	duck
Nceh1	0.14	1.00000000	duck
Dcaf13	-0.01	1.00000000	duck
Cdk5r1	0.09	1.00000000	duck
Ivd	-0.21	1.00000000	duck
Pnrc2	0.32	1.00000000	duck
Dync1h1	0.04	1.00000000	duck
Lasp1	0.32	1.00000000	duck
Hdac7	0.59	1.00000000	duck
Sap130	0.73	1.00000000	duck
Phf5a	0.17	1.00000000	duck
Rnpepl1	0.21	1.00000000	duck
Snap47	0.54	1.00000000	duck
Scly	0.03	1.00000000	duck
Timm22	0.53	1.00000000	duck
Aup1	0.25	1.00000000	duck
Vps53	-0.24	1.00000000	duck
Hspa9	0.19	1.00000000	duck
Nptn	-0.22	1.00000000	duck
Trip4	0.08	1.00000000	duck
Cops3	-0.11	1.00000000	duck
Gid8	0.05	1.00000000	duck
Nfya	0.37	1.00000000	duck
Ptch1	0.57	1.00000000	duck
Aldoc	0.50	1.00000000	duck
Git2	0.40	1.00000000	duck
Recql5	0.20	1.00000000	duck
Disc1	0.37	1.00000000	duck
Nip7	0.19	1.00000000	duck
Ssr2	0.33	1.00000000	duck

Nrbp1	0.28	1.00000000	duck
Trak2	0.50	1.00000000	duck
Ptges3	0.24	1.00000000	duck
Erh	0.72	1.00000000	duck
Xrcc6	0.03	1.00000000	duck
Ncbp1	0.62	1.00000000	duck
Ifngr1	0.39	1.00000000	duck
Ptpn6	-0.13	1.00000000	duck
Clk4	0.19	1.00000000	duck
Prkcb	0.37	1.00000000	duck
Exoc5	0.09	1.00000000	duck
Sar1a	0.02	1.00000000	duck
Nfia	-0.14	1.00000000	duck
Ube3b	-0.14	1.00000000	duck
Ywhag	0.42	1.00000000	duck
Asb7	0.08	1.00000000	duck
Sulf2	0.49	1.00000000	duck
Zfand5	0.97	1.00000000	duck
Surf2	-0.18	1.00000000	duck
Hspa4l	0.04	1.00000000	duck
Poldip3	0.69	1.00000000	duck
Rsl24d1	0.00	1.00000000	duck
Usp44	0.85	1.00000000	duck
Ndufa6	0.19	1.00000000	duck
Fbxo45	0.12	1.00000000	duck
Gtf2a1	0.03	1.00000000	duck
Tmtc3	-0.29	1.00000000	duck
Ppwd1	-0.08	1.00000000	duck
Samm50	0.42	1.00000000	duck
Arl1	0.10	1.00000000	duck
Fcho2	0.61	1.00000000	duck
Psmd4	-0.08	1.00000000	duck
Pgpep1	0.36	1.00000000	duck
Wipi1	0.04	1.00000000	duck
Cdc40	0.29	1.00000000	duck
Nek6	-0.20	1.00000000	duck
Dnajc17	-0.04	1.00000000	duck
Ndufs7	0.10	1.00000000	duck
Ctdspl	0.35	1.00000000	duck
Ei24	0.25	1.00000000	duck
Slk	-0.24	1.00000000	duck
Slc35f5	0.14	1.00000000	duck
Snrpc	0.37	1.00000000	duck
Kmt5b	-0.13	1.00000000	duck
Ubap2l	0.27	1.00000000	duck
Atxn7l1	0.37	1.00000000	duck

Tex11	0.41	1.00000000	duck
Plagl1	0.15	1.00000000	duck
Rab9b	0.15	1.00000000	duck
Commd4	0.15	1.00000000	duck
Acot7	0.12	1.00000000	duck
Abl1	0.73	1.00000000	duck
Itpr1	0.18	1.00000000	duck
Rhof	0.36	1.00000000	duck
Kif3a	0.23	1.00000000	duck
Ppm1f	-0.08	1.00000000	duck
Cops7a	0.28	1.00000000	duck
Pik3cb	-0.02	1.00000000	duck
Jak1	0.27	1.00000000	duck
Ndufaf3	0.22	1.00000000	duck
1110059G10Rik	0.16	1.00000000	duck
Ociad1	0.71	1.00000000	duck
Siah1b	0.53	1.00000000	duck
Zfand4	0.15	1.00000000	duck
Usp5	-0.04	1.00000000	duck
Snx18	0.03	1.00000000	duck
Jmjd1c	0.16	1.00000000	duck
Snrnp200	0.69	1.00000000	duck
Dapk3	0.31	1.00000000	duck
Dhx15	0.50	1.00000000	duck
Gadl1	0.25	1.00000000	duck
Cdk5rap3	0.04	1.00000000	duck
Slc45a4	0.53	1.00000000	duck
Kras	0.49	1.00000000	duck
Eif3g	0.35	1.00000000	duck
Ndufb1	0.36	1.00000000	duck
Golga3	0.36	1.00000000	duck
Ahcyl1	1.34	1.00000000	duck
Pdia3	0.61	1.00000000	duck
Invs	0.03	1.00000000	duck
Ebag9	-0.02	1.00000000	duck
Picalm	0.38	1.00000000	duck
Thoc5	0.23	1.00000000	duck
Zmpste24	0.69	1.00000000	duck
Inpp5a	0.14	1.00000000	duck
Gpi1	0.16	1.00000000	duck
Tsr1	0.27	1.00000000	duck
Epc1	0.26	1.00000000	duck
Ndufb10	0.23	1.00000000	duck
Rab39b	0.27	1.00000000	duck
Stim1	0.48	1.00000000	duck
Dyrk1a	0.08	1.00000000	duck

Parp10	-0.03	1.00000000	duck
Trip12	0.01	1.00000000	duck
Serinc1	0.40	1.00000000	duck
Cggbp1	0.65	1.00000000	duck
Snx2	0.66	1.00000000	duck
Crybg3	-0.72	1.00000000	duck
Smad7	0.89	1.00000000	duck
Fbxo32	0.04	1.00000000	duck
Klhl36	0.26	1.00000000	duck
Pde8a	0.30	1.00000000	duck
Scoc	0.59	1.00000000	duck
Pfdn4	0.00	1.00000000	duck
Ubn2	0.26	1.00000000	duck
Tmem70	-0.04	1.00000000	duck
Asap1	0.43	1.00000000	duck
Fbxw11	0.23	1.00000000	duck
Cept1	0.56	1.00000000	duck
Dnajb14	0.28	1.00000000	duck
Pcmt1	0.95	1.00000000	duck
Chchd5	0.33	1.00000000	duck
Prdm11	0.62	1.00000000	duck
Lsg1	0.90	1.00000000	duck
Ndufa5	0.21	1.00000000	duck
Zcchc10	0.11	1.00000000	duck
Trafd1	0.29	1.00000000	duck
Slc9a5	0.03	1.00000000	duck
Strip1	0.10	1.00000000	duck
Atp5c1	0.21	1.00000000	duck
Agl	-0.47	1.00000000	duck
Ap1g1	0.33	1.00000000	duck
Rnf11	0.37	1.00000000	duck
Rab3gap1	0.63	1.00000000	duck
Slc25a25	0.17	1.00000000	duck
Hs6st3	0.23	1.00000000	duck
Sec16a	0.31	1.00000000	duck
Crim1	0.75	1.00000000	duck
Asxl2	0.09	1.00000000	duck
Zfp384	0.31	1.00000000	duck
Plcg2	0.27	1.00000000	duck
Kif1b	0.14	1.00000000	duck
Garre1	-0.04	1.00000000	duck
Ndufs3	0.45	1.00000000	duck
Tmem97	0.09	1.00000000	duck
Psmc3	0.17	1.00000000	duck
Atp11a	0.90	1.00000000	duck
Fbxl18	0.51	1.00000000	duck

Zpr1	0.45	1.00000000	duck
Emc3	0.38	1.00000000	duck
Abca2	0.07	1.00000000	duck
Phkb	0.14	1.00000000	duck
Fam168a	0.06	1.00000000	duck
Ythdc2	0.39	1.00000000	duck
Vps11	0.27	1.00000000	duck
Dhx8	0.38	1.00000000	duck
Trim33	0.21	1.00000000	duck
Dapk2	0.18	1.00000000	duck
Ppm1a	0.09	1.00000000	duck
Erc1	0.30	1.00000000	duck
Aurkaip1	0.48	1.00000000	duck
Nipa2	0.42	1.00000000	duck
Ndufs5	0.15	1.00000000	duck
Stam	0.05	1.00000000	duck
Ppcdc	0.26	1.00000000	duck
Ppp1cb	-0.02	1.00000000	duck
Plagl2	0.29	1.00000000	duck
Vta1	0.66	1.00000000	duck
Arhgap10	0.14	1.00000000	duck
Hagh	-0.24	1.00000000	duck
Cdc37l1	0.34	1.00000000	duck
Dctn6	0.16	1.00000000	duck
Rcc1l	0.27	1.00000000	duck
Polr2h	0.32	1.00000000	duck
Rab10	0.19	1.00000000	duck
Usp15	0.46	1.00000000	duck
Nipbl	0.67	1.00000000	duck
Tef	0.63	1.00000000	duck
Xrn1	0.63	1.00000000	duck
Ndufaf2	0.18	1.00000000	duck
Rbm22	0.22	1.00000000	duck
Tom1	0.27	1.00000000	duck
Smad2	0.29	1.00000000	duck
Wwp1	0.79	1.00000000	duck
Ipmk	-0.73	1.00000000	duck
Dph3	0.12	1.00000000	duck
Golga1	-0.06	1.00000000	duck
Dgkh	-0.09	1.00000000	duck
Psme3ip1	0.20	1.00000000	duck
Tle3	0.07	1.00000000	duck
Zdhhc14	0.51	1.00000000	duck
Snx17	0.26	1.00000000	duck
Sub1	0.41	1.00000000	duck
Klhl28	0.01	1.00000000	duck

Dnajc21	0.12	1.00000000	duck
Atp5j	0.23	1.00000000	duck
Tra2b	0.52	1.00000000	duck
Brix1	0.04	1.00000000	duck
Flii	0.16	1.00000000	duck
Sgsm3	0.20	1.00000000	duck
Ctsh	0.24	1.00000000	duck
Arid4a	0.58	1.00000000	duck
Usp8	0.52	1.00000000	duck
Rpf2	0.22	1.00000000	duck
Pip5k1b	0.31	1.00000000	duck
Lrrk2	0.24	1.00000000	duck
Gtf2h5	0.40	1.00000000	duck
Cd247	-0.04	1.00000000	duck
Dctn5	0.29	1.00000000	duck
Nudt21	0.17	1.00000000	duck
Sec61a1	0.20	1.00000000	duck
Gucd1	0.32	1.00000000	duck
Ostc	0.12	1.00000000	duck
Slc15a4	0.67	1.00000000	duck
Etv6	-0.11	1.00000000	duck
Chka	0.76	1.00000000	duck
Psma7	0.40	1.00000000	duck
B3gnt4	0.12	1.00000000	duck
Trim59	0.16	1.00000000	duck
Herpud2	0.35	1.00000000	duck
Yme1l1	0.45	1.00000000	duck
Dram2	0.82	1.00000000	duck
Selenof	0.49	1.00000000	duck
Tmf1	0.09	1.00000000	duck
Ost4	0.15	1.00000000	duck
Ubxn2b	0.15	1.00000000	duck
Sp4	0.44	1.00000000	duck
Azin1	0.47	1.00000000	duck
Arih2	-0.10	1.00000000	duck
Slc35a3	0.36	1.00000000	duck
Setx	0.16	1.00000000	duck
Fkbp5	0.42	1.00000000	duck
Psma6	0.38	1.00000000	duck
Adar	1.01	1.00000000	duck
Srp19	0.26	1.00000000	duck
Zbtb8os	0.20	1.00000000	duck
Synrg	0.49	1.00000000	duck
Ptpa	0.10	1.00000000	duck
Ranbp17	-0.26	1.00000000	duck
Sppl2a	0.32	1.00000000	duck

Hif1a	0.31	1.00000000	duck
Nup35	0.05	1.00000000	duck
Slc20a2	-0.06	1.00000000	duck
Vti1a	0.31	1.00000000	duck
Tspan4	0.02	1.00000000	duck
Hspa14	0.28	1.00000000	duck
Otulin	0.03	1.00000000	duck
Ypel1	0.21	1.00000000	duck
Sec14l1	-0.02	1.00000000	duck
Srp54a	0.20	1.00000000	duck
Got1	-0.05	1.00000000	duck
Arl15	0.02	1.00000000	duck
Tbc1d9b	0.36	1.00000000	duck
Rap1b	0.30	1.00000000	duck
Nr4a2	0.08	1.00000000	duck
Prkrip1	0.36	1.00000000	duck
Ccnb2	0.23	1.00000000	duck
Nisch	-0.17	1.00000000	duck
Rbl2	0.22	1.00000000	duck
Golt1b	0.12	1.00000000	duck
Tcp11l2	0.13	1.00000000	duck
Spsb1	0.24	1.00000000	duck
Tpm1	0.30	1.00000000	duck
Dbr1	0.23	1.00000000	duck
Ndufv2	0.58	1.00000000	duck
Iqgap1	0.20	1.00000000	duck
Lsm8	0.45	1.00000000	duck
Nxt2	0.19	1.00000000	duck
Acad9	0.21	1.00000000	duck
Ypel5	0.50	1.00000000	duck
Eif2b5	0.20	1.00000000	duck
Ufd1	0.24	1.00000000	duck
Nup88	0.05	1.00000000	duck
Herc4	0.43	1.00000000	duck
Stx6	0.35	1.00000000	duck
Fig4	0.25	1.00000000	duck
Pdia6	0.41	1.00000000	duck
Ipo9	0.86	1.00000000	duck
Farp2	0.04	1.00000000	duck
Skap2	0.03	1.00000000	duck
Fbxo9	0.59	1.00000000	duck
Larp1	0.24	1.00000000	duck
Yaf2	0.05	1.00000000	duck
Zdhhc12	-0.24	1.00000000	duck
Dctn4	0.17	1.00000000	duck
Bst1	0.17	1.00000000	duck

Csnk1g2	0.61	1.00000000	duck
Tmem208	0.05	1.00000000	duck
Kctd10	0.22	1.00000000	duck
Utp6	0.11	1.00000000	duck
Tmed2	0.39	1.00000000	duck
Entpd4	-0.12	1.00000000	duck
Dhx40	0.25	1.00000000	duck
Canx	0.22	1.00000000	duck
Commd1b	0.50	1.00000000	duck
Ahsa1	0.26	1.00000000	duck
Usp38	-0.20	1.00000000	duck
Hexb	0.14	1.00000000	duck
Abcd3	0.51	1.00000000	duck
Dcps	0.10	1.00000000	duck
Polr2e	0.69	1.00000000	duck
Sod1	0.62	1.00000000	duck
Nono	0.65	1.00000000	duck
Cpeb2	0.06	1.00000000	duck
Abcc5	0.72	1.00000000	duck
Rab8b	0.35	1.00000000	duck
Chd1	0.54	1.00000000	duck
Klhl21	0.15	1.00000000	duck
Nfrkb	0.14	1.00000000	duck
Tmem33	0.42	1.00000000	duck
Rexo2	0.13	1.00000000	duck
Zbtb21	0.37	1.00000000	duck
Sar1b	0.03	1.00000000	duck
Ywhaq	0.06	1.00000000	duck
Rtn4	0.07	1.00000000	duck
Sbno1	0.09	1.00000000	duck
Gnpat	0.49	1.00000000	duck
Psmf1	-0.07	1.00000000	duck
Brca2	0.03	1.00000000	duck
Zmiz1	0.56	1.00000000	duck
C1galt1	0.10	1.00000000	duck
Far1	0.25	1.00000000	duck
Psme3	0.19	1.00000000	duck
Polr2d	0.71	1.00000000	duck
Nod1	0.04	1.00000000	duck
Hsd17b4	-0.06	1.00000000	duck
Rars2	0.45	1.00000000	duck
Pop4	0.68	1.00000000	duck
Fyttd1	0.52	1.00000000	duck
Slc30a9	0.27	1.00000000	duck
Etfrf1	0.10	1.00000000	duck
Exosc10	0.16	1.00000000	duck

Tsc22d3	0.18	1.00000000	duck
Tmem248	0.63	1.00000000	duck
Slc39a11	0.08	1.00000000	duck
Rab29	0.39	1.00000000	duck
Rsu1	0.39	1.00000000	duck
Cwc15	0.03	1.00000000	duck
Rufy1	0.15	1.00000000	duck
Phactr2	0.23	1.00000000	duck
Dcun1d5	0.55	1.00000000	duck
Fbxo8	0.44	1.00000000	duck
Stk40	0.67	1.00000000	duck
Edrf1	0.63	1.00000000	duck
Wdr47	-0.08	1.00000000	duck
Abhd5	0.24	1.00000000	duck
Bnc2	-0.11	1.00000000	duck
Arfgap1	0.12	1.00000000	duck
Kansl3	0.11	1.00000000	duck
Bnip2	0.02	1.00000000	duck
Shc1	0.25	1.00000000	duck
B4galt1	0.08	1.00000000	duck
Lfng	0.25	1.00000000	duck
Kif5b	0.54	1.00000000	duck
Stt3a	0.41	1.00000000	duck
Ube2r2	0.22	1.00000000	duck
Gga3	0.54	1.00000000	duck
Epas1	-0.25	1.00000000	duck
Chordc1	0.35	1.00000000	duck
Sephs1	0.37	1.00000000	duck
Cct4	0.09	1.00000000	duck
Txn2	0.43	1.00000000	duck
Rprd1a	0.32	1.00000000	duck
Bckdhb	-0.20	1.00000000	duck
Rnf20	0.20	1.00000000	duck
Cables2	0.13	1.00000000	duck
Cbl	0.29	1.00000000	duck
Lamtor5	0.20	1.00000000	duck
Cdk14	-2.55	0.00000343	duck
Inpp5d	-2.66	0.00000677	duck
Sptan1	-2.17	0.00000951	duck
Ubl3	-2.53	0.00001096	duck
Rapgef2	-2.37	0.00003953	duck
Prr5	-2.54	0.00003981	duck
Aff2	-2.62	0.00004177	duck
Cerk	-2.17	0.00005598	duck
Vps13c	-2.83	0.00012870	duck
Ebf1	-2.27	0.00015570	duck

Tnrc6c	-1.89	0.00019316	duck
Ppm1e	-2.18	0.00035367	duck
Evl	-1.60	0.00043471	duck
Snx9	-1.89	0.00050030	duck
Slc38a1	-1.87	0.00050143	duck
Carhsp1	-1.93	0.00050309	duck
Eif2ak3	-1.98	0.00052796	duck
Bcat1	-3.02	0.00059133	duck
Tet1	-1.90	0.00071316	duck
Dennd1b	-1.88	0.00077850	duck
Arhgap24	-1.68	0.00092026	duck
Tmem163	-2.15	0.00092111	duck
Cd74	-1.44	0.00109719	duck
Casd1	-1.77	0.00131845	duck
Aftph	-1.45	0.00196912	duck
Zmynd8	-1.74	0.00248173	duck
Snx13	-1.60	0.00282921	duck
Pik3r1	-1.76	0.00371867	duck
Stag1	-2.12	0.00432694	duck
Tspan5	-1.53	0.00477568	duck
Osbpl11	-1.45	0.00535095	duck
Ncoa2	-1.66	0.00580198	duck
Pax5	-1.55	0.00612573	duck
Lrba	-1.74	0.00709343	duck
Klf13	-1.64	0.00756811	duck
Zeb1	-1.70	0.00809240	duck
Rasa3	-1.64	0.00855801	duck
Cdk13	-1.46	0.00887708	duck
Ranbp9	-1.85	0.00915969	duck
Atp9b	-1.90	0.00933315	duck
Ppp2r2d	-1.37	0.00977645	duck
Ankrd44	-1.82	0.00996339	duck
Epb41	-1.94	0.01131152	duck
Runx3	-1.42	0.01134005	duck
Nfatc3	-1.43	0.01267881	duck
Nmt2	-1.77	0.01323911	duck
Cry1	-1.46	0.01432980	duck
Bcl11a	-1.13	0.01513014	duck
Itga4	-1.61	0.01570779	duck
Cd82	-1.21	0.01623397	duck
Armc2	-1.52	0.01641427	duck
Slc4a7	-1.54	0.01843706	duck
Ralgapa2	-1.41	0.02015672	duck
Abcc1	-1.86	0.02102415	duck
Stk38l	-1.93	0.02443772	duck
Glcci1	-1.07	0.02541178	duck

Slc46a3	-1.51	0.02563469	duck
Grk3	-1.51	0.02696811	duck
Rftn1	-1.71	0.02720889	duck
Nap1l4	-1.52	0.02947494	duck
Plcl2	-1.53	0.03078064	duck
Prrc2b	-1.32	0.03101221	duck
Cblb	-2.39	0.03174277	duck
Stat4	-2.02	0.03708789	duck
Dennd1a	-1.61	0.04063187	duck
Prex1	-1.80	0.04119002	duck
Cnot6	-1.30	0.04444718	duck
Chchd3	-2.01	0.04458495	duck
Kdm4b	-1.46	0.04790664	duck
Tdrd3	-1.71	0.05387900	duck
Ehmt1	-1.47	0.05481961	duck
Sult4a1	-1.55	0.05873028	duck
Swap70	-1.95	0.05995783	duck
Tcf4	-1.12	0.06388957	duck
Arid1b	-1.15	0.06646432	duck
Septin6	-1.56	0.07275792	duck
Gabpa	-1.38	0.07463958	duck
Dyrk2	-1.22	0.07607370	duck
Stx7	-1.27	0.09755061	duck
Tpt1	5.80	0.00000000	pigeons
Rgs2	4.50	0.00000000	pigeons
Rab33a	4.32	0.00000000	pigeons
Cyria	4.26	0.00000000	pigeons
Adss	3.44	0.00000000	pigeons
Arpc2	3.08	0.00000000	pigeons
Dars	3.22	0.00000000	pigeons
Cmip	3.90	0.00000000	pigeons
Atp5b	2.53	0.00000000	pigeons
Lrmp	3.44	0.00000000	pigeons
Cxcr4	2.13	0.00000000	pigeons
Atp5g3	2.39	0.00000000	pigeons
Niban1	3.10	0.00000000	pigeons
Dido1	2.31	0.00000000	pigeons
U2surp	2.26	0.00000000	pigeons
Rgs1	2.03	0.00000000	pigeons
Sap18	2.24	0.00000000	pigeons
Vim	2.62	0.00000000	pigeons
Tiparp	2.50	0.00000000	pigeons
Cnbp	2.21	0.00000000	pigeons
Pcf11	2.16	0.00000000	pigeons
Phip	2.27	0.00000000	pigeons
Txnip	2.18	0.00000000	pigeons

Oga	2.11	0.00000000	pigeons
Cklf	1.96	0.00000000	pigeons
App	2.77	0.00000000	pigeons
Ankhd1	2.03	0.00000000	pigeons
Wtap	1.92	0.00000000	pigeons
Zdhhc20	2.51	0.00000000	pigeons
Anapc15-ps	1.68	0.00000000	pigeons
Sdcbp	1.78	0.00000000	pigeons
Atf7ip	2.13	0.00000000	pigeons
Arid5b	1.66	0.00000000	pigeons
Zc3h12d	1.58	0.00000000	pigeons
Pomp	1.55	0.00000000	pigeons
Syncrip	1.69	0.00000000	pigeons
Trpc4ap	1.60	0.00000000	pigeons
Lnpep	1.66	0.00000000	pigeons
Ccz1	1.46	0.00000000	pigeons
Evi2a	1.21	0.00000000	pigeons
Prep	1.72	0.00000000	pigeons
Cdc37	1.38	0.00000000	pigeons
Sulf2	1.50	0.00000000	pigeons
Evl	1.12	0.00000000	pigeons
Plcg2	1.20	0.00000000	pigeons
Kif5c	1.28	0.00000000	pigeons
Vcp	1.33	0.00000000	pigeons
Cars	1.65	0.00000000	pigeons
Adam9	1.51	0.00000000	pigeons
Snrpd2	1.31	0.00000000	pigeons
Ncoa7	1.03	0.00000000	pigeons
Rab30	1.46	0.00000000	pigeons
Ccdc25	1.36	0.00000000	pigeons
Ndufa1	1.33	0.00000000	pigeons
Eif4b	1.30	0.00000000	pigeons
Tgfbr1	1.49	0.00000000	pigeons
Rad51b	1.61	0.00000000	pigeons
Strap	1.29	0.00000000	pigeons
Cacybp	1.40	0.00000000	pigeons
Adnp	1.37	0.00000000	pigeons
Rftn1	1.66	0.00000000	pigeons
Sdhc	1.25	0.00000000	pigeons
Lpp	1.79	0.00000000	pigeons
Atp5k	1.31	0.00000000	pigeons
Csnk1d	1.26	0.00000000	pigeons
Rtf2	1.24	0.00000000	pigeons
Pde4b	1.01	0.00000000	pigeons
Blcap	1.26	0.00000000	pigeons
Brd8	1.25	0.00000000	pigeons

Golm2	1.21	0.00000000	pigeons
C1qbp	1.21	0.00000000	pigeons
Enox2	1.20	0.00000000	pigeons
Rhob	1.41	0.00000000	pigeons
Ankrd44	1.06	0.00000000	pigeons
Kars	1.17	0.00000000	pigeons
Pcnp	1.15	0.00000000	pigeons
Phax	1.12	0.00000000	pigeons
Apbb1ip	1.36	0.00000000	pigeons
Atp5c1	1.12	0.00000000	pigeons
Slc12a7	1.39	0.00000000	pigeons
Nop56	1.14	0.00000000	pigeons
Kifbp	1.08	0.00000000	pigeons
Adam23	1.20	0.00000000	pigeons
Abca1	1.03	0.00000000	pigeons
Eprs	1.21	0.00000000	pigeons
Fgl2	1.42	0.00000000	pigeons
Fbh1	1.23	0.00000000	pigeons
Ano5	1.02	0.00000000	pigeons
Trank1	1.20	0.00000000	pigeons
Sinhcaf	1.29	0.00000000	pigeons
Slirp	1.08	0.00000000	pigeons
Trrap	1.07	0.00000000	pigeons
Eif2ak3	1.06	0.00000000	pigeons
Ezr	1.09	0.00000000	pigeons
Tollip	1.03	0.00000000	pigeons
Foxn2	1.13	0.00000000	pigeons
Nars	1.01	0.00000000	pigeons
Ptger4	1.17	0.00000000	pigeons
Dntt	1.08	0.00000000	pigeons
Sh3pxd2a	1.54	0.00000000	pigeons
Dtd1	1.01	0.00000000	pigeons
Clec16a	1.08	0.00000000	pigeons
Cxcl13	2.28	0.00000000	pigeons
Rnf38	1.21	0.00000000	pigeons
Scd2	1.13	0.00000000	pigeons
Syk	1.06	0.00000000	pigeons
Ahsa2	1.15	0.00000000	pigeons
Basp1	1.04	0.00000000	pigeons
Cd2ap	1.19	0.00000000	pigeons
Bach2	1.09	0.00000000	pigeons
Klhl6	1.01	0.00000000	pigeons
Cd83	1.41	0.00000000	pigeons
Emp1	1.23	0.00000000	pigeons
Nebl	1.16	0.00000000	pigeons
Rin2	1.16	0.00000000	pigeons

Fgd4	1.01	0.00000001	pigeons
Nfatc1	1.19	0.00000001	pigeons
Ikzf1	1.12	0.00000002	pigeons
Adgrg6	1.10	0.00000005	pigeons
Spock1	1.81	0.00000007	pigeons
Ctla4	1.03	0.00000023	pigeons
Necab1	1.02	0.00000081	pigeons
Otud7a	1.12	0.00000102	pigeons
Il1b	1.07	0.00000639	pigeons
Thsd4	1.05	0.00013523	pigeons
Arhgap15	0.99	0.00000000	pigeons
B2m	-0.69	0.00000000	pigeons
Ankrd10	1.00	0.00000000	pigeons
Gna13	0.87	0.00000000	pigeons
Rel	0.96	0.00000000	pigeons
Cdk5r1	0.99	0.00000000	pigeons
Spcs1	0.94	0.00000000	pigeons
Polr1f	1.00	0.00000000	pigeons
Ssh2	0.74	0.00000000	pigeons
Sarnp	0.99	0.00000000	pigeons
Tut7	0.99	0.00000000	pigeons
Tdpoz8	0.99	0.00000000	pigeons
Ankrd17	0.86	0.00000000	pigeons
Tnfrsf13c	0.88	0.00000000	pigeons
Ptprc	0.85	0.00000000	pigeons
Rars	0.93	0.00000000	pigeons
Rfx7	0.90	0.00000000	pigeons
Pabpc1	0.65	0.00000000	pigeons
Sfswap	0.95	0.00000000	pigeons
Kctd20	0.94	0.00000000	pigeons
Bcl11a	0.85	0.00000000	pigeons
Btg1b	0.69	0.00000000	pigeons
Pik3ap1	0.81	0.00000000	pigeons
Crip2	0.93	0.00000000	pigeons
Lamp3	0.92	0.00000000	pigeons
Zbtb38	0.76	0.00000000	pigeons
Stk38	0.81	0.00000000	pigeons
Ccny	0.94	0.00000000	pigeons
Wipf1	0.89	0.00000000	pigeons
Arhgap12	0.90	0.00000000	pigeons
Tars	0.88	0.00000000	pigeons
Tmbim1	0.95	0.00000000	pigeons
Ergic1	0.68	0.00000000	pigeons
Rgs9bp	0.96	0.00000000	pigeons
Crtc1	0.83	0.00000000	pigeons
Icosl	0.90	0.00000000	pigeons

Nfil3	0.79	0.00000000	pigeons
Glrx3	0.78	0.00000000	pigeons
Enah	-0.99	0.00000000	pigeons
Rai14	-0.90	0.00000000	pigeons
Naa20	0.80	0.00000000	pigeons
Tmem154	0.88	0.00000000	pigeons
Bccip	0.78	0.00000000	pigeons
Scaf8	0.68	0.00000000	pigeons
Gpatch8	0.65	0.00000000	pigeons
Ramp3	-0.80	0.00000000	pigeons
Brap	0.82	0.00000000	pigeons
Wwc2	-0.82	0.00000000	pigeons
Jchain	0.73	0.00000000	pigeons
Scube3	-0.89	0.00000000	pigeons
Pim1	0.87	0.00000000	pigeons
Tfpi	-0.92	0.00000000	pigeons
Atp5j2	0.76	0.00000000	pigeons
Tnfaip8	0.79	0.00000000	pigeons
Nr4a1	0.84	0.00000000	pigeons
Nlk	0.82	0.00000000	pigeons
Tardbp	0.85	0.00000000	pigeons
Apip	0.78	0.00000000	pigeons
Slc4a7	0.74	0.00000000	pigeons
Ptpn1	0.76	0.00000000	pigeons
Swap70	0.76	0.00000000	pigeons
Itpr2	0.65	0.00000000	pigeons
Dock11	0.60	0.00000000	pigeons
Polr2i	0.76	0.00000000	pigeons
Sap30bp	0.81	0.00000000	pigeons
Siah2	0.72	0.00000000	pigeons
Gnai1	0.90	0.00000000	pigeons
Bri3bp	0.79	0.00000000	pigeons
Nfatc3	0.69	0.00000000	pigeons
Egfl7	-0.97	0.00000000	pigeons
Stat4	0.88	0.00000000	pigeons
Prrg1	-0.85	0.00000000	pigeons
Pggt1b	0.88	0.00000000	pigeons
Bach1	0.76	0.00000000	pigeons
Fbxw11	0.62	0.00000000	pigeons
Ehbp1	0.70	0.00000000	pigeons
Zfp777	0.73	0.00000000	pigeons
Arfip1	0.74	0.00000000	pigeons
Ctdp1	0.72	0.00000000	pigeons
Rffl	0.70	0.00000000	pigeons
Gpr160	0.88	0.00000000	pigeons
Xpo6	0.71	0.00000000	pigeons

Bcat1	0.83	0.00000000	pigeons
Psme3ip1	0.72	0.00000000	pigeons
Camk2d	0.66	0.00000000	pigeons
Abcg1	0.88	0.00000001	pigeons
Rad23b	0.71	0.00000001	pigeons
Sp4	0.66	0.00000001	pigeons
Cntrl	0.64	0.00000001	pigeons
F8	-0.89	0.00000001	pigeons
Cdkn2aip	0.71	0.00000001	pigeons
Smad1	-0.92	0.00000001	pigeons
Drc7	-0.92	0.00000001	pigeons
Kdelr3	0.71	0.00000001	pigeons
Arhgap25	0.61	0.00000001	pigeons
Ccng2	0.72	0.00000002	pigeons
Prr5	0.61	0.00000002	pigeons
Arl1	0.63	0.00000002	pigeons
Zfp706	0.38	0.00000002	pigeons
Nasp	0.76	0.00000002	pigeons
Pfkp	0.83	0.00000002	pigeons
Far1	0.74	0.00000003	pigeons
Ikzf3	0.54	0.00000003	pigeons
Cald1	-0.93	0.00000003	pigeons
Rock1	0.69	0.00000003	pigeons
Trim28	0.67	0.00000003	pigeons
Fam53a	0.78	0.00000003	pigeons
Ttl	0.67	0.00000004	pigeons
Spag6l	0.92	0.00000004	pigeons
Chst11	0.98	0.00000004	pigeons
Emp2	-0.88	0.00000005	pigeons
Casp3	0.77	0.00000005	pigeons
Irf4	0.86	0.00000005	pigeons
Gcdh	0.68	0.00000005	pigeons
Hpgd	-0.97	0.00000006	pigeons
Zfp608	0.82	0.00000008	pigeons
Gpcpd1	0.62	0.00000008	pigeons
Dock8	0.70	0.00000010	pigeons
Idh3b	0.63	0.00000011	pigeons
Sars	0.60	0.00000013	pigeons
Cbx6	0.64	0.00000014	pigeons
Grk3	0.51	0.00000018	pigeons
Nrp1	-0.90	0.00000019	pigeons
Ndel1	0.59	0.00000021	pigeons
Wdr82	0.51	0.00000023	pigeons
Cytip	0.66	0.00000026	pigeons
Gpr18	0.77	0.00000029	pigeons
Smap2	0.49	0.00000030	pigeons

Rgs18	0.76	0.00000031	pigeons
Dock2	0.91	0.00000033	pigeons
Ptpn6	0.51	0.00000037	pigeons
Szrd1	0.49	0.00000038	pigeons
Usp10	0.38	0.00000039	pigeons
Oxr1	0.78	0.00000042	pigeons
Srsf11	0.56	0.00000046	pigeons
Ctbp1	0.62	0.00000048	pigeons
Txndc11	0.65	0.00000052	pigeons
Sla	0.56	0.00000053	pigeons
Amotl2	-0.98	0.00000054	pigeons
Soga1	0.57	0.00000056	pigeons
Gpd1l	0.69	0.00000060	pigeons
Dok2	0.65	0.00000067	pigeons
Ndufb1	0.65	0.00000075	pigeons
Tagap1	0.79	0.00000086	pigeons
Cstf3	0.61	0.00000088	pigeons
Ddx3y	0.41	0.00000092	pigeons
Rubcnl	0.63	0.00000104	pigeons
Tmem131l	0.64	0.00000113	pigeons
Rab35	0.50	0.00000114	pigeons
Klhl24	0.59	0.00000116	pigeons
Pin4	0.54	0.00000116	pigeons
Pde4d	0.60	0.00000118	pigeons
Bckdhb	0.84	0.00000122	pigeons
Dusp1	-0.98	0.00000140	pigeons
Helz	0.48	0.00000142	pigeons
Tent4a	0.68	0.00000143	pigeons
Zmynd8	0.68	0.00000150	pigeons
Slmap	0.69	0.00000200	pigeons
Kras	0.62	0.00000213	pigeons
Slc17a9	0.61	0.00000213	pigeons
Zhx2	0.55	0.00000215	pigeons
Trpv2	0.70	0.00000236	pigeons
Yars	0.60	0.00000236	pigeons
Tbp	0.58	0.00000236	pigeons
Cd47	0.47	0.00000239	pigeons
Pik3cg	0.59	0.00000258	pigeons
Osbpl8	0.53	0.00000263	pigeons
Borcs5	0.68	0.00000277	pigeons
Itprip	0.61	0.00000279	pigeons
Sowahc	0.76	0.00000287	pigeons
Naa15	0.70	0.00000311	pigeons
Gadd45b	0.70	0.00000389	pigeons
Pstpip1	0.62	0.00000394	pigeons
Sh3bp5	0.65	0.00000395	pigeons

Tnrc6c	0.50	0.00000403	pigeons
Stx17	0.58	0.00000447	pigeons
Psmg2	0.39	0.00000455	pigeons
Polr1d	0.60	0.00000542	pigeons
Kdm3a	0.63	0.00000542	pigeons
Snrpc	0.56	0.00000571	pigeons
Atad1	0.54	0.00000649	pigeons
Thyn1	0.67	0.00000683	pigeons
Arl6ip5	0.51	0.00000685	pigeons
Cnot1	0.41	0.00000686	pigeons
Ankrd11	0.39	0.00000702	pigeons
Clk1	0.60	0.00000708	pigeons
Fam107b	0.54	0.00000744	pigeons
Sat1	0.57	0.00000884	pigeons
Nsd3	0.33	0.00000911	pigeons
Fam110b	0.84	0.00000943	pigeons
Cab39	0.33	0.00000982	pigeons
Setd2	0.48	0.00001031	pigeons
Rac2	0.53	0.00001193	pigeons
Tkt	0.62	0.00001194	pigeons
Ube2j1	0.61	0.00001262	pigeons
Fth1	-0.53	0.00001318	pigeons
Fbxo11	0.40	0.00001340	pigeons
L3mbtl3	0.52	0.00001404	pigeons
Atp2a3	0.31	0.00001462	pigeons
Selenot	0.41	0.00001582	pigeons
Rab11fip4	0.70	0.00001596	pigeons
Rgs4	0.84	0.00001867	pigeons
Kdm3b	0.32	0.00001954	pigeons
Slc41a1	0.60	0.00002075	pigeons
Ndufa13	0.51	0.00002104	pigeons
Hax1	0.49	0.00002129	pigeons
Cyfip2	0.57	0.00002157	pigeons
Runx3	0.50	0.00002221	pigeons
Zc3h12c	0.52	0.00002278	pigeons
Adcy7	0.55	0.00002318	pigeons
Arf6	0.47	0.00002327	pigeons
Rbmx2	0.55	0.00002367	pigeons
Ankmy2	0.62	0.00002574	pigeons
Wdr26	0.04	0.00002761	pigeons
Dapk1	0.59	0.00002815	pigeons
Ric8b	0.60	0.00002835	pigeons
Golga1	0.62	0.00002974	pigeons
Nmrk2	0.81	0.00003054	pigeons
Sesn1	0.49	0.00003108	pigeons
Tnip2	0.63	0.00003220	pigeons

Srsf3	0.38	0.00003375	pigeons
Stat1	0.53	0.00003633	pigeons
Jak1	0.46	0.00003942	pigeons
Nsmaf	0.57	0.00004037	pigeons
Prpf8	0.40	0.00004075	pigeons
Trim24	0.77	0.00004124	pigeons
Dscam	0.74	0.00004175	pigeons
Inpp5k	-0.84	0.00004468	pigeons
Ubtf	0.52	0.00004515	pigeons
Asb8	0.48	0.00004515	pigeons
Vgll4	0.40	0.00004548	pigeons
Wac	0.34	0.00004588	pigeons
Tgfbr2	0.49	0.00004812	pigeons
Eif2s3y	0.37	0.00004875	pigeons
Spi1	0.72	0.00004957	pigeons
Rnf139	0.56	0.00005020	pigeons
Hace1	0.77	0.00005070	pigeons
Rap1gds1	0.37	0.00005164	pigeons
Usp15	0.35	0.00005775	pigeons
Ube2h	0.41	0.00005984	pigeons
Dnpep	0.48	0.00006224	pigeons
Khdc4	0.51	0.00006224	pigeons
Pfdn4	0.29	0.00006270	pigeons
Tln2	0.72	0.00006412	pigeons
Plk2	-0.76	0.00006829	pigeons
Pcid2	0.50	0.00007303	pigeons
Psmc3ip	0.51	0.00007303	pigeons
Bcl7a	0.57	0.00007344	pigeons
Psme3	0.43	0.00007482	pigeons
Lpxn	0.55	0.00007775	pigeons
Wdr44	0.41	0.00007834	pigeons
Aftph	0.44	0.00007977	pigeons
Vapb	0.38	0.00008131	pigeons
Rnf34	0.61	0.00008407	pigeons
Gars	0.52	0.00008567	pigeons
Sytl3	0.68	0.00008567	pigeons
Fndc3a	0.67	0.00008712	pigeons
Pus10	0.51	0.00008919	pigeons
Enpp4	0.69	0.00009094	pigeons
Ankrd49	0.41	0.00009206	pigeons
Nr3c1	0.47	0.00009249	pigeons
Abcc1	0.50	0.00009328	pigeons
Cdc42se2	0.66	0.00009962	pigeons
Cog3	0.52	0.00010046	pigeons
Tk2	0.47	0.00011086	pigeons
Trmt10c	0.53	0.00011499	pigeons

Gjd4	0.59	0.00011712	pigeons
Hif1a	0.44	0.00011881	pigeons
Tbk1	0.50	0.00011897	pigeons
Phf12	0.52	0.00012055	pigeons
Dffb	0.53	0.00012527	pigeons
Zeb1	0.48	0.00012825	pigeons
Nop14	0.48	0.00012972	pigeons
Lrrk1	0.56	0.00013333	pigeons
Ckb	-0.87	0.00013436	pigeons
Rnf103	0.60	0.00013800	pigeons
Rxylt1	0.55	0.00013800	pigeons
Arhgap30	0.51	0.00013800	pigeons
Ebf1	0.41	0.00014042	pigeons
Ppig	0.35	0.00014530	pigeons
Rhoh	0.41	0.00014700	pigeons
Notch2	0.53	0.00015223	pigeons
Chfr	0.49	0.00015961	pigeons
Sec23ip	0.48	0.00016165	pigeons
Ahi1	0.52	0.00016259	pigeons
Jmjd1c	0.53	0.00016476	pigeons
Hectd1	0.49	0.00016539	pigeons
Pdpk1	0.44	0.00016566	pigeons
Lrpprc	0.60	0.00016852	pigeons
Rcsd1	0.65	0.00018690	pigeons
Dnajc1	0.75	0.00018960	pigeons
Usp25	0.56	0.00020001	pigeons
Slc43a2	0.56	0.00021795	pigeons
Banp	0.69	0.00022161	pigeons
Cd3g	0.59	0.00022161	pigeons
LTO1	0.56	0.00022161	pigeons
Ywhaz	0.39	0.00023019	pigeons
Tada1	0.47	0.00023204	pigeons
Cxcr5	0.45	0.00023229	pigeons
Etnk1	0.41	0.00023623	pigeons
Nol9	0.44	0.00023885	pigeons
Hspa4	0.48	0.00025922	pigeons
Lhpp	0.56	0.00025934	pigeons
Eif2b4	0.45	0.00025934	pigeons
Hook1	0.50	0.00025952	pigeons
Rgs3	-0.86	0.00026818	pigeons
Sfmbt1	0.57	0.00026936	pigeons
Lrrfip1	0.43	0.00027872	pigeons
Chd9	0.48	0.00028410	pigeons
Stk10	0.35	0.00029834	pigeons
Sgms1	0.36	0.00030029	pigeons
Eapp	0.53	0.00030339	pigeons

Stat2	0.51	0.00034633	pigeons
Dedd	0.47	0.00035482	pigeons
Rnaset2b	0.45	0.00035586	pigeons
Emc2	0.42	0.00036862	pigeons
Pde7a	0.46	0.00036880	pigeons
Nfkbie	0.40	0.00039785	pigeons
Itpr1	0.46	0.00040160	pigeons
Trerf1	0.54	0.00044027	pigeons
Sec62	0.30	0.00044146	pigeons
Ppa2	0.22	0.00044322	pigeons
Zfp207	0.34	0.00044634	pigeons
Rbm26	0.19	0.00046282	pigeons
Cep170	0.39	0.00046579	pigeons
Ncoa3	0.38	0.00047717	pigeons
Aars	0.49	0.00048480	pigeons
Npepps	0.41	0.00048819	pigeons
Atxn7l1	0.47	0.00049228	pigeons
Kdm5b	0.55	0.00049584	pigeons
Rictor	0.43	0.00054254	pigeons
Btk	0.51	0.00056421	pigeons
Ccm2	0.43	0.00056641	pigeons
Dis3l2	0.39	0.00056988	pigeons
Ube2f	0.52	0.00058409	pigeons
Bhlhe41	0.68	0.00059736	pigeons
Dapp1	0.58	0.00060684	pigeons
Rasgef1a	0.82	0.00061614	pigeons
Asap1	0.61	0.00064264	pigeons
Gpr171	0.47	0.00065335	pigeons
Hmces	0.60	0.00065471	pigeons
Dcbld2	0.43	0.00065929	pigeons
Nanp	0.50	0.00066154	pigeons
Rnf19b	0.55	0.00067442	pigeons
Tnfaip3	0.31	0.00069211	pigeons
Crem	0.55	0.00071996	pigeons
Zfp644	0.28	0.00072588	pigeons
Chst2	0.51	0.00072859	pigeons
Slc9a7	0.40	0.00073194	pigeons
Slc7a5	0.68	0.00075310	pigeons
Ddx6	0.39	0.00079295	pigeons
Hbp1	0.43	0.00079677	pigeons
Ctsc	0.54	0.00080740	pigeons
Ppp4r3a	0.21	0.00080898	pigeons
Primpol	0.46	0.00086570	pigeons
Adam19	0.53	0.00087099	pigeons
Slc16a6	0.61	0.00088136	pigeons
Cerk	0.41	0.00089606	pigeons

Vbp1	0.43	0.00090155	pigeons
Kcnk1	0.68	0.00091225	pigeons
Ubac2	0.43	0.00096250	pigeons
Zcchc2	0.47	0.00097461	pigeons
Glcci1	0.66	0.00097625	pigeons
Il16	0.36	0.00103135	pigeons
Irf2	0.33	0.00113742	pigeons
Ddx42	0.45	0.00114157	pigeons
Cnot2	0.34	0.00118172	pigeons
Fdx1	0.28	0.00119758	pigeons
St8sia6	0.59	0.00123770	pigeons
Pxk	0.40	0.00123893	pigeons
Gnb4	0.39	0.00125002	pigeons
Ip6k2	0.25	0.00128799	pigeons
Rundc3b	0.71	0.00131634	pigeons
Atxn2	0.38	0.00141029	pigeons
Dusp23	0.28	0.00141419	pigeons
Xpo4	0.59	0.00153832	pigeons
Tlr7	0.55	0.00154611	pigeons
Zfp831	0.47	0.00156410	pigeons
Rpap3	0.40	0.00160695	pigeons
Tbc1d4	0.59	0.00165537	pigeons
Terf2ip	0.48	0.00166795	pigeons
Aifm1	0.40	0.00166795	pigeons
Paip2	0.39	0.00166795	pigeons
Xpo7	0.40	0.00169387	pigeons
Rpn1	0.36	0.00170054	pigeons
Btbd9	0.32	0.00170883	pigeons
Phldb2	-0.96	0.00174368	pigeons
Nsrp1	0.34	0.00179912	pigeons
Jarid2	0.55	0.00180016	pigeons
Vezt	0.56	0.00181972	pigeons
Rasa2	0.28	0.00183795	pigeons
Spopl	0.31	0.00184189	pigeons
Arap2	0.59	0.00184428	pigeons
Pou2af1	0.45	0.00187075	pigeons
Braf	0.29	0.00190645	pigeons
Ddx1	0.45	0.00191087	pigeons
Stk11ip	0.41	0.00194374	pigeons
Pdk3	0.39	0.00197567	pigeons
Herpud2	0.30	0.00200060	pigeons
Epn2	0.20	0.00203141	pigeons
Chd2	0.33	0.00205107	pigeons
Asb2	0.54	0.00208679	pigeons
Rdx	0.34	0.00209656	pigeons
Pnisr	0.21	0.00226216	pigeons

Cherp	0.42	0.00226441	pigeons
Zbtb21	0.23	0.00236216	pigeons
Vta1	0.43	0.00238772	pigeons
Grb2	0.42	0.00241124	pigeons
Atf2	0.29	0.00247186	pigeons
Klf13	0.33	0.00249750	pigeons
Prkdc	0.44	0.00258040	pigeons
Ptpn2	0.35	0.00258956	pigeons
Fbxl21	0.46	0.00263716	pigeons
Vav3	0.39	0.00267771	pigeons
Pdap1	0.38	0.00278756	pigeons
Susd6	0.41	0.00280969	pigeons
Rbm33	0.24	0.00288737	pigeons
Capzb	0.34	0.00301608	pigeons
Cd28	0.60	0.00307032	pigeons
Cnot6	0.34	0.00317011	pigeons
Sf3a3	0.36	0.00317744	pigeons
Nr2c2	0.30	0.00330211	pigeons
Arl3	0.32	0.00340540	pigeons
Fbxo31	0.44	0.00348463	pigeons
Aimp1	0.39	0.00352347	pigeons
Fnip1	0.46	0.00358419	pigeons
Utp20	0.48	0.00365850	pigeons
Pip4k2a	-0.48	0.00381898	pigeons
Gtf2f2	0.47	0.00386414	pigeons
Snhg4	0.30	0.00405464	pigeons
Ciao3	0.39	0.00415790	pigeons
Letm1	0.38	0.00427303	pigeons
Cct8	0.35	0.00441410	pigeons
Pank3	0.54	0.00450956	pigeons
Slc12a4	0.30	0.00456921	pigeons
Syne3	0.30	0.00461019	pigeons
Gtpbp4	0.33	0.00463490	pigeons
Lrch2	0.48	0.00469874	pigeons
Gpr68	0.36	0.00477116	pigeons
Atp11a	0.37	0.00477164	pigeons
Csta1	0.44	0.00483637	pigeons
Hdac3	0.39	0.00483638	pigeons
Tspan13	0.37	0.00490048	pigeons
Pcnx	0.30	0.00494982	pigeons
Srp14	0.44	0.00502673	pigeons
Parp8	0.63	0.00515612	pigeons
Emc6	0.21	0.00544345	pigeons
Slc41a2	0.59	0.00550645	pigeons
Trim36	0.48	0.00562384	pigeons
Cul4a	0.45	0.00562384	pigeons

Pudp	0.60	0.00562384	pigeons
Rfx3	0.53	0.00569909	pigeons
Ube2l3	0.30	0.00585780	pigeons
Pcmtd1	0.38	0.00594079	pigeons
Rfx2	0.40	0.00595189	pigeons
Gnal	0.60	0.00604942	pigeons
Tmem63a	0.57	0.00623266	pigeons
Fbxo33	0.34	0.00627026	pigeons
Zbed4	0.48	0.00631620	pigeons
Taf15	0.29	0.00633536	pigeons
Zdhhc18	0.51	0.00657430	pigeons
Rlim	0.38	0.00666997	pigeons
Lats1	0.17	0.00674566	pigeons
Rexo2	0.34	0.00678878	pigeons
Rab8a	0.35	0.00684023	pigeons
Pex13	0.48	0.00686011	pigeons
Erp29	0.38	0.00699472	pigeons
Cdv3	0.28	0.00752887	pigeons
Ddx28	0.35	0.00767164	pigeons
Abi1	0.20	0.00789291	pigeons
Senp5	0.35	0.00830157	pigeons
Gnptab	0.40	0.00831669	pigeons
Samd4	0.62	0.00845383	pigeons
Nmt2	0.49	0.00865037	pigeons
Dazap1	0.27	0.00871110	pigeons
Sbno2	0.43	0.00878446	pigeons
Tapbpl	0.38	0.00882643	pigeons
Stambp	0.37	0.00882643	pigeons
Dohh	0.38	0.00882643	pigeons
Bbx	0.37	0.00884793	pigeons
Cdc42	0.34	0.00885860	pigeons
Pgam1	0.26	0.00888410	pigeons
Utrn	-0.95	0.00898539	pigeons
Lmbrd1	0.40	0.00901505	pigeons
Pacsin2	0.28	0.00915619	pigeons
Il10ra	0.43	0.00949682	pigeons
Cep350	0.27	0.00952925	pigeons
Utp25	0.39	0.01025122	pigeons
Tent5a	0.49	0.01025122	pigeons
Seh1l	0.45	0.01044596	pigeons
Ncoa5	0.28	0.01057604	pigeons
Emc8	0.37	0.01068156	pigeons
Tlk1	0.28	0.01104469	pigeons
Ran	0.25	0.01167457	pigeons
Arid5a	0.38	0.01171843	pigeons
Erlec1	0.31	0.01172850	pigeons

Sec24d	0.42	0.01190247	pigeons
Ccdc58	0.40	0.01190248	pigeons
Eif2b2	0.43	0.01194025	pigeons
Zfp318	0.44	0.01203940	pigeons
Ppia	0.34	0.01208819	pigeons
St8sia4	0.50	0.01222698	pigeons
Kmt2e	0.30	0.01229769	pigeons
Acsl3	0.45	0.01263812	pigeons
Traf3ip3	0.32	0.01264250	pigeons
Ganc	0.34	0.01266380	pigeons
Xpot	0.41	0.01311558	pigeons
Zfp518a	0.59	0.01364078	pigeons
Senp1	0.37	0.01381564	pigeons
Ddx17	0.10	0.01396321	pigeons
Cul3	0.26	0.01399691	pigeons
Gzf1	0.34	0.01417287	pigeons
Tmem87a	0.17	0.01430384	pigeons
Psip1	0.34	0.01443728	pigeons
Rsbn1l	0.26	0.01466351	pigeons
Atp11b	0.46	0.01473586	pigeons
Snrnp35	0.52	0.01475982	pigeons
Zbtb1	0.33	0.01476743	pigeons
Vps37b	0.35	0.01487019	pigeons
Orai1	0.32	0.01499953	pigeons
Zcchc8	0.25	0.01518252	pigeons
Utp3	0.30	0.01582977	pigeons
Zfp516	0.55	0.01611267	pigeons
Btbd3	0.40	0.01615661	pigeons
Atf6	0.37	0.01623297	pigeons
Cbfa2t3	0.32	0.01646109	pigeons
Xrn2	0.29	0.01666121	pigeons
Pag1	0.56	0.01676619	pigeons
Zc3h18	0.35	0.01686813	pigeons
Eml6	-0.76	0.01714718	pigeons
Dicer1	0.27	0.01729414	pigeons
Secisbp2	0.34	0.01747304	pigeons
Capn3	0.34	0.01781620	pigeons
Cd82	0.31	0.01811268	pigeons
Clcn1	-0.56	0.01812903	pigeons
Ivns1abp	0.37	0.01859760	pigeons
Qars	0.40	0.01859760	pigeons
Kdm4b	0.29	0.01860373	pigeons
Akap9	0.41	0.01909791	pigeons
Unc119	0.52	0.01926936	pigeons
Snx29	0.23	0.02061003	pigeons
Dmac2l	0.36	0.02156803	pigeons

Pym1	0.38	0.02156804	pigeons
Csk	0.35	0.02189797	pigeons
Smc6	0.12	0.02324180	pigeons
Rnf111	0.27	0.02388127	pigeons
Rasa3	0.42	0.02411819	pigeons
Umad1	0.47	0.02432954	pigeons
Srpk1	0.33	0.02440080	pigeons
Riok2	0.38	0.02500573	pigeons
Arl14ep	0.35	0.02500573	pigeons
Crcp	0.37	0.02500573	pigeons
Bcl2l11	0.43	0.02515440	pigeons
Farsb	0.31	0.02529737	pigeons
Prkd3	0.26	0.02539050	pigeons
Auh	0.42	0.02578997	pigeons
Strn	0.21	0.02606422	pigeons
C2cd5	0.34	0.02672675	pigeons
Pknox1	0.23	0.02682006	pigeons
Arih2	0.24	0.02695741	pigeons
Atxn7	0.49	0.02709546	pigeons
Tpd52l2	0.28	0.02731816	pigeons
Ppil4	0.10	0.02827474	pigeons
Tctn1	0.44	0.02840463	pigeons
Ncbp1	0.49	0.02972155	pigeons
Ypel1	0.15	0.02981242	pigeons
Ambra1	0.18	0.03007011	pigeons
Ralgapb	0.29	0.03029709	pigeons
Ip6k1	0.39	0.03109760	pigeons
Anks1	-0.97	0.03124294	pigeons
Hs1bp3	0.22	0.03135945	pigeons
Papss1	0.23	0.03191290	pigeons
Tmem192	0.26	0.03202980	pigeons
Ercc6	0.44	0.03210450	pigeons
Tmem123	0.28	0.03290775	pigeons
Zyx	0.39	0.03325579	pigeons
Prpsap2	0.44	0.03341138	pigeons
Pnpla8	0.21	0.03348377	pigeons
Pip4p2	0.42	0.03358344	pigeons
Dap	0.46	0.03358344	pigeons
Tmsb4x	0.27	0.03397106	pigeons
Zdhhc3	0.28	0.03426204	pigeons
Ift46	0.38	0.03433593	pigeons
Arhgdib	0.27	0.03446904	pigeons
Phc3	0.25	0.03468715	pigeons
Dda1	0.14	0.03476448	pigeons
Dennd5b	0.90	0.03531515	pigeons
Lrrc8d	0.36	0.03541213	pigeons

Tns3	-0.76	0.03571266	pigeons
Smc4	0.29	0.03653673	pigeons
Arpp19	0.18	0.03664589	pigeons
Kcnk5	0.55	0.03666506	pigeons
Fbxo42	0.36	0.03696059	pigeons
Plcl2	0.72	0.03774630	pigeons
Naa25	0.49	0.03846851	pigeons
Gal	-0.65	0.03888788	pigeons
Supt6	0.14	0.03959411	pigeons
Pdia6	0.15	0.04009662	pigeons
Cnn2	0.39	0.04220417	pigeons
Cmtm7	0.32	0.04228701	pigeons
Ssrp1	0.29	0.04246632	pigeons
Wdr20	0.26	0.04283681	pigeons
Ptbp2	0.17	0.04284811	pigeons
Sema4g	0.40	0.04323941	pigeons
Serpine2	0.35	0.04437854	pigeons
Runx1	0.41	0.04438222	pigeons
Fsbp	0.32	0.04505267	pigeons
Tnfrsf18	0.66	0.04538946	pigeons
Ankrd27	0.30	0.04573861	pigeons
Ncoa2	0.32	0.04718174	pigeons
Ddx46	0.27	0.04934882	pigeons
Stk17b	0.09	0.05082844	pigeons
Ubap2l	0.41	0.05163392	pigeons
Traf6	0.24	0.05206051	pigeons
Lrr1	0.33	0.05215987	pigeons
Sfxn3	0.40	0.05215987	pigeons
Unc119b	0.22	0.05231134	pigeons
Slc25a43	0.36	0.05359655	pigeons
Susd3	0.45	0.05430961	pigeons
Sytl1	0.19	0.05511099	pigeons
4833439L19Rik	0.34	0.05620563	pigeons
Pkn2	0.14	0.05644553	pigeons
Aip	0.38	0.06037156	pigeons
Eif4h	0.24	0.06222050	pigeons
Stxbp6	-0.82	0.06254393	pigeons
Anapc1	0.38	0.06334495	pigeons
Stx7	0.40	0.06594912	pigeons
Psmd1	0.08	0.06763471	pigeons
Snrpd1	0.21	0.06889814	pigeons
Rnpc3	0.37	0.06965340	pigeons
Gabrr2	0.42	0.06985679	pigeons
R3hdm1	0.29	0.07134344	pigeons
Tax1bp3	0.30	0.07388572	pigeons
Pxdc1	-0.88	0.07728532	pigeons

Grk4	0.30	0.07786520	pigeons
Rbm12	0.27	0.08402482	pigeons
Nf1	0.12	0.08570324	pigeons
Il27ra	0.28	0.08586104	pigeons
Gkap1	0.35	0.08586627	pigeons
Ccdc88c	0.37	0.08625563	pigeons
Gpbp1l1	0.22	0.08642153	pigeons
Fbxl3	0.17	0.08733384	pigeons
Galnt9	0.55	0.08845467	pigeons
Nsfl1c	0.23	0.08947576	pigeons
Ube3c	0.30	0.09017497	pigeons
Uhrf1bp1l	0.39	0.09057157	pigeons
Dus2	0.30	0.09221065	pigeons
Aamp	0.34	0.09345634	pigeons
Nfic	0.33	0.09345634	pigeons
Sp1	0.33	0.09345638	pigeons
Pdhx	0.17	0.09354889	pigeons
Adnp2	0.33	0.09458676	pigeons
Psme4	0.20	0.09465362	pigeons
Kansl1	0.27	0.09488876	pigeons
Capza2	-0.04	0.09807433	pigeons
Wdr37	0.22	0.09834406	pigeons
Vopp1	0.26	0.10006870	pigeons
Lct	0.54	0.10035452	pigeons
Nfe2l1	0.27	0.10132989	pigeons
Lonp1	0.19	0.10170590	pigeons
Ptpn9	0.36	0.10214874	pigeons
Rad51c	0.37	0.10253067	pigeons
Rab27a	0.18	0.10670601	pigeons
Klhl7	0.31	0.10712232	pigeons
Lcp2	0.31	0.10725974	pigeons
Clk3	0.31	0.10916836	pigeons
Ccdc88a	0.36	0.10997493	pigeons
Tsen15	0.37	0.11081825	pigeons
Glyr1	0.21	0.11269488	pigeons
Ttc17	0.14	0.11430904	pigeons
Pmp22	-1.48	0.11693588	pigeons
Psma1	0.18	0.12034668	pigeons
Rbm34	0.32	0.12063217	pigeons
Ube2v1	0.34	0.12101358	pigeons
Gfod1	0.29	0.12136250	pigeons
Lrch3	0.13	0.12154937	pigeons
Sec61g	0.22	0.12176214	pigeons
Arpc5	0.28	0.12274231	pigeons
Kdelr2	0.22	0.12291192	pigeons
Ipo7	0.12	0.12457677	pigeons

Zbtb7a	0.22	0.12479953	pigeons
Ccdc85a	-1.09	0.12542258	pigeons
Lpgat1	0.32	0.12658836	pigeons
Stau2	0.42	0.12704352	pigeons
Plekha8	0.34	0.12775867	pigeons
Irf7	0.41	0.12965628	pigeons
Klhl15	0.26	0.12984630	pigeons
Pgpep1	0.45	0.13105944	pigeons
Etv6	-1.30	0.13119686	pigeons
Rbm15	0.25	0.13708793	pigeons
Spata5	0.55	0.13813951	pigeons
Agpat5	0.39	0.13965238	pigeons
Ube3a	0.18	0.13993607	pigeons
Yaf2	0.33	0.14243324	pigeons
Sh3kbp1	0.21	0.14740216	pigeons
Plekhb2	0.17	0.14794212	pigeons
Trpm1	0.52	0.14848673	pigeons
Krit1	0.16	0.14862473	pigeons
Prdm15	0.32	0.14881460	pigeons
Atp6v0b	0.16	0.15249127	pigeons
Rasal2	-0.97	0.15301145	pigeons
Itpk1	-1.35	0.15471211	pigeons
Ncoa6	0.09	0.15579108	pigeons
Nmi	0.16	0.15622491	pigeons
Dst	-0.44	0.15729499	pigeons
Ccdc6	0.12	0.15816507	pigeons
Ica1	-0.72	0.15918908	pigeons
Tmcc1	0.22	0.15935118	pigeons
Ankib1	0.43	0.15958821	pigeons
Snx20	0.38	0.15990524	pigeons
Eif3b	0.18	0.16508673	pigeons
Arhgef7	0.31	0.16544284	pigeons
Ddx51	0.35	0.16670948	pigeons
Hnrnpa1	0.30	0.16673246	pigeons
Rybp	0.43	0.16673253	pigeons
Plekha1	-1.04	0.16788538	pigeons
Pign	0.43	0.16947090	pigeons
Exoc6	0.20	0.16976234	pigeons
Nr4a3	0.43	0.17447691	pigeons
Cdh2	-0.92	0.17755407	pigeons
Smcr8	0.32	0.18167357	pigeons
Hyou1	0.20	0.18216016	pigeons
Kpna1	0.03	0.18284655	pigeons
Rab21	0.11	0.18609564	pigeons
Grsf1	0.27	0.18853537	pigeons
Fam120a	0.13	0.18960055	pigeons

Dnm1l	0.38	0.19043250	pigeons
Themis	0.40	0.19131914	pigeons
Fbxl5	0.10	0.19245851	pigeons
Polr1b	0.30	0.19257050	pigeons
Bcl2	0.84	0.19445129	pigeons
Eif3m	0.30	0.19496462	pigeons
Prkcb	0.21	0.19551458	pigeons
Hivep2	0.29	0.19930648	pigeons
Tra2b	-0.01	0.20174086	pigeons
Setd5	0.20	0.20298614	pigeons
Fggy	0.26	0.20639145	pigeons
Stk3	0.26	0.20678986	pigeons
Lcp1	0.19	0.21215035	pigeons
Gins2	0.41	0.21287100	pigeons
Rrp1b	0.36	0.21645951	pigeons
Virma	0.13	0.21770689	pigeons
Rnf213	0.21	0.22085097	pigeons
Prcp	0.30	0.22235551	pigeons
Ikbip	0.33	0.22235551	pigeons
Rufy1	0.18	0.22404079	pigeons
Xpc	0.23	0.22746392	pigeons
Anxa11	0.13	0.22791219	pigeons
Arhgef3	-1.09	0.22823679	pigeons
Sec24b	0.17	0.22880627	pigeons
Commd7	0.26	0.23766754	pigeons
Pias1	0.06	0.24018023	pigeons
Osbpl11	0.21	0.24549652	pigeons
Ptpn7	0.41	0.24568928	pigeons
Snrpe	0.07	0.24875585	pigeons
B4galt5	0.32	0.25081030	pigeons
Kdm7a	0.12	0.25417424	pigeons
Ankrd13a	0.30	0.25631976	pigeons
Sae1	0.29	0.25668214	pigeons
Tnpo3	0.30	0.25668225	pigeons
Inip	0.28	0.25668225	pigeons
Porcn	0.28	0.25668225	pigeons
Dctn2	0.28	0.25668225	pigeons
Adprs	0.32	0.25668225	pigeons
Prpf3	0.40	0.25738748	pigeons
Nt5c3b	0.28	0.25837324	pigeons
Nedd9	0.25	0.25999682	pigeons
Ube2g1	0.05	0.26190100	pigeons
Lsm14a	0.10	0.26468829	pigeons
Ccnd1	0.31	0.26597450	pigeons
Ercc4	0.03	0.26868747	pigeons
Dnajc7	0.11	0.27197217	pigeons

Tm9sf3	0.26	0.27254964	pigeons
Nubp1	0.32	0.27263576	pigeons
Usp44	0.26	0.27774797	pigeons
Ostf1	0.14	0.27795148	pigeons
Zfp148	0.38	0.28547617	pigeons
Bag5	0.12	0.28594706	pigeons
Gpr137b	0.32	0.28707231	pigeons
Ptk2b	0.29	0.28954160	pigeons
Polk	0.15	0.29448999	pigeons
Ap2a1	0.10	0.29573265	pigeons
Eif2b3	0.33	0.29597857	pigeons
Plscr1	0.31	0.29623385	pigeons
Zranb2	0.16	0.29922426	pigeons
Washc4	0.24	0.30475455	pigeons
Smarcb1	0.28	0.30626845	pigeons
Sgms2	0.37	0.31303669	pigeons
Irf8	0.39	0.31484164	pigeons
Zc3h7a	0.11	0.31521204	pigeons
Herc1	0.09	0.32359479	pigeons
Ssu72	0.21	0.32623141	pigeons
Slc39a9	0.29	0.32687351	pigeons
Dcaf1	0.16	0.33178448	pigeons
S1pr1	-1.39	0.33319820	pigeons
Laptm5	0.20	0.33464035	pigeons
Pafah1b2	0.15	0.33495099	pigeons
Cpt1a	0.33	0.33607375	pigeons
Nudt3	0.06	0.34163483	pigeons
Nrap	0.33	0.34179491	pigeons
Zfand1	0.44	0.34744154	pigeons
Wdr12	0.30	0.34822327	pigeons
Luc7l3	0.06	0.35335125	pigeons
Wasf2	0.16	0.35386166	pigeons
Tec	0.58	0.35850140	pigeons
Dmxl1	0.13	0.35899415	pigeons
Taf2	0.19	0.35934588	pigeons
Tbl1xr1	0.16	0.36468534	pigeons
Abcb7	0.02	0.36740977	pigeons
Btaf1	0.06	0.36768468	pigeons
Hps3	0.40	0.37583687	pigeons
Tacc1	0.30	0.38783623	pigeons
Rnf138	0.18	0.39191503	pigeons
Smim14	0.15	0.39248469	pigeons
Il1rap	0.53	0.39426636	pigeons
Atrip	0.32	0.39426636	pigeons
Trappc5	0.29	0.39426636	pigeons
Slc39a6	0.26	0.39692602	pigeons

Akt1	0.14	0.39874816	pigeons
Cipc	0.23	0.40123044	pigeons
Cast	0.38	0.40150718	pigeons
Prdm2	0.32	0.40333026	pigeons
Tapt1	0.08	0.41315835	pigeons
Ccnk	0.10	0.41440845	pigeons
Fam13b	0.03	0.41595936	pigeons
Oxnad1	0.18	0.41779547	pigeons
Cep112	0.20	0.43254210	pigeons
Ap1g1	0.15	0.43266291	pigeons
Ppp6r2	0.19	0.43295144	pigeons
Elavl1	0.21	0.43424348	pigeons
Lmo3	-1.02	0.43432826	pigeons
Lcor	0.21	0.43520033	pigeons
Exoc3	0.33	0.43804329	pigeons
Capn7	0.35	0.43971988	pigeons
Klhl5	0.33	0.44161795	pigeons
Clasp1	0.07	0.44566942	pigeons
Znfx1	0.22	0.44939442	pigeons
Atp1b1	0.27	0.45387290	pigeons
Vps16	0.29	0.45468236	pigeons
Ilkap	0.29	0.45468255	pigeons
Kbtbd8	0.29	0.45554973	pigeons
Usp3	0.22	0.46235759	pigeons
Cnih1	0.28	0.46589404	pigeons
Got2	0.29	0.47397146	pigeons
Pds5a	0.06	0.47891274	pigeons
Ep300	0.22	0.47927055	pigeons
Snx9	0.15	0.48433438	pigeons
Nemf	0.25	0.49797078	pigeons
Gramd4	0.41	0.50442752	pigeons
Snap23	0.08	0.50527664	pigeons
Scaf4	0.21	0.51305711	pigeons
Cyld	0.14	0.51363478	pigeons
Brd7	0.25	0.52167738	pigeons
Tcof1	0.13	0.52407030	pigeons
Ppp2r2a	0.08	0.53814696	pigeons
Rbm17	0.36	0.54303816	pigeons
Slf2	0.20	0.54458879	pigeons
Aasdh	0.43	0.54577045	pigeons
Vps4b	0.22	0.55438343	pigeons
Copb2	0.21	0.55467971	pigeons
Cwc25	0.21	0.55641813	pigeons
Taf4b	0.44	0.55662539	pigeons
Setbp1	0.29	0.55973601	pigeons
Tnfaip8l1	0.30	0.56584525	pigeons

Ttc27	0.27	0.56776272	pigeons
Nudt16l1	0.17	0.56861646	pigeons
Yrdc	0.27	0.57037098	pigeons
Edem1	0.19	0.57046384	pigeons
Rgs9	0.32	0.58094079	pigeons
Tial1	0.07	0.58353842	pigeons
Ddit4	-0.67	0.58722054	pigeons
Hars	0.27	0.60427344	pigeons
Dhx15	0.12	0.61628859	pigeons
Ftsj3	0.35	0.62592618	pigeons
Ndfip1	-1.01	0.64101852	pigeons
Actr8	0.22	0.64245583	pigeons
Ppid	0.32	0.64977552	pigeons
Guk1	0.25	0.64991182	pigeons
Rab5a	0.23	0.66015755	pigeons
Pi4k2b	0.12	0.66356310	pigeons
Rcc1	0.25	0.66402620	pigeons
Ikbkb	0.34	0.68050697	pigeons
Rnaseh2b	0.24	0.68543273	pigeons
Acp1	0.12	0.70014862	pigeons
Pigb	0.17	0.70016607	pigeons
Yme1l1	0.22	0.71642805	pigeons
Fam8a1	0.27	0.72127816	pigeons
Eif2s2	0.07	0.72558132	pigeons
Tcea1	0.13	0.72928573	pigeons
Prelid3b	0.25	0.73759792	pigeons
Hivep1	0.21	0.74179974	pigeons
Pik3cd	0.18	0.74266806	pigeons
Ppp1r9a	0.34	0.75743649	pigeons
Immt	0.17	0.77317084	pigeons
4932438A13Rik	-0.03	0.77587066	pigeons
Cpq	0.26	0.77863297	pigeons
Tmem131	0.14	0.78631067	pigeons
Gapvd1	0.33	0.79549287	pigeons
Vps29	0.25	0.80862047	pigeons
Tmem222	0.27	0.82019001	pigeons
Nhsl2	0.24	0.82955531	pigeons
Brd3	0.23	0.83484628	pigeons
Ttbk1	0.19	0.84019821	pigeons
Pigt	0.15	0.86411603	pigeons
Slc25a16	-0.01	0.87029818	pigeons
Exosc9	0.36	0.87161475	pigeons
Rab7	0.06	0.88378966	pigeons
Dnajb4	0.22	0.89394757	pigeons
Ndufv3	0.09	0.89496335	pigeons
Dnaja4	0.20	0.91766579	pigeons

Exoc2	0.08	0.91974508	pigeons
Atp1b3	-1.48	0.91984279	pigeons
Foxo1	0.27	0.92005554	pigeons
Gpr34	0.35	0.92594989	pigeons
Rabggtb	0.34	0.94235474	pigeons
Tab2	0.18	0.95310258	pigeons
Rrp12	0.30	0.95512790	pigeons
Rpa2	0.16	0.95589295	pigeons
Ypel2	0.33	0.96941188	pigeons
Tmed2	0.14	0.98600545	pigeons
Zbtb2	0.15	0.98619650	pigeons
Atrn	-1.30	0.99490625	pigeons
Rbl2	0.26	0.99675773	pigeons
Cdc73	0.09	0.99899984	pigeons
Rfc2	0.28	1.00000000	pigeons
Park7	0.30	1.00000000	pigeons
Ctr9	0.24	1.00000000	pigeons
Uchl5	0.28	1.00000000	pigeons
Rpf2	0.26	1.00000000	pigeons
Uhmk1	0.31	1.00000000	pigeons
Urm1	0.16	1.00000000	pigeons
Rassf5	0.36	1.00000000	pigeons
Oma1	0.24	1.00000000	pigeons
Ints13	0.14	1.00000000	pigeons
Lrrc59	0.32	1.00000000	pigeons
Arih1	0.09	1.00000000	pigeons
Unc93b1	0.27	1.00000000	pigeons
Gnas	0.29	1.00000000	pigeons
Ppp4r1	-0.04	1.00000000	pigeons
Hbs1l	0.07	1.00000000	pigeons
Nup85	0.30	1.00000000	pigeons
Lepr	0.30	1.00000000	pigeons
Epb41l4a	0.26	1.00000000	pigeons
Ankrd13d	0.11	1.00000000	pigeons
Hipk2	-1.04	1.00000000	pigeons
Uri1	0.16	1.00000000	pigeons
Cct4	0.14	1.00000000	pigeons
Zbtb14	0.27	1.00000000	pigeons
Slc52a3	0.27	1.00000000	pigeons
Clip1	0.15	1.00000000	pigeons
Dok3	0.21	1.00000000	pigeons
Tpr	0.24	1.00000000	pigeons
Card11	0.39	1.00000000	pigeons
Fkbp3	0.21	1.00000000	pigeons
Klhl36	0.30	1.00000000	pigeons
Ube2d2a	0.03	1.00000000	pigeons

Cabin1	0.23	1.00000000	pigeons
Rhof	0.16	1.00000000	pigeons
Atf3	-1.34	1.00000000	pigeons
Slc25a28	0.25	1.00000000	pigeons
Tgfbrap1	0.29	1.00000000	pigeons
Erlin2	0.21	1.00000000	pigeons
Trio	-1.21	1.00000000	pigeons
Ankle2	0.25	1.00000000	pigeons
Ddx52	0.28	1.00000000	pigeons
Atp6v1e2	0.09	1.00000000	pigeons
Pes1	0.31	1.00000000	pigeons
Polr1c	0.26	1.00000000	pigeons
Dhx38	0.23	1.00000000	pigeons
Gnl2	0.20	1.00000000	pigeons
Hnrnpll	0.02	1.00000000	pigeons
Uck2	0.30	1.00000000	pigeons
Blk	0.37	1.00000000	pigeons
Fam222b	0.34	1.00000000	pigeons
Slc15a4	0.30	1.00000000	pigeons
Git2	0.13	1.00000000	pigeons
Lpin2	0.17	1.00000000	pigeons
Sync	0.28	1.00000000	pigeons
Lyrm7	0.28	1.00000000	pigeons
Caprin1	0.15	1.00000000	pigeons
Tfpi2	-1.21	1.00000000	pigeons
Ak1	-1.02	1.00000000	pigeons
Luzp1	-1.22	1.00000000	pigeons
Nudc	0.15	1.00000000	pigeons
Hipk1	-0.02	1.00000000	pigeons
Cdc16	0.30	1.00000000	pigeons
Tulp3	0.20	1.00000000	pigeons
Def6	0.29	1.00000000	pigeons
Scfd2	0.35	1.00000000	pigeons
Sccpdh	0.26	1.00000000	pigeons
Srp9	0.11	1.00000000	pigeons
Clk4	-0.07	1.00000000	pigeons
Usp45	-0.03	1.00000000	pigeons
Ankra2	0.05	1.00000000	pigeons
Pigf	0.34	1.00000000	pigeons
Ewsr1	0.20	1.00000000	pigeons
Fez2	0.23	1.00000000	pigeons
Luc7l	0.11	1.00000000	pigeons
Arrdc3	0.35	1.00000000	pigeons
Ppp1r26	0.19	1.00000000	pigeons
Dennd2d	0.33	1.00000000	pigeons
Gpc4	0.33	1.00000000	pigeons

Rab8b	0.23	1.00000000	pigeons
Ctdspl2	0.39	1.00000000	pigeons
Ift122	0.28	1.00000000	pigeons
Bzw1	0.32	1.00000000	pigeons
Utp6	0.34	1.00000000	pigeons
Cdc42ep3	0.27	1.00000000	pigeons
Lag3	0.30	1.00000000	pigeons
Bms1	0.23	1.00000000	pigeons
Osbpl9	0.32	1.00000000	pigeons
Ehd3	0.31	1.00000000	pigeons
Slu7	-0.05	1.00000000	pigeons
Ik	-0.02	1.00000000	pigeons
Gdap2	-0.03	1.00000000	pigeons
Hint1	0.36	1.00000000	pigeons
Eif5	0.01	1.00000000	pigeons
Tspan18	-0.89	1.00000000	pigeons
Gatad2b	0.18	1.00000000	pigeons
Flii	0.15	1.00000000	pigeons
Sdhaf4	0.11	1.00000000	pigeons
Sbno1	0.10	1.00000000	pigeons
Rab23	0.34	1.00000000	pigeons
Sin3a	0.17	1.00000000	pigeons
Whamm	0.25	1.00000000	pigeons
Rnmt	0.22	1.00000000	pigeons
Ppcs	0.23	1.00000000	pigeons
Tmco4	0.28	1.00000000	pigeons
Cbx7	0.37	1.00000000	pigeons
Itpa	0.32	1.00000000	pigeons
Scaf11	0.01	1.00000000	pigeons
Tia1	0.13	1.00000000	pigeons
Dennd5a	0.14	1.00000000	pigeons
Get4	0.07	1.00000000	pigeons
Sass6	0.25	1.00000000	pigeons
Sec16a	0.10	1.00000000	pigeons
Atl2	0.04	1.00000000	pigeons
Pdcd11	0.12	1.00000000	pigeons
Ythdf3	0.02	1.00000000	pigeons
Pyroxd1	0.26	1.00000000	pigeons
Cdk19	0.12	1.00000000	pigeons
Ash1l	-0.05	1.00000000	pigeons
Ddx27	0.12	1.00000000	pigeons
Gnai2	0.21	1.00000000	pigeons
Nek9	0.20	1.00000000	pigeons
Nlrc3	0.32	1.00000000	pigeons
Trp73	0.29	1.00000000	pigeons
Ccnd2	0.25	1.00000000	pigeons

Cct7	0.16	1.00000000	pigeons
Ndufab1	0.20	1.00000000	pigeons
Usp7	-0.01	1.00000000	pigeons
Eif3f	0.17	1.00000000	pigeons
Snrk	0.05	1.00000000	pigeons
Fam32a	0.12	1.00000000	pigeons
Fhl3	0.20	1.00000000	pigeons
Fbxo9	0.23	1.00000000	pigeons
Hspa4l	0.13	1.00000000	pigeons
Tmem242	0.17	1.00000000	pigeons
Cflar	0.14	1.00000000	pigeons
Foxk2	0.06	1.00000000	pigeons
Hcfc2	0.17	1.00000000	pigeons
Rufy3	0.25	1.00000000	pigeons
Wdr91	0.21	1.00000000	pigeons
Sf3b5	0.13	1.00000000	pigeons
Cep104	0.28	1.00000000	pigeons
Cep85l	0.26	1.00000000	pigeons
2210408I21Rik	0.22	1.00000000	pigeons
Fbxo34	0.15	1.00000000	pigeons
Stox2	-1.01	1.00000000	pigeons
Otulin	0.08	1.00000000	pigeons
Eif4a2	0.24	1.00000000	pigeons
Synj1	-0.09	1.00000000	pigeons
Ccnd3	0.29	1.00000000	pigeons
Vps54	-0.88	1.00000000	pigeons
Hddc2	0.20	1.00000000	pigeons
Neil1	0.40	1.00000000	pigeons
Ube2w	0.11	1.00000000	pigeons
Xpa	0.11	1.00000000	pigeons
Eif3d	0.17	1.00000000	pigeons
Itch	0.23	1.00000000	pigeons
Btbd11	0.36	1.00000000	pigeons
Dus3l	0.29	1.00000000	pigeons
Diablo	0.32	1.00000000	pigeons
Lias	0.10	1.00000000	pigeons
Pcif1	0.16	1.00000000	pigeons
Ppm1b	0.03	1.00000000	pigeons
Plagl2	0.26	1.00000000	pigeons
Rsu1	0.04	1.00000000	pigeons
C1galt1	0.27	1.00000000	pigeons
Trmt11	0.16	1.00000000	pigeons
Pdxk	0.31	1.00000000	pigeons
Agfg1	0.09	1.00000000	pigeons
Scamp2	-0.18	1.00000000	pigeons
Rbbp4	0.32	1.00000000	pigeons

Ccser2	0.03	1.00000000	pigeons
Srsf5	0.14	1.00000000	pigeons
Nsun2	0.18	1.00000000	pigeons
Eif2ak2	0.22	1.00000000	pigeons
Foxn3	0.15	1.00000000	pigeons
Ogfr	0.22	1.00000000	pigeons
Edc3	0.08	1.00000000	pigeons
Fam98a	0.21	1.00000000	pigeons
Sucla2	0.19	1.00000000	pigeons
Dtwd2	0.21	1.00000000	pigeons
Derl1	0.06	1.00000000	pigeons
Usp24	-0.18	1.00000000	pigeons
Pkp4	0.23	1.00000000	pigeons
Ap3b2	0.17	1.00000000	pigeons
Rprd1b	0.08	1.00000000	pigeons
Atm	0.25	1.00000000	pigeons
Ikzf5	0.21	1.00000000	pigeons
Aldh18a1	0.29	1.00000000	pigeons
Abce1	0.07	1.00000000	pigeons
Dnajc13	0.21	1.00000000	pigeons
Wnk1	0.16	1.00000000	pigeons
Sacs	0.27	1.00000000	pigeons
Zfp36l1	0.28	1.00000000	pigeons
Kpna6	0.06	1.00000000	pigeons
Nfyc	0.17	1.00000000	pigeons
Ing4	0.31	1.00000000	pigeons
Ubxn11	0.25	1.00000000	pigeons
Suclg1	0.35	1.00000000	pigeons
Xpnpep1	0.18	1.00000000	pigeons
Ythdf2	0.02	1.00000000	pigeons
Dnajc18	0.20	1.00000000	pigeons
Wasl	0.20	1.00000000	pigeons
Hmgcs1	0.24	1.00000000	pigeons
Raf1	0.11	1.00000000	pigeons
Amd2	0.16	1.00000000	pigeons
Srsf1	0.12	1.00000000	pigeons
Naa50	0.05	1.00000000	pigeons
Lrp12	0.15	1.00000000	pigeons
Pafah1b1	0.03	1.00000000	pigeons
Tra2a	-0.14	1.00000000	pigeons
Lrig2	0.28	1.00000000	pigeons
Vmp1	0.12	1.00000000	pigeons
Ppp2r3c	0.25	1.00000000	pigeons
Csnk1a1	0.23	1.00000000	pigeons
Ccdc137	0.25	1.00000000	pigeons
Rap2c	-0.04	1.00000000	pigeons

Sh3bgrl3	0.03	1.00000000	pigeons
Ndufs2	0.01	1.00000000	pigeons
Tarbp1	0.23	1.00000000	pigeons
Zcchc10	0.23	1.00000000	pigeons
Ss18	0.06	1.00000000	pigeons
Sec23b	0.13	1.00000000	pigeons
Dok1	0.15	1.00000000	pigeons
Epcam	0.28	1.00000000	pigeons
Jade2	-0.01	1.00000000	pigeons
Spred2	-1.29	1.00000000	pigeons
Arid2	0.11	1.00000000	pigeons
Gemin4	0.25	1.00000000	pigeons
Thada	0.19	1.00000000	pigeons
Polr1e	0.17	1.00000000	pigeons
Rad54b	0.13	1.00000000	pigeons
Vav2	0.15	1.00000000	pigeons
Nipa1	0.23	1.00000000	pigeons
Bmt2	0.20	1.00000000	pigeons
Helq	0.14	1.00000000	pigeons
Wdfy2	0.01	1.00000000	pigeons
Lrrc8b	0.16	1.00000000	pigeons
Rc3h1	-0.02	1.00000000	pigeons
Asns	0.29	1.00000000	pigeons
Sart3	0.14	1.00000000	pigeons
Pla2g6	0.24	1.00000000	pigeons
Tfg	0.09	1.00000000	pigeons
Zbtb48	0.14	1.00000000	pigeons
Ctbp2	0.00	1.00000000	pigeons
Atad5	0.32	1.00000000	pigeons
Ulk1	-0.98	1.00000000	pigeons
Rit1	0.08	1.00000000	pigeons
C2cd2l	0.22	1.00000000	pigeons
Tspan2	0.21	1.00000000	pigeons
Cbx3	0.12	1.00000000	pigeons
Pnrc2	0.17	1.00000000	pigeons
Ctsz	0.09	1.00000000	pigeons
Iqgap2	0.17	1.00000000	pigeons
Parp1	0.18	1.00000000	pigeons
Itga4	0.17	1.00000000	pigeons
Sag	-0.59	1.00000000	pigeons
Lrrc47	0.21	1.00000000	pigeons
Dennd4a	-1.08	1.00000000	pigeons
Ptp4a2	0.08	1.00000000	pigeons
Chic2	0.00	1.00000000	pigeons
Atp13a3	0.34	1.00000000	pigeons
Nbn	0.20	1.00000000	pigeons

Usp33	0.32	1.00000000	pigeons
Zfat	0.15	1.00000000	pigeons
Usp16	0.06	1.00000000	pigeons
Suds3	0.34	1.00000000	pigeons
Dkc1	0.28	1.00000000	pigeons
Ext1	-1.23	1.00000000	pigeons
Agk	0.19	1.00000000	pigeons
Prpf4b	0.04	1.00000000	pigeons
Otud4	0.31	1.00000000	pigeons
Sesn3	0.24	1.00000000	pigeons
Eif2a	0.18	1.00000000	pigeons
Tbc1d5	0.08	1.00000000	pigeons
Zfp326	0.16	1.00000000	pigeons
Gpalpp1	-0.14	1.00000000	pigeons
Traf3	0.20	1.00000000	pigeons
Ubap1	0.00	1.00000000	pigeons
Strn3	0.02	1.00000000	pigeons
Sh3bp2	0.20	1.00000000	pigeons
Psmd13	0.18	1.00000000	pigeons
Pola1	0.21	1.00000000	pigeons
Ubn2	0.03	1.00000000	pigeons
Rab3gap2	0.11	1.00000000	pigeons
Pisd	0.05	1.00000000	pigeons
Ppp1cc	0.16	1.00000000	pigeons
Polr2k	0.25	1.00000000	pigeons
Gnai3	-0.08	1.00000000	pigeons
Tubgcp5	0.20	1.00000000	pigeons
Togaram1	0.14	1.00000000	pigeons
Necap2	0.12	1.00000000	pigeons
Trit1	0.14	1.00000000	pigeons
Snrpb2	0.19	1.00000000	pigeons
Upf1	0.10	1.00000000	pigeons
Atg13	0.18	1.00000000	pigeons
Tgs1	0.20	1.00000000	pigeons
Ywhah	0.23	1.00000000	pigeons
Kif21b	0.24	1.00000000	pigeons
Dock10	0.08	1.00000000	pigeons
Prrc1	0.28	1.00000000	pigeons
Nucb2	0.16	1.00000000	pigeons
Ndufaf4	0.13	1.00000000	pigeons
Ankrd46	0.31	1.00000000	pigeons
Ndufa2	0.08	1.00000000	pigeons
Ubash3b	0.27	1.00000000	pigeons
Ppp1r27	0.17	1.00000000	pigeons
Puf60	0.21	1.00000000	pigeons
Ncoa1	0.16	1.00000000	pigeons

Spag9	-0.14	1.00000000	pigeons
Ythdf1	0.12	1.00000000	pigeons
Alcam	0.33	1.00000000	pigeons
Rab11a	0.12	1.00000000	pigeons
Cog7	0.15	1.00000000	pigeons
Syne1	0.10	1.00000000	pigeons
Ei24	0.20	1.00000000	pigeons
Rmnd1	0.28	1.00000000	pigeons
Zbtb6	0.31	1.00000000	pigeons
Igf1r	0.23	1.00000000	pigeons
Becn2	0.14	1.00000000	pigeons
Rspry1	0.24	1.00000000	pigeons
Itgb1bp1	0.29	1.00000000	pigeons
Lasp1	0.18	1.00000000	pigeons
Srp72	0.14	1.00000000	pigeons
Ptdss1	0.21	1.00000000	pigeons
Arhgap5	-0.94	1.00000000	pigeons
Surf6	0.24	1.00000000	pigeons
Gns	0.08	1.00000000	pigeons
Hsph1	0.37	1.00000000	pigeons
Rilpl2	0.12	1.00000000	pigeons
Traf1	0.15	1.00000000	pigeons
Nipa2	0.12	1.00000000	pigeons
Snw1	0.16	1.00000000	pigeons
Zfp292	0.15	1.00000000	pigeons
Zfand3	0.14	1.00000000	pigeons
Acaca	0.12	1.00000000	pigeons
Kctd3	0.24	1.00000000	pigeons
Ino80c	0.25	1.00000000	pigeons
Tvp23a	0.27	1.00000000	pigeons
Rprd2	0.11	1.00000000	pigeons
Rnf126	0.13	1.00000000	pigeons
Ndufs8	0.12	1.00000000	pigeons
Ube2b	-0.01	1.00000000	pigeons
Xpr1	0.33	1.00000000	pigeons
Slc30a4	0.27	1.00000000	pigeons
Gcc1	0.12	1.00000000	pigeons
Traf2	0.28	1.00000000	pigeons
Adprm	0.24	1.00000000	pigeons
Pmm2	0.09	1.00000000	pigeons
Limk1	0.05	1.00000000	pigeons
Rnasel	0.27	1.00000000	pigeons
Reps1	0.17	1.00000000	pigeons
Cops2	0.07	1.00000000	pigeons
Stx2	0.05	1.00000000	pigeons
Rundc1	0.23	1.00000000	pigeons

Atp6v1d	0.01	1.00000000	pigeons
Comtd1	0.21	1.00000000	pigeons
Sin3b	0.13	1.00000000	pigeons
4933400A11Rik	0.22	1.00000000	pigeons
Psmd3	0.29	1.00000000	pigeons
Wbp2	0.06	1.00000000	pigeons
Ralgps1	0.28	1.00000000	pigeons
Dpy19l3	0.22	1.00000000	pigeons
Pwwp2a	0.14	1.00000000	pigeons
Usp46	-0.15	1.00000000	pigeons
Nol10	0.12	1.00000000	pigeons
Itfg1	0.09	1.00000000	pigeons
Oat	0.18	1.00000000	pigeons
Tcp1	-0.03	1.00000000	pigeons
Cep44	0.16	1.00000000	pigeons
Pusl1	0.20	1.00000000	pigeons
Csgalnact2	0.20	1.00000000	pigeons
Taf8	0.17	1.00000000	pigeons
Chst15	0.19	1.00000000	pigeons
Azi2	0.08	1.00000000	pigeons
Iws1	0.00	1.00000000	pigeons
Chaf1a	0.17	1.00000000	pigeons
Snx27	0.23	1.00000000	pigeons
Psma4	0.13	1.00000000	pigeons
Pcyt1a	0.15	1.00000000	pigeons
Slc25a26	0.24	1.00000000	pigeons
Rpp40	0.20	1.00000000	pigeons
Slain2	0.02	1.00000000	pigeons
Rlf	0.13	1.00000000	pigeons
Crbn	0.13	1.00000000	pigeons
Ilf3	-0.09	1.00000000	pigeons
Cramp1	-0.09	1.00000000	pigeons
Tepsin	0.18	1.00000000	pigeons
Sdf2l1	0.12	1.00000000	pigeons
Cpsf3	0.15	1.00000000	pigeons
Ppp1ca	0.20	1.00000000	pigeons
Cpsf2	0.24	1.00000000	pigeons
Tiprl	0.20	1.00000000	pigeons
Cwc15	-0.09	1.00000000	pigeons
Fam168b	-0.11	1.00000000	pigeons
Galnt10	0.24	1.00000000	pigeons
Selenof	0.11	1.00000000	pigeons
Ptprj	0.20	1.00000000	pigeons
Efl1	0.23	1.00000000	pigeons
Kpnb1	0.15	1.00000000	pigeons
Abracl	-0.05	1.00000000	pigeons

Eif3g	0.02	1.00000000	pigeons
Ndufa4	0.13	1.00000000	pigeons
Stx16	0.15	1.00000000	pigeons
Ranbp9	0.01	1.00000000	pigeons
Hacd2	0.08	1.00000000	pigeons
Gosr1	0.08	1.00000000	pigeons
Irak1bp1	0.23	1.00000000	pigeons
Nup88	0.10	1.00000000	pigeons
Idua	0.18	1.00000000	pigeons
Ctnna1	-0.09	1.00000000	pigeons
Rassf2	0.27	1.00000000	pigeons
Atp6v0a4	0.25	1.00000000	pigeons
Ehmt1	-0.08	1.00000000	pigeons
Ssbp3	0.11	1.00000000	pigeons
Jmjd7	0.15	1.00000000	pigeons
Trappc4	0.20	1.00000000	pigeons
Denr	0.13	1.00000000	pigeons
G3bp1	0.21	1.00000000	pigeons
Prkaa1	0.11	1.00000000	pigeons
Aig1	0.19	1.00000000	pigeons
Klhl28	0.22	1.00000000	pigeons
Lrrc31	0.21	1.00000000	pigeons
Sbds	-0.03	1.00000000	pigeons
Parp6	0.29	1.00000000	pigeons
Carmil2	0.17	1.00000000	pigeons
Caps2	0.20	1.00000000	pigeons
Xbp1	0.01	1.00000000	pigeons
Tnks2	0.15	1.00000000	pigeons
Heatr6	0.29	1.00000000	pigeons
Cct2	-0.10	1.00000000	pigeons
Dhx37	0.29	1.00000000	pigeons
Itm2a	-0.78	1.00000000	pigeons
Arid1b	0.22	1.00000000	pigeons
Odf2	0.19	1.00000000	pigeons
Ccdc47	0.28	1.00000000	pigeons
Timmdc1	0.09	1.00000000	pigeons
Tcirg1	0.32	1.00000000	pigeons
Prpf6	-0.03	1.00000000	pigeons
Pde7b	0.51	1.00000000	pigeons
Pdpr	0.25	1.00000000	pigeons
Cpsf4l	0.11	1.00000000	pigeons
Prdx4	0.19	1.00000000	pigeons
Dlgap4	0.06	1.00000000	pigeons
Pak2	0.11	1.00000000	pigeons
Cap1	0.25	1.00000000	pigeons
Hint3	0.23	1.00000000	pigeons

Fam168a	-0.98	1.00000000	pigeons
Psmd2	0.19	1.00000000	pigeons
Rnf114	0.14	1.00000000	pigeons
Prrc2c	0.15	1.00000000	pigeons
Igf2bp3	0.30	1.00000000	pigeons
Pdhb	0.29	1.00000000	pigeons
Prox1	0.32	1.00000000	pigeons
Atg12	0.21	1.00000000	pigeons
Pdgfb	-0.73	1.00000000	pigeons
Eea1	-0.03	1.00000000	pigeons
Ublcp1	0.03	1.00000000	pigeons
Cdk17	-1.05	1.00000000	pigeons
Csnk1e	0.02	1.00000000	pigeons
Antxr2	0.32	1.00000000	pigeons
Stoml2	0.16	1.00000000	pigeons
Tmem258	0.02	1.00000000	pigeons
Fkbp1a	-1.09	1.00000000	pigeons
Cds2	-0.11	1.00000000	pigeons
Ppib	-0.02	1.00000000	pigeons
Lmbr1	0.11	1.00000000	pigeons
Zmat5	0.17	1.00000000	pigeons
Vps13d	0.09	1.00000000	pigeons
Fbxo21	0.13	1.00000000	pigeons
Wdr1	-0.15	1.00000000	pigeons
Zdhhc17	0.12	1.00000000	pigeons
Socs1	0.24	1.00000000	pigeons
Nus1	0.17	1.00000000	pigeons
Zfp711	0.00	1.00000000	pigeons
Csnk2a2	0.09	1.00000000	pigeons
Wdr77	0.20	1.00000000	pigeons
Ascc1	-0.03	1.00000000	pigeons
Bicd2	-0.07	1.00000000	pigeons
Orc3	0.17	1.00000000	pigeons
Clp1	0.10	1.00000000	pigeons
Tcerg1	0.00	1.00000000	pigeons
Slc25a36	0.09	1.00000000	pigeons
Nrros	0.30	1.00000000	pigeons
Vezf1	0.17	1.00000000	pigeons
Hipk3	0.28	1.00000000	pigeons
Jagn1	0.19	1.00000000	pigeons
Dgkh	-1.04	1.00000000	pigeons
Lig3	0.23	1.00000000	pigeons
Cdkn1b	0.07	1.00000000	pigeons
Frs2	0.11	1.00000000	pigeons
Lpcat3	0.10	1.00000000	pigeons
Zc3h15	0.01	1.00000000	pigeons

Omg	0.28	1.00000000	pigeons
Angel2	0.19	1.00000000	pigeons
Psmg3	0.12	1.00000000	pigeons
Dram2	0.06	1.00000000	pigeons
Ubr5	-0.10	1.00000000	pigeons
Actl6a	0.27	1.00000000	pigeons
Decr1	0.21	1.00000000	pigeons
Capn15	0.14	1.00000000	pigeons
Ranbp17	-0.87	1.00000000	pigeons
Prrc2b	-0.01	1.00000000	pigeons
Ptges3	-0.05	1.00000000	pigeons
Smarcc1	0.13	1.00000000	pigeons
Nrd1	0.12	1.00000000	pigeons
Txnrd1	-1.13	1.00000000	pigeons
Tango2	0.25	1.00000000	pigeons
Susd1	-0.69	1.00000000	pigeons
Hspa9	-0.01	1.00000000	pigeons
Dynlrb1	0.20	1.00000000	pigeons
Fas	0.00	1.00000000	pigeons
Gramd1c	0.05	1.00000000	pigeons
Gpr155	0.10	1.00000000	pigeons
Adipor1	0.25	1.00000000	pigeons
Spen	0.18	1.00000000	pigeons
Bivm	0.31	1.00000000	pigeons
Limd2	0.13	1.00000000	pigeons
Dstn	-0.32	1.00000000	pigeons
Ppm1a	-0.04	1.00000000	pigeons
Haus2	0.15	1.00000000	pigeons
Sirt6	0.19	1.00000000	pigeons
Cog2	0.11	1.00000000	pigeons
Dnaja3	0.17	1.00000000	pigeons
Ugcg	0.15	1.00000000	pigeons
Zmynd11	0.02	1.00000000	pigeons
Nufip2	-0.18	1.00000000	pigeons
Slc23a2	-0.04	1.00000000	pigeons
Ndufb8	0.11	1.00000000	pigeons
Ypel5	-0.22	1.00000000	pigeons
Aebp2	0.09	1.00000000	pigeons
Pgap2	0.18	1.00000000	pigeons
Tpp2	-0.01	1.00000000	pigeons
Oaz1	0.10	1.00000000	pigeons
Lrba	0.10	1.00000000	pigeons
Pdk1	0.11	1.00000000	pigeons
Pik3cb	0.02	1.00000000	pigeons
Epb41l3	-0.39	1.00000000	pigeons
Ovch2	0.27	1.00000000	pigeons

Psmc2	0.12	1.00000000	pigeons
Tmem41b	0.18	1.00000000	pigeons
Ipo5	0.07	1.00000000	pigeons
Nrbp1	0.02	1.00000000	pigeons
Ube4a	0.04	1.00000000	pigeons
Kctd10	0.20	1.00000000	pigeons
Plk3	0.01	1.00000000	pigeons
B4galt4	-0.52	1.00000000	pigeons
Add1	0.13	1.00000000	pigeons
Smarca2	0.14	1.00000000	pigeons
Lingo2	0.74	1.00000000	pigeons
Ibtk	0.07	1.00000000	pigeons
Klf12	0.20	1.00000000	pigeons
Pbrm1	-0.06	1.00000000	pigeons
Ccnh	0.12	1.00000000	pigeons
Abhd17c	-0.05	1.00000000	pigeons
Ccdc91	0.32	1.00000000	pigeons
Wdr4	0.27	1.00000000	pigeons
Adipor2	0.03	1.00000000	pigeons
Pphln1	0.04	1.00000000	pigeons
Zmynd19	0.14	1.00000000	pigeons
Tfdp1	0.05	1.00000000	pigeons
Zrsr1	0.00	1.00000000	pigeons
Smg1	0.01	1.00000000	pigeons
Ric1	0.09	1.00000000	pigeons
Acss2	0.26	1.00000000	pigeons
Rbm6	-0.06	1.00000000	pigeons
Rala	-0.01	1.00000000	pigeons
Ube2j2	0.23	1.00000000	pigeons
Nek7	0.10	1.00000000	pigeons
Nr2c1	-0.09	1.00000000	pigeons
Blvra	0.22	1.00000000	pigeons
Qrich1	0.12	1.00000000	pigeons
Btbd2	0.19	1.00000000	pigeons
Ash2l	0.16	1.00000000	pigeons
Pdia4	-0.10	1.00000000	pigeons
Wsb2	0.05	1.00000000	pigeons
Ppp1r8	0.23	1.00000000	pigeons
Arel1	0.28	1.00000000	pigeons
Ndufs3	0.05	1.00000000	pigeons
Fh1	-0.03	1.00000000	pigeons
Ccdc66	0.17	1.00000000	pigeons
Arhgap45	0.17	1.00000000	pigeons
Scarb2	-1.15	1.00000000	pigeons
Atg2b	0.01	1.00000000	pigeons
Rc3h2	0.19	1.00000000	pigeons

Syngr3	0.09	1.00000000	pigeons
Htra2	0.16	1.00000000	pigeons
Eif4e	0.02	1.00000000	pigeons
Erbin	0.07	1.00000000	pigeons
Nup205	0.21	1.00000000	pigeons
Acbd5	0.14	1.00000000	pigeons
Nudt14	0.17	1.00000000	pigeons
Ccdc117	0.13	1.00000000	pigeons
Cbl	0.18	1.00000000	pigeons
Leo1	0.21	1.00000000	pigeons
Gtpbp1	0.29	1.00000000	pigeons
Cul2	0.09	1.00000000	pigeons
Fam126a	0.33	1.00000000	pigeons
Tmem181a	0.23	1.00000000	pigeons
Dnmt1	-0.03	1.00000000	pigeons
Arid4b	0.08	1.00000000	pigeons
Lcmt2	0.10	1.00000000	pigeons
Hmg20b	0.22	1.00000000	pigeons
Champ1	0.18	1.00000000	pigeons
Sufu	0.18	1.00000000	pigeons
Nfx1	0.08	1.00000000	pigeons
Chst10	0.19	1.00000000	pigeons
Khdrbs1	0.01	1.00000000	pigeons
Galnt12	0.31	1.00000000	pigeons
Uxs1	0.09	1.00000000	pigeons
Tdrd3	0.00	1.00000000	pigeons
Sppl3	0.02	1.00000000	pigeons
Pdss1	0.20	1.00000000	pigeons
Ttc21b	0.08	1.00000000	pigeons
Tmem263	0.30	1.00000000	pigeons
Arhgap21	-1.46	1.00000000	pigeons
Copa	0.22	1.00000000	pigeons
Otud7b	0.13	1.00000000	pigeons
Usp48	0.06	1.00000000	pigeons
Dynlt3	0.09	1.00000000	pigeons
Itsn2	0.07	1.00000000	pigeons
Agtpbp1	0.26	1.00000000	pigeons
Urb1	0.19	1.00000000	pigeons
Elovl5	0.02	1.00000000	pigeons
Xrn1	-0.03	1.00000000	pigeons
Pus7l	0.24	1.00000000	pigeons
Fbxw8	0.19	1.00000000	pigeons
Eef1e1	0.00	1.00000000	pigeons
Ranbp3	0.04	1.00000000	pigeons
Psmd12	0.08	1.00000000	pigeons
Ccnc	0.25	1.00000000	pigeons

Anapc2	0.17	1.00000000	pigeons
Pld4	0.10	1.00000000	pigeons
Fbxo32	0.21	1.00000000	pigeons
Hic2	0.26	1.00000000	pigeons
Kif13a	0.14	1.00000000	pigeons
Zfp639	0.11	1.00000000	pigeons
Clpx	-0.06	1.00000000	pigeons
Phf2	-0.06	1.00000000	pigeons
Ppp2r2d	0.04	1.00000000	pigeons
Bmf	0.27	1.00000000	pigeons
Nsun5	0.19	1.00000000	pigeons
Ipo8	-0.02	1.00000000	pigeons
Thrap3	0.05	1.00000000	pigeons
Pum2	-0.03	1.00000000	pigeons
Cnksr2	0.35	1.00000000	pigeons
Ppil3	-0.07	1.00000000	pigeons
Npepl1	0.22	1.00000000	pigeons
Rab9b	0.21	1.00000000	pigeons
Srpr	0.08	1.00000000	pigeons
Zfp330	0.01	1.00000000	pigeons
Sun2	0.15	1.00000000	pigeons
Akap11	-0.15	1.00000000	pigeons
Tmem230	0.11	1.00000000	pigeons
Usf3	0.24	1.00000000	pigeons
Nfxl1	0.18	1.00000000	pigeons
Nudcd3	0.23	1.00000000	pigeons
Commd2	0.11	1.00000000	pigeons
Rasgef1b	0.33	1.00000000	pigeons
Ccr7	0.11	1.00000000	pigeons
Smchd1	0.10	1.00000000	pigeons
Fam162a	0.18	1.00000000	pigeons
Nucks1	-0.16	1.00000000	pigeons
Hscb	0.09	1.00000000	pigeons
Gcnt4	-0.02	1.00000000	pigeons
Cog6	0.04	1.00000000	pigeons
Eif1ad12	0.16	1.00000000	pigeons
Ndfip2	0.03	1.00000000	pigeons
Pop4	0.14	1.00000000	pigeons
Tnfrsf21	0.10	1.00000000	pigeons
Tmem18	0.15	1.00000000	pigeons
Lamtor2	-0.01	1.00000000	pigeons
Rfc1	-0.01	1.00000000	pigeons
Tomm70a	-0.03	1.00000000	pigeons
Rtf1	-0.10	1.00000000	pigeons
Rab22a	0.07	1.00000000	pigeons
Cd81	0.20	1.00000000	pigeons

Spz1	-0.07	1.00000000	pigeons
Rcor3	0.19	1.00000000	pigeons
Srsf10	-0.01	1.00000000	pigeons
Akt3	0.15	1.00000000	pigeons
Fastkd3	0.15	1.00000000	pigeons
Atg5	0.07	1.00000000	pigeons
Ddrgk1	0.14	1.00000000	pigeons
Pm20d2	-0.54	1.00000000	pigeons
Elmod2	0.09	1.00000000	pigeons
Gng2	0.17	1.00000000	pigeons
Psmc1	-0.02	1.00000000	pigeons
Acvr1	0.27	1.00000000	pigeons
Sun1	-0.11	1.00000000	pigeons
Fubp3	0.07	1.00000000	pigeons
Polr2e	0.04	1.00000000	pigeons
Tprgl	0.16	1.00000000	pigeons
Nxpe3	0.17	1.00000000	pigeons
Add3	0.11	1.00000000	pigeons
Nhp2	0.06	1.00000000	pigeons
Scyl2	-0.08	1.00000000	pigeons
Stk39	-0.01	1.00000000	pigeons
Zc3h7b	0.09	1.00000000	pigeons
Socs6	-0.05	1.00000000	pigeons
Acvr2a	0.09	1.00000000	pigeons
Katnbl1	0.06	1.00000000	pigeons
Ets1	-0.06	1.00000000	pigeons
Chmp5	-0.13	1.00000000	pigeons
Ube2d1	0.10	1.00000000	pigeons
Tex10	0.32	1.00000000	pigeons
Acsl4	0.09	1.00000000	pigeons
Cltc	-0.33	1.00000000	pigeons
Sertad2	-0.06	1.00000000	pigeons
Ufm1	-0.06	1.00000000	pigeons
Ruvbl1	0.12	1.00000000	pigeons
Lyn	0.24	1.00000000	pigeons
Ube2a	0.04	1.00000000	pigeons
Larp1b	-0.12	1.00000000	pigeons
Ddx23	-0.24	1.00000000	pigeons
Nelfa	0.11	1.00000000	pigeons
Rasgrp3	0.06	1.00000000	pigeons
Gtf3a	0.14	1.00000000	pigeons
Pdlim5	0.07	1.00000000	pigeons
Atp9b	-0.01	1.00000000	pigeons
Rab43	0.23	1.00000000	pigeons
Rpn2	0.05	1.00000000	pigeons
Cenpt	0.11	1.00000000	pigeons

Ccnl2	-0.10	1.00000000	pigeons
Gyg	-0.04	1.00000000	pigeons
Dap3	0.19	1.00000000	pigeons
Tcf3	-0.20	1.00000000	pigeons
Sec61a2	0.18	1.00000000	pigeons
Snx5	0.20	1.00000000	pigeons
Smtnl2	0.14	1.00000000	pigeons
Desi2	0.08	1.00000000	pigeons
Tnrc6a	-0.12	1.00000000	pigeons
Sash3	0.16	1.00000000	pigeons
Vapa	-0.03	1.00000000	pigeons
Atp6v0e	0.14	1.00000000	pigeons
Ube2k	-0.17	1.00000000	pigeons
Sdf4	0.07	1.00000000	pigeons
Zfp384	0.04	1.00000000	pigeons
Tpra1	0.02	1.00000000	pigeons
Paxbp1	0.04	1.00000000	pigeons
Eif3i	-0.45	1.00000000	pigeons
Supt4a	0.02	1.00000000	pigeons
Pnpt1	0.13	1.00000000	pigeons
Ccni	0.03	1.00000000	pigeons
Rer1	0.05	1.00000000	pigeons
Klhl22	0.02	1.00000000	pigeons
Eif6	0.10	1.00000000	pigeons
Vash1	0.08	1.00000000	pigeons
Polr2d	0.21	1.00000000	pigeons
Pebp1	-0.01	1.00000000	pigeons
Cntln	0.22	1.00000000	pigeons
Copg2	0.21	1.00000000	pigeons
Ttc7	0.15	1.00000000	pigeons
Sh3rf1	0.20	1.00000000	pigeons
Odc1	-0.03	1.00000000	pigeons
Taok3	0.06	1.00000000	pigeons
Tmem11	-0.09	1.00000000	pigeons
Rreb1	0.09	1.00000000	pigeons
Stk4	0.22	1.00000000	pigeons
Prkd1	0.21	1.00000000	pigeons
Bcas2	0.11	1.00000000	pigeons
Nbas	0.13	1.00000000	pigeons
Fto	0.13	1.00000000	pigeons
Smarce1	-0.03	1.00000000	pigeons
Cul5	-0.05	1.00000000	pigeons
Lonrf2	0.03	1.00000000	pigeons
Slc38a10	0.20	1.00000000	pigeons
Nup214	-0.11	1.00000000	pigeons
St6gal1	0.13	1.00000000	pigeons

Fxr1	-0.09	1.00000000	pigeons
Ciapin1	-0.09	1.00000000	pigeons
Ddx49	0.14	1.00000000	pigeons
Tnip1	-0.21	1.00000000	pigeons
Slc22a15	0.20	1.00000000	pigeons
Zc3h14	0.13	1.00000000	pigeons
Ormdl1	0.04	1.00000000	pigeons
Il18	0.22	1.00000000	pigeons
Sanbr	0.18	1.00000000	pigeons
Nop16	0.15	1.00000000	pigeons
Wbp1l	0.06	1.00000000	pigeons
Sppl2a	0.02	1.00000000	pigeons
Cops7a	-0.06	1.00000000	pigeons
Ap1m1	0.20	1.00000000	pigeons
Zbtb49	0.19	1.00000000	pigeons
Zfp618	0.14	1.00000000	pigeons
Appbp2	0.17	1.00000000	pigeons
Dync1i2	-0.02	1.00000000	pigeons
Cotl1	0.16	1.00000000	pigeons
Prkar1a	0.08	1.00000000	pigeons
Arl5a	0.06	1.00000000	pigeons
Eif2ak1	0.21	1.00000000	pigeons
Fem1a	0.03	1.00000000	pigeons
Sptan1	0.15	1.00000000	pigeons
Sltm	0.03	1.00000000	pigeons
Slc35b2	0.21	1.00000000	pigeons
Anxa6	-0.01	1.00000000	pigeons
Eml4	0.25	1.00000000	pigeons
Ptges2	0.07	1.00000000	pigeons
Cnot7	0.03	1.00000000	pigeons
Sugp1	0.20	1.00000000	pigeons
Trip4	0.15	1.00000000	pigeons
Samhd1	0.17	1.00000000	pigeons
Dhx8	-0.07	1.00000000	pigeons
Rnf216	0.10	1.00000000	pigeons
Ppp2ca	0.05	1.00000000	pigeons
Ercc8	0.12	1.00000000	pigeons
Hnrnph3	0.01	1.00000000	pigeons
Zfp143	0.01	1.00000000	pigeons
Carhsp1	0.14	1.00000000	pigeons
Slc25a24	0.04	1.00000000	pigeons
Smurf2	-0.77	1.00000000	pigeons
St6galnac4	0.09	1.00000000	pigeons
Spata2	0.10	1.00000000	pigeons
Grpel1	0.19	1.00000000	pigeons
Naa35	0.22	1.00000000	pigeons

Sec13	-0.07	1.00000000	pigeons
Ppp3ca	-0.16	1.00000000	pigeons
Nol11	0.06	1.00000000	pigeons
Btf3l4	-0.09	1.00000000	pigeons
Grhl1	0.28	1.00000000	pigeons
Nav3	0.00	1.00000000	pigeons
Phb2	0.04	1.00000000	pigeons
Exoc5	0.05	1.00000000	pigeons
Farp2	-0.49	1.00000000	pigeons
Sms	0.13	1.00000000	pigeons
Cul1	0.06	1.00000000	pigeons
Ndufa8	-0.12	1.00000000	pigeons
Tbc1d15	-0.01	1.00000000	pigeons
Gnl3	0.16	1.00000000	pigeons
Ifih1	0.09	1.00000000	pigeons
Fam13a	-0.71	1.00000000	pigeons
Ythdc2	0.09	1.00000000	pigeons
Chsy3	0.13	1.00000000	pigeons
Dalrd3	0.25	1.00000000	pigeons
Pabpc4	0.15	1.00000000	pigeons
Pspc1	-0.04	1.00000000	pigeons
Eif3j2	0.02	1.00000000	pigeons
Prkrip1	-0.02	1.00000000	pigeons
Fam104a	0.02	1.00000000	pigeons
Sf3a1	0.09	1.00000000	pigeons
Ap2b1	-0.02	1.00000000	pigeons
Xpo5	0.00	1.00000000	pigeons
Tox	0.53	1.00000000	pigeons
Jakmip2	0.00	1.00000000	pigeons
Sprtn	0.16	1.00000000	pigeons
Crnkl1	0.06	1.00000000	pigeons
E2f6	0.09	1.00000000	pigeons
Fmr1	-0.09	1.00000000	pigeons
Ifnar1	0.07	1.00000000	pigeons
Polr2f	0.09	1.00000000	pigeons
Ube2o	0.15	1.00000000	pigeons
Slc1a4	0.00	1.00000000	pigeons
Ubfd1	0.19	1.00000000	pigeons
Rsl1d1	0.00	1.00000000	pigeons
Cdk7	-0.10	1.00000000	pigeons
Ech1	0.13	1.00000000	pigeons
Ube2q1	-0.08	1.00000000	pigeons
Naf1	0.13	1.00000000	pigeons
Plrg1	0.00	1.00000000	pigeons
Dpm2	-0.05	1.00000000	pigeons
Fryl	-0.66	1.00000000	pigeons

Fam114a2	0.17	1.00000000	pigeons
Nkrf	0.13	1.00000000	pigeons
Eny2	0.03	1.00000000	pigeons
Dcaf13	0.08	1.00000000	pigeons
Slc7a3	0.19	1.00000000	pigeons
Abi2	0.12	1.00000000	pigeons
Ccdc82	0.21	1.00000000	pigeons
Zranb1	-0.10	1.00000000	pigeons
Secisbp2l	-0.20	1.00000000	pigeons
Crk	0.02	1.00000000	pigeons
Dclre1a	0.14	1.00000000	pigeons
Herpud1	-0.03	1.00000000	pigeons
Cdpf1	-0.33	1.00000000	pigeons
Ptpra	0.05	1.00000000	pigeons
Eif2b1	0.02	1.00000000	pigeons
Slc25a15	0.06	1.00000000	pigeons
Hmox2	0.17	1.00000000	pigeons
Lysmd2	-0.02	1.00000000	pigeons
Lbr	0.08	1.00000000	pigeons
Rsl24d1	0.07	1.00000000	pigeons
Parp9	0.16	1.00000000	pigeons
Pdcd4	0.16	1.00000000	pigeons
Trappc13	0.18	1.00000000	pigeons
Kin	-0.01	1.00000000	pigeons
Zfand5	0.13	1.00000000	pigeons
Ino80d	0.12	1.00000000	pigeons
Unk	-0.11	1.00000000	pigeons
Tmem50b	0.09	1.00000000	pigeons
Rnf13	0.09	1.00000000	pigeons
Cnot8	0.13	1.00000000	pigeons
Irak4	0.08	1.00000000	pigeons
Hlf	-0.80	1.00000000	pigeons
Sumf2	0.18	1.00000000	pigeons
Rere	0.05	1.00000000	pigeons
Dhrs7b	0.17	1.00000000	pigeons
Vps35	-0.16	1.00000000	pigeons
Nabp1	0.10	1.00000000	pigeons
Sel1l	0.12	1.00000000	pigeons
Ndufb9	-0.17	1.00000000	pigeons
Tob2	-0.13	1.00000000	pigeons
Wdr3	0.09	1.00000000	pigeons
Pdzd8	0.19	1.00000000	pigeons
Fyttd1	-0.06	1.00000000	pigeons
Exoc8	-0.03	1.00000000	pigeons
Gid8	-0.11	1.00000000	pigeons
Usp8	-0.02	1.00000000	pigeons

Zzz3	-0.07	1.00000000	pigeons
Prkar2b	0.20	1.00000000	pigeons
Naxd	0.06	1.00000000	pigeons
Il17ra	0.22	1.00000000	pigeons
Aff4	-0.07	1.00000000	pigeons
Ing3	0.05	1.00000000	pigeons
Pex14	0.13	1.00000000	pigeons
Bbs2	0.09	1.00000000	pigeons
Ralgapa1	-0.04	1.00000000	pigeons
Atp6v0d1	0.08	1.00000000	pigeons
Ap3m1	0.11	1.00000000	pigeons
Psmd6	0.11	1.00000000	pigeons
Plekha2	0.26	1.00000000	pigeons
Zbtb43	0.00	1.00000000	pigeons
Slc35b1	0.10	1.00000000	pigeons
Stag2	0.07	1.00000000	pigeons
Gtf3c1	0.20	1.00000000	pigeons
Hectd4	0.11	1.00000000	pigeons
Psma7	0.09	1.00000000	pigeons
Lmo2	-0.74	1.00000000	pigeons
Necap1	0.18	1.00000000	pigeons
Fbxo22	0.11	1.00000000	pigeons
Tsen2	0.15	1.00000000	pigeons
Arglu1	-0.04	1.00000000	pigeons
Akirin2	-0.16	1.00000000	pigeons
Celf2	0.10	1.00000000	pigeons
Dek	0.12	1.00000000	pigeons
Rabep1	0.07	1.00000000	pigeons
Ncald	0.24	1.00000000	pigeons
Fbxw7	0.10	1.00000000	pigeons
Dvl1	0.10	1.00000000	pigeons
Usp47	-0.05	1.00000000	pigeons
Tango6	0.06	1.00000000	pigeons
Sec22c	0.11	1.00000000	pigeons
Atp23	0.20	1.00000000	pigeons
Rars2	0.20	1.00000000	pigeons
Nolc1	0.21	1.00000000	pigeons
Rbm38	0.20	1.00000000	pigeons
Zfp319	0.03	1.00000000	pigeons
Klhdc2	0.14	1.00000000	pigeons
Wipi1	0.16	1.00000000	pigeons
Asxl1	0.10	1.00000000	pigeons
Dtd2	0.09	1.00000000	pigeons
Psmc3	0.00	1.00000000	pigeons
Asf1a	0.16	1.00000000	pigeons
Qser1	-0.05	1.00000000	pigeons

Ubr1	0.03	1.00000000	pigeons
Ell	0.08	1.00000000	pigeons
Pias4	-0.03	1.00000000	pigeons
Elf2	-0.07	1.00000000	pigeons
Kansl3	0.21	1.00000000	pigeons
Snip1	0.05	1.00000000	pigeons
Bsdc1	0.08	1.00000000	pigeons
Vps26a	0.10	1.00000000	pigeons
Strada	0.19	1.00000000	pigeons
Nmd3	0.08	1.00000000	pigeons
Yeats2	0.15	1.00000000	pigeons
Srfbp1	0.02	1.00000000	pigeons
Rsbn1	0.09	1.00000000	pigeons
Aqr	-0.06	1.00000000	pigeons
Tmem33	0.11	1.00000000	pigeons
Sdhaf2	0.09	1.00000000	pigeons
Pofut1	0.20	1.00000000	pigeons
Nipal3	-0.08	1.00000000	pigeons
Tnik	-0.09	1.00000000	pigeons
Pno1	0.22	1.00000000	pigeons
Dync1li2	-0.09	1.00000000	pigeons
Fam135a	0.01	1.00000000	pigeons
Rbpj	-0.33	1.00000000	pigeons
Ireb2	0.01	1.00000000	pigeons
Gabpa	-0.05	1.00000000	pigeons
Cnot4	-0.06	1.00000000	pigeons
Dpy19l4	0.29	1.00000000	pigeons
Cdc42bpb	-0.20	1.00000000	pigeons
Ranbp1	-0.18	1.00000000	pigeons
Sdf2	0.16	1.00000000	pigeons
Tsnax	-0.02	1.00000000	pigeons
Brwd1	-0.12	1.00000000	pigeons
Lsm8	-0.06	1.00000000	pigeons
Prr14l	0.06	1.00000000	pigeons
Cers5	0.14	1.00000000	pigeons
Ttll12	0.01	1.00000000	pigeons
Galc	0.21	1.00000000	pigeons
Spg21	0.12	1.00000000	pigeons
F2r	-0.84	1.00000000	pigeons
Thumpd3	-0.19	1.00000000	pigeons
Sde2	-0.01	1.00000000	pigeons
Cbfb	-0.27	1.00000000	pigeons
Ttc33	0.02	1.00000000	pigeons
Zfp512b	0.16	1.00000000	pigeons
Nono	0.07	1.00000000	pigeons
Naa30	-0.03	1.00000000	pigeons

Vps11	-0.21	1.00000000	pigeons
Ndufb10	-0.16	1.00000000	pigeons
Iars2	0.05	1.00000000	pigeons
Chmp7	-0.01	1.00000000	pigeons
Rexo1	0.09	1.00000000	pigeons
Osbpl3	0.03	1.00000000	pigeons
Prmt3	0.15	1.00000000	pigeons
Uba3	0.17	1.00000000	pigeons
Psmd14	0.02	1.00000000	pigeons
Tnpo1	-0.07	1.00000000	pigeons
Gtf2b	-0.06	1.00000000	pigeons
Tm9sf2	-0.22	1.00000000	pigeons
Zfp609	0.26	1.00000000	pigeons
Fuca2	0.17	1.00000000	pigeons
Oard1	0.02	1.00000000	pigeons
Arhgef18	0.11	1.00000000	pigeons
Pde3b	0.17	1.00000000	pigeons
Gab3	0.29	1.00000000	pigeons
Phf21a	0.07	1.00000000	pigeons
Bloc1s6	-0.16	1.00000000	pigeons
Tlk2	-0.02	1.00000000	pigeons
Slc35b3	0.12	1.00000000	pigeons
Cry2	0.14	1.00000000	pigeons
Pms2	0.22	1.00000000	pigeons
Cbr4	0.05	1.00000000	pigeons
Psma6	0.06	1.00000000	pigeons
Lat2	0.12	1.00000000	pigeons
Eri3	0.05	1.00000000	pigeons
Lonp2	0.06	1.00000000	pigeons
Pcnx2	-0.04	1.00000000	pigeons
Rad54l2	-0.07	1.00000000	pigeons
Zfp654	-0.07	1.00000000	pigeons
Esrp2	0.08	1.00000000	pigeons
Sike1	0.03	1.00000000	pigeons
Stim1	0.05	1.00000000	pigeons
Nup93	0.26	1.00000000	pigeons
Stk38l	0.21	1.00000000	pigeons
Esf1	-0.16	1.00000000	pigeons
Shisa5	0.07	1.00000000	pigeons
Zfp512	0.01	1.00000000	pigeons
Dnajc15	0.03	1.00000000	pigeons
Dgkq	0.06	1.00000000	pigeons
Dpf3	0.00	1.00000000	pigeons
Dhx40	0.17	1.00000000	pigeons
Psmc5	0.00	1.00000000	pigeons
Ogfrl1	0.12	1.00000000	pigeons

Tmco1	0.09	1.00000000	pigeons
Slf1	-0.22	1.00000000	pigeons
Dcp1a	0.17	1.00000000	pigeons
Invs	0.10	1.00000000	pigeons
Serac1	0.03	1.00000000	pigeons
Usp22	-0.88	1.00000000	pigeons
Ippk	-0.15	1.00000000	pigeons
Wdr70	-0.06	1.00000000	pigeons
Prdx3	-0.05	1.00000000	pigeons
Ubn1	-0.11	1.00000000	pigeons
Cenpc1	0.00	1.00000000	pigeons
Trp53rka	0.17	1.00000000	pigeons
Tbxa2r	0.07	1.00000000	pigeons
Slc25a13	0.09	1.00000000	pigeons
Tspan3	-0.95	1.00000000	pigeons
Ifnar2	-0.12	1.00000000	pigeons
Eif4g1	0.10	1.00000000	pigeons
Npc2	-0.82	1.00000000	pigeons
Smg7	0.08	1.00000000	pigeons
Napepld	-0.10	1.00000000	pigeons
Noc3l	0.06	1.00000000	pigeons
Dpy30	0.17	1.00000000	pigeons
Got1	0.20	1.00000000	pigeons
Ap1s3	0.06	1.00000000	pigeons
Trmt6	0.07	1.00000000	pigeons
Txlna	0.15	1.00000000	pigeons
Ywhaq	-0.51	1.00000000	pigeons
Sik3	-0.02	1.00000000	pigeons
Psma3	-0.15	1.00000000	pigeons
Senp6	0.00	1.00000000	pigeons
Aph1a	0.07	1.00000000	pigeons
Insr	-0.19	1.00000000	pigeons
Gpn3	0.01	1.00000000	pigeons
Aspscr1	-0.02	1.00000000	pigeons
Clasp2	0.11	1.00000000	pigeons
Poldip3	0.12	1.00000000	pigeons
Coro1c	0.21	1.00000000	pigeons
Zcchc7	0.17	1.00000000	pigeons
Inpp5a	-0.62	1.00000000	pigeons
Tcf25	0.13	1.00000000	pigeons
Gcn1	0.01	1.00000000	pigeons
Tfb1m	0.18	1.00000000	pigeons
Rnf20	0.09	1.00000000	pigeons
Erh	0.05	1.00000000	pigeons
Bicral	-0.25	1.00000000	pigeons
Clptm1l	-0.19	1.00000000	pigeons

Pdf	0.04	1.00000000	pigeons
Ccne1	0.12	1.00000000	pigeons
Rbm15b	-0.01	1.00000000	pigeons
Commd6	0.11	1.00000000	pigeons
Phf10	0.02	1.00000000	pigeons
Fyco1	0.02	1.00000000	pigeons
Zbtb41	0.05	1.00000000	pigeons
Hdac1	0.16	1.00000000	pigeons
Spg11	-0.05	1.00000000	pigeons
Acvr1b	0.14	1.00000000	pigeons
Slc9a8	0.19	1.00000000	pigeons
Lamtor3	-0.08	1.00000000	pigeons
Tatdn1	0.16	1.00000000	pigeons
Itsn1	0.19	1.00000000	pigeons
Rab10	-0.10	1.00000000	pigeons
Fancd2	0.10	1.00000000	pigeons
Uggt1	-0.05	1.00000000	pigeons
Lnpk	0.09	1.00000000	pigeons
Abcc4	0.16	1.00000000	pigeons
Phkb	0.03	1.00000000	pigeons
Crebrf	-0.18	1.00000000	pigeons
Kdm4c	0.10	1.00000000	pigeons
Cwc27	0.10	1.00000000	pigeons
Cggbp1	0.02	1.00000000	pigeons
Pmpcb	0.15	1.00000000	pigeons
Fam3c	0.15	1.00000000	pigeons
Ngly1	-0.18	1.00000000	pigeons
Aup1	0.17	1.00000000	pigeons
Dync1li1	-0.04	1.00000000	pigeons
Arxes2	-0.22	1.00000000	pigeons
Anapc5	0.08	1.00000000	pigeons
Pank1	0.34	1.00000000	pigeons
Exosc7	0.16	1.00000000	pigeons
Trip12	-0.31	1.00000000	pigeons
Klhl2	0.19	1.00000000	pigeons
Wdr89	0.21	1.00000000	pigeons
Parn	-0.10	1.00000000	pigeons
U2af1	-0.03	1.00000000	pigeons
Ddb1	0.12	1.00000000	pigeons
Phf13	0.15	1.00000000	pigeons
Bdp1	-0.03	1.00000000	pigeons
Phf20l1	0.13	1.00000000	pigeons
Ints1	0.06	1.00000000	pigeons
Pls3	-1.21	1.00000000	pigeons
Arcn1	-0.06	1.00000000	pigeons
Ube2g2	-0.08	1.00000000	pigeons

Tmem138	0.05	1.00000000	pigeons
Socs7	0.24	1.00000000	pigeons
Nhlrc2	0.25	1.00000000	pigeons
Asb3	-0.11	1.00000000	pigeons
Drg2	-0.04	1.00000000	pigeons
Thoc1	-0.23	1.00000000	pigeons
Gpi1	-0.08	1.00000000	pigeons
Vti1b	-0.10	1.00000000	pigeons
Suclg2	0.21	1.00000000	pigeons
Snrpa1	-0.13	1.00000000	pigeons
Zmat2	0.09	1.00000000	pigeons
Prkab1	0.11	1.00000000	pigeons
Sod2	0.02	1.00000000	pigeons
Pigk	-0.03	1.00000000	pigeons
Trappc12	-0.11	1.00000000	pigeons
Eif5a2	0.01	1.00000000	pigeons
Cacna1d	0.51	1.00000000	pigeons
Rabgap1	-0.07	1.00000000	pigeons
Apoo	0.04	1.00000000	pigeons
Kmt2c	0.04	1.00000000	pigeons
Pik3r4	0.15	1.00000000	pigeons
Rnf4	0.10	1.00000000	pigeons
Tmem65	0.12	1.00000000	pigeons
Trabd	0.03	1.00000000	pigeons
Setx	0.03	1.00000000	pigeons
Fam184a	0.23	1.00000000	pigeons
Agps	0.05	1.00000000	pigeons
Vps41	0.00	1.00000000	pigeons
Upf3b	0.05	1.00000000	pigeons
Bcl6	0.21	1.00000000	pigeons
Ppp2r5c	-0.05	1.00000000	pigeons
Ercc3	-0.15	1.00000000	pigeons
Nfatc2	0.00	1.00000000	pigeons
Sirt1	-0.09	1.00000000	pigeons
Hmbox1	0.10	1.00000000	pigeons
Nt5c3	0.05	1.00000000	pigeons
Rpap2	-0.05	1.00000000	pigeons
Eftud2	0.13	1.00000000	pigeons
Prkag2	0.08	1.00000000	pigeons
Esd	-0.01	1.00000000	pigeons
Tmem35b	-0.03	1.00000000	pigeons
Haao	-0.04	1.00000000	pigeons
Scp2	-0.01	1.00000000	pigeons
Tulp4	0.12	1.00000000	pigeons
Hook3	-0.16	1.00000000	pigeons
Dennd1a	-0.02	1.00000000	pigeons

Tlnrd1	-0.86	1.00000000	pigeons
Ddhd2	0.16	1.00000000	pigeons
Baz1b	-0.11	1.00000000	pigeons
Dr1	-0.07	1.00000000	pigeons
Upf2	-0.17	1.00000000	pigeons
Pten	0.00	1.00000000	pigeons
Tmx1	-0.15	1.00000000	pigeons
Pikfyve	0.07	1.00000000	pigeons
Tsn	0.00	1.00000000	pigeons
Pgs1	0.06	1.00000000	pigeons
Rab9	0.09	1.00000000	pigeons
Rab14	-0.02	1.00000000	pigeons
Slk	-0.13	1.00000000	pigeons
Nfyb	0.03	1.00000000	pigeons
Atp2b1	-0.08	1.00000000	pigeons
Dnajb12	-0.09	1.00000000	pigeons
Rab3gap1	0.05	1.00000000	pigeons
Actr1a	-0.02	1.00000000	pigeons
Arid3b	0.04	1.00000000	pigeons
Sft2d1	-0.15	1.00000000	pigeons
Washc3	0.04	1.00000000	pigeons
Rtcb	0.11	1.00000000	pigeons
F5	-0.98	1.00000000	pigeons
Ints8	-0.02	1.00000000	pigeons
Dffa	0.06	1.00000000	pigeons
Itgav	0.08	1.00000000	pigeons
Rnf149	-0.58	1.00000000	pigeons
Nudcd1	0.24	1.00000000	pigeons
Timm13	-0.04	1.00000000	pigeons
Flnb	-0.18	1.00000000	pigeons
Trappc3	0.10	1.00000000	pigeons
Rabepk	-0.12	1.00000000	pigeons
Pank4	0.10	1.00000000	pigeons
Rnf141	0.05	1.00000000	pigeons
Casp2	0.07	1.00000000	pigeons
Svil	-0.64	1.00000000	pigeons
Hspa14	0.17	1.00000000	pigeons
Smarcc2	0.17	1.00000000	pigeons
Drosha	0.13	1.00000000	pigeons
Yod1	0.20	1.00000000	pigeons
Ube2v2	0.05	1.00000000	pigeons
Ubxn6	0.13	1.00000000	pigeons
Sap30l	0.10	1.00000000	pigeons
Rhot1	0.01	1.00000000	pigeons
Suco	0.00	1.00000000	pigeons
Acox1	-0.03	1.00000000	pigeons

Ccdc127	0.04	1.00000000	pigeons
Snx13	-0.05	1.00000000	pigeons
Pacs2	0.12	1.00000000	pigeons
Vps39	0.15	1.00000000	pigeons
Tbpl1	-0.08	1.00000000	pigeons
Ybx1	-0.35	1.00000000	pigeons
Atp13a1	0.03	1.00000000	pigeons
Ppip5k2	0.17	1.00000000	pigeons
Cc2d1b	0.06	1.00000000	pigeons
Rbfox2	-0.93	1.00000000	pigeons
St3gal1	-0.76	1.00000000	pigeons
Yipf4	-0.03	1.00000000	pigeons
Plbd1	0.12	1.00000000	pigeons
Txndc12	-0.02	1.00000000	pigeons
Cisd1	-0.01	1.00000000	pigeons
Ryk	0.11	1.00000000	pigeons
Aco2	0.02	1.00000000	pigeons
Rab11fip2	0.04	1.00000000	pigeons
Ep400	-0.13	1.00000000	pigeons
Trappc8	-0.11	1.00000000	pigeons
Ctso	0.14	1.00000000	pigeons
Zfp106	0.19	1.00000000	pigeons
Sec11c	-0.07	1.00000000	pigeons
Exoc1	-0.09	1.00000000	pigeons
Cpeb2	0.14	1.00000000	pigeons
Fgd6	0.14	1.00000000	pigeons
Wdr25	0.06	1.00000000	pigeons
Wwc1	0.18	1.00000000	pigeons
St7l	0.02	1.00000000	pigeons
Rubcn	0.17	1.00000000	pigeons
Erich1	-0.04	1.00000000	pigeons
Eif2d	0.03	1.00000000	pigeons
Cdc40	-0.04	1.00000000	pigeons
Lactb	0.09	1.00000000	pigeons
Nlrc5	0.01	1.00000000	pigeons
Ddx24	0.06	1.00000000	pigeons
Taf5	0.11	1.00000000	pigeons
Ubxn7	0.14	1.00000000	pigeons
R3hdm4	0.17	1.00000000	pigeons
Kdm5a	-0.01	1.00000000	pigeons
Tiam2	-0.17	1.00000000	pigeons
Lyplal1	-0.04	1.00000000	pigeons
Alg3	0.14	1.00000000	pigeons
Kidins220	-0.25	1.00000000	pigeons
E2f5	0.19	1.00000000	pigeons
Utp18	0.06	1.00000000	pigeons

Acp6	0.13	1.00000000	pigeons
Nr1h3	-0.14	1.00000000	pigeons
Cubn	0.17	1.00000000	pigeons
Cdc34	-0.07	1.00000000	pigeons
Etf1	-0.12	1.00000000	pigeons
Ctps2	0.03	1.00000000	pigeons
Bop1	-0.01	1.00000000	pigeons
Ubp1	-0.14	1.00000000	pigeons
Dnajc17	0.12	1.00000000	pigeons
Adi1	0.03	1.00000000	pigeons
Hvcn1	0.10	1.00000000	pigeons
Pum3	0.09	1.00000000	pigeons
Cln5	0.03	1.00000000	pigeons
Sumo1	-0.06	1.00000000	pigeons
Zdhhc13	0.13	1.00000000	pigeons
Ints10	0.06	1.00000000	pigeons
Zbtb46	-0.04	1.00000000	pigeons
Pop5	0.18	1.00000000	pigeons
Dbndd2	0.17	1.00000000	pigeons
Xrcc5	0.08	1.00000000	pigeons
Lrpap1	-0.10	1.00000000	pigeons
Nup98	-0.18	1.00000000	pigeons
Tmem9b	-0.08	1.00000000	pigeons
Ppm1g	0.11	1.00000000	pigeons
Ung	0.14	1.00000000	pigeons
Crlf3	0.12	1.00000000	pigeons
Entpd4	-0.02	1.00000000	pigeons
Stk11	0.19	1.00000000	pigeons
Dus1l	0.16	1.00000000	pigeons
Ppp1r13b	-0.03	1.00000000	pigeons
Dhx29	0.01	1.00000000	pigeons
Ufl1	-0.14	1.00000000	pigeons
Thoc2	-0.06	1.00000000	pigeons
Nfat5	-0.25	1.00000000	pigeons
Lemd3	-0.09	1.00000000	pigeons
Acadl	-0.13	1.00000000	pigeons
Snrnp25	0.07	1.00000000	pigeons
Dph5	0.06	1.00000000	pigeons
Rrn3	-0.06	1.00000000	pigeons
Cnot6l	0.06	1.00000000	pigeons
G3bp2	0.00	1.00000000	pigeons
Dip2a	0.11	1.00000000	pigeons
Lap3	0.06	1.00000000	pigeons
Sav1	-0.07	1.00000000	pigeons
Dut	-0.03	1.00000000	pigeons
Plcb2	0.13	1.00000000	pigeons

Usp37	0.03	1.00000000	pigeons
Sec24a	-0.05	1.00000000	pigeons
Gnpda2	-0.05	1.00000000	pigeons
Stx12	-0.30	1.00000000	pigeons
Riok1	0.04	1.00000000	pigeons
Rnaseh1	-0.09	1.00000000	pigeons
Prpf39	-0.17	1.00000000	pigeons
Chuk	0.04	1.00000000	pigeons
Nup54	0.04	1.00000000	pigeons
Timeless	0.12	1.00000000	pigeons
Gemin2	0.19	1.00000000	pigeons
Abcd2	-0.22	1.00000000	pigeons
Cul4b	-0.16	1.00000000	pigeons
Ndufs1	0.08	1.00000000	pigeons
Smarca5	-0.26	1.00000000	pigeons
Ing1	-0.01	1.00000000	pigeons
Dnajc19	-0.03	1.00000000	pigeons
Lrch1	0.13	1.00000000	pigeons
Atad3a	0.17	1.00000000	pigeons
Dhx30	0.09	1.00000000	pigeons
Nfs1	0.20	1.00000000	pigeons
Stim2	-0.22	1.00000000	pigeons
Txndc9	-0.10	1.00000000	pigeons
Eif1	0.16	1.00000000	pigeons
Dnaja1	0.00	1.00000000	pigeons
Foxk1	-0.11	1.00000000	pigeons
Cnot11	0.07	1.00000000	pigeons
Ndufb3	-0.08	1.00000000	pigeons
Adam17	0.03	1.00000000	pigeons
Dis3	-0.07	1.00000000	pigeons
Rbx1	0.00	1.00000000	pigeons
Slc25a3	-0.12	1.00000000	pigeons
Bhlhe40	-0.73	1.00000000	pigeons
Tdg	-0.05	1.00000000	pigeons
Hdgfl3	-0.08	1.00000000	pigeons
Vamp3	0.07	1.00000000	pigeons
Itfg2	-0.27	1.00000000	pigeons
Zfp276	0.03	1.00000000	pigeons
Gabpb1	0.04	1.00000000	pigeons
Eci1	0.12	1.00000000	pigeons
Esyt2	0.01	1.00000000	pigeons
Snx1	-0.05	1.00000000	pigeons
Cog1	0.15	1.00000000	pigeons
Gcc2	0.02	1.00000000	pigeons
Dusp7	-0.30	1.00000000	pigeons
Tacc3	0.08	1.00000000	pigeons

Rpgrip1l	0.03	1.00000000	pigeons
Wdr36	-0.02	1.00000000	pigeons
Shoc2	-0.11	1.00000000	pigeons
Rheb	-0.33	1.00000000	pigeons
Gnpat	0.12	1.00000000	pigeons
Eefsec	-0.06	1.00000000	pigeons
Prkca	0.11	1.00000000	pigeons
Gabarapl2	-0.02	1.00000000	pigeons
Dip2b	0.19	1.00000000	pigeons
Gpsm2	-0.05	1.00000000	pigeons
Tomm20	-0.26	1.00000000	pigeons
Zfc3h1	-0.15	1.00000000	pigeons
Ly96	0.04	1.00000000	pigeons
Fam177a	-0.97	1.00000000	pigeons
Hdac2	-0.20	1.00000000	pigeons
Vamp4	0.11	1.00000000	pigeons
Cc2d2a	-0.02	1.00000000	pigeons
Snx4	0.09	1.00000000	pigeons
Amdhd2	-0.48	1.00000000	pigeons
Tnks	-0.09	1.00000000	pigeons
Selenok	0.00	1.00000000	pigeons
Acaa2	-0.09	1.00000000	pigeons
Rpa3	0.10	1.00000000	pigeons
Dusp5	0.10	1.00000000	pigeons
Armc8	-0.11	1.00000000	pigeons
Cyth3	-0.79	1.00000000	pigeons
Eef2	0.07	1.00000000	pigeons
Taf12	0.03	1.00000000	pigeons
Ttf1	-0.03	1.00000000	pigeons
Tcf20	0.05	1.00000000	pigeons
Trmt2a	0.08	1.00000000	pigeons
Sqstm1	-0.12	1.00000000	pigeons
Cct6b	-0.10	1.00000000	pigeons
Cnst	0.03	1.00000000	pigeons
Csde1	-0.07	1.00000000	pigeons
Rpa1	-0.04	1.00000000	pigeons
Tshz2	0.08	1.00000000	pigeons
Sema4b	0.11	1.00000000	pigeons
Yeats4	0.11	1.00000000	pigeons
Commd1b	-0.05	1.00000000	pigeons
Usp6nl	-0.13	1.00000000	pigeons
Trub1	0.05	1.00000000	pigeons
Plin2	0.04	1.00000000	pigeons
Rrbp1	0.18	1.00000000	pigeons
Fam234b	0.10	1.00000000	pigeons
Nup210	0.02	1.00000000	pigeons

Klhl8	-0.06	1.00000000	pigeons
Ap3b1	-0.10	1.00000000	pigeons
Klhl18	0.14	1.00000000	pigeons
Uba6	0.09	1.00000000	pigeons
Nap1l4	0.02	1.00000000	pigeons
Rnf11	-0.14	1.00000000	pigeons
Coq9	0.13	1.00000000	pigeons
Dennd1b	0.00	1.00000000	pigeons
2510039O18Rik	0.05	1.00000000	pigeons
Brix1	0.04	1.00000000	pigeons
Nsl1	-0.02	1.00000000	pigeons
Fbxl4	-0.02	1.00000000	pigeons
Rbm31y	-0.47	1.00000000	pigeons
Tfdp2	0.00	1.00000000	pigeons
Usb1	0.10	1.00000000	pigeons
Fbxo38	0.04	1.00000000	pigeons
Lrif1	0.08	1.00000000	pigeons
Dync1h1	-0.35	1.00000000	pigeons
Supt3	0.02	1.00000000	pigeons
Ubr2	-0.03	1.00000000	pigeons
Kat14	0.01	1.00000000	pigeons
Zfp469	0.01	1.00000000	pigeons
Klhdc4	-0.03	1.00000000	pigeons
Nip7	0.08	1.00000000	pigeons
Txndc17	-0.02	1.00000000	pigeons
Dpysl2	-0.84	1.00000000	pigeons
Taf11	-0.04	1.00000000	pigeons
Aurkaip1	-0.11	1.00000000	pigeons
Acsl1	0.16	1.00000000	pigeons
Srebf2	-0.06	1.00000000	pigeons
Lemd2	-0.10	1.00000000	pigeons
Tex9	0.11	1.00000000	pigeons
Lamc1	-0.80	1.00000000	pigeons
Llph	-0.13	1.00000000	pigeons
Bud31	-0.04	1.00000000	pigeons
Nxt2	0.07	1.00000000	pigeons
Asxl3	-0.57	1.00000000	pigeons
Tsr3	-0.05	1.00000000	pigeons
Rexo5	0.17	1.00000000	pigeons
Spryd7	0.15	1.00000000	pigeons
Rnf145	0.14	1.00000000	pigeons
Rabl3	0.10	1.00000000	pigeons
Tnr	0.18	1.00000000	pigeons
Arid4a	-0.09	1.00000000	pigeons
Larp1	0.05	1.00000000	pigeons
Gosr2	0.12	1.00000000	pigeons

Cnpy3	-0.10	1.00000000	pigeons
Jun	-0.69	1.00000000	pigeons
Nek6	0.01	1.00000000	pigeons
Ctnnb1	-0.71	1.00000000	pigeons
Serinc5	-0.16	1.00000000	pigeons
Wipi2	0.06	1.00000000	pigeons
Erp44	-0.15	1.00000000	pigeons
Jak2	-0.12	1.00000000	pigeons
Borcs7	0.06	1.00000000	pigeons
Hcrt	0.17	1.00000000	pigeons
Gtf2a1	-0.27	1.00000000	pigeons
Echdc2	0.12	1.00000000	pigeons
Wwox	0.08	1.00000000	pigeons
Stam	-0.10	1.00000000	pigeons
Syap1	-0.21	1.00000000	pigeons
Thoc5	-0.10	1.00000000	pigeons
Dynlt1a	0.12	1.00000000	pigeons
Pdxdc1	-0.10	1.00000000	pigeons
Nelfcd	0.00	1.00000000	pigeons
Sbf2	-0.13	1.00000000	pigeons
Wbp2nl	-0.07	1.00000000	pigeons
Prdx1	0.04	1.00000000	pigeons
Polr2h	0.15	1.00000000	pigeons
Rnf8	0.09	1.00000000	pigeons
Tcf7	0.39	1.00000000	pigeons
Dtx1	0.16	1.00000000	pigeons
1700034I23Rik	0.14	1.00000000	pigeons
Dcps	-0.02	1.00000000	pigeons
Fam149b	0.09	1.00000000	pigeons
Slc2a8	0.05	1.00000000	pigeons
Pitpna	-0.25	1.00000000	pigeons
Eif3h	0.03	1.00000000	pigeons
Ppp6r3	-0.03	1.00000000	pigeons
Kat6a	-0.06	1.00000000	pigeons
Utp11	-0.01	1.00000000	pigeons
Arpc5l	0.07	1.00000000	pigeons
Usp14	-0.25	1.00000000	pigeons
Hadhb	0.03	1.00000000	pigeons
Ttc39a	0.03	1.00000000	pigeons
Gatad2a	-0.05	1.00000000	pigeons
Kcnq5	0.24	1.00000000	pigeons
Tmem167	-0.04	1.00000000	pigeons
Csnk2a1	-0.19	1.00000000	pigeons
Acp2	0.13	1.00000000	pigeons
Fxn	0.09	1.00000000	pigeons
Ppp1cb	-0.01	1.00000000	pigeons

Anxa2	0.03	1.00000000	pigeons
Tbc1d22b	0.07	1.00000000	pigeons
Spx	0.08	1.00000000	pigeons
Noct	-0.22	1.00000000	pigeons
Elac2	0.08	1.00000000	pigeons
Phtf2	-0.21	1.00000000	pigeons
Hsbp1	-0.22	1.00000000	pigeons
Angel1	-0.10	1.00000000	pigeons
Idh2	-0.11	1.00000000	pigeons
Zdhhc9	0.02	1.00000000	pigeons
Uchl3	0.14	1.00000000	pigeons
Anapc7	0.00	1.00000000	pigeons
Ubap2	-0.06	1.00000000	pigeons
Tmem245	0.06	1.00000000	pigeons
Elp4	0.02	1.00000000	pigeons
Dctn4	-0.01	1.00000000	pigeons
Riok3	-0.10	1.00000000	pigeons
Api5	-0.05	1.00000000	pigeons
Zfp236	-0.01	1.00000000	pigeons
Eif3l	-0.06	1.00000000	pigeons
Cluh	0.02	1.00000000	pigeons
Nup37	0.08	1.00000000	pigeons
Tmem161b	-0.06	1.00000000	pigeons
Robo2	-0.78	1.00000000	pigeons
Ppil1	0.02	1.00000000	pigeons
Nbr1	-0.03	1.00000000	pigeons
Cd79b	0.06	1.00000000	pigeons
Gne	-0.08	1.00000000	pigeons
Adat1	0.02	1.00000000	pigeons
Avl9	-0.07	1.00000000	pigeons
Bend5	-0.17	1.00000000	pigeons
Sf3b1	-0.16	1.00000000	pigeons
Plaa	-0.09	1.00000000	pigeons
Rad21	-0.24	1.00000000	pigeons
Ptpn22	-0.07	1.00000000	pigeons
Gtdc1	-0.10	1.00000000	pigeons
Prpf4	0.03	1.00000000	pigeons
Ascc2	-0.02	1.00000000	pigeons
Gtf2h5	-0.06	1.00000000	pigeons
Glo1	0.06	1.00000000	pigeons
Chchd4	0.08	1.00000000	pigeons
Snapc1l	0.11	1.00000000	pigeons
Btbd7	0.09	1.00000000	pigeons
Gtpbp10	0.01	1.00000000	pigeons
Parp16	0.11	1.00000000	pigeons
Pdcd5	-0.18	1.00000000	pigeons

Irf1	0.00	1.00000000	pigeons
Plekho1	-0.02	1.00000000	pigeons
Brf1	-0.03	1.00000000	pigeons
Camk1d	0.07	1.00000000	pigeons
Aff2	0.15	1.00000000	pigeons
Tank	-0.15	1.00000000	pigeons
Iqgap1	-0.14	1.00000000	pigeons
Psmb3	0.00	1.00000000	pigeons
Cript	0.08	1.00000000	pigeons
Gatad1	0.01	1.00000000	pigeons
Tmem135	-0.08	1.00000000	pigeons
Snx19	-0.08	1.00000000	pigeons
Dnajc5	-0.07	1.00000000	pigeons
Crot	-0.09	1.00000000	pigeons
Rab6a	-0.13	1.00000000	pigeons
Zfand6	-0.26	1.00000000	pigeons
Wdr43	0.11	1.00000000	pigeons
Zcrb1	-0.11	1.00000000	pigeons
Kat7	-0.11	1.00000000	pigeons
Rabl6	-0.02	1.00000000	pigeons
Ghitm	0.00	1.00000000	pigeons
Ywhae	-0.31	1.00000000	pigeons
Ext2	-0.05	1.00000000	pigeons
Yes1	-0.20	1.00000000	pigeons
Tmem164	0.12	1.00000000	pigeons
Focad	-0.32	1.00000000	pigeons
Parvg	-0.11	1.00000000	pigeons
Trafd1	-0.09	1.00000000	pigeons
Nme6	0.02	1.00000000	pigeons
Pdcl3	-0.02	1.00000000	pigeons
Ttc37	-0.28	1.00000000	pigeons
Helz2	-0.14	1.00000000	pigeons
Pum1	-0.07	1.00000000	pigeons
Ska2	-0.12	1.00000000	pigeons
Eif2ak4	-0.05	1.00000000	pigeons
Cndp2	0.09	1.00000000	pigeons
Lmo4	0.01	1.00000000	pigeons
Atf1	-0.04	1.00000000	pigeons
Camkmt	0.23	1.00000000	pigeons
Pcm1	-0.09	1.00000000	pigeons
Stx8	-0.01	1.00000000	pigeons
Ccnyl1	-0.17	1.00000000	pigeons
Zbtb44	0.10	1.00000000	pigeons
Nubpl	0.07	1.00000000	pigeons
Samsn1	0.06	1.00000000	pigeons
Aprt	0.11	1.00000000	pigeons

S100a11	-0.03	1.00000000	pigeons
Nprl3	0.06	1.00000000	pigeons
Sdad1	0.01	1.00000000	pigeons
Cops5	-0.08	1.00000000	pigeons
Sar1b	0.00	1.00000000	pigeons
Actr6	0.06	1.00000000	pigeons
Igf2r	-0.08	1.00000000	pigeons
Rad17	-0.19	1.00000000	pigeons
Ubqln4	-0.02	1.00000000	pigeons
Dnajc2	-0.11	1.00000000	pigeons
Ncbp3	0.06	1.00000000	pigeons
Tfb2m	0.11	1.00000000	pigeons
Fbxl14	-0.06	1.00000000	pigeons
Vps36	-0.01	1.00000000	pigeons
Smap1	0.05	1.00000000	pigeons
Zcchc17	0.02	1.00000000	pigeons
Fig4	-0.01	1.00000000	pigeons
Ripk1	-0.34	1.00000000	pigeons
Ppp1r12a	0.02	1.00000000	pigeons
Psma2	-0.15	1.00000000	pigeons
Ndufaf7	0.09	1.00000000	pigeons
Serinc1	-0.06	1.00000000	pigeons
Smurf1	-0.04	1.00000000	pigeons
Rai2	-0.11	1.00000000	pigeons
Fzr1	0.08	1.00000000	pigeons
Zranb3	-0.04	1.00000000	pigeons
Pdrg1	-0.01	1.00000000	pigeons
Atp6v1c1	-0.57	1.00000000	pigeons
Ahsa1	-0.20	1.00000000	pigeons
Usp34	-0.22	1.00000000	pigeons
Cdkal1	-0.03	1.00000000	pigeons
Csrp1	-0.56	1.00000000	pigeons
Sp3	-0.08	1.00000000	pigeons
Ccdc134	-0.03	1.00000000	pigeons
Paxip1	-0.01	1.00000000	pigeons
Kif2a	-0.16	1.00000000	pigeons
Tm6sf1	-0.16	1.00000000	pigeons
Oser1	0.05	1.00000000	pigeons
Tma16	0.03	1.00000000	pigeons
Scfd1	-0.02	1.00000000	pigeons
Lin7a	0.18	1.00000000	pigeons
Fbxl17	-0.05	1.00000000	pigeons
Ppp1r7	0.06	1.00000000	pigeons
Ppp1r2	-0.07	1.00000000	pigeons
Hadha	0.09	1.00000000	pigeons
Phf3	-0.04	1.00000000	pigeons

Rbm45	0.09	1.00000000	pigeons
Faf2	0.04	1.00000000	pigeons
Arhgap6	0.25	1.00000000	pigeons
Copb1	-0.10	1.00000000	pigeons
Lef1	0.13	1.00000000	pigeons
Rnf146	0.00	1.00000000	pigeons
Snx10	-0.32	1.00000000	pigeons
Usp38	-0.12	1.00000000	pigeons
Pcgf5	-0.08	1.00000000	pigeons
Iah1	0.02	1.00000000	pigeons
Brd1	-0.21	1.00000000	pigeons
Lrrfip2	-0.11	1.00000000	pigeons
Top1	-0.17	1.00000000	pigeons
Celf1	-0.07	1.00000000	pigeons
Bfar	-0.01	1.00000000	pigeons
Tmem106b	-0.01	1.00000000	pigeons
Exosc1	0.08	1.00000000	pigeons
Cstf2	-0.01	1.00000000	pigeons
Pcgf6	0.11	1.00000000	pigeons
Stx18	0.18	1.00000000	pigeons
Coq2	0.10	1.00000000	pigeons
Afg3l2	-0.17	1.00000000	pigeons
Cse1l	-0.01	1.00000000	pigeons
Armt1	-0.17	1.00000000	pigeons
Thoc7	0.02	1.00000000	pigeons
Klf6	-0.16	1.00000000	pigeons
Bmp2k	-0.16	1.00000000	pigeons
Sri	-0.22	1.00000000	pigeons
Immp1l	0.06	1.00000000	pigeons
Btg2	-0.21	1.00000000	pigeons
AF366264	0.07	1.00000000	pigeons
Nutf2	-0.14	1.00000000	pigeons
Trappc11	0.14	1.00000000	pigeons
Cops4	-0.12	1.00000000	pigeons
Sec61a1	0.01	1.00000000	pigeons
Vcl	-0.96	1.00000000	pigeons
Slc37a4	0.07	1.00000000	pigeons
Chmp1a	-0.12	1.00000000	pigeons
Rragc	0.01	1.00000000	pigeons
Dctn3	-0.10	1.00000000	pigeons
Atg101	-0.09	1.00000000	pigeons
Sap130	0.06	1.00000000	pigeons
Ptpre	-0.81	1.00000000	pigeons
Nedd4	0.07	1.00000000	pigeons
Pcsk7	0.09	1.00000000	pigeons
Prkx	-0.23	1.00000000	pigeons

Yipf1	-0.17	1.00000000	pigeons
Gid4	0.15	1.00000000	pigeons
Cebpz	-0.09	1.00000000	pigeons
Dennd4c	-0.22	1.00000000	pigeons
Nob1	0.10	1.00000000	pigeons
Tmem19	-0.07	1.00000000	pigeons
Zfp438	0.21	1.00000000	pigeons
Dcaf5	-0.36	1.00000000	pigeons
Ncln	0.17	1.00000000	pigeons
Trnt1	-0.04	1.00000000	pigeons
Ncor2	-0.17	1.00000000	pigeons
Etfrf1	0.14	1.00000000	pigeons
Snx3	-0.49	1.00000000	pigeons
Sf3a2	0.00	1.00000000	pigeons
Actr2	-0.08	1.00000000	pigeons
Tbrg4	0.17	1.00000000	pigeons
Alg2	0.00	1.00000000	pigeons
Nrf1	0.01	1.00000000	pigeons
Hsf2	-0.09	1.00000000	pigeons
Tm9sf4	0.05	1.00000000	pigeons
Opa1	-0.12	1.00000000	pigeons
Snx7	0.09	1.00000000	pigeons
Rgp1	0.04	1.00000000	pigeons
Golga5	-0.04	1.00000000	pigeons
Nol8	-0.11	1.00000000	pigeons
Topbp1	-0.01	1.00000000	pigeons
Cdk12	-0.14	1.00000000	pigeons
Arpc1a	-0.08	1.00000000	pigeons
Sos2	-0.23	1.00000000	pigeons
Hspd1	0.01	1.00000000	pigeons
Rasgrp1	-0.05	1.00000000	pigeons
Klhdc10	-0.04	1.00000000	pigeons
Eif3e	0.06	1.00000000	pigeons
Gpatch1	-0.28	1.00000000	pigeons
Bcl2l13	-0.15	1.00000000	pigeons
Txlng	0.06	1.00000000	pigeons
Pogz	0.07	1.00000000	pigeons
Brpf3	-0.02	1.00000000	pigeons
Glg1	-0.12	1.00000000	pigeons
Slc35f5	-0.07	1.00000000	pigeons
Pdzd11	0.04	1.00000000	pigeons
Timm21	-0.06	1.00000000	pigeons
Zfp598	0.03	1.00000000	pigeons
Tbc1d1	-0.08	1.00000000	pigeons
Scaper	-0.14	1.00000000	pigeons
Tecpr2	-0.13	1.00000000	pigeons

Ndufs4	0.06	1.00000000	pigeons
Rapgef1	-0.27	1.00000000	pigeons
Ddx5	-0.20	1.00000000	pigeons
Srek1ip1	-0.11	1.00000000	pigeons
Ndufa10	-0.09	1.00000000	pigeons
Pfdn1	-0.22	1.00000000	pigeons
Zfp335	-0.09	1.00000000	pigeons
Nktr	-0.36	1.00000000	pigeons
Ilf2	0.02	1.00000000	pigeons
Ccndbp1	0.07	1.00000000	pigeons
Cbll1	-0.01	1.00000000	pigeons
Hagh	-0.07	1.00000000	pigeons
Eif4a3l1	-0.03	1.00000000	pigeons
Taldo1	-0.16	1.00000000	pigeons
Ebna1bp2	0.00	1.00000000	pigeons
Hyi	-0.39	1.00000000	pigeons
Rnf214	-0.15	1.00000000	pigeons
Pot1b	0.01	1.00000000	pigeons
Ppie	-0.13	1.00000000	pigeons
Ctss	-0.02	1.00000000	pigeons
Rab2a	-0.10	1.00000000	pigeons
Wdr7	-0.05	1.00000000	pigeons
Rprd1a	0.01	1.00000000	pigeons
Dnajc10	-0.06	1.00000000	pigeons
Retsat	0.05	1.00000000	pigeons
Tbcd	0.03	1.00000000	pigeons
Ddx18	0.12	1.00000000	pigeons
Cebpg	0.03	1.00000000	pigeons
Kif1b	-0.07	1.00000000	pigeons
Ndufaf2	0.07	1.00000000	pigeons
Acap2	-0.08	1.00000000	pigeons
Sf3b4	0.12	1.00000000	pigeons
Akap1	0.02	1.00000000	pigeons
Setdb1	-0.03	1.00000000	pigeons
Slx4	-0.01	1.00000000	pigeons
Prdm10	0.04	1.00000000	pigeons
Rp2	-0.17	1.00000000	pigeons
Irak2	-0.16	1.00000000	pigeons
Ppp3cb	-0.07	1.00000000	pigeons
Urb2	0.01	1.00000000	pigeons
Tpi1	-0.20	1.00000000	pigeons
Elp6	-0.02	1.00000000	pigeons
Hspa13	0.03	1.00000000	pigeons
Camsap2	-0.85	1.00000000	pigeons
Pptc7	0.03	1.00000000	pigeons
Hnrnpm	-0.14	1.00000000	pigeons

Clta	0.08	1.00000000	pigeons
Ccdc43	0.00	1.00000000	pigeons
Slc10a7	-0.15	1.00000000	pigeons
N4bp2l2	-0.16	1.00000000	pigeons
Apaf1	0.04	1.00000000	pigeons
Brcc3	-0.18	1.00000000	pigeons
Exosc3	-0.10	1.00000000	pigeons
Yars2	-0.08	1.00000000	pigeons
Naxe	-0.06	1.00000000	pigeons
Dnajb6	-0.07	1.00000000	pigeons
Slc35c1	0.11	1.00000000	pigeons
Emc7	-0.25	1.00000000	pigeons
Ctnnbl1	-0.07	1.00000000	pigeons
Srsf2	-0.28	1.00000000	pigeons
Pde12	-0.02	1.00000000	pigeons
Hsdl2	-0.03	1.00000000	pigeons
Cmtr1	-0.14	1.00000000	pigeons
Plpp7	0.06	1.00000000	pigeons
Exosc2	0.05	1.00000000	pigeons
Pld1	0.00	1.00000000	pigeons
Nptn	-0.29	1.00000000	pigeons
Nusap1	-0.44	1.00000000	pigeons
Clk2	-0.14	1.00000000	pigeons
Atxn10	-0.13	1.00000000	pigeons
Stard3nl	-0.14	1.00000000	pigeons
Ostc	-0.09	1.00000000	pigeons
Commd9	-0.02	1.00000000	pigeons
Zfp652	-0.16	1.00000000	pigeons
Arv1	-0.02	1.00000000	pigeons
Tfip11	0.13	1.00000000	pigeons
Atg3	0.11	1.00000000	pigeons
Tspan5	0.00	1.00000000	pigeons
Rab18	-0.34	1.00000000	pigeons
Lamp2	-0.16	1.00000000	pigeons
Edc4	0.09	1.00000000	pigeons
Ap1s2	-0.14	1.00000000	pigeons
Pak3	-0.07	1.00000000	pigeons
Nup155	0.16	1.00000000	pigeons
Dcp1b	-0.12	1.00000000	pigeons
Hhex	-0.14	1.00000000	pigeons
Lrrk2	0.07	1.00000000	pigeons
Dyrk1a	-0.11	1.00000000	pigeons
Hsdl1	-0.09	1.00000000	pigeons
Gtf2h3	0.05	1.00000000	pigeons
Pdcd6	-0.23	1.00000000	pigeons
C1d	-0.11	1.00000000	pigeons

Kdsr	-0.10	1.00000000	pigeons
Armc1	-0.23	1.00000000	pigeons
Atp8a1	-0.71	1.00000000	pigeons
Pibf1	0.09	1.00000000	pigeons
Guf1	0.00	1.00000000	pigeons
Fkbp8	0.03	1.00000000	pigeons
Traf5	0.08	1.00000000	pigeons
Fyn	-0.02	1.00000000	pigeons
Usp53	0.06	1.00000000	pigeons
Lrrc57	0.06	1.00000000	pigeons
Zfhx3	-0.13	1.00000000	pigeons
Abr	0.00	1.00000000	pigeons
Phka2	0.06	1.00000000	pigeons
Fry	-0.03	1.00000000	pigeons
Jade3	-0.73	1.00000000	pigeons
Fkbp11	-0.22	1.00000000	pigeons
Hat1	-0.03	1.00000000	pigeons
Aagab	-0.02	1.00000000	pigeons
Ogdh	-0.07	1.00000000	pigeons
Ddost	0.06	1.00000000	pigeons
Lrp3	-0.05	1.00000000	pigeons
Ttc1	0.00	1.00000000	pigeons
Azin1	-0.12	1.00000000	pigeons
Gtpbp8	-0.02	1.00000000	pigeons
Tdp1	0.04	1.00000000	pigeons
Timm22	0.12	1.00000000	pigeons
Ube2e3	-0.12	1.00000000	pigeons
Exoc4	-0.04	1.00000000	pigeons
Pik3r5	-0.11	1.00000000	pigeons
Zzef1	0.13	1.00000000	pigeons
Ssb	-0.19	1.00000000	pigeons
Nsmce2	-0.19	1.00000000	pigeons
Zfp622	-0.18	1.00000000	pigeons
Ywhab	-0.16	1.00000000	pigeons
Rb1	-0.15	1.00000000	pigeons
Fmnl1	0.06	1.00000000	pigeons
Lrrc28	-0.14	1.00000000	pigeons
Pi4kb	-0.11	1.00000000	pigeons
Sacm1l	-0.17	1.00000000	pigeons
Ggnbp2	-0.22	1.00000000	pigeons
Ccdc59	-0.07	1.00000000	pigeons
Elf1	0.04	1.00000000	pigeons
Srp54a	-0.10	1.00000000	pigeons
Kdm1a	-0.26	1.00000000	pigeons
Anapc10	0.11	1.00000000	pigeons
Spcs2	-0.19	1.00000000	pigeons

Timm44	0.04	1.00000000	pigeons
Zfyve21	-0.02	1.00000000	pigeons
Pan3	-0.11	1.00000000	pigeons
Arpc3	-0.03	1.00000000	pigeons
Rragd	0.06	1.00000000	pigeons
Ide	-0.15	1.00000000	pigeons
Las1l	0.00	1.00000000	pigeons
Chmp4b	-0.17	1.00000000	pigeons
Slc25a20	-0.20	1.00000000	pigeons
Uap1	-0.04	1.00000000	pigeons
Nfrkb	0.04	1.00000000	pigeons
Ift27	0.00	1.00000000	pigeons
Gpatch2	-0.24	1.00000000	pigeons
Arfgap2	0.03	1.00000000	pigeons
Rbm12b2	-0.05	1.00000000	pigeons
Nif3l1	0.07	1.00000000	pigeons
Utp23	0.12	1.00000000	pigeons
Tor4a	-0.09	1.00000000	pigeons
Pdcd10	-0.15	1.00000000	pigeons
Trappc2	-0.18	1.00000000	pigeons
Smpd2	-0.03	1.00000000	pigeons
Pank2	-0.02	1.00000000	pigeons
Wdr35	-0.43	1.00000000	pigeons
Pparg	0.17	1.00000000	pigeons
Arhgef12	-0.41	1.00000000	pigeons
Uckl1	0.01	1.00000000	pigeons
Ints2	-0.12	1.00000000	pigeons
Rptor	-0.01	1.00000000	pigeons
Btf3	-0.19	1.00000000	pigeons
Nudt21	0.02	1.00000000	pigeons
Wdr59	-0.03	1.00000000	pigeons
Zbtb24	-0.12	1.00000000	pigeons
Arnt	-0.15	1.00000000	pigeons
Tmem60	0.00	1.00000000	pigeons
Slc30a5	0.02	1.00000000	pigeons
Stambpl1	-0.03	1.00000000	pigeons
Ccpg1	-0.12	1.00000000	pigeons
Borcs8	-0.03	1.00000000	pigeons
Arfgap1	-0.12	1.00000000	pigeons
Fbxw2	-0.19	1.00000000	pigeons
Gpr107	-0.28	1.00000000	pigeons
Etfa	-0.08	1.00000000	pigeons
Alas1	-0.04	1.00000000	pigeons
Htatsf1	-0.24	1.00000000	pigeons
Rapgef2	-1.22	1.00000000	pigeons
Pou2f1	-0.08	1.00000000	pigeons

Ascc3	-0.10	1.00000000	pigeons
Fut8	-0.13	1.00000000	pigeons
Ddx20	-0.06	1.00000000	pigeons
Zcchc14	0.05	1.00000000	pigeons
Asb5	-0.04	1.00000000	pigeons
Tmem185a	-0.15	1.00000000	pigeons
Zbtb33	0.00	1.00000000	pigeons
Ap3s1	-0.77	1.00000000	pigeons
Sec63	-0.22	1.00000000	pigeons
Cltb	-0.22	1.00000000	pigeons
Skp1	-0.25	1.00000000	pigeons
Bcl9l	-0.01	1.00000000	pigeons
9930021J03Rik	-0.06	1.00000000	pigeons
Ldlrad4	-0.06	1.00000000	pigeons
Pik3c3	-0.15	1.00000000	pigeons
Pcgf3	0.00	1.00000000	pigeons
Lrwd1	0.02	1.00000000	pigeons
Elp2	0.02	1.00000000	pigeons
Vps53	-0.14	1.00000000	pigeons
Smu1	-0.38	1.00000000	pigeons
Rnf144b	0.12	1.00000000	pigeons
Rexo4	0.01	1.00000000	pigeons
Nrde2	-0.01	1.00000000	pigeons
Pin1	-0.18	1.00000000	pigeons
Rwdd2b	-0.06	1.00000000	pigeons
Arhgap17	-0.02	1.00000000	pigeons
Bzw2	-0.18	1.00000000	pigeons
Fndc3b	0.05	1.00000000	pigeons
Ssbp1	-0.15	1.00000000	pigeons
Zdhhc6	-0.45	1.00000000	pigeons
Itga1	-0.08	1.00000000	pigeons
Phf6	-0.18	1.00000000	pigeons
Ttc3	-0.31	1.00000000	pigeons
Ofd1	-0.01	1.00000000	pigeons
Pgap1	-0.08	1.00000000	pigeons
Chsy1	-0.04	1.00000000	pigeons
Git1	-0.14	1.00000000	pigeons
Ints11	0.03	1.00000000	pigeons
Ogt	-0.11	1.00000000	pigeons
Akap7	-0.19	1.00000000	pigeons
Cd74	0.10	1.00000000	pigeons
Pwwp2b	-0.17	1.00000000	pigeons
Lta4h	-0.03	1.00000000	pigeons
Fubp1	-0.18	1.00000000	pigeons
Galnt1	-0.18	1.00000000	pigeons
Acad9	-0.24	1.00000000	pigeons

Zfyve26	-0.05	1.00000000	pigeons
Polr2l	-0.03	1.00000000	pigeons
Akap10	-0.11	1.00000000	pigeons
Exoc6b	-1.01	1.00000000	pigeons
Dync2h1	0.04	1.00000000	pigeons
Larp4b	-0.12	1.00000000	pigeons
Taf3	-0.37	1.00000000	pigeons
Drg1	-0.02	1.00000000	pigeons
Tbc1d24	0.11	1.00000000	pigeons
Gsn	-0.90	1.00000000	pigeons
Crkl	-0.12	1.00000000	pigeons
Rbm19	-0.09	1.00000000	pigeons
Nfu1	-0.13	1.00000000	pigeons
Ifngr1	-0.26	1.00000000	pigeons
Lrmda	-0.60	1.00000000	pigeons
Fbxl20	-0.08	1.00000000	pigeons
2610507B11Rik	0.02	1.00000000	pigeons
Rpap1	-0.25	1.00000000	pigeons
Emc3	-0.23	1.00000000	pigeons
Kbtbd2	-0.23	1.00000000	pigeons
Pwp1	-0.12	1.00000000	pigeons
Rab4a	-0.05	1.00000000	pigeons
Uba5	-0.04	1.00000000	pigeons
Pdia3	-0.08	1.00000000	pigeons
Eepd1	-0.33	1.00000000	pigeons
Diaph2	-0.19	1.00000000	pigeons
Bphl	-0.16	1.00000000	pigeons
Zbtb11	-0.41	1.00000000	pigeons
Bnip2	-0.19	1.00000000	pigeons
Trim25	-0.01	1.00000000	pigeons
Nup50	-0.03	1.00000000	pigeons
Fktn	0.03	1.00000000	pigeons
Lyar	-0.10	1.00000000	pigeons
Gem	-0.03	1.00000000	pigeons
Dld	-0.02	1.00000000	pigeons
Notch1	-0.36	1.00000000	pigeons
Psmd7	-0.16	1.00000000	pigeons
Rhoa	-0.24	1.00000000	pigeons
Nifk	-0.14	1.00000000	pigeons
Cope	-0.14	1.00000000	pigeons
Fam91a1	0.06	1.00000000	pigeons
Bnip1	0.07	1.00000000	pigeons
Fosl2	-0.23	1.00000000	pigeons
Rab5c	0.02	1.00000000	pigeons
Nek10	-0.49	1.00000000	pigeons
Larp4	-0.02	1.00000000	pigeons

D630045J12Rik	-0.55	1.00000000	pigeons
Tmem208	-0.13	1.00000000	pigeons
Ammecr1	-0.12	1.00000000	pigeons
Dcun1d4	-0.15	1.00000000	pigeons
Washc5	-0.07	1.00000000	pigeons
Ckap5	-0.36	1.00000000	pigeons
Exosc8	0.06	1.00000000	pigeons
Proser1	-0.10	1.00000000	pigeons
Kmt5b	-0.48	1.00000000	pigeons
Zc3h13	-0.13	1.00000000	pigeons
Dcp2	-0.45	1.00000000	pigeons
Ago2	-0.18	1.00000000	pigeons
Gsr	-0.02	1.00000000	pigeons
Acsbg2	-0.13	1.00000000	pigeons
Apba3	0.10	1.00000000	pigeons
Lmbrd2	0.00	1.00000000	pigeons
Ylpm1	-0.03	1.00000000	pigeons
Rmdn3	-0.13	1.00000000	pigeons
Actb	-0.15	1.00000000	pigeons
Tceanc2	0.10	1.00000000	pigeons
Uhrf2	-0.24	1.00000000	pigeons
Txnl4b	-0.03	1.00000000	pigeons
Suv39h2	0.17	1.00000000	pigeons
Gemin8	0.04	1.00000000	pigeons
Rbsn	-0.18	1.00000000	pigeons
Ctcf	-0.10	1.00000000	pigeons
Pald1	-0.34	1.00000000	pigeons
Fam76b	-0.07	1.00000000	pigeons
Fam133b	-0.29	1.00000000	pigeons
Arl15	0.04	1.00000000	pigeons
Cenpu	0.16	1.00000000	pigeons
Itga9	-0.48	1.00000000	pigeons
Tmx3	-0.17	1.00000000	pigeons
Large1	-0.19	1.00000000	pigeons
Hmgxb3	-0.06	1.00000000	pigeons
Senp7	-0.29	1.00000000	pigeons
Dtx2	-0.42	1.00000000	pigeons
Adsl	-0.02	1.00000000	pigeons
Ndufa6	-0.58	1.00000000	pigeons
Vcpip1	-0.47	1.00000000	pigeons
Saraf	-0.16	1.00000000	pigeons
Sdha	-0.14	1.00000000	pigeons
Xylb	0.00	1.00000000	pigeons
Sec31a	-0.96	1.00000000	pigeons
Cwf19l2	0.00	1.00000000	pigeons
Vti1a	-0.24	1.00000000	pigeons

Pds5b	-0.19	1.00000000	pigeons
Wars2	0.09	1.00000000	pigeons
Prmt9	-0.20	1.00000000	pigeons
Elp3	-0.05	1.00000000	pigeons
Fkbp15	-0.15	1.00000000	pigeons
Znrf1	-0.19	1.00000000	pigeons
Chrac1	-0.12	1.00000000	pigeons
Efcab14	-0.45	1.00000000	pigeons
Epc2	-0.28	1.00000000	pigeons
Brox	-0.17	1.00000000	pigeons
Cog5	-0.21	1.00000000	pigeons
Heatr3	-0.02	1.00000000	pigeons
Krr1	-0.08	1.00000000	pigeons
Tmem168	-0.18	1.00000000	pigeons
Acer2	-0.40	1.00000000	pigeons
Rab24	-0.04	1.00000000	pigeons
Cmtm3	-0.03	1.00000000	pigeons
Arl6ip4	-0.11	1.00000000	pigeons
Sfi1	-0.08	1.00000000	pigeons
Trak1	-0.36	1.00000000	pigeons
Ntan1	-0.24	1.00000000	pigeons
Trdmt1	-0.14	1.00000000	pigeons
Ube4b	-0.14	1.00000000	pigeons
Shprh	-0.12	1.00000000	pigeons
Leprot	-0.63	1.00000000	pigeons
Tecpr1	0.06	1.00000000	pigeons
Herc3	-0.11	1.00000000	pigeons
Syt11	-0.11	1.00000000	pigeons
Kif13b	-0.21	1.00000000	pigeons
Nub1	-0.08	1.00000000	pigeons
Rnf220	-0.31	1.00000000	pigeons
Rsrc2	-0.19	1.00000000	pigeons
Snx12	-0.08	1.00000000	pigeons
Nr4a2	-0.07	1.00000000	pigeons
Optn	-0.36	1.00000000	pigeons
Hdac4	-0.78	1.00000000	pigeons
Cd63	-0.11	1.00000000	pigeons
Pitrm1	-0.08	1.00000000	pigeons
Deaf1	-0.03	1.00000000	pigeons
Reep2	-0.12	1.00000000	pigeons
Rnf130	0.00	1.00000000	pigeons
Zmpste24	-0.05	1.00000000	pigeons
Slc6a6	-0.85	1.00000000	pigeons
Chd7	-0.07	1.00000000	pigeons
Ctu2	0.03	1.00000000	pigeons
Casp7	-0.37	1.00000000	pigeons

Arhgap24	-0.18	1.00000000	pigeons
Trp53bp2	-0.40	1.00000000	pigeons
Slc25a12	-0.04	1.00000000	pigeons
Ciao1	-0.13	1.00000000	pigeons
H13	-0.16	1.00000000	pigeons
Skap2	-0.53	1.00000000	pigeons
Ctsh	-0.13	1.00000000	pigeons
Epc1	-0.20	1.00000000	pigeons
Rnf6	0.01	1.00000000	pigeons
Ppfibp1	-0.49	1.00000000	pigeons
Sfxn4	0.08	1.00000000	pigeons
Samm50	-0.11	1.00000000	pigeons
Phlpp1	-0.24	1.00000000	pigeons
Ero1b	-0.12	1.00000000	pigeons
Srp68	-0.11	1.00000000	pigeons
Capn11	-0.10	1.00000000	pigeons
Chordc1	-0.13	1.00000000	pigeons
Aatf	-0.08	1.00000000	pigeons
Fbxo48	-0.01	1.00000000	pigeons
Crebl2	-0.03	1.00000000	pigeons
Fam114a1	-0.03	1.00000000	pigeons
Plcb4	-0.17	1.00000000	pigeons
Tatdn3	-0.06	1.00000000	pigeons
Tbc1d2b	-0.09	1.00000000	pigeons
Arf4	-0.35	1.00000000	pigeons
Pepd	-0.19	1.00000000	pigeons
Supt20	-0.15	1.00000000	pigeons
Csrnp1	-0.25	1.00000000	pigeons
Acbd6	0.02	1.00000000	pigeons
Cisd2	-0.02	1.00000000	pigeons
Rnf19a	-0.04	1.00000000	pigeons
Rbfa	-0.74	1.00000000	pigeons
Tceanc	0.10	1.00000000	pigeons
Utp4	-0.12	1.00000000	pigeons
Itpka	-0.20	1.00000000	pigeons
Cops3	-0.26	1.00000000	pigeons
Aimp2	-0.04	1.00000000	pigeons
Hp1bp3	-0.26	1.00000000	pigeons
Paip1	-0.05	1.00000000	pigeons
Cdca7l	-0.62	1.00000000	pigeons
Uimc1	0.00	1.00000000	pigeons
Fundc1	-0.05	1.00000000	pigeons
Ankrd54	-0.06	1.00000000	pigeons
Gpatch11	-0.17	1.00000000	pigeons
Vdac2	-0.09	1.00000000	pigeons
Copg1	-0.07	1.00000000	pigeons

Gpr65	-0.24	1.00000000	pigeons
Sugt1	-0.06	1.00000000	pigeons
Slc35c2	-0.23	1.00000000	pigeons
Slc30a7	-0.23	1.00000000	pigeons
Srsf4	-0.03	1.00000000	pigeons
Asxl2	-0.02	1.00000000	pigeons
Clock	-0.20	1.00000000	pigeons
Sil1	-0.13	1.00000000	pigeons
Itgb1	-0.88	1.00000000	pigeons
Stat3	-0.04	1.00000000	pigeons
Ola1	-0.16	1.00000000	pigeons
Plekha3	-0.09	1.00000000	pigeons
Rcor1	-0.27	1.00000000	pigeons
Rad18	0.00	1.00000000	pigeons
Usp54	-0.84	1.00000000	pigeons
P4ha1	-0.28	1.00000000	pigeons
Ppil2	-0.14	1.00000000	pigeons
Ncstn	0.10	1.00000000	pigeons
Trib1	-0.18	1.00000000	pigeons
Idh3a	0.02	1.00000000	pigeons
Yipf6	-0.11	1.00000000	pigeons
Chtf8	-0.11	1.00000000	pigeons
Spg7	-0.03	1.00000000	pigeons
Setd3	-0.24	1.00000000	pigeons
Gphn	0.01	1.00000000	pigeons
Lsm1	-0.10	1.00000000	pigeons
Lin52	-0.25	1.00000000	pigeons
Pfkfb3	-0.45	1.00000000	pigeons
Rsf1	-0.23	1.00000000	pigeons
Cactin	-0.31	1.00000000	pigeons
Sgip1	-0.28	1.00000000	pigeons
Rangap1	-0.07	1.00000000	pigeons
Dytn	-0.14	1.00000000	pigeons
Sec22a	-0.09	1.00000000	pigeons
Chid1	0.04	1.00000000	pigeons
Tmem170	0.04	1.00000000	pigeons
Gprin3	0.02	1.00000000	pigeons
Fuca1	-0.03	1.00000000	pigeons
Abtb1	-0.21	1.00000000	pigeons
Kpna4	-0.16	1.00000000	pigeons
Aldh1a2	-0.31	1.00000000	pigeons
Tm2d1	-0.09	1.00000000	pigeons
Alg12	-0.08	1.00000000	pigeons
Fer	-0.27	1.00000000	pigeons
Nbeal1	0.00	1.00000000	pigeons
Slc25a25	-0.14	1.00000000	pigeons

Polr2b	-0.15	1.00000000	pigeons
Zer1	-0.27	1.00000000	pigeons
Tmbim6	-0.32	1.00000000	pigeons
Ap1ar	-0.16	1.00000000	pigeons
Ythdc1	-0.12	1.00000000	pigeons
Lztfl1	0.04	1.00000000	pigeons
Prkar2a	-0.22	1.00000000	pigeons
Dhx36	-0.36	1.00000000	pigeons
Ltn1	-0.19	1.00000000	pigeons
Cpsf4	0.00	1.00000000	pigeons
Zbtb37	-0.16	1.00000000	pigeons
Slc39a10	-0.14	1.00000000	pigeons
Stxbp1	0.01	1.00000000	pigeons
Dph6	-0.11	1.00000000	pigeons
Ifi35	-0.19	1.00000000	pigeons
Bpnt1	0.05	1.00000000	pigeons
Tmem70	-0.17	1.00000000	pigeons
Gemin6	0.05	1.00000000	pigeons
Fkbp4	-0.08	1.00000000	pigeons
Sos1	-0.12	1.00000000	pigeons
Btrc	-0.26	1.00000000	pigeons
Ptp4a1	-0.28	1.00000000	pigeons
Wdfy1	-0.06	1.00000000	pigeons
Prpf40a	-0.09	1.00000000	pigeons
Zc2hc1a	-0.07	1.00000000	pigeons
Bub3	-0.18	1.00000000	pigeons
Tob1	-0.44	1.00000000	pigeons
Ints4	-0.09	1.00000000	pigeons
Atp2a2	-0.38	1.00000000	pigeons
Ptpn4	-0.30	1.00000000	pigeons
Snx2	-0.12	1.00000000	pigeons
Natd1	0.00	1.00000000	pigeons
Dlst	-0.09	1.00000000	pigeons
Dnajc9	-0.50	1.00000000	pigeons
Acbd3	-0.30	1.00000000	pigeons
Nab1	-0.28	1.00000000	pigeons
Eri1	-0.03	1.00000000	pigeons
Slc35a1	-0.45	1.00000000	pigeons
Cst7	-0.04	1.00000000	pigeons
Hnrnpr	-0.21	1.00000000	pigeons
Hspa8	-0.45	1.00000000	pigeons
Phb	-0.14	1.00000000	pigeons
Zc3h12b	-0.14	1.00000000	pigeons
Stom	-0.32	1.00000000	pigeons
Zfp830	-0.32	1.00000000	pigeons
Cdk9	-0.12	1.00000000	pigeons

Ints6	-1.07	1.00000000	pigeons
Cpne8	0.12	1.00000000	pigeons
Gramd3	-0.44	1.00000000	pigeons
Ankfy1	-0.19	1.00000000	pigeons
Enpp2	-0.23	1.00000000	pigeons
Cdc23	0.05	1.00000000	pigeons
Gclc	-0.09	1.00000000	pigeons
Fhit	-0.49	1.00000000	pigeons
Tmem248	-0.28	1.00000000	pigeons
Yipf3	-0.04	1.00000000	pigeons
Agl	-0.02	1.00000000	pigeons
Creb1	-0.02	1.00000000	pigeons
Gfra3	-0.15	1.00000000	pigeons
Slc9a9	-0.07	1.00000000	pigeons
Bbs9	0.08	1.00000000	pigeons
Tmem30a	-0.38	1.00000000	pigeons
Exosc10	-0.05	1.00000000	pigeons
Arfgap3	-0.19	1.00000000	pigeons
Paqr3	-0.10	1.00000000	pigeons
Asah1	-0.23	1.00000000	pigeons
Dtnbp1	-0.05	1.00000000	pigeons
Supv3l1	-0.02	1.00000000	pigeons
Inpp5d	-0.60	1.00000000	pigeons
Rbm22	-0.31	1.00000000	pigeons
Fam204a	-0.17	1.00000000	pigeons
Inpp4a	0.01	1.00000000	pigeons
Psen1	-0.50	1.00000000	pigeons
Ppp2r5d	-0.20	1.00000000	pigeons
Cgrrf1	0.04	1.00000000	pigeons
Arrdc1	-0.07	1.00000000	pigeons
Pde8a	-0.46	1.00000000	pigeons
Aar2	0.06	1.00000000	pigeons
Vps13c	-0.20	1.00000000	pigeons
Itgb2l	-0.22	1.00000000	pigeons
B9d1	0.05	1.00000000	pigeons
Tbc1d23	-0.25	1.00000000	pigeons
Pinx1	-0.08	1.00000000	pigeons
Bcr	-0.17	1.00000000	pigeons
Atf4	-0.37	1.00000000	pigeons
Scoc	-0.70	1.00000000	pigeons
Pias2	0.08	1.00000000	pigeons
Eif4enif1	-0.05	1.00000000	pigeons
Golt1b	-0.04	1.00000000	pigeons
Arl16	0.10	1.00000000	pigeons
Naaa	-0.12	1.00000000	pigeons
Cops8	-0.38	1.00000000	pigeons

G2e3	0.05	1.00000000	pigeons
Tpmt	-0.02	1.00000000	pigeons
Yipf5	-0.08	1.00000000	pigeons
Rab12	-0.37	1.00000000	pigeons
Snx17	-0.75	1.00000000	pigeons
Ankrd28	-0.30	1.00000000	pigeons
Lzic	-0.03	1.00000000	pigeons
Nr1d2	-0.64	1.00000000	pigeons
Napg	-0.31	1.00000000	pigeons
Rif1	-0.12	1.00000000	pigeons
Eci3	-0.14	1.00000000	pigeons
Eif4e2	-0.01	1.00000000	pigeons
Lztr1	-0.12	1.00000000	pigeons
Spry2	-0.02	1.00000000	pigeons
Vps25	-0.01	1.00000000	pigeons
Kdm2a	0.08	1.00000000	pigeons
Hmgb1	-0.29	1.00000000	pigeons
Otud3	-0.08	1.00000000	pigeons
Prex1	-0.11	1.00000000	pigeons
Ostm1	-0.20	1.00000000	pigeons
Ppt1	-0.23	1.00000000	pigeons
Pink1	-0.05	1.00000000	pigeons
Eed	-0.08	1.00000000	pigeons
Ccdc93	-0.35	1.00000000	pigeons
Trim33	-0.18	1.00000000	pigeons
Sdr42e1	0.06	1.00000000	pigeons
Arhgdia	-0.20	1.00000000	pigeons
Sf3b3	-0.20	1.00000000	pigeons
Gnptg	0.02	1.00000000	pigeons
Smyd2	-0.06	1.00000000	pigeons
Dnajb2	-0.23	1.00000000	pigeons
Nfe2l2	-0.57	1.00000000	pigeons
Sash1	0.24	1.00000000	pigeons
Ipo9	-0.01	1.00000000	pigeons
Borcs6	-0.49	1.00000000	pigeons
Ppm1d	-0.13	1.00000000	pigeons
Tmem183a	-0.12	1.00000000	pigeons
Gtf2a2	-0.17	1.00000000	pigeons
Fbxo8	-0.14	1.00000000	pigeons
Tmem184b	-0.39	1.00000000	pigeons
Rell1	-0.24	1.00000000	pigeons
Rock2	-0.14	1.00000000	pigeons
Sik1	-0.25	1.00000000	pigeons
Wapl	-0.22	1.00000000	pigeons
Stt3b	-0.19	1.00000000	pigeons
Clip4	-0.05	1.00000000	pigeons

Gstcd	-0.35	1.00000000	pigeons
Stk26	-0.25	1.00000000	pigeons
Zfp507	-0.23	1.00000000	pigeons
Osbpl5	-0.39	1.00000000	pigeons
Nr3c2	-0.19	1.00000000	pigeons
Tasp1	-0.17	1.00000000	pigeons
Sh3bgrl	-0.21	1.00000000	pigeons
Stk40	-0.36	1.00000000	pigeons
Reck	-0.02	1.00000000	pigeons
Pdss2	-0.14	1.00000000	pigeons
Zfp352	-0.63	1.00000000	pigeons
Acot13	-0.40	1.00000000	pigeons
Wdr61	-0.05	1.00000000	pigeons
Setd7	-0.27	1.00000000	pigeons
Dcaf17	0.02	1.00000000	pigeons
Acot8	-0.08	1.00000000	pigeons
Polg2	-0.29	1.00000000	pigeons
Zfp423	-0.26	1.00000000	pigeons
Cdc27	-0.17	1.00000000	pigeons
Alkbh5	-0.15	1.00000000	pigeons
Ctns	0.10	1.00000000	pigeons
Dbr1	-0.02	1.00000000	pigeons
Fbxo7	-0.11	1.00000000	pigeons
Sfmbt2	0.14	1.00000000	pigeons
Tmco3	-0.22	1.00000000	pigeons
Commd10	-0.27	1.00000000	pigeons
Cdc14a	-0.45	1.00000000	pigeons
Fam98b	-0.02	1.00000000	pigeons
Thsd7b	-0.19	1.00000000	pigeons
Wsb1	0.10	1.00000000	pigeons
Ghdc	-0.13	1.00000000	pigeons
Gpx4	-0.08	1.00000000	pigeons
Tbl3	0.05	1.00000000	pigeons
Pip5k1a	-0.61	1.00000000	pigeons
Pcbd2	-0.15	1.00000000	pigeons
Cdk8	-0.24	1.00000000	pigeons
Sf3b6	-0.11	1.00000000	pigeons
Cstf1	-0.06	1.00000000	pigeons
Clns1a	-0.14	1.00000000	pigeons
Phkg1	-0.10	1.00000000	pigeons
Tbc1d12	-0.17	1.00000000	pigeons
Fbxl22	0.04	1.00000000	pigeons
Smarcal1	0.04	1.00000000	pigeons
Rab40c	-0.07	1.00000000	pigeons
Ccng1	0.02	1.00000000	pigeons
Surf2	-0.11	1.00000000	pigeons

Ppp1r16b	-0.18	1.00000000	pigeons
Bag1	-0.30	1.00000000	pigeons
Dgkz	0.05	1.00000000	pigeons
Bud13	-0.04	1.00000000	pigeons
Nsa2	-0.17	1.00000000	pigeons
Unc45a	-0.05	1.00000000	pigeons
Ctsb	-0.47	1.00000000	pigeons
Aoah	-0.25	1.00000000	pigeons
Lca5	-0.11	1.00000000	pigeons
Cacul1	-0.54	1.00000000	pigeons
Pcmtd2	-0.32	1.00000000	pigeons
Smad5	-0.44	1.00000000	pigeons
Ssr3	-0.11	1.00000000	pigeons
Ptcd3	-0.07	1.00000000	pigeons
Sema7a	-0.58	1.00000000	pigeons
Chd1l	-0.11	1.00000000	pigeons
Tmem59	-0.41	1.00000000	pigeons
Dag1	0.04	1.00000000	pigeons
Taok1	-0.08	1.00000000	pigeons
Fbxo28	-0.19	1.00000000	pigeons
Nfkb2	-0.18	1.00000000	pigeons
Eif2s1	-0.15	1.00000000	pigeons
Pam	-0.01	1.00000000	pigeons
Sh3glb1	-0.36	1.00000000	pigeons
Ybx3	-0.12	1.00000000	pigeons
Arfrp1	-0.07	1.00000000	pigeons
Tbc1d22a	-0.18	1.00000000	pigeons
Trap1	-0.06	1.00000000	pigeons
Pgd	-0.20	1.00000000	pigeons
Cd44	-0.29	1.00000000	pigeons
Pak1ip1	0.02	1.00000000	pigeons
Osgepl1	-0.03	1.00000000	pigeons
Fbxl18	-0.32	1.00000000	pigeons
Acaa1b	-0.53	1.00000000	pigeons
Kpna3	-0.43	1.00000000	pigeons
Ube2i	-0.17	1.00000000	pigeons
Pdcd7	-0.13	1.00000000	pigeons
Jazf1	-0.05	1.00000000	pigeons
Prss45	-0.47	1.00000000	pigeons
Telo2	-0.13	1.00000000	pigeons
Hsd17b2	0.03	1.00000000	pigeons
Hnrnpu	-0.21	1.00000000	pigeons
Zbtb8os	0.09	1.00000000	pigeons
Usp1	-0.14	1.00000000	pigeons
Gse1	-0.18	1.00000000	pigeons
Sema4d	-0.05	1.00000000	pigeons

Rnft1	0.00	1.00000000	pigeons
Dnajc3	-0.31	1.00000000	pigeons
Nvl	-0.44	1.00000000	pigeons
Cacna2d2	-0.18	1.00000000	pigeons
Bloc1s2	-0.26	1.00000000	pigeons
Dbi	-0.28	1.00000000	pigeons
Trp53bp1	-0.33	1.00000000	pigeons
Gtf2e1	-0.08	1.00000000	pigeons
Ahr	-0.16	1.00000000	pigeons
Atrx	-0.23	1.00000000	pigeons
Nupl1	-0.05	1.00000000	pigeons
Abhd11	-0.03	1.00000000	pigeons
Gbe1	-0.08	1.00000000	pigeons
Pfn2	0.05	1.00000000	pigeons
Bcap29	-0.51	1.00000000	pigeons
Ppp3r1	-0.38	1.00000000	pigeons
Ntmt1	0.01	1.00000000	pigeons
Ndufa12	-0.18	1.00000000	pigeons
Reps2	-0.24	1.00000000	pigeons
Timp2	-0.33	1.00000000	pigeons
Gpatch2l	-0.04	1.00000000	pigeons
B3galnt2	-0.22	1.00000000	pigeons
Ice2	-0.05	1.00000000	pigeons
Scpep1	-0.43	1.00000000	pigeons
Pigx	-0.27	1.00000000	pigeons
Impdh2	-0.08	1.00000000	pigeons
Nop58	-0.21	1.00000000	pigeons
Heca	-0.41	1.00000000	pigeons
Stau1	-0.18	1.00000000	pigeons
Edf1	-0.14	1.00000000	pigeons
Oxsr1	-0.88	1.00000000	pigeons
Slc25a46	-0.19	1.00000000	pigeons
Phf20	-0.14	1.00000000	pigeons
Kiz	0.03	1.00000000	pigeons
Hikeshi	-0.17	1.00000000	pigeons
Arl6ip1	-0.49	1.00000000	pigeons
Aldh4a1	-0.09	1.00000000	pigeons
Ranbp10	-0.13	1.00000000	pigeons
Lman2	-0.31	1.00000000	pigeons
Tax1bp1	-0.33	1.00000000	pigeons
Fam185a	-0.01	1.00000000	pigeons
Tmem50a	-0.68	1.00000000	pigeons
Chpt1	-0.01	1.00000000	pigeons
Nup107	-0.57	1.00000000	pigeons
Pi4ka	-0.30	1.00000000	pigeons
Tbc1d9b	-0.22	1.00000000	pigeons

Sec11a	-0.13	1.00000000	pigeons
Nfkb1	-0.41	1.00000000	pigeons
Fbxo25	-0.08	1.00000000	pigeons
Dcun1d1	-0.27	1.00000000	pigeons
Nop2	-0.06	1.00000000	pigeons
Surf4	-0.11	1.00000000	pigeons
Bet1	-0.13	1.00000000	pigeons
Snapc4	0.06	1.00000000	pigeons
Hadh	-0.22	1.00000000	pigeons
Ppa1	-0.14	1.00000000	pigeons
Tln1	-0.08	1.00000000	pigeons
Tnrc6b	-0.62	1.00000000	pigeons
Edrf1	-0.17	1.00000000	pigeons
Ttc7b	-0.45	1.00000000	pigeons
Psmb1	-0.25	1.00000000	pigeons
Casd1	-0.19	1.00000000	pigeons
Gatb	-0.07	1.00000000	pigeons
Esrrg	-0.16	1.00000000	pigeons
Limd1	-0.33	1.00000000	pigeons
Tspan9	-0.56	1.00000000	pigeons
Chchd1	-0.18	1.00000000	pigeons
Klhl14	-0.50	1.00000000	pigeons
Amfr	-0.08	1.00000000	pigeons
Anks6	-0.11	1.00000000	pigeons
Rev1	-0.19	1.00000000	pigeons
Xrcc4	-0.12	1.00000000	pigeons
Ssr2	-0.20	1.00000000	pigeons
Tmf1	-0.22	1.00000000	pigeons
Txn2	-0.02	1.00000000	pigeons
Rfng	-0.22	1.00000000	pigeons
Uso1	-0.37	1.00000000	pigeons
Dnajc11	-0.05	1.00000000	pigeons
Ebag9	-0.32	1.00000000	pigeons
Esco1	-0.06	1.00000000	pigeons
Usp40	-0.08	1.00000000	pigeons
Ube2z	0.05	1.00000000	pigeons
Pik3ca	-0.88	1.00000000	pigeons
Hmbs	-0.09	1.00000000	pigeons
Ggps1	-0.44	1.00000000	pigeons
Chmp2b	-0.07	1.00000000	pigeons
Ttyh3	-0.43	1.00000000	pigeons
Tor1b	-0.16	1.00000000	pigeons
Aven	-0.24	1.00000000	pigeons
Spidr	-0.08	1.00000000	pigeons
Gorasp2	-0.18	1.00000000	pigeons
2900026A02Rik	-0.45	1.00000000	pigeons

Srrm1	-0.07	1.00000000	pigeons
Ccdc50	-0.20	1.00000000	pigeons
Ralb	-0.32	1.00000000	pigeons
Wdr11	0.02	1.00000000	pigeons
Hpcal1	-0.95	1.00000000	pigeons
Il6st	-0.32	1.00000000	pigeons
AW554918	-0.26	1.00000000	pigeons
Rcbtb1	-0.68	1.00000000	pigeons
Psmc6	-0.36	1.00000000	pigeons
Ppp3cc	-0.10	1.00000000	pigeons
Stxbp5	-0.58	1.00000000	pigeons
Gnb1	-0.31	1.00000000	pigeons
Zfp131	-0.09	1.00000000	pigeons
Gigyf2	-0.18	1.00000000	pigeons
Atg16l1	-0.19	1.00000000	pigeons
St3gal6	-0.57	1.00000000	pigeons
Scyl3	0.09	1.00000000	pigeons
Txnrd3	-0.14	1.00000000	pigeons
Vps45	-0.18	1.00000000	pigeons
Rassf3	-0.31	1.00000000	pigeons
Blmh	-0.13	1.00000000	pigeons
Lbh	-0.30	1.00000000	pigeons
Prelid1	-0.09	1.00000000	pigeons
Gpd2	-0.30	1.00000000	pigeons
Aplf	-0.32	1.00000000	pigeons
Cnot10	-0.10	1.00000000	pigeons
Gna11	-0.22	1.00000000	pigeons
Pax5	-0.14	1.00000000	pigeons
Ptrh2	-0.07	1.00000000	pigeons
Fars2	-0.13	1.00000000	pigeons
Spata20	-0.15	1.00000000	pigeons
Fasn	-0.09	1.00000000	pigeons
Hsd17b12	-1.04	1.00000000	pigeons
Clcn6	-0.14	1.00000000	pigeons
Hells	-0.11	1.00000000	pigeons
Slc36a4	-0.12	1.00000000	pigeons
Arg2	-0.06	1.00000000	pigeons
Sec31b	-0.18	1.00000000	pigeons
Gps1	-0.05	1.00000000	pigeons
Pik3r1	-0.01	1.00000000	pigeons
Erc1	-0.42	1.00000000	pigeons
Dtx3l	-0.20	1.00000000	pigeons
Polr3b	-0.69	1.00000000	pigeons
Klf7	-0.39	1.00000000	pigeons
Dck	0.03	1.00000000	pigeons
Snrpd3	-0.14	1.00000000	pigeons

Sergef	-0.29	1.00000000	pigeons
Atxn1	-0.20	1.00000000	pigeons
Lclat1	-0.33	1.00000000	pigeons
Rpusd4	0.08	1.00000000	pigeons
Prpf38a	-0.01	1.00000000	pigeons
Adk	-0.52	1.00000000	pigeons
Ctnnbip1	-0.17	1.00000000	pigeons
Smg6	-0.40	1.00000000	pigeons
Tpk1	-0.11	1.00000000	pigeons
Fam76a	-0.14	1.00000000	pigeons
Nuak1	-0.19	1.00000000	pigeons
Jade1	-0.45	1.00000000	pigeons
Atmin	-0.13	1.00000000	pigeons
Rabgap1l	0.02	1.00000000	pigeons
Zdhhc21	-0.22	1.00000000	pigeons
Plek	-0.09	1.00000000	pigeons
Adrm1	-0.32	1.00000000	pigeons
Ppme1	-0.16	1.00000000	pigeons
Dpp8	0.02	1.00000000	pigeons
Tmed10	-0.10	1.00000000	pigeons
Pcyt1b	0.00	1.00000000	pigeons
Klf3	-0.56	1.00000000	pigeons
Slc35a5	-0.13	1.00000000	pigeons
Rnps1	-0.05	1.00000000	pigeons
Ttc14	-0.17	1.00000000	pigeons
Ccdc174	-0.03	1.00000000	pigeons
B3gnt5	-0.05	1.00000000	pigeons
Stx11	-0.23	1.00000000	pigeons
Brms1l	-0.20	1.00000000	pigeons
Cdk6	-0.13	1.00000000	pigeons
Slc7a6os	-0.23	1.00000000	pigeons
Ppwd1	-0.16	1.00000000	pigeons
Tubgcp3	-0.10	1.00000000	pigeons
Stam2	-0.46	1.00000000	pigeons
Patj	-0.18	1.00000000	pigeons
Vpreb3	0.38	1.00000000	pigeons
Cep57	-0.22	1.00000000	pigeons
Tab3	-0.24	1.00000000	pigeons
Fcf1	0.01	1.00000000	pigeons
Cpne1	-0.42	1.00000000	pigeons
Tet2	0.04	1.00000000	pigeons
Dusp15	-0.15	1.00000000	pigeons
Hexim1	-0.09	1.00000000	pigeons
Ugp2	-0.27	1.00000000	pigeons
Usp28	-0.23	1.00000000	pigeons
B3gntl1	-0.05	1.00000000	pigeons

Gpn1	0.02	1.00000000	pigeons
Syf2	-0.26	1.00000000	pigeons
Rev3l	-0.16	1.00000000	pigeons
Dnajb14	0.08	1.00000000	pigeons
Vwa8	-0.07	1.00000000	pigeons
Homer1	-0.51	1.00000000	pigeons
Trim54	-0.25	1.00000000	pigeons
Ddhd1	-0.12	1.00000000	pigeons
Smndc1	-0.33	1.00000000	pigeons
Isca1	-0.32	1.00000000	pigeons
N4bp1	-0.29	1.00000000	pigeons
Poldip2	0.02	1.00000000	pigeons
Tada3	0.04	1.00000000	pigeons
Sptlc2	-0.39	1.00000000	pigeons
Tanc1	-0.23	1.00000000	pigeons
Arhgap10	-0.44	1.00000000	pigeons
Eef1d	0.05	1.00000000	pigeons
Hif1an	-0.18	1.00000000	pigeons
Zfp592	-0.20	1.00000000	pigeons
Pard3	-0.66	1.00000000	pigeons
Cd9	-0.20	1.00000000	pigeons
Ndufa9	-0.14	1.00000000	pigeons
Rmdn1	-0.29	1.00000000	pigeons
Psmd9	-0.47	1.00000000	pigeons
Zswim7	0.05	1.00000000	pigeons
Smim8	-0.22	1.00000000	pigeons
Lsm5	-0.24	1.00000000	pigeons
Tmem141	-0.15	1.00000000	pigeons
Ldb1	-0.10	1.00000000	pigeons
Hsd17b4	-0.48	1.00000000	pigeons
Golim4	-0.29	1.00000000	pigeons
Dctn1	-0.45	1.00000000	pigeons
Naca	-0.16	1.00000000	pigeons
Swi5	-0.34	1.00000000	pigeons
Ddx54	0.00	1.00000000	pigeons
Tmc7	-0.45	1.00000000	pigeons
Alad	-0.17	1.00000000	pigeons
Ap2m1	-0.31	1.00000000	pigeons
Ptpn11	-0.25	1.00000000	pigeons
Stk24	-0.62	1.00000000	pigeons
Nadk	-0.27	1.00000000	pigeons
Rwdd4a	-0.12	1.00000000	pigeons
Pnn	-0.42	1.00000000	pigeons
Tcn2	-0.28	1.00000000	pigeons
Prkcd	-0.23	1.00000000	pigeons
Peak1	-0.58	1.00000000	pigeons

Phf5a	-0.17	1.00000000	pigeons
Reep5	-0.10	1.00000000	pigeons
Rbm18	-0.23	1.00000000	pigeons
Tgif1	-0.14	1.00000000	pigeons
Rbms1	-0.69	1.00000000	pigeons
Arhgef6	-0.44	1.00000000	pigeons
Cldn12	-0.22	1.00000000	pigeons
Usp12	-0.50	1.00000000	pigeons
Camk2g	-0.55	1.00000000	pigeons
Acat1	-0.23	1.00000000	pigeons
Immp2l	-0.31	1.00000000	pigeons
Trmt13	-0.26	1.00000000	pigeons
Dusp10	-0.45	1.00000000	pigeons
Pmepa1	-0.18	1.00000000	pigeons
Chmp6	-0.19	1.00000000	pigeons
Elmo1	-0.12	1.00000000	pigeons
Htt	-0.72	1.00000000	pigeons
Cdh17	0.01	1.00000000	pigeons
Ptk2	-0.85	1.00000000	pigeons
Efr3a	-0.28	1.00000000	pigeons
Ndufb5	-0.27	1.00000000	pigeons
Phactr2	-0.47	1.00000000	pigeons
Armc2	-0.24	1.00000000	pigeons
Disp1	-0.84	1.00000000	pigeons
Tspan14	-0.78	1.00000000	pigeons
Snx24	-0.24	1.00000000	pigeons
Zfp410	-0.17	1.00000000	pigeons
Rfxank	-0.15	1.00000000	pigeons
Cpne3	-0.41	1.00000000	pigeons
Usp4	-0.21	1.00000000	pigeons
Tmpo	-0.38	1.00000000	pigeons
Snap29	-0.41	1.00000000	pigeons
Nap1l1	-0.28	1.00000000	pigeons
Pcna	-0.33	1.00000000	pigeons
Sdhd	-0.20	1.00000000	pigeons
S100a10	-0.54	1.00000000	pigeons
Chp1	-0.55	1.00000000	pigeons
Rpia	-0.47	1.00000000	pigeons
Gpx1	-0.09	1.00000000	pigeons
Heatr5b	-0.18	1.00000000	pigeons
Homer2	-0.33	1.00000000	pigeons
Ubqln1	-0.15	1.00000000	pigeons
Dmap1	-0.69	1.00000000	pigeons
Cdca4	-0.46	1.00000000	pigeons
Ccser1	-0.61	1.00000000	pigeons
Dgkd	-0.37	1.00000000	pigeons

Rnf10	-0.31	1.00000000	pigeons
Dtwd1	-0.40	1.00000000	pigeons
Elk4	-0.39	1.00000000	pigeons
Glipr2	-0.26	1.00000000	pigeons
Atp6v1g1	-0.24	1.00000000	pigeons
Zcchc4	-0.20	1.00000000	pigeons
Fxyd6	-0.36	1.00000000	pigeons
Sfpq	-0.14	1.00000000	pigeons
Pccb	-0.18	1.00000000	pigeons
Use1	-0.09	1.00000000	pigeons
Angptl2	-0.43	1.00000000	pigeons
Psmd4	-0.09	1.00000000	pigeons
Ino80	-0.33	1.00000000	pigeons
Rgl1	-0.34	1.00000000	pigeons
Jmy	-0.45	1.00000000	pigeons
Rsrp1	-0.66	1.00000000	pigeons
Zfp821	-0.07	1.00000000	pigeons
Fem1b	-0.03	1.00000000	pigeons
Appl2	-0.28	1.00000000	pigeons
Rbl1	-0.29	1.00000000	pigeons
Zbtb26	-0.17	1.00000000	pigeons
Samd11	-0.41	1.00000000	pigeons
Tef	-0.40	1.00000000	pigeons
Rae1	-0.10	1.00000000	pigeons
Dnaja2	-0.35	1.00000000	pigeons
Nrip1	-0.42	1.00000000	pigeons
Eif4ebp1	-0.58	1.00000000	pigeons
Sox5	0.00	1.00000000	pigeons
Adam10	-0.35	1.00000000	pigeons
Eef1b2	-0.05	1.00000000	pigeons
Ccdc186	-0.33	1.00000000	pigeons
Dctn6	-0.51	1.00000000	pigeons
Hmgb2	-0.69	1.00000000	pigeons
Tpm4	-0.38	1.00000000	pigeons
Bend3	-0.50	1.00000000	pigeons
Cd247	-0.35	1.00000000	pigeons
Uvrag	-0.34	1.00000000	pigeons
Nelfb	-0.32	1.00000000	pigeons
Arhgap8	-0.45	1.00000000	pigeons
Rnh1	-0.16	1.00000000	pigeons
Sgpp1	-0.29	1.00000000	pigeons
Sec23a	-0.33	1.00000000	pigeons
Rtn4	-0.60	1.00000000	pigeons
Tmem266	-0.35	1.00000000	pigeons
Lfng	-0.76	1.00000000	pigeons
Nkiras2	-0.29	1.00000000	pigeons

Srpk2	-0.43	1.00000000	pigeons
Osbpl1a	-0.73	1.00000000	pigeons
Casp8ap2	-0.11	1.00000000	pigeons
Serinc3	-0.48	1.00000000	pigeons
Aak1	-0.39	1.00000000	pigeons
Eif5b	-0.21	1.00000000	pigeons
Clcn3	-0.39	1.00000000	pigeons
Ankrd39	-0.27	1.00000000	pigeons
Pts	-0.10	1.00000000	pigeons
Slc38a2	-0.23	1.00000000	pigeons
Taf13	-0.22	1.00000000	pigeons
Poc1b	-0.48	1.00000000	pigeons
Irf2bpl	-0.20	1.00000000	pigeons
Clybl	-0.22	1.00000000	pigeons
Lpcat1	-0.22	1.00000000	pigeons
Hmgcr	-0.30	1.00000000	pigeons
Golga4	-0.41	1.00000000	pigeons
Ndufs7	-0.31	1.00000000	pigeons
Ldah	-0.41	1.00000000	pigeons
Lrrcc1	-0.40	1.00000000	pigeons
Tpm1	-0.31	1.00000000	pigeons
Epb41	-0.45	1.00000000	pigeons
Slc2a13	-0.23	1.00000000	pigeons
Rpgr	-0.22	1.00000000	pigeons
Uspl1	-0.25	1.00000000	pigeons
Ulk2	-0.17	1.00000000	pigeons
Sh3gl1	-0.62	1.00000000	pigeons
Ehd4	-0.62	1.00000000	pigeons
Psmf1	-0.18	1.00000000	pigeons
Rft1	-0.24	1.00000000	pigeons
Slc4a11	-0.33	1.00000000	pigeons
Ubl3	-0.32	1.00000000	pigeons
Unc50	-0.20	1.00000000	pigeons
Tle3	-0.49	1.00000000	pigeons
Ppfia1	-0.21	1.00000000	pigeons
Ifrd1	-0.67	1.00000000	pigeons
Polr3a	-0.18	1.00000000	pigeons
Nipsnap2	-0.43	1.00000000	pigeons
Adcy9	-0.68	1.00000000	pigeons
Trim8	-0.52	1.00000000	pigeons
Phlpp2	-0.10	1.00000000	pigeons
Abcb10	-0.39	1.00000000	pigeons
Appl1	-0.18	1.00000000	pigeons
Chgb	-0.17	1.00000000	pigeons
Trip11	-0.72	1.00000000	pigeons
Acot10	-0.06	1.00000000	pigeons

Slc5a1	-0.47	1.00000000	pigeons
D5Ertd579e	-0.79	1.00000000	pigeons
Pard3b	-0.57	1.00000000	pigeons
Atp6ap2	-0.36	1.00000000	pigeons
Fgfr1op2	-0.24	1.00000000	pigeons
Uba2	-0.36	1.00000000	pigeons
Actn1	-0.35	1.00000000	pigeons
Per3	-0.29	1.00000000	pigeons
Wdsub1	-0.27	1.00000000	pigeons
Tbc1d14	-0.39	1.00000000	pigeons
Sod1	-0.24	1.00000000	pigeons
Hnrnpa2b1	-0.14	1.00000000	pigeons
Larp7	-0.25	1.00000000	pigeons
Slc30a9	-0.56	1.00000000	pigeons
Ppard	-0.35	1.00000000	pigeons
Rap1b	-0.50	1.00000000	pigeons
Atp2c1	-0.57	1.00000000	pigeons
Apoa1	-0.97	1.00000000	pigeons
Smyd3	-0.24	1.00000000	pigeons
Ipo11	-0.15	1.00000000	pigeons
Zdhhc7	-0.05	1.00000000	pigeons
Fam102a	-0.22	1.00000000	pigeons
Atp6v1h	-0.46	1.00000000	pigeons
Arhgap26	-0.81	1.00000000	pigeons
Itpkb	-0.49	1.00000000	pigeons
Fancl	-0.18	1.00000000	pigeons
Actg1	-0.07	1.00000000	pigeons
Ergic2	-0.17	1.00000000	pigeons
Ly75	-0.43	1.00000000	pigeons
Rmnd5a	-0.30	1.00000000	pigeons
Qdpr	-0.19	1.00000000	pigeons
Polr2c	-0.10	1.00000000	pigeons
Ppm1f	-0.45	1.00000000	pigeons
Ncoa4	-0.16	1.00000000	pigeons
Pcdh7	0.22	1.00000000	pigeons
Ralbp1	-0.61	1.00000000	pigeons
Nrbf2	-0.03	1.00000000	pigeons
Xpo1	-0.55	1.00000000	pigeons
Tert	-0.16	1.00000000	pigeons
Znhit6	-0.31	1.00000000	pigeons
Atp6v0a2	-0.36	1.00000000	pigeons
Fbxo30	-0.35	1.00000000	pigeons
Jakmip1	-0.36	1.00000000	pigeons
Kcnq1	-0.49	1.00000000	pigeons
Ift140	-0.34	1.00000000	pigeons
Ddx31	-0.22	1.00000000	pigeons

Atp7a	-0.41	1.00000000	pigeons
Ptpn12	-0.53	1.00000000	pigeons
Nudt19	-0.33	1.00000000	pigeons
Axin1	-0.05	1.00000000	pigeons
Birc6	-0.17	1.00000000	pigeons
Eif4g2	-0.34	1.00000000	pigeons
Crip1	-0.09	1.00000000	pigeons
Arl8b	-0.23	1.00000000	pigeons
Hdac7	-0.44	1.00000000	pigeons
Rcn2	-0.19	1.00000000	pigeons
Hnrnpab	-0.18	1.00000000	pigeons
Znrf3	-0.27	1.00000000	pigeons
Tfrc	-0.56	1.00000000	pigeons
Zdhhc14	-0.50	1.00000000	pigeons
Edem3	-0.33	1.00000000	pigeons
Alg6	-0.09	1.00000000	pigeons
Atp1a1	-0.54	1.00000000	pigeons
Hlcs	-0.37	1.00000000	pigeons
Commd4	-0.32	1.00000000	pigeons
Zfp518b	-0.39	1.00000000	pigeons
Nek1	-0.33	1.00000000	pigeons
Rilpl1	-0.51	1.00000000	pigeons
Top2b	-0.27	1.00000000	pigeons
Atp6v0c	-0.42	1.00000000	pigeons
Ginm1	-0.39	1.00000000	pigeons
Tmppe	-0.10	1.00000000	pigeons
Cask	-0.53	1.00000000	pigeons
Rrp15	-0.06	1.00000000	pigeons
Rfc5	-0.11	1.00000000	pigeons
Abhd13	-0.21	1.00000000	pigeons
Fchsd2	-0.55	1.00000000	pigeons
Bnip3l	-0.71	1.00000000	pigeons
Rb1cc1	-0.34	1.00000000	pigeons
Nisch	-0.67	1.00000000	pigeons
Fmnl2	-0.73	1.00000000	pigeons
Pex7	-0.38	1.00000000	pigeons
Rbm39	-0.34	1.00000000	pigeons
Gtf2h1	-0.45	1.00000000	pigeons
Psma5	-0.35	1.00000000	pigeons
Ate1	-0.59	1.00000000	pigeons
Nploc4	-0.07	1.00000000	pigeons
Trpm7	-0.44	1.00000000	pigeons
Klhdc1	-0.26	1.00000000	pigeons
Uprt	-0.22	1.00000000	pigeons
Oaz2	-0.14	1.00000000	pigeons
Tmed5	-0.49	1.00000000	pigeons

Vrk1	-0.38	1.00000000	pigeons
Oxct1	-0.37	1.00000000	pigeons
Tdp2	-0.18	1.00000000	pigeons
Zw10	-0.27	1.00000000	pigeons
Kat6b	-0.50	1.00000000	pigeons
Cpeb3	-0.21	1.00000000	pigeons
Bcor	-0.57	1.00000000	pigeons
Hspb11	-0.30	1.00000000	pigeons
Cnih4	-0.06	1.00000000	pigeons
Hspa5	-0.61	1.00000000	pigeons
Nufip1	-0.13	1.00000000	pigeons
Hnrnpa3	0.00	1.00000000	pigeons
Srp19	-0.24	1.00000000	pigeons
Cct5	-0.40	1.00000000	pigeons
Psmb7	-0.20	1.00000000	pigeons
Exoc7	-0.17	1.00000000	pigeons
Slc48a1	-0.23	1.00000000	pigeons
Fam199x	-0.17	1.00000000	pigeons
Rpp30	-0.15	1.00000000	pigeons
Hmgcl	-0.13	1.00000000	pigeons
Ppm1k	-0.20	1.00000000	pigeons
Prelid3a	-0.45	1.00000000	pigeons
Fndc5	-0.05	1.00000000	pigeons
Ttc28	-0.68	1.00000000	pigeons
Actr10	-0.18	1.00000000	pigeons
Abcc5	-0.19	1.00000000	pigeons
Creld2	-0.09	1.00000000	pigeons
Amot	-0.52	1.00000000	pigeons
Kif5b	-0.57	1.00000000	pigeons
Tanc2	-0.53	1.00000000	pigeons
B4galt1	-0.24	1.00000000	pigeons
Heatr5a	-0.36	1.00000000	pigeons
Noa1	-0.19	1.00000000	pigeons
Faf1	-0.76	1.00000000	pigeons
Nup153	-0.47	1.00000000	pigeons
Slc30a1	-0.26	1.00000000	pigeons
Ndufb6	-0.26	1.00000000	pigeons
Rfc4	-0.27	1.00000000	pigeons
Fbrsl1	-0.07	1.00000000	pigeons
Arl8a	-0.38	1.00000000	pigeons
Lsg1	-0.17	1.00000000	pigeons
Bap1	-0.06	1.00000000	pigeons
Lsm6	-0.14	1.00000000	pigeons
Id2	-0.16	1.00000000	pigeons
Pef1	-0.37	1.00000000	pigeons
Chchd5	-0.30	1.00000000	pigeons

Twf2	-0.29	1.00000000	pigeons
Fzd6	-0.26	1.00000000	pigeons
Dnmt3a	-0.22	1.00000000	pigeons
Rac1	-0.40	1.00000000	pigeons
Acly	-0.35	1.00000000	pigeons
Wwtr1	-0.18	1.00000000	pigeons
Dlg1	-0.50	1.00000000	pigeons
Pla2g12a	-0.27	1.00000000	pigeons
Gbf1	-0.39	1.00000000	pigeons
Parg	-0.36	1.00000000	pigeons
Cnnm2	-0.36	1.00000000	pigeons
Ccnl1	-0.07	1.00000000	pigeons
Nf2	-0.24	1.00000000	pigeons
Arpc1b	-0.12	1.00000000	pigeons
Bin3	-0.53	1.00000000	pigeons
Ak2	-0.45	1.00000000	pigeons
Xylt1	-0.23	1.00000000	pigeons
Ezh2	-0.47	1.00000000	pigeons
Snx14	-0.46	1.00000000	pigeons
Kansl1l	-0.51	1.00000000	pigeons
Nfkbiz	-0.73	1.00000000	pigeons
Per2	-0.39	1.00000000	pigeons
Cfdp1	-0.46	1.00000000	pigeons
Btbd1	-0.14	1.00000000	pigeons
Dennd3	-0.27	1.00000000	pigeons
Fam172a	-0.27	1.00000000	pigeons
Pla2g7	-0.41	1.00000000	pigeons
Atp6v0a1	-0.66	1.00000000	pigeons
Picalm	-0.61	1.00000000	pigeons
Clcc1	-0.36	1.00000000	pigeons
Ing5	-0.35	1.00000000	pigeons
Anp32e	-0.43	1.00000000	pigeons
Nde1	-0.34	1.00000000	pigeons
Ndufv2	-0.21	1.00000000	pigeons
Fnbp1	-0.42	1.00000000	pigeons
Ifngr2	-0.58	1.00000000	pigeons
Aplp2	-0.57	1.00000000	pigeons
Cpeb4	-0.66	1.00000000	pigeons
Tmem165	-0.18	1.00000000	pigeons
Ndufs6	-0.43	1.00000000	pigeons
Taf5l	-0.49	1.00000000	pigeons
Canx	-0.24	1.00000000	pigeons
Adgra1	-0.24	1.00000000	pigeons
Noc2l	-0.17	1.00000000	pigeons
Ifi30	-0.49	1.00000000	pigeons
Ssr1	-0.20	1.00000000	pigeons

Ube2q2	-0.40	1.00000000	pigeons
Rwdd1	-0.22	1.00000000	pigeons
Ppp2r5a	-0.58	1.00000000	pigeons
Tcf12	-0.23	1.00000000	pigeons
Pitpnb	-0.44	1.00000000	pigeons
Hmgb3	-0.38	1.00000000	pigeons
Rsrc1	-0.44	1.00000000	pigeons
Rbbp6	-0.11	1.00000000	pigeons
Cmss1	-0.45	1.00000000	pigeons
Dot1l	-0.34	1.00000000	pigeons
Smc3	-0.37	1.00000000	pigeons
9130409I23Rik	-0.66	1.00000000	pigeons
Cir1	-0.38	1.00000000	pigeons
Abcf2	-0.30	1.00000000	pigeons
Lin54	-0.23	1.00000000	pigeons
Rcan1	-0.85	1.00000000	pigeons
Kcnab2	-0.24	1.00000000	pigeons
Ist1	-0.22	1.00000000	pigeons
Hsp90b1	-0.30	1.00000000	pigeons
Ltf	-0.39	1.00000000	pigeons
Clint1	-0.43	1.00000000	pigeons
Nsmce1	-0.19	1.00000000	pigeons
Slc13a1	-0.17	1.00000000	pigeons
Aida	-0.37	1.00000000	pigeons
Pik3c2a	-0.45	1.00000000	pigeons
Adar	-0.35	1.00000000	pigeons
Zhx1	-0.76	1.00000000	pigeons
Vps37a	-0.32	1.00000000	pigeons
Cry1	-0.45	1.00000000	pigeons
Ptbp1	-0.33	1.00000000	pigeons
Psmb4	-0.47	1.00000000	pigeons
Eno1b	-0.38	1.00000000	pigeons
Nans	-0.58	1.00000000	pigeons
Hsp90aa1	0.72	1.00000000	pigeons
Sub1	-0.37	1.00000000	pigeons
Vps50	-0.20	1.00000000	pigeons
Rbbp8	-0.16	1.00000000	pigeons
Pbx1	-0.59	1.00000000	pigeons
B3gnt2	-0.43	1.00000000	pigeons
Slc30a6	-0.37	1.00000000	pigeons
Timm10	-0.23	1.00000000	pigeons
Ift20	-0.37	1.00000000	pigeons
Pcmt1	-0.43	1.00000000	pigeons
Elovl6	-0.32	1.00000000	pigeons
Hdx	-0.68	1.00000000	pigeons
Ets2	-0.52	1.00000000	pigeons

Pik3ip1	-0.37	1.00000000	pigeons
Smad3	-0.59	1.00000000	pigeons
Zfp407	-0.22	1.00000000	pigeons
Slc12a2	-0.31	1.00000000	pigeons
Arhgap29	-0.63	1.00000000	pigeons
Ipmk	-0.50	1.00000000	pigeons
Bclaf1	-0.42	1.00000000	pigeons
Kdm2b	-0.37	1.00000000	pigeons
Ddx55	-0.33	1.00000000	pigeons
Dym	-0.67	1.00000000	pigeons
Sipa1l1	-0.32	1.00000000	pigeons
Dcun1d5	-0.39	1.00000000	pigeons
Prkn	-0.43	1.00000000	pigeons
Nnt	-0.14	1.00000000	pigeons
Fgd3	-0.42	1.00000000	pigeons
Saal1	-0.32	1.00000000	pigeons
Isca2	-0.15	1.00000000	pigeons
Serbp1	-0.03	1.00000000	pigeons
Trib2	-0.75	1.00000000	pigeons
Cd99l2	-0.37	1.00000000	pigeons
Zfyve1	-0.47	1.00000000	pigeons
Ubac1	-0.38	1.00000000	pigeons
Zc3h12a	-0.35	1.00000000	pigeons
Wdr47	-0.21	1.00000000	pigeons
Rnf144a	-0.29	1.00000000	pigeons
Raph1	-0.52	1.00000000	pigeons
Scrn3	-0.19	1.00000000	pigeons
Cutc	-0.05	1.00000000	pigeons
Disc1	-0.35	1.00000000	pigeons
Atad2b	-0.43	1.00000000	pigeons
Ndufa5	-0.39	1.00000000	pigeons
Dpp9	-0.19	1.00000000	pigeons
Btbd10	-0.31	1.00000000	pigeons
Sec14l1	-0.70	1.00000000	pigeons
Ppp2r5e	-0.38	1.00000000	pigeons
Ldlrad3	-0.38	1.00000000	pigeons
Ecd	-0.18	1.00000000	pigeons
Smim4	-0.14	1.00000000	pigeons
Nme7	-0.40	1.00000000	pigeons
Arntl	-0.23	1.00000000	pigeons
Lamp1	-0.47	1.00000000	pigeons
Wdr48	-0.20	1.00000000	pigeons
Cmtm4	-0.41	1.00000000	pigeons
Utp15	-0.12	1.00000000	pigeons
Sipa1l2	-0.51	1.00000000	pigeons
Wbp4	-0.40	1.00000000	pigeons

Hdac11	-0.22	1.00000000	pigeons
Ttll5	-0.43	1.00000000	pigeons
Pigyl	-0.15	1.00000000	pigeons
Snx25	-0.20	1.00000000	pigeons
Psmb2	-0.20	1.00000000	pigeons
Atp11c	-0.35	1.00000000	pigeons
Eif4g3	-0.42	1.00000000	pigeons
Ptprm	-4.18	0.00000000	pigeons
Aqp1	-4.30	0.00000000	pigeons
Cfd	-4.57	0.00000000	pigeons
Igfbp7	-3.66	0.00000000	pigeons
Pecam1	-3.45	0.00000000	pigeons
Timp3	-3.67	0.00000000	pigeons
Epas1	-3.86	0.00000000	pigeons
Sparc	-2.70	0.00000000	pigeons
Rbms3	-3.33	0.00000000	pigeons
Tmtc1	-3.12	0.00000000	pigeons
Ptprb	-3.00	0.00000000	pigeons
Calcrl	-3.16	0.00000000	pigeons
Adgrf5	-2.74	0.00000000	pigeons
Adgrl4	-2.83	0.00000000	pigeons
Adgrl2	-2.79	0.00000000	pigeons
Hecw2	-3.49	0.00000000	pigeons
Dock1	-2.34	0.00000000	pigeons
Akap12	-2.48	0.00000000	pigeons
Plcb1	-2.45	0.00000000	pigeons
Ldb2	-2.97	0.00000000	pigeons
Cavin2	-2.41	0.00000000	pigeons
Syne2	-2.88	0.00000000	pigeons
Fam189a1	-2.39	0.00000000	pigeons
Tjp1	-2.85	0.00000000	pigeons
Podxl	-2.22	0.00000000	pigeons
Arhgef9	-2.58	0.00000000	pigeons
Fos	-3.38	0.00000000	pigeons
Npr3	-2.36	0.00000000	pigeons
Tmem100	-2.80	0.00000000	pigeons
Nova1	-2.77	0.00000000	pigeons
Sox6	-2.51	0.00000000	pigeons
Plekhg1	-2.22	0.00000000	pigeons
Stard13	-2.63	0.00000000	pigeons
Cav1	-1.73	0.00000000	pigeons
Sdc4	-2.66	0.00000000	pigeons
Kcne3	-2.26	0.00000000	pigeons
Rapgef5	-2.91	0.00000000	pigeons
Fermt2	-1.81	0.00000000	pigeons
Zfp366	-1.79	0.00000000	pigeons

Arhgap31	-2.18	0.00000000	pigeons
Nfia	-2.13	0.00000000	pigeons
Sdk1	-2.52	0.00000000	pigeons
Dll4	-1.94	0.00000000	pigeons
Gpx3	-2.41	0.00000000	pigeons
Prex2	-1.85	0.00000000	pigeons
Cdh5	-1.79	0.00000000	pigeons
Bicd1	-1.94	0.00000000	pigeons
Heg1	-1.96	0.00000000	pigeons
Tmem204	-1.72	0.00000000	pigeons
Apcdd1	-1.45	0.00000000	pigeons
Sema6a	-1.81	0.00000000	pigeons
Jcad	-1.83	0.00000000	pigeons
Tead4	-2.01	0.00000000	pigeons
Crim1	-2.68	0.00000000	pigeons
Efnb2	-1.85	0.00000000	pigeons
Rhoj	-1.51	0.00000000	pigeons
Smad6	-2.08	0.00000000	pigeons
Galnt18	-1.71	0.00000000	pigeons
Zbtb20	-2.13	0.00000000	pigeons
Dclk1	-1.79	0.00000000	pigeons
Tbx3	-1.59	0.00000000	pigeons
Synm	-1.55	0.00000000	pigeons
Fgd5	-1.66	0.00000000	pigeons
Rgcc	-1.90	0.00000000	pigeons
Col4a1	-1.85	0.00000000	pigeons
Dennd2a	-1.51	0.00000000	pigeons
Pde10a	-1.92	0.00000000	pigeons
Sptbn1	-1.55	0.00000000	pigeons
Egr1	-2.67	0.00000000	pigeons
Lpl	-1.83	0.00000000	pigeons
Ephb1	-1.50	0.00000000	pigeons
Slc9a3r2	-1.71	0.00000000	pigeons
Rasgrf2	-1.15	0.00000000	pigeons
Bmpr2	-2.16	0.00000000	pigeons
Dock9	-2.45	0.00000000	pigeons
Afdn	-2.08	0.00000000	pigeons
Tek	-1.39	0.00000000	pigeons
Arl4a	-2.57	0.00000000	pigeons
Laptm4a	-1.99	0.00000000	pigeons
Fgfr2	-1.51	0.00000000	pigeons
Kcnc1	-1.54	0.00000000	pigeons
Acvrl1	-1.40	0.00000000	pigeons
4932411N23Rik	-2.95	0.00000000	pigeons
Auts2	-1.62	0.00000000	pigeons
Foxf1	-1.17	0.00000000	pigeons

Efemp1	-1.52	0.00000000	pigeons
Colec10	-1.06	0.00000000	pigeons
Cadps2	-1.21	0.00000000	pigeons
Skap1	-2.13	0.00000000	pigeons
Rassf9	-1.28	0.00000000	pigeons
Sdc2	-1.22	0.00000000	pigeons
Zfp521	-1.07	0.00000000	pigeons
Gsk3b	-1.64	0.00000000	pigeons
Ramp2	-1.04	0.00000000	pigeons
Cobll1	-1.06	0.00000000	pigeons
Caskin2	-1.05	0.00000000	pigeons
Cgnl1	-1.00	0.00000000	pigeons
Corin	-1.88	0.00000000	pigeons
Dach1	-1.51	0.00000000	pigeons
Npas3	-1.95	0.00000000	pigeons
Neo1	-1.57	0.00000000	pigeons
Wdfy3	-1.68	0.00000000	pigeons
Ptprn2	-1.44	0.00000000	pigeons
Sftpc	-2.13	0.00000000	pigeons
Ptpn14	-1.54	0.00000000	pigeons
Cnn3	-1.49	0.00000000	pigeons
Sorbs1	-2.05	0.00000000	pigeons
Nckap1	-1.21	0.00000000	pigeons
Erg	-1.18	0.00000000	pigeons
Spred1	-1.18	0.00000000	pigeons
Bmp6	-1.08	0.00000000	pigeons
Twsg1	-1.19	0.00000000	pigeons
Runx1t1	-1.51	0.00000000	pigeons
Kcnj5	-1.82	0.00000000	pigeons
Cmtm8	-1.09	0.00000000	pigeons
Pim3	-1.94	0.00000000	pigeons
Kdr	-1.05	0.00000000	pigeons
Lifr	-1.23	0.00000000	pigeons
Tjp2	-1.15	0.00000000	pigeons
Cyfip1	-1.25	0.00000000	pigeons
Agbl1	-1.05	0.00000000	pigeons
Tcf7l2	-1.32	0.00000001	pigeons
Ctdspl	-1.94	0.00000001	pigeons
Pcsk2	-1.15	0.00000001	pigeons
Tmcc3	-1.65	0.00000004	pigeons
Ralgps2	-1.85	0.00000008	pigeons
Col22a1	-1.01	0.00000011	pigeons
Serpind1	-1.16	0.00000011	pigeons
Prkce	-1.06	0.00000025	pigeons
Yap1	-1.20	0.00000026	pigeons
Thbs1	-1.05	0.00000034	pigeons

Creb5	-1.38	0.00000039	pigeons
Dpyd	-1.54	0.00000065	pigeons
Zswim8	-1.31	0.00000090	pigeons
S100a13	-1.37	0.00000100	pigeons
Neurl1b	-1.78	0.00000138	pigeons
Aff1	-1.32	0.00000158	pigeons
Ablim1	-1.22	0.00000209	pigeons
Hs6st1	-1.24	0.00000246	pigeons
Anxa5	-1.28	0.00000312	pigeons
Fli1	-1.75	0.00000391	pigeons
Cttnbp2nl	-1.28	0.00000397	pigeons
Etl4	-1.51	0.00000609	pigeons
Itm2b	-1.65	0.00000680	pigeons
Ssbp2	-1.27	0.00000688	pigeons
Cdc42bpa	-1.27	0.00000698	pigeons
Tgfbr3	-1.17	0.00002592	pigeons
Tns1	-1.56	0.00002992	pigeons
Sh2d1a	-1.10	0.00003171	pigeons
Gata3	-1.18	0.00004452	pigeons
Ripor3	-1.15	0.00004704	pigeons
Baz2b	-1.54	0.00005389	pigeons
Qk	-1.20	0.00005686	pigeons
Tex2	-1.53	0.00010514	pigeons
Ski	-1.31	0.00015668	pigeons
Zeb2	-1.66	0.00017862	pigeons
Fnbp1l	-1.83	0.00018632	pigeons
Cep83	-1.47	0.00019104	pigeons
Dcn	-1.33	0.00024411	pigeons
Adamts2	-1.22	0.00027559	pigeons
Hoxa3	-1.02	0.00031406	pigeons
Dlc1	-1.20	0.00036715	pigeons
Dock4	-1.04	0.00036887	pigeons
Trps1	-1.42	0.00133739	pigeons
Lims2	-1.32	0.00137652	pigeons
Gk5	-1.02	0.00153505	pigeons
Clic4	-1.45	0.00264883	pigeons
Osbpl10	-1.09	0.00271587	pigeons
Creg1	-1.41	0.00384340	pigeons
Bsg	-1.83	0.00640488	pigeons
Agap1	-1.11	0.00897981	pigeons
Spty2d1	-1.29	0.01285088	pigeons
Numb	-1.79	0.01867816	pigeons
Arvcf	-1.39	0.04894093	pigeons
Ifi27l2a	2.69	0.00000000	mice
A330040F15Rik	1.83	0.00000000	mice
Irf7	1.40	0.00000000	mice

Trim30a	1.70	0.00000000	mice
Stat1	1.52	0.00000000	mice
Ifi203	1.78	0.00000000	mice
Ly6a	1.80	0.00000000	mice
Rnf213	1.61	0.00000000	mice
Zbp1	1.28	0.00000000	mice
Ifi214	1.49	0.00000000	mice
Shisa5	1.06	0.00000000	mice
Parp14	1.54	0.00000000	mice
Ifit3	1.20	0.00000000	mice
Smchd1	1.21	0.00000000	mice
Isg15	1.07	0.00000000	mice
Herc6	1.47	0.00000000	mice
Ifi47	1.19	0.00000000	mice
Ifi209	1.17	0.00000000	mice
Parp9	1.17	0.00000000	mice
Cybb	1.22	0.00000000	mice
Nlrc5	1.22	0.00000000	mice
Oasl1	1.01	0.00000000	mice
Slfn5	1.23	0.00000000	mice
Irgm1	1.07	0.00000000	mice
Ifitm3	1.04	0.00000000	mice
Pml	1.09	0.00000000	mice
Epsti1	1.04	0.00000000	mice
Samhd1	1.07	0.00000000	mice
Pkib	1.06	0.00000000	mice
Rtp4	0.90	0.00000000	mice
B2m	0.78	0.00000000	mice
Syk	-0.93	0.00000000	mice
Bst2	0.90	0.00000000	mice
A930037H05Rik	0.94	0.00000000	mice
Ighm	-0.99	0.00000000	mice
Usp18	0.83	0.00000000	mice
Xaf1	0.74	0.00000000	mice
Ly6c2	0.82	0.00000000	mice
Fau	0.43	0.00000000	mice
H2-K1	0.61	0.00000000	mice
Slfn8	0.93	0.00000000	mice
Tap1	0.80	0.00000000	mice
Ifi44	0.98	0.00000000	mice
Ifi206	0.73	0.00000000	mice
Samd9l	1.00	0.00000000	mice
Ifit1	0.85	0.00000000	mice
Sp100	0.88	0.00000000	mice
Foxo1	-0.82	0.00000000	mice
Tor3a	1.00	0.00000000	mice

Ccm2	-0.97	0.00000000	mice
Phf11b	0.78	0.00000000	mice
Slfn2	0.97	0.00000000	mice
Rel	0.82	0.00000000	mice
Tmsb10	0.59	0.00000000	mice
Pik3ip1	-0.97	0.00000000	mice
Dgkd	-0.72	0.00000000	mice
Aff3	-0.68	0.00000000	mice
Fam107b	-0.67	0.00000000	mice
Tsc22d3	-0.98	0.00000000	mice
H2-Aa	0.61	0.00000000	mice
Tpt1	0.36	0.00000000	mice
Igtp	0.63	0.00000000	mice
H2-T23	0.74	0.00000000	mice
Ccl4	0.58	0.00000000	mice
Rsad2	0.65	0.00000000	mice
Ifit2	0.75	0.00000000	mice
Cd83	0.84	0.00000000	mice
H2-Eb1	0.59	0.00000000	mice
H3f3a	-0.46	0.00000000	mice
Helz2	0.75	0.00000000	mice
Ebf1	-0.56	0.00000000	mice
Stat2	0.87	0.00000000	mice
AY036118	0.74	0.00000000	mice
Cd79a	-0.52	0.00000000	mice
Ifi208	0.56	0.00000000	mice
Sorl1	-0.74	0.00000000	mice
Gramd4	-0.94	0.00000000	mice
Trim30c	0.69	0.00000000	mice
Vps37b	0.84	0.00000000	mice
Gbp7	0.84	0.00000000	mice
Cd86	0.88	0.00000000	mice
Atp1b1	-0.97	0.00000000	mice
Nsf	-0.67	0.00000000	mice
Inpp5d	-0.55	0.00000000	mice
Cd79b	-0.80	0.00000000	mice
Dtx3l	0.66	0.00000000	mice
Zup1	0.91	0.00000000	mice
Trim11	-0.83	0.00000000	mice
Scaf11	-0.70	0.00000000	mice
Irf9	0.61	0.00000000	mice
Psmb9	0.68	0.00000000	mice
Serpina3g	0.79	0.00000000	mice
Ly86	0.91	0.00000000	mice
H2-Ab1	0.54	0.00000000	mice
Irf2bp2	-0.83	0.00000000	mice

Ccnd3	-0.79	0.00000000	mice
Elmo1	-0.68	0.00000000	mice
Uba52	0.65	0.00000000	mice
Cblb	0.72	0.00000000	mice
Cxcl10	0.48	0.00000000	mice
Ddx24	0.64	0.00000000	mice
Smad7	-0.73	0.00000000	mice
Ddx58	0.77	0.00000000	mice
Eif2ak2	0.64	0.00000000	mice
Atp5e	0.60	0.00000000	mice
Gne	-0.84	0.00000000	mice
Prkcd	-0.83	0.00000000	mice
Tlr7	0.49	0.00000000	mice
Arl5c	-0.93	0.00000000	mice
Foxp1	-0.43	0.00000000	mice
Tspan14	-0.93	0.00000000	mice
Fam129c	-0.83	0.00000000	mice
Serinc3	-0.47	0.00000000	mice
Cux1	-0.74	0.00000000	mice
Scgb1a1	0.81	0.00000000	mice
Fryl	-0.59	0.00000000	mice
Setbp1	0.69	0.00000000	mice
Il1b	0.60	0.00000000	mice
Nr4a1	0.79	0.00000000	mice
Sik1	-0.69	0.00000000	mice
Ifi35	0.63	0.00000000	mice
Dennd4a	0.62	0.00000000	mice
Bcr	-0.89	0.00000000	mice
Clec2d	0.48	0.00000000	mice
Rell1	-0.98	0.00000000	mice
Ddx60	0.61	0.00000000	mice
Cxcl2	0.76	0.00000000	mice
Sell	0.63	0.00000000	mice
Cd52	0.54	0.00000000	mice
Plek	0.81	0.00000000	mice
Zc3hav1	0.61	0.00000000	mice
Ucp2	-0.64	0.00000000	mice
Inpp4b	0.64	0.00000000	mice
Ly6e	0.56	0.00000000	mice
Setdb2	0.76	0.00000000	mice
Trib2	-0.76	0.00000000	mice
Jund	-0.54	0.00000000	mice
Isg20	0.46	0.00000000	mice
Gimap6	0.60	0.00000000	mice
Cdyl2	-0.88	0.00000000	mice
Oas2	0.38	0.00000000	mice

Cxcr5	0.66	0.00000000	mice
Ifit3b	0.34	0.00000000	mice
H2-Oa	0.55	0.00000000	mice
Gbp4	0.53	0.00000000	mice
Gvin1	0.40	0.00000000	mice
Ifi30	-0.83	0.00000000	mice
Fam53b	-0.69	0.00000000	mice
Psme1	0.61	0.00000000	mice
Inpp5a	-0.71	0.00000000	mice
9930111J21Rik2	0.65	0.00000000	mice
Fam117a	-0.81	0.00000000	mice
H2-D1	0.36	0.00000000	mice
Ftl1	-0.42	0.00000000	mice
Chd7	0.62	0.00000000	mice
H2-T22	0.54	0.00000000	mice
Atp2a3	-0.69	0.00000000	mice
Ppp1r21	-0.57	0.00000000	mice
Stxbp1	-0.75	0.00000000	mice
Arhgef18	-0.70	0.00000000	mice
Ralgps2	-0.59	0.00000000	mice
Iglc1	-0.94	0.00000000	mice
Lrmp	-0.81	0.00000000	mice
Prkcg	-0.62	0.00000000	mice
Daxx	0.54	0.00000000	mice
Stat4	0.67	0.00000000	mice
Tpst1	0.72	0.00000000	mice
Etnk1	0.60	0.00000000	mice
Foxn3	-0.60	0.00000000	mice
Plac8	0.84	0.00000000	mice
Ifih1	0.49	0.00000000	mice
Blnk	-0.64	0.00000000	mice
Clint1	-0.64	0.00000000	mice
Tmem131l	-0.52	0.00000000	mice
Psmb8	0.58	0.00000000	mice
Crem	0.60	0.00000000	mice
Fam111a	0.61	0.00000000	mice
Snx5	-0.49	0.00000000	mice
Sec61g	0.52	0.00000000	mice
Spib	-0.72	0.00000000	mice
Baz1a	0.52	0.00000000	mice
Slc12a6	-0.51	0.00000000	mice
Ctss	0.50	0.00000000	mice
C130026I21Rik	0.59	0.00000000	mice
Arhgap17	-0.63	0.00000000	mice
Arhgap25	-0.60	0.00000000	mice
Pten	-0.53	0.00000000	mice

Adar	0.49	0.00000000	mice
Plekha2	-0.48	0.00000000	mice
Vav2	-0.66	0.00000000	mice
Snrpg	0.48	0.00000000	mice
Ascc3	0.70	0.00000000	mice
Ighd	0.48	0.00000000	mice
H2-T24	0.36	0.00000000	mice
Eef1a1	0.31	0.00000000	mice
Cmpk2	0.33	0.00000000	mice
Dtx1	-0.70	0.00000000	mice
AW112010	0.46	0.00000000	mice
Tle3	-0.65	0.00000000	mice
5330438D12Rik	0.56	0.00000000	mice
Il7r	-0.65	0.00000000	mice
Rhoh	-0.73	0.00000000	mice
Ankrd55	-0.68	0.00000000	mice
Chst3	-0.66	0.00000000	mice
Sp140	0.49	0.00000000	mice
Sp110	0.52	0.00000000	mice
Stap1	0.64	0.00000000	mice
Trp53i11	-0.64	0.00000000	mice
Tdrd7	0.45	0.00000000	mice
Rnf114	0.47	0.00000000	mice
Gnas	-0.45	0.00000000	mice
Trafd1	0.43	0.00000000	mice
Immp2l	0.61	0.00000000	mice
Hsh2d	0.43	0.00000000	mice
Arid1b	-0.55	0.00000000	mice
Irf1	0.72	0.00000000	mice
Trp53inp1	-0.60	0.00000000	mice
BE692007	0.55	0.00000000	mice
Arhgap15	-0.42	0.00000000	mice
Dhx58	0.30	0.00000000	mice
Tnfrsf13c	-0.56	0.00000000	mice
Plekho1	-0.53	0.00000000	mice
Rasgef1b	0.62	0.00000000	mice
Cerk	-0.46	0.00000000	mice
Cst3	-0.56	0.00000000	mice
Pax5	-0.47	0.00000000	mice
Nampt	0.51	0.00000000	mice
Gns	-0.61	0.00000000	mice
Irgm2	0.30	0.00000000	mice
Ccl2	0.23	0.00000000	mice
Gbp5	0.32	0.00000000	mice
Lyz2	0.69	0.00000000	mice
Cxcr4	-0.48	0.00000000	mice

Cxcl1	0.31	0.00000000	mice
Ppa1	0.50	0.00000000	mice
Ifi204	0.30	0.00000000	mice
Ptk2b	-0.49	0.00000000	mice
Camk1d	-0.48	0.00000000	mice
Sh3kbp1	-0.60	0.00000000	mice
Lgals3bp	0.21	0.00000000	mice
Napsa	-0.50	0.00000000	mice
Bcl2a1b	0.47	0.00000000	mice
P2ry10	0.50	0.00000000	mice
Cxxc5	-0.52	0.00000000	mice
Zfp706	-0.48	0.00000000	mice
H3f3b	-0.32	0.00000000	mice
Cyfip2	-0.51	0.00000000	mice
Tfeb	-0.52	0.00000000	mice
Ankrd12	-0.51	0.00000000	mice
Fli1	-0.43	0.00000000	mice
Fyn	0.45	0.00000000	mice
Siglecg	-0.55	0.00000000	mice
Gpr132	0.47	0.00000000	mice
Fut8	-0.59	0.00000000	mice
Rasgrp3	0.56	0.00000000	mice
Wdfy4	-0.54	0.00000000	mice
Cplx2	-0.45	0.00000000	mice
Itpr2	-0.53	0.00000000	mice
Arhgap24	-0.77	0.00000000	mice
Lef1	-0.53	0.00000000	mice
Ikzf1	-0.63	0.00000000	mice
Fbxo11	-0.44	0.00000000	mice
Prkar2b	-0.47	0.00000000	mice
Isy1	0.50	0.00000000	mice
Rragd	-0.44	0.00000000	mice
Gimap4	0.38	0.00000000	mice
Smc6	-0.40	0.00000000	mice
Ogdh	-0.51	0.00000000	mice
Rreb1	-0.55	0.00000000	mice
Cbfa2t3	-0.51	0.00000000	mice
Plcg2	-0.52	0.00000000	mice
Casp4	0.48	0.00000000	mice
Ccr6	0.50	0.00000000	mice
Ube2h	-0.62	0.00000000	mice
Tapbp	0.50	0.00000000	mice
Dennd3	-0.49	0.00000000	mice
Cd24a	-0.64	0.00000000	mice
Gbp2b	0.38	0.00000000	mice
Ccrl2	0.30	0.00000000	mice

Fcer2a	0.46	0.00000000	mice
Psme2	0.48	0.00000000	mice
Herpud1	-0.53	0.00000000	mice
Kmt2c	-0.45	0.00000000	mice
Gpd1l	-0.49	0.00000000	mice
Elmsan1	-0.54	0.00000000	mice
Dusp6	-0.42	0.00000000	mice
Antxr2	0.53	0.00000000	mice
Satb1	0.41	0.00000000	mice
Slc38a1	-0.52	0.00000000	mice
Ly6c1	0.29	0.00000000	mice
Csprs	0.20	0.00000000	mice
Klhl6	-0.60	0.00000000	mice
Phf11c	0.30	0.00000000	mice
Ptpn18	0.44	0.00000000	mice
Akap13	-0.42	0.00000000	mice
Abcg1	-0.55	0.00000000	mice
Arf4	0.48	0.00000000	mice
Nuak1	-0.44	0.00000000	mice
Adam10	-0.75	0.00000000	mice
Hspa5	0.42	0.00000000	mice
Galnt7	-0.56	0.00000000	mice
Srsf7	0.41	0.00000000	mice
Gbp2	0.31	0.00000000	mice
Ptma	-0.28	0.00000000	mice
Gbp9	0.33	0.00000000	mice
Pnpt1	0.37	0.00000000	mice
Sgms1	-0.56	0.00000000	mice
Osbpl8	-0.54	0.00000000	mice
Pkig	-0.46	0.00000000	mice
Pde2a	-0.61	0.00000000	mice
Cd2	-0.50	0.00000000	mice
Slco4a1	-0.38	0.00000000	mice
Edaradd	-0.50	0.00000000	mice
Eif5b	0.40	0.00000000	mice
Fcrla	-0.49	0.00000000	mice
Zdhhc18	0.38	0.00000000	mice
Cd37	-0.39	0.00000000	mice
B4galt1	-0.54	0.00000000	mice
Fchsd2	0.46	0.00000000	mice
Litaf	0.44	0.00000000	mice
Cr2	0.40	0.00000000	mice
Eif2ak3	-0.60	0.00000000	mice
Stk4	-0.43	0.00000000	mice
E330020D12Rik	-0.59	0.00000000	mice
Pde4c	-0.33	0.00000000	mice

Hivep3	-0.63	0.00000000	mice
Cobll1	-0.60	0.00000000	mice
Slc25a18	-0.45	0.00000000	mice
Nceh1	0.47	0.00000000	mice
5830418P13Rik	-0.57	0.00000000	mice
Tfam	0.40	0.00000000	mice
N4bp2	-0.51	0.00000000	mice
Strbp	-0.56	0.00000000	mice
Ecpas	-0.47	0.00000000	mice
Bank1	0.37	0.00000000	mice
Kat6b	-0.66	0.00000000	mice
Egfl6	-0.38	0.00000000	mice
Sept11	-0.59	0.00000000	mice
Gpr183	0.69	0.00000000	mice
Lgals9	0.49	0.00000000	mice
Gpam	-0.34	0.00000000	mice
Zcchc2	0.46	0.00000000	mice
Fnip1	-0.41	0.00000000	mice
Rmnd5b	-0.43	0.00000000	mice
Rhoq	-0.53	0.00000000	mice
Pi4ka	-0.46	0.00000000	mice
5031439G07Rik	-0.45	0.00000000	mice
Ddx5	0.26	0.00000000	mice
Dynll1	0.39	0.00000000	mice
Commd10	-0.60	0.00000000	mice
Pip5k1c	-0.41	0.00000000	mice
Ptges3	0.34	0.00000000	mice
Hsp90b1	-0.45	0.00000000	mice
Tmem140	0.30	0.00000000	mice
Prex1	-0.50	0.00000000	mice
Ier5	0.42	0.00000000	mice
Irs2	-0.50	0.00000000	mice
Unc93b1	-0.49	0.00000000	mice
Eps8	-0.56	0.00000000	mice
Coro7	-0.50	0.00000000	mice
Rcsd1	-0.47	0.00000000	mice
St3gal1	-0.51	0.00000000	mice
Trim12c	0.41	0.00000000	mice
Wasf2	-0.40	0.00000000	mice
Ubn1	-0.41	0.00000000	mice
Foxn2	-0.54	0.00000000	mice
Ikzf3	-0.49	0.00000000	mice
Cox6c	0.38	0.00000000	mice
Fcer1g	0.31	0.00000000	mice
Crebrf	-0.42	0.00000000	mice
Smap2	0.34	0.00000000	mice

Klhl24	-0.41	0.00000000	mice
Apoe	0.51	0.00000000	mice
Id3	-0.52	0.00000000	mice
Sdc4	-0.50	0.00000000	mice
Tomm7	0.36	0.00000000	mice
Sesn1	-0.53	0.00000000	mice
Ptk2	0.37	0.00000000	mice
Arid5b	0.47	0.00000000	mice
Klf13	-0.38	0.00000000	mice
Gbp3	0.23	0.00000000	mice
Ppp1r16b	0.38	0.00000000	mice
Pde7a	-0.31	0.00000000	mice
Irf2	0.38	0.00000000	mice
Dok3	-0.46	0.00000000	mice
Ypel3	-0.46	0.00000000	mice
Ogfr	0.32	0.00000000	mice
Polm	-0.39	0.00000000	mice
Cdc42ep3	-0.43	0.00000000	mice
Rbl1	0.33	0.00000000	mice
Aig1	0.48	0.00000000	mice
Pik3cd	-0.53	0.00000000	mice
Ppp2r5a	-0.42	0.00000000	mice
Bmf	-0.35	0.00000000	mice
Tgfbr2	-0.35	0.00000000	mice
Fgd2	-0.39	0.00000000	mice
AC149090.1	0.39	0.00000000	mice
Slc30a5	-0.47	0.00000000	mice
Zdhhc14	-0.66	0.00000000	mice
Cd47	0.38	0.00000000	mice
Rasgrp2	-0.39	0.00000000	mice
Wfdc17	0.20	0.00000000	mice
Cox17	0.35	0.00000000	mice
Cd72	-0.58	0.00000000	mice
Cmss1	0.38	0.00000000	mice
Tmsb4x	0.23	0.00000000	mice
Cnot6l	0.36	0.00000000	mice
Iglc2	-0.51	0.00000000	mice
Atp13a2	-0.37	0.00000000	mice
Cep170	-0.50	0.00000000	mice
Gpr174	0.31	0.00000000	mice
Tcf3	-0.39	0.00000000	mice
Sbk1	-0.45	0.00000000	mice
Asph	0.52	0.00000000	mice
Fbxo33	-0.42	0.00000000	mice
Cox7c	0.33	0.00000000	mice
Gbp8	0.27	0.00000000	mice

Coq7	-0.36	0.00000000	mice
Sh3pxd2a	-0.51	0.00000000	mice
Nmi	0.38	0.00000000	mice
Iqsec1	-0.45	0.00000000	mice
Psd3	-0.56	0.00000000	mice
Adam19	-0.46	0.00000000	mice
Pafah1b3	-0.41	0.00000000	mice
Ubl5	0.32	0.00000000	mice
Insyn2b	0.37	0.00000000	mice
Tank	0.43	0.00000000	mice
4933406I18Rik	0.47	0.00000000	mice
Sox4	-0.36	0.00000000	mice
Pgm2l1	-0.40	0.00000000	mice
Orai2	-0.38	0.00000000	mice
Nedd4	-0.34	0.00000000	mice
Gimap8	0.32	0.00000000	mice
Ctsh	0.31	0.00000000	mice
Pnpla7	-0.42	0.00000000	mice
Nedd4l	-0.49	0.00000000	mice
Erp29	-0.39	0.00000000	mice
Acss1	-0.47	0.00000000	mice
Xpr1	-0.49	0.00000000	mice
Ahnak	-0.43	0.00000000	mice
Ppm1k	0.33	0.00000000	mice
Arpc5l	-0.41	0.00000000	mice
Nrgn	-0.33	0.00000000	mice
Chd6	-0.38	0.00000000	mice
Ube2j1	-0.38	0.00000000	mice
Cpt1a	-0.38	0.00000000	mice
Prkce	-0.38	0.00000000	mice
Traf1	0.28	0.00000000	mice
Olfr56	0.25	0.00000000	mice
Rai1	-0.47	0.00000000	mice
Rab3a	-0.26	0.00000000	mice
Heatr6	-0.37	0.00000000	mice
Eef2k	-0.30	0.00000000	mice
Rack1	0.23	0.00000000	mice
Lamb3	0.21	0.00000000	mice
Il6st	-0.49	0.00000000	mice
H2-Q4	0.26	0.00000000	mice
Cuedc1	-0.30	0.00000000	mice
Acp5	-0.37	0.00000000	mice
Tspo	0.36	0.00000000	mice
Junb	0.35	0.00000000	mice
St6gal1	-0.62	0.00000000	mice
Susd3	-0.35	0.00000000	mice

Tor1aip1	0.43	0.00000000	mice
Son	0.27	0.00000000	mice
A930015D03Rik	0.35	0.00000000	mice
Hvcn1	-0.39	0.00000000	mice
Rnf122	-0.30	0.00000000	mice
Ero1lb	-0.39	0.00000000	mice
5430427O19Rik	0.34	0.00000000	mice
Tfrc	-0.45	0.00000000	mice
Stt3b	-0.40	0.00000000	mice
Filip1l	0.33	0.00000000	mice
Ppm1e	-0.69	0.00000000	mice
Xrn2	0.34	0.00000000	mice
Dusp10	-0.44	0.00000000	mice
Fbxl5	-0.39	0.00000000	mice
Tpm4	0.32	0.00000000	mice
2900026A02Rik	-0.28	0.00000000	mice
Csk	-0.34	0.00000000	mice
Aida	0.34	0.00000000	mice
Arhgap45	-0.37	0.00000000	mice
Ten1	-0.39	0.00000000	mice
Uqcrb	0.31	0.00000000	mice
Sik3	-0.34	0.00000000	mice
Cyba	-0.43	0.00000000	mice
Pfdn5	0.29	0.00000000	mice
Usp12	-0.40	0.00000000	mice
Gpat3	-0.33	0.00000000	mice
Akap17b	-0.31	0.00000000	mice
Ttc7	-0.41	0.00000000	mice
Tacc1	-0.41	0.00000000	mice
Slc36a4	-0.29	0.00000000	mice
Slc38a9	-0.45	0.00000000	mice
Pced1b	0.42	0.00000000	mice
H2-Ob	0.33	0.00000000	mice
Icosl	0.24	0.00000000	mice
Kras	0.24	0.00000000	mice
Gatad2b	-0.31	0.00000000	mice
Desi1	-0.45	0.00000000	mice
Sec24a	-0.44	0.00000000	mice
Tmc6	-0.36	0.00000000	mice
Ets1	-0.23	0.00000000	mice
Rere	-0.37	0.00000000	mice
Rb1	-0.38	0.00000001	mice
Ccnd2	0.38	0.00000001	mice
Ier2	0.35	0.00000001	mice
Tma7	0.31	0.00000001	mice
Zfp318	0.37	0.00000001	mice

Atad2b	-0.33	0.00000001	mice
Rev3l	-0.39	0.00000001	mice
Xrcc6	-0.40	0.00000001	mice
Tubb2b	-0.30	0.00000001	mice
Svil	-0.47	0.00000001	mice
Nfkb1	0.29	0.00000001	mice
Sun2	-0.36	0.00000001	mice
Fam49b	-0.28	0.00000001	mice
Ccdc50	0.34	0.00000001	mice
Wipf1	-0.45	0.00000001	mice
Nipbl	-0.30	0.00000001	mice
Usp3	-0.43	0.00000001	mice
Ndufb1-ps	0.29	0.00000001	mice
Sftpc	-0.08	0.00000001	mice
Trim56	0.29	0.00000002	mice
Ubash3b	-0.56	0.00000002	mice
Srpk2	-0.40	0.00000002	mice
Hip1r	-0.35	0.00000002	mice
Slc43a2	-0.38	0.00000002	mice
Sema4d	-0.53	0.00000002	mice
Tuba1b	0.27	0.00000003	mice
Pou2f2	-0.48	0.00000003	mice
Itpr3	-0.34	0.00000003	mice
Hbp1	-0.34	0.00000003	mice
Ndufa2	0.32	0.00000003	mice
Syngr2	0.27	0.00000003	mice
Patj	0.38	0.00000003	mice
Arl8a	-0.33	0.00000003	mice
Gse1	-0.42	0.00000004	mice
Bhlhe41	-0.34	0.00000004	mice
Cep78	-0.28	0.00000004	mice
Pld4	-0.36	0.00000004	mice
Tnfrsf1b	0.26	0.00000004	mice
Slc44a2	-0.44	0.00000004	mice
Runx3	0.28	0.00000004	mice
Ndufa13	0.27	0.00000004	mice
Atp6ap2	-0.34	0.00000005	mice
Ankfy1	0.34	0.00000005	mice
Aldh7a1	0.26	0.00000005	mice
Gstt2	-0.29	0.00000005	mice
1700017B05Rik	-0.30	0.00000006	mice
Ptprj	-0.35	0.00000006	mice
Ptpn6	-0.38	0.00000007	mice
Pdxk	-0.31	0.00000007	mice
Gga2	-0.37	0.00000008	mice
Ptprcap	-0.40	0.00000008	mice

Rcc2	-0.33	0.00000009	mice
Arid3a	-0.29	0.00000010	mice
Ccng2	-0.28	0.00000010	mice
Zmiz1	-0.40	0.00000011	mice
Tram2	-0.37	0.00000012	mice
Znfx1	0.30	0.00000012	mice
Smco4	-0.21	0.00000012	mice
Calm2	-0.33	0.00000013	mice
Purb	-0.33	0.00000013	mice
Zfp827	-0.38	0.00000014	mice
Usp25	0.45	0.00000014	mice
H2-Q7	0.26	0.00000014	mice
Smurf1	-0.38	0.00000015	mice
Kdm1b	-0.26	0.00000016	mice
Ddx54	-0.37	0.00000016	mice
Csrp2	-0.30	0.00000016	mice
Tnrc6b	-0.30	0.00000017	mice
Ifnar1	-0.32	0.00000017	mice
Romo1	0.27	0.00000017	mice
Dip2c	-0.58	0.00000018	mice
Rbm38	-0.35	0.00000018	mice
Tra2b	0.28	0.00000020	mice
Ypel5	-0.32	0.00000022	mice
Slf2	-0.37	0.00000023	mice
Tor1aip2	0.32	0.00000023	mice
Tmem243	0.34	0.00000024	mice
N4bp3	-0.19	0.00000024	mice
Grk6	-0.32	0.00000026	mice
Dusp2	-0.35	0.00000028	mice
Atp6v0d1	-0.34	0.00000029	mice
Nfkbiz	0.30	0.00000031	mice
Hs2st1	-0.47	0.00000032	mice
Clec2i	0.21	0.00000034	mice
Ostf1	-0.33	0.00000034	mice
Elf2	-0.35	0.00000034	mice
Hist1h1c	-0.28	0.00000035	mice
Tom1	-0.39	0.00000035	mice
Slc24a1	0.17	0.00000035	mice
Serpini1	0.32	0.00000036	mice
Cdc25b	-0.23	0.00000038	mice
Cib1	-0.34	0.00000040	mice
Kdm3a	-0.28	0.00000042	mice
Zfp951	-0.33	0.00000045	mice
Tapt1	-0.37	0.00000045	mice
Rab43	0.33	0.00000051	mice
Eif2s2	0.27	0.00000056	mice

Pip5k1b	-0.49	0.00000067	mice
Kctd3	-0.34	0.00000067	mice
Afap1	-0.37	0.00000072	mice
Vps18	-0.28	0.00000073	mice
Ralgps1	-0.34	0.00000073	mice
S100a6	0.35	0.00000083	mice
Capzb	-0.29	0.00000088	mice
Evl	0.31	0.00000090	mice
Limd1	-0.35	0.00000100	mice
Ccl6	0.30	0.00000106	mice
Ago2	-0.37	0.00000108	mice
Bcl2a1d	0.18	0.00000109	mice
Srsf3	-0.28	0.00000110	mice
Zmynd11	-0.33	0.00000111	mice
Atp5mpl	0.30	0.00000125	mice
Blk	-0.37	0.00000127	mice
Cyfip1	0.29	0.00000131	mice
Ilrun	0.31	0.00000138	mice
Atp6v1b2	-0.36	0.00000140	mice
Lrrc8c	-0.45	0.00000140	mice
Atp5j	0.23	0.00000142	mice
Txndc11	-0.40	0.00000144	mice
Snx30	-0.31	0.00000154	mice
Gfi1	-0.22	0.00000162	mice
Gna11	-0.28	0.00000184	mice
Atp6v0e	-0.28	0.00000198	mice
Casp8	0.27	0.00000211	mice
Psap	-0.30	0.00000223	mice
Pik3cg	-0.33	0.00000240	mice
Fig4	-0.36	0.00000254	mice
Sppl2a	0.29	0.00000258	mice
Coro1c	-0.39	0.00000270	mice
Arfgef2	-0.35	0.00000284	mice
Dleu2	-0.45	0.00000284	mice
Oga	-0.28	0.00000288	mice
Cfap43	-0.40	0.00000298	mice
Zfp292	-0.29	0.00000303	mice
Wdr81	-0.22	0.00000314	mice
Bcor	-0.42	0.00000335	mice
Pfkfb3	-0.45	0.00000360	mice
Zhx2	-0.29	0.00000361	mice
Frat2	-0.24	0.00000385	mice
Gnpda1	-0.26	0.00000396	mice
Cirbp	-0.30	0.00000434	mice
Cmc2	-0.24	0.00000437	mice
Abi3	-0.30	0.00000462	mice

Pakap.1	-0.48	0.00000464	mice
Lacc1	0.16	0.00000498	mice
Rragc	-0.33	0.00000527	mice
Eif4g3	-0.30	0.00000584	mice
Emid1	-0.27	0.00000586	mice
Ptpn22	-0.37	0.00000637	mice
Nsd3	0.33	0.00000642	mice
Alcam	0.25	0.00000671	mice
Btrc	-0.35	0.00000674	mice
Usp50	0.18	0.00000691	mice
Pde4b	0.28	0.00000752	mice
Rasal1	-0.28	0.00000765	mice
Sh3bp2	0.21	0.00000772	mice
Dip2b	-0.28	0.00000812	mice
Natd1	-0.28	0.00000891	mice
Slirp	0.19	0.00000941	mice
Galk1	-0.19	0.00001077	mice
Rb1cc1	-0.31	0.00001234	mice
Trim14	0.29	0.00001239	mice
Phf6	0.29	0.00001262	mice
Snrpf	0.26	0.00001361	mice
Rnf157	0.47	0.00001366	mice
Ppp1cc	-0.31	0.00001380	mice
Dnah8	-0.38	0.00001393	mice
4930444A19Rik	0.19	0.00001558	mice
Hmgn1	-0.28	0.00001594	mice
Inpp5k	-0.34	0.00001612	mice
Ssbp2	-0.43	0.00001614	mice
Cspp1	-0.36	0.00001647	mice
Bzw2	-0.34	0.00001657	mice
Arhgap12	-0.32	0.00001657	mice
Rest	-0.30	0.00001670	mice
Phf2	-0.27	0.00001690	mice
Gsap	0.20	0.00001706	mice
Ddx21	0.27	0.00001899	mice
Xiap	0.33	0.00002233	mice
Cox7b	0.26	0.00002253	mice
Ddx6	0.22	0.00002263	mice
Tsix	0.27	0.00002400	mice
Slc1a5	-0.38	0.00002414	mice
Lyst	-0.39	0.00002471	mice
Cdkn1b	-0.32	0.00002606	mice
Wwp2	-0.33	0.00002621	mice
Znrf3	-0.37	0.00002687	mice
Sec11a	-0.30	0.00002700	mice
Pnpla8	-0.27	0.00002743	mice

Zbtb2	-0.44	0.00002789	mice
Jmjd1c	-0.24	0.00002802	mice
Hck	-0.52	0.00002892	mice
Rab30	-0.44	0.00002974	mice
Paip2	-0.25	0.00003031	mice
Cmtr1	0.24	0.00003053	mice
Btg2	-0.29	0.00003077	mice
Brwd1	-0.38	0.00003201	mice
Rpia	-0.28	0.00003438	mice
Fars2	-0.39	0.00003542	mice
Tsc1	-0.29	0.00003568	mice
Cyth1	-0.22	0.00003662	mice
Ubb	0.17	0.00003684	mice
Cd69	0.41	0.00003690	mice
Uba7	0.16	0.00003701	mice
Stat3	0.28	0.00003743	mice
Nsmce2	-0.33	0.00003911	mice
Srpk3	-0.25	0.00003935	mice
Slc49a4	-0.38	0.00004088	mice
Eef1g	0.25	0.00004291	mice
Tspan13	-0.25	0.00004643	mice
Ywhaz	-0.26	0.00004704	mice
H2-Eb2	0.14	0.00004744	mice
Cd93	-0.26	0.00004952	mice
Ezr	0.22	0.00005174	mice
Ctdp1	-0.30	0.00005296	mice
Neurl3	0.22	0.00005664	mice
Sh2d4b	-0.27	0.00005874	mice
Eef1b2	0.20	0.00005902	mice
Ahdc1	-0.28	0.00005908	mice
Pet100	0.19	0.00005960	mice
Sub1	0.26	0.00006179	mice
Smarca4	-0.34	0.00006576	mice
Pqlc2	-0.24	0.00006807	mice
4930522L14Rik	-0.30	0.00006841	mice
Cmtm7	-0.34	0.00006953	mice
Retreg3	-0.27	0.00007115	mice
Dlg1	-0.32	0.00008095	mice
Tgfbr1	-0.37	0.00008556	mice
Rabgap1l	-0.31	0.00008691	mice
Rnf31	0.17	0.00008804	mice
Raet1e	0.27	0.00008863	mice
Bckdha	-0.29	0.00008869	mice
Chmp4b	0.31	0.00009360	mice
Vps13b	-0.28	0.00009448	mice
Atp5l	0.22	0.00009563	mice

Hnrnpab	0.24	0.00009594	mice
Cd14	0.14	0.00009784	mice
B4galt5	0.27	0.00010025	mice
Ncl	0.24	0.00010647	mice
Plaat3	0.16	0.00010725	mice
Kcnq5	-0.40	0.00010736	mice
Sesn3	0.27	0.00011006	mice
Adamts6	-0.49	0.00011020	mice
Rgs2	-0.37	0.00011033	mice
Usf2	-0.25	0.00011210	mice
Tsga10	-0.27	0.00011235	mice
Limk2	-0.31	0.00011549	mice
Retreg1	0.17	0.00012032	mice
Diaph2	0.31	0.00012869	mice
Exoc4	-0.29	0.00013552	mice
Pdpk1	-0.30	0.00013914	mice
Bcar3	-0.40	0.00014259	mice
Zfp36l1	0.24	0.00014505	mice
Far1os	0.22	0.00014772	mice
Arhgap35	-0.32	0.00014988	mice
Ap3b1	-0.30	0.00015105	mice
Relb	-0.33	0.00015337	mice
Vim	-0.43	0.00015422	mice
Ctsz	-0.28	0.00016998	mice
Chst7	-0.21	0.00017117	mice
Cflar	0.23	0.00017907	mice
Pik3c2b	-0.30	0.00018393	mice
Scaf8	-0.27	0.00018413	mice
Iqgap1	-0.23	0.00019470	mice
Pmepa1	0.31	0.00019765	mice
Eif4ebp1	-0.19	0.00019883	mice
Nr4a3	0.37	0.00020001	mice
Tiparp	-0.39	0.00020279	mice
Rab31	-0.36	0.00021194	mice
Kmt2e	-0.20	0.00021703	mice
Nsmaf	0.21	0.00022100	mice
Cd44	-0.32	0.00023569	mice
Cox7a2	0.23	0.00023747	mice
Sipa1l1	0.32	0.00024215	mice
Ncoa1	-0.29	0.00024647	mice
Gimap3	0.18	0.00026471	mice
Nsa2	0.23	0.00027963	mice
5430405H02Rik	0.19	0.00028614	mice
Tet3	-0.29	0.00030979	mice
Tmem184b	0.25	0.00031481	mice
Cd180	-0.38	0.00031598	mice

C1galt1	-0.36	0.00032056	mice
Per1	-0.26	0.00032976	mice
Ripor1	-0.21	0.00033059	mice
Bri3	-0.25	0.00035469	mice
Limd2	-0.33	0.00036766	mice
Kynu	0.29	0.00037614	mice
Cic	-0.24	0.00039254	mice
Trak1	-0.32	0.00040318	mice
Etv6	0.39	0.00042020	mice
Ghitm	0.23	0.00043645	mice
Tnrc6c	-0.31	0.00044393	mice
Erbin	-0.27	0.00044705	mice
Bbx	0.44	0.00044907	mice
Fgd3	-0.32	0.00044986	mice
Tram1	-0.33	0.00049841	mice
Wdr89	0.17	0.00052832	mice
Clcn5	-0.29	0.00053204	mice
Qk	-0.27	0.00055470	mice
Chka	-0.36	0.00060466	mice
Tubb4b	0.21	0.00062489	mice
Clta	-0.26	0.00065553	mice
Chchd10	-0.26	0.00069073	mice
Il12a	-0.27	0.00069727	mice
Atrnl1	-0.30	0.00070247	mice
S100a9	0.16	0.00070520	mice
Lcp1	0.24	0.00075356	mice
Txnip	-0.30	0.00079583	mice
4930426D05Rik	-0.20	0.00082630	mice
Fxyd5	0.20	0.00083452	mice
H2-Q6	0.17	0.00091098	mice
Phf3	-0.25	0.00092417	mice
2310001H17Rik	0.16	0.00092809	mice
Plekhf2	-0.26	0.00093772	mice
Hist3h2a	-0.23	0.00097331	mice
9430038I01Rik	-0.28	0.00099236	mice
Rras2	0.23	0.00099641	mice
Naa40	-0.25	0.00101381	mice
Rsrp1	0.25	0.00102567	mice
Sem1	0.22	0.00106943	mice
Lpin2	-0.33	0.00107100	mice
4930597A21Rik	-0.26	0.00107682	mice
Vps35	-0.25	0.00111621	mice
Tab2	-0.27	0.00112071	mice
Soat1	0.19	0.00112158	mice
Kdm6b	0.31	0.00112700	mice
Kpna1	0.23	0.00115045	mice

Cfl1	-0.23	0.00116501	mice
Plcl2	-0.28	0.00117009	mice
Tbc1d15	-0.26	0.00121962	mice
Snx2	0.28	0.00123216	mice
Hcst	0.15	0.00124943	mice
Baz2a	-0.32	0.00131160	mice
Tagln2	-0.42	0.00135066	mice
Cd274	0.41	0.00139865	mice
Tnfrsf13b	-0.30	0.00141039	mice
Gas5	0.27	0.00144818	mice
Jmy	-0.26	0.00145305	mice
Hmgb1	-0.24	0.00146560	mice
Lcorl	-0.33	0.00153577	mice
Clasp1	-0.32	0.00155602	mice
Cpm	-0.48	0.00169132	mice
Nap1l1	0.22	0.00177875	mice
Ppp3ca	0.24	0.00187860	mice
Igf1r	-0.33	0.00193952	mice
Galnt11	0.23	0.00199975	mice
Sfi1	0.25	0.00202587	mice
Serbp1	0.22	0.00202707	mice
4930523C07Rik	0.20	0.00202739	mice
Exoc2	-0.33	0.00204519	mice
Scd2	-0.21	0.00207778	mice
Rapgef2	-0.33	0.00208096	mice
Rsbn1l	0.25	0.00215346	mice
Ell2	0.28	0.00227039	mice
Ptp4a3	-0.30	0.00231606	mice
Ndufa3	0.21	0.00234005	mice
Nrm	-0.25	0.00236963	mice
Lgals3	0.21	0.00241834	mice
Bcl2l1	-0.31	0.00243747	mice
Atp6v0b	-0.28	0.00244291	mice
Snn	-0.29	0.00253748	mice
Patz1	-0.26	0.00256683	mice
Zfp362	-0.19	0.00257049	mice
Ddx18	0.19	0.00259433	mice
Abcd1	-0.20	0.00263048	mice
Atg7	-0.28	0.00273328	mice
Ppp1r15a	0.27	0.00281505	mice
Grasp	0.22	0.00290971	mice
Zfas1	0.21	0.00296833	mice
Hnrnpl	-0.23	0.00300024	mice
Stk24	-0.25	0.00302134	mice
Paxx	-0.19	0.00302343	mice
Hist1h1e	-0.18	0.00305013	mice

Wapl	0.20	0.00317833	mice
Ube2g2	-0.24	0.00318425	mice
Bicra	-0.29	0.00321288	mice
Wdr7	-0.30	0.00327872	mice
Srrm2	0.19	0.00329049	mice
Ifrd1	0.23	0.00331596	mice
Gabarap	-0.23	0.00332191	mice
Clcn6	-0.22	0.00335356	mice
Ripk1	0.22	0.00340932	mice
Nup98	0.22	0.00354449	mice
Atf6	-0.31	0.00354575	mice
Themis2	0.20	0.00355688	mice
Pcyt1a	-0.28	0.00380132	mice
Ndufa1	0.21	0.00383534	mice
Nucks1	0.20	0.00385634	mice
Tcp11l2	-0.26	0.00398885	mice
Sclt1	0.23	0.00410282	mice
Rasgrp1	-0.26	0.00416693	mice
Chd3	-0.26	0.00430990	mice
Dek	0.27	0.00433603	mice
Lrrfip1	0.22	0.00436840	mice
Rab8a	0.20	0.00438104	mice
Arpc1a	-0.24	0.00438119	mice
Tent2	-0.30	0.00453885	mice
Cdkl1	-0.18	0.00453995	mice
Dusp3	-0.24	0.00467604	mice
Zmat2	0.21	0.00469195	mice
Rftn1	-0.24	0.00477428	mice
Aff1	-0.34	0.00509690	mice
1810037I17Rik	0.19	0.00511496	mice
Klhl7	-0.23	0.00514163	mice
Ttyh3	-0.20	0.00525676	mice
Otud7b	-0.32	0.00529558	mice
Vps26a	-0.24	0.00538302	mice
Vezf1	-0.25	0.00539178	mice
Pcgf5	0.35	0.00547985	mice
Zfp944	-0.23	0.00570370	mice
Tbc1d22a	-0.32	0.00574910	mice
Cyp39a1	-0.25	0.00609082	mice
Nsd1	-0.27	0.00611281	mice
Pxk	-0.28	0.00630896	mice
Bmp2k	0.22	0.00667664	mice
Traf3ip3	0.23	0.00684089	mice
Rae1	-0.25	0.00707136	mice
Rapgef4	-0.42	0.00718070	mice
Kidins220	-0.31	0.00765796	mice

Ehbp1l1	-0.23	0.00772377	mice
Dnajc21	-0.29	0.00781938	mice
Tfdp2	-0.34	0.00798368	mice
Lamtor1	-0.26	0.00812241	mice
Lbh	-0.26	0.00820657	mice
Uggt1	-0.23	0.00842361	mice
Sys1	-0.24	0.00864250	mice
Lpxn	0.24	0.00886778	mice
Bcl2	0.19	0.00905543	mice
St6galnac3	0.29	0.00944670	mice
Dennd1b	-0.23	0.00968464	mice
Atp6v1g1	-0.20	0.00997403	mice
Synj1	-0.22	0.00998394	mice
Vps41	-0.33	0.01005108	mice
H2afv	-0.27	0.01006556	mice
Sppl3	-0.25	0.01007757	mice
Uqcr10	0.25	0.01037092	mice
Trim8	-0.21	0.01040161	mice
Rnf19a	-0.24	0.01137538	mice
Atp5k	0.19	0.01177589	mice
Epb41l5	-0.24	0.01224395	mice
Ilf3	-0.23	0.01238620	mice
Psat1	-0.18	0.01248100	mice
Id2	-0.34	0.01280391	mice
Nfx1	-0.25	0.01298213	mice
Ttc7b	-0.26	0.01303881	mice
Nt5c3	0.28	0.01340129	mice
Atp8a1	-0.22	0.01354647	mice
Fcmr	0.16	0.01365008	mice
Tespa1	0.14	0.01437997	mice
Atxn1	0.34	0.01442933	mice
Lsp1	-0.27	0.01447784	mice
Plekha5	-0.24	0.01507878	mice
Colgalt1	-0.35	0.01517691	mice
Dnajb6	0.23	0.01519970	mice
Ttc39b	0.23	0.01532843	mice
Tifa	-0.20	0.01580305	mice
Ifnar2	-0.39	0.01593702	mice
1700029H14Rik	-0.15	0.01594895	mice
Eapp	0.19	0.01598661	mice
Rnf144a	0.18	0.01599100	mice
Slc25a36	-0.23	0.01620714	mice
Ccny	-0.24	0.01679152	mice
Top2b	-0.26	0.01776165	mice
Pabpc1	0.16	0.01779537	mice
Crbn	0.24	0.01821933	mice

B3gnt7	0.16	0.01870611	mice
Synrg	-0.26	0.01887157	mice
Rnf145	-0.28	0.01928680	mice
Edem3	-0.22	0.01982550	mice
Srek1	-0.27	0.02014989	mice
Rmc1	-0.17	0.02028618	mice
Fbxo3	-0.17	0.02086227	mice
Atp13a3	-0.27	0.02089434	mice
Hs3st1	0.21	0.02148324	mice
Cnbp	0.16	0.02159433	mice
Hdac7	-0.21	0.02195645	mice
Smim20	-0.20	0.02218432	mice
Usp15	-0.25	0.02235922	mice
Elk3	-0.31	0.02252741	mice
Dgke	-0.30	0.02304863	mice
Eif4a1	0.23	0.02311218	mice
E130102H24Rik	0.20	0.02311717	mice
Rapgef6	0.24	0.02480957	mice
Anapc13	0.13	0.02536492	mice
Il2rg	0.19	0.02549804	mice
Ube2e3	-0.29	0.02556042	mice
Cdk9	-0.26	0.02560930	mice
Daam1	-0.24	0.02567414	mice
Kdm4a	-0.20	0.02644646	mice
Ctps2	-0.34	0.02652230	mice
Ric1	-0.31	0.02660808	mice
Xpo1	-0.24	0.02663928	mice
Abcc1	0.23	0.02712443	mice
Rbl2	-0.26	0.02720695	mice
Notch2	0.19	0.02769022	mice
E330009J07Rik	0.22	0.02780518	mice
Pag1	-0.27	0.02832614	mice
Tap2	0.19	0.03007275	mice
Ivns1abp	-0.24	0.03023577	mice
Gdi2	-0.20	0.03037846	mice
Adam17	0.25	0.03039619	mice
Ttc28	0.05	0.03076986	mice
Lztfl1	-0.33	0.03108523	mice
Rbm4b	0.20	0.03145358	mice
Tmem123	-0.22	0.03221481	mice
Sh2b3	-0.23	0.03281609	mice
Uqcr11	0.15	0.03301405	mice
Pla2g12a	-0.17	0.03364086	mice
Tlk1	-0.26	0.03371258	mice
Sfxn1	-0.24	0.03487075	mice
Cdv3	-0.25	0.03504956	mice

Lclat1	-0.30	0.03531832	mice
Kcnk10	0.22	0.03547032	mice
Hnrnpa0	-0.18	0.03614225	mice
Ndufb2	0.18	0.03642898	mice
Sft2d1	-0.22	0.03730664	mice
Fam168b	-0.23	0.03768002	mice
Cep128	-0.30	0.03832774	mice
Git2	-0.28	0.03870778	mice
Pacsin1	0.14	0.04101545	mice
Lmo4	-0.30	0.04106251	mice
Rmnd5a	-0.25	0.04159299	mice
Bptf	-0.21	0.04185771	mice
Pdcd10	0.24	0.04291608	mice
Samsn1	0.36	0.04304816	mice
Nptn	-0.29	0.04370600	mice
Rbm28	-0.25	0.04406903	mice
Tpp1	-0.19	0.04507087	mice
Otulin	-0.29	0.04507820	mice
Zfpm1	-0.16	0.04526529	mice
Card11	-0.39	0.04555229	mice
Zfp608	-0.32	0.04603425	mice
Zfp644	-0.26	0.04641144	mice
Washc2	-0.20	0.04732086	mice
Arl6ip1	0.21	0.04825458	mice
Tent4a	0.15	0.05148551	mice
Itgal	0.15	0.05395459	mice
Tgif1	0.17	0.05407465	mice
Tmem258	0.19	0.05426151	mice
Osgep	-0.25	0.05633217	mice
Dock2	-0.16	0.05739438	mice
Arid2	-0.29	0.05842681	mice
Ubac2	-0.29	0.05860284	mice
Tbcel	-0.22	0.05901056	mice
Arid3b	-0.25	0.05951335	mice
Evi5	-0.26	0.05952543	mice
Tbl1x	-0.29	0.05959245	mice
Xrn1	0.29	0.05975682	mice
Cyp51	0.14	0.06050725	mice
Gtf3c1	-0.25	0.06054755	mice
Tes	0.19	0.06066598	mice
Pkn1	-0.22	0.06068359	mice
Pold1	-0.19	0.06124549	mice
Lgals1	0.12	0.06202069	mice
Adgre5	-0.20	0.06295034	mice
Cebpz	0.20	0.06314229	mice
Acsl1	-0.23	0.06353057	mice

Sptan1	-0.27	0.06410531	mice
Oaz1	-0.16	0.06443970	mice
Parp11	0.25	0.06520209	mice
Ctsa	-0.22	0.06650595	mice
Fuca1	-0.24	0.06701332	mice
Xylt1	-0.25	0.06819335	mice
Lonp2	-0.24	0.06855068	mice
Carmil1	0.22	0.06859813	mice
Fam126a	-0.27	0.07506606	mice
Ccdc88c	-0.25	0.07531903	mice
Grcc10	0.19	0.07630477	mice
Smarca5	0.19	0.07660812	mice
Flcn	-0.21	0.07705126	mice
Rev1	-0.24	0.07800320	mice
Bola2	0.17	0.07917136	mice
Zfp1	-0.24	0.07948729	mice
Plekhm3	0.17	0.08075722	mice
Syne1	-0.26	0.08097263	mice
Erp44	0.16	0.08182288	mice
Slc35c2	-0.16	0.08442551	mice
Eya1	-0.24	0.08451589	mice
Pip4p1	-0.21	0.08493316	mice
Tent5a	0.16	0.08633626	mice
Ktn1	-0.22	0.08751388	mice
Klf12	0.19	0.08893458	mice
Eea1	-0.28	0.08917854	mice
Ano6	-0.35	0.08923562	mice
Gpr18	0.15	0.09155575	mice
Ppp1r11	0.18	0.09223507	mice
Arpp19	0.18	0.09321727	mice
D030056L22Rik	0.09	0.09555953	mice
Heatr5a	-0.25	0.09713061	mice
Kdm7a	-0.27	0.09907324	mice
Pdcd1lg2	0.13	0.10315501	mice
Rapgef1	-0.24	0.10367027	mice
Smim14	-0.21	0.10552165	mice
Selenow	0.21	0.10592742	mice
Rsbn1	0.19	0.10750162	mice
Ptprc	-0.16	0.10942000	mice
Lpp	-0.31	0.11177053	mice
Ttc1	0.15	0.11300255	mice
Whrn	-0.27	0.11696368	mice
Pitpnm2	-0.20	0.11810304	mice
Cnp	-0.29	0.11965345	mice
Rchy1	-0.24	0.12012484	mice
Psmb10	0.23	0.12215507	mice

Lyl1	-0.19	0.12277359	mice
Vars	-0.23	0.12714186	mice
Rp9	-0.25	0.12729101	mice
Pde7b	0.42	0.12740543	mice
Nop58	0.16	0.12778854	mice
Lrrc4	0.16	0.12799381	mice
Igf2r	-0.14	0.12863296	mice
Ankrd28	-0.33	0.12866981	mice
Rab7	-0.23	0.12885748	mice
Slc2a13	0.39	0.12924266	mice
Atg16l2	-0.26	0.13006062	mice
Prkag2	-0.15	0.13456895	mice
Atp5md	0.23	0.13577378	mice
Trim65	-0.18	0.13613928	mice
Ranbp2	0.25	0.13796293	mice
Cdyl	-0.26	0.13867468	mice
Csnk1e	-0.19	0.13879113	mice
Fkbp3	0.19	0.14073095	mice
Trps1	0.42	0.14363689	mice
Sh2b2	0.15	0.14439824	mice
Zfp182	-0.24	0.14582248	mice
Rfc1	-0.25	0.14703005	mice
Chmp2a	0.16	0.15142784	mice
Ssbp3	-0.23	0.15471492	mice
Hpse	-0.27	0.15480100	mice
Sat1	0.20	0.15637967	mice
Chchd1	0.15	0.15785045	mice
Hmox2	0.23	0.15879969	mice
Ube3a	0.22	0.16576031	mice
Gigyf1	-0.19	0.17794985	mice
Asxl1	-0.27	0.18058732	mice
Traf6	-0.28	0.18298323	mice
C2cd5	-0.27	0.18577729	mice
Gpbp1	0.14	0.18688764	mice
Snrpd2	0.20	0.18870249	mice
Zfand3	-0.27	0.18909339	mice
Trio	-0.29	0.19206873	mice
Atg5	-0.23	0.19265311	mice
Tmed8	-0.22	0.19316138	mice
Ptpn14	0.18	0.19353619	mice
Tbcd	-0.23	0.19618316	mice
Slc3a2	0.23	0.21430178	mice
Rheb	0.19	0.21502064	mice
Il1rap	0.17	0.21566880	mice
Acox3	-0.23	0.21595562	mice
Capg	0.21	0.21664779	mice

Tnik	0.21	0.22656865	mice
Dusp5	-0.22	0.22672983	mice
Cdk4	-0.19	0.23166112	mice
Pds5b	-0.26	0.23415191	mice
Cables1	0.15	0.24076672	mice
Eif2ak1	-0.23	0.24088048	mice
Cenpa	-0.23	0.24145383	mice
Ppargc1a	-0.16	0.24331630	mice
Snupn	0.12	0.24355592	mice
Cpd	-0.22	0.24936769	mice
Nudt3	-0.24	0.25084378	mice
Herc3	0.24	0.25740961	mice
Sos2	-0.24	0.26122759	mice
Arf5	-0.17	0.26151649	mice
Arhgap1	-0.16	0.27282282	mice
Kdm3b	-0.21	0.27502301	mice
Cab39l	0.21	0.28350960	mice
Ddx1	0.15	0.29147886	mice
Rtraf	0.18	0.29178742	mice
Atl2	-0.24	0.30083686	mice
Pdhb	-0.19	0.30169731	mice
Gtf2h1	-0.23	0.30694484	mice
Phyh	0.13	0.30732925	mice
Top1	0.18	0.32003466	mice
Il21r	-0.30	0.32492714	mice
Trmt10c	0.11	0.32684434	mice
Cdkn2d	-0.19	0.33039708	mice
Lrch3	-0.31	0.33250985	mice
Hdac9	-0.57	0.33373110	mice
Asb13	0.16	0.34124036	mice
Cln5	-0.15	0.34177981	mice
Qtrt1	-0.15	0.34435825	mice
Ciita	-0.34	0.36257654	mice
Lrrc8b	-0.17	0.36277508	mice
Camk4	-0.37	0.36442271	mice
Lrig2	-0.25	0.36600064	mice
Tcirg1	-0.17	0.37009122	mice
Eef1d	0.18	0.37160403	mice
Asxl2	-0.22	0.37464749	mice
Eif4ebp2	-0.19	0.37561198	mice
Lamp1	-0.18	0.37726019	mice
A930007I19Rik	-0.28	0.38578601	mice
Cry1	-0.21	0.38872298	mice
Dnajc7	-0.18	0.39482236	mice
Napa	-0.20	0.40277267	mice
Tmpo	0.14	0.40976489	mice

Xist	-0.22	0.41918558	mice
Helq	-0.16	0.42232587	mice
Casc4	-0.24	0.42351410	mice
Zswim7	-0.16	0.42516511	mice
Kat6a	0.19	0.42731239	mice
Hexa	-0.23	0.43726426	mice
Slc2a1	-0.22	0.43954888	mice
Slc10a7	-0.29	0.44675040	mice
Gnai2	-0.19	0.45011280	mice
Ccdc25	0.12	0.45237643	mice
Tob2	0.24	0.45468833	mice
Zzef1	-0.20	0.45691418	mice
Rfx3	-0.29	0.45784503	mice
Pou6f1	0.19	0.46191243	mice
Ptpa	-0.18	0.46656174	mice
Hdac4	-0.25	0.47587329	mice
Crybg1	0.16	0.49600328	mice
Tomm70a	0.16	0.50645628	mice
Rala	-0.26	0.50887082	mice
2010309G21Rik	-0.30	0.52039081	mice
Prdx1	0.17	0.52374084	mice
Wars	0.17	0.53410764	mice
Slc23a2	-0.25	0.53678552	mice
Pip4k2c	-0.20	0.53744547	mice
Rabgap1	-0.23	0.55925179	mice
Sash3	-0.25	0.56202968	mice
Hsp90aa1	0.13	0.56358330	mice
Eif3i	0.17	0.56461457	mice
Tef	-0.19	0.56651585	mice
Lsm4	0.16	0.57220979	mice
Atp5j2	0.18	0.57346325	mice
Hivep2	0.20	0.59046600	mice
Nop53	0.18	0.59632015	mice
Jade1	-0.19	0.59828272	mice
Rassf5	-0.19	0.60281336	mice
Eml5	-0.22	0.60300472	mice
Cfdp1	0.16	0.61464441	mice
Lmbr1l	-0.19	0.61623331	mice
Gpcpd1	-0.13	0.63425208	mice
Fam32a	0.15	0.66992926	mice
Itprid2	-0.23	0.68466054	mice
Arfgef1	0.19	0.68671845	mice
Plgrkt	-0.22	0.69673773	mice
Tmem251	-0.19	0.70568029	mice
Tubb5	0.16	0.71450984	mice
Reep5	-0.18	0.71686569	mice

Sipa1l2	-0.20	0.72405935	mice
Tprgl	-0.14	0.72851714	mice
Zdhhc20	-0.25	0.73800047	mice
Osbpl2	-0.18	0.74363738	mice
Abr	-0.31	0.75037189	mice
Atm	-0.23	0.76265806	mice
Fbl	0.16	0.76472125	mice
Ubald1	-0.17	0.76524258	mice
Lax1	0.12	0.76553532	mice
Tsc22d1	-0.18	0.78516580	mice
Thap2	0.12	0.78746929	mice
Donson	0.14	0.78782722	mice
Gpatch2l	-0.21	0.78954871	mice
Coq10b	0.20	0.79468858	mice
Psma5	0.13	0.79706772	mice
Trappc13	0.08	0.80500136	mice
B3gnt5	0.16	0.81334309	mice
Prrc2b	-0.22	0.82948999	mice
Tbc1d10a	-0.16	0.83244354	mice
Pdk3	0.19	0.85066447	mice
Taf4b	0.21	0.85945161	mice
Fbxo4	0.12	0.86195804	mice
Pcsk7	-0.19	0.87296646	mice
Cbx4	-0.22	0.88617370	mice
Ephx1	-0.18	0.88739886	mice
Il10ra	-0.24	0.88768222	mice
R3hdm4	-0.18	0.90112847	mice
Slc14a1	-0.19	0.90569073	mice
Sae1	-0.23	0.91519784	mice
Reep3	0.23	0.94426757	mice
Proser1	-0.20	0.94779370	mice
B230307C23Rik	0.17	0.96430471	mice
Cmas	-0.15	0.97456458	mice
Rnf113a2	-0.14	0.97510660	mice
Nr3c1	-0.22	0.97958089	mice
Slc30a4	-0.13	0.98054438	mice
Arhgap21	-0.23	0.98790884	mice
Atl3	0.20	0.99976875	mice
Serf2	0.16	1.00000000	mice
Hnrnpa2b1	0.16	1.00000000	mice
Atp6v1d	-0.20	1.00000000	mice
Fam222a	-0.11	1.00000000	mice
Zmynd8	-0.23	1.00000000	mice
Trp53inp2	-0.19	1.00000000	mice
Prrc1	-0.21	1.00000000	mice
Ccnt1	0.12	1.00000000	mice

U2af1	-0.23	1.00000000	mice
Iglc3	-0.33	1.00000000	mice
Rnf185	-0.22	1.00000000	mice
Ptbp2	-0.23	1.00000000	mice
Zfp398	-0.23	1.00000000	mice
Tgfb1	-0.21	1.00000000	mice
Psma1	0.13	1.00000000	mice
Ly75	0.11	1.00000000	mice
Gnaq	-0.30	1.00000000	mice
Dtnbp1	-0.26	1.00000000	mice
Klf10	-0.20	1.00000000	mice
Hat1	0.15	1.00000000	mice
Fus	0.14	1.00000000	mice
Atp6v0c	-0.21	1.00000000	mice
Dhx40	-0.23	1.00000000	mice
Wipi2	-0.20	1.00000000	mice
Stx8	-0.31	1.00000000	mice
Taok1	-0.18	1.00000000	mice
Snrnp25	0.11	1.00000000	mice
D6Wsu163e	-0.14	1.00000000	mice
Add1	-0.22	1.00000000	mice
Vapa	-0.22	1.00000000	mice
H2afy	-0.19	1.00000000	mice
Stag2	0.21	1.00000000	mice
Tmem38b	-0.14	1.00000000	mice
Rtf1	0.19	1.00000000	mice
Vac14	-0.18	1.00000000	mice
Fam76a	-0.19	1.00000000	mice
Glyr1	-0.22	1.00000000	mice
Kdm2b	0.23	1.00000000	mice
Ttc32	-0.18	1.00000000	mice
Zeb2	-0.18	1.00000000	mice
Spin1	-0.22	1.00000000	mice
Chst15	-0.18	1.00000000	mice
Erh	-0.14	1.00000000	mice
Pcnt	-0.19	1.00000000	mice
Gimap9	0.15	1.00000000	mice
Rnf111	-0.21	1.00000000	mice
Phykpl	0.11	1.00000000	mice
Gna13	0.18	1.00000000	mice
Hif1a	-0.21	1.00000000	mice
Kdm4b	-0.17	1.00000000	mice
Trappc8	-0.21	1.00000000	mice
Kdsr	-0.16	1.00000000	mice
Dock10	-0.22	1.00000000	mice
Zc3h15	0.18	1.00000000	mice

Gpr107	-0.22	1.00000000	mice
Blmh	-0.22	1.00000000	mice
Prkag1	-0.20	1.00000000	mice
Gimap1	0.17	1.00000000	mice
Nasp	0.14	1.00000000	mice
Cactin	-0.14	1.00000000	mice
Rnf103	-0.22	1.00000000	mice
Tmem135	-0.25	1.00000000	mice
Bcl6	0.18	1.00000000	mice
Fam102a	0.10	1.00000000	mice
Bnip3l	-0.20	1.00000000	mice
Zfr	0.22	1.00000000	mice
Lrrk2	-0.21	1.00000000	mice
Arid4b	0.16	1.00000000	mice
Fry	-0.23	1.00000000	mice
Akr7a5	-0.16	1.00000000	mice
Impact	0.16	1.00000000	mice
Cd74	0.12	1.00000000	mice
A930024E05Rik	0.12	1.00000000	mice
Dbnl	-0.16	1.00000000	mice
Ash1l	-0.17	1.00000000	mice
Tbl1xr1	-0.21	1.00000000	mice
Spg21	-0.18	1.00000000	mice
Ubr3	-0.22	1.00000000	mice
Trim59	0.15	1.00000000	mice
Rrbp1	-0.24	1.00000000	mice
St3gal3	-0.23	1.00000000	mice
Asb3	-0.21	1.00000000	mice
Zbtb11	0.18	1.00000000	mice
Picalm	-0.17	1.00000000	mice
Exoc6	-0.23	1.00000000	mice
Copg2	-0.25	1.00000000	mice
2310058D17Rik	-0.17	1.00000000	mice
Rubcnl	-0.21	1.00000000	mice
Brox	-0.19	1.00000000	mice
Abcb9	0.10	1.00000000	mice
Smg1	0.17	1.00000000	mice
Calhm2	-0.15	1.00000000	mice
Phc2	0.16	1.00000000	mice
Prpf38a	0.14	1.00000000	mice
Terf2	-0.23	1.00000000	mice
Sarnp	0.13	1.00000000	mice
Gramd3	0.05	1.00000000	mice
Tasor2	0.21	1.00000000	mice
Rnf14	-0.19	1.00000000	mice
Zfp958	0.10	1.00000000	mice

Selenot	0.17	1.00000000	mice
Tcf20	0.14	1.00000000	mice
Stk10	-0.21	1.00000000	mice
Casp8ap2	0.13	1.00000000	mice
Pde8a	0.11	1.00000000	mice
Leprotl1	-0.17	1.00000000	mice
Eif3e	0.16	1.00000000	mice
Cyb5a	-0.18	1.00000000	mice
Tbc1d17	-0.13	1.00000000	mice
Ttc3	-0.24	1.00000000	mice
Cldnd1	0.12	1.00000000	mice
Crlf3	0.13	1.00000000	mice
Epg5	-0.17	1.00000000	mice
Iscu	-0.20	1.00000000	mice
Zbtb7a	-0.18	1.00000000	mice
Akna	-0.18	1.00000000	mice
Hcfc2	-0.16	1.00000000	mice
Zfp653	-0.13	1.00000000	mice
Utp14b	-0.17	1.00000000	mice
Brd1	-0.18	1.00000000	mice
Slain1	-0.16	1.00000000	mice
Ly6d	-0.27	1.00000000	mice
Trappc10	-0.19	1.00000000	mice
Gtf2i	-0.16	1.00000000	mice
Ldlrad3	-0.17	1.00000000	mice
Cnot8	-0.20	1.00000000	mice
Nfkbid	0.13	1.00000000	mice
Kansl1	-0.16	1.00000000	mice
Cdip1	-0.20	1.00000000	mice
Trim25	-0.27	1.00000000	mice
Cdk17	0.18	1.00000000	mice
Txndc9	0.14	1.00000000	mice
Akap9	-0.21	1.00000000	mice
Gpn2	0.11	1.00000000	mice
Yeats4	-0.20	1.00000000	mice
Usp22	-0.13	1.00000000	mice
Use1	0.14	1.00000000	mice
Smc3	-0.18	1.00000000	mice
Farp2	0.13	1.00000000	mice
Entpd5	-0.14	1.00000000	mice
Cuedc2	-0.17	1.00000000	mice
Prmt9	-0.17	1.00000000	mice
Stard9	-0.16	1.00000000	mice
Rnf166	-0.16	1.00000000	mice
Rufy1	-0.23	1.00000000	mice
Wdr44	0.16	1.00000000	mice

Bcl2l11	-0.13	1.00000000	mice
Esr1	-0.23	1.00000000	mice
Taf12	0.13	1.00000000	mice
Xbp1	-0.16	1.00000000	mice
Smg6	-0.19	1.00000000	mice
Hibadh	-0.28	1.00000000	mice
Unk	-0.17	1.00000000	mice
Lck	0.13	1.00000000	mice
Hmces	0.16	1.00000000	mice
Elof1	-0.18	1.00000000	mice
Vps8	-0.18	1.00000000	mice
Usp47	-0.23	1.00000000	mice
Zfp942	-0.19	1.00000000	mice
Edem1	-0.18	1.00000000	mice
Rab3gap2	-0.23	1.00000000	mice
Yipf3	-0.11	1.00000000	mice
Egr3	0.31	1.00000000	mice
Tcof1	0.18	1.00000000	mice
Pnp	0.10	1.00000000	mice
Prkcb	-0.19	1.00000000	mice
Npc1	-0.19	1.00000000	mice
Tpm3	0.12	1.00000000	mice
Ncoa2	-0.24	1.00000000	mice
Ddx55	-0.16	1.00000000	mice
Ccpg1	-0.21	1.00000000	mice
Ttc5	-0.21	1.00000000	mice
Laptm5	-0.15	1.00000000	mice
Ugcg	0.17	1.00000000	mice
Gpr137c	0.20	1.00000000	mice
Zswim6	0.13	1.00000000	mice
Anp32a	-0.17	1.00000000	mice
Zcchc7	0.14	1.00000000	mice
Zdhhc13	-0.19	1.00000000	mice
Tk2	-0.19	1.00000000	mice
Setx	-0.18	1.00000000	mice
Ptms	0.10	1.00000000	mice
Atp9b	-0.23	1.00000000	mice
Fam13b	-0.21	1.00000000	mice
Prpf6	0.10	1.00000000	mice
Pecam1	0.11	1.00000000	mice
Smarcc1	-0.19	1.00000000	mice
Ormdl2	0.12	1.00000000	mice
Eloc	0.12	1.00000000	mice
Pop5	0.11	1.00000000	mice
Tet2	-0.17	1.00000000	mice
Socs1	0.23	1.00000000	mice

Ctsc	0.16	1.00000000	mice
Dcaf5	-0.28	1.00000000	mice
Cop1	-0.21	1.00000000	mice
Rab2a	-0.18	1.00000000	mice
Cog5	-0.16	1.00000000	mice
Tdg	-0.14	1.00000000	mice
Dhx9	-0.17	1.00000000	mice
Cnot2	0.16	1.00000000	mice
Alkbh8	-0.17	1.00000000	mice
Sde2	0.15	1.00000000	mice
Denr	0.13	1.00000000	mice
Smim11	0.11	1.00000000	mice
Ube2b	-0.15	1.00000000	mice
Arhgap30	0.19	1.00000000	mice
Eif2ak4	-0.24	1.00000000	mice
Zfp367	-0.19	1.00000000	mice
Tmed5	0.15	1.00000000	mice
Lysmd4	0.07	1.00000000	mice
Homer1	0.13	1.00000000	mice
Clic1	-0.18	1.00000000	mice
Bin1	-0.18	1.00000000	mice
Ddrgk1	0.10	1.00000000	mice
Syvn1	-0.17	1.00000000	mice
Dusp16	0.18	1.00000000	mice
Rbm26	-0.18	1.00000000	mice
Nr6a1	-0.32	1.00000000	mice
Prkd3	-0.23	1.00000000	mice
Psmg4	0.10	1.00000000	mice
Ppib	0.15	1.00000000	mice
Slc44a1	-0.15	1.00000000	mice
Jakmip1	-0.19	1.00000000	mice
Arl4a	-0.11	1.00000000	mice
Phf14	-0.18	1.00000000	mice
Il10rb	-0.18	1.00000000	mice
Sla	0.17	1.00000000	mice
Apbb1ip	-0.19	1.00000000	mice
Ost4	0.16	1.00000000	mice
Slc38a10	-0.14	1.00000000	mice
Phf23	-0.12	1.00000000	mice
Pex19	-0.12	1.00000000	mice
Sept1	-0.17	1.00000000	mice
Snrnp27	0.11	1.00000000	mice
Rhbdd1	-0.20	1.00000000	mice
Osbpl9	-0.13	1.00000000	mice
Fbxl3	-0.17	1.00000000	mice
Gle1	-0.21	1.00000000	mice

Ankrd44	-0.17	1.00000000	mice
Atf1	-0.18	1.00000000	mice
Neat1	-0.20	1.00000000	mice
Smim3	-0.12	1.00000000	mice
Pibf1	0.17	1.00000000	mice
Fbxw4	0.12	1.00000000	mice
Ssr3	0.13	1.00000000	mice
Cdkn2aipnl	-0.17	1.00000000	mice
Wtap	-0.17	1.00000000	mice
Slamf7	0.14	1.00000000	mice
Rbm25	0.12	1.00000000	mice
Nek7	-0.20	1.00000000	mice
Nars2	-0.21	1.00000000	mice
Fgf13	0.26	1.00000000	mice
Egr1	0.32	1.00000000	mice
Resf1	0.17	1.00000000	mice
Glul	-0.16	1.00000000	mice
Zranb2	0.12	1.00000000	mice
Smim4	0.08	1.00000000	mice
Fndc9	0.15	1.00000000	mice
Scap	-0.17	1.00000000	mice
Rgs1	-0.14	1.00000000	mice
Vti1a	-0.31	1.00000000	mice
Btg1	-0.09	1.00000000	mice
Amfr	-0.21	1.00000000	mice
Socs3	0.06	1.00000000	mice
Prkacb	-0.18	1.00000000	mice
Crnkl1	0.09	1.00000000	mice
Icam1	0.16	1.00000000	mice
Kcnq1ot1	-0.24	1.00000000	mice
Trp53bp1	0.08	1.00000000	mice
Snx11	0.07	1.00000000	mice
Iars2	0.12	1.00000000	mice
Pik3ca	-0.19	1.00000000	mice
Arhgef2	-0.15	1.00000000	mice
Ubxn1	-0.20	1.00000000	mice
Rasa1	-0.19	1.00000000	mice
Vcpip1	0.11	1.00000000	mice
Fam43a	-0.19	1.00000000	mice
Ranbp9	-0.24	1.00000000	mice
1110008P14Rik	-0.17	1.00000000	mice
Dnaja1	0.17	1.00000000	mice
Cpsf6	-0.16	1.00000000	mice
Trmt11	0.17	1.00000000	mice
Insr	-0.27	1.00000000	mice
Yaf2	-0.17	1.00000000	mice

Lactb2	-0.11	1.00000000	mice
Slc25a3	0.14	1.00000000	mice
Rnf125	-0.16	1.00000000	mice
Eml4	-0.19	1.00000000	mice
Pex14	-0.22	1.00000000	mice
Pole4	0.13	1.00000000	mice
Sptssa	-0.16	1.00000000	mice
Atp6ap1	-0.19	1.00000000	mice
Set	0.14	1.00000000	mice
Abhd17a	-0.16	1.00000000	mice
Flnb	0.16	1.00000000	mice
AU020206	0.13	1.00000000	mice
Cct4	0.12	1.00000000	mice
Grk2	-0.15	1.00000000	mice
Azi2	0.11	1.00000000	mice
Gfod1	0.19	1.00000000	mice
Cox8a	0.15	1.00000000	mice
Calcoco1	-0.13	1.00000000	mice
Vamp8	-0.19	1.00000000	mice
Pbrm1	-0.17	1.00000000	mice
Ppp2r2a	0.12	1.00000000	mice
Zyg11b	-0.21	1.00000000	mice
Ythdf2	0.12	1.00000000	mice
Epc1	-0.15	1.00000000	mice
Slbp	0.14	1.00000000	mice
Stk3	-0.21	1.00000000	mice
Dcaf7	0.09	1.00000000	mice
Idnk	0.13	1.00000000	mice
Ankrd10	-0.20	1.00000000	mice
Eif2s1	0.12	1.00000000	mice
Ubr5	-0.18	1.00000000	mice
Smad4	-0.20	1.00000000	mice
Snap23	0.13	1.00000000	mice
Slc25a37	-0.11	1.00000000	mice
Dcaf6	-0.27	1.00000000	mice
Cfap20	-0.17	1.00000000	mice
Dars	0.11	1.00000000	mice
Nmt2	-0.23	1.00000000	mice
Tgoln1	0.10	1.00000000	mice
Pcbd2	0.08	1.00000000	mice
D16Ertd472e	0.15	1.00000000	mice
Gsk3b	-0.19	1.00000000	mice
Dstyk	-0.18	1.00000000	mice
Zdhhc3	-0.17	1.00000000	mice
Rlf	-0.20	1.00000000	mice
Znrf1	-0.20	1.00000000	mice

Pcif1	-0.17	1.00000000	mice
Phkb	-0.23	1.00000000	mice
Uvrag	-0.15	1.00000000	mice
Yars	-0.14	1.00000000	mice
Lsm6	0.12	1.00000000	mice
Cd84	0.07	1.00000000	mice
Lcor	-0.16	1.00000000	mice
Stag1	-0.13	1.00000000	mice
Ube2w	-0.22	1.00000000	mice
Pnn	0.14	1.00000000	mice
Sirt7	-0.14	1.00000000	mice
Rasa3	-0.28	1.00000000	mice
Nfkbia	0.14	1.00000000	mice
Rbfa	-0.14	1.00000000	mice
Rsf1os1	0.11	1.00000000	mice
Sec11c	-0.22	1.00000000	mice
Trpv2	-0.17	1.00000000	mice
Ndor1	-0.15	1.00000000	mice
Aldh2	-0.18	1.00000000	mice
Arf6	-0.16	1.00000000	mice
Cbx5	0.12	1.00000000	mice
Srp9	-0.14	1.00000000	mice
Ccdc82	0.11	1.00000000	mice
Ier5l	-0.13	1.00000000	mice
Sbds	0.10	1.00000000	mice
Pbx1	-0.27	1.00000000	mice
Cycs	0.12	1.00000000	mice
AI837181	0.09	1.00000000	mice
Fam214a	-0.17	1.00000000	mice
Emb	-0.29	1.00000000	mice
Slc41a2	-0.20	1.00000000	mice
Cc2d1b	-0.12	1.00000000	mice
Rassf3	-0.18	1.00000000	mice
Pitpna	-0.14	1.00000000	mice
Fbrsl1	-0.16	1.00000000	mice
Pard3b	-0.21	1.00000000	mice
Actb	-0.08	1.00000000	mice
Nck2	-0.23	1.00000000	mice
Chfr	-0.18	1.00000000	mice
Irf5	-0.16	1.00000000	mice
Slc9a8	-0.15	1.00000000	mice
Rsu1	0.09	1.00000000	mice
Skil	0.13	1.00000000	mice
Pcnx	-0.21	1.00000000	mice
Lpgat1	0.16	1.00000000	mice
Snapc1	0.10	1.00000000	mice

Isca1	0.11	1.00000000	mice
Gab3	-0.25	1.00000000	mice
Ehd1	0.11	1.00000000	mice
Pank4	-0.12	1.00000000	mice
Cradd	-0.20	1.00000000	mice
Nckap1l	-0.16	1.00000000	mice
Zfp207	0.10	1.00000000	mice
Zfand6	-0.17	1.00000000	mice
Cd19	-0.19	1.00000000	mice
Rab18	0.13	1.00000000	mice
Tmem156	-0.16	1.00000000	mice
Pdap1	0.11	1.00000000	mice
Angptl1	0.12	1.00000000	mice
Snx12	-0.13	1.00000000	mice
Acat2	0.11	1.00000000	mice
Stx11	0.10	1.00000000	mice
Pabpn1	0.12	1.00000000	mice
Sfpq	0.10	1.00000000	mice
Zc3h10	0.09	1.00000000	mice
Trappc3	-0.12	1.00000000	mice
Hes6	-0.11	1.00000000	mice
Cpeb2	-0.23	1.00000000	mice
Tex264	-0.08	1.00000000	mice
Pcna	0.08	1.00000000	mice
Enox2	-0.27	1.00000000	mice
Sdcbp	0.14	1.00000000	mice
Dyrk1a	-0.14	1.00000000	mice
Eif1	0.08	1.00000000	mice
Cdadc1	-0.14	1.00000000	mice
Nr2c2	-0.16	1.00000000	mice
Auh	-0.14	1.00000000	mice
Prpsap2	-0.19	1.00000000	mice
Rassf2	-0.19	1.00000000	mice
Chst11	0.13	1.00000000	mice
Lyn	0.19	1.00000000	mice
Ehmt1	-0.17	1.00000000	mice
Fkbp8	-0.17	1.00000000	mice
Arid1a	-0.18	1.00000000	mice
Acly	-0.19	1.00000000	mice
Spred2	-0.24	1.00000000	mice
Chst12	-0.10	1.00000000	mice
Pstpip1	0.09	1.00000000	mice
Gimap5	0.09	1.00000000	mice
Fmnl1	-0.18	1.00000000	mice
Chd9	-0.20	1.00000000	mice
Selenoi	0.10	1.00000000	mice

Nop10	0.13	1.00000000	mice
Hmgcr	0.11	1.00000000	mice
Hnrnpd	-0.12	1.00000000	mice
Gars	-0.18	1.00000000	mice
Eif3k	-0.15	1.00000000	mice
Adnp	-0.14	1.00000000	mice
Ap1ar	-0.15	1.00000000	mice
Sh2d3c	-0.17	1.00000000	mice
Heg1	-0.23	1.00000000	mice
Chd4	-0.14	1.00000000	mice
Smad2	-0.19	1.00000000	mice
Hepacam2	0.10	1.00000000	mice
Pias2	-0.23	1.00000000	mice
Gtpbp4	0.12	1.00000000	mice
Cdc42se2	-0.14	1.00000000	mice
Runx1	-0.13	1.00000000	mice
Zbed4	-0.13	1.00000000	mice
Hdac1	0.10	1.00000000	mice
S100a8	0.15	1.00000000	mice
Tinf2	0.08	1.00000000	mice
Pex1	-0.13	1.00000000	mice
Ier3ip1	0.11	1.00000000	mice
Cox7a2l	0.13	1.00000000	mice
Tex30	-0.16	1.00000000	mice
Kpnb1	0.14	1.00000000	mice
Aopep	-0.21	1.00000000	mice
Fabp5	0.14	1.00000000	mice
Tsc22d4	0.12	1.00000000	mice
Zxdc	-0.12	1.00000000	mice
Raly	-0.13	1.00000000	mice
Bcl10	0.14	1.00000000	mice
Ppfibp2	0.17	1.00000000	mice
Dck	-0.15	1.00000000	mice
Ubap2	0.14	1.00000000	mice
Vrk2	0.12	1.00000000	mice
Ercc5	-0.11	1.00000000	mice
Erlec1	-0.12	1.00000000	mice
Timm10	0.10	1.00000000	mice
Sar1b	-0.17	1.00000000	mice
Itm2b	0.11	1.00000000	mice
Stk11	0.12	1.00000000	mice
2610020C07Rik	0.11	1.00000000	mice
Krtcap2	0.14	1.00000000	mice
Rptor	-0.20	1.00000000	mice
Krcc1	0.11	1.00000000	mice
Sugt1	0.11	1.00000000	mice

Gnb1	-0.18	1.00000000	mice
Tln1	-0.18	1.00000000	mice
Pfkp	0.06	1.00000000	mice
Arhgap6	0.09	1.00000000	mice
Ifngr2	-0.15	1.00000000	mice
Rabac1	0.15	1.00000000	mice
Slc25a5	-0.16	1.00000000	mice
Cox18	0.05	1.00000000	mice
Tapbpl	0.06	1.00000000	mice
Sirt2	-0.16	1.00000000	mice
Npepps	-0.15	1.00000000	mice
Zrsr2	0.11	1.00000000	mice
Fmnl3	0.09	1.00000000	mice
Gem	0.18	1.00000000	mice
Ptrhd1	0.06	1.00000000	mice
Selplg	-0.13	1.00000000	mice
Ifitm2	0.11	1.00000000	mice
Chchd4	0.06	1.00000000	mice
Arhgdia	-0.15	1.00000000	mice
Hspbap1	-0.17	1.00000000	mice
Wbp2	-0.17	1.00000000	mice
Zadh2	-0.16	1.00000000	mice
Supt5	0.09	1.00000000	mice
Ctbp1	-0.15	1.00000000	mice
Dhx8	0.11	1.00000000	mice
Vps13a	-0.18	1.00000000	mice
Nfia	-0.27	1.00000000	mice
Glcci1	-0.15	1.00000000	mice
Cipc	0.08	1.00000000	mice
Retreg2	-0.12	1.00000000	mice
Snrpd1	0.12	1.00000000	mice
Gatd1	-0.09	1.00000000	mice
Hsdl1	-0.13	1.00000000	mice
Spata2	0.09	1.00000000	mice
Fbxl2	0.08	1.00000000	mice
Chmp5	0.09	1.00000000	mice
Tmem65	-0.11	1.00000000	mice
Ddx27	0.11	1.00000000	mice
Arhgap4	-0.16	1.00000000	mice
Spidr	0.10	1.00000000	mice
Cpsf7	-0.17	1.00000000	mice
Trappc6b	0.11	1.00000000	mice
Gngt2	0.08	1.00000000	mice
Tubb2a	-0.10	1.00000000	mice
Nop56	0.10	1.00000000	mice
Ncor1	-0.15	1.00000000	mice

Tmem192	-0.16	1.00000000	mice
Fos	0.25	1.00000000	mice
Neu1	-0.16	1.00000000	mice
Ppig	0.15	1.00000000	mice
Rnf19b	0.10	1.00000000	mice
Dedd2	-0.12	1.00000000	mice
Dennd1a	-0.15	1.00000000	mice
Aim2	0.12	1.00000000	mice
Pou2f1	0.05	1.00000000	mice
Rab8b	0.18	1.00000000	mice
Smyd3	0.13	1.00000000	mice
Zfp740	-0.14	1.00000000	mice
Depdc5	-0.16	1.00000000	mice
Cyb5b	-0.18	1.00000000	mice
Clic4	0.15	1.00000000	mice
Lnpep	0.16	1.00000000	mice
Tnrc6a	-0.16	1.00000000	mice
Zfp384	-0.13	1.00000000	mice
Sec63	-0.16	1.00000000	mice
Qrich1	-0.14	1.00000000	mice
Traf3	-0.21	1.00000000	mice
1810058I24Rik	-0.13	1.00000000	mice
Impad1	0.05	1.00000000	mice
Fbxo8	-0.14	1.00000000	mice
Smim10l1	-0.14	1.00000000	mice
Pikfyve	0.13	1.00000000	mice
Desi2	-0.20	1.00000000	mice
Trmt6	-0.18	1.00000000	mice
Aftph	-0.18	1.00000000	mice
Tubgcp2	0.10	1.00000000	mice
Echdc1	0.10	1.00000000	mice
Acat1	-0.16	1.00000000	mice
Tomm20	0.09	1.00000000	mice
Tax1bp1	-0.10	1.00000000	mice
Ube2d1	0.14	1.00000000	mice
Sept6	0.11	1.00000000	mice
Cebpb	0.06	1.00000000	mice
Rmdn1	-0.15	1.00000000	mice
Ptpra	-0.21	1.00000000	mice
Narf	-0.10	1.00000000	mice
Zcchc9	0.09	1.00000000	mice
Dcaf11	0.11	1.00000000	mice
Bex3	-0.14	1.00000000	mice
Scarb2	-0.14	1.00000000	mice
2310033P09Rik	0.12	1.00000000	mice
Phkg2	-0.15	1.00000000	mice

Cdk12	0.13	1.00000000	mice
Hira	-0.19	1.00000000	mice
Zfp53	-0.16	1.00000000	mice
Flna	-0.15	1.00000000	mice
Cpeb4	-0.17	1.00000000	mice
Ambra1	-0.15	1.00000000	mice
Dyrk2	-0.13	1.00000000	mice
Dbt	-0.09	1.00000000	mice
Stx12	0.10	1.00000000	mice
Rubcn	-0.20	1.00000000	mice
Suclg2	-0.20	1.00000000	mice
Exoc5	-0.19	1.00000000	mice
Cyb561a3	-0.17	1.00000000	mice
Psmd4	0.13	1.00000000	mice
2010315B03Rik	0.12	1.00000000	mice
Pdpr	-0.16	1.00000000	mice
Atp6v1h	-0.20	1.00000000	mice
A530040E14Rik	0.07	1.00000000	mice
Dgat1	0.05	1.00000000	mice
Fnta	-0.15	1.00000000	mice
Rraga	0.06	1.00000000	mice
Gpn3	0.07	1.00000000	mice
Hdac8	-0.27	1.00000000	mice
Insig1	-0.13	1.00000000	mice
Grn	-0.16	1.00000000	mice
Nf1	0.07	1.00000000	mice
Alg14	0.08	1.00000000	mice
Pdss2	0.09	1.00000000	mice
E030030I06Rik	-0.11	1.00000000	mice
Actl6a	-0.15	1.00000000	mice
Fbxo34	0.07	1.00000000	mice
Ppp1r13b	-0.17	1.00000000	mice
Wdr59	-0.13	1.00000000	mice
Pja2	-0.20	1.00000000	mice
Snx13	-0.17	1.00000000	mice
Ndufc1	0.10	1.00000000	mice
Actg1	-0.14	1.00000000	mice
Srp72	-0.13	1.00000000	mice
Ttpal	0.11	1.00000000	mice
Zfp746	-0.13	1.00000000	mice
Derl1	-0.18	1.00000000	mice
Polr2h	-0.10	1.00000000	mice
Dpm3	0.09	1.00000000	mice
Ogt	0.11	1.00000000	mice
Acyp2	-0.11	1.00000000	mice
Zfp36	-0.12	1.00000000	mice

Coro1a	-0.10	1.00000000	mice
C330007P06Rik	-0.18	1.00000000	mice
Oxr1	-0.21	1.00000000	mice
Tmem59	-0.14	1.00000000	mice
Lncpint	0.14	1.00000000	mice
Eya3	-0.16	1.00000000	mice
Haus2	-0.14	1.00000000	mice
Ewsr1	-0.18	1.00000000	mice
Plp2	-0.13	1.00000000	mice
Gclc	-0.20	1.00000000	mice
Rrnad1	0.08	1.00000000	mice
Hspa4l	-0.14	1.00000000	mice
Ncf1	0.10	1.00000000	mice
Washc5	-0.15	1.00000000	mice
Safb	0.10	1.00000000	mice
Ehd4	0.12	1.00000000	mice
Glrx3	-0.17	1.00000000	mice
Necap2	-0.13	1.00000000	mice
Tra2a	0.09	1.00000000	mice
Apc	-0.24	1.00000000	mice
Pcca	0.10	1.00000000	mice
Kmt2a	-0.17	1.00000000	mice
Tanc2	-0.17	1.00000000	mice
Cdc14a	-0.17	1.00000000	mice
Ints6	0.16	1.00000000	mice
Pebp1	-0.15	1.00000000	mice
Lrrc58	-0.16	1.00000000	mice
Ext1	-0.38	1.00000000	mice
Rbm17	0.12	1.00000000	mice
Rfx7	-0.17	1.00000000	mice
Rapgef5	-0.19	1.00000000	mice
Phtf2	0.14	1.00000000	mice
Kctd20	-0.12	1.00000000	mice
Nkap	0.07	1.00000000	mice
Fam3c	-0.21	1.00000000	mice
Siah1a	-0.20	1.00000000	mice
Ggta1	-0.21	1.00000000	mice
Kmo	0.14	1.00000000	mice
Puf60	-0.16	1.00000000	mice
Ergic1	-0.12	1.00000000	mice
Tcf4	0.11	1.00000000	mice
Nme1	-0.14	1.00000000	mice
Asah1	-0.12	1.00000000	mice
Nop14	0.10	1.00000000	mice
Gtpbp2	-0.15	1.00000000	mice
Snrpb2	0.11	1.00000000	mice

Rab10	0.12	1.00000000	mice
Cited2	-0.11	1.00000000	mice
Pias4	-0.12	1.00000000	mice
Dido1	-0.18	1.00000000	mice
Ube2d3	-0.12	1.00000000	mice
Abcf1	0.08	1.00000000	mice
Tceal9	-0.19	1.00000000	mice
Snhg8	0.10	1.00000000	mice
Tm2d2	-0.12	1.00000000	mice
Dipk1a	-0.16	1.00000000	mice
Dera	-0.12	1.00000000	mice
Rrad	0.12	1.00000000	mice
Cnksr3	-0.13	1.00000000	mice
Rab24	-0.14	1.00000000	mice
Gpatch11	0.08	1.00000000	mice
Taf15	0.11	1.00000000	mice
Sin3a	-0.14	1.00000000	mice
Sucla2	0.04	1.00000000	mice
Frg1	0.11	1.00000000	mice
Itga6	-0.13	1.00000000	mice
Ngly1	-0.13	1.00000000	mice
Peli1	-0.15	1.00000000	mice
Synj2bp	0.07	1.00000000	mice
Lman1	0.08	1.00000000	mice
Prrc2c	0.12	1.00000000	mice
Pin4	0.09	1.00000000	mice
Pcnx3	-0.10	1.00000000	mice
Sgpl1	-0.23	1.00000000	mice
Ninj1	-0.10	1.00000000	mice
Ddhd1	-0.16	1.00000000	mice
Gemin7	-0.11	1.00000000	mice
Lamc1	-0.12	1.00000000	mice
Zranb1	-0.15	1.00000000	mice
Brf1	-0.15	1.00000000	mice
Clasp2	-0.20	1.00000000	mice
Shkbp1	-0.11	1.00000000	mice
Ccr7	0.10	1.00000000	mice
Bcas3	-0.23	1.00000000	mice
Fam133b	0.10	1.00000000	mice
Amn1	0.10	1.00000000	mice
Dnajc19	0.11	1.00000000	mice
Nbr1	-0.13	1.00000000	mice
Scaf1	-0.13	1.00000000	mice
Tspan3	-0.10	1.00000000	mice
Srp54a	0.06	1.00000000	mice
Brms1l	0.10	1.00000000	mice

Tcea1	-0.12	1.00000000	mice
Armc9	0.06	1.00000000	mice
Phf12	-0.20	1.00000000	mice
Btg3	-0.15	1.00000000	mice
Chst10	0.11	1.00000000	mice
Amz2	0.09	1.00000000	mice
Ppm1l	0.16	1.00000000	mice
Sec61b	0.11	1.00000000	mice
Zfp655	-0.12	1.00000000	mice
Ssh1	-0.10	1.00000000	mice
Dnaja2	0.12	1.00000000	mice
Slc15a3	-0.10	1.00000000	mice
Nhlrc2	-0.22	1.00000000	mice
Entpd7	-0.12	1.00000000	mice
Dnajc1	-0.20	1.00000000	mice
Psme4	-0.14	1.00000000	mice
Stau2	-0.10	1.00000000	mice
Phf20	-0.15	1.00000000	mice
Rufy3	0.03	1.00000000	mice
Ilkap	0.08	1.00000000	mice
Chd2	-0.15	1.00000000	mice
Taz	0.12	1.00000000	mice
Acap2	-0.13	1.00000000	mice
Zfp831	-0.16	1.00000000	mice
Eed	0.07	1.00000000	mice
Ubl3	-0.13	1.00000000	mice
Nup160	0.10	1.00000000	mice
Ube2r2	-0.21	1.00000000	mice
Rnasek	-0.15	1.00000000	mice
Cd81	-0.15	1.00000000	mice
Zfp821	-0.13	1.00000000	mice
Znhit6	0.06	1.00000000	mice
Crtc1	-0.09	1.00000000	mice
Kansl1l	-0.24	1.00000000	mice
Eif2b4	0.09	1.00000000	mice
Hspa14	-0.14	1.00000000	mice
Xpnpep3	-0.13	1.00000000	mice
Lin54	0.12	1.00000000	mice
Dclre1c	-0.23	1.00000000	mice
Nab1	0.13	1.00000000	mice
B3galnt2	0.09	1.00000000	mice
Fubp1	0.09	1.00000000	mice
Dusp11	0.12	1.00000000	mice
Cdk2ap2	0.10	1.00000000	mice
Cept1	0.09	1.00000000	mice
Zfp148	-0.13	1.00000000	mice

Dcaf8	-0.13	1.00000000	mice
Nln	-0.15	1.00000000	mice
Atp6v1c1	-0.15	1.00000000	mice
Scml4	-0.18	1.00000000	mice
As3mt	-0.11	1.00000000	mice
Tmem41b	-0.12	1.00000000	mice
Osgin2	0.08	1.00000000	mice
Taf10	0.11	1.00000000	mice
Kat14	-0.08	1.00000000	mice
Ubr7	-0.13	1.00000000	mice
Rap1gds1	0.09	1.00000000	mice
Pds5a	-0.17	1.00000000	mice
Rassf1	-0.13	1.00000000	mice
Phf8	-0.13	1.00000000	mice
1810013L24Rik	-0.11	1.00000000	mice
Las1l	0.09	1.00000000	mice
Jak1	0.05	1.00000000	mice
Smg7	0.17	1.00000000	mice
Ybx1	-0.09	1.00000000	mice
Trim35	-0.10	1.00000000	mice
Usp6nl	-0.12	1.00000000	mice
Rab3gap1	0.13	1.00000000	mice
Acap1	0.10	1.00000000	mice
Appl1	-0.15	1.00000000	mice
Pdcd11	0.08	1.00000000	mice
Rhobtb2	-0.17	1.00000000	mice
Serpinb1a	-0.15	1.00000000	mice
Coq8a	-0.16	1.00000000	mice
Klf9	-0.09	1.00000000	mice
Cmip	-0.11	1.00000000	mice
Tmed7	-0.16	1.00000000	mice
Cnr2	-0.18	1.00000000	mice
Ndufa6	0.13	1.00000000	mice
Esrra	-0.13	1.00000000	mice
Cuta	-0.14	1.00000000	mice
Zfp280d	0.11	1.00000000	mice
Tmem147	-0.13	1.00000000	mice
Rab11b	-0.13	1.00000000	mice
Arl15	-0.28	1.00000000	mice
Kmt2d	0.09	1.00000000	mice
Usp48	-0.14	1.00000000	mice
Celf1	-0.13	1.00000000	mice
Rrp1	-0.12	1.00000000	mice
Psmc3	0.07	1.00000000	mice
Paip2b	0.09	1.00000000	mice
Cenps	-0.12	1.00000000	mice

Apobec3	0.07	1.00000000	mice
Wdr91	-0.10	1.00000000	mice
Tbc1d22b	-0.10	1.00000000	mice
Srsf2	0.12	1.00000000	mice
Zfp568	-0.14	1.00000000	mice
Scmh1	-0.21	1.00000000	mice
Glb1	-0.14	1.00000000	mice
Acaa2	-0.11	1.00000000	mice
Nisch	-0.10	1.00000000	mice
N4bp1	0.10	1.00000000	mice
Tuba1a	0.06	1.00000000	mice
Gpr137b	-0.10	1.00000000	mice
Drg1	0.09	1.00000000	mice
Arhgef7	-0.13	1.00000000	mice
Psen2	-0.14	1.00000000	mice
Surf6	0.09	1.00000000	mice
Ifi27	0.09	1.00000000	mice
Smarcd2	-0.14	1.00000000	mice
Arf3	-0.18	1.00000000	mice
Pes1	-0.13	1.00000000	mice
2310011J03Rik	-0.11	1.00000000	mice
Utp11	0.10	1.00000000	mice
Rnps1	0.09	1.00000000	mice
Ptpn1	-0.09	1.00000000	mice
Dcun1d3	-0.10	1.00000000	mice
Ate1	0.08	1.00000000	mice
Pdha1	-0.13	1.00000000	mice
Trappc5	-0.11	1.00000000	mice
Shq1	0.10	1.00000000	mice
Tulp4	-0.15	1.00000000	mice
Nfatc3	-0.09	1.00000000	mice
Spast	-0.15	1.00000000	mice
Glud1	-0.17	1.00000000	mice
Ptpn23	-0.09	1.00000000	mice
Slc17a5	-0.13	1.00000000	mice
Klf6	0.07	1.00000000	mice
Cd82	-0.14	1.00000000	mice
Tmem131	-0.12	1.00000000	mice
Ndufa4	0.11	1.00000000	mice
Lmo2	0.08	1.00000000	mice
Ndufb4	0.08	1.00000000	mice
Prdx2	0.08	1.00000000	mice
Cd48	0.10	1.00000000	mice
Tceanc2	0.07	1.00000000	mice
Entr1	-0.09	1.00000000	mice
Atg2b	-0.14	1.00000000	mice

Tmem50b	0.07	1.00000000	mice
Dusp22	-0.13	1.00000000	mice
Nup93	0.07	1.00000000	mice
Akt3	0.13	1.00000000	mice
Pdcd2l	-0.10	1.00000000	mice
Gcn1	-0.12	1.00000000	mice
a	0.10	1.00000000	mice
Snap29	-0.15	1.00000000	mice
Pcnp	0.12	1.00000000	mice
Dhx38	-0.11	1.00000000	mice
Phb2	-0.12	1.00000000	mice
Lgals8	0.11	1.00000000	mice
Rrm2b	-0.13	1.00000000	mice
Brip1os	0.08	1.00000000	mice
Skap2	-0.17	1.00000000	mice
Gpx4	-0.14	1.00000000	mice
Atp7a	-0.13	1.00000000	mice
Borcs8	0.07	1.00000000	mice
Riok2	0.08	1.00000000	mice
Atrx	-0.09	1.00000000	mice
Snu13	-0.11	1.00000000	mice
Opa3	0.07	1.00000000	mice
Cbwd1	0.08	1.00000000	mice
Eif4a2	0.10	1.00000000	mice
Rnf7	-0.13	1.00000000	mice
Oat	0.07	1.00000000	mice
Zcrb1	0.05	1.00000000	mice
Pacc1	-0.09	1.00000000	mice
Ncbp3	-0.11	1.00000000	mice
Cd55	0.11	1.00000000	mice
Nme2	0.16	1.00000000	mice
Fam91a1	-0.12	1.00000000	mice
Csnk2b	-0.14	1.00000000	mice
Tnpo1	-0.14	1.00000000	mice
Rnf38	-0.19	1.00000000	mice
Aplp2	-0.15	1.00000000	mice
Tomm34	0.07	1.00000000	mice
Uqcrq	0.10	1.00000000	mice
Dnmbp	-0.11	1.00000000	mice
Hes1	-0.07	1.00000000	mice
St3gal6	0.08	1.00000000	mice
Uqcrh	0.11	1.00000000	mice
Nrbp1	0.10	1.00000000	mice
Rnpc3	-0.14	1.00000000	mice
Ppard	-0.13	1.00000000	mice
Zfp472	-0.12	1.00000000	mice

Ndufaf4	0.06	1.00000000	mice
Rbm4	0.07	1.00000000	mice
Ppp1r37	-0.10	1.00000000	mice
Secisbp2	-0.15	1.00000000	mice
1600020E01Rik	-0.18	1.00000000	mice
Zfp868	0.07	1.00000000	mice
Armc1	0.06	1.00000000	mice
Jun	0.10	1.00000000	mice
Smurf2	-0.13	1.00000000	mice
Eif3d	0.07	1.00000000	mice
Cct3	0.10	1.00000000	mice
C1qbp	-0.11	1.00000000	mice
Larp7	0.09	1.00000000	mice
Yeats2	-0.13	1.00000000	mice
Fbxl14	-0.09	1.00000000	mice
Ran	0.11	1.00000000	mice
Smarcad1	-0.12	1.00000000	mice
Ndufa5	0.09	1.00000000	mice
Uqcc2	0.07	1.00000000	mice
Fam193b	-0.13	1.00000000	mice
Polg2	0.07	1.00000000	mice
Cyp20a1	-0.10	1.00000000	mice
Unc119b	-0.11	1.00000000	mice
Prpf8	-0.11	1.00000000	mice
Kiz	-0.17	1.00000000	mice
Cct5	0.08	1.00000000	mice
Crtc3	-0.16	1.00000000	mice
Rab4b	-0.13	1.00000000	mice
Nup107	0.01	1.00000000	mice
Ccdc77	0.08	1.00000000	mice
Prr3	-0.11	1.00000000	mice
Htt	0.06	1.00000000	mice
Fmc1	0.07	1.00000000	mice
Pptc7	-0.12	1.00000000	mice
Rnft1	0.07	1.00000000	mice
Bmyc	0.07	1.00000000	mice
Asnsd1	0.07	1.00000000	mice
Ccdc71l	-0.09	1.00000000	mice
Actr1b	-0.09	1.00000000	mice
Psmb2	-0.13	1.00000000	mice
B4galnt1	0.07	1.00000000	mice
Ergic3	-0.11	1.00000000	mice
Prr5	-0.10	1.00000000	mice
Birc2	0.13	1.00000000	mice
Treml2	-0.13	1.00000000	mice
Afg1l	-0.15	1.00000000	mice

Usp49	-0.15	1.00000000	mice
Abcf3	0.05	1.00000000	mice
Rbms1	0.13	1.00000000	mice
Larp4	-0.13	1.00000000	mice
Nsrp1	0.10	1.00000000	mice
Ano10	-0.17	1.00000000	mice
Ttc17	-0.17	1.00000000	mice
Epb41l4aos	0.08	1.00000000	mice
Ptpn2	-0.15	1.00000000	mice
Zmpste24	-0.12	1.00000000	mice
Sdhb	-0.13	1.00000000	mice
Srbd1	0.08	1.00000000	mice
Clptm1l	-0.13	1.00000000	mice
Smap1	0.10	1.00000000	mice
Higd1a	0.09	1.00000000	mice
Fbxw11	0.08	1.00000000	mice
E130307A14Rik	-0.16	1.00000000	mice
Hdac5	-0.12	1.00000000	mice
Lamtor4	-0.09	1.00000000	mice
Ndufaf2	0.08	1.00000000	mice
Dlgap4	0.09	1.00000000	mice
Scfd2	-0.19	1.00000000	mice
Ccnc	0.11	1.00000000	mice
Txnrd1	0.06	1.00000000	mice
Prr14l	-0.12	1.00000000	mice
Ube2s	0.11	1.00000000	mice
Sipa1	-0.14	1.00000000	mice
Rilpl2	-0.08	1.00000000	mice
Pms2	-0.14	1.00000000	mice
Pld3	-0.10	1.00000000	mice
Sapcd1	0.05	1.00000000	mice
Apopt1	0.09	1.00000000	mice
Dnajc3	-0.10	1.00000000	mice
Primpol	-0.08	1.00000000	mice
Add3	0.01	1.00000000	mice
Trrap	-0.11	1.00000000	mice
Fth1	-0.06	1.00000000	mice
Aff4	0.11	1.00000000	mice
Rrp1b	-0.11	1.00000000	mice
Bclaf1	-0.12	1.00000000	mice
Kcmf1	0.08	1.00000000	mice
Btf3	0.08	1.00000000	mice
Ethe1	0.04	1.00000000	mice
Sh3bp5	0.13	1.00000000	mice
Polr2c	0.06	1.00000000	mice
Ptrh2	0.04	1.00000000	mice

Nadk2	-0.10	1.00000000	mice
Nars	0.12	1.00000000	mice
Dpm1	-0.16	1.00000000	mice
Lrpprc	-0.16	1.00000000	mice
Ntmt1	-0.09	1.00000000	mice
Rp2	-0.11	1.00000000	mice
Ube2m	-0.12	1.00000000	mice
Smc4	0.10	1.00000000	mice
Nxt1	0.04	1.00000000	mice
Nfyb	-0.07	1.00000000	mice
Ndc1	-0.12	1.00000000	mice
Unc50	0.08	1.00000000	mice
Ap3s1	-0.13	1.00000000	mice
Prkca	-0.11	1.00000000	mice
Tbc1d14	-0.14	1.00000000	mice
Dynlt3	0.09	1.00000000	mice
Glg1	-0.15	1.00000000	mice
Nol10	0.08	1.00000000	mice
Bfsp2	-0.15	1.00000000	mice
Acbd5	-0.19	1.00000000	mice
Uri1	-0.16	1.00000000	mice
Cbl	-0.14	1.00000000	mice
Tmed9	-0.12	1.00000000	mice
Ptbp1	-0.15	1.00000000	mice
Adcy7	-0.16	1.00000000	mice
Timm8b	0.05	1.00000000	mice
Ebna1bp2	0.07	1.00000000	mice
Tfg	-0.16	1.00000000	mice
Thumpd3	0.08	1.00000000	mice
Psmg2	0.07	1.00000000	mice
Ube4b	-0.13	1.00000000	mice
Snx4	-0.15	1.00000000	mice
Nme7	0.08	1.00000000	mice
Cops9	0.11	1.00000000	mice
Gng12	-0.11	1.00000000	mice
Nfatc1	0.15	1.00000000	mice
Psmd9	-0.11	1.00000000	mice
Sacm1l	-0.13	1.00000000	mice
Ireb2	-0.18	1.00000000	mice
Smim19	0.06	1.00000000	mice
Ctnna1	0.07	1.00000000	mice
Uimc1	-0.09	1.00000000	mice
Taok3	-0.11	1.00000000	mice
Rmnd1	-0.11	1.00000000	mice
Ermard	-0.08	1.00000000	mice
Wdr43	0.10	1.00000000	mice

Cops3	-0.13	1.00000000	mice
Dcun1d4	0.05	1.00000000	mice
Pnkp	-0.12	1.00000000	mice
Usp9x	0.16	1.00000000	mice
Cab39	-0.17	1.00000000	mice
Cdc37	0.10	1.00000000	mice
AI413582	-0.10	1.00000000	mice
Galnt2	-0.09	1.00000000	mice
Sar1a	0.07	1.00000000	mice
Ssb	0.09	1.00000000	mice
Dennd4b	-0.11	1.00000000	mice
Phlpp2	0.06	1.00000000	mice
Ube2a	-0.13	1.00000000	mice
Tubgcp5	-0.13	1.00000000	mice
Pip5k1a	-0.16	1.00000000	mice
Tnks2	-0.16	1.00000000	mice
Rnf168	0.08	1.00000000	mice
Gfpt1	0.08	1.00000000	mice
R3hdm2	-0.16	1.00000000	mice
2610008E11Rik	-0.11	1.00000000	mice
Nlrc4	0.09	1.00000000	mice
Clk1	0.11	1.00000000	mice
Ndufs4	-0.16	1.00000000	mice
Pan3	-0.10	1.00000000	mice
BC052040	0.08	1.00000000	mice
Rbm41	0.04	1.00000000	mice
Scpep1	-0.13	1.00000000	mice
Pmvk	0.08	1.00000000	mice
Tex10	-0.12	1.00000000	mice
Uap1	0.08	1.00000000	mice
Fem1b	0.06	1.00000000	mice
Akap10	-0.19	1.00000000	mice
Vamp3	-0.10	1.00000000	mice
Senp5	0.12	1.00000000	mice
Kif3b	-0.10	1.00000000	mice
Bckdhb	0.07	1.00000000	mice
Traf4	0.09	1.00000000	mice
Pias1	0.13	1.00000000	mice
Ncbp2	0.04	1.00000000	mice
Atg16l1	-0.10	1.00000000	mice
Luc7l2	-0.07	1.00000000	mice
Tmem134	0.08	1.00000000	mice
Ywhaq	-0.14	1.00000000	mice
Esf1	0.06	1.00000000	mice
Sugp2	-0.09	1.00000000	mice
Tmem160	0.09	1.00000000	mice

Phlpp1	0.14	1.00000000	mice
Cytip	-0.06	1.00000000	mice
Sfswap	-0.12	1.00000000	mice
Ccdc115	-0.11	1.00000000	mice
Psmd2	0.06	1.00000000	mice
Snx1	-0.12	1.00000000	mice
Gphn	0.12	1.00000000	mice
Sp1	0.10	1.00000000	mice
Rbis	0.06	1.00000000	mice
Zfp62	-0.13	1.00000000	mice
0610010K14Rik	0.06	1.00000000	mice
Ciao2b	0.08	1.00000000	mice
Pbx3	0.16	1.00000000	mice
Nck1	0.11	1.00000000	mice
Pigk	-0.14	1.00000000	mice
Swt1	0.08	1.00000000	mice
Dmtf1	-0.12	1.00000000	mice
Lims1	-0.21	1.00000000	mice
Anp32e	-0.16	1.00000000	mice
Dennd5a	-0.13	1.00000000	mice
4932438A13Rik	0.10	1.00000000	mice
Dock9	-0.20	1.00000000	mice
Ubn2	-0.13	1.00000000	mice
Rab3ip	-0.15	1.00000000	mice
Gng2	-0.20	1.00000000	mice
Fosl2	-0.13	1.00000000	mice
Sec23b	0.10	1.00000000	mice
Vps13c	-0.17	1.00000000	mice
Elf1	0.12	1.00000000	mice
Pus3	0.05	1.00000000	mice
Gtf2e2	-0.15	1.00000000	mice
Ssbp1	0.08	1.00000000	mice
Zfp64	-0.15	1.00000000	mice
Stim2	0.08	1.00000000	mice
Adrb2	-0.07	1.00000000	mice
Golga4	-0.12	1.00000000	mice
Khsrp	-0.09	1.00000000	mice
Arhgap26	-0.15	1.00000000	mice
Golt1b	0.06	1.00000000	mice
Ppp1r14b	-0.14	1.00000000	mice
Sec62	0.08	1.00000000	mice
Rbks	0.11	1.00000000	mice
Fam120b	0.06	1.00000000	mice
Commd1	0.05	1.00000000	mice
Zfp91	-0.15	1.00000000	mice
Ezh2	-0.13	1.00000000	mice

Taf1d	0.13	1.00000000	mice
Adh5	-0.08	1.00000000	mice
Bin2	0.09	1.00000000	mice
Fgd6	-0.17	1.00000000	mice
Trmt112	-0.12	1.00000000	mice
Fbxo32	-0.11	1.00000000	mice
Erap1	0.12	1.00000000	mice
Dnttip2	0.08	1.00000000	mice
Alyref	-0.09	1.00000000	mice
Atg13	-0.13	1.00000000	mice
Cops8	0.05	1.00000000	mice
Atxn10	-0.15	1.00000000	mice
Tcerg1	0.07	1.00000000	mice
Rab2b	0.07	1.00000000	mice
Actn4	0.08	1.00000000	mice
D1Ertd622e	0.12	1.00000000	mice
Smarcb1	-0.15	1.00000000	mice
Ppt1	-0.09	1.00000000	mice
Fam207a	-0.10	1.00000000	mice
Papss1	0.03	1.00000000	mice
Txndc5	-0.13	1.00000000	mice
Tsnax	-0.10	1.00000000	mice
Agps	-0.12	1.00000000	mice
Fam174a	0.11	1.00000000	mice
Nr4a2	0.14	1.00000000	mice
Dynlrb1	0.05	1.00000000	mice
Psd4	-0.14	1.00000000	mice
Ubxn4	-0.17	1.00000000	mice
Ywhab	0.09	1.00000000	mice
Hnrnpc	0.10	1.00000000	mice
Slc25a4	-0.13	1.00000000	mice
Pim3	0.08	1.00000000	mice
Fcho2	-0.13	1.00000000	mice
Pold4	-0.13	1.00000000	mice
Irak1	0.09	1.00000000	mice
Bsdc1	-0.11	1.00000000	mice
Abl2	-0.17	1.00000000	mice
Ccdc174	0.05	1.00000000	mice
Stat5b	-0.20	1.00000000	mice
Rsf1	-0.11	1.00000000	mice
Dpp3	-0.09	1.00000000	mice
Pkd1	0.03	1.00000000	mice
Atraid	-0.10	1.00000000	mice
Atf7ip	0.13	1.00000000	mice
Smc1a	0.12	1.00000000	mice
Rspry1	0.07	1.00000000	mice

Bfar	0.04	1.00000000	mice
Tob1	-0.09	1.00000000	mice
Slc9a9	-0.12	1.00000000	mice
Suclg1	0.08	1.00000000	mice
Ilf2	-0.14	1.00000000	mice
E2f4	-0.09	1.00000000	mice
Cpq	0.07	1.00000000	mice
Gabarapl2	0.05	1.00000000	mice
Wdr74	-0.09	1.00000000	mice
Naa20	0.10	1.00000000	mice
Rac2	-0.10	1.00000000	mice
Actr10	0.07	1.00000000	mice
Strn4	-0.11	1.00000000	mice
Vdac1	-0.12	1.00000000	mice
Anks1	-0.10	1.00000000	mice
Aar2	0.06	1.00000000	mice
Tom1l2	-0.20	1.00000000	mice
Dph5	0.08	1.00000000	mice
Taf1	0.12	1.00000000	mice
Dusp12	0.05	1.00000000	mice
Snrpb	-0.08	1.00000000	mice
4632427E13Rik	0.05	1.00000000	mice
Pank2	0.11	1.00000000	mice
Emd	0.09	1.00000000	mice
Orai1	0.12	1.00000000	mice
Ablim1	-0.11	1.00000000	mice
Pde3b	0.10	1.00000000	mice
St7	-0.13	1.00000000	mice
Camk2d	-0.10	1.00000000	mice
Stk11ip	-0.08	1.00000000	mice
Lsm5	0.10	1.00000000	mice
Zfp943	0.06	1.00000000	mice
Ccdc134	-0.09	1.00000000	mice
Spg7	0.06	1.00000000	mice
Zer1	-0.05	1.00000000	mice
Gtf2h2	-0.09	1.00000000	mice
Slc4a7	0.10	1.00000000	mice
Upf3b	0.11	1.00000000	mice
Pink1	-0.11	1.00000000	mice
Usp53	-0.06	1.00000000	mice
Rnf169	-0.14	1.00000000	mice
Dnajb9	-0.12	1.00000000	mice
Supt3	0.06	1.00000000	mice
Ahi1	0.10	1.00000000	mice
Bmt2	-0.16	1.00000000	mice
AI662270	0.08	1.00000000	mice

Hps4	0.05	1.00000000	mice
Polr1c	0.06	1.00000000	mice
Cdc5l	0.09	1.00000000	mice
Txndc12	-0.10	1.00000000	mice
Gosr2	-0.10	1.00000000	mice
Ppp2r1a	-0.12	1.00000000	mice
Ppp4r3b	0.12	1.00000000	mice
Anapc4	-0.12	1.00000000	mice
Epb41	0.05	1.00000000	mice
Asrgl1	-0.11	1.00000000	mice
Ube2l3	0.07	1.00000000	mice
Ahctf1	0.09	1.00000000	mice
Gnl1	0.06	1.00000000	mice
Eif2b3	0.06	1.00000000	mice
Arpc3	0.10	1.00000000	mice
Larp4b	-0.13	1.00000000	mice
Poc1b	-0.14	1.00000000	mice
Snhg12	0.08	1.00000000	mice
1110004F10Rik	0.09	1.00000000	mice
Bcl2l13	-0.11	1.00000000	mice
Bsg	0.07	1.00000000	mice
Rpp14	0.07	1.00000000	mice
Zfp282	-0.06	1.00000000	mice
Zfp800	0.08	1.00000000	mice
Cap1	-0.14	1.00000000	mice
Derl2	0.09	1.00000000	mice
Specc1l	-0.15	1.00000000	mice
Appbp2	-0.08	1.00000000	mice
Abhd2	0.07	1.00000000	mice
Tango6	-0.15	1.00000000	mice
Pskh1	-0.06	1.00000000	mice
Rap2c	0.07	1.00000000	mice
Vav3	0.16	1.00000000	mice
Gps2	0.10	1.00000000	mice
Grk5	-0.12	1.00000000	mice
Shprh	-0.17	1.00000000	mice
Prdm11	-0.13	1.00000000	mice
Cisd1	0.05	1.00000000	mice
Gan	-0.09	1.00000000	mice
Spns1	-0.09	1.00000000	mice
Gdpd3	-0.08	1.00000000	mice
Haus3	0.08	1.00000000	mice
Rap1a	-0.09	1.00000000	mice
Socs5	0.09	1.00000000	mice
Dazap1	-0.12	1.00000000	mice
Ttc13	-0.12	1.00000000	mice

Snx8	-0.18	1.00000000	mice
Pnisr	0.08	1.00000000	mice
Atp2b1	-0.09	1.00000000	mice
Pgap1	-0.12	1.00000000	mice
Sertad1	0.06	1.00000000	mice
Rbms2	0.07	1.00000000	mice
Sap30bp	-0.10	1.00000000	mice
Naca	0.07	1.00000000	mice
Ssrp1	0.09	1.00000000	mice
Hprt	0.08	1.00000000	mice
Ubr4	0.09	1.00000000	mice
Eefsec	0.08	1.00000000	mice
Ing1	-0.11	1.00000000	mice
Hadhb	0.05	1.00000000	mice
Gapvd1	0.05	1.00000000	mice
Cir1	-0.12	1.00000000	mice
AI506816	0.05	1.00000000	mice
Usp24	-0.12	1.00000000	mice
Parp8	0.08	1.00000000	mice
Dap	-0.08	1.00000000	mice
Oser1	-0.12	1.00000000	mice
Rhog	0.07	1.00000000	mice
Reps1	-0.15	1.00000000	mice
Coa7	-0.09	1.00000000	mice
Pycard	0.07	1.00000000	mice
Lsm3	0.08	1.00000000	mice
2510039O18Rik	-0.11	1.00000000	mice
Uso1	-0.12	1.00000000	mice
Rbmxl1	0.05	1.00000000	mice
Gadd45gip1	0.05	1.00000000	mice
Sltm	0.07	1.00000000	mice
Spen	-0.15	1.00000000	mice
Trp53	-0.11	1.00000000	mice
Dmac2	-0.09	1.00000000	mice
Phospho2	0.05	1.00000000	mice
Xpo6	-0.13	1.00000000	mice
Cstf2	-0.09	1.00000000	mice
Sf3a3	0.09	1.00000000	mice
Dctn5	-0.08	1.00000000	mice
Arhgef1	-0.08	1.00000000	mice
Rbm45	-0.11	1.00000000	mice
Hnrnpul1	-0.11	1.00000000	mice
Dhx15	0.10	1.00000000	mice
B3gat3	0.04	1.00000000	mice
Ccdc59	-0.10	1.00000000	mice
Ergic2	-0.10	1.00000000	mice

Arpc4	-0.11	1.00000000	mice
Lman2	-0.12	1.00000000	mice
Trp53bp2	-0.07	1.00000000	mice
Lmbrd1	0.08	1.00000000	mice
Ndufb7	0.07	1.00000000	mice
1110038F14Rik	0.08	1.00000000	mice
Tmem14c	-0.09	1.00000000	mice
Ap1b1	-0.13	1.00000000	mice
Rbm15	0.06	1.00000000	mice
Btla	-0.19	1.00000000	mice
Eif3j1	0.06	1.00000000	mice
Ralgapa2	-0.17	1.00000000	mice
Parp4	0.06	1.00000000	mice
Ddi2	-0.13	1.00000000	mice
Dpy19l3	-0.12	1.00000000	mice
Slc7a6	-0.09	1.00000000	mice
Btbd10	0.05	1.00000000	mice
Ankrd13c	0.06	1.00000000	mice
Lpcat3	-0.16	1.00000000	mice
Rxrb	-0.10	1.00000000	mice
Frs2	-0.18	1.00000000	mice
Cisd2	0.09	1.00000000	mice
Trpm7	-0.12	1.00000000	mice
Ebag9	0.04	1.00000000	mice
Psen1	-0.15	1.00000000	mice
Prorsd1	-0.07	1.00000000	mice
Acbd6	0.06	1.00000000	mice
Commd2	-0.10	1.00000000	mice
Cnn2	-0.09	1.00000000	mice
Ubtf	-0.07	1.00000000	mice
Pdia6	-0.15	1.00000000	mice
Scfd1	0.07	1.00000000	mice
Arid4a	-0.12	1.00000000	mice
Klf3	-0.15	1.00000000	mice
Rsrc2	0.08	1.00000000	mice
Vmp1	0.03	1.00000000	mice
AW549877	-0.07	1.00000000	mice
Bin3	-0.13	1.00000000	mice
Cep44	-0.10	1.00000000	mice
Zkscan1	0.07	1.00000000	mice
Sod1	0.07	1.00000000	mice
Rars2	-0.09	1.00000000	mice
Kansl2	-0.09	1.00000000	mice
Nipsnap2	-0.11	1.00000000	mice
Pum1	0.09	1.00000000	mice
Ago1	-0.14	1.00000000	mice

Spag9	0.10	1.00000000	mice
Slc15a4	-0.12	1.00000000	mice
Celf2	-0.15	1.00000000	mice
Imp4	-0.07	1.00000000	mice
Cbx1	0.08	1.00000000	mice
Papolg	0.06	1.00000000	mice
Tox4	0.07	1.00000000	mice
Wdr1	-0.11	1.00000000	mice
Rabif	-0.12	1.00000000	mice
Ubxn8	0.06	1.00000000	mice
Anxa7	0.07	1.00000000	mice
Tmed2	-0.09	1.00000000	mice
Nbeal1	-0.17	1.00000000	mice
Slmap	-0.10	1.00000000	mice
Cdk8	0.10	1.00000000	mice
Zc3h3	-0.11	1.00000000	mice
Ncf4	-0.14	1.00000000	mice
Hax1	0.05	1.00000000	mice
Ccnyl1	0.04	1.00000000	mice
Rgl1	0.07	1.00000000	mice
Dnajb2	0.07	1.00000000	mice
Selenok	-0.08	1.00000000	mice
Ostc	-0.10	1.00000000	mice
Lamtor2	0.05	1.00000000	mice
Ctsb	0.11	1.00000000	mice
Ap2a2	0.05	1.00000000	mice
Pik3r1	-0.15	1.00000000	mice
Pitpnc1	-0.25	1.00000000	mice
Zfp984	-0.08	1.00000000	mice
Tasor	0.11	1.00000000	mice
Ppp1r7	0.09	1.00000000	mice
Ranbp1	0.07	1.00000000	mice
Unkl	-0.11	1.00000000	mice
A330023F24Rik	0.05	1.00000000	mice
Blvrb	0.06	1.00000000	mice
Golga1	-0.06	1.00000000	mice
Cdc42se1	-0.09	1.00000000	mice
Commd3	-0.10	1.00000000	mice
Sh3gl1	0.09	1.00000000	mice
Lamtor3	-0.12	1.00000000	mice
Rnf123	-0.11	1.00000000	mice
Peak1	0.09	1.00000000	mice
Trnau1ap	0.05	1.00000000	mice
Apex1	-0.11	1.00000000	mice
Ildr1	0.03	1.00000000	mice
Ncapd3	-0.09	1.00000000	mice

Atp6v0a1	-0.13	1.00000000	mice
Psmb4	0.10	1.00000000	mice
Xab2	-0.07	1.00000000	mice
Tjap1	0.08	1.00000000	mice
Srd5a3	-0.10	1.00000000	mice
Sh3glb1	-0.13	1.00000000	mice
Gtf2a2	-0.11	1.00000000	mice
Cacna1i	-0.10	1.00000000	mice
St13	-0.09	1.00000000	mice
Tbc1d5	0.04	1.00000000	mice
Asna1	-0.11	1.00000000	mice
Pim2	0.06	1.00000000	mice
Skiv2l	-0.09	1.00000000	mice
Usp28	-0.13	1.00000000	mice
Ramac	-0.13	1.00000000	mice
Nab2	0.05	1.00000000	mice
Trappc11	-0.08	1.00000000	mice
Ppp2cb	0.08	1.00000000	mice
Lats2	0.09	1.00000000	mice
Alg5	0.04	1.00000000	mice
Usp32	-0.11	1.00000000	mice
Dhdds	0.05	1.00000000	mice
Timm17b	-0.09	1.00000000	mice
Stx7	-0.15	1.00000000	mice
Slc37a3	0.05	1.00000000	mice
Ndufb3	0.07	1.00000000	mice
Zfp52	0.02	1.00000000	mice
Hipk3	0.09	1.00000000	mice
Lpcat1	0.07	1.00000000	mice
Calcrl	0.08	1.00000000	mice
Dtnb	-0.16	1.00000000	mice
Poc5	-0.08	1.00000000	mice
Slc25a53	-0.12	1.00000000	mice
Elp2	-0.08	1.00000000	mice
Pxdc1	0.05	1.00000000	mice
Taf4	-0.10	1.00000000	mice
Nmrk1	-0.08	1.00000000	mice
Ap2a1	-0.06	1.00000000	mice
Nacc1	0.08	1.00000000	mice
Yju2	-0.10	1.00000000	mice
Trnt1	0.08	1.00000000	mice
Srpr	0.06	1.00000000	mice
Fkbp4	-0.09	1.00000000	mice
Ap5m1	0.06	1.00000000	mice
Katna1	0.09	1.00000000	mice
Smarcc2	-0.12	1.00000000	mice

Spint2	0.06	1.00000000	mice
Anapc2	-0.08	1.00000000	mice
Cnnm4	0.05	1.00000000	mice
Ctr9	0.05	1.00000000	mice
Lbr	-0.12	1.00000000	mice
Clcn7	-0.06	1.00000000	mice
Sri	-0.10	1.00000000	mice
Wasl	-0.14	1.00000000	mice
S1pr4	-0.08	1.00000000	mice
Arhgef12	0.06	1.00000000	mice
Tmod3	-0.14	1.00000000	mice
Ptbp3	-0.10	1.00000000	mice
Slk	-0.10	1.00000000	mice
Serp1	-0.10	1.00000000	mice
2810013P06Rik	-0.10	1.00000000	mice
Tmem248	0.05	1.00000000	mice
Hectd4	-0.13	1.00000000	mice
Ap2m1	-0.08	1.00000000	mice
Zfyve1	-0.13	1.00000000	mice
Ap2s1	-0.09	1.00000000	mice
Nap1l4	-0.10	1.00000000	mice
Lars	0.08	1.00000000	mice
Ecsit	-0.10	1.00000000	mice
Agpat3	-0.13	1.00000000	mice
Itch	-0.11	1.00000000	mice
Atg101	-0.08	1.00000000	mice
Ift52	0.02	1.00000000	mice
Slc25a28	-0.09	1.00000000	mice
Fbxl20	-0.14	1.00000000	mice
Atp5b	0.09	1.00000000	mice
Gpd2	0.11	1.00000000	mice
Ubald2	0.11	1.00000000	mice
Lrrc8dos	0.07	1.00000000	mice
Jtb	-0.11	1.00000000	mice
Rab5if	0.08	1.00000000	mice
Rmi1	0.06	1.00000000	mice
Srsf4	-0.11	1.00000000	mice
Neil1	-0.10	1.00000000	mice
Zfp326	-0.11	1.00000000	mice
Pex5	-0.08	1.00000000	mice
Aldh3a2	-0.07	1.00000000	mice
Eif3a	0.08	1.00000000	mice
Rbm5	0.10	1.00000000	mice
Lrmda	-0.31	1.00000000	mice
Parp1	-0.08	1.00000000	mice
Itpkb	-0.02	1.00000000	mice

Osbp	-0.10	1.00000000	mice
Dcun1d1	0.06	1.00000000	mice
Dctn3	-0.11	1.00000000	mice
Ttc4	-0.07	1.00000000	mice
Bag4	0.07	1.00000000	mice
Flii	-0.12	1.00000000	mice
Ostm1	-0.10	1.00000000	mice
Slx4ip	0.08	1.00000000	mice
Tmem164	-0.09	1.00000000	mice
Ufd1	0.09	1.00000000	mice
Pgls	-0.10	1.00000000	mice
Zfp397	0.06	1.00000000	mice
Cul2	0.11	1.00000000	mice
Spop	-0.14	1.00000000	mice
Clcc1	0.05	1.00000000	mice
Epc2	-0.10	1.00000000	mice
Sumo2	0.03	1.00000000	mice
Grina	-0.10	1.00000000	mice
S100pbp	-0.09	1.00000000	mice
Srsf5	-0.07	1.00000000	mice
Akirin1	-0.08	1.00000000	mice
Nfe2l2	-0.08	1.00000000	mice
Il16	-0.10	1.00000000	mice
Plk4	0.03	1.00000000	mice
Hp1bp3	-0.12	1.00000000	mice
Polr2k	0.06	1.00000000	mice
Toe1	0.03	1.00000000	mice
Kdelr2	0.04	1.00000000	mice
Vcp	0.04	1.00000000	mice
Kdm2a	-0.12	1.00000000	mice
Dph3	0.07	1.00000000	mice
Lin9	0.07	1.00000000	mice
Cct7	0.07	1.00000000	mice
Acot8	-0.08	1.00000000	mice
Sgf29	0.02	1.00000000	mice
Tubgcp3	-0.12	1.00000000	mice
Ppm1g	0.12	1.00000000	mice
Dlst	-0.15	1.00000000	mice
Arhgap39	-0.08	1.00000000	mice
Stk17b	0.05	1.00000000	mice
Cetn2	0.07	1.00000000	mice
Rexo4	0.05	1.00000000	mice
Snhg6	0.04	1.00000000	mice
Ssr4	0.09	1.00000000	mice
Icam2	-0.08	1.00000000	mice
Clcf1	-0.07	1.00000000	mice

Jpx	0.07	1.00000000	mice
Pbxip1	-0.14	1.00000000	mice
Klhdc10	-0.18	1.00000000	mice
Fbxo22	-0.11	1.00000000	mice
Zc3h12d	-0.16	1.00000000	mice
Asap1	-0.17	1.00000000	mice
Atpif1	0.05	1.00000000	mice
Dcp2	0.05	1.00000000	mice
Usp4	0.04	1.00000000	mice
Stk26	-0.06	1.00000000	mice
Xrcc1	-0.10	1.00000000	mice
Atp5h	0.08	1.00000000	mice
BC003965	-0.09	1.00000000	mice
Trim26	0.09	1.00000000	mice
Cox4i1	0.05	1.00000000	mice
Fbxo28	0.05	1.00000000	mice
Ddit3	-0.07	1.00000000	mice
Wbp4	0.04	1.00000000	mice
Pitpnb	-0.12	1.00000000	mice
Dnajc14	-0.07	1.00000000	mice
Polr2a	-0.11	1.00000000	mice
Dhps	-0.12	1.00000000	mice
Smc2	0.04	1.00000000	mice
Bhlhe40	-0.05	1.00000000	mice
Angel2	-0.10	1.00000000	mice
Rbm3	0.06	1.00000000	mice
Haao	-0.12	1.00000000	mice
Gak	-0.13	1.00000000	mice
Usp34	0.08	1.00000000	mice
Dgcr2	-0.12	1.00000000	mice
Usf3	0.07	1.00000000	mice
Clcn4	-0.09	1.00000000	mice
Fam136a	0.04	1.00000000	mice
BC028528	-0.09	1.00000000	mice
Ccz1	0.05	1.00000000	mice
Simc1	-0.12	1.00000000	mice
Grpel2	-0.09	1.00000000	mice
Trip11	0.05	1.00000000	mice
Wdhd1	-0.06	1.00000000	mice
Prkx	-0.08	1.00000000	mice
Nup214	-0.10	1.00000000	mice
Ndufaf8	0.06	1.00000000	mice
Ap3d1	-0.11	1.00000000	mice
Srpk1	0.04	1.00000000	mice
G3bp2	0.11	1.00000000	mice
Prpf38b	0.08	1.00000000	mice

Ppdpf	0.10	1.00000000	mice
Xpo7	-0.12	1.00000000	mice
Bnip2	0.07	1.00000000	mice
Hsd17b11	0.05	1.00000000	mice
Tm2d3	-0.08	1.00000000	mice
2300009A05Rik	0.06	1.00000000	mice
Cmc1	-0.08	1.00000000	mice
Acvr2a	-0.10	1.00000000	mice
Pnpla2	0.07	1.00000000	mice
Uhrf2	0.12	1.00000000	mice
Sin3b	-0.07	1.00000000	mice
Ppp1r12c	-0.10	1.00000000	mice
Cep83	-0.13	1.00000000	mice
Kdm4c	-0.12	1.00000000	mice
Zfyve16	-0.08	1.00000000	mice
Syne3	-0.11	1.00000000	mice
Itsn1	-0.10	1.00000000	mice
Irf4	-0.22	1.00000000	mice
Eif4g1	-0.10	1.00000000	mice
Dbi	0.05	1.00000000	mice
Ap1s2	-0.12	1.00000000	mice
4921524J17Rik	-0.08	1.00000000	mice
Fancc	0.06	1.00000000	mice
Cnot1	-0.08	1.00000000	mice
Dnase2a	0.05	1.00000000	mice
Snx20	0.07	1.00000000	mice
Ppip5k2	-0.13	1.00000000	mice
Lypla1	-0.14	1.00000000	mice
Aagab	-0.08	1.00000000	mice
Gdap2	-0.10	1.00000000	mice
Nipal3	-0.08	1.00000000	mice
Lsm7	0.06	1.00000000	mice
Rnaset2a	-0.09	1.00000000	mice
Kpna3	-0.10	1.00000000	mice
Exosc7	-0.09	1.00000000	mice
Klf2	-0.12	1.00000000	mice
Usp16	0.07	1.00000000	mice
Abhd10	-0.09	1.00000000	mice
Sav1	-0.11	1.00000000	mice
Eef1e1	0.06	1.00000000	mice
Selenof	-0.12	1.00000000	mice
Invs	0.07	1.00000000	mice
Cfap97	0.06	1.00000000	mice
Ap3s2	-0.14	1.00000000	mice
Zmym4	-0.14	1.00000000	mice
Impdh1	-0.07	1.00000000	mice

AI987944	0.04	1.00000000	mice
Slc35d1	-0.09	1.00000000	mice
Crcp	-0.05	1.00000000	mice
Spty2d1	0.08	1.00000000	mice
Zeb1	-0.05	1.00000000	mice
Glrx2	-0.11	1.00000000	mice
2410002F23Rik	-0.10	1.00000000	mice
Ufc1	-0.11	1.00000000	mice
S100a11	0.05	1.00000000	mice
Dicer1	-0.11	1.00000000	mice
Ndst1	-0.09	1.00000000	mice
Pigt	-0.08	1.00000000	mice
Irak4	0.02	1.00000000	mice
Chic2	-0.10	1.00000000	mice
Ddx39	0.04	1.00000000	mice
Tomm6	0.07	1.00000000	mice
Ccdc127	0.05	1.00000000	mice
Psmf1	-0.09	1.00000000	mice
Nipsnap3b	-0.10	1.00000000	mice
Klhl28	0.04	1.00000000	mice
Abracl	0.09	1.00000000	mice
Otud1	-0.08	1.00000000	mice
Cep152	-0.07	1.00000000	mice
Hnrnpr	0.10	1.00000000	mice
Vav1	-0.14	1.00000000	mice
Ddx17	-0.14	1.00000000	mice
Iqcb1	0.11	1.00000000	mice
Ipmk	0.03	1.00000000	mice
Nt5dc3	-0.11	1.00000000	mice
Rpf2	0.03	1.00000000	mice
Elf4	-0.12	1.00000000	mice
Zbtb4	-0.10	1.00000000	mice
Nfrkb	-0.14	1.00000000	mice
Hmgcs1	0.04	1.00000000	mice
Rnf216	-0.10	1.00000000	mice
Ppm1a	-0.09	1.00000000	mice
C2cd3	0.06	1.00000000	mice
Pnrc2	-0.11	1.00000000	mice
Parg	-0.09	1.00000000	mice
Cep192	-0.10	1.00000000	mice
Xpnpep1	-0.07	1.00000000	mice
Rprd1a	0.04	1.00000000	mice
Cdk5rap3	-0.06	1.00000000	mice
Rcl1	-0.12	1.00000000	mice
Hnrnpu	0.09	1.00000000	mice
Aldh16a1	-0.05	1.00000000	mice

Stxbp3	-0.06	1.00000000	mice
Irak3	0.05	1.00000000	mice
9930021J03Rik	-0.16	1.00000000	mice
Zbtb10	0.11	1.00000000	mice
Slc16a10	0.20	1.00000000	mice
Kif20b	-0.07	1.00000000	mice
Zbtb17	-0.08	1.00000000	mice
Hps3	-0.07	1.00000000	mice
Ncor2	-0.11	1.00000000	mice
Ubr1	-0.10	1.00000000	mice
Hsd17b12	0.04	1.00000000	mice
Timmdc1	0.02	1.00000000	mice
Tut7	0.12	1.00000000	mice
Hars	0.05	1.00000000	mice
Nrd1	0.03	1.00000000	mice
Utp20	0.00	1.00000000	mice
Ndufs8	-0.12	1.00000000	mice
Rsrc1	-0.15	1.00000000	mice
Psmc6	0.02	1.00000000	mice
Upf3a	0.05	1.00000000	mice
Nt5c2	-0.13	1.00000000	mice
Pfdn2	0.07	1.00000000	mice
Atf4	-0.10	1.00000000	mice
Cul1	0.07	1.00000000	mice
Twsg1	-0.10	1.00000000	mice
Paxip1	-0.12	1.00000000	mice
Zswim4	0.04	1.00000000	mice
Nifk	0.04	1.00000000	mice
Anapc5	-0.10	1.00000000	mice
Bcas2	-0.10	1.00000000	mice
Atp6v1a	-0.16	1.00000000	mice
Pus10	0.08	1.00000000	mice
Os9	-0.10	1.00000000	mice
Tcp1	0.07	1.00000000	mice
Supv3l1	-0.07	1.00000000	mice
Arap1	-0.12	1.00000000	mice
Nus1	-0.08	1.00000000	mice
Cers5	-0.12	1.00000000	mice
Traf3ip2	-0.09	1.00000000	mice
Polr3a	-0.07	1.00000000	mice
Vwa8	0.05	1.00000000	mice
Slain2	-0.09	1.00000000	mice
Klhl18	0.04	1.00000000	mice
Tbrg1	0.06	1.00000000	mice
Gfm2	0.04	1.00000000	mice
Aamp	0.07	1.00000000	mice

Nup205	-0.08	1.00000000	mice
Eif3g	0.06	1.00000000	mice
Tsc22d2	-0.07	1.00000000	mice
Arfrp1	-0.07	1.00000000	mice
Foxo3	-0.14	1.00000000	mice
Bag1	-0.12	1.00000000	mice
Nvl	0.02	1.00000000	mice
Bdp1	0.07	1.00000000	mice
Rsl24d1	0.05	1.00000000	mice
Sbno2	-0.10	1.00000000	mice
Ankra2	0.05	1.00000000	mice
Dnmt3a	0.02	1.00000000	mice
Dync1li1	-0.15	1.00000000	mice
Tex261	-0.10	1.00000000	mice
Lats1	0.02	1.00000000	mice
Stx18	-0.11	1.00000000	mice
Polr2e	-0.08	1.00000000	mice
Ppp2r5c	-0.11	1.00000000	mice
Ppp1ca	-0.09	1.00000000	mice
Psme3	-0.09	1.00000000	mice
Polr2d	-0.10	1.00000000	mice
Cenpc1	-0.14	1.00000000	mice
Cnot7	-0.08	1.00000000	mice
Noc3l	0.06	1.00000000	mice
Slc7a1	0.09	1.00000000	mice
Fra10ac1	0.03	1.00000000	mice
Klf7	-0.11	1.00000000	mice
Snrnp48	0.06	1.00000000	mice
Rbm6	-0.10	1.00000000	mice
Trmt2b	0.03	1.00000000	mice
Gyg	-0.09	1.00000000	mice
Def6	-0.10	1.00000000	mice
Gtf2h5	-0.10	1.00000000	mice
Emc7	0.06	1.00000000	mice
Epn1	-0.09	1.00000000	mice
Etv3	0.07	1.00000000	mice
Tbca	0.05	1.00000000	mice
Psmb1	0.06	1.00000000	mice
Tmem30a	0.07	1.00000000	mice
Emp3	0.02	1.00000000	mice
Copz1	-0.05	1.00000000	mice
Rfc3	0.09	1.00000000	mice
Hps1	-0.06	1.00000000	mice
Ppp4r1	-0.08	1.00000000	mice
Ammecr1	-0.11	1.00000000	mice
Wdr12	-0.11	1.00000000	mice

Cggbp1	0.05	1.00000000	mice
Galk2	0.05	1.00000000	mice
Slc35b3	-0.08	1.00000000	mice
Ric8b	-0.09	1.00000000	mice
Spindoc	-0.07	1.00000000	mice
Fndc3a	-0.05	1.00000000	mice
Card6	0.08	1.00000000	mice
Anapc10	0.04	1.00000000	mice
Wdr36	-0.11	1.00000000	mice
Pola2	-0.06	1.00000000	mice
Bnip1	-0.07	1.00000000	mice
Cetn3	0.07	1.00000000	mice
Scd1	-0.07	1.00000000	mice
Cox6b1	0.08	1.00000000	mice
Fgfr1op	-0.07	1.00000000	mice
Nae1	0.12	1.00000000	mice
Pnrc1	-0.06	1.00000000	mice
Tecr	-0.11	1.00000000	mice
Gpatch2	-0.09	1.00000000	mice
Atxn7	-0.10	1.00000000	mice
Nudcd3	0.04	1.00000000	mice
Naxd	-0.07	1.00000000	mice
Kdm5a	-0.08	1.00000000	mice
Gstcd	0.02	1.00000000	mice
Abhd6	-0.11	1.00000000	mice
Atxn7l3b	-0.12	1.00000000	mice
Gprasp1	0.10	1.00000000	mice
Cds2	-0.13	1.00000000	mice
Zfp609	-0.19	1.00000000	mice
Wiz	-0.06	1.00000000	mice
Armcx5	-0.09	1.00000000	mice
Ldah	0.05	1.00000000	mice
Dpy19l4	-0.09	1.00000000	mice
Cabin1	-0.10	1.00000000	mice
Calm3	0.05	1.00000000	mice
Golph3l	-0.08	1.00000000	mice
Wdr3	-0.07	1.00000000	mice
Phactr4	0.01	1.00000000	mice
Bach1	-0.14	1.00000000	mice
Ssu72	-0.12	1.00000000	mice
Cdc27	-0.10	1.00000000	mice
Slamf6	-0.02	1.00000000	mice
Efhd2	0.05	1.00000000	mice
Ubp1	-0.11	1.00000000	mice
Gnptab	-0.10	1.00000000	mice
Coq5	0.08	1.00000000	mice

Rfc2	0.06	1.00000000	mice
Fancm	0.06	1.00000000	mice
Wars2	0.02	1.00000000	mice
Larp1	-0.07	1.00000000	mice
Sidt2	-0.10	1.00000000	mice
Rictor	-0.08	1.00000000	mice
Ppm1d	-0.13	1.00000000	mice
Pan2	0.05	1.00000000	mice
Klc1	-0.07	1.00000000	mice
Snip1	-0.06	1.00000000	mice
Tmem127	-0.10	1.00000000	mice
Anapc16	-0.08	1.00000000	mice
Fxr1	0.07	1.00000000	mice
Nfkbib	0.03	1.00000000	mice
Apaf1	-0.10	1.00000000	mice
Cnot4	-0.07	1.00000000	mice
Slc31a1	0.04	1.00000000	mice
Sp4	0.07	1.00000000	mice
Ppp1r12a	-0.05	1.00000000	mice
Pex7	0.05	1.00000000	mice
Pcbp1	-0.11	1.00000000	mice
Kdm5b	-0.10	1.00000000	mice
Rfwd3	0.06	1.00000000	mice
8030462N17Rik	-0.11	1.00000000	mice
Hint3	0.04	1.00000000	mice
E130308A19Rik	-0.15	1.00000000	mice
Aatf	-0.12	1.00000000	mice
Rif1	-0.11	1.00000000	mice
Ciapin1	0.04	1.00000000	mice
Sec23a	-0.10	1.00000000	mice
Dnm1l	0.03	1.00000000	mice
Srgn	0.06	1.00000000	mice
Csnk1d	-0.07	1.00000000	mice
Ndufb10	-0.07	1.00000000	mice
Akr1b3	-0.11	1.00000000	mice
Sh3bgrl3	-0.07	1.00000000	mice
Usp37	0.04	1.00000000	mice
Acp1	-0.07	1.00000000	mice
Pik3ap1	0.10	1.00000000	mice
Rexo2	0.05	1.00000000	mice
Tor1a	-0.06	1.00000000	mice
Ccdc12	0.05	1.00000000	mice
Armc8	-0.10	1.00000000	mice
Yme1l1	0.04	1.00000000	mice
Emc8	-0.09	1.00000000	mice
Nsmce1	-0.07	1.00000000	mice

1700084C06Rik	0.06	1.00000000	mice
Edf1	0.06	1.00000000	mice
Eri3	-0.11	1.00000000	mice
Tle5	-0.07	1.00000000	mice
Ppia	-0.05	1.00000000	mice
Ncf2	0.12	1.00000000	mice
Anks3	-0.07	1.00000000	mice
Cul4a	-0.10	1.00000000	mice
Rbm34	0.06	1.00000000	mice
Ciao2a	-0.10	1.00000000	mice
Srfbp1	-0.07	1.00000000	mice
S100a10	-0.11	1.00000000	mice
Pura	0.05	1.00000000	mice
Irf8	0.00	1.00000000	mice
Ltb	0.03	1.00000000	mice
Dffa	-0.08	1.00000000	mice
Ap3m2	0.07	1.00000000	mice
Exosc9	0.07	1.00000000	mice
Ap1s3	-0.10	1.00000000	mice
Eps15l1	-0.09	1.00000000	mice
Adam9	0.04	1.00000000	mice
AU040320	-0.16	1.00000000	mice
Psmd1	0.06	1.00000000	mice
Psmd5	-0.07	1.00000000	mice
Rnf130	-0.12	1.00000000	mice
1700109H08Rik	-0.09	1.00000000	mice
Brd4	-0.09	1.00000000	mice
Golga5	-0.12	1.00000000	mice
Strada	0.05	1.00000000	mice
Camkmt	0.15	1.00000000	mice
Ccdc9	-0.07	1.00000000	mice
Stau1	0.01	1.00000000	mice
Sepsecs	-0.09	1.00000000	mice
Card19	-0.06	1.00000000	mice
Nemp2	-0.06	1.00000000	mice
Nxpe3	0.05	1.00000000	mice
Ncoa5	0.02	1.00000000	mice
Phf21a	0.01	1.00000000	mice
Scai	-0.18	1.00000000	mice
Fbxo38	0.04	1.00000000	mice
Rnf6	0.04	1.00000000	mice
Dock11	-0.08	1.00000000	mice
Ssh2	0.07	1.00000000	mice
Ppp2r3a	-0.08	1.00000000	mice
Zfand5	-0.09	1.00000000	mice
Ddb2	0.04	1.00000000	mice

Ldha	0.10	1.00000000	mice
Hexb	-0.13	1.00000000	mice
Wdr48	-0.10	1.00000000	mice
Brd8	-0.10	1.00000000	mice
Rhoa	-0.06	1.00000000	mice
Prkdc	-0.17	1.00000000	mice
Zc3h12a	0.05	1.00000000	mice
Ipo5	-0.09	1.00000000	mice
Tbck	0.03	1.00000000	mice
Ythdf1	0.06	1.00000000	mice
Zfp281	0.04	1.00000000	mice
Cryzl1	-0.07	1.00000000	mice
Emc10	-0.07	1.00000000	mice
Scaf4	0.05	1.00000000	mice
Asf1a	-0.06	1.00000000	mice
Eif3m	0.05	1.00000000	mice
Twistnb	-0.10	1.00000000	mice
Fbxl17	-0.15	1.00000000	mice
Elovl5	-0.06	1.00000000	mice
Rap1b	0.05	1.00000000	mice
Yipf1	-0.13	1.00000000	mice
Tspyl2	-0.06	1.00000000	mice
Rexo1	-0.09	1.00000000	mice
Cox10	-0.12	1.00000000	mice
Arpc1b	-0.08	1.00000000	mice
Fcgr2b	-0.12	1.00000000	mice
B4galt7	-0.05	1.00000000	mice
Cfap36	0.04	1.00000000	mice
Aspscr1	-0.08	1.00000000	mice
Tatdn2	-0.07	1.00000000	mice
Nup188	-0.09	1.00000000	mice
Pmpcb	0.05	1.00000000	mice
Tyw1	-0.10	1.00000000	mice
Coq2	-0.11	1.00000000	mice
Snrpa1	0.04	1.00000000	mice
S1pr1	-0.04	1.00000000	mice
Txndc16	-0.13	1.00000000	mice
Dad1	0.04	1.00000000	mice
Tpi1	0.02	1.00000000	mice
Aup1	0.03	1.00000000	mice
Lrba	-0.12	1.00000000	mice
Cog3	-0.10	1.00000000	mice
Eaf1	-0.07	1.00000000	mice
Helz	-0.13	1.00000000	mice
Tcf12	-0.06	1.00000000	mice
Tmem209	-0.06	1.00000000	mice

Tgs1	0.06	1.00000000	mice
Prelid2	-0.09	1.00000000	mice
Cemip2	0.04	1.00000000	mice
Anxa11	-0.10	1.00000000	mice
Pigs	0.01	1.00000000	mice
Pogz	-0.09	1.00000000	mice
Fkbp5	-0.09	1.00000000	mice
Ccdc93	-0.09	1.00000000	mice
Pcmtd1	-0.06	1.00000000	mice
Nufip1	-0.11	1.00000000	mice
Agap2	-0.06	1.00000000	mice
Lamtor5	-0.11	1.00000000	mice
Ranbp3	-0.07	1.00000000	mice
Csde1	0.03	1.00000000	mice
Usp7	-0.07	1.00000000	mice
Slc25a51	-0.08	1.00000000	mice
Csnk1a1	-0.04	1.00000000	mice
Rpf1	-0.07	1.00000000	mice
Abce1	0.03	1.00000000	mice
Sf1	0.07	1.00000000	mice
Ptpn4	-0.13	1.00000000	mice
Rnf41	-0.12	1.00000000	mice
Gramd1a	0.05	1.00000000	mice
Dnajc2	-0.07	1.00000000	mice
Eid1	-0.06	1.00000000	mice
Cul5	-0.13	1.00000000	mice
Arl8b	-0.09	1.00000000	mice
Gspt1	0.02	1.00000000	mice
Zc3h18	-0.08	1.00000000	mice
Nrip1	0.02	1.00000000	mice
Ssna1	-0.08	1.00000000	mice
Exoc1	-0.10	1.00000000	mice
Casp3	0.04	1.00000000	mice
Fam204a	-0.11	1.00000000	mice
Rnf13	-0.06	1.00000000	mice
Ndufs7	0.04	1.00000000	mice
Bud23	-0.06	1.00000000	mice
Psmc1	0.07	1.00000000	mice
Wbp11	0.05	1.00000000	mice
Sufu	-0.07	1.00000000	mice
Tmem9b	-0.07	1.00000000	mice
St8sia6	0.00	1.00000000	mice
Rnf149	-0.07	1.00000000	mice
Ece1	0.01	1.00000000	mice
Elob	0.09	1.00000000	mice
Dnajb11	-0.08	1.00000000	mice

Pfdn6	-0.04	1.00000000	mice
Lrif1	0.08	1.00000000	mice
Zc3h14	-0.07	1.00000000	mice
Rcn2	0.04	1.00000000	mice
Erg28	0.03	1.00000000	mice
Fbxo9	0.05	1.00000000	mice
Pdzd2	-0.19	1.00000000	mice
Ngrn	0.02	1.00000000	mice
Tmbim4	0.04	1.00000000	mice
Rnf20	-0.11	1.00000000	mice
Eef2	-0.05	1.00000000	mice
Ctso	0.03	1.00000000	mice
Tmem19	-0.07	1.00000000	mice
Hltf	-0.11	1.00000000	mice
Sumo1	0.02	1.00000000	mice
Txn1	0.04	1.00000000	mice
Exoc3	-0.09	1.00000000	mice
Stx5a	-0.06	1.00000000	mice
Strap	-0.08	1.00000000	mice
Suz12	-0.09	1.00000000	mice
Relch	-0.09	1.00000000	mice
Tecpr1	-0.07	1.00000000	mice
Pot1a	0.04	1.00000000	mice
Nup88	-0.10	1.00000000	mice
Slc25a16	-0.05	1.00000000	mice
Lrrc57	0.03	1.00000000	mice
Usp8	0.06	1.00000000	mice
Cfl2	0.04	1.00000000	mice
Vkorc1l1	-0.10	1.00000000	mice
Tec	0.13	1.00000000	mice
Trpc4ap	-0.07	1.00000000	mice
Wdr70	-0.09	1.00000000	mice
Zc3h4	-0.09	1.00000000	mice
Anapc1	-0.09	1.00000000	mice
Qser1	-0.01	1.00000000	mice
Sc5d	-0.07	1.00000000	mice
Ppwd1	0.06	1.00000000	mice
Ero1l	0.02	1.00000000	mice
Cope	0.03	1.00000000	mice
Nkapd1	0.06	1.00000000	mice
Kif13b	-0.14	1.00000000	mice
Stk38	-0.11	1.00000000	mice
Senp3	-0.06	1.00000000	mice
D430042O09Rik	-0.10	1.00000000	mice
Wwox	-0.37	1.00000000	mice
Fam167a	0.09	1.00000000	mice

Thada	-0.13	1.00000000	mice
Trim23	-0.08	1.00000000	mice
Npm1	0.08	1.00000000	mice
Zfp266	-0.08	1.00000000	mice
Prpf4	0.04	1.00000000	mice
Araf	-0.08	1.00000000	mice
Boll	0.02	1.00000000	mice
Sorbs1	-0.10	1.00000000	mice
Cep85l	0.06	1.00000000	mice
Snx18	0.06	1.00000000	mice
Tspan32	0.03	1.00000000	mice
Tipin	0.04	1.00000000	mice
Eci1	0.03	1.00000000	mice
Ndel1	0.05	1.00000000	mice
Utp14a	0.04	1.00000000	mice
Aqr	0.04	1.00000000	mice
Abcc4	-0.10	1.00000000	mice
Rsl1d1	0.03	1.00000000	mice
Dph6	0.02	1.00000000	mice
Anapc7	-0.08	1.00000000	mice
Plk2	-0.11	1.00000000	mice
Stxbp2	-0.06	1.00000000	mice
Zfp422	-0.02	1.00000000	mice
Acer3	0.04	1.00000000	mice
Lactb	-0.09	1.00000000	mice
Rasa2	-0.11	1.00000000	mice
Fam49a	-0.06	1.00000000	mice
Rbm22	-0.08	1.00000000	mice
Armh3	-0.14	1.00000000	mice
Ccdc88a	-0.10	1.00000000	mice
Crls1	0.04	1.00000000	mice
Itgav	-0.07	1.00000000	mice
Pfdn4	0.03	1.00000000	mice
Tmem106b	-0.08	1.00000000	mice
Pdia4	0.04	1.00000000	mice
Btaf1	0.07	1.00000000	mice
Ttll3	-0.06	1.00000000	mice
Hmg20a	-0.09	1.00000000	mice
Rbck1	0.04	1.00000000	mice
Xndc1	0.04	1.00000000	mice
Kansl3	0.08	1.00000000	mice
Ciz1	-0.07	1.00000000	mice
Setd1a	-0.07	1.00000000	mice
Sept7	0.04	1.00000000	mice
Dmxl1	-0.01	1.00000000	mice
Scyl3	-0.07	1.00000000	mice

Zc3h6	0.07	1.00000000	mice
Crkl	-0.02	1.00000000	mice
Bms1	-0.08	1.00000000	mice
Tm9sf1	0.02	1.00000000	mice
Ctnnbl1	-0.10	1.00000000	mice
Ell	-0.12	1.00000000	mice
Setd2	0.04	1.00000000	mice
Tmem259	-0.06	1.00000000	mice
Slc9a1	-0.09	1.00000000	mice
Grpel1	0.05	1.00000000	mice
Srcap	-0.06	1.00000000	mice
Plekhj1	-0.07	1.00000000	mice
Naa35	-0.06	1.00000000	mice
Dut	0.01	1.00000000	mice
Tnks	-0.10	1.00000000	mice
Zfp622	0.04	1.00000000	mice
Ralgapb	-0.10	1.00000000	mice
Dcp1b	-0.09	1.00000000	mice
Tspan5	-0.11	1.00000000	mice
Rtf2	-0.08	1.00000000	mice
Chp1	0.01	1.00000000	mice
Piezo1	-0.11	1.00000000	mice
Zfp950	0.01	1.00000000	mice
Umad1	-0.07	1.00000000	mice
Nt5c	-0.07	1.00000000	mice
Creg1	0.05	1.00000000	mice
Cenpx	-0.06	1.00000000	mice
Sra1	0.07	1.00000000	mice
Phc3	-0.10	1.00000000	mice
Crebbp	0.04	1.00000000	mice
Acin1	-0.07	1.00000000	mice
Dnaja3	0.02	1.00000000	mice
Sil1	-0.14	1.00000000	mice
Exosc4	0.03	1.00000000	mice
Prkcsh	-0.05	1.00000000	mice
Akt1	-0.05	1.00000000	mice
Rrm1	0.04	1.00000000	mice
Rbm7	0.05	1.00000000	mice
Tigd2	-0.06	1.00000000	mice
2610037D02Rik	-0.15	1.00000000	mice
Lmtk2	-0.11	1.00000000	mice
Fam120a	-0.03	1.00000000	mice
Cdc73	0.05	1.00000000	mice
Dcaf13	-0.05	1.00000000	mice
Saraf	-0.08	1.00000000	mice
Rdm1	-0.13	1.00000000	mice

Vps13d	-0.09	1.00000000	mice
Gdi1	-0.09	1.00000000	mice
Dclk2	-0.03	1.00000000	mice
Ptger4	-0.10	1.00000000	mice
Asb8	-0.05	1.00000000	mice
Zbtb18	-0.09	1.00000000	mice
Wnk1	-0.05	1.00000000	mice
Rab33b	-0.08	1.00000000	mice
Ncaph2	-0.07	1.00000000	mice
Gid4	0.03	1.00000000	mice
Eif2a	-0.10	1.00000000	mice
Slc6a6	-0.09	1.00000000	mice
Slf1	0.08	1.00000000	mice
Scamp2	-0.10	1.00000000	mice
Prep	-0.12	1.00000000	mice
Aco2	-0.07	1.00000000	mice
Atxn2	-0.15	1.00000000	mice
BC002059	0.01	1.00000000	mice
Dapp1	-0.16	1.00000000	mice
Ftx	-0.11	1.00000000	mice
Ice1	-0.08	1.00000000	mice
Trabd	-0.07	1.00000000	mice
Wdr4	0.01	1.00000000	mice
Rpn2	0.04	1.00000000	mice
S100a13	0.03	1.00000000	mice
Atp1a1	0.01	1.00000000	mice
Ube2v2	0.01	1.00000000	mice
Clock	0.04	1.00000000	mice
Gbe1	-0.10	1.00000000	mice
Tpk1	0.00	1.00000000	mice
Tmem68	0.03	1.00000000	mice
2900097C17Rik	-0.09	1.00000000	mice
Sympk	0.05	1.00000000	mice
Stambpl1	-0.10	1.00000000	mice
Sptbn1	-0.07	1.00000000	mice
Hace1	-0.16	1.00000000	mice
Hnrnpa1	0.06	1.00000000	mice
Rprd1b	-0.08	1.00000000	mice
Copa	-0.07	1.00000000	mice
Preb	-0.05	1.00000000	mice
Chkb	-0.07	1.00000000	mice
Galnt10	-0.06	1.00000000	mice
Cript	-0.09	1.00000000	mice
Lsg1	-0.08	1.00000000	mice
Nbas	0.04	1.00000000	mice
Herc2	-0.08	1.00000000	mice

Cct6a	0.05	1.00000000	mice
Cox6a1	0.02	1.00000000	mice
Gnai3	-0.09	1.00000000	mice
Carm1	-0.09	1.00000000	mice
Ddx41	0.02	1.00000000	mice
Jhy	-0.11	1.00000000	mice
Tpst2	0.02	1.00000000	mice
Xrcc4	-0.15	1.00000000	mice
Slc16a6	-0.08	1.00000000	mice
Camsap1	-0.01	1.00000000	mice
Ndufv3	0.04	1.00000000	mice
Top3a	-0.09	1.00000000	mice
Sbf2	-0.14	1.00000000	mice
Pex3	0.05	1.00000000	mice
Recql5	-0.01	1.00000000	mice
Tmx3	0.02	1.00000000	mice
Fcrl1	-0.09	1.00000000	mice
Sap30l	0.04	1.00000000	mice
Ikzf5	0.05	1.00000000	mice
Slu7	0.02	1.00000000	mice
Aifm1	0.05	1.00000000	mice
Dennd4c	0.00	1.00000000	mice
Swi5	0.04	1.00000000	mice
Scand1	-0.08	1.00000000	mice
Tnfaip3	-0.07	1.00000000	mice
Plekho2	-0.08	1.00000000	mice
Thoc5	-0.10	1.00000000	mice
Rint1	-0.03	1.00000000	mice
Usp40	0.06	1.00000000	mice
Dhx33	-0.06	1.00000000	mice
Prpf18	-0.09	1.00000000	mice
Uxt	-0.08	1.00000000	mice
Trmt10a	0.05	1.00000000	mice
Utp4	0.02	1.00000000	mice
Brd3	-0.08	1.00000000	mice
Nfu1	-0.07	1.00000000	mice
Itprip	0.05	1.00000000	mice
Atrn	-0.05	1.00000000	mice
Brd9	0.02	1.00000000	mice
Gng5	0.06	1.00000000	mice
Lemd3	0.07	1.00000000	mice
Wdr75	0.04	1.00000000	mice
Arl6ip5	0.01	1.00000000	mice
Tnpo3	-0.10	1.00000000	mice
Ubc	0.05	1.00000000	mice
Dnm2	-0.12	1.00000000	mice

Zfp445	0.03	1.00000000	mice
Eif1a	0.09	1.00000000	mice
Cdk6	0.19	1.00000000	mice
Igkc	-0.12	1.00000000	mice
Gps1	-0.07	1.00000000	mice
0610012G03Rik	-0.07	1.00000000	mice
Cd2ap	0.06	1.00000000	mice
Wbp11	-0.10	1.00000000	mice
Itgb7	0.03	1.00000000	mice
Cnpy3	-0.07	1.00000000	mice
Nrbf2	0.01	1.00000000	mice
Dync1li2	-0.07	1.00000000	mice
Ttf1	-0.07	1.00000000	mice
Atp2a2	-0.05	1.00000000	mice
Rbm10	-0.10	1.00000000	mice
Sf3b3	0.05	1.00000000	mice
Gon4l	-0.07	1.00000000	mice
Gtpbp1	0.04	1.00000000	mice
Klhdc2	-0.07	1.00000000	mice
Ppp1r2	-0.07	1.00000000	mice
E4f1	0.04	1.00000000	mice
Kif1b	0.05	1.00000000	mice
Fam168a	-0.10	1.00000000	mice
Ctse	-0.12	1.00000000	mice
Arpc2	0.05	1.00000000	mice
Npat	-0.04	1.00000000	mice
Rpa1	0.02	1.00000000	mice
Borcs7	-0.06	1.00000000	mice
Senp7	0.09	1.00000000	mice
Glod4	0.00	1.00000000	mice
Abca7	-0.07	1.00000000	mice
Capn7	-0.09	1.00000000	mice
Socs4	-0.09	1.00000000	mice
Nudc	0.04	1.00000000	mice
Arhgef6	0.07	1.00000000	mice
Dcaf12	0.04	1.00000000	mice
Cpsf4	-0.05	1.00000000	mice
Grap	0.03	1.00000000	mice
Plekhm1	0.05	1.00000000	mice
Agtpbp1	-0.10	1.00000000	mice
Adpgk	-0.09	1.00000000	mice
Hook3	-0.10	1.00000000	mice
Tpd52	-0.09	1.00000000	mice
Hspa9	0.05	1.00000000	mice
B230219D22Rik	0.03	1.00000000	mice
Ube3b	-0.05	1.00000000	mice

Fam162a	0.02	1.00000000	mice
A530032D15Rik	0.06	1.00000000	mice
Khnyn	0.04	1.00000000	mice
Dazap2	-0.08	1.00000000	mice
9430091E24Rik	-0.10	1.00000000	mice
Tuba4a	0.04	1.00000000	mice
Snd1	0.02	1.00000000	mice
Zdhhc17	-0.04	1.00000000	mice
Ptpn9	-0.12	1.00000000	mice
Cep63	-0.07	1.00000000	mice
Nudt13	0.04	1.00000000	mice
Zfp110	0.04	1.00000000	mice
Ascc2	-0.05	1.00000000	mice
Psma3	0.06	1.00000000	mice
Arfgap2	-0.10	1.00000000	mice
Vps4a	0.02	1.00000000	mice
Pno1	0.02	1.00000000	mice
Gnl2	0.03	1.00000000	mice
Ndrg3	0.04	1.00000000	mice
Pomp	0.05	1.00000000	mice
Vps28	0.06	1.00000000	mice
Tollip	-0.07	1.00000000	mice
Snrnp70	-0.08	1.00000000	mice
Gnl3	0.07	1.00000000	mice
Cyth2	-0.07	1.00000000	mice
Dag1	0.06	1.00000000	mice
Nmt1	0.04	1.00000000	mice
Hectd1	0.06	1.00000000	mice
Tstd2	-0.03	1.00000000	mice
Rars	0.05	1.00000000	mice
Casc3	0.00	1.00000000	mice
Strn3	-0.08	1.00000000	mice
Eif3c	0.02	1.00000000	mice
Nsun2	0.05	1.00000000	mice
Ube2z	-0.08	1.00000000	mice
Vps33a	-0.10	1.00000000	mice
Atox1	0.03	1.00000000	mice
Plpbp	-0.08	1.00000000	mice
Pvt1	-0.01	1.00000000	mice
Birc3	0.04	1.00000000	mice
Prps1	-0.06	1.00000000	mice
Ddx52	0.05	1.00000000	mice
Kdelr1	-0.08	1.00000000	mice
Ddx10	-0.08	1.00000000	mice
Ppm1b	-0.08	1.00000000	mice
Washc3	0.00	1.00000000	mice

Swap70	-0.15	1.00000000	mice
Zfp26	-0.05	1.00000000	mice
Jade2	0.02	1.00000000	mice
Ccdc58	0.05	1.00000000	mice
Smdt1	-0.06	1.00000000	mice
Pfdn1	0.01	1.00000000	mice
Rnf44	-0.06	1.00000000	mice
Gatad1	0.06	1.00000000	mice
Hook1	-0.05	1.00000000	mice
Zzz3	-0.08	1.00000000	mice
Ccnl2	0.05	1.00000000	mice
Cd164	-0.01	1.00000000	mice
Sdhaf2	-0.09	1.00000000	mice
Togaram1	-0.10	1.00000000	mice
Lmna	0.01	1.00000000	mice
6720427I07Rik	0.04	1.00000000	mice
Snw1	0.03	1.00000000	mice
Trim33	-0.10	1.00000000	mice
L3mbtl3	0.00	1.00000000	mice
Parl	-0.08	1.00000000	mice
Npc2	-0.05	1.00000000	mice
Ppil3	0.03	1.00000000	mice
Rnf4	0.03	1.00000000	mice
Zfp438	-0.08	1.00000000	mice
Bloc1s2	-0.09	1.00000000	mice
Comt	-0.05	1.00000000	mice
Riok1	0.02	1.00000000	mice
Atp5a1	-0.05	1.00000000	mice
Erc1	-0.14	1.00000000	mice
Plbd2	-0.07	1.00000000	mice
Itpr1	-0.06	1.00000000	mice
Foxj3	-0.12	1.00000000	mice
Ankrd40	0.02	1.00000000	mice
Klhdc3	-0.06	1.00000000	mice
Ndufs5	0.03	1.00000000	mice
Eif4h	-0.06	1.00000000	mice
Kmt5b	-0.04	1.00000000	mice
Plaur	-0.03	1.00000000	mice
Prpf39	0.02	1.00000000	mice
Gucd1	0.02	1.00000000	mice
Arcn1	0.04	1.00000000	mice
Adrm1	-0.04	1.00000000	mice
Psmd14	0.01	1.00000000	mice
Hnrnpm	0.06	1.00000000	mice
Elavl1	-0.10	1.00000000	mice
Ncoa6	-0.10	1.00000000	mice

Ppp1r15b	-0.07	1.00000000	mice
Ppp1r12b	-0.13	1.00000000	mice
Syncrip	0.05	1.00000000	mice
Sumo3	-0.07	1.00000000	mice
Zfp654	-0.03	1.00000000	mice
Lypla2	-0.07	1.00000000	mice
Kctd12	0.02	1.00000000	mice
Zfp518a	-0.07	1.00000000	mice
Emsy	0.00	1.00000000	mice
Rbbp7	0.02	1.00000000	mice
R3hcc1l	0.02	1.00000000	mice
Fopnl	-0.05	1.00000000	mice
Lin7c	0.05	1.00000000	mice
Lnx2	-0.08	1.00000000	mice
Fbrs	-0.08	1.00000000	mice
Zfp664	0.04	1.00000000	mice
Brk1	-0.06	1.00000000	mice
Polr2g	-0.06	1.00000000	mice
Arrdc3	-0.03	1.00000000	mice
Abraxas2	-0.08	1.00000000	mice
Ankrd49	0.02	1.00000000	mice
Fam118b	0.05	1.00000000	mice
Slc25a30	-0.07	1.00000000	mice
Ddx39b	-0.08	1.00000000	mice
Fam114a2	-0.06	1.00000000	mice
Senp6	-0.06	1.00000000	mice
Stimate	-0.12	1.00000000	mice
Gna12	-0.09	1.00000000	mice
Ripk2	0.05	1.00000000	mice
Parp2	-0.07	1.00000000	mice
Tars	0.03	1.00000000	mice
Washc4	-0.08	1.00000000	mice
Phf10	0.04	1.00000000	mice
Fancl	-0.09	1.00000000	mice
Zfp346	-0.08	1.00000000	mice
Supt6	0.03	1.00000000	mice
Heca	-0.08	1.00000000	mice
Atp6v1e1	-0.08	1.00000000	mice
Cstf3	0.04	1.00000000	mice
Camta1	-0.10	1.00000000	mice
Prdm4	-0.05	1.00000000	mice
Abt1	0.04	1.00000000	mice
Rabep2	0.03	1.00000000	mice
Gpkow	0.03	1.00000000	mice
Herc4	-0.08	1.00000000	mice
Tmem87b	-0.06	1.00000000	mice

Prr14	-0.06	1.00000000	mice
Urb2	0.01	1.00000000	mice
Nup50	0.05	1.00000000	mice
1110059E24Rik	0.00	1.00000000	mice
Ubqln1	-0.07	1.00000000	mice
Tfip11	0.01	1.00000000	mice
Srp14	-0.08	1.00000000	mice
2510002D24Rik	0.03	1.00000000	mice
Scaper	-0.08	1.00000000	mice
Nsmce3	0.01	1.00000000	mice
Tma16	0.05	1.00000000	mice
Zfp592	-0.11	1.00000000	mice
Pde4dip	-0.07	1.00000000	mice
Sypl	0.02	1.00000000	mice
Aars	0.02	1.00000000	mice
Zbtb20	-0.08	1.00000000	mice
Smim7	-0.05	1.00000000	mice
BC005561	0.04	1.00000000	mice
N4bp2l2	0.04	1.00000000	mice
Adss	0.08	1.00000000	mice
Haus6	-0.07	1.00000000	mice
Tmem208	0.03	1.00000000	mice
Bag6	0.04	1.00000000	mice
Canx	-0.09	1.00000000	mice
Fbxl4	-0.07	1.00000000	mice
Taf3	0.04	1.00000000	mice
Far1	-0.07	1.00000000	mice
Eif3b	0.00	1.00000000	mice
Supt4a	-0.05	1.00000000	mice
Zfp830	0.02	1.00000000	mice
Zfp729b	-0.09	1.00000000	mice
Rrp36	-0.08	1.00000000	mice
Tdp2	-0.08	1.00000000	mice
Keap1	-0.05	1.00000000	mice
Utp15	0.02	1.00000000	mice
Nras	0.04	1.00000000	mice
Zbtb25	0.07	1.00000000	mice
Chrac1	-0.06	1.00000000	mice
U2af2	0.04	1.00000000	mice
Armc10	0.04	1.00000000	mice
Herpud2	0.04	1.00000000	mice
Sec22b	0.05	1.00000000	mice
Hpgds	0.05	1.00000000	mice
Tmem245	-0.09	1.00000000	mice
Sidt1	-0.02	1.00000000	mice
Tnfaip8	0.02	1.00000000	mice

Atp11c	-0.09	1.00000000	mice
Cherp	0.01	1.00000000	mice
Poldip3	-0.10	1.00000000	mice
Fcf1	-0.04	1.00000000	mice
Hells	0.02	1.00000000	mice
Lrrc47	0.00	1.00000000	mice
Snrpe	0.06	1.00000000	mice
Prcp	0.03	1.00000000	mice
Faf2	-0.06	1.00000000	mice
Zfp638	0.07	1.00000000	mice
Ago3	0.00	1.00000000	mice
Klhdc4	-0.04	1.00000000	mice
Prrc2a	-0.06	1.00000000	mice
Dync1i2	0.05	1.00000000	mice
Atp2c1	-0.10	1.00000000	mice
Rex1bd	0.02	1.00000000	mice
Xpot	-0.07	1.00000000	mice
Casd1	-0.08	1.00000000	mice
Ncoa7	0.00	1.00000000	mice
Golgb1	0.05	1.00000000	mice
Eif2s3x	0.06	1.00000000	mice
Tlnrd1	-0.07	1.00000000	mice
Nsun6	0.00	1.00000000	mice
Srsf11	-0.05	1.00000000	mice
Sdhd	-0.08	1.00000000	mice
Dapk3	-0.03	1.00000000	mice
Helb	-0.10	1.00000000	mice
Sirt1	-0.03	1.00000000	mice
Pgrmc2	-0.04	1.00000000	mice
Hdac3	-0.08	1.00000000	mice
AW554918	-0.18	1.00000000	mice
Rrp7a	0.02	1.00000000	mice
Bicral	-0.08	1.00000000	mice
Cpsf2	0.04	1.00000000	mice
Impdh2	0.05	1.00000000	mice
Tsn	-0.07	1.00000000	mice
Hjurp	-0.05	1.00000000	mice
Cbx3	-0.11	1.00000000	mice
Ccdc117	0.02	1.00000000	mice
BC035044	-0.09	1.00000000	mice
Psmd13	-0.09	1.00000000	mice
Tmem222	-0.04	1.00000000	mice
Gsn	0.02	1.00000000	mice
Senp2	-0.08	1.00000000	mice
Cd38	-0.04	1.00000000	mice
Rnmt	0.04	1.00000000	mice

Pygb	-0.06	1.00000000	mice
Adsl	0.04	1.00000000	mice
Utp6	0.03	1.00000000	mice
Rtn3	0.11	1.00000000	mice
Nr1h2	-0.06	1.00000000	mice
Tmco1	0.03	1.00000000	mice
Zfp236	-0.07	1.00000000	mice
Stx4a	-0.09	1.00000000	mice
Spag7	-0.08	1.00000000	mice
Tbce	-0.07	1.00000000	mice
Dgkz	-0.09	1.00000000	mice
Cnot9	0.03	1.00000000	mice
Ddt	0.02	1.00000000	mice
Josd1	-0.05	1.00000000	mice
Cpsf3	0.00	1.00000000	mice
Prdx3	0.04	1.00000000	mice
Arntl	0.07	1.00000000	mice
Cenpq	-0.01	1.00000000	mice
Rnf219	0.02	1.00000000	mice
Atg3	-0.08	1.00000000	mice
Vps4b	0.03	1.00000000	mice
Wls	-0.07	1.00000000	mice
Zfp322a	0.02	1.00000000	mice
Polr3e	-0.07	1.00000000	mice
Ppp1cb	-0.04	1.00000000	mice
Fam126b	-0.10	1.00000000	mice
Ubxn6	-0.04	1.00000000	mice
Pi4kb	-0.07	1.00000000	mice
Polr1d	-0.05	1.00000000	mice
Nfatc2ip	0.03	1.00000000	mice
Aldoa	-0.09	1.00000000	mice
Zfp777	-0.07	1.00000000	mice
Selenos	-0.09	1.00000000	mice
Polr3g	-0.06	1.00000000	mice
Tmem39a	-0.03	1.00000000	mice
Tmem241	-0.11	1.00000000	mice
D130040H23Rik	-0.06	1.00000000	mice
Ip6k1	-0.06	1.00000000	mice
Trit1	-0.08	1.00000000	mice
Birc6	-0.07	1.00000000	mice
Higd2a	0.03	1.00000000	mice
Clasrp	-0.08	1.00000000	mice
Ppp2r2d	-0.05	1.00000000	mice
Ube2e1	-0.13	1.00000000	mice
Rnf40	0.01	1.00000000	mice
Cbfb	-0.10	1.00000000	mice

Rbbp8	0.10	1.00000000	mice
Srrt	0.03	1.00000000	mice
Irak2	0.01	1.00000000	mice
Bag5	0.00	1.00000000	mice
Foxk2	0.01	1.00000000	mice
Wdr45b	-0.03	1.00000000	mice
Jpt1	-0.06	1.00000000	mice
Ywhah	-0.06	1.00000000	mice
Supt16	0.01	1.00000000	mice
Twf1	-0.06	1.00000000	mice
Glrx5	-0.07	1.00000000	mice
Vipas39	-0.05	1.00000000	mice
Nin	-0.01	1.00000000	mice
Cs	-0.08	1.00000000	mice
Htatsf1	-0.07	1.00000000	mice
Rdh14	-0.05	1.00000000	mice
Agk	0.03	1.00000000	mice
Pik3c2a	-0.12	1.00000000	mice
Atg2a	-0.07	1.00000000	mice
Ube2q2	-0.08	1.00000000	mice
Hk1	-0.02	1.00000000	mice
Galnt1	-0.06	1.00000000	mice
Hspd1	-0.05	1.00000000	mice
Eif4enif1	0.02	1.00000000	mice
Cyth3	-0.10	1.00000000	mice
Tnrc18	0.06	1.00000000	mice
Hivep1	0.02	1.00000000	mice
Cep350	0.00	1.00000000	mice
Ubfd1	0.02	1.00000000	mice
Atp5c1	-0.08	1.00000000	mice
Asb7	0.03	1.00000000	mice
Rac1	-0.05	1.00000000	mice
Uqcrc2	-0.09	1.00000000	mice
Cdc16	-0.05	1.00000000	mice
Eny2	0.03	1.00000000	mice
Wdr47	-0.06	1.00000000	mice
Rbbp6	0.03	1.00000000	mice
4930402H24Rik	-0.12	1.00000000	mice
Zfp106	-0.01	1.00000000	mice
Tomm40	-0.08	1.00000000	mice
Dop1b	-0.09	1.00000000	mice
Zc3h7a	0.06	1.00000000	mice
Avl9	-0.07	1.00000000	mice
Pmm2	0.01	1.00000000	mice
Psmb5	0.04	1.00000000	mice
Pknox1	-0.04	1.00000000	mice

Adipor2	-0.07	1.00000000	mice
Gatad2a	0.05	1.00000000	mice
Bcl7c	0.00	1.00000000	mice
Tent4b	-0.11	1.00000000	mice
Srsf9	0.03	1.00000000	mice
Hagh	-0.10	1.00000000	mice
Fam98b	0.02	1.00000000	mice
Trappc12	-0.07	1.00000000	mice
Cwc22	-0.06	1.00000000	mice
Fip1l1	-0.07	1.00000000	mice
Srgap2	0.06	1.00000000	mice
Cse1l	0.01	1.00000000	mice
Ankle2	-0.10	1.00000000	mice
4933434E20Rik	-0.07	1.00000000	mice
Emc2	0.02	1.00000000	mice
Lrrk1	-0.01	1.00000000	mice
Tmem33	0.00	1.00000000	mice
Txlng	0.01	1.00000000	mice
Surf4	-0.06	1.00000000	mice
Rbm42	0.01	1.00000000	mice
Rnf2	-0.08	1.00000000	mice
Polr2i	0.03	1.00000000	mice
Pik3r4	0.04	1.00000000	mice
Psmd6	0.02	1.00000000	mice
Timm17a	0.03	1.00000000	mice
Dctn2	-0.08	1.00000000	mice
Serinc1	-0.07	1.00000000	mice
Wdr33	-0.10	1.00000000	mice
Slc38a2	-0.07	1.00000000	mice
Sft2d2	-0.07	1.00000000	mice
Zmiz2	0.02	1.00000000	mice
Ctdsp2	-0.07	1.00000000	mice
Pign	0.00	1.00000000	mice
Fyttd1	0.02	1.00000000	mice
Dync1h1	0.06	1.00000000	mice
Capza1	0.00	1.00000000	mice
Chtop	0.05	1.00000000	mice
Cep68	0.03	1.00000000	mice
Tex2	-0.06	1.00000000	mice
G3bp1	0.04	1.00000000	mice
Smarcd1	-0.09	1.00000000	mice
Vps16	0.03	1.00000000	mice
Tab3	-0.08	1.00000000	mice
Fam173a	0.03	1.00000000	mice
Btk	-0.03	1.00000000	mice
Ing4	-0.05	1.00000000	mice

Akirin2	-0.05	1.00000000	mice
Tmem64	0.02	1.00000000	mice
Sbno1	-0.05	1.00000000	mice
Hmgb2	-0.08	1.00000000	mice
Ammecr1l	-0.06	1.00000000	mice
Anxa5	0.02	1.00000000	mice
Uba1	-0.04	1.00000000	mice
Ppp3r1	-0.08	1.00000000	mice
Cd9	0.07	1.00000000	mice
Syf2	0.03	1.00000000	mice
Crebzf	0.04	1.00000000	mice
Zswim8	-0.06	1.00000000	mice
Gcnt1	0.07	1.00000000	mice
Plagl2	-0.04	1.00000000	mice
Abtb2	-0.01	1.00000000	mice
Siah2	-0.06	1.00000000	mice
Sdhaf1	0.01	1.00000000	mice
Pde4d	0.04	1.00000000	mice
Grsf1	-0.05	1.00000000	mice
Ivd	-0.10	1.00000000	mice
Nup133	0.03	1.00000000	mice
Prcc	0.04	1.00000000	mice
Smad5	-0.05	1.00000000	mice
Caprin1	-0.02	1.00000000	mice
Atp5d	0.04	1.00000000	mice
Naa16	0.01	1.00000000	mice
Ppat	0.06	1.00000000	mice
Lzic	0.01	1.00000000	mice
Ppp4c	-0.08	1.00000000	mice
Nudt4	-0.08	1.00000000	mice
Tkt	0.03	1.00000000	mice
Pcgf6	0.01	1.00000000	mice
Ssr2	-0.05	1.00000000	mice
Ctdnep1	-0.08	1.00000000	mice
Vps11	-0.06	1.00000000	mice
Cnn3	0.04	1.00000000	mice
Ints4	-0.07	1.00000000	mice
Letm1	0.02	1.00000000	mice
Ccdc6	0.00	1.00000000	mice
Zbtb43	-0.04	1.00000000	mice
Glmp	-0.06	1.00000000	mice
Hs6st1	-0.07	1.00000000	mice
Kdm5c	0.05	1.00000000	mice
Bscl2	-0.03	1.00000000	mice
Atad2	-0.09	1.00000000	mice
Top3b	-0.05	1.00000000	mice

Sqstm1	-0.06	1.00000000	mice
Trim37	-0.04	1.00000000	mice
Tmem170b	-0.02	1.00000000	mice
Arhgef11	-0.10	1.00000000	mice
Snapc5	-0.05	1.00000000	mice
Actr5	-0.03	1.00000000	mice
Tmem234	0.03	1.00000000	mice
Aldh9a1	-0.05	1.00000000	mice
Fdx2	-0.06	1.00000000	mice
Uba3	-0.06	1.00000000	mice
Khdc4	0.00	1.00000000	mice
Cdk19	-0.07	1.00000000	mice
Gabpa	-0.09	1.00000000	mice
Trappc6a	-0.03	1.00000000	mice
Pdzd8	-0.09	1.00000000	mice
Gxylt1	-0.10	1.00000000	mice
Ddx19a	-0.05	1.00000000	mice
Taf5	-0.02	1.00000000	mice
Bcl7b	-0.08	1.00000000	mice
Prkar1a	-0.05	1.00000000	mice
Strip1	-0.07	1.00000000	mice
Lias	-0.08	1.00000000	mice
Atf7	-0.06	1.00000000	mice
Hps5	-0.05	1.00000000	mice
Inip	-0.08	1.00000000	mice
Tmbim6	0.07	1.00000000	mice
Pxn	0.01	1.00000000	mice
Ik	0.04	1.00000000	mice
1110059G10Rik	-0.01	1.00000000	mice
Sergef	-0.12	1.00000000	mice
Arih1	-0.05	1.00000000	mice
Pcmt1	-0.09	1.00000000	mice
Ccser2	0.05	1.00000000	mice
Wdr26	0.03	1.00000000	mice
Ift140	0.12	1.00000000	mice
Prr13	0.03	1.00000000	mice
Fundc2	0.03	1.00000000	mice
Prdx6	-0.07	1.00000000	mice
Ints6l	-0.06	1.00000000	mice
Nfkb2	0.02	1.00000000	mice
Ntan1	0.00	1.00000000	mice
Urgcp	-0.04	1.00000000	mice
B3glct	-0.07	1.00000000	mice
Rela	0.02	1.00000000	mice
Plaa	-0.10	1.00000000	mice
Zfp639	0.01	1.00000000	mice

Carnmt1	-0.06	1.00000000	mice
Coa5	0.02	1.00000000	mice
Igbp1	0.01	1.00000000	mice
Ggps1	-0.01	1.00000000	mice
Ino80	-0.10	1.00000000	mice
Smarce1	-0.05	1.00000000	mice
Cnppd1	-0.04	1.00000000	mice
Tmem183a	0.01	1.00000000	mice
Ccni	-0.08	1.00000000	mice
Fam160b1	-0.05	1.00000000	mice
Raf1	-0.08	1.00000000	mice
D10Wsu102e	-0.06	1.00000000	mice
Srrd	0.01	1.00000000	mice
Lsm1	-0.07	1.00000000	mice
Spata5	0.04	1.00000000	mice
Park7	0.05	1.00000000	mice
Cox5b	0.06	1.00000000	mice
5031425E22Rik	0.03	1.00000000	mice
Casp2	0.04	1.00000000	mice
N4bp2l1	0.02	1.00000000	mice
Tpp2	0.04	1.00000000	mice
Clp1	0.01	1.00000000	mice
Tusc3	0.01	1.00000000	mice
Bcap29	-0.11	1.00000000	mice
Gsdme	0.03	1.00000000	mice
Aimp1	0.03	1.00000000	mice
Ftsj3	0.00	1.00000000	mice
Psmc5	0.04	1.00000000	mice
Uba5	-0.06	1.00000000	mice
Cd53	-0.03	1.00000000	mice
Scyl2	0.02	1.00000000	mice
E2f5	-0.05	1.00000000	mice
Zfp871	0.04	1.00000000	mice
Snx14	0.04	1.00000000	mice
Zc3h7b	-0.03	1.00000000	mice
Snrnp200	0.03	1.00000000	mice
Zdhhc6	-0.01	1.00000000	mice
Skp1a	-0.04	1.00000000	mice
St8sia4	0.09	1.00000000	mice
Csrp1	0.05	1.00000000	mice
Nedd8	0.03	1.00000000	mice
Zfp410	-0.04	1.00000000	mice
Agpat5	-0.04	1.00000000	mice
Fam50a	-0.03	1.00000000	mice
Prelid3b	0.03	1.00000000	mice
Bcap31	0.05	1.00000000	mice

Npepl1	0.02	1.00000000	mice
Lrrc28	0.04	1.00000000	mice
Pak1ip1	-0.06	1.00000000	mice
Lrch1	-0.09	1.00000000	mice
Clip1	0.02	1.00000000	mice
Oxct1	-0.08	1.00000000	mice
Itga4	-0.05	1.00000000	mice
Ltn1	-0.07	1.00000000	mice
Thap12	0.00	1.00000000	mice
Zcchc17	0.03	1.00000000	mice
Atg4b	-0.05	1.00000000	mice
Ppp2r3c	-0.06	1.00000000	mice
Akap8	-0.04	1.00000000	mice
Rnf214	-0.06	1.00000000	mice
Atxn1l	0.04	1.00000000	mice
Ube2f	-0.07	1.00000000	mice
Ing5	0.02	1.00000000	mice
Slc20a1	-0.06	1.00000000	mice
Brwd3	0.02	1.00000000	mice
Ola1	-0.08	1.00000000	mice
Zgpat	-0.04	1.00000000	mice
Rhot1	-0.01	1.00000000	mice
Caml	0.00	1.00000000	mice
Psma7	0.07	1.00000000	mice
Dimt1	0.02	1.00000000	mice
Zfp787	-0.04	1.00000000	mice
Ube4a	-0.11	1.00000000	mice
Eif4b	-0.09	1.00000000	mice
Cers4	-0.02	1.00000000	mice
Tm9sf3	0.01	1.00000000	mice
Ncoa4	0.02	1.00000000	mice
Edem2	-0.05	1.00000000	mice
Itgb1	-0.06	1.00000000	mice
Trmt13	-0.08	1.00000000	mice
Sms	0.01	1.00000000	mice
Fh1	0.05	1.00000000	mice
Ubl7	-0.07	1.00000000	mice
Rrn3	0.01	1.00000000	mice
Commd7	-0.05	1.00000000	mice
Sdha	0.01	1.00000000	mice
Wwp1	-0.06	1.00000000	mice
Rnf167	0.06	1.00000000	mice
Prps2	-0.06	1.00000000	mice
Guk1	0.02	1.00000000	mice
Ipo11	0.04	1.00000000	mice
Klhl20	-0.07	1.00000000	mice

Eprs	-0.03	1.00000000	mice
Ppan	0.02	1.00000000	mice
Bccip	0.03	1.00000000	mice
Rab40c	-0.12	1.00000000	mice
Pinx1	-0.04	1.00000000	mice
Prxl2a	-0.05	1.00000000	mice
Psenen	-0.06	1.00000000	mice
Arv1	-0.05	1.00000000	mice
Pepd	-0.09	1.00000000	mice
Arl6ip4	0.01	1.00000000	mice
Ccng1	-0.01	1.00000000	mice
Polr3d	0.03	1.00000000	mice
Ep400	0.05	1.00000000	mice
Pim1	0.09	1.00000000	mice
Hnrnph3	-0.07	1.00000000	mice
2700049A03Rik	0.01	1.00000000	mice
Rer1	-0.06	1.00000000	mice
Baz2b	-0.05	1.00000000	mice
Psmd12	0.04	1.00000000	mice
Ing2	-0.04	1.00000000	mice
Ppp2ca	-0.06	1.00000000	mice
Nelfb	0.04	1.00000000	mice
Fbxw7	-0.05	1.00000000	mice
Ldb1	-0.05	1.00000000	mice
Cyhr1	-0.04	1.00000000	mice
Atp5g1	0.02	1.00000000	mice
Zfp408	-0.02	1.00000000	mice
Rnase6	-0.07	1.00000000	mice
Pik3c3	-0.07	1.00000000	mice
Il17ra	-0.07	1.00000000	mice
Pom121	0.02	1.00000000	mice
Nup153	0.04	1.00000000	mice
Ankrd16	-0.07	1.00000000	mice
Vps37a	-0.05	1.00000000	mice
Ercc4	-0.04	1.00000000	mice
Leng1	0.02	1.00000000	mice
Jkamp	0.01	1.00000000	mice
Rnaseh2c	0.04	1.00000000	mice
Eif2b2	0.01	1.00000000	mice
Foxj2	0.00	1.00000000	mice
Nploc4	-0.08	1.00000000	mice
Phf20l1	-0.08	1.00000000	mice
Pacs1	-0.03	1.00000000	mice
Eif1ax	0.03	1.00000000	mice
Hmgn5	0.03	1.00000000	mice
Smarca2	-0.07	1.00000000	mice

Uqcc3	-0.06	1.00000000	mice
Exosc5	-0.07	1.00000000	mice
Brpf3	-0.04	1.00000000	mice
Pcyt2	-0.04	1.00000000	mice
Ipo8	0.02	1.00000000	mice
Golga3	-0.03	1.00000000	mice
Csnk2a1	0.04	1.00000000	mice
Trmt1	0.02	1.00000000	mice
Ndufb8	-0.07	1.00000000	mice
Ttll5	-0.11	1.00000000	mice
Tshz1	-0.11	1.00000000	mice
Prpf4b	0.04	1.00000000	mice
Pgap2	0.00	1.00000000	mice
Ngdn	0.02	1.00000000	mice
Zcchc10	-0.05	1.00000000	mice
Elk4	-0.07	1.00000000	mice
Crk	0.04	1.00000000	mice
Acsl5	-0.01	1.00000000	mice
Psma4	-0.01	1.00000000	mice
Hspa8	0.06	1.00000000	mice
Tatdn1	-0.01	1.00000000	mice
4931406P16Rik	-0.07	1.00000000	mice
Tmem168	-0.09	1.00000000	mice
Fibp	-0.06	1.00000000	mice
Ppih	-0.05	1.00000000	mice
Nfat5	0.03	1.00000000	mice
Cnst	0.05	1.00000000	mice
Gnpat	-0.04	1.00000000	mice
Col4a3bp	-0.08	1.00000000	mice
Rab6a	-0.06	1.00000000	mice
Zc3h13	0.03	1.00000000	mice
Ints11	0.02	1.00000000	mice
Cpne3	0.05	1.00000000	mice
Pcid2	0.02	1.00000000	mice
Dap3	0.02	1.00000000	mice
Cltc	-0.02	1.00000000	mice
Riox2	-0.05	1.00000000	mice
Plcxd2	0.00	1.00000000	mice
Hgs	0.01	1.00000000	mice
Hnrnpf	0.01	1.00000000	mice
Cdc34	-0.06	1.00000000	mice
Sec22a	-0.02	1.00000000	mice
Hint1	0.02	1.00000000	mice
Slc25a11	-0.05	1.00000000	mice
Ttc19	-0.08	1.00000000	mice
Gadd45b	0.14	1.00000000	mice

Atr	0.03	1.00000000	mice
Snhg3	-0.05	1.00000000	mice
Hspe1	0.03	1.00000000	mice
Tmem219	-0.03	1.00000000	mice
Tmco4	0.02	1.00000000	mice
Abl1	-0.03	1.00000000	mice
Leo1	0.03	1.00000000	mice
Prelid1	-0.06	1.00000000	mice
Inpp5f	0.03	1.00000000	mice
Ube2d2a	-0.04	1.00000000	mice
Pabpc4	-0.07	1.00000000	mice
Dhx36	0.02	1.00000000	mice
Sel1l	0.04	1.00000000	mice
Nlk	0.04	1.00000000	mice
Ywhae	0.04	1.00000000	mice
Setd5	0.04	1.00000000	mice
Aggf1	-0.10	1.00000000	mice
Lrrfip2	-0.12	1.00000000	mice
Hif1an	-0.04	1.00000000	mice
Sp2	0.02	1.00000000	mice
Ern1	-0.06	1.00000000	mice
Tvp23b	0.01	1.00000000	mice
Cndp2	-0.07	1.00000000	mice
Ythdc1	0.00	1.00000000	mice
Trappc4	-0.05	1.00000000	mice
Cyb5r4	0.00	1.00000000	mice
Ibtk	-0.01	1.00000000	mice
2810004N23Rik	0.04	1.00000000	mice
Nxn	0.07	1.00000000	mice
Itsn2	-0.03	1.00000000	mice
Numa1	-0.06	1.00000000	mice
Fam53a	-0.04	1.00000000	mice
Cand1	-0.03	1.00000000	mice
Khdrbs1	-0.07	1.00000000	mice
Zfp646	-0.06	1.00000000	mice
Rad50	-0.07	1.00000000	mice
Nudt21	0.03	1.00000000	mice
Utp23	-0.02	1.00000000	mice
Nrros	-0.08	1.00000000	mice
Slc25a33	0.02	1.00000000	mice
Sars	-0.08	1.00000000	mice
Itgb1bp1	-0.04	1.00000000	mice
Ptp4a2	0.03	1.00000000	mice
Sec23ip	-0.04	1.00000000	mice
Rara	0.05	1.00000000	mice
Gapdh	-0.01	1.00000000	mice

Rab35	-0.05	1.00000000	mice
Zfp507	-0.02	1.00000000	mice
Rab22a	-0.04	1.00000000	mice
Itfg2	-0.09	1.00000000	mice
Polr2m	0.02	1.00000000	mice
BC005624	0.02	1.00000000	mice
Apool	-0.05	1.00000000	mice
Fam149b	0.03	1.00000000	mice
Bloc1s6	-0.05	1.00000000	mice
Zbtb1	0.06	1.00000000	mice
Nmd3	-0.01	1.00000000	mice
Ggct	0.02	1.00000000	mice
Trim28	-0.04	1.00000000	mice
Copg1	-0.04	1.00000000	mice
Zfp652	-0.07	1.00000000	mice
C87436	-0.01	1.00000000	mice
Vamp2	-0.06	1.00000000	mice
Prdm2	0.01	1.00000000	mice
Cdc123	-0.01	1.00000000	mice
Dpy30	0.02	1.00000000	mice
Tfb2m	-0.04	1.00000000	mice
Nucb2	0.02	1.00000000	mice
Vasp	0.00	1.00000000	mice
Naa30	-0.01	1.00000000	mice
Serpinb6a	-0.03	1.00000000	mice
Crl1	0.01	1.00000000	mice
Ube2k	-0.09	1.00000000	mice
Fgfr2	-0.06	1.00000000	mice
Snx19	-0.06	1.00000000	mice
Cox20	0.02	1.00000000	mice
Anxa6	-0.03	1.00000000	mice
Brms1	0.00	1.00000000	mice
Cnot10	-0.05	1.00000000	mice
Fermt3	-0.06	1.00000000	mice
Ociad1	0.03	1.00000000	mice
Orc5	-0.05	1.00000000	mice
Ccdc186	-0.04	1.00000000	mice
Opa1	0.02	1.00000000	mice
Zfp180	-0.03	1.00000000	mice
Zfx	-0.02	1.00000000	mice
Lin52	-0.09	1.00000000	mice
Tmed10	-0.05	1.00000000	mice
Hsbp1	0.03	1.00000000	mice
Kctd18	-0.01	1.00000000	mice
Nipa2	-0.02	1.00000000	mice
Dohh	-0.04	1.00000000	mice

Dda1	0.02	1.00000000	mice
Pcm1	-0.13	1.00000000	mice
Tbc1d12	-0.01	1.00000000	mice
Zfp512	-0.08	1.00000000	mice
Agpat4	-0.11	1.00000000	mice
1110038B12Rik	-0.01	1.00000000	mice
Gpatch4	0.00	1.00000000	mice
Ppp2r3d	-0.03	1.00000000	mice
Cops2	0.00	1.00000000	mice
Ube3c	-0.11	1.00000000	mice
Prdm10	0.01	1.00000000	mice
Vbp1	0.03	1.00000000	mice
Psma2	0.05	1.00000000	mice
Uchl3	0.02	1.00000000	mice
Sart1	0.01	1.00000000	mice
Srp19	0.03	1.00000000	mice
Slc9a7	-0.02	1.00000000	mice
Zmym5	0.03	1.00000000	mice
Cdipt	0.02	1.00000000	mice
Rufy2	-0.03	1.00000000	mice
Ube2i	-0.07	1.00000000	mice
Ndufb9	0.01	1.00000000	mice
Paf1	0.00	1.00000000	mice
Hnrnpk	-0.06	1.00000000	mice
Usp1	-0.04	1.00000000	mice
Eps15	0.04	1.00000000	mice
Gpr171	-0.01	1.00000000	mice
Cul3	-0.03	1.00000000	mice
Stip1	0.04	1.00000000	mice
Lsm8	0.03	1.00000000	mice
Ckap5	-0.01	1.00000000	mice
Eif1ad	-0.06	1.00000000	mice
Ncstn	0.01	1.00000000	mice
Tmem60	-0.04	1.00000000	mice
Utrn	-0.06	1.00000000	mice
Cks2	0.03	1.00000000	mice
Otud5	0.04	1.00000000	mice
Idh3g	-0.04	1.00000000	mice
Gfm1	0.02	1.00000000	mice
Hccs	-0.05	1.00000000	mice
Rnf121	-0.05	1.00000000	mice
Ppie	-0.04	1.00000000	mice
Gclm	-0.02	1.00000000	mice
Wdpcp	-0.08	1.00000000	mice
Hsp90ab1	0.00	1.00000000	mice
Frmd6	-0.02	1.00000000	mice

Rbm19	-0.06	1.00000000	mice
Pdss1	0.02	1.00000000	mice
Slc35a3	-0.01	1.00000000	mice
Nuak2	0.01	1.00000000	mice
Polr2f	0.04	1.00000000	mice
Akr1a1	-0.08	1.00000000	mice
Cox14	-0.01	1.00000000	mice
Usp38	0.03	1.00000000	mice
Nf2	0.02	1.00000000	mice
Atxn7l3	-0.04	1.00000000	mice
Dennd1c	-0.01	1.00000000	mice
Npm3	-0.03	1.00000000	mice
Sdf4	0.02	1.00000000	mice
Hmbox1	-0.07	1.00000000	mice
Atf2	-0.08	1.00000000	mice
Hnrnph1	-0.03	1.00000000	mice
Impa1	-0.03	1.00000000	mice
Seh1l	0.01	1.00000000	mice
Pdcd7	0.02	1.00000000	mice
Sec61a2	0.00	1.00000000	mice
Fmr1	-0.07	1.00000000	mice
Aph1a	-0.02	1.00000000	mice
Ctdsp1	0.00	1.00000000	mice
Ift20	0.03	1.00000000	mice
Brap	-0.05	1.00000000	mice
Cox5a	0.01	1.00000000	mice
Zfp593	0.00	1.00000000	mice
H2afz	-0.05	1.00000000	mice
Krit1	-0.05	1.00000000	mice
Vti1b	-0.02	1.00000000	mice
Samm50	0.03	1.00000000	mice
Ctps	-0.01	1.00000000	mice
Ank	0.12	1.00000000	mice
Crlf2	0.05	1.00000000	mice
Arnt	-0.01	1.00000000	mice
Chd1	-0.01	1.00000000	mice
Pfkl	-0.04	1.00000000	mice
Commd8	0.00	1.00000000	mice
Smn1	0.03	1.00000000	mice
Atp5g2	0.02	1.00000000	mice
Luc7l	0.01	1.00000000	mice
Babam2	-0.03	1.00000000	mice
Dnajc13	-0.05	1.00000000	mice
Orc2	0.08	1.00000000	mice
Srsf10	-0.02	1.00000000	mice
Chd1l	-0.04	1.00000000	mice

Spi1	0.00	1.00000000	mice
Dhrs7b	-0.04	1.00000000	mice
Egln2	-0.06	1.00000000	mice
Grap2	-0.07	1.00000000	mice
Gch1	-0.06	1.00000000	mice
Tmem165	-0.05	1.00000000	mice
Cog1	-0.04	1.00000000	mice
Gpat4	0.04	1.00000000	mice
Hipk2	-0.10	1.00000000	mice
Ddx46	-0.07	1.00000000	mice
Arf1	-0.04	1.00000000	mice
Sf3b4	0.01	1.00000000	mice
Sun1	-0.06	1.00000000	mice
1500011B03Rik	-0.02	1.00000000	mice
Foxk1	-0.05	1.00000000	mice
Vrk3	0.01	1.00000000	mice
Rbm8a	-0.07	1.00000000	mice
Tmx4	-0.08	1.00000000	mice
Zdhhc21	-0.09	1.00000000	mice
Ccnh	-0.09	1.00000000	mice
Clk2	-0.06	1.00000000	mice
Aarsd1	0.02	1.00000000	mice
Prkd2	-0.05	1.00000000	mice
Rbpj	0.04	1.00000000	mice
Cnep1r1	-0.05	1.00000000	mice
Sbf1	-0.05	1.00000000	mice
Vta1	-0.05	1.00000000	mice
Elp4	-0.12	1.00000000	mice
Cntrl	0.05	1.00000000	mice
Panx1	-0.05	1.00000000	mice
Hdgfl2	0.03	1.00000000	mice
Rnf220	0.03	1.00000000	mice
Txn2	-0.04	1.00000000	mice
Snrpa	0.02	1.00000000	mice
Polr3h	0.05	1.00000000	mice
Efl1	-0.08	1.00000000	mice
Rnpepl1	-0.04	1.00000000	mice
Fen1	0.02	1.00000000	mice
Ndufs1	0.01	1.00000000	mice
Esyt1	-0.06	1.00000000	mice
Cdc26	-0.05	1.00000000	mice
Yipf5	0.00	1.00000000	mice
Rpap2	-0.03	1.00000000	mice
Clk3	-0.05	1.00000000	mice
Zbtb37	0.01	1.00000000	mice
Rab5c	0.02	1.00000000	mice

Cnih1	-0.06	1.00000000	mice
Ubxn7	-0.06	1.00000000	mice
Jak2	0.08	1.00000000	mice
Usf1	-0.06	1.00000000	mice
Sec16a	-0.04	1.00000000	mice
L3mbtl2	-0.03	1.00000000	mice
Bcl9	-0.03	1.00000000	mice
Zfp961	0.03	1.00000000	mice
Capza2	0.00	1.00000000	mice
Fnbp4	0.03	1.00000000	mice
Topbp1	-0.04	1.00000000	mice
Otud6b	0.01	1.00000000	mice
Bud31	-0.04	1.00000000	mice
Tbk1	-0.09	1.00000000	mice
Psmc2	0.04	1.00000000	mice
B3gnt2	-0.08	1.00000000	mice
Rpe	-0.02	1.00000000	mice
Lmnb1	0.05	1.00000000	mice
Kctd5	0.00	1.00000000	mice
Sptlc2	-0.12	1.00000000	mice
Zcchc8	-0.06	1.00000000	mice
Kmt5a	-0.08	1.00000000	mice
Ubap1	0.03	1.00000000	mice
Polr1a	0.00	1.00000000	mice
Ltv1	0.02	1.00000000	mice
Ddx47	-0.05	1.00000000	mice
Chd8	-0.09	1.00000000	mice
Fam172a	-0.08	1.00000000	mice
Taf1a	0.01	1.00000000	mice
Znrd1	0.01	1.00000000	mice
Il6ra	0.00	1.00000000	mice
Srrm1	-0.05	1.00000000	mice
Pde12	0.02	1.00000000	mice
Eipr1	-0.05	1.00000000	mice
Srsf1	-0.01	1.00000000	mice
Odc1	0.02	1.00000000	mice
Eif3h	-0.04	1.00000000	mice
P2ry10b	0.03	1.00000000	mice
Ugp2	-0.05	1.00000000	mice
Psmb7	-0.04	1.00000000	mice
Cd40	-0.10	1.00000000	mice
Fam76b	0.04	1.00000000	mice
Emc4	0.03	1.00000000	mice
Tut4	0.02	1.00000000	mice
Usp42	-0.07	1.00000000	mice
Snhg4	0.00	1.00000000	mice

Gtf3c6	-0.01	1.00000000	mice
Tpd52l2	0.01	1.00000000	mice
Exosc8	0.00	1.00000000	mice
Wdr61	-0.05	1.00000000	mice
Rbm48	-0.01	1.00000000	mice
Rhob	-0.03	1.00000000	mice
Vcl	-0.11	1.00000000	mice
Ppcdc	0.03	1.00000000	mice
Prdx5	-0.06	1.00000000	mice
Sec31a	-0.07	1.00000000	mice
Nop9	-0.04	1.00000000	mice
Nup210l	0.00	1.00000000	mice
Prpf3	-0.08	1.00000000	mice
Pigu	-0.05	1.00000000	mice
Pcf11	0.02	1.00000000	mice
Hcfc1r1	0.01	1.00000000	mice
Tmc8	0.00	1.00000000	mice
Nubp2	-0.05	1.00000000	mice
Ppp3cb	0.04	1.00000000	mice
Dld	0.04	1.00000000	mice
Lars2	0.04	1.00000000	mice
Isca2	-0.05	1.00000000	mice
Dcps	-0.02	1.00000000	mice
Sod2	0.02	1.00000000	mice
Kat8	0.00	1.00000000	mice
Zfp983	-0.04	1.00000000	mice
Prkaa1	-0.03	1.00000000	mice
Ankrd13a	-0.09	1.00000000	mice
Stxbp5	0.05	1.00000000	mice
Lrpap1	0.00	1.00000000	mice
Gtf2a1	0.00	1.00000000	mice
Idh3b	0.03	1.00000000	mice
Gpbp1l1	-0.06	1.00000000	mice
Pdcd6ip	-0.05	1.00000000	mice
Cnot6	-0.10	1.00000000	mice
Csnk2a2	-0.06	1.00000000	mice
Dr1	0.01	1.00000000	mice
Ppid	0.01	1.00000000	mice
Shc1	0.00	1.00000000	mice
Dcp1a	-0.02	1.00000000	mice
Atp5g3	-0.03	1.00000000	mice
Abca3	-0.04	1.00000000	mice
Zfp84	0.02	1.00000000	mice
Rabl6	-0.07	1.00000000	mice
Rbm39	-0.02	1.00000000	mice
Vdac3	-0.04	1.00000000	mice

Nfs1	0.01	1.00000000	mice
Supt20	-0.07	1.00000000	mice
Rbx1	-0.01	1.00000000	mice
Smim12	0.01	1.00000000	mice
Ttc37	-0.01	1.00000000	mice
Ankrd11	-0.04	1.00000000	mice
Zfp933	-0.02	1.00000000	mice
Parp6	-0.04	1.00000000	mice
Crim1	0.04	1.00000000	mice
Tmem179b	-0.04	1.00000000	mice
Gigyf2	-0.01	1.00000000	mice
Immt	0.02	1.00000000	mice
Uba2	-0.04	1.00000000	mice
Baiap2	-0.06	1.00000000	mice
Dock8	0.01	1.00000000	mice
Rbm14	0.01	1.00000000	mice
Tmem189	-0.06	1.00000000	mice
Ip6k2	-0.02	1.00000000	mice
Lrp10	-0.04	1.00000000	mice
Sdccag8	-0.07	1.00000000	mice
Llph	0.02	1.00000000	mice
Pde6d	0.02	1.00000000	mice
Rnf8	-0.03	1.00000000	mice
Ppp1r8	-0.05	1.00000000	mice
Zw10	0.01	1.00000000	mice
Abcf2	0.02	1.00000000	mice
Usp45	-0.04	1.00000000	mice
Dtd1	0.02	1.00000000	mice
Hmgn2	-0.01	1.00000000	mice
Rabep1	-0.01	1.00000000	mice
2210016F16Rik	-0.04	1.00000000	mice
Fez2	-0.03	1.00000000	mice
BC031181	-0.05	1.00000000	mice
Eif4a3	-0.01	1.00000000	mice
Klhl12	-0.03	1.00000000	mice
Ets2	-0.03	1.00000000	mice
Prdx4	0.01	1.00000000	mice
Fem1c	0.02	1.00000000	mice
Vamp4	-0.03	1.00000000	mice
Gde1	-0.06	1.00000000	mice
Rad17	0.01	1.00000000	mice
Tm2d1	0.03	1.00000000	mice
Zfp516	-0.08	1.00000000	mice
Ss18	-0.01	1.00000000	mice
Rab5b	0.00	1.00000000	mice
Prmt1	0.01	1.00000000	mice

Phka1	-0.02	1.00000000	mice
Zfp335os	-0.03	1.00000000	mice
Txnl1	-0.03	1.00000000	mice
Pola1	-0.09	1.00000000	mice
Golga7	-0.07	1.00000000	mice
Laptm4a	0.04	1.00000000	mice
Alkbh1	0.01	1.00000000	mice
Snapc3	-0.04	1.00000000	mice
Ptcd3	-0.05	1.00000000	mice
Ube2o	-0.05	1.00000000	mice
Gtf3c2	-0.04	1.00000000	mice
Usp10	-0.01	1.00000000	mice
Sh3bgrl	-0.03	1.00000000	mice
Ctnnb1	-0.01	1.00000000	mice
Samd8	-0.04	1.00000000	mice
Clpx	-0.01	1.00000000	mice
Arap2	0.02	1.00000000	mice
Rundc3b	-0.03	1.00000000	mice
Whamm	-0.01	1.00000000	mice
Tia1	-0.07	1.00000000	mice
Gripap1	0.04	1.00000000	mice
Ppm1m	-0.03	1.00000000	mice
Sec61a1	-0.03	1.00000000	mice
Cmpk1	-0.05	1.00000000	mice
Arglu1	0.01	1.00000000	mice
Rfk	0.00	1.00000000	mice
Heatr1	-0.04	1.00000000	mice
Hmgcl	-0.04	1.00000000	mice
Rmdn3	0.02	1.00000000	mice
Ubqln2	0.02	1.00000000	mice
Btbd9	0.08	1.00000000	mice
P4ha1	-0.01	1.00000000	mice
Emc3	0.04	1.00000000	mice
Rcbtb1	-0.06	1.00000000	mice
Ubr2	-0.01	1.00000000	mice
Vapb	-0.04	1.00000000	mice
Nans	0.00	1.00000000	mice
Eaf2	-0.08	1.00000000	mice
Dpp8	-0.06	1.00000000	mice
Zfp560	-0.02	1.00000000	mice
Cops4	-0.01	1.00000000	mice
Nhp2	-0.04	1.00000000	mice
Uba6	0.00	1.00000000	mice
Atpaf1	-0.01	1.00000000	mice
Hpf1	-0.01	1.00000000	mice
Tmf1	0.02	1.00000000	mice

Pgk1	-0.01	1.00000000	mice
Trappc2l	-0.07	1.00000000	mice
Phtf1	-0.05	1.00000000	mice
Ranbp10	0.01	1.00000000	mice
Slc30a9	0.02	1.00000000	mice
Cox16	-0.03	1.00000000	mice
Numb	-0.04	1.00000000	mice
Tmcc1	-0.11	1.00000000	mice
Ppp1r10	-0.04	1.00000000	mice
Sart3	-0.06	1.00000000	mice
Smg9	-0.07	1.00000000	mice
Aven	-0.08	1.00000000	mice
Ech1	-0.03	1.00000000	mice
Apip	-0.05	1.00000000	mice
Fbxo42	-0.01	1.00000000	mice
Gid8	-0.02	1.00000000	mice
Tmem167	-0.04	1.00000000	mice
2610001J05Rik	-0.04	1.00000000	mice
Rnf34	-0.01	1.00000000	mice
Gpatch8	0.04	1.00000000	mice
Slc20a2	0.01	1.00000000	mice
Prpf31	-0.01	1.00000000	mice
Atad1	-0.03	1.00000000	mice
Styx	-0.05	1.00000000	mice
Bax	0.01	1.00000000	mice
Dctn6	-0.03	1.00000000	mice
Rnf187	-0.04	1.00000000	mice
Dnajc24	-0.06	1.00000000	mice
Taf13	-0.05	1.00000000	mice
Sdhc	0.00	1.00000000	mice
Ddb1	0.02	1.00000000	mice
Polr3b	-0.10	1.00000000	mice
Pot1b	-0.08	1.00000000	mice
Braf	-0.03	1.00000000	mice
Usp31	-0.08	1.00000000	mice
Tbc1d20	0.01	1.00000000	mice
Naf1	-0.06	1.00000000	mice
Psip1	0.01	1.00000000	mice
Setd7	-0.11	1.00000000	mice
Iars	0.01	1.00000000	mice
Pkn2	0.02	1.00000000	mice
Wdr5	0.02	1.00000000	mice
Rc3h1	-0.02	1.00000000	mice
Rnf25	-0.03	1.00000000	mice
Tmem263	0.01	1.00000000	mice
Slc38a6	-0.10	1.00000000	mice

Phip	0.11	1.00000000	mice
Zfp729a	-0.02	1.00000000	mice
Slc39a9	-0.04	1.00000000	mice
Sf3b6	-0.06	1.00000000	mice
Camsap2	0.01	1.00000000	mice
Zfp131	0.02	1.00000000	mice
Alkbh3	-0.09	1.00000000	mice
Bbip1	0.00	1.00000000	mice
Tmem126a	-0.01	1.00000000	mice
Traf5	0.05	1.00000000	mice
Diaph3	-0.07	1.00000000	mice
Pdcd5	-0.05	1.00000000	mice
Dop1a	-0.05	1.00000000	mice
Huwe1	-0.04	1.00000000	mice
Gpr89	-0.01	1.00000000	mice
Taf1b	-0.07	1.00000000	mice
Terf1	-0.04	1.00000000	mice
Cct8	0.05	1.00000000	mice
Nufip2	0.02	1.00000000	mice
Pank3	0.01	1.00000000	mice
Acsf2	0.03	1.00000000	mice
Eif2b5	-0.04	1.00000000	mice
Zfp869	0.05	1.00000000	mice
Ahcyl1	-0.04	1.00000000	mice
Erlin1	-0.04	1.00000000	mice
Lonp1	-0.04	1.00000000	mice
Banp	0.03	1.00000000	mice
Blzf1	0.01	1.00000000	mice
Atxn7l1	0.03	1.00000000	mice
Ufl1	-0.08	1.00000000	mice
Gar1	-0.06	1.00000000	mice
Capns1	-0.05	1.00000000	mice
Ndufa7	-0.01	1.00000000	mice
Dmac1	-0.04	1.00000000	mice
Gpi1	0.02	1.00000000	mice
Rpa2	-0.06	1.00000000	mice
Atp6v0a2	-0.04	1.00000000	mice
Tardbp	0.00	1.00000000	mice
Krr1	0.02	1.00000000	mice
Rgs19	-0.04	1.00000000	mice
4833439L19Rik	0.00	1.00000000	mice
Phrf1	-0.01	1.00000000	mice
Eri1	0.01	1.00000000	mice
Cklf	0.01	1.00000000	mice
Pin1	-0.07	1.00000000	mice
Zfc3h1	-0.04	1.00000000	mice

Zfp277	-0.12	1.00000000	mice
Degs1	-0.03	1.00000000	mice
Atp1b3	-0.08	1.00000000	mice
2410004B18Rik	-0.02	1.00000000	mice
Pak2	0.04	1.00000000	mice
Ctsl	-0.02	1.00000000	mice
Rad51d	-0.03	1.00000000	mice
Chmp3	-0.04	1.00000000	mice
Slc28a2	0.00	1.00000000	mice
Rab5a	-0.03	1.00000000	mice
Orc4	-0.03	1.00000000	mice
Smndc1	0.00	1.00000000	mice
Acd	-0.02	1.00000000	mice
Diaph1	-0.04	1.00000000	mice
Terf2ip	-0.02	1.00000000	mice
Ppp4r2	0.02	1.00000000	mice
Lgmn	0.01	1.00000000	mice
Zfp451	-0.05	1.00000000	mice
Atp13a1	-0.04	1.00000000	mice
Fam20b	-0.06	1.00000000	mice
Orc6	-0.01	1.00000000	mice
Parn	-0.05	1.00000000	mice
Lrrc8d	-0.04	1.00000000	mice
Rock2	-0.06	1.00000000	mice
Dnmt1	-0.01	1.00000000	mice
Acadm	-0.03	1.00000000	mice
Eif4e2	-0.03	1.00000000	mice
Ints8	0.01	1.00000000	mice
Polr2b	-0.02	1.00000000	mice
Efr3a	0.01	1.00000000	mice
Gls	-0.05	1.00000000	mice
Bach2	-0.04	1.00000000	mice
Eif4e	0.00	1.00000000	mice
Arpc5	-0.05	1.00000000	mice
Optn	-0.02	1.00000000	mice
Eif3l	-0.06	1.00000000	mice
Otud4	-0.06	1.00000000	mice
Zfp212	0.01	1.00000000	mice
Zfp160	-0.04	1.00000000	mice
Timm8a1	-0.02	1.00000000	mice
Rab21	0.00	1.00000000	mice
Ankib1	0.01	1.00000000	mice
Uhrf1bp1l	0.03	1.00000000	mice
Plekha3	-0.05	1.00000000	mice
Cebpg	0.00	1.00000000	mice
Naa15	0.04	1.00000000	mice

Clns1a	-0.03	1.00000000	mice
Dram2	0.03	1.00000000	mice
Arel1	-0.10	1.00000000	mice
Papola	0.04	1.00000000	mice
Cyb5r3	0.00	1.00000000	mice
Cybc1	-0.01	1.00000000	mice
Srebf2	-0.05	1.00000000	mice
Got2	-0.04	1.00000000	mice
Arfgap1	-0.04	1.00000000	mice
Lrrc59	-0.03	1.00000000	mice
Usp39	0.02	1.00000000	mice
Secisbp2l	0.02	1.00000000	mice
Dnajc5	-0.06	1.00000000	mice
Akap11	-0.01	1.00000000	mice
Pdxdc1	0.00	1.00000000	mice
Kdm6a	-0.01	1.00000000	mice
Ino80b	0.00	1.00000000	mice
Crip1	-0.06	1.00000000	mice
Lamp2	0.02	1.00000000	mice
Dbf4	0.00	1.00000000	mice
P4hb	-0.01	1.00000000	mice
Clpb	-0.01	1.00000000	mice
Heatr3	0.03	1.00000000	mice
Entpd1	-0.10	1.00000000	mice
Vps35l	-0.06	1.00000000	mice
Zfp369	0.00	1.00000000	mice
Gtf3c3	-0.02	1.00000000	mice
Smim15	-0.02	1.00000000	mice
CAAA01118383.1	0.00	1.00000000	mice
Psmd3	-0.05	1.00000000	mice
Faim	-0.04	1.00000000	mice
D230025D16Rik	0.00	1.00000000	mice
Timm22	-0.02	1.00000000	mice
Azin1	-0.06	1.00000000	mice
Stub1	0.04	1.00000000	mice
Hcls1	-0.02	1.00000000	mice
Ndufs6	0.04	1.00000000	mice
Ndfip1	-0.03	1.00000000	mice
Traf7	-0.02	1.00000000	mice
Hscb	-0.04	1.00000000	mice
Scamp3	-0.01	1.00000000	mice
Ppp3cc	-0.05	1.00000000	mice
Amd1	0.00	1.00000000	mice
Sp3os	0.00	1.00000000	mice
Snhg1	0.02	1.00000000	mice
Necap1	-0.05	1.00000000	mice

Phf5a	-0.04	1.00000000	mice
Faf1	-0.05	1.00000000	mice
Ei24	0.03	1.00000000	mice
Vps50	0.01	1.00000000	mice
Nsd2	0.01	1.00000000	mice
Wdr82	-0.01	1.00000000	mice
Wdr20	-0.07	1.00000000	mice
Stx16	-0.01	1.00000000	mice
Sgk3	-0.01	1.00000000	mice
BC004004	0.01	1.00000000	mice
Pdcl3	-0.03	1.00000000	mice
Ly9	-0.03	1.00000000	mice
Rangap1	-0.06	1.00000000	mice
Ufm1	-0.05	1.00000000	mice
Clptm1	-0.05	1.00000000	mice
Timm44	-0.04	1.00000000	mice
Cd2bp2	0.00	1.00000000	mice
Cyld	0.02	1.00000000	mice
Snrpc	-0.03	1.00000000	mice
Klhl2	-0.09	1.00000000	mice
B9d2	-0.04	1.00000000	mice
Acot2	-0.03	1.00000000	mice
Rybp	0.00	1.00000000	mice
Actr2	-0.01	1.00000000	mice
Tubgcp4	-0.02	1.00000000	mice
Rcc1l	0.01	1.00000000	mice
Tmem87a	-0.01	1.00000000	mice
Cdk7	-0.03	1.00000000	mice
Fam193a	-0.07	1.00000000	mice
Ap2b1	-0.04	1.00000000	mice
Sipa1l3	-0.01	1.00000000	mice
Rwdd4a	0.01	1.00000000	mice
Pcmtd2	0.01	1.00000000	mice
Rdx	-0.02	1.00000000	mice
Ppp1r18	-0.02	1.00000000	mice
Ppil4	0.02	1.00000000	mice
Zfp263	-0.01	1.00000000	mice
Immp1l	0.01	1.00000000	mice
Acox1	-0.06	1.00000000	mice
Trim27	0.04	1.00000000	mice
Nadk	0.00	1.00000000	mice
Ccdc47	-0.01	1.00000000	mice
Ndufa8	-0.02	1.00000000	mice
Triap1	-0.01	1.00000000	mice
Polr3c	0.01	1.00000000	mice
Grb2	-0.02	1.00000000	mice

Vdac2	-0.06	1.00000000	mice
Itfg1	-0.03	1.00000000	mice
Rttn	0.04	1.00000000	mice
Pi4k2a	0.00	1.00000000	mice
Spata6	-0.04	1.00000000	mice
Phgdh	-0.03	1.00000000	mice
Pdia3	-0.06	1.00000000	mice
Brix1	0.01	1.00000000	mice
Rnf138	0.03	1.00000000	mice
Gtf2f2	-0.04	1.00000000	mice
Stk40	-0.07	1.00000000	mice
Zfp280c	-0.06	1.00000000	mice
Tmem29	-0.02	1.00000000	mice
Eif4g2	0.00	1.00000000	mice
Cdkal1	-0.08	1.00000000	mice
Rad23a	-0.03	1.00000000	mice
Rab1b	0.00	1.00000000	mice
Ahcyl2	-0.15	1.00000000	mice
Fosb	0.08	1.00000000	mice
Slc4a1ap	-0.05	1.00000000	mice
Nfe2l1	-0.01	1.00000000	mice
Scpep1os	0.02	1.00000000	mice
Rwdd1	0.02	1.00000000	mice
Tmx1	0.02	1.00000000	mice
Sf3b1	0.01	1.00000000	mice
Rab1a	-0.01	1.00000000	mice
Svbp	-0.02	1.00000000	mice
Glce	0.00	1.00000000	mice
Hypk	-0.01	1.00000000	mice
Ankrd17	0.01	1.00000000	mice
Oard1	-0.03	1.00000000	mice
Tnip1	-0.01	1.00000000	mice
Pgrmc1	-0.07	1.00000000	mice
Ube2v1	0.00	1.00000000	mice
Fam222b	-0.02	1.00000000	mice
Tcf25	0.02	1.00000000	mice
Trub2	-0.02	1.00000000	mice
Pgam1	-0.06	1.00000000	mice
Atg12	-0.07	1.00000000	mice
4833420G17Rik	-0.07	1.00000000	mice
Atp5f1	-0.03	1.00000000	mice
Hnrnph2	0.01	1.00000000	mice
Ddx19b	0.00	1.00000000	mice
Ankrd33b	-0.04	1.00000000	mice
Gpx1	0.04	1.00000000	mice
Pithd1	0.01	1.00000000	mice

Galns	-0.05	1.00000000	mice
Rbm27	0.00	1.00000000	mice
Rc3h2	-0.08	1.00000000	mice
Nek9	-0.09	1.00000000	mice
D5Ertd579e	-0.03	1.00000000	mice
Katnbl1	0.00	1.00000000	mice
H2afx	-0.04	1.00000000	mice
Smc5	-0.01	1.00000000	mice
Hadha	-0.06	1.00000000	mice
Esyt2	0.01	1.00000000	mice
Slc25a19	-0.05	1.00000000	mice
Zbtb44	0.02	1.00000000	mice
Pphln1	-0.01	1.00000000	mice
Cers6	0.01	1.00000000	mice
Rin3	-0.06	1.00000000	mice
Pfn1	0.00	1.00000000	mice
Chsy1	0.01	1.00000000	mice
Ksr1	0.02	1.00000000	mice
Ap4e1	-0.04	1.00000000	mice
Dtymk	-0.03	1.00000000	mice
Camkk2	-0.04	1.00000000	mice
Klf4	0.01	1.00000000	mice
Nol9	-0.02	1.00000000	mice
Axin1	0.02	1.00000000	mice
Rbm18	-0.06	1.00000000	mice
Pex2	-0.05	1.00000000	mice
Cops5	0.03	1.00000000	mice
Setd1b	-0.01	1.00000000	mice
Rnf11	-0.02	1.00000000	mice
Abhd17b	0.01	1.00000000	mice
Tiprl	-0.01	1.00000000	mice
Knop1	-0.01	1.00000000	mice
Prim2	0.01	1.00000000	mice
Inpp4a	-0.06	1.00000000	mice
Tbpl1	-0.01	1.00000000	mice
Dcaf1	0.02	1.00000000	mice
Slc25a25	-0.03	1.00000000	mice
Fbxw8	-0.07	1.00000000	mice
Rbmx	-0.01	1.00000000	mice
Ifngr1	-0.07	1.00000000	mice
Fis1	-0.03	1.00000000	mice
Calm1	-0.01	1.00000000	mice
Cep120	-0.06	1.00000000	mice
Tyrobp	0.03	1.00000000	mice
Txndc17	0.01	1.00000000	mice
Triobp	-0.01	1.00000000	mice

Jmjd6	0.01	1.00000000	mice
Wsb1	-0.01	1.00000000	mice
Cstb	-0.03	1.00000000	mice
Il4ra	0.01	1.00000000	mice
Slc25a20	0.00	1.00000000	mice
Tm6sf1	-0.01	1.00000000	mice
Casz1	-0.01	1.00000000	mice
Itgb3	-0.03	1.00000000	mice
Cdk5rap2	-0.06	1.00000000	mice
Taok2	-0.03	1.00000000	mice
Fam241a	-0.04	1.00000000	mice
Zwint	-0.01	1.00000000	mice
Ctsd	0.04	1.00000000	mice
Ep300	0.03	1.00000000	mice
Hdlbp	-0.02	1.00000000	mice
Prpf19	0.02	1.00000000	mice
Gadd45a	0.01	1.00000000	mice
Cep295	-0.01	1.00000000	mice
Ybx3	-0.04	1.00000000	mice
Rtcb	0.02	1.00000000	mice
P2rx4	0.02	1.00000000	mice
Aebp2	0.00	1.00000000	mice
Bet1	0.01	1.00000000	mice
Thumpd1	0.00	1.00000000	mice
Pum3	0.04	1.00000000	mice
0610030E20Rik	0.01	1.00000000	mice
Ubap2l	0.01	1.00000000	mice
Exosc1	0.01	1.00000000	mice
Fbxo7	0.01	1.00000000	mice
Zfp809	0.01	1.00000000	mice
Nr2c2ap	0.00	1.00000000	mice
Dis3l2	-0.08	1.00000000	mice
Atg10	-0.03	1.00000000	mice
Slc25a26	-0.06	1.00000000	mice
Uspl1	-0.05	1.00000000	mice
Rad18	-0.04	1.00000000	mice
Tmem184c	-0.03	1.00000000	mice
Sf3b5	-0.03	1.00000000	mice
Itgb2	0.00	1.00000000	mice
Dpysl2	-0.06	1.00000000	mice
Slc30a7	0.00	1.00000000	mice
Srp68	-0.01	1.00000000	mice
Yipf6	0.00	1.00000000	mice
Ezh1	-0.04	1.00000000	mice
Afg3l2	-0.01	1.00000000	mice
Ndufa12	-0.01	1.00000000	mice

Scp2	-0.01	1.00000000	mice
Fkbp15	-0.08	1.00000000	mice
Adprhl2	0.00	1.00000000	mice
Spryd3	-0.01	1.00000000	mice
Fbxo30	0.01	1.00000000	mice
Zc3hc1	-0.03	1.00000000	mice
Naa60	-0.05	1.00000000	mice
Dgcr8	-0.02	1.00000000	mice
Efcab2	0.01	1.00000000	mice
Unc45a	0.00	1.00000000	mice
Uxs1	-0.03	1.00000000	mice
Glo1	-0.06	1.00000000	mice
Ccdc86	-0.03	1.00000000	mice
Cdk2ap1	-0.03	1.00000000	mice
Nfyc	-0.05	1.00000000	mice
Qars	-0.04	1.00000000	mice
Cdc42	-0.03	1.00000000	mice
Ccnl1	-0.01	1.00000000	mice
Trap1	-0.07	1.00000000	mice
Ripor2	-0.06	1.00000000	mice
Utp18	-0.06	1.00000000	mice
Got1	0.01	1.00000000	mice
Zfp260	0.02	1.00000000	mice
Pold3	-0.01	1.00000000	mice
Sos1	-0.03	1.00000000	mice
Senp1	-0.04	1.00000000	mice
Aurkaip1	-0.05	1.00000000	mice
Snrk	-0.02	1.00000000	mice
Ndufa9	-0.03	1.00000000	mice
Copb1	-0.04	1.00000000	mice
Rnh1	-0.03	1.00000000	mice
Snf8	-0.04	1.00000000	mice
Gng10	0.01	1.00000000	mice
Ilk	-0.02	1.00000000	mice
Ganab	-0.03	1.00000000	mice
Taf2	-0.06	1.00000000	mice
Golph3	-0.01	1.00000000	mice
Ak2	-0.03	1.00000000	mice
Ino80e	-0.04	1.00000000	mice
Rab28	-0.05	1.00000000	mice
Pcgf3	-0.07	1.00000000	mice
Dusp1	-0.03	1.00000000	mice
Rnf115	-0.01	1.00000000	mice
Topors	-0.02	1.00000000	mice
Sf3b2	-0.02	1.00000000	mice
Imp3	-0.04	1.00000000	mice

Snx25	-0.07	1.00000000	mice
Edc3	-0.03	1.00000000	mice
Stt3a	-0.01	1.00000000	mice
Aprt	0.00	1.00000000	mice
Cystm1	0.04	1.00000000	mice
Hnrnpul2	0.00	1.00000000	mice
Ercc6	-0.07	1.00000000	mice
Ap4s1	-0.05	1.00000000	mice
Ddx20	-0.02	1.00000000	mice
Eif5	0.01	1.00000000	mice
Sgta	-0.04	1.00000000	mice
Akt2	-0.07	1.00000000	mice
Dnajc10	-0.02	1.00000000	mice
Eif6	-0.04	1.00000000	mice
Lemd2	-0.03	1.00000000	mice
Actr1a	-0.03	1.00000000	mice
Agfg1	-0.01	1.00000000	mice
Csnk1g3	-0.01	1.00000000	mice
Isg20l2	-0.01	1.00000000	mice
Hipk1	-0.06	1.00000000	mice
Timm10b	0.00	1.00000000	mice
Notch1	0.01	1.00000000	mice
Rnaseh1	-0.03	1.00000000	mice
Sinhcaf	-0.01	1.00000000	mice
Ptpn11	-0.05	1.00000000	mice
Srprb	0.02	1.00000000	mice
Hlcs	-0.11	1.00000000	mice
Rfxap	0.00	1.00000000	mice
Bola3	0.02	1.00000000	mice
Cat	-0.02	1.00000000	mice
Dennd6a	-0.04	1.00000000	mice
Klhl15	0.01	1.00000000	mice
Pa2g4	-0.04	1.00000000	mice
Furin	0.01	1.00000000	mice
Ndufv1	0.00	1.00000000	mice
Gla	0.02	1.00000000	mice
Vezt	-0.03	1.00000000	mice
Ndufb5	-0.01	1.00000000	mice
Arl6ip6	-0.03	1.00000000	mice
Nup35	-0.03	1.00000000	mice
Chchd3	-0.04	1.00000000	mice
Cdk13	0.01	1.00000000	mice
2610507B11Rik	0.01	1.00000000	mice
Tnfaip8l2	-0.03	1.00000000	mice
Snrnp40	0.01	1.00000000	mice
Pex13	0.02	1.00000000	mice

Tbc1d1	0.10	1.00000000	mice
Rabgef1	-0.05	1.00000000	mice
St6galnac4	0.00	1.00000000	mice
Ccdc43	-0.05	1.00000000	mice
Bclaf3	-0.02	1.00000000	mice
Prxl2c	-0.02	1.00000000	mice
Eno1	-0.06	1.00000000	mice
Cep162	0.05	1.00000000	mice
Fam185a	-0.07	1.00000000	mice
Twf2	0.00	1.00000000	mice
Fndc3b	-0.02	1.00000000	mice
Ttc27	-0.04	1.00000000	mice
Uck2	0.08	1.00000000	mice
Tut1	-0.04	1.00000000	mice
Elp5	-0.01	1.00000000	mice
Ptdss1	-0.04	1.00000000	mice
Hexim1	0.00	1.00000000	mice
Carhsp1	0.01	1.00000000	mice
Tmem256	-0.03	1.00000000	mice
Acbd3	-0.03	1.00000000	mice
Snapin	-0.04	1.00000000	mice
Naa50	-0.01	1.00000000	mice
Hikeshi	-0.01	1.00000000	mice
Prmt3	-0.02	1.00000000	mice
Ints7	-0.05	1.00000000	mice
Pbdc1	-0.02	1.00000000	mice
Dnajc11	-0.01	1.00000000	mice
Sap130	-0.08	1.00000000	mice
Cmtm6	-0.01	1.00000000	mice
Arl13b	0.00	1.00000000	mice
Atg4d	-0.06	1.00000000	mice
Gt(ROSA)26Sor	-0.01	1.00000000	mice
Lnpk	-0.03	1.00000000	mice
Plekhm2	0.00	1.00000000	mice
Szrd1	-0.07	1.00000000	mice
Ppme1	-0.01	1.00000000	mice
Cotl1	-0.07	1.00000000	mice
Cwc25	-0.01	1.00000000	mice
Pum2	-0.04	1.00000000	mice
Caap1	-0.07	1.00000000	mice
Ehmt2	-0.04	1.00000000	mice
Elp1	0.03	1.00000000	mice
Upf2	-0.05	1.00000000	mice
Taldo1	-0.02	1.00000000	mice
Gtf2ird2	-0.04	1.00000000	mice
Bbs9	-0.03	1.00000000	mice

Rab9	-0.04	1.00000000	mice
Cog4	-0.05	1.00000000	mice
Napg	-0.04	1.00000000	mice
Naa25	-0.01	1.00000000	mice
Esco1	0.04	1.00000000	mice
Cbll1	-0.05	1.00000000	mice
Ndufab1	-0.01	1.00000000	mice
Sap18	-0.02	1.00000000	mice
Traf2	-0.01	1.00000000	mice
Psmd7	-0.02	1.00000000	mice
Smg5	-0.06	1.00000000	mice
Farsb	-0.01	1.00000000	mice
Wdtc1	0.00	1.00000000	mice
Afmid	-0.03	1.00000000	mice
Tial1	-0.07	1.00000000	mice
Pspc1	0.01	1.00000000	mice
A630001G21Rik	0.03	1.00000000	mice
Noc2l	-0.01	1.00000000	mice
Nfkbie	-0.01	1.00000000	mice
Stim1	-0.02	1.00000000	mice
Stmn1	-0.02	1.00000000	mice
Clcn3	0.00	1.00000000	mice
Ralgapa1	0.02	1.00000000	mice
Pgam5	-0.02	1.00000000	mice
Rtn4	0.01	1.00000000	mice
Slc9a3r1	-0.05	1.00000000	mice
Kpna4	-0.01	1.00000000	mice
Gabpb2	-0.02	1.00000000	mice
Nktr	-0.02	1.00000000	mice
Dot1l	0.00	1.00000000	mice
Shoc2	-0.03	1.00000000	mice
Patl1	-0.05	1.00000000	mice
Tdrd3	0.00	1.00000000	mice
Dpp4	0.06	1.00000000	mice
Abhd12	-0.11	1.00000000	mice
Pggt1b	-0.06	1.00000000	mice
Utp3	0.00	1.00000000	mice
Atxn2l	-0.06	1.00000000	mice
Cdc40	0.00	1.00000000	mice
Kbtbd2	-0.02	1.00000000	mice
Ccl25	-0.05	1.00000000	mice
Zfp386	-0.03	1.00000000	mice
Ndufv2	-0.01	1.00000000	mice
Sec13	-0.01	1.00000000	mice
2410006H16Rik	0.01	1.00000000	mice
Ppp4r3a	0.00	1.00000000	mice

Rcor1	-0.09	1.00000000	mice
Timm23	-0.02	1.00000000	mice
Hdgf	-0.04	1.00000000	mice
Snx15	-0.02	1.00000000	mice
Cast	-0.03	1.00000000	mice
Ncoa3	0.01	1.00000000	mice
Dguok	-0.04	1.00000000	mice
Exosc3	0.00	1.00000000	mice
Ppp2r1b	-0.03	1.00000000	mice
Hspa4	0.01	1.00000000	mice
Ddost	-0.01	1.00000000	mice
Ythdc2	-0.07	1.00000000	mice
Brcc3	-0.03	1.00000000	mice
Nom1	0.00	1.00000000	mice
Atxn3	-0.02	1.00000000	mice
Rrs1	-0.01	1.00000000	mice
Slc25a17	-0.05	1.00000000	mice
Clec16a	-0.01	1.00000000	mice
Tomm22	-0.04	1.00000000	mice
Chuk	-0.05	1.00000000	mice
Eftud2	0.00	1.00000000	mice
Cdt1	-0.02	1.00000000	mice
Ddx42	-0.02	1.00000000	mice
Slc35b1	0.02	1.00000000	mice
Rpp21	-0.01	1.00000000	mice
Gosr1	0.00	1.00000000	mice
Nup210	-0.01	1.00000000	mice
Tsr1	-0.04	1.00000000	mice
Safb2	-0.03	1.00000000	mice
Dnttip1	-0.02	1.00000000	mice
Hnrnpdl	-0.03	1.00000000	mice
Hsf1	-0.05	1.00000000	mice
Bcl11a	-0.11	1.00000000	mice
Fyco1	0.00	1.00000000	mice
Adk	-0.15	1.00000000	mice
Zdhhc5	0.02	1.00000000	mice
Sec24c	-0.01	1.00000000	mice
Arid5a	-0.01	1.00000000	mice
Urm1	-0.04	1.00000000	mice
Fiz1	-0.01	1.00000000	mice
Syap1	0.00	1.00000000	mice
Iah1	-0.04	1.00000000	mice
Anapc11	-0.03	1.00000000	mice
Rbbp4	-0.03	1.00000000	mice
Pdhx	-0.06	1.00000000	mice
U2surp	0.00	1.00000000	mice

Itpk1	-0.05	1.00000000	mice
Apobec1	-0.08	1.00000000	mice
Riok3	-0.04	1.00000000	mice
Il5ra	-0.06	1.00000000	mice
Yrdc	0.00	1.00000000	mice
Ralbp1	-0.03	1.00000000	mice
Suds3	-0.02	1.00000000	mice
Osbpl11	-0.05	1.00000000	mice
Txnl4a	-0.02	1.00000000	mice
Wdr37	-0.01	1.00000000	mice
Calu	-0.03	1.00000000	mice
Cxxc1	-0.02	1.00000000	mice
Btbd7	-0.03	1.00000000	mice
Plekha1	-0.07	1.00000000	mice
Timm13	-0.02	1.00000000	mice
Rffl	-0.01	1.00000000	mice
Rnf139	0.01	1.00000000	mice
Dnajc8	-0.06	1.00000000	mice
Cul4b	-0.05	1.00000000	mice
Rab11fip2	-0.05	1.00000000	mice
Acsl3	-0.01	1.00000000	mice
Cbfa2t2	-0.05	1.00000000	mice
Egln1	0.00	1.00000000	mice
Insig2	-0.08	1.00000000	mice
Cramp1l	-0.07	1.00000000	mice
Vps39	0.00	1.00000000	mice
Etfa	-0.03	1.00000000	mice
Anp32b	-0.01	1.00000000	mice
Nol12	0.00	1.00000000	mice
Phax	-0.04	1.00000000	mice
Banf1	-0.02	1.00000000	mice
Pus7	0.00	1.00000000	mice
Yipf4	-0.06	1.00000000	mice
Fto	-0.03	1.00000000	mice
Gbf1	-0.04	1.00000000	mice
Atp5o	-0.03	1.00000000	mice
Zpr1	-0.04	1.00000000	mice
Ddx23	0.01	1.00000000	mice
Ppil2	0.00	1.00000000	mice
Foxp4	-0.10	1.00000000	mice
Uqcc1	-0.03	1.00000000	mice
Idh3a	0.01	1.00000000	mice
Csrnp1	0.03	1.00000000	mice
Hacd3	-0.05	1.00000000	mice
Copb2	-0.02	1.00000000	mice
Rbm33	0.00	1.00000000	mice

Trappc9	0.00	1.00000000	mice
Pmpca	-0.02	1.00000000	mice
Dpm2	-0.02	1.00000000	mice
Fxr2	-0.02	1.00000000	mice
Pou2af1	0.02	1.00000000	mice
Pprc1	-0.01	1.00000000	mice
Zfp169	-0.03	1.00000000	mice
Dtx2	-0.04	1.00000000	mice
Actr3	0.01	1.00000000	mice
Gsk3a	0.01	1.00000000	mice
Becn1	-0.03	1.00000000	mice
Pttg1ip	-0.04	1.00000000	mice
Wsb2	-0.02	1.00000000	mice
Farsa	-0.03	1.00000000	mice
Xrcc5	-0.01	1.00000000	mice
Odf2	-0.05	1.00000000	mice
Dnajc9	-0.03	1.00000000	mice
Polh	0.01	1.00000000	mice
Hnrnpa3	0.01	1.00000000	mice
Aasdhppt	-0.03	1.00000000	mice
Arrdc1	-0.03	1.00000000	mice
Eloa	-0.05	1.00000000	mice
Prpf40a	-0.03	1.00000000	mice
Chchd7	-0.01	1.00000000	mice
AB124611	-0.02	1.00000000	mice
Zcchc4	-0.03	1.00000000	mice
Ddx31	0.01	1.00000000	mice
Gnl3l	0.01	1.00000000	mice
Iws1	0.00	1.00000000	mice
Pisd	-0.05	1.00000000	mice
Pip4k2a	-0.07	1.00000000	mice
Pym1	-0.05	1.00000000	mice
Diablo	0.00	1.00000000	mice
Trak2	-0.07	1.00000000	mice
Ppp2r5e	-0.02	1.00000000	mice
Zbtb24	-0.01	1.00000000	mice
Sf3a1	-0.04	1.00000000	mice
Ccnk	-0.01	1.00000000	mice
Ipo9	-0.05	1.00000000	mice
Churc1	0.00	1.00000000	mice
Dgkh	0.00	1.00000000	mice
Trim44	0.00	1.00000000	mice
Nsmce4a	-0.03	1.00000000	mice
Psmb6	0.03	1.00000000	mice
Nfya	-0.01	1.00000000	mice
Nsun4	-0.01	1.00000000	mice

Taf8	-0.04	1.00000000	mice
Stard5	-0.03	1.00000000	mice
Arhgef3	0.02	1.00000000	mice
Dhx57	-0.06	1.00000000	mice
Rprd2	-0.02	1.00000000	mice
Luc7l3	-0.02	1.00000000	mice
Eif5a	0.00	1.00000000	mice
H2afj	0.00	1.00000000	mice
Gtdc1	-0.06	1.00000000	mice
Fam98a	-0.01	1.00000000	mice
Alg9	-0.04	1.00000000	mice
Nol7	-0.01	1.00000000	mice
Afg3l1	0.01	1.00000000	mice
Timm9	-0.03	1.00000000	mice
Fam104a	0.02	1.00000000	mice
Cnot11	-0.01	1.00000000	mice
Cd22	-0.07	1.00000000	mice
Polr3f	0.02	1.00000000	mice
Yy1	-0.01	1.00000000	mice
Taf7	-0.04	1.00000000	mice
Clk4	0.01	1.00000000	mice
Sharpin	-0.03	1.00000000	mice
Cwc27	-0.08	1.00000000	mice
Zfyve26	-0.02	1.00000000	mice
Pts	-0.02	1.00000000	mice
N6amt1	-0.01	1.00000000	mice
Ski	-0.08	1.00000000	mice
Ppp6r3	-0.05	1.00000000	mice
Snx27	-0.04	1.00000000	mice
Ap3m1	-0.01	1.00000000	mice
Zmym2	0.01	1.00000000	mice
Ppp6r2	-0.06	1.00000000	mice
Slc46a2	0.02	1.00000000	mice
Ndufs2	-0.03	1.00000000	mice
Sec24b	-0.03	1.00000000	mice
Efcab14	-0.04	1.00000000	mice
Wdr18	-0.02	1.00000000	mice
Cacybp	-0.06	1.00000000	mice
Abcb7	-0.05	1.00000000	mice
Ccndbp1	-0.02	1.00000000	mice
Samd4b	0.00	1.00000000	mice
5830408C22Rik	-0.02	1.00000000	mice
Nol8	0.00	1.00000000	mice
Por	0.00	1.00000000	mice
Smad3	-0.05	1.00000000	mice
Tle4	0.00	1.00000000	mice

Rnf181	-0.04	1.00000000	mice
Tusc2	-0.04	1.00000000	mice
Fgfr1op2	-0.06	1.00000000	mice
Dnajb1	-0.03	1.00000000	mice
Dgka	-0.08	1.00000000	mice
H13	-0.03	1.00000000	mice
Dhx16	-0.02	1.00000000	mice
Thrap3	0.03	1.00000000	mice
Sacs	0.02	1.00000000	mice
Irf3	0.00	1.00000000	mice
Vps29	-0.02	1.00000000	mice
Bet1l	0.00	1.00000000	mice
Dcun1d5	0.00	1.00000000	mice
Dhx29	0.04	1.00000000	mice
Thoc7	-0.01	1.00000000	mice
Lrp6	-0.06	1.00000000	mice
Adipor1	-0.04	1.00000000	mice
Ahsa1	-0.02	1.00000000	mice
Dus2	0.03	1.00000000	mice
Taf11	0.02	1.00000000	mice
Ndufa10	-0.04	1.00000000	mice
Tango2	-0.04	1.00000000	mice
Siva1	-0.01	1.00000000	mice
Nxf1	-0.03	1.00000000	mice
Babam1	-0.04	1.00000000	mice
Sik2	0.00	1.00000000	mice
Dnajb12	-0.03	1.00000000	mice
Pgp	-0.03	1.00000000	mice
Vma21	-0.03	1.00000000	mice
Nubp1	-0.04	1.00000000	mice
Pus1	-0.06	1.00000000	mice
Gltp	-0.04	1.00000000	mice
Cplane1	-0.02	1.00000000	mice
Stam2	-0.07	1.00000000	mice
Wac	-0.03	1.00000000	mice
Psmd11	-0.05	1.00000000	mice
Ulk2	-0.03	1.00000000	mice
Tpm3-rs7	0.02	1.00000000	mice
Kif1bp	-0.02	1.00000000	mice
Rab10os	0.01	1.00000000	mice
Nelfa	-0.02	1.00000000	mice
Ext2	-0.03	1.00000000	mice
Gabpb1	0.02	1.00000000	mice
Tmem128	0.00	1.00000000	mice
Ube2j2	0.01	1.00000000	mice
Lat2	-0.01	1.00000000	mice

Susd6	-0.02	1.00000000	mice
4930556J24Rik	0.02	1.00000000	mice
Zfyve27	-0.02	1.00000000	mice
Brd2	0.00	1.00000000	mice
Zfp68	0.00	1.00000000	mice
Rasal3	0.00	1.00000000	mice
Cluap1	-0.03	1.00000000	mice
Ruvbl1	0.00	1.00000000	mice
Thoc1	-0.05	1.00000000	mice
Ensa	-0.03	1.00000000	mice
Edrf1	-0.05	1.00000000	mice
Snx6	-0.01	1.00000000	mice
Cep57	0.00	1.00000000	mice
Polg	-0.04	1.00000000	mice
Commd6	-0.03	1.00000000	mice
Dkc1	-0.02	1.00000000	mice
Dpf2	-0.01	1.00000000	mice
Nrf1	-0.01	1.00000000	mice
Rngtt	-0.05	1.00000000	mice
Oxa1l	-0.02	1.00000000	mice
Calr	-0.04	1.00000000	mice
Cct2	-0.01	1.00000000	mice
Cdc37l1	0.00	1.00000000	mice
Pafah1b1	-0.03	1.00000000	mice
Bloc1s4	0.00	1.00000000	mice
Spata13	-0.08	1.00000000	mice
Erich1	-0.04	1.00000000	mice
Ubxn2a	-0.03	1.00000000	mice
Hmgxb3	0.00	1.00000000	mice
Stat6	-0.02	1.00000000	mice
Kpna6	-0.02	1.00000000	mice
Nolc1	-0.04	1.00000000	mice
Hdac2	-0.02	1.00000000	mice
Chchd2	0.01	1.00000000	mice
Ublcp1	-0.02	1.00000000	mice
Cask	0.08	1.00000000	mice
Fam192a	-0.06	1.00000000	mice
Ndufs3	-0.04	1.00000000	mice
Cwc15	-0.01	1.00000000	mice
Spg11	-0.06	1.00000000	mice
Slc25a46	-0.03	1.00000000	mice
Lsm2	0.00	1.00000000	mice
Slc7a6os	0.00	1.00000000	mice
Usp19	-0.01	1.00000000	mice
Lmbrd2	-0.07	1.00000000	mice
Stx17	-0.01	1.00000000	mice

Clpp	0.00	1.00000000	mice
Stard7	0.01	1.00000000	mice
Txlna	-0.03	1.00000000	mice
Nosip	-0.02	1.00000000	mice
Nsfl1c	0.00	1.00000000	mice
Bcl3	-0.03	1.00000000	mice
Stk19	-0.01	1.00000000	mice
Pkm	-0.01	1.00000000	mice
Lyar	-0.02	1.00000000	mice
Rbbp5	0.00	1.00000000	mice
Etf1	-0.02	1.00000000	mice
1810030O07Rik	0.00	1.00000000	mice
Als2	-0.03	1.00000000	mice
Tex14	-0.02	1.00000000	mice
Trim24	-0.01	1.00000000	mice
Gadd45g	0.01	1.00000000	mice
Gga1	-0.02	1.00000000	mice
Zfp330	-0.02	1.00000000	mice
Ythdf3	-0.01	1.00000000	mice
Etfdh	-0.02	1.00000000	mice
Cables2	-0.02	1.00000000	mice
Emc6	-0.03	1.00000000	mice
Rpp30	-0.02	1.00000000	mice
Vps36	-0.01	1.00000000	mice
Virma	-0.03	1.00000000	mice
Dync2h1	-0.01	1.00000000	mice
Gtf2b	-0.01	1.00000000	mice
Hacd2	0.00	1.00000000	mice
Upf1	-0.03	1.00000000	mice
Ginm1	0.01	1.00000000	mice
Rab14	-0.01	1.00000000	mice
Tmem161b	-0.04	1.00000000	mice
Kin	-0.03	1.00000000	mice
Hspbp1	0.00	1.00000000	mice
Zfp710	-0.10	1.00000000	mice
Atg4a	-0.06	1.00000000	mice
Utp25	0.00	1.00000000	mice
Uhmk1	-0.03	1.00000000	mice
Nol11	0.00	1.00000000	mice
Pqbp1	-0.01	1.00000000	mice
Uqcrfs1	-0.04	1.00000000	mice
Ints9	-0.04	1.00000000	mice
Nedd1	0.02	1.00000000	mice
Gart	-0.02	1.00000000	mice
Ddx50	-0.02	1.00000000	mice
Hhex	0.03	1.00000000	mice

Larp1b	-0.04	1.00000000	mice
Psmc4	-0.03	1.00000000	mice
Usp14	-0.03	1.00000000	mice
Bod1l	-0.01	1.00000000	mice
C1d	-0.03	1.00000000	mice
Arhgdib	-0.01	1.00000000	mice
Sdf2	-0.06	1.00000000	mice
Atp6v1f	-0.01	1.00000000	mice
Zscan26	-0.02	1.00000000	mice
Socs7	-0.03	1.00000000	mice
Znrf2	-0.05	1.00000000	mice
Ercc6l2	-0.04	1.00000000	mice
Ldlrad4	0.01	1.00000000	mice
Zbtb5	-0.02	1.00000000	mice
Kmt2b	-0.01	1.00000000	mice
Tmem50a	-0.05	1.00000000	mice
Tsg101	0.02	1.00000000	mice
Abhd16a	-0.02	1.00000000	mice
Kdm1a	-0.02	1.00000000	mice
Capn15	0.00	1.00000000	mice
Gpalpp1	-0.02	1.00000000	mice
Ikbkb	-0.06	1.00000000	mice
Spcs3	0.01	1.00000000	mice
Tbc1d9b	0.00	1.00000000	mice
Rnf10	-0.02	1.00000000	mice
Abi1	-0.04	1.00000000	mice
Prkrip1	0.00	1.00000000	mice
Ocel1	-0.02	1.00000000	mice
BC005537	-0.01	1.00000000	mice
Tuba1c	-0.01	1.00000000	mice
Nt5dc1	-0.05	1.00000000	mice
Dlat	-0.05	1.00000000	mice
1810026B05Rik	-0.01	1.00000000	mice
Hmgxb4	-0.03	1.00000000	mice
Fam169b	-0.04	1.00000000	mice
Ndufb11	-0.02	1.00000000	mice
Dctn4	-0.04	1.00000000	mice
Rrp15	-0.01	1.00000000	mice
Kif21b	-0.04	1.00000000	mice
Bzw1	-0.02	1.00000000	mice
Brpf1	-0.02	1.00000000	mice
Extl3	-0.01	1.00000000	mice
Ppfia1	-0.05	1.00000000	mice
Fam117b	0.00	1.00000000	mice
Strn	0.01	1.00000000	mice
Anapc15	-0.01	1.00000000	mice

Hsd17b10	-0.05	1.00000000	mice
Arl1	0.00	1.00000000	mice
Usp36	-0.03	1.00000000	mice
Cog8	-0.03	1.00000000	mice
Pdlim5	0.03	1.00000000	mice
Pafah1b2	-0.03	1.00000000	mice
Setdb1	-0.08	1.00000000	mice
Ppp6r1	-0.01	1.00000000	mice
Cops6	-0.01	1.00000000	mice
Ganc	0.00	1.00000000	mice
Cmah	-0.06	1.00000000	mice
Tm9sf4	-0.02	1.00000000	mice
Stk25	-0.02	1.00000000	mice
Rab11a	-0.02	1.00000000	mice
Pgs1	-0.01	1.00000000	mice
Thoc3	-0.05	1.00000000	mice
Cnih4	-0.03	1.00000000	mice
Pdcd6	-0.03	1.00000000	mice
Ccnt2	-0.06	1.00000000	mice
Kri1	-0.01	1.00000000	mice
Akap8l	-0.08	1.00000000	mice
Slc50a1	-0.04	1.00000000	mice
Cep104	-0.03	1.00000000	mice
Vamp1	-0.03	1.00000000	mice
Kat2b	-0.11	1.00000000	mice
D8Ertd738e	0.00	1.00000000	mice
Phc1	-0.01	1.00000000	mice
Ndufa11	-0.03	1.00000000	mice
Dym	-0.07	1.00000000	mice
Csnk1g1	-0.05	1.00000000	mice
Rad23b	-0.04	1.00000000	mice
Polr3k	-0.02	1.00000000	mice
Stam	-0.01	1.00000000	mice
Cpne1	0.00	1.00000000	mice
Rxylt1	0.00	1.00000000	mice
Sumf1	-0.04	1.00000000	mice
Atic	0.02	1.00000000	mice
Cdkn2aip	-0.04	1.00000000	mice
Ash2l	-0.03	1.00000000	mice
Gskip	0.00	1.00000000	mice
Ube2g1	-0.04	1.00000000	mice
Tspyl1	0.00	1.00000000	mice
Creb1	0.02	1.00000000	mice
Snrpd3	0.02	1.00000000	mice
Pi4k2b	-0.02	1.00000000	mice
Acadl	-0.06	1.00000000	mice

Fbxw2	0.00	1.00000000	mice
Acot9	0.01	1.00000000	mice
Leprot	-0.02	1.00000000	mice
Taf5l	-0.01	1.00000000	mice
Ncbp1	-0.04	1.00000000	mice
Rad54l2	-0.04	1.00000000	mice
Zscan21	-0.01	1.00000000	mice
Gorasp2	-0.01	1.00000000	mice
Hcfc1	-0.01	1.00000000	mice
Snx3	-0.04	1.00000000	mice
Stx6	-0.05	1.00000000	mice
Cep135	0.03	1.00000000	mice
Pacsin2	-0.06	1.00000000	mice
Wrn	-0.07	1.00000000	mice
Cers2	-0.02	1.00000000	mice
Wdfy2	-0.04	1.00000000	mice
Disp1	-0.01	1.00000000	mice
Sfmbt1	-0.07	1.00000000	mice
Trip12	0.00	1.00000000	mice
Thoc2	-0.02	1.00000000	mice
Ggnbp2	-0.01	1.00000000	mice
Ube2n	0.00	1.00000000	mice
Zbtb38	-0.01	1.00000000	mice
Eif1b	-0.04	1.00000000	mice
Sertad2	0.03	1.00000000	mice
A930005H10Rik	0.02	1.00000000	mice
Stk38l	0.09	1.00000000	mice
Cog2	-0.04	1.00000000	mice
Rock1	0.02	1.00000000	mice
Tpm1	0.03	1.00000000	mice
Psmd8	-0.03	1.00000000	mice
Hinfp	-0.01	1.00000000	mice
Uchl5	0.00	1.00000000	mice
Arl5a	0.01	1.00000000	mice
Pon2	0.00	1.00000000	mice
Csnk1g2	-0.01	1.00000000	mice
1700123O20Rik	-0.02	1.00000000	mice
Ddhd2	-0.04	1.00000000	mice
Gcc2	-0.08	1.00000000	mice
Zfp24	-0.04	1.00000000	mice
Fnbp1	0.02	1.00000000	mice
Arl5b	-0.03	1.00000000	mice
Nr1d2	-0.04	1.00000000	mice
Tbc1d23	-0.04	1.00000000	mice
5530601H04Rik	-0.04	1.00000000	mice
Tcaim	0.01	1.00000000	mice

Dcaf17	-0.04	1.00000000	mice
Ctcf	-0.03	1.00000000	mice
Heatr5b	0.01	1.00000000	mice
Atf6b	-0.02	1.00000000	mice
Spata1	-0.04	1.00000000	mice
Lrrc41	-0.02	1.00000000	mice
Pop7	-0.01	1.00000000	mice
Aak1	-0.02	1.00000000	mice
Pwwp2a	-0.04	1.00000000	mice
Lysmd3	-0.05	1.00000000	mice
Plxnc1	0.01	1.00000000	mice
Polb	0.01	1.00000000	mice
Csgalnact2	0.00	1.00000000	mice
Tmcc3	-0.03	1.00000000	mice
Sptlc1	-0.05	1.00000000	mice
Was	-0.01	1.00000000	mice
Orc3	-0.01	1.00000000	mice
Sfr1	-0.05	1.00000000	mice
Alkbh5	-0.01	1.00000000	mice
Zkscan3	-0.02	1.00000000	mice
Ykt6	-0.01	1.00000000	mice
Xpa	-0.06	1.00000000	mice
Btf3l4	-0.01	1.00000000	mice
Pofut2	0.00	1.00000000	mice
Oip5os1	-0.03	1.00000000	mice
Ipo7	0.00	1.00000000	mice
Rlim	-0.04	1.00000000	mice
Pam16	-0.01	1.00000000	mice
Tgif2	0.00	1.00000000	mice
Cacul1	-0.01	1.00000000	mice
Psma6	-0.06	1.00000000	mice
Ddx3x	0.02	1.00000000	mice
Aip	-0.03	1.00000000	mice
Arl2bp	-0.02	1.00000000	mice
Brd7	0.01	1.00000000	mice
Setd3	-0.04	1.00000000	mice
Zfp407	-0.03	1.00000000	mice
Vps72	-0.06	1.00000000	mice
Ccdc124	-0.03	1.00000000	mice
Zhx1	-0.01	1.00000000	mice
Hmg20b	-0.03	1.00000000	mice
Tmem11	0.00	1.00000000	mice
Btbd1	-0.01	1.00000000	mice
Esd	0.01	1.00000000	mice
Tlk2	-0.01	1.00000000	mice
Rab12	-0.02	1.00000000	mice

Tasp1	-0.05	1.00000000	mice
Cyp4f18	0.01	1.00000000	mice
Gopc	-0.05	1.00000000	mice
Zmat5	-0.01	1.00000000	mice
Uqcrc1	0.00	1.00000000	mice
Prpsap1	0.00	1.00000000	mice
Trim7	-0.09	1.00000000	mice
Ing3	-0.05	1.00000000	mice
Cyth4	-0.03	1.00000000	mice
Fam120c	-0.03	1.00000000	mice
Exoc6b	-0.03	1.00000000	mice
Ap1m1	-0.04	1.00000000	mice
Lsm12	-0.03	1.00000000	mice
Ywhag	-0.02	1.00000000	mice
Kif2a	-0.04	1.00000000	mice
Dexi	0.05	1.00000000	mice
Lsm14a	-0.06	1.00000000	mice
Acsl4	-0.06	1.00000000	mice
Dedd	-0.03	1.00000000	mice
Tbc1d10c	-0.04	1.00000000	mice
Ndufc2	-0.02	1.00000000	mice
Ecd	0.01	1.00000000	mice
Ints13	-0.05	1.00000000	mice
Jarid2	0.06	1.00000000	mice
Rnf146	-0.01	1.00000000	mice
Cep164	-0.01	1.00000000	mice
Tpr	0.01	1.00000000	mice
Ist1	-0.03	1.00000000	mice
Chmp2b	-0.03	1.00000000	mice
Cyc1	0.00	1.00000000	mice
Fam45a	-0.01	1.00000000	mice
Dhx30	-0.03	1.00000000	mice
Tomm5	-0.01	1.00000000	mice
Rad21	-0.03	1.00000000	mice
H2-Ke6	0.00	1.00000000	mice
Trir	0.01	1.00000000	mice
Sp3	-0.03	1.00000000	mice
Hbs1l	-0.01	1.00000000	mice
Usp33	-0.08	1.00000000	mice
Epb41l2	-0.05	1.00000000	mice
Ino80d	-0.02	1.00000000	mice
R3hdm1	-0.03	1.00000000	mice
Crybg3	-0.02	1.00000000	mice
Shld2	-0.05	1.00000000	mice
Odr4	0.00	1.00000000	mice
Sugp1	-0.06	1.00000000	mice

Exosc10	-0.03	1.00000000	mice
Ercc3	-0.04	1.00000000	mice
Atp11b	-0.01	1.00000000	mice
Ofd1	0.02	1.00000000	mice
Fkbp1a	-0.04	1.00000000	mice
Xpo5	-0.04	1.00000000	mice
Trip4	-0.06	1.00000000	mice
Cdk11b	-0.01	1.00000000	mice
Otub1	-0.01	1.00000000	mice
Wipf2	-0.02	1.00000000	mice
Rhof	-0.05	1.00000000	mice
Lasp1	-0.04	1.00000000	mice
Pgd	-0.01	1.00000000	mice
Sf3a2	-0.01	1.00000000	mice
Paics	-0.03	1.00000000	mice
Ndufb6	-0.01	1.00000000	mice
Suco	0.00	1.00000000	mice
Cox11	-0.01	1.00000000	mice
Nub1	-0.03	1.00000000	mice
Tbcb	0.00	1.00000000	mice
Ctdspl2	-0.05	1.00000000	mice
Slc25a12	-0.03	1.00000000	mice
Tle1	-0.01	1.00000000	mice
Tmem175	-0.01	1.00000000	mice
Ptar1	-0.02	1.00000000	mice
Snx17	-0.02	1.00000000	mice
Nat10	-0.02	1.00000000	mice
Ints14	0.03	1.00000000	mice
Fbxl12	-0.03	1.00000000	mice
Kif5b	-0.02	1.00000000	mice
Sdad1	-0.03	1.00000000	mice
Leng8	0.00	1.00000000	mice
Vrk1	-0.02	1.00000000	mice
Ndfip2	0.01	1.00000000	mice
Chordc1	-0.01	1.00000000	mice
Kat7	-0.03	1.00000000	mice
Ppp6c	-0.08	1.00000000	mice
Gpsm3	-0.01	1.00000000	mice
Nedd9	-0.02	1.00000000	mice
Snx9	-0.02	1.00000000	mice
Paip1	-0.03	1.00000000	mice
Cnot3	0.00	1.00000000	mice
Tmem223	0.00	1.00000000	mice
Stat5a	-0.02	1.00000000	mice
Ankhd1	0.02	1.00000000	mice
Psmb3	-0.01	1.00000000	mice

Snhg15	0.01	1.00000000	mice
Baz1b	-0.04	1.00000000	mice
Drap1	0.00	1.00000000	mice
Abca1	-0.05	1.00000000	mice
Tug1	-0.03	1.00000000	mice
Nob1	-0.05	1.00000000	mice
Srsf6	-0.01	1.00000000	mice
4930453N24Rik	0.01	1.00000000	mice
Ylpm1	-0.04	1.00000000	mice
Gtf2f1	-0.03	1.00000000	mice
Pmf1	-0.03	1.00000000	mice
Tm9sf2	-0.05	1.00000000	mice
Xpo4	-0.08	1.00000000	mice
Ssr1	-0.02	1.00000000	mice
Zfp317	0.01	1.00000000	mice
Nemf	-0.03	1.00000000	mice
Smu1	-0.03	1.00000000	mice
Api5	-0.06	1.00000000	mice
Dennd5b	-0.14	1.00000000	mice
Kxd1	-0.05	1.00000000	mice
Nip7	-0.02	1.00000000	mice
Herc1	-0.01	1.00000000	mice
Vps54	-0.02	1.00000000	mice
Slc35e2	-0.01	1.00000000	mice
Bub3	-0.01	1.00000000	mice
Zbtb8os	0.01	1.00000000	mice
Emg1	0.00	1.00000000	mice
Ttc14	-0.03	1.00000000	mice
Coa3	-0.01	1.00000000	mice
Eif3f	-0.01	1.00000000	mice
Vps53	-0.01	1.00000000	mice
Ap1g1	-0.04	1.00000000	mice
Polr2j	0.01	1.00000000	mice
Rpn1	-0.01	1.00000000	mice
Paxbp1	-0.03	1.00000000	mice
Prmt7	-0.02	1.00000000	mice
3110056K07Rik	-0.01	1.00000000	mice
Rbsn	-0.03	1.00000000	mice
9530068E07Rik	-0.02	1.00000000	mice
Chm	-0.01	1.00000000	mice
Ccar1	-0.03	1.00000000	mice
Chmp1b	-0.04	1.00000000	mice
Cacna1e	-0.11	1.00000000	mice
2210016L21Rik	-0.01	1.00000000	mice
Kars	-0.01	1.00000000	mice
Ankrd52	-0.02	1.00000000	mice

Nup155	-0.01	1.00000000	mice
Commd4	-0.03	1.00000000	mice
Selenoh	-0.04	1.00000000	mice
Smim8	0.00	1.00000000	mice
Kif16b	0.02	1.00000000	mice
Pcbp2	0.00	1.00000000	mice
Spopl	-0.04	1.00000000	mice
Spcs1	-0.04	1.00000000	mice
Ptpn12	0.01	1.00000000	mice
Bop1	0.00	1.00000000	mice
Spcs2	-0.03	1.00000000	mice
Acaca	-0.05	1.00000000	mice
Cwf19l2	0.02	1.00000000	mice
Arih2	0.02	1.00000000	mice
Nono	-0.01	1.00000000	mice
Ddx49	-0.02	1.00000000	mice
Gpn1	-0.01	1.00000000	mice
Sike1	-0.04	1.00000000	mice
Dcaf10	-0.02	1.00000000	mice
Rpa3	-0.01	1.00000000	mice
Gnb2	0.01	1.00000000	mice
Wdfy1	-0.01	1.00000000	mice
Ccdc32	-0.03	1.00000000	mice
Ube2q1	-0.02	1.00000000	mice
Nup54	-0.01	1.00000000	mice
Zfp36l2	-1.11	0.00000000	mice
Cecr2	-1.42	0.00000000	mice
Snx29	-1.04	0.00000000	mice
Tmem108	-1.65	0.00000000	mice
Pdcd4	-1.07	0.00000000	mice
Agbl1	-1.96	0.00000000	mice
Sox5	-1.75	0.00000000	mice
Vpreb3	-1.12	0.00000000	mice
Sema4b	-1.01	0.00000000	mice
Serinc5	-1.08	0.00000000	mice
Akap12	-1.23	0.00000000	mice
Tcf7l2	-1.32	0.00000000	mice
Tmem163	-1.15	0.00000000	mice
Bcl7a	-1.04	0.00000000	mice
Vgll4	-1.01	0.00000000	mice
Susd1	-1.00	0.00000000	mice
Klhl14	-1.16	0.00000000	mice
Gab2	-1.04	0.00000000	mice
Fhit	-1.11	0.00000000	mice
Slc8a1	-1.26	0.00000000	mice
Saa3	1.95	0.00000000	pigs

Ago2	1.10	0.00000000	pigs
Prkce	1.16	0.00000000	pigs
Lrrc8c	1.28	0.00000000	pigs
Gcnt2	1.41	0.00000000	pigs
Hsph1	1.08	0.00000084	pigs
Abtb2	1.55	0.00000128	pigs
Crim1	1.18	0.00007963	pigs
Slamf7	1.08	0.00578400	pigs
Tpt1	0.58	0.00000000	pigs
Stk24	0.97	0.00000000	pigs
Tpi1	0.90	0.00000000	pigs
Lnx2	0.93	0.00000000	pigs
Traf5	0.87	0.00000000	pigs
Ccr7	0.92	0.00000000	pigs
Cyth1	0.64	0.00000000	pigs
Dusp4	0.95	0.00000000	pigs
Eef1g	0.67	0.00000000	pigs
Uvrag	0.92	0.00000000	pigs
Atp6v1c2	-0.79	0.00000000	pigs
Vps37b	0.97	0.00000001	pigs
Timp1	0.86	0.00000001	pigs
Rack1	0.58	0.00000001	pigs
Tsen2	0.19	0.00000001	pigs
Rcor1	0.87	0.00000001	pigs
Pcnx	0.92	0.00000002	pigs
Atp1a1	0.70	0.00000002	pigs
Sfmbt2	0.71	0.00000007	pigs
Ccnd2	0.90	0.00000007	pigs
Arid1b	0.75	0.00000009	pigs
Fam3c	0.76	0.00000013	pigs
Ncf1	0.67	0.00000020	pigs
Havcr2	0.83	0.00000034	pigs
Runx3	0.75	0.00000036	pigs
Tet2	0.59	0.00000040	pigs
Tcf4	0.78	0.00000042	pigs
Smap2	0.72	0.00000044	pigs
Inpp5d	0.88	0.00000047	pigs
Ranbp9	0.73	0.00000057	pigs
Traf3	0.71	0.00000094	pigs
Wnk1	0.63	0.00000104	pigs
Pik3r5	0.90	0.00000106	pigs
Arhgap24	0.82	0.00000134	pigs
Pml	0.72	0.00000208	pigs
Stim1	0.74	0.00000219	pigs
Slc30a5	0.70	0.00000251	pigs
Laptm5	0.63	0.00000300	pigs

Sh3bgrl3	-0.88	0.00000321	pigs
Zdhhc20	0.79	0.00000338	pigs
Eif4g3	0.67	0.00000423	pigs
Cmip	0.66	0.00000441	pigs
Hspa8	0.65	0.00000484	pigs
Ctsc	0.67	0.00000485	pigs
Eif4a1	0.55	0.00000489	pigs
Irf8	0.62	0.00000495	pigs
Fads3	-0.86	0.00000561	pigs
Chd7	0.66	0.00000708	pigs
Fam102a	0.73	0.00000732	pigs
Bnip3	0.66	0.00000808	pigs
Snx9	0.75	0.00000842	pigs
Hsp90aa1	0.74	0.00001051	pigs
Gls	0.57	0.00001085	pigs
Zswim6	0.64	0.00001449	pigs
Ttc39c	0.81	0.00001586	pigs
Sqle	0.87	0.00001806	pigs
Ugcg	0.47	0.00002034	pigs
Rac1	0.60	0.00002124	pigs
Tnfaip8	0.80	0.00002139	pigs
Top3a	0.49	0.00002369	pigs
Elovl5	0.65	0.00002999	pigs
Cd79b	-0.82	0.00003083	pigs
Sema4d	0.54	0.00003509	pigs
Scarb1	0.56	0.00003620	pigs
Ftl1-ps1	0.63	0.00003809	pigs
Sik3	0.68	0.00003899	pigs
Rhoq	0.66	0.00003984	pigs
Arrdc2	0.48	0.00006920	pigs
Chsy1	0.61	0.00008452	pigs
Vav2	0.67	0.00009070	pigs
Eif3g	0.52	0.00009778	pigs
Lrrc8d	0.70	0.00010215	pigs
Ybey	0.52	0.00010861	pigs
Glrx3	0.54	0.00012915	pigs
Ido1	0.69	0.00015505	pigs
Pde8a	0.71	0.00016994	pigs
Eif3a	0.55	0.00017213	pigs
Lbr	-0.71	0.00017560	pigs
Ubl5	-0.68	0.00018183	pigs
Lgmn	0.63	0.00018707	pigs
Ubc	0.54	0.00022718	pigs
Ubap2	0.60	0.00024240	pigs
Hspe1-rs1	0.59	0.00029899	pigs
S100a6	-0.73	0.00030462	pigs

Plgrkt	0.47	0.00031421	pigs
Stard4	0.63	0.00038424	pigs
Csnk1a1	0.52	0.00039229	pigs
Cd80	0.61	0.00041515	pigs
Sptan1	0.54	0.00041702	pigs
Cct8	0.51	0.00045134	pigs
Sestd1	-0.72	0.00051557	pigs
Ankrd11	0.59	0.00053697	pigs
Polr1d	0.53	0.00055862	pigs
Prkca	0.88	0.00056995	pigs
H2-Ob	0.50	0.00072438	pigs
Prkn	-0.79	0.00084250	pigs
Prex1	0.71	0.00103482	pigs
Hk2	0.46	0.00124724	pigs
Runx2	0.58	0.00128437	pigs
Zfp326	0.49	0.00132502	pigs
Dennd5a	0.23	0.00135731	pigs
Fgd2	0.57	0.00150212	pigs
Pam	0.62	0.00150572	pigs
Arhgap25	0.48	0.00150726	pigs
Naf1	0.56	0.00178542	pigs
Ccdc149	-0.36	0.00195578	pigs
Fyn	0.74	0.00211716	pigs
St8sia4	0.62	0.00214874	pigs
Dmxl1	0.57	0.00217798	pigs
Ptbp2	0.53	0.00224150	pigs
Hmgxb3	0.43	0.00246266	pigs
Kpna1	0.47	0.00246923	pigs
Eef2	0.41	0.00248869	pigs
Cdyl	0.55	0.00249481	pigs
Furin	0.50	0.00262604	pigs
Xpr1	0.59	0.00302440	pigs
Rfx2	0.59	0.00307563	pigs
Bhlhe40	0.59	0.00313395	pigs
Cbll1	0.36	0.00351756	pigs
Ptpn18	-0.57	0.00417249	pigs
Slc15a4	0.46	0.00431964	pigs
Csrnp1	0.47	0.00434718	pigs
Rell1	0.60	0.00446248	pigs
Ankrd17	0.52	0.00446466	pigs
Sema7a	0.38	0.00466682	pigs
Snapc1l	0.43	0.00492990	pigs
Trim75	0.46	0.00501114	pigs
Prdm2	0.46	0.00518012	pigs
Gla	0.44	0.00520473	pigs
C1qa	0.39	0.00546920	pigs

Prpf40a	0.41	0.00580585	pigs
Fez2	0.47	0.00587114	pigs
Pnp	0.32	0.00636167	pigs
Szrd1	0.43	0.00661181	pigs
Tom1	0.39	0.00703880	pigs
Hk1	0.40	0.00771228	pigs
Cyth3	0.58	0.00816494	pigs
Chd1	0.46	0.00818021	pigs
Prkcq	0.45	0.00835352	pigs
Zfp36l2	-0.59	0.00882313	pigs
Birc6	0.50	0.00897383	pigs
Car8	0.59	0.00905978	pigs
Elf4	0.36	0.00913419	pigs
Insig1	0.73	0.00921039	pigs
Susd6	0.49	0.00962394	pigs
Ssbp3	0.70	0.01083235	pigs
Septin11	0.53	0.01161566	pigs
Lman2	0.34	0.01276055	pigs
Rrp15	0.38	0.01290242	pigs
Jmjd1c	0.55	0.01299082	pigs
Trps1	0.53	0.01307327	pigs
Bmp2k	0.61	0.01326632	pigs
Gnb1	0.36	0.01329379	pigs
Ptpn1	0.54	0.01341493	pigs
Rabepk	0.35	0.01350410	pigs
Nek7	0.50	0.01493695	pigs
Cd53	0.36	0.01525870	pigs
Vcp	0.43	0.01581481	pigs
Tamalin	0.46	0.01615504	pigs
Cd300c	0.34	0.01630835	pigs
Hspa9	0.36	0.01778695	pigs
Actg1	0.57	0.01841199	pigs
Prrc2b	0.46	0.01930666	pigs
Srpk2	0.53	0.02039248	pigs
Fcer2a	-0.68	0.02082505	pigs
Fkbp5	0.48	0.02091940	pigs
A4galt	0.42	0.02232869	pigs
Rassf1	0.45	0.02325692	pigs
Cry2	0.57	0.02350610	pigs
Zhx2	0.65	0.02457676	pigs
Gpi1	0.44	0.02484977	pigs
Arid4b	0.42	0.02497121	pigs
Aimp2	0.40	0.02573064	pigs
Twf2	0.36	0.02598773	pigs
Nfat5	0.47	0.02682299	pigs
Tab2	0.45	0.02685189	pigs

Fbxl5	0.43	0.02803213	pigs
Rheb	0.36	0.02816526	pigs
Crybg1	0.44	0.02828964	pigs
Slc4a7	0.49	0.02847031	pigs
Tmem181a	0.31	0.02931401	pigs
Stat5a	0.36	0.03277811	pigs
Anks1	0.57	0.03516890	pigs
Rnf19a	0.52	0.03595079	pigs
Irf2bp2	0.43	0.03622000	pigs
St6galnac3	-1.00	0.03904328	pigs
Cops2	0.35	0.04081739	pigs
Nr2f6	0.28	0.04088378	pigs
Crem	0.41	0.04229158	pigs
Ppp3ca	-0.75	0.04275356	pigs
Plcg2	0.48	0.04410014	pigs
Eif4b	0.34	0.04640708	pigs
Fh1	0.21	0.04723625	pigs
Rtn4	0.51	0.04789115	pigs
Chordc1	0.40	0.04896649	pigs
Rala	0.50	0.04984077	pigs
Nck2	0.57	0.05445138	pigs
Trappc9	0.40	0.05471739	pigs
Fundc1	0.34	0.05560903	pigs
Yipf5	0.39	0.05572259	pigs
Ccnl1	0.34	0.05636593	pigs
Igf2r	0.36	0.05808609	pigs
Foxk1	0.38	0.06025719	pigs
Epsti1	0.46	0.06431018	pigs
Gtf3c1	0.45	0.06507505	pigs
Atp2b1	0.47	0.06521149	pigs
Cnot9	0.53	0.06569843	pigs
Itgb1bp1	0.31	0.06677620	pigs
Slc7a1	0.34	0.06973680	pigs
Qk	0.56	0.07014523	pigs
N4bp1	0.36	0.07020634	pigs
Cstf3	0.37	0.07040493	pigs
Kpnb1	0.42	0.07212530	pigs
Fam117b	0.57	0.07226576	pigs
Ctif	0.38	0.07281429	pigs
Atf6	0.42	0.07479146	pigs
Cdk13	0.42	0.07738365	pigs
Anxa6	0.28	0.07843923	pigs
Cd79a	-0.57	0.07863931	pigs
Icam1	0.60	0.07892006	pigs
Eif3e	0.38	0.07919169	pigs
Ifngr2	0.50	0.07933777	pigs

Prkar1a	0.34	0.08151939	pigs
Usp7	0.28	0.08991569	pigs
Fbxw7	0.42	0.09084597	pigs
Adamts12	0.37	0.09102351	pigs
Syne3	0.44	0.09393961	pigs
Card11	0.59	0.09712280	pigs
Wdr1	0.40	0.10144254	pigs
Eef1b2	0.35	0.10244105	pigs
Ppm1k	0.37	0.10724926	pigs
Hck	-0.82	0.10926972	pigs
Ube2h	0.36	0.11100530	pigs
Sytl3	0.56	0.11317208	pigs
Ppp6r3	0.40	0.12072243	pigs
Rel	0.36	0.12326782	pigs
Supt3	0.35	0.12523367	pigs
Bcl2a1d	0.55	0.12581631	pigs
Hspb1	0.55	0.12648321	pigs
Ncf4	0.33	0.12948092	pigs
Itpr2	0.43	0.13034821	pigs
Rbm26	0.31	0.13676848	pigs
Braf	0.38	0.13882480	pigs
Lrrk1	0.69	0.14426279	pigs
Ubald2	0.44	0.14655400	pigs
Camsap2	0.46	0.15695676	pigs
Fbxl3	0.31	0.15886199	pigs
Smad7	0.36	0.15902188	pigs
Rab10	0.48	0.15960264	pigs
Pgap1	0.44	0.16371809	pigs
Zranb1	0.27	0.16458336	pigs
Tbc1d22a	0.27	0.16578999	pigs
Slc20a1	0.33	0.16837989	pigs
Sqstm1	0.25	0.16904488	pigs
Ccny	0.52	0.16991827	pigs
Ric1	0.56	0.17442264	pigs
Bms1	0.36	0.17752044	pigs
Efr3a	0.31	0.17893110	pigs
Atp11a	0.54	0.18058661	pigs
Tnfrsf13b	0.31	0.18683422	pigs
Psmd11	0.31	0.18690904	pigs
Ppp2r5c	0.34	0.19697249	pigs
Gpr183	0.36	0.19770613	pigs
Fes	0.32	0.19779874	pigs
Aebp2	0.41	0.20236580	pigs
Hsd17b4	0.43	0.20900322	pigs
Eif3f	0.43	0.21141739	pigs
C4bp	-0.77	0.21409848	pigs

Cdk8	0.33	0.21501008	pigs
Nfatc1	0.31	0.21869534	pigs
Dnajc2	0.28	0.22253378	pigs
Ehbp1	0.44	0.22675978	pigs
Tcf7	0.25	0.22884192	pigs
Xkr6	0.51	0.22919850	pigs
P4ha1	0.45	0.22969889	pigs
Ubr2	0.39	0.23112489	pigs
Btbd8	-0.49	0.23366600	pigs
Fam120a	0.31	0.24403053	pigs
Aldh3a2	0.38	0.24616881	pigs
Id2	0.75	0.25030006	pigs
Caprin1	0.40	0.25523696	pigs
Eif3l	0.34	0.25773398	pigs
Brd4	0.38	0.25838201	pigs
Ppard	0.37	0.25860244	pigs
Prkd3	0.35	0.27542735	pigs
Rnf138	0.28	0.28485236	pigs
Itga2	-0.81	0.28490783	pigs
Zeb1	0.61	0.28606686	pigs
Elf1	0.36	0.28935177	pigs
Csnk1g2	0.22	0.29031491	pigs
Sec24b	0.17	0.29849109	pigs
Rgs16	0.39	0.30546537	pigs
Hivep2	0.46	0.30819023	pigs
Fosl2	0.29	0.31030246	pigs
Cyth4	0.46	0.31160919	pigs
Phc2	0.23	0.31235023	pigs
Nt5c3	0.39	0.31275596	pigs
Otulin	0.38	0.31758724	pigs
Alcam	0.61	0.36133708	pigs
Arg1	0.39	0.37454529	pigs
Acaca	0.42	0.37550025	pigs
Gpbp1	0.36	0.38417280	pigs
Ppp4r3a	0.32	0.38861862	pigs
Uap1	0.27	0.39566749	pigs
Spock2	0.22	0.40288115	pigs
Frmpd4	-0.76	0.42542997	pigs
Arhgap9	0.33	0.43793963	pigs
Nemp2	0.24	0.43982681	pigs
Eif2ak3	0.31	0.46076354	pigs
Cert1	0.35	0.46320295	pigs
Klf10	0.37	0.47673570	pigs
Rgs1	0.13	0.47972318	pigs
Txndc5	0.20	0.50934342	pigs
Klhl5	-0.91	0.51347363	pigs

Rhof	0.26	0.53595841	pigs
Npepps	0.27	0.53765103	pigs
Rnf139	0.27	0.54075491	pigs
Xpo5	0.30	0.54389631	pigs
Ttc23l	0.26	0.55034769	pigs
Tmem11	0.21	0.55213984	pigs
Rara	0.39	0.55437131	pigs
Acat3	0.28	0.55708351	pigs
Diaph1	0.26	0.56114704	pigs
Umps	0.24	0.56220876	pigs
Cfl1	0.26	0.58162535	pigs
Ggh	0.24	0.60132277	pigs
Tma7	-0.55	0.60187610	pigs
Ppp2r1b	0.32	0.60489024	pigs
Wdr76	-0.59	0.61052777	pigs
Dnajb14	0.28	0.65814813	pigs
Spp1	0.39	0.66102167	pigs
Eif5b	0.29	0.67037001	pigs
B3gnt2	0.33	0.67715571	pigs
Sdc2	0.28	0.68337484	pigs
Slc41a2	0.32	0.69179427	pigs
Rab9	0.34	0.69474586	pigs
Atp6v0b	0.35	0.69510560	pigs
Ctdsp1	-0.50	0.70776992	pigs
Scpep1	0.46	0.70948055	pigs
Rnf216	0.28	0.71167963	pigs
Plcl2	-0.70	0.72543575	pigs
Shisa2	0.42	0.72571497	pigs
Cdk16	0.25	0.72863852	pigs
Rasa3	-0.72	0.72878580	pigs
Itgal	0.23	0.73208296	pigs
Plekho1	0.13	0.74140728	pigs
Tox	1.03	0.75150583	pigs
Lrmda	-0.53	0.75837260	pigs
Etv6	0.38	0.76232192	pigs
Cox17	-0.66	0.77360813	pigs
Tpp2	0.38	0.77887908	pigs
Cyp51	0.44	0.77959212	pigs
Stag2	0.33	0.79086188	pigs
Kidins220	0.41	0.79231529	pigs
4932411N23Rik	0.23	0.79297699	pigs
Pla2g7	-0.34	0.79681949	pigs
Nampt	0.38	0.80870655	pigs
Pced1b	0.44	0.82125561	pigs
Sec61g	-0.58	0.82770597	pigs
Pum1	0.34	0.86057433	pigs

Tspan31	0.27	0.87155766	pigs
Serf2	-0.43	0.87894339	pigs
Cct3	0.28	0.89585417	pigs
Dmap1	0.26	0.89867473	pigs
Dyrk1a	0.40	0.90168086	pigs
Zdhhc17	0.32	0.90366286	pigs
Pus7	0.38	0.90709813	pigs
Plekhf2	-0.61	0.91881342	pigs
Luc7l3	0.38	0.92027060	pigs
Ahsa1	0.20	0.94418753	pigs
Smg1	0.43	0.94543267	pigs
Ptdss1	0.36	0.96148353	pigs
Kynu	0.35	0.97759153	pigs
Cdyl2	0.33	1.00000000	pigs
Sec14l1	0.42	1.00000000	pigs
Csnk1d	0.29	1.00000000	pigs
Fmnl2	0.68	1.00000000	pigs
Sat1	0.19	1.00000000	pigs
Abcb9	0.14	1.00000000	pigs
Cflar	0.45	1.00000000	pigs
Jak2	0.40	1.00000000	pigs
Sun1	0.17	1.00000000	pigs
Il21r	0.45	1.00000000	pigs
Rad23b	0.26	1.00000000	pigs
Rab3gap2	0.26	1.00000000	pigs
Lpin2	0.33	1.00000000	pigs
Pgk1	0.29	1.00000000	pigs
Pwwp2a	0.28	1.00000000	pigs
Actn1	0.24	1.00000000	pigs
Pxn	-0.41	1.00000000	pigs
Sf3b3	0.20	1.00000000	pigs
Hif1a	0.53	1.00000000	pigs
Rexo1	0.28	1.00000000	pigs
Zfp644	0.35	1.00000000	pigs
Lamp2	0.29	1.00000000	pigs
Tuba4a	0.22	1.00000000	pigs
1810037I17Rik	0.24	1.00000000	pigs
Ctu2	0.18	1.00000000	pigs
Kdm6a	0.29	1.00000000	pigs
Ilrun	0.34	1.00000000	pigs
Daam1	0.17	1.00000000	pigs
Kpna3	0.29	1.00000000	pigs
Pknox1	0.34	1.00000000	pigs
Naa16	0.17	1.00000000	pigs
Stat3	0.42	1.00000000	pigs
Prr29	0.16	1.00000000	pigs

Prkar2a	0.45	1.00000000	pigs
Tia1	0.19	1.00000000	pigs
Ints14	0.27	1.00000000	pigs
Plagl1	0.24	1.00000000	pigs
Trim2	0.36	1.00000000	pigs
Fnip1	0.43	1.00000000	pigs
Abhd6	0.23	1.00000000	pigs
Kcnc3	0.28	1.00000000	pigs
Xpnpep1	0.20	1.00000000	pigs
Ap1ar	0.33	1.00000000	pigs
Tkt	0.16	1.00000000	pigs
Rsf1	0.34	1.00000000	pigs
Ubn1	0.20	1.00000000	pigs
Prrg4	0.24	1.00000000	pigs
Fhit	0.54	1.00000000	pigs
Exo1	0.47	1.00000000	pigs
Abl2	0.29	1.00000000	pigs
Clasp1	0.32	1.00000000	pigs
Pop7	0.25	1.00000000	pigs
Btbd9	0.38	1.00000000	pigs
Dr1	0.26	1.00000000	pigs
Ywhab	-0.48	1.00000000	pigs
Srsf4	0.21	1.00000000	pigs
Kdm5c	0.26	1.00000000	pigs
Esf1	0.28	1.00000000	pigs
Idi1	0.26	1.00000000	pigs
Nf1	0.27	1.00000000	pigs
Stk17b	0.38	1.00000000	pigs
Primpol	-0.58	1.00000000	pigs
Ntan1	-0.66	1.00000000	pigs
Cltc	0.32	1.00000000	pigs
Tpst1	0.44	1.00000000	pigs
Smc4	0.33	1.00000000	pigs
Stt3b	0.33	1.00000000	pigs
Rsbn1	0.24	1.00000000	pigs
Tbrg4	0.31	1.00000000	pigs
Abhd5	0.33	1.00000000	pigs
Bnip3l	0.19	1.00000000	pigs
Smap1	0.28	1.00000000	pigs
Ybx3	0.27	1.00000000	pigs
Txnip	-0.43	1.00000000	pigs
Ric8b	0.26	1.00000000	pigs
Smurf2	0.34	1.00000000	pigs
Tiparp	0.48	1.00000000	pigs
Wars	0.24	1.00000000	pigs
Rc3h2	0.19	1.00000000	pigs

Tnfrsf1b	0.38	1.00000000	pigs
Tbc1d1	0.19	1.00000000	pigs
Tmed8	0.30	1.00000000	pigs
Pgs1	0.32	1.00000000	pigs
Tsc1	0.19	1.00000000	pigs
Ubr5	0.29	1.00000000	pigs
Hivep1	0.40	1.00000000	pigs
Nkg7	0.27	1.00000000	pigs
Glud1	0.28	1.00000000	pigs
Dhx36	0.27	1.00000000	pigs
Dkc1	0.28	1.00000000	pigs
Selenoi	0.40	1.00000000	pigs
Ewsr1	0.30	1.00000000	pigs
Coro1a	0.35	1.00000000	pigs
Uggt1	0.25	1.00000000	pigs
Tgfb1	0.21	1.00000000	pigs
Fosb	0.50	1.00000000	pigs
Phc3	0.20	1.00000000	pigs
Sh2b2	0.19	1.00000000	pigs
Ndrg1	0.31	1.00000000	pigs
Npc2	0.25	1.00000000	pigs
Nol7	-0.58	1.00000000	pigs
Pitpnm2	0.28	1.00000000	pigs
Zfr	0.35	1.00000000	pigs
Arid3a	0.23	1.00000000	pigs
Ppif	0.42	1.00000000	pigs
Qsox2	0.20	1.00000000	pigs
Socs3	0.14	1.00000000	pigs
Atp2a2	0.34	1.00000000	pigs
Rgcc	0.11	1.00000000	pigs
Kdm3a	0.27	1.00000000	pigs
Lmtk2	-0.90	1.00000000	pigs
Rnf122	0.23	1.00000000	pigs
Dedd	0.24	1.00000000	pigs
Gigyf2	0.33	1.00000000	pigs
Odc1	0.35	1.00000000	pigs
Atp8a1	-0.50	1.00000000	pigs
Atp5k	-0.68	1.00000000	pigs
Orc4	0.34	1.00000000	pigs
Psmb7	0.27	1.00000000	pigs
Rlf	0.35	1.00000000	pigs
Drap1	0.25	1.00000000	pigs
Traf7	0.20	1.00000000	pigs
Ncoa3	0.39	1.00000000	pigs
Narf	0.30	1.00000000	pigs
Isg20	0.28	1.00000000	pigs

Zcchc14	0.28	1.00000000	pigs
Chchd5	0.26	1.00000000	pigs
Eif3m	0.17	1.00000000	pigs
Pdha1	0.29	1.00000000	pigs
Gapvd1	0.31	1.00000000	pigs
Ccnc	0.26	1.00000000	pigs
Opa1	0.25	1.00000000	pigs
Fbh1	0.28	1.00000000	pigs
Rasgef1b	0.16	1.00000000	pigs
Cast	-0.56	1.00000000	pigs
Ints6	0.43	1.00000000	pigs
Eif1	-0.33	1.00000000	pigs
Steep1	0.18	1.00000000	pigs
Tent4a	0.22	1.00000000	pigs
Ikbkg	0.08	1.00000000	pigs
Pfkfb3	0.29	1.00000000	pigs
Tnip2	0.32	1.00000000	pigs
Zfp2	0.15	1.00000000	pigs
Cnot7	0.25	1.00000000	pigs
Galnt10	0.39	1.00000000	pigs
Pfkl	0.24	1.00000000	pigs
Pax5	-0.62	1.00000000	pigs
Klhl15	0.53	1.00000000	pigs
Etf1	0.27	1.00000000	pigs
Lap3	0.16	1.00000000	pigs
Ogdh	0.26	1.00000000	pigs
Pxylp1	0.30	1.00000000	pigs
Zfp106	0.31	1.00000000	pigs
Tfg	0.08	1.00000000	pigs
Yae1d1	0.20	1.00000000	pigs
Atp2c1	0.17	1.00000000	pigs
Tapt1	0.29	1.00000000	pigs
Jarid2	0.30	1.00000000	pigs
Nr4a3	0.29	1.00000000	pigs
Ube2k	0.20	1.00000000	pigs
Usp10	0.35	1.00000000	pigs
Elovl1	0.20	1.00000000	pigs
Stat1	0.31	1.00000000	pigs
Abcg4	0.16	1.00000000	pigs
Ktn1	0.20	1.00000000	pigs
Ing1	0.05	1.00000000	pigs
Lyst	-0.70	1.00000000	pigs
Polh	0.27	1.00000000	pigs
Cox7a2l	0.25	1.00000000	pigs
Pik3r1	0.28	1.00000000	pigs
Ndfip1	0.43	1.00000000	pigs

Washc5	0.20	1.00000000	pigs
Fam172a	-0.60	1.00000000	pigs
Npat	0.32	1.00000000	pigs
Ankib1	0.25	1.00000000	pigs
Tuba1b	0.16	1.00000000	pigs
Txndc12	0.25	1.00000000	pigs
Polr2b	0.25	1.00000000	pigs
Gpbp1l1	0.26	1.00000000	pigs
Emp3	-0.52	1.00000000	pigs
Tsc22d2	0.26	1.00000000	pigs
Sos1	0.31	1.00000000	pigs
Cybb	0.28	1.00000000	pigs
Pik3ca	0.15	1.00000000	pigs
Setd5	0.30	1.00000000	pigs
Piezo1	0.06	1.00000000	pigs
Sccpdh	0.36	1.00000000	pigs
Ier5	0.31	1.00000000	pigs
Khdrbs1	0.12	1.00000000	pigs
Hipk3	0.24	1.00000000	pigs
Anxa11	0.21	1.00000000	pigs
Nop58	0.20	1.00000000	pigs
Polr1a	0.12	1.00000000	pigs
Dync1h1	0.20	1.00000000	pigs
Nr2c1	0.11	1.00000000	pigs
Tbc1d5	-0.52	1.00000000	pigs
Prkag2	0.39	1.00000000	pigs
Rae1	0.28	1.00000000	pigs
Ugp2	0.23	1.00000000	pigs
Hes1	-0.68	1.00000000	pigs
Smc3	0.27	1.00000000	pigs
Zfp622	0.23	1.00000000	pigs
B4galt5	0.33	1.00000000	pigs
Ikzf1	0.33	1.00000000	pigs
Psmd1	0.20	1.00000000	pigs
Wdr47	0.26	1.00000000	pigs
Itpr1	0.32	1.00000000	pigs
Arhgdib	0.25	1.00000000	pigs
Dennd2c	0.35	1.00000000	pigs
Ezh2	0.42	1.00000000	pigs
Phactr3	-0.62	1.00000000	pigs
Spsb1	0.24	1.00000000	pigs
Snx25	0.15	1.00000000	pigs
Smarcb1	0.16	1.00000000	pigs
Prep	0.26	1.00000000	pigs
Cyfip2	0.33	1.00000000	pigs
Rbmx	0.23	1.00000000	pigs

Cdkn1a	0.17	1.00000000	pigs
Arhgap18	0.19	1.00000000	pigs
Rpap3	0.20	1.00000000	pigs
Ncoa4	0.21	1.00000000	pigs
Papolg	0.18	1.00000000	pigs
Eef1d	0.29	1.00000000	pigs
Slain2	0.28	1.00000000	pigs
Snrnp48	0.32	1.00000000	pigs
Ifnar1	0.20	1.00000000	pigs
Tlk2	0.24	1.00000000	pigs
Crebbp	0.26	1.00000000	pigs
Sec24a	0.09	1.00000000	pigs
Golga7	0.19	1.00000000	pigs
Rab5a	0.19	1.00000000	pigs
Pxk	0.26	1.00000000	pigs
Tmem259	0.17	1.00000000	pigs
Rab1a	0.25	1.00000000	pigs
Rnf2	0.35	1.00000000	pigs
Crip3	-0.26	1.00000000	pigs
Scarb2	0.29	1.00000000	pigs
Irak2	0.31	1.00000000	pigs
Fbxo7	0.19	1.00000000	pigs
Clec16a	0.22	1.00000000	pigs
Slc44a1	-0.71	1.00000000	pigs
Kpna6	0.16	1.00000000	pigs
Pea15a	0.27	1.00000000	pigs
Taf1a	0.23	1.00000000	pigs
Blk	0.26	1.00000000	pigs
Eif2ak2	0.23	1.00000000	pigs
Rbm18	0.21	1.00000000	pigs
Cntln	0.40	1.00000000	pigs
Cep83	0.23	1.00000000	pigs
Xpo1	0.28	1.00000000	pigs
Rnf121	0.37	1.00000000	pigs
Cir1	0.21	1.00000000	pigs
Lasp1	0.19	1.00000000	pigs
Ubqln1	0.32	1.00000000	pigs
Ralgapa1	0.38	1.00000000	pigs
Rela	0.21	1.00000000	pigs
Pfdn4	0.22	1.00000000	pigs
Ncor2	0.18	1.00000000	pigs
Ecpas	0.22	1.00000000	pigs
Cul1	0.18	1.00000000	pigs
Ndufs5	0.24	1.00000000	pigs
Lcor	0.35	1.00000000	pigs
Fabp5	0.32	1.00000000	pigs

Abhd13	0.09	1.00000000	pigs
Tmem154	-0.65	1.00000000	pigs
Cops8	0.26	1.00000000	pigs
Unc119	0.20	1.00000000	pigs
Psap	0.31	1.00000000	pigs
Tnip3	0.44	1.00000000	pigs
Bmpr1a	0.31	1.00000000	pigs
Sugct	0.31	1.00000000	pigs
Trim25	-0.01	1.00000000	pigs
Vmp1	0.30	1.00000000	pigs
Nup98	0.29	1.00000000	pigs
Aste1	-0.30	1.00000000	pigs
Strn3	0.29	1.00000000	pigs
Ola1	0.32	1.00000000	pigs
Cst3	-2.42	1.00000000	pigs
Slc25a5	0.19	1.00000000	pigs
Pcgf5	0.28	1.00000000	pigs
Arfgef1	0.23	1.00000000	pigs
Arhgef18	-0.67	1.00000000	pigs
Lix1l	0.13	1.00000000	pigs
Ipo7	0.30	1.00000000	pigs
Hbp1	0.18	1.00000000	pigs
Dusp11	0.33	1.00000000	pigs
Urm1	0.15	1.00000000	pigs
Nktr	0.19	1.00000000	pigs
Ptpn22	0.35	1.00000000	pigs
Jak3	0.17	1.00000000	pigs
Kdm7a	0.21	1.00000000	pigs
Smg5	0.19	1.00000000	pigs
Pdcd1lg2	0.47	1.00000000	pigs
S100a8	-0.71	1.00000000	pigs
Ahr	0.25	1.00000000	pigs
Dhx33	0.22	1.00000000	pigs
Dnajc3	0.12	1.00000000	pigs
Ubr4	0.16	1.00000000	pigs
Txlng	0.18	1.00000000	pigs
Otud4	0.20	1.00000000	pigs
Sorl1	0.02	1.00000000	pigs
Secisbp2l	0.20	1.00000000	pigs
Cep95	0.16	1.00000000	pigs
Bach1	0.27	1.00000000	pigs
Sde2	0.19	1.00000000	pigs
Dctn4	0.27	1.00000000	pigs
Pdcd7	0.14	1.00000000	pigs
Zfand5	0.23	1.00000000	pigs
Lrp10	0.17	1.00000000	pigs

Napsa	0.33	1.00000000	pigs
Arhgap12	0.21	1.00000000	pigs
Camta2	0.16	1.00000000	pigs
Adss	0.24	1.00000000	pigs
Slc25a36	0.27	1.00000000	pigs
Pafah1b1	0.32	1.00000000	pigs
Clcc1	0.17	1.00000000	pigs
Cops4	0.15	1.00000000	pigs
Ruvbl1	0.25	1.00000000	pigs
Tm2d3	0.15	1.00000000	pigs
Rrn3	0.30	1.00000000	pigs
Iws1	0.26	1.00000000	pigs
Kmt2c	0.26	1.00000000	pigs
Pmepa1	0.35	1.00000000	pigs
Add3	-0.37	1.00000000	pigs
Plekhb2	0.20	1.00000000	pigs
Ak3	0.21	1.00000000	pigs
Ppp1ca	0.20	1.00000000	pigs
Sod2	0.40	1.00000000	pigs
Parp1	0.09	1.00000000	pigs
Pptc7	0.23	1.00000000	pigs
Bud23	0.13	1.00000000	pigs
Irf4	0.21	1.00000000	pigs
Wdr12	0.26	1.00000000	pigs
Tagap1	0.33	1.00000000	pigs
Cfp	0.14	1.00000000	pigs
Bloc1s6	0.18	1.00000000	pigs
Ppfia1	0.29	1.00000000	pigs
Txlna	0.11	1.00000000	pigs
Tbca	0.14	1.00000000	pigs
Ksr1	0.33	1.00000000	pigs
Slc23a2	0.13	1.00000000	pigs
Pde7a	0.37	1.00000000	pigs
Larp1	0.21	1.00000000	pigs
Tec	0.28	1.00000000	pigs
Elob	-0.49	1.00000000	pigs
Zmym2	0.20	1.00000000	pigs
Stk26	0.31	1.00000000	pigs
Gramd1a	0.15	1.00000000	pigs
Tap1	0.08	1.00000000	pigs
Echdc1	0.21	1.00000000	pigs
Baz1a	0.29	1.00000000	pigs
Zfp280c	0.27	1.00000000	pigs
Srgn	0.08	1.00000000	pigs
Nle1	0.19	1.00000000	pigs
Sipa1	0.16	1.00000000	pigs

Tsr1	0.11	1.00000000	pigs
Akr1a1	0.12	1.00000000	pigs
G3bp2	0.23	1.00000000	pigs
Gpnmb	0.26	1.00000000	pigs
Ddx17	0.18	1.00000000	pigs
Usp11	0.23	1.00000000	pigs
Slc9a8	0.24	1.00000000	pigs
Sdhb	0.20	1.00000000	pigs
Stx4a	0.17	1.00000000	pigs
Amfr	0.18	1.00000000	pigs
Abcg3	-0.53	1.00000000	pigs
Ambn	0.17	1.00000000	pigs
Gemin4	0.19	1.00000000	pigs
Ddx47	0.15	1.00000000	pigs
Ptp4a1	0.16	1.00000000	pigs
Hspa4	0.08	1.00000000	pigs
Taf1d	0.25	1.00000000	pigs
Nfil3	0.21	1.00000000	pigs
Usp25	0.16	1.00000000	pigs
Dnajb6	0.20	1.00000000	pigs
Nedd4l	0.37	1.00000000	pigs
Wdfy1	0.27	1.00000000	pigs
Tgfbr2	0.25	1.00000000	pigs
Rest	0.27	1.00000000	pigs
Dnajc13	0.28	1.00000000	pigs
Pola1	0.19	1.00000000	pigs
Atg16l1	0.20	1.00000000	pigs
Ndufv1	0.17	1.00000000	pigs
Cblb	-0.59	1.00000000	pigs
Kcns3	0.19	1.00000000	pigs
Copb1	0.07	1.00000000	pigs
Abca1	0.34	1.00000000	pigs
Tmem183a	0.12	1.00000000	pigs
Slc38a2	0.21	1.00000000	pigs
Adamts6	-0.25	1.00000000	pigs
Atl2	0.17	1.00000000	pigs
Gspt1	0.23	1.00000000	pigs
Tm7sf3	0.20	1.00000000	pigs
Arf5	0.19	1.00000000	pigs
Wac	0.27	1.00000000	pigs
Thoc2	0.28	1.00000000	pigs
Wbp11	0.27	1.00000000	pigs
Zbtb1	0.21	1.00000000	pigs
Rasa2	0.27	1.00000000	pigs
Clic5	0.61	1.00000000	pigs
Phactr1	-0.53	1.00000000	pigs

Golm2	0.21	1.00000000	pigs
Ankrd55	-0.56	1.00000000	pigs
Setd3	0.12	1.00000000	pigs
Evl	0.18	1.00000000	pigs
Entpd4	0.15	1.00000000	pigs
Slc19a2	0.23	1.00000000	pigs
Eif3d	0.19	1.00000000	pigs
Fam110a	0.21	1.00000000	pigs
Luc7l	0.15	1.00000000	pigs
Klhdc10	0.40	1.00000000	pigs
Qars	0.04	1.00000000	pigs
Fmnl3	0.24	1.00000000	pigs
Ppp2r2a	0.29	1.00000000	pigs
Rfxank	0.23	1.00000000	pigs
Rnf10	0.10	1.00000000	pigs
Fdft1	0.30	1.00000000	pigs
Lrp8	0.14	1.00000000	pigs
Pias2	0.20	1.00000000	pigs
Oaz1	-0.44	1.00000000	pigs
Rnf157	0.36	1.00000000	pigs
Trappc4	-0.50	1.00000000	pigs
Calr	0.29	1.00000000	pigs
Ubl7	0.25	1.00000000	pigs
Aplf	0.24	1.00000000	pigs
Atf4	0.24	1.00000000	pigs
Cbfb	0.18	1.00000000	pigs
Golga5	0.14	1.00000000	pigs
Huwe1	0.32	1.00000000	pigs
Ccdc32	0.17	1.00000000	pigs
Tnks2	0.22	1.00000000	pigs
Ankrd12	0.34	1.00000000	pigs
Gramd3	0.28	1.00000000	pigs
Prcc	0.13	1.00000000	pigs
Cdc34	0.20	1.00000000	pigs
Gng10	0.18	1.00000000	pigs
Vkorc1l1	0.13	1.00000000	pigs
Tlk1	0.29	1.00000000	pigs
4933434E20Rik	0.16	1.00000000	pigs
Vps54	0.33	1.00000000	pigs
Psme4	0.25	1.00000000	pigs
Tspan14	-0.02	1.00000000	pigs
Csrnp2	0.14	1.00000000	pigs
Exoc5	0.13	1.00000000	pigs
Dctn2	0.25	1.00000000	pigs
Snx13	0.14	1.00000000	pigs
Gpr82	0.17	1.00000000	pigs

Emc10	0.21	1.00000000	pigs
Pdp2	0.20	1.00000000	pigs
Tnpo1	0.17	1.00000000	pigs
Foxs1	0.17	1.00000000	pigs
Fam126a	0.21	1.00000000	pigs
Zfp831	0.28	1.00000000	pigs
Kars	0.22	1.00000000	pigs
Tmub1	0.20	1.00000000	pigs
Bst2	0.19	1.00000000	pigs
Rasal1	0.35	1.00000000	pigs
Ap1s1	0.25	1.00000000	pigs
Gatad2a	0.21	1.00000000	pigs
Tifa	0.14	1.00000000	pigs
Usp42	0.20	1.00000000	pigs
Armc1	0.07	1.00000000	pigs
Cacna1e	0.14	1.00000000	pigs
Sacs	0.14	1.00000000	pigs
Dcaf1	0.08	1.00000000	pigs
Csgalnact2	0.25	1.00000000	pigs
Acsf2	0.18	1.00000000	pigs
Cabyr	0.23	1.00000000	pigs
Dap	-0.51	1.00000000	pigs
Rock2	0.10	1.00000000	pigs
Adgb	0.17	1.00000000	pigs
Ipo9	0.15	1.00000000	pigs
Edem1	0.17	1.00000000	pigs
Ly75	0.15	1.00000000	pigs
Ap5m1	0.30	1.00000000	pigs
Rpp40	0.17	1.00000000	pigs
Llgl1	0.16	1.00000000	pigs
Avpi1	0.10	1.00000000	pigs
Hmces	-0.69	1.00000000	pigs
Brwd3	0.16	1.00000000	pigs
Tmub2	0.15	1.00000000	pigs
Rp2	0.30	1.00000000	pigs
Batf3	0.30	1.00000000	pigs
Pecam1	-0.66	1.00000000	pigs
Rcc2	0.23	1.00000000	pigs
Btf3	0.13	1.00000000	pigs
Txnl1	0.15	1.00000000	pigs
Polr1c	0.13	1.00000000	pigs
Ppa1	0.11	1.00000000	pigs
Pcf11	0.20	1.00000000	pigs
Ptges3	0.13	1.00000000	pigs
Psmc6	0.18	1.00000000	pigs
Nufip2	0.19	1.00000000	pigs

Copa	0.23	1.00000000	pigs
Aplp2	0.25	1.00000000	pigs
Kdm6b	0.25	1.00000000	pigs
Ptk2	0.17	1.00000000	pigs
Nek6	0.28	1.00000000	pigs
Ints13	0.21	1.00000000	pigs
Dguok	0.19	1.00000000	pigs
Ulk2	0.22	1.00000000	pigs
Acap2	0.35	1.00000000	pigs
Vav1	0.20	1.00000000	pigs
Safb	0.17	1.00000000	pigs
Ube2d3	0.23	1.00000000	pigs
Bzw1	-0.30	1.00000000	pigs
Sugp1	0.16	1.00000000	pigs
Nudt21	0.09	1.00000000	pigs
Ripk2	0.23	1.00000000	pigs
Zc3hav1	0.32	1.00000000	pigs
Cyld	0.21	1.00000000	pigs
Slc5a10	0.14	1.00000000	pigs
Larp4	0.24	1.00000000	pigs
Tcp11l1	0.18	1.00000000	pigs
Dynlt1a	0.14	1.00000000	pigs
Nipsnap3b	0.14	1.00000000	pigs
Atp6ap2	0.05	1.00000000	pigs
Zc3h7a	0.17	1.00000000	pigs
Ndufa13	-0.57	1.00000000	pigs
Gnptab	0.33	1.00000000	pigs
Ddx20	0.18	1.00000000	pigs
Notch2	-0.45	1.00000000	pigs
Lat2	0.11	1.00000000	pigs
Pacsin2	0.21	1.00000000	pigs
Tead3	0.24	1.00000000	pigs
Cct7	0.16	1.00000000	pigs
Ctsa	0.15	1.00000000	pigs
Abraxas2	0.16	1.00000000	pigs
Wdr3	0.17	1.00000000	pigs
Lpin1	0.26	1.00000000	pigs
Cdv3	0.19	1.00000000	pigs
Araf	0.12	1.00000000	pigs
Cdk9	0.04	1.00000000	pigs
Rab30	0.38	1.00000000	pigs
Rngtt	0.18	1.00000000	pigs
Tmcc3	0.29	1.00000000	pigs
Ulk4	-0.38	1.00000000	pigs
Rufy1	-0.68	1.00000000	pigs
Nt5c2	0.20	1.00000000	pigs

Rlim	0.22	1.00000000	pigs
Lrrd1	-0.25	1.00000000	pigs
Aspscr1	0.05	1.00000000	pigs
Slc5a3	0.11	1.00000000	pigs
Crebzf	0.14	1.00000000	pigs
9130409I23Rik	0.12	1.00000000	pigs
Ube2r2	0.19	1.00000000	pigs
Tm2d2	0.28	1.00000000	pigs
Slc7a5	0.19	1.00000000	pigs
Atp5pb	0.19	1.00000000	pigs
Cd180	0.22	1.00000000	pigs
Fam78a	0.11	1.00000000	pigs
Saraf	0.19	1.00000000	pigs
Abhd18	0.29	1.00000000	pigs
Urb2	0.21	1.00000000	pigs
Bach2	-0.80	1.00000000	pigs
Cda	-0.67	1.00000000	pigs
Ptpn11	0.16	1.00000000	pigs
Per1	0.31	1.00000000	pigs
Pnkd	-0.49	1.00000000	pigs
Cenpm	0.32	1.00000000	pigs
Tcerg1	0.12	1.00000000	pigs
Wsb2	0.16	1.00000000	pigs
Rab11fip2	0.22	1.00000000	pigs
Zfp410	0.29	1.00000000	pigs
Epb41	0.20	1.00000000	pigs
Eogt	0.17	1.00000000	pigs
Trap1	0.21	1.00000000	pigs
Ptpn12	0.21	1.00000000	pigs
Itgb1	0.20	1.00000000	pigs
Arhgef3	0.19	1.00000000	pigs
Pnn	0.16	1.00000000	pigs
Dhrs3	0.19	1.00000000	pigs
Api5	0.23	1.00000000	pigs
Ppp3cb	0.20	1.00000000	pigs
Arid5a	0.03	1.00000000	pigs
Il1a	0.42	1.00000000	pigs
Hmgxb4	0.12	1.00000000	pigs
Snd1	0.16	1.00000000	pigs
Snx3	0.11	1.00000000	pigs
Clpx	0.23	1.00000000	pigs
F5	-0.37	1.00000000	pigs
Ddx54	0.18	1.00000000	pigs
Lamtor1	0.15	1.00000000	pigs
Naa25	0.20	1.00000000	pigs
Apbb1ip	-0.57	1.00000000	pigs

Comt	0.12	1.00000000	pigs
Akirin1	0.20	1.00000000	pigs
Clock	0.23	1.00000000	pigs
Tmem165	0.10	1.00000000	pigs
Tcof1	0.15	1.00000000	pigs
Pigt	0.18	1.00000000	pigs
Ralgps2	-0.36	1.00000000	pigs
Tnpo3	0.23	1.00000000	pigs
Hmgcr	0.22	1.00000000	pigs
Ncln	0.08	1.00000000	pigs
Trafd1	0.12	1.00000000	pigs
Azi2	0.20	1.00000000	pigs
Hacd3	0.19	1.00000000	pigs
Bphl	0.13	1.00000000	pigs
Ccdc174	0.15	1.00000000	pigs
Rnf103	0.03	1.00000000	pigs
Ap1s2	0.13	1.00000000	pigs
Zfp236	0.17	1.00000000	pigs
Uqcrc1	0.21	1.00000000	pigs
Osbpl8	0.27	1.00000000	pigs
Nsun2	0.19	1.00000000	pigs
Zswim8	0.10	1.00000000	pigs
Fbxo28	0.23	1.00000000	pigs
Tnfaip6	0.14	1.00000000	pigs
Glul	0.16	1.00000000	pigs
Ccdc142	0.09	1.00000000	pigs
Rhbdd1	0.15	1.00000000	pigs
Cnot6l	0.29	1.00000000	pigs
Ppp2r5e	0.23	1.00000000	pigs
Asxl1	0.32	1.00000000	pigs
Esyt2	0.13	1.00000000	pigs
Ghitm	0.10	1.00000000	pigs
Gpr107	0.18	1.00000000	pigs
Antxr2	-0.05	1.00000000	pigs
Tnip1	0.20	1.00000000	pigs
Eaf1	0.17	1.00000000	pigs
E2f3	0.12	1.00000000	pigs
Nsmce1	0.10	1.00000000	pigs
Pdxk	0.09	1.00000000	pigs
Nop2	0.14	1.00000000	pigs
Ago1	0.08	1.00000000	pigs
Tmem189	0.15	1.00000000	pigs
Rnf115	0.30	1.00000000	pigs
Fryl	0.28	1.00000000	pigs
Itpk1	0.21	1.00000000	pigs
Cenpj	0.16	1.00000000	pigs

Trnau1ap	0.26	1.00000000	pigs
Nup43	0.12	1.00000000	pigs
Rbm38	0.14	1.00000000	pigs
Cox7c	-0.38	1.00000000	pigs
Rcl1	0.29	1.00000000	pigs
Stx11	0.29	1.00000000	pigs
Srp19	0.00	1.00000000	pigs
Nol10	0.11	1.00000000	pigs
Rsrc2	0.18	1.00000000	pigs
Trim28	0.18	1.00000000	pigs
Ppp1cc	0.17	1.00000000	pigs
Usp18	0.06	1.00000000	pigs
Tet3	0.26	1.00000000	pigs
Tsr2	0.17	1.00000000	pigs
8030462N17Rik	0.15	1.00000000	pigs
Tmem184b	0.15	1.00000000	pigs
Zpr1	0.13	1.00000000	pigs
Fxr1	0.20	1.00000000	pigs
Cep63	0.13	1.00000000	pigs
Hsp90ab1	0.22	1.00000000	pigs
Supt4a	0.10	1.00000000	pigs
Arap2	0.33	1.00000000	pigs
Ska3	0.11	1.00000000	pigs
Hspb11	-0.31	1.00000000	pigs
Spag9	0.06	1.00000000	pigs
Hsf2	0.18	1.00000000	pigs
Nek9	0.26	1.00000000	pigs
Ccdc88c	0.19	1.00000000	pigs
Egr1	-0.71	1.00000000	pigs
Cacybp	0.19	1.00000000	pigs
Ube2q1	-0.50	1.00000000	pigs
Trp53	0.23	1.00000000	pigs
Rnf145	0.19	1.00000000	pigs
Septin2	0.19	1.00000000	pigs
Sumo2	-0.36	1.00000000	pigs
Zfp516	0.15	1.00000000	pigs
Wtap	0.21	1.00000000	pigs
Rnf144b	-0.49	1.00000000	pigs
Ptp4a2	0.19	1.00000000	pigs
Atad2b	0.16	1.00000000	pigs
Axin1	0.19	1.00000000	pigs
Vps53	0.18	1.00000000	pigs
Eps15	0.26	1.00000000	pigs
Clcn7	0.08	1.00000000	pigs
Slc35f2	0.19	1.00000000	pigs
Slc38a10	0.15	1.00000000	pigs

Fndc3a	0.33	1.00000000	pigs
Fnbp1	0.30	1.00000000	pigs
Ice2	0.25	1.00000000	pigs
Sin3a	0.09	1.00000000	pigs
Crebrf	0.15	1.00000000	pigs
Uck2	0.30	1.00000000	pigs
Isg15	0.28	1.00000000	pigs
Dcun1d5	0.06	1.00000000	pigs
Prkx	0.11	1.00000000	pigs
Rrp1b	0.10	1.00000000	pigs
Phf6	0.27	1.00000000	pigs
Foxo1	0.26	1.00000000	pigs
Serac1	0.07	1.00000000	pigs
Qdpr	-0.56	1.00000000	pigs
Sik2	0.25	1.00000000	pigs
Ero1b	0.23	1.00000000	pigs
Tyk2	0.16	1.00000000	pigs
Dhrs7b	0.16	1.00000000	pigs
Ifrd2	0.15	1.00000000	pigs
Aff1	0.24	1.00000000	pigs
Tbc1d23	0.27	1.00000000	pigs
Hadhb	0.17	1.00000000	pigs
Pik3r6	0.14	1.00000000	pigs
Becn1	0.15	1.00000000	pigs
Dusp10	0.06	1.00000000	pigs
Ipo8	0.08	1.00000000	pigs
Pard6g	-0.26	1.00000000	pigs
Resf1	0.18	1.00000000	pigs
Wsb1	0.20	1.00000000	pigs
Dnajc11	0.17	1.00000000	pigs
Atxn1	0.19	1.00000000	pigs
Rprd1a	0.15	1.00000000	pigs
Stx17	0.10	1.00000000	pigs
Hemgn	-0.58	1.00000000	pigs
Zbtb10	0.22	1.00000000	pigs
Oxct1	-0.55	1.00000000	pigs
Sbds	0.16	1.00000000	pigs
Retreg3	0.21	1.00000000	pigs
Ik	-0.55	1.00000000	pigs
Phkg2	0.28	1.00000000	pigs
Smarca4	0.13	1.00000000	pigs
Ccnt2	0.14	1.00000000	pigs
Fbxl15	0.15	1.00000000	pigs
Atp11c	0.17	1.00000000	pigs
Nip7	0.10	1.00000000	pigs
Sypl	0.03	1.00000000	pigs

Ndel1	0.11	1.00000000	pigs
Osbpl9	0.26	1.00000000	pigs
Lsm14a	-0.04	1.00000000	pigs
Srsf11	0.12	1.00000000	pigs
Psip1	0.18	1.00000000	pigs
Ammecr1l	0.06	1.00000000	pigs
Papola	0.15	1.00000000	pigs
Cnpy3	0.03	1.00000000	pigs
Acer3	0.14	1.00000000	pigs
Larp1b	0.16	1.00000000	pigs
Cdc27	0.09	1.00000000	pigs
Lpxn	0.16	1.00000000	pigs
Cox18	0.07	1.00000000	pigs
Srpk1	0.24	1.00000000	pigs
Hexb	0.15	1.00000000	pigs
Dusp16	0.16	1.00000000	pigs
Naa15	0.15	1.00000000	pigs
Smg7	0.21	1.00000000	pigs
Ppp1r8	0.17	1.00000000	pigs
Nat10	0.11	1.00000000	pigs
Nop53	0.04	1.00000000	pigs
Thyn1	0.13	1.00000000	pigs
Gca	0.08	1.00000000	pigs
Plscr3	0.14	1.00000000	pigs
Gtf2h1	0.18	1.00000000	pigs
Usp39	0.16	1.00000000	pigs
Plagl2	0.13	1.00000000	pigs
Naaa	-0.01	1.00000000	pigs
Sun2	-0.60	1.00000000	pigs
Tnik	0.32	1.00000000	pigs
Dlst	0.12	1.00000000	pigs
Kdelr2	0.12	1.00000000	pigs
Akap11	0.10	1.00000000	pigs
Elmo2	0.14	1.00000000	pigs
Rad51b	0.21	1.00000000	pigs
Ift52	0.06	1.00000000	pigs
Atm	-0.39	1.00000000	pigs
Ctdspl2	0.20	1.00000000	pigs
Fbxo11	0.18	1.00000000	pigs
Prdm1	0.12	1.00000000	pigs
Kat6b	0.28	1.00000000	pigs
Dnajc14	0.14	1.00000000	pigs
Rep15	0.15	1.00000000	pigs
Rrp7a	0.14	1.00000000	pigs
Tasor2	0.13	1.00000000	pigs
Htatsf1	0.18	1.00000000	pigs

Ddx3x	0.19	1.00000000	pigs
Il27ra	0.06	1.00000000	pigs
Dnmt3a	0.04	1.00000000	pigs
Sesn3	0.15	1.00000000	pigs
Dennd11	-0.79	1.00000000	pigs
Hprt	0.10	1.00000000	pigs
Zfand6	0.17	1.00000000	pigs
Zc3h18	0.23	1.00000000	pigs
Rpn2	0.08	1.00000000	pigs
Cd44	0.11	1.00000000	pigs
Sclt1	0.32	1.00000000	pigs
Bag6	0.07	1.00000000	pigs
Leo1	-0.42	1.00000000	pigs
Nufip1	0.19	1.00000000	pigs
Akt3	0.39	1.00000000	pigs
Ppp2ca	0.16	1.00000000	pigs
Ankrd28	0.20	1.00000000	pigs
Rbm39	0.14	1.00000000	pigs
Lemd2	0.11	1.00000000	pigs
Wdr74	0.13	1.00000000	pigs
Ncf2	0.16	1.00000000	pigs
Ndufb9	-0.45	1.00000000	pigs
Ncoa6	0.15	1.00000000	pigs
Ino80c	0.14	1.00000000	pigs
Nsd1	-0.57	1.00000000	pigs
Hsd17b12	0.18	1.00000000	pigs
Bcl2l13	0.09	1.00000000	pigs
Tmem263	0.07	1.00000000	pigs
Rufy3	0.20	1.00000000	pigs
Dennd4a	0.23	1.00000000	pigs
Psmd3	0.08	1.00000000	pigs
Inpp5a	0.13	1.00000000	pigs
Psma5	0.18	1.00000000	pigs
Cpm	0.32	1.00000000	pigs
Rhoa	0.23	1.00000000	pigs
Kdm4a	0.09	1.00000000	pigs
Pgam5	-0.02	1.00000000	pigs
Hdac5	0.10	1.00000000	pigs
Usp37	0.26	1.00000000	pigs
Rbm12b2	0.11	1.00000000	pigs
Abcc1	0.17	1.00000000	pigs
Zfp277	0.26	1.00000000	pigs
Pan3	0.24	1.00000000	pigs
Adam17	0.08	1.00000000	pigs
Lrrc57	0.10	1.00000000	pigs
Ube2z	0.07	1.00000000	pigs

Bicra	0.15	1.00000000	pigs
Gars	0.13	1.00000000	pigs
Chd1l	0.19	1.00000000	pigs
Numa1	0.08	1.00000000	pigs
Cep68	-0.05	1.00000000	pigs
Cpsf7	0.20	1.00000000	pigs
Tnfaip3	0.41	1.00000000	pigs
Rnase6	-0.47	1.00000000	pigs
Rap1gap2	-0.43	1.00000000	pigs
Fkbp4	0.18	1.00000000	pigs
Tial1	0.09	1.00000000	pigs
Lrrk2	-1.00	1.00000000	pigs
Isoc1	0.09	1.00000000	pigs
9930104L06Rik	0.12	1.00000000	pigs
Flnb	0.16	1.00000000	pigs
Irf2	-0.50	1.00000000	pigs
Gan	0.24	1.00000000	pigs
Chd6	0.06	1.00000000	pigs
Bclaf1	0.21	1.00000000	pigs
Pias1	0.14	1.00000000	pigs
Tgif1	0.28	1.00000000	pigs
Ubp1	0.01	1.00000000	pigs
Cnih4	0.06	1.00000000	pigs
Dpyd	0.19	1.00000000	pigs
Orai1	0.11	1.00000000	pigs
Kansl2	0.16	1.00000000	pigs
Armc8	0.12	1.00000000	pigs
Rabgap1l	0.17	1.00000000	pigs
Nfxl1	0.18	1.00000000	pigs
Zbtb8os	0.17	1.00000000	pigs
Nr1h2	0.09	1.00000000	pigs
Psmd13	0.15	1.00000000	pigs
Cnot2	0.16	1.00000000	pigs
Chka	0.19	1.00000000	pigs
Pkn1	0.14	1.00000000	pigs
Sptlc2	0.30	1.00000000	pigs
1110004F10Rik	-0.40	1.00000000	pigs
Cc2d1b	0.09	1.00000000	pigs
Slc6a9	0.16	1.00000000	pigs
Pin1	0.15	1.00000000	pigs
Ptbp3	0.20	1.00000000	pigs
Taf4	0.19	1.00000000	pigs
Taf9b	0.18	1.00000000	pigs
Ppm1m	0.07	1.00000000	pigs
Twsg1	0.15	1.00000000	pigs
Clk4	0.05	1.00000000	pigs

Laptm4a	0.14	1.00000000	pigs
Sufu	0.27	1.00000000	pigs
Glyr1	0.18	1.00000000	pigs
Tlr7	0.02	1.00000000	pigs
Tmem87a	0.07	1.00000000	pigs
Slc12a2	0.21	1.00000000	pigs
Atp5d	0.04	1.00000000	pigs
Ncapg	0.11	1.00000000	pigs
Rabl6	0.08	1.00000000	pigs
Polr2g	0.03	1.00000000	pigs
H1f4	-0.49	1.00000000	pigs
Vopp1	0.14	1.00000000	pigs
Os9	0.16	1.00000000	pigs
Cmpk2	0.14	1.00000000	pigs
Atp6ap1	0.11	1.00000000	pigs
Ssbp2	-0.73	1.00000000	pigs
Zbtb44	0.21	1.00000000	pigs
Klhl36	0.00	1.00000000	pigs
Zbp1	0.15	1.00000000	pigs
Phtf1	0.27	1.00000000	pigs
Smad3	0.25	1.00000000	pigs
Pphln1	0.06	1.00000000	pigs
Acly	0.07	1.00000000	pigs
Igf2bp3	0.05	1.00000000	pigs
Cd9	-0.37	1.00000000	pigs
Arrdc4	0.66	1.00000000	pigs
Dock8	-0.42	1.00000000	pigs
Endov	0.20	1.00000000	pigs
Tmem19	0.08	1.00000000	pigs
Lamp1	0.13	1.00000000	pigs
1700025G04Rik	0.16	1.00000000	pigs
Uba3	0.05	1.00000000	pigs
Gtf2e2	0.15	1.00000000	pigs
Sco1	0.11	1.00000000	pigs
Fndc7	0.13	1.00000000	pigs
Cdc37	0.17	1.00000000	pigs
Cct5	0.15	1.00000000	pigs
Upf3b	0.10	1.00000000	pigs
Calm1	-0.40	1.00000000	pigs
Ninj1	0.22	1.00000000	pigs
Cct4	0.14	1.00000000	pigs
Fcmr	-0.43	1.00000000	pigs
Plpp1	0.26	1.00000000	pigs
Gbp2b	0.06	1.00000000	pigs
Anapc7	0.17	1.00000000	pigs
Nupl1	0.14	1.00000000	pigs

Ube3c	0.07	1.00000000	pigs
Tmem39a	0.24	1.00000000	pigs
Usp45	0.10	1.00000000	pigs
Scaf4	-0.01	1.00000000	pigs
Gns	0.20	1.00000000	pigs
Churc1	0.07	1.00000000	pigs
Grk2	0.14	1.00000000	pigs
Cnst	0.15	1.00000000	pigs
Tmem30a	-0.04	1.00000000	pigs
Snrpa1	0.22	1.00000000	pigs
Esco1	0.15	1.00000000	pigs
Il7	-0.35	1.00000000	pigs
Lyl1	0.08	1.00000000	pigs
Snx14	0.12	1.00000000	pigs
Uros	0.08	1.00000000	pigs
Usp12	0.11	1.00000000	pigs
Ttc1	0.10	1.00000000	pigs
Il10	0.19	1.00000000	pigs
Ccdc58	0.16	1.00000000	pigs
Arl5a	-0.02	1.00000000	pigs
Oxsr1	0.06	1.00000000	pigs
Agpat4	0.15	1.00000000	pigs
Dennd3	0.08	1.00000000	pigs
Fam193a	0.09	1.00000000	pigs
Hnrnph1	0.15	1.00000000	pigs
Brd2	0.09	1.00000000	pigs
Smcr8	0.18	1.00000000	pigs
Peli1	0.27	1.00000000	pigs
Tnrc6b	-0.36	1.00000000	pigs
App	0.12	1.00000000	pigs
Rif1	-0.05	1.00000000	pigs
Cd22	0.10	1.00000000	pigs
Rtraf	0.09	1.00000000	pigs
Kif18a	0.14	1.00000000	pigs
Zfp64	0.20	1.00000000	pigs
Wbp2	-0.02	1.00000000	pigs
Lrpprc	0.07	1.00000000	pigs
Cep192	0.12	1.00000000	pigs
Septin6	0.04	1.00000000	pigs
Gpaa1	0.10	1.00000000	pigs
Dld	0.12	1.00000000	pigs
Aida	0.13	1.00000000	pigs
Ccdc117	0.08	1.00000000	pigs
Ascc3	-0.05	1.00000000	pigs
Ndufb1	-0.60	1.00000000	pigs
Afmid	0.14	1.00000000	pigs

Fam91a1	0.12	1.00000000	pigs
Uspl1	0.26	1.00000000	pigs
Gtpbp1	0.05	1.00000000	pigs
Stxbp2	0.11	1.00000000	pigs
Prdx6	0.10	1.00000000	pigs
Arid4a	0.26	1.00000000	pigs
Sh3kbp1	0.33	1.00000000	pigs
Oser1	0.15	1.00000000	pigs
Sbf1	0.05	1.00000000	pigs
Gfpt1	0.04	1.00000000	pigs
Pcm1	0.02	1.00000000	pigs
Osgepl1	0.17	1.00000000	pigs
Vars	0.10	1.00000000	pigs
Qrsl1	0.12	1.00000000	pigs
Rassf2	0.16	1.00000000	pigs
F13a1	0.12	1.00000000	pigs
Plxnc1	0.14	1.00000000	pigs
Rai1	0.24	1.00000000	pigs
Ndufaf5	0.16	1.00000000	pigs
B2m	0.16	1.00000000	pigs
Bag3	0.47	1.00000000	pigs
Ankhd1	0.29	1.00000000	pigs
Rab11fip3	0.13	1.00000000	pigs
Cpt1a	0.12	1.00000000	pigs
Ldb1	0.17	1.00000000	pigs
Npm3	0.20	1.00000000	pigs
Ptprj	-0.54	1.00000000	pigs
Pafah1b2	0.05	1.00000000	pigs
1110065P20Rik	0.10	1.00000000	pigs
Yeats2	0.11	1.00000000	pigs
Sgta	0.04	1.00000000	pigs
Cdc25b	-0.25	1.00000000	pigs
Snap29	0.21	1.00000000	pigs
Septin1	0.07	1.00000000	pigs
Srm	0.19	1.00000000	pigs
Trmt11	0.13	1.00000000	pigs
Ssr3	0.09	1.00000000	pigs
Phactr4	0.18	1.00000000	pigs
Poc1a	0.07	1.00000000	pigs
Add1	-0.62	1.00000000	pigs
Pitpnb	0.10	1.00000000	pigs
Rnf34	0.16	1.00000000	pigs
Tfeb	-0.02	1.00000000	pigs
Cenpc1	0.16	1.00000000	pigs
Ganab	0.10	1.00000000	pigs
Gfod1	0.07	1.00000000	pigs

Sh3glb1	-0.41	1.00000000	pigs
Kctd20	-0.40	1.00000000	pigs
Hint3	0.10	1.00000000	pigs
Slc17a5	0.14	1.00000000	pigs
Prpf39	0.14	1.00000000	pigs
Etnk1	0.04	1.00000000	pigs
9530068E07Rik	0.14	1.00000000	pigs
Ccni	0.16	1.00000000	pigs
Sh3bp2	0.15	1.00000000	pigs
Cep152	0.11	1.00000000	pigs
Tmed10	-0.05	1.00000000	pigs
Fam174a	0.03	1.00000000	pigs
Inpp4a	0.33	1.00000000	pigs
Slc35c2	0.06	1.00000000	pigs
Slc30a1	0.07	1.00000000	pigs
Zfand2a	0.11	1.00000000	pigs
Stat6	0.06	1.00000000	pigs
Eed	0.17	1.00000000	pigs
Ipo5	0.13	1.00000000	pigs
Clk1	0.17	1.00000000	pigs
Pitpnm1	0.06	1.00000000	pigs
Usp9x	0.18	1.00000000	pigs
Chmp4b	-0.06	1.00000000	pigs
Rictor	0.07	1.00000000	pigs
Shkbp1	0.15	1.00000000	pigs
Itprid2	0.16	1.00000000	pigs
Ell2	0.20	1.00000000	pigs
Zbtb18	-0.02	1.00000000	pigs
Txndc15	0.04	1.00000000	pigs
Zfp830	0.09	1.00000000	pigs
Shc1	0.11	1.00000000	pigs
Thy1	-0.39	1.00000000	pigs
Pacc1	0.16	1.00000000	pigs
Lig1	0.16	1.00000000	pigs
Pcbd1	0.07	1.00000000	pigs
Sash3	0.11	1.00000000	pigs
Poc1b	0.08	1.00000000	pigs
Zkscan1	0.12	1.00000000	pigs
Trim24	0.14	1.00000000	pigs
Nkrf	0.12	1.00000000	pigs
Ubald1	0.02	1.00000000	pigs
Smndc1	0.11	1.00000000	pigs
Pdzd8	0.10	1.00000000	pigs
Cdc73	0.12	1.00000000	pigs
Ccdc22	0.16	1.00000000	pigs
Gbf1	0.17	1.00000000	pigs

Cpne3	0.03	1.00000000	pigs
Klf13	0.12	1.00000000	pigs
Ddx51	0.16	1.00000000	pigs
Anapc1	0.24	1.00000000	pigs
Ralgapa2	-0.27	1.00000000	pigs
E2f4	0.07	1.00000000	pigs
Klhl24	0.05	1.00000000	pigs
Ssrp1	0.11	1.00000000	pigs
Gpr108	0.11	1.00000000	pigs
Rap2c	0.09	1.00000000	pigs
Heatr1	0.14	1.00000000	pigs
Ube2j1	0.02	1.00000000	pigs
Ndufa9	0.03	1.00000000	pigs
Fbxo8	0.11	1.00000000	pigs
Vta1	0.23	1.00000000	pigs
Serp1	0.07	1.00000000	pigs
Ddx39a	0.14	1.00000000	pigs
Ddx5	0.18	1.00000000	pigs
Rfx7	0.17	1.00000000	pigs
Pum2	0.20	1.00000000	pigs
Rangap1	0.13	1.00000000	pigs
Bcat2	0.03	1.00000000	pigs
Rras	0.08	1.00000000	pigs
Cenpp	0.13	1.00000000	pigs
Hyou1	0.11	1.00000000	pigs
Emb	0.22	1.00000000	pigs
Kdm2b	0.21	1.00000000	pigs
Txndc11	-0.03	1.00000000	pigs
Nolc1	0.20	1.00000000	pigs
Sdhd	0.09	1.00000000	pigs
Rasal2	-0.36	1.00000000	pigs
Cnep1r1	0.04	1.00000000	pigs
Arpc5	0.19	1.00000000	pigs
Usp15	0.09	1.00000000	pigs
Znrf2	0.18	1.00000000	pigs
Grn	0.13	1.00000000	pigs
Pomgnt1	0.15	1.00000000	pigs
Poldip3	0.12	1.00000000	pigs
Ctsh	-0.41	1.00000000	pigs
Slc1a5	0.11	1.00000000	pigs
Phf12	0.15	1.00000000	pigs
Rcbtb2	0.13	1.00000000	pigs
Irf7	-0.01	1.00000000	pigs
Syk	-0.39	1.00000000	pigs
Thumpd1	0.14	1.00000000	pigs
Lpcat3	0.02	1.00000000	pigs

Crkl	0.01	1.00000000	pigs
Cldn15	0.10	1.00000000	pigs
Ermp1	0.19	1.00000000	pigs
Cluh	0.13	1.00000000	pigs
Foxn3	0.20	1.00000000	pigs
Slc38a6	0.25	1.00000000	pigs
Psmb10	0.09	1.00000000	pigs
Flii	0.06	1.00000000	pigs
Il4i1	0.28	1.00000000	pigs
Gtf3c4	0.13	1.00000000	pigs
Herpud1	0.04	1.00000000	pigs
Xab2	-0.01	1.00000000	pigs
Banp	0.05	1.00000000	pigs
Rev3l	0.11	1.00000000	pigs
Naa50	0.09	1.00000000	pigs
Vps36	-0.47	1.00000000	pigs
Efl1	0.02	1.00000000	pigs
Dhrs9	0.16	1.00000000	pigs
Rnf19b	0.17	1.00000000	pigs
Gnl3	0.17	1.00000000	pigs
Uso1	-0.01	1.00000000	pigs
Tmem268	-0.33	1.00000000	pigs
Kbtbd8	0.05	1.00000000	pigs
Atrx	0.19	1.00000000	pigs
Tesk1	-0.15	1.00000000	pigs
Rab35	0.16	1.00000000	pigs
Prkcsh	0.07	1.00000000	pigs
Cwc15	0.07	1.00000000	pigs
Lipa	-0.05	1.00000000	pigs
Phlpp1	0.24	1.00000000	pigs
Helz	0.06	1.00000000	pigs
Dnajb4	0.23	1.00000000	pigs
Txnl4a	0.04	1.00000000	pigs
Rpp30	0.20	1.00000000	pigs
Xpo7	0.14	1.00000000	pigs
9430038I01Rik	0.04	1.00000000	pigs
Aimp1	0.07	1.00000000	pigs
Nup214	-0.04	1.00000000	pigs
Aldoart2	0.11	1.00000000	pigs
Brd7	-0.47	1.00000000	pigs
Fgr	0.23	1.00000000	pigs
Fam53c	0.17	1.00000000	pigs
Ralgapb	0.18	1.00000000	pigs
Ap1b1	0.11	1.00000000	pigs
Gripap1	0.09	1.00000000	pigs
Wdr48	0.12	1.00000000	pigs

Fam168b	0.18	1.00000000	pigs
Gnpda1	0.16	1.00000000	pigs
Arel1	0.15	1.00000000	pigs
Irf3	0.08	1.00000000	pigs
Ddx52	0.13	1.00000000	pigs
Nmral1	0.03	1.00000000	pigs
Zcrb1	0.13	1.00000000	pigs
Kansl1	0.13	1.00000000	pigs
Tm9sf2	0.20	1.00000000	pigs
Dipk2a	0.07	1.00000000	pigs
Bckdhb	0.15	1.00000000	pigs
Adam9	0.24	1.00000000	pigs
Zfp128	0.13	1.00000000	pigs
Cyb5a	0.12	1.00000000	pigs
Pspc1	0.21	1.00000000	pigs
Pcbp4	0.07	1.00000000	pigs
Asap1	0.19	1.00000000	pigs
Themis2	-0.02	1.00000000	pigs
Tmf1	0.05	1.00000000	pigs
Utp18	0.08	1.00000000	pigs
Ctnnb1	0.09	1.00000000	pigs
Sdf4	0.15	1.00000000	pigs
Alas1	0.08	1.00000000	pigs
Hsf1	0.00	1.00000000	pigs
Ppp2r5a	0.10	1.00000000	pigs
Prpf4b	-0.55	1.00000000	pigs
Gin1	-0.14	1.00000000	pigs
Clspn	-0.43	1.00000000	pigs
Tex10	0.09	1.00000000	pigs
Hace1	0.13	1.00000000	pigs
Aatf	0.17	1.00000000	pigs
Crtc3	0.11	1.00000000	pigs
Asb1	0.09	1.00000000	pigs
Jmjd8	0.09	1.00000000	pigs
Rnf44	0.11	1.00000000	pigs
Safb2	0.13	1.00000000	pigs
Nelfa	0.15	1.00000000	pigs
Slc9a6	0.06	1.00000000	pigs
Lmna	0.14	1.00000000	pigs
Akt2	0.07	1.00000000	pigs
Ints2	0.07	1.00000000	pigs
Zfp207	0.06	1.00000000	pigs
Pdcd4	0.09	1.00000000	pigs
Zc3h12d	0.19	1.00000000	pigs
Cstb	0.20	1.00000000	pigs
Auts2	-0.35	1.00000000	pigs

Vdac3	0.02	1.00000000	pigs
Actb	-0.37	1.00000000	pigs
C5ar1	-0.62	1.00000000	pigs
Serpinb9	0.13	1.00000000	pigs
Pde4d	-0.25	1.00000000	pigs
Ints11	0.11	1.00000000	pigs
Yrdc	0.00	1.00000000	pigs
Smarce1	-0.47	1.00000000	pigs
Sec23ip	0.01	1.00000000	pigs
Hmgcs1	0.14	1.00000000	pigs
Cd164	0.18	1.00000000	pigs
Cdca4	0.12	1.00000000	pigs
Ap3d1	0.03	1.00000000	pigs
Topors	0.07	1.00000000	pigs
Hnrnpa0	0.04	1.00000000	pigs
Kdm2a	0.21	1.00000000	pigs
Pik3ap1	0.21	1.00000000	pigs
Vars2	0.14	1.00000000	pigs
Tom1l2	0.11	1.00000000	pigs
Zbtb7c	-0.57	1.00000000	pigs
Ddhd1	0.15	1.00000000	pigs
Ppm1a	0.10	1.00000000	pigs
Srp72	0.11	1.00000000	pigs
Slc7a11	0.26	1.00000000	pigs
Foxp4	0.08	1.00000000	pigs
Rnf144a	-0.19	1.00000000	pigs
Gatad2b	0.15	1.00000000	pigs
Pes1	-0.43	1.00000000	pigs
Ppp2r1a	0.07	1.00000000	pigs
Copg2	0.15	1.00000000	pigs
Trerf1	0.26	1.00000000	pigs
Ogt	0.15	1.00000000	pigs
Tmem33	0.05	1.00000000	pigs
Leng8	0.08	1.00000000	pigs
Ufl1	0.06	1.00000000	pigs
Zbtb41	-0.69	1.00000000	pigs
Agpat2	0.11	1.00000000	pigs
Rbm25	0.13	1.00000000	pigs
Arhgef39	0.07	1.00000000	pigs
Alkbh1	0.15	1.00000000	pigs
Chmp2b	0.01	1.00000000	pigs
Ranbp2	0.13	1.00000000	pigs
Sh3bp5	-0.46	1.00000000	pigs
Sdhc	0.12	1.00000000	pigs
Ist1	-0.04	1.00000000	pigs
Tfdp1	0.13	1.00000000	pigs

Hipk2	0.14	1.00000000	pigs
Yaf2	0.12	1.00000000	pigs
Lcorl	-0.08	1.00000000	pigs
Fbxl20	0.08	1.00000000	pigs
Ypel5	0.11	1.00000000	pigs
St13	0.08	1.00000000	pigs
Bicd2	0.11	1.00000000	pigs
Tmem101	0.12	1.00000000	pigs
Nsf	0.35	1.00000000	pigs
Niban1	0.03	1.00000000	pigs
Appbp2	0.12	1.00000000	pigs
Snrpa	0.05	1.00000000	pigs
Rpn1	0.12	1.00000000	pigs
Arl8b	0.05	1.00000000	pigs
Tra2a	0.16	1.00000000	pigs
Agpat5	0.09	1.00000000	pigs
Fbxw11	0.19	1.00000000	pigs
Pnpla8	-0.52	1.00000000	pigs
Osbpl11	0.21	1.00000000	pigs
Ano6	-0.54	1.00000000	pigs
Tmem59	-0.22	1.00000000	pigs
Thop1	0.13	1.00000000	pigs
Ppme1	0.08	1.00000000	pigs
Zfyve1	0.11	1.00000000	pigs
Rrp8	-0.34	1.00000000	pigs
Zfp800	0.13	1.00000000	pigs
Sfswap	0.06	1.00000000	pigs
Eps15l1	0.18	1.00000000	pigs
Cse1l	0.16	1.00000000	pigs
Pus7l	-0.25	1.00000000	pigs
Ddx23	0.12	1.00000000	pigs
Atxn2l	0.13	1.00000000	pigs
Foxk2	0.15	1.00000000	pigs
Trmt2a	0.10	1.00000000	pigs
Nde1	0.27	1.00000000	pigs
Uox	-0.38	1.00000000	pigs
Tmed7	0.09	1.00000000	pigs
Ctdnep1	-0.05	1.00000000	pigs
Shq1	0.08	1.00000000	pigs
Frmd8	0.07	1.00000000	pigs
Slc41a1	0.11	1.00000000	pigs
Nob1	0.18	1.00000000	pigs
Rsl1d1	0.10	1.00000000	pigs
Papss1	0.12	1.00000000	pigs
Dctn6	0.09	1.00000000	pigs
Ndfip2	0.23	1.00000000	pigs

Eif2a	0.14	1.00000000	pigs
Zfp248	0.11	1.00000000	pigs
Zbtb24	0.08	1.00000000	pigs
Ap4e1	0.07	1.00000000	pigs
Ufd1	0.00	1.00000000	pigs
Drosha	0.11	1.00000000	pigs
Nisch	0.13	1.00000000	pigs
Hint1	-0.03	1.00000000	pigs
Tasl	0.11	1.00000000	pigs
Smyd2	0.12	1.00000000	pigs
Stau1	-0.02	1.00000000	pigs
Hnrnpu	0.15	1.00000000	pigs
Btg3	0.09	1.00000000	pigs
Lin54	0.10	1.00000000	pigs
Golt1b	0.10	1.00000000	pigs
Ppp2r5d	0.13	1.00000000	pigs
Patl1	0.17	1.00000000	pigs
Pik3c2b	0.18	1.00000000	pigs
Acsl3	-0.07	1.00000000	pigs
Idh3g	0.11	1.00000000	pigs
Atp6v1b2	0.05	1.00000000	pigs
Lsg1	0.09	1.00000000	pigs
Il4ra	0.29	1.00000000	pigs
Ube2l6	0.05	1.00000000	pigs
Rwdd4a	0.13	1.00000000	pigs
Creb1	0.19	1.00000000	pigs
Rfk	-0.01	1.00000000	pigs
Hbq1b	-0.18	1.00000000	pigs
Lysmd3	0.09	1.00000000	pigs
Ect2	0.12	1.00000000	pigs
Usp14	0.16	1.00000000	pigs
Ric8a	0.09	1.00000000	pigs
Spen	0.19	1.00000000	pigs
Nfkb2	0.07	1.00000000	pigs
Pakap	0.17	1.00000000	pigs
Tln1	-0.45	1.00000000	pigs
Aco2	0.03	1.00000000	pigs
Cndp2	0.10	1.00000000	pigs
Fcer1g	-0.11	1.00000000	pigs
Pnkp	0.14	1.00000000	pigs
Idh3a	0.00	1.00000000	pigs
U2af1l4	-0.54	1.00000000	pigs
Btbd11	0.18	1.00000000	pigs
Bhlhe41	0.13	1.00000000	pigs
Pdlim5	-0.03	1.00000000	pigs
Pcid2	0.10	1.00000000	pigs

Xpc	0.06	1.00000000	pigs
Dcun1d4	0.11	1.00000000	pigs
R3hdm2	-0.03	1.00000000	pigs
Idh3b	0.09	1.00000000	pigs
Rftn1	0.20	1.00000000	pigs
Smarcd2	0.12	1.00000000	pigs
Bpnt1	0.21	1.00000000	pigs
Ppp6c	0.06	1.00000000	pigs
Sharpin	0.09	1.00000000	pigs
Pms2	0.10	1.00000000	pigs
Ylpm1	-0.02	1.00000000	pigs
Chtop	0.07	1.00000000	pigs
Sec31a	0.00	1.00000000	pigs
Scamp2	0.11	1.00000000	pigs
Pdxdc1	0.14	1.00000000	pigs
Slc25a38	-0.36	1.00000000	pigs
Rbbp6	0.19	1.00000000	pigs
Clcn5	0.13	1.00000000	pigs
Adam10	-0.02	1.00000000	pigs
Riok1	0.06	1.00000000	pigs
Ctsb	0.16	1.00000000	pigs
Ddb1	0.07	1.00000000	pigs
Taf8	0.05	1.00000000	pigs
Ess2	0.12	1.00000000	pigs
Higd1a	0.08	1.00000000	pigs
Pip4p1	0.17	1.00000000	pigs
Rasal3	0.02	1.00000000	pigs
Nlk	0.14	1.00000000	pigs
Tns3	0.07	1.00000000	pigs
Pfkp	0.21	1.00000000	pigs
Vapb	0.04	1.00000000	pigs
Aars	0.10	1.00000000	pigs
Pitpna	0.12	1.00000000	pigs
Bicral	0.12	1.00000000	pigs
Dnm2	0.09	1.00000000	pigs
Lamtor2	0.14	1.00000000	pigs
Wdr41	0.13	1.00000000	pigs
Rora	0.78	1.00000000	pigs
Sertad1	-0.03	1.00000000	pigs
Thoc7	-0.54	1.00000000	pigs
Stk40	0.06	1.00000000	pigs
Synj1	0.13	1.00000000	pigs
Chchd6	0.14	1.00000000	pigs
Pop1	-0.01	1.00000000	pigs
Lsm14b	0.16	1.00000000	pigs
Dlg1	0.20	1.00000000	pigs

Zc3h12a	0.11	1.00000000	pigs
Apoo	-0.03	1.00000000	pigs
Rab7	0.25	1.00000000	pigs
Dync1li1	0.03	1.00000000	pigs
Sec23a	0.20	1.00000000	pigs
Ttbk2	0.13	1.00000000	pigs
Tmem135	0.12	1.00000000	pigs
Ccnh	0.09	1.00000000	pigs
Cstf1	0.14	1.00000000	pigs
Wapl	0.14	1.00000000	pigs
Cox8a	-0.42	1.00000000	pigs
Nthl1	0.10	1.00000000	pigs
Tmem106b	0.11	1.00000000	pigs
Sirt1	0.11	1.00000000	pigs
Stx12	0.06	1.00000000	pigs
S100a4	-0.67	1.00000000	pigs
Clic4	0.17	1.00000000	pigs
Hdac4	0.20	1.00000000	pigs
Wdr89	0.06	1.00000000	pigs
Git2	0.19	1.00000000	pigs
Ostf1	-0.39	1.00000000	pigs
Heatr6	0.09	1.00000000	pigs
Tfe3	0.12	1.00000000	pigs
Ccnk	0.05	1.00000000	pigs
Cog6	0.03	1.00000000	pigs
Gfm1	0.14	1.00000000	pigs
Fbxo22	0.19	1.00000000	pigs
Actr1b	0.11	1.00000000	pigs
Uri1	0.18	1.00000000	pigs
Ubfd1	0.03	1.00000000	pigs
Unkl	0.10	1.00000000	pigs
Vim	-0.62	1.00000000	pigs
Brms1	0.05	1.00000000	pigs
Ell	0.19	1.00000000	pigs
Elp6	-0.02	1.00000000	pigs
Ddx27	0.17	1.00000000	pigs
Gemin5	0.11	1.00000000	pigs
Hpf1	0.04	1.00000000	pigs
Slc36a1	0.13	1.00000000	pigs
Itpa	0.04	1.00000000	pigs
Bri3	0.13	1.00000000	pigs
G3bp1	0.16	1.00000000	pigs
Stk25	0.06	1.00000000	pigs
Rfc1	-0.38	1.00000000	pigs
Sae1	0.07	1.00000000	pigs
Cnot1	0.10	1.00000000	pigs

Smim14	-0.39	1.00000000	pigs
Slc66a2	0.02	1.00000000	pigs
Ilf3	0.19	1.00000000	pigs
Dmac2	0.09	1.00000000	pigs
Lin7c	0.04	1.00000000	pigs
Polr3e	0.03	1.00000000	pigs
Trmt10a	-0.01	1.00000000	pigs
Uba6	0.14	1.00000000	pigs
Ifit2	-0.46	1.00000000	pigs
Il34	0.22	1.00000000	pigs
Sh2d2a	0.12	1.00000000	pigs
Ilkap	0.06	1.00000000	pigs
Nasp	0.04	1.00000000	pigs
Ntmt1	0.07	1.00000000	pigs
Dpm2	-0.08	1.00000000	pigs
Wdr45	-0.01	1.00000000	pigs
Jmjd6	0.05	1.00000000	pigs
Csk	0.07	1.00000000	pigs
Pex13	0.08	1.00000000	pigs
Cmah	0.15	1.00000000	pigs
Emsy	0.09	1.00000000	pigs
4930523C07Rik	-0.30	1.00000000	pigs
Chchd4	0.07	1.00000000	pigs
Ccnt1	0.01	1.00000000	pigs
Slmap	0.09	1.00000000	pigs
Tollip	0.09	1.00000000	pigs
Ftsj3	0.08	1.00000000	pigs
Eipr1	0.12	1.00000000	pigs
Tcaim	0.12	1.00000000	pigs
Dph1	0.09	1.00000000	pigs
Zc3h3	0.05	1.00000000	pigs
Psmd2	0.00	1.00000000	pigs
Rpap2	0.01	1.00000000	pigs
Nap1l4	0.06	1.00000000	pigs
Nkapd1	0.17	1.00000000	pigs
Hdac2	0.00	1.00000000	pigs
Nagk	0.05	1.00000000	pigs
Usp46	0.12	1.00000000	pigs
Anxa7	-0.06	1.00000000	pigs
Ireb2	-0.02	1.00000000	pigs
Exosc3	0.00	1.00000000	pigs
Yy1	0.09	1.00000000	pigs
Zcchc2	-0.02	1.00000000	pigs
Copg1	0.01	1.00000000	pigs
Sap18	0.08	1.00000000	pigs
Rbm6	0.11	1.00000000	pigs

Senp5	-0.13	1.00000000	pigs
Acsl5	0.12	1.00000000	pigs
Lrch1	0.21	1.00000000	pigs
Prdx1	0.15	1.00000000	pigs
Cpped1	0.11	1.00000000	pigs
Lnpep	0.06	1.00000000	pigs
Hexim1	0.11	1.00000000	pigs
Fam193b	0.11	1.00000000	pigs
Syap1	0.02	1.00000000	pigs
Ddost	0.05	1.00000000	pigs
Txnrd1	0.02	1.00000000	pigs
Gpat4	-0.06	1.00000000	pigs
Heatr3	0.11	1.00000000	pigs
Dgkz	-0.01	1.00000000	pigs
Slc37a2	0.14	1.00000000	pigs
Ly96	0.04	1.00000000	pigs
Nrf1	0.10	1.00000000	pigs
Picalm	0.05	1.00000000	pigs
Timmdc1	0.10	1.00000000	pigs
5730455P16Rik	0.10	1.00000000	pigs
Tmem129	0.05	1.00000000	pigs
Apoe	0.12	1.00000000	pigs
Pogz	0.13	1.00000000	pigs
Sdf2	0.07	1.00000000	pigs
Spryd3	0.09	1.00000000	pigs
Rnf114	0.01	1.00000000	pigs
Plin2	0.14	1.00000000	pigs
Stx7	-0.13	1.00000000	pigs
Gna13	0.11	1.00000000	pigs
Tmem243	0.10	1.00000000	pigs
Casp6	0.03	1.00000000	pigs
Gemin6	0.08	1.00000000	pigs
Tmem161b	0.01	1.00000000	pigs
Cacul1	0.18	1.00000000	pigs
Agk	0.05	1.00000000	pigs
Hook2	-0.17	1.00000000	pigs
Far1	0.04	1.00000000	pigs
Ankrd42	0.13	1.00000000	pigs
Rnmt	0.01	1.00000000	pigs
Selenos	-0.08	1.00000000	pigs
Vti1b	0.12	1.00000000	pigs
Kmo	0.18	1.00000000	pigs
Ruvbl2	0.07	1.00000000	pigs
Slc10a3	0.04	1.00000000	pigs
Psme3	0.01	1.00000000	pigs
Filip1l	-0.07	1.00000000	pigs

Zfp263	0.04	1.00000000	pigs
Tm9sf3	0.13	1.00000000	pigs
Ndst1	0.00	1.00000000	pigs
Cep85l	0.15	1.00000000	pigs
Ap2b1	0.01	1.00000000	pigs
Tmem106c	0.05	1.00000000	pigs
Rbm41	0.14	1.00000000	pigs
Uqcc1	-0.21	1.00000000	pigs
Klf16	0.08	1.00000000	pigs
H2aj	-0.03	1.00000000	pigs
Bri3bp	0.09	1.00000000	pigs
Tango2	0.11	1.00000000	pigs
Nfkbiz	0.17	1.00000000	pigs
Frat2	0.13	1.00000000	pigs
Spag8	-0.29	1.00000000	pigs
Frem2	-0.27	1.00000000	pigs
Cd2ap	0.04	1.00000000	pigs
Scap	-0.30	1.00000000	pigs
Pycrl	0.14	1.00000000	pigs
Drg1	0.08	1.00000000	pigs
Sh2d3c	0.10	1.00000000	pigs
Ifi44l	0.19	1.00000000	pigs
Trim26	0.08	1.00000000	pigs
Il17ra	0.02	1.00000000	pigs
Scnm1	0.09	1.00000000	pigs
Hadha	0.06	1.00000000	pigs
Otulinl	0.03	1.00000000	pigs
Cemip2	-0.47	1.00000000	pigs
Fbxo31	0.10	1.00000000	pigs
Vav3	-0.30	1.00000000	pigs
Glipr2	-0.43	1.00000000	pigs
Rptor	0.10	1.00000000	pigs
Kdsr	-0.70	1.00000000	pigs
Clip1	0.03	1.00000000	pigs
Zfp90	0.09	1.00000000	pigs
Cmss1	0.19	1.00000000	pigs
Sertad3	0.05	1.00000000	pigs
Clint1	-0.08	1.00000000	pigs
Pygb	0.11	1.00000000	pigs
Acaa1b	0.02	1.00000000	pigs
Nsdhl	0.19	1.00000000	pigs
Tmem184c	0.14	1.00000000	pigs
Ubiad1	0.02	1.00000000	pigs
Actr6	0.00	1.00000000	pigs
Slc16a3	0.07	1.00000000	pigs
Wdr37	0.11	1.00000000	pigs

Ppp1cb	0.04	1.00000000	pigs
Rrp1	0.11	1.00000000	pigs
Nop10	0.01	1.00000000	pigs
Psmd10	0.01	1.00000000	pigs
Nudcd1	0.12	1.00000000	pigs
Psmb4	0.01	1.00000000	pigs
Trmt1	0.00	1.00000000	pigs
Tmx3	0.10	1.00000000	pigs
Whamm	0.05	1.00000000	pigs
Paxip1	0.11	1.00000000	pigs
Csde1	0.09	1.00000000	pigs
Cul3	0.14	1.00000000	pigs
Nadk2	0.17	1.00000000	pigs
Ndufa4	-0.41	1.00000000	pigs
Agps	0.10	1.00000000	pigs
Pcnx4	0.14	1.00000000	pigs
Csnk2a2	0.12	1.00000000	pigs
Aven	0.24	1.00000000	pigs
Srebf1	0.01	1.00000000	pigs
Dram1	0.14	1.00000000	pigs
Smyd3	0.10	1.00000000	pigs
Irgq	-0.03	1.00000000	pigs
Itm2b	0.06	1.00000000	pigs
Armc10	0.03	1.00000000	pigs
Rybp	0.09	1.00000000	pigs
Anp32e	0.03	1.00000000	pigs
Pdss1	0.02	1.00000000	pigs
Zcchc17	0.02	1.00000000	pigs
Eloa	0.15	1.00000000	pigs
Nubpl	0.05	1.00000000	pigs
Tbc1d4	0.11	1.00000000	pigs
Uchl3	-0.01	1.00000000	pigs
Uhrf2	-0.20	1.00000000	pigs
Chd3	0.04	1.00000000	pigs
Glb1	-0.47	1.00000000	pigs
Lrch3	0.04	1.00000000	pigs
Spi1	-0.39	1.00000000	pigs
Drg2	0.01	1.00000000	pigs
Suz12	0.11	1.00000000	pigs
Plekha3	0.04	1.00000000	pigs
Rab31	-0.01	1.00000000	pigs
Ern1	0.00	1.00000000	pigs
Dis3	0.05	1.00000000	pigs
Slc9a9	0.05	1.00000000	pigs
Ankfy1	0.15	1.00000000	pigs
Gclc	0.05	1.00000000	pigs

Wdr55	0.12	1.00000000	pigs
Lrp2bp	-0.24	1.00000000	pigs
Rbm17	0.03	1.00000000	pigs
Sf3b6	-0.01	1.00000000	pigs
Dbndd2	0.11	1.00000000	pigs
Rapgef1	0.11	1.00000000	pigs
Son	-0.26	1.00000000	pigs
Ddx41	0.10	1.00000000	pigs
Fam210b	0.05	1.00000000	pigs
Cinp	0.09	1.00000000	pigs
Gemin7	0.01	1.00000000	pigs
Ppp1r3b	0.08	1.00000000	pigs
Aff4	0.08	1.00000000	pigs
Srrt	0.08	1.00000000	pigs
Ndufs1	0.10	1.00000000	pigs
Zdhhc21	0.02	1.00000000	pigs
Ccdc115	0.09	1.00000000	pigs
Serpinb1a	-0.64	1.00000000	pigs
Ahcyl1	0.09	1.00000000	pigs
Prkag1	0.05	1.00000000	pigs
Anapc5	-0.33	1.00000000	pigs
Pik3cd	0.04	1.00000000	pigs
Wipi1	0.11	1.00000000	pigs
Lrrfip2	0.09	1.00000000	pigs
Gorasp2	-0.01	1.00000000	pigs
Polr2i	-0.14	1.00000000	pigs
Jade3	0.22	1.00000000	pigs
Tcf3	0.05	1.00000000	pigs
Spag1	-0.10	1.00000000	pigs
Zfc3h1	0.02	1.00000000	pigs
Slc20a2	0.22	1.00000000	pigs
Psmd4	-0.03	1.00000000	pigs
C1qb	0.17	1.00000000	pigs
Pmm1	0.05	1.00000000	pigs
Ctr9	0.10	1.00000000	pigs
Eif3j2	0.03	1.00000000	pigs
Bad	-0.34	1.00000000	pigs
Gtdc1	0.11	1.00000000	pigs
Sdad1	0.07	1.00000000	pigs
Eif2b2	0.18	1.00000000	pigs
Borcs5	0.16	1.00000000	pigs
Exosc2	0.12	1.00000000	pigs
Asxl2	0.14	1.00000000	pigs
Wdr20	0.02	1.00000000	pigs
Dnajc5	-0.06	1.00000000	pigs
Ttc7	0.09	1.00000000	pigs

Carhsp1	-0.10	1.00000000	pigs
Nptn	0.06	1.00000000	pigs
Zbtb11	0.08	1.00000000	pigs
Rhoc	-0.01	1.00000000	pigs
Ypel3	-0.37	1.00000000	pigs
Slc7a6os	0.13	1.00000000	pigs
Sugt1	0.01	1.00000000	pigs
Ell3	-0.11	1.00000000	pigs
Pdlim7	0.04	1.00000000	pigs
Soat1	-0.20	1.00000000	pigs
Peak1	-0.02	1.00000000	pigs
Tsr3	-0.06	1.00000000	pigs
Ccdc69	0.14	1.00000000	pigs
Vasp	0.10	1.00000000	pigs
Creb3	0.11	1.00000000	pigs
Taf5l	-0.01	1.00000000	pigs
Nepro	0.08	1.00000000	pigs
Ldlrad4	0.16	1.00000000	pigs
Vps13b	0.19	1.00000000	pigs
Polb	0.05	1.00000000	pigs
Umad1	0.00	1.00000000	pigs
Ash1l	0.15	1.00000000	pigs
Hscb	0.07	1.00000000	pigs
Zcchc24	0.17	1.00000000	pigs
Pik3r4	0.13	1.00000000	pigs
Eml4	0.00	1.00000000	pigs
Pank3	-0.07	1.00000000	pigs
Setd7	0.07	1.00000000	pigs
Pgap3	0.09	1.00000000	pigs
Dnase2a	-0.43	1.00000000	pigs
Tcea1	-0.56	1.00000000	pigs
Fndc3b	-0.35	1.00000000	pigs
Surf2	0.02	1.00000000	pigs
Qser1	-0.03	1.00000000	pigs
Zcchc8	0.10	1.00000000	pigs
Ctdp1	0.09	1.00000000	pigs
Fam168a	0.18	1.00000000	pigs
Polr2m	-0.04	1.00000000	pigs
Klhl28	0.08	1.00000000	pigs
Yipf3	0.06	1.00000000	pigs
Bnip1	0.08	1.00000000	pigs
Slc39a10	-0.51	1.00000000	pigs
Smarca2	0.17	1.00000000	pigs
Lmnb2	0.14	1.00000000	pigs
Ralb	-0.35	1.00000000	pigs
Eef1akmt3	0.04	1.00000000	pigs

Cox6a2	0.06	1.00000000	pigs
Cnn2	-0.04	1.00000000	pigs
Trappc5	-0.03	1.00000000	pigs
Psme1	-0.42	1.00000000	pigs
Atp2b4	-0.21	1.00000000	pigs
Elk3	-0.04	1.00000000	pigs
Vps13d	-0.03	1.00000000	pigs
Btf3l4	-0.02	1.00000000	pigs
Tmem127	0.01	1.00000000	pigs
Fubp3	-0.07	1.00000000	pigs
Kcnab2	0.09	1.00000000	pigs
Fcrl1	-0.23	1.00000000	pigs
Lats1	-0.14	1.00000000	pigs
Trip13	-0.44	1.00000000	pigs
Gadd45b	0.20	1.00000000	pigs
Scg5	-0.06	1.00000000	pigs
Zbtb40	0.03	1.00000000	pigs
Rabif	0.10	1.00000000	pigs
Gna12	0.02	1.00000000	pigs
Psmd7	-0.06	1.00000000	pigs
Ddx6	0.05	1.00000000	pigs
Ttc14	0.05	1.00000000	pigs
Rassf5	0.10	1.00000000	pigs
Xiap	-0.02	1.00000000	pigs
Trit1	-0.02	1.00000000	pigs
Esd	0.02	1.00000000	pigs
Ormdl1	0.07	1.00000000	pigs
Rgs18	0.07	1.00000000	pigs
Gcn1	0.04	1.00000000	pigs
Lfng	-0.05	1.00000000	pigs
Ttc27	0.11	1.00000000	pigs
Smg6	0.03	1.00000000	pigs
Smad5	0.01	1.00000000	pigs
Ehd3	-0.22	1.00000000	pigs
Tubgcp3	0.08	1.00000000	pigs
Sirt7	0.10	1.00000000	pigs
Taf5	0.12	1.00000000	pigs
Pno1	0.04	1.00000000	pigs
Lrrc45	-0.03	1.00000000	pigs
Cggbp1	-0.35	1.00000000	pigs
Topbp1	0.04	1.00000000	pigs
Bub3	-0.03	1.00000000	pigs
Atad3a	0.11	1.00000000	pigs
Samm50	0.02	1.00000000	pigs
Celf1	0.06	1.00000000	pigs
Usp16	0.02	1.00000000	pigs

Zfp672	-0.34	1.00000000	pigs
2410004B18Rik	0.00	1.00000000	pigs
Fbxo39	-0.08	1.00000000	pigs
Samd8	0.03	1.00000000	pigs
Ctss	-0.56	1.00000000	pigs
Osbpl3	-0.04	1.00000000	pigs
Nin	-0.50	1.00000000	pigs
Pmpca	0.08	1.00000000	pigs
Ticam1	-0.02	1.00000000	pigs
Ppp1r13b	0.12	1.00000000	pigs
Iars	0.10	1.00000000	pigs
Ide	0.07	1.00000000	pigs
Ints6l	-0.02	1.00000000	pigs
Usf1	0.08	1.00000000	pigs
Gadd45g	0.09	1.00000000	pigs
Arl8a	0.02	1.00000000	pigs
Kat14	0.04	1.00000000	pigs
Tdrd3	0.10	1.00000000	pigs
Nars	0.05	1.00000000	pigs
Ppcs	0.10	1.00000000	pigs
Tmod3	-0.02	1.00000000	pigs
Hspbap1	0.04	1.00000000	pigs
Pip5k1a	0.02	1.00000000	pigs
Lpgat1	0.10	1.00000000	pigs
Ccdc12	-0.03	1.00000000	pigs
4933400A11Rik	0.15	1.00000000	pigs
Rapgef5	-0.53	1.00000000	pigs
Fmo4	0.04	1.00000000	pigs
Tomm20	-0.03	1.00000000	pigs
Fam222a	0.10	1.00000000	pigs
Gpx1	-0.23	1.00000000	pigs
Dnph1	-0.32	1.00000000	pigs
BC005537	0.02	1.00000000	pigs
Cop1	0.11	1.00000000	pigs
Glt8d1	0.07	1.00000000	pigs
Camta1	0.14	1.00000000	pigs
Fbxw4	0.07	1.00000000	pigs
Rgs3	-0.03	1.00000000	pigs
Ykt6	0.04	1.00000000	pigs
Ogfod1	0.09	1.00000000	pigs
Fyco1	0.15	1.00000000	pigs
Nfkbia	0.38	1.00000000	pigs
Lgals1	-1.37	1.00000000	pigs
S1pr1	0.13	1.00000000	pigs
Dnajc18	0.02	1.00000000	pigs
Bod1l	0.04	1.00000000	pigs

Gnl1	0.08	1.00000000	pigs
Wdr82	0.07	1.00000000	pigs
Nup88	-0.15	1.00000000	pigs
Arhgef33	-0.20	1.00000000	pigs
Cubn	-0.35	1.00000000	pigs
Slc25a25	0.07	1.00000000	pigs
Gpr171	0.04	1.00000000	pigs
Tardbp	0.10	1.00000000	pigs
Blnk	0.23	1.00000000	pigs
Aifm1	0.02	1.00000000	pigs
Rgs14	-0.26	1.00000000	pigs
Ube2b	-0.34	1.00000000	pigs
Cep44	0.02	1.00000000	pigs
Oxa1l	0.07	1.00000000	pigs
Aig1	0.14	1.00000000	pigs
Plekha8	0.16	1.00000000	pigs
Zdhhc5	0.04	1.00000000	pigs
Uchl5	-0.07	1.00000000	pigs
Coro1c	0.10	1.00000000	pigs
Blmh	0.04	1.00000000	pigs
Galk2	-0.14	1.00000000	pigs
Tmem9	-0.31	1.00000000	pigs
Sap30bp	0.04	1.00000000	pigs
Rnaseh2c	-0.08	1.00000000	pigs
Sgf29	0.10	1.00000000	pigs
Eif4enif1	0.01	1.00000000	pigs
Stpg4	-0.23	1.00000000	pigs
Rbks	0.02	1.00000000	pigs
Golgb1	-0.45	1.00000000	pigs
Enoph1	0.03	1.00000000	pigs
Pnisr	0.12	1.00000000	pigs
Rtf2	0.09	1.00000000	pigs
Nub1	0.14	1.00000000	pigs
Gcc1	-0.04	1.00000000	pigs
Rsl24d1	0.00	1.00000000	pigs
Trim35	-0.02	1.00000000	pigs
Eif4e2	-0.07	1.00000000	pigs
Usp28	0.02	1.00000000	pigs
Ppp1r12a	0.32	1.00000000	pigs
Clasrp	0.07	1.00000000	pigs
Senp6	0.00	1.00000000	pigs
Trim33	0.08	1.00000000	pigs
Stip1	0.05	1.00000000	pigs
Commd4	-0.13	1.00000000	pigs
Ubqln4	0.08	1.00000000	pigs
Nfkbie	0.04	1.00000000	pigs

Fam13b	-0.65	1.00000000	pigs
Dnajb12	0.04	1.00000000	pigs
Kbtbd7	0.13	1.00000000	pigs
Casc3	0.10	1.00000000	pigs
Srsf1	0.06	1.00000000	pigs
Hook3	0.10	1.00000000	pigs
Bid	-0.01	1.00000000	pigs
Prkaca	0.11	1.00000000	pigs
Lrrc42	0.00	1.00000000	pigs
BC048507	0.02	1.00000000	pigs
Armc7	-0.05	1.00000000	pigs
Brox	0.04	1.00000000	pigs
Rabgap1	0.10	1.00000000	pigs
Sinhcaf	0.22	1.00000000	pigs
Nucks1	0.10	1.00000000	pigs
Nsun3	0.06	1.00000000	pigs
Tubb5	0.06	1.00000000	pigs
AA467197	0.08	1.00000000	pigs
Usp6nl	0.03	1.00000000	pigs
Vps35	-0.05	1.00000000	pigs
Pik3ip1	0.10	1.00000000	pigs
Slc50a1	0.09	1.00000000	pigs
Ggps1	-0.09	1.00000000	pigs
Ankrd27	0.14	1.00000000	pigs
Vps13c	-0.51	1.00000000	pigs
Ly9	0.15	1.00000000	pigs
Nup205	0.02	1.00000000	pigs
Secisbp2	-0.46	1.00000000	pigs
Znrf3	-0.19	1.00000000	pigs
Stmn1	-0.05	1.00000000	pigs
5031439G07Rik	0.09	1.00000000	pigs
Fabp3	-0.04	1.00000000	pigs
Cd86	0.26	1.00000000	pigs
Zfp292	0.16	1.00000000	pigs
Tmem134	0.02	1.00000000	pigs
U2surp	0.07	1.00000000	pigs
Cybc1	0.06	1.00000000	pigs
Tstd2	0.07	1.00000000	pigs
Dcp1a	-0.06	1.00000000	pigs
Eepd1	0.33	1.00000000	pigs
Trip12	0.05	1.00000000	pigs
Rnaseh2b	-0.03	1.00000000	pigs
Lgals3	0.10	1.00000000	pigs
Zxdc	0.07	1.00000000	pigs
Tgds	0.06	1.00000000	pigs
Aurkaip1	-0.34	1.00000000	pigs

Itprip	0.01	1.00000000	pigs
Zmiz1	0.08	1.00000000	pigs
Tbc1d10a	0.02	1.00000000	pigs
Katna1	0.04	1.00000000	pigs
Pdpk1	-0.05	1.00000000	pigs
Cops5	0.03	1.00000000	pigs
Rfx5	-0.35	1.00000000	pigs
Hcfc2	-0.05	1.00000000	pigs
Desi1	0.00	1.00000000	pigs
Gnb2	-0.37	1.00000000	pigs
Paxbp1	0.00	1.00000000	pigs
Sesn1	-0.07	1.00000000	pigs
Ap2m1	0.04	1.00000000	pigs
Lrrc1	0.11	1.00000000	pigs
Ccdc137	0.10	1.00000000	pigs
Ankrd10	0.05	1.00000000	pigs
Utp15	0.07	1.00000000	pigs
Ubap1	-0.03	1.00000000	pigs
Bzw2	0.05	1.00000000	pigs
Sirt5	0.03	1.00000000	pigs
Lyrm1	0.03	1.00000000	pigs
C2cd5	0.04	1.00000000	pigs
Ube2a	0.05	1.00000000	pigs
Bptf	0.04	1.00000000	pigs
Pfdn2	-0.04	1.00000000	pigs
Ergic3	0.05	1.00000000	pigs
Nrde2	0.08	1.00000000	pigs
Ndufa10	-0.09	1.00000000	pigs
Snx8	0.02	1.00000000	pigs
Abcd3	0.01	1.00000000	pigs
Sec23b	-0.09	1.00000000	pigs
Fam162a	-0.01	1.00000000	pigs
Ccar1	0.08	1.00000000	pigs
Extl3	0.13	1.00000000	pigs
Ssr1	-0.01	1.00000000	pigs
Agfg1	0.05	1.00000000	pigs
Trabd	0.02	1.00000000	pigs
Yipf6	-0.02	1.00000000	pigs
Polk	-0.18	1.00000000	pigs
Ndufc2	-0.50	1.00000000	pigs
Dbi	-0.35	1.00000000	pigs
Skil	0.15	1.00000000	pigs
Scml4	0.16	1.00000000	pigs
Bdh2	-0.32	1.00000000	pigs
Dot1l	0.12	1.00000000	pigs
Ccdc107	-0.27	1.00000000	pigs

Tmem163	-0.35	1.00000000	pigs
Rbm10	-0.08	1.00000000	pigs
Csnk1g3	0.11	1.00000000	pigs
Ppp4c	0.10	1.00000000	pigs
Dennd2d	0.18	1.00000000	pigs
Sec61a1	-0.03	1.00000000	pigs
Vps51	0.04	1.00000000	pigs
Chst11	0.05	1.00000000	pigs
Pgpep1	0.09	1.00000000	pigs
Tmem87b	0.04	1.00000000	pigs
Sertad2	-0.10	1.00000000	pigs
Zfp512b	-0.08	1.00000000	pigs
Gcnt1	0.06	1.00000000	pigs
Glrx5	0.00	1.00000000	pigs
Dhx30	0.00	1.00000000	pigs
Btbd10	0.10	1.00000000	pigs
Prrc2c	0.08	1.00000000	pigs
Tbcd	0.09	1.00000000	pigs
Kyat3	0.04	1.00000000	pigs
Hnrnpdl	-0.35	1.00000000	pigs
Sms	-0.09	1.00000000	pigs
Ttpal	-0.08	1.00000000	pigs
Aacs	0.07	1.00000000	pigs
Stoml2	-0.14	1.00000000	pigs
Jmy	0.02	1.00000000	pigs
Hnrnpul1	0.01	1.00000000	pigs
Cnp	-0.01	1.00000000	pigs
Ltn1	-0.04	1.00000000	pigs
Zbtb20	-0.32	1.00000000	pigs
Klf9	0.14	1.00000000	pigs
Rnh1	-0.11	1.00000000	pigs
Gart	0.03	1.00000000	pigs
Pstpip1	-0.04	1.00000000	pigs
Ccdc127	-0.13	1.00000000	pigs
Raf1	0.06	1.00000000	pigs
Ate1	0.02	1.00000000	pigs
L3mbtl3	-0.35	1.00000000	pigs
Folr1	-0.59	1.00000000	pigs
Gas7	-0.31	1.00000000	pigs
Acrbp	0.07	1.00000000	pigs
Atp13a3	0.05	1.00000000	pigs
Uhrf1bp1	-0.30	1.00000000	pigs
Rmc1	0.02	1.00000000	pigs
Wwox	0.07	1.00000000	pigs
Specc1	-0.03	1.00000000	pigs
Rbm14	0.07	1.00000000	pigs

Shmt2	0.08	1.00000000	pigs
Fchsd2	0.17	1.00000000	pigs
Zdhhc7	0.00	1.00000000	pigs
Rfwd3	-0.41	1.00000000	pigs
Pds5b	0.08	1.00000000	pigs
Fam126b	0.06	1.00000000	pigs
Gtf2i	-0.02	1.00000000	pigs
Arhgap26	0.13	1.00000000	pigs
H6pd	0.04	1.00000000	pigs
Stam2	-0.10	1.00000000	pigs
Ubtd2	0.23	1.00000000	pigs
Bmp4	0.01	1.00000000	pigs
Rnf40	0.04	1.00000000	pigs
Btbd1	0.06	1.00000000	pigs
Slc39a14	0.17	1.00000000	pigs
Atp6v0d1	-0.10	1.00000000	pigs
Rhbdf2	0.14	1.00000000	pigs
Sike1	0.07	1.00000000	pigs
Prkra	0.05	1.00000000	pigs
Cdk5rap2	0.06	1.00000000	pigs
Orc2	-0.32	1.00000000	pigs
Trdmt1	0.20	1.00000000	pigs
Trp53bp1	0.12	1.00000000	pigs
Grcc10	-0.46	1.00000000	pigs
Zfp451	0.03	1.00000000	pigs
Fnta	0.16	1.00000000	pigs
Pde4b	-0.21	1.00000000	pigs
Ublcp1	-0.39	1.00000000	pigs
Trmt10b	0.03	1.00000000	pigs
Plekhm1	0.13	1.00000000	pigs
Fbl	0.03	1.00000000	pigs
Chic1	0.05	1.00000000	pigs
Grwd1	0.12	1.00000000	pigs
Uba1	0.06	1.00000000	pigs
Ehbp1l1	0.07	1.00000000	pigs
Rap1gds1	-0.02	1.00000000	pigs
Ninl	0.07	1.00000000	pigs
Cds2	-0.11	1.00000000	pigs
Trip11	0.04	1.00000000	pigs
Bcl3	-0.14	1.00000000	pigs
Exoc1	-0.05	1.00000000	pigs
Xrn2	0.10	1.00000000	pigs
Pcmtd1	0.06	1.00000000	pigs
Ythdf2	0.12	1.00000000	pigs
Scfd1	-0.01	1.00000000	pigs
Eif1b	-0.36	1.00000000	pigs

Nup153	0.03	1.00000000	pigs
Dcaf5	0.04	1.00000000	pigs
Bscl2	0.03	1.00000000	pigs
Prdm11	0.19	1.00000000	pigs
Uxs1	0.12	1.00000000	pigs
Rspry1	0.09	1.00000000	pigs
Inpp5b	0.18	1.00000000	pigs
Pmpcb	0.01	1.00000000	pigs
Entpd1	-0.03	1.00000000	pigs
Fkbp8	-0.01	1.00000000	pigs
Katnal1	0.03	1.00000000	pigs
Tsfm	0.03	1.00000000	pigs
Simc1	0.22	1.00000000	pigs
Gak	0.12	1.00000000	pigs
Dram2	0.05	1.00000000	pigs
Tubb4b	-0.07	1.00000000	pigs
Rnf126	0.06	1.00000000	pigs
Tnf	-0.30	1.00000000	pigs
Rab22a	0.04	1.00000000	pigs
Fyttd1	-0.10	1.00000000	pigs
Rc3h1	-0.06	1.00000000	pigs
Arf1	0.03	1.00000000	pigs
Naxd	0.06	1.00000000	pigs
Vezf1	0.01	1.00000000	pigs
Pprc1	-0.04	1.00000000	pigs
Rapgef2	-0.16	1.00000000	pigs
Nup107	0.04	1.00000000	pigs
Itsn2	0.15	1.00000000	pigs
Katnbl1	-0.05	1.00000000	pigs
Nrbp1	0.04	1.00000000	pigs
Cenpi	0.06	1.00000000	pigs
Arl1	-0.02	1.00000000	pigs
Ccdc85b	-0.08	1.00000000	pigs
Dhx15	-0.06	1.00000000	pigs
Ammecr1	0.12	1.00000000	pigs
Cpeb4	0.06	1.00000000	pigs
Capg	-0.07	1.00000000	pigs
Bcl11a	0.01	1.00000000	pigs
Ssr4	-0.24	1.00000000	pigs
Brk1	-0.54	1.00000000	pigs
Dido1	0.06	1.00000000	pigs
Togaram1	-0.09	1.00000000	pigs
Stk35	-0.07	1.00000000	pigs
Pycard	0.04	1.00000000	pigs
Nop56	0.19	1.00000000	pigs
Ndufaf4	0.03	1.00000000	pigs

Rpf1	-0.02	1.00000000	pigs
Llph	0.02	1.00000000	pigs
U2af2	0.03	1.00000000	pigs
Ambra1	0.03	1.00000000	pigs
Nup50	0.07	1.00000000	pigs
Gdi2	0.01	1.00000000	pigs
Gpatch4	-0.05	1.00000000	pigs
Atp6v1c1	-0.03	1.00000000	pigs
Pcif1	-0.01	1.00000000	pigs
Surf4	0.00	1.00000000	pigs
Ndufs6	-0.02	1.00000000	pigs
Col1a1	0.03	1.00000000	pigs
Alkbh6	0.08	1.00000000	pigs
Dhx34	-0.28	1.00000000	pigs
Atp5c1	-0.03	1.00000000	pigs
Garem1	-0.30	1.00000000	pigs
Iffo1	-0.29	1.00000000	pigs
Rab5c	0.11	1.00000000	pigs
Ints9	0.14	1.00000000	pigs
Psmc3	-0.04	1.00000000	pigs
Pak1ip1	0.06	1.00000000	pigs
Rnf111	0.05	1.00000000	pigs
Cyc1	0.01	1.00000000	pigs
Krt10	-0.45	1.00000000	pigs
Adal	0.12	1.00000000	pigs
Arhgef2	0.03	1.00000000	pigs
Zfp143	0.06	1.00000000	pigs
Gabarapl1	-0.09	1.00000000	pigs
Rhbdd2	-0.21	1.00000000	pigs
Trmt13	-0.02	1.00000000	pigs
Cmtm7	-0.41	1.00000000	pigs
P4hb	0.11	1.00000000	pigs
Arhgap39	-0.08	1.00000000	pigs
Krr1	-0.10	1.00000000	pigs
Cdc42se2	0.04	1.00000000	pigs
Arhgdia	0.01	1.00000000	pigs
Ankrd49	0.11	1.00000000	pigs
Cd47	0.16	1.00000000	pigs
Ip6k1	0.01	1.00000000	pigs
2510039O18Rik	0.05	1.00000000	pigs
Ing5	0.04	1.00000000	pigs
Rhot1	-0.03	1.00000000	pigs
Bin1	-0.09	1.00000000	pigs
Frs2	-0.05	1.00000000	pigs
Prkab1	-0.03	1.00000000	pigs
Aup1	0.01	1.00000000	pigs

Slc39a1	0.01	1.00000000	pigs
Cd3eap	-0.06	1.00000000	pigs
Zfp777	0.04	1.00000000	pigs
Wdr91	0.01	1.00000000	pigs
Stard3nl	-0.37	1.00000000	pigs
Dnajc7	0.03	1.00000000	pigs
Ranbp1	0.06	1.00000000	pigs
Nus1	-0.03	1.00000000	pigs
Armcx3	-0.06	1.00000000	pigs
Emc3	-0.07	1.00000000	pigs
Rsu1	0.01	1.00000000	pigs
Necap2	0.04	1.00000000	pigs
Cops7b	0.03	1.00000000	pigs
Ctnnbl1	0.06	1.00000000	pigs
Sf3b2	0.00	1.00000000	pigs
Rrbp1	0.07	1.00000000	pigs
Traf2	-0.02	1.00000000	pigs
Ifi35	-0.09	1.00000000	pigs
Rabl3	0.02	1.00000000	pigs
Saysd1	-0.24	1.00000000	pigs
Abhd12	0.12	1.00000000	pigs
Sbno1	0.10	1.00000000	pigs
Pih1d1	0.14	1.00000000	pigs
Iqgap2	-0.46	1.00000000	pigs
Psmb1	-0.13	1.00000000	pigs
Tspo	-0.52	1.00000000	pigs
Gpatch2	-0.32	1.00000000	pigs
Nup160	-0.08	1.00000000	pigs
Slc45a4	0.05	1.00000000	pigs
Ube2o	0.08	1.00000000	pigs
Puf60	0.03	1.00000000	pigs
Dpp3	0.01	1.00000000	pigs
Cep120	-0.31	1.00000000	pigs
Col19a1	-0.53	1.00000000	pigs
Btk	-0.35	1.00000000	pigs
Ggcx	0.03	1.00000000	pigs
Prkcz	-0.30	1.00000000	pigs
Snap23	0.03	1.00000000	pigs
Atp6v1d	-0.05	1.00000000	pigs
Gtf2b	-0.07	1.00000000	pigs
Atg5	0.03	1.00000000	pigs
Rab43	0.05	1.00000000	pigs
Urb1	0.10	1.00000000	pigs
Gch1	-0.24	1.00000000	pigs
Azin1	0.20	1.00000000	pigs
Csf2rb2	-0.09	1.00000000	pigs

Abraxas1	-0.45	1.00000000	pigs
Serinc1	0.02	1.00000000	pigs
Retreg1	-0.45	1.00000000	pigs
Lyn	0.14	1.00000000	pigs
Ints8	-0.09	1.00000000	pigs
Cd82	0.09	1.00000000	pigs
Slc35b3	-0.03	1.00000000	pigs
Klb	-0.16	1.00000000	pigs
Cmtm3	0.05	1.00000000	pigs
Tlr4	0.19	1.00000000	pigs
Ranbp3	-0.01	1.00000000	pigs
Ccser1	0.16	1.00000000	pigs
Fmnl1	-0.01	1.00000000	pigs
Wrnip1	-0.05	1.00000000	pigs
Arih2	0.04	1.00000000	pigs
Acss1	0.06	1.00000000	pigs
Sart3	0.03	1.00000000	pigs
Abhd3	-0.05	1.00000000	pigs
Wdr70	-0.01	1.00000000	pigs
Cd38	0.04	1.00000000	pigs
Traf1	0.06	1.00000000	pigs
Ppp1r9b	0.06	1.00000000	pigs
S100a10	-0.86	1.00000000	pigs
Jazf1	-0.06	1.00000000	pigs
Rtcb	-0.10	1.00000000	pigs
Hmox2	-0.07	1.00000000	pigs
Ndufa2	-0.37	1.00000000	pigs
Eif4ebp2	0.08	1.00000000	pigs
Odf2	0.11	1.00000000	pigs
Slc30a9	0.02	1.00000000	pigs
Polm	-0.49	1.00000000	pigs
Trappc6b	-0.05	1.00000000	pigs
Bccip	-0.02	1.00000000	pigs
Cyb5b	0.00	1.00000000	pigs
Dusp14	0.05	1.00000000	pigs
Serinc3	0.06	1.00000000	pigs
Psmd8	-0.15	1.00000000	pigs
Actr2	0.07	1.00000000	pigs
Nuggc	-0.07	1.00000000	pigs
Frmd6	0.08	1.00000000	pigs
Wdr61	0.00	1.00000000	pigs
Cab39	0.05	1.00000000	pigs
Zzef1	-0.04	1.00000000	pigs
Gbe1	0.01	1.00000000	pigs
Rubcnl	-0.02	1.00000000	pigs
Eif2s1	-0.12	1.00000000	pigs

Srebf2	0.09	1.00000000	pigs
Gar1	0.10	1.00000000	pigs
Lxn	0.04	1.00000000	pigs
Zfp408	-0.02	1.00000000	pigs
Gyg	-0.02	1.00000000	pigs
Clk3	-0.03	1.00000000	pigs
Dym	0.10	1.00000000	pigs
Slc17a9	0.03	1.00000000	pigs
Rangrf	-0.13	1.00000000	pigs
Zmynd8	-0.01	1.00000000	pigs
Cxcr4	0.01	1.00000000	pigs
Cmc1	0.08	1.00000000	pigs
Itpr3	0.01	1.00000000	pigs
Ubxn1	0.06	1.00000000	pigs
Paf1	0.06	1.00000000	pigs
Dcaf13	-0.04	1.00000000	pigs
Cactin	0.01	1.00000000	pigs
Xrcc5	-0.23	1.00000000	pigs
Dtx3	0.04	1.00000000	pigs
Gcsam	-0.45	1.00000000	pigs
Atf6b	0.04	1.00000000	pigs
Snx10	0.09	1.00000000	pigs
Strada	0.04	1.00000000	pigs
Rab4a	-0.33	1.00000000	pigs
Grsf1	0.09	1.00000000	pigs
Kcmf1	0.03	1.00000000	pigs
Cpsf2	0.06	1.00000000	pigs
Flot1	-0.06	1.00000000	pigs
Ccser2	-0.09	1.00000000	pigs
Cul4b	-0.11	1.00000000	pigs
Dnajc10	-0.40	1.00000000	pigs
Ebag9	0.06	1.00000000	pigs
Eif2ak4	0.07	1.00000000	pigs
Cant1	0.04	1.00000000	pigs
Fbxo21	-0.13	1.00000000	pigs
Tmem63a	0.08	1.00000000	pigs
Dcakd	-0.03	1.00000000	pigs
Shld1	-0.38	1.00000000	pigs
Adh5	-0.03	1.00000000	pigs
Pbdc1	-0.13	1.00000000	pigs
Reps1	-0.02	1.00000000	pigs
Sco2	-0.11	1.00000000	pigs
Abi1	0.10	1.00000000	pigs
Tut4	-0.60	1.00000000	pigs
Slc9a3r1	-0.02	1.00000000	pigs
Ptcd3	0.20	1.00000000	pigs

Dnajc17	0.06	1.00000000	pigs
Usp48	0.02	1.00000000	pigs
Ptprc	-0.12	1.00000000	pigs
Tmem128	0.01	1.00000000	pigs
Pdss2	-0.15	1.00000000	pigs
Spire1	-0.04	1.00000000	pigs
Akna	0.06	1.00000000	pigs
Hsbp1	0.01	1.00000000	pigs
Snrpe	-0.36	1.00000000	pigs
Vcpip1	0.03	1.00000000	pigs
Stam	-0.17	1.00000000	pigs
Trp53inp1	-0.35	1.00000000	pigs
Crlf3	0.08	1.00000000	pigs
Ehd1	0.02	1.00000000	pigs
Dock9	0.11	1.00000000	pigs
Zfp341	0.05	1.00000000	pigs
Utp25	0.03	1.00000000	pigs
Zfp628	-0.36	1.00000000	pigs
Zc3h6	-0.09	1.00000000	pigs
Adprh	-0.05	1.00000000	pigs
Bcar3	0.08	1.00000000	pigs
Eri1	0.00	1.00000000	pigs
Fam227b	-0.23	1.00000000	pigs
Setdb2	0.05	1.00000000	pigs
Smu1	-0.13	1.00000000	pigs
Alpk2	-0.21	1.00000000	pigs
Ehd4	0.11	1.00000000	pigs
Cytip	-0.07	1.00000000	pigs
Eif4ebp1	0.02	1.00000000	pigs
Gpn1	0.06	1.00000000	pigs
Uhrf1bp1l	-0.08	1.00000000	pigs
Prmt5	0.00	1.00000000	pigs
Tnfsf12	-0.22	1.00000000	pigs
Fbxo6	0.16	1.00000000	pigs
Pigf	-0.36	1.00000000	pigs
Il1rap	-0.21	1.00000000	pigs
Snrk	-0.01	1.00000000	pigs
Slc35a1	-0.03	1.00000000	pigs
Zc3h14	0.08	1.00000000	pigs
Rab6a	0.01	1.00000000	pigs
Tmem68	0.02	1.00000000	pigs
Tbl2	0.02	1.00000000	pigs
Cat	0.03	1.00000000	pigs
Arl5b	-0.02	1.00000000	pigs
Nox1	-0.16	1.00000000	pigs
Etfa	0.00	1.00000000	pigs

Hip1r	0.09	1.00000000	pigs
Ptpn23	0.05	1.00000000	pigs
Pskh1	0.09	1.00000000	pigs
Bend7	-0.05	1.00000000	pigs
Hadh	0.09	1.00000000	pigs
Pet100	-0.32	1.00000000	pigs
Fam53b	-0.02	1.00000000	pigs
Dynlrb2	-0.32	1.00000000	pigs
Polr3b	0.02	1.00000000	pigs
Rad52	-0.28	1.00000000	pigs
Coq2	0.09	1.00000000	pigs
Abcf1	-0.06	1.00000000	pigs
Was	-0.36	1.00000000	pigs
Gle1	0.09	1.00000000	pigs
Cib1	-0.14	1.00000000	pigs
Rnf217	0.05	1.00000000	pigs
Pdcl	-0.30	1.00000000	pigs
Hspbp1	-0.07	1.00000000	pigs
Ddb2	0.07	1.00000000	pigs
Pgm2	-0.06	1.00000000	pigs
Zmpste24	0.04	1.00000000	pigs
Ogfrl1	-0.03	1.00000000	pigs
Phf5a	-0.50	1.00000000	pigs
Krcc1	-0.23	1.00000000	pigs
Eefsec	0.03	1.00000000	pigs
Polr2a	-0.08	1.00000000	pigs
Tmem131	0.07	1.00000000	pigs
Oga	0.06	1.00000000	pigs
Hbb-bh2	-0.41	1.00000000	pigs
Cops6	-0.07	1.00000000	pigs
Polr1b	0.01	1.00000000	pigs
Ddx31	0.11	1.00000000	pigs
Podn	0.01	1.00000000	pigs
Cog3	-0.09	1.00000000	pigs
Erbin	0.09	1.00000000	pigs
Pole4	-0.11	1.00000000	pigs
Wdr26	0.05	1.00000000	pigs
Sft2d1	0.03	1.00000000	pigs
Ythdc2	0.05	1.00000000	pigs
Lgals3bp	0.02	1.00000000	pigs
Ubash3b	-0.14	1.00000000	pigs
Fhod1	-0.02	1.00000000	pigs
Crtc1	0.14	1.00000000	pigs
Dhx32	-0.02	1.00000000	pigs
Hspa5	0.01	1.00000000	pigs
Nol4l	-0.42	1.00000000	pigs

Thap11	0.04	1.00000000	pigs
Rmnd5a	0.05	1.00000000	pigs
Rbmx2	0.00	1.00000000	pigs
Osbpl2	-0.27	1.00000000	pigs
Rnf4	-0.01	1.00000000	pigs
Slc43a2	0.07	1.00000000	pigs
Itgb5	-0.26	1.00000000	pigs
Nrip1	-0.53	1.00000000	pigs
Bloc1s5	0.01	1.00000000	pigs
Irak1	-0.01	1.00000000	pigs
Eif4g1	0.06	1.00000000	pigs
Nedd4	-0.30	1.00000000	pigs
Bud31	-0.02	1.00000000	pigs
Wipi2	0.00	1.00000000	pigs
Pacs1	-0.42	1.00000000	pigs
Pex1	0.06	1.00000000	pigs
Exosc10	0.02	1.00000000	pigs
Sntb2	0.04	1.00000000	pigs
Ppp1r16b	-0.36	1.00000000	pigs
Cxcr5	0.01	1.00000000	pigs
Casd1	0.03	1.00000000	pigs
Dad1	-0.08	1.00000000	pigs
Smim7	-0.04	1.00000000	pigs
Crls1	0.00	1.00000000	pigs
Rgs10	0.10	1.00000000	pigs
Fbxo4	0.05	1.00000000	pigs
Eloc	0.02	1.00000000	pigs
Supt20	-0.05	1.00000000	pigs
Clp1	0.08	1.00000000	pigs
Acot13	-0.05	1.00000000	pigs
Arfgap3	0.04	1.00000000	pigs
Litaf	-0.07	1.00000000	pigs
Otud5	0.03	1.00000000	pigs
Rbx1	-0.38	1.00000000	pigs
Ezh1	0.02	1.00000000	pigs
Tpm4	0.06	1.00000000	pigs
Shoc2	-0.39	1.00000000	pigs
Fdx1	-0.08	1.00000000	pigs
Slco3a1	0.07	1.00000000	pigs
Jade2	0.06	1.00000000	pigs
Rab3gap1	0.13	1.00000000	pigs
Irs2	-0.37	1.00000000	pigs
Taf4b	0.15	1.00000000	pigs
Btg1b	-0.17	1.00000000	pigs
Romo1	-0.33	1.00000000	pigs
Fam98c	0.06	1.00000000	pigs

Dusp5	0.06	1.00000000	pigs
Alg5	0.08	1.00000000	pigs
Tram1	-0.08	1.00000000	pigs
Tab3	-0.05	1.00000000	pigs
Unc45a	0.08	1.00000000	pigs
Aga	-0.04	1.00000000	pigs
Rhoh	0.14	1.00000000	pigs
Piga	0.07	1.00000000	pigs
Arid2	0.04	1.00000000	pigs
Rubcn	-0.03	1.00000000	pigs
Kras	0.05	1.00000000	pigs
Rbck1	-0.03	1.00000000	pigs
Pot1b	-0.09	1.00000000	pigs
Ap2a2	0.01	1.00000000	pigs
Ccdc62	0.03	1.00000000	pigs
Gsap	0.01	1.00000000	pigs
Ahi1	0.11	1.00000000	pigs
Slc16a1	-0.04	1.00000000	pigs
Usf3	0.01	1.00000000	pigs
Atxn2	0.09	1.00000000	pigs
Eny2	-0.05	1.00000000	pigs
Rab29	-0.04	1.00000000	pigs
Tubb2b	-0.03	1.00000000	pigs
Vps39	0.02	1.00000000	pigs
Virma	-0.14	1.00000000	pigs
Adipor2	0.04	1.00000000	pigs
Ptpn9	-0.33	1.00000000	pigs
Phf10	0.04	1.00000000	pigs
Acin1	0.05	1.00000000	pigs
Tmem115	-0.06	1.00000000	pigs
Smad4	0.04	1.00000000	pigs
Tmem260	-0.42	1.00000000	pigs
Ube2g2	0.05	1.00000000	pigs
Fmr1	0.05	1.00000000	pigs
Ccdc66	-0.01	1.00000000	pigs
Alkbh5	0.01	1.00000000	pigs
Tyrobp	-0.13	1.00000000	pigs
Irak3	-0.27	1.00000000	pigs
Tbl3	0.03	1.00000000	pigs
Eif4h	0.03	1.00000000	pigs
Yipf4	-0.03	1.00000000	pigs
Lss	0.00	1.00000000	pigs
Satb1	0.10	1.00000000	pigs
Zfand3	-0.05	1.00000000	pigs
Arhgap35	0.01	1.00000000	pigs
Ring1	0.09	1.00000000	pigs

Lrrc58	0.07	1.00000000	pigs
Got1	-0.04	1.00000000	pigs
Ppm1d	-0.11	1.00000000	pigs
Dnttip2	0.05	1.00000000	pigs
Thumpd2	-0.35	1.00000000	pigs
Socs1	-0.10	1.00000000	pigs
LTO1	-0.25	1.00000000	pigs
Ppp5c	0.05	1.00000000	pigs
Bank1	0.07	1.00000000	pigs
Ndufs2	0.00	1.00000000	pigs
Prmt2	0.07	1.00000000	pigs
Sp3	-0.18	1.00000000	pigs
H13	0.02	1.00000000	pigs
Dennd1a	-0.03	1.00000000	pigs
Tbcb	-0.05	1.00000000	pigs
Srprb	0.06	1.00000000	pigs
Kbtbd2	0.04	1.00000000	pigs
Pim3	-0.05	1.00000000	pigs
Tfb1m	0.06	1.00000000	pigs
Nup54	0.10	1.00000000	pigs
Cobll1	-0.36	1.00000000	pigs
Kmt5a	0.08	1.00000000	pigs
Anapc2	0.03	1.00000000	pigs
Lcp2	-0.23	1.00000000	pigs
Acaa2	-0.26	1.00000000	pigs
Tnrc18	0.03	1.00000000	pigs
Nap1l1	-0.21	1.00000000	pigs
Spast	0.03	1.00000000	pigs
Hspa14	0.02	1.00000000	pigs
Surf6	0.05	1.00000000	pigs
Lsm2	-0.05	1.00000000	pigs
Hnrnpa3	0.03	1.00000000	pigs
Zhx1	-0.01	1.00000000	pigs
Prlr	-0.23	1.00000000	pigs
Uqcrc2	-0.05	1.00000000	pigs
Cd2bp2	-0.01	1.00000000	pigs
Atg101	-0.11	1.00000000	pigs
Pdhx	0.03	1.00000000	pigs
Edc3	0.08	1.00000000	pigs
Prpf31	-0.03	1.00000000	pigs
Herc6	0.23	1.00000000	pigs
Apip	-0.05	1.00000000	pigs
Exosc5	-0.06	1.00000000	pigs
Cln3	0.00	1.00000000	pigs
Fubp1	0.06	1.00000000	pigs
Xpo4	0.09	1.00000000	pigs

Exosc9	-0.09	1.00000000	pigs
Rad17	-0.14	1.00000000	pigs
Snrnp40	0.01	1.00000000	pigs
0610030E20Rik	0.00	1.00000000	pigs
Wdhd1	-0.26	1.00000000	pigs
Cbfa2t2	-0.17	1.00000000	pigs
Card19	0.06	1.00000000	pigs
Ets2	0.00	1.00000000	pigs
Lamtor3	-0.04	1.00000000	pigs
Gorab	-0.12	1.00000000	pigs
Utp6	-0.12	1.00000000	pigs
Erc1	-0.04	1.00000000	pigs
Ralbp1	-0.07	1.00000000	pigs
Arl6ip4	-0.19	1.00000000	pigs
Nfu1	-0.05	1.00000000	pigs
Capza2	-0.09	1.00000000	pigs
Pomp	-0.09	1.00000000	pigs
Parvb	0.07	1.00000000	pigs
Il2ra	0.09	1.00000000	pigs
Tle5	-0.41	1.00000000	pigs
Tfdp2	-0.28	1.00000000	pigs
Pip5k1c	-0.02	1.00000000	pigs
Ppp1r12c	0.10	1.00000000	pigs
Fem1b	-0.02	1.00000000	pigs
Trpv2	0.01	1.00000000	pigs
Timm8a2	0.06	1.00000000	pigs
Arhgap45	-0.01	1.00000000	pigs
Tpk1	0.02	1.00000000	pigs
Nbeal1	-0.14	1.00000000	pigs
Nupl2	-0.27	1.00000000	pigs
Arhgap31	-0.04	1.00000000	pigs
Tmcc1	0.00	1.00000000	pigs
Twf1	0.01	1.00000000	pigs
DPCD	0.09	1.00000000	pigs
Jak1	0.10	1.00000000	pigs
Atf1	0.04	1.00000000	pigs
Tmem245	-0.06	1.00000000	pigs
Eif4e3	-0.40	1.00000000	pigs
Pkn2	0.03	1.00000000	pigs
Cage1	-0.43	1.00000000	pigs
Timm10	-0.43	1.00000000	pigs
Gfus	0.00	1.00000000	pigs
Deaf1	-0.06	1.00000000	pigs
Ppil1	-0.08	1.00000000	pigs
Dnase1l1	-0.05	1.00000000	pigs
Ube2m	0.02	1.00000000	pigs

Inppl1	0.02	1.00000000	pigs
Bmt2	-0.06	1.00000000	pigs
Sgpl1	0.07	1.00000000	pigs
Rnf150	-0.22	1.00000000	pigs
Snx6	0.09	1.00000000	pigs
Fut8	-0.26	1.00000000	pigs
Rabep2	-0.07	1.00000000	pigs
Fli1	-0.36	1.00000000	pigs
Hddc2	0.00	1.00000000	pigs
St6gal1	-0.21	1.00000000	pigs
Zdhhc18	-0.02	1.00000000	pigs
Kctd9	-0.02	1.00000000	pigs
Vapa	-0.01	1.00000000	pigs
Ndufb4	-0.32	1.00000000	pigs
Olfml3	-0.05	1.00000000	pigs
Smarca5	0.08	1.00000000	pigs
Tgfbr1	-0.02	1.00000000	pigs
Iqsec1	0.13	1.00000000	pigs
Spg11	-0.28	1.00000000	pigs
Invs	-0.40	1.00000000	pigs
Nudc	0.07	1.00000000	pigs
Edem3	-0.05	1.00000000	pigs
Sdccag8	-0.08	1.00000000	pigs
Eif2b5	-0.05	1.00000000	pigs
Coq4	-0.05	1.00000000	pigs
Polr1e	0.00	1.00000000	pigs
Pcca	-0.22	1.00000000	pigs
Rfc3	0.12	1.00000000	pigs
Snx17	-0.07	1.00000000	pigs
Phrf1	0.05	1.00000000	pigs
Slc35b2	-0.09	1.00000000	pigs
Haus1	0.03	1.00000000	pigs
Nudcd3	0.01	1.00000000	pigs
Plpbp	-0.07	1.00000000	pigs
Ccdc15	-0.35	1.00000000	pigs
Tctn2	-0.28	1.00000000	pigs
Ncbp3	-0.03	1.00000000	pigs
Hexim2	-0.28	1.00000000	pigs
Atg2b	0.02	1.00000000	pigs
Gabpb1	-0.09	1.00000000	pigs
Ripk1	-0.24	1.00000000	pigs
Irf5	0.01	1.00000000	pigs
Ubac2	0.18	1.00000000	pigs
Elk4	0.12	1.00000000	pigs
Tbl1xr1	0.01	1.00000000	pigs
Etv3	-0.03	1.00000000	pigs

Ncbp1	-0.03	1.00000000	pigs
Fam222b	-0.55	1.00000000	pigs
Pef1	-0.07	1.00000000	pigs
Psmd14	-0.05	1.00000000	pigs
H2-Ea	-0.23	1.00000000	pigs
Ypel2	-0.02	1.00000000	pigs
Gde1	0.06	1.00000000	pigs
Fbxo42	0.06	1.00000000	pigs
Nudt13	-0.41	1.00000000	pigs
Usp34	0.09	1.00000000	pigs
Rpf2	-0.05	1.00000000	pigs
Cdk4	-0.01	1.00000000	pigs
Eif4e	-0.04	1.00000000	pigs
Vamp5	-0.06	1.00000000	pigs
Pus3	-0.01	1.00000000	pigs
Ubr3	-0.16	1.00000000	pigs
Akip1	-0.04	1.00000000	pigs
Stx8	-0.06	1.00000000	pigs
Ccng1	-0.08	1.00000000	pigs
Qtrt2	0.01	1.00000000	pigs
Cebpb	-0.40	1.00000000	pigs
Abhd17b	-0.08	1.00000000	pigs
Dhx9	0.03	1.00000000	pigs
Sbno2	0.01	1.00000000	pigs
Herc2	-0.07	1.00000000	pigs
Elp1	-0.06	1.00000000	pigs
Galnt2	0.02	1.00000000	pigs
Cspp1	0.14	1.00000000	pigs
Dctn5	0.00	1.00000000	pigs
Dnttip1	-0.01	1.00000000	pigs
Rrp9	0.05	1.00000000	pigs
Kif2a	0.05	1.00000000	pigs
Prpf8	0.02	1.00000000	pigs
Fnbp4	0.02	1.00000000	pigs
Stxbp3	-0.06	1.00000000	pigs
Cdkl5	-0.23	1.00000000	pigs
Dnpep	-0.07	1.00000000	pigs
Nfkb1	0.11	1.00000000	pigs
Npc1	0.16	1.00000000	pigs
Dynlrb1	-0.11	1.00000000	pigs
Kctd10	-0.10	1.00000000	pigs
Atf2	0.05	1.00000000	pigs
Srpr	-0.05	1.00000000	pigs
Sppl3	0.06	1.00000000	pigs
Siah1b	0.02	1.00000000	pigs
Phf1	0.02	1.00000000	pigs

Pigb	0.09	1.00000000	pigs
Apmap	0.01	1.00000000	pigs
Rprd1b	-0.02	1.00000000	pigs
Polr2f	-0.10	1.00000000	pigs
Cers2	-0.03	1.00000000	pigs
Utrn	-0.03	1.00000000	pigs
Slc6a3	-0.26	1.00000000	pigs
Hdac9	-0.34	1.00000000	pigs
Naa30	-0.11	1.00000000	pigs
Gab2	-0.35	1.00000000	pigs
Tdp2	0.04	1.00000000	pigs
Sdcbp	-0.04	1.00000000	pigs
Canx	0.01	1.00000000	pigs
Flvcr1	-0.08	1.00000000	pigs
Desi2	-0.05	1.00000000	pigs
Tcf20	-0.02	1.00000000	pigs
Nifk	0.05	1.00000000	pigs
Ncstn	-0.06	1.00000000	pigs
Zbtb43	0.09	1.00000000	pigs
Bloc1s2	-0.16	1.00000000	pigs
Herc4	0.07	1.00000000	pigs
Slc10a1	-0.13	1.00000000	pigs
Dhx38	-0.01	1.00000000	pigs
H2-Ke6	-0.05	1.00000000	pigs
Lrba	-0.30	1.00000000	pigs
Ttc37	0.12	1.00000000	pigs
Ttf2	0.06	1.00000000	pigs
Cox4i1	-0.19	1.00000000	pigs
Sbf2	-0.35	1.00000000	pigs
Eif2b1	-0.40	1.00000000	pigs
Prdm15	0.03	1.00000000	pigs
Kif20b	-0.01	1.00000000	pigs
Ikbkb	-0.30	1.00000000	pigs
Trappc13	-0.09	1.00000000	pigs
Denr	-0.06	1.00000000	pigs
Tbpl1	-0.05	1.00000000	pigs
Rcan3	-0.10	1.00000000	pigs
Dnmbp	-0.20	1.00000000	pigs
Pds5a	0.00	1.00000000	pigs
Smpd2	-0.06	1.00000000	pigs
Scamp4	-0.22	1.00000000	pigs
Rtf1	-0.06	1.00000000	pigs
Ep300	0.09	1.00000000	pigs
Nsfl1c	-0.05	1.00000000	pigs
Sh3gl1	-0.05	1.00000000	pigs
Zfand2b	0.01	1.00000000	pigs

Tex14	-0.08	1.00000000	pigs
Rnf38	-0.02	1.00000000	pigs
Calcoco2	-0.06	1.00000000	pigs
Trim23	0.01	1.00000000	pigs
B3gat3	0.02	1.00000000	pigs
Fbxo38	-0.01	1.00000000	pigs
Apobec3	0.01	1.00000000	pigs
Rap1a	-0.01	1.00000000	pigs
Zc3h8	-0.02	1.00000000	pigs
Cdc37l1	-0.03	1.00000000	pigs
Ddx39b	-0.33	1.00000000	pigs
Ifih1	-0.06	1.00000000	pigs
Ddrgk1	0.00	1.00000000	pigs
Atp6v1f	-0.02	1.00000000	pigs
Emilin2	0.08	1.00000000	pigs
Sumf1	-0.11	1.00000000	pigs
Prmt1	-0.02	1.00000000	pigs
Capzb	0.02	1.00000000	pigs
Wipf2	-0.02	1.00000000	pigs
Cdk17	0.04	1.00000000	pigs
Clptm1	0.01	1.00000000	pigs
Dcps	-0.18	1.00000000	pigs
Camk1d	0.18	1.00000000	pigs
Hipk1	-0.04	1.00000000	pigs
Taldo1	-0.12	1.00000000	pigs
Vps16	-0.02	1.00000000	pigs
Dnajc24	-0.26	1.00000000	pigs
Polr2d	-0.50	1.00000000	pigs
Hrh4	-0.23	1.00000000	pigs
Usp22	-0.01	1.00000000	pigs
Lin37	-0.03	1.00000000	pigs
Slc39a7	-0.05	1.00000000	pigs
Zdhhc3	-0.04	1.00000000	pigs
D8Ertd738e	-0.12	1.00000000	pigs
H2-Ab1	0.06	1.00000000	pigs
Zc3h7b	-0.04	1.00000000	pigs
Dcaf10	-0.14	1.00000000	pigs
Cdipt	-0.10	1.00000000	pigs
Tst	0.04	1.00000000	pigs
Borcs6	0.05	1.00000000	pigs
Gid4	0.04	1.00000000	pigs
Tmem147	-0.07	1.00000000	pigs
Znhit6	0.02	1.00000000	pigs
Adcy7	0.00	1.00000000	pigs
Optn	0.03	1.00000000	pigs
Avl9	-0.12	1.00000000	pigs

Cmtr1	-0.41	1.00000000	pigs
Zbtb25	0.02	1.00000000	pigs
Ccnb1ip1	-0.08	1.00000000	pigs
Ptk2b	-0.01	1.00000000	pigs
Capn15	0.05	1.00000000	pigs
Kcnab3	-0.12	1.00000000	pigs
Naa20	-0.06	1.00000000	pigs
Klhl18	-0.19	1.00000000	pigs
Ftsj1	-0.01	1.00000000	pigs
B4galt3	0.10	1.00000000	pigs
Tnk2	0.03	1.00000000	pigs
Aip	-0.06	1.00000000	pigs
Rdh13	-0.35	1.00000000	pigs
Gnl3l	-0.01	1.00000000	pigs
Ext1	-0.08	1.00000000	pigs
Cd200	-0.14	1.00000000	pigs
Glmn	-0.40	1.00000000	pigs
Grk6	-0.31	1.00000000	pigs
Atp6v1h	-0.01	1.00000000	pigs
Dnajb9	0.07	1.00000000	pigs
Tsc22d1	0.02	1.00000000	pigs
Zfp146	-0.07	1.00000000	pigs
Rbbp8	-0.01	1.00000000	pigs
Nlrc3	-0.20	1.00000000	pigs
Grk4	-0.19	1.00000000	pigs
Uhmk1	0.07	1.00000000	pigs
Cand1	-0.02	1.00000000	pigs
Spcs2	0.06	1.00000000	pigs
Pik3cg	-0.03	1.00000000	pigs
Galk1	0.01	1.00000000	pigs
Plod3	-0.02	1.00000000	pigs
Hnrnpm	0.02	1.00000000	pigs
Plec	-0.02	1.00000000	pigs
Slc16a10	0.11	1.00000000	pigs
Fxr2	0.00	1.00000000	pigs
Gspt2	-0.36	1.00000000	pigs
Erlec1	0.02	1.00000000	pigs
Ccdc25	-0.09	1.00000000	pigs
Pip4k2a	0.00	1.00000000	pigs
Ciao2b	-0.02	1.00000000	pigs
Phf14	-0.08	1.00000000	pigs
Tesc	-0.30	1.00000000	pigs
Psmg3	0.00	1.00000000	pigs
Ube3a	0.05	1.00000000	pigs
Golim4	0.06	1.00000000	pigs
Surf1	-0.27	1.00000000	pigs

Chst7	-0.12	1.00000000	pigs
Tax1bp1	0.04	1.00000000	pigs
Nfya	-0.15	1.00000000	pigs
Polr1h	-0.26	1.00000000	pigs
Psme2	-0.07	1.00000000	pigs
Ttc17	-0.12	1.00000000	pigs
Sprtn	-0.07	1.00000000	pigs
Slc44a2	-0.02	1.00000000	pigs
Rab11a	-0.09	1.00000000	pigs
Dele1	0.06	1.00000000	pigs
Pinx1	-0.07	1.00000000	pigs
Pim1	-0.04	1.00000000	pigs
Pgam2	0.02	1.00000000	pigs
Zeb2	0.24	1.00000000	pigs
Nudt15	-0.31	1.00000000	pigs
Slc35b1	-0.07	1.00000000	pigs
Eapp	-0.37	1.00000000	pigs
Cdc40	-0.06	1.00000000	pigs
Tfip11	-0.18	1.00000000	pigs
Rassf3	-0.23	1.00000000	pigs
Cul5	-0.09	1.00000000	pigs
Cldnd1	0.03	1.00000000	pigs
Slc3a2	0.05	1.00000000	pigs
Srp68	-0.13	1.00000000	pigs
Ak6	0.00	1.00000000	pigs
Dhx40	-0.14	1.00000000	pigs
Cops7a	-0.20	1.00000000	pigs
Cisd2	-0.12	1.00000000	pigs
Fam89a	-0.30	1.00000000	pigs
Slamf6	0.05	1.00000000	pigs
Arf4	0.01	1.00000000	pigs
Dicer1	-0.11	1.00000000	pigs
Arhgef26	-0.27	1.00000000	pigs
Arsa	-0.21	1.00000000	pigs
Nnt	-0.37	1.00000000	pigs
Rbis	-0.05	1.00000000	pigs
Cmas	-0.01	1.00000000	pigs
Tmtc3	0.01	1.00000000	pigs
Tufm	0.02	1.00000000	pigs
Zmat5	0.08	1.00000000	pigs
Wwp2	-0.35	1.00000000	pigs
Klf6	-0.32	1.00000000	pigs
Acadm	-0.06	1.00000000	pigs
Bsdc1	-0.01	1.00000000	pigs
Usp1	-0.01	1.00000000	pigs
Nans	-0.01	1.00000000	pigs

Prkaa1	-0.09	1.00000000	pigs
A130010J15Rik	-0.11	1.00000000	pigs
Cep135	-0.18	1.00000000	pigs
Pgp	0.02	1.00000000	pigs
Atrn	-0.11	1.00000000	pigs
Itfg1	-0.36	1.00000000	pigs
Thrap3	0.08	1.00000000	pigs
Epn1	-0.02	1.00000000	pigs
Tbc1d22b	0.02	1.00000000	pigs
Exosc1	0.02	1.00000000	pigs
Cep162	-0.22	1.00000000	pigs
Slc6a6	0.02	1.00000000	pigs
Skp2	0.05	1.00000000	pigs
Ing2	0.06	1.00000000	pigs
Paip1	-0.05	1.00000000	pigs
Abhd15	0.00	1.00000000	pigs
Traf6	-0.08	1.00000000	pigs
Uba5	-0.04	1.00000000	pigs
Lrrcc1	0.03	1.00000000	pigs
Zfp318	-0.33	1.00000000	pigs
Limd2	-0.30	1.00000000	pigs
Ap3m1	0.01	1.00000000	pigs
Phtf2	-0.36	1.00000000	pigs
Alox5ap	-0.24	1.00000000	pigs
Rgs2	-0.05	1.00000000	pigs
Nr4a1	-0.23	1.00000000	pigs
Stk19	-0.01	1.00000000	pigs
Osgep	-0.01	1.00000000	pigs
Aen	0.00	1.00000000	pigs
Scamp3	-0.02	1.00000000	pigs
Rfc4	-0.31	1.00000000	pigs
Casp8	-0.06	1.00000000	pigs
Ostc	-0.08	1.00000000	pigs
4833439L19Rik	-0.06	1.00000000	pigs
Oas2	0.08	1.00000000	pigs
Wdfy2	-0.20	1.00000000	pigs
Sgpp1	0.05	1.00000000	pigs
Sf3a3	-0.05	1.00000000	pigs
Tmx4	0.07	1.00000000	pigs
Zbtb7a	0.03	1.00000000	pigs
Dync1li2	-0.02	1.00000000	pigs
Usp8	-0.11	1.00000000	pigs
Nbn	-0.02	1.00000000	pigs
Nipbl	0.12	1.00000000	pigs
Stag1	-0.23	1.00000000	pigs
Bpnt2	-0.22	1.00000000	pigs

Fam120c	0.03	1.00000000	pigs
Ddx18	0.05	1.00000000	pigs
Ptar1	0.08	1.00000000	pigs
Ilk	-0.07	1.00000000	pigs
Bin2	-0.38	1.00000000	pigs
Selenof	-0.27	1.00000000	pigs
L2hgdh	-0.13	1.00000000	pigs
Cd24a	-0.23	1.00000000	pigs
Ufc1	-0.01	1.00000000	pigs
Wdr5	-0.01	1.00000000	pigs
Casp8ap2	-0.23	1.00000000	pigs
Eftud2	-0.13	1.00000000	pigs
Eif2b4	0.10	1.00000000	pigs
Pepd	-0.02	1.00000000	pigs
Aopep	-0.29	1.00000000	pigs
Dnaja3	-0.07	1.00000000	pigs
Gnb1l	-0.02	1.00000000	pigs
Fbxo3	0.02	1.00000000	pigs
Ccnq	-0.07	1.00000000	pigs
Trub2	-0.06	1.00000000	pigs
Smarcc1	-0.03	1.00000000	pigs
Pbx3	-0.02	1.00000000	pigs
Poglut1	-0.02	1.00000000	pigs
Ivns1abp	-0.12	1.00000000	pigs
Naa35	0.00	1.00000000	pigs
Bin3	0.03	1.00000000	pigs
Acot10	-0.07	1.00000000	pigs
Ppp1r10	-0.45	1.00000000	pigs
Glce	0.06	1.00000000	pigs
Ssr2	-0.03	1.00000000	pigs
Tsen34	-0.05	1.00000000	pigs
Tnrc6a	-0.13	1.00000000	pigs
Rrm1	0.06	1.00000000	pigs
Coil	-0.05	1.00000000	pigs
Ccpg1	-0.35	1.00000000	pigs
Washc1	0.03	1.00000000	pigs
Tnks	0.01	1.00000000	pigs
Zfp787	0.01	1.00000000	pigs
Dock10	-0.52	1.00000000	pigs
Fbxo25	-0.17	1.00000000	pigs
Ube2v2	-0.08	1.00000000	pigs
Pum3	0.01	1.00000000	pigs
Tomm40	-0.02	1.00000000	pigs
Xpa	-0.29	1.00000000	pigs
Ppp1r35	-0.01	1.00000000	pigs
Cep78	-0.08	1.00000000	pigs

Fgfr1op2	-0.18	1.00000000	pigs
Rnft1	0.06	1.00000000	pigs
Ptpn4	0.01	1.00000000	pigs
Nrg1	-0.65	1.00000000	pigs
Vcpkmt	-0.04	1.00000000	pigs
Cables1	0.23	1.00000000	pigs
Sigirr	-0.24	1.00000000	pigs
Cherp	-0.10	1.00000000	pigs
Dph6	0.08	1.00000000	pigs
Brms1l	-0.11	1.00000000	pigs
Tlcd2	-0.05	1.00000000	pigs
Hif1an	-0.09	1.00000000	pigs
Haus6	-0.06	1.00000000	pigs
Rad21	-0.01	1.00000000	pigs
Rnf169	0.07	1.00000000	pigs
Col3a1	0.10	1.00000000	pigs
Ecsit	-0.15	1.00000000	pigs
Ptpdc1	-0.09	1.00000000	pigs
Zhx3	0.14	1.00000000	pigs
Rars	-0.01	1.00000000	pigs
Tmem123	-0.19	1.00000000	pigs
Glmp	-0.07	1.00000000	pigs
Zc3h15	-0.08	1.00000000	pigs
Lrpap1	-0.01	1.00000000	pigs
Pelo	-0.07	1.00000000	pigs
Wdfy3	-0.32	1.00000000	pigs
Parp14	0.07	1.00000000	pigs
Commd8	0.07	1.00000000	pigs
Kansl1l	0.07	1.00000000	pigs
Nr4a2	-0.11	1.00000000	pigs
Tmem248	0.02	1.00000000	pigs
1110059G10Rik	0.01	1.00000000	pigs
Zfp384	-0.21	1.00000000	pigs
Pou2f1	-0.02	1.00000000	pigs
Sar1b	-0.08	1.00000000	pigs
6030458C11Rik	-0.03	1.00000000	pigs
Tmed9	-0.11	1.00000000	pigs
Atp2a3	-0.05	1.00000000	pigs
Zdhhc9	0.04	1.00000000	pigs
Elp5	0.00	1.00000000	pigs
Brap	-0.03	1.00000000	pigs
Derpc	-0.01	1.00000000	pigs
Phf8	0.09	1.00000000	pigs
Rbm5	0.01	1.00000000	pigs
Exoc4	0.05	1.00000000	pigs
Tmc1	-0.39	1.00000000	pigs

Pja2	-0.07	1.00000000	pigs
Ncald	0.10	1.00000000	pigs
Trim38	-0.27	1.00000000	pigs
Qrich1	0.13	1.00000000	pigs
Atp5g3	-0.39	1.00000000	pigs
Cul4a	-0.08	1.00000000	pigs
Gtpbp4	-0.12	1.00000000	pigs
Fam8a1	0.00	1.00000000	pigs
Rsrp1	0.06	1.00000000	pigs
Raly	0.10	1.00000000	pigs
Snrpf	-0.05	1.00000000	pigs
Usp3	0.04	1.00000000	pigs
Nmi	0.10	1.00000000	pigs
Cdc123	-0.08	1.00000000	pigs
Tspan3	-0.38	1.00000000	pigs
Zfp518b	0.07	1.00000000	pigs
Zc3h13	0.08	1.00000000	pigs
Rbm15	-0.06	1.00000000	pigs
Ung	-0.16	1.00000000	pigs
Srsf7	-0.05	1.00000000	pigs
Strn4	0.06	1.00000000	pigs
Pdrg1	0.00	1.00000000	pigs
Ikzf2	0.20	1.00000000	pigs
Arrdc3	0.18	1.00000000	pigs
Nprl3	0.03	1.00000000	pigs
Pex3	-0.34	1.00000000	pigs
Taok3	-0.09	1.00000000	pigs
Rgl2	-0.17	1.00000000	pigs
Phf23	-0.03	1.00000000	pigs
Noct	-0.04	1.00000000	pigs
Bcas3	0.09	1.00000000	pigs
Rasgrp3	-0.33	1.00000000	pigs
Arglu1	-0.32	1.00000000	pigs
Ndufs3	-0.06	1.00000000	pigs
Ppp1r15a	0.22	1.00000000	pigs
Unc50	-0.04	1.00000000	pigs
Ggnbp2	-0.06	1.00000000	pigs
Gga2	-0.04	1.00000000	pigs
Psmd12	-0.06	1.00000000	pigs
Paip2	-0.21	1.00000000	pigs
Ibtk	-0.12	1.00000000	pigs
Tti2	0.06	1.00000000	pigs
Ncapd2	0.02	1.00000000	pigs
Chpf2	-0.18	1.00000000	pigs
Slc38a9	-0.40	1.00000000	pigs
Ubr1	0.01	1.00000000	pigs

Gpatch11	0.01	1.00000000	pigs
Ginm1	-0.32	1.00000000	pigs
Thap7	0.04	1.00000000	pigs
Cops3	-0.08	1.00000000	pigs
Kdm1a	-0.01	1.00000000	pigs
Socs6	0.07	1.00000000	pigs
Snapc2	0.03	1.00000000	pigs
Dhrs11	-0.06	1.00000000	pigs
Gck	-0.21	1.00000000	pigs
Hmgb2	-0.31	1.00000000	pigs
Selenow	-0.12	1.00000000	pigs
Zbtb22	-0.06	1.00000000	pigs
Sec13	-0.11	1.00000000	pigs
Sin3b	0.00	1.00000000	pigs
Eprs	0.00	1.00000000	pigs
Rmdn1	-0.09	1.00000000	pigs
Slc35b4	0.02	1.00000000	pigs
Inpp1	-0.18	1.00000000	pigs
G6pc3	0.07	1.00000000	pigs
Ccdc59	0.06	1.00000000	pigs
Tpr	0.05	1.00000000	pigs
Tmem65	0.12	1.00000000	pigs
Tmem251	-0.10	1.00000000	pigs
Daglb	-0.05	1.00000000	pigs
Ndufaf2	0.14	1.00000000	pigs
Ankle2	0.03	1.00000000	pigs
Ttc33	-0.04	1.00000000	pigs
Eea1	-0.26	1.00000000	pigs
Spata21	-0.05	1.00000000	pigs
Nfyc	-0.13	1.00000000	pigs
Sit1	-0.09	1.00000000	pigs
Ubap2l	0.04	1.00000000	pigs
Wdfy4	-0.33	1.00000000	pigs
Zbtb32	0.16	1.00000000	pigs
Cad	0.02	1.00000000	pigs
Zfyve9	-0.26	1.00000000	pigs
Ddhd2	-0.01	1.00000000	pigs
Supv3l1	-0.04	1.00000000	pigs
Lin9	-0.11	1.00000000	pigs
Parn	0.02	1.00000000	pigs
Proser1	-0.11	1.00000000	pigs
Gtf2f1	-0.04	1.00000000	pigs
Vps13a	0.01	1.00000000	pigs
2210016L21Rik	0.06	1.00000000	pigs
Vrk1	-0.11	1.00000000	pigs
Hectd1	-0.06	1.00000000	pigs

Ppip5k2	-0.09	1.00000000	pigs
Yipf1	-0.41	1.00000000	pigs
Xylt1	0.11	1.00000000	pigs
Polr2h	-0.12	1.00000000	pigs
Atp5md	-0.46	1.00000000	pigs
Tlr6	-0.01	1.00000000	pigs
Traf3ip2	-0.16	1.00000000	pigs
Zcchc7	-0.33	1.00000000	pigs
Notch1	-0.07	1.00000000	pigs
Wdr7	0.00	1.00000000	pigs
Lmbr1l	-0.13	1.00000000	pigs
Hspa13	-0.07	1.00000000	pigs
Nsd2	-0.12	1.00000000	pigs
Smdt1	-0.41	1.00000000	pigs
Tmco3	-0.04	1.00000000	pigs
Faf2	-0.10	1.00000000	pigs
Cbr4	0.03	1.00000000	pigs
Srp14	-0.20	1.00000000	pigs
Ddi2	0.04	1.00000000	pigs
Kctd13	0.05	1.00000000	pigs
Nipal3	-0.04	1.00000000	pigs
Srsf5	0.00	1.00000000	pigs
Rad18	0.08	1.00000000	pigs
Napa	-0.02	1.00000000	pigs
Supt7l	0.06	1.00000000	pigs
Tcf25	-0.21	1.00000000	pigs
Hk3	-0.04	1.00000000	pigs
Scyl2	0.02	1.00000000	pigs
Donson	0.03	1.00000000	pigs
Srp9	-0.03	1.00000000	pigs
Nfyb	0.03	1.00000000	pigs
Selenot	-0.08	1.00000000	pigs
Immt	0.10	1.00000000	pigs
Tamm41	0.01	1.00000000	pigs
Fam133b	-0.06	1.00000000	pigs
H1f3	-0.16	1.00000000	pigs
L3mbtl1	0.02	1.00000000	pigs
Oard1	-0.01	1.00000000	pigs
Cnot11	-0.12	1.00000000	pigs
Slc25a3	0.05	1.00000000	pigs
Guf1	-0.02	1.00000000	pigs
Rcc1l	-0.04	1.00000000	pigs
Snrpd1	-0.32	1.00000000	pigs
Elf2	-0.07	1.00000000	pigs
Nadk	-0.07	1.00000000	pigs
Fam185a	-0.09	1.00000000	pigs

Kif21b	-0.05	1.00000000	pigs
Cdk2ap2	-0.31	1.00000000	pigs
1810013L24Rik	-0.03	1.00000000	pigs
Akap3	-0.21	1.00000000	pigs
Cox10	0.06	1.00000000	pigs
Ufsp2	0.07	1.00000000	pigs
Lrrc41	0.10	1.00000000	pigs
Flna	-0.32	1.00000000	pigs
Dip2b	-0.04	1.00000000	pigs
Actr3	-0.07	1.00000000	pigs
Rpa3	0.06	1.00000000	pigs
Itsn1	0.03	1.00000000	pigs
Slc39a9	-0.04	1.00000000	pigs
Sptlc1	-0.04	1.00000000	pigs
Hspd1	0.21	1.00000000	pigs
Slc25a24	-0.32	1.00000000	pigs
Nipsnap2	-0.21	1.00000000	pigs
Ifi30	-0.48	1.00000000	pigs
Limd1	-0.04	1.00000000	pigs
Snx27	-0.15	1.00000000	pigs
Btbd19	-0.07	1.00000000	pigs
Ccl5	-0.35	1.00000000	pigs
Gne	-0.06	1.00000000	pigs
Trrap	0.01	1.00000000	pigs
4930453N24Rik	-0.06	1.00000000	pigs
Pus10	-0.48	1.00000000	pigs
Atmin	0.03	1.00000000	pigs
Sidt2	-0.13	1.00000000	pigs
Golm1	0.03	1.00000000	pigs
Ppm1g	0.08	1.00000000	pigs
Zdhhc14	-0.12	1.00000000	pigs
Zbtb2	0.11	1.00000000	pigs
Bola1	-0.44	1.00000000	pigs
Rint1	-0.07	1.00000000	pigs
Prr14l	0.06	1.00000000	pigs
Nudt1	-0.28	1.00000000	pigs
Nup210	0.01	1.00000000	pigs
Higd2a	-0.07	1.00000000	pigs
Cenpw	-0.44	1.00000000	pigs
Tmem60	0.02	1.00000000	pigs
Diablo	-0.10	1.00000000	pigs
S100a11	-0.28	1.00000000	pigs
Aldh18a1	0.01	1.00000000	pigs
Arfgef2	-0.03	1.00000000	pigs
Dcaf15	0.00	1.00000000	pigs
Tssc4	0.02	1.00000000	pigs

Fer	-0.14	1.00000000	pigs
Arcn1	-0.04	1.00000000	pigs
Khsrp	-0.08	1.00000000	pigs
Cep85	0.00	1.00000000	pigs
Tubgcp4	0.06	1.00000000	pigs
Cdk19	-0.26	1.00000000	pigs
Dnmt1	-0.09	1.00000000	pigs
Wdr81	0.04	1.00000000	pigs
Flad1	-0.10	1.00000000	pigs
Ero1a	0.03	1.00000000	pigs
Ikzf5	-0.35	1.00000000	pigs
Upf1	-0.04	1.00000000	pigs
Ptgr1	0.02	1.00000000	pigs
Ckap5	-0.08	1.00000000	pigs
Poldip2	0.03	1.00000000	pigs
Pi4kb	0.05	1.00000000	pigs
Dennd10	-0.01	1.00000000	pigs
Kpna4	-0.04	1.00000000	pigs
Prkcb	0.09	1.00000000	pigs
Bcap31	-0.10	1.00000000	pigs
Ctdsp2	-0.07	1.00000000	pigs
Parp8	-0.14	1.00000000	pigs
Taf15	0.03	1.00000000	pigs
Ptcd2	-0.16	1.00000000	pigs
Fus	-0.22	1.00000000	pigs
Ankmy2	-0.12	1.00000000	pigs
Hcls1	-0.30	1.00000000	pigs
Rnf166	-0.07	1.00000000	pigs
Nmd3	-0.21	1.00000000	pigs
Tars	0.00	1.00000000	pigs
Pdia4	0.05	1.00000000	pigs
Dnajb2	0.00	1.00000000	pigs
Fam89b	-0.19	1.00000000	pigs
Bfar	-0.03	1.00000000	pigs
Srrm1	-0.20	1.00000000	pigs
Angptl4	0.00	1.00000000	pigs
Slc25a37	0.05	1.00000000	pigs
Rhob	0.08	1.00000000	pigs
Top2a	-0.10	1.00000000	pigs
Ubb	-0.18	1.00000000	pigs
Dclre1c	-0.12	1.00000000	pigs
Btaf1	0.07	1.00000000	pigs
Nabp1	-0.05	1.00000000	pigs
Polr1f	-0.10	1.00000000	pigs
Siah2	0.00	1.00000000	pigs
Arv1	-0.13	1.00000000	pigs

Ap2a1	0.06	1.00000000	pigs
Tatdn1	-0.30	1.00000000	pigs
Pir	0.01	1.00000000	pigs
Btbd7	0.01	1.00000000	pigs
Yipf2	-0.31	1.00000000	pigs
Tmem39b	-0.11	1.00000000	pigs
Snw1	-0.17	1.00000000	pigs
Bud13	-0.04	1.00000000	pigs
Tpp1	0.00	1.00000000	pigs
Cnot4	0.00	1.00000000	pigs
Coq5	-0.04	1.00000000	pigs
Lman1	-0.06	1.00000000	pigs
Rbms1	-0.17	1.00000000	pigs
Snrpd2	-0.26	1.00000000	pigs
Dus1l	-0.04	1.00000000	pigs
B3glct	0.07	1.00000000	pigs
Evi2	-0.01	1.00000000	pigs
Zfp664	-0.02	1.00000000	pigs
Reep3	-0.04	1.00000000	pigs
Dazap1	-0.41	1.00000000	pigs
Psme3ip1	-0.27	1.00000000	pigs
Psmc2	-0.34	1.00000000	pigs
Dus4l	-0.04	1.00000000	pigs
Phactr2	-0.35	1.00000000	pigs
Cdc42se1	-0.05	1.00000000	pigs
Svil	-0.20	1.00000000	pigs
Rasgrp1	-0.16	1.00000000	pigs
Slc25a4	-0.21	1.00000000	pigs
Kat8	-0.06	1.00000000	pigs
Ptprcap	-0.34	1.00000000	pigs
Cd81	0.04	1.00000000	pigs
Acads	0.04	1.00000000	pigs
Tbc1d8	0.16	1.00000000	pigs
Oaz2	-0.07	1.00000000	pigs
Arl13b	-0.04	1.00000000	pigs
Brpf3	-0.01	1.00000000	pigs
Taf3	0.00	1.00000000	pigs
Cebpd	-0.22	1.00000000	pigs
Cd274	0.02	1.00000000	pigs
Sh2b3	0.00	1.00000000	pigs
Net1	0.01	1.00000000	pigs
Gabpb2	-0.01	1.00000000	pigs
Phf3	-0.11	1.00000000	pigs
Osgin2	-0.07	1.00000000	pigs
Sergef	0.02	1.00000000	pigs
Hs6st1	-0.08	1.00000000	pigs

Cyhr1	-0.15	1.00000000	pigs
Ccdc9	-0.09	1.00000000	pigs
Fbxo34	-0.08	1.00000000	pigs
Trip4	0.05	1.00000000	pigs
Trmt10c	-0.03	1.00000000	pigs
Tcp11l2	-0.05	1.00000000	pigs
Ppib	-0.08	1.00000000	pigs
Ciz1	-0.20	1.00000000	pigs
Acadl	0.05	1.00000000	pigs
Dlgap4	-0.01	1.00000000	pigs
Timm17b	-0.35	1.00000000	pigs
Tmco1	-0.39	1.00000000	pigs
Fuom	-0.24	1.00000000	pigs
Ss18	-0.20	1.00000000	pigs
Synrg	-0.10	1.00000000	pigs
Gpr18	0.07	1.00000000	pigs
Plk3	-0.05	1.00000000	pigs
Cpne1	-0.12	1.00000000	pigs
Abl1	-0.06	1.00000000	pigs
Tes	-0.05	1.00000000	pigs
Sp1	-0.18	1.00000000	pigs
Ppp1r15b	0.06	1.00000000	pigs
Cfap20dc	-0.06	1.00000000	pigs
Spin1	0.05	1.00000000	pigs
Ppp1r11	-0.21	1.00000000	pigs
Nol11	-0.21	1.00000000	pigs
Gnai3	-0.27	1.00000000	pigs
Casp1	-0.01	1.00000000	pigs
Gdi1	0.01	1.00000000	pigs
Supt6	-0.03	1.00000000	pigs
Fam32a	-0.08	1.00000000	pigs
Nsd3	-0.04	1.00000000	pigs
Faxc	-0.16	1.00000000	pigs
Rnf5	-0.16	1.00000000	pigs
Exoc3	-0.09	1.00000000	pigs
Cars	0.06	1.00000000	pigs
Zmym4	-0.37	1.00000000	pigs
Lonp2	-0.23	1.00000000	pigs
Pitpnm3	0.00	1.00000000	pigs
Ttc28	-0.25	1.00000000	pigs
Siva1	-0.37	1.00000000	pigs
Dusp2	-0.27	1.00000000	pigs
Ddx46	-0.24	1.00000000	pigs
Gabpa	-0.36	1.00000000	pigs
Prkdc	-0.07	1.00000000	pigs
Kcnj13	-0.05	1.00000000	pigs

Pex7	0.03	1.00000000	pigs
Otud7b	-0.05	1.00000000	pigs
Nfe2l1	-0.03	1.00000000	pigs
Ep400	0.00	1.00000000	pigs
Glipr1	-0.01	1.00000000	pigs
Slc35e1	-0.02	1.00000000	pigs
Vrk3	-0.18	1.00000000	pigs
Prdm10	0.01	1.00000000	pigs
Tex30	0.03	1.00000000	pigs
Sipa1l1	-0.30	1.00000000	pigs
Adam8	0.08	1.00000000	pigs
Spred2	-0.02	1.00000000	pigs
Phf21a	0.01	1.00000000	pigs
Pam16	-0.08	1.00000000	pigs
Arg2	-0.01	1.00000000	pigs
Tex264	-0.09	1.00000000	pigs
Bckdk	0.02	1.00000000	pigs
Smc1a	-0.34	1.00000000	pigs
Sltm	-0.06	1.00000000	pigs
Pgrmc1	-0.10	1.00000000	pigs
Arnt	0.03	1.00000000	pigs
Gadd45gip1	-0.19	1.00000000	pigs
Letm1	-0.04	1.00000000	pigs
Brd1	0.00	1.00000000	pigs
Ccdc125	-0.05	1.00000000	pigs
Zfp438	-0.26	1.00000000	pigs
Arl14ep	-0.24	1.00000000	pigs
Evi5	-0.38	1.00000000	pigs
Apex1	0.03	1.00000000	pigs
Polr2c	-0.04	1.00000000	pigs
Fam71e1	-0.17	1.00000000	pigs
Por	-0.06	1.00000000	pigs
Cep295	-0.07	1.00000000	pigs
Parm1	0.12	1.00000000	pigs
Xrcc1	0.00	1.00000000	pigs
Zkscan6	-0.08	1.00000000	pigs
Zdbf2	-0.39	1.00000000	pigs
Nrbf2	-0.04	1.00000000	pigs
Diaph2	-0.22	1.00000000	pigs
Arfip1	-0.08	1.00000000	pigs
Ncoa5	0.02	1.00000000	pigs
Plekhm3	-0.29	1.00000000	pigs
Bag5	0.00	1.00000000	pigs
Lrrc59	-0.19	1.00000000	pigs
Arhgap15	-0.16	1.00000000	pigs
Srp54a	-0.07	1.00000000	pigs

Rbm42	-0.07	1.00000000	pigs
Erbb4	0.10	1.00000000	pigs
Sap130	-0.02	1.00000000	pigs
Utp20	-0.09	1.00000000	pigs
Acp1	-0.10	1.00000000	pigs
Gnpda2	-0.26	1.00000000	pigs
Arf6	-0.16	1.00000000	pigs
Fcho2	-0.19	1.00000000	pigs
Dapp1	-0.22	1.00000000	pigs
Dbnl	-0.15	1.00000000	pigs
Perp	0.07	1.00000000	pigs
B3gntl1	-0.01	1.00000000	pigs
Rfesd	-0.01	1.00000000	pigs
Usp5	0.03	1.00000000	pigs
Dazap2	-0.04	1.00000000	pigs
Dusp1	-0.45	1.00000000	pigs
Ap1s3	-0.10	1.00000000	pigs
Babam2	-0.09	1.00000000	pigs
Rab8b	-0.28	1.00000000	pigs
Zranb2	-0.06	1.00000000	pigs
Dennd1c	0.08	1.00000000	pigs
Rfx1	0.01	1.00000000	pigs
Dennd1b	-0.03	1.00000000	pigs
Cry1	-0.33	1.00000000	pigs
Ptpn6	-0.34	1.00000000	pigs
Abca3	-0.02	1.00000000	pigs
Dbt	0.09	1.00000000	pigs
Kctd3	0.04	1.00000000	pigs
Trim27	-0.09	1.00000000	pigs
Golga4	-0.02	1.00000000	pigs
Rin3	-0.16	1.00000000	pigs
Htt	-0.09	1.00000000	pigs
Cwc27	-0.18	1.00000000	pigs
Foxj3	-0.11	1.00000000	pigs
Hus1	-0.18	1.00000000	pigs
Atg3	-0.05	1.00000000	pigs
Vps41	-0.33	1.00000000	pigs
Iars2	-0.02	1.00000000	pigs
Creld2	-0.18	1.00000000	pigs
St3gal3	-0.16	1.00000000	pigs
Gid8	-0.21	1.00000000	pigs
Ppp6r2	-0.17	1.00000000	pigs
Hdac7	0.06	1.00000000	pigs
Telo2	0.01	1.00000000	pigs
Pcna	-0.12	1.00000000	pigs
Tor1a	-0.05	1.00000000	pigs

Plaa	-0.01	1.00000000	pigs
Cd40	-0.11	1.00000000	pigs
Ndufa1	-0.37	1.00000000	pigs
Tmem41b	0.03	1.00000000	pigs
Psmb2	-0.22	1.00000000	pigs
Odr4	0.02	1.00000000	pigs
Vps52	0.09	1.00000000	pigs
Shld2	0.10	1.00000000	pigs
Smurf1	-0.08	1.00000000	pigs
Dynll2	-0.06	1.00000000	pigs
Srrm2	-0.13	1.00000000	pigs
Trpc4ap	-0.06	1.00000000	pigs
Hvcn1	-0.52	1.00000000	pigs
Zbtb17	0.01	1.00000000	pigs
Smarcad1	-0.37	1.00000000	pigs
Scaper	0.02	1.00000000	pigs
Taok1	-0.09	1.00000000	pigs
Phb	-0.07	1.00000000	pigs
Pbxip1	-0.07	1.00000000	pigs
Atg13	-0.04	1.00000000	pigs
Idh2	-0.18	1.00000000	pigs
Nfic	-0.19	1.00000000	pigs
Upp1	-0.03	1.00000000	pigs
Als2	-0.14	1.00000000	pigs
Rtn2	0.06	1.00000000	pigs
Clns1a	-0.03	1.00000000	pigs
Cd302	-0.15	1.00000000	pigs
Clptm1l	-0.05	1.00000000	pigs
Obi1	0.00	1.00000000	pigs
Hes6	-0.01	1.00000000	pigs
Prdx3	-0.16	1.00000000	pigs
Cox16	-0.11	1.00000000	pigs
Suclg1	-0.24	1.00000000	pigs
Imp4	-0.12	1.00000000	pigs
Hsd17b11	0.01	1.00000000	pigs
Yars	-0.06	1.00000000	pigs
Carm1	-0.08	1.00000000	pigs
Aph1a	-0.30	1.00000000	pigs
Klf12	0.08	1.00000000	pigs
Ccdc47	-0.05	1.00000000	pigs
Elp4	0.02	1.00000000	pigs
Ube4b	-0.20	1.00000000	pigs
Stk4	-0.19	1.00000000	pigs
N6amt1	-0.12	1.00000000	pigs
Wdr18	0.00	1.00000000	pigs
Fam118b	0.04	1.00000000	pigs

Kcng3	-0.53	1.00000000	pigs
Cox5b-ps	-0.32	1.00000000	pigs
Nipa2	-0.06	1.00000000	pigs
Ascc2	0.01	1.00000000	pigs
Hps3	-0.11	1.00000000	pigs
Vcl	-0.27	1.00000000	pigs
Tmem168	-0.10	1.00000000	pigs
Sec62	0.01	1.00000000	pigs
Crbn	-0.25	1.00000000	pigs
Klhl2	-0.33	1.00000000	pigs
Trib1	-0.25	1.00000000	pigs
Prr7	-0.21	1.00000000	pigs
Klf2	-0.34	1.00000000	pigs
Utp23	-0.07	1.00000000	pigs
Cebpz	0.00	1.00000000	pigs
Pbrm1	-0.31	1.00000000	pigs
Slc10a7	0.09	1.00000000	pigs
Ccsap	-0.30	1.00000000	pigs
Rsbn1l	-0.08	1.00000000	pigs
Snx4	0.11	1.00000000	pigs
Rev1	0.14	1.00000000	pigs
Slc37a1	-0.05	1.00000000	pigs
Nkap	-0.01	1.00000000	pigs
Acot7	-0.02	1.00000000	pigs
Cnot6	-0.20	1.00000000	pigs
Lmo2	-0.05	1.00000000	pigs
Tbc1d15	-0.09	1.00000000	pigs
C9orf72	-0.02	1.00000000	pigs
Irak4	-0.02	1.00000000	pigs
Atr	-0.39	1.00000000	pigs
Kansl3	-0.08	1.00000000	pigs
Ctsz	-0.04	1.00000000	pigs
Cab39l	-0.28	1.00000000	pigs
Unc93b1	-0.07	1.00000000	pigs
Cdk12	-0.10	1.00000000	pigs
Zfp131	-0.55	1.00000000	pigs
Nme6	-0.06	1.00000000	pigs
Ccm2	0.02	1.00000000	pigs
Sec16a	-0.03	1.00000000	pigs
Fos	-0.30	1.00000000	pigs
Cept1	-0.26	1.00000000	pigs
Rnpep	-0.28	1.00000000	pigs
Ncoa7	-0.24	1.00000000	pigs
Asb3	-0.04	1.00000000	pigs
Atg12	0.03	1.00000000	pigs
Vps35l	-0.19	1.00000000	pigs

Chp1	-0.18	1.00000000	pigs
Ifngr1	-0.07	1.00000000	pigs
Wdr75	0.03	1.00000000	pigs
Gnpnat1	-0.09	1.00000000	pigs
Zc3h4	-0.16	1.00000000	pigs
Snx18	-0.13	1.00000000	pigs
Psma4	-0.40	1.00000000	pigs
Cpsf6	-0.01	1.00000000	pigs
Vbp1	-0.10	1.00000000	pigs
Gpkow	-0.20	1.00000000	pigs
Wwp1	-0.04	1.00000000	pigs
Ppp4r3b	-0.01	1.00000000	pigs
Nbas	-0.08	1.00000000	pigs
Dhrs1	-0.21	1.00000000	pigs
Smpd1	-0.01	1.00000000	pigs
Ghsr	-0.27	1.00000000	pigs
Glg1	-0.05	1.00000000	pigs
Rp9	-0.12	1.00000000	pigs
Utp4	0.06	1.00000000	pigs
Zfp592	-0.16	1.00000000	pigs
Khnyn	-0.07	1.00000000	pigs
Gins2	0.06	1.00000000	pigs
Kcp	-0.08	1.00000000	pigs
Ppp1r12b	-0.33	1.00000000	pigs
Ss18l2	-0.02	1.00000000	pigs
Ywhaq	-0.05	1.00000000	pigs
Zfp1	-0.13	1.00000000	pigs
Aftph	-0.04	1.00000000	pigs
Tigar	0.04	1.00000000	pigs
Atpaf2	-0.10	1.00000000	pigs
Rcan1	0.03	1.00000000	pigs
Ppid	0.01	1.00000000	pigs
Dtx2	-0.24	1.00000000	pigs
Ercc8	-0.06	1.00000000	pigs
Slc25a13	-0.13	1.00000000	pigs
Tomm70a	0.00	1.00000000	pigs
Acsf3	-0.08	1.00000000	pigs
Ociad2	-0.16	1.00000000	pigs
Wbp4	-0.07	1.00000000	pigs
Colgalt1	-0.01	1.00000000	pigs
Taf12	-0.32	1.00000000	pigs
Adgrg5	0.11	1.00000000	pigs
Nelfcd	0.06	1.00000000	pigs
Ap5z1	-0.08	1.00000000	pigs
Ccdc90b	-0.22	1.00000000	pigs
Gcc2	-0.08	1.00000000	pigs

Anapc13	-0.13	1.00000000	pigs
Atp10d	-0.30	1.00000000	pigs
Prpf3	-0.09	1.00000000	pigs
Fam114a2	-0.10	1.00000000	pigs
Tyw5	0.02	1.00000000	pigs
Exoc6	-0.30	1.00000000	pigs
Lnpk	0.04	1.00000000	pigs
Pde6d	0.09	1.00000000	pigs
Ostm1	-0.19	1.00000000	pigs
Prmt9	0.04	1.00000000	pigs
Aqr	-0.04	1.00000000	pigs
Sppl2a	-0.20	1.00000000	pigs
Bap1	-0.13	1.00000000	pigs
Stxbp5	0.03	1.00000000	pigs
Wasf2	0.02	1.00000000	pigs
Rbm8a2	-0.23	1.00000000	pigs
Txndc16	0.04	1.00000000	pigs
Tsn	-0.13	1.00000000	pigs
Pcbp1	-0.13	1.00000000	pigs
Ocel1	-0.06	1.00000000	pigs
S100pbp	-0.13	1.00000000	pigs
Utp3	0.01	1.00000000	pigs
Ccdc18	-0.26	1.00000000	pigs
Prmt7	-0.11	1.00000000	pigs
Syf2	-0.34	1.00000000	pigs
Acbd3	-0.09	1.00000000	pigs
Anxa5	-0.04	1.00000000	pigs
Prpf6	-0.08	1.00000000	pigs
Phlda2	-0.10	1.00000000	pigs
Zmym1	-0.27	1.00000000	pigs
Tbc1d9	-0.14	1.00000000	pigs
Parg	-0.28	1.00000000	pigs
Pop4	-0.13	1.00000000	pigs
Unk	-0.01	1.00000000	pigs
Ipo11	-0.06	1.00000000	pigs
Cuta	-0.17	1.00000000	pigs
Ncoa2	0.04	1.00000000	pigs
Stx6	0.06	1.00000000	pigs
Ehmt1	-0.08	1.00000000	pigs
Stk10	-0.08	1.00000000	pigs
Tmx1	-0.16	1.00000000	pigs
Atxn7l3	-0.07	1.00000000	pigs
Glcci1	-0.28	1.00000000	pigs
Id3	-0.56	1.00000000	pigs
Gps1	-0.04	1.00000000	pigs
Klf11	-0.25	1.00000000	pigs

Imp3	-0.04	1.00000000	pigs
Anapc15-ps	-0.17	1.00000000	pigs
Rfc2	-0.07	1.00000000	pigs
Nfkbid	-0.04	1.00000000	pigs
Kdm8	-0.17	1.00000000	pigs
Dennd4c	-0.17	1.00000000	pigs
Rabep1	-0.08	1.00000000	pigs
Dctn3	-0.11	1.00000000	pigs
Tle4	-0.18	1.00000000	pigs
Arhgef12	-0.06	1.00000000	pigs
Sgms1	-0.31	1.00000000	pigs
Angel2	-0.12	1.00000000	pigs
Acbd5	-0.16	1.00000000	pigs
Jkamp	-0.13	1.00000000	pigs
Nhlrc3	0.05	1.00000000	pigs
Epm2aip1	-0.07	1.00000000	pigs
Tmem164	-0.06	1.00000000	pigs
Uprt	-0.20	1.00000000	pigs
Ccndbp1	0.05	1.00000000	pigs
Socs7	0.04	1.00000000	pigs
St6galnac4	-0.29	1.00000000	pigs
Ppp1r14bl	0.02	1.00000000	pigs
Exoc2	-0.11	1.00000000	pigs
Noa1	-0.20	1.00000000	pigs
Ifitm6	-0.20	1.00000000	pigs
Gtpbp10	0.03	1.00000000	pigs
Ikbke	-0.05	1.00000000	pigs
Tmem192	0.04	1.00000000	pigs
Ablim1	-0.31	1.00000000	pigs
Slc5a1	-0.30	1.00000000	pigs
Syne2	0.03	1.00000000	pigs
Pik3cb	0.01	1.00000000	pigs
Atf7ip2	-0.10	1.00000000	pigs
Tprkb	-0.05	1.00000000	pigs
Cers5	-0.14	1.00000000	pigs
Cwf19l1	-0.09	1.00000000	pigs
Park7	-0.24	1.00000000	pigs
Hbs1l	-0.03	1.00000000	pigs
Tesk2	0.10	1.00000000	pigs
Bcl10	-0.22	1.00000000	pigs
Dusp28	-0.29	1.00000000	pigs
Ttll12	-0.05	1.00000000	pigs
Atxn7	-0.02	1.00000000	pigs
Tfcp2	0.00	1.00000000	pigs
Nadsyn1	0.04	1.00000000	pigs
Prr14	0.04	1.00000000	pigs

Gsr	-0.04	1.00000000	pigs
Tbc1d20	-0.07	1.00000000	pigs
Snrpc	-0.04	1.00000000	pigs
Derl1	-0.12	1.00000000	pigs
Snrnp70	-0.35	1.00000000	pigs
Appl1	-0.28	1.00000000	pigs
Uqcc3	-0.13	1.00000000	pigs
Arpc5l	-0.09	1.00000000	pigs
Lclat1	-0.23	1.00000000	pigs
Zfp518a	-0.03	1.00000000	pigs
Nudcd2	-0.19	1.00000000	pigs
Tmed3	-0.04	1.00000000	pigs
Nup93	-0.08	1.00000000	pigs
Nxt1	-0.08	1.00000000	pigs
Car9	-0.20	1.00000000	pigs
Emc9	0.07	1.00000000	pigs
Ska2	-0.10	1.00000000	pigs
Lekr1	-0.04	1.00000000	pigs
Tgfbrap1	0.03	1.00000000	pigs
Hras	0.00	1.00000000	pigs
Rbpj	-0.12	1.00000000	pigs
Acadvl	-0.09	1.00000000	pigs
Baz2b	-0.01	1.00000000	pigs
Tmem238	-0.35	1.00000000	pigs
Iqgap1	0.02	1.00000000	pigs
Dohh	0.03	1.00000000	pigs
Tfam	-0.06	1.00000000	pigs
Dennd6a	-0.26	1.00000000	pigs
H2-Oa	-0.17	1.00000000	pigs
Zfp39	-0.07	1.00000000	pigs
Uimc1	-0.13	1.00000000	pigs
Emc4	-0.04	1.00000000	pigs
Zadh2	-0.15	1.00000000	pigs
Erg28	-0.11	1.00000000	pigs
Necap1	-0.28	1.00000000	pigs
Scaf11	-0.04	1.00000000	pigs
Kri1	0.04	1.00000000	pigs
Plscr1	0.03	1.00000000	pigs
Tbc1d9b	-0.08	1.00000000	pigs
Kin	-0.30	1.00000000	pigs
Edf1	-0.28	1.00000000	pigs
Cfap97	-0.18	1.00000000	pigs
Dyrk2	-0.04	1.00000000	pigs
Dcaf11	0.02	1.00000000	pigs
Srfbp1	-0.10	1.00000000	pigs
Cdca7l	-0.40	1.00000000	pigs

Tbc1d7	-0.06	1.00000000	pigs
Dyrk1b	-0.06	1.00000000	pigs
Fkbp3	-0.27	1.00000000	pigs
Kat7	-0.03	1.00000000	pigs
Ube2cbp	-0.12	1.00000000	pigs
Spidr	-0.07	1.00000000	pigs
Ctbp1	-0.10	1.00000000	pigs
Fam76b	-0.25	1.00000000	pigs
Srsf3	-0.12	1.00000000	pigs
Ankrd46	-0.08	1.00000000	pigs
Ddx50	-0.12	1.00000000	pigs
Zdhhc6	-0.04	1.00000000	pigs
Nlrc5	-0.13	1.00000000	pigs
Plekhm2	-0.12	1.00000000	pigs
Slc25a28	-0.02	1.00000000	pigs
Vstm4	-0.14	1.00000000	pigs
Ubxn2a	-0.20	1.00000000	pigs
Zfp28	-0.03	1.00000000	pigs
Pou2af1	0.04	1.00000000	pigs
Dek	-0.20	1.00000000	pigs
Elavl1	-0.10	1.00000000	pigs
Abce1	-0.05	1.00000000	pigs
Eaf2	-0.44	1.00000000	pigs
Cetn3	-0.13	1.00000000	pigs
Nelfe	-0.34	1.00000000	pigs
Foxn2	0.01	1.00000000	pigs
Gpank1	0.04	1.00000000	pigs
Cmbl	-0.37	1.00000000	pigs
Cd48	-0.14	1.00000000	pigs
Rac2	0.03	1.00000000	pigs
Shisa5	-0.06	1.00000000	pigs
Rpap1	-0.02	1.00000000	pigs
Rragb	-0.39	1.00000000	pigs
Rexo5	-0.06	1.00000000	pigs
Fam122b	0.04	1.00000000	pigs
Atg14	-0.37	1.00000000	pigs
Cdkal1	-0.05	1.00000000	pigs
Fam76a	-0.28	1.00000000	pigs
Zfp282	-0.02	1.00000000	pigs
Prrc2a	-0.12	1.00000000	pigs
Adk	-0.27	1.00000000	pigs
Eif2s2	-0.16	1.00000000	pigs
Ebf1	0.09	1.00000000	pigs
Ccdc150	-0.05	1.00000000	pigs
Tmco4	0.03	1.00000000	pigs
Fxn	-0.06	1.00000000	pigs

Timm50	-0.10	1.00000000	pigs
Limk2	-0.23	1.00000000	pigs
Yif1a	-0.16	1.00000000	pigs
Trappc3	-0.07	1.00000000	pigs
Ufm1	0.00	1.00000000	pigs
Setbp1	-0.34	1.00000000	pigs
Alg8	-0.17	1.00000000	pigs
Sec11a	-0.30	1.00000000	pigs
Atf3	-0.09	1.00000000	pigs
Gse1	-0.15	1.00000000	pigs
Rnf220	-0.18	1.00000000	pigs
Ahctf1	-0.40	1.00000000	pigs
Ccdc50	-0.43	1.00000000	pigs
Atp6v0a1	-0.03	1.00000000	pigs
Slc9a7	-0.06	1.00000000	pigs
Tmem126a	0.05	1.00000000	pigs
Emp1	-0.23	1.00000000	pigs
Commd5	-0.06	1.00000000	pigs
Ski	0.00	1.00000000	pigs
Prdx4	-0.08	1.00000000	pigs
Adgre5	0.15	1.00000000	pigs
Cbx4	-0.17	1.00000000	pigs
Nubp2	0.00	1.00000000	pigs
Arntl	-0.18	1.00000000	pigs
Ptpa	0.02	1.00000000	pigs
Ino80d	-0.12	1.00000000	pigs
Orc5	-0.01	1.00000000	pigs
Gpatch1	-0.11	1.00000000	pigs
Arsb	-0.01	1.00000000	pigs
Zfp407	-0.26	1.00000000	pigs
Vps8	-0.17	1.00000000	pigs
Prorp	-0.18	1.00000000	pigs
Thoc6	-0.11	1.00000000	pigs
Orc6	-0.17	1.00000000	pigs
Baz1b	-0.21	1.00000000	pigs
Cux1	-0.14	1.00000000	pigs
Arl6ip1	-0.16	1.00000000	pigs
Lancl1	-0.12	1.00000000	pigs
Ascc1	-0.04	1.00000000	pigs
Nfe2l3	-0.13	1.00000000	pigs
Pwwp3a	-0.13	1.00000000	pigs
Hsdl2	-0.07	1.00000000	pigs
Zfp24	-0.11	1.00000000	pigs
Rmnd5b	-0.06	1.00000000	pigs
Cdk14	-0.23	1.00000000	pigs
Rbm48	-0.04	1.00000000	pigs

Slc25a17	-0.18	1.00000000	pigs
9030025P20Rik	0.02	1.00000000	pigs
Nit2	-0.08	1.00000000	pigs
Agfg2	-0.06	1.00000000	pigs
Trnt1	-0.10	1.00000000	pigs
Nfkbil1	-0.05	1.00000000	pigs
Nfe2l2	-0.08	1.00000000	pigs
Ndufaf7	-0.09	1.00000000	pigs
Cryl1	-0.02	1.00000000	pigs
Pigu	-0.11	1.00000000	pigs
Rnf20	-0.06	1.00000000	pigs
BC004004	0.02	1.00000000	pigs
2210408I21Rik	-0.21	1.00000000	pigs
Emg1	-0.01	1.00000000	pigs
Fig4	-0.21	1.00000000	pigs
Clk2	-0.04	1.00000000	pigs
Ranbp17	-0.14	1.00000000	pigs
Zfp30	-0.04	1.00000000	pigs
Limk1	-0.04	1.00000000	pigs
Eif1ad	0.03	1.00000000	pigs
Hnrnpk	-0.17	1.00000000	pigs
Lancl2	-0.10	1.00000000	pigs
Cabin1	-0.01	1.00000000	pigs
Chmp5	-0.19	1.00000000	pigs
Acot4	-0.25	1.00000000	pigs
Keap1	-0.09	1.00000000	pigs
Snip1	-0.26	1.00000000	pigs
Galnt7	-0.01	1.00000000	pigs
Rida	-0.24	1.00000000	pigs
Hira	-0.09	1.00000000	pigs
Rprd2	-0.27	1.00000000	pigs
Pdpr	-0.03	1.00000000	pigs
St3gal4	0.01	1.00000000	pigs
Efcab14	-0.20	1.00000000	pigs
Atp13a2	-0.09	1.00000000	pigs
Rwdd1	-0.08	1.00000000	pigs
Gtf2ird1	-0.06	1.00000000	pigs
Dcp2	0.00	1.00000000	pigs
Bcap29	-0.25	1.00000000	pigs
Paxx	-0.14	1.00000000	pigs
Slc9a1	-0.36	1.00000000	pigs
Slc46a3	0.00	1.00000000	pigs
Adnp2	-0.07	1.00000000	pigs
Setx	0.02	1.00000000	pigs
Pi4k2b	-0.04	1.00000000	pigs
Ogfod3	0.00	1.00000000	pigs

Lypla1	-0.08	1.00000000	pigs
Pabpc1	-0.06	1.00000000	pigs
Fcrla	-0.05	1.00000000	pigs
Gadd45a	0.01	1.00000000	pigs
Rpusd1	-0.03	1.00000000	pigs
H2ac20	-0.39	1.00000000	pigs
Ndufa11	-0.16	1.00000000	pigs
Spring1	-0.11	1.00000000	pigs
Kdm5a	-0.26	1.00000000	pigs
P3h1	-0.10	1.00000000	pigs
Tlr2	0.08	1.00000000	pigs
Tgif2	-0.02	1.00000000	pigs
Lig3	-0.01	1.00000000	pigs
Dhx8	-0.22	1.00000000	pigs
Cilk1	-0.02	1.00000000	pigs
9930021J03Rik	-0.18	1.00000000	pigs
Wdr11	-0.03	1.00000000	pigs
Cenph	-0.18	1.00000000	pigs
Fam50a	0.00	1.00000000	pigs
Ankrd39	-0.04	1.00000000	pigs
Scly	-0.15	1.00000000	pigs
Gss	-0.01	1.00000000	pigs
Dpf3	-0.22	1.00000000	pigs
Zc3hc1	-0.07	1.00000000	pigs
Tjp1	-0.03	1.00000000	pigs
Tcp1	-0.03	1.00000000	pigs
Slc4a1ap	-0.10	1.00000000	pigs
Socs4	-0.06	1.00000000	pigs
Traf4	-0.02	1.00000000	pigs
Prcp	-0.15	1.00000000	pigs
Aamp	-0.10	1.00000000	pigs
Slc35d1	-0.27	1.00000000	pigs
Zfat	0.03	1.00000000	pigs
Ythdc1	-0.10	1.00000000	pigs
Tmem160	-0.31	1.00000000	pigs
Scaf8	-0.11	1.00000000	pigs
Bclaf3	-0.06	1.00000000	pigs
Slf1	-0.38	1.00000000	pigs
Atp6v0a2	-0.21	1.00000000	pigs
Cwc22	0.01	1.00000000	pigs
Xpo6	-0.11	1.00000000	pigs
Dhx29	-0.23	1.00000000	pigs
Camk2g	-0.01	1.00000000	pigs
Il16	-0.19	1.00000000	pigs
Dera	-0.23	1.00000000	pigs
Cyba	-0.08	1.00000000	pigs

Xkrx	-0.14	1.00000000	pigs
Guk1	-0.30	1.00000000	pigs
Usp31	-0.12	1.00000000	pigs
Serpini1	-0.50	1.00000000	pigs
Orai3	0.03	1.00000000	pigs
Ubxn8	-0.23	1.00000000	pigs
Nol9	-0.17	1.00000000	pigs
Ythdf3	-0.01	1.00000000	pigs
Zfyve26	-0.14	1.00000000	pigs
Smim15	-0.12	1.00000000	pigs
Nuak2	-0.13	1.00000000	pigs
R3hdm4	-0.14	1.00000000	pigs
Rnf13	-0.16	1.00000000	pigs
Lonrf1	-0.06	1.00000000	pigs
Nmt1	-0.18	1.00000000	pigs
Cass4	-0.16	1.00000000	pigs
Adi1	-0.29	1.00000000	pigs
Snx16	0.03	1.00000000	pigs
Il6ra	-0.37	1.00000000	pigs
Btn2a2	-0.23	1.00000000	pigs
Ankrd44	-0.01	1.00000000	pigs
Elovl6	-0.10	1.00000000	pigs
Aff2	-0.25	1.00000000	pigs
Dnlz	-0.22	1.00000000	pigs
Ptrh2	0.00	1.00000000	pigs
Smchd1	0.03	1.00000000	pigs
Pdcd10	-0.17	1.00000000	pigs
Ccnl2	-0.29	1.00000000	pigs
Zfp639	-0.03	1.00000000	pigs
Dtnbp1	-0.02	1.00000000	pigs
Edrf1	-0.08	1.00000000	pigs
Ippk	-0.13	1.00000000	pigs
Chml	-0.13	1.00000000	pigs
Rnf149	-0.25	1.00000000	pigs
Prim2	-0.09	1.00000000	pigs
Nab2	-0.14	1.00000000	pigs
Tinf2	-0.13	1.00000000	pigs
Sema4a	0.05	1.00000000	pigs
Klhdc2	-0.25	1.00000000	pigs
Nupr1	0.01	1.00000000	pigs
Heca	-0.30	1.00000000	pigs
Wdr44	0.03	1.00000000	pigs
Ccdc126	-0.03	1.00000000	pigs
Slamf1	0.05	1.00000000	pigs
Clpb	0.03	1.00000000	pigs
Zfp369	-0.21	1.00000000	pigs

Ly86	-0.09	1.00000000	pigs
Hgs	-0.27	1.00000000	pigs
C1qbp	-0.19	1.00000000	pigs
Pex2	0.03	1.00000000	pigs
Taf11	-0.34	1.00000000	pigs
Fam20b	0.00	1.00000000	pigs
Grk5	-0.17	1.00000000	pigs
Dpy19l1	0.02	1.00000000	pigs
Atp5a1	-0.04	1.00000000	pigs
Slc4a4	-0.34	1.00000000	pigs
Rtca	-0.08	1.00000000	pigs
Tango6	-0.01	1.00000000	pigs
Stradb	-0.17	1.00000000	pigs
Hsp90b1	-0.15	1.00000000	pigs
Chic2	-0.16	1.00000000	pigs
Usp33	-0.06	1.00000000	pigs
Pgls	-0.06	1.00000000	pigs
Snx30	-0.17	1.00000000	pigs
Dpy30	-0.36	1.00000000	pigs
Dhx57	-0.30	1.00000000	pigs
Plekhg1	0.10	1.00000000	pigs
Ccne2	-0.31	1.00000000	pigs
Slu7	-0.08	1.00000000	pigs
Ezr	0.00	1.00000000	pigs
Igfbp7	0.05	1.00000000	pigs
Alg14	-0.05	1.00000000	pigs
Actr1a	-0.07	1.00000000	pigs
Nup188	-0.07	1.00000000	pigs
Pold1	-0.18	1.00000000	pigs
Stat2	-0.03	1.00000000	pigs
Kmt5b	-0.02	1.00000000	pigs
Rbak	0.00	1.00000000	pigs
Lars	-0.13	1.00000000	pigs
Riox2	-0.13	1.00000000	pigs
Recql	-0.26	1.00000000	pigs
Abhd17a	-0.21	1.00000000	pigs
Nsa2	-0.05	1.00000000	pigs
Ccdc28b	-0.08	1.00000000	pigs
Uck1	0.00	1.00000000	pigs
Tab1	-0.03	1.00000000	pigs
Pign	-0.09	1.00000000	pigs
Stau2	-0.37	1.00000000	pigs
Nelfb	-0.02	1.00000000	pigs
Caml	-0.22	1.00000000	pigs
Strap	-0.18	1.00000000	pigs
Chrac1	-0.31	1.00000000	pigs

Sart1	-0.11	1.00000000	pigs
Ift88	-0.15	1.00000000	pigs
Hsd17b10	-0.21	1.00000000	pigs
Arid1a	-0.14	1.00000000	pigs
Ppp1r18	-0.32	1.00000000	pigs
Rnf146	-0.19	1.00000000	pigs
Nup85	-0.23	1.00000000	pigs
Tdg	-0.33	1.00000000	pigs
Nfs1	-0.08	1.00000000	pigs
Nrd1	0.03	1.00000000	pigs
Immp1l	-0.15	1.00000000	pigs
Casp3	-0.56	1.00000000	pigs
Topaz1	-0.18	1.00000000	pigs
Hp1bp3	-0.31	1.00000000	pigs
Usp47	-0.15	1.00000000	pigs
Aldh16a1	-0.06	1.00000000	pigs
Tmem104	0.00	1.00000000	pigs
Ddx55	-0.08	1.00000000	pigs
Hdac3	-0.07	1.00000000	pigs
Ing3	-0.02	1.00000000	pigs
Spats2	-0.03	1.00000000	pigs
Crk	-0.12	1.00000000	pigs
Trmt112	-0.28	1.00000000	pigs
Dcaf8	-0.02	1.00000000	pigs
Rragc	-0.11	1.00000000	pigs
Kmt2b	-0.15	1.00000000	pigs
Tasor	-0.11	1.00000000	pigs
Stk3	0.02	1.00000000	pigs
Shfl	-0.04	1.00000000	pigs
Usf2	-0.07	1.00000000	pigs
Ogfr	-0.20	1.00000000	pigs
Psmb9	-0.19	1.00000000	pigs
Tfb2m	-0.10	1.00000000	pigs
Ccdc6	-0.05	1.00000000	pigs
Trak1	-0.04	1.00000000	pigs
Zzz3	-0.04	1.00000000	pigs
Nme1	-0.06	1.00000000	pigs
Slc35f5	-0.07	1.00000000	pigs
Tle3	-0.14	1.00000000	pigs
Auh	-0.35	1.00000000	pigs
Ngly1	-0.27	1.00000000	pigs
Ust	-0.23	1.00000000	pigs
Pomt2	-0.05	1.00000000	pigs
Abhd2	-0.27	1.00000000	pigs
Sp4	-0.31	1.00000000	pigs
Scyl3	-0.13	1.00000000	pigs

Gpx4	-0.25	1.00000000	pigs
Samd4b	-0.06	1.00000000	pigs
Aar2	-0.01	1.00000000	pigs
Sec63	-0.17	1.00000000	pigs
Egfl8	-0.05	1.00000000	pigs
Gpn3	-0.04	1.00000000	pigs
Nfx1	-0.08	1.00000000	pigs
Zfp445	-0.10	1.00000000	pigs
Wasl	-0.23	1.00000000	pigs
Sigmar1	-0.01	1.00000000	pigs
Fbxo48	-0.26	1.00000000	pigs
Las1l	-0.02	1.00000000	pigs
Cd2	-0.11	1.00000000	pigs
Hpcal1	-0.19	1.00000000	pigs
Bicdl1	-0.23	1.00000000	pigs
Ndufb11b	-0.18	1.00000000	pigs
Exosc7	-0.08	1.00000000	pigs
Snrpn	-0.35	1.00000000	pigs
Ssbp4	-0.02	1.00000000	pigs
Icmt	-0.08	1.00000000	pigs
Rex1bd	-0.38	1.00000000	pigs
Itga4	-0.32	1.00000000	pigs
Usp20	-0.09	1.00000000	pigs
Skap2	-0.24	1.00000000	pigs
Dcaf12	0.01	1.00000000	pigs
Rock1	-0.04	1.00000000	pigs
Khk	-0.13	1.00000000	pigs
Tada1	0.00	1.00000000	pigs
Nme7	0.02	1.00000000	pigs
Nt5c3b	-0.09	1.00000000	pigs
Apbb3	-0.15	1.00000000	pigs
Agl	-0.04	1.00000000	pigs
Rbm28	-0.03	1.00000000	pigs
Ccs	-0.14	1.00000000	pigs
Snrnp200	-0.20	1.00000000	pigs
Paqr3	-0.03	1.00000000	pigs
Ivd	-0.03	1.00000000	pigs
Arap1	-0.32	1.00000000	pigs
Lpp	-0.46	1.00000000	pigs
Tkfc	-0.03	1.00000000	pigs
Ddx42	-0.32	1.00000000	pigs
Cdkn2aip	-0.16	1.00000000	pigs
Prpf38a	-0.11	1.00000000	pigs
Cap1	-0.09	1.00000000	pigs
Lmf1	-0.10	1.00000000	pigs
Aspdh	-0.14	1.00000000	pigs

B3gnt5	-0.15	1.00000000	pigs
Rilpl2	-0.01	1.00000000	pigs
Fermt3	-0.15	1.00000000	pigs
St3gal1	-0.05	1.00000000	pigs
Dynlt3	0.02	1.00000000	pigs
Atrip	0.01	1.00000000	pigs
Sass6	0.00	1.00000000	pigs
Pccb	-0.03	1.00000000	pigs
Cuedc2	-0.16	1.00000000	pigs
Susd1	-0.11	1.00000000	pigs
Trp53bp2	-0.02	1.00000000	pigs
Cbx1	-0.18	1.00000000	pigs
Aif1	-0.10	1.00000000	pigs
Golga3	-0.23	1.00000000	pigs
Ube2e3	-0.08	1.00000000	pigs
Plek	-0.23	1.00000000	pigs
Clcn3	-0.22	1.00000000	pigs
Pak2	-0.07	1.00000000	pigs
Dpp8	-0.24	1.00000000	pigs
Sucla2	-0.06	1.00000000	pigs
2700049A03Rik	-0.08	1.00000000	pigs
Stat4	-0.29	1.00000000	pigs
Fundc2	-0.20	1.00000000	pigs
Psmc5	-0.07	1.00000000	pigs
Iah1	-0.27	1.00000000	pigs
Psma7	-0.03	1.00000000	pigs
Sptbn1	-0.30	1.00000000	pigs
Gigyf1	-0.20	1.00000000	pigs
Prdx5	-0.24	1.00000000	pigs
Kdm4c	-0.02	1.00000000	pigs
Pwp1	-0.32	1.00000000	pigs
Clasp2	-0.06	1.00000000	pigs
Dnajb1	0.41	1.00000000	pigs
Ttc13	-0.23	1.00000000	pigs
Ift20	-0.12	1.00000000	pigs
Toe1	-0.03	1.00000000	pigs
Fbp1	-0.10	1.00000000	pigs
Ciao1	-0.06	1.00000000	pigs
Irf1	-0.33	1.00000000	pigs
Rnpepl1	-0.09	1.00000000	pigs
B3galnt1	-0.08	1.00000000	pigs
Lemd3	-0.22	1.00000000	pigs
Scfd2	-0.44	1.00000000	pigs
Nsmce2	-0.11	1.00000000	pigs
Hacd2	-0.10	1.00000000	pigs
Pdia6	-0.01	1.00000000	pigs

Lsm3	-0.22	1.00000000	pigs
Kif3b	-0.04	1.00000000	pigs
Stk11	-0.09	1.00000000	pigs
Elp2	-0.17	1.00000000	pigs
Tob2	-0.15	1.00000000	pigs
Arpc3	-0.12	1.00000000	pigs
Eif2d	-0.08	1.00000000	pigs
Sec22a	-0.36	1.00000000	pigs
Areg	0.06	1.00000000	pigs
Pgd	0.00	1.00000000	pigs
P2ry14	-0.09	1.00000000	pigs
Klc2	-0.29	1.00000000	pigs
Ndufb3	-0.23	1.00000000	pigs
Trio	-0.04	1.00000000	pigs
Pdk3	-0.23	1.00000000	pigs
Anxa4	0.15	1.00000000	pigs
Rrm2b	-0.18	1.00000000	pigs
Derl2	-0.13	1.00000000	pigs
Hnrnpd	-0.11	1.00000000	pigs
Agpat1	-0.01	1.00000000	pigs
Slc24a1	-0.14	1.00000000	pigs
Tmem107	-0.23	1.00000000	pigs
Nsrp1	-0.27	1.00000000	pigs
Got2	-0.03	1.00000000	pigs
Sds	-0.58	1.00000000	pigs
Rap2a	-0.06	1.00000000	pigs
Sod1	-0.32	1.00000000	pigs
Thoc1	-0.12	1.00000000	pigs
Kiz	-0.11	1.00000000	pigs
Gosr1	-0.16	1.00000000	pigs
Sec22b	-0.02	1.00000000	pigs
Osbpl1a	-0.26	1.00000000	pigs
Appl2	-0.16	1.00000000	pigs
Disp1	-0.06	1.00000000	pigs
Gnai2	-0.09	1.00000000	pigs
1810030O07Rik	-0.12	1.00000000	pigs
Nutf2	-0.08	1.00000000	pigs
Slc25a33	-0.02	1.00000000	pigs
Plekha2	-0.13	1.00000000	pigs
Ccdc186	-0.35	1.00000000	pigs
Emc2	-0.11	1.00000000	pigs
Dhx35	-0.10	1.00000000	pigs
Gnal	-0.05	1.00000000	pigs
Tmem222	-0.11	1.00000000	pigs
Pnrc1	-0.10	1.00000000	pigs
Pithd1	-0.21	1.00000000	pigs

Rufy2	-0.01	1.00000000	pigs
1600012H06Rik	-0.13	1.00000000	pigs
Otud3	-0.05	1.00000000	pigs
Hlcs	-0.01	1.00000000	pigs
Dffa	-0.12	1.00000000	pigs
Aldh1l2	-0.13	1.00000000	pigs
Jchain	-0.43	1.00000000	pigs
Abr	-0.10	1.00000000	pigs
Thumpd3	-0.05	1.00000000	pigs
Ppm1l	-0.21	1.00000000	pigs
Jade1	-0.05	1.00000000	pigs
Lrig2	-0.16	1.00000000	pigs
Dag1	-0.05	1.00000000	pigs
Impa1	0.01	1.00000000	pigs
G2e3	-0.05	1.00000000	pigs
Pop5	-0.26	1.00000000	pigs
Camkk2	-0.17	1.00000000	pigs
Sdhaf2	-0.03	1.00000000	pigs
Cerk	-0.28	1.00000000	pigs
Ranbp10	-0.04	1.00000000	pigs
Oxnad1	-0.17	1.00000000	pigs
Gnl2	-0.08	1.00000000	pigs
Atp8b4	-0.10	1.00000000	pigs
Sumf2	-0.08	1.00000000	pigs
Lhpp	-0.38	1.00000000	pigs
Fbxo30	-0.23	1.00000000	pigs
Bbs9	-0.15	1.00000000	pigs
Nono	-0.15	1.00000000	pigs
Phf2	-0.08	1.00000000	pigs
Cdc26	-0.21	1.00000000	pigs
Preb	-0.32	1.00000000	pigs
Inip	-0.17	1.00000000	pigs
Slc16a6	-0.14	1.00000000	pigs
Taf10	-0.16	1.00000000	pigs
Cxcr3	-0.15	1.00000000	pigs
Htatip2	-0.17	1.00000000	pigs
Cpt1b	0.06	1.00000000	pigs
Nvl	-0.13	1.00000000	pigs
Pcdhga4	-0.18	1.00000000	pigs
Pygl	-0.22	1.00000000	pigs
Pibf1	-0.11	1.00000000	pigs
Tgs1	-0.27	1.00000000	pigs
Pdk1	-0.11	1.00000000	pigs
Recql5	-0.05	1.00000000	pigs
Polrmt	-0.13	1.00000000	pigs
Timm22	-0.14	1.00000000	pigs

Zranb3	-0.12	1.00000000	pigs
Pgm3	0.00	1.00000000	pigs
Psmd6	-0.14	1.00000000	pigs
Fgd3	-0.22	1.00000000	pigs
Yju2	-0.19	1.00000000	pigs
Ro60	-0.22	1.00000000	pigs
Dnaaf2	-0.13	1.00000000	pigs
Acap1	-0.16	1.00000000	pigs
Csf3r	-0.14	1.00000000	pigs
Grb2	-0.02	1.00000000	pigs
Cdkn2aipnl	-0.18	1.00000000	pigs
Sepsecs	-0.05	1.00000000	pigs
Hypk	-0.30	1.00000000	pigs
Ppp4r1	-0.10	1.00000000	pigs
Ppan	-0.20	1.00000000	pigs
Nras	-0.19	1.00000000	pigs
Ap2s1	-0.20	1.00000000	pigs
Cep104	-0.27	1.00000000	pigs
Inpp5f	-0.28	1.00000000	pigs
Naga	-0.17	1.00000000	pigs
Ndufs4	-0.18	1.00000000	pigs
Vps37c	-0.14	1.00000000	pigs
Arxes2	-0.12	1.00000000	pigs
Tmem255b	-0.05	1.00000000	pigs
Terf1	-0.18	1.00000000	pigs
Wdr59	-0.05	1.00000000	pigs
Golga1	-0.14	1.00000000	pigs
Ndufb2	-0.09	1.00000000	pigs
Nsl1	-0.15	1.00000000	pigs
Ghrl	-0.59	1.00000000	pigs
Antkmt	0.02	1.00000000	pigs
Pggt1b	0.05	1.00000000	pigs
Pcgf6	-0.06	1.00000000	pigs
Tubgcp2	-0.12	1.00000000	pigs
Daxx	-0.06	1.00000000	pigs
Amd2	-0.20	1.00000000	pigs
Nedd8	-0.34	1.00000000	pigs
Ebna1bp2	-0.25	1.00000000	pigs
Fbrs	0.00	1.00000000	pigs
Pou2f2	-0.23	1.00000000	pigs
Psen1	-0.14	1.00000000	pigs
Cep290	0.00	1.00000000	pigs
Kif5b	-0.11	1.00000000	pigs
Gpr174	-0.35	1.00000000	pigs
Spg21	-0.05	1.00000000	pigs
Ssh1	-0.22	1.00000000	pigs

Cramp1	-0.08	1.00000000	pigs
Zfp335	-0.15	1.00000000	pigs
Vdac2	-0.08	1.00000000	pigs
Dpp9	-0.05	1.00000000	pigs
Atg16l2	0.01	1.00000000	pigs
Phkb	-0.23	1.00000000	pigs
Lmnb1	-0.22	1.00000000	pigs
Nab1	0.02	1.00000000	pigs
Helq	0.01	1.00000000	pigs
Trappc11	-0.24	1.00000000	pigs
Junb	-0.27	1.00000000	pigs
Tmem38b	-0.04	1.00000000	pigs
Thg1l	-0.04	1.00000000	pigs
Sowahd	-0.01	1.00000000	pigs
Cks2	-0.38	1.00000000	pigs
Vgll4	-0.16	1.00000000	pigs
Ttyh2	0.09	1.00000000	pigs
Uqcr10	-0.09	1.00000000	pigs
Tshz1	-0.05	1.00000000	pigs
Txndc17	-0.20	1.00000000	pigs
Galnt11	0.00	1.00000000	pigs
Rexo2	-0.08	1.00000000	pigs
Syvn1	-0.04	1.00000000	pigs
Cwf19l2	-0.13	1.00000000	pigs
Ciao2a	-0.08	1.00000000	pigs
Nploc4	-0.07	1.00000000	pigs
Camkmt	0.17	1.00000000	pigs
Aasdh	-0.24	1.00000000	pigs
Fbxl14	-0.05	1.00000000	pigs
Lmbrd1	-0.24	1.00000000	pigs
Fam189a1	-0.36	1.00000000	pigs
Tprgl	-0.10	1.00000000	pigs
Ssna1	-0.19	1.00000000	pigs
Spcs1	-0.39	1.00000000	pigs
Pqbp1	-0.09	1.00000000	pigs
Ndufv2	-0.29	1.00000000	pigs
Atg4b	-0.24	1.00000000	pigs
Ankrd13c	-0.18	1.00000000	pigs
Ndufaf3	-0.28	1.00000000	pigs
Ywhaz	0.03	1.00000000	pigs
Kif13a	-0.14	1.00000000	pigs
Kif1b	-0.18	1.00000000	pigs
Ubn2	-0.14	1.00000000	pigs
Sp110	-0.16	1.00000000	pigs
Cacnb4	0.02	1.00000000	pigs
Dctd	-0.18	1.00000000	pigs

Tada3	-0.06	1.00000000	pigs
Zfp36	-0.39	1.00000000	pigs
Dipk1a	-0.26	1.00000000	pigs
Cdin1	-0.09	1.00000000	pigs
Pdcd6	-0.04	1.00000000	pigs
Rbm45	-0.16	1.00000000	pigs
Alkbh7	-0.27	1.00000000	pigs
Cox6a1	-0.27	1.00000000	pigs
Tasp1	-0.02	1.00000000	pigs
Tut7	-0.13	1.00000000	pigs
Fbxo33	-0.09	1.00000000	pigs
Vti1a	-0.37	1.00000000	pigs
Serinc5	-0.32	1.00000000	pigs
Agpat3	-0.34	1.00000000	pigs
Cfap20	-0.21	1.00000000	pigs
Tnfsf4	-0.08	1.00000000	pigs
Klf4	-0.18	1.00000000	pigs
Fmc1	-0.06	1.00000000	pigs
Accsl	-0.21	1.00000000	pigs
Zbtb38	-0.40	1.00000000	pigs
Cstf2	-0.06	1.00000000	pigs
Arfrp1	-0.04	1.00000000	pigs
Ccdc97	-0.01	1.00000000	pigs
Hnrnph2	-0.15	1.00000000	pigs
Spopl	-0.13	1.00000000	pigs
Cdc42	-0.09	1.00000000	pigs
Ddit4	-0.25	1.00000000	pigs
Cyria	-0.28	1.00000000	pigs
Dyrk3	-0.16	1.00000000	pigs
Htra2	0.02	1.00000000	pigs
Dcaf6	-0.04	1.00000000	pigs
Trp53rka	-0.12	1.00000000	pigs
Hat1	-0.13	1.00000000	pigs
Aak1	-0.23	1.00000000	pigs
Hmg20b	-0.12	1.00000000	pigs
Cisd3	-0.30	1.00000000	pigs
Sc5d	-0.08	1.00000000	pigs
Ndufb10	-0.25	1.00000000	pigs
Taf7	-0.29	1.00000000	pigs
Dap3	-0.23	1.00000000	pigs
Ecd	-0.14	1.00000000	pigs
Aasdhppt	-0.15	1.00000000	pigs
Capn7	-0.12	1.00000000	pigs
Hells	-0.10	1.00000000	pigs
Skp1	-0.13	1.00000000	pigs
Abca5	-0.16	1.00000000	pigs

Rnls	0.02	1.00000000	pigs
Chd8	-0.05	1.00000000	pigs
Grk3	-0.23	1.00000000	pigs
Rasgrp2	-0.28	1.00000000	pigs
Ahcyl2	-0.30	1.00000000	pigs
Itm2c	-0.14	1.00000000	pigs
Znhit1	-0.14	1.00000000	pigs
Rbm19	-0.15	1.00000000	pigs
Apc	-0.17	1.00000000	pigs
Eef2k	-0.03	1.00000000	pigs
Arl6ip5	-0.12	1.00000000	pigs
Arid5b	-0.03	1.00000000	pigs
Usp38	0.06	1.00000000	pigs
Smc2	-0.07	1.00000000	pigs
Emc7	-0.24	1.00000000	pigs
Crnkl1	-0.09	1.00000000	pigs
Nxf1	0.01	1.00000000	pigs
Ralgps1	-0.05	1.00000000	pigs
Gtf2a1	-0.09	1.00000000	pigs
Riok3	-0.20	1.00000000	pigs
Ssb	0.00	1.00000000	pigs
Sgsm2	-0.17	1.00000000	pigs
Sars	-0.23	1.00000000	pigs
Ndufb7	-0.35	1.00000000	pigs
Lars2	-0.07	1.00000000	pigs
Snx12	0.02	1.00000000	pigs
Eif3b	0.06	1.00000000	pigs
Arid3b	-0.28	1.00000000	pigs
Pin4	-0.03	1.00000000	pigs
Fibp	-0.25	1.00000000	pigs
Ppp2r3c	-0.12	1.00000000	pigs
Sephs1	-0.24	1.00000000	pigs
R3hdm1	-0.18	1.00000000	pigs
Acsl1	-0.32	1.00000000	pigs
Tor4a	-0.15	1.00000000	pigs
Zcchc9	-0.06	1.00000000	pigs
Sbk1	-0.27	1.00000000	pigs
Synpo2	0.09	1.00000000	pigs
Zdhhc15	-0.13	1.00000000	pigs
Cnot8	-0.30	1.00000000	pigs
4921524J17Rik	-0.33	1.00000000	pigs
Dock11	-0.03	1.00000000	pigs
Dok1	-0.18	1.00000000	pigs
Arfgap2	-0.08	1.00000000	pigs
Sap30l	-0.09	1.00000000	pigs
Trpm7	-0.14	1.00000000	pigs

Cox7a2	-0.32	1.00000000	pigs
Katnip	-0.03	1.00000000	pigs
Osbpl10	-0.16	1.00000000	pigs
Stim2	-0.15	1.00000000	pigs
Sh3tc1	-0.22	1.00000000	pigs
Dtymk	-0.07	1.00000000	pigs
Ercc6	-0.08	1.00000000	pigs
Commd3	-0.13	1.00000000	pigs
Fam102b	-0.08	1.00000000	pigs
Sox5	-0.30	1.00000000	pigs
Dgke	-0.07	1.00000000	pigs
Aagab	-0.12	1.00000000	pigs
Ccdc167	-0.02	1.00000000	pigs
Ttc4	-0.16	1.00000000	pigs
2810004N23Rik	-0.19	1.00000000	pigs
Isg20l2	-0.13	1.00000000	pigs
Asprv1	-0.08	1.00000000	pigs
Epc2	-0.24	1.00000000	pigs
Tsc22d4	-0.10	1.00000000	pigs
Tbc1d10c	-0.16	1.00000000	pigs
Ankra2	-0.14	1.00000000	pigs
Smim19	-0.17	1.00000000	pigs
Bysl	-0.23	1.00000000	pigs
Sacm1l	-0.27	1.00000000	pigs
Csrp1	-0.25	1.00000000	pigs
Rfx3	-0.18	1.00000000	pigs
Cfap36	-0.04	1.00000000	pigs
Zfp330	-0.19	1.00000000	pigs
Atp9b	-0.08	1.00000000	pigs
Ndufb8	-0.17	1.00000000	pigs
Thap3	-0.03	1.00000000	pigs
Pex16	-0.25	1.00000000	pigs
Lrrc47	-0.07	1.00000000	pigs
Supt16	-0.11	1.00000000	pigs
Terf2ip	-0.16	1.00000000	pigs
Brd8	-0.22	1.00000000	pigs
Dpy19l4	-0.12	1.00000000	pigs
Srsf9	-0.16	1.00000000	pigs
Dnajc15	-0.19	1.00000000	pigs
Zmynd11	-0.18	1.00000000	pigs
Eif4g2	-0.08	1.00000000	pigs
Itfg2	-0.12	1.00000000	pigs
Ipo4	-0.23	1.00000000	pigs
Fcho1	-0.15	1.00000000	pigs
Akap9	-0.25	1.00000000	pigs
Xrn1	-0.05	1.00000000	pigs

Ankrd13a	-0.10	1.00000000	pigs
Dpp7	-0.12	1.00000000	pigs
Psmc4	-0.27	1.00000000	pigs
Hdgf	-0.15	1.00000000	pigs
Ccdc124	-0.17	1.00000000	pigs
Wrap53	-0.10	1.00000000	pigs
Tmem203	-0.27	1.00000000	pigs
Tmem9b	-0.10	1.00000000	pigs
Cnih1	-0.06	1.00000000	pigs
Scimp	-0.11	1.00000000	pigs
Ubac1	-0.14	1.00000000	pigs
Golph3l	-0.17	1.00000000	pigs
Stx18	-0.02	1.00000000	pigs
Tbck	-0.12	1.00000000	pigs
Arrb2	-0.03	1.00000000	pigs
Dgkd	-0.17	1.00000000	pigs
Tmem50a	-0.27	1.00000000	pigs
Uxt	-0.09	1.00000000	pigs
Pias4	-0.03	1.00000000	pigs
L3mbtl2	-0.05	1.00000000	pigs
Ctps2	-0.11	1.00000000	pigs
Thap1	-0.15	1.00000000	pigs
B230219D22Rik	-0.14	1.00000000	pigs
Chid1	-0.21	1.00000000	pigs
Gatd1	-0.02	1.00000000	pigs
Tmed4	-0.22	1.00000000	pigs
Ttc3	-0.12	1.00000000	pigs
Zfp148	-0.11	1.00000000	pigs
Atl3	-0.07	1.00000000	pigs
Pabpc4	0.02	1.00000000	pigs
Bbx	0.00	1.00000000	pigs
Pank2	-0.13	1.00000000	pigs
Atxn7l1	-0.17	1.00000000	pigs
Paqr8	-0.27	1.00000000	pigs
Nfatc2	-0.23	1.00000000	pigs
Ryk	-0.11	1.00000000	pigs
Ubxn6	-0.18	1.00000000	pigs
Stk38	-0.25	1.00000000	pigs
Ptpn2	-0.08	1.00000000	pigs
Dock2	0.03	1.00000000	pigs
Tbrg1	-0.23	1.00000000	pigs
3110040N11Rik	-0.25	1.00000000	pigs
Snrnp25	-0.29	1.00000000	pigs
Rdx	-0.51	1.00000000	pigs
Dhx16	-0.04	1.00000000	pigs
Usp44	-0.21	1.00000000	pigs

Tecpr1	-0.14	1.00000000	pigs
Zfp12	-0.09	1.00000000	pigs
Tm9sf4	-0.11	1.00000000	pigs
Atxn10	-0.17	1.00000000	pigs
Coq10b	-0.10	1.00000000	pigs
Rcsd1	-0.29	1.00000000	pigs
Rnf14	-0.18	1.00000000	pigs
Rnf214	-0.09	1.00000000	pigs
Tada2a	-0.17	1.00000000	pigs
Prr5	-0.16	1.00000000	pigs
D16Ertd472e	-0.08	1.00000000	pigs
Bora	-0.15	1.00000000	pigs
Ssbp1	-0.18	1.00000000	pigs
Chuk	-0.05	1.00000000	pigs
Akap7	-0.25	1.00000000	pigs
1700123O20Rik	-0.20	1.00000000	pigs
Tceanc	-0.13	1.00000000	pigs
Anxa1	-0.31	1.00000000	pigs
Tnfrsf13c	-0.09	1.00000000	pigs
Rpp21	-0.38	1.00000000	pigs
Zfp827	-0.17	1.00000000	pigs
Cradd	-0.22	1.00000000	pigs
Hnrnph3	-0.11	1.00000000	pigs
Slc39a6	-0.10	1.00000000	pigs
Cep128	-0.06	1.00000000	pigs
Arpc1b	-0.05	1.00000000	pigs
Rbl2	-0.14	1.00000000	pigs
Lrrc18	-0.13	1.00000000	pigs
Stambpl1	0.02	1.00000000	pigs
Bdp1	-0.12	1.00000000	pigs
Reep5	-0.04	1.00000000	pigs
Tmem208	-0.30	1.00000000	pigs
Btbd2	-0.04	1.00000000	pigs
Lrrc28	-0.22	1.00000000	pigs
Bnip2	-0.06	1.00000000	pigs
Cebpg	-0.23	1.00000000	pigs
Fem1c	-0.03	1.00000000	pigs
Ndufaf1	-0.04	1.00000000	pigs
Czib	-0.14	1.00000000	pigs
Lyar	-0.10	1.00000000	pigs
Polr3f	-0.08	1.00000000	pigs
Arl6ip6	-0.21	1.00000000	pigs
Hnrnpr	-0.18	1.00000000	pigs
Cdadc1	-0.18	1.00000000	pigs
Ethe1	-0.18	1.00000000	pigs
Nfatc3	-0.20	1.00000000	pigs

Tyw3	-0.15	1.00000000	pigs
Ranbp6	-0.51	1.00000000	pigs
Dstyk	-0.23	1.00000000	pigs
Cgas	-0.10	1.00000000	pigs
Dnaja2	-0.13	1.00000000	pigs
Ndufab1	-0.28	1.00000000	pigs
Relb	-0.15	1.00000000	pigs
Stk38l	-0.11	1.00000000	pigs
Srbd1	-0.15	1.00000000	pigs
Nck1	-0.31	1.00000000	pigs
Tpst2	-0.06	1.00000000	pigs
St3gal2	-0.03	1.00000000	pigs
Dync1i2	-0.09	1.00000000	pigs
Swap70	-0.09	1.00000000	pigs
Cox5a	-0.24	1.00000000	pigs
Dop1a	-0.19	1.00000000	pigs
Tk1	-0.02	1.00000000	pigs
Gpd2	-0.09	1.00000000	pigs
Xbp1	-0.21	1.00000000	pigs
Wdtc1	0.03	1.00000000	pigs
Nedd9	-0.24	1.00000000	pigs
Tcf12	-0.17	1.00000000	pigs
Ddx1	-0.06	1.00000000	pigs
Herc1	-0.08	1.00000000	pigs
Pde3b	-0.13	1.00000000	pigs
Tmem120b	-0.14	1.00000000	pigs
Dennd6b	-0.10	1.00000000	pigs
Xrcc6	-0.10	1.00000000	pigs
Prss55	-0.12	1.00000000	pigs
St7l	-0.17	1.00000000	pigs
Zfp652	0.00	1.00000000	pigs
Leng1	-0.12	1.00000000	pigs
Orc3	-0.10	1.00000000	pigs
Foxj2	-0.13	1.00000000	pigs
Kdm5b	-0.27	1.00000000	pigs
Epb41l5	-0.08	1.00000000	pigs
Ccdc82	-0.25	1.00000000	pigs
Nek1	-0.06	1.00000000	pigs
3110082I17Rik	-0.28	1.00000000	pigs
Pmaip1	-0.01	1.00000000	pigs
Erp44	-0.11	1.00000000	pigs
Smarcd1	-0.20	1.00000000	pigs
Snrpb2	-0.12	1.00000000	pigs
Psma3	-0.10	1.00000000	pigs
Bcl2l12	-0.15	1.00000000	pigs
Etfrf1	-0.13	1.00000000	pigs

Pld1	-0.09	1.00000000	pigs
Gimap4	-0.16	1.00000000	pigs
Pdhb	-0.06	1.00000000	pigs
Atp6v1a	-0.18	1.00000000	pigs
Ptpre	-0.44	1.00000000	pigs
Snrnp35	-0.03	1.00000000	pigs
Ndufa8	-0.19	1.00000000	pigs
Lcp1	-0.18	1.00000000	pigs
Sympk	0.01	1.00000000	pigs
Vps4b	-0.11	1.00000000	pigs
Phip	-0.15	1.00000000	pigs
Arhgap4	-0.17	1.00000000	pigs
Trmt6	-0.25	1.00000000	pigs
Faf1	-0.07	1.00000000	pigs
Tarbp1	-0.23	1.00000000	pigs
Nup133	-0.03	1.00000000	pigs
Cog5	-0.11	1.00000000	pigs
Bcl7c	-0.12	1.00000000	pigs
Def8	-0.07	1.00000000	pigs
Ndufb6	-0.19	1.00000000	pigs
Stambp	-0.08	1.00000000	pigs
Pcyt1a	-0.15	1.00000000	pigs
H2az2	-0.19	1.00000000	pigs
Pus1	-0.08	1.00000000	pigs
Sel1l	-0.10	1.00000000	pigs
Anapc16	-0.09	1.00000000	pigs
Cwc25	-0.15	1.00000000	pigs
Capns1	-0.16	1.00000000	pigs
Tfec	-0.10	1.00000000	pigs
Nsmaf	-0.25	1.00000000	pigs
Cdkn3	-0.33	1.00000000	pigs
Insyn2b	-0.22	1.00000000	pigs
Sugp2	-0.07	1.00000000	pigs
Terb1	-0.16	1.00000000	pigs
Akr1b3	-0.09	1.00000000	pigs
4932438A13Rik	-0.13	1.00000000	pigs
Arhgap27	-0.22	1.00000000	pigs
Rpia	-0.08	1.00000000	pigs
Acbd6	-0.20	1.00000000	pigs
Arhgef6	-0.08	1.00000000	pigs
Nosip	-0.17	1.00000000	pigs
Cxxc5	-0.20	1.00000000	pigs
Brd3	-0.13	1.00000000	pigs
Dusp22	-0.14	1.00000000	pigs
Lsm6	-0.29	1.00000000	pigs
Ddx56	-0.08	1.00000000	pigs

Taok2	-0.21	1.00000000	pigs
Rbl1	-0.18	1.00000000	pigs
Slc7a7	-0.24	1.00000000	pigs
Ptpmt1	-0.12	1.00000000	pigs
Pafah1b3	-0.24	1.00000000	pigs
Atp5j	-0.27	1.00000000	pigs
Adcy3	-0.05	1.00000000	pigs
Itgb2l	-0.03	1.00000000	pigs
Capn2	-0.26	1.00000000	pigs
Setd2	-0.01	1.00000000	pigs
Zfp654	-0.08	1.00000000	pigs
Polr3a	-0.19	1.00000000	pigs
Eif2ak1	-0.16	1.00000000	pigs
Smc6	0.00	1.00000000	pigs
Cpeb3	-0.18	1.00000000	pigs
Plaat3	-0.11	1.00000000	pigs
Acat1	-0.23	1.00000000	pigs
Tk2	-0.07	1.00000000	pigs
Clta	-0.09	1.00000000	pigs
Ccp110	-0.12	1.00000000	pigs
Cpsf3	-0.18	1.00000000	pigs
Pcmt1	-0.08	1.00000000	pigs
Usp24	-0.17	1.00000000	pigs
Armh3	-0.22	1.00000000	pigs
Tmem156	-0.11	1.00000000	pigs
Tfpt	-0.02	1.00000000	pigs
Ddx49	-0.20	1.00000000	pigs
Pafah2	-0.04	1.00000000	pigs
Pelp1	-0.04	1.00000000	pigs
Josd1	-0.27	1.00000000	pigs
Carnmt1	-0.07	1.00000000	pigs
Ap1m1	-0.19	1.00000000	pigs
Eif3h	-0.23	1.00000000	pigs
Vps50	-0.14	1.00000000	pigs
Cep250	-0.32	1.00000000	pigs
Ift57	-0.18	1.00000000	pigs
Tmem62	-0.04	1.00000000	pigs
Rab28	-0.20	1.00000000	pigs
Ccng2	-0.25	1.00000000	pigs
Rsrc1	-0.19	1.00000000	pigs
Uqcrq	-0.24	1.00000000	pigs
Cdk6	-0.26	1.00000000	pigs
Coq9	-0.17	1.00000000	pigs
Wrap73	-0.18	1.00000000	pigs
Rpp38	-0.08	1.00000000	pigs
Dcun1d3	0.01	1.00000000	pigs

Sri	-0.27	1.00000000	pigs
Cic	-0.19	1.00000000	pigs
BC005624	-0.13	1.00000000	pigs
Slc26a2	-0.11	1.00000000	pigs
Lmbrd2	-0.15	1.00000000	pigs
Kat5	-0.03	1.00000000	pigs
Asns	-0.18	1.00000000	pigs
Gpatch8	-0.14	1.00000000	pigs
Vps26a	0.01	1.00000000	pigs
Tm2d1	-0.18	1.00000000	pigs
Rab27a	-0.31	1.00000000	pigs
Gsk3b	-0.07	1.00000000	pigs
Epc1	-0.10	1.00000000	pigs
Txndc9	-0.05	1.00000000	pigs
Rpain	-0.11	1.00000000	pigs
Cltb	-0.14	1.00000000	pigs
Dbr1	-0.04	1.00000000	pigs
Nudt5	-0.06	1.00000000	pigs
Enox2	-0.01	1.00000000	pigs
Ipmk	-0.44	1.00000000	pigs
Tradd	-0.10	1.00000000	pigs
Il12rb1	-0.03	1.00000000	pigs
Chmp2a	-0.21	1.00000000	pigs
Vps26c	-0.04	1.00000000	pigs
Tra2b	0.01	1.00000000	pigs
Angptl1	-0.12	1.00000000	pigs
Osbp	-0.01	1.00000000	pigs
Eif2b3	-0.10	1.00000000	pigs
Ddx24	-0.23	1.00000000	pigs
Prkcd	-0.04	1.00000000	pigs
Pnpo	-0.14	1.00000000	pigs
Homer1	-0.09	1.00000000	pigs
Haus2	0.00	1.00000000	pigs
Zfp524	-0.09	1.00000000	pigs
Napepld	-0.06	1.00000000	pigs
Zfp317	-0.13	1.00000000	pigs
Zmym5	-0.14	1.00000000	pigs
Acox1	-0.14	1.00000000	pigs
Rrs1	-0.03	1.00000000	pigs
Csnk1g1	-0.11	1.00000000	pigs
Atpif1	-0.33	1.00000000	pigs
Ints3	-0.02	1.00000000	pigs
Rap1b	0.05	1.00000000	pigs
Zc2hc1a	-0.08	1.00000000	pigs
Slc15a2	-0.18	1.00000000	pigs
Itgb7	-0.13	1.00000000	pigs

Spsb3	-0.12	1.00000000	pigs
Psmd5	-0.09	1.00000000	pigs
Atf7ip	-0.22	1.00000000	pigs
Nbr1	-0.08	1.00000000	pigs
Dip2a	-0.18	1.00000000	pigs
Chm	-0.18	1.00000000	pigs
Lactb	-0.21	1.00000000	pigs
Chmp1a	-0.08	1.00000000	pigs
Slc30a7	-0.10	1.00000000	pigs
C030006K11Rik	-0.03	1.00000000	pigs
Srek1ip1	-0.26	1.00000000	pigs
Fto	0.05	1.00000000	pigs
Upf2	-0.08	1.00000000	pigs
Suco	-0.09	1.00000000	pigs
Rars2	0.03	1.00000000	pigs
Cers4	0.02	1.00000000	pigs
Ergic2	-0.16	1.00000000	pigs
Ubxn4	-0.16	1.00000000	pigs
Zer1	-0.35	1.00000000	pigs
Eya3	-0.23	1.00000000	pigs
Ccnd3	-0.19	1.00000000	pigs
Bcl6	-0.15	1.00000000	pigs
Adnp	-0.25	1.00000000	pigs
Sgk1	-0.18	1.00000000	pigs
Echs1	-0.16	1.00000000	pigs
Wars2	-0.16	1.00000000	pigs
Top2b	-0.14	1.00000000	pigs
Snf8	-0.19	1.00000000	pigs
Ankrd54	-0.05	1.00000000	pigs
Wrn	-0.13	1.00000000	pigs
Pgap2	-0.14	1.00000000	pigs
Spag16	-0.16	1.00000000	pigs
Hsd17b7	-0.11	1.00000000	pigs
Sap30	-0.30	1.00000000	pigs
Selenop	-0.01	1.00000000	pigs
Dnajc8	-0.19	1.00000000	pigs
Plekhj1	-0.13	1.00000000	pigs
Phax	-0.22	1.00000000	pigs
Arpc4	-0.05	1.00000000	pigs
Naa60	-0.25	1.00000000	pigs
Tma16	-0.18	1.00000000	pigs
Ints12	-0.10	1.00000000	pigs
Snap47	-0.19	1.00000000	pigs
Hdac1	-0.11	1.00000000	pigs
Exoc7	-0.09	1.00000000	pigs
Ube3b	-0.12	1.00000000	pigs

Copb2	-0.03	1.00000000	pigs
Arpc2	-0.08	1.00000000	pigs
Rgs19	-0.08	1.00000000	pigs
Fancm	-0.11	1.00000000	pigs
Aco1	-0.09	1.00000000	pigs
Relch	-0.10	1.00000000	pigs
Phf20l1	-0.26	1.00000000	pigs
Ciita	-0.08	1.00000000	pigs
Psmf1	-0.16	1.00000000	pigs
Kif3a	0.03	1.00000000	pigs
Nsun6	-0.05	1.00000000	pigs
Nr3c1	0.00	1.00000000	pigs
Ppa2	-0.09	1.00000000	pigs
Rad23a	-0.08	1.00000000	pigs
Ctcf	-0.22	1.00000000	pigs
Creld1	0.00	1.00000000	pigs
Ywhah	-0.24	1.00000000	pigs
Krtcap2	-0.27	1.00000000	pigs
Fbxo9	-0.10	1.00000000	pigs
Trp53i11	-0.11	1.00000000	pigs
Trak2	-0.16	1.00000000	pigs
Abhd11	-0.05	1.00000000	pigs
Gk5	-0.27	1.00000000	pigs
Ap4s1	-0.17	1.00000000	pigs
Terf2	-0.10	1.00000000	pigs
Zfp507	-0.11	1.00000000	pigs
Plaur	-0.19	1.00000000	pigs
Nedd1	-0.09	1.00000000	pigs
Adar	-0.04	1.00000000	pigs
Akap6	-0.17	1.00000000	pigs
Sec11c	-0.24	1.00000000	pigs
Rrp36	-0.11	1.00000000	pigs
Wbp1	-0.11	1.00000000	pigs
Klf3	-0.16	1.00000000	pigs
Setdb1	-0.03	1.00000000	pigs
Septin7	-0.01	1.00000000	pigs
Cecr2	-0.22	1.00000000	pigs
Fadd	-0.25	1.00000000	pigs
Ccdc28a	-0.12	1.00000000	pigs
Adpgk	-0.27	1.00000000	pigs
Tbc1d17	-0.16	1.00000000	pigs
Fam214a	0.07	1.00000000	pigs
Il10ra	-0.19	1.00000000	pigs
Ptpra	-0.12	1.00000000	pigs
Vps28	-0.28	1.00000000	pigs
Cog4	-0.03	1.00000000	pigs

Cyb561d2	-0.08	1.00000000	pigs
Actl6a	-0.07	1.00000000	pigs
Trim37	-0.16	1.00000000	pigs
Srsf10	-0.18	1.00000000	pigs
Tomm7	-0.18	1.00000000	pigs
Cdkn1b	-0.15	1.00000000	pigs
Slc35a5	-0.07	1.00000000	pigs
Alms1	-0.11	1.00000000	pigs
Enpp4	-0.06	1.00000000	pigs
Sell	-0.31	1.00000000	pigs
Zfp511	-0.33	1.00000000	pigs
Ercc3	-0.05	1.00000000	pigs
Senp7	-0.29	1.00000000	pigs
Ankrd33b	-0.04	1.00000000	pigs
Vcan	-0.03	1.00000000	pigs
Tmbim6	-0.15	1.00000000	pigs
Sh3glb2	-0.04	1.00000000	pigs
Bcat1	-0.15	1.00000000	pigs
Nt5c	-0.14	1.00000000	pigs
Chchd3	-0.07	1.00000000	pigs
Ppp2r3a	-0.27	1.00000000	pigs
Prmt3	-0.11	1.00000000	pigs
Afg1l	-0.09	1.00000000	pigs
Wdr77	-0.19	1.00000000	pigs
Eif6	-0.23	1.00000000	pigs
Cd19	-0.13	1.00000000	pigs
Herpud2	-0.14	1.00000000	pigs
Fasn	-0.24	1.00000000	pigs
Ago4	-0.16	1.00000000	pigs
Ikzf3	-0.15	1.00000000	pigs
Traf3ip3	-0.24	1.00000000	pigs
Gps2	-0.22	1.00000000	pigs
Yeats4	-0.15	1.00000000	pigs
Actr10	-0.09	1.00000000	pigs
Utp11	-0.23	1.00000000	pigs
Slc39a11	-0.19	1.00000000	pigs
Ddx21	-0.15	1.00000000	pigs
Fancd2	-0.15	1.00000000	pigs
Chchd1	-0.16	1.00000000	pigs
Cox11	-0.21	1.00000000	pigs
Zfp638	-0.21	1.00000000	pigs
Smg9	-0.20	1.00000000	pigs
Slc35a3	-0.12	1.00000000	pigs
Sar1a	-0.24	1.00000000	pigs
Socs5	-0.14	1.00000000	pigs
Btrc	-0.09	1.00000000	pigs

Arhgap17	-0.11	1.00000000	pigs
Akap10	-0.25	1.00000000	pigs
Star	-0.15	1.00000000	pigs
Cnot10	-0.20	1.00000000	pigs
Cdk7	-0.05	1.00000000	pigs
Prdx2	-0.38	1.00000000	pigs
Zfp395	-0.25	1.00000000	pigs
Zw10	-0.10	1.00000000	pigs
Erp29	-0.08	1.00000000	pigs
Tor1aip1	-0.20	1.00000000	pigs
Fam98a	-0.33	1.00000000	pigs
Gpcpd1	-0.20	1.00000000	pigs
Cd69	-0.28	1.00000000	pigs
Rbm22	-0.09	1.00000000	pigs
Chfr	-0.14	1.00000000	pigs
Psmb8	-0.05	1.00000000	pigs
Ifi204	-0.23	1.00000000	pigs
Tmem241	-0.06	1.00000000	pigs
Commd2	-0.16	1.00000000	pigs
Get3	-0.19	1.00000000	pigs
Psmb3	-0.23	1.00000000	pigs
Gdap2	-0.18	1.00000000	pigs
Gnpat	-0.03	1.00000000	pigs
Exosc4	-0.12	1.00000000	pigs
Nubp1	-0.18	1.00000000	pigs
Shprh	-0.10	1.00000000	pigs
Rnf125	-0.11	1.00000000	pigs
Txk	-0.06	1.00000000	pigs
Suclg2	-0.03	1.00000000	pigs
Commd10	-0.26	1.00000000	pigs
Tmem14a	-0.12	1.00000000	pigs
Psmc1	-0.17	1.00000000	pigs
Zfp609	-0.30	1.00000000	pigs
Capn10	-0.02	1.00000000	pigs
Tmem50b	-0.23	1.00000000	pigs
Rnf6	-0.18	1.00000000	pigs
Tdpoz8	-0.18	1.00000000	pigs
Pnpt1	0.00	1.00000000	pigs
Tcp11	-0.05	1.00000000	pigs
Crtc2	-0.22	1.00000000	pigs
Klhdc4	-0.16	1.00000000	pigs
Oxr1	-0.21	1.00000000	pigs
Tipin	-0.13	1.00000000	pigs
Fam3a	-0.11	1.00000000	pigs
Lsm8	-0.30	1.00000000	pigs
Ino80	-0.13	1.00000000	pigs

Acp5	-0.18	1.00000000	pigs
Epg5	-0.21	1.00000000	pigs
Top1	-0.11	1.00000000	pigs
Spint2	-0.34	1.00000000	pigs
Rer1	-0.21	1.00000000	pigs
Pygm	-0.11	1.00000000	pigs
Cdc16	-0.16	1.00000000	pigs
Abracl	-0.09	1.00000000	pigs
Fbrsl1	-0.19	1.00000000	pigs
Zfp422	-0.15	1.00000000	pigs
Smpd4	-0.10	1.00000000	pigs
Adsl	-0.10	1.00000000	pigs
Ppp1r21	-0.11	1.00000000	pigs
Focad	-0.16	1.00000000	pigs
Tent2	-0.27	1.00000000	pigs
Itgae	-0.14	1.00000000	pigs
Cxxc1	-0.12	1.00000000	pigs
Ets1	-0.08	1.00000000	pigs
Blzf1	-0.10	1.00000000	pigs
Samhd1	-0.21	1.00000000	pigs
Nat9	-0.05	1.00000000	pigs
Sf3a1	-0.31	1.00000000	pigs
Slc30a6	-0.13	1.00000000	pigs
Prox1	-0.23	1.00000000	pigs
Rrp12	-0.13	1.00000000	pigs
Insig2	-0.14	1.00000000	pigs
Xxylt1	-0.03	1.00000000	pigs
Helb	-0.10	1.00000000	pigs
Rchy1	-0.13	1.00000000	pigs
Taf1b	-0.02	1.00000000	pigs
Fam177a	-0.20	1.00000000	pigs
Ppt1	-0.12	1.00000000	pigs
Edem2	0.00	1.00000000	pigs
Pag1	0.05	1.00000000	pigs
Erh	-0.23	1.00000000	pigs
Pik3c2a	-0.13	1.00000000	pigs
Numb	-0.38	1.00000000	pigs
Phf11a	-0.15	1.00000000	pigs
Brcc3	-0.28	1.00000000	pigs
Zfp169	-0.07	1.00000000	pigs
Sf3b1	-0.07	1.00000000	pigs
Arl15	-0.39	1.00000000	pigs
Exosc8	-0.14	1.00000000	pigs
Pex14	-0.15	1.00000000	pigs
Acsl4	-0.31	1.00000000	pigs
Cyb5r4	-0.14	1.00000000	pigs

Cdc14a	-0.16	1.00000000	pigs
Trmt11	-0.24	1.00000000	pigs
Atg7	-0.26	1.00000000	pigs
Fggy	-0.06	1.00000000	pigs
Ppil4	-0.20	1.00000000	pigs
Xpot	-0.01	1.00000000	pigs
Vezt	-0.01	1.00000000	pigs
Gltp	-0.10	1.00000000	pigs
Phykpl	-0.23	1.00000000	pigs
Pdia3	-0.11	1.00000000	pigs
Pld4	-0.16	1.00000000	pigs
Erlin2	-0.01	1.00000000	pigs
Ankrd37	-0.09	1.00000000	pigs
Tiprl	-0.20	1.00000000	pigs
Tex9	-0.22	1.00000000	pigs
Tpd52l2	-0.29	1.00000000	pigs
Ubtf	-0.27	1.00000000	pigs
Kdm1b	-0.17	1.00000000	pigs
Bbs7	-0.18	1.00000000	pigs
Rab18	-0.23	1.00000000	pigs
Gimap8	-0.07	1.00000000	pigs
Pik3c3	-0.11	1.00000000	pigs
Ssx2ip	-0.03	1.00000000	pigs
Vwa8	-0.16	1.00000000	pigs
Rb1cc1	-0.18	1.00000000	pigs
Ndufa5	-0.19	1.00000000	pigs
Prkrip1	-0.21	1.00000000	pigs
Bag4	-0.22	1.00000000	pigs
Polr3d	-0.11	1.00000000	pigs
Cdip1	-0.20	1.00000000	pigs
Ccdc43	-0.13	1.00000000	pigs
Fbxl17	-0.15	1.00000000	pigs
Septin9	-0.15	1.00000000	pigs
Ddx59	-0.08	1.00000000	pigs
Lsp1	-0.12	1.00000000	pigs
Tnrc6c	-0.11	1.00000000	pigs
Ergic1	-0.09	1.00000000	pigs
Prpf19	-0.12	1.00000000	pigs
Slc15a3	-0.01	1.00000000	pigs
Prkacb	-0.21	1.00000000	pigs
Pon2	-0.11	1.00000000	pigs
Derl3	-0.39	1.00000000	pigs
Tram2	-0.36	1.00000000	pigs
Cyb561a3	-0.16	1.00000000	pigs
Ppp2cb	-0.11	1.00000000	pigs
Snx2	-0.03	1.00000000	pigs

Kifap3	-0.10	1.00000000	pigs
Noc3l	-0.27	1.00000000	pigs
Ube2e2	-0.02	1.00000000	pigs
Prkd2	-0.25	1.00000000	pigs
Zbtb49	-0.04	1.00000000	pigs
Pdcd5	-0.30	1.00000000	pigs
Ptger4	-0.15	1.00000000	pigs
Dscc1	-0.14	1.00000000	pigs
Immp2l	-0.13	1.00000000	pigs
Senp1	-0.03	1.00000000	pigs
Pqlc3	-0.24	1.00000000	pigs
Rcn2	-0.19	1.00000000	pigs
Pms1	-0.25	1.00000000	pigs
Ap3b1	-0.12	1.00000000	pigs
Abhd16a	-0.20	1.00000000	pigs
Rbm47	-0.22	1.00000000	pigs
Nf2	-0.15	1.00000000	pigs
Ints7	-0.17	1.00000000	pigs
Sec24d	-0.20	1.00000000	pigs
B4galt1	-0.21	1.00000000	pigs
Ilf2	-0.19	1.00000000	pigs
Sf3b4	-0.20	1.00000000	pigs
Washc4	-0.31	1.00000000	pigs
Entr1	-0.13	1.00000000	pigs
Swt1	-0.15	1.00000000	pigs
Rcor3	-0.14	1.00000000	pigs
Gtpbp8	-0.24	1.00000000	pigs
Gtf2h3	-0.31	1.00000000	pigs
Cxcl3	-0.62	1.00000000	pigs
Zyx	-0.23	1.00000000	pigs
Brwd1	-0.15	1.00000000	pigs
Gtf2f2	-0.18	1.00000000	pigs
Qtrt1	-0.03	1.00000000	pigs
Vps25	-0.18	1.00000000	pigs
Nemf	-0.15	1.00000000	pigs
Use1	-0.13	1.00000000	pigs
Dalrd3	-0.26	1.00000000	pigs
Ciapin1	-0.18	1.00000000	pigs
Otud6b	-0.22	1.00000000	pigs
Pigk	-0.11	1.00000000	pigs
Commd9	-0.06	1.00000000	pigs
Acadsb	-0.10	1.00000000	pigs
Slc12a6	-0.07	1.00000000	pigs
Pold2	-0.10	1.00000000	pigs
Naxe	-0.27	1.00000000	pigs
Exoc6b	0.00	1.00000000	pigs

Akap8	-0.15	1.00000000	pigs
BC003965	-0.21	1.00000000	pigs
Aprt	-0.14	1.00000000	pigs
Uqcrfs1	-0.38	1.00000000	pigs
Arhgap30	-0.12	1.00000000	pigs
Nhlrc2	-0.27	1.00000000	pigs
Dis3l2	-0.24	1.00000000	pigs
Galnt1	-0.20	1.00000000	pigs
Dars	-0.26	1.00000000	pigs
Casp7	-0.10	1.00000000	pigs
AU040320	-0.05	1.00000000	pigs
Pigc	-0.14	1.00000000	pigs
Psmg4	-0.21	1.00000000	pigs
Cog2	-0.14	1.00000000	pigs
Kifbp	-0.18	1.00000000	pigs
Gpatch3	-0.15	1.00000000	pigs
Dnajc19	-0.26	1.00000000	pigs
Otub1	-0.15	1.00000000	pigs
Fam204a	-0.10	1.00000000	pigs
Cmpk1	-0.23	1.00000000	pigs
Itch	-0.13	1.00000000	pigs
Tmed5	-0.11	1.00000000	pigs
Wdr36	-0.02	1.00000000	pigs
Calu	0.00	1.00000000	pigs
Scand1	-0.21	1.00000000	pigs
Spata5	-0.06	1.00000000	pigs
Dctpp1	-0.41	1.00000000	pigs
Pyroxd1	-0.02	1.00000000	pigs
Tsc22d3	-0.23	1.00000000	pigs
Pfdn1	-0.09	1.00000000	pigs
Cep170	-0.20	1.00000000	pigs
Nfrkb	-0.17	1.00000000	pigs
Pikfyve	-0.05	1.00000000	pigs
Fam117a	-0.22	1.00000000	pigs
Pfdn6	-0.17	1.00000000	pigs
Ifit3	-0.41	1.00000000	pigs
Rasa1	-0.17	1.00000000	pigs
Rb1	-0.15	1.00000000	pigs
Pip5k1b	-0.10	1.00000000	pigs
Nup155	-0.16	1.00000000	pigs
Slk	-0.26	1.00000000	pigs
Abcb7	-0.22	1.00000000	pigs
Ccdc93	-0.27	1.00000000	pigs
Dut	0.05	1.00000000	pigs
Hibch	-0.17	1.00000000	pigs
Vrk2	-0.26	1.00000000	pigs

Pdgfd	-0.36	1.00000000	pigs
Fam120b	-0.20	1.00000000	pigs
Akap8l	-0.06	1.00000000	pigs
Ube2w	-0.04	1.00000000	pigs
Stk39	-0.11	1.00000000	pigs
Strn	-0.14	1.00000000	pigs
Hectd4	-0.14	1.00000000	pigs
Taf13	-0.11	1.00000000	pigs
2700062C07Rik	-0.14	1.00000000	pigs
Nars2	-0.07	1.00000000	pigs
Chmp6	-0.10	1.00000000	pigs
Gsn	-0.13	1.00000000	pigs
Nr2c2	-0.33	1.00000000	pigs
Smim4	-0.13	1.00000000	pigs
Zfp217	-0.25	1.00000000	pigs
Cul2	-0.13	1.00000000	pigs
Rbbp5	-0.13	1.00000000	pigs
Pram1	-0.07	1.00000000	pigs
Zmym6	-0.03	1.00000000	pigs
Exoc8	0.02	1.00000000	pigs
Eri3	-0.05	1.00000000	pigs
Tmem131l	-0.14	1.00000000	pigs
Gtf3c6	-0.05	1.00000000	pigs
Nup35	-0.09	1.00000000	pigs
Lyrm7	-0.11	1.00000000	pigs
Amz2	-0.24	1.00000000	pigs
Ttc38	-0.09	1.00000000	pigs
Lzic	-0.05	1.00000000	pigs
Rbsn	-0.07	1.00000000	pigs
Emc1	-0.07	1.00000000	pigs
Slc25a39	-0.18	1.00000000	pigs
Cd37	-0.12	1.00000000	pigs
Gpsm3	-0.13	1.00000000	pigs
Parp12	-0.25	1.00000000	pigs
Senp2	-0.14	1.00000000	pigs
Tmem97	-0.06	1.00000000	pigs
Vsir	-0.17	1.00000000	pigs
Ip6k2	-0.03	1.00000000	pigs
Napg	-0.07	1.00000000	pigs
Kcnq5	0.00	1.00000000	pigs
Taco1	-0.19	1.00000000	pigs
Tlnrd1	-0.24	1.00000000	pigs
Zdhhc13	-0.09	1.00000000	pigs
Slc25a26	-0.24	1.00000000	pigs
Cfdp1	-0.14	1.00000000	pigs
Ncoa1	-0.09	1.00000000	pigs

Cbl	-0.12	1.00000000	pigs
Polr2e	-0.20	1.00000000	pigs
N4bp2	-0.13	1.00000000	pigs
Atpaf1	-0.13	1.00000000	pigs
Heatr5b	-0.25	1.00000000	pigs
Rab40b	-0.28	1.00000000	pigs
Amh	-0.14	1.00000000	pigs
Ube2j2	-0.31	1.00000000	pigs
Rpa2	-0.25	1.00000000	pigs
Bag1	-0.40	1.00000000	pigs
Commd1b	-0.20	1.00000000	pigs
Vps29	-0.29	1.00000000	pigs
Prelid1	-0.35	1.00000000	pigs
Stub1	-0.21	1.00000000	pigs
Ppig	-0.19	1.00000000	pigs
Runx1	-0.01	1.00000000	pigs
Dhx37	-0.23	1.00000000	pigs
Acp6	-0.05	1.00000000	pigs
Gtpbp2	-0.02	1.00000000	pigs
Kmt2e	-0.03	1.00000000	pigs
Vamp4	-0.14	1.00000000	pigs
Jpt2	-0.16	1.00000000	pigs
Hars	-0.11	1.00000000	pigs
Chd9	-0.13	1.00000000	pigs
Slc25a12	-0.22	1.00000000	pigs
Prpf4	-0.05	1.00000000	pigs
Tex2	-0.03	1.00000000	pigs
Sfpq	-0.19	1.00000000	pigs
Etfdh	-0.02	1.00000000	pigs
Rfc5	-0.17	1.00000000	pigs
Fam98b	-0.12	1.00000000	pigs
Dock7	-0.10	1.00000000	pigs
Ccdc61	-0.10	1.00000000	pigs
P2ry10	-0.11	1.00000000	pigs
Vps37a	-0.21	1.00000000	pigs
Ndufaf6	-0.17	1.00000000	pigs
Spg7	-0.10	1.00000000	pigs
Itgam	-0.22	1.00000000	pigs
Gramd1b	-0.15	1.00000000	pigs
Timm8b	-0.29	1.00000000	pigs
Znhit3	-0.19	1.00000000	pigs
Snx29	-0.10	1.00000000	pigs
1700037H04Rik	-0.24	1.00000000	pigs
Calm3	-0.24	1.00000000	pigs
Synj2bp	-0.11	1.00000000	pigs
Slirp	-0.05	1.00000000	pigs

Cd84	-0.14	1.00000000	pigs
Pnrc2	-0.26	1.00000000	pigs
Ucp2	-0.18	1.00000000	pigs
Pdap1	-0.16	1.00000000	pigs
Rpa1	-0.13	1.00000000	pigs
Asb7	-0.19	1.00000000	pigs
Dpf2	-0.13	1.00000000	pigs
Vps72	-0.25	1.00000000	pigs
Cox20	-0.16	1.00000000	pigs
Clstn1	-0.14	1.00000000	pigs
Dennd5b	-0.19	1.00000000	pigs
Lypla2	-0.18	1.00000000	pigs
Tspan5	-0.02	1.00000000	pigs
Triobp	-0.13	1.00000000	pigs
Tmem223	-0.28	1.00000000	pigs
Trank1	-0.17	1.00000000	pigs
Wdr33	-0.09	1.00000000	pigs
Asrgl1	-0.18	1.00000000	pigs
Btd	-0.27	1.00000000	pigs
Lrrc14	-0.10	1.00000000	pigs
Washc3	-0.31	1.00000000	pigs
Rab2a	-0.13	1.00000000	pigs
C2cd3	-0.19	1.00000000	pigs
Pdcd11	-0.20	1.00000000	pigs
Ap1g1	-0.18	1.00000000	pigs
Pcmtd2	-0.06	1.00000000	pigs
Ncl	0.05	1.00000000	pigs
Hltf	-0.25	1.00000000	pigs
Erap1	-0.11	1.00000000	pigs
Dnajc1	-0.19	1.00000000	pigs
Hps1	-0.05	1.00000000	pigs
Cpeb2	-0.20	1.00000000	pigs
Sfxn4	-0.13	1.00000000	pigs
Pdcl3	-0.27	1.00000000	pigs
Tfrc	-0.23	1.00000000	pigs
Capn1	-0.17	1.00000000	pigs
2610507B11Rik	-0.15	1.00000000	pigs
Serbp1	-0.03	1.00000000	pigs
Lsm7	-0.32	1.00000000	pigs
Strbp	-0.02	1.00000000	pigs
Flot2	-0.09	1.00000000	pigs
Abt1	-0.15	1.00000000	pigs
Klhl20	-0.28	1.00000000	pigs
Hmg20a	-0.09	1.00000000	pigs
Gtf3a	-0.05	1.00000000	pigs
Pi4ka	-0.21	1.00000000	pigs

Ppil3	-0.17	1.00000000	pigs
Gcfc2	-0.06	1.00000000	pigs
Sil1	-0.13	1.00000000	pigs
Rbbp7	-0.11	1.00000000	pigs
Tor3a	-0.12	1.00000000	pigs
Timm17a	-0.13	1.00000000	pigs
Brd9	-0.15	1.00000000	pigs
Apaf1	-0.21	1.00000000	pigs
2310009B15Rik	-0.13	1.00000000	pigs
Fam149b	-0.35	1.00000000	pigs
Usp19	-0.15	1.00000000	pigs
Tmc6	-0.18	1.00000000	pigs
Prepl	-0.16	1.00000000	pigs
Stap1	-0.24	1.00000000	pigs
Fkbp15	-0.10	1.00000000	pigs
Atad1	-0.19	1.00000000	pigs
Septin3	-0.23	1.00000000	pigs
Slc1a2	-0.61	1.00000000	pigs
Zfp36l1	-0.25	1.00000000	pigs
Ppil2	-0.16	1.00000000	pigs
Znfx1	-0.23	1.00000000	pigs
Gusb	-0.22	1.00000000	pigs
G6pdx	-0.13	1.00000000	pigs
AI597479	-0.19	1.00000000	pigs
Cdk5rap3	-0.12	1.00000000	pigs
Hmbox1	-0.07	1.00000000	pigs
Eif4a3l1	-0.24	1.00000000	pigs
Acad11	0.05	1.00000000	pigs
Scai	-0.20	1.00000000	pigs
Exog	-0.35	1.00000000	pigs
Ccar2	-0.07	1.00000000	pigs
Usp40	-0.06	1.00000000	pigs
G0s2	-0.31	1.00000000	pigs
Pcgf3	-0.12	1.00000000	pigs
Smad2	-0.36	1.00000000	pigs
Kdm4b	-0.21	1.00000000	pigs
Ampd3	0.03	1.00000000	pigs
Lmbr1	-0.12	1.00000000	pigs
Txnl4b	-0.09	1.00000000	pigs
Hyal2	-0.14	1.00000000	pigs
Leprotl1	-0.17	1.00000000	pigs
Adrm1	-0.11	1.00000000	pigs
Cfd	-2.03	0.00000000	pigs
