# Supplementary material for: Heterogeneous viral-host response at single-cell resolution and viral adaptive mutational atlas in birds and mammals upon H5 influenza virus infection
Source: Natl Sci Rev. 2026 Jun 25;13(14):nwag380. doi: 10.1093/nsr/nwag380 (PMC13458601; doi:10.1093/nsr/nwag380)
Supplement: Supplementary file 1 [file nwag380_supplemental_files.zip › Supplementary Data 16.pdf]

pplementary Data 16. Differentially expressed genes of dendritic across different species upon H5 HPAIV infectio

gene	log2FoldChange	p value	host
Ifit2	3.73	0.00000072	chicken
Hpgd	2.17	0.00000626	chicken
Epn2	1.57	0.00000958	chicken
Rack1	-2.22	0.00003223	chicken
Tpt1	-1.74	0.00013521	chicken
Zbtb46	1.95	0.00015743	chicken
Zfp385b	2.67	0.00016101	chicken
Piezo2	1.81	0.00019684	chicken
Smarcal1	-1.79	0.00022753	chicken
Irf1	1.73	0.00049127	chicken
Oasl2	1.81	0.00056877	chicken
Plxna1	1.63	0.00060079	chicken
Gpr183	2.15	0.00071823	chicken
Ptpn12	1.77	0.00079556	chicken
Rgs1	1.77	0.00083914	chicken
Angpt1	1.11	0.00122164	chicken
Eef1d	-1.73	0.00186704	chicken
Stat1	2.02	0.00207593	chicken
Eef1b2	-1.66	0.00357966	chicken
Pacsin2	1.48	0.00414059	chicken
Birc2	2.11	0.00472252	chicken
Rnf213	1.70	0.00500182	chicken
Nme2	-1.58	0.00633742	chicken
Znfx1	1.20	0.01134805	chicken
Rasgef1a	2.28	0.01582363	chicken
Saraf	1.10	0.01774977	chicken
Coro1c	-1.31	0.01839144	chicken
Ppm1h	2.81	0.02254468	chicken
Cmip	1.08	0.02706478	chicken
Lrp12	1.53	0.02755866	chicken
Cmpk2	0.57	0.03871048	chicken
Creb5	2.09	0.03915393	chicken
Gna13	1.44	0.04106014	chicken
Dusp1	1.69	0.04532241	chicken
H3f3a	-1.36	0.05547100	chicken
Ackr4	0.54	0.05583718	chicken
Ppp1r3b	0.71	0.05583718	chicken
Olfml1	0.89	0.05583718	chicken
Prss45	1.07	0.05583718	chicken
Ston2	1.68	0.05706121	chicken
Btg2	1.53	0.06274898	chicken

Ugcg	1.43	0.08857587	chicken
Ddx60	0.98	0.08991588	chicken
Usp25	1.40	0.09661912	chicken
Gpx1	-2.04	0.09704831	chicken
Lmbrd1	1.51	0.09883561	chicken
Rsad2	1.20	0.10952607	chicken
Fau	-1.24	0.11592432	chicken
Ppp3r1	0.94	0.11608835	chicken
Pabpc1	-0.87	0.12985237	chicken
Dusp5	1.12	0.13877546	chicken
Ybx1	-1.12	0.14018579	chicken
Ndel1	0.95	0.16262319	chicken
Sema3c	2.73	0.19094875	chicken
Cdc42ep4	0.72	0.21765935	chicken
Tnip2	1.81	0.23551450	chicken
Azin1	0.94	0.24704391	chicken
Klhl18	1.23	0.27029351	chicken
Ndufa10	-1.17	0.29658044	chicken
Hmgcs1	2.19	0.30975708	chicken
Eef2	-1.13	0.35083197	chicken
Htr7	0.89	0.37583044	chicken
Tnfsf13b	1.04	0.39044498	chicken
Trio	1.41	0.39943919	chicken
Eif3e	-1.18	0.43341427	chicken
Trank1	1.52	0.46109306	chicken
Stat2	1.00	0.48570618	chicken
Zzef1	0.96	0.49445139	chicken
Top2b	-1.25	0.52334215	chicken
Ifih1	1.79	0.53735726	chicken
Klf6	1.37	0.57315008	chicken
Ly86	-2.64	0.60420075	chicken
Baz2b	1.85	0.61904533	chicken
Rassf5	1.02	0.65425463	chicken
Dock10	1.28	0.72998561	chicken
D16Ertd472e	1.13	0.78274913	chicken
Traf3	1.46	0.80210615	chicken
Slk	-1.25	0.82490694	chicken
Neurl1a	1.49	0.84786863	chicken
Fth1	-1.19	0.84825366	chicken
Rybp	1.17	0.86725925	chicken
Tnfaip3	1.52	0.87751995	chicken
Zfp36l1	0.90	0.88328683	chicken
Cflar	2.26	0.92798272	chicken
Rubcnl	0.76	0.95423649	chicken
Ptpn2	0.81	0.96494661	chicken
Hivep1	1.13	1.00000000	chicken

Ly6e	1.17	1.00000000	chicken
Rapsn	1.01	1.00000000	chicken
Hif1a	1.27	1.00000000	chicken
Tut4	0.92	1.00000000	chicken
Tuba1b	0.64	1.00000000	chicken
Ass1	1.67	1.00000000	chicken
Nfkb1	0.99	1.00000000	chicken
Pik3r5	1.23	1.00000000	chicken
Gls	1.16	1.00000000	chicken
Eif4a2	-0.91	1.00000000	chicken
Rnf19b	0.71	1.00000000	chicken
Lemd3	0.79	1.00000000	chicken
Nos2	1.90	1.00000000	chicken
Jund	0.71	1.00000000	chicken
Gpr82	0.32	1.00000000	chicken
Rab30	1.24	1.00000000	chicken
Ttc7b	1.06	1.00000000	chicken
Ercc6	-1.20	1.00000000	chicken
Wnk1	1.26	1.00000000	chicken
Fosb	0.73	1.00000000	chicken
Hnrnpa1	-1.23	1.00000000	chicken
Nkain3	1.25	1.00000000	chicken
Osbpl9	0.82	1.00000000	chicken
Dot1l	0.95	1.00000000	chicken
Vdr	0.33	1.00000000	chicken
Pik3r1	1.51	1.00000000	chicken
Pik3cb	1.37	1.00000000	chicken
Wdfy2	0.92	1.00000000	chicken
Abcc1	1.25	1.00000000	chicken
Ube2v1	0.72	1.00000000	chicken
Tax1bp1	1.16	1.00000000	chicken
Syt14	2.11	1.00000000	chicken
Hipk3	1.02	1.00000000	chicken
Atp10a	1.17	1.00000000	chicken
Actg1	-0.90	1.00000000	chicken
Snx14	-1.27	1.00000000	chicken
Rgs18	1.09	1.00000000	chicken
Cnot2	0.98	1.00000000	chicken
Esd	-0.75	1.00000000	chicken
Cd44	1.09	1.00000000	chicken
Rel	1.24	1.00000000	chicken
Rock1	1.02	1.00000000	chicken
Ddx3y	-0.70	1.00000000	chicken
Rftn1	1.39	1.00000000	chicken
Tomm20	-1.11	1.00000000	chicken
Pkib	1.97	1.00000000	chicken

Cox6a1	-1.08	1.00000000	chicken
Atp11c	0.74	1.00000000	chicken
Fbxo11	1.39	1.00000000	chicken
Evi2a	0.82	1.00000000	chicken
Sash1	1.61	1.00000000	chicken
Itm2a	-1.03	1.00000000	chicken
Bhlhe41	-1.45	1.00000000	chicken
Sgk1	1.73	1.00000000	chicken
Jpt1	0.67	1.00000000	chicken
Arhgef6	-1.11	1.00000000	chicken
Aspg	1.50	1.00000000	chicken
Tpd52l2	0.92	1.00000000	chicken
Dnase1l3	1.57	1.00000000	chicken
Plppr5	0.98	1.00000000	chicken
Sh3bp2	0.37	1.00000000	chicken
Dnajb9	0.55	1.00000000	chicken
Pim3	0.65	1.00000000	chicken
St8sia4	1.18	1.00000000	chicken
Foxn3	0.82	1.00000000	chicken
Thsd4	1.58	1.00000000	chicken
Snx10	0.90	1.00000000	chicken
Syngr3	0.85	1.00000000	chicken
Ets2	1.20	1.00000000	chicken
Akap9	1.03	1.00000000	chicken
Cd83	1.31	1.00000000	chicken
Ppp1r10	0.78	1.00000000	chicken
Sdc4	1.30	1.00000000	chicken
Fmnl2	0.50	1.00000000	chicken
Lsp1	-1.44	1.00000000	chicken
Ccser1	-1.25	1.00000000	chicken
Eif3m	-1.09	1.00000000	chicken
Pde4b	0.85	1.00000000	chicken
Pakap	0.72	1.00000000	chicken
Fbxw7	-1.73	1.00000000	chicken
Prpf39	0.68	1.00000000	chicken
Cd200r1	0.48	1.00000000	chicken
Dpys	0.56	1.00000000	chicken
Dll1	0.63	1.00000000	chicken
Rgs13	0.47	1.00000000	chicken
Kazn	0.44	1.00000000	chicken
Nampt	0.91	1.00000000	chicken
Il2ra	0.94	1.00000000	chicken
Ube2d2a	0.63	1.00000000	chicken
Numb	-1.01	1.00000000	chicken
Btk	-0.96	1.00000000	chicken
Zmiz1	-1.77	1.00000000	chicken

Sipa1l2	1.68	1.00000000	chicken
Gpcpd1	0.68	1.00000000	chicken
P2ry6	-0.72	1.00000000	chicken
Synj1	0.38	1.00000000	chicken
Eepd1	1.31	1.00000000	chicken
Cbl	0.67	1.00000000	chicken
Jak2	1.01	1.00000000	chicken
Lrch1	0.88	1.00000000	chicken
Prdm1	1.57	1.00000000	chicken
Limd1	0.90	1.00000000	chicken
Rpn2	-0.93	1.00000000	chicken
Aif1l	-1.69	1.00000000	chicken
Pde4d	1.28	1.00000000	chicken
Cox7b	-1.03	1.00000000	chicken
Sestd1	1.18	1.00000000	chicken
H3f3b	-0.75	1.00000000	chicken
Vps50	1.62	1.00000000	chicken
Pafah1b1	0.79	1.00000000	chicken
Dpp8	0.57	1.00000000	chicken
Gzma	1.01	1.00000000	chicken
Ergic1	0.85	1.00000000	chicken
5031439G07Rik	1.45	1.00000000	chicken
Rspry1	0.82	1.00000000	chicken
Ifi30	-1.07	1.00000000	chicken
Tomm6	-0.96	1.00000000	chicken
P2rx7	0.54	1.00000000	chicken
Rfc1	0.38	1.00000000	chicken
Il10ra	0.73	1.00000000	chicken
Prpsap1	0.62	1.00000000	chicken
Far1	0.86	1.00000000	chicken
Ccnd2	-1.06	1.00000000	chicken
Zfp438	0.63	1.00000000	chicken
Gnb1	0.75	1.00000000	chicken
Amn1	0.71	1.00000000	chicken
Cisd2	0.75	1.00000000	chicken
Ptafr	1.09	1.00000000	chicken
Eif3f	-0.93	1.00000000	chicken
Pxn	0.98	1.00000000	chicken
Bms1	-0.99	1.00000000	chicken
Aldh5a1	0.59	1.00000000	chicken
Id3	0.60	1.00000000	chicken
Tex10	0.63	1.00000000	chicken
Aff4	0.62	1.00000000	chicken
Dusp16	0.69	1.00000000	chicken
Ndufb4	-0.86	1.00000000	chicken
Fndc3a	0.85	1.00000000	chicken

Pgm2	0.38	1.00000000	chicken
Dennd5b	1.27	1.00000000	chicken
Lama3	1.39	1.00000000	chicken
Etv6	1.05	1.00000000	chicken
Arf4	0.76	1.00000000	chicken
Hnrnpa2b1	-0.70	1.00000000	chicken
Slbp	0.60	1.00000000	chicken
Sap18	-0.76	1.00000000	chicken
Svbp	-1.00	1.00000000	chicken
Ndufb5	-0.59	1.00000000	chicken
Tmed10	-0.66	1.00000000	chicken
Sting1	0.37	1.00000000	chicken
Fnbp1	0.88	1.00000000	chicken
Focad	0.37	1.00000000	chicken
Pfkp	1.18	1.00000000	chicken
Eif2ak2	0.43	1.00000000	chicken
Atp7a	0.83	1.00000000	chicken
Rbpj	0.71	1.00000000	chicken
Cstf3	0.78	1.00000000	chicken
Ptprj	1.01	1.00000000	chicken
Urm1	-0.94	1.00000000	chicken
Pebp1	-0.89	1.00000000	chicken
Cotl1	-1.10	1.00000000	chicken
Traf5	0.66	1.00000000	chicken
Batf3	0.72	1.00000000	chicken
Erp29	-0.83	1.00000000	chicken
Phactr3	1.45	1.00000000	chicken
Nr4a3	1.01	1.00000000	chicken
Hic1	-0.95	1.00000000	chicken
Il6	1.50	1.00000000	chicken
Tdrp	0.41	1.00000000	chicken
Bend6	0.64	1.00000000	chicken
Tent5d	0.28	1.00000000	chicken
Samd8	0.28	1.00000000	chicken
Kcnt2	0.79	1.00000000	chicken
Slc24a1	0.44	1.00000000	chicken
Kcnmb1	0.33	1.00000000	chicken
Rrn3	0.27	1.00000000	chicken
Smurf1	0.36	1.00000000	chicken
Uckl1	0.23	1.00000000	chicken
Plekhg5	0.37	1.00000000	chicken
Rbm7	0.20	1.00000000	chicken
Azin2	0.30	1.00000000	chicken
Ndufb10	-0.90	1.00000000	chicken
Ptprs	-1.08	1.00000000	chicken
Susd6	0.74	1.00000000	chicken

Zbtb16	0.98	1.00000000	chicken
Rcan1	0.31	1.00000000	chicken
Ell	0.50	1.00000000	chicken
Adss	0.66	1.00000000	chicken
Nfkbiz	1.40	1.00000000	chicken
Tmtc1	2.83	1.00000000	chicken
Elf1	0.43	1.00000000	chicken
Oat	-0.83	1.00000000	chicken
Kbtbd8	-0.58	1.00000000	chicken
Eif4b	-0.88	1.00000000	chicken
Tbl1xr1	0.73	1.00000000	chicken
Atf7ip	0.63	1.00000000	chicken
Cnot7	-1.10	1.00000000	chicken
Pcnp	0.86	1.00000000	chicken
Uba2	0.78	1.00000000	chicken
Tcf12	1.40	1.00000000	chicken
Rnmt	-1.25	1.00000000	chicken
Irf4	0.86	1.00000000	chicken
Bcor	-0.80	1.00000000	chicken
Ctnna1	0.57	1.00000000	chicken
Gng2	-0.88	1.00000000	chicken
Wasl	0.51	1.00000000	chicken
Sec24b	0.97	1.00000000	chicken
H2az2	-1.04	1.00000000	chicken
Jarid2	0.42	1.00000000	chicken
Vps13a	0.91	1.00000000	chicken
Stim2	-0.98	1.00000000	chicken
Anxa11	-0.77	1.00000000	chicken
Atic	-0.90	1.00000000	chicken
D5Ertd579e	1.05	1.00000000	chicken
Uvrag	1.18	1.00000000	chicken
Pgd	-1.08	1.00000000	chicken
Alcam	1.91	1.00000000	chicken
Parp12	0.67	1.00000000	chicken
Ctsa	-1.16	1.00000000	chicken
Slc10a7	-1.04	1.00000000	chicken
Tenm3	1.57	1.00000000	chicken
Slc25a20	-0.70	1.00000000	chicken
Slc9a9	-0.95	1.00000000	chicken
Osbpl5	1.02	1.00000000	chicken
Grap2	-1.16	1.00000000	chicken
Lrmda	0.83	1.00000000	chicken
Pla2g6	0.48	1.00000000	chicken
Trap1	0.56	1.00000000	chicken
Celf2	0.65	1.00000000	chicken
Dusp10	0.57	1.00000000	chicken

Dock4	1.08	1.00000000	chicken
Ints14	0.74	1.00000000	chicken
Septin10	0.59	1.00000000	chicken
Sh2b3	0.54	1.00000000	chicken
Sla	1.57	1.00000000	chicken
Zswim5	0.86	1.00000000	chicken
Ssr3	-0.57	1.00000000	chicken
Dennd4a	1.09	1.00000000	chicken
Romo1	-0.68	1.00000000	chicken
Per2	0.69	1.00000000	chicken
Kif9	0.74	1.00000000	chicken
C9orf72	0.51	1.00000000	chicken
Cpne7	0.29	1.00000000	chicken
Pim1	0.74	1.00000000	chicken
Pphln1	0.45	1.00000000	chicken
Atp5pb	-0.69	1.00000000	chicken
Skap2	1.12	1.00000000	chicken
Rsu1	0.64	1.00000000	chicken
Il1rap	-1.11	1.00000000	chicken
Itpr2	-0.85	1.00000000	chicken
Ss18l2	-0.87	1.00000000	chicken
Zfp593	-0.86	1.00000000	chicken
Brip1	-0.88	1.00000000	chicken
Xdh	0.56	1.00000000	chicken
Susd3	-0.63	1.00000000	chicken
Sgcd	1.48	1.00000000	chicken
Fap	0.47	1.00000000	chicken
Rbm39	-0.76	1.00000000	chicken
Crip1	-0.79	1.00000000	chicken
Rab22a	0.73	1.00000000	chicken
Eipr1	-1.26	1.00000000	chicken
Pgls	-0.87	1.00000000	chicken
Tcf7	-2.13	1.00000000	chicken
Pknox1	0.38	1.00000000	chicken
Il21r	0.44	1.00000000	chicken
Rimklb	-0.73	1.00000000	chicken
Kctd17	0.35	1.00000000	chicken
Zc3h12c	0.84	1.00000000	chicken
Jam2	0.54	1.00000000	chicken
Sbf1	0.64	1.00000000	chicken
Ide	0.84	1.00000000	chicken
Dip2b	0.96	1.00000000	chicken
Vps54	0.54	1.00000000	chicken
Slc2a8	-0.65	1.00000000	chicken
Fmnl1	-0.95	1.00000000	chicken
Tpr	-0.84	1.00000000	chicken

Zcchc7	-0.69	1.00000000	chicken
Zranb1	0.28	1.00000000	chicken
Vmp1	0.94	1.00000000	chicken
Fgd4	1.09	1.00000000	chicken
Eif2s2	0.44	1.00000000	chicken
Strn	-0.79	1.00000000	chicken
Gpatch3	0.27	1.00000000	chicken
Dhx36	-0.94	1.00000000	chicken
Ttc17	0.73	1.00000000	chicken
Add3	-0.66	1.00000000	chicken
Dock9	0.58	1.00000000	chicken
Spi1	1.33	1.00000000	chicken
Utrn	0.37	1.00000000	chicken
Taldo1	-0.88	1.00000000	chicken
Pitpna	0.83	1.00000000	chicken
Dock7	0.76	1.00000000	chicken
Ubl3	-1.17	1.00000000	chicken
Ddhd1	-0.64	1.00000000	chicken
Cx3cr1	-0.80	1.00000000	chicken
Hmces	-0.63	1.00000000	chicken
Hmgb3	-0.67	1.00000000	chicken
Arhgap12	1.54	1.00000000	chicken
Kctd20	0.62	1.00000000	chicken
Tnpo1	0.76	1.00000000	chicken
Stat5b	0.82	1.00000000	chicken
Skil	0.57	1.00000000	chicken
Bcl2a1d	0.70	1.00000000	chicken
Cep192	0.95	1.00000000	chicken
Plod2	1.46	1.00000000	chicken
Plekho1	-0.81	1.00000000	chicken
Snx2	-0.71	1.00000000	chicken
Pik3ca	0.51	1.00000000	chicken
Abcb8	-0.34	1.00000000	chicken
Psmg4	-0.52	1.00000000	chicken
Bnip3	-0.62	1.00000000	chicken
Kirrel	-0.79	1.00000000	chicken
Poldip3	0.44	1.00000000	chicken
Fam13a	0.96	1.00000000	chicken
Rab8b	0.38	1.00000000	chicken
Sclt1	-1.00	1.00000000	chicken
Idh2	-0.76	1.00000000	chicken
Inpp5k	0.41	1.00000000	chicken
Spty2d1	0.38	1.00000000	chicken
Adam10	0.71	1.00000000	chicken
Upf2	0.44	1.00000000	chicken
Il18r1	0.83	1.00000000	chicken

Nsa2	-0.65	1.00000000	chicken
Hnrnpul1	-0.76	1.00000000	chicken
Rtn4	0.94	1.00000000	chicken
Rasgrp3	1.30	1.00000000	chicken
Chsy1	1.69	1.00000000	chicken
Cmtm7	0.44	1.00000000	chicken
Stx17	0.51	1.00000000	chicken
H2ac20	0.51	1.00000000	chicken
Atf3	1.33	1.00000000	chicken
Wsb1	0.47	1.00000000	chicken
Slco3a1	1.13	1.00000000	chicken
Herc1	0.47	1.00000000	chicken
Tmtc2	1.96	1.00000000	chicken
Spen	-0.72	1.00000000	chicken
Apbb1ip	0.55	1.00000000	chicken
Arxes2	-0.76	1.00000000	chicken
Cd81	-0.80	1.00000000	chicken
Galns	0.54	1.00000000	chicken
Dr1	-0.48	1.00000000	chicken
Abhd14b	-0.56	1.00000000	chicken
Gclc	-1.02	1.00000000	chicken
Slc41a1	0.74	1.00000000	chicken
Fbxo34	0.71	1.00000000	chicken
Sinhcaf	0.64	1.00000000	chicken
Psmg3	-0.55	1.00000000	chicken
Hnrnpa3	-0.39	1.00000000	chicken
Ski	0.83	1.00000000	chicken
Prkce	-0.93	1.00000000	chicken
Sp3	-0.64	1.00000000	chicken
Otud7b	-0.59	1.00000000	chicken
Oaz2	-0.58	1.00000000	chicken
Hspa5	1.02	1.00000000	chicken
Sema7a	0.66	1.00000000	chicken
Tgfbi	-1.21	1.00000000	chicken
Use1	0.39	1.00000000	chicken
Nap1l4	-0.72	1.00000000	chicken
Ptgs2	0.38	1.00000000	chicken
Eif4g2	-0.52	1.00000000	chicken
Tnik	-1.28	1.00000000	chicken
Cyba	-0.82	1.00000000	chicken
Borcs5	0.44	1.00000000	chicken
Cdc42bpb	0.60	1.00000000	chicken
Tfdp2	-1.18	1.00000000	chicken
Akirin2	0.53	1.00000000	chicken
Scin	-0.96	1.00000000	chicken
Ncald	0.93	1.00000000	chicken

Eif3d	-0.76	1.00000000	chicken
Ikzf1	0.77	1.00000000	chicken
Lman2	-0.58	1.00000000	chicken
Cass4	0.46	1.00000000	chicken
Gfod1	0.81	1.00000000	chicken
Abi1	0.75	1.00000000	chicken
Ilrun	0.50	1.00000000	chicken
Cnot6l	0.57	1.00000000	chicken
Apeh	0.32	1.00000000	chicken
Rer1	-0.43	1.00000000	chicken
Sap30	-0.69	1.00000000	chicken
Ivns1abp	-0.84	1.00000000	chicken
Tasl	1.20	1.00000000	chicken
Arl2bp	0.30	1.00000000	chicken
Arhgdib	-0.85	1.00000000	chicken
Sdhaf2	-0.44	1.00000000	chicken
Ldlrad4	0.48	1.00000000	chicken
Parp4	-0.63	1.00000000	chicken
Pdcd4	-0.79	1.00000000	chicken
Rab29	0.54	1.00000000	chicken
Abraxas1	-0.50	1.00000000	chicken
Has2	0.73	1.00000000	chicken
St6galnac3	-1.51	1.00000000	chicken
Gsn	-0.82	1.00000000	chicken
Yeats2	-0.87	1.00000000	chicken
Nbeal2	0.42	1.00000000	chicken
Tmem123	0.49	1.00000000	chicken
Insr	0.91	1.00000000	chicken
Sgms1	0.37	1.00000000	chicken
Supt4a	-0.72	1.00000000	chicken
BC016579	0.31	1.00000000	chicken
Vldlr	0.39	1.00000000	chicken
G2e3	0.26	1.00000000	chicken
Trpv2	0.36	1.00000000	chicken
Nemp1	0.40	1.00000000	chicken
Ckap2l	0.31	1.00000000	chicken
Qk	0.95	1.00000000	chicken
Oit3	0.45	1.00000000	chicken
Ttc28	0.57	1.00000000	chicken
Tada1	0.46	1.00000000	chicken
Bok	0.47	1.00000000	chicken
Clec16a	0.64	1.00000000	chicken
Sntb1	0.72	1.00000000	chicken
Pdgfb	0.64	1.00000000	chicken
Vps53	0.37	1.00000000	chicken
Susd1	0.23	1.00000000	chicken

Got1	0.24	1.00000000	chicken
Glipr2	0.56	1.00000000	chicken
Kcnq5	-0.58	1.00000000	chicken
Enpp4	-0.42	1.00000000	chicken
Slc40a1	-0.50	1.00000000	chicken
Smarcb1	-0.39	1.00000000	chicken
Smarca4	-0.64	1.00000000	chicken
Ttc27	0.74	1.00000000	chicken
Zfand6	0.92	1.00000000	chicken
Tmsb4x	-0.54	1.00000000	chicken
Kcnma1	1.08	1.00000000	chicken
Stap1	0.46	1.00000000	chicken
Reps1	0.32	1.00000000	chicken
Irf8	0.94	1.00000000	chicken
Tmcc1	0.41	1.00000000	chicken
Wrap73	0.54	1.00000000	chicken
Nin	0.44	1.00000000	chicken
Carnmt1	0.78	1.00000000	chicken
Wdr92	0.30	1.00000000	chicken
Nfyc	-0.42	1.00000000	chicken
Lamp3	1.02	1.00000000	chicken
Pinx1	0.50	1.00000000	chicken
Wdfy3	2.03	1.00000000	chicken
Plpp1	0.51	1.00000000	chicken
Ttf1	-0.71	1.00000000	chicken
Pggt1b	0.57	1.00000000	chicken
Stag2	-0.74	1.00000000	chicken
Lrig2	0.38	1.00000000	chicken
Lamtor2	-0.48	1.00000000	chicken
Faf1	0.82	1.00000000	chicken
Smoc2	0.57	1.00000000	chicken
Fli1	0.78	1.00000000	chicken
Dnajb12	0.40	1.00000000	chicken
Ldha	-0.58	1.00000000	chicken
Rin2	-0.46	1.00000000	chicken
Tuba1a	0.63	1.00000000	chicken
Sirt1	-0.70	1.00000000	chicken
Dlst	0.28	1.00000000	chicken
Crispld2	0.43	1.00000000	chicken
Vps35l	-0.76	1.00000000	chicken
Gid8	0.40	1.00000000	chicken
Cd164	0.58	1.00000000	chicken
Kcnip1	0.23	1.00000000	chicken
Emb	-0.72	1.00000000	chicken
Cul1	0.19	1.00000000	chicken
Garem1	0.69	1.00000000	chicken

Vgll4	0.77	1.00000000	chicken
Cdca4	0.45	1.00000000	chicken
Coq8a	-0.57	1.00000000	chicken
Rap1gds1	0.62	1.00000000	chicken
Zfp423	0.39	1.00000000	chicken
Ndufc2	-0.54	1.00000000	chicken
Tmem165	-0.56	1.00000000	chicken
Cnot9	-0.53	1.00000000	chicken
Zfand2a	0.26	1.00000000	chicken
Ube2e3	-0.51	1.00000000	chicken
Shtn1	0.92	1.00000000	chicken
Afg1l	0.31	1.00000000	chicken
Rapgef2	0.70	1.00000000	chicken
Cebpz	-0.99	1.00000000	chicken
Glt1d1	0.14	1.00000000	chicken
Chst10	0.15	1.00000000	chicken
Gnb5	0.20	1.00000000	chicken
Ptbp1	-0.49	1.00000000	chicken
Bbs7	-0.43	1.00000000	chicken
Rasa2	-0.82	1.00000000	chicken
Usp15	0.71	1.00000000	chicken
Fbxw11	0.52	1.00000000	chicken
Itgav	0.84	1.00000000	chicken
Prelid3b	0.39	1.00000000	chicken
Ssbp1	-0.60	1.00000000	chicken
Abhd5	0.64	1.00000000	chicken
Kif13b	0.65	1.00000000	chicken
Il13ra1	0.68	1.00000000	chicken
Itfg1	0.64	1.00000000	chicken
Dgkh	1.13	1.00000000	chicken
Cct2	-0.57	1.00000000	chicken
Hsp90aa1	0.32	1.00000000	chicken
Abce1	-0.68	1.00000000	chicken
Il18	0.62	1.00000000	chicken
Sf3b5	0.43	1.00000000	chicken
Clock	0.46	1.00000000	chicken
Ncoa7	1.33	1.00000000	chicken
Cpeb4	0.36	1.00000000	chicken
Inpp5a	-0.83	1.00000000	chicken
Srsf4	-0.57	1.00000000	chicken
Carf	-0.44	1.00000000	chicken
Icos	-0.93	1.00000000	chicken
Nmi	0.63	1.00000000	chicken
Snip1	0.48	1.00000000	chicken
Rnf19a	0.68	1.00000000	chicken
Invs	0.40	1.00000000	chicken

Etv3	0.42	1.00000000	chicken
Atrx	0.61	1.00000000	chicken
Fas	0.08	1.00000000	chicken
Trim59	0.56	1.00000000	chicken
Rbm47	1.13	1.00000000	chicken
Dnajc19	-0.50	1.00000000	chicken
Hmbs	-0.56	1.00000000	chicken
Sdcbp	-0.62	1.00000000	chicken
Bmi1	-0.70	1.00000000	chicken
Bloc1s2	-0.41	1.00000000	chicken
Sdhc	-0.67	1.00000000	chicken
Bmpr2	-0.70	1.00000000	chicken
Hmgb1	-0.62	1.00000000	chicken
Cpsf4l	-1.11	1.00000000	chicken
Eif3l	-0.61	1.00000000	chicken
Arglu1	-0.59	1.00000000	chicken
Slc25a3	-0.53	1.00000000	chicken
Virma	-0.49	1.00000000	chicken
Kdm7a	0.76	1.00000000	chicken
Bach1	0.98	1.00000000	chicken
Nav3	0.93	1.00000000	chicken
Dld	0.17	1.00000000	chicken
Rnf166	-0.59	1.00000000	chicken
Napa	0.41	1.00000000	chicken
Ythdc1	-0.66	1.00000000	chicken
Sec11c	0.38	1.00000000	chicken
Tbc1d8	2.03	1.00000000	chicken
Ptpn6	0.67	1.00000000	chicken
Zcrb1	-0.52	1.00000000	chicken
Qpctl	-0.46	1.00000000	chicken
Syap1	-0.52	1.00000000	chicken
Dennd1a	0.53	1.00000000	chicken
Pisd	0.39	1.00000000	chicken
Zfp706	-0.81	1.00000000	chicken
Ncbp3	0.52	1.00000000	chicken
Cirbp	-0.71	1.00000000	chicken
Arsg	0.95	1.00000000	chicken
Raf1	0.49	1.00000000	chicken
Psmd1	0.54	1.00000000	chicken
Cpne8	1.07	1.00000000	chicken
Slc49a4	0.46	1.00000000	chicken
Polg2	-0.54	1.00000000	chicken
Arhgap10	1.50	1.00000000	chicken
Slc30a7	-0.74	1.00000000	chicken
Actr2	-0.75	1.00000000	chicken
Ptger4	0.53	1.00000000	chicken

Bcl6	-0.77	1.00000000	chicken
Hacd3	0.40	1.00000000	chicken
Nbas	0.37	1.00000000	chicken
Syne3	0.64	1.00000000	chicken
Psen2	-0.52	1.00000000	chicken
B4galt2	-0.41	1.00000000	chicken
Txnrd1	-0.64	1.00000000	chicken
Gpr157	0.43	1.00000000	chicken
Strap	-0.63	1.00000000	chicken
Flt3	-0.48	1.00000000	chicken
Acsl3	-0.79	1.00000000	chicken
Ptk2	1.22	1.00000000	chicken
St13	-0.51	1.00000000	chicken
Ogfrl1	-0.33	1.00000000	chicken
Ankrd39	-0.58	1.00000000	chicken
Tent5a	-0.57	1.00000000	chicken
Hdac4	1.28	1.00000000	chicken
Cdk17	0.85	1.00000000	chicken
Rab7	-0.62	1.00000000	chicken
Ripk1	0.60	1.00000000	chicken
Ssr1	-0.59	1.00000000	chicken
Exoc4	0.72	1.00000000	chicken
9930021J03Rik	-0.75	1.00000000	chicken
Arl4c	0.67	1.00000000	chicken
Dram1	0.37	1.00000000	chicken
Dcun1d3	0.28	1.00000000	chicken
Itgb1bp1	-0.35	1.00000000	chicken
Tbc1d19	-0.59	1.00000000	chicken
Fermt2	-0.35	1.00000000	chicken
Ing5	-0.40	1.00000000	chicken
Polr2d	-0.33	1.00000000	chicken
Eif4e3	-0.57	1.00000000	chicken
Tvp23a	-0.33	1.00000000	chicken
Hira	-0.35	1.00000000	chicken
Zap70	-0.60	1.00000000	chicken
Plbd1	1.51	1.00000000	chicken
Ccnl1	-0.47	1.00000000	chicken
Btf3	-0.47	1.00000000	chicken
Cul2	0.60	1.00000000	chicken
Esyt2	0.50	1.00000000	chicken
Septin2	-0.49	1.00000000	chicken
Zfp326	-0.73	1.00000000	chicken
Ahr	0.83	1.00000000	chicken
Cox7a2l	-0.38	1.00000000	chicken
Prdx1	-0.46	1.00000000	chicken
Gatad2b	0.22	1.00000000	chicken

Pds5b	-1.01	1.00000000	chicken
Cdc42se1	0.24	1.00000000	chicken
Zdhhc20	0.51	1.00000000	chicken
Spsb1	0.42	1.00000000	chicken
Efl1	0.93	1.00000000	chicken
Prkcd	0.43	1.00000000	chicken
Pdlim5	1.62	1.00000000	chicken
Arhgef7	-0.45	1.00000000	chicken
Cnot1	-0.63	1.00000000	chicken
St3gal6	0.50	1.00000000	chicken
Slc66a1	0.38	1.00000000	chicken
Adam19	0.56	1.00000000	chicken
Dpf3	0.62	1.00000000	chicken
Adcy7	-0.76	1.00000000	chicken
Wac	0.44	1.00000000	chicken
Cnnm2	0.66	1.00000000	chicken
Cd47	0.48	1.00000000	chicken
Tapbp	0.56	1.00000000	chicken
Spry2	0.26	1.00000000	chicken
Gabarapl1	-0.62	1.00000000	chicken
Atp8a1	0.35	1.00000000	chicken
Cep164	-0.79	1.00000000	chicken
Hdgf	-0.63	1.00000000	chicken
Tnfrsf18	0.37	1.00000000	chicken
Swap70	-0.63	1.00000000	chicken
Plk3	0.74	1.00000000	chicken
Tmed2	-0.43	1.00000000	chicken
Exosc10	-0.45	1.00000000	chicken
Prkar1a	0.61	1.00000000	chicken
Desi2	-0.79	1.00000000	chicken
Wdr48	-0.39	1.00000000	chicken
Riok3	0.13	1.00000000	chicken
Il20ra	-0.96	1.00000000	chicken
Naaa	-0.43	1.00000000	chicken
Cep85l	0.68	1.00000000	chicken
Tmem120b	0.26	1.00000000	chicken
Eef2k	0.58	1.00000000	chicken
Rap2c	0.58	1.00000000	chicken
Lamtor3	0.49	1.00000000	chicken
Cd86	0.77	1.00000000	chicken
Ubfd1	0.26	1.00000000	chicken
Pacs2	-0.59	1.00000000	chicken
Yeats4	-0.50	1.00000000	chicken
Zcchc17	-0.67	1.00000000	chicken
Lin54	0.50	1.00000000	chicken
Rev3l	0.61	1.00000000	chicken

Snrpd1	-0.60	1.00000000	chicken
Gdi2	-0.73	1.00000000	chicken
Prcp	-0.42	1.00000000	chicken
Chchd10	-0.50	1.00000000	chicken
Prkcb	0.58	1.00000000	chicken
Traf2	0.39	1.00000000	chicken
Rcor1	0.84	1.00000000	chicken
Tm9sf5	-0.21	1.00000000	chicken
Hmgcr	-0.07	1.00000000	chicken
Arpc4	-0.79	1.00000000	chicken
Taok1	0.68	1.00000000	chicken
Tub	0.55	1.00000000	chicken
Grap	-0.53	1.00000000	chicken
Ube2j2	-0.52	1.00000000	chicken
Tomm70a	0.45	1.00000000	chicken
Eif1	-0.41	1.00000000	chicken
Hyal6	0.90	1.00000000	chicken
Polr1d	-0.30	1.00000000	chicken
Rilp	-0.44	1.00000000	chicken
Hdac7	-0.58	1.00000000	chicken
Czib	0.25	1.00000000	chicken
Ralbp1	-0.63	1.00000000	chicken
Rab14	-0.44	1.00000000	chicken
Bcl11b	-1.75	1.00000000	chicken
Rab5a	-0.71	1.00000000	chicken
Hnrnph2	-0.46	1.00000000	chicken
Cnot4	0.46	1.00000000	chicken
Fryl	0.46	1.00000000	chicken
Setdb2	-0.52	1.00000000	chicken
Eif4e	0.21	1.00000000	chicken
Esco1	0.38	1.00000000	chicken
Smg1	0.46	1.00000000	chicken
Rc3h1	0.45	1.00000000	chicken
Parvb	-0.20	1.00000000	chicken
Elovl1	0.27	1.00000000	chicken
Zdhhc14	1.05	1.00000000	chicken
Ifnar1	0.67	1.00000000	chicken
Aopep	0.34	1.00000000	chicken
Slco4a1	0.33	1.00000000	chicken
Cars2	-0.86	1.00000000	chicken
Rngtt	0.59	1.00000000	chicken
Bcap29	0.57	1.00000000	chicken
Akt3	0.76	1.00000000	chicken
Ncapg	0.59	1.00000000	chicken
Calr	0.44	1.00000000	chicken
Itsn2	0.50	1.00000000	chicken

Kcnq1	-3.35	1.00000000	chicken
Ripk2	0.35	1.00000000	chicken
Lmnb1	0.46	1.00000000	chicken
Tpm3-rs7	-0.25	1.00000000	chicken
Fes	0.34	1.00000000	chicken
Tomm22	-0.59	1.00000000	chicken
Dlg1	0.63	1.00000000	chicken
Fam162a	-0.28	1.00000000	chicken
Acaa1b	-0.32	1.00000000	chicken
Cdk14	-0.71	1.00000000	chicken
Ptpn14	-0.68	1.00000000	chicken
Pigyl	-0.35	1.00000000	chicken
Stn1	-0.52	1.00000000	chicken
Entpd1	-0.48	1.00000000	chicken
Efcab14	-0.35	1.00000000	chicken
Lrp3	-0.28	1.00000000	chicken
Slc29a4	-0.50	1.00000000	chicken
Hagh	-0.32	1.00000000	chicken
Tnfsf8	-0.54	1.00000000	chicken
Tanc2	-0.37	1.00000000	chicken
Setdb1	-0.47	1.00000000	chicken
Rhoa	-0.46	1.00000000	chicken
Wwox	-0.78	1.00000000	chicken
Sppl2a	0.09	1.00000000	chicken
Plscr1	0.85	1.00000000	chicken
Dnajc5	0.62	1.00000000	chicken
Ptprc	0.36	1.00000000	chicken
Ufm1	0.24	1.00000000	chicken
Fgd3	-0.47	1.00000000	chicken
P2rx4	0.64	1.00000000	chicken
Nbn	0.71	1.00000000	chicken
Ostm1	0.34	1.00000000	chicken
Igdcc4	0.68	1.00000000	chicken
Slc25a25	-0.52	1.00000000	chicken
Cd3g	-0.07	1.00000000	chicken
Tgfb1	-0.51	1.00000000	chicken
Slu7	0.10	1.00000000	chicken
Nsmce2	0.41	1.00000000	chicken
Itch	0.64	1.00000000	chicken
Usp38	-0.64	1.00000000	chicken
Slc8a1	0.69	1.00000000	chicken
Thrb	0.67	1.00000000	chicken
Tmem268	0.31	1.00000000	chicken
Atp5k	-0.52	1.00000000	chicken
Hcfc2	0.32	1.00000000	chicken
Lsmem1	0.74	1.00000000	chicken

Usp4	-0.42	1.00000000	chicken
Phf2	0.90	1.00000000	chicken
Pak3	0.83	1.00000000	chicken
Ric8b	0.29	1.00000000	chicken
Csrp2	-0.23	1.00000000	chicken
Ndufs8	-0.45	1.00000000	chicken
Pgk1	-0.66	1.00000000	chicken
Psmb4	-0.48	1.00000000	chicken
C2cd2	0.17	1.00000000	chicken
Zfp512	0.21	1.00000000	chicken
Rhobtb2	0.14	1.00000000	chicken
Sil1	-0.38	1.00000000	chicken
Ddrgk1	-0.65	1.00000000	chicken
Golgb1	-0.70	1.00000000	chicken
Pla2g4a	0.61	1.00000000	chicken
Utp15	0.13	1.00000000	chicken
Dstn	-0.55	1.00000000	chicken
Gnai3	-0.62	1.00000000	chicken
Atp6v0d1	0.33	1.00000000	chicken
Narf	0.57	1.00000000	chicken
Phf10	-0.53	1.00000000	chicken
Commd3	-0.33	1.00000000	chicken
Setd7	-0.35	1.00000000	chicken
Naif1	-0.37	1.00000000	chicken
Flot2	-0.46	1.00000000	chicken
Rap2a	0.28	1.00000000	chicken
Apaf1	0.22	1.00000000	chicken
Rasgef1b	0.31	1.00000000	chicken
Sirt6	0.24	1.00000000	chicken
Casp1	0.88	1.00000000	chicken
Eif3i	-0.48	1.00000000	chicken
Polr2h	-0.45	1.00000000	chicken
Prep	-0.77	1.00000000	chicken
Tmed5	-0.63	1.00000000	chicken
Klhl5	0.30	1.00000000	chicken
Id2	0.62	1.00000000	chicken
Zdhhc3	-0.39	1.00000000	chicken
Fbxw8	-0.54	1.00000000	chicken
Coro7	-0.69	1.00000000	chicken
Hmgn3	-0.61	1.00000000	chicken
Bicral	0.28	1.00000000	chicken
Asl	0.52	1.00000000	chicken
Tmem140	0.23	1.00000000	chicken
Cog3	0.20	1.00000000	chicken
Polr2m	0.39	1.00000000	chicken
Erbin	0.64	1.00000000	chicken

Acadl	-0.65	1.00000000	chicken
Ifnar2	0.43	1.00000000	chicken
Socs1	0.08	1.00000000	chicken
Usp14	0.47	1.00000000	chicken
Supt3	0.62	1.00000000	chicken
Ptp4a2	-0.64	1.00000000	chicken
Ndufa5	-0.44	1.00000000	chicken
Sorcs2	-1.28	1.00000000	chicken
Ndufs1	-0.27	1.00000000	chicken
Dgat2	0.18	1.00000000	chicken
Cyp51	-0.32	1.00000000	chicken
Bend7	0.50	1.00000000	chicken
Brwd1	-0.46	1.00000000	chicken
Dst	0.86	1.00000000	chicken
Ppm1e	0.44	1.00000000	chicken
Exoc5	-0.54	1.00000000	chicken
Ndufaf4	0.26	1.00000000	chicken
8030462N17Rik	0.26	1.00000000	chicken
Rbms1	0.69	1.00000000	chicken
Sat1	0.86	1.00000000	chicken
Dbi	-0.94	1.00000000	chicken
Usp10	0.42	1.00000000	chicken
Hras	0.31	1.00000000	chicken
Fam168b	-0.82	1.00000000	chicken
Lars	0.48	1.00000000	chicken
Tmem245	-0.67	1.00000000	chicken
Canx	0.42	1.00000000	chicken
Ubtd2	0.29	1.00000000	chicken
Ggps1	-0.67	1.00000000	chicken
Necap1	-0.48	1.00000000	chicken
Tia1	0.54	1.00000000	chicken
Rbis	-0.47	1.00000000	chicken
Creg1	-0.69	1.00000000	chicken
Cox4i1	-0.67	1.00000000	chicken
Fam204a	-0.46	1.00000000	chicken
Atp11a	0.51	1.00000000	chicken
Katna1	0.27	1.00000000	chicken
Nudt13	0.28	1.00000000	chicken
Clec2h	0.37	1.00000000	chicken
Gtf2f2	-0.71	1.00000000	chicken
Tex264	-0.39	1.00000000	chicken
Surf1	-0.30	1.00000000	chicken
Slc35e2	0.50	1.00000000	chicken
Ctdspl	0.82	1.00000000	chicken
Nanp	0.55	1.00000000	chicken
Borcs6	0.29	1.00000000	chicken

Inpp5d	0.27	1.00000000	chicken
Gfpt2	-0.21	1.00000000	chicken
Scaf11	-0.53	1.00000000	chicken
Rwdd4a	-0.23	1.00000000	chicken
Sptlc2	-0.76	1.00000000	chicken
Papola	-0.64	1.00000000	chicken
Helb	0.24	1.00000000	chicken
Churc1	-0.57	1.00000000	chicken
Ssh2	-0.34	1.00000000	chicken
Cdkal1	0.54	1.00000000	chicken
Kmt2c	0.72	1.00000000	chicken
4930453N24Rik	0.30	1.00000000	chicken
Nt5e	0.55	1.00000000	chicken
Tmem43	0.28	1.00000000	chicken
Adcy9	0.33	1.00000000	chicken
Septin12	0.24	1.00000000	chicken
Arrdc1	0.24	1.00000000	chicken
Lsm14a	-0.61	1.00000000	chicken
Nlrc5	0.20	1.00000000	chicken
Prkn	-1.03	1.00000000	chicken
Cnot8	-0.60	1.00000000	chicken
Rgs3	0.81	1.00000000	chicken
Grn	-1.08	1.00000000	chicken
Tbc1d5	0.48	1.00000000	chicken
Itga4	-0.29	1.00000000	chicken
Grm8	-0.58	1.00000000	chicken
Hal	-0.24	1.00000000	chicken
Trak1	0.36	1.00000000	chicken
Steap3	-0.24	1.00000000	chicken
Rwdd3	-0.33	1.00000000	chicken
Car5b	-0.29	1.00000000	chicken
Thg1l	-0.20	1.00000000	chicken
Sco1	-0.30	1.00000000	chicken
Ypel2	-0.37	1.00000000	chicken
Ndufv2	-0.37	1.00000000	chicken
4933427D14Rik	-0.47	1.00000000	chicken
Kmt5b	-0.69	1.00000000	chicken
Ing4	-0.43	1.00000000	chicken
Tmem9b	-0.23	1.00000000	chicken
Cmtm3	-0.52	1.00000000	chicken
Rabggtb	0.38	1.00000000	chicken
Grb2	0.66	1.00000000	chicken
Sos2	0.20	1.00000000	chicken
Atp6v1d	-0.40	1.00000000	chicken
Prkaa1	0.17	1.00000000	chicken
Slc15a4	0.19	1.00000000	chicken

Stx5a	0.14	1.00000000	chicken
Pak2	-0.58	1.00000000	chicken
Dmxl1	-0.50	1.00000000	chicken
Rnf220	-0.64	1.00000000	chicken
Uhrf1	0.48	1.00000000	chicken
Trip12	0.38	1.00000000	chicken
Scp2	-0.27	1.00000000	chicken
Abhd6	0.48	1.00000000	chicken
Cttnbp2nl	0.47	1.00000000	chicken
Litaf	0.36	1.00000000	chicken
Ip6k2	0.37	1.00000000	chicken
Baz1b	0.61	1.00000000	chicken
Snrpe	-0.58	1.00000000	chicken
Acsbg2	0.47	1.00000000	chicken
Sdhb	-0.51	1.00000000	chicken
Gpc5	0.64	1.00000000	chicken
Enpp2	0.52	1.00000000	chicken
Avl9	0.22	1.00000000	chicken
Fasl	0.27	1.00000000	chicken
Egr1	0.81	1.00000000	chicken
Plcg2	0.38	1.00000000	chicken
Atp5d	-0.60	1.00000000	chicken
Zfyve1	-0.58	1.00000000	chicken
Eif3a	-0.57	1.00000000	chicken
Eif3g	-0.57	1.00000000	chicken
Serbp1	-0.42	1.00000000	chicken
Orc1	0.18	1.00000000	chicken
Cox7a2	-0.70	1.00000000	chicken
Kpna4	0.31	1.00000000	chicken
Scaper	-0.69	1.00000000	chicken
Rev1	-0.47	1.00000000	chicken
Lasp1	-0.46	1.00000000	chicken
Fam102a	-0.96	1.00000000	chicken
Ppm1d	0.62	1.00000000	chicken
Gda	0.22	1.00000000	chicken
Lhpp	0.28	1.00000000	chicken
Samd13	0.10	1.00000000	chicken
Neurl1b	0.30	1.00000000	chicken
Ddx46	-1.08	1.00000000	chicken
Sbf2	0.40	1.00000000	chicken
Agl	-0.53	1.00000000	chicken
Pus10	0.54	1.00000000	chicken
Ccni	-0.29	1.00000000	chicken
Lyn	0.59	1.00000000	chicken
Smap2	-0.70	1.00000000	chicken
1810058I24Rik	-0.38	1.00000000	chicken

Txndc9	-0.30	1.00000000	chicken
Cep250	-0.53	1.00000000	chicken
Lgals8	0.57	1.00000000	chicken
Wdsub1	0.33	1.00000000	chicken
Nap1l1	-0.71	1.00000000	chicken
Cnih1	-0.31	1.00000000	chicken
Dnajb4	0.62	1.00000000	chicken
Phf13	0.09	1.00000000	chicken
Epb41	-0.51	1.00000000	chicken
Tgfbr2	0.35	1.00000000	chicken
Abcg1	-0.73	1.00000000	chicken
Eny2	-0.34	1.00000000	chicken
Parp1	-0.40	1.00000000	chicken
Plagl1	-0.53	1.00000000	chicken
Uqcrb	-0.79	1.00000000	chicken
Arid1b	-0.60	1.00000000	chicken
Psmc3	-0.39	1.00000000	chicken
Ube2a	-0.43	1.00000000	chicken
Utp25	0.31	1.00000000	chicken
Dnajc13	0.55	1.00000000	chicken
Nufip1	-0.42	1.00000000	chicken
Snrpg	-0.57	1.00000000	chicken
Il1b	1.46	1.00000000	chicken
Hpse	0.15	1.00000000	chicken
Exosc8	0.30	1.00000000	chicken
Nubp1	0.26	1.00000000	chicken
Unc93b1	-0.41	1.00000000	chicken
Ptdss1	0.42	1.00000000	chicken
Ndfip2	0.82	1.00000000	chicken
Lrrc8d	0.46	1.00000000	chicken
Kdm2a	-0.32	1.00000000	chicken
Fbxo33	-0.52	1.00000000	chicken
Foxn2	0.56	1.00000000	chicken
E2f5	0.33	1.00000000	chicken
Tax1bp3	0.40	1.00000000	chicken
Rangap1	0.42	1.00000000	chicken
Gbp8	0.58	1.00000000	chicken
Uqcrh	-0.53	1.00000000	chicken
Arfgap1	0.23	1.00000000	chicken
Ilf2	-0.51	1.00000000	chicken
2510002D24Rik	-0.22	1.00000000	chicken
Cmklr1	-0.28	1.00000000	chicken
Sirt5	0.38	1.00000000	chicken
Cpne4	-1.05	1.00000000	chicken
Asb5	-0.63	1.00000000	chicken
Ostc	-0.59	1.00000000	chicken

Capn2	0.12	1.00000000	chicken
Uchl5	-0.38	1.00000000	chicken
Hsbp1	-0.53	1.00000000	chicken
Pak1ip1	-0.32	1.00000000	chicken
Snx17	-0.35	1.00000000	chicken
Psmd2	-0.56	1.00000000	chicken
Csnk1a1	0.50	1.00000000	chicken
Cib1	-0.37	1.00000000	chicken
Pard3	-0.17	1.00000000	chicken
Ess2	0.22	1.00000000	chicken
Prdx4	-0.79	1.00000000	chicken
Napepld	-0.06	1.00000000	chicken
Nckipsd	-0.35	1.00000000	chicken
Lao1	0.82	1.00000000	chicken
Golga4	0.68	1.00000000	chicken
Ube2e2	1.08	1.00000000	chicken
AI597479	0.41	1.00000000	chicken
Tceanc2	0.26	1.00000000	chicken
Tkfc	-0.23	1.00000000	chicken
Prkab1	-0.42	1.00000000	chicken
Jade3	0.26	1.00000000	chicken
Tubb6	0.42	1.00000000	chicken
Pi4ka	-0.72	1.00000000	chicken
Jun	1.01	1.00000000	chicken
Zc3h11a	0.23	1.00000000	chicken
Asxl2	0.58	1.00000000	chicken
Ccdc126	-0.48	1.00000000	chicken
Klhl7	-0.46	1.00000000	chicken
U2af2	-0.41	1.00000000	chicken
Rara	-0.42	1.00000000	chicken
Pde6d	0.31	1.00000000	chicken
Paip1	0.33	1.00000000	chicken
Smad1	0.31	1.00000000	chicken
Rell1	-0.68	1.00000000	chicken
Ly96	0.37	1.00000000	chicken
Rapgef1	0.53	1.00000000	chicken
Rpap2	0.09	1.00000000	chicken
Sh2d1a	-1.14	1.00000000	chicken
Snx21	-0.34	1.00000000	chicken
Rit1	-0.60	1.00000000	chicken
Ogfr	0.25	1.00000000	chicken
Klhl13	-0.43	1.00000000	chicken
Cd63	-0.52	1.00000000	chicken
Slc16a7	1.11	1.00000000	chicken
Slc39a6	-0.43	1.00000000	chicken
Foxk1	0.22	1.00000000	chicken

Glrx5	-0.34	1.00000000	chicken
Ptpn11	0.31	1.00000000	chicken
Dad1	-0.48	1.00000000	chicken
Dcaf13	-0.45	1.00000000	chicken
Gpbp1l1	0.46	1.00000000	chicken
Ubn1	-0.37	1.00000000	chicken
Dnm1l	0.33	1.00000000	chicken
Ddx24	0.38	1.00000000	chicken
Il6st	0.16	1.00000000	chicken
Snap29	-0.47	1.00000000	chicken
Als2cl	0.64	1.00000000	chicken
Stard3nl	0.38	1.00000000	chicken
Selenoi	0.20	1.00000000	chicken
Ints10	0.17	1.00000000	chicken
Cox19	-0.33	1.00000000	chicken
Il6ra	-0.43	1.00000000	chicken
Paics	-0.43	1.00000000	chicken
Wdr91	-0.36	1.00000000	chicken
Acap1	-0.61	1.00000000	chicken
Atp5b	-0.48	1.00000000	chicken
Erh	-0.50	1.00000000	chicken
Ctss	-0.54	1.00000000	chicken
Gpx4	-0.45	1.00000000	chicken
Ywhab	-0.43	1.00000000	chicken
Efr3a	0.72	1.00000000	chicken
Adk	-1.12	1.00000000	chicken
Slc12a4	0.72	1.00000000	chicken
Scfd1	-0.50	1.00000000	chicken
Ubqln4	-0.38	1.00000000	chicken
Rgs9	-0.58	1.00000000	chicken
Efhd1	-0.67	1.00000000	chicken
Gigyf2	0.85	1.00000000	chicken
Cul4b	0.34	1.00000000	chicken
Tmem154	-0.41	1.00000000	chicken
Crem	0.67	1.00000000	chicken
Prnp	0.39	1.00000000	chicken
Kdm3a	0.10	1.00000000	chicken
Cpeb2	0.93	1.00000000	chicken
Slc30a4	-0.08	1.00000000	chicken
Akap8l	-0.41	1.00000000	chicken
Ear1	0.69	1.00000000	chicken
Psmc2	-0.44	1.00000000	chicken
Arih1	0.32	1.00000000	chicken
Fhod1	0.40	1.00000000	chicken
Dcaf1	-0.39	1.00000000	chicken
Abhd12	-0.34	1.00000000	chicken

Zdhhc6	-0.49	1.00000000	chicken
Pdhb	-0.34	1.00000000	chicken
Nfkbie	0.19	1.00000000	chicken
Fam124b	0.08	1.00000000	chicken
Ippk	0.19	1.00000000	chicken
Chchd3	0.70	1.00000000	chicken
Npc2	-0.53	1.00000000	chicken
Hnrnpu	-0.51	1.00000000	chicken
Wsb2	-0.32	1.00000000	chicken
Phf11a	0.40	1.00000000	chicken
Uspl1	0.12	1.00000000	chicken
Parp9	0.33	1.00000000	chicken
Tex12	0.34	1.00000000	chicken
Smap1	0.51	1.00000000	chicken
Prelid1	-0.48	1.00000000	chicken
Psmd6	-0.32	1.00000000	chicken
Cope	-0.51	1.00000000	chicken
Xpo7	-0.69	1.00000000	chicken
Emc6	0.33	1.00000000	chicken
Rtf1	-0.29	1.00000000	chicken
S100a6	-1.00	1.00000000	chicken
Tpd52l1	-0.55	1.00000000	chicken
Ckb	-0.59	1.00000000	chicken
Lta4h	-0.73	1.00000000	chicken
Tacc3	0.23	1.00000000	chicken
Aldh18a1	-0.52	1.00000000	chicken
Ndufb2	-0.37	1.00000000	chicken
Rtcb	-0.34	1.00000000	chicken
Khdrbs3	-0.51	1.00000000	chicken
Aqr	-0.35	1.00000000	chicken
Slc7a6os	-0.27	1.00000000	chicken
Ranbp17	-0.32	1.00000000	chicken
Ss18l1	-0.32	1.00000000	chicken
Ap1b1	-0.25	1.00000000	chicken
Lpar5	-0.47	1.00000000	chicken
Lsamp	-1.03	1.00000000	chicken
Ift27	0.24	1.00000000	chicken
Slc7a3	0.18	1.00000000	chicken
Spata7	0.38	1.00000000	chicken
Ap2m1	-0.50	1.00000000	chicken
Sh3bgrl	0.39	1.00000000	chicken
Gnal	0.33	1.00000000	chicken
Sf3b3	-0.61	1.00000000	chicken
Tmem214	0.24	1.00000000	chicken
Gnb1l	-0.55	1.00000000	chicken
Tdrd3	0.26	1.00000000	chicken

Ubr5	0.04	1.00000000	chicken
Ankrd13d	-0.37	1.00000000	chicken
Ash1l	0.37	1.00000000	chicken
Bmf	-0.54	1.00000000	chicken
Sgf29	-0.32	1.00000000	chicken
Eif5	0.37	1.00000000	chicken
Chic2	-0.53	1.00000000	chicken
Paip2	-0.62	1.00000000	chicken
Ccnk	-0.69	1.00000000	chicken
Ei24	0.22	1.00000000	chicken
Fez2	-0.59	1.00000000	chicken
Lgals2	-0.53	1.00000000	chicken
Ikzf3	-0.84	1.00000000	chicken
Pid1	1.15	1.00000000	chicken
Ndufa2	0.38	1.00000000	chicken
Pdss2	-0.68	1.00000000	chicken
Arl8b	-0.43	1.00000000	chicken
Rpia	-0.22	1.00000000	chicken
Gtf3c1	0.74	1.00000000	chicken
Chp1	-0.29	1.00000000	chicken
Spag9	0.60	1.00000000	chicken
Impact	0.26	1.00000000	chicken
Acap3	0.49	1.00000000	chicken
Lnpep	-0.83	1.00000000	chicken
Ctsh	0.54	1.00000000	chicken
Bcas2	0.17	1.00000000	chicken
Sh3glb1	0.01	1.00000000	chicken
Fam120a	0.46	1.00000000	chicken
Fus	0.17	1.00000000	chicken
Phkg1	-0.13	1.00000000	chicken
Ppia	-0.48	1.00000000	chicken
Timp3	0.45	1.00000000	chicken
Sec24d	0.44	1.00000000	chicken
Primpol	0.22	1.00000000	chicken
Sfmbt1	0.21	1.00000000	chicken
Rxra	-0.55	1.00000000	chicken
Rnpep	-0.29	1.00000000	chicken
Cacybp	0.55	1.00000000	chicken
Kidins220	0.56	1.00000000	chicken
Relch	0.17	1.00000000	chicken
Rnf214	-0.86	1.00000000	chicken
Ppp1ca	-0.53	1.00000000	chicken
Arf1	-0.50	1.00000000	chicken
Tmem229b	0.29	1.00000000	chicken
Cbfb	0.57	1.00000000	chicken
Sh3bgrl3	-0.47	1.00000000	chicken

Pfdn6	-0.38	1.00000000	chicken
Cstf1	-0.41	1.00000000	chicken
Larp4	0.13	1.00000000	chicken
Cst3	-0.85	1.00000000	chicken
Tagap1	0.24	1.00000000	chicken
Kcna3	0.31	1.00000000	chicken
Bri3	-0.56	1.00000000	chicken
Tle5	-0.54	1.00000000	chicken
Pde3b	0.54	1.00000000	chicken
Bpnt2	-0.30	1.00000000	chicken
Unk	-0.77	1.00000000	chicken
Arhgap24	1.09	1.00000000	chicken
Nelfe	-0.37	1.00000000	chicken
Tra2b	-0.18	1.00000000	chicken
Ndufa4	-0.47	1.00000000	chicken
Rftn2	0.56	1.00000000	chicken
Hhex	-0.36	1.00000000	chicken
Heca	0.30	1.00000000	chicken
Cog7	0.25	1.00000000	chicken
Irf2	-0.90	1.00000000	chicken
Fgl2	0.85	1.00000000	chicken
Asb7	0.25	1.00000000	chicken
Slc38a10	0.14	1.00000000	chicken
Alox5	0.76	1.00000000	chicken
Kpnb1	0.07	1.00000000	chicken
Zfyve26	-0.49	1.00000000	chicken
Pfdn4	-0.41	1.00000000	chicken
Fbp1	-0.48	1.00000000	chicken
Nop53	0.19	1.00000000	chicken
F10	-0.54	1.00000000	chicken
Abca2	-0.50	1.00000000	chicken
Cpeb3	0.12	1.00000000	chicken
Arhgef18	0.46	1.00000000	chicken
Vdac2	-0.53	1.00000000	chicken
Osbpl11	-0.22	1.00000000	chicken
Cmtr1	-0.04	1.00000000	chicken
Srp14	-0.22	1.00000000	chicken
Tfec	1.19	1.00000000	chicken
Rad51b	0.52	1.00000000	chicken
Ppfibp1	0.27	1.00000000	chicken
Rad23b	-0.45	1.00000000	chicken
Blcap	0.38	1.00000000	chicken
Hspa9	-0.53	1.00000000	chicken
Lpin2	-0.37	1.00000000	chicken
Dhx29	0.42	1.00000000	chicken
Slc12a7	-0.41	1.00000000	chicken

Exosc7	-0.31	1.00000000	chicken
E2f3	-0.32	1.00000000	chicken
Rnf150	-0.65	1.00000000	chicken
Rif1	-0.41	1.00000000	chicken
Svil	-0.95	1.00000000	chicken
Plcxd1	0.52	1.00000000	chicken
Acvr2a	-0.49	1.00000000	chicken
Psap	-0.69	1.00000000	chicken
Bicd2	0.20	1.00000000	chicken
Sytl1	-0.63	1.00000000	chicken
Ogdh	0.54	1.00000000	chicken
Hddc2	-0.28	1.00000000	chicken
Atg16l1	0.19	1.00000000	chicken
Anapc7	0.13	1.00000000	chicken
Pdgfa	-0.63	1.00000000	chicken
Rlim	0.35	1.00000000	chicken
Nubpl	-0.70	1.00000000	chicken
Sfxn4	-0.33	1.00000000	chicken
Lpcat3	-0.62	1.00000000	chicken
Timm10	-0.40	1.00000000	chicken
Rad18	-0.86	1.00000000	chicken
H13	-0.54	1.00000000	chicken
Hsf2	-0.40	1.00000000	chicken
Pbrm1	-0.53	1.00000000	chicken
Tnfrsf21	-0.58	1.00000000	chicken
Tlk2	0.46	1.00000000	chicken
Grk3	-0.24	1.00000000	chicken
Rtraf	-0.30	1.00000000	chicken
Tor1a	-0.09	1.00000000	chicken
Gpr89	0.36	1.00000000	chicken
Igsf3	0.31	1.00000000	chicken
Cyth1	0.18	1.00000000	chicken
Smarcc1	-0.31	1.00000000	chicken
Gpd2	0.18	1.00000000	chicken
Cpm	0.37	1.00000000	chicken
Clcc1	0.23	1.00000000	chicken
Stoml1	0.32	1.00000000	chicken
Zbtb8b	0.29	1.00000000	chicken
Nmrk2	0.26	1.00000000	chicken
Snhg4	0.18	1.00000000	chicken
Nat8f7	-0.22	1.00000000	chicken
Tasor2	0.43	1.00000000	chicken
Ckap4	-0.43	1.00000000	chicken
Naxe	-0.28	1.00000000	chicken
Sec61a1	-0.50	1.00000000	chicken
Gnptab	-0.66	1.00000000	chicken

Copg1	-0.37	1.00000000	chicken
Hemk1	-0.50	1.00000000	chicken
Higd1a	-0.51	1.00000000	chicken
Bcr	0.20	1.00000000	chicken
Stat3	-0.55	1.00000000	chicken
Rsrc2	0.14	1.00000000	chicken
Yy2	0.47	1.00000000	chicken
Vta1	0.47	1.00000000	chicken
Ccl9	1.33	1.00000000	chicken
Cherp	-0.25	1.00000000	chicken
Sertad2	0.49	1.00000000	chicken
Polr2e	-0.41	1.00000000	chicken
Arl15	0.28	1.00000000	chicken
Chd1	0.15	1.00000000	chicken
Ostf1	-0.61	1.00000000	chicken
Rab5c	-0.47	1.00000000	chicken
Arl6ip4	0.42	1.00000000	chicken
Acot7	0.32	1.00000000	chicken
Trib1	0.25	1.00000000	chicken
Cfd	0.20	1.00000000	chicken
Gstm4	-0.36	1.00000000	chicken
Slc6a6	0.63	1.00000000	chicken
Rfx7	-0.28	1.00000000	chicken
Edf1	-0.54	1.00000000	chicken
C1qa	0.26	1.00000000	chicken
Prkd3	-0.61	1.00000000	chicken
Slc16a6	0.29	1.00000000	chicken
Arpc1b	-0.60	1.00000000	chicken
Eno1b	-0.38	1.00000000	chicken
Ust	-1.06	1.00000000	chicken
Nfkbib	-0.36	1.00000000	chicken
Cox5a	-0.54	1.00000000	chicken
Prdm11	-0.28	1.00000000	chicken
Utp18	-0.35	1.00000000	chicken
Tnfrsf9	-0.41	1.00000000	chicken
Antxr2	0.85	1.00000000	chicken
Appl2	0.36	1.00000000	chicken
Lrguk	0.32	1.00000000	chicken
Zswim7	0.18	1.00000000	chicken
Etnk1	0.26	1.00000000	chicken
Dedd	-0.43	1.00000000	chicken
Celf1	0.84	1.00000000	chicken
Psme3	-0.31	1.00000000	chicken
Arhgdia	-0.43	1.00000000	chicken
Slc39a10	0.54	1.00000000	chicken
Sdc2	0.69	1.00000000	chicken

Ncoa3	-0.03	1.00000000	chicken
Wdr3	-0.61	1.00000000	chicken
Carhsp1	-0.59	1.00000000	chicken
Tmem101	0.05	1.00000000	chicken
Tm9sf3	-0.45	1.00000000	chicken
Tbc1d7	-0.28	1.00000000	chicken
Ugdh	-0.28	1.00000000	chicken
Errfi1	-0.46	1.00000000	chicken
Ube2k	0.32	1.00000000	chicken
Arid4a	-0.21	1.00000000	chicken
Copg2	0.28	1.00000000	chicken
Smad2	-0.14	1.00000000	chicken
Nr4a2	0.27	1.00000000	chicken
Oxnad1	0.13	1.00000000	chicken
Ppp2r2c	-0.35	1.00000000	chicken
Fam177a	-0.35	1.00000000	chicken
Rae1	-0.17	1.00000000	chicken
Abcd2	-0.56	1.00000000	chicken
Casp8	0.61	1.00000000	chicken
Smarcd2	-0.30	1.00000000	chicken
Arrdc2	0.08	1.00000000	chicken
Phka2	-0.54	1.00000000	chicken
Itpr1	-0.21	1.00000000	chicken
Optn	0.46	1.00000000	chicken
Apba1	0.42	1.00000000	chicken
Zfp511	0.25	1.00000000	chicken
Lysmd2	0.14	1.00000000	chicken
Rfx2	0.97	1.00000000	chicken
Vma21	-0.53	1.00000000	chicken
Sbno2	0.22	1.00000000	chicken
Suco	0.20	1.00000000	chicken
Gabrr2	0.31	1.00000000	chicken
Zfat	0.48	1.00000000	chicken
Dennd1b	0.38	1.00000000	chicken
Hoxa7	-0.17	1.00000000	chicken
Iah1	-0.15	1.00000000	chicken
Plekhd1	-0.25	1.00000000	chicken
Smpd3	-0.16	1.00000000	chicken
Rabepk	-0.16	1.00000000	chicken
Eml3	0.23	1.00000000	chicken
Acss2	0.38	1.00000000	chicken
L3mbtl3	0.07	1.00000000	chicken
Atxn7l1	0.98	1.00000000	chicken
Ap5m1	-0.28	1.00000000	chicken
Cdk2ap2	-0.28	1.00000000	chicken
Zfp608	-0.47	1.00000000	chicken

Arl6	0.28	1.00000000	chicken
Osbpl10	0.42	1.00000000	chicken
Gpr68	0.30	1.00000000	chicken
Btbd6	0.19	1.00000000	chicken
Pdcl	0.19	1.00000000	chicken
Ahrr	-0.55	1.00000000	chicken
Arhgap18	-0.22	1.00000000	chicken
Nfatc3	-0.53	1.00000000	chicken
Ccnd3	0.50	1.00000000	chicken
Scpep1	-0.45	1.00000000	chicken
Ddx19a	-0.50	1.00000000	chicken
Anxa6	0.28	1.00000000	chicken
Fam13b	0.43	1.00000000	chicken
Alkbh1	0.25	1.00000000	chicken
Golim4	-0.35	1.00000000	chicken
Cndp2	0.04	1.00000000	chicken
Slc38a1	0.40	1.00000000	chicken
Yipf4	-0.27	1.00000000	chicken
Sqstm1	0.71	1.00000000	chicken
Nasp	-0.65	1.00000000	chicken
Galnt2	0.24	1.00000000	chicken
Commd7	-0.34	1.00000000	chicken
Adh5	-0.25	1.00000000	chicken
Asb8	-0.32	1.00000000	chicken
Slc35b3	0.49	1.00000000	chicken
Psmf1	-0.25	1.00000000	chicken
Cemip2	0.37	1.00000000	chicken
Larp1	0.33	1.00000000	chicken
Zfp318	0.33	1.00000000	chicken
Tsg101	0.46	1.00000000	chicken
Nfx1	0.32	1.00000000	chicken
Sel1l	0.46	1.00000000	chicken
Elp4	0.41	1.00000000	chicken
Plagl2	0.18	1.00000000	chicken
Lyrm9	0.19	1.00000000	chicken
C1qc	0.36	1.00000000	chicken
Crlf3	-0.68	1.00000000	chicken
Jagn1	-0.39	1.00000000	chicken
Cd2ap	-0.48	1.00000000	chicken
Rab9	0.26	1.00000000	chicken
Hnrnpd	-0.55	1.00000000	chicken
Il16	0.09	1.00000000	chicken
Dcun1d5	0.33	1.00000000	chicken
Utp11	-0.33	1.00000000	chicken
Setd5	0.15	1.00000000	chicken
Reep2	-0.60	1.00000000	chicken

Psma1	0.13	1.00000000	chicken
Pfkfb3	0.46	1.00000000	chicken
Ptpre	0.61	1.00000000	chicken
Ndufb1	-0.49	1.00000000	chicken
Smarca5	-0.47	1.00000000	chicken
Chd7	0.50	1.00000000	chicken
Ppp1cb	-0.49	1.00000000	chicken
Pex16	-0.45	1.00000000	chicken
B3gnt3	-0.38	1.00000000	chicken
2310022B05Rik	0.34	1.00000000	chicken
Dnajc24	0.09	1.00000000	chicken
Sag	0.34	1.00000000	chicken
Rarres1	0.19	1.00000000	chicken
Fbxl18	0.15	1.00000000	chicken
Dnajc15	-0.31	1.00000000	chicken
Zfp821	-0.32	1.00000000	chicken
Itpkb	0.20	1.00000000	chicken
Snx1	-0.30	1.00000000	chicken
Lactb	-0.39	1.00000000	chicken
Cpt1a	0.63	1.00000000	chicken
Srpr	0.34	1.00000000	chicken
Nadk	0.37	1.00000000	chicken
Cox10	0.52	1.00000000	chicken
Fadd	-0.49	1.00000000	chicken
Il2rb	1.57	1.00000000	chicken
Xrcc4	0.17	1.00000000	chicken
Ptp4a1	-0.56	1.00000000	chicken
Batf	0.50	1.00000000	chicken
Hps3	-0.69	1.00000000	chicken
Rnf10	0.26	1.00000000	chicken
Hsf1	-0.50	1.00000000	chicken
Rsrp1	0.26	1.00000000	chicken
Tmem64	-0.22	1.00000000	chicken
Ufd1	-0.25	1.00000000	chicken
Dsn1	-0.28	1.00000000	chicken
Eif2ak1	0.36	1.00000000	chicken
Crebbp	-0.44	1.00000000	chicken
Hunk	-0.48	1.00000000	chicken
Hpf1	-0.22	1.00000000	chicken
Fam174a	-0.21	1.00000000	chicken
Eml1	-0.56	1.00000000	chicken
Cyld	0.30	1.00000000	chicken
Nlk	-0.60	1.00000000	chicken
Dok6	0.35	1.00000000	chicken
Pex1	0.24	1.00000000	chicken
Ttc21b	0.17	1.00000000	chicken

Tal1	0.37	1.00000000	chicken
B3gnt5	0.17	1.00000000	chicken
Wdr6	0.14	1.00000000	chicken
Psmd12	0.23	1.00000000	chicken
Helz2	0.33	1.00000000	chicken
Sf3a1	-0.40	1.00000000	chicken
Lonp2	0.47	1.00000000	chicken
Ptges3	0.18	1.00000000	chicken
AU040320	0.41	1.00000000	chicken
Lcp1	-0.57	1.00000000	chicken
Erich1	-0.63	1.00000000	chicken
Arpc2	-0.42	1.00000000	chicken
Chordc1	-0.45	1.00000000	chicken
4932411N23Rik	-0.38	1.00000000	chicken
Heatr6	0.49	1.00000000	chicken
Tlk1	-0.58	1.00000000	chicken
Prelid3a	0.39	1.00000000	chicken
Tollip	-0.37	1.00000000	chicken
Rbm12	-0.32	1.00000000	chicken
Larp4b	0.26	1.00000000	chicken
Bzw2	0.73	1.00000000	chicken
Gadd45g	0.10	1.00000000	chicken
Twnk	0.12	1.00000000	chicken
Fbxl21	-0.08	1.00000000	chicken
Ldlrap1	0.15	1.00000000	chicken
Arhgap45	-0.33	1.00000000	chicken
Washc1	-0.20	1.00000000	chicken
Txn2	-0.30	1.00000000	chicken
Eloa	0.20	1.00000000	chicken
Uhrf1bp1l	0.53	1.00000000	chicken
Cdk19	0.22	1.00000000	chicken
Tbc1d9	0.40	1.00000000	chicken
Wdr5b	-0.24	1.00000000	chicken
Fhl3	-0.46	1.00000000	chicken
Nudt16l1	-0.42	1.00000000	chicken
Cadm1	0.72	1.00000000	chicken
Tnrc6a	0.27	1.00000000	chicken
Ybx3	-0.50	1.00000000	chicken
Itga1	0.92	1.00000000	chicken
Ube3a	0.28	1.00000000	chicken
R3hdm4	0.16	1.00000000	chicken
Fxn	0.11	1.00000000	chicken
Ube4a	-0.45	1.00000000	chicken
Pbxip1	-0.35	1.00000000	chicken
Stxbp6	0.36	1.00000000	chicken
Srfbp1	-0.38	1.00000000	chicken

Ezr	0.73	1.00000000	chicken
Tubgcp5	0.21	1.00000000	chicken
Wdr47	-0.27	1.00000000	chicken
Ptges	-0.39	1.00000000	chicken
Spidr	1.40	1.00000000	chicken
Ifngr2	0.36	1.00000000	chicken
Tor1b	0.30	1.00000000	chicken
Inpp5b	-0.30	1.00000000	chicken
Opa3	-0.36	1.00000000	chicken
Vipr1	-0.76	1.00000000	chicken
Stoml2	-0.23	1.00000000	chicken
Dph6	0.35	1.00000000	chicken
Erp44	0.55	1.00000000	chicken
Slc30a6	-0.35	1.00000000	chicken
Ptcd3	-0.32	1.00000000	chicken
E130309D02Rik	-0.28	1.00000000	chicken
Limd2	-0.41	1.00000000	chicken
Pum1	-0.36	1.00000000	chicken
Lap3	-0.25	1.00000000	chicken
Erlin2	-0.38	1.00000000	chicken
4932438A13Rik	-0.18	1.00000000	chicken
Tab3	-0.28	1.00000000	chicken
Zc3h14	0.22	1.00000000	chicken
Tnks	0.18	1.00000000	chicken
Sf3b1	-0.58	1.00000000	chicken
Rab11fip4	-0.30	1.00000000	chicken
Unc13d	-0.74	1.00000000	chicken
Tnfsf11	0.07	1.00000000	chicken
Taf11	-0.40	1.00000000	chicken
Ndufs3	-0.23	1.00000000	chicken
Tm2d2	-0.24	1.00000000	chicken
Nisch	-0.33	1.00000000	chicken
Sdk1	-0.08	1.00000000	chicken
Hspa13	0.10	1.00000000	chicken
Cish	0.00	1.00000000	chicken
Crtam	0.09	1.00000000	chicken
Psmd9	-0.37	1.00000000	chicken
Kpna1	0.14	1.00000000	chicken
Idh1	0.30	1.00000000	chicken
Txnip	0.10	1.00000000	chicken
Ndufa6	-0.14	1.00000000	chicken
Cenpc1	0.25	1.00000000	chicken
Habp4	-0.21	1.00000000	chicken
Cdin1	0.49	1.00000000	chicken
Bbip1	-0.20	1.00000000	chicken
Uck1	-0.20	1.00000000	chicken

Dffa	-0.23	1.00000000	chicken
Nmt2	-0.80	1.00000000	chicken
Fbxo38	0.21	1.00000000	chicken
Hexim1	-0.61	1.00000000	chicken
Pdcl3	-0.27	1.00000000	chicken
Dock5	0.93	1.00000000	chicken
Snu13	-0.27	1.00000000	chicken
Pygb	-0.15	1.00000000	chicken
BC048403	-0.30	1.00000000	chicken
Fh1	-0.17	1.00000000	chicken
St8sia1	0.09	1.00000000	chicken
Stk3	-0.23	1.00000000	chicken
Calcb	-0.42	1.00000000	chicken
Borcs8	-0.20	1.00000000	chicken
Ubqln1	0.46	1.00000000	chicken
Dgkd	0.29	1.00000000	chicken
Nek10	1.66	1.00000000	chicken
Ndufs2	-0.40	1.00000000	chicken
Snx24	0.74	1.00000000	chicken
Copz1	0.20	1.00000000	chicken
P4htm	-0.30	1.00000000	chicken
Hscb	-0.35	1.00000000	chicken
Nup214	0.36	1.00000000	chicken
Card11	-0.41	1.00000000	chicken
Slc24a4	0.58	1.00000000	chicken
Hspe1-rs1	-0.21	1.00000000	chicken
Commd2	-0.25	1.00000000	chicken
Sumo3	-0.47	1.00000000	chicken
Srebf1	-0.21	1.00000000	chicken
Sarnp	-0.50	1.00000000	chicken
Tmem255a	0.17	1.00000000	chicken
Enpp6	-0.16	1.00000000	chicken
Hccs	-0.21	1.00000000	chicken
Osbp2	0.49	1.00000000	chicken
Samd11	-0.09	1.00000000	chicken
Zfand3	0.55	1.00000000	chicken
Gas7	-0.67	1.00000000	chicken
Atp6ap2	0.33	1.00000000	chicken
Smim4	-0.28	1.00000000	chicken
Ppat	-0.23	1.00000000	chicken
Ankle2	-0.35	1.00000000	chicken
Anapc15-ps	-0.32	1.00000000	chicken
Eloc	0.22	1.00000000	chicken
Cdc42ep3	0.10	1.00000000	chicken
Abcb7	-0.72	1.00000000	chicken
Pfdn2	-0.42	1.00000000	chicken

Slc25a5	-0.23	1.00000000	chicken
Ndrg1	0.29	1.00000000	chicken
Nrde2	0.24	1.00000000	chicken
Socs3	0.20	1.00000000	chicken
Ube2q1	-0.52	1.00000000	chicken
Hnrnph3	-0.52	1.00000000	chicken
Spopl	0.40	1.00000000	chicken
Ptpn21	0.33	1.00000000	chicken
Snorc	0.48	1.00000000	chicken
Cbx4	-0.37	1.00000000	chicken
Ech1	-0.24	1.00000000	chicken
Gyg	0.25	1.00000000	chicken
Uprt	-0.05	1.00000000	chicken
Pmpcb	-0.29	1.00000000	chicken
Arpc1a	-0.42	1.00000000	chicken
Sdhd	-0.12	1.00000000	chicken
Ttll5	-0.80	1.00000000	chicken
Coq9	-0.15	1.00000000	chicken
Agtrap	-0.22	1.00000000	chicken
Arhgap29	-0.95	1.00000000	chicken
Prox1	0.52	1.00000000	chicken
Nktr	-0.07	1.00000000	chicken
Cybc1	-0.27	1.00000000	chicken
Actb	-0.70	1.00000000	chicken
Ccdc85c	0.83	1.00000000	chicken
Hsp90ab1	-0.44	1.00000000	chicken
Stim1	0.18	1.00000000	chicken
Rab5if	-0.30	1.00000000	chicken
Eftud2	-0.27	1.00000000	chicken
Ppfibp2	0.22	1.00000000	chicken
Zdhhc5	-0.30	1.00000000	chicken
Sltm	-0.44	1.00000000	chicken
Cyfip1	0.35	1.00000000	chicken
Lef1	-0.52	1.00000000	chicken
Stk26	-0.33	1.00000000	chicken
Anapc10	1.23	1.00000000	chicken
Gtpbp4	-0.50	1.00000000	chicken
Nt5c3b	0.45	1.00000000	chicken
Slc2a3	-0.32	1.00000000	chicken
Ets1	-0.76	1.00000000	chicken
Chuk	-0.42	1.00000000	chicken
Aldh7a1	0.13	1.00000000	chicken
Tnfrsf26	-0.38	1.00000000	chicken
Tmem30a	-0.32	1.00000000	chicken
Ranbp3	-0.20	1.00000000	chicken
Runx2	0.20	1.00000000	chicken

Rnf146	-0.46	1.00000000	chicken
Dis3l	-0.38	1.00000000	chicken
Ago4	-0.34	1.00000000	chicken
Wbp4	-0.20	1.00000000	chicken
Dut	-0.23	1.00000000	chicken
Timm13	-0.34	1.00000000	chicken
Hectd1	0.21	1.00000000	chicken
Rabgap1l	-0.27	1.00000000	chicken
Csrnp1	0.18	1.00000000	chicken
Cox7c	-0.44	1.00000000	chicken
Frs2	0.29	1.00000000	chicken
Rrp7a	0.16	1.00000000	chicken
Arhgap15	-0.23	1.00000000	chicken
Tmem204	-0.59	1.00000000	chicken
Rhbg	-0.18	1.00000000	chicken
Sccpdh	-0.33	1.00000000	chicken
Ercc3	-0.05	1.00000000	chicken
Ccdc91	0.15	1.00000000	chicken
Sptbn1	-0.76	1.00000000	chicken
Arhgap17	0.11	1.00000000	chicken
Brcc3	0.34	1.00000000	chicken
Aco2	-0.22	1.00000000	chicken
Scamp2	-0.21	1.00000000	chicken
Zfp207	-0.32	1.00000000	chicken
Fer	0.43	1.00000000	chicken
Tfrc	-0.51	1.00000000	chicken
Ascc3	0.40	1.00000000	chicken
Scd2	-0.34	1.00000000	chicken
Ero1b	-0.37	1.00000000	chicken
Dynll2	-0.28	1.00000000	chicken
Srsf6	-0.25	1.00000000	chicken
Pdss1	0.18	1.00000000	chicken
Taf5	0.37	1.00000000	chicken
Phactr2	-0.12	1.00000000	chicken
Arfgap2	-0.50	1.00000000	chicken
Eapp	0.20	1.00000000	chicken
Trdmt1	0.22	1.00000000	chicken
Gemin6	-0.21	1.00000000	chicken
Rbmx2	-0.20	1.00000000	chicken
D2hgdh	-0.20	1.00000000	chicken
Yju2	-0.28	1.00000000	chicken
Cd40	0.42	1.00000000	chicken
Gpn1	-0.23	1.00000000	chicken
Sec14l5	-0.16	1.00000000	chicken
Syncrip	-0.61	1.00000000	chicken
Lzic	-0.31	1.00000000	chicken

Pcgf6	-0.40	1.00000000	chicken
Pik3r4	0.33	1.00000000	chicken
Fnip1	0.11	1.00000000	chicken
Zzz3	0.40	1.00000000	chicken
Srgap3	-0.36	1.00000000	chicken
Armh3	-0.38	1.00000000	chicken
Rab20	0.58	1.00000000	chicken
Csf1r	-0.49	1.00000000	chicken
Ano5	0.40	1.00000000	chicken
Ap4b1	0.15	1.00000000	chicken
Wdr4	0.38	1.00000000	chicken
Cep76	0.24	1.00000000	chicken
Rdx	0.38	1.00000000	chicken
Spg20	-0.23	1.00000000	chicken
Hibch	-0.17	1.00000000	chicken
Ralgps2	-0.23	1.00000000	chicken
Sar1b	-0.16	1.00000000	chicken
Elp6	-0.54	1.00000000	chicken
Bdh2	-0.31	1.00000000	chicken
C77080	-0.49	1.00000000	chicken
Ftsj3	-0.29	1.00000000	chicken
Iba57	-0.32	1.00000000	chicken
Arl1	-0.61	1.00000000	chicken
Arl6ip5	-0.40	1.00000000	chicken
Ewsr1	-0.30	1.00000000	chicken
Tbc1d1	0.45	1.00000000	chicken
Anks1	0.16	1.00000000	chicken
Cast	-0.31	1.00000000	chicken
Sh3bp5	0.53	1.00000000	chicken
Galm	-0.26	1.00000000	chicken
Osbp	0.14	1.00000000	chicken
Hspa8	0.02	1.00000000	chicken
Ubiad1	0.15	1.00000000	chicken
Ythdf1	-0.18	1.00000000	chicken
Arl6ip1	-0.29	1.00000000	chicken
Rimbp2	0.73	1.00000000	chicken
Cnot6	0.06	1.00000000	chicken
Elavl1	0.25	1.00000000	chicken
Zbtb21	0.28	1.00000000	chicken
Aff2	0.25	1.00000000	chicken
Nqo2	-0.42	1.00000000	chicken
Pms1	-0.25	1.00000000	chicken
Tnn	-0.20	1.00000000	chicken
Ift52	-0.20	1.00000000	chicken
Rcan3	-0.37	1.00000000	chicken
Srsf2	0.21	1.00000000	chicken

Rnf114	-0.46	1.00000000	chicken
Ptrh2	-0.48	1.00000000	chicken
Flvcr2	0.42	1.00000000	chicken
Acaca	-0.43	1.00000000	chicken
Flii	0.19	1.00000000	chicken
Snx6	0.20	1.00000000	chicken
Pofut1	-0.38	1.00000000	chicken
Lrrk1	0.12	1.00000000	chicken
Guk1	-0.30	1.00000000	chicken
Fbh1	-0.17	1.00000000	chicken
Cmc4	0.14	1.00000000	chicken
Fzr1	0.08	1.00000000	chicken
Zmat2	-0.31	1.00000000	chicken
Ubac2	0.47	1.00000000	chicken
Odc1	-0.29	1.00000000	chicken
Fam3c	-0.68	1.00000000	chicken
Chd2	0.29	1.00000000	chicken
Afg3l2	0.44	1.00000000	chicken
Dcn	-0.35	1.00000000	chicken
Hspa14	-0.26	1.00000000	chicken
Tshz1	-0.59	1.00000000	chicken
Skp2	-0.42	1.00000000	chicken
Dtymk	-0.14	1.00000000	chicken
Fgf12	-0.22	1.00000000	chicken
Cdk6	1.05	1.00000000	chicken
Psma2	0.15	1.00000000	chicken
Ube2o	-0.34	1.00000000	chicken
Acad9	-0.34	1.00000000	chicken
Pcdh10	0.46	1.00000000	chicken
Prdx3	-0.40	1.00000000	chicken
Dock8	-0.23	1.00000000	chicken
B2m	0.42	1.00000000	chicken
Ahctf1	0.33	1.00000000	chicken
Disp1	0.09	1.00000000	chicken
Gfi1	0.19	1.00000000	chicken
Rrp15	-0.45	1.00000000	chicken
Vegfc	0.30	1.00000000	chicken
Denr	-0.27	1.00000000	chicken
Hp1bp3	-0.29	1.00000000	chicken
Htra2	-0.43	1.00000000	chicken
Parp8	0.35	1.00000000	chicken
Ndufaf6	-0.19	1.00000000	chicken
Coq6	-0.27	1.00000000	chicken
Hyi	-0.17	1.00000000	chicken
Clcn7	-0.24	1.00000000	chicken
2610507B11Rik	-0.21	1.00000000	chicken

Fbxl20	-0.25	1.00000000	chicken
Rcor3	-0.37	1.00000000	chicken
Ninj1	0.32	1.00000000	chicken
Usp33	-0.41	1.00000000	chicken
Snx9	0.40	1.00000000	chicken
Upf1	-0.47	1.00000000	chicken
Rhob	0.36	1.00000000	chicken
Asf1a	0.22	1.00000000	chicken
Tsnax	-0.33	1.00000000	chicken
Zfp827	0.11	1.00000000	chicken
Appbp2	0.11	1.00000000	chicken
Cops3	0.10	1.00000000	chicken
Amd2	-0.06	1.00000000	chicken
Kansl3	0.20	1.00000000	chicken
Prex1	0.52	1.00000000	chicken
Hmgb2	-0.37	1.00000000	chicken
Ptpn1	-0.40	1.00000000	chicken
Orai1	-0.08	1.00000000	chicken
Rad21	-0.42	1.00000000	chicken
Atp6v0e	-0.51	1.00000000	chicken
Plcb2	-0.59	1.00000000	chicken
Gorasp2	-0.32	1.00000000	chicken
Slc7a5	-0.67	1.00000000	chicken
Nr4a1	0.14	1.00000000	chicken
Shoc2	0.47	1.00000000	chicken
Eya3	0.22	1.00000000	chicken
Gpd1l	-0.30	1.00000000	chicken
Cdc25a	-0.22	1.00000000	chicken
Ints7	-0.32	1.00000000	chicken
Polr1c	-0.55	1.00000000	chicken
Kif27	-0.14	1.00000000	chicken
Gemin2	-0.19	1.00000000	chicken
Gse1	-0.21	1.00000000	chicken
Trp53rka	-0.16	1.00000000	chicken
Stambp	-0.25	1.00000000	chicken
Pmvk	-0.20	1.00000000	chicken
Hsph1	0.20	1.00000000	chicken
Taf4b	-0.53	1.00000000	chicken
Bend3	-0.62	1.00000000	chicken
Nmrk1	0.24	1.00000000	chicken
Pla2r1	0.02	1.00000000	chicken
Stab1	-0.62	1.00000000	chicken
Irf5	0.22	1.00000000	chicken
Ciz1	0.18	1.00000000	chicken
Vamp4	-0.12	1.00000000	chicken
Eif1ad12	-0.36	1.00000000	chicken

Spast	0.36	1.00000000	chicken
Brd1	-0.30	1.00000000	chicken
Ubr7	-0.25	1.00000000	chicken
Stk17b	0.21	1.00000000	chicken
Creb3l1	0.07	1.00000000	chicken
Igf1r	-0.44	1.00000000	chicken
Pdap1	-0.23	1.00000000	chicken
Cbfa2t3	0.40	1.00000000	chicken
Ikzf2	1.08	1.00000000	chicken
Elmo1	-0.42	1.00000000	chicken
Tomm40l	-0.50	1.00000000	chicken
Tubb1	-0.37	1.00000000	chicken
Atp23	-0.24	1.00000000	chicken
Dnpep	-0.24	1.00000000	chicken
Tnks2	-0.32	1.00000000	chicken
Eif5a2	-0.34	1.00000000	chicken
Vps8	-0.19	1.00000000	chicken
Ap1s2	-0.38	1.00000000	chicken
Zdhhc17	0.57	1.00000000	chicken
Gramd1b	0.03	1.00000000	chicken
Eif3k	0.52	1.00000000	chicken
Ube2cbp	0.36	1.00000000	chicken
Rab39b	-0.36	1.00000000	chicken
Glyr1	0.47	1.00000000	chicken
Plekhm2	0.02	1.00000000	chicken
Adsl	-0.46	1.00000000	chicken
Eif5b	0.26	1.00000000	chicken
Cars	0.31	1.00000000	chicken
Rptor	-0.53	1.00000000	chicken
Polr1f	-0.33	1.00000000	chicken
Hdgfl3	-0.45	1.00000000	chicken
Crk	0.17	1.00000000	chicken
Glrx3	-0.45	1.00000000	chicken
Commd9	-0.06	1.00000000	chicken
Nfu1	-0.05	1.00000000	chicken
Stk40	0.18	1.00000000	chicken
Dock2	0.22	1.00000000	chicken
Atxn2	0.44	1.00000000	chicken
Ankrd17	0.18	1.00000000	chicken
Vamp3	0.16	1.00000000	chicken
Nfe2l2	0.38	1.00000000	chicken
Disc1	0.21	1.00000000	chicken
Zpld1	0.92	1.00000000	chicken
Emg1	0.20	1.00000000	chicken
Fem1c	0.26	1.00000000	chicken
Zfhx3	-0.53	1.00000000	chicken

Thumpd3	-0.42	1.00000000	chicken
Hcls1	-0.31	1.00000000	chicken
Nckap1l	-0.30	1.00000000	chicken
Elp2	0.04	1.00000000	chicken
Wwp1	1.16	1.00000000	chicken
Chmp2a	0.12	1.00000000	chicken
Impg2	-0.18	1.00000000	chicken
Tmem19	0.14	1.00000000	chicken
Acyp2	-0.59	1.00000000	chicken
Prdm2	-0.09	1.00000000	chicken
Tnfaip8	0.25	1.00000000	chicken
Sdhaf4	-0.33	1.00000000	chicken
Rbsn	-0.31	1.00000000	chicken
Otud4	0.26	1.00000000	chicken
Slc37a2	-0.30	1.00000000	chicken
Bcl9l	-0.41	1.00000000	chicken
Anp32e	-0.05	1.00000000	chicken
Smpd1	-0.28	1.00000000	chicken
Naa50	0.16	1.00000000	chicken
Glo1	-0.20	1.00000000	chicken
Slc9a3r1	-0.37	1.00000000	chicken
Usp3	-0.04	1.00000000	chicken
Csnk1d	0.36	1.00000000	chicken
Lancl2	0.25	1.00000000	chicken
Rbks	0.25	1.00000000	chicken
Rbm33	0.15	1.00000000	chicken
Ttc37	-0.26	1.00000000	chicken
Slf2	0.20	1.00000000	chicken
Arhgef26	-0.15	1.00000000	chicken
Ypel1	0.11	1.00000000	chicken
Tbc1d15	0.15	1.00000000	chicken
Akr1e1	-0.24	1.00000000	chicken
Dpysl2	0.30	1.00000000	chicken
Gpc4	-0.27	1.00000000	chicken
Cpne3	-0.35	1.00000000	chicken
E2f6	-0.26	1.00000000	chicken
Jpt2	-0.09	1.00000000	chicken
Smu1	0.22	1.00000000	chicken
Ttc3	0.08	1.00000000	chicken
Gadd45b	-0.40	1.00000000	chicken
Rbm24	0.17	1.00000000	chicken
Fosl2	0.42	1.00000000	chicken
Atp5c1	-0.51	1.00000000	chicken
Git2	-0.41	1.00000000	chicken
Exosc2	-0.29	1.00000000	chicken
Lman1	0.17	1.00000000	chicken

Rnh1	0.32	1.00000000	chicken
Spcs1	0.31	1.00000000	chicken
Katnbl1	-0.26	1.00000000	chicken
Zfp592	-0.21	1.00000000	chicken
Scaf8	0.19	1.00000000	chicken
Noct	0.19	1.00000000	chicken
Cdk5rap3	0.16	1.00000000	chicken
Scamp4	0.13	1.00000000	chicken
Hmgxb3	0.28	1.00000000	chicken
Dcp1b	-0.12	1.00000000	chicken
Sec24a	0.33	1.00000000	chicken
Snapin	-0.37	1.00000000	chicken
Atp1a1	0.44	1.00000000	chicken
Srp72	-0.25	1.00000000	chicken
Zbtb1	0.29	1.00000000	chicken
Ints13	0.43	1.00000000	chicken
Atf2	0.42	1.00000000	chicken
2900026A02Rik	0.22	1.00000000	chicken
Asns	-0.34	1.00000000	chicken
Uqcrc1	0.21	1.00000000	chicken
Pus7	0.40	1.00000000	chicken
Ppp4r1	-0.20	1.00000000	chicken
Unc50	-0.15	1.00000000	chicken
Tmem168	0.12	1.00000000	chicken
Ldah	-0.58	1.00000000	chicken
Fam241a	-0.31	1.00000000	chicken
Elmod2	-0.13	1.00000000	chicken
Vrk1	-0.28	1.00000000	chicken
Ggnbp2	0.22	1.00000000	chicken
Ube2w	0.33	1.00000000	chicken
Sbno1	0.13	1.00000000	chicken
Ppp6r3	0.02	1.00000000	chicken
Pecam1	0.13	1.00000000	chicken
Alg12	0.21	1.00000000	chicken
Chrm4	0.19	1.00000000	chicken
Nol8	0.15	1.00000000	chicken
Bcdin3d	0.13	1.00000000	chicken
Tacc1	-0.40	1.00000000	chicken
Nono	0.26	1.00000000	chicken
Wipf1	0.22	1.00000000	chicken
Stk4	0.46	1.00000000	chicken
Irs4	-0.17	1.00000000	chicken
Polr2i	-0.23	1.00000000	chicken
Otulinl	-0.60	1.00000000	chicken
Klhl36	-0.23	1.00000000	chicken
Ube2i	0.45	1.00000000	chicken

Arl5a	-0.43	1.00000000	chicken
Camsap2	0.62	1.00000000	chicken
Nfil3	0.45	1.00000000	chicken
Srsf7	-0.28	1.00000000	chicken
Slamf8	-0.15	1.00000000	chicken
Tbk1	-0.38	1.00000000	chicken
Snx13	0.11	1.00000000	chicken
Ccz1	-0.53	1.00000000	chicken
Commd4	-0.21	1.00000000	chicken
Park7	0.00	1.00000000	chicken
Rbms3	0.19	1.00000000	chicken
Fto	0.27	1.00000000	chicken
Ctns	0.16	1.00000000	chicken
Smyd4	0.21	1.00000000	chicken
Farsa	0.14	1.00000000	chicken
Naxd	-0.34	1.00000000	chicken
Atg12	-0.42	1.00000000	chicken
Crb1	-0.31	1.00000000	chicken
Zbtb7a	0.21	1.00000000	chicken
Csnk1g1	0.06	1.00000000	chicken
Tulp3	-0.38	1.00000000	chicken
Edc4	0.32	1.00000000	chicken
Atp5g3	-0.24	1.00000000	chicken
Rassf1	-0.47	1.00000000	chicken
Hipk2	0.58	1.00000000	chicken
Xpo6	-0.45	1.00000000	chicken
Dhx30	0.19	1.00000000	chicken
Ube2d1	0.28	1.00000000	chicken
Atad5	0.38	1.00000000	chicken
Dtnbp1	0.09	1.00000000	chicken
Anapc2	-0.09	1.00000000	chicken
Snrk	-0.03	1.00000000	chicken
Foxj2	-0.43	1.00000000	chicken
Rasgrp1	-0.03	1.00000000	chicken
Tmem131	-0.43	1.00000000	chicken
Prdx6	-0.23	1.00000000	chicken
Fam114a1	0.20	1.00000000	chicken
Ddb2	0.16	1.00000000	chicken
Dph1	0.16	1.00000000	chicken
Txndc12	-0.33	1.00000000	chicken
Drg1	-0.37	1.00000000	chicken
Abhd17a	-0.28	1.00000000	chicken
Tmcc3	-0.12	1.00000000	chicken
Lrif1	-0.15	1.00000000	chicken
Cramp1	-0.40	1.00000000	chicken
Ppp1r2	-0.41	1.00000000	chicken

Trim25	0.50	1.00000000	chicken
Rassf2	0.30	1.00000000	chicken
Sacm1l	0.33	1.00000000	chicken
Camk1d	0.23	1.00000000	chicken
Smcr8	0.14	1.00000000	chicken
Ywhah	0.20	1.00000000	chicken
Klf10	0.23	1.00000000	chicken
Lrrfip2	0.33	1.00000000	chicken
Bin1	-0.23	1.00000000	chicken
Insig2	0.35	1.00000000	chicken
U2surp	0.26	1.00000000	chicken
Ociad1	-0.31	1.00000000	chicken
Suclg1	-0.15	1.00000000	chicken
Rab21	0.31	1.00000000	chicken
Wmp	0.07	1.00000000	chicken
Crlf2	0.07	1.00000000	chicken
Rnf2	0.22	1.00000000	chicken
Fn3krp	0.17	1.00000000	chicken
Cbx3	-0.37	1.00000000	chicken
Nme7	-0.33	1.00000000	chicken
Atp2a2	0.16	1.00000000	chicken
Sult4a1	0.22	1.00000000	chicken
9130409I23Rik	0.10	1.00000000	chicken
Snw1	0.17	1.00000000	chicken
Cdk11b	0.26	1.00000000	chicken
Atp2b4	0.00	1.00000000	chicken
Itpk1	-0.07	1.00000000	chicken
Crtc1	0.01	1.00000000	chicken
Inhba	0.92	1.00000000	chicken
Ccnyl1	0.16	1.00000000	chicken
Wbp2	0.16	1.00000000	chicken
Plin2	0.39	1.00000000	chicken
Dhx8	-0.34	1.00000000	chicken
Nxt2	0.11	1.00000000	chicken
Bcl11a	0.70	1.00000000	chicken
Slc30a5	-0.38	1.00000000	chicken
Rabl6	0.31	1.00000000	chicken
Arhgap27	-0.20	1.00000000	chicken
Armc1	-0.06	1.00000000	chicken
Nupl1	0.10	1.00000000	chicken
Tob2	-0.56	1.00000000	chicken
Trnt1	-0.32	1.00000000	chicken
Pcdhgb4	-0.41	1.00000000	chicken
Cbfa2t2	-0.42	1.00000000	chicken
Kansl1l	0.33	1.00000000	chicken
Deptor	-0.08	1.00000000	chicken

Fam133b	0.07	1.00000000	chicken
Usp46	-0.07	1.00000000	chicken
Reep5	0.29	1.00000000	chicken
R3hdm1	0.08	1.00000000	chicken
Glipr1l1	0.36	1.00000000	chicken
Ddost	0.23	1.00000000	chicken
Pudp	0.24	1.00000000	chicken
Brms1l	0.21	1.00000000	chicken
Sra1	0.32	1.00000000	chicken
Ccng2	0.72	1.00000000	chicken
Kcnk13	-0.06	1.00000000	chicken
Cat	-0.21	1.00000000	chicken
Tiparp	0.27	1.00000000	chicken
Eif3h	-0.32	1.00000000	chicken
Nae1	0.21	1.00000000	chicken
C1galt1	-0.22	1.00000000	chicken
Sf3a3	-0.27	1.00000000	chicken
Phf6	0.30	1.00000000	chicken
Nars	-0.40	1.00000000	chicken
S100a10	-0.27	1.00000000	chicken
Sos1	-0.27	1.00000000	chicken
Soat1	0.15	1.00000000	chicken
Wbp11	0.17	1.00000000	chicken
Dicer1	0.19	1.00000000	chicken
Cltc	0.19	1.00000000	chicken
Cep83	-0.26	1.00000000	chicken
Lgr4	-0.26	1.00000000	chicken
Ahsa1	-0.29	1.00000000	chicken
Cyp2j12	-0.24	1.00000000	chicken
Kat2b	-0.16	1.00000000	chicken
Prkx	0.46	1.00000000	chicken
Taf13	-0.16	1.00000000	chicken
Ifi35	0.10	1.00000000	chicken
Il27ra	-0.18	1.00000000	chicken
Abcc5	-0.18	1.00000000	chicken
C3	-0.20	1.00000000	chicken
Prkar2a	-0.83	1.00000000	chicken
Emp1	0.24	1.00000000	chicken
Chpt1	-0.33	1.00000000	chicken
Ubash3a	0.20	1.00000000	chicken
Utp20	0.05	1.00000000	chicken
Tmem53	0.07	1.00000000	chicken
BC055324	0.03	1.00000000	chicken
Rnf168	0.06	1.00000000	chicken
Tradd	0.05	1.00000000	chicken
Tubb5	0.07	1.00000000	chicken

Tubb2a	0.47	1.00000000	chicken
Znrf3	0.33	1.00000000	chicken
Fam122b	0.33	1.00000000	chicken
Emc8	0.28	1.00000000	chicken
Arfrp1	0.21	1.00000000	chicken
Dync1i2	0.18	1.00000000	chicken
Ncoa2	-0.50	1.00000000	chicken
Atad2b	0.02	1.00000000	chicken
Pef1	-0.18	1.00000000	chicken
Acp2	0.18	1.00000000	chicken
Srrm1	-0.40	1.00000000	chicken
Vwa8	-0.39	1.00000000	chicken
Elp3	-0.33	1.00000000	chicken
Ndst4	-0.44	1.00000000	chicken
Npepl1	-0.36	1.00000000	chicken
Rasal2	-0.47	1.00000000	chicken
Gapvd1	0.26	1.00000000	chicken
Stmn1	-0.69	1.00000000	chicken
Lpcat1	0.34	1.00000000	chicken
Spata5	0.58	1.00000000	chicken
Atl2	-0.58	1.00000000	chicken
Fgd6	-0.31	1.00000000	chicken
Ywhaz	0.20	1.00000000	chicken
Slc35b2	0.04	1.00000000	chicken
Rbm5	0.18	1.00000000	chicken
Rassf3	0.57	1.00000000	chicken
Tns3	0.26	1.00000000	chicken
Slc17a5	0.00	1.00000000	chicken
Qrich1	0.01	1.00000000	chicken
Itgb2l	-0.45	1.00000000	chicken
Tbcd	0.08	1.00000000	chicken
Uqcr11	-0.35	1.00000000	chicken
Kat6b	-0.62	1.00000000	chicken
Utp6	-0.04	1.00000000	chicken
Lipa	0.26	1.00000000	chicken
Pias1	0.15	1.00000000	chicken
Ttc39b	-0.28	1.00000000	chicken
Osbpl2	-0.21	1.00000000	chicken
Oxsr1	0.50	1.00000000	chicken
BC005537	0.21	1.00000000	chicken
Rsbn1l	0.05	1.00000000	chicken
Sesn1	-0.02	1.00000000	chicken
Ipo9	-0.32	1.00000000	chicken
Blk	-0.67	1.00000000	chicken
Abhd13	0.13	1.00000000	chicken
Sephs1	0.23	1.00000000	chicken

Tcirg1	0.27	1.00000000	chicken
Uso1	0.10	1.00000000	chicken
Zc3hav1l	0.06	1.00000000	chicken
Rac1	-0.03	1.00000000	chicken
Stk11ip	0.16	1.00000000	chicken
Acsl4	-0.30	1.00000000	chicken
Ppp1r7	-0.48	1.00000000	chicken
Fbxl2	-0.57	1.00000000	chicken
Fahd2a	-0.20	1.00000000	chicken
4933400A11Rik	0.16	1.00000000	chicken
Dvl1	-0.29	1.00000000	chicken
Inip	-0.19	1.00000000	chicken
Pdia5	-0.27	1.00000000	chicken
Armh1	-0.20	1.00000000	chicken
Ppil1	-0.20	1.00000000	chicken
Traf3ip3	-0.12	1.00000000	chicken
Hexb	-0.51	1.00000000	chicken
Arid5b	-0.57	1.00000000	chicken
Atad1	-0.03	1.00000000	chicken
Atp6v0c	-0.37	1.00000000	chicken
Btbd9	0.11	1.00000000	chicken
Tmx3	0.22	1.00000000	chicken
Cyria	0.67	1.00000000	chicken
Hspd1	0.02	1.00000000	chicken
Lrba	-0.59	1.00000000	chicken
Tpp1	-0.22	1.00000000	chicken
Pdk3	0.15	1.00000000	chicken
Uqcrfs1	-0.42	1.00000000	chicken
Gdap2	-0.24	1.00000000	chicken
Snrpb2	-0.28	1.00000000	chicken
Rfc5	-0.29	1.00000000	chicken
Nup88	-0.25	1.00000000	chicken
Babam2	-0.03	1.00000000	chicken
Cdc14a	-0.42	1.00000000	chicken
BC048507	0.43	1.00000000	chicken
Ubxn2a	-0.06	1.00000000	chicken
Hmox1	-0.04	1.00000000	chicken
Ilkap	-0.12	1.00000000	chicken
Il1r2	0.64	1.00000000	chicken
Fmc1	0.16	1.00000000	chicken
Lclat1	-0.17	1.00000000	chicken
Usp34	0.24	1.00000000	chicken
Rcn2	-0.32	1.00000000	chicken
Akap1	-0.35	1.00000000	chicken
Rasa3	-0.05	1.00000000	chicken
Gnai2	-0.27	1.00000000	chicken

Uhrf2	-0.41	1.00000000	chicken
Cyth3	-0.25	1.00000000	chicken
Uqcc1	0.38	1.00000000	chicken
Vps45	0.39	1.00000000	chicken
Chchd5	0.12	1.00000000	chicken
Slc35e3	0.30	1.00000000	chicken
Nfat5	0.23	1.00000000	chicken
Xiap	-0.34	1.00000000	chicken
Vps13c	0.28	1.00000000	chicken
Gfpt1	-0.06	1.00000000	chicken
Cpsf3	-0.27	1.00000000	chicken
Kif11	-0.26	1.00000000	chicken
Prkca	-0.32	1.00000000	chicken
Fkbp15	-0.21	1.00000000	chicken
Klhl42	-0.39	1.00000000	chicken
Ppp1r21	0.38	1.00000000	chicken
Rbm17	0.32	1.00000000	chicken
Lgmn	0.19	1.00000000	chicken
Sf3a2	0.27	1.00000000	chicken
Parvg	-0.16	1.00000000	chicken
Anxa2	0.31	1.00000000	chicken
Sbds	-0.22	1.00000000	chicken
Foxk2	0.37	1.00000000	chicken
Phtf2	0.40	1.00000000	chicken
Tmem181a	-0.45	1.00000000	chicken
Nsf	0.09	1.00000000	chicken
Gpr137b	0.15	1.00000000	chicken
Dnah12	-0.13	1.00000000	chicken
Tbc1d23	-0.10	1.00000000	chicken
Gpat4	0.20	1.00000000	chicken
Ccr7	0.31	1.00000000	chicken
Gtf2h1	-0.45	1.00000000	chicken
Cd82	-0.63	1.00000000	chicken
Ap1ar	-0.48	1.00000000	chicken
Terf2	-0.51	1.00000000	chicken
Ift20	-0.53	1.00000000	chicken
Cbx1	-0.23	1.00000000	chicken
Tbc1d9b	0.15	1.00000000	chicken
Stom	0.11	1.00000000	chicken
Banp	0.41	1.00000000	chicken
Nolc1	-0.19	1.00000000	chicken
Fkbp1a	0.18	1.00000000	chicken
Selenot	-0.46	1.00000000	chicken
Sec14l1	0.26	1.00000000	chicken
Ckap5	0.37	1.00000000	chicken
Slc39a9	-0.41	1.00000000	chicken

Ndfip1	-0.14	1.00000000	chicken
Prpf4	0.02	1.00000000	chicken
Tdg	-0.47	1.00000000	chicken
Colgalt1	-0.25	1.00000000	chicken
Pou2f1	0.56	1.00000000	chicken
Acbd6	-0.16	1.00000000	chicken
Nup210	0.33	1.00000000	chicken
Rpf2	0.06	1.00000000	chicken
Pla2g4f	0.27	1.00000000	chicken
Clk4	-0.53	1.00000000	chicken
Lgals3	-0.15	1.00000000	chicken
Nrbf2	-0.13	1.00000000	chicken
Tmem184b	0.17	1.00000000	chicken
Smyd3	-0.67	1.00000000	chicken
Nt5dc2	-0.20	1.00000000	chicken
Diaph2	-0.19	1.00000000	chicken
Rbm6	0.08	1.00000000	chicken
Dph3	0.29	1.00000000	chicken
Snrpc	-0.41	1.00000000	chicken
Uqcrc2	-0.20	1.00000000	chicken
Xpc	-0.12	1.00000000	chicken
Rrs1	-0.26	1.00000000	chicken
Ubl5b	-0.29	1.00000000	chicken
Thoc7	-0.19	1.00000000	chicken
Gtf2a1	0.26	1.00000000	chicken
Ntan1	-0.56	1.00000000	chicken
Nelfcd	-0.26	1.00000000	chicken
Cops5	-0.29	1.00000000	chicken
Rundc1	-0.28	1.00000000	chicken
Slain2	-0.48	1.00000000	chicken
Snx3	-0.30	1.00000000	chicken
Plekha1	-0.17	1.00000000	chicken
Slc4a7	0.35	1.00000000	chicken
Actn1	-0.26	1.00000000	chicken
Arpp19	-0.42	1.00000000	chicken
Gabpb2	0.15	1.00000000	chicken
Dhdds	-0.28	1.00000000	chicken
Cir1	-0.01	1.00000000	chicken
Abat	-0.32	1.00000000	chicken
Rilpl1	-0.03	1.00000000	chicken
Rnf34	0.37	1.00000000	chicken
Hdac2	-0.19	1.00000000	chicken
Sipa1l1	-0.52	1.00000000	chicken
Eif2s3y	-0.31	1.00000000	chicken
Slc9a8	-0.06	1.00000000	chicken
Npepps	0.21	1.00000000	chicken

Cdc37	-0.30	1.00000000	chicken
Camkmt	-0.50	1.00000000	chicken
Exoc8	-0.21	1.00000000	chicken
Kras	0.16	1.00000000	chicken
Sema4d	-0.35	1.00000000	chicken
Ccser2	-0.11	1.00000000	chicken
C4b	-0.33	1.00000000	chicken
Snapc1l	0.19	1.00000000	chicken
Hnrnpab	0.08	1.00000000	chicken
Ctdp1	0.40	1.00000000	chicken
Cmtm6	-0.22	1.00000000	chicken
Rfx3	-0.54	1.00000000	chicken
Otud7a	0.14	1.00000000	chicken
Socs6	0.15	1.00000000	chicken
Fkbp11	0.07	1.00000000	chicken
Igfbp3	-0.11	1.00000000	chicken
Imp4	-0.10	1.00000000	chicken
Ctdspl2	-0.02	1.00000000	chicken
Kctd2	-0.08	1.00000000	chicken
Fam32a	-0.07	1.00000000	chicken
Ppp2r5c	-0.41	1.00000000	chicken
Rmnd5a	-0.26	1.00000000	chicken
Gxylt1	-0.27	1.00000000	chicken
Aagab	0.06	1.00000000	chicken
Srsf11	-0.36	1.00000000	chicken
Arhgap30	-0.57	1.00000000	chicken
Grpel1	-0.21	1.00000000	chicken
Rab24	-0.28	1.00000000	chicken
Dhx35	-0.25	1.00000000	chicken
Brpf3	-0.49	1.00000000	chicken
Hspa4l	-0.18	1.00000000	chicken
Lrrfip1	0.61	1.00000000	chicken
Slc20a2	0.05	1.00000000	chicken
Creb3	-0.25	1.00000000	chicken
Ccdc43	-0.24	1.00000000	chicken
Clns1a	-0.21	1.00000000	chicken
Lbr	-0.29	1.00000000	chicken
Nrip1	-0.57	1.00000000	chicken
Srsf3	-0.45	1.00000000	chicken
Ttc39c	0.45	1.00000000	chicken
Sde2	-0.05	1.00000000	chicken
Ndrg3	0.27	1.00000000	chicken
Ak2	-0.17	1.00000000	chicken
Htatsf1	-0.38	1.00000000	chicken
Snx22	0.16	1.00000000	chicken
Cpsf2	0.16	1.00000000	chicken

Ppp1r16b	-0.27	1.00000000	chicken
Actr10	-0.26	1.00000000	chicken
Dusp14	-0.33	1.00000000	chicken
Ciao2b	0.21	1.00000000	chicken
Wdr43	0.09	1.00000000	chicken
Slc36a4	0.03	1.00000000	chicken
Cdk7	-0.01	1.00000000	chicken
Kmt2e	-0.21	1.00000000	chicken
Atp5j2	-0.25	1.00000000	chicken
Nmt1	0.43	1.00000000	chicken
Top1	0.04	1.00000000	chicken
Cd3e	-0.30	1.00000000	chicken
Sart3	0.29	1.00000000	chicken
Slc25a24	-0.55	1.00000000	chicken
Pcbp2	-0.38	1.00000000	chicken
Ubac1	-0.23	1.00000000	chicken
Cyb5r2	0.08	1.00000000	chicken
Sdf4	-0.17	1.00000000	chicken
Galnt7	-0.56	1.00000000	chicken
Dennd4c	-0.29	1.00000000	chicken
Dek	-0.32	1.00000000	chicken
Ptpa	0.07	1.00000000	chicken
Ermp1	-0.44	1.00000000	chicken
Zfp410	-0.41	1.00000000	chicken
Hk2	0.07	1.00000000	chicken
Usp47	-0.06	1.00000000	chicken
Siah2	-0.31	1.00000000	chicken
Zfp711	0.29	1.00000000	chicken
Tma16	0.18	1.00000000	chicken
Vkorc1l1	0.23	1.00000000	chicken
Ddx5	-0.12	1.00000000	chicken
Actr1a	0.01	1.00000000	chicken
Trib2	0.26	1.00000000	chicken
Vav3	0.84	1.00000000	chicken
Stau1	-0.38	1.00000000	chicken
Cilk1	0.16	1.00000000	chicken
Sae1	0.13	1.00000000	chicken
Ppt1	0.50	1.00000000	chicken
Hook3	0.19	1.00000000	chicken
Scap	0.09	1.00000000	chicken
Elmo2	-0.08	1.00000000	chicken
Rbm34	-0.33	1.00000000	chicken
Cdc14b	0.28	1.00000000	chicken
Rhot1	0.28	1.00000000	chicken
Agfg1	0.27	1.00000000	chicken
Arfgef1	-0.03	1.00000000	chicken

Slc43a2	0.20	1.00000000	chicken
Ebna1bp2	-0.12	1.00000000	chicken
Fkbp5	0.30	1.00000000	chicken
Skp1	-0.23	1.00000000	chicken
Cyb5a	-0.30	1.00000000	chicken
Tpmt	-0.31	1.00000000	chicken
B4galt4	-0.25	1.00000000	chicken
Srp54a	-0.25	1.00000000	chicken
Usp24	0.43	1.00000000	chicken
Zfp451	-0.08	1.00000000	chicken
Usp37	0.18	1.00000000	chicken
Tom1l2	0.10	1.00000000	chicken
Rnf103	0.38	1.00000000	chicken
Rnps1	0.16	1.00000000	chicken
Hipk1	0.07	1.00000000	chicken
Dcbld2	0.30	1.00000000	chicken
Lamtor1	0.21	1.00000000	chicken
Cep44	0.19	1.00000000	chicken
Rubcn	0.11	1.00000000	chicken
Nudt3	-0.01	1.00000000	chicken
Lrwd1	0.44	1.00000000	chicken
Ppil2	-0.36	1.00000000	chicken
Zcchc2	0.47	1.00000000	chicken
Phf5a	-0.16	1.00000000	chicken
Nudt14	0.36	1.00000000	chicken
Ap2a1	-0.16	1.00000000	chicken
Yaf2	0.13	1.00000000	chicken
Tns1	-0.49	1.00000000	chicken
Ctcf	-0.11	1.00000000	chicken
Setx	0.09	1.00000000	chicken
Eif4ebp1	-0.26	1.00000000	chicken
Edem1	0.15	1.00000000	chicken
Spred1	0.48	1.00000000	chicken
Zeb1	0.41	1.00000000	chicken
Cecr2	-0.60	1.00000000	chicken
Pald1	-0.43	1.00000000	chicken
Gabarapl2	-0.12	1.00000000	chicken
Capn15	-0.19	1.00000000	chicken
Cdc26	-0.23	1.00000000	chicken
Ptpn4	-0.58	1.00000000	chicken
Polr2l	-0.24	1.00000000	chicken
Ost4	0.29	1.00000000	chicken
Themis2	0.42	1.00000000	chicken
Bbx	0.46	1.00000000	chicken
Bank1	0.72	1.00000000	chicken
Zc3h12a	0.46	1.00000000	chicken

Dync1li1	-0.34	1.00000000	chicken
Gcn1	0.02	1.00000000	chicken
Ints6l	0.38	1.00000000	chicken
Smn1	-0.14	1.00000000	chicken
Cd200	0.49	1.00000000	chicken
Pigx	0.16	1.00000000	chicken
Trim52	0.30	1.00000000	chicken
Krit1	0.41	1.00000000	chicken
Zdhhc8	0.56	1.00000000	chicken
Pdha1	-0.31	1.00000000	chicken
Orc5	-0.07	1.00000000	chicken
Ppp1r12a	0.26	1.00000000	chicken
Cstb	0.46	1.00000000	chicken
Zfp64	0.21	1.00000000	chicken
Pgam1	-0.21	1.00000000	chicken
Rilpl2	-0.16	1.00000000	chicken
Dctn5	-0.26	1.00000000	chicken
Strada	0.24	1.00000000	chicken
Gpsm1	-0.25	1.00000000	chicken
Fasn	-0.07	1.00000000	chicken
Ppp2ca	-0.53	1.00000000	chicken
Zadh2	-0.30	1.00000000	chicken
Arhgap5	-0.43	1.00000000	chicken
Serpine2	-0.51	1.00000000	chicken
Adipor1	-0.18	1.00000000	chicken
Lrrc34	-1.03	1.00000000	chicken
Lrch2	-0.40	1.00000000	chicken
Pola1	-0.13	1.00000000	chicken
Otud6b	-0.22	1.00000000	chicken
Vim	-0.27	1.00000000	chicken
Copb2	0.15	1.00000000	chicken
2210016F16Rik	-0.02	1.00000000	chicken
Atg4a-ps	0.13	1.00000000	chicken
Scamp1	0.11	1.00000000	chicken
Fmn1	0.41	1.00000000	chicken
Lonrf2	-0.26	1.00000000	chicken
Carmil1	0.14	1.00000000	chicken
Ppa1	0.30	1.00000000	chicken
Fbxl17	0.24	1.00000000	chicken
Lonrf1	0.24	1.00000000	chicken
Nub1	0.10	1.00000000	chicken
Ythdf3	-0.58	1.00000000	chicken
Wdr36	-0.26	1.00000000	chicken
Parg	-0.25	1.00000000	chicken
Samsn1	0.69	1.00000000	chicken
Cdyl2	-0.39	1.00000000	chicken

Basp1	-0.06	1.00000000	chicken
Ttc33	0.16	1.00000000	chicken
Zfp352	0.27	1.00000000	chicken
Vac14	0.26	1.00000000	chicken
Senp6	0.35	1.00000000	chicken
Ttll12	-0.17	1.00000000	chicken
Rrbp1	-0.29	1.00000000	chicken
Dcaf12	-0.21	1.00000000	chicken
Itga6	-0.20	1.00000000	chicken
Ecd	-0.29	1.00000000	chicken
Noc3l	-0.19	1.00000000	chicken
Gspt1	0.13	1.00000000	chicken
Nnt	-0.30	1.00000000	chicken
Atrn	-0.42	1.00000000	chicken
Pdia4	0.16	1.00000000	chicken
Las1l	0.16	1.00000000	chicken
S100a4	0.60	1.00000000	chicken
Clk2	0.15	1.00000000	chicken
Sec31a	0.28	1.00000000	chicken
Pikfyve	-0.54	1.00000000	chicken
Lfng	0.39	1.00000000	chicken
Galc	-0.30	1.00000000	chicken
Dkc1	0.24	1.00000000	chicken
Lmna	-0.39	1.00000000	chicken
Ppfia1	0.07	1.00000000	chicken
Ankrd10	0.11	1.00000000	chicken
Slc30a9	0.19	1.00000000	chicken
Fam160b1	0.11	1.00000000	chicken
Zbtb45	0.12	1.00000000	chicken
Sppl3	-0.24	1.00000000	chicken
Tra2a	0.29	1.00000000	chicken
Tln1	-0.39	1.00000000	chicken
Phf12	-0.07	1.00000000	chicken
Notch1	-0.45	1.00000000	chicken
Edc3	-0.48	1.00000000	chicken
Btbd11	-0.21	1.00000000	chicken
Hlx	0.46	1.00000000	chicken
Snrpb	-0.14	1.00000000	chicken
Tubgcp3	-0.15	1.00000000	chicken
Krr1	-0.10	1.00000000	chicken
Slc25a30	-0.16	1.00000000	chicken
Ppil3	-0.30	1.00000000	chicken
Cln6	-0.15	1.00000000	chicken
Fem1b	-0.27	1.00000000	chicken
Ampd2	-0.20	1.00000000	chicken
Dnase2b	0.23	1.00000000	chicken

Herpud1	-0.10	1.00000000	chicken
Rab40b	-0.43	1.00000000	chicken
Arpc5	-0.35	1.00000000	chicken
Nelfa	0.20	1.00000000	chicken
Scml2	0.28	1.00000000	chicken
Wdr75	0.40	1.00000000	chicken
Nsrp1	-0.28	1.00000000	chicken
Cert1	0.02	1.00000000	chicken
Papss1	-0.21	1.00000000	chicken
Lamp2	0.06	1.00000000	chicken
Capza2	0.47	1.00000000	chicken
Fbxo28	0.12	1.00000000	chicken
Jakmip1	0.38	1.00000000	chicken
Fancm	0.02	1.00000000	chicken
Pigt	0.07	1.00000000	chicken
Extl3	-0.69	1.00000000	chicken
Nbr1	-0.11	1.00000000	chicken
Sel1l3	0.08	1.00000000	chicken
Arhgap11a	-0.62	1.00000000	chicken
Rmnd1	0.35	1.00000000	chicken
Srsf1	-0.09	1.00000000	chicken
Dip2a	-0.74	1.00000000	chicken
Ylpm1	0.10	1.00000000	chicken
Ndufb8	-0.26	1.00000000	chicken
Herpud2	-0.22	1.00000000	chicken
Cnpy3	0.04	1.00000000	chicken
Pwp1	-0.05	1.00000000	chicken
Rbm22	-0.45	1.00000000	chicken
Fndc3b	0.48	1.00000000	chicken
Socs2	-0.20	1.00000000	chicken
Adgrg6	-0.17	1.00000000	chicken
Tmem254a	-0.04	1.00000000	chicken
Zbtb17	-0.18	1.00000000	chicken
Csde1	-0.27	1.00000000	chicken
Rpap3	0.15	1.00000000	chicken
Tmco1	0.26	1.00000000	chicken
Ankib1	0.21	1.00000000	chicken
Usp7	-0.31	1.00000000	chicken
Ccnd1	-0.37	1.00000000	chicken
Syk	-0.57	1.00000000	chicken
Samhd1	0.19	1.00000000	chicken
Zranb2	0.00	1.00000000	chicken
Acbd5	0.31	1.00000000	chicken
Naa30	0.21	1.00000000	chicken
Nup50	-0.06	1.00000000	chicken
Atxn1	-0.52	1.00000000	chicken

Fbxo3	0.16	1.00000000	chicken
Dnm2	0.09	1.00000000	chicken
Ecpas	-0.13	1.00000000	chicken
Golga3	0.32	1.00000000	chicken
Npas3	0.95	1.00000000	chicken
Dus2	-0.18	1.00000000	chicken
Rab31	-0.12	1.00000000	chicken
Cog2	-0.28	1.00000000	chicken
Dtwd2	-0.51	1.00000000	chicken
Ppip5k2	-0.05	1.00000000	chicken
Ruvbl2	-0.16	1.00000000	chicken
Ubap2l	0.42	1.00000000	chicken
Psmb1	0.03	1.00000000	chicken
Arcn1	-0.36	1.00000000	chicken
Npc1	0.16	1.00000000	chicken
Fbxo30	-0.34	1.00000000	chicken
Cpsf4	-0.36	1.00000000	chicken
Naa15	-0.40	1.00000000	chicken
Stard5	0.35	1.00000000	chicken
Gpalpp1	0.20	1.00000000	chicken
Acp1	-0.12	1.00000000	chicken
Pcnx	0.14	1.00000000	chicken
Pnpla8	0.05	1.00000000	chicken
Eif4g3	-0.21	1.00000000	chicken
Gpr141	0.05	1.00000000	chicken
Hsd17b4	0.09	1.00000000	chicken
Smarcd1	0.11	1.00000000	chicken
Zfp148	-0.07	1.00000000	chicken
Ncs1	-0.31	1.00000000	chicken
Uxs1	-0.62	1.00000000	chicken
Pum3	-0.52	1.00000000	chicken
Ndufv3	-0.11	1.00000000	chicken
Riok1	-0.20	1.00000000	chicken
Sec31b	-0.21	1.00000000	chicken
Pink1	-0.14	1.00000000	chicken
Plpp5	-0.18	1.00000000	chicken
Nemf	-0.14	1.00000000	chicken
Taok3	0.24	1.00000000	chicken
Nt5c3	0.24	1.00000000	chicken
Pms2	0.11	1.00000000	chicken
Gpatch1	-0.31	1.00000000	chicken
Zfp652	-0.09	1.00000000	chicken
Cdk5rap1	1.23	1.00000000	chicken
Pdxdc1	-0.04	1.00000000	chicken
Lin7c	-0.38	1.00000000	chicken
Zfand5	-0.12	1.00000000	chicken

Pard3b	0.81	1.00000000	chicken
Rmdn3	-0.41	1.00000000	chicken
Ndufaf8	-0.27	1.00000000	chicken
Bcat1	-0.27	1.00000000	chicken
Cisd1	0.04	1.00000000	chicken
Evi5	0.07	1.00000000	chicken
Tardbp	0.30	1.00000000	chicken
Hnrnpll	-0.38	1.00000000	chicken
Kansl1	0.16	1.00000000	chicken
Kbtbd2	-0.18	1.00000000	chicken
Smarcad1	-0.11	1.00000000	chicken
Luc7l	-0.40	1.00000000	chicken
Brd2	0.22	1.00000000	chicken
Sqle	-0.36	1.00000000	chicken
Sik2	-0.27	1.00000000	chicken
Nifk	-0.05	1.00000000	chicken
Shld2	0.23	1.00000000	chicken
Reep3	0.35	1.00000000	chicken
Gabpb1	0.25	1.00000000	chicken
Trrap	0.17	1.00000000	chicken
Nol9	-0.40	1.00000000	chicken
Psma6	-0.29	1.00000000	chicken
Arntl2	-0.25	1.00000000	chicken
Napg	-0.30	1.00000000	chicken
Slc22a3	-0.65	1.00000000	chicken
Slc25a13	0.50	1.00000000	chicken
Ssb	-0.37	1.00000000	chicken
Auh	-0.49	1.00000000	chicken
Ypel5	-0.15	1.00000000	chicken
Zc3h15	-1.03	1.00000000	chicken
Ap3b1	0.24	1.00000000	chicken
Ube2e1	0.37	1.00000000	chicken
Acot10	-0.02	1.00000000	chicken
Fgr	-0.08	1.00000000	chicken
Snx20	-0.08	1.00000000	chicken
Zmynd8	0.19	1.00000000	chicken
Hyou1	-0.41	1.00000000	chicken
Orc3	0.05	1.00000000	chicken
Dnajc1	-0.46	1.00000000	chicken
Khdrbs1	0.30	1.00000000	chicken
Lmbr1	0.28	1.00000000	chicken
Rap2b	0.16	1.00000000	chicken
Bsg	-0.23	1.00000000	chicken
Cyrib	0.28	1.00000000	chicken
Ankrd44	0.14	1.00000000	chicken
Psmb2	-0.38	1.00000000	chicken

Nop58	-0.13	1.00000000	chicken
Fam91a1	-0.07	1.00000000	chicken
Umps	0.02	1.00000000	chicken
Hnrnpk	-0.26	1.00000000	chicken
Ift80	-0.01	1.00000000	chicken
Timm50	0.04	1.00000000	chicken
Erlin1	0.14	1.00000000	chicken
Trabd	0.25	1.00000000	chicken
Kif13a	0.18	1.00000000	chicken
Qser1	-0.65	1.00000000	chicken
Hk1	0.17	1.00000000	chicken
Eif2b3	-0.21	1.00000000	chicken
Taz	-0.15	1.00000000	chicken
Lrpap1	-0.29	1.00000000	chicken
Tbc1d12	-0.23	1.00000000	chicken
Zbtb8os	-0.18	1.00000000	chicken
Adar	-0.33	1.00000000	chicken
Cradd	0.46	1.00000000	chicken
Hdx	0.21	1.00000000	chicken
Xylt1	0.12	1.00000000	chicken
Itm2b	-0.62	1.00000000	chicken
Arl4a	0.41	1.00000000	chicken
Atxn7	0.10	1.00000000	chicken
Ncbp1	-0.14	1.00000000	chicken
As3mt	-0.01	1.00000000	chicken
Amfr	-0.07	1.00000000	chicken
Ints11	-0.02	1.00000000	chicken
Grcc10	0.08	1.00000000	chicken
Akt1	0.58	1.00000000	chicken
Cdv3	-0.33	1.00000000	chicken
Abitram	-0.19	1.00000000	chicken
Cox16	-0.48	1.00000000	chicken
Brsk2	-0.41	1.00000000	chicken
Ralb	0.30	1.00000000	chicken
Acot8	-0.12	1.00000000	chicken
Nhlrc2	0.02	1.00000000	chicken
Pcmtd1	-0.38	1.00000000	chicken
Xbp1	-0.23	1.00000000	chicken
Nol10	0.32	1.00000000	chicken
Ndufa9	-0.47	1.00000000	chicken
Ppp1r13b	0.07	1.00000000	chicken
Pfdn1	-0.27	1.00000000	chicken
Ywhag	0.53	1.00000000	chicken
Smndc1	-0.14	1.00000000	chicken
Plcl2	0.13	1.00000000	chicken
Fam122a	-0.03	1.00000000	chicken

Znrf2	-0.26	1.00000000	chicken
Rpusd2	0.11	1.00000000	chicken
Tbl3	0.00	1.00000000	chicken
Slc16a1	0.07	1.00000000	chicken
Cfap20	0.38	1.00000000	chicken
Trip11	-0.19	1.00000000	chicken
Trpm7	0.01	1.00000000	chicken
Hexa	0.14	1.00000000	chicken
Lats1	0.16	1.00000000	chicken
Pde8a	0.50	1.00000000	chicken
Uba3	-0.11	1.00000000	chicken
Tagln2	-0.14	1.00000000	chicken
Nras	-0.23	1.00000000	chicken
Ibtk	0.17	1.00000000	chicken
Pls3	-0.19	1.00000000	chicken
Exoc6b	-0.03	1.00000000	chicken
St6gal1	-0.15	1.00000000	chicken
Usp6nl	-0.19	1.00000000	chicken
Sesn3	-0.06	1.00000000	chicken
Wdfy1	-0.13	1.00000000	chicken
Gosr2	-0.23	1.00000000	chicken
Cox20	-0.07	1.00000000	chicken
Fam172a	0.04	1.00000000	chicken
Prrc2b	-0.08	1.00000000	chicken
Hnrnpr	-0.12	1.00000000	chicken
Lsm5	0.16	1.00000000	chicken
Fig4	0.08	1.00000000	chicken
Tmem266	0.11	1.00000000	chicken
Rundc3b	0.27	1.00000000	chicken
Bend5	0.42	1.00000000	chicken
Nek6	0.29	1.00000000	chicken
D630045J12Rik	-0.49	1.00000000	chicken
Pde7a	0.41	1.00000000	chicken
Thada	-0.22	1.00000000	chicken
Tmem50a	0.23	1.00000000	chicken
Prpf3	-0.17	1.00000000	chicken
Csf2ra	0.40	1.00000000	chicken
Snrpf	-0.25	1.00000000	chicken
Def6	-0.30	1.00000000	chicken
Snn	0.18	1.00000000	chicken
Eif4h	-0.14	1.00000000	chicken
Rpn1	-0.13	1.00000000	chicken
Insig1	-0.03	1.00000000	chicken
Ndufab1	0.17	1.00000000	chicken
Arrdc3	-0.15	1.00000000	chicken
Rb1	0.44	1.00000000	chicken

Sytl3	-0.43	1.00000000	chicken
Atg2b	-0.37	1.00000000	chicken
Slc25a16	0.12	1.00000000	chicken
Sem1	0.11	1.00000000	chicken
Chd9	-0.13	1.00000000	chicken
Derl3	-0.09	1.00000000	chicken
Urb2	-0.14	1.00000000	chicken
Nabp1	-0.04	1.00000000	chicken
Lmf2	-0.18	1.00000000	chicken
Dcun1d4	-0.25	1.00000000	chicken
Peli2	0.12	1.00000000	chicken
Bfar	0.12	1.00000000	chicken
Tmem33	0.18	1.00000000	chicken
Cadps2	0.29	1.00000000	chicken
Sppl2b	0.09	1.00000000	chicken
Slc16a3	0.17	1.00000000	chicken
Pik3r6	0.49	1.00000000	chicken
Eci3	-0.29	1.00000000	chicken
Dnajc7	0.13	1.00000000	chicken
Tomm7	-0.02	1.00000000	chicken
Rheb	-0.03	1.00000000	chicken
Tlr4	0.15	1.00000000	chicken
Ppp2r2d	0.20	1.00000000	chicken
Cse1l	0.04	1.00000000	chicken
Cyp20a1	-0.20	1.00000000	chicken
Ddx59	-0.56	1.00000000	chicken
Psma5	-0.18	1.00000000	chicken
Aig1	-0.26	1.00000000	chicken
Fars2	-0.04	1.00000000	chicken
Bmp2k	0.16	1.00000000	chicken
P2ry4	0.44	1.00000000	chicken
Herc3	0.00	1.00000000	chicken
Phlpp2	0.07	1.00000000	chicken
Ecsit	-0.06	1.00000000	chicken
Ankrd52	0.00	1.00000000	chicken
Pccb	-0.01	1.00000000	chicken
Eif6	-0.07	1.00000000	chicken
Nrd1	-0.12	1.00000000	chicken
Fyttd1	-0.56	1.00000000	chicken
Kif21b	0.03	1.00000000	chicken
Nbeal1	-0.16	1.00000000	chicken
Ptbp2	-0.17	1.00000000	chicken
Tesk2	0.19	1.00000000	chicken
Ppp1r9a	-0.76	1.00000000	chicken
BC005624	-0.18	1.00000000	chicken
Psmc1	-0.22	1.00000000	chicken

Ccdc50	-0.16	1.00000000	chicken
Lamp1	0.02	1.00000000	chicken
Abcd3	-0.15	1.00000000	chicken
Gdpd5	0.30	1.00000000	chicken
Ccr4	0.12	1.00000000	chicken
Dap3	0.04	1.00000000	chicken
P4hb	-0.11	1.00000000	chicken
Atp6v1g3	0.18	1.00000000	chicken
Kdm1a	0.19	1.00000000	chicken
Psmd7	0.24	1.00000000	chicken
Tcea1	-0.22	1.00000000	chicken
Cd247	0.31	1.00000000	chicken
Zfp236	-0.22	1.00000000	chicken
Znhit3	0.15	1.00000000	chicken
Ddx51	-0.07	1.00000000	chicken
Ptch1	-0.25	1.00000000	chicken
Samm50	-0.20	1.00000000	chicken
Rab3gap1	-0.34	1.00000000	chicken
Sdf2	-0.13	1.00000000	chicken
Prkag1	-0.14	1.00000000	chicken
Washc2	-0.30	1.00000000	chicken
Seh1l	0.14	1.00000000	chicken
Gtdc1	-0.56	1.00000000	chicken
Lpgat1	-0.25	1.00000000	chicken
AW554918	-0.37	1.00000000	chicken
Slc12a2	-0.94	1.00000000	chicken
Spata2	-0.20	1.00000000	chicken
Atp2b1	0.28	1.00000000	chicken
P4ha1	-0.17	1.00000000	chicken
Afdn	0.08	1.00000000	chicken
Ccdc88c	-0.30	1.00000000	chicken
Nkrf	-0.29	1.00000000	chicken
Shisa5	0.09	1.00000000	chicken
Kdm4b	-0.38	1.00000000	chicken
Ube2v2	0.27	1.00000000	chicken
Txndc17	-0.17	1.00000000	chicken
Gsdme	-0.26	1.00000000	chicken
Rffl	0.05	1.00000000	chicken
Dpyd	0.64	1.00000000	chicken
Smchd1	-0.06	1.00000000	chicken
Cers5	0.11	1.00000000	chicken
Plekha5	-0.22	1.00000000	chicken
Uggt1	-0.08	1.00000000	chicken
Scn8a	0.00	1.00000000	chicken
Hprt	0.05	1.00000000	chicken
Traf7	-0.30	1.00000000	chicken

Cltb	-0.23	1.00000000	chicken
Usp19	0.28	1.00000000	chicken
Wtap	-0.43	1.00000000	chicken
Bop1	-0.11	1.00000000	chicken
Abi2	0.19	1.00000000	chicken
N4bp2l2	-0.17	1.00000000	chicken
Drosha	-0.40	1.00000000	chicken
Nenf	-0.15	1.00000000	chicken
Lrrc1	-0.49	1.00000000	chicken
Rab11fip5	-0.24	1.00000000	chicken
Rpap1	-0.30	1.00000000	chicken
Abcf3	-0.13	1.00000000	chicken
Taf4	-0.21	1.00000000	chicken
Arvcf	-0.27	1.00000000	chicken
Disp3	-0.22	1.00000000	chicken
Gbe1	0.05	1.00000000	chicken
Hectd4	0.21	1.00000000	chicken
Usp49	0.21	1.00000000	chicken
Rbm14	-0.26	1.00000000	chicken
Sfswap	-0.59	1.00000000	chicken
Gab3	0.18	1.00000000	chicken
Capn7	-0.11	1.00000000	chicken
Cep43	-0.13	1.00000000	chicken
Tmem230	-0.12	1.00000000	chicken
Gpatch2	-0.39	1.00000000	chicken
Pdia6	-0.44	1.00000000	chicken
Ext1	-0.22	1.00000000	chicken
Srf	-0.23	1.00000000	chicken
Gzf1	-0.41	1.00000000	chicken
Slmap	-0.16	1.00000000	chicken
Btf3l4	-0.27	1.00000000	chicken
Phip	-0.36	1.00000000	chicken
Asb2	0.11	1.00000000	chicken
Dctn1	-0.04	1.00000000	chicken
Klf7	-0.07	1.00000000	chicken
Dtx3l	-0.19	1.00000000	chicken
Sike1	-0.12	1.00000000	chicken
Tex9	-0.21	1.00000000	chicken
Ift22	0.09	1.00000000	chicken
Rab18	0.26	1.00000000	chicken
Hsd17b10	0.39	1.00000000	chicken
Ppard	0.35	1.00000000	chicken
Tmem259	-0.19	1.00000000	chicken
Tgfbr1	0.22	1.00000000	chicken
Ccdc25	0.21	1.00000000	chicken
Phf21a	0.04	1.00000000	chicken

Arfip2	-0.26	1.00000000	chicken
Cdc40	-0.41	1.00000000	chicken
Snrnp25	-0.26	1.00000000	chicken
Chka	0.16	1.00000000	chicken
Gpatch8	0.07	1.00000000	chicken
Rcbtb1	0.06	1.00000000	chicken
Pdpr	-0.07	1.00000000	chicken
Lrch3	0.56	1.00000000	chicken
Cbwd1	-0.16	1.00000000	chicken
Pno1	-0.10	1.00000000	chicken
Wdr12	-0.14	1.00000000	chicken
Pla2g15	-0.19	1.00000000	chicken
4833439L19Rik	-0.17	1.00000000	chicken
Rdh5	-0.20	1.00000000	chicken
Scaf4	-0.01	1.00000000	chicken
Camk2g	0.41	1.00000000	chicken
Ube2b	-0.30	1.00000000	chicken
Sap30bp	-0.34	1.00000000	chicken
Nudcd1	0.10	1.00000000	chicken
Ubxn7	-0.17	1.00000000	chicken
Zbed4	0.20	1.00000000	chicken
Appl1	0.11	1.00000000	chicken
Sh3pxd2a	0.03	1.00000000	chicken
Fubp1	0.18	1.00000000	chicken
Atp11b	-0.02	1.00000000	chicken
Psma7	0.06	1.00000000	chicken
Pnisr	-0.28	1.00000000	chicken
Fbxo9	-0.13	1.00000000	chicken
Sav1	-0.20	1.00000000	chicken
Atp6v1e2	-0.38	1.00000000	chicken
Traf1	0.07	1.00000000	chicken
Rgs10	0.34	1.00000000	chicken
Nrbp1	-0.15	1.00000000	chicken
Gramd4	-0.06	1.00000000	chicken
Ppig	-0.27	1.00000000	chicken
Hdgfl2	-0.19	1.00000000	chicken
Clcn3	-0.23	1.00000000	chicken
Pi4k2b	0.40	1.00000000	chicken
Zfp639	-0.13	1.00000000	chicken
Arid3b	-0.19	1.00000000	chicken
Neil1	-0.09	1.00000000	chicken
Sin3a	-0.19	1.00000000	chicken
Rnft1	-0.08	1.00000000	chicken
Wdr77	-0.15	1.00000000	chicken
Dalrd3	0.26	1.00000000	chicken
Sfpq	-0.29	1.00000000	chicken

Sufu	-0.16	1.00000000	chicken
Slc45a3	-0.27	1.00000000	chicken
Igf2bp2	-0.21	1.00000000	chicken
Strn3	-0.36	1.00000000	chicken
Heatr5b	0.06	1.00000000	chicken
Senp1	0.06	1.00000000	chicken
Ldb1	-0.70	1.00000000	chicken
Senp7	-0.41	1.00000000	chicken
C1d	-0.27	1.00000000	chicken
Zfpm1	0.40	1.00000000	chicken
Casc3	0.05	1.00000000	chicken
Pcid2	-0.19	1.00000000	chicken
Zyx	0.12	1.00000000	chicken
St3gal2	-0.26	1.00000000	chicken
Bap1	-0.03	1.00000000	chicken
Pithd1	-0.10	1.00000000	chicken
Itpripl2	0.05	1.00000000	chicken
Decr1	-0.19	1.00000000	chicken
Trmt11	-0.23	1.00000000	chicken
Tatdn1	-0.19	1.00000000	chicken
Spire1	-0.21	1.00000000	chicken
Cep68	-0.09	1.00000000	chicken
Anapc1	0.23	1.00000000	chicken
Ublcp1	-0.15	1.00000000	chicken
Gtf2h3	-0.12	1.00000000	chicken
Adamtsl2	0.44	1.00000000	chicken
Pogz	-0.10	1.00000000	chicken
Sik1	0.02	1.00000000	chicken
Ankrd6	0.85	1.00000000	chicken
Sec23b	-0.35	1.00000000	chicken
Azi2	0.18	1.00000000	chicken
Sugt1	0.28	1.00000000	chicken
Dctn3	-0.14	1.00000000	chicken
Ncln	0.23	1.00000000	chicken
Psmb7	-0.44	1.00000000	chicken
Bzw1	-0.22	1.00000000	chicken
Fbrsl1	-0.56	1.00000000	chicken
Rnf113a1	0.02	1.00000000	chicken
Xpr1	-0.21	1.00000000	chicken
Poc1b	0.63	1.00000000	chicken
Abhd2	0.14	1.00000000	chicken
B4galt1	0.23	1.00000000	chicken
Pwwp2b	0.10	1.00000000	chicken
Nfatc1	-0.20	1.00000000	chicken
Fyco1	1.40	1.00000000	chicken
Ddx27	0.18	1.00000000	chicken

Ppm1a	-0.48	1.00000000	chicken
Ppp2r5e	0.61	1.00000000	chicken
Rnaseh1	0.17	1.00000000	chicken
Cep128	0.49	1.00000000	chicken
Aar2	-0.22	1.00000000	chicken
9430015G10Rik	-0.17	1.00000000	chicken
Tsc22d2	0.06	1.00000000	chicken
Atp6v1c1	0.14	1.00000000	chicken
Ezh2	-0.18	1.00000000	chicken
Jmjd1c	0.35	1.00000000	chicken
Wrn	-0.10	1.00000000	chicken
Znhit6	-0.01	1.00000000	chicken
Ubp1	-0.51	1.00000000	chicken
Ahsa2	-0.06	1.00000000	chicken
Ndufs4	0.07	1.00000000	chicken
Srp19	-0.27	1.00000000	chicken
Gnl2	-0.20	1.00000000	chicken
Tent2	-0.06	1.00000000	chicken
Tmem39a	0.05	1.00000000	chicken
Rft1	0.19	1.00000000	chicken
Nploc4	0.22	1.00000000	chicken
Timm44	-0.21	1.00000000	chicken
Fam53a	0.14	1.00000000	chicken
Arhgef17	-0.04	1.00000000	chicken
Phtf1	-0.11	1.00000000	chicken
Lpcat2b	0.06	1.00000000	chicken
Phkb	-0.07	1.00000000	chicken
Arid2	0.32	1.00000000	chicken
Rwdd1	0.18	1.00000000	chicken
Leprotl1	0.08	1.00000000	chicken
Tspan18	0.52	1.00000000	chicken
Ndufs5	0.13	1.00000000	chicken
Psmb6	-0.11	1.00000000	chicken
Dnmt3a	-0.33	1.00000000	chicken
Ascc1	-0.35	1.00000000	chicken
Hsf3	0.03	1.00000000	chicken
Prpf40a	-0.25	1.00000000	chicken
Rgl1	0.04	1.00000000	chicken
Nptx2	0.18	1.00000000	chicken
Surf4	-0.24	1.00000000	chicken
Bnip3l	-0.17	1.00000000	chicken
Paxip1	0.07	1.00000000	chicken
Rpp14	-0.11	1.00000000	chicken
Nedd9	0.24	1.00000000	chicken
Sacs	-0.01	1.00000000	chicken
Ncor2	-0.41	1.00000000	chicken

Itgb1	0.58	1.00000000	chicken
H2az1	0.16	1.00000000	chicken
Phb2	-0.31	1.00000000	chicken
Gnpat	-0.11	1.00000000	chicken
Prdm10	-0.13	1.00000000	chicken
Herc2	0.00	1.00000000	chicken
Psma3	0.01	1.00000000	chicken
Dnlz	-0.04	1.00000000	chicken
Plac8	0.09	1.00000000	chicken
Reck	-0.41	1.00000000	chicken
Ago3	-0.01	1.00000000	chicken
Ahi1	0.21	1.00000000	chicken
Usb1	-0.04	1.00000000	chicken
Wipi1	0.09	1.00000000	chicken
Wbp1	0.00	1.00000000	chicken
Ppie	-0.17	1.00000000	chicken
Arhgap6	0.58	1.00000000	chicken
1600014C10Rik	0.21	1.00000000	chicken
Qdpr	-0.36	1.00000000	chicken
Crym	-0.09	1.00000000	chicken
Rfxap	0.18	1.00000000	chicken
Bcl2l1	0.20	1.00000000	chicken
4933434E20Rik	0.18	1.00000000	chicken
Kdm4c	0.19	1.00000000	chicken
Pik3ap1	0.09	1.00000000	chicken
Siah1b	0.31	1.00000000	chicken
Eif2a	-0.31	1.00000000	chicken
Tmem65	-0.31	1.00000000	chicken
Dock11	0.49	1.00000000	chicken
Btg1b	-0.19	1.00000000	chicken
Kdelr2	-0.35	1.00000000	chicken
Gosr1	-0.22	1.00000000	chicken
Ulbp1	-0.15	1.00000000	chicken
Rcsd1	-0.82	1.00000000	chicken
Hivep2	0.15	1.00000000	chicken
Trmt11	-0.24	1.00000000	chicken
Ada	-0.09	1.00000000	chicken
Ncapd2	-0.43	1.00000000	chicken
Stx18	0.02	1.00000000	chicken
Cdc27	-0.02	1.00000000	chicken
Insc	0.44	1.00000000	chicken
Ifrd1	0.36	1.00000000	chicken
Rab12	0.03	1.00000000	chicken
Gabpa	-0.28	1.00000000	chicken
Nr1h3	-0.32	1.00000000	chicken
Clptm1l	-0.38	1.00000000	chicken

Plxnc1	0.16	1.00000000	chicken
Rbm19	-0.22	1.00000000	chicken
Usp48	0.11	1.00000000	chicken
Ndufs6	-0.05	1.00000000	chicken
Fcf1	-0.06	1.00000000	chicken
Sf3b6	0.30	1.00000000	chicken
Trim8	-0.48	1.00000000	chicken
Lnpk	-0.30	1.00000000	chicken
Bak1	0.19	1.00000000	chicken
Jmy	0.37	1.00000000	chicken
Ppp2r3c	0.06	1.00000000	chicken
Selenof	-0.29	1.00000000	chicken
Rdm1	-0.30	1.00000000	chicken
Tubb4b	0.34	1.00000000	chicken
Bloc1s6	-0.08	1.00000000	chicken
U2af1	-0.18	1.00000000	chicken
Ubap1	-0.03	1.00000000	chicken
Nkap	-0.25	1.00000000	chicken
Ttc7	0.52	1.00000000	chicken
Cnn2	-0.16	1.00000000	chicken
Zc3h13	-0.08	1.00000000	chicken
Slc25a36	0.12	1.00000000	chicken
Nr2c2	0.34	1.00000000	chicken
Stk11	-0.39	1.00000000	chicken
Zfp143	-0.03	1.00000000	chicken
Ncoa1	0.17	1.00000000	chicken
Clcn5	0.08	1.00000000	chicken
Hint1	0.02	1.00000000	chicken
Secisbp2l	-0.19	1.00000000	chicken
Rras2	0.69	1.00000000	chicken
Wnt5b	0.86	1.00000000	chicken
Sucla2	0.03	1.00000000	chicken
Ids	-0.05	1.00000000	chicken
Gnpnat1	0.08	1.00000000	chicken
Polr2c	0.08	1.00000000	chicken
Cabin1	0.14	1.00000000	chicken
Tmem164	0.40	1.00000000	chicken
Ptpra	0.09	1.00000000	chicken
Isca1	-0.10	1.00000000	chicken
Gstm5	-0.11	1.00000000	chicken
Wapl	-0.20	1.00000000	chicken
Tfam	-0.25	1.00000000	chicken
Ptpn22	0.24	1.00000000	chicken
Ilf3	-0.10	1.00000000	chicken
Anapc16	-0.22	1.00000000	chicken
Vbp1	-0.01	1.00000000	chicken

Klhl6	-0.28	1.00000000	chicken
Rcl1	-0.12	1.00000000	chicken
Cd300c	0.00	1.00000000	chicken
Usp22	-0.42	1.00000000	chicken
Esf1	0.36	1.00000000	chicken
Plekhb2	-0.14	1.00000000	chicken
Necap2	-0.03	1.00000000	chicken
Rap1b	0.14	1.00000000	chicken
Was	-0.16	1.00000000	chicken
Helz	0.15	1.00000000	chicken
Cyb5r4	-0.41	1.00000000	chicken
Hsd17b12	0.16	1.00000000	chicken
Dync1h1	-0.11	1.00000000	chicken
St14	-0.33	1.00000000	chicken
Arfgap3	-0.03	1.00000000	chicken
Prpf6	-0.10	1.00000000	chicken
Camk2d	-0.19	1.00000000	chicken
Cdkn2aip	0.05	1.00000000	chicken
Pex14	0.39	1.00000000	chicken
Iars2	0.13	1.00000000	chicken
Ube4b	-0.28	1.00000000	chicken
Acvrl1	-0.21	1.00000000	chicken
Gpr155	-0.33	1.00000000	chicken
Rreb1	-0.14	1.00000000	chicken
Eif4a3l1	-0.23	1.00000000	chicken
Pmf1	-0.13	1.00000000	chicken
Gsk3b	0.09	1.00000000	chicken
Ints4	-0.25	1.00000000	chicken
Qpct	-0.05	1.00000000	chicken
Trim33	-0.22	1.00000000	chicken
Adrb2	0.21	1.00000000	chicken
Sptan1	-0.06	1.00000000	chicken
Nucb2	-0.09	1.00000000	chicken
Fgfr1op2	0.06	1.00000000	chicken
St7	0.17	1.00000000	chicken
Ppa2	-0.29	1.00000000	chicken
B3gnt4	0.15	1.00000000	chicken
Gnl3	-0.09	1.00000000	chicken
Ran	0.05	1.00000000	chicken
Tbxas1	0.24	1.00000000	chicken
Prkdc	-0.02	1.00000000	chicken
Chst13	0.11	1.00000000	chicken
Exosc1	-0.25	1.00000000	chicken
Zbtb41	0.10	1.00000000	chicken
Aars	-0.17	1.00000000	chicken
Stub1	0.14	1.00000000	chicken

Ap3m2	-0.39	1.00000000	chicken
Itprid2	0.01	1.00000000	chicken
Nop14	-0.18	1.00000000	chicken
Rnf38	-0.14	1.00000000	chicken
Slc2a1	-0.06	1.00000000	chicken
Osbpl3	0.73	1.00000000	chicken
Zfp598	0.14	1.00000000	chicken
Nol11	-0.42	1.00000000	chicken
Dgkz	-0.18	1.00000000	chicken
Dnajc18	0.14	1.00000000	chicken
Gtf2b	-0.24	1.00000000	chicken
Gatad2a	-0.44	1.00000000	chicken
Sar1a	0.06	1.00000000	chicken
Crim1	0.08	1.00000000	chicken
Btbd7	-0.30	1.00000000	chicken
Capn10	0.12	1.00000000	chicken
Fbxo21	-0.26	1.00000000	chicken
Kcnj5	0.37	1.00000000	chicken
Hao1	0.18	1.00000000	chicken
Tln2	0.04	1.00000000	chicken
Tubg1	-0.15	1.00000000	chicken
Fyb	0.35	1.00000000	chicken
Twsg1	0.09	1.00000000	chicken
Tecpr1	0.32	1.00000000	chicken
Bcl7b	0.04	1.00000000	chicken
Spns3	0.04	1.00000000	chicken
Igf2bp3	0.25	1.00000000	chicken
Fyn	-0.05	1.00000000	chicken
Ctsc	-0.06	1.00000000	chicken
Fbxl5	0.01	1.00000000	chicken
Btrc	0.09	1.00000000	chicken
Fmr1	-0.14	1.00000000	chicken
Cct7	0.00	1.00000000	chicken
Galk2	0.11	1.00000000	chicken
Yipf1	0.03	1.00000000	chicken
Cebpd	0.12	1.00000000	chicken
Bivm	0.12	1.00000000	chicken
Dnajc16	-0.16	1.00000000	chicken
Ino80c	-0.25	1.00000000	chicken
Nsmaf	-0.05	1.00000000	chicken
Dbf4	0.04	1.00000000	chicken
Hif1an	0.08	1.00000000	chicken
Leprot	-0.19	1.00000000	chicken
Them4	-0.10	1.00000000	chicken
Kif5c	-0.15	1.00000000	chicken
Rnf6	0.05	1.00000000	chicken

9030025P20Rik	-0.07	1.00000000	chicken
Tnip1	0.18	1.00000000	chicken
Rnf144b	0.32	1.00000000	chicken
Thoc1	0.06	1.00000000	chicken
Sec11a	0.00	1.00000000	chicken
Rictor	0.12	1.00000000	chicken
Adam17	-0.33	1.00000000	chicken
Jade1	0.03	1.00000000	chicken
Gga3	-0.01	1.00000000	chicken
Rnf20	-0.13	1.00000000	chicken
Scfd2	0.11	1.00000000	chicken
Ncoa6	0.17	1.00000000	chicken
Runx3	0.12	1.00000000	chicken
Pptc7	0.03	1.00000000	chicken
Insrr	-0.57	1.00000000	chicken
Erlec1	0.05	1.00000000	chicken
Inpp4b	0.21	1.00000000	chicken
Nup205	-0.14	1.00000000	chicken
D430041D05Rik	0.24	1.00000000	chicken
Thap4	-0.10	1.00000000	chicken
Fam214a	0.01	1.00000000	chicken
Nup93	0.06	1.00000000	chicken
Rexo5	-0.19	1.00000000	chicken
Decr2	-0.23	1.00000000	chicken
Bcl7a	-0.21	1.00000000	chicken
Cul3	0.10	1.00000000	chicken
Add1	-0.63	1.00000000	chicken
Eea1	-0.21	1.00000000	chicken
Atp2a3	-0.10	1.00000000	chicken
Ikbkb	-0.19	1.00000000	chicken
Dync1li2	0.03	1.00000000	chicken
Smg7	0.00	1.00000000	chicken
Exoc6	0.28	1.00000000	chicken
Cryzl1	0.07	1.00000000	chicken
Ing1	0.16	1.00000000	chicken
Arih2	0.08	1.00000000	chicken
Pdcd6	0.18	1.00000000	chicken
Dhx38	0.05	1.00000000	chicken
Dgki	-0.35	1.00000000	chicken
Actr6	-0.17	1.00000000	chicken
Fam199x	0.08	1.00000000	chicken
Wdr26	-0.61	1.00000000	chicken
Tec	0.01	1.00000000	chicken
Pkm	-0.19	1.00000000	chicken
Nans	0.01	1.00000000	chicken
Rbl2	0.08	1.00000000	chicken

Tiam1	0.47	1.00000000	chicken
Far2	0.15	1.00000000	chicken
Fam120b	0.00	1.00000000	chicken
Cracr2b	0.23	1.00000000	chicken
Sun1	0.30	1.00000000	chicken
Aplp2	0.14	1.00000000	chicken
Rbbp6	-0.10	1.00000000	chicken
Syne1	-0.48	1.00000000	chicken
Pxk	-0.13	1.00000000	chicken
Vezt	0.20	1.00000000	chicken
Edem3	-0.46	1.00000000	chicken
Slc23a2	-0.30	1.00000000	chicken
Notch2	-0.37	1.00000000	chicken
Iqgap2	0.05	1.00000000	chicken
Nup153	0.01	1.00000000	chicken
Ern1	-0.26	1.00000000	chicken
Timm17a	-0.10	1.00000000	chicken
Eefsec	0.48	1.00000000	chicken
Wasf2	0.29	1.00000000	chicken
Trappc11	0.09	1.00000000	chicken
Cop1	-0.01	1.00000000	chicken
Dnajc2	0.16	1.00000000	chicken
Tom1	-0.13	1.00000000	chicken
Plekhm1	-0.04	1.00000000	chicken
Zc3h7a	-0.35	1.00000000	chicken
Vps29	-0.06	1.00000000	chicken
Tmem223	-0.03	1.00000000	chicken
Arnt	-0.22	1.00000000	chicken
Naa20	0.13	1.00000000	chicken
Tpk1	0.01	1.00000000	chicken
Ralgapb	0.11	1.00000000	chicken
Sap130	0.10	1.00000000	chicken
Arfip1	0.13	1.00000000	chicken
Ext2	0.01	1.00000000	chicken
Csrp1	-0.08	1.00000000	chicken
B3gntl1	0.13	1.00000000	chicken
Fkbp8	-0.11	1.00000000	chicken
Tctn3	-0.01	1.00000000	chicken
Setd3	-0.13	1.00000000	chicken
Ssbp3	-0.26	1.00000000	chicken
N4bp1	-0.17	1.00000000	chicken
Ssu72	0.29	1.00000000	chicken
Cetn4	0.18	1.00000000	chicken
Tial1	-0.11	1.00000000	chicken
Emc2	-0.39	1.00000000	chicken
Polr2b	-0.20	1.00000000	chicken

Hdac3	-0.16	1.00000000	chicken
Dnajb14	0.01	1.00000000	chicken
Tpp2	-0.51	1.00000000	chicken
Fam168a	-0.09	1.00000000	chicken
Angel1	-0.19	1.00000000	chicken
Eif4ebp3	-0.27	1.00000000	chicken
Chst2	0.04	1.00000000	chicken
Ubl7	0.11	1.00000000	chicken
Cbll1	-0.11	1.00000000	chicken
Zfp618	0.06	1.00000000	chicken
Ndufa1	-0.23	1.00000000	chicken
Clint1	-0.06	1.00000000	chicken
Ttl	-0.18	1.00000000	chicken
Shprh	-0.04	1.00000000	chicken
Taf5l	0.03	1.00000000	chicken
Pdhx	0.14	1.00000000	chicken
Hivep3	0.37	1.00000000	chicken
Btaf1	0.02	1.00000000	chicken
Dynlrb1	-0.02	1.00000000	chicken
Tmx4	0.10	1.00000000	chicken
Stam2	-0.36	1.00000000	chicken
Ctsb	0.11	1.00000000	chicken
Rac2	0.01	1.00000000	chicken
Dazap2	-0.28	1.00000000	chicken
Fhit	0.52	1.00000000	chicken
Cyfip2	-0.22	1.00000000	chicken
Tmem258	-0.11	1.00000000	chicken
Bnip2	0.12	1.00000000	chicken
Tmem50b	-0.36	1.00000000	chicken
Dtd1	-0.34	1.00000000	chicken
Gnpda1	-0.22	1.00000000	chicken
Pip5k1c	0.16	1.00000000	chicken
Fggy	-0.26	1.00000000	chicken
Ulk2	-0.62	1.00000000	chicken
Abraxas2	-0.08	1.00000000	chicken
Gpat3	-0.07	1.00000000	chicken
Cwc15	-0.25	1.00000000	chicken
Alox5ap	0.08	1.00000000	chicken
Smad6	0.12	1.00000000	chicken
Hbs1l	-0.35	1.00000000	chicken
Pfdn5	-0.17	1.00000000	chicken
Txndc5	0.04	1.00000000	chicken
Fancd2	-0.07	1.00000000	chicken
Pcbd2	0.11	1.00000000	chicken
Phb	0.01	1.00000000	chicken
Rcc2	-0.11	1.00000000	chicken

Srp9	-0.18	1.00000000	chicken
Bnc2	-1.42	1.00000000	chicken
Atp6v0a2	-0.04	1.00000000	chicken
Naa40	0.15	1.00000000	chicken
Lcor	-0.04	1.00000000	chicken
Rabgap1	-0.02	1.00000000	chicken
Hopx	-0.02	1.00000000	chicken
Arhgap1	-0.11	1.00000000	chicken
Becn2	-0.28	1.00000000	chicken
Zfp292	0.06	1.00000000	chicken
Rab11a	-0.05	1.00000000	chicken
Stk38l	-0.24	1.00000000	chicken
Gtf2h5	-0.19	1.00000000	chicken
Pum2	-0.26	1.00000000	chicken
Lrpprc	0.09	1.00000000	chicken
Stx8	0.29	1.00000000	chicken
Ppp4r3a	0.09	1.00000000	chicken
Tbck	0.48	1.00000000	chicken
Klf11	-0.15	1.00000000	chicken
Taf6	0.01	1.00000000	chicken
Smg6	-0.12	1.00000000	chicken
Rpa3	0.00	1.00000000	chicken
Haus2	-0.12	1.00000000	chicken
Itm2c	-0.05	1.00000000	chicken
Rnf41	-0.08	1.00000000	chicken
Rrp1b	-0.08	1.00000000	chicken
Suclg2	-0.14	1.00000000	chicken
Derl1	-0.27	1.00000000	chicken
Csnk2a1	-0.14	1.00000000	chicken
Pmp22	0.30	1.00000000	chicken
Dnaja1	0.01	1.00000000	chicken
Ep300	-0.24	1.00000000	chicken
Zcchc8	-0.16	1.00000000	chicken
Psmd14	-0.11	1.00000000	chicken
Nucks1	-0.20	1.00000000	chicken
Rtf2	-0.11	1.00000000	chicken
Rnf145	-0.15	1.00000000	chicken
Ncbp2	-0.19	1.00000000	chicken
Prpsap2	-0.06	1.00000000	chicken
2700049A03Rik	0.61	1.00000000	chicken
Dohh	0.03	1.00000000	chicken
Asah1	-0.24	1.00000000	chicken
Cd74	-0.04	1.00000000	chicken
Setd1b	0.05	1.00000000	chicken
Il17ra	0.03	1.00000000	chicken
Snrpd2	-0.02	1.00000000	chicken

Ctsz	0.00	1.00000000	chicken
Scyl2	-0.17	1.00000000	chicken
Gata3	0.00	1.00000000	chicken
Slc25a14	-0.04	1.00000000	chicken
Guf1	0.05	1.00000000	chicken
Ube2f	-0.44	1.00000000	chicken
Ppib	-0.31	1.00000000	chicken
Prr5l	-0.39	1.00000000	chicken
Gars	-0.12	1.00000000	chicken
Snx30	-0.13	1.00000000	chicken
Sec23ip	-0.05	1.00000000	chicken
Prpf38a	-0.11	1.00000000	chicken
Fbxo45	-0.08	1.00000000	chicken
Vapa	-0.13	1.00000000	chicken
Ebag9	-0.17	1.00000000	chicken
Tprkb	0.18	1.00000000	chicken
Immp2l	0.30	1.00000000	chicken
Abhd18	-0.32	1.00000000	chicken
Proser1	0.13	1.00000000	chicken
Vdac1	0.06	1.00000000	chicken
Osbpl8	-0.14	1.00000000	chicken
Zfp384	-0.33	1.00000000	chicken
Adarb1	-0.34	1.00000000	chicken
Dctn2	-0.13	1.00000000	chicken
Sptssa	-0.13	1.00000000	chicken
Dcp1a	0.00	1.00000000	chicken
Tprgl	-0.10	1.00000000	chicken
Atp6v0a1	0.14	1.00000000	chicken
Nelfb	0.04	1.00000000	chicken
Dars	-0.43	1.00000000	chicken
Rp2	-0.20	1.00000000	chicken
Psen1	-0.06	1.00000000	chicken
Zswim8	-0.13	1.00000000	chicken
Champ1	-0.07	1.00000000	chicken
Eml6	-0.21	1.00000000	chicken
Dda1	-0.34	1.00000000	chicken
Pip4k2a	0.04	1.00000000	chicken
Zeb2	0.03	1.00000000	chicken
Ccdc93	-0.03	1.00000000	chicken
Chchd1	-0.12	1.00000000	chicken
Tshz2	-1.23	1.00000000	chicken
Sec62	-0.22	1.00000000	chicken
Nuak1	-0.13	1.00000000	chicken
Adgrg5	0.52	1.00000000	chicken
Tmem192	-0.26	1.00000000	chicken
Clta	-0.30	1.00000000	chicken

Nup155	-0.18	1.00000000	chicken
Adap2	-0.10	1.00000000	chicken
Lamtor5	0.03	1.00000000	chicken
Ndufs7	0.28	1.00000000	chicken
Nol7	-0.22	1.00000000	chicken
Ccnc	-0.08	1.00000000	chicken
Wdr45b	-0.24	1.00000000	chicken
Cog5	-0.08	1.00000000	chicken
Rpp30	0.05	1.00000000	chicken
Tmem104	-0.08	1.00000000	chicken
Gpr34	-0.34	1.00000000	chicken
Api5	0.11	1.00000000	chicken
Pik3cg	-0.09	1.00000000	chicken
Dera	0.19	1.00000000	chicken
Tspo	0.15	1.00000000	chicken
Trafd1	-0.03	1.00000000	chicken
Chmp5	-0.12	1.00000000	chicken
Ankrd13a	-0.36	1.00000000	chicken
Tmem87a	-0.24	1.00000000	chicken
Akap11	-0.15	1.00000000	chicken
Ap2b1	0.05	1.00000000	chicken
Tent4b	-0.33	1.00000000	chicken
Tmem106b	-0.33	1.00000000	chicken
Arhgef9	-0.14	1.00000000	chicken
Lyrm1	-0.04	1.00000000	chicken
Fbxw5	-0.06	1.00000000	chicken
Ubr1	0.15	1.00000000	chicken
Nek9	-0.45	1.00000000	chicken
Ppp6r2	0.13	1.00000000	chicken
Pigk	-0.33	1.00000000	chicken
Prpf19	0.06	1.00000000	chicken
Eif3j2	0.00	1.00000000	chicken
Nod1	0.15	1.00000000	chicken
Iars	0.38	1.00000000	chicken
Leo1	0.15	1.00000000	chicken
Gss	0.17	1.00000000	chicken
Cdk12	0.01	1.00000000	chicken
Sfxn1	-0.05	1.00000000	chicken
Tbp	-0.28	1.00000000	chicken
Rnaseh2b	-0.06	1.00000000	chicken
Taf2	0.21	1.00000000	chicken
Uchl3	-0.02	1.00000000	chicken
Snap47	0.12	1.00000000	chicken
Pdcd5	-0.11	1.00000000	chicken
Atp6v1a	-0.29	1.00000000	chicken
Ywhae	-0.22	1.00000000	chicken

Tapt1	-0.31	1.00000000	chicken
Klhl15	-0.05	1.00000000	chicken
Tmem59	-0.17	1.00000000	chicken
Trerf1	0.26	1.00000000	chicken
Zdhhc9	0.02	1.00000000	chicken
Trim24	0.07	1.00000000	chicken
Tgs1	-0.19	1.00000000	chicken
Tmem141	-0.10	1.00000000	chicken
Braf	-0.09	1.00000000	chicken
Pnrc2	0.11	1.00000000	chicken
Adat1	-0.32	1.00000000	chicken
Pmepa1	-0.30	1.00000000	chicken
Cdc25b	0.08	1.00000000	chicken
Smad5	-0.03	1.00000000	chicken
Ralgapa1	-0.11	1.00000000	chicken
Zcchc10	0.07	1.00000000	chicken
Tbc1d14	-0.17	1.00000000	chicken
Pcsk7	-0.07	1.00000000	chicken
Dip2c	0.39	1.00000000	chicken
Pld4	-0.10	1.00000000	chicken
Ciao1	0.09	1.00000000	chicken
Atp5j	-0.27	1.00000000	chicken
Gpr107	0.29	1.00000000	chicken
Csnk1e	0.06	1.00000000	chicken
Tmod3	0.27	1.00000000	chicken
Paf1	0.05	1.00000000	chicken
Hs6st1	-0.89	1.00000000	chicken
Pitpnb	-0.06	1.00000000	chicken
Setbp1	-0.71	1.00000000	chicken
Sirt2	0.05	1.00000000	chicken
Fn1	-0.10	1.00000000	chicken
Rock2	0.11	1.00000000	chicken
Esrrg	0.00	1.00000000	chicken
Cops4	-0.05	1.00000000	chicken
Gtpbp1	-0.13	1.00000000	chicken
Eri3	-0.09	1.00000000	chicken
Pdcd11	0.14	1.00000000	chicken
Brd7	-0.14	1.00000000	chicken
Brox	0.00	1.00000000	chicken
Acox1	0.08	1.00000000	chicken
Peli1	-0.11	1.00000000	chicken
Birc6	-0.18	1.00000000	chicken
Pik3cd	0.09	1.00000000	chicken
Ranbp10	-0.37	1.00000000	chicken
Steep1	0.16	1.00000000	chicken
Cct5	-0.19	1.00000000	chicken

Phf20	0.06	1.00000000	chicken
Oxr1	0.28	1.00000000	chicken
Tle1	-0.01	1.00000000	chicken
Rab35	-0.12	1.00000000	chicken
Senp5	-0.03	1.00000000	chicken
Ttbk1	0.18	1.00000000	chicken
1810013L24Rik	0.20	1.00000000	chicken
Oxct1	-0.46	1.00000000	chicken
Zfp638	-0.15	1.00000000	chicken
Omg	0.22	1.00000000	chicken
Eif4g1	-0.15	1.00000000	chicken
Arhgap26	-0.20	1.00000000	chicken
Tkt	0.22	1.00000000	chicken
Polr2k	-0.17	1.00000000	chicken
Apmap	-0.11	1.00000000	chicken
Smurf2	-0.26	1.00000000	chicken
Rpa1	-0.08	1.00000000	chicken
Dym	-0.19	1.00000000	chicken
Cebpg	-0.08	1.00000000	chicken
Sars	0.03	1.00000000	chicken
Rars	-0.14	1.00000000	chicken
Vps39	0.19	1.00000000	chicken
Slirp	0.05	1.00000000	chicken
Agps	0.10	1.00000000	chicken
Spg7	0.21	1.00000000	chicken
Cmss1	-0.28	1.00000000	chicken
Btbd10	-0.20	1.00000000	chicken
Thyn1	-0.03	1.00000000	chicken
Luc7l2	-0.02	1.00000000	chicken
Snap91	0.40	1.00000000	chicken
Epc2	-0.24	1.00000000	chicken
Get4	-0.11	1.00000000	chicken
Anxa5	0.18	1.00000000	chicken
Pter	0.08	1.00000000	chicken
Rab3gap2	0.15	1.00000000	chicken
Ccdc171	0.13	1.00000000	chicken
Kat7	0.00	1.00000000	chicken
Cab39l	-0.04	1.00000000	chicken
Fbxo22	0.26	1.00000000	chicken
Stat4	0.46	1.00000000	chicken
Ddx17	-0.35	1.00000000	chicken
Tle4	0.32	1.00000000	chicken
Ubn2	-0.12	1.00000000	chicken
Timmdc1	-0.17	1.00000000	chicken
Hadh	0.27	1.00000000	chicken
Ltn1	-0.08	1.00000000	chicken

Nup133	-0.13	1.00000000	chicken
Rgs14	-0.27	1.00000000	chicken
Snf8	-0.09	1.00000000	chicken
Fbxw2	-0.08	1.00000000	chicken
Ythdf2	-0.35	1.00000000	chicken
Atp9b	0.02	1.00000000	chicken
Usp45	0.17	1.00000000	chicken
Alas1	0.15	1.00000000	chicken
Ppcs	0.09	1.00000000	chicken
Zfp512b	-0.16	1.00000000	chicken
Szrd1	-0.35	1.00000000	chicken
Psma4	0.07	1.00000000	chicken
Ctr9	-0.34	1.00000000	chicken
Arid4b	0.09	1.00000000	chicken
Spg11	-0.25	1.00000000	chicken
Kpna6	-0.05	1.00000000	chicken
Armc8	-0.19	1.00000000	chicken
Gemin4	0.13	1.00000000	chicken
Elk4	0.15	1.00000000	chicken
Dtx2	-0.31	1.00000000	chicken
Nup107	-0.45	1.00000000	chicken
Fam126a	0.07	1.00000000	chicken
Bdp1	0.03	1.00000000	chicken
Capzb	-0.11	1.00000000	chicken
Sec16a	-0.04	1.00000000	chicken
Ermn	-0.17	1.00000000	chicken
Rsl24d1	-0.27	1.00000000	chicken
Vps33b	-0.09	1.00000000	chicken
Vrk2	-0.10	1.00000000	chicken
Tut7	-0.09	1.00000000	chicken
Arntl	-0.06	1.00000000	chicken
Tle3	-0.10	1.00000000	chicken
Snapc4	-0.11	1.00000000	chicken
Dpagt1	-0.11	1.00000000	chicken
Arl8a	-0.07	1.00000000	chicken
Cct6b	0.00	1.00000000	chicken
Pdzd8	0.18	1.00000000	chicken
Ipo7	-0.12	1.00000000	chicken
Calm2	-0.30	1.00000000	chicken
Pdxk	-0.19	1.00000000	chicken
Gemin8	0.22	1.00000000	chicken
Ndufaf2	0.20	1.00000000	chicken
Nip7	-0.11	1.00000000	chicken
Aven	0.37	1.00000000	chicken
Stk10	0.21	1.00000000	chicken
Polr2f	-0.07	1.00000000	chicken

Pag1	0.71	1.00000000	chicken
Ube2g1	0.42	1.00000000	chicken
Fbxl22	-0.25	1.00000000	chicken
Pcmt1	-0.16	1.00000000	chicken
Srgap1	0.42	1.00000000	chicken
Arpc3	-0.01	1.00000000	chicken
Crot	0.36	1.00000000	chicken
Sp4	-0.56	1.00000000	chicken
Lamc1	0.04	1.00000000	chicken
Ist1	-0.05	1.00000000	chicken
Fam107b	0.19	1.00000000	chicken
Ythdc2	-0.33	1.00000000	chicken
Epc1	0.07	1.00000000	chicken
Pten	-0.12	1.00000000	chicken
Sgsm2	-0.01	1.00000000	chicken
Cdkn1b	-0.11	1.00000000	chicken
Ddx1	-0.24	1.00000000	chicken
Prkd1	-0.48	1.00000000	chicken
Kars	-0.33	1.00000000	chicken
Sharpin	-0.14	1.00000000	chicken
Exoc7	-0.05	1.00000000	chicken
Ipo8	0.12	1.00000000	chicken
Lin52	0.05	1.00000000	chicken
Dgcr6	0.04	1.00000000	chicken
Ogfod3	0.16	1.00000000	chicken
Baiap2	0.05	1.00000000	chicken
Strip1	0.11	1.00000000	chicken
Lmo2	-0.07	1.00000000	chicken
Golga7	-0.07	1.00000000	chicken
Tmem167	0.02	1.00000000	chicken
Tanc1	-0.02	1.00000000	chicken
Tfg	-0.21	1.00000000	chicken
Cry1	-0.74	1.00000000	chicken
Paxbp1	-0.48	1.00000000	chicken
Gnb4	-0.15	1.00000000	chicken
Pepd	-0.08	1.00000000	chicken
Cwf19l2	-0.05	1.00000000	chicken
Tpd52	0.05	1.00000000	chicken
Atp10d	0.03	1.00000000	chicken
Slc29a1	0.03	1.00000000	chicken
Iqgap1	0.13	1.00000000	chicken
Bclaf1	-0.15	1.00000000	chicken
Xpot	-0.15	1.00000000	chicken
Grb10	0.43	1.00000000	chicken
Tcerg1	-0.23	1.00000000	chicken
Wbp2nl	-0.20	1.00000000	chicken

Smad3	0.41	1.00000000	chicken
Rere	-0.03	1.00000000	chicken
Icosl	0.08	1.00000000	chicken
Trpc4ap	-0.20	1.00000000	chicken
Ppm1b	-0.34	1.00000000	chicken
Vcp	-0.06	1.00000000	chicken
Tmtc4	-0.22	1.00000000	chicken
Tbc1d4	-0.10	1.00000000	chicken
Agap1	0.89	1.00000000	chicken
Umad1	0.54	1.00000000	chicken
Casp3	-0.15	1.00000000	chicken
Vps4b	-0.25	1.00000000	chicken
Eed	0.01	1.00000000	chicken
Cubn	0.15	1.00000000	chicken
Rnf185	0.00	1.00000000	chicken
Ptpn7	0.04	1.00000000	chicken
Tnrc6b	0.24	1.00000000	chicken
Ahdc1	-0.01	1.00000000	chicken
Ppif	-0.10	1.00000000	chicken
Erc1	-0.38	1.00000000	chicken
Eif2ak3	-0.51	1.00000000	chicken
Fbxo25	-0.31	1.00000000	chicken
Chmp7	-0.17	1.00000000	chicken
Phf14	0.12	1.00000000	chicken
Rprd1a	0.00	1.00000000	chicken
Fkbp3	-0.14	1.00000000	chicken
Ghitm	-0.11	1.00000000	chicken
Laptm4a	-0.17	1.00000000	chicken
Runx1	0.05	1.00000000	chicken
Cox5b-ps	-0.37	1.00000000	chicken
Srsf5	-0.32	1.00000000	chicken
Ube2q2	-0.13	1.00000000	chicken
Klhdc1	0.01	1.00000000	chicken
Noc2l	0.37	1.00000000	chicken
Hadhb	-0.10	1.00000000	chicken
Nfatc2	-0.12	1.00000000	chicken
Rab3a	0.10	1.00000000	chicken
Sap30l	0.06	1.00000000	chicken
Klf13	-0.39	1.00000000	chicken
Fanci	0.01	1.00000000	chicken
Erc2	-0.08	1.00000000	chicken
Arid5a	-0.25	1.00000000	chicken
Osgin2	0.00	1.00000000	chicken
Irf7	0.01	1.00000000	chicken
Nipa2	0.40	1.00000000	chicken
Phaf1	-0.36	1.00000000	chicken

Otub1	0.29	1.00000000	chicken
Xpo1	0.23	1.00000000	chicken
1110004F10Rik	0.19	1.00000000	chicken
Ppil4	-0.07	1.00000000	chicken
Cap1	-0.13	1.00000000	chicken
Rad54l2	-0.01	1.00000000	chicken
Cytip	-0.26	1.00000000	chicken
Tpra1	-0.12	1.00000000	chicken
Pank3	0.11	1.00000000	chicken
Pcgf5	-0.06	1.00000000	chicken
Txndc11	0.03	1.00000000	chicken
Ankhd1	0.17	1.00000000	chicken
Gpr18	-0.15	1.00000000	chicken
Pcgf3	-0.10	1.00000000	chicken
Szt2	0.06	1.00000000	chicken
Abcf2	-0.03	1.00000000	chicken
Ssr2	-0.35	1.00000000	chicken
Cdc42	0.09	1.00000000	chicken
Akr1a1	0.10	1.00000000	chicken
Psme3ip1	-0.21	1.00000000	chicken
Gnas	0.01	1.00000000	chicken
Cept1	-0.01	1.00000000	chicken
Pik3c2a	-0.65	1.00000000	chicken
Srm	-0.04	1.00000000	chicken
Hdac11	0.29	1.00000000	chicken
Dapk2	0.09	1.00000000	chicken
Ndufa8	0.02	1.00000000	chicken
Trappc8	0.15	1.00000000	chicken
Vps13d	0.01	1.00000000	chicken
Tmem183a	-0.15	1.00000000	chicken
Orai2	-0.33	1.00000000	chicken
Sh3kbp1	-0.26	1.00000000	chicken
Wdr7	0.10	1.00000000	chicken
Nr2c1	0.09	1.00000000	chicken
Dcaf7	0.12	1.00000000	chicken
Alg6	-0.48	1.00000000	chicken
Rufy3	-0.25	1.00000000	chicken
Psmc5	-0.05	1.00000000	chicken
Ginm1	0.26	1.00000000	chicken
Ammecr1	0.12	1.00000000	chicken
Klf12	0.08	1.00000000	chicken
Rnf13	-0.09	1.00000000	chicken
Prmt9	-0.49	1.00000000	chicken
Zfp654	-0.01	1.00000000	chicken
Gatd3a	-0.03	1.00000000	chicken
Wdr81	0.07	1.00000000	chicken

Ndufaf1	0.05	1.00000000	chicken
Atxn3	0.01	1.00000000	chicken
Desi1	-0.08	1.00000000	chicken
Hibadh	0.04	1.00000000	chicken
Secisbp2	-0.11	1.00000000	chicken
Spats2	0.04	1.00000000	chicken
Ndufb3	-0.15	1.00000000	chicken
Impa2	-0.09	1.00000000	chicken
Gns	0.07	1.00000000	chicken
Suds3	0.47	1.00000000	chicken
Ormdl2	-0.03	1.00000000	chicken
Hace1	-0.06	1.00000000	chicken
Txlng	-0.09	1.00000000	chicken
Zrsr1	-0.11	1.00000000	chicken
Nrp1	-0.33	1.00000000	chicken
Nipal3	-0.15	1.00000000	chicken
Wdr44	-0.14	1.00000000	chicken
Utp3	-0.17	1.00000000	chicken
Atp5a1	-0.21	1.00000000	chicken
Hspa4	-0.24	1.00000000	chicken
Fbxo8	0.21	1.00000000	chicken
Rbl1	-0.19	1.00000000	chicken
Serp1	-0.11	1.00000000	chicken
Ube2h	0.08	1.00000000	chicken
Trappc12	-0.14	1.00000000	chicken
Ltf	-0.02	1.00000000	chicken
Eaf1	0.07	1.00000000	chicken
Nek7	0.00	1.00000000	chicken
Ice2	-0.02	1.00000000	chicken
Fbxo42	-0.03	1.00000000	chicken
Tbc1d25	-0.04	1.00000000	chicken
Rlf	-0.08	1.00000000	chicken
Tcf20	0.39	1.00000000	chicken
Nab1	-0.07	1.00000000	chicken
Gpatch4	-0.12	1.00000000	chicken
Stxbp1	-0.17	1.00000000	chicken
Atp6v1h	0.03	1.00000000	chicken
Efcab1	0.11	1.00000000	chicken
Rc3h2	0.01	1.00000000	chicken
Uqcrq	0.16	1.00000000	chicken
Zcchc14	-0.28	1.00000000	chicken
Rnf217	0.47	1.00000000	chicken
Cct8	-0.08	1.00000000	chicken
Creb1	-0.18	1.00000000	chicken
Rpf1	-0.04	1.00000000	chicken
Tmbim6	-0.10	1.00000000	chicken

Snap23	0.20	1.00000000	chicken
Tfip11	-0.02	1.00000000	chicken
Yipf3	-0.25	1.00000000	chicken
Rab6a	-0.02	1.00000000	chicken
Snx12	-0.16	1.00000000	chicken
Akap8	0.03	1.00000000	chicken
Stx7	-0.15	1.00000000	chicken
Fbln5	0.06	1.00000000	chicken
Tecpr2	0.10	1.00000000	chicken
Atg7	-0.03	1.00000000	chicken
Slc38a2	0.17	1.00000000	chicken
Septin9	-0.01	1.00000000	chicken
Pgap1	0.01	1.00000000	chicken
Dennd5a	-0.18	1.00000000	chicken
Enah	-0.08	1.00000000	chicken
Ncf2	-0.20	1.00000000	chicken
Bcl2	-0.13	1.00000000	chicken
Nop56	-0.38	1.00000000	chicken
Ccdc59	-0.06	1.00000000	chicken
Vps37a	-0.15	1.00000000	chicken
Lrp1	0.02	1.00000000	chicken
Tmtc3	-0.06	1.00000000	chicken
Bub3	-0.05	1.00000000	chicken
Serinc3	0.03	1.00000000	chicken
Sft2d1	0.00	1.00000000	chicken
Ndufa12	0.15	1.00000000	chicken
Ercc8	0.07	1.00000000	chicken
Ifngr1	-0.15	1.00000000	chicken
Lonp1	0.13	1.00000000	chicken
Vps26a	-0.60	1.00000000	chicken
Cc2d1b	-0.35	1.00000000	chicken
Flot1	-0.28	1.00000000	chicken
Dcp2	0.16	1.00000000	chicken
Trpc1	-0.19	1.00000000	chicken
Atf4	0.07	1.00000000	chicken
Nde1	-0.07	1.00000000	chicken
Efhd2	-0.04	1.00000000	chicken
Gtf3c3	0.08	1.00000000	chicken
Derl2	0.01	1.00000000	chicken
Pxdc1	-0.05	1.00000000	chicken
Rnf4	-0.05	1.00000000	chicken
Snx29	-0.21	1.00000000	chicken
Septin14	-0.01	1.00000000	chicken
Trps1	-0.63	1.00000000	chicken
Ttc4	-0.03	1.00000000	chicken
Dazap1	0.16	1.00000000	chicken

Fxr1	0.01	1.00000000	chicken
Dglucy	-0.04	1.00000000	chicken
Tmpo	0.39	1.00000000	chicken
Atp6v1b2	-0.03	1.00000000	chicken
G3bp2	0.00	1.00000000	chicken
Commd8	-0.10	1.00000000	chicken
Lbh	-0.40	1.00000000	chicken
Aimp1	-0.33	1.00000000	chicken
Aup1	-0.11	1.00000000	chicken
Cul5	-0.12	1.00000000	chicken
Zc3h6	0.16	1.00000000	chicken
Wdr70	0.50	1.00000000	chicken
Tasp1	0.13	1.00000000	chicken
Dnaja4	-0.03	1.00000000	chicken
Cops2	-0.05	1.00000000	chicken
Calm1	-0.03	1.00000000	chicken
Eif3b	0.02	1.00000000	chicken
Xrn2	-0.19	1.00000000	chicken
Exoc2	0.14	1.00000000	chicken
Nfkb2	0.03	1.00000000	chicken
Igsf6	0.04	1.00000000	chicken
Poldip2	-0.07	1.00000000	chicken
Kdm5b	-0.09	1.00000000	chicken
Ddx20	-0.17	1.00000000	chicken
Csl	-0.12	1.00000000	chicken
Deaf1	0.03	1.00000000	chicken
Lrrc28	0.26	1.00000000	chicken
Rsl1d1	0.18	1.00000000	chicken
Dus1l	0.04	1.00000000	chicken
Gne	-0.34	1.00000000	chicken
Ddx6	-0.11	1.00000000	chicken
Prdm15	0.01	1.00000000	chicken
Stard8	-0.19	1.00000000	chicken
Ncoa4	-0.30	1.00000000	chicken
Zmpste24	0.04	1.00000000	chicken
Phf3	0.03	1.00000000	chicken
Sh3pxd2b	-0.77	1.00000000	chicken
Nrp2	-0.03	1.00000000	chicken
Pmm2	-0.28	1.00000000	chicken
Txn1	0.07	1.00000000	chicken
Rbx1	0.06	1.00000000	chicken
Dnajc3	-0.15	1.00000000	chicken
Ube3c	-0.36	1.00000000	chicken
Prkag2	-0.33	1.00000000	chicken
C2cd2l	-0.23	1.00000000	chicken
Jak1	0.14	1.00000000	chicken

Tmem135	0.20	1.00000000	chicken
Abcc4	0.61	1.00000000	chicken
Cited4	0.16	1.00000000	chicken
Tspan14	0.00	1.00000000	chicken
Supt20	-0.09	1.00000000	chicken
Zfand4	0.05	1.00000000	chicken
Tirap	0.27	1.00000000	chicken
Tfdp1	0.22	1.00000000	chicken
Vti1b	-0.11	1.00000000	chicken
Poll	0.17	1.00000000	chicken
Tmem115	-0.10	1.00000000	chicken
Prmt7	-0.16	1.00000000	chicken
Rhof	-0.10	1.00000000	chicken
Card9	-0.05	1.00000000	chicken
Tbc1d22a	0.14	1.00000000	chicken
Emc1	-0.17	1.00000000	chicken
Chmp4b	-0.22	1.00000000	chicken
Ap1g1	0.07	1.00000000	chicken
Ccny	0.43	1.00000000	chicken
Ubap2	0.08	1.00000000	chicken
Dido1	-0.06	1.00000000	chicken
Xpo5	-0.21	1.00000000	chicken
Elf2	0.09	1.00000000	chicken
Smc3	0.80	1.00000000	chicken
Stt3b	-0.27	1.00000000	chicken
Pank4	-0.25	1.00000000	chicken
Pkn2	0.16	1.00000000	chicken
Bhlhe40	0.51	1.00000000	chicken
Wdr61	-0.17	1.00000000	chicken
Tex2	0.04	1.00000000	chicken
Fam104a	0.02	1.00000000	chicken
Nsd3	-0.33	1.00000000	chicken
Tcof1	-0.02	1.00000000	chicken
Cep170	-0.12	1.00000000	chicken
Ssrp1	0.08	1.00000000	chicken
Ppp6c	0.01	1.00000000	chicken
Alkbh5	-0.08	1.00000000	chicken
Dusp23	-0.02	1.00000000	chicken
Ddx31	-0.16	1.00000000	chicken
Rb1cc1	-0.34	1.00000000	chicken
Ccdc6	0.02	1.00000000	chicken
Hmbox1	-0.44	1.00000000	chicken
Eef1e1	0.11	1.00000000	chicken
Cnot11	-0.13	1.00000000	chicken
Foxp1	-0.19	1.00000000	chicken
Dap	-0.12	1.00000000	chicken

Dbr1	-0.07	1.00000000	chicken
Apoo	0.11	1.00000000	chicken
Tbrg4	0.01	1.00000000	chicken
Srebf2	-0.11	1.00000000	chicken
Slc16a10	-0.35	1.00000000	chicken
Nr3c2	0.08	1.00000000	chicken
Lmbrd2	0.00	1.00000000	chicken
Zfp365	-0.21	1.00000000	chicken
Plek	-0.06	1.00000000	chicken
Cped1	-0.16	1.00000000	chicken
Tor4a	-0.02	1.00000000	chicken
Niban1	0.05	1.00000000	chicken
Tgif1	-0.03	1.00000000	chicken
Ppp2r5a	-0.43	1.00000000	chicken
Fchsd2	0.29	1.00000000	chicken
Ddx18	0.06	1.00000000	chicken
Wdr11	0.27	1.00000000	chicken
Rabep1	0.17	1.00000000	chicken
Slc35b1	0.28	1.00000000	chicken
Pcna	0.10	1.00000000	chicken
Homer1	0.64	1.00000000	chicken
Tmem18	-0.05	1.00000000	chicken
Cog4	-0.12	1.00000000	chicken
Hars	-0.10	1.00000000	chicken
Spdl1	-0.24	1.00000000	chicken
Slc25a1	-0.15	1.00000000	chicken
Cdk9	-0.14	1.00000000	chicken
Akap10	-0.09	1.00000000	chicken
Plod1	-0.10	1.00000000	chicken
Pafah1b2	0.06	1.00000000	chicken
Dcun1d1	-0.39	1.00000000	chicken
D6Wsu163e	0.60	1.00000000	chicken
Sumo1	-0.07	1.00000000	chicken
Tmem263	0.01	1.00000000	chicken
Rragd	0.02	1.00000000	chicken
E2f4	0.08	1.00000000	chicken
Vapb	-0.39	1.00000000	chicken
Hook1	-0.22	1.00000000	chicken
Dck	0.28	1.00000000	chicken
Rchy1	-0.15	1.00000000	chicken
Commd5	-0.02	1.00000000	chicken
Nedd4	-0.04	1.00000000	chicken
Ccdc174	-0.13	1.00000000	chicken
Fuca1	-0.04	1.00000000	chicken
Nfyb	-0.23	1.00000000	chicken
Dnajb6	0.01	1.00000000	chicken

Igf2r	0.14	1.00000000	chicken
2210408I21Rik	-0.24	1.00000000	chicken
Rexo1	0.15	1.00000000	chicken
Zbtb38	-0.08	1.00000000	chicken
Asap1	-0.16	1.00000000	chicken
Ahcyl1	-0.13	1.00000000	chicken
Uhmk1	-0.31	1.00000000	chicken
Ergic2	0.08	1.00000000	chicken
Letm1	0.18	1.00000000	chicken
Ovch2	0.34	1.00000000	chicken
Psme4	-0.59	1.00000000	chicken
Zfp644	0.11	1.00000000	chicken
Clk1	-0.24	1.00000000	chicken
Gtf2e1	0.17	1.00000000	chicken
Unc45a	-0.12	1.00000000	chicken
Rab1a	-0.02	1.00000000	chicken
Pspc1	0.11	1.00000000	chicken
Rnf126	0.13	1.00000000	chicken
Abracl	-0.07	1.00000000	chicken
Ankrd11	-0.02	1.00000000	chicken
Prkrip1	-0.14	1.00000000	chicken
Sdha	0.08	1.00000000	chicken
Satb1	0.43	1.00000000	chicken
Rbm45	-0.23	1.00000000	chicken
Ambra1	0.00	1.00000000	chicken
Fam135a	0.17	1.00000000	chicken
Ehmt1	-0.33	1.00000000	chicken
Ppp2r2a	0.13	1.00000000	chicken
Polr1e	-0.05	1.00000000	chicken
Smarca1	-0.04	1.00000000	chicken
Trak2	-0.21	1.00000000	chicken
Usp1	-0.18	1.00000000	chicken
Lyrm4	-0.25	1.00000000	chicken
Tspan3	0.05	1.00000000	chicken
Cask	0.08	1.00000000	chicken
Abca1	-0.35	1.00000000	chicken
Ndufa13	-0.13	1.00000000	chicken
Lrrc59	0.11	1.00000000	chicken
Spz1	-0.19	1.00000000	chicken
Eif1b	-0.19	1.00000000	chicken
Gcnt4	0.20	1.00000000	chicken
Cdk8	-0.50	1.00000000	chicken
Cst7	-0.05	1.00000000	chicken
Fdx1	-0.10	1.00000000	chicken
Pign	-0.06	1.00000000	chicken
Garre1	-0.12	1.00000000	chicken

Gan	-0.15	1.00000000	chicken
Vps35	-0.04	1.00000000	chicken
Bri3bp	-0.10	1.00000000	chicken
Ctnnb1	0.06	1.00000000	chicken
Glb1	0.06	1.00000000	chicken
Zfyve21	0.03	1.00000000	chicken
Fgf2	0.08	1.00000000	chicken
Wdr74	0.07	1.00000000	chicken
Vps18	-0.14	1.00000000	chicken
Ric1	-0.22	1.00000000	chicken
Pdcd10	0.04	1.00000000	chicken
Creb3l2	0.11	1.00000000	chicken
Slc9a6	-0.18	1.00000000	chicken
Fbxo32	-0.12	1.00000000	chicken
Ip6k1	-0.28	1.00000000	chicken
Ptpn9	-0.07	1.00000000	chicken
Snrnp35	-0.37	1.00000000	chicken
Wdr37	0.08	1.00000000	chicken
Fut8	0.25	1.00000000	chicken
Abtb1	-0.05	1.00000000	chicken
Nfs1	0.01	1.00000000	chicken
Sdf2l1	-0.13	1.00000000	chicken
Cblb	0.17	1.00000000	chicken
Pdpk1	0.07	1.00000000	chicken
Serinc1	0.13	1.00000000	chicken
Glg1	-0.16	1.00000000	chicken
Itsn1	0.03	1.00000000	chicken
3110082I17Rik	-0.01	1.00000000	chicken
Psmd5	0.18	1.00000000	chicken
Ipo5	-0.03	1.00000000	chicken
Ube2j1	-0.16	1.00000000	chicken
Adnp	-0.04	1.00000000	chicken
Vps11	-0.13	1.00000000	chicken
1110059G10Rik	-0.05	1.00000000	chicken
Dctn4	-0.04	1.00000000	chicken
Clip1	0.15	1.00000000	chicken
Wipi2	0.00	1.00000000	chicken
Pgs1	-0.09	1.00000000	chicken
Ctnnbl1	-0.04	1.00000000	chicken
Nr3c1	-0.22	1.00000000	chicken
Crebrf	-0.13	1.00000000	chicken
Hlcs	0.22	1.00000000	chicken
Rnf111	0.12	1.00000000	chicken
Naca	0.01	1.00000000	chicken
Ciapin1	0.15	1.00000000	chicken
G3bp1	-0.20	1.00000000	chicken

Auts2	0.51	1.00000000	chicken
Slc37a3	0.08	1.00000000	chicken
Epcam	-0.13	1.00000000	chicken
Iqsec1	0.06	1.00000000	chicken
Reps2	-0.06	1.00000000	chicken
Sms	-0.04	1.00000000	chicken
Rala	0.06	1.00000000	chicken
Prrc1	-0.01	1.00000000	chicken
Nup85	0.03	1.00000000	chicken
Usp36	-0.06	1.00000000	chicken
Zfc3h1	0.08	1.00000000	chicken
Oaz1	0.11	1.00000000	chicken
Pds5a	-0.43	1.00000000	chicken
Fos	0.40	1.00000000	chicken
Trp53inp1	-0.05	1.00000000	chicken
Drg2	-0.08	1.00000000	chicken
Pnpla7	0.05	1.00000000	chicken
Zbtb48	-0.04	1.00000000	chicken
Clptm1	-0.03	1.00000000	chicken
Sec61g	0.01	1.00000000	chicken
Sgk3	-0.04	1.00000000	chicken
Sod1	0.16	1.00000000	chicken
Sfmbt2	0.20	1.00000000	chicken
Ncf4	0.12	1.00000000	chicken
Caprin1	0.02	1.00000000	chicken
Eif2b4	-0.01	1.00000000	chicken
Dag1	-0.14	1.00000000	chicken
Ccdc88a	0.26	1.00000000	chicken
S100a11	-0.04	1.00000000	chicken
Ntmt1	0.16	1.00000000	chicken
Atp5h	0.07	1.00000000	chicken
Phpt1	-0.04	1.00000000	chicken
Hs6st2	-0.26	1.00000000	chicken
Nob1	-0.09	1.00000000	chicken
Chd5	-0.13	1.00000000	chicken
Slc6a1	-0.44	1.00000000	chicken
Slc9a7	-0.26	1.00000000	chicken
Prpf8	-0.07	1.00000000	chicken
Smc4	-0.25	1.00000000	chicken
App	-0.32	1.00000000	chicken
Sub1	-0.03	1.00000000	chicken
Ugp2	0.00	1.00000000	chicken
Nus1	-0.03	1.00000000	chicken
Cep350	-0.24	1.00000000	chicken
Serpinb8	-0.12	1.00000000	chicken
Scoc	0.03	1.00000000	chicken

Tmem208	0.08	1.00000000	chicken
Rab2a	0.02	1.00000000	chicken
Clasp2	-0.27	1.00000000	chicken
Zc3h12d	0.24	1.00000000	chicken
Cnot10	-0.22	1.00000000	chicken
Tm9sf2	-0.25	1.00000000	chicken
Irak2	0.07	1.00000000	chicken
Kif1b	0.00	1.00000000	chicken
Large1	-0.02	1.00000000	chicken
LTO1	0.02	1.00000000	chicken
Ilvbl	-0.08	1.00000000	chicken
Brf1	-0.02	1.00000000	chicken
Bin2	-0.15	1.00000000	chicken
Elk3	0.00	1.00000000	chicken
Rsrc1	-0.09	1.00000000	chicken
Atp1b3	-0.19	1.00000000	chicken
Pnpt1	-0.32	1.00000000	chicken
Inpp4a	-0.62	1.00000000	chicken
Cerk	-0.11	1.00000000	chicken
Lat2	0.00	1.00000000	chicken
Nav1	-0.01	1.00000000	chicken
Fubp3	-0.14	1.00000000	chicken
Slc38a6	0.16	1.00000000	chicken
Usf3	-0.02	1.00000000	chicken
Coa5	-0.04	1.00000000	chicken
Vps16	0.01	1.00000000	chicken
Sass6	0.40	1.00000000	chicken
Csnk2a2	0.15	1.00000000	chicken
Dcaf5	0.02	1.00000000	chicken
Acvr1	-0.06	1.00000000	chicken
Pbx1	-0.14	1.00000000	chicken
Tmed7	-0.12	1.00000000	chicken
Rbmxl1	-0.34	1.00000000	chicken
Rab27a	0.07	1.00000000	chicken
Psmd4	0.04	1.00000000	chicken
Tmem241	-0.08	1.00000000	chicken
Lsm14b	-0.08	1.00000000	chicken
Cct4	-0.03	1.00000000	chicken
Trp53bp2	0.03	1.00000000	chicken
Slco4c1	-0.01	1.00000000	chicken
Vezf1	-0.06	1.00000000	chicken
Ints3	-0.10	1.00000000	chicken
Aak1	-0.05	1.00000000	chicken
Eif2ak4	-0.23	1.00000000	chicken
Elovl5	-0.08	1.00000000	chicken
Tdpoz8	-0.09	1.00000000	chicken

Ogt	-0.54	1.00000000	chicken
Stx2	-0.03	1.00000000	chicken
Upf3b	0.23	1.00000000	chicken
Eml4	-0.01	1.00000000	chicken
Gcc2	0.15	1.00000000	chicken
Bcar3	-0.16	1.00000000	chicken
Chmp2b	-0.07	1.00000000	chicken
Lars2	-0.07	1.00000000	chicken
Ppid	0.08	1.00000000	chicken
Cplane2	-0.18	1.00000000	chicken
Pm20d1	0.03	1.00000000	chicken
Prpf4b	-0.03	1.00000000	chicken
Bcas3	0.05	1.00000000	chicken
Cnbp	-0.10	1.00000000	chicken
C2cd5	-0.08	1.00000000	chicken
Anxa1	0.17	1.00000000	chicken
Cluh	-0.04	1.00000000	chicken
Zmynd11	0.08	1.00000000	chicken
Rprd2	-0.06	1.00000000	chicken
Ngly1	-0.20	1.00000000	chicken
Bahd1	-0.18	1.00000000	chicken
Laptm5	0.41	1.00000000	chicken
Ctps	0.08	1.00000000	chicken
Nf1	0.08	1.00000000	chicken
Blmh	0.04	1.00000000	chicken
Atad3a	0.09	1.00000000	chicken
Gpatch2l	0.01	1.00000000	chicken
Sgsh	-0.06	1.00000000	chicken
Exoc1	-0.20	1.00000000	chicken
Ube2g2	-0.19	1.00000000	chicken
Spg21	0.25	1.00000000	chicken
Rexo2	0.33	1.00000000	chicken
Arhgef3	0.16	1.00000000	chicken
Etf1	-0.02	1.00000000	chicken
Usp8	0.01	1.00000000	chicken
Cep112	-0.08	1.00000000	chicken
Nutf2	-0.05	1.00000000	chicken
Rpa2	0.07	1.00000000	chicken
Ttc13	0.13	1.00000000	chicken
Evl	0.26	1.00000000	chicken
Gsr	-0.10	1.00000000	chicken
Cops7a	-0.13	1.00000000	chicken
Chchd2	-0.17	1.00000000	chicken
Pnpla2	0.06	1.00000000	chicken
Rad23a	-0.19	1.00000000	chicken
Brix1	-0.12	1.00000000	chicken

Rnf130	0.22	1.00000000	chicken
Nxpe3	-0.14	1.00000000	chicken
Dennd3	0.11	1.00000000	chicken
Smc6	-0.18	1.00000000	chicken
Plxnd1	-0.12	1.00000000	chicken
Stau2	-0.04	1.00000000	chicken
B3gnt2	0.21	1.00000000	chicken
Psip1	-0.54	1.00000000	chicken
Slc2a13	-0.16	1.00000000	chicken
Nfe2l1	0.00	1.00000000	chicken
Cxcr4	0.14	1.00000000	chicken
Tasor	-0.07	1.00000000	chicken
Zfp407	-0.24	1.00000000	chicken
Psmg2	-0.02	1.00000000	chicken
Ywhaq	0.06	1.00000000	chicken
Xpa	-0.02	1.00000000	chicken
Sec63	-0.29	1.00000000	chicken
Kdm3b	-0.05	1.00000000	chicken
Fdft1	0.12	1.00000000	chicken
Txnrd3	-0.27	1.00000000	chicken
Ly75	0.27	1.00000000	chicken
Chm	-0.27	1.00000000	chicken
Arhgef12	0.19	1.00000000	chicken
Net1	0.10	1.00000000	chicken
Agpat4	-0.14	1.00000000	chicken
Nfrkb	0.06	1.00000000	chicken
Rab3il1	0.16	1.00000000	chicken
Golga1	-0.07	1.00000000	chicken
Dis3	-0.08	1.00000000	chicken
Slc45a4	0.03	1.00000000	chicken
Tmem161b	-0.03	1.00000000	chicken
Scube2	0.05	1.00000000	chicken
Rbbp5	0.08	1.00000000	chicken
Ranbp9	0.22	1.00000000	chicken
Togaram1	0.06	1.00000000	chicken
Aco1	0.18	1.00000000	chicken
Srpk2	-0.01	1.00000000	chicken
Rbm26	-0.15	1.00000000	chicken
Kdm5a	-0.12	1.00000000	chicken
Pdia3	0.17	1.00000000	chicken
Rbbp4	-0.11	1.00000000	chicken
Actr3	-0.02	1.00000000	chicken
Bag1	-0.04	1.00000000	chicken
Cwc25	-0.05	1.00000000	chicken
Prr14l	-0.39	1.00000000	chicken
Ctbp1	0.16	1.00000000	chicken

Zc3h18	-0.08	1.00000000	chicken
Bach2	-0.37	1.00000000	chicken
Snta1	-0.14	1.00000000	chicken
Gorab	0.00	1.00000000	chicken
Abhd17c	0.00	1.00000000	chicken
Ino80	-0.27	1.00000000	chicken
Cyth4	0.08	1.00000000	chicken
Tob1	0.08	1.00000000	chicken
Nck2	-0.17	1.00000000	chicken
Cul4a	-0.07	1.00000000	chicken
Srp68	-0.01	1.00000000	chicken
Eps15l1	-0.02	1.00000000	chicken
Limk1	-0.07	1.00000000	chicken
Cab39	-0.25	1.00000000	chicken
Snrpd3	0.08	1.00000000	chicken
Crnkl1	-0.08	1.00000000	chicken
Ino80d	-0.16	1.00000000	chicken
Dusp7	0.30	1.00000000	chicken
Tvp23b	0.23	1.00000000	chicken
Usp20	0.10	1.00000000	chicken
Pnn	-0.33	1.00000000	chicken
Ireb2	-0.06	1.00000000	chicken
Dhx15	-0.26	1.00000000	chicken
Per3	0.35	1.00000000	chicken
Nrf1	-0.12	1.00000000	chicken
Tmem79	-0.05	1.00000000	chicken
Ank	0.47	1.00000000	chicken
Bud31	-0.08	1.00000000	chicken
Ints6	0.21	1.00000000	chicken
Smdt1	-0.10	1.00000000	chicken
Rgs19	-0.32	1.00000000	chicken
Tspan4	-0.22	1.00000000	chicken
Chchd4	0.21	1.00000000	chicken
Taf3	-0.28	1.00000000	chicken
Golm2	-0.12	1.00000000	chicken
Ghdc	0.04	1.00000000	chicken
Sync	0.14	1.00000000	chicken
Smarca2	-0.36	1.00000000	chicken
Ube2l3	-0.01	1.00000000	chicken
Rnaset2b	0.09	1.00000000	chicken
Uqcr10	-0.16	1.00000000	chicken
Vps41	-0.26	1.00000000	chicken
Fzd6	0.09	1.00000000	chicken
Xcr1	0.17	1.00000000	chicken
Cdc73	-0.22	1.00000000	chicken
Ppp1cc	-0.25	1.00000000	chicken

Hsp90b1	0.29	1.00000000	chicken
Dlgap4	-0.08	1.00000000	chicken
Ppp3ca	0.33	1.00000000	chicken
Dennd6a	-0.49	1.00000000	chicken
Ikbke	-0.11	1.00000000	chicken
Stt3a	0.17	1.00000000	chicken
Ipmk	-0.27	1.00000000	chicken
Gtf2a2	-0.15	1.00000000	chicken
Adrm1	-0.12	1.00000000	chicken
Hypk	0.11	1.00000000	chicken
Rbm31y	-0.15	1.00000000	chicken
Hbp1	0.14	1.00000000	chicken
Ttyh3	-0.27	1.00000000	chicken
Sp1	-0.16	1.00000000	chicken
Eif4ebp2	0.07	1.00000000	chicken
Ddx23	-0.09	1.00000000	chicken
Spryd7	0.00	1.00000000	chicken
Scamp5	0.16	1.00000000	chicken
Dynlt1a	-0.18	1.00000000	chicken
Arf6	-0.10	1.00000000	chicken
Casp8ap2	-0.05	1.00000000	chicken
Qsox1	0.04	1.00000000	chicken
Srsf10	-0.18	1.00000000	chicken
Dok1	-0.01	1.00000000	chicken
Thoc5	-0.04	1.00000000	chicken
Ap3s1	-0.08	1.00000000	chicken
Tnfrsf11a	0.28	1.00000000	chicken
Csgalnact2	-0.05	1.00000000	chicken
Pan3	-0.03	1.00000000	chicken
Jdp2	0.19	1.00000000	chicken
Pcf11	0.14	1.00000000	chicken
Ube2r2	0.07	1.00000000	chicken
Rin3	0.27	1.00000000	chicken
Sod2	0.03	1.00000000	chicken
Dnaja2	-0.20	1.00000000	chicken
Grk4	-0.41	1.00000000	chicken
Grk2	-0.12	1.00000000	chicken
Lhfp	0.20	1.00000000	chicken
Gtf3a	0.24	1.00000000	chicken
Iws1	0.10	1.00000000	chicken
Erap1	0.17	1.00000000	chicken
Twf2	0.07	1.00000000	chicken
Specc1l	0.20	1.00000000	chicken
Crygn	0.10	1.00000000	chicken
Pnrc1	0.22	1.00000000	chicken
Pcm1	-0.06	1.00000000	chicken

Zfp704	0.11	1.00000000	chicken
Vash1	-0.02	1.00000000	chicken
Rufy1	-0.05	1.00000000	chicken
Brd8	-0.01	1.00000000	chicken
Pes1	0.08	1.00000000	chicken
F5	0.76	1.00000000	chicken
Ppp3cb	0.06	1.00000000	chicken
Tnnt3	-0.03	1.00000000	chicken
Zfp609	-0.12	1.00000000	chicken
Qars	0.08	1.00000000	chicken
Wdr20	-0.34	1.00000000	chicken
Txlna	-0.13	1.00000000	chicken
Lig3	-0.15	1.00000000	chicken
Dyrk1a	-0.04	1.00000000	chicken
Hvcn1	-0.14	1.00000000	chicken
Vti1a	-0.13	1.00000000	chicken
Cfdp1	-0.17	1.00000000	chicken
Tef	-0.11	1.00000000	chicken
Rab10	-0.12	1.00000000	chicken
Rhoh	-0.08	1.00000000	chicken
Ankrd28	-0.26	1.00000000	chicken
Slc66a2	-0.01	1.00000000	chicken
Phlpp1	-0.89	1.00000000	chicken
Rprd1b	-0.19	1.00000000	chicken
Eprs	-0.40	1.00000000	chicken
Kif2a	-0.21	1.00000000	chicken
Gnai1	0.02	1.00000000	chicken
Zfp131	-0.08	1.00000000	chicken
Gpi1	-0.14	1.00000000	chicken
Srpk1	-0.09	1.00000000	chicken
Ilk	-0.02	1.00000000	chicken
Pin4	-0.16	1.00000000	chicken
Clasp1	-0.08	1.00000000	chicken
Stxbp5	0.21	1.00000000	chicken
Gpr65	-0.35	1.00000000	chicken
Klhl24	-0.18	1.00000000	chicken
Slc1a4	-0.20	1.00000000	chicken
Immt	-0.05	1.00000000	chicken
Hps5	-0.12	1.00000000	chicken
Ss18	-0.13	1.00000000	chicken
Snx4	-0.08	1.00000000	chicken
Hdac1	-0.10	1.00000000	chicken
Fkbp4	-0.37	1.00000000	chicken
Zfp516	-0.01	1.00000000	chicken
Golt1b	-0.19	1.00000000	chicken
Dusp6	0.16	1.00000000	chicken

Prkar1b	-0.14	1.00000000	chicken
Coq5	0.37	1.00000000	chicken
Ubash3b	-0.32	1.00000000	chicken
Tnrc6c	-0.13	1.00000000	chicken
Atp13a3	0.06	1.00000000	chicken
Oga	-0.18	1.00000000	chicken
Trappc9	-0.24	1.00000000	chicken
Clstn1	-0.06	1.00000000	chicken
Prcc	-0.05	1.00000000	chicken
Isg20l2	-0.01	1.00000000	chicken
Smim14	-0.16	1.00000000	chicken
4921524J17Rik	0.01	1.00000000	chicken
Tet2	0.49	1.00000000	chicken
Pea15a	-0.01	1.00000000	chicken
Siva1	-0.14	1.00000000	chicken
Taf15	0.29	1.00000000	chicken
Fry	-0.68	1.00000000	chicken
Bmt2	-0.29	1.00000000	chicken
Dus3l	-0.05	1.00000000	chicken
Nr1d2	0.11	1.00000000	chicken
Klhdc4	-0.06	1.00000000	chicken
Smarce1	-0.03	1.00000000	chicken
Iqcb1	-0.09	1.00000000	chicken
Arl3	-0.04	1.00000000	chicken
Arfgef2	-0.08	1.00000000	chicken
Sec13	-0.14	1.00000000	chicken
Clpx	0.11	1.00000000	chicken
Acbd3	-0.02	1.00000000	chicken
Eps15	-0.02	1.00000000	chicken
Hnrnpm	-0.01	1.00000000	chicken
Pstpip1	0.14	1.00000000	chicken
Aftph	0.14	1.00000000	chicken
Gbf1	-0.19	1.00000000	chicken
Ola1	0.20	1.00000000	chicken
Ing3	-0.13	1.00000000	chicken
Ldhb	-0.13	1.00000000	chicken
Atg3	-0.05	1.00000000	chicken
Lsm4	0.01	1.00000000	chicken
Bcap31	-0.09	1.00000000	chicken
Etfa	-0.03	1.00000000	chicken
Rnf141	-0.16	1.00000000	chicken
Copa	0.00	1.00000000	chicken
Wdr82	-0.03	1.00000000	chicken
Hpcal1	-0.25	1.00000000	chicken
Supt6	0.08	1.00000000	chicken
Lsm1	-0.16	1.00000000	chicken

9530068E07Rik	-0.07	1.00000000	chicken
Stk24	-0.09	1.00000000	chicken
Osbpl1a	0.00	1.00000000	chicken
Idh3a	0.04	1.00000000	chicken
Opa1	-0.08	1.00000000	chicken
Btbd1	0.04	1.00000000	chicken
Aifm1	-0.16	1.00000000	chicken
Plrg1	-0.41	1.00000000	chicken
Cwc22	-0.11	1.00000000	chicken
Kat6a	-0.13	1.00000000	chicken
Wdr41	-0.17	1.00000000	chicken
Htt	-0.22	1.00000000	chicken
Tst	0.01	1.00000000	chicken
Snx27	0.08	1.00000000	chicken
Atp6v0b	0.02	1.00000000	chicken
Ccpg1	0.29	1.00000000	chicken
Emc3	-0.13	1.00000000	chicken
Lpp	-0.09	1.00000000	chicken
Foxo1	-0.39	1.00000000	chicken
Thrap3	-0.10	1.00000000	chicken
Zfp830	0.07	1.00000000	chicken
Itpka	-0.30	1.00000000	chicken
Smarcc2	-0.31	1.00000000	chicken
Usp16	0.40	1.00000000	chicken
Rnf216	-0.17	1.00000000	chicken
Ufl1	-0.13	1.00000000	chicken
Glcci1	0.03	1.00000000	chicken
Cacul1	0.21	1.00000000	chicken
Washc4	-0.17	1.00000000	chicken
Socs5	-0.22	1.00000000	chicken
Gphn	-0.35	1.00000000	chicken
Ssbp2	1.20	1.00000000	chicken
Sptlc1	0.03	1.00000000	chicken
Trmt10c	0.00	1.00000000	chicken
Adprh	-0.06	1.00000000	chicken
Stx12	-0.02	1.00000000	chicken
Ccnl2	0.09	1.00000000	chicken
Wdr1	-0.15	1.00000000	chicken
Tsn	-0.10	1.00000000	chicken
Pxylp1	-0.30	1.00000000	chicken
Ubox5	0.10	1.00000000	chicken
Plekhf2	0.06	1.00000000	chicken
Bin3	0.06	1.00000000	chicken
Vcpip1	-0.06	1.00000000	chicken
Prmt3	0.16	1.00000000	chicken
Nemp2	0.11	1.00000000	chicken

Farp1	0.36	1.00000000	chicken
Tgfbrap1	-0.17	1.00000000	chicken
Tmem185a	-0.18	1.00000000	chicken
Aebp2	0.05	1.00000000	chicken
Flnb	0.11	1.00000000	chicken
Tram1l1	0.02	1.00000000	chicken
Ddx54	0.10	1.00000000	chicken
Picalm	-0.27	1.00000000	chicken
Yipf5	-0.16	1.00000000	chicken
Bclaf3	-0.17	1.00000000	chicken
Ccndbp1	-0.16	1.00000000	chicken
Atg5	0.09	1.00000000	chicken
Cnst	0.12	1.00000000	chicken
Dis3l2	0.11	1.00000000	chicken
Ryk	-0.23	1.00000000	chicken
Uri1	-0.07	1.00000000	chicken
Adipor2	-0.06	1.00000000	chicken
Ccdc47	-0.29	1.00000000	chicken
Smim7	-0.11	1.00000000	chicken
Tnfrsf1a	0.27	1.00000000	chicken
Nhp2	0.16	1.00000000	chicken
Aoah	-0.12	1.00000000	chicken
Tpm1	-0.01	1.00000000	chicken
Luc7l3	-0.16	1.00000000	chicken
Syf2	-0.03	1.00000000	chicken
Nipsnap2	0.08	1.00000000	chicken
Copb1	-0.09	1.00000000	chicken
Cyb5b	-0.08	1.00000000	chicken
Pomp	0.07	1.00000000	chicken
Acap2	-0.04	1.00000000	chicken
Aff1	0.07	1.00000000	chicken
Dapp1	0.23	1.00000000	chicken
Idh3b	0.00	1.00000000	chicken
AA467197	0.14	1.00000000	chicken
Lrmp	0.02	1.00000000	chicken
BC004004	-0.11	1.00000000	chicken
Surf6	0.09	1.00000000	chicken
Kif5b	-0.22	1.00000000	chicken
Isca2	-0.22	1.00000000	chicken
Dop1a	-0.28	1.00000000	chicken
Psmb3	0.07	1.00000000	chicken
Dclk3	0.14	1.00000000	chicken
Thoc2	-0.03	1.00000000	chicken
Elof1	0.00	1.00000000	chicken
Khdc4	-0.17	1.00000000	chicken
Radx	0.25	1.00000000	chicken

Timp2	0.14	1.00000000	chicken
Ehd3	-0.18	1.00000000	chicken
Tmem131l	-0.33	1.00000000	chicken
Pgrmc2	0.06	1.00000000	chicken
Ubtf	-0.15	1.00000000	chicken
Aldoc	0.10	1.00000000	chicken
Dnaaf9	0.23	1.00000000	chicken
Ipo11	0.09	1.00000000	chicken
Lpxn	0.20	1.00000000	chicken
Jade2	-0.25	1.00000000	chicken
Naa35	-0.27	1.00000000	chicken
Gk5	-0.02	1.00000000	chicken
Sulf2	0.02	1.00000000	chicken
Rab11fip1	-0.25	1.00000000	chicken
Wdr13	-0.02	1.00000000	chicken
Pfn2	0.07	1.00000000	chicken
Yars	0.04	1.00000000	chicken
Ubr2	-0.11	1.00000000	chicken
Atp9a	-0.40	1.00000000	chicken
Slc25a22	0.10	1.00000000	chicken
Cops8	0.01	1.00000000	chicken
Atf1	-0.03	1.00000000	chicken
Lcp2	0.20	1.00000000	chicken
Sgpl1	0.22	1.00000000	chicken
Ppm1k	-0.05	1.00000000	chicken
Kpna3	0.18	1.00000000	chicken
Arap2	0.42	1.00000000	chicken
Ppp1r8	-0.07	1.00000000	chicken
Atp1b1	-0.06	1.00000000	chicken
Tsc22d3	-0.16	1.00000000	chicken
Plcd1	-0.02	1.00000000	chicken
Cpne1	-0.01	1.00000000	chicken
Capn11	-0.13	1.00000000	chicken
Commd10	0.03	1.00000000	chicken
Peak1	-0.10	1.00000000	chicken
Nup98	-0.17	1.00000000	chicken
Vcl	0.13	1.00000000	chicken
Pde7b	-0.14	1.00000000	chicken
C1qbp	-0.16	1.00000000	chicken
Cntrl	0.22	1.00000000	chicken
Cmtm4	-0.07	1.00000000	chicken
Tpi1	0.04	1.00000000	chicken
Exoc3	-0.10	1.00000000	chicken
Sik3	-0.14	1.00000000	chicken
Psmc6	-0.09	1.00000000	chicken
Cnp	0.03	1.00000000	chicken

Bud13	-0.10	1.00000000	chicken
Aktip	-0.14	1.00000000	chicken
Aurkaip1	-0.12	1.00000000	chicken
Traf6	-0.14	1.00000000	chicken
Arid3a	-0.21	1.00000000	chicken
Pibf1	-0.03	1.00000000	chicken
Smim19	-0.14	1.00000000	chicken
Ndufb9	-0.07	1.00000000	chicken
Commd1b	0.65	1.00000000	chicken
Atp2c1	-0.04	1.00000000	chicken
Rsf1	0.14	1.00000000	chicken
Prrc2c	-0.05	1.00000000	chicken
Spred2	0.08	1.00000000	chicken
Irf2bp2	-0.25	1.00000000	chicken
Llph	-0.13	1.00000000	chicken
Dhx37	-0.05	1.00000000	chicken
Cactin	0.02	1.00000000	chicken
Ktn1	0.12	1.00000000	chicken
Prr5	-0.03	1.00000000	chicken
Nfxl1	-0.24	1.00000000	chicken
Thra	-0.01	1.00000000	chicken
Ankmy2	0.28	1.00000000	chicken
Anapc13	0.09	1.00000000	chicken
B230219D22Rik	-0.21	1.00000000	chicken
Tab2	0.12	1.00000000	chicken
Larp7	0.21	1.00000000	chicken
Haghl	0.06	1.00000000	chicken
Arhgap21	0.04	1.00000000	chicken
Hadha	0.04	1.00000000	chicken
Cebpzos	-0.06	1.00000000	chicken
Gab1	-0.45	1.00000000	chicken
Selenok	-0.05	1.00000000	chicken
Oser1	-0.05	1.00000000	chicken
Unc119b	-0.02	1.00000000	chicken
Nudc	0.06	1.00000000	chicken
Psmd13	-0.01	1.00000000	chicken
Zbtb2	-0.43	1.00000000	chicken
Atf6	-0.07	1.00000000	chicken
Ints2	-0.13	1.00000000	chicken
Impdh2	-0.09	1.00000000	chicken
Eif2s1	-0.15	1.00000000	chicken
Tcp1	0.03	1.00000000	chicken
Xrn1	-0.17	1.00000000	chicken
Galnt1	0.06	1.00000000	chicken
Ndufb6	-0.05	1.00000000	chicken
Rnf11	-0.22	1.00000000	chicken

Tmf1	-0.01	1.00000000	chicken
Tcf3	0.14	1.00000000	chicken
Jph1	-0.22	1.00000000	chicken
Eif4e2	-0.14	1.00000000	chicken
Sri	-0.26	1.00000000	chicken
Lsm12	0.11	1.00000000	chicken
Setd2	0.17	1.00000000	chicken
Phax	-0.07	1.00000000	chicken
Xpo4	-0.18	1.00000000	chicken
Ensa	0.00	1.00000000	chicken
Naa25	-0.28	1.00000000	chicken
Rnpc3	-0.07	1.00000000	chicken
Ddx42	0.17	1.00000000	chicken
Lemd2	0.02	1.00000000	chicken
Numa1	-0.18	1.00000000	chicken
Prkar2b	0.05	1.00000000	chicken
Cep120	-0.10	1.00000000	chicken
Ttc1	-0.11	1.00000000	chicken
Rab8a	-0.11	1.00000000	chicken
Nedd8	-0.06	1.00000000	chicken
Ndufb7	0.00	1.00000000	chicken
Gltp	0.22	1.00000000	chicken
Atxn10	-0.09	1.00000000	chicken
Washc5	-0.14	1.00000000	chicken
Inpp5f	-0.35	1.00000000	chicken
Kifbp	-0.20	1.00000000	chicken
Pld1	-0.24	1.00000000	chicken
Nop2	0.00	1.00000000	chicken
Pcnt	-0.17	1.00000000	chicken
Tcp11l2	-0.12	1.00000000	chicken
Ranbp1	-0.04	1.00000000	chicken
Slc44a1	-0.06	1.00000000	chicken
Atp6v1g1	-0.08	1.00000000	chicken
Yme1l1	-0.27	1.00000000	chicken
Tcf25	0.03	1.00000000	chicken
Dapk1	-0.66	1.00000000	chicken
Cdc42se2	-0.62	1.00000000	chicken
Vps26c	-0.08	1.00000000	chicken
Phf20l1	-0.03	1.00000000	chicken
Klhl2	-0.14	1.00000000	chicken
Ccdc32	-0.01	1.00000000	chicken
Heatr5a	-0.33	1.00000000	chicken
Ap3m1	-0.03	1.00000000	chicken
Lrp8	0.01	1.00000000	chicken
Nptn	-0.18	1.00000000	chicken
Ik	0.10	1.00000000	chicken

Faf2	-0.03	1.00000000	chicken
Ep400	-0.38	1.00000000	chicken
Garnl3	-0.03	1.00000000	chicken
Synrg	-0.04	1.00000000	chicken
Tmem120a	0.03	1.00000000	chicken
Psmd3	-0.07	1.00000000	chicken
Rarg	-0.05	1.00000000	chicken
Hacd4	1.18	0.02783331	duck
Plac8	1.62	0.11088622	duck
Xpa	1.03	0.18620480	duck
Dctn3	1.04	0.32342798	duck
Nfam1	0.86	0.38998561	duck
Acer3	0.59	0.51911883	duck
Ctsb	1.13	0.66601425	duck
Lmna	1.39	0.73819193	duck
Abi1	0.94	0.80775315	duck
Hfm1	-1.38	1.00000000	duck
Atp5b	1.11	1.00000000	duck
Rgs19	0.72	1.00000000	duck
Lpar1	2.13	1.00000000	duck
Brsk2	1.34	1.00000000	duck
Erh	0.97	1.00000000	duck
Rsrp1	-2.11	1.00000000	duck
Nup93	0.69	1.00000000	duck
Kif21a	0.73	1.00000000	duck
Cobl	1.44	1.00000000	duck
Crispld2	0.85	1.00000000	duck
Srgn	0.67	1.00000000	duck
Snx13	1.13	1.00000000	duck
Epb41	-2.33	1.00000000	duck
Nos2	1.08	1.00000000	duck
Dram2	0.57	1.00000000	duck
Slc1a2	1.41	1.00000000	duck
Fermt1	1.01	1.00000000	duck
Ston2	0.88	1.00000000	duck
Vps37c	0.41	1.00000000	duck
Cdk5rap3	0.45	1.00000000	duck
Sox6	-4.21	1.00000000	duck
Rapgef2	1.90	1.00000000	duck
Anxa2	1.11	1.00000000	duck
Ctnna1	0.90	1.00000000	duck
Plaa	0.62	1.00000000	duck
Igsf9	1.01	1.00000000	duck
Usp48	-1.37	1.00000000	duck
Tmem203	0.95	1.00000000	duck
Clic4	1.31	1.00000000	duck

Psmb2	0.55	1.00000000	duck
Ahcyl2	0.93	1.00000000	duck
Tmem35b	1.18	1.00000000	duck
Epsti1	0.35	1.00000000	duck
Fam174a	0.94	1.00000000	duck
Wee1	0.68	1.00000000	duck
Tmem199	0.67	1.00000000	duck
Dcaf6	1.30	1.00000000	duck
St8sia4	1.57	1.00000000	duck
2210408I21Rik	0.84	1.00000000	duck
Top2b	-1.44	1.00000000	duck
Sh3kbp1	1.33	1.00000000	duck
Dennd5b	2.14	1.00000000	duck
Lmo7	0.69	1.00000000	duck
Sgce	0.66	1.00000000	duck
Haao	0.22	1.00000000	duck
Klf15	0.42	1.00000000	duck
Noxa1	0.19	1.00000000	duck
Llph	0.54	1.00000000	duck
Esyt2	0.96	1.00000000	duck
Gpcpd1	-1.31	1.00000000	duck
Radx	1.12	1.00000000	duck
Sgms2	1.53	1.00000000	duck
Synm	-3.68	1.00000000	duck
Sod1	0.63	1.00000000	duck
Clta	0.77	1.00000000	duck
Iqck	0.42	1.00000000	duck
Rundc1	0.81	1.00000000	duck
Zbtb16	1.63	1.00000000	duck
Focad	1.06	1.00000000	duck
Pim3	1.24	1.00000000	duck
Pepd	0.53	1.00000000	duck
Plcl1	1.03	1.00000000	duck
Asb2	1.00	1.00000000	duck
Clcn7	0.56	1.00000000	duck
Fam168b	-1.02	1.00000000	duck
Lmf2	0.89	1.00000000	duck
Fbrsl1	1.08	1.00000000	duck
Hivep3	1.17	1.00000000	duck
Stat1	1.30	1.00000000	duck
Ubr2	-1.68	1.00000000	duck
Serpine2	1.06	1.00000000	duck
Grb10	0.61	1.00000000	duck
S100a11	1.34	1.00000000	duck
Swap70	1.26	1.00000000	duck
Satb2	0.52	1.00000000	duck

Igf2bp3	1.04	1.00000000	duck
Chka	0.84	1.00000000	duck
Ift74	-0.09	1.00000000	duck
Rnf216	0.78	1.00000000	duck
Vps13b	-2.11	1.00000000	duck
Dapk3	-0.44	1.00000000	duck
Sec24b	-1.59	1.00000000	duck
Pid1	1.71	1.00000000	duck
Arih1	-1.47	1.00000000	duck
Psma5	0.95	1.00000000	duck
Itch	-1.54	1.00000000	duck
Inpp5a	0.58	1.00000000	duck
Zswim6	0.76	1.00000000	duck
Klf7	0.74	1.00000000	duck
Fnbp1l	0.49	1.00000000	duck
Prrc2c	-1.01	1.00000000	duck
Sub1	0.86	1.00000000	duck
Jmjd1c	-0.91	1.00000000	duck
Psmb4	0.83	1.00000000	duck
Sppl3	0.79	1.00000000	duck
H13	0.48	1.00000000	duck
Tubb4b	0.88	1.00000000	duck
Cnpy3	0.75	1.00000000	duck
Efl1	0.58	1.00000000	duck
Prdm15	0.68	1.00000000	duck
Vgll4	0.85	1.00000000	duck
Adora2b	0.67	1.00000000	duck
Rev1	-1.32	1.00000000	duck
Rfesd	-2.87	1.00000000	duck
Ostm1	0.64	1.00000000	duck
Nfkbiz	0.49	1.00000000	duck
Slc8a1	0.95	1.00000000	duck
Cldnd1	0.53	1.00000000	duck
Nrip1	1.02	1.00000000	duck
Pard3	0.30	1.00000000	duck
Blnk	0.57	1.00000000	duck
Pip5k1b	0.95	1.00000000	duck
Cish	-1.26	1.00000000	duck
Bambi	0.49	1.00000000	duck
Smad6	0.83	1.00000000	duck
Cnot9	0.63	1.00000000	duck
Rnf130	1.11	1.00000000	duck
Adcy7	0.83	1.00000000	duck
Syngr2	0.78	1.00000000	duck
Zfand5	0.56	1.00000000	duck
Srp19	0.73	1.00000000	duck

Cnksr2	0.83	1.00000000	duck
Kcnq5	1.53	1.00000000	duck
Cep57	0.23	1.00000000	duck
Gas6	0.29	1.00000000	duck
Dcn	0.38	1.00000000	duck
Rbfox2	1.09	1.00000000	duck
Runx1t1	1.03	1.00000000	duck
Tfpi2	0.60	1.00000000	duck
Slc35b2	0.39	1.00000000	duck
Rassf6	0.21	1.00000000	duck
Fgfr3	0.80	1.00000000	duck
Sema6a	0.76	1.00000000	duck
Nrip3	0.26	1.00000000	duck
Inhbb	0.37	1.00000000	duck
Tysnd1	0.22	1.00000000	duck
Gfpt2	0.16	1.00000000	duck
Ranbp17	0.41	1.00000000	duck
Insyn2b	1.03	1.00000000	duck
Ptges	0.52	1.00000000	duck
Lrrc47	0.16	1.00000000	duck
Sh3tc2	0.24	1.00000000	duck
Lox	0.37	1.00000000	duck
Tmem131	0.64	1.00000000	duck
Ndufb6	0.63	1.00000000	duck
Aimp2	0.32	1.00000000	duck
Ddost	0.58	1.00000000	duck
Flnb	0.67	1.00000000	duck
Ginm1	0.98	1.00000000	duck
Bri3	0.31	1.00000000	duck
Atp5g3	0.92	1.00000000	duck
Tm9sf2	-1.11	1.00000000	duck
Nudt5	0.34	1.00000000	duck
Smad3	0.39	1.00000000	duck
Itih4	0.43	1.00000000	duck
Tnpo1	0.26	1.00000000	duck
Romo1	0.79	1.00000000	duck
Pigf	0.51	1.00000000	duck
Golph3	0.43	1.00000000	duck
Sptlc1	-0.65	1.00000000	duck
C1qa	1.24	1.00000000	duck
Timm8a2	0.72	1.00000000	duck
Nucb2	-1.42	1.00000000	duck
Snrpg	0.95	1.00000000	duck
Slc26a11	0.29	1.00000000	duck
Dnah17	0.89	1.00000000	duck
Atrx	-1.69	1.00000000	duck

Ost4	1.38	1.00000000	duck
Gnl2	0.62	1.00000000	duck
Zswim5	0.62	1.00000000	duck
Ctsc	1.29	1.00000000	duck
Pkm	0.48	1.00000000	duck
Cdk7	0.39	1.00000000	duck
Pde4b	0.75	1.00000000	duck
Lpar5	0.74	1.00000000	duck
Ankrd33b	1.46	1.00000000	duck
Bmp5	0.94	1.00000000	duck
Ccdc124	0.27	1.00000000	duck
Ubl5b	0.61	1.00000000	duck
Slc35f1	0.66	1.00000000	duck
Lyplal1	0.32	1.00000000	duck
Tesk1	0.46	1.00000000	duck
Plpp1	0.59	1.00000000	duck
Cep83	0.70	1.00000000	duck
Tmem39b	0.21	1.00000000	duck
Psma7	0.74	1.00000000	duck
Psma3	0.95	1.00000000	duck
Slc22a23	1.05	1.00000000	duck
Tox	1.33	1.00000000	duck
Prdm1	0.79	1.00000000	duck
Pradc1	0.18	1.00000000	duck
Dusp8	0.31	1.00000000	duck
Snrk	-1.36	1.00000000	duck
Nhs	0.77	1.00000000	duck
Ptchd3	0.31	1.00000000	duck
Igf1r	0.97	1.00000000	duck
Ier5	1.19	1.00000000	duck
Dennd4c	0.75	1.00000000	duck
Fhl5	0.33	1.00000000	duck
Clstn1	0.29	1.00000000	duck
Ube2r2	0.21	1.00000000	duck
Rpia	-0.76	1.00000000	duck
Helq	-0.75	1.00000000	duck
Reep5	0.72	1.00000000	duck
Noc3l	0.66	1.00000000	duck
P4htm	0.27	1.00000000	duck
Ikzf1	1.57	1.00000000	duck
Hdx	-0.75	1.00000000	duck
Atf3	1.18	1.00000000	duck
Ankrd12	-1.94	1.00000000	duck
Fam3c	0.85	1.00000000	duck
9930021J03Rik	0.06	1.00000000	duck
Cilk1	-1.20	1.00000000	duck

Anp32e	-0.47	1.00000000	duck
Nbr1	-1.48	1.00000000	duck
Stxbp1	0.87	1.00000000	duck
Cdk6	0.88	1.00000000	duck
Itgb6	1.74	1.00000000	duck
Rpp30	0.48	1.00000000	duck
Yeats4	0.68	1.00000000	duck
Tbk1	0.44	1.00000000	duck
Ramp1	0.06	1.00000000	duck
Bcor	0.72	1.00000000	duck
Pip4k2a	0.72	1.00000000	duck
Arpc1b	0.92	1.00000000	duck
Wdr82	0.64	1.00000000	duck
Srsf10	-1.17	1.00000000	duck
Arpc5	0.69	1.00000000	duck
Rap2b	0.92	1.00000000	duck
Rngtt	1.35	1.00000000	duck
Rab11fip1	-0.93	1.00000000	duck
Arf6	0.72	1.00000000	duck
Bcl2a1d	1.00	1.00000000	duck
Edrf1	-1.65	1.00000000	duck
Arid4b	-0.99	1.00000000	duck
Fgfr1op2	-1.18	1.00000000	duck
Lhfpl2	1.36	1.00000000	duck
Snrnp40	0.55	1.00000000	duck
Bmt2	0.15	1.00000000	duck
Herc1	-1.19	1.00000000	duck
Kdm2a	0.81	1.00000000	duck
Arhgdib	0.60	1.00000000	duck
Dcaf12	0.56	1.00000000	duck
Ssr3	0.69	1.00000000	duck
Tnfaip8	0.93	1.00000000	duck
Smurf2	1.45	1.00000000	duck
Ccdc12	0.58	1.00000000	duck
Csrp1	0.70	1.00000000	duck
Smpd1	0.84	1.00000000	duck
Cep89	0.93	1.00000000	duck
Zbtb20	1.53	1.00000000	duck
Tmem104	0.26	1.00000000	duck
Gfod1	0.04	1.00000000	duck
Ndufa6	1.03	1.00000000	duck
Grap2	1.25	1.00000000	duck
Suco	-1.69	1.00000000	duck
Elavl1	-0.88	1.00000000	duck
Trappc3	0.58	1.00000000	duck
Cttn	0.63	1.00000000	duck

Cpt1a	0.63	1.00000000	duck
Als2cl	0.69	1.00000000	duck
Ccnt1	-1.04	1.00000000	duck
Ptpn4	-0.82	1.00000000	duck
Gfi1	0.80	1.00000000	duck
Fkbp7	0.27	1.00000000	duck
Kdm2b	0.28	1.00000000	duck
Arpc3	0.83	1.00000000	duck
Traf1	0.54	1.00000000	duck
Gtf2b	0.37	1.00000000	duck
Add1	-1.54	1.00000000	duck
Brix1	0.50	1.00000000	duck
Ktn1	0.64	1.00000000	duck
Zfand2b	0.48	1.00000000	duck
Tasor	0.65	1.00000000	duck
Dusp7	0.66	1.00000000	duck
Tubb5	0.88	1.00000000	duck
Runx1	0.65	1.00000000	duck
Qser1	0.59	1.00000000	duck
Ankdd1a	0.37	1.00000000	duck
Ube2i	0.14	1.00000000	duck
Scn3b	-0.27	1.00000000	duck
Tcirg1	0.95	1.00000000	duck
Echdc2	0.68	1.00000000	duck
Arrdc3	1.88	1.00000000	duck
Nprl3	0.22	1.00000000	duck
Irf2bpl	0.94	1.00000000	duck
Dram1	0.71	1.00000000	duck
Plcg2	1.23	1.00000000	duck
Dusp5	0.38	1.00000000	duck
Btaf1	-1.24	1.00000000	duck
Lpcat2b	0.78	1.00000000	duck
Atf1	-1.07	1.00000000	duck
Sh3bp2	0.81	1.00000000	duck
Tbl1xr1	0.60	1.00000000	duck
Cpb2	0.75	1.00000000	duck
Neil1	0.35	1.00000000	duck
Raph1	0.83	1.00000000	duck
Ptprs	0.61	1.00000000	duck
Prkd3	0.51	1.00000000	duck
Smarcb1	0.57	1.00000000	duck
G3bp2	-0.06	1.00000000	duck
Fdps	0.78	1.00000000	duck
Adgrg6	0.96	1.00000000	duck
Hipk2	1.10	1.00000000	duck
Ugdh	0.20	1.00000000	duck

Nat9	0.35	1.00000000	duck
Sema4a	0.76	1.00000000	duck
Blk	0.60	1.00000000	duck
Jund	0.92	1.00000000	duck
Decr2	0.91	1.00000000	duck
Rnf220	0.84	1.00000000	duck
Ppm1k	0.36	1.00000000	duck
Hsbp1	0.52	1.00000000	duck
Dgkh	1.27	1.00000000	duck
Slc16a9	0.28	1.00000000	duck
Kif1b	0.11	1.00000000	duck
Apaf1	0.54	1.00000000	duck
Ybx3	-1.03	1.00000000	duck
Polr2d	0.47	1.00000000	duck
Rpa1	-0.41	1.00000000	duck
Galnt6	0.54	1.00000000	duck
P2rx7	0.26	1.00000000	duck
Cblb	0.78	1.00000000	duck
Ankrd10	0.49	1.00000000	duck
Snx16	-0.57	1.00000000	duck
Ormdl2	0.56	1.00000000	duck
Pdrg1	0.79	1.00000000	duck
Rad18	1.13	1.00000000	duck
Aimp1	0.32	1.00000000	duck
Sec23a	-0.11	1.00000000	duck
Tdrd7	0.15	1.00000000	duck
Galnt9	1.20	1.00000000	duck
Zcchc7	0.91	1.00000000	duck
Sdhb	0.55	1.00000000	duck
Gnai2	0.65	1.00000000	duck
Nuak1	0.45	1.00000000	duck
Nhsl2	0.45	1.00000000	duck
Smim20	0.76	1.00000000	duck
Stk17b	-1.05	1.00000000	duck
Pik3c2a	-1.49	1.00000000	duck
Selenoo	-1.05	1.00000000	duck
Hoxa7	-0.32	1.00000000	duck
Odf2	-0.25	1.00000000	duck
Dcun1d4	-1.32	1.00000000	duck
Bcap29	0.32	1.00000000	duck
Ifngr2	0.77	1.00000000	duck
Slc39a8	0.97	1.00000000	duck
Galnt7	0.76	1.00000000	duck
Bcl7a	-0.60	1.00000000	duck
Etf1	-0.81	1.00000000	duck
Prdx1	0.59	1.00000000	duck

Adi1	0.26	1.00000000	duck
Pgbd5	0.47	1.00000000	duck
Ighmbp2	0.27	1.00000000	duck
Ttc21b	0.18	1.00000000	duck
Stn1	0.25	1.00000000	duck
Cebpa	0.64	1.00000000	duck
Hspa8	0.55	1.00000000	duck
Trmt10c	0.44	1.00000000	duck
Rab9	0.02	1.00000000	duck
Sgsm3	0.19	1.00000000	duck
Hic2	0.32	1.00000000	duck
Nfil3	1.85	1.00000000	duck
Sirt5	0.47	1.00000000	duck
Gna13	-0.80	1.00000000	duck
Stxbp4	0.47	1.00000000	duck
Bnip3l	-1.14	1.00000000	duck
Fbxl14	0.28	1.00000000	duck
Ntng1	0.45	1.00000000	duck
Txn1	0.74	1.00000000	duck
Rhoa	0.49	1.00000000	duck
Ccnl2	-0.91	1.00000000	duck
Rnpepl1	0.39	1.00000000	duck
Immp2l	0.41	1.00000000	duck
Fubp1	0.66	1.00000000	duck
Nsmf	0.36	1.00000000	duck
Cdc14a	-2.00	1.00000000	duck
Atp5a1	0.60	1.00000000	duck
Slc30a9	-1.46	1.00000000	duck
Yae1d1	0.56	1.00000000	duck
Sec61g	0.44	1.00000000	duck
Ddx60	-0.61	1.00000000	duck
Azin1	0.39	1.00000000	duck
Uvrag	0.33	1.00000000	duck
Arhgap12	0.86	1.00000000	duck
Elovl6	1.07	1.00000000	duck
Utp6	0.33	1.00000000	duck
Pafah1b2	0.22	1.00000000	duck
Snrpf	0.73	1.00000000	duck
Fli1	0.79	1.00000000	duck
Slc6a6	-0.88	1.00000000	duck
Pcmtd2	0.18	1.00000000	duck
Polr1e	0.21	1.00000000	duck
Dbf4	0.15	1.00000000	duck
Dcun1d2	-1.53	1.00000000	duck
Slc66a2	-0.44	1.00000000	duck
Slc16a10	-0.90	1.00000000	duck

Prkar1b	-0.92	1.00000000	duck
Lars2	0.75	1.00000000	duck
Sfmbt1	0.34	1.00000000	duck
Acod1	0.43	1.00000000	duck
Snapc3	0.22	1.00000000	duck
Crebl2	0.36	1.00000000	duck
Fam83f	0.56	1.00000000	duck
Stard13	1.82	1.00000000	duck
Gata6	0.45	1.00000000	duck
Acat3	0.16	1.00000000	duck
Tnip3	1.77	1.00000000	duck
Brcc3	0.25	1.00000000	duck
Nsdhl	0.57	1.00000000	duck
Nudt2	0.13	1.00000000	duck
Ablim1	0.62	1.00000000	duck
Pdlim1	0.75	1.00000000	duck
Snx7	0.62	1.00000000	duck
Frmd4b	0.25	1.00000000	duck
Htr4	0.25	1.00000000	duck
Syt17	0.21	1.00000000	duck
St6galnac1	0.56	1.00000000	duck
Kirrel3	0.66	1.00000000	duck
Pgls	0.21	1.00000000	duck
Itpr3	0.23	1.00000000	duck
Dnajb12	-0.57	1.00000000	duck
Cdk5rap2	-1.47	1.00000000	duck
Paip1	0.55	1.00000000	duck
Slf1	0.54	1.00000000	duck
Rexo2	0.63	1.00000000	duck
Slc35e3	-0.16	1.00000000	duck
Tbc1d2	0.69	1.00000000	duck
Slc25a13	-0.08	1.00000000	duck
Usp34	0.32	1.00000000	duck
Camk2g	-1.04	1.00000000	duck
Lrch3	-1.40	1.00000000	duck
Aopep	0.83	1.00000000	duck
Wapl	-0.86	1.00000000	duck
Tmem259	0.26	1.00000000	duck
Pdcd5	-0.65	1.00000000	duck
Rrbp1	0.53	1.00000000	duck
Cdo1	0.40	1.00000000	duck
Nudt12	0.22	1.00000000	duck
Zranb3	0.17	1.00000000	duck
Necab2	0.24	1.00000000	duck
Cyth3	0.31	1.00000000	duck
Ebag9	-0.50	1.00000000	duck

Ncoa3	0.61	1.00000000	duck
Hypk	0.62	1.00000000	duck
Disc1	0.87	1.00000000	duck
Trappc11	-1.12	1.00000000	duck
Hat1	0.35	1.00000000	duck
Vps54	0.57	1.00000000	duck
Atg16l1	0.08	1.00000000	duck
Sorbs1	0.41	1.00000000	duck
Stk26	-1.24	1.00000000	duck
Sp4	-0.81	1.00000000	duck
Larp1b	-2.06	1.00000000	duck
Abhd13	-0.67	1.00000000	duck
Eef1b2	-0.51	1.00000000	duck
Pten	0.37	1.00000000	duck
Wdr47	0.38	1.00000000	duck
Prkce	0.76	1.00000000	duck
Unc13b	0.43	1.00000000	duck
Exd1	0.19	1.00000000	duck
Tmem80	0.30	1.00000000	duck
Ptgs2	0.57	1.00000000	duck
Wdr53	0.10	1.00000000	duck
Csf3r	0.33	1.00000000	duck
Foxo4	0.07	1.00000000	duck
Sltm	-0.68	1.00000000	duck
Fxn	0.36	1.00000000	duck
Sdc2	0.84	1.00000000	duck
Furin	-0.40	1.00000000	duck
Tacc1	-0.31	1.00000000	duck
Sptan1	-1.84	1.00000000	duck
Slc15a4	0.39	1.00000000	duck
Nab1	0.43	1.00000000	duck
Scyl2	-0.67	1.00000000	duck
Sema4d	0.27	1.00000000	duck
Nipbl	0.27	1.00000000	duck
Eif3b	-0.83	1.00000000	duck
Yap1	0.87	1.00000000	duck
Ints11	0.78	1.00000000	duck
Erp44	0.16	1.00000000	duck
Scube2	1.22	1.00000000	duck
Ssb	0.45	1.00000000	duck
Phyh	-0.10	1.00000000	duck
Slc25a33	0.11	1.00000000	duck
Ikbkb	0.51	1.00000000	duck
Papola	-1.23	1.00000000	duck
Tspan3	0.05	1.00000000	duck
Ergic1	0.66	1.00000000	duck

Stap1	0.52	1.00000000	duck
Ttc33	0.07	1.00000000	duck
Pik3r4	-0.26	1.00000000	duck
Scamp4	0.27	1.00000000	duck
Dip2c	1.42	1.00000000	duck
Zmym3	0.71	1.00000000	duck
Hmgb2	0.00	1.00000000	duck
Ift88	0.30	1.00000000	duck
Gadd45g	0.70	1.00000000	duck
Pcmtd1	-1.58	1.00000000	duck
Pnrc1	0.28	1.00000000	duck
Tomm20	0.45	1.00000000	duck
Nsg1	-0.50	1.00000000	duck
Dstyk	-0.33	1.00000000	duck
Tasor2	-0.58	1.00000000	duck
Abcc8	-0.34	1.00000000	duck
Umps	-0.19	1.00000000	duck
Usp31	-0.82	1.00000000	duck
Cert1	0.29	1.00000000	duck
Tmem41b	0.55	1.00000000	duck
Rnf139	0.59	1.00000000	duck
Zbtb43	-1.21	1.00000000	duck
Specc1	0.82	1.00000000	duck
Crot	0.46	1.00000000	duck
Ccndbp1	-0.65	1.00000000	duck
Kif2a	0.71	1.00000000	duck
Ulk4	-0.51	1.00000000	duck
Arsk	-1.17	1.00000000	duck
Cstf2	0.30	1.00000000	duck
Cnppd1	0.33	1.00000000	duck
Ccr7	-0.23	1.00000000	duck
Setd1b	0.41	1.00000000	duck
Prpsap1	-0.65	1.00000000	duck
Trmt1l	-0.45	1.00000000	duck
Zfp131	-0.20	1.00000000	duck
Cask	0.62	1.00000000	duck
Ndufb2	1.06	1.00000000	duck
Rac2	0.81	1.00000000	duck
Dpagt1	0.20	1.00000000	duck
Trak1	0.82	1.00000000	duck
Fam172a	0.62	1.00000000	duck
Pfn2	0.85	1.00000000	duck
Pcnx2	0.67	1.00000000	duck
Chst12	0.36	1.00000000	duck
Gpr65	0.55	1.00000000	duck
Vps26c	-0.44	1.00000000	duck

Ppm1h	0.92	1.00000000	duck
Zfp318	-0.89	1.00000000	duck
Tmem245	1.21	1.00000000	duck
Kars	0.47	1.00000000	duck
Star	0.67	1.00000000	duck
Dennd2c	0.30	1.00000000	duck
Trip11	-1.59	1.00000000	duck
Tgfbr2	-0.55	1.00000000	duck
Lztfl1	0.48	1.00000000	duck
Trim8	0.44	1.00000000	duck
Uck2	0.32	1.00000000	duck
Oxnad1	-0.69	1.00000000	duck
Tubd1	-0.58	1.00000000	duck
Erap1	0.49	1.00000000	duck
Exosc7	0.20	1.00000000	duck
Rbbp6	-0.85	1.00000000	duck
Ppm1f	0.50	1.00000000	duck
Katnip	-0.23	1.00000000	duck
Rac1	0.56	1.00000000	duck
Trappc12	1.02	1.00000000	duck
Sgsh	0.42	1.00000000	duck
Cebpb	0.90	1.00000000	duck
Aptx	0.27	1.00000000	duck
Snorc	0.23	1.00000000	duck
Spag9	-0.89	1.00000000	duck
Prkag2	1.15	1.00000000	duck
Ppp1ca	0.62	1.00000000	duck
Tkt	0.87	1.00000000	duck
Adssl1	-0.09	1.00000000	duck
Sf3a2	0.41	1.00000000	duck
Ube2j2	-0.81	1.00000000	duck
Yme1l1	-0.61	1.00000000	duck
Rere	0.76	1.00000000	duck
Nhlrc2	-0.72	1.00000000	duck
Ero1b	-0.48	1.00000000	duck
Glrx	0.71	1.00000000	duck
Rbks	0.42	1.00000000	duck
Grid1	0.76	1.00000000	duck
Samd13	0.28	1.00000000	duck
Itsn1	1.21	1.00000000	duck
Cd44	0.86	1.00000000	duck
Rbbp7	-0.47	1.00000000	duck
Lipc	-0.35	1.00000000	duck
Slc30a5	0.04	1.00000000	duck
Bod1l	-1.53	1.00000000	duck
Angel2	-0.66	1.00000000	duck

Itga1	-0.48	1.00000000	duck
Slc38a1	0.61	1.00000000	duck
Stambpl1	0.39	1.00000000	duck
Upf2	-0.71	1.00000000	duck
Sh2b3	-1.23	1.00000000	duck
Pum1	0.06	1.00000000	duck
Papss1	1.01	1.00000000	duck
Psmb3	0.24	1.00000000	duck
Prkx	0.85	1.00000000	duck
Fndc3b	0.13	1.00000000	duck
Sash1	-1.48	1.00000000	duck
Pgm1	-0.31	1.00000000	duck
Ralb	0.39	1.00000000	duck
Ppp2r2a	-0.72	1.00000000	duck
Snrpd1	0.59	1.00000000	duck
Sbno2	0.31	1.00000000	duck
Gabpb1	-1.07	1.00000000	duck
Pwwp2a	0.09	1.00000000	duck
Gpbp1l1	-0.90	1.00000000	duck
Sema3d	-2.16	1.00000000	duck
Qrfpr	-0.25	1.00000000	duck
Pomgnt1	-0.19	1.00000000	duck
Rgs5	-0.87	1.00000000	duck
Capn10	-0.29	1.00000000	duck
Fchsd1	-0.50	1.00000000	duck
Cbx4	-0.19	1.00000000	duck
Sugp2	-0.15	1.00000000	duck
Frmpd2	-0.27	1.00000000	duck
Tmf1	-1.34	1.00000000	duck
Ccdc117	-1.78	1.00000000	duck
Grk4	0.58	1.00000000	duck
Lrmda	0.37	1.00000000	duck
Arhgef6	0.50	1.00000000	duck
Pmp22	0.88	1.00000000	duck
Arfip1	-0.58	1.00000000	duck
Tmem59	0.36	1.00000000	duck
Atrnl1	1.05	1.00000000	duck
Lrmp	0.21	1.00000000	duck
Cndp2	0.75	1.00000000	duck
Srp68	-0.27	1.00000000	duck
Nsun2	-0.72	1.00000000	duck
Evl	0.55	1.00000000	duck
Tdg	-0.26	1.00000000	duck
Srpk2	0.87	1.00000000	duck
Tsr3	0.18	1.00000000	duck
Arfrp1	-0.20	1.00000000	duck

Ppm1e	0.48	1.00000000	duck
Btbd9	-1.35	1.00000000	duck
Pla2g4a	1.31	1.00000000	duck
Cpeb3	0.79	1.00000000	duck
Ran	0.56	1.00000000	duck
Eef2	-0.43	1.00000000	duck
Plcxd1	0.38	1.00000000	duck
Itm2c	-0.36	1.00000000	duck
Rell1	-0.63	1.00000000	duck
Sash3	0.36	1.00000000	duck
Nme7	0.47	1.00000000	duck
Dscc1	0.36	1.00000000	duck
Rubcn	0.29	1.00000000	duck
Rpusd1	0.77	1.00000000	duck
Lsp1	0.64	1.00000000	duck
Btbd10	0.61	1.00000000	duck
Fxr1	-1.13	1.00000000	duck
Ilrun	-1.19	1.00000000	duck
Capg	-0.17	1.00000000	duck
Hdac1	0.53	1.00000000	duck
Tmem33	0.87	1.00000000	duck
Rxra	1.05	1.00000000	duck
Psmc1	0.90	1.00000000	duck
Gtdc1	0.72	1.00000000	duck
Ranbp3	-0.15	1.00000000	duck
Phactr2	0.59	1.00000000	duck
Tssc4	0.36	1.00000000	duck
Top2a	0.82	1.00000000	duck
Golph3l	0.50	1.00000000	duck
Bcl6	0.43	1.00000000	duck
Usp25	0.60	1.00000000	duck
Laptm5	0.67	1.00000000	duck
Epc1	0.24	1.00000000	duck
Galnt11	-0.29	1.00000000	duck
Pag1	-0.40	1.00000000	duck
Pgap4	0.48	1.00000000	duck
Eny2	0.37	1.00000000	duck
Avl9	-0.09	1.00000000	duck
Tmem230	0.58	1.00000000	duck
Slc25a26	0.57	1.00000000	duck
Fam214b	-0.27	1.00000000	duck
Tle1	0.31	1.00000000	duck
Sos2	-0.63	1.00000000	duck
Pex7	0.33	1.00000000	duck
Tmcc3	1.48	1.00000000	duck
Id2	0.95	1.00000000	duck

Nfatc1	0.96	1.00000000	duck
Cflar	0.02	1.00000000	duck
Atp5c1	0.64	1.00000000	duck
Ptprm	0.88	1.00000000	duck
St3gal2	0.25	1.00000000	duck
Cep97	0.23	1.00000000	duck
Il6ra	0.57	1.00000000	duck
Exoc7	0.11	1.00000000	duck
Cope	0.55	1.00000000	duck
Bloc1s2	-0.03	1.00000000	duck
Ipo11	0.52	1.00000000	duck
Cnot6l	-1.13	1.00000000	duck
Ntmt1	0.50	1.00000000	duck
Topbp1	0.54	1.00000000	duck
Aig1	0.53	1.00000000	duck
Osbpl1a	-1.58	1.00000000	duck
Bola3	0.27	1.00000000	duck
Tent5a	1.24	1.00000000	duck
Sema3c	1.60	1.00000000	duck
Klhl18	0.24	1.00000000	duck
Pdzd11	0.21	1.00000000	duck
Tubgcp4	0.42	1.00000000	duck
Gps1	-0.15	1.00000000	duck
Trim25	0.47	1.00000000	duck
Irf7	0.66	1.00000000	duck
Stt3a	0.38	1.00000000	duck
Tet2	0.33	1.00000000	duck
Ncf1	0.63	1.00000000	duck
Poc1b	0.50	1.00000000	duck
Trpc3	-0.35	1.00000000	duck
Ccdc32	-0.18	1.00000000	duck
Cat	-0.92	1.00000000	duck
Wdr75	-0.38	1.00000000	duck
Chchd1	-0.17	1.00000000	duck
Glce	-0.18	1.00000000	duck
Klhdc4	-0.17	1.00000000	duck
Ccnf	-0.39	1.00000000	duck
Pgap6	-0.35	1.00000000	duck
Pbx4	-1.60	1.00000000	duck
Itk	-0.86	1.00000000	duck
Smad2	-0.32	1.00000000	duck
Atg12	0.68	1.00000000	duck
Ubac2	0.61	1.00000000	duck
Slf2	-0.52	1.00000000	duck
Znrf2	0.49	1.00000000	duck
Peli1	-0.71	1.00000000	duck

Sfxn5	0.32	1.00000000	duck
Rarres1	0.34	1.00000000	duck
Acaa2	0.30	1.00000000	duck
Snap47	0.17	1.00000000	duck
Tst	0.71	1.00000000	duck
Gdpd5	1.23	1.00000000	duck
Cmtm6	0.35	1.00000000	duck
Lactb	0.55	1.00000000	duck
Thap12	-0.36	1.00000000	duck
Cpm	-0.27	1.00000000	duck
Jak1	-0.56	1.00000000	duck
Pm20d1	0.38	1.00000000	duck
Ttc4	-0.58	1.00000000	duck
Litaf	0.46	1.00000000	duck
Polk	-0.77	1.00000000	duck
Lgals3	0.71	1.00000000	duck
Cryl1	-0.38	1.00000000	duck
Ppib	0.69	1.00000000	duck
Tmbim6	0.45	1.00000000	duck
Ipo7	-0.65	1.00000000	duck
Orc1	-0.18	1.00000000	duck
Kctd7	0.37	1.00000000	duck
Gale	0.27	1.00000000	duck
Snrpd3	0.58	1.00000000	duck
Lrch1	-0.21	1.00000000	duck
Slc25a22	0.48	1.00000000	duck
Smarca5	-0.73	1.00000000	duck
Kctd2	0.43	1.00000000	duck
Spopl	-1.05	1.00000000	duck
Panx2	0.59	1.00000000	duck
Ncf2	0.19	1.00000000	duck
Sulf2	2.30	1.00000000	duck
Rpap1	-0.10	1.00000000	duck
Dhrs3	0.38	1.00000000	duck
Slc25a15	-0.21	1.00000000	duck
Zc3hc1	0.41	1.00000000	duck
Pcyox1	0.37	1.00000000	duck
Slc25a3	0.39	1.00000000	duck
Glrx5	-0.97	1.00000000	duck
Depdc7	-0.07	1.00000000	duck
Brms1l	0.99	1.00000000	duck
Wac	-0.89	1.00000000	duck
Dctn4	0.21	1.00000000	duck
Bcl11a	0.27	1.00000000	duck
Nup210	-0.47	1.00000000	duck
Zfr	0.32	1.00000000	duck

Klhl15	-0.88	1.00000000	duck
Gnal	0.41	1.00000000	duck
Fem1b	-1.11	1.00000000	duck
Ncstn	0.50	1.00000000	duck
Insr	0.60	1.00000000	duck
Ranbp1	0.40	1.00000000	duck
Cpe	-0.57	1.00000000	duck
Rpgrip1l	-1.08	1.00000000	duck
Kank4	-0.21	1.00000000	duck
Prkcq	-0.63	1.00000000	duck
Fnip1	0.03	1.00000000	duck
Lmbrd1	0.16	1.00000000	duck
Bach1	-0.79	1.00000000	duck
Slc46a3	-1.39	1.00000000	duck
Arfgap1	0.26	1.00000000	duck
Lzic	0.52	1.00000000	duck
Lars	-0.19	1.00000000	duck
Cyria	0.11	1.00000000	duck
Ppp3ca	0.56	1.00000000	duck
Arl15	0.40	1.00000000	duck
Tbc1d23	-0.38	1.00000000	duck
Gnai3	-0.61	1.00000000	duck
Kat6a	-1.16	1.00000000	duck
Zeb2	0.74	1.00000000	duck
B4galt3	0.06	1.00000000	duck
Spred1	0.78	1.00000000	duck
Setd3	0.55	1.00000000	duck
Cwc15	0.67	1.00000000	duck
Vapb	0.19	1.00000000	duck
Inpp5d	-0.75	1.00000000	duck
Alcam	0.80	1.00000000	duck
Aff4	-0.97	1.00000000	duck
Ubxn2a	-0.49	1.00000000	duck
Pak4	0.30	1.00000000	duck
Sypl2	-0.12	1.00000000	duck
Ppil4	0.60	1.00000000	duck
Adipor2	-0.94	1.00000000	duck
Gfm1	0.23	1.00000000	duck
Rsrc1	0.34	1.00000000	duck
Ap3m2	-0.26	1.00000000	duck
Bdkrb2	0.71	1.00000000	duck
Rnf213	0.55	1.00000000	duck
Dars2	0.13	1.00000000	duck
Bspry	0.56	1.00000000	duck
Psen1	-0.65	1.00000000	duck
Prr5l	0.33	1.00000000	duck

Rictor	0.02	1.00000000	duck
Larp4b	0.50	1.00000000	duck
Scarb2	0.45	1.00000000	duck
Ssr2	0.49	1.00000000	duck
Pdxdc1	-0.09	1.00000000	duck
Hmgn3	0.40	1.00000000	duck
Hmces	0.43	1.00000000	duck
Rab20	0.56	1.00000000	duck
Csnk1g3	0.50	1.00000000	duck
Dusp22	-0.90	1.00000000	duck
Rsl1d1	0.80	1.00000000	duck
Tmem223	0.57	1.00000000	duck
Vmp1	0.57	1.00000000	duck
Gpr171	-1.63	1.00000000	duck
Slc25a37	-1.40	1.00000000	duck
Cdc42se2	0.62	1.00000000	duck
Itgb2l	-0.43	1.00000000	duck
Il21r	0.64	1.00000000	duck
Pcgf3	0.03	1.00000000	duck
Usp4	0.06	1.00000000	duck
Ptrhd1	0.26	1.00000000	duck
Erbin	0.25	1.00000000	duck
Nck2	0.13	1.00000000	duck
Sf3b3	-0.23	1.00000000	duck
Pot1b	-0.38	1.00000000	duck
A4galt	-0.18	1.00000000	duck
Tmem209	-0.17	1.00000000	duck
Oit3	-0.46	1.00000000	duck
Serpinc1	-0.12	1.00000000	duck
Adamts9	-0.37	1.00000000	duck
Pde12	-0.40	1.00000000	duck
Gas8	-0.18	1.00000000	duck
Nrn1l	-0.47	1.00000000	duck
Zhx3	-0.20	1.00000000	duck
Ank1	-1.54	1.00000000	duck
Gcfc2	-0.12	1.00000000	duck
Ndfip2	0.25	1.00000000	duck
Rnf128	0.62	1.00000000	duck
Stoml1	0.18	1.00000000	duck
Prelid1	0.58	1.00000000	duck
5031439G07Rik	0.52	1.00000000	duck
Dync1h1	-0.57	1.00000000	duck
Cggbp1	-0.33	1.00000000	duck
Klf13	-0.58	1.00000000	duck
Slc7a6	0.46	1.00000000	duck
Lsm8	0.38	1.00000000	duck

Snx10	1.42	1.00000000	duck
Endov	0.13	1.00000000	duck
Ccdc28a	-0.72	1.00000000	duck
Nrbf2	-0.26	1.00000000	duck
Ece1	-0.24	1.00000000	duck
Eif5a2	-0.61	1.00000000	duck
Ankrd17	-0.72	1.00000000	duck
Trps1	0.52	1.00000000	duck
Hras	0.34	1.00000000	duck
Chek2	0.08	1.00000000	duck
Lrig2	-1.14	1.00000000	duck
Osbpl8	-0.54	1.00000000	duck
Adsl	0.37	1.00000000	duck
Rab3gap1	0.43	1.00000000	duck
Cds2	0.45	1.00000000	duck
Pbx1	-0.37	1.00000000	duck
Parva	0.24	1.00000000	duck
Samd8	0.20	1.00000000	duck
Snrnp35	0.18	1.00000000	duck
Washc4	-0.30	1.00000000	duck
Nfyb	0.41	1.00000000	duck
Riox2	0.17	1.00000000	duck
Efnb2	1.18	1.00000000	duck
Wdr35	0.12	1.00000000	duck
Dync2li1	0.21	1.00000000	duck
Tmem38b	0.23	1.00000000	duck
Gsc	0.38	1.00000000	duck
Slc29a3	0.16	1.00000000	duck
Urod	0.19	1.00000000	duck
Ptx3	0.37	1.00000000	duck
Gemin4	0.13	1.00000000	duck
Cfd	0.26	1.00000000	duck
Plek	0.35	1.00000000	duck
Pdia4	0.51	1.00000000	duck
Adam19	0.50	1.00000000	duck
Kpna3	0.21	1.00000000	duck
Sdad1	0.73	1.00000000	duck
Ndufa10	0.67	1.00000000	duck
Nsmaf	0.45	1.00000000	duck
Nf1	0.53	1.00000000	duck
Lpxn	0.18	1.00000000	duck
Parp8	0.50	1.00000000	duck
Aldh5a1	-0.37	1.00000000	duck
Dnajc11	-0.24	1.00000000	duck
Rpn1	0.44	1.00000000	duck
Nadk2	-0.70	1.00000000	duck

Cdca4	0.17	1.00000000	duck
Ensa	-0.30	1.00000000	duck
Sepsecs	0.24	1.00000000	duck
Dsel	0.23	1.00000000	duck
Arvcf	1.20	1.00000000	duck
Tlk2	-0.49	1.00000000	duck
Anapc2	0.77	1.00000000	duck
Sin3a	-0.37	1.00000000	duck
Cbx1	-0.23	1.00000000	duck
Hdgfl3	0.62	1.00000000	duck
Ext1	0.64	1.00000000	duck
Zdhhc21	0.24	1.00000000	duck
Sync	-0.17	1.00000000	duck
Tatdn1	0.26	1.00000000	duck
Dcaf13	0.14	1.00000000	duck
Qtrt2	0.17	1.00000000	duck
Nfia	1.64	1.00000000	duck
Slc9a8	-0.05	1.00000000	duck
Ncoa2	0.59	1.00000000	duck
Aup1	0.86	1.00000000	duck
Trip4	0.05	1.00000000	duck
Aff2	0.94	1.00000000	duck
Arid2	0.46	1.00000000	duck
Ddb1	-1.00	1.00000000	duck
P2ry1	-0.38	1.00000000	duck
Ttc39a	0.39	1.00000000	duck
Bhlhe40	0.65	1.00000000	duck
Gpr180	-0.09	1.00000000	duck
Casp3	-0.08	1.00000000	duck
Pdia3	0.53	1.00000000	duck
Cwf19l2	0.45	1.00000000	duck
Ppp1r26	-0.30	1.00000000	duck
Zc3h12b	0.63	1.00000000	duck
Plcb1	0.97	1.00000000	duck
Chrm3	0.98	1.00000000	duck
Rnf165	0.14	1.00000000	duck
Tnr	0.16	1.00000000	duck
Fgf12	0.32	1.00000000	duck
Sik2	0.27	1.00000000	duck
Tex14	0.20	1.00000000	duck
Tmem218	0.18	1.00000000	duck
Cog1	0.28	1.00000000	duck
Sf3b6	0.43	1.00000000	duck
Bpnt2	-0.43	1.00000000	duck
Ppp2ca	-0.60	1.00000000	duck
Timm17a	0.36	1.00000000	duck

Pparg	-0.24	1.00000000	duck
Dennd1b	0.46	1.00000000	duck
Eogt	-0.42	1.00000000	duck
Kdm4b	-0.61	1.00000000	duck
Klhdc2	0.12	1.00000000	duck
Pdxk	-0.92	1.00000000	duck
Tpp2	-0.69	1.00000000	duck
Reep2	0.17	1.00000000	duck
Aagab	0.25	1.00000000	duck
Tedc1	0.40	1.00000000	duck
Heatr6	0.43	1.00000000	duck
Taf13	0.35	1.00000000	duck
Cep20	-0.61	1.00000000	duck
Gatad2a	0.17	1.00000000	duck
Deaf1	0.27	1.00000000	duck
Oxsm	0.15	1.00000000	duck
Cidea	0.89	1.00000000	duck
Klhdc3	0.12	1.00000000	duck
Rgs4	0.17	1.00000000	duck
Slc41a3	0.12	1.00000000	duck
Ctu2	0.10	1.00000000	duck
Tada2a	0.15	1.00000000	duck
Anxa11	0.30	1.00000000	duck
Rbpj	0.28	1.00000000	duck
Fchsd2	0.46	1.00000000	duck
Pitpna	-0.47	1.00000000	duck
Cfap97	-0.55	1.00000000	duck
Trp53bp1	-0.24	1.00000000	duck
Rcbtb1	0.31	1.00000000	duck
Pdp1	-0.76	1.00000000	duck
Chaf1a	-0.45	1.00000000	duck
Hook1	-0.40	1.00000000	duck
Etv3	0.39	1.00000000	duck
1700034I23Rik	0.40	1.00000000	duck
Ppard	-0.50	1.00000000	duck
Ehd4	0.29	1.00000000	duck
Atp5j	0.46	1.00000000	duck
Scaper	0.00	1.00000000	duck
Arhgef18	0.27	1.00000000	duck
Fbxw11	-0.63	1.00000000	duck
Dcp1b	0.30	1.00000000	duck
Ptpn12	-0.06	1.00000000	duck
Setdb1	-0.66	1.00000000	duck
Cbl	-0.71	1.00000000	duck
Calm1	0.52	1.00000000	duck
Slc25a25	0.38	1.00000000	duck

Agpat4	0.19	1.00000000	duck
Actb	0.59	1.00000000	duck
Polr3b	0.05	1.00000000	duck
Tgm4	0.65	1.00000000	duck
Abl2	0.09	1.00000000	duck
Naaladl2	0.45	1.00000000	duck
Nde1	0.17	1.00000000	duck
Tmem35a	0.26	1.00000000	duck
Gnat2	0.17	1.00000000	duck
Creb5	0.61	1.00000000	duck
Casr	-0.25	1.00000000	duck
Smchd1	-0.11	1.00000000	duck
Dtnbp1	0.40	1.00000000	duck
Rtcb	0.23	1.00000000	duck
Ccdc137	0.37	1.00000000	duck
Lmnb1	0.51	1.00000000	duck
Arfgef2	0.68	1.00000000	duck
Ppp1cb	-0.85	1.00000000	duck
Esrrg	0.80	1.00000000	duck
Tmed5	-0.66	1.00000000	duck
Tmem11	-0.20	1.00000000	duck
Hsd17b4	-0.14	1.00000000	duck
Zbtb38	-0.01	1.00000000	duck
Tfrc	-1.52	1.00000000	duck
Tmem250-ps	-0.06	1.00000000	duck
Il17ra	0.51	1.00000000	duck
Golgb1	-0.98	1.00000000	duck
Ldb1	-0.37	1.00000000	duck
Dnajb6	-0.28	1.00000000	duck
Dusp23	0.31	1.00000000	duck
Kdm1b	-0.51	1.00000000	duck
Psd3	0.76	1.00000000	duck
Lasp1	0.57	1.00000000	duck
Npc2	0.48	1.00000000	duck
Per2	-1.40	1.00000000	duck
Rab39	-0.39	1.00000000	duck
Pp2d1	-0.15	1.00000000	duck
Tmppe	-0.15	1.00000000	duck
Fkbp9	-0.16	1.00000000	duck
Pnpla2	-0.14	1.00000000	duck
Xrcc3	-0.31	1.00000000	duck
Lrrc34	-0.18	1.00000000	duck
Rnf14	-0.39	1.00000000	duck
Gfer	-0.32	1.00000000	duck
Anks3	-0.14	1.00000000	duck
Serpind1	-0.12	1.00000000	duck

Cmklr1	-0.21	1.00000000	duck
Pik3cd	-0.35	1.00000000	duck
Tekt2	-0.14	1.00000000	duck
Wdr55	-0.10	1.00000000	duck
Sh3gl2	-0.18	1.00000000	duck
Washc3	-0.16	1.00000000	duck
Gramd4	-0.46	1.00000000	duck
Il13ra1	0.63	1.00000000	duck
Scaf8	-0.90	1.00000000	duck
Satb1	1.17	1.00000000	duck
Dhx40	-0.29	1.00000000	duck
Chd1l	0.70	1.00000000	duck
Ikzf2	1.22	1.00000000	duck
Nob1	0.61	1.00000000	duck
Emc3	0.20	1.00000000	duck
Dgkd	0.37	1.00000000	duck
Poldip3	0.15	1.00000000	duck
Slc30a10	-0.20	1.00000000	duck
Ptpn14	0.10	1.00000000	duck
Cog5	0.11	1.00000000	duck
Ap1b1	-0.22	1.00000000	duck
Eif4a2	-0.33	1.00000000	duck
Phtf1	-0.38	1.00000000	duck
Ptbp2	0.17	1.00000000	duck
Ndufs5	0.50	1.00000000	duck
Ppm1m	0.48	1.00000000	duck
Ntpcr	0.15	1.00000000	duck
Prkg2	1.12	1.00000000	duck
Itpa	0.20	1.00000000	duck
Cpsf4	0.39	1.00000000	duck
Pacc1	-0.30	1.00000000	duck
Crls1	0.17	1.00000000	duck
Tnks2	-1.40	1.00000000	duck
Stam2	-0.83	1.00000000	duck
Palld	-2.47	1.00000000	duck
Kmt2e	-0.33	1.00000000	duck
Acadl	-0.42	1.00000000	duck
Flrt3	-0.46	1.00000000	duck
Hspa4	-0.71	1.00000000	duck
Foxo1	0.21	1.00000000	duck
Zbtb41	-0.93	1.00000000	duck
Fyb	-0.14	1.00000000	duck
Smarca1	-0.72	1.00000000	duck
Hivep2	-0.75	1.00000000	duck
St3gal6	0.21	1.00000000	duck
Cap1	-0.45	1.00000000	duck

Usp44	0.68	1.00000000	duck
Chaf1b	0.47	1.00000000	duck
Zfp608	1.31	1.00000000	duck
Phlpp2	0.42	1.00000000	duck
Hk2	-0.17	1.00000000	duck
Pwwp2b	0.03	1.00000000	duck
Ndufs6	-0.73	1.00000000	duck
Ap1ar	-0.20	1.00000000	duck
Gnptab	1.06	1.00000000	duck
Anxa4	0.30	1.00000000	duck
Fen1	0.15	1.00000000	duck
Actl6a	0.34	1.00000000	duck
Dpp8	0.18	1.00000000	duck
Hcn4	0.85	1.00000000	duck
Tcaim	-0.23	1.00000000	duck
Aasdh	-0.65	1.00000000	duck
Fam8a1	-0.72	1.00000000	duck
Tnrc6b	-2.27	1.00000000	duck
Fam214a	0.26	1.00000000	duck
Commd2	-0.54	1.00000000	duck
B4galt1	0.58	1.00000000	duck
Ulk2	-1.40	1.00000000	duck
Tomm7	0.42	1.00000000	duck
Nr4a2	0.67	1.00000000	duck
Znrf3	0.25	1.00000000	duck
Adprm	0.27	1.00000000	duck
Pdgfa	-0.31	1.00000000	duck
Ing4	0.33	1.00000000	duck
Tpm2	0.57	1.00000000	duck
Vamp4	0.27	1.00000000	duck
Cdc26	0.50	1.00000000	duck
Ptpn6	-0.50	1.00000000	duck
Acsf3	0.71	1.00000000	duck
Dlst	-0.14	1.00000000	duck
Chchd3	0.32	1.00000000	duck
Rgp1	0.20	1.00000000	duck
Rnf123	0.15	1.00000000	duck
Txndc15	0.19	1.00000000	duck
Notch2	-0.25	1.00000000	duck
Sec14l1	0.30	1.00000000	duck
Adss	0.32	1.00000000	duck
Zbtb21	-0.52	1.00000000	duck
Erlin2	-0.82	1.00000000	duck
Plekhj1	0.31	1.00000000	duck
Stag1	0.49	1.00000000	duck
LTO1	0.27	1.00000000	duck

Tm6sf1	-0.25	1.00000000	duck
Ccng1	0.72	1.00000000	duck
Klf6	0.01	1.00000000	duck
Cyth1	-0.31	1.00000000	duck
Ddx5	-0.44	1.00000000	duck
Gramd2	0.25	1.00000000	duck
Itpr2	0.48	1.00000000	duck
Pmpcb	-0.17	1.00000000	duck
Gar1	-0.10	1.00000000	duck
Ssbp1	0.65	1.00000000	duck
Smarcc1	-0.45	1.00000000	duck
Traf6	0.04	1.00000000	duck
Cpeb4	0.22	1.00000000	duck
Chac2	0.57	1.00000000	duck
Fam13c	0.62	1.00000000	duck
Serpini1	0.11	1.00000000	duck
Fam76b	-0.18	1.00000000	duck
Hmgcs1	-0.13	1.00000000	duck
Adap2	0.23	1.00000000	duck
AU040320	-0.23	1.00000000	duck
Kdelr2	0.28	1.00000000	duck
Commd8	0.65	1.00000000	duck
Rnf144b	0.17	1.00000000	duck
Upf1	0.28	1.00000000	duck
Tfdp1	-0.65	1.00000000	duck
Lyrm1	-0.79	1.00000000	duck
Prpf4b	-0.51	1.00000000	duck
R3hdm1	0.36	1.00000000	duck
Dvl1	-0.06	1.00000000	duck
Gatad1	0.27	1.00000000	duck
Cstf1	-0.36	1.00000000	duck
Ascc2	0.41	1.00000000	duck
Slc43a2	-0.20	1.00000000	duck
Fam126a	0.26	1.00000000	duck
Ell2	-0.38	1.00000000	duck
Jmjd8	0.38	1.00000000	duck
Psmb1	0.25	1.00000000	duck
Adrm1	-0.26	1.00000000	duck
4930523C07Rik	0.56	1.00000000	duck
Sipa1l1	0.94	1.00000000	duck
Tenm3	0.52	1.00000000	duck
Fam13a	0.37	1.00000000	duck
Ambra1	0.16	1.00000000	duck
Tmem131l	0.12	1.00000000	duck
Angptl2	-0.43	1.00000000	duck
Dyrk2	0.47	1.00000000	duck

Scamp3	0.47	1.00000000	duck
Abhd2	-0.39	1.00000000	duck
Diaph2	0.27	1.00000000	duck
Fnbp1	0.46	1.00000000	duck
Rpusd2	-0.16	1.00000000	duck
Nabp1	0.60	1.00000000	duck
Prkcd	0.44	1.00000000	duck
Ipo5	-0.43	1.00000000	duck
Cltc	-0.66	1.00000000	duck
AW554918	0.49	1.00000000	duck
Gns	0.20	1.00000000	duck
N4bp2	0.44	1.00000000	duck
Nudt21	0.10	1.00000000	duck
Ss18	-0.70	1.00000000	duck
Dnajc5	0.64	1.00000000	duck
C2cd5	-0.40	1.00000000	duck
Pgap2	-0.11	1.00000000	duck
Dync2i1	0.22	1.00000000	duck
Pam	0.12	1.00000000	duck
Snx24	0.53	1.00000000	duck
Nlrc5	0.20	1.00000000	duck
Ephx1	0.61	1.00000000	duck
Fabp5	0.58	1.00000000	duck
Tmem165	-0.44	1.00000000	duck
Ppm1l	0.55	1.00000000	duck
Sap130	-0.48	1.00000000	duck
Ercc6	-0.22	1.00000000	duck
Agl	-0.25	1.00000000	duck
Rasgef1b	0.85	1.00000000	duck
Puf60	-0.73	1.00000000	duck
Kdm3b	-0.49	1.00000000	duck
Stum	0.50	1.00000000	duck
Strn3	-0.14	1.00000000	duck
Smurf1	0.92	1.00000000	duck
Exoc6b	0.38	1.00000000	duck
Ythdf3	0.21	1.00000000	duck
Plekhm2	0.39	1.00000000	duck
Yeats2	0.33	1.00000000	duck
Fryl	-1.82	1.00000000	duck
Atp2b4	0.53	1.00000000	duck
Rnf145	-0.12	1.00000000	duck
Ncln	0.42	1.00000000	duck
Psmc3	0.45	1.00000000	duck
Eif2b2	-0.04	1.00000000	duck
Ints1	0.27	1.00000000	duck
Tle4	0.95	1.00000000	duck

Ttc39b	0.59	1.00000000	duck
Rcn2	0.46	1.00000000	duck
Lbh	0.73	1.00000000	duck
Tbxas1	-0.52	1.00000000	duck
Nfx1	-0.48	1.00000000	duck
Pin1	0.68	1.00000000	duck
Ski	0.35	1.00000000	duck
Slc35a3	0.18	1.00000000	duck
Smpdl3a	0.34	1.00000000	duck
Ccdc125	0.18	1.00000000	duck
Scamp5	-0.14	1.00000000	duck
Phf12	-0.80	1.00000000	duck
Zdhhc12	-0.36	1.00000000	duck
Snu13	0.23	1.00000000	duck
Dynlt3	0.35	1.00000000	duck
Trip12	-0.68	1.00000000	duck
Tmcc1	-1.17	1.00000000	duck
Fbxo46	-0.17	1.00000000	duck
Txnl1	-0.30	1.00000000	duck
Cnot7	-1.24	1.00000000	duck
Fbxo38	0.44	1.00000000	duck
Rmi2	0.27	1.00000000	duck
Dhx29	0.11	1.00000000	duck
Samd11	0.67	1.00000000	duck
Sbno1	-0.89	1.00000000	duck
Zfp827	0.30	1.00000000	duck
Iqcb1	0.35	1.00000000	duck
Srp14	0.14	1.00000000	duck
Bank1	0.47	1.00000000	duck
Pik3r1	0.12	1.00000000	duck
Gabarapl1	-0.65	1.00000000	duck
Plec	-0.69	1.00000000	duck
Zfp639	0.05	1.00000000	duck
Tcf3	0.11	1.00000000	duck
Gsdme	-0.18	1.00000000	duck
Zbtb33	-0.14	1.00000000	duck
Elmod2	-0.14	1.00000000	duck
B4galt5	-0.34	1.00000000	duck
Golm2	-0.72	1.00000000	duck
Gclc	0.32	1.00000000	duck
Ggcx	0.11	1.00000000	duck
Dennd5a	0.18	1.00000000	duck
Vdr	-0.50	1.00000000	duck
Creg1	-2.43	1.00000000	duck
Ppp2r5c	-1.42	1.00000000	duck
Rbms1	-0.20	1.00000000	duck

Zbtb18	0.35	1.00000000	duck
St13	-0.20	1.00000000	duck
Hmgb3	0.39	1.00000000	duck
Spred2	-0.28	1.00000000	duck
Rer1	0.10	1.00000000	duck
Atp6v0e	0.34	1.00000000	duck
Ndfip1	0.40	1.00000000	duck
Net1	-0.47	1.00000000	duck
Nup107	-1.07	1.00000000	duck
Slc7a5	0.51	1.00000000	duck
Farp2	-0.22	1.00000000	duck
Dym	0.25	1.00000000	duck
Wdr36	0.76	1.00000000	duck
Gata3	1.22	1.00000000	duck
Pln	-0.34	1.00000000	duck
Cry2	-0.13	1.00000000	duck
Hyi	-0.14	1.00000000	duck
B4galt2	0.26	1.00000000	duck
Uhrf1bp1	-0.30	1.00000000	duck
Armh3	-0.52	1.00000000	duck
Dbnl	0.53	1.00000000	duck
Jun	0.20	1.00000000	duck
Taok3	-0.65	1.00000000	duck
Dnm1l	-0.86	1.00000000	duck
Nipal3	-0.22	1.00000000	duck
Rbm22	0.13	1.00000000	duck
Aars	0.24	1.00000000	duck
Pcnt	-0.75	1.00000000	duck
Rexo1	-0.91	1.00000000	duck
Smarca2	-0.06	1.00000000	duck
Ino80d	0.51	1.00000000	duck
Anapc7	-0.35	1.00000000	duck
Erlin1	-0.04	1.00000000	duck
Gprin3	-0.62	1.00000000	duck
Dpp9	-0.16	1.00000000	duck
Vapa	0.31	1.00000000	duck
Herpud2	0.29	1.00000000	duck
Slc9a7	-0.49	1.00000000	duck
Eif3g	0.34	1.00000000	duck
Osbpl11	-0.02	1.00000000	duck
Camk1d	0.58	1.00000000	duck
Rbl2	-0.81	1.00000000	duck
Stk38	-0.78	1.00000000	duck
Slc48a1	0.43	1.00000000	duck
Slc25a38	-0.01	1.00000000	duck
Lsm3	0.40	1.00000000	duck

Paip2	-0.46	1.00000000	duck
Farsb	0.36	1.00000000	duck
Srf	0.35	1.00000000	duck
Snx20	-0.11	1.00000000	duck
Wars	-0.19	1.00000000	duck
Cdk12	0.54	1.00000000	duck
Fam135a	0.74	1.00000000	duck
Rprd1b	-0.55	1.00000000	duck
Nyx	0.31	1.00000000	duck
Ncor2	0.35	1.00000000	duck
Psmb7	-0.11	1.00000000	duck
Hdac11	-0.74	1.00000000	duck
Asf1a	-0.82	1.00000000	duck
Sh3bp4	0.16	1.00000000	duck
Abhd17c	-0.39	1.00000000	duck
Psmd6	0.38	1.00000000	duck
Chpf2	0.11	1.00000000	duck
Slc25a5	0.17	1.00000000	duck
Ska2	-0.05	1.00000000	duck
Crem	-0.38	1.00000000	duck
Btf3	0.57	1.00000000	duck
Smim19	-0.39	1.00000000	duck
Ddx23	-0.18	1.00000000	duck
Tbc1d15	-0.48	1.00000000	duck
Acsl1	0.43	1.00000000	duck
Ywhah	0.56	1.00000000	duck
Arhgap22	-0.50	1.00000000	duck
Tubgcp5	-0.35	1.00000000	duck
Rabggtb	-0.45	1.00000000	duck
Celf2	0.40	1.00000000	duck
Prkcb	-0.54	1.00000000	duck
Cdc42bpb	-0.26	1.00000000	duck
Klf9	0.15	1.00000000	duck
Bahd1	-0.20	1.00000000	duck
Slc7a11	1.10	1.00000000	duck
Slc25a47	0.13	1.00000000	duck
Lekr1	0.53	1.00000000	duck
Gpr155	-0.12	1.00000000	duck
Dop1b	0.11	1.00000000	duck
Trp53inp2	-0.16	1.00000000	duck
Hint1	0.50	1.00000000	duck
Celf1	-0.25	1.00000000	duck
Thoc7	0.25	1.00000000	duck
Txlng	-0.98	1.00000000	duck
Snx17	0.38	1.00000000	duck
Eri3	-0.09	1.00000000	duck

Sh3bgrl3	0.41	1.00000000	duck
Senp7	0.05	1.00000000	duck
Srek1ip1	0.24	1.00000000	duck
Rsu1	0.55	1.00000000	duck
Coq7	-0.18	1.00000000	duck
Hlf	0.36	1.00000000	duck
Tmem123	0.18	1.00000000	duck
Usp7	-0.96	1.00000000	duck
Snrpb	0.49	1.00000000	duck
Ctif	0.30	1.00000000	duck
Cradd	0.06	1.00000000	duck
Hbp1	-0.67	1.00000000	duck
Glis2	0.59	1.00000000	duck
Gpatch1	-0.43	1.00000000	duck
Eif3a	0.30	1.00000000	duck
Taf3	0.29	1.00000000	duck
Ampd2	-0.26	1.00000000	duck
Opa1	-0.06	1.00000000	duck
Pex14	0.36	1.00000000	duck
Vezt	-0.61	1.00000000	duck
Acp2	0.45	1.00000000	duck
Usp6nl	-0.88	1.00000000	duck
Slc45a4	-0.66	1.00000000	duck
Nsmce2	0.36	1.00000000	duck
Rnh1	0.57	1.00000000	duck
Ubac1	0.36	1.00000000	duck
Tsr1	-0.09	1.00000000	duck
Ywhaq	-0.31	1.00000000	duck
Dhx38	-0.38	1.00000000	duck
Ing5	-0.20	1.00000000	duck
Csgalnact2	-0.56	1.00000000	duck
Pdcd11	-0.26	1.00000000	duck
Flot2	-0.45	1.00000000	duck
Scamp2	0.64	1.00000000	duck
Dhx36	-0.38	1.00000000	duck
Rab22a	-0.62	1.00000000	duck
Tmem138	-0.18	1.00000000	duck
Rag2	-1.49	1.00000000	duck
Oser1	0.14	1.00000000	duck
Rala	-0.31	1.00000000	duck
Cenpu	0.69	1.00000000	duck
Rasa2	-0.43	1.00000000	duck
Tbc1d9	0.65	1.00000000	duck
Irak2	0.55	1.00000000	duck
Zcchc2	0.09	1.00000000	duck
Spry1	1.60	1.00000000	duck

Cep135	0.72	1.00000000	duck
Ssu72	-0.61	1.00000000	duck
Stk24	-0.35	1.00000000	duck
Ogfr	0.29	1.00000000	duck
Ctdspl	-0.22	1.00000000	duck
Eif4g3	0.22	1.00000000	duck
Sowahc	0.25	1.00000000	duck
Gkap1	-0.48	1.00000000	duck
Pcyt1a	0.25	1.00000000	duck
Casp9	-0.08	1.00000000	duck
Tada1	0.00	1.00000000	duck
Emg1	0.58	1.00000000	duck
Acyp2	0.69	1.00000000	duck
Frmd7	0.47	1.00000000	duck
Sav1	0.37	1.00000000	duck
Senp8	0.34	1.00000000	duck
Wdr24	0.16	1.00000000	duck
Spout1	0.22	1.00000000	duck
Gdpd1	0.62	1.00000000	duck
Hdac2	0.34	1.00000000	duck
Cct2	0.82	1.00000000	duck
Pelo	0.05	1.00000000	duck
Brd3	-0.81	1.00000000	duck
Gpsm1	-0.24	1.00000000	duck
Hdac4	0.21	1.00000000	duck
Timp2	0.02	1.00000000	duck
Polr2e	0.51	1.00000000	duck
Polr2h	0.45	1.00000000	duck
Adam8	-0.43	1.00000000	duck
Fem1a	-0.17	1.00000000	duck
Tm2d2	0.33	1.00000000	duck
Tarsl2	0.35	1.00000000	duck
Ilvbl	0.58	1.00000000	duck
Alg13	0.17	1.00000000	duck
Zzz3	0.05	1.00000000	duck
Hsp90b1	0.20	1.00000000	duck
Pcyt2	0.25	1.00000000	duck
Sdf4	-0.46	1.00000000	duck
Dynll2	0.41	1.00000000	duck
Rspry1	-0.56	1.00000000	duck
Eif4ebp1	0.34	1.00000000	duck
Rock2	0.18	1.00000000	duck
Htra1	-0.71	1.00000000	duck
Rel	0.36	1.00000000	duck
Rfng	-0.18	1.00000000	duck
Cntn1	-0.83	1.00000000	duck

Rnls	0.56	1.00000000	duck
Gtf2a2	0.57	1.00000000	duck
Kdm1a	-1.09	1.00000000	duck
Ndufb4	0.68	1.00000000	duck
Tapt1	-1.60	1.00000000	duck
Ints9	-0.36	1.00000000	duck
Ublcp1	0.08	1.00000000	duck
Tbl2	-0.09	1.00000000	duck
Elmo2	-0.46	1.00000000	duck
Zfp438	-1.09	1.00000000	duck
Prmt9	0.17	1.00000000	duck
Fpgt	0.14	1.00000000	duck
Cited4	0.73	1.00000000	duck
Ube2q2	-0.48	1.00000000	duck
Ppp2r5a	-0.61	1.00000000	duck
Ide	0.64	1.00000000	duck
Atl2	-0.02	1.00000000	duck
Cep104	0.26	1.00000000	duck
Slc16a6	-0.85	1.00000000	duck
Supt7l	-1.29	1.00000000	duck
Cdk5r1	-0.20	1.00000000	duck
Rora	-0.01	1.00000000	duck
Prex1	0.45	1.00000000	duck
Znfx1	0.08	1.00000000	duck
Fars2	0.58	1.00000000	duck
Ano10	0.37	1.00000000	duck
Fcf1	0.69	1.00000000	duck
Zfp638	-0.75	1.00000000	duck
Tspan12	-0.20	1.00000000	duck
Exosc2	-0.17	1.00000000	duck
Trib2	0.20	1.00000000	duck
Hnrnpr	0.19	1.00000000	duck
Atxn1	0.28	1.00000000	duck
Wdr44	-0.43	1.00000000	duck
Btbd7	-0.36	1.00000000	duck
Spic	-0.37	1.00000000	duck
Acss1	-0.27	1.00000000	duck
Prpf8	-0.57	1.00000000	duck
Rasal2	0.59	1.00000000	duck
Dennd6a	-1.03	1.00000000	duck
Fam53b	0.22	1.00000000	duck
Tubgcp2	0.34	1.00000000	duck
Pias4	0.18	1.00000000	duck
Arl6ip5	0.26	1.00000000	duck
Cop1	-0.90	1.00000000	duck
Apoo	0.45	1.00000000	duck

Alg8	0.15	1.00000000	duck
Diaph3	0.35	1.00000000	duck
Spire1	0.64	1.00000000	duck
Depdc1b	0.20	1.00000000	duck
Tox2	0.65	1.00000000	duck
Pi4ka	-0.70	1.00000000	duck
Rnf19a	0.20	1.00000000	duck
Slc30a4	0.11	1.00000000	duck
Fes	-0.27	1.00000000	duck
Bfar	-0.20	1.00000000	duck
Septin12	0.35	1.00000000	duck
Unc119	-0.48	1.00000000	duck
Sdf2	0.18	1.00000000	duck
Herc2	-1.28	1.00000000	duck
Rfx3	0.32	1.00000000	duck
Acsl4	-0.41	1.00000000	duck
Emc7	-0.81	1.00000000	duck
Pitpnm3	-0.14	1.00000000	duck
Nans	0.22	1.00000000	duck
Ube2d1	-0.69	1.00000000	duck
Gpi1	0.60	1.00000000	duck
Nxt2	-0.58	1.00000000	duck
Lmbrd2	0.01	1.00000000	duck
Amfr	-0.34	1.00000000	duck
Kidins220	-0.50	1.00000000	duck
Gpatch8	-0.72	1.00000000	duck
Cadm1	0.58	1.00000000	duck
Serinc3	0.25	1.00000000	duck
Bud31	-0.08	1.00000000	duck
Sdf2l1	0.54	1.00000000	duck
Sigmar1	0.17	1.00000000	duck
Ccne1	0.29	1.00000000	duck
Srm	0.19	1.00000000	duck
Crtam	0.61	1.00000000	duck
Acox1	-0.57	1.00000000	duck
Ppic	0.91	1.00000000	duck
Aldh18a1	0.32	1.00000000	duck
Lrrc42	0.36	1.00000000	duck
Socs3	0.22	1.00000000	duck
Rfc2	0.13	1.00000000	duck
Dffa	0.41	1.00000000	duck
Fam162a	0.28	1.00000000	duck
Urb1	0.14	1.00000000	duck
Eef1akmt1	0.20	1.00000000	duck
Acot13	0.26	1.00000000	duck
Rbm47	0.92	1.00000000	duck

Slco4c1	0.14	1.00000000	duck
Plbd2	0.12	1.00000000	duck
Ak3	-0.16	1.00000000	duck
Rchy1	0.21	1.00000000	duck
Zfand6	0.36	1.00000000	duck
Prdx4	-0.07	1.00000000	duck
Nubp1	-0.24	1.00000000	duck
Rassf3	0.39	1.00000000	duck
Slc25a24	-0.81	1.00000000	duck
Rabep1	0.12	1.00000000	duck
Kdsr	0.10	1.00000000	duck
Rad17	0.12	1.00000000	duck
Plekhn1	-0.01	1.00000000	duck
Cebpz	0.19	1.00000000	duck
Dip2b	-0.19	1.00000000	duck
Fyn	0.59	1.00000000	duck
Abcg1	0.71	1.00000000	duck
Ldah	0.31	1.00000000	duck
Gna11	-0.23	1.00000000	duck
Qk	0.19	1.00000000	duck
Ecpas	0.34	1.00000000	duck
Gpr85	-0.39	1.00000000	duck
Haus3	-0.14	1.00000000	duck
Tecpr1	-0.17	1.00000000	duck
Rufy3	0.47	1.00000000	duck
Capn7	-1.84	1.00000000	duck
Zfp652	-0.72	1.00000000	duck
Tcf7l2	-0.09	1.00000000	duck
Rnft1	-0.12	1.00000000	duck
Ralgapa1	-0.76	1.00000000	duck
Ctss	0.43	1.00000000	duck
Cnot1	-0.56	1.00000000	duck
Tdp2	0.11	1.00000000	duck
F13a1	0.27	1.00000000	duck
Efhc1	0.17	1.00000000	duck
Nbn	0.10	1.00000000	duck
Irf4	0.34	1.00000000	duck
Lyar	0.18	1.00000000	duck
Hnrnph3	-0.70	1.00000000	duck
Pxylp1	-0.19	1.00000000	duck
Isoc1	-0.20	1.00000000	duck
Rest	-0.82	1.00000000	duck
Supt4a	0.44	1.00000000	duck
Ing1	-0.21	1.00000000	duck
Tmc7	-0.22	1.00000000	duck
Psmg4	0.45	1.00000000	duck

Ptpn9	-1.10	1.00000000	duck
Pfdn4	-0.15	1.00000000	duck
Cdc42se1	0.53	1.00000000	duck
Nfkb2	-0.07	1.00000000	duck
Tars	0.36	1.00000000	duck
Grsf1	0.34	1.00000000	duck
Dusp10	0.49	1.00000000	duck
Stoml3	-0.16	1.00000000	duck
Arel1	-0.11	1.00000000	duck
Golm1	-0.05	1.00000000	duck
Papolg	0.48	1.00000000	duck
Tnrc6c	-0.43	1.00000000	duck
Nup35	0.12	1.00000000	duck
Rfc4	0.32	1.00000000	duck
Acot7	0.10	1.00000000	duck
Rab11fip2	0.82	1.00000000	duck
Srsf5	-0.38	1.00000000	duck
Nudcd1	-0.37	1.00000000	duck
Erich6	0.00	1.00000000	duck
Wdr48	0.15	1.00000000	duck
Rab14	-0.52	1.00000000	duck
Zmym2	0.67	1.00000000	duck
Dapp1	-0.56	1.00000000	duck
Trib1	0.41	1.00000000	duck
Gpalpp1	0.45	1.00000000	duck
Hivep1	0.22	1.00000000	duck
Pde6d	0.25	1.00000000	duck
Ppcs	-0.10	1.00000000	duck
Lztr1	0.25	1.00000000	duck
Commd4	0.45	1.00000000	duck
Tmem154	0.25	1.00000000	duck
Smad5	-0.36	1.00000000	duck
Il1rl1	0.37	1.00000000	duck
Pde9a	-0.03	1.00000000	duck
Setbp1	0.83	1.00000000	duck
Cep170b	0.25	1.00000000	duck
Bmp2k	0.40	1.00000000	duck
Uggt1	-0.86	1.00000000	duck
Pdk3	-1.22	1.00000000	duck
Tmx3	-0.31	1.00000000	duck
Nr1h3	-0.33	1.00000000	duck
Atxn2	-0.50	1.00000000	duck
Adprh	-0.16	1.00000000	duck
Agap1	1.46	1.00000000	duck
Ctps2	-0.63	1.00000000	duck
Tmed2	-0.26	1.00000000	duck

Zswim8	-0.65	1.00000000	duck
Cd47	0.13	1.00000000	duck
Slc23a2	-0.08	1.00000000	duck
Chp1	0.51	1.00000000	duck
Eif3h	-0.50	1.00000000	duck
Rnf150	0.53	1.00000000	duck
Wfs1	0.13	1.00000000	duck
Aspg	0.36	1.00000000	duck
Sgpl1	0.34	1.00000000	duck
Gtf3c1	-0.62	1.00000000	duck
Cnot8	0.37	1.00000000	duck
Rhog	0.49	1.00000000	duck
Zfp207	-0.92	1.00000000	duck
Nipsnap1	-0.19	1.00000000	duck
Zfp706	0.34	1.00000000	duck
Ezh2	-0.63	1.00000000	duck
Rnf20	0.37	1.00000000	duck
Ints6	-0.57	1.00000000	duck
Necap1	-0.32	1.00000000	duck
Rnpc3	-0.36	1.00000000	duck
Yipf4	-0.75	1.00000000	duck
Cpeb2	-0.50	1.00000000	duck
Ptp4a2	0.13	1.00000000	duck
Rida	-0.05	1.00000000	duck
Fam53a	-0.86	1.00000000	duck
Commd10	0.12	1.00000000	duck
Ddx31	-0.85	1.00000000	duck
Timm13	0.19	1.00000000	duck
Plk2	-0.05	1.00000000	duck
Dock3	-0.39	1.00000000	duck
Drg2	-0.16	1.00000000	duck
Nfya	-0.83	1.00000000	duck
Tmco1	0.32	1.00000000	duck
Tnni2	0.19	1.00000000	duck
Tom1l2	-0.17	1.00000000	duck
Nfs1	-0.08	1.00000000	duck
Drd4	-0.26	1.00000000	duck
Nmd3	-0.47	1.00000000	duck
Tmem231	-0.16	1.00000000	duck
Eri2	0.13	1.00000000	duck
Uba2	-0.92	1.00000000	duck
Vash1	-1.05	1.00000000	duck
Prdm2	-0.27	1.00000000	duck
Fam185a	-0.43	1.00000000	duck
Ap2b1	0.06	1.00000000	duck
Zfyve1	-0.95	1.00000000	duck

Heatr5a	0.18	1.00000000	duck
Pdia6	0.48	1.00000000	duck
Zmpste24	-0.26	1.00000000	duck
Tmem19	-0.12	1.00000000	duck
Acsl3	-0.14	1.00000000	duck
Ccp110	-1.15	1.00000000	duck
Sf3a3	0.20	1.00000000	duck
Taf5l	-0.34	1.00000000	duck
Tmed3	-0.23	1.00000000	duck
Ncoa7	-0.18	1.00000000	duck
Dapk2	0.20	1.00000000	duck
Impa1	0.50	1.00000000	duck
Nup205	-0.17	1.00000000	duck
Pars2	0.52	1.00000000	duck
Nlk	-0.70	1.00000000	duck
Klf11	0.25	1.00000000	duck
Ap5z1	0.18	1.00000000	duck
Nfrkb	-0.07	1.00000000	duck
B9d1	0.17	1.00000000	duck
Gnb1l	0.22	1.00000000	duck
Creb1	-0.30	1.00000000	duck
Ropn1l	0.07	1.00000000	duck
Nkd1	0.07	1.00000000	duck
Pdcd6	-0.24	1.00000000	duck
Psma6	0.48	1.00000000	duck
Slc35b3	0.39	1.00000000	duck
Rasgef1a	0.65	1.00000000	duck
Plbd1	0.69	1.00000000	duck
Acad9	0.16	1.00000000	duck
Rtraf	0.44	1.00000000	duck
Bnip1	-0.30	1.00000000	duck
Gins4	-0.14	1.00000000	duck
Plrg1	-0.01	1.00000000	duck
Akap8	0.57	1.00000000	duck
Syne3	0.25	1.00000000	duck
Nbeal2	0.33	1.00000000	duck
Lin54	0.23	1.00000000	duck
Ngly1	-0.54	1.00000000	duck
Rwdd1	0.26	1.00000000	duck
Ldlrad3	-0.19	1.00000000	duck
Trabd	0.21	1.00000000	duck
Akt3	0.68	1.00000000	duck
Bsdc1	0.42	1.00000000	duck
Lonp2	0.02	1.00000000	duck
Actr6	-0.80	1.00000000	duck
Ncbp3	0.10	1.00000000	duck

Nfkb1	0.32	1.00000000	duck
Trank1	-0.17	1.00000000	duck
Zfp711	-0.82	1.00000000	duck
Rasa1	0.51	1.00000000	duck
Alox5	-1.43	1.00000000	duck
Ppp3cb	0.09	1.00000000	duck
Gpd2	-0.14	1.00000000	duck
Zc3h3	0.13	1.00000000	duck
Gng2	-0.19	1.00000000	duck
Kat6b	0.22	1.00000000	duck
Adpgk	0.09	1.00000000	duck
Bzw1	-0.48	1.00000000	duck
Acot10	0.11	1.00000000	duck
Lims2	-0.53	1.00000000	duck
Ahcyl1	0.22	1.00000000	duck
Lpin2	-0.96	1.00000000	duck
Klhdc1	0.37	1.00000000	duck
Dtymk	0.03	1.00000000	duck
Rapsn	0.21	1.00000000	duck
Dclk3	-0.28	1.00000000	duck
Gli3	0.31	1.00000000	duck
Arf4	-0.41	1.00000000	duck
Rlim	0.28	1.00000000	duck
Samm50	-0.52	1.00000000	duck
Eif5	-0.01	1.00000000	duck
Rmdn1	-0.10	1.00000000	duck
Utp23	-0.11	1.00000000	duck
Pigx	-0.11	1.00000000	duck
Palb2	-0.12	1.00000000	duck
Slc16a3	-0.12	1.00000000	duck
Slc11a2	-0.47	1.00000000	duck
Gpatch3	-0.12	1.00000000	duck
Sag	0.16	1.00000000	duck
Nkrf	0.41	1.00000000	duck
Gpn1	-0.60	1.00000000	duck
Spata2	-0.10	1.00000000	duck
Axin1	-1.00	1.00000000	duck
Hadha	0.35	1.00000000	duck
Nlrx1	0.23	1.00000000	duck
Thap1	-0.72	1.00000000	duck
Insig2	-0.18	1.00000000	duck
B2m	0.24	1.00000000	duck
Ccdc88a	-0.98	1.00000000	duck
Tm9sf4	-0.39	1.00000000	duck
Usp20	-0.34	1.00000000	duck
Plcl2	0.55	1.00000000	duck

Psmd7	-0.12	1.00000000	duck
Get4	0.11	1.00000000	duck
Tmem50a	0.24	1.00000000	duck
Phf21a	0.23	1.00000000	duck
Nav3	0.36	1.00000000	duck
Rabepk	0.34	1.00000000	duck
Cubn	-0.64	1.00000000	duck
Rnf144a	-0.28	1.00000000	duck
Arhgap18	-0.21	1.00000000	duck
Atp11b	-0.24	1.00000000	duck
Babam2	0.39	1.00000000	duck
Fam102b	-0.73	1.00000000	duck
Gbe1	0.41	1.00000000	duck
Cwc27	-0.01	1.00000000	duck
Ust	0.04	1.00000000	duck
Slc25a16	-0.09	1.00000000	duck
Rnf126	-0.19	1.00000000	duck
Hmgcr	0.65	1.00000000	duck
Dnajc24	0.23	1.00000000	duck
Ripor3	-2.48	1.00000000	duck
Usb1	0.43	1.00000000	duck
Cars2	-0.15	1.00000000	duck
Dhrs7b	-0.35	1.00000000	duck
Sec11a	0.30	1.00000000	duck
Far1	0.55	1.00000000	duck
Plcb2	-0.80	1.00000000	duck
Ppif	0.64	1.00000000	duck
Ly86	-0.61	1.00000000	duck
Nr2c2	-0.79	1.00000000	duck
Pi4kb	0.20	1.00000000	duck
Dync1li2	-0.60	1.00000000	duck
Slc39a6	0.20	1.00000000	duck
Cbx6	0.26	1.00000000	duck
Pgap1	0.08	1.00000000	duck
Creld2	-0.40	1.00000000	duck
Wrn	-0.81	1.00000000	duck
Tcerg1	-0.03	1.00000000	duck
Fh1	0.44	1.00000000	duck
Coro1b	-0.03	1.00000000	duck
Arl4a	0.31	1.00000000	duck
Fto	-1.11	1.00000000	duck
Kdm7a	-0.43	1.00000000	duck
Basp1	0.61	1.00000000	duck
Lamtor2	0.46	1.00000000	duck
Ptpn22	0.02	1.00000000	duck
Suv39h2	-0.08	1.00000000	duck

Eme1	0.05	1.00000000	duck
Unc13d	-0.01	1.00000000	duck
Gpc5	0.83	1.00000000	duck
Oga	-0.80	1.00000000	duck
Sh2b2	0.22	1.00000000	duck
Cyrib	0.18	1.00000000	duck
Derl3	0.41	1.00000000	duck
Arsg	-0.18	1.00000000	duck
E4f1	-0.58	1.00000000	duck
Polr2l	-0.33	1.00000000	duck
Chuk	0.26	1.00000000	duck
Borcs5	0.22	1.00000000	duck
Tcea1	-0.19	1.00000000	duck
Ankrd28	0.32	1.00000000	duck
Ash2l	-0.08	1.00000000	duck
Nfyc	0.19	1.00000000	duck
Rnf13	-0.58	1.00000000	duck
Larp1	0.25	1.00000000	duck
Ptpn1	0.26	1.00000000	duck
Wbp2	-0.29	1.00000000	duck
Oat	0.35	1.00000000	duck
Eif3d	0.69	1.00000000	duck
Prpf40a	0.48	1.00000000	duck
Pdss1	0.42	1.00000000	duck
Sdhaf4	0.64	1.00000000	duck
Aurkaip1	0.22	1.00000000	duck
Slc2a1	0.43	1.00000000	duck
Dnase2b	0.39	1.00000000	duck
Arhgap25	0.27	1.00000000	duck
Apool	-0.32	1.00000000	duck
Lman1	-0.36	1.00000000	duck
Iqsec1	-0.46	1.00000000	duck
Utp11	-0.16	1.00000000	duck
Fbxl22	-0.55	1.00000000	duck
Ada	-0.18	1.00000000	duck
Dicer1	-0.95	1.00000000	duck
Zfp236	-0.85	1.00000000	duck
Arid1b	-0.77	1.00000000	duck
Pygl	-0.06	1.00000000	duck
Fhod1	0.15	1.00000000	duck
Zdhhc13	0.43	1.00000000	duck
Rai2	1.04	1.00000000	duck
Esd	0.16	1.00000000	duck
Arid3b	0.26	1.00000000	duck
Rab19	-0.12	1.00000000	duck
Chsy1	0.62	1.00000000	duck

Lrp8	0.17	1.00000000	duck
Abcd3	-0.15	1.00000000	duck
Slx4	-0.02	1.00000000	duck
Kras	0.32	1.00000000	duck
Pik3r6	0.40	1.00000000	duck
Vsir	0.47	1.00000000	duck
Has2	0.28	1.00000000	duck
Hspa9	-0.31	1.00000000	duck
Hemk1	-0.78	1.00000000	duck
Zfand3	-0.24	1.00000000	duck
Cct4	0.20	1.00000000	duck
Clk2	-0.49	1.00000000	duck
Pef1	0.26	1.00000000	duck
Ebna1bp2	0.31	1.00000000	duck
Pex10	-0.49	1.00000000	duck
Zfp507	-0.14	1.00000000	duck
Laptm4a	0.51	1.00000000	duck
Gpatch11	-0.18	1.00000000	duck
Ap3b1	-0.11	1.00000000	duck
Atp5pb	0.20	1.00000000	duck
Pkib	-0.59	1.00000000	duck
Lcp2	1.78	1.00000000	duck
Pla2g4f	-0.70	1.00000000	duck
Rad21	0.24	1.00000000	duck
Bcat1	0.71	1.00000000	duck
Ppp4r1	0.61	1.00000000	duck
Pi4k2b	-0.64	1.00000000	duck
Rbm31y	0.17	1.00000000	duck
Rabgap1l	0.11	1.00000000	duck
Ndufb9	0.41	1.00000000	duck
Tmem183a	-0.65	1.00000000	duck
Bhlhe41	-0.49	1.00000000	duck
Cep170	-0.77	1.00000000	duck
Pptc7	-0.39	1.00000000	duck
Golt1b	0.17	1.00000000	duck
Rnf11	0.21	1.00000000	duck
Cct7	0.22	1.00000000	duck
C1qb	0.33	1.00000000	duck
Gpbp1	-0.70	1.00000000	duck
Tef	-0.84	1.00000000	duck
Dnajc3	-0.14	1.00000000	duck
Shprh	-0.76	1.00000000	duck
Slc30a6	-0.37	1.00000000	duck
Trpc1	-0.17	1.00000000	duck
St3gal4	-0.09	1.00000000	duck
Reps2	-0.84	1.00000000	duck

Lcmt1	-0.08	1.00000000	duck
Trio	0.54	1.00000000	duck
Scn8a	-0.16	1.00000000	duck
Kifap3	-0.20	1.00000000	duck
Tfip11	-0.62	1.00000000	duck
Rnf214	-0.38	1.00000000	duck
Prkci	-0.44	1.00000000	duck
Arhgap21	1.57	1.00000000	duck
Gnptg	0.60	1.00000000	duck
Cdc34	-0.09	1.00000000	duck
Timmdc1	-0.06	1.00000000	duck
Gpx1	0.29	1.00000000	duck
Krr1	0.47	1.00000000	duck
Zmynd8	-0.31	1.00000000	duck
Sparc	-0.26	1.00000000	duck
Snx4	0.68	1.00000000	duck
Wiz	0.08	1.00000000	duck
Naa30	-0.36	1.00000000	duck
Raver2	0.45	1.00000000	duck
Trim24	0.36	1.00000000	duck
Tmem167	0.16	1.00000000	duck
Ttc1	0.31	1.00000000	duck
Csnk2a2	0.27	1.00000000	duck
Picalm	-0.18	1.00000000	duck
Wdr3	-0.33	1.00000000	duck
Omg	-0.17	1.00000000	duck
Phf3	-0.85	1.00000000	duck
Srbd1	0.01	1.00000000	duck
Tm9sf3	0.20	1.00000000	duck
Bcl2	0.31	1.00000000	duck
Psmd14	0.21	1.00000000	duck
Ppp1r3b	-0.35	1.00000000	duck
Zbtb48	-0.37	1.00000000	duck
Idh3a	0.22	1.00000000	duck
Clk4	0.12	1.00000000	duck
Tiprl	0.16	1.00000000	duck
Rtf2	-0.36	1.00000000	duck
Tshz2	0.27	1.00000000	duck
Smndc1	-0.81	1.00000000	duck
Bpnt1	0.32	1.00000000	duck
Arntl	0.18	1.00000000	duck
Fbxo45	-0.18	1.00000000	duck
Ccdc171	-0.10	1.00000000	duck
Eya3	0.00	1.00000000	duck
Fgd2	-0.38	1.00000000	duck
Zbed4	-0.28	1.00000000	duck

Zfp704	0.03	1.00000000	duck
Cdc37l1	-1.06	1.00000000	duck
Fmr1	-0.27	1.00000000	duck
Cetn3	0.66	1.00000000	duck
Pus7l	-0.63	1.00000000	duck
Fbxo30	-0.07	1.00000000	duck
Arhgap17	-0.56	1.00000000	duck
Caap1	-0.16	1.00000000	duck
Fbxo34	-0.11	1.00000000	duck
Ube2h	-0.22	1.00000000	duck
Ppwd1	-0.05	1.00000000	duck
Vrk2	0.23	1.00000000	duck
Immt	0.03	1.00000000	duck
Cdk19	-0.63	1.00000000	duck
Uxs1	0.22	1.00000000	duck
Tyw3	0.14	1.00000000	duck
Poc1a	0.21	1.00000000	duck
Rnf115	0.15	1.00000000	duck
Fmnl2	0.82	1.00000000	duck
Smyd3	-0.93	1.00000000	duck
Pald1	-0.20	1.00000000	duck
Oaz2	-0.11	1.00000000	duck
Snrpc	0.53	1.00000000	duck
Tfam	-0.44	1.00000000	duck
Sf3b4	-0.28	1.00000000	duck
Ets1	-0.58	1.00000000	duck
Fosb	0.90	1.00000000	duck
Itpr1	-0.89	1.00000000	duck
Atp13a1	0.25	1.00000000	duck
Agtpbp1	0.42	1.00000000	duck
Borcs6	0.21	1.00000000	duck
Smarcd2	0.29	1.00000000	duck
Ppp1r8	0.60	1.00000000	duck
Cep162	0.20	1.00000000	duck
Ophn1	0.13	1.00000000	duck
Htr7	0.42	1.00000000	duck
Crybb1	0.20	1.00000000	duck
Enpp4	-0.69	1.00000000	duck
Foxk1	0.00	1.00000000	duck
Fbxo3	-1.10	1.00000000	duck
Fbf1	0.17	1.00000000	duck
Pop4	0.02	1.00000000	duck
Cyld	-0.58	1.00000000	duck
Rc3h2	0.31	1.00000000	duck
Ap4e1	-0.66	1.00000000	duck
Septin8	-0.18	1.00000000	duck

Lfng	0.48	1.00000000	duck
Fkbp14	-0.09	1.00000000	duck
Exoc3	-0.98	1.00000000	duck
Pdk4	-0.56	1.00000000	duck
Tbpl2	-0.14	1.00000000	duck
Rhbdd1	-0.17	1.00000000	duck
Khdc4	-0.20	1.00000000	duck
Camsap1	0.20	1.00000000	duck
Eps15l1	-0.52	1.00000000	duck
Cramp1	0.24	1.00000000	duck
Lonrf2	0.44	1.00000000	duck
Abraxas2	-0.43	1.00000000	duck
Leo1	-0.53	1.00000000	duck
Ndufaf3	0.43	1.00000000	duck
Ncoa1	0.60	1.00000000	duck
Itgav	-0.57	1.00000000	duck
Gpr34	0.28	1.00000000	duck
Pla2g6	0.20	1.00000000	duck
Ttk	0.10	1.00000000	duck
Ankrd9	0.15	1.00000000	duck
Itgb5	0.35	1.00000000	duck
Chst3	0.19	1.00000000	duck
Cacna1d	0.65	1.00000000	duck
Tpst2	0.36	1.00000000	duck
Cant1	0.18	1.00000000	duck
Tmem141	0.58	1.00000000	duck
Slc37a4	0.28	1.00000000	duck
Dlg2	0.25	1.00000000	duck
Tra2a	-0.17	1.00000000	duck
Hnrnpm	0.01	1.00000000	duck
Akap13	0.21	1.00000000	duck
Zfp532	0.60	1.00000000	duck
Ahsa1	-0.47	1.00000000	duck
Ift46	0.18	1.00000000	duck
Pank3	-0.31	1.00000000	duck
Traf5	-0.98	1.00000000	duck
Cdc27	-0.22	1.00000000	duck
Cenpw	0.37	1.00000000	duck
Wdr61	0.08	1.00000000	duck
Nr6a1	-0.12	1.00000000	duck
Acadm	-0.22	1.00000000	duck
Isca1	-0.67	1.00000000	duck
Selenok	-0.03	1.00000000	duck
Bcas3	0.74	1.00000000	duck
Lyn	0.21	1.00000000	duck
Zcchc17	0.34	1.00000000	duck

Nsmce1	-0.57	1.00000000	duck
Pphln1	0.00	1.00000000	duck
Spg11	-0.81	1.00000000	duck
Rrp1b	-0.77	1.00000000	duck
Hnrnpk	0.13	1.00000000	duck
Snx21	-0.07	1.00000000	duck
Tti2	-0.32	1.00000000	duck
Edc3	0.19	1.00000000	duck
Ctcf	-0.70	1.00000000	duck
Tmem65	0.20	1.00000000	duck
P2rx1	-0.25	1.00000000	duck
Rpa3	0.04	1.00000000	duck
Dtx4	0.20	1.00000000	duck
Bud23	0.16	1.00000000	duck
Zfp292	-0.44	1.00000000	duck
Bbs4	-0.18	1.00000000	duck
Gng7	0.11	1.00000000	duck
Nolc1	-0.34	1.00000000	duck
Etfrf1	-0.11	1.00000000	duck
Npc1	0.16	1.00000000	duck
Hsf2	0.29	1.00000000	duck
Blzf1	0.33	1.00000000	duck
Sec22c	0.11	1.00000000	duck
Orai2	0.14	1.00000000	duck
Wdfy3	0.32	1.00000000	duck
Tfec	2.23	1.00000000	duck
Hps6	0.19	1.00000000	duck
Nrde2	0.21	1.00000000	duck
Rxylt1	0.13	1.00000000	duck
Tub	0.22	1.00000000	duck
Nol9	0.10	1.00000000	duck
Kbtbd8	-0.38	1.00000000	duck
Zfp598	-0.44	1.00000000	duck
Snap29	-0.46	1.00000000	duck
Dhdds	-0.51	1.00000000	duck
Ywhaz	-0.55	1.00000000	duck
Rai1	-0.60	1.00000000	duck
Vdac3	0.40	1.00000000	duck
Eipr1	-1.44	1.00000000	duck
Dennd2a	0.22	1.00000000	duck
Ankrd52	0.42	1.00000000	duck
Ints13	0.17	1.00000000	duck
Gpr107	-0.57	1.00000000	duck
Eif5b	-1.03	1.00000000	duck
Stat3	-0.42	1.00000000	duck
Tent4a	-0.58	1.00000000	duck

Rab9b	-0.15	1.00000000	duck
Dph5	-0.14	1.00000000	duck
Sec63	-0.45	1.00000000	duck
Dennd11	-0.11	1.00000000	duck
Rbm25	-0.38	1.00000000	duck
Xrcc6	0.16	1.00000000	duck
Sumo1	0.05	1.00000000	duck
Dock4	0.14	1.00000000	duck
Utp18	-0.06	1.00000000	duck
Slc35c2	-0.73	1.00000000	duck
Slc4a8	0.76	1.00000000	duck
Plxnb2	0.51	1.00000000	duck
Tasl	-0.36	1.00000000	duck
Ccdc127	0.26	1.00000000	duck
Zbtb10	-0.27	1.00000000	duck
Rbm19	0.26	1.00000000	duck
Arhgap45	-0.31	1.00000000	duck
Alg12	-0.50	1.00000000	duck
Smg1	0.20	1.00000000	duck
Cep85l	-0.38	1.00000000	duck
Wipi2	-1.24	1.00000000	duck
Ncbp1	-0.45	1.00000000	duck
Zfyve28	0.10	1.00000000	duck
Pdss2	-0.43	1.00000000	duck
Eif4ebp2	0.18	1.00000000	duck
Fubp3	0.14	1.00000000	duck
Gtpbp6	0.09	1.00000000	duck
Rbms3	1.44	1.00000000	duck
Fam171a1	0.36	1.00000000	duck
Epcam	0.20	1.00000000	duck
Pdf	0.21	1.00000000	duck
Ift81	0.16	1.00000000	duck
Abhd11	0.10	1.00000000	duck
Sec11c	0.15	1.00000000	duck
Ppid	0.33	1.00000000	duck
Uba3	-0.34	1.00000000	duck
Stt3b	-0.58	1.00000000	duck
Dcaf1	-0.24	1.00000000	duck
Elf1	0.15	1.00000000	duck
Acbd3	0.39	1.00000000	duck
Atmin	-0.34	1.00000000	duck
Pds5b	0.33	1.00000000	duck
Zfp143	-0.01	1.00000000	duck
Wdr77	-0.10	1.00000000	duck
Kcnab2	0.21	1.00000000	duck
Thyn1	0.16	1.00000000	duck

Exoc1	0.39	1.00000000	duck
Zfp346	-0.30	1.00000000	duck
Fbxo21	-0.07	1.00000000	duck
Homer1	0.28	1.00000000	duck
Kat14	0.15	1.00000000	duck
Abtb1	-0.03	1.00000000	duck
Kansl3	-0.04	1.00000000	duck
Plin2	0.73	1.00000000	duck
Sar1b	-0.04	1.00000000	duck
Ccdc71l	0.24	1.00000000	duck
Ankfy1	-0.08	1.00000000	duck
Appbp2	-0.39	1.00000000	duck
Vps53	0.30	1.00000000	duck
Fbxo33	0.31	1.00000000	duck
Xrcc5	0.28	1.00000000	duck
Phf13	0.10	1.00000000	duck
Tex11	0.24	1.00000000	duck
St8sia1	0.23	1.00000000	duck
Runx3	0.00	1.00000000	duck
Ormdl3	-0.27	1.00000000	duck
Hsf1	0.28	1.00000000	duck
Yipf3	0.83	1.00000000	duck
Lsg1	0.17	1.00000000	duck
Reep3	-0.65	1.00000000	duck
Agpat5	-0.19	1.00000000	duck
Elp1	-0.35	1.00000000	duck
Tmem42	0.10	1.00000000	duck
Armt1	-0.10	1.00000000	duck
Gtf3c6	0.69	1.00000000	duck
Ereg	0.56	1.00000000	duck
Tada2b	0.31	1.00000000	duck
Tmem8b	0.10	1.00000000	duck
Pdzd2	0.27	1.00000000	duck
Rgs3	0.17	1.00000000	duck
Srcin1	0.09	1.00000000	duck
Impa2	-0.07	1.00000000	duck
Nol7	0.24	1.00000000	duck
Pgpep1	0.00	1.00000000	duck
Hectd4	-0.23	1.00000000	duck
Reps1	-0.42	1.00000000	duck
Osbpl3	0.09	1.00000000	duck
Eml4	0.37	1.00000000	duck
Nifk	0.26	1.00000000	duck
Rnf121	0.35	1.00000000	duck
Ubqln1	0.00	1.00000000	duck
Dgke	-0.28	1.00000000	duck

Vps35	0.32	1.00000000	duck
Usp22	-0.62	1.00000000	duck
Phc1	0.04	1.00000000	duck
Pde10a	1.82	1.00000000	duck
Rapgef1	0.05	1.00000000	duck
Fdx1	0.07	1.00000000	duck
Taldo1	0.24	1.00000000	duck
Gcn1	-0.44	1.00000000	duck
Nptn	0.06	1.00000000	duck
Cog7	0.56	1.00000000	duck
Clcc1	-0.79	1.00000000	duck
Hcfc2	-0.45	1.00000000	duck
Nceh1	-0.25	1.00000000	duck
Cst7	-0.42	1.00000000	duck
Taf6l	-0.05	1.00000000	duck
Naa60	-0.17	1.00000000	duck
Lsm7	-0.12	1.00000000	duck
Bop1	0.14	1.00000000	duck
Dcaf10	0.11	1.00000000	duck
Ndc1	0.19	1.00000000	duck
Ppcdc	0.08	1.00000000	duck
Pus3	0.06	1.00000000	duck
Rhobtb2	0.27	1.00000000	duck
Kcne2	0.18	1.00000000	duck
Tgs1	0.27	1.00000000	duck
Efnb1	-0.17	1.00000000	duck
Hnrnpa0	0.35	1.00000000	duck
Ddx1	0.65	1.00000000	duck
Chordc1	0.73	1.00000000	duck
Dnajb9	-0.33	1.00000000	duck
Zfhx3	-0.40	1.00000000	duck
Pnisr	0.25	1.00000000	duck
Ccdc186	-0.09	1.00000000	duck
Adal	0.07	1.00000000	duck
Dcun1d5	-0.39	1.00000000	duck
Pou2f1	0.01	1.00000000	duck
Tra2b	0.14	1.00000000	duck
Fbxo8	-0.43	1.00000000	duck
Adnp2	-0.90	1.00000000	duck
Rfc1	-0.35	1.00000000	duck
Sirt1	-0.73	1.00000000	duck
Ip6k1	-0.63	1.00000000	duck
Dck	-0.25	1.00000000	duck
Cul1	-0.83	1.00000000	duck
Vps4b	-0.52	1.00000000	duck
Usp45	-0.01	1.00000000	duck

Tubgcp6	0.24	1.00000000	duck
Cdc25a	-1.11	1.00000000	duck
Zup1	-0.14	1.00000000	duck
Guf1	-0.37	1.00000000	duck
Ndufaf2	-0.44	1.00000000	duck
Sort1	-0.15	1.00000000	duck
Suclg1	-0.11	1.00000000	duck
Taf12	0.04	1.00000000	duck
Slc9a3r1	0.23	1.00000000	duck
Bend7	-0.16	1.00000000	duck
Arg2	0.24	1.00000000	duck
Atrn	1.15	1.00000000	duck
Gch1	-0.18	1.00000000	duck
Kif13a	-0.14	1.00000000	duck
Rasgrp1	-0.16	1.00000000	duck
Ube2b	0.16	1.00000000	duck
Rfc5	0.37	1.00000000	duck
Fth1	0.15	1.00000000	duck
Fnip2	0.23	1.00000000	duck
Naa25	0.06	1.00000000	duck
Txndc12	0.19	1.00000000	duck
Phka1	0.13	1.00000000	duck
Lias	0.07	1.00000000	duck
Brat1	0.08	1.00000000	duck
Disp3	0.25	1.00000000	duck
Dapk1	0.48	1.00000000	duck
Tmem248	0.11	1.00000000	duck
Pmvk	0.11	1.00000000	duck
Ilk	-0.04	1.00000000	duck
Ccnt2	-0.69	1.00000000	duck
Bend6	0.31	1.00000000	duck
Josd1	0.11	1.00000000	duck
Ripor1	0.10	1.00000000	duck
Rhbdf2	-0.02	1.00000000	duck
Ak2	-1.13	1.00000000	duck
Zmat3	-0.11	1.00000000	duck
Polr2k	0.23	1.00000000	duck
Arhgap1	0.19	1.00000000	duck
Zcchc14	0.31	1.00000000	duck
Cmtr1	-0.75	1.00000000	duck
Dip2a	0.31	1.00000000	duck
Sacm1l	-0.36	1.00000000	duck
Pisd	-0.14	1.00000000	duck
Fasn	0.55	1.00000000	duck
Ist1	0.16	1.00000000	duck
Wdr26	-0.34	1.00000000	duck

Gsn	0.00	1.00000000	duck
Adgrg2	-0.05	1.00000000	duck
Rbm39	-0.17	1.00000000	duck
Rb1	0.06	1.00000000	duck
Katnbl1	-0.24	1.00000000	duck
Nek6	0.51	1.00000000	duck
Epas1	-1.36	1.00000000	duck
Rbl1	-0.14	1.00000000	duck
Aatf	-0.10	1.00000000	duck
Ube2j1	0.10	1.00000000	duck
Tnfaip3	0.32	1.00000000	duck
Bcr	0.74	1.00000000	duck
Copb2	-0.28	1.00000000	duck
Plxnc1	-0.40	1.00000000	duck
Pnpla8	-0.13	1.00000000	duck
Exoc5	-0.77	1.00000000	duck
Ankrd44	-0.51	1.00000000	duck
Ralgapb	0.14	1.00000000	duck
Purb	-0.04	1.00000000	duck
Ccdc174	-0.32	1.00000000	duck
Ckap5	-0.22	1.00000000	duck
Nktr	-1.02	1.00000000	duck
Rheb	-1.04	1.00000000	duck
Snrnp48	-0.32	1.00000000	duck
Pla2g12a	-0.35	1.00000000	duck
Arl3	0.30	1.00000000	duck
Rexo5	-0.64	1.00000000	duck
Slc3a1	-0.12	1.00000000	duck
Nrros	0.05	1.00000000	duck
Xxylt1	0.43	1.00000000	duck
Ttll10	0.59	1.00000000	duck
Anks1	0.51	1.00000000	duck
Gpr18	0.15	1.00000000	duck
Asb7	0.34	1.00000000	duck
Sars	0.06	1.00000000	duck
Uhrf1	0.42	1.00000000	duck
Zmynd11	-0.11	1.00000000	duck
Aff1	0.22	1.00000000	duck
Kcnk5	0.17	1.00000000	duck
Rabl6	-0.13	1.00000000	duck
Plod1	-0.38	1.00000000	duck
Gnl3	-0.20	1.00000000	duck
Cdyl2	-0.38	1.00000000	duck
Egln3	0.25	1.00000000	duck
Sema7a	0.22	1.00000000	duck
Dnaja3	0.18	1.00000000	duck

Klf4	0.90	1.00000000	duck
Usp16	-0.50	1.00000000	duck
Add3	-0.16	1.00000000	duck
Edem1	0.22	1.00000000	duck
Srgap3	0.46	1.00000000	duck
Rock1	0.04	1.00000000	duck
Ccnh	0.41	1.00000000	duck
Nedd4l	-0.81	1.00000000	duck
Flvcr2	-0.25	1.00000000	duck
Nudcd2	-0.08	1.00000000	duck
Aar2	-0.10	1.00000000	duck
Fmn2	-0.83	1.00000000	duck
Gpx2	-0.68	1.00000000	duck
Hadh	-0.07	1.00000000	duck
Ino80c	-0.11	1.00000000	duck
Cdin1	-0.24	1.00000000	duck
Actn1	0.23	1.00000000	duck
Pik3ap1	-0.55	1.00000000	duck
Gpn3	-0.37	1.00000000	duck
Cfl2	-1.16	1.00000000	duck
Trak2	-0.68	1.00000000	duck
Epg5	-0.01	1.00000000	duck
Chic2	-0.63	1.00000000	duck
Acvr2a	-0.19	1.00000000	duck
Trmt13	-0.33	1.00000000	duck
Parp11	0.37	1.00000000	duck
Triap1	0.41	1.00000000	duck
Dars	0.08	1.00000000	duck
Ly96	0.26	1.00000000	duck
Txlna	-0.18	1.00000000	duck
Zcchc8	0.11	1.00000000	duck
Cep350	-0.81	1.00000000	duck
Lamp1	0.30	1.00000000	duck
Epc2	0.12	1.00000000	duck
Stmn1	-0.69	1.00000000	duck
Lpl	-0.10	1.00000000	duck
Pacsin3	0.15	1.00000000	duck
Guk1	0.38	1.00000000	duck
Ripk1	0.02	1.00000000	duck
Larp4	-0.25	1.00000000	duck
Ptbp3	0.32	1.00000000	duck
Far2	0.15	1.00000000	duck
Pigw	0.10	1.00000000	duck
Lsm14b	0.06	1.00000000	duck
Gnpnat1	0.57	1.00000000	duck
Akap8l	0.27	1.00000000	duck

Helz	-0.19	1.00000000	duck
Rnf10	-0.76	1.00000000	duck
Smdt1	0.53	1.00000000	duck
Dctn6	0.32	1.00000000	duck
Atg2b	-0.58	1.00000000	duck
Zw10	-0.23	1.00000000	duck
Usp53	0.33	1.00000000	duck
Yars2	-0.25	1.00000000	duck
Aoah	0.13	1.00000000	duck
Vps39	-0.41	1.00000000	duck
Dkk3	0.23	1.00000000	duck
Ammecr1l	-0.04	1.00000000	duck
Sgsm2	-0.15	1.00000000	duck
Pex13	-0.33	1.00000000	duck
Snx30	-0.09	1.00000000	duck
Gins2	-0.38	1.00000000	duck
Evi5	-0.75	1.00000000	duck
Stk10	0.41	1.00000000	duck
Tmem106b	-0.56	1.00000000	duck
Lysmd3	-0.27	1.00000000	duck
Cxxc5	-0.13	1.00000000	duck
Cbfa2t2	-0.13	1.00000000	duck
Dusp14	-0.08	1.00000000	duck
Aldh9a1	0.67	1.00000000	duck
Heatr1	-0.15	1.00000000	duck
Ptcd3	-0.26	1.00000000	duck
Lrrc8a	-0.37	1.00000000	duck
Ube3c	-0.87	1.00000000	duck
Btg1b	-0.12	1.00000000	duck
Fam107b	-0.13	1.00000000	duck
Adcy9	-1.54	1.00000000	duck
Zfp609	-0.32	1.00000000	duck
Emc8	0.40	1.00000000	duck
Ciao2a	0.22	1.00000000	duck
Ubap2	0.15	1.00000000	duck
Fam13b	0.25	1.00000000	duck
Commd6	-0.35	1.00000000	duck
Ugp2	-0.44	1.00000000	duck
Asap2	1.33	1.00000000	duck
Sult4a1	-0.11	1.00000000	duck
Arl9	-0.27	1.00000000	duck
Wars2	0.46	1.00000000	duck
Ddx47	0.22	1.00000000	duck
Rabif	0.15	1.00000000	duck
Eif4h	-0.37	1.00000000	duck
Ccny	0.76	1.00000000	duck

Plekhf2	0.16	1.00000000	duck
Chst2	0.32	1.00000000	duck
Herpud1	-0.38	1.00000000	duck
Slc35b1	-0.34	1.00000000	duck
Pcnx	-0.47	1.00000000	duck
Fmnl1	-0.13	1.00000000	duck
Kansl2	-0.06	1.00000000	duck
Hmbs	-0.30	1.00000000	duck
Eefsec	0.56	1.00000000	duck
Adck1	-0.16	1.00000000	duck
Zfp511	-0.09	1.00000000	duck
Cdc20	-0.13	1.00000000	duck
Cep131	-0.08	1.00000000	duck
Evi5l	-0.08	1.00000000	duck
Adm	-0.09	1.00000000	duck
Adam10	-0.37	1.00000000	duck
Rnf166	0.40	1.00000000	duck
Parp10	0.21	1.00000000	duck
Pno1	0.28	1.00000000	duck
Fbxo22	0.07	1.00000000	duck
Rcsd1	0.11	1.00000000	duck
Steep1	-0.68	1.00000000	duck
4933400A11Rik	0.44	1.00000000	duck
Xrn2	0.45	1.00000000	duck
Gosr1	-0.81	1.00000000	duck
Kcnq1	1.57	1.00000000	duck
Fam199x	-0.24	1.00000000	duck
Coasy	0.08	1.00000000	duck
Dus3l	-0.17	1.00000000	duck
Dcp1a	-0.01	1.00000000	duck
Gas7	-0.27	1.00000000	duck
Scfd1	-0.38	1.00000000	duck
Cir1	-0.16	1.00000000	duck
Rae1	-1.08	1.00000000	duck
Hsd17b12	0.47	1.00000000	duck
Luc7l3	-0.10	1.00000000	duck
Wdr37	0.33	1.00000000	duck
Leprotl1	-0.09	1.00000000	duck
Saal1	0.57	1.00000000	duck
Ddx55	0.36	1.00000000	duck
Rsbn1	-0.08	1.00000000	duck
Pdcd4	-0.33	1.00000000	duck
Ppp1r21	-0.75	1.00000000	duck
Bin2	0.08	1.00000000	duck
Trp53inp1	-0.08	1.00000000	duck
Vps29	0.16	1.00000000	duck

Ppp1r12a	-0.49	1.00000000	duck
Klhl28	-0.07	1.00000000	duck
Psd	-0.18	1.00000000	duck
Iqgap2	0.51	1.00000000	duck
Ctbp1	0.11	1.00000000	duck
Ppp3cc	-0.29	1.00000000	duck
Yaf2	0.27	1.00000000	duck
Lsm6	0.16	1.00000000	duck
Stau2	0.52	1.00000000	duck
Nipa2	0.41	1.00000000	duck
Nub1	-0.42	1.00000000	duck
Naa15	-0.33	1.00000000	duck
Klhdc10	0.12	1.00000000	duck
Ppm1a	0.01	1.00000000	duck
Sh3glb1	-0.79	1.00000000	duck
Top3a	-0.05	1.00000000	duck
Slc17a9	-0.07	1.00000000	duck
Pcsk7	-0.05	1.00000000	duck
Bace1	-0.03	1.00000000	duck
Ern2	-0.07	1.00000000	duck
Srgap1	-0.20	1.00000000	duck
Zbtb49	-0.06	1.00000000	duck
Asph	0.38	1.00000000	duck
Lix1	-1.04	1.00000000	duck
Cbx3	-0.54	1.00000000	duck
Zfp516	0.53	1.00000000	duck
Taf15	0.27	1.00000000	duck
Bak1	0.35	1.00000000	duck
Atf4	0.17	1.00000000	duck
Anxa1	0.56	1.00000000	duck
Efhd2	-0.11	1.00000000	duck
Uprt	0.56	1.00000000	duck
Cdkn1a	0.37	1.00000000	duck
Rfx2	-0.39	1.00000000	duck
Ccdc93	-0.36	1.00000000	duck
Zbtb5	-0.07	1.00000000	duck
Pkp4	-0.08	1.00000000	duck
Scly	-0.11	1.00000000	duck
Slc9a1	0.04	1.00000000	duck
Rdx	0.21	1.00000000	duck
Afap1l2	-0.67	1.00000000	duck
Polr1a	-0.41	1.00000000	duck
Cnot11	-0.46	1.00000000	duck
Galc	-0.92	1.00000000	duck
Stub1	0.19	1.00000000	duck
Supt3	0.11	1.00000000	duck

Nt5c3	-0.68	1.00000000	duck
Ulk1	-0.26	1.00000000	duck
Zmat2	-0.11	1.00000000	duck
Tab1	0.00	1.00000000	duck
Hsdl1	-0.18	1.00000000	duck
Usp10	-0.37	1.00000000	duck
Api5	-0.51	1.00000000	duck
Septin7	-0.09	1.00000000	duck
Ppm1g	0.15	1.00000000	duck
Samd4	0.34	1.00000000	duck
Tmem163	0.33	1.00000000	duck
Ppa1	0.20	1.00000000	duck
Ywhab	-0.44	1.00000000	duck
Atp2c1	-0.29	1.00000000	duck
Coq2	0.22	1.00000000	duck
Ilf2	0.54	1.00000000	duck
Kif5b	-0.09	1.00000000	duck
Atp13a3	-0.46	1.00000000	duck
Slc66a1	0.32	1.00000000	duck
Ric8b	-0.46	1.00000000	duck
Ogt	-1.06	1.00000000	duck
Xcr1	0.42	1.00000000	duck
Dynlrb1	-0.05	1.00000000	duck
Nfkbie	0.11	1.00000000	duck
Akr1a1	-0.40	1.00000000	duck
Synrg	0.11	1.00000000	duck
Paqr7	0.14	1.00000000	duck
Cpq	-0.04	1.00000000	duck
Alpk1	0.11	1.00000000	duck
Ercc8	-0.06	1.00000000	duck
Cdk10	-0.04	1.00000000	duck
Sil1	0.02	1.00000000	duck
Usp28	-0.04	1.00000000	duck
Btrc	-0.47	1.00000000	duck
Arid4a	-0.17	1.00000000	duck
Sms	0.20	1.00000000	duck
Ralbp1	-0.37	1.00000000	duck
Gtf2h5	-0.01	1.00000000	duck
Gabarapl2	-0.18	1.00000000	duck
Cers5	0.24	1.00000000	duck
Nrp2	0.16	1.00000000	duck
Cdpf1	0.14	1.00000000	duck
Slc51a	0.13	1.00000000	duck
Atp6ap2	0.47	1.00000000	duck
Fam114a2	0.15	1.00000000	duck
Nsd1	0.22	1.00000000	duck

Ppargc1a	0.47	1.00000000	duck
Ubash3b	0.37	1.00000000	duck
Dstn	0.15	1.00000000	duck
Rreb1	0.24	1.00000000	duck
Atg4b	-0.04	1.00000000	duck
Tmem208	-0.05	1.00000000	duck
Entpd4	-0.28	1.00000000	duck
Tspan8	-0.32	1.00000000	duck
Nol8	-0.90	1.00000000	duck
Ddx41	-0.08	1.00000000	duck
Arv1	-0.37	1.00000000	duck
Camsap2	-0.78	1.00000000	duck
Acap2	-0.53	1.00000000	duck
Lrrc59	0.20	1.00000000	duck
Emp1	0.24	1.00000000	duck
P3h1	-0.34	1.00000000	duck
Sun1	-1.38	1.00000000	duck
Rnf34	-0.20	1.00000000	duck
Ascc3	-0.62	1.00000000	duck
Polg2	-0.28	1.00000000	duck
Tpd52l2	-0.21	1.00000000	duck
Psmc2	0.18	1.00000000	duck
Surf4	-0.32	1.00000000	duck
Prmt3	-0.02	1.00000000	duck
Rcor1	0.05	1.00000000	duck
Acaca	0.37	1.00000000	duck
Nol10	0.04	1.00000000	duck
Cep250	-0.02	1.00000000	duck
Tgif1	0.27	1.00000000	duck
Sugct	-1.01	1.00000000	duck
Unk	-0.47	1.00000000	duck
Fbxw2	0.07	1.00000000	duck
Cebpd	1.61	1.00000000	duck
Nupl1	0.12	1.00000000	duck
Gzf1	-0.56	1.00000000	duck
Cwc25	-0.13	1.00000000	duck
Thap4	0.03	1.00000000	duck
Ubqln4	0.20	1.00000000	duck
Eif2ak3	0.15	1.00000000	duck
Hnrnpd	0.15	1.00000000	duck
Gpr82	0.12	1.00000000	duck
Pofut2	-0.26	1.00000000	duck
Prune1	-0.04	1.00000000	duck
Tpmt	0.27	1.00000000	duck
Arhgap29	0.45	1.00000000	duck
Serinc5	0.59	1.00000000	duck

Zmat5	0.16	1.00000000	duck
Camta1	-0.05	1.00000000	duck
Nudt1	-0.09	1.00000000	duck
Eif2a	-0.55	1.00000000	duck
Rb1cc1	-0.81	1.00000000	duck
Tfcp2	-0.11	1.00000000	duck
Ninj1	0.31	1.00000000	duck
Sesn1	-0.29	1.00000000	duck
Nfe2l2	-1.16	1.00000000	duck
Ankrd13a	-0.24	1.00000000	duck
Tor2a	-0.26	1.00000000	duck
Fos	0.39	1.00000000	duck
Gpr157	0.18	1.00000000	duck
Sri	1.22	1.00000000	duck
Pcif1	-0.26	1.00000000	duck
Qars	0.14	1.00000000	duck
Rufy1	-0.10	1.00000000	duck
Fam76a	0.15	1.00000000	duck
Sesn3	-0.13	1.00000000	duck
Wbp2nl	0.28	1.00000000	duck
Ube2o	0.02	1.00000000	duck
Sat1	0.17	1.00000000	duck
Susd3	-0.05	1.00000000	duck
Arf1	0.09	1.00000000	duck
Amd2	-0.26	1.00000000	duck
Cep164	0.26	1.00000000	duck
Jpt1	0.12	1.00000000	duck
Aldh7a1	0.09	1.00000000	duck
Fgd4	0.20	1.00000000	duck
Vav3	-0.35	1.00000000	duck
Bst1	-0.26	1.00000000	duck
Tom1l1	0.31	1.00000000	duck
Polr3k	-0.11	1.00000000	duck
Sel1l3	-0.06	1.00000000	duck
Vwa8	0.48	1.00000000	duck
Dpp10	0.44	1.00000000	duck
Pip4p2	-0.05	1.00000000	duck
Sugp1	-0.31	1.00000000	duck
Rhof	0.03	1.00000000	duck
Usp33	0.19	1.00000000	duck
Cnih1	0.38	1.00000000	duck
Bend5	0.59	1.00000000	duck
Crbn	-0.40	1.00000000	duck
Cdc123	-0.43	1.00000000	duck
Rnf185	-0.12	1.00000000	duck
Sinhcaf	-0.23	1.00000000	duck

Zfp385b	0.16	1.00000000	duck
Psmd2	-0.24	1.00000000	duck
Exosc9	0.26	1.00000000	duck
Scrn3	0.33	1.00000000	duck
B3gntl1	0.59	1.00000000	duck
Tsc22d2	-0.80	1.00000000	duck
Noc2l	-0.78	1.00000000	duck
Btg2	-0.06	1.00000000	duck
Nectin3	0.25	1.00000000	duck
Chd7	-0.11	1.00000000	duck
Rabl3	-0.12	1.00000000	duck
Dock7	0.24	1.00000000	duck
Tulp4	-0.51	1.00000000	duck
Zc3h18	0.00	1.00000000	duck
Naa50	-0.44	1.00000000	duck
Arhgap31	0.21	1.00000000	duck
Abl1	0.35	1.00000000	duck
Abcb7	-0.75	1.00000000	duck
Glcci1	-0.98	1.00000000	duck
Rab33b	-0.03	1.00000000	duck
Rpap3	-0.10	1.00000000	duck
Usp5	0.00	1.00000000	duck
Rogdi	-0.07	1.00000000	duck
Dhx37	0.30	1.00000000	duck
Edem3	0.03	1.00000000	duck
Ppp2r5e	-0.40	1.00000000	duck
Carhsp1	0.14	1.00000000	duck
Pfkfb3	-0.42	1.00000000	duck
Psmg2	0.18	1.00000000	duck
Sde2	-0.70	1.00000000	duck
Pacs2	0.30	1.00000000	duck
Itgb8	0.01	1.00000000	duck
Glyr1	-0.79	1.00000000	duck
Washc5	-0.26	1.00000000	duck
Clptm1l	-0.14	1.00000000	duck
Ccdc50	0.05	1.00000000	duck
Kin	0.15	1.00000000	duck
Riok1	-0.30	1.00000000	duck
Pgrmc2	-0.21	1.00000000	duck
Osbpl2	-0.71	1.00000000	duck
Prkag1	-0.03	1.00000000	duck
Uso1	-0.70	1.00000000	duck
Phc2	0.16	1.00000000	duck
Atg5	-0.59	1.00000000	duck
Ptafr	0.13	1.00000000	duck
Irf2	-0.37	1.00000000	duck

Qsox2	-0.15	1.00000000	duck
Hsf5	0.56	1.00000000	duck
Tob2	0.31	1.00000000	duck
Ndufb3	-0.11	1.00000000	duck
Usp8	-0.56	1.00000000	duck
Nek9	0.31	1.00000000	duck
Atp6v0d1	0.19	1.00000000	duck
Arhgap28	0.27	1.00000000	duck
Pdlim5	0.30	1.00000000	duck
Atp2a3	0.08	1.00000000	duck
Lman2	-0.13	1.00000000	duck
Kctd5	-0.70	1.00000000	duck
Ggh	-0.63	1.00000000	duck
Tmem175	-0.31	1.00000000	duck
Slc27a1	-0.10	1.00000000	duck
Pls3	0.42	1.00000000	duck
Senp6	-0.34	1.00000000	duck
Ndufs4	0.36	1.00000000	duck
Cgrrf1	-0.39	1.00000000	duck
Sucla2	-0.31	1.00000000	duck
Rangap1	-0.19	1.00000000	duck
Ubap1	0.20	1.00000000	duck
Eif4enif1	-0.28	1.00000000	duck
Sirt6	0.12	1.00000000	duck
Atp5j2	-0.03	1.00000000	duck
Nup50	0.01	1.00000000	duck
Cept1	-0.43	1.00000000	duck
Retreg1	0.31	1.00000000	duck
Fam177a	0.58	1.00000000	duck
G0s2	0.58	1.00000000	duck
Cdc16	-0.11	1.00000000	duck
Gga3	-0.03	1.00000000	duck
Klhl12	0.02	1.00000000	duck
Pxdc1	-0.06	1.00000000	duck
Syap1	-0.36	1.00000000	duck
Rab35	-0.16	1.00000000	duck
Gpd1l	-0.12	1.00000000	duck
Pxmp4	-0.09	1.00000000	duck
Sdk1	0.48	1.00000000	duck
Lmnb2	0.10	1.00000000	duck
Tomm22	0.19	1.00000000	duck
Ccpg1	-1.16	1.00000000	duck
Igf2bp2	-0.06	1.00000000	duck
Tle5	0.16	1.00000000	duck
Smcr8	-0.38	1.00000000	duck
Bcas2	-0.01	1.00000000	duck

Secisbp2l	-0.37	1.00000000	duck
Rad51b	0.10	1.00000000	duck
Efr3b	-0.16	1.00000000	duck
Lgals8	-0.07	1.00000000	duck
Batf	-0.25	1.00000000	duck
Hacd2	-0.07	1.00000000	duck
Ccnb2	-0.09	1.00000000	duck
Cbx8	-0.06	1.00000000	duck
Cobll1	-1.32	1.00000000	duck
Zbtb2	0.18	1.00000000	duck
Gpr68	0.32	1.00000000	duck
Dvl3	0.00	1.00000000	duck
Eif2ak2	0.05	1.00000000	duck
Klhl21	0.01	1.00000000	duck
Lmtk2	-0.56	1.00000000	duck
Csde1	-0.49	1.00000000	duck
Fam91a1	0.06	1.00000000	duck
Taf5	0.17	1.00000000	duck
Dcp2	0.50	1.00000000	duck
Pip5k1c	-0.21	1.00000000	duck
Serbp1	-0.09	1.00000000	duck
Tspan14	-0.04	1.00000000	duck
C1galt1	-0.03	1.00000000	duck
Sppl2b	0.27	1.00000000	duck
Galnt2	0.43	1.00000000	duck
Lrba	-0.32	1.00000000	duck
Acot11	0.22	1.00000000	duck
Utp15	0.52	1.00000000	duck
Rrp12	0.13	1.00000000	duck
Ntan1	0.16	1.00000000	duck
Rlf	-0.55	1.00000000	duck
Cdc73	-0.51	1.00000000	duck
Jarid2	0.21	1.00000000	duck
Rab5a	0.18	1.00000000	duck
Ppt1	0.20	1.00000000	duck
Fbxl5	-0.97	1.00000000	duck
Cdk13	-0.57	1.00000000	duck
Vps35l	0.01	1.00000000	duck
Pigt	0.29	1.00000000	duck
Ip6k2	0.04	1.00000000	duck
Hip1	0.30	1.00000000	duck
Tmem98	0.20	1.00000000	duck
Tmem251	0.40	1.00000000	duck
Slco3a1	0.02	1.00000000	duck
Stk32c	-0.17	1.00000000	duck
Ddx20	-0.02	1.00000000	duck

Dclre1a	-0.08	1.00000000	duck
Pkn2	-0.29	1.00000000	duck
Jak2	-0.93	1.00000000	duck
Gars	0.36	1.00000000	duck
Ppil3	0.25	1.00000000	duck
Strn	-0.30	1.00000000	duck
Rmc1	-0.34	1.00000000	duck
Abca2	-0.10	1.00000000	duck
Immp1l	-0.28	1.00000000	duck
Itpka	-1.72	1.00000000	duck
Ndufa4	0.26	1.00000000	duck
Tmem242	0.25	1.00000000	duck
Impdh2	0.24	1.00000000	duck
Slc17a5	-0.40	1.00000000	duck
Phkb	-0.24	1.00000000	duck
Itprid2	0.08	1.00000000	duck
Rprd1a	-0.60	1.00000000	duck
Exosc3	-0.16	1.00000000	duck
Usp38	-0.11	1.00000000	duck
Txndc9	-1.45	1.00000000	duck
Nrp1	0.59	1.00000000	duck
Smu1	-0.24	1.00000000	duck
Dhx30	0.21	1.00000000	duck
Slc39a13	0.22	1.00000000	duck
Vps36	0.12	1.00000000	duck
Noc4l	0.06	1.00000000	duck
Tlr4	0.13	1.00000000	duck
Polr2f	0.15	1.00000000	duck
Kif15	0.97	1.00000000	duck
Plekha7	-0.13	1.00000000	duck
Katnb1	-0.05	1.00000000	duck
Dele1	-0.06	1.00000000	duck
Slc41a2	0.23	1.00000000	duck
Polr1c	0.08	1.00000000	duck
Gda	-0.02	1.00000000	duck
Pitpnm2	-0.57	1.00000000	duck
Xpr1	-0.80	1.00000000	duck
Hspa2	-0.80	1.00000000	duck
Ipo9	0.30	1.00000000	duck
Neurl1b	0.71	1.00000000	duck
Kazn	0.32	1.00000000	duck
Ddx54	0.26	1.00000000	duck
Lta4h	-0.15	1.00000000	duck
Lrpap1	0.17	1.00000000	duck
Nr3c1	-0.65	1.00000000	duck
Ino80	-0.39	1.00000000	duck

Kpnb1	0.24	1.00000000	duck
Ctnnal1	-0.29	1.00000000	duck
Sccpdh	0.08	1.00000000	duck
Fkbp3	0.00	1.00000000	duck
Cdv3	-0.03	1.00000000	duck
Ptges3	-0.75	1.00000000	duck
Sec13	-0.08	1.00000000	duck
Sertad2	0.35	1.00000000	duck
Ostc	0.16	1.00000000	duck
Slc45a1	-0.56	1.00000000	duck
Pde7b	-0.64	1.00000000	duck
Fbxl17	0.47	1.00000000	duck
Pacsin2	-0.22	1.00000000	duck
Zbtb7c	-0.06	1.00000000	duck
Polr3d	-0.04	1.00000000	duck
Cd151	-0.03	1.00000000	duck
Zbtb14	0.12	1.00000000	duck
Gpr75	0.06	1.00000000	duck
Otud4	0.20	1.00000000	duck
Brca1	0.09	1.00000000	duck
Tmem185a	0.23	1.00000000	duck
Tyms	-0.01	1.00000000	duck
Grk2	0.00	1.00000000	duck
Cops8	-0.05	1.00000000	duck
Fosl2	-0.20	1.00000000	duck
Zfp414	0.18	1.00000000	duck
Tmem87a	0.06	1.00000000	duck
Rnf146	-0.28	1.00000000	duck
St6gal1	-0.06	1.00000000	duck
Rftn1	-0.62	1.00000000	duck
Cul5	-0.14	1.00000000	duck
Hmgxb4	0.20	1.00000000	duck
Ric1	-0.84	1.00000000	duck
Slu7	-0.92	1.00000000	duck
Emc6	0.01	1.00000000	duck
Pstpip1	0.40	1.00000000	duck
Akap10	-0.34	1.00000000	duck
Tbc1d4	-0.08	1.00000000	duck
Tpi1	0.38	1.00000000	duck
Snx12	0.19	1.00000000	duck
Smg7	-0.45	1.00000000	duck
Csnk1a1	-0.43	1.00000000	duck
Emb	-0.31	1.00000000	duck
Fndc3a	-0.78	1.00000000	duck
Pard3b	-0.12	1.00000000	duck
Tbc1d14	0.00	1.00000000	duck

Tanc1	0.17	1.00000000	duck
Pqlc3	-0.06	1.00000000	duck
Smyd2	0.21	1.00000000	duck
Wwp1	-0.03	1.00000000	duck
Ppp6r3	-0.60	1.00000000	duck
Seh1l	0.58	1.00000000	duck
Pxn	0.46	1.00000000	duck
Eif1b	0.10	1.00000000	duck
Slc4a2	0.38	1.00000000	duck
Cd83	0.00	1.00000000	duck
Oxr1	-0.78	1.00000000	duck
Kndc1	-0.33	1.00000000	duck
Cmtm3	0.22	1.00000000	duck
Vkorc1l1	-0.65	1.00000000	duck
Efcab14	0.18	1.00000000	duck
Tnrc18	-0.65	1.00000000	duck
Coro1c	0.19	1.00000000	duck
Tlnrd1	0.33	1.00000000	duck
Trappc2l	0.12	1.00000000	duck
Zrsr1	-0.36	1.00000000	duck
Aven	0.23	1.00000000	duck
Spsb3	-0.18	1.00000000	duck
Fam102a	0.10	1.00000000	duck
Bach2	-0.22	1.00000000	duck
Dnm3	-1.30	1.00000000	duck
Cttnbp2nl	0.21	1.00000000	duck
Tfg	-0.14	1.00000000	duck
Cisd2	-0.41	1.00000000	duck
Clk1	-0.56	1.00000000	duck
Rbbp4	0.23	1.00000000	duck
Disp1	-0.42	1.00000000	duck
E2f6	-0.12	1.00000000	duck
Ddx49	-0.52	1.00000000	duck
Naxd	0.09	1.00000000	duck
Klhl5	0.47	1.00000000	duck
Lin7c	-0.24	1.00000000	duck
Rcbtb2	0.45	1.00000000	duck
Cdc40	-0.40	1.00000000	duck
Asns	0.42	1.00000000	duck
Slc31a1	0.19	1.00000000	duck
Slk	-0.40	1.00000000	duck
Uhrf2	-0.61	1.00000000	duck
Fbxo11	-0.20	1.00000000	duck
Exoc8	-0.05	1.00000000	duck
Sf3b1	-0.62	1.00000000	duck
Abitram	-0.09	1.00000000	duck

Tmem14a	0.01	1.00000000	duck
Cyfip2	-0.03	1.00000000	duck
Ndufs7	0.24	1.00000000	duck
Pfdn1	0.08	1.00000000	duck
Rint1	0.45	1.00000000	duck
Ampd3	-0.04	1.00000000	duck
Rrn3	0.15	1.00000000	duck
Lemd3	0.19	1.00000000	duck
Rbm33	-0.24	1.00000000	duck
Rgs1	-0.70	1.00000000	duck
Epb41l2	-0.09	1.00000000	duck
Arhgef3	0.60	1.00000000	duck
Bdp1	-0.61	1.00000000	duck
Fam131b	-0.20	1.00000000	duck
Ndufaf6	-1.01	1.00000000	duck
Acaa1b	-0.10	1.00000000	duck
Tspan15	0.10	1.00000000	duck
Tmem43	-0.28	1.00000000	duck
Susd1	-1.27	1.00000000	duck
Actr2	0.15	1.00000000	duck
Top1	-0.04	1.00000000	duck
Pex5	-0.10	1.00000000	duck
Cutc	0.12	1.00000000	duck
Ap1s3	0.15	1.00000000	duck
Ciapin1	0.04	1.00000000	duck
Gtf3c4	0.07	1.00000000	duck
Ern1	-0.89	1.00000000	duck
Dalrd3	0.49	1.00000000	duck
Ggnbp2	-0.05	1.00000000	duck
Iqgap1	0.17	1.00000000	duck
Xpot	-0.41	1.00000000	duck
Ndufs3	0.02	1.00000000	duck
Pstpip2	0.19	1.00000000	duck
Cd81	0.05	1.00000000	duck
P4ha1	-0.86	1.00000000	duck
Rsl24d1	0.30	1.00000000	duck
Csnk1g2	0.48	1.00000000	duck
Nek7	-0.17	1.00000000	duck
Rab7	0.14	1.00000000	duck
Ccdc134	-0.19	1.00000000	duck
Esf1	0.31	1.00000000	duck
Nfat5	-0.05	1.00000000	duck
Zc3h12a	0.03	1.00000000	duck
Relch	-0.27	1.00000000	duck
Jade1	-1.39	1.00000000	duck
Vps8	0.21	1.00000000	duck

Trappc4	-0.54	1.00000000	duck
Btbd2	-0.12	1.00000000	duck
Fastkd2	0.09	1.00000000	duck
Fxyd6	-0.01	1.00000000	duck
Filip1l	0.35	1.00000000	duck
Vdac2	-0.14	1.00000000	duck
Thoc2	-0.18	1.00000000	duck
Ago4	0.25	1.00000000	duck
Cln6	0.33	1.00000000	duck
Efhd1	0.23	1.00000000	duck
Gpsm2	-0.40	1.00000000	duck
Uspl1	0.16	1.00000000	duck
Jdp2	-0.32	1.00000000	duck
Slc5a1	0.38	1.00000000	duck
Ttf1	0.17	1.00000000	duck
Bcl9l	0.08	1.00000000	duck
Nup214	-0.44	1.00000000	duck
Scd2	-0.80	1.00000000	duck
Agfg1	0.00	1.00000000	duck
Telo2	0.02	1.00000000	duck
Dynlt1a	0.35	1.00000000	duck
Ube2k	-0.04	1.00000000	duck
Zfp277	0.08	1.00000000	duck
Aamdc	-0.10	1.00000000	duck
Rcan2	0.32	1.00000000	duck
Btbd6	0.42	1.00000000	duck
Scp2	0.06	1.00000000	duck
Ptger4	0.39	1.00000000	duck
Ppip5k1	-0.21	1.00000000	duck
Itpkb	0.20	1.00000000	duck
Cd86	-0.49	1.00000000	duck
Sugt1	-0.53	1.00000000	duck
Cacna1i	0.24	1.00000000	duck
Sh3bp5	-0.30	1.00000000	duck
Fam204a	-0.20	1.00000000	duck
Calm2	-0.14	1.00000000	duck
Rnd3	0.32	1.00000000	duck
Abat	0.00	1.00000000	duck
Per3	-0.10	1.00000000	duck
Cstf3	-0.34	1.00000000	duck
Aplp2	0.45	1.00000000	duck
Psmd12	-0.75	1.00000000	duck
Copb1	0.18	1.00000000	duck
Cks1brt	1.08	1.00000000	duck
Tbc1d1	0.21	1.00000000	duck
Naa16	-0.33	1.00000000	duck

Crim1	-1.60	1.00000000	duck
Dcps	-0.07	1.00000000	duck
Snap91	-0.57	1.00000000	duck
Fam78a	-0.07	1.00000000	duck
Smim14	-0.01	1.00000000	duck
Wasf2	0.00	1.00000000	duck
Cul3	0.17	1.00000000	duck
Lyrm2	-0.30	1.00000000	duck
Zbtb44	-0.69	1.00000000	duck
Pgrmc1	0.14	1.00000000	duck
Ercc3	0.05	1.00000000	duck
Parn	-0.40	1.00000000	duck
Atg14	-0.32	1.00000000	duck
Plpbp	0.05	1.00000000	duck
Chmp4b	0.13	1.00000000	duck
Nbas	0.38	1.00000000	duck
Entpd1	-0.25	1.00000000	duck
Fam98a	0.15	1.00000000	duck
Pdpk1	0.24	1.00000000	duck
Rsbn1l	-0.35	1.00000000	duck
Sec23b	0.03	1.00000000	duck
Fam126b	-0.67	1.00000000	duck
Arhgap42	0.14	1.00000000	duck
Hsdl2	0.10	1.00000000	duck
Ctbs	0.13	1.00000000	duck
Bmf	0.30	1.00000000	duck
Ccdc92	0.17	1.00000000	duck
Asah1	-0.23	1.00000000	duck
Afg1l	-0.07	1.00000000	duck
Dgkq	-0.05	1.00000000	duck
Strada	-0.08	1.00000000	duck
Adk	0.57	1.00000000	duck
Errfi1	-0.26	1.00000000	duck
Txndc17	0.14	1.00000000	duck
Arl4c	0.17	1.00000000	duck
Fry	-0.44	1.00000000	duck
Siah1b	-0.24	1.00000000	duck
Pbdc1	0.00	1.00000000	duck
Hs6st3	-1.05	1.00000000	duck
N6amt1	-0.05	1.00000000	duck
Zfp830	-0.10	1.00000000	duck
Cep44	-0.08	1.00000000	duck
Spata7	-0.28	1.00000000	duck
DPCD	0.39	1.00000000	duck
Slc7a6os	0.04	1.00000000	duck
Gprc5c	-0.09	1.00000000	duck

Ctnnbip1	0.21	1.00000000	duck
Otud7b	-0.41	1.00000000	duck
St3gal5	-0.86	1.00000000	duck
Septin11	0.41	1.00000000	duck
Unc119b	-0.37	1.00000000	duck
Stim1	0.25	1.00000000	duck
Septin9	-0.21	1.00000000	duck
Atp6v0c	0.06	1.00000000	duck
Eif4e	-0.61	1.00000000	duck
Snapc1l	0.17	1.00000000	duck
Zfand2a	0.61	1.00000000	duck
Sgpp1	0.22	1.00000000	duck
Shisa5	-0.22	1.00000000	duck
Cog6	-0.24	1.00000000	duck
Rufy2	0.06	1.00000000	duck
Rmnd1	-0.33	1.00000000	duck
Zfp36l2	0.54	1.00000000	duck
Ivd	-0.18	1.00000000	duck
Sh3pxd2a	0.07	1.00000000	duck
Eif3e	-0.32	1.00000000	duck
Eif3i	0.24	1.00000000	duck
Plcb4	0.82	1.00000000	duck
Atxn10	0.21	1.00000000	duck
Sspn	0.04	1.00000000	duck
B3glct	-0.07	1.00000000	duck
Jchain	0.01	1.00000000	duck
Raly	-0.04	1.00000000	duck
Extl3	-0.20	1.00000000	duck
Cpne3	-0.23	1.00000000	duck
Cct3	-0.03	1.00000000	duck
Gper1	-0.15	1.00000000	duck
Ptpn11	-0.40	1.00000000	duck
Paxbp1	-0.98	1.00000000	duck
Tmem64	0.31	1.00000000	duck
Eef1a1	-0.12	1.00000000	duck
Rbbp5	-0.25	1.00000000	duck
Vcl	-0.66	1.00000000	duck
Rilpl2	0.14	1.00000000	duck
Acp6	-0.04	1.00000000	duck
Wdr86	-0.08	1.00000000	duck
Nvl	-0.09	1.00000000	duck
Ints12	-0.01	1.00000000	duck
Trim23	-0.47	1.00000000	duck
Actr10	-0.08	1.00000000	duck
Comt	-0.19	1.00000000	duck
Clasp2	0.01	1.00000000	duck

Cops3	-0.33	1.00000000	duck
Khdrbs1	0.01	1.00000000	duck
Zdhhc15	0.44	1.00000000	duck
Epn2	-0.51	1.00000000	duck
Fam117b	-0.70	1.00000000	duck
Pms1	-0.50	1.00000000	duck
Czib	-0.07	1.00000000	duck
Prkcz	-0.65	1.00000000	duck
Spg21	0.26	1.00000000	duck
Gpr137c	-0.08	1.00000000	duck
Traf7	-0.14	1.00000000	duck
Nudt3	-0.66	1.00000000	duck
Pnpla7	-1.56	1.00000000	duck
Kifbp	-0.82	1.00000000	duck
Wdr91	-0.16	1.00000000	duck
Vezf1	-0.68	1.00000000	duck
Canx	0.18	1.00000000	duck
Stx7	0.10	1.00000000	duck
Pola1	-0.09	1.00000000	duck
Itga4	0.07	1.00000000	duck
Lbr	-0.38	1.00000000	duck
Utp20	0.57	1.00000000	duck
Raf1	-0.57	1.00000000	duck
Prkd1	-0.25	1.00000000	duck
Zbtb34	-0.26	1.00000000	duck
Zfp36l1	-0.30	1.00000000	duck
Dcaf5	0.01	1.00000000	duck
Zfyve9	0.15	1.00000000	duck
Cnn3	0.04	1.00000000	duck
Ift122	0.03	1.00000000	duck
Gnas	-0.26	1.00000000	duck
Crebrf	-0.86	1.00000000	duck
Lamp2	-0.29	1.00000000	duck
Ints8	0.02	1.00000000	duck
Arih2	-0.16	1.00000000	duck
Zfp687	0.01	1.00000000	duck
Nr2c1	-0.31	1.00000000	duck
Tut7	-0.01	1.00000000	duck
Snx5	-0.32	1.00000000	duck
Cpsf2	-0.64	1.00000000	duck
AF366264	-0.35	1.00000000	duck
Copg1	0.13	1.00000000	duck
Dock9	-0.69	1.00000000	duck
Styk1	-0.44	1.00000000	duck
Alas1	-0.07	1.00000000	duck
Batf3	0.22	1.00000000	duck

Tmem86a	0.48	1.00000000	duck
Atp7a	-0.61	1.00000000	duck
Tnfrsf21	0.64	1.00000000	duck
Ythdc2	-0.32	1.00000000	duck
Gpt2	0.42	1.00000000	duck
Flt3	-0.57	1.00000000	duck
Slc38a10	0.19	1.00000000	duck
Rtf1	-0.40	1.00000000	duck
Ammecr1	-0.84	1.00000000	duck
Ppig	-0.84	1.00000000	duck
Vps13d	0.04	1.00000000	duck
Atp6v1h	-0.26	1.00000000	duck
Zpr1	0.00	1.00000000	duck
Faf2	-0.05	1.00000000	duck
Galns	0.18	1.00000000	duck
Xrn1	-0.16	1.00000000	duck
Usp14	-0.27	1.00000000	duck
Nsl1	0.13	1.00000000	duck
Cdk5rap1	0.23	1.00000000	duck
Sf3a1	0.19	1.00000000	duck
Runx2	0.31	1.00000000	duck
Scamp1	-0.32	1.00000000	duck
Zcrb1	0.18	1.00000000	duck
Ryk	0.14	1.00000000	duck
Park7	0.23	1.00000000	duck
Pofut1	-0.33	1.00000000	duck
Pank4	0.13	1.00000000	duck
Serp1	0.26	1.00000000	duck
Rab27a	0.23	1.00000000	duck
Ube2e2	0.78	1.00000000	duck
Eif3j2	0.02	1.00000000	duck
Stag2	-0.68	1.00000000	duck
Anln	-0.10	1.00000000	duck
Ylpm1	-0.16	1.00000000	duck
Hsd11b2	0.57	1.00000000	duck
Fam117a	-0.41	1.00000000	duck
St7l	-0.13	1.00000000	duck
Nudt15	-0.04	1.00000000	duck
Decr1	-0.03	1.00000000	duck
Trmt10a	-0.22	1.00000000	duck
Gusb	-0.03	1.00000000	duck
Ubr3	-0.30	1.00000000	duck
Zyx	0.17	1.00000000	duck
Phf6	-0.87	1.00000000	duck
Gne	0.63	1.00000000	duck
Cog2	-0.68	1.00000000	duck

Csnk1g1	-0.55	1.00000000	duck
Ralgps2	0.04	1.00000000	duck
Clip2	-0.14	1.00000000	duck
Fig4	-0.33	1.00000000	duck
Ankmy2	-0.03	1.00000000	duck
Gnb1	-0.04	1.00000000	duck
Fkbp4	0.12	1.00000000	duck
Trdmt1	-0.33	1.00000000	duck
Slc1a4	-0.25	1.00000000	duck
Acox3	-1.21	1.00000000	duck
Tfb1m	-0.31	1.00000000	duck
Arhgap24	0.60	1.00000000	duck
P2rx4	0.53	1.00000000	duck
Cct5	0.19	1.00000000	duck
Yipf5	0.11	1.00000000	duck
Arhgap5	0.01	1.00000000	duck
Dcun1d3	-0.31	1.00000000	duck
Sec22a	-0.08	1.00000000	duck
Htt	-0.62	1.00000000	duck
Ptdss1	-0.37	1.00000000	duck
Btbd11	0.25	1.00000000	duck
Atp6v0a2	-0.74	1.00000000	duck
Rp2	-0.23	1.00000000	duck
Hdc	-0.06	1.00000000	duck
Slc44a1	-0.33	1.00000000	duck
Dut	0.08	1.00000000	duck
Irf1	-0.28	1.00000000	duck
Bsg	-0.15	1.00000000	duck
Ndufv2	-0.14	1.00000000	duck
Herc4	0.25	1.00000000	duck
Gtf2e2	-0.11	1.00000000	duck
Edc4	-0.29	1.00000000	duck
Sstr4	0.47	1.00000000	duck
Ap3s1	0.31	1.00000000	duck
Ipo8	-0.39	1.00000000	duck
Hnrnpa2b1	0.15	1.00000000	duck
Aida	0.17	1.00000000	duck
Nol4l	0.18	1.00000000	duck
Cep192	-0.24	1.00000000	duck
Galnt13	-0.15	1.00000000	duck
Gfod2	-0.38	1.00000000	duck
Gemin5	-0.21	1.00000000	duck
Ptpa	-0.03	1.00000000	duck
Srp54a	0.07	1.00000000	duck
Sod2	-0.08	1.00000000	duck
Serinc1	-0.01	1.00000000	duck

Gsg1	-0.21	1.00000000	duck
Naaa	-0.01	1.00000000	duck
Ube2v2	-0.22	1.00000000	duck
Polr2c	0.18	1.00000000	duck
Kif13b	0.15	1.00000000	duck
Timp3	-1.25	1.00000000	duck
Bclaf3	0.31	1.00000000	duck
Cdh23	0.23	1.00000000	duck
Sec22b	0.21	1.00000000	duck
Golga3	-0.22	1.00000000	duck
Tent2	-0.38	1.00000000	duck
Stx17	-0.40	1.00000000	duck
Xpo5	-0.31	1.00000000	duck
Septin2	0.49	1.00000000	duck
Parp16	0.08	1.00000000	duck
Strip1	0.20	1.00000000	duck
Fyttd1	-0.34	1.00000000	duck
Atxn7l1	-0.16	1.00000000	duck
Pcmt1	-0.82	1.00000000	duck
Cnot4	-0.64	1.00000000	duck
Prelid3a	-0.30	1.00000000	duck
Arhgef7	0.51	1.00000000	duck
Chmp1a	-0.20	1.00000000	duck
Saraf	0.13	1.00000000	duck
Prdx6	0.22	1.00000000	duck
Ipmk	-0.69	1.00000000	duck
Brwd3	-0.32	1.00000000	duck
Pdap1	-0.45	1.00000000	duck
Hipk1	-0.33	1.00000000	duck
Sfxn1	-0.11	1.00000000	duck
Insig1	-0.07	1.00000000	duck
Cep95	-0.29	1.00000000	duck
Ppp6c	0.20	1.00000000	duck
Psme3	0.01	1.00000000	duck
Ccng2	-0.36	1.00000000	duck
Hddc2	0.26	1.00000000	duck
Bcl2l13	-0.46	1.00000000	duck
Vcpip1	-1.00	1.00000000	duck
Qrich1	-0.10	1.00000000	duck
Ubn2	0.01	1.00000000	duck
Atp2b1	-0.59	1.00000000	duck
Heph	-0.25	1.00000000	duck
Lap3	0.39	1.00000000	duck
Slc25a29	0.06	1.00000000	duck
Hscb	-0.12	1.00000000	duck
Dhx8	-0.47	1.00000000	duck

Rgs9	0.15	1.00000000	duck
Slc7a3	0.13	1.00000000	duck
Bclaf1	-0.57	1.00000000	duck
Dyrk3	-0.32	1.00000000	duck
Ppp2cb	-0.16	1.00000000	duck
Dnajc21	-0.33	1.00000000	duck
Gk5	0.07	1.00000000	duck
Ehd3	-0.29	1.00000000	duck
Bin1	-0.32	1.00000000	duck
Psmd10	-0.06	1.00000000	duck
Slc16a1	-0.09	1.00000000	duck
Atp6v1b2	-0.31	1.00000000	duck
Tet1	-0.63	1.00000000	duck
Tatdn3	-0.03	1.00000000	duck
Zbtb1	0.00	1.00000000	duck
Asxl2	-0.27	1.00000000	duck
Tiam1	-0.56	1.00000000	duck
Sec31b	0.27	1.00000000	duck
Lxn	0.11	1.00000000	duck
Cpd	0.12	1.00000000	duck
Rasa3	-0.22	1.00000000	duck
Rap1b	-0.42	1.00000000	duck
Cnot6	0.03	1.00000000	duck
Pdcd10	-0.17	1.00000000	duck
Cnst	-0.28	1.00000000	duck
Acat1	-0.16	1.00000000	duck
Emsy	-0.39	1.00000000	duck
Sh3bgrl	-0.19	1.00000000	duck
Ing3	-0.72	1.00000000	duck
Tasp1	-0.19	1.00000000	duck
Wnk1	0.11	1.00000000	duck
Arhgdia	0.22	1.00000000	duck
Fam20b	0.83	1.00000000	duck
Ncoa6	-0.04	1.00000000	duck
Ube2e3	-0.21	1.00000000	duck
Smarcad1	0.05	1.00000000	duck
Atad2b	-1.05	1.00000000	duck
Gpat4	0.28	1.00000000	duck
Prpf3	-0.35	1.00000000	duck
Lrp12	0.20	1.00000000	duck
Rap1gds1	-0.16	1.00000000	duck
Rnf4	-0.14	1.00000000	duck
Peak1	0.05	1.00000000	duck
Lima1	-0.01	1.00000000	duck
Clasp1	0.96	1.00000000	duck
Ints4	-0.05	1.00000000	duck

Ankra2	-0.32	1.00000000	duck
Appl1	0.10	1.00000000	duck
Rap2a	-0.03	1.00000000	duck
Tepsin	-0.01	1.00000000	duck
Elk4	-0.34	1.00000000	duck
Psmc6	-0.52	1.00000000	duck
Gpatch2l	-0.77	1.00000000	duck
Ppat	0.15	1.00000000	duck
Gipc2	0.34	1.00000000	duck
Rnf168	-0.33	1.00000000	duck
Ostf1	-0.01	1.00000000	duck
Pik3ca	-1.18	1.00000000	duck
Gak	-0.03	1.00000000	duck
Aplf	-0.46	1.00000000	duck
Atp8a2	0.52	1.00000000	duck
Pias2	0.02	1.00000000	duck
Twsg1	0.25	1.00000000	duck
Cep63	-0.48	1.00000000	duck
Dipk2a	-1.08	1.00000000	duck
Cops7b	0.08	1.00000000	duck
B3gnt4	-0.34	1.00000000	duck
Rcc1l	0.36	1.00000000	duck
Rtn4	-0.28	1.00000000	duck
Prdx3	0.23	1.00000000	duck
Uck1	-0.13	1.00000000	duck
Hmox1	-0.15	1.00000000	duck
Ptbp1	-0.35	1.00000000	duck
Rmnd5a	-0.27	1.00000000	duck
Samhd1	0.12	1.00000000	duck
Hdac7	0.04	1.00000000	duck
Ybx1	-0.16	1.00000000	duck
Gstk1	0.39	1.00000000	duck
Eif4g2	-0.32	1.00000000	duck
Kpna4	-1.00	1.00000000	duck
Plekha1	0.05	1.00000000	duck
Reck	0.14	1.00000000	duck
Fam167b	0.26	1.00000000	duck
Hgsnat	0.01	1.00000000	duck
Gorasp2	-0.19	1.00000000	duck
Ubap2l	-0.22	1.00000000	duck
Ttc17	-0.09	1.00000000	duck
Trappc9	-0.08	1.00000000	duck
Ifnar1	-1.34	1.00000000	duck
Zcchc4	0.05	1.00000000	duck
Abhd5	0.10	1.00000000	duck
Ankrd13c	-0.80	1.00000000	duck

Sp3	-0.85	1.00000000	duck
Zmiz1	-0.78	1.00000000	duck
Eif2b1	0.05	1.00000000	duck
Fjx1	0.36	1.00000000	duck
Ythdf1	0.00	1.00000000	duck
Plekha3	0.10	1.00000000	duck
Atp6v1c1	0.11	1.00000000	duck
Rab6a	-0.49	1.00000000	duck
Dock11	-0.53	1.00000000	duck
Shld2	0.27	1.00000000	duck
Cbfa2t3	-0.32	1.00000000	duck
Gnpat	0.22	1.00000000	duck
Ero1a	0.19	1.00000000	duck
Nop14	-0.02	1.00000000	duck
Rbm12b2	-0.26	1.00000000	duck
Tnfaip1	-0.21	1.00000000	duck
Stk25	-0.10	1.00000000	duck
Ddx28	-0.05	1.00000000	duck
Ptpn3	0.31	1.00000000	duck
Lifr	0.23	1.00000000	duck
Plek2	-0.05	1.00000000	duck
Tex264	0.10	1.00000000	duck
Lclat1	-1.18	1.00000000	duck
Lonp1	0.51	1.00000000	duck
Ccdc59	0.14	1.00000000	duck
Gphn	-0.28	1.00000000	duck
P4hb	0.01	1.00000000	duck
Ogdh	-0.08	1.00000000	duck
Rbm34	-0.73	1.00000000	duck
Gfpt1	-0.19	1.00000000	duck
Wdr33	-0.69	1.00000000	duck
Ttll1	0.02	1.00000000	duck
Zc3h15	-0.13	1.00000000	duck
Chmp5	-0.08	1.00000000	duck
Hace1	-0.48	1.00000000	duck
Cmss1	0.07	1.00000000	duck
Sphk1	0.14	1.00000000	duck
Wdr1	-0.16	1.00000000	duck
Dot1l	0.19	1.00000000	duck
Tmtc1	-0.55	1.00000000	duck
Casd1	-0.60	1.00000000	duck
Cops4	-0.41	1.00000000	duck
Spag1	0.21	1.00000000	duck
Gpr137b	-0.32	1.00000000	duck
Eif2d	-0.36	1.00000000	duck
N4bp2l1	-0.07	1.00000000	duck

Dph3	-0.11	1.00000000	duck
Stom	-0.03	1.00000000	duck
Lrrc8c	0.14	1.00000000	duck
Abcc5	-0.58	1.00000000	duck
Tnrc6a	-0.37	1.00000000	duck
Lpar6	-0.17	1.00000000	duck
Galnt15	0.23	1.00000000	duck
2610507B11Rik	0.52	1.00000000	duck
Pld1	-0.36	1.00000000	duck
Lrp6	0.47	1.00000000	duck
Fzd6	0.16	1.00000000	duck
Trmt6	-0.06	1.00000000	duck
Dtx2	-0.03	1.00000000	duck
Rc3h1	-0.91	1.00000000	duck
Lrrcc1	0.10	1.00000000	duck
Abca5	-0.02	1.00000000	duck
Zhx1	0.07	1.00000000	duck
Stim2	0.19	1.00000000	duck
Tbc1d9b	-0.11	1.00000000	duck
Pex2	0.20	1.00000000	duck
Yipf6	0.01	1.00000000	duck
Parp9	0.02	1.00000000	duck
Denr	0.19	1.00000000	duck
Brox	0.33	1.00000000	duck
Prrc1	0.38	1.00000000	duck
Lpcat3	-0.27	1.00000000	duck
Cab39	-0.15	1.00000000	duck
Spi1	0.05	1.00000000	duck
Akap1	-0.03	1.00000000	duck
Unc50	0.17	1.00000000	duck
Kbtbd2	-0.07	1.00000000	duck
Vps18	-0.15	1.00000000	duck
Ccdc43	0.26	1.00000000	duck
Cry1	-0.52	1.00000000	duck
Atp10d	-0.17	1.00000000	duck
Snx25	-0.60	1.00000000	duck
Pdzd8	-0.21	1.00000000	duck
Ppara	-0.06	1.00000000	duck
Tmem63b	0.49	1.00000000	duck
Dtd2	0.08	1.00000000	duck
Lnpk	0.09	1.00000000	duck
Csrnp1	-0.48	1.00000000	duck
Frs2	-0.41	1.00000000	duck
Fgd6	0.20	1.00000000	duck
Akt1	0.13	1.00000000	duck
Cse1l	0.37	1.00000000	duck

Golga1	-0.24	1.00000000	duck
Itpripl2	0.09	1.00000000	duck
Crkl	-0.59	1.00000000	duck
Urm1	0.40	1.00000000	duck
Plekhb2	-0.04	1.00000000	duck
Arhgap15	0.22	1.00000000	duck
Glb1	0.20	1.00000000	duck
Prpf4	0.11	1.00000000	duck
Prpf38a	0.11	1.00000000	duck
Ahdc1	0.59	1.00000000	duck
Cdk9	0.08	1.00000000	duck
Hnrnpu	-0.27	1.00000000	duck
Gnb4	-0.18	1.00000000	duck
Shoc2	-0.15	1.00000000	duck
C1d	-0.16	1.00000000	duck
Ints10	-0.35	1.00000000	duck
Ypel1	-0.83	1.00000000	duck
Atxn7l2	0.02	1.00000000	duck
Plekha2	0.38	1.00000000	duck
Htatsf1	-0.41	1.00000000	duck
Scai	0.20	1.00000000	duck
Vps37b	-0.10	1.00000000	duck
Selenop	-0.48	1.00000000	duck
Bicd2	-0.37	1.00000000	duck
Zzef1	-0.29	1.00000000	duck
Stk40	-0.06	1.00000000	duck
Eif2s2	0.30	1.00000000	duck
Otud7a	1.10	1.00000000	duck
Garre1	0.22	1.00000000	duck
Vdac1	0.43	1.00000000	duck
Tspan2	0.33	1.00000000	duck
Erp29	0.12	1.00000000	duck
Bptf	-0.64	1.00000000	duck
Rab39b	-0.10	1.00000000	duck
Fam120a	0.07	1.00000000	duck
Dnajc2	-0.42	1.00000000	duck
Tbc1d31	-0.06	1.00000000	duck
Lactb2	-0.07	1.00000000	duck
Dusp4	-0.23	1.00000000	duck
Sema4g	-0.08	1.00000000	duck
Eif2ak1	-0.05	1.00000000	duck
Prkab1	-0.09	1.00000000	duck
Timm22	-0.07	1.00000000	duck
Pigl	-0.32	1.00000000	duck
Ralgps1	0.03	1.00000000	duck
Ripk2	0.55	1.00000000	duck

Nampt	0.03	1.00000000	duck
Trmu	0.09	1.00000000	duck
Ppp1r1c	0.33	1.00000000	duck
Eef2k	-0.08	1.00000000	duck
Pusl1	0.06	1.00000000	duck
Pikfyve	-0.66	1.00000000	duck
Pbrm1	-0.15	1.00000000	duck
Eloc	0.17	1.00000000	duck
Srp9	-0.50	1.00000000	duck
Klhl7	-0.05	1.00000000	duck
Tspan5	0.74	1.00000000	duck
Abca3	0.29	1.00000000	duck
Scfd2	-0.06	1.00000000	duck
Dctn5	0.38	1.00000000	duck
Slc35e1	0.11	1.00000000	duck
Phf5a	-0.01	1.00000000	duck
Flii	0.21	1.00000000	duck
Dnaja1	-0.20	1.00000000	duck
Srsf6	-0.37	1.00000000	duck
Chchd5	-0.04	1.00000000	duck
Nfatc2	0.58	1.00000000	duck
Sec16a	-0.34	1.00000000	duck
Rit1	-0.75	1.00000000	duck
Klf12	0.31	1.00000000	duck
Dock10	0.24	1.00000000	duck
Natd1	-0.07	1.00000000	duck
Tmem39a	0.14	1.00000000	duck
Ppp1cc	-0.21	1.00000000	duck
Fut11	0.05	1.00000000	duck
Arrdc1	0.06	1.00000000	duck
Slc30a7	-0.01	1.00000000	duck
Tfpi	-0.27	1.00000000	duck
Phf2	-0.12	1.00000000	duck
Zdhhc14	0.15	1.00000000	duck
Serac1	0.18	1.00000000	duck
Zfyve26	-0.22	1.00000000	duck
Ibtk	-0.15	1.00000000	duck
Tmed10	0.02	1.00000000	duck
Nufip1	-0.09	1.00000000	duck
Rsf1	-0.40	1.00000000	duck
Ifngr1	-0.44	1.00000000	duck
Gramd1b	-0.79	1.00000000	duck
Zfat	-0.22	1.00000000	duck
Synj2	-0.05	1.00000000	duck
Etnk1	0.36	1.00000000	duck
Kif5c	-0.08	1.00000000	duck

Wdr20	-0.28	1.00000000	duck
Sirt7	-0.29	1.00000000	duck
Spast	-0.84	1.00000000	duck
Cotl1	0.23	1.00000000	duck
Fgd5	-0.05	1.00000000	duck
Anapc5	-0.15	1.00000000	duck
Skil	0.04	1.00000000	duck
Gpatch2	0.11	1.00000000	duck
Tut4	-0.56	1.00000000	duck
Traf2	-0.22	1.00000000	duck
Robo2	0.45	1.00000000	duck
Coq10b	0.07	1.00000000	duck
Dipk1a	-0.22	1.00000000	duck
Arhgef26	0.10	1.00000000	duck
Chd1	-0.05	1.00000000	duck
Phf14	-0.18	1.00000000	duck
Adarb1	-0.58	1.00000000	duck
Got2	-0.11	1.00000000	duck
Napg	-0.24	1.00000000	duck
Cnih4	0.44	1.00000000	duck
Fkbp15	0.03	1.00000000	duck
Surf6	-0.24	1.00000000	duck
Scyl3	-0.06	1.00000000	duck
Spsb4	0.14	1.00000000	duck
Terf2	0.05	1.00000000	duck
Sap30l	0.20	1.00000000	duck
Rexo4	0.21	1.00000000	duck
Srebf2	0.00	1.00000000	duck
Nckipsd	0.15	1.00000000	duck
Uggt2	-0.27	1.00000000	duck
Dis3	-0.36	1.00000000	duck
Golim4	-0.30	1.00000000	duck
Srsf4	-0.17	1.00000000	duck
Hs6st1	0.29	1.00000000	duck
Elovl1	0.14	1.00000000	duck
Eea1	-0.03	1.00000000	duck
Rbm6	-0.09	1.00000000	duck
Hpse	-0.10	1.00000000	duck
Hmox2	0.20	1.00000000	duck
Eif4a3l1	-0.12	1.00000000	duck
Ccser1	-0.30	1.00000000	duck
Eif3m	-0.05	1.00000000	duck
Sec61a1	-0.16	1.00000000	duck
Kansl1	-0.88	1.00000000	duck
Emc2	-0.01	1.00000000	duck
Sar1a	0.24	1.00000000	duck

Rnf114	-0.03	1.00000000	duck
Scmh1	-0.45	1.00000000	duck
Xbp1	-0.32	1.00000000	duck
Acvr1b	0.31	1.00000000	duck
Gcnt4	0.22	1.00000000	duck
Gfm2	-0.04	1.00000000	duck
Txndc16	-0.13	1.00000000	duck
Als2	-0.27	1.00000000	duck
Rab40c	-0.62	1.00000000	duck
Ccser2	0.62	1.00000000	duck
Pdia5	-0.41	1.00000000	duck
Srp72	-0.06	1.00000000	duck
Pum2	-0.16	1.00000000	duck
Sla	-0.46	1.00000000	duck
Scaf11	-0.53	1.00000000	duck
Ints2	0.00	1.00000000	duck
Ndufa5	-0.05	1.00000000	duck
Atad1	-0.39	1.00000000	duck
Ubn1	-0.26	1.00000000	duck
Rnf41	0.39	1.00000000	duck
Ate1	-0.17	1.00000000	duck
Rad51ap1	0.05	1.00000000	duck
Vrk1	0.05	1.00000000	duck
Gapvd1	-0.56	1.00000000	duck
Crybg3	-0.88	1.00000000	duck
C2cd2	-0.46	1.00000000	duck
Usp12	-0.63	1.00000000	duck
Nin	-0.21	1.00000000	duck
Zc3h14	-0.11	1.00000000	duck
Hspa5	-0.14	1.00000000	duck
Snx18	-0.15	1.00000000	duck
Fam193a	-0.22	1.00000000	duck
Sel1l	0.00	1.00000000	duck
Snx14	-0.21	1.00000000	duck
Spg7	-0.07	1.00000000	duck
Cnot2	-0.19	1.00000000	duck
Tia1	0.07	1.00000000	duck
Vps26a	-0.38	1.00000000	duck
Uimc1	-0.41	1.00000000	duck
Uhmk1	-0.15	1.00000000	duck
Nelfb	-0.23	1.00000000	duck
Arid5b	-0.15	1.00000000	duck
Ppil1	0.42	1.00000000	duck
Atp23	0.25	1.00000000	duck
St3gal1	-0.67	1.00000000	duck
Rab11a	-0.33	1.00000000	duck

Ggps1	-0.11	1.00000000	duck
Sec14l5	-0.02	1.00000000	duck
Cep128	-0.61	1.00000000	duck
Arl1	0.09	1.00000000	duck
Hes6	-0.32	1.00000000	duck
Tmem260	-0.03	1.00000000	duck
Iftap	-0.02	1.00000000	duck
Dbr1	-0.06	1.00000000	duck
Ppp6r2	-0.31	1.00000000	duck
Golga7	-0.24	1.00000000	duck
Clint1	-0.35	1.00000000	duck
Selenoi	-0.02	1.00000000	duck
Tbc1d2b	-0.15	1.00000000	duck
Pafah1b1	0.13	1.00000000	duck
Kcnk13	0.19	1.00000000	duck
Hnrnpll	-0.04	1.00000000	duck
Ube3a	-0.16	1.00000000	duck
Rnf141	0.02	1.00000000	duck
Phlda1	0.23	1.00000000	duck
Shisa2	0.20	1.00000000	duck
Sntb1	-0.21	1.00000000	duck
Tmem192	-0.14	1.00000000	duck
Epb41l5	0.10	1.00000000	duck
Emcn	0.28	1.00000000	duck
Trmt44	-0.55	1.00000000	duck
Nae1	0.02	1.00000000	duck
Niban1	-0.15	1.00000000	duck
Gigyf2	-0.56	1.00000000	duck
Psmf1	0.04	1.00000000	duck
Txnrd1	-0.59	1.00000000	duck
Ahctf1	-0.41	1.00000000	duck
Eif2s1	-0.10	1.00000000	duck
Tcea2	-0.23	1.00000000	duck
Dda1	-0.16	1.00000000	duck
Ddx10	-0.42	1.00000000	duck
Ubxn7	-0.88	1.00000000	duck
Fam122b	-0.42	1.00000000	duck
Ctr9	-0.53	1.00000000	duck
Antkmt	-0.02	1.00000000	duck
Pknox1	-1.70	1.00000000	duck
Ift27	0.04	1.00000000	duck
Bysl	0.00	1.00000000	duck
Usp47	0.04	1.00000000	duck
Ywhag	0.08	1.00000000	duck
Cacul1	-0.95	1.00000000	duck
Prdm11	-0.25	1.00000000	duck

Cdk17	-0.43	1.00000000	duck
Tax1bp3	-0.07	1.00000000	duck
Elf2	0.05	1.00000000	duck
Gna12	-0.43	1.00000000	duck
Acmsd	-0.45	1.00000000	duck
Ice2	-0.95	1.00000000	duck
Acbd5	0.04	1.00000000	duck
Lpgat1	0.30	1.00000000	duck
Jpt2	0.04	1.00000000	duck
Zfp326	-0.71	1.00000000	duck
Bzw2	0.26	1.00000000	duck
Eri1	-0.56	1.00000000	duck
Gorasp1	-0.04	1.00000000	duck
Amot	-0.17	1.00000000	duck
Hpgd	0.13	1.00000000	duck
Kif3b	-0.74	1.00000000	duck
Asxl1	-0.20	1.00000000	duck
Trim37	-0.02	1.00000000	duck
Kdm4c	-0.22	1.00000000	duck
Cmtm7	0.00	1.00000000	duck
Proser1	-0.02	1.00000000	duck
Acadsb	-0.13	1.00000000	duck
Orai1	-0.14	1.00000000	duck
Fam98b	-0.02	1.00000000	duck
Vps45	0.13	1.00000000	duck
Smc3	0.07	1.00000000	duck
1110059G10Rik	0.14	1.00000000	duck
Snx9	-0.44	1.00000000	duck
Pyroxd1	-0.15	1.00000000	duck
Phospho1	-0.23	1.00000000	duck
Plgrkt	0.00	1.00000000	duck
Tbc1d5	-0.11	1.00000000	duck
Pcyt1b	0.20	1.00000000	duck
Cxcr4	-0.69	1.00000000	duck
Zbtb7a	0.17	1.00000000	duck
Foxj2	-0.26	1.00000000	duck
Adgrl2	0.50	1.00000000	duck
Zfp618	-0.39	1.00000000	duck
Cep43	-0.70	1.00000000	duck
Ugcg	0.25	1.00000000	duck
Tpm4	0.40	1.00000000	duck
Dok2	-0.51	1.00000000	duck
Nadk	-0.85	1.00000000	duck
Irf8	-1.17	1.00000000	duck
Vps50	-0.16	1.00000000	duck
Nup98	-0.71	1.00000000	duck

Nup85	-0.57	1.00000000	duck
Fbxw8	-0.09	1.00000000	duck
Ttc39c	-0.22	1.00000000	duck
Nrbp1	-0.49	1.00000000	duck
Bdh1	0.04	1.00000000	duck
Tpcn1	0.24	1.00000000	duck
Cwf19l1	0.07	1.00000000	duck
Nelfcd	-0.30	1.00000000	duck
Fbxo32	-0.39	1.00000000	duck
Pdp2	-0.05	1.00000000	duck
Nudt7	0.15	1.00000000	duck
Ncaph	0.20	1.00000000	duck
Actn4	-0.04	1.00000000	duck
Ap3s2	-0.01	1.00000000	duck
Stard5	-0.10	1.00000000	duck
Tpra1	-0.18	1.00000000	duck
Cd247	-0.29	1.00000000	duck
Stx18	-0.08	1.00000000	duck
Rars	-0.03	1.00000000	duck
Gan	0.05	1.00000000	duck
Rab10	0.09	1.00000000	duck
Fbxo28	0.32	1.00000000	duck
Cebpg	-0.24	1.00000000	duck
Pip5k1a	-0.15	1.00000000	duck
Atic	0.03	1.00000000	duck
Taok1	-0.17	1.00000000	duck
Galm	0.12	1.00000000	duck
Igfbp7	0.20	1.00000000	duck
Ascc1	0.08	1.00000000	duck
Vps11	0.06	1.00000000	duck
Nip7	-0.03	1.00000000	duck
Samd12	-0.11	1.00000000	duck
Akirin2	-0.07	1.00000000	duck
Fbxl18	0.07	1.00000000	duck
Kdm5a	-0.10	1.00000000	duck
Vps41	-0.11	1.00000000	duck
Lats1	-0.14	1.00000000	duck
Cdc42	-0.16	1.00000000	duck
Ell	-0.03	1.00000000	duck
Ahrr	-0.05	1.00000000	duck
Ddx6	-0.40	1.00000000	duck
Ciao2b	0.03	1.00000000	duck
Pithd1	0.34	1.00000000	duck
Hdac10	0.28	1.00000000	duck
Ttc32	-0.08	1.00000000	duck
Tmem9b	-0.13	1.00000000	duck

Cops7a	-0.02	1.00000000	duck
Tecpr2	-0.03	1.00000000	duck
Slc35f5	0.04	1.00000000	duck
Rab21	-0.28	1.00000000	duck
Slc39a11	0.37	1.00000000	duck
Nr1d2	-0.26	1.00000000	duck
Rhpn2	-0.12	1.00000000	duck
Ext2	0.16	1.00000000	duck
Limd2	-0.41	1.00000000	duck
Tspan4	-0.48	1.00000000	duck
Clec16a	-0.63	1.00000000	duck
Wsb1	-0.20	1.00000000	duck
Gxylt1	0.14	1.00000000	duck
Swi5	-0.11	1.00000000	duck
Ttc7	0.30	1.00000000	duck
Fbxl2	0.07	1.00000000	duck
Ackr3	0.00	1.00000000	duck
Bnip3	-0.07	1.00000000	duck
Stx8	-0.68	1.00000000	duck
Srpr	0.25	1.00000000	duck
Auh	0.48	1.00000000	duck
Ints7	-0.19	1.00000000	duck
Ttbk2	0.39	1.00000000	duck
Fcho2	-0.41	1.00000000	duck
Plekha8	-0.14	1.00000000	duck
Xpc	0.02	1.00000000	duck
Nudt19	0.09	1.00000000	duck
Snx8	-0.10	1.00000000	duck
Llgl1	-0.01	1.00000000	duck
Hic1	0.10	1.00000000	duck
Atm	-0.06	1.00000000	duck
Derl1	-0.01	1.00000000	duck
Atf6	-0.37	1.00000000	duck
Pdcl3	0.22	1.00000000	duck
Supv3l1	-0.22	1.00000000	duck
Trafd1	-0.04	1.00000000	duck
Stau1	-0.36	1.00000000	duck
Rragd	-0.10	1.00000000	duck
Cul2	0.18	1.00000000	duck
Slc2a8	0.01	1.00000000	duck
Vps26b	0.01	1.00000000	duck
Srpk1	-0.12	1.00000000	duck
Eprs	-0.12	1.00000000	duck
Rnf169	0.10	1.00000000	duck
Themis2	-0.04	1.00000000	duck
Dhx15	-0.34	1.00000000	duck

Snx6	-0.34	1.00000000	duck
Rrp15	-0.01	1.00000000	duck
Kcnmb4	-0.39	1.00000000	duck
4833439L19Rik	0.01	1.00000000	duck
Dnaaf5	-0.40	1.00000000	duck
Slc9a5	0.32	1.00000000	duck
Icmt	0.12	1.00000000	duck
Sap30	-0.32	1.00000000	duck
Ptpn2	-0.60	1.00000000	duck
Znhit6	0.06	1.00000000	duck
Crnkl1	-0.01	1.00000000	duck
Acss2	0.13	1.00000000	duck
Dcun1d1	0.91	1.00000000	duck
P2ry13	-0.01	1.00000000	duck
Ywhae	0.08	1.00000000	duck
Alg5	0.06	1.00000000	duck
Hadhb	-0.02	1.00000000	duck
Etfdh	-0.21	1.00000000	duck
Gng10	0.08	1.00000000	duck
Pik3cg	-0.03	1.00000000	duck
Zc3h7a	-0.30	1.00000000	duck
Pla2g15	-0.33	1.00000000	duck
Adap1	-0.04	1.00000000	duck
Gtf2f2	-0.65	1.00000000	duck
Gab3	-0.62	1.00000000	duck
Spty2d1	-0.31	1.00000000	duck
Tbck	-0.16	1.00000000	duck
Trmt2a	0.23	1.00000000	duck
Chmp2b	-0.37	1.00000000	duck
Ndufa8	-0.10	1.00000000	duck
Gyg	-0.06	1.00000000	duck
Hagh	-0.87	1.00000000	duck
Sestd1	-0.03	1.00000000	duck
Lrrc45	0.00	1.00000000	duck
Kat7	0.17	1.00000000	duck
Ppip5k2	0.44	1.00000000	duck
Rpa2	0.29	1.00000000	duck
Spryd7	0.01	1.00000000	duck
Cd276	0.21	1.00000000	duck
Bcar1	0.31	1.00000000	duck
Tab2	-0.25	1.00000000	duck
Tram2	0.30	1.00000000	duck
Dnajb14	-0.13	1.00000000	duck
Glod4	0.10	1.00000000	duck
Larp7	-0.13	1.00000000	duck
Eif4g1	-0.08	1.00000000	duck

Dennd4a	-0.20	1.00000000	duck
Sgms1	-0.48	1.00000000	duck
Dmxl1	-0.07	1.00000000	duck
Rmdn3	-0.66	1.00000000	duck
Atxn7	-0.09	1.00000000	duck
Sft2d1	0.08	1.00000000	duck
Vopp1	0.39	1.00000000	duck
Snx29	-0.02	1.00000000	duck
Dennd1a	-0.25	1.00000000	duck
Cops2	-0.27	1.00000000	duck
Bloc1s6	0.06	1.00000000	duck
Sephs1	0.28	1.00000000	duck
Pgd	0.02	1.00000000	duck
Pheta2	0.20	1.00000000	duck
Atg13	0.37	1.00000000	duck
Slc25a20	-0.10	1.00000000	duck
Psmd13	-0.23	1.00000000	duck
Caml	-0.19	1.00000000	duck
Naga	-0.04	1.00000000	duck
Capzb	0.26	1.00000000	duck
Cnot10	0.00	1.00000000	duck
Irak4	-0.23	1.00000000	duck
Nat10	-0.06	1.00000000	duck
Zfyve27	0.22	1.00000000	duck
Pak2	0.01	1.00000000	duck
Tmem222	0.11	1.00000000	duck
Ndufs1	-0.26	1.00000000	duck
Suox	0.05	1.00000000	duck
Desi1	-0.25	1.00000000	duck
Ccdc126	-0.27	1.00000000	duck
Git2	0.19	1.00000000	duck
Tcp11l2	-0.22	1.00000000	duck
Rap2c	0.16	1.00000000	duck
St3gal3	-0.39	1.00000000	duck
Nsa2	0.26	1.00000000	duck
Rab11fip4	-0.14	1.00000000	duck
Ndufb1	0.14	1.00000000	duck
Traf3	0.00	1.00000000	duck
Odr4	-0.53	1.00000000	duck
Hprt	-0.99	1.00000000	duck
Tesk2	-0.06	1.00000000	duck
Zfp622	0.17	1.00000000	duck
Xylt2	0.15	1.00000000	duck
Ctns	0.50	1.00000000	duck
Frs3	0.11	1.00000000	duck
Fam222b	-0.71	1.00000000	duck

Otulinl	-0.11	1.00000000	duck
Hif1a	-0.41	1.00000000	duck
Wdr19	-0.02	1.00000000	duck
Whamm	-0.10	1.00000000	duck
Cdkal1	0.01	1.00000000	duck
Utp25	-0.03	1.00000000	duck
Prkdc	-0.77	1.00000000	duck
Nol11	0.34	1.00000000	duck
Dusp1	1.09	1.00000000	duck
Dpm2	0.20	1.00000000	duck
2510039O18Rik	0.19	1.00000000	duck
Tma16	0.05	1.00000000	duck
Stard4	-0.01	1.00000000	duck
Ttc19	0.25	1.00000000	duck
Tsc1	-0.22	1.00000000	duck
Tnfrsf11a	-0.31	1.00000000	duck
Pds5a	0.28	1.00000000	duck
4932411N23Rik	-0.69	1.00000000	duck
Pogz	-0.12	1.00000000	duck
Pspc1	-0.23	1.00000000	duck
Clybl	0.20	1.00000000	duck
Dach2	0.25	1.00000000	duck
Rgl1	-0.04	1.00000000	duck
Pskh1	-0.06	1.00000000	duck
Ssbp2	-0.73	1.00000000	duck
Usf3	0.15	1.00000000	duck
Poldip2	0.06	1.00000000	duck
Prdm10	-0.20	1.00000000	duck
Tpk1	-0.14	1.00000000	duck
Nt5dc1	-0.08	1.00000000	duck
Narf	-0.72	1.00000000	duck
Parp1	-0.25	1.00000000	duck
Tial1	-0.56	1.00000000	duck
Ndufb10	-0.04	1.00000000	duck
Tmtc3	-0.22	1.00000000	duck
Ifih1	-0.29	1.00000000	duck
Galt	0.14	1.00000000	duck
Aco1	0.02	1.00000000	duck
Setx	-0.28	1.00000000	duck
Dbi	0.34	1.00000000	duck
Atp1b3	-0.21	1.00000000	duck
Pwp1	0.27	1.00000000	duck
Cd99l2	-0.09	1.00000000	duck
Spata5	-0.24	1.00000000	duck
Gid8	-0.40	1.00000000	duck
Tmem70	0.03	1.00000000	duck

Slc25a46	-0.66	1.00000000	duck
Tbc1d22a	-0.21	1.00000000	duck
Arap3	0.04	1.00000000	duck
Pigg	-0.04	1.00000000	duck
Sptlc2	-0.86	1.00000000	duck
Npepl1	-0.47	1.00000000	duck
Drg1	-0.03	1.00000000	duck
Ccdc47	-0.47	1.00000000	duck
Kdm5b	-0.09	1.00000000	duck
Crtc1	-0.15	1.00000000	duck
Spats2	-0.09	1.00000000	duck
Hs1bp3	0.27	1.00000000	duck
Slc30a1	0.02	1.00000000	duck
Papss2	1.94	1.00000000	duck
Armc9	-0.23	1.00000000	duck
Acads	0.07	1.00000000	duck
Gpatch4	0.00	1.00000000	duck
Commd9	-0.13	1.00000000	duck
Nars	0.11	1.00000000	duck
Jade2	-0.65	1.00000000	duck
Nup133	-0.37	1.00000000	duck
Cass4	-0.05	1.00000000	duck
Zc3h13	-0.18	1.00000000	duck
Smarce1	0.07	1.00000000	duck
Rnps1	0.04	1.00000000	duck
Gsr	-0.23	1.00000000	duck
Suclg2	0.19	1.00000000	duck
Smarcal1	-0.01	1.00000000	duck
Wbp4	-1.43	1.00000000	duck
G3bp1	-0.60	1.00000000	duck
Faf1	-0.58	1.00000000	duck
Abtb2	0.82	1.00000000	duck
Zbtb37	-0.37	1.00000000	duck
Wsb2	0.13	1.00000000	duck
Wdr81	0.08	1.00000000	duck
Fer	0.35	1.00000000	duck
Dazap1	-0.41	1.00000000	duck
Ago2	-0.46	1.00000000	duck
P2ry6	-0.04	1.00000000	duck
Cfap36	-0.60	1.00000000	duck
Crb1	0.05	1.00000000	duck
Lrrk1	-0.07	1.00000000	duck
Arpp19	0.23	1.00000000	duck
Sox5	-0.53	1.00000000	duck
Zcchc10	-0.05	1.00000000	duck
Anxa5	0.19	1.00000000	duck

Hspa14	0.09	1.00000000	duck
Agpat3	0.00	1.00000000	duck
Pgm2	0.02	1.00000000	duck
Tmem258	0.20	1.00000000	duck
Slc25a36	-0.31	1.00000000	duck
Ncoa4	-0.74	1.00000000	duck
Orc3	0.03	1.00000000	duck
Snapc4	-0.27	1.00000000	duck
Tnfsf11	0.04	1.00000000	duck
Tbc1d12	-0.32	1.00000000	duck
Ap1s2	0.24	1.00000000	duck
Zfp644	-0.44	1.00000000	duck
Pdgfb	0.31	1.00000000	duck
Spata6	-0.17	1.00000000	duck
Chchd7	-0.23	1.00000000	duck
Stat4	-0.26	1.00000000	duck
Recql	0.27	1.00000000	duck
Dnase1l3	0.13	1.00000000	duck
Dhrs7c	-0.09	1.00000000	duck
Dnajc7	-0.56	1.00000000	duck
Ttc13	-0.94	1.00000000	duck
Dnttip2	-0.36	1.00000000	duck
Snw1	-0.12	1.00000000	duck
Clpx	-0.18	1.00000000	duck
Ppp2r2d	-0.25	1.00000000	duck
Gtf2e1	-0.99	1.00000000	duck
Psmg3	-0.21	1.00000000	duck
Chpt1	0.17	1.00000000	duck
Foxk2	-0.62	1.00000000	duck
Fzr1	-0.14	1.00000000	duck
Cers2	0.28	1.00000000	duck
Stam	0.10	1.00000000	duck
Pla2r1	0.27	1.00000000	duck
Phb2	0.07	1.00000000	duck
Igf2r	-0.92	1.00000000	duck
Cisd1	0.20	1.00000000	duck
Ranbp9	0.14	1.00000000	duck
Carf	-0.48	1.00000000	duck
Hacd3	0.16	1.00000000	duck
Zbtb6	0.26	1.00000000	duck
Ubox5	0.21	1.00000000	duck
Tmem51	0.07	1.00000000	duck
Tbc1d8	-0.45	1.00000000	duck
Ncald	0.16	1.00000000	duck
Slc12a4	-0.66	1.00000000	duck
Dclre1c	-0.01	1.00000000	duck

Qdpr	-1.25	1.00000000	duck
Trappc6b	-0.05	1.00000000	duck
Smpd4	-0.46	1.00000000	duck
Adar	0.07	1.00000000	duck
Lpcat1	-0.20	1.00000000	duck
Hipk3	0.09	1.00000000	duck
Gtpbp1	-0.36	1.00000000	duck
Carnmt1	0.30	1.00000000	duck
Dnaja2	-0.10	1.00000000	duck
Phlpp1	0.24	1.00000000	duck
Eci3	-0.19	1.00000000	duck
Pdhx	0.10	1.00000000	duck
St6galnac4	-0.04	1.00000000	duck
Dusp11	-0.20	1.00000000	duck
B3gnt5	-0.31	1.00000000	duck
Thumpd3	-0.01	1.00000000	duck
Tax1bp1	-0.08	1.00000000	duck
Siah2	0.07	1.00000000	duck
Stk38l	0.10	1.00000000	duck
Rab12	-0.24	1.00000000	duck
Hikeshi	0.11	1.00000000	duck
Btbd1	0.11	1.00000000	duck
Exoc6	-0.05	1.00000000	duck
Trnt1	-0.33	1.00000000	duck
Dlgap4	-0.18	1.00000000	duck
Cct8	-0.21	1.00000000	duck
Nop58	0.02	1.00000000	duck
Lonrf1	-0.13	1.00000000	duck
Ncoa5	-0.19	1.00000000	duck
Pgam5	-0.09	1.00000000	duck
Gnpda2	-0.15	1.00000000	duck
Copg2	-0.16	1.00000000	duck
Soat1	0.01	1.00000000	duck
Vegfc	-0.59	1.00000000	duck
Itfg1	-0.36	1.00000000	duck
Otulin	0.07	1.00000000	duck
Tango2	0.08	1.00000000	duck
Zer1	-0.27	1.00000000	duck
Pdcd2	0.07	1.00000000	duck
Uchl3	-0.07	1.00000000	duck
Ubtd2	-0.06	1.00000000	duck
Nmt2	0.01	1.00000000	duck
Nipsnap2	0.02	1.00000000	duck
Dennd6b	0.10	1.00000000	duck
Dlat	0.10	1.00000000	duck
Cmas	-0.02	1.00000000	duck

Tlr7	-0.02	1.00000000	duck
Rdh10	-0.52	1.00000000	duck
Kcnk1	0.01	1.00000000	duck
Zfp330	-0.05	1.00000000	duck
Fam114a1	-0.23	1.00000000	duck
Stk39	-0.07	1.00000000	duck
Sh3gl3	0.05	1.00000000	duck
Actr8	-0.38	1.00000000	duck
Fanci	-0.23	1.00000000	duck
Fuca2	0.07	1.00000000	duck
Arl8b	-0.32	1.00000000	duck
Iars	0.10	1.00000000	duck
Bcl7b	-0.34	1.00000000	duck
Aco2	-0.11	1.00000000	duck
Cntrl	-0.23	1.00000000	duck
Arl5b	0.32	1.00000000	duck
Dtx3l	-0.57	1.00000000	duck
Cactin	-0.36	1.00000000	duck
Pemt	0.17	1.00000000	duck
Ago3	-0.28	1.00000000	duck
Slc26a5	-0.53	1.00000000	duck
Etv6	-0.27	1.00000000	duck
Zdhhc3	0.44	1.00000000	duck
Klhl2	-0.04	1.00000000	duck
Atxn1l	-0.01	1.00000000	duck
Prrc2b	-0.32	1.00000000	duck
Zdhhc7	0.07	1.00000000	duck
Gpx3	0.44	1.00000000	duck
Wdr45b	0.16	1.00000000	duck
Cbx7	0.12	1.00000000	duck
Rab3il1	0.18	1.00000000	duck
Rab24	0.11	1.00000000	duck
Slain2	0.06	1.00000000	duck
Setd7	0.22	1.00000000	duck
Rab8b	0.08	1.00000000	duck
Ap3d1	-0.64	1.00000000	duck
Appl2	-0.01	1.00000000	duck
Fem1c	-0.22	1.00000000	duck
Hsph1	-0.12	1.00000000	duck
Wdsub1	0.18	1.00000000	duck
Lsm11	-0.05	1.00000000	duck
Sh3gl1	0.11	1.00000000	duck
Rab2a	-0.48	1.00000000	duck
Aebp2	-0.57	1.00000000	duck
Riok3	-0.44	1.00000000	duck
Itgb1	-0.15	1.00000000	duck

Dpy30	-0.13	1.00000000	duck
Ube2a	-0.08	1.00000000	duck
Kansl1l	-0.39	1.00000000	duck
Exog	0.06	1.00000000	duck
Tns1	0.30	1.00000000	duck
Trp53rka	-0.18	1.00000000	duck
Wdr43	-0.06	1.00000000	duck
Abracl	0.15	1.00000000	duck
Lca5	-0.46	1.00000000	duck
Ccni	-0.15	1.00000000	duck
Tiam2	-0.91	1.00000000	duck
Lysmd2	-0.43	1.00000000	duck
Rnf19b	0.11	1.00000000	duck
Hectd1	0.05	1.00000000	duck
Tex10	0.07	1.00000000	duck
Rftn2	-0.05	1.00000000	duck
Chd5	0.04	1.00000000	duck
Slc45a3	0.02	1.00000000	duck
Cracr2a	-0.04	1.00000000	duck
Eif4e2	-0.09	1.00000000	duck
Tcf25	0.22	1.00000000	duck
Pdcl	0.23	1.00000000	duck
Cdk8	-0.52	1.00000000	duck
Ociad1	-0.08	1.00000000	duck
Eif3l	-0.02	1.00000000	duck
Prpf39	-0.80	1.00000000	duck
Arl6ip4	0.05	1.00000000	duck
Plekho1	-0.04	1.00000000	duck
Polr2b	0.01	1.00000000	duck
Cep152	-0.06	1.00000000	duck
Zfp592	-0.76	1.00000000	duck
Gcc2	-0.14	1.00000000	duck
Hps3	0.18	1.00000000	duck
Zdhhc9	0.10	1.00000000	duck
Ndrg3	-1.02	1.00000000	duck
Smim13	-0.08	1.00000000	duck
Trim59	-0.06	1.00000000	duck
Hmg20a	0.10	1.00000000	duck
Atg7	0.12	1.00000000	duck
Nfe2l1	-0.12	1.00000000	duck
Exosc10	0.08	1.00000000	duck
Grpel1	0.10	1.00000000	duck
Pik3c3	0.07	1.00000000	duck
Osgin2	-0.01	1.00000000	duck
Zfp654	-0.38	1.00000000	duck
Deptor	-0.32	1.00000000	duck

Abhd17a	0.21	1.00000000	duck
Pccb	-0.06	1.00000000	duck
Ufm1	-0.08	1.00000000	duck
Tns3	1.59	1.00000000	duck
Slc37a2	0.12	1.00000000	duck
Xpo4	-0.48	1.00000000	duck
Rassf5	0.40	1.00000000	duck
Tln1	0.10	1.00000000	duck
Vps13a	-0.79	1.00000000	duck
Sprtn	-0.75	1.00000000	duck
Vat1l	-0.05	1.00000000	duck
Ppp1r13b	0.14	1.00000000	duck
Pold2	0.47	1.00000000	duck
Rragc	-0.25	1.00000000	duck
Dsn1	0.19	1.00000000	duck
C1qc	0.37	1.00000000	duck
Gss	0.07	1.00000000	duck
Baz1b	0.12	1.00000000	duck
Tnip1	-0.21	1.00000000	duck
Snrnp200	0.22	1.00000000	duck
Usp1	0.09	1.00000000	duck
Cdkn1b	-0.16	1.00000000	duck
Prkar1a	-0.25	1.00000000	duck
Scin	0.55	1.00000000	duck
Tlr2	0.00	1.00000000	duck
Sclt1	-0.55	1.00000000	duck
Socs4	-0.01	1.00000000	duck
Erg28	-0.40	1.00000000	duck
Smad4	-0.03	1.00000000	duck
Selenof	0.19	1.00000000	duck
Cux1	-0.41	1.00000000	duck
Cluh	-0.11	1.00000000	duck
Ro60	-0.03	1.00000000	duck
Nup88	-0.02	1.00000000	duck
Phf10	-0.03	1.00000000	duck
Brd1	-0.40	1.00000000	duck
Obi1	0.11	1.00000000	duck
Bend3	-0.24	1.00000000	duck
Trpm7	-0.43	1.00000000	duck
Pigh	-0.16	1.00000000	duck
Gtf2h1	-0.23	1.00000000	duck
Nudc	0.04	1.00000000	duck
Atp11c	0.35	1.00000000	duck
Armc1	-0.83	1.00000000	duck
Rnaseh2b	0.12	1.00000000	duck
Eftud2	-0.01	1.00000000	duck

Wmp	-0.06	1.00000000	duck
Esco1	-0.45	1.00000000	duck
Fgd3	0.08	1.00000000	duck
Trerf1	-0.18	1.00000000	duck
Rnf149	-0.53	1.00000000	duck
Dag1	0.07	1.00000000	duck
Tnfsf15	0.00	1.00000000	duck
Pak1ip1	-0.32	1.00000000	duck
Anapc13	0.27	1.00000000	duck
Ogfrl1	-0.11	1.00000000	duck
Letm1	-0.07	1.00000000	duck
Dek	0.04	1.00000000	duck
Rab30	0.01	1.00000000	duck
Klhl11	0.08	1.00000000	duck
Lcor	0.06	1.00000000	duck
Ctdspl2	-0.39	1.00000000	duck
Cyth4	0.12	1.00000000	duck
Pitpnm1	0.18	1.00000000	duck
Zc3h12c	-0.06	1.00000000	duck
Fam32a	-0.26	1.00000000	duck
Lancl2	0.44	1.00000000	duck
Ptdss2	0.50	1.00000000	duck
Plagl1	0.11	1.00000000	duck
Slc4a3	0.01	1.00000000	duck
Sfr1	0.00	1.00000000	duck
Arrdc4	0.04	1.00000000	duck
Adat1	0.00	1.00000000	duck
St14	0.03	1.00000000	duck
Ptpre	-0.06	1.00000000	duck
Nif3l1	0.39	1.00000000	duck
Kpna6	-0.32	1.00000000	duck
N4bp1	-0.24	1.00000000	duck
Galnt12	-0.07	1.00000000	duck
Top1mt	0.11	1.00000000	duck
Itgb1bp1	0.19	1.00000000	duck
Nup43	-0.05	1.00000000	duck
Fbxl4	-0.74	1.00000000	duck
Tacc3	-0.31	1.00000000	duck
Ehmt1	0.11	1.00000000	duck
Baz1a	-0.41	1.00000000	duck
Spen	-0.37	1.00000000	duck
Nek1	-0.83	1.00000000	duck
Elk3	0.56	1.00000000	duck
Chfr	-0.15	1.00000000	duck
Anxa7	-0.06	1.00000000	duck
Syngr3	0.13	1.00000000	duck

Srsf11	0.05	1.00000000	duck
Prkrip1	-0.22	1.00000000	duck
Abhd12	-0.32	1.00000000	duck
Hmbox1	-0.13	1.00000000	duck
Virma	-0.15	1.00000000	duck
Timm44	0.08	1.00000000	duck
Tom1	0.02	1.00000000	duck
Kit	0.24	1.00000000	duck
Dgkz	0.17	1.00000000	duck
Ppp2r3c	-0.01	1.00000000	duck
Nono	-0.08	1.00000000	duck
Rabgap1	-0.13	1.00000000	duck
Uap1l1	0.16	1.00000000	duck
Pank2	0.30	1.00000000	duck
9130409I23Rik	0.14	1.00000000	duck
Lsm14a	-0.44	1.00000000	duck
Tes	0.09	1.00000000	duck
Pcca	-0.07	1.00000000	duck
Ubfd1	-0.67	1.00000000	duck
Phb	0.04	1.00000000	duck
Nap1l1	-0.23	1.00000000	duck
Vma21	0.17	1.00000000	duck
Dtwd1	0.16	1.00000000	duck
Fam168a	-0.27	1.00000000	duck
Dmxl2	-0.19	1.00000000	duck
Hibch	0.12	1.00000000	duck
Pes1	0.16	1.00000000	duck
Hormad1	0.14	1.00000000	duck
Zfp148	-0.21	1.00000000	duck
Nrf1	-0.25	1.00000000	duck
C9orf72	-0.40	1.00000000	duck
Osbp2	-0.12	1.00000000	duck
Ufd1	0.19	1.00000000	duck
Tec	0.11	1.00000000	duck
Ecd	-0.03	1.00000000	duck
Armc6	0.00	1.00000000	duck
Hells	0.09	1.00000000	duck
Wdtc1	-0.10	1.00000000	duck
Adam17	-0.59	1.00000000	duck
Leprot	-0.23	1.00000000	duck
Kmt5b	-0.51	1.00000000	duck
Slc44a2	0.29	1.00000000	duck
Copa	0.26	1.00000000	duck
Pik3r5	-0.23	1.00000000	duck
Il1rapl2	0.21	1.00000000	duck
Ggt7	0.02	1.00000000	duck

Slc12a2	0.37	1.00000000	duck
Tmem184b	-0.47	1.00000000	duck
Zeb1	-0.08	1.00000000	duck
Abce1	-0.15	1.00000000	duck
Lrrk2	0.03	1.00000000	duck
Skp1	-0.24	1.00000000	duck
Slc4a4	0.10	1.00000000	duck
Dnajc19	0.05	1.00000000	duck
Dnmt1	-0.05	1.00000000	duck
Pitpnb	-0.06	1.00000000	duck
Tlk1	-0.05	1.00000000	duck
Kctd10	-0.16	1.00000000	duck
Rap1gap2	-0.23	1.00000000	duck
Pinx1	-0.64	1.00000000	duck
Zfp217	-0.07	1.00000000	duck
Dpp7	0.07	1.00000000	duck
Rbx1	-0.01	1.00000000	duck
Prpf6	-0.36	1.00000000	duck
Dyrk1a	-0.69	1.00000000	duck
Pld4	0.07	1.00000000	duck
Nutf2	0.02	1.00000000	duck
Tmem30a	0.41	1.00000000	duck
Abhd6	0.54	1.00000000	duck
Psip1	-0.15	1.00000000	duck
Ssh2	0.07	1.00000000	duck
Atf2	-0.24	1.00000000	duck
Actr3	0.07	1.00000000	duck
Ppfia1	-0.37	1.00000000	duck
Dclk2	-0.14	1.00000000	duck
Trmt5	0.12	1.00000000	duck
Snrpb2	-0.06	1.00000000	duck
Csl	-0.04	1.00000000	duck
Tpm1	0.03	1.00000000	duck
Zfp512	-0.30	1.00000000	duck
Tmem50b	-0.03	1.00000000	duck
As3mt	0.11	1.00000000	duck
Rcl1	-0.04	1.00000000	duck
Lrrc43	-0.15	1.00000000	duck
Ttbk1	0.16	1.00000000	duck
Ttc28	-0.26	1.00000000	duck
Nnt	-0.18	1.00000000	duck
Rnf111	-0.17	1.00000000	duck
Caprin1	-0.13	1.00000000	duck
Numb	-0.28	1.00000000	duck
Ankrd27	-0.31	1.00000000	duck
Pcgf6	-0.59	1.00000000	duck

Lcp1	-0.07	1.00000000	duck
Susd6	-0.24	1.00000000	duck
Tusc2	-0.25	1.00000000	duck
Dpysl2	-0.07	1.00000000	duck
Rif1	-0.01	1.00000000	duck
Slc2a6	0.16	1.00000000	duck
Pmpca	0.06	1.00000000	duck
Zmynd19	0.09	1.00000000	duck
Lmbr1l	0.06	1.00000000	duck
Cul4b	-0.54	1.00000000	duck
Ubl3	0.29	1.00000000	duck
Tmem164	0.16	1.00000000	duck
Ctnnb1	-0.04	1.00000000	duck
Dync1li1	0.01	1.00000000	duck
Ccnl1	-0.43	1.00000000	duck
Senp5	-0.25	1.00000000	duck
Aftph	0.03	1.00000000	duck
Zfp513	0.08	1.00000000	duck
Stx6	0.14	1.00000000	duck
Ube4a	0.14	1.00000000	duck
Efr3a	-0.15	1.00000000	duck
Pfpl	-0.09	1.00000000	duck
Inpp4a	-0.31	1.00000000	duck
Smg5	0.14	1.00000000	duck
Dnajc1	-0.53	1.00000000	duck
Xrcc2	0.03	1.00000000	duck
Prdm16	0.36	1.00000000	duck
Sin3b	0.07	1.00000000	duck
Exoc2	0.07	1.00000000	duck
Ppme1	-0.23	1.00000000	duck
Il6st	0.24	1.00000000	duck
Hvcn1	-0.04	1.00000000	duck
Slc35e4	0.22	1.00000000	duck
Nkiras2	0.16	1.00000000	duck
L3mbtl3	-0.10	1.00000000	duck
Haus2	0.10	1.00000000	duck
Fancg	0.53	1.00000000	duck
Wdr12	0.14	1.00000000	duck
Wdr4	-0.03	1.00000000	duck
Dusp6	0.33	1.00000000	duck
Kat2b	-0.16	1.00000000	duck
Gucd1	0.17	1.00000000	duck
Pus10	0.98	1.00000000	duck
Bcl2l1	-0.69	1.00000000	duck
Snf8	0.01	1.00000000	duck
Foxn2	-0.02	1.00000000	duck

D5Ertd579e	-1.51	1.00000000	duck
Capn15	-0.09	1.00000000	duck
Shc1	0.27	1.00000000	duck
Taf4	-1.00	1.00000000	duck
Cipc	0.09	1.00000000	duck
Dock1	0.41	1.00000000	duck
Nuf2	0.22	1.00000000	duck
Prr7	0.24	1.00000000	duck
Arhgef12	-0.37	1.00000000	duck
Siva1	0.10	1.00000000	duck
Cd82	0.00	1.00000000	duck
Kctd20	0.27	1.00000000	duck
Akap11	-0.38	1.00000000	duck
Bmpr2	0.07	1.00000000	duck
Lmf1	0.05	1.00000000	duck
Gdi2	-0.06	1.00000000	duck
Lrrc8d	-0.30	1.00000000	duck
Tank	-0.06	1.00000000	duck
Snrpa1	0.15	1.00000000	duck
Elovl5	-0.19	1.00000000	duck
Wdr11	-0.11	1.00000000	duck
Eed	0.07	1.00000000	duck
Asap1	0.70	1.00000000	duck
Clcn3	-0.71	1.00000000	duck
Cars	0.05	1.00000000	duck
Lrrc1	-0.20	1.00000000	duck
Gpr141	0.17	1.00000000	duck
Isca2	0.02	1.00000000	duck
Usp24	-0.25	1.00000000	duck
Parg	-0.75	1.00000000	duck
Arhgap26	-0.65	1.00000000	duck
Septin6	0.14	1.00000000	duck
Cables2	-0.21	1.00000000	duck
Cep290	-0.29	1.00000000	duck
Dcbld2	0.23	1.00000000	duck
Grk3	-0.06	1.00000000	duck
Tln2	-0.38	1.00000000	duck
Anapc1	0.33	1.00000000	duck
Snx2	0.14	1.00000000	duck
Sidt2	0.07	1.00000000	duck
Tcp1	-0.10	1.00000000	duck
Hook3	-0.29	1.00000000	duck
Hexim1	-0.09	1.00000000	duck
Jmjd4	-0.01	1.00000000	duck
Rpf1	0.05	1.00000000	duck
Optn	0.19	1.00000000	duck

Zc3h12d	-0.44	1.00000000	duck
Jade3	-0.10	1.00000000	duck
Klf10	0.26	1.00000000	duck
Nedd9	0.11	1.00000000	duck
Tmx1	0.01	1.00000000	duck
Rhoh	-0.23	1.00000000	duck
Lmo2	-1.24	1.00000000	duck
Gorab	-0.01	1.00000000	duck
Pxk	-0.23	1.00000000	duck
Rbsn	-0.26	1.00000000	duck
Aak1	-0.22	1.00000000	duck
Slc35b4	0.03	1.00000000	duck
Rab31	0.01	1.00000000	duck
Ralgapa2	-0.72	1.00000000	duck
Txn2	0.26	1.00000000	duck
Zfp518a	-0.48	1.00000000	duck
Poglut2	-0.23	1.00000000	duck
Socs5	0.06	1.00000000	duck
Pcnx4	-0.13	1.00000000	duck
Gsk3b	-0.25	1.00000000	duck
Clock	-0.42	1.00000000	duck
Jag2	-0.43	1.00000000	duck
Rpap2	-0.02	1.00000000	duck
Psmd5	-0.30	1.00000000	duck
Ippk	-0.11	1.00000000	duck
Rbm26	-0.26	1.00000000	duck
Trappc13	0.41	1.00000000	duck
Nol6	0.10	1.00000000	duck
Enpp6	0.11	1.00000000	duck
Tbc1d16	0.05	1.00000000	duck
Zranb1	0.11	1.00000000	duck
Zfp362	0.09	1.00000000	duck
Tpr	-0.18	1.00000000	duck
Iffo2	-0.34	1.00000000	duck
Hif1an	-0.08	1.00000000	duck
Thoc5	-0.09	1.00000000	duck
Csf1r	-0.45	1.00000000	duck
Pcgf5	-0.09	1.00000000	duck
Phtf2	-0.41	1.00000000	duck
Chic1	-0.74	1.00000000	duck
Lig3	-0.02	1.00000000	duck
Yy2	-0.41	1.00000000	duck
Usp15	-0.17	1.00000000	duck
Osbpl9	-0.58	1.00000000	duck
Ap1m1	-0.24	1.00000000	duck
Rab8a	-0.15	1.00000000	duck

Fbxo9	-0.29	1.00000000	duck
Atg4c	-0.58	1.00000000	duck
Uri1	-0.32	1.00000000	duck
Zdhhc17	-0.73	1.00000000	duck
Tcf4	-0.36	1.00000000	duck
Ctdp1	-0.22	1.00000000	duck
Acvr1	0.34	1.00000000	duck
Lamc1	-0.24	1.00000000	duck
Oard1	-0.14	1.00000000	duck
Fundc1	0.13	1.00000000	duck
Slc41a1	0.14	1.00000000	duck
Diablo	-0.05	1.00000000	duck
Bicral	-0.45	1.00000000	duck
Dlg1	0.34	1.00000000	duck
Ube4b	-0.09	1.00000000	duck
Luzp1	0.11	1.00000000	duck
Phf20l1	-0.51	1.00000000	duck
Dusp16	0.09	1.00000000	duck
Eaf1	0.01	1.00000000	duck
Dnajb2	0.17	1.00000000	duck
Flot1	0.15	1.00000000	duck
Sipa1l2	0.35	1.00000000	duck
Zdhhc18	0.31	1.00000000	duck
Il16	0.05	1.00000000	duck
Scaf4	-0.62	1.00000000	duck
Tppp3	0.06	1.00000000	duck
Arcn1	-0.34	1.00000000	duck
Golga4	-0.14	1.00000000	duck
Capn2	-0.15	1.00000000	duck
Arsb	0.18	1.00000000	duck
Fggy	0.02	1.00000000	duck
Sart3	0.16	1.00000000	duck
Pgghg	-0.02	1.00000000	duck
Nsfl1c	0.14	1.00000000	duck
Trim33	-0.38	1.00000000	duck
Pnrc2	-0.05	1.00000000	duck
Ap3m1	0.23	1.00000000	duck
Pak3	-0.13	1.00000000	duck
Itm2a	-0.60	1.00000000	duck
Arhgap6	0.18	1.00000000	duck
Rab1a	-0.05	1.00000000	duck
Setd5	-0.34	1.00000000	duck
Plxnd1	0.61	1.00000000	duck
Rnf217	-0.25	1.00000000	duck
Ddx27	0.27	1.00000000	duck
Tmem168	-0.77	1.00000000	duck

Lonrf3	0.30	1.00000000	duck
Rassf2	0.07	1.00000000	duck
Unc93b1	0.18	1.00000000	duck
Lin52	0.03	1.00000000	duck
Smap1	0.08	1.00000000	duck
Tsc2	0.11	1.00000000	duck
Cryz	-0.21	1.00000000	duck
Ankle2	0.06	1.00000000	duck
Ccnk	-0.15	1.00000000	duck
Idh2	0.22	1.00000000	duck
Ola1	0.14	1.00000000	duck
Spsb1	-0.23	1.00000000	duck
Osbpl5	-0.57	1.00000000	duck
Ankrd54	-0.22	1.00000000	duck
Tdrd3	-0.52	1.00000000	duck
Scoc	-0.15	1.00000000	duck
Dnaja4	-1.05	1.00000000	duck
Dnajc30	0.21	1.00000000	duck
Parp4	-0.02	1.00000000	duck
Ppa2	-0.50	1.00000000	duck
Ric8a	-0.01	1.00000000	duck
Entpd5	-0.43	1.00000000	duck
Poli	-0.03	1.00000000	duck
Ythdc1	-0.32	1.00000000	duck
Spcs2	0.12	1.00000000	duck
Enah	-0.36	1.00000000	duck
Suz12	-0.09	1.00000000	duck
Eps15	-0.03	1.00000000	duck
Ttc37	-0.27	1.00000000	duck
Ppm1d	-0.27	1.00000000	duck
Slc2a13	-1.04	1.00000000	duck
Utp3	-0.01	1.00000000	duck
Tmem161b	-0.70	1.00000000	duck
Prpf19	0.05	1.00000000	duck
Slc38a2	0.30	1.00000000	duck
Prkar2a	-0.35	1.00000000	duck
Thada	0.14	1.00000000	duck
Eif3f	-0.05	1.00000000	duck
Atp9b	-0.05	1.00000000	duck
Cpsf7	0.07	1.00000000	duck
Ruvbl1	-0.16	1.00000000	duck
Ccdc6	-0.28	1.00000000	duck
Xpo6	-0.41	1.00000000	duck
Eif1	-0.15	1.00000000	duck
Gga2	-0.19	1.00000000	duck
Thumpd2	0.03	1.00000000	duck

Desi2	-0.09	1.00000000	duck
Cfap20	-0.36	1.00000000	duck
Bbx	0.11	1.00000000	duck
Fgl2	-0.22	1.00000000	duck
Hyou1	-0.04	1.00000000	duck
Prkar2b	0.31	1.00000000	duck
Phactr4	0.04	1.00000000	duck
Ikzf5	0.07	1.00000000	duck
Braf	-0.24	1.00000000	duck
Xylt1	-0.49	1.00000000	duck
Fut8	0.38	1.00000000	duck
Ehbp1	0.09	1.00000000	duck
Vps72	-0.18	1.00000000	duck
Otud6b	0.06	1.00000000	duck
Creb3l2	0.22	1.00000000	duck
Ndrg1	-0.03	1.00000000	duck
Cops5	-0.41	1.00000000	duck
Ei24	0.05	1.00000000	duck
Nom1	0.01	1.00000000	duck
Purg	-1.00	1.00000000	duck
Hectd2	0.16	1.00000000	duck
Nt5m	-0.02	1.00000000	duck
Ikbke	0.02	1.00000000	duck
Bloc1s4	-0.11	1.00000000	duck
Itpk1	-1.29	1.00000000	duck
Fbxo25	-0.40	1.00000000	duck
Ufsp2	-0.17	1.00000000	duck
Papln	-0.13	1.00000000	duck
Ddx18	0.02	1.00000000	duck
Rubcnl	-0.04	1.00000000	duck
Bnip2	-0.18	1.00000000	duck
Dazap2	0.35	1.00000000	duck
Pex1	0.10	1.00000000	duck
Ppie	-0.20	1.00000000	duck
Cfap45	0.08	1.00000000	duck
Nup155	0.38	1.00000000	duck
Plekhm3	0.05	1.00000000	duck
Rpn2	-0.25	1.00000000	duck
Galk1	0.08	1.00000000	duck
Zfp384	-0.33	1.00000000	duck
Gtf3c3	-0.06	1.00000000	duck
Xpnpep1	0.25	1.00000000	duck
Hbs1l	-0.28	1.00000000	duck
Cd74	-0.14	1.00000000	duck
Naa35	-0.32	1.00000000	duck
Arl5a	0.03	1.00000000	duck

Xpo7	0.10	1.00000000	duck
Sec62	-0.14	1.00000000	duck
Golga5	-0.98	1.00000000	duck
Chst7	-0.05	1.00000000	duck
Commd1b	0.29	1.00000000	duck
Sppl2a	-0.17	1.00000000	duck
Blmh	-0.17	1.00000000	duck
Abca1	0.71	1.00000000	duck
Rab29	0.05	1.00000000	duck
Taf10	0.12	1.00000000	duck
Wdfy2	-0.51	1.00000000	duck
Imp3	-0.56	1.00000000	duck
Pik3cb	-0.45	1.00000000	duck
Bin3	0.05	1.00000000	duck
Umad1	-0.23	1.00000000	duck
Arid1a	0.01	1.00000000	duck
Stk4	-0.37	1.00000000	duck
Psmd4	-0.37	1.00000000	duck
Rimbp2	0.08	1.00000000	duck
Gtf2h3	-0.20	1.00000000	duck
Nucks1	-0.03	1.00000000	duck
Vta1	-0.43	1.00000000	duck
Ddx24	-0.15	1.00000000	duck
Tsn	-0.13	1.00000000	duck
Slc4a7	-0.17	1.00000000	duck
Azi2	-0.31	1.00000000	duck
Tbpl1	-0.25	1.00000000	duck
Pgs1	0.18	1.00000000	duck
Ergic2	-0.04	1.00000000	duck
C2cd3	0.06	1.00000000	duck
Trim3	0.04	1.00000000	duck
Ttc27	0.05	1.00000000	duck
Tmem268	0.15	1.00000000	duck
Chst13	0.14	1.00000000	duck
Lyrm7	-0.21	1.00000000	duck
Hspa4l	-0.63	1.00000000	duck
Plpp7	0.03	1.00000000	duck
Lsm4	0.09	1.00000000	duck
Crk	0.15	1.00000000	duck
Ssrp1	0.17	1.00000000	duck
Erlec1	0.04	1.00000000	duck
Scpep1	0.32	1.00000000	duck
Rbis	-0.29	1.00000000	duck
Lypla1	0.07	1.00000000	duck
Ccdc82	-0.02	1.00000000	duck
Zfp366	-0.08	1.00000000	duck

Nsd2	-0.05	1.00000000	duck
Atp6v1a	-0.02	1.00000000	duck
Syf2	-0.27	1.00000000	duck
Nelfa	0.02	1.00000000	duck
Hecw2	0.17	1.00000000	duck
Naf1	0.17	1.00000000	duck
Cep112	0.03	1.00000000	duck
Atp6v1c2	0.15	1.00000000	duck
Dcaf17	-0.13	1.00000000	duck
Cnnm2	-1.32	1.00000000	duck
Nras	0.56	1.00000000	duck
Phrf1	-0.33	1.00000000	duck
Nemp1	0.27	1.00000000	duck
Ypel5	-0.29	1.00000000	duck
Eepd1	-0.57	1.00000000	duck
Slc25a44	-0.01	1.00000000	duck
Aldoc	0.31	1.00000000	duck
Fkbp5	-0.21	1.00000000	duck
Hmg20b	0.11	1.00000000	duck
Antxr2	-0.16	1.00000000	duck
Gga1	-0.11	1.00000000	duck
Tle3	0.11	1.00000000	duck
Idh1	-0.23	1.00000000	duck
Sufu	-0.65	1.00000000	duck
Ano5	0.18	1.00000000	duck
Galnt1	-0.09	1.00000000	duck
Gpn2	0.00	1.00000000	duck
Srrm1	-0.17	1.00000000	duck
Hnrnpab	-0.41	1.00000000	duck
Arfip2	0.11	1.00000000	duck
Tmem241	0.06	1.00000000	duck
Lgmn	0.04	1.00000000	duck
Daglb	-0.17	1.00000000	duck
Adipor1	-0.03	1.00000000	duck
Dync1i2	-0.20	1.00000000	duck
Pcm1	-0.51	1.00000000	duck
Ap2m1	-0.15	1.00000000	duck
Fbxl20	-0.21	1.00000000	duck
Tsc22d3	-0.29	1.00000000	duck
Nmt1	0.08	1.00000000	duck
Nr3c2	-0.04	1.00000000	duck
Cpne1	-0.34	1.00000000	duck
Phka2	0.00	1.00000000	duck
Atp1a1	-0.15	1.00000000	duck
Wipi1	-0.25	1.00000000	duck
Erc1	-0.43	1.00000000	duck

Rhot1	-0.14	1.00000000	duck
Zbtb7b	0.12	1.00000000	duck
Coq5	0.07	1.00000000	duck
Pus1	-0.01	1.00000000	duck
Fbxw5	0.03	1.00000000	duck
Smap2	-0.22	1.00000000	duck
B3gnt2	-0.42	1.00000000	duck
Plekhh3	0.27	1.00000000	duck
Sec31a	0.29	1.00000000	duck
Rbm5	-0.20	1.00000000	duck
Ghitm	-0.57	1.00000000	duck
Glipr2	-0.40	1.00000000	duck
Plod2	0.20	1.00000000	duck
Snap23	-0.46	1.00000000	duck
Fbh1	-0.01	1.00000000	duck
Aprt	-0.04	1.00000000	duck
Ciao3	-0.13	1.00000000	duck
Adora2a	0.06	1.00000000	duck
Sc5d	0.37	1.00000000	duck
Nod1	0.03	1.00000000	duck
Hexb	0.00	1.00000000	duck
Gtf2a1	-0.33	1.00000000	duck
Aqr	0.15	1.00000000	duck
Stk11	-0.10	1.00000000	duck
Entpd7	0.25	1.00000000	duck
Smad7	0.04	1.00000000	duck
Tent4b	-0.92	1.00000000	duck
Rgs10	0.19	1.00000000	duck
Stradb	-0.23	1.00000000	duck
Calcrl	-0.07	1.00000000	duck
Pole4	0.02	1.00000000	duck
Ctnnbl1	-0.32	1.00000000	duck
Ppfibp2	0.00	1.00000000	duck
Bub3	0.23	1.00000000	duck
Rsrc2	-0.46	1.00000000	duck
Pan3	-0.55	1.00000000	duck
Pmm2	-0.01	1.00000000	duck
Nap1l4	-0.32	1.00000000	duck
Iws1	-0.73	1.00000000	duck
Rras2	-0.24	1.00000000	duck
Inpp5k	-0.78	1.00000000	duck
Ankrd11	-0.12	1.00000000	duck
Ube2g1	-0.29	1.00000000	duck
Psma4	-0.28	1.00000000	duck
Gab1	-0.70	1.00000000	duck
Rcc2	0.14	1.00000000	duck

Abhd17b	0.18	1.00000000	duck
Elp3	0.13	1.00000000	duck
Nudt14	0.10	1.00000000	duck
Clip1	-0.21	1.00000000	duck
Wdr70	-1.63	1.00000000	duck
Svil	-0.38	1.00000000	duck
Hgs	0.03	1.00000000	duck
Abcc4	-0.12	1.00000000	duck
Slc9a6	-0.27	1.00000000	duck
Vps13c	-1.05	1.00000000	duck
Invs	-0.05	1.00000000	duck
Skap2	-0.17	1.00000000	duck
Ccnyl1	0.17	1.00000000	duck
Scrib	-0.10	1.00000000	duck
Arnt	-0.49	1.00000000	duck
Txnrd3	0.34	1.00000000	duck
Socs7	-0.06	1.00000000	duck
Taf2	0.10	1.00000000	duck
Ddhd2	-0.01	1.00000000	duck
Wdr41	-0.01	1.00000000	duck
Amacr	0.04	1.00000000	duck
Lrrc40	0.03	1.00000000	duck
Zbtb26	0.05	1.00000000	duck
Eef1d	0.16	1.00000000	duck
Lrrfip2	0.22	1.00000000	duck
Ppil2	0.13	1.00000000	duck
Casp8ap2	0.11	1.00000000	duck
Ccdc66	0.52	1.00000000	duck
Ddx46	0.03	1.00000000	duck
Tor4a	0.05	1.00000000	duck
Sos1	-0.52	1.00000000	duck
Vti1a	-0.54	1.00000000	duck
Wdr7	-0.04	1.00000000	duck
Dnajc16	0.06	1.00000000	duck
Arglu1	-0.47	1.00000000	duck
Ppp4r3a	-0.01	1.00000000	duck
Gse1	-0.13	1.00000000	duck
Tgds	0.00	1.00000000	duck
Csgalnact1	-0.13	1.00000000	duck
Phc3	0.01	1.00000000	duck
Nf2	-0.18	1.00000000	duck
Ubiad1	-0.24	1.00000000	duck
Inpp5b	0.10	1.00000000	duck
Dagla	0.02	1.00000000	duck
Tk2	0.19	1.00000000	duck
Gpc1	-0.32	1.00000000	duck

Lipa	0.26	1.00000000	duck
Arntl2	-0.58	1.00000000	duck
Atp10a	-0.63	1.00000000	duck
Tnip2	0.32	1.00000000	duck
Baiap2l1	0.70	1.00000000	duck
Echs1	0.04	1.00000000	duck
Paxip1	-0.46	1.00000000	duck
Parvg	-0.02	1.00000000	duck
Ssr1	-0.12	1.00000000	duck
Rnmt	0.18	1.00000000	duck
Hbegf	0.17	1.00000000	duck
Rab3gap2	-0.62	1.00000000	duck
Trp53bp2	0.22	1.00000000	duck
Rad54l2	0.06	1.00000000	duck
Suds3	-0.64	1.00000000	duck
Rpf2	0.42	1.00000000	duck
Sdc1	-0.18	1.00000000	duck
Agps	-0.03	1.00000000	duck
Bms1	0.00	1.00000000	duck
Noct	0.19	1.00000000	duck
Slc37a1	-0.24	1.00000000	duck
Zc2hc1a	-0.01	1.00000000	duck
Tm2d1	0.02	1.00000000	duck
Ube2g2	0.04	1.00000000	duck
Tob1	-0.24	1.00000000	duck
Hp1bp3	0.03	1.00000000	duck
Alg2	-0.18	1.00000000	duck
Cnep1r1	-0.17	1.00000000	duck
Abcd2	0.18	1.00000000	duck
Stxbp5	0.10	1.00000000	duck
Arxes2	0.02	1.00000000	duck
Klhl24	-0.12	1.00000000	duck
Ubl7	0.02	1.00000000	duck
Tnks	-0.32	1.00000000	duck
Dnajb4	-0.78	1.00000000	duck
Rhoq	-0.23	1.00000000	duck
Bcl2l11	-0.01	1.00000000	duck
Kpna1	-0.47	1.00000000	duck
Anxa6	0.05	1.00000000	duck
Smc4	-0.32	1.00000000	duck
Crlf3	-0.18	1.00000000	duck
Tab3	-0.14	1.00000000	duck
Zfp800	0.04	1.00000000	duck
Derl2	-0.48	1.00000000	duck
Prelid3b	0.07	1.00000000	duck
Pros1	0.39	1.00000000	duck

Clp1	-0.16	1.00000000	duck
Vamp3	-0.40	1.00000000	duck
Tmx4	0.18	1.00000000	duck
Klf3	-0.21	1.00000000	duck
Ythdf2	-0.08	1.00000000	duck
Gabpa	-0.27	1.00000000	duck
Ufl1	-0.55	1.00000000	duck
Atp11a	-0.02	1.00000000	duck
Thrap3	0.04	1.00000000	duck
Atad3a	0.07	1.00000000	duck
Cript	-0.44	1.00000000	duck
Fez2	-0.18	1.00000000	duck
Vcpkmt	0.07	1.00000000	duck
Tubgcp3	0.07	1.00000000	duck
Rfc3	0.03	1.00000000	duck
Iyd	0.15	1.00000000	duck
Ephb1	0.07	1.00000000	duck
Rpp14	0.00	1.00000000	duck
Kif3a	0.03	1.00000000	duck
Tbl3	0.50	1.00000000	duck
Surf2	0.04	1.00000000	duck
Nedd4	-0.06	1.00000000	duck
Lmbr1	-0.41	1.00000000	duck
Atp1b1	0.23	1.00000000	duck
Smc6	-0.29	1.00000000	duck
Zdhhc6	-0.02	1.00000000	duck
Nop2	0.60	1.00000000	duck
Tti1	-0.05	1.00000000	duck
Cib1	0.33	1.00000000	duck
Rnf6	-0.59	1.00000000	duck
Zdhhc8	0.11	1.00000000	duck
Acsf2	-0.07	1.00000000	duck
Ubp1	-0.15	1.00000000	duck
Fbxw7	0.38	1.00000000	duck
Odc1	0.32	1.00000000	duck
Rassf8	0.57	1.00000000	duck
Thoc1	0.27	1.00000000	duck
Secisbp2	-0.55	1.00000000	duck
Tfdp2	0.00	1.00000000	duck
Baiap2	0.15	1.00000000	duck
Srfbp1	0.07	1.00000000	duck
Psmd1	-0.71	1.00000000	duck
Acsbg2	0.08	1.00000000	duck
Ssbp3	-0.56	1.00000000	duck
Heatr5b	-0.11	1.00000000	duck
Cbfb	-0.73	1.00000000	duck

Ddx17	-0.63	1.00000000	duck
Pitrm1	-0.49	1.00000000	duck
Erich1	-0.57	1.00000000	duck
Dop1a	-0.28	1.00000000	duck
Zranb2	-0.64	1.00000000	duck
Swt1	0.00	1.00000000	duck
Ube3b	-0.20	1.00000000	duck
Zc3h7b	-0.13	1.00000000	duck
Yars	0.42	1.00000000	duck
Zfp407	0.12	1.00000000	duck
Slc35a1	0.03	1.00000000	duck
Wwox	-0.39	1.00000000	duck
Npepps	-0.22	1.00000000	duck
Vti1b	0.23	1.00000000	duck
Rbm17	-0.12	1.00000000	duck
Pcna	0.35	1.00000000	duck
Uhrf1bp1l	-0.01	1.00000000	duck
Zfand4	-0.55	1.00000000	duck
Acbd6	0.01	1.00000000	duck
Ranbp10	-0.42	1.00000000	duck
Afg3l2	-0.48	1.00000000	duck
Stk3	-0.24	1.00000000	duck
Cerk	-0.18	1.00000000	duck
Luc7l	0.02	1.00000000	duck
Pde8a	0.30	1.00000000	duck
Abcc1	-0.55	1.00000000	duck
Grb2	-0.21	1.00000000	duck
Slc49a4	0.25	1.00000000	duck
Thoc3	-0.17	1.00000000	duck
Smarcd1	0.14	1.00000000	duck
Lamtor5	0.34	1.00000000	duck
2700049A03Rik	-0.66	1.00000000	duck
Il18	-0.12	1.00000000	duck
Prkaa1	-0.46	1.00000000	duck
Dhcr24	0.20	1.00000000	duck
Phf20	-0.35	1.00000000	duck
Vps37a	-0.58	1.00000000	duck
Nisch	-0.29	1.00000000	duck
Sacs	0.29	1.00000000	duck
Rnf138	0.32	1.00000000	duck
Tbcd	-0.21	1.00000000	duck
Slc39a9	0.15	1.00000000	duck
Nr4a1	-0.17	1.00000000	duck
Cntln	-0.05	1.00000000	duck
Polr3a	-0.23	1.00000000	duck
Card11	0.01	1.00000000	duck

Pan2	0.00	1.00000000	duck
Zbtb8os	-0.01	1.00000000	duck
Tube1	-0.40	1.00000000	duck
Trappc8	-0.62	1.00000000	duck
Itm2b	-0.04	1.00000000	duck
Atp6v1d	-0.04	1.00000000	duck
Nr4a3	-0.58	1.00000000	duck
Nfatc3	-0.17	1.00000000	duck
Hnrnpa3	0.02	1.00000000	duck
Iars2	-0.36	1.00000000	duck
Cemip2	0.35	1.00000000	duck
Hibadh	-0.21	1.00000000	duck
Ap1g1	-0.10	1.00000000	duck
Supt6	-0.27	1.00000000	duck
Ash1l	-0.67	1.00000000	duck
Oaz1	-0.04	1.00000000	duck
Ptch1	-0.31	1.00000000	duck
Dnajb5	0.09	1.00000000	duck
Ttc14	0.07	1.00000000	duck
Usp19	0.09	1.00000000	duck
Pigs	0.04	1.00000000	duck
Sirt2	0.04	1.00000000	duck
Lrp3	-0.11	1.00000000	duck
Ift80	0.03	1.00000000	duck
Yod1	0.06	1.00000000	duck
Xrcc4	-0.90	1.00000000	duck
Nav1	0.15	1.00000000	duck
Trim71	-0.23	1.00000000	duck
Npl	-0.37	1.00000000	duck
Thsd4	0.07	1.00000000	duck
Arfgap2	0.17	1.00000000	duck
Dmap1	0.30	1.00000000	duck
Eif2b5	0.13	1.00000000	duck
Fuca1	-0.16	1.00000000	duck
Slc10a7	0.37	1.00000000	duck
Lrif1	-0.18	1.00000000	duck
Ireb2	-0.21	1.00000000	duck
Samsn1	-0.33	1.00000000	duck
Uchl5	-0.51	1.00000000	duck
Snx1	-0.14	1.00000000	duck
Brd7	-0.21	1.00000000	duck
Supt5	-0.13	1.00000000	duck
Lemd2	0.27	1.00000000	duck
Stx2	0.00	1.00000000	duck
Btf3l4	0.34	1.00000000	duck
Ep300	-0.57	1.00000000	duck

Tpm3-rs7	-0.08	1.00000000	duck
Capza2	-0.04	1.00000000	duck
Agk	0.01	1.00000000	duck
Rrm2b	0.06	1.00000000	duck
Uba5	0.05	1.00000000	duck
Ppp2r5d	0.09	1.00000000	duck
Nus1	-0.22	1.00000000	duck
Ccnc	0.05	1.00000000	duck
Birc6	-0.25	1.00000000	duck
Rwdd4a	0.03	1.00000000	duck
Abraxas1	0.14	1.00000000	duck
Cep120	0.21	1.00000000	duck
Fnta	0.09	1.00000000	duck
Sergef	0.03	1.00000000	duck
Wbp1l	-0.87	1.00000000	duck
Pigk	0.07	1.00000000	duck
Ctsh	0.13	1.00000000	duck
Zfp821	0.36	1.00000000	duck
Psme3ip1	0.04	1.00000000	duck
Chd9	-0.46	1.00000000	duck
Pmepa1	-0.06	1.00000000	duck
Atp2a2	-0.21	1.00000000	duck
Recql5	-0.12	1.00000000	duck
Auts2	0.11	1.00000000	duck
Nsrp1	-0.03	1.00000000	duck
Tnfaip8l1	0.38	1.00000000	duck
Ube2q1	-0.01	1.00000000	duck
Hdac9	-0.22	1.00000000	duck
Tpt1	5.66	0.00000000	pigeons
Arpc2	3.81	0.00000000	pigeons
Cyria	4.50	0.00000000	pigeons
Rab33a	4.73	0.00000000	pigeons
Niban1	3.89	0.00000000	pigeons
Cmip	3.86	0.00000000	pigeons
Vim	4.09	0.00000001	pigeons
Atp5b	2.37	0.00000001	pigeons
Dars	2.57	0.00000005	pigeons
Cnbp	2.21	0.00000005	pigeons
Cklf	2.35	0.00000005	pigeons
Dido1	2.58	0.00000005	pigeons
Pcf11	2.19	0.00000020	pigeons
Pomp	1.92	0.00000020	pigeons
Adss	2.39	0.00000020	pigeons
Vcp	1.59	0.00000020	pigeons
Atp5k	1.74	0.00000020	pigeons
Atp5g3	2.38	0.00000020	pigeons

Trpc4ap	2.06	0.00000020	pigeons
Sap18	2.35	0.00000075	pigeons
Phip	2.33	0.00000075	pigeons
Tiparp	2.62	0.00000075	pigeons
Ankhd1	2.38	0.00000075	pigeons
Cfd	-4.53	0.00000188	pigeons
App	3.44	0.00000273	pigeons
Atf7ip	2.15	0.00000273	pigeons
Syncrip	1.64	0.00000273	pigeons
Fyb	2.59	0.00000273	pigeons
Adnp	1.48	0.00000273	pigeons
Txnip	1.86	0.00000273	pigeons
Oga	1.77	0.00000965	pigeons
Timp3	-3.14	0.00001632	pigeons
Igfbp7	-3.70	0.00003127	pigeons
Anapc15-ps	1.54	0.00003300	pigeons
Sdcbp	1.83	0.00003300	pigeons
Lnpep	1.61	0.00003300	pigeons
U2surp	1.75	0.00003300	pigeons
Lpp	1.85	0.00003300	pigeons
Fos	-3.22	0.00010531	pigeons
Zdhhc20	2.18	0.00010925	pigeons
Tgfbr1	2.26	0.00010925	pigeons
Apbb1ip	1.99	0.00010925	pigeons
Ndufa1	1.29	0.00010925	pigeons
Cacybp	1.87	0.00010925	pigeons
Pim1	1.79	0.00021727	pigeons
Aqp1	-4.19	0.00032277	pigeons
Pcnp	1.19	0.00035067	pigeons
Rin2	2.10	0.00035067	pigeons
Wtap	1.82	0.00035067	pigeons
Eprs	1.75	0.00035067	pigeons
Cars	1.76	0.00035067	pigeons
Tmbim1	1.61	0.00035067	pigeons
Golm2	1.15	0.00035067	pigeons
Spcs1	1.17	0.00035067	pigeons
Kars	1.19	0.00035067	pigeons
Trrap	1.24	0.00035067	pigeons
Il1b	2.83	0.00035067	pigeons
Arhgap15	2.48	0.00035633	pigeons
Pecam1	-3.16	0.00040190	pigeons
Atp11b	1.72	0.00082018	pigeons
Evl	2.01	0.00084980	pigeons
Sh3kbp1	1.84	0.00091524	pigeons
Ccdc25	1.12	0.00109225	pigeons
Tollip	1.24	0.00109225	pigeons

Il1rap	1.75	0.00109225	pigeons
C1qbp	0.96	0.00109225	pigeons
Eif4b	1.53	0.00109225	pigeons
Sdhc	1.19	0.00109225	pigeons
Cxcr4	2.41	0.00159383	pigeons
Epas1	-3.67	0.00170543	pigeons
Fam120a	1.17	0.00182458	pigeons
Fbh1	1.50	0.00330472	pigeons
Prep	1.22	0.00330472	pigeons
Rnf38	1.23	0.00330472	pigeons
Phax	0.94	0.00330472	pigeons
Rtf2	1.13	0.00330472	pigeons
Tdpoz8	1.20	0.00330472	pigeons
Tardbp	0.97	0.00330472	pigeons
Cdc37	1.28	0.00330472	pigeons
Snrpd2	1.36	0.00330472	pigeons
Rftn1	2.51	0.00363849	pigeons
Jak1	1.47	0.00476125	pigeons
Ptprm	-4.41	0.00476647	pigeons
Adnp2	1.07	0.00781288	pigeons
Sinhcaf	1.92	0.00972244	pigeons
Alox5ap	1.05	0.00972244	pigeons
Slc12a7	1.85	0.00972244	pigeons
Cd2ap	1.35	0.00972244	pigeons
Enox2	1.12	0.00972244	pigeons
Brd8	1.17	0.00972244	pigeons
Sap30bp	0.97	0.00972244	pigeons
Gadd45b	1.33	0.00972244	pigeons
Gzf1	1.04	0.01151125	pigeons
Cep170	1.98	0.01262601	pigeons
Crim1	-3.56	0.01297355	pigeons
Klhl5	1.83	0.01552759	pigeons
Itpr2	1.83	0.02401564	pigeons
Rgs9	1.67	0.02550778	pigeons
Rasa3	1.80	0.02553014	pigeons
Supt6	0.94	0.02567513	pigeons
Strap	0.92	0.02784108	pigeons
Lrmp	2.12	0.02784108	pigeons
Csta1	2.04	0.02784108	pigeons
Slirp	1.04	0.02784108	pigeons
Apip	0.75	0.02784108	pigeons
Kifbp	1.15	0.02784108	pigeons
Ccz1	0.76	0.02784108	pigeons
Sarnp	1.02	0.02784108	pigeons
Trim28	0.97	0.02784108	pigeons
Rbms3	-3.43	0.02848765	pigeons

Lamp3	1.37	0.03082278	pigeons
Slc4a7	1.50	0.03155862	pigeons
Cbx6	1.70	0.03163719	pigeons
Adgrl4	-3.33	0.03389613	pigeons
Prkce	-2.68	0.03602565	pigeons
Kif5c	1.50	0.03714376	pigeons
Zc3h12d	2.07	0.03772128	pigeons
Gnpda2	0.87	0.03879787	pigeons
Rhoh	1.47	0.04279627	pigeons
Tmem100	-3.31	0.04747174	pigeons
Plcg2	1.67	0.04935276	pigeons
Sox6	-3.35	0.05019976	pigeons
Dapp1	1.48	0.05647967	pigeons
Tmtc1	-4.11	0.06564775	pigeons
Bcat1	1.22	0.06717385	pigeons
Prkcb	1.18	0.06806328	pigeons
Rab30	1.55	0.07576095	pigeons
Cdkn2aip	0.80	0.07768243	pigeons
Rnf103	0.69	0.07768243	pigeons
Bccip	0.68	0.07768243	pigeons
Psme3ip1	0.88	0.07768243	pigeons
Bri3bp	0.67	0.07768243	pigeons
Csnk1d	0.82	0.07768243	pigeons
Oxr1	0.86	0.08115913	pigeons
Cd83	1.63	0.09176278	pigeons
Rgs1	2.12	0.09198582	pigeons
Sparc	-3.06	0.09429872	pigeons
Ptprb	-3.20	0.09590799	pigeons
Stk40	0.99	0.09849238	pigeons
Nfe2l1	0.91	0.10170586	pigeons
Prpf4b	0.98	0.10356166	pigeons
Slc15a4	1.04	0.10356166	pigeons
Ptger4	1.22	0.11566178	pigeons
Pusl1	1.02	0.13567835	pigeons
Ptprc	1.49	0.13615109	pigeons
Far1	1.10	0.13648117	pigeons
Spi1	1.44	0.13974730	pigeons
Bmp2k	1.15	0.14266165	pigeons
Rasgef1a	2.21	0.14704045	pigeons
Ddrgk1	1.03	0.14826501	pigeons
Tjp1	-3.64	0.14826501	pigeons
Pik3ap1	1.77	0.15133896	pigeons
Antxr2	1.47	0.16288858	pigeons
Chd9	1.44	0.17123063	pigeons
Nova1	-3.28	0.17927401	pigeons
Ankrd44	1.83	0.18143341	pigeons

Eif2ak3	1.18	0.18418478	pigeons
Enpp4	1.10	0.18896222	pigeons
Adgrf5	-3.35	0.19092158	pigeons
Ldb2	-3.25	0.19182105	pigeons
Gpatch8	1.23	0.19182105	pigeons
Ndel1	1.21	0.19919606	pigeons
Atp5c1	1.01	0.21142601	pigeons
Tent4a	0.92	0.21142601	pigeons
Ezr	0.81	0.21142601	pigeons
Dtd1	1.09	0.21142601	pigeons
Apmap	0.69	0.21142601	pigeons
Ahsa2	1.06	0.21142601	pigeons
Tbp	0.81	0.21142601	pigeons
Jchain	1.75	0.21142601	pigeons
Nars	0.80	0.21142601	pigeons
Tut7	0.88	0.21142601	pigeons
Gch1	1.12	0.21142601	pigeons
Atp5j2	0.93	0.21142601	pigeons
Dohh	0.67	0.21142601	pigeons
Btf3	0.88	0.21802324	pigeons
Clec16a	1.19	0.22127690	pigeons
Wipf1	1.20	0.23732063	pigeons
Arfip1	1.45	0.25014864	pigeons
Lrrfip1	1.35	0.25765362	pigeons
Ptdss1	0.86	0.26879108	pigeons
Usp7	0.56	0.27448840	pigeons
Ikzf1	1.58	0.27811536	pigeons
Pik3cd	0.81	0.28217705	pigeons
Abcc1	1.28	0.28237511	pigeons
Epb41	1.24	0.29675335	pigeons
Spopl	1.17	0.31813611	pigeons
Rassf5	1.10	0.32906413	pigeons
Adgrl2	-3.37	0.33525976	pigeons
Rbm34	0.91	0.34487831	pigeons
Nsd3	1.13	0.36570417	pigeons
Pag1	1.04	0.36794799	pigeons
Calcrl	-3.05	0.37188277	pigeons
Zfp36l1	1.26	0.37388106	pigeons
Nfia	-3.44	0.37617804	pigeons
Rgs4	1.12	0.38016807	pigeons
Ttbk1	0.85	0.38258870	pigeons
Dock10	1.66	0.39331865	pigeons
Ddit4	-2.02	0.40712191	pigeons
Rfx7	1.92	0.41760654	pigeons
Pacsin2	1.21	0.41940386	pigeons
Ypel5	0.84	0.42645488	pigeons

Chst2	1.22	0.43661790	pigeons
Chsy1	1.59	0.44309510	pigeons
Rhbdf2	0.83	0.46226404	pigeons
Nf1	0.54	0.47360095	pigeons
Notch2	1.28	0.49508552	pigeons
Rab11fip4	1.27	0.52378638	pigeons
Hck	0.75	0.54621104	pigeons
Prcp	0.88	0.56194168	pigeons
Gnai1	0.97	0.56194168	pigeons
Dap	1.20	0.56194168	pigeons
Zfp777	0.69	0.56194168	pigeons
Sec24d	0.86	0.56194168	pigeons
Kdm3a	0.81	0.56194168	pigeons
Rad23b	0.93	0.56194168	pigeons
Sec23ip	0.62	0.56194168	pigeons
Sars	0.73	0.56194168	pigeons
Atp13a1	0.67	0.56194168	pigeons
Pde7b	1.62	0.60446395	pigeons
Rnf213	0.94	0.62871852	pigeons
Gpd2	1.00	0.63766902	pigeons
Osbpl8	1.03	0.64429693	pigeons
Hbegf	1.01	0.68550469	pigeons
Ccdc88a	1.32	0.69236058	pigeons
Tnpo1	0.64	0.71458821	pigeons
Tial1	0.66	0.73379449	pigeons
Pik3r5	1.53	0.73591360	pigeons
Vopp1	0.99	0.73703866	pigeons
Slc9a9	1.43	0.77151826	pigeons
Chic2	0.80	0.77336469	pigeons
Cnot6	0.65	0.77654665	pigeons
Phc3	1.40	0.78665345	pigeons
Rhof	1.05	0.81210421	pigeons
Scfd1	0.48	0.82635695	pigeons
Cavin2	-2.95	0.83518779	pigeons
Zfp711	1.08	0.85672949	pigeons
Zfp352	-2.37	0.90792750	pigeons
Bcr	0.89	0.90860564	pigeons
Pde4b	1.15	0.93635169	pigeons
Rab5a	0.82	0.93976761	pigeons
Bag5	0.79	0.94883011	pigeons
Cyld	0.88	0.94898290	pigeons
Plcb1	-3.08	0.96617855	pigeons
Ctdp1	1.13	0.97336573	pigeons
Rad51b	1.52	0.97336573	pigeons
Slc41a1	0.92	0.99928240	pigeons
Snx9	1.06	1.00000000	pigeons

Adam19	1.02	1.00000000	pigeons
Sin3a	1.30	1.00000000	pigeons
Rabggtb	0.51	1.00000000	pigeons
Tmem164	1.34	1.00000000	pigeons
Ewsr1	1.09	1.00000000	pigeons
Kctd20	1.11	1.00000000	pigeons
Iqgap2	1.22	1.00000000	pigeons
Cmtm3	0.97	1.00000000	pigeons
Wdr91	0.73	1.00000000	pigeons
Tnfaip8l1	0.74	1.00000000	pigeons
Il27ra	0.61	1.00000000	pigeons
Lrrk1	0.94	1.00000000	pigeons
Rfx2	1.48	1.00000000	pigeons
Unc119b	0.97	1.00000000	pigeons
Podxl	-2.84	1.00000000	pigeons
Camk1d	1.60	1.00000000	pigeons
Aspscr1	1.40	1.00000000	pigeons
Rnaset2b	1.61	1.00000000	pigeons
Fgl2	0.95	1.00000000	pigeons
Rhob	0.78	1.00000000	pigeons
Gabrr2	1.14	1.00000000	pigeons
Naa20	0.88	1.00000000	pigeons
Nasp	0.65	1.00000000	pigeons
Rgs18	0.89	1.00000000	pigeons
Gabpb1	0.66	1.00000000	pigeons
Banp	0.87	1.00000000	pigeons
Porcn	0.69	1.00000000	pigeons
Blcap	0.73	1.00000000	pigeons
Adam9	1.26	1.00000000	pigeons
Zc3h12c	1.44	1.00000000	pigeons
Capn3	1.20	1.00000000	pigeons
Traf6	0.71	1.00000000	pigeons
Lrch2	1.23	1.00000000	pigeons
Itgb2l	1.08	1.00000000	pigeons
Limk1	0.68	1.00000000	pigeons
Ptges3	0.89	1.00000000	pigeons
Swap70	1.32	1.00000000	pigeons
Egr1	-2.61	1.00000000	pigeons
Ugcg	0.82	1.00000000	pigeons
Zhx2	0.73	1.00000000	pigeons
Eef1e1	1.39	1.00000000	pigeons
Lpcat2b	0.27	1.00000000	pigeons
Ypel1	1.03	1.00000000	pigeons
Ergic1	1.00	1.00000000	pigeons
Ptp4a1	0.60	1.00000000	pigeons
Acsl4	1.13	1.00000000	pigeons

Rcsd1	0.83	1.00000000	pigeons
Slc25a36	0.68	1.00000000	pigeons
Pfdn1	1.17	1.00000000	pigeons
Jarid2	1.19	1.00000000	pigeons
Ssu72	0.65	1.00000000	pigeons
Uhrf1bp1l	0.55	1.00000000	pigeons
Rgl1	0.07	1.00000000	pigeons
Nt5c3b	1.03	1.00000000	pigeons
Commd6	0.92	1.00000000	pigeons
Zfp512b	0.62	1.00000000	pigeons
Smarcc1	1.07	1.00000000	pigeons
Usp6nl	0.78	1.00000000	pigeons
Stat1	0.93	1.00000000	pigeons
Cul3	0.67	1.00000000	pigeons
Stk38	0.94	1.00000000	pigeons
Lpgat1	0.52	1.00000000	pigeons
Ptpn6	0.78	1.00000000	pigeons
Stx12	1.12	1.00000000	pigeons
Ubac2	1.20	1.00000000	pigeons
Fam189a1	-2.64	1.00000000	pigeons
Bcl2l11	0.92	1.00000000	pigeons
Scd2	1.18	1.00000000	pigeons
Zeb1	1.16	1.00000000	pigeons
Arl4a	-3.25	1.00000000	pigeons
Laptm4a	-2.02	1.00000000	pigeons
Sfi1	1.32	1.00000000	pigeons
Klhl6	1.11	1.00000000	pigeons
Atg13	0.59	1.00000000	pigeons
Psmd3	0.74	1.00000000	pigeons
Acadl	1.20	1.00000000	pigeons
Fbxw11	0.95	1.00000000	pigeons
Wmp	1.45	1.00000000	pigeons
Xpo7	0.50	1.00000000	pigeons
Irf7	0.65	1.00000000	pigeons
Setd2	0.79	1.00000000	pigeons
Spag6l	0.85	1.00000000	pigeons
Hecw2	-2.96	1.00000000	pigeons
Cep85l	0.48	1.00000000	pigeons
Sowahc	1.06	1.00000000	pigeons
Rbx1	1.12	1.00000000	pigeons
Capn7	1.03	1.00000000	pigeons
Tmem123	0.95	1.00000000	pigeons
Wdr20	0.69	1.00000000	pigeons
Itga4	0.93	1.00000000	pigeons
Dok1	0.86	1.00000000	pigeons
Zdhhc3	0.91	1.00000000	pigeons

Tmem87a	0.81	1.00000000	pigeons
Slc35f5	0.75	1.00000000	pigeons
Tpd52l2	0.79	1.00000000	pigeons
Ttl	0.68	1.00000000	pigeons
Nop56	1.01	1.00000000	pigeons
Primpol	1.22	1.00000000	pigeons
Ifih1	0.55	1.00000000	pigeons
Glrx3	0.81	1.00000000	pigeons
Ivns1abp	0.76	1.00000000	pigeons
Snx1	0.48	1.00000000	pigeons
Slmap	0.67	1.00000000	pigeons
Gfpt2	2.04	1.00000000	pigeons
Adrb2	0.64	1.00000000	pigeons
Sfswap	0.49	1.00000000	pigeons
Atxn7l3	0.51	1.00000000	pigeons
Polr2i	0.56	1.00000000	pigeons
Naa15	0.12	1.00000000	pigeons
Dock8	0.81	1.00000000	pigeons
Lcor	0.44	1.00000000	pigeons
Pdk3	0.38	1.00000000	pigeons
Tspan2	0.77	1.00000000	pigeons
Sdk1	-2.55	1.00000000	pigeons
Zc3h12b	0.72	1.00000000	pigeons
Ankrd13a	0.69	1.00000000	pigeons
Dffb	0.56	1.00000000	pigeons
Tns1	-2.50	1.00000000	pigeons
Rasgef1b	0.75	1.00000000	pigeons
Egln3	1.62	1.00000000	pigeons
Otud7a	2.17	1.00000000	pigeons
Ankrd27	0.70	1.00000000	pigeons
Rpn1	0.58	1.00000000	pigeons
Plekhg1	-2.95	1.00000000	pigeons
Synm	-2.26	1.00000000	pigeons
Cwc27	1.02	1.00000000	pigeons
Smg1	0.44	1.00000000	pigeons
Champ1	0.67	1.00000000	pigeons
Stam2	0.75	1.00000000	pigeons
Lrch3	0.42	1.00000000	pigeons
Bmpr2	-2.92	1.00000000	pigeons
Dek	0.72	1.00000000	pigeons
Shisa5	0.56	1.00000000	pigeons
Nr4a1	0.81	1.00000000	pigeons
Nubp1	0.46	1.00000000	pigeons
Eif3b	0.68	1.00000000	pigeons
Commd2	0.76	1.00000000	pigeons
Spg11	0.68	1.00000000	pigeons

Dip2b	0.64	1.00000000	pigeons
Gna13	0.97	1.00000000	pigeons
Pde10a	-3.35	1.00000000	pigeons
Ctdspl	-2.26	1.00000000	pigeons
Acsl3	0.94	1.00000000	pigeons
Ptprj	0.88	1.00000000	pigeons
Ehbp1	1.19	1.00000000	pigeons
Kcne3	-2.64	1.00000000	pigeons
Vps4b	0.50	1.00000000	pigeons
Herpud2	0.57	1.00000000	pigeons
Eif1ad12	0.37	1.00000000	pigeons
Sptbn1	-2.38	1.00000000	pigeons
Ctsh	0.83	1.00000000	pigeons
Afdn	-2.72	1.00000000	pigeons
Atg2b	0.81	1.00000000	pigeons
Xrn2	1.13	1.00000000	pigeons
Susd6	0.90	1.00000000	pigeons
Sf3b1	1.04	1.00000000	pigeons
Cpsf3	0.97	1.00000000	pigeons
Vps13c	1.10	1.00000000	pigeons
Kdelr3	1.10	1.00000000	pigeons
Copb1	0.67	1.00000000	pigeons
Sfmbt1	0.73	1.00000000	pigeons
Tank	0.74	1.00000000	pigeons
Ehd3	1.56	1.00000000	pigeons
Clk4	0.10	1.00000000	pigeons
Fnip1	0.77	1.00000000	pigeons
Sema6a	-2.73	1.00000000	pigeons
Sp3	0.67	1.00000000	pigeons
Itfg2	0.55	1.00000000	pigeons
Zfp654	0.29	1.00000000	pigeons
Cdk5r1	1.02	1.00000000	pigeons
Snx20	0.72	1.00000000	pigeons
Arap2	0.50	1.00000000	pigeons
Bach2	1.54	1.00000000	pigeons
Pisd	0.70	1.00000000	pigeons
Clp1	0.69	1.00000000	pigeons
Pabpc1	0.82	1.00000000	pigeons
Ppp6r3	0.76	1.00000000	pigeons
Prr5	1.31	1.00000000	pigeons
Acvr1b	0.74	1.00000000	pigeons
Prkaa1	0.66	1.00000000	pigeons
Zbtb20	-1.79	1.00000000	pigeons
Tbc1d8	0.77	1.00000000	pigeons
Kdm4b	0.82	1.00000000	pigeons
Abca1	0.56	1.00000000	pigeons

Evi2a	0.56	1.00000000	pigeons
Ddx6	1.04	1.00000000	pigeons
Grk3	0.99	1.00000000	pigeons
Vps41	0.73	1.00000000	pigeons
Rabgap1l	1.95	1.00000000	pigeons
Pfkp	1.55	1.00000000	pigeons
Il22ra2	0.46	1.00000000	pigeons
Sgk1	0.70	1.00000000	pigeons
Las1l	0.47	1.00000000	pigeons
Rbmx2	0.90	1.00000000	pigeons
Syk	0.80	1.00000000	pigeons
Rgs2	3.11	1.00000000	pigeons
Crip2	1.05	1.00000000	pigeons
Fbxl21	0.51	1.00000000	pigeons
Scarb1	0.44	1.00000000	pigeons
Lrrc45	0.48	1.00000000	pigeons
Ubtf	0.43	1.00000000	pigeons
Yars	0.40	1.00000000	pigeons
Stambp	0.47	1.00000000	pigeons
Dedd	0.53	1.00000000	pigeons
Tada1	0.27	1.00000000	pigeons
Ercc6	0.90	1.00000000	pigeons
Asap1	1.02	1.00000000	pigeons
Pdcd10	0.31	1.00000000	pigeons
Fli1	-2.83	1.00000000	pigeons
Ech1	0.97	1.00000000	pigeons
Ralgps2	-2.52	1.00000000	pigeons
Ltn1	-0.12	1.00000000	pigeons
1700034I23Rik	1.07	1.00000000	pigeons
Erlec1	0.96	1.00000000	pigeons
Nfkb1	0.90	1.00000000	pigeons
Rbm45	0.29	1.00000000	pigeons
Gtf2f2	0.86	1.00000000	pigeons
Rasa2	1.09	1.00000000	pigeons
Letm1	0.46	1.00000000	pigeons
Cep44	0.48	1.00000000	pigeons
Tmem50b	1.01	1.00000000	pigeons
Iars2	1.11	1.00000000	pigeons
Tor4a	0.91	1.00000000	pigeons
Ptprs	0.74	1.00000000	pigeons
Acvr2a	0.08	1.00000000	pigeons
Tcerg1	0.59	1.00000000	pigeons
Fam53a	0.84	1.00000000	pigeons
Usp47	0.61	1.00000000	pigeons
Prkn	0.57	1.00000000	pigeons
Prmt9	0.43	1.00000000	pigeons

Gpr137b	0.99	1.00000000	pigeons
Rac2	0.65	1.00000000	pigeons
Nr2c2	0.67	1.00000000	pigeons
Clcn3	0.56	1.00000000	pigeons
Syne2	-2.48	1.00000000	pigeons
Psmd1	0.83	1.00000000	pigeons
Ogfr	0.69	1.00000000	pigeons
Tnip1	0.81	1.00000000	pigeons
Acp1	0.74	1.00000000	pigeons
Rapgef1	0.69	1.00000000	pigeons
Cast	0.62	1.00000000	pigeons
Prdx4	0.78	1.00000000	pigeons
Pcif1	0.19	1.00000000	pigeons
Hbp1	0.58	1.00000000	pigeons
Surf4	0.70	1.00000000	pigeons
Zbtb21	0.24	1.00000000	pigeons
Stard3nl	0.08	1.00000000	pigeons
Cntrl	0.82	1.00000000	pigeons
Commd7	0.79	1.00000000	pigeons
Nmt2	0.43	1.00000000	pigeons
Ginm1	0.57	1.00000000	pigeons
Tacc3	0.67	1.00000000	pigeons
Pin4	0.43	1.00000000	pigeons
Wdr44	0.35	1.00000000	pigeons
Eif3f	0.57	1.00000000	pigeons
Atl2	0.69	1.00000000	pigeons
Stat2	0.28	1.00000000	pigeons
Lmbrd1	1.06	1.00000000	pigeons
E2f6	0.99	1.00000000	pigeons
Arl1	0.79	1.00000000	pigeons
Ptpn9	0.59	1.00000000	pigeons
Gtf3c1	0.52	1.00000000	pigeons
Osbpl11	0.94	1.00000000	pigeons
Xpot	0.25	1.00000000	pigeons
Ccdc6	0.86	1.00000000	pigeons
Hook1	0.84	1.00000000	pigeons
Nptn	0.51	1.00000000	pigeons
Sf3a2	0.37	1.00000000	pigeons
Kmt2e	0.71	1.00000000	pigeons
Ormdl1	0.46	1.00000000	pigeons
Lrif1	0.65	1.00000000	pigeons
Tomm70a	0.73	1.00000000	pigeons
Sec24a	0.55	1.00000000	pigeons
Rfc2	0.57	1.00000000	pigeons
Sucla2	0.39	1.00000000	pigeons
Nfkb2	0.54	1.00000000	pigeons

Rab27a	0.84	1.00000000	pigeons
Sesn3	1.17	1.00000000	pigeons
Bivm	0.64	1.00000000	pigeons
Kbtbd8	1.12	1.00000000	pigeons
Adcy7	0.87	1.00000000	pigeons
Tnfaip3	1.04	1.00000000	pigeons
Pcmtd2	0.05	1.00000000	pigeons
Rc3h1	-0.33	1.00000000	pigeons
Atp2b1	0.52	1.00000000	pigeons
Scaf4	0.13	1.00000000	pigeons
Tab2	0.50	1.00000000	pigeons
Zbtb46	1.46	1.00000000	pigeons
Usp10	0.76	1.00000000	pigeons
Exoc4	0.42	1.00000000	pigeons
Paip1	0.12	1.00000000	pigeons
Parp6	0.47	1.00000000	pigeons
Sbno2	0.77	1.00000000	pigeons
Fbxo11	0.48	1.00000000	pigeons
Dcbld2	1.34	1.00000000	pigeons
Klhl7	0.47	1.00000000	pigeons
Sf3b6	0.25	1.00000000	pigeons
Smap2	0.67	1.00000000	pigeons
Nfkbiz	1.03	1.00000000	pigeons
Arhgap31	-1.89	1.00000000	pigeons
Hspa8	-1.41	1.00000000	pigeons
Psmg3	0.67	1.00000000	pigeons
Smc3	0.86	1.00000000	pigeons
S1pr1	-2.42	1.00000000	pigeons
Zc3h7a	0.44	1.00000000	pigeons
Gpx3	-2.50	1.00000000	pigeons
Gsk3b	-2.01	1.00000000	pigeons
Ankrd17	0.94	1.00000000	pigeons
Nsfl1c	0.47	1.00000000	pigeons
Slc43a2	0.53	1.00000000	pigeons
Erh	0.39	1.00000000	pigeons
Nup210	0.77	1.00000000	pigeons
Nup93	0.61	1.00000000	pigeons
Oma1	0.64	1.00000000	pigeons
Ovch2	0.58	1.00000000	pigeons
Pik3r1	1.40	1.00000000	pigeons
Braf	0.46	1.00000000	pigeons
Il10ra	0.71	1.00000000	pigeons
Psmc6	0.79	1.00000000	pigeons
Wipi2	0.23	1.00000000	pigeons
Ddx1	0.45	1.00000000	pigeons
Plek	0.80	1.00000000	pigeons

Rufy3	0.98	1.00000000	pigeons
Slc16a6	0.95	1.00000000	pigeons
Htr4	0.80	1.00000000	pigeons
Entpd7	0.28	1.00000000	pigeons
Zfp523	0.98	1.00000000	pigeons
Ttc17	0.93	1.00000000	pigeons
Ssbp2	-2.39	1.00000000	pigeons
Dock2	1.27	1.00000000	pigeons
Srp14	0.26	1.00000000	pigeons
Siah2	0.84	1.00000000	pigeons
Zbtb7a	0.03	1.00000000	pigeons
Sun2	0.61	1.00000000	pigeons
Tbc1d1	0.65	1.00000000	pigeons
Sema4d	0.66	1.00000000	pigeons
Zmynd8	0.87	1.00000000	pigeons
Nfyb	0.54	1.00000000	pigeons
Irak4	0.41	1.00000000	pigeons
Cog3	0.45	1.00000000	pigeons
Tapbpl	0.42	1.00000000	pigeons
Rnf6	0.59	1.00000000	pigeons
Pudp	0.82	1.00000000	pigeons
Pip4p2	0.47	1.00000000	pigeons
Otulinl	0.40	1.00000000	pigeons
Zpbp	0.38	1.00000000	pigeons
Ccm2	0.50	1.00000000	pigeons
Wdr43	0.36	1.00000000	pigeons
Eif2b4	0.50	1.00000000	pigeons
Polr1d	0.45	1.00000000	pigeons
Tars	0.70	1.00000000	pigeons
LTO1	0.57	1.00000000	pigeons
Lgmn	0.50	1.00000000	pigeons
Csgalnact2	0.51	1.00000000	pigeons
Dntt	1.70	1.00000000	pigeons
Rpf1	0.96	1.00000000	pigeons
Alpk3	0.57	1.00000000	pigeons
Tsen2	0.56	1.00000000	pigeons
Terf2	0.47	1.00000000	pigeons
Hdac3	0.42	1.00000000	pigeons
Rars	0.50	1.00000000	pigeons
Naa60	0.91	1.00000000	pigeons
Sh2b3	0.51	1.00000000	pigeons
Brap	0.46	1.00000000	pigeons
Trpv2	0.54	1.00000000	pigeons
Cep104	0.52	1.00000000	pigeons
Zdhhc18	1.01	1.00000000	pigeons
Arhgap30	0.43	1.00000000	pigeons

Khdc4	0.41	1.00000000	pigeons
Gcdh	0.47	1.00000000	pigeons
Senp6	0.33	1.00000000	pigeons
Dscam	2.89	1.00000000	pigeons
Stom	1.37	1.00000000	pigeons
Zbtb38	1.14	1.00000000	pigeons
Tmem168	0.46	1.00000000	pigeons
Csf1r	2.06	1.00000000	pigeons
Ppp1r9a	0.62	1.00000000	pigeons
Cav1	-2.54	1.00000000	pigeons
Tmem138	0.56	1.00000000	pigeons
Ube2j1	0.56	1.00000000	pigeons
Ccng2	0.86	1.00000000	pigeons
Gtpbp4	0.08	1.00000000	pigeons
Spen	0.40	1.00000000	pigeons
Arhgap24	1.62	1.00000000	pigeons
Traf2	-0.01	1.00000000	pigeons
Chd1l	0.67	1.00000000	pigeons
Ppp1r12a	0.70	1.00000000	pigeons
Fbxo32	1.24	1.00000000	pigeons
Plk3	0.51	1.00000000	pigeons
Lpl	-2.53	1.00000000	pigeons
Ndrg3	0.04	1.00000000	pigeons
Smndc1	0.51	1.00000000	pigeons
Dusp1	-1.52	1.00000000	pigeons
Prpf40a	0.52	1.00000000	pigeons
Brd3	0.71	1.00000000	pigeons
Arl6ip5	0.50	1.00000000	pigeons
Zranb3	0.56	1.00000000	pigeons
Cish	0.86	1.00000000	pigeons
Abhd17c	0.67	1.00000000	pigeons
Tfg	0.74	1.00000000	pigeons
Rragd	0.77	1.00000000	pigeons
Nek6	0.38	1.00000000	pigeons
Ipo5	0.44	1.00000000	pigeons
Aagab	0.61	1.00000000	pigeons
Pnisr	0.77	1.00000000	pigeons
Tln1	0.45	1.00000000	pigeons
Pdcd11	0.47	1.00000000	pigeons
Rnf34	0.62	1.00000000	pigeons
Atf2	0.19	1.00000000	pigeons
Scyl2	0.74	1.00000000	pigeons
Snrpe	0.67	1.00000000	pigeons
Ero1b	0.35	1.00000000	pigeons
Dync1i2	0.42	1.00000000	pigeons
Ncoa6	0.59	1.00000000	pigeons

Dock11	0.93	1.00000000	pigeons
Spata5	0.93	1.00000000	pigeons
Jun	-1.45	1.00000000	pigeons
Ccdc93	0.85	1.00000000	pigeons
Pik3r4	0.46	1.00000000	pigeons
Thada	0.78	1.00000000	pigeons
Parg	0.63	1.00000000	pigeons
Amfr	0.30	1.00000000	pigeons
Hnrnpa3	0.78	1.00000000	pigeons
Gns	0.80	1.00000000	pigeons
Usp3	0.88	1.00000000	pigeons
Xpo6	0.23	1.00000000	pigeons
Nrf1	0.17	1.00000000	pigeons
Rapgef5	-1.21	1.00000000	pigeons
Fam13b	-0.01	1.00000000	pigeons
Atg5	0.51	1.00000000	pigeons
Amd2	0.65	1.00000000	pigeons
Btaf1	0.52	1.00000000	pigeons
Srsf5	0.56	1.00000000	pigeons
Npc1	0.42	1.00000000	pigeons
Zmynd19	0.14	1.00000000	pigeons
Ptpn22	0.80	1.00000000	pigeons
Gapvd1	0.08	1.00000000	pigeons
Ccnl2	0.03	1.00000000	pigeons
Dcaf13	0.39	1.00000000	pigeons
Rragc	0.61	1.00000000	pigeons
Eif3l	0.67	1.00000000	pigeons
Sec14l1	-2.34	1.00000000	pigeons
Hipk3	1.27	1.00000000	pigeons
Frs2	0.24	1.00000000	pigeons
Capza2	0.49	1.00000000	pigeons
Ep300	0.22	1.00000000	pigeons
Sgpl1	0.49	1.00000000	pigeons
Utp3	0.54	1.00000000	pigeons
Akap12	-2.36	1.00000000	pigeons
Arih1	0.00	1.00000000	pigeons
Angel1	0.50	1.00000000	pigeons
Sf3a3	0.73	1.00000000	pigeons
Ccdc88c	1.40	1.00000000	pigeons
Gtpbp1	0.16	1.00000000	pigeons
Fam8a1	0.55	1.00000000	pigeons
Vav3	0.44	1.00000000	pigeons
Rnf19b	0.44	1.00000000	pigeons
Capzb	0.67	1.00000000	pigeons
Dennd1b	1.24	1.00000000	pigeons
Sap130	0.33	1.00000000	pigeons

Fkbp8	0.81	1.00000000	pigeons
Aldh18a1	0.66	1.00000000	pigeons
Acaca	0.34	1.00000000	pigeons
Srsf11	0.58	1.00000000	pigeons
Prpsap2	0.48	1.00000000	pigeons
Rfxank	0.55	1.00000000	pigeons
Rnf216	0.65	1.00000000	pigeons
Hmg20b	0.36	1.00000000	pigeons
Xpr1	0.84	1.00000000	pigeons
Slco3a1	1.37	1.00000000	pigeons
Rbm6	0.25	1.00000000	pigeons
Desi2	0.65	1.00000000	pigeons
Usp8	0.05	1.00000000	pigeons
Trank1	-0.47	1.00000000	pigeons
Washc4	0.31	1.00000000	pigeons
Clptm1l	0.80	1.00000000	pigeons
Sec23b	0.94	1.00000000	pigeons
Adam17	0.58	1.00000000	pigeons
Zc4h2	0.52	1.00000000	pigeons
Zbtb37	0.49	1.00000000	pigeons
R3hdm4	0.65	1.00000000	pigeons
Fbxl17	0.39	1.00000000	pigeons
Zcchc8	0.64	1.00000000	pigeons
Etnk1	0.50	1.00000000	pigeons
Sgms1	0.40	1.00000000	pigeons
Eif3g	0.52	1.00000000	pigeons
Rlim	0.05	1.00000000	pigeons
G3bp1	0.21	1.00000000	pigeons
Zfp207	-0.06	1.00000000	pigeons
Fbxl5	0.35	1.00000000	pigeons
Tgfbrap1	0.76	1.00000000	pigeons
Rab9b	0.59	1.00000000	pigeons
Bach1	0.77	1.00000000	pigeons
Apcdd1	-2.16	1.00000000	pigeons
Ablim1	-1.71	1.00000000	pigeons
Cep128	0.67	1.00000000	pigeons
Pknox1	1.05	1.00000000	pigeons
Spg21	0.75	1.00000000	pigeons
Nfu1	0.72	1.00000000	pigeons
Ppp6r2	0.54	1.00000000	pigeons
Txndc17	0.13	1.00000000	pigeons
Tmcc3	-1.97	1.00000000	pigeons
Dcaf1	0.46	1.00000000	pigeons
Usp15	-0.37	1.00000000	pigeons
Klhl22	0.51	1.00000000	pigeons
Sike1	0.59	1.00000000	pigeons

Tmem181a	0.58	1.00000000	pigeons
Pkp4	0.55	1.00000000	pigeons
Arhgap45	0.73	1.00000000	pigeons
Samhd1	0.32	1.00000000	pigeons
Pank3	0.79	1.00000000	pigeons
Efnb2	-2.16	1.00000000	pigeons
Tnfrsf18	1.53	1.00000000	pigeons
Actr2	0.81	1.00000000	pigeons
Hnrnpm	0.45	1.00000000	pigeons
Csnk1e	0.09	1.00000000	pigeons
Klhl15	0.71	1.00000000	pigeons
Nfat5	0.95	1.00000000	pigeons
Cyfip2	0.80	1.00000000	pigeons
Helz	-0.02	1.00000000	pigeons
Zfp516	0.49	1.00000000	pigeons
Ganc	0.24	1.00000000	pigeons
Trim33	0.35	1.00000000	pigeons
Edem3	1.26	1.00000000	pigeons
Ibtk	-0.04	1.00000000	pigeons
Efcab14	0.50	1.00000000	pigeons
Fbxo38	0.48	1.00000000	pigeons
Ing5	0.17	1.00000000	pigeons
Stat4	0.87	1.00000000	pigeons
Kcnj5	-2.49	1.00000000	pigeons
Klhdc1	0.16	1.00000000	pigeons
Atp6ap2	0.71	1.00000000	pigeons
Ptbp1	0.60	1.00000000	pigeons
Cab39	0.46	1.00000000	pigeons
Tbk1	0.44	1.00000000	pigeons
Sdf2l1	0.54	1.00000000	pigeons
Rab14	0.35	1.00000000	pigeons
Cenpu	0.77	1.00000000	pigeons
Cbx3	0.66	1.00000000	pigeons
Ndufaf2	0.57	1.00000000	pigeons
Nfatc3	0.63	1.00000000	pigeons
Ccdc85a	-2.52	1.00000000	pigeons
Neo1	-2.44	1.00000000	pigeons
Ipo7	0.47	1.00000000	pigeons
Lrp3	0.63	1.00000000	pigeons
Ctnna1	0.74	1.00000000	pigeons
Ddx27	0.68	1.00000000	pigeons
Appl2	1.18	1.00000000	pigeons
Hif1a	0.99	1.00000000	pigeons
Erp44	0.55	1.00000000	pigeons
Pnn	0.51	1.00000000	pigeons
Epc2	0.33	1.00000000	pigeons

Ptgfrn	0.65	1.00000000	pigeons
Crtc1	0.51	1.00000000	pigeons
Kcnk1	0.32	1.00000000	pigeons
Eif2s3y	0.20	1.00000000	pigeons
Scaf8	0.28	1.00000000	pigeons
Trerf1	0.28	1.00000000	pigeons
Ubn2	0.52	1.00000000	pigeons
Aebp2	0.80	1.00000000	pigeons
Ino80	0.18	1.00000000	pigeons
Syne3	0.58	1.00000000	pigeons
Lemd3	0.43	1.00000000	pigeons
Rnf139	0.86	1.00000000	pigeons
Rxylt1	0.33	1.00000000	pigeons
Abcg1	0.75	1.00000000	pigeons
Ankrd54	0.28	1.00000000	pigeons
Polr1f	0.32	1.00000000	pigeons
Uba5	0.33	1.00000000	pigeons
Kdm1b	0.55	1.00000000	pigeons
Serpinb1a	0.40	1.00000000	pigeons
Vip	0.54	1.00000000	pigeons
Tagap1	0.35	1.00000000	pigeons
Acyp2	0.29	1.00000000	pigeons
Idh3b	0.44	1.00000000	pigeons
Vps16	0.35	1.00000000	pigeons
Slc25a43	0.47	1.00000000	pigeons
Vsig4	0.99	1.00000000	pigeons
Aifm1	0.25	1.00000000	pigeons
Aip	0.35	1.00000000	pigeons
Eapp	0.32	1.00000000	pigeons
Cd28	0.69	1.00000000	pigeons
Stk11ip	0.44	1.00000000	pigeons
Pnkd	0.34	1.00000000	pigeons
Lancl1	0.29	1.00000000	pigeons
Itprip	0.55	1.00000000	pigeons
Ntng1	0.84	1.00000000	pigeons
Alg14	0.33	1.00000000	pigeons
Qars	0.40	1.00000000	pigeons
Pop4	0.34	1.00000000	pigeons
Rfwd3	0.56	1.00000000	pigeons
Sec14l5	0.37	1.00000000	pigeons
Tesc	0.54	1.00000000	pigeons
Spata20	0.32	1.00000000	pigeons
Sp1	0.65	1.00000000	pigeons
Lman2l	0.41	1.00000000	pigeons
Nfic	0.54	1.00000000	pigeons
Trappc5	0.34	1.00000000	pigeons

Atp6v1h	1.00	1.00000000	pigeons
Yod1	0.12	1.00000000	pigeons
Gng2	0.49	1.00000000	pigeons
Nfkbie	0.64	1.00000000	pigeons
Slf2	0.61	1.00000000	pigeons
Dicer1	0.29	1.00000000	pigeons
Sulf2	1.56	1.00000000	pigeons
Uri1	0.37	1.00000000	pigeons
Atxn2	0.62	1.00000000	pigeons
Xpnpep1	0.63	1.00000000	pigeons
Celf1	0.45	1.00000000	pigeons
Slc9a7	1.32	1.00000000	pigeons
Itsn1	0.86	1.00000000	pigeons
Sp4	0.71	1.00000000	pigeons
Rbm12	0.29	1.00000000	pigeons
L3mbtl3	0.63	1.00000000	pigeons
Fyn	0.78	1.00000000	pigeons
Fars2	0.73	1.00000000	pigeons
Gng10	0.75	1.00000000	pigeons
Sdf2	0.37	1.00000000	pigeons
C3	0.53	1.00000000	pigeons
Rilpl2	0.65	1.00000000	pigeons
Qk	-0.87	1.00000000	pigeons
Pdpk1	0.39	1.00000000	pigeons
4932411N23Rik	-3.61	1.00000000	pigeons
Arhgef9	-1.98	1.00000000	pigeons
Atp2a2	-0.16	1.00000000	pigeons
Cdk8	0.39	1.00000000	pigeons
Adprm	0.48	1.00000000	pigeons
Cxcr5	0.99	1.00000000	pigeons
Ppid	0.32	1.00000000	pigeons
Rad54l2	0.30	1.00000000	pigeons
Gpr157	0.57	1.00000000	pigeons
Ppm1a	0.40	1.00000000	pigeons
Vash1	0.24	1.00000000	pigeons
Pcbd1	0.27	1.00000000	pigeons
Herpud1	0.14	1.00000000	pigeons
Nploc4	0.09	1.00000000	pigeons
Dock9	-2.80	1.00000000	pigeons
Sbno1	0.33	1.00000000	pigeons
Plod1	0.26	1.00000000	pigeons
Brd7	0.33	1.00000000	pigeons
Akap11	0.63	1.00000000	pigeons
Poldip3	0.09	1.00000000	pigeons
Rbm19	0.77	1.00000000	pigeons
Ostf1	0.42	1.00000000	pigeons

Raf1	0.52	1.00000000	pigeons
Luc7l3	0.50	1.00000000	pigeons
Dock5	0.86	1.00000000	pigeons
Vps37a	0.11	1.00000000	pigeons
Rhoa	-0.73	1.00000000	pigeons
Npr3	-2.84	1.00000000	pigeons
Grpel1	0.26	1.00000000	pigeons
Rbm26	0.33	1.00000000	pigeons
Tmem39a	0.56	1.00000000	pigeons
Pigb	0.77	1.00000000	pigeons
Bcl11a	1.59	1.00000000	pigeons
Cflar	0.34	1.00000000	pigeons
Dclk1	-2.53	1.00000000	pigeons
Corin	-2.45	1.00000000	pigeons
Tek	-1.88	1.00000000	pigeons
Sftpc	-3.41	1.00000000	pigeons
Igf1r	0.96	1.00000000	pigeons
Slc35c2	0.03	1.00000000	pigeons
Plcl2	1.40	1.00000000	pigeons
Atp23	0.19	1.00000000	pigeons
Rabgap1	0.35	1.00000000	pigeons
Senp5	0.50	1.00000000	pigeons
Eif3e	0.59	1.00000000	pigeons
Eif2ak2	0.19	1.00000000	pigeons
Ppp1cb	0.23	1.00000000	pigeons
Ddx17	0.29	1.00000000	pigeons
Ctbp1	0.88	1.00000000	pigeons
Rpn2	0.12	1.00000000	pigeons
Tm2d1	0.32	1.00000000	pigeons
Runx3	1.13	1.00000000	pigeons
Cdkal1	0.85	1.00000000	pigeons
Odf2	0.56	1.00000000	pigeons
Arih2	0.60	1.00000000	pigeons
Bap1	0.75	1.00000000	pigeons
Arpc5l	0.47	1.00000000	pigeons
Zfand5	0.26	1.00000000	pigeons
Rfc1	0.45	1.00000000	pigeons
Tmem131l	0.37	1.00000000	pigeons
N4bp1	0.16	1.00000000	pigeons
Acsl1	1.38	1.00000000	pigeons
Tmem183a	0.18	1.00000000	pigeons
Polr2b	-0.11	1.00000000	pigeons
Themis	0.83	1.00000000	pigeons
Nudt16l1	0.18	1.00000000	pigeons
Ube3a	0.74	1.00000000	pigeons
Prrc2c	0.59	1.00000000	pigeons

Eea1	0.14	1.00000000	pigeons
Btbd11	1.43	1.00000000	pigeons
Cobll1	-2.16	1.00000000	pigeons
Acp2	0.14	1.00000000	pigeons
Aifm2	0.35	1.00000000	pigeons
Tasp1	0.23	1.00000000	pigeons
Ttc33	0.36	1.00000000	pigeons
Usp46	0.64	1.00000000	pigeons
Ccr7	0.17	1.00000000	pigeons
Psmd12	0.36	1.00000000	pigeons
Atp1b1	0.42	1.00000000	pigeons
Pias2	0.49	1.00000000	pigeons
Grb2	0.74	1.00000000	pigeons
Stard13	-2.95	1.00000000	pigeons
Khdrbs1	0.02	1.00000000	pigeons
Acbd3	0.13	1.00000000	pigeons
Coq9	0.83	1.00000000	pigeons
Creb3l2	1.17	1.00000000	pigeons
Akap9	0.25	1.00000000	pigeons
Dach1	-2.34	1.00000000	pigeons
Ptprn2	-2.17	1.00000000	pigeons
Appbp2	0.06	1.00000000	pigeons
Fam214a	0.32	1.00000000	pigeons
Elmo1	0.70	1.00000000	pigeons
Ninj1	0.66	1.00000000	pigeons
Ube2v2	0.00	1.00000000	pigeons
Cdc42	0.51	1.00000000	pigeons
Pnpla8	0.49	1.00000000	pigeons
Pi4k2b	0.56	1.00000000	pigeons
Hps3	0.55	1.00000000	pigeons
Fkbp15	0.46	1.00000000	pigeons
Oaz1	0.60	1.00000000	pigeons
Prrc2b	0.14	1.00000000	pigeons
Erp29	0.73	1.00000000	pigeons
Wbp11	0.30	1.00000000	pigeons
Arcn1	0.23	1.00000000	pigeons
Naa35	0.28	1.00000000	pigeons
Smad6	-2.67	1.00000000	pigeons
Fbxo42	0.62	1.00000000	pigeons
Dtnbp1	0.67	1.00000000	pigeons
Bmf	0.52	1.00000000	pigeons
Rftn2	0.76	1.00000000	pigeons
Gpcpd1	0.64	1.00000000	pigeons
Cnot2	0.41	1.00000000	pigeons
Znfx1	0.81	1.00000000	pigeons
Nfatc1	1.00	1.00000000	pigeons

Tnfrsf21	0.77	1.00000000	pigeons
Pnpt1	0.34	1.00000000	pigeons
Ttc13	0.39	1.00000000	pigeons
Exosc10	0.71	1.00000000	pigeons
Fbxo9	0.44	1.00000000	pigeons
Iffo2	0.52	1.00000000	pigeons
Phf3	0.31	1.00000000	pigeons
Traf3	0.33	1.00000000	pigeons
Zbtb1	0.64	1.00000000	pigeons
Tbc1d23	0.12	1.00000000	pigeons
Prex1	0.56	1.00000000	pigeons
Secisbp2	0.35	1.00000000	pigeons
Cerk	0.89	1.00000000	pigeons
Fkbp3	0.49	1.00000000	pigeons
Btbd10	0.33	1.00000000	pigeons
Ncoa2	1.01	1.00000000	pigeons
Tram2	0.36	1.00000000	pigeons
Dcp1a	0.38	1.00000000	pigeons
Smpd2	0.53	1.00000000	pigeons
Gsn	0.93	1.00000000	pigeons
Ccnd2	0.74	1.00000000	pigeons
Ubr2	0.12	1.00000000	pigeons
Traf1	0.66	1.00000000	pigeons
Gnai2	0.68	1.00000000	pigeons
Taok3	0.38	1.00000000	pigeons
Arhgap25	0.81	1.00000000	pigeons
Zfhx3	0.02	1.00000000	pigeons
Irf4	0.65	1.00000000	pigeons
Srf	0.09	1.00000000	pigeons
Jmjd1c	0.88	1.00000000	pigeons
Creb5	-1.70	1.00000000	pigeons
Usp44	0.52	1.00000000	pigeons
Ctcf	0.71	1.00000000	pigeons
Vapb	0.16	1.00000000	pigeons
Rbm33	0.02	1.00000000	pigeons
Fbxl20	0.36	1.00000000	pigeons
Ypel2	0.66	1.00000000	pigeons
Ranbp9	0.12	1.00000000	pigeons
Cog6	0.41	1.00000000	pigeons
Aplf	0.54	1.00000000	pigeons
Gpr171	0.60	1.00000000	pigeons
Lat2	0.66	1.00000000	pigeons
Twf2	0.38	1.00000000	pigeons
Telo2	0.31	1.00000000	pigeons
Arglu1	0.25	1.00000000	pigeons
Sdhaf2	0.46	1.00000000	pigeons

Taf13	0.44	1.00000000	pigeons
Fbxo33	0.29	1.00000000	pigeons
Pwwp2a	0.25	1.00000000	pigeons
Zfand3	0.65	1.00000000	pigeons
Wbp2nl	0.36	1.00000000	pigeons
Zmat2	-0.16	1.00000000	pigeons
Sh3bp2	0.82	1.00000000	pigeons
Ccnk	0.32	1.00000000	pigeons
Sema7a	1.13	1.00000000	pigeons
Ccdc82	0.46	1.00000000	pigeons
Ankrd6	0.67	1.00000000	pigeons
Tdp1	0.45	1.00000000	pigeons
Cpne1	0.38	1.00000000	pigeons
Ranbp3	0.21	1.00000000	pigeons
Btk	0.61	1.00000000	pigeons
Kansl1	0.46	1.00000000	pigeons
Zfp326	0.72	1.00000000	pigeons
Pdia4	0.50	1.00000000	pigeons
Tecpr2	0.33	1.00000000	pigeons
Epb41l3	-0.95	1.00000000	pigeons
Trmt10c	0.27	1.00000000	pigeons
Fam126a	0.74	1.00000000	pigeons
Serac1	0.35	1.00000000	pigeons
Sorbs1	-2.57	1.00000000	pigeons
Arpc3	0.56	1.00000000	pigeons
Gpr155	0.27	1.00000000	pigeons
Cd74	0.44	1.00000000	pigeons
Scaper	0.02	1.00000000	pigeons
Itpr1	0.77	1.00000000	pigeons
Glg1	0.19	1.00000000	pigeons
Rassf9	-2.18	1.00000000	pigeons
Fat4	-1.82	1.00000000	pigeons
Pdlim1	-1.78	1.00000000	pigeons
Sox18	-1.93	1.00000000	pigeons
Copg2	0.02	1.00000000	pigeons
Eny2	0.26	1.00000000	pigeons
Commd4	0.07	1.00000000	pigeons
Hmbox1	1.25	1.00000000	pigeons
Copa	0.34	1.00000000	pigeons
Uap1	0.71	1.00000000	pigeons
Larp4	0.55	1.00000000	pigeons
Nudcd3	0.18	1.00000000	pigeons
Heg1	-2.31	1.00000000	pigeons
Rab3gap1	0.20	1.00000000	pigeons
Gorasp2	0.26	1.00000000	pigeons
Chd2	0.07	1.00000000	pigeons

Itm2b	-1.54	1.00000000	pigeons
Stox2	-1.94	1.00000000	pigeons
Zc3h15	0.24	1.00000000	pigeons
Stx8	0.19	1.00000000	pigeons
Sdhaf4	0.31	1.00000000	pigeons
Armc1	0.27	1.00000000	pigeons
Cstf3	0.42	1.00000000	pigeons
Stk38l	0.45	1.00000000	pigeons
Ptbp2	0.16	1.00000000	pigeons
Bahd1	0.35	1.00000000	pigeons
Hif1an	0.45	1.00000000	pigeons
Ascc2	-0.06	1.00000000	pigeons
Npas3	-2.07	1.00000000	pigeons
Rab24	0.09	1.00000000	pigeons
Tnip2	1.00	1.00000000	pigeons
Impg2	0.61	1.00000000	pigeons
Ccdc90b	0.31	1.00000000	pigeons
Caprin2	0.35	1.00000000	pigeons
Pcid2	0.43	1.00000000	pigeons
Pla2g6	0.21	1.00000000	pigeons
Asmt	0.33	1.00000000	pigeons
Drosha	0.36	1.00000000	pigeons
Abitram	0.45	1.00000000	pigeons
Fam91a1	0.36	1.00000000	pigeons
Tgm4	3.32	1.00000000	pigeons
Wrnip1	0.32	1.00000000	pigeons
Krit1	0.39	1.00000000	pigeons
Grhl1	0.40	1.00000000	pigeons
Sytl3	1.01	1.00000000	pigeons
Birc5	0.52	1.00000000	pigeons
Lca5	0.38	1.00000000	pigeons
Zdhhc15	0.38	1.00000000	pigeons
Gpc3	2.00	1.00000000	pigeons
Tada2b	0.29	1.00000000	pigeons
Elp1	0.24	1.00000000	pigeons
Rcl1	0.23	1.00000000	pigeons
Hacd4	0.30	1.00000000	pigeons
Fbp1	0.58	1.00000000	pigeons
Fam219a	0.35	1.00000000	pigeons
Tnnt3	0.57	1.00000000	pigeons
Cinp	0.27	1.00000000	pigeons
Cytip	0.28	1.00000000	pigeons
Aamp	0.36	1.00000000	pigeons
Ddx18	0.29	1.00000000	pigeons
Tm4sf19	0.54	1.00000000	pigeons
Plscr1	0.48	1.00000000	pigeons

Xxylt1	0.43	1.00000000	pigeons
Thsd4	0.49	1.00000000	pigeons
Crbn	0.22	1.00000000	pigeons
Glt8d1	0.36	1.00000000	pigeons
Rgs9bp	0.35	1.00000000	pigeons
Dcun1d3	0.83	1.00000000	pigeons
Cyth1	0.27	1.00000000	pigeons
Adap2	0.33	1.00000000	pigeons
Cnp	0.32	1.00000000	pigeons
Psmc3ip	0.29	1.00000000	pigeons
Itgb3	0.31	1.00000000	pigeons
Pym1	0.37	1.00000000	pigeons
Hnrnpa1	0.27	1.00000000	pigeons
Slc66a1	0.35	1.00000000	pigeons
Chmp2a	0.26	1.00000000	pigeons
Ahdc1	0.39	1.00000000	pigeons
Brpf3	0.21	1.00000000	pigeons
Kif21b	0.33	1.00000000	pigeons
Rnpep	0.23	1.00000000	pigeons
Rbbp5	0.36	1.00000000	pigeons
Cherp	0.38	1.00000000	pigeons
Tcf25	-0.04	1.00000000	pigeons
Supt3	0.71	1.00000000	pigeons
Ylpm1	0.10	1.00000000	pigeons
Vps35	0.14	1.00000000	pigeons
Tead4	-2.03	1.00000000	pigeons
Farsb	0.74	1.00000000	pigeons
Cdh5	-2.12	1.00000000	pigeons
Tmem204	-2.29	1.00000000	pigeons
Cops4	0.57	1.00000000	pigeons
Tia1	-0.12	1.00000000	pigeons
Ncoa5	0.38	1.00000000	pigeons
Slc35a5	0.33	1.00000000	pigeons
Hecw1	0.60	1.00000000	pigeons
Ank2	0.66	1.00000000	pigeons
Atg9a	0.39	1.00000000	pigeons
Fam114a2	0.44	1.00000000	pigeons
Nde1	0.43	1.00000000	pigeons
Ptp4a2	0.52	1.00000000	pigeons
Agtpbp1	0.05	1.00000000	pigeons
Rnf10	-0.02	1.00000000	pigeons
Ssh2	0.31	1.00000000	pigeons
Ttc21b	0.34	1.00000000	pigeons
Tk2	0.59	1.00000000	pigeons
Btbd9	0.64	1.00000000	pigeons
Dnajb12	0.29	1.00000000	pigeons

Rlf	-0.04	1.00000000	pigeons
Zfp292	0.08	1.00000000	pigeons
Nr3c1	0.37	1.00000000	pigeons
Cdc42se2	0.40	1.00000000	pigeons
Ube2f	0.88	1.00000000	pigeons
Pip4k2a	-0.97	1.00000000	pigeons
Bmp6	-1.81	1.00000000	pigeons
Tfpi2	-2.00	1.00000000	pigeons
Atg12	0.98	1.00000000	pigeons
Zer1	0.30	1.00000000	pigeons
Baz1b	0.41	1.00000000	pigeons
Ampd3	0.41	1.00000000	pigeons
Tecpr1	0.15	1.00000000	pigeons
Gosr2	0.37	1.00000000	pigeons
Fchsd2	0.67	1.00000000	pigeons
Clip4	0.28	1.00000000	pigeons
Prox1	0.30	1.00000000	pigeons
Tmem185a	0.26	1.00000000	pigeons
Ttc1	0.14	1.00000000	pigeons
Sik3	0.10	1.00000000	pigeons
Ythdf3	-0.11	1.00000000	pigeons
Smcr8	0.62	1.00000000	pigeons
Nelfb	0.39	1.00000000	pigeons
Usp38	-0.12	1.00000000	pigeons
Pofut1	0.09	1.00000000	pigeons
Ptpn1	0.56	1.00000000	pigeons
Kidins220	0.52	1.00000000	pigeons
Pik3cb	0.77	1.00000000	pigeons
Cbfb	0.72	1.00000000	pigeons
Tulp3	0.46	1.00000000	pigeons
Git2	0.34	1.00000000	pigeons
Xbp1	-0.01	1.00000000	pigeons
Tm9sf2	0.29	1.00000000	pigeons
Dll4	-2.97	1.00000000	pigeons
Fgd4	0.69	1.00000000	pigeons
Sun1	0.13	1.00000000	pigeons
Hexb	0.29	1.00000000	pigeons
Drg1	0.09	1.00000000	pigeons
Wdr82	0.41	1.00000000	pigeons
Setx	0.47	1.00000000	pigeons
Rangap1	0.18	1.00000000	pigeons
Smarce1	0.40	1.00000000	pigeons
Lrrc8d	1.15	1.00000000	pigeons
Lrpprc	0.39	1.00000000	pigeons
Tmem208	0.48	1.00000000	pigeons
Nhp2	0.42	1.00000000	pigeons

Kbtbd2	-0.07	1.00000000	pigeons
Vezt	0.39	1.00000000	pigeons
Ppm1k	0.13	1.00000000	pigeons
Tmem35b	0.24	1.00000000	pigeons
Ugp2	0.35	1.00000000	pigeons
Paqr3	0.36	1.00000000	pigeons
Arhgap1	0.40	1.00000000	pigeons
Plxnc1	0.57	1.00000000	pigeons
Ogt	-0.01	1.00000000	pigeons
Sesn1	0.47	1.00000000	pigeons
Ercc3	0.36	1.00000000	pigeons
Pyroxd1	0.10	1.00000000	pigeons
Ss18	0.48	1.00000000	pigeons
Ntan1	-0.06	1.00000000	pigeons
Cnot1	0.25	1.00000000	pigeons
Osbpl9	0.64	1.00000000	pigeons
Dazap1	-0.03	1.00000000	pigeons
Glyr1	0.16	1.00000000	pigeons
Rsbn1l	-0.08	1.00000000	pigeons
Sema3c	1.84	1.00000000	pigeons
Sdc4	-1.69	1.00000000	pigeons
Hddc2	0.52	1.00000000	pigeons
Pfdn4	0.28	1.00000000	pigeons
Bcl2a1d	0.90	1.00000000	pigeons
Tnfsf13b	1.05	1.00000000	pigeons
Crlf2	0.74	1.00000000	pigeons
Serinc3	0.30	1.00000000	pigeons
Ranbp1	0.28	1.00000000	pigeons
Snhg4	0.43	1.00000000	pigeons
Esrp2	1.11	1.00000000	pigeons
Rreb1	-0.02	1.00000000	pigeons
Crlf3	0.29	1.00000000	pigeons
Tlr7	0.02	1.00000000	pigeons
Psmb7	-0.12	1.00000000	pigeons
Lnpk	0.28	1.00000000	pigeons
Zdhhc21	0.29	1.00000000	pigeons
Tnik	0.58	1.00000000	pigeons
Galns	0.07	1.00000000	pigeons
Zfp831	1.07	1.00000000	pigeons
Ippk	0.18	1.00000000	pigeons
Srsf10	0.20	1.00000000	pigeons
Zbtb2	0.16	1.00000000	pigeons
Epc1	0.02	1.00000000	pigeons
Cap1	0.48	1.00000000	pigeons
Rasgrp1	0.30	1.00000000	pigeons
Ddx5	0.30	1.00000000	pigeons

Tprgl	0.64	1.00000000	pigeons
Arid5b	1.16	1.00000000	pigeons
Wbp2	0.56	1.00000000	pigeons
Plcb2	0.74	1.00000000	pigeons
B3gntl1	0.30	1.00000000	pigeons
Rxra	0.56	1.00000000	pigeons
Tbc1d22a	-0.08	1.00000000	pigeons
Ccpg1	0.13	1.00000000	pigeons
Ube2v1	0.01	1.00000000	pigeons
Tomm20	0.32	1.00000000	pigeons
Pdap1	0.50	1.00000000	pigeons
Wdr26	0.09	1.00000000	pigeons
Zfp609	0.14	1.00000000	pigeons
Gtdc1	0.50	1.00000000	pigeons
Arpc1b	0.77	1.00000000	pigeons
Cul1	0.17	1.00000000	pigeons
Zfp518a	0.65	1.00000000	pigeons
Rapgef2	-1.55	1.00000000	pigeons
Ppp3r1	0.16	1.00000000	pigeons
Slc6a6	-1.09	1.00000000	pigeons
Rbfa	-1.90	1.00000000	pigeons
Cgnl1	-1.83	1.00000000	pigeons
Lta4h	0.63	1.00000000	pigeons
Ube2i	0.27	1.00000000	pigeons
Chpt1	0.35	1.00000000	pigeons
Fam53b	0.37	1.00000000	pigeons
Tango6	0.37	1.00000000	pigeons
Nadk	0.71	1.00000000	pigeons
Serbp1	0.35	1.00000000	pigeons
Lpcat3	0.25	1.00000000	pigeons
Hmbs	0.53	1.00000000	pigeons
Cacul1	-1.61	1.00000000	pigeons
Jcad	-2.06	1.00000000	pigeons
Prex2	-1.89	1.00000000	pigeons
S100a13	-2.09	1.00000000	pigeons
Ext2	0.36	1.00000000	pigeons
Bcl2l13	0.36	1.00000000	pigeons
Rexo1	0.22	1.00000000	pigeons
Oard1	0.72	1.00000000	pigeons
Nucb2	0.08	1.00000000	pigeons
Cdk12	0.21	1.00000000	pigeons
Dnajc13	0.25	1.00000000	pigeons
Ppp2ca	-0.24	1.00000000	pigeons
Sync	0.23	1.00000000	pigeons
Nxpe3	0.39	1.00000000	pigeons
Tmem128	0.03	1.00000000	pigeons

Clk3	0.27	1.00000000	pigeons
Ercc4	0.46	1.00000000	pigeons
Npepl1	0.00	1.00000000	pigeons
Naa30	0.58	1.00000000	pigeons
Lasp1	0.04	1.00000000	pigeons
Dusp23	0.80	1.00000000	pigeons
Ndufa12	0.45	1.00000000	pigeons
Wipi1	0.86	1.00000000	pigeons
Cd47	0.94	1.00000000	pigeons
Fto	0.66	1.00000000	pigeons
Flii	0.20	1.00000000	pigeons
Dnajb9	0.54	1.00000000	pigeons
Ciz1	1.12	1.00000000	pigeons
Gnl2	0.37	1.00000000	pigeons
Wac	0.23	1.00000000	pigeons
Tnfaip8	0.83	1.00000000	pigeons
Zrsr1	0.11	1.00000000	pigeons
Slc13a1	0.04	1.00000000	pigeons
Nrros	0.34	1.00000000	pigeons
Fbxl3	-0.04	1.00000000	pigeons
Arf4	0.39	1.00000000	pigeons
Armc8	0.49	1.00000000	pigeons
Atad1	0.08	1.00000000	pigeons
Caprin1	0.41	1.00000000	pigeons
Sf3a1	0.35	1.00000000	pigeons
Reps2	0.62	1.00000000	pigeons
Slc35b3	0.39	1.00000000	pigeons
Tcf3	0.22	1.00000000	pigeons
Cpt1a	0.59	1.00000000	pigeons
Ebf1	2.17	1.00000000	pigeons
Vdr	0.87	1.00000000	pigeons
Smc4	0.48	1.00000000	pigeons
Lyn	0.51	1.00000000	pigeons
Chmp1a	0.30	1.00000000	pigeons
St7l	0.20	1.00000000	pigeons
Psme4	-0.14	1.00000000	pigeons
Ccdc134	0.99	1.00000000	pigeons
Zc3h7b	0.90	1.00000000	pigeons
Dclk3	0.56	1.00000000	pigeons
Fxn	0.29	1.00000000	pigeons
Gpsm2	0.42	1.00000000	pigeons
Alg3	0.26	1.00000000	pigeons
Trpm1	1.25	1.00000000	pigeons
Prdm10	0.40	1.00000000	pigeons
Gramd1b	0.37	1.00000000	pigeons
Itgav	1.20	1.00000000	pigeons

Ano5	0.74	1.00000000	pigeons
Tpr	0.24	1.00000000	pigeons
Dcaf5	-0.30	1.00000000	pigeons
Ube2h	0.27	1.00000000	pigeons
Osbpl10	-2.31	1.00000000	pigeons
Hpgd	-1.72	1.00000000	pigeons
Fermt2	-2.12	1.00000000	pigeons
Kcnc1	-2.09	1.00000000	pigeons
Fgd5	-2.13	1.00000000	pigeons
Rufy1	0.79	1.00000000	pigeons
Aak1	0.16	1.00000000	pigeons
Ap2a1	-0.02	1.00000000	pigeons
Acer3	0.45	1.00000000	pigeons
Rnasel	0.93	1.00000000	pigeons
Cep350	0.25	1.00000000	pigeons
Cacnb4	1.65	1.00000000	pigeons
Edem1	0.19	1.00000000	pigeons
Kin	-0.05	1.00000000	pigeons
Eif2a	0.51	1.00000000	pigeons
Noct	0.31	1.00000000	pigeons
Necap2	0.38	1.00000000	pigeons
Lrp12	0.20	1.00000000	pigeons
Ap1s2	0.43	1.00000000	pigeons
Dennd5a	-0.25	1.00000000	pigeons
Sdha	0.30	1.00000000	pigeons
Cops2	0.30	1.00000000	pigeons
Zbtb14	0.53	1.00000000	pigeons
Rnh1	0.31	1.00000000	pigeons
AF366264	0.58	1.00000000	pigeons
Synrg	0.49	1.00000000	pigeons
Fmnl1	0.45	1.00000000	pigeons
Lats1	0.21	1.00000000	pigeons
Zc3h12a	0.27	1.00000000	pigeons
Dmxl1	-0.48	1.00000000	pigeons
Rab11a	-0.18	1.00000000	pigeons
Orc3	0.33	1.00000000	pigeons
Ankrd49	0.16	1.00000000	pigeons
Osbpl3	1.11	1.00000000	pigeons
Cd86	0.79	1.00000000	pigeons
Vta1	0.43	1.00000000	pigeons
Atg14	0.43	1.00000000	pigeons
Foxn2	0.58	1.00000000	pigeons
Atp6v0b	0.27	1.00000000	pigeons
Ankle2	0.20	1.00000000	pigeons
Cep112	0.28	1.00000000	pigeons
St8sia4	0.39	1.00000000	pigeons

Gpatch2	-0.02	1.00000000	pigeons
Celf2	0.28	1.00000000	pigeons
Hsp90aa1	-0.53	1.00000000	pigeons
Sdf4	0.21	1.00000000	pigeons
Grk4	0.79	1.00000000	pigeons
Urb2	0.30	1.00000000	pigeons
Ddah1	1.21	1.00000000	pigeons
Phlpp2	0.41	1.00000000	pigeons
Ncln	0.48	1.00000000	pigeons
Ccni	0.85	1.00000000	pigeons
Thyn1	0.34	1.00000000	pigeons
Golga5	0.25	1.00000000	pigeons
Rbbp6	0.58	1.00000000	pigeons
Helz2	0.25	1.00000000	pigeons
Emc6	0.36	1.00000000	pigeons
Sec61a1	0.48	1.00000000	pigeons
Pabpc4	0.58	1.00000000	pigeons
Rdx	0.31	1.00000000	pigeons
Cd63	0.23	1.00000000	pigeons
Tbc1d4	0.73	1.00000000	pigeons
Rpf2	0.64	1.00000000	pigeons
Elovl5	-0.12	1.00000000	pigeons
Sh3bp5	0.03	1.00000000	pigeons
Atg16l1	-0.44	1.00000000	pigeons
Dcakd	0.30	1.00000000	pigeons
Rmnd5a	-0.18	1.00000000	pigeons
Sec63	0.18	1.00000000	pigeons
Setd3	0.40	1.00000000	pigeons
Smurf1	0.44	1.00000000	pigeons
Tbc1d22b	0.20	1.00000000	pigeons
Urm1	-0.17	1.00000000	pigeons
Plekha8	0.00	1.00000000	pigeons
Ranbp10	-0.29	1.00000000	pigeons
Adgrg6	0.86	1.00000000	pigeons
Syne1	0.66	1.00000000	pigeons
Csrnp1	0.23	1.00000000	pigeons
Nup153	0.31	1.00000000	pigeons
Fosl2	0.24	1.00000000	pigeons
Xpo4	0.24	1.00000000	pigeons
Ski	-0.76	1.00000000	pigeons
Inpp4a	-0.92	1.00000000	pigeons
Sfpq	0.25	1.00000000	pigeons
Tmem258	0.23	1.00000000	pigeons
Top1	0.47	1.00000000	pigeons
Uba3	0.30	1.00000000	pigeons
Rassf2	0.37	1.00000000	pigeons

Actl6a	0.27	1.00000000	pigeons
Ptrh2	0.46	1.00000000	pigeons
Tada2a	0.32	1.00000000	pigeons
Ikzf3	0.89	1.00000000	pigeons
Trappc4	0.86	1.00000000	pigeons
Arhgdib	0.30	1.00000000	pigeons
Avl9	0.05	1.00000000	pigeons
Pgs1	0.45	1.00000000	pigeons
Haao	0.91	1.00000000	pigeons
Tbc1d12	-0.08	1.00000000	pigeons
Lcp1	0.41	1.00000000	pigeons
Dnajc17	0.44	1.00000000	pigeons
Pten	0.55	1.00000000	pigeons
Dnajb4	0.33	1.00000000	pigeons
Oser1	0.53	1.00000000	pigeons
Ube2w	0.49	1.00000000	pigeons
Tbc1d15	0.66	1.00000000	pigeons
Dtwd1	0.08	1.00000000	pigeons
Ap1ar	0.19	1.00000000	pigeons
Zfp276	0.29	1.00000000	pigeons
Fbxl22	0.43	1.00000000	pigeons
Nfatc2	0.54	1.00000000	pigeons
Capn11	0.08	1.00000000	pigeons
Ccdc191	0.25	1.00000000	pigeons
Abhd10	0.28	1.00000000	pigeons
Caps2	0.21	1.00000000	pigeons
Pgap2	0.37	1.00000000	pigeons
Hal	0.25	1.00000000	pigeons
Calu	0.40	1.00000000	pigeons
Scube1	0.71	1.00000000	pigeons
Tnfrsf13c	0.73	1.00000000	pigeons
Trim13	0.21	1.00000000	pigeons
Cyth4	0.30	1.00000000	pigeons
Gsap	0.38	1.00000000	pigeons
Scap	0.35	1.00000000	pigeons
Dsel	0.41	1.00000000	pigeons
Gcnt2	0.21	1.00000000	pigeons
Pex2	0.31	1.00000000	pigeons
Rpp38	0.20	1.00000000	pigeons
Utp25	0.25	1.00000000	pigeons
Lyrm2	0.32	1.00000000	pigeons
Pgbd5	0.33	1.00000000	pigeons
Tmem144	0.23	1.00000000	pigeons
Inpp4b	0.94	1.00000000	pigeons
Dimt1	0.25	1.00000000	pigeons
Pggt1b	0.16	1.00000000	pigeons

Lyrm7	0.19	1.00000000	pigeons
Jkamp	0.27	1.00000000	pigeons
Harbi1	0.81	1.00000000	pigeons
Cpsf7	0.19	1.00000000	pigeons
Dmac2l	0.38	1.00000000	pigeons
Tcp11l1	0.19	1.00000000	pigeons
Syt8	0.21	1.00000000	pigeons
Batf	0.29	1.00000000	pigeons
Ikzf2	0.22	1.00000000	pigeons
Dcaf17	0.21	1.00000000	pigeons
Tesk2	0.24	1.00000000	pigeons
Abl2	0.29	1.00000000	pigeons
Atg4b	0.29	1.00000000	pigeons
Pid1	0.32	1.00000000	pigeons
Adamts17	0.94	1.00000000	pigeons
Sema4b	0.33	1.00000000	pigeons
Ap3b2	0.37	1.00000000	pigeons
Arid3b	0.46	1.00000000	pigeons
Adal	0.23	1.00000000	pigeons
Apeh	0.37	1.00000000	pigeons
Fbxo31	0.42	1.00000000	pigeons
Aars	0.24	1.00000000	pigeons
Cog4	0.30	1.00000000	pigeons
Phykpl	0.46	1.00000000	pigeons
Spock1	0.57	1.00000000	pigeons
Slc23a1	0.42	1.00000000	pigeons
Simc1	0.32	1.00000000	pigeons
Chst12	0.19	1.00000000	pigeons
Ap5z1	0.20	1.00000000	pigeons
Cass4	0.48	1.00000000	pigeons
Edem2	0.26	1.00000000	pigeons
Slc17a9	0.25	1.00000000	pigeons
Smim5	0.33	1.00000000	pigeons
Sgsh	0.20	1.00000000	pigeons
Ppp1r26	0.30	1.00000000	pigeons
Fut7	0.26	1.00000000	pigeons
Akap1	0.21	1.00000000	pigeons
Sgsm2	0.40	1.00000000	pigeons
Cers5	0.22	1.00000000	pigeons
Senp1	0.30	1.00000000	pigeons
Nlrx1	0.24	1.00000000	pigeons
Cd3g	0.58	1.00000000	pigeons
Clptm1	0.18	1.00000000	pigeons
Zbtb8os	0.28	1.00000000	pigeons
Tekt2	0.22	1.00000000	pigeons
Dus3l	0.26	1.00000000	pigeons

Armc6	0.77	1.00000000	pigeons
Jund	0.26	1.00000000	pigeons
Gatad2b	0.22	1.00000000	pigeons
Tyk2	0.25	1.00000000	pigeons
Pfkfb1	0.22	1.00000000	pigeons
Cbll1	0.43	1.00000000	pigeons
Fam110b	0.65	1.00000000	pigeons
Ufsp2	0.33	1.00000000	pigeons
Aldh7a1	0.23	1.00000000	pigeons
Rnf168	0.28	1.00000000	pigeons
Cog1	0.36	1.00000000	pigeons
Ubqln4	0.24	1.00000000	pigeons
Gtf2a1	-0.01	1.00000000	pigeons
Kdm3b	0.05	1.00000000	pigeons
Larp7	0.25	1.00000000	pigeons
Exoc2	0.31	1.00000000	pigeons
Tmed3	0.47	1.00000000	pigeons
Fggy	0.78	1.00000000	pigeons
Prdm2	0.63	1.00000000	pigeons
Epn2	0.22	1.00000000	pigeons
Psma5	0.15	1.00000000	pigeons
Foxk2	0.10	1.00000000	pigeons
Ints13	0.22	1.00000000	pigeons
Ppm1b	0.36	1.00000000	pigeons
Pspc1	0.18	1.00000000	pigeons
Dnajc1	0.46	1.00000000	pigeons
Heatr5b	0.30	1.00000000	pigeons
Rassf8	-0.02	1.00000000	pigeons
Xpo5	0.21	1.00000000	pigeons
Psmd6	0.74	1.00000000	pigeons
Pdpr	-0.05	1.00000000	pigeons
Snw1	0.27	1.00000000	pigeons
Emc2	0.26	1.00000000	pigeons
Cipc	0.55	1.00000000	pigeons
Galnt7	0.42	1.00000000	pigeons
Kif3a	0.38	1.00000000	pigeons
Fbxl4	-0.03	1.00000000	pigeons
Ube3c	0.45	1.00000000	pigeons
Hectd4	0.29	1.00000000	pigeons
Vps53	0.27	1.00000000	pigeons
Srp72	0.29	1.00000000	pigeons
Cltc	0.15	1.00000000	pigeons
Pgam1	0.31	1.00000000	pigeons
Cryzl1	0.17	1.00000000	pigeons
Tmem47	-1.42	1.00000000	pigeons
Eaf1	0.24	1.00000000	pigeons

Nrp1	-1.55	1.00000000	pigeons
Itgb1bp1	-0.36	1.00000000	pigeons
Katna1	-0.10	1.00000000	pigeons
Gtpbp2	0.27	1.00000000	pigeons
Sh2d1a	-1.67	1.00000000	pigeons
Unc5c	-1.77	1.00000000	pigeons
Tmem184c	-0.09	1.00000000	pigeons
Rai14	-1.59	1.00000000	pigeons
Lifr	-1.64	1.00000000	pigeons
Ncoa4	0.17	1.00000000	pigeons
Pld1	0.14	1.00000000	pigeons
Nr2f2	-1.59	1.00000000	pigeons
Apba2	-1.64	1.00000000	pigeons
Prkab1	0.22	1.00000000	pigeons
Hlf	-1.59	1.00000000	pigeons
Zbtb26	-0.21	1.00000000	pigeons
Golph3l	0.17	1.00000000	pigeons
Cfap45	-1.64	1.00000000	pigeons
Nemf	-0.13	1.00000000	pigeons
Ctso	0.09	1.00000000	pigeons
Zyx	0.03	1.00000000	pigeons
Senp7	0.09	1.00000000	pigeons
Ndfip2	0.42	1.00000000	pigeons
Sccpdh	0.31	1.00000000	pigeons
Sacs	0.48	1.00000000	pigeons
Efemp1	-2.02	1.00000000	pigeons
Atxn7l1	0.61	1.00000000	pigeons
Pgpep1	0.34	1.00000000	pigeons
Zfp131	0.55	1.00000000	pigeons
Ubl7	0.25	1.00000000	pigeons
Rad18	0.47	1.00000000	pigeons
Bzw1	0.40	1.00000000	pigeons
Ctsz	0.44	1.00000000	pigeons
Inhba	1.07	1.00000000	pigeons
Cnih1	0.23	1.00000000	pigeons
Tm9sf3	0.51	1.00000000	pigeons
Dot1l	0.49	1.00000000	pigeons
Pan3	0.32	1.00000000	pigeons
Upf1	0.11	1.00000000	pigeons
Tmcc1	-0.02	1.00000000	pigeons
Ncoa3	0.33	1.00000000	pigeons
Atf6	0.27	1.00000000	pigeons
Ptk2b	0.78	1.00000000	pigeons
Unc93b1	0.33	1.00000000	pigeons
Kcnq1	-1.81	1.00000000	pigeons
Stk10	0.27	1.00000000	pigeons

Sri	0.39	1.00000000	pigeons
Ctsb	0.23	1.00000000	pigeons
Rsrp1	0.05	1.00000000	pigeons
Trim37	0.39	1.00000000	pigeons
C2cd2l	0.39	1.00000000	pigeons
Rabep1	0.23	1.00000000	pigeons
Kras	0.00	1.00000000	pigeons
Pkn2	-0.32	1.00000000	pigeons
Ctsa	0.40	1.00000000	pigeons
Zzz3	0.18	1.00000000	pigeons
Dbndd2	0.31	1.00000000	pigeons
Txlna	-0.16	1.00000000	pigeons
Arhgef18	0.16	1.00000000	pigeons
Rffl	0.66	1.00000000	pigeons
Poc1b	0.34	1.00000000	pigeons
Dhcr24	0.75	1.00000000	pigeons
Kat7	0.37	1.00000000	pigeons
Cnot10	0.19	1.00000000	pigeons
Elmod2	0.04	1.00000000	pigeons
Clk1	0.04	1.00000000	pigeons
Ddx28	0.42	1.00000000	pigeons
Pmepa1	0.62	1.00000000	pigeons
Prpf8	0.21	1.00000000	pigeons
Ilf3	0.03	1.00000000	pigeons
Ahi1	0.42	1.00000000	pigeons
Dram2	0.52	1.00000000	pigeons
Ndrg1	0.42	1.00000000	pigeons
Phb	0.26	1.00000000	pigeons
Rab12	-0.45	1.00000000	pigeons
Pdzd11	0.31	1.00000000	pigeons
Cutc	0.45	1.00000000	pigeons
Eral1	0.32	1.00000000	pigeons
Ubap2l	0.37	1.00000000	pigeons
Irf2	-0.41	1.00000000	pigeons
Suds3	0.58	1.00000000	pigeons
Nr4a3	0.89	1.00000000	pigeons
Usp25	0.48	1.00000000	pigeons
Srpk1	0.07	1.00000000	pigeons
Ndufb1	0.21	1.00000000	pigeons
Luc7l	-0.16	1.00000000	pigeons
Lrrc28	0.11	1.00000000	pigeons
Tpi1	0.31	1.00000000	pigeons
Dusp5	-0.33	1.00000000	pigeons
Rit1	0.50	1.00000000	pigeons
Arpc5	0.62	1.00000000	pigeons
Lclat1	0.64	1.00000000	pigeons

Bcl7a	0.59	1.00000000	pigeons
Fasn	0.40	1.00000000	pigeons
Cdc42bpa	-1.92	1.00000000	pigeons
Kat6a	-0.08	1.00000000	pigeons
Slc25a46	0.31	1.00000000	pigeons
Nup88	0.24	1.00000000	pigeons
Rundc1	-0.03	1.00000000	pigeons
Cwc25	0.59	1.00000000	pigeons
Tmem135	0.44	1.00000000	pigeons
Upf2	0.14	1.00000000	pigeons
Dram1	0.26	1.00000000	pigeons
Prdm15	0.11	1.00000000	pigeons
Slc39a9	-0.08	1.00000000	pigeons
Arfgap1	-0.05	1.00000000	pigeons
Ppib	0.27	1.00000000	pigeons
Ppil1	0.19	1.00000000	pigeons
Vdac2	0.04	1.00000000	pigeons
Ppil4	-0.26	1.00000000	pigeons
Rspry1	0.40	1.00000000	pigeons
Sdc3	0.57	1.00000000	pigeons
Ndufb6	0.20	1.00000000	pigeons
Ccdc47	0.31	1.00000000	pigeons
Ran	0.12	1.00000000	pigeons
Nup85	-0.19	1.00000000	pigeons
Yap1	-1.50	1.00000000	pigeons
Acvrl1	-1.48	1.00000000	pigeons
Ppp1r27	0.51	1.00000000	pigeons
Slc41a2	0.56	1.00000000	pigeons
Zfp384	0.49	1.00000000	pigeons
Hspa4	0.38	1.00000000	pigeons
Ammecr1	-0.25	1.00000000	pigeons
Zfp608	0.56	1.00000000	pigeons
Jdp2	0.24	1.00000000	pigeons
Lrrc8b	0.22	1.00000000	pigeons
Rpap2	0.15	1.00000000	pigeons
Pmm2	0.14	1.00000000	pigeons
Ppp3cb	0.45	1.00000000	pigeons
Ppa2	0.36	1.00000000	pigeons
Syngr3	0.31	1.00000000	pigeons
Ube2l3	-0.14	1.00000000	pigeons
Tulp4	-0.11	1.00000000	pigeons
Btbd7	0.22	1.00000000	pigeons
4933400A11Rik	0.22	1.00000000	pigeons
Tcof1	0.46	1.00000000	pigeons
Lbr	-0.06	1.00000000	pigeons
Plxna1	-0.25	1.00000000	pigeons

Il17ra	0.49	1.00000000	pigeons
Sema3d	1.93	1.00000000	pigeons
Elac2	0.06	1.00000000	pigeons
Ankrd11	0.51	1.00000000	pigeons
Gata3	-1.50	1.00000000	pigeons
Ifnar1	0.15	1.00000000	pigeons
Soga1	0.30	1.00000000	pigeons
Arhgap21	-1.85	1.00000000	pigeons
Laptm5	0.55	1.00000000	pigeons
Skap1	-1.57	1.00000000	pigeons
Rab21	0.24	1.00000000	pigeons
Apaf1	0.19	1.00000000	pigeons
Slc7a5	0.59	1.00000000	pigeons
Cggbp1	0.39	1.00000000	pigeons
Zfp512	0.23	1.00000000	pigeons
Sys1	-0.01	1.00000000	pigeons
Ddx51	0.35	1.00000000	pigeons
Rnf220	0.42	1.00000000	pigeons
Hax1	0.24	1.00000000	pigeons
Vmp1	0.16	1.00000000	pigeons
Dbi	0.11	1.00000000	pigeons
Ncf2	0.59	1.00000000	pigeons
Ptpn2	-0.35	1.00000000	pigeons
Snapc1l	0.34	1.00000000	pigeons
Kpna3	-0.12	1.00000000	pigeons
Taf2	0.18	1.00000000	pigeons
Derl1	0.08	1.00000000	pigeons
Ndufa8	0.19	1.00000000	pigeons
Agpat3	-1.26	1.00000000	pigeons
Ash1l	-0.02	1.00000000	pigeons
Ythdc1	0.12	1.00000000	pigeons
Dok2	0.00	1.00000000	pigeons
Col4a1	-2.18	1.00000000	pigeons
Sdc2	-2.25	1.00000000	pigeons
Icosl	0.78	1.00000000	pigeons
Hook3	0.05	1.00000000	pigeons
Traf3ip3	0.20	1.00000000	pigeons
Hyou1	0.14	1.00000000	pigeons
Spic	0.82	1.00000000	pigeons
Dag1	0.21	1.00000000	pigeons
Lcmt1	0.49	1.00000000	pigeons
Numb	-1.69	1.00000000	pigeons
Cltb	0.27	1.00000000	pigeons
Aimp2	-0.12	1.00000000	pigeons
Runx1	0.60	1.00000000	pigeons
Rel	0.78	1.00000000	pigeons

Vps37b	0.30	1.00000000	pigeons
Ubr1	0.55	1.00000000	pigeons
Borcs8	0.34	1.00000000	pigeons
Lsm8	0.40	1.00000000	pigeons
Fcf1	0.26	1.00000000	pigeons
Dhx37	0.47	1.00000000	pigeons
Ip6k1	0.52	1.00000000	pigeons
Rpp30	0.17	1.00000000	pigeons
Ppard	0.29	1.00000000	pigeons
Scaf11	0.44	1.00000000	pigeons
Ube2d2a	0.11	1.00000000	pigeons
Syap1	0.07	1.00000000	pigeons
Casp3	0.46	1.00000000	pigeons
Disc1	0.64	1.00000000	pigeons
Dhx15	0.42	1.00000000	pigeons
Syf2	0.29	1.00000000	pigeons
Hipk1	-0.27	1.00000000	pigeons
Grb10	0.75	1.00000000	pigeons
Ctu2	0.31	1.00000000	pigeons
Nup50	0.02	1.00000000	pigeons
Gyg	0.37	1.00000000	pigeons
Fnbp1	0.73	1.00000000	pigeons
Wrn	0.13	1.00000000	pigeons
Pes1	0.28	1.00000000	pigeons
Kpna4	0.01	1.00000000	pigeons
Aftph	-0.02	1.00000000	pigeons
Gpbp1l1	0.29	1.00000000	pigeons
Ei24	0.33	1.00000000	pigeons
Abracl	0.25	1.00000000	pigeons
Atxn1	0.10	1.00000000	pigeons
Rap1gds1	0.70	1.00000000	pigeons
Nbas	-0.34	1.00000000	pigeons
Lrrfip2	0.50	1.00000000	pigeons
Diaph2	0.66	1.00000000	pigeons
Rrp1b	0.17	1.00000000	pigeons
Tmem222	0.24	1.00000000	pigeons
Zdhhc13	0.24	1.00000000	pigeons
Dusp16	0.60	1.00000000	pigeons
Stx18	0.68	1.00000000	pigeons
Ptgs2	0.61	1.00000000	pigeons
Rpgrip1l	0.26	1.00000000	pigeons
Lcmt2	0.86	1.00000000	pigeons
Slc30a5	0.31	1.00000000	pigeons
Angptl2	0.42	1.00000000	pigeons
Kctd10	0.21	1.00000000	pigeons
Sppl3	0.40	1.00000000	pigeons

Smurf2	-1.77	1.00000000	pigeons
Upf3b	-0.50	1.00000000	pigeons
Srp19	-0.94	1.00000000	pigeons
Cdca4	0.11	1.00000000	pigeons
Arid1b	0.48	1.00000000	pigeons
Phb2	0.03	1.00000000	pigeons
Pdia6	-0.08	1.00000000	pigeons
Dera	-0.76	1.00000000	pigeons
Dcn	-1.63	1.00000000	pigeons
Ncf4	0.39	1.00000000	pigeons
Gabbr2	1.59	1.00000000	pigeons
Asxl3	-1.25	1.00000000	pigeons
Sash3	0.24	1.00000000	pigeons
Zfp488	0.38	1.00000000	pigeons
Rnf123	0.88	1.00000000	pigeons
Caskin2	-1.62	1.00000000	pigeons
Dhrs3	-1.29	1.00000000	pigeons
Scube3	-1.48	1.00000000	pigeons
Gpatch4	0.30	1.00000000	pigeons
Usf3	0.08	1.00000000	pigeons
Prpf3	0.11	1.00000000	pigeons
Xrcc4	0.33	1.00000000	pigeons
Dcps	0.05	1.00000000	pigeons
AU040320	0.07	1.00000000	pigeons
Bysl	0.13	1.00000000	pigeons
Rbfox2	-1.78	1.00000000	pigeons
Arvcf	-2.18	1.00000000	pigeons
Ccdc40	0.37	1.00000000	pigeons
Sat1	0.42	1.00000000	pigeons
Qser1	0.03	1.00000000	pigeons
Gtf2a2	0.16	1.00000000	pigeons
Tlk1	0.19	1.00000000	pigeons
Ctbs	0.15	1.00000000	pigeons
Oat	0.28	1.00000000	pigeons
Lamtor3	-0.28	1.00000000	pigeons
Bclaf1	-0.01	1.00000000	pigeons
Rnf149	0.74	1.00000000	pigeons
Arhgap6	1.22	1.00000000	pigeons
Vps25	-0.27	1.00000000	pigeons
Pitpna	0.09	1.00000000	pigeons
Bcl2	0.74	1.00000000	pigeons
Herc3	0.38	1.00000000	pigeons
Rexo5	0.25	1.00000000	pigeons
Ralgps1	0.42	1.00000000	pigeons
Nudcd1	0.30	1.00000000	pigeons
Tmem41b	0.32	1.00000000	pigeons

Pigx	0.40	1.00000000	pigeons
Gucd1	0.08	1.00000000	pigeons
Ankrd52	0.30	1.00000000	pigeons
Dap3	0.26	1.00000000	pigeons
Dis3l2	0.02	1.00000000	pigeons
Aven	-0.18	1.00000000	pigeons
Prkd1	-0.23	1.00000000	pigeons
Brms1l	-0.11	1.00000000	pigeons
Utp6	-0.02	1.00000000	pigeons
Pcnx	0.01	1.00000000	pigeons
Wdfy1	0.01	1.00000000	pigeons
Pum1	-0.10	1.00000000	pigeons
Pnrc2	-0.11	1.00000000	pigeons
Grsf1	0.33	1.00000000	pigeons
Ssbp3	0.27	1.00000000	pigeons
Ppp1r7	0.04	1.00000000	pigeons
Nbn	-0.16	1.00000000	pigeons
Azin1	0.13	1.00000000	pigeons
Uck2	0.22	1.00000000	pigeons
Pus7l	0.16	1.00000000	pigeons
Rab9	-0.04	1.00000000	pigeons
Adamtsl2	0.72	1.00000000	pigeons
Pcgf5	0.31	1.00000000	pigeons
Ankib1	-0.22	1.00000000	pigeons
Sub1	0.04	1.00000000	pigeons
Strn3	-0.28	1.00000000	pigeons
Snx13	0.34	1.00000000	pigeons
Rmnd1	-0.08	1.00000000	pigeons
Hivep1	0.72	1.00000000	pigeons
Tmx3	0.95	1.00000000	pigeons
Pus7	0.26	1.00000000	pigeons
Rbm48	0.22	1.00000000	pigeons
Prag1	0.26	1.00000000	pigeons
Fktn	0.44	1.00000000	pigeons
Eif4g1	0.39	1.00000000	pigeons
Wdr59	0.27	1.00000000	pigeons
Eya2	0.39	1.00000000	pigeons
Zbtb44	0.46	1.00000000	pigeons
Dym	-0.35	1.00000000	pigeons
Gbf1	0.00	1.00000000	pigeons
Dnajc7	0.04	1.00000000	pigeons
Nus1	0.01	1.00000000	pigeons
Smarca5	-0.13	1.00000000	pigeons
Ptpra	0.37	1.00000000	pigeons
Rnf13	0.28	1.00000000	pigeons
Fzd6	-0.07	1.00000000	pigeons

Brox	0.27	1.00000000	pigeons
Lysmd2	0.11	1.00000000	pigeons
Nlk	0.20	1.00000000	pigeons
Ufl1	-0.01	1.00000000	pigeons
Etfa	0.33	1.00000000	pigeons
Nop58	0.07	1.00000000	pigeons
Wsb1	0.78	1.00000000	pigeons
Ap3m1	0.12	1.00000000	pigeons
Ggps1	-0.03	1.00000000	pigeons
Asb5	0.13	1.00000000	pigeons
Fads2b	0.42	1.00000000	pigeons
Zfp598	-0.05	1.00000000	pigeons
Zcchc2	-0.15	1.00000000	pigeons
Zfp236	0.38	1.00000000	pigeons
Ctbp2	0.52	1.00000000	pigeons
Ankfy1	0.35	1.00000000	pigeons
Fem1a	0.21	1.00000000	pigeons
Galc	1.29	1.00000000	pigeons
Bsg	-1.59	1.00000000	pigeons
Fbxw7	0.46	1.00000000	pigeons
Hnrnpll	0.15	1.00000000	pigeons
Cnot7	0.35	1.00000000	pigeons
Top2b	-0.07	1.00000000	pigeons
Kifap3	0.87	1.00000000	pigeons
Acox1	0.91	1.00000000	pigeons
E2f5	0.32	1.00000000	pigeons
Tfb1m	0.33	1.00000000	pigeons
Ints9	0.28	1.00000000	pigeons
Setbp1	1.11	1.00000000	pigeons
Fam98b	0.18	1.00000000	pigeons
Saal1	0.33	1.00000000	pigeons
Lrrc56	0.32	1.00000000	pigeons
Wdr12	0.35	1.00000000	pigeons
Plod2	0.28	1.00000000	pigeons
Csk	0.39	1.00000000	pigeons
Recql5	0.21	1.00000000	pigeons
Fam168b	0.42	1.00000000	pigeons
Tnfrsf11a	0.85	1.00000000	pigeons
Selenot	-0.08	1.00000000	pigeons
Dr1	-0.02	1.00000000	pigeons
Xpa	0.17	1.00000000	pigeons
Ppig	0.03	1.00000000	pigeons
Zfp521	-1.82	1.00000000	pigeons
Tmem9b	-0.05	1.00000000	pigeons
Rnaseh2b	0.02	1.00000000	pigeons
Tjp2	-1.82	1.00000000	pigeons

Egfl7	-1.46	1.00000000	pigeons
Inpp5k	-0.89	1.00000000	pigeons
Pign	0.19	1.00000000	pigeons
Hdac1	-0.13	1.00000000	pigeons
Sos2	-0.10	1.00000000	pigeons
Vrk1	0.43	1.00000000	pigeons
Gid8	0.04	1.00000000	pigeons
Trim8	-0.22	1.00000000	pigeons
Atg3	-0.29	1.00000000	pigeons
Chfr	0.30	1.00000000	pigeons
Asb3	0.53	1.00000000	pigeons
Chmp6	0.08	1.00000000	pigeons
Unc119	0.45	1.00000000	pigeons
Spcs2	0.21	1.00000000	pigeons
Dgat2	0.21	1.00000000	pigeons
Ctnnal1	0.49	1.00000000	pigeons
Elp6	0.23	1.00000000	pigeons
Traf5	0.23	1.00000000	pigeons
Sanbr	0.33	1.00000000	pigeons
Gpc4	0.44	1.00000000	pigeons
Chst15	0.61	1.00000000	pigeons
Pskh1	0.32	1.00000000	pigeons
Nup98	-0.19	1.00000000	pigeons
Lims2	-1.51	1.00000000	pigeons
Eml4	0.90	1.00000000	pigeons
Tnrc6c	0.47	1.00000000	pigeons
Usp4	-0.30	1.00000000	pigeons
Ankrd10	0.74	1.00000000	pigeons
Pim3	-2.09	1.00000000	pigeons
U2af1	-0.05	1.00000000	pigeons
Sh3bp4	-1.53	1.00000000	pigeons
Htra1	-1.69	1.00000000	pigeons
Glul	-1.02	1.00000000	pigeons
Ephb1	-1.32	1.00000000	pigeons
Sag	-0.83	1.00000000	pigeons
Rbbp4	0.11	1.00000000	pigeons
Fxyd6	-0.07	1.00000000	pigeons
Adipor2	0.22	1.00000000	pigeons
Agpat2	0.41	1.00000000	pigeons
Sod2	0.33	1.00000000	pigeons
Atrx	-0.28	1.00000000	pigeons
Ptpn4	-0.15	1.00000000	pigeons
Adsl	0.30	1.00000000	pigeons
Tm6sf1	0.31	1.00000000	pigeons
Sertad2	0.15	1.00000000	pigeons
Carhsp1	0.39	1.00000000	pigeons

Lpin2	0.33	1.00000000	pigeons
Znhit6	0.13	1.00000000	pigeons
Apoo	0.08	1.00000000	pigeons
Lbh	0.03	1.00000000	pigeons
Taf4	-0.01	1.00000000	pigeons
Iws1	-0.04	1.00000000	pigeons
Pik3cg	-0.03	1.00000000	pigeons
Slc19a2	0.01	1.00000000	pigeons
Gpd1l	0.21	1.00000000	pigeons
Lztfl1	0.02	1.00000000	pigeons
Rundc3b	0.35	1.00000000	pigeons
Pex1	-0.07	1.00000000	pigeons
Polr3f	-0.15	1.00000000	pigeons
Fam184a	0.17	1.00000000	pigeons
Borcs6	0.10	1.00000000	pigeons
Aga	0.18	1.00000000	pigeons
Plaa	0.15	1.00000000	pigeons
Stradb	0.04	1.00000000	pigeons
Gpr39	0.29	1.00000000	pigeons
Bicc1	0.38	1.00000000	pigeons
Kcnt2	0.16	1.00000000	pigeons
Dis3l	0.20	1.00000000	pigeons
Rft1	0.07	1.00000000	pigeons
Zdhhc7	-0.23	1.00000000	pigeons
Zbtb43	0.01	1.00000000	pigeons
Pigs	-0.16	1.00000000	pigeons
Ccnt1	-0.12	1.00000000	pigeons
Cebpz	0.51	1.00000000	pigeons
Zdhhc23	0.15	1.00000000	pigeons
Aldh1l2	0.17	1.00000000	pigeons
Ift57	0.14	1.00000000	pigeons
Cul4a	0.18	1.00000000	pigeons
Lrig3	0.35	1.00000000	pigeons
Cacna1c	0.16	1.00000000	pigeons
Rhog	0.21	1.00000000	pigeons
Cln5	0.13	1.00000000	pigeons
Tnpo3	0.20	1.00000000	pigeons
Ddx47	0.19	1.00000000	pigeons
Ccdc58	0.12	1.00000000	pigeons
Pdp1	0.18	1.00000000	pigeons
Fsbp	0.13	1.00000000	pigeons
Cep76	0.19	1.00000000	pigeons
Piezo2	0.25	1.00000000	pigeons
Kif9	0.15	1.00000000	pigeons
Tmem241	0.26	1.00000000	pigeons
Rnf182	0.32	1.00000000	pigeons

St8sia6	0.33	1.00000000	pigeons
Plagl1	0.18	1.00000000	pigeons
Tmem214	0.26	1.00000000	pigeons
Agbl5	0.11	1.00000000	pigeons
Kif3c	0.15	1.00000000	pigeons
Polr1b	0.12	1.00000000	pigeons
Hhipl2	0.14	1.00000000	pigeons
Tent5a	0.43	1.00000000	pigeons
Rab23	0.22	1.00000000	pigeons
Pigm	0.17	1.00000000	pigeons
Chac2	0.18	1.00000000	pigeons
Pax1	0.14	1.00000000	pigeons
Klf8	0.14	1.00000000	pigeons
Trmt10a	0.13	1.00000000	pigeons
Cenpe	0.24	1.00000000	pigeons
Lnx1	0.22	1.00000000	pigeons
Lpar4	0.19	1.00000000	pigeons
Slc25a14	0.17	1.00000000	pigeons
Wdr17	0.22	1.00000000	pigeons
Ptpn13	0.24	1.00000000	pigeons
Gpat3	0.28	1.00000000	pigeons
Acox3	0.22	1.00000000	pigeons
Ccdc125	0.10	1.00000000	pigeons
Inip	0.20	1.00000000	pigeons
Trim36	0.29	1.00000000	pigeons
Ccdc171	0.14	1.00000000	pigeons
Apba1	0.21	1.00000000	pigeons
Alpk2	0.19	1.00000000	pigeons
S1pr3	0.13	1.00000000	pigeons
Stard4	0.15	1.00000000	pigeons
Creb3	0.33	1.00000000	pigeons
Idua	0.18	1.00000000	pigeons
Tmem38b	0.13	1.00000000	pigeons
Tmem267	0.18	1.00000000	pigeons
Parp8	0.62	1.00000000	pigeons
Kbtbd4	0.14	1.00000000	pigeons
Osbp	0.14	1.00000000	pigeons
Socs4	0.23	1.00000000	pigeons
Vps37c	0.18	1.00000000	pigeons
Lrr1	0.19	1.00000000	pigeons
Chst14	0.20	1.00000000	pigeons
Ttbk2	0.15	1.00000000	pigeons
Arl14ep	0.19	1.00000000	pigeons
Arg2	0.22	1.00000000	pigeons
Vipas39	0.16	1.00000000	pigeons
Cntf	0.24	1.00000000	pigeons

Unc79	0.25	1.00000000	pigeons
Slc25a12	0.13	1.00000000	pigeons
Lrp2	0.20	1.00000000	pigeons
Dnpep	0.21	1.00000000	pigeons
Zfand2b	0.23	1.00000000	pigeons
Iqcb1	0.19	1.00000000	pigeons
Sfxn3	0.20	1.00000000	pigeons
Lhpp	0.19	1.00000000	pigeons
Fut11	0.19	1.00000000	pigeons
Alox5	0.25	1.00000000	pigeons
Lrrc8c	0.18	1.00000000	pigeons
Hpdl	0.16	1.00000000	pigeons
Efcab7	0.17	1.00000000	pigeons
F3	0.12	1.00000000	pigeons
Wdr53	0.31	1.00000000	pigeons
Ilkap	0.24	1.00000000	pigeons
Abcf3	0.13	1.00000000	pigeons
Kif23	0.21	1.00000000	pigeons
Cd276	0.16	1.00000000	pigeons
Rybp	0.30	1.00000000	pigeons
Ptpdc1	0.15	1.00000000	pigeons
Pde12	0.22	1.00000000	pigeons
Dpy19l3	0.32	1.00000000	pigeons
Terf2ip	0.37	1.00000000	pigeons
Carmil2	0.32	1.00000000	pigeons
Fhod1	0.29	1.00000000	pigeons
Aktip	0.21	1.00000000	pigeons
Hars	0.16	1.00000000	pigeons
Paip2	0.23	1.00000000	pigeons
Adra1b	0.18	1.00000000	pigeons
Zfp346	0.23	1.00000000	pigeons
Ublcp1	0.11	1.00000000	pigeons
Tmem130	0.24	1.00000000	pigeons
Pgp	0.27	1.00000000	pigeons
Tbc1d20	0.16	1.00000000	pigeons
Rbm38	0.48	1.00000000	pigeons
Fam217b	0.31	1.00000000	pigeons
Glt1d1	0.16	1.00000000	pigeons
Tmem132b	0.27	1.00000000	pigeons
Lrrc43	0.19	1.00000000	pigeons
Dolpp1	0.16	1.00000000	pigeons
Dab2ip	0.36	1.00000000	pigeons
Dnm1	0.46	1.00000000	pigeons
Zdhhc12	0.14	1.00000000	pigeons
Dhx40	0.24	1.00000000	pigeons
Tubd1	0.21	1.00000000	pigeons

Spns3	0.35	1.00000000	pigeons
Rad51d	0.25	1.00000000	pigeons
Crcp	0.18	1.00000000	pigeons
Scrn2	0.15	1.00000000	pigeons
Socs7	0.25	1.00000000	pigeons
Jup	0.30	1.00000000	pigeons
Klhl11	0.23	1.00000000	pigeons
Zbtb39	0.11	1.00000000	pigeons
Asb8	0.36	1.00000000	pigeons
Copz1	0.11	1.00000000	pigeons
Acot7	0.31	1.00000000	pigeons
Chd5	0.20	1.00000000	pigeons
Pink1	0.10	1.00000000	pigeons
Klhl12	0.13	1.00000000	pigeons
Tmem127	0.12	1.00000000	pigeons
Slc20a1	0.10	1.00000000	pigeons
Capg	0.13	1.00000000	pigeons
Ppox	0.16	1.00000000	pigeons
Wdr48	0.43	1.00000000	pigeons
Creb1	0.14	1.00000000	pigeons
Usp28	-1.45	1.00000000	pigeons
Ide	-0.28	1.00000000	pigeons
Dstn	0.50	1.00000000	pigeons
Nsmaf	0.43	1.00000000	pigeons
Rnf146	0.12	1.00000000	pigeons
Exosc9	0.09	1.00000000	pigeons
Ubfd1	0.24	1.00000000	pigeons
Slf1	0.59	1.00000000	pigeons
Sec61g	0.37	1.00000000	pigeons
Psma3	-0.07	1.00000000	pigeons
Anxa2	0.21	1.00000000	pigeons
Nipa2	-0.42	1.00000000	pigeons
Tmem42	0.35	1.00000000	pigeons
C1galt1c1	0.31	1.00000000	pigeons
Itgb5	0.46	1.00000000	pigeons
Itgb1	-1.33	1.00000000	pigeons
Kcnk5	0.13	1.00000000	pigeons
Trip12	0.25	1.00000000	pigeons
Cbl	0.14	1.00000000	pigeons
Bud31	-0.11	1.00000000	pigeons
Nufip1	0.09	1.00000000	pigeons
Hsf2	0.01	1.00000000	pigeons
Dus2	0.25	1.00000000	pigeons
Dennd1a	-0.04	1.00000000	pigeons
Psmb1	0.05	1.00000000	pigeons
Scamp2	0.09	1.00000000	pigeons

Ell	0.02	1.00000000	pigeons
Rpa2	-0.12	1.00000000	pigeons
Ifi30	0.03	1.00000000	pigeons
Hvcn1	1.97	1.00000000	pigeons
Zmat5	0.29	1.00000000	pigeons
Tmem30a	-0.09	1.00000000	pigeons
Polk	0.07	1.00000000	pigeons
Gpn3	0.19	1.00000000	pigeons
Rab22a	-0.15	1.00000000	pigeons
Ttc3	0.22	1.00000000	pigeons
Spryd7	0.39	1.00000000	pigeons
Cd44	0.97	1.00000000	pigeons
Gtpbp8	0.87	1.00000000	pigeons
Dclk2	0.64	1.00000000	pigeons
Coq2	0.50	1.00000000	pigeons
Slc35c1	0.21	1.00000000	pigeons
Klhl28	0.30	1.00000000	pigeons
Gpat4	0.46	1.00000000	pigeons
Cul2	-0.15	1.00000000	pigeons
Hmgb3	0.10	1.00000000	pigeons
Cd99l2	0.27	1.00000000	pigeons
Pstpip2	-0.11	1.00000000	pigeons
Becn2	0.19	1.00000000	pigeons
Adipor1	-0.12	1.00000000	pigeons
Ppia	0.52	1.00000000	pigeons
Umad1	0.28	1.00000000	pigeons
Rgcc	-1.70	1.00000000	pigeons
Canx	0.57	1.00000000	pigeons
Pibf1	0.57	1.00000000	pigeons
Prkar2a	0.05	1.00000000	pigeons
Wdr37	-0.01	1.00000000	pigeons
Nek9	0.58	1.00000000	pigeons
Cct5	-0.02	1.00000000	pigeons
Spsb4	-0.77	1.00000000	pigeons
Crnkl1	-0.14	1.00000000	pigeons
Ppm1f	-1.72	1.00000000	pigeons
Clns1a	0.23	1.00000000	pigeons
Ogfrl1	0.25	1.00000000	pigeons
Ppp2r2d	-0.26	1.00000000	pigeons
Pgd	0.22	1.00000000	pigeons
Stambpl1	0.28	1.00000000	pigeons
Snrk	-0.25	1.00000000	pigeons
Bag1	-0.12	1.00000000	pigeons
Auh	0.42	1.00000000	pigeons
Tgif1	0.09	1.00000000	pigeons
Lrrc59	0.58	1.00000000	pigeons

Setd5	0.25	1.00000000	pigeons
Uso1	0.32	1.00000000	pigeons
Ap3b1	0.03	1.00000000	pigeons
Erbin	-0.29	1.00000000	pigeons
Nedd4	0.31	1.00000000	pigeons
Vps29	0.37	1.00000000	pigeons
Fh1	0.03	1.00000000	pigeons
Utrn	-0.86	1.00000000	pigeons
Xpc	0.64	1.00000000	pigeons
Lrwd1	0.58	1.00000000	pigeons
Zfp639	0.24	1.00000000	pigeons
Prelid3b	0.41	1.00000000	pigeons
Pacs2	0.34	1.00000000	pigeons
Il21r	-0.01	1.00000000	pigeons
Rsf1	0.16	1.00000000	pigeons
Sirt1	-0.39	1.00000000	pigeons
Herc1	0.18	1.00000000	pigeons
Rpa3	0.28	1.00000000	pigeons
Dip2a	0.11	1.00000000	pigeons
Il16	0.50	1.00000000	pigeons
Ube2q1	-0.19	1.00000000	pigeons
Fam172a	-0.07	1.00000000	pigeons
Hectd1	-0.23	1.00000000	pigeons
Lman2	0.23	1.00000000	pigeons
Sbf1	0.51	1.00000000	pigeons
Paxip1	0.15	1.00000000	pigeons
Arel1	0.24	1.00000000	pigeons
Zfp319	0.40	1.00000000	pigeons
Cse1l	-0.16	1.00000000	pigeons
Sart3	-0.04	1.00000000	pigeons
Wdtc1	0.00	1.00000000	pigeons
Plekha2	0.35	1.00000000	pigeons
Arid5a	0.55	1.00000000	pigeons
Rp2	0.04	1.00000000	pigeons
Zfp148	0.10	1.00000000	pigeons
Nufip2	0.29	1.00000000	pigeons
Spag9	0.20	1.00000000	pigeons
Stk24	-0.96	1.00000000	pigeons
Eps8	-1.54	1.00000000	pigeons
Gata6	-1.26	1.00000000	pigeons
Ncapg2	-0.42	1.00000000	pigeons
Jakmip1	-1.41	1.00000000	pigeons
Cetn3	-0.30	1.00000000	pigeons
Rhoj	-1.11	1.00000000	pigeons
Hspa12a	-1.12	1.00000000	pigeons
Fgfr2	-1.41	1.00000000	pigeons

Slco2a1	-1.20	1.00000000	pigeons
Epha4	-1.27	1.00000000	pigeons
Tex264	-0.42	1.00000000	pigeons
Tubb1	-0.45	1.00000000	pigeons
Gab3	0.25	1.00000000	pigeons
Bbx	0.08	1.00000000	pigeons
Virma	0.16	1.00000000	pigeons
Wdr70	0.04	1.00000000	pigeons
Nbeal1	0.04	1.00000000	pigeons
Golga1	-0.03	1.00000000	pigeons
Cpsf2	0.06	1.00000000	pigeons
Usp33	-0.18	1.00000000	pigeons
Akt1	0.25	1.00000000	pigeons
Dnase1l3	0.92	1.00000000	pigeons
Plag1	-0.05	1.00000000	pigeons
Aasdh	-0.13	1.00000000	pigeons
Cacna2d2	0.16	1.00000000	pigeons
Plpp7	0.22	1.00000000	pigeons
Ghdc	-0.08	1.00000000	pigeons
Ralgapa1	0.23	1.00000000	pigeons
Plekhb2	0.65	1.00000000	pigeons
Eif2b3	0.13	1.00000000	pigeons
Coro1c	0.47	1.00000000	pigeons
Nav3	0.19	1.00000000	pigeons
F5	0.99	1.00000000	pigeons
Sft2d1	-0.08	1.00000000	pigeons
Zfp385b	0.64	1.00000000	pigeons
Cdc123	0.36	1.00000000	pigeons
Exoc1	0.49	1.00000000	pigeons
Helq	0.33	1.00000000	pigeons
Fam162a	0.04	1.00000000	pigeons
Atp2a3	0.74	1.00000000	pigeons
Cds2	0.21	1.00000000	pigeons
Arhgef6	0.13	1.00000000	pigeons
Zfand6	0.15	1.00000000	pigeons
Anxa11	0.22	1.00000000	pigeons
Parp9	0.10	1.00000000	pigeons
Psmc5	-0.31	1.00000000	pigeons
Cct8	-0.21	1.00000000	pigeons
Chmp2b	0.23	1.00000000	pigeons
Slc25a15	-0.28	1.00000000	pigeons
Gemin8	0.17	1.00000000	pigeons
Cpeb3	0.24	1.00000000	pigeons
Ccdc66	-0.13	1.00000000	pigeons
Eya3	-0.11	1.00000000	pigeons
Plpbp	0.27	1.00000000	pigeons

Ankrd39	0.07	1.00000000	pigeons
Stoml2	0.19	1.00000000	pigeons
Pla2g4a	0.63	1.00000000	pigeons
Top3b	0.58	1.00000000	pigeons
Topbp1	0.09	1.00000000	pigeons
Klhl2	0.14	1.00000000	pigeons
Tada3	0.21	1.00000000	pigeons
Pcbd2	0.34	1.00000000	pigeons
Hcrt	0.18	1.00000000	pigeons
Snrpc	0.23	1.00000000	pigeons
Arf6	0.42	1.00000000	pigeons
Ccdc50	0.60	1.00000000	pigeons
Ppt1	0.13	1.00000000	pigeons
Stk26	-0.21	1.00000000	pigeons
Slc25a24	-0.29	1.00000000	pigeons
Bhlhe40	0.59	1.00000000	pigeons
Psma6	0.09	1.00000000	pigeons
Spred2	-1.19	1.00000000	pigeons
Pafah1b1	0.17	1.00000000	pigeons
Ankmy2	0.34	1.00000000	pigeons
Esf1	0.39	1.00000000	pigeons
Igsf6	0.38	1.00000000	pigeons
Borcs5	0.75	1.00000000	pigeons
Dtx3l	0.23	1.00000000	pigeons
Ppp2r5e	0.09	1.00000000	pigeons
Atxn7	0.54	1.00000000	pigeons
Sec24b	0.38	1.00000000	pigeons
Rbm12b2	-0.72	1.00000000	pigeons
Raph1	0.06	1.00000000	pigeons
Gtf2h1	0.04	1.00000000	pigeons
Nipa1	0.54	1.00000000	pigeons
Ift122	0.14	1.00000000	pigeons
Coq7	0.26	1.00000000	pigeons
Axin1	-0.01	1.00000000	pigeons
Ada	0.12	1.00000000	pigeons
P2rx4	0.21	1.00000000	pigeons
Fam222b	0.23	1.00000000	pigeons
Crebrf	-0.26	1.00000000	pigeons
9930021J03Rik	0.10	1.00000000	pigeons
Gemin4	0.33	1.00000000	pigeons
Poldip2	0.10	1.00000000	pigeons
Polr2l	0.00	1.00000000	pigeons
Pde4d	0.78	1.00000000	pigeons
Nap1l4	0.25	1.00000000	pigeons
Park7	-0.58	1.00000000	pigeons
Eif5a2	0.23	1.00000000	pigeons

Phf20l1	-0.54	1.00000000	pigeons
Srrm1	-0.28	1.00000000	pigeons
Srebf2	0.91	1.00000000	pigeons
Ndufb5	0.17	1.00000000	pigeons
Nipal3	0.43	1.00000000	pigeons
Psip1	0.05	1.00000000	pigeons
Ywhae	-0.38	1.00000000	pigeons
Ncbp1	-0.28	1.00000000	pigeons
Vamp4	0.08	1.00000000	pigeons
Phf2	-0.19	1.00000000	pigeons
Ep400	0.03	1.00000000	pigeons
Slc22a23	0.23	1.00000000	pigeons
Ltf	0.26	1.00000000	pigeons
Kdm5b	-0.20	1.00000000	pigeons
Gcc2	0.16	1.00000000	pigeons
Ostc	-0.08	1.00000000	pigeons
Oxnad1	-0.22	1.00000000	pigeons
Ikzf5	0.18	1.00000000	pigeons
Nxt2	0.18	1.00000000	pigeons
Dph6	0.43	1.00000000	pigeons
Smg7	0.27	1.00000000	pigeons
2610507B11Rik	-0.06	1.00000000	pigeons
Nudt3	0.03	1.00000000	pigeons
Galnt18	-1.55	1.00000000	pigeons
Specc1l	-1.50	1.00000000	pigeons
Uba2	0.50	1.00000000	pigeons
Cnot4	-0.53	1.00000000	pigeons
Eif2s1	0.47	1.00000000	pigeons
Irf1	-0.24	1.00000000	pigeons
Nfil3	-0.03	1.00000000	pigeons
Foxo1	0.57	1.00000000	pigeons
Ripk1	0.11	1.00000000	pigeons
Ostm1	-0.14	1.00000000	pigeons
Spidr	0.84	1.00000000	pigeons
Dnajc5	0.04	1.00000000	pigeons
Ndufs3	0.17	1.00000000	pigeons
Pdhx	-0.20	1.00000000	pigeons
Tlnrd1	-1.25	1.00000000	pigeons
Wdr4	0.14	1.00000000	pigeons
Nupl1	0.37	1.00000000	pigeons
C1galt1	0.35	1.00000000	pigeons
Ndufaf4	0.32	1.00000000	pigeons
Lemd2	0.71	1.00000000	pigeons
Cdkn1b	0.05	1.00000000	pigeons
Uvrag	0.21	1.00000000	pigeons
Nfx1	-0.20	1.00000000	pigeons

Cald1	-1.42	1.00000000	pigeons
Als2cl	-1.47	1.00000000	pigeons
Rmdn3	0.21	1.00000000	pigeons
Auts2	-1.53	1.00000000	pigeons
Sumf2	0.03	1.00000000	pigeons
Psmd13	-0.15	1.00000000	pigeons
Plekho1	0.32	1.00000000	pigeons
Tmem64	0.11	1.00000000	pigeons
Smc6	0.48	1.00000000	pigeons
Prkrip1	0.05	1.00000000	pigeons
Exoc6	-0.18	1.00000000	pigeons
Dennd2a	-1.78	1.00000000	pigeons
Luzp1	-2.07	1.00000000	pigeons
Ifnar2	0.26	1.00000000	pigeons
Ttyh3	0.49	1.00000000	pigeons
Hspa13	0.34	1.00000000	pigeons
Trappc11	0.35	1.00000000	pigeons
Dctn3	-0.07	1.00000000	pigeons
Lig3	0.37	1.00000000	pigeons
Rab29	0.29	1.00000000	pigeons
Srp54a	-0.45	1.00000000	pigeons
Ndufa2	0.18	1.00000000	pigeons
Tet2	0.57	1.00000000	pigeons
Cul4b	0.14	1.00000000	pigeons
Psmd9	0.22	1.00000000	pigeons
Aplp2	-0.12	1.00000000	pigeons
Fam104a	0.27	1.00000000	pigeons
Creld2	0.00	1.00000000	pigeons
Xrcc6	0.21	1.00000000	pigeons
Pno1	0.30	1.00000000	pigeons
Pik3ca	-0.22	1.00000000	pigeons
Snx17	-0.44	1.00000000	pigeons
Gpatch11	-0.14	1.00000000	pigeons
Rab20	0.79	1.00000000	pigeons
Slc39a8	0.10	1.00000000	pigeons
Nhlrc2	0.20	1.00000000	pigeons
Chst13	-0.03	1.00000000	pigeons
Nae1	-0.19	1.00000000	pigeons
Sntb2	-0.09	1.00000000	pigeons
Reep2	0.05	1.00000000	pigeons
Abca3	-0.53	1.00000000	pigeons
Baiap2l1	-0.23	1.00000000	pigeons
Slc2a6	0.16	1.00000000	pigeons
Pithd1	-0.11	1.00000000	pigeons
Slc4a4	0.02	1.00000000	pigeons
Sgip1	-0.27	1.00000000	pigeons

Rbm15b	-0.43	1.00000000	pigeons
Btg1b	0.63	1.00000000	pigeons
Tmx1	-0.06	1.00000000	pigeons
Ndufa13	-0.06	1.00000000	pigeons
Rrn3	0.18	1.00000000	pigeons
Chordc1	-0.07	1.00000000	pigeons
Cnpy3	0.22	1.00000000	pigeons
Klhl18	-0.06	1.00000000	pigeons
Csnk2a1	-0.13	1.00000000	pigeons
Zfp106	0.15	1.00000000	pigeons
Cnppd1	0.23	1.00000000	pigeons
Wsb2	0.01	1.00000000	pigeons
Elp4	0.07	1.00000000	pigeons
Zdhhc9	-0.44	1.00000000	pigeons
Thrap3	0.16	1.00000000	pigeons
Kdm4c	0.76	1.00000000	pigeons
Etf1	0.08	1.00000000	pigeons
B2m	-0.30	1.00000000	pigeons
Arid4a	0.16	1.00000000	pigeons
Rap2c	0.27	1.00000000	pigeons
Uimc1	-0.13	1.00000000	pigeons
Tm9sf4	0.27	1.00000000	pigeons
Thoc5	0.26	1.00000000	pigeons
Ppm1d	0.19	1.00000000	pigeons
Osbpl1a	-2.14	1.00000000	pigeons
Rgs10	0.45	1.00000000	pigeons
Gnb4	-0.16	1.00000000	pigeons
Ppp4r3a	-0.08	1.00000000	pigeons
Pde6d	0.02	1.00000000	pigeons
Tmem230	-0.42	1.00000000	pigeons
Klhl8	-0.09	1.00000000	pigeons
Baz2b	-1.40	1.00000000	pigeons
Btf3l4	0.08	1.00000000	pigeons
Ube2d1	0.36	1.00000000	pigeons
Cln6	0.34	1.00000000	pigeons
Cope	0.51	1.00000000	pigeons
Tmco1	0.18	1.00000000	pigeons
Txndc11	-0.10	1.00000000	pigeons
Dcun1d4	0.00	1.00000000	pigeons
Haus2	-0.23	1.00000000	pigeons
Zzef1	-0.02	1.00000000	pigeons
Tom1	0.15	1.00000000	pigeons
Tgds	0.78	1.00000000	pigeons
L3mbtl2	0.17	1.00000000	pigeons
Has2	0.67	1.00000000	pigeons
Tigd5	0.22	1.00000000	pigeons

Papolg	0.30	1.00000000	pigeons
Etfdh	0.26	1.00000000	pigeons
Pax5	1.09	1.00000000	pigeons
Gpr137c	0.21	1.00000000	pigeons
Jag2	0.36	1.00000000	pigeons
Dytn	0.31	1.00000000	pigeons
Adcy6	0.13	1.00000000	pigeons
Spata6	0.23	1.00000000	pigeons
Chchd4	0.29	1.00000000	pigeons
Aar2	0.28	1.00000000	pigeons
B3gnt4	0.31	1.00000000	pigeons
Tpst2	0.24	1.00000000	pigeons
Rnf215	0.40	1.00000000	pigeons
Sik2	0.20	1.00000000	pigeons
Ubash3b	0.60	1.00000000	pigeons
Ampd2	0.21	1.00000000	pigeons
Dusp10	-0.11	1.00000000	pigeons
Ip6k2	0.08	1.00000000	pigeons
Zc3h18	-0.32	1.00000000	pigeons
Actr1a	0.19	1.00000000	pigeons
Pphln1	0.21	1.00000000	pigeons
Cndp2	0.31	1.00000000	pigeons
Stx16	0.07	1.00000000	pigeons
Cyth3	-1.69	1.00000000	pigeons
Ddost	0.22	1.00000000	pigeons
Btbd2	-0.03	1.00000000	pigeons
Hace1	-0.07	1.00000000	pigeons
Rasal2	-1.25	1.00000000	pigeons
Ergic2	0.00	1.00000000	pigeons
Nr4a2	0.29	1.00000000	pigeons
Ints4	0.25	1.00000000	pigeons
Tmem19	-0.24	1.00000000	pigeons
Zfand1	0.16	1.00000000	pigeons
Dpy19l4	-0.08	1.00000000	pigeons
Tmem18	-0.23	1.00000000	pigeons
Ttc37	-0.41	1.00000000	pigeons
Timm10	0.19	1.00000000	pigeons
Anxa6	-0.02	1.00000000	pigeons
Tbc1d9	0.71	1.00000000	pigeons
Tgfbr3	-2.06	1.00000000	pigeons
Cul5	0.02	1.00000000	pigeons
Dnajc2	0.14	1.00000000	pigeons
Txnl4b	0.12	1.00000000	pigeons
Dennd3	0.47	1.00000000	pigeons
Usp14	0.05	1.00000000	pigeons
Chtf8	0.25	1.00000000	pigeons

Smg6	-0.12	1.00000000	pigeons
Dnajb6	0.02	1.00000000	pigeons
Cdpf1	0.29	1.00000000	pigeons
Kcnq5	0.84	1.00000000	pigeons
Cenph	0.37	1.00000000	pigeons
Rgp1	0.25	1.00000000	pigeons
Deaf1	0.41	1.00000000	pigeons
Zfp410	0.32	1.00000000	pigeons
Borcs7	0.44	1.00000000	pigeons
Neurl1a	0.60	1.00000000	pigeons
Rab11fip2	0.18	1.00000000	pigeons
Lepr	0.42	1.00000000	pigeons
Lsm14b	0.23	1.00000000	pigeons
Slc31a1	0.25	1.00000000	pigeons
Ctnnbip1	0.19	1.00000000	pigeons
Sugp1	0.76	1.00000000	pigeons
Napepld	-0.42	1.00000000	pigeons
Timm21	-0.51	1.00000000	pigeons
Txndc12	0.10	1.00000000	pigeons
Lcp2	0.60	1.00000000	pigeons
Hspa9	-0.21	1.00000000	pigeons
Ckap5	-0.11	1.00000000	pigeons
Tmed10	0.14	1.00000000	pigeons
Ccnh	0.01	1.00000000	pigeons
Trp53bp1	-0.33	1.00000000	pigeons
Srsf4	-0.30	1.00000000	pigeons
Pfkfb3	-0.14	1.00000000	pigeons
G3bp2	0.09	1.00000000	pigeons
Ap1g1	-0.51	1.00000000	pigeons
Tmed2	-0.05	1.00000000	pigeons
Gpi1	0.57	1.00000000	pigeons
Usb1	-0.03	1.00000000	pigeons
Slc38a10	0.30	1.00000000	pigeons
Prmt3	0.25	1.00000000	pigeons
Actr8	0.22	1.00000000	pigeons
Tsr3	0.34	1.00000000	pigeons
Igf2r	0.21	1.00000000	pigeons
Papss1	0.06	1.00000000	pigeons
Cwf19l2	0.63	1.00000000	pigeons
Dennd4c	-0.08	1.00000000	pigeons
B3gnt2	-0.03	1.00000000	pigeons
St6gal1	0.58	1.00000000	pigeons
Alg12	-0.15	1.00000000	pigeons
Cnst	0.11	1.00000000	pigeons
Scfd2	0.25	1.00000000	pigeons
Smarcb1	-0.04	1.00000000	pigeons

Sytl1	0.16	1.00000000	pigeons
Btrc	0.05	1.00000000	pigeons
Nr1h3	-0.36	1.00000000	pigeons
Rell1	0.25	1.00000000	pigeons
Tcf7l2	-1.86	1.00000000	pigeons
Nit2	0.27	1.00000000	pigeons
Slc38a1	0.13	1.00000000	pigeons
Tceanc	0.75	1.00000000	pigeons
Asns	0.45	1.00000000	pigeons
Necab1	0.30	1.00000000	pigeons
Triqk	0.16	1.00000000	pigeons
Cbr4	0.62	1.00000000	pigeons
Rwdd4a	0.17	1.00000000	pigeons
Ermp1	0.13	1.00000000	pigeons
Large2	0.32	1.00000000	pigeons
Usp30	0.19	1.00000000	pigeons
Atad5	0.31	1.00000000	pigeons
Ccnd3	0.25	1.00000000	pigeons
Plpp5	0.15	1.00000000	pigeons
Cd82	-0.40	1.00000000	pigeons
Rpap3	0.48	1.00000000	pigeons
Gpc6	0.47	1.00000000	pigeons
Slc26a5	0.21	1.00000000	pigeons
Tpd52	0.19	1.00000000	pigeons
Csnk2a2	-0.08	1.00000000	pigeons
Smyd3	0.22	1.00000000	pigeons
Dda1	0.25	1.00000000	pigeons
Rnf145	-0.42	1.00000000	pigeons
Spz1	-0.88	1.00000000	pigeons
Limd2	0.34	1.00000000	pigeons
Pou2f1	0.32	1.00000000	pigeons
Rab6a	-0.22	1.00000000	pigeons
Atp6v1d	-0.10	1.00000000	pigeons
Rrbp1	-0.02	1.00000000	pigeons
Cnih4	-0.18	1.00000000	pigeons
Commd9	0.27	1.00000000	pigeons
Ulk2	0.37	1.00000000	pigeons
Pdzd8	-0.35	1.00000000	pigeons
Gpx1	0.44	1.00000000	pigeons
Eif5	-0.18	1.00000000	pigeons
Cdc27	-0.37	1.00000000	pigeons
Slc7a11	0.72	1.00000000	pigeons
Secisbp2l	0.16	1.00000000	pigeons
Nkrf	-0.04	1.00000000	pigeons
Eif4e2	0.02	1.00000000	pigeons
Blmh	0.19	1.00000000	pigeons

Fgr	0.07	1.00000000	pigeons
Eif2s2	-0.03	1.00000000	pigeons
Abcc4	0.47	1.00000000	pigeons
Rnf111	-0.20	1.00000000	pigeons
Samsn1	0.51	1.00000000	pigeons
Atp6v0a4	0.12	1.00000000	pigeons
Rnf121	0.16	1.00000000	pigeons
Gtpbp6	0.20	1.00000000	pigeons
Slc45a4	0.15	1.00000000	pigeons
Ccnc	0.15	1.00000000	pigeons
Pdgfc	0.28	1.00000000	pigeons
Tmem260	0.25	1.00000000	pigeons
Soat1	0.34	1.00000000	pigeons
Nceh1	0.26	1.00000000	pigeons
Lysmd4	0.16	1.00000000	pigeons
Bco1	0.25	1.00000000	pigeons
Gins2	0.21	1.00000000	pigeons
Rgs14	0.22	1.00000000	pigeons
Dctn4	0.19	1.00000000	pigeons
Nfya	0.14	1.00000000	pigeons
Crem	-0.94	1.00000000	pigeons
Adam10	0.35	1.00000000	pigeons
Utp18	0.03	1.00000000	pigeons
Zfp652	-0.29	1.00000000	pigeons
Sptlc2	0.03	1.00000000	pigeons
Ndufa10	-0.01	1.00000000	pigeons
Itfg1	-0.16	1.00000000	pigeons
Rock2	-0.01	1.00000000	pigeons
Tra2b	-0.12	1.00000000	pigeons
Nsmce1	0.31	1.00000000	pigeons
Acp6	0.34	1.00000000	pigeons
Fadd	0.34	1.00000000	pigeons
Arhgdia	0.26	1.00000000	pigeons
Prpsap1	0.27	1.00000000	pigeons
Ube2t	0.96	1.00000000	pigeons
Frs3	0.76	1.00000000	pigeons
Cadm1	-0.70	1.00000000	pigeons
Pum2	-0.15	1.00000000	pigeons
Agps	0.24	1.00000000	pigeons
Kif1b	0.11	1.00000000	pigeons
Cdk6	0.21	1.00000000	pigeons
Fnbp1l	-1.57	1.00000000	pigeons
Csde1	-0.40	1.00000000	pigeons
Impdh2	-0.06	1.00000000	pigeons
Acap3	0.04	1.00000000	pigeons
Gnai3	0.00	1.00000000	pigeons

Atf1	0.05	1.00000000	pigeons
Bmt2	-0.04	1.00000000	pigeons
Psma2	0.35	1.00000000	pigeons
Qrich1	0.04	1.00000000	pigeons
Znrf1	-0.07	1.00000000	pigeons
Ice2	0.20	1.00000000	pigeons
Kdm5a	-0.25	1.00000000	pigeons
Tmem167	-0.06	1.00000000	pigeons
Snrpa1	-0.06	1.00000000	pigeons
Snrpd1	0.08	1.00000000	pigeons
Itpk1	-1.63	1.00000000	pigeons
Arl13b	-0.30	1.00000000	pigeons
Donson	0.00	1.00000000	pigeons
Adgrg2	0.25	1.00000000	pigeons
Csmd3	0.10	1.00000000	pigeons
Prkag2	0.11	1.00000000	pigeons
Cmpk2	-0.02	1.00000000	pigeons
Slc35a1	0.02	1.00000000	pigeons
Qpct	0.15	1.00000000	pigeons
Galnt2	0.04	1.00000000	pigeons
Gatb	-0.09	1.00000000	pigeons
Cat	0.06	1.00000000	pigeons
Wdr11	0.12	1.00000000	pigeons
Oit3	0.15	1.00000000	pigeons
Ch25h	0.16	1.00000000	pigeons
Eri3	0.04	1.00000000	pigeons
Trmt1l	0.18	1.00000000	pigeons
Hexa	0.11	1.00000000	pigeons
Abhd6	-0.22	1.00000000	pigeons
Atmin	-0.11	1.00000000	pigeons
Gan	-0.06	1.00000000	pigeons
Fabp6	-0.07	1.00000000	pigeons
Kcnq2	0.07	1.00000000	pigeons
Pigl	0.05	1.00000000	pigeons
Rad51c	0.13	1.00000000	pigeons
Cluh	0.00	1.00000000	pigeons
Ube2z	-0.62	1.00000000	pigeons
Ppcs	0.09	1.00000000	pigeons
Pou2af1	0.13	1.00000000	pigeons
Lsm10	-0.07	1.00000000	pigeons
Rsbn1	-0.09	1.00000000	pigeons
Ddx49	0.06	1.00000000	pigeons
Get4	-0.12	1.00000000	pigeons
Pxdc1	-1.48	1.00000000	pigeons
D5Ertd579e	-0.56	1.00000000	pigeons
Dync1h1	-0.14	1.00000000	pigeons

Aif1l	0.09	1.00000000	pigeons
Rpa1	-0.29	1.00000000	pigeons
Lct	0.89	1.00000000	pigeons
Szt2	0.39	1.00000000	pigeons
Rbm18	0.23	1.00000000	pigeons
Oxct1	0.41	1.00000000	pigeons
Cops7a	-0.17	1.00000000	pigeons
Chmp7	-0.27	1.00000000	pigeons
Atp6v0e	0.09	1.00000000	pigeons
Ascc3	0.07	1.00000000	pigeons
Uchl3	0.43	1.00000000	pigeons
Cenpc1	0.04	1.00000000	pigeons
Lpxn	-0.06	1.00000000	pigeons
Srp9	-0.23	1.00000000	pigeons
Zdhhc6	-0.44	1.00000000	pigeons
Dnajc3	-0.08	1.00000000	pigeons
Hsp90b1	0.10	1.00000000	pigeons
Zranb1	-0.55	1.00000000	pigeons
Arl3	0.22	1.00000000	pigeons
Atm	0.08	1.00000000	pigeons
Hs1bp3	0.16	1.00000000	pigeons
Rai2	0.02	1.00000000	pigeons
Zfp330	0.03	1.00000000	pigeons
Ryk	0.41	1.00000000	pigeons
Tfip11	0.12	1.00000000	pigeons
Ythdf2	-0.06	1.00000000	pigeons
Slc37a2	-0.04	1.00000000	pigeons
Lyar	-0.89	1.00000000	pigeons
Ubtd2	0.31	1.00000000	pigeons
Ttc19	0.33	1.00000000	pigeons
Ptpru	0.18	1.00000000	pigeons
Yaf2	-0.33	1.00000000	pigeons
Cadps2	-1.96	1.00000000	pigeons
Ywhab	-0.15	1.00000000	pigeons
Dgkz	-0.11	1.00000000	pigeons
Selenof	-0.54	1.00000000	pigeons
Fam204a	0.67	1.00000000	pigeons
Tbc1d9b	-0.06	1.00000000	pigeons
Nudc	-0.43	1.00000000	pigeons
Gnas	0.19	1.00000000	pigeons
Nedd9	-0.08	1.00000000	pigeons
Lamp2	-0.44	1.00000000	pigeons
Tlk2	-0.04	1.00000000	pigeons
Dnajc10	-0.03	1.00000000	pigeons
Wnk1	-0.01	1.00000000	pigeons
Akirin2	0.22	1.00000000	pigeons

Pcyt1a	-0.27	1.00000000	pigeons
Ncor2	0.17	1.00000000	pigeons
Pcmtd1	0.31	1.00000000	pigeons
Hsd17b12	-1.36	1.00000000	pigeons
Kpna1	0.02	1.00000000	pigeons
Stx7	-0.19	1.00000000	pigeons
Shoc2	-0.05	1.00000000	pigeons
Bcl6	0.28	1.00000000	pigeons
Ndufa6	0.09	1.00000000	pigeons
Uhrf2	0.29	1.00000000	pigeons
Ttll7	0.55	1.00000000	pigeons
Ino80d	-0.26	1.00000000	pigeons
Nip7	0.22	1.00000000	pigeons
Aff4	-0.44	1.00000000	pigeons
Dffa	0.33	1.00000000	pigeons
Elp2	0.37	1.00000000	pigeons
Serinc5	0.25	1.00000000	pigeons
Itga6	0.75	1.00000000	pigeons
Stard5	0.24	1.00000000	pigeons
Gid4	0.21	1.00000000	pigeons
Esyt2	-0.07	1.00000000	pigeons
Tbl1xr1	0.25	1.00000000	pigeons
Kdm7a	0.65	1.00000000	pigeons
Atp6v1c1	0.19	1.00000000	pigeons
Pkib	0.34	1.00000000	pigeons
Psma1	-0.35	1.00000000	pigeons
Ankrd28	0.08	1.00000000	pigeons
Tmem184b	-0.20	1.00000000	pigeons
Azi2	-0.24	1.00000000	pigeons
Rock1	0.30	1.00000000	pigeons
Bloc1s6	-0.08	1.00000000	pigeons
Ino80c	-0.13	1.00000000	pigeons
Hps5	-0.06	1.00000000	pigeons
Eef1b2	0.32	1.00000000	pigeons
Pwwp2b	-0.21	1.00000000	pigeons
Abr	-0.04	1.00000000	pigeons
Cd81	-0.92	1.00000000	pigeons
5031439G07Rik	0.58	1.00000000	pigeons
Edc3	0.16	1.00000000	pigeons
Ripor3	-1.54	1.00000000	pigeons
Ndufab1	-0.28	1.00000000	pigeons
Prpf4	0.80	1.00000000	pigeons
Tnrc6a	-0.12	1.00000000	pigeons
Sh3gl1	-0.67	1.00000000	pigeons
Pot1b	0.21	1.00000000	pigeons
Hoxa5	-1.26	1.00000000	pigeons

Colec10	-1.36	1.00000000	pigeons
Ramp3	-1.30	1.00000000	pigeons
Sulf1	-1.59	1.00000000	pigeons
Aig1	0.19	1.00000000	pigeons
Klhdc2	0.03	1.00000000	pigeons
Zc3h14	0.26	1.00000000	pigeons
Foxf1	-1.15	1.00000000	pigeons
Rnf166	0.22	1.00000000	pigeons
Arap3	-1.64	1.00000000	pigeons
Slc9a3r2	-1.47	1.00000000	pigeons
Serpind1	-1.42	1.00000000	pigeons
Tbx3	-1.72	1.00000000	pigeons
Plekhm2	0.09	1.00000000	pigeons
Gdap2	0.04	1.00000000	pigeons
Prelid3a	0.24	1.00000000	pigeons
Lmbr1	-0.38	1.00000000	pigeons
Phf6	0.42	1.00000000	pigeons
Cotl1	-0.20	1.00000000	pigeons
Eif4enif1	-0.48	1.00000000	pigeons
Rbpj	0.32	1.00000000	pigeons
Cpsf4l	-0.38	1.00000000	pigeons
Usp24	0.04	1.00000000	pigeons
Kansl3	0.36	1.00000000	pigeons
Runx1t1	-1.44	1.00000000	pigeons
Dnaja4	0.35	1.00000000	pigeons
Lactb	0.06	1.00000000	pigeons
Thoc2	-0.05	1.00000000	pigeons
Birc6	0.23	1.00000000	pigeons
Sec62	-0.07	1.00000000	pigeons
Zbtb49	0.25	1.00000000	pigeons
Nrbf2	0.16	1.00000000	pigeons
Crkl	0.08	1.00000000	pigeons
Yeats2	-0.42	1.00000000	pigeons
Arhgef12	0.24	1.00000000	pigeons
Wdfy2	0.07	1.00000000	pigeons
Klhdc10	-0.10	1.00000000	pigeons
Rad54b	0.76	1.00000000	pigeons
Rbsn	0.19	1.00000000	pigeons
Kif3b	-0.19	1.00000000	pigeons
Tiprl	0.18	1.00000000	pigeons
Clic4	-1.40	1.00000000	pigeons
Arhgap12	0.15	1.00000000	pigeons
Atp13a3	0.11	1.00000000	pigeons
Iqsec1	0.66	1.00000000	pigeons
Itch	0.17	1.00000000	pigeons
Dpm2	-0.23	1.00000000	pigeons

Cabin1	0.25	1.00000000	pigeons
Shprh	0.00	1.00000000	pigeons
Rnf130	0.47	1.00000000	pigeons
Hbs1l	-0.16	1.00000000	pigeons
Ddx42	0.07	1.00000000	pigeons
Ppm1g	-0.23	1.00000000	pigeons
Arl8a	0.13	1.00000000	pigeons
Aco2	0.18	1.00000000	pigeons
Tpmt	0.31	1.00000000	pigeons
Trdmt1	0.21	1.00000000	pigeons
Sdad1	0.65	1.00000000	pigeons
Vti1b	0.08	1.00000000	pigeons
Ctdspl2	-0.13	1.00000000	pigeons
Blk	0.62	1.00000000	pigeons
Rnf19a	0.16	1.00000000	pigeons
Prr14l	-0.41	1.00000000	pigeons
Abcd2	-0.04	1.00000000	pigeons
Unc50	0.64	1.00000000	pigeons
Yars2	0.18	1.00000000	pigeons
Prkdc	0.08	1.00000000	pigeons
Lrrc57	-0.05	1.00000000	pigeons
Zfyve21	0.04	1.00000000	pigeons
Slc30a7	-0.03	1.00000000	pigeons
Nfs1	-0.07	1.00000000	pigeons
Card9	0.15	1.00000000	pigeons
Kat6b	-1.43	1.00000000	pigeons
Tpm1	-0.38	1.00000000	pigeons
Gsr	0.28	1.00000000	pigeons
Nnt	0.00	1.00000000	pigeons
Bhlhe41	0.34	1.00000000	pigeons
Bnip3l	-0.51	1.00000000	pigeons
Prkd3	-0.17	1.00000000	pigeons
Zranb2	-0.05	1.00000000	pigeons
Nsmce2	-0.04	1.00000000	pigeons
Vrk2	-0.17	1.00000000	pigeons
Fbxw8	-0.13	1.00000000	pigeons
Csnk1a1	0.14	1.00000000	pigeons
Pias1	0.23	1.00000000	pigeons
Kif2a	-0.32	1.00000000	pigeons
Nsa2	0.13	1.00000000	pigeons
Fxr1	-0.19	1.00000000	pigeons
Reps1	-0.30	1.00000000	pigeons
Chgb	0.47	1.00000000	pigeons
Ccp110	-0.59	1.00000000	pigeons
Derl2	0.17	1.00000000	pigeons
Il6st	-0.59	1.00000000	pigeons

Tmem116	-0.82	1.00000000	pigeons
Tspan14	-0.51	1.00000000	pigeons
Sec11a	-0.05	1.00000000	pigeons
Ybx3	0.31	1.00000000	pigeons
Arntl2	0.32	1.00000000	pigeons
Slc36a4	0.11	1.00000000	pigeons
Gpn1	-0.10	1.00000000	pigeons
Rmdn2	0.13	1.00000000	pigeons
Inppl1	0.16	1.00000000	pigeons
Insig2	0.14	1.00000000	pigeons
Chchd1	0.02	1.00000000	pigeons
Suclg2	-0.16	1.00000000	pigeons
Ccdc92	-0.67	1.00000000	pigeons
Abca5	0.03	1.00000000	pigeons
Dnajc16	-0.17	1.00000000	pigeons
C2cd5	-0.08	1.00000000	pigeons
N4bp2l2	-0.52	1.00000000	pigeons
Gclc	-0.03	1.00000000	pigeons
Ebna1bp2	-0.48	1.00000000	pigeons
Alcam	0.08	1.00000000	pigeons
Pin1	0.12	1.00000000	pigeons
Kpnb1	-0.09	1.00000000	pigeons
Tbc1d5	0.34	1.00000000	pigeons
Zfyve1	-0.18	1.00000000	pigeons
Gatad1	0.09	1.00000000	pigeons
Trmt2a	0.27	1.00000000	pigeons
Ncstn	0.23	1.00000000	pigeons
Robo2	0.68	1.00000000	pigeons
Pde3b	0.31	1.00000000	pigeons
Usp22	-1.77	1.00000000	pigeons
Klhdc4	0.02	1.00000000	pigeons
Zfp821	0.04	1.00000000	pigeons
Zswim7	0.05	1.00000000	pigeons
Uxs1	0.13	1.00000000	pigeons
Dapk1	0.38	1.00000000	pigeons
Ppfibp1	0.04	1.00000000	pigeons
Tmf1	0.06	1.00000000	pigeons
Ing4	-0.11	1.00000000	pigeons
Psmd2	0.40	1.00000000	pigeons
Hadhb	-0.40	1.00000000	pigeons
Sec61a2	0.40	1.00000000	pigeons
Hdgfl3	0.37	1.00000000	pigeons
4833439L19Rik	0.32	1.00000000	pigeons
Psmd14	0.31	1.00000000	pigeons
Pdrg1	0.46	1.00000000	pigeons
Faf2	-0.24	1.00000000	pigeons

Camk2d	0.08	1.00000000	pigeons
Ptk7	0.06	1.00000000	pigeons
Ptch1	-0.25	1.00000000	pigeons
Dok3	0.29	1.00000000	pigeons
Atp6v0a2	0.17	1.00000000	pigeons
Ap1m1	-0.03	1.00000000	pigeons
Vps26a	0.21	1.00000000	pigeons
Pde8a	0.08	1.00000000	pigeons
Nr2c1	-0.11	1.00000000	pigeons
Fryl	-0.91	1.00000000	pigeons
Elp3	0.17	1.00000000	pigeons
Dalrd3	0.16	1.00000000	pigeons
Bicral	-0.47	1.00000000	pigeons
Ndufb9	-0.03	1.00000000	pigeons
Phf20	-0.37	1.00000000	pigeons
Rprd1b	0.01	1.00000000	pigeons
Slc39a10	-0.32	1.00000000	pigeons
Vcl	-1.57	1.00000000	pigeons
Nup54	0.17	1.00000000	pigeons
Ppic	-0.22	1.00000000	pigeons
Tnks	0.11	1.00000000	pigeons
Gbe1	0.03	1.00000000	pigeons
Zw10	-0.27	1.00000000	pigeons
Fbxl14	-0.63	1.00000000	pigeons
Ube2g2	-0.22	1.00000000	pigeons
Ndufa5	-0.24	1.00000000	pigeons
Yipf4	0.15	1.00000000	pigeons
Dpyd	-1.14	1.00000000	pigeons
Ppara	-0.14	1.00000000	pigeons
B3glct	-0.26	1.00000000	pigeons
Snx16	-0.29	1.00000000	pigeons
Ddhd1	-0.34	1.00000000	pigeons
Glmn	-0.04	1.00000000	pigeons
Vps8	-0.21	1.00000000	pigeons
Ndst1	-0.20	1.00000000	pigeons
Ezh1	-0.09	1.00000000	pigeons
Trit1	0.07	1.00000000	pigeons
Fam110d	-0.19	1.00000000	pigeons
Polr2c	-0.23	1.00000000	pigeons
Ssb	-0.02	1.00000000	pigeons
Ndufb8	0.06	1.00000000	pigeons
Tsen15	0.10	1.00000000	pigeons
Rbm22	0.21	1.00000000	pigeons
Ppp3cc	0.15	1.00000000	pigeons
Pdss2	-0.15	1.00000000	pigeons
Nrbp1	0.04	1.00000000	pigeons

Ugdh	-0.19	1.00000000	pigeons
Comtd1	0.07	1.00000000	pigeons
Hexim1	0.06	1.00000000	pigeons
Grn	0.11	1.00000000	pigeons
Hmgcl	0.14	1.00000000	pigeons
Def6	0.28	1.00000000	pigeons
Snap23	-0.45	1.00000000	pigeons
Sde2	-0.24	1.00000000	pigeons
Zfyve26	0.06	1.00000000	pigeons
Tcf20	-0.32	1.00000000	pigeons
Ube2b	-0.36	1.00000000	pigeons
Tex2	-2.02	1.00000000	pigeons
Sdhd	-0.31	1.00000000	pigeons
Rgs6	0.53	1.00000000	pigeons
Cwc15	-0.15	1.00000000	pigeons
H13	-0.19	1.00000000	pigeons
Afg1l	-0.23	1.00000000	pigeons
Rhbdd1	-0.11	1.00000000	pigeons
Tdrd3	-0.65	1.00000000	pigeons
Hnrnpab	-0.12	1.00000000	pigeons
Dld	-0.27	1.00000000	pigeons
Ap1s3	0.23	1.00000000	pigeons
Ube2o	-0.32	1.00000000	pigeons
Fbxw2	0.14	1.00000000	pigeons
Tor2a	0.25	1.00000000	pigeons
Lrba	0.29	1.00000000	pigeons
Tshz2	-1.12	1.00000000	pigeons
Sms	0.16	1.00000000	pigeons
Cdc40	-0.22	1.00000000	pigeons
Pdlim5	-0.07	1.00000000	pigeons
Zcrb1	0.01	1.00000000	pigeons
Rcor1	-0.23	1.00000000	pigeons
Gse1	0.10	1.00000000	pigeons
Psme3	0.15	1.00000000	pigeons
Rexo2	-0.12	1.00000000	pigeons
Noc3l	0.42	1.00000000	pigeons
Bst1	0.06	1.00000000	pigeons
Tmed5	-0.17	1.00000000	pigeons
Polr2k	-0.03	1.00000000	pigeons
Zfp335	0.10	1.00000000	pigeons
Nelfcd	0.22	1.00000000	pigeons
Ggnbp2	-0.20	1.00000000	pigeons
Ulk1	-1.83	1.00000000	pigeons
Pip5k1a	-0.50	1.00000000	pigeons
Aimp1	-0.04	1.00000000	pigeons
Tnks2	-0.11	1.00000000	pigeons

Sap30l	-0.09	1.00000000	pigeons
Ctps2	-0.08	1.00000000	pigeons
Fbxo25	-0.66	1.00000000	pigeons
C1d	0.14	1.00000000	pigeons
Prim2	-0.14	1.00000000	pigeons
Hsdl2	-0.03	1.00000000	pigeons
Pigk	-0.25	1.00000000	pigeons
Prmt7	-0.20	1.00000000	pigeons
Cdc23	-0.17	1.00000000	pigeons
Eif3d	-0.04	1.00000000	pigeons
Wasf2	-0.58	1.00000000	pigeons
Skp1	-0.23	1.00000000	pigeons
Acap2	-0.07	1.00000000	pigeons
Rab8b	0.16	1.00000000	pigeons
Dhx30	-0.61	1.00000000	pigeons
Tbpl1	0.08	1.00000000	pigeons
Zfp827	0.19	1.00000000	pigeons
Eif2b1	-0.01	1.00000000	pigeons
Fancd2	-0.40	1.00000000	pigeons
Tcp1	-0.18	1.00000000	pigeons
Rab35	-0.38	1.00000000	pigeons
Actr6	-0.11	1.00000000	pigeons
Gosr1	-0.09	1.00000000	pigeons
Gigyf2	-0.39	1.00000000	pigeons
Ddx23	-0.20	1.00000000	pigeons
Atp2c1	-0.54	1.00000000	pigeons
Stt3b	-0.42	1.00000000	pigeons
Rnf11	0.17	1.00000000	pigeons
Ctsc	-0.07	1.00000000	pigeons
Cct2	0.04	1.00000000	pigeons
Pikfyve	-0.28	1.00000000	pigeons
Dst	-0.50	1.00000000	pigeons
Cbx7	0.03	1.00000000	pigeons
Alg5	0.01	1.00000000	pigeons
Bub3	-0.09	1.00000000	pigeons
Glce	-0.05	1.00000000	pigeons
Agrn	0.16	1.00000000	pigeons
Nin	0.09	1.00000000	pigeons
Cant1	-0.20	1.00000000	pigeons
Arl5a	0.02	1.00000000	pigeons
Wdfy3	0.53	1.00000000	pigeons
Plekha1	-0.02	1.00000000	pigeons
Nt5dc1	-0.39	1.00000000	pigeons
Ptcd3	-0.04	1.00000000	pigeons
Immp1l	0.38	1.00000000	pigeons
Susd3	0.43	1.00000000	pigeons

Rsl24d1	-0.12	1.00000000	pigeons
Arid4b	-0.14	1.00000000	pigeons
Atp6v0d1	-0.08	1.00000000	pigeons
Copb2	-0.24	1.00000000	pigeons
Gtf2h5	-0.36	1.00000000	pigeons
Agfg1	-0.32	1.00000000	pigeons
Cnot11	-0.01	1.00000000	pigeons
Hagh	-0.08	1.00000000	pigeons
Tpcn1	-0.19	1.00000000	pigeons
Gas7	0.08	1.00000000	pigeons
Ftsj3	-0.21	1.00000000	pigeons
Tlr3	0.01	1.00000000	pigeons
Capn15	0.15	1.00000000	pigeons
Dcp2	0.07	1.00000000	pigeons
Adk	-0.52	1.00000000	pigeons
Ano10	-1.20	1.00000000	pigeons
Kdm2a	-0.07	1.00000000	pigeons
Gulp1	-1.37	1.00000000	pigeons
Gatad2a	-0.57	1.00000000	pigeons
Ppp1r13b	-1.01	1.00000000	pigeons
Slu7	-0.07	1.00000000	pigeons
Parvb	0.52	1.00000000	pigeons
Neurl1b	-1.17	1.00000000	pigeons
Fubp3	0.13	1.00000000	pigeons
Wdr7	0.04	1.00000000	pigeons
Srsf1	-0.25	1.00000000	pigeons
Xylt1	0.10	1.00000000	pigeons
Pde7a	0.04	1.00000000	pigeons
Snx4	0.31	1.00000000	pigeons
Skap2	0.19	1.00000000	pigeons
Tmem59	-0.16	1.00000000	pigeons
Smap1	-0.03	1.00000000	pigeons
Usp53	-0.11	1.00000000	pigeons
Gramd3	-0.22	1.00000000	pigeons
Nol8	-0.16	1.00000000	pigeons
Elmo2	0.16	1.00000000	pigeons
Vapa	0.08	1.00000000	pigeons
Cmtm7	0.30	1.00000000	pigeons
Snrpd3	0.34	1.00000000	pigeons
Crot	-0.20	1.00000000	pigeons
Prdm1	0.00	1.00000000	pigeons
Sufu	-0.14	1.00000000	pigeons
Sil1	-0.13	1.00000000	pigeons
Kit	0.38	1.00000000	pigeons
Txnrd1	-1.65	1.00000000	pigeons
Commd1b	-0.09	1.00000000	pigeons

Aqr	-0.17	1.00000000	pigeons
Sos1	-0.07	1.00000000	pigeons
Tmem242	0.07	1.00000000	pigeons
Esd	0.16	1.00000000	pigeons
Emc8	0.02	1.00000000	pigeons
Pigt	-0.17	1.00000000	pigeons
Naaa	0.11	1.00000000	pigeons
Rcc1	-0.27	1.00000000	pigeons
Asap2	0.06	1.00000000	pigeons
Nmi	-0.17	1.00000000	pigeons
Adam23	0.16	1.00000000	pigeons
Ubap2	-0.28	1.00000000	pigeons
Selenoo	0.13	1.00000000	pigeons
Tmtc4	0.24	1.00000000	pigeons
Diaph3	0.28	1.00000000	pigeons
Gas2l3	0.26	1.00000000	pigeons
Alg11	0.28	1.00000000	pigeons
Tmppe	0.11	1.00000000	pigeons
9530077C05Rik	0.18	1.00000000	pigeons
Ndufaf5	0.21	1.00000000	pigeons
Il13ra2	0.15	1.00000000	pigeons
Epha5	0.39	1.00000000	pigeons
Ercc8	0.73	1.00000000	pigeons
Trappc13	0.11	1.00000000	pigeons
Fancm	0.20	1.00000000	pigeons
Flvcr2	0.41	1.00000000	pigeons
Chuk	0.17	1.00000000	pigeons
Kank4	0.20	1.00000000	pigeons
Swt1	0.42	1.00000000	pigeons
Sass6	0.44	1.00000000	pigeons
Pfkl	0.11	1.00000000	pigeons
Dvl3	0.11	1.00000000	pigeons
Gpr160	0.64	1.00000000	pigeons
Scamp5	0.15	1.00000000	pigeons
Selenok	0.12	1.00000000	pigeons
Slc7a6os	0.12	1.00000000	pigeons
Dctn5	0.27	1.00000000	pigeons
Txnrd2	0.28	1.00000000	pigeons
Pgam5	0.17	1.00000000	pigeons
Tmc6	0.17	1.00000000	pigeons
Sirt7	0.16	1.00000000	pigeons
P2rx5	0.17	1.00000000	pigeons
Atcay	0.21	1.00000000	pigeons
Ncoa7	1.74	1.00000000	pigeons
Abcc5	0.43	1.00000000	pigeons
Tspan9	-1.42	1.00000000	pigeons

Arl6ip1	0.15	1.00000000	pigeons
Phldb2	-0.16	1.00000000	pigeons
Trp53bp2	-1.14	1.00000000	pigeons
F2r	-1.51	1.00000000	pigeons
Epb41l4a	-0.38	1.00000000	pigeons
Tspan18	-1.12	1.00000000	pigeons
Lmo4	-0.76	1.00000000	pigeons
Dars2	-0.13	1.00000000	pigeons
Exoc7	-0.19	1.00000000	pigeons
Pik3c2a	-0.29	1.00000000	pigeons
Tab3	0.15	1.00000000	pigeons
Tax1bp1	-0.31	1.00000000	pigeons
Prelid1	0.07	1.00000000	pigeons
Arpc1a	-0.08	1.00000000	pigeons
Taf12	0.19	1.00000000	pigeons
Phtf2	-0.28	1.00000000	pigeons
Tcea1	0.22	1.00000000	pigeons
Btbd1	-0.18	1.00000000	pigeons
Afg3l2	-0.85	1.00000000	pigeons
Psmc1	-0.06	1.00000000	pigeons
Acot10	-0.86	1.00000000	pigeons
Stx17	-0.16	1.00000000	pigeons
Cttnbp2	-1.27	1.00000000	pigeons
Tmem117	-1.19	1.00000000	pigeons
Pdgfb	-1.02	1.00000000	pigeons
Cmtm8	-1.15	1.00000000	pigeons
Adcy8	-1.18	1.00000000	pigeons
Lonrf3	-1.00	1.00000000	pigeons
Dlc1	-1.03	1.00000000	pigeons
Anapc10	-0.39	1.00000000	pigeons
Chac1	-1.05	1.00000000	pigeons
Scly	-0.22	1.00000000	pigeons
Ccdc174	-0.59	1.00000000	pigeons
Prr7	-0.83	1.00000000	pigeons
Ddx46	-0.32	1.00000000	pigeons
Plin2	-0.16	1.00000000	pigeons
Zbtb11	-0.38	1.00000000	pigeons
Zfp618	-0.71	1.00000000	pigeons
Rbl2	-0.03	1.00000000	pigeons
Stau2	0.00	1.00000000	pigeons
Srp68	0.23	1.00000000	pigeons
Ccdc59	0.06	1.00000000	pigeons
Ccdc43	-0.01	1.00000000	pigeons
P2ry6	0.14	1.00000000	pigeons
Nme6	0.14	1.00000000	pigeons
Dusp12	0.17	1.00000000	pigeons

Rttn	0.16	1.00000000	pigeons
Gar1	0.24	1.00000000	pigeons
Tenm3	0.40	1.00000000	pigeons
Cep120	0.13	1.00000000	pigeons
Ttc8	0.13	1.00000000	pigeons
Ramp1	0.17	1.00000000	pigeons
Raver2	0.13	1.00000000	pigeons
Trp63	0.97	1.00000000	pigeons
Acot8	0.28	1.00000000	pigeons
Nat9	0.70	1.00000000	pigeons
Pik3r6	0.50	1.00000000	pigeons
Nacc2	0.15	1.00000000	pigeons
Vat1	0.14	1.00000000	pigeons
P3h1	0.21	1.00000000	pigeons
Stt3a	0.16	1.00000000	pigeons
Dkc1	0.23	1.00000000	pigeons
Ing1	-0.14	1.00000000	pigeons
Arhgap10	-0.15	1.00000000	pigeons
Slc37a4	-0.31	1.00000000	pigeons
Pds5a	-0.34	1.00000000	pigeons
Irf2bpl	-0.27	1.00000000	pigeons
Ahr	0.40	1.00000000	pigeons
Kmt5b	-0.94	1.00000000	pigeons
Guk1	0.28	1.00000000	pigeons
Tmem192	0.39	1.00000000	pigeons
E2f1	0.34	1.00000000	pigeons
Ik	-0.13	1.00000000	pigeons
Suclg1	-0.23	1.00000000	pigeons
Fkbp1a	-1.52	1.00000000	pigeons
Cisd2	-0.22	1.00000000	pigeons
Bcas2	-0.13	1.00000000	pigeons
Plbd1	0.06	1.00000000	pigeons
Wwp1	-0.70	1.00000000	pigeons
Carmil1	-0.77	1.00000000	pigeons
Gab1	-0.94	1.00000000	pigeons
Kdr	-1.04	1.00000000	pigeons
Nat10	-0.49	1.00000000	pigeons
Wdr89	-0.57	1.00000000	pigeons
Cnnm2	-1.05	1.00000000	pigeons
Zcchc24	-0.69	1.00000000	pigeons
Entpd1	-0.44	1.00000000	pigeons
Eci1	-0.45	1.00000000	pigeons
Ttc27	-0.17	1.00000000	pigeons
Pdxdc1	-0.45	1.00000000	pigeons
Cstf1	0.21	1.00000000	pigeons
Uba6	-0.20	1.00000000	pigeons

Nudt21	0.16	1.00000000	pigeons
Arfgap3	-0.17	1.00000000	pigeons
Cpne3	-0.61	1.00000000	pigeons
Commd10	-0.25	1.00000000	pigeons
Serpine2	-0.01	1.00000000	pigeons
Abcf2	0.08	1.00000000	pigeons
Sf3b5	0.03	1.00000000	pigeons
Slc25a16	-0.47	1.00000000	pigeons
Bdp1	-0.06	1.00000000	pigeons
Polr2f	-0.50	1.00000000	pigeons
Stau1	0.34	1.00000000	pigeons
Zfat	-0.19	1.00000000	pigeons
Tspan12	0.14	1.00000000	pigeons
Cep57	0.56	1.00000000	pigeons
Grip1	0.20	1.00000000	pigeons
Sgsm3	0.27	1.00000000	pigeons
Scin	0.54	1.00000000	pigeons
Ndc80	0.23	1.00000000	pigeons
Nqo2	0.17	1.00000000	pigeons
Nebl	0.34	1.00000000	pigeons
Fuca2	0.16	1.00000000	pigeons
Snx5	0.20	1.00000000	pigeons
Rab28	0.71	1.00000000	pigeons
Sergef	0.15	1.00000000	pigeons
Asb2	0.20	1.00000000	pigeons
Acadsb	0.13	1.00000000	pigeons
Ubxn7	0.08	1.00000000	pigeons
Tctn1	0.21	1.00000000	pigeons
Pcyt2	0.09	1.00000000	pigeons
Dph7	0.12	1.00000000	pigeons
Naglu	0.10	1.00000000	pigeons
Uhrf1bp1	0.10	1.00000000	pigeons
Rhobtb2	0.18	1.00000000	pigeons
Nmrk2	0.16	1.00000000	pigeons
Gatd1	0.42	1.00000000	pigeons
Agap1	-1.36	1.00000000	pigeons
St3gal6	-0.62	1.00000000	pigeons
Saraf	-0.42	1.00000000	pigeons
Tnfrsf8	-0.35	1.00000000	pigeons
St7	-0.87	1.00000000	pigeons
Zfp438	0.39	1.00000000	pigeons
Erlin2	-0.58	1.00000000	pigeons
Vps45	-0.07	1.00000000	pigeons
Dlgap4	-0.19	1.00000000	pigeons
Dennd5b	0.90	1.00000000	pigeons
Anks1b	0.50	1.00000000	pigeons

Gpr82	0.22	1.00000000	pigeons
Tprkb	0.18	1.00000000	pigeons
Arfgap2	0.27	1.00000000	pigeons
Nav2	1.11	1.00000000	pigeons
Prr5l	0.27	1.00000000	pigeons
Trmt13	-0.67	1.00000000	pigeons
Rab11fip3	-0.59	1.00000000	pigeons
Lztr1	0.35	1.00000000	pigeons
Sltm	-0.61	1.00000000	pigeons
Hspa4l	-0.23	1.00000000	pigeons
Trip4	-0.19	1.00000000	pigeons
Lrpap1	-0.13	1.00000000	pigeons
Sh3glb1	-0.47	1.00000000	pigeons
Nbeal2	0.70	1.00000000	pigeons
Smarca2	-0.24	1.00000000	pigeons
Fbxl18	-0.20	1.00000000	pigeons
Supt4a	-0.10	1.00000000	pigeons
Rbl1	0.28	1.00000000	pigeons
Api5	-0.23	1.00000000	pigeons
Wapl	-0.04	1.00000000	pigeons
Ahsa1	0.28	1.00000000	pigeons
Zfp622	-0.47	1.00000000	pigeons
Pak3	-0.17	1.00000000	pigeons
Aamdc	0.21	1.00000000	pigeons
Gcc1	0.30	1.00000000	pigeons
Cript	0.32	1.00000000	pigeons
Srek1ip1	0.01	1.00000000	pigeons
Fam32a	-0.33	1.00000000	pigeons
Oxsr1	-1.66	1.00000000	pigeons
Npepps	-0.08	1.00000000	pigeons
Bet1	0.33	1.00000000	pigeons
Rab8a	0.20	1.00000000	pigeons
Nr3c2	-1.49	1.00000000	pigeons
Nsun3	-0.03	1.00000000	pigeons
Snapc3	-0.11	1.00000000	pigeons
Nhlrc3	0.09	1.00000000	pigeons
Phex	-0.41	1.00000000	pigeons
Styk1	-0.35	1.00000000	pigeons
Lrrc58	-0.11	1.00000000	pigeons
Ankrd33b	-0.22	1.00000000	pigeons
Fastkd3	-0.14	1.00000000	pigeons
Rida	-0.22	1.00000000	pigeons
Anks6	0.02	1.00000000	pigeons
Snap47	0.07	1.00000000	pigeons
Tmx4	0.07	1.00000000	pigeons
Ephx2	0.09	1.00000000	pigeons

Vegfa	-0.20	1.00000000	pigeons
Ntpcr	0.10	1.00000000	pigeons
Amer1	-0.22	1.00000000	pigeons
Tspan5	-0.77	1.00000000	pigeons
Sh3rf1	-0.04	1.00000000	pigeons
Anapc4	-0.22	1.00000000	pigeons
Sfxn5	0.07	1.00000000	pigeons
Ppwd1	-0.17	1.00000000	pigeons
Nln	-0.22	1.00000000	pigeons
Cdc14b	-0.30	1.00000000	pigeons
Il7r	-0.22	1.00000000	pigeons
Zfyve19	-0.03	1.00000000	pigeons
Ndufaf1	0.07	1.00000000	pigeons
Prpf39	-0.16	1.00000000	pigeons
Ptgr2	-0.39	1.00000000	pigeons
Vps51	-0.19	1.00000000	pigeons
Trak2	0.05	1.00000000	pigeons
Tsn	0.06	1.00000000	pigeons
Thsd7b	0.04	1.00000000	pigeons
Atrnl1	-0.01	1.00000000	pigeons
Chst3	-0.17	1.00000000	pigeons
Cops7b	-0.14	1.00000000	pigeons
Capn10	0.07	1.00000000	pigeons
Prkci	-0.40	1.00000000	pigeons
Wwtr1	-0.08	1.00000000	pigeons
Ccdc71	-0.23	1.00000000	pigeons
Edc4	0.03	1.00000000	pigeons
Bbs2	0.11	1.00000000	pigeons
Lsm11	-0.21	1.00000000	pigeons
Nptx2	0.06	1.00000000	pigeons
Top3a	0.07	1.00000000	pigeons
Ears2	-0.07	1.00000000	pigeons
Epb41l1	-0.40	1.00000000	pigeons
Dgke	-0.17	1.00000000	pigeons
Ass1	-0.11	1.00000000	pigeons
Pdk2	0.11	1.00000000	pigeons
Vamp3	-0.12	1.00000000	pigeons
Prkcz	-0.12	1.00000000	pigeons
Ubiad1	-0.23	1.00000000	pigeons
Cdc42se1	-0.38	1.00000000	pigeons
Trio	-0.62	1.00000000	pigeons
Lamc1	-1.25	1.00000000	pigeons
Net1	-0.81	1.00000000	pigeons
Abtb1	-0.01	1.00000000	pigeons
Naa25	0.41	1.00000000	pigeons
Slc7a3	0.35	1.00000000	pigeons

Dpp8	0.27	1.00000000	pigeons
Sgcd	0.37	1.00000000	pigeons
Dhrs7b	0.21	1.00000000	pigeons
Daglb	0.11	1.00000000	pigeons
Ipo9	-0.21	1.00000000	pigeons
Pole4	0.31	1.00000000	pigeons
Ints6	-1.92	1.00000000	pigeons
Sumo1	-0.40	1.00000000	pigeons
Oaz2	-0.30	1.00000000	pigeons
Pebp1	0.29	1.00000000	pigeons
Tubb6	-0.16	1.00000000	pigeons
Gpatch1	-0.23	1.00000000	pigeons
Rsrc1	0.00	1.00000000	pigeons
Phf12	-0.14	1.00000000	pigeons
Taf3	-0.48	1.00000000	pigeons
Rprd2	-0.22	1.00000000	pigeons
Ube2k	-0.17	1.00000000	pigeons
Snap91	0.36	1.00000000	pigeons
Tnfsf15	0.25	1.00000000	pigeons
Aff2	0.59	1.00000000	pigeons
Idh3a	0.31	1.00000000	pigeons
Tfec	1.02	1.00000000	pigeons
Stx11	1.05	1.00000000	pigeons
Pstk	0.34	1.00000000	pigeons
Homer2	0.35	1.00000000	pigeons
Gps1	0.32	1.00000000	pigeons
Trib1	-0.62	1.00000000	pigeons
Atp2b4	-0.19	1.00000000	pigeons
Zdhhc17	-0.41	1.00000000	pigeons
Tbxas1	1.10	1.00000000	pigeons
Immt	0.09	1.00000000	pigeons
Riok3	-0.82	1.00000000	pigeons
Nup214	-0.17	1.00000000	pigeons
Phka2	0.07	1.00000000	pigeons
Snx10	-0.13	1.00000000	pigeons
Fndc3a	-0.06	1.00000000	pigeons
Ric8b	0.18	1.00000000	pigeons
Dnaja1	-0.02	1.00000000	pigeons
Snap29	-0.33	1.00000000	pigeons
Usp48	-0.31	1.00000000	pigeons
Pmp22	-1.32	1.00000000	pigeons
Tle3	-0.26	1.00000000	pigeons
Nek10	0.74	1.00000000	pigeons
Hs6st2	0.26	1.00000000	pigeons
Pofut2	0.19	1.00000000	pigeons
Tepsin	0.23	1.00000000	pigeons

Alg2	0.18	1.00000000	pigeons
Fmr1	-0.60	1.00000000	pigeons
Nuak1	0.08	1.00000000	pigeons
Samd4	0.34	1.00000000	pigeons
Gusb	0.22	1.00000000	pigeons
Snx3	0.37	1.00000000	pigeons
Zcchc14	-0.08	1.00000000	pigeons
Yipf1	-0.17	1.00000000	pigeons
Tbc1d14	0.40	1.00000000	pigeons
Anxa5	-1.07	1.00000000	pigeons
Slc30a9	-0.16	1.00000000	pigeons
Llph	-0.06	1.00000000	pigeons
Gpr107	0.04	1.00000000	pigeons
Casp8ap2	-0.11	1.00000000	pigeons
Zmynd11	-0.04	1.00000000	pigeons
Fyttd1	-0.20	1.00000000	pigeons
Timm13	-0.39	1.00000000	pigeons
Ppp2r2a	-0.48	1.00000000	pigeons
Cnn3	-1.30	1.00000000	pigeons
Rala	0.01	1.00000000	pigeons
Zfp277	0.03	1.00000000	pigeons
Sult4a1	0.21	1.00000000	pigeons
Rint1	0.12	1.00000000	pigeons
Gtf3a	0.13	1.00000000	pigeons
Ints8	-0.02	1.00000000	pigeons
Bloc1s4	-0.26	1.00000000	pigeons
Blvra	0.24	1.00000000	pigeons
Tcirg1	0.24	1.00000000	pigeons
Vps18	-0.01	1.00000000	pigeons
Phospho2	0.12	1.00000000	pigeons
Tctn3	0.27	1.00000000	pigeons
Fas	-0.08	1.00000000	pigeons
Dnajc19	0.11	1.00000000	pigeons
Dusp15	0.16	1.00000000	pigeons
Acads	-0.34	1.00000000	pigeons
Kctd9	0.05	1.00000000	pigeons
Ndufaf7	0.29	1.00000000	pigeons
Akr1a1	0.18	1.00000000	pigeons
Bcl9l	0.20	1.00000000	pigeons
Ptafr	0.24	1.00000000	pigeons
Slc38a2	0.24	1.00000000	pigeons
Dennd6b	0.16	1.00000000	pigeons
Chka	-0.60	1.00000000	pigeons
Ralb	-0.07	1.00000000	pigeons
Ppie	-0.35	1.00000000	pigeons
Klf12	-0.96	1.00000000	pigeons

Rin3	-0.14	1.00000000	pigeons
Akap10	-0.15	1.00000000	pigeons
Cry1	-0.27	1.00000000	pigeons
Ebag9	-0.17	1.00000000	pigeons
Dlg1	-0.34	1.00000000	pigeons
Idh2	0.01	1.00000000	pigeons
Pld4	0.26	1.00000000	pigeons
Disp1	-0.85	1.00000000	pigeons
Lonp1	0.25	1.00000000	pigeons
Galnt1	-0.21	1.00000000	pigeons
Golim4	-0.12	1.00000000	pigeons
Eif4h	-0.43	1.00000000	pigeons
Rubcnl	0.00	1.00000000	pigeons
Slc12a4	-0.09	1.00000000	pigeons
Rwdd1	0.04	1.00000000	pigeons
Stxbp6	0.42	1.00000000	pigeons
Usp37	-0.09	1.00000000	pigeons
Ube4a	0.07	1.00000000	pigeons
Abce1	-0.37	1.00000000	pigeons
Eri1	-1.09	1.00000000	pigeons
Naxd	-0.41	1.00000000	pigeons
Plxnb2	0.39	1.00000000	pigeons
Eya1	0.50	1.00000000	pigeons
Apool	0.12	1.00000000	pigeons
Nup155	-0.15	1.00000000	pigeons
Pus1	0.16	1.00000000	pigeons
Adora2b	-0.23	1.00000000	pigeons
Sbds	0.26	1.00000000	pigeons
Nemp1	0.03	1.00000000	pigeons
Cmas	-0.18	1.00000000	pigeons
Elovl6	0.05	1.00000000	pigeons
St3gal5	-0.12	1.00000000	pigeons
Rpp25l	-0.04	1.00000000	pigeons
Tln2	0.32	1.00000000	pigeons
Tcf7	0.60	1.00000000	pigeons
Asxl1	0.11	1.00000000	pigeons
P4hb	0.20	1.00000000	pigeons
Aldh4a1	0.12	1.00000000	pigeons
Fkbp5	-0.13	1.00000000	pigeons
Etv6	-0.34	1.00000000	pigeons
Ola1	-0.29	1.00000000	pigeons
Iqgap1	0.14	1.00000000	pigeons
Tfdp1	0.06	1.00000000	pigeons
Pdia3	-0.25	1.00000000	pigeons
Abcb7	-0.46	1.00000000	pigeons
Clta	-0.36	1.00000000	pigeons

Brwd1	-0.16	1.00000000	pigeons
Tdg	-0.07	1.00000000	pigeons
Thumpd3	-0.21	1.00000000	pigeons
Gfod1	-0.33	1.00000000	pigeons
Prpf6	-0.39	1.00000000	pigeons
Nsrp1	-0.39	1.00000000	pigeons
Pus10	0.09	1.00000000	pigeons
Vkorc1l1	0.03	1.00000000	pigeons
Trim24	0.07	1.00000000	pigeons
Eif4a3l1	-0.08	1.00000000	pigeons
Fbxo28	-0.03	1.00000000	pigeons
Cfap36	0.14	1.00000000	pigeons
Brix1	-0.06	1.00000000	pigeons
Prpf38a	0.12	1.00000000	pigeons
Itm2c	0.01	1.00000000	pigeons
Ogfod3	0.00	1.00000000	pigeons
Zmpste24	0.06	1.00000000	pigeons
Trappc3	-0.04	1.00000000	pigeons
Ankrd46	-0.81	1.00000000	pigeons
Atp6v1b2	0.11	1.00000000	pigeons
Arl8b	-1.02	1.00000000	pigeons
Cdk17	0.02	1.00000000	pigeons
Ptk2	-0.04	1.00000000	pigeons
Fundc1	0.10	1.00000000	pigeons
Rmdn1	0.11	1.00000000	pigeons
Dhx29	-0.07	1.00000000	pigeons
Rnls	-0.03	1.00000000	pigeons
Brpf1	-0.04	1.00000000	pigeons
Gga3	0.08	1.00000000	pigeons
Ak1	0.11	1.00000000	pigeons
Errfi1	-0.39	1.00000000	pigeons
Ctps	-0.09	1.00000000	pigeons
Dennd2d	0.10	1.00000000	pigeons
Eif3j2	-0.32	1.00000000	pigeons
Fmnl2	-0.43	1.00000000	pigeons
Ndfip1	-0.35	1.00000000	pigeons
Ssr2	-0.21	1.00000000	pigeons
Tmem33	0.02	1.00000000	pigeons
Copg1	0.21	1.00000000	pigeons
Cog7	0.02	1.00000000	pigeons
Yeats4	0.01	1.00000000	pigeons
Cct7	0.24	1.00000000	pigeons
Ube2j2	0.08	1.00000000	pigeons
4932438A13Rik	-0.50	1.00000000	pigeons
Exosc7	0.14	1.00000000	pigeons
Stam	-0.05	1.00000000	pigeons

Crls1	0.42	1.00000000	pigeons
Dgkh	-1.23	1.00000000	pigeons
Dnaja3	-0.81	1.00000000	pigeons
Nucks1	-0.01	1.00000000	pigeons
Atg101	-0.60	1.00000000	pigeons
R3hdm1	0.67	1.00000000	pigeons
Gpx4	-0.14	1.00000000	pigeons
Ubap1	-0.24	1.00000000	pigeons
Ube2a	-0.08	1.00000000	pigeons
Necap1	-0.31	1.00000000	pigeons
Supt20	0.00	1.00000000	pigeons
Tatdn3	0.01	1.00000000	pigeons
Plekhm3	-0.18	1.00000000	pigeons
Osbpl2	0.23	1.00000000	pigeons
Timp2	-0.20	1.00000000	pigeons
Timm22	0.12	1.00000000	pigeons
Nav1	0.14	1.00000000	pigeons
Clk2	-0.14	1.00000000	pigeons
Cct4	-0.48	1.00000000	pigeons
Psma4	-0.37	1.00000000	pigeons
Flt3	0.36	1.00000000	pigeons
Uprt	0.05	1.00000000	pigeons
Trmt61a	-0.19	1.00000000	pigeons
Cpne2	0.11	1.00000000	pigeons
Tbcd	0.07	1.00000000	pigeons
Gpatch3	-0.09	1.00000000	pigeons
2510039O18Rik	-0.28	1.00000000	pigeons
Bcap29	-0.21	1.00000000	pigeons
Zbtb33	0.32	1.00000000	pigeons
Kat2b	-0.02	1.00000000	pigeons
Acad9	0.11	1.00000000	pigeons
Ccdc91	-0.75	1.00000000	pigeons
Tbc1d32	0.37	1.00000000	pigeons
Fam234a	-0.08	1.00000000	pigeons
Tti2	0.20	1.00000000	pigeons
Ndufb10	-0.38	1.00000000	pigeons
Ythdf1	-0.36	1.00000000	pigeons
Ngly1	-0.94	1.00000000	pigeons
Atp7a	-0.35	1.00000000	pigeons
Naa50	0.22	1.00000000	pigeons
Srsf3	-0.01	1.00000000	pigeons
Helb	-0.10	1.00000000	pigeons
Sugt1	0.01	1.00000000	pigeons
Sec22c	-0.35	1.00000000	pigeons
Eif1b	-0.33	1.00000000	pigeons
Bphl	0.12	1.00000000	pigeons

Pigyl	0.13	1.00000000	pigeons
Fbxo3	-0.10	1.00000000	pigeons
Hmces	-0.17	1.00000000	pigeons
Ubn1	0.07	1.00000000	pigeons
Tmem94	-0.15	1.00000000	pigeons
Ptges2	0.18	1.00000000	pigeons
Taf11	0.16	1.00000000	pigeons
Gpr34	0.55	1.00000000	pigeons
Proser1	0.04	1.00000000	pigeons
Satb1	-0.10	1.00000000	pigeons
Lrmda	0.21	1.00000000	pigeons
Fgd3	-0.21	1.00000000	pigeons
Ddb1	0.00	1.00000000	pigeons
Katnbl1	-0.03	1.00000000	pigeons
Slc25a28	0.22	1.00000000	pigeons
Noc2l	0.04	1.00000000	pigeons
Ssbp1	0.13	1.00000000	pigeons
Tmem65	-0.34	1.00000000	pigeons
Plekha5	-0.30	1.00000000	pigeons
Bend7	-0.02	1.00000000	pigeons
Iffo1	-0.20	1.00000000	pigeons
Tubgcp5	-0.18	1.00000000	pigeons
Xylb	-0.10	1.00000000	pigeons
Lypla1	-0.21	1.00000000	pigeons
Trmt11	-0.29	1.00000000	pigeons
Cwh43	-0.07	1.00000000	pigeons
Sptlc1	-0.28	1.00000000	pigeons
Itpka	-0.52	1.00000000	pigeons
G2e3	-0.09	1.00000000	pigeons
Nubpl	-0.62	1.00000000	pigeons
Tfpi	0.11	1.00000000	pigeons
Dclre1a	-0.17	1.00000000	pigeons
Echdc2	-0.16	1.00000000	pigeons
Gng12	-0.35	1.00000000	pigeons
Tpx2	-0.20	1.00000000	pigeons
Derl3	-0.03	1.00000000	pigeons
Tbl2	-0.10	1.00000000	pigeons
Tox	-1.04	1.00000000	pigeons
Dynlt1a	-0.05	1.00000000	pigeons
Snx19	-0.15	1.00000000	pigeons
Sirt6	0.00	1.00000000	pigeons
Sqstm1	-0.19	1.00000000	pigeons
Nckap1	-1.38	1.00000000	pigeons
Per2	-0.02	1.00000000	pigeons
Bckdhb	0.16	1.00000000	pigeons
Lonp2	-0.14	1.00000000	pigeons

Agpat5	-0.56	1.00000000	pigeons
Tmem248	-0.20	1.00000000	pigeons
Pls3	0.05	1.00000000	pigeons
Dync2h1	-0.35	1.00000000	pigeons
Etfrf1	-0.08	1.00000000	pigeons
Popdc2	0.13	1.00000000	pigeons
Taf5l	-0.93	1.00000000	pigeons
Ap2m1	-0.49	1.00000000	pigeons
Rnpc3	0.00	1.00000000	pigeons
Rtca	-0.34	1.00000000	pigeons
Pstpip1	0.00	1.00000000	pigeons
Ints2	-0.09	1.00000000	pigeons
Cpsf4	-0.22	1.00000000	pigeons
Surf6	-0.17	1.00000000	pigeons
Eif4ebp1	-0.36	1.00000000	pigeons
Ndufs2	-0.21	1.00000000	pigeons
Unk	-0.62	1.00000000	pigeons
Srfbp1	-0.67	1.00000000	pigeons
Slk	-0.41	1.00000000	pigeons
Parvg	0.08	1.00000000	pigeons
Psmg1	-0.13	1.00000000	pigeons
Zbtb24	-0.23	1.00000000	pigeons
Zcchc7	0.02	1.00000000	pigeons
Cc2d1b	-0.06	1.00000000	pigeons
Rc3h2	-0.32	1.00000000	pigeons
Il18	-0.11	1.00000000	pigeons
Ehmt1	-0.49	1.00000000	pigeons
Cisd1	-0.37	1.00000000	pigeons
Kmt2c	0.01	1.00000000	pigeons
Ago2	-0.99	1.00000000	pigeons
Pola1	-0.03	1.00000000	pigeons
Acaa2	-0.01	1.00000000	pigeons
Dhx38	-0.44	1.00000000	pigeons
Clcc1	-0.42	1.00000000	pigeons
Gnpnat1	-0.19	1.00000000	pigeons
Adat1	0.10	1.00000000	pigeons
Zbtb48	-0.16	1.00000000	pigeons
Dpp9	-0.25	1.00000000	pigeons
Reep5	0.12	1.00000000	pigeons
Arhgef3	-1.55	1.00000000	pigeons
Tnrc6b	-1.27	1.00000000	pigeons
Hnrnpr	-0.19	1.00000000	pigeons
Adcy9	-1.20	1.00000000	pigeons
Cnn2	0.03	1.00000000	pigeons
Parp16	-0.33	1.00000000	pigeons
Dnm1l	-0.48	1.00000000	pigeons

Ints1	-0.06	1.00000000	pigeons
Fam107b	0.08	1.00000000	pigeons
Psmg2	-0.42	1.00000000	pigeons
Asxl2	-0.42	1.00000000	pigeons
Ccdc186	-0.34	1.00000000	pigeons
Notch1	-0.39	1.00000000	pigeons
Sh3bgrl3	0.00	1.00000000	pigeons
Ndufb3	-0.42	1.00000000	pigeons
Naxe	0.11	1.00000000	pigeons
Fbrsl1	0.26	1.00000000	pigeons
Dyrk1a	-0.35	1.00000000	pigeons
2210408I21Rik	-0.02	1.00000000	pigeons
Thoc7	0.24	1.00000000	pigeons
Pdk4	-0.11	1.00000000	pigeons
Spry1	0.06	1.00000000	pigeons
Brip1	0.07	1.00000000	pigeons
Vps39	0.08	1.00000000	pigeons
Opa1	0.07	1.00000000	pigeons
Blzf1	-0.14	1.00000000	pigeons
Hsph1	0.04	1.00000000	pigeons
Rabl3	-0.17	1.00000000	pigeons
Decr1	-0.03	1.00000000	pigeons
Tdp2	-0.17	1.00000000	pigeons
Hadha	0.10	1.00000000	pigeons
Rnps1	-0.06	1.00000000	pigeons
Sla	0.50	1.00000000	pigeons
Emc7	-0.39	1.00000000	pigeons
Esco1	-0.05	1.00000000	pigeons
Yme1l1	-0.33	1.00000000	pigeons
Fbxo48	0.14	1.00000000	pigeons
Hacd2	-0.50	1.00000000	pigeons
Elavl1	-0.31	1.00000000	pigeons
Ankrd13d	-0.24	1.00000000	pigeons
Gnl3	-0.48	1.00000000	pigeons
Tmem154	0.76	1.00000000	pigeons
Ube2g1	-0.63	1.00000000	pigeons
Foxn3	0.53	1.00000000	pigeons
Nans	-0.19	1.00000000	pigeons
Zc2hc1a	-0.96	1.00000000	pigeons
Asb14	-0.20	1.00000000	pigeons
Fbxo21	-0.32	1.00000000	pigeons
Nectin1	-0.54	1.00000000	pigeons
Ogdh	0.24	1.00000000	pigeons
Ttll12	-0.28	1.00000000	pigeons
Vps36	-0.24	1.00000000	pigeons
Zbtb18	-0.09	1.00000000	pigeons

Prrc1	-0.54	1.00000000	pigeons
Prkar1a	-0.44	1.00000000	pigeons
Stk3	-0.60	1.00000000	pigeons
Golt1b	-0.37	1.00000000	pigeons
Hsd17b4	-0.16	1.00000000	pigeons
Zc3h13	-0.51	1.00000000	pigeons
Ly86	-0.11	1.00000000	pigeons
B4galt1	-0.23	1.00000000	pigeons
Tanc2	-0.22	1.00000000	pigeons
Bicd1	-1.21	1.00000000	pigeons
Kdelr2	-0.17	1.00000000	pigeons
Tspan13	-0.18	1.00000000	pigeons
Ssrp1	-0.21	1.00000000	pigeons
Ascc1	-0.17	1.00000000	pigeons
Ctss	-0.54	1.00000000	pigeons
Irf8	-1.04	1.00000000	pigeons
Phf13	0.28	1.00000000	pigeons
Smchd1	-0.39	1.00000000	pigeons
Casp2	-0.71	1.00000000	pigeons
Slc16a10	-0.45	1.00000000	pigeons
Tle1	-0.49	1.00000000	pigeons
Rilpl1	-0.76	1.00000000	pigeons
Slc23a2	-0.28	1.00000000	pigeons
Sh3bgrl	-0.37	1.00000000	pigeons
Zfp830	-0.17	1.00000000	pigeons
Smu1	-0.18	1.00000000	pigeons
Klhl24	0.00	1.00000000	pigeons
Slc2a8	-0.11	1.00000000	pigeons
Aurkaip1	-0.26	1.00000000	pigeons
Phf10	0.01	1.00000000	pigeons
Slc25a5	-0.06	1.00000000	pigeons
Cntln	0.01	1.00000000	pigeons
Uggt1	-0.16	1.00000000	pigeons
Tra2a	-0.66	1.00000000	pigeons
P4ha1	-0.52	1.00000000	pigeons
Pts	0.10	1.00000000	pigeons
Ccnd1	0.38	1.00000000	pigeons
Hlcs	0.33	1.00000000	pigeons
Eno1b	-0.08	1.00000000	pigeons
Ezh2	-0.58	1.00000000	pigeons
Vps54	-0.12	1.00000000	pigeons
Rfc5	-0.24	1.00000000	pigeons
Erg	-1.25	1.00000000	pigeons
Pdcl	-0.16	1.00000000	pigeons
Suco	-0.34	1.00000000	pigeons
Paqr7	-0.15	1.00000000	pigeons

Nsl1	-0.49	1.00000000	pigeons
Scpep1	-0.26	1.00000000	pigeons
Stx2	-0.51	1.00000000	pigeons
Kdm1a	-0.22	1.00000000	pigeons
Ppp3ca	-0.21	1.00000000	pigeons
Homer1	-0.09	1.00000000	pigeons
Fam76a	-0.40	1.00000000	pigeons
Emp1	0.45	1.00000000	pigeons
Dpysl2	-0.40	1.00000000	pigeons
Flnb	-1.33	1.00000000	pigeons
Lsm14a	-0.13	1.00000000	pigeons
Rbm17	0.29	1.00000000	pigeons
Ly96	-0.13	1.00000000	pigeons
Sec13	0.04	1.00000000	pigeons
Vcpip1	-0.10	1.00000000	pigeons
Vezf1	-0.04	1.00000000	pigeons
Nfyc	0.01	1.00000000	pigeons
Riok1	-0.29	1.00000000	pigeons
Cep162	-0.69	1.00000000	pigeons
Dync1li2	-1.00	1.00000000	pigeons
Kctd12	-0.32	1.00000000	pigeons
Srgap3	0.03	1.00000000	pigeons
Kdm2b	-0.29	1.00000000	pigeons
Rnf20	-0.21	1.00000000	pigeons
Slc22a15	0.83	1.00000000	pigeons
S100a10	-0.64	1.00000000	pigeons
Psen1	-0.01	1.00000000	pigeons
Tiam1	0.74	1.00000000	pigeons
Usp45	-0.31	1.00000000	pigeons
Actb	0.15	1.00000000	pigeons
Usp16	-0.24	1.00000000	pigeons
Rsu1	-0.28	1.00000000	pigeons
Jak2	-0.36	1.00000000	pigeons
Klf6	-0.20	1.00000000	pigeons
Arid2	-0.54	1.00000000	pigeons
Ccser2	-0.12	1.00000000	pigeons
Tacc1	-0.65	1.00000000	pigeons
Rab3gap2	-0.80	1.00000000	pigeons
Ndufs4	-0.30	1.00000000	pigeons
Sppl2a	-0.05	1.00000000	pigeons
Gjd4	-0.35	1.00000000	pigeons
Hnrnpu	-0.56	1.00000000	pigeons
Npc2	-0.06	1.00000000	pigeons
Pak1ip1	-0.22	1.00000000	pigeons
Zcchc17	0.27	1.00000000	pigeons
Tnfaip2	1.93	1.00000000	pigeons

Erc1	-1.48	1.00000000	pigeons
Larp4b	-0.81	1.00000000	pigeons
Slc9a6	0.16	1.00000000	pigeons
Psmc3	0.07	1.00000000	pigeons
Picalm	-0.57	1.00000000	pigeons
Cd9	-0.17	1.00000000	pigeons
Nabp1	0.23	1.00000000	pigeons
Srsf2	-0.53	1.00000000	pigeons
Tkt	-0.05	1.00000000	pigeons
Eef2	0.14	1.00000000	pigeons
Inpp5d	-0.02	1.00000000	pigeons
Larp1b	-0.24	1.00000000	pigeons
Trak1	-0.21	1.00000000	pigeons
Efhd1	0.09	1.00000000	pigeons
Smad5	-1.52	1.00000000	pigeons
Lin54	-0.63	1.00000000	pigeons
Dennd6a	-0.70	1.00000000	pigeons
Sprtn	-0.56	1.00000000	pigeons
Commd8	-0.39	1.00000000	pigeons
Gabarapl2	-0.30	1.00000000	pigeons
Gnal	0.51	1.00000000	pigeons
Ddx3y	-0.29	1.00000000	pigeons
Bcor	0.13	1.00000000	pigeons
Atp6v0a1	-0.32	1.00000000	pigeons
Ints10	0.27	1.00000000	pigeons
Ppm1j	0.19	1.00000000	pigeons
Abhd17a	0.18	1.00000000	pigeons
Ghitm	-0.52	1.00000000	pigeons
Sipa1l1	-0.92	1.00000000	pigeons
Htatsf1	0.09	1.00000000	pigeons
Zfp469	-0.83	1.00000000	pigeons
Yrdc	-0.78	1.00000000	pigeons
Btg2	0.20	1.00000000	pigeons
Polr2e	-0.31	1.00000000	pigeons
Optn	-0.08	1.00000000	pigeons
Hat1	0.01	1.00000000	pigeons
Itsn2	-0.97	1.00000000	pigeons
Rabl6	-0.45	1.00000000	pigeons
Hsbp1	-0.89	1.00000000	pigeons
Slc39a6	-0.32	1.00000000	pigeons
Nisch	-0.64	1.00000000	pigeons
Tcn2	-0.15	1.00000000	pigeons
Pex14	-0.01	1.00000000	pigeons
Vps26b	0.14	1.00000000	pigeons
Elf1	-0.13	1.00000000	pigeons
Pdk1	-0.05	1.00000000	pigeons

Pdcd4	0.19	1.00000000	pigeons
Dnmt3a	-0.44	1.00000000	pigeons
Fhit	-0.29	1.00000000	pigeons
Pef1	-0.47	1.00000000	pigeons
Seh1l	0.01	1.00000000	pigeons
Tfdp2	-0.05	1.00000000	pigeons
Lamtor2	-0.20	1.00000000	pigeons
Vps50	0.77	1.00000000	pigeons
Iah1	0.45	1.00000000	pigeons
Arfrp1	-0.04	1.00000000	pigeons
Puf60	-0.34	1.00000000	pigeons
Eif3m	0.11	1.00000000	pigeons
Zfp366	-1.04	1.00000000	pigeons
Phactr2	-1.26	1.00000000	pigeons
Enah	-0.78	1.00000000	pigeons
Pccb	-0.29	1.00000000	pigeons
Pak2	-0.56	1.00000000	pigeons
Clpx	-0.27	1.00000000	pigeons
Eif1	0.26	1.00000000	pigeons
Ecd	0.13	1.00000000	pigeons
Slc25a20	0.02	1.00000000	pigeons
Ncoa1	-0.26	1.00000000	pigeons
Angel2	-0.43	1.00000000	pigeons
Snx25	-0.44	1.00000000	pigeons
Abhd13	-0.08	1.00000000	pigeons
Pank4	-1.02	1.00000000	pigeons
Ppp2r5a	-0.94	1.00000000	pigeons
Hsd17b2	0.44	1.00000000	pigeons
Pms2	0.28	1.00000000	pigeons
Slc16a3	0.14	1.00000000	pigeons
Tapt1	-0.23	1.00000000	pigeons
Fam3c	-0.77	1.00000000	pigeons
Fam76b	0.14	1.00000000	pigeons
Tmem60	0.21	1.00000000	pigeons
Adgra1	0.70	1.00000000	pigeons
Rnf114	0.03	1.00000000	pigeons
Ptpre	-0.01	1.00000000	pigeons
Txndc9	-0.68	1.00000000	pigeons
Lonrf2	-0.05	1.00000000	pigeons
Ccdc117	0.05	1.00000000	pigeons
Cryl1	0.13	1.00000000	pigeons
Vps13d	-0.27	1.00000000	pigeons
Rab2a	-0.72	1.00000000	pigeons
Ncapd2	0.22	1.00000000	pigeons
Slc7a1	0.21	1.00000000	pigeons
Trmt44	0.17	1.00000000	pigeons

Nif3l1	0.17	1.00000000	pigeons
Aqp9	0.16	1.00000000	pigeons
Elmo3	0.19	1.00000000	pigeons
Pycr2	0.47	1.00000000	pigeons
Tmem39b	0.17	1.00000000	pigeons
Clasp1	-0.25	1.00000000	pigeons
Cbfa2t3	-0.07	1.00000000	pigeons
Ppp2r3c	0.22	1.00000000	pigeons
Pank1	0.51	1.00000000	pigeons
Bbs4	0.16	1.00000000	pigeons
Synj1	-0.30	1.00000000	pigeons
Ift27	0.19	1.00000000	pigeons
Pfn2	0.08	1.00000000	pigeons
Gtf2h3	-0.11	1.00000000	pigeons
Dhx8	0.06	1.00000000	pigeons
Cyfip1	0.31	1.00000000	pigeons
Fam135a	-0.89	1.00000000	pigeons
Adamts2	-0.96	1.00000000	pigeons
Ptpn14	-1.31	1.00000000	pigeons
Dock7	-0.78	1.00000000	pigeons
Ppil2	-0.04	1.00000000	pigeons
Tpp2	-0.34	1.00000000	pigeons
Atf3	0.65	1.00000000	pigeons
Tmtc3	0.00	1.00000000	pigeons
Dnajc15	-0.03	1.00000000	pigeons
Lias	-0.16	1.00000000	pigeons
Aprt	0.09	1.00000000	pigeons
Stk4	0.22	1.00000000	pigeons
Cramp1	-0.73	1.00000000	pigeons
Dnaja2	-0.16	1.00000000	pigeons
Add3	0.22	1.00000000	pigeons
Dcun1d5	-0.24	1.00000000	pigeons
Fkbp14	-0.37	1.00000000	pigeons
Klf13	-0.75	1.00000000	pigeons
Denr	0.05	1.00000000	pigeons
Frmd4a	-1.40	1.00000000	pigeons
Slc3a1	0.05	1.00000000	pigeons
Slc35b2	0.15	1.00000000	pigeons
Ankrd50	-0.94	1.00000000	pigeons
Naf1	0.20	1.00000000	pigeons
Flt4	-1.25	1.00000000	pigeons
Arhgap17	0.17	1.00000000	pigeons
Emp2	-1.58	1.00000000	pigeons
Rap1gap2	0.15	1.00000000	pigeons
Ramp2	-1.09	1.00000000	pigeons
Cdc34	0.16	1.00000000	pigeons

Pi4kb	-0.08	1.00000000	pigeons
Gramd1c	0.20	1.00000000	pigeons
Rwdd2b	0.37	1.00000000	pigeons
Tfb2m	0.13	1.00000000	pigeons
Dusp4	0.31	1.00000000	pigeons
Ivd	0.10	1.00000000	pigeons
Got1	0.15	1.00000000	pigeons
Fes	0.14	1.00000000	pigeons
Clcn7	0.28	1.00000000	pigeons
Zbtb6	0.18	1.00000000	pigeons
Abca2	0.10	1.00000000	pigeons
Pmpcb	-0.18	1.00000000	pigeons
Nudt14	0.19	1.00000000	pigeons
Snx8	0.20	1.00000000	pigeons
Alad	-0.07	1.00000000	pigeons
Nek7	-0.24	1.00000000	pigeons
Rheb	-0.37	1.00000000	pigeons
Gcnt4	-0.10	1.00000000	pigeons
Fhl3	-0.20	1.00000000	pigeons
Rbm15	-0.04	1.00000000	pigeons
Sin3b	-0.34	1.00000000	pigeons
Nifk	0.07	1.00000000	pigeons
Fubp1	-0.26	1.00000000	pigeons
Lrch1	0.24	1.00000000	pigeons
Rptor	0.10	1.00000000	pigeons
Ift46	0.29	1.00000000	pigeons
Nono	-0.43	1.00000000	pigeons
Scp2	-0.21	1.00000000	pigeons
Itpkb	-1.03	1.00000000	pigeons
Trib2	-1.32	1.00000000	pigeons
Crip1	0.10	1.00000000	pigeons
Sel1l	-0.63	1.00000000	pigeons
Crk	-1.21	1.00000000	pigeons
Psmb2	-0.46	1.00000000	pigeons
Cubn	0.07	1.00000000	pigeons
Nhsl2	0.08	1.00000000	pigeons
Rtf1	-0.07	1.00000000	pigeons
Ywhaz	-0.10	1.00000000	pigeons
Jade1	0.20	1.00000000	pigeons
AW554918	0.19	1.00000000	pigeons
Cgrrf1	0.22	1.00000000	pigeons
Dner	-0.89	1.00000000	pigeons
Hacd3	-0.07	1.00000000	pigeons
Tmem186	-0.20	1.00000000	pigeons
Znrf3	-0.72	1.00000000	pigeons
Omg	0.13	1.00000000	pigeons

Lyrm9	-0.44	1.00000000	pigeons
Dnajc11	0.05	1.00000000	pigeons
Sptan1	-0.11	1.00000000	pigeons
Ggh	0.11	1.00000000	pigeons
Sash1	0.29	1.00000000	pigeons
Cnot6l	-0.07	1.00000000	pigeons
Dcun1d1	0.03	1.00000000	pigeons
Aff1	-1.37	1.00000000	pigeons
Ppp1r8	-0.06	1.00000000	pigeons
Cab39l	0.09	1.00000000	pigeons
Etv1	0.16	1.00000000	pigeons
Tbrg4	0.12	1.00000000	pigeons
Acbd6	0.17	1.00000000	pigeons
Sdr42e1	0.22	1.00000000	pigeons
Cables2	0.15	1.00000000	pigeons
Dcxr	0.14	1.00000000	pigeons
Ccdc137	0.22	1.00000000	pigeons
Surf2	0.07	1.00000000	pigeons
Aoah	0.11	1.00000000	pigeons
Ppil3	-0.35	1.00000000	pigeons
Ift20	-0.43	1.00000000	pigeons
Zfc3h1	-0.41	1.00000000	pigeons
Ppp1ca	-0.47	1.00000000	pigeons
Plbd2	-0.56	1.00000000	pigeons
Ralgapb	-0.23	1.00000000	pigeons
Glcci1	0.01	1.00000000	pigeons
Gpalpp1	-0.52	1.00000000	pigeons
Nup205	-0.09	1.00000000	pigeons
Chchd3	0.11	1.00000000	pigeons
Tex10	-0.24	1.00000000	pigeons
Eif2b2	0.59	1.00000000	pigeons
Ldb1	-0.32	1.00000000	pigeons
Ints11	0.05	1.00000000	pigeons
Ddx20	-0.17	1.00000000	pigeons
Rab10	-0.53	1.00000000	pigeons
Leo1	-0.23	1.00000000	pigeons
Acat1	-0.12	1.00000000	pigeons
Rpgr	-0.47	1.00000000	pigeons
Abcb10	-0.32	1.00000000	pigeons
Lamp1	-0.41	1.00000000	pigeons
Rnmt	0.15	1.00000000	pigeons
Sap30	-0.12	1.00000000	pigeons
Slc25a22	-0.12	1.00000000	pigeons
Foxk1	-0.33	1.00000000	pigeons
Sec16a	-0.24	1.00000000	pigeons
Faf1	-0.63	1.00000000	pigeons

Cenpj	-0.67	1.00000000	pigeons
Utp20	-0.10	1.00000000	pigeons
Nhs	0.22	1.00000000	pigeons
Utp23	-0.09	1.00000000	pigeons
Ttc7	0.14	1.00000000	pigeons
Hint1	0.15	1.00000000	pigeons
Nadk2	0.18	1.00000000	pigeons
Dbr1	-0.27	1.00000000	pigeons
Pdf	-0.43	1.00000000	pigeons
Nf2	-0.13	1.00000000	pigeons
Tmem203	-0.77	1.00000000	pigeons
Slc25a34	-0.24	1.00000000	pigeons
Timm44	-0.07	1.00000000	pigeons
Gemin6	-0.37	1.00000000	pigeons
Jade2	-0.80	1.00000000	pigeons
Chst11	0.09	1.00000000	pigeons
Il18r1	-0.04	1.00000000	pigeons
Ndufaf6	0.17	1.00000000	pigeons
Abhd12	-0.08	1.00000000	pigeons
Ccdc170	-0.13	1.00000000	pigeons
Gins1	0.06	1.00000000	pigeons
Gng4	-0.18	1.00000000	pigeons
Otud4	-0.06	1.00000000	pigeons
Qdpr	0.07	1.00000000	pigeons
Rnf170	-0.36	1.00000000	pigeons
Idh1	0.00	1.00000000	pigeons
Whamm	0.10	1.00000000	pigeons
Cdk10	-0.10	1.00000000	pigeons
Cnot8	0.06	1.00000000	pigeons
Eif2ak1	0.06	1.00000000	pigeons
Slc9a3r1	-0.09	1.00000000	pigeons
Nos2	0.14	1.00000000	pigeons
4933427D14Rik	0.08	1.00000000	pigeons
Tmem199	-0.21	1.00000000	pigeons
Otud7b	-0.28	1.00000000	pigeons
Pdcd6	-0.42	1.00000000	pigeons
Vgll4	-0.35	1.00000000	pigeons
Cd247	-0.84	1.00000000	pigeons
Pak1	0.03	1.00000000	pigeons
Scoc	-0.19	1.00000000	pigeons
Pcgf3	-0.11	1.00000000	pigeons
Ambra1	-0.20	1.00000000	pigeons
Ldlrad3	-0.78	1.00000000	pigeons
Tns3	0.11	1.00000000	pigeons
Tmem63a	-0.18	1.00000000	pigeons
Washc5	-0.22	1.00000000	pigeons

Vps33b	0.06	1.00000000	pigeons
Pepd	0.09	1.00000000	pigeons
Slc9a5	-0.03	1.00000000	pigeons
Card11	-0.17	1.00000000	pigeons
Stmn1	0.24	1.00000000	pigeons
Prdx3	-0.29	1.00000000	pigeons
Ndufs6	-0.44	1.00000000	pigeons
Atp10a	0.78	1.00000000	pigeons
Napg	-0.29	1.00000000	pigeons
Hyi	-0.08	1.00000000	pigeons
Stxbp1	-0.54	1.00000000	pigeons
Camsap2	-1.44	1.00000000	pigeons
Ssr3	-0.56	1.00000000	pigeons
Tspan3	-1.40	1.00000000	pigeons
Farp2	0.05	1.00000000	pigeons
Klf3	-0.73	1.00000000	pigeons
Ncbp3	-0.51	1.00000000	pigeons
Fbxo7	-0.70	1.00000000	pigeons
Txn2	0.11	1.00000000	pigeons
Atp6v1a	0.11	1.00000000	pigeons
Tatdn1	-0.11	1.00000000	pigeons
Nek1	-0.21	1.00000000	pigeons
Epb41l5	-0.68	1.00000000	pigeons
Blm	-0.18	1.00000000	pigeons
Ripor1	-0.56	1.00000000	pigeons
Acss2	0.11	1.00000000	pigeons
Trafd1	-0.34	1.00000000	pigeons
Arrdc1	-0.08	1.00000000	pigeons
Samd11	0.09	1.00000000	pigeons
Ubxn6	0.00	1.00000000	pigeons
Pm20d2	-0.44	1.00000000	pigeons
Cep83	-1.17	1.00000000	pigeons
Ndufs8	-0.57	1.00000000	pigeons
Pam	-1.09	1.00000000	pigeons
Trabd	-0.13	1.00000000	pigeons
Rnaseh1	-0.12	1.00000000	pigeons
Ldah	-0.06	1.00000000	pigeons
Poc1a	0.08	1.00000000	pigeons
B4galt5	0.14	1.00000000	pigeons
Cactin	-0.24	1.00000000	pigeons
Polr3b	-0.61	1.00000000	pigeons
Pdcd5	-0.02	1.00000000	pigeons
Slc35e3	0.04	1.00000000	pigeons
Arhgef17	0.07	1.00000000	pigeons
Wdr92	-0.15	1.00000000	pigeons
Slc35f1	-0.08	1.00000000	pigeons

Gpr63	-0.06	1.00000000	pigeons
Fam120b	-0.19	1.00000000	pigeons
Arsb	-0.20	1.00000000	pigeons
Nudt12	-0.22	1.00000000	pigeons
Tyw5	-0.11	1.00000000	pigeons
Fastkd1	0.09	1.00000000	pigeons
Adam8	0.13	1.00000000	pigeons
Tmem53	-0.11	1.00000000	pigeons
Anapc13	0.14	1.00000000	pigeons
Dapk2	0.01	1.00000000	pigeons
Zfp423	-0.17	1.00000000	pigeons
Sfxn1	-0.16	1.00000000	pigeons
Tango2	0.07	1.00000000	pigeons
Cdc42ep4	-0.11	1.00000000	pigeons
Exosc2	-0.16	1.00000000	pigeons
Smtnl2	-0.08	1.00000000	pigeons
Aldoc	0.15	1.00000000	pigeons
Nsf	0.05	1.00000000	pigeons
Fam118b	0.08	1.00000000	pigeons
Zfp706	-0.26	1.00000000	pigeons
Xpo1	-0.68	1.00000000	pigeons
Wdr3	0.04	1.00000000	pigeons
Cmtr1	-0.05	1.00000000	pigeons
Tmem11	-0.58	1.00000000	pigeons
Ipo8	-0.04	1.00000000	pigeons
Pcyt1b	-0.05	1.00000000	pigeons
Alkbh5	-0.04	1.00000000	pigeons
Ntmt1	0.07	1.00000000	pigeons
Pcsk7	-0.14	1.00000000	pigeons
Hells	-0.50	1.00000000	pigeons
Atp1a1	-0.87	1.00000000	pigeons
Hdac2	-0.51	1.00000000	pigeons
Cdv3	-0.38	1.00000000	pigeons
Shtn1	-0.29	1.00000000	pigeons
Ybx1	-0.60	1.00000000	pigeons
Ric1	-0.08	1.00000000	pigeons
Rictor	-0.82	1.00000000	pigeons
Cops8	-0.48	1.00000000	pigeons
Tfrc	-1.41	1.00000000	pigeons
Nolc1	-0.19	1.00000000	pigeons
Pwp1	0.10	1.00000000	pigeons
Suv39h2	-0.34	1.00000000	pigeons
Dynlt3	0.69	1.00000000	pigeons
Gtf2e1	0.07	1.00000000	pigeons
Wdr35	-1.12	1.00000000	pigeons
Bpnt1	-0.23	1.00000000	pigeons

Rnf8	-0.33	1.00000000	pigeons
Abhd18	-0.36	1.00000000	pigeons
Tmem229b	-0.32	1.00000000	pigeons
Pdia5	-0.19	1.00000000	pigeons
Taf5	-0.50	1.00000000	pigeons
D2hgdh	-0.33	1.00000000	pigeons
Zfpm1	-0.40	1.00000000	pigeons
Grk6	-0.49	1.00000000	pigeons
Thg1l	-0.10	1.00000000	pigeons
Tnfrsf25	-0.56	1.00000000	pigeons
Apba3	-0.34	1.00000000	pigeons
Trappc12	-0.44	1.00000000	pigeons
Heatr3	0.16	1.00000000	pigeons
Fzr1	-0.59	1.00000000	pigeons
Ipmk	-0.65	1.00000000	pigeons
Slc37a3	-0.16	1.00000000	pigeons
Acot13	0.05	1.00000000	pigeons
Dph5	-0.23	1.00000000	pigeons
Fndc3b	-0.21	1.00000000	pigeons
Lrrc8a	-0.26	1.00000000	pigeons
Strn	-0.19	1.00000000	pigeons
Wasl	-0.22	1.00000000	pigeons
Dusp7	-0.45	1.00000000	pigeons
Thap12	-0.19	1.00000000	pigeons
Usp5	-0.24	1.00000000	pigeons
Slc46a3	0.00	1.00000000	pigeons
Pitrm1	-0.02	1.00000000	pigeons
Ccdc28a	-0.06	1.00000000	pigeons
Smox	0.01	1.00000000	pigeons
Aup1	-0.39	1.00000000	pigeons
Vil1	-0.12	1.00000000	pigeons
Sec22a	-0.05	1.00000000	pigeons
DPCD	0.06	1.00000000	pigeons
Ddx59	0.06	1.00000000	pigeons
Gpc1	-0.14	1.00000000	pigeons
Wdr81	-0.14	1.00000000	pigeons
Srm	-0.32	1.00000000	pigeons
Rab7b	0.06	1.00000000	pigeons
Fbxo22	-0.53	1.00000000	pigeons
Ncald	-0.17	1.00000000	pigeons
Abhd2	0.00	1.00000000	pigeons
Ppm1e	-0.06	1.00000000	pigeons
St3gal3	-0.02	1.00000000	pigeons
Syt11	0.05	1.00000000	pigeons
Lmo3	-0.18	1.00000000	pigeons
Cpq	-0.26	1.00000000	pigeons

Armt1	-0.14	1.00000000	pigeons
Fam98a	-0.25	1.00000000	pigeons
Lef1	-0.24	1.00000000	pigeons
Utp15	-0.36	1.00000000	pigeons
Lgals3	0.06	1.00000000	pigeons
Nrxn3	0.05	1.00000000	pigeons
Btbd6	-0.27	1.00000000	pigeons
Inhbb	-0.72	1.00000000	pigeons
Osgepl1	0.05	1.00000000	pigeons
Stoml1	0.12	1.00000000	pigeons
Ern1	-0.47	1.00000000	pigeons
Dhx36	-0.53	1.00000000	pigeons
Zbtb41	-0.46	1.00000000	pigeons
Prkx	-0.12	1.00000000	pigeons
Pla2r1	-0.54	1.00000000	pigeons
Faap100	-0.24	1.00000000	pigeons
Lrig2	-0.29	1.00000000	pigeons
Stim1	-0.25	1.00000000	pigeons
Phyh	-0.30	1.00000000	pigeons
Dpf3	-1.00	1.00000000	pigeons
Ctnnbl1	-0.52	1.00000000	pigeons
Spata2	-0.42	1.00000000	pigeons
Usp34	-0.24	1.00000000	pigeons
Tmsb4x	-0.11	1.00000000	pigeons
Clasp2	-0.47	1.00000000	pigeons
Cfap97	-0.52	1.00000000	pigeons
St6galnac4	-0.23	1.00000000	pigeons
Atp8a1	-0.88	1.00000000	pigeons
Amn1	-0.21	1.00000000	pigeons
Samd12	-0.46	1.00000000	pigeons
Dscc1	0.02	1.00000000	pigeons
Cdk14	-0.38	1.00000000	pigeons
Lin9	-0.18	1.00000000	pigeons
Col12a1	-0.31	1.00000000	pigeons
Napb	-0.16	1.00000000	pigeons
Tbc1d8b	-0.42	1.00000000	pigeons
Lonrf1	-0.45	1.00000000	pigeons
Evc	-0.47	1.00000000	pigeons
Pigh	-0.35	1.00000000	pigeons
Abi2	-0.32	1.00000000	pigeons
Umps	-0.02	1.00000000	pigeons
Trim59	-0.41	1.00000000	pigeons
Prtg	-0.49	1.00000000	pigeons
Pparg	-0.12	1.00000000	pigeons
Yipf5	-0.13	1.00000000	pigeons
Plagl2	-0.17	1.00000000	pigeons

Ddx55	-0.34	1.00000000	pigeons
Tbc1d13	-0.46	1.00000000	pigeons
Rpusd4	-0.37	1.00000000	pigeons
Brf2	-0.35	1.00000000	pigeons
Wars2	-0.19	1.00000000	pigeons
Erich1	-0.23	1.00000000	pigeons
Heph	-0.19	1.00000000	pigeons
Isca2	-0.11	1.00000000	pigeons
Fan1	-0.16	1.00000000	pigeons
Txnrd3	-0.16	1.00000000	pigeons
Lrp1	-0.04	1.00000000	pigeons
Arhgap5	-0.84	1.00000000	pigeons
Togaram1	0.26	1.00000000	pigeons
Exoc3	-0.45	1.00000000	pigeons
Amot	-0.73	1.00000000	pigeons
Polr3a	-0.23	1.00000000	pigeons
Gdpd5	-0.27	1.00000000	pigeons
Bop1	0.05	1.00000000	pigeons
Tnni2	-0.15	1.00000000	pigeons
Scrn3	-0.36	1.00000000	pigeons
Zdhhc14	-0.32	1.00000000	pigeons
Ly75	-0.65	1.00000000	pigeons
Uchl5	-0.36	1.00000000	pigeons
Actn1	-0.30	1.00000000	pigeons
Fdx1	-0.43	1.00000000	pigeons
Paxbp1	-0.89	1.00000000	pigeons
Dock1	-0.99	1.00000000	pigeons
Eif3h	0.02	1.00000000	pigeons
Chmp4b	-0.40	1.00000000	pigeons
Polr3e	-0.45	1.00000000	pigeons
Snapc4	-0.42	1.00000000	pigeons
Lsm1	-0.24	1.00000000	pigeons
Pds5b	-0.24	1.00000000	pigeons
Nup107	-0.41	1.00000000	pigeons
Stag2	-0.53	1.00000000	pigeons
Fig4	-0.27	1.00000000	pigeons
Dnajb14	0.00	1.00000000	pigeons
Bcar3	-0.11	1.00000000	pigeons
Chek2	-0.13	1.00000000	pigeons
Taok1	-0.23	1.00000000	pigeons
Gramd4	0.24	1.00000000	pigeons
Nvl	-0.38	1.00000000	pigeons
Arhgef7	-0.53	1.00000000	pigeons
Phlpp1	-0.56	1.00000000	pigeons
Ireb2	-0.15	1.00000000	pigeons
Dtx2	-0.69	1.00000000	pigeons

Pi4ka	-0.61	1.00000000	pigeons
Elf2	-0.61	1.00000000	pigeons
Hpcal1	-0.29	1.00000000	pigeons
Pex7	-0.79	1.00000000	pigeons
Pigf	-0.94	1.00000000	pigeons
Tma16	-0.15	1.00000000	pigeons
Cdc37l1	-0.58	1.00000000	pigeons
Fa2h	-0.36	1.00000000	pigeons
Ccng1	-0.28	1.00000000	pigeons
Snx29	-0.51	1.00000000	pigeons
Ilf2	-0.13	1.00000000	pigeons
Ap3s1	-1.14	1.00000000	pigeons
Atp9b	-0.90	1.00000000	pigeons
Dync1li1	-0.43	1.00000000	pigeons
Dctn1	-0.30	1.00000000	pigeons
Acly	-0.66	1.00000000	pigeons
Wdr36	-0.16	1.00000000	pigeons
Nub1	-0.42	1.00000000	pigeons
Glo1	-0.67	1.00000000	pigeons
Ndufs7	-0.21	1.00000000	pigeons
Ppp1r2	-0.45	1.00000000	pigeons
Pafah1b2	-0.04	1.00000000	pigeons
Chd7	0.50	1.00000000	pigeons
Arhgap18	-0.12	1.00000000	pigeons
Bfar	-0.54	1.00000000	pigeons
Tpm4	-0.50	1.00000000	pigeons
Acsbg2	-0.84	1.00000000	pigeons
Desi1	-0.41	1.00000000	pigeons
Tmem106b	-0.52	1.00000000	pigeons
Steap4	-0.66	1.00000000	pigeons
Tpm3-rs7	-0.40	1.00000000	pigeons
Bcl11b	-0.25	1.00000000	pigeons
Stk39	-0.17	1.00000000	pigeons
Stab1	-0.52	1.00000000	pigeons
Pdhb	-0.66	1.00000000	pigeons
Trnt1	0.04	1.00000000	pigeons
Eif6	-0.45	1.00000000	pigeons
Nktr	-0.70	1.00000000	pigeons
Smarcal1	-0.30	1.00000000	pigeons
Bnip2	-0.42	1.00000000	pigeons
Pdcl3	-0.24	1.00000000	pigeons
Fbxo34	0.17	1.00000000	pigeons
Gcn1	-0.38	1.00000000	pigeons
Shroom2	-1.21	1.00000000	pigeons
Ppp1cc	-0.64	1.00000000	pigeons
Parp1	-0.32	1.00000000	pigeons

Col22a1	-1.40	1.00000000	pigeons
Rnf141	-0.43	1.00000000	pigeons
Acvr1	-0.56	1.00000000	pigeons
Phkg1	-0.40	1.00000000	pigeons
Nfxl1	-0.43	1.00000000	pigeons
Hscb	0.00	1.00000000	pigeons
Cpe	0.60	1.00000000	pigeons
Actr10	-0.10	1.00000000	pigeons
Tspan8	-0.50	1.00000000	pigeons
Psmb3	-0.19	1.00000000	pigeons
Dnajc9	-0.90	1.00000000	pigeons
Wdr47	-0.29	1.00000000	pigeons
Otud3	-0.76	1.00000000	pigeons
Gk5	-1.13	1.00000000	pigeons
Ifi35	-0.15	1.00000000	pigeons
Ttc39a	-0.32	1.00000000	pigeons
Wdr61	-0.42	1.00000000	pigeons
Otulin	-0.49	1.00000000	pigeons
Pde1c	-0.96	1.00000000	pigeons
Focad	-0.36	1.00000000	pigeons
Ciao1	-0.03	1.00000000	pigeons
Ddx52	-0.16	1.00000000	pigeons
Hipk2	-0.74	1.00000000	pigeons
Hikeshi	-0.07	1.00000000	pigeons
Aco1	-0.48	1.00000000	pigeons
Rev3l	-0.36	1.00000000	pigeons
Dennd4a	-1.54	1.00000000	pigeons
Txlng	-0.78	1.00000000	pigeons
Urb1	-1.09	1.00000000	pigeons
Svil	-0.57	1.00000000	pigeons
Rnf150	-0.46	1.00000000	pigeons
Sh3pxd2b	-0.50	1.00000000	pigeons
Nol11	-0.02	1.00000000	pigeons
Tmem50a	-0.48	1.00000000	pigeons
Slc12a2	-1.45	1.00000000	pigeons
Slc25a25	-0.77	1.00000000	pigeons
Rad21	-0.62	1.00000000	pigeons
Atp11c	-0.42	1.00000000	pigeons
Fgd6	-0.09	1.00000000	pigeons
Hoxa7	-0.43	1.00000000	pigeons
Cask	-0.20	1.00000000	pigeons
Sft2d2	-0.51	1.00000000	pigeons
Rnf144a	-1.17	1.00000000	pigeons
Fth1	0.15	1.00000000	pigeons
Jade3	-0.47	1.00000000	pigeons
Fam168a	-1.37	1.00000000	pigeons

Atp6v0c	-0.42	1.00000000	pigeons
Gcsh	-0.24	1.00000000	pigeons
Arnt	-0.15	1.00000000	pigeons
Bzw2	-0.69	1.00000000	pigeons
Tmbim6	-0.45	1.00000000	pigeons
Immp2l	0.06	1.00000000	pigeons
Kif13a	-0.76	1.00000000	pigeons
Nrd1	-0.04	1.00000000	pigeons
Ets2	-1.37	1.00000000	pigeons
Setd7	-0.70	1.00000000	pigeons
Hnrnph3	-0.66	1.00000000	pigeons
Eif3i	-0.48	1.00000000	pigeons
Smad3	-0.80	1.00000000	pigeons
Kctd3	-0.43	1.00000000	pigeons
Rassf3	-0.29	1.00000000	pigeons
Hoxa3	-0.87	1.00000000	pigeons
Arrdc3	-0.46	1.00000000	pigeons
Pptc7	-0.19	1.00000000	pigeons
Tax1bp3	-0.47	1.00000000	pigeons
Txndc5	0.68	1.00000000	pigeons
Jagn1	0.04	1.00000000	pigeons
Nudt1	0.46	1.00000000	pigeons
Ccndbp1	-0.14	1.00000000	pigeons
Supv3l1	0.16	1.00000000	pigeons
Golga4	-0.58	1.00000000	pigeons
Gnptab	-0.13	1.00000000	pigeons
Nol9	-0.54	1.00000000	pigeons
Phf5a	0.21	1.00000000	pigeons
Nbr1	-0.19	1.00000000	pigeons
Chp1	-1.27	1.00000000	pigeons
Zfp644	-0.68	1.00000000	pigeons
Exoc6b	-0.94	1.00000000	pigeons
Ptpn12	-0.30	1.00000000	pigeons
Ttc7b	-0.34	1.00000000	pigeons
Srpr	-0.47	1.00000000	pigeons
Naca	-0.23	1.00000000	pigeons
Hmgcs1	0.29	1.00000000	pigeons
Dusp6	0.72	1.00000000	pigeons
Ppm1h	0.42	1.00000000	pigeons
Clip1	-0.62	1.00000000	pigeons
Atp6v1g1	-0.22	1.00000000	pigeons
Zhx1	-0.77	1.00000000	pigeons
Uhmk1	-0.63	1.00000000	pigeons
Ube2q2	-0.39	1.00000000	pigeons
Stk17b	0.00	1.00000000	pigeons
Gnpat	-0.30	1.00000000	pigeons

Tmem268	0.04	1.00000000	pigeons
Tecta	-0.14	1.00000000	pigeons
Fermt1	-0.21	1.00000000	pigeons
Ap2b1	0.02	1.00000000	pigeons
Gabpa	-0.60	1.00000000	pigeons
Nr1d2	-1.31	1.00000000	pigeons
Zeb2	0.06	1.00000000	pigeons
Rere	-0.46	1.00000000	pigeons
Anapc1	-0.52	1.00000000	pigeons
Odc1	-0.48	1.00000000	pigeons
Slc10a7	-0.25	1.00000000	pigeons
Arhgap29	-1.20	1.00000000	pigeons
Bin3	-1.11	1.00000000	pigeons
Ppme1	-0.80	1.00000000	pigeons
Cebpg	-0.68	1.00000000	pigeons
Tob2	-0.71	1.00000000	pigeons
Leprot	-0.61	1.00000000	pigeons
Snip1	-0.72	1.00000000	pigeons
Psmb4	-0.82	1.00000000	pigeons
Tob1	-0.73	1.00000000	pigeons
Tiam2	-0.91	1.00000000	pigeons
Ubr5	-0.36	1.00000000	pigeons
Inpp5a	-0.59	1.00000000	pigeons
Etl4	-0.42	1.00000000	pigeons
Basp1	0.04	1.00000000	pigeons
Arhgap26	-0.23	1.00000000	pigeons
St3gal1	-1.23	1.00000000	pigeons
Exoc8	0.09	1.00000000	pigeons
Snx18	-0.08	1.00000000	pigeons
Dck	-0.09	1.00000000	pigeons
Rab7	-0.94	1.00000000	pigeons
Diablo	0.06	1.00000000	pigeons
Hdx	-0.49	1.00000000	pigeons
Nutf2	0.52	1.00000000	pigeons
Plrg1	-0.66	1.00000000	pigeons
B9d1	0.18	1.00000000	pigeons
Narf	-0.37	1.00000000	pigeons
Ehd4	-0.71	1.00000000	pigeons
Nol10	-0.46	1.00000000	pigeons
Zbed4	-0.67	1.00000000	pigeons
Rev1	-0.64	1.00000000	pigeons
Add1	-0.57	1.00000000	pigeons
Cdc42bpb	-0.32	1.00000000	pigeons
Ywhaq	-0.32	1.00000000	pigeons
Cttnbp2nl	-0.81	1.00000000	pigeons
Abi1	-0.13	1.00000000	pigeons

Skil	-0.32	1.00000000	pigeons
Ccnyl1	-0.45	1.00000000	pigeons
Cct6b	-0.71	1.00000000	pigeons
Casr	-0.24	1.00000000	pigeons
Timmdc1	-0.14	1.00000000	pigeons
Pbx1	-0.82	1.00000000	pigeons
Snx14	-0.84	1.00000000	pigeons
Ppfibp2	0.13	1.00000000	pigeons
Rab40c	0.06	1.00000000	pigeons
Ap1b1	0.10	1.00000000	pigeons
Cog2	-0.95	1.00000000	pigeons
Smco4	0.12	1.00000000	pigeons
Exosc8	0.15	1.00000000	pigeons
Kat14	0.71	1.00000000	pigeons
Vstm4	0.28	1.00000000	pigeons
Ift80	0.83	1.00000000	pigeons
Ciapin1	0.10	1.00000000	pigeons
Ufm1	0.01	1.00000000	pigeons
Yipf3	-0.46	1.00000000	pigeons
Htt	-0.88	1.00000000	pigeons
Irak2	-0.52	1.00000000	pigeons
Kif13b	-0.53	1.00000000	pigeons
Lzic	-0.48	1.00000000	pigeons
Hspd1	-0.59	1.00000000	pigeons
Pex13	-0.67	1.00000000	pigeons
Kif5b	-0.90	1.00000000	pigeons
Cfdp1	-0.51	1.00000000	pigeons
Lrrk2	-0.54	1.00000000	pigeons
Acbd5	-0.36	1.00000000	pigeons
Snrnp35	-0.02	1.00000000	pigeons
Coq5	0.05	1.00000000	pigeons
Rfx3	0.03	1.00000000	pigeons
Tmem170	-0.01	1.00000000	pigeons
Dnmt1	-0.54	1.00000000	pigeons
Ldlrad4	-0.69	1.00000000	pigeons
Arv1	0.02	1.00000000	pigeons
Dus1l	-0.52	1.00000000	pigeons
Fam149b	-0.70	1.00000000	pigeons
Rfc3	0.22	1.00000000	pigeons
Fdft1	0.16	1.00000000	pigeons
Cir1	-0.86	1.00000000	pigeons
Pik3ip1	-0.92	1.00000000	pigeons
Cpeb2	-0.87	1.00000000	pigeons
Eftud2	-0.71	1.00000000	pigeons
Efl1	-0.59	1.00000000	pigeons
Pard3b	-1.09	1.00000000	pigeons

Limd1	-0.45	1.00000000	pigeons
Hmgb2	-0.09	1.00000000	pigeons
Cdk7	-0.62	1.00000000	pigeons
Tbc1d2b	-0.31	1.00000000	pigeons
Ikbkb	-0.52	1.00000000	pigeons
Akt3	-0.28	1.00000000	pigeons
Ahcyl2	0.07	1.00000000	pigeons
Dip2c	-0.09	1.00000000	pigeons
Adi1	-0.17	1.00000000	pigeons
Traf3ip1	0.09	1.00000000	pigeons
Tmem43	0.09	1.00000000	pigeons
Sf3b4	0.05	1.00000000	pigeons
Hnrnpa2b1	-0.13	1.00000000	pigeons
Tgs1	-0.59	1.00000000	pigeons
Zfp318	-0.30	1.00000000	pigeons
Wwox	-0.14	1.00000000	pigeons
Rsrc2	-0.54	1.00000000	pigeons
Taf15	-0.10	1.00000000	pigeons
Id2	-0.54	1.00000000	pigeons
Il13ra1	-0.19	1.00000000	pigeons
Erlin1	-0.17	1.00000000	pigeons
Plekhf2	-0.12	1.00000000	pigeons
Ssr1	-0.32	1.00000000	pigeons
Cst7	-0.23	1.00000000	pigeons
Colgalt2	-0.43	1.00000000	pigeons
Ece1	-0.82	1.00000000	pigeons
Snrnp25	-0.74	1.00000000	pigeons
Adrm1	-0.28	1.00000000	pigeons
Dock4	-0.93	1.00000000	pigeons
Ofd1	0.20	1.00000000	pigeons
Trmt2b	0.44	1.00000000	pigeons
Pank2	0.14	1.00000000	pigeons
Trim25	0.09	1.00000000	pigeons
Use1	0.24	1.00000000	pigeons
Hivep2	-0.24	1.00000000	pigeons
Sav1	-0.41	1.00000000	pigeons
Ppfia1	-0.27	1.00000000	pigeons
Dnajb2	-0.22	1.00000000	pigeons
Tmem165	-0.70	1.00000000	pigeons
Tanc1	-0.98	1.00000000	pigeons
Slc40a1	-0.46	1.00000000	pigeons
Ndufv2	-0.22	1.00000000	pigeons
Strada	-0.52	1.00000000	pigeons
Zmat4	-0.48	1.00000000	pigeons
Nfe2l2	-0.79	1.00000000	pigeons
Ruvbl1	-0.68	1.00000000	pigeons

Tubgcp3	-0.44	1.00000000	pigeons
Psen2	-0.40	1.00000000	pigeons
Unc45a	-0.46	1.00000000	pigeons
Ppp1r16b	-1.13	1.00000000	pigeons
Abl1	0.05	1.00000000	pigeons
Snx27	-0.47	1.00000000	pigeons
Samm50	-0.38	1.00000000	pigeons
Exoc5	-0.98	1.00000000	pigeons
Dut	-0.42	1.00000000	pigeons
Ifngr1	-0.64	1.00000000	pigeons
Ttc28	-1.17	1.00000000	pigeons
Arntl	-0.14	1.00000000	pigeons
Pitpnm3	0.03	1.00000000	pigeons
Ctnnb1	-0.94	1.00000000	pigeons
Nab1	-0.64	1.00000000	pigeons
Chmp5	-0.57	1.00000000	pigeons
Rtn4	-0.07	1.00000000	pigeons
Sec31b	-0.37	1.00000000	pigeons
Appl1	-0.25	1.00000000	pigeons
Kif11	0.08	1.00000000	pigeons
Pxylp1	0.12	1.00000000	pigeons
Cenpt	0.13	1.00000000	pigeons
Hmgxb3	0.27	1.00000000	pigeons
Anapc7	0.12	1.00000000	pigeons
Sgpp1	-0.53	1.00000000	pigeons
Eif5b	-0.83	1.00000000	pigeons
Polr1e	-0.30	1.00000000	pigeons
Wdr25	-0.31	1.00000000	pigeons
Tvp23a	-0.29	1.00000000	pigeons
Cdc42ep3	-1.00	1.00000000	pigeons
Rab18	-0.20	1.00000000	pigeons
Atad2b	-0.31	1.00000000	pigeons
Xrcc5	-0.19	1.00000000	pigeons
Dnajc18	-0.36	1.00000000	pigeons
Slc2a13	-0.16	1.00000000	pigeons
Ppp2r5d	-0.55	1.00000000	pigeons
Acyp1	-0.66	1.00000000	pigeons
Slc24a4	-0.08	1.00000000	pigeons
Sh3pxd2a	-0.76	1.00000000	pigeons
Lxn	-0.25	1.00000000	pigeons
Eefsec	-0.31	1.00000000	pigeons
Pip5k1c	-0.26	1.00000000	pigeons
Eif4g2	-0.35	1.00000000	pigeons
Arxes2	-0.42	1.00000000	pigeons
Pcmt1	-0.74	1.00000000	pigeons
Sbf2	-0.84	1.00000000	pigeons

Taldo1	-0.21	1.00000000	pigeons
Eif4e	-0.28	1.00000000	pigeons
Ndufv3	-0.51	1.00000000	pigeons
Ak2	-0.38	1.00000000	pigeons
Parn	-0.29	1.00000000	pigeons
Stat3	-0.59	1.00000000	pigeons
Phc1	0.04	1.00000000	pigeons
Cpne8	0.08	1.00000000	pigeons
Impa1	0.10	1.00000000	pigeons
Sqle	0.12	1.00000000	pigeons
Slc30a6	-0.02	1.00000000	pigeons
Scamp1	0.11	1.00000000	pigeons
Rpap1	0.03	1.00000000	pigeons
Pcgf6	0.57	1.00000000	pigeons
Naaladl2	-0.16	1.00000000	pigeons
B4galt7	0.60	1.00000000	pigeons
Kdm8	0.07	1.00000000	pigeons
Orai1	0.07	1.00000000	pigeons
Snf8	0.05	1.00000000	pigeons
Aph1a	0.05	1.00000000	pigeons
Pbx4	0.22	1.00000000	pigeons
Rsl1d1	-0.39	1.00000000	pigeons
Sar1b	-0.32	1.00000000	pigeons
Rpia	-0.95	1.00000000	pigeons
Rbm31y	-0.49	1.00000000	pigeons
Atxn10	-0.21	1.00000000	pigeons
Snx2	-0.11	1.00000000	pigeons
Lmbrd2	-0.37	1.00000000	pigeons
Anxa7	-0.17	1.00000000	pigeons
Pinx1	-0.29	1.00000000	pigeons
Atp6v1e2	-0.51	1.00000000	pigeons
Isca1	-0.30	1.00000000	pigeons
Lrrcc1	-0.17	1.00000000	pigeons
Klf10	-0.38	1.00000000	pigeons
Lsm5	-0.28	1.00000000	pigeons
Lap3	-0.24	1.00000000	pigeons
Slc35g2	-0.39	1.00000000	pigeons
Slc25a26	-0.54	1.00000000	pigeons
Utp11	0.01	1.00000000	pigeons
Washc3	0.20	1.00000000	pigeons
Cdk19	-0.25	1.00000000	pigeons
2900026A02Rik	-1.03	1.00000000	pigeons
Sec23a	-0.39	1.00000000	pigeons
Pcm1	-0.53	1.00000000	pigeons
Trappc8	-0.85	1.00000000	pigeons
Brf1	-0.64	1.00000000	pigeons

Scarb2	-0.86	1.00000000	pigeons
Arl15	-0.90	1.00000000	pigeons
Thoc1	-0.94	1.00000000	pigeons
Hmgcr	-0.83	1.00000000	pigeons
Zfp518b	-0.50	1.00000000	pigeons
Spg7	0.17	1.00000000	pigeons
Sod1	-0.25	1.00000000	pigeons
Slc25a3	-0.53	1.00000000	pigeons
Tubgcp6	0.04	1.00000000	pigeons
Nkiras1	0.09	1.00000000	pigeons
Enoph1	-0.25	1.00000000	pigeons
Haus1	-0.14	1.00000000	pigeons
Lin7c	-0.20	1.00000000	pigeons
Gal	-0.17	1.00000000	pigeons
Sfxn4	-0.22	1.00000000	pigeons
Slc30a4	-0.18	1.00000000	pigeons
Anapc2	0.02	1.00000000	pigeons
Ap4b1	0.12	1.00000000	pigeons
Entpd4	-0.65	1.00000000	pigeons
Fam177a	-1.15	1.00000000	pigeons
Cpeb4	-1.04	1.00000000	pigeons
Cldnd1	0.02	1.00000000	pigeons
Sntb1	-0.13	1.00000000	pigeons
Ust	-0.51	1.00000000	pigeons
Asf1a	-0.54	1.00000000	pigeons
Esrrg	-0.05	1.00000000	pigeons
Cry2	-0.50	1.00000000	pigeons
Casp7	-0.41	1.00000000	pigeons
Scyl3	-0.25	1.00000000	pigeons
Arhgef26	-0.53	1.00000000	pigeons
Nckipsd	-0.34	1.00000000	pigeons
Ranbp17	-1.15	1.00000000	pigeons
Stk11	-0.56	1.00000000	pigeons
Ubl3	-0.75	1.00000000	pigeons
Actg1	-0.08	1.00000000	pigeons
Fut8	-0.88	1.00000000	pigeons
Krr1	-0.38	1.00000000	pigeons
Pard3	0.07	1.00000000	pigeons
Atrn	-0.33	1.00000000	pigeons
Itm2a	-0.39	1.00000000	pigeons
Rac1	-0.43	1.00000000	pigeons
Nrp2	0.01	1.00000000	pigeons
Xrn1	-0.64	1.00000000	pigeons
Spty2d1	-0.76	1.00000000	pigeons
Trim54	-0.21	1.00000000	pigeons
Rb1cc1	-0.66	1.00000000	pigeons

Atp1b3	-0.99	1.00000000	pigeons
Insr	-0.51	1.00000000	pigeons
Ppp2r5c	-0.22	1.00000000	pigeons
Ttll1	-0.14	1.00000000	pigeons
Aldh9a1	-0.76	1.00000000	pigeons
Polg2	-0.69	1.00000000	pigeons
Gtf2b	-0.98	1.00000000	pigeons
Iqub	-0.24	1.00000000	pigeons
Pgm2l1	-0.36	1.00000000	pigeons
Elk3	-0.21	1.00000000	pigeons
Bend3	-0.54	1.00000000	pigeons
Ints7	-0.51	1.00000000	pigeons
Ophn1	-0.33	1.00000000	pigeons
Gpatch2l	-0.33	1.00000000	pigeons
Usp1	-0.63	1.00000000	pigeons
Hdac11	-0.56	1.00000000	pigeons
Gnptg	-0.15	1.00000000	pigeons
Rexo4	-0.46	1.00000000	pigeons
Camsap1	-0.12	1.00000000	pigeons
Zbtb34	-0.15	1.00000000	pigeons
Heatr6	-0.16	1.00000000	pigeons
Bud13	-0.50	1.00000000	pigeons
Slc45a3	-0.24	1.00000000	pigeons
Tmco3	-1.22	1.00000000	pigeons
Ripk2	-0.65	1.00000000	pigeons
Hibadh	-0.78	1.00000000	pigeons
Fnip2	-0.52	1.00000000	pigeons
Zfand4	-0.35	1.00000000	pigeons
Raly	-0.42	1.00000000	pigeons
Ptpn7	-0.54	1.00000000	pigeons
Gnb1	-0.55	1.00000000	pigeons
Gpr68	-0.66	1.00000000	pigeons
Rfc4	-0.62	1.00000000	pigeons
Cmtm4	-0.77	1.00000000	pigeons
Clint1	-0.77	1.00000000	pigeons
Emc3	-0.57	1.00000000	pigeons
Large1	-0.60	1.00000000	pigeons
Nt5c3	-0.60	1.00000000	pigeons
Serpini1	-0.80	1.00000000	pigeons
Srebf1	-0.69	1.00000000	pigeons
Eif4g3	-0.51	1.00000000	pigeons
Zfp592	-0.67	1.00000000	pigeons
Ext1	-0.14	1.00000000	pigeons
Rab5c	-0.59	1.00000000	pigeons
Trp53inp1	-0.45	1.00000000	pigeons
Ndst4	-0.53	1.00000000	pigeons

Fbxo8	-0.56	1.00000000	pigeons
Aspm	-0.27	1.00000000	pigeons
Agl	-0.49	1.00000000	pigeons
Snx7	-0.11	1.00000000	pigeons
Tpra1	-0.60	1.00000000	pigeons
Nt5dc2	-0.58	1.00000000	pigeons
Ska2	-0.17	1.00000000	pigeons
Chchd5	-0.04	1.00000000	pigeons
Fkbp11	-0.75	1.00000000	pigeons
Fbxo30	0.02	1.00000000	pigeons
Eml6	-0.62	1.00000000	pigeons
Dgkq	-0.09	1.00000000	pigeons
Ap4s1	-0.05	1.00000000	pigeons
Samd8	-0.22	1.00000000	pigeons
Rab43	-0.32	1.00000000	pigeons
Usp31	-0.44	1.00000000	pigeons
Fabp3	-0.62	1.00000000	pigeons
Tmem161a	-0.44	1.00000000	pigeons
Gtpbp10	-0.14	1.00000000	pigeons
Acaa1b	-0.43	1.00000000	pigeons
Glipr2	-0.20	1.00000000	pigeons
Rb1	-0.55	1.00000000	pigeons
Farp1	-0.69	1.00000000	pigeons
Plcb4	-0.76	1.00000000	pigeons
Wdr45b	-0.30	1.00000000	pigeons
Atf4	-0.25	1.00000000	pigeons
Fyco1	-0.70	1.00000000	pigeons
Hadh	-0.37	1.00000000	pigeons
Trps1	0.15	1.00000000	pigeons
Sik1	-0.78	1.00000000	pigeons
Ndufs1	-0.55	1.00000000	pigeons
Tfap2b	-0.07	1.00000000	pigeons
Zmym3	-0.55	1.00000000	pigeons
Jmy	-0.51	1.00000000	pigeons
Bsdc1	-0.65	1.00000000	pigeons
Rnf4	-0.40	1.00000000	pigeons
Tmem70	-0.60	1.00000000	pigeons
Spry2	-0.27	1.00000000	pigeons
Tspo	-0.47	1.00000000	pigeons
Ttf1	-0.62	1.00000000	pigeons
Dpp7	-0.55	1.00000000	pigeons
Per3	-0.47	1.00000000	pigeons
Kpna6	-0.54	1.00000000	pigeons
Ubp1	-0.50	1.00000000	pigeons
Trappc2	-0.49	1.00000000	pigeons
Atad3a	-0.40	1.00000000	pigeons

Usp12	-0.93	1.00000000	pigeons
Exosc1	-0.46	1.00000000	pigeons
Psmd7	-0.75	1.00000000	pigeons
Pcna	-0.54	1.00000000	pigeons
Dgkd	-0.93	1.00000000	pigeons
Ppp4r1	-0.42	1.00000000	pigeons
D630045J12Rik	-0.31	1.00000000	pigeons
Dcun1d2	-2.04	1.00000000	pigeons
Adgra3	-0.64	1.00000000	pigeons
Pik3c3	-0.33	1.00000000	pigeons
Dlst	-0.05	1.00000000	pigeons
Rfng	-0.48	1.00000000	pigeons
Nap1l1	-0.69	1.00000000	pigeons
Slain2	-0.25	1.00000000	pigeons
Jmjd7	-0.10	1.00000000	pigeons
Anp32e	-0.78	1.00000000	pigeons
Bms1	-1.08	1.00000000	pigeons
Sf3b3	-0.45	1.00000000	pigeons
Twsg1	-0.88	1.00000000	pigeons
Smad1	-0.74	1.00000000	pigeons
Cstf2	-0.36	1.00000000	pigeons
Polr3h	-0.37	1.00000000	pigeons
Pdss1	-0.49	1.00000000	pigeons
Nelfa	-0.37	1.00000000	pigeons
Lpar1	-0.48	1.00000000	pigeons
Dlg5	-0.53	1.00000000	pigeons
Abca4	-0.48	1.00000000	pigeons
Alas1	-0.47	1.00000000	pigeons
Sema3g	-0.54	1.00000000	pigeons
Traf7	-0.18	1.00000000	pigeons
Rhot1	-0.82	1.00000000	pigeons
Tsnax	-0.12	1.00000000	pigeons
Usp20	-0.11	1.00000000	pigeons
Tgfbr2	-0.35	1.00000000	pigeons
Ndufa4	-0.17	1.00000000	pigeons
Fer	-0.30	1.00000000	pigeons
Kansl1l	-1.14	1.00000000	pigeons
Aacs	-0.74	1.00000000	pigeons
Rad17	0.16	1.00000000	pigeons
Tmem266	-0.45	1.00000000	pigeons
Pbrm1	-1.08	1.00000000	pigeons
Tcaim	-0.03	1.00000000	pigeons
Ccdc149	-0.18	1.00000000	pigeons
Psmc2	-0.45	1.00000000	pigeons
Hspa5	-0.65	1.00000000	pigeons
Psma7	-0.38	1.00000000	pigeons

Osbpl5	-0.41	1.00000000	pigeons
Sipa1l2	-0.89	1.00000000	pigeons
Zfyve9	-0.86	1.00000000	pigeons
Pla2g7	-0.69	1.00000000	pigeons
Rnft1	0.07	1.00000000	pigeons
Baiap2	-0.88	1.00000000	pigeons
Snrpb2	-0.94	1.00000000	pigeons
Fkbp4	-0.62	1.00000000	pigeons
Slc25a13	-0.57	1.00000000	pigeons
Ube2e3	0.03	1.00000000	pigeons
Bloc1s2	0.09	1.00000000	pigeons
Atp11a	0.73	1.00000000	pigeons
Sec11c	-0.53	1.00000000	pigeons
Wdr1	-0.48	1.00000000	pigeons
Ube4b	-0.65	1.00000000	pigeons
Ralbp1	-0.68	1.00000000	pigeons
Ttll5	-0.08	1.00000000	pigeons
Bend5	-0.57	1.00000000	pigeons
Polr2d	-0.48	1.00000000	pigeons
Lyplal1	-0.10	1.00000000	pigeons
Efr3a	-0.43	1.00000000	pigeons
Ate1	-0.50	1.00000000	pigeons
Tes	-0.72	1.00000000	pigeons
Galm	-0.28	1.00000000	pigeons
Chid1	-0.43	1.00000000	pigeons
B3gnt5	-0.39	1.00000000	pigeons
Nipsnap2	-0.16	1.00000000	pigeons
Camk2g	-1.50	1.00000000	pigeons
Sestd1	0.21	1.00000000	pigeons
Srpk2	-1.08	1.00000000	pigeons
Hs6st1	-0.66	1.00000000	pigeons
Edf1	-0.06	1.00000000	pigeons
Rnf214	0.39	1.00000000	pigeons
Apoa1	-1.63	1.00000000	pigeons
Dtwd2	0.11	1.00000000	pigeons
Pals1	-0.14	1.00000000	pigeons
Ndc1	0.00	1.00000000	pigeons
Nme7	-0.25	1.00000000	pigeons
Pla2g12a	-0.52	1.00000000	pigeons
Ddx24	-0.38	1.00000000	pigeons
Edrf1	-0.69	1.00000000	pigeons
Pxk	-0.21	1.00000000	pigeons
Ddx31	-0.33	1.00000000	pigeons
Hcfc2	-0.57	1.00000000	pigeons
Eif4a2	-0.09	1.00000000	pigeons
Cmss1	-0.96	1.00000000	pigeons

Aida	-0.66	1.00000000	pigeons
Nusap1	-0.88	1.00000000	pigeons
Wbp4	-1.47	1.00000000	pigeons
Arl4c	-0.73	1.00000000	pigeons
Dynll2	-0.67	1.00000000	pigeons
Dpy30	-0.48	1.00000000	pigeons
Gphn	-0.42	1.00000000	pigeons
Spred1	0.44	1.00000000	pigeons
Nod1	-0.16	1.00000000	pigeons
Lrrc1	0.07	1.00000000	pigeons
Ccser1	-0.71	1.00000000	pigeons
C1qa	-0.05	1.00000000	pigeons
Sacm1l	-0.94	1.00000000	pigeons
Nsun2	-0.43	1.00000000	pigeons
Arpp19	-0.47	1.00000000	pigeons
B4galt2	-0.03	1.00000000	pigeons
Brd1	-0.46	1.00000000	pigeons
Nkiras2	0.11	1.00000000	pigeons
Heca	-0.92	1.00000000	pigeons
Eef1d	-0.21	1.00000000	pigeons
Drg2	-0.49	1.00000000	pigeons
Larp1	-0.30	1.00000000	pigeons
Eif2ak4	-0.80	1.00000000	pigeons
Hdac4	-1.12	1.00000000	pigeons
Sec31a	-0.66	1.00000000	pigeons
Gna11	-0.98	1.00000000	pigeons
Gpr18	-0.48	1.00000000	pigeons
Ppa1	-0.86	1.00000000	pigeons
Fam133b	-0.23	1.00000000	pigeons
Nop16	-0.40	1.00000000	pigeons
Tor1b	-0.11	1.00000000	pigeons
Crebl2	-0.01	1.00000000	pigeons
Pald1	-0.44	1.00000000	pigeons
Rbm39	-0.63	1.00000000	pigeons
Swi5	-0.61	1.00000000	pigeons
Aldh1a2	-0.39	1.00000000	pigeons
Tmem141	-0.09	1.00000000	pigeons
Psmd4	-0.45	1.00000000	pigeons
Rcan1	-0.80	1.00000000	pigeons
Fez2	-0.86	1.00000000	pigeons
Kctd6	0.08	1.00000000	pigeons
Dynlrb1	-0.58	1.00000000	pigeons
Dctn6	-0.23	1.00000000	pigeons
Tmpo	-0.78	1.00000000	pigeons
Cops3	-0.49	1.00000000	pigeons
Trim35	-0.30	1.00000000	pigeons

Vps11	-0.56	1.00000000	pigeons
St14	-0.40	1.00000000	pigeons
Serinc1	-0.58	1.00000000	pigeons
Cdc14a	-0.23	1.00000000	pigeons
B3galnt2	-0.56	1.00000000	pigeons
Dmap1	-0.83	1.00000000	pigeons
Lfng	-0.09	1.00000000	pigeons
Nop14	-0.54	1.00000000	pigeons
Fgfr1op2	0.10	1.00000000	pigeons
Il20ra	0.34	1.00000000	pigeons
Pitpnb	-0.85	1.00000000	pigeons
Atp8a2	0.10	1.00000000	pigeons
Arl5b	-0.06	1.00000000	pigeons
Tmem161b	0.01	1.00000000	pigeons
Heatr5a	-0.24	1.00000000	pigeons
Galnt3	0.08	1.00000000	pigeons
Pias4	0.00	1.00000000	pigeons
Dis3	-0.40	1.00000000	pigeons
Amdhd2	-0.20	1.00000000	pigeons
Vwa8	-0.51	1.00000000	pigeons
Comt	-0.60	1.00000000	pigeons
S100a11	-0.68	1.00000000	pigeons
Rnf24	-0.45	1.00000000	pigeons
Ist1	-0.58	1.00000000	pigeons
Prkca	-0.28	1.00000000	pigeons
Vti1a	-0.50	1.00000000	pigeons
Fam185a	-0.33	1.00000000	pigeons
Creg1	-1.01	1.00000000	pigeons
Fry	-0.10	1.00000000	pigeons
Runx2	-0.73	1.00000000	pigeons
Plekha3	-0.35	1.00000000	pigeons
Pdxk	-0.62	1.00000000	pigeons
Brca2	0.05	1.00000000	pigeons
Ipo11	-0.53	1.00000000	pigeons
Hdac7	-1.03	1.00000000	pigeons
Fem1b	-0.27	1.00000000	pigeons
Klf7	-0.76	1.00000000	pigeons
Rcn2	-0.20	1.00000000	pigeons
Ywhah	-1.02	1.00000000	pigeons
Tpk1	-0.69	1.00000000	pigeons
Rap1b	-0.37	1.00000000	pigeons
Cdca7l	-0.94	1.00000000	pigeons
Tcf12	0.01	1.00000000	pigeons
Lpcat1	-0.17	1.00000000	pigeons
Lsm6	-0.66	1.00000000	pigeons
Psmf1	-0.79	1.00000000	pigeons

Adar	-0.25	1.00000000	pigeons
Bicd2	-0.64	1.00000000	pigeons
Enox1	-0.15	1.00000000	pigeons
Twf1	-0.71	1.00000000	pigeons
Nrip1	0.09	1.00000000	pigeons
St8sia1	-0.75	1.00000000	pigeons
Eif2b5	-0.10	1.00000000	pigeons
Prkar1b	-0.27	1.00000000	pigeons
Trpm7	-0.92	1.00000000	pigeons
Rbms1	-0.66	1.00000000	pigeons
Ccnl1	-0.69	1.00000000	pigeons
Prkcd	-0.83	1.00000000	pigeons
Phf21a	-0.59	1.00000000	pigeons
Rnf138	-0.13	1.00000000	pigeons
Slc35b1	-0.67	1.00000000	pigeons
Ets1	-1.30	1.00000000	pigeons
Ifrd1	-0.38	1.00000000	pigeons
Rae1	-0.31	1.00000000	pigeons
Cops5	-0.54	1.00000000	pigeons
Tmem263	-0.79	1.00000000	pigeons
Sgce	-0.78	1.00000000	pigeons
9130409I23Rik	-1.11	1.00000000	pigeons
Uspl1	-0.56	1.00000000	pigeons
Peak1	-0.78	1.00000000	pigeons
Ifngr2	-0.48	1.00000000	pigeons
Eepd1	-0.39	1.00000000	pigeons
Pdcd7	-0.80	1.00000000	pigeons
Cdc73	-0.76	1.00000000	pigeons
Gnb5	-0.75	1.00000000	pigeons
Trip11	-0.66	1.00000000	pigeons
Lmo2	-1.10	1.00000000	pigeons
Ccny	-0.64	1.00000000	pigeons
Reck	-0.15	1.00000000	pigeons
Rcor3	-0.30	1.00000000	pigeons
Rasgrp3	-0.92	1.00000000	pigeons
Ubqln1	-0.59	1.00000000	pigeons
Ctr9	-0.79	1.00000000	pigeons
Trap1	-0.08	1.00000000	pigeons
Vav2	-0.76	1.00000000	pigeons
Nfrkb	-0.52	1.00000000	pigeons
Cog5	-0.64	1.00000000	pigeons
Cradd	-0.52	1.00000000	pigeons
Yes1	-0.07	1.00000000	pigeons
Eci3	0.14	1.00000000	pigeons
Elovl1	-0.56	1.00000000	pigeons
Atg4c	-0.29	1.00000000	pigeons

Eif2d	-0.69	1.00000000	pigeons
Invs	-0.76	1.00000000	pigeons
Zfp143	-0.67	1.00000000	pigeons
Zfp407	-0.71	1.00000000	pigeons
Arhgap8	-0.60	1.00000000	pigeons
Plcd1	-1.02	1.00000000	pigeons
Slc30a1	-0.99	1.00000000	pigeons
Bin1	-0.61	1.00000000	pigeons
Hhex	-0.67	1.00000000	pigeons
Ppip5k2	-0.27	1.00000000	pigeons
Tbl3	-0.24	1.00000000	pigeons
Rgs3	-0.83	1.00000000	pigeons
Tef	-0.55	1.00000000	pigeons
Htra2	-0.39	1.00000000	pigeons
Hmgb1	-0.26	1.00000000	pigeons
Galnt12	-0.49	1.00000000	pigeons
Socs6	-0.48	1.00000000	pigeons
Nom1	-0.58	1.00000000	pigeons
Tarbp1	-0.52	1.00000000	pigeons
Nup133	-0.33	1.00000000	pigeons
Fam199x	-0.01	1.00000000	pigeons
Rbm47	-0.28	1.00000000	pigeons
Sgms2	-0.24	1.00000000	pigeons
Ero1a	0.02	1.00000000	pigeons
Spats2l	-0.55	1.00000000	pigeons
Ccne1	-0.43	1.00000000	pigeons
Txndc15	-0.01	1.00000000	pigeons
Dhx35	-0.27	1.00000000	pigeons
Timeless	-0.40	1.00000000	pigeons
Pogz	-0.51	1.00000000	pigeons
Rer1	-0.09	1.00000000	pigeons
Stxbp5	-0.16	1.00000000	pigeons
Tmem131	-0.82	1.00000000	pigeons
Zswim8	-0.63	1.00000000	pigeons
Csrp1	-0.97	1.00000000	pigeons
Ephx1	-0.60	1.00000000	pigeons
Snx12	-0.76	1.00000000	pigeons
Cfl2	-0.58	1.00000000	pigeons
Snapc5	-0.38	1.00000000	pigeons
Cdc16	-0.15	1.00000000	pigeons
Camkmt	-0.14	1.00000000	pigeons
Gpr65	0.17	1.00000000	pigeons
Phkb	-0.37	1.00000000	pigeons
Hspb11	-0.73	1.00000000	pigeons
Rabepk	-0.63	1.00000000	pigeons
Rcbtb1	-0.77	1.00000000	pigeons

Prdx1	-0.83	1.00000000	pigeons
Ing3	-0.52	1.00000000	pigeons
Rprd1a	-0.29	1.00000000	pigeons
E2f3	-0.36	1.00000000	pigeons
Polr1c	-0.02	1.00000000	pigeons
Asah1	-0.40	1.00000000	pigeons
Clock	-0.75	1.00000000	pigeons
Slc20a2	-0.55	1.00000000	pigeons
Smim14	-0.41	1.00000000	pigeons
Stim2	-0.92	1.00000000	pigeons
Pum3	-0.21	1.00000000	pigeons
Wdsub1	-0.15	1.00000000	pigeons
Usp54	-0.71	1.00000000	pigeons
Lsg1	-0.16	1.00000000	pigeons
Neu2	-0.30	1.00000000	pigeons
Gas8	-0.03	1.00000000	pigeons
Wwc1	-0.15	1.00000000	pigeons
Ptpn11	-0.21	1.00000000	pigeons
Pop5	-0.81	1.00000000	pigeons
Flot2	-0.67	1.00000000	pigeons
Szrd1	-0.07	1.00000000	pigeons
Hp1bp3	-0.44	1.00000000	pigeons
Fuca1	-0.43	1.00000000	pigeons
Grhl3	-0.11	1.00000000	pigeons
Wdr77	-0.01	1.00000000	pigeons
Ash2l	-0.45	1.00000000	pigeons
Chaf1a	-0.44	1.00000000	pigeons
Setdb1	-0.02	1.00000000	pigeons
Irf7	2.96	0.00000000	mice
Isg15	3.61	0.00000000	mice
B2m	1.74	0.00000000	mice
Ifi44	2.37	0.00000000	mice
Ifi27l2a	3.31	0.00000000	mice
Ly6e	2.50	0.00000000	mice
Ly6a	4.18	0.00000000	mice
Stat1	1.86	0.00000000	mice
Zbp1	1.59	0.00000000	mice
Usp18	1.65	0.00000000	mice
Oasl2	1.61	0.00000000	mice
H2-K1	1.48	0.00000000	mice
Ifi44l	1.15	0.00000000	mice
Phf11b	1.85	0.00000000	mice
Rnf213	1.80	0.00000000	mice
Slfn5	1.79	0.00000000	mice
Ifi209	1.55	0.00000000	mice
Isg20	1.31	0.00000000	mice

Parp14	1.60	0.00000000	mice
Trim30d	1.31	0.00000000	mice
Ahnak	-1.39	0.00000000	mice
Ifih1	1.51	0.00000000	mice
Rsad2	2.05	0.00000000	mice
Trim30a	1.40	0.00000000	mice
Herc6	1.57	0.00000000	mice
Ifi204	1.50	0.00000000	mice
Xaf1	1.20	0.00000000	mice
Sp110	1.02	0.00000000	mice
Tspo	1.87	0.00000000	mice
Sp140	1.27	0.00000000	mice
Ifitm3	2.39	0.00000000	mice
Ctsz	1.09	0.00000000	mice
H2-T23	1.01	0.00000000	mice
Klrk1	1.25	0.00000000	mice
H2-D1	1.15	0.00000000	mice
Psme2	1.27	0.00000000	mice
Lgals3bp	1.00	0.00000000	mice
Psme1	1.03	0.00000000	mice
Parp9	1.04	0.00000000	mice
Samhd1	1.26	0.00000000	mice
Ube2l6	1.24	0.00000000	mice
C130026I21Rik	1.05	0.00000000	mice
St6gal1	-1.15	0.00000000	mice
Tapbp	0.83	0.00000000	mice
Stat2	1.28	0.00000000	mice
Ifit2	1.47	0.00000000	mice
Dtx3l	0.88	0.00000000	mice
Ifi202b	1.34	0.00000000	mice
Kdr	1.32	0.00000000	mice
Psmb8	0.97	0.00000000	mice
Eef1b2	-0.93	0.00000000	mice
Nlrc5	1.12	0.00000000	mice
Psma7	1.06	0.00000001	mice
Psmb9	1.18	0.00000001	mice
Eif2ak2	0.94	0.00000001	mice
Npc2	1.25	0.00000001	mice
Cox17	0.91	0.00000002	mice
Phf11a	0.92	0.00000003	mice
Ifit1	1.33	0.00000004	mice
Ndufb1-ps	0.86	0.00000005	mice
Plac8	1.99	0.00000006	mice
Acadl	1.15	0.00000013	mice
Dhx58	0.62	0.00000016	mice
Phf11d	0.96	0.00000017	mice

Ifi203	1.36	0.00000017	mice
Kctd12	-1.01	0.00000023	mice
Ifi47	1.01	0.00000024	mice
Gbp4	0.98	0.00000026	mice
Sult1a1	-0.55	0.00000028	mice
Ddx60	0.96	0.00000029	mice
Pfn1	0.88	0.00000030	mice
Oas3	0.67	0.00000033	mice
Slfn1	0.99	0.00000033	mice
Ifit3	0.98	0.00000033	mice
Frmd4a	1.15	0.00000045	mice
Pml	0.84	0.00000056	mice
Tor3a	0.89	0.00000057	mice
Nmi	0.70	0.00000063	mice
Arhgap15	-0.89	0.00000072	mice
Trafd1	0.88	0.00000076	mice
Rtp4	0.96	0.00000090	mice
Daxx	0.98	0.00000122	mice
Gbp2b	1.20	0.00000194	mice
Irgm1	0.88	0.00000223	mice
Rack1	-0.60	0.00000242	mice
Oas1a	0.61	0.00000289	mice
Ext1	-1.96	0.00000296	mice
Ifi214	0.77	0.00000431	mice
Oasl1	1.36	0.00000574	mice
Bst2	0.97	0.00000576	mice
Sp100	0.85	0.00000591	mice
Nt5c3	0.76	0.00000828	mice
Samd9l	0.86	0.00000884	mice
Fgfr1	-0.69	0.00000990	mice
Sgms1	-0.92	0.00001001	mice
Elmo1	-0.69	0.00001079	mice
Arpc5	0.74	0.00001132	mice
Hepacam2	-0.49	0.00001162	mice
Eef2	-0.60	0.00001312	mice
Psma4	0.75	0.00001324	mice
Helz2	0.77	0.00001388	mice
Idi1	0.88	0.00001404	mice
Gbp5	0.88	0.00001528	mice
Pcdh7	-1.38	0.00001624	mice
Ly6c2	1.72	0.00001736	mice
Cdk14	-1.62	0.00001813	mice
Fam117b	-0.84	0.00001868	mice
Slfn8	0.59	0.00002144	mice
Psmb10	0.67	0.00002443	mice
Tor1aip1	0.76	0.00002885	mice

Ifi35	0.70	0.00003188	mice
Ifi206	0.57	0.00003732	mice
Syngr2	0.87	0.00004077	mice
Jund	-0.89	0.00004453	mice
Slc9a9	-1.10	0.00004551	mice
Ctsh	0.90	0.00004894	mice
Atp5md	0.71	0.00005054	mice
Stxbp3	0.99	0.00005277	mice
Tap1	0.73	0.00005395	mice
AW112010	1.45	0.00007047	mice
H2-T22	0.81	0.00007510	mice
Pira2	0.86	0.00007786	mice
Igf2r	-0.88	0.00008859	mice
Lgals9	0.69	0.00011049	mice
Tjp2	-0.54	0.00011604	mice
Lmna	-1.05	0.00014248	mice
Nampt	0.97	0.00015241	mice
Actb	0.92	0.00017586	mice
Rnf114	0.70	0.00019338	mice
Lamp1	0.93	0.00021871	mice
Znfx1	0.72	0.00023119	mice
Sh3tc1	-0.48	0.00024482	mice
Ifi207	1.11	0.00024557	mice
Fli1	-0.83	0.00025184	mice
Il2rg	0.64	0.00029760	mice
Irf9	0.53	0.00029807	mice
Rasa4	0.77	0.00034481	mice
Hdac4	-1.01	0.00037076	mice
H2-Q7	0.88	0.00037290	mice
Foxp1	-0.95	0.00037677	mice
Psmb3	0.50	0.00042427	mice
Txn1	0.95	0.00042912	mice
Filip1l	0.94	0.00047722	mice
Zfhx3	-0.79	0.00049170	mice
Pgap2	0.61	0.00051211	mice
Etnk1	0.74	0.00052015	mice
Ltb4r1	-0.36	0.00055125	mice
Lst1	1.18	0.00056026	mice
Igtp	0.49	0.00062774	mice
Trf	-1.00	0.00068088	mice
Csrp1	0.85	0.00070967	mice
Cd207	-1.20	0.00071607	mice
Gbp2	0.99	0.00080190	mice
Pabpc1	-0.59	0.00085916	mice
Eef1a1	-0.51	0.00090235	mice
Usp25	0.93	0.00090590	mice

Bckdhb	-0.66	0.00103554	mice
Ccl4	1.43	0.00109537	mice
Psmd8	0.69	0.00115143	mice
Zmiz1	-0.73	0.00121964	mice
Ctss	0.81	0.00141168	mice
Cfb	0.88	0.00184501	mice
Iigp1	1.04	0.00206527	mice
Cytip	-0.66	0.00209955	mice
Afmid	-0.67	0.00281862	mice
Necap2	0.66	0.00282093	mice
Cops9	0.57	0.00301528	mice
Picalm	-0.85	0.00303354	mice
Calm1	0.96	0.00303819	mice
Dennd4b	-0.42	0.00334656	mice
Hmgcs1	0.72	0.00349631	mice
Epsti1	0.94	0.00354881	mice
Irf1	0.77	0.00432378	mice
Fcgr2b	-0.55	0.00432725	mice
Psma5	0.57	0.00436925	mice
Zbtb20	-0.73	0.00471289	mice
Sdc3	0.68	0.00474045	mice
Gfra2	-0.37	0.00475797	mice
Ciao2b	0.51	0.00477178	mice
Cldn1	-0.93	0.00490349	mice
Il21r	0.94	0.00490530	mice
Csprs	0.39	0.00522296	mice
Bak1	0.42	0.00580331	mice
Immp2l	-1.17	0.00598213	mice
Cxcl10	1.60	0.00607014	mice
Tmem106a	0.48	0.00642578	mice
Socs2	1.23	0.00642763	mice
Vwa5a	1.07	0.00654518	mice
Cpd	-0.55	0.00671947	mice
Arhgap18	-1.00	0.00697455	mice
Fkbp5	-0.84	0.00731640	mice
Fcgr1	0.69	0.00741862	mice
AY036118	0.64	0.00829576	mice
A930037H05Rik	0.44	0.00834346	mice
Lrp1	-0.85	0.00842979	mice
P3h2	-0.82	0.00853401	mice
Ly6c1	0.86	0.00866444	mice
Wdfy4	-1.03	0.00926850	mice
Nop10	-0.44	0.00929230	mice
Arhgap30	0.60	0.00963586	mice
Cd274	1.39	0.00967602	mice
Sct	1.65	0.01016278	mice

9930111J21Rik2	0.63	0.01128743	mice
Apod	0.98	0.01193815	mice
Jdp2	-1.14	0.01210434	mice
Aff3	-0.89	0.01343574	mice
Apobec1	0.58	0.01430211	mice
Kdm7a	-0.70	0.01455326	mice
Frmd5	-0.86	0.01484367	mice
Ywhae	0.45	0.01570971	mice
Glul	-0.41	0.01586991	mice
S100a6	1.26	0.01592669	mice
Ostf1	1.00	0.01607182	mice
Gpr68	-0.75	0.01615430	mice
Oas2	0.41	0.01621771	mice
Slfn4	0.80	0.01621771	mice
Arpc5l	0.55	0.01673275	mice
Casp3	0.52	0.01676564	mice
Actr2	0.49	0.01725375	mice
Foxo1	-0.81	0.01736641	mice
Il4i1	1.22	0.01812766	mice
Adgre5	-0.62	0.02004434	mice
AW011738	0.75	0.02018686	mice
Tmem123	0.94	0.02037229	mice
Hck	0.84	0.02045050	mice
Fto	-0.56	0.02099626	mice
Arhgap26	-1.27	0.02181482	mice
Cd200	1.14	0.02206055	mice
Tbc1d5	-0.77	0.02229353	mice
Stk10	-0.92	0.02251440	mice
Ifngr1	-0.58	0.02382282	mice
Vps13b	-0.57	0.02395461	mice
Naalad2	-0.37	0.02461050	mice
Arhgap21	-0.59	0.02464164	mice
Ptafr	0.81	0.02465155	mice
Cd9	-1.09	0.02551252	mice
Rtl8a	-0.53	0.02574619	mice
Eps8	-1.82	0.02587064	mice
Smyd3	-0.84	0.02703209	mice
Synj2	-0.38	0.02804704	mice
Pnp	0.86	0.02920110	mice
Scand1	0.57	0.02996140	mice
Foxn3	-0.69	0.03063859	mice
Cybb	0.80	0.03200020	mice
Adar	0.54	0.03250002	mice
H2-Q4	0.47	0.03623307	mice
Xylt1	-0.97	0.03625819	mice
Il1rn	0.97	0.03779116	mice

Resf1	1.19	0.03939234	mice
Ddx58	0.62	0.04046585	mice
Cdc42bpg	-0.46	0.04463422	mice
Cxcl1	0.85	0.04598629	mice
Hdac9	-1.12	0.04827336	mice
Cox5b	0.50	0.04866289	mice
Dynll1	0.56	0.04929321	mice
Sdcbp2	-0.53	0.04933157	mice
Lgmn	1.15	0.04960727	mice
Adap1	0.89	0.05004962	mice
Entpd1	-0.52	0.05006701	mice
Apool	0.45	0.05239008	mice
Ahr	-0.68	0.05367412	mice
BC147527	0.41	0.05445562	mice
Dock5	-0.42	0.05892149	mice
Slfn2	0.95	0.05906482	mice
Rab7b	-0.65	0.06023258	mice
Arg2	0.80	0.06183739	mice
Elmsan1	-0.69	0.06224857	mice
Fam129b	-0.55	0.06230162	mice
Enthd1	-0.63	0.06248244	mice
Fscn1	1.65	0.06620272	mice
Snrnp27	0.42	0.07017962	mice
Itm2b	0.51	0.07219019	mice
Slc22a15	-0.60	0.07309414	mice
Inpp5a	-0.57	0.07413692	mice
Gbp7	0.76	0.07723979	mice
Cxcl2	-0.20	0.07968608	mice
Gngt2	0.64	0.07995588	mice
Zup1	0.49	0.08141704	mice
Airn	-1.86	0.08246296	mice
Itgae	-0.58	0.08255458	mice
Elmod3	-0.33	0.08346165	mice
Ptpn22	-0.78	0.08567779	mice
Clec12a	-0.55	0.08613416	mice
Antxr2	-1.04	0.08706934	mice
Pmvk	0.53	0.09010531	mice
Il18bp	0.34	0.09016995	mice
Cox7c	0.41	0.09025030	mice
Hmgcr	0.36	0.09102155	mice
Ptpn6	0.69	0.09507487	mice
Krcc1	0.56	0.09560063	mice
Cacnb3	0.97	0.09619147	mice
Zeb1	-1.16	0.09679268	mice
Serpina3g	0.85	0.09682160	mice
Agpat4	-0.93	0.09743030	mice

Tomm7	0.50	0.09843130	mice
Spon1	1.00	0.09863658	mice
Aph1c	-0.90	0.09907681	mice
Khdrbs3	-0.34	0.09938971	mice
Fam149a	-0.19	0.10230766	mice
Tor1aip2	0.58	0.10303940	mice
Ogfrl1	0.64	0.10572749	mice
Runx1	-0.47	0.10722640	mice
Dck	0.46	0.10885230	mice
Tmem219	0.52	0.11279939	mice
Lpp	0.76	0.11674729	mice
Gpbp1	0.46	0.12050782	mice
Cd36	-0.24	0.12092861	mice
Ndufa2	0.43	0.12193642	mice
Stk19	0.48	0.12246750	mice
Sptbn1	-0.51	0.12498394	mice
Pdcd4	-0.81	0.12892049	mice
Psmg4	0.45	0.12957278	mice
Casp1	0.34	0.13187533	mice
Pcgf5	1.08	0.14035218	mice
Gdi2	-0.49	0.14056388	mice
Pcyt1a	0.79	0.14304799	mice
Rin3	-0.72	0.14686932	mice
Clec2d	0.50	0.14721238	mice
Syf2	0.32	0.15990129	mice
Ccl5	2.19	0.16006850	mice
Shisa5	0.62	0.16639785	mice
Rbx1	0.44	0.16643997	mice
Ncoa4	0.38	0.17055151	mice
Rap2c	0.41	0.17377007	mice
Slirp	0.36	0.17564723	mice
Ube2l3	0.49	0.17650914	mice
Tmem135	-0.75	0.18050740	mice
Scgb1a1	0.45	0.18152227	mice
Eapp	0.42	0.18419500	mice
Gnptab	0.78	0.19069985	mice
Vkorc1	-0.26	0.19180200	mice
Rhog	0.47	0.19182187	mice
Vav3	-0.78	0.19347744	mice
Gbp8	0.40	0.19731245	mice
Selenow	0.70	0.20192753	mice
Cadm1	-0.74	0.20559076	mice
Cfh	-0.50	0.20562404	mice
Zmiz2	0.52	0.21312355	mice
Gnb4	0.76	0.21582858	mice
Ddx3x	-0.49	0.22491542	mice

Kcnk13	-0.54	0.22584919	mice
Cd81	-0.65	0.24222742	mice
Serpinb6b	1.27	0.24671642	mice
Ogfr	0.48	0.24725502	mice
Stat4	1.40	0.24846244	mice
Plcl1	1.05	0.24885413	mice
Irf8	0.97	0.25095983	mice
Casp4	0.64	0.25118002	mice
Il1rap	-0.77	0.25626218	mice
Sec61g	0.48	0.26297625	mice
Vrk2	0.83	0.26619287	mice
Gramd1b	0.94	0.27094157	mice
Sinhcaf	0.37	0.27716947	mice
Prkcd	0.70	0.27816600	mice
Prkar2a	-0.57	0.29071730	mice
Gpd2	-0.65	0.29725341	mice
Atp5k	0.40	0.30028710	mice
Rsrp1	0.48	0.30212464	mice
Rptor	-0.70	0.30380026	mice
Ddx39	0.29	0.30764658	mice
Snrpc	0.37	0.31095332	mice
Grap	-0.30	0.31700811	mice
St8sia1	0.51	0.31933858	mice
Lacc1	0.42	0.32232759	mice
Arhgap25	-0.78	0.33294971	mice
Gpr141b	-0.19	0.34038276	mice
Zdhhc18	0.67	0.34482527	mice
Irs2	-0.51	0.34612663	mice
Inpp5d	-0.71	0.34621622	mice
Edem1	0.54	0.35298645	mice
Cd2bp2	0.28	0.35546154	mice
Gbp6	0.33	0.36008660	mice
Ifit3b	0.36	0.36008660	mice
Tm6sf1	-0.66	0.36455159	mice
Btbd9	-0.36	0.37438005	mice
Wnt11	-0.29	0.39064705	mice
Glipr2	0.78	0.39129147	mice
Chsy3	-0.76	0.40163112	mice
Dop1b	0.44	0.40627780	mice
Katna1	0.34	0.40923641	mice
Nck1	0.47	0.40986438	mice
Ptprj	-0.70	0.41594548	mice
Sipa1l2	-0.47	0.43397944	mice
Bcor	-0.48	0.43933470	mice
Tsc22d3	-0.63	0.44619888	mice
Acsf3	-0.27	0.46078291	mice

Gpsm3	0.35	0.47352487	mice
Il15	0.99	0.47923178	mice
Vim	-0.61	0.49165562	mice
Dcp2	0.41	0.52659039	mice
Il6	0.62	0.52780451	mice
Oas1g	0.24	0.52780451	mice
Cd14	-1.31	0.53469367	mice
A530032D15Rik	0.42	0.53736698	mice
Clec2i	0.54	0.53791556	mice
Arf4	0.50	0.54356711	mice
Orai1	-0.44	0.55866946	mice
Gbp9	0.30	0.57043635	mice
Cd24a	-0.43	0.57415907	mice
AA467197	0.94	0.58799240	mice
Frmd4b	-1.07	0.60197910	mice
Chn2	-0.63	0.60901773	mice
Ltb	0.61	0.61420689	mice
Pde1b	-0.43	0.63302018	mice
Csnk2b	0.28	0.63916707	mice
Ldlrad4	-0.79	0.64441066	mice
Fnbp1	-0.72	0.64486889	mice
Slc36a4	-0.25	0.64636197	mice
Pde6d	0.21	0.65639976	mice
Ghitm	0.49	0.65938972	mice
Car13	0.67	0.67281974	mice
Sec24d	-0.66	0.68085812	mice
Ccnd2	0.90	0.68098934	mice
Trim12c	0.27	0.68172613	mice
Tmem173	0.33	0.69345141	mice
Svbp	0.83	0.73152684	mice
Sept3	-0.57	0.73283354	mice
Klri1	0.67	0.74968312	mice
Cyp8b1	-0.11	0.75551599	mice
Chd7	-0.63	0.76488576	mice
Zfp608	-0.49	0.78351825	mice
Cyp51	0.40	0.81967859	mice
Atp6v1g1	0.41	0.82546635	mice
Aldoc	-0.11	0.82824712	mice
Atp5e	0.41	0.83604465	mice
Sco1	0.38	0.84959858	mice
Ncf1	0.61	0.86819265	mice
Spg11	0.47	0.87311904	mice
Entpd5	-0.12	0.88710081	mice
Nav2	-0.53	0.89385512	mice
Rab22a	0.61	0.89665716	mice
Nckap1l	-0.82	0.90010880	mice

Tmem128	0.31	0.90618213	mice
Rras2	0.63	0.92304902	mice
Arhgap28	0.71	0.93191404	mice
H2-T24	0.28	0.93702739	mice
Fhit	-0.69	0.96280262	mice
Rcsd1	0.91	0.96282235	mice
Tiam1	-0.78	0.98128514	mice
Coa5	0.33	0.98473812	mice
Uba7	0.33	1.00000000	mice
Rab7	-0.51	1.00000000	mice
Dapk1	-1.05	1.00000000	mice
Atf6	-0.62	1.00000000	mice
Chmp4b	0.43	1.00000000	mice
Cyb5a	-0.44	1.00000000	mice
Sem1	0.37	1.00000000	mice
Ifi213	0.28	1.00000000	mice
Dock7	-0.48	1.00000000	mice
Adk	-0.58	1.00000000	mice
Klri2	0.34	1.00000000	mice
Dst	-1.74	1.00000000	mice
Psmc3	0.34	1.00000000	mice
Lrmp	-0.63	1.00000000	mice
Tapbpl	0.29	1.00000000	mice
Cd40	1.17	1.00000000	mice
Prdx6	-0.40	1.00000000	mice
E030030I06Rik	-0.19	1.00000000	mice
Dek	0.53	1.00000000	mice
Afdn	-0.47	1.00000000	mice
Parp12	0.49	1.00000000	mice
P4htm	-0.30	1.00000000	mice
Pabpc4	-0.24	1.00000000	mice
Itgb7	-0.43	1.00000000	mice
Fdps	0.56	1.00000000	mice
S100a10	-0.48	1.00000000	mice
Stard3	0.28	1.00000000	mice
Rtl8b	-0.26	1.00000000	mice
Ckb	-1.10	1.00000000	mice
Serpinb9	1.40	1.00000000	mice
Pan3	-0.44	1.00000000	mice
Ccser1	-0.93	1.00000000	mice
Arl5a	0.76	1.00000000	mice
Syap1	0.30	1.00000000	mice
Setdb2	0.45	1.00000000	mice
Fam102a	-0.25	1.00000000	mice
Xcr1	-0.47	1.00000000	mice
Zfas1	0.70	1.00000000	mice

Samsn1	1.10	1.00000000	mice
Twf2	0.37	1.00000000	mice
Plce1	-0.26	1.00000000	mice
Fosb	-0.89	1.00000000	mice
Ankrd33b	1.08	1.00000000	mice
Sys1	0.43	1.00000000	mice
Otof	-0.10	1.00000000	mice
Tpt1	-0.34	1.00000000	mice
Rbl1	0.27	1.00000000	mice
Vcpip1	0.45	1.00000000	mice
Psmb7	0.39	1.00000000	mice
Btg1	-0.45	1.00000000	mice
Ndufb11	-0.31	1.00000000	mice
Psmc5	0.35	1.00000000	mice
Arap1	-0.34	1.00000000	mice
Axl	0.52	1.00000000	mice
H2-Q6	0.36	1.00000000	mice
Ndufs5	0.32	1.00000000	mice
Tmem167	0.37	1.00000000	mice
Xist	-0.48	1.00000000	mice
Iffo2	0.42	1.00000000	mice
Tulp4	-0.59	1.00000000	mice
Supt4a	0.45	1.00000000	mice
Rbm47	-0.65	1.00000000	mice
Ddit3	0.33	1.00000000	mice
Ctsd	0.35	1.00000000	mice
Psmc2	0.38	1.00000000	mice
Dcun1d4	-0.14	1.00000000	mice
Fbl	-0.42	1.00000000	mice
Jmjd1c	-0.54	1.00000000	mice
Chd2	-0.54	1.00000000	mice
Ifitm6	0.41	1.00000000	mice
Tfdp2	-0.17	1.00000000	mice
Adora2a	0.60	1.00000000	mice
Tmbim6	0.38	1.00000000	mice
Irgm2	0.20	1.00000000	mice
Cfl1	0.32	1.00000000	mice
Pglyrp1	-0.22	1.00000000	mice
Smpdl3a	-0.35	1.00000000	mice
Ccser2	0.83	1.00000000	mice
Zmat2	0.33	1.00000000	mice
Ldah	0.24	1.00000000	mice
Ccdc149	-0.20	1.00000000	mice
Tdrd7	0.33	1.00000000	mice
Cstb	0.64	1.00000000	mice
Lncpint	-0.51	1.00000000	mice

Ust	-0.75	1.00000000	mice
Atp5j2	0.42	1.00000000	mice
Vti1b	0.38	1.00000000	mice
Parp11	0.40	1.00000000	mice
Tmem184b	0.39	1.00000000	mice
Psmb2	0.37	1.00000000	mice
Ctnnd2	-0.54	1.00000000	mice
S100a1	-0.28	1.00000000	mice
Cysltr1	-0.28	1.00000000	mice
Pttg1	0.26	1.00000000	mice
Slc29a3	0.53	1.00000000	mice
Dbnl	0.42	1.00000000	mice
Anxa1	-0.76	1.00000000	mice
Bend6	-0.21	1.00000000	mice
Arsb	-0.72	1.00000000	mice
Sept6	-0.47	1.00000000	mice
Cd69	0.56	1.00000000	mice
Arrb1	-0.24	1.00000000	mice
Rab21	0.54	1.00000000	mice
Net1	0.57	1.00000000	mice
Slc44a5	0.65	1.00000000	mice
Cd74	-0.63	1.00000000	mice
Dpysl2	-0.45	1.00000000	mice
Ndufv2	0.34	1.00000000	mice
Lrp8	0.49	1.00000000	mice
Gabarap	0.44	1.00000000	mice
Tctex1d2	0.60	1.00000000	mice
Plxna2	-0.33	1.00000000	mice
Psmd2	0.34	1.00000000	mice
Xdh	0.35	1.00000000	mice
Acot11	-0.19	1.00000000	mice
Slc9a4	-0.16	1.00000000	mice
Stxbp6	-0.85	1.00000000	mice
Dok1	0.35	1.00000000	mice
Nr4a2	-0.64	1.00000000	mice
Pi4k2a	-0.54	1.00000000	mice
Cers6	0.91	1.00000000	mice
Chst11	-1.10	1.00000000	mice
Birc5	-0.23	1.00000000	mice
Rbpj	-1.00	1.00000000	mice
Zfp36l2	-0.60	1.00000000	mice
Arih1	-0.54	1.00000000	mice
Vcl	-0.65	1.00000000	mice
Ddt	0.74	1.00000000	mice
Cs	0.26	1.00000000	mice
Pdap1	0.34	1.00000000	mice

Psma2	0.33	1.00000000	mice
Ciao2a	0.39	1.00000000	mice
Fndc3b	-0.82	1.00000000	mice
Ube2i	0.43	1.00000000	mice
Il2ra	0.82	1.00000000	mice
Naaa	-0.84	1.00000000	mice
Gnaq	-0.28	1.00000000	mice
Ski	-0.48	1.00000000	mice
Chmp5	0.35	1.00000000	mice
Fbxo11	-0.43	1.00000000	mice
Ccnd1	0.99	1.00000000	mice
Dnajc7	0.84	1.00000000	mice
Psmc6	0.27	1.00000000	mice
Wdfy3	-0.59	1.00000000	mice
Keap1	0.28	1.00000000	mice
Rab32	-0.24	1.00000000	mice
Sh2d4b	-0.77	1.00000000	mice
Slc11a2	-0.18	1.00000000	mice
H2-Ab1	-0.52	1.00000000	mice
S100a8	0.55	1.00000000	mice
Xlr	-0.40	1.00000000	mice
Sftpc	-0.27	1.00000000	mice
Gramd2	-0.12	1.00000000	mice
Naga	-0.40	1.00000000	mice
Sumf1	0.29	1.00000000	mice
Naa38	0.21	1.00000000	mice
Ddx24	0.33	1.00000000	mice
Furin	-0.67	1.00000000	mice
Smarce1	0.48	1.00000000	mice
Tafa1	-0.42	1.00000000	mice
Atp5b	0.40	1.00000000	mice
Plpp1	-0.62	1.00000000	mice
Itgav	-1.05	1.00000000	mice
Baz2b	-0.57	1.00000000	mice
Nrg1	0.73	1.00000000	mice
Wsb1	-0.37	1.00000000	mice
Cd44	-0.35	1.00000000	mice
Abca9	-0.10	1.00000000	mice
Itpkc	-0.20	1.00000000	mice
Alas1	0.28	1.00000000	mice
Dennd4a	-0.41	1.00000000	mice
Snx29	-0.48	1.00000000	mice
Nfatc2	-0.60	1.00000000	mice
Golga5	0.44	1.00000000	mice
Slc25a22	0.18	1.00000000	mice
Arhgdib	-0.29	1.00000000	mice

Cmpk2	0.34	1.00000000	mice
Ptk2	-0.42	1.00000000	mice
Orai3	0.23	1.00000000	mice
Pnpt1	0.24	1.00000000	mice
Bach2	-0.77	1.00000000	mice
Cd101	-0.28	1.00000000	mice
Pet100	0.29	1.00000000	mice
Lrrc4	0.39	1.00000000	mice
Sfxn3	-0.25	1.00000000	mice
Uhrf1bp1l	-0.53	1.00000000	mice
Esyt2	-0.60	1.00000000	mice
Ubl5	0.38	1.00000000	mice
Gsdmd	0.28	1.00000000	mice
Thoc6	0.22	1.00000000	mice
Capza2	0.43	1.00000000	mice
Iqsec1	-0.48	1.00000000	mice
Abcc4	-0.82	1.00000000	mice
Gpr183	1.35	1.00000000	mice
Cd300c2	-0.36	1.00000000	mice
Tmem86a	0.26	1.00000000	mice
Naca	-0.31	1.00000000	mice
Cd79a	-0.75	1.00000000	mice
Phf11c	0.25	1.00000000	mice
Dram1	0.39	1.00000000	mice
Sat1	0.59	1.00000000	mice
Ccz1	0.35	1.00000000	mice
Slc2a6	0.21	1.00000000	mice
Impdh1	-0.28	1.00000000	mice
Arpp19	0.29	1.00000000	mice
9130230L23Rik	-0.12	1.00000000	mice
Cast	-0.46	1.00000000	mice
Tomm70a	0.43	1.00000000	mice
Tead1	-0.51	1.00000000	mice
Cxcl9	1.12	1.00000000	mice
Nmt1	0.37	1.00000000	mice
Impdh2	-0.35	1.00000000	mice
Tmem14c	0.32	1.00000000	mice
Nectin2	0.23	1.00000000	mice
Inpp5b	0.49	1.00000000	mice
Rgs2	-1.13	1.00000000	mice
Ttc28	-0.37	1.00000000	mice
3830406C13Rik	0.25	1.00000000	mice
Nupr1	0.30	1.00000000	mice
Rhof	-0.64	1.00000000	mice
Itga1	-0.12	1.00000000	mice
Cog2	0.22	1.00000000	mice

Cndp2	0.20	1.00000000	mice
Acer2	-0.11	1.00000000	mice
Srsf5	-0.31	1.00000000	mice
Grn	0.71	1.00000000	mice
Ccnd3	-0.80	1.00000000	mice
Fbrs	0.28	1.00000000	mice
Plbd1	-0.84	1.00000000	mice
Kcnn4	-0.51	1.00000000	mice
Ccl3	0.81	1.00000000	mice
G3bp1	-0.41	1.00000000	mice
Nedd4l	-0.57	1.00000000	mice
App	-0.51	1.00000000	mice
Traf1	0.82	1.00000000	mice
Plcb4	-0.60	1.00000000	mice
Ptpn18	-0.36	1.00000000	mice
Lgals1	0.69	1.00000000	mice
Fam32a	0.71	1.00000000	mice
Elob	0.29	1.00000000	mice
Parp10	0.24	1.00000000	mice
Agpat3	-0.49	1.00000000	mice
Dynlrb1	0.31	1.00000000	mice
Commd3	0.25	1.00000000	mice
5730522E02Rik	-0.34	1.00000000	mice
Cd300lf	0.32	1.00000000	mice
Pdgfb	-0.20	1.00000000	mice
Sap30	0.27	1.00000000	mice
Nptn	-0.35	1.00000000	mice
Kcnk6	0.30	1.00000000	mice
Ndufa11	0.35	1.00000000	mice
Cd209a	-0.74	1.00000000	mice
Plekhg3	-0.29	1.00000000	mice
Snx25	-0.38	1.00000000	mice
Pdlim1	-0.42	1.00000000	mice
Psmd11	0.41	1.00000000	mice
B4galt1	-0.65	1.00000000	mice
Rab18	-0.42	1.00000000	mice
Cyba	0.50	1.00000000	mice
Itpr2	-0.50	1.00000000	mice
Asb8	0.20	1.00000000	mice
Ifi211	0.47	1.00000000	mice
Plxnc1	0.51	1.00000000	mice
Atp6v0c	0.40	1.00000000	mice
Ing1	0.24	1.00000000	mice
Irf2bp2	-0.52	1.00000000	mice
Smg9	-0.36	1.00000000	mice
Clk4	-0.31	1.00000000	mice

Gpr4	-0.22	1.00000000	mice
Znrf2	-0.42	1.00000000	mice
Stt3b	-0.41	1.00000000	mice
Atp6v1c1	0.27	1.00000000	mice
Slc25a17	-0.21	1.00000000	mice
Prkch	-0.78	1.00000000	mice
Esyt3	-0.24	1.00000000	mice
Chd6	-0.37	1.00000000	mice
Tespa1	0.26	1.00000000	mice
Birc2	0.83	1.00000000	mice
Ttc39b	-0.55	1.00000000	mice
Phf20	0.32	1.00000000	mice
Actn1	0.76	1.00000000	mice
Pygm	-0.22	1.00000000	mice
Lgals3	0.85	1.00000000	mice
A630001O12Rik	-0.16	1.00000000	mice
Gadd45b	0.81	1.00000000	mice
Tmem19	0.32	1.00000000	mice
Sbno2	-0.40	1.00000000	mice
Prex1	0.46	1.00000000	mice
Cap1	0.33	1.00000000	mice
Gusb	-0.26	1.00000000	mice
Asb13	0.22	1.00000000	mice
Npepl1	-0.21	1.00000000	mice
Lag3	0.26	1.00000000	mice
Tm4sf5	0.58	1.00000000	mice
Prdx1	0.44	1.00000000	mice
Pam	-0.39	1.00000000	mice
Nudt16l1	0.17	1.00000000	mice
Anxa4	0.70	1.00000000	mice
Socs1	0.28	1.00000000	mice
Ubash3b	-0.80	1.00000000	mice
Lamtor5	0.31	1.00000000	mice
Dhx40	-1.28	1.00000000	mice
Ndufa3	0.28	1.00000000	mice
Csnk1g2	0.26	1.00000000	mice
Slc2a1	-0.42	1.00000000	mice
Gas2l3	-0.43	1.00000000	mice
Cdk9	0.24	1.00000000	mice
Rnase6	0.97	1.00000000	mice
Slc22a21	-0.18	1.00000000	mice
Psmd4	0.42	1.00000000	mice
Pde4b	-0.57	1.00000000	mice
Plcxd2	-0.18	1.00000000	mice
Usp6nl	-0.54	1.00000000	mice
Frg1	0.28	1.00000000	mice

Sec31a	-0.38	1.00000000	mice
Klrb1b	-0.31	1.00000000	mice
Clnk	-0.06	1.00000000	mice
E130102H24Rik	0.17	1.00000000	mice
Tmem256	0.33	1.00000000	mice
Rasgrp1	0.15	1.00000000	mice
Cd59a	0.30	1.00000000	mice
A530040E14Rik	0.24	1.00000000	mice
Prrc2c	0.38	1.00000000	mice
Smagp	-0.16	1.00000000	mice
Rfx7	-0.46	1.00000000	mice
Ipo5	-0.29	1.00000000	mice
Emid1	0.49	1.00000000	mice
Fdft1	0.25	1.00000000	mice
Sorbs1	-0.21	1.00000000	mice
Hsdl1	0.23	1.00000000	mice
Ccrl2	0.68	1.00000000	mice
Spop	0.58	1.00000000	mice
Timp2	-0.21	1.00000000	mice
Vcp	0.31	1.00000000	mice
Psmb6	0.36	1.00000000	mice
Thap3	0.14	1.00000000	mice
E230029C05Rik	-0.65	1.00000000	mice
Jarid2	-0.77	1.00000000	mice
Nmrk1	0.41	1.00000000	mice
Slamf9	0.66	1.00000000	mice
Srek1	-0.42	1.00000000	mice
Cycs	0.64	1.00000000	mice
Arl5c	0.82	1.00000000	mice
Zcchc4	-0.18	1.00000000	mice
Pde3b	-0.51	1.00000000	mice
Pank2	0.28	1.00000000	mice
Arl15	-0.54	1.00000000	mice
Eif2b2	0.18	1.00000000	mice
BC028528	-0.43	1.00000000	mice
Usp9x	-0.39	1.00000000	mice
Cd244a	-0.39	1.00000000	mice
Hebp2	-0.09	1.00000000	mice
Psmb1	0.30	1.00000000	mice
Cdc42se1	0.39	1.00000000	mice
Glrx5	-0.25	1.00000000	mice
Ndufa13	0.35	1.00000000	mice
Zfp809	0.30	1.00000000	mice
Fgr	0.69	1.00000000	mice
Arpc3	0.25	1.00000000	mice
AI662270	0.27	1.00000000	mice

Plk3	-0.17	1.00000000	mice
Oat	-0.26	1.00000000	mice
Pdcd1lg2	0.44	1.00000000	mice
Emc8	0.41	1.00000000	mice
Olfm1	-0.48	1.00000000	mice
Cgref1	-0.10	1.00000000	mice
Sgcb	0.24	1.00000000	mice
Plekho2	-0.45	1.00000000	mice
Cbx5	0.22	1.00000000	mice
2610301B20Rik	0.16	1.00000000	mice
Plaat3	0.37	1.00000000	mice
Stk39	0.61	1.00000000	mice
Parvb	-0.25	1.00000000	mice
Ier5	0.44	1.00000000	mice
Pigyl	0.17	1.00000000	mice
Atp6v0b	0.49	1.00000000	mice
Emp1	-0.42	1.00000000	mice
Ppdpf	0.48	1.00000000	mice
Srsf9	0.28	1.00000000	mice
Selplg	0.29	1.00000000	mice
Smox	0.43	1.00000000	mice
Traf3	0.70	1.00000000	mice
Batf	-0.34	1.00000000	mice
Fhad1	-0.23	1.00000000	mice
Fabp5	1.19	1.00000000	mice
Havcr2	-0.56	1.00000000	mice
Lrrfip1	-0.53	1.00000000	mice
Tnfaip2	0.57	1.00000000	mice
Adss	-0.34	1.00000000	mice
Rnf145	-0.27	1.00000000	mice
Scimp	0.53	1.00000000	mice
Pomp	0.34	1.00000000	mice
Ttll3	-0.24	1.00000000	mice
Syn3	0.44	1.00000000	mice
A630012P03Rik	0.27	1.00000000	mice
Ttc1	0.23	1.00000000	mice
Aif1	0.64	1.00000000	mice
Olfr920	0.30	1.00000000	mice
Fam107b	-0.68	1.00000000	mice
Cd37	-0.40	1.00000000	mice
Eif3h	-0.28	1.00000000	mice
Ifi208	0.18	1.00000000	mice
Pgm2l1	-0.30	1.00000000	mice
Aacs	0.19	1.00000000	mice
Dync1i2	0.23	1.00000000	mice
Dnajb6	0.28	1.00000000	mice

Puf60	-0.38	1.00000000	mice
Son	0.27	1.00000000	mice
Adam17	0.31	1.00000000	mice
Fkbp1b	-0.21	1.00000000	mice
Traf2	0.29	1.00000000	mice
Slc38a2	0.48	1.00000000	mice
A330023F24Rik	-0.31	1.00000000	mice
Ccdc138	0.53	1.00000000	mice
Gca	0.26	1.00000000	mice
Tns3	-0.75	1.00000000	mice
Prxl2c	-0.25	1.00000000	mice
Ist1	0.28	1.00000000	mice
1110004F10Rik	0.22	1.00000000	mice
Ift22	0.17	1.00000000	mice
Blk	-0.36	1.00000000	mice
Il12a	-0.24	1.00000000	mice
Lilra6	0.18	1.00000000	mice
Ctnnd1	-0.23	1.00000000	mice
2310009A05Rik	0.15	1.00000000	mice
Adam15	-0.16	1.00000000	mice
Nsf	-0.51	1.00000000	mice
Serping1	0.15	1.00000000	mice
Rchy1	0.28	1.00000000	mice
Tmcc3	1.30	1.00000000	mice
Lsr	-0.16	1.00000000	mice
Zbtb44	-0.43	1.00000000	mice
Chmp2a	0.33	1.00000000	mice
Diaph3	-0.30	1.00000000	mice
Pabpn1	0.28	1.00000000	mice
Psma1	0.34	1.00000000	mice
Ptpn3	0.31	1.00000000	mice
4732471J01Rik	-0.25	1.00000000	mice
C3	0.14	1.00000000	mice
Nup210l	-0.74	1.00000000	mice
Rab8a	0.36	1.00000000	mice
4930445E18Rik	-0.15	1.00000000	mice
Rarb	-0.26	1.00000000	mice
Pianp	-0.12	1.00000000	mice
Nop9	0.20	1.00000000	mice
Lamp2	0.34	1.00000000	mice
Etfa	0.32	1.00000000	mice
Pisd	-0.26	1.00000000	mice
Afap1l2	-0.20	1.00000000	mice
2510002D24Rik	-0.14	1.00000000	mice
Ppp2ca	-0.27	1.00000000	mice
Zfp871	0.27	1.00000000	mice

Pag1	-0.19	1.00000000	mice
Cope	0.26	1.00000000	mice
Pirb	0.43	1.00000000	mice
Cyb5r4	0.24	1.00000000	mice
Prelid3b	0.21	1.00000000	mice
Bclaf3	0.32	1.00000000	mice
Slc15a4	0.31	1.00000000	mice
Serf2	0.28	1.00000000	mice
Krt80	-0.13	1.00000000	mice
Aaas	0.12	1.00000000	mice
Ifit1bl1	0.19	1.00000000	mice
Vdac3	0.20	1.00000000	mice
Ccdc12	0.34	1.00000000	mice
Akt3	0.53	1.00000000	mice
Limd2	0.21	1.00000000	mice
Cd63	1.22	1.00000000	mice
Gdpd3	-0.23	1.00000000	mice
N4bp1	-0.56	1.00000000	mice
Lims1	0.39	1.00000000	mice
Psma6	0.36	1.00000000	mice
H2afx	-0.29	1.00000000	mice
Ppfibp2	-0.61	1.00000000	mice
Notch2	-0.50	1.00000000	mice
Pot1b	0.52	1.00000000	mice
Cmip	-0.38	1.00000000	mice
Fam219a	0.14	1.00000000	mice
Tifab	0.21	1.00000000	mice
Crtam	-0.32	1.00000000	mice
Senp6	-0.22	1.00000000	mice
Gde1	-0.22	1.00000000	mice
Apobec3	0.27	1.00000000	mice
Slc35e1	0.04	1.00000000	mice
Fam220a.1	0.25	1.00000000	mice
Uqcrq	0.28	1.00000000	mice
Hat1	0.38	1.00000000	mice
Eif1	-0.17	1.00000000	mice
Camk1d	-0.34	1.00000000	mice
1700109H08Rik	-0.23	1.00000000	mice
Urb1	-0.11	1.00000000	mice
Cpne1	0.18	1.00000000	mice
Eif2s2	0.29	1.00000000	mice
Psmd10	0.20	1.00000000	mice
Ranbp2	0.70	1.00000000	mice
Pttg1ip	-0.34	1.00000000	mice
Ppp1r1c	-0.05	1.00000000	mice
Ripk2	0.54	1.00000000	mice

Polg	-0.13	1.00000000	mice
Wdr18	-0.26	1.00000000	mice
Nipsnap3b	0.24	1.00000000	mice
Lrrc58	-0.30	1.00000000	mice
2610001J05Rik	0.23	1.00000000	mice
Alyref2	-0.15	1.00000000	mice
Farp2	-0.15	1.00000000	mice
Fgf13	-0.44	1.00000000	mice
Rhoa	0.22	1.00000000	mice
Eloa	0.24	1.00000000	mice
Trappc2l	0.26	1.00000000	mice
Npm1	-0.38	1.00000000	mice
Ap3m1	0.19	1.00000000	mice
Lamc1	-0.46	1.00000000	mice
Lrch4	0.19	1.00000000	mice
Bbip1	0.17	1.00000000	mice
Poglut1	0.81	1.00000000	mice
Fcer1g	0.75	1.00000000	mice
Col3a1	0.17	1.00000000	mice
Rsl1d1	-0.30	1.00000000	mice
Copz1	0.30	1.00000000	mice
Lrrc8d	-0.46	1.00000000	mice
Hmgn3	0.46	1.00000000	mice
Ctnnb1	0.34	1.00000000	mice
S100a4	0.86	1.00000000	mice
Xiap	0.24	1.00000000	mice
1600014C10Rik	0.18	1.00000000	mice
Tmem176a	0.62	1.00000000	mice
Pnrc2	0.18	1.00000000	mice
Tmem238	-0.16	1.00000000	mice
Gosr1	0.15	1.00000000	mice
Slco4a1	0.25	1.00000000	mice
Pbx1	-1.49	1.00000000	mice
Ifitm2	0.77	1.00000000	mice
Ctnnbip1	-0.23	1.00000000	mice
Fth1	-0.57	1.00000000	mice
A430005L14Rik	0.15	1.00000000	mice
Sowahc	0.40	1.00000000	mice
Dennd1b	-0.49	1.00000000	mice
Chpt1	0.33	1.00000000	mice
Cox6c	0.30	1.00000000	mice
Adgrg6	1.42	1.00000000	mice
Ccr7	1.75	1.00000000	mice
LTO1	0.18	1.00000000	mice
Rab29	0.20	1.00000000	mice
Top1	-0.35	1.00000000	mice

Derl2	0.20	1.00000000	mice
H1f0	0.29	1.00000000	mice
Prkab2	-0.15	1.00000000	mice
Borcs8	0.26	1.00000000	mice
Ints10	0.11	1.00000000	mice
Tcea1	0.34	1.00000000	mice
Anxa3	0.53	1.00000000	mice
Ppif	0.26	1.00000000	mice
Atp5g2	-0.22	1.00000000	mice
Atp5j	0.26	1.00000000	mice
Ankrd28	-0.35	1.00000000	mice
Prps1l3	-0.10	1.00000000	mice
Aagab	0.25	1.00000000	mice
Trerf1	-0.46	1.00000000	mice
Ubtd1	0.21	1.00000000	mice
Tarbp1	-0.23	1.00000000	mice
Vdac2	0.32	1.00000000	mice
Satb2	-0.47	1.00000000	mice
Idh1	0.17	1.00000000	mice
Dxo	0.16	1.00000000	mice
Lsmem1	-0.36	1.00000000	mice
Treml2	0.22	1.00000000	mice
Smad4	-0.21	1.00000000	mice
Snrpb2	0.29	1.00000000	mice
9330175E14Rik	0.13	1.00000000	mice
Dgkd	-0.46	1.00000000	mice
Arhgef2	0.26	1.00000000	mice
Cttnbp2nl	0.31	1.00000000	mice
Usf1	0.25	1.00000000	mice
Crip2	-0.18	1.00000000	mice
Neurl3	-0.32	1.00000000	mice
Saa3	0.24	1.00000000	mice
Hax1	0.23	1.00000000	mice
Serpinf1	-0.13	1.00000000	mice
Akr1a1	0.44	1.00000000	mice
Dnttip1	0.13	1.00000000	mice
Rgs19	0.15	1.00000000	mice
Coa3	0.11	1.00000000	mice
Washc1	0.23	1.00000000	mice
Gtf2a1	0.51	1.00000000	mice
Ahcyl1	0.32	1.00000000	mice
Pde1c	-0.51	1.00000000	mice
Crim1	-0.68	1.00000000	mice
Zcchc2	0.26	1.00000000	mice
Relb	0.67	1.00000000	mice
Pdlim5	-0.37	1.00000000	mice

Htra2	0.33	1.00000000	mice
Ppia	0.23	1.00000000	mice
Btbd1	-0.33	1.00000000	mice
D1Ertd622e	0.23	1.00000000	mice
Tsr3	0.13	1.00000000	mice
Clasp2	-0.27	1.00000000	mice
Bach1	-0.59	1.00000000	mice
Pepd	-0.27	1.00000000	mice
Ndufs7	0.24	1.00000000	mice
Rgs9	-0.13	1.00000000	mice
Ndufa7	0.19	1.00000000	mice
Cd226	-0.19	1.00000000	mice
Arhgef18	-0.31	1.00000000	mice
Eml6	0.82	1.00000000	mice
Hdlbp	-0.26	1.00000000	mice
Hlcs	-0.21	1.00000000	mice
Psma3	0.26	1.00000000	mice
Lrrc49	-0.16	1.00000000	mice
Pgd	0.20	1.00000000	mice
Sox5	-0.29	1.00000000	mice
Fam120b	0.23	1.00000000	mice
Arl14ep	0.13	1.00000000	mice
Runx2	0.71	1.00000000	mice
Cyth1	-0.34	1.00000000	mice
Tap2	0.39	1.00000000	mice
Col27a1	-0.11	1.00000000	mice
Pfdn1	0.18	1.00000000	mice
Pus10	0.27	1.00000000	mice
4930595D18Rik	0.18	1.00000000	mice
Dtd2	0.13	1.00000000	mice
Usp28	-0.39	1.00000000	mice
Iscu	0.58	1.00000000	mice
G3bp2	0.34	1.00000000	mice
Osbpl2	-0.20	1.00000000	mice
Cpeb2	-0.39	1.00000000	mice
Rnf24	0.39	1.00000000	mice
Hhex	0.19	1.00000000	mice
Ostm1	0.18	1.00000000	mice
Eya3	0.50	1.00000000	mice
Ppt1	-0.50	1.00000000	mice
Pacsin2	-0.59	1.00000000	mice
Tgm2	0.39	1.00000000	mice
Gnai2	0.26	1.00000000	mice
Clec4b1	-0.14	1.00000000	mice
Unkl	-0.29	1.00000000	mice
Ift46	-0.14	1.00000000	mice

Dcstamp	-0.26	1.00000000	mice
Slc1a5	-0.23	1.00000000	mice
Fam192a	0.19	1.00000000	mice
Akirin1	0.26	1.00000000	mice
Slc38a1	-0.40	1.00000000	mice
Plxna1	-0.09	1.00000000	mice
Ccdc122	0.17	1.00000000	mice
Tmem140	0.16	1.00000000	mice
Rnf38	-0.34	1.00000000	mice
Peg13	-0.17	1.00000000	mice
Tma7	0.24	1.00000000	mice
Dnase1l3	0.73	1.00000000	mice
Plcl2	0.71	1.00000000	mice
Nub1	0.27	1.00000000	mice
Atl3	0.38	1.00000000	mice
Ndufaf3	0.12	1.00000000	mice
Slc22a5	-0.14	1.00000000	mice
Arhgap4	-0.10	1.00000000	mice
Usp15	-0.28	1.00000000	mice
Itsn2	-0.43	1.00000000	mice
Svil	-0.52	1.00000000	mice
Pik3c2a	-0.20	1.00000000	mice
Casr	-0.43	1.00000000	mice
Atp5l	0.27	1.00000000	mice
Nsdhl	0.17	1.00000000	mice
Baiap2	-0.35	1.00000000	mice
Tshz1	-0.21	1.00000000	mice
Nectin4	0.16	1.00000000	mice
Bex6	0.18	1.00000000	mice
Trnt1	0.18	1.00000000	mice
Src	-0.27	1.00000000	mice
Ccny	-0.36	1.00000000	mice
Ndufv3	0.24	1.00000000	mice
Xpo1	0.31	1.00000000	mice
Klf13	-0.51	1.00000000	mice
Pink1	-0.20	1.00000000	mice
Slc14a1	-0.13	1.00000000	mice
Cdip1	0.39	1.00000000	mice
Prpf38b	0.32	1.00000000	mice
Hnrnpul1	0.33	1.00000000	mice
Calm3	0.41	1.00000000	mice
Acp1	0.15	1.00000000	mice
Bola1	0.13	1.00000000	mice
Cnp	0.40	1.00000000	mice
Calhm6	0.24	1.00000000	mice
Serpine1	0.19	1.00000000	mice

Galnt7	-0.66	1.00000000	mice
Ppp3r1	0.16	1.00000000	mice
Fig4	-0.32	1.00000000	mice
Polr3c	0.23	1.00000000	mice
Cntrl	0.20	1.00000000	mice
Tom1l2	-0.28	1.00000000	mice
Casp9	0.11	1.00000000	mice
Atg101	0.20	1.00000000	mice
Ero1lb	-0.59	1.00000000	mice
Hsd11b1	0.37	1.00000000	mice
4930520O04Rik	0.28	1.00000000	mice
Oxct1	-0.27	1.00000000	mice
Dhrs7	-0.25	1.00000000	mice
Pnpo	-0.19	1.00000000	mice
Gng10	-0.20	1.00000000	mice
Cript	0.19	1.00000000	mice
Ass1	0.65	1.00000000	mice
Rnf6	0.44	1.00000000	mice
Ccl17	-0.55	1.00000000	mice
Ptgs2	0.75	1.00000000	mice
Cited2	0.52	1.00000000	mice
Rbm42	0.24	1.00000000	mice
Rasgrp3	-0.36	1.00000000	mice
Xrn1	0.32	1.00000000	mice
Cdc16	0.19	1.00000000	mice
Gpank1	0.17	1.00000000	mice
Prep	-0.26	1.00000000	mice
Hmg20b	0.24	1.00000000	mice
Trim56	0.16	1.00000000	mice
Psmd6	0.17	1.00000000	mice
A330040F15Rik	0.17	1.00000000	mice
Rogdi	0.55	1.00000000	mice
Tgtp2	0.15	1.00000000	mice
Pxk	-0.22	1.00000000	mice
Sec13	0.18	1.00000000	mice
Carhsp1	0.23	1.00000000	mice
Clec9a	-0.35	1.00000000	mice
Slc20a1	-0.25	1.00000000	mice
Akap10	0.35	1.00000000	mice
Slc25a12	0.56	1.00000000	mice
Upf3a	0.19	1.00000000	mice
Carmil1	0.99	1.00000000	mice
Trim30b	0.12	1.00000000	mice
Zmynd8	0.28	1.00000000	mice
Wars	0.14	1.00000000	mice
Ceacam16	0.17	1.00000000	mice

Atp8b4	-0.49	1.00000000	mice
Tnfrsf11a	0.40	1.00000000	mice
Sppl2a	0.32	1.00000000	mice
Sdhb	0.29	1.00000000	mice
Aff1	-0.75	1.00000000	mice
Zfp131	0.26	1.00000000	mice
Aurkaip1	0.23	1.00000000	mice
Plbd2	0.21	1.00000000	mice
Creb5	0.79	1.00000000	mice
Pdzd11	0.14	1.00000000	mice
Ap1b1	-0.32	1.00000000	mice
Tcn2	0.16	1.00000000	mice
Tsc22d4	0.25	1.00000000	mice
Psmd14	0.26	1.00000000	mice
Dnm1l	0.38	1.00000000	mice
Rras	0.20	1.00000000	mice
Fgd3	-0.23	1.00000000	mice
Cdkn2aip	-0.16	1.00000000	mice
Asb6	0.15	1.00000000	mice
Polr3f	0.10	1.00000000	mice
Polr2k	0.24	1.00000000	mice
Cdipt	0.19	1.00000000	mice
2310011J03Rik	0.17	1.00000000	mice
Utp14a	-0.21	1.00000000	mice
Tmub1	0.11	1.00000000	mice
Pid1	-0.62	1.00000000	mice
Ppp1cb	0.35	1.00000000	mice
Fgd6	-0.20	1.00000000	mice
Fam89a	0.31	1.00000000	mice
Gbp3	0.31	1.00000000	mice
Cox7b	0.31	1.00000000	mice
A3galt2	-0.20	1.00000000	mice
Utp3	0.26	1.00000000	mice
Csf2rb	0.46	1.00000000	mice
Ube2q1	0.27	1.00000000	mice
Eif3f	-0.27	1.00000000	mice
Dgka	-0.05	1.00000000	mice
Fbxl20	0.32	1.00000000	mice
Top2b	-0.24	1.00000000	mice
Siah2	-0.27	1.00000000	mice
Ppp4r2	0.40	1.00000000	mice
Phf14	0.23	1.00000000	mice
Pard3b	-0.62	1.00000000	mice
Csnk1d	0.22	1.00000000	mice
Hist1h2bc	0.26	1.00000000	mice
Phyh	0.26	1.00000000	mice

Smurf2	-0.37	1.00000000	mice
Nolc1	-0.40	1.00000000	mice
Commd7	0.17	1.00000000	mice
Tmem167b	0.12	1.00000000	mice
Atp2a2	-0.30	1.00000000	mice
A930007I19Rik	0.20	1.00000000	mice
Hk2	-0.45	1.00000000	mice
AI837181	0.18	1.00000000	mice
Sec24a	-0.27	1.00000000	mice
Tmub2	0.12	1.00000000	mice
Clec4a3	0.40	1.00000000	mice
Rnf135	0.13	1.00000000	mice
Uqcrc2	0.22	1.00000000	mice
Fam114a2	0.13	1.00000000	mice
1700025G04Rik	-0.24	1.00000000	mice
Amz1	-0.19	1.00000000	mice
Cnr2	-0.11	1.00000000	mice
Adam19	-0.36	1.00000000	mice
Kif1b	0.29	1.00000000	mice
Satb1	-0.84	1.00000000	mice
Fbxl17	-0.27	1.00000000	mice
Fcgrt	-0.21	1.00000000	mice
Dtnbp1	-0.47	1.00000000	mice
Sqle	0.20	1.00000000	mice
4933406I18Rik	-0.29	1.00000000	mice
Snx2	0.38	1.00000000	mice
Ndufb9	0.25	1.00000000	mice
Dock4	-0.48	1.00000000	mice
Zfp414	0.12	1.00000000	mice
Elof1	0.21	1.00000000	mice
Ssr3	0.17	1.00000000	mice
Brwd1	-0.23	1.00000000	mice
Lamtor1	0.29	1.00000000	mice
Hebp1	0.19	1.00000000	mice
Atxn1	0.43	1.00000000	mice
AI987944	0.13	1.00000000	mice
Abhd2	0.25	1.00000000	mice
Hspa9	-0.31	1.00000000	mice
Rab5if	0.28	1.00000000	mice
Atp11c	-0.26	1.00000000	mice
Eif1ax	0.28	1.00000000	mice
Rnh1	0.36	1.00000000	mice
Atrnl1	0.54	1.00000000	mice
Gcn1	-0.23	1.00000000	mice
Ascc3	0.36	1.00000000	mice
Reps1	-0.58	1.00000000	mice

Ppp3ca	-0.33	1.00000000	mice
Tmem242	0.15	1.00000000	mice
Gypc	0.65	1.00000000	mice
Aup1	0.17	1.00000000	mice
Ubxn2a	0.33	1.00000000	mice
Lrrc41	0.18	1.00000000	mice
Gnai3	0.32	1.00000000	mice
Clec10a	0.15	1.00000000	mice
5031439G07Rik	-0.23	1.00000000	mice
Usp36	-0.14	1.00000000	mice
Psme2b	0.14	1.00000000	mice
Sap18	0.24	1.00000000	mice
Rab11fip4	-0.14	1.00000000	mice
Ube2d2a	0.19	1.00000000	mice
Rab14	0.26	1.00000000	mice
Slc22a4	-0.32	1.00000000	mice
Nop58	-0.31	1.00000000	mice
Cdk6	-0.43	1.00000000	mice
Arrb2	0.28	1.00000000	mice
Cmpk1	0.22	1.00000000	mice
H3f3b	0.30	1.00000000	mice
Cops5	0.19	1.00000000	mice
Banf1	0.23	1.00000000	mice
Tenm4	-0.24	1.00000000	mice
Tsix	0.17	1.00000000	mice
Inpp5f	0.12	1.00000000	mice
Acvrl1	-0.18	1.00000000	mice
Atp6v1b2	0.32	1.00000000	mice
Ints4	0.15	1.00000000	mice
Dhrs9	-0.13	1.00000000	mice
Atp6v1d	0.30	1.00000000	mice
Pde7b	0.87	1.00000000	mice
Zkscan17	-0.19	1.00000000	mice
Ctsb	0.30	1.00000000	mice
Lasp1	0.21	1.00000000	mice
Phldb1	-0.35	1.00000000	mice
Olfr1459	-0.08	1.00000000	mice
Hspa1a	0.10	1.00000000	mice
Wbp4	0.16	1.00000000	mice
Kit	-0.42	1.00000000	mice
Dym	-0.35	1.00000000	mice
Rassf3	0.28	1.00000000	mice
Plxnd1	0.42	1.00000000	mice
Srp19	0.28	1.00000000	mice
Hivep2	-0.48	1.00000000	mice
Uxt	0.11	1.00000000	mice

Ppm1b	-0.33	1.00000000	mice
Ilrun	0.36	1.00000000	mice
Ints7	-0.31	1.00000000	mice
Ppp1r11	0.28	1.00000000	mice
Cfap43	-0.33	1.00000000	mice
Cox5a	0.30	1.00000000	mice
Pin4	0.25	1.00000000	mice
Rbm19	-0.19	1.00000000	mice
Dicer1	-0.31	1.00000000	mice
Smim20	0.15	1.00000000	mice
Rnf25	0.16	1.00000000	mice
Ankrd46	-0.12	1.00000000	mice
Il13ra1	-0.19	1.00000000	mice
Nudt17	0.30	1.00000000	mice
Tbc1d22a	-0.43	1.00000000	mice
Lpin2	-0.42	1.00000000	mice
Cd8a	0.53	1.00000000	mice
Camsap1	0.15	1.00000000	mice
Flad1	0.08	1.00000000	mice
D030056L22Rik	0.15	1.00000000	mice
2610507B11Rik	0.15	1.00000000	mice
Dync1li1	0.19	1.00000000	mice
Cd79b	-0.36	1.00000000	mice
H2-Aa	-0.33	1.00000000	mice
Tle1	-0.18	1.00000000	mice
Krr1	-0.21	1.00000000	mice
Abce1	-0.19	1.00000000	mice
Ints14	-0.63	1.00000000	mice
Tnfaip8	-0.50	1.00000000	mice
Tle3	-0.35	1.00000000	mice
Vdac1	0.21	1.00000000	mice
Phip	0.43	1.00000000	mice
Dusp7	-0.08	1.00000000	mice
1810064F22Rik	0.45	1.00000000	mice
Bmpr2	-0.39	1.00000000	mice
Flt3	-0.44	1.00000000	mice
Unc119b	-0.19	1.00000000	mice
Eif3c	0.27	1.00000000	mice
Tmem243	-0.31	1.00000000	mice
Tnrc6c	-0.34	1.00000000	mice
Exoc4	-0.26	1.00000000	mice
Eef1akmt2	0.08	1.00000000	mice
C2cd5	0.19	1.00000000	mice
Pxn	-0.36	1.00000000	mice
Eno3	0.30	1.00000000	mice
Nectin1	-0.54	1.00000000	mice

Cenpv	-0.10	1.00000000	mice
Usp2	0.09	1.00000000	mice
Pdcd6	-0.17	1.00000000	mice
Trir	0.22	1.00000000	mice
Senp2	-0.40	1.00000000	mice
Itga6	-0.17	1.00000000	mice
Abcb1a	0.20	1.00000000	mice
Naa30	-0.17	1.00000000	mice
Gcnt2	0.41	1.00000000	mice
Ccdc88b	0.12	1.00000000	mice
Klhl28	0.17	1.00000000	mice
Rbis	0.20	1.00000000	mice
Rnaseh2b	0.25	1.00000000	mice
Tmem234	0.27	1.00000000	mice
Notch4	-0.10	1.00000000	mice
Gyg	0.51	1.00000000	mice
Atp9b	-0.30	1.00000000	mice
Dnajc10	0.34	1.00000000	mice
Sin3b	0.27	1.00000000	mice
Errfi1	-0.37	1.00000000	mice
Capn15	-0.24	1.00000000	mice
Ccdc57	-0.08	1.00000000	mice
Cox4i1	0.28	1.00000000	mice
Ddx41	0.15	1.00000000	mice
Nsun6	-0.23	1.00000000	mice
Ints11	0.11	1.00000000	mice
Lrrc8b	-0.24	1.00000000	mice
Sod1	0.31	1.00000000	mice
Fuca1	-0.28	1.00000000	mice
Clcn6	0.17	1.00000000	mice
Pkp3	0.19	1.00000000	mice
Erc1	-0.55	1.00000000	mice
Smim3	-0.38	1.00000000	mice
Tmco3	-0.38	1.00000000	mice
Dleu2	-0.33	1.00000000	mice
Ankrd27	0.14	1.00000000	mice
1700056N10Rik	-0.08	1.00000000	mice
Atp6v0a1	0.46	1.00000000	mice
Ago4	0.17	1.00000000	mice
Naa15	-0.26	1.00000000	mice
Lpcat4	-0.12	1.00000000	mice
Slc15a3	0.38	1.00000000	mice
Pim1	0.47	1.00000000	mice
Zfp984	0.16	1.00000000	mice
Cd209d	-0.43	1.00000000	mice
Sufu	-0.31	1.00000000	mice

Trim16	-0.09	1.00000000	mice
Ebpl	-0.15	1.00000000	mice
Slc3a2	0.33	1.00000000	mice
Psd3	-0.23	1.00000000	mice
Cox7a2l	-0.19	1.00000000	mice
Tmpo	0.31	1.00000000	mice
Zfp626	0.11	1.00000000	mice
Rab5a	-0.19	1.00000000	mice
Suclg1	0.19	1.00000000	mice
St3gal6	-0.18	1.00000000	mice
Fbxl2	0.08	1.00000000	mice
Cox16	-0.30	1.00000000	mice
Larp4b	-0.37	1.00000000	mice
Rbm18	-0.15	1.00000000	mice
Stk4	0.38	1.00000000	mice
Smim4	0.16	1.00000000	mice
Ppp1r7	0.17	1.00000000	mice
Cpq	-0.24	1.00000000	mice
Selenok	0.19	1.00000000	mice
Tmem138	0.15	1.00000000	mice
Rnf126	-0.15	1.00000000	mice
Eef1d	-0.17	1.00000000	mice
Il12b	2.40	1.00000000	mice
Brap	0.17	1.00000000	mice
St3gal1	-0.61	1.00000000	mice
Ddrgk1	-0.21	1.00000000	mice
Tspan5	-0.45	1.00000000	mice
Wnk1	-0.20	1.00000000	mice
Gramd1a	-0.24	1.00000000	mice
Plekhm2	0.60	1.00000000	mice
Sla	-0.31	1.00000000	mice
Klhl12	-0.10	1.00000000	mice
Vps13a	-0.44	1.00000000	mice
Hist1h1c	0.61	1.00000000	mice
1700123O20Rik	0.19	1.00000000	mice
Slco3a1	-0.36	1.00000000	mice
Prrc2b	-0.32	1.00000000	mice
Chmp6	0.12	1.00000000	mice
Ier3ip1	0.23	1.00000000	mice
Ap2a1	0.17	1.00000000	mice
Uqcr10	0.23	1.00000000	mice
B3galnt2	-0.18	1.00000000	mice
Tmem160	0.23	1.00000000	mice
Pfkp	-0.45	1.00000000	mice
Zdhhc13	0.17	1.00000000	mice
Pmaip1	0.30	1.00000000	mice

Qdpr	-0.19	1.00000000	mice
Klf2	-0.43	1.00000000	mice
B4galt4	0.11	1.00000000	mice
Galns	0.12	1.00000000	mice
Cox7a2	0.22	1.00000000	mice
Rnf34	0.17	1.00000000	mice
Ppil3	0.15	1.00000000	mice
Arl6ip1	0.25	1.00000000	mice
Slc30a5	-0.23	1.00000000	mice
Ggnbp2	-0.29	1.00000000	mice
D730003I15Rik	0.09	1.00000000	mice
Aopep	-0.30	1.00000000	mice
Eya1	-0.46	1.00000000	mice
Evi2a	0.30	1.00000000	mice
Dusp11	0.23	1.00000000	mice
Vapb	0.19	1.00000000	mice
Dock11	0.37	1.00000000	mice
Eaf1	-0.16	1.00000000	mice
Psap	-0.26	1.00000000	mice
Stau1	-0.17	1.00000000	mice
AU040320	0.30	1.00000000	mice
Ptpro	-0.24	1.00000000	mice
Huwe1	-0.31	1.00000000	mice
Pnkp	0.17	1.00000000	mice
Nlrp3	-0.66	1.00000000	mice
Scyl2	0.24	1.00000000	mice
Cwc15	0.20	1.00000000	mice
Ndufa12	-0.19	1.00000000	mice
P2ry6	-0.24	1.00000000	mice
Cd2ap	0.38	1.00000000	mice
Slc25a1	0.16	1.00000000	mice
Gxylt1	-0.17	1.00000000	mice
Utp6	-0.21	1.00000000	mice
Pltp	0.82	1.00000000	mice
Fam214b	-0.14	1.00000000	mice
Adprm	0.18	1.00000000	mice
Grcc10	-0.17	1.00000000	mice
Pip4k2c	0.16	1.00000000	mice
Timm10b	0.31	1.00000000	mice
Lnpep	0.34	1.00000000	mice
Tyrobp	0.32	1.00000000	mice
Atg14	0.15	1.00000000	mice
Txndc17	0.51	1.00000000	mice
Tstd2	0.11	1.00000000	mice
Abcf1	0.31	1.00000000	mice
Clip1	0.33	1.00000000	mice

Sart1	0.18	1.00000000	mice
1110038F14Rik	0.16	1.00000000	mice
Snrpe	0.25	1.00000000	mice
Nr1d2	-0.14	1.00000000	mice
Prkacb	-0.27	1.00000000	mice
Ndrg1	0.59	1.00000000	mice
Zc3hav1l	-0.19	1.00000000	mice
Nostrin	0.48	1.00000000	mice
Tcf20	0.22	1.00000000	mice
Ncoa7	0.67	1.00000000	mice
Lrrk2	-0.78	1.00000000	mice
Tnfsf13b	-0.07	1.00000000	mice
Gtf2h4	0.06	1.00000000	mice
Trpm7	-0.32	1.00000000	mice
Helq	0.11	1.00000000	mice
Cbx4	0.20	1.00000000	mice
Wars2	-0.29	1.00000000	mice
Jmy	-0.27	1.00000000	mice
Abcc1	-0.33	1.00000000	mice
Eif4b	-0.27	1.00000000	mice
Hdac1	0.24	1.00000000	mice
Ets2	0.34	1.00000000	mice
Bnip2	0.26	1.00000000	mice
Pgm1	0.14	1.00000000	mice
Chmp1b	0.18	1.00000000	mice
Ptpn12	-0.19	1.00000000	mice
Dock2	-0.30	1.00000000	mice
Clint1	-0.30	1.00000000	mice
Git2	-0.32	1.00000000	mice
Smarcd2	0.17	1.00000000	mice
Ago2	-0.49	1.00000000	mice
Paip1	0.10	1.00000000	mice
Daglb	-0.12	1.00000000	mice
Bco2	-0.24	1.00000000	mice
Odf2	-0.28	1.00000000	mice
Napsa	0.39	1.00000000	mice
Tmsb10	0.63	1.00000000	mice
Plagl2	0.19	1.00000000	mice
Otulin	0.07	1.00000000	mice
Rap1gds1	-0.38	1.00000000	mice
Zdhhc20	-0.30	1.00000000	mice
N4bp2l1	0.26	1.00000000	mice
Ehmt2	0.17	1.00000000	mice
Hikeshi	0.13	1.00000000	mice
Otulinl	-0.32	1.00000000	mice
Tspan9	-0.18	1.00000000	mice

Sema4f	-0.12	1.00000000	mice
Ankdd1a	-0.21	1.00000000	mice
Hsp90b1	-0.15	1.00000000	mice
Ndufab1	0.27	1.00000000	mice
Gng5	0.23	1.00000000	mice
Asl	0.14	1.00000000	mice
Cog4	0.28	1.00000000	mice
Dnajc8	0.23	1.00000000	mice
Ybx1	-0.22	1.00000000	mice
Smarca2	-0.23	1.00000000	mice
Aebp2	0.58	1.00000000	mice
Pex13	0.23	1.00000000	mice
Pitpnb	-0.20	1.00000000	mice
Insl6	0.31	1.00000000	mice
Babam1	0.13	1.00000000	mice
Ilf2	-0.27	1.00000000	mice
Clec2g	0.15	1.00000000	mice
Aph1b	-0.13	1.00000000	mice
Cep128	-0.16	1.00000000	mice
Dnah2	0.22	1.00000000	mice
Ppfibp1	-0.32	1.00000000	mice
Parp3	0.12	1.00000000	mice
Ubxn7	0.21	1.00000000	mice
Slc23a2	-0.29	1.00000000	mice
Cdkn2b	0.40	1.00000000	mice
Gabarapl1	0.15	1.00000000	mice
Rnf31	0.12	1.00000000	mice
Slc25a26	-0.13	1.00000000	mice
Nrde2	0.23	1.00000000	mice
Vps50	0.16	1.00000000	mice
BC031181	-0.08	1.00000000	mice
Kcnq1ot1	-0.46	1.00000000	mice
Lynx1	0.10	1.00000000	mice
Klrb1c	-0.25	1.00000000	mice
Eif3d	0.14	1.00000000	mice
Snx10	-0.23	1.00000000	mice
Selenop	-0.26	1.00000000	mice
Cd164	0.35	1.00000000	mice
Adrm1	0.17	1.00000000	mice
Nudcd3	0.35	1.00000000	mice
Polr1c	0.17	1.00000000	mice
Atg2b	-0.13	1.00000000	mice
Tesk1	0.15	1.00000000	mice
Iqcb1	-0.23	1.00000000	mice
Il15ra	0.47	1.00000000	mice
Ankrd50	-0.10	1.00000000	mice

St3gal5	-0.09	1.00000000	mice
Zbtb5	0.18	1.00000000	mice
Dglucy	-0.07	1.00000000	mice
Fam169b	0.42	1.00000000	mice
Nono	0.22	1.00000000	mice
Ccng2	0.22	1.00000000	mice
Tmem70	0.18	1.00000000	mice
Polr2g	0.33	1.00000000	mice
Eif3k	-0.27	1.00000000	mice
Ankfy1	0.29	1.00000000	mice
Camkk2	-0.26	1.00000000	mice
Tmem67	0.13	1.00000000	mice
Prkcg	-0.36	1.00000000	mice
Rnf214	0.16	1.00000000	mice
Hnrnpdl	0.15	1.00000000	mice
Lactb	0.69	1.00000000	mice
Slc39a11	-0.20	1.00000000	mice
Cnih4	0.21	1.00000000	mice
Atp6v1e1	0.18	1.00000000	mice
Supt5	0.14	1.00000000	mice
Tgfbr2	-0.46	1.00000000	mice
Tmc8	0.12	1.00000000	mice
Ppa1	0.26	1.00000000	mice
Golph3	-0.25	1.00000000	mice
Per1	-0.17	1.00000000	mice
Lzic	0.10	1.00000000	mice
Cib1	0.22	1.00000000	mice
Ubxn1	0.20	1.00000000	mice
Fam49a	0.74	1.00000000	mice
Ikzf3	-0.37	1.00000000	mice
Epn1	-0.24	1.00000000	mice
4930599N23Rik	0.25	1.00000000	mice
Spns1	0.16	1.00000000	mice
Cox6a1	0.16	1.00000000	mice
Cops4	-0.19	1.00000000	mice
4930523C07Rik	0.50	1.00000000	mice
Lrba	-0.28	1.00000000	mice
Isg20l2	-0.20	1.00000000	mice
Nudt22	0.08	1.00000000	mice
Ank2	0.04	1.00000000	mice
Vti1a	0.39	1.00000000	mice
Psmd7	0.29	1.00000000	mice
Atg16l1	-0.25	1.00000000	mice
Nup88	0.18	1.00000000	mice
Tmem131	1.04	1.00000000	mice
Ocel1	0.18	1.00000000	mice

Pafah1b1	0.25	1.00000000	mice
Epb41l2	-0.57	1.00000000	mice
Tubb5	0.41	1.00000000	mice
Itpr1	-0.26	1.00000000	mice
Dse	-0.16	1.00000000	mice
Tsc1	0.24	1.00000000	mice
Ikbkg	0.11	1.00000000	mice
Hsf2	0.38	1.00000000	mice
Bag6	-0.17	1.00000000	mice
Cnn2	0.31	1.00000000	mice
Abcf3	0.10	1.00000000	mice
Fbxw11	-0.22	1.00000000	mice
Ivns1abp	-0.26	1.00000000	mice
Rrn3	-0.14	1.00000000	mice
Cox11	0.16	1.00000000	mice
Rfc1	0.24	1.00000000	mice
Fnbp1l	-0.46	1.00000000	mice
Sri	0.29	1.00000000	mice
Csnk1g3	-0.35	1.00000000	mice
Bdp1	0.28	1.00000000	mice
Csgalnact2	-0.16	1.00000000	mice
Ubr1	-0.27	1.00000000	mice
Ramp1	-0.42	1.00000000	mice
Trim14	0.24	1.00000000	mice
Tnf	0.94	1.00000000	mice
Plekhb2	0.25	1.00000000	mice
Apol8	-0.15	1.00000000	mice
Ppp1ca	0.18	1.00000000	mice
Parvg	-0.14	1.00000000	mice
Wdtc1	0.23	1.00000000	mice
Edil3	-0.29	1.00000000	mice
Ckap4	0.14	1.00000000	mice
Snrpd1	0.16	1.00000000	mice
Sppl3	0.22	1.00000000	mice
Cd300a	0.70	1.00000000	mice
Zbtb10	-0.24	1.00000000	mice
Sh2d1b1	0.07	1.00000000	mice
Rlim	-0.27	1.00000000	mice
Erp44	0.16	1.00000000	mice
Ccdc6	0.28	1.00000000	mice
Ndufb3	0.23	1.00000000	mice
Tsta3	0.11	1.00000000	mice
Ica1l	-0.08	1.00000000	mice
Cers4	-0.13	1.00000000	mice
Sft2d1	-0.21	1.00000000	mice
Noa1	0.03	1.00000000	mice

Pot1a	0.07	1.00000000	mice
Ktn1	0.98	1.00000000	mice
Hs6st1	0.15	1.00000000	mice
Surf4	0.24	1.00000000	mice
Esyt1	0.14	1.00000000	mice
Tasor	0.24	1.00000000	mice
Smim24	0.11	1.00000000	mice
Bms1	0.13	1.00000000	mice
Aida	0.23	1.00000000	mice
Tnnt3	-0.11	1.00000000	mice
Tmem241	-0.18	1.00000000	mice
Nup210	-0.20	1.00000000	mice
Fibp	0.06	1.00000000	mice
Ptk2b	-0.41	1.00000000	mice
Ctbp1	-0.31	1.00000000	mice
Samd4b	0.04	1.00000000	mice
Crcp	0.09	1.00000000	mice
Cant1	0.07	1.00000000	mice
4930579C12Rik	0.15	1.00000000	mice
Ppp1r13b	-0.20	1.00000000	mice
Aff4	-0.23	1.00000000	mice
Nvl	0.15	1.00000000	mice
Nsd3	0.24	1.00000000	mice
Vps36	-0.26	1.00000000	mice
Stard8	-0.09	1.00000000	mice
Csnk1g1	0.23	1.00000000	mice
Sugt1	0.33	1.00000000	mice
E2f2	-0.11	1.00000000	mice
Yme1l1	0.23	1.00000000	mice
Psmb4	0.19	1.00000000	mice
Rreb1	-0.12	1.00000000	mice
Slc25a45	0.11	1.00000000	mice
Trmt1	-0.15	1.00000000	mice
Slc25a47	-0.10	1.00000000	mice
Pkn2	0.24	1.00000000	mice
Ank1	-0.10	1.00000000	mice
Slc22a23	0.80	1.00000000	mice
Ppp3cb	0.28	1.00000000	mice
Yipf4	0.10	1.00000000	mice
Pacs1	-0.12	1.00000000	mice
Pitpnc1	-0.45	1.00000000	mice
Ssna1	0.10	1.00000000	mice
Cep57	0.19	1.00000000	mice
Rgp1	0.11	1.00000000	mice
Dgat1	0.25	1.00000000	mice
Ssb	0.24	1.00000000	mice

Gnpda2	0.10	1.00000000	mice
Vrk3	0.20	1.00000000	mice
Psmc4	0.17	1.00000000	mice
Xpo5	0.12	1.00000000	mice
Ablim1	-0.70	1.00000000	mice
Arrdc2	0.11	1.00000000	mice
Commd6	0.18	1.00000000	mice
Afg3l2	0.14	1.00000000	mice
Cdc73	-0.38	1.00000000	mice
Nudt2	0.13	1.00000000	mice
Chkb	-0.15	1.00000000	mice
Nipal1	0.14	1.00000000	mice
Alkbh7	0.05	1.00000000	mice
Atf7	-0.34	1.00000000	mice
4933421O10Rik	-0.06	1.00000000	mice
Atf7ip	0.28	1.00000000	mice
Sh3glb1	0.34	1.00000000	mice
Elmo2	0.09	1.00000000	mice
Copg2	0.14	1.00000000	mice
Snx6	0.26	1.00000000	mice
Pfkfb3	0.43	1.00000000	mice
Clcn5	0.37	1.00000000	mice
Uqcrfs1	-0.26	1.00000000	mice
BC004004	-0.20	1.00000000	mice
Nop16	0.16	1.00000000	mice
P2rx5	-0.10	1.00000000	mice
Plin2	0.25	1.00000000	mice
Tgfbr3	-0.07	1.00000000	mice
Usp24	-0.25	1.00000000	mice
Lmo2	0.43	1.00000000	mice
Sh3bp5	-0.37	1.00000000	mice
Zfp513	0.15	1.00000000	mice
Pik3cg	-0.20	1.00000000	mice
Sdc4	0.45	1.00000000	mice
Ano6	-0.34	1.00000000	mice
Hsbp1	0.23	1.00000000	mice
Pmpca	0.11	1.00000000	mice
Numb	-0.28	1.00000000	mice
S1pr1	-0.12	1.00000000	mice
Tent4a	0.11	1.00000000	mice
Neat1	-0.43	1.00000000	mice
Lyrm1	0.18	1.00000000	mice
Cyp7b1	0.50	1.00000000	mice
Papss2	0.26	1.00000000	mice
Brd3	0.18	1.00000000	mice
Hnrnpd	0.22	1.00000000	mice

Ecpas	-0.39	1.00000000	mice
2810004N23Rik	0.10	1.00000000	mice
Cep290	0.13	1.00000000	mice
Vps13d	-0.14	1.00000000	mice
Immp1l	0.16	1.00000000	mice
Sgsm3	-0.17	1.00000000	mice
Ccdc25	0.17	1.00000000	mice
Suv39h2	0.09	1.00000000	mice
Adcy7	-0.18	1.00000000	mice
Asb3	0.07	1.00000000	mice
Wdr3	-0.17	1.00000000	mice
Prkn	-0.14	1.00000000	mice
Flcn	0.15	1.00000000	mice
Vps26b	0.09	1.00000000	mice
Fam177a	0.16	1.00000000	mice
Cep85l	0.45	1.00000000	mice
C87436	-0.21	1.00000000	mice
Tspan33	0.29	1.00000000	mice
Capn9	0.10	1.00000000	mice
Ucp2	-0.20	1.00000000	mice
Kdm4a	-0.38	1.00000000	mice
Ap3d1	0.24	1.00000000	mice
Rnf130	-0.22	1.00000000	mice
Synpo2	0.25	1.00000000	mice
Htatip2	0.14	1.00000000	mice
Ctps2	-0.19	1.00000000	mice
Cd28	-0.15	1.00000000	mice
Phlda1	-0.59	1.00000000	mice
Smchd1	0.48	1.00000000	mice
Hk1	-0.44	1.00000000	mice
Tmem216	0.08	1.00000000	mice
B3glct	-0.16	1.00000000	mice
Ncor2	-0.26	1.00000000	mice
P2rx7	-0.17	1.00000000	mice
Luc7l3	0.21	1.00000000	mice
Ccdc32	0.11	1.00000000	mice
Lrsam1	-0.05	1.00000000	mice
Riok3	-0.40	1.00000000	mice
Herc1	-0.32	1.00000000	mice
Tpm3	0.28	1.00000000	mice
Grina	0.41	1.00000000	mice
Tpcn1	-0.09	1.00000000	mice
Vps72	0.08	1.00000000	mice
Smpd4	0.08	1.00000000	mice
Cnn3	0.19	1.00000000	mice
Itga3	-0.18	1.00000000	mice

Slc30a4	-0.10	1.00000000	mice
Snx8	-0.20	1.00000000	mice
Grk2	0.25	1.00000000	mice
Arhgap27	0.17	1.00000000	mice
Card11	0.73	1.00000000	mice
Arglu1	0.23	1.00000000	mice
Arpc4	0.19	1.00000000	mice
Ptcd2	0.22	1.00000000	mice
Usb1	0.12	1.00000000	mice
Gvin1	0.15	1.00000000	mice
Rtn4	0.30	1.00000000	mice
Car2	0.53	1.00000000	mice
Pcsk7	0.22	1.00000000	mice
Oxr1	-0.45	1.00000000	mice
Nsrp1	0.17	1.00000000	mice
Cerk	-0.43	1.00000000	mice
Ryr1	-0.19	1.00000000	mice
Pex16	0.14	1.00000000	mice
Abtb2	0.05	1.00000000	mice
Nelfe	0.15	1.00000000	mice
Yeats2	-0.13	1.00000000	mice
Pigp	-0.16	1.00000000	mice
E530011L22Rik	0.07	1.00000000	mice
Arl8a	0.16	1.00000000	mice
AI413582	0.14	1.00000000	mice
Rilpl1	0.17	1.00000000	mice
Cdk4	-0.19	1.00000000	mice
1110002J07Rik	-0.03	1.00000000	mice
Tmem101	0.06	1.00000000	mice
Qtrt1	-0.18	1.00000000	mice
Cdk17	0.00	1.00000000	mice
Cdkn1a	0.47	1.00000000	mice
Stard7	0.26	1.00000000	mice
Pakap.1	0.34	1.00000000	mice
Ldha	-0.12	1.00000000	mice
Klhl6	-0.31	1.00000000	mice
Ciz1	0.12	1.00000000	mice
Exosc4	0.07	1.00000000	mice
Ppp1r2	-0.27	1.00000000	mice
1700029H14Rik	-0.18	1.00000000	mice
Hipk3	-0.32	1.00000000	mice
Prr7	-0.09	1.00000000	mice
Yif1b	0.14	1.00000000	mice
Grap2	-0.35	1.00000000	mice
Tnfrsf1b	0.34	1.00000000	mice
Dcun1d3	0.19	1.00000000	mice

Nxn	-0.28	1.00000000	mice
Bag3	0.13	1.00000000	mice
Ly9	0.12	1.00000000	mice
Zfp592	0.24	1.00000000	mice
Ier2	-0.23	1.00000000	mice
Ubl4a	0.12	1.00000000	mice
Prickle1	-0.55	1.00000000	mice
Tmem8	0.10	1.00000000	mice
Gipc1	0.09	1.00000000	mice
Snhg1	0.24	1.00000000	mice
Ndufa4	-0.24	1.00000000	mice
Ndufc1	0.15	1.00000000	mice
Gsto1	0.13	1.00000000	mice
Prpf8	-0.21	1.00000000	mice
Acot8	0.13	1.00000000	mice
Pdcd10	0.22	1.00000000	mice
Jpt2	0.11	1.00000000	mice
Agap3	-0.19	1.00000000	mice
Ubqln4	-0.09	1.00000000	mice
Dbn1	-0.18	1.00000000	mice
Tubb2a	0.27	1.00000000	mice
Palld	0.41	1.00000000	mice
B4galnt1	0.23	1.00000000	mice
Pelp1	0.07	1.00000000	mice
Tspan31	0.18	1.00000000	mice
Lonp1	0.07	1.00000000	mice
Ppan	-0.14	1.00000000	mice
Tnfrsf9	0.65	1.00000000	mice
1110059G10Rik	0.09	1.00000000	mice
Srfbp1	0.10	1.00000000	mice
A530064D06Rik	0.11	1.00000000	mice
Gltp	-0.27	1.00000000	mice
Fars2	-0.23	1.00000000	mice
Pam16	0.13	1.00000000	mice
Smdt1	0.29	1.00000000	mice
Psmd12	0.23	1.00000000	mice
Rnf216	-0.22	1.00000000	mice
Ccl7	-0.03	1.00000000	mice
Ikzf2	-0.21	1.00000000	mice
Gsdme	-0.12	1.00000000	mice
Ptpra	0.16	1.00000000	mice
Gtf2f1	-0.03	1.00000000	mice
Tanc1	-0.01	1.00000000	mice
Tnfaip3	-0.29	1.00000000	mice
Pprc1	-0.04	1.00000000	mice
Tbc1d15	-0.17	1.00000000	mice

Tapt1	-0.15	1.00000000	mice
Cep170	-0.39	1.00000000	mice
Nedd8	0.16	1.00000000	mice
Raph1	-0.29	1.00000000	mice
Apip	0.14	1.00000000	mice
Adgrg5	0.35	1.00000000	mice
Ndel1	-0.44	1.00000000	mice
Emb	-0.26	1.00000000	mice
Pias4	0.12	1.00000000	mice
Fyn	-0.16	1.00000000	mice
Hspa8	0.25	1.00000000	mice
Nop2	0.08	1.00000000	mice
4833438C02Rik	-0.08	1.00000000	mice
Pikfyve	0.17	1.00000000	mice
Dnal1	-0.20	1.00000000	mice
Plekhf2	0.23	1.00000000	mice
Fiz1	0.12	1.00000000	mice
Tmem260	0.18	1.00000000	mice
4931406C07Rik	0.10	1.00000000	mice
Rnf217	0.35	1.00000000	mice
Akap13	-0.28	1.00000000	mice
Smim11	-0.23	1.00000000	mice
Naxd	0.17	1.00000000	mice
Tmem176b	0.49	1.00000000	mice
Dstn	0.22	1.00000000	mice
Nacc1	0.16	1.00000000	mice
Polb	0.22	1.00000000	mice
Ezr	-0.28	1.00000000	mice
Spopl	-0.02	1.00000000	mice
Vegfb	-0.09	1.00000000	mice
Ube2k	-0.22	1.00000000	mice
Zfp64	-0.29	1.00000000	mice
Smad2	0.23	1.00000000	mice
Car4	0.10	1.00000000	mice
Prpf38a	0.19	1.00000000	mice
Arel1	0.13	1.00000000	mice
Prkce	-0.56	1.00000000	mice
Dcaf15	0.09	1.00000000	mice
Exoc7	0.06	1.00000000	mice
Spen	-0.18	1.00000000	mice
Use1	0.20	1.00000000	mice
Bin1	-0.35	1.00000000	mice
Btbd10	-0.15	1.00000000	mice
Adap2	0.20	1.00000000	mice
Herc4	0.40	1.00000000	mice
Dusp16	0.21	1.00000000	mice

Fam126b	-0.18	1.00000000	mice
Nosip	0.13	1.00000000	mice
Plxdc2	-0.39	1.00000000	mice
Lypla2	0.19	1.00000000	mice
Dusp4	-0.05	1.00000000	mice
Gadd45gip1	0.08	1.00000000	mice
Washc3	0.12	1.00000000	mice
Zfp667	-0.18	1.00000000	mice
Sestd1	-0.35	1.00000000	mice
Apobr	-0.18	1.00000000	mice
Timm44	0.12	1.00000000	mice
Kat7	-0.16	1.00000000	mice
Fam160b2	0.09	1.00000000	mice
Snx3	-0.19	1.00000000	mice
Slc25a53	-0.18	1.00000000	mice
Luc7l2	-0.25	1.00000000	mice
Yju2	-0.23	1.00000000	mice
Nfkb2	0.32	1.00000000	mice
Atp5g3	0.14	1.00000000	mice
Ube2d3	0.18	1.00000000	mice
Nol7	0.19	1.00000000	mice
Xpnpep1	0.15	1.00000000	mice
Nhlrc2	0.31	1.00000000	mice
Suds3	0.16	1.00000000	mice
Themis2	0.21	1.00000000	mice
Cpne8	-0.67	1.00000000	mice
Zfand2a	-0.16	1.00000000	mice
Dnaja3	0.08	1.00000000	mice
Tmod3	0.25	1.00000000	mice
Tmem184c	-0.11	1.00000000	mice
AB124611	-0.09	1.00000000	mice
E2f4	0.11	1.00000000	mice
Pum2	0.22	1.00000000	mice
Zfp119a	0.13	1.00000000	mice
Trappc8	0.20	1.00000000	mice
Nrbf2	0.32	1.00000000	mice
Heatr5a	-0.23	1.00000000	mice
Tppp3	0.15	1.00000000	mice
Piezo1	-0.37	1.00000000	mice
Vps13c	-0.20	1.00000000	mice
Ilkap	0.15	1.00000000	mice
Zfp787	-0.11	1.00000000	mice
Gstt3	0.19	1.00000000	mice
Stat6	0.27	1.00000000	mice
Dedd	0.12	1.00000000	mice
Syk	-0.20	1.00000000	mice

Faf1	-0.51	1.00000000	mice
Fam102b	-0.15	1.00000000	mice
Zfp810	0.19	1.00000000	mice
Acss1	-0.12	1.00000000	mice
Gid4	0.13	1.00000000	mice
Sdf2l1	-0.22	1.00000000	mice
Tob2	-0.19	1.00000000	mice
Adap2os	0.12	1.00000000	mice
Itpa	0.06	1.00000000	mice
Lpin1	0.14	1.00000000	mice
Tmem127	0.11	1.00000000	mice
Cbll1	0.13	1.00000000	mice
Tbc1d2b	0.12	1.00000000	mice
Igfbp7	0.14	1.00000000	mice
Tmem106c	-0.09	1.00000000	mice
Rapgef1	-0.23	1.00000000	mice
Plekha8	-0.06	1.00000000	mice
Ezh1	-0.18	1.00000000	mice
Csf1	0.10	1.00000000	mice
Ap2s1	0.18	1.00000000	mice
Znrf1	0.30	1.00000000	mice
Arap2	-0.41	1.00000000	mice
Acot13	0.09	1.00000000	mice
Sap30bp	0.12	1.00000000	mice
Cdc42ep3	0.28	1.00000000	mice
Suco	-0.31	1.00000000	mice
Tnni2	0.35	1.00000000	mice
Cltc	-0.18	1.00000000	mice
Pld3	0.14	1.00000000	mice
Gpr160	0.09	1.00000000	mice
Urm1	0.12	1.00000000	mice
Sephs2	0.07	1.00000000	mice
Plec	-0.24	1.00000000	mice
A930015D03Rik	0.24	1.00000000	mice
Rubcn	0.09	1.00000000	mice
Pqlc3	0.22	1.00000000	mice
Tmem39a	0.86	1.00000000	mice
Psen2	0.13	1.00000000	mice
Tmem183a	0.08	1.00000000	mice
Coa4	0.08	1.00000000	mice
Pdik1l	0.06	1.00000000	mice
Spib	0.50	1.00000000	mice
Sf1	-0.21	1.00000000	mice
Pebp1	-0.23	1.00000000	mice
Sumo3	0.19	1.00000000	mice
Capg	0.31	1.00000000	mice

Dclre1c	0.22	1.00000000	mice
Ttc17	0.12	1.00000000	mice
Zfhx2	-0.11	1.00000000	mice
Cacna1e	-0.58	1.00000000	mice
Rab1b	0.17	1.00000000	mice
Dad1	-0.19	1.00000000	mice
Fbxo6	0.14	1.00000000	mice
Rpain	0.08	1.00000000	mice
Pdss1	-0.16	1.00000000	mice
Tardbp	0.15	1.00000000	mice
Notch1	-0.22	1.00000000	mice
Psmd13	0.13	1.00000000	mice
Zfp326	-0.15	1.00000000	mice
Topbp1	-0.18	1.00000000	mice
Kmt2b	0.16	1.00000000	mice
Edf1	0.20	1.00000000	mice
Alms1	-0.08	1.00000000	mice
Ywhab	0.18	1.00000000	mice
Zdhhc3	0.17	1.00000000	mice
Hnrnpa3	0.24	1.00000000	mice
Ncstn	0.20	1.00000000	mice
Lrrfip2	-0.38	1.00000000	mice
Uba1	0.11	1.00000000	mice
Rbm5	0.21	1.00000000	mice
Ubtf	0.14	1.00000000	mice
Il6ra	-0.17	1.00000000	mice
Emc4	0.14	1.00000000	mice
Nsun3	0.11	1.00000000	mice
Rad21	0.40	1.00000000	mice
Uvrag	-0.24	1.00000000	mice
Snx1	0.09	1.00000000	mice
Gab2	-0.34	1.00000000	mice
Pgap1	-0.13	1.00000000	mice
Slc30a1	0.10	1.00000000	mice
Nt5dc3	-0.15	1.00000000	mice
Polr1d	-0.25	1.00000000	mice
Atp11b	0.34	1.00000000	mice
Ubtd2	-0.16	1.00000000	mice
F2r	-0.19	1.00000000	mice
Ndufaf4	-0.12	1.00000000	mice
Lyst	0.34	1.00000000	mice
Lilrb4a	0.53	1.00000000	mice
Asf1a	0.10	1.00000000	mice
Ttf1	0.11	1.00000000	mice
Nkiras2	0.10	1.00000000	mice
Tax1bp3	0.14	1.00000000	mice

Hdgf	-0.25	1.00000000	mice
Rab9	0.17	1.00000000	mice
Tasp1	-0.27	1.00000000	mice
Fbxw8	-0.19	1.00000000	mice
Ssu72	0.20	1.00000000	mice
Wdr61	0.16	1.00000000	mice
Evl	-0.23	1.00000000	mice
Cdk5	0.09	1.00000000	mice
Uqcrb	0.23	1.00000000	mice
Spag9	-0.42	1.00000000	mice
Numa1	-0.27	1.00000000	mice
Gnl3	-0.16	1.00000000	mice
Spire1	-0.15	1.00000000	mice
Tuba1a	0.35	1.00000000	mice
Sco2	-0.08	1.00000000	mice
Hipk1	-0.28	1.00000000	mice
Zranb2	0.15	1.00000000	mice
Aco2	0.17	1.00000000	mice
Hspa1b	0.38	1.00000000	mice
Stub1	0.15	1.00000000	mice
Trim44	-0.34	1.00000000	mice
Osm	0.14	1.00000000	mice
Rragc	0.29	1.00000000	mice
Itgal	-0.06	1.00000000	mice
Kat2b	0.13	1.00000000	mice
Stx2	0.06	1.00000000	mice
Zfp983	0.11	1.00000000	mice
Nabp1	0.33	1.00000000	mice
C330007P06Rik	-0.21	1.00000000	mice
Rnf180	-0.57	1.00000000	mice
Ccnl1	-0.08	1.00000000	mice
Nfe2l1	0.24	1.00000000	mice
Agtrap	0.09	1.00000000	mice
Slc39a7	0.13	1.00000000	mice
Cyb5r1	-0.08	1.00000000	mice
Hgsnat	-0.28	1.00000000	mice
Pard6a	0.09	1.00000000	mice
Grasp	0.30	1.00000000	mice
Slc9a8	-0.15	1.00000000	mice
Aip	0.13	1.00000000	mice
Tada3	0.08	1.00000000	mice
Pdzd8	0.21	1.00000000	mice
Trmt61b	-0.19	1.00000000	mice
Elf1	0.13	1.00000000	mice
Csrp2	-0.03	1.00000000	mice
Lyz2	-0.97	1.00000000	mice

Arfgap3	-0.18	1.00000000	mice
Ino80dos	0.14	1.00000000	mice
Fads3	0.14	1.00000000	mice
Grpel1	0.16	1.00000000	mice
Olfr56	0.12	1.00000000	mice
Ino80c	0.10	1.00000000	mice
Tk2	0.12	1.00000000	mice
Otud6b	-0.17	1.00000000	mice
Klhl9	0.21	1.00000000	mice
Rassf8	-0.13	1.00000000	mice
Tmem80	0.07	1.00000000	mice
Rtn4rl1	0.22	1.00000000	mice
Cass4	-0.37	1.00000000	mice
Thoc7	0.20	1.00000000	mice
Copg1	0.09	1.00000000	mice
Fas	0.35	1.00000000	mice
Egln1	-0.12	1.00000000	mice
Acox1	-0.20	1.00000000	mice
Nsmce1	0.20	1.00000000	mice
Rbm26	-0.35	1.00000000	mice
Rgs10	0.46	1.00000000	mice
Gng4	0.23	1.00000000	mice
Plxnb2	0.10	1.00000000	mice
Fam241a	0.35	1.00000000	mice
Nars2	-0.36	1.00000000	mice
Zfp217	0.28	1.00000000	mice
Id3	0.13	1.00000000	mice
Il1rl2	-0.09	1.00000000	mice
Slbp	0.28	1.00000000	mice
Cecr2	-0.50	1.00000000	mice
Prpf31	0.17	1.00000000	mice
Hrh1	-0.10	1.00000000	mice
Pip4p2	-0.08	1.00000000	mice
Flna	-0.26	1.00000000	mice
Tdg	0.11	1.00000000	mice
Plxdc1	0.52	1.00000000	mice
Elovl1	0.11	1.00000000	mice
Polr3k	0.07	1.00000000	mice
Srrm1	0.16	1.00000000	mice
Trmt11	0.14	1.00000000	mice
Sdf2	0.10	1.00000000	mice
Atp10a	0.34	1.00000000	mice
Tsen34	0.06	1.00000000	mice
Dgke	-0.17	1.00000000	mice
Itpkb	-0.15	1.00000000	mice
2310015A10Rik	0.11	1.00000000	mice

Ino80b	0.09	1.00000000	mice
Faf2	0.18	1.00000000	mice
Zfp703	-0.28	1.00000000	mice
Iah1	0.09	1.00000000	mice
Prkag2	-0.02	1.00000000	mice
BC005561	0.15	1.00000000	mice
Ogfod2	0.08	1.00000000	mice
Hook3	-0.23	1.00000000	mice
Ccdc127	0.08	1.00000000	mice
Rpa1	0.15	1.00000000	mice
Tasor2	0.24	1.00000000	mice
Smim10l1	-0.26	1.00000000	mice
Rab11fip1	-0.56	1.00000000	mice
Sar1a	0.22	1.00000000	mice
Hivep1	0.22	1.00000000	mice
Tnpo3	-0.14	1.00000000	mice
Pus7	-0.23	1.00000000	mice
Trim28	-0.09	1.00000000	mice
Phb2	0.12	1.00000000	mice
Prr14	0.21	1.00000000	mice
Zc3hav1	0.13	1.00000000	mice
Tgfbi	-0.07	1.00000000	mice
Gpr174	-0.15	1.00000000	mice
Exoc3	0.16	1.00000000	mice
Ndufa1	0.13	1.00000000	mice
Snn	0.18	1.00000000	mice
Arhgap1	0.15	1.00000000	mice
Cox20	0.14	1.00000000	mice
Zfp568	-0.13	1.00000000	mice
Amn1	0.13	1.00000000	mice
Zfp362	0.15	1.00000000	mice
Ssbp1	0.13	1.00000000	mice
Rsbn1l	0.15	1.00000000	mice
Stoml1	0.11	1.00000000	mice
Yy1	0.20	1.00000000	mice
Nhlrc3	0.09	1.00000000	mice
Dnpep	0.10	1.00000000	mice
Sh2b1	-0.14	1.00000000	mice
Lrrc45	-0.09	1.00000000	mice
Brpf1	0.13	1.00000000	mice
Golm1	-0.11	1.00000000	mice
Cep120	-0.33	1.00000000	mice
Plekha5	-0.22	1.00000000	mice
Litaf	0.22	1.00000000	mice
Atxn1l	0.14	1.00000000	mice
Hsh2d	0.22	1.00000000	mice

Tlr7	0.29	1.00000000	mice
Aldh1a2	0.18	1.00000000	mice
Setd7	-0.23	1.00000000	mice
Taf2	-0.17	1.00000000	mice
Ssbp4	-0.10	1.00000000	mice
Cluh	-0.21	1.00000000	mice
Tuba1c	-0.28	1.00000000	mice
Pank3	0.14	1.00000000	mice
Exoc5	-0.13	1.00000000	mice
Hnrnpa2b1	0.21	1.00000000	mice
Rhoc	0.21	1.00000000	mice
Ctns	0.08	1.00000000	mice
Uqcr11	0.31	1.00000000	mice
Lpar6	-0.13	1.00000000	mice
Samd1	-0.10	1.00000000	mice
Taf4b	-0.11	1.00000000	mice
Napg	0.19	1.00000000	mice
Zfp943	0.10	1.00000000	mice
Ly86	0.28	1.00000000	mice
Kdm6a	0.23	1.00000000	mice
Tk1	0.09	1.00000000	mice
Pwwp3a	0.07	1.00000000	mice
Tmem185a	-0.15	1.00000000	mice
Slc10a7	0.06	1.00000000	mice
Zfp719	0.06	1.00000000	mice
Ypel1	-0.07	1.00000000	mice
Baz2a	0.35	1.00000000	mice
Tusc3	-0.12	1.00000000	mice
Anxa11os	0.08	1.00000000	mice
Angel2	0.12	1.00000000	mice
Polr2a	-0.31	1.00000000	mice
Tacc2	-0.06	1.00000000	mice
Bicra	0.24	1.00000000	mice
Hfe	-0.16	1.00000000	mice
Lrrc57	0.11	1.00000000	mice
Cops8	0.17	1.00000000	mice
Hacd2	-0.21	1.00000000	mice
AI467606	0.11	1.00000000	mice
Chd9	-0.18	1.00000000	mice
Rad17	0.11	1.00000000	mice
Cdk13	-0.40	1.00000000	mice
Csad	-0.11	1.00000000	mice
Tmem168	0.18	1.00000000	mice
Pmpcb	0.08	1.00000000	mice
Wdr5	-0.17	1.00000000	mice
Vps4b	0.31	1.00000000	mice

Emc3	0.16	1.00000000	mice
Nras	0.13	1.00000000	mice
Osbpl8	0.30	1.00000000	mice
Ythdc1	-0.14	1.00000000	mice
Ctsc	0.26	1.00000000	mice
Sun2	-0.14	1.00000000	mice
Rab43	-0.30	1.00000000	mice
Eif4h	0.15	1.00000000	mice
Socs6	0.19	1.00000000	mice
Eml5	-0.27	1.00000000	mice
2610020C07Rik	-0.11	1.00000000	mice
Cep350	0.34	1.00000000	mice
Ubn1	-0.03	1.00000000	mice
Armcx2	-0.11	1.00000000	mice
Ttc39a	-0.03	1.00000000	mice
Ptdss2	0.09	1.00000000	mice
Jun	-0.25	1.00000000	mice
Kdelr1	0.17	1.00000000	mice
Fubp1	-0.24	1.00000000	mice
Cybc1	0.12	1.00000000	mice
Rabac1	-0.29	1.00000000	mice
Sema7a	0.43	1.00000000	mice
Tnks	-0.31	1.00000000	mice
Rnaset2a	-0.16	1.00000000	mice
Fgfr1op2	0.28	1.00000000	mice
Timmdc1	0.26	1.00000000	mice
9530068E07Rik	0.18	1.00000000	mice
Herc3	0.00	1.00000000	mice
Trim26	0.17	1.00000000	mice
Gapvd1	-0.29	1.00000000	mice
Tpgs1	0.09	1.00000000	mice
Ints9	0.11	1.00000000	mice
Polr2c	0.10	1.00000000	mice
Wapl	0.28	1.00000000	mice
Slc35d1	-0.12	1.00000000	mice
Apbb2	-0.27	1.00000000	mice
Creb3	0.10	1.00000000	mice
Tmx3	0.12	1.00000000	mice
Naa20	0.24	1.00000000	mice
Ssh2	-0.46	1.00000000	mice
Ikzf4	0.24	1.00000000	mice
Fads1	-0.18	1.00000000	mice
Dnajc2	0.20	1.00000000	mice
Rgmb	0.16	1.00000000	mice
Papola	0.18	1.00000000	mice
Gosr2	0.20	1.00000000	mice

Snrpa	0.15	1.00000000	mice
Psmd1	0.18	1.00000000	mice
Igf1r	-0.51	1.00000000	mice
U2surp	0.12	1.00000000	mice
Iqgap2	-0.30	1.00000000	mice
Sdhd	0.10	1.00000000	mice
Pofut1	-0.11	1.00000000	mice
Cpne2	0.19	1.00000000	mice
Stap1	0.37	1.00000000	mice
Pycr2	-0.16	1.00000000	mice
Fam98a	-0.13	1.00000000	mice
Rbms1	-0.40	1.00000000	mice
Ppp4r3b	0.14	1.00000000	mice
Naf1	-0.12	1.00000000	mice
Vps26c	0.08	1.00000000	mice
Uhmk1	0.17	1.00000000	mice
Cd8b1	0.25	1.00000000	mice
Ttc9c	0.13	1.00000000	mice
Esr1	0.13	1.00000000	mice
Rev3l	-0.28	1.00000000	mice
Azi2	0.17	1.00000000	mice
Erbin	-0.24	1.00000000	mice
Castor2	0.11	1.00000000	mice
Trio	-0.25	1.00000000	mice
Pdxdc1	0.19	1.00000000	mice
Cep192	0.42	1.00000000	mice
Fnip2	-0.30	1.00000000	mice
Golgb1	-0.18	1.00000000	mice
Aamdc	-0.10	1.00000000	mice
Kif2a	-0.29	1.00000000	mice
Ola1	0.12	1.00000000	mice
Tbc1d8	0.32	1.00000000	mice
Cuta	0.12	1.00000000	mice
Neu1	0.16	1.00000000	mice
Dcxr	0.07	1.00000000	mice
Uqcc3	0.11	1.00000000	mice
Gna13	-0.19	1.00000000	mice
Hspa13	0.11	1.00000000	mice
Cwc25	0.18	1.00000000	mice
Nfkbiz	0.53	1.00000000	mice
Kifap3	-0.10	1.00000000	mice
Cdkn2aipnl	-0.22	1.00000000	mice
Sept2	0.08	1.00000000	mice
Pex3	-0.14	1.00000000	mice
Fkbp4	-0.28	1.00000000	mice
Arhgef12	-0.30	1.00000000	mice

Cd300c	-0.06	1.00000000	mice
Lta4h	0.14	1.00000000	mice
Thap1	0.07	1.00000000	mice
Zcrb1	0.13	1.00000000	mice
Clptm1	0.23	1.00000000	mice
Ncl	-0.16	1.00000000	mice
Ilvbl	0.08	1.00000000	mice
Rnf181	0.11	1.00000000	mice
Arfgap2	0.16	1.00000000	mice
Txn2	-0.19	1.00000000	mice
Zfand6	0.35	1.00000000	mice
Chst15	0.11	1.00000000	mice
Htt	-0.18	1.00000000	mice
Sh3bp4	-0.08	1.00000000	mice
Pak1ip1	0.25	1.00000000	mice
Zc2hc1a	0.16	1.00000000	mice
Gucd1	0.20	1.00000000	mice
Ccni	0.13	1.00000000	mice
Usp8	-0.22	1.00000000	mice
Usp1	0.02	1.00000000	mice
Fgl2	0.68	1.00000000	mice
Plekha2	-0.51	1.00000000	mice
Plscr1	0.44	1.00000000	mice
Cfap97	0.06	1.00000000	mice
Ruvbl2	-0.12	1.00000000	mice
Nktr	0.19	1.00000000	mice
Cbx1	0.12	1.00000000	mice
Gnb1	0.25	1.00000000	mice
Ly75	-0.51	1.00000000	mice
Brca1	0.12	1.00000000	mice
Slc9a1	0.30	1.00000000	mice
Emsy	-0.23	1.00000000	mice
Srsf4	-0.25	1.00000000	mice
Lair1	0.81	1.00000000	mice
Wdr36	-0.12	1.00000000	mice
Dstyk	0.12	1.00000000	mice
Bloc1s2	0.23	1.00000000	mice
Ccdc97	0.08	1.00000000	mice
Stard4	0.15	1.00000000	mice
Sfpq	-0.15	1.00000000	mice
Tspan17	0.10	1.00000000	mice
Tsc22d2	-0.29	1.00000000	mice
Ndufs4	0.11	1.00000000	mice
Vdr	-0.16	1.00000000	mice
Fcrla	0.01	1.00000000	mice
Kmo	0.33	1.00000000	mice

Dhrs7b	0.06	1.00000000	mice
Dhcr24	0.12	1.00000000	mice
Senp1	0.16	1.00000000	mice
Hspa5	-0.15	1.00000000	mice
Ckap5	0.18	1.00000000	mice
Hist3h2a	-0.16	1.00000000	mice
Lrmda	0.20	1.00000000	mice
Slc35a4	-0.10	1.00000000	mice
Zfp800	0.28	1.00000000	mice
Cdc37l1	0.06	1.00000000	mice
Kdm5b	-0.34	1.00000000	mice
Ctif	-0.26	1.00000000	mice
Ppp1r16a	0.09	1.00000000	mice
Zfp715	0.06	1.00000000	mice
Acaa1a	0.13	1.00000000	mice
Id2	-0.47	1.00000000	mice
Ap2a2	-0.19	1.00000000	mice
Paf1	-0.23	1.00000000	mice
Pold2	0.07	1.00000000	mice
Uck2	-0.20	1.00000000	mice
Rrm1	0.06	1.00000000	mice
Snw1	0.23	1.00000000	mice
Vps37c	0.21	1.00000000	mice
Akna	0.17	1.00000000	mice
Ssbp3	-0.39	1.00000000	mice
Cebpz	-0.24	1.00000000	mice
Snrpd2	0.16	1.00000000	mice
Rasip1	0.11	1.00000000	mice
Plgrkt	0.13	1.00000000	mice
Kmt5b	0.16	1.00000000	mice
Tspyl2	-0.09	1.00000000	mice
Bex3	-0.27	1.00000000	mice
Atxn10	-0.30	1.00000000	mice
Cul2	-0.13	1.00000000	mice
Adrb2	-0.27	1.00000000	mice
Cklf	0.16	1.00000000	mice
Zdhhc7	0.02	1.00000000	mice
Ttc27	-0.16	1.00000000	mice
Vps29	0.13	1.00000000	mice
Gtpbp8	0.10	1.00000000	mice
Tob1	-0.30	1.00000000	mice
Zfp438	-0.22	1.00000000	mice
Fndc7	0.18	1.00000000	mice
Sf3a3	0.10	1.00000000	mice
Nup54	0.18	1.00000000	mice
Scaf8	-0.20	1.00000000	mice

Gclm	-0.11	1.00000000	mice
Chchd3	-0.25	1.00000000	mice
Vps53	0.04	1.00000000	mice
Gtf3c2	-0.16	1.00000000	mice
Tmem258	0.19	1.00000000	mice
Lnx2	-0.17	1.00000000	mice
Yipf1	0.16	1.00000000	mice
Zfp346	0.02	1.00000000	mice
Nrip1	0.16	1.00000000	mice
Slc33a1	0.20	1.00000000	mice
Stam2	0.17	1.00000000	mice
Chd1l	-0.11	1.00000000	mice
Elk4	-0.26	1.00000000	mice
Smad5	-0.08	1.00000000	mice
Eef1g	-0.18	1.00000000	mice
Appl1	-0.20	1.00000000	mice
Rbm8a	0.17	1.00000000	mice
Cdc14a	-0.30	1.00000000	mice
Trbc2	-0.24	1.00000000	mice
Tcp1	0.12	1.00000000	mice
Imp3	-0.14	1.00000000	mice
Ankrd11	-0.16	1.00000000	mice
Eif2b1	0.09	1.00000000	mice
Sumo2	0.22	1.00000000	mice
Alpk2	0.23	1.00000000	mice
Arfip2	-0.14	1.00000000	mice
Triobp	0.14	1.00000000	mice
Fam91a1	-0.24	1.00000000	mice
Irf2	0.27	1.00000000	mice
Lrp5	-0.11	1.00000000	mice
Bsdc1	0.24	1.00000000	mice
Fmr1	0.14	1.00000000	mice
Rufy3	0.35	1.00000000	mice
Cux1	-0.26	1.00000000	mice
Cd86	0.48	1.00000000	mice
Rbm39	-0.12	1.00000000	mice
Pvt1	-0.40	1.00000000	mice
Bend4	0.08	1.00000000	mice
Rab20	0.19	1.00000000	mice
Cacna1s	0.01	1.00000000	mice
Cyb561d2	0.04	1.00000000	mice
S100a9	0.54	1.00000000	mice
Wdr91	-0.25	1.00000000	mice
Nrros	0.22	1.00000000	mice
Pten	-0.20	1.00000000	mice
Ccl9	0.06	1.00000000	mice

Tpm4	0.19	1.00000000	mice
D6Wsu163e	0.08	1.00000000	mice
Rfx2	-0.14	1.00000000	mice
Prkdc	-0.25	1.00000000	mice
Atp2b1	-0.21	1.00000000	mice
Actr1b	-0.17	1.00000000	mice
Nme2	-0.15	1.00000000	mice
Timm9	0.11	1.00000000	mice
St3gal3	-0.37	1.00000000	mice
Trim37	0.09	1.00000000	mice
Slc25a11	0.19	1.00000000	mice
Afg3l1	0.05	1.00000000	mice
Grk5	-0.18	1.00000000	mice
Ppm1l	0.53	1.00000000	mice
Gpr137b	-0.17	1.00000000	mice
Ndfip2	-0.21	1.00000000	mice
Psmf1	-0.06	1.00000000	mice
Capns1	-0.14	1.00000000	mice
Slc44a1	-0.48	1.00000000	mice
Fundc1	0.13	1.00000000	mice
Poldip2	0.10	1.00000000	mice
Hsd17b10	-0.11	1.00000000	mice
Hmox1	-0.24	1.00000000	mice
Rnf115	0.33	1.00000000	mice
Pip5k1c	-0.25	1.00000000	mice
Slc20a2	-0.13	1.00000000	mice
Tent4b	0.15	1.00000000	mice
Ap5m1	0.09	1.00000000	mice
Phb	-0.08	1.00000000	mice
Fam43a	-0.13	1.00000000	mice
Bcas3	-0.29	1.00000000	mice
Anxa5	0.32	1.00000000	mice
Rad23b	0.23	1.00000000	mice
Nr2c2ap	0.02	1.00000000	mice
Josd1	0.11	1.00000000	mice
Pdcd11	0.12	1.00000000	mice
Rsf1	0.08	1.00000000	mice
Dennd1c	0.13	1.00000000	mice
Gatm	-0.13	1.00000000	mice
Retreg1	-0.26	1.00000000	mice
Ccl2	-0.11	1.00000000	mice
Shtn1	-0.19	1.00000000	mice
Chdh	0.21	1.00000000	mice
Ints12	0.10	1.00000000	mice
Olr1	0.09	1.00000000	mice
Prkar2b	-0.14	1.00000000	mice

Atp6v1a	0.09	1.00000000	mice
Bod1l	0.23	1.00000000	mice
Psmd9	0.10	1.00000000	mice
Egr3	-0.11	1.00000000	mice
Suox	-0.06	1.00000000	mice
Rnf169	0.15	1.00000000	mice
Dock8	0.40	1.00000000	mice
Tada2b	-0.08	1.00000000	mice
Top3b	-0.14	1.00000000	mice
Plekha3	0.07	1.00000000	mice
Cr1l	-0.20	1.00000000	mice
Aco1	0.10	1.00000000	mice
Fem1c	-0.23	1.00000000	mice
Etv3	0.26	1.00000000	mice
Egln2	-0.11	1.00000000	mice
Gaa	0.11	1.00000000	mice
Rabgap1	0.18	1.00000000	mice
Nmral1	0.11	1.00000000	mice
Tuba1b	0.09	1.00000000	mice
Ifi30	0.58	1.00000000	mice
1600012H06Rik	0.08	1.00000000	mice
Prmt3	-0.28	1.00000000	mice
Thumpd1	-0.22	1.00000000	mice
2900060B14Rik	-0.05	1.00000000	mice
Cnnm3	-0.07	1.00000000	mice
Cep295	0.10	1.00000000	mice
Prpf6	0.11	1.00000000	mice
Dctn2	0.21	1.00000000	mice
Rfwd3	0.04	1.00000000	mice
Ubqln2	0.12	1.00000000	mice
Irak2	-0.13	1.00000000	mice
Fxr1	0.16	1.00000000	mice
Pdia3	-0.15	1.00000000	mice
0610030E20Rik	0.10	1.00000000	mice
Camsap2	-0.34	1.00000000	mice
Phtf2	-0.30	1.00000000	mice
Xxylt1	-0.08	1.00000000	mice
Acad9	-0.12	1.00000000	mice
Zscan21	0.10	1.00000000	mice
Cpne3	0.11	1.00000000	mice
Fam133b	0.10	1.00000000	mice
H2-Eb1	-0.23	1.00000000	mice
Trappc12	0.12	1.00000000	mice
Alox5ap	-0.12	1.00000000	mice
C1galt1	0.23	1.00000000	mice
Srpk1	-0.25	1.00000000	mice

Sh3pxd2b	-0.35	1.00000000	mice
Rfxank	0.08	1.00000000	mice
Arhgap9	0.06	1.00000000	mice
Zfp369	-0.08	1.00000000	mice
Clec4e	0.14	1.00000000	mice
Gsn	-0.02	1.00000000	mice
Ssh1	0.19	1.00000000	mice
Hpse	0.69	1.00000000	mice
Dnlz	0.08	1.00000000	mice
Ptdss1	-0.26	1.00000000	mice
2410006H16Rik	0.27	1.00000000	mice
Plk2	0.10	1.00000000	mice
Slc24a1	-0.07	1.00000000	mice
Pgam1	-0.18	1.00000000	mice
Cisd2	0.05	1.00000000	mice
Nsfl1c	0.16	1.00000000	mice
Apc	0.35	1.00000000	mice
Cdh5	0.18	1.00000000	mice
Ahctf1	-0.08	1.00000000	mice
Hint1	-0.12	1.00000000	mice
B3gat3	0.10	1.00000000	mice
Trim30c	0.23	1.00000000	mice
Fam83f	0.05	1.00000000	mice
Spata1	0.04	1.00000000	mice
Srp72	-0.18	1.00000000	mice
Drg2	-0.10	1.00000000	mice
Ccdc59	0.09	1.00000000	mice
Fbxl5	-0.21	1.00000000	mice
Fosl2	-0.15	1.00000000	mice
Ddr1	0.26	1.00000000	mice
Zdhhc16	0.05	1.00000000	mice
Chchd2	0.13	1.00000000	mice
Plekha1	0.14	1.00000000	mice
Arl4c	-0.33	1.00000000	mice
Triap1	0.10	1.00000000	mice
Twf1	0.09	1.00000000	mice
Smim27	0.09	1.00000000	mice
Tm9sf2	0.09	1.00000000	mice
Wdr70	-0.22	1.00000000	mice
Slc39a3	0.06	1.00000000	mice
Ndufb7	0.19	1.00000000	mice
Zc3h14	-0.21	1.00000000	mice
Itln1	-0.10	1.00000000	mice
Jaml	0.39	1.00000000	mice
Srgap3	0.38	1.00000000	mice
Nagk	0.10	1.00000000	mice

Zfp597	-0.09	1.00000000	mice
Rab39	0.10	1.00000000	mice
Fance	-0.18	1.00000000	mice
Foxn2	0.25	1.00000000	mice
Tmbim1	-0.21	1.00000000	mice
Casp8	0.22	1.00000000	mice
Ado	0.08	1.00000000	mice
Cyc1	0.14	1.00000000	mice
Srpr	0.13	1.00000000	mice
Exoc6b	-0.31	1.00000000	mice
Ctsa	0.14	1.00000000	mice
Scamp2	0.17	1.00000000	mice
Ppp1r12b	-0.22	1.00000000	mice
Nr1h2	0.12	1.00000000	mice
Sfmbt1	-0.18	1.00000000	mice
Zfc3h1	0.51	1.00000000	mice
Vgll4	-0.24	1.00000000	mice
AI506816	-0.06	1.00000000	mice
Cct4	0.13	1.00000000	mice
Slc12a9	0.12	1.00000000	mice
Ccdc71l	0.07	1.00000000	mice
Trim36	-0.07	1.00000000	mice
Ccpg1	-0.26	1.00000000	mice
Gba	0.13	1.00000000	mice
Larp4	-0.19	1.00000000	mice
Borcs7	0.08	1.00000000	mice
Tbc1d10b	0.10	1.00000000	mice
Fra10ac1	0.11	1.00000000	mice
Chmp7	0.09	1.00000000	mice
Pkig	0.39	1.00000000	mice
Taldo1	0.41	1.00000000	mice
Dcaf5	0.13	1.00000000	mice
Higd1a	-0.28	1.00000000	mice
Apaf1	0.40	1.00000000	mice
Trim7	-0.03	1.00000000	mice
Nudt16	0.09	1.00000000	mice
Rdm1	-0.21	1.00000000	mice
Bcl2a1b	0.45	1.00000000	mice
Hsp90ab1	0.19	1.00000000	mice
Pfkfb4	0.12	1.00000000	mice
Tmem141	-0.08	1.00000000	mice
Ankrd49	0.11	1.00000000	mice
R3hdm2	0.16	1.00000000	mice
Hsdl2	0.17	1.00000000	mice
Armh3	-0.05	1.00000000	mice
Bcar3	-0.22	1.00000000	mice

Eif2ak3	-0.24	1.00000000	mice
Taf1a	-0.23	1.00000000	mice
Sbk1	-0.18	1.00000000	mice
Dpy19l1	0.15	1.00000000	mice
R3hcc1l	0.23	1.00000000	mice
Polr3b	-0.03	1.00000000	mice
I830077J02Rik	0.20	1.00000000	mice
Rfx3	-0.39	1.00000000	mice
Rbm3	0.06	1.00000000	mice
Tiparp	-0.28	1.00000000	mice
Fam222b	-0.32	1.00000000	mice
Tipin	0.06	1.00000000	mice
Tmem120a	0.08	1.00000000	mice
Wdr7	0.12	1.00000000	mice
Crtc2	-0.18	1.00000000	mice
Rfc2	0.13	1.00000000	mice
Rundc3b	0.15	1.00000000	mice
Snhg15	0.33	1.00000000	mice
Dmxl1	-0.30	1.00000000	mice
Fam3a	-0.08	1.00000000	mice
Itk	-0.16	1.00000000	mice
Phf21a	0.36	1.00000000	mice
Zzef1	-0.24	1.00000000	mice
Tbcel	-0.24	1.00000000	mice
Abhd5	0.12	1.00000000	mice
1600002K03Rik	0.07	1.00000000	mice
Alyref	-0.21	1.00000000	mice
Crkl	0.12	1.00000000	mice
Zfp266	0.16	1.00000000	mice
2410131K14Rik	0.04	1.00000000	mice
Hccs	0.11	1.00000000	mice
Trps1	-0.27	1.00000000	mice
Upb1	-0.28	1.00000000	mice
Gps2	0.05	1.00000000	mice
Smg6	0.11	1.00000000	mice
Zfp951	0.34	1.00000000	mice
Vamp2	0.04	1.00000000	mice
Ergic2	-0.09	1.00000000	mice
Pi4k2b	0.27	1.00000000	mice
Ap1s1	0.10	1.00000000	mice
Rab30	0.24	1.00000000	mice
Rex1bd	0.19	1.00000000	mice
Rnf19a	0.12	1.00000000	mice
Diaph2	0.19	1.00000000	mice
Uxs1	-0.24	1.00000000	mice
Taf1	-0.27	1.00000000	mice

Kif21b	-0.22	1.00000000	mice
Zfyve16	0.13	1.00000000	mice
Irf2bp1	0.05	1.00000000	mice
Adam8	-0.19	1.00000000	mice
Pced1b	-0.07	1.00000000	mice
3110082I17Rik	-0.28	1.00000000	mice
Cnnm4	-0.24	1.00000000	mice
Ciita	-0.22	1.00000000	mice
Reep4	0.08	1.00000000	mice
Tars	0.07	1.00000000	mice
Itgb1	0.19	1.00000000	mice
Ucp1	-0.07	1.00000000	mice
Pigc	0.06	1.00000000	mice
Zfp207	0.15	1.00000000	mice
Tgs1	0.22	1.00000000	mice
2010315B03Rik	0.09	1.00000000	mice
Gtf2f2	-0.20	1.00000000	mice
Slc49a4	-0.33	1.00000000	mice
Rbmx2	0.08	1.00000000	mice
Capzb	0.14	1.00000000	mice
Trak1	-0.29	1.00000000	mice
Dnajc14	0.12	1.00000000	mice
Sltm	0.20	1.00000000	mice
Rnf40	0.09	1.00000000	mice
Taz	0.08	1.00000000	mice
Lima1	0.17	1.00000000	mice
Elf2	-0.29	1.00000000	mice
Cnot11	0.10	1.00000000	mice
Fdx2	0.03	1.00000000	mice
Tkt	0.12	1.00000000	mice
Lmo1	-0.14	1.00000000	mice
Braf	-0.13	1.00000000	mice
Erg28	0.04	1.00000000	mice
Nr4a3	0.31	1.00000000	mice
CAAA01118383.1	0.08	1.00000000	mice
Cd53	-0.22	1.00000000	mice
Sec24c	0.17	1.00000000	mice
Kif13a	-0.21	1.00000000	mice
Nbeal2	-0.05	1.00000000	mice
Insyn2b	0.16	1.00000000	mice
Shq1	-0.23	1.00000000	mice
Dpp4	0.36	1.00000000	mice
Etv5	-0.06	1.00000000	mice
Sdhaf1	0.19	1.00000000	mice
Il6st	-0.16	1.00000000	mice
Ociad1	0.12	1.00000000	mice

Slc4a1ap	0.07	1.00000000	mice
Zc3h12a	-0.23	1.00000000	mice
Rapgef6	-0.04	1.00000000	mice
Cxcr5	0.14	1.00000000	mice
Sphk2	0.08	1.00000000	mice
Ube2q2	-0.29	1.00000000	mice
Crebrf	0.17	1.00000000	mice
Trappc1	0.12	1.00000000	mice
Cops3	0.12	1.00000000	mice
Gale	0.08	1.00000000	mice
Pi4ka	-0.45	1.00000000	mice
Gpi1	-0.20	1.00000000	mice
Cars2	0.07	1.00000000	mice
Arl2	0.07	1.00000000	mice
Lipa	0.00	1.00000000	mice
Cyfip2	-0.19	1.00000000	mice
Slc27a3	-0.07	1.00000000	mice
Slc25a16	0.07	1.00000000	mice
BC002059	0.06	1.00000000	mice
Parg	-0.14	1.00000000	mice
Cstf2t	0.05	1.00000000	mice
Knop1	0.07	1.00000000	mice
Sdcbp	0.20	1.00000000	mice
0610010F05Rik	-0.03	1.00000000	mice
Ncapd3	-0.09	1.00000000	mice
Lysmd4	0.16	1.00000000	mice
Tex30	-0.14	1.00000000	mice
Selenoi	-0.10	1.00000000	mice
Ubr5	-0.20	1.00000000	mice
Nfkbie	0.12	1.00000000	mice
Esd	0.13	1.00000000	mice
Zfp655	-0.16	1.00000000	mice
Yif1a	0.08	1.00000000	mice
Ly96	0.22	1.00000000	mice
Hip1	-0.06	1.00000000	mice
Xbp1	0.67	1.00000000	mice
Ppil2	0.08	1.00000000	mice
Rfk	0.10	1.00000000	mice
Deaf1	0.13	1.00000000	mice
Tmem165	0.09	1.00000000	mice
Cox4i2	-0.03	1.00000000	mice
St13	0.23	1.00000000	mice
Hmgxb4	0.08	1.00000000	mice
Lifr	-0.35	1.00000000	mice
Gtf2b	0.17	1.00000000	mice
Gng2	-0.14	1.00000000	mice

Rab2a	-0.18	1.00000000	mice
Nup107	-0.24	1.00000000	mice
Inpp1	-0.06	1.00000000	mice
Eif2ak1	-0.22	1.00000000	mice
Ccng1	-0.10	1.00000000	mice
Nubp2	0.10	1.00000000	mice
Usp40	0.08	1.00000000	mice
Klf16	0.05	1.00000000	mice
Snx11	0.12	1.00000000	mice
Atxn7l1	0.33	1.00000000	mice
Ctla2a	0.01	1.00000000	mice
Utp18	0.13	1.00000000	mice
Panx1	0.11	1.00000000	mice
Pdpk1	0.19	1.00000000	mice
Dhx57	0.14	1.00000000	mice
Gpm6b	0.38	1.00000000	mice
Actr10	0.08	1.00000000	mice
Srsf11	-0.16	1.00000000	mice
Uap1l1	0.07	1.00000000	mice
Pak1	-0.31	1.00000000	mice
H2-Ke6	-0.15	1.00000000	mice
Zbtb18	0.25	1.00000000	mice
Sik3	0.20	1.00000000	mice
Syne2	-0.26	1.00000000	mice
Rac2	0.42	1.00000000	mice
Rbm4	0.15	1.00000000	mice
Tubgcp4	0.11	1.00000000	mice
Slc4a7	-0.27	1.00000000	mice
Fh1	0.21	1.00000000	mice
Zbtb11	-0.24	1.00000000	mice
Ptpn11	-0.17	1.00000000	mice
Ppm1k	0.08	1.00000000	mice
Cdc26	0.13	1.00000000	mice
Fbf1	-0.08	1.00000000	mice
Higd2a	-0.10	1.00000000	mice
Bcl7a	-0.16	1.00000000	mice
Rangap1	-0.17	1.00000000	mice
Gstcd	-0.11	1.00000000	mice
Sass6	0.05	1.00000000	mice
Snapc5	0.10	1.00000000	mice
Chil3	0.07	1.00000000	mice
A730081D07Rik	0.07	1.00000000	mice
Nup93	-0.13	1.00000000	mice
Jakmip1	-0.36	1.00000000	mice
Skp1a	0.16	1.00000000	mice
Ewsr1	-0.20	1.00000000	mice

Hivep3	-0.04	1.00000000	mice
Col4a3bp	-0.34	1.00000000	mice
Spcs3	0.12	1.00000000	mice
Pdhx	0.06	1.00000000	mice
Xpo4	-0.17	1.00000000	mice
Eed	-0.24	1.00000000	mice
Wtap	0.06	1.00000000	mice
Tmem134	0.16	1.00000000	mice
Fyco1	-0.26	1.00000000	mice
Prr3	-0.07	1.00000000	mice
Klhl5	0.29	1.00000000	mice
Pmm2	0.03	1.00000000	mice
Otud4	0.05	1.00000000	mice
Ikzf5	0.07	1.00000000	mice
Cep68	0.11	1.00000000	mice
Rassf4	-0.19	1.00000000	mice
Anks3	-0.12	1.00000000	mice
Il18	-0.12	1.00000000	mice
Slc7a5	0.30	1.00000000	mice
Atp5a1	0.15	1.00000000	mice
Lrp4	0.20	1.00000000	mice
St3gal2	0.07	1.00000000	mice
Cdc34	0.10	1.00000000	mice
Kif23	-0.10	1.00000000	mice
Guca1a	-0.06	1.00000000	mice
Acsl1	0.26	1.00000000	mice
Slc31a2	0.11	1.00000000	mice
Pycard	0.15	1.00000000	mice
Usp4	0.15	1.00000000	mice
Palm	0.11	1.00000000	mice
1810037I17Rik	0.27	1.00000000	mice
Afap1	-0.09	1.00000000	mice
Sh2b2	0.11	1.00000000	mice
Zmym4	0.10	1.00000000	mice
Hmgn5	-0.24	1.00000000	mice
Syne1	-0.08	1.00000000	mice
Prpf39	-0.13	1.00000000	mice
Lyrm2	0.12	1.00000000	mice
Cplane1	0.14	1.00000000	mice
Hnrnpul2	0.16	1.00000000	mice
Galc	-0.26	1.00000000	mice
Nelfcd	0.07	1.00000000	mice
Clca3a1	0.11	1.00000000	mice
Ppp1r14b	-0.24	1.00000000	mice
Pop7	0.06	1.00000000	mice
Hectd4	0.13	1.00000000	mice

Ccl6	-0.52	1.00000000	mice
Aprt	-0.17	1.00000000	mice
Terf2	0.17	1.00000000	mice
Cgas	0.11	1.00000000	mice
Prim2	0.21	1.00000000	mice
Zbtb2	-0.18	1.00000000	mice
Hs3st1	-0.11	1.00000000	mice
Jade2	-0.11	1.00000000	mice
Ctse	-0.39	1.00000000	mice
Fam207a	0.12	1.00000000	mice
Enpp4	0.13	1.00000000	mice
Zfp106	-0.09	1.00000000	mice
4921511I17Rik	0.04	1.00000000	mice
Ccdc162	0.08	1.00000000	mice
Lap3	0.07	1.00000000	mice
Polg2	-0.12	1.00000000	mice
Acox3	-0.10	1.00000000	mice
Washc4	0.07	1.00000000	mice
Slc8a1	0.13	1.00000000	mice
Otud7b	-0.19	1.00000000	mice
Sepsecs	0.17	1.00000000	mice
N4bp2	-0.27	1.00000000	mice
Nudt18	0.09	1.00000000	mice
Hs3st3b1	-0.19	1.00000000	mice
Elf4	0.24	1.00000000	mice
Hps6	-0.05	1.00000000	mice
Fancc	-0.19	1.00000000	mice
Sept7	0.09	1.00000000	mice
Taf12	0.05	1.00000000	mice
Snhg6	0.06	1.00000000	mice
Zfp777	-0.09	1.00000000	mice
Ndufaf7	-0.08	1.00000000	mice
Bcl11a	0.30	1.00000000	mice
Hpf1	0.15	1.00000000	mice
Klf9	-0.15	1.00000000	mice
Card10	-0.11	1.00000000	mice
Cmah	-0.13	1.00000000	mice
Srd5a3	0.11	1.00000000	mice
Trappc3	0.14	1.00000000	mice
Ldlr	0.26	1.00000000	mice
Plekhm1	-0.09	1.00000000	mice
Cbr3	-0.09	1.00000000	mice
Ift57	0.18	1.00000000	mice
Tmem126b	0.05	1.00000000	mice
Capza1	0.11	1.00000000	mice
Ipo11	-0.12	1.00000000	mice

Dcun1d2	-0.11	1.00000000	mice
Abi3	0.18	1.00000000	mice
Trim23	-0.10	1.00000000	mice
Ninj1	0.17	1.00000000	mice
Git1	0.12	1.00000000	mice
Casp7	0.10	1.00000000	mice
Fermt3	0.24	1.00000000	mice
Ppp2r3c	0.09	1.00000000	mice
Ppp2r2d	0.05	1.00000000	mice
Ubxn6	-0.07	1.00000000	mice
Sharpin	0.08	1.00000000	mice
Flot1	0.07	1.00000000	mice
Ppp6r1	0.14	1.00000000	mice
Kbtbd2	-0.12	1.00000000	mice
Sdhc	0.11	1.00000000	mice
Stxbp1	-0.14	1.00000000	mice
Il7r	0.24	1.00000000	mice
Cdh22	0.09	1.00000000	mice
Lcp2	0.18	1.00000000	mice
Prpf19	-0.09	1.00000000	mice
Lgals8	0.10	1.00000000	mice
Was	0.03	1.00000000	mice
Bud13	0.05	1.00000000	mice
Atxn7l3	0.06	1.00000000	mice
Stom	0.05	1.00000000	mice
Dusp1	-0.32	1.00000000	mice
Zeb2os	0.09	1.00000000	mice
Sf3a2	0.04	1.00000000	mice
Fam114a1	-0.07	1.00000000	mice
Spi1	0.30	1.00000000	mice
Coro7	0.22	1.00000000	mice
Cyhr1	0.10	1.00000000	mice
Tollip	0.07	1.00000000	mice
Rhbdf2	0.43	1.00000000	mice
Dnajc24	0.07	1.00000000	mice
Selenom	-0.04	1.00000000	mice
Cd84	-0.07	1.00000000	mice
Ints6	0.18	1.00000000	mice
Snhg4	-0.09	1.00000000	mice
Fbxo21	-0.08	1.00000000	mice
Prmt9	0.05	1.00000000	mice
Stox1	-0.21	1.00000000	mice
Ccdc90b	0.11	1.00000000	mice
Rrp36	0.12	1.00000000	mice
Zrsr2	0.15	1.00000000	mice
Dhps	0.11	1.00000000	mice

Got2	-0.13	1.00000000	mice
Slc43a2	-0.07	1.00000000	mice
Apex1	0.06	1.00000000	mice
Ppcdc	-0.08	1.00000000	mice
Dnajc13	0.36	1.00000000	mice
Eci2	0.10	1.00000000	mice
Zswim6	-0.37	1.00000000	mice
Pcmtd1	0.16	1.00000000	mice
Klk1b27	0.29	1.00000000	mice
Cd72	0.12	1.00000000	mice
Ttc39c	0.11	1.00000000	mice
Ak2	0.05	1.00000000	mice
Noct	-0.29	1.00000000	mice
Rsbn1	-0.21	1.00000000	mice
Bad	0.05	1.00000000	mice
Atg5	-0.22	1.00000000	mice
Pde12	0.06	1.00000000	mice
Fbxw17	0.11	1.00000000	mice
Faim	0.18	1.00000000	mice
Gatad2a	-0.13	1.00000000	mice
Rrs1	-0.22	1.00000000	mice
Ebna1bp2	0.10	1.00000000	mice
Cdc42	0.15	1.00000000	mice
N6amt1	0.05	1.00000000	mice
Dennd6a	0.06	1.00000000	mice
Cst3	-0.41	1.00000000	mice
Pfkl	-0.20	1.00000000	mice
Inafm2	0.07	1.00000000	mice
Bnip3l	-0.20	1.00000000	mice
A930024E05Rik	-0.09	1.00000000	mice
Aldh3b1	-0.08	1.00000000	mice
Pole3	0.06	1.00000000	mice
Abhd17c	-0.12	1.00000000	mice
Zfand2b	0.09	1.00000000	mice
Acod1	0.19	1.00000000	mice
Traf6	0.32	1.00000000	mice
Slc44a2	0.32	1.00000000	mice
Cct6a	-0.13	1.00000000	mice
Rab24	0.09	1.00000000	mice
Gpr146	0.18	1.00000000	mice
Synj1	0.29	1.00000000	mice
Ric1	0.53	1.00000000	mice
Plekho1	0.22	1.00000000	mice
Sema4d	0.13	1.00000000	mice
Ralgps2	-0.21	1.00000000	mice
Hddc2	0.06	1.00000000	mice

Exosc3	0.09	1.00000000	mice
Hspa14	-0.14	1.00000000	mice
Acyp1	-0.06	1.00000000	mice
H2-Ob	-0.07	1.00000000	mice
Creg1	0.03	1.00000000	mice
Gspt1	-0.13	1.00000000	mice
Fkbp3	0.08	1.00000000	mice
Srp68	0.09	1.00000000	mice
Dipk2a	-0.16	1.00000000	mice
Dgcr2	-0.14	1.00000000	mice
Vrk1	-0.04	1.00000000	mice
2310022A10Rik	0.10	1.00000000	mice
Ndst1	0.11	1.00000000	mice
Hcls1	0.16	1.00000000	mice
Gpr157	0.15	1.00000000	mice
Srf	0.04	1.00000000	mice
Tomm20	-0.16	1.00000000	mice
Ufd1	0.14	1.00000000	mice
Slc25a3	0.31	1.00000000	mice
Pip5k1a	0.13	1.00000000	mice
Sptssa	-0.07	1.00000000	mice
Dlat	-0.14	1.00000000	mice
Usp7	0.18	1.00000000	mice
Nt5c	0.11	1.00000000	mice
Tgfbr1	0.11	1.00000000	mice
Frs2	0.20	1.00000000	mice
Eri1	0.10	1.00000000	mice
8030462N17Rik	-0.18	1.00000000	mice
Prelid1	0.16	1.00000000	mice
Gpalpp1	0.12	1.00000000	mice
Akirin2	0.06	1.00000000	mice
Lactb2	0.11	1.00000000	mice
Orc3	0.06	1.00000000	mice
Slmap	0.20	1.00000000	mice
Cep85	-0.07	1.00000000	mice
Smc1a	0.14	1.00000000	mice
Gadd45g	-0.21	1.00000000	mice
Arl13b	-0.02	1.00000000	mice
Gtpbp1	0.16	1.00000000	mice
Abracl	0.21	1.00000000	mice
Il1b	0.20	1.00000000	mice
Ube2b	0.05	1.00000000	mice
Pdcd5	0.10	1.00000000	mice
Wbp11	-0.18	1.00000000	mice
Tmem108	-0.60	1.00000000	mice
Hcst	0.05	1.00000000	mice

Itprid2	-0.25	1.00000000	mice
Slc7a1	-0.26	1.00000000	mice
Acin1	0.05	1.00000000	mice
Dusp18	-0.07	1.00000000	mice
Pcyt2	-0.15	1.00000000	mice
Sacm1l	-0.25	1.00000000	mice
Vps45	0.09	1.00000000	mice
Ubac1	0.08	1.00000000	mice
Zfp91	0.14	1.00000000	mice
Abhd13	0.07	1.00000000	mice
Eif4g2	0.03	1.00000000	mice
Casz1	0.13	1.00000000	mice
Scfd2	-0.26	1.00000000	mice
Cox18	0.07	1.00000000	mice
Rbm27	0.23	1.00000000	mice
Coq10b	0.26	1.00000000	mice
Ccdc191	0.09	1.00000000	mice
Cflar	0.44	1.00000000	mice
Rpn2	-0.15	1.00000000	mice
Rab5b	0.12	1.00000000	mice
Zfp593	-0.07	1.00000000	mice
Khnyn	0.09	1.00000000	mice
Acap1	0.08	1.00000000	mice
Apoo	0.07	1.00000000	mice
Pacc1	0.09	1.00000000	mice
Ripk1	0.16	1.00000000	mice
Dot1l	-0.13	1.00000000	mice
Twsg1	-0.12	1.00000000	mice
1700016P03Rik	-0.02	1.00000000	mice
Dnaja1	0.20	1.00000000	mice
Rrad	0.34	1.00000000	mice
Fasn	-0.16	1.00000000	mice
Tmed9	0.10	1.00000000	mice
Foxo3	0.09	1.00000000	mice
Cbfa2t3	-0.27	1.00000000	mice
Gas7	-0.36	1.00000000	mice
Fbxo45	0.12	1.00000000	mice
Kdm2b	-0.30	1.00000000	mice
Ubc	0.23	1.00000000	mice
Ddx49	0.11	1.00000000	mice
Wdr92	-0.12	1.00000000	mice
Ap3s2	0.01	1.00000000	mice
Traf3ip2	-0.24	1.00000000	mice
Cog1	0.07	1.00000000	mice
Spata5	-0.21	1.00000000	mice
Kcnab2	-0.07	1.00000000	mice

Slc35e4	-0.07	1.00000000	mice
Haus4	-0.20	1.00000000	mice
Slamf8	-0.10	1.00000000	mice
Acadm	0.31	1.00000000	mice
Rab33b	0.39	1.00000000	mice
Bcl2l11	-0.31	1.00000000	mice
Cd47	0.10	1.00000000	mice
Stx18	-0.12	1.00000000	mice
Ash2l	0.08	1.00000000	mice
Hmg20a	-0.17	1.00000000	mice
Atp13a3	-0.02	1.00000000	mice
Lrwd1	0.10	1.00000000	mice
H2-Q1	0.20	1.00000000	mice
Ell2	-0.22	1.00000000	mice
Dap3	0.10	1.00000000	mice
Ankrd16	-0.09	1.00000000	mice
Nans	0.14	1.00000000	mice
Srsf7	0.15	1.00000000	mice
Polr2i	0.13	1.00000000	mice
Rgcc	-0.18	1.00000000	mice
A430093F15Rik	-0.08	1.00000000	mice
Wdr45	0.04	1.00000000	mice
Otub1	0.01	1.00000000	mice
Pan2	0.10	1.00000000	mice
Nln	-0.18	1.00000000	mice
Ppip5k2	0.10	1.00000000	mice
Strn3	-0.27	1.00000000	mice
Nxf1	0.15	1.00000000	mice
Focad	-0.12	1.00000000	mice
Oma1	0.13	1.00000000	mice
Ift52	0.08	1.00000000	mice
Zfp408	0.07	1.00000000	mice
Alg13	0.07	1.00000000	mice
Usp11	0.09	1.00000000	mice
Cdk2ap1	-0.25	1.00000000	mice
Plp2	-0.19	1.00000000	mice
Dnaaf2	-0.07	1.00000000	mice
Praf2	-0.06	1.00000000	mice
Gpatch2	-0.07	1.00000000	mice
Upf3b	0.05	1.00000000	mice
Znhit1	0.05	1.00000000	mice
Zfpl1	0.05	1.00000000	mice
Zfp398	-0.14	1.00000000	mice
Klf12	0.24	1.00000000	mice
Zbtb6	0.04	1.00000000	mice
Arnt	-0.15	1.00000000	mice

Ermard	0.10	1.00000000	mice
B230219D22Rik	0.05	1.00000000	mice
Rnf150	-0.09	1.00000000	mice
Fam168a	-0.38	1.00000000	mice
Card19	-0.11	1.00000000	mice
Ddb1	0.08	1.00000000	mice
Vamp8	0.17	1.00000000	mice
Scpep1	0.19	1.00000000	mice
Taco1	-0.08	1.00000000	mice
Nab1	0.13	1.00000000	mice
Ift43	0.05	1.00000000	mice
Auts2	-0.16	1.00000000	mice
Rnf10	0.09	1.00000000	mice
Zbtb8os	-0.13	1.00000000	mice
Sgf29	-0.09	1.00000000	mice
Hspa2	0.07	1.00000000	mice
Eps15	0.23	1.00000000	mice
Cystm1	-0.06	1.00000000	mice
Cebpg	0.15	1.00000000	mice
Pak2	-0.21	1.00000000	mice
Gda	-0.26	1.00000000	mice
Tank	0.45	1.00000000	mice
Tmem251	-0.14	1.00000000	mice
Tm2d3	0.04	1.00000000	mice
Guk1	-0.09	1.00000000	mice
Atf5	0.10	1.00000000	mice
Taf5	0.04	1.00000000	mice
Timm17b	-0.12	1.00000000	mice
Xab2	0.05	1.00000000	mice
Atp6ap1	0.08	1.00000000	mice
Tg	-0.16	1.00000000	mice
Aftph	0.27	1.00000000	mice
Kin	0.10	1.00000000	mice
Dcaf8	-0.16	1.00000000	mice
Rab11fip5	0.08	1.00000000	mice
Ube3a	0.04	1.00000000	mice
Hdac3	0.05	1.00000000	mice
Cat	-0.11	1.00000000	mice
AU022252	0.07	1.00000000	mice
Xpnpep3	-0.27	1.00000000	mice
Tmem175	0.11	1.00000000	mice
Cebpa	-0.09	1.00000000	mice
Foxk1	0.07	1.00000000	mice
Rasa3	-0.55	1.00000000	mice
Rilpl2	0.26	1.00000000	mice
Timm13	0.09	1.00000000	mice

Prcp	0.22	1.00000000	mice
Dnajc9	-0.11	1.00000000	mice
Rasa2	0.59	1.00000000	mice
Zfp236	0.11	1.00000000	mice
Ankrd54	0.07	1.00000000	mice
Zfp426	0.05	1.00000000	mice
Tbrg4	-0.13	1.00000000	mice
Smarcc2	-0.13	1.00000000	mice
Sav1	0.17	1.00000000	mice
Nsa2	0.13	1.00000000	mice
Il18rap	-0.01	1.00000000	mice
Naip5	-0.05	1.00000000	mice
Btaf1	-0.21	1.00000000	mice
Bag1	0.15	1.00000000	mice
Ibtk	-0.14	1.00000000	mice
Asxl1	-0.18	1.00000000	mice
Khdc4	0.13	1.00000000	mice
Qrsl1	-0.09	1.00000000	mice
Cdc37	0.17	1.00000000	mice
Sos2	-0.23	1.00000000	mice
Vat1	-0.07	1.00000000	mice
Wwox	-0.48	1.00000000	mice
Stx6	0.19	1.00000000	mice
Champ1	-0.05	1.00000000	mice
Nfkbia	-0.15	1.00000000	mice
Rab31	-0.27	1.00000000	mice
Atxn7l3b	0.10	1.00000000	mice
Fau	-0.08	1.00000000	mice
Ate1	0.06	1.00000000	mice
Eif3g	-0.18	1.00000000	mice
Npc1	0.17	1.00000000	mice
Cpsf2	0.07	1.00000000	mice
Adprh	0.17	1.00000000	mice
Blvra	0.12	1.00000000	mice
Lpcat2	-0.11	1.00000000	mice
Ep400	0.14	1.00000000	mice
Cd83	0.35	1.00000000	mice
Pgk1	-0.16	1.00000000	mice
Epas1	-0.08	1.00000000	mice
Phgdh	-0.10	1.00000000	mice
Nomo1	-0.08	1.00000000	mice
Dusp5	0.34	1.00000000	mice
Nubp1	0.16	1.00000000	mice
Cyp20a1	-0.14	1.00000000	mice
Noc4l	0.10	1.00000000	mice
Ube2m	0.13	1.00000000	mice

Umad1	0.13	1.00000000	mice
Slain1	-0.04	1.00000000	mice
Rybp	-0.12	1.00000000	mice
Tram2	0.14	1.00000000	mice
Siva1	0.09	1.00000000	mice
Cry2	-0.07	1.00000000	mice
Zbtb38	-0.12	1.00000000	mice
Ppfia1	0.08	1.00000000	mice
Rnpep	0.01	1.00000000	mice
Ets1	-0.59	1.00000000	mice
Gatad1	0.10	1.00000000	mice
Zfp609	0.12	1.00000000	mice
Cyth4	0.28	1.00000000	mice
Chpf2	-0.08	1.00000000	mice
Eef2kmt	-0.07	1.00000000	mice
Cd180	-0.26	1.00000000	mice
Erlec1	0.12	1.00000000	mice
Ntan1	0.14	1.00000000	mice
Tet2	-0.22	1.00000000	mice
Rpn1	-0.14	1.00000000	mice
Prdm15	0.32	1.00000000	mice
Chrac1	0.08	1.00000000	mice
Tmem221	0.06	1.00000000	mice
Hcfc1	-0.02	1.00000000	mice
Tm2d1	0.10	1.00000000	mice
Bfar	0.15	1.00000000	mice
Dnajb12	0.16	1.00000000	mice
Ufm1	0.12	1.00000000	mice
Aig1	-0.01	1.00000000	mice
Aldh2	-0.19	1.00000000	mice
Fam3c	0.23	1.00000000	mice
Zfp180	0.08	1.00000000	mice
Arcn1	0.13	1.00000000	mice
Ndufc2	0.17	1.00000000	mice
Ssbp2	-0.63	1.00000000	mice
Zfp445	-0.17	1.00000000	mice
Bmi1	0.03	1.00000000	mice
Tfdp1	0.11	1.00000000	mice
Arhgef10	0.10	1.00000000	mice
Rasa1	-0.23	1.00000000	mice
Nme7	0.05	1.00000000	mice
Oxa1l	0.07	1.00000000	mice
Serbp1	-0.07	1.00000000	mice
Dcaf12	-0.19	1.00000000	mice
Gas8	0.04	1.00000000	mice
Tpm3-rs7	0.05	1.00000000	mice

Ptpn1	0.06	1.00000000	mice
Capn2	-0.19	1.00000000	mice
Arih2	-0.27	1.00000000	mice
Pdia6	0.21	1.00000000	mice
Ctso	0.12	1.00000000	mice
Celf1	-0.08	1.00000000	mice
Stox2	-0.36	1.00000000	mice
Ncoa3	0.10	1.00000000	mice
Crtc1	-0.09	1.00000000	mice
Rp2	0.20	1.00000000	mice
Zfand5	0.06	1.00000000	mice
Atp6v1f	0.16	1.00000000	mice
Zfp771	0.09	1.00000000	mice
Cacybp	0.11	1.00000000	mice
4732440D04Rik	-0.08	1.00000000	mice
Kti12	0.05	1.00000000	mice
Seh1l	0.13	1.00000000	mice
Dnajc3	-0.13	1.00000000	mice
Pros1	-0.01	1.00000000	mice
Itfg1	0.11	1.00000000	mice
Traf5	-0.28	1.00000000	mice
Cyth2	0.10	1.00000000	mice
Cc2d1a	0.06	1.00000000	mice
Skiv2l	0.07	1.00000000	mice
Naa10	-0.06	1.00000000	mice
Shprh	-0.24	1.00000000	mice
Sh3glb2	-0.05	1.00000000	mice
Arhgap32	0.15	1.00000000	mice
Ep300	0.12	1.00000000	mice
Rasgrp2	-0.08	1.00000000	mice
Rasgrp4	-0.07	1.00000000	mice
Purb	0.15	1.00000000	mice
Il17ra	-0.21	1.00000000	mice
Imp4	0.07	1.00000000	mice
Lsp1	0.20	1.00000000	mice
Nfkbil1	0.09	1.00000000	mice
Lrrc42	0.03	1.00000000	mice
Dag1	0.11	1.00000000	mice
Rfxap	0.08	1.00000000	mice
Ing4	0.10	1.00000000	mice
1810013L24Rik	-0.04	1.00000000	mice
Btla	-0.27	1.00000000	mice
Ypel5	0.17	1.00000000	mice
Rpap2	0.08	1.00000000	mice
Stambp	0.05	1.00000000	mice
Zfp397	0.17	1.00000000	mice

Nt5c3b	-0.09	1.00000000	mice
Pex11b	0.04	1.00000000	mice
Vkorc1l1	-0.20	1.00000000	mice
Pla1a	0.17	1.00000000	mice
Lsm12	0.10	1.00000000	mice
Ap4s1	0.12	1.00000000	mice
Saysd1	-0.08	1.00000000	mice
Fam111a	0.11	1.00000000	mice
Pkd1	-0.09	1.00000000	mice
4632427E13Rik	-0.02	1.00000000	mice
Bcl2a1d	0.31	1.00000000	mice
Timm17a	0.07	1.00000000	mice
Lsm10	0.04	1.00000000	mice
Laptm4b	0.37	1.00000000	mice
Klf11	0.06	1.00000000	mice
Pigq	0.07	1.00000000	mice
Tmem229b	0.43	1.00000000	mice
Cdyl2	-0.28	1.00000000	mice
Wdr73	0.06	1.00000000	mice
Zfat	-0.09	1.00000000	mice
St8sia4	-0.16	1.00000000	mice
Scmh1	-0.26	1.00000000	mice
Ccdc28b	0.06	1.00000000	mice
Evi2	0.06	1.00000000	mice
Gpr137	0.03	1.00000000	mice
Kat6b	-0.08	1.00000000	mice
Tpk1	-0.21	1.00000000	mice
Atad2b	0.19	1.00000000	mice
Dera	0.10	1.00000000	mice
Prps2	-0.08	1.00000000	mice
Atpaf2	0.03	1.00000000	mice
Taf8	-0.05	1.00000000	mice
Amdhd2	0.11	1.00000000	mice
Psmb5	0.15	1.00000000	mice
Arhgap6	-0.10	1.00000000	mice
Pdcd2l	0.04	1.00000000	mice
Cxxc5	-0.28	1.00000000	mice
Ppp1r8	0.17	1.00000000	mice
Cnot10	-0.10	1.00000000	mice
Sirt7	0.08	1.00000000	mice
Dlg4	0.05	1.00000000	mice
Hagh	-0.18	1.00000000	mice
Cacnb2	-0.28	1.00000000	mice
Dock1	0.08	1.00000000	mice
Cdh1	0.27	1.00000000	mice
Kcmf1	0.19	1.00000000	mice

Usp42	0.06	1.00000000	mice
Tbc1d14	-0.11	1.00000000	mice
E2f5	0.11	1.00000000	mice
Emc10	-0.06	1.00000000	mice
Hmces	-0.13	1.00000000	mice
1500015A07Rik	0.03	1.00000000	mice
Ubl7	0.12	1.00000000	mice
Klf10	-0.09	1.00000000	mice
Golga3	0.02	1.00000000	mice
Timd4	0.00	1.00000000	mice
Idh3g	0.11	1.00000000	mice
Usp19	0.10	1.00000000	mice
Atn1	0.05	1.00000000	mice
Tmem50a	-0.11	1.00000000	mice
Pon3	0.18	1.00000000	mice
Nadk	-0.12	1.00000000	mice
Xpa	0.05	1.00000000	mice
Nufip1	-0.08	1.00000000	mice
Suclg2	-0.12	1.00000000	mice
Rbm22	0.05	1.00000000	mice
Cul4b	0.08	1.00000000	mice
Unc45a	-0.14	1.00000000	mice
Zfp770	0.10	1.00000000	mice
Rasal3	0.09	1.00000000	mice
Avl9	-0.18	1.00000000	mice
Ndufb6	0.12	1.00000000	mice
Sec23b	0.01	1.00000000	mice
Heatr6	-0.21	1.00000000	mice
Pqlc1	-0.15	1.00000000	mice
Kpna3	0.25	1.00000000	mice
Pdp2	0.09	1.00000000	mice
Vapa	0.06	1.00000000	mice
Abcg1	-0.46	1.00000000	mice
Rft1	0.06	1.00000000	mice
Ttc37	0.08	1.00000000	mice
Leprot	0.11	1.00000000	mice
Gch1	0.31	1.00000000	mice
Sde2	0.18	1.00000000	mice
Prmt7	0.07	1.00000000	mice
Rdx	0.07	1.00000000	mice
Tm9sf3	-0.14	1.00000000	mice
Crppa	-0.12	1.00000000	mice
Arl1	0.13	1.00000000	mice
Uqcc2	0.11	1.00000000	mice
Rnf44	0.10	1.00000000	mice
Cdkn2c	-0.03	1.00000000	mice

Glyr1	0.11	1.00000000	mice
Slk	-0.19	1.00000000	mice
Eid3	-0.08	1.00000000	mice
Serinc5	0.30	1.00000000	mice
Lcorl	-0.15	1.00000000	mice
Tent5a	0.15	1.00000000	mice
Rubcnl	-0.14	1.00000000	mice
Cxcr4	-0.07	1.00000000	mice
Sh3kbp1	0.09	1.00000000	mice
Rcl1	-0.08	1.00000000	mice
Pde2a	-0.19	1.00000000	mice
Zfp516	-0.32	1.00000000	mice
Acsl3	-0.08	1.00000000	mice
Lrrcc1	0.07	1.00000000	mice
Tecpr2	0.11	1.00000000	mice
Ube2s	-0.11	1.00000000	mice
Tlr1	0.05	1.00000000	mice
Erf	-0.06	1.00000000	mice
Usf3	0.09	1.00000000	mice
Agap1	-0.11	1.00000000	mice
Ugdh	0.10	1.00000000	mice
1810030O07Rik	-0.06	1.00000000	mice
Diablo	0.11	1.00000000	mice
Bin2	-0.11	1.00000000	mice
Gopc	0.03	1.00000000	mice
Gripap1	-0.21	1.00000000	mice
Atp6v0e	0.14	1.00000000	mice
Eloc	0.41	1.00000000	mice
Sc5d	0.11	1.00000000	mice
Tram1	0.25	1.00000000	mice
Rai1	-0.28	1.00000000	mice
Cmtm7	-0.14	1.00000000	mice
Gsk3a	0.05	1.00000000	mice
Foxred1	0.01	1.00000000	mice
Ubfd1	0.08	1.00000000	mice
Sclt1	-0.11	1.00000000	mice
Actr6	-0.02	1.00000000	mice
Rgl2	0.06	1.00000000	mice
Galk1	0.05	1.00000000	mice
Usp14	0.11	1.00000000	mice
Anxa11	-0.13	1.00000000	mice
Ak6	0.06	1.00000000	mice
Glipr1	-0.18	1.00000000	mice
Ccdc71	-0.23	1.00000000	mice
Zfp672	0.07	1.00000000	mice
Taok2	0.08	1.00000000	mice

Caprin1	0.17	1.00000000	mice
Copb1	0.01	1.00000000	mice
Edem3	-0.10	1.00000000	mice
Zhx3	0.12	1.00000000	mice
Parp1	-0.17	1.00000000	mice
Zfp622	-0.04	1.00000000	mice
Lrp8os2	0.06	1.00000000	mice
Ptrhd1	-0.12	1.00000000	mice
Gtf3c5	-0.07	1.00000000	mice
Ndufa9	0.08	1.00000000	mice
Tgfb1	-0.23	1.00000000	mice
Sesn2	0.12	1.00000000	mice
Zfyve26	0.12	1.00000000	mice
Tlk1	-0.21	1.00000000	mice
Gars	0.08	1.00000000	mice
Lin7c	-0.16	1.00000000	mice
Anp32e	-0.11	1.00000000	mice
Dpagt1	0.04	1.00000000	mice
Xpot	0.02	1.00000000	mice
Rce1	-0.24	1.00000000	mice
Tdrd3	-0.30	1.00000000	mice
Zfp691	0.12	1.00000000	mice
Slc25a5	-0.09	1.00000000	mice
Fndc3a	0.22	1.00000000	mice
Brd8	0.22	1.00000000	mice
Zfand3	-0.26	1.00000000	mice
Abhd17b	0.04	1.00000000	mice
AI504432	-0.12	1.00000000	mice
Phlpp2	-0.15	1.00000000	mice
Ankle2	0.10	1.00000000	mice
Zfp335os	-0.07	1.00000000	mice
Atpaf1	0.07	1.00000000	mice
Abt1	0.04	1.00000000	mice
Gpbp1l1	0.15	1.00000000	mice
Gpr108	-0.16	1.00000000	mice
Ehd1	-0.24	1.00000000	mice
Ifnar1	0.25	1.00000000	mice
Cntln	0.16	1.00000000	mice
Emilin2	0.01	1.00000000	mice
Aimp1	0.14	1.00000000	mice
Fchsd2	0.44	1.00000000	mice
Ndfip1	-0.16	1.00000000	mice
Plek	-0.26	1.00000000	mice
Tnfrsf21	-0.14	1.00000000	mice
Bcat2	0.10	1.00000000	mice
Peak1	-0.28	1.00000000	mice

Klhdc2	-0.07	1.00000000	mice
Gon4l	-0.06	1.00000000	mice
Ensa	0.14	1.00000000	mice
Las1l	-0.18	1.00000000	mice
Brms1	0.14	1.00000000	mice
Abhd16a	0.13	1.00000000	mice
Irf2bpl	-0.02	1.00000000	mice
Slc11a1	-0.11	1.00000000	mice
Rap2a	-0.20	1.00000000	mice
Phf6	0.18	1.00000000	mice
Pola1	-0.09	1.00000000	mice
Cyb5b	0.10	1.00000000	mice
Dtnb	-0.14	1.00000000	mice
Oxsr1	0.12	1.00000000	mice
Cks2	0.06	1.00000000	mice
Tmem126a	0.03	1.00000000	mice
Rock1	-0.20	1.00000000	mice
Pphln1	0.02	1.00000000	mice
Csk	0.07	1.00000000	mice
Zyx	0.00	1.00000000	mice
Cops7b	0.03	1.00000000	mice
Tsn	0.11	1.00000000	mice
Rdh14	-0.08	1.00000000	mice
Pggt1b	0.07	1.00000000	mice
Slc7a8	0.17	1.00000000	mice
Pitpna	-0.12	1.00000000	mice
Slf1	-0.18	1.00000000	mice
Naip6	-0.09	1.00000000	mice
Srpk2	-0.15	1.00000000	mice
Zswim8	-0.10	1.00000000	mice
Cct5	-0.12	1.00000000	mice
Rspry1	0.11	1.00000000	mice
Ric8a	0.04	1.00000000	mice
Ern1	-0.43	1.00000000	mice
Alg5	-0.08	1.00000000	mice
Smpdl3b	-0.04	1.00000000	mice
Herc2	0.08	1.00000000	mice
Fads2	-0.23	1.00000000	mice
Clec1a	0.11	1.00000000	mice
Ccdc186	0.19	1.00000000	mice
Tango2	0.07	1.00000000	mice
Rer1	0.10	1.00000000	mice
Ofd1	-0.09	1.00000000	mice
Sik1	-0.44	1.00000000	mice
Slc41a3	0.05	1.00000000	mice
Zfp366	-0.68	1.00000000	mice

Tsg101	0.13	1.00000000	mice
Odc1	-0.41	1.00000000	mice
Klrd1	-0.29	1.00000000	mice
Smcr8	0.04	1.00000000	mice
Cfap20	0.08	1.00000000	mice
Ahdc1	0.06	1.00000000	mice
Ranbp10	0.00	1.00000000	mice
Grsf1	-0.12	1.00000000	mice
Ceacam1	0.22	1.00000000	mice
Gapt	0.03	1.00000000	mice
Itpripl1	-0.02	1.00000000	mice
Abhd12	0.06	1.00000000	mice
Serinc3	-0.06	1.00000000	mice
Ankrd44	-0.12	1.00000000	mice
Nbn	0.02	1.00000000	mice
Gtf2i	-0.29	1.00000000	mice
Nsmce3	0.15	1.00000000	mice
Supt16	-0.04	1.00000000	mice
Eif4ebp1	0.10	1.00000000	mice
Dcaf13	0.07	1.00000000	mice
Eif4enif1	0.11	1.00000000	mice
Trip10	0.06	1.00000000	mice
Ythdc2	-0.11	1.00000000	mice
Tango6	-0.02	1.00000000	mice
Arfgap1	0.03	1.00000000	mice
Trpv2	-0.18	1.00000000	mice
Jak1	-0.21	1.00000000	mice
Fbh1	0.06	1.00000000	mice
Ercc6l2	0.10	1.00000000	mice
Cfp	0.40	1.00000000	mice
Ddx23	0.06	1.00000000	mice
Chmp3	0.07	1.00000000	mice
Cpsf3	-0.09	1.00000000	mice
Glis3	-0.25	1.00000000	mice
Ddx19b	0.03	1.00000000	mice
Spn	0.08	1.00000000	mice
Castor1	0.07	1.00000000	mice
Sema6d	-0.23	1.00000000	mice
Phf2	-0.08	1.00000000	mice
Cct8	0.10	1.00000000	mice
Sra1	0.10	1.00000000	mice
Arfgef1	0.07	1.00000000	mice
Adcy6	0.36	1.00000000	mice
Fbxo31	-0.05	1.00000000	mice
Sec11c	-0.07	1.00000000	mice
Acads	0.04	1.00000000	mice

Tspoap1	-0.11	1.00000000	mice
Smc2	0.08	1.00000000	mice
Kat8	-0.09	1.00000000	mice
Rnf8	-0.21	1.00000000	mice
Llph	-0.17	1.00000000	mice
Pnpla8	-0.14	1.00000000	mice
Vwa8	-0.05	1.00000000	mice
Atp13a2	0.17	1.00000000	mice
Cdk10	-0.05	1.00000000	mice
Nr4a1	-0.02	1.00000000	mice
Avpi1	-0.13	1.00000000	mice
Timm22	0.08	1.00000000	mice
Cxxc1	0.05	1.00000000	mice
Clic4	-0.13	1.00000000	mice
Arrdc1	0.10	1.00000000	mice
Snap29	0.10	1.00000000	mice
Eif3b	-0.14	1.00000000	mice
Rcbtb1	-0.03	1.00000000	mice
Thap12	0.22	1.00000000	mice
Gbe1	-0.42	1.00000000	mice
Dctn4	0.11	1.00000000	mice
Rac1	0.17	1.00000000	mice
Arhgap24	-0.84	1.00000000	mice
Npat	0.29	1.00000000	mice
Rora	0.27	1.00000000	mice
6030458C11Rik	0.07	1.00000000	mice
Rae1	0.06	1.00000000	mice
Hnrnpu	0.14	1.00000000	mice
Ncoa6	0.00	1.00000000	mice
Trit1	0.12	1.00000000	mice
Ttc3	0.08	1.00000000	mice
Pde10a	-0.39	1.00000000	mice
Scap	-0.12	1.00000000	mice
Pi4kb	-0.22	1.00000000	mice
Bloc1s4	0.05	1.00000000	mice
Patj	-0.16	1.00000000	mice
Stk3	-0.04	1.00000000	mice
Txnl1	0.10	1.00000000	mice
Nudt14	-0.02	1.00000000	mice
Strada	-0.22	1.00000000	mice
BC003965	-0.15	1.00000000	mice
Rell1	0.31	1.00000000	mice
Bcl9l	0.12	1.00000000	mice
Lpar3	-0.05	1.00000000	mice
Khk	-0.08	1.00000000	mice
Ehd4	0.02	1.00000000	mice

AU020206	0.19	1.00000000	mice
Bmyc	-0.16	1.00000000	mice
Aatf	-0.25	1.00000000	mice
Fancl	-0.18	1.00000000	mice
Psmd3	0.11	1.00000000	mice
Pmepa1	-0.15	1.00000000	mice
Fam168b	0.11	1.00000000	mice
Naglu	-0.06	1.00000000	mice
Ppp1r16b	-0.48	1.00000000	mice
Efna5	0.22	1.00000000	mice
Dvl3	0.08	1.00000000	mice
Dnajc4	0.07	1.00000000	mice
Smc4	-0.09	1.00000000	mice
Ripor2	-0.09	1.00000000	mice
Pacsin1	0.47	1.00000000	mice
Gcc2	-0.12	1.00000000	mice
Ppp1r18	-0.04	1.00000000	mice
Dennd5a	0.40	1.00000000	mice
Ddx6	-0.18	1.00000000	mice
Firre	0.07	1.00000000	mice
Brk1	0.17	1.00000000	mice
Usp53	-0.03	1.00000000	mice
Rhoh	-0.15	1.00000000	mice
Prkx	0.14	1.00000000	mice
Eif3m	-0.21	1.00000000	mice
Smarca5	0.06	1.00000000	mice
Suz12	0.10	1.00000000	mice
Ncln	-0.05	1.00000000	mice
Ythdf3	0.07	1.00000000	mice
Ppp1cc	0.04	1.00000000	mice
Zfp865	0.05	1.00000000	mice
Bri3	0.20	1.00000000	mice
Ocrl	0.18	1.00000000	mice
Exosc9	0.06	1.00000000	mice
Asrgl1	0.12	1.00000000	mice
Tpd52	-0.12	1.00000000	mice
Nae1	0.15	1.00000000	mice
Yes1	-0.07	1.00000000	mice
Cdk11b	0.06	1.00000000	mice
Ppwd1	-0.02	1.00000000	mice
Nars	0.15	1.00000000	mice
Pcnt	-0.15	1.00000000	mice
Tmem248	-0.22	1.00000000	mice
Gramd4	-0.10	1.00000000	mice
Txndc16	-0.13	1.00000000	mice
E330009J07Rik	0.11	1.00000000	mice

D930030I03Rik	-0.02	1.00000000	mice
Zdhhc4	0.06	1.00000000	mice
Tbl3	-0.08	1.00000000	mice
Comt	0.06	1.00000000	mice
Naxe	0.20	1.00000000	mice
Rere	-0.04	1.00000000	mice
Zcchc10	0.07	1.00000000	mice
Nckipsd	-0.07	1.00000000	mice
Ube2j2	0.19	1.00000000	mice
Pank4	-0.07	1.00000000	mice
2510017J16Rik	-0.05	1.00000000	mice
Gprc5a	-0.07	1.00000000	mice
E330020D12Rik	0.02	1.00000000	mice
Snx15	-0.06	1.00000000	mice
Sptlc2	0.22	1.00000000	mice
Wipi2	-0.16	1.00000000	mice
Dnajc25	0.08	1.00000000	mice
Dnajb11	0.06	1.00000000	mice
Wdr1	0.19	1.00000000	mice
Clk2	0.06	1.00000000	mice
Gatad2b	-0.22	1.00000000	mice
Fkbp15	-0.12	1.00000000	mice
Rnf113a2	0.07	1.00000000	mice
Zyg11b	-0.16	1.00000000	mice
Nr2f6	0.05	1.00000000	mice
Ccdc47	0.12	1.00000000	mice
Fmnl3	-0.11	1.00000000	mice
Tbc1d20	0.18	1.00000000	mice
Exd1	-0.06	1.00000000	mice
Dus1l	0.04	1.00000000	mice
Osbpl11	0.18	1.00000000	mice
Cdc123	0.12	1.00000000	mice
Nfyb	0.05	1.00000000	mice
Ankra2	0.10	1.00000000	mice
Zfp821	0.08	1.00000000	mice
Atp6v0d1	0.16	1.00000000	mice
Klf4	-0.19	1.00000000	mice
Csf1r	0.07	1.00000000	mice
Txnrd1	-0.36	1.00000000	mice
Fxyd5	-0.05	1.00000000	mice
Nrbp1	0.06	1.00000000	mice
Nav1	0.35	1.00000000	mice
Rtraf	0.10	1.00000000	mice
Aggf1	-0.19	1.00000000	mice
Atf3	0.45	1.00000000	mice
Baiap2l1	-0.10	1.00000000	mice

Fosl1	-0.08	1.00000000	mice
Bcl7b	0.06	1.00000000	mice
Tbck	0.03	1.00000000	mice
Tex10	0.11	1.00000000	mice
Slc30a9	0.16	1.00000000	mice
Cdk8	0.05	1.00000000	mice
Hbs1l	-0.15	1.00000000	mice
Ccdc167	0.08	1.00000000	mice
Leng1	0.03	1.00000000	mice
Als2	0.21	1.00000000	mice
Otud5	0.10	1.00000000	mice
Coro1c	-0.25	1.00000000	mice
Hspa4l	-0.02	1.00000000	mice
Tmem120b	0.33	1.00000000	mice
Arf5	-0.06	1.00000000	mice
Cyb561a3	0.55	1.00000000	mice
Fam199x	-0.04	1.00000000	mice
Pex6	-0.06	1.00000000	mice
Hint3	0.02	1.00000000	mice
Vbp1	0.06	1.00000000	mice
Itgb3	-0.16	1.00000000	mice
Rwdd1	0.16	1.00000000	mice
Jade1	-0.05	1.00000000	mice
Tmem11	-0.06	1.00000000	mice
Egr1	0.42	1.00000000	mice
Wdr44	0.01	1.00000000	mice
Dcp1a	0.05	1.00000000	mice
Gramd3	0.01	1.00000000	mice
Anp32a	-0.10	1.00000000	mice
Glrx3	0.16	1.00000000	mice
Cox6b1	0.07	1.00000000	mice
Zbtb7b	-0.05	1.00000000	mice
Pqbp1	0.06	1.00000000	mice
Caap1	0.11	1.00000000	mice
a	-0.10	1.00000000	mice
Gtf3c6	0.09	1.00000000	mice
Ergic1	0.37	1.00000000	mice
Fez2	0.11	1.00000000	mice
Denr	0.12	1.00000000	mice
Tubb4b	0.11	1.00000000	mice
Sp1	0.16	1.00000000	mice
Stard3nl	-0.06	1.00000000	mice
Nrp1	0.24	1.00000000	mice
Cpt1a	0.09	1.00000000	mice
Ralgapa1	-0.19	1.00000000	mice
Ppp1r15b	0.02	1.00000000	mice

Ctu2	0.06	1.00000000	mice
Wdr12	-0.14	1.00000000	mice
Srgap1	0.08	1.00000000	mice
Zfp35	0.04	1.00000000	mice
Hnrnpa1	-0.05	1.00000000	mice
Isyna1	-0.10	1.00000000	mice
Dennd1a	-0.09	1.00000000	mice
Hnrnpm	-0.01	1.00000000	mice
Lin54	-0.14	1.00000000	mice
Fam135a	0.06	1.00000000	mice
Hsd17b7	0.07	1.00000000	mice
Armc1	0.01	1.00000000	mice
Spred2	0.06	1.00000000	mice
Setd3	0.12	1.00000000	mice
Cks1b	0.02	1.00000000	mice
Cradd	-0.22	1.00000000	mice
Zdhhc17	-0.10	1.00000000	mice
Gigyf1	-0.09	1.00000000	mice
Srm	0.02	1.00000000	mice
Nudt8	-0.06	1.00000000	mice
Snx12	-0.15	1.00000000	mice
Rap1a	0.14	1.00000000	mice
Ube2v1	0.14	1.00000000	mice
Pias1	-0.09	1.00000000	mice
Slc16a3	0.18	1.00000000	mice
Frmd6	-0.07	1.00000000	mice
Zfp36l1	0.26	1.00000000	mice
Ncf4	-0.14	1.00000000	mice
Usp31	-0.02	1.00000000	mice
Zbtb1	0.06	1.00000000	mice
Cdk12	0.20	1.00000000	mice
Tmx4	-0.20	1.00000000	mice
Abcb1b	0.31	1.00000000	mice
Insr	-0.20	1.00000000	mice
Pcnp	0.14	1.00000000	mice
Dlg1	-0.26	1.00000000	mice
Ttll5	-0.13	1.00000000	mice
Spata6	-0.04	1.00000000	mice
Casp2	0.02	1.00000000	mice
Tsc22d1	-0.29	1.00000000	mice
Copa	-0.07	1.00000000	mice
Wdfy2	-0.14	1.00000000	mice
Lsm1	0.04	1.00000000	mice
Gpr35	0.07	1.00000000	mice
Rb1cc1	-0.15	1.00000000	mice
Ier3	-0.25	1.00000000	mice

Wls	0.07	1.00000000	mice
Nudt19	0.07	1.00000000	mice
Eif1b	0.07	1.00000000	mice
Sos1	0.18	1.00000000	mice
Klra17	-0.20	1.00000000	mice
Dcaf11	0.09	1.00000000	mice
Scfd1	0.02	1.00000000	mice
Nrd1	0.06	1.00000000	mice
Zfp638	0.24	1.00000000	mice
Vps51	0.06	1.00000000	mice
Slc39a9	0.00	1.00000000	mice
Ncor1	0.01	1.00000000	mice
Sdad1	0.00	1.00000000	mice
Rnaseh1	0.02	1.00000000	mice
Nipsnap2	0.06	1.00000000	mice
Prmt1	-0.08	1.00000000	mice
Phf3	-0.13	1.00000000	mice
Kif5b	0.15	1.00000000	mice
Gapdh	0.23	1.00000000	mice
Rfc3	0.06	1.00000000	mice
Dcps	0.08	1.00000000	mice
Safb2	0.10	1.00000000	mice
Tusc1	0.04	1.00000000	mice
Fam210a	-0.04	1.00000000	mice
Kctd2	0.06	1.00000000	mice
Ylpm1	-0.23	1.00000000	mice
Cltb	0.10	1.00000000	mice
Ndufb5	-0.07	1.00000000	mice
Serpinb6a	0.02	1.00000000	mice
Banp	0.08	1.00000000	mice
Simc1	-0.10	1.00000000	mice
Ddx5	-0.07	1.00000000	mice
Zfp62	0.15	1.00000000	mice
Rel	0.30	1.00000000	mice
Slc12a6	-0.18	1.00000000	mice
Dop1a	-0.17	1.00000000	mice
Pbxip1	0.11	1.00000000	mice
Cd52	0.44	1.00000000	mice
Surf1	0.07	1.00000000	mice
Vps41	0.07	1.00000000	mice
Fam120a	-0.09	1.00000000	mice
Prelid2	-0.03	1.00000000	mice
Adsl	0.05	1.00000000	mice
Golga4	-0.16	1.00000000	mice
Senp3	0.07	1.00000000	mice
Slamf7	0.34	1.00000000	mice

Gpkow	0.07	1.00000000	mice
Oaz1	-0.11	1.00000000	mice
L3mbtl2	-0.06	1.00000000	mice
Nsun4	0.12	1.00000000	mice
Rnf139	0.09	1.00000000	mice
Cdkl5	0.15	1.00000000	mice
Ifitm1	0.60	1.00000000	mice
Noc2l	-0.16	1.00000000	mice
Sod2	-0.16	1.00000000	mice
Slc48a1	-0.14	1.00000000	mice
Ilk	0.13	1.00000000	mice
Tmem156	-0.01	1.00000000	mice
Thap7	0.08	1.00000000	mice
Urod	-0.07	1.00000000	mice
Bcl2l1	-0.51	1.00000000	mice
Echs1	0.05	1.00000000	mice
Nek7	0.10	1.00000000	mice
Dtx4	-0.06	1.00000000	mice
Atp6v0a2	0.05	1.00000000	mice
Scamp1	-0.10	1.00000000	mice
Nemp1	0.02	1.00000000	mice
Qars	0.02	1.00000000	mice
Herpud2	-0.09	1.00000000	mice
N4bp2l2	0.01	1.00000000	mice
Qk	-0.19	1.00000000	mice
Impa1	0.01	1.00000000	mice
Gfpt1	-0.20	1.00000000	mice
Cstf1	0.05	1.00000000	mice
Aamp	0.17	1.00000000	mice
A630001G21Rik	-0.14	1.00000000	mice
Kdm3b	0.02	1.00000000	mice
Capn1	0.09	1.00000000	mice
Actr3	0.08	1.00000000	mice
Arid5a	-0.14	1.00000000	mice
Zmynd11	0.08	1.00000000	mice
Atmin	0.11	1.00000000	mice
Olfr433	-0.06	1.00000000	mice
Adi1	-0.09	1.00000000	mice
Dip2a	0.04	1.00000000	mice
4932438A13Rik	-0.11	1.00000000	mice
Thoc5	0.05	1.00000000	mice
Plod1	-0.05	1.00000000	mice
Erh	-0.03	1.00000000	mice
Enox2	0.23	1.00000000	mice
Snhg12	-0.08	1.00000000	mice
Cdk16	-0.02	1.00000000	mice

Phc3	-0.15	1.00000000	mice
Ndufs1	-0.02	1.00000000	mice
Dazap1	0.06	1.00000000	mice
Daam1	0.04	1.00000000	mice
Ube2e1	0.05	1.00000000	mice
Anapc13	0.08	1.00000000	mice
Pwwp2a	0.08	1.00000000	mice
Pptc7	-0.25	1.00000000	mice
Rasal2	0.40	1.00000000	mice
Wdr82	0.05	1.00000000	mice
Sertad3	0.07	1.00000000	mice
Bop1	-0.18	1.00000000	mice
Acat1	0.09	1.00000000	mice
Slc35e2	0.18	1.00000000	mice
Crtc3	0.27	1.00000000	mice
Nipal3	-0.06	1.00000000	mice
Rgs14	0.07	1.00000000	mice
Rrp12	-0.06	1.00000000	mice
D5Ertd579e	0.08	1.00000000	mice
Kdm6bos	0.08	1.00000000	mice
Yae1d1	0.00	1.00000000	mice
P2ry12	-0.20	1.00000000	mice
Ipcef1	-0.06	1.00000000	mice
Hmgxb3	-0.05	1.00000000	mice
Baz1a	-0.14	1.00000000	mice
Arntl	-0.19	1.00000000	mice
Ggh	0.09	1.00000000	mice
Rnf138	0.13	1.00000000	mice
Ralbp1	0.15	1.00000000	mice
Eif4ebp2	-0.05	1.00000000	mice
Hs2st1	-0.24	1.00000000	mice
Ercc6	-0.09	1.00000000	mice
Cnot2	0.11	1.00000000	mice
Disp1	-0.16	1.00000000	mice
Camk4	-0.03	1.00000000	mice
Rars2	0.02	1.00000000	mice
Cox14	0.10	1.00000000	mice
Ankrd13a	0.11	1.00000000	mice
Laptm5	0.11	1.00000000	mice
Ap1ar	-0.01	1.00000000	mice
Add3	-0.24	1.00000000	mice
Crebl2	0.09	1.00000000	mice
Snx16	0.10	1.00000000	mice
Cebpd	-0.08	1.00000000	mice
Dnase2a	0.05	1.00000000	mice
Phka2	-0.09	1.00000000	mice

Entr1	-0.09	1.00000000	mice
Blmh	-0.02	1.00000000	mice
Slc35b3	-0.11	1.00000000	mice
Thoc2	-0.08	1.00000000	mice
Pspc1	-0.18	1.00000000	mice
Pfdn2	-0.14	1.00000000	mice
Aars	0.06	1.00000000	mice
Spty2d1	0.18	1.00000000	mice
St6galnac4	0.06	1.00000000	mice
Ppid	0.05	1.00000000	mice
Ggact	0.09	1.00000000	mice
Tmem209	0.06	1.00000000	mice
Zfp467	0.05	1.00000000	mice
Wdr89	-0.08	1.00000000	mice
4930503L19Rik	-0.07	1.00000000	mice
Sdha	0.14	1.00000000	mice
Cog3	-0.11	1.00000000	mice
Rnasel	0.09	1.00000000	mice
Tbxa2r	0.13	1.00000000	mice
Eif2b4	0.07	1.00000000	mice
Gsr	-0.24	1.00000000	mice
Ddit4	0.13	1.00000000	mice
Slc26a2	0.11	1.00000000	mice
Elp2	0.06	1.00000000	mice
Lbh	-0.12	1.00000000	mice
Shc1	0.08	1.00000000	mice
Rc3h2	0.11	1.00000000	mice
Asah1	-0.16	1.00000000	mice
Pstpip2	-0.07	1.00000000	mice
Naip2	0.07	1.00000000	mice
Eva1b	0.09	1.00000000	mice
Zbtb7a	0.11	1.00000000	mice
Tmem63b	0.10	1.00000000	mice
Ccs	-0.09	1.00000000	mice
Bsg	0.09	1.00000000	mice
Spata13	-0.30	1.00000000	mice
Tcf3	0.06	1.00000000	mice
Rbbp6	-0.20	1.00000000	mice
Slc7a2	0.06	1.00000000	mice
Scamp3	0.05	1.00000000	mice
Hspa4	-0.16	1.00000000	mice
Ldhb	0.04	1.00000000	mice
Cpeb4	0.09	1.00000000	mice
Ormdl2	0.07	1.00000000	mice
Dync1h1	0.15	1.00000000	mice
Dkc1	0.07	1.00000000	mice

Ftl1-ps1	0.06	1.00000000	mice
Snx18	-0.19	1.00000000	mice
Pno1	0.05	1.00000000	mice
Pde4dip	-0.16	1.00000000	mice
Skil	-0.19	1.00000000	mice
Pdia5	-0.07	1.00000000	mice
Ubxn2b	0.07	1.00000000	mice
Plod2	0.09	1.00000000	mice
Sulf2	0.09	1.00000000	mice
Tut7	-0.08	1.00000000	mice
Il12rb2	0.28	1.00000000	mice
Rnf187	0.05	1.00000000	mice
Smad1	-0.03	1.00000000	mice
Tbc1d10c	0.08	1.00000000	mice
Eef1akmt1	0.07	1.00000000	mice
Ppm1m	-0.12	1.00000000	mice
Osgin2	0.24	1.00000000	mice
Tbc1d13	0.06	1.00000000	mice
Nck2	-0.19	1.00000000	mice
Tspyl4	0.03	1.00000000	mice
Npm3	-0.12	1.00000000	mice
Fam98c	-0.08	1.00000000	mice
Rbm48	-0.07	1.00000000	mice
Tpst1	0.10	1.00000000	mice
Dhx9	-0.18	1.00000000	mice
Phf5a	0.02	1.00000000	mice
Ccnk	0.08	1.00000000	mice
Sap30l	0.00	1.00000000	mice
Glmp	-0.05	1.00000000	mice
Kmt2a	0.05	1.00000000	mice
Snx27	-0.12	1.00000000	mice
Cep162	0.04	1.00000000	mice
Sipa1l3	-0.19	1.00000000	mice
Lyn	-0.06	1.00000000	mice
Wfdc17	-0.11	1.00000000	mice
Hace1	-0.17	1.00000000	mice
Tubgcp2	-0.15	1.00000000	mice
2300009A05Rik	0.09	1.00000000	mice
Slx4ip	0.15	1.00000000	mice
Aven	-0.10	1.00000000	mice
Wdyhv1	0.06	1.00000000	mice
Nmnat3	-0.08	1.00000000	mice
Leprotl1	0.22	1.00000000	mice
Txnl4a	0.10	1.00000000	mice
Cdc42ep2	0.02	1.00000000	mice
Kmt2e	-0.13	1.00000000	mice

Os9	0.14	1.00000000	mice
Nup155	0.05	1.00000000	mice
Selenof	-0.08	1.00000000	mice
Rttn	-0.13	1.00000000	mice
Rpp14	-0.08	1.00000000	mice
Anapc5	-0.17	1.00000000	mice
Desi2	0.18	1.00000000	mice
U2af1l4	0.08	1.00000000	mice
Prpsap2	0.09	1.00000000	mice
Dedd2	-0.06	1.00000000	mice
Nbdy	-0.06	1.00000000	mice
Tpi1	-0.06	1.00000000	mice
Nxpe3	0.12	1.00000000	mice
Ublcp1	0.02	1.00000000	mice
Trmt13	0.10	1.00000000	mice
Edc3	0.04	1.00000000	mice
Echdc1	0.05	1.00000000	mice
Rpp21	-0.09	1.00000000	mice
Uhrf2	0.10	1.00000000	mice
Cdt1	0.05	1.00000000	mice
Spdl1	0.06	1.00000000	mice
Coasy	0.05	1.00000000	mice
Arhgap27os2	0.16	1.00000000	mice
2410004B18Rik	0.07	1.00000000	mice
Rnf168	0.04	1.00000000	mice
Pcgf3	0.10	1.00000000	mice
Tspan14	-0.32	1.00000000	mice
Plat	-0.15	1.00000000	mice
Eif3e	-0.14	1.00000000	mice
Ccdc102a	0.05	1.00000000	mice
Hcfc1r1	0.11	1.00000000	mice
Hacd3	-0.33	1.00000000	mice
Rnf215	0.06	1.00000000	mice
Sh3bgrl3	-0.13	1.00000000	mice
Hspbap1	0.13	1.00000000	mice
Strip1	-0.05	1.00000000	mice
Syncrip	-0.11	1.00000000	mice
Ccdc66	0.06	1.00000000	mice
Brip1os	0.09	1.00000000	mice
Lbr	0.09	1.00000000	mice
Rmc1	-0.09	1.00000000	mice
Zcchc8	-0.09	1.00000000	mice
Ascc1	0.03	1.00000000	mice
Cryl1	-0.08	1.00000000	mice
Evi5l	-0.07	1.00000000	mice
Zfp169	0.02	1.00000000	mice

Phkb	0.12	1.00000000	mice
Slc30a7	0.16	1.00000000	mice
Trp53bp2	0.02	1.00000000	mice
Acbd5	0.07	1.00000000	mice
Pik3c3	0.02	1.00000000	mice
Setd1b	0.12	1.00000000	mice
Cdh23	0.12	1.00000000	mice
Lage3	-0.11	1.00000000	mice
Abca3	-0.03	1.00000000	mice
0610040J01Rik	-0.03	1.00000000	mice
Actr5	-0.19	1.00000000	mice
Ran	0.10	1.00000000	mice
Klf3	-0.20	1.00000000	mice
Trim33	0.09	1.00000000	mice
Kmt5c	0.05	1.00000000	mice
Pin1	-0.09	1.00000000	mice
Rab5c	0.10	1.00000000	mice
Cnih1	0.07	1.00000000	mice
Zfp385a	-0.14	1.00000000	mice
Dhx16	0.07	1.00000000	mice
Ccdc115	0.00	1.00000000	mice
Fbxo33	0.03	1.00000000	mice
Tifa	0.13	1.00000000	mice
Stard9	-0.31	1.00000000	mice
Cfap298	-0.07	1.00000000	mice
Paics	0.09	1.00000000	mice
Hipk2	0.05	1.00000000	mice
Stimate	0.13	1.00000000	mice
Usp12	-0.08	1.00000000	mice
Rbfa	0.05	1.00000000	mice
Arhgef40	0.20	1.00000000	mice
Acvr2a	-0.34	1.00000000	mice
Pogk	-0.07	1.00000000	mice
Clasrp	-0.09	1.00000000	mice
Scpep1os	0.06	1.00000000	mice
Commd10	-0.15	1.00000000	mice
Rffl	-0.21	1.00000000	mice
Slc37a3	-0.11	1.00000000	mice
Xpr1	-0.12	1.00000000	mice
Ap1g2	0.02	1.00000000	mice
Slc9a7	0.08	1.00000000	mice
Impa2	0.07	1.00000000	mice
Tbxas1	-0.04	1.00000000	mice
Zcchc17	-0.14	1.00000000	mice
Nedd4	-0.14	1.00000000	mice
Nek9	-0.14	1.00000000	mice

Dr1	0.12	1.00000000	mice
Tpr	-0.08	1.00000000	mice
Slc25a25	-0.35	1.00000000	mice
Bbx	-0.11	1.00000000	mice
Ppp4r1	-0.13	1.00000000	mice
Rp9	-0.17	1.00000000	mice
Cnnm2	-0.10	1.00000000	mice
Srsf2	-0.12	1.00000000	mice
Tex9	0.04	1.00000000	mice
Zfp830	0.02	1.00000000	mice
Bcl6	-0.27	1.00000000	mice
Ttc14	0.10	1.00000000	mice
Ppm1h	-0.23	1.00000000	mice
Tex261	0.04	1.00000000	mice
Atp2c1	-0.14	1.00000000	mice
Dhrs3	-0.21	1.00000000	mice
Pwp1	-0.04	1.00000000	mice
Zfp52	-0.07	1.00000000	mice
Rin2	0.16	1.00000000	mice
Fmnl1	0.06	1.00000000	mice
Tcf4	-0.06	1.00000000	mice
Cd68	-0.06	1.00000000	mice
Raf1	0.22	1.00000000	mice
Dnase1l1	0.07	1.00000000	mice
Bcap31	-0.05	1.00000000	mice
Cd200r1	0.08	1.00000000	mice
Pik3r5	0.30	1.00000000	mice
Pold3	0.04	1.00000000	mice
Nprl3	0.05	1.00000000	mice
Zc3h10	0.06	1.00000000	mice
Trappc4	0.09	1.00000000	mice
Ccm2	-0.07	1.00000000	mice
Trappc6b	0.11	1.00000000	mice
Eif3l	-0.10	1.00000000	mice
Cmtr1	-0.08	1.00000000	mice
Ptp4a2	-0.16	1.00000000	mice
Scarb2	0.00	1.00000000	mice
Susd6	-0.24	1.00000000	mice
Dctn6	0.06	1.00000000	mice
Psmc1	0.12	1.00000000	mice
Bphl	-0.07	1.00000000	mice
Elp4	-0.29	1.00000000	mice
Tfip11	0.03	1.00000000	mice
Scarb1	-0.05	1.00000000	mice
Gfm1	0.07	1.00000000	mice
Usp22	-0.09	1.00000000	mice

Wdr11	-0.10	1.00000000	mice
Rbl2	0.05	1.00000000	mice
Sgpl1	0.22	1.00000000	mice
Ywhaq	0.08	1.00000000	mice
Orc4	0.07	1.00000000	mice
Kctd10	0.07	1.00000000	mice
Cyb5r3	0.03	1.00000000	mice
Tcaim	0.08	1.00000000	mice
Gnptg	0.04	1.00000000	mice
Lmtk2	-0.15	1.00000000	mice
Cdk2ap2	-0.12	1.00000000	mice
Irf3	0.04	1.00000000	mice
Ube2e2	0.26	1.00000000	mice
Gcsam	-0.04	1.00000000	mice
E130308A19Rik	0.09	1.00000000	mice
Canx	0.15	1.00000000	mice
Rnf41	0.06	1.00000000	mice
Dctn5	0.05	1.00000000	mice
Cenpc1	0.11	1.00000000	mice
Ptprf	0.17	1.00000000	mice
Zfp639	-0.14	1.00000000	mice
AW549877	0.03	1.00000000	mice
Vps4a	0.01	1.00000000	mice
Scaper	-0.17	1.00000000	mice
Klhdc3	-0.07	1.00000000	mice
Carm1	-0.09	1.00000000	mice
Cdc25a	0.04	1.00000000	mice
Cenpl	0.06	1.00000000	mice
Retreg3	0.08	1.00000000	mice
Sept1	-0.12	1.00000000	mice
Prkaa1	0.07	1.00000000	mice
Rtn1	-0.02	1.00000000	mice
Eng	0.08	1.00000000	mice
Hmox2	0.07	1.00000000	mice
Klhl2	-0.19	1.00000000	mice
Srsf6	-0.12	1.00000000	mice
Zw10	0.05	1.00000000	mice
Eif4e	-0.20	1.00000000	mice
Rnf13	0.15	1.00000000	mice
Pla2g7	0.10	1.00000000	mice
Irf5	-0.25	1.00000000	mice
Pfdn5	-0.12	1.00000000	mice
Ddx46	0.12	1.00000000	mice
Ebi3	0.36	1.00000000	mice
Stk11	0.07	1.00000000	mice
Nos1ap	-0.02	1.00000000	mice

Pitrm1	-0.10	1.00000000	mice
Tigd2	-0.06	1.00000000	mice
Fis1	0.09	1.00000000	mice
Flrt3	-0.11	1.00000000	mice
Kcnq5	-1.17	1.00000000	mice
Pcif1	-0.02	1.00000000	mice
Elovl6	0.12	1.00000000	mice
Naa35	-0.16	1.00000000	mice
Tbc1d32	0.07	1.00000000	mice
Dtx3	0.05	1.00000000	mice
Serac1	0.02	1.00000000	mice
Dbi	0.07	1.00000000	mice
Zfyve1	0.10	1.00000000	mice
Cc2d1b	-0.06	1.00000000	mice
Dab2ip	-0.06	1.00000000	mice
Gabpb1	-0.12	1.00000000	mice
Wdr41	0.07	1.00000000	mice
Scyl1	0.04	1.00000000	mice
Btbd7	0.06	1.00000000	mice
Dhx35	0.03	1.00000000	mice
Tradd	0.03	1.00000000	mice
Celf2	-0.01	1.00000000	mice
Rc3h1	0.10	1.00000000	mice
Nmd3	0.07	1.00000000	mice
Prkrip1	0.07	1.00000000	mice
Setd1a	-0.09	1.00000000	mice
Stx3	-0.15	1.00000000	mice
Tmem163	-0.31	1.00000000	mice
Fam122a	-0.07	1.00000000	mice
Hnrnpll	0.22	1.00000000	mice
Ccdc124	0.05	1.00000000	mice
Yipf6	-0.06	1.00000000	mice
H2afj	0.15	1.00000000	mice
Zbtb21	-0.04	1.00000000	mice
Srxn1	-0.18	1.00000000	mice
Tanc2	-0.43	1.00000000	mice
Hmgb2	-0.11	1.00000000	mice
Nup62	0.09	1.00000000	mice
Ubac2	-0.12	1.00000000	mice
Ppt2	0.01	1.00000000	mice
Hras	0.07	1.00000000	mice
Srp9	-0.15	1.00000000	mice
Tnip3	-0.10	1.00000000	mice
Nelfb	0.04	1.00000000	mice
Nfu1	0.08	1.00000000	mice
Rela	-0.19	1.00000000	mice

Rrp15	-0.09	1.00000000	mice
Paqr5	0.18	1.00000000	mice
Rexo1	-0.07	1.00000000	mice
Kansl3	0.09	1.00000000	mice
Cdc5l	0.00	1.00000000	mice
Ppp3cc	-0.12	1.00000000	mice
Sigmar1	0.03	1.00000000	mice
Dpy30	0.04	1.00000000	mice
Akap11	-0.01	1.00000000	mice
Habp4	-0.04	1.00000000	mice
Ints1	-0.12	1.00000000	mice
Exoc2	-0.11	1.00000000	mice
Bclaf1	-0.09	1.00000000	mice
Ndor1	-0.13	1.00000000	mice
Cyth3	-0.04	1.00000000	mice
Vars	0.07	1.00000000	mice
Fundc2	-0.13	1.00000000	mice
Nfatc3	-0.23	1.00000000	mice
B4galt5	0.16	1.00000000	mice
Arhgef3	0.14	1.00000000	mice
Optn	-0.10	1.00000000	mice
Rbm14	-0.03	1.00000000	mice
Nip7	0.04	1.00000000	mice
Creld2	0.07	1.00000000	mice
Sgta	-0.03	1.00000000	mice
Pdhb	-0.09	1.00000000	mice
C1qbp	-0.01	1.00000000	mice
Snhg8	-0.11	1.00000000	mice
Smap1	0.10	1.00000000	mice
Ncapg2	0.11	1.00000000	mice
Fam172a	0.17	1.00000000	mice
Ppm1g	0.14	1.00000000	mice
Fam118b	0.02	1.00000000	mice
Wsb2	0.08	1.00000000	mice
Akap9	-0.11	1.00000000	mice
Golga7	-0.08	1.00000000	mice
Gid8	0.00	1.00000000	mice
Dhrs1	0.03	1.00000000	mice
Lsamp	-0.36	1.00000000	mice
Racgap1	0.08	1.00000000	mice
Yrdc	-0.07	1.00000000	mice
Acp2	0.03	1.00000000	mice
Rabgef1	-0.17	1.00000000	mice
Rheb	-0.08	1.00000000	mice
Gna15	0.14	1.00000000	mice
Nol10	-0.25	1.00000000	mice

Fbxl4	0.05	1.00000000	mice
Snrpf	0.10	1.00000000	mice
Pip4k2a	-0.10	1.00000000	mice
Nfx1	-0.16	1.00000000	mice
Dnmt3a	-0.02	1.00000000	mice
Lrp6	-0.11	1.00000000	mice
Rbm15	0.02	1.00000000	mice
Bcl2l13	0.06	1.00000000	mice
Nme1	-0.13	1.00000000	mice
Efl1	0.04	1.00000000	mice
Wwp1	-0.25	1.00000000	mice
P2ry10	-0.15	1.00000000	mice
Arf1	0.08	1.00000000	mice
St3gal4	-0.08	1.00000000	mice
U2af1	0.12	1.00000000	mice
Zdhhc5	0.07	1.00000000	mice
Ccndbp1	0.07	1.00000000	mice
Pik3ca	-0.27	1.00000000	mice
Slc5a3	0.12	1.00000000	mice
Rab27a	0.08	1.00000000	mice
Rbpms	0.26	1.00000000	mice
Fam104a	-0.16	1.00000000	mice
Chst12	0.11	1.00000000	mice
Lrif1	0.11	1.00000000	mice
Sfswap	-0.07	1.00000000	mice
Tcirg1	0.23	1.00000000	mice
Atp5g1	0.12	1.00000000	mice
Zfx	0.05	1.00000000	mice
Srek1ip1	0.02	1.00000000	mice
Apoe	-0.17	1.00000000	mice
Hsp90aa1	0.11	1.00000000	mice
Nrm	-0.18	1.00000000	mice
L3mbtl3	-0.14	1.00000000	mice
Ttll4	-0.05	1.00000000	mice
Tm9sf4	-0.18	1.00000000	mice
Arpc1a	-0.12	1.00000000	mice
4921524J17Rik	0.10	1.00000000	mice
Abcd1	0.07	1.00000000	mice
Papolg	0.14	1.00000000	mice
Phf23	0.10	1.00000000	mice
Araf	-0.09	1.00000000	mice
Nup153	-0.07	1.00000000	mice
Fam110a	0.19	1.00000000	mice
Chchd4	-0.10	1.00000000	mice
Top3a	-0.08	1.00000000	mice
Cnksr3	-0.16	1.00000000	mice

Tcf25	0.08	1.00000000	mice
Zmat1	-0.04	1.00000000	mice
Pef1	-0.03	1.00000000	mice
Atad1	-0.09	1.00000000	mice
Abl2	-0.06	1.00000000	mice
Prdm2	0.15	1.00000000	mice
Dhx37	-0.01	1.00000000	mice
Selenot	0.17	1.00000000	mice
Oip5os1	0.06	1.00000000	mice
Ddx42	0.08	1.00000000	mice
Usp34	-0.10	1.00000000	mice
Dda1	0.08	1.00000000	mice
Ppp1r9b	-0.09	1.00000000	mice
Ahcyl2	0.34	1.00000000	mice
Rexo4	0.06	1.00000000	mice
Fxr2	0.11	1.00000000	mice
Cnot3	0.10	1.00000000	mice
Atp5mpl	0.16	1.00000000	mice
Smg5	0.07	1.00000000	mice
Lztr1	-0.06	1.00000000	mice
Ift20	0.03	1.00000000	mice
Gtf2e1	-0.03	1.00000000	mice
Ythdf1	0.05	1.00000000	mice
Srsf1	0.13	1.00000000	mice
Tmem222	-0.07	1.00000000	mice
Washc5	-0.06	1.00000000	mice
Fam49b	-0.17	1.00000000	mice
Wdr43	-0.13	1.00000000	mice
Zfp69	0.01	1.00000000	mice
Emp2	-0.07	1.00000000	mice
Sh3bgrl	0.20	1.00000000	mice
Sdhaf4	0.08	1.00000000	mice
Kctd9	-0.04	1.00000000	mice
Stag2	0.10	1.00000000	mice
Pxmp4	0.05	1.00000000	mice
Pgm2	0.05	1.00000000	mice
Ykt6	0.06	1.00000000	mice
Aktip	0.02	1.00000000	mice
Gas5	0.20	1.00000000	mice
Cask	0.16	1.00000000	mice
Tmem203	0.00	1.00000000	mice
Tmem30a	0.11	1.00000000	mice
Dguok	-0.07	1.00000000	mice
Sil1	0.10	1.00000000	mice
Rbmxl1	0.11	1.00000000	mice
Nol8	-0.04	1.00000000	mice

Rock2	-0.15	1.00000000	mice
Exosc1	0.04	1.00000000	mice
Sort1	0.11	1.00000000	mice
Atp6ap2	-0.09	1.00000000	mice
Uba52	0.08	1.00000000	mice
Ccdc174	0.08	1.00000000	mice
Setbp1	-0.52	1.00000000	mice
Pdk3	0.12	1.00000000	mice
Stx12	0.12	1.00000000	mice
Igkc	-0.88	1.00000000	mice
Ino80d	0.14	1.00000000	mice
Drg1	0.01	1.00000000	mice
Nfkb1	-0.10	1.00000000	mice
Sp3os	0.03	1.00000000	mice
Fam174a	0.06	1.00000000	mice
Kctd18	0.07	1.00000000	mice
Pfdn4	0.02	1.00000000	mice
Tmem181a	-0.07	1.00000000	mice
Ppp1r37	-0.08	1.00000000	mice
Cog8	-0.05	1.00000000	mice
Anp32b	-0.26	1.00000000	mice
Ticam1	-0.03	1.00000000	mice
Ttc4	-0.03	1.00000000	mice
Nfkbib	0.21	1.00000000	mice
Arid3b	0.11	1.00000000	mice
Ttc19	-0.24	1.00000000	mice
Tbk1	0.02	1.00000000	mice
Rest	0.23	1.00000000	mice
Relch	0.13	1.00000000	mice
Stx17	-0.10	1.00000000	mice
Slc6a6	-0.19	1.00000000	mice
Gle1	-0.11	1.00000000	mice
Fdxacb1	-0.03	1.00000000	mice
Uchl3	0.11	1.00000000	mice
Nuak2	0.17	1.00000000	mice
Efr3a	-0.32	1.00000000	mice
Ctdspl2	-0.04	1.00000000	mice
Polr2f	0.09	1.00000000	mice
Tnpo1	0.10	1.00000000	mice
Vps18	0.05	1.00000000	mice
Rcn2	0.07	1.00000000	mice
Vamp3	0.08	1.00000000	mice
Reep3	-0.18	1.00000000	mice
Klf6	0.38	1.00000000	mice
Arid2	-0.11	1.00000000	mice
Wwc2	0.09	1.00000000	mice

Kdm5a	-0.16	1.00000000	mice
Gan	-0.21	1.00000000	mice
Gabpb2	0.11	1.00000000	mice
Actl6a	-0.06	1.00000000	mice
Flt1	0.04	1.00000000	mice
Slc7a11	0.12	1.00000000	mice
Prpf18	-0.10	1.00000000	mice
Kdsr	-0.14	1.00000000	mice
Kmt2d	0.07	1.00000000	mice
Tspan3	0.44	1.00000000	mice
Tcf12	0.89	1.00000000	mice
Chst3	-0.22	1.00000000	mice
Sema4c	0.10	1.00000000	mice
Golph3l	0.12	1.00000000	mice
Zc3h13	-0.07	1.00000000	mice
Trrap	-0.08	1.00000000	mice
Pcna	0.15	1.00000000	mice
Spef2	0.06	1.00000000	mice
Ppp2r3a	-0.18	1.00000000	mice
Plin3	0.08	1.00000000	mice
Ascc2	-0.15	1.00000000	mice
Pnisr	-0.11	1.00000000	mice
Cggbp1	0.07	1.00000000	mice
Dab2	0.04	1.00000000	mice
Idh2	0.07	1.00000000	mice
Ndufs8	0.10	1.00000000	mice
5830432E09Rik	-0.02	1.00000000	mice
Acly	-0.13	1.00000000	mice
Spcs2	0.12	1.00000000	mice
Psip1	-0.12	1.00000000	mice
Jak3	0.04	1.00000000	mice
Gstt2	0.10	1.00000000	mice
B9d2	-0.07	1.00000000	mice
Hmgn1	-0.15	1.00000000	mice
Ube2e3	-0.08	1.00000000	mice
Ncbp3	-0.01	1.00000000	mice
Fes	-0.11	1.00000000	mice
Ptar1	-0.11	1.00000000	mice
Ifi27	-0.10	1.00000000	mice
Rbbp8	0.29	1.00000000	mice
Kctd3	0.00	1.00000000	mice
Arhgef6	-0.16	1.00000000	mice
Ift140	0.07	1.00000000	mice
Rcor3	0.02	1.00000000	mice
2210016F16Rik	0.06	1.00000000	mice
Yipf3	0.07	1.00000000	mice

Ddx10	-0.20	1.00000000	mice
Wdr47	-0.05	1.00000000	mice
Zfp472	-0.14	1.00000000	mice
Prr14l	0.02	1.00000000	mice
Serpinh1	0.08	1.00000000	mice
Aasdhppt	0.03	1.00000000	mice
1110038B12Rik	0.05	1.00000000	mice
Ermp1	0.00	1.00000000	mice
Nlk	0.00	1.00000000	mice
Scn1b	-0.06	1.00000000	mice
Hars	-0.14	1.00000000	mice
Hsph1	-0.02	1.00000000	mice
Pigm	0.04	1.00000000	mice
Pex5	-0.08	1.00000000	mice
Blnk	-0.83	1.00000000	mice
Taf7	-0.07	1.00000000	mice
Bmt2	0.20	1.00000000	mice
Hadh	-0.01	1.00000000	mice
Edrf1	0.03	1.00000000	mice
Ttyh3	0.18	1.00000000	mice
Ccdc88c	-0.01	1.00000000	mice
Gabbr1	-0.10	1.00000000	mice
Letm2	0.01	1.00000000	mice
Rpa3	0.02	1.00000000	mice
Psenen	0.10	1.00000000	mice
Ncbp2	0.03	1.00000000	mice
Anxa6	-0.02	1.00000000	mice
Gkap1	-0.08	1.00000000	mice
Anapc11	-0.12	1.00000000	mice
Pdxk	-0.14	1.00000000	mice
Taf13	0.05	1.00000000	mice
Poc5	-0.07	1.00000000	mice
Itga5	0.02	1.00000000	mice
Hgs	0.09	1.00000000	mice
Stn1	0.05	1.00000000	mice
Runx2os1	0.04	1.00000000	mice
C9orf72	-0.06	1.00000000	mice
Cipc	0.07	1.00000000	mice
Stx11	0.21	1.00000000	mice
Zc3h4	-0.10	1.00000000	mice
Sorl1	-0.33	1.00000000	mice
Zkscan1	0.05	1.00000000	mice
Abhd4	-0.09	1.00000000	mice
Smad3	-0.16	1.00000000	mice
Ppme1	-0.10	1.00000000	mice
Cops6	0.11	1.00000000	mice

Uck1	0.05	1.00000000	mice
Smndc1	0.06	1.00000000	mice
Tmem29	0.08	1.00000000	mice
Hint2	0.07	1.00000000	mice
Hp1bp3	-0.10	1.00000000	mice
Nsmaf	-0.13	1.00000000	mice
Spcs1	0.09	1.00000000	mice
Ifngr2	0.16	1.00000000	mice
Fopnl	-0.04	1.00000000	mice
Procr	0.33	1.00000000	mice
Tvp23b	-0.08	1.00000000	mice
Slc46a3	-0.05	1.00000000	mice
Hspb11	-0.06	1.00000000	mice
Tpm1	0.13	1.00000000	mice
Lamtor3	0.04	1.00000000	mice
Sipa1	-0.20	1.00000000	mice
Sf3a1	0.01	1.00000000	mice
Stx4a	0.04	1.00000000	mice
Rint1	0.01	1.00000000	mice
Cln8	0.02	1.00000000	mice
Cpsf6	-0.11	1.00000000	mice
Txndc15	-0.15	1.00000000	mice
Prorsd1	-0.10	1.00000000	mice
Thoc3	-0.08	1.00000000	mice
Plk4	-0.04	1.00000000	mice
Capn5	-0.05	1.00000000	mice
Cx3cl1	-0.10	1.00000000	mice
Tmc6	-0.15	1.00000000	mice
Slpi	0.12	1.00000000	mice
Rnd3	-0.24	1.00000000	mice
Tfb2m	-0.06	1.00000000	mice
Ing3	-0.14	1.00000000	mice
Tug1	-0.05	1.00000000	mice
Vps39	0.13	1.00000000	mice
Ctnna1	-0.12	1.00000000	mice
Inpp5k	0.11	1.00000000	mice
Lrpprc	-0.22	1.00000000	mice
Dph3	-0.07	1.00000000	mice
Crebzf	-0.03	1.00000000	mice
Tma16	-0.18	1.00000000	mice
Rusc1	0.05	1.00000000	mice
Thoc1	-0.22	1.00000000	mice
Gk	0.12	1.00000000	mice
Il3ra	0.10	1.00000000	mice
Hps4	-0.06	1.00000000	mice
Krtcap2	-0.07	1.00000000	mice

Uggt1	-0.22	1.00000000	mice
Gnas	-0.08	1.00000000	mice
Ptbp2	-0.16	1.00000000	mice
Ubn2	-0.28	1.00000000	mice
Hibadh	0.09	1.00000000	mice
Alkbh6	0.06	1.00000000	mice
Pkm	-0.09	1.00000000	mice
4930444A19Rik	-0.01	1.00000000	mice
Serinc1	-0.04	1.00000000	mice
Nop56	-0.05	1.00000000	mice
Pla2g4a	-0.16	1.00000000	mice
Lrig2	0.06	1.00000000	mice
Ormdl3	0.05	1.00000000	mice
Lars	0.08	1.00000000	mice
Adgrl2	0.08	1.00000000	mice
Rabggtb	0.04	1.00000000	mice
Setd2	-0.01	1.00000000	mice
Ak8	0.05	1.00000000	mice
Snx13	0.09	1.00000000	mice
Tut4	-0.08	1.00000000	mice
Enc1	-0.01	1.00000000	mice
Rrp9	0.02	1.00000000	mice
Ggta1	0.00	1.00000000	mice
Vopp1	0.09	1.00000000	mice
Zcchc9	0.04	1.00000000	mice
Ppp1r12a	0.07	1.00000000	mice
Fkbp2	0.04	1.00000000	mice
Tmbim4	0.09	1.00000000	mice
Crem	-0.04	1.00000000	mice
Dis3l2	-0.19	1.00000000	mice
Itgb1bp1	0.01	1.00000000	mice
Enoph1	-0.03	1.00000000	mice
Llgl1	0.03	1.00000000	mice
Anpep	-0.03	1.00000000	mice
Bmp2k	0.25	1.00000000	mice
Dtx2	0.09	1.00000000	mice
Slc31a1	0.06	1.00000000	mice
Nap1l1	0.31	1.00000000	mice
Ttc33	0.03	1.00000000	mice
Kbtbd11	0.06	1.00000000	mice
Phka1	-0.06	1.00000000	mice
Zfp280c	-0.08	1.00000000	mice
Bud31	-0.12	1.00000000	mice
Igbp1	-0.11	1.00000000	mice
Polr2j	-0.07	1.00000000	mice
Bcl10	0.08	1.00000000	mice

Osbpl1a	0.03	1.00000000	mice
1110032A03Rik	0.11	1.00000000	mice
Ilf3	-0.05	1.00000000	mice
Aptx	-0.05	1.00000000	mice
Ahi1	-0.10	1.00000000	mice
Slc41a1	-0.16	1.00000000	mice
Nap1l4	0.01	1.00000000	mice
Utp11	0.09	1.00000000	mice
Dnaja2	0.10	1.00000000	mice
Atp5h	-0.10	1.00000000	mice
Gdap2	0.02	1.00000000	mice
Txlng	-0.12	1.00000000	mice
Dohh	0.04	1.00000000	mice
Donson	0.00	1.00000000	mice
Taf15	0.07	1.00000000	mice
Lpxn	0.00	1.00000000	mice
Trmt10c	0.01	1.00000000	mice
Ndufaf8	0.09	1.00000000	mice
Dexi	0.19	1.00000000	mice
Dnajc1	-0.24	1.00000000	mice
Naa16	0.04	1.00000000	mice
Rbm34	0.03	1.00000000	mice
5830408C22Rik	0.00	1.00000000	mice
Eid1	0.07	1.00000000	mice
Gar1	-0.11	1.00000000	mice
Dhdds	0.03	1.00000000	mice
Sparc	0.06	1.00000000	mice
Gtpbp2	0.05	1.00000000	mice
Tbcb	0.11	1.00000000	mice
Zfp24	0.05	1.00000000	mice
Pus1	-0.02	1.00000000	mice
Snrk	0.00	1.00000000	mice
Cep44	0.00	1.00000000	mice
Gpr107	0.08	1.00000000	mice
Ppa2	0.04	1.00000000	mice
Glb1	-0.11	1.00000000	mice
Tmem63a	0.07	1.00000000	mice
Ramac	0.05	1.00000000	mice
Tnfrsf13b	0.08	1.00000000	mice
Uap1	0.48	1.00000000	mice
Ston2	0.35	1.00000000	mice
Kdm1a	0.02	1.00000000	mice
Pnkd	0.00	1.00000000	mice
Cmss1	0.29	1.00000000	mice
Copb2	0.04	1.00000000	mice
Ikzf1	-0.09	1.00000000	mice

Pde8a	-0.06	1.00000000	mice
Gnl3l	-0.06	1.00000000	mice
Patz1	-0.11	1.00000000	mice
Dhx38	0.03	1.00000000	mice
Atrn	-0.18	1.00000000	mice
Snrnp70	-0.11	1.00000000	mice
Coro1a	0.14	1.00000000	mice
Zfp706	-0.12	1.00000000	mice
Tjap1	0.03	1.00000000	mice
Klhdc4	0.03	1.00000000	mice
Bcap29	-0.20	1.00000000	mice
Gpr18	0.09	1.00000000	mice
2900097C17Rik	0.09	1.00000000	mice
Ramp3	0.30	1.00000000	mice
Prorp	-0.18	1.00000000	mice
Rab10	-0.32	1.00000000	mice
Dip2c	-0.22	1.00000000	mice
Sae1	-0.13	1.00000000	mice
Tmco4	0.02	1.00000000	mice
Stambpl1	-0.11	1.00000000	mice
Cdyl	-0.10	1.00000000	mice
Grpel2	0.02	1.00000000	mice
Zkscan6	0.02	1.00000000	mice
Zfp658	0.05	1.00000000	mice
Cenpq	0.04	1.00000000	mice
Zdhhc8	-0.03	1.00000000	mice
Eif2d	0.01	1.00000000	mice
Cramp1l	-0.02	1.00000000	mice
Tmem106b	0.03	1.00000000	mice
Sugp1	0.01	1.00000000	mice
Nkap	0.05	1.00000000	mice
Zfp386	-0.07	1.00000000	mice
Uqcrh	-0.06	1.00000000	mice
Pbdc1	-0.20	1.00000000	mice
Ung	0.02	1.00000000	mice
Jak2	-0.32	1.00000000	mice
Ints8	-0.01	1.00000000	mice
Gtf2ird2	-0.05	1.00000000	mice
Ankrd10	-0.11	1.00000000	mice
Tmem64	-0.11	1.00000000	mice
4833420G17Rik	-0.08	1.00000000	mice
Glcci1	-0.56	1.00000000	mice
Smim14	0.07	1.00000000	mice
Haus8	0.04	1.00000000	mice
Prdx2	0.09	1.00000000	mice
Nsmce2	-0.21	1.00000000	mice

Paxx	-0.06	1.00000000	mice
Pts	-0.05	1.00000000	mice
Ptcd3	-0.04	1.00000000	mice
Lipo3	-0.08	1.00000000	mice
Camta1	-0.17	1.00000000	mice
Impad1	0.02	1.00000000	mice
Lmbrd1	0.01	1.00000000	mice
Creb3l2	-0.14	1.00000000	mice
P4hb	-0.11	1.00000000	mice
Rdh11	0.04	1.00000000	mice
Zmat5	0.05	1.00000000	mice
Smad7	-0.25	1.00000000	mice
Nfam1	-0.01	1.00000000	mice
Gpat3	-0.04	1.00000000	mice
C1d	0.04	1.00000000	mice
Ehmt1	-0.07	1.00000000	mice
Chd8	0.06	1.00000000	mice
Hinfp	0.07	1.00000000	mice
Alcam	-0.17	1.00000000	mice
Nsmce4a	0.01	1.00000000	mice
Rab11fip2	0.03	1.00000000	mice
Ext2	-0.11	1.00000000	mice
5031425E22Rik	-0.17	1.00000000	mice
Dpf2	0.06	1.00000000	mice
Tspyl1	-0.11	1.00000000	mice
Osbpl9	-0.24	1.00000000	mice
Kpna4	-0.33	1.00000000	mice
Get4	0.05	1.00000000	mice
Cebpb	0.11	1.00000000	mice
Lyrm4	-0.06	1.00000000	mice
Pdha1	-0.05	1.00000000	mice
Pelo	0.02	1.00000000	mice
Usp45	-0.13	1.00000000	mice
Slu7	0.11	1.00000000	mice
Smap2	-0.04	1.00000000	mice
Eif4a1	-0.05	1.00000000	mice
Abr	-0.25	1.00000000	mice
Gphn	0.01	1.00000000	mice
Camta2	0.04	1.00000000	mice
Tmtc2	-0.09	1.00000000	mice
Wipf1	-0.09	1.00000000	mice
Pms2	-0.12	1.00000000	mice
Ndufb2	0.03	1.00000000	mice
Ppm1d	0.03	1.00000000	mice
Otud3	0.06	1.00000000	mice
Dcaf17	0.03	1.00000000	mice

Tsfm	0.02	1.00000000	mice
Nup98	0.01	1.00000000	mice
Gpr171	0.23	1.00000000	mice
Pbx3	-0.20	1.00000000	mice
Smu1	-0.03	1.00000000	mice
Cyp27a1	-0.10	1.00000000	mice
Sqstm1	0.00	1.00000000	mice
Dctpp1	-0.08	1.00000000	mice
Limk1	-0.09	1.00000000	mice
Arhgap12	0.10	1.00000000	mice
Tcp11l2	-0.25	1.00000000	mice
Atg4d	0.05	1.00000000	mice
Ppib	0.06	1.00000000	mice
Leng8	0.06	1.00000000	mice
Isoc1	0.06	1.00000000	mice
Pdss2	-0.03	1.00000000	mice
Zmynd15	0.58	1.00000000	mice
Prkar1a	0.11	1.00000000	mice
Zgpat	0.05	1.00000000	mice
Zfp182	-0.10	1.00000000	mice
Uso1	-0.10	1.00000000	mice
Ost4	0.10	1.00000000	mice
Ppil4	-0.09	1.00000000	mice
Fam117a	0.05	1.00000000	mice
Sirpa	-0.57	1.00000000	mice
Tmcc1	0.02	1.00000000	mice
4930402H24Rik	0.04	1.00000000	mice
Cpsf1	0.00	1.00000000	mice
Tinf2	0.03	1.00000000	mice
Ppp2r5c	0.05	1.00000000	mice
Ivd	0.04	1.00000000	mice
Brms1l	-0.06	1.00000000	mice
Fbxo22	0.02	1.00000000	mice
Atp5o	0.11	1.00000000	mice
Cnpy2	-0.02	1.00000000	mice
Lzts1	-0.41	1.00000000	mice
Rnasek	0.12	1.00000000	mice
Tyw5	-0.02	1.00000000	mice
Eif4a2	-0.04	1.00000000	mice
Snip1	0.03	1.00000000	mice
Kdm1b	0.04	1.00000000	mice
Aph1a	0.08	1.00000000	mice
Isca2	-0.12	1.00000000	mice
Pyroxd1	-0.09	1.00000000	mice
Cacul1	-0.09	1.00000000	mice
Arhgdia	-0.06	1.00000000	mice

Unc50	0.06	1.00000000	mice
Arfrp1	-0.04	1.00000000	mice
Slc39a10	-0.06	1.00000000	mice
Sp4	-0.07	1.00000000	mice
Cfap36	0.09	1.00000000	mice
Tmx1	-0.05	1.00000000	mice
Ercc3	-0.06	1.00000000	mice
Fubp3	-0.01	1.00000000	mice
Lemd3	-0.08	1.00000000	mice
Taf11	0.04	1.00000000	mice
Kdm6b	-0.09	1.00000000	mice
Lin52	-0.21	1.00000000	mice
Erich1	0.05	1.00000000	mice
5330438D12Rik	0.07	1.00000000	mice
Ptprs	-0.26	1.00000000	mice
S100pbp	-0.03	1.00000000	mice
Ing2	-0.15	1.00000000	mice
Tecr	0.08	1.00000000	mice
Plcg1	-0.05	1.00000000	mice
Timm8a1	-0.01	1.00000000	mice
Osbp	-0.04	1.00000000	mice
Hnrnpk	0.03	1.00000000	mice
Rrp1	-0.01	1.00000000	mice
Ffar4	-0.08	1.00000000	mice
Gbf1	0.01	1.00000000	mice
Tle4	-0.10	1.00000000	mice
Tnfrsf13c	-0.09	1.00000000	mice
Rexo2	0.07	1.00000000	mice
Atl2	-0.09	1.00000000	mice
Nus1	-0.13	1.00000000	mice
Capn7	-0.08	1.00000000	mice
Gas2l1	0.15	1.00000000	mice
Cherp	-0.08	1.00000000	mice
Sike1	-0.09	1.00000000	mice
Naa25	0.07	1.00000000	mice
Itpr3	-0.04	1.00000000	mice
Gak	-0.21	1.00000000	mice
Slc25a36	-0.13	1.00000000	mice
Azin1	-0.12	1.00000000	mice
Faap20	-0.03	1.00000000	mice
Tnfsf12	-0.02	1.00000000	mice
Sesn1	0.08	1.00000000	mice
Serp1	0.00	1.00000000	mice
Hvcn1	0.09	1.00000000	mice
Calcoco1	0.09	1.00000000	mice
Ago3	-0.22	1.00000000	mice

Prkra	0.10	1.00000000	mice
Hook2	0.07	1.00000000	mice
Zbtb37	-0.05	1.00000000	mice
Ppp1r35	0.00	1.00000000	mice
Ten1	0.08	1.00000000	mice
Thyn1	0.04	1.00000000	mice
Gpat4	0.04	1.00000000	mice
Eml3	-0.16	1.00000000	mice
Ide	-0.05	1.00000000	mice
Tbpl1	-0.07	1.00000000	mice
B3gnt5	0.15	1.00000000	mice
Clcf1	0.09	1.00000000	mice
Lpl	-0.01	1.00000000	mice
Nsd2	-0.10	1.00000000	mice
Eif4a3	0.08	1.00000000	mice
Cd1d1	0.21	1.00000000	mice
Urgcp	0.04	1.00000000	mice
Polr2d	0.04	1.00000000	mice
Eps15l1	-0.01	1.00000000	mice
Zxdc	-0.08	1.00000000	mice
D430042O09Rik	-0.08	1.00000000	mice
Jup	0.03	1.00000000	mice
Ctbp2	-0.08	1.00000000	mice
Prdx3	-0.04	1.00000000	mice
Dyrk2	0.24	1.00000000	mice
Rftn1	-0.22	1.00000000	mice
Anapc10	0.03	1.00000000	mice
Fam76b	0.04	1.00000000	mice
Commd2	0.05	1.00000000	mice
Arpin	0.05	1.00000000	mice
Dcaf7	0.03	1.00000000	mice
Emd	0.11	1.00000000	mice
2510039O18Rik	0.08	1.00000000	mice
Lman1	-0.07	1.00000000	mice
Slc35b1	0.04	1.00000000	mice
Prpf4b	-0.08	1.00000000	mice
Luzp1	-0.10	1.00000000	mice
Wdr74	-0.09	1.00000000	mice
Vta1	-0.02	1.00000000	mice
Txndc12	-0.09	1.00000000	mice
Dll4	0.02	1.00000000	mice
Ipo8	-0.11	1.00000000	mice
Trmt6	-0.16	1.00000000	mice
Arhgef11	-0.09	1.00000000	mice
Nt5c2	-0.05	1.00000000	mice
Fem1b	-0.11	1.00000000	mice

Plrg1	-0.02	1.00000000	mice
Ddx31	0.01	1.00000000	mice
Chsy1	-0.24	1.00000000	mice
Stx8	0.07	1.00000000	mice
Atg4a	0.06	1.00000000	mice
Arl2bp	-0.12	1.00000000	mice
Chic2	0.17	1.00000000	mice
Ebag9	-0.11	1.00000000	mice
Zfp280d	0.03	1.00000000	mice
Nat10	-0.04	1.00000000	mice
Itgb2	-0.11	1.00000000	mice
Rap2b	0.30	1.00000000	mice
Lats2	0.09	1.00000000	mice
Gns	0.25	1.00000000	mice
Glo1	-0.06	1.00000000	mice
Cetn3	0.03	1.00000000	mice
Ccdc117	0.10	1.00000000	mice
Isy1	0.01	1.00000000	mice
Nfyc	-0.13	1.00000000	mice
Srsf3	-0.14	1.00000000	mice
Eprs	-0.13	1.00000000	mice
Sirt3	0.01	1.00000000	mice
Utp25	-0.04	1.00000000	mice
Zswim4	-0.09	1.00000000	mice
Ptma	-0.08	1.00000000	mice
Pnrc1	-0.07	1.00000000	mice
Brwd3	0.10	1.00000000	mice
B3gnt2	0.07	1.00000000	mice
5430427O19Rik	0.06	1.00000000	mice
Vegfa	0.04	1.00000000	mice
Ubr2	0.01	1.00000000	mice
Hectd1	-0.10	1.00000000	mice
Zscan25	-0.06	1.00000000	mice
Dusp2	0.17	1.00000000	mice
Bbs9	-0.10	1.00000000	mice
Cenpb	0.02	1.00000000	mice
Tnfrsf12a	-0.04	1.00000000	mice
Nol12	-0.01	1.00000000	mice
Phospho2	-0.09	1.00000000	mice
Armc7	0.18	1.00000000	mice
Desi1	-0.04	1.00000000	mice
Ptrh2	-0.03	1.00000000	mice
Nemp2	0.02	1.00000000	mice
Cyp4v3	0.06	1.00000000	mice
Kdelr2	0.05	1.00000000	mice
Fbxo34	0.06	1.00000000	mice

Heg1	0.19	1.00000000	mice
Clic1	0.14	1.00000000	mice
Nol4l	0.11	1.00000000	mice
Bet1	-0.03	1.00000000	mice
Vamp7	0.05	1.00000000	mice
Ube4b	0.06	1.00000000	mice
Brd1	0.02	1.00000000	mice
Slc25a4	-0.13	1.00000000	mice
Nipbl	-0.09	1.00000000	mice
Edem2	0.08	1.00000000	mice
Abraxas2	0.05	1.00000000	mice
Atg4b	0.05	1.00000000	mice
Pgp	0.01	1.00000000	mice
Nol9	-0.03	1.00000000	mice
Cblb	0.42	1.00000000	mice
Trim59	-0.09	1.00000000	mice
Erlin2	0.06	1.00000000	mice
Wdr37	0.06	1.00000000	mice
Rcan3	-0.02	1.00000000	mice
Tmem65	-0.10	1.00000000	mice
Hexa	-0.16	1.00000000	mice
Siah1a	0.09	1.00000000	mice
Ezh2	0.11	1.00000000	mice
Strn	-0.08	1.00000000	mice
Slc50a1	-0.12	1.00000000	mice
Tacc1	-0.14	1.00000000	mice
Ginm1	-0.16	1.00000000	mice
Ctdsp1	-0.05	1.00000000	mice
Hdhd2	-0.01	1.00000000	mice
Tmed4	0.05	1.00000000	mice
Cdk5rap3	0.01	1.00000000	mice
Sdccag8	-0.10	1.00000000	mice
4930581F22Rik	0.05	1.00000000	mice
Zfp36	0.10	1.00000000	mice
Zfp143	0.06	1.00000000	mice
Nfatc1	-0.21	1.00000000	mice
Dpp3	0.06	1.00000000	mice
Tpp2	-0.11	1.00000000	mice
Psd4	0.16	1.00000000	mice
Papss1	-0.06	1.00000000	mice
Dut	0.00	1.00000000	mice
Spast	-0.07	1.00000000	mice
Anapc4	0.03	1.00000000	mice
Epb41l4aos	0.02	1.00000000	mice
Trip11	0.05	1.00000000	mice
Gcc1	0.03	1.00000000	mice

Cep135	0.02	1.00000000	mice
Spg21	-0.02	1.00000000	mice
Depdc5	0.05	1.00000000	mice
Prdm10	-0.11	1.00000000	mice
Churc1	-0.07	1.00000000	mice
Pqlc2	0.04	1.00000000	mice
Swap70	0.15	1.00000000	mice
Ntpcr	-0.05	1.00000000	mice
Sun1	-0.05	1.00000000	mice
Inpp4a	0.17	1.00000000	mice
Styx	0.09	1.00000000	mice
Ak3	-0.04	1.00000000	mice
Clec4n	-0.13	1.00000000	mice
Fam53b	0.06	1.00000000	mice
Ddx56	0.02	1.00000000	mice
Cstf3	0.03	1.00000000	mice
Srp14	0.02	1.00000000	mice
H2-Eb2	-0.04	1.00000000	mice
Abi1	0.01	1.00000000	mice
Rbm4b	-0.02	1.00000000	mice
Dip2b	0.12	1.00000000	mice
2310001H17Rik	0.08	1.00000000	mice
Atp1a3	0.11	1.00000000	mice
Dhx36	0.07	1.00000000	mice
Snrpb	0.04	1.00000000	mice
Pes1	0.03	1.00000000	mice
Smarcal1	0.02	1.00000000	mice
Apbb1ip	0.01	1.00000000	mice
Prkaca	-0.05	1.00000000	mice
Ss18l2	-0.06	1.00000000	mice
Plpbp	0.02	1.00000000	mice
Jmjd6	0.01	1.00000000	mice
Trappc5	-0.01	1.00000000	mice
Htatsf1	-0.13	1.00000000	mice
Ctbs	0.04	1.00000000	mice
Ears2	0.03	1.00000000	mice
Chka	0.03	1.00000000	mice
C2cd2	-0.03	1.00000000	mice
Rad54l2	0.02	1.00000000	mice
Icam1	0.10	1.00000000	mice
Upf1	-0.11	1.00000000	mice
Arpc2	0.10	1.00000000	mice
Yars	0.01	1.00000000	mice
Fam173a	0.05	1.00000000	mice
Cxcl16	0.26	1.00000000	mice
Ptpre	-0.15	1.00000000	mice

Tbcc	-0.04	1.00000000	mice
Dnajc12	0.15	1.00000000	mice
Inf2	-0.01	1.00000000	mice
Pip4k2b	-0.12	1.00000000	mice
Uqcrc1	0.03	1.00000000	mice
Eif5a	-0.12	1.00000000	mice
Ddx19a	0.07	1.00000000	mice
Arhgap17	0.13	1.00000000	mice
Prdx4	0.13	1.00000000	mice
Calr	0.03	1.00000000	mice
Clp1	0.03	1.00000000	mice
Sema4a	0.11	1.00000000	mice
Ddi2	-0.17	1.00000000	mice
Soga1	-0.10	1.00000000	mice
Rabepk	-0.04	1.00000000	mice
Nr2c2	0.07	1.00000000	mice
Tti1	0.06	1.00000000	mice
Fam160b1	-0.01	1.00000000	mice
Sh2d3c	0.09	1.00000000	mice
Trappc9	-0.27	1.00000000	mice
Arhgef10l	0.03	1.00000000	mice
Hps5	0.07	1.00000000	mice
Scyl3	0.05	1.00000000	mice
Tbcd	-0.04	1.00000000	mice
Pik3cd	-0.17	1.00000000	mice
Tnfrsf18	0.07	1.00000000	mice
Cdc42se2	-0.10	1.00000000	mice
Nup50	-0.06	1.00000000	mice
Limk2	-0.15	1.00000000	mice
Rpf2	-0.04	1.00000000	mice
Eif4g3	-0.07	1.00000000	mice
Traf4	0.23	1.00000000	mice
Heca	0.00	1.00000000	mice
Ndst2	0.02	1.00000000	mice
Tnpo2	0.03	1.00000000	mice
Bzw2	-0.17	1.00000000	mice
Hyou1	-0.08	1.00000000	mice
Syne3	-0.11	1.00000000	mice
Thumpd3	0.01	1.00000000	mice
Sema4b	0.13	1.00000000	mice
Cenpx	0.06	1.00000000	mice
Spsb2	-0.06	1.00000000	mice
Khsrp	-0.05	1.00000000	mice
Ptpn9	0.05	1.00000000	mice
Clec4a2	-0.06	1.00000000	mice
Ddx39b	-0.06	1.00000000	mice

Gtf3c1	-0.10	1.00000000	mice
Ankib1	0.03	1.00000000	mice
Tmem189	0.02	1.00000000	mice
Trib1	-0.09	1.00000000	mice
Sbno1	0.09	1.00000000	mice
Ganab	-0.07	1.00000000	mice
Nfat5	0.21	1.00000000	mice
Usp50	0.05	1.00000000	mice
St7l	-0.11	1.00000000	mice
Bhlhe40	0.17	1.00000000	mice
Plekhn1	0.03	1.00000000	mice
Tgfbrap1	0.02	1.00000000	mice
Aspscr1	-0.05	1.00000000	mice
Tiprl	0.03	1.00000000	mice
Spin1	-0.08	1.00000000	mice
Tmed8	0.00	1.00000000	mice
Uba3	0.01	1.00000000	mice
Akap8l	0.05	1.00000000	mice
Dusp10	0.12	1.00000000	mice
Rad18	-0.04	1.00000000	mice
Klhl18	0.08	1.00000000	mice
Gng12	0.28	1.00000000	mice
Eldr	-0.08	1.00000000	mice
Hnrnpa0	-0.18	1.00000000	mice
A430035B10Rik	-0.12	1.00000000	mice
Snx17	0.08	1.00000000	mice
Tmem147	-0.06	1.00000000	mice
Anapc2	0.05	1.00000000	mice
Rab6a	0.02	1.00000000	mice
Cd33	0.08	1.00000000	mice
Bckdk	0.00	1.00000000	mice
Rnf4	0.03	1.00000000	mice
Calm2	-0.07	1.00000000	mice
Cdkn1b	0.10	1.00000000	mice
Pnpla2	0.07	1.00000000	mice
Zc3h3	-0.04	1.00000000	mice
Scaf11	-0.13	1.00000000	mice
Wasf2	0.08	1.00000000	mice
Ints3	0.08	1.00000000	mice
Pgs1	-0.03	1.00000000	mice
Crebbp	0.04	1.00000000	mice
Irf4	0.03	1.00000000	mice
Ube2n	0.09	1.00000000	mice
Gne	0.08	1.00000000	mice
Cnot1	-0.08	1.00000000	mice
Fam53c	0.00	1.00000000	mice

P2ry14	0.02	1.00000000	mice
Sla2	0.23	1.00000000	mice
Fam204a	0.03	1.00000000	mice
Ugcg	-0.07	1.00000000	mice
Virma	-0.09	1.00000000	mice
Nat9	0.02	1.00000000	mice
Pafah1b3	0.09	1.00000000	mice
Trim3	0.01	1.00000000	mice
Cd2	-0.01	1.00000000	mice
Wdr46	-0.08	1.00000000	mice
Atp2b4	-0.19	1.00000000	mice
Nit1	0.03	1.00000000	mice
Glrx2	0.04	1.00000000	mice
Ppie	-0.07	1.00000000	mice
Plcg2	-0.09	1.00000000	mice
Smarcc1	-0.16	1.00000000	mice
Rabep2	0.04	1.00000000	mice
Calhm2	0.07	1.00000000	mice
Cdkn2d	0.19	1.00000000	mice
Abca7	0.03	1.00000000	mice
Znrd2	0.04	1.00000000	mice
Galnt12	0.02	1.00000000	mice
Sbf1	-0.06	1.00000000	mice
Paxip1	-0.08	1.00000000	mice
Nom1	0.02	1.00000000	mice
Stx16	0.05	1.00000000	mice
Iars	0.03	1.00000000	mice
Nceh1	-0.42	1.00000000	mice
Drosha	-0.08	1.00000000	mice
Pold4	-0.06	1.00000000	mice
Ttpal	0.00	1.00000000	mice
Ube4a	0.12	1.00000000	mice
Xpo7	-0.09	1.00000000	mice
Fut8	0.12	1.00000000	mice
Tmem179b	0.02	1.00000000	mice
Dnajb1	0.06	1.00000000	mice
Rpp30	-0.02	1.00000000	mice
Ip6k2	0.06	1.00000000	mice
Ptges3	0.05	1.00000000	mice
Tuba4a	0.03	1.00000000	mice
Runx3	0.07	1.00000000	mice
Dmac2	0.02	1.00000000	mice
Fgfr2	-0.08	1.00000000	mice
Fkbp1a	-0.11	1.00000000	mice
Snx5	0.07	1.00000000	mice
Rassf2	0.24	1.00000000	mice

Tarbp2	0.01	1.00000000	mice
Lamtor4	0.05	1.00000000	mice
Dock9	0.04	1.00000000	mice
Chmp1a	-0.11	1.00000000	mice
Atf4	-0.10	1.00000000	mice
Tspan2	0.20	1.00000000	mice
Rundc1	0.01	1.00000000	mice
Far1os	0.04	1.00000000	mice
Patl1	0.06	1.00000000	mice
Brpf3	0.06	1.00000000	mice
Zfp704	-0.24	1.00000000	mice
Nfia	-0.16	1.00000000	mice
Bcl9	0.05	1.00000000	mice
Cox10	0.04	1.00000000	mice
2310058D17Rik	-0.09	1.00000000	mice
Lman2	0.02	1.00000000	mice
Aldh3a2	-0.01	1.00000000	mice
Arhgap23	-0.07	1.00000000	mice
Paip2b	-0.04	1.00000000	mice
Stk17b	0.02	1.00000000	mice
Plod3	0.02	1.00000000	mice
Herpud1	-0.10	1.00000000	mice
Bola3	-0.02	1.00000000	mice
Rgs18	0.10	1.00000000	mice
Whrn	-0.05	1.00000000	mice
Kansl1l	0.00	1.00000000	mice
Fem1a	-0.06	1.00000000	mice
Prune1	-0.07	1.00000000	mice
Pitpnm1	0.06	1.00000000	mice
Fcf1	0.04	1.00000000	mice
Tmem41b	0.08	1.00000000	mice
Ssrp1	-0.03	1.00000000	mice
Cetn2	0.00	1.00000000	mice
Pir	0.34	1.00000000	mice
Ago1	0.01	1.00000000	mice
Wwp2	-0.06	1.00000000	mice
Secisbp2l	-0.09	1.00000000	mice
Card6	0.03	1.00000000	mice
Zc3h15	0.05	1.00000000	mice
Sell	-0.05	1.00000000	mice
Nt5dc1	-0.01	1.00000000	mice
Naa40	0.03	1.00000000	mice
Tmem161b	0.06	1.00000000	mice
Rbm12b2	-0.07	1.00000000	mice
Csnk2a1	0.01	1.00000000	mice
Ngdn	-0.05	1.00000000	mice

Wipf2	0.08	1.00000000	mice
Cebpzos	0.01	1.00000000	mice
Gpatch1	-0.02	1.00000000	mice
Snx19	-0.01	1.00000000	mice
Atad2	-0.04	1.00000000	mice
Tmem59	0.07	1.00000000	mice
Rnaseh2c	-0.04	1.00000000	mice
Zc3h12b	-0.21	1.00000000	mice
Acvr1	-0.02	1.00000000	mice
Shoc2	0.04	1.00000000	mice
Armc10	0.04	1.00000000	mice
Zbtb32	0.12	1.00000000	mice
Spint2	0.15	1.00000000	mice
Tagln2	0.16	1.00000000	mice
Abl1	0.03	1.00000000	mice
Tes	0.20	1.00000000	mice
Arfgef2	-0.08	1.00000000	mice
Ndufa6	0.04	1.00000000	mice
Ccl22	0.78	1.00000000	mice
Cd80	0.16	1.00000000	mice
Thrap3	0.03	1.00000000	mice
Smyd1	0.02	1.00000000	mice
Cln5	0.04	1.00000000	mice
Ncf2	-0.18	1.00000000	mice
Zmym5	0.16	1.00000000	mice
Trappc11	0.04	1.00000000	mice
Rcor1	0.05	1.00000000	mice
Rwdd4a	0.09	1.00000000	mice
Supv3l1	-0.05	1.00000000	mice
Sgk1	-0.20	1.00000000	mice
Sh3pxd2a	-0.26	1.00000000	mice
Cspp1	-0.10	1.00000000	mice
Sprtn	0.01	1.00000000	mice
Fam126a	0.04	1.00000000	mice
Smg1	-0.16	1.00000000	mice
Tmem245	-0.05	1.00000000	mice
Bcl2	-0.07	1.00000000	mice
Stxbp2	0.01	1.00000000	mice
C1galt1c1	0.03	1.00000000	mice
Erp29	-0.04	1.00000000	mice
Sptan1	0.12	1.00000000	mice
Disc1	0.00	1.00000000	mice
Cdadc1	0.00	1.00000000	mice
Rab2b	-0.10	1.00000000	mice
Tmem86b	-0.06	1.00000000	mice
Pbrm1	0.06	1.00000000	mice

Ltv1	0.04	1.00000000	mice
Ankrd40	-0.10	1.00000000	mice
Ufsp2	0.07	1.00000000	mice
Btf3	0.05	1.00000000	mice
Ctnnbl1	-0.08	1.00000000	mice
Srp54a	0.03	1.00000000	mice
Il1r2	0.61	1.00000000	mice
6530409C15Rik	0.00	1.00000000	mice
Atxn7	0.09	1.00000000	mice
Cenpa	-0.04	1.00000000	mice
Large1	-0.24	1.00000000	mice
Cd302	0.08	1.00000000	mice
Tgoln1	0.02	1.00000000	mice
Ppp4c	0.10	1.00000000	mice
Pkp4	0.02	1.00000000	mice
Dynll2	0.09	1.00000000	mice
Smc5	0.09	1.00000000	mice
Rhob	0.03	1.00000000	mice
Nup35	0.04	1.00000000	mice
1700096K18Rik	-0.08	1.00000000	mice
2210408I21Rik	0.03	1.00000000	mice
Tmem223	0.01	1.00000000	mice
Lonp2	0.00	1.00000000	mice
Parl	-0.03	1.00000000	mice
Eef1e1	-0.06	1.00000000	mice
Sirt1	0.03	1.00000000	mice
Cish	0.08	1.00000000	mice
Rbbp5	0.01	1.00000000	mice
Pde4a	-0.01	1.00000000	mice
Fyttd1	-0.11	1.00000000	mice
E130309D02Rik	-0.03	1.00000000	mice
Def6	0.03	1.00000000	mice
Hps1	-0.01	1.00000000	mice
Atox1	0.14	1.00000000	mice
Xpo6	-0.07	1.00000000	mice
Nop14	-0.04	1.00000000	mice
Ldlrad3	-0.29	1.00000000	mice
Pcmt1	-0.13	1.00000000	mice
Phf12	0.09	1.00000000	mice
Tnfaip8l2	0.04	1.00000000	mice
Nbl1	0.11	1.00000000	mice
Atg3	-0.09	1.00000000	mice
Synj2bp	0.01	1.00000000	mice
Plpp5	-0.03	1.00000000	mice
Apmap	-0.07	1.00000000	mice
Trim65	-0.08	1.00000000	mice

Thg1l	0.04	1.00000000	mice
Sec62	-0.02	1.00000000	mice
Amfr	-0.10	1.00000000	mice
Rcbtb2	0.04	1.00000000	mice
Ccdc9	-0.02	1.00000000	mice
Erlin1	-0.02	1.00000000	mice
Ahsa2	0.05	1.00000000	mice
Lrrc63	-0.08	1.00000000	mice
Swt1	0.03	1.00000000	mice
Atp2a1	0.06	1.00000000	mice
Vav1	0.10	1.00000000	mice
Ipo7	-0.10	1.00000000	mice
Rab4b	0.06	1.00000000	mice
Relt	-0.05	1.00000000	mice
Arf2	-0.07	1.00000000	mice
Fam8a1	-0.04	1.00000000	mice
Pigf	0.01	1.00000000	mice
Esrra	0.08	1.00000000	mice
Emc6	0.08	1.00000000	mice
Wbp11	0.05	1.00000000	mice
Trmt2a	-0.04	1.00000000	mice
Pofut2	-0.04	1.00000000	mice
Tfeb	0.06	1.00000000	mice
Scoc	0.04	1.00000000	mice
Asb7	0.00	1.00000000	mice
Fbxo38	0.08	1.00000000	mice
Lsm14a	-0.09	1.00000000	mice
Eno1	-0.01	1.00000000	mice
Zdhhc14	0.58	1.00000000	mice
Tbrg1	0.10	1.00000000	mice
Cmtm3	-0.04	1.00000000	mice
Agpat1	0.05	1.00000000	mice
Glce	-0.09	1.00000000	mice
H3f3a	-0.04	1.00000000	mice
Hexb	-0.08	1.00000000	mice
Golga2	0.00	1.00000000	mice
Srebf2	-0.09	1.00000000	mice
Khdrbs1	0.05	1.00000000	mice
Anapc15	0.04	1.00000000	mice
Tagap	-0.16	1.00000000	mice
Gpn1	0.02	1.00000000	mice
Atp7a	-0.15	1.00000000	mice
Commd8	-0.11	1.00000000	mice
Emc2	0.04	1.00000000	mice
Itsn1	-0.21	1.00000000	mice
Utp20	-0.10	1.00000000	mice

Fbxw4	-0.06	1.00000000	mice
Adamts6	-0.30	1.00000000	mice
Hmgcl	-0.10	1.00000000	mice
Noc3l	-0.03	1.00000000	mice
Cmtm4	-0.05	1.00000000	mice
Akr7a5	-0.04	1.00000000	mice
Rab35	0.03	1.00000000	mice
Tmem38b	-0.09	1.00000000	mice
Ebp	0.02	1.00000000	mice
Kat14	-0.04	1.00000000	mice
Gfod1	-0.04	1.00000000	mice
Birc3	-0.09	1.00000000	mice
Ammecr1l	-0.09	1.00000000	mice
Ap2m1	-0.07	1.00000000	mice
Acot9	0.02	1.00000000	mice
Psmd5	-0.10	1.00000000	mice
Dipk1a	0.12	1.00000000	mice
Dcaf1	0.08	1.00000000	mice
Lrrc1	-0.01	1.00000000	mice
Ndrg3	-0.03	1.00000000	mice
Gnb2	0.10	1.00000000	mice
Cisd1	0.00	1.00000000	mice
Smim5	0.32	1.00000000	mice
Nudcd2	-0.04	1.00000000	mice
Hmbox1	0.05	1.00000000	mice
Rbm25	0.05	1.00000000	mice
Becn1	0.09	1.00000000	mice
Trim11	-0.13	1.00000000	mice
Ddx17	0.07	1.00000000	mice
Fam98b	0.08	1.00000000	mice
Crybg1	0.07	1.00000000	mice
Zfp367	-0.04	1.00000000	mice
B230307C23Rik	-0.08	1.00000000	mice
Ank	-0.02	1.00000000	mice
Pcmtd2	-0.03	1.00000000	mice
Smn1	-0.01	1.00000000	mice
Kpna6	-0.08	1.00000000	mice
Rtcb	-0.11	1.00000000	mice
Spryd3	0.03	1.00000000	mice
Psen1	-0.22	1.00000000	mice
Katnbl1	-0.09	1.00000000	mice
Srrm2	-0.05	1.00000000	mice
Glt8d1	0.03	1.00000000	mice
Preb	-0.10	1.00000000	mice
Gga3	0.06	1.00000000	mice
Coq8b	0.04	1.00000000	mice

Homer1	0.12	1.00000000	mice
Supt3	-0.16	1.00000000	mice
Ndufs3	-0.12	1.00000000	mice
Lcp1	0.14	1.00000000	mice
Kpnb1	0.06	1.00000000	mice
Lrp12	0.06	1.00000000	mice
Sms	0.04	1.00000000	mice
Slc9a6	0.02	1.00000000	mice
Ywhah	0.10	1.00000000	mice
Gcnt7	0.00	1.00000000	mice
F11r	0.12	1.00000000	mice
Hbp1	0.03	1.00000000	mice
Itgam	-0.19	1.00000000	mice
Pdzd4	0.13	1.00000000	mice
9430038I01Rik	-0.04	1.00000000	mice
Tbc1d12	-0.14	1.00000000	mice
Evi5	0.03	1.00000000	mice
Gtdc1	-0.35	1.00000000	mice
Jtb	-0.06	1.00000000	mice
Cep95	0.00	1.00000000	mice
Fen1	0.16	1.00000000	mice
Ccr5	0.21	1.00000000	mice
Ptp4a3	0.01	1.00000000	mice
Rnf185	-0.05	1.00000000	mice
Zfp710	-0.07	1.00000000	mice
Tnrc18	0.06	1.00000000	mice
Uba6	0.06	1.00000000	mice
Slc9a3r1	-0.04	1.00000000	mice
Tom1	0.02	1.00000000	mice
Brd7	-0.02	1.00000000	mice
Pik3r4	-0.01	1.00000000	mice
Brf1	-0.08	1.00000000	mice
Endod1	0.04	1.00000000	mice
Aga	-0.04	1.00000000	mice
Cyren	0.06	1.00000000	mice
Ppig	0.02	1.00000000	mice
Arhgap45	-0.08	1.00000000	mice
Hscb	0.03	1.00000000	mice
Ypel3	-0.12	1.00000000	mice
Otud1	0.09	1.00000000	mice
Ccnl2	0.10	1.00000000	mice
Galnt10	0.05	1.00000000	mice
Bid	-0.01	1.00000000	mice
Rbm7	-0.04	1.00000000	mice
Ppm1a	0.03	1.00000000	mice
Tmem170b	0.07	1.00000000	mice

Ctdsp2	-0.08	1.00000000	mice
Aldoa	0.12	1.00000000	mice
Nudt13	-0.01	1.00000000	mice
Dap	0.00	1.00000000	mice
Gtf2h5	-0.11	1.00000000	mice
Zfr	0.10	1.00000000	mice
Bin3	0.08	1.00000000	mice
Slc35f6	-0.07	1.00000000	mice
Haghl	-0.02	1.00000000	mice
Glod4	0.01	1.00000000	mice
Chd3	0.05	1.00000000	mice
Tmem115	0.02	1.00000000	mice
Csnk2a2	0.09	1.00000000	mice
Nabp2	-0.18	1.00000000	mice
Vipas39	0.01	1.00000000	mice
Ece1	-0.11	1.00000000	mice
Eipr1	-0.02	1.00000000	mice
Snrnp48	0.10	1.00000000	mice
Ankmy2	-0.09	1.00000000	mice
Cab39l	-0.18	1.00000000	mice
Cx3cr1	-0.13	1.00000000	mice
Psmg3	0.04	1.00000000	mice
Pygo2	0.02	1.00000000	mice
Pea15a	0.06	1.00000000	mice
Ugp2	-0.16	1.00000000	mice
Atf1	-0.09	1.00000000	mice
Rufy1	-0.03	1.00000000	mice
Zfp160	0.02	1.00000000	mice
Dpp9	0.04	1.00000000	mice
Atp5d	0.12	1.00000000	mice
Zfp729b	-0.11	1.00000000	mice
Fam214a	0.16	1.00000000	mice
Hsd17b11	0.00	1.00000000	mice
Surf6	-0.03	1.00000000	mice
Acsl5	-0.01	1.00000000	mice
Arid1b	0.08	1.00000000	mice
Taf3	-0.12	1.00000000	mice
Stx5a	-0.11	1.00000000	mice
Snapc3	-0.08	1.00000000	mice
Lsm5	-0.02	1.00000000	mice
Actr1a	-0.11	1.00000000	mice
Zfp933	-0.05	1.00000000	mice
Cul5	0.03	1.00000000	mice
Klhl26	0.07	1.00000000	mice
Tmem161a	0.02	1.00000000	mice
Alg10b	0.03	1.00000000	mice

Vps35l	0.02	1.00000000	mice
Rcc2	-0.10	1.00000000	mice
Phactr4	-0.14	1.00000000	mice
Hsf1	-0.05	1.00000000	mice
Sfr1	0.07	1.00000000	mice
Gzf1	-0.03	1.00000000	mice
Cand1	-0.06	1.00000000	mice
Ubxn4	-0.02	1.00000000	mice
Itm2c	0.00	1.00000000	mice
Arf6	-0.06	1.00000000	mice
Dlgap4	-0.18	1.00000000	mice
Slc39a13	0.02	1.00000000	mice
Crbn	-0.03	1.00000000	mice
Scml4	-0.14	1.00000000	mice
Ppp5c	-0.05	1.00000000	mice
Pccb	0.05	1.00000000	mice
Elp5	0.06	1.00000000	mice
Stac2	-0.02	1.00000000	mice
Ctcf	-0.12	1.00000000	mice
Snx4	0.00	1.00000000	mice
Ttc7b	-0.10	1.00000000	mice
Ubr7	-0.06	1.00000000	mice
Zbtb43	0.02	1.00000000	mice
Ppp4r3a	0.02	1.00000000	mice
Poc1b	-0.10	1.00000000	mice
Orc2	-0.07	1.00000000	mice
Cbfb	-0.16	1.00000000	mice
Ccnt1	0.04	1.00000000	mice
Tex2	-0.19	1.00000000	mice
Gsap	-0.17	1.00000000	mice
Prr5	0.02	1.00000000	mice
Serhl	0.04	1.00000000	mice
Nde1	0.03	1.00000000	mice
Aifm1	-0.01	1.00000000	mice
Cnpy3	0.09	1.00000000	mice
Tceanc2	0.07	1.00000000	mice
Ino80	-0.06	1.00000000	mice
Fcho2	0.22	1.00000000	mice
Nrxn2	0.08	1.00000000	mice
Tatdn1	0.01	1.00000000	mice
Pla2g12a	-0.10	1.00000000	mice
Ccnt2	-0.04	1.00000000	mice
Tnfsf9	0.33	1.00000000	mice
Sntb2	-0.11	1.00000000	mice
Sec61a1	0.02	1.00000000	mice
Adssl1	0.05	1.00000000	mice

Hmgn2	-0.06	1.00000000	mice
Anapc16	0.01	1.00000000	mice
Srrd	-0.04	1.00000000	mice
Foxj3	-0.02	1.00000000	mice
Eif5	-0.07	1.00000000	mice
4933434E20Rik	0.10	1.00000000	mice
Dmtf1	-0.18	1.00000000	mice
Rnft1	0.05	1.00000000	mice
Sergef	0.06	1.00000000	mice
Tox4	0.11	1.00000000	mice
Sumo1	0.06	1.00000000	mice
Nudt3	0.07	1.00000000	mice
Pigt	0.05	1.00000000	mice
2310022B05Rik	-0.04	1.00000000	mice
Zfp729a	0.02	1.00000000	mice
Bri3bp	-0.05	1.00000000	mice
Acadvl	0.06	1.00000000	mice
Eif4g1	-0.09	1.00000000	mice
Ethe1	-0.03	1.00000000	mice
Znrd1	-0.12	1.00000000	mice
Park7	-0.01	1.00000000	mice
Pde7a	-0.20	1.00000000	mice
Ybx3	0.04	1.00000000	mice
Phkg2	0.04	1.00000000	mice
Zc3h12c	-0.16	1.00000000	mice
Ptov1	0.15	1.00000000	mice
Nsun2	-0.07	1.00000000	mice
Zbtb4	-0.06	1.00000000	mice
Rnpepl1	0.01	1.00000000	mice
Hsd3b7	0.02	1.00000000	mice
Tmem60	0.00	1.00000000	mice
Alg1	0.02	1.00000000	mice
Cd48	0.10	1.00000000	mice
Fry	-0.08	1.00000000	mice
Scin	0.38	1.00000000	mice
Bicd2	-0.07	1.00000000	mice
Tbca	0.03	1.00000000	mice
Ogt	0.00	1.00000000	mice
Zfp407	0.05	1.00000000	mice
Il16	-0.06	1.00000000	mice
Zfp84	-0.03	1.00000000	mice
Pecam1	-0.13	1.00000000	mice
Aldh9a1	-0.06	1.00000000	mice
Vezt	-0.09	1.00000000	mice
Cep164	-0.06	1.00000000	mice
Gtf2a2	-0.05	1.00000000	mice

Taf5l	-0.03	1.00000000	mice
Dnajb4	0.01	1.00000000	mice
Slc25a20	-0.03	1.00000000	mice
AC149090.1	0.08	1.00000000	mice
Bloc1s1	0.04	1.00000000	mice
Slc16a2	-0.01	1.00000000	mice
Anapc7	0.04	1.00000000	mice
Cct3	-0.06	1.00000000	mice
Rsrc2	0.10	1.00000000	mice
Rpia	-0.04	1.00000000	mice
Atp1b1	0.29	1.00000000	mice
Lat2	-0.04	1.00000000	mice
Erap1	0.02	1.00000000	mice
Slc39a1	-0.05	1.00000000	mice
Atg16l2	-0.08	1.00000000	mice
Lfng	0.03	1.00000000	mice
Tmem192	0.05	1.00000000	mice
Dntt	0.02	1.00000000	mice
Ppih	0.02	1.00000000	mice
Ddx1	-0.05	1.00000000	mice
Nudt4	-0.10	1.00000000	mice
Recql5	-0.07	1.00000000	mice
Cop1	0.00	1.00000000	mice
Idnk	-0.02	1.00000000	mice
Spr	0.07	1.00000000	mice
Cic	-0.11	1.00000000	mice
Nucks1	-0.03	1.00000000	mice
E4f1	-0.04	1.00000000	mice
Tbc1d23	0.05	1.00000000	mice
Gemin7	0.02	1.00000000	mice
Zxdb	-0.03	1.00000000	mice
Aarsd1	-0.01	1.00000000	mice
Fnip1	-0.18	1.00000000	mice
Ythdf2	0.10	1.00000000	mice
Nt5m	0.02	1.00000000	mice
Lancl1	-0.10	1.00000000	mice
Cul4a	0.04	1.00000000	mice
Rmnd5b	-0.09	1.00000000	mice
Ap3b1	-0.01	1.00000000	mice
Ddx27	-0.02	1.00000000	mice
Rmdn3	-0.01	1.00000000	mice
Eif6	-0.08	1.00000000	mice
4931406P16Rik	-0.10	1.00000000	mice
Acer3	0.12	1.00000000	mice
Ptgr2	0.03	1.00000000	mice
Fbxo4	-0.06	1.00000000	mice

Fnbp4	0.00	1.00000000	mice
Adh5	0.00	1.00000000	mice
Helb	-0.01	1.00000000	mice
Emp3	-0.03	1.00000000	mice
Sec61b	0.08	1.00000000	mice
Snapc1	0.02	1.00000000	mice
Letm1	-0.02	1.00000000	mice
Tfg	0.04	1.00000000	mice
Trip12	0.08	1.00000000	mice
Abhd17a	0.00	1.00000000	mice
Etv6	-0.05	1.00000000	mice
Tnfrsf1a	0.09	1.00000000	mice
Ildr1	0.09	1.00000000	mice
G6pc3	0.02	1.00000000	mice
Fuca2	-0.01	1.00000000	mice
Fbxl12	-0.04	1.00000000	mice
Ppp2r2a	-0.08	1.00000000	mice
Frmd8	-0.04	1.00000000	mice
Xrn2	0.02	1.00000000	mice
Aplp2	-0.15	1.00000000	mice
Ccdc107	-0.09	1.00000000	mice
Tbl1xr1	-0.13	1.00000000	mice
Rab3gap2	-0.10	1.00000000	mice
Tln1	0.11	1.00000000	mice
Trim8	-0.04	1.00000000	mice
Coa6	-0.03	1.00000000	mice
Edc4	-0.03	1.00000000	mice
Ints13	-0.13	1.00000000	mice
Stt3a	-0.16	1.00000000	mice
Rsl24d1	-0.16	1.00000000	mice
Snd1	0.05	1.00000000	mice
1700084C06Rik	-0.03	1.00000000	mice
Slc4a2	0.03	1.00000000	mice
A530017D24Rik	0.05	1.00000000	mice
Hspd1	-0.17	1.00000000	mice
Slf2	-0.18	1.00000000	mice
Trub2	-0.04	1.00000000	mice
Hdac5	-0.07	1.00000000	mice
Rapgef2	-0.12	1.00000000	mice
Trp53i11	0.06	1.00000000	mice
Ggps1	0.01	1.00000000	mice
Agl	0.01	1.00000000	mice
Tyw1	0.00	1.00000000	mice
Agap2	0.01	1.00000000	mice
Grm8	0.01	1.00000000	mice
Ecsit	0.04	1.00000000	mice

Arl5b	0.10	1.00000000	mice
Ube2h	0.17	1.00000000	mice
St8sia6	-0.10	1.00000000	mice
Scaf1	0.03	1.00000000	mice
Zc3h12d	0.04	1.00000000	mice
Slc17a5	-0.01	1.00000000	mice
Snu13	-0.05	1.00000000	mice
Upf2	-0.05	1.00000000	mice
Traf7	0.02	1.00000000	mice
Topors	-0.08	1.00000000	mice
Trip4	-0.07	1.00000000	mice
H13	0.00	1.00000000	mice
Pik3r1	-0.04	1.00000000	mice
Znhit2	-0.07	1.00000000	mice
Osbpl7	-0.02	1.00000000	mice
Socs7	-0.01	1.00000000	mice
Rab3gap1	0.09	1.00000000	mice
Trabd	-0.02	1.00000000	mice
Atxn3	-0.05	1.00000000	mice
Yeats4	-0.09	1.00000000	mice
Tomm40	-0.04	1.00000000	mice
0610010K14Rik	-0.05	1.00000000	mice
Gatd1	0.02	1.00000000	mice
Insig2	0.03	1.00000000	mice
Nckap1	0.09	1.00000000	mice
Pop4	0.03	1.00000000	mice
Zfp87	-0.04	1.00000000	mice
Sec23a	0.00	1.00000000	mice
Lsm2	-0.09	1.00000000	mice
Tecpr1	0.01	1.00000000	mice
Stk16	0.04	1.00000000	mice
Dhx8	-0.21	1.00000000	mice
Uqcc1	0.01	1.00000000	mice
Slc35a2	0.01	1.00000000	mice
Zbtb40	-0.01	1.00000000	mice
Srprb	0.00	1.00000000	mice
Tm9sf1	-0.04	1.00000000	mice
Adck1	0.02	1.00000000	mice
Rsrc1	0.09	1.00000000	mice
Rprd1a	0.04	1.00000000	mice
Tspan13	-0.06	1.00000000	mice
Ear2	-0.30	1.00000000	mice
Zfp410	-0.05	1.00000000	mice
Arrdc3	-0.07	1.00000000	mice
Hnrnph2	0.05	1.00000000	mice
Dph6	-0.02	1.00000000	mice

Pfdn6	-0.29	1.00000000	mice
Snapin	0.02	1.00000000	mice
Cir1	0.04	1.00000000	mice
Ptms	0.22	1.00000000	mice
Rabif	-0.03	1.00000000	mice
Ccdc34	0.00	1.00000000	mice
Impact	-0.15	1.00000000	mice
Zwint	-0.01	1.00000000	mice
Alkbh5	-0.02	1.00000000	mice
Dgkh	0.04	1.00000000	mice
Kdm2a	-0.14	1.00000000	mice
Arid3a	0.10	1.00000000	mice
Adora2b	0.01	1.00000000	mice
Tpst2	-0.08	1.00000000	mice
Efhd2	-0.08	1.00000000	mice
Klhl20	0.08	1.00000000	mice
Cdc23	-0.01	1.00000000	mice
Plekhj1	0.04	1.00000000	mice
Dnm2	-0.10	1.00000000	mice
Eif2ak4	-0.05	1.00000000	mice
Btrc	-0.07	1.00000000	mice
Gga1	-0.03	1.00000000	mice
Stam	-0.01	1.00000000	mice
Pdcd6ip	-0.04	1.00000000	mice
Cdv3	-0.06	1.00000000	mice
Bicral	0.05	1.00000000	mice
Pex7	0.03	1.00000000	mice
Itch	-0.06	1.00000000	mice
Chid1	0.00	1.00000000	mice
Clec16a	-0.14	1.00000000	mice
Dram2	-0.07	1.00000000	mice
Dnmbp	-0.11	1.00000000	mice
Arhgap35	0.00	1.00000000	mice
Usp20	0.08	1.00000000	mice
Adipor1	-0.02	1.00000000	mice
Tufm	-0.02	1.00000000	mice
Armc8	-0.01	1.00000000	mice
Ltn1	-0.08	1.00000000	mice
Uchl5	-0.14	1.00000000	mice
Cd3eap	0.02	1.00000000	mice
Pdcd7	0.02	1.00000000	mice
Uckl1	-0.05	1.00000000	mice
Slc25a33	-0.07	1.00000000	mice
Dnmt1	0.05	1.00000000	mice
Rnf141	-0.02	1.00000000	mice
Srsf10	-0.05	1.00000000	mice

Snx9	-0.17	1.00000000	mice
Vps33a	0.02	1.00000000	mice
Dph5	0.00	1.00000000	mice
Rtf1	-0.05	1.00000000	mice
Tmed2	-0.06	1.00000000	mice
Lrch1	0.08	1.00000000	mice
Slc25a24	0.02	1.00000000	mice
Bcr	-0.05	1.00000000	mice
Gtf2h1	0.11	1.00000000	mice
Zkscan14	0.03	1.00000000	mice
Slc25a38	-0.11	1.00000000	mice
Cdk19	-0.02	1.00000000	mice
Usp37	0.08	1.00000000	mice
Rassf1	-0.04	1.00000000	mice
Caml	-0.02	1.00000000	mice
Kynu	-0.20	1.00000000	mice
Irak4	-0.10	1.00000000	mice
Gpx4	0.05	1.00000000	mice
Hes6	0.02	1.00000000	mice
Eefsec	-0.03	1.00000000	mice
Cacfd1	0.04	1.00000000	mice
Glud1	-0.08	1.00000000	mice
Cbx3	-0.06	1.00000000	mice
Alpk1	-0.02	1.00000000	mice
Hpgds	-0.14	1.00000000	mice
Plaa	0.05	1.00000000	mice
Lrrk1	-0.01	1.00000000	mice
Pcm1	0.09	1.00000000	mice
Rnf170	-0.04	1.00000000	mice
2310039H08Rik	0.00	1.00000000	mice
Tmsb4x	0.05	1.00000000	mice
Timeless	0.11	1.00000000	mice
Kxd1	0.07	1.00000000	mice
Cmc1	0.03	1.00000000	mice
Usp30	0.08	1.00000000	mice
Rnf157	0.13	1.00000000	mice
BC005537	0.10	1.00000000	mice
Slc39a6	0.02	1.00000000	mice
Ptbp1	-0.18	1.00000000	mice
Ube2f	-0.01	1.00000000	mice
Ap1s3	-0.06	1.00000000	mice
Etfb	-0.03	1.00000000	mice
Dennd3	-0.13	1.00000000	mice
Nipa2	-0.03	1.00000000	mice
Cpeb3	0.14	1.00000000	mice
Coil	-0.05	1.00000000	mice

Zfp26	0.05	1.00000000	mice
Esf1	-0.07	1.00000000	mice
Appbp2	0.08	1.00000000	mice
AC125149.3	0.07	1.00000000	mice
Ifi205	0.08	1.00000000	mice
Pak4	-0.03	1.00000000	mice
Eaf2	-0.09	1.00000000	mice
Rictor	-0.04	1.00000000	mice
Slc27a1	0.04	1.00000000	mice
Acsf2	-0.05	1.00000000	mice
Eci1	0.04	1.00000000	mice
Ctr9	-0.09	1.00000000	mice
Ctdp1	-0.08	1.00000000	mice
Slain2	-0.06	1.00000000	mice
Anapc1	-0.09	1.00000000	mice
Dnajc19	0.04	1.00000000	mice
Epg5	-0.07	1.00000000	mice
Rxrb	0.01	1.00000000	mice
Sh3bgr	-0.06	1.00000000	mice
Susd3	-0.05	1.00000000	mice
Rara	0.02	1.00000000	mice
Wdr45b	0.03	1.00000000	mice
Gtf2e2	0.06	1.00000000	mice
Ccr2	-0.05	1.00000000	mice
5530601H04Rik	-0.04	1.00000000	mice
U2af2	-0.02	1.00000000	mice
Ufl1	-0.02	1.00000000	mice
Tnrc6b	-0.27	1.00000000	mice
Utp4	-0.01	1.00000000	mice
Ftsj3	0.00	1.00000000	mice
1110065P20Rik	0.02	1.00000000	mice
Fam136a	0.03	1.00000000	mice
Zfp945	0.00	1.00000000	mice
Sfi1	0.01	1.00000000	mice
Washc2	-0.01	1.00000000	mice
Trmt11	-0.04	1.00000000	mice
Chtop	-0.09	1.00000000	mice
Usp38	0.02	1.00000000	mice
Btg2	-0.05	1.00000000	mice
Rtf2	0.04	1.00000000	mice
Casp8ap2	0.06	1.00000000	mice
Rmdn1	0.02	1.00000000	mice
Tial1	0.01	1.00000000	mice
Prpf40a	0.01	1.00000000	mice
Klhl8	-0.01	1.00000000	mice
Ssr2	0.07	1.00000000	mice

Smim15	0.03	1.00000000	mice
Slc35a1	-0.05	1.00000000	mice
Swi5	-0.03	1.00000000	mice
Zc3hc1	-0.06	1.00000000	mice
Pcf11	0.06	1.00000000	mice
Zbtb46	-0.13	1.00000000	mice
Pskh1	-0.04	1.00000000	mice
Dcun1d1	0.00	1.00000000	mice
Cog6	0.01	1.00000000	mice
Pum1	-0.10	1.00000000	mice
Eif2b5	-0.09	1.00000000	mice
Stk40	-0.14	1.00000000	mice
B930036N10Rik	-0.32	1.00000000	mice
Prnp	0.18	1.00000000	mice
Pcnx	-0.37	1.00000000	mice
Phtf1	-0.19	1.00000000	mice
Sertad2	-0.10	1.00000000	mice
Trp53inp2	0.01	1.00000000	mice
Ddx52	-0.02	1.00000000	mice
Esco1	0.04	1.00000000	mice
Nup160	-0.12	1.00000000	mice
Lpgat1	0.49	1.00000000	mice
Heatr5b	-0.05	1.00000000	mice
Eef2k	-0.03	1.00000000	mice
Hnrnph3	0.08	1.00000000	mice
Igsf8	0.07	1.00000000	mice
Ppp2r5a	-0.19	1.00000000	mice
Cep152	-0.05	1.00000000	mice
Rnaset2b	-0.05	1.00000000	mice
Pik3ap1	0.13	1.00000000	mice
Tmem33	-0.06	1.00000000	mice
Usp49	0.03	1.00000000	mice
Spindoc	-0.08	1.00000000	mice
Calu	-0.05	1.00000000	mice
Slc38a9	-0.12	1.00000000	mice
Fbxo3	0.01	1.00000000	mice
Arid4a	0.17	1.00000000	mice
Nol11	-0.11	1.00000000	mice
Il10rb	0.13	1.00000000	mice
Kctd20	0.01	1.00000000	mice
Uvssa	-0.07	1.00000000	mice
Kptn	-0.05	1.00000000	mice
H2afv	-0.09	1.00000000	mice
Phf1	0.01	1.00000000	mice
Larp7	-0.03	1.00000000	mice
Cse1l	-0.07	1.00000000	mice

Dynlt3	0.03	1.00000000	mice
Tlr2	0.06	1.00000000	mice
Laptm4a	0.01	1.00000000	mice
Cep250	0.04	1.00000000	mice
Btg3	-0.09	1.00000000	mice
Sgsm2	0.01	1.00000000	mice
Acd	-0.01	1.00000000	mice
Pdrg1	0.00	1.00000000	mice
Ruvbl1	-0.05	1.00000000	mice
Polr3gl	0.02	1.00000000	mice
Strap	-0.11	1.00000000	mice
Clec4d	0.06	1.00000000	mice
Ldb1	-0.07	1.00000000	mice
Ing5	0.03	1.00000000	mice
Slc37a2	-0.01	1.00000000	mice
Frrs1	-0.01	1.00000000	mice
Gpcpd1	-0.09	1.00000000	mice
Cables1	-0.42	1.00000000	mice
Tor1b	-0.04	1.00000000	mice
Sucla2	-0.02	1.00000000	mice
Arpc1b	0.05	1.00000000	mice
Fryl	-0.24	1.00000000	mice
Epb41	0.26	1.00000000	mice
Pnpla7	-0.15	1.00000000	mice
Gnl2	0.00	1.00000000	mice
Anxa2	0.13	1.00000000	mice
R3hdm1	-0.09	1.00000000	mice
Luc7l	-0.13	1.00000000	mice
Rbm38	0.02	1.00000000	mice
Parp4	0.14	1.00000000	mice
Ncdn	0.01	1.00000000	mice
Paip2	0.02	1.00000000	mice
Bysl	-0.04	1.00000000	mice
Bckdha	-0.01	1.00000000	mice
Aldh16a1	-0.04	1.00000000	mice
Sf3b2	0.12	1.00000000	mice
Nufip2	-0.14	1.00000000	mice
Rasgef1b	0.31	1.00000000	mice
Stx7	0.01	1.00000000	mice
Brd2	0.05	1.00000000	mice
Cnbd2	0.01	1.00000000	mice
Atg2a	-0.09	1.00000000	mice
Lnpk	-0.13	1.00000000	mice
Cab39	-0.05	1.00000000	mice
Clta	0.09	1.00000000	mice
Zfp1	-0.07	1.00000000	mice

Dnajb2	0.07	1.00000000	mice
Tax1bp1	0.01	1.00000000	mice
Iars2	0.03	1.00000000	mice
Epcam	-0.13	1.00000000	mice
Fgfr1op	-0.10	1.00000000	mice
Invs	0.01	1.00000000	mice
Zdhhc23	-0.02	1.00000000	mice
Faap100	0.03	1.00000000	mice
Atp11a	0.06	1.00000000	mice
Degs1	0.10	1.00000000	mice
Dpm2	0.03	1.00000000	mice
Brcc3	-0.08	1.00000000	mice
H2-Q10	0.02	1.00000000	mice
Ik	-0.03	1.00000000	mice
Kpna1	-0.19	1.00000000	mice
Tubb6	-0.06	1.00000000	mice
Sptlc1	0.02	1.00000000	mice
Ube2a	0.05	1.00000000	mice
Stim1	-0.09	1.00000000	mice
Npepps	-0.03	1.00000000	mice
Klra3	0.12	1.00000000	mice
Rnaseh2a	0.02	1.00000000	mice
Cpox	-0.05	1.00000000	mice
Cnot6l	-0.09	1.00000000	mice
Rad23a	0.04	1.00000000	mice
Eftud2	-0.01	1.00000000	mice
Dnajc5	0.06	1.00000000	mice
Crnkl1	0.09	1.00000000	mice
Fbxl14	0.04	1.00000000	mice
Recql	0.01	1.00000000	mice
Orc6	-0.06	1.00000000	mice
Xrcc4	0.03	1.00000000	mice
Cracr2a	-0.19	1.00000000	mice
Ankrd13c	-0.09	1.00000000	mice
Tsga10	0.02	1.00000000	mice
Tfpt	0.01	1.00000000	mice
Neurl4	-0.04	1.00000000	mice
Ntmt1	-0.12	1.00000000	mice
Slc38a6	0.11	1.00000000	mice
Hexim1	-0.22	1.00000000	mice
Agps	-0.09	1.00000000	mice
Pigs	-0.05	1.00000000	mice
1500011B03Rik	-0.03	1.00000000	mice
Nenf	-0.13	1.00000000	mice
Ccdc58	0.03	1.00000000	mice
Bcl2a1a	0.07	1.00000000	mice

Ncaph2	-0.03	1.00000000	mice
Dmac1	0.02	1.00000000	mice
Itga4	0.49	1.00000000	mice
Lrp10	-0.08	1.00000000	mice
Ccdc134	-0.05	1.00000000	mice
Srgap2	0.06	1.00000000	mice
Tbc1d9	-0.21	1.00000000	mice
Nek6	-0.08	1.00000000	mice
Epc1	0.21	1.00000000	mice
Acsl4	0.07	1.00000000	mice
Cnot6	-0.02	1.00000000	mice
Dtymk	0.02	1.00000000	mice
Rhot1	-0.17	1.00000000	mice
Cinp	0.02	1.00000000	mice
2310033P09Rik	0.02	1.00000000	mice
Snrnp40	-0.09	1.00000000	mice
Naa60	0.04	1.00000000	mice
Fbxo28	0.06	1.00000000	mice
Sf3b6	-0.08	1.00000000	mice
Pinx1	-0.06	1.00000000	mice
Cul1	-0.15	1.00000000	mice
Far1	-0.03	1.00000000	mice
Blcap	-0.02	1.00000000	mice
Rbck1	0.02	1.00000000	mice
Cwf19l2	-0.13	1.00000000	mice
Zfp53	-0.11	1.00000000	mice
Sik2	0.06	1.00000000	mice
Clcn7	0.01	1.00000000	mice
Fam45a	0.01	1.00000000	mice
Adpgk	0.08	1.00000000	mice
Spag7	0.05	1.00000000	mice
Hnrnpl	-0.13	1.00000000	mice
Xrcc1	-0.06	1.00000000	mice
Ube2r2	-0.08	1.00000000	mice
Rnf11	-0.01	1.00000000	mice
Eif2s1	-0.13	1.00000000	mice
Ddx18	-0.13	1.00000000	mice
Dusp12	-0.07	1.00000000	mice
Emg1	-0.05	1.00000000	mice
Actn4	0.04	1.00000000	mice
Nelfa	-0.01	1.00000000	mice
Armcx3	-0.05	1.00000000	mice
Phf20l1	0.07	1.00000000	mice
Zfp330	-0.05	1.00000000	mice
Baz1b	-0.04	1.00000000	mice
Bbc3	-0.04	1.00000000	mice

Ccdc91	0.00	1.00000000	mice
Gt(ROSA)26Sor	0.10	1.00000000	mice
Pias2	-0.08	1.00000000	mice
Sf3b3	-0.12	1.00000000	mice
Stoml2	0.02	1.00000000	mice
Ammecr1	0.02	1.00000000	mice
Tia1	0.06	1.00000000	mice
Trap1	-0.08	1.00000000	mice
Plaur	-0.12	1.00000000	mice
Srgn	-0.01	1.00000000	mice
Setd5	-0.07	1.00000000	mice
Ndufv1	-0.01	1.00000000	mice
Il10ra	0.21	1.00000000	mice
Ech1	0.12	1.00000000	mice
Add1	-0.13	1.00000000	mice
Wdr33	-0.04	1.00000000	mice
Trnau1ap	0.01	1.00000000	mice
Sec63	-0.02	1.00000000	mice
Ppfia4	0.04	1.00000000	mice
Lancl2	-0.03	1.00000000	mice
Ppp6r2	-0.04	1.00000000	mice
Eml2	-0.05	1.00000000	mice
Crk	0.07	1.00000000	mice
Grk6	-0.07	1.00000000	mice
Trim35	0.02	1.00000000	mice
Poldip3	0.00	1.00000000	mice
Tbc1d17	0.02	1.00000000	mice
Lrch3	0.05	1.00000000	mice
Wrn	0.02	1.00000000	mice
Sft2d2	0.11	1.00000000	mice
Whamm	-0.07	1.00000000	mice
Wasl	0.14	1.00000000	mice
Gnl1	-0.15	1.00000000	mice
Soat1	0.12	1.00000000	mice
Kri1	-0.01	1.00000000	mice
Cog7	-0.02	1.00000000	mice
Taf1d	-0.06	1.00000000	mice
Rab3d	0.01	1.00000000	mice
Arhgap27os3	0.02	1.00000000	mice
Thap2	0.22	1.00000000	mice
Decr1	0.01	1.00000000	mice
Actr8	-0.03	1.00000000	mice
Smc6	-0.15	1.00000000	mice
Ppp2r1b	0.04	1.00000000	mice
Hdac2	-0.02	1.00000000	mice
Rbm6	-0.12	1.00000000	mice

Fam193b	0.00	1.00000000	mice
Zfp277	-0.09	1.00000000	mice
Cnot7	-0.08	1.00000000	mice
Zbed4	0.10	1.00000000	mice
Pvr	0.00	1.00000000	mice
Krit1	0.03	1.00000000	mice
Arf3	-0.04	1.00000000	mice
Tbc1d9b	-0.03	1.00000000	mice
Chfr	-0.03	1.00000000	mice
Dpy19l4	0.04	1.00000000	mice
Ndufs6	0.07	1.00000000	mice
Fbxo42	0.03	1.00000000	mice
Prdx5	0.15	1.00000000	mice
Tmem263	-0.04	1.00000000	mice
Cobll1	-0.04	1.00000000	mice
Rbm15b	-0.09	1.00000000	mice
Tmem131l	0.16	1.00000000	mice
Vmp1	-0.05	1.00000000	mice
Togaram1	0.08	1.00000000	mice
Sdhaf2	0.05	1.00000000	mice
Klk1	0.50	1.00000000	mice
Chm	0.05	1.00000000	mice
Setdb1	0.21	1.00000000	mice
Pura	0.03	1.00000000	mice
Scai	-0.08	1.00000000	mice
Nup188	0.01	1.00000000	mice
Acbd6	-0.09	1.00000000	mice
Rit1	0.01	1.00000000	mice
Glrx	-0.13	1.00000000	mice
Ncoa5	0.04	1.00000000	mice
Rsu1	0.02	1.00000000	mice
Pxdc1	-0.07	1.00000000	mice
Ndnf	-0.17	1.00000000	mice
Ikbkb	-0.01	1.00000000	mice
Tmem199	0.02	1.00000000	mice
Dpp8	-0.50	1.00000000	mice
Rab10os	-0.07	1.00000000	mice
Nf2	-0.02	1.00000000	mice
Crot	-0.03	1.00000000	mice
Ppp1r10	0.13	1.00000000	mice
Sgk3	0.19	1.00000000	mice
Dzip3	-0.08	1.00000000	mice
Kctd13	0.01	1.00000000	mice
Prkca	-0.40	1.00000000	mice
Sp3	0.02	1.00000000	mice
Bag4	0.01	1.00000000	mice

BC005624	-0.03	1.00000000	mice
Farsb	-0.11	1.00000000	mice
Ttc5	-0.17	1.00000000	mice
Commd4	0.02	1.00000000	mice
Polr1a	-0.10	1.00000000	mice
Gga2	-0.08	1.00000000	mice
Ftx	0.11	1.00000000	mice
Zfp598	-0.04	1.00000000	mice
Zeb2	-0.06	1.00000000	mice
Fmc1	-0.08	1.00000000	mice
Cops7a	0.01	1.00000000	mice
Zkscan5	0.03	1.00000000	mice
Trpc4ap	0.00	1.00000000	mice
Arhgef1	0.12	1.00000000	mice
Ube3b	-0.01	1.00000000	mice
Exosc8	-0.07	1.00000000	mice
Arid5b	-0.22	1.00000000	mice
Chuk	-0.07	1.00000000	mice
Kif13b	0.08	1.00000000	mice
Ap3m2	-0.08	1.00000000	mice
Cdkal1	-0.03	1.00000000	mice
1810026B05Rik	-0.27	1.00000000	mice
Junb	0.17	1.00000000	mice
Dync2h1	-0.06	1.00000000	mice
Zfp955b	-0.03	1.00000000	mice
Zer1	-0.04	1.00000000	mice
Wdr20	0.04	1.00000000	mice
Ndufaf2	0.03	1.00000000	mice
Ciapin1	-0.12	1.00000000	mice
Nol6	0.01	1.00000000	mice
Snx24	-0.08	1.00000000	mice
Stk35	-0.02	1.00000000	mice
Arhgap22	0.40	1.00000000	mice
Asnsd1	0.03	1.00000000	mice
Vps52	-0.01	1.00000000	mice
Nasp	-0.05	1.00000000	mice
Usp32	-0.15	1.00000000	mice
Rnf2	-0.01	1.00000000	mice
Stim2	-0.01	1.00000000	mice
Nrg2	0.07	1.00000000	mice
Cpped1	-0.03	1.00000000	mice
Arrdc4	-0.05	1.00000000	mice
Gpr89	-0.02	1.00000000	mice
Secisbp2	0.00	1.00000000	mice
Smurf1	-0.02	1.00000000	mice
Trappc6a	-0.01	1.00000000	mice

Dcp1b	-0.12	1.00000000	mice
Dna2	0.01	1.00000000	mice
Tmed10	-0.09	1.00000000	mice
Exoc1	0.02	1.00000000	mice
Chp1	0.00	1.00000000	mice
Cryzl1	0.05	1.00000000	mice
Rnps1	0.04	1.00000000	mice
Ndufb10	-0.03	1.00000000	mice
Senp5	-0.11	1.00000000	mice
Dock10	-0.08	1.00000000	mice
Lcor	-0.12	1.00000000	mice
Capn10	0.01	1.00000000	mice
Coq7	0.00	1.00000000	mice
Kdm5c	0.15	1.00000000	mice
Flvcr1	0.00	1.00000000	mice
Ubald1	0.00	1.00000000	mice
Spns3	-0.13	1.00000000	mice
Bax	0.03	1.00000000	mice
Dnajb14	-0.03	1.00000000	mice
Hdgfl2	0.04	1.00000000	mice
Rab12	0.05	1.00000000	mice
Prrc2a	0.01	1.00000000	mice
Kdm4c	-0.19	1.00000000	mice
Pds5a	-0.14	1.00000000	mice
Rnf149	-0.10	1.00000000	mice
Gna11	0.09	1.00000000	mice
Slc45a4	-0.01	1.00000000	mice
Ripor1	0.02	1.00000000	mice
Snx20	-0.04	1.00000000	mice
Asap2	0.00	1.00000000	mice
Ssr4	0.01	1.00000000	mice
Ube2w	0.17	1.00000000	mice
Slc2a3	-0.11	1.00000000	mice
Zranb1	-0.13	1.00000000	mice
Pds5b	-0.10	1.00000000	mice
3110056K07Rik	-0.11	1.00000000	mice
Pdcd2	0.01	1.00000000	mice
Kcnip4	-0.05	1.00000000	mice
Gigyf2	-0.05	1.00000000	mice
Traf3ip3	-0.01	1.00000000	mice
Pex14	-0.07	1.00000000	mice
Tor2a	-0.02	1.00000000	mice
Sart3	-0.13	1.00000000	mice
Tatdn2	0.03	1.00000000	mice
Tpd52l2	0.01	1.00000000	mice
Tns1	0.22	1.00000000	mice

Parp2	-0.02	1.00000000	mice
Bola2	0.01	1.00000000	mice
Josd2	0.01	1.00000000	mice
Ankhd1	0.00	1.00000000	mice
Snrpg	-0.08	1.00000000	mice
Fkbp8	0.00	1.00000000	mice
Pacs2	-0.16	1.00000000	mice
AW554918	-0.08	1.00000000	mice
Ei24	0.02	1.00000000	mice
Api5	0.08	1.00000000	mice
Nop53	-0.11	1.00000000	mice
Zc3h6	0.05	1.00000000	mice
Fdx1	-0.05	1.00000000	mice
AI480526	0.00	1.00000000	mice
4931406G06Rik	0.01	1.00000000	mice
Fam76a	0.02	1.00000000	mice
Ambra1	0.05	1.00000000	mice
Nbas	-0.03	1.00000000	mice
Gpatch8	0.07	1.00000000	mice
Ecd	-0.13	1.00000000	mice
Fam129a	0.32	1.00000000	mice
Ccl25	0.01	1.00000000	mice
Cbfa2t2	0.01	1.00000000	mice
Prpf3	-0.03	1.00000000	mice
Ptpn23	-0.02	1.00000000	mice
Supt6	0.10	1.00000000	mice
Irak1	0.07	1.00000000	mice
Nbr1	0.06	1.00000000	mice
Crip1	0.29	1.00000000	mice
Cox19	0.00	1.00000000	mice
Ptger4	0.10	1.00000000	mice
Basp1	0.05	1.00000000	mice
Anks1	-0.16	1.00000000	mice
Btbd2	-0.01	1.00000000	mice
Zfp110	-0.05	1.00000000	mice
Vps8	-0.10	1.00000000	mice
Tmed3	-0.05	1.00000000	mice
Stag1	0.00	1.00000000	mice
Alkbh8	0.05	1.00000000	mice
Igfbp4	0.09	1.00000000	mice
Rbmx	-0.05	1.00000000	mice
Dyrk1a	0.04	1.00000000	mice
Pogz	0.03	1.00000000	mice
Rbm28	0.02	1.00000000	mice
Zfp260	-0.08	1.00000000	mice
Txndc11	-0.19	1.00000000	mice

Pex2	0.00	1.00000000	mice
Sfxn1	-0.02	1.00000000	mice
Aqr	0.00	1.00000000	mice
Atp8a1	0.10	1.00000000	mice
Polr3e	-0.03	1.00000000	mice
Fam193a	-0.13	1.00000000	mice
Tusc2	-0.06	1.00000000	mice
Camkmt	0.06	1.00000000	mice
Ctsl	0.20	1.00000000	mice
Zpr1	-0.02	1.00000000	mice
Hdac8	-0.23	1.00000000	mice
Usp16	-0.05	1.00000000	mice
Btf3l4	-0.09	1.00000000	mice
Cd82	0.13	1.00000000	mice
Ints2	0.04	1.00000000	mice
Ubap1	0.06	1.00000000	mice
Ring1	0.02	1.00000000	mice
Pgam5	0.00	1.00000000	mice
Ddx20	-0.04	1.00000000	mice
Lmf1	-0.01	1.00000000	mice
Zfp664	-0.06	1.00000000	mice
Asna1	-0.03	1.00000000	mice
Ap4e1	-0.08	1.00000000	mice
Atm	-0.05	1.00000000	mice
Dlst	-0.04	1.00000000	mice
Atpsckmt	0.01	1.00000000	mice
Nploc4	0.03	1.00000000	mice
Dgkz	0.09	1.00000000	mice
Acp5	0.15	1.00000000	mice
Iqgap1	-0.03	1.00000000	mice
Phc2	0.10	1.00000000	mice
D16Ertd472e	0.00	1.00000000	mice
Dhx30	-0.09	1.00000000	mice
Gabpa	-0.01	1.00000000	mice
Jpt1	-0.14	1.00000000	mice
Larp1	-0.06	1.00000000	mice
Csf2ra	-0.07	1.00000000	mice
Sash3	-0.16	1.00000000	mice
Ccr9	0.32	1.00000000	mice
Rpgrip1	0.29	1.00000000	mice
Pum3	0.00	1.00000000	mice
Arl3	0.02	1.00000000	mice
Hltf	-0.04	1.00000000	mice
Fam234a	0.03	1.00000000	mice
Tdp2	-0.05	1.00000000	mice
Paxbp1	0.04	1.00000000	mice

Ube2g2	-0.09	1.00000000	mice
Atg13	-0.16	1.00000000	mice
Polr2l	0.08	1.00000000	mice
Sntb1	0.18	1.00000000	mice
Inppl1	-0.02	1.00000000	mice
Zfp281	0.02	1.00000000	mice
Cnot8	-0.01	1.00000000	mice
Stk38l	0.14	1.00000000	mice
Extl3	0.01	1.00000000	mice
Polr3a	0.06	1.00000000	mice
Dnajc11	-0.03	1.00000000	mice
P4ha1	0.05	1.00000000	mice
Dus3l	-0.01	1.00000000	mice
Asph	0.14	1.00000000	mice
Prr5l	-0.03	1.00000000	mice
Ccdc88a	0.36	1.00000000	mice
Got1	0.01	1.00000000	mice
Rpp25l	0.04	1.00000000	mice
Tex14	0.48	1.00000000	mice
Eif4e2	0.07	1.00000000	mice
Zfp318	-0.03	1.00000000	mice
Prmt5	-0.11	1.00000000	mice
5730480H06Rik	-0.04	1.00000000	mice
Tsr1	-0.05	1.00000000	mice
Smim8	0.04	1.00000000	mice
Tbp	0.00	1.00000000	mice
Sdf4	-0.03	1.00000000	mice
Ube2j1	-0.05	1.00000000	mice
Slc38a10	0.00	1.00000000	mice
Nab2	-0.06	1.00000000	mice
Ubqln1	-0.06	1.00000000	mice
Lrrc59	-0.03	1.00000000	mice
Ltbr	0.00	1.00000000	mice
Pdlim7	0.01	1.00000000	mice
Rnf111	0.10	1.00000000	mice
Acyp2	-0.13	1.00000000	mice
Ulk2	-0.09	1.00000000	mice
Apba1	-0.32	1.00000000	mice
Csnk1e	0.02	1.00000000	mice
Rbks	-0.05	1.00000000	mice
Iws1	-0.03	1.00000000	mice
Ankrd12	-0.14	1.00000000	mice
Pgls	0.17	1.00000000	mice
Cept1	0.00	1.00000000	mice
Zfp451	0.01	1.00000000	mice
Klhdc10	-0.18	1.00000000	mice

Spsb1	-0.13	1.00000000	mice
Grb2	0.06	1.00000000	mice
Lrrc14	-0.02	1.00000000	mice
Vps28	0.00	1.00000000	mice
Prkcb	0.35	1.00000000	mice
Kansl2	0.00	1.00000000	mice
Eif4e3	-0.02	1.00000000	mice
Sipa1l1	0.25	1.00000000	mice
Tm7sf3	0.02	1.00000000	mice
Kmt2c	-0.05	1.00000000	mice
Smarcb1	-0.02	1.00000000	mice
Clptm1l	-0.05	1.00000000	mice
Borcs6	0.01	1.00000000	mice
Fbxo8	-0.15	1.00000000	mice
Carnmt1	-0.02	1.00000000	mice
Retreg2	-0.07	1.00000000	mice
Lmo4	0.09	1.00000000	mice
Rpe	-0.06	1.00000000	mice
Trp53i13	-0.14	1.00000000	mice
Oser1	-0.04	1.00000000	mice
Sec14l1	0.00	1.00000000	mice
Cep104	-0.05	1.00000000	mice
Cox6a2	0.24	1.00000000	mice
Abhd11	-0.02	1.00000000	mice
Dcakd	0.00	1.00000000	mice
Opa1	-0.12	1.00000000	mice
Nudt5	-0.06	1.00000000	mice
Stip1	0.00	1.00000000	mice
Birc6	0.00	1.00000000	mice
Oaz2	-0.03	1.00000000	mice
Dhx15	0.05	1.00000000	mice
Lztfl1	0.05	1.00000000	mice
Pgrmc2	-0.01	1.00000000	mice
Kctd5	0.00	1.00000000	mice
Prrc1	-0.02	1.00000000	mice
Srebf1	-0.01	1.00000000	mice
Tmx2	-0.03	1.00000000	mice
Uba5	-0.03	1.00000000	mice
Cgrrf1	-0.18	1.00000000	mice
Tssc4	-0.03	1.00000000	mice
Immt	0.07	1.00000000	mice
Usp47	-0.01	1.00000000	mice
Spryd7	-0.01	1.00000000	mice
Dpy19l3	0.05	1.00000000	mice
Zfp958	-0.02	1.00000000	mice
Nfkbid	-0.08	1.00000000	mice

Usp33	-0.07	1.00000000	mice
Rbm33	-0.02	1.00000000	mice
Wdr53	0.01	1.00000000	mice
Haus6	-0.01	1.00000000	mice
Naa50	-0.16	1.00000000	mice
Rnf146	-0.09	1.00000000	mice
Gins4	0.01	1.00000000	mice
Spata2	0.02	1.00000000	mice
Taf4	-0.12	1.00000000	mice
Fastk	0.02	1.00000000	mice
Rabep1	0.01	1.00000000	mice
Sf3b4	-0.10	1.00000000	mice
Oga	0.00	1.00000000	mice
Agpat5	0.01	1.00000000	mice
Sh2b3	-0.05	1.00000000	mice
Havcr1	0.12	1.00000000	mice
Vps26a	-0.08	1.00000000	mice
Nr3c1	0.28	1.00000000	mice
Fam234b	0.05	1.00000000	mice
Lemd2	-0.01	1.00000000	mice
2410002F23Rik	-0.05	1.00000000	mice
Snrpd3	0.04	1.00000000	mice
Raly	0.04	1.00000000	mice
Gpatch11	-0.02	1.00000000	mice
Ehf	-0.17	1.00000000	mice
Thbs1	-0.02	1.00000000	mice
Atic	-0.03	1.00000000	mice
Pmf1	-0.06	1.00000000	mice
Flnb	0.10	1.00000000	mice
D8Ertd738e	-0.01	1.00000000	mice
Ids	-0.06	1.00000000	mice
Fam162a	-0.06	1.00000000	mice
Ralgapb	0.11	1.00000000	mice
Timm23	0.04	1.00000000	mice
Ap1s2	-0.02	1.00000000	mice
Ndufa10	0.05	1.00000000	mice
Eif3j2	-0.03	1.00000000	mice
Commd1	0.02	1.00000000	mice
Zfp740	-0.09	1.00000000	mice
Dazap2	-0.07	1.00000000	mice
Smim12	0.00	1.00000000	mice
Pnn	-0.11	1.00000000	mice
Sbf2	0.14	1.00000000	mice
Elmod2	0.04	1.00000000	mice
Gpatch4	0.01	1.00000000	mice
Hnrnpc	0.06	1.00000000	mice

Nfic	-0.10	1.00000000	mice
Dcaf10	-0.06	1.00000000	mice
Eri3	-0.01	1.00000000	mice
Fam50a	-0.02	1.00000000	mice
Rsf1os2	-0.03	1.00000000	mice
Epc2	-0.09	1.00000000	mice
Tmem185b	-0.04	1.00000000	mice
Pom121	0.02	1.00000000	mice
Arl6ip5	0.08	1.00000000	mice
Bahd1	0.02	1.00000000	mice
Zscan26	-0.05	1.00000000	mice
Dnajc21	-0.06	1.00000000	mice
Cep83	-0.09	1.00000000	mice
Tmem87b	-0.04	1.00000000	mice
Lsm3	-0.05	1.00000000	mice
Exoc6	-0.05	1.00000000	mice
Yipf5	-0.06	1.00000000	mice
Flot2	-0.06	1.00000000	mice
Apopt1	0.06	1.00000000	mice
Hif1a	-0.03	1.00000000	mice
Zbtb25	-0.06	1.00000000	mice
Kremen1	-0.05	1.00000000	mice
Rnf5	0.05	1.00000000	mice
Reep5	0.00	1.00000000	mice
Polr3d	0.00	1.00000000	mice
Rnf7	-0.06	1.00000000	mice
Clcn3	0.01	1.00000000	mice
Ippk	-0.04	1.00000000	mice
Chchd10	-0.09	1.00000000	mice
Kdm4b	-0.09	1.00000000	mice
Zfp282	-0.02	1.00000000	mice
Snhg3	0.02	1.00000000	mice
Pld4	0.39	1.00000000	mice
Tle5	-0.03	1.00000000	mice
Tcp11l1	0.08	1.00000000	mice
Tlnrd1	-0.15	1.00000000	mice
Parn	0.03	1.00000000	mice
Vamp4	-0.01	1.00000000	mice
Wee1	0.02	1.00000000	mice
1110059E24Rik	-0.04	1.00000000	mice
Stat5b	-0.10	1.00000000	mice
2210016L21Rik	-0.07	1.00000000	mice
Pa2g4	0.03	1.00000000	mice
Pcbd2	0.00	1.00000000	mice
Phactr2	0.05	1.00000000	mice
Chd1	-0.06	1.00000000	mice

Atrx	0.09	1.00000000	mice
Kansl1	-0.05	1.00000000	mice
Pop5	0.04	1.00000000	mice
Plet1	-0.02	1.00000000	mice
Cdk5rap2	-0.01	1.00000000	mice
G6pdx	0.02	1.00000000	mice
Lsm8	-0.08	1.00000000	mice
Nucb1	-0.10	1.00000000	mice
Nf1	-0.09	1.00000000	mice
Klhl42	-0.11	1.00000000	mice
Wdr13	0.00	1.00000000	mice
Zc3h18	0.03	1.00000000	mice
Cwc22	0.07	1.00000000	mice
Atf2	-0.06	1.00000000	mice
Trim25	-0.15	1.00000000	mice
Ano10	0.00	1.00000000	mice
Polr2h	-0.08	1.00000000	mice
Dynlt1f	0.00	1.00000000	mice
Vac14	-0.02	1.00000000	mice
Ppp6c	-0.04	1.00000000	mice
Vps11	0.01	1.00000000	mice
Gdi1	0.02	1.00000000	mice
Nfya	0.11	1.00000000	mice
Camk2d	0.12	1.00000000	mice
Ankrd52	-0.02	1.00000000	mice
Nrp2	0.33	1.00000000	mice
Rev1	-0.04	1.00000000	mice
Spred1	0.03	1.00000000	mice
Klhl24	-0.13	1.00000000	mice
Tbl1x	-0.01	1.00000000	mice
Arl6ip6	0.07	1.00000000	mice
Fbxo9	0.01	1.00000000	mice
Kiz	0.01	1.00000000	mice
Elp1	0.00	1.00000000	mice
Uba2	0.00	1.00000000	mice
Usp5	-0.01	1.00000000	mice
Tmem51	0.16	1.00000000	mice
Kif9	0.00	1.00000000	mice
Prkd3	0.00	1.00000000	mice
Saraf	-0.06	1.00000000	mice
Casc3	-0.09	1.00000000	mice
Lig3	0.00	1.00000000	mice
Csf2rb2	-0.03	1.00000000	mice
Cep70	-0.04	1.00000000	mice
Sh3gl1	0.09	1.00000000	mice
Ccdc43	-0.02	1.00000000	mice

Sbds	-0.08	1.00000000	mice
Farsa	-0.02	1.00000000	mice
Ppp2r3d	-0.02	1.00000000	mice
Fam13b	0.03	1.00000000	mice
Zfp322a	-0.02	1.00000000	mice
Atp2a3	0.13	1.00000000	mice
Urb2	0.04	1.00000000	mice
Eif2a	-0.14	1.00000000	mice
Ighm	-0.08	1.00000000	mice
Cox8a	0.02	1.00000000	mice
Pgrmc1	-0.04	1.00000000	mice
Utp15	-0.09	1.00000000	mice
Lats1	0.03	1.00000000	mice
1110008P14Rik	-0.03	1.00000000	mice
Atg12	0.03	1.00000000	mice
Bccip	-0.08	1.00000000	mice
Pole4	0.02	1.00000000	mice
Jagn1	-0.05	1.00000000	mice
Cops2	0.06	1.00000000	mice
Ccdc28a	0.01	1.00000000	mice
Zbtb17	0.06	1.00000000	mice
Ly6d	0.38	1.00000000	mice
Abhd15	0.06	1.00000000	mice
Dctn3	-0.07	1.00000000	mice
Haao	0.05	1.00000000	mice
Nfs1	-0.04	1.00000000	mice
Bcl7c	0.01	1.00000000	mice
Trmt10a	-0.01	1.00000000	mice
Slc25a13	0.00	1.00000000	mice
Renbp	-0.03	1.00000000	mice
Ncoa1	0.15	1.00000000	mice
Prkci	0.00	1.00000000	mice
Arl6ip4	-0.02	1.00000000	mice
4930453N24Rik	-0.03	1.00000000	mice
Ptpn2	0.11	1.00000000	mice
Rpf1	0.02	1.00000000	mice
Zfp868	0.00	1.00000000	mice
Rbm10	-0.07	1.00000000	mice
Plekhg2	-0.05	1.00000000	mice
Chchd6	0.05	1.00000000	mice
Slc16a10	-0.19	1.00000000	mice
Ciao1	-0.05	1.00000000	mice
Adipor2	0.03	1.00000000	mice
Nek1	-0.01	1.00000000	mice
Nedd9	-0.07	1.00000000	mice
Acbd3	0.10	1.00000000	mice

Rbm17	0.03	1.00000000	mice
Drap1	0.04	1.00000000	mice
1600020E01Rik	-0.19	1.00000000	mice
Atp6v1h	0.02	1.00000000	mice
Taok1	-0.04	1.00000000	mice
Lsm6	-0.05	1.00000000	mice
P2rx4	0.14	1.00000000	mice
Tab3	0.02	1.00000000	mice
Coq9	0.01	1.00000000	mice
Zfp653	0.04	1.00000000	mice
Eea1	0.01	1.00000000	mice
Inip	-0.09	1.00000000	mice
Brix1	-0.06	1.00000000	mice
Clcc1	-0.05	1.00000000	mice
Specc1	0.48	1.00000000	mice
9930021J03Rik	0.10	1.00000000	mice
Cpsf7	0.05	1.00000000	mice
Wdr83	-0.01	1.00000000	mice
Cers5	-0.14	1.00000000	mice
Camk2g	-0.07	1.00000000	mice
Ccr1	0.06	1.00000000	mice
Zfp560	0.02	1.00000000	mice
Socs5	0.01	1.00000000	mice
Nr2c1	-0.02	1.00000000	mice
Pdcl3	0.03	1.00000000	mice
Rprd1b	-0.06	1.00000000	mice
Ubl3	0.00	1.00000000	mice
Sec61a2	-0.07	1.00000000	mice
Gnpda1	0.03	1.00000000	mice
Coro2a	0.07	1.00000000	mice
Tfam	-0.14	1.00000000	mice
Unc13d	0.01	1.00000000	mice
Wdr81	-0.06	1.00000000	mice
Rnf20	-0.04	1.00000000	mice
1810058I24Rik	0.01	1.00000000	mice
Zbtb24	0.00	1.00000000	mice
Cds2	0.00	1.00000000	mice
Ccdc85b	-0.01	1.00000000	mice
Arid4b	0.02	1.00000000	mice
Pou2f1	0.14	1.00000000	mice
Adam10	-0.12	1.00000000	mice
Tmem68	0.04	1.00000000	mice
Pcbp2	0.02	1.00000000	mice
Sap130	-0.07	1.00000000	mice
Vps37a	0.08	1.00000000	mice
Nxt1	-0.05	1.00000000	mice

Chordc1	-0.05	1.00000000	mice
Entpd7	0.05	1.00000000	mice
4833439L19Rik	0.03	1.00000000	mice
Acap2	-0.08	1.00000000	mice
Wdr75	0.03	1.00000000	mice
Ccdc82	0.02	1.00000000	mice
Scp2	-0.02	1.00000000	mice
Tmem109	-0.05	1.00000000	mice
Elavl1	0.09	1.00000000	mice
Chmp2b	0.01	1.00000000	mice
Clcn4	-0.04	1.00000000	mice
Rgs12	0.01	1.00000000	mice
Lman2l	0.00	1.00000000	mice
Trp53	0.02	1.00000000	mice
Cirbp	-0.07	1.00000000	mice
Cnppd1	0.04	1.00000000	mice
Gprasp1	-0.03	1.00000000	mice
Acot7	0.00	1.00000000	mice
Zscan29	0.02	1.00000000	mice
Safb	0.00	1.00000000	mice
Zfp148	-0.05	1.00000000	mice
Tomm6	-0.03	1.00000000	mice
Tnrc6a	-0.11	1.00000000	mice
Aim2	-0.12	1.00000000	mice
Dcaf6	-0.12	1.00000000	mice
Babam2	-0.09	1.00000000	mice
Lin37	0.02	1.00000000	mice
Pbx2	0.02	1.00000000	mice
Strip2	0.30	1.00000000	mice
Adam23	-0.63	1.00000000	mice
Cuedc2	0.01	1.00000000	mice
Crls1	-0.01	1.00000000	mice
Fbxw7	-0.23	1.00000000	mice
Galnt1	-0.04	1.00000000	mice
Sec22b	-0.09	1.00000000	mice
Cfl2	-0.08	1.00000000	mice
Zfp652	-0.08	1.00000000	mice
Fbxo7	0.02	1.00000000	mice
Tgif2	0.00	1.00000000	mice
Trappc13	-0.06	1.00000000	mice
Usp3	-0.01	1.00000000	mice
Kat6a	0.08	1.00000000	mice
Rbsn	0.00	1.00000000	mice
Wfs1	0.00	1.00000000	mice
Tnfaip1	0.03	1.00000000	mice
Ralgps1	0.09	1.00000000	mice

Rhbdd1	-0.08	1.00000000	mice
P2ry10b	-0.03	1.00000000	mice
Rars	0.02	1.00000000	mice
Taf10	-0.07	1.00000000	mice
Pkn1	0.06	1.00000000	mice
Peli2	-0.09	1.00000000	mice
Rhobtb2	0.04	1.00000000	mice
Gna12	-0.07	1.00000000	mice
Gpx1	-0.09	1.00000000	mice
Lipe	0.03	1.00000000	mice
Batf3	0.09	1.00000000	mice
Scly	-0.01	1.00000000	mice
3110040N11Rik	-0.01	1.00000000	mice
Ttc7	-0.09	1.00000000	mice
Gla	0.36	1.00000000	mice
Slc35b2	-0.03	1.00000000	mice
Aak1	-0.01	1.00000000	mice
Rab8b	-0.16	1.00000000	mice
Snrnp35	-0.06	1.00000000	mice
Zfp646	-0.03	1.00000000	mice
Proser1	-0.07	1.00000000	mice
Trim27	0.03	1.00000000	mice
Glg1	-0.06	1.00000000	mice
Polr2m	-0.03	1.00000000	mice
Pym1	0.00	1.00000000	mice
Tor1a	0.01	1.00000000	mice
Ulk1	-0.04	1.00000000	mice
Rrbp1	0.00	1.00000000	mice
Cnbp	-0.13	1.00000000	mice
Nhp2	-0.05	1.00000000	mice
Cacna1a	-0.24	1.00000000	mice
Adam9	-0.04	1.00000000	mice
Rab3ip	0.00	1.00000000	mice
Pithd1	-0.04	1.00000000	mice
Atp13a1	0.00	1.00000000	mice
Zdhhc21	-0.07	1.00000000	mice
Phf10	-0.07	1.00000000	mice
Specc1l	0.03	1.00000000	mice
Terf1	-0.04	1.00000000	mice
Slc26a11	-0.05	1.00000000	mice
Tceal8	-0.07	1.00000000	mice
Pign	0.05	1.00000000	mice
Coq5	0.01	1.00000000	mice
Hadha	-0.02	1.00000000	mice
Rif1	0.03	1.00000000	mice
Ccdc50	0.11	1.00000000	mice

Crybg3	-0.03	1.00000000	mice
Dapp1	0.00	1.00000000	mice
Rgs3	-0.01	1.00000000	mice
Slc7a7	-0.01	1.00000000	mice
Atad5	-0.03	1.00000000	mice
Fip1l1	0.02	1.00000000	mice
Unk	-0.10	1.00000000	mice
Pstpip1	0.03	1.00000000	mice
Acadsb	-0.03	1.00000000	mice
Plscr3	0.03	1.00000000	mice
Zfp384	0.00	1.00000000	mice
Ppp1r21	-0.03	1.00000000	mice
Pih1d1	-0.01	1.00000000	mice
Hjurp	-0.05	1.00000000	mice
Sept9	0.13	1.00000000	mice
Ranbp3	0.11	1.00000000	mice
Cnot9	0.00	1.00000000	mice
Auh	-0.09	1.00000000	mice
H2-Oa	-0.03	1.00000000	mice
Wdr83os	-0.02	1.00000000	mice
BC029722	0.00	1.00000000	mice
Eif3a	-0.05	1.00000000	mice
Ergic3	-0.06	1.00000000	mice
Sp2	-0.01	1.00000000	mice
Dpm1	0.01	1.00000000	mice
Nit2	-0.03	1.00000000	mice
Cd7	-0.22	1.00000000	mice
Ppp2r5e	0.02	1.00000000	mice
Tab2	-0.19	1.00000000	mice
Acaa2	-0.01	1.00000000	mice
Eif1ad	0.04	1.00000000	mice
Bap1	-0.01	1.00000000	mice
Utp23	-0.03	1.00000000	mice
Rbm41	0.01	1.00000000	mice
Rala	-0.11	1.00000000	mice
Tpgs2	-0.03	1.00000000	mice
Arhgap5	0.14	1.00000000	mice
Sh3bp2	-0.03	1.00000000	mice
Ptbp3	0.02	1.00000000	mice
Ptpmt1	0.01	1.00000000	mice
Dnajc17	-0.02	1.00000000	mice
Alkbh1	0.00	1.00000000	mice
Ralgds	0.09	1.00000000	mice
Srrt	-0.05	1.00000000	mice
Zfp65	-0.01	1.00000000	mice
Snrnp200	-0.04	1.00000000	mice

Phykpl	-0.03	1.00000000	mice
Ddost	0.05	1.00000000	mice
Zfp869	-0.04	1.00000000	mice
Tbc1d4	0.20	1.00000000	mice
Ptpa	-0.12	1.00000000	mice
Ifrd1	0.07	1.00000000	mice
4833407H14Rik	0.04	1.00000000	mice
Pik3ip1	-0.04	1.00000000	mice
Psph	-0.04	1.00000000	mice
Nup214	0.01	1.00000000	mice
Rgl1	-0.15	1.00000000	mice
Clk3	-0.02	1.00000000	mice
Tlcd2	0.01	1.00000000	mice
Shkbp1	-0.01	1.00000000	mice
Stat3	0.07	1.00000000	mice
Txlna	-0.07	1.00000000	mice
Smarcd1	-0.01	1.00000000	mice
Tlk2	-0.05	1.00000000	mice
Slc8b1	0.05	1.00000000	mice
Cpsf4	-0.13	1.00000000	mice
Blvrb	-0.05	1.00000000	mice
Jkamp	-0.02	1.00000000	mice
Galk2	-0.08	1.00000000	mice
Bzw1	-0.01	1.00000000	mice
Depdc7	0.07	1.00000000	mice
Clpp	0.01	1.00000000	mice
Zfp521	0.01	1.00000000	mice
Utrn	0.05	1.00000000	mice
Tnip2	-0.09	1.00000000	mice
Stard5	0.09	1.00000000	mice
Nfil3	0.03	1.00000000	mice
Slc41a2	0.35	1.00000000	mice
Arid1a	-0.03	1.00000000	mice
Tbc1d10a	0.03	1.00000000	mice
Efcab14	-0.11	1.00000000	mice
Sar1b	-0.09	1.00000000	mice
Gclc	-0.23	1.00000000	mice
Gart	-0.08	1.00000000	mice
Dnajc15	0.01	1.00000000	mice
Exosc5	-0.03	1.00000000	mice
Clpb	-0.02	1.00000000	mice
Stk24	-0.14	1.00000000	mice
Polr2b	-0.10	1.00000000	mice
Dbr1	-0.05	1.00000000	mice
Cnep1r1	-0.02	1.00000000	mice
Dhx29	-0.05	1.00000000	mice

Tceal9	0.01	1.00000000	mice
Rnf220	-0.63	1.00000000	mice
Ctdnep1	-0.05	1.00000000	mice
Arl8b	-0.10	1.00000000	mice
Phf8	0.07	1.00000000	mice
Tmem230	0.02	1.00000000	mice
Txnip	0.17	1.00000000	mice
Lysmd3	-0.01	1.00000000	mice
Uhrf1bp1	0.01	1.00000000	mice
Nudt9	0.04	1.00000000	mice
Sec23ip	-0.07	1.00000000	mice
Rrnad1	-0.02	1.00000000	mice
Smim7	-0.01	1.00000000	mice
Aar2	0.01	1.00000000	mice
Galnt6	-0.11	1.00000000	mice
Tomm5	0.00	1.00000000	mice
Gtf2h2	0.03	1.00000000	mice
Ahsa1	-0.17	1.00000000	mice
Selenoh	0.03	1.00000000	mice
Fnta	-0.13	1.00000000	mice
Zc3h7a	0.00	1.00000000	mice
Ttc13	0.01	1.00000000	mice
Parp6	0.01	1.00000000	mice
H2afy	0.10	1.00000000	mice
Riok1	0.00	1.00000000	mice
1700017B05Rik	0.11	1.00000000	mice
Sars	0.04	1.00000000	mice
Timm8b	-0.04	1.00000000	mice
Ube2o	-0.01	1.00000000	mice
Tmem259	0.00	1.00000000	mice
Ogfod1	-0.01	1.00000000	mice
Zbtb33	-0.05	1.00000000	mice
Ndufb4	-0.04	1.00000000	mice
Ell	0.01	1.00000000	mice
Nfrkb	-0.09	1.00000000	mice
Cyp4f13	0.04	1.00000000	mice
Cabin1	-0.07	1.00000000	mice
6720427I07Rik	0.07	1.00000000	mice
Hpcal1	0.00	1.00000000	mice
Lmbrd2	-0.08	1.00000000	mice
Hip1r	-0.10	1.00000000	mice
Exosc7	0.00	1.00000000	mice
Kars	-0.08	1.00000000	mice
Nup205	-0.01	1.00000000	mice
Ppp6r3	-0.01	1.00000000	mice
Hnrnpab	-0.07	1.00000000	mice

Ptch1	-0.03	1.00000000	mice
Stau2	-0.05	1.00000000	mice
Ube3c	-0.09	1.00000000	mice
Ndufa8	-0.01	1.00000000	mice
Bloc1s6	0.04	1.00000000	mice
Clk1	-0.04	1.00000000	mice
Rhoq	-0.11	1.00000000	mice
Riok2	0.07	1.00000000	mice
Ip6k1	-0.10	1.00000000	mice
Oard1	-0.03	1.00000000	mice
2510009E07Rik	0.06	1.00000000	mice
Cdc14b	-0.06	1.00000000	mice
Ddx21	0.02	1.00000000	mice
Gpn2	-0.01	1.00000000	mice
Agfg1	-0.11	1.00000000	mice
Eif2s3x	-0.04	1.00000000	mice
Sesn3	-0.10	1.00000000	mice
Samd8	-0.09	1.00000000	mice
Ccnh	-0.05	1.00000000	mice
Pigu	-0.02	1.00000000	mice
Golga1	-0.05	1.00000000	mice
Gria3	-0.06	1.00000000	mice
Slc25a19	0.01	1.00000000	mice
Ice1	-0.10	1.00000000	mice
Tcerg1	-0.01	1.00000000	mice
Pibf1	-0.09	1.00000000	mice
Cep63	-0.04	1.00000000	mice
Chchd7	0.00	1.00000000	mice
Trp53bp1	-0.02	1.00000000	mice
Rnf19b	-0.06	1.00000000	mice
Ercc5	-0.04	1.00000000	mice
Prkcsh	0.01	1.00000000	mice
Clasp1	-0.07	1.00000000	mice
Vps37b	-0.07	1.00000000	mice
Bud23	-0.05	1.00000000	mice
Tada1	0.01	1.00000000	mice
Hadhb	0.00	1.00000000	mice
Nucb2	0.13	1.00000000	mice
Epha2	0.10	1.00000000	mice
Nr6a1	-0.19	1.00000000	mice
Tnks2	0.03	1.00000000	mice
Ncbp1	-0.02	1.00000000	mice
Adnp	0.02	1.00000000	mice
Taok3	-0.17	1.00000000	mice
Creb1	-0.09	1.00000000	mice
D10Wsu102e	-0.23	1.00000000	mice

Gem	0.13	1.00000000	mice
Ttl	-0.01	1.00000000	mice
Usp48	-0.09	1.00000000	mice
Rnf167	-0.03	1.00000000	mice
Tgif1	0.13	1.00000000	mice
Zhx2	0.15	1.00000000	mice
Trmt112	0.03	1.00000000	mice
Ift172	-0.07	1.00000000	mice
Slc25a46	-0.04	1.00000000	mice
Lclat1	-0.10	1.00000000	mice
Zfyve27	-0.01	1.00000000	mice
Lrrc8c	-0.07	1.00000000	mice
Cds1	-0.09	1.00000000	mice
Rab40c	-0.05	1.00000000	mice
Sh3bp1	0.02	1.00000000	mice
Smim13	0.01	1.00000000	mice
Pim3	-0.05	1.00000000	mice
Tyk2	-0.01	1.00000000	mice
Nudt21	0.00	1.00000000	mice
Ngly1	-0.11	1.00000000	mice
Prr13	0.04	1.00000000	mice
Btk	-0.12	1.00000000	mice
Fbxl6	-0.11	1.00000000	mice
Slc7a6os	-0.01	1.00000000	mice
Sec16a	-0.02	1.00000000	mice
Lpcat3	-0.13	1.00000000	mice
Ubr3	-0.09	1.00000000	mice
Alkbh3	-0.03	1.00000000	mice
Eny2	-0.12	1.00000000	mice
Axin1	-0.06	1.00000000	mice
Eepd1	-0.06	1.00000000	mice
Ddx55	-0.03	1.00000000	mice
Dnttip2	-0.10	1.00000000	mice
Slc16a6	0.04	1.00000000	mice
Alg9	-0.01	1.00000000	mice
Sel1l	0.06	1.00000000	mice
Pls3	-0.03	1.00000000	mice
Ptprcap	0.00	1.00000000	mice
Arhgef7	-0.06	1.00000000	mice
Acaca	-0.16	1.00000000	mice
Atp1b3	0.11	1.00000000	mice
Tec	-0.09	1.00000000	mice
Diaph1	-0.02	1.00000000	mice
Ddhd1	-0.11	1.00000000	mice
Rnpc3	0.01	1.00000000	mice
Plppr4	0.35	1.00000000	mice

Kifc3	-0.13	1.00000000	mice
Pex19	-0.04	1.00000000	mice
Dhrs4	0.02	1.00000000	mice
Tpp1	-0.03	1.00000000	mice
Nifk	-0.01	1.00000000	mice
Lsg1	-0.07	1.00000000	mice
Slc25a30	-0.05	1.00000000	mice
Por	0.03	1.00000000	mice
Atp5f1	0.00	1.00000000	mice
Ogdh	-0.13	1.00000000	mice
Slc25a39	-0.01	1.00000000	mice
Iqsec2	0.09	1.00000000	mice
Zfp422	-0.03	1.00000000	mice
Lrrc25	0.01	1.00000000	mice
Usf2	0.02	1.00000000	mice
Jpx	-0.07	1.00000000	mice
Nfe2l2	-0.04	1.00000000	mice
4932438H23Rik	0.05	1.00000000	mice
Rraga	-0.06	1.00000000	mice
Cfdp1	-0.04	1.00000000	mice
Pdlim2	0.02	1.00000000	mice
Wdr26	-0.09	1.00000000	mice
Csde1	-0.03	1.00000000	mice
Tmem208	-0.03	1.00000000	mice
E130307A14Rik	0.04	1.00000000	mice
Klc1	-0.01	1.00000000	mice
Tet3	-0.05	1.00000000	mice
Wrnip1	0.00	1.00000000	mice
Ubp1	-0.03	1.00000000	mice
Pld2	-0.02	1.00000000	mice
S1pr4	0.05	1.00000000	mice
Helz	0.09	1.00000000	mice
Ostc	0.05	1.00000000	mice
Ap1m1	-0.05	1.00000000	mice
Ipmk	0.08	1.00000000	mice
Foxj2	0.06	1.00000000	mice
Trim24	-0.06	1.00000000	mice
Ypel2	-0.07	1.00000000	mice
Dync1li2	-0.02	1.00000000	mice
Pafah1b2	-0.05	1.00000000	mice
Zzz3	0.03	1.00000000	mice
Ndufs2	0.01	1.00000000	mice
Pdgfa	0.07	1.00000000	mice
Stxbp5	-0.11	1.00000000	mice
Sidt2	-0.06	1.00000000	mice
Tmem87a	-0.07	1.00000000	mice

Ptpn4	0.04	1.00000000	mice
Ubap2l	-0.04	1.00000000	mice
Nkapd1	0.03	1.00000000	mice
Ube2v2	0.05	1.00000000	mice
Ankrd17	0.00	1.00000000	mice
Nrf1	-0.23	1.00000000	mice
Prpsap1	-0.02	1.00000000	mice
Ndufa5	0.01	1.00000000	mice
Emc7	0.01	1.00000000	mice
Ppp1r15a	0.01	1.00000000	mice
Taf1b	-0.09	1.00000000	mice
Ube2d1	0.04	1.00000000	mice
Sec22a	0.03	1.00000000	mice
Srcap	0.12	1.00000000	mice
Abhd6	-0.07	1.00000000	mice
Agtpbp1	0.07	1.00000000	mice
Csrnp1	0.15	1.00000000	mice
Ppp2cb	-0.10	1.00000000	mice
Gpr141	0.17	1.00000000	mice
Vps16	-0.03	1.00000000	mice
Lsm4	0.00	1.00000000	mice
Arhgap39	0.01	1.00000000	mice
Rnf166	-0.07	1.00000000	mice
Shb	0.24	1.00000000	mice
Ralgapa2	0.05	1.00000000	mice
Ccdc86	-0.03	1.00000000	mice
Nin	-0.10	1.00000000	mice
Hnrnpf	-0.09	1.00000000	mice
Rprd2	-0.04	1.00000000	mice
Cog5	-0.04	1.00000000	mice
St6galnac6	-0.01	1.00000000	mice
Pde4d	0.01	1.00000000	mice
Znrf3	-0.11	1.00000000	mice
Ifnar2	0.15	1.00000000	mice
Adprhl2	0.01	1.00000000	mice
Phc1	0.03	1.00000000	mice
Fus	-0.03	1.00000000	mice
Gsk3b	-0.15	1.00000000	mice
Tbce	-0.05	1.00000000	mice
Bub3	0.01	1.00000000	mice
Snrpa1	-0.10	1.00000000	mice
Atxn2l	-0.07	1.00000000	mice
Insig1	0.06	1.00000000	mice
Osbpl3	0.03	1.00000000	mice
Cemip2	0.07	1.00000000	mice
Nagpa	-0.01	1.00000000	mice

Lefty1	-0.06	1.00000000	mice
Gpaa1	0.00	1.00000000	mice
Vhl	-0.02	1.00000000	mice
Pygb	-0.01	1.00000000	mice
Wiz	-0.01	1.00000000	mice
Strn4	0.01	1.00000000	mice
Ftl1	0.14	1.00000000	mice
BC035044	0.12	1.00000000	mice
Lrrc47	-0.07	1.00000000	mice
Lrrc28	0.01	1.00000000	mice
Tsr2	0.00	1.00000000	mice
Clip2	0.01	1.00000000	mice
Atr	-0.01	1.00000000	mice
Hsd17b4	-0.03	1.00000000	mice
Blzf1	-0.02	1.00000000	mice
Pdia4	0.06	1.00000000	mice
Tbc1d16	0.02	1.00000000	mice
Tmem214	0.02	1.00000000	mice
Galnt11	0.01	1.00000000	mice
Ccnyl1	0.05	1.00000000	mice
Siglech	-0.06	1.00000000	mice
Nob1	-0.03	1.00000000	mice
Abca5	-0.04	1.00000000	mice
Lias	-0.01	1.00000000	mice
Actg1	0.08	1.00000000	mice
Ap1g1	-0.14	1.00000000	mice
Ywhag	-0.04	1.00000000	mice
Gfer	-0.04	1.00000000	mice
Czib	0.00	1.00000000	mice
Rbms2	0.02	1.00000000	mice
Ranbp1	0.09	1.00000000	mice
H2afz	0.02	1.00000000	mice
Cstf2	-0.02	1.00000000	mice
Szrd1	-0.03	1.00000000	mice
Fos	-0.02	1.00000000	mice
Prps1	-0.01	1.00000000	mice
Ubap2	-0.13	1.00000000	mice
Anxa7	0.00	1.00000000	mice
Kif16b	0.15	1.00000000	mice
Gabarapl2	0.03	1.00000000	mice
Rb1	-0.14	1.00000000	mice
Atp5c1	0.04	1.00000000	mice
Rbbp4	-0.03	1.00000000	mice
Bag5	0.00	1.00000000	mice
Amz2	-0.09	1.00000000	mice
Sarnp	0.04	1.00000000	mice

Ubr4	0.07	1.00000000	mice
Dnajb9	-0.06	1.00000000	mice
Dgcr8	-0.01	1.00000000	mice
Ralb	-0.10	1.00000000	mice
Pou2f2	-0.02	1.00000000	mice
Rnf103	-0.06	1.00000000	mice
Zmpste24	-0.01	1.00000000	mice
Zswim7	-0.03	1.00000000	mice
Selenos	-0.04	1.00000000	mice
Fyb	0.03	1.00000000	mice
Prdm1	-0.13	1.00000000	mice
B4galt6	0.00	1.00000000	mice
Aen	-0.01	1.00000000	mice
Eif5b	-0.02	1.00000000	mice
Tnip1	-0.13	1.00000000	mice
R3hdm4	-0.05	1.00000000	mice
Timm50	0.00	1.00000000	mice
Zfp512	-0.02	1.00000000	mice
Snf8	-0.02	1.00000000	mice
Asxl2	0.02	1.00000000	mice
Agk	-0.15	1.00000000	mice
Elovl5	-0.02	1.00000000	mice
Asap1	0.26	1.00000000	mice
Slc39a14	0.05	1.00000000	mice
Pja2	-0.04	1.00000000	mice
Gps1	-0.01	1.00000000	mice
Fam53a	-0.03	1.00000000	mice
Twistnb	0.01	1.00000000	mice
Cct2	-0.04	1.00000000	mice
Polr2e	0.08	1.00000000	mice
Pigx	0.03	1.00000000	mice
Cyp4f16	0.05	1.00000000	mice
Lars2	0.11	1.00000000	mice
Polk	0.04	1.00000000	mice
Lpcat1	0.03	1.00000000	mice
Fbxo30	0.04	1.00000000	mice
Cmc2	0.11	1.00000000	mice
Ufc1	-0.06	1.00000000	mice
Wac	-0.09	1.00000000	mice
Gfm2	0.00	1.00000000	mice
Rgs1	0.87	1.00000000	mice
Ube2z	0.06	1.00000000	mice
As3mt	0.02	1.00000000	mice
Fzr1	0.00	1.00000000	mice
Gls	-0.01	1.00000000	mice
Tmf1	0.01	1.00000000	mice

Dimt1	0.02	1.00000000	mice
Zkscan3	0.01	1.00000000	mice
Nemf	0.07	1.00000000	mice
Necap1	-0.07	1.00000000	mice
Larp1b	-0.21	1.00000000	mice
Haus2	0.03	1.00000000	mice
Nisch	-0.06	1.00000000	mice
Gse1	0.14	1.00000000	mice
Prpf4	0.01	1.00000000	mice
Zfp518a	-0.05	1.00000000	mice
Grk3	-0.06	1.00000000	mice
Dbf4	0.02	1.00000000	mice
Abcb7	0.03	1.00000000	mice
Rabl3	0.02	1.00000000	mice
Cdc42ep4	-0.05	1.00000000	mice
Rragd	0.01	1.00000000	mice
Rab28	-0.02	1.00000000	mice
Il1a	-0.08	1.00000000	mice
Senp7	-0.07	1.00000000	mice
Pdcl	0.01	1.00000000	mice
Lcmt1	-0.03	1.00000000	mice
Rhno1	-0.03	1.00000000	mice
Gatd3a	-0.02	1.00000000	mice
Akap7	0.00	1.00000000	mice
Gskip	-0.17	1.00000000	mice
Hps3	0.03	1.00000000	mice
Rap1gap2	0.05	1.00000000	mice
Usp10	-0.07	1.00000000	mice
Itpripl2	0.01	1.00000000	mice
Aldh1b1	0.01	1.00000000	mice
Kmt5a	-0.02	1.00000000	mice
Fbrsl1	-0.04	1.00000000	mice
Heatr1	-0.05	1.00000000	mice
Ino80e	-0.02	1.00000000	mice
Lrpap1	-0.01	1.00000000	mice
Flii	0.03	1.00000000	mice
Hira	-0.10	1.00000000	mice
Sh3rf1	-0.19	1.00000000	mice
Atp1a1	0.04	1.00000000	mice
Zfp511	-0.02	1.00000000	mice
Vma21	-0.07	1.00000000	mice
Rad51d	-0.02	1.00000000	mice
Cnot4	0.07	1.00000000	mice
Ndufb8	0.00	1.00000000	mice
Spata32	0.07	1.00000000	mice
2810013P06Rik	-0.03	1.00000000	mice

Ipo9	0.01	1.00000000	mice
Akr1b3	-0.02	1.00000000	mice
Sirt2	0.01	1.00000000	mice
0610012G03Rik	-0.03	1.00000000	mice
Hprt	0.00	1.00000000	mice
Zfp746	-0.05	1.00000000	mice
Pola2	-0.05	1.00000000	mice
Sertad1	0.02	1.00000000	mice
Akap8	-0.08	1.00000000	mice
Hspe1	-0.03	1.00000000	mice
Wbp2	-0.03	1.00000000	mice
Cwc27	-0.13	1.00000000	mice
Rlf	-0.17	1.00000000	mice
Sec24b	0.03	1.00000000	mice
Calcrl	0.01	1.00000000	mice
Eif2b3	-0.07	1.00000000	mice
Gtpbp6	0.00	1.00000000	mice
Vsir	-0.05	1.00000000	mice
Rxylt1	-0.03	1.00000000	mice
Zdhhc9	-0.01	1.00000000	mice
Tmem9b	-0.08	1.00000000	mice
Smg7	-0.12	1.00000000	mice
Trak2	-0.06	1.00000000	mice
Thada	0.14	1.00000000	mice
Socs4	0.00	1.00000000	mice
Raet1e	-0.05	1.00000000	mice
Wdfy1	-0.01	1.00000000	mice
Cry1	-0.06	1.00000000	mice
Plcb2	0.00	1.00000000	mice
Ranbp9	0.20	1.00000000	mice
Etf1	-0.03	1.00000000	mice
Wdpcp	-0.13	1.00000000	mice
Lmbr1l	-0.02	1.00000000	mice
Itgax	-0.16	1.00000000	mice
Kat5	0.03	1.00000000	mice
Tfe3	-0.12	1.00000000	mice
Klf8	-0.05	1.00000000	mice
Usp39	0.04	1.00000000	mice
Tent2	0.01	1.00000000	mice
Abcf2	-0.04	1.00000000	mice
Pcca	0.05	1.00000000	mice
Phlpp1	0.09	1.00000000	mice
Rnf123	-0.01	1.00000000	mice
2610037D02Rik	-0.03	1.00000000	mice
Qsox1	0.05	1.00000000	mice
Hilpda	-0.26	1.00000000	mice

Crlf3	0.08	1.00000000	mice
Amd1	-0.01	1.00000000	mice
Fam149b	0.01	1.00000000	mice
Ero1l	-0.14	1.00000000	mice
Slc25a28	0.01	1.00000000	mice
Pdpr	0.02	1.00000000	mice
Txndc5	0.16	1.00000000	mice
Tbc1d1	-0.14	1.00000000	mice
Pik3cb	-0.09	1.00000000	mice
Psme4	0.02	1.00000000	mice
Akt1	-0.10	1.00000000	mice
Ncoa2	0.13	1.00000000	mice
Ash1l	-0.01	1.00000000	mice
Rab11b	-0.02	1.00000000	mice
Kif1c	-0.03	1.00000000	mice
Prickle3	0.00	1.00000000	mice
Hsd17b12	-0.01	1.00000000	mice
Uri1	-0.03	1.00000000	mice
Spg7	-0.07	1.00000000	mice
Unc119	0.04	1.00000000	mice
Ube2g1	-0.06	1.00000000	mice
Slc25a44	-0.16	1.00000000	mice
Nbeal1	0.02	1.00000000	mice
Unc93b1	-0.02	1.00000000	mice
Zfp617	-0.12	1.00000000	mice
Apex2	-0.01	1.00000000	mice
Dffa	-0.01	1.00000000	mice
Ephb2	0.02	1.00000000	mice
Arhgap31	0.12	1.00000000	mice
Eif3j1	-0.06	1.00000000	mice
Nmt2	0.01	1.00000000	mice
Uimc1	-0.05	1.00000000	mice
Dnajc30	-0.02	1.00000000	mice
Tcof1	-0.04	1.00000000	mice
Rnf121	-0.03	1.00000000	mice
Tmem164	-0.02	1.00000000	mice
Rnf122	0.06	1.00000000	mice
Hnrnph1	-0.12	1.00000000	mice
Rnase4	0.01	1.00000000	mice
Znhit6	-0.01	1.00000000	mice
Sgpp1	0.00	1.00000000	mice
Atpif1	0.07	1.00000000	mice
Zfp654	0.01	1.00000000	mice
Ppp1r12c	-0.02	1.00000000	mice
Lsm7	-0.08	1.00000000	mice
Tubgcp3	0.16	1.00000000	mice

Iglc3	0.16	1.00000000	mice
Zfp574	-0.04	1.00000000	mice
Dbndd2	0.00	1.00000000	mice
Wdr48	-0.06	1.00000000	mice
N4bp3	0.08	1.00000000	mice
Odr4	0.01	1.00000000	mice
Nod2	0.04	1.00000000	mice
Cluap1	-0.06	1.00000000	mice
Dapk3	0.00	1.00000000	mice
Bcas2	0.04	1.00000000	mice
Ss18	0.01	1.00000000	mice
Gtpbp4	0.00	1.00000000	mice
Stk25	-0.01	1.00000000	mice
Cct7	0.00	1.00000000	mice
Gemin5	0.05	1.00000000	mice
Smim19	-0.12	1.00000000	mice
S100a11	0.24	1.00000000	mice
Rab1a	-0.02	1.00000000	mice
Zfp950	-0.04	1.00000000	mice
Pip4p1	-0.09	1.00000000	mice
Zmym2	-0.15	1.00000000	mice
Cd38	0.02	1.00000000	mice
Samm50	0.06	1.00000000	mice
Tm2d2	-0.01	1.00000000	mice
Kras	0.08	1.00000000	mice
Tex264	-0.02	1.00000000	mice
Rab11fip3	-0.07	1.00000000	mice
Napa	-0.01	1.00000000	mice
Rassf5	-0.08	1.00000000	mice
Setx	-0.02	1.00000000	mice
Snx30	0.12	1.00000000	mice
Ppat	-0.07	1.00000000	mice
Lypla1	-0.01	1.00000000	mice
Tprgl	-0.04	1.00000000	mice
Tmed5	0.06	1.00000000	mice
Icosl	0.00	1.00000000	mice
Coq10a	0.11	1.00000000	mice
Bcl3	0.10	1.00000000	mice
Ccdc93	0.02	1.00000000	mice
Clpx	-0.01	1.00000000	mice
Tep1	0.00	1.00000000	mice
Arl4a	-0.01	1.00000000	mice
Tmem50b	-0.03	1.00000000	mice
Atg7	0.02	1.00000000	mice
Tra2a	0.16	1.00000000	mice
Fam89b	-0.02	1.00000000	mice

Clock	-0.13	1.00000000	mice
Vav2	0.06	1.00000000	mice
Snupn	0.02	1.00000000	mice
Rad52	-0.04	1.00000000	mice
E2f3	-0.05	1.00000000	mice
Cdc42bpb	0.03	1.00000000	mice
Rad51b	0.13	1.00000000	mice
Ccnc	-0.01	1.00000000	mice
Rrp1b	-0.02	1.00000000	mice
Vps54	0.05	1.00000000	mice
Eml4	0.06	1.00000000	mice
Tra2b	-0.01	1.00000000	mice
Pou6f1	-0.04	1.00000000	mice
Ehbp1l1	-0.14	1.00000000	mice
Timm10	-0.05	1.00000000	mice
Zfp142	-0.03	1.00000000	mice
Leo1	-0.02	1.00000000	mice
Zfp212	-0.01	1.00000000	mice
Lmnb1	0.00	1.00000000	mice
Tubgcp5	0.26	1.00000000	mice
Hypk	0.01	1.00000000	mice
Trp53inp1	0.23	1.00000000	mice
Il4ra	0.10	1.00000000	mice
Rbm45	-0.07	1.00000000	mice
Gpr55	0.02	1.00000000	mice
Kdm3a	0.02	1.00000000	mice
Lilr4b	0.05	1.00000000	mice
Ppard	-0.03	1.00000000	mice
Zcchc7	0.09	1.00000000	mice
Cdk7	-0.02	1.00000000	mice
Brd9	-0.01	1.00000000	mice
Fbxl3	-0.01	1.00000000	mice
Htr7	-0.23	1.00000000	mice
Inpp4b	0.13	1.00000000	mice
Rnf14	-0.03	1.00000000	mice
Cbl	-0.04	1.00000000	mice
Nedd1	-0.09	1.00000000	mice
Smarcad1	-0.02	1.00000000	mice
Hnrnpr	0.01	1.00000000	mice
Stat5a	0.07	1.00000000	mice
Pcbp1	0.03	1.00000000	mice
Supt20	-0.06	1.00000000	mice
Pon2	0.06	1.00000000	mice
Uspl1	-0.03	1.00000000	mice
4933423P22Rik	0.00	1.00000000	mice
Synrg	0.00	1.00000000	mice

Zfp68	0.01	1.00000000	mice
Ddx50	-0.03	1.00000000	mice
Ywhaz	-0.04	1.00000000	mice
Rmnd5a	-0.08	1.00000000	mice
Pcnx3	-0.15	1.00000000	mice
Cactin	0.00	1.00000000	mice
Casd1	-0.05	1.00000000	mice
Pip5k1b	-0.19	1.00000000	mice
Brox	0.00	1.00000000	mice
Tmtc3	-0.03	1.00000000	mice
Gpr65	-0.09	1.00000000	mice
Scd2	0.00	1.00000000	mice
Vasp	0.02	1.00000000	mice
Csnk1a1	-0.05	1.00000000	mice
Cldnd1	-0.08	1.00000000	mice
Slc35c2	-0.10	1.00000000	mice
Snx14	-0.02	1.00000000	mice
Ddx54	0.02	1.00000000	mice
Ppm1e	-0.08	1.00000000	mice
Atxn2	0.06	1.00000000	mice
Casp6	0.00	1.00000000	mice
Rtca	0.02	1.00000000	mice
Slc35a3	0.02	1.00000000	mice
Spidr	0.02	1.00000000	mice
5430405H02Rik	-0.02	1.00000000	mice
Zfp292	-0.11	1.00000000	mice
Bet1l	0.04	1.00000000	mice
Cyld	-0.06	1.00000000	mice
Cotl1	-0.10	1.00000000	mice
Kif3b	0.03	1.00000000	mice
Ccar1	-0.02	1.00000000	mice
Dusp3	0.05	1.00000000	mice
Ppp2r1a	-0.12	1.00000000	mice
Kidins220	0.06	1.00000000	mice
Borcs5	-0.02	1.00000000	mice
Ubb	0.02	1.00000000	mice
Snap23	-0.02	1.00000000	mice
Lamtor2	-0.03	1.00000000	mice
Fgd2	-0.05	1.00000000	mice
Dido1	-0.01	1.00000000	mice
Rtn3	-0.10	1.00000000	mice
Ptprc	0.07	1.00000000	mice
Orai2	0.04	1.00000000	mice
Dhx33	0.01	1.00000000	mice
Elk3	-0.12	1.00000000	mice
Ndufaf5	-0.06	1.00000000	mice

Parp8	0.01	1.00000000	mice
Vps35	-0.08	1.00000000	mice
Yaf2	0.03	1.00000000	mice
Zhx1	-0.01	1.00000000	mice
Rrp7a	-0.02	1.00000000	mice
Isca1	-0.06	1.00000000	mice
Hspbp1	-0.01	1.00000000	mice
Dgcr6	-0.01	1.00000000	mice
Ncald	0.14	1.00000000	mice
Tmco1	0.05	1.00000000	mice
Pknox1	0.04	1.00000000	mice
Exosc10	0.01	1.00000000	mice
Tomm34	-0.04	1.00000000	mice
Rabl6	0.06	1.00000000	mice
Tbc1d22b	0.02	1.00000000	mice
Cbwd1	0.00	1.00000000	mice
2210408F21Rik	-0.04	1.00000000	mice
Tmco6	-0.01	1.00000000	mice
Adnp2	-0.02	1.00000000	mice
Tfrc	-0.01	1.00000000	mice
S100a13	-0.13	1.00000000	mice
2310010J17Rik	-0.01	1.00000000	mice
Opa3	-0.02	1.00000000	mice
Klhl7	-0.06	1.00000000	mice
Pcid2	-0.07	1.00000000	mice
Qser1	0.02	1.00000000	mice
Osgep	0.03	1.00000000	mice
Cyfip1	0.03	1.00000000	mice
Cmtm6	0.01	1.00000000	mice
Pold1	-0.01	1.00000000	mice
Tcf7l2	0.40	1.00000000	mice
Nsd1	0.04	1.00000000	mice
Foxk2	-0.01	1.00000000	mice
Cmas	0.09	1.00000000	mice
Dpm3	0.00	1.00000000	mice
Golt1b	-0.03	1.00000000	mice
Cers2	0.02	1.00000000	mice
St7	0.00	1.00000000	mice
Ercc4	-0.01	1.00000000	mice
Smarca4	-0.05	1.00000000	mice
Set	-0.11	1.00000000	mice
Smc3	0.01	1.00000000	mice
Primpol	0.00	1.00000000	mice
Idh3b	-0.04	1.00000000	mice
Klhl15	-0.10	1.00000000	mice
Stk38	-0.03	1.00000000	mice

Serpinb1a	0.31	1.00000000	mice
Rmnd1	0.01	1.00000000	mice
Dctn1	-0.01	1.00000000	mice
Rrm2b	0.02	1.00000000	mice
Cul3	-0.04	1.00000000	mice
Pgm3	-0.02	1.00000000	mice
Cdc40	-0.05	1.00000000	mice
Fbxw2	-0.03	1.00000000	mice
Heatr3	-0.07	1.00000000	mice
Hist1h1e	-0.04	1.00000000	mice
Akt2	0.00	1.00000000	mice
Txndc9	-0.03	1.00000000	mice
Eif3i	-0.02	1.00000000	mice
Nfix	-0.07	1.00000000	mice
Dcun1d5	-0.02	1.00000000	mice
Ap2b1	-0.01	1.00000000	mice
Atg10	-0.09	1.00000000	mice
Trim41	-0.01	1.00000000	mice
Ssr1	-0.02	1.00000000	mice
Ric8b	-0.04	1.00000000	mice
Ubxn8	0.00	1.00000000	mice
Sympk	-0.01	1.00000000	mice
Gorasp2	-0.02	1.00000000	mice
Foxp4	0.00	1.00000000	mice
Chd4	-0.07	1.00000000	mice
Pigk	-0.06	1.00000000	mice
Rhbdd2	0.03	1.00000000	mice
Slc7a6	-0.05	1.00000000	mice
Zcchc24	0.00	1.00000000	mice
Ercc8	-0.04	1.00000000	mice
Pik3r6	-0.14	1.00000000	mice
Ddx47	-0.10	1.00000000	mice
Gtf3a	-0.05	1.00000000	mice
Tbc1d31	0.01	1.00000000	mice
Cd4	0.11	1.00000000	mice
Srbd1	0.02	1.00000000	mice
Ddhd2	0.08	1.00000000	mice
Cars	-0.04	1.00000000	mice
Pkib	0.01	1.00000000	mice
Tmed7	0.01	1.00000000	mice
Nudc	-0.17	1.00000000	mice
L1cam	-0.10	1.00000000	mice
Rnmt	-0.07	1.00000000	mice
Dennd4c	0.07	1.00000000	mice
Sephs1	-0.02	1.00000000	mice
Crlf2	-0.02	1.00000000	mice

Gpatch2l	-0.03	1.00000000	mice
Colgalt1	-0.05	1.00000000	mice
Bptf	-0.02	1.00000000	mice
Skap2	0.01	1.00000000	mice
Bnip1	0.02	1.00000000	mice
Rfx1	0.01	1.00000000	mice
Etfdh	0.04	1.00000000	mice
Tmem104	-0.04	1.00000000	mice
Ccdc22	-0.02	1.00000000	mice
Sec11a	0.00	1.00000000	mice
Limd1	-0.17	1.00000000	mice
Prdm4	-0.03	1.00000000	mice
Fmnl2	0.01	1.00000000	mice
Zfp263	0.12	1.00000000	mice
Rabgap1l	0.12	1.00000000	mice
Phrf1	-0.01	1.00000000	mice
Strbp	0.01	1.00000000	mice
Sypl	-0.07	1.00000000	mice
Qrich1	-0.07	1.00000000	mice
Trappc10	0.05	1.00000000	mice
Rad50	-0.01	1.00000000	mice
Itpk1	-0.05	1.00000000	mice
Psme3	-0.03	1.00000000	mice
Lyar	0.00	1.00000000	mice
Gpd1l	-0.04	1.00000000	mice
Rsf1os1	-0.01	1.00000000	mice
Prcc	-0.04	1.00000000	mice
Vezf1	-0.06	1.00000000	mice
Plekhm3	0.15	1.00000000	mice
Cdc27	0.06	1.00000000	mice
Cul9	-0.09	1.00000000	mice
Rngtt	0.14	1.00000000	mice
Sf3b1	0.01	1.00000000	mice
Haus3	-0.02	1.00000000	mice
Rbbp7	-0.04	1.00000000	mice
Kif1bp	0.05	1.00000000	mice
2310009B15Rik	0.00	1.00000000	mice
Rap1b	-0.06	1.00000000	mice
Clns1a	0.01	1.00000000	mice
Scaf4	-0.03	1.00000000	mice
Cmklr1	-0.06	1.00000000	mice
Galnt2	-0.06	1.00000000	mice
Zfp942	-0.01	1.00000000	mice
Ngrn	0.00	1.00000000	mice
Klf7	-0.33	1.00000000	mice
Hmgb1	0.11	1.00000000	mice

Bscl2	-0.06	1.00000000	mice
Dld	-0.02	1.00000000	mice
Tubb2b	0.01	1.00000000	mice
Rab11a	0.01	1.00000000	mice
Abi2	-0.03	1.00000000	mice
Sf3b5	-0.03	1.00000000	mice
Eif1a	-0.15	1.00000000	mice
Gpr132	-0.02	1.00000000	mice
Sub1	-0.06	1.00000000	mice
Golim4	-0.03	1.00000000	mice
Slc25a51	-0.03	1.00000000	mice
Socs3	0.09	1.00000000	mice
Wbp1	-0.04	1.00000000	mice
Gpn3	-0.01	1.00000000	mice
Atf6b	0.05	1.00000000	mice
Sin3a	0.05	1.00000000	mice
Idh3a	-0.03	1.00000000	mice
Ntng2	-0.01	1.00000000	mice
Tomm22	0.03	1.00000000	mice
Zdhhc6	-0.06	1.00000000	mice
Chchd1	0.03	1.00000000	mice
Ubald2	-0.02	1.00000000	mice
Coq2	-0.08	1.00000000	mice
Phax	-0.10	1.00000000	mice
Gtf3c3	0.00	1.00000000	mice
Snrnp25	0.00	1.00000000	mice
C2cd3	0.02	1.00000000	mice
Spp1	0.02	1.00000000	mice
Prkag1	-0.06	1.00000000	mice
Zfp644	-0.04	1.00000000	mice
Atp8b2	-0.15	1.00000000	mice
Siglecg	0.04	1.00000000	mice
Atraid	-0.07	1.00000000	mice
Derl1	-0.01	1.00000000	mice
Brd4	-0.06	1.00000000	mice
Syvn1	-0.04	1.00000000	mice
Cln3	0.03	1.00000000	mice
Ap3s1	0.01	1.00000000	mice
Ireb2	0.00	1.00000000	mice
Peli1	0.09	1.00000000	mice
Ints6l	0.00	1.00000000	mice
Dars	0.01	1.00000000	mice
Fam20b	0.03	1.00000000	mice
Gcsh	0.01	1.00000000	mice
Romo1	0.03	1.00000000	mice
Dusp22	0.02	1.00000000	mice

Sept11	-0.04	1.00000000	mice
Ifi27	-3.33	0.00000000	pigs
Antxr2	1.46	0.00000000	pigs
Saa3	2.88	0.00000000	pigs
Cyth1	1.21	0.00000000	pigs
Timp1	2.75	0.00000000	pigs
Rubcnl	-1.41	0.00000000	pigs
Ago2	1.40	0.00000000	pigs
Plxnc1	1.54	0.00000000	pigs
Cfd	-2.06	0.00000000	pigs
Xylt1	1.67	0.00000000	pigs
Ell2	1.37	0.00000000	pigs
Cmip	1.02	0.00000000	pigs
Asap1	1.93	0.00000000	pigs
Bhlhe40	1.10	0.00000000	pigs
Gnat3	-0.94	0.00000000	pigs
Pcnx	1.36	0.00000000	pigs
Vav3	-1.09	0.00000000	pigs
Ahi1	1.21	0.00000000	pigs
Rac1	0.81	0.00000000	pigs
Ifitm6	1.79	0.00000000	pigs
Derl3	-1.39	0.00000000	pigs
Pmepa1	1.02	0.00000000	pigs
Atp1a1	1.21	0.00000000	pigs
Prkn	-1.53	0.00000000	pigs
Ssr4	-0.73	0.00000000	pigs
Hpse	1.06	0.00000001	pigs
Sik3	0.96	0.00000001	pigs
Hdac4	1.25	0.00000001	pigs
Ndel1	1.14	0.00000001	pigs
Gpr137b	1.82	0.00000001	pigs
Cd36	-1.24	0.00000001	pigs
Cdca7l	-0.93	0.00000002	pigs
Fryl	-1.15	0.00000002	pigs
Cobll1	-1.58	0.00000002	pigs
Cux1	1.00	0.00000003	pigs
Atp5k	-0.88	0.00000006	pigs
H2-Ea	1.54	0.00000006	pigs
Arhgap15	-1.20	0.00000006	pigs
Apbb1ip	-1.11	0.00000015	pigs
Sec61g	-0.99	0.00000015	pigs
Nfkbie	0.70	0.00000017	pigs
Ccdc50	-1.07	0.00000018	pigs
Bnip3l	-1.01	0.00000018	pigs
Niban1	1.45	0.00000020	pigs
Ybey	0.55	0.00000027	pigs

Itsn1	-1.33	0.00000029	pigs
Cdkn1a	0.90	0.00000031	pigs
Syne3	0.79	0.00000035	pigs
Rgcc	1.17	0.00000045	pigs
Cst7	-1.00	0.00000046	pigs
Gcnt2	1.35	0.00000049	pigs
Dennd4a	0.77	0.00000060	pigs
Klf12	-1.21	0.00000061	pigs
Ctss	-1.17	0.00000068	pigs
Pim1	0.56	0.00000070	pigs
Adamts12	-1.26	0.00000074	pigs
Id2	0.81	0.00000076	pigs
Sec14l1	0.59	0.00000082	pigs
Inpp5d	1.16	0.00000108	pigs
Esyt2	1.00	0.00000110	pigs
Cst3	-0.92	0.00000120	pigs
Arpc1b	0.80	0.00000127	pigs
Pdxk	1.09	0.00000146	pigs
Arid4a	0.95	0.00000154	pigs
Smap2	0.91	0.00000163	pigs
Acer3	1.20	0.00000186	pigs
Nell2	-1.24	0.00000197	pigs
Tapt1	0.88	0.00000228	pigs
Cd4	-1.04	0.00000256	pigs
Nlrc5	-0.98	0.00000258	pigs
Ptprk	-1.00	0.00000260	pigs
Elob	-0.68	0.00000264	pigs
Atrnl1	-1.05	0.00000270	pigs
Ubl5	-0.78	0.00000274	pigs
Krt10	-0.76	0.00000331	pigs
Ranbp9	0.96	0.00000391	pigs
Nfkbia	1.29	0.00000411	pigs
Plek	-1.06	0.00000484	pigs
Spcs1	-0.86	0.00000618	pigs
Ttyh2	0.64	0.00000685	pigs
Lpin2	0.93	0.00000744	pigs
Tenm2	-0.54	0.00000837	pigs
Bcl2a1d	1.61	0.00000864	pigs
Rybp	1.19	0.00000940	pigs
Cox7c	-0.54	0.00000947	pigs
Capzb	0.57	0.00000982	pigs
Zfand5	0.66	0.00001049	pigs
Serpinb1a	1.24	0.00001117	pigs
Pde3b	-0.98	0.00001130	pigs
Zswim6	0.95	0.00001185	pigs
Rasgef1b	0.65	0.00001225	pigs

Atp13a2	-0.96	0.00001313	pigs
H2-Ab1	1.66	0.00001317	pigs
Ckb	1.37	0.00001472	pigs
Tk1	-0.72	0.00001527	pigs
Rhoc	0.75	0.00001582	pigs
Ubr3	0.98	0.00001609	pigs
Plgrkt	0.54	0.00001794	pigs
Cstb	0.76	0.00001823	pigs
Icam1	0.94	0.00001901	pigs
Pdlim5	0.98	0.00001994	pigs
Abhd5	0.76	0.00002016	pigs
Top3a	0.52	0.00002070	pigs
Hip1	-0.95	0.00002102	pigs
Upp1	1.14	0.00002153	pigs
Tamalin	1.12	0.00002170	pigs
Abtb2	1.78	0.00002306	pigs
Hsbp1	-0.77	0.00002709	pigs
Cap1	0.61	0.00002868	pigs
Fgfr1	-0.96	0.00003013	pigs
Irak3	0.80	0.00003554	pigs
Gpbp1	0.57	0.00003851	pigs
Asap2	1.59	0.00003965	pigs
Atp2b4	-0.85	0.00004250	pigs
Plagl1	1.10	0.00004285	pigs
Pan3	0.81	0.00004736	pigs
Spp1	0.34	0.00004910	pigs
Ttll7	-0.74	0.00004950	pigs
Il21r	0.94	0.00005082	pigs
Sh3rf1	1.25	0.00005615	pigs
Hsph1	1.04	0.00006099	pigs
Spsb1	0.68	0.00006738	pigs
Tsen2	0.17	0.00006963	pigs
Ywhah	0.56	0.00007504	pigs
Ehd1	0.66	0.00007629	pigs
Cry2	0.92	0.00008156	pigs
Tmem59	-1.01	0.00008382	pigs
Iqgap2	-0.75	0.00008806	pigs
Per1	0.48	0.00009333	pigs
Fbxw7	0.64	0.00009347	pigs
H2-Oa	0.53	0.00009498	pigs
Blnk	-1.13	0.00009692	pigs
Rnf19b	1.01	0.00009911	pigs
Phactr1	0.83	0.00010991	pigs
Hspb1	0.92	0.00011090	pigs
Plekha2	-0.81	0.00011355	pigs
Relt	0.43	0.00011812	pigs

Anxa6	0.50	0.00012547	pigs
Arntl	0.74	0.00013638	pigs
Jak2	1.14	0.00015208	pigs
Odc1	0.72	0.00015364	pigs
Nck2	1.01	0.00015668	pigs
Scpep1	0.74	0.00016081	pigs
Ogfrl1	-0.76	0.00016140	pigs
Jarid2	0.60	0.00017095	pigs
Cd40	0.99	0.00017096	pigs
Abr	1.04	0.00017182	pigs
Aebp2	0.81	0.00017491	pigs
Epha4	-0.74	0.00017523	pigs
Runx3	0.95	0.00017948	pigs
Ndufb9	-0.78	0.00019080	pigs
Tiparp	0.89	0.00020348	pigs
Nampt	0.77	0.00028239	pigs
Atp6v1b2	0.55	0.00029346	pigs
P4ha1	0.96	0.00031522	pigs
Eef2	0.52	0.00031680	pigs
Eif4a1	0.58	0.00031764	pigs
Zmiz1	0.84	0.00031976	pigs
Irak2	0.99	0.00032446	pigs
Nfkb2	0.49	0.00034954	pigs
Adam8	0.51	0.00036811	pigs
Ezh2	0.60	0.00039993	pigs
Tnip1	0.64	0.00040504	pigs
Il4i1	1.22	0.00040505	pigs
Kdm6b	0.46	0.00041924	pigs
Szrd1	0.53	0.00042703	pigs
Safb	0.46	0.00042708	pigs
Fyb	-0.89	0.00046911	pigs
Ptpn1	0.66	0.00047611	pigs
Abcc1	1.16	0.00049779	pigs
Ccr7	1.45	0.00050321	pigs
Tspan13	0.84	0.00051117	pigs
Prdx2	-0.75	0.00054180	pigs
Klf4	0.77	0.00059514	pigs
Hbp1	0.46	0.00077125	pigs
Parm1	0.80	0.00090569	pigs
Enah	1.10	0.00094330	pigs
Lat2	0.70	0.00096755	pigs
Lgmn	0.71	0.00097011	pigs
Srgn	1.63	0.00097430	pigs
Usp3	0.87	0.00101987	pigs
Evi5	0.63	0.00102173	pigs
Wnk1	0.80	0.00103904	pigs

Aff1	-0.84	0.00104277	pigs
Dennd3	0.37	0.00105233	pigs
Prdm2	0.76	0.00105385	pigs
Sv2c	1.08	0.00109594	pigs
Ppard	0.71	0.00124120	pigs
Ntn1	1.66	0.00126636	pigs
Tuba1b	0.84	0.00127754	pigs
Actg1	1.13	0.00131526	pigs
Ramp2	0.98	0.00131904	pigs
Rhob	0.59	0.00134731	pigs
Ifi30	1.21	0.00139026	pigs
Tcea1	-0.57	0.00139920	pigs
Grb2	0.84	0.00143363	pigs
Ninj1	0.79	0.00148747	pigs
Ctsz	0.69	0.00149072	pigs
Napsa	0.58	0.00161263	pigs
Ndufa4	-0.61	0.00163499	pigs
Rnh1	0.71	0.00173824	pigs
Csf1r	0.85	0.00177060	pigs
Pik3c2b	-0.52	0.00179687	pigs
Skap1	-1.00	0.00187516	pigs
U2af1l4	-0.66	0.00192951	pigs
Spi1	0.67	0.00200912	pigs
Zfp804a	0.47	0.00212307	pigs
Nfkb1	1.01	0.00239508	pigs
Tpi1	0.52	0.00240572	pigs
Ugcg	-0.87	0.00267050	pigs
Dek	-0.58	0.00269861	pigs
Tcf12	-0.76	0.00271668	pigs
Tesk2	1.09	0.00273306	pigs
Uvrag	0.67	0.00288323	pigs
Ecm1	0.80	0.00303463	pigs
Nrxn1	-1.12	0.00311830	pigs
Itpk1	0.68	0.00326253	pigs
Ephb2	-0.72	0.00327334	pigs
Csnk1a1	0.50	0.00329591	pigs
Smad7	0.43	0.00332350	pigs
Usp46	-0.77	0.00334005	pigs
Ranbp2	0.51	0.00334404	pigs
Ddx5	0.34	0.00337893	pigs
Baiap2	0.30	0.00359206	pigs
Pik3r5	1.01	0.00360371	pigs
St6galnac4	-0.80	0.00365338	pigs
Por	0.58	0.00383612	pigs
Ttc22	-0.75	0.00385373	pigs
Snx10	0.66	0.00387014	pigs

Rrbp1	-0.75	0.00407962	pigs
Hdgf	-0.68	0.00408981	pigs
Traf3	0.83	0.00414526	pigs
Cd53	0.62	0.00444656	pigs
Atp6v0b	0.47	0.00459005	pigs
Cnot6l	0.67	0.00460176	pigs
Sh3bp2	0.52	0.00470499	pigs
Dot1l	0.45	0.00480334	pigs
Epb41l2	0.60	0.00492933	pigs
Rassf2	0.59	0.00496103	pigs
Crybg1	1.20	0.00515341	pigs
S1pr2	0.34	0.00515643	pigs
St6gal1	0.80	0.00520076	pigs
Zbtb46	0.66	0.00526323	pigs
Lix1l	0.38	0.00546435	pigs
Eng	0.34	0.00555929	pigs
Tanc2	1.13	0.00617552	pigs
Tmem19	0.55	0.00641730	pigs
Il1a	0.78	0.00647257	pigs
Rufy1	-0.58	0.00700327	pigs
Bicc1	1.03	0.00700779	pigs
Aanat	-0.34	0.00763290	pigs
Csgalnact2	0.54	0.00858558	pigs
Ldlrad3	0.89	0.00865424	pigs
Kmt2e	0.49	0.00877190	pigs
Kirrel	1.06	0.00883562	pigs
Acot7	0.39	0.00886336	pigs
Lonrf1	0.27	0.00921087	pigs
Ptpn18	-0.58	0.00938007	pigs
Flt3	0.75	0.00978781	pigs
Khdrbs3	-0.45	0.01025969	pigs
Rnf149	0.67	0.01041550	pigs
Rnf216	-0.65	0.01049531	pigs
Rcor1	0.87	0.01053000	pigs
Ftl1-ps1	1.18	0.01059396	pigs
Igf2r	0.77	0.01087203	pigs
Irs2	0.62	0.01123528	pigs
Smim5	-0.66	0.01129745	pigs
Vcp	0.50	0.01167842	pigs
Ciita	0.65	0.01189542	pigs
Xpo7	0.33	0.01286273	pigs
H2-Ob	0.44	0.01305588	pigs
Icmt	-0.35	0.01329846	pigs
Arhgap6	0.69	0.01331241	pigs
Sbno1	0.48	0.01397778	pigs
Orai1	0.29	0.01413798	pigs

Gpx4	0.54	0.01475915	pigs
Plekhb2	0.63	0.01496166	pigs
Stk24	0.73	0.01513598	pigs
Serf2	-0.48	0.01522661	pigs
Vash1	0.26	0.01538184	pigs
Arrdc2	0.34	0.01559906	pigs
Cd300c	0.91	0.01659569	pigs
Rala	0.94	0.01748339	pigs
Stat5a	0.62	0.01756321	pigs
Soat1	0.77	0.01838818	pigs
Eif1b	-0.63	0.01879529	pigs
Vasp	0.53	0.01934735	pigs
Irf8	-0.54	0.02041493	pigs
Slc25a38	-0.61	0.02044561	pigs
Osbpl3	1.36	0.02117490	pigs
Socs1	-0.67	0.02136322	pigs
Ncf4	-0.83	0.02184890	pigs
Gpr82	0.34	0.02225322	pigs
Gaa	0.28	0.02274817	pigs
Fosb	0.41	0.02292588	pigs
Arg1	0.94	0.02298401	pigs
Stk3	0.71	0.02325657	pigs
Hsdl1	-0.40	0.02483136	pigs
Gnb1	0.46	0.02489439	pigs
Nr4a3	0.83	0.02502930	pigs
Ccdc186	-0.57	0.02605283	pigs
Dusp22	-0.60	0.02625083	pigs
Gpi1	0.40	0.02691594	pigs
Bid	-0.62	0.02839352	pigs
Cd84	-0.55	0.02865536	pigs
Lcor	0.51	0.02928348	pigs
Gpat4	0.54	0.02932100	pigs
Sh3bgrl3	-0.63	0.03008738	pigs
Cd82	0.38	0.03038900	pigs
Hbegf	0.65	0.03084150	pigs
Smurf1	0.77	0.03249704	pigs
Atp5md	-0.56	0.03252368	pigs
Tmf1	0.34	0.03261568	pigs
Erp29	-0.55	0.03285669	pigs
Atl3	-0.62	0.03333885	pigs
Piezo1	0.29	0.03342380	pigs
Eml4	0.42	0.03446467	pigs
Spon2	0.36	0.03519989	pigs
Itm2c	-0.57	0.03560826	pigs
Eri1	0.32	0.03577157	pigs
Vps8	-0.66	0.03669150	pigs

Kif27	0.26	0.03747229	pigs
Stambpl1	-0.70	0.03747291	pigs
Ap2m1	0.40	0.03751077	pigs
Ppp3ca	-0.61	0.03760839	pigs
Fosl2	0.35	0.03828208	pigs
Rhoq	0.76	0.03845549	pigs
Ptbp2	0.49	0.03871351	pigs
Tmcc3	1.08	0.03937148	pigs
Nkg7	0.45	0.04066768	pigs
Lrp8	-0.92	0.04128080	pigs
Septin11	-0.57	0.04129374	pigs
Batf3	0.96	0.04142916	pigs
Rrp8	-0.41	0.04154203	pigs
Zdhhc14	-0.96	0.04190527	pigs
Zdhhc20	0.58	0.04229018	pigs
Rnf150	0.73	0.04264909	pigs
Snx8	0.53	0.04266730	pigs
Prxl2a	-0.64	0.04337328	pigs
Slc6a12	0.27	0.04498149	pigs
Baz1a	0.83	0.04547871	pigs
Rel	0.85	0.04760562	pigs
Arhgap31	0.96	0.04851128	pigs
Hsd3b7	0.41	0.04902336	pigs
Dennd1a	0.57	0.04924988	pigs
Crim1	1.16	0.04988102	pigs
Disc1	-0.68	0.05163739	pigs
Arid4b	0.52	0.05509040	pigs
Dipk1a	0.72	0.05612376	pigs
Nfe2l2	0.62	0.05656536	pigs
Igsf8	-0.60	0.05695778	pigs
Fhl3	-0.75	0.05724474	pigs
Rell1	0.35	0.05753709	pigs
Tap1	0.57	0.05973730	pigs
Ubc	0.55	0.06040326	pigs
Rnf19a	0.85	0.06188876	pigs
Cndp2	0.58	0.06271786	pigs
Pik3r1	1.19	0.06301369	pigs
Slc16a3	0.24	0.06318392	pigs
Adgrg6	0.67	0.06346137	pigs
Irf2	-0.85	0.06409563	pigs
Rb1cc1	0.44	0.06597791	pigs
Sesn3	0.78	0.06655024	pigs
Plekho1	0.41	0.06692450	pigs
Pkib	0.65	0.06979204	pigs
Kdm6a	0.61	0.07232391	pigs
C1qa	0.39	0.07267157	pigs

Tom1	0.54	0.07316830	pigs
Gimap4	0.53	0.07497029	pigs
Nin	-0.49	0.07835766	pigs
Pdcd1lg2	0.83	0.07904565	pigs
Ppfibp2	0.75	0.08051254	pigs
Traf5	0.55	0.08179198	pigs
Rab2a	-0.39	0.08205922	pigs
Tgfb1	0.63	0.08828182	pigs
Yaf2	0.46	0.09090194	pigs
Slc4a4	-0.77	0.09124045	pigs
Ppp1r12a	0.52	0.09396695	pigs
Rnf114	0.33	0.09437438	pigs
Pik3cg	0.60	0.09760156	pigs
H2az2	-0.39	0.09800615	pigs
Wdfy3	0.90	0.10001606	pigs
Rack1	0.46	0.10029518	pigs
Grk3	0.68	0.10426850	pigs
Aak1	-0.58	0.10464393	pigs
Gpr35	0.22	0.10562255	pigs
Casp3	-0.61	0.10608883	pigs
Zranb1	0.37	0.10929141	pigs
Foxo1	0.64	0.10973650	pigs
Ccni	0.49	0.11048896	pigs
Ostc	-0.57	0.11145066	pigs
Gadd45b	0.55	0.11430125	pigs
Nfam1	0.22	0.11756605	pigs
Ppp1r14a	0.46	0.11870656	pigs
Ndufb1	-0.52	0.12019794	pigs
Krtcap2	-0.64	0.12740025	pigs
Slco3a1	0.73	0.12978455	pigs
Ppp1r14bl	-0.50	0.13014114	pigs
Ssr2	-0.45	0.13105718	pigs
Rnf169	0.79	0.13113886	pigs
Furin	0.42	0.13579120	pigs
Cct7	0.40	0.13647878	pigs
Psmg3	0.24	0.13778979	pigs
Ubald2	-0.66	0.14007022	pigs
Ppp1r16b	0.47	0.14029115	pigs
Fut8	-0.93	0.14250083	pigs
Nol11	0.78	0.14366861	pigs
Trip10	0.16	0.14367984	pigs
Grcc10	-0.58	0.14384050	pigs
Podxl2	-0.50	0.14529676	pigs
Hint3	0.38	0.14698732	pigs
Rev1	-0.58	0.14892136	pigs
Vegfa	0.46	0.15267651	pigs

Peli1	0.54	0.15409181	pigs
Areg	1.45	0.15690328	pigs
Larp1	0.34	0.15700681	pigs
Dnah17	0.28	0.15849390	pigs
N4bp1	0.59	0.15936392	pigs
Nfil3	0.57	0.16445191	pigs
Ier5	0.44	0.16586692	pigs
Rtn4	0.42	0.16597034	pigs
Ddx3x	0.40	0.16616103	pigs
Eps8	-0.71	0.18292400	pigs
Tpra1	-0.50	0.18391275	pigs
Il4ra	0.50	0.19047616	pigs
Tex30	-0.63	0.19214921	pigs
AA467197	0.47	0.19239072	pigs
Extl3	-0.62	0.19495331	pigs
Cyld	0.51	0.19884216	pigs
Xcl1	-0.68	0.20150906	pigs
Havcr2	0.88	0.20367396	pigs
Csf3r	0.40	0.21256156	pigs
Rasl11a	0.61	0.21359624	pigs
Plekhf2	-0.61	0.21582393	pigs
Ifi44	-0.42	0.21594087	pigs
Comt	0.34	0.21860328	pigs
Rab3c	-0.87	0.22022960	pigs
Bri3	0.35	0.23112726	pigs
Plaur	0.88	0.23846306	pigs
Selenoi	0.66	0.23856220	pigs
Picalm	0.85	0.23866258	pigs
Ampd3	0.30	0.24194575	pigs
Hivep2	0.71	0.24584329	pigs
Jup	0.19	0.25023509	pigs
Gpr174	-0.57	0.25108895	pigs
Ppp1cb	0.46	0.26030877	pigs
Pdcd4	-0.62	0.26414675	pigs
Bag2	-0.67	0.27010607	pigs
Selenop	0.26	0.27144802	pigs
Ppp6r3	0.38	0.27714344	pigs
Apba1	-0.76	0.27910054	pigs
Csnk1g3	0.77	0.28523469	pigs
Prdx1	0.69	0.29137132	pigs
Cep120	0.81	0.29306061	pigs
Slc7a11	0.86	0.29632692	pigs
Rassf1	0.41	0.29942101	pigs
Uchl5	-0.52	0.30001134	pigs
Grhpr	0.22	0.30153607	pigs
Rnd2	-0.31	0.30644446	pigs

Traf7	0.26	0.31127309	pigs
Pip4k2a	-0.52	0.31308522	pigs
Prex1	0.75	0.31627384	pigs
Pdlim7	0.39	0.31995223	pigs
Ubr5	0.49	0.32710507	pigs
Bag3	0.32	0.32781924	pigs
Ido1	1.04	0.33251419	pigs
Zbtb20	-0.64	0.33658014	pigs
3110040N11Rik	-0.54	0.33685768	pigs
Tnni2	0.41	0.33928065	pigs
Dcxr	0.32	0.34292574	pigs
Prkar2a	0.44	0.34496493	pigs
Tubb5	0.55	0.34521987	pigs
Rap1gds1	0.41	0.34826979	pigs
Plin2	-1.06	0.35825006	pigs
Ppif	-0.99	0.36238611	pigs
Ngly1	0.23	0.37844308	pigs
Rab5c	0.34	0.37978483	pigs
Ndrg1	0.64	0.39088699	pigs
Sestd1	0.06	0.39278085	pigs
Upf3b	-0.50	0.39463280	pigs
Tyrobp	0.55	0.39745872	pigs
Actn1	0.73	0.39846730	pigs
Dnajc10	-0.45	0.40117259	pigs
Stim1	0.60	0.40397956	pigs
Cacna1d	-0.61	0.40506572	pigs
Gse1	0.63	0.41127162	pigs
Chst15	-0.69	0.42754298	pigs
Nrip1	0.43	0.43539262	pigs
Arxes2	-0.51	0.44764758	pigs
Tmed5	-0.47	0.45004539	pigs
Tma7	-0.51	0.45454950	pigs
Sqstm1	0.48	0.45650926	pigs
Atp6v1c1	-0.44	0.46482519	pigs
Chrnb1	0.24	0.46515466	pigs
Bach1	0.44	0.47374351	pigs
Ywhaq	0.46	0.47550852	pigs
Axl	0.66	0.49017514	pigs
Nedd9	-0.59	0.49617406	pigs
Ybx3	0.49	0.49809941	pigs
Lrp6	0.29	0.50089756	pigs
Serpinb10	-0.64	0.50777772	pigs
Ophn1	-0.62	0.50897241	pigs
Kpnb1	0.45	0.51178697	pigs
Efr3a	0.40	0.51229717	pigs
Camk1d	0.31	0.51378193	pigs

Yipf1	-0.57	0.51936508	pigs
Trim75	-0.50	0.52025672	pigs
Snapc1l	0.33	0.52083640	pigs
Kpna4	0.64	0.52089549	pigs
Usp7	0.34	0.53137031	pigs
Braf	0.57	0.53554831	pigs
Lrrfip2	0.66	0.54907296	pigs
Nfat5	0.44	0.55965342	pigs
Ppm1j	0.29	0.56436313	pigs
Traf1	0.21	0.56813947	pigs
Cd101	-0.32	0.57431144	pigs
Chordc1	0.39	0.59870014	pigs
Fgr	0.41	0.60129681	pigs
Usp22	0.42	0.60324149	pigs
Aqp3	0.81	0.60610094	pigs
Col18a1	-0.22	0.60758140	pigs
Arhgap20	0.35	0.61367780	pigs
Scimp	0.22	0.61367780	pigs
Arhgap25	0.88	0.61507965	pigs
Tpt1	0.30	0.62784712	pigs
St18	1.04	0.63242111	pigs
Aldh2	0.26	0.64492994	pigs
Bmp2k	0.56	0.64667076	pigs
Rassf4	0.35	0.64857965	pigs
Spaca3	-0.47	0.65024312	pigs
Acss3	-0.71	0.65534938	pigs
Abl2	0.59	0.65692352	pigs
Dnmbp	-0.57	0.67576931	pigs
Gramd1b	-0.53	0.67860285	pigs
Ptprm	0.09	0.67960371	pigs
Nsd2	0.26	0.68354779	pigs
Selenow	-0.62	0.69159918	pigs
Tbc1d9	0.22	0.69776420	pigs
Rgs10	0.49	0.70347930	pigs
Serpinb9	0.59	0.70353084	pigs
Negr1	1.06	0.71366890	pigs
Gns	0.25	0.71663329	pigs
Nav1	0.65	0.71740494	pigs
Tmem50a	-0.40	0.72396923	pigs
Samhd1	0.35	0.72731605	pigs
Tet2	0.55	0.72782406	pigs
Flna	0.38	0.73555074	pigs
Hr	0.23	0.74040459	pigs
Tgm2	0.30	0.76797357	pigs
Brd4	0.45	0.77309458	pigs
Ptafr	0.27	0.79159756	pigs

Tmem35b	0.22	0.79588719	pigs
Pard6g	-0.24	0.80798415	pigs
Kdm5b	0.50	0.84813009	pigs
Gab2	0.13	0.86327367	pigs
Spen	0.48	0.86708803	pigs
Bicd2	0.42	0.88484734	pigs
Cnn2	0.36	0.90854153	pigs
Pafah1b3	0.28	0.90964657	pigs
Cdyl	0.54	0.90972463	pigs
Trio	0.65	0.93273360	pigs
Rasgrp1	0.60	0.94299616	pigs
Itga4	-0.54	0.94998446	pigs
Slc1a4	-0.41	0.95801452	pigs
Cd38	-0.51	0.97549018	pigs
Trp53bp2	0.37	0.98584138	pigs
Dync1i1	-0.49	1.00000000	pigs
Slc30a4	0.21	1.00000000	pigs
Cd163	0.46	1.00000000	pigs
Lrmda	1.25	1.00000000	pigs
Erp44	-0.46	1.00000000	pigs
Zdhhc4	-0.40	1.00000000	pigs
Cd86	0.90	1.00000000	pigs
Lgals1	-1.03	1.00000000	pigs
Rgs9	0.42	1.00000000	pigs
Gabpb1	0.31	1.00000000	pigs
Cog5	-0.72	1.00000000	pigs
Rbm39	0.30	1.00000000	pigs
Ccr2	-0.38	1.00000000	pigs
Sesn2	0.40	1.00000000	pigs
Bcl9	0.17	1.00000000	pigs
Akap11	0.33	1.00000000	pigs
Lrrc8c	0.46	1.00000000	pigs
Ube2q1	-0.47	1.00000000	pigs
Dazap1	-0.46	1.00000000	pigs
Cul1	0.84	1.00000000	pigs
Tex2	-0.49	1.00000000	pigs
Pgls	-0.50	1.00000000	pigs
Ghrl	-0.12	1.00000000	pigs
Ctsa	0.35	1.00000000	pigs
Gsap	0.47	1.00000000	pigs
Prkar1a	0.43	1.00000000	pigs
Rcvrn	-0.34	1.00000000	pigs
Galk1	-0.46	1.00000000	pigs
Stom	0.30	1.00000000	pigs
Igsf6	0.30	1.00000000	pigs
Lsp1	0.45	1.00000000	pigs

Smad3	0.48	1.00000000	pigs
Pgs1	0.37	1.00000000	pigs
Fcer1g	-0.54	1.00000000	pigs
Tg	0.33	1.00000000	pigs
P2ry14	-0.56	1.00000000	pigs
Tmem147	-0.53	1.00000000	pigs
Tmem109	-0.51	1.00000000	pigs
Vav1	0.41	1.00000000	pigs
Parvb	0.37	1.00000000	pigs
Psmb8	0.41	1.00000000	pigs
Casp7	0.27	1.00000000	pigs
Fam110a	0.81	1.00000000	pigs
Stx11	0.50	1.00000000	pigs
Rara	0.35	1.00000000	pigs
Gnai1	0.41	1.00000000	pigs
Pgm2	0.47	1.00000000	pigs
Pde8a	0.49	1.00000000	pigs
Pdgfb	0.46	1.00000000	pigs
Slamf8	0.21	1.00000000	pigs
Akirin1	0.37	1.00000000	pigs
Rarres1	0.61	1.00000000	pigs
Kpna1	0.43	1.00000000	pigs
Pfkp	0.32	1.00000000	pigs
Cox7a2	-0.51	1.00000000	pigs
Iffo2	0.31	1.00000000	pigs
Phf23	0.33	1.00000000	pigs
Atf6	0.49	1.00000000	pigs
Smg7	0.36	1.00000000	pigs
Ddx17	0.31	1.00000000	pigs
Poc1b	0.25	1.00000000	pigs
Arhgap27	-0.47	1.00000000	pigs
Ppp1r13b	0.50	1.00000000	pigs
Laptm5	0.47	1.00000000	pigs
Usp48	0.34	1.00000000	pigs
Osbpl8	0.56	1.00000000	pigs
Ywhaz	0.37	1.00000000	pigs
Azin1	0.40	1.00000000	pigs
Smurf2	0.47	1.00000000	pigs
Calcrl	-0.68	1.00000000	pigs
Cdip1	-0.36	1.00000000	pigs
Necap2	0.55	1.00000000	pigs
Vrk2	-0.53	1.00000000	pigs
Rae1	0.32	1.00000000	pigs
Gdi2	0.42	1.00000000	pigs
Lamp2	-0.51	1.00000000	pigs
Dntt	-0.38	1.00000000	pigs

Flnb	0.33	1.00000000	pigs
Prr14	0.18	1.00000000	pigs
Lactb	0.39	1.00000000	pigs
Slamf7	0.72	1.00000000	pigs
Zcchc7	-0.58	1.00000000	pigs
Agpat5	-0.47	1.00000000	pigs
Crebzf	0.32	1.00000000	pigs
Phlda2	0.22	1.00000000	pigs
Dnajb1	0.60	1.00000000	pigs
Pacsin2	0.44	1.00000000	pigs
Cd247	-0.38	1.00000000	pigs
Secisbp2	-0.46	1.00000000	pigs
Aoah	-0.24	1.00000000	pigs
Ppil3	-0.40	1.00000000	pigs
Tax1bp3	0.23	1.00000000	pigs
Sap30bp	-0.50	1.00000000	pigs
Cdc42ep4	0.18	1.00000000	pigs
Tnfrsf1a	0.17	1.00000000	pigs
Dip2b	0.46	1.00000000	pigs
Srxn1	0.12	1.00000000	pigs
Top1	-0.40	1.00000000	pigs
Btbd9	0.76	1.00000000	pigs
Tnip2	0.38	1.00000000	pigs
Bcap29	-0.55	1.00000000	pigs
Hnrnph2	-0.49	1.00000000	pigs
Npc2	-0.49	1.00000000	pigs
Ndst1	0.29	1.00000000	pigs
Vdr	0.35	1.00000000	pigs
Cdc42	0.28	1.00000000	pigs
Fhod1	0.13	1.00000000	pigs
Kbtbd2	0.19	1.00000000	pigs
Bach2	1.23	1.00000000	pigs
Pdpk1	0.39	1.00000000	pigs
Rabgap1l	-0.39	1.00000000	pigs
Nupl1	0.26	1.00000000	pigs
Scfd2	-0.59	1.00000000	pigs
F11r	0.09	1.00000000	pigs
Tmem176a	0.45	1.00000000	pigs
Cnot9	0.45	1.00000000	pigs
Dyrk3	0.18	1.00000000	pigs
Epas1	0.40	1.00000000	pigs
Rsf1	0.40	1.00000000	pigs
Cd2ap	-0.38	1.00000000	pigs
Relb	0.41	1.00000000	pigs
Chst11	0.99	1.00000000	pigs
Eif4e	0.39	1.00000000	pigs

Cd79a	-0.58	1.00000000	pigs
Csrnp1	0.36	1.00000000	pigs
Cmklr1	-0.44	1.00000000	pigs
Wnk2	0.87	1.00000000	pigs
Sdc2	-0.69	1.00000000	pigs
Ubn1	0.29	1.00000000	pigs
Tead3	0.70	1.00000000	pigs
Rab23	-0.54	1.00000000	pigs
Snx29	-0.40	1.00000000	pigs
Notch3	-0.18	1.00000000	pigs
Hsf2	0.20	1.00000000	pigs
Ankrd29	-0.37	1.00000000	pigs
Shmt1	0.47	1.00000000	pigs
Thyn1	0.23	1.00000000	pigs
Sik2	0.52	1.00000000	pigs
Nebl	0.74	1.00000000	pigs
Zyx	0.27	1.00000000	pigs
Ppp1r10	-0.46	1.00000000	pigs
Adgb	0.47	1.00000000	pigs
Apoe	0.18	1.00000000	pigs
Nedd4l	0.56	1.00000000	pigs
Dazap2	0.19	1.00000000	pigs
Edem1	0.37	1.00000000	pigs
St6galnac3	0.27	1.00000000	pigs
Slc5a3	0.17	1.00000000	pigs
Lrp2bp	-0.34	1.00000000	pigs
Rab5a	0.38	1.00000000	pigs
Htt	-0.49	1.00000000	pigs
Dnah11	-0.23	1.00000000	pigs
Spint2	0.78	1.00000000	pigs
Ccl22	0.47	1.00000000	pigs
Csnk1d	0.32	1.00000000	pigs
Rhbdf2	-0.51	1.00000000	pigs
Ncoa1	0.37	1.00000000	pigs
Rab20	0.14	1.00000000	pigs
Zfand6	0.50	1.00000000	pigs
Slc12a9	-0.27	1.00000000	pigs
Rgs2	-0.29	1.00000000	pigs
Pou2f1	0.31	1.00000000	pigs
Siah1b	0.29	1.00000000	pigs
Pik3r6	0.12	1.00000000	pigs
Bin1	0.34	1.00000000	pigs
Catsperg1	0.17	1.00000000	pigs
Tifa	0.17	1.00000000	pigs
Pcbp1	0.33	1.00000000	pigs
Grpel2	0.24	1.00000000	pigs

Tcf4	-0.44	1.00000000	pigs
Spata5	-0.55	1.00000000	pigs
Rpusd3	0.13	1.00000000	pigs
Gpatch3	0.23	1.00000000	pigs
Gclc	0.37	1.00000000	pigs
Tdrd1	-0.26	1.00000000	pigs
Sos1	0.42	1.00000000	pigs
Zfp292	0.33	1.00000000	pigs
Dimt1	0.15	1.00000000	pigs
Ahcyl2	-0.79	1.00000000	pigs
Chd1	0.27	1.00000000	pigs
Qk	0.40	1.00000000	pigs
Pnp	0.62	1.00000000	pigs
Sdf4	0.35	1.00000000	pigs
Usp37	0.43	1.00000000	pigs
Supt4a	0.32	1.00000000	pigs
Hif1a	0.35	1.00000000	pigs
Slc22a3	0.22	1.00000000	pigs
Ifitm5	0.38	1.00000000	pigs
Arl13b	0.33	1.00000000	pigs
Cyp2j13	0.19	1.00000000	pigs
Farp1	0.43	1.00000000	pigs
Srebf2	-0.34	1.00000000	pigs
St8sia4	0.66	1.00000000	pigs
Tnfaip3	0.62	1.00000000	pigs
Hacd1	0.26	1.00000000	pigs
Actr2	0.33	1.00000000	pigs
Prrg4	0.37	1.00000000	pigs
Brd2	0.31	1.00000000	pigs
Eif4h	0.34	1.00000000	pigs
Plcd1	0.15	1.00000000	pigs
Zdhhc2	0.18	1.00000000	pigs
Appl2	-0.29	1.00000000	pigs
Pvrig	-0.15	1.00000000	pigs
Ahr	0.41	1.00000000	pigs
Arl8b	0.36	1.00000000	pigs
Armc9	0.13	1.00000000	pigs
Vash2	-0.33	1.00000000	pigs
Lrrk1	0.59	1.00000000	pigs
Arl8a	0.27	1.00000000	pigs
Ikbkb	-0.41	1.00000000	pigs
Spcs2	-0.36	1.00000000	pigs
Rapgef1	0.69	1.00000000	pigs
Arrb1	0.49	1.00000000	pigs
Ankrd13a	0.32	1.00000000	pigs
Cfl1	0.37	1.00000000	pigs

Stx7	-0.49	1.00000000	pigs
Tmtc2	-0.29	1.00000000	pigs
Ccng2	0.35	1.00000000	pigs
Plxna2	0.65	1.00000000	pigs
Dusp4	0.17	1.00000000	pigs
Jak1	0.29	1.00000000	pigs
Ptcra	-0.18	1.00000000	pigs
Cnp	-0.41	1.00000000	pigs
Zfand3	0.42	1.00000000	pigs
Ada	0.15	1.00000000	pigs
Ppfia1	0.37	1.00000000	pigs
Ankrd11	0.44	1.00000000	pigs
Slc39a8	0.28	1.00000000	pigs
Arfgap3	0.67	1.00000000	pigs
Parp16	0.15	1.00000000	pigs
Tmem223	0.22	1.00000000	pigs
Tnfrsf1b	0.48	1.00000000	pigs
Tfeb	0.14	1.00000000	pigs
Tpst1	0.36	1.00000000	pigs
Rnf2	0.31	1.00000000	pigs
Esco1	0.38	1.00000000	pigs
Otulinl	0.47	1.00000000	pigs
Phactr2	0.34	1.00000000	pigs
Slk	0.28	1.00000000	pigs
Hck	0.26	1.00000000	pigs
Trafd1	0.42	1.00000000	pigs
Slc25a28	0.17	1.00000000	pigs
Nudcd3	0.22	1.00000000	pigs
Pitrm1	0.12	1.00000000	pigs
Herc6	0.68	1.00000000	pigs
Cpsf6	0.68	1.00000000	pigs
Ppp5c	0.13	1.00000000	pigs
Tlr8	0.27	1.00000000	pigs
Itfg1	-0.52	1.00000000	pigs
Ablim1	-0.44	1.00000000	pigs
Cfp	0.43	1.00000000	pigs
Letm1	0.13	1.00000000	pigs
Exoc6b	0.76	1.00000000	pigs
Bnip3	0.43	1.00000000	pigs
Ifnar1	-0.51	1.00000000	pigs
Gorasp1	-0.27	1.00000000	pigs
Fndc3b	0.19	1.00000000	pigs
Adamtsl4	0.16	1.00000000	pigs
Senp6	0.45	1.00000000	pigs
Dmxl2	0.29	1.00000000	pigs
Stard4	0.26	1.00000000	pigs

Stat4	0.69	1.00000000	pigs
Slc50a1	-0.51	1.00000000	pigs
Pim3	0.39	1.00000000	pigs
Slc43a2	0.61	1.00000000	pigs
Nptx2	0.53	1.00000000	pigs
Gbp2b	0.26	1.00000000	pigs
Slc45a4	0.23	1.00000000	pigs
Lipa	0.30	1.00000000	pigs
Ubr2	0.42	1.00000000	pigs
Erc1	0.42	1.00000000	pigs
Colgalt1	-0.47	1.00000000	pigs
Gpatch2	-0.72	1.00000000	pigs
Phlpp2	0.18	1.00000000	pigs
Ahrr	0.32	1.00000000	pigs
Klhl42	0.26	1.00000000	pigs
Ppm1k	0.32	1.00000000	pigs
Pik3ca	0.43	1.00000000	pigs
Pdgfrb	0.20	1.00000000	pigs
Il13ra1	0.16	1.00000000	pigs
Kars	0.32	1.00000000	pigs
B3galnt1	-0.29	1.00000000	pigs
Tmco1	-0.39	1.00000000	pigs
Prdm1	0.23	1.00000000	pigs
Snx24	0.36	1.00000000	pigs
F2rl1	0.21	1.00000000	pigs
Cinp	0.21	1.00000000	pigs
Glmp	0.15	1.00000000	pigs
Snap23	0.27	1.00000000	pigs
Sulf1	-0.93	1.00000000	pigs
Zfp64	0.11	1.00000000	pigs
Rab3gap2	0.29	1.00000000	pigs
Txndc17	-0.41	1.00000000	pigs
Epsti1	-0.49	1.00000000	pigs
Nav3	0.96	1.00000000	pigs
Acsf3	0.16	1.00000000	pigs
Btg3	0.22	1.00000000	pigs
Zfp128	0.16	1.00000000	pigs
Fabp6	-0.24	1.00000000	pigs
Nfasc	-0.49	1.00000000	pigs
Acap1	0.16	1.00000000	pigs
Sgms2	0.16	1.00000000	pigs
Acsl1	0.06	1.00000000	pigs
Gnl1	0.21	1.00000000	pigs
Atp6v0d1	0.27	1.00000000	pigs
Stat6	0.25	1.00000000	pigs
Sppl2a	-0.40	1.00000000	pigs

Epb41l3	0.67	1.00000000	pigs
Iqsec1	0.27	1.00000000	pigs
Selenbp2	-0.44	1.00000000	pigs
Tnfsf12	0.11	1.00000000	pigs
1700025G04Rik	-0.39	1.00000000	pigs
Il1rapl2	0.13	1.00000000	pigs
Atp6ap1	0.27	1.00000000	pigs
Fam174a	0.27	1.00000000	pigs
Lyrm1	0.14	1.00000000	pigs
Rin3	0.80	1.00000000	pigs
Zeb1	-0.37	1.00000000	pigs
Tmbim6	-0.38	1.00000000	pigs
Cmtm7	-0.44	1.00000000	pigs
Ppp1r3b	0.23	1.00000000	pigs
Dbi	-0.43	1.00000000	pigs
Clec16a	0.21	1.00000000	pigs
Aars	0.19	1.00000000	pigs
Chchd5	0.30	1.00000000	pigs
Rest	0.27	1.00000000	pigs
Son	-0.31	1.00000000	pigs
Cyb561a3	-0.45	1.00000000	pigs
Ets2	0.28	1.00000000	pigs
Reep5	-0.32	1.00000000	pigs
Plekhm3	0.51	1.00000000	pigs
Zfp516	0.31	1.00000000	pigs
Gng10	-0.40	1.00000000	pigs
Pqlc3	-0.52	1.00000000	pigs
Osbpl2	0.18	1.00000000	pigs
Deaf1	-0.48	1.00000000	pigs
Tm9sf4	-0.39	1.00000000	pigs
Pgap1	0.31	1.00000000	pigs
Rnase6	0.24	1.00000000	pigs
Snrpd2	-0.39	1.00000000	pigs
Hnrnpk	0.28	1.00000000	pigs
Naaa	0.35	1.00000000	pigs
Ctsh	-0.40	1.00000000	pigs
Vim	0.43	1.00000000	pigs
Syngr2	0.13	1.00000000	pigs
Ppp6r1	0.15	1.00000000	pigs
Pitpna	0.32	1.00000000	pigs
Etv3	0.64	1.00000000	pigs
Pfdn1	0.52	1.00000000	pigs
Oas1e	0.16	1.00000000	pigs
H13	-0.46	1.00000000	pigs
Ggh	0.26	1.00000000	pigs
Smim14	-0.46	1.00000000	pigs

Rac2	-0.38	1.00000000	pigs
Rabep1	0.49	1.00000000	pigs
Dyrk1a	0.36	1.00000000	pigs
Capns1	0.23	1.00000000	pigs
Uqcrq	-0.41	1.00000000	pigs
Atxn7l1	-0.47	1.00000000	pigs
Hells	-0.32	1.00000000	pigs
B3glct	-0.37	1.00000000	pigs
Cdk6	0.98	1.00000000	pigs
Slc22a15	0.58	1.00000000	pigs
Traf6	0.41	1.00000000	pigs
Nadsyn1	0.16	1.00000000	pigs
Ssr3	-0.40	1.00000000	pigs
Ksr1	-0.50	1.00000000	pigs
Prdx6	0.32	1.00000000	pigs
Rpia	0.35	1.00000000	pigs
Hcfc2	0.20	1.00000000	pigs
Tmem40	0.26	1.00000000	pigs
Kctd20	0.30	1.00000000	pigs
Hnrnpdl	-0.33	1.00000000	pigs
Ppib	-0.31	1.00000000	pigs
Tmem181a	0.29	1.00000000	pigs
Ptp4a2	0.27	1.00000000	pigs
Fmnl1	0.49	1.00000000	pigs
Oxr1	-0.53	1.00000000	pigs
Cd80	0.59	1.00000000	pigs
Arpc5	0.36	1.00000000	pigs
Dip2c	0.52	1.00000000	pigs
Parn	-0.39	1.00000000	pigs
Gpr4	-0.02	1.00000000	pigs
Kcnq5	-0.77	1.00000000	pigs
Ulk2	0.32	1.00000000	pigs
F5	-0.25	1.00000000	pigs
Commd3	-0.38	1.00000000	pigs
Pitpnm1	-0.01	1.00000000	pigs
Wdr47	0.33	1.00000000	pigs
Tubb2b	0.53	1.00000000	pigs
Eed	0.39	1.00000000	pigs
Tmem107	-0.70	1.00000000	pigs
Clock	0.25	1.00000000	pigs
Higd1a	0.33	1.00000000	pigs
Ly86	0.24	1.00000000	pigs
Hdac9	-0.35	1.00000000	pigs
Pbx4	0.03	1.00000000	pigs
Nsmce1	0.34	1.00000000	pigs
Cog6	0.27	1.00000000	pigs

S100z	-0.37	1.00000000	pigs
Gpr18	0.16	1.00000000	pigs
Haao	0.08	1.00000000	pigs
Psap	0.51	1.00000000	pigs
Chmp2a	0.27	1.00000000	pigs
Slc30a5	0.43	1.00000000	pigs
Pgk1	0.32	1.00000000	pigs
Gapvd1	0.32	1.00000000	pigs
Lyplal1	0.15	1.00000000	pigs
Cwc27	-0.52	1.00000000	pigs
Arhgef10l	0.17	1.00000000	pigs
Kdm1a	0.16	1.00000000	pigs
Nrg1	0.68	1.00000000	pigs
Rfxank	0.23	1.00000000	pigs
Timm44	-0.19	1.00000000	pigs
Hoga1	0.13	1.00000000	pigs
Tmem88	0.11	1.00000000	pigs
Cdk5rap2	0.14	1.00000000	pigs
Urm1	0.13	1.00000000	pigs
Glipr2	0.16	1.00000000	pigs
Ptprcap	-0.43	1.00000000	pigs
Opa1	0.49	1.00000000	pigs
Dock1	-0.48	1.00000000	pigs
Eef1g	0.33	1.00000000	pigs
Eea1	-0.46	1.00000000	pigs
Srp14	-0.42	1.00000000	pigs
Cnot4	0.31	1.00000000	pigs
Pdhx	0.20	1.00000000	pigs
Rap2c	0.24	1.00000000	pigs
Ahsa1	0.24	1.00000000	pigs
Trim28	0.14	1.00000000	pigs
Lfng	0.04	1.00000000	pigs
Cir1	0.22	1.00000000	pigs
Fam222a	0.15	1.00000000	pigs
Fchsd2	-0.43	1.00000000	pigs
Arnt	-0.38	1.00000000	pigs
Galns	0.13	1.00000000	pigs
Scmh1	0.14	1.00000000	pigs
Nrtn	-0.19	1.00000000	pigs
Atp13a3	-0.53	1.00000000	pigs
Foxj2	0.19	1.00000000	pigs
Plxna1	0.16	1.00000000	pigs
Ttc3	0.14	1.00000000	pigs
Usp8	0.15	1.00000000	pigs
Smpdl3a	0.21	1.00000000	pigs
Tnf	-1.23	1.00000000	pigs

Usp12	0.34	1.00000000	pigs
Clcn5	-0.47	1.00000000	pigs
Rab7b	0.32	1.00000000	pigs
Lrp10	0.25	1.00000000	pigs
Egln3	0.11	1.00000000	pigs
Ctbp2	0.27	1.00000000	pigs
Eloa	0.18	1.00000000	pigs
Tmem165	0.20	1.00000000	pigs
Plaat3	-0.27	1.00000000	pigs
Tnfsf13b	0.53	1.00000000	pigs
Cebpb	0.29	1.00000000	pigs
Flii	0.29	1.00000000	pigs
Ndufb3	-0.38	1.00000000	pigs
Tollip	0.17	1.00000000	pigs
Qser1	0.00	1.00000000	pigs
Usf1	0.16	1.00000000	pigs
Ccdc82	0.35	1.00000000	pigs
Gimap8	0.15	1.00000000	pigs
Paf1	0.21	1.00000000	pigs
Cyp2s1	0.22	1.00000000	pigs
Pkn2	-0.44	1.00000000	pigs
Acvr1	0.24	1.00000000	pigs
Aicda	-0.19	1.00000000	pigs
Add1	0.68	1.00000000	pigs
Lasp1	0.22	1.00000000	pigs
Cep95	0.29	1.00000000	pigs
Gca	0.16	1.00000000	pigs
Irf2bp2	0.38	1.00000000	pigs
Golm1	-0.33	1.00000000	pigs
Idh3a	0.20	1.00000000	pigs
Rab40b	0.23	1.00000000	pigs
Txnrd1	0.58	1.00000000	pigs
Snx25	0.19	1.00000000	pigs
Slc44a2	-0.46	1.00000000	pigs
Armcx3	-0.35	1.00000000	pigs
Tep1	0.15	1.00000000	pigs
Tmem160	-0.36	1.00000000	pigs
Sec62	-0.41	1.00000000	pigs
Fbxo22	0.33	1.00000000	pigs
Ppcs	0.16	1.00000000	pigs
Vps13c	-0.47	1.00000000	pigs
Rbm47	-0.23	1.00000000	pigs
4833439L19Rik	0.24	1.00000000	pigs
Prim2	-0.48	1.00000000	pigs
Akr1b3	-0.44	1.00000000	pigs
Csrp1	0.31	1.00000000	pigs

Snrpn	-0.48	1.00000000	pigs
Ilf3	0.18	1.00000000	pigs
Rragc	-0.44	1.00000000	pigs
Shkbp1	0.16	1.00000000	pigs
Sppl3	0.52	1.00000000	pigs
Ssna1	-0.28	1.00000000	pigs
Ssh1	0.44	1.00000000	pigs
Dhx38	0.13	1.00000000	pigs
Klhl13	-0.48	1.00000000	pigs
Yy1	0.33	1.00000000	pigs
Usp39	0.17	1.00000000	pigs
Phactr4	0.22	1.00000000	pigs
Phip	0.39	1.00000000	pigs
Spag9	0.36	1.00000000	pigs
Zfyve26	-0.46	1.00000000	pigs
Prdx4	-0.30	1.00000000	pigs
Cxcl10	0.08	1.00000000	pigs
Galnt3	0.22	1.00000000	pigs
Psma4	-0.47	1.00000000	pigs
Cyba	0.34	1.00000000	pigs
Tmem184b	0.12	1.00000000	pigs
Lmna	0.12	1.00000000	pigs
Slamf9	0.09	1.00000000	pigs
Sh3glb1	-0.31	1.00000000	pigs
Gcnt7	-0.18	1.00000000	pigs
C1qb	0.51	1.00000000	pigs
Slc17a9	0.14	1.00000000	pigs
Fam193b	0.17	1.00000000	pigs
Eif3l	0.26	1.00000000	pigs
Utp18	0.10	1.00000000	pigs
Cytip	0.28	1.00000000	pigs
Unc93b1	-0.42	1.00000000	pigs
Slc35b2	0.13	1.00000000	pigs
Tbc1d8	0.15	1.00000000	pigs
Ccnl1	0.29	1.00000000	pigs
Hacd4	0.20	1.00000000	pigs
Hltf	0.28	1.00000000	pigs
Arap1	0.14	1.00000000	pigs
Plxna4	0.80	1.00000000	pigs
Pcdhga4	-0.38	1.00000000	pigs
Spire1	0.20	1.00000000	pigs
Hipk1	0.18	1.00000000	pigs
Gab1	-0.41	1.00000000	pigs
Kif1a	0.24	1.00000000	pigs
Ing2	0.11	1.00000000	pigs
Slc36a4	0.35	1.00000000	pigs

Tsc22d3	0.26	1.00000000	pigs
Qrich1	0.20	1.00000000	pigs
Chid1	-0.41	1.00000000	pigs
Ext1	-0.34	1.00000000	pigs
Smcr8	0.28	1.00000000	pigs
Ptk2	0.33	1.00000000	pigs
Eps15	0.37	1.00000000	pigs
Apbb2	0.31	1.00000000	pigs
Cherp	0.16	1.00000000	pigs
Pwwp2b	0.13	1.00000000	pigs
Zbtb11	0.26	1.00000000	pigs
Plaa	0.34	1.00000000	pigs
Recql5	-0.38	1.00000000	pigs
Tmem260	-0.30	1.00000000	pigs
Scarb2	-0.41	1.00000000	pigs
Kidins220	0.23	1.00000000	pigs
Gas7	-0.22	1.00000000	pigs
Bicra	0.30	1.00000000	pigs
Siah2	0.15	1.00000000	pigs
Rac3	0.09	1.00000000	pigs
Klf9	0.17	1.00000000	pigs
Creg1	0.14	1.00000000	pigs
Armt1	0.09	1.00000000	pigs
Slc25a25	0.16	1.00000000	pigs
Phc2	0.34	1.00000000	pigs
Tpd52l2	0.25	1.00000000	pigs
Galnt7	0.57	1.00000000	pigs
Atf7ip	-0.39	1.00000000	pigs
Hsp90ab1	0.37	1.00000000	pigs
Nptn	0.24	1.00000000	pigs
Rasa2	0.40	1.00000000	pigs
Ap1g1	0.32	1.00000000	pigs
Nmral1	-0.26	1.00000000	pigs
Raf1	0.24	1.00000000	pigs
Tns3	0.33	1.00000000	pigs
Coro1b	0.43	1.00000000	pigs
Alox5	0.00	1.00000000	pigs
Atxn1	0.51	1.00000000	pigs
Cttnbp2nl	0.09	1.00000000	pigs
Tgfbr2	-0.49	1.00000000	pigs
Hexb	0.22	1.00000000	pigs
Rnf145	0.26	1.00000000	pigs
Clk3	0.15	1.00000000	pigs
Tstd1	0.11	1.00000000	pigs
Abtb1	-0.27	1.00000000	pigs
Pim2	0.12	1.00000000	pigs

Hspd1	0.33	1.00000000	pigs
Tnip3	0.16	1.00000000	pigs
Zdbf2	0.23	1.00000000	pigs
Wdr1	0.35	1.00000000	pigs
Ust	-0.43	1.00000000	pigs
Phlpp1	0.16	1.00000000	pigs
Ncoa3	0.34	1.00000000	pigs
Luzp1	0.32	1.00000000	pigs
Sp110	-0.35	1.00000000	pigs
Hipk3	0.37	1.00000000	pigs
Slc4a7	0.25	1.00000000	pigs
Fuca2	0.16	1.00000000	pigs
Skap2	0.25	1.00000000	pigs
Tcp11l2	0.18	1.00000000	pigs
Terb1	0.17	1.00000000	pigs
Psmd3	0.17	1.00000000	pigs
Ncoa7	0.95	1.00000000	pigs
Zfyve21	0.13	1.00000000	pigs
Trim44	-0.54	1.00000000	pigs
Ghitm	0.26	1.00000000	pigs
Gsn	0.27	1.00000000	pigs
R3hdm1	-0.43	1.00000000	pigs
Chrac1	-0.35	1.00000000	pigs
C4bp	0.11	1.00000000	pigs
Bzw1	-0.23	1.00000000	pigs
Glrx3	0.27	1.00000000	pigs
Pbxip1	0.14	1.00000000	pigs
Foxn3	-0.27	1.00000000	pigs
Itgb2l	0.36	1.00000000	pigs
Aifm2	0.18	1.00000000	pigs
1700102P08Rik	-0.15	1.00000000	pigs
Carm1	0.22	1.00000000	pigs
Cntfr	0.19	1.00000000	pigs
Tram1	-0.37	1.00000000	pigs
Dr1	0.30	1.00000000	pigs
Atp5g3	-0.37	1.00000000	pigs
Ppp2r1a	0.23	1.00000000	pigs
Tgif2	0.17	1.00000000	pigs
Cd81	0.37	1.00000000	pigs
Trim47	0.13	1.00000000	pigs
Ttc39c	0.65	1.00000000	pigs
Tmem11	0.22	1.00000000	pigs
Hsf5	-0.17	1.00000000	pigs
Tent4a	0.20	1.00000000	pigs
Cd164	0.27	1.00000000	pigs
Psmb2	-0.28	1.00000000	pigs

Cerk	-0.37	1.00000000	pigs
Alas1	0.13	1.00000000	pigs
Pxk	0.37	1.00000000	pigs
Cct8	0.23	1.00000000	pigs
Tmem203	-0.29	1.00000000	pigs
Rab11fip5	0.14	1.00000000	pigs
Trp53bp1	0.15	1.00000000	pigs
Chmp2b	0.16	1.00000000	pigs
Atp8a1	-0.24	1.00000000	pigs
Ykt6	0.30	1.00000000	pigs
Pomt1	0.10	1.00000000	pigs
Dhrs3	0.23	1.00000000	pigs
Tmx4	0.26	1.00000000	pigs
Dcaf5	0.23	1.00000000	pigs
Hadhb	0.15	1.00000000	pigs
Akt2	0.17	1.00000000	pigs
Adnp2	-0.36	1.00000000	pigs
Atp2b1	0.19	1.00000000	pigs
Susd1	-0.44	1.00000000	pigs
Arpc2	0.32	1.00000000	pigs
Prcc	0.18	1.00000000	pigs
Vpreb1	-0.58	1.00000000	pigs
Rnf115	0.20	1.00000000	pigs
Btaf1	-0.39	1.00000000	pigs
Psme1	-0.32	1.00000000	pigs
Tbc1d23	0.29	1.00000000	pigs
Slc6a9	0.25	1.00000000	pigs
Pdcd5	-0.43	1.00000000	pigs
Idua	0.13	1.00000000	pigs
Llph	0.25	1.00000000	pigs
Ttc21a	-0.18	1.00000000	pigs
Il6st	0.42	1.00000000	pigs
Tmod3	0.17	1.00000000	pigs
Ankrd37	0.20	1.00000000	pigs
Fam185a	0.10	1.00000000	pigs
Ssbp2	0.69	1.00000000	pigs
Aldh3a2	0.09	1.00000000	pigs
Smarcc1	0.27	1.00000000	pigs
Nxn	1.71	1.00000000	pigs
Pnpla8	0.25	1.00000000	pigs
Zfp408	0.11	1.00000000	pigs
Lrrc8d	0.58	1.00000000	pigs
Lrif1	-0.18	1.00000000	pigs
Atg4d	0.17	1.00000000	pigs
Zdhhc18	0.06	1.00000000	pigs
Shfl	0.09	1.00000000	pigs

Lhfpl2	0.27	1.00000000	pigs
Disp1	-0.43	1.00000000	pigs
Suco	0.48	1.00000000	pigs
Hat1	0.27	1.00000000	pigs
Brd1	0.21	1.00000000	pigs
Ash1l	0.25	1.00000000	pigs
Dffa	0.12	1.00000000	pigs
Prkg1	0.66	1.00000000	pigs
Pla2g4a	-0.35	1.00000000	pigs
Caml	0.24	1.00000000	pigs
Aopep	-0.48	1.00000000	pigs
Prdx5	0.11	1.00000000	pigs
Ube2z	0.18	1.00000000	pigs
Ppm1a	0.14	1.00000000	pigs
Dhrs11	0.17	1.00000000	pigs
Rnf122	0.20	1.00000000	pigs
Nadk	0.12	1.00000000	pigs
Pfkfb4	0.20	1.00000000	pigs
Nipa2	-0.35	1.00000000	pigs
Khdrbs1	0.31	1.00000000	pigs
Zfp36	-0.25	1.00000000	pigs
Dcbld2	0.19	1.00000000	pigs
Cd274	0.36	1.00000000	pigs
Leprotl1	-0.43	1.00000000	pigs
Agpat4	0.31	1.00000000	pigs
Folr1	0.73	1.00000000	pigs
Pmaip1	0.37	1.00000000	pigs
Pir	-0.34	1.00000000	pigs
Plekha1	0.23	1.00000000	pigs
Dbnl	0.26	1.00000000	pigs
Psmb4	0.27	1.00000000	pigs
Pcdh7	0.66	1.00000000	pigs
Zc3h7a	0.25	1.00000000	pigs
Tmppe	0.09	1.00000000	pigs
Dad1	-0.36	1.00000000	pigs
Fam102b	0.39	1.00000000	pigs
Dnase1l1	-0.48	1.00000000	pigs
Wee1	0.26	1.00000000	pigs
Fnip1	0.30	1.00000000	pigs
Btg1b	-0.34	1.00000000	pigs
Ap3d1	0.23	1.00000000	pigs
Ccdc167	0.14	1.00000000	pigs
Phkb	-0.41	1.00000000	pigs
Zfp647	0.09	1.00000000	pigs
Diaph1	0.30	1.00000000	pigs
Sptan1	0.01	1.00000000	pigs

Galc	0.12	1.00000000	pigs
Alkbh4	0.10	1.00000000	pigs
Adap2	0.18	1.00000000	pigs
Pdcd7	0.15	1.00000000	pigs
Adgrg5	-0.37	1.00000000	pigs
Osbpl11	-0.41	1.00000000	pigs
Adck5	0.13	1.00000000	pigs
Caprin1	0.26	1.00000000	pigs
Cxcr3	-0.26	1.00000000	pigs
Hspe1-rs1	0.32	1.00000000	pigs
Ccnt2	0.27	1.00000000	pigs
Nqo2	0.07	1.00000000	pigs
Asxl1	0.25	1.00000000	pigs
Crtc3	0.36	1.00000000	pigs
Cd44	-0.48	1.00000000	pigs
Sulf2	0.37	1.00000000	pigs
Sec24d	-0.47	1.00000000	pigs
Capg	0.89	1.00000000	pigs
Gba	0.21	1.00000000	pigs
Lhpp	0.09	1.00000000	pigs
Ahctf1	0.18	1.00000000	pigs
Il1r2	0.30	1.00000000	pigs
Bex2	-0.33	1.00000000	pigs
Zfp382	0.16	1.00000000	pigs
Nisch	0.24	1.00000000	pigs
Cd48	0.33	1.00000000	pigs
Fgd2	0.12	1.00000000	pigs
Vgll4	0.35	1.00000000	pigs
Pecam1	0.39	1.00000000	pigs
Eif3m	-0.34	1.00000000	pigs
Fnip2	0.19	1.00000000	pigs
Sbf2	-0.41	1.00000000	pigs
Il12rb1	0.14	1.00000000	pigs
Pign	0.26	1.00000000	pigs
Cmah	-0.38	1.00000000	pigs
Ccdc117	0.17	1.00000000	pigs
Xpr1	0.43	1.00000000	pigs
Ormdl3	-0.28	1.00000000	pigs
Cstf1	-0.28	1.00000000	pigs
Atp8b4	0.34	1.00000000	pigs
Rnft1	-0.35	1.00000000	pigs
Nol10	0.22	1.00000000	pigs
Eef1d	0.31	1.00000000	pigs
Acly	0.19	1.00000000	pigs
Smim15	-0.36	1.00000000	pigs
Rgl1	0.85	1.00000000	pigs

Glyr1	0.32	1.00000000	pigs
Psmd2	0.19	1.00000000	pigs
Drg1	0.22	1.00000000	pigs
Arhgap18	0.10	1.00000000	pigs
Hbs1l	-0.34	1.00000000	pigs
Acss1	0.06	1.00000000	pigs
Pcyt1a	0.19	1.00000000	pigs
Serpinf1	0.14	1.00000000	pigs
C2cd3	-0.48	1.00000000	pigs
Acsl4	0.09	1.00000000	pigs
Surf1	-0.29	1.00000000	pigs
Gpr162	-0.18	1.00000000	pigs
Ttc7	-0.26	1.00000000	pigs
Lnpk	0.27	1.00000000	pigs
Lamtor1	0.19	1.00000000	pigs
Bag5	0.21	1.00000000	pigs
Prmt1	0.21	1.00000000	pigs
Prpf31	0.10	1.00000000	pigs
Gpd2	0.40	1.00000000	pigs
Usp42	0.17	1.00000000	pigs
Slc7a1	-0.43	1.00000000	pigs
Ramp1	0.18	1.00000000	pigs
Snx3	0.33	1.00000000	pigs
Rab10	0.50	1.00000000	pigs
Sema4d	0.16	1.00000000	pigs
Fance	0.16	1.00000000	pigs
Narf	0.13	1.00000000	pigs
Ap2a2	0.14	1.00000000	pigs
S100a11	0.13	1.00000000	pigs
Pex13	0.14	1.00000000	pigs
Chodl	-0.24	1.00000000	pigs
Plekhh2	-0.17	1.00000000	pigs
Ncoa2	-0.46	1.00000000	pigs
Smarcd2	0.11	1.00000000	pigs
Glul	0.04	1.00000000	pigs
Tmem131l	0.42	1.00000000	pigs
Ola1	0.17	1.00000000	pigs
Cd69	-0.42	1.00000000	pigs
Thg1l	0.10	1.00000000	pigs
Tmco4	-0.36	1.00000000	pigs
Acbd6	-0.34	1.00000000	pigs
Daam1	0.43	1.00000000	pigs
Poldip3	0.20	1.00000000	pigs
Polr3h	0.09	1.00000000	pigs
Rbm26	0.35	1.00000000	pigs
Etnk1	0.37	1.00000000	pigs

Rbpj	0.24	1.00000000	pigs
Ranbp3	0.23	1.00000000	pigs
Stx6	0.19	1.00000000	pigs
Ik	-0.32	1.00000000	pigs
Agpat2	-0.41	1.00000000	pigs
Fbxl17	-0.33	1.00000000	pigs
Pqbp1	0.13	1.00000000	pigs
Kctd12	-0.31	1.00000000	pigs
Epb41	0.43	1.00000000	pigs
Ddit4	0.61	1.00000000	pigs
Pde7a	0.84	1.00000000	pigs
Gramd1a	0.16	1.00000000	pigs
Nat10	-0.29	1.00000000	pigs
Phf12	0.18	1.00000000	pigs
Glrx5	0.04	1.00000000	pigs
Ankhd1	0.33	1.00000000	pigs
Ddx21	-0.32	1.00000000	pigs
Lrrc57	0.13	1.00000000	pigs
Chp1	0.25	1.00000000	pigs
Atad2b	0.28	1.00000000	pigs
Pfkfb3	0.41	1.00000000	pigs
Arl5a	-0.39	1.00000000	pigs
Anxa11	0.23	1.00000000	pigs
Spata6	-0.42	1.00000000	pigs
Clint1	0.26	1.00000000	pigs
Btbd6	0.06	1.00000000	pigs
Pop7	0.17	1.00000000	pigs
Vcam1	0.30	1.00000000	pigs
Trmt112	-0.35	1.00000000	pigs
G3bp2	0.22	1.00000000	pigs
Klhl21	0.11	1.00000000	pigs
Creb3l2	-0.37	1.00000000	pigs
Fastk	0.11	1.00000000	pigs
Limk1	0.15	1.00000000	pigs
Zdhhc5	0.17	1.00000000	pigs
Acap2	0.34	1.00000000	pigs
Sfswap	0.21	1.00000000	pigs
Smad4	0.27	1.00000000	pigs
B4galt2	0.08	1.00000000	pigs
Lcp2	0.11	1.00000000	pigs
Rab11a	0.26	1.00000000	pigs
Arhgap1	0.27	1.00000000	pigs
D3Ertd751e	-0.20	1.00000000	pigs
Tec	0.75	1.00000000	pigs
Ptpn11	0.22	1.00000000	pigs
Clptm1l	0.17	1.00000000	pigs

Ndfip1	-0.30	1.00000000	pigs
Slc31a2	0.17	1.00000000	pigs
Tcf7l2	0.45	1.00000000	pigs
Polr1c	0.15	1.00000000	pigs
Tmtc3	0.17	1.00000000	pigs
Cyria	0.22	1.00000000	pigs
Rfk	0.15	1.00000000	pigs
Cep164	-0.24	1.00000000	pigs
Septin7	-0.22	1.00000000	pigs
Sema6b	-0.29	1.00000000	pigs
Nt5dc1	-0.48	1.00000000	pigs
Cmpk2	-0.25	1.00000000	pigs
Atf3	-0.15	1.00000000	pigs
Sap130	0.17	1.00000000	pigs
Vopp1	0.27	1.00000000	pigs
Flt1	0.51	1.00000000	pigs
Ptrh1	0.08	1.00000000	pigs
Cbl	0.32	1.00000000	pigs
Trappc9	0.32	1.00000000	pigs
Nufip2	0.30	1.00000000	pigs
Nob1	0.14	1.00000000	pigs
Sh2b2	0.11	1.00000000	pigs
Ezr	-0.31	1.00000000	pigs
Socs3	0.40	1.00000000	pigs
Nod2	0.12	1.00000000	pigs
Zfp787	0.12	1.00000000	pigs
Coq7	-0.23	1.00000000	pigs
Stxbp2	-0.36	1.00000000	pigs
Paxx	-0.34	1.00000000	pigs
Nmnat1	0.05	1.00000000	pigs
Cd2	0.34	1.00000000	pigs
Hivep1	0.44	1.00000000	pigs
Deptor	0.26	1.00000000	pigs
Cd3eap	-0.02	1.00000000	pigs
Gpx1	0.43	1.00000000	pigs
St3gal5	0.26	1.00000000	pigs
Elk3	0.22	1.00000000	pigs
Ppp2r3a	-0.42	1.00000000	pigs
Thoc7	-0.39	1.00000000	pigs
Ilrun	0.38	1.00000000	pigs
Gnai2	0.26	1.00000000	pigs
P2ry12	-0.56	1.00000000	pigs
Nagk	0.07	1.00000000	pigs
Rhoh	-0.32	1.00000000	pigs
Eif3g	0.26	1.00000000	pigs
Slc20a1	0.17	1.00000000	pigs

Pter	-0.09	1.00000000	pigs
Frrs1	-0.21	1.00000000	pigs
Bex4	-0.24	1.00000000	pigs
Bcl6	0.09	1.00000000	pigs
Gapt	-0.34	1.00000000	pigs
Dcbld1	0.10	1.00000000	pigs
Ubp1	0.29	1.00000000	pigs
Otud7b	0.09	1.00000000	pigs
Tspo	0.23	1.00000000	pigs
BC005624	-0.36	1.00000000	pigs
Crlf3	0.43	1.00000000	pigs
Eif2ak2	-0.35	1.00000000	pigs
Idh3g	0.18	1.00000000	pigs
Nr4a2	0.40	1.00000000	pigs
Fbxo39	-0.36	1.00000000	pigs
Kcnq1	0.16	1.00000000	pigs
Acvr1b	0.09	1.00000000	pigs
Tnfaip8l2	0.11	1.00000000	pigs
Slc9a3r1	-0.27	1.00000000	pigs
Ccpg1	-0.28	1.00000000	pigs
Atp23	0.06	1.00000000	pigs
Erlec1	-0.42	1.00000000	pigs
Rasgrp4	0.07	1.00000000	pigs
Egr1	-0.56	1.00000000	pigs
Rogdi	0.07	1.00000000	pigs
Afg1l	-0.43	1.00000000	pigs
Secisbp2l	0.14	1.00000000	pigs
Rasa3	-0.37	1.00000000	pigs
Rbpms	0.09	1.00000000	pigs
Usp9x	0.27	1.00000000	pigs
St3gal1	0.22	1.00000000	pigs
Slc1a2	-0.46	1.00000000	pigs
Slc16a6	0.08	1.00000000	pigs
Tubb4b	-0.15	1.00000000	pigs
Dmac2	0.14	1.00000000	pigs
Tkt	0.20	1.00000000	pigs
Klf6	-0.20	1.00000000	pigs
Atxn7	0.31	1.00000000	pigs
Scand1	-0.33	1.00000000	pigs
Acsl3	0.15	1.00000000	pigs
Rasal3	0.10	1.00000000	pigs
Klf10	0.35	1.00000000	pigs
Ccnq	0.14	1.00000000	pigs
Unc119	0.17	1.00000000	pigs
Tiam2	0.14	1.00000000	pigs
Slc39a13	0.05	1.00000000	pigs

Lin52	0.07	1.00000000	pigs
H1f4	-0.37	1.00000000	pigs
Lpgat1	-0.48	1.00000000	pigs
Zfp326	0.19	1.00000000	pigs
Niban2	0.15	1.00000000	pigs
Otud4	0.15	1.00000000	pigs
Eya3	0.25	1.00000000	pigs
Ankrd33b	0.80	1.00000000	pigs
Utp11	-0.31	1.00000000	pigs
Sin3a	0.23	1.00000000	pigs
Rev3l	0.48	1.00000000	pigs
Agpat3	-0.56	1.00000000	pigs
Ccl17	1.93	1.00000000	pigs
Gnb5	0.12	1.00000000	pigs
Slamf6	-0.23	1.00000000	pigs
Fbxo7	0.15	1.00000000	pigs
Huwe1	0.22	1.00000000	pigs
Cxcr4	0.58	1.00000000	pigs
Csad	-0.18	1.00000000	pigs
Ssbp3	0.48	1.00000000	pigs
Card11	0.54	1.00000000	pigs
Dcun1d4	0.20	1.00000000	pigs
Itgb1bp1	0.14	1.00000000	pigs
Srpr	-0.38	1.00000000	pigs
Ecpas	0.24	1.00000000	pigs
Ndufa1	-0.47	1.00000000	pigs
Xcr1	0.43	1.00000000	pigs
Dbndd2	0.20	1.00000000	pigs
Abcc2	-0.26	1.00000000	pigs
Smim26	-0.16	1.00000000	pigs
Fnbp1l	0.16	1.00000000	pigs
Tmed4	-0.35	1.00000000	pigs
Atp2a2	0.25	1.00000000	pigs
Spred1	0.20	1.00000000	pigs
Fbrsl1	-0.26	1.00000000	pigs
Fam219b	0.09	1.00000000	pigs
Pigk	-0.28	1.00000000	pigs
Tmem189	0.21	1.00000000	pigs
Snx11	0.06	1.00000000	pigs
Nuak2	-0.51	1.00000000	pigs
Gpr34	-0.34	1.00000000	pigs
Cish	0.16	1.00000000	pigs
Slmap	0.06	1.00000000	pigs
Sugct	0.36	1.00000000	pigs
Phrf1	0.19	1.00000000	pigs
Rbis	-0.33	1.00000000	pigs

Foxk2	0.22	1.00000000	pigs
Ndufa8	0.10	1.00000000	pigs
Crem	0.31	1.00000000	pigs
Ppp1r3d	0.10	1.00000000	pigs
Slc66a2	0.22	1.00000000	pigs
Bud23	-0.06	1.00000000	pigs
Phf21a	-0.53	1.00000000	pigs
Cdk14	-0.55	1.00000000	pigs
Taok1	0.28	1.00000000	pigs
Ppp4r3a	0.26	1.00000000	pigs
Slc66a1	0.11	1.00000000	pigs
Rgs18	0.46	1.00000000	pigs
Efna1	-0.23	1.00000000	pigs
Prkab2	0.13	1.00000000	pigs
Socs5	0.13	1.00000000	pigs
Sgpp1	0.18	1.00000000	pigs
Tbc1d10b	0.10	1.00000000	pigs
2300009A05Rik	-0.19	1.00000000	pigs
Tgfbi	-0.31	1.00000000	pigs
Rab35	0.17	1.00000000	pigs
Dmap1	0.10	1.00000000	pigs
Pip5k1a	0.29	1.00000000	pigs
Cltc	0.22	1.00000000	pigs
Optn	-0.24	1.00000000	pigs
Zfp512b	0.14	1.00000000	pigs
Fam91a1	0.18	1.00000000	pigs
Tysnd1	-0.21	1.00000000	pigs
Rora	0.16	1.00000000	pigs
Ptpdc1	-0.38	1.00000000	pigs
Rbak	0.10	1.00000000	pigs
Spin1	0.13	1.00000000	pigs
Fbxw11	0.28	1.00000000	pigs
Crhbp	-0.08	1.00000000	pigs
Stk25	0.10	1.00000000	pigs
Pkd1	0.10	1.00000000	pigs
Hus1	0.13	1.00000000	pigs
Gpr107	0.24	1.00000000	pigs
Npepps	0.18	1.00000000	pigs
Oaz1	-0.29	1.00000000	pigs
Rufy2	-0.30	1.00000000	pigs
Alox5ap	-0.08	1.00000000	pigs
Gna13	0.32	1.00000000	pigs
Notch2	0.34	1.00000000	pigs
Il17ra	0.13	1.00000000	pigs
Smarcad1	-0.34	1.00000000	pigs
Fam3a	0.09	1.00000000	pigs

Dnmt1	0.23	1.00000000	pigs
Cox20	-0.34	1.00000000	pigs
Elf1	0.30	1.00000000	pigs
9130409I23Rik	0.31	1.00000000	pigs
Sap30	0.12	1.00000000	pigs
Rftn1	0.44	1.00000000	pigs
Capn7	-0.18	1.00000000	pigs
Ehbp1l1	0.19	1.00000000	pigs
Dtx3	-0.18	1.00000000	pigs
Zfp687	0.08	1.00000000	pigs
Lama3	-0.22	1.00000000	pigs
Acads	0.24	1.00000000	pigs
Tm7sf3	0.12	1.00000000	pigs
Nup153	0.28	1.00000000	pigs
Tbck	-0.32	1.00000000	pigs
Fam111a	0.11	1.00000000	pigs
Plekhg3	0.17	1.00000000	pigs
Cdk16	0.13	1.00000000	pigs
Serping1	0.31	1.00000000	pigs
Prrc1	-0.28	1.00000000	pigs
Sorbs1	0.20	1.00000000	pigs
Smndc1	0.35	1.00000000	pigs
Dcun1d3	0.38	1.00000000	pigs
Iqgap1	0.30	1.00000000	pigs
Wasl	0.27	1.00000000	pigs
Vbp1	0.28	1.00000000	pigs
Ccser1	0.46	1.00000000	pigs
Pawr	0.10	1.00000000	pigs
Creb5	0.34	1.00000000	pigs
Xpnpep1	0.13	1.00000000	pigs
Ly96	0.23	1.00000000	pigs
Scnm1	0.07	1.00000000	pigs
Cars	0.13	1.00000000	pigs
Scly	0.22	1.00000000	pigs
Creld1	0.13	1.00000000	pigs
Cd14	0.26	1.00000000	pigs
Rap1b	0.20	1.00000000	pigs
Dhx15	0.25	1.00000000	pigs
Ivns1abp	0.24	1.00000000	pigs
Rnf138	0.25	1.00000000	pigs
Atp6v1a	0.04	1.00000000	pigs
Herc4	0.10	1.00000000	pigs
Pgam2	0.33	1.00000000	pigs
Gpnmb	0.15	1.00000000	pigs
Tnrc18	0.18	1.00000000	pigs
Ell	0.33	1.00000000	pigs

Card9	0.13	1.00000000	pigs
Zswim7	-0.23	1.00000000	pigs
Pdap1	-0.34	1.00000000	pigs
Lcorl	0.31	1.00000000	pigs
Prkce	0.47	1.00000000	pigs
Glb1	0.28	1.00000000	pigs
Dnajc19	-0.27	1.00000000	pigs
Ndufaf8	-0.22	1.00000000	pigs
Topaz1	-0.11	1.00000000	pigs
Utp23	-0.24	1.00000000	pigs
Polr3a	-0.29	1.00000000	pigs
Gsdme	-0.12	1.00000000	pigs
Sel1l	0.17	1.00000000	pigs
Arhgef26	-0.26	1.00000000	pigs
Fig4	-0.39	1.00000000	pigs
Il12rb2	0.34	1.00000000	pigs
Exph5	-0.23	1.00000000	pigs
Dpm2	-0.29	1.00000000	pigs
Fam168b	0.34	1.00000000	pigs
Grina	0.08	1.00000000	pigs
Nedd8	-0.33	1.00000000	pigs
Tlr4	0.23	1.00000000	pigs
Dusp23	0.07	1.00000000	pigs
Ikzf1	-0.34	1.00000000	pigs
Cep43	0.14	1.00000000	pigs
Fam172a	-0.30	1.00000000	pigs
Zhx2	0.49	1.00000000	pigs
Snx21	0.08	1.00000000	pigs
Usf2	0.15	1.00000000	pigs
Fxr1	0.17	1.00000000	pigs
Galnt10	0.23	1.00000000	pigs
Rbbp6	0.28	1.00000000	pigs
Hebp1	-0.17	1.00000000	pigs
Acadl	-0.22	1.00000000	pigs
Clec7a	0.11	1.00000000	pigs
Dhrs9	-0.37	1.00000000	pigs
Kdm5a	0.19	1.00000000	pigs
Prnp	0.04	1.00000000	pigs
Sod1	-0.10	1.00000000	pigs
Entpd4	0.18	1.00000000	pigs
Prkcsh	0.17	1.00000000	pigs
Dync1li1	0.56	1.00000000	pigs
Zfp410	0.10	1.00000000	pigs
Ttc17	-0.28	1.00000000	pigs
Tspan5	0.46	1.00000000	pigs
Dtnbp1	0.23	1.00000000	pigs

Zfp827	0.04	1.00000000	pigs
Vps37a	0.10	1.00000000	pigs
C330018D20Rik	0.07	1.00000000	pigs
Ap1ar	0.11	1.00000000	pigs
Gna12	0.18	1.00000000	pigs
Alkbh5	0.12	1.00000000	pigs
Exo5	-0.17	1.00000000	pigs
Gale	0.06	1.00000000	pigs
Palb2	0.04	1.00000000	pigs
Lsm14b	0.13	1.00000000	pigs
Itgam	0.15	1.00000000	pigs
Zfp518b	0.07	1.00000000	pigs
Fam118b	0.15	1.00000000	pigs
Rexo2	-0.31	1.00000000	pigs
Prpf4b	-0.23	1.00000000	pigs
B4galt5	0.15	1.00000000	pigs
Fkbp3	-0.26	1.00000000	pigs
Acer2	0.10	1.00000000	pigs
Slc19a2	0.06	1.00000000	pigs
Dpysl2	0.25	1.00000000	pigs
Nt5c	0.10	1.00000000	pigs
Ethe1	-0.33	1.00000000	pigs
Rnf220	-0.26	1.00000000	pigs
Tbc1d31	-0.15	1.00000000	pigs
Capn2	0.15	1.00000000	pigs
Cstf3	0.21	1.00000000	pigs
Eif3b	0.14	1.00000000	pigs
Osbpl10	-0.33	1.00000000	pigs
Nup88	0.11	1.00000000	pigs
Aldh7a1	-0.36	1.00000000	pigs
Slc35f5	0.18	1.00000000	pigs
Adora2a	-0.03	1.00000000	pigs
Slco5a1	0.09	1.00000000	pigs
Zbtb7a	0.28	1.00000000	pigs
Tex10	0.10	1.00000000	pigs
Anxa7	0.15	1.00000000	pigs
Akt3	0.31	1.00000000	pigs
Sypl	-0.28	1.00000000	pigs
Atg16l1	0.34	1.00000000	pigs
Zfp131	0.17	1.00000000	pigs
Hnrnpul1	0.04	1.00000000	pigs
Ccdc9	0.24	1.00000000	pigs
Gucy1a1	-0.40	1.00000000	pigs
Igf1r	0.79	1.00000000	pigs
Golga7	0.19	1.00000000	pigs
Psmg4	-0.36	1.00000000	pigs

Snx15	-0.25	1.00000000	pigs
Nucks1	0.26	1.00000000	pigs
Rnf157	-0.25	1.00000000	pigs
Slc8a1	-0.31	1.00000000	pigs
Pef1	0.19	1.00000000	pigs
Slc9a7	0.28	1.00000000	pigs
Dync1h1	0.18	1.00000000	pigs
Abca1	0.24	1.00000000	pigs
Plekha7	0.25	1.00000000	pigs
Pakap	0.30	1.00000000	pigs
Ndufs4	-0.30	1.00000000	pigs
Fbxo42	0.16	1.00000000	pigs
Wwp2	0.21	1.00000000	pigs
Ankrd17	0.28	1.00000000	pigs
Gtf3c4	0.09	1.00000000	pigs
Ncr1	-0.30	1.00000000	pigs
C2	-0.23	1.00000000	pigs
Fzd5	0.11	1.00000000	pigs
Ubr4	0.23	1.00000000	pigs
Rnf139	0.23	1.00000000	pigs
Ska2	0.07	1.00000000	pigs
Emc7	-0.35	1.00000000	pigs
Slc35c2	-0.26	1.00000000	pigs
Diaph2	-0.24	1.00000000	pigs
Ube3a	0.32	1.00000000	pigs
Bcl2l14	0.28	1.00000000	pigs
Tor3a	0.06	1.00000000	pigs
Shisa5	0.13	1.00000000	pigs
Fam210b	0.07	1.00000000	pigs
Ogdh	0.26	1.00000000	pigs
Sqle	0.24	1.00000000	pigs
Swap70	-0.38	1.00000000	pigs
Psmb10	0.19	1.00000000	pigs
Tra2b	-0.22	1.00000000	pigs
Tmem144	-0.34	1.00000000	pigs
Apc	-0.35	1.00000000	pigs
Cspp1	0.26	1.00000000	pigs
Tnpo1	0.41	1.00000000	pigs
Canx	-0.21	1.00000000	pigs
Abcg1	0.14	1.00000000	pigs
Cd8b1	-0.17	1.00000000	pigs
Thap7	0.12	1.00000000	pigs
Asxl2	0.14	1.00000000	pigs
Tcf20	0.16	1.00000000	pigs
Syk	-0.20	1.00000000	pigs
Hint1	0.27	1.00000000	pigs

Acaca	0.22	1.00000000	pigs
Sema7a	0.14	1.00000000	pigs
Tasp1	-0.17	1.00000000	pigs
Thoc6	0.13	1.00000000	pigs
Hspa8	0.32	1.00000000	pigs
Stt3b	0.33	1.00000000	pigs
Ufsp2	-0.04	1.00000000	pigs
Rgs19	0.25	1.00000000	pigs
Ndufa5	-0.23	1.00000000	pigs
Piga	0.07	1.00000000	pigs
Immt	0.19	1.00000000	pigs
Tut4	-0.41	1.00000000	pigs
AI597479	0.05	1.00000000	pigs
Psmd14	0.24	1.00000000	pigs
Ninl	-0.41	1.00000000	pigs
Slc35a1	0.13	1.00000000	pigs
Ctnna3	-0.61	1.00000000	pigs
Sh3bp5	-0.43	1.00000000	pigs
Abcb10	-0.28	1.00000000	pigs
Pum2	0.21	1.00000000	pigs
Rnf10	0.28	1.00000000	pigs
Got1	0.12	1.00000000	pigs
Tom1l2	0.11	1.00000000	pigs
Ube2r2	0.31	1.00000000	pigs
Trim14	-0.17	1.00000000	pigs
Pacs2	0.17	1.00000000	pigs
Clec5a	0.08	1.00000000	pigs
S100a10	-0.10	1.00000000	pigs
Rtraf	0.13	1.00000000	pigs
Nvl	-0.28	1.00000000	pigs
Hs6st1	0.17	1.00000000	pigs
Orai2	0.09	1.00000000	pigs
Zc3h12a	0.01	1.00000000	pigs
Hmces	-0.37	1.00000000	pigs
Arpc4	0.25	1.00000000	pigs
Xpo1	0.08	1.00000000	pigs
Sat1	0.19	1.00000000	pigs
Psmb7	0.29	1.00000000	pigs
Unc5c	-0.38	1.00000000	pigs
Nav2	0.09	1.00000000	pigs
Synrg	-0.31	1.00000000	pigs
Ror2	-0.32	1.00000000	pigs
Epc2	-0.44	1.00000000	pigs
Rnf38	0.31	1.00000000	pigs
Tmem64	0.04	1.00000000	pigs
Trmt10b	-0.16	1.00000000	pigs

Pkn1	0.12	1.00000000	pigs
Cdkn1c	0.32	1.00000000	pigs
Cdk8	0.21	1.00000000	pigs
Slc30a1	0.19	1.00000000	pigs
1810030O07Rik	0.07	1.00000000	pigs
Coro1c	0.24	1.00000000	pigs
Tsen34	0.12	1.00000000	pigs
Stk32b	-0.38	1.00000000	pigs
Ostf1	0.37	1.00000000	pigs
Gpr157	0.00	1.00000000	pigs
Dohh	0.13	1.00000000	pigs
Nek9	0.14	1.00000000	pigs
Nsdhl	0.04	1.00000000	pigs
Sec11a	-0.28	1.00000000	pigs
Qdpr	-0.26	1.00000000	pigs
Snrnp48	0.20	1.00000000	pigs
Parp8	-0.10	1.00000000	pigs
Hspa9	0.20	1.00000000	pigs
Robo2	-0.53	1.00000000	pigs
Gtf2b	-0.33	1.00000000	pigs
Svil	0.06	1.00000000	pigs
Bloc1s5	0.13	1.00000000	pigs
Pias2	0.23	1.00000000	pigs
Anxa5	0.18	1.00000000	pigs
Ppara	-0.28	1.00000000	pigs
Tmem70	0.07	1.00000000	pigs
Dmxl1	0.19	1.00000000	pigs
Wfs1	0.08	1.00000000	pigs
Cyhr1	0.09	1.00000000	pigs
Fbxo11	0.34	1.00000000	pigs
DPCD	0.02	1.00000000	pigs
Rela	0.12	1.00000000	pigs
Atp5d	0.18	1.00000000	pigs
Ncor2	0.19	1.00000000	pigs
Adipor2	-0.36	1.00000000	pigs
Fyco1	-0.23	1.00000000	pigs
Smarca5	0.28	1.00000000	pigs
Tsc22d2	0.38	1.00000000	pigs
Enpp4	0.11	1.00000000	pigs
Dpy19l3	0.15	1.00000000	pigs
Pitpnb	0.26	1.00000000	pigs
Cox17	-0.27	1.00000000	pigs
Gdi1	0.08	1.00000000	pigs
Zfr	0.19	1.00000000	pigs
Wbp2	0.12	1.00000000	pigs
Klhl36	0.13	1.00000000	pigs

Sbf1	0.07	1.00000000	pigs
Otulin	0.22	1.00000000	pigs
Ric1	0.38	1.00000000	pigs
Camsap2	0.34	1.00000000	pigs
Ube2a	0.24	1.00000000	pigs
Fubp1	0.13	1.00000000	pigs
Immp1l	-0.40	1.00000000	pigs
Pdxdc1	0.20	1.00000000	pigs
Gls	0.20	1.00000000	pigs
Nrap	0.06	1.00000000	pigs
Cad	0.07	1.00000000	pigs
Akna	-0.41	1.00000000	pigs
Tk2	-0.18	1.00000000	pigs
Cers6	0.32	1.00000000	pigs
Depdc7	0.06	1.00000000	pigs
Brms1	0.15	1.00000000	pigs
Papss1	0.13	1.00000000	pigs
Dis3l	0.11	1.00000000	pigs
Ttc13	-0.37	1.00000000	pigs
Inpp4b	-0.47	1.00000000	pigs
Dgkh	-0.02	1.00000000	pigs
Eif2d	-0.31	1.00000000	pigs
Safb2	0.04	1.00000000	pigs
Gucy2c	-0.22	1.00000000	pigs
Itm2b	0.23	1.00000000	pigs
B4galt4	0.14	1.00000000	pigs
Urgcp	0.12	1.00000000	pigs
Zswim4	0.09	1.00000000	pigs
Gatad2a	0.16	1.00000000	pigs
Add3	-0.36	1.00000000	pigs
Prkx	0.28	1.00000000	pigs
Naa50	0.20	1.00000000	pigs
Cebpz	-0.28	1.00000000	pigs
Tvp23a	0.13	1.00000000	pigs
Hsd17b11	0.14	1.00000000	pigs
Ripk1	0.11	1.00000000	pigs
Nnt	-0.44	1.00000000	pigs
Llgl1	0.15	1.00000000	pigs
Uxt	-0.30	1.00000000	pigs
Phf14	-0.43	1.00000000	pigs
Oser1	0.17	1.00000000	pigs
Timm22	-0.22	1.00000000	pigs
Ift57	0.05	1.00000000	pigs
Dnaja2	-0.25	1.00000000	pigs
Slc35b4	0.03	1.00000000	pigs
Polr2a	0.16	1.00000000	pigs

Pou6f1	-0.16	1.00000000	pigs
Stk17b	0.26	1.00000000	pigs
Snx30	0.09	1.00000000	pigs
Cbx4	-0.24	1.00000000	pigs
Pdss1	-0.42	1.00000000	pigs
Plekha5	0.80	1.00000000	pigs
Helb	-0.35	1.00000000	pigs
Impa1	-0.20	1.00000000	pigs
Osbp	-0.18	1.00000000	pigs
Gatad2b	0.25	1.00000000	pigs
Gk5	0.25	1.00000000	pigs
Atg2a	0.01	1.00000000	pigs
Oxa1l	0.17	1.00000000	pigs
Farp2	0.10	1.00000000	pigs
Sbno2	0.12	1.00000000	pigs
Cd34	0.11	1.00000000	pigs
Ivd	-0.26	1.00000000	pigs
Zfc3h1	0.27	1.00000000	pigs
Zbtb22	0.09	1.00000000	pigs
Il1rap	0.44	1.00000000	pigs
Akr1a1	0.23	1.00000000	pigs
Pea15a	0.22	1.00000000	pigs
Rnf4	0.22	1.00000000	pigs
Pgm3	-0.16	1.00000000	pigs
Hacd3	-0.27	1.00000000	pigs
Bend7	-0.34	1.00000000	pigs
Zfp62	-0.19	1.00000000	pigs
Tmem268	-0.21	1.00000000	pigs
Stpg4	-0.19	1.00000000	pigs
Trim33	0.23	1.00000000	pigs
Ccng1	0.17	1.00000000	pigs
Htatip2	-0.25	1.00000000	pigs
Adamts6	-0.35	1.00000000	pigs
Dynlt1a	0.16	1.00000000	pigs
Hs3st1	0.28	1.00000000	pigs
Fam222b	-0.34	1.00000000	pigs
Ppp1r12c	0.31	1.00000000	pigs
Iars	-0.25	1.00000000	pigs
Tlcd2	0.10	1.00000000	pigs
Invs	-0.30	1.00000000	pigs
Tram2	-0.37	1.00000000	pigs
Akap8l	0.07	1.00000000	pigs
Ino80c	0.06	1.00000000	pigs
Gpr45	-0.19	1.00000000	pigs
Gsto1	0.18	1.00000000	pigs
Fmnl2	-0.34	1.00000000	pigs

Psmc4	0.16	1.00000000	pigs
Cd93	-0.16	1.00000000	pigs
Eif1ad	0.05	1.00000000	pigs
Pik3c3	-0.25	1.00000000	pigs
1110065P20Rik	0.09	1.00000000	pigs
Arl14ep	0.19	1.00000000	pigs
Psma5	0.14	1.00000000	pigs
Leprot	0.13	1.00000000	pigs
Dnajc15	-0.23	1.00000000	pigs
Sec23ip	0.17	1.00000000	pigs
Usp15	-0.22	1.00000000	pigs
Xpo5	-0.05	1.00000000	pigs
Elp5	0.11	1.00000000	pigs
Sipa1l1	0.40	1.00000000	pigs
Vps37b	-0.04	1.00000000	pigs
Orc2	-0.16	1.00000000	pigs
Snrnp35	-0.21	1.00000000	pigs
Bcl11a	-0.23	1.00000000	pigs
Heatr1	0.07	1.00000000	pigs
Crebrf	0.14	1.00000000	pigs
Hprt	0.13	1.00000000	pigs
Cep170	0.30	1.00000000	pigs
Tmem50b	0.19	1.00000000	pigs
Tuba4a	-0.10	1.00000000	pigs
Rbms1	0.39	1.00000000	pigs
Gpsm3	0.12	1.00000000	pigs
Rnpep	0.15	1.00000000	pigs
Whamm	0.14	1.00000000	pigs
Kdm3a	0.05	1.00000000	pigs
Ccnh	0.08	1.00000000	pigs
Pnkp	0.17	1.00000000	pigs
2610507B11Rik	0.14	1.00000000	pigs
Rab7	0.21	1.00000000	pigs
Lad1	0.12	1.00000000	pigs
Lonrf3	0.25	1.00000000	pigs
Slc5a6	0.06	1.00000000	pigs
Aff2	0.07	1.00000000	pigs
Imp3	-0.32	1.00000000	pigs
Slc35d1	0.17	1.00000000	pigs
Gtf2f2	-0.28	1.00000000	pigs
Tnfrsf9	-0.01	1.00000000	pigs
Wars	0.10	1.00000000	pigs
Zkscan6	-0.27	1.00000000	pigs
Tet1	-0.20	1.00000000	pigs
Snrpb2	-0.29	1.00000000	pigs
Fli1	-0.24	1.00000000	pigs

Pex5	0.07	1.00000000	pigs
Zfp90	0.13	1.00000000	pigs
Adcy6	0.09	1.00000000	pigs
Rnf14	-0.32	1.00000000	pigs
Ncl	-0.17	1.00000000	pigs
Spsb3	0.12	1.00000000	pigs
Odf2	0.10	1.00000000	pigs
Lrp11	-0.12	1.00000000	pigs
Arl15	-0.33	1.00000000	pigs
Clasp1	0.26	1.00000000	pigs
Cluh	0.08	1.00000000	pigs
Vamp5	0.10	1.00000000	pigs
Ern1	-0.27	1.00000000	pigs
Actr1a	0.06	1.00000000	pigs
Ktn1	-0.32	1.00000000	pigs
Itgav	0.27	1.00000000	pigs
Hfm1	-0.19	1.00000000	pigs
Rnf121	1.08	1.00000000	pigs
Tmed9	-0.30	1.00000000	pigs
Plekhg5	-0.25	1.00000000	pigs
Ano6	0.38	1.00000000	pigs
Dnajc9	0.10	1.00000000	pigs
Coq2	-0.37	1.00000000	pigs
Nr4a1	0.15	1.00000000	pigs
Lpcat1	0.06	1.00000000	pigs
Pcm1	0.11	1.00000000	pigs
Bcat1	0.13	1.00000000	pigs
Ccdc88c	-0.24	1.00000000	pigs
Lins1	-0.12	1.00000000	pigs
Gna15	0.12	1.00000000	pigs
Ski	0.11	1.00000000	pigs
Tle3	0.15	1.00000000	pigs
Aimp2	0.32	1.00000000	pigs
Tmem140	0.12	1.00000000	pigs
Pam16	-0.32	1.00000000	pigs
Nol4l	0.20	1.00000000	pigs
Sytl3	0.23	1.00000000	pigs
Eif5b	0.32	1.00000000	pigs
Polr2h	0.06	1.00000000	pigs
Rad17	-0.22	1.00000000	pigs
Crtc2	0.10	1.00000000	pigs
Rab1a	0.25	1.00000000	pigs
Osgin2	0.11	1.00000000	pigs
Hlx	0.09	1.00000000	pigs
Zfp146	0.01	1.00000000	pigs
Luc7l3	0.20	1.00000000	pigs

Abraxas2	0.13	1.00000000	pigs
Rsl1d1	-0.17	1.00000000	pigs
Tnpo3	0.34	1.00000000	pigs
Jag1	-0.23	1.00000000	pigs
Junb	0.03	1.00000000	pigs
Jak3	0.07	1.00000000	pigs
Hipk2	0.77	1.00000000	pigs
Nf2	0.09	1.00000000	pigs
Unc13b	0.09	1.00000000	pigs
Kcna3	0.19	1.00000000	pigs
Ncs1	0.18	1.00000000	pigs
Nt5dc2	0.05	1.00000000	pigs
Aldoart2	0.06	1.00000000	pigs
Nutf2	0.11	1.00000000	pigs
Tbl2	0.12	1.00000000	pigs
Ralb	0.15	1.00000000	pigs
U2surp	0.29	1.00000000	pigs
Acp2	0.07	1.00000000	pigs
Ubash3b	0.47	1.00000000	pigs
Wrnip1	0.17	1.00000000	pigs
Adam28	-0.08	1.00000000	pigs
Selenos	-0.31	1.00000000	pigs
Ppp2r3c	0.13	1.00000000	pigs
Arhgap22	0.28	1.00000000	pigs
Ccdc85a	-0.37	1.00000000	pigs
Araf	0.15	1.00000000	pigs
Naa16	0.23	1.00000000	pigs
Commd4	0.15	1.00000000	pigs
Rngtt	-0.30	1.00000000	pigs
Klf7	0.17	1.00000000	pigs
Zc3h7b	0.10	1.00000000	pigs
Tmigd3	0.12	1.00000000	pigs
Dock4	0.15	1.00000000	pigs
Cisd3	0.05	1.00000000	pigs
Smyd2	0.01	1.00000000	pigs
Atp9b	-0.35	1.00000000	pigs
Churc1	0.07	1.00000000	pigs
Uck1	0.07	1.00000000	pigs
Rhot1	-0.38	1.00000000	pigs
Eif4e3	-0.29	1.00000000	pigs
Wdfy4	-0.63	1.00000000	pigs
Fxn	-0.17	1.00000000	pigs
Sf3b3	0.16	1.00000000	pigs
Cox8a	-0.27	1.00000000	pigs
Slc16a1	0.03	1.00000000	pigs
Bhlha15	0.32	1.00000000	pigs

Txndc9	-0.28	1.00000000	pigs
Ier3	0.13	1.00000000	pigs
Dph6	-0.27	1.00000000	pigs
Klhl5	0.04	1.00000000	pigs
Ccdc28a	-0.28	1.00000000	pigs
Supt3	0.20	1.00000000	pigs
Wdfy1	0.18	1.00000000	pigs
Klhl24	0.12	1.00000000	pigs
Unkl	0.08	1.00000000	pigs
Tubgcp3	0.08	1.00000000	pigs
Zbtb40	0.15	1.00000000	pigs
Arhgef6	-0.32	1.00000000	pigs
Tomm7	-0.26	1.00000000	pigs
Amfr	0.11	1.00000000	pigs
St3gal3	-0.24	1.00000000	pigs
Ttll12	0.06	1.00000000	pigs
Pxdn	-0.18	1.00000000	pigs
Git2	0.16	1.00000000	pigs
Ubiad1	0.07	1.00000000	pigs
Ptpn9	0.18	1.00000000	pigs
Pank3	-0.44	1.00000000	pigs
Panx1	0.15	1.00000000	pigs
Dock8	0.62	1.00000000	pigs
Chpt1	0.06	1.00000000	pigs
Epm2aip1	-0.19	1.00000000	pigs
Rapgef2	0.16	1.00000000	pigs
Arsg	0.06	1.00000000	pigs
Cdc34	0.03	1.00000000	pigs
Smim19	-0.37	1.00000000	pigs
Net1	0.29	1.00000000	pigs
Sf3a3	-0.21	1.00000000	pigs
Septin3	-0.17	1.00000000	pigs
Lzts1	-0.12	1.00000000	pigs
Farsa	0.09	1.00000000	pigs
Polh	0.09	1.00000000	pigs
Nfatc3	0.13	1.00000000	pigs
Uqcrc2	0.18	1.00000000	pigs
Ilvbl	0.06	1.00000000	pigs
Gars	0.13	1.00000000	pigs
Tsr2	0.11	1.00000000	pigs
Cdc27	0.09	1.00000000	pigs
Nme7	-0.22	1.00000000	pigs
Tbrg4	0.13	1.00000000	pigs
Anks1	-0.17	1.00000000	pigs
Pmpcb	0.09	1.00000000	pigs
Snx4	-0.35	1.00000000	pigs

Psmd8	0.19	1.00000000	pigs
Cdc37l1	0.18	1.00000000	pigs
Pmm1	0.17	1.00000000	pigs
Terf1	-0.29	1.00000000	pigs
Kdm8	-0.13	1.00000000	pigs
Upk3a	0.10	1.00000000	pigs
Pip5k1c	0.09	1.00000000	pigs
Arl6ip4	0.05	1.00000000	pigs
Atp9a	0.18	1.00000000	pigs
P3h1	0.22	1.00000000	pigs
Cybb	-0.23	1.00000000	pigs
Glg1	-0.19	1.00000000	pigs
Sf3b1	0.19	1.00000000	pigs
Ufm1	-0.30	1.00000000	pigs
Dhx32	0.04	1.00000000	pigs
Ppp2r5d	0.14	1.00000000	pigs
Cadm1	0.57	1.00000000	pigs
Ltbr	0.09	1.00000000	pigs
Sh3kbp1	0.36	1.00000000	pigs
Calr	0.30	1.00000000	pigs
Ccdc91	-0.32	1.00000000	pigs
Gadd45g	0.16	1.00000000	pigs
Ddx56	0.05	1.00000000	pigs
Eps8l2	-0.16	1.00000000	pigs
Vps13b	-0.30	1.00000000	pigs
Fnbp4	0.26	1.00000000	pigs
Hmgxb3	0.20	1.00000000	pigs
Hsd17b12	0.10	1.00000000	pigs
Zfp467	-0.16	1.00000000	pigs
Slc16a10	-0.11	1.00000000	pigs
Rictor	0.16	1.00000000	pigs
Esd	-0.23	1.00000000	pigs
Ifit3	-0.63	1.00000000	pigs
Atf7ip2	-0.02	1.00000000	pigs
Ube2o	0.11	1.00000000	pigs
Haus6	-0.22	1.00000000	pigs
Kif26b	0.13	1.00000000	pigs
Khsrp	0.16	1.00000000	pigs
Vps36	-0.27	1.00000000	pigs
Fasn	0.01	1.00000000	pigs
Ndufs6	0.22	1.00000000	pigs
Ago1	0.23	1.00000000	pigs
Eef2k	0.04	1.00000000	pigs
Actr3	0.29	1.00000000	pigs
Yipf3	0.05	1.00000000	pigs
Eny2	-0.31	1.00000000	pigs

Frmpd4	0.54	1.00000000	pigs
Zfp639	0.09	1.00000000	pigs
Arid5a	-0.27	1.00000000	pigs
Adat2	0.04	1.00000000	pigs
Zc3h3	0.12	1.00000000	pigs
Tspyl2	0.10	1.00000000	pigs
Lpin1	0.11	1.00000000	pigs
Brk1	-0.35	1.00000000	pigs
Stmn3	0.10	1.00000000	pigs
Pacc1	0.51	1.00000000	pigs
Cog1	0.08	1.00000000	pigs
Fkbp4	0.17	1.00000000	pigs
Tlr2	0.12	1.00000000	pigs
Pi4kb	0.03	1.00000000	pigs
Slc35a4	0.10	1.00000000	pigs
Anapc15-ps	0.10	1.00000000	pigs
Ammecr1	-0.35	1.00000000	pigs
Dapk1	-0.24	1.00000000	pigs
Csf2rb2	-0.24	1.00000000	pigs
Rhpn2	0.11	1.00000000	pigs
Suv39h1	0.09	1.00000000	pigs
Creb1	0.18	1.00000000	pigs
Fbxl3	0.25	1.00000000	pigs
Pde4b	0.07	1.00000000	pigs
Hps1	-0.22	1.00000000	pigs
Armc5	0.09	1.00000000	pigs
Etfrf1	-0.19	1.00000000	pigs
Tox	0.26	1.00000000	pigs
Fbxo6	0.22	1.00000000	pigs
Xbp1	-0.29	1.00000000	pigs
Anpep	0.08	1.00000000	pigs
Tpp2	0.12	1.00000000	pigs
Pgam5	0.10	1.00000000	pigs
Snrpf	-0.24	1.00000000	pigs
Il22ra2	0.33	1.00000000	pigs
Tgfbrap1	0.09	1.00000000	pigs
Kpna6	0.17	1.00000000	pigs
Bclaf3	0.10	1.00000000	pigs
Nrbp1	0.14	1.00000000	pigs
Erh	-0.24	1.00000000	pigs
Pin1	-0.25	1.00000000	pigs
Emg1	-0.25	1.00000000	pigs
Emc1	0.06	1.00000000	pigs
Tnfaip2	0.07	1.00000000	pigs
Ddb1	-0.29	1.00000000	pigs
Dock10	-0.18	1.00000000	pigs

Clrn3	-0.10	1.00000000	pigs
Iars2	0.12	1.00000000	pigs
Rbm18	-0.25	1.00000000	pigs
Acox1	0.19	1.00000000	pigs
Abcd1	0.07	1.00000000	pigs
Rc3h2	0.15	1.00000000	pigs
Sertad1	0.14	1.00000000	pigs
Fcrla	-0.21	1.00000000	pigs
Bud13	0.09	1.00000000	pigs
Blk	0.28	1.00000000	pigs
Fam8a1	0.13	1.00000000	pigs
Strn3	0.22	1.00000000	pigs
Ddx54	0.10	1.00000000	pigs
Rock1	0.17	1.00000000	pigs
Phkg2	0.18	1.00000000	pigs
Tceanc2	-0.20	1.00000000	pigs
Ptpn2	0.21	1.00000000	pigs
Ppp2r5e	0.28	1.00000000	pigs
Cebpa	0.00	1.00000000	pigs
B3galnt2	0.19	1.00000000	pigs
Agap3	0.17	1.00000000	pigs
Lrch3	0.18	1.00000000	pigs
Dock9	-0.19	1.00000000	pigs
Wdr26	0.20	1.00000000	pigs
Gins4	0.06	1.00000000	pigs
Exosc9	-0.24	1.00000000	pigs
Uhrf1	-0.14	1.00000000	pigs
Galnt2	0.07	1.00000000	pigs
Nudt14	0.09	1.00000000	pigs
Papss2	0.48	1.00000000	pigs
Heatr3	0.37	1.00000000	pigs
Tor1aip1	0.17	1.00000000	pigs
Usp38	0.11	1.00000000	pigs
Ubxn2b	0.10	1.00000000	pigs
Dnase2a	0.33	1.00000000	pigs
Nab1	0.13	1.00000000	pigs
Ldlrad4	0.28	1.00000000	pigs
Prr5l	0.02	1.00000000	pigs
Cct4	0.20	1.00000000	pigs
Lmbrd2	-0.31	1.00000000	pigs
Tex14	-0.36	1.00000000	pigs
Arhgef33	-0.19	1.00000000	pigs
Dym	0.17	1.00000000	pigs
Ciao2b	0.06	1.00000000	pigs
Slc39a14	0.12	1.00000000	pigs
Hgs	0.08	1.00000000	pigs

Fbxo25	-0.15	1.00000000	pigs
Ift20	0.01	1.00000000	pigs
Ascc2	-0.15	1.00000000	pigs
Pld4	-0.24	1.00000000	pigs
Ston2	0.38	1.00000000	pigs
Pias1	0.17	1.00000000	pigs
Prickle3	0.08	1.00000000	pigs
Btrc	-0.35	1.00000000	pigs
Arl4c	-0.20	1.00000000	pigs
Bex3	-0.21	1.00000000	pigs
Spg21	0.07	1.00000000	pigs
Skiv2l	0.07	1.00000000	pigs
L3mbtl1	0.22	1.00000000	pigs
Nr3c2	-0.19	1.00000000	pigs
Tnfsf4	-0.21	1.00000000	pigs
Uck2	-0.48	1.00000000	pigs
Idh2	0.35	1.00000000	pigs
Pnpt1	-0.19	1.00000000	pigs
Coa4	0.07	1.00000000	pigs
Acot13	-0.30	1.00000000	pigs
Gabpb2	0.07	1.00000000	pigs
Trmt1	0.06	1.00000000	pigs
Smc4	0.16	1.00000000	pigs
Jchain	0.02	1.00000000	pigs
Kcp	0.06	1.00000000	pigs
Dars2	-0.13	1.00000000	pigs
Bst2	0.13	1.00000000	pigs
Zfp644	-0.28	1.00000000	pigs
Il2ra	0.03	1.00000000	pigs
Taf1d	-0.27	1.00000000	pigs
Larp1b	0.16	1.00000000	pigs
Cxcl3	0.16	1.00000000	pigs
Gcnt1	0.28	1.00000000	pigs
Katnbl1	0.24	1.00000000	pigs
Arhgap24	-0.16	1.00000000	pigs
Pxylp1	0.34	1.00000000	pigs
Plcl2	0.39	1.00000000	pigs
Cdc42bpa	0.09	1.00000000	pigs
Cnpy3	-0.33	1.00000000	pigs
Vps50	-0.15	1.00000000	pigs
Plvap	0.10	1.00000000	pigs
Phf8	0.11	1.00000000	pigs
Snx9	0.24	1.00000000	pigs
Tmem245	-0.01	1.00000000	pigs
BC048507	0.20	1.00000000	pigs
Dennd5a	0.27	1.00000000	pigs

Ankrd42	0.20	1.00000000	pigs
Ccdc85b	0.13	1.00000000	pigs
Slc22a23	0.19	1.00000000	pigs
Reps1	0.12	1.00000000	pigs
Ccdc6	0.46	1.00000000	pigs
Cybc1	0.08	1.00000000	pigs
Atf5	0.37	1.00000000	pigs
Wbp11	0.16	1.00000000	pigs
Ehf	0.02	1.00000000	pigs
Phex	0.14	1.00000000	pigs
Cybrd1	0.20	1.00000000	pigs
Haus1	0.05	1.00000000	pigs
Atp8b2	0.08	1.00000000	pigs
Cd300lb	0.13	1.00000000	pigs
Naa25	0.22	1.00000000	pigs
Asf1a	0.06	1.00000000	pigs
Scaf8	0.11	1.00000000	pigs
Trmt44	0.05	1.00000000	pigs
Rras	0.11	1.00000000	pigs
Ino80e	0.08	1.00000000	pigs
Fhit	0.41	1.00000000	pigs
Atp5pb	0.19	1.00000000	pigs
Ppp4c	0.10	1.00000000	pigs
Tm9sf2	-0.10	1.00000000	pigs
Ywhab	-0.28	1.00000000	pigs
Tdrd3	-0.20	1.00000000	pigs
Kdm5c	0.06	1.00000000	pigs
Ipo7	0.18	1.00000000	pigs
Polr2i	0.09	1.00000000	pigs
Rab9	0.32	1.00000000	pigs
Galnt1	-0.22	1.00000000	pigs
Lrba	-0.20	1.00000000	pigs
Ptpro	0.09	1.00000000	pigs
Sfmbt1	0.27	1.00000000	pigs
Grk5	0.12	1.00000000	pigs
Tmem87b	-0.23	1.00000000	pigs
Cdk9	0.21	1.00000000	pigs
Brpf3	0.01	1.00000000	pigs
Hk3	0.18	1.00000000	pigs
Syn1	0.08	1.00000000	pigs
Hk2	0.09	1.00000000	pigs
Tm9sf3	-0.19	1.00000000	pigs
Rin2	-0.27	1.00000000	pigs
Tpp1	0.06	1.00000000	pigs
B4galt1	-0.21	1.00000000	pigs
Sec22c	0.08	1.00000000	pigs

Ankrd28	-0.21	1.00000000	pigs
Stard5	0.05	1.00000000	pigs
Gtf2i	-0.32	1.00000000	pigs
Eef1akmt3	-0.10	1.00000000	pigs
Filip1l	-0.47	1.00000000	pigs
Dynlrb1	0.15	1.00000000	pigs
Ly75	-0.15	1.00000000	pigs
Nmt1	0.14	1.00000000	pigs
Pygb	-0.23	1.00000000	pigs
Cox4i1	0.16	1.00000000	pigs
Tmem106b	-0.26	1.00000000	pigs
Slc7a7	0.09	1.00000000	pigs
Gna11	0.05	1.00000000	pigs
Smg1	0.15	1.00000000	pigs
Exosc7	0.10	1.00000000	pigs
Scrn3	0.06	1.00000000	pigs
Nudt21	0.16	1.00000000	pigs
Slc25a39	-0.32	1.00000000	pigs
Sntb2	0.25	1.00000000	pigs
Cmbl	-0.37	1.00000000	pigs
Zbtb17	0.01	1.00000000	pigs
C1qc	0.06	1.00000000	pigs
Nol8	0.04	1.00000000	pigs
Kpna7	0.06	1.00000000	pigs
Ghdc	0.04	1.00000000	pigs
Anapc13	0.08	1.00000000	pigs
Akap6	-0.30	1.00000000	pigs
Pccb	-0.24	1.00000000	pigs
Wwc2	0.24	1.00000000	pigs
Zfp12	0.09	1.00000000	pigs
Syt14	-0.29	1.00000000	pigs
Pde4a	0.09	1.00000000	pigs
Trit1	0.10	1.00000000	pigs
Stk26	0.16	1.00000000	pigs
Trmt6	-0.26	1.00000000	pigs
Ipo4	-0.29	1.00000000	pigs
Tlr7	0.20	1.00000000	pigs
Cert1	0.14	1.00000000	pigs
Rbm44	-0.24	1.00000000	pigs
Sp3	-0.33	1.00000000	pigs
Pet100	-0.31	1.00000000	pigs
Clcn7	0.05	1.00000000	pigs
Ctdsp2	0.07	1.00000000	pigs
Kmt5a	0.08	1.00000000	pigs
Pag1	-0.23	1.00000000	pigs
Preb	0.07	1.00000000	pigs

Rab3il1	-0.33	1.00000000	pigs
Nmi	-0.29	1.00000000	pigs
Herpud2	0.46	1.00000000	pigs
Prdm11	-0.07	1.00000000	pigs
Tspan14	0.42	1.00000000	pigs
Limd1	0.29	1.00000000	pigs
Gtpbp1	0.14	1.00000000	pigs
Qsox2	-0.16	1.00000000	pigs
Flot1	0.11	1.00000000	pigs
Adar	-0.28	1.00000000	pigs
Ndor1	-0.01	1.00000000	pigs
Ctnnd1	-0.04	1.00000000	pigs
Paip2	-0.22	1.00000000	pigs
Vapa	0.14	1.00000000	pigs
Rbsn	0.08	1.00000000	pigs
Bcl7c	0.16	1.00000000	pigs
Nup54	0.12	1.00000000	pigs
Ganab	0.08	1.00000000	pigs
Sdc1	-0.28	1.00000000	pigs
Ugdh	-0.09	1.00000000	pigs
Arrdc3	0.30	1.00000000	pigs
Pcbd1	0.01	1.00000000	pigs
Zbtb41	0.12	1.00000000	pigs
Osbpl1a	0.10	1.00000000	pigs
Ppp1r15a	-0.36	1.00000000	pigs
Fabp3	0.07	1.00000000	pigs
Peak1	0.18	1.00000000	pigs
Sirt1	-0.01	1.00000000	pigs
Pptc7	0.20	1.00000000	pigs
Plagl2	0.07	1.00000000	pigs
Tmem127	0.14	1.00000000	pigs
Sh2d4b	0.25	1.00000000	pigs
Hnrnpa0	0.06	1.00000000	pigs
Stard7	0.04	1.00000000	pigs
Prkcb	-0.16	1.00000000	pigs
Fbxo38	0.11	1.00000000	pigs
Napepld	0.06	1.00000000	pigs
Exoc8	0.09	1.00000000	pigs
Cdr2	-0.18	1.00000000	pigs
Tasor2	0.02	1.00000000	pigs
Gan	0.15	1.00000000	pigs
Rexo4	-0.16	1.00000000	pigs
Ddx47	0.05	1.00000000	pigs
Lss	0.20	1.00000000	pigs
Khk	0.06	1.00000000	pigs
Rars	0.10	1.00000000	pigs

Zbtb7c	0.41	1.00000000	pigs
Fbp1	-0.32	1.00000000	pigs
Dhrs1	0.08	1.00000000	pigs
Tmem168	0.09	1.00000000	pigs
Etv6	0.50	1.00000000	pigs
Arhgap17	0.10	1.00000000	pigs
B4galt3	0.09	1.00000000	pigs
Sorl1	-0.44	1.00000000	pigs
Tmprss13	0.04	1.00000000	pigs
Pdxp	-0.21	1.00000000	pigs
Lrrc41	0.16	1.00000000	pigs
Alkbh6	0.03	1.00000000	pigs
Trerf1	0.10	1.00000000	pigs
Jpt2	0.07	1.00000000	pigs
Dnajc24	-0.21	1.00000000	pigs
Traf3ip3	-0.25	1.00000000	pigs
Rad21	0.11	1.00000000	pigs
Rab11fip4	0.01	1.00000000	pigs
Tnfrsf13c	-0.11	1.00000000	pigs
Trim26	-0.24	1.00000000	pigs
Dhrs7b	0.05	1.00000000	pigs
Kcnab2	0.20	1.00000000	pigs
Lyst	0.27	1.00000000	pigs
Gnpat	0.05	1.00000000	pigs
Dram2	-0.14	1.00000000	pigs
Gnptab	0.26	1.00000000	pigs
Dzip3	-0.15	1.00000000	pigs
Arg2	0.08	1.00000000	pigs
Kif3a	0.04	1.00000000	pigs
Ero1a	0.27	1.00000000	pigs
Anks3	0.09	1.00000000	pigs
Dbr1	0.06	1.00000000	pigs
Satb1	0.48	1.00000000	pigs
Rmnd5b	0.09	1.00000000	pigs
Clk1	-0.24	1.00000000	pigs
Yipf5	0.11	1.00000000	pigs
Ss18	0.26	1.00000000	pigs
Dna2	-0.12	1.00000000	pigs
Eif6	-0.33	1.00000000	pigs
Lta4h	-0.40	1.00000000	pigs
Ccnt1	-0.24	1.00000000	pigs
Wscd2	-0.11	1.00000000	pigs
Kansl1	0.21	1.00000000	pigs
Parp3	0.03	1.00000000	pigs
Il7r	0.29	1.00000000	pigs
Tmem120b	0.21	1.00000000	pigs

Fkbp15	0.17	1.00000000	pigs
Grn	-0.09	1.00000000	pigs
Baz1b	0.18	1.00000000	pigs
Tarbp1	-0.20	1.00000000	pigs
Adam15	0.05	1.00000000	pigs
Eif4b	0.15	1.00000000	pigs
Nop14	-0.13	1.00000000	pigs
Vps54	0.36	1.00000000	pigs
Fabp5	0.58	1.00000000	pigs
Zzz3	-0.33	1.00000000	pigs
Acsf2	0.18	1.00000000	pigs
Atf4	0.19	1.00000000	pigs
Prkd3	0.13	1.00000000	pigs
Strip1	0.06	1.00000000	pigs
Slc39a1	0.06	1.00000000	pigs
Ubxn4	-0.27	1.00000000	pigs
Actr10	-0.23	1.00000000	pigs
Casp6	0.06	1.00000000	pigs
Stat1	-0.15	1.00000000	pigs
Banp	0.04	1.00000000	pigs
Nit2	0.09	1.00000000	pigs
Eif3f	0.12	1.00000000	pigs
Armc8	-0.16	1.00000000	pigs
G6pc3	-0.38	1.00000000	pigs
R3hdm2	-0.34	1.00000000	pigs
C2cd5	0.08	1.00000000	pigs
Atrn	0.02	1.00000000	pigs
Dph1	0.05	1.00000000	pigs
Nub1	0.26	1.00000000	pigs
Ctnnbl1	-0.33	1.00000000	pigs
Rfx5	0.06	1.00000000	pigs
1700017B05Rik	0.08	1.00000000	pigs
Fam133b	-0.28	1.00000000	pigs
Endov	0.14	1.00000000	pigs
Pwp1	-0.20	1.00000000	pigs
Cpne1	-0.24	1.00000000	pigs
Ints6	0.06	1.00000000	pigs
Zfp236	0.08	1.00000000	pigs
Ric8b	0.18	1.00000000	pigs
Rpa3	-0.33	1.00000000	pigs
Snx7	-0.17	1.00000000	pigs
Tmem33	0.03	1.00000000	pigs
Ift27	0.04	1.00000000	pigs
Stx3	0.10	1.00000000	pigs
Tgfbr1	-0.19	1.00000000	pigs
Rusf1	-0.20	1.00000000	pigs

Dele1	0.05	1.00000000	pigs
Ndrg4	-0.21	1.00000000	pigs
E2f6	0.05	1.00000000	pigs
Traf2	0.05	1.00000000	pigs
Uap1	-0.09	1.00000000	pigs
Slc17a5	0.14	1.00000000	pigs
Lrrc25	0.13	1.00000000	pigs
Primpol	-0.32	1.00000000	pigs
Wipf2	-0.01	1.00000000	pigs
Fbxo34	0.06	1.00000000	pigs
Timm8b	-0.31	1.00000000	pigs
Thumpd2	0.07	1.00000000	pigs
Npr3	0.08	1.00000000	pigs
Tmem131	-0.14	1.00000000	pigs
Slc12a7	0.04	1.00000000	pigs
Katnal1	0.32	1.00000000	pigs
Srsf3	0.18	1.00000000	pigs
Kynu	0.24	1.00000000	pigs
Pradc1	0.06	1.00000000	pigs
Fibp	-0.25	1.00000000	pigs
Chst12	-0.02	1.00000000	pigs
Slc15a4	0.40	1.00000000	pigs
Zxdc	0.01	1.00000000	pigs
Ncf1	0.37	1.00000000	pigs
Stap1	0.03	1.00000000	pigs
Nudt6	-0.16	1.00000000	pigs
Tcp1	-0.20	1.00000000	pigs
Sf3b2	0.15	1.00000000	pigs
Tcp11	0.06	1.00000000	pigs
Cul5	-0.04	1.00000000	pigs
Sugp1	0.06	1.00000000	pigs
Ipo9	0.13	1.00000000	pigs
Slc9a9	0.06	1.00000000	pigs
Acd	0.07	1.00000000	pigs
Cers2	-0.19	1.00000000	pigs
Hdac6	0.11	1.00000000	pigs
Fam53b	0.22	1.00000000	pigs
Thumpd1	-0.22	1.00000000	pigs
Usp34	-0.22	1.00000000	pigs
Sumo2	-0.19	1.00000000	pigs
Adgrl2	0.42	1.00000000	pigs
Aspscr1	0.10	1.00000000	pigs
Adamts2	-0.03	1.00000000	pigs
Bin2	-0.21	1.00000000	pigs
Phf11a	0.21	1.00000000	pigs
1810013L24Rik	0.07	1.00000000	pigs

Rsrc1	-0.32	1.00000000	pigs
Xrcc6	0.02	1.00000000	pigs
Snrnp70	-0.20	1.00000000	pigs
Usp14	0.16	1.00000000	pigs
Calm3	0.23	1.00000000	pigs
Atpif1	-0.22	1.00000000	pigs
Xrcc5	0.30	1.00000000	pigs
Ythdf1	0.05	1.00000000	pigs
Spout1	-0.10	1.00000000	pigs
Scarf1	0.04	1.00000000	pigs
Bclaf1	0.17	1.00000000	pigs
Dctn3	-0.28	1.00000000	pigs
Nmd3	-0.28	1.00000000	pigs
Copb1	-0.22	1.00000000	pigs
Gpr52	0.10	1.00000000	pigs
Dst	0.51	1.00000000	pigs
Lin7c	0.09	1.00000000	pigs
Paxip1	-0.33	1.00000000	pigs
Kbtbd8	-0.12	1.00000000	pigs
Efna2	-0.13	1.00000000	pigs
Ap4s1	0.19	1.00000000	pigs
Gnb4	-0.26	1.00000000	pigs
Gxylt1	0.11	1.00000000	pigs
Itgb7	0.24	1.00000000	pigs
Nrros	-0.18	1.00000000	pigs
Tcp11l1	0.15	1.00000000	pigs
Rcc2	0.10	1.00000000	pigs
Ndufab1	-0.19	1.00000000	pigs
Cat	-0.18	1.00000000	pigs
Rsrc2	0.23	1.00000000	pigs
Ehmt1	0.14	1.00000000	pigs
Egfl7	0.00	1.00000000	pigs
Cwc22	-0.22	1.00000000	pigs
Syap1	-0.19	1.00000000	pigs
Ptcd3	0.11	1.00000000	pigs
Gatd1	0.05	1.00000000	pigs
Blzf1	0.12	1.00000000	pigs
Suclg2	-0.21	1.00000000	pigs
Slc35b1	-0.16	1.00000000	pigs
Sh2d2a	-0.08	1.00000000	pigs
Fam76b	-0.14	1.00000000	pigs
Ube3b	-0.19	1.00000000	pigs
Rnpepl1	-0.22	1.00000000	pigs
Cxcl14	0.53	1.00000000	pigs
Ndufa9	0.03	1.00000000	pigs
Rxylt1	0.00	1.00000000	pigs

Ccdc25	-0.24	1.00000000	pigs
Qpctl	0.11	1.00000000	pigs
Commd9	0.06	1.00000000	pigs
Sidt2	-0.27	1.00000000	pigs
Ythdf3	-0.33	1.00000000	pigs
Tiprl	0.12	1.00000000	pigs
Nfs1	0.04	1.00000000	pigs
Pecr	0.04	1.00000000	pigs
Aldh4a1	0.05	1.00000000	pigs
Zfp148	-0.18	1.00000000	pigs
Gpr161	-0.27	1.00000000	pigs
Usp6nl	0.34	1.00000000	pigs
Bri3bp	0.06	1.00000000	pigs
Gramd3	0.09	1.00000000	pigs
Ube2j2	0.15	1.00000000	pigs
Fbxo48	-0.13	1.00000000	pigs
Tmem126a	0.06	1.00000000	pigs
Cry1	0.17	1.00000000	pigs
Cd200	0.19	1.00000000	pigs
G2e3	0.11	1.00000000	pigs
Trim8	-0.14	1.00000000	pigs
Pbx1	-0.12	1.00000000	pigs
D11Wsu47e	0.02	1.00000000	pigs
Pdcl3	0.01	1.00000000	pigs
Snx17	0.13	1.00000000	pigs
Actl6a	0.04	1.00000000	pigs
Atp6v0a2	0.26	1.00000000	pigs
Senp1	0.08	1.00000000	pigs
Heca	0.15	1.00000000	pigs
Sptbn1	-0.19	1.00000000	pigs
Tradd	0.02	1.00000000	pigs
Wipi1	0.14	1.00000000	pigs
Ccny	0.34	1.00000000	pigs
Atp11c	0.05	1.00000000	pigs
Glud1	0.23	1.00000000	pigs
Cstf2	0.02	1.00000000	pigs
Zfp638	0.07	1.00000000	pigs
Gfod1	0.20	1.00000000	pigs
Wbp4	0.20	1.00000000	pigs
Washc4	-0.32	1.00000000	pigs
Trmt10a	0.04	1.00000000	pigs
Dlg2	-0.44	1.00000000	pigs
Txnl4b	-0.12	1.00000000	pigs
Cabyr	-0.49	1.00000000	pigs
Vps26b	0.08	1.00000000	pigs
Dhrs4	0.05	1.00000000	pigs

Gpr108	0.09	1.00000000	pigs
Trp53inp1	0.06	1.00000000	pigs
Kctd3	0.12	1.00000000	pigs
Prkra	0.11	1.00000000	pigs
Cetn3	-0.24	1.00000000	pigs
Fbxl15	0.06	1.00000000	pigs
Reep3	0.31	1.00000000	pigs
Cdc26	0.05	1.00000000	pigs
Slc9b1	0.14	1.00000000	pigs
S100a6	-0.88	1.00000000	pigs
Kif2a	0.23	1.00000000	pigs
Bag4	-0.02	1.00000000	pigs
Cdca4	0.06	1.00000000	pigs
Tctn2	0.14	1.00000000	pigs
Ttll1	0.04	1.00000000	pigs
Wac	0.18	1.00000000	pigs
Smc6	0.20	1.00000000	pigs
Tln1	0.23	1.00000000	pigs
Rcsd1	-0.27	1.00000000	pigs
Senp7	-0.27	1.00000000	pigs
Timmdc1	0.06	1.00000000	pigs
Sgk1	-0.01	1.00000000	pigs
Tbc1d17	0.09	1.00000000	pigs
Stk11	0.08	1.00000000	pigs
Pex7	-0.40	1.00000000	pigs
Laptm4a	0.16	1.00000000	pigs
Dnajb12	0.05	1.00000000	pigs
Alg14	-0.23	1.00000000	pigs
Usp45	0.14	1.00000000	pigs
Srp19	-0.24	1.00000000	pigs
Prkcq	-0.16	1.00000000	pigs
Pbld2	0.10	1.00000000	pigs
Rpa2	0.07	1.00000000	pigs
Ubxn2a	0.16	1.00000000	pigs
Oxsr1	0.07	1.00000000	pigs
Polr1e	0.01	1.00000000	pigs
Soga1	-0.15	1.00000000	pigs
Phf1	0.09	1.00000000	pigs
Kif21b	0.22	1.00000000	pigs
Snrk	0.11	1.00000000	pigs
Fez2	0.06	1.00000000	pigs
Ccndbp1	-0.26	1.00000000	pigs
Nyap2	0.94	1.00000000	pigs
Prcp	0.30	1.00000000	pigs
Gne	0.18	1.00000000	pigs
Recql	-0.22	1.00000000	pigs

Cradd	0.15	1.00000000	pigs
4933400A11Rik	0.21	1.00000000	pigs
Nop2	0.05	1.00000000	pigs
Ints13	0.13	1.00000000	pigs
Ccdc66	-0.38	1.00000000	pigs
Ggps1	0.00	1.00000000	pigs
Tnfaip6	0.19	1.00000000	pigs
Cox11	-0.13	1.00000000	pigs
Ccl5	-0.33	1.00000000	pigs
Fermt3	0.19	1.00000000	pigs
Lrsam1	0.06	1.00000000	pigs
Slc16a4	0.06	1.00000000	pigs
Bnip2	0.04	1.00000000	pigs
Hk1	-0.29	1.00000000	pigs
Ttbk2	-0.06	1.00000000	pigs
Xxylt1	-0.36	1.00000000	pigs
Tmem97	-0.36	1.00000000	pigs
Tfip11	0.05	1.00000000	pigs
Slc9a8	-0.28	1.00000000	pigs
Txndc12	0.00	1.00000000	pigs
Vmp1	0.21	1.00000000	pigs
Kctd10	0.01	1.00000000	pigs
Gas2l3	0.02	1.00000000	pigs
Entpd7	0.12	1.00000000	pigs
Tia1	0.11	1.00000000	pigs
Adap1	0.08	1.00000000	pigs
Atpaf1	-0.11	1.00000000	pigs
Fbxo28	0.21	1.00000000	pigs
Nt5c2	0.02	1.00000000	pigs
Lats1	0.09	1.00000000	pigs
Exoc4	-0.22	1.00000000	pigs
Cage1	-0.24	1.00000000	pigs
Snx22	0.02	1.00000000	pigs
Dipk2a	-0.03	1.00000000	pigs
Foxk1	0.22	1.00000000	pigs
Itsn2	0.10	1.00000000	pigs
Prkag1	0.03	1.00000000	pigs
Trim24	-0.13	1.00000000	pigs
Rrn3	0.07	1.00000000	pigs
Ruvbl2	0.05	1.00000000	pigs
Dgke	0.05	1.00000000	pigs
Camkk2	0.07	1.00000000	pigs
Zbp1	-0.08	1.00000000	pigs
Dlst	0.12	1.00000000	pigs
Serpine2	-0.22	1.00000000	pigs
Zfand2b	-0.12	1.00000000	pigs

Rabepk	0.05	1.00000000	pigs
B3gnt5	0.09	1.00000000	pigs
Cd320	0.16	1.00000000	pigs
Clic4	-0.27	1.00000000	pigs
Rnf126	0.12	1.00000000	pigs
Tfrc	0.18	1.00000000	pigs
Rbm15	0.06	1.00000000	pigs
Podn	-0.17	1.00000000	pigs
Csrnp2	-0.03	1.00000000	pigs
Trp53i11	-0.20	1.00000000	pigs
Ifi44l	-0.20	1.00000000	pigs
Spats2	-0.23	1.00000000	pigs
Tshz1	-0.13	1.00000000	pigs
Fes	0.09	1.00000000	pigs
Yeats4	-0.20	1.00000000	pigs
Baz2b	-0.53	1.00000000	pigs
Zcchc2	0.04	1.00000000	pigs
Sugt1	0.11	1.00000000	pigs
Smc1b	0.06	1.00000000	pigs
Pms1	-0.17	1.00000000	pigs
Insig1	0.16	1.00000000	pigs
Psd3	0.40	1.00000000	pigs
Pip4k2b	-0.03	1.00000000	pigs
Shisa4	0.07	1.00000000	pigs
Cop1	-0.25	1.00000000	pigs
Edrf1	0.15	1.00000000	pigs
Cwc15	-0.14	1.00000000	pigs
Casp8	-0.24	1.00000000	pigs
Far1	0.17	1.00000000	pigs
3110082I17Rik	0.20	1.00000000	pigs
Inka2	0.11	1.00000000	pigs
Fam120b	0.00	1.00000000	pigs
Kctd2	0.04	1.00000000	pigs
Ankrd27	0.05	1.00000000	pigs
Def8	0.04	1.00000000	pigs
Atp5j	-0.18	1.00000000	pigs
Emsy	0.10	1.00000000	pigs
Nfkbid	0.06	1.00000000	pigs
Patl1	0.09	1.00000000	pigs
Pum1	0.16	1.00000000	pigs
Fundc1	-0.19	1.00000000	pigs
Setd3	0.14	1.00000000	pigs
Atp11a	0.21	1.00000000	pigs
Cyb5a	0.13	1.00000000	pigs
Dbt	0.09	1.00000000	pigs
Spata5l1	0.08	1.00000000	pigs

Rbm42	0.00	1.00000000	pigs
Cd5	0.24	1.00000000	pigs
Nacc2	0.08	1.00000000	pigs
Wdr44	0.15	1.00000000	pigs
App	0.38	1.00000000	pigs
Adat1	-0.01	1.00000000	pigs
Smarce1	-0.19	1.00000000	pigs
Rcan3	-0.13	1.00000000	pigs
Srprb	-0.21	1.00000000	pigs
Dus4l	0.03	1.00000000	pigs
Trpm7	-0.19	1.00000000	pigs
Glipr1	-0.28	1.00000000	pigs
Rapgef5	-0.12	1.00000000	pigs
Kif13a	0.05	1.00000000	pigs
Abcd3	0.08	1.00000000	pigs
Pex14	0.03	1.00000000	pigs
BC004004	0.05	1.00000000	pigs
Pum3	0.01	1.00000000	pigs
Wsb2	0.05	1.00000000	pigs
Coq10b	0.10	1.00000000	pigs
Sec13	0.17	1.00000000	pigs
Ndufs2	0.12	1.00000000	pigs
Sppl2b	0.06	1.00000000	pigs
Npl	0.09	1.00000000	pigs
Arf4	-0.15	1.00000000	pigs
Hsd17b10	0.10	1.00000000	pigs
Jade3	0.15	1.00000000	pigs
Brd7	-0.24	1.00000000	pigs
Ppp2r1b	0.12	1.00000000	pigs
Psmd6	0.08	1.00000000	pigs
Appbp2	0.07	1.00000000	pigs
Lcp1	-0.19	1.00000000	pigs
Trub2	0.10	1.00000000	pigs
Ccm2	0.21	1.00000000	pigs
Cdkal1	-0.29	1.00000000	pigs
Rab18	0.12	1.00000000	pigs
Pip5k1b	-0.23	1.00000000	pigs
Srpk2	-0.22	1.00000000	pigs
4930523C07Rik	0.08	1.00000000	pigs
Slc15a3	0.10	1.00000000	pigs
Thoc1	-0.20	1.00000000	pigs
Fam71e1	0.04	1.00000000	pigs
Dab2	0.12	1.00000000	pigs
Thap1	0.09	1.00000000	pigs
Fdps	0.03	1.00000000	pigs
Snx18	0.20	1.00000000	pigs

Stag2	-0.21	1.00000000	pigs
Gnl3	0.04	1.00000000	pigs
Dusp11	0.14	1.00000000	pigs
Xrn2	-0.18	1.00000000	pigs
Samd4b	0.06	1.00000000	pigs
Tbcb	0.05	1.00000000	pigs
Rnaseh2a	-0.15	1.00000000	pigs
Aktip	0.05	1.00000000	pigs
Pdik1l	-0.01	1.00000000	pigs
Slc6a6	0.11	1.00000000	pigs
Wrap53	0.05	1.00000000	pigs
Aph1a	-0.16	1.00000000	pigs
Bccip	0.06	1.00000000	pigs
Haus7	-0.02	1.00000000	pigs
Terf2ip	-0.30	1.00000000	pigs
Zc3h8	0.07	1.00000000	pigs
Tbc1d2b	0.12	1.00000000	pigs
Ap1b1	0.14	1.00000000	pigs
Cdkn2aip	0.09	1.00000000	pigs
Plin3	-0.16	1.00000000	pigs
Zadh2	0.09	1.00000000	pigs
Samm50	0.06	1.00000000	pigs
Scamp3	-0.17	1.00000000	pigs
Pcgf5	0.24	1.00000000	pigs
Ctnnb1	0.17	1.00000000	pigs
Zfp654	0.15	1.00000000	pigs
Bloc1s6	0.08	1.00000000	pigs
Htr7	0.14	1.00000000	pigs
Dcaf15	0.07	1.00000000	pigs
Cmtm8	0.06	1.00000000	pigs
Slc37a4	0.05	1.00000000	pigs
Trip11	0.05	1.00000000	pigs
Nupr1	-0.01	1.00000000	pigs
Sfmbt2	0.18	1.00000000	pigs
Themis2	0.09	1.00000000	pigs
Cox6a2	-0.28	1.00000000	pigs
Riok1	0.16	1.00000000	pigs
Supt7l	0.00	1.00000000	pigs
Vps45	0.02	1.00000000	pigs
Kansl3	0.06	1.00000000	pigs
Copa	-0.17	1.00000000	pigs
Srgap3	-0.04	1.00000000	pigs
Slc2a13	0.09	1.00000000	pigs
Creb3	0.08	1.00000000	pigs
H6pd	0.05	1.00000000	pigs
Tab2	0.22	1.00000000	pigs

Lman1l	0.02	1.00000000	pigs
Cited2	-0.21	1.00000000	pigs
Fam120c	0.06	1.00000000	pigs
Ddx39b	-0.14	1.00000000	pigs
Tubb6	0.09	1.00000000	pigs
Slc24a1	-0.01	1.00000000	pigs
Srsf11	0.08	1.00000000	pigs
Azi2	0.07	1.00000000	pigs
Atm	-0.28	1.00000000	pigs
Plekhm1	-0.21	1.00000000	pigs
Cops2	0.13	1.00000000	pigs
Tm2d2	-0.38	1.00000000	pigs
Phf2	-0.27	1.00000000	pigs
Slc35e1	0.09	1.00000000	pigs
Zfyve27	0.05	1.00000000	pigs
Zfp189	0.01	1.00000000	pigs
Dynlt3	-0.02	1.00000000	pigs
Clic5	0.45	1.00000000	pigs
Zfp518a	-0.14	1.00000000	pigs
Gigyf1	-0.01	1.00000000	pigs
Ctsb	0.21	1.00000000	pigs
Wdr12	-0.25	1.00000000	pigs
Txnl4a	-0.23	1.00000000	pigs
Lca5l	-0.14	1.00000000	pigs
Agap1	-0.22	1.00000000	pigs
Nr3c1	0.06	1.00000000	pigs
Cracd	-0.10	1.00000000	pigs
Arhgdib	0.20	1.00000000	pigs
Armc10	0.11	1.00000000	pigs
Ptpra	-0.11	1.00000000	pigs
Tm2d3	0.12	1.00000000	pigs
Txndc15	0.06	1.00000000	pigs
Slc25a20	-0.15	1.00000000	pigs
Plpp5	-0.10	1.00000000	pigs
Rbm38	-0.16	1.00000000	pigs
Casp1	0.13	1.00000000	pigs
Utp4	0.09	1.00000000	pigs
Qrsl1	0.00	1.00000000	pigs
Ppp2ca	0.11	1.00000000	pigs
Snap29	0.14	1.00000000	pigs
Lmnb2	0.07	1.00000000	pigs
Fcer1a	0.01	1.00000000	pigs
Zfp831	0.26	1.00000000	pigs
Hmgcr	0.11	1.00000000	pigs
Senp8	-0.11	1.00000000	pigs
Setd5	0.18	1.00000000	pigs

Dhx16	-0.20	1.00000000	pigs
Tm2d1	-0.25	1.00000000	pigs
Saysd1	-0.14	1.00000000	pigs
Uhmk1	0.08	1.00000000	pigs
Tagln	-0.10	1.00000000	pigs
Smad2	0.13	1.00000000	pigs
Grip1	-0.24	1.00000000	pigs
Capn10	-0.19	1.00000000	pigs
Ube2e2	-0.26	1.00000000	pigs
Gdpd5	0.10	1.00000000	pigs
Acot8	0.08	1.00000000	pigs
Ctnna1	0.11	1.00000000	pigs
Spats2l	-0.23	1.00000000	pigs
Plcg2	-0.24	1.00000000	pigs
Bms1	0.16	1.00000000	pigs
Oma1	-0.14	1.00000000	pigs
Adam9	-0.39	1.00000000	pigs
Dync1i2	0.08	1.00000000	pigs
Ucp2	-0.03	1.00000000	pigs
Spidr	-0.33	1.00000000	pigs
Runx2	-0.05	1.00000000	pigs
Psmb9	0.18	1.00000000	pigs
Lars	-0.24	1.00000000	pigs
Tmem263	0.08	1.00000000	pigs
Slc25a22	0.02	1.00000000	pigs
Pcf11	0.15	1.00000000	pigs
Cmss1	-0.23	1.00000000	pigs
Homer1	-0.05	1.00000000	pigs
Plec	0.05	1.00000000	pigs
Slc3a2	0.12	1.00000000	pigs
Sparc	0.03	1.00000000	pigs
Gorasp2	0.07	1.00000000	pigs
Dvl3	0.05	1.00000000	pigs
Capn1	0.06	1.00000000	pigs
Zbtb37	-0.19	1.00000000	pigs
Nsl1	-0.29	1.00000000	pigs
Rnmt	0.10	1.00000000	pigs
Ncoa5	-0.15	1.00000000	pigs
Shtn1	-0.29	1.00000000	pigs
Nfe2l3	-0.20	1.00000000	pigs
Anapc7	-0.09	1.00000000	pigs
Dusp5	-0.03	1.00000000	pigs
Tnrc6b	0.21	1.00000000	pigs
Pdgfd	-0.18	1.00000000	pigs
Zfp771	-0.09	1.00000000	pigs
Arfgef2	0.17	1.00000000	pigs

Ppp4r3b	0.08	1.00000000	pigs
Pde7b	-0.06	1.00000000	pigs
Ap4e1	0.09	1.00000000	pigs
Tcof1	0.08	1.00000000	pigs
Fam120a	0.22	1.00000000	pigs
Ndufb4	-0.25	1.00000000	pigs
Faap20	-0.11	1.00000000	pigs
Klhl18	0.11	1.00000000	pigs
Adcy7	0.06	1.00000000	pigs
Tpcn1	0.07	1.00000000	pigs
Ube2d3	0.14	1.00000000	pigs
Zfp784	-0.08	1.00000000	pigs
Srsf1	0.08	1.00000000	pigs
Tlk2	0.10	1.00000000	pigs
Znrf2	-0.12	1.00000000	pigs
Dnajc8	-0.21	1.00000000	pigs
Lgals8	0.00	1.00000000	pigs
Dpp8	-0.27	1.00000000	pigs
Ttc9c	0.08	1.00000000	pigs
Socs7	0.08	1.00000000	pigs
Nol6	0.02	1.00000000	pigs
Eml3	-0.04	1.00000000	pigs
Psmc1	0.05	1.00000000	pigs
Nprl3	-0.11	1.00000000	pigs
Nagpa	0.05	1.00000000	pigs
Strap	-0.22	1.00000000	pigs
Cacna1e	0.09	1.00000000	pigs
Ciao2a	-0.17	1.00000000	pigs
Cdc42se2	0.15	1.00000000	pigs
Ccdc137	0.06	1.00000000	pigs
Clp1	0.03	1.00000000	pigs
Nrde2	0.04	1.00000000	pigs
Cdkl5	0.00	1.00000000	pigs
Pla2g7	-0.10	1.00000000	pigs
Fam20c	0.16	1.00000000	pigs
Get3	0.05	1.00000000	pigs
Fnta	-0.20	1.00000000	pigs
Atxn2	0.12	1.00000000	pigs
Tmem39b	-0.18	1.00000000	pigs
BC005537	-0.38	1.00000000	pigs
Clec12a	-0.10	1.00000000	pigs
Aspdh	0.04	1.00000000	pigs
Ccdc142	0.07	1.00000000	pigs
Bcas1	0.26	1.00000000	pigs
Hormad1	-0.13	1.00000000	pigs
Dnmt3a	0.00	1.00000000	pigs

Pik3cb	-0.04	1.00000000	pigs
Dut	0.22	1.00000000	pigs
Bcas3	-0.29	1.00000000	pigs
Tifab	0.25	1.00000000	pigs
Exosc1	0.07	1.00000000	pigs
Tmem14a	-0.16	1.00000000	pigs
Ccdc68	0.08	1.00000000	pigs
Numa1	0.15	1.00000000	pigs
C9orf72	-0.25	1.00000000	pigs
Irf3	0.09	1.00000000	pigs
Brwd1	0.08	1.00000000	pigs
Adal	-0.11	1.00000000	pigs
Ints6l	0.07	1.00000000	pigs
Trappc3	0.09	1.00000000	pigs
Cadps2	0.08	1.00000000	pigs
Lsm2	-0.37	1.00000000	pigs
Vps28	0.03	1.00000000	pigs
Hspa14	-0.06	1.00000000	pigs
Ranbp10	0.05	1.00000000	pigs
Ankfy1	0.20	1.00000000	pigs
Amigo2	-0.06	1.00000000	pigs
Lrpap1	0.07	1.00000000	pigs
Cic	0.08	1.00000000	pigs
Sertad2	0.16	1.00000000	pigs
Slc35f6	0.05	1.00000000	pigs
Cpeb2	-0.23	1.00000000	pigs
Pdia5	0.11	1.00000000	pigs
Rbms3	-0.10	1.00000000	pigs
Snx20	-0.01	1.00000000	pigs
Sema4b	-0.09	1.00000000	pigs
Bsdc1	0.07	1.00000000	pigs
Fbxo33	0.09	1.00000000	pigs
Slc41a2	0.30	1.00000000	pigs
Il10ra	-0.29	1.00000000	pigs
Zfp395	-0.14	1.00000000	pigs
Litaf	0.19	1.00000000	pigs
Tmem43	-0.04	1.00000000	pigs
Cdk5rap3	0.05	1.00000000	pigs
Dcaf10	0.12	1.00000000	pigs
Sfxn4	0.05	1.00000000	pigs
Rnf111	0.16	1.00000000	pigs
Thap11	0.06	1.00000000	pigs
Elovl6	0.22	1.00000000	pigs
Btbd1	-0.23	1.00000000	pigs
Eif3j2	0.08	1.00000000	pigs
Tmem135	0.33	1.00000000	pigs

Utp6	-0.18	1.00000000	pigs
Fam189a1	-0.24	1.00000000	pigs
Idh3b	0.05	1.00000000	pigs
Vti1a	-0.20	1.00000000	pigs
Sec23b	0.08	1.00000000	pigs
Bcl2l13	0.01	1.00000000	pigs
Psmd10	0.06	1.00000000	pigs
Simc1	0.07	1.00000000	pigs
Nop53	0.08	1.00000000	pigs
Ppil4	0.07	1.00000000	pigs
Sar1b	0.11	1.00000000	pigs
Srp54a	-0.19	1.00000000	pigs
Aplp2	0.29	1.00000000	pigs
1810037I17Rik	-0.12	1.00000000	pigs
Gadd45a	0.35	1.00000000	pigs
Pml	0.11	1.00000000	pigs
Zfp30	-0.12	1.00000000	pigs
Atp5a1	0.17	1.00000000	pigs
Clip1	0.09	1.00000000	pigs
Palld	0.03	1.00000000	pigs
Prkdc	-0.18	1.00000000	pigs
Fadd	0.03	1.00000000	pigs
Zmat3	0.07	1.00000000	pigs
Ubtd1	0.10	1.00000000	pigs
Cnih4	-0.21	1.00000000	pigs
Hspa5	-0.14	1.00000000	pigs
Ruvbl1	0.14	1.00000000	pigs
Cdpf1	-0.18	1.00000000	pigs
Bcl2l12	-0.12	1.00000000	pigs
Dhx29	-0.34	1.00000000	pigs
Fkbp14	-0.16	1.00000000	pigs
Cenph	-0.19	1.00000000	pigs
Pdk3	0.08	1.00000000	pigs
Psen1	0.27	1.00000000	pigs
Syvn1	0.04	1.00000000	pigs
Col1a2	0.02	1.00000000	pigs
Slc24a4	0.46	1.00000000	pigs
Arhgef39	0.03	1.00000000	pigs
Ago4	-0.20	1.00000000	pigs
Tmem134	-0.29	1.00000000	pigs
Ing3	-0.12	1.00000000	pigs
Gcdh	0.05	1.00000000	pigs
Srp9	-0.04	1.00000000	pigs
Nus1	0.08	1.00000000	pigs
Slc25a4	0.02	1.00000000	pigs
Trim39	0.05	1.00000000	pigs

Hnrnpa3	0.16	1.00000000	pigs
Cyc1	0.04	1.00000000	pigs
Tkfc	-0.23	1.00000000	pigs
Acp6	0.08	1.00000000	pigs
Slc4a8	-0.15	1.00000000	pigs
Cabin1	0.05	1.00000000	pigs
Bscl2	0.01	1.00000000	pigs
Dop1a	0.01	1.00000000	pigs
Ptpn4	0.24	1.00000000	pigs
Nudt15	-0.12	1.00000000	pigs
Rnf166	0.05	1.00000000	pigs
Dop1b	0.12	1.00000000	pigs
Bphl	0.05	1.00000000	pigs
Kcnk6	0.08	1.00000000	pigs
Ptdss1	0.07	1.00000000	pigs
Gch1	0.13	1.00000000	pigs
Scarb1	0.12	1.00000000	pigs
Clasp2	-0.15	1.00000000	pigs
Ndufa13	-0.19	1.00000000	pigs
Prkcd	-0.21	1.00000000	pigs
Cbfb	0.19	1.00000000	pigs
Ppp2r2a	0.14	1.00000000	pigs
Accs	0.02	1.00000000	pigs
Slc49a4	0.04	1.00000000	pigs
Hip1r	0.08	1.00000000	pigs
St3gal4	-0.30	1.00000000	pigs
Zbtb43	0.05	1.00000000	pigs
Lrrc47	0.03	1.00000000	pigs
Dars	-0.35	1.00000000	pigs
Prkrip1	0.09	1.00000000	pigs
Eef1b2	0.16	1.00000000	pigs
Chd7	0.14	1.00000000	pigs
Bcar3	-0.11	1.00000000	pigs
Zfp664	0.05	1.00000000	pigs
Pex2	-0.10	1.00000000	pigs
Ube2j1	0.11	1.00000000	pigs
Eif2ak1	0.10	1.00000000	pigs
Ntpcr	0.11	1.00000000	pigs
Retsat	0.03	1.00000000	pigs
Taf7	-0.21	1.00000000	pigs
Atxn10	-0.18	1.00000000	pigs
Zfp280c	0.20	1.00000000	pigs
Krr1	0.03	1.00000000	pigs
Vcpkmt	0.04	1.00000000	pigs
Ap4m1	-0.05	1.00000000	pigs
Hpf1	-0.18	1.00000000	pigs

Cox6a1	-0.12	1.00000000	pigs
Pram1	0.04	1.00000000	pigs
Itgal	-0.13	1.00000000	pigs
Pbx3	0.14	1.00000000	pigs
Glce	-0.44	1.00000000	pigs
Tbc1d20	0.07	1.00000000	pigs
Nfkbiz	-0.04	1.00000000	pigs
Sfpq	-0.14	1.00000000	pigs
Tcaim	0.07	1.00000000	pigs
Jdp2	0.08	1.00000000	pigs
Utrn	-0.31	1.00000000	pigs
Diablo	-0.24	1.00000000	pigs
Crtap	-0.04	1.00000000	pigs
Rwdd1	-0.19	1.00000000	pigs
Nr2c1	0.08	1.00000000	pigs
Col27a1	-0.12	1.00000000	pigs
Nsa2	-0.13	1.00000000	pigs
Bcat2	0.14	1.00000000	pigs
Zfp36l1	0.19	1.00000000	pigs
Cdh2	-0.24	1.00000000	pigs
Src	0.06	1.00000000	pigs
Riok3	0.12	1.00000000	pigs
Smc3	0.04	1.00000000	pigs
Abhd2	-0.49	1.00000000	pigs
Kcnj13	-0.10	1.00000000	pigs
Pcyt2	0.04	1.00000000	pigs
Camta2	0.04	1.00000000	pigs
Ei24	-0.12	1.00000000	pigs
Zdhhc13	-0.16	1.00000000	pigs
Nup37	0.08	1.00000000	pigs
Klhdc4	0.06	1.00000000	pigs
Ndufa2	0.03	1.00000000	pigs
Pik3cd	-0.30	1.00000000	pigs
Glt8d1	-0.19	1.00000000	pigs
Ppp2r5c	0.24	1.00000000	pigs
Lsm6	-0.17	1.00000000	pigs
Zfp330	0.10	1.00000000	pigs
Osbpl9	-0.23	1.00000000	pigs
Ilk	0.09	1.00000000	pigs
Tmem175	-0.15	1.00000000	pigs
Sltm	0.13	1.00000000	pigs
Eaf1	-0.15	1.00000000	pigs
Pcgf3	0.08	1.00000000	pigs
Swt1	0.14	1.00000000	pigs
Ginm1	-0.20	1.00000000	pigs
Slain2	0.05	1.00000000	pigs

Hnrnpm	0.14	1.00000000	pigs
Tardbp	0.08	1.00000000	pigs
Elf2	0.21	1.00000000	pigs
Atp6v0a1	0.14	1.00000000	pigs
Eif3e	0.16	1.00000000	pigs
Cyfip2	-0.23	1.00000000	pigs
Hnrnpu	0.14	1.00000000	pigs
Nlk	0.10	1.00000000	pigs
Sms	-0.04	1.00000000	pigs
Nfatc1	-0.19	1.00000000	pigs
Gstk1	0.03	1.00000000	pigs
Ilkap	0.03	1.00000000	pigs
Arhgef11	0.33	1.00000000	pigs
Golm2	-0.23	1.00000000	pigs
Cilk1	-0.21	1.00000000	pigs
Uchl3	-0.24	1.00000000	pigs
Fbxo21	-0.06	1.00000000	pigs
Emilin2	0.21	1.00000000	pigs
Ing1	0.00	1.00000000	pigs
Bloc1s2	0.03	1.00000000	pigs
Vapb	0.12	1.00000000	pigs
Hsd17b4	0.22	1.00000000	pigs
Timm50	0.00	1.00000000	pigs
Gatad1	-0.13	1.00000000	pigs
Erlin2	-0.13	1.00000000	pigs
Sdccag8	0.08	1.00000000	pigs
Twf2	0.16	1.00000000	pigs
Shq1	0.12	1.00000000	pigs
Lysmd3	0.13	1.00000000	pigs
Blmh	0.14	1.00000000	pigs
Ogt	-0.15	1.00000000	pigs
Ddx41	0.03	1.00000000	pigs
Isoc1	0.04	1.00000000	pigs
Fam102a	-0.12	1.00000000	pigs
Smg5	0.05	1.00000000	pigs
Cd22	0.07	1.00000000	pigs
Wdr73	-0.21	1.00000000	pigs
Cenpc1	-0.19	1.00000000	pigs
Spag16	-0.12	1.00000000	pigs
Aldh1l2	0.06	1.00000000	pigs
Tnrc6a	-0.32	1.00000000	pigs
Pprc1	0.00	1.00000000	pigs
Chchd3	0.07	1.00000000	pigs
Dcaf11	0.10	1.00000000	pigs
Pdk1	0.07	1.00000000	pigs
Ssrp1	0.10	1.00000000	pigs

Rb1	0.08	1.00000000	pigs
Tmem222	-0.11	1.00000000	pigs
Ppan	-0.08	1.00000000	pigs
Car9	-0.05	1.00000000	pigs
Gpatch11	0.04	1.00000000	pigs
Daxx	-0.15	1.00000000	pigs
Cdk17	0.17	1.00000000	pigs
Tfam	-0.14	1.00000000	pigs
Pigx	-0.12	1.00000000	pigs
Riox2	0.02	1.00000000	pigs
Rpusd4	0.03	1.00000000	pigs
Hcls1	0.03	1.00000000	pigs
Npm3	0.07	1.00000000	pigs
Ddost	0.12	1.00000000	pigs
Tmem183a	0.08	1.00000000	pigs
Lrrc1	0.10	1.00000000	pigs
Ncapg2	0.10	1.00000000	pigs
Egf	-0.31	1.00000000	pigs
Skil	-0.21	1.00000000	pigs
Ugp2	0.13	1.00000000	pigs
Api5	0.16	1.00000000	pigs
Cd302	0.09	1.00000000	pigs
Cep20	-0.13	1.00000000	pigs
Cyp51	0.12	1.00000000	pigs
Atp7a	0.03	1.00000000	pigs
B3gat3	0.08	1.00000000	pigs
Rbm10	0.15	1.00000000	pigs
Pgrmc1	0.03	1.00000000	pigs
Spock2	0.06	1.00000000	pigs
Stk35	0.01	1.00000000	pigs
Dennd2c	-0.10	1.00000000	pigs
Ccdc22	0.06	1.00000000	pigs
Ttc28	0.04	1.00000000	pigs
Yeats2	-0.03	1.00000000	pigs
Scube3	0.01	1.00000000	pigs
Tirap	0.03	1.00000000	pigs
Arrb2	-0.15	1.00000000	pigs
Cd37	-0.31	1.00000000	pigs
Hexim2	-0.13	1.00000000	pigs
Iws1	-0.01	1.00000000	pigs
Csnk2a2	0.04	1.00000000	pigs
Setd2	-0.08	1.00000000	pigs
Ssr1	0.15	1.00000000	pigs
Nosip	0.08	1.00000000	pigs
Corin	-0.12	1.00000000	pigs
Ube2k	0.16	1.00000000	pigs

Pnpo	0.07	1.00000000	pigs
Clstn1	0.03	1.00000000	pigs
Rp2	0.05	1.00000000	pigs
Smap1	-0.22	1.00000000	pigs
Pou2f2	0.07	1.00000000	pigs
Tor1a	0.02	1.00000000	pigs
Rgs3	-0.03	1.00000000	pigs
Zbtb8os	-0.17	1.00000000	pigs
Pfkl	0.11	1.00000000	pigs
Ndufv2	-0.30	1.00000000	pigs
Zfp277	-0.01	1.00000000	pigs
Hddc2	0.04	1.00000000	pigs
Senp2	-0.18	1.00000000	pigs
Ccar1	0.01	1.00000000	pigs
Pelo	-0.02	1.00000000	pigs
B2m	0.16	1.00000000	pigs
Dctn4	0.24	1.00000000	pigs
Tmem106a	0.00	1.00000000	pigs
Plpbp	-0.12	1.00000000	pigs
Pus10	0.02	1.00000000	pigs
Fam32a	0.08	1.00000000	pigs
Kctd4	-0.12	1.00000000	pigs
A830018L16Rik	-0.38	1.00000000	pigs
Dusp6	-0.06	1.00000000	pigs
Cenpu	-0.14	1.00000000	pigs
Rbl1	0.07	1.00000000	pigs
Aurkaip1	-0.17	1.00000000	pigs
9930104L06Rik	0.04	1.00000000	pigs
Rbm45	0.09	1.00000000	pigs
Ppp2r5b	-0.20	1.00000000	pigs
Itpr1	0.16	1.00000000	pigs
Ero1b	0.16	1.00000000	pigs
Exosc2	0.04	1.00000000	pigs
Fam193a	0.15	1.00000000	pigs
Polr3k	0.05	1.00000000	pigs
Cgas	-0.13	1.00000000	pigs
Vps13d	-0.09	1.00000000	pigs
Itpa	-0.19	1.00000000	pigs
Fnbp1	0.05	1.00000000	pigs
Cpne3	-0.10	1.00000000	pigs
Ly6d	-0.19	1.00000000	pigs
Pank2	0.02	1.00000000	pigs
Prpf6	0.04	1.00000000	pigs
Tmem259	0.02	1.00000000	pigs
Rnf144b	-0.01	1.00000000	pigs
Ppcdc	-0.10	1.00000000	pigs

Tmx1	-0.15	1.00000000	pigs
Ints1	0.05	1.00000000	pigs
Was	-0.13	1.00000000	pigs
Gtdc1	-0.07	1.00000000	pigs
Ptgr1	-0.01	1.00000000	pigs
Stmn1	0.14	1.00000000	pigs
Atad5	-0.08	1.00000000	pigs
Asb7	0.04	1.00000000	pigs
Zfp263	0.02	1.00000000	pigs
Slc12a6	-0.24	1.00000000	pigs
Srsf5	-0.08	1.00000000	pigs
Slc25a26	-0.23	1.00000000	pigs
Ptgs2	0.13	1.00000000	pigs
Pdia4	0.05	1.00000000	pigs
Ubqln1	0.04	1.00000000	pigs
Fem1b	0.07	1.00000000	pigs
Exoc1	0.09	1.00000000	pigs
Ipo5	0.14	1.00000000	pigs
Cpeb4	0.09	1.00000000	pigs
Tprkb	0.02	1.00000000	pigs
Rbm48	-0.20	1.00000000	pigs
Tada3	0.02	1.00000000	pigs
Auts2	0.08	1.00000000	pigs
Nifk	-0.16	1.00000000	pigs
Pcbp4	0.04	1.00000000	pigs
Zranb3	0.06	1.00000000	pigs
Cutc	0.05	1.00000000	pigs
Cnot8	-0.65	1.00000000	pigs
Fam117a	0.02	1.00000000	pigs
Tbl3	0.02	1.00000000	pigs
Tmem42	-0.16	1.00000000	pigs
Ndufb2	0.03	1.00000000	pigs
Ccdc174	-0.13	1.00000000	pigs
Stab1	0.06	1.00000000	pigs
Fchsd1	0.12	1.00000000	pigs
Lztfl1	0.10	1.00000000	pigs
Eefsec	0.01	1.00000000	pigs
Ftsj1	0.04	1.00000000	pigs
Nt5c3	0.06	1.00000000	pigs
Golim4	-0.19	1.00000000	pigs
Klhl11	-0.07	1.00000000	pigs
Lnx2	0.06	1.00000000	pigs
Ccdc171	-0.10	1.00000000	pigs
Cdc37	0.04	1.00000000	pigs
Hdac3	-0.19	1.00000000	pigs
Ppfia4	0.05	1.00000000	pigs

Psme4	0.11	1.00000000	pigs
Psip1	-0.16	1.00000000	pigs
Zcchc14	-0.11	1.00000000	pigs
Snrnp25	-0.20	1.00000000	pigs
Klhl2	-0.01	1.00000000	pigs
Shoc2	0.17	1.00000000	pigs
Trappc4	-0.18	1.00000000	pigs
Jade2	-0.08	1.00000000	pigs
Slc10a1	-0.31	1.00000000	pigs
Srebf1	0.05	1.00000000	pigs
Bckdk	0.04	1.00000000	pigs
Supt16	-0.22	1.00000000	pigs
Rsbn1	0.04	1.00000000	pigs
Zfyve1	-0.02	1.00000000	pigs
Sike1	0.09	1.00000000	pigs
Pigt	0.11	1.00000000	pigs
L3mbtl3	-0.24	1.00000000	pigs
Cdc123	0.01	1.00000000	pigs
Hmgb2	0.01	1.00000000	pigs
Phf3	-0.17	1.00000000	pigs
Cnot1	0.03	1.00000000	pigs
Evl	0.13	1.00000000	pigs
Prdx3	0.01	1.00000000	pigs
Ankrd49	-0.20	1.00000000	pigs
Ndufs3	0.09	1.00000000	pigs
Bag6	0.08	1.00000000	pigs
Lin37	-0.23	1.00000000	pigs
Hdac10	-0.11	1.00000000	pigs
Smg6	-0.26	1.00000000	pigs
Acaa2	-0.15	1.00000000	pigs
Csnk1g1	-0.06	1.00000000	pigs
Wdr3	0.03	1.00000000	pigs
Fcho1	0.08	1.00000000	pigs
Akap3	-0.17	1.00000000	pigs
Pnn	-0.18	1.00000000	pigs
Spag8	-0.24	1.00000000	pigs
Cryz	-0.15	1.00000000	pigs
Nr1h2	0.02	1.00000000	pigs
Slc11a2	0.03	1.00000000	pigs
Cldn15	0.07	1.00000000	pigs
Keap1	0.06	1.00000000	pigs
Gpr171	-0.23	1.00000000	pigs
Pcid2	0.09	1.00000000	pigs
Pafah2	-0.01	1.00000000	pigs
Pkia	0.07	1.00000000	pigs
Daglb	0.04	1.00000000	pigs

Nap1l4	0.05	1.00000000	pigs
Scaper	-0.28	1.00000000	pigs
Zranb2	-0.18	1.00000000	pigs
Sf3b4	0.03	1.00000000	pigs
Ric8a	0.05	1.00000000	pigs
Retreg3	-0.21	1.00000000	pigs
Dnttip2	0.12	1.00000000	pigs
Aida	-0.06	1.00000000	pigs
Dda1	0.07	1.00000000	pigs
Cep135	-0.14	1.00000000	pigs
Kcnt1	0.04	1.00000000	pigs
Borcs6	0.03	1.00000000	pigs
Runx1	0.20	1.00000000	pigs
Ogfod1	0.04	1.00000000	pigs
Zc3h14	-0.02	1.00000000	pigs
Uba1	0.06	1.00000000	pigs
Rbx1	-0.22	1.00000000	pigs
Fto	-0.14	1.00000000	pigs
Abhd17b	0.06	1.00000000	pigs
Zfp217	-0.24	1.00000000	pigs
Gga3	0.02	1.00000000	pigs
Stk38	0.02	1.00000000	pigs
Adprh	0.01	1.00000000	pigs
Tmc1	-0.10	1.00000000	pigs
Hdac5	0.07	1.00000000	pigs
Gle1	0.02	1.00000000	pigs
Tent5a	-0.17	1.00000000	pigs
Xab2	0.04	1.00000000	pigs
F13a1	0.62	1.00000000	pigs
Abhd3	0.02	1.00000000	pigs
Gps1	0.07	1.00000000	pigs
Fam20a	0.01	1.00000000	pigs
Wls	0.08	1.00000000	pigs
Rab11fip3	0.01	1.00000000	pigs
Tspan15	0.04	1.00000000	pigs
Ankmy2	0.07	1.00000000	pigs
S100a4	-1.43	1.00000000	pigs
Kat2a	-0.07	1.00000000	pigs
Zfp704	-0.11	1.00000000	pigs
Vezf1	-0.11	1.00000000	pigs
Rexo1	0.09	1.00000000	pigs
Pik3c2a	-0.32	1.00000000	pigs
Oas2	0.19	1.00000000	pigs
Ehd4	0.09	1.00000000	pigs
Rab43	0.14	1.00000000	pigs
Lonp2	-0.17	1.00000000	pigs

Lemd2	-0.04	1.00000000	pigs
Vps13a	-0.10	1.00000000	pigs
Wdhd1	0.06	1.00000000	pigs
Romo1	-0.21	1.00000000	pigs
Rab3gap1	-0.19	1.00000000	pigs
Naa35	0.06	1.00000000	pigs
Csde1	0.15	1.00000000	pigs
Ss18l1	0.09	1.00000000	pigs
Prdm10	0.00	1.00000000	pigs
Zfp688	-0.15	1.00000000	pigs
Nubp1	0.01	1.00000000	pigs
Rhbdl2	0.14	1.00000000	pigs
Ccdc127	0.01	1.00000000	pigs
Nap1l1	-0.25	1.00000000	pigs
Gatb	-0.07	1.00000000	pigs
Pigg	-0.12	1.00000000	pigs
Ddx1	-0.17	1.00000000	pigs
Smpd4	0.04	1.00000000	pigs
Tars	0.02	1.00000000	pigs
Gclm	0.10	1.00000000	pigs
Cables2	0.03	1.00000000	pigs
Rcbtb2	0.03	1.00000000	pigs
Agl	-0.13	1.00000000	pigs
Uggt1	0.23	1.00000000	pigs
Gramd4	-0.08	1.00000000	pigs
Vldlr	0.13	1.00000000	pigs
Ccnd3	-0.09	1.00000000	pigs
Ssb	-0.17	1.00000000	pigs
Aco1	0.19	1.00000000	pigs
Crkl	0.13	1.00000000	pigs
Rsu1	-0.13	1.00000000	pigs
Tcta	-0.05	1.00000000	pigs
Anln	-0.14	1.00000000	pigs
Hibch	-0.08	1.00000000	pigs
Tac1	-0.07	1.00000000	pigs
Ttc37	-0.24	1.00000000	pigs
Arpc3	-0.03	1.00000000	pigs
Psma3	-0.26	1.00000000	pigs
Chka	0.26	1.00000000	pigs
Odf2l	0.03	1.00000000	pigs
Sptlc2	0.26	1.00000000	pigs
Plxdc2	-0.49	1.00000000	pigs
Ddi2	0.02	1.00000000	pigs
Rbmx	0.08	1.00000000	pigs
Arid2	0.02	1.00000000	pigs
Clybl	0.08	1.00000000	pigs

Eml2	-0.06	1.00000000	pigs
Spopl	0.05	1.00000000	pigs
Yipf4	-0.14	1.00000000	pigs
Fancb	-0.32	1.00000000	pigs
Zfp622	-0.03	1.00000000	pigs
Clcn3	0.08	1.00000000	pigs
Il16	-0.11	1.00000000	pigs
Dpf3	0.12	1.00000000	pigs
Arcn1	-0.18	1.00000000	pigs
Stx8	0.42	1.00000000	pigs
9530068E07Rik	-0.13	1.00000000	pigs
Ncapd3	-0.01	1.00000000	pigs
Sil1	-0.26	1.00000000	pigs
Exosc5	-0.25	1.00000000	pigs
Arl6ip5	0.06	1.00000000	pigs
Ndufv1	0.04	1.00000000	pigs
Ano10	0.11	1.00000000	pigs
Xpo4	-0.18	1.00000000	pigs
Acbd3	0.02	1.00000000	pigs
Abhd17c	0.10	1.00000000	pigs
Rtf2	0.34	1.00000000	pigs
Cep76	0.05	1.00000000	pigs
Hmox1	0.12	1.00000000	pigs
Arih2	0.12	1.00000000	pigs
Rbks	0.08	1.00000000	pigs
Cct5	0.13	1.00000000	pigs
2410004B18Rik	0.09	1.00000000	pigs
Nxf1	-0.19	1.00000000	pigs
Ezh1	0.01	1.00000000	pigs
Rtn2	-0.05	1.00000000	pigs
Nol9	-0.15	1.00000000	pigs
Cox10	0.05	1.00000000	pigs
Hps3	-0.01	1.00000000	pigs
Hibadh	0.02	1.00000000	pigs
Plcb1	0.16	1.00000000	pigs
Trim36	0.12	1.00000000	pigs
Vta1	0.08	1.00000000	pigs
Gpr155	-0.28	1.00000000	pigs
Gfm1	0.07	1.00000000	pigs
Wasf2	0.09	1.00000000	pigs
Upf1	0.04	1.00000000	pigs
Pih1d1	0.02	1.00000000	pigs
Uhrf1bp1	0.09	1.00000000	pigs
Phtf2	0.08	1.00000000	pigs
Brms1l	0.01	1.00000000	pigs
Plod3	0.07	1.00000000	pigs

Psmc3	0.06	1.00000000	pigs
Alkbh1	0.00	1.00000000	pigs
Slc7a5	-0.17	1.00000000	pigs
Pds5b	0.10	1.00000000	pigs
Kpna3	-0.33	1.00000000	pigs
1700123O20Rik	0.02	1.00000000	pigs
Taf4	0.04	1.00000000	pigs
Gadd45gip1	-0.24	1.00000000	pigs
Otud6b	-0.15	1.00000000	pigs
Hmg20a	-0.30	1.00000000	pigs
Sinhcaf	0.21	1.00000000	pigs
Zdhhc7	0.07	1.00000000	pigs
Tgs1	-0.17	1.00000000	pigs
Setbp1	0.17	1.00000000	pigs
Serp1	-0.24	1.00000000	pigs
Trim25	0.15	1.00000000	pigs
Ncf2	0.27	1.00000000	pigs
Atp6v1d	0.00	1.00000000	pigs
Papola	-0.17	1.00000000	pigs
Scamp2	0.15	1.00000000	pigs
Adam17	0.14	1.00000000	pigs
Ptpa	0.05	1.00000000	pigs
Pcyox1	0.03	1.00000000	pigs
Cmtm3	0.01	1.00000000	pigs
Nsf	-0.16	1.00000000	pigs
Tab1	0.06	1.00000000	pigs
Wsb1	0.08	1.00000000	pigs
Pparg	0.10	1.00000000	pigs
Samd12	0.06	1.00000000	pigs
Anp32e	0.09	1.00000000	pigs
Nhej1	0.04	1.00000000	pigs
Rxra	-0.01	1.00000000	pigs
Rheb	-0.14	1.00000000	pigs
Rttn	-0.09	1.00000000	pigs
Dcn	-0.06	1.00000000	pigs
Adrm1	0.08	1.00000000	pigs
Paip1	-0.20	1.00000000	pigs
Col3a1	0.02	1.00000000	pigs
Entpd6	-0.08	1.00000000	pigs
Ccdc88a	0.07	1.00000000	pigs
Heatr5b	-0.17	1.00000000	pigs
Smarca2	0.08	1.00000000	pigs
Large1	-0.24	1.00000000	pigs
Cnot2	0.15	1.00000000	pigs
Cib1	-0.03	1.00000000	pigs
Slc38a9	-0.18	1.00000000	pigs

Rgs14	-0.10	1.00000000	pigs
Dctn2	0.07	1.00000000	pigs
Cog2	0.02	1.00000000	pigs
Lmbr1l	0.04	1.00000000	pigs
Sting1	-0.18	1.00000000	pigs
Gxylt2	-0.14	1.00000000	pigs
Akr1d1	-0.16	1.00000000	pigs
Hdgfl2	-0.05	1.00000000	pigs
Inpp5a	0.04	1.00000000	pigs
Slc23a2	0.21	1.00000000	pigs
Eprs	0.11	1.00000000	pigs
Puf60	0.06	1.00000000	pigs
Gpatch4	-0.21	1.00000000	pigs
Lrch1	0.17	1.00000000	pigs
Zeb2	-0.19	1.00000000	pigs
Txlng	0.09	1.00000000	pigs
Fbxo9	-0.17	1.00000000	pigs
Avl9	0.05	1.00000000	pigs
Akap7	-0.24	1.00000000	pigs
Pnisr	-0.06	1.00000000	pigs
Reep4	0.01	1.00000000	pigs
Exosc3	-0.19	1.00000000	pigs
Rad52	-0.26	1.00000000	pigs
Dscc1	0.03	1.00000000	pigs
Kdm4a	0.00	1.00000000	pigs
Synpo2	-0.04	1.00000000	pigs
D8Ertd738e	-0.24	1.00000000	pigs
Ndufaf6	-0.09	1.00000000	pigs
Ipmk	-0.12	1.00000000	pigs
Kdm2b	0.19	1.00000000	pigs
Tbc1d22b	0.05	1.00000000	pigs
Fam13a	-0.25	1.00000000	pigs
Lig4	0.07	1.00000000	pigs
Kctd6	0.05	1.00000000	pigs
Fam98b	0.08	1.00000000	pigs
Kdsr	0.09	1.00000000	pigs
Supt6	0.07	1.00000000	pigs
Dtwd2	0.02	1.00000000	pigs
Phc3	0.06	1.00000000	pigs
Ppa1	0.30	1.00000000	pigs
Stard3nl	-0.18	1.00000000	pigs
Pmpca	0.04	1.00000000	pigs
Asb1	-0.14	1.00000000	pigs
Abcg4	0.03	1.00000000	pigs
Nf1	0.15	1.00000000	pigs
Arl1	-0.06	1.00000000	pigs

Rnf34	0.05	1.00000000	pigs
Stk16	0.04	1.00000000	pigs
Nudcd2	-0.17	1.00000000	pigs
Usp31	0.03	1.00000000	pigs
Rep15	0.03	1.00000000	pigs
Pknox1	0.06	1.00000000	pigs
Clpx	0.00	1.00000000	pigs
Commd1b	-0.22	1.00000000	pigs
Wdr41	-0.20	1.00000000	pigs
Slc2a6	0.11	1.00000000	pigs
Cdon	0.07	1.00000000	pigs
Stat3	-0.22	1.00000000	pigs
Lpxn	0.05	1.00000000	pigs
Ip6k2	0.00	1.00000000	pigs
1110032A03Rik	0.03	1.00000000	pigs
Nsd1	-0.27	1.00000000	pigs
Sart1	0.03	1.00000000	pigs
Fbxo46	0.06	1.00000000	pigs
Bmf	-0.20	1.00000000	pigs
Fam214b	0.02	1.00000000	pigs
Fbxl12	-0.11	1.00000000	pigs
Adi1	-0.13	1.00000000	pigs
Fam110b	0.10	1.00000000	pigs
Ticam1	0.02	1.00000000	pigs
Slc38a10	0.01	1.00000000	pigs
Pgm2l1	-0.12	1.00000000	pigs
Nox1	-0.13	1.00000000	pigs
Spns1	0.04	1.00000000	pigs
Ptp4a1	0.09	1.00000000	pigs
Polm	-0.21	1.00000000	pigs
Hemgn	-0.20	1.00000000	pigs
Hmgxb4	0.16	1.00000000	pigs
Resf1	0.09	1.00000000	pigs
Pds5a	0.15	1.00000000	pigs
Hectd3	0.00	1.00000000	pigs
Ppp1r15b	0.09	1.00000000	pigs
Zcchc8	0.02	1.00000000	pigs
Slc30a6	-0.19	1.00000000	pigs
Gnal	0.01	1.00000000	pigs
Cep44	0.06	1.00000000	pigs
Zfand2a	0.02	1.00000000	pigs
Prmt3	-0.21	1.00000000	pigs
Nudt7	0.00	1.00000000	pigs
Xkr8	-0.09	1.00000000	pigs
Evi5l	-0.15	1.00000000	pigs
Pus7l	-0.13	1.00000000	pigs

Wapl	0.10	1.00000000	pigs
Tnrc6c	-0.26	1.00000000	pigs
Rab37	0.07	1.00000000	pigs
Arap2	0.35	1.00000000	pigs
AU040320	-0.22	1.00000000	pigs
Tmem129	0.07	1.00000000	pigs
Snrpc	0.06	1.00000000	pigs
Aftph	0.15	1.00000000	pigs
Sod2	0.29	1.00000000	pigs
Plpp7	0.03	1.00000000	pigs
Atp10d	0.21	1.00000000	pigs
Cenpw	-0.14	1.00000000	pigs
Tsc22d4	-0.05	1.00000000	pigs
Aldh16a1	0.03	1.00000000	pigs
Ppil2	0.03	1.00000000	pigs
Setx	-0.20	1.00000000	pigs
Spryd3	0.01	1.00000000	pigs
Hnmt	-0.07	1.00000000	pigs
Sft2d1	-0.13	1.00000000	pigs
4932411N23Rik	0.05	1.00000000	pigs
Vti1b	0.07	1.00000000	pigs
Hsdl2	0.07	1.00000000	pigs
Chd3	0.01	1.00000000	pigs
Frmd4a	-0.29	1.00000000	pigs
Eif4g2	0.04	1.00000000	pigs
Ankrd10	0.26	1.00000000	pigs
Stxbp3	0.13	1.00000000	pigs
Arfgap2	0.05	1.00000000	pigs
Skp1	0.10	1.00000000	pigs
Ppp1r3e	0.01	1.00000000	pigs
Zfp407	-0.29	1.00000000	pigs
Polr3b	-0.13	1.00000000	pigs
Kansl1l	0.09	1.00000000	pigs
Hsf1	0.04	1.00000000	pigs
Dnase1l3	0.20	1.00000000	pigs
Pla2r1	0.17	1.00000000	pigs
Snx2	0.14	1.00000000	pigs
Rbm14	0.04	1.00000000	pigs
Zfp281	-0.13	1.00000000	pigs
Nek6	0.19	1.00000000	pigs
Zfp383	0.05	1.00000000	pigs
Thap3	-0.13	1.00000000	pigs
Tmem156	0.14	1.00000000	pigs
Gga2	0.14	1.00000000	pigs
Zdhhc3	0.06	1.00000000	pigs
Zbtb38	0.07	1.00000000	pigs

Trim37	-0.14	1.00000000	pigs
Cep104	0.12	1.00000000	pigs
Dnajb14	0.04	1.00000000	pigs
Rab28	0.11	1.00000000	pigs
Slc36a1	0.07	1.00000000	pigs
Sharpin	0.04	1.00000000	pigs
Gtf3c3	0.05	1.00000000	pigs
Parg	-0.16	1.00000000	pigs
Abraxas1	0.12	1.00000000	pigs
Rassf8	0.05	1.00000000	pigs
Nasp	-0.39	1.00000000	pigs
Rasal1	0.12	1.00000000	pigs
Nfic	0.06	1.00000000	pigs
Arpc5l	-0.20	1.00000000	pigs
Eif3d	0.03	1.00000000	pigs
Hscb	0.04	1.00000000	pigs
Prpf3	-0.07	1.00000000	pigs
Polr2b	0.09	1.00000000	pigs
Qars	0.08	1.00000000	pigs
Ncbp3	0.08	1.00000000	pigs
Nlrp3	0.02	1.00000000	pigs
Pbrm1	-0.17	1.00000000	pigs
Rufy3	0.15	1.00000000	pigs
Tmem126b	-0.18	1.00000000	pigs
Wdr89	-0.13	1.00000000	pigs
Rwdd3	-0.18	1.00000000	pigs
Xpc	0.05	1.00000000	pigs
Tecpr1	0.06	1.00000000	pigs
Tmem9b	0.00	1.00000000	pigs
Eif2b2	0.04	1.00000000	pigs
Ldah	-0.20	1.00000000	pigs
Sacm1l	0.05	1.00000000	pigs
Slc25a12	-0.49	1.00000000	pigs
Sf3b6	-0.13	1.00000000	pigs
Ddx31	0.09	1.00000000	pigs
Elovl5	0.07	1.00000000	pigs
Elp4	0.02	1.00000000	pigs
Ift52	-0.14	1.00000000	pigs
Gbe1	0.22	1.00000000	pigs
Nelfcd	0.04	1.00000000	pigs
Ppp6c	0.08	1.00000000	pigs
Tmcc2	-0.08	1.00000000	pigs
Taf6	0.03	1.00000000	pigs
Atp2c1	0.02	1.00000000	pigs
Kdm4c	0.07	1.00000000	pigs
Hypk	0.14	1.00000000	pigs

Pcmt1	0.01	1.00000000	pigs
Gspt1	-0.22	1.00000000	pigs
Csk	0.08	1.00000000	pigs
Adrb2	-0.31	1.00000000	pigs
2810004N23Rik	-0.12	1.00000000	pigs
Ddx51	-0.17	1.00000000	pigs
Copb2	-0.17	1.00000000	pigs
Sco2	-0.07	1.00000000	pigs
Noct	-0.05	1.00000000	pigs
Agk	0.09	1.00000000	pigs
Vezt	0.01	1.00000000	pigs
Frmd4b	0.24	1.00000000	pigs
Itprid2	0.00	1.00000000	pigs
Eif2b5	-0.06	1.00000000	pigs
Emp3	-0.09	1.00000000	pigs
Frs2	0.13	1.00000000	pigs
Tpst2	0.14	1.00000000	pigs
Zc3h15	-0.13	1.00000000	pigs
Necap1	-0.21	1.00000000	pigs
Lemd3	-0.12	1.00000000	pigs
Szt2	-0.10	1.00000000	pigs
Ankrd12	-0.08	1.00000000	pigs
Cda	-0.56	1.00000000	pigs
Lmnb1	-0.26	1.00000000	pigs
Fam177a	0.14	1.00000000	pigs
Tsc1	0.06	1.00000000	pigs
Eci3	-0.18	1.00000000	pigs
Rabl6	0.00	1.00000000	pigs
Dcp1b	0.04	1.00000000	pigs
Terf2	-0.12	1.00000000	pigs
Btk	-0.10	1.00000000	pigs
Stk39	0.00	1.00000000	pigs
Rint1	-0.09	1.00000000	pigs
Dtx4	-0.10	1.00000000	pigs
Pop4	-0.01	1.00000000	pigs
Sec16a	0.02	1.00000000	pigs
Sucla2	0.09	1.00000000	pigs
Rdx	-0.33	1.00000000	pigs
Brd8	-0.19	1.00000000	pigs
Hgh1	0.00	1.00000000	pigs
Tcerg1	0.03	1.00000000	pigs
Arf5	0.01	1.00000000	pigs
Plxnd1	0.07	1.00000000	pigs
Plekha3	0.00	1.00000000	pigs
Arf1	-0.10	1.00000000	pigs
Tbc1d15	0.15	1.00000000	pigs

Zfp276	0.05	1.00000000	pigs
Lig3	-0.13	1.00000000	pigs
Birc5	0.08	1.00000000	pigs
Lgals3bp	0.08	1.00000000	pigs
Sart3	0.02	1.00000000	pigs
Sema4a	-0.05	1.00000000	pigs
Sde2	-0.01	1.00000000	pigs
Ubtd2	0.10	1.00000000	pigs
Rmnd5a	0.09	1.00000000	pigs
Cflar	-0.04	1.00000000	pigs
Fam126b	0.03	1.00000000	pigs
Atp13a1	-0.06	1.00000000	pigs
Tmem60	0.00	1.00000000	pigs
Nfkbil1	0.05	1.00000000	pigs
H1f3	0.14	1.00000000	pigs
H2ac20	-0.33	1.00000000	pigs
Vsir	0.03	1.00000000	pigs
Arrdc4	0.05	1.00000000	pigs
Tmem87a	0.06	1.00000000	pigs
Tax1bp1	-0.17	1.00000000	pigs
Ndufa11	-0.24	1.00000000	pigs
Vps35l	-0.13	1.00000000	pigs
Lancl1	-0.08	1.00000000	pigs
Fbxo3	0.04	1.00000000	pigs
Ttc4	-0.10	1.00000000	pigs
Clk4	0.03	1.00000000	pigs
Ndufb10	0.05	1.00000000	pigs
Stk40	0.10	1.00000000	pigs
Ddx39a	0.07	1.00000000	pigs
Zfp207	0.05	1.00000000	pigs
Washc3	0.05	1.00000000	pigs
Fer	-0.20	1.00000000	pigs
Prr5	-0.09	1.00000000	pigs
Ermp1	0.02	1.00000000	pigs
Srgap1	0.20	1.00000000	pigs
Cpt1b	0.07	1.00000000	pigs
Greb1l	0.32	1.00000000	pigs
Trmt5	0.07	1.00000000	pigs
Hnrnpd	0.08	1.00000000	pigs
Dtwd1	-0.08	1.00000000	pigs
Abhd11	0.01	1.00000000	pigs
Dld	0.03	1.00000000	pigs
Zmym1	-0.19	1.00000000	pigs
Nkapd1	-0.14	1.00000000	pigs
Dync1li2	0.11	1.00000000	pigs
Zfp1	0.06	1.00000000	pigs

Fkbp8	-0.03	1.00000000	pigs
Drosha	-0.15	1.00000000	pigs
Eloc	-0.23	1.00000000	pigs
Arid3b	-0.21	1.00000000	pigs
Edf1	0.02	1.00000000	pigs
Entpd1	-0.18	1.00000000	pigs
Cttnbp2	-0.05	1.00000000	pigs
Slc10a7	-0.24	1.00000000	pigs
Ctdp1	-0.17	1.00000000	pigs
Cenpm	0.05	1.00000000	pigs
Aasdh	-0.33	1.00000000	pigs
Ppt1	0.07	1.00000000	pigs
Obi1	-0.17	1.00000000	pigs
Bud31	0.06	1.00000000	pigs
Slc5a1	-0.20	1.00000000	pigs
Zfp317	0.08	1.00000000	pigs
Specc1	0.37	1.00000000	pigs
Deup1	0.07	1.00000000	pigs
Vkorc1l1	0.07	1.00000000	pigs
Cpsf7	-0.22	1.00000000	pigs
Borcs5	-0.01	1.00000000	pigs
Rgs1	0.13	1.00000000	pigs
Ttf2	0.07	1.00000000	pigs
Rhbdd2	0.01	1.00000000	pigs
Pcmtd2	0.06	1.00000000	pigs
Kcmf1	0.12	1.00000000	pigs
Galk2	-0.12	1.00000000	pigs
Ift88	0.03	1.00000000	pigs
Zc3h13	-0.25	1.00000000	pigs
Guk1	-0.21	1.00000000	pigs
Fh1	0.09	1.00000000	pigs
Cfap20	-0.11	1.00000000	pigs
Slc35c1	-0.19	1.00000000	pigs
Sertad3	-0.02	1.00000000	pigs
Dusp16	-0.10	1.00000000	pigs
Serinc1	0.06	1.00000000	pigs
Otub1	0.02	1.00000000	pigs
Ifnlr1	-0.22	1.00000000	pigs
Znfx1	-0.26	1.00000000	pigs
Pibf1	-0.15	1.00000000	pigs
Rab31	-0.02	1.00000000	pigs
Tex9	0.01	1.00000000	pigs
Ppp6r2	0.10	1.00000000	pigs
Nup214	-0.14	1.00000000	pigs
Lrriq3	-0.20	1.00000000	pigs
Taf12	0.00	1.00000000	pigs

Nras	-0.19	1.00000000	pigs
Znhit6	0.02	1.00000000	pigs
Csnk1g2	0.05	1.00000000	pigs
Cggbp1	-0.17	1.00000000	pigs
Acot4	0.05	1.00000000	pigs
Cfap36	-0.12	1.00000000	pigs
Uri1	-0.17	1.00000000	pigs
Pitpnm3	-0.08	1.00000000	pigs
Rpp40	-0.10	1.00000000	pigs
Itgae	0.04	1.00000000	pigs
Ppp2cb	-0.03	1.00000000	pigs
Pced1b	-0.32	1.00000000	pigs
Tfe3	0.01	1.00000000	pigs
Ctdsp1	-0.11	1.00000000	pigs
Trex1	0.02	1.00000000	pigs
Polr1f	0.03	1.00000000	pigs
Dennd10	0.01	1.00000000	pigs
Gfus	-0.21	1.00000000	pigs
Gtf2f1	-0.29	1.00000000	pigs
Nbas	-0.21	1.00000000	pigs
Itch	-0.16	1.00000000	pigs
Tsn	-0.07	1.00000000	pigs
Angel2	0.12	1.00000000	pigs
Vrk1	-0.23	1.00000000	pigs
Arfgef1	0.15	1.00000000	pigs
Fxr2	0.04	1.00000000	pigs
Amh	0.01	1.00000000	pigs
Ubac1	0.04	1.00000000	pigs
Il18	-0.17	1.00000000	pigs
Tmx3	0.03	1.00000000	pigs
2210016L21Rik	-0.07	1.00000000	pigs
Erg28	0.00	1.00000000	pigs
Lin54	0.17	1.00000000	pigs
Noa1	-0.05	1.00000000	pigs
Ppp1r9b	0.04	1.00000000	pigs
Dhx57	-0.09	1.00000000	pigs
Adora2b	0.21	1.00000000	pigs
Fam181b	-0.12	1.00000000	pigs
Cox5a	0.08	1.00000000	pigs
Dcps	0.02	1.00000000	pigs
Polr3d	-0.21	1.00000000	pigs
Smu1	0.05	1.00000000	pigs
Acat1	-0.18	1.00000000	pigs
Nfu1	-0.21	1.00000000	pigs
Aamp	0.03	1.00000000	pigs
Sarm1	0.07	1.00000000	pigs

Avpi1	0.01	1.00000000	pigs
Cwf19l1	-0.20	1.00000000	pigs
Eftud2	-0.07	1.00000000	pigs
Ebi3	0.19	1.00000000	pigs
Cyb5r4	-0.17	1.00000000	pigs
Rrp36	-0.20	1.00000000	pigs
Wdr70	-0.13	1.00000000	pigs
Trip13	-0.08	1.00000000	pigs
Tsen15	0.06	1.00000000	pigs
Lrrcc1	-0.13	1.00000000	pigs
Ttc14	0.00	1.00000000	pigs
Stam	0.04	1.00000000	pigs
Plcb4	-0.29	1.00000000	pigs
Nipbl	0.10	1.00000000	pigs
Smpd2	0.05	1.00000000	pigs
Cenpj	-0.03	1.00000000	pigs
Prepl	0.10	1.00000000	pigs
Tbc1d9b	0.10	1.00000000	pigs
Akr7a5	0.07	1.00000000	pigs
Rpf2	0.06	1.00000000	pigs
Lrrk2	-0.80	1.00000000	pigs
Nxpe3	-0.02	1.00000000	pigs
Rbck1	-0.20	1.00000000	pigs
Prep	0.18	1.00000000	pigs
Blm	0.05	1.00000000	pigs
Cactin	-0.03	1.00000000	pigs
Rwdd4a	-0.02	1.00000000	pigs
Ctps	0.05	1.00000000	pigs
Ncln	-0.16	1.00000000	pigs
Hp1bp3	-0.18	1.00000000	pigs
Tmem53	-0.11	1.00000000	pigs
S100pbp	0.07	1.00000000	pigs
Cdc40	0.00	1.00000000	pigs
Crat	0.03	1.00000000	pigs
Usp33	0.12	1.00000000	pigs
Lman1	-0.12	1.00000000	pigs
Arhgef2	0.03	1.00000000	pigs
Dpy19l4	-0.11	1.00000000	pigs
Zfp422	0.09	1.00000000	pigs
Erg	-0.07	1.00000000	pigs
Itga9	0.18	1.00000000	pigs
Aig1	-0.20	1.00000000	pigs
Rchy1	-0.02	1.00000000	pigs
Faf1	0.13	1.00000000	pigs
Nif3l1	0.06	1.00000000	pigs
Wdr20	0.00	1.00000000	pigs

Tmed10	0.08	1.00000000	pigs
Rad23b	-0.14	1.00000000	pigs
Agfg2	-0.10	1.00000000	pigs
Ptpre	-0.14	1.00000000	pigs
Lbr	-0.19	1.00000000	pigs
Pigf	-0.10	1.00000000	pigs
Pcna	0.02	1.00000000	pigs
Gspt2	-0.13	1.00000000	pigs
Rpap2	0.10	1.00000000	pigs
Ccdc14	-0.06	1.00000000	pigs
Lnpep	0.03	1.00000000	pigs
Gpsm2	0.00	1.00000000	pigs
Cbll1	0.03	1.00000000	pigs
2700062C07Rik	-0.17	1.00000000	pigs
Tssk4	-0.09	1.00000000	pigs
Atad1	-0.18	1.00000000	pigs
Ubald1	-0.09	1.00000000	pigs
Hnrnpr	-0.15	1.00000000	pigs
Cops7b	0.09	1.00000000	pigs
St3gal6	-0.09	1.00000000	pigs
Tgds	0.03	1.00000000	pigs
Hyou1	0.06	1.00000000	pigs
St3gal2	0.05	1.00000000	pigs
Il1rl1	-0.11	1.00000000	pigs
Chmp4b	-0.12	1.00000000	pigs
Rccd1	0.03	1.00000000	pigs
Crnkl1	0.05	1.00000000	pigs
Papolg	0.07	1.00000000	pigs
Brd3	-0.05	1.00000000	pigs
Prkag2	0.11	1.00000000	pigs
Ret	0.13	1.00000000	pigs
Actr1b	-0.02	1.00000000	pigs
Cog4	0.02	1.00000000	pigs
Slc25a3	0.08	1.00000000	pigs
Kif3c	0.08	1.00000000	pigs
Pnkd	-0.18	1.00000000	pigs
Usp30	0.03	1.00000000	pigs
Sit1	-0.20	1.00000000	pigs
Nup188	0.01	1.00000000	pigs
Rfc2	0.11	1.00000000	pigs
Aatf	0.02	1.00000000	pigs
Cpped1	-0.02	1.00000000	pigs
G6pdx	-0.01	1.00000000	pigs
Hras	-0.01	1.00000000	pigs
Agfg1	0.06	1.00000000	pigs
Gpn3	-0.19	1.00000000	pigs

Rrp1b	0.04	1.00000000	pigs
Chd1l	-0.06	1.00000000	pigs
Ehbp1	0.13	1.00000000	pigs
Elmod3	-0.01	1.00000000	pigs
Cul4a	0.07	1.00000000	pigs
Qrich2	-0.03	1.00000000	pigs
Tufm	0.04	1.00000000	pigs
Golt1b	-0.18	1.00000000	pigs
Acad11	-0.10	1.00000000	pigs
Vars	0.04	1.00000000	pigs
Nup155	-0.13	1.00000000	pigs
Kat5	0.01	1.00000000	pigs
Thoc2	0.08	1.00000000	pigs
Dnajc16	0.05	1.00000000	pigs
Cse1l	0.04	1.00000000	pigs
Ogfod3	-0.18	1.00000000	pigs
Klhdc3	0.03	1.00000000	pigs
Chm	0.06	1.00000000	pigs
Sec24a	0.19	1.00000000	pigs
Spast	-0.20	1.00000000	pigs
Wdfy2	0.29	1.00000000	pigs
Oscar	0.02	1.00000000	pigs
Bmpr2	0.10	1.00000000	pigs
Sc5d	0.01	1.00000000	pigs
Bysl	-0.14	1.00000000	pigs
Ifngr2	0.18	1.00000000	pigs
Ccdc124	0.04	1.00000000	pigs
Dpp9	-0.16	1.00000000	pigs
Tek	0.11	1.00000000	pigs
Prkca	-0.07	1.00000000	pigs
B3gntl1	-0.01	1.00000000	pigs
Pcca	0.06	1.00000000	pigs
Napg	-0.22	1.00000000	pigs
Sumf2	0.00	1.00000000	pigs
Ap1s1	0.06	1.00000000	pigs
Ipo13	0.02	1.00000000	pigs
Ufd1	0.06	1.00000000	pigs
Bap1	-0.08	1.00000000	pigs
Xrn1	-0.16	1.00000000	pigs
Sass6	-0.01	1.00000000	pigs
Dsn1	0.05	1.00000000	pigs
Ide	0.08	1.00000000	pigs
Pld1	0.01	1.00000000	pigs
Polr2d	0.09	1.00000000	pigs
Rpn1	0.15	1.00000000	pigs
Sphk2	-0.09	1.00000000	pigs

Aco2	0.03	1.00000000	pigs
Dhx37	-0.01	1.00000000	pigs
Pfdn4	-0.29	1.00000000	pigs
Arid5b	-0.07	1.00000000	pigs
Coq9	-0.17	1.00000000	pigs
Rbfox1	0.02	1.00000000	pigs
Dalrd3	0.01	1.00000000	pigs
Nars2	-0.12	1.00000000	pigs
Pole4	-0.22	1.00000000	pigs
Lrig2	0.05	1.00000000	pigs
Scyl1	0.04	1.00000000	pigs
Kif3b	-0.17	1.00000000	pigs
Trnt1	0.08	1.00000000	pigs
Cops3	0.07	1.00000000	pigs
Cdkn1b	0.02	1.00000000	pigs
Cox7a2l	0.12	1.00000000	pigs
Itga6	0.16	1.00000000	pigs
Cab39l	-0.01	1.00000000	pigs
Ubap1	0.08	1.00000000	pigs
Tbc1d22a	-0.12	1.00000000	pigs
Glcci1	-0.12	1.00000000	pigs
Sap30l	0.05	1.00000000	pigs
Thrap3	-0.11	1.00000000	pigs
Iqcg	0.00	1.00000000	pigs
2310009B15Rik	-0.03	1.00000000	pigs
Fbxl5	-0.16	1.00000000	pigs
C3	-0.22	1.00000000	pigs
Entr1	0.08	1.00000000	pigs
Ube2l6	-0.07	1.00000000	pigs
Prr12	-0.01	1.00000000	pigs
Gkap1	-0.21	1.00000000	pigs
Ube2w	-0.25	1.00000000	pigs
Tmem30a	0.05	1.00000000	pigs
Eepd1	0.94	1.00000000	pigs
Tpm1	0.01	1.00000000	pigs
St7l	-0.01	1.00000000	pigs
Gpatch1	-0.03	1.00000000	pigs
Casd1	0.01	1.00000000	pigs
Trim23	0.04	1.00000000	pigs
Ulk1	-0.06	1.00000000	pigs
Sgsm2	0.08	1.00000000	pigs
Ireb2	0.00	1.00000000	pigs
Ppp4r1	-0.02	1.00000000	pigs
Nubpl	0.09	1.00000000	pigs
Rrp9	-0.10	1.00000000	pigs
Acbd5	-0.01	1.00000000	pigs

Slc37a1	-0.12	1.00000000	pigs
Ttc1	0.06	1.00000000	pigs
Foxj3	0.07	1.00000000	pigs
Dnttip1	0.07	1.00000000	pigs
Bfar	-0.43	1.00000000	pigs
Nelfa	-0.03	1.00000000	pigs
Cuta	-0.19	1.00000000	pigs
Alg5	-0.14	1.00000000	pigs
Zbtb7b	0.05	1.00000000	pigs
Zw10	0.01	1.00000000	pigs
Dach1	-0.17	1.00000000	pigs
Lrrc59	0.06	1.00000000	pigs
Dusp10	-0.11	1.00000000	pigs
Arid1b	0.07	1.00000000	pigs
Pdzd8	0.03	1.00000000	pigs
Nup93	0.04	1.00000000	pigs
Hectd4	0.19	1.00000000	pigs
Eif4g3	-0.07	1.00000000	pigs
Prmt5	0.02	1.00000000	pigs
Acadsb	-0.16	1.00000000	pigs
Slc46a3	0.26	1.00000000	pigs
Lrrc45	0.04	1.00000000	pigs
Rhod	-0.15	1.00000000	pigs
Eif3a	0.14	1.00000000	pigs
Hnrnph3	0.02	1.00000000	pigs
Prpf4	0.03	1.00000000	pigs
Dock2	-0.17	1.00000000	pigs
Cep68	-0.07	1.00000000	pigs
Rragb	-0.01	1.00000000	pigs
Accsl	0.07	1.00000000	pigs
Psmf1	0.06	1.00000000	pigs
Nexn	-0.14	1.00000000	pigs
Cln3	0.06	1.00000000	pigs
Zscan12	-0.09	1.00000000	pigs
Tst	0.10	1.00000000	pigs
Arl6ip1	-0.20	1.00000000	pigs
Tlr3	0.01	1.00000000	pigs
Unc13d	0.04	1.00000000	pigs
Gtf3c6	0.00	1.00000000	pigs
Tfpt	-0.01	1.00000000	pigs
Zbtb34	0.00	1.00000000	pigs
Tmpo	-0.24	1.00000000	pigs
Rangrf	0.02	1.00000000	pigs
Cask	0.17	1.00000000	pigs
Acin1	0.02	1.00000000	pigs
Imp4	-0.25	1.00000000	pigs

Nars	0.04	1.00000000	pigs
Pycrl	0.01	1.00000000	pigs
Sergef	0.07	1.00000000	pigs
Frem2	-0.11	1.00000000	pigs
Tmem209	-0.09	1.00000000	pigs
Limk2	-0.07	1.00000000	pigs
Tmub1	-0.02	1.00000000	pigs
Paqr5	-0.20	1.00000000	pigs
Atg16l2	0.00	1.00000000	pigs
Amd2	0.03	1.00000000	pigs
Ppp3cb	0.04	1.00000000	pigs
Nudt5	0.05	1.00000000	pigs
Nenf	0.00	1.00000000	pigs
Tob2	0.05	1.00000000	pigs
Eps15l1	0.09	1.00000000	pigs
Tmem101	-0.13	1.00000000	pigs
Wdr11	0.05	1.00000000	pigs
Dcun1d5	-0.16	1.00000000	pigs
Arel1	0.00	1.00000000	pigs
Ung	-0.14	1.00000000	pigs
Hmox2	-0.19	1.00000000	pigs
Cdk10	0.00	1.00000000	pigs
Fancd2	0.07	1.00000000	pigs
Gdap2	0.06	1.00000000	pigs
Zhx1	-0.05	1.00000000	pigs
Dnpep	0.05	1.00000000	pigs
Ccdc59	-0.02	1.00000000	pigs
Tasor	-0.28	1.00000000	pigs
Gosr1	0.00	1.00000000	pigs
Dnajb2	0.10	1.00000000	pigs
Cdkn2aipnl	-0.15	1.00000000	pigs
Faf2	0.00	1.00000000	pigs
Wrn	-0.14	1.00000000	pigs
Gorab	0.00	1.00000000	pigs
Dcaf17	0.02	1.00000000	pigs
Tspan17	0.00	1.00000000	pigs
Isca2	-0.14	1.00000000	pigs
Tmem192	-0.10	1.00000000	pigs
Dicer1	0.01	1.00000000	pigs
Tmtc1	0.47	1.00000000	pigs
Cdc16	-0.11	1.00000000	pigs
Usp16	-0.17	1.00000000	pigs
Sephs1	-0.04	1.00000000	pigs
Rc3h1	-0.15	1.00000000	pigs
Ssx2ip	0.01	1.00000000	pigs
Fdx1	-0.19	1.00000000	pigs

Cep192	0.02	1.00000000	pigs
Abcb7	0.00	1.00000000	pigs
Cfdp1	-0.20	1.00000000	pigs
Isg20l2	0.05	1.00000000	pigs
Fbxo45	-0.11	1.00000000	pigs
Kbtbd7	0.13	1.00000000	pigs
Bmp4	-0.14	1.00000000	pigs
Dolpp1	0.01	1.00000000	pigs
Gpaa1	-0.03	1.00000000	pigs
Cul4b	0.02	1.00000000	pigs
Tex264	-0.13	1.00000000	pigs
Prrc2a	-0.04	1.00000000	pigs
Dguok	0.00	1.00000000	pigs
Wasf3	0.10	1.00000000	pigs
Pcnx4	-0.05	1.00000000	pigs
Vcl	-0.13	1.00000000	pigs
Ptpn12	0.08	1.00000000	pigs
Yae1d1	0.04	1.00000000	pigs
Coq5	0.01	1.00000000	pigs
Atg7	0.08	1.00000000	pigs
Tnfsf8	0.01	1.00000000	pigs
Ppp1r37	0.07	1.00000000	pigs
Nup205	0.07	1.00000000	pigs
9030025P20Rik	0.02	1.00000000	pigs
Ecd	0.04	1.00000000	pigs
Dera	0.12	1.00000000	pigs
Adck2	0.01	1.00000000	pigs
Fmn1	-0.10	1.00000000	pigs
Lpcat3	-0.23	1.00000000	pigs
Tubg1	0.08	1.00000000	pigs
Ppm1l	-0.28	1.00000000	pigs
Ccdc47	0.01	1.00000000	pigs
Tmem39a	-0.02	1.00000000	pigs
Ttc33	-0.10	1.00000000	pigs
Prdm4	-0.24	1.00000000	pigs
Fus	-0.14	1.00000000	pigs
Dstn	0.08	1.00000000	pigs
Cdkn3	0.00	1.00000000	pigs
Actr5	0.02	1.00000000	pigs
Ascc1	-0.27	1.00000000	pigs
Egfl8	0.05	1.00000000	pigs
Ppp1r11	0.00	1.00000000	pigs
Bhlhe41	0.08	1.00000000	pigs
Pygm	0.08	1.00000000	pigs
Plk3	0.07	1.00000000	pigs
Carnmt1	-0.14	1.00000000	pigs

Scaf4	0.03	1.00000000	pigs
Psmd1	0.05	1.00000000	pigs
Fbh1	-0.15	1.00000000	pigs
Nrp2	0.60	1.00000000	pigs
Tmem104	-0.11	1.00000000	pigs
Ergic3	-0.13	1.00000000	pigs
Epn1	0.06	1.00000000	pigs
Stx4a	0.00	1.00000000	pigs
Grk2	-0.09	1.00000000	pigs
Emc4	-0.11	1.00000000	pigs
Hook3	0.15	1.00000000	pigs
Tomm70a	0.03	1.00000000	pigs
Uba5	-0.21	1.00000000	pigs
Zcchc9	-0.16	1.00000000	pigs
Fkbp5	0.26	1.00000000	pigs
Txlna	0.05	1.00000000	pigs
Srbd1	-0.24	1.00000000	pigs
Sdhb	-0.08	1.00000000	pigs
Pdcd6	-0.19	1.00000000	pigs
Pot1b	-0.14	1.00000000	pigs
Dact1	-0.08	1.00000000	pigs
Creld2	-0.16	1.00000000	pigs
Zfp777	0.07	1.00000000	pigs
Suv39h2	0.02	1.00000000	pigs
Eapp	-0.13	1.00000000	pigs
Hbb-bh2	-0.39	1.00000000	pigs
Kat2b	0.06	1.00000000	pigs
Nono	-0.16	1.00000000	pigs
Nelfb	-0.02	1.00000000	pigs
Tsr3	-0.22	1.00000000	pigs
Prkaa1	0.04	1.00000000	pigs
Slc26a6	-0.02	1.00000000	pigs
Zmynd11	-0.34	1.00000000	pigs
Plekha8	-0.03	1.00000000	pigs
Sirt5	-0.07	1.00000000	pigs
Fdft1	-0.20	1.00000000	pigs
Fggy	0.13	1.00000000	pigs
Tpmt	0.08	1.00000000	pigs
Noc3l	-0.12	1.00000000	pigs
Atl2	-0.04	1.00000000	pigs
Sash3	-0.18	1.00000000	pigs
Ripor1	-0.17	1.00000000	pigs
Npat	0.02	1.00000000	pigs
Pogz	-0.02	1.00000000	pigs
P2ry10	0.03	1.00000000	pigs
Cyth4	-0.04	1.00000000	pigs

Surf4	-0.21	1.00000000	pigs
Dnajb4	-0.09	1.00000000	pigs
Dyrk1b	-0.20	1.00000000	pigs
Wdr61	-0.21	1.00000000	pigs
Usp40	-0.11	1.00000000	pigs
Scamp1	0.07	1.00000000	pigs
Gtf2h1	-0.13	1.00000000	pigs
Nat9	0.09	1.00000000	pigs
Hsp90b1	-0.05	1.00000000	pigs
Trappc6b	-0.18	1.00000000	pigs
Itfg2	-0.06	1.00000000	pigs
Camk2g	0.02	1.00000000	pigs
Grwd1	-0.07	1.00000000	pigs
Ddhd2	-0.11	1.00000000	pigs
Psmd11	0.02	1.00000000	pigs
Sptlc1	-0.15	1.00000000	pigs
Zfp9	0.00	1.00000000	pigs
Vars2	0.00	1.00000000	pigs
Zkscan1	0.05	1.00000000	pigs
Smpd1	0.02	1.00000000	pigs
Ints3	0.00	1.00000000	pigs
Golga4	0.07	1.00000000	pigs
Fmnl3	0.04	1.00000000	pigs
Kdm7a	0.11	1.00000000	pigs
Gpt	-0.12	1.00000000	pigs
Phf5a	-0.16	1.00000000	pigs
Birc6	0.09	1.00000000	pigs
Pfdn6	-0.16	1.00000000	pigs
Uprt	-0.03	1.00000000	pigs
Derpc	0.00	1.00000000	pigs
Ube4b	-0.16	1.00000000	pigs
Ebna1bp2	-0.03	1.00000000	pigs
Srsf7	-0.06	1.00000000	pigs
Hyi	0.00	1.00000000	pigs
Tstd2	-0.10	1.00000000	pigs
Slc30a7	-0.18	1.00000000	pigs
Chmp6	-0.14	1.00000000	pigs
Klf3	0.08	1.00000000	pigs
Ube2cbp	-0.11	1.00000000	pigs
Chd9	0.09	1.00000000	pigs
Irak4	0.02	1.00000000	pigs
Nipsnap2	0.04	1.00000000	pigs
Ammecr1l	-0.22	1.00000000	pigs
Sae1	0.06	1.00000000	pigs
Fam162a	-0.21	1.00000000	pigs
Gtf2a1	-0.14	1.00000000	pigs

Ddx6	0.08	1.00000000	pigs
Trappc14	0.02	1.00000000	pigs
Slc39a6	-0.02	1.00000000	pigs
Lap3	0.06	1.00000000	pigs
Umad1	0.01	1.00000000	pigs
Socs6	-0.27	1.00000000	pigs
Psmb3	-0.03	1.00000000	pigs
Lypla2	0.06	1.00000000	pigs
Dpp10	-0.60	1.00000000	pigs
Slc9a1	-0.02	1.00000000	pigs
Fam149b	-0.19	1.00000000	pigs
Lancl2	0.13	1.00000000	pigs
Bbx	0.10	1.00000000	pigs
Thbd	-0.11	1.00000000	pigs
Vps41	-0.16	1.00000000	pigs
Ctsc	0.18	1.00000000	pigs
Nde1	0.14	1.00000000	pigs
Tbc1d5	-0.11	1.00000000	pigs
Paqr3	-0.08	1.00000000	pigs
Osgepl1	-0.09	1.00000000	pigs
Rpp30	0.07	1.00000000	pigs
Tmed8	-0.11	1.00000000	pigs
Vps4b	-0.09	1.00000000	pigs
Ppig	0.02	1.00000000	pigs
5730455P16Rik	0.02	1.00000000	pigs
Stim2	-0.05	1.00000000	pigs
Kras	-0.08	1.00000000	pigs
Sell	-0.14	1.00000000	pigs
Clnk	0.14	1.00000000	pigs
Tlnrd1	0.04	1.00000000	pigs
Hpcal1	0.12	1.00000000	pigs
Pogk	0.02	1.00000000	pigs
Rprd1b	0.08	1.00000000	pigs
Uqcc3	0.00	1.00000000	pigs
Katna1	-0.11	1.00000000	pigs
Rock2	0.13	1.00000000	pigs
Rnf40	0.04	1.00000000	pigs
Capza2	-0.14	1.00000000	pigs
Cd24a	-0.28	1.00000000	pigs
Lclat1	0.00	1.00000000	pigs
Slc25a19	0.03	1.00000000	pigs
Ranbp6	-0.10	1.00000000	pigs
Ccdc97	0.00	1.00000000	pigs
Ifi35	0.04	1.00000000	pigs
Dcp2	-0.15	1.00000000	pigs
Plk2	-0.28	1.00000000	pigs

Polr1a	0.00	1.00000000	pigs
Gba2	0.07	1.00000000	pigs
Ddx49	0.01	1.00000000	pigs
Gabbr1	0.00	1.00000000	pigs
Utp20	-0.19	1.00000000	pigs
Ccdc12	-0.09	1.00000000	pigs
Rasgef1a	-0.02	1.00000000	pigs
Sh2b3	-0.08	1.00000000	pigs
Zfp740	-0.07	1.00000000	pigs
Coil	-0.14	1.00000000	pigs
Ftsj3	0.01	1.00000000	pigs
Pkig	-0.10	1.00000000	pigs
Pkp4	-0.01	1.00000000	pigs
Trib1	-0.14	1.00000000	pigs
Pak2	-0.13	1.00000000	pigs
Pus7	-0.07	1.00000000	pigs
Cdc14a	-0.01	1.00000000	pigs
Wdr59	0.07	1.00000000	pigs
Zfp800	0.09	1.00000000	pigs
Arv1	-0.10	1.00000000	pigs
Cacul1	0.12	1.00000000	pigs
Chmp7	0.03	1.00000000	pigs
Sec61a1	0.08	1.00000000	pigs
Brwd3	0.05	1.00000000	pigs
Bcorl1	0.03	1.00000000	pigs
Lmtk2	0.03	1.00000000	pigs
Taldo1	-0.18	1.00000000	pigs
Nploc4	-0.15	1.00000000	pigs
Lgals3	-0.05	1.00000000	pigs
Zfp628	0.01	1.00000000	pigs
Ipp	-0.09	1.00000000	pigs
Rnf214	-0.13	1.00000000	pigs
Stoml2	-0.04	1.00000000	pigs
Psmd7	-0.04	1.00000000	pigs
Elovl1	0.05	1.00000000	pigs
Kcnip1	-0.44	1.00000000	pigs
Itpr2	-0.07	1.00000000	pigs
Slc9b2	-0.06	1.00000000	pigs
Pi4k2b	0.04	1.00000000	pigs
Ets1	-0.17	1.00000000	pigs
Tbca	-0.20	1.00000000	pigs
Rnf13	-0.16	1.00000000	pigs
Hacd2	0.02	1.00000000	pigs
Nsun3	-0.09	1.00000000	pigs
Iffo1	-0.09	1.00000000	pigs
Ipo11	-0.23	1.00000000	pigs

Cnot3	0.03	1.00000000	pigs
Vstm4	-0.04	1.00000000	pigs
Uxs1	-0.04	1.00000000	pigs
Fem1c	-0.16	1.00000000	pigs
Zmym2	-0.06	1.00000000	pigs
Zswim8	0.01	1.00000000	pigs
Asrgl1	-0.06	1.00000000	pigs
Armc1	0.10	1.00000000	pigs
Poglut1	0.09	1.00000000	pigs
Nfyb	-0.10	1.00000000	pigs
Prox1	0.05	1.00000000	pigs
Gtpbp3	0.02	1.00000000	pigs
Lsg1	0.05	1.00000000	pigs
Rcn2	-0.20	1.00000000	pigs
Acvr2a	0.01	1.00000000	pigs
Ddx24	-0.15	1.00000000	pigs
Plbd2	-0.18	1.00000000	pigs
Gpr137	-0.13	1.00000000	pigs
Snrnp40	0.02	1.00000000	pigs
Wdr36	-0.02	1.00000000	pigs
Srrm1	0.04	1.00000000	pigs
Zfp2	0.02	1.00000000	pigs
Tial1	0.06	1.00000000	pigs
Pla2g6	0.03	1.00000000	pigs
Brox	-0.02	1.00000000	pigs
Slc8b1	-0.04	1.00000000	pigs
Ifrd2	0.05	1.00000000	pigs
Slc44a1	-0.21	1.00000000	pigs
Uevld	-0.22	1.00000000	pigs
Klf13	-0.07	1.00000000	pigs
Cltb	0.11	1.00000000	pigs
Fam98c	0.03	1.00000000	pigs
Slc25a16	0.04	1.00000000	pigs
Acrbp	-0.01	1.00000000	pigs
Emc9	-0.14	1.00000000	pigs
Ccdc32	0.01	1.00000000	pigs
Ccdc18	-0.17	1.00000000	pigs
Tmem184c	-0.13	1.00000000	pigs
Cpsf3	0.02	1.00000000	pigs
Snx27	0.06	1.00000000	pigs
Rsrp1	-0.03	1.00000000	pigs
Slc39a10	0.00	1.00000000	pigs
Snrpa	-0.14	1.00000000	pigs
Ptprc	-0.08	1.00000000	pigs
Zfp592	0.09	1.00000000	pigs
Hax1	-0.02	1.00000000	pigs

Zfp618	-0.20	1.00000000	pigs
Pnpla2	-0.17	1.00000000	pigs
Zfp672	-0.21	1.00000000	pigs
Dlgap4	-0.13	1.00000000	pigs
Aip	-0.05	1.00000000	pigs
Als2	-0.03	1.00000000	pigs
Dnm2	0.08	1.00000000	pigs
Stag3	0.11	1.00000000	pigs
C5ar1	-0.17	1.00000000	pigs
Dusp28	0.07	1.00000000	pigs
Exog	-0.03	1.00000000	pigs
Ephx2	-0.08	1.00000000	pigs
Aifm1	-0.03	1.00000000	pigs
Clptm1	0.13	1.00000000	pigs
Rpusd1	-0.09	1.00000000	pigs
Rab6a	0.13	1.00000000	pigs
Fam227b	-0.21	1.00000000	pigs
Cbfa2t2	-0.12	1.00000000	pigs
Aagab	-0.15	1.00000000	pigs
Tnik	0.28	1.00000000	pigs
Dcp1a	-0.14	1.00000000	pigs
Nol7	0.04	1.00000000	pigs
Scn4a	-0.15	1.00000000	pigs
Ciao1	0.04	1.00000000	pigs
Ate1	0.02	1.00000000	pigs
Tfb1m	-0.23	1.00000000	pigs
Arhgef3	-0.05	1.00000000	pigs
Clk2	-0.02	1.00000000	pigs
Naa15	0.00	1.00000000	pigs
Zc3hc1	-0.10	1.00000000	pigs
Zc2hc1a	0.01	1.00000000	pigs
Nubp2	-0.15	1.00000000	pigs
Acaa1b	0.06	1.00000000	pigs
Rnf6	0.11	1.00000000	pigs
Zpr1	-0.16	1.00000000	pigs
Sec31a	0.07	1.00000000	pigs
Ptar1	0.04	1.00000000	pigs
Plpp1	-0.15	1.00000000	pigs
Gabpa	-0.24	1.00000000	pigs
Erap1	0.04	1.00000000	pigs
Saraf	-0.23	1.00000000	pigs
Cpne2	-0.05	1.00000000	pigs
Zcrb1	-0.18	1.00000000	pigs
Arhgap35	-0.15	1.00000000	pigs
Rex1bd	-0.17	1.00000000	pigs
Ap2a1	0.03	1.00000000	pigs

Syf2	-0.13	1.00000000	pigs
Casp8ap2	0.02	1.00000000	pigs
Ccdc150	0.00	1.00000000	pigs
Xkr6	0.03	1.00000000	pigs
Uqcr10	-0.16	1.00000000	pigs
Cep128	0.28	1.00000000	pigs
Fam20b	-0.01	1.00000000	pigs
Taf11	-0.11	1.00000000	pigs
Lzic	-0.02	1.00000000	pigs
Cnih1	-0.17	1.00000000	pigs
Bcl10	0.06	1.00000000	pigs
Ddx11	0.03	1.00000000	pigs
Skp2	0.11	1.00000000	pigs
Cdk4	0.08	1.00000000	pigs
Polr2e	0.03	1.00000000	pigs
Tcf3	-0.06	1.00000000	pigs
Denr	-0.16	1.00000000	pigs
Dpp3	-0.16	1.00000000	pigs
Glyctk	0.01	1.00000000	pigs
Vdac2	-0.18	1.00000000	pigs
Wdr75	-0.14	1.00000000	pigs
Rtca	0.03	1.00000000	pigs
Sigmar1	0.02	1.00000000	pigs
Taf8	-0.04	1.00000000	pigs
Ccdc43	0.00	1.00000000	pigs
Pno1	-0.14	1.00000000	pigs
Gin1	-0.23	1.00000000	pigs
Rpp21	-0.15	1.00000000	pigs
Eif2s1	0.08	1.00000000	pigs
Slirp	-0.17	1.00000000	pigs
Habp4	-0.03	1.00000000	pigs
Apmap	-0.10	1.00000000	pigs
Tsen54	-0.03	1.00000000	pigs
Bin3	0.03	1.00000000	pigs
Surf6	-0.11	1.00000000	pigs
Dhx40	-0.20	1.00000000	pigs
Tbcd	0.03	1.00000000	pigs
Sgpl1	0.05	1.00000000	pigs
Taok3	-0.19	1.00000000	pigs
Timm10	-0.18	1.00000000	pigs
Ppip5k2	0.11	1.00000000	pigs
Usp18	-0.06	1.00000000	pigs
Pik3ap1	0.13	1.00000000	pigs
Zfp14	0.05	1.00000000	pigs
1600012H06Rik	-0.22	1.00000000	pigs
Ndufaf1	-0.09	1.00000000	pigs

Cds2	-0.14	1.00000000	pigs
Apip	-0.12	1.00000000	pigs
Atf6b	0.01	1.00000000	pigs
Ndufb6	-0.16	1.00000000	pigs
Dpyd	0.24	1.00000000	pigs
Psme3	0.00	1.00000000	pigs
Ankib1	-0.19	1.00000000	pigs
Rhoa	0.05	1.00000000	pigs
Sp4	-0.15	1.00000000	pigs
Isg20	0.06	1.00000000	pigs
Zfp335	0.01	1.00000000	pigs
Trim27	-0.03	1.00000000	pigs
AI837181	-0.09	1.00000000	pigs
Zfp362	0.02	1.00000000	pigs
Slc39a9	-0.05	1.00000000	pigs
Atp1b1	-0.23	1.00000000	pigs
Epha2	-0.16	1.00000000	pigs
Sort1	0.04	1.00000000	pigs
Tpgs1	-0.01	1.00000000	pigs
Dnajc3	0.07	1.00000000	pigs
Desi1	0.15	1.00000000	pigs
Nip7	-0.07	1.00000000	pigs
Pyroxd1	-0.08	1.00000000	pigs
Gemin7	-0.10	1.00000000	pigs
Psmd13	-0.06	1.00000000	pigs
Eri3	0.14	1.00000000	pigs
Uba6	0.03	1.00000000	pigs
Nupl2	-0.16	1.00000000	pigs
Septin2	-0.16	1.00000000	pigs
Dock11	-0.03	1.00000000	pigs
Rprd1a	-0.01	1.00000000	pigs
Ulk4	-0.08	1.00000000	pigs
Cep250	-0.09	1.00000000	pigs
Rbm25	0.04	1.00000000	pigs
Lsm8	-0.04	1.00000000	pigs
Dcaf6	-0.10	1.00000000	pigs
Dkc1	-0.13	1.00000000	pigs
Grb10	0.09	1.00000000	pigs
Cab39	-0.18	1.00000000	pigs
Tubd1	0.03	1.00000000	pigs
Rbm22	0.03	1.00000000	pigs
Xpo6	-0.04	1.00000000	pigs
Rrp7a	0.01	1.00000000	pigs
Ripk2	-0.11	1.00000000	pigs
Capn15	0.01	1.00000000	pigs
Zc3h4	-0.09	1.00000000	pigs

Sumf1	-0.01	1.00000000	pigs
Abcf3	0.00	1.00000000	pigs
Ddx46	-0.15	1.00000000	pigs
Tcfl5	-0.06	1.00000000	pigs
Ddx27	-0.20	1.00000000	pigs
Glmn	-0.11	1.00000000	pigs
Rad23a	0.09	1.00000000	pigs
Hdac2	0.06	1.00000000	pigs
Zfp36l2	0.09	1.00000000	pigs
Exoc5	-0.01	1.00000000	pigs
Rrm1	0.02	1.00000000	pigs
Kifbp	0.02	1.00000000	pigs
Serpini1	-0.18	1.00000000	pigs
Serbp1	-0.04	1.00000000	pigs
Ypel3	-0.16	1.00000000	pigs
Gsr	-0.04	1.00000000	pigs
Bdp1	0.04	1.00000000	pigs
Wdr25	-0.13	1.00000000	pigs
Apobec3	0.01	1.00000000	pigs
Nsrp1	-0.16	1.00000000	pigs
Gnai3	0.06	1.00000000	pigs
Uqcrc1	0.09	1.00000000	pigs
Traf4	-0.20	1.00000000	pigs
Atg13	0.12	1.00000000	pigs
Cc2d1b	0.03	1.00000000	pigs
Aacs	-0.09	1.00000000	pigs
Osr1	0.08	1.00000000	pigs
Spring1	-0.03	1.00000000	pigs
Fam168a	-0.13	1.00000000	pigs
Taok2	-0.06	1.00000000	pigs
Asah1	0.04	1.00000000	pigs
Poldip2	-0.11	1.00000000	pigs
Scap	0.05	1.00000000	pigs
Tor1aip2	0.01	1.00000000	pigs
Car2	-0.05	1.00000000	pigs
Slc9a6	-0.03	1.00000000	pigs
Cnot7	-0.10	1.00000000	pigs
Ube2e3	-0.12	1.00000000	pigs
Ttc21b	-0.06	1.00000000	pigs
Ufc1	-0.15	1.00000000	pigs
Tbc1d7	0.02	1.00000000	pigs
Sash1	-0.03	1.00000000	pigs
Snapc5	-0.06	1.00000000	pigs
Uhrf1bp1l	0.10	1.00000000	pigs
Ifi204	-0.15	1.00000000	pigs
Psme3ip1	-0.07	1.00000000	pigs

Sgf29	-0.12	1.00000000	pigs
Btf3	0.03	1.00000000	pigs
Hspb11	0.00	1.00000000	pigs
Etfa	0.02	1.00000000	pigs
Ibtk	0.02	1.00000000	pigs
Donson	0.03	1.00000000	pigs
Sh3tc1	-0.17	1.00000000	pigs
Pgp	0.00	1.00000000	pigs
Edem3	0.01	1.00000000	pigs
Srek1ip1	0.02	1.00000000	pigs
Rfc5	0.01	1.00000000	pigs
Dtx3l	-0.12	1.00000000	pigs
Sipa1	0.07	1.00000000	pigs
Phax	0.03	1.00000000	pigs
Usp10	0.10	1.00000000	pigs
Trpc4ap	0.04	1.00000000	pigs
Ctcf	-0.12	1.00000000	pigs
Ints2	-0.10	1.00000000	pigs
Zfp39	0.00	1.00000000	pigs
Ubr1	-0.23	1.00000000	pigs
Slc25a33	-0.20	1.00000000	pigs
Ilf2	0.00	1.00000000	pigs
Fbxo30	-0.10	1.00000000	pigs
Anapc1	-0.11	1.00000000	pigs
Oard1	0.04	1.00000000	pigs
Stau2	-0.08	1.00000000	pigs
Trp53i13	-0.05	1.00000000	pigs
Ccdc93	-0.20	1.00000000	pigs
Phyh	0.00	1.00000000	pigs
Bnc2	0.02	1.00000000	pigs
Brpf1	0.03	1.00000000	pigs
Dennd5b	0.15	1.00000000	pigs
Gnb1l	-0.11	1.00000000	pigs
Slc35a3	0.00	1.00000000	pigs
Slit3	-0.13	1.00000000	pigs
Pip4p1	0.01	1.00000000	pigs
Tsnax	0.02	1.00000000	pigs
Pygl	0.05	1.00000000	pigs
2700049A03Rik	-0.15	1.00000000	pigs
S100a8	0.01	1.00000000	pigs
Zfp428	-0.10	1.00000000	pigs
Racgap1	0.02	1.00000000	pigs
Dnajc18	0.07	1.00000000	pigs
Dlg4	-0.10	1.00000000	pigs
Eif2ak3	0.21	1.00000000	pigs
Nrbp2	0.04	1.00000000	pigs

Lpp	-0.05	1.00000000	pigs
Cdv3	0.09	1.00000000	pigs
Atrx	-0.06	1.00000000	pigs
Tmem47	-0.25	1.00000000	pigs
Dnaaf2	-0.04	1.00000000	pigs
Ggnbp2	0.09	1.00000000	pigs
Uqcrfs1	0.07	1.00000000	pigs
Sars	0.08	1.00000000	pigs
Axin1	-0.03	1.00000000	pigs
Plekhm2	0.01	1.00000000	pigs
Umps	-0.16	1.00000000	pigs
Gabarapl1	0.02	1.00000000	pigs
Pes1	-0.14	1.00000000	pigs
Pwwp2a	0.04	1.00000000	pigs
Tmem69	-0.10	1.00000000	pigs
LTO1	-0.10	1.00000000	pigs
Alg2	0.00	1.00000000	pigs
Slc35a5	-0.10	1.00000000	pigs
Fads2	-0.04	1.00000000	pigs
Irf1	-0.25	1.00000000	pigs
Cyp2e1	-0.09	1.00000000	pigs
Scfd1	-0.07	1.00000000	pigs
Herc1	-0.02	1.00000000	pigs
Haus2	0.02	1.00000000	pigs
Esf1	-0.02	1.00000000	pigs
Ttyh3	-0.07	1.00000000	pigs
Rars2	0.10	1.00000000	pigs
Rbbp8	-0.11	1.00000000	pigs
Prr14l	-0.17	1.00000000	pigs
Naa20	-0.07	1.00000000	pigs
Kat6b	0.05	1.00000000	pigs
Acat3	0.03	1.00000000	pigs
Gnl2	0.03	1.00000000	pigs
Aasdhppt	-0.10	1.00000000	pigs
Tmem38b	-0.05	1.00000000	pigs
Pikfyve	0.01	1.00000000	pigs
Hspbap1	-0.07	1.00000000	pigs
Hmgcs1	-0.18	1.00000000	pigs
Ddx42	0.03	1.00000000	pigs
Rfx7	-0.15	1.00000000	pigs
Usp47	0.02	1.00000000	pigs
Slc29a1	-0.13	1.00000000	pigs
Ip6k1	-0.16	1.00000000	pigs
Thumpd3	-0.15	1.00000000	pigs
Utp25	0.05	1.00000000	pigs
Dhdds	-0.02	1.00000000	pigs

Smyd3	0.07	1.00000000	pigs
Stau1	0.00	1.00000000	pigs
Acad9	-0.12	1.00000000	pigs
Tab3	-0.04	1.00000000	pigs
Vps72	0.09	1.00000000	pigs
Exoc3	-0.12	1.00000000	pigs
Topors	0.05	1.00000000	pigs
Ano8	-0.25	1.00000000	pigs
Ro60	-0.18	1.00000000	pigs
Polr3e	0.02	1.00000000	pigs
Arhgap9	-0.13	1.00000000	pigs
Trabd	-0.01	1.00000000	pigs
Stt3a	0.00	1.00000000	pigs
Jmjd6	-0.05	1.00000000	pigs
Nhlrc2	-0.07	1.00000000	pigs
Prorp	0.08	1.00000000	pigs
Ergic2	-0.20	1.00000000	pigs
Tomm40	0.06	1.00000000	pigs
Taf10	-0.14	1.00000000	pigs
Dnajc14	-0.03	1.00000000	pigs
Slc12a4	-0.02	1.00000000	pigs
Trak1	-0.08	1.00000000	pigs
Fanci	-0.08	1.00000000	pigs
Unk	-0.10	1.00000000	pigs
Rfwd3	-0.04	1.00000000	pigs
Pdcd11	0.00	1.00000000	pigs
Usp1	-0.14	1.00000000	pigs
Hadha	0.02	1.00000000	pigs
Nt5c3b	0.00	1.00000000	pigs
Sgsm3	0.00	1.00000000	pigs
Atg14	-0.06	1.00000000	pigs
Arhgap10	0.25	1.00000000	pigs
Adam10	-0.11	1.00000000	pigs
Zc3h12c	-0.14	1.00000000	pigs
Fam122b	-0.17	1.00000000	pigs
Rptor	0.12	1.00000000	pigs
Luc7l	0.11	1.00000000	pigs
Ctsf	-0.06	1.00000000	pigs
Lrp12	0.13	1.00000000	pigs
Gpbp1l1	0.06	1.00000000	pigs
Prpf8	-0.13	1.00000000	pigs
Tmem238	-0.10	1.00000000	pigs
Ints14	0.04	1.00000000	pigs
Tspan18	0.02	1.00000000	pigs
4921524J17Rik	-0.09	1.00000000	pigs
Gna14	0.16	1.00000000	pigs

Dgat2	-0.03	1.00000000	pigs
Chpf2	0.02	1.00000000	pigs
Scoc	-0.08	1.00000000	pigs
Nudc	0.02	1.00000000	pigs
Pde6d	0.01	1.00000000	pigs
Sun2	-0.07	1.00000000	pigs
Bcap31	0.06	1.00000000	pigs
Abcg3	-0.03	1.00000000	pigs
Cand1	0.07	1.00000000	pigs
Jmjd1c	0.24	1.00000000	pigs
Rgl2	-0.15	1.00000000	pigs
Tmem214	-0.12	1.00000000	pigs
Twsg1	-0.03	1.00000000	pigs
Rnaseh2b	-0.19	1.00000000	pigs
Lamp1	0.11	1.00000000	pigs
Klhl20	-0.24	1.00000000	pigs
Vps16	-0.09	1.00000000	pigs
Ndufaf7	-0.06	1.00000000	pigs
Actr6	-0.06	1.00000000	pigs
Rbm17	0.00	1.00000000	pigs
Ints9	0.00	1.00000000	pigs
Kat8	-0.04	1.00000000	pigs
Psmc6	0.00	1.00000000	pigs
Fam53c	0.05	1.00000000	pigs
Pde5a	-0.30	1.00000000	pigs
Acp5	0.00	1.00000000	pigs
Pmvk	0.00	1.00000000	pigs
Cpt1a	0.11	1.00000000	pigs
Tomm20	-0.11	1.00000000	pigs
Parp4	-0.18	1.00000000	pigs
Tmem150a	-0.05	1.00000000	pigs
Zfp28	-0.13	1.00000000	pigs
Efcab14	0.07	1.00000000	pigs
Hars	-0.01	1.00000000	pigs
Aga	0.14	1.00000000	pigs
Wrap73	-0.03	1.00000000	pigs
Ppp1ca	0.06	1.00000000	pigs
Dcaf12	0.00	1.00000000	pigs
Dhx9	0.08	1.00000000	pigs
Ddx20	0.00	1.00000000	pigs
Desi2	0.02	1.00000000	pigs
Pon2	-0.02	1.00000000	pigs
Rfx3	-0.31	1.00000000	pigs
Chmp1a	-0.21	1.00000000	pigs
Napa	-0.07	1.00000000	pigs
BC003965	0.02	1.00000000	pigs

Ccs	-0.08	1.00000000	pigs
Ddrgk1	-0.07	1.00000000	pigs
Dcakd	-0.02	1.00000000	pigs
Cpq	-0.11	1.00000000	pigs
Abcc5	-0.03	1.00000000	pigs
Dock7	-0.02	1.00000000	pigs
Tmem128	-0.12	1.00000000	pigs
Kyat3	-0.08	1.00000000	pigs
Dok1	-0.08	1.00000000	pigs
Gabbr2	-0.21	1.00000000	pigs
Rspry1	0.07	1.00000000	pigs
Efl1	-0.35	1.00000000	pigs
Ascc3	-0.04	1.00000000	pigs
Nrf1	0.17	1.00000000	pigs
Parp12	-0.06	1.00000000	pigs
Olfml3	-0.03	1.00000000	pigs
Cryl1	-0.06	1.00000000	pigs
Rif1	0.01	1.00000000	pigs
Pias4	-0.01	1.00000000	pigs
Kat7	0.01	1.00000000	pigs
Rcc1l	-0.05	1.00000000	pigs
Sec11c	-0.16	1.00000000	pigs
Ifit2	-0.42	1.00000000	pigs
Echdc1	-0.13	1.00000000	pigs
Gyg	-0.19	1.00000000	pigs
AW549877	-0.01	1.00000000	pigs
Arhgap21	-0.01	1.00000000	pigs
Zbtb2	0.02	1.00000000	pigs
Ccdc28b	0.03	1.00000000	pigs
Nop10	-0.08	1.00000000	pigs
Gemin2	0.01	1.00000000	pigs
Pnrc1	0.12	1.00000000	pigs
Nudt19	-0.08	1.00000000	pigs
Nup35	0.02	1.00000000	pigs
Serpinb8	0.00	1.00000000	pigs
Ndufc2	-0.16	1.00000000	pigs
Tigar	0.05	1.00000000	pigs
Tmem161a	0.00	1.00000000	pigs
Adnp	0.07	1.00000000	pigs
Prelid1	0.05	1.00000000	pigs
Ewsr1	-0.09	1.00000000	pigs
Orc3	-0.11	1.00000000	pigs
Fbl	-0.10	1.00000000	pigs
Fam214a	0.55	1.00000000	pigs
Dpp7	-0.18	1.00000000	pigs
Ikbkg	-0.08	1.00000000	pigs

Flot2	0.02	1.00000000	pigs
Pick1	0.05	1.00000000	pigs
Cacna2d4	0.26	1.00000000	pigs
Arhgap30	0.06	1.00000000	pigs
Dnajc17	-0.01	1.00000000	pigs
Cks2	-0.06	1.00000000	pigs
Tet3	-0.20	1.00000000	pigs
Sec22a	-0.04	1.00000000	pigs
Commd8	0.05	1.00000000	pigs
Zmynd8	-0.01	1.00000000	pigs
Pex16	-0.17	1.00000000	pigs
Inip	-0.02	1.00000000	pigs
Cpeb3	0.00	1.00000000	pigs
Stx12	0.05	1.00000000	pigs
Dpagt1	0.01	1.00000000	pigs
Rer1	0.01	1.00000000	pigs
Prrc2c	-0.14	1.00000000	pigs
Wdtc1	-0.06	1.00000000	pigs
Bbs9	0.08	1.00000000	pigs
Rrp1	-0.10	1.00000000	pigs
Tbl1xr1	-0.10	1.00000000	pigs
Crls1	-0.03	1.00000000	pigs
Ttc23l	0.02	1.00000000	pigs
Fbxo8	0.00	1.00000000	pigs
Enkur	0.01	1.00000000	pigs
Ntan1	-0.08	1.00000000	pigs
Slc26a2	-0.20	1.00000000	pigs
Eif5a2	-0.01	1.00000000	pigs
Rida	0.00	1.00000000	pigs
Dedd	0.01	1.00000000	pigs
Pgap2	-0.08	1.00000000	pigs
Dis3	0.09	1.00000000	pigs
Ifih1	-0.19	1.00000000	pigs
L2hgdh	-0.04	1.00000000	pigs
Tfdp2	0.01	1.00000000	pigs
Ubl7	-0.12	1.00000000	pigs
Bag1	0.00	1.00000000	pigs
Cops4	0.01	1.00000000	pigs
Icos	-0.04	1.00000000	pigs
Ralgapb	-0.07	1.00000000	pigs
Polrmt	0.02	1.00000000	pigs
Whrn	0.59	1.00000000	pigs
Cd47	-0.10	1.00000000	pigs
Ep300	-0.09	1.00000000	pigs
Gpr183	0.21	1.00000000	pigs
Gab3	-0.05	1.00000000	pigs

Gphn	0.10	1.00000000	pigs
Oaz2	-0.01	1.00000000	pigs
Uso1	0.04	1.00000000	pigs
Prkacb	-0.01	1.00000000	pigs
Ankrd40	0.00	1.00000000	pigs
E2f3	-0.06	1.00000000	pigs
Ifngr1	0.12	1.00000000	pigs
Cars2	0.00	1.00000000	pigs
Retreg2	0.06	1.00000000	pigs
Pspc1	0.03	1.00000000	pigs
Gbf1	0.02	1.00000000	pigs
Rtn4ip1	-0.02	1.00000000	pigs
Srp68	-0.02	1.00000000	pigs
Eif3h	-0.07	1.00000000	pigs
Alg12	0.03	1.00000000	pigs
Zfp94	-0.05	1.00000000	pigs
Rab11fip2	0.03	1.00000000	pigs
Ptov1	-0.02	1.00000000	pigs
Il6ra	0.03	1.00000000	pigs
Aqr	-0.16	1.00000000	pigs
Dennd4b	-0.23	1.00000000	pigs
Zbtb1	-0.04	1.00000000	pigs
Vwa8	-0.12	1.00000000	pigs
Rbm12b2	0.00	1.00000000	pigs
Tprgl	-0.10	1.00000000	pigs
Sdad1	0.01	1.00000000	pigs
Ecsit	-0.04	1.00000000	pigs
Wdr13	-0.14	1.00000000	pigs
Ociad2	-0.09	1.00000000	pigs
Anapc5	-0.01	1.00000000	pigs
Rtcb	-0.11	1.00000000	pigs
Cmc1	-0.16	1.00000000	pigs
Cwc25	-0.13	1.00000000	pigs
Tmem242	-0.03	1.00000000	pigs
Tra2a	-0.11	1.00000000	pigs
AW554918	0.00	1.00000000	pigs
Orc5	-0.01	1.00000000	pigs
Kdm2a	0.26	1.00000000	pigs
Prmt9	0.01	1.00000000	pigs
Ikzf2	0.02	1.00000000	pigs
Stx17	-0.18	1.00000000	pigs
Ptbp3	-0.23	1.00000000	pigs
Rangap1	-0.02	1.00000000	pigs
Alms1	-0.04	1.00000000	pigs
Tiam1	0.00	1.00000000	pigs
Pbdc1	-0.07	1.00000000	pigs

Tmem248	-0.07	1.00000000	pigs
Etfdh	-0.12	1.00000000	pigs
Usb1	0.01	1.00000000	pigs
Aldh18a1	0.04	1.00000000	pigs
Gfpt1	0.05	1.00000000	pigs
Rxrb	0.00	1.00000000	pigs
Commd2	-0.18	1.00000000	pigs
Naxd	-0.04	1.00000000	pigs
Lxn	0.00	1.00000000	pigs
Ikzf5	-0.11	1.00000000	pigs
Hccs	-0.04	1.00000000	pigs
Gsdmd	-0.02	1.00000000	pigs
Scyl2	-0.25	1.00000000	pigs
Lyn	-0.01	1.00000000	pigs
Dlat	-0.13	1.00000000	pigs
Kctd13	-0.02	1.00000000	pigs
Slc4a2	-0.09	1.00000000	pigs
Suds3	-0.04	1.00000000	pigs
Ddx50	-0.15	1.00000000	pigs
Slc7a6os	0.01	1.00000000	pigs
Nedd1	0.00	1.00000000	pigs
Sesn1	0.00	1.00000000	pigs
Vps39	0.08	1.00000000	pigs
Rap2a	-0.06	1.00000000	pigs
Ppil1	-0.04	1.00000000	pigs
Iah1	-0.07	1.00000000	pigs
Zcchc17	-0.08	1.00000000	pigs
Stam2	-0.12	1.00000000	pigs
Gga1	-0.02	1.00000000	pigs
Brap	-0.09	1.00000000	pigs
Golgb1	-0.09	1.00000000	pigs
Lman2	0.08	1.00000000	pigs
Zdhhc16	-0.04	1.00000000	pigs
Cops5	-0.03	1.00000000	pigs
Dap	-0.10	1.00000000	pigs
Alg8	0.02	1.00000000	pigs
Got2	-0.15	1.00000000	pigs
Hmg20b	-0.12	1.00000000	pigs
Cep152	0.04	1.00000000	pigs
Pop5	-0.17	1.00000000	pigs
Lrrc4	-0.06	1.00000000	pigs
Strn	0.01	1.00000000	pigs
Comtd1	-0.02	1.00000000	pigs
Cep83	-0.04	1.00000000	pigs
D030056L22Rik	-0.06	1.00000000	pigs
Orc4	-0.05	1.00000000	pigs

Zfhx3	-0.09	1.00000000	pigs
Nolc1	0.05	1.00000000	pigs
Dnajc5	-0.01	1.00000000	pigs
Ippk	0.04	1.00000000	pigs
Hmbs	0.01	1.00000000	pigs
Acadm	-0.08	1.00000000	pigs
Bod1l	-0.02	1.00000000	pigs
Edc3	0.00	1.00000000	pigs
Atpaf2	-0.13	1.00000000	pigs
Ppme1	-0.02	1.00000000	pigs
Trap1	-0.18	1.00000000	pigs
Slc4a1ap	0.00	1.00000000	pigs
Iqsec2	-0.01	1.00000000	pigs
Slc39a11	-0.02	1.00000000	pigs
Taf15	-0.13	1.00000000	pigs
Nek7	-0.08	1.00000000	pigs
Atg101	-0.01	1.00000000	pigs
Lrrc14	-0.12	1.00000000	pigs
Smchd1	-0.32	1.00000000	pigs
Coro1a	0.14	1.00000000	pigs
Nbn	-0.19	1.00000000	pigs
Lrrc42	0.04	1.00000000	pigs
Otud3	0.02	1.00000000	pigs
Txndc5	-0.05	1.00000000	pigs
Hdac1	0.16	1.00000000	pigs
Lrpprc	-0.19	1.00000000	pigs
Tmem65	-0.17	1.00000000	pigs
Kifap3	-0.24	1.00000000	pigs
Gcn1	0.03	1.00000000	pigs
Abhd15	-0.07	1.00000000	pigs
Fkbp9	-0.03	1.00000000	pigs
Pus3	-0.05	1.00000000	pigs
Srpk1	0.16	1.00000000	pigs
Rassf3	0.17	1.00000000	pigs
Clasrp	-0.19	1.00000000	pigs
Anxa1	-0.05	1.00000000	pigs
Pdpr	-0.01	1.00000000	pigs
Zcchc24	0.05	1.00000000	pigs
Nudt1	-0.14	1.00000000	pigs
Kri1	0.06	1.00000000	pigs
Cibar1	-0.16	1.00000000	pigs
Ctdnep1	0.00	1.00000000	pigs
Adh5	-0.19	1.00000000	pigs
Parp1	0.08	1.00000000	pigs
Tnks	0.02	1.00000000	pigs
Trmu	0.01	1.00000000	pigs

Gart	0.05	1.00000000	pigs
Lrrc58	0.01	1.00000000	pigs
Kin	-0.22	1.00000000	pigs
Arsa	-0.09	1.00000000	pigs
Cfap410	0.00	1.00000000	pigs
Ulk3	-0.11	1.00000000	pigs
Naxe	-0.13	1.00000000	pigs
Thap4	0.01	1.00000000	pigs
Spata21	-0.06	1.00000000	pigs
Rnf20	-0.07	1.00000000	pigs
Ubxn1	-0.02	1.00000000	pigs
Snx13	0.01	1.00000000	pigs
Trem1	0.00	1.00000000	pigs
Taf1a	-0.05	1.00000000	pigs
Tsc2	-0.03	1.00000000	pigs
Trpc1	0.01	1.00000000	pigs
Prkaca	-0.11	1.00000000	pigs
Serinc5	0.01	1.00000000	pigs
Cyb5d2	0.03	1.00000000	pigs
Slc25a13	0.09	1.00000000	pigs
Sec63	-0.14	1.00000000	pigs
Timm29	-0.08	1.00000000	pigs
Abracl	0.02	1.00000000	pigs
Polb	0.13	1.00000000	pigs
Gemin6	0.00	1.00000000	pigs
Casp9	-0.02	1.00000000	pigs
Ubxn8	-0.18	1.00000000	pigs
Nup50	-0.08	1.00000000	pigs
Rai1	0.22	1.00000000	pigs
Elp2	-0.03	1.00000000	pigs
Smim7	-0.04	1.00000000	pigs
Wwox	0.31	1.00000000	pigs
Rcc1	-0.06	1.00000000	pigs
Gps2	-0.08	1.00000000	pigs
Actb	-0.09	1.00000000	pigs
Use1	0.02	1.00000000	pigs
Bcl2l1	0.06	1.00000000	pigs
Prrc2b	0.02	1.00000000	pigs
Irak1	-0.05	1.00000000	pigs
Elmo2	0.08	1.00000000	pigs
Znhit1	-0.04	1.00000000	pigs
St14	0.02	1.00000000	pigs
Ap5m1	-0.03	1.00000000	pigs
Tceanc	0.00	1.00000000	pigs
Ubfd1	0.02	1.00000000	pigs
Klc2	0.01	1.00000000	pigs

Nsmaf	0.05	1.00000000	pigs
Rab22a	0.17	1.00000000	pigs
Arhgap32	-0.15	1.00000000	pigs
Zcchc4	-0.02	1.00000000	pigs
Acp1	-0.16	1.00000000	pigs
Paxbp1	-0.07	1.00000000	pigs
Herc2	-0.05	1.00000000	pigs
Cmas	-0.13	1.00000000	pigs
Slc30a9	-0.07	1.00000000	pigs
Drap1	0.05	1.00000000	pigs
Vps52	-0.03	1.00000000	pigs
Usp25	-0.19	1.00000000	pigs
Arhgap5	-0.21	1.00000000	pigs
Gsk3b	-0.02	1.00000000	pigs
Cnst	-0.01	1.00000000	pigs
Dram1	0.01	1.00000000	pigs
Dmd	-0.01	1.00000000	pigs
Snapc4	-0.04	1.00000000	pigs
Ubn2	-0.03	1.00000000	pigs
Txndc16	0.05	1.00000000	pigs
Dctn5	-0.12	1.00000000	pigs
Tuba8	-0.02	1.00000000	pigs
Wdr82	-0.01	1.00000000	pigs
Tbc1d10a	0.09	1.00000000	pigs
Tspan33	-0.09	1.00000000	pigs
Tle5	-0.01	1.00000000	pigs
Abhd17a	-0.02	1.00000000	pigs
Slc12a2	-0.13	1.00000000	pigs
Nup133	-0.08	1.00000000	pigs
Shld1	-0.26	1.00000000	pigs
Ranbp1	-0.15	1.00000000	pigs
2310033P09Rik	-0.02	1.00000000	pigs
Zfp24	-0.07	1.00000000	pigs
Rce1	-0.02	1.00000000	pigs
Trps1	0.28	1.00000000	pigs
Asprv1	-0.02	1.00000000	pigs
Pafah1b2	-0.04	1.00000000	pigs
Rab33b	-0.12	1.00000000	pigs
Nfya	0.08	1.00000000	pigs
Zc3h6	-0.12	1.00000000	pigs
Psmd12	-0.03	1.00000000	pigs
Ubap2	-0.02	1.00000000	pigs
Ino80d	0.01	1.00000000	pigs
Sntb1	-0.27	1.00000000	pigs
Syne2	-0.13	1.00000000	pigs
Cul2	-0.09	1.00000000	pigs

Ncoa4	0.09	1.00000000	pigs
Gemin4	0.08	1.00000000	pigs
Zdhhc6	-0.14	1.00000000	pigs
Tcf7	0.00	1.00000000	pigs
Sh2d3c	0.04	1.00000000	pigs
Eif1	-0.13	1.00000000	pigs
Rmdn3	-0.04	1.00000000	pigs
Acss2	0.03	1.00000000	pigs
Nsmce2	-0.11	1.00000000	pigs
Togaram1	-0.07	1.00000000	pigs
Brcc3	-0.12	1.00000000	pigs
Kat14	-0.05	1.00000000	pigs
Sdcbp	-0.12	1.00000000	pigs
Zfp583	-0.14	1.00000000	pigs
Ppp1r8	-0.14	1.00000000	pigs
Prr7	-0.02	1.00000000	pigs
Col19a1	0.05	1.00000000	pigs
Golga5	-0.20	1.00000000	pigs
Polr2g	-0.05	1.00000000	pigs
Cox7a1	-0.02	1.00000000	pigs
Sh3glb2	-0.03	1.00000000	pigs
Tyw3	-0.01	1.00000000	pigs
Atp6v1f	0.02	1.00000000	pigs
Nckap5l	-0.08	1.00000000	pigs
Tlk1	-0.13	1.00000000	pigs
Pomt2	-0.02	1.00000000	pigs
Htra2	-0.01	1.00000000	pigs
Trip12	-0.20	1.00000000	pigs
Polr3f	-0.08	1.00000000	pigs
Fbxo4	0.01	1.00000000	pigs
Sco1	-0.01	1.00000000	pigs
Pts	-0.11	1.00000000	pigs
Fos	0.37	1.00000000	pigs
Ppm1g	-0.21	1.00000000	pigs
Josd1	-0.01	1.00000000	pigs
Numb	-0.20	1.00000000	pigs
Fbrs	0.02	1.00000000	pigs
Rnase4	-0.05	1.00000000	pigs
Pcif1	-0.05	1.00000000	pigs
Apaf1	-0.21	1.00000000	pigs
Rexo5	0.05	1.00000000	pigs
Ergic1	0.00	1.00000000	pigs
Jrkl	-0.06	1.00000000	pigs
Exoc6	-0.13	1.00000000	pigs
Btbd2	0.02	1.00000000	pigs
Chic2	-0.02	1.00000000	pigs

Snf8	-0.09	1.00000000	pigs
Tcf25	-0.21	1.00000000	pigs
Gucy1b1	-0.07	1.00000000	pigs
E2f2	0.01	1.00000000	pigs
Emb	-0.17	1.00000000	pigs
Helq	-0.08	1.00000000	pigs
Cebpg	0.06	1.00000000	pigs
Ttpal	0.03	1.00000000	pigs
Shmt2	-0.02	1.00000000	pigs
Ap2s1	-0.07	1.00000000	pigs
Bpnt2	0.07	1.00000000	pigs
Dhx34	-0.12	1.00000000	pigs
Sh3gl1	0.02	1.00000000	pigs
Pigc	-0.02	1.00000000	pigs
Tgif1	-0.26	1.00000000	pigs
Vamp4	-0.20	1.00000000	pigs
Ints12	-0.04	1.00000000	pigs
Kdelr2	-0.07	1.00000000	pigs
Pnrc2	0.05	1.00000000	pigs
Kmt2c	0.09	1.00000000	pigs
Bmt2	0.21	1.00000000	pigs
Tmem170b	-0.09	1.00000000	pigs
Drg2	-0.10	1.00000000	pigs
Slc39a7	-0.10	1.00000000	pigs
Nhsl1	-0.14	1.00000000	pigs
Oip5	-0.09	1.00000000	pigs
Cops8	-0.01	1.00000000	pigs
Ankrd46	0.07	1.00000000	pigs
Txndc11	0.00	1.00000000	pigs
Cbr4	0.00	1.00000000	pigs
Hspa13	-0.15	1.00000000	pigs
Cfap20dc	-0.02	1.00000000	pigs
1110004F10Rik	-0.13	1.00000000	pigs
Nceh1	-0.03	1.00000000	pigs
Nedd4	-0.09	1.00000000	pigs
Gltp	-0.02	1.00000000	pigs
Dhx36	0.05	1.00000000	pigs
Pcmtd1	-0.10	1.00000000	pigs
Trim11	-0.03	1.00000000	pigs
Fbxl20	-0.06	1.00000000	pigs
Sbds	0.06	1.00000000	pigs
Abcf1	-0.04	1.00000000	pigs
Cnot11	-0.12	1.00000000	pigs
Pigb	0.11	1.00000000	pigs
Nrp1	0.32	1.00000000	pigs
Ocel1	-0.09	1.00000000	pigs

Pi4ka	-0.03	1.00000000	pigs
Grk4	-0.02	1.00000000	pigs
Ak6	-0.12	1.00000000	pigs
Poglut2	-0.01	1.00000000	pigs
Zbtb25	-0.07	1.00000000	pigs
Rfc4	0.01	1.00000000	pigs
Il34	-0.09	1.00000000	pigs
Slco4c1	-0.02	1.00000000	pigs
Id3	-0.17	1.00000000	pigs
Ppp1r35	-0.08	1.00000000	pigs
Arl4a	-0.22	1.00000000	pigs
Nlrc3	-0.02	1.00000000	pigs
Zfp282	-0.04	1.00000000	pigs
Impg2	-0.10	1.00000000	pigs
St13	-0.07	1.00000000	pigs
Ap3b1	-0.11	1.00000000	pigs
Fndc7	0.02	1.00000000	pigs
Klf2	0.28	1.00000000	pigs
Ptrh2	-0.06	1.00000000	pigs
Klhl15	0.06	1.00000000	pigs
Ufl1	-0.07	1.00000000	pigs
Tmco3	-0.03	1.00000000	pigs
Htatsf1	-0.15	1.00000000	pigs
Kiz	-0.05	1.00000000	pigs
Xiap	0.09	1.00000000	pigs
Taf1b	-0.04	1.00000000	pigs
N6amt1	-0.04	1.00000000	pigs
Zfp358	-0.07	1.00000000	pigs
Pabpc1	0.20	1.00000000	pigs
Enoph1	-0.08	1.00000000	pigs
Nipsnap3b	-0.15	1.00000000	pigs
Tmem63b	0.04	1.00000000	pigs
Pomgnt1	0.03	1.00000000	pigs
Stip1	-0.03	1.00000000	pigs
Lonp1	0.01	1.00000000	pigs
Spryd7	-0.12	1.00000000	pigs
Ptger4	-0.08	1.00000000	pigs
Sccpdh	-0.20	1.00000000	pigs
Cast	-0.06	1.00000000	pigs
Pxn	-0.10	1.00000000	pigs
Dock5	0.01	1.00000000	pigs
D6Wsu163e	-0.03	1.00000000	pigs
Cuedc2	-0.06	1.00000000	pigs
Pycard	0.02	1.00000000	pigs
Psmc2	0.03	1.00000000	pigs
Rprd2	-0.13	1.00000000	pigs

Wdr53	-0.04	1.00000000	pigs
Crybg3	0.02	1.00000000	pigs
Uimc1	-0.02	1.00000000	pigs
Snx14	0.03	1.00000000	pigs
Septin9	0.09	1.00000000	pigs
Cep85l	-0.03	1.00000000	pigs
Zfp366	-0.07	1.00000000	pigs
Cubn	0.03	1.00000000	pigs
Cryba1	-0.01	1.00000000	pigs
Rabl3	-0.02	1.00000000	pigs
P4hb	0.05	1.00000000	pigs
Insig2	-0.01	1.00000000	pigs
Toe1	-0.06	1.00000000	pigs
Fmc1	-0.09	1.00000000	pigs
Ube2b	0.01	1.00000000	pigs
Copg2	-0.07	1.00000000	pigs
Tmem68	-0.16	1.00000000	pigs
Arrdc1	-0.12	1.00000000	pigs
Tigd2	-0.07	1.00000000	pigs
Akip1	0.01	1.00000000	pigs
Epb41l5	-0.05	1.00000000	pigs
Pstk	-0.11	1.00000000	pigs
Notch1	-0.10	1.00000000	pigs
Adss	-0.10	1.00000000	pigs
Taf5	-0.05	1.00000000	pigs
Rtf1	-0.07	1.00000000	pigs
Arhgap12	-0.08	1.00000000	pigs
Zbtb10	0.00	1.00000000	pigs
Trappc13	-0.01	1.00000000	pigs
Chml	-0.04	1.00000000	pigs
Arhgdia	-0.04	1.00000000	pigs
Gnpda2	-0.06	1.00000000	pigs
Nsd3	0.03	1.00000000	pigs
Fmr1	-0.04	1.00000000	pigs
Fan1	-0.07	1.00000000	pigs
Fbxl6	-0.06	1.00000000	pigs
Sp1	-0.01	1.00000000	pigs
Zbtb33	-0.06	1.00000000	pigs
Lin7a	-0.03	1.00000000	pigs
Nxt2	-0.02	1.00000000	pigs
Blvra	-0.04	1.00000000	pigs
Ccdc34	-0.06	1.00000000	pigs
Decr1	-0.01	1.00000000	pigs
Eif2b1	-0.05	1.00000000	pigs
Lamtor3	0.01	1.00000000	pigs
Slc35a2	0.00	1.00000000	pigs

Lsm7	-0.13	1.00000000	pigs
Gfm2	-0.09	1.00000000	pigs
Ypel2	-0.06	1.00000000	pigs
Oga	0.00	1.00000000	pigs
Chtop	-0.10	1.00000000	pigs
Tspan3	0.10	1.00000000	pigs
Usp28	0.01	1.00000000	pigs
Vrk3	-0.12	1.00000000	pigs
Ddx18	-0.05	1.00000000	pigs
Synj2bp	-0.05	1.00000000	pigs
Ipo8	-0.06	1.00000000	pigs
Vdac3	-0.05	1.00000000	pigs
Adcy3	0.03	1.00000000	pigs
Arid1a	-0.19	1.00000000	pigs
6030458C11Rik	0.04	1.00000000	pigs
Lmf1	-0.02	1.00000000	pigs
Kcnab1	-0.01	1.00000000	pigs
Atrip	-0.01	1.00000000	pigs
Ormdl1	-0.14	1.00000000	pigs
Dhx33	0.02	1.00000000	pigs
Hectd2	-0.11	1.00000000	pigs
Serhl	0.08	1.00000000	pigs
Klhdc1	-0.01	1.00000000	pigs
G3bp1	0.06	1.00000000	pigs
Hadh	-0.12	1.00000000	pigs
Cant1	-0.05	1.00000000	pigs
Serpine1	-0.23	1.00000000	pigs
Cnep1r1	-0.14	1.00000000	pigs
Pdcd10	-0.06	1.00000000	pigs
Smad5	0.10	1.00000000	pigs
Zwilch	0.00	1.00000000	pigs
H2bc14	-0.12	1.00000000	pigs
Snap47	-0.08	1.00000000	pigs
Tdp2	-0.01	1.00000000	pigs
Abt1	-0.04	1.00000000	pigs
Zfp438	-0.22	1.00000000	pigs
Xirp2	-0.26	1.00000000	pigs
Gcc1	-0.07	1.00000000	pigs
Ece1	-0.10	1.00000000	pigs
Cd151	-0.05	1.00000000	pigs
Pde4d	-0.01	1.00000000	pigs
Zfp384	-0.09	1.00000000	pigs
Vps11	-0.03	1.00000000	pigs
Tipin	-0.13	1.00000000	pigs
Atf2	-0.05	1.00000000	pigs
Elp1	-0.06	1.00000000	pigs

Cops6	-0.07	1.00000000	pigs
Ctif	-0.03	1.00000000	pigs
Shc1	-0.06	1.00000000	pigs
Sap18	-0.13	1.00000000	pigs
Pwwp3a	-0.03	1.00000000	pigs
Ylpm1	-0.11	1.00000000	pigs
Gtf3c1	0.08	1.00000000	pigs
Dido1	0.03	1.00000000	pigs
Tle4	-0.10	1.00000000	pigs
Fbxw5	0.00	1.00000000	pigs
Nrd1	-0.10	1.00000000	pigs
Zfp106	-0.13	1.00000000	pigs
Rhof	0.00	1.00000000	pigs
Nup107	-0.13	1.00000000	pigs
Ankra2	0.01	1.00000000	pigs
Cep162	0.00	1.00000000	pigs
Rad18	0.00	1.00000000	pigs
Nelfe	-0.04	1.00000000	pigs
Ciz1	-0.05	1.00000000	pigs
Gnpda1	-0.19	1.00000000	pigs
Chchd6	-0.03	1.00000000	pigs
Fam199x	0.03	1.00000000	pigs
Ddb2	0.04	1.00000000	pigs
Ttc7b	-0.19	1.00000000	pigs
Chfr	0.03	1.00000000	pigs
Ythdf2	0.02	1.00000000	pigs
2310011J03Rik	-0.14	1.00000000	pigs
Cblb	0.12	1.00000000	pigs
Tmem132a	0.00	1.00000000	pigs
Isg15	-0.57	1.00000000	pigs
Fuca1	-0.11	1.00000000	pigs
Tti2	0.00	1.00000000	pigs
Slc37a2	-0.08	1.00000000	pigs
Ficd	0.00	1.00000000	pigs
Crbn	-0.01	1.00000000	pigs
Ppp1r21	0.01	1.00000000	pigs
Klf11	-0.06	1.00000000	pigs
Cnnm4	-0.04	1.00000000	pigs
Tmem251	-0.14	1.00000000	pigs
Evi2	0.04	1.00000000	pigs
Amigo3	0.01	1.00000000	pigs
Exosc4	-0.09	1.00000000	pigs
Arhgef18	0.04	1.00000000	pigs
Rrm2b	0.04	1.00000000	pigs
Sar1a	-0.08	1.00000000	pigs
Rnf170	-0.07	1.00000000	pigs

Ankrd39	-0.03	1.00000000	pigs
Pin4	-0.05	1.00000000	pigs
Fam89b	-0.03	1.00000000	pigs
Kif5b	-0.01	1.00000000	pigs
Ambra1	0.05	1.00000000	pigs
Gar1	-0.17	1.00000000	pigs
Tdpoz8	-0.12	1.00000000	pigs
Ppm1m	0.05	1.00000000	pigs
Btbd10	-0.02	1.00000000	pigs
Pabpc4	-0.03	1.00000000	pigs
Aar2	-0.07	1.00000000	pigs
Pex3	0.00	1.00000000	pigs
Spag1	-0.06	1.00000000	pigs
Acadvl	-0.07	1.00000000	pigs
Ccdc58	-0.16	1.00000000	pigs
Rcbtb1	-0.10	1.00000000	pigs
Abce1	-0.10	1.00000000	pigs
Elavl1	-0.11	1.00000000	pigs
Inpp5f	0.11	1.00000000	pigs
Dclk1	0.00	1.00000000	pigs
Ect2	-0.13	1.00000000	pigs
Col1a1	-0.07	1.00000000	pigs
Bdh2	-0.12	1.00000000	pigs
Ap5z1	-0.06	1.00000000	pigs
Pask	-0.14	1.00000000	pigs
Purb	-0.03	1.00000000	pigs
R3hdm4	-0.13	1.00000000	pigs
Rab14	-0.03	1.00000000	pigs
Slc38a2	-0.11	1.00000000	pigs
Elf4	-0.08	1.00000000	pigs
Cers5	-0.17	1.00000000	pigs
Naa30	-0.03	1.00000000	pigs
Rbm6	-0.11	1.00000000	pigs
Stambp	-0.08	1.00000000	pigs
Grsf1	-0.18	1.00000000	pigs
Rasgrp2	-0.01	1.00000000	pigs
Pdia6	-0.06	1.00000000	pigs
Gtpbp8	-0.09	1.00000000	pigs
Rab4a	-0.11	1.00000000	pigs
Cacybp	-0.09	1.00000000	pigs
Aff4	0.00	1.00000000	pigs
Rasa1	0.05	1.00000000	pigs
Prdm15	-0.02	1.00000000	pigs
Btd	-0.02	1.00000000	pigs
Clta	-0.02	1.00000000	pigs
Zc3h18	0.01	1.00000000	pigs

Snw1	0.03	1.00000000	pigs
Wdr74	-0.27	1.00000000	pigs
Bola1	-0.19	1.00000000	pigs
Coq4	-0.06	1.00000000	pigs
Rbbp5	-0.06	1.00000000	pigs
Zfp609	-0.34	1.00000000	pigs
Fam204a	-0.01	1.00000000	pigs
9930021J03Rik	-0.13	1.00000000	pigs
Lamtor2	0.06	1.00000000	pigs
Psma7	-0.05	1.00000000	pigs
Uox	-0.01	1.00000000	pigs
Ralgapa1	0.04	1.00000000	pigs
Cdk2ap2	0.12	1.00000000	pigs
Exd2	0.00	1.00000000	pigs
Hnrnpll	-0.21	1.00000000	pigs
Cdk13	0.02	1.00000000	pigs
Izumo4	-0.01	1.00000000	pigs
Carmil2	0.00	1.00000000	pigs
Camk4	0.18	1.00000000	pigs
Zdhhc17	-0.07	1.00000000	pigs
Wdr33	0.03	1.00000000	pigs
Topbp1	0.03	1.00000000	pigs
Fgl2	0.03	1.00000000	pigs
Dnajb6	0.04	1.00000000	pigs
Ddhd1	0.18	1.00000000	pigs
Pold1	-0.01	1.00000000	pigs
Ube2v2	-0.14	1.00000000	pigs
Hif1an	-0.11	1.00000000	pigs
Eipr1	-0.01	1.00000000	pigs
Dctpp1	-0.21	1.00000000	pigs
4930453N24Rik	-0.04	1.00000000	pigs
Ctps2	-0.04	1.00000000	pigs
Ccnl2	-0.08	1.00000000	pigs
Rpap3	0.00	1.00000000	pigs
Slc1a5	-0.14	1.00000000	pigs
Cog7	-0.10	1.00000000	pigs
Prpf40a	0.01	1.00000000	pigs
Xpot	0.01	1.00000000	pigs
Chchd1	-0.08	1.00000000	pigs
Snrpa1	-0.08	1.00000000	pigs
Gtpbp10	-0.12	1.00000000	pigs
Rpf1	-0.07	1.00000000	pigs
Nans	0.00	1.00000000	pigs
Ppm1d	0.00	1.00000000	pigs
Kif1b	0.06	1.00000000	pigs
Rnf217	-0.07	1.00000000	pigs

Tent2	0.02	1.00000000	pigs
Bcl3	0.02	1.00000000	pigs
Dtd2	-0.03	1.00000000	pigs
Rab8b	-0.03	1.00000000	pigs
Tango2	-0.02	1.00000000	pigs
Plscr1	-0.07	1.00000000	pigs
Armh3	-0.20	1.00000000	pigs
Rab27a	-0.02	1.00000000	pigs
Klhdc2	0.06	1.00000000	pigs
Herpud1	-0.17	1.00000000	pigs
Snx12	-0.01	1.00000000	pigs
Elk4	-0.08	1.00000000	pigs
Rfesd	-0.04	1.00000000	pigs
Commd10	0.09	1.00000000	pigs
Ccdc180	-0.07	1.00000000	pigs
Dpp4	-0.10	1.00000000	pigs
Tmem115	-0.06	1.00000000	pigs
Usp20	0.04	1.00000000	pigs
Yrdc	-0.09	1.00000000	pigs
Nhlrc3	0.11	1.00000000	pigs
Tsr1	-0.02	1.00000000	pigs
Usp11	-0.01	1.00000000	pigs
Spindoc	-0.01	1.00000000	pigs
Trmt1l	-0.05	1.00000000	pigs
Rab12	-0.01	1.00000000	pigs
Dclre1c	-0.11	1.00000000	pigs
Oxld1	-0.02	1.00000000	pigs
Slc25a17	-0.06	1.00000000	pigs
Ebpl	-0.04	1.00000000	pigs
Gtf3a	-0.02	1.00000000	pigs
Vcan	-0.13	1.00000000	pigs
Tle1	0.01	1.00000000	pigs
Ptpn23	-0.02	1.00000000	pigs
Ndufs5	0.01	1.00000000	pigs
Yif1b	-0.14	1.00000000	pigs
Ercc4	-0.01	1.00000000	pigs
Ap3m1	-0.14	1.00000000	pigs
Eif2b3	-0.03	1.00000000	pigs
Rbl2	-0.15	1.00000000	pigs
Phtf1	-0.03	1.00000000	pigs
Ubqln2	0.00	1.00000000	pigs
Ccdc134	0.01	1.00000000	pigs
Fbxw4	-0.10	1.00000000	pigs
Klhl7	-0.05	1.00000000	pigs
N4bp2	-0.11	1.00000000	pigs
Rilpl2	-0.02	1.00000000	pigs

Klf16	0.02	1.00000000	pigs
4933434E20Rik	-0.15	1.00000000	pigs
Ercc3	-0.06	1.00000000	pigs
Vps9d1	-0.06	1.00000000	pigs
Gla	0.07	1.00000000	pigs
Ppp1cc	-0.14	1.00000000	pigs
Pdcl	-0.14	1.00000000	pigs
Cdc73	0.05	1.00000000	pigs
Phf10	-0.05	1.00000000	pigs
Armcx4	0.01	1.00000000	pigs
Kdm1b	0.03	1.00000000	pigs
Eogt	0.04	1.00000000	pigs
Tasl	0.10	1.00000000	pigs
Tsfm	-0.01	1.00000000	pigs
Tmem185a	-0.06	1.00000000	pigs
Atxn2l	-0.09	1.00000000	pigs
Zfp667	-0.11	1.00000000	pigs
Clns1a	-0.03	1.00000000	pigs
Taf13	-0.09	1.00000000	pigs
Card19	0.01	1.00000000	pigs
Zfat	-0.05	1.00000000	pigs
Kcnab3	0.03	1.00000000	pigs
Impdh1	-0.05	1.00000000	pigs
Tmem208	-0.06	1.00000000	pigs
Orc6	-0.20	1.00000000	pigs
Hes6	-0.09	1.00000000	pigs
Ndufaf2	0.00	1.00000000	pigs
Pgd	0.02	1.00000000	pigs
Fkbp7	-0.07	1.00000000	pigs
Tgfbr3	0.44	1.00000000	pigs
Fgd3	0.03	1.00000000	pigs
Ptpn6	-0.22	1.00000000	pigs
Trmt13	-0.16	1.00000000	pigs
Dhx8	-0.03	1.00000000	pigs
Sec22b	-0.09	1.00000000	pigs
Aplf	-0.03	1.00000000	pigs
Chd6	0.02	1.00000000	pigs
Strada	0.02	1.00000000	pigs
Commd5	-0.12	1.00000000	pigs
Pacs1	-0.11	1.00000000	pigs
Proser1	-0.10	1.00000000	pigs
Usf3	-0.06	1.00000000	pigs
Uhrf2	-0.06	1.00000000	pigs
4931414P19Rik	-0.10	1.00000000	pigs
H2aj	-0.06	1.00000000	pigs
Dnajc2	-0.03	1.00000000	pigs

Derl2	-0.18	1.00000000	pigs
Rrp15	-0.08	1.00000000	pigs
Bub3	-0.04	1.00000000	pigs
Ublcp1	-0.09	1.00000000	pigs
Gck	-0.03	1.00000000	pigs
Zfp369	0.01	1.00000000	pigs
Ing5	-0.01	1.00000000	pigs
Nup210	0.01	1.00000000	pigs
Tmem154	-0.22	1.00000000	pigs
Rabep2	-0.01	1.00000000	pigs
Pggt1b	-0.06	1.00000000	pigs
Slc10a3	-0.13	1.00000000	pigs
Ralgapa2	-0.20	1.00000000	pigs
B3gnt2	-0.01	1.00000000	pigs
Ska3	-0.05	1.00000000	pigs
Eif4enif1	-0.03	1.00000000	pigs
Gemin5	0.03	1.00000000	pigs
Ptch1	-0.11	1.00000000	pigs
Ndufb7	-0.16	1.00000000	pigs
Acot10	-0.15	1.00000000	pigs
Washc5	0.00	1.00000000	pigs
Rcl1	0.05	1.00000000	pigs
Rhobtb1	0.02	1.00000000	pigs
Phf20l1	0.12	1.00000000	pigs
Leo1	-0.11	1.00000000	pigs
Vcpip1	-0.07	1.00000000	pigs
Dus1l	-0.06	1.00000000	pigs
Eif4ebp1	-0.12	1.00000000	pigs
Tma16	-0.01	1.00000000	pigs
Igf2bp3	-0.10	1.00000000	pigs
Cdk19	-0.17	1.00000000	pigs
Dennd1b	0.47	1.00000000	pigs
Inpp4a	-0.06	1.00000000	pigs
Ice2	0.05	1.00000000	pigs
Dpy19l1	-0.24	1.00000000	pigs
Tnfaip8	-0.07	1.00000000	pigs
Rfc1	0.04	1.00000000	pigs
Sugp2	-0.04	1.00000000	pigs
Lypla1	-0.17	1.00000000	pigs
Arhgap4	-0.10	1.00000000	pigs
Oaf	-0.02	1.00000000	pigs
Gtpbp2	0.00	1.00000000	pigs
Dcaf13	-0.06	1.00000000	pigs
Srsf10	0.00	1.00000000	pigs
Osbpl5	0.13	1.00000000	pigs
Ttc27	-0.10	1.00000000	pigs

Tyw5	-0.04	1.00000000	pigs
Guf1	-0.02	1.00000000	pigs
Slc31a1	-0.03	1.00000000	pigs
Stk19	-0.07	1.00000000	pigs
Sds	-0.21	1.00000000	pigs
Atg2b	-0.14	1.00000000	pigs
Selenof	-0.07	1.00000000	pigs
Zfp213	0.01	1.00000000	pigs
Aven	-0.16	1.00000000	pigs
Gnl3l	-0.02	1.00000000	pigs
Wtap	-0.01	1.00000000	pigs
Cdk7	0.01	1.00000000	pigs
Snupn	-0.06	1.00000000	pigs
Insyn2b	-0.09	1.00000000	pigs
Arfrp1	-0.05	1.00000000	pigs
Limd2	-0.08	1.00000000	pigs
Alg6	-0.03	1.00000000	pigs
Pfdn2	-0.14	1.00000000	pigs
Kdm4b	-0.66	1.00000000	pigs
Rlf	0.01	1.00000000	pigs
Atg4a-ps	-0.03	1.00000000	pigs
Zc3h12d	-0.03	1.00000000	pigs
Fam78a	-0.08	1.00000000	pigs
Parp2	-0.03	1.00000000	pigs
Sec24b	0.01	1.00000000	pigs
Wipi2	0.04	1.00000000	pigs
Tspan31	-0.08	1.00000000	pigs
Fndc3a	-0.07	1.00000000	pigs
Plekhj1	-0.08	1.00000000	pigs
Etf1	-0.06	1.00000000	pigs
Ctbp1	-0.06	1.00000000	pigs
Tbc1d13	-0.04	1.00000000	pigs
Ralgps2	0.18	1.00000000	pigs
Pepd	0.02	1.00000000	pigs
Spata2	-0.05	1.00000000	pigs
Gpatch8	0.01	1.00000000	pigs
Chmp5	-0.17	1.00000000	pigs
Calu	-0.02	1.00000000	pigs
Sri	-0.12	1.00000000	pigs
Gcc2	0.01	1.00000000	pigs
Cd2bp2	-0.03	1.00000000	pigs
Asb3	0.02	1.00000000	pigs
Qtrt1	-0.02	1.00000000	pigs
Gpcpd1	-0.06	1.00000000	pigs
Cmtr1	-0.08	1.00000000	pigs
Fam76a	-0.01	1.00000000	pigs

Cisd2	-0.15	1.00000000	pigs
Zmym4	-0.12	1.00000000	pigs
Tpr	-0.12	1.00000000	pigs
Ogfr	-0.02	1.00000000	pigs
Tmtc4	0.01	1.00000000	pigs
Tsga10	-0.05	1.00000000	pigs
Supv3l1	-0.03	1.00000000	pigs
Suz12	-0.07	1.00000000	pigs
Ankle2	-0.04	1.00000000	pigs
Chuk	0.00	1.00000000	pigs
Vps25	-0.14	1.00000000	pigs
0610030E20Rik	0.00	1.00000000	pigs
Ap1m1	-0.06	1.00000000	pigs
Bicral	-0.05	1.00000000	pigs
Polr2c	-0.03	1.00000000	pigs
Abcc10	-0.07	1.00000000	pigs
Eif4ebp2	0.09	1.00000000	pigs
Xkrx	-0.03	1.00000000	pigs
Angptl1	0.02	1.00000000	pigs
Atp6v1h	0.00	1.00000000	pigs
Trappc11	-0.10	1.00000000	pigs
Btbd3	-0.09	1.00000000	pigs
Ccnd2	0.09	1.00000000	pigs
Prkar2b	-0.05	1.00000000	pigs
F2r	0.46	1.00000000	pigs
Scyl3	0.02	1.00000000	pigs
Rnf125	-0.04	1.00000000	pigs
Ptges3	0.04	1.00000000	pigs
Pithd1	-0.12	1.00000000	pigs
Prpf38a	-0.14	1.00000000	pigs
Tnfrsf21	0.62	1.00000000	pigs
Dnajc7	-0.11	1.00000000	pigs
Ccnk	-0.06	1.00000000	pigs
Atmin	-0.07	1.00000000	pigs
Smarcal1	-0.06	1.00000000	pigs
Tpm4	-0.05	1.00000000	pigs
Abhd6	-0.04	1.00000000	pigs
Slc25a1	-0.09	1.00000000	pigs
Fyttd1	-0.07	1.00000000	pigs
Nudcd1	-0.09	1.00000000	pigs
Calcoco2	-0.05	1.00000000	pigs
Nemp2	0.05	1.00000000	pigs
Prkab1	-0.04	1.00000000	pigs
U2af2	-0.08	1.00000000	pigs
Exosc8	-0.09	1.00000000	pigs
Dcaf1	-0.08	1.00000000	pigs

Tubgcp2	-0.08	1.00000000	pigs
Ankrd44	0.14	1.00000000	pigs
Rassf5	0.03	1.00000000	pigs
Foxp4	-0.04	1.00000000	pigs
Arl6ip6	-0.08	1.00000000	pigs
Eif4e2	0.00	1.00000000	pigs
Snx19	-0.03	1.00000000	pigs
Rsl24d1	-0.13	1.00000000	pigs
Ntmt1	-0.06	1.00000000	pigs
Tfdp1	-0.08	1.00000000	pigs
Itgb4	0.10	1.00000000	pigs
Slu7	-0.11	1.00000000	pigs
Smg9	0.00	1.00000000	pigs
Arfip1	-0.16	1.00000000	pigs
Tbc1d4	0.19	1.00000000	pigs
Apbb3	-0.05	1.00000000	pigs
Aimp1	-0.01	1.00000000	pigs
Srfbp1	-0.04	1.00000000	pigs
Tssc4	-0.02	1.00000000	pigs
Lrrc28	-0.11	1.00000000	pigs
Rfc3	-0.10	1.00000000	pigs
Xrcc2	0.01	1.00000000	pigs
Strbp	-0.40	1.00000000	pigs
Focad	0.03	1.00000000	pigs
Ncaph2	-0.05	1.00000000	pigs
Psmb1	-0.04	1.00000000	pigs
Trp53	-0.02	1.00000000	pigs
Aup1	-0.05	1.00000000	pigs
Kmt2b	-0.14	1.00000000	pigs
Fyn	0.22	1.00000000	pigs
Anapc16	-0.10	1.00000000	pigs
Srm	0.01	1.00000000	pigs
Wdr4	0.00	1.00000000	pigs
Cul3	0.08	1.00000000	pigs
Alkbh7	-0.15	1.00000000	pigs
Akap10	-0.11	1.00000000	pigs
Crk	-0.09	1.00000000	pigs
A4galt	-0.05	1.00000000	pigs
Ube3c	-0.01	1.00000000	pigs
Ddx52	-0.04	1.00000000	pigs
Snx6	0.04	1.00000000	pigs
Tmed3	-0.08	1.00000000	pigs
Ccdc69	-0.05	1.00000000	pigs
Cct3	0.00	1.00000000	pigs
Eif2a	-0.09	1.00000000	pigs
Ldb1	-0.06	1.00000000	pigs

Zdhhc9	0.07	1.00000000	pigs
Alcam	0.26	1.00000000	pigs
Yars	-0.13	1.00000000	pigs
Cpox	0.00	1.00000000	pigs
2210016F16Rik	-0.03	1.00000000	pigs
Fancm	-0.02	1.00000000	pigs
Rnaseh2c	-0.10	1.00000000	pigs
Sorbs3	0.03	1.00000000	pigs
Dctn6	-0.04	1.00000000	pigs
Stag1	-0.03	1.00000000	pigs
Ube2g2	0.01	1.00000000	pigs
Poc1a	-0.01	1.00000000	pigs
Sufu	0.02	1.00000000	pigs
Rcor3	-0.13	1.00000000	pigs
Ccser2	-0.14	1.00000000	pigs
Orai3	-0.01	1.00000000	pigs
Xpnpep3	-0.07	1.00000000	pigs
Tesk1	-0.06	1.00000000	pigs
Selenot	0.01	1.00000000	pigs
Fam126a	-0.10	1.00000000	pigs
Galnt6	-0.23	1.00000000	pigs
Bptf	0.07	1.00000000	pigs
Gak	0.08	1.00000000	pigs
Ring1	-0.10	1.00000000	pigs
Nxt1	-0.12	1.00000000	pigs
Ubxn6	0.00	1.00000000	pigs
Sav1	-0.02	1.00000000	pigs
Anxa4	-0.09	1.00000000	pigs
Dnaja3	-0.11	1.00000000	pigs
Ltn1	-0.12	1.00000000	pigs
Fam189a2	-0.06	1.00000000	pigs
Plau	-0.25	1.00000000	pigs
Setdb2	-0.05	1.00000000	pigs
Spg7	0.01	1.00000000	pigs
Rbm41	-0.19	1.00000000	pigs
Siva1	-0.02	1.00000000	pigs
Dlg1	0.08	1.00000000	pigs
Abl1	-0.13	1.00000000	pigs
Cdyl2	-0.04	1.00000000	pigs
Ak3	-0.06	1.00000000	pigs
Tmem63a	-0.04	1.00000000	pigs
Purg	-0.14	1.00000000	pigs
Hmbox1	-0.18	1.00000000	pigs
Trank1	-0.07	1.00000000	pigs
Ccr5	-0.01	1.00000000	pigs
Becn1	-0.11	1.00000000	pigs

Pinx1	0.00	1.00000000	pigs
Btbd7	-0.03	1.00000000	pigs
Dennd11	-0.37	1.00000000	pigs
Armc7	-0.12	1.00000000	pigs
Ctu2	-0.02	1.00000000	pigs
Tnks2	-0.05	1.00000000	pigs
Enox2	-0.12	1.00000000	pigs
Agtpbp1	-0.05	1.00000000	pigs
Gusb	-0.02	1.00000000	pigs
Dnajc13	0.12	1.00000000	pigs
Nudt22	-0.12	1.00000000	pigs
Snapc2	-0.07	1.00000000	pigs
Prf1	0.09	1.00000000	pigs
Rap2b	-0.04	1.00000000	pigs
Tada2a	-0.02	1.00000000	pigs
Las1l	-0.05	1.00000000	pigs
Dag1	0.06	1.00000000	pigs
Nme1	-0.04	1.00000000	pigs
Dennd4c	0.09	1.00000000	pigs
Dnajc1	-0.03	1.00000000	pigs
Ctdspl2	-0.01	1.00000000	pigs
Zer1	-0.13	1.00000000	pigs
Txnl1	-0.09	1.00000000	pigs
Zfp652	-0.26	1.00000000	pigs
Sirt7	-0.08	1.00000000	pigs
Arhgap39	-0.08	1.00000000	pigs
Slain1	0.07	1.00000000	pigs
Tnfrsf11a	-0.05	1.00000000	pigs
Tmem86a	-0.01	1.00000000	pigs
Septin6	0.03	1.00000000	pigs
Slc35b3	0.02	1.00000000	pigs
Ist1	-0.13	1.00000000	pigs
Polr1h	-0.01	1.00000000	pigs
Rnls	-0.07	1.00000000	pigs
Zdhhc15	0.00	1.00000000	pigs
Lars2	-0.13	1.00000000	pigs
Ccnc	-0.13	1.00000000	pigs
Clspn	-0.05	1.00000000	pigs
Cog3	0.00	1.00000000	pigs
AB124611	-0.07	1.00000000	pigs
Pafah1b1	0.01	1.00000000	pigs
Cops7a	-0.11	1.00000000	pigs
Ndufa6	-0.08	1.00000000	pigs
Stk4	0.17	1.00000000	pigs
Taco1	-0.05	1.00000000	pigs
Rad51b	-0.25	1.00000000	pigs

Slc20a2	-0.20	1.00000000	pigs
Ccdc61	-0.02	1.00000000	pigs
A130010J15Rik	-0.04	1.00000000	pigs
Park7	-0.09	1.00000000	pigs
Ncapd2	0.01	1.00000000	pigs
Tubgcp4	-0.06	1.00000000	pigs
Fmo4	-0.05	1.00000000	pigs
Rnf146	-0.06	1.00000000	pigs
Rbm8a2	-0.03	1.00000000	pigs
Celf1	-0.03	1.00000000	pigs
Vps26a	-0.04	1.00000000	pigs
Dgkz	-0.10	1.00000000	pigs
Khnyn	-0.03	1.00000000	pigs
Kmo	-0.12	1.00000000	pigs
BC028528	-0.26	1.00000000	pigs
Ercc8	-0.12	1.00000000	pigs
Trnau1ap	-0.02	1.00000000	pigs
Stxbp5	-0.17	1.00000000	pigs
Ssbp4	0.01	1.00000000	pigs
Ap2b1	-0.14	1.00000000	pigs
Tada1	-0.09	1.00000000	pigs
Polk	-0.12	1.00000000	pigs
Klhl32	-0.05	1.00000000	pigs
Urb2	-0.06	1.00000000	pigs
Eif2ak4	-0.12	1.00000000	pigs
Klhl8	0.00	1.00000000	pigs
Zmiz2	-0.12	1.00000000	pigs
Rfng	-0.04	1.00000000	pigs
Abcd4	-0.07	1.00000000	pigs
Cass4	0.04	1.00000000	pigs
Ndufaf3	-0.16	1.00000000	pigs
Vps37c	-0.04	1.00000000	pigs
Adsl	-0.14	1.00000000	pigs
Klhl28	-0.19	1.00000000	pigs
Anapc2	-0.08	1.00000000	pigs
Stradb	0.06	1.00000000	pigs
Ly9	0.07	1.00000000	pigs
Stx18	-0.10	1.00000000	pigs
Ncstn	-0.15	1.00000000	pigs
Zbtb18	-0.14	1.00000000	pigs
Dlg5	-0.06	1.00000000	pigs
Tatdn1	-0.09	1.00000000	pigs
Fam98a	-0.04	1.00000000	pigs
Nkrf	-0.01	1.00000000	pigs
Rpn2	-0.10	1.00000000	pigs
Sh3bp5l	-0.09	1.00000000	pigs

Adprm	-0.06	1.00000000	pigs
Fbxo31	-0.05	1.00000000	pigs
Cox5b-ps	-0.13	1.00000000	pigs
Zmat5	-0.10	1.00000000	pigs
Ess2	-0.02	1.00000000	pigs
Cep295	-0.04	1.00000000	pigs
Tinf2	-0.03	1.00000000	pigs
Golga1	-0.05	1.00000000	pigs
Nabp1	-0.24	1.00000000	pigs
Zfp445	-0.03	1.00000000	pigs
E2f4	-0.06	1.00000000	pigs
Idnk	-0.02	1.00000000	pigs
Akap9	-0.12	1.00000000	pigs
Tnk2	-0.06	1.00000000	pigs
Zfp830	0.00	1.00000000	pigs
Uba3	-0.10	1.00000000	pigs
Nsun6	-0.14	1.00000000	pigs
Amn1	-0.11	1.00000000	pigs
Apoo	0.12	1.00000000	pigs
L3mbtl2	-0.05	1.00000000	pigs
Nfrkb	-0.19	1.00000000	pigs
Cp	-0.06	1.00000000	pigs
Txnip	0.07	1.00000000	pigs
Utp3	-0.02	1.00000000	pigs
Zmym6	-0.09	1.00000000	pigs
Smarcb1	-0.07	1.00000000	pigs
Zzef1	-0.16	1.00000000	pigs
Wbp1	-0.06	1.00000000	pigs
Ypel5	-0.03	1.00000000	pigs
Rab29	-0.01	1.00000000	pigs
Zfp251	-0.12	1.00000000	pigs
Timm8a2	-0.04	1.00000000	pigs
Rpap1	-0.01	1.00000000	pigs
Znhit3	-0.05	1.00000000	pigs
Dusp2	-0.24	1.00000000	pigs
Galnt11	-0.06	1.00000000	pigs
Dcaf8	-0.07	1.00000000	pigs
Sfxn1	-0.13	1.00000000	pigs
B230219D22Rik	-0.02	1.00000000	pigs
Emc10	-0.03	1.00000000	pigs
Kctd9	-0.03	1.00000000	pigs
Irf5	-0.06	1.00000000	pigs
Fam114a2	0.00	1.00000000	pigs
Leng1	-0.05	1.00000000	pigs
Uros	0.02	1.00000000	pigs
Ndufa10	-0.10	1.00000000	pigs

Selenoo	-0.01	1.00000000	pigs
Cep63	-0.16	1.00000000	pigs
Atg12	-0.17	1.00000000	pigs
Tmem164	0.05	1.00000000	pigs
Cacfd1	-0.04	1.00000000	pigs
Ppid	-0.01	1.00000000	pigs
Pdp1	-0.04	1.00000000	pigs
Itgb1	-0.01	1.00000000	pigs
Tfec	-0.06	1.00000000	pigs
Zc3hav1	0.13	1.00000000	pigs
Ubqln4	-0.04	1.00000000	pigs
Yju2	-0.04	1.00000000	pigs
Atr	-0.13	1.00000000	pigs
Setd7	-0.02	1.00000000	pigs
Odr4	-0.09	1.00000000	pigs
Taf4b	0.23	1.00000000	pigs
Zdhhc21	-0.05	1.00000000	pigs
Tyk2	-0.24	1.00000000	pigs
Sgta	-0.04	1.00000000	pigs
Stxbp6	-0.17	1.00000000	pigs
Lsm3	-0.01	1.00000000	pigs
Cdipt	-0.04	1.00000000	pigs
Nova1	-0.03	1.00000000	pigs
Lrrc8a	-0.06	1.00000000	pigs
Gtpbp4	-0.04	1.00000000	pigs
Ube2h	-0.07	1.00000000	pigs
Pald1	0.01	1.00000000	pigs
Tmub2	-0.01	1.00000000	pigs
Timm17a	-0.06	1.00000000	pigs
Rasal2	0.19	1.00000000	pigs
Il27ra	-0.11	1.00000000	pigs
Stk38l	0.09	1.00000000	pigs
Czib	-0.12	1.00000000	pigs
Tbrg1	-0.11	1.00000000	pigs
Cebpd	0.03	1.00000000	pigs
Jazf1	-0.08	1.00000000	pigs
Ccnb1ip1	-0.08	1.00000000	pigs
Cyb5b	-0.10	1.00000000	pigs
Supt20	-0.07	1.00000000	pigs
Nup160	0.01	1.00000000	pigs
Wwp1	-0.09	1.00000000	pigs
Nop56	-0.10	1.00000000	pigs
Ints7	-0.10	1.00000000	pigs
Dynll2	-0.05	1.00000000	pigs
Dtx1	-0.01	1.00000000	pigs
Twf1	-0.09	1.00000000	pigs

Atxn7l3	-0.06	1.00000000	pigs
Foxn2	0.11	1.00000000	pigs
Rabgap1	-0.04	1.00000000	pigs
Tor4a	-0.09	1.00000000	pigs
Trappc12	-0.05	1.00000000	pigs
Akap8	-0.14	1.00000000	pigs
Trappc5	-0.09	1.00000000	pigs
Hdgfl3	-0.17	1.00000000	pigs
Ddx59	-0.07	1.00000000	pigs
Stat2	-0.03	1.00000000	pigs
Pus1	0.00	1.00000000	pigs
Fpgs	-0.36	1.00000000	pigs
Alg3	-0.06	1.00000000	pigs
5031439G07Rik	-0.03	1.00000000	pigs
Ccnd1	-0.04	1.00000000	pigs
Exoc7	-0.05	1.00000000	pigs
Katnip	0.01	1.00000000	pigs
Shprh	-0.08	1.00000000	pigs
Ccdc15	-0.06	1.00000000	pigs
Tmcc1	0.02	1.00000000	pigs
8030462N17Rik	-0.08	1.00000000	pigs
Prkci	-0.07	1.00000000	pigs
Neu1	-0.02	1.00000000	pigs
Dis3l2	-0.13	1.00000000	pigs
Hdhd2	0.02	1.00000000	pigs
Nfe2l1	-0.10	1.00000000	pigs
Ppm1h	-0.01	1.00000000	pigs
Rbm19	-0.04	1.00000000	pigs
Tmem106c	-0.15	1.00000000	pigs
Smc1a	-0.11	1.00000000	pigs
Rpain	-0.11	1.00000000	pigs
Sdhaf2	-0.05	1.00000000	pigs
Wdr77	-0.02	1.00000000	pigs
Zfp169	0.01	1.00000000	pigs
G0s2	0.05	1.00000000	pigs
1110059G10Rik	-0.03	1.00000000	pigs
Atp2a3	-0.02	1.00000000	pigs
Unc50	-0.10	1.00000000	pigs
Ndfip2	-0.07	1.00000000	pigs
Snrpe	-0.04	1.00000000	pigs
Cwf19l2	-0.07	1.00000000	pigs
Lig1	0.03	1.00000000	pigs
Gigyf2	-0.09	1.00000000	pigs
Higd2a	0.02	1.00000000	pigs
Inppl1	-0.03	1.00000000	pigs
Ankzf1	-0.02	1.00000000	pigs

Car13	-0.02	1.00000000	pigs
Synj1	-0.07	1.00000000	pigs
Cnot10	0.02	1.00000000	pigs
Septin1	-0.04	1.00000000	pigs
Dstyk	-0.08	1.00000000	pigs
Serac1	-0.02	1.00000000	pigs
Cdc42se1	0.00	1.00000000	pigs
Vps29	-0.08	1.00000000	pigs
Slc38a6	-0.03	1.00000000	pigs
Vat1	-0.04	1.00000000	pigs
Ino80	-0.10	1.00000000	pigs
Tut7	0.05	1.00000000	pigs
Kmt5b	-0.19	1.00000000	pigs
Wdr37	0.00	1.00000000	pigs
Amz2	-0.08	1.00000000	pigs
Taf5l	-0.10	1.00000000	pigs
Brd9	-0.17	1.00000000	pigs
Wdr48	0.00	1.00000000	pigs
Prmt7	-0.05	1.00000000	pigs
Trrap	-0.17	1.00000000	pigs
Dnajb9	-0.05	1.00000000	pigs
Ap1s2	0.18	1.00000000	pigs
Slc25a5	-0.06	1.00000000	pigs
Star	-0.11	1.00000000	pigs
Pofut1	-0.05	1.00000000	pigs
Atxn1l	-0.02	1.00000000	pigs
Smdt1	-0.02	1.00000000	pigs
Nfyc	-0.14	1.00000000	pigs
Smarcd1	-0.16	1.00000000	pigs
Chsy1	-0.07	1.00000000	pigs
Suclg1	-0.17	1.00000000	pigs
Rap1gap2	0.06	1.00000000	pigs
Cd180	0.00	1.00000000	pigs
Cept1	-0.04	1.00000000	pigs
Shld2	-0.15	1.00000000	pigs
Telo2	0.00	1.00000000	pigs
Rcan1	-0.01	1.00000000	pigs
Adgre5	-0.14	1.00000000	pigs
Rab30	0.36	1.00000000	pigs
Pja2	-0.13	1.00000000	pigs
Zfp511	-0.02	1.00000000	pigs
Fads3	-0.05	1.00000000	pigs
Rmi2	0.01	1.00000000	pigs
Trmt10c	-0.05	1.00000000	pigs
Ppa2	-0.05	1.00000000	pigs
Raly	0.02	1.00000000	pigs

Camkmt	-0.05	1.00000000	pigs
Oxct1	0.12	1.00000000	pigs
Zbtb24	-0.10	1.00000000	pigs
Arid3a	-0.08	1.00000000	pigs
Taf9b	-0.14	1.00000000	pigs
Osgep	-0.02	1.00000000	pigs
Atg3	-0.12	1.00000000	pigs
Krcc1	-0.07	1.00000000	pigs
Bora	-0.05	1.00000000	pigs
Antkmt	-0.16	1.00000000	pigs
Pcsk7	-0.01	1.00000000	pigs
Foxred1	-0.01	1.00000000	pigs
Nipal3	-0.08	1.00000000	pigs
Gnpnat1	-0.09	1.00000000	pigs
Nemf	-0.07	1.00000000	pigs
Leng8	0.04	1.00000000	pigs
Atg5	-0.08	1.00000000	pigs
Gid4	-0.07	1.00000000	pigs
Snip1	-0.07	1.00000000	pigs
Jade1	0.23	1.00000000	pigs
Serinc3	-0.12	1.00000000	pigs
Fbxl14	-0.02	1.00000000	pigs
Zbtb44	-0.14	1.00000000	pigs
Eif2s2	0.02	1.00000000	pigs
Slc25a24	-0.02	1.00000000	pigs
Afmid	-0.18	1.00000000	pigs
Sclt1	0.35	1.00000000	pigs
Kif18a	-0.05	1.00000000	pigs
Apex1	-0.14	1.00000000	pigs
Vps26c	-0.10	1.00000000	pigs
Faxc	-0.11	1.00000000	pigs
Lrrc18	-0.18	1.00000000	pigs
Nek1	0.03	1.00000000	pigs
Ccdc90b	-0.09	1.00000000	pigs
Flvcr1	0.31	1.00000000	pigs
Dph7	-0.06	1.00000000	pigs
Nbeal1	-0.06	1.00000000	pigs
Carhsp1	-0.08	1.00000000	pigs
Fastkd2	0.00	1.00000000	pigs
Fam114a1	-0.07	1.00000000	pigs
Vps51	-0.09	1.00000000	pigs
Gtf2e2	-0.12	1.00000000	pigs
Tmem123	0.06	1.00000000	pigs
Zmpste24	-0.05	1.00000000	pigs
Cdk12	-0.11	1.00000000	pigs
Bckdhb	-0.08	1.00000000	pigs

Calm1	-0.02	1.00000000	pigs
Tecta	-0.08	1.00000000	pigs
Trim13	-0.01	1.00000000	pigs
C1qbp	-0.13	1.00000000	pigs
Gid8	-0.12	1.00000000	pigs
Gins2	-0.07	1.00000000	pigs
Ranbp17	-0.01	1.00000000	pigs
Smarca4	-0.01	1.00000000	pigs
Hexdc	-0.05	1.00000000	pigs
Zfpl1	0.01	1.00000000	pigs
Rhbdd1	-0.01	1.00000000	pigs
Rbm5	-0.01	1.00000000	pigs
Rbbp9	-0.06	1.00000000	pigs
Nck1	-0.02	1.00000000	pigs
Tamm41	-0.06	1.00000000	pigs
Kansl2	-0.09	1.00000000	pigs
Cldnd1	-0.13	1.00000000	pigs
Cbfa2t3	-0.05	1.00000000	pigs
Tbpl1	-0.03	1.00000000	pigs
Gss	-0.04	1.00000000	pigs
Dnlz	-0.09	1.00000000	pigs
AI413582	-0.09	1.00000000	pigs
Ubb	0.02	1.00000000	pigs
Tbxas1	-0.06	1.00000000	pigs
Tagap1	-0.26	1.00000000	pigs
Fubp3	-0.11	1.00000000	pigs
Gcfc2	-0.22	1.00000000	pigs
Strn4	-0.05	1.00000000	pigs
Ndufs1	-0.03	1.00000000	pigs
Os9	-0.01	1.00000000	pigs
Lyar	-0.05	1.00000000	pigs
Ythdc1	-0.08	1.00000000	pigs
Gnb2	-0.13	1.00000000	pigs
Gpank1	0.00	1.00000000	pigs
Hectd1	-0.07	1.00000000	pigs
Elmod2	-0.02	1.00000000	pigs
Arhgap45	-0.09	1.00000000	pigs
Xpa	0.00	1.00000000	pigs
Ube2m	-0.04	1.00000000	pigs
Rbm28	-0.10	1.00000000	pigs
Sympk	-0.03	1.00000000	pigs
Top2b	-0.07	1.00000000	pigs
Eif4g1	-0.07	1.00000000	pigs
Srsf4	0.08	1.00000000	pigs
Cxxc1	-0.10	1.00000000	pigs
Aprt	-0.11	1.00000000	pigs

Rnf44	-0.05	1.00000000	pigs
Lpcat2	-0.02	1.00000000	pigs
Atp6ap2	0.01	1.00000000	pigs
Uqcc1	-0.02	1.00000000	pigs
Clcc1	0.00	1.00000000	pigs
Hyal2	-0.06	1.00000000	pigs
Agpat1	-0.04	1.00000000	pigs
1700037H04Rik	-0.08	1.00000000	pigs
Ndufb11b	-0.14	1.00000000	pigs
Golga3	-0.04	1.00000000	pigs
Ndufb8	-0.01	1.00000000	pigs
Tmem205	-0.01	1.00000000	pigs
Pgpep1	0.02	1.00000000	pigs
Adamts17	-0.04	1.00000000	pigs
Ppm1f	0.01	1.00000000	pigs
C030006K11Rik	-0.01	1.00000000	pigs
Trmt11	-0.05	1.00000000	pigs
Flad1	-0.09	1.00000000	pigs
Uspl1	-0.05	1.00000000	pigs
Snrpd1	-0.08	1.00000000	pigs
Ggcx	-0.08	1.00000000	pigs
Pomp	-0.06	1.00000000	pigs
Ptprj	0.24	1.00000000	pigs
Virma	-0.04	1.00000000	pigs
Pura	-0.02	1.00000000	pigs
Auh	0.02	1.00000000	pigs
Sun1	-0.04	1.00000000	pigs
Vps35	-0.09	1.00000000	pigs
Ctr9	-0.08	1.00000000	pigs
Rpa1	-0.04	1.00000000	pigs
Cyb561d1	-0.06	1.00000000	pigs
Fam135a	-0.20	1.00000000	pigs
Irf4	0.16	1.00000000	pigs
Relch	0.05	1.00000000	pigs
Rbmx2	-0.03	1.00000000	pigs
Hira	-0.03	1.00000000	pigs
Abhd16a	-0.10	1.00000000	pigs
Yipf2	-0.13	1.00000000	pigs
Yif1a	-0.09	1.00000000	pigs
Lmbrd1	0.01	1.00000000	pigs
Pam	0.03	1.00000000	pigs
Hcfc1r1	-0.03	1.00000000	pigs
Hspa4	0.05	1.00000000	pigs
Ahcyl1	-0.16	1.00000000	pigs
Polg	0.00	1.00000000	pigs
Acox3	-0.04	1.00000000	pigs

Dnajc11	-0.08	1.00000000	pigs
Dpy30	-0.12	1.00000000	pigs
Gpkow	-0.02	1.00000000	pigs
Btf3l4	-0.06	1.00000000	pigs
Dhx30	-0.09	1.00000000	pigs
Cmpk1	-0.20	1.00000000	pigs
Cep290	-0.04	1.00000000	pigs
Sf3a1	-0.05	1.00000000	pigs
Taf3	0.00	1.00000000	pigs
Appl1	-0.07	1.00000000	pigs
Nrbf2	-0.02	1.00000000	pigs
Zfp341	-0.05	1.00000000	pigs
Cd8a	0.02	1.00000000	pigs
Ciapin1	-0.14	1.00000000	pigs
Abhd18	-0.05	1.00000000	pigs
Tango6	-0.01	1.00000000	pigs
Naga	-0.11	1.00000000	pigs
Abhd12	-0.18	1.00000000	pigs
Nsmf	-0.06	1.00000000	pigs
Trpv2	-0.17	1.00000000	pigs
Grk6	-0.05	1.00000000	pigs
Klhdc10	-0.02	1.00000000	pigs
Sh2b1	-0.11	1.00000000	pigs
Nadk2	-0.07	1.00000000	pigs
Itgb5	0.16	1.00000000	pigs
Igfbp7	-0.06	1.00000000	pigs
Eif2b4	-0.07	1.00000000	pigs
Gtf2h3	-0.02	1.00000000	pigs
Stk10	0.00	1.00000000	pigs
Klhl9	-0.03	1.00000000	pigs
Oxnad1	0.00	1.00000000	pigs
Nfx1	-0.18	1.00000000	pigs
Fam13b	-0.10	1.00000000	pigs
Ltbp1	0.06	1.00000000	pigs
Arl5b	-0.11	1.00000000	pigs
Dgkd	0.12	1.00000000	pigs
Insr	0.21	1.00000000	pigs
Pard3b	0.02	1.00000000	pigs
Sec23a	-0.10	1.00000000	pigs
Rnf103	-0.01	1.00000000	pigs
Usp24	0.38	1.00000000	pigs
Tmem161b	-0.13	1.00000000	pigs
Derl1	-0.03	1.00000000	pigs
Ints8	-0.15	1.00000000	pigs
Idh1	-0.13	1.00000000	pigs
Cpsf2	-0.03	1.00000000	pigs

Tfcp2	-0.04	1.00000000	pigs
Trak2	-0.01	1.00000000	pigs
Sprtn	-0.02	1.00000000	pigs
Fgfr1op2	-0.08	1.00000000	pigs
Atg4c	-0.04	1.00000000	pigs
Timm17b	-0.06	1.00000000	pigs
Tfg	-0.06	1.00000000	pigs
Bank1	-0.08	1.00000000	pigs
Hnrnph1	0.00	1.00000000	pigs
Ostm1	0.08	1.00000000	pigs
Arhgap26	-0.07	1.00000000	pigs
Pdia3	0.02	1.00000000	pigs
Hlcs	-0.05	1.00000000	pigs
Cysltr1	-0.02	1.00000000	pigs
Txnrd3	-0.04	1.00000000	pigs
Asns	-0.03	1.00000000	pigs
Cbx1	0.01	1.00000000	pigs
Helz	-0.11	1.00000000	pigs
Polr1d	-0.02	1.00000000	pigs
Cep78	-0.04	1.00000000	pigs
Pola1	-0.15	1.00000000	pigs
Inpp5b	-0.09	1.00000000	pigs
Lsm14a	-0.01	1.00000000	pigs
Phc1	0.03	1.00000000	pigs
Ubtf	-0.14	1.00000000	pigs
Emc3	-0.05	1.00000000	pigs
Srrm2	-0.05	1.00000000	pigs
Emp1	-0.02	1.00000000	pigs
Pik3r4	-0.02	1.00000000	pigs
Ubap2l	-0.12	1.00000000	pigs
Nfia	-0.13	1.00000000	pigs
Dhx35	-0.02	1.00000000	pigs
Tmem243	-0.13	1.00000000	pigs
Tfb2m	0.00	1.00000000	pigs
Calhm6	-0.10	1.00000000	pigs
Sdhd	-0.04	1.00000000	pigs
Pelp1	-0.14	1.00000000	pigs
Lman2l	-0.06	1.00000000	pigs
Lactb2	-0.08	1.00000000	pigs
Srrt	-0.19	1.00000000	pigs
Pdrg1	-0.01	1.00000000	pigs
Wdr45	-0.02	1.00000000	pigs
Crebbp	-0.02	1.00000000	pigs
Tmed7	-0.05	1.00000000	pigs
Hsd17b7	-0.15	1.00000000	pigs
Chchd4	-0.12	1.00000000	pigs

Yipf6	-0.12	1.00000000	pigs
Wdr7	-0.07	1.00000000	pigs
Gpn1	-0.02	1.00000000	pigs
Slc25a36	-0.14	1.00000000	pigs
Pdzd11	-0.11	1.00000000	pigs
Snx16	-0.03	1.00000000	pigs
Dmac2l	-0.08	1.00000000	pigs
Ppp1r18	-0.05	1.00000000	pigs
Dtymk	-0.08	1.00000000	pigs
9430038I01Rik	-0.02	1.00000000	pigs
Prkd2	0.02	1.00000000	pigs
Rap1a	0.04	1.00000000	pigs
Sdhc	0.03	1.00000000	pigs
Napb	-0.01	1.00000000	pigs
Jmjd8	-0.03	1.00000000	pigs
Prmt2	-0.25	1.00000000	pigs
Polr2m	-0.09	1.00000000	pigs
Sgms1	-0.10	1.00000000	pigs
Nthl1	-0.03	1.00000000	pigs
Cnot6	-0.07	1.00000000	pigs
D16Ertd472e	-0.10	1.00000000	pigs
Samd8	-0.02	1.00000000	pigs
Irf7	0.12	1.00000000	pigs
Usp5	-0.01	1.00000000	pigs
Casc3	-0.07	1.00000000	pigs
Gde1	-0.05	1.00000000	pigs
Ddx23	-0.08	1.00000000	pigs
Pard6a	0.00	1.00000000	pigs
Rlim	-0.06	1.00000000	pigs
Nkap	-0.07	1.00000000	pigs
Steep1	-0.03	1.00000000	pigs
Ythdc2	-0.06	1.00000000	pigs
Nudt13	-0.02	1.00000000	pigs
Rsbn1l	0.10	1.00000000	pigs
Fcho2	-0.18	1.00000000	pigs
Dpf2	-0.10	1.00000000	pigs
Ankrd13c	0.11	1.00000000	pigs
Spata1	-0.03	1.00000000	pigs
Zfp451	-0.07	1.00000000	pigs
Ssbp1	-0.07	1.00000000	pigs
Naa40	-0.02	1.00000000	pigs
Dhx58	0.00	1.00000000	pigs
Nme6	-0.09	1.00000000	pigs
Psmd4	-0.07	1.00000000	pigs
Ncbp1	-0.03	1.00000000	pigs
Prss55	0.06	1.00000000	pigs

Idi1	-0.06	1.00000000	pigs
Trip4	-0.05	1.00000000	pigs
Clpb	-0.02	1.00000000	pigs
Psme2	-0.05	1.00000000	pigs
Ptk2b	0.10	1.00000000	pigs
Cfap97	-0.01	1.00000000	pigs
Cdadc1	0.07	1.00000000	pigs
Ccdc115	-0.05	1.00000000	pigs
Prpf39	0.01	1.00000000	pigs
Lmo2	-0.05	1.00000000	pigs
Hexim1	-0.06	1.00000000	pigs
Echs1	-0.09	1.00000000	pigs
Sin3b	-0.07	1.00000000	pigs
Nfatc2	-0.06	1.00000000	pigs
Zhx3	-0.07	1.00000000	pigs
Pms2	-0.06	1.00000000	pigs
Cemip2	-0.09	1.00000000	pigs
Epc1	-0.01	1.00000000	pigs
Trim38	-0.05	1.00000000	pigs
Exoc2	-0.12	1.00000000	pigs
Scai	-0.12	1.00000000	pigs
Hrh4	-0.02	1.00000000	pigs
Camta1	-0.06	1.00000000	pigs
Rmc1	-0.12	1.00000000	pigs
Polr2f	-0.05	1.00000000	pigs
Prr29	-0.10	1.00000000	pigs
Zfand1	-0.04	1.00000000	pigs
Pphln1	-0.09	1.00000000	pigs
Nup85	-0.05	1.00000000	pigs
Slc5a10	0.11	1.00000000	pigs
Dap3	-0.06	1.00000000	pigs
Abhd13	-0.04	1.00000000	pigs
Snd1	0.00	1.00000000	pigs
Socs4	-0.04	1.00000000	pigs
Nfxl1	0.02	1.00000000	pigs
Bnip1	0.00	1.00000000	pigs
Emc2	-0.09	1.00000000	pigs
Elp6	-0.03	1.00000000	pigs
Pskh1	0.01	1.00000000	pigs
Usp19	-0.06	1.00000000	pigs
Phb	-0.06	1.00000000	pigs
Tpk1	-0.01	1.00000000	pigs
Retreg1	0.15	1.00000000	pigs
Upf2	-0.15	1.00000000	pigs
Otud5	-0.03	1.00000000	pigs
Polr1b	-0.03	1.00000000	pigs

Psmd5	0.00	1.00000000	pigs
Bahd1	-0.12	1.00000000	pigs
Zfp248	0.10	1.00000000	pigs
Dip2a	0.01	1.00000000	pigs
Ciart	-0.22	1.00000000	pigs
Fam50a	-0.10	1.00000000	pigs
Srp72	-0.03	1.00000000	pigs
Cdin1	0.01	1.00000000	pigs
Pigu	-0.13	1.00000000	pigs
Ubac2	-0.13	1.00000000	pigs
Pdha1	-0.10	1.00000000	pigs
B9d2	-0.01	1.00000000	pigs
Nsfl1c	-0.01	1.00000000	pigs
Wdr18	-0.04	1.00000000	pigs
4932438A13Rik	-0.10	1.00000000	pigs
Rmdn1	-0.09	1.00000000	pigs
Eif4a3l1	-0.06	1.00000000	pigs
Edem2	-0.10	1.00000000	pigs
Utp15	-0.07	1.00000000	pigs
Babam2	0.07	1.00000000	pigs
Phf6	-0.01	1.00000000	pigs
Pitpnm2	0.10	1.00000000	pigs
Tbc1d1	-0.07	1.00000000	pigs
Senp5	-0.04	1.00000000	pigs
Parp14	-0.04	1.00000000	pigs
Rims2	-0.42	1.00000000	pigs
Wdr55	-0.07	1.00000000	pigs
Zfp524	-0.03	1.00000000	pigs
Zmym5	-0.08	1.00000000	pigs
Nsun2	-0.10	1.00000000	pigs
Cnppd1	0.01	1.00000000	pigs
Nup98	-0.14	1.00000000	pigs
Chd8	-0.05	1.00000000	pigs
Paqr8	-0.07	1.00000000	pigs
Ppp1r12b	0.01	1.00000000	pigs
Scaf11	0.08	1.00000000	pigs
Atf1	-0.04	1.00000000	pigs
Spred2	0.08	1.00000000	pigs
Ebag9	-0.16	1.00000000	pigs
Aif1	-0.06	1.00000000	pigs
Tsc22d1	-0.12	1.00000000	pigs
Pex1	-0.14	1.00000000	pigs
Ralbp1	-0.08	1.00000000	pigs
Cxxc5	-0.08	1.00000000	pigs
Ckap5	-0.07	1.00000000	pigs
Cyb561d2	-0.13	1.00000000	pigs

Epg5	-0.09	1.00000000	pigs
Dennd1c	-0.04	1.00000000	pigs
Smc2	-0.04	1.00000000	pigs
Rbbp7	-0.11	1.00000000	pigs
Coasy	-0.01	1.00000000	pigs
Hsp90aa1	0.22	1.00000000	pigs
P2ry6	0.02	1.00000000	pigs
Fam3c	-0.06	1.00000000	pigs
Trmt2a	-0.04	1.00000000	pigs
2510039O18Rik	-0.10	1.00000000	pigs
Ptpn22	-0.02	1.00000000	pigs
Prpf19	-0.05	1.00000000	pigs
Clpp	-0.06	1.00000000	pigs
Cox18	-0.06	1.00000000	pigs
Acsl5	0.07	1.00000000	pigs
Hook2	-0.09	1.00000000	pigs
Triobp	-0.20	1.00000000	pigs
Trim3	-0.10	1.00000000	pigs
Psmc5	-0.09	1.00000000	pigs
Ep400	-0.06	1.00000000	pigs
Pde12	-0.09	1.00000000	pigs
Adpgk	-0.06	1.00000000	pigs
Jkamp	-0.04	1.00000000	pigs
Ncoa6	-0.06	1.00000000	pigs
Cox16	-0.07	1.00000000	pigs
Scaf1	-0.02	1.00000000	pigs
Adk	-0.24	1.00000000	pigs
Cd9	-0.24	1.00000000	pigs
Pdhb	-0.05	1.00000000	pigs
Acp4	-0.04	1.00000000	pigs
Smim4	-0.15	1.00000000	pigs
Cnrip1	-0.04	1.00000000	pigs
Galm	-0.07	1.00000000	pigs
Nmnat2	-0.04	1.00000000	pigs
Vps53	-0.02	1.00000000	pigs
Plcb2	-0.11	1.00000000	pigs
Trdmt1	0.04	1.00000000	pigs
Ppwd1	-0.05	1.00000000	pigs
Tjap1	-0.04	1.00000000	pigs
Jmy	-0.07	1.00000000	pigs
Ap1s3	-0.01	1.00000000	pigs
Alad	-0.03	1.00000000	pigs
Zfp143	-0.04	1.00000000	pigs
Copg1	-0.02	1.00000000	pigs
Ppp2r5a	0.10	1.00000000	pigs
Dusp1	0.01	1.00000000	pigs

Rubcn	-0.06	1.00000000	pigs
Tdg	-0.11	1.00000000	pigs
Nufip1	-0.05	1.00000000	pigs
Wfdc2	-0.02	1.00000000	pigs
Ss18l2	-0.08	1.00000000	pigs
Serpinb6b	0.02	1.00000000	pigs
Dtx2	-0.03	1.00000000	pigs
Ddx55	-0.08	1.00000000	pigs
Wdr5	-0.06	1.00000000	pigs
Immp2l	0.09	1.00000000	pigs
Qtrt2	-0.10	1.00000000	pigs
Nr2c2	-0.09	1.00000000	pigs
Bad	-0.05	1.00000000	pigs
Gripap1	-0.03	1.00000000	pigs
Hace1	-0.08	1.00000000	pigs
Ttc38	-0.01	1.00000000	pigs
Rfx1	-0.08	1.00000000	pigs
Ints11	-0.02	1.00000000	pigs
Cyth3	0.08	1.00000000	pigs
Wdr91	-0.05	1.00000000	pigs
4930503L19Rik	0.00	1.00000000	pigs
Tmem41b	-0.07	1.00000000	pigs
Nop58	-0.05	1.00000000	pigs
Lmbr1	0.01	1.00000000	pigs
Ccdc191	-0.05	1.00000000	pigs
Rrs1	-0.04	1.00000000	pigs
Cenpp	-0.25	1.00000000	pigs
Nr2f6	-0.05	1.00000000	pigs
Ccdc107	-0.06	1.00000000	pigs
Ccar2	-0.10	1.00000000	pigs
Fundc2	-0.12	1.00000000	pigs
Nbr1	-0.21	1.00000000	pigs
Far2	-0.04	1.00000000	pigs
Dapp1	0.00	1.00000000	pigs
Atp5c1	-0.06	1.00000000	pigs
Sdf2	-0.14	1.00000000	pigs
Bzw2	-0.07	1.00000000	pigs
Snrnp200	-0.08	1.00000000	pigs
Srsf9	-0.11	1.00000000	pigs
Abi1	-0.09	1.00000000	pigs
Kazn	0.06	1.00000000	pigs
Wars2	-0.02	1.00000000	pigs
Cnnm3	-0.08	1.00000000	pigs
Brf1	-0.01	1.00000000	pigs
Fra10ac1	-0.07	1.00000000	pigs
Arglu1	0.02	1.00000000	pigs

Bpnt1	-0.06	1.00000000	pigs
Rnf5	-0.04	1.00000000	pigs
Susd6	0.30	1.00000000	pigs
H2-Ke6	-0.11	1.00000000	pigs
Larp4	-0.07	1.00000000	pigs
Agps	-0.14	1.00000000	pigs
Rp9	-0.05	1.00000000	pigs
Cramp1	-0.13	1.00000000	pigs
Stub1	-0.16	1.00000000	pigs
Gucd1	-0.08	1.00000000	pigs
Fbxl2	-0.06	1.00000000	pigs
Heatr6	-0.06	1.00000000	pigs
Exosc10	-0.04	1.00000000	pigs
Naf1	-0.04	1.00000000	pigs
Nepro	-0.01	1.00000000	pigs
Naa60	-0.02	1.00000000	pigs
Fam117b	0.03	1.00000000	pigs
Pak1ip1	-0.11	1.00000000	pigs
Nktr	-0.02	1.00000000	pigs
Arf6	-0.04	1.00000000	pigs
Atg4b	-0.04	1.00000000	pigs
Erbin	-0.11	1.00000000	pigs
Npc1	-0.08	1.00000000	pigs
Epb41l4a	-0.12	1.00000000	pigs
Spg11	-0.10	1.00000000	pigs
Dennd6a	-0.13	1.00000000	pigs
Crcp	-0.05	1.00000000	pigs
Thop1	-0.03	1.00000000	pigs
Thap2	-0.03	1.00000000	pigs
