# Supplementary material for: Heterogeneous viral-host response at single-cell resolution and viral adaptive mutational atlas in birds and mammals upon H5 influenza virus infection
Source: Natl Sci Rev. 2026 Jun 25;13(14):nwag380. doi: 10.1093/nsr/nwag380 (PMC13458601; doi:10.1093/nsr/nwag380)
Supplement: Supplementary file 1 [file nwag380_supplemental_files.zip › Supplementary Data 17.pdf]

Supplementary Data 17. Differentially expressed genes of epithelial across different sp

gene	log2FoldChange	p value
Smarcal1	-2.465762028	9.17621277419417e-22
Oasl2	2.62968846281708	1.0733010496555e-20
Tpt1	-2.277241752	2.0070154087538e-20
Ifit2	3.05657587745231	5.63591076218639e-19
Ddx60	2.62652711105718	9.64455354016912e-19
Fau	-1.954357874	2.65755376530622e-18
Rnf213	2.10969352988085	1.98085006602356e-17
Smchd1	2.00384304508096	1.31588087111105e-16
Rsad2	2.69874453317533	2.18093220353821e-16
Rack1	-2.222001561	5.13824091890487e-16
Eef1b2	-1.946187177	8.41533017981513e-16
Trank1	2.25226186426653	3.9689820460172e-14
Tox3	2.72623934977753	1.41960411697312e-13
Pabpc1	-1.625860808	1.67093334871135e-13
Plac8	2.42988402067378	2.05886000001579e-13
Ybx1	-1.733034923	5.76782528615827e-13
Eef1d	-1.605360003	1.43387938758805e-11
Phf11a	1.57220989177785	9.34774927172724e-11
Tmem164	2.462531326	1.99557021998733e-10
Eef2	-1.460586035	3.2538074708596e-10
Nme2	-1.80917258	4.36282865664048e-10
Fth1	-1.170565917	5.12597543549569e-10
H3f3a	-1.510343724	5.46411857078904e-10
Hmgb1	-1.653168564	6.02837443727467e-10
Ifih1	2.03668094967131	2.33009980651153e-09
Cox6a1	-1.416578156	1.48168901274667e-08
Zfp593	-1.363190432	2.83641641817751e-08
Nucks1	-1.319610205	3.4061745480083e-08
H2az2	-1.605843431	6.54986747556831e-08
Ppia	-1.268406581	7.62942384078205e-08
Unc5c	1.50821377149928	2.07868558069722e-07
Ass1	1.12862697554933	4.96524429550039e-07
Trafd1	1.28836308022083	7.10825735690107e-07
Dusp1	1.80654385475883	8.33914747716817e-07
Tomm20	-1.399937812	8.71686996687258e-07
Ly6e	1.94072186069963	1.05788466347704e-06
Urm1	-1.42441056	1.70695210725946e-06
Sting1	1.23315567090657	2.3644310908278e-06
Jun	1.06097778168757	5.02220112664184e-06
Cox7c	-1.132536249	1.01870734679818e-05
Rnf19b	0.837832374775876	1.1386628536521e-05

Lrrfip1	1.60673097060013	1.36013241445113e-05
Nol4	2.46772105427062	2.2223379025427e-05
Bsg	-1.24855573	2.37457418688303e-05
Eif4b	-1.329128697	7.45559507509449e-05
Zc3hav1l	1.26192352761604	8.51150084975046e-05
Eif3m	-1.245271003	9.76162444672832e-05
Ndufb5	-1.146733166	0.000100098815740418
Eif3e	-1.137616367	0.000121441799584098
Per2	1.42333386988324	0.000139476175895526
Akap9	1.01469212700702	0.000198614008083641
Cmtr1	1.19441896137354	0.000232362378536307
Phlda2	1.6940226588573	0.000252759829085723
Pebp1	-1.21736804	0.000312334660450331
Ankrd39	-1.023103846	0.000334831673565553
Serbp1	-0.968736273	0.000356137527444092
Spidr	1.92916602910373	0.000415634548555933
Hivep1	1.54390180553035	0.00041826280214312
Irf1	1.88253815758723	0.000442737286257672
Nt5c3b	0.737680202956188	0.000503326199850178
Svbp	-0.929366884	0.000568064613609278
H3f3b	-0.782443984	0.000648794605791534
Cd81	-0.931958844	0.000656781356909368
Uqcrh	-1.068628632	0.000674105469576308
Nmi	0.980254517022103	0.000737162907098506
Hdgf	-0.974760994	0.000832261048306619
Cox7b	-1.100719181	0.000859154721066697
Eif5a2	-1.17432398	0.000933594774530965
Uvrag	1.39300382180827	0.000969032010559694
Atp5j2	-1.100689558	0.00134671982559902
Cd3g	-1.18223827	0.00189229194829461
Itpr2	1.4042154209144	0.00228484980402778
Trak1	1.32642236439622	0.00263698457553313
Pms2	1.26866914160926	0.00276096857905244
Etv6	0.957454846637545	0.00299419767888098
Tm4sf1	-1.077729081	0.00322000056128862
Ndufb1	-1.099534553	0.00341169239360249
Eif4a2	-0.757467028	0.00352875009353655
Susd6	1.70811965717849	0.00373927814016037
Arxes2	-0.981141988	0.00380773714131274
Cox4i1	-0.976484811	0.00403758375263486
Eif3l	-0.759685853	0.00418083453776576
Fosb	0.958002645606203	0.00424144267689819
Ndufc2	-0.847276866	0.00443385015273305
Usp25	1.22226290679645	0.00454422975095884
Rfx2	2.43530892711887	0.00485500858935533
Ppib	-1.156037068	0.00490992892022523

Ndufa4	-0.978258552	0.0050801841662996
Fkbp1a	-0.973126898	0.00565243886131827
Glo1	-1.048036222	0.00604923640440535
Crip1	-1.282818507	0.00608243984780876
Upf2	0.732793441535342	0.00673367903119862
Fgd3	-1.138705752	0.00753347709909639
Calm2	-1.07660144	0.008643702527028
Znfx1	1.06634408593009	0.0097549636529224
Trim25	1.0261407403273	0.0111406753180964
Tgfb1	-0.936153072	0.0117244552469507
Creb5	1.26853838361506	0.0128732840704884
Tcf7	-1.909688466	0.0132448265181414
Hnrnpa1	-0.850523075	0.0142569332193199
Zfp706	-1.018849592	0.0155214327517359
Stmn1	-1.359815978	0.0163406308731666
Nap1l1	-0.875710896	0.0275139057350451
Cbx3	-0.961275767	0.0307585299547249
Hbegf	1.20792430693435	0.0342110429443081
Arpp19	-0.81193443	0.034814128455117
Dusp16	1.47228349378069	0.035059135663645
Birc2	1.19191082059587	0.0350989828042949
Syt16	1.38139077441534	0.0356989397991979
Fos	1.00579728599792	0.0362957571830911
Atp5d	-0.800120365	0.0380312865353208
Cxxc4	-1.000835284	0.040028540866107
Gng13	-2.163164541	0.0429617793962115
Romo1	-1.024794488	0.0450300810108142
Dstn	-0.680296849	0.0523336382646519
Cox7a2	-0.86250907	0.0549515825283002
Cox5a	-0.67801245	0.0591484522850004
Lrrc8a	0.94065802502133	0.0597552718008488
Smad1	2.67816731107216	0.0639377637436513
Fyttd1	0.8568229896066	0.0640226917257714
Atf3	0.884950913116272	0.0643403880905027
Pgls	-0.964583618	0.0648936257576386
Ndufb2	-1.052340814	0.0656229790459738
Ccl9	1.98724407109819	0.067878673452142
Sf3b1	-1.063911458	0.0736343516816238
Plk3	1.07515367667748	0.0738013806919731
Hipk2	1.18789265245844	0.0747968200013944
Nbn	0.743974151143219	0.078094976027567
H13	-0.924253894	0.0819872765913619
Il27ra	-0.784882028	0.0852674694792347
4932438A13Rik	1.14530060004614	0.0933376440513189
Klf6	1.25794131625905	0.0950970415214983
Uqcr10	-0.99209916	0.0977368389795187

Cdhr1	0.524427421680504	0.106308574776737
Dbi	-1.118331547	0.107318783455835
Ndufa13	-0.938704977	0.111177059676231
Ets2	1.14517101907644	0.113991594595418
Skp1	-0.851001238	0.115019714417378
Hmgb2	-1.106401252	0.117989080798913
Vim	-1.342135039	0.119561284373817
Itm2a	-0.775866846	0.119974096368047
Hspa4	-0.745242958	0.1227385619078
Eif3f	-0.775009456	0.122914453359476
Eif3d	-0.83337214	0.124677913987039
Hs6st1	1.36259998140844	0.124993464905513
AU040320	1.02934473673381	0.126172917662414
Snrk	0.965223966258315	0.126545924345346
Shroom3	1.2933815936111	0.127259424848598
Ccni	-0.967348717	0.127319664960694
Dennd3	1.24794625794747	0.129615149859777
Cd3e	-0.915646967	0.139926451058317
Ctnna1	0.988507294114212	0.140744835968026
Acad9	0.93773553375774	0.149877483287933
Ndufab1	-0.939026572	0.156588843032195
Cst3	-1.667063644	0.158726442950245
Snrpd3	-0.780664454	0.165367185533932
Atp5a1	-0.853933293	0.168374413440771
Sumo3	-0.956982718	0.168553724288831
Cct5	-0.996305159	0.169921938518439
Parp12	0.91898102048788	0.179063168578865
Snx10	1.00768249905644	0.180383019502263
Rab8b	-0.684664222	0.188140921975294
Vdac2	-0.804687169	0.19450344493944
Spag16	3.12024565726975	0.2048915381224
Dlgap2	1.54751791722251	0.211254613731385
Cdh4	1.89541735960671	0.224695380879684
Eif2ak2	0.695562266713978	0.243413437906221
Sap18	-0.681353265	0.244738834770908
Atp5g3	-0.671290406	0.244983058384552
Slc9a3r1	-1.006289077	0.248677138162786
P2rx7	0.994882156125908	0.255034483015043
Nsa2	-0.804468837	0.269982408784135
Hivep2	1.32420394811518	0.27026692525823
Tut4	0.794095713386246	0.288755159960691
Tec	1.3074689615903	0.314968366801675
Stat1	0.884229018780361	0.318408615121642
B2m	0.978782600362707	0.334850101075429
Obp2a	3.26044203660479	0.34056104480052
Ggt5	1.09547763070562	0.351815205576515

Eif3a	-0.871914961	0.356574300407305
Jpt1	-0.728140514	0.367087490593701
Arhgap15	-1.793356622	0.3700212390735
Helz2	0.563117613369938	0.371342811971125
Park7	-0.736961387	0.425978421758836
Smim20	-0.682523014	0.445843423351102
Hnrnpa2b1	-0.703325419	0.456682122372408
Ascc3	1.04345305754846	0.479207179544604
Ube2j1	0.740044348716545	0.490896157980335
Satb1	-1.058940625	0.516909396633628
Cdc26	-0.687164973	0.518970994362906
Was	-0.828139381	0.542422199630977
Slc20a2	0.962193290970401	0.568421685781665
Atp5pb	-0.547328867	0.579196506772793
Psma4	-0.688291759	0.601150436164554
Eny2	-0.628423188	0.622323101070288
Cops7a	-0.786134768	0.638564147541112
Churc1	-0.667477365	0.685994144204084
Tmsb4x	-0.673516382	0.703288804467529
Timm13	-0.887825519	0.711644110729649
Foxn3	0.784424094363951	0.733901445991437
Itgb6	1.19272931869626	0.743941644925458
Wbp4	-1.10248224	0.74422677662056
Rb1cc1	1.1111776400854	0.758935247439034
Uqcrfs1	-0.798822834	0.769884152825127
Csrnp1	1.08928518710291	0.80422307817809
Agpat5	-0.941423323	0.82215436680024
Plekhg1	0.744209002221017	0.85755109110337
Foxp1	1.13473108699629	0.876877986272434
Cmip	0.936929512060257	0.898899112428876
Epc1	0.680240405675082	0.935614870896278
Nfe2l2	0.839941622095734	0.973437072173061
Hsp90aa1	-1.70299819	0.988229185574461
Lrmp	1.86706247814461	1
Snrpd1	-0.930565838	1
Ypel2	-1.101378032	1
Dlec1	0.722173913107934	1
Ddx5	-0.611004674	1
Wbp1	-0.733587475	1
3425401B19Rik	0.839089605102297	1
Atp5c1	-0.623696345	1
Ctsc	1.37873890318188	1
Tmed2	-0.689766906	1
Sult1b1	0.486464551357898	1
Ndufa1	-0.765237436	1
Tomm7	-0.820615843	1

1600014C10Rik	1.05461376	1
Stat2	0.547844546539696	1
Gimap5	0.485968901068816	1
Slc25a29	0.412864116774119	1
Adk	-0.843710883	1
Lbr	-0.704490882	1
Fdps	-0.647301175	1
Angpt1	1.36536629898942	1
Stxbp6	1.08888666199681	1
Abhd14b	-0.750813265	1
Rab27a	-0.669731121	1
Dot1l	0.689277278688585	1
Anxa6	-0.665510065	1
Ssr2	-0.736812534	1
Tomm6	-0.895678153	1
Cmpk2	0.756525535474151	1
Tox	1.2586091495258	1
Camk1d	0.906428586146619	1
Kcng2	0.558547323939231	1
Snrnp25	-0.801948296	1
Stk17b	-0.600699153	1
Chd7	1.40911617256765	1
Eif4ebp1	-0.891383193	1
Crtc1	-0.683553269	1
Sppl2a	-0.694264897	1
Lpp	0.9435349410183	1
Krt18	-0.924992621	1
Ankrd28	1.29589077496564	1
Nos2	1.58305089784418	1
Tfam	-0.682189584	1
Samhd1	1.54983049858224	1
Bach1	1.12799900477302	1
Phf2	1.04996231222369	1
Eif3h	-0.707405599	1
Lamtor2	-0.77818015	1
Emp2	-0.387482336	1
Ankrd66	-0.753047236	1
Agl	-0.923806316	1
Hivep3	1.33631718876859	1
Dio2	1.82681908325764	1
Orc5	0.752736660156228	1
Nox4	1.35239729176706	1
Phf20l1	-0.928275182	1
Elf3	1.04362634836684	1
Acsl3	-0.690385557	1
Runx3	-0.830796569	1

Ndufb3	-0.724303466	1
Fmc1	-0.530746749	1
Eif2s3y	-0.768612551	1
Ppp1ca	-0.753777172	1
Hspd1	-0.746275699	1
Ikzf1	-0.994073897	1
Twf1	-0.873340274	1
Il6	0.906362784777454	1
B3gnt7	1.23902752350619	1
Ostc	-0.508667915	1
Diaph2	0.924839129976697	1
Ifi35	0.704139189963219	1
Csnk1a1	0.737880758562189	1
Rtf1	-0.792855195	1
Sf3b6	-0.729916878	1
Ssbp1	-0.529599317	1
Psmb1	-0.764554206	1
Fbxl17	0.563842031161005	1
Asl	0.295280805927098	1
Tshz2	-1.142069733	1
Lef1	-0.596455317	1
Dync1h1	-0.621848117	1
Ndufs5	-0.500055542	1
Ap3s1	-0.664165692	1
Aspg	0.639830675757966	1
Ndufb4	-0.60839328	1
Nop58	-0.53127415	1
B230219D22Rik	-0.721284431	1
Arsj	1.09292889935788	1
Plscr1	0.665490843217213	1
Far2	0.934654253410301	1
Lgals1	-0.589464965	1
Ppara	-0.796596581	1
Sfrp2	-1.003507552	1
Parp9	0.808071021812107	1
Stat4	-0.691328062	1
Fto	-0.769489178	1
Pard3b	0.969841037113466	1
Serpinb5	1.26931315618686	1
Afap1	-0.596612887	1
Cox7a2l	-0.542249842	1
Txndc17	-0.748758437	1
H2az1	-0.934762481	1
Nacc2	0.610936134939185	1
C1galt1c1	-0.395812864	1
Wdfy2	1.09235381713836	1

Tjp2	1.09933042106057	1
Npas2	1.99798807700055	1
Jak2	0.901401552989586	1
Dock2	-1.309058068	1
Sema3b	0.678863760222873	1
Canx	-0.72145507	1
G2e3	-0.8561984	1
Rnf24	0.566535130646449	1
Tmem14a	-0.513505169	1
Dnlz	-0.530871955	1
Hectd1	0.860294795685157	1
Serpine2	1.66399182813299	1
Dtna	0.763826375605922	1
Ndufb10	-0.775457689	1
Ndufv2	-0.665003796	1
Igfbp4	-0.560529024	1
Hnrnpab	-0.65953906	1
Ube2q1	-0.769672187	1
Akr7a5	-0.462380269	1
Rgs17	0.723948035623624	1
Rpn1	-0.689583883	1
Tnfaip3	0.861725127944808	1
Pdia4	-0.880898466	1
Grn	-1.073942382	1
Atp5j	-0.613134994	1
2510002D24Rik	-0.541115692	1
Ccdc6	-0.721637882	1
Fap	0.974937119945966	1
Tc2n	1.10165288223285	1
Pm20d2	0.799232992864363	1
Angptl2	-0.472372509	1
Cat	-0.647696018	1
Ufl1	-0.525178019	1
Galnt12	0.957094954746765	1
Atp5b	-0.602000709	1
Slc16a6	0.576015144146564	1
Vapb	-0.542874356	1
Tmbim6	-0.694776394	1
Pxk	0.782698016540592	1
Ptpn12	0.820813782732839	1
Kif1b	-0.642704813	1
Arpc1a	-0.543247603	1
Cbr1	-0.659680622	1
Ssr3	-0.48925704	1
Prpf38a	-0.447675278	1
Stk10	0.968368298364606	1

Cd82	-0.537143423	1
Emc3	-0.406180592	1
Trp53inp1	-0.785282139	1
Fasn	-0.738707495	1
Jund	0.665221962646188	1
Commd7	-0.486506496	1
Fmnl1	-0.449281529	1
Elf1	0.721194602785649	1
Ate1	-0.521985481	1
Ddt	-0.596378505	1
Zbtb16	0.84871834910997	1
Pnrc2	-0.785436196	1
Dnaja4	-1.227921592	1
Bri3	-0.826206184	1
Noc2l	-0.550973861	1
Sec61g	-0.693770649	1
Ptrh2	-0.623160858	1
Fnbp1l	0.731597564807162	1
Rbm38	-1.346497396	1
Spryd7	-0.567912378	1
Ssb	-0.572482203	1
Ints13	-0.686056468	1
Eml4	1.05748223039249	1
Tmem108	-0.878340864	1
Psmg4	-0.409326135	1
Sod1	-0.756786806	1
Uqcrq	-0.742409764	1
Copb2	-0.510742631	1
Rnf4	0.541385084359173	1
Pparg	0.879574557537089	1
Ndufb9	-0.51699647	1
Tnfrsf9	-0.43986991	1
Hdgfl3	-0.66310529	1
Cib1	-0.62838054	1
Hadhb	0.715828558717379	1
Ndufaf8	-0.581858674	1
Fmn1	0.648471413306014	1
Ezr	1.20424862198957	1
Wls	-0.525534531	1
Cstb	-0.548114907	1
Actr10	-0.422456135	1
G6pc2	0.436081224870223	1
Ptpn2	0.879255513918181	1
Itga4	-1.046350888	1
Atp5h	-0.780848684	1
Smc3	-0.511631262	1

Pls3	-0.603261122	1
Fdft1	-0.642261463	1
Atrnl1	-1.440724865	1
Dpf3	0.983753666689646	1
Arhgef18	0.599555398752908	1
Tubb2a	-0.668531959	1
Cxcr4	-0.618784961	1
Arid4b	0.739589153149099	1
Utp11	-0.484039187	1
Coasy	-0.405830157	1
Cdc42ep4	0.531546643714239	1
Srsf3	-0.855695685	1
Eif1	-0.667815166	1
Sema6d	1.12763017654205	1
Sox8	-1.036318819	1
Acly	-0.548884309	1
Prelid1	-0.484142486	1
Ctss	1.06911578390226	1
Zc3h12a	0.58772741624711	1
Dnajc3	-0.561764093	1
Slc38a6	-0.480622841	1
Terf2	-0.470639211	1
Spdl1	-0.673037592	1
Cox5b-ps	-0.568953633	1
Zfp516	0.91751214020382	1
Dolpp1	-0.286441143	1
Wac	0.658240568631022	1
Utrn	0.997304375895649	1
Ap1s3	0.63354947667133	1
Acat3	-0.545193232	1
Vti1a	0.838817359350799	1
Grap2	-0.627773527	1
Trib2	0.564827841391126	1
Anp32e	-0.546523597	1
Zfp644	0.708056655885399	1
Rmdn3	-0.610297831	1
2900026A02Rik	-0.846067841	1
Snrpg	-0.554281571	1
Lmna	-0.778399717	1
Syne1	0.898277042065611	1
Zfp207	-0.663517805	1
Traf3	0.819558812824673	1
Sec13	-0.436025293	1
Brd3	-0.712137986	1
Hmga2	0.69761997737805	1
Cope	-0.588598099	1

Tpm3-rs7	-0.789582603	1
Vav2	0.681701480838474	1
Gcc2	-0.776246692	1
Eif3b	-0.595595316	1
Zc3h15	-0.583553139	1
Acvr2a	0.975491205486651	1
Slc1a2	0.970747147208889	1
Gsn	-0.559658168	1
Eif3g	-0.607421762	1
Ubl5b	-0.605108488	1
Plekha5	1.36613000565515	1
Dusp23	-0.495332445	1
Actg1	-0.437847464	1
Id3	-0.819066845	1
Elmo2	-0.486864742	1
Socs3	1.12846993543085	1
Vps13d	-0.780254318	1
Psmd5	-0.307293259	1
Dad1	-0.706729439	1
Cpt1a	0.622291492979602	1
Clta	-0.495734849	1
Decr2	-0.593792903	1
Jarid2	-0.802447197	1
Hsbp1	-0.670975531	1
Galnt1	-0.722085557	1
Phf6	-0.405570724	1
Pcyt1a	0.794957180455445	1
Stard8	0.430900404761523	1
Pdhb	-0.521683352	1
Fndc3a	0.630847348398794	1
Ptprz1	0.780479473642249	1
Ldhb	-0.711271379	1
BC016579	1.45573315154806	1
Fgd4	0.903469354611699	1
Esd	-0.647558521	1
Cdo1	-0.687436157	1
Il7r	-0.45444332	1
Arhgap30	-0.477684353	1
Rel	0.840574669798306	1
Borcs5	0.575239467389977	1
Trabd2b	1.20997306643454	1
Ankrd6	0.435039597599965	1
Cd247	-0.803439707	1
Prpf4b	-0.707558454	1
Bzw1	-0.320129407	1
G3bp1	-0.510637157	1

Optn	0.655346393875419	1
Vgll4	0.796717379982081	1
Atp10b	0.97427845269374	1
Hnrnpu	-0.381700958	1
Rcan1	1.64207467988813	1
Nkiras2	-0.466443107	1
Nudt16l1	-0.618542237	1
Ptk2	0.861430062232129	1
Hipk3	-0.319535337	1
Samm50	-0.328533993	1
Lrba	0.559680522541265	1
Polr1d	-0.529465649	1
Dip2b	0.627245034372435	1
Hyi	-0.59050749	1
Serinc1	-0.688791294	1
Erh	-0.614072335	1
Dap	-0.7365848	1
Ctsz	-0.816266011	1
Ptprc	-1.016890387	1
Etfa	-0.572019366	1
Grcc10	-0.584443275	1
Lao1	-0.450992075	1
Thoc7	-0.570582023	1
Acot7	-0.355916599	1
Eif4g2	-0.523726924	1
Epas1	1.14606263987474	1
Grin2a	-1.052359713	1
Cstf3	0.711042497077683	1
Lap3	-0.353536047	1
Arhgap29	-0.743432536	1
Lamtor3	-0.579351713	1
Snrpf	-0.708576793	1
Garem1	0.785060563420239	1
Tex264	-0.365696119	1
Cc2d1b	0.787093276365013	1
Rbm31y	-0.583210319	1
Btf3	-0.500466593	1
Bcl11b	-0.68078018	1
Tmem138	-0.584198297	1
Eif3i	-0.468703326	1
Snx14	-0.667484188	1
Spns2	1.00318569898093	1
Rida	-0.633652973	1
Gtpbp4	-0.519120384	1
Gatd3a	-0.512037193	1
Laptm5	-0.616446466	1

Tbxas1	1.07346710309283	1
Syap1	-0.539849146	1
Dpyd	0.921549233118101	1
Acot10	1.03867831842624	1
Rab5if	-0.533551464	1
Rac2	-0.344531566	1
Pag1	-0.712678264	1
Smim8	-0.28111783	1
Pigyl	-0.39246042	1
Neil1	-0.342645031	1
Unc13d	-0.427946354	1
Top2a	-0.470023228	1
Bnip3l	-0.456452224	1
Setx	0.778865985402954	1
Dpagt1	-0.537508674	1
Etv4	0.340842674047092	1
Rad23a	-0.726567597	1
Atad2b	1.05227581810991	1
Arhgap11a	1.04023637320781	1
Cyb5a	-0.464275846	1
Luc7l3	-0.468915917	1
Smarca2	-0.370147851	1
Glis3	1.03327082340678	1
Dab2	1.27469904311709	1
Fkbp8	-0.475422651	1
Slc25a3	-0.615020809	1
Sap30l	-0.399413469	1
Ergic1	0.859356243501935	1
Aacs	-0.504721906	1
Pea15a	0.302743637589234	1
Exoc4	0.785957196448083	1
Lamp2	-0.630130822	1
Sgsm2	-0.535395786	1
Rbm43	0.483046774957137	1
Fam162a	-0.511882804	1
Denr	-0.496077032	1
Prmt8	0.412737334749187	1
Arnt2	0.32584603037832	1
Rfxap	-0.340897819	1
Lipa	0.651068152352628	1
Brms1l	-0.46604681	1
Stx2	-0.669508568	1
Slc22a3	-0.566936598	1
Kctd1	0.594344839646862	1
Tnik	-0.446447287	1
Tef	-0.562399239	1

BC048507	-0.557205814	1
Slc22a23	0.915592209682196	1
Trio	0.684129271238407	1
Gpx1	-0.592257545	1
Rnf19a	0.508563131748794	1
Slco4c1	0.854928791593022	1
Hnrnpul1	-0.605618462	1
D630045J12Rik	0.631468751082398	1
Traf5	-0.52873586	1
Slc25a24	0.484309431660736	1
Itgb1bp1	-0.38026176	1
Lman2	-0.483878009	1
Fkbp3	-0.517434438	1
Eif1ad12	-0.361475938	1
Coq7	-0.341842484	1
Hspe1-rs1	-0.539307177	1
Creg1	-0.486804891	1
Tst	-0.663725658	1
Stard3nl	0.377799665574944	1
Ndufa12	-0.381483564	1
Srp72	-0.581052529	1
Serp1	-0.553403916	1
Snrpc	-0.373623711	1
Gsap	0.716119584263991	1
Rilpl2	-0.388649234	1
Ndufb8	-0.338990303	1
Hccs	-0.517352718	1
Sox6	0.937223089720885	1
Ptpn11	-0.603104564	1
Pmf1	-0.471126062	1
Adgrg2	1.08843213750016	1
Hexim1	-0.656363533	1
Klf11	0.372936210020976	1
Ecpas	0.715165445278565	1
Setbp1	1.11780191409363	1
Lsm14a	-0.639138491	1
Pwwp2a	-0.397125058	1
Cpeb4	1.09148562158488	1
Ndufaf1	-0.461648774	1
Hnrnph2	-0.400702047	1
Srpk2	-0.498245061	1
Idh2	-0.480047787	1
Hspa9	-0.620708507	1
Acadl	-0.587211527	1
Igfbp7	-0.889232717	1
Kat7	-0.484125871	1

Ezh2	-0.667456268	1
Sparc	-0.355827522	1
Gnai1	-0.75179213	1
Kdm4c	1.05005014933003	1
Ccdc47	-0.65213655	1
1700025G04Rik	1.40351766888001	1
Camk2b	0.0940511587221504	1
Vps35l	-0.51492675	1
Slirp	-0.555971902	1
Sel1l3	-0.613211287	1
Polr2b	-0.556273131	1
Ccdc85c	1.07458454214271	1
Atp2a2	0.914232391749154	1
Shroom2	0.845752478068695	1
Akap1	-0.351570912	1
Dnaaf4	-0.3750008	1
Etfrf1	-0.118853093	1
Bhlhe41	-0.59624599	1
Shisa2	-0.375593001	1
Inip	-0.391724971	1
Skap1	-0.431450145	1
Eif2d	-0.368003391	1
Lrmda	1.05146590282513	1
Bms1	-0.753168059	1
Tpp1	-0.576093418	1
Sumo1	-0.3899444	1
Atp6ap2	-0.418315589	1
Aco2	-0.487941513	1
Ccdc28a	-0.393709523	1
Bcl11a	-0.580700525	1
Btg2	0.801069292330772	1
Dclk3	0.603905002463162	1
Rbbp4	-0.628171612	1
Palb2	0.32684224183521	1
Impa1	-0.4187361	1
Bbs9	1.27460211565772	1
Gkap1	0.490371258914781	1
Rcor3	-0.328321226	1
Acot11	0.558314357786962	1
Dnajb1	-0.576092768	1
Rffl	0.360816026702551	1
Trmt10c	-0.27620028	1
Hvcn1	-0.710812742	1
Tcp1	-0.550620602	1
Tpi1	-0.647871093	1
Slc2a3	-0.559405452	1

Smad6	-0.608922053	1
Cacybp	-0.402430199	1
Gltp	-0.432966475	1
Dalrd3	0.435168546851753	1
Crls1	-0.421698635	1
Ap2b1	-0.326568211	1
Psmd2	-0.579784906	1
Nln	0.224157753866233	1
Cct7	-0.508097232	1
Srsf11	-0.573603743	1
Efna5	-0.800839676	1
Ndufa5	-0.427316865	1
Rhpn1	0.402923317613918	1
Taf3	0.800911756150787	1
Nbea	1.07285170793985	1
Sla	-1.015893018	1
Rab8a	-0.258891398	1
Ptafr	0.413153096851245	1
Fam76b	-0.420381115	1
Cyth3	-0.482482532	1
Cuedc1	0.574222142047569	1
Bbs4	-0.646493522	1
Jmy	-0.448015419	1
Ptprj	0.725461099611937	1
Gata6	1.30322638370752	1
Klf13	0.687248269776511	1
Xrn1	0.408940883498337	1
S100a6	-0.427620016	1
Sec63	-0.553127448	1
Ank2	-0.744766505	1
Asf1a	-0.333056284	1
Lasp1	-0.23436461	1
Rad21	-0.357505963	1
Cobl	0.697837353203554	1
Hnrnpll	-0.486252607	1
Dynlrb1	-0.387243445	1
Sh3bgrl3	-0.535494073	1
Gstm4	-0.57823138	1
Hsp90b1	-0.478442012	1
Nars	-0.463545008	1
Bcap29	0.923815262410664	1
Eif3k	0.719020942225744	1
Unc79	-0.229433771	1
Cct2	-0.514226344	1
Ndufb7	-0.621259764	1
Foxo1	-1.078015232	1

Rngtt	0.652424634046118	1
Inpp4b	0.913155035570369	1
Prrg3	-0.370576966	1
Hsd17b4	-0.669791484	1
Sirt1	-0.341907183	1
Rbmxl1	-0.495310923	1
Vtn	0.316548491096771	1
Sgms2	0.804170953542598	1
Timm10	-0.427837848	1
Hsp90ab1	-0.625288509	1
Ssbp2	0.82781437709586	1
Vwc2l	0.606294539271024	1
Top2b	-0.246556483	1
Sh3bgrl	-0.693024915	1
Cdv3	-0.513267895	1
Hecw2	0.728134455217671	1
Sgms1	0.756443229632836	1
Nsun3	0.367004289615913	1
Rabif	-0.323774943	1
Gstk1	-0.563019157	1
Czib	-0.317860487	1
Tspan3	-0.586744314	1
Samd4	0.645836796766732	1
Tmem97	-0.411026914	1
Tnfsf15	0.42050806097103	1
Insr	0.693017628402457	1
Uqcrb	-0.224393709	1
Tnfsf8	-0.500339567	1
Ptpn22	-0.474768387	1
Rbms3	0.576647326392935	1
Cltb	-0.383841902	1
Ccno	-0.888230354	1
Vapa	-0.511658789	1
Cth	-0.346011976	1
Ak3	-0.64459331	1
Ror1	-0.390532071	1
Epb41l4a	-0.601805318	1
Arf6	-0.442473852	1
Pla2g4a	0.80062538991799	1
Pgap2	-0.474787489	1
Fam219a	0.326862849577375	1
Fbxo9	-0.424175119	1
B4galt2	-0.422702655	1
Pak1ip1	-0.268552889	1
Pdap1	-0.407285783	1
Bcl6	0.507405009006788	1

Abcf2	-0.432095259	1
Ndufs3	-0.347588623	1
Sh3bgrl2	-0.335380113	1
Cdkn1b	-0.648654724	1
Tmem258	-0.604727817	1
Erlin1	-0.581109338	1
Ppat	-0.540983235	1
Pdlim5	0.693128951929866	1
Rpia	-0.384919966	1
Pcnx	0.737438935046085	1
Pds5b	-0.474565077	1
Kyat3	-0.446668308	1
Ndufs1	-0.366991403	1
Twsg1	-0.4111654	1
Surf4	-0.380783485	1
Sgf29	-0.359457925	1
Frrs1	-0.466091472	1
Vnn3	0.763712879868739	1
Nat10	-0.340381948	1
Cytip	-0.3707566	1
Sra1	-0.429045281	1
Tia1	0.580897448967793	1
Smg7	-0.474702826	1
Scp2	-0.477167242	1
Atp6v1g1	-0.572423374	1
Gstm5	-0.440404869	1
Necap1	-0.614167337	1
Cacnb4	-0.281943399	1
Ankrd44	-0.51766169	1
Rfx3	1.91014764511273	1
4930579G24Rik	-0.187797356	1
Ros1	1.34578198011039	1
Xpo7	-0.568170077	1
Lamtor5	-0.56039321	1
Srebf2	-0.567764156	1
Neurl1a	1.46818301455327	1
Aga	-0.319416529	1
Sbds	-0.500422358	1
Utp18	-0.381743307	1
Ube2g2	-0.558043566	1
Cyth1	-0.371784427	1
Yeats2	-0.486197865	1
Hk1	-0.516986538	1
Yaf2	-0.489454199	1
Egr1	0.389906217157756	1
Slbp	0.510666207193689	1

Dcun1d2	0.458917228249259	1
Tenm3	-0.515474565	1
Camk2d	0.968327920672078	1
Ndc1	-0.331494293	1
Fkbp4	-0.534752894	1
Gnptab	-0.554997784	1
Ptgs2	1.64107151842652	1
Flad1	-0.396740944	1
Rlf	-0.235539616	1
Nenf	-0.360438034	1
Htra1	-0.34838207	1
Upf3b	-0.438801038	1
Ybx3	-0.338129962	1
Sos1	-0.551385575	1
Psma5	-0.562480959	1
Cct8	-0.482871146	1
Copg1	-0.388165905	1
Ireb2	-0.895837996	1
Taz	-0.39454326	1
Ndufv3	-0.530531024	1
Atg101	-0.387941424	1
Atp6v0e	-0.413052417	1
Zdhhc20	0.718910964049093	1
Ubald1	0.0868258359950062	1
Psip1	-0.499354052	1
Celf2	1.73109083487914	1
Otub1	-0.625390638	1
Bahcc1	-0.49755588	1
Srsf7	-0.45752484	1
Fam168b	-0.560705704	1
Coa5	-0.275414614	1
Pus1	-0.278709592	1
Ext1	-0.646983247	1
Chka	1.00058483239202	1
Plek2	1.04863507950407	1
Ttll12	-0.460778435	1
Irf2	0.592981681745704	1
Hk2	0.58157890012646	1
Gtf2h5	-0.432588533	1
Serpinf2	-0.649591604	1
Ptgfrn	0.26941025844698	1
Asrgl1	-0.326550598	1
Aebp2	-0.547097776	1
Cmc4	-0.469001556	1
Eif5	-0.456155913	1
Nsfl1c	-0.399912385	1

Osbpl5	1.12610982916126	1
Sh3pxd2a	-0.309582978	1
Cebpzos	-0.397199265	1
Naca	-0.403470753	1
Cpeb2	1.01570012534204	1
Polr2l	-0.468340004	1
Phax	-0.17454605	1
Khdrbs1	-0.414455421	1
Uqcrc2	-0.36176934	1
Rnf103	0.448338805743731	1
Tmc5	0.624232163979914	1
Ogfod3	0.309673672305966	1
Ncor2	0.44088698904436	1
1110004F10Rik	-0.308061602	1
Fam177a	-0.43790374	1
Tbl1xr1	0.775145497393256	1
Erap1	0.619296057252887	1
Casp8ap2	-0.389720869	1
Ifi30	-0.342939527	1
Ncoa7	0.729490136708937	1
Xpr1	0.612603312600169	1
Slc29a4	-0.249319092	1
Fam118b	0.242918489098489	1
Atxn3	-0.306362356	1
Ly75	1.0738210557539	1
Rnf130	-0.462621058	1
Hibch	-0.365472194	1
Cap1	-0.239326302	1
Bcas3	-0.319213194	1
BC048403	-0.137502461	1
Dhcr24	-0.556362735	1
Wnt11	-0.288491268	1
Tubb4b	0.419115481745958	1
Btg1b	-0.246250785	1
Acaca	-0.781256141	1
Arhgap26	0.696819142336206	1
Ccnb2	-0.55795236	1
Shisa5	0.361834765232851	1
Usp45	-0.739368473	1
Fbxo22	-0.476260216	1
Commd3	-0.347730435	1
Nsrp1	-0.546674117	1
Iqsec1	1.27037889142851	1
Gipc2	-0.42095935	1
Soat1	0.642928985891547	1
Spty2d1	-0.607442255	1

Idh3a	-0.455393309	1
Hps3	-0.613200712	1
Zfp423	-0.457495617	1
Antkmt	-0.513371435	1
Rnf41	-0.332649721	1
Ppp1cc	-0.394120512	1
Gnl3	-0.480385305	1
Cct6b	-0.414725003	1
Ctdspl2	-0.471231259	1
Gpatch2	0.540512850814853	1
Evl	-0.598858636	1
Ndufs8	-0.383377246	1
Rmdn2	0.251860446235818	1
Btf3l4	-0.463626319	1
Gnal	0.799054137171216	1
Vmp1	0.912758800945805	1
Acbd3	-0.297333917	1
Ankrd13a	-0.509707496	1
Nf2	-0.302052201	1
Gdpd5	0.740452034404218	1
Hint1	-0.305306004	1
Tspan1	-0.472259948	1
Cdkal1	0.650194149477751	1
Irf7	0.623022567452871	1
Dhx37	-0.453724814	1
Capn8	-0.312700078	1
Pak2	-0.519371698	1
Cnih1	-0.328484184	1
Nfil3	0.633574396269507	1
Ppcs	-0.391426297	1
Lrrn4	-0.488392285	1
Arhgef7	0.576094943960531	1
Tiam2	0.706759512899125	1
Tgm4	0.492893569620877	1
Nop56	-0.513460906	1
Dock3	1.0892293024815	1
Ciao1	-0.295390142	1
Sccpdh	-0.441698865	1
C1s1	0.39706494915031	1
Rasgrf2	1.38703863883998	1
Arhgdia	-0.474447256	1
Angel1	-0.439761821	1
Shank2	-0.465963688	1
Plekhm3	-0.714922041	1
Glul	0.650611034461575	1
Ints6l	0.544846007968612	1

Pdcl3	-0.391742305	1
Arl3	-0.179413586	1
Nfia	0.674251244868222	1
Prdx3	-0.524659625	1
Igf1r	0.581543327305028	1
Flvcr2	0.975468781214865	1
Ppp1r12a	0.735024917470208	1
Rnmt	-0.353932307	1
Cirbp	-0.546333246	1
Polr2e	-0.467213505	1
Parp8	0.778504328429304	1
Cyfip1	0.871917530969813	1
Camk2g	-0.415723767	1
Pxdn	0.668793039056181	1
Crmp1	-0.504030977	1
Zfand6	0.606869454330453	1
Rchy1	-0.379645947	1
Exoc2	0.666581274372459	1
Synj2	1.44272185785327	1
Fbxl3	-0.330743967	1
Ccdc90b	-0.288764855	1
Cotl1	-0.333130122	1
Slc2a13	-0.386088124	1
Nup85	-0.555876209	1
Ppil4	-0.503203776	1
Sars	-0.226967736	1
Nmral1	-0.419134507	1
Syf2	-0.313362827	1
Pla2r1	0.515381598036332	1
Klhdc2	-0.392536567	1
Hypk	-0.505210291	1
Atic	-0.474295958	1
Lgi3	-0.881922637	1
Baz2b	0.544441952334297	1
Dazap1	-0.266584633	1
Gtpbp1	-0.320562853	1
Vkorc1l1	-0.226495751	1
Hook2	-0.296714328	1
Bmpr2	-0.274301766	1
Pde4d	1.08660175522356	1
Pank1	-0.27076427	1
Atp6v1c1	-0.444411387	1
Sdhb	-0.458716873	1
C1qbp	-0.393457521	1
Akr1e1	-0.454278121	1
Kalrn	0.763827036922326	1

Osbpl8	-0.196962191	1
Selenot	-0.466243976	1
Eif6	-0.368531919	1
Prpf6	-0.562069288	1
Cep83	-0.362497494	1
Pop5	-0.205391891	1
Acsf2	-0.265259992	1
Gls	0.857674544778886	1
Ttc3	0.533099455644285	1
Gpalpp1	-0.588979749	1
Irs4	0.577557797828523	1
Sigirr	-0.271382903	1
Gng4	-0.331715587	1
Trim62	-0.279681177	1
Dhx36	-0.479596302	1
Samsn1	-0.598059146	1
Sh3bp5	0.705297243113456	1
Rnf220	-0.545946457	1
Ndufa6	-0.463132649	1
Xpo1	-0.65956947	1
Polr1f	-0.324866393	1
Tox2	-0.356283626	1
Slc25a5	-0.309217163	1
Nfu1	-0.257625796	1
St8sia1	-0.422595803	1
Sdhd	-0.468883962	1
Atp6v0a1	0.550071604449242	1
Ttc37	-0.485313334	1
Ibtk	-0.298210813	1
Kcnt2	-0.988943345	1
Sap30	-0.451034734	1
Naf1	-0.32324406	1
Fam114a2	-0.342934883	1
Coa7	-0.26625103	1
Cd276	-0.302636647	1
Zmat2	-0.173357785	1
Lpin1	0.940831196373842	1
Taf13	-0.233990654	1
Bcat1	-0.72910027	1
Tnip3	1.45468185108126	1
Pfdn4	-0.555839482	1
Il20ra	-0.749902823	1
Kcnj16	0.526633698669524	1
Tmem69	-0.261688473	1
Ndfip1	-0.55048576	1
Impdh2	-0.291159205	1

Snx30	-0.447730027	1
Rbm14	-0.495023749	1
Syt14	-0.295323707	1
Arl14ep	-0.282601434	1
Syne2	0.837960136489432	1
Smc6	0.459379965540917	1
Fxyd6	-0.619393009	1
Txndc5	-0.188728765	1
Hdac4	0.704504206121363	1
Prkar1a	-0.532547963	1
Fabp3	-1.076897117	1
Cpne4	-0.485507171	1
B3galnt2	-0.180669036	1
Ncapd2	-0.310967338	1
Thop1	-0.261632769	1
Trps1	-0.785923073	1
Saraf	0.920265344367431	1
Sdc1	0.895723010123087	1
Dtd2	-0.455119707	1
Zadh2	-0.390021897	1
Rasa3	1.60848567988367	1
F13a1	0.495694548425262	1
Lamp3	-1.215613154	1
Krt19	-0.230954155	1
Prpf19	-0.40678729	1
Htra2	-0.32669777	1
Alox5ap	-1.185198774	1
Gab1	0.726911009313354	1
Psmb2	-0.347025104	1
Asap1	-0.357694274	1
Hsd17b10	-0.318840039	1
Virma	-0.340684932	1
Hadha	0.547688527803528	1
Ccnh	-0.389794479	1
Pten	-0.4900041	1
Pcnt	-0.480258804	1
Itprid2	1.41453326670303	1
Cfd	-0.430014633	1
Tiparp	0.621099106459236	1
2210016F16Rik	-0.318364942	1
Anapc15-ps	-0.329892383	1
Uqcr11	-0.525980469	1
Scin	-0.501410558	1
Otud4	0.521798191310163	1
Znhit3	-0.292089544	1
Cyp51	-0.500460227	1

Tmem63b	-0.598661613	1
Tctn3	-0.514951389	1
Cep170	-0.289221078	1
Iws1	-0.364565909	1
Fat1	-0.529681219	1
Prkcd	0.563089776916469	1
Slco3a1	0.773174964276706	1
Uchl5	-0.661442119	1
Lhfpl2	1.30035265897285	1
Zdhhc9	-0.258068447	1
Dapk2	0.315280575421433	1
Smc4	-0.324602639	1
Snrpb	-0.380205312	1
Cers6	0.551360590676766	1
Bbip1	-0.298578726	1
Slc35b3	-0.297359284	1
Amn	-0.462452434	1
Plxna2	0.148753003017031	1
Plxdc2	1.2264698925247	1
Robo2	0.812554350008897	1
Smarce1	-0.521673577	1
Cadm1	0.985980636180149	1
Dnajc18	-0.295841277	1
Ctr9	-0.348902327	1
Kdm5b	-0.492069508	1
Scel	-0.590955922	1
Reln	-0.911674547	1
Adi1	-0.331842907	1
Thumpd2	-0.452891407	1
Rab5c	-0.468065419	1
Chn1	-0.821110245	1
Actb	-0.541300313	1
Nfic	0.384359521372168	1
Tex10	-0.368824164	1
Nr4a3	0.750945557062942	1
Nptn	-0.459903815	1
Igf2bp3	0.672462977553866	1
Auts2	-0.277944085	1
Apbb1ip	-0.410194962	1
Use1	-0.257954219	1
Frk	0.150596631204248	1
Gpx4	-0.413149479	1
9530068E07Rik	-0.386670933	1
Fus	0.391641589300092	1
Rtcb	-0.23036378	1
Khdrbs3	-0.446049782	1

Wipf1	-0.387776302	1
Usp54	0.749767962991382	1
Rnf216	0.223501591813534	1
Cnn3	0.636563854318073	1
Naa40	-0.562577267	1
Smad5	-0.767449388	1
Kif3a	-0.47616925	1
Edf1	-0.382224818	1
Rprd1b	-0.554996822	1
Nop16	-0.260573978	1
Lin9	-0.44201728	1
Sfxn1	-0.376468787	1
Ebna1bp2	-0.411094034	1
Sec22b	-0.343036323	1
Bend3	-0.307108997	1
Zfyve9	-0.221757335	1
Sephs1	-0.441362709	1
Csnk2a2	-0.448285857	1
Dcaf12	-0.358824439	1
Stau1	-0.388279169	1
Zfp511	-0.382898552	1
Ulbp1	0.447455936572418	1
Sema3c	1.23548864399175	1
Timm44	-0.399141203	1
Ep400	-0.385601996	1
Elovl7	-0.291693868	1
Ctsh	-0.356699062	1
Plbd1	-0.577909458	1
Npepl1	-0.222331211	1
Acadsb	-0.242214398	1
Dtx3l	0.301178903994147	1
Rala	-0.274001003	1
Ccdc174	-0.333956741	1
Dhdds	-0.205100123	1
Akt1	0.260949221673659	1
Tmem220	-0.258889601	1
Nelfb	-0.257143804	1
Fhl4	-0.512560244	1
Tasor2	0.351014862064058	1
Wwox	-0.028789615	1
Slc39a8	0.378876037571546	1
Strn3	-0.297230512	1
Srsf1	-0.463982553	1
Tmed7	-0.352609981	1
Dll1	-0.727977188	1
Eif4ebp2	-0.340521007	1

Tbk1	-0.345593792	1
Hmgb3	-0.288937969	1
Pdcd10	0.329768005199221	1
Dock10	-0.650736629	1
Cdc37	-0.325502551	1
Tbc1d4	-0.475167777	1
Papola	-0.225008079	1
Vamp3	-0.335240847	1
Tmpo	-0.549633073	1
Abtb1	-0.186129495	1
Sos2	0.596024577027074	1
Rrs1	-0.339903287	1
Kmt5b	-0.48244856	1
Qdpr	-0.146506284	1
Slc35c2	-0.361756968	1
Wdr36	-0.217304809	1
Sun1	-0.442071168	1
Calm1	-0.512394379	1
Grpel1	-0.27538912	1
Gxylt2	-0.376970841	1
Rsf1	-0.514045246	1
P4hb	-0.514293599	1
Adsl	-0.36919322	1
Oard1	-0.304332253	1
Zfp326	-0.23351687	1
Eloc	-0.380426292	1
Pcnp	-0.616291184	1
Larp1	-0.412932927	1
Enpp2	0.806336685022666	1
Nr2f2	-0.483762094	1
Snx3	-0.320255866	1
Irf8	0.405001210870941	1
Abca2	-0.287652082	1
Rbx1	-0.435384669	1
Pfn2	-0.502749174	1
Lonrf1	0.432908016327518	1
Rab3gap1	-0.332746333	1
Psma6	-0.512088393	1
Irak2	-0.362366404	1
Usp15	0.54014661283553	1
Ano5	0.497261499611019	1
Pcca	0.766809671939721	1
Gadd45a	-0.588785743	1
Ppp4r3a	-0.405677596	1
Dhx38	-0.207420673	1
Slu7	-0.523676333	1

Rsrp1	-0.719449024	1
Ddx19a	-0.305725297	1
Vps33b	-0.295652581	1
Hsph1	-0.396682325	1
Dmxl1	0.517337267095608	1
Rasgef1b	1.00281322246651	1
Fubp1	-0.412197006	1
Zfp512	-0.257676003	1
Capza2	-0.446480777	1
E2f5	0.238827398413704	1
P4ha1	0.50260621555091	1
Yap1	0.614686042356175	1
Cd28	-0.418101161	1
Tcim	0.561910420535919	1
Tekt1	0.141210545769511	1
Ruvbl2	-0.375810124	1
Fam185a	0.268813781152495	1
Six1	-0.629481509	1
Gpat3	0.499600878458743	1
Bnip3	-0.175364757	1
Rpap3	-0.351437117	1
Sec61a1	-0.599973068	1
Psmb4	-0.248684118	1
Hectd4	-0.389381668	1
S100a11	-0.737575268	1
Tlr2	0.19173338192801	1
Gpatch11	-0.513565382	1
Lcp2	-0.398206003	1
Psmg3	-0.330560347	1
Ndfip2	0.587296221333143	1
C1qtnf12	-0.223100371	1
Cfap20	-0.480859236	1
Slc16a10	-0.375005127	1
Limd1	-0.254436666	1
Dtwd1	-0.292077153	1
Fhod1	0.496309066851165	1
Tuba1b	-0.668694629	1
Parg	-0.228648828	1
Hyou1	-0.25578497	1
Ago2	0.634340689839291	1
C4b	-0.229327513	1
Ywhaq	-0.281950684	1
Tspan9	0.633821541117997	1
Card11	-0.155777103	1
Cd63	-0.56102373	1
Foxk2	0.453160600437776	1

Aplp2	-0.620347367	1
Cdcp1	0.441915360030353	1
Wasl	-0.266654171	1
Nono	-0.447295926	1
Ankrd50	0.87796299981177	1
Sil1	-0.202245022	1
Pink1	-0.302962216	1
Arpc5	-0.38089357	1
Gnpat	-0.410998567	1
Sel1l	-0.458104391	1
Tmem41b	0.310246960198687	1
Cacng2	0.234558096623123	1
Relch	-0.552595214	1
Ppig	-0.076091741	1
Osbpl1a	-0.424320048	1
Batf3	0.426311741472239	1
Pdcd4	-0.501643605	1
Chl1	0.224715288661278	1
Sart3	0.291534689731169	1
Coa6	-0.337970323	1
Acer3	0.372078744880109	1
Apool	-0.375608334	1
Ece1	-0.238352992	1
Ndufa10	-0.450170018	1
Kbtbd4	0.256433913561811	1
Ank3	0.972127452602976	1
Rwdd3	-0.376217399	1
Cyba	-0.347886014	1
Chordc1	-0.283465505	1
Ppp2r2d	-0.344657138	1
Atp2b4	-0.294936973	1
Tspo	0.725884702457139	1
Tcerg1l	0.738960696371835	1
Nubp1	-0.298095867	1
Aldh1a1	-0.623938357	1
Ddx1	-0.201669116	1
Tpd52	0.668163428006672	1
Tcaim	-0.403287539	1
Glt1d1	-0.266558587	1
Raf1	0.358752739319263	1
Atg4c	-0.360197025	1
Prkab1	-0.325580343	1
Tmx3	-0.350074017	1
Dek	-0.478173503	1
Fam174a	-0.270466214	1
Ipmk	-0.286969514	1

Ntmt1	-0.303859442	1
Cdh15	-0.240071201	1
Adh5	-0.398697665	1
Tmem241	-0.185720789	1
Washc2	-0.343849775	1
Bend5	-0.49284623	1
BC005624	-0.394805048	1
Rnaseh1	-0.527055302	1
Rad18	-0.367407189	1
Dnajc2	-0.435067253	1
Ralbp1	-0.398143036	1
Eif4g3	0.353306814460691	1
Ube2a	-0.470068982	1
Rasd1	0.898437769572268	1
Sf3a3	-0.266411168	1
Ifnar2	0.378653205684592	1
Sertad2	-0.334697755	1
Rims2	-0.60780758	1
Ppp4r4	0.474619919867179	1
Sbno2	0.397813880752924	1
Chid1	0.256502930711351	1
Dscam	0.844159541955738	1
Snw1	-0.384679955	1
Clcn5	-0.305570732	1
Sf3a1	-0.379368796	1
Tet2	0.62423866215683	1
Crlf1	-0.310883752	1
Ubac2	0.236105024282263	1
Lysmd2	-0.271662226	1
Def6	-0.251088675	1
Rcbtb1	-0.475640156	1
Rnf114	-0.437556763	1
Fbxo7	-0.325193816	1
Idh1	-0.308400941	1
Psmc6	-0.362225295	1
Tesc	-0.386807013	1
Rab39b	-0.272835595	1
Tapbp	0.501153752239045	1
Zfp438	0.510321478967166	1
Hnrnpa3	-0.318232586	1
Slc12a2	-0.430630188	1
Dnajc5	-0.382743576	1
Cpne3	-0.376055294	1
Zfp821	-0.278582834	1
Cep85l	0.51512000827351	1
Kat6a	-0.450114088	1

Gab2	0.408127918182303	1
Flnb	1.25474582364546	1
Sptssa	-0.250942293	1
Pno1	-0.234575046	1
Pcbd2	-0.220412375	1
Txndc9	-0.292331549	1
Ikzf5	-0.43272373	1
Pitpnb	-0.178018371	1
N6amt1	-0.179529441	1
Cbx1	-0.306328287	1
Reep5	-0.249864305	1
Cstf1	-0.230341127	1
Dkk3	-0.46801626	1
Tmem106b	-0.295676872	1
Plin2	0.944740876638482	1
Dennd4a	0.543165863778335	1
Abi2	-0.451634487	1
Smim7	-0.291627202	1
Ptk7	-0.329221238	1
Lyg2	1.42277474766781	1
Sik1	0.416811853433085	1
Fbxo25	-0.450656878	1
Zfp292	-0.605825054	1
Prkag1	-0.311076651	1
Tjp1	1.42956888753933	1
Kifap3	-0.432318248	1
Chtf18	-0.514299342	1
Atrx	-0.339765296	1
Tsnax	-0.292932597	1
Ip6k2	0.414308469955549	1
Leo1	-0.479314643	1
Rbl2	-0.262357539	1
Rassf2	-0.300348626	1
Add3	-0.274594081	1
Hras	-0.475182956	1
Prdx4	-0.524029496	1
Trib1	0.292370865263128	1
Tmem161b	-0.15990449	1
Grik4	-0.441036827	1
Zfp131	0.409551772612824	1
Akap8l	-0.373091848	1
Sesn3	-0.479136876	1
Lin7a	-0.582930245	1
Pank3	-0.38682506	1
Ccp110	-0.596518777	1
Itsn1	-0.081916262	1

Lrrc42	-0.302212436	1
Insig1	-0.521813786	1
Srsf4	-0.390251448	1
Acox1	0.646370141931943	1
C3	0.213607281248635	1
Ost4	-0.251489052	1
Gcsh	-0.491051271	1
Tenm2	0.565644800141049	1
Fh1	-0.378045444	1
Cops2	-0.456765094	1
Yipf1	-0.397346993	1
Sh2d4b	-0.130981551	1
Pdk1	-0.400827885	1
Exosc7	-0.252066983	1
Napg	-0.304688842	1
Rabggtb	-0.352688558	1
Tmem43	-0.29812321	1
Cped1	-0.359953862	1
Rdx	-0.344062929	1
Parn	-0.310161239	1
Vrk2	0.330355392503955	1
Tpr	-0.586500438	1
Eif1b	-0.311051616	1
Lig3	-0.136411865	1
Atxn7l1	0.599616569796208	1
Ap3m1	-0.213885895	1
Actr1a	-0.342305202	1
Gsta4	-0.406204646	1
Pxn	0.269453171281562	1
Rpn2	-0.250816215	1
Ttc26	-0.412926956	1
Ndufs7	-0.430210839	1
Lrch2	-0.259936664	1
Hikeshi	-0.378595161	1
Gtf3a	-0.236468295	1
Pfdn6	-0.29799598	1
Rtf2	-0.379349919	1
Pik3r5	-0.399815191	1
Gramd1c	0.629399854601319	1
Oprm1	-0.080762724	1
Ing1	-0.538498967	1
Pptc7	0.544401864724415	1
Ppp1r1c	1.24059245229844	1
Tmem183a	-0.353571741	1
Tcf7l2	0.764309572675631	1
Bdnf	-0.563666705	1

Sod3	0.825122151720672	1
Rexo2	-0.35058038	1
Srrm1	-0.434937059	1
Tfdp2	-0.312211549	1
Sipa1l2	0.867305203086697	1
Kpna4	-0.369185867	1
Tuft1	0.58903342526257	1
Cish	-0.298217653	1
Fbxo39	-0.308850016	1
Ptgr1	0.389027415518631	1
Pex6	-0.205486867	1
Dctn6	-0.252893208	1
Wdr74	-0.06893554	1
Aig1	0.61033625620081	1
Sem1	-0.239591665	1
Esf1	-0.267586842	1
Dpy30	-0.435061991	1
Arhgap8	0.333615197278987	1
Hnrnph3	-0.430789115	1
Dgkz	-0.442619006	1
Nat8f7	-0.164235844	1
Fosl2	0.532649439494728	1
Tle5	-0.438501423	1
Zcrb1	-0.216963343	1
Tmem131l	-0.446623845	1
Phkg1	-0.306147525	1
Jup	-0.303664413	1
Nav2	0.72887906328807	1
Tmem175	-0.225471661	1
Gins3	-0.233521775	1
Arpc4	-0.48460519	1
Cct4	-0.448786172	1
Ppp1r7	-0.363497276	1
Ralyl	1.18259039726283	1
Cherp	-0.290480683	1
Tbc1d8	0.455502286113003	1
Tmed5	-0.34742713	1
Thoc2	0.221398353841835	1
Anapc2	-0.332278071	1
Ace2	-0.30429683	1
Itga3	-0.246510295	1
Tubb6	0.516309901709442	1
Hsf3	-0.320446628	1
Gnas	0.253467449613235	1
Tmem192	-0.228898069	1
Zfand5	0.427765000831872	1

Exoc6b	0.403368336458118	1
Fbxo34	0.355826070783047	1
Tle4	0.667301455645218	1
Armc8	-0.340320543	1
Atp8a1	0.488973962354343	1
Polr2i	-0.318275798	1
Ash2l	-0.271779509	1
Ift52	-0.308540451	1
Tprgl	-0.207932198	1
Ing4	-0.445532434	1
Usp22	0.331302289044585	1
Timmdc1	-0.333204628	1
Ddrgk1	-0.442989274	1
Ehf	0.531348650724897	1
Srp14	-0.163496775	1
Ciao2b	-0.353527044	1
Klhl24	-0.656967469	1
Ipo5	-0.275973548	1
Shprh	-0.178304051	1
Kazn	0.354233725069594	1
Sh3yl1	-0.227343389	1
Adar	0.280276258944581	1
Raph1	0.567311682939912	1
Spcs1	-0.439284365	1
Eif2s1	-0.39537525	1
Cript	-0.233813348	1
Wdr5b	-0.246636705	1
U2af1	-0.319024496	1
Tsg101	-0.254228093	1
Glrx3	-0.263196187	1
Rimklb	-0.644787849	1
Nampt	0.47768042390026	1
Ywhag	-0.606069774	1
Golm2	-0.464885757	1
Dnajc19	-0.399752315	1
Ghdc	0.329671753714948	1
Cdk7	-0.267441332	1
Nop14	-0.42262828	1
Plagl1	0.463401925744634	1
Efcab2	-0.148890789	1
Fam199x	-0.147527176	1
Ipo11	-0.20351617	1
Rnh1	-0.320039956	1
Snapin	-0.433341861	1
Zfp64	-0.39839796	1
Pgk1	-0.432107762	1

Ift74	-0.335512224	1
Fnbp1	-0.763515856	1
Usp8	-0.266328028	1
Phb2	-0.222184701	1
Drosha	-0.254992322	1
Col14a1	-0.382170046	1
Coq3	-0.272561698	1
Gnpda2	-0.230401596	1
Cdc25b	-0.345620919	1
Dkc1	-0.395305593	1
Creld2	-0.268419349	1
Znrf1	-0.255736028	1
Fam8a1	0.227087927388778	1
Zbtb24	-0.245239406	1
Snrpe	-0.38998328	1
Dlgap1	1.65156450321275	1
Aimp1	-0.327652884	1
Gnai3	-0.279397195	1
Zfhx4	0.844398528285434	1
Sdf4	-0.280909077	1
Pgm3	-0.188784915	1
Adam23	-0.581012442	1
Atp13a3	0.637711886898971	1
Rttn	0.655883584436182	1
Rtca	-0.374524822	1
Nrxn3	0.762569823264908	1
Slc10a7	0.631302614112682	1
Nbr1	-0.418766589	1
Samd12	0.439457837545994	1
Epb41	-0.422826984	1
Psma1	-0.343825821	1
Slc25a36	-0.223001261	1
Nucb2	-0.317520625	1
BC005537	0.275019004443501	1
Lrrc75a	-0.595488528	1
Opa1	-0.242390517	1
Ric8b	-0.357040726	1
Ssrp1	-0.146613771	1
Ppil1	-0.276234765	1
Galnt2	0.453933093843164	1
Plod2	0.687479669435845	1
Trappc2l	-0.245640523	1
Wdr43	-0.431034914	1
Cdc40	-0.265383149	1
Snap29	-0.336149038	1
Napa	0.21229533776818	1

Pnpla3	-0.270274348	1
Ahsa1	-0.316516456	1
Timp3	0.441654688338411	1
Dnajc12	-0.601780393	1
Zbtb21	0.252250807775669	1
Tmem259	-0.309439301	1
Ftdc1	-0.265726793	1
Pacsin2	0.478625175208828	1
Cog1	-0.170595001	1
Wdr61	-0.87737883	1
Sacm1l	-0.256441815	1
Rwdd1	-0.348063386	1
Tram1l1	-0.278151647	1
Grhl1	0.400155566184191	1
Rtkn2	-0.377574435	1
Steap4	0.516756976888772	1
D5Ertd579e	0.472588458747215	1
Dnajc1	-0.348105366	1
Stx17	0.292259676131965	1
Bpnt2	-0.214773913	1
Pcdh10	-0.515731281	1
Ska2	-0.259215223	1
Fam102a	0.425847441122327	1
Polr3b	-0.312174151	1
Cep20	-0.370906799	1
Cnpy3	-0.385995315	1
Vegfc	-0.534893361	1
Tmem165	-0.357649327	1
Rnf13	-0.26463477	1
Tln1	-0.412023277	1
Aldh9a1	-0.259350461	1
Sdhaf2	-0.209317371	1
Ciao2a	-0.168159999	1
Ddx24	-0.311993267	1
Bst1	0.446654027593219	1
Fundc1	-0.240919771	1
Trp53rka	-0.249529497	1
Smarcb1	-0.267116422	1
Ssu72	0.296996559526998	1
Prex1	-0.631610762	1
Lmo7	2.24949635256034	1
Afg3l2	-0.486923746	1
Cecr2	-0.353093001	1
Sphkap	-0.912772842	1
Ddx41	-0.347001397	1
Lsamp	-0.291655861	1

Nktr	0.532786611271604	1
Lin52	0.491675633789043	1
Vdac1	0.291886066009949	1
Ing3	0.298593732558668	1
Tmem223	-0.374655617	1
Antxr2	-0.014867134	1
Fcf1	-0.157276036	1
Btbd9	0.386711580746117	1
Zc3h13	-0.327497204	1
Trmt13	-0.295663911	1
Suclg1	-0.240775483	1
Hnf1b	-0.329904582	1
4933427D14Rik	-0.267562422	1
Actr2	-0.470356705	1
Urah	-0.147851161	1
Atp5k	-0.379094797	1
Hs2st1	-0.403250006	1
Pou2f3	0.485568693119391	1
Wdr82	-0.433851478	1
Tmod3	-0.155802608	1
Ipo7	-0.468571857	1
Ptcd2	-0.419509023	1
Golgb1	-0.52832928	1
Ift20	-0.342309977	1
Tmem230	-0.266586879	1
Tfdp1	-0.363767915	1
Abraxas2	-0.250854439	1
Orc1	0.365134549343964	1
U2surp	-0.372799057	1
Thumpd3	-0.41185115	1
Samd13	-0.286057853	1
Cep192	0.342087646222184	1
Akap7	-0.277501443	1
Kat14	-0.250647071	1
Zfand1	-0.336047373	1
Ube2cbp	0.967712528847217	1
Ddx17	0.317911929796472	1
Psmb7	-0.223997447	1
Tmem263	-0.21810241	1
Il17ra	0.436396453934964	1
Jagn1	-0.399370019	1
Dcp1a	-0.228962907	1
Endov	0.334793777673703	1
Ddx46	-0.55989407	1
Tbc1d5	0.662671255087304	1
Slc9a1	-0.22662583	1

Tyk2	0.200120280160098	1
Smarca1	-0.570774119	1
Pdpk1	-0.229802246	1
Coro7	-0.285471922	1
Tiprl	-0.246585609	1
Slc35c1	-0.165929702	1
Ogfr	0.543028821387535	1
Ovch2	0.351079303345983	1
Dock5	-0.270122846	1
Tctn2	-0.204399264	1
Dclk1	1.01936328634886	1
Kdelr2	-0.280673762	1
Ctdp1	-0.314350408	1
Sec62	-0.346923108	1
Gyg	-0.462345371	1
Dcaf13	-0.234788966	1
Cse1l	-0.259998386	1
Rassf8	0.336531293559064	1
Gfpt1	-0.360780208	1
Ptpn13	0.5002528869952	1
Aqr	-0.315294779	1
Srsf6	-0.458565976	1
Gatad2b	0.225380044219833	1
Arcn1	-0.269104102	1
Dnajb2	-0.281060069	1
Ctbp1	-0.530096007	1
Anapc10	0.578592865868973	1
Dync2i1	0.220794971299447	1
Rif1	-0.267829648	1
Fam241a	-0.346778263	1
Abi1	0.581113845709963	1
Ndufa2	-0.370393022	1
4921524J17Rik	-0.500871957	1
Cul3	-0.379286583	1
Ift57	-0.375804382	1
Dmd	0.71005978282427	1
Hpgd	0.258393310795663	1
Exoc5	-0.364128645	1
Gpr37l1	0.193411702590876	1
Baiap2l1	0.503729310606568	1
Lrp4	-0.538744324	1
Efhd1	0.508872920914528	1
Nisch	-0.373940162	1
Cplane2	-0.273761958	1
Rpp14	-0.25922008	1
Plcd1	0.599244553975158	1

Dip2c	0.408322774661836	1
Arfgap3	0.491053925727206	1
Errfi1	0.656032801678881	1
Fkbp11	-0.481851191	1
Smap1	-0.320540297	1
Itfg1	0.336021214957369	1
Tomm40l	-0.254552757	1
Aldh1a3	1.11463005386866	1
Chmp4c	-0.085096994	1
Ndrg3	-0.218381998	1
Smyd3	-0.2579873	1
Sass6	-0.351235023	1
Ubxn7	-0.22942114	1
Suds3	-0.4545609	1
Dhrs2	-0.240655369	1
Ccdc32	-0.279111659	1
Ilf2	-0.380914492	1
Odf2	-0.291470323	1
Ubap2	0.470861323712664	1
Nr3c2	0.622540417229291	1
Hspa8	-1.024964655	1
Pi4ka	-0.4398304	1
Per3	0.358227864254732	1
Ddx52	-0.444906364	1
Tuba1a	-0.5704074	1
Stx16	-0.308595517	1
Trim3	0.164531968071527	1
Grk5	-0.361876393	1
Dcn	-0.269364072	1
Ndufaf6	0.278968035301415	1
F2rl1	0.232649267730382	1
Epha7	0.93255748675639	1
Sms	-0.253386126	1
Ccdc25	-0.19260849	1
Dag1	-0.462912639	1
Ppp1r26	-0.321235816	1
Etnk1	0.349973916795245	1
F8	-0.343801791	1
Rubcn	0.183135086429194	1
Reps1	-0.224798055	1
Vps50	0.491836836910703	1
Tm2d2	-0.249309932	1
Arhgef28	-0.139902	1
Pbxip1	-0.558988785	1
Pdia6	-0.369865259	1
Plekha6	-0.329357334	1

Grk3	-0.689024097	1
Dut	-0.234478554	1
Thg1l	-0.251456909	1
Dynlt3	0.00283906360495156	1
Armc2	0.654161078439145	1
Rpgrip1l	-0.420825033	1
Tmem170b	0.253238905864473	1
Fbxl5	0.504391754473147	1
Rab11a	-0.25114188	1
Klhl20	0.163972389836552	1
Chrac1	-0.197845827	1
Sri	-0.339161351	1
Ldb2	-0.484453007	1
Chrna5	-0.446842163	1
Parp1	-0.022788341	1
Nfyc	-0.327140117	1
Rtraf	-0.261269918	1
Sp3	-0.125271	1
Apaf1	0.284151424476739	1
Crlf3	-0.341666687	1
Ncbp3	-0.533872197	1
Paics	-0.282228814	1
Fermt1	-0.143895035	1
Nkx2-1	-0.568142397	1
Aox3	-0.361240924	1
Ets1	-0.370822437	1
Nr1d2	0.762091297015225	1
4932411N23Rik	-0.365104812	1
Clk1	-0.37156922	1
Atp11b	-0.294845888	1
Gtf2ird1	-0.290976358	1
Nt5dc1	0.492051521708184	1
Nup88	-0.320643052	1
Colgalt1	-0.238821115	1
Aip	0.10524615520103	1
Hdac2	-0.342155754	1
Rnf150	-0.632579223	1
Nap1l4	-0.387774713	1
Stoml2	-0.242827152	1
Tmem168	0.151602111319059	1
Wbp2	0.293476831197028	1
Lrpap1	-0.202620493	1
Cacul1	0.517873574101473	1
Champ1	-0.204941867	1
Arf1	-0.291067691	1
Carhsp1	-0.834747166	1

Usp6nl	0.599999867517226	1
Trp53bp2	0.688044258898194	1
Anxa2	0.908436017566881	1
Rarb	0.133012046579032	1
Pde1a	-0.336311808	1
Cracr2b	0.492820891481245	1
Rorb	0.433847273092451	1
Adrb2	0.276555336415674	1
Rnf185	-0.245235982	1
Peli1	0.464228609420174	1
Nfe2l1	-0.240865567	1
Ndufs2	-0.127299322	1
Ube4a	-0.09451235	1
Gna13	0.400093716575171	1
Plrg1	-0.242394258	1
Commd8	0.445556546588693	1
Tspan13	-0.39780224	1
St18	0.756455155259783	1
Adcy7	-0.317808132	1
Epb41l5	0.319283765762658	1
Bfar	0.280386632265791	1
Hmgn3	-0.417694337	1
Smn1	-0.270642488	1
Nedd8	-0.236157315	1
Chuk	-0.325007867	1
Smtn	0.0874447621421583	1
Ppp3cb	-0.334809653	1
Uba2	0.284862870125729	1
Coq8a	-0.301272866	1
Naxe	-0.18878096	1
Zeb1	-0.057282652	1
Rb1	0.522381237933714	1
Wdr13	-0.415676414	1
Rbms1	0.352611159246226	1
Arhgap45	-0.198831695	1
Perp	-0.506552867	1
Alox5	0.432535382587713	1
Sh3d19	0.809284408871298	1
Exoc3	0.573868134722668	1
Ist1	-0.407766883	1
Pde10a	1.31848928782991	1
Hp1bp3	-0.286197775	1
Pik3ca	0.510678787815715	1
Phpt1	-0.337387994	1
Bcar3	-0.785347103	1
Ssh2	0.274841257833569	1

Tenm4	-0.453839127	1
Ciz1	-0.274958429	1
Lin7c	-0.115994854	1
Rnf168	0.324380367645496	1
Pomp	-0.289593439	1
Lrrc31	-0.280043585	1
Casd1	-0.360689837	1
Ltbp1	-0.406018177	1
Ugp2	0.650973402106846	1
Kif13b	0.511138123675091	1
Ppm1h	-0.278181824	1
Zfp830	-0.401267273	1
Tusc3	0.387642332855947	1
Niban1	-0.318444261	1
Fam189a1	-0.358606956	1
Ush2a	-0.328301017	1
Disp1	0.518641119085189	1
Ube2i	-0.179682822	1
Emp1	-0.991907815	1
Sh3rf1	0.611980513214882	1
Ahsa2	-0.383463799	1
Tmem116	0.604414134004745	1
Kif23	-0.57739777	1
Pkm	-0.200697455	1
Paip2	-0.30849826	1
Naif1	-0.209692737	1
Fam107b	0.0444421403100437	1
Ppp1r13b	-0.135857186	1
Scnn1a	0.253715583376363	1
Phb	-0.307132846	1
Wdr4	-0.20885891	1
Asb5	-0.246759661	1
Srp54a	-0.438311422	1
Ica1	0.601146613225797	1
Cars	0.488932744651719	1
Casp7	0.352725882982959	1
Pcdh15	-0.27176907	1
Ube2b	-0.296983866	1
Pus7	-0.113430634	1
Pdha1	-0.257622973	1
Tmem254a	-0.270371224	1
Pemt	-0.235859674	1
Fam126a	-0.21741113	1
Elof1	-0.190805848	1
Dnm1l	0.486986941073724	1
Wfdc2	-0.375359002	1

Rbm22	-0.2504211	1
Cdk2ap2	-0.215927502	1
Pnisr	0.2726449780875	1
Ranbp1	-0.29658951	1
Agpat4	-0.528820277	1
Rnf20	-0.331158932	1
Cntn5	0.90496654389987	1
Nae1	-0.089598197	1
Sytl1	-0.314087868	1
Slc39a13	-0.252671208	1
Alad	-0.225214872	1
R3hdm1	-0.327531049	1
Gtf2h1	0.329404996543188	1
Faf2	0.290471260272345	1
Htt	-0.196120193	1
Vcl	0.959031228164867	1
Triap1	-0.222841209	1
Sdhaf4	-0.053713137	1
Slc30a9	0.19836882984484	1
Piga	0.13355574812843	1
Id4	-0.672012871	1
Aak1	0.151016225727301	1
Rfc1	0.2710143434523	1
Commd4	-0.220788517	1
Cox19	-0.350531865	1
Snx9	-0.277402963	1
Serinc3	-0.136217252	1
Suclg2	0.308981271883882	1
Cp	-0.187584304	1
Nadk	0.418413553826866	1
Sftpa1	1.70072260812187	1
Rfk	-0.170677024	1
Ubtf	-0.253873033	1
Sgk1	0.796189441127814	1
Cnksr2	0.783125922702982	1
Sbf2	0.629885223948475	1
Srp19	-0.20029044	1
Trim24	-0.133646806	1
Ndufa8	-0.192349714	1
Pkdcc	-0.589641618	1
Papss1	-0.248471613	1
Plat	-0.268596264	1
Dnpep	-0.118251311	1
Plpp3	0.225489426661371	1
St3gal1	-0.287295611	1
Arhgef12	-0.347558572	1

Ilrun	0.529909879135691	1
Ubr2	-0.318100297	1
Acat1	-0.486706041	1
Chchd2	-0.272552447	1
Pip4k2a	-0.442966558	1
Atp1b1	0.438885187060151	1
Cog5	0.368654905424679	1
Tmem167	-0.122879548	1
Ctps2	0.706154040034528	1
Ckap4	-0.268215367	1
Cldn3	-0.392137129	1
Prkn	0.919618954553486	1
Cep162	-0.031107093	1
Etl4	0.543323436705528	1
Exosc9	-0.168679454	1
Uqcc1	-0.20317628	1
Eefsec	0.59673196079759	1
Ecd	-0.318468287	1
Wdr3	-0.20503079	1
Sall4	0.215816601938672	1
Polr2c	-0.180575678	1
Coq9	-0.151140679	1
Pcsk5	1.15426911878957	1
Hnf4g	0.362578674117398	1
Syncrip	-0.334279229	1
Nfat5	0.098844533777295	1
Nvl	-0.245722292	1
Col1a1	-0.377387993	1
Tomm22	-0.271310651	1
Dcps	-0.325783762	1
Pxmp4	-0.219487032	1
Llph	-0.162770067	1
Lsm1	-0.16804389	1
Fhod3	0.748682169303319	1
Cisd1	-0.085767284	1
Rnf126	-0.143071883	1
Pde4b	0.385715819314978	1
Klf10	0.353209571934442	1
Lcmt2	-0.245412464	1
Ophn1	-0.329950734	1
Hdac1	-0.319874093	1
Avl9	0.707912167273393	1
Fam83h	0.217444694571222	1
Ttyh3	-0.406429373	1
Ccng2	-0.227644173	1
Lgr4	-0.480705232	1

Anxa7	-0.293056088	1
Slc9a6	-0.429484542	1
Patj	0.532903712398739	1
Zbed4	-0.069438368	1
Uqcc2	-0.206445895	1
Epn3	0.65513523705579	1
Psme3ip1	-0.387571224	1
Ptges3	-0.162874322	1
Chmp2a	-0.387459016	1
Prr5l	-0.363803998	1
Prtg	0.3111607383094	1
Supt4a	-0.19359363	1
Gxylt1	-0.278862741	1
Strada	-0.256550742	1
Stk11	-0.255183156	1
Nxt2	-0.222293043	1
Trmt1l	-0.387021113	1
Zbtb46	0.692229305743666	1
Nudcd3	-0.153940199	1
Slc25a12	-0.124546524	1
Gramd4	-0.374435403	1
Prkca	0.363241903665542	1
Psap	0.206837082032081	1
Arid5b	0.348590065370115	1
Scg5	1.40432113280098	1
Plekha3	-0.34306151	1
Eif2s2	-0.193472795	1
Usp34	0.0681122736440343	1
Akr1a1	0.250116132971071	1
Kif26b	-0.0840347	1
Trpm5	0.340811943053454	1
Secisbp2l	0.550678372447032	1
Dnase2b	-0.328805622	1
Daglb	-0.330782552	1
Zdhhc6	-0.122966839	1
Tlr3	0.270568213366757	1
Ptpra	0.554652235717539	1
Serpinf1	-0.249544647	1
Anapc13	-0.441794339	1
Prelid3b	0.318183401047433	1
Ythdf2	-0.33553655	1
Commd2	-0.320313563	1
Phaf1	0.403451268981238	1
Zranb2	-0.394057738	1
Hexb	-0.225745893	1
Tomm70a	-0.220065815	1

Rsu1	-0.162825628	1
Tmtc2	-0.304921806	1
Zbtb20	0.224253061081914	1
Nav3	-0.38755959	1
Cpne8	1.0590877959487	1
Cdkl5	-0.39896771	1
Ran	-0.522828642	1
Actl6a	-0.230976371	1
Yrdc	-0.284391953	1
Fam3b	-0.307990644	1
Aars	-0.171643572	1
Sde2	-0.276378691	1
Adal	-0.251281214	1
Rras2	-0.183975345	1
Clec16a	-0.355846751	1
St14	0.105896514995848	1
Exosc1	-0.317830933	1
Lzic	-0.264053646	1
Atg4b	-0.164343045	1
Ubr7	-0.16855517	1
Hells	-0.25049014	1
Peak1	0.35566304263311	1
Aff2	-0.548748106	1
Fahd2a	-0.354420968	1
Nsmf	0.359666324765393	1
Numb	-0.364754786	1
Nek1	-0.255835013	1
Cops8	-0.220258824	1
Scamp4	-0.210919159	1
Pdgfa	0.744325014194442	1
Fgfr2	-0.438429323	1
Rad54l2	-0.391061506	1
Steap3	0.251300545263942	1
Dtd1	-0.237014855	1
Ift122	-0.254934157	1
Cdk17	0.784035182814179	1
Aida	-0.153878814	1
Bpnt1	-0.13074384	1
Upf1	0.486921652109488	1
Ppm1d	0.361177182814705	1
Glrx5	-0.156602768	1
Tom1	-0.23732027	1
Aplf	-0.162316545	1
Six4	-0.351565322	1
5730455P16Rik	-0.261620764	1
Rpa2	-0.328335241	1

Cep68	0.395405586422243	1
Nudt7	-0.269883577	1
Kansl1l	0.314252954832345	1
Rnf111	0.564294397838964	1
Bmp2k	0.592544068327546	1
Smarcc1	-0.391642101	1
Fkbp9	0.212550459259817	1
Commd6	0.278368315697526	1
Slc25a32	-0.193452439	1
Hesx1	-0.208858849	1
Ddx31	-0.428195249	1
Ikbke	0.250679837387979	1
Arl6ip1	-0.378010858	1
Chst5	-0.296480918	1
Stard4	-0.206136637	1
Ipo9	-0.227346149	1
D16Ertd472e	0.297394826379601	1
Clpx	0.418611394428314	1
Socs1	0.490551364028414	1
Grb14	-0.204285723	1
Ccdc43	-0.129901677	1
Farsb	-0.162443141	1
Ywhah	0.228849782427843	1
Clptm1l	-0.269360835	1
Galnt9	0.359120802325727	1
Atp6v1b2	-0.218597451	1
Erlin2	-0.377215138	1
Ago3	-0.420012984	1
Ankrd17	0.0323799028461731	1
Ctnnd1	0.240312718810323	1
Rap2a	-0.304877304	1
Camsap1	-0.257517282	1
Fzr1	0.141495602181752	1
Fam204a	-0.222242644	1
Agap1	0.522354553239873	1
Lrwd1	-0.312988467	1
Gfod1	0.41895782751868	1
Noct	0.237769268389311	1
Zcchc10	-0.31850751	1
Wdr89	-0.197224037	1
Copz1	-0.365748206	1
Cdc42	-0.371180719	1
Otud3	0.21740580979716	1
Taf2	-0.179768972	1
Nebl	0.537810994365032	1
Ctcf	-0.453279335	1

Psmc3	-0.133786508	1
Lypla1	-0.15001613	1
St7l	-0.297717991	1
Usp7	-0.304969889	1
Wdr48	-0.281483359	1
Atxn1	0.355467455110931	1
Tdg	-0.36083825	1
Zmynd8	-0.436189342	1
Rbks	0.322931079526406	1
Ap3m2	-0.237833239	1
Anapc16	-0.261724597	1
Ei24	-0.401267565	1
Snrpb2	-0.284453997	1
Stk11ip	-0.212581031	1
Ghr	-0.278169844	1
Dctn4	-0.143715535	1
Gart	-0.226169428	1
Tubg1	0.250172413370599	1
Prelid3a	0.117362825502863	1
Stx7	0.331228585448872	1
Ube2r2	-0.138516412	1
Wwc2	-0.422675428	1
Hectd2	-0.30625711	1
Klhl13	-0.244090445	1
Tm9sf5	-0.243497153	1
Taf8	0.0831349888194308	1
Dync1i2	-0.426915569	1
Rhof	-0.068400811	1
Crim1	-0.261356499	1
Arhgef9	0.298571440024514	1
Nans	-0.143909488	1
Bnip2	-0.29353578	1
Thyn1	-0.250366254	1
Sdf2	-0.252075384	1
Tm9sf4	-0.145024573	1
Dsn1	-0.193528516	1
Eif4e2	-0.138216317	1
Rarres2	-0.304299831	1
Septin9	0.374258111316666	1
Bach2	-0.237445248	1
Lrrc38	0.221899212451565	1
Prpf4	-0.293236602	1
Vbp1	-0.0825694	1
Riok3	0.579976760085495	1
Kpnb1	-0.19862409	1
Rsph3b	0.201332158801217	1

Ptpn14	-0.108203095	1
Ebag9	-0.426478263	1
Plxna1	0.256698998539636	1
Ago1	0.154861035572845	1
Clip1	-0.463749369	1
Esyt2	-0.511412183	1
Reps2	-0.319263931	1
Tax1bp3	-0.440895029	1
Nsd3	-0.359424013	1
Dock7	0.451892668785569	1
Pfkfb3	0.27310437511088	1
Tpmt	-0.171804564	1
Cetn1	-0.182657837	1
Katnbl1	-0.103116943	1
Rnf113a1	-0.164560008	1
Cttnbp2nl	0.410517938794439	1
Vezf1	-0.222350647	1
Dcp2	-0.094465879	1
Pecam1	-0.661055239	1
Gsr	-0.10334793	1
Rab9	-0.344977981	1
Cep120	-0.165090622	1
Ift27	0.143391252713582	1
Hlf	-0.30422533	1
Nasp	-0.3204119	1
Rftn1	-0.319345832	1
Ythdc1	-0.413987153	1
Acp1	-0.285688185	1
Iqcb1	0.482840174975942	1
Bud23	-0.174505517	1
Dnajb6	-0.272253927	1
Fbxo45	-0.383513262	1
Clstn1	-0.262611936	1
Smim12	-0.324860003	1
Cdc42se2	-0.730279111	1
Hao1	0.142437908577287	1
Rab11fip2	-0.168026619	1
Lamc1	-0.320936565	1
Prdx6	0.411760271026732	1
Slk	-0.046583821	1
Tspan4	-0.014592946	1
Xrn2	0.112552088477394	1
Ablim2	1.08283490621485	1
Ska3	0.312175064339934	1
Dhx15	-0.372559494	1
Arfip2	-0.021792565	1

Dync2h1	-0.374022411	1
Ptpn21	0.336087581140583	1
Pik3cb	0.651212792641227	1
Ufd1	-0.233690253	1
Foxp4	0.317932221858423	1
Gpn1	0.0226850563516959	1
Eif4e3	0.0538689253959144	1
Ap2m1	-0.073556418	1
Tpp2	-0.220865397	1
Zfpm1	0.102489064744807	1
Foxn2	0.327154055927875	1
Bccip	-0.09231782	1
Zfp608	-0.146632624	1
Afg1l	0.276030318643869	1
Golga3	0.435914001167342	1
Klhl15	-0.14150462	1
Ccdc66	-0.116932402	1
Bmt2	0.281303143452376	1
Kif5c	0.224044103367348	1
Bdh2	-0.079157118	1
Sugt1	-0.218868162	1
Jade3	-0.186416269	1
Pof1b	-0.106623816	1
Plpbp	-0.110099454	1
Bud13	-0.305188935	1
Rbbp7	-0.18313176	1
Dock11	-0.183032352	1
Ckap5	-0.304398239	1
Ik	-0.195878395	1
Tmem87a	-0.108993993	1
Slco4a1	0.297532292669952	1
C2cd2l	-0.51585774	1
Arhgap12	0.365841709208266	1
Anxa11	-0.341863731	1
Galnt7	-0.47137541	1
Lpin2	-0.312661878	1
Psma3	-0.268040258	1
Nkap	-0.383501168	1
Sc5d	-0.316546503	1
Phip	0.192726442751499	1
Kat2a	-0.306780629	1
Pgrmc2	-0.276953514	1
Etv5	-0.318130286	1
Fam98a	-0.169798778	1
Gigyf2	0.242972068406691	1
Chat	-0.210862259	1

Snf8	0.0568084596683702	1
Acap2	-0.158289813	1
Pex1	-0.231759127	1
Snx1	-0.289158753	1
Tmem250-ps	-0.176722823	1
Phf20	0.405286836545299	1
Hira	-0.298061527	1
Opa3	-0.214007715	1
Stard13	0.473458919490115	1
Csl	-0.293679386	1
Cachd1	-0.506753749	1
Cbx4	-0.321225616	1
Letm1	-0.161072879	1
Snrnp40	-0.197254419	1
Atxn7	0.422716455118892	1
Acss2	0.469281067	1
Cd74	0.409884792997898	1
Psmb6	-0.349539854	1
Ube4b	-0.166442421	1
Pithd1	-0.185131385	1
Hnrnpd	-0.368154803	1
Dido1	0.267206365745698	1
Ulk2	-0.338294553	1
Sco1	-0.287651908	1
Tlk1	-0.260586461	1
Slit3	-0.109775146	1
Sema4g	0.214988081339503	1
Il6st	0.334470693170818	1
Pdcd6	-0.376744994	1
Apoa1	0.712346455611073	1
Psmd12	-0.272186837	1
Dis3l2	0.235139753092379	1
St3gal6	0.253518765327486	1
Nudc	-0.302222158	1
Surf6	-0.228797655	1
Fktn	0.0296812434826121	1
Rmnd5a	0.291994549143933	1
Tmem50a	0.0499647599301952	1
St6gal1	-0.415153595	1
Slc44a5	-0.33823771	1
Atp6v1d	-0.007498936	1
Eya4	-0.902641165	1
Ppil3	-0.25178545	1
Eipr1	-0.38200765	1
Ccpg1	-0.373922366	1
Pdss2	-0.289878	1

Pknox1	-0.362222012	1
Swt1	0.289138965326395	1
Csde1	-0.398350541	1
Nrf1	-0.198246219	1
Akirin2	-0.228110938	1
Golga5	-0.081534876	1
Ing5	-0.218331201	1
Cisd2	-0.086100614	1
Zmiz1	0.395781621965255	1
Pgd	-0.141081978	1
Spink8	-0.540277993	1
Ash1l	0.232103691673164	1
Pak4	-0.227678548	1
Sf3b5	-0.225845178	1
Utp6	-0.052418285	1
Rab14	-0.521990711	1
Dlst	-0.22623194	1
Zcchc17	-0.18282439	1
Ckap2l	0.4528632852996	1
Vill	0.506682084726333	1
Them4	-0.084301189	1
Desi1	-0.168809096	1
Cln6	-0.245218645	1
Luzp1	-0.279199525	1
Tgs1	-0.193490987	1
Ythdf3	-0.245996535	1
Rab4a	0.199019796167504	1
Sec23ip	-0.342230366	1
Sh3gl3	-0.317628177	1
Eno1b	-0.113385484	1
Tcf25	-0.337655282	1
Fyb2	0.242459649447686	1
Rc3h1	-0.208638073	1
Pigx	-0.362853353	1
Pudp	0.407068511599554	1
Rapgef2	0.865747662784638	1
Lsp1	0.000292556558142421	1
Rabgap1l	-0.421635555	1
Rasa2	-0.321195335	1
Nudt3	0.272091055753972	1
Iqgap2	0.243964136285481	1
Ccdc88a	-0.094211704	1
Cfap97	-0.229378507	1
Arid2	0.458859228087822	1
Prdm16	0.636192455483911	1
Palmd	-0.485071261	1

Cog2	0.61397436078305	1
D430041D05Rik	0.353683682394299	1
Bag1	-0.303224714	1
Pnpt1	-0.164605171	1
Rnf144b	0.279645075410083	1
Snapc4	-0.206137674	1
Rbbp6	0.77477649930321	1
Azi2	0.246361618070191	1
Bud31	-0.305942111	1
Htatsf1	-0.355232424	1
Tprkb	-0.159269818	1
Dnmbp	-0.148512631	1
Snrpd2	-0.164271544	1
Pcm1	-0.43395506	1
Sclt1	-0.41950914	1
Sox9	-0.346220353	1
Ccnd1	-0.270319201	1
Slc40a1	0.444002131101248	1
Slc16a7	-0.224713431	1
Khdc4	0.486714371297195	1
Pxylp1	0.503015841895682	1
Atp6v0a2	-0.181612489	1
Prdm10	-0.178017726	1
Trit1	-0.251656578	1
Sdk1	0.967005420434071	1
Bhlhe40	-0.267094558	1
Ppp3ca	0.526436816404904	1
Arl1	-0.083025527	1
Pum2	0.357569798079417	1
Zfand2a	0.0846082313789617	1
Eif2ak4	-0.156544201	1
Babam2	0.318394033796749	1
Arntl2	-0.208158396	1
Tfg	-0.262469215	1
Zfp622	-0.273237749	1
Vrk1	-0.231984382	1
Usb1	-0.181393284	1
Ccdc59	-0.380177945	1
Arih1	-0.355299859	1
Zfc3h1	-0.373999841	1
Ern1	-0.257756642	1
Rhoa	-0.150418812	1
Arhgap10	-0.170224052	1
Spsb1	0.496242002102601	1
Nudcd1	-0.207474115	1
Hbs1l	0.273382453014883	1

Lyrm4	-0.288780022	1
Carnmt1	-0.449219279	1
Qk	0.487400379875391	1
Kif13a	-0.251215041	1
Kcnk5	0.463739319055975	1
Plppr4	-0.147008543	1
Sec23b	0.381843914471681	1
Adam10	0.329562440357725	1
Rbm5	-0.253322592	1
Osbpl10	0.539781430927105	1
Trappc2	-0.115741422	1
Invs	-0.230950104	1
2410004B18Rik	-0.133541763	1
Tgif1	0.130354566307487	1
Dis3	-0.176324101	1
Btbd1	-0.142733491	1
Znhit6	-0.186977137	1
Dipk1a	-0.149894809	1
Eif3j2	-0.328556656	1
Cdk6	-0.423132732	1
Fam135a	-0.552785709	1
Ehbp1	-0.269824036	1
Xiap	-0.310377672	1
St13	-0.174113776	1
Elavl1	-0.184917895	1
Api5	-0.313277802	1
Heatr5a	0.549874637437483	1
Nhlrc2	-0.208042122	1
Gcn1	-0.349635622	1
Cnot9	-0.333623061	1
Uprt	-0.159183487	1
Ccng1	-0.090304032	1
Bysl	-0.158051874	1
Acsl4	0.361347183203483	1
Pdcd5	-0.212074007	1
Irf6	-0.255514088	1
Sema5a	-0.037895678	1
Cplane1	0.576077249574151	1
Phf5a	-0.375860179	1
E2f4	0.174672972474063	1
Pip4p2	-0.160697455	1
Ercc8	-0.148445021	1
Agtpbp1	-0.133347043	1
Homer2	-0.19369026	1
Traf6	0.151725179297123	1
Abhd18	-0.276569505	1

Sgpl1	-0.266910415	1
Secisbp2	-0.376920207	1
Ptprk	0.866993930218335	1
Ankrd10	0.383019598551604	1
Uqcrc1	-0.098657134	1
Pacc1	0.400887021231391	1
Pwwp2b	-0.177933743	1
Hmbs	-0.379891123	1
Stambpl1	-0.227896962	1
Trappc3	-0.009448172	1
Ddost	-0.208727243	1
Fbxl2	-0.502427434	1
Ptbp3	0.477964697726606	1
Dicer1	-0.233336288	1
Fbxo8	0.217522371587321	1
Pdxk	0.481301647136101	1
Rbsn	-0.286309354	1
Pef1	-0.184222599	1
Snx27	-0.133333177	1
Vegfa	0.270367963539006	1
Ppm1g	-0.073361034	1
Prr14l	-0.284725778	1
Abcb8	-0.142589231	1
Zfp143	0.110733120357345	1
Alkbh5	-0.261404658	1
Atxn2	-0.45855745	1
Pafah1b1	-0.282637049	1
Smim4	-0.090979949	1
Sat1	0.930724011473616	1
Gpam	0.195096320854001	1
Strip1	-0.171821853	1
Higd1a	-0.218063161	1
Zc4h2	-0.225308495	1
Plcg2	-0.801900994	1
Stat3	-0.331394297	1
Nbas	0.338278804901151	1
Srp9	-0.277868396	1
Sorbs2	0.551600993234167	1
Xpo4	-0.202460371	1
Rrad	0.429471513451886	1
Cd47	0.393395109110006	1
Top1	-0.132876159	1
Ifrd1	0.407106253060237	1
Ap2a1	-0.43459757	1
Noc4l	-0.150620803	1
Igfbp3	-0.340145122	1

Dnajc15	-0.175378897	1
Gli3	-0.19790696	1
Cgnl1	0.295385969951064	1
Arntl	0.0548926613208687	1
Basp1	-0.650639492	1
Fbxo30	0.272837284882836	1
Get4	-0.298868985	1
Isca1	-0.055772375	1
Tsc22d3	0.215216221451149	1
Ppid	-0.252130049	1
Rasal2	-0.324546396	1
Pdia3	-0.278524271	1
Hddc2	-0.068469768	1
Cwc15	-0.159847744	1
Supt6	0.285662910221846	1
Atp6v1c2	-0.071195	1
Glg1	-0.325658808	1
Ubl7	-0.053051986	1
Sharpin	0.199683688038941	1
Lamb2	-0.214973955	1
Hdgfl2	-0.200587093	1
Cd164	0.19359524257594	1
Gsto2	-0.358861196	1
G3bp2	-0.1716495	1
Tppp	1.12728826344964	1
Tbc1d15	-0.178016011	1
Thnsl2	0.17290708752049	1
Traf7	0.253873149264314	1
Rbpj	0.327019023940499	1
Galntl6	0.172666266361802	1
Dtnbp1	-0.061290729	1
Nelfa	-0.199438819	1
4930453N24Rik	0.00997876861739294	1
Sult4a1	-0.277193265	1
Ss18l2	-0.31942548	1
Slc36a4	0.213127464867771	1
Atrn	-0.314715001	1
DPCD	-0.306539951	1
Arhgap17	0.57916009084149	1
Afdn	1.06092364776413	1
Ehd3	0.441050405984341	1
Nckap1	0.422322112430102	1
Pcif1	-0.249105085	1
Stk4	0.273242438184708	1
Spag6l	0.394234950693508	1
Lnpep	-0.217307094	1

Notch1	-0.26387706	1
Dpp8	-0.186246712	1
Gramd1b	0.391761268627837	1
Pak3	0.163870687844922	1
Slc35f3	-0.281376586	1
Ttc1	-0.140616699	1
Bivm	-0.370738143	1
Stxbp1	-0.196541423	1
Ctsa	-0.081685664	1
Cnbp	-0.18511584	1
Cdk1	-0.597146954	1
Grid2	0.492014299427641	1
Nlk	-0.214715299	1
Daam1	-0.594894095	1
Slc38a1	0.430701563939695	1
Pik3cg	-0.383740341	1
Nr6a1	-0.317467586	1
Usp47	0.252181536958725	1
Slc35b2	-0.142889825	1
Lurap1l	-0.11366589	1
Colec11	-0.06778574	1
Klf5	0.330915264966172	1
Crcp	-0.236563255	1
Dusp6	0.0833303624753838	1
Ttc4	-0.273424562	1
Atf4	-0.261686523	1
Krt23	0.344713868517668	1
Actn1	0.601354109993333	1
Smarcd2	-0.211614206	1
Pik3r1	-0.324826838	1
Skiv2l	-0.158097566	1
Tmf1	-0.193715145	1
Ptprf	0.452926125606647	1
Cd44	0.536707567454514	1
Tor1b	-0.412714886	1
Pcf11	0.266180531635951	1
Pam	0.10933732528937	1
Arhgap35	0.215346782222555	1
Ddx6	-0.247720489	1
Stx18	0.289988372112687	1
Yars	0.145942104086344	1
Pdgfb	-0.108707449	1
Ubash3b	0.514054615810918	1
Tnfrsf11a	0.316912662446633	1
Clic6	0.192404615594682	1
Calcr	0.451487150869949	1

Efr3a	0.285087355828844	1
Cog3	0.217575730822282	1
Dctn2	-0.178161294	1
Umad1	-0.229890918	1
Apmap	-0.194304884	1
Spata6	-0.324913282	1
Trp53bp1	-0.1904437	1
Ints3	-0.282406626	1
Drg1	-0.249947879	1
Rufy1	-0.229230089	1
Adcy6	0.22713262473272	1
Tnks	0.344575578094948	1
Erc2	0.0255977810006588	1
Sltm	0.0893919918165026	1
Sybu	0.0689815557940305	1
Frmd3	0.249095436009315	1
Erlec1	-0.264711174	1
Inpp5f	0.0367575869172145	1
Rnf166	-0.193890312	1
Abhd17a	-0.291555006	1
Arhgef10	0.424967614486436	1
Ganc	0.131959515275227	1
Abcb11	0.172144103211924	1
Tub	0.364647346000831	1
Dars	-0.108262616	1
Nek9	0.242838516030024	1
Osbpl3	-0.120568051	1
Ythdf1	-0.261777022	1
Bbs2	0.0480766846010426	1
Slc30a5	-0.313148596	1
Reep2	-0.234131416	1
P2rx4	0.124609932248308	1
Usp33	0.264869152839443	1
Slc25a25	0.33288757710244	1
Ubr1	-0.111124549	1
Oat	-0.177799006	1
Fgf9	0.331154853689355	1
Ephb3	-0.243257015	1
Vps13a	0.250216890738044	1
Vwa8	0.540575369743007	1
Plekha1	-0.181079814	1
Phlpp2	0.0954954785934779	1
Pnpla8	0.247964839054357	1
Pnn	-0.155311412	1
Ppp1r2	-0.157624954	1
Senp6	-0.292565173	1

Zbtb48	-0.216081535	1
Abhd5	0.808898999172216	1
Fam98b	0.147354258808431	1
Socs6	0.248383214840593	1
Ube2v1	0.336098536752207	1
Rnps1	0.138831730074378	1
Asah1	0.00363008136775034	1
Kat6b	-0.297823619	1
Arrdc3	0.0718569586355143	1
Rcc2	-0.319888829	1
Usp4	-0.416978088	1
Tars	-0.223356519	1
Nub1	-0.12100172	1
Tmem51	0.105254098803058	1
Fam169a	-0.349030913	1
Elmo1	0.496605165647704	1
Cavin2	-0.263066721	1
Hibadh	-0.261485864	1
Arl15	0.419697570144992	1
Eci3	-0.116360305	1
Cdkn2aip	-0.268238636	1
Diablo	-0.295643825	1
Sqle	-0.168600937	1
Grk2	0.121978663488855	1
Sppl2b	0.113324211808332	1
Pgm2	0.167587068045406	1
Esrp2	0.966343361842219	1
Jade1	-0.344900238	1
Cilk1	-0.133580658	1
Prkd1	0.560483262938503	1
Nol9	-0.30659541	1
Nbeal2	0.108026057163462	1
Ube3a	-0.262278148	1
Slc66a1	0.095989789242661	1
Amfr	-0.122469276	1
Fig4	-0.159163613	1
Ahcyl1	-0.157548379	1
Dnajc13	0.192266290155611	1
Cfap36	-0.161934945	1
Coq5	-0.277063852	1
Spats2l	0.442037060478526	1
Ivns1abp	0.587790948028457	1
Cdc42bpa	-0.328987219	1
Raly	-0.205984516	1
Itga1	-0.303162198	1
Psmc2	-0.082971233	1

Igf2r	-0.407938715	1
Ciapin1	-0.142848914	1
Prpsap1	0.0726380948575529	1
Parp16	-0.148694147	1
Pstpip1	-0.287699571	1
Spen	0.209672024410377	1
Ndufa9	-0.020314829	1
Rtn1	0.300343027056446	1
Rcn2	-0.216362272	1
Agrn	-0.542025525	1
Hsd17b2	0.102628876291213	1
Tob1	-0.180390161	1
Nde1	-0.134508187	1
Wdr44	-0.114074688	1
Stoml1	0.0713075490079215	1
Cask	-0.336371965	1
Sox4	-0.17678261	1
Helb	0.216622005302772	1
Tob2	-0.020394327	1
Grip1	0.574215948116252	1
Slc22a15	0.125274560446707	1
Lcp1	-0.021138307	1
Bbs7	-0.13356939	1
Rbis	-0.150593941	1
Lrif1	0.19008881832331	1
Adnp	-0.165657721	1
Psmd9	-0.265670746	1
Bbx	0.289935307082252	1
Rbm19	-0.486273664	1
Agpat3	-0.375402532	1
Fyn	-0.255169978	1
Ddx47	-0.145866529	1
Sh2d1a	-0.239680602	1
Gnpda1	-0.130475057	1
Slc31a1	-0.104199662	1
Tmco3	0.213449054046828	1
Tmem150c	-0.038424403	1
Nfx1	-0.308848173	1
Ociad1	-0.29420149	1
Ripk2	0.345096274510571	1
Gpr107	-0.305618806	1
Rsl24d1	-0.125250604	1
Pebp4	-0.289215604	1
Esrrg	0.588106882845458	1
Tmed3	-0.254076422	1
Ankrd52	-0.120827011	1

Siva1	0.0886149586489219	1
Caprin1	-0.031354086	1
Rab22a	-0.219773919	1
Llgl2	0.216934050927896	1
Rhot1	-0.117413553	1
Dsg2	0.765206863102899	1
Zswim5	0.28731667989582	1
Gdap2	-0.180535357	1
1810013L24Rik	-0.076214663	1
Itsn2	0.361808961477772	1
Gorasp1	-0.031164175	1
Cert1	-0.342855855	1
Tsn	-0.121378869	1
Cyb5r4	-0.145591814	1
H1f6	-0.107321986	1
Trap1	-0.158217549	1
Arid4a	-0.113290838	1
Gna11	-0.316063751	1
Gse1	-0.406527823	1
Suv39h2	-0.248721408	1
Fryl	-0.282691258	1
Appl1	-0.245479416	1
Nedd9	0.658813654421871	1
Ppfibp2	0.358080637969434	1
Bop1	0.0453640135850585	1
Dglucy	0.0786883355578644	1
Rgs7	-0.294098283	1
Polr2d	0.0180368631426402	1
Gemin4	-0.240916674	1
Ugcg	0.299223606501339	1
Tm9sf3	-0.045703089	1
Psma2	-0.223905701	1
Zfp839	-0.133622854	1
Arfgef1	0.0490526763727737	1
Wdr91	-0.334686929	1
Snx29	-0.211415276	1
Commd5	-0.176271654	1
Rrp15	0.0062876032968967	1
Fbxl20	0.101314930419094	1
Snx16	0.287086162073105	1
Bub3	-0.275696072	1
Slc9a2	0.302254116365326	1
Tmem115	-0.286699399	1
Tbrg4	-0.142653907	1
Polr3f	-0.063046942	1
Syt8	-0.159586681	1

Ascc2	-0.135720712	1
Rab11fip3	0.219487395743819	1
Cdc23	-0.099176782	1
Fli1	-0.174415787	1
Epc2	-0.324441822	1
Bdh1	-0.182236984	1
Vps35	-0.072509864	1
Gnpnat1	-0.3080662	1
Syngr2	-0.289497422	1
Slc25a13	0.238213936258499	1
Ttc27	0.29050281804087	1
Cblb	-0.367769343	1
Tubgcp5	-0.100884957	1
Fnip1	0.385439908565726	1
Tmem59	-0.241295863	1
Chsy1	-0.111267941	1
Rgs6	-0.018610184	1
Sipa1l1	-0.288460788	1
Nudt14	-0.06701092	1
Fam13a	0.656703737779667	1
Ghitm	-0.14217699	1
Pacs2	-0.176873021	1
Stim1	-0.317954855	1
Dr1	-0.153811268	1
Arhgap18	0.463014217711064	1
Polr2m	-0.174390271	1
Aff4	-0.182771789	1
Crispld1	0.498657355604166	1
Asns	-0.004342509	1
Ifngr2	-0.203758433	1
Prpf8	-0.18352173	1
Ripk1	-0.237034225	1
Sec11a	0.0803096919644704	1
Rbm26	-0.212106861	1
Ccdc82	-0.091444448	1
Jam2	0.368497431308197	1
Capn2	0.26406594237605	1
Ptprm	-0.155038266	1
Shtn1	0.411727287703266	1
Snu13	-0.148861617	1
Gsto1	-0.380858142	1
Smad3	0.323981733104566	1
Fars2	0.444774699665654	1
Pgm1	-0.079468812	1
Ap1g1	0.553665154110291	1
Arid3a	-0.342364928	1

Ctsb	-0.217008985	1
Srpr	0.457315037554336	1
Dynlt1a	-0.374877878	1
Slc39a11	-0.238303745	1
Nek6	0.249631621691488	1
Naa50	0.34851455340431	1
Rab31	0.0769650805978424	1
Id2	0.127046904143463	1
Vma21	-0.23541508	1
Usf3	0.215956578768965	1
Psen2	-0.103987733	1
Bcap31	-0.244904864	1
Ddx23	-0.226381202	1
Arid1b	0.384573781829204	1
Nupl1	0.164415717148539	1
Oser1	0.1378686119432	1
Rbm33	-0.26662313	1
Stx6	-0.276363184	1
Eea1	0.310023880064821	1
N4bp2l2	0.0890332455152502	1
Smad2	-0.414757162	1
Ctps	-0.080178549	1
Iqch	-0.356139144	1
Tor1a	0.161215985543761	1
Fastk	-0.132871351	1
Dnajc11	0.114320517614381	1
Edem3	0.178670485800955	1
Dus3l	0.194518181104963	1
Taok1	-0.281367425	1
Hsf1	-0.129427133	1
Gcdh	-0.256144293	1
Hnrnpr	-0.155107039	1
Ap5s1	-0.352138022	1
Tdrd3	0.231779912485573	1
Ndufb6	-0.262881007	1
Bend6	-0.268332168	1
Rad17	-0.262173262	1
Prox1	-0.405229846	1
Cept1	0.189187988425206	1
Rpf1	-0.287904806	1
Prdm2	0.324520703349215	1
Srfbp1	-0.104792234	1
Tnrc6c	0.284251109010312	1
Dip2a	-0.166241033	1
Pon1	-0.216108562	1
Wars	0.10146464162967	1

Ift80	-0.156454379	1
Appl2	0.248219410345138	1
Tpk1	0.455816414975096	1
Tbl2	-0.340466927	1
Smdt1	-0.058100382	1
Yme1l1	-0.137580264	1
Phlpp1	0.0633730622819648	1
Ptp4a1	-0.289269083	1
Styk1	-0.068971406	1
Adss	0.514096736851372	1
Ext2	0.343482430656305	1
Ergic3	-0.443126383	1
Tedc1	-0.018569615	1
Fam122a	-0.145420399	1
Ndufaf2	-0.318340939	1
Polr2h	-0.270380651	1
Pcmt1	-0.25483662	1
Pim3	0.105368031661232	1
Tmem35b	0.456958393931806	1
Foxj1	-0.180362978	1
Zfp618	0.0406666185810169	1
Rbm34	-0.163871876	1
Kcnk1	0.440225868531597	1
Ola1	-0.081197835	1
Lyar	-0.254797326	1
Brix1	-0.119058828	1
Lbh	-0.206802232	1
Clmn	-0.355863732	1
Ewsr1	-0.154060579	1
Snx4	-0.203347087	1
Rgs2	0.0923053288409678	1
Sdha	-0.213310934	1
Katna1	-0.332831617	1
Wnt5a	-0.20951726	1
Herc1	0.491802297069286	1
Sorcs2	-0.153612242	1
Ldb1	-0.142917657	1
Rbm17	-0.310072712	1
Sftpc	-0.94348513	1
Aagab	-0.568319083	1
Hid1	0.357387549467946	1
Nedd4	0.308658111184774	1
Ttf1	-0.166360682	1
Nmt2	-0.231741127	1
Scrn1	-0.13612069	1
Stam2	-0.217919185	1

Ube2e2	0.944938960133622	1
Zfp654	0.299061411381971	1
Dock1	0.466177850220024	1
Arpc2	-0.295369423	1
Smpdl3a	0.257387975042512	1
Hacd2	-0.14932051	1
Cdin1	-0.495043311	1
Rundc1	-0.294800276	1
Wsb1	0.357025346501496	1
Rbl1	-0.081729529	1
Rcsd1	-0.441278057	1
Acbd5	-0.078408324	1
Eef1e1	-0.07180856	1
Slc49a4	-0.187371836	1
Adamts20	-0.339903422	1
Ostf1	-0.134182829	1
Ndnf	0.208223654179507	1
Gemin8	-0.288508563	1
Wdr24	-0.218091193	1
Tmem123	0.225328004530787	1
Cgas	0.24384884604909	1
Gpm6b	0.180307338412007	1
Nsmce2	-0.062189579	1
Tmem30a	-0.093364559	1
Hopx	-0.159639318	1
Ube2l3	-0.102564488	1
Steep1	-0.148551124	1
Psmb3	-0.162688362	1
Als2	-0.156576268	1
Ctnnbl1	-0.248657332	1
Pla2g6	0.25678668227107	1
Farp2	0.140528275447759	1
Trerf1	0.286768466854403	1
Abhd17c	0.0953497215920005	1
Yeats4	-0.246602578	1
Itgb8	-0.18484947	1
Nsun2	-0.160537088	1
Ap1ar	-0.074255987	1
Nrip3	0.134573751890843	1
Hnrnpm	0.321647089227876	1
Unk	0.202989901651923	1
Polr2k	-0.348147527	1
Casp1	0.663029739280691	1
Adamts6	0.819582355136777	1
Hagh	0.0704975899343533	1
Rint1	0.434992909608963	1

Vps26b	-0.202801098	1
Vti1b	-0.12051022	1
Zfyve26	-0.191579925	1
Btbd10	-0.191755522	1
Brd8	-0.021979813	1
Oxct1	-0.062455017	1
Psen1	-0.076683827	1
Ywhab	-0.254006187	1
Ube3c	0.223522270928324	1
Fam104a	-0.228456872	1
Togaram1	0.270183632193178	1
Prkx	0.755114740190176	1
Snx13	0.226901653807383	1
Dgkh	1.4871738851581	1
Qpctl	-0.174487974	1
Slc9a7	-0.199212732	1
Sobp	-0.220372586	1
Cul4a	-0.208201617	1
Oaz2	-0.526528982	1
Stom	0.344740626222334	1
Gabpa	-0.149694208	1
Vipr1	0.406552326681533	1
Tmem184b	0.116488171185869	1
Naa20	-0.2697709	1
Txnrd1	0.442723813355279	1
Thap7	-0.147426669	1
Gcnt4	-0.133070719	1
Azin1	-0.041827468	1
Vps11	-0.37909615	1
Pcgf5	0.134781007091081	1
Trrap	-0.246007764	1
Lonp1	-0.072409204	1
Sec16a	0.176831115988136	1
Irak1bp1	0.159705255267104	1
Nob1	-0.101756208	1
Specc1l	-0.243286723	1
Pnpla2	0.678662221475308	1
Colgalt2	-0.227302931	1
Psmd14	0.392886580614019	1
C3ar1	0.134529154459289	1
Unc45a	-0.095174682	1
Gtf2e2	-0.111521051	1
Pm20d1	-0.130192981	1
Lemd2	-0.094235527	1
Usp38	-0.140717499	1
Ano10	0.117130931452126	1

Eif2ak3	-0.307118114	1
Swap70	0.231167990563628	1
Pepd	-0.119654388	1
Gns	0.869126810558593	1
Tmem79	-0.117468977	1
Fry	0.604989988796765	1
Ckb	-0.605458827	1
Anks1	-0.137612176	1
Zfp318	-0.278134835	1
Arhgef26	0.294530234658177	1
Ppa1	-0.102774399	1
Tbc1d12	-0.030529311	1
Golga4	-0.084242243	1
Nrbf2	-0.129221506	1
Bcl2l13	-0.004231433	1
Wnk1	0.386331473127757	1
Serinc2	0.225340259891573	1
Itpk1	0.237523673464723	1
Lrig1	0.357548936109663	1
Tmem208	-0.048687786	1
Zfp598	-0.136676538	1
Tab3	-0.141510187	1
Atg7	0.480610288205849	1
Dbf4	-0.148268751	1
Fbxl21	-0.142874975	1
Apbb1	1.69569525401813	1
Trpc1	-0.234228826	1
Ppa2	-0.089508575	1
Ncoa1	0.285762277683438	1
Eprs	-0.228640097	1
Ramp1	0.242808595228831	1
Adck1	0.110745234856341	1
Nectin1	-0.191825839	1
Eif4g1	0.0306714984649296	1
Vps13c	0.343319345787543	1
Herc2	-0.419885348	1
Tagap1	0.0833007067028688	1
Dcbld1	0.46439059237449	1
Numa1	-0.204247654	1
Nfrkb	0.19981820900183	1
Otud7b	0.182840545119838	1
Neo1	0.378748836469233	1
Rdm1	-0.034115164	1
Pim1	-0.23613546	1
Arhgdib	-0.117445198	1
Ift43	-0.156999819	1

Rab1a	0.217501260961489	1
Ywhae	-0.283764391	1
Stxbp5	-0.489816315	1
Dnajb9	0.22128125854909	1
Entpd1	-0.164582078	1
Dennd5a	0.325817637867976	1
Enox2	-0.238354287	1
Cttn	-0.148046404	1
Csnk1g1	-0.223819475	1
Lias	-0.087319824	1
Slc4a4	1.53743079015916	1
C130074G19Rik	-0.072644528	1
Ncbp2	0.0163729481257564	1
Strn	-0.196550383	1
Cul2	-0.079324858	1
Ppp6c	0.0803868271914779	1
Sf3a2	-0.161225108	1
Ikzf2	-0.375847734	1
Alkbh1	-0.090594731	1
Slc1a4	0.0648412379183747	1
Uhrf2	-0.232605284	1
Slc25a1	-0.213785608	1
Atp9a	0.41360913569243	1
Osbpl2	-0.126549206	1
Pinx1	0.104927384386092	1
Lsm5	-0.027124696	1
Igfbp1	-0.126296895	1
Hif1an	-0.131150208	1
Crispld2	-0.171995482	1
Abcc1	0.255653884048263	1
Pals1	-0.326798084	1
Spg11	-0.068365448	1
Xpo5	-0.060422243	1
Sfpq	-0.13402129	1
Tmem140	0.00780867006374125	1
Plod1	0.223025119363329	1
Scfd2	0.390858339690895	1
Rbbp5	-0.202480901	1
Nfs1	-0.128106267	1
Tnfaip8	-0.202571467	1
Hacd3	0.290346785278226	1
Zdhhc8	-0.020700782	1
Slc30a4	0.215838941713527	1
Fam76a	0.173635399831493	1
Ngly1	0.339903266184947	1
Cpsf3	-0.099312055	1

Cdh1	0.0542086632169572	1
Fam168a	-0.039321564	1
Itpripl2	-0.270217471	1
1810058I24Rik	-0.005002728	1
Kdm3a	-0.216419067	1
Stox2	-0.17093553	1
Smpd4	0.139363679358933	1
Plppr5	0.19711922721731	1
Armc6	-0.109106029	1
Ralgapa1	0.0381929470280347	1
Trabd	0.166590138072064	1
Rora	-0.267869264	1
Fam13b	-0.439396915	1
Pou3f2	0.197760512220232	1
Prpf40a	-0.317560436	1
Rhob	-0.576172126	1
Hdac7	-0.119552578	1
Runx1t1	-0.045500132	1
Wdr92	-0.062452712	1
Cmas	-0.027970015	1
Fgfr4	-0.276998425	1
Reck	-0.153001712	1
Cyyr1	-0.238797855	1
Golph3l	0.196537350460846	1
Eloa	0.0942951934934991	1
Nol8	-0.178368877	1
Golga1	-0.070901752	1
Ccdc125	-0.337826586	1
Crbn	-0.036092208	1
Nol7	-0.03362853	1
Smox	0.255946215235408	1
Septin14	-0.302485109	1
Ccdc88c	-0.058536209	1
Tanc2	0.133926298467617	1
Aldh7a1	-0.133896316	1
Ube2f	0.27972913078735	1
Eif4a3l1	0.0438282932017788	1
Akr1c13	-0.266090872	1
Lmbrd1	0.446450992746794	1
Ndrg1	0.0937912720756846	1
Dscaml1	-0.093768804	1
Nuak1	-0.282784577	1
Uri1	-0.166139571	1
Kctd3	-0.177272201	1
Lancl1	0.0410038225353667	1
Srpk1	0.0169313393522816	1

Imp4	0.183470717552915	1
Gzf1	-0.252340869	1
Wdsub1	-0.210840664	1
Nme7	0.167088992762192	1
Rbm45	-0.365067182	1
Chchd4	-0.022460839	1
Uspl1	-0.277836151	1
Runx2	-0.176827922	1
Nup50	-0.187740779	1
Nemf	-0.344372227	1
Pde5a	0.150187335479782	1
Vps26a	-0.273913541	1
Kdm2a	-0.089565795	1
Immp2l	-0.254990065	1
Sfmbt2	0.0721245376769928	1
Ammecr1	0.303357623297775	1
Dock4	-0.352224362	1
Gpi1	-0.028916928	1
Pcmtd2	-0.140043517	1
Ppp6r3	0.421682207453903	1
Tada1	0.326858110779405	1
Ttc13	0.0669196277321637	1
Gadd45b	0.10509278456812	1
Exosc10	-0.017676166	1
Cndp2	-0.312359164	1
Ypel1	-0.216266053	1
Ttll7	-0.258850082	1
Poc1a	0.296831407721296	1
Zfp711	-0.346657155	1
Heatr5b	-0.135044392	1
Lclat1	0.686022463350706	1
Crppa	0.0669020104362488	1
Chchd5	0.0782414150539135	1
Rnf141	0.574651003273213	1
Lyn	-0.0648608	1
Wdfy1	0.237813893841821	1
Rab21	-0.002496144	1
Csnk1e	-0.058694969	1
Gfod2	-0.046901652	1
Stc2	-0.095159978	1
Dlgap4	-0.370081127	1
Creb3l2	0.307276650542452	1
Thoc1	-0.107150371	1
Tnr	0.0904418024259445	1
Clk3	0.0468312591152175	1
Ppp2r5c	0.411604887887524	1

Lancl2	-0.225883544	1
C77080	-0.351281585	1
Dld	-0.016950438	1
Pbrm1	-0.260434261	1
Lgmn	-0.152661578	1
Erich1	0.148587977334784	1
Tatdn3	-0.250065943	1
Cpsf2	-0.29691734	1
Ubxn2a	0.106669804157412	1
Phtf2	0.110189362326495	1
Aspscr1	-0.228098727	1
Dlg1	0.223424380044053	1
Rbm47	0.424494149534349	1
Tdpoz8	-0.070307367	1
Oxr1	0.184535590556038	1
Atg10	0.358363465367782	1
Wdr7	-0.010990088	1
Dph3	-0.177436338	1
Fbxo32	0.0469740896158956	1
Nnt	-0.142779877	1
Sec24a	0.0837215368335198	1
Pde3b	-0.346695143	1
Orai1	-0.256755101	1
Plcb1	0.372158756070259	1
Nom1	0.161400112191515	1
Wdfy3	0.344763005468547	1
Siah1b	0.223908423911063	1
Arl8a	-0.079386533	1
Dync1li2	0.273156908947379	1
Tmem144	-0.038500502	1
Arhgef10l	-0.167963115	1
Ogdh	-0.248468328	1
Zfp36l1	0.112367998135417	1
Serpinh1	-0.107252748	1
Dennd6b	0.0844242783439173	1
Slit2	1.2237526079033	1
Gfpt2	0.418532206447486	1
Pikfyve	-0.128775327	1
Vps29	-0.136487285	1
Ppp4r1	0.0252648542421485	1
Lnx2	-0.124758621	1
P4ha2	0.0663608785607203	1
Eps8	0.983976235691238	1
Rbm12	-0.108696249	1
Polr2f	-0.204661816	1
Bmpr1a	0.176117646654116	1

Stt3a	-0.141281687	1
Fancm	-0.142769149	1
Ptger4	0.143180408675044	1
Atg4a-ps	-0.108555179	1
Ppm1l	0.359258775536082	1
H2ac20	0.18369063816683	1
Ccdc58	-0.113769724	1
Rnpc3	-0.084484291	1
Tdp2	-0.008213244	1
Gdi2	0.128788219601507	1
Nfatc2	0.563021336896165	1
Tbc1d25	-0.130625147	1
Impg2	0.211945674755539	1
Tmed10	-0.181316597	1
Nup93	-0.409460178	1
Gatad2a	-0.151822916	1
Tmem9b	-0.136326745	1
Hmgcs1	0.8241557024207	1
Kiz	-0.122499988	1
Lmo1	0.0406559548392478	1
Adamts1	0.113994135688617	1
Abl2	0.294691135061214	1
Slc24a3	-0.291607629	1
Csnk2a1	-0.093800993	1
Dctn3	-0.349663495	1
Arhgef6	-0.224126461	1
Fra10ac1	-0.147450819	1
Pfdn2	-0.126370294	1
Tnip2	0.201440415617792	1
Cdc42bpb	-0.353187946	1
Golt1b	0.149700279029451	1
Tead1	0.373469926621538	1
Szt2	-0.155321393	1
Hpf1	-0.09753309	1
Dpp10	0.548843137164206	1
Stk39	0.369172163127475	1
Scrn3	-0.128142765	1
Cdc14a	-0.524925359	1
Tnfsf10	0.101388801438966	1
Itgb1	-0.082609823	1
Far1	0.177276724227451	1
Cyp2d22	-0.120107534	1
Rfc4	-0.18431512	1
Ptpn9	-0.205511695	1
Rragd	0.00917939137970236	1
Fbxw8	-0.254927796	1

Adgrg6	-0.195172152	1
Wtap	0.147435638808393	1
Ccnc	-0.203974815	1
Stx8	-0.158045392	1
Cacna2d1	0.455656006202618	1
Inpp4a	-0.216739442	1
Atp1b3	-0.015322167	1
Bdp1	-0.172170511	1
0610040J01Rik	-0.274221573	1
Scamp1	0.264855921413711	1
Unc119b	-0.037293995	1
Large1	0.364521587015375	1
Atp2c1	-0.046333256	1
Usp53	0.103842357577278	1
Fgfrl1	-0.658006958	1
Nupl2	-0.191177777	1
Ptprs	-0.291670175	1
Wsb2	-0.231279604	1
Ube2j2	-0.02899982	1
Nufip1	-0.252705212	1
Kbtbd8	0.094684833963716	1
Echdc2	-0.088129006	1
Iffo2	0.0290394782115415	1
Hmox2	-0.118723539	1
Prim2	-0.195620279	1
Hmcn1	-0.279223981	1
Tcerg1	0.208363888538187	1
Arfgef3	0.186280223110487	1
Hlcs	0.408988947251856	1
Tax1bp1	0.509645855126067	1
Pik3c3	-0.336557937	1
Cox10	-0.257623666	1
Lin54	0.302411576924235	1
Oma1	-0.175284039	1
Sar1a	0.0553990259649539	1
Rprd1a	0.146864373143669	1
Apoo	0.0624021189444381	1
Pde7a	0.113319328568208	1
Hnrnpk	-0.26339085	1
Cpne1	-0.101784944	1
Hook3	-0.110269031	1
Wdr26	-0.310996579	1
Arhgap5	0.326207518756501	1
Usp24	-0.362811168	1
Trim2	-0.074247131	1
Synm	0.163720489095243	1

Cfap45	-0.185698911	1
Nipal3	0.466102435919748	1
Slc43a2	-0.118099765	1
Scaf8	0.00724575780192127	1
Tex2	0.245849503319678	1
Rab12	0.0170995737796367	1
Grb2	-0.253755259	1
Cnot6	-0.347038494	1
Wdr81	-0.127550189	1
Paf1	0.0830826921674718	1
Isca2	-0.093281282	1
Tmem185a	-0.133801417	1
Cryl1	-0.096060745	1
Dnmt3a	-0.325053711	1
Rab40c	-0.09927587	1
Phactr2	-0.136745729	1
Gabrr2	-0.016215994	1
Slc25a20	-0.207072373	1
Nop53	-0.136631006	1
Sgsh	0.153854709296312	1
Pspc1	0.152438925870173	1
Zfp407	0.11091284332695	1
Gbf1	0.0365562904156822	1
Ncoa2	-0.130678424	1
Lamb1	-0.118146634	1
Alas1	-0.133745939	1
Irx5	-0.348968532	1
Cldn1	0.617565768458443	1
Usp20	-0.019853781	1
Tgfbr2	-0.264790523	1
Fyco1	-0.028313041	1
Tvp23a	-0.120973198	1
Blmh	-0.125588753	1
Creld1	-0.235835306	1
Cul4b	0.085744662730844	1
Lyrm1	-0.092158002	1
Borcs8	-0.05143996	1
Slc25a16	-0.278233854	1
Zfp148	-0.145025838	1
Slmap	0.21050798437913	1
Fbxo38	-0.170923261	1
Prkar1b	-0.203296778	1
Dhx8	-0.136187676	1
Nfyb	0.276301694884507	1
Utp3	-0.219839244	1
Atg12	-0.190380314	1

Dusp7	-0.39087705	1
Npepps	-0.125707399	1
Fggy	0.370735272028591	1
Bcl2	-0.149467925	1
Atp6v1e2	-0.149984014	1
Prkd3	-0.093099217	1
Yipf5	-0.107637978	1
Ankle2	-0.217795469	1
Fam160a1	0.209215072909961	1
Pcgf3	0.236569998211195	1
Arrdc1	0.145386444712172	1
Eml1	0.223103124332769	1
Dpp7	-0.079063209	1
Ufm1	-0.280115845	1
Tulp3	0.105529013758205	1
Rock1	0.214049233197564	1
Cox16	0.135701395056479	1
Cep164	-0.006934442	1
Areg	1.0220460339847	1
Ropn1l	0.170983318258475	1
Necab2	0.22887673641781	1
Polg2	-0.084507163	1
Tpst1	-0.11540445	1
Tbc1d23	-0.098814481	1
Ncapg	-0.215425509	1
Srgap1	0.0835987905638318	1
Etf1	-0.131754736	1
Ntan1	-0.062431419	1
Anxa1	-0.091734093	1
Prkrip1	-0.070238562	1
Chmp7	-0.066592812	1
Senp5	-0.267332494	1
Smoc2	0.231693532346369	1
Pla2g7	-0.258401066	1
Pphln1	0.143848569867548	1
Sestd1	-0.059473481	1
Supv3l1	-0.342663837	1
Spz1	-0.100295796	1
Cdc27	-0.011296655	1
Plag1	0.0211818218526407	1
Katnip	0.344620997495004	1
Spire1	0.537887788402199	1
Snhg4	-0.066038347	1
Clint1	0.231642485298695	1
Angptl3	0.144215241789883	1
Hs6st2	-0.430832176	1

Xkr4	0.100229106797383	1
Rnd3	0.366505579147689	1
Hic2	0.22571835751618	1
Kank1	0.141384437478909	1
Cpq	0.291116001490113	1
Wdr53	-0.184180674	1
Cramp1	-0.125091916	1
Spg7	-0.136547669	1
Kpna3	-0.263050941	1
Smim19	-0.065384315	1
Kank3	0.151997087023566	1
Cnot7	-0.26613055	1
Tshz1	-0.178226746	1
Hace1	-0.02460496	1
Epha4	0.108996726297776	1
Notch2	-0.174595798	1
Tagln2	0.0533814827153857	1
Ergic2	-0.104772152	1
Scnn1b	-0.207109339	1
Dohh	-0.164717989	1
Atf2	0.236232701956277	1
Ube2o	-0.165733097	1
Slc2a8	-0.054359356	1
Kat2b	-0.243598857	1
Nolc1	-0.106905868	1
Snip1	-0.108805418	1
Ldlrad3	0.0359930772002943	1
Enkur	0.0790574320376596	1
Arl6ip4	-0.402379673	1
Cog6	-0.124168034	1
Rab20	0.504787638759914	1
Uggt1	-0.041757188	1
Rnf11	0.486922820484847	1
Il13ra1	0.0885325849738952	1
Scamp2	-0.10746875	1
Taf5	0.147065928347903	1
Gstp3	0.0101614295248129	1
Rrbp1	0.166490296185437	1
Commd1b	-0.039715071	1
Odad2	-0.092265387	1
Bnc2	0.100740091646985	1
Ercc3	0.134749088454211	1
Col5a2	-0.092319738	1
Ccdc91	0.509241618130518	1
Il1b	0.357453401340106	1
Rnf144a	0.474997378145066	1

Prickle2	-0.270278927	1
Prpf3	-0.114517508	1
Cadps2	0.602960856059665	1
Twf2	-0.244719634	1
Nrip1	0.109958820932986	1
Trip11	0.209884326427464	1
Prr16	0.288630293438611	1
Rad51b	0.0103112377287431	1
Pafah1b2	0.055021976940919	1
Ptdss1	0.311840907383084	1
Rnf128	0.440301036747704	1
Fnip2	0.45925033239668	1
Ptges	-0.295098301	1
Vamp4	-0.308160298	1
Yju2	-0.127031979	1
Tns1	-0.143265428	1
Tmtc3	-0.032744007	1
Gpsm2	-0.095254856	1
Brap	0.0162482406491454	1
Hbp1	0.141491907834449	1
Trappc9	0.41419631461509	1
Wasf2	0.0580099011695427	1
Tns3	0.168908642230036	1
Pin4	-0.155649314	1
Enpp4	-0.269588189	1
Rtn4ip1	0.0979581439505474	1
Skap2	0.216719791360773	1
Grk4	-0.096638761	1
AI597479	-0.057926033	1
Washc1	-0.012378243	1
Espnl	-0.332760925	1
Fkbp5	0.736177692872279	1
Chfr	0.121072298407994	1
Nrd1	-0.022485081	1
Ankib1	-0.344827872	1
Lrp6	0.084584428329425	1
Stau2	-0.023227771	1
Chp1	0.579019550240923	1
Cnnm2	0.0567500092607043	1
Arl6ip5	-0.224606497	1
Ltbp2	0.0854373465844967	1
Eftud2	-0.106913056	1
Capn5	-0.038908103	1
Ddx59	-0.254665483	1
Pak5	0.575124269885019	1
Dnajc10	0.260957346478764	1

Slc41a2	0.163656350226528	1
Ubn2	-0.190741649	1
Poldip2	-0.063307286	1
Shld2	0.413762183771993	1
Tacc3	0.0169394483331994	1
Rev3l	-0.012261107	1
Ranbp3	0.0635468753655446	1
Hpgds	0.433372510432532	1
St8sia4	-0.27680925	1
Npas3	0.204808634478103	1
Zfp451	-0.226727911	1
Kctd6	-0.085780628	1
Hmgcr	0.10949045106933	1
Btbd3	0.0193195538921214	1
Ilf3	-0.1565147	1
Ccdc149	-0.013982884	1
Zcchc7	-0.476415315	1
Sbf1	-0.02571957	1
Gpx3	-0.138436316	1
St7	0.436425228331286	1
Dhrs9	-0.17476151	1
Dpysl2	0.282449143580811	1
Stub1	-0.196258906	1
Rapsn	0.00876086366090889	1
Prdx1	0.341359660140792	1
Ginm1	-0.128181799	1
Prlr	0.216768307785145	1
Gpr68	0.0195512318206637	1
Cyb561	-0.099702273	1
Tmem141	-0.093305599	1
Slc16a1	0.195887429946659	1
Rabgap1	0.0686972531580647	1
Casc3	-0.055638105	1
Txlna	0.079250623555735	1
Ddx55	-0.370800819	1
Adarb1	-0.116168579	1
Stim2	0.0718740580620643	1
Blcap	0.196666873485384	1
Snx24	0.059716014821387	1
Atf1	-0.160469509	1
Tardbp	-0.027003024	1
Agbl1	0.207350804521282	1
Ubr5	0.147382231062871	1
Fbxo28	-0.07275694	1
Tcp11l2	-0.256065352	1
Setd2	-0.309036116	1

Rab29	0.210655463791121	1
Acsl1	0.245569561747945	1
Itgav	0.175745230999102	1
Tmem199	-0.110380658	1
Plch1	-0.33963802	1
Ormdl2	-0.114662975	1
Rer1	-0.216216485	1
Fam53a	-0.208961738	1
Epcam	0.027740408953365	1
Ntng1	0.826445453455918	1
Xpo6	-0.191193578	1
Sik2	-0.19662018	1
Psmf1	-0.095950125	1
Sash1	-0.022100031	1
Ppp2r5a	0.036690199686038	1
Zfand4	-0.174913105	1
Galk2	-0.097780492	1
Rheb	-0.025579306	1
Lman1	-0.127317587	1
Tmem33	-0.292784495	1
Fam32a	0.12476986900184	1
Plekhg5	-0.137864969	1
Abcd2	0.102229289550937	1
Fbxo3	0.0330336536093349	1
Prrc2b	-0.184912797	1
Ppp1cb	0.0751646974968947	1
Aktip	-0.068145295	1
Fam174c	-0.212191214	1
Arpc3	-0.281301946	1
Rev1	-0.192446471	1
Ildr1	-0.208735557	1
Wwp1	0.643441097767466	1
Slc35a3	0.168390376791906	1
Ilkap	-0.051473431	1
Rasgef1a	0.419979153157056	1
Odc1	-0.257999188	1
Tlcd4	-0.271384485	1
Dpp4	0.377553898411693	1
Wdr47	-0.054487612	1
Fancd2	-0.176329297	1
Lpar2	-0.107356896	1
Drd3	0.118397577482452	1
Aff1	0.243767818945682	1
Gpd2	0.0865281226837168	1
Aopep	0.755435386197554	1
Ccdc92	-0.048319074	1

Klf7	0.479261353431787	1
Adamts15	0.173917977561407	1
Ppp2r3c	-0.030137872	1
Cd2ap	0.398807126924388	1
Ttc17	-0.304968019	1
Eif2a	-0.592819596	1
Npy	0.50986870261691	1
Ap4e1	-0.102418915	1
Anxa5	-0.218907877	1
Arid3b	0.036445741596102	1
Usp44	0.0208149137915301	1
Xrcc3	0.0661285765012718	1
Ogfrl1	-0.052025504	1
Itpkb	-0.119392594	1
Xdh	-0.005862047	1
Kif5b	-0.090652426	1
Efcab14	-0.166208553	1
Amot	-0.044229362	1
Rnf38	-0.116270055	1
Tex9	0.0257888652975602	1
Snap91	0.0102419196697354	1
Noc3l	-0.128970143	1
Glyr1	-0.029402048	1
Pan2	-0.165098793	1
Rae1	0.160690485239279	1
Usp14	0.0969299767866766	1
Ttll5	0.103351334262545	1
Retreg1	0.139773290898035	1
Pdia5	-0.215421726	1
Fez2	-0.276790486	1
Egfr	0.427521770174183	1
Slc25a21	0.019420209337814	1
Tsen54	0.00772317561616581	1
Dph6	0.246171479861893	1
Otud6b	-0.067704425	1
Nifk	-0.079265149	1
Hmces	-0.052626888	1
Haus6	-0.045580785	1
Letm2	0.0272016838814773	1
Nt5c3	0.360639791414407	1
Spred2	-0.163324696	1
Plcb4	0.104950118402425	1
Dennd1a	0.310912758844685	1
Chchd3	0.262105532746986	1
Ccnl2	0.113167011808744	1
Arfip1	0.204056874197075	1

Clcn3	0.266528099884508	1
Fmo3	-0.443630367	1
Tubb1	-0.048086374	1
Edem1	-0.182120401	1
Thada	0.0546651473731564	1
Slitrk6	-0.042765266	1
Sh3gl1	0.240732820564111	1
Rpgr	0.255902878581121	1
Gpatch2l	0.154186429766654	1
Ap1s2	0.176298968049326	1
Rhou	0.424631029411685	1
Nhsl1	0.334742768411052	1
Ggt7	-0.194021144	1
Taf12	-0.058299657	1
Serpini1	-0.116119983	1
Aldh18a1	-0.005429796	1
Gabarapl2	-0.253435259	1
Igsf3	0.0580806727523351	1
Ranbp17	0.241763548265233	1
Elovl6	-0.104975728	1
Unc93b1	0.0119997222725359	1
Nploc4	-0.196252935	1
Lsm6	-0.071711926	1
Fam20c	-0.049784206	1
Dysf	0.134385475575125	1
Hmg20a	0.0493724685969242	1
Dennd6a	0.036859172647273	1
Dpm2	0.0383984341831894	1
Ppp1r12b	-0.069517605	1
Bloc1s4	-0.139144075	1
Trmt6	-0.102807369	1
Tsc22d2	-0.155705707	1
Plcb2	-0.274238667	1
Senp7	0.275637639158018	1
Gnb1	0.319192103544908	1
Agpat2	-0.061128654	1
Rabep1	-0.139859197	1
Dcun1d1	-0.037639957	1
Trappc6b	-0.005752073	1
Clock	-0.023350238	1
Lpgat1	0.0133925615895605	1
Zdhhc23	0.244878167785629	1
Pkd1	0.0106525690079871	1
Sf3b3	-0.170515377	1
2700049A03Rik	0.146433027015491	1
Stag2	-0.099255554	1

Dnajb14	0.0442297933411103	1
Cntrl	-0.02104695	1
Jpt2	-0.248106776	1
Vps45	0.0539940626148592	1
Tmx4	-0.108059731	1
Cmtm7	-0.148338843	1
Tfpi	-0.052648883	1
Helz	0.151486018964739	1
Enah	0.23874201637998	1
Ptbp1	-0.055628193	1
Vcp	0.172169128391673	1
Slc38a2	0.549784187147967	1
Srp68	-0.161938913	1
Ttc33	-0.311201746	1
Cyrib	-0.307295577	1
Snx33	-0.159794258	1
Rsbn1l	-0.129048174	1
Cavin1	-0.133351447	1
Dap3	-0.124088209	1
Reep3	-0.057109404	1
Nlrc5	0.356562971482865	1
Ippk	0.00059819260772398	1
Snx12	-0.102078007	1
Jak1	-0.096010488	1
Osgin2	-0.17158274	1
Rpap1	-0.030604166	1
Nrbp1	0.241638614171862	1
Stxbp3	0.0229421440746707	1
Osbpl6	1.24388909123468	1
Plekha2	-0.009078721	1
Pus10	0.287948675625653	1
Cobll1	-0.032488417	1
Arhgef3	0.0896693126645718	1
Tnfrsf21	-0.040570784	1
Slc35e2	-0.201294999	1
Tmem181a	0.194545180570772	1
Bcl2l1	-0.018060519	1
Ranbp10	0.0331010280890465	1
Bclaf1	-0.07956536	1
Pkn2	-0.018243406	1
Trappc8	0.0201717868594051	1
Pnrc1	0.154960026038945	1
Nr3c1	0.179718084695849	1
Washc5	-0.159712071	1
Usp36	-0.196410634	1
Pdcl	-0.168012726	1

Fbxo33	-0.089615421	1
Akap11	-0.156212679	1
Uba3	-0.183357014	1
Dlg3	-0.362486115	1
Sae1	-0.075326285	1
Glb1	-0.024369831	1
Txnrd2	0.0810782285390954	1
Faf1	0.116947895135201	1
Cbfb	0.0672045208105272	1
Rhov	-0.221906954	1
Dcxr	0.0847803949582309	1
Rab2a	-0.11182284	1
Gatb	0.103429658253833	1
Sesn1	-0.162219169	1
Tbc1d32	-0.124768099	1
F3	0.690315889118721	1
Gnb4	-0.077931969	1
Ncoa6	-0.143384461	1
Osbpl9	0.0115628270393562	1
Lactb	0.0683643677864875	1
Dbr1	-0.217530306	1
Sec31a	0.175146297942157	1
Lsm4	0.230545014548848	1
Phf12	-0.25681427	1
Amd2	0.168226518032726	1
Cpeb3	-0.001681636	1
U2af2	0.0760514212329392	1
Spata5	0.405366368723848	1
Ino80c	-0.103683106	1
Nup133	-0.1082401	1
Trub1	0.177983301246611	1
Rgs7bp	0.274533657310841	1
Brd1	-0.117415721	1
Hadh	-0.165649904	1
Slc34a2	-0.09388262	1
Ruvbl1	-0.063883927	1
Ccnyl1	0.083860685547632	1
Sorl1	-0.233600849	1
Slc35b1	-0.036890856	1
Tmc7	0.0617761528596096	1
Pan3	0.398456753598817	1
Atp10a	0.731614471553228	1
Rrp1b	0.0438571724326992	1
BC051665	-0.120666563	1
Ythdc2	-0.162515209	1
Sh3kbp1	0.23289609079271	1

Abhd2	0.176315852573817	1
Slc37a1	-0.012071081	1
Ttc32	-0.092789202	1
Spopl	-0.377613058	1
Scaf11	-0.082435546	1
Trh	-0.584372989	1
Nup107	-0.06014359	1
Nras	-0.075264129	1
Actr8	-0.091911018	1
Eif5b	-0.09014904	1
Exoc6	0.161260003767736	1
Tmem101	0.0291603010480123	1
App	0.134923694233555	1
Naaladl2	0.843394086712376	1
Chd2	0.275048061245023	1
Nfib	0.126362158244259	1
Dcaf5	0.0262268798257019	1
B3gntl1	0.147567335380346	1
Tmem135	0.405202805159217	1
Comt	0.0583853334488233	1
Smarcd1	-0.121569625	1
Xrcc4	0.234978349793638	1
Ppp1r10	-0.25733568	1
Stk40	0.285528296201552	1
Prmt9	0.278005503114633	1
Naa15	-0.179549116	1
Ktn1	-0.205496768	1
Ryk	0.137450665635202	1
Unc50	-0.071871735	1
Qars	-0.073177845	1
Clic3	0.234240360573266	1
B3gnt5	0.0189015385436017	1
Rab6a	-0.040752118	1
Cep112	0.0501857281459136	1
Rnf145	-0.117967662	1
Tnks2	0.0993549765425353	1
Gtdc1	0.077074123807497	1
Csnk1d	-0.008861404	1
Larp7	-0.041671766	1
Rgs1	0.643882504436969	1
Chd1	0.12272139388083	1
Nexmif	0.129013348312408	1
Zbtb38	-0.369221807	1
Eri3	0.106839304002498	1
Bend7	0.10637655678597	1
Prep	-0.129106412	1

Sema4d	0.0544781069417672	1
Adgrl2	0.386036752422102	1
Glcci1	0.459252538550742	1
Abcb7	0.0666791729344804	1
Arl4c	0.273130102883059	1
Arpc1b	-0.217987112	1
Nol11	-0.35984402	1
Zdhhc7	-0.207950497	1
Lrpprc	-0.239299918	1
Ankmy2	-0.008132408	1
Pum1	0.131991097346368	1
Ppfia1	0.348485064528896	1
Frmpd4	0.133224300008302	1
Hprt	-0.199794931	1
Vps54	-0.192650559	1
Usp49	-0.026422545	1
Pou2f1	0.481480530789218	1
Slc2a1	-0.075329023	1
Tmem184c	-0.138389794	1
Abca1	0.0604412272190807	1
Ccz1	-0.111016764	1
Gclc	0.372947878889333	1
Dhrs3	0.525143588179559	1
Ppp2r2a	0.124482420907196	1
Ptbp2	-0.015667751	1
Slc30a7	-0.172238217	1
Pde6d	-0.19457473	1
Ano6	0.228861737751164	1
Luc7l	-0.174789121	1
Oxnad1	-0.202490854	1
Dgkd	-0.18467761	1
Setd5	0.0273686929346215	1
Zc3h14	0.0115984238027645	1
Stk38l	-0.056780137	1
Chic2	-0.073255255	1
Flot2	0.204606038853813	1
Tmem11	0.055670874200668	1
Cdk19	-0.104515753	1
Zcchc2	0.0165076887250346	1
Rin2	-0.105121678	1
Wdr59	-0.056843966	1
Rap1gds1	-0.155546825	1
Cpne2	-0.38512784	1
Ubap2l	-0.060060924	1
Dipk2a	-0.124258758	1
Tpd52l2	-0.003808415	1

Tent4b	-0.009228642	1
Rap1gap2	-0.088016798	1
Rarg	-0.210850533	1
Arl8b	-0.195815862	1
Sqstm1	-0.206521445	1
Ddx51	0.00197803616818792	1
Rab3a	-0.116456048	1
Rpap2	-0.033727178	1
Pdzd8	0.206039738384072	1
Dync1i1	0.0578155559322899	1
Ints2	-0.027268059	1
Spry2	-0.12369046	1
Cd83	0.19664774888399	1
Eepd1	0.0563823349304401	1
Zfp512b	-0.067688692	1
Ncam1	-0.091696787	1
Utp23	-0.122392345	1
Ccdc18	-0.134867493	1
Inpp5a	0.302617744139506	1
Slc4a1ap	0.0928856337135246	1
Tes	0.288954165793952	1
Plxnb2	0.314393012445622	1
Zrsr1	-0.019816145	1
Rpa3	-0.017237615	1
4933400A11Rik	-0.002364705	1
Fndc3b	0.323519718422724	1
Adam9	0.0413564913755224	1
Cltc	-0.033480496	1
Gas7	-0.101969607	1
Golim4	0.429238600904492	1
Rnf14	0.0695696654957771	1
Clasp1	0.148302773731245	1
Rnf34	-0.089689042	1
Zbtb8b	-0.018230151	1
Slc35e3	-0.162095463	1
Cdc34	-0.187335349	1
Cemip2	0.187841468379547	1
Elovl5	0.020001927026207	1
Dhx29	0.0614914143111017	1
Esco1	0.0968348943110284	1
Tcf3	-0.025693912	1
Ripk4	-0.237396286	1
Gars	-0.231821892	1
Tmem231	-0.172967208	1
Acyp2	0.0137068719984318	1
Actr3	0.190522817421054	1

Dctn5	0.1481537092391	1
Strap	-0.05154253	1
Txndc11	-0.104232966	1
Btrc	0.00151823747196311	1
Pcbp2	-0.161334619	1
Cwc22	-0.041633471	1
Snx8	0.0633654219477479	1
Snx17	-0.185668863	1
Sergef	0.27772545222401	1
Fes	0.116455106722519	1
Fbxw2	-0.142262736	1
Wdr20	0.374805341061978	1
Cnp	0.0202197399923187	1
Sumf2	-0.059343673	1
Calcrl	-0.434989243	1
Atg9a	-0.080177518	1
Zhx1	0.00505658396227282	1
Gne	0.602573501074138	1
Ensa	0.0103935860558501	1
Nr1h3	0.0736865024564208	1
Serinc5	-0.061385216	1
Ppfibp1	-0.173469258	1
Ppie	0.145102803895025	1
Scyl2	-0.017532864	1
Jazf1	-0.070472317	1
Limk2	-0.124466965	1
Zbtb8os	-0.04415582	1
Nars2	-0.017827239	1
Sgpp1	0.0902397688396661	1
Washc3	-0.066899055	1
Dcaf7	-0.164285141	1
Farp1	0.621094483747235	1
Ehmt1	-0.106962231	1
Krit1	-0.250065924	1
Zc3h7b	-0.051217561	1
Vac14	-0.114547726	1
Galnt11	0.024848712135787	1
Ccl20	0.827472318854944	1
Kmt2e	0.222430811355111	1
LTO1	-0.079480761	1
Tapt1	0.0222593125178574	1
Rmc1	0.0148002173337827	1
Smim14	0.0114339249987783	1
Rassf1	-0.059655024	1
Plch2	0.0384464709925934	1
Heca	0.666026048798288	1

Dlg5	0.133107471471436	1
Chm	0.131773278647004	1
Pard6g	0.322881774217395	1
Ankrd54	-0.004062438	1
Srsf2	-0.260760951	1
Slc9a8	0.161193526045181	1
Commd10	0.472206181000461	1
Col4a1	-0.267477321	1
Rbbp8	0.333665477487724	1
Fbxo21	0.00784686877379054	1
Zmat5	0.0630141865575866	1
Cyb5r2	-0.135242428	1
Als2cl	0.133675673322693	1
Prkcz	0.119785501095364	1
Stt3b	0.270353211722037	1
Zfp462	-0.067684386	1
Smap2	0.0956696944937867	1
Rnf214	-0.072147482	1
Calr	-0.051185079	1
Rbm6	0.0343305162235045	1
Ndufs4	0.276861305985457	1
Cry2	0.115481232654154	1
Suco	-0.062807921	1
Atp9b	-0.060593948	1
Dctn1	-0.173856416	1
Wnt5b	0.0757510865279392	1
Agps	-0.184996994	1
Nr2c2	-0.137605284	1
Lonrf3	0.186451587135001	1
Zc3h11a	-0.093199624	1
Herpud2	0.180964637496036	1
Hscb	0.0528674685979788	1
Klhdc1	-0.036538001	1
Cnot11	0.0837354440753942	1
Stx12	0.0367266682828202	1
Rap1b	-0.157014705	1
Nus1	-0.088374192	1
Tmem50b	0.260502635366499	1
Acvr2b	-0.154175031	1
Polr3a	-0.098309866	1
Aqp1	-1.310513879	1
Nhs	0.483168564872107	1
Kifbp	-0.093938222	1
Ifnar1	-0.150970465	1
Emc6	0.0137677008736492	1
Aco1	0.171480742939202	1

Tbc1d22a	0.132271329428935	1
Dnah12	0.11887030369786	1
Prrc2c	0.22732693343126	1
Psmc5	-0.020887521	1
Bcas2	-0.115055185	1
Pip5k1b	0.175942002758445	1
Ddx27	-0.03276864	1
Dnajb12	0.0911402545560451	1
R3hdm4	-0.146039934	1
Parp4	-0.155399941	1
St3gal3	-0.17446269	1
D630039A03Rik	-0.089011989	1
Whamm	-0.046552843	1
Galnt18	0.0723188714426423	1
Kitl	0.280478903551511	1
Arhgef38	0.379403675906079	1
Zfpm2	0.763067461191991	1
Emilin2	0.175098492447167	1
Cds1	0.0994297866832373	1
Selenof	0.105945108637635	1
Prpf39	-0.234991994	1
Trim71	0.1708112601296	1
Zbtb49	0.0241654238444927	1
Rab10	0.210503848875103	1
Hsbp1l1	-0.141112534	1
Abcd3	0.222095023133582	1
Tmtc1	0.0632838873386445	1
Zbtb7a	-0.018548646	1
Sucla2	0.200254771248386	1
Ptprd	0.623739491385034	1
Greb1l	-0.220107802	1
Ctnnbip1	-0.164175188	1
Emc8	-0.035071156	1
Gcnt1	0.199399799549384	1
Nmt1	0.0611651180465086	1
Tesk2	-0.124502555	1
L3mbtl3	0.268751681827403	1
Paxbp1	-0.005498049	1
Pcid2	-0.157423874	1
Pip5k1c	-0.053179587	1
Copa	-0.086749801	1
Dnaja2	0.0723503458572583	1
Erp44	-0.117583558	1
Lonrf2	0.0956957442999533	1
Jade2	-0.17394338	1
Slc6a6	0.443476913542411	1

Stk24	-0.091754428	1
Ctbs	-0.049203817	1
Hdac11	-0.189548851	1
Wapl	0.280944918644301	1
Rock2	0.0958906929500116	1
Tab2	-0.07397937	1
Pias2	-0.135789346	1
Hdac3	0.0192982730822884	1
Exoc7	0.168122738587456	1
Kcnn2	0.167221774096004	1
Ftsj3	0.119961441065361	1
Ap3b1	0.0456281983358886	1
Agr2	-0.334178392	1
Svep1	-0.080392031	1
Uxs1	0.188379030722773	1
Tpm1	-0.009322214	1
Cab39l	0.207541722691289	1
Brd7	-0.05107553	1
Prmt7	-0.331577624	1
Sntb1	0.786076755265619	1
Iars	0.194023289329358	1
Tk2	0.0670294700158107	1
Gfm1	-0.192891879	1
Efnb2	0.127767537405433	1
P4htm	-0.040803602	1
Rapgef5	0.26853200442009	1
Arsg	-0.245334048	1
Cdc73	0.0312871313371981	1
Chmp5	-0.23959636	1
Cactin	0.0948417510682683	1
Capn7	0.0880860305187778	1
Sh3bp4	0.121882784126037	1
Hmbox1	0.0365174556937183	1
Slc23a2	-0.18655603	1
Sod2	-0.146119812	1
Nab1	-0.214428137	1
Pacrg	0.500959025364157	1
C2cd5	0.0713472050882811	1
Ncald	0.371632757243583	1
Dazap2	-0.103480315	1
Git2	-0.288258519	1
Atxn10	0.174129213844214	1
Nol10	0.0688920606007101	1
Ube2h	-0.00687113	1
Rufy3	0.117389015923457	1
Scfd1	0.0737948725543534	1

Pde3a	0.560820795306256	1
Acta2	0.313167302673227	1
Nanp	-0.024568799	1
Pcsk7	-0.059145388	1
Phldb2	0.254420101317797	1
Sptbn1	0.0704555756715557	1
Brip1	-0.118416389	1
9930021J03Rik	-0.19022179	1
Nutf2	-0.073990897	1
Ube2w	0.405183099345161	1
Ubqln1	-0.092430993	1
Pum3	-0.239047569	1
Casp6	0.0231553742159697	1
C1d	-0.034047079	1
Prkcsh	-0.10918033	1
Dcp1b	-0.196537128	1
Taf15	-0.125907177	1
Zzz3	-0.25838024	1
Skil	0.161121346115693	1
Ip6k1	0.100701642255122	1
Pdhx	0.0309809272737991	1
Slc25a14	0.063953900042691	1
Ube2k	-0.014694584	1
Ube2d2a	0.0227543361855136	1
Acvr1	0.1026168795014	1
Plekha7	0.273312790800991	1
Cers5	-0.142247164	1
Nr4a1	0.0100293952272944	1
Aldh3a2	-0.084998928	1
Fbn1	-0.129018339	1
Pex14	-0.128500937	1
Xylt1	0.653380780576122	1
Lonp2	0.255312059556151	1
Camsap2	0.171231199364126	1
Kmt2c	-0.215570477	1
Kif2a	0.0818367417969939	1
Extl3	-0.077125802	1
Vps37b	0.140939686047163	1
Acads	0.0635365671821314	1
Tmbim1	-0.152784751	1
Chchd10	0.0924659056266535	1
Capn11	0.00082446974623146	1
Fbxw7	-0.151003795	1
Rit1	0.141623028122949	1
Nip7	-0.080834164	1
Ppp1r8	-0.051009473	1

Zfhx3	-0.103992672	1
Sdhc	-0.020937648	1
Psmd7	-0.086966734	1
Tbck	-0.162497911	1
Cdc37l1	-0.042282586	1
Castor2	-0.199926005	1
Ccn1	0.514299984540778	1
Gpatch8	-0.316884492	1
Cradd	0.271096878091268	1
Tmem245	0.0752653078503918	1
Zdhhc5	-0.034936217	1
Terf2ip	-0.162153397	1
Pofut1	-0.091679442	1
Foxp2	0.0519627362170112	1
Cmss1	-0.014607636	1
Ppargc1a	0.139148753035161	1
Krt20	-0.036048458	1
Txn2	-0.04213715	1
Lrrc28	-0.275077968	1
Gata3	-0.42276904	1
Naxd	-0.058780304	1
Prkdc	0.0264476210487746	1
Hspa5	0.276764027670136	1
Kcnq5	0.517704022113731	1
Anks1b	0.312935763722763	1
Ctnnb1	0.225820127284464	1
Cnot2	0.165333667615732	1
Tm9sf2	-0.257322863	1
Stx19	0.562918359304951	1
Zc3h6	0.0400282072802878	1
Ice2	0.0120643453054727	1
Sgk3	0.274792403723197	1
Tnrc6a	0.209623985094487	1
Ube2e3	0.129498627175844	1
Bicd1	-0.094393077	1
Bmx	0.159675195261062	1
E2f6	0.114235518005338	1
Fbxo42	-0.127035686	1
Sirt6	0.175234079199008	1
Erbb3	0.0109145465806515	1
Adamts3	-0.311270058	1
Pbx1	-0.012100734	1
Ndel1	0.222799508781739	1
Yes1	0.0413647366309444	1
Sema5b	0.325722390815927	1
Arhgef11	-0.135843601	1

Trip12	-0.031854276	1
Slc39a10	-0.188225621	1
Ube2e1	-0.127525994	1
Rabl6	0.102621537074418	1
Tut7	-0.133591161	1
Ccdc186	0.131413612907809	1
Supt3	0.415328851431178	1
Socs5	-0.076990143	1
Ppl	0.0202861503028384	1
Sorbs1	-0.186000302	1
Capn15	-0.085898231	1
Hs1bp3	0.0377288029444879	1
Cfap298	0.016835851664354	1
Tra2a	0.0133154093400023	1
Phf10	0.0344732858753185	1
Snx25	0.178972614102514	1
Pdk4	0.404767709902569	1
Gphn	0.360317915076233	1
Dera	-0.108061645	1
Dop1b	-0.097848509	1
Cog7	-0.094548035	1
Klhdc4	0.0475214876005733	1
Srf	0.00589423358706997	1
Kctd2	-0.025629812	1
Timm17a	0.0316643028104854	1
Wrn	-0.194242507	1
Tyro3	0.0189648702123344	1
Capsl	-0.296627722	1
Nsf	-0.080331496	1
Runx1	-0.062566127	1
Fam20b	-0.184485899	1
Sp1	0.183221266999356	1
Ttc39c	-0.097914301	1
Pkib	0.370956975211767	1
Creb3	0.0101778294032459	1
Lhfp	-0.012212429	1
Nhp2	-0.017955924	1
Ppp3r1	0.135867691149993	1
Wwc1	-0.121477438	1
Ahr	-0.087700818	1
Ints8	-0.14647101	1
Ahctf1	-0.118503386	1
1110059G10Rik	-0.200861284	1
Lmo4	-0.110402459	1
Ubn1	-0.005091828	1
Slc45a3	-0.147397871	1

Bicc1	0.118466792922424	1
Sptan1	0.182778956972497	1
Inpp5d	-0.012437366	1
Ttc39a	-0.283329812	1
Tanc1	0.24584434375314	1
Sptlc1	0.0723957229971139	1
Psmc1	0.129325433955622	1
Zfp385b	-0.015084851	1
Neurl1b	-0.664358978	1
Efnb1	-0.071080061	1
Selenbp2	0.307055445063152	1
Adcy9	0.0100079031720373	1
Kif21a	0.173383472144439	1
Sanbr	-0.210122583	1
Taf11	-0.05305672	1
Lsm8	-0.081634692	1
Cnot1	0.09465916746181	1
Itga6	-0.085542347	1
Col12a1	0.0798446941320462	1
Ilk	-0.258996954	1
Acot8	-0.113442475	1
Tm4sf4	-0.419055359	1
Dennd4c	0.343000873240122	1
Cerk	-0.179266803	1
Klhl5	0.195338848039991	1
Hepacam2	-0.10363096	1
Trpc4ap	-0.265276552	1
Gtf3c1	-0.256548781	1
Ptpn6	0.0364153316772606	1
Rcor1	0.0795069641775475	1
Stard3	0.0252879740852738	1
Guk1	-0.085176666	1
AW554918	-0.252137734	1
Ino80d	-0.059592804	1
Psmd6	-0.010076714	1
Pdxdc1	-0.108734719	1
Synrg	-0.145476323	1
Fzd6	-0.289314143	1
Lmbrd2	-0.186026422	1
Radx	-0.111939942	1
Thsd4	0.298614744554818	1
Tlk2	0.0483714885072168	1
Dlc1	0.403956637881997	1
Hip1r	-0.052865702	1
Gorab	0.00378208067195002	1
B3gnt2	-0.076275584	1

Rac1	-0.000348734	1
Dach1	0.36985443479251	1
Cdk12	-0.233973845	1
Fam122b	0.214611382308335	1
2610507B11Rik	-0.032058288	1
Elf2	-0.127239553	1
Gtf3c6	-0.16177444	1
Tgfbr3	0.10511908081665	1
Prpsap2	-0.028327436	1
Sbno1	0.181873664873063	1
Tial1	-0.055479838	1
Wdr37	0.152958862467408	1
Lemd3	0.229595728464674	1
Iba57	-0.104589847	1
Synj1	0.104525883030029	1
Pfdn1	-0.139843817	1
Sap30bp	0.377444895629903	1
Yipf4	-0.056096101	1
Smu1	-0.173588035	1
Slc9a9	-0.051477758	1
Tollip	-0.124191575	1
Usp16	0.166456796696319	1
Eif4e	-0.03453281	1
Ccser2	-0.125657012	1
Ikbkb	0.0588728454877115	1
Tgfb2	0.0275254395593402	1
Tada3	-0.028757415	1
Eef2k	-0.008166431	1
Kansl2	0.054343900095716	1
Xbp1	-0.016614472	1
Cul1	0.171187355109219	1
Zswim7	0.030593835023873	1
Kdm4b	0.0196638052862379	1
Rexo1	0.139225132558044	1
Sufu	0.177179675135761	1
2310033P09Rik	-0.166590881	1
Pmp22	0.143327934490563	1
Oaz1	-0.155913844	1
Tbc1d9	-0.027069407	1
Bcor	-0.175689245	1
Fubp3	-0.112940999	1
Tkt	0.219969271386349	1
Srsf5	-0.13876528	1
Atp2a3	-0.174399869	1
Rsbn1	-0.000669076	1
Carf	0.32495605871488	1

Wdr11	0.0935363115031631	1
Slc5a8	0.0311173823391196	1
Wdr12	0.00645873786152218	1
Selenok	-0.028269294	1
Fam120a	0.159237104678399	1
Jag2	-0.195115597	1
Clip4	0.142051497875787	1
Ankrd46	0.00856599557683968	1
Bzw2	0.386086374007458	1
Cops3	0.0908844806927597	1
Cflar	0.842705195959287	1
Smpd1	-0.098935701	1
Tmem131	-0.116499554	1
Zfp384	0.0147696508525903	1
Stk3	0.296468645123462	1
Grb10	0.327527034371999	1
Il12rb2	0.0345296112479505	1
Garnl3	-0.216281514	1
Cry1	0.229940437443583	1
Anpep	-0.060197044	1
Wtip	0.149976930446154	1
Cep128	-0.251840155	1
Eps15l1	0.135159974567262	1
Ldah	0.219409282493919	1
Armc1	-0.118870305	1
Pitpna	0.0254610382040864	1
Cir1	-0.290511433	1
Actn4	-0.008534827	1
Smarca4	0.132680158680627	1
Lars	0.118315103524824	1
Vps53	0.0107020553180111	1
Bmi1	0.0170940068139976	1
Ppm1k	0.0487583184323949	1
Arih2	0.138129415435747	1
Klf3	-0.029245585	1
Sfswap	0.0238521642198195	1
Rapgef1	0.0477776352786796	1
Gpr137b	-0.013276146	1
Clk4	-0.191329863	1
Prdm11	-0.126113532	1
Arhgap42	0.364734337016128	1
Tmem104	-0.00100728	1
Btbd7	-0.279016983	1
Cdpf1	0.0130406729891245	1
Sp4	0.0352327678449633	1
Snx7	-0.046675049	1

Bicd2	-0.204423112	1
Atg5	-0.075060709	1
Sec24b	0.0494846551062125	1
Phkb	-0.134176117	1
Denn2b	0.0219649944854859	1
Usp19	-0.007946642	1
Tor4a	0.0273436353952019	1
Tpd52l1	0.000933248523027119	1
AA467197	0.368761502681126	1
Fuca2	-0.096567757	1
Fbrsl1	0.0345417029800027	1
Cdyl2	-0.002846282	1
Washc4	0.186254109344647	1
Nprl2	-0.096724921	1
Ralgapb	0.0181304043370283	1
Taldo1	0.107615127082641	1
Pfkp	0.326982406984887	1
Rhbdd1	-0.164870268	1
Bckdhb	-0.018509063	1
Dock8	0.00565902220954767	1
Ndufs6	-0.083569113	1
Zmpste24	-0.114372819	1
Nbl1	0.103854300400555	1
Evi5	-0.082537626	1
Lima1	0.0816534894139804	1
Aamp	0.0968289636696704	1
Cwh43	0.0239532590553657	1
Brox	-0.02457072	1
Nfatc1	-0.039349003	1
Rbm39	-0.244794057	1
Kcnma1	0.139963365882096	1
Dock9	0.0440268232903807	1
Pfdn5	-0.026966062	1
Vps4b	-0.042605404	1
Sh2d4a	-0.182291764	1
Gnb1l	0.0337685301730951	1
Ric8a	0.143519905183526	1
Acsl5	0.119063011010096	1
Fuca1	0.00381422907940604	1
Pkp4	-0.080394223	1
Dapk1	-0.025796334	1
Slc12a4	0.232402962718013	1
Por	-0.091407721	1
Stbd1	-0.162062851	1
Frrs1l	-0.008157598	1
Ints10	0.192320798593726	1

Ubqln4	0.020472145649491	1
Tctn1	0.0842384688838247	1
Aup1	-0.050124464	1
Abcc4	0.323207604725999	1
Apip	-0.004482139	1
Fgfr1	-0.27575149	1
Prkag2	0.463617903202305	1
Poc1b	0.0479413199058444	1
Ptpn1	-0.069336346	1
Gatd1	-0.23070017	1
Elk3	0.0795504684333462	1
Nmrk1	-0.149618417	1
Sar1b	-0.08451345	1
Hspa4l	0.0441605311356583	1
Daam2	0.107019649802483	1
Tfb1m	0.0545192733045321	1
Zc3h12b	0.11072211687071	1
Slain2	-0.117882657	1
Atp1a1	0.220773294776934	1
Hif1a	0.214897709154952	1
Gtf2a1	-0.332256268	1
Tln2	-0.305220776	1
Nelfe	-0.222455537	1
Gypc	-0.039073301	1
Ints6	0.428406546257304	1
Dym	0.108126601388698	1
Zdhhc3	-0.203922617	1
Adipor2	0.103213372510667	1
Vps41	-0.226919149	1
Sdc2	0.710142655833402	1
Nipsnap2	0.187390871261752	1
Ddx20	-0.053680633	1
Ttc7b	0.109137926072537	1
Fam184a	-0.299946079	1
Znrf3	0.0982088801784183	1
Rnf149	0.213733518778988	1
Adam17	0.229102262571741	1
Braf	0.351772756312797	1
Btbd11	0.154952074745506	1
Vta1	0.161181751849605	1
Dcun1d4	0.0153358886203947	1
Supt20	0.00932822632949432	1
Appbp2	-0.003295517	1
Zfp352	-0.104267687	1
Vps36	0.0218449372277535	1
Spag9	0.0797738428924611	1

Txnrd3	0.229903043618918	1
Akt3	-0.113786666	1
Dync1li1	0.172732191450451	1
Arfgap2	-0.074675222	1
Smg1	0.0932939718239858	1
Cd9	0.126214187171223	1
Rilpl1	0.244965178145635	1
Sec14l1	0.238808991544515	1
Atp12a	0.0236145492572972	1
Creb1	-0.189465346	1
Naa25	-0.015818004	1
Prr5	0.122921506432219	1
Pms1	0.0167887275169087	1
Nrp2	0.0371594260100864	1
Exoc1	0.180582652278371	1
Snap47	-0.034166953	1
Bin1	-0.014920356	1
Armh3	-0.093696269	1
Ugt8a	0.272127123351259	1
Pcnx2	0.174041606727785	1
Sox5	0.0490827308764774	1
Fam214a	0.119114721885753	1
Snx6	-0.009575975	1
Rbm18	0.118381622986714	1
Brsk2	0.196402271337711	1
Cldn18	-0.107796196	1
Ccdc50	-0.060182476	1
Eya2	0.420421844342039	1
Cnot6l	0.170609534194895	1
Tmem229b	-0.239892161	1
Lyrm9	0.027764305516118	1
Emc1	-0.099165527	1
Coq10b	0.0644178601763137	1
Tvp23b	-0.025097898	1
Scai	0.0557611693552745	1
Rsrc1	0.00951313524923192	1
Flot1	0.0103587725467686	1
Ralb	0.175004607157986	1
Cttnbp2	0.133521341857987	1
Chmp2b	-0.017900108	1
Wwtr1	0.133638659647756	1
Zyx	0.173192351519111	1
Rnf146	0.0307262365221685	1
Nusap1	-0.305722324	1
Shoc2	0.117143108137628	1
Usp37	0.020375343404996	1

Itpka	0.0486682882418399	1
Gabrb2	0.294410782820067	1
Napepld	-0.070036999	1
Ankrd11	-0.028286488	1
Tasp1	-0.099601276	1
Tmem243	0.163213844540322	1
Smg6	-0.072616468	1
Dhx30	-0.196787676	1
Psme3	-0.113073119	1
Thra	0.163573926220723	1
Dnajb4	-0.092818558	1
Lars2	-0.025722588	1
Sptlc2	-0.042174906	1
Dennd1b	-0.460218055	1
Tnrc6b	0.142230933062485	1
Ift172	0.0936721717274416	1
Atg13	-0.088494985	1
Wipi2	-0.051236766	1
Add1	0.194578922603711	1
Gpatch4	0.00422243131827715	1
Golga7	-0.103656219	1
Bcl9	0.0673249419305482	1
Arhgap24	-0.261649762	1
Ssr1	-0.154033695	1
Pdk3	0.392265317332697	1
Nfya	-0.072501337	1
Fbxw11	-0.009845318	1
Trim59	0.428000977904946	1
Gpatch1	0.0286849558519744	1
Taok3	0.0907354061087793	1
Aldh5a1	0.0258640952145972	1
Inpp5b	-0.252656809	1
Dyrk2	0.00999696184400922	1
Gtf2e1	-0.172042275	1
Zfp236	-0.123325708	1
Senp1	-0.169603291	1
Ubl3	0.064589184444753	1
Acss3	-0.333087667	1
Heatr6	-0.041676168	1
Orai2	0.0798837948891033	1
Sema4b	-0.114984026	1
Glipr2	0.00936977047999249	1
Unc13b	-0.148757442	1
Syne3	0.314758018324967	1
Eps15	-0.102024063	1
Las1l	0.208568549631292	1

Tubb5	-0.158486708	1
Chd5	0.0088702464908324	1
Cebpz	0.179970872028993	1
Grid1	0.0908644437451485	1
Atp6v1a	0.000678678307425185	1
Fer	0.107668481511288	1
Pgrmc1	0.0389759367306701	1
Naa35	-0.029976099	1
Ppm1b	-0.183107913	1
Erbin	0.0955165005795038	1
Ric1	0.00227421985176302	1
Nup153	0.0388317850455832	1
Arhgap1	0.121695936663173	1
Brpf3	-0.153430813	1
Bag3	-0.512211616	1
Phf14	0.142857292422459	1
Herc3	0.135267612096328	1
Ercc6	-0.072901808	1
Chchd1	0.0154413522036912	1
As3mt	0.0319156763046282	1
Cop1	-0.107910053	1
Xpa	-0.083760511	1
Smarca5	-0.025570352	1
Elp3	0.117990098366515	1
Lsg1	0.0138453075744796	1
Tfrc	-0.178170825	1
Bace2	0.0249580779172119	1
Dsp	0.16418038079278	1
Gga3	0.0916573398718041	1
Lactb2	0.192611075779053	1
Derl2	0.133455955566606	1
Atp2b1	0.487133696140909	1
Septin10	-0.036120041	1
Zswim8	-0.194257785	1
Oxsr1	0.147707336388673	1
Fam120b	0.114614294870923	1
Strbp	0.157197988066988	1
Itm2b	-0.048291346	1
Adrm1	-0.070249636	1
Ssbp3	0.0383766340185869	1
Zranb1	-0.118004639	1
Svil	-0.062622851	1
Ahdc1	-0.105083521	1
Atg2b	-0.12546418	1
Nek10	0.581320556619143	1
Parva	0.0635727817761965	1

Cabin1	-0.024724806	1
Zdhhc17	-0.055496014	1
Abl1	0.11925312948455	1
Zbtb2	0.0282336212688067	1
Cbll1	0.0292638612013427	1
Cntln	0.023048566324621 8	1
Glis1	-0.083185333	1
Lcor	0.00395154356616945	1
Usp10	-0.133337362	1
Coro2a	0.0235259163713862	1
Fam133b	-0.102445211	1
Cep350	0.172647288331999	1
Traf2	0.102319844099592	1
Atp11a	0.135597607848541	1
Ddx42	-0.135233842	1
Nfatc3	-0.01507024	1
Slc3a1	-0.149697836	1
Ddhd1	0.404619771676749	1
Asap2	-0.005399472	1
Spred1	0.327989815363832	1
Ctns	-0.055696565	1
Plxnc1	0.367168279664284	1
Brwd1	-0.187710005	1
Lamp1	0.0831692215684112	1
Traf3ip3	-0.059196224	1
Plcl2	0.313126394380006	1
Poll	-0.050502538	1
Frs2	-0.152558032	1
Psmd4	-0.046348493	1
Trpm6	0.0632287357474302	1
Rell1	-0.031090111	1
Tasor	0.434596088197428	1
Gbe1	0.177280189222719	1
Laptm4a	0.220939136385737	1
Tubgcp3	-0.000134731	1
Spast	-0.135406039	1
Cbwd1	-0.130824939	1
Nfkbib	0.118291025334714	1
Sdf2l1	0.303314704497718	1
Brd2	-0.268729979	1
Ak2	-0.072120381	1
Alg5	-0.060935026	1
Gk5	0.612812716138511	1
Ccnl1	0.050136303436723	1
Crem	-0.229574777	1
Ncoa3	0.444259144705176	1

Gtf2a2	-0.121078487	1
Dgcr6	0.191569623129191	1
Ift22	-0.045920368	1
Edc3	0.0431075807205336	1
Ints14	0.0257349264223369	1
Nipal1	-0.003088258	1
Atp7a	0.126794162502301	1
Txlng	0.141718332620455	1
Banp	-0.07764229	1
Actr6	0.0289848557535543	1
Vps8	0.00678439795191194	1
Ralgps1	-0.025101591	1
Npc2	0.243528704772513	1
Tgfbrap1	-0.096413008	1
Cwc25	0.347239026571637	1
Rnf10	0.0488935829141741	1
Cacna1d	-0.303119996	1
Ripor3	0.139569311178786	1
Rgs13	-0.042052676	1
Raver2	0.0277952232491821	1
Immp1l	-0.008150158	1
Eif2ak1	-0.059116797	1
Cdk11b	0.115159834904517	1
Erp29	0.0962736887604366	1
Sin3a	-0.084097505	1
Epha1	-0.07046059	1
Sugct	0.0774932730994017	1
Gstcd	-0.073175819	1
Ttc7	0.495965317675677	1
Plekhf2	0.0543769682825372	1
Epm2a	0.127468383988991	1
Phf3	-0.024274083	1
Tnks1bp1	-0.135945734	1
Tmem39a	-0.116005404	1
Slf2	0.10535516247571	1
Scaper	0.0873949105631877	1
S100a10	0.0171478109471872	1
Chd9	-0.054165704	1
Arnt	0.02755623384207	1
Rab28	0.102270400585037	1
Kansl1	-0.154336736	1
Eva1c	0.776136493400732	1
Arglu1	-0.199661144	1
Slc45a4	-0.082056581	1
Erc1	0.268359976991901	1
Abitram	-0.045057049	1

Shisa9	0.232153478879153	1
Aftph	-0.063393174	1
Tmem65	-0.12039388	1
Ppp1r9b	-0.032311396	1
Cul5	0.235663096002363	1
Pde7b	-0.319347187	1
Dcun1d3	0.186291672311983	1
2210408I21Rik	0.393089278318386	1
Foxj2	-0.008378734	1
Gpr176	0.186958184461931	1
Timm50	-0.096321353	1
Capzb	0.0128608022292727	1
Serpinb8	0.333180758915711	1
Kif27	-0.091760782	1
Ube2q2	0.0830903295865251	1
Kras	0.2480967743014	1
Trmt11	0.144670645560629	1
Paxip1	0.087266183323804	1
Tppp3	-0.084318487	1
Arl5b	0.138881079250497	1
Xylb	-0.02866248	1
Calcb	-0.536581357	1
Ino80	0.181779681461407	1
Fmr1	0.0880632252554443	1
Slc4a10	0.15050449260638	1
Dop1a	0.394292440069303	1
Gosr2	0.0253152046210526	1
BC004004	0.101176626233202	1
Paip1	-0.103397771	1
Dram1	-0.021884862	1
Cldnd1	0.0127891342471279	1
Tom1l1	0.137181545037333	1
Ccny	-0.223229415	1
Zfp609	0.190466464498439	1
Crnkl1	-0.02098225	1
Hdx	0.0102046426398235	1
Atp8b1	0.0854678484726593	1
Dst	-0.273709799	1
Arap2	0.233803982321672	1
Rdh10	0.0206856462370173	1
Ttc39b	0.00682544225949527	1
Arf4	0.309660170776268	1
Sap130	-0.06791531	1
Slc35f5	0.155201250476941	1
Rasgrp1	0.122849084922957	1
Fermt2	0.0566012826151738	1

Lmbr1	-0.138989165	1
Dedd	0.0437827582779811	1
Rapgef4	-0.254174873	1
Setd3	0.298508599811173	1
Poldip3	0.161302251858917	1
Sppl3	-0.037317233	1
Ppp6r2	0.00966838083092991	1
Galnt3	-0.032068646	1
Chst10	0.0914852121247071	1
Pccb	0.282876784751601	1
Cpsf4l	0.157697777762698	1
Lama3	1.28204847183941	1
Focad	0.11816182087417	1
Galnt16	0.158220593761004	1
1110059E24Rik	0.377503992036093	1
Cd151	0.0633267726040714	1
Ppm1a	0.133050020363566	1
Kdm3b	-0.112793684	1
Pgam1	-0.040725421	1
Hipk1	-0.28783121	1
Zfand3	0.20720598129422	1
Kdm5a	0.0194908590258507	1
Lgals8	0.0278879709357454	1
Gnl2	-0.035142387	1
Dcaf1	-0.090386884	1
Ptpn4	-0.114675822	1
Glipr1l1	0.031993066843348	1
Txndc12	0.0149620573252355	1
Psme4	0.0392313856703397	1
Lca5	0.0427676680995794	1
Wdpcp	0.22455869368715	1
Rreb1	0.196454769583566	1
9130409I23Rik	0.0777029777043642	1
Spp1	0.560030466132102	1
Cebpd	-0.10218571	1
Sarnp	-0.141037362	1
Xpot	-0.009244543	1
Rmnd1	-0.126813768	1
Akap8	-0.04342719	1
Ppil2	-0.006079517	1
Cebpg	0.194646216644424	1
Bin3	-0.03853754	1
Zeb2	0.37267001108742	1
Aven	0.0794886744042691	1
Prdm15	0.186434602596878	1
Tspan14	0.0643634918820614	1

Ralgps2	0.304342224620528	1
Senp8	-0.043254005	1
Ldlrad4	0.298420110526758	1
Ube2g1	-0.078019445	1
Iars2	-0.15985033	1
Cyld	0.103275203374215	1
Slc25a22	0.0416423569898809	1
Trim33	-0.122003267	1
Pmepa1	0.311024546495166	1
Atm	0.181567671714973	1
Pomk	-0.016897225	1
Pik3c2a	-0.110224236	1
Slx4	0.0700083285044452	1
Slc9a3r2	0.126793712122457	1
Ldha	-0.469564369	1
Akap6	-0.135735351	1
Osbp	0.0519862512902119	1
Vav3	0.160017663058639	1
Prrg4	0.161314287791705	1
Zfyve21	-0.141864302	1
Clasp2	0.192395850913681	1
Zzef1	0.0209515672532622	1
Dok6	0.34121834729937	1
Atf6	-0.060597637	1
Zc3h18	0.011940402867703	1
Cdk14	0.515591771756856	1
Ell	-0.104957828	1
Tle1	0.164428549106048	1
Lmo2	0.152300758260821	1
Gabarapl1	-0.103612767	1
Foxa1	0.0555386283920684	1
Desi2	-0.267860465	1
Naa30	0.111087787665136	1
Tra2b	0.107373060077645	1
Nubpl	0.0291842353301772	1
Dab2ip	-0.017412985	1
Cracdl	0.0772135624853418	1
Apba1	0.147216567316739	1
Nbeal1	-0.172778093	1
Crebl2	-0.017344369	1
Kars	-0.031941337	1
Psca	-0.407477491	1
Nup98	-0.079110047	1
Nfkb1	0.0261425171309155	1
Pcna	-0.330918987	1
Kpna1	0.104179306171802	1

Trim8	0.0291245734194796	1
Gadd45g	0.172599392118227	1
Dennd11	0.00243950618746236	1
Gpr89	0.0168813742077122	1
Trim36	0.0297615572506529	1
Hps5	-0.087658643	1
Dusp5	0.217942312638792	1
Sh3pxd2b	-0.06281193	1
Sirt2	0.020493078614781	1
Srbd1	0.00489890959673001	1
Pdlim1	0.285896381040837	1
Igf2bp2	0.00451283681525139	1
Kcnq1	0.182398341564737	1
Lifr	0.273176402402344	1
Zfp704	0.0524424369950018	1
Zbtb1	0.17576860101537	1
Tcf12	0.105744503071627	1
Rars	-0.103000439	1
Ppp1r21	-0.063191567	1
Ppp2ca	0.311931391806845	1
Pds5a	-0.122085712	1
Entpd5	0.0646714323701399	1
1810020O05Rik	-0.235763882	1
Prmt3	0.00455514734187218	1
Nfkbiz	0.0349747567301464	1
Ccdc93	0.0487519969613864	1
Yipf3	0.112211089468901	1
Nrp1	0.0879801383999427	1
Ywhaz	0.180876614679903	1
Insig2	0.01615598588905	1
Ankrd13d	-0.077275189	1
Prrg1	-0.121406295	1
Baiap2	-0.043962967	1
Gorasp2	-0.063132315	1
Yy2	-0.163557	1
Bclaf3	-0.06946931	1
Copb1	0.0373918232958104	1
Ehd2	0.0056181405624578	1
Rptor	-0.003151568	1
Tent4a	0.254680658286086	1
Ptp4a2	0.055382933855362	1
Fgf18	-0.08585143	1
Nr2c1	0.170235537122565	1
Lats1	-0.142105352	1
Sdc4	0.28818370945636	1
Slc66a2	-0.050072366	1

Ubap1	0.000205074816607698	1
Crebbp	-0.029351116	1
Selenoi	-0.041452575	1
Abce1	0.0404723033558338	1
Gpr39	0.237387982229493	1
Atf7ip	-0.191706325	1
Pmpcb	0.00900301990417585	1
Itch	-0.193945089	1
Rybp	-0.019370857	1
Psmg1	0.0932633304119448	1
Cdca4	-0.02042588	1
Zfat	-0.241227798	1
Stxbp4	-0.057317834	1
Psma7	-0.034962936	1
Zfyve1	0.0114728160184953	1
Gpc5	0.66760413341339	1
Pex2	-0.100605655	1
Wdr1	-0.054964055	1
Foxk1	-0.061995149	1
Peli2	0.288784165920769	1
Pard3	0.285730472421595	1
Bok	-0.033939081	1
Dcbld2	0.0477127903232537	1
Pcmtd1	0.119411537984002	1
Qser1	0.043033339877792	1
Dvl3	0.0257242505819115	1
Decr1	-0.120597896	1
Setd1b	-0.237942074	1
Anapc7	-0.103477264	1
Vps25	0.0635602241917615	1
Gsk3b	0.0449808916292289	1
Rictor	0.0178161207461882	1
Eif2b3	-0.082927503	1
Nup205	0.236265104262207	1
Septin8	-0.041542108	1
Arvcf	0.000250720405225335	1
Trpm7	0.00446518049250266	1
Klf12	0.321546766354458	1
Arl4a	-0.096504351	1
Ddx3y	0.378851129700821	1
Il1rap	-0.011649892	1
Traf3ip1	0.0372352985183634	1
Wbp2nl	0.0541840819798377	1
Fgfr3	0.282802227762597	1
Tma16	-0.168508282	1
Scube2	-0.058024446	1

Aspm	-0.082529089	1
Herpud1	-0.128874188	1
Iqgap1	-0.015475421	1
Ylpm1	-0.106122765	1
Foxa2	0.257180459877656	1
Scaf4	0.0790757158515019	1
Cdc42se1	-0.043187286	1
Zdhhc14	0.370292639312271	1
Smndc1	-0.076405139	1
Rab11fip1	-0.08707446	1
Ebf1	-0.055425219	1
Psmg2	0.105741059853551	1
Pcgf6	0.0435033256519783	1
Riok1	0.0667754854989576	1
Cast	0.148185409726107	1
Cmtm4	0.091312021068839	1
Fam172a	-0.091452986	1
Hook1	0.382015777389779	1
Sgcd	0.25321052772505	1
Cited4	0.338727834066949	1
Efl1	0.0217924567934623	1
N4bp1	0.168800558061794	1
Alcam	0.42441610861318	1
Rab3gap2	-0.130371596	1
Sinhcaf	-0.070577983	1
Hs3st1	0.153953970766148	1
Nyap2	-0.010918577	1
Prrc1	-0.180825999	1
Epn2	-0.123587171	1
Plekhb2	-0.012557754	1
Casp3	-0.174063357	1
Stard5	0.281885258684441	1
Gpcpd1	0.190995709394647	1
Fgfr1op2	-0.11913617	1
Vps16	0.078189735369207	1
Cab39	0.0677648200327516	1
Rbm12b2	-0.051106923	1
Atp6v0d1	0.0502145992802567	1
Tcof1	-0.096934272	1
Eci1	-0.060586896	1
Robo1	-0.053637916	1
Deaf1	-0.130422276	1
Nup214	-0.072297591	1
Adat1	-0.121617758	1
Nck2	0.21531678620009	1
Crot	0.0303954947958092	1

Casp8	0.329549897533799	1
Rnf139	-0.066696991	1
Fadd	0.0261578854289209	1
Slc44a1	0.102947939937571	1
Rspry1	0.0533809554106117	1
Ift140	0.107412163072378	1
Nrtn	0.220821104140181	1
Rab35	0.181457507434508	1
Gng12	-0.084004841	1
Rab5a	-0.055885075	1
Zmynd11	-0.042335992	1
Rab11fip4	0.141412089740913	1
Sdr16c5	0.108821595396277	1
Rassf9	0.103079979121784	1
Usp1	0.174976964079244	1
Ppp2r5e	0.258268890613572	1
C1galt1	-0.027741601	1
Agfg1	0.31691445227216	1
Sik3	-0.01218071	1
Chmp6	0.00998250226182551	1
Rc3h2	-0.052239069	1
Timp4	0.0109447469568511	1
Ahi1	0.0850664429199124	1
Cdc14b	0.0396797124653661	1
Derl1	0.0334785791125168	1
Tbcd	0.477086763690629	1
Wee1	0.145637780234097	1
Slc38a10	0.284410387617212	1
Slc4a2	-0.029664812	1
Atp6v0c	0.357116180847671	1
Zfp638	-0.096632279	1
Ppp2cb	0.100746409359965	1
Eya3	0.108716237230591	1
Dusp10	0.454663094052786	1
Impa2	0.155082106081207	1
Tcf20	0.0632906902795487	1
Rad23b	-0.008861452	1
Lrp12	0.278878384367274	1
Pigk	0.245861863754263	1
Nrde2	0.111949031003165	1
Lrrfip2	0.32264075078735	1
Lrrc1	0.183420260465017	1
5031439G07Rik	-0.121526751	1
Cmtm3	0.0459743212855501	1
Fign	-0.249543461	1
Ube2d1	0.151693208759957	1

Wdr45b	0.037898544183239	1
Tnpo1	0.0905259108472576	1
Ift81	0.0997307462803307	1
Pde8a	-0.133778233	1
Arhgap6	0.297987877847207	1
Fbxl22	0.138754638760373	1
Ankhd1	-0.14554197	1
Srsf10	0.0773639522527058	1
Camkmt	0.469793857141884	1
Gatad1	0.030258210102305	1
Pard6b	-0.043614707	1
Smurf2	-0.152167671	1
Ptch1	-0.014558298	1
Slc44a3	0.135890275207286	1
Dyrk1a	-0.216694796	1
Rxra	0.104788710563503	1
Ccndbp1	0.197167411573892	1
Seh1l	0.0350041811411602	1
Rhbg	-0.071242503	1
Gspt1	-0.091188262	1
Sh3bp2	0.457190297913573	1
Fxr1	-0.149189831	1
Baz1b	-0.036399324	1
Pex16	0.0663086151793278	1
Wdr70	0.18668394860172	1
Txnip	-0.098587921	1
Klhl7	0.176071046787752	1
Leprot	-0.054482924	1
Kidins220	0.030194403728349	1
Ski	0.524285814965688	1
Bmpr1b	0.13662010771597	1
Polk	0.0435430952073138	1
Jmjd1c	0.0365504401872192	1
Rprd2	-0.024449998	1
Nabp1	-0.001022137	1
Rnf217	0.342208722458018	1
Rlim	-0.068525668	1
Ss18	0.0253152958460965	1
Snapc1l	-0.058427218	1
Ttll9	-0.035280217	1
Sav1	-0.055705786	1
Rubcnl	0.516664817942246	1
Ascc1	0.0215878579510882	1
Klhl2	-0.226650827	1
Alpk1	0.132486949398725	1
Slc25a26	0.0823994263453247	1

Slc4a7	0.313101332064153	1
Rap2c	0.101748235243908	1
Zfand2b	-0.033945855	1
Lsmem1	0.0561663865752171	1
Xpnpep1	-0.095223433	1
Ogt	-0.225123692	1
Cdc42ep3	0.192605933206791	1
Ambra1	0.138230136561696	1
Rbfox2	-0.041360158	1
Ppip5k2	-0.006578042	1
Atad1	0.0333870526133517	1
Atp6v0b	0.186177481537987	1
Rpf2	-0.001034989	1
Idh3b	-0.022071747	1
Larp4b	-0.157199804	1
Cops4	0.0354721009964284	1
Snx2	-0.032112683	1
Fhl3	-0.067421999	1
Itpr1	0.261303780135867	1
Rpp40	0.00155689461418107	1
Nf1	0.0399677091529229	1
Ide	-0.080613111	1
Ccnd2	0.197703050083588	1
Nkain2	0.340902093534746	1
Crk	-0.03274223	1
Dvl1	-0.056638981	1
Tcea1	-0.020646931	1
Zc3h7a	-0.133627881	1
Frmd4b	0.131312857988961	1
Carmil1	0.639137433508424	1
Rangap1	-0.034657991	1
Aldoc	-0.058544638	1
Rsl1d1	-0.007929221	1
Cars2	0.00620192414157983	1
Pola1	0.286067701800458	1
Ust	0.239600001319284	1
Tmco1	-0.015654779	1
Eif4h	0.100578450925969	1
Pawr	-0.117934533	1
Cnot10	0.133236998342264	1
Atp11c	-0.321750578	1
Lrch3	0.022517052261304	1
Jph1	0.186258970394337	1
Irf2bp2	0.000985233877639491	1
Usp3	0.211098180206544	1
Dda1	0.155868119541943	1

Taf4b	-0.177415127	1
Bap1	-0.15386475	1
Gtf2b	0.136116484703863	1
Elp4	0.469962011658457	1
Zfp827	0.623822606983007	1
Ppfia2	-0.465759977	1
Chmp4b	0.0827473646282044	1
Adprh	-0.062721993	1
Tent5a	0.107570156095289	1
Nek7	0.0738637330460371	1
Rab18	-0.08405689	1
Ppard	0.15303219573179	1
Prkci	0.073946316601347	1
Septin11	0.104711574587197	1
Sema7a	-0.11311003	1
Gtf2f2	0.19490606185375	1
Aurkaip1	0.0817062083090369	1
Uso1	0.0432486116440951	1
Tfcp2l1	-0.126247465	1
Rimbp2	-0.053252424	1
Ptpn3	-0.27815961	1
Klhl18	-0.150573009	1
Gulp1	0.093276727223931	1
Atp6v1h	-0.057629834	1
Angel2	-0.017586306	1
Kpna6	-0.019119797	1
Snap23	0.0249414073985734	1
Wdr35	-0.147362391	1
Lrrc8d	0.396798428354277	1
Prune2	0.141385709856356	1
Tarbp1	0.116515794865878	1
Emc2	0.208969412272661	1
Sec11c	-0.104951399	1
Clns1a	0.135296469848753	1
Emc7	0.0864470165391987	1
Gapvd1	-0.052238699	1
Gab3	-0.097603487	1
Cfdp1	-0.13867343	1
Larp1b	0.00365390486080874	1
Hcfc2	-0.038606652	1
Fbxo11	0.555485075025689	1
Tnip1	0.103870197028019	1
Birc6	-0.036405037	1
Ttc14	-0.0325158	1
Gosr1	0.0352101353512909	1
Ggps1	-0.015344783	1

Usp48	0.0359736935480193	1
Abcc5	-0.133178868	1
Psmd3	0.116633115097477	1
Clk2	0.0390585477352427	1
Rsrc2	-0.016508722	1
Susd4	0.176560396630794	1
Sh3glb1	0.0785987951331701	1
Rassf3	0.0612869798716287	1
Tgfbr1	0.184854515205079	1
Rara	-0.199253073	1
Ypel5	-0.083026243	1
Ctdspl	-0.01730271	1
Tom1l2	-0.025381529	1
Nipa2	0.0642052672168439	1
Nsmaf	-0.095068885	1
Acbd6	-0.111995127	1
Brf1	-0.614853575	1
Crebrf	-0.180608934	1
Psmd13	0.0817069958645433	1
Ddx54	0.00701360970815568	1
Ccser1	0.269328737652732	1
Uhmk1	-0.00499192	1
Hpcal1	0.340535648270683	1
Tbc1d14	0.0770741540839652	1
Ncstn	0.202898439948319	1
Cwc27	0.0720264411082737	1
Cdk8	0.1316065921584	1
Pi4k2b	0.290292861045981	1
Scoc	0.0541507054714735	1
Frem2	0.199056504968435	1
Ppp1r9a	0.137603237784346	1
Celf1	0.156582565288303	1
Acp2	0.098170745088125	1
Sdcbp	0.0783150583659238	1
Jdp2	0.169343808054771	1
Saal1	-0.193752577	1
Qrich1	0.0449449768083335	1
Adipor1	-0.02496764	1
Sub1	0.0160669174472816	1
Ccnk	-0.024225169	1
Nelfcd	-0.00370166	1
Hsd17b12	-0.001904711	1
Fut8	0.105872477364077	1
Kctd20	0.17862820110554	1
Ank	-0.125574651	1
Ulk1	0.223038294308061	1

Ttc28	-0.140932184	1
Btaf1	0.0294236412270851	1
Phka2	0.209804934476086	1
Zcchc8	-0.051418733	1
Pacsin3	0.128500241954373	1
Tmem19	0.116952638178607	1
Lta4h	0.0123810759133826	1
Rtn4	0.0922099749231222	1
Pmm2	0.0491934777636127	1
Tssc4	0.0995003931823915	1
Lpcat3	-0.114978398	1
Prom1	-0.032784541	1
Pank4	-0.101321811	1
Cxadr	0.229951928597089	1
Pias1	0.1153205729292	1
Atl2	0.0686848874748789	1
Tmcc1	0.113807456471239	1
Thrap3	0.0852088819497065	1
Rai2	0.0147479321071944	1
Stk26	0.0840029011624885	1
Asxl2	-0.008496492	1
Kdm7a	-0.052652316	1
Utp20	0.0335942225861466	1
Gabpb1	0.0210529614572172	1
Iqck	0.150459108186746	1
Prkar2a	0.0417239369654827	1
Garre1	0.337909530854797	1
Tent2	-0.103003668	1
Pnpla7	-0.093429171	1
Gpbp1l1	0.00994737736432527	1
Lamtor1	0.103976021527746	1
Larp4	-0.189135336	1
Trappc12	0.09980932178359	1
Bicral	0.229938051480637	1
Kdm1a	-0.029477468	1
Taf6	0.214100929467064	1
Vezt	0.130901356309132	1
Pkd2	0.224064074595805	1
Pak1	0.00444658655844599	1
Coro1c	0.0526734981840404	1
Cep76	0.109807534695093	1
Dnajc7	-0.028695797	1
Lrguk	0.17459206037931	1
Dnaja1	-0.159673738	1
Lpcat1	-0.317447747	1
Ubtd2	0.0263353257654299	1

Rcan3	0.0214616577756851	1
Gucy1a1	-0.08146361	1
Ep300	-0.153997366	1
Spon1	0.711509004567447	1
Gtf3c3	-0.118166009	1
Prkce	0.334965615726023	1
Rab7	0.319191286522786	1
Tulp4	0.202065439516168	1
Ube2v2	0.140197661291799	1
Tatdn1	0.0330581791463433	1
4933434E20Rik	-0.011575319	1
Arhgap21	0.170233019357453	1
Ggnbp2	-0.046588417	1
Disc1	0.200093176397839	1
Picalm	0.505322885111711	1
Trappc4	-0.032674267	1
Ranbp9	0.231736591368166	1
Ago4	-0.012016361	1
Qsox1	0.085710030050525	1
Bcl2l15	0.145682733871112	1
Gata5	-0.138856686	1
Cdkl2	0.139519073652198	1
Lsm12	0.0874516445446514	1
Luc7l2	0.00208709576640387	1
Plekhm2	-0.04799703	1
Slc39a9	0.0281912202469989	1
Git1	0.048218431882219	1
Becn2	0.00287249725412642	1
Wbp11	0.0247758992690392	1
Elmod2	-0.016764298	1
Tspan12	0.0413762827197502	1
Bcl9l	0.0285641066434771	1
Phf21a	0.167456027698602	1
Fam3c	0.120880563057266	1
Zfp518a	-0.135547196	1
Pwp1	0.138091407782664	1
Cwf19l2	0.0875857942502212	1
Szrd1	-0.129074804	1
Nop2	-0.049906109	1
Ptpru	0.323391295837095	1
Tbc1d1	0.11270310736054	1
Txn1	0.204387262921206	1
Clic4	-0.116247943	1
Galns	-0.124706945	1
Cops5	0.165127648529447	1
Gid8	0.119319731626905	1

Cnot4	0.165629116340418	1
Zbtb41	-0.055716969	1
Sft2d1	0.0794381677853662	1
Ncoa4	0.256636540059279	1
Elovl1	0.0436013062418227	1
Cyria	0.261923174204934	1
Cyb5b	-0.007225991	1
Cidea	-0.095699548	1
Axin1	0.0674521869887561	1
Pik3ap1	0.100106834310656	1
Oga	-0.018947612	1
Gfer	-0.083141079	1
Fhit	0.313671189818978	1
Auh	0.0511487123766607	1
Tmprss2	0.0744808020045817	1
Vcpip1	0.0573825508014855	1
Ston2	0.0438362174632607	1
Fam160b1	0.0204728371273014	1
Cnot8	-0.26658213	1
Ccdc171	0.217540835787728	1
Gnai2	0.0772477516406784	1
Rnaseh2b	0.0238270000401883	1
Psmd1	0.240368801405639	1
Dusp14	-0.040505264	1
Ubp1	0.187329688079778	1
Bak1	0.00285464198982049	1
Rere	-0.107160022	1
B4galt5	-0.012228532	1
Bcr	0.301141078046951	1
Immt	0.0940403037874143	1
Lrch1	-0.231974887	1
Uchl3	-0.029844909	1
Arl13b	-0.082398128	1
Acaa2	0.0668416805816529	1
Slc11a2	-0.022102774	1
Ifngr1	-0.048403569	1
Slc12a7	0.0300467946146086	1
Net1	-0.003679113	1
Atg3	0.136195009848353	1
Thap4	0.0360127462186621	1
Nr4a2	-0.334093244	1
Znrf2	0.270595221165382	1
Fchsd2	0.0948891137394795	1
Fmnl2	0.139031328127289	1
Hpx	1.91	0.00
Nfil3	1.47	0.00

Epsti1	1.18	0.00
Stat1	0.96	0.00
B2m	0.91	0.00
Znfx1	1.09	0.00
Irs2	1.52	0.00
Pdzrn3	1.33	0.00
Igf1r	0.95	0.00
Cebpb	1.09	0.00
Dusp7	1.04	0.00
Hspa2	2.16	0.00
Rundc1	0.82	0.00
Samhd1	1.06	0.00
Rufy3	1.15	0.00
Tox2	1.39	0.00
Txn1	0.84	0.00
Edem1	1.06	0.00
Epb41l4a	1.14	0.00
Arrdc3	0.87	0.00
Ddx60	0.80	0.00
Fgfr3	1.27	0.00
Plac8	0.98	0.00
Kirrel3	1.72	0.00
B3gnt7	0.93	0.00
Arg2	0.95	0.00
Slc38a2	0.79	0.00
Ppargc1a	1.06	0.00
Rnf213	0.97	0.00
Nrg1	1.29	0.00
Xpa	0.66	0.00
Sri	0.53	0.00
C1s1	0.64	0.00
Tenm2	-1.31	0.00
Eif2ak2	0.69	0.00
Ctss	0.61	0.00
Arl9	0.61	0.00
Sdc2	1.07	0.00
Veph1	0.92	0.00
Vmp1	0.67	0.00
Erap1	0.61	0.00
Tmem116	0.74	0.00
Hecw2	1.05	0.00
Tmem158	0.63	0.00
Jazf1	1.16	0.00
Gpd2	0.73	0.00
Elavl1	0.87	0.00
Kcnip1	0.43	0.00

C3	0.70	0.00
Gpx1	1.02	0.00
Zbtb16	0.77	0.00
Slc1a2	1.16	0.00
Spred2	0.67	0.00
Etl4	0.89	0.00
Slc39a8	0.69	0.00
Riok3	0.68	0.00
Egfr	0.85	0.00
Trim25	0.62	0.00
Klf4	1.03	0.00
Dctn3	0.51	0.00
Tox3	1.09	0.00
Jun	0.74	0.00
Ttc39b	0.73	0.00
Shcbp1	0.58	0.00
Btf3	0.46	0.00
Itsn1	1.04	0.00
Vsnl1	1.17	0.00
Csrnp3	0.88	0.00
Notch2	0.67	0.00
Cdo1	0.80	0.00
Snx10	0.92	0.00
Hdac9	-0.98	0.00
Dcbld1	0.90	0.00
Sh3bgrl3	0.70	0.00
Ppm1e	1.03	0.00
Arhgdib	0.85	0.00
Cd151	0.61	0.00
Cert1	0.67	0.00
Igf2bp3	0.69	0.00
Enpp1	0.79	0.00
Foxp2	0.63	0.00
Gxylt1	0.60	0.00
5031439G07Rik	0.57	0.00
Lcor	0.70	0.00
Tmem132c	-1.05	0.00
Nipbl	0.57	0.00
Aptx	0.53	0.00
Atp1a1	0.76	0.00
Capn7	-1.34	0.00
Prkcd	0.53	0.00
Xdh	0.61	0.00
Cpm	0.89	0.00
Tpmt	0.53	0.00
Tdrd7	0.48	0.00

Acvr1	0.63	0.00
Ptpn3	0.81	0.00
Hdac4	0.64	0.00
Esrrg	0.84	0.00
Fkbp5	0.45	0.00
Ptbp2	0.61	0.00
Stom	0.49	0.00
Adamts8	1.01	0.00
Smad7	0.59	0.00
Tle4	0.95	0.00
Acaca	0.65	0.00
Slc9a8	0.57	0.00
Polr2d	0.57	0.00
Laptm5	0.93	0.00
Dclk1	-1.29	0.00
Nos2	1.18	0.00
Tnfsf10	0.72	0.00
Slc43a2	0.43	0.00
Trafd1	0.52	0.00
Ubap2	0.43	0.00
Zswim6	0.77	0.00
Cfd	0.80	0.00
Cndp2	0.57	0.00
Klf15	0.41	0.00
Wwc2	0.89	0.00
Hipk3	0.65	0.00
Arhgap24	0.78	0.00
Smpd1	0.45	0.00
Togaram2	0.62	0.00
Pou2f1	0.64	0.00
Carmil1	0.73	0.00
Nfkbiz	0.61	0.00
Uvrag	0.69	0.00
Sytl5	0.77	0.00
Ctdspl	0.70	0.00
Nhs	0.77	0.00
Tmem50a	0.49	0.00
Ndufs4	0.50	0.00
Dhx58	0.42	0.00
Rab2a	0.46	0.00
Bckdhb	0.61	0.00
Hsdl2	0.45	0.00
Nfia	0.58	0.00
Tmem63b	0.67	0.00
Slc35f3	0.62	0.00
Srp19	0.55	0.00

Fndc3b	0.87	0.00
Otud1	0.71	0.00
Fam172a	0.61	0.00
Celf2	0.61	0.00
Nudt1	0.42	0.00
Slc39a9	0.40	0.00
Atp2b2	1.01	0.00
Odc1	0.38	0.00
Efna5	0.65	0.00
Rasef	0.58	0.00
Rictor	0.57	0.00
Slc20a2	0.63	0.00
Runx1t1	0.76	0.00
Pxk	0.47	0.00
Ptprr	0.64	0.00
Cpt1a	0.36	0.00
Cd99l2	-1.02	0.00
Psma7	0.44	0.00
Npc1	0.61	0.00
Pdgfrb	0.47	0.00
Anxa1	0.95	0.00
Ica1	0.52	0.00
Optn	0.52	0.00
Hdac7	0.64	0.00
Ttc33	0.61	0.00
Efnb1	0.51	0.00
Ndufb6	0.36	0.00
Timp3	0.70	0.00
Dusp1	0.58	0.00
Zfyve28	0.61	0.00
Btg1b	0.43	0.00
Alas1	0.57	0.00
Stx19	0.58	0.00
Akirin2	0.38	0.00
Irf7	0.57	0.00
Pard3	0.57	0.00
Arpc1b	0.81	0.00
Bcl6	0.45	0.00
Stt3a	0.35	0.00
Ier5	0.46	0.00
Arpc3	0.35	0.00
Fzd9	0.54	0.00
Commd10	0.58	0.00
Pcdh15	0.57	0.00
Atrn	0.40	0.00
Ppic	0.44	0.00

Rbm47	0.55	0.00
Pdgfb	0.54	0.00
Lipg	0.51	0.00
Hif1a	0.45	0.00
Tfpi2	0.54	0.00
Zfp706	0.37	0.00
Adar	0.31	0.00
Wdr91	0.31	0.00
Robo2	-0.66	0.00
Sox5	0.66	0.00
Ogfr	0.42	0.00
Cpeb2	0.54	0.00
Diaph3	-0.43	0.00
Smad3	0.20	0.00
Tspan14	0.22	0.00
Rxra	0.61	0.00
Tnfaip3	0.67	0.00
Igfbp1	0.90	0.00
Ncoa2	0.48	0.00
C1galt1	0.49	0.00
Parp9	0.29	0.00
Ubl5b	0.41	0.00
Tcf7l2	0.42	0.00
Traf2	0.51	0.00
Tmem243	0.65	0.00
Eif4a2	0.33	0.00
Jmjd8	0.44	0.00
Npc2	0.20	0.00
Cebpd	0.44	0.00
Asxl2	0.40	0.00
Igf2bp1	0.76	0.00
Fbxl17	0.55	0.00
Zfr	0.41	0.00
Lamtor5	0.34	0.00
Atxn10	0.54	0.00
Gadd45g	0.69	0.00
Glrx	0.62	0.00
Sema5b	0.67	0.00
Ube2b	0.50	0.00
Dennd11	0.24	0.00
Pbx1	0.48	0.00
Rnf19b	0.38	0.00
Hsd17b12	0.40	0.00
Lrp5	0.58	0.00
Rasgef1a	0.18	0.00
Nadk	0.51	0.00

Ppfibp2	0.36	0.00
Pik3ap1	0.42	0.00
Irx3	0.33	0.00
Cmtr1	0.34	0.00
Vipr2	-0.70	0.00
Cldn18	0.54	0.00
Atp6v0e	0.32	0.00
Ptpn21	0.65	0.00
Adamts2	0.68	0.00
Snx8	0.49	0.00
Tub	0.48	0.00
Lonrf3	0.55	0.00
Fst	0.39	0.00
Cables1	0.50	0.00
Utp20	0.33	0.00
Sub1	0.33	0.00
Hlf	0.50	0.00
Nkd1	0.38	0.00
Rac2	0.93	0.00
Igfbp3	0.53	0.00
Cdkl2	-0.41	0.00
Crebl2	0.32	0.00
Ctbs	0.40	0.00
Ptger4	0.20	0.00
Xxylt1	0.31	0.00
Snrpf	0.22	0.00
Bri3	0.34	0.00
Romo1	0.34	0.00
Dnaja1	0.39	0.00
Dgkd	0.53	0.00
Atl2	0.30	0.00
Stc2	0.78	0.00
Kazn	0.56	0.00
9130409I23Rik	0.30	0.00
Foxa1	0.53	0.00
Sox9	0.39	0.00
Atp5g3	0.37	0.00
Tmem248	0.24	0.00
Capn8	-1.00	0.00
Pigo	0.27	0.00
Disc1	0.51	0.00
Cps1	0.64	0.00
Pip5k1b	0.25	0.00
Ric1	0.18	0.00
Ccnh	0.24	0.00
Auh	0.50	0.00

Ccdc85c	0.57	0.00
Nme3	0.35	0.00
Rfx3	0.72	0.00
Ptafr	0.50	0.00
Kpna1	0.44	0.00
Smim20	0.49	0.00
Pgap2	0.37	0.00
Fbrsl1	0.35	0.00
Tbl1xr1	0.43	0.00
Rab27a	0.44	0.00
Ankdd1b	0.34	0.00
Rnf19a	0.34	0.00
Sema5a	-0.99	0.00
Hexim1	0.38	0.00
Gng13	-0.75	0.00
Arhgdia	0.34	0.00
Timm13	0.46	0.00
Hmox1	0.42	0.00
Sdhb	0.30	0.00
Ccng2	0.29	0.00
Rfesd	-0.68	0.00
Abca3	0.37	0.00
Kcnk5	0.44	0.00
Adgrg2	0.34	0.00
Plbd1	0.29	0.00
Eif4ebp1	0.28	0.00
Pelo	0.24	0.00
Steap4	0.39	0.00
Ap3b1	0.19	0.00
Sh3kbp1	0.53	0.00
Ptch1	0.56	0.00
Cdh1	0.33	0.00
Arfgef3	0.23	0.00
Atp6v1h	0.44	0.00
Sppl3	0.52	0.00
Sntb1	0.44	0.00
Dda1	0.20	0.00
Gulp1	0.42	0.00
Ndufb3	0.37	0.00
F13a1	0.46	0.00
Trank1	0.14	0.00
Klf7	0.38	0.00
Flvcr1	0.31	0.00
Tbc1d2	0.39	0.00
Itpka	0.36	0.00
Fam43a	0.38	0.00

Slc49a4	0.35	0.00
Sh3bgrl	0.38	0.00
Sptlc3	0.67	0.00
Wdr37	0.50	0.00
Cdc42ep3	0.36	0.00
Fars2	0.56	0.00
Srfbp1	0.41	0.00
Zbtb20	0.34	0.00
Gch1	0.39	0.00
Vps13a	0.34	0.00
Pid1	0.65	0.00
Zhx3	0.29	0.00
Btg2	0.22	0.00
Tcf7	-1.11	0.00
Nipa2	0.20	0.00
Slc22a16	-0.27	0.00
Actb	0.77	0.00
Nr4a2	0.30	0.00
Adamts3	0.49	0.00
Acvr2a	0.47	0.00
Acvr1b	0.24	0.00
Cmtm6	0.43	0.00
Slc38a10	0.10	0.00
Emc8	0.28	0.00
Clip1	0.32	0.00
Oat	0.32	0.00
Slc35b1	0.22	0.00
Nsa2	0.24	0.00
Pdp1	0.36	0.00
Tmem117	0.41	0.00
Rpn2	0.26	0.00
Errfi1	0.41	0.00
Ano3	-0.83	0.00
Fxn	0.37	0.00
Ost4	0.25	0.00
Impa2	0.26	0.00
Arl6ip5	0.19	0.00
Sorcs2	0.83	0.00
Hnrnpk	0.04	0.00
Pgrmc1	0.34	0.00
Snrpg	0.32	0.00
Zfp438	0.36	0.00
Tubb5	0.39	0.00
Fchsd2	0.40	0.00
Pigx	0.36	0.00
Lpcat2b	-0.48	0.00

Fndc5	0.24	0.00
Plag1	0.29	0.00
Stxbp6	0.54	0.00
Snta1	0.36	0.00
Ppp1ca	0.29	0.00
Cat	0.34	0.00
Eif4g2	-0.54	0.00
Cdc42se2	0.14	0.00
Eef1a1	-0.35	0.00
Dazap2	0.32	0.00
Smdt1	0.07	0.00
Dcaf6	0.41	0.00
Emg1	0.44	0.00
Tgm4	0.71	0.00
Cep89	0.41	0.00
Trabd2b	0.55	0.00
Adm	0.44	0.00
Plbd2	0.22	0.00
Fam174a	0.28	0.00
Nfam1	0.55	0.00
Atg12	0.25	0.00
Zfp36l2	0.33	0.00
Insig1	0.40	0.00
Rbfox2	0.47	0.00
Rassf2	0.49	0.00
Pik3r1	0.32	0.00
Exoc6b	0.35	0.00
Nabp1	0.38	0.00
Pmepa1	0.33	0.00
Cwc27	0.38	0.00
Rab31	0.11	0.00
Polr2k	0.27	0.00
Limk2	0.41	0.00
Ube2j1	0.16	0.00
Rreb1	0.41	0.00
Cmtm7	-0.47	0.00
Yaf2	0.26	0.00
Nfkb2	0.41	0.00
Ptx3	0.21	0.00
Cdc34	0.26	0.00
Psmb2	0.23	0.00
Nsmaf	0.39	0.00
Rnf20	0.22	0.00
Cacul1	0.17	0.00
Picalm	0.32	0.00
Hibadh	0.33	0.00

Wdr70	0.28	0.00
Dram1	0.20	0.00
Acsl4	-0.66	0.00
P2rx4	0.15	0.00
Plek	0.30	0.00
Psmd2	0.28	0.00
Atrx	-0.70	0.00
4932411N23Rik	0.38	0.00
Wdr20	0.34	0.00
Siva1	0.27	0.00
Hnrnpll	0.06	0.00
Acox1	0.26	0.00
H13	0.23	0.00
Sumo1	0.26	0.00
Atp1b4	0.36	0.00
Klf12	0.29	0.00
Acod1	0.50	0.00
Rasa1	0.24	0.00
Adrm1	0.25	0.00
Lhfpl3	-1.10	0.00
Elf1	0.20	0.00
Ly96	0.30	0.00
Ccdc171	0.37	0.00
Sorbs2	-0.55	0.00
Zc3h12a	0.35	0.00
Neurod6	0.28	0.00
Ptbp3	0.30	0.00
Actn1	0.30	0.00
Batf3	0.24	0.00
Cd82	-0.05	0.00
Cachd1	0.52	0.00
Sorbs3	0.22	0.00
Als2cl	0.60	0.00
Ptp4a1	0.28	0.00
Chchd4	0.22	0.00
Snrpd1	0.32	0.00
Hmgcr	0.37	0.00
Atp5a1	0.34	0.00
Cited4	0.34	0.00
Tacc2	-0.72	0.00
Cnot9	0.19	0.00
Bmpr1b	0.76	0.00
Dtx3l	0.22	0.00
Etv4	0.28	0.00
Sergef	0.43	0.00
Ap1ar	0.22	0.00

Itga1	0.25	0.00
Helz	0.29	0.00
Tspan3	0.27	0.00
C1ra	0.18	0.00
Usp10	0.22	0.00
Oaz1	0.31	0.00
Cd74	0.32	0.00
Casp6	-0.34	0.00
Tpk1	0.44	0.00
Rhpn1	0.38	0.00
Atp6ap2	0.20	0.00
Ank	0.30	0.00
G0s2	0.69	0.00
Spi1	0.60	0.00
Wdr36	0.38	0.00
Gpr137b	0.31	0.00
Deptor	0.37	0.00
Syngr2	0.19	0.00
Extl3	0.36	0.00
Tmx3	0.21	0.00
Sertad2	0.30	0.00
Lmnb1	0.48	0.00
Cidea	0.40	0.00
Hspa8	0.86	0.00
Ndufb2	0.20	0.00
Ube2r2	0.23	0.00
Sf3b4	0.36	0.00
Cd83	0.64	0.00
Tmem218	0.28	0.00
Aldoc	0.29	0.00
Comt	0.30	0.00
Rdh10	0.29	0.00
Sec61g	0.27	0.00
Adgrf5	-0.67	0.00
Slc46a2	0.38	0.00
Fam117b	0.38	0.00
Sptan1	-0.76	0.00
Babam2	0.27	0.00
Hmgb2	0.26	0.00
Gpx3	0.32	0.00
Cpq	0.57	0.00
Ifngr1	0.25	0.00
Atp6v0d1	0.28	0.00
Laptm4a	0.37	0.00
Ifngr2	0.26	0.00
Hepacam2	-0.53	0.00

Kctd2	0.21	0.00
Ctnnal1	-0.28	0.00
Psmf1	0.32	0.00
Galnt7	0.32	0.00
Lgi3	0.21	0.00
Tut7	0.05	0.00
Arhgap21	0.46	0.00
Sema4d	0.38	0.00
Sod3	0.24	0.00
Chst13	0.44	0.00
Bcl2l15	0.21	0.00
Osmr	0.31	0.00
Syt8	0.08	0.00
Crebrf	0.04	0.00
Tor2a	0.24	0.00
Crot	0.24	0.00
Fstl4	0.28	0.00
Alcam	-0.62	0.00
Dhrs3	0.33	0.00
Spata5	0.53	0.00
Chordc1	0.37	0.00
Slc4a10	-0.54	0.00
Fosb	0.27	0.00
Angptl4	0.33	0.00
Txn2	0.36	0.00
Tcim	0.44	0.00
Ubqln1	0.26	0.00
Ncf1	0.26	0.00
Fos	0.53	0.00
Arhgef7	0.33	0.00
Ctbp1	0.32	0.00
Rbm31y	0.33	0.00
Fam13a	0.35	0.00
Adi1	0.32	0.00
Cyria	0.35	0.00
Rnf223	0.33	0.00
Lpcat3	0.30	0.00
Taf13	0.29	0.00
Dctn6	0.22	0.00
Srgn	0.67	0.00
Kdm4c	0.30	0.00
Snrpc	0.20	0.00
2210408I21Rik	0.36	0.00
Nyap2	-0.46	0.00
Synm	-1.53	0.00
Agr2	0.52	0.00

Pinx1	0.62	0.00
Psmc6	0.18	0.00
Suco	0.26	0.00
Kdelr2	0.22	0.00
Klhl32	-0.20	0.00
Acaa1b	0.21	0.00
Snrpd3	0.15	0.00
Ccdc12	0.22	0.00
Dvl1	0.31	0.00
Atp6v1b2	0.31	0.00
Scarb1	0.12	0.00
Azin1	0.33	0.00
Fam53b	0.29	0.00
Coro1c	0.28	0.00
Cct7	0.22	0.00
Dach2	0.70	0.00
Ostf1	0.19	0.00
Lyrm7	0.35	0.00
Hagh	0.25	0.00
Ap1s2	0.19	0.00
Park7	0.19	0.00
Immp2l	0.42	0.00
Angpt1	0.38	0.00
Fam32a	0.20	0.00
Cd44	0.79	0.01
Il17ra	0.24	0.01
Carnmt1	0.33	0.01
Crispld2	0.42	0.01
Fam222b	0.31	0.01
Lrrc1	0.35	0.01
Parp10	0.23	0.01
Twsg1	0.13	0.01
Trim14	0.30	0.01
Slc9a3r1	0.33	0.01
Slc7a6	0.27	0.01
Hormad1	0.05	0.01
Zyx	0.20	0.01
Paip1	0.22	0.01
Ptbp1	0.30	0.01
Chsy1	0.28	0.01
Taldo1	0.25	0.01
Pes1	0.23	0.01
Blnk	0.43	0.01
Anxa11	0.21	0.01
Dcp1a	0.41	0.01
Rai2	0.21	0.01

Peli2	0.40	0.01
AW554918	0.34	0.01
Crem	0.21	0.01
Elk4	0.30	0.01
Ell	0.25	0.01
Gnb3	-0.22	0.01
Sptlc1	0.33	0.01
Eaf2	0.14	0.01
Elovl3	0.14	0.01
Itfg1	0.31	0.01
Srek1ip1	0.25	0.01
Galnt10	0.25	0.01
Erh	0.14	0.01
Gdpd5	0.04	0.01
Zc3h7b	0.29	0.01
Cpb2	0.32	0.01
Sema4a	0.22	0.01
Lancl1	0.30	0.01
Ralbp1	0.50	0.01
Hspa12a	0.34	0.01
Snapc5	0.30	0.01
Fgf2	0.39	0.01
Sc5d	0.19	0.01
Polr2f	0.27	0.01
Tspo	0.07	0.01
Fem1c	0.26	0.01
Pxn	0.30	0.01
Fbxl7	0.35	0.01
Irx5	0.28	0.01
Prrg3	0.10	0.01
Trpm6	0.60	0.01
Cstf2	0.23	0.01
Igf2r	0.13	0.01
Tex14	0.19	0.01
Slc10a7	0.41	0.01
Abl1	0.11	0.01
Hnrnpa0	0.24	0.01
Pef1	0.17	0.01
Txnrd3	0.04	0.01
Slc30a5	0.30	0.01
Yap1	0.34	0.01
Sox6	-1.56	0.01
Hsbp1	0.22	0.01
Lipa	0.29	0.01
Slc9a3r2	0.25	0.02
Kctd1	0.35	0.02

Acadl	0.06	0.02
Ripk2	0.32	0.02
Atic	0.16	0.02
Ensa	0.19	0.02
Gab1	0.22	0.02
Ppp1r3b	0.23	0.02
Snx25	0.38	0.02
Atp13a3	0.18	0.02
Gsdme	-0.30	0.02
Akt1	0.18	0.02
Hdac1	0.12	0.02
Atp5b	0.19	0.02
Agap1	0.32	0.02
Aven	0.34	0.02
Rpain	0.21	0.02
Psma6	0.23	0.02
Ndufb1	0.05	0.02
Prok2	0.29	0.02
Ptgfrn	0.38	0.02
Actr2	0.19	0.02
Igf2bp2	0.09	0.02
Hsph1	0.82	0.02
Nsmce2	0.38	0.02
Gnmt	0.25	0.02
Bcor	0.29	0.02
Nrp2	0.03	0.02
Bud31	0.17	0.02
Tax1bp1	0.31	0.02
Nek7	0.33	0.02
Ssr2	0.05	0.02
Tst	0.28	0.02
Ugcg	-0.08	0.02
Trappc3	0.37	0.02
Cnot2	0.07	0.02
Slc35f5	-0.74	0.02
Depdc7	0.34	0.02
Adora2b	0.57	0.02
Lsm8	0.28	0.02
Fam193a	0.20	0.02
Lmnb2	0.08	0.02
Nhsl1	0.46	0.02
Ptpn6	0.31	0.02
Snapc3	0.21	0.02
Sf3b6	0.22	0.02
Rnf14	0.26	0.02
Furin	0.15	0.02

Stx17	0.29	0.03
Spred1	0.19	0.03
Ino80d	0.17	0.03
Ccnl1	-0.66	0.03
Sil1	0.18	0.03
Nr2f6	-0.14	0.03
Tex9	0.27	0.03
Garem1	0.17	0.03
Lgmn	0.35	0.03
Tpm2	0.44	0.03
Hspb8	0.13	0.03
Pold2	0.28	0.03
Slc25a36	0.22	0.03
Naaladl2	0.37	0.03
Clta	0.23	0.03
Chic2	0.27	0.03
Echdc2	0.25	0.03
Slc25a22	0.28	0.03
Arsb	0.22	0.03
Tmed3	0.11	0.03
Supt5	0.16	0.03
Lcp1	0.85	0.03
Fzd8	0.22	0.03
Acsf3	0.21	0.03
Sh2d4a	0.33	0.04
Sdhaf4	0.14	0.04
Ddost	0.03	0.04
Lactb	0.18	0.04
Eif1	0.23	0.04
Gtf2h5	0.13	0.04
Rhoa	-0.35	0.04
Kcnk2	-0.50	0.04
Pkm	0.11	0.04
Flii	0.20	0.04
Ppp1r3g	-0.17	0.04
Gak	-0.20	0.04
Cnksr2	-0.55	0.04
Abca1	0.35	0.04
Slc16a5	0.14	0.04
Tomm7	0.15	0.04
Psmb1	0.19	0.04
Tmem74	0.32	0.04
Upk1b	0.27	0.04
Rpa3	0.23	0.04
Nppc	0.67	0.04
Rnd3	0.28	0.04

Cttn	-0.81	0.05
Guk1	0.12	0.05
Ldlrad3	0.11	0.05
Brix1	0.24	0.05
Herpud2	0.05	0.05
Bcr	0.13	0.05
Vma21	0.04	0.05
Rsl1d1	0.32	0.05
Lmo7	0.37	0.05
Naa25	0.10	0.05
Rbm39	0.35	0.05
Saraf	0.12	0.05
Erp44	0.15	0.05
Ikbke	0.19	0.05
Cbl	0.02	0.06
Pigg	0.22	0.06
Dnajb5	0.25	0.06
Traf3	0.53	0.06
Hid1	0.19	0.06
Tulp1	0.07	0.06
Wnt5b	0.38	0.06
Snx2	0.05	0.06
Nus1	0.22	0.06
Klf10	0.28	0.06
Plcl1	0.30	0.06
Tlcd1	0.13	0.06
Arf4	0.25	0.06
Trim8	0.02	0.06
Rhov	0.23	0.06
Polr1e	0.31	0.07
Eps8	0.64	0.07
Pla2g6	0.12	0.07
Slc24a5	-0.12	0.07
Hint1	0.22	0.07
Tusc3	0.31	0.07
Ptdss1	0.24	0.07
Il1rapl2	0.42	0.07
Nme7	0.23	0.07
Ptpru	0.26	0.07
Nucb2	-0.63	0.07
Socs3	0.42	0.07
Gng7	0.19	0.07
Usp47	0.20	0.07
9930021J03Rik	0.13	0.08
Podxl	0.36	0.08
Sec63	0.27	0.08

Smox	0.08	0.08
Pard3b	0.33	0.08
Cds1	0.09	0.08
Fbh1	0.21	0.08
Kcnrg	0.25	0.08
Stim2	-0.59	0.08
Luc7l	0.21	0.08
Itga8	0.27	0.08
H6pd	0.18	0.08
Bach1	0.16	0.08
Vwa8	0.24	0.08
Arl8b	0.19	0.09
Snrpb	0.27	0.09
Lmbrd2	0.43	0.09
Ppm1l	0.32	0.09
Bcap29	0.11	0.09
Pan3	0.11	0.09
Zmat2	0.20	0.09
Gtf2b	0.23	0.09
Slc16a6	0.30	0.09
Lsm4	0.07	0.09
Polr2e	0.08	0.10
Ripor3	-1.18	0.10
Cep120	0.28	0.10
Arhgap28	0.16	0.10
Ppie	0.29	0.10
Golga7	0.11	0.10
Pds5a	0.15	0.10
Rhog	0.20	0.10
Xrcc4	0.22	0.10
Dcn	0.25	0.10
Robo1	0.61	0.11
Six4	0.23	0.11
Ddx10	0.21	0.11
Aldh7a1	0.16	0.11
Ran	0.08	0.11
Skap2	0.24	0.11
St8sia2	-0.15	0.12
Inpp5d	-1.01	0.12
Pex16	0.29	0.12
Zc3h3	0.39	0.12
Tdrd3	0.14	0.12
Zcchc7	0.03	0.12
Fdps	0.25	0.13
Fibin	0.16	0.13
Klf9	-0.41	0.13

Rsad2	0.08	0.13
Epha5	0.21	0.13
Gna13	0.17	0.13
Zcrb1	0.16	0.13
Rbks	0.32	0.14
Sgsh	0.26	0.14
Phlpp2	0.26	0.15
Nop14	0.19	0.15
Fgfbp1	0.54	0.15
Urod	0.22	0.15
Zfp36l1	0.14	0.16
Hivep2	-0.46	0.16
Diablo	0.11	0.16
Ergic2	0.05	0.16
Braf	0.19	0.16
Icmt	0.22	0.16
Fez2	0.22	0.16
Nlrc5	0.12	0.17
Ndufs5	0.15	0.17
Otulinl	0.29	0.17
Vsir	0.24	0.17
Frmd4b	0.10	0.17
Tmem199	0.06	0.17
Psmb3	0.25	0.17
Litaf	0.06	0.17
Pcmtd1	0.08	0.18
Tomm22	0.20	0.18
Zfp516	0.30	0.18
Celsr1	0.30	0.18
Ergic1	0.12	0.18
Kras	0.24	0.18
Aopep	0.50	0.18
Hrh3	0.26	0.18
Lsm3	0.19	0.18
Arpc5	0.10	0.18
Star	0.27	0.19
Tbc1d16	0.34	0.19
Slc26a9	0.11	0.19
Galm	0.03	0.19
Ext2	0.24	0.19
B3gnt5	0.18	0.20
Plcb2	-0.91	0.20
Arvcf	0.22	0.20
Osbp2	0.31	0.20
Acvr1c	0.31	0.20
Aprt	0.26	0.20

1700034I23Rik	0.06	0.20
Agtpbp1	0.39	0.21
Smad6	0.39	0.21
Fam107b	0.44	0.21
Ski	0.37	0.21
Rbm8a2	-0.12	0.21
Psma3	0.20	0.21
Lclat1	0.09	0.21
Eefsec	0.33	0.21
Rims1	0.11	0.21
Dbnl	0.28	0.21
Ano6	0.29	0.22
Cir1	0.37	0.22
Aff1	0.05	0.22
Rab1a	0.07	0.22
Crlf1	0.19	0.22
Irx2	0.26	0.22
Dusp5	0.33	0.22
Lmf2	0.12	0.22
Syf2	0.22	0.23
Plch1	-0.56	0.23
Brsk2	0.30	0.23
Tmem108	0.55	0.23
Ankra2	0.27	0.23
Rgs3	0.22	0.23
Kctd20	0.10	0.23
Alpk1	0.22	0.24
Arrdc4	0.15	0.24
Scfd2	0.28	0.24
Emc6	0.22	0.24
Pdzd8	0.13	0.24
Lsmem1	0.30	0.24
Lpar1	0.57	0.25
Zfand5	0.28	0.25
Fgfrl1	0.30	0.25
Dennd4c	0.07	0.25
Dtnbp1	0.07	0.25
Slc39a13	0.40	0.25
Lsp1	0.15	0.26
Oga	0.07	0.26
Cfap410	0.24	0.26
Btf3l4	0.10	0.26
Tmem11	0.25	0.26
Tpi1	0.20	0.26
Cables2	0.20	0.26
Isca1	0.21	0.26

Cibar1	-0.19	0.27
Ubr3	0.19	0.27
Pfkl	0.23	0.27
Fancm	0.24	0.27
Pspc1	0.04	0.28
Fer	0.26	0.28
Appl2	0.24	0.28
Cpeb3	0.04	0.29
Cass4	0.16	0.29
Wdr89	0.22	0.29
Aldh9a1	0.19	0.29
Sgpl1	0.25	0.30
Tmem179b	0.20	0.30
Limd2	0.11	0.31
Reep5	0.16	0.31
Slc35e1	0.26	0.32
Psd	0.24	0.32
Kalrn	0.08	0.33
Foxo4	0.11	0.33
Lrig1	0.20	0.33
Exosc6	0.16	0.34
Soat1	0.16	0.34
Usp12	-0.57	0.34
Rel	0.21	0.34
Bfsp2	0.15	0.35
Phlda2	0.37	0.36
Cd47	0.19	0.36
Septin11	0.38	0.36
Ndufb4	0.04	0.36
Nifk	0.09	0.37
Nek9	0.15	0.37
Snrk	-0.84	0.37
Gsr	0.20	0.37
Tars	0.07	0.38
Ftcd	0.27	0.38
Recql5	0.30	0.38
Cacna1e	0.22	0.38
Nav2	-0.58	0.39
Sec23a	0.21	0.39
Pde10a	0.40	0.39
Dennd1a	0.25	0.39
Lrba	-0.40	0.39
Plcxd2	0.23	0.39
Krt20	-1.43	0.40
Trpm5	-1.05	0.40
Vegfc	0.52	0.40

Ssr1	0.03	0.40
Nrip1	0.17	0.41
Ptprs	0.19	0.41
Isoc1	0.13	0.41
Cmss1	0.19	0.41
Gpbp1	0.12	0.42
Snx17	0.23	0.42
Scly	0.13	0.42
Pdia3	0.14	0.42
Ranbp9	0.08	0.42
Ppa1	0.29	0.43
Ciao2b	0.28	0.43
Relch	0.18	0.43
Lonrf1	0.16	0.44
Pdss1	0.23	0.44
Tkt	0.08	0.44
Setbp1	0.24	0.44
Ros1	0.19	0.45
B4galt1	0.22	0.45
Csf3r	0.12	0.45
Efhd2	0.26	0.45
Trak1	0.25	0.45
Sash1	-0.15	0.46
Osbpl2	0.09	0.46
Rap2b	0.16	0.46
Pafah1b2	0.04	0.46
Naa35	-0.01	0.47
Esd	0.29	0.47
Nudt2	0.17	0.48
Acbd6	0.21	0.48
Vwf	-0.69	0.48
Nup210	-0.01	0.50
Tshz1	0.22	0.50
Plxdc2	0.25	0.50
Phf5a	0.15	0.51
Rcbtb2	0.21	0.51
Aspg	0.28	0.51
Pim3	-0.04	0.51
Fem1a	0.16	0.51
Sema6a	0.23	0.51
Cct2	0.20	0.51
Cflar	-0.10	0.52
Prkce	0.21	0.53
Tnip2	0.01	0.53
Pmvk	0.05	0.53
Ddc	0.22	0.53

Aldh1a3	-0.64	0.54
Rbm6	0.18	0.54
Puf60	0.22	0.55
Chmp4b	0.15	0.56
Stx8	0.09	0.56
Pgm1	0.04	0.56
Clybl	0.23	0.57
Cfap97	0.02	0.57
Ssrp1	0.07	0.58
Spty2d1	0.18	0.58
Arhgap15	0.66	0.58
Pdlim1	0.25	0.59
Parn	0.20	0.59
Rb1cc1	0.17	0.59
Fam126a	-0.55	0.59
Dusp6	0.09	0.60
Tnfaip2	0.35	0.60
Cbfb	0.20	0.61
Rtf2	0.18	0.61
Rsbn1l	0.02	0.62
Plec	-0.82	0.62
Cacng4	-0.27	0.62
Ccn1	0.28	0.62
Tnrc6c	0.22	0.63
Irak2	0.18	0.63
Fbxo11	0.12	0.64
Pisd	0.27	0.65
Slc4a11	0.00	0.65
Tbcd	0.13	0.66
Srsf5	0.20	0.66
Abca12	0.19	0.66
Ggt7	0.25	0.67
Raf1	0.06	0.68
Dnaja4	0.27	0.68
Txnrd1	0.32	0.68
Ptk7	0.11	0.69
Sgpp1	0.07	0.69
Hypk	0.31	0.70
Purg	-0.30	0.72
Gabarapl2	0.12	0.73
Grm7	0.19	0.73
Als2	0.21	0.74
Echs1	0.21	0.74
Lasp1	0.13	0.75
Clic3	0.22	0.75
Psip1	0.34	0.76

Dock10	0.13	0.77
Hadhb	0.09	0.77
Smoc1	0.20	0.78
Bend4	-0.25	0.79
Bcl7b	0.20	0.79
Chpt1	0.05	0.80
Prrc1	0.16	0.80
Ecpas	0.18	0.81
Fancg	0.17	0.81
Cxxc4	0.32	0.81
Tmem268	0.25	0.82
Ubtd2	0.11	0.83
St6galnac1	-0.79	0.83
Sfxn2	0.14	0.83
Casp7	0.11	0.84
Ciao2a	0.13	0.84
Tcerg1	0.23	0.84
Rsu1	0.11	0.85
Dipk1c	0.14	0.85
Cib1	0.05	0.85
Atp2a3	-0.62	0.85
Snrpb2	0.13	0.86
Farp2	0.22	0.87
Antkmt	0.13	0.87
Rexo2	0.18	0.87
Stk26	-0.63	0.88
Rai14	0.34	0.89
Arhgap26	0.06	0.89
Kansl1	0.13	0.89
Tnfrsf11a	0.06	0.92
Arhgef26	0.06	0.92
Cav2	-0.18	0.93
Lratd1	-0.39	0.93
Cdc42bpa	0.18	0.94
Ilf2	0.20	0.94
Drd4	0.18	0.94
Ext1	0.18	0.96
Dagla	0.24	0.97
Zranb2	0.20	0.97
Fxr1	-0.75	0.97
Rad51d	0.17	0.97
Entpd3	0.17	0.97
Chid1	0.35	0.98
Wdr82	0.06	0.98
Ncf2	0.15	0.99
Abtb2	0.40	1.00

Acsbg2	0.15	1.00
Tbc1d12	0.22	1.00
Dgkq	0.19	1.00
Angel2	-0.68	1.00
Endog	0.11	1.00
Gngt2	-0.31	1.00
Ywhaq	-0.42	1.00
Cbx4	0.28	1.00
Phb2	0.19	1.00
Nol6	0.12	1.00
Rapgef2	-0.38	1.00
Thbs1	0.55	1.00
Dlgap4	0.17	1.00
Emc2	0.17	1.00
Rnf130	-0.11	1.00
Pou6f2	-0.71	1.00
Commd8	-0.18	1.00
Afap1l2	0.22	1.00
Itpkb	0.25	1.00
Ppp3cb	0.22	1.00
Bmp3	-1.11	1.00
Prkag2	0.11	1.00
Tcirg1	0.10	1.00
Pink1	0.20	1.00
Cep170	-0.60	1.00
Gli3	0.38	1.00
Pla2g12a	-0.47	1.00
Chrac1	0.11	1.00
Rnf122	0.09	1.00
Kif13a	0.22	1.00
Tmem86a	0.10	1.00
Cbfa2t2	0.21	1.00
Pdrg1	0.15	1.00
Ccdc91	0.40	1.00
Tnfaip8l1	0.23	1.00
Fgl2	0.16	1.00
Ssu72	0.08	1.00
Klf13	-0.88	1.00
Fosl2	0.11	1.00
Ptpn4	0.00	1.00
Rer1	-0.02	1.00
Pdxk	-0.09	1.00
Etv3	0.16	1.00
Zfand3	0.18	1.00
Pou2f3	-0.19	1.00
Supt4a	0.18	1.00

Lfng	0.18	1.00
Pgm2	0.26	1.00
Derl1	-0.01	1.00
Sacm1l	0.06	1.00
Slc25a3	0.10	1.00
Fgd5	0.05	1.00
Trappc9	0.13	1.00
Baiap2l1	0.29	1.00
Rpa2	0.30	1.00
Vill	0.01	1.00
Cdc73	-0.06	1.00
Dpagt1	0.23	1.00
Ostc	0.00	1.00
Ak2	-0.08	1.00
Piezo2	0.44	1.00
Tmem98	0.14	1.00
Col9a2	-0.05	1.00
Eif4e	0.01	1.00
Tnfsf15	0.31	1.00
Zranb1	0.01	1.00
Prdm11	0.25	1.00
Rbx1	0.12	1.00
Ttc7b	0.28	1.00
Zcchc17	0.29	1.00
Rpn1	0.06	1.00
Pag1	0.44	1.00
Bmp2k	0.09	1.00
Pwp1	0.23	1.00
Cav1	-0.10	1.00
Adprh	-0.38	1.00
Naga	0.05	1.00
Cdc20	-0.05	1.00
Nudt21	0.01	1.00
Ptprm	0.36	1.00
Aplp2	-0.45	1.00
Yeats4	0.26	1.00
Arhgap18	-0.47	1.00
Zfp395	0.20	1.00
Pigl	0.19	1.00
Tent5a	0.27	1.00
Psmd14	0.15	1.00
Slc45a3	0.14	1.00
Sh3tc2	0.10	1.00
Tmem230	0.11	1.00
Bin3	0.18	1.00
Lemd2	0.05	1.00

Taf2	0.21	1.00
Plaa	-0.17	1.00
Nfkbie	0.25	1.00
Chmp1a	0.17	1.00
Herc2	-0.60	1.00
Krr1	0.13	1.00
Sdf2	0.20	1.00
Adgrl2	0.00	1.00
Bcl9l	0.22	1.00
Tmed5	-0.05	1.00
Bbs4	0.17	1.00
Sulf1	0.29	1.00
Dgat2	0.16	1.00
Abl2	0.20	1.00
Nckap1	-0.69	1.00
Ppp2r5c	0.16	1.00
Wbp11	0.14	1.00
Tasp1	0.14	1.00
Ube3c	0.13	1.00
Unc13d	0.21	1.00
Nxt2	-0.82	1.00
Ppargc1b	0.26	1.00
Zfp536	-0.54	1.00
Akap12	0.03	1.00
Foxa2	0.10	1.00
Snu13	0.14	1.00
Tmem161b	0.20	1.00
Fam20c	0.23	1.00
Tmem241	0.05	1.00
Il13ra2	-0.67	1.00
Grid1	0.48	1.00
Basp1	-0.96	1.00
Pof1b	-0.42	1.00
Shisa5	-0.01	1.00
Ube2k	0.03	1.00
Thsd7a	0.27	1.00
Cpne8	0.03	1.00
Arhgap10	-0.08	1.00
Lrrc8a	0.04	1.00
Hmgxb4	0.07	1.00
Cd109	-0.38	1.00
Ccdc92b	0.11	1.00
Ssb	0.12	1.00
Aak1	0.07	1.00
Kars	0.16	1.00
Syn3	0.16	1.00

Atxn7l1	0.11	1.00
Pcyox1	0.12	1.00
Spopl	0.03	1.00
Decr2	0.06	1.00
Ctbp2	-0.29	1.00
Pik3ip1	0.25	1.00
Creb5	-0.06	1.00
Itga6	0.17	1.00
Gucy1b1	-0.15	1.00
Fdx1	0.12	1.00
Fmnl1	0.18	1.00
Tle1	0.18	1.00
Pcca	0.20	1.00
Derl3	0.09	1.00
Sufu	0.01	1.00
Rerg	-0.16	1.00
Rcbtb1	-0.03	1.00
Rab3il1	0.26	1.00
Erg28	0.09	1.00
Prelid1	0.07	1.00
Psmc2	-0.04	1.00
Rubcnl	0.17	1.00
Ap2m1	0.05	1.00
Psmd13	0.16	1.00
Psen1	0.09	1.00
Septin9	0.19	1.00
Afap1	0.36	1.00
Emc7	0.04	1.00
Dpp7	0.21	1.00
Kctd8	0.21	1.00
Elp1	0.11	1.00
Ncbp1	0.25	1.00
Sgsm3	0.19	1.00
Ddr2	0.16	1.00
Gdpd1	-0.96	1.00
Fam171b	0.11	1.00
Dcp2	-0.07	1.00
Znrf3	0.26	1.00
Gatad2a	0.12	1.00
Tmcc1	0.22	1.00
Adsl	0.23	1.00
Ccdc134	0.07	1.00
Hnrnpa2b1	-0.33	1.00
Psmb7	0.11	1.00
Zdhhc15	0.13	1.00
Gnai2	0.03	1.00

Fam107a	0.18	1.00
Wwp1	0.26	1.00
Selenof	0.01	1.00
Tmem242	0.09	1.00
Lrpap1	0.05	1.00
Fkbp9	-0.32	1.00
Arl3	0.15	1.00
Dennd2a	0.13	1.00
Tenm3	0.21	1.00
Poc1a	0.02	1.00
Ebf1	0.22	1.00
Uaca	-0.76	1.00
Sh3bgrl2	-0.54	1.00
Nfkb1	0.22	1.00
Lims2	0.11	1.00
Spats2	0.25	1.00
Scfd1	0.24	1.00
9530077C05Rik	0.18	1.00
Atp2c2	-0.12	1.00
Ndufb9	0.08	1.00
Dusp15	0.05	1.00
Osgin2	-0.58	1.00
Rngtt	0.03	1.00
Snx4	0.08	1.00
Rapgef1	0.17	1.00
Fam177a	0.09	1.00
Eif3i	0.12	1.00
Hmces	0.19	1.00
Foxk1	0.01	1.00
Suclg2	0.04	1.00
Bmx	-1.24	1.00
Tob2	0.22	1.00
Tm2d2	0.00	1.00
Baiap2	0.21	1.00
Rragd	0.08	1.00
Psmb4	0.08	1.00
Csmd2	0.27	1.00
Calm2	0.14	1.00
Kif15	0.07	1.00
Kctd19	0.33	1.00
Zdhhc14	0.07	1.00
Pig	0.18	1.00
Herpud1	0.20	1.00
Ndufa8	0.07	1.00
Tmem9b	0.06	1.00
Vps54	0.10	1.00

Adipor1	0.18	1.00
Bdkrb2	0.14	1.00
Pdf	0.13	1.00
Pparg	0.01	1.00
Bmpr1a	0.10	1.00
Dync1h1	-0.54	1.00
Ash2l	0.10	1.00
Entpd5	-0.62	1.00
Ets1	-0.01	1.00
Spsb1	0.19	1.00
Rnf185	0.05	1.00
Rcan2	-0.27	1.00
Zc3h15	0.25	1.00
Ttc39c	0.06	1.00
Zfpm1	0.19	1.00
Ifih1	0.18	1.00
Psmg4	0.21	1.00
Tmem104	0.20	1.00
Vdr	0.02	1.00
Klf6	0.13	1.00
Wbp2	0.26	1.00
Zfp143	0.10	1.00
Clic6	-0.64	1.00
Rassf6	0.15	1.00
Cript	-0.06	1.00
Jdp2	0.32	1.00
Nob1	0.17	1.00
Arl4c	-0.37	1.00
Ppl	-0.58	1.00
Suds3	0.07	1.00
Phb	0.16	1.00
Faf2	0.04	1.00
Fggy	0.20	1.00
Tgds	0.22	1.00
Oxr1	0.12	1.00
Ttf1	-0.01	1.00
Zbtb25	-0.11	1.00
Ssh2	-0.06	1.00
Vav3	0.96	1.00
Snx12	0.21	1.00
Frmd3	0.22	1.00
Gclc	-0.18	1.00
Snx30	0.16	1.00
Hivep1	0.13	1.00
Borcs6	0.14	1.00
Stard4	0.16	1.00

Nsun3	0.21	1.00
Serpine2	0.29	1.00
Etv1	0.35	1.00
Kdm4b	0.21	1.00
Kit	-0.50	1.00
Rcsd1	-0.02	1.00
Lamc2	-0.08	1.00
Fam3d	0.06	1.00
Nup98	0.07	1.00
Chka	0.15	1.00
Gpalpp1	0.06	1.00
Hacd4	0.21	1.00
Hip1	-0.08	1.00
Scamp1	0.07	1.00
Jund	0.14	1.00
Ostm1	0.24	1.00
Lars2	0.16	1.00
Psma5	-0.01	1.00
Wdr61	0.22	1.00
Ypel1	0.17	1.00
Vps41	0.14	1.00
Bend7	0.13	1.00
Slc23a2	0.90	1.00
Rnf11	0.05	1.00
Slf1	0.11	1.00
Surf4	-0.15	1.00
Borcs5	0.27	1.00
Tnpo1	0.02	1.00
Rnf216	0.11	1.00
Zdhhc3	0.06	1.00
Tysnd1	0.11	1.00
Slc16a14	-0.03	1.00
Acsl1	0.54	1.00
Amotl1	0.23	1.00
Noc3l	0.17	1.00
Usp7	0.09	1.00
Dvl3	0.14	1.00
Vopp1	0.07	1.00
Surf6	0.09	1.00
Ell2	0.08	1.00
Ncoa7	0.22	1.00
Zfand2a	0.09	1.00
Ppil2	0.14	1.00
Tmem184c	0.18	1.00
Got2	0.24	1.00
Ankrd12	-0.58	1.00

Atp12a	0.20	1.00
Rspo2	0.26	1.00
Zbtb10	0.04	1.00
Serp1	0.02	1.00
Nipal4	0.05	1.00
Cdc42se1	0.21	1.00
Lrp2	-0.31	1.00
Ncoa1	0.13	1.00
Vash1	-0.56	1.00
Tsn	0.00	1.00
Aplf	0.18	1.00
Vti1b	0.05	1.00
Wnt5a	-0.01	1.00
Rabgap1l	0.02	1.00
Ano10	0.29	1.00
Fcf1	0.08	1.00
Gpi1	-0.05	1.00
Epb41l4b	0.12	1.00
Prkag1	0.11	1.00
Ntmt1	0.26	1.00
Cacna1c	0.03	1.00
Ube4a	0.25	1.00
Dnajc19	0.18	1.00
Eef2	-0.44	1.00
Ranbp17	0.21	1.00
Pdia2	0.12	1.00
Tpra1	0.12	1.00
Bpnt2	-0.59	1.00
Ncor2	0.12	1.00
Acad8	0.08	1.00
Slc44a5	-0.66	1.00
Zfyve27	0.11	1.00
Peli1	-0.02	1.00
Etnk1	-0.42	1.00
Nelfcd	0.27	1.00
Ephx1	0.12	1.00
Sft2d1	0.01	1.00
Gpm6b	-0.49	1.00
Fam120b	0.17	1.00
Septin8	0.10	1.00
Slc16a7	0.11	1.00
Stx6	0.01	1.00
Noct	0.17	1.00
Ephb3	0.27	1.00
Efna1	0.08	1.00
Ilvbl	0.22	1.00

Fuca2	0.17	1.00
Ptges	-0.29	1.00
Vamp3	0.09	1.00
Dcaf10	0.09	1.00
Tusc2	0.22	1.00
Llph	0.11	1.00
Prkn	0.35	1.00
Btbd9	0.11	1.00
Atoh8	0.29	1.00
Lysmd3	0.12	1.00
Epc1	-0.01	1.00
Plagl1	0.01	1.00
Ppip5k2	0.20	1.00
Rad51b	0.15	1.00
Sdf2l1	0.03	1.00
Hspa14	0.25	1.00
Elf3	0.10	1.00
Ube2i	0.20	1.00
Phactr2	-0.22	1.00
Utp3	0.16	1.00
Ebag9	0.21	1.00
Cry2	0.14	1.00
Efhd1	0.04	1.00
Plppr1	-0.27	1.00
Rbm19	0.17	1.00
1110059G10Rik	0.06	1.00
Eif5	0.07	1.00
Pofut2	0.11	1.00
Col12a1	0.20	1.00
Pole4	0.07	1.00
Pm20d1	0.17	1.00
Arsk	-0.04	1.00
Ginm1	-0.05	1.00
Tom1l2	0.15	1.00
Ptpre	0.17	1.00
Actr3	0.19	1.00
Ttpa	0.18	1.00
Trerf1	-0.05	1.00
Ank2	-0.13	1.00
Plcl2	0.11	1.00
Pam	0.00	1.00
Galnt16	0.14	1.00
Ikzf2	0.31	1.00
Helz2	0.16	1.00
Dck	-0.25	1.00
Rpf2	0.08	1.00

Arfip2	-0.05	1.00
Rpgrip1l	0.28	1.00
Cnot4	0.07	1.00
Rhoq	-0.26	1.00
Ndufa10	0.04	1.00
Prdx1	0.02	1.00
Hpse	0.21	1.00
Sdk1	-1.35	1.00
Npl	0.27	1.00
Pdss2	0.23	1.00
Usp44	-0.08	1.00
Ripor1	0.30	1.00
Sms	0.09	1.00
Sh3bp2	-0.03	1.00
Rint1	0.19	1.00
Cdk5rap3	0.09	1.00
Acp2	0.00	1.00
Gpr39	-0.27	1.00
Papss2	0.14	1.00
Gfra1	0.23	1.00
Ppm1h	-0.65	1.00
Hnrnpd	0.27	1.00
Tmem167	0.14	1.00
Zdhhc6	0.08	1.00
Gsg1	-0.63	1.00
Adgrv1	0.11	1.00
Susd6	0.17	1.00
Nudt3	-0.14	1.00
Egln3	0.04	1.00
Nfyc	0.13	1.00
Osbpl9	-0.01	1.00
Fa2h	0.12	1.00
Slc1a4	-0.03	1.00
Ckap4	-0.38	1.00
Pafah2	0.16	1.00
Prdx3	0.16	1.00
Spry1	-0.02	1.00
Cdc42	0.04	1.00
Rnf149	0.01	1.00
Ndrg3	0.24	1.00
Ugdh	0.08	1.00
Slc39a6	-0.02	1.00
Dstn	-0.18	1.00
Klhl24	-0.07	1.00
Dtwd2	0.02	1.00
Ptpn14	0.18	1.00

Txndc9	-0.52	1.00
Slc34a2	-0.05	1.00
Pias2	0.10	1.00
Kdm2a	0.00	1.00
Slc48a1	0.17	1.00
Secisbp2	-0.01	1.00
Npr3	0.24	1.00
Lrrc41	0.11	1.00
Aldh6a1	0.04	1.00
Smu1	0.03	1.00
Dhx35	0.40	1.00
Nr4a1	0.22	1.00
Sh3gl3	-0.02	1.00
Ccdc50	0.25	1.00
Trib1	-0.10	1.00
Sh3d19	-0.08	1.00
Siah1b	0.22	1.00
Cdkal1	0.16	1.00
Kcnu1	0.06	1.00
Pradc1	0.18	1.00
Scn8a	0.15	1.00
Slc30a6	0.17	1.00
Pde5a	-0.55	1.00
Tspan5	0.18	1.00
Dusp16	0.06	1.00
Snx24	0.15	1.00
Clmn	0.01	1.00
Gjc2	-0.41	1.00
Prkg1	0.13	1.00
Srp68	0.11	1.00
Eya3	-0.02	1.00
Slc40a1	-0.11	1.00
Dhx29	0.02	1.00
Txndc17	0.05	1.00
N4bp1	-0.02	1.00
Ngly1	0.06	1.00
Nrf1	0.20	1.00
Atad3a	0.30	1.00
Ammecr1l	-0.03	1.00
Erlec1	-0.21	1.00
Slc12a2	0.04	1.00
Pak4	0.07	1.00
Sptlc2	0.11	1.00
Slco4a1	-0.29	1.00
Npas2	0.11	1.00
Igfbp7	0.12	1.00

Scamp5	0.08	1.00
Polr2b	-0.08	1.00
Ncoa3	0.19	1.00
Ackr4	-0.01	1.00
S100a11	0.17	1.00
Slc52a3	0.08	1.00
Ivd	0.20	1.00
Hnrnpm	0.15	1.00
Klhl21	0.16	1.00
Lrrc8d	0.03	1.00
Dusp4	0.19	1.00
Slc45a1	0.20	1.00
Clint1	0.10	1.00
Sirt5	0.17	1.00
Tsr3	0.22	1.00
Elf2	0.06	1.00
Hspbap1	0.21	1.00
Ptpn1	0.03	1.00
Tmem140	0.05	1.00
Tle5	0.16	1.00
Afg3l2	0.05	1.00
Lin52	0.04	1.00
Zeb1	-0.16	1.00
Galnt17	0.31	1.00
Psmd6	0.11	1.00
Nme6	0.16	1.00
Ccn2	-0.05	1.00
Plxnc1	-0.18	1.00
Fkbp15	-0.36	1.00
Crhr2	-0.49	1.00
Tmem97	0.13	1.00
Tjp3	0.13	1.00
Fli1	-0.01	1.00
Tafa4	0.25	1.00
Itga9	0.16	1.00
Nutf2	0.15	1.00
Lsm14a	0.20	1.00
Pdlim5	0.25	1.00
Nans	-0.39	1.00
Ogfrl1	-0.15	1.00
Ggcx	0.10	1.00
Golph3	0.00	1.00
Large1	0.19	1.00
Nubp2	0.09	1.00
Srf	0.16	1.00
Tor3a	0.27	1.00

Fam110a	0.20	1.00
Ptpa	0.14	1.00
Tmem59	0.07	1.00
Uhrf2	0.21	1.00
Pfdn4	0.04	1.00
Ndufa6	0.16	1.00
Nudt12	0.07	1.00
Ryr2	-0.86	1.00
Rnf145	0.04	1.00
Tll1	0.32	1.00
Gda	0.01	1.00
Uhrf1bp1l	0.05	1.00
Pigk	0.08	1.00
Dhrs9	-0.29	1.00
Ap3m1	-0.01	1.00
Nuak1	-0.32	1.00
Tmem175	0.13	1.00
Sigmar1	0.20	1.00
Zfhx3	0.21	1.00
Txnrd2	-0.04	1.00
Lonrf2	-0.01	1.00
Uap1	0.16	1.00
Polr2h	-0.01	1.00
Sdad1	-0.97	1.00
Cyyr1	0.05	1.00
Gramd1c	-0.13	1.00
Itga4	-0.97	1.00
Ralgps1	-0.09	1.00
Rhou	-0.67	1.00
Abtb1	0.20	1.00
Kdm2b	-0.02	1.00
Cenpu	0.15	1.00
Naxd	0.08	1.00
Top2a	-0.33	1.00
Tomm20	0.17	1.00
Klhl5	-0.01	1.00
Arhgef3	0.15	1.00
Gpr137c	0.15	1.00
Podxl2	-0.09	1.00
Afg1l	0.28	1.00
Lrrc15	0.16	1.00
Acot11	-0.36	1.00
Sf3a1	0.08	1.00
Rtn1	0.08	1.00
Cnih4	0.02	1.00
Rbms3	0.30	1.00

Dcun1d3	0.15	1.00
Plek2	0.05	1.00
Fbxo34	0.05	1.00
Acbd3	0.06	1.00
G2e3	-0.50	1.00
Dgkz	-0.33	1.00
Fam110b	0.24	1.00
Nsrp1	0.23	1.00
Prr5	0.24	1.00
Dpyd	0.19	1.00
Ttc27	0.23	1.00
Zfat	0.20	1.00
Abcd3	0.04	1.00
Sel1l3	0.03	1.00
Rabepk	0.04	1.00
Aimp1	0.03	1.00
Tiam2	0.15	1.00
Slc35e3	0.15	1.00
Exosc9	-0.04	1.00
Cth	0.19	1.00
Arhgap29	0.17	1.00
Tasor	0.02	1.00
Ccdc28a	0.01	1.00
Shprh	-0.13	1.00
Wdr12	0.09	1.00
Lpl	-0.25	1.00
Adam17	0.03	1.00
Plcb1	-0.67	1.00
Agpat3	-1.18	1.00
Sox11	0.07	1.00
Ube2e2	0.16	1.00
Taf15	-0.01	1.00
Gabra3	-0.31	1.00
Rrp15	0.17	1.00
Net1	-0.07	1.00
Arid3b	0.07	1.00
Foxj2	0.24	1.00
Sod1	-0.05	1.00
Vapa	-0.03	1.00
Gnas	0.10	1.00
D430041D05Rik	0.20	1.00
Sh3yl1	0.01	1.00
Slc25a26	0.20	1.00
Fkbp3	0.13	1.00
Ube2e3	0.16	1.00
Dynlt3	-0.07	1.00

Phkb	-0.02	1.00
Tmem250-ps	0.07	1.00
Neurl1a	-0.58	1.00
Tanc1	0.06	1.00
Dnajc7	-0.09	1.00
Erbin	0.29	1.00
Gtdc1	0.23	1.00
Gigyf2	-0.74	1.00
Htatip2	0.11	1.00
Capza2	0.03	1.00
Slc35a5	-0.09	1.00
Sec31a	0.11	1.00
Fbxl4	0.04	1.00
Arid1a	0.05	1.00
Sap30	-0.01	1.00
Pard6b	0.03	1.00
Focad	-0.01	1.00
Gabarapl1	-0.09	1.00
Lsm7	0.14	1.00
Pcyt2	0.02	1.00
Vash2	0.09	1.00
Tent2	0.15	1.00
Igsf9	0.10	1.00
Rab40c	0.05	1.00
Btbd11	-0.04	1.00
Nktr	-0.03	1.00
Eif4e2	0.13	1.00
Ppm1a	0.05	1.00
Tnip1	0.08	1.00
Pkdcc	0.18	1.00
Rubcn	-0.39	1.00
Tent5b	0.05	1.00
Cdk8	-0.03	1.00
Creld2	0.02	1.00
Nsfl1c	0.24	1.00
Lhfp	-0.04	1.00
Oser1	0.01	1.00
Phf20l1	0.09	1.00
Ctcf	0.08	1.00
Hspb11	0.25	1.00
Rhbdf2	0.06	1.00
Ccdc71l	0.16	1.00
Rflnb	-0.43	1.00
Flvcr2	-0.88	1.00
Sec11c	-0.20	1.00
Cse1l	-0.58	1.00

Gcn1	0.16	1.00
Tmem35b	0.03	1.00
Rfc5	0.22	1.00
Tmem42	-0.02	1.00
Hdx	-0.34	1.00
Ago3	0.01	1.00
Khdrbs3	0.11	1.00
Snx29	0.17	1.00
Arf6	-0.01	1.00
Ppp2r5e	0.16	1.00
Nt5m	0.19	1.00
Atg7	0.08	1.00
Exosc7	0.15	1.00
Rnf139	0.04	1.00
Kctd14	0.25	1.00
Adra2a	0.11	1.00
Ska2	-0.01	1.00
Supt3	0.26	1.00
Arap2	0.12	1.00
L3mbtl3	0.01	1.00
Cry1	0.04	1.00
Trappc13	0.14	1.00
Wdr33	-0.06	1.00
Nthl1	0.12	1.00
Ssr3	0.06	1.00
Disp1	-0.10	1.00
Hexb	0.00	1.00
Elovl5	-0.01	1.00
Dtx2	0.13	1.00
Itgb6	-0.11	1.00
Stat3	0.02	1.00
Psmc1	0.12	1.00
Homer1	0.15	1.00
Ttc17	-0.32	1.00
Gpat4	0.18	1.00
Ythdc2	0.20	1.00
Pxmp4	0.12	1.00
Cept1	0.08	1.00
Ubl7	0.05	1.00
Shisa3	0.19	1.00
Arhgef18	0.02	1.00
Sec22a	0.07	1.00
Cope	-0.02	1.00
Neurl1b	-1.21	1.00
Ntng1	0.27	1.00
Gnal	-0.98	1.00

Pigt	0.07	1.00
Tab3	0.17	1.00
Rsl24d1	-0.07	1.00
Anapc2	0.16	1.00
Pum3	0.11	1.00
Cnpy3	0.07	1.00
Atp2b4	-0.52	1.00
Cited2	0.17	1.00
Scamp2	-0.08	1.00
Cul1	0.08	1.00
Ctif	0.01	1.00
Samd4	0.13	1.00
Lamp1	0.13	1.00
Scamp4	-0.01	1.00
Ddx49	0.12	1.00
Scrib	0.11	1.00
Vps37c	0.05	1.00
Itpr1	0.00	1.00
Rnf10	0.09	1.00
Carf	0.11	1.00
Shld1	0.09	1.00
Nras	-0.18	1.00
Ghdc	0.09	1.00
G3bp1	-0.01	1.00
Prelid3b	-0.06	1.00
Acadm	0.02	1.00
Setd1b	-0.09	1.00
Capg	0.26	1.00
Tpgs2	0.17	1.00
Snrnp200	0.15	1.00
Large2	0.15	1.00
Chst10	0.07	1.00
Atp6v1a	0.10	1.00
Avl9	0.18	1.00
Apoo	-0.03	1.00
Dock3	0.20	1.00
Nsun5	0.11	1.00
Cttnbp2nl	0.00	1.00
Galnt1	0.19	1.00
Abhd13	0.04	1.00
Snrnp40	0.23	1.00
Tspan1	-0.36	1.00
Acsl5	0.05	1.00
Fbxo22	-0.08	1.00
Sgce	-0.74	1.00
Car8	0.17	1.00

Phf6	0.05	1.00
Rnf166	0.11	1.00
Numb	0.25	1.00
Tia1	0.05	1.00
Arl6ip4	0.14	1.00
Creb3l2	0.05	1.00
Stk35	-0.01	1.00
Tipin	0.12	1.00
Sema3f	0.18	1.00
Dctn4	0.03	1.00
Sgms1	0.03	1.00
Lamp2	0.04	1.00
Bst1	0.25	1.00
Sort1	0.01	1.00
Atp5j	-0.02	1.00
Scarb2	-0.18	1.00
Polg2	0.14	1.00
Saal1	0.47	1.00
Wdr75	0.14	1.00
Prkaa1	0.03	1.00
Rfx2	-0.77	1.00
Ybx1	-0.32	1.00
Fam8a1	0.11	1.00
Fzd3	0.10	1.00
Zcchc10	0.18	1.00
Rbm17	0.09	1.00
Dtymk	0.18	1.00
Entpd8	0.03	1.00
Pik3c3	-0.12	1.00
Ccnc	0.12	1.00
Ip6k2	0.08	1.00
Gpc5	0.18	1.00
Tmco1	-0.07	1.00
Nr4a3	0.03	1.00
Pign	0.18	1.00
Smim12	-0.01	1.00
Zdhhc21	0.11	1.00
St7l	-0.03	1.00
Epha1	0.15	1.00
Id2	0.25	1.00
N4bp2	0.06	1.00
Glipr2	0.19	1.00
Smad2	-0.24	1.00
Ppp2r2a	-0.21	1.00
Axin1	0.04	1.00
Samm50	-0.07	1.00

Tfdp2	-0.06	1.00
Nipa1	-0.60	1.00
Nt5dc1	0.05	1.00
Uchl3	0.02	1.00
Pus7	0.13	1.00
Papln	-0.26	1.00
Bcas1	0.16	1.00
Larp1	-0.06	1.00
Fcho2	-0.17	1.00
Got1	0.03	1.00
Tet1	-0.50	1.00
2610507B11Rik	0.07	1.00
Arl15	0.17	1.00
Id4	0.18	1.00
Ift74	0.08	1.00
Prelid2	0.13	1.00
Adcy1	0.14	1.00
Cryl1	0.04	1.00
Lmf1	-0.02	1.00
Zmat5	0.10	1.00
Il6ra	0.19	1.00
Ptpn22	-0.20	1.00
Lamtor2	-0.06	1.00
Ddo	-0.11	1.00
Stam	0.11	1.00
Btbd6	0.19	1.00
Cbx7	0.07	1.00
Thada	0.10	1.00
Rock2	0.02	1.00
Gtf2a2	0.04	1.00
Ctso	-0.25	1.00
Tpm1	-0.14	1.00
Shld2	0.17	1.00
Tulp3	0.17	1.00
Dusp10	-0.04	1.00
Hook1	-0.48	1.00
Sf3b3	0.06	1.00
Syne3	-0.03	1.00
Cmtm8	-0.02	1.00
Pex10	0.14	1.00
Nudcd2	0.15	1.00
Cnot6	-0.45	1.00
Ugt8a	-0.27	1.00
Uggt1	-0.14	1.00
Tshz2	-1.02	1.00
Smarcd2	0.15	1.00

Cdk7	0.10	1.00
Lamb3	0.12	1.00
Pip5k1c	0.19	1.00
Rbm22	0.01	1.00
Tmed10	-0.02	1.00
Entpd4	0.05	1.00
Adarb1	0.17	1.00
Acadsb	-0.45	1.00
Bud23	0.02	1.00
Rras2	-0.20	1.00
Eif3j2	0.07	1.00
Ube2g1	0.01	1.00
Exosc8	0.13	1.00
Usp28	0.18	1.00
B4galt7	0.09	1.00
Amfr	0.19	1.00
Tlnrd1	-0.11	1.00
Serinc3	-0.23	1.00
Trappc12	0.12	1.00
Ccdc137	0.10	1.00
Smurf2	0.01	1.00
Ttc38	-0.14	1.00
Tsc22d2	-0.01	1.00
Edc3	0.18	1.00
Synrg	0.09	1.00
Ecd	0.16	1.00
Ctns	0.06	1.00
Slc35a3	0.15	1.00
Arid4a	0.05	1.00
Bysl	0.07	1.00
Cnot8	0.16	1.00
Ubfd1	0.19	1.00
Timm17a	0.07	1.00
Unc119b	0.13	1.00
Shtn1	0.03	1.00
Lrwd1	-0.13	1.00
Rasgrf1	-0.32	1.00
Ggh	0.03	1.00
Slc26a11	0.04	1.00
Rasgef1b	0.06	1.00
Ap2b1	0.13	1.00
Papss1	-0.09	1.00
AU040320	0.06	1.00
Psd3	0.11	1.00
Etfrf1	-0.24	1.00
Spag1	0.11	1.00

Gars	-0.04	1.00
Itpk1	-0.81	1.00
Suclg1	0.09	1.00
Atxn1	0.24	1.00
Prkab1	-0.04	1.00
Sema3e	-0.31	1.00
Phf2	0.05	1.00
Crmp1	-0.37	1.00
Arl13b	0.09	1.00
Actn4	0.14	1.00
Ankrd22	-0.23	1.00
Wars	0.02	1.00
Nol7	0.13	1.00
Spdef	0.04	1.00
Snap47	0.13	1.00
Sowaha	0.15	1.00
Grhl2	-0.01	1.00
Hipk2	-0.07	1.00
Tfpi	0.34	1.00
Elp3	0.01	1.00
Pdxdc1	-0.01	1.00
Alg3	0.13	1.00
Jade3	0.04	1.00
Agfg1	-0.04	1.00
Vps13d	0.00	1.00
Serac1	0.06	1.00
Phlpp1	0.15	1.00
Rrm2b	0.11	1.00
Psph	-0.26	1.00
2700049A03Rik	0.07	1.00
Aoah	-0.04	1.00
Fzd1	-0.21	1.00
Utp15	0.07	1.00
Sh3rf1	0.08	1.00
Bcas3	0.12	1.00
Rgs1	-0.36	1.00
Ap3s1	0.01	1.00
Fam3b	-0.24	1.00
Derl2	0.12	1.00
Pde3a	0.44	1.00
Nol9	0.02	1.00
Ntan1	0.13	1.00
Pdhx	0.10	1.00
Ywhah	-0.04	1.00
Septin2	0.13	1.00
Ptchd1	0.19	1.00

Ppp1cb	-0.20	1.00
Tatdn1	0.10	1.00
Gnptg	-0.07	1.00
Pde12	0.15	1.00
Lad1	0.00	1.00
Nnt	-0.51	1.00
Pxdc1	0.04	1.00
Nfyb	0.21	1.00
Dlg1	0.18	1.00
Fbxo4	0.15	1.00
Ncln	-0.10	1.00
Nrbp2	-0.32	1.00
Arsg	-0.07	1.00
Wipi2	0.03	1.00
Zdhhc9	0.00	1.00
Cdin1	0.15	1.00
Fbxl2	-0.01	1.00
Pithd1	0.21	1.00
Aass	0.07	1.00
Ctnnb1	-0.15	1.00
Scpep1	0.16	1.00
Pik3cg	-0.18	1.00
Polk	0.19	1.00
Taf3	-0.05	1.00
Rab25	0.10	1.00
Arhgap12	-0.06	1.00
Calm1	-0.36	1.00
Slc16a10	-0.94	1.00
Aox1	0.02	1.00
Hadh	0.05	1.00
Imp3	-0.15	1.00
Dlst	-0.05	1.00
Zmynd11	-0.07	1.00
Serhl	-0.11	1.00
Dop1b	-0.14	1.00
Smc6	0.07	1.00
Ddx18	0.05	1.00
Anxa13	-0.20	1.00
Dcun1d5	0.06	1.00
Ccdc125	-0.42	1.00
Gpr155	-0.52	1.00
Tfrc	-0.72	1.00
Scaf8	-0.08	1.00
Osgin1	-0.02	1.00
Csnk1g2	0.11	1.00
Cog5	-0.01	1.00

Sall4	0.17	1.00
Gne	-0.01	1.00
Klf3	0.17	1.00
Zc3h12c	0.17	1.00
Reep3	0.00	1.00
Ascc3	0.00	1.00
Eif3g	0.02	1.00
Wrnip1	0.10	1.00
Ptges3	-0.08	1.00
Tmem33	-0.05	1.00
Dnajb12	0.20	1.00
Sh3bp4	-0.04	1.00
Lrig3	-0.01	1.00
Tmem50b	-0.11	1.00
Helq	-0.04	1.00
Pros1	-0.36	1.00
Slc22a23	0.11	1.00
Exoc7	-0.04	1.00
Prkab2	0.09	1.00
Vps45	0.15	1.00
Smarca2	0.09	1.00
Cerk	-0.39	1.00
Pgd	-0.02	1.00
Lurap1l	0.05	1.00
Tmem39a	0.11	1.00
Bola3	-0.05	1.00
Pcmtd2	0.01	1.00
Gdi2	0.07	1.00
Glis2	-0.03	1.00
Fam76b	0.09	1.00
Hsp90b1	0.26	1.00
Fzr1	0.08	1.00
Bhlhe40	0.15	1.00
Stmn1	-0.43	1.00
Tnrc6b	-0.87	1.00
Slc25a38	0.19	1.00
Bcas2	0.03	1.00
Chd1	-0.10	1.00
Cep112	0.02	1.00
Vstm4	-0.19	1.00
Irf6	0.19	1.00
Ormdl2	0.04	1.00
Arl1	-0.10	1.00
Plcb4	-0.36	1.00
Susd3	0.07	1.00
Fabp3	-0.11	1.00

Efna2	0.16	1.00
Ncbp3	-0.04	1.00
Heph	0.06	1.00
Ccny	0.08	1.00
Ppm1m	0.05	1.00
Cpd	-0.33	1.00
Utp18	0.01	1.00
Fbxw7	0.08	1.00
Unc13b	0.16	1.00
Sidt2	0.20	1.00
Rnf115	0.07	1.00
Tns1	-0.39	1.00
Bcl9	0.15	1.00
Rhpn2	0.09	1.00
Fam126b	0.05	1.00
Abracl	0.10	1.00
Antxr1	-0.17	1.00
Aldh1a1	-0.56	1.00
Gpc4	0.17	1.00
Exd2	0.09	1.00
Atp6v0c	0.12	1.00
Rilpl2	-0.03	1.00
Golt1b	0.08	1.00
Tecpr2	0.13	1.00
Ube4b	0.12	1.00
Cdcp1	-0.20	1.00
Tdp2	0.15	1.00
Hlcs	0.16	1.00
Ntpcr	0.06	1.00
Pdcd2	0.08	1.00
Abcg1	0.09	1.00
Hsd17b7	0.11	1.00
Acbd5	0.06	1.00
Ankrd13c	0.01	1.00
Alox5	-0.88	1.00
Rrp12	0.12	1.00
Csnk1g3	0.22	1.00
Stag1	0.07	1.00
Eny2	0.14	1.00
Kifap3	0.06	1.00
Ube2h	-0.41	1.00
Hmgcs1	0.02	1.00
Cdk5r1	-0.08	1.00
Ist1	0.12	1.00
Ccng1	-0.35	1.00
Thsd7b	0.11	1.00

Irx1	0.11	1.00
Ero1a	0.12	1.00
Fnip1	0.14	1.00
Fhod1	0.13	1.00
Atp2a2	0.03	1.00
Cpne3	-0.01	1.00
Pitpnb	-0.01	1.00
Ndufs3	0.05	1.00
Rarres1	-0.17	1.00
Sec11a	0.05	1.00
Lsg1	0.05	1.00
Swi5	0.02	1.00
Abhd5	-0.07	1.00
Cbx3	0.05	1.00
Nt5dc2	-0.07	1.00
Eif2b2	0.07	1.00
Znrf2	-0.04	1.00
Ifitm10	0.01	1.00
Btrc	0.08	1.00
Rad21	-0.08	1.00
Grtp1	0.01	1.00
Cask	-0.01	1.00
Adamts9	-0.56	1.00
Pld1	-0.11	1.00
Lgr5	-0.42	1.00
Slc35c1	0.10	1.00
Slc45a4	-0.26	1.00
Ints14	0.01	1.00
Gpr63	-0.47	1.00
Irag1	0.05	1.00
Csgalnact2	-0.11	1.00
Snx20	0.12	1.00
Rpf1	0.08	1.00
Trmt9b	0.12	1.00
Rab3gap1	0.16	1.00
Arxes2	-0.04	1.00
Serpini1	0.06	1.00
Lpin2	-0.05	1.00
Hbp1	0.02	1.00
Rassf7	0.01	1.00
Rnls	0.09	1.00
Zmiz1	0.06	1.00
Sod2	-0.18	1.00
Foxn2	-0.01	1.00
Radx	-0.14	1.00
Leo1	-0.03	1.00

Abitram	0.05	1.00
Bsdc1	0.11	1.00
Gns	-0.04	1.00
Psmg3	0.04	1.00
Dars	-0.09	1.00
Slc41a1	0.13	1.00
Setd7	-0.09	1.00
Hnf1b	0.27	1.00
Adgrg6	-0.72	1.00
Atg5	0.05	1.00
Copg1	-0.09	1.00
Tnfrsf19	0.09	1.00
Cebpa	0.01	1.00
Txndc12	-0.05	1.00
Dbi	-0.06	1.00
Plk2	0.27	1.00
Ap5z1	0.06	1.00
Elovl6	-0.38	1.00
Dsel	0.06	1.00
Dennd2d	0.16	1.00
Stk40	-0.02	1.00
Nudt15	0.09	1.00
Tgfbr2	-0.75	1.00
Epg5	-0.02	1.00
Ppig	-0.01	1.00
Gramd2	0.13	1.00
Rmdn3	0.10	1.00
Ereg	-0.16	1.00
Ddhd2	0.11	1.00
Zcchc9	0.13	1.00
Slc9a1	0.03	1.00
Hmx3	-0.08	1.00
Psma4	-0.50	1.00
Rap1gds1	0.06	1.00
Gk5	-0.04	1.00
Yars	0.06	1.00
Aebp2	-0.02	1.00
Xrcc5	0.09	1.00
Dnajb9	0.20	1.00
Yipf4	-0.07	1.00
Tra2b	-0.04	1.00
Sardh	0.14	1.00
Invs	-0.17	1.00
Stk16	0.15	1.00
Tmem41b	-0.16	1.00
Eif3f	-0.01	1.00

Coq5	0.08	1.00
Armc10	-0.38	1.00
Xpo7	0.01	1.00
Ampd2	0.11	1.00
Bmpr2	-0.33	1.00
Bicc1	0.18	1.00
Slc25a13	-0.05	1.00
Gal3st1	-0.09	1.00
Trappc4	0.16	1.00
Purb	0.00	1.00
Hs6st1	-0.39	1.00
Armh3	0.00	1.00
Shc1	0.17	1.00
Ddx54	0.11	1.00
Rtraf	-0.11	1.00
Arhgap17	0.11	1.00
Slc7a3	0.09	1.00
Atp13a1	0.13	1.00
Usp38	-0.13	1.00
Tes	0.19	1.00
Polg	-0.06	1.00
As3mt	0.05	1.00
Cdyl2	0.23	1.00
Ubqln4	0.05	1.00
Tti2	-0.02	1.00
Cgnl1	-0.66	1.00
Ints6	-0.40	1.00
Rev1	0.08	1.00
Dcaf5	0.02	1.00
Slc9a2	-0.23	1.00
Dnai2	-0.15	1.00
Lrrn4	-0.54	1.00
Ldah	0.03	1.00
Prdm4	-0.22	1.00
Stag2	-0.43	1.00
Cops7a	0.03	1.00
Hdhd2	0.02	1.00
Ppa2	-0.22	1.00
Atp10d	0.14	1.00
Poldip3	0.00	1.00
Cstf3	0.01	1.00
Gtf2f2	0.11	1.00
Sec13	0.04	1.00
Nkx2-9	-0.09	1.00
Plekha5	0.17	1.00
Nrxn3	0.08	1.00

Tshz3	0.14	1.00
Nop58	0.01	1.00
Srpr	-0.09	1.00
Sirt6	0.15	1.00
Hspa9	-0.15	1.00
Ppp3ca	-0.01	1.00
Tmbim6	-0.10	1.00
Kcnq5	-1.32	1.00
Acox3	0.15	1.00
Klf5	0.05	1.00
Osbpl6	-0.03	1.00
Fbxw5	0.13	1.00
Rab8b	-0.04	1.00
Pdp2	-0.19	1.00
Atp5c1	0.00	1.00
Rftn1	0.06	1.00
Lancl2	0.00	1.00
Wdr59	0.05	1.00
Tubb1	0.03	1.00
Dapp1	0.21	1.00
Edrf1	0.12	1.00
Lmbr1	0.05	1.00
Nr1d2	0.11	1.00
Kdsr	-0.16	1.00
Psmc3	0.20	1.00
Slc30a1	0.06	1.00
Runx1	0.12	1.00
Rp2	-0.01	1.00
Dcps	0.09	1.00
Bsg	-0.04	1.00
Pex2	0.23	1.00
Ints2	0.13	1.00
Aco1	0.00	1.00
Ola1	0.08	1.00
Rundc3b	-0.15	1.00
Eps8l2	-0.25	1.00
Ndufs7	-0.01	1.00
Coro2a	-0.05	1.00
Ryk	0.01	1.00
Hk2	-0.13	1.00
Insig2	0.09	1.00
Dcp1b	-0.57	1.00
Adnp2	0.11	1.00
Iars2	-0.11	1.00
Neil1	0.03	1.00
Rspry1	0.07	1.00

Lcp2	-0.06	1.00
Pxdn	-0.43	1.00
Fasn	-0.71	1.00
Cwf19l2	-0.01	1.00
B3gat2	0.22	1.00
Tmem259	-0.04	1.00
Eif4a3l1	0.02	1.00
Ufd1	-0.37	1.00
Creg1	-1.16	1.00
Cops3	-0.16	1.00
Senp7	-0.08	1.00
Tgif1	0.08	1.00
Rnf138	0.11	1.00
N4bp2l1	-0.11	1.00
Ern1	0.05	1.00
Kdf1	0.11	1.00
Plpp2	-0.02	1.00
Larp4	-0.06	1.00
Ash1l	-0.39	1.00
Pfkfb3	-0.09	1.00
Acss2	0.05	1.00
Ndufaf2	0.12	1.00
Acyp2	0.19	1.00
Tapt1	-0.54	1.00
Ap1m1	0.13	1.00
Dimt1	0.13	1.00
Wasl	-0.01	1.00
Hook3	-0.02	1.00
Pstpip1	-0.28	1.00
Nudc	0.02	1.00
Irak4	-0.08	1.00
Kcnq1	-0.47	1.00
Rad51ap1	0.09	1.00
Slc30a7	-0.12	1.00
Kif1b	-0.28	1.00
Chst9	0.02	1.00
Rcor1	0.16	1.00
Vps26a	-0.50	1.00
Id3	0.07	1.00
Emc3	-0.06	1.00
Acer2	0.07	1.00
Pdcd6	0.11	1.00
Areg	-0.23	1.00
Idua	0.02	1.00
Ddx5	-0.37	1.00
DPCD	0.05	1.00

Top2b	-0.46	1.00
Fsip1	0.13	1.00
Cops4	0.07	1.00
Jarid2	0.25	1.00
Slc41a3	-0.10	1.00
Scd2	-0.20	1.00
Yipf3	-0.14	1.00
Epb41l3	-0.04	1.00
Lrrc8c	-0.23	1.00
Tpst1	0.08	1.00
Kif5b	-0.55	1.00
Riok1	0.06	1.00
Pxylp1	-0.14	1.00
Pank3	-0.59	1.00
Paxbp1	-0.28	1.00
Htra1	0.08	1.00
Klhl22	0.00	1.00
Ehf	-1.07	1.00
Slit2	0.26	1.00
Enah	-0.10	1.00
Slco4c1	0.17	1.00
Nfatc2	-0.36	1.00
Syt17	-0.15	1.00
Ppm1d	0.02	1.00
Ddb1	0.13	1.00
Get4	0.00	1.00
Ppp1r8	0.08	1.00
Adk	-0.04	1.00
Fam241a	-0.34	1.00
Ubash3b	0.24	1.00
Bicral	-0.23	1.00
Prelid3a	0.20	1.00
Epb41l5	0.02	1.00
Rangap1	-0.09	1.00
Psmd7	-0.08	1.00
Fzd6	-0.03	1.00
St8sia4	-0.24	1.00
Nup93	0.22	1.00
Caap1	0.04	1.00
Isca2	0.16	1.00
Cog7	-0.02	1.00
Tom1l1	0.06	1.00
Sat1	0.09	1.00
Panx2	0.14	1.00
Prim2	0.18	1.00
Pcbd2	-0.18	1.00

Ctsc	0.07	1.00
Il17b	0.13	1.00
Prmt7	-0.07	1.00
Dock4	-0.09	1.00
Ino80c	-0.02	1.00
Coq9	-0.06	1.00
Chchd7	-0.22	1.00
Fam122b	-0.38	1.00
Heatr1	-0.31	1.00
Trp53rka	-0.03	1.00
Eif4g3	0.00	1.00
P2ry1	0.09	1.00
Agrn	-0.43	1.00
Rac1	-0.48	1.00
N6amt1	0.13	1.00
Dner	-0.43	1.00
Cpeb4	-0.19	1.00
Nedd4	0.07	1.00
Htt	0.10	1.00
Ankrd27	-0.58	1.00
Nptx2	-0.31	1.00
Ap4b1	0.09	1.00
Elovl7	-0.76	1.00
Trp53inp1	-0.42	1.00
Copa	-0.10	1.00
Pdzd11	0.04	1.00
Rgp1	0.00	1.00
Lrp4	0.02	1.00
Rcc1l	0.15	1.00
Trmt13	-0.05	1.00
Vcpkmt	0.07	1.00
Dcun1d1	-0.07	1.00
Slc2a13	0.10	1.00
Specc1	-0.64	1.00
Dek	0.07	1.00
Ralgps2	-0.50	1.00
Ndufb10	-0.06	1.00
Clcc1	-0.01	1.00
Zpr1	0.19	1.00
Ctnnbl1	-0.08	1.00
Gfm1	-0.05	1.00
Snf8	0.15	1.00
Ldb1	0.02	1.00
Colec12	0.13	1.00
Tmem141	0.04	1.00
Tsc22d3	-0.03	1.00

Txndc5	-0.26	1.00
Tfg	0.06	1.00
B3gnt2	-0.04	1.00
Amotl2	0.04	1.00
Dcaf12	-0.06	1.00
Plpp1	-0.13	1.00
Zfp511	-0.03	1.00
Aup1	0.02	1.00
Strn3	0.00	1.00
Prrg4	0.13	1.00
Fermt2	0.10	1.00
Ppil1	0.04	1.00
Bcar1	0.15	1.00
Zbtb24	0.17	1.00
Luzp1	-0.48	1.00
Dkk3	-0.03	1.00
Gpr176	0.28	1.00
Ilrun	-0.12	1.00
Ppp1r26	-0.22	1.00
Prpsap1	0.13	1.00
Cd36	-0.66	1.00
Kcnk1	-0.11	1.00
Slc35b3	0.00	1.00
Ufm1	0.21	1.00
Dnmt1	-0.25	1.00
Homer2	-0.40	1.00
Nckipsd	0.17	1.00
Jmjd4	0.16	1.00
Rab20	0.22	1.00
Rit1	-0.16	1.00
Tcf25	0.08	1.00
Lats1	-0.38	1.00
Ppm1k	0.07	1.00
Acap2	-0.20	1.00
Parp16	-0.04	1.00
Sh3bp5	-0.11	1.00
Fmr1	0.08	1.00
Zfp207	-0.49	1.00
Anxa2	-0.29	1.00
Polr2c	-0.03	1.00
Reep2	0.08	1.00
Lsm5	-0.02	1.00
Ptgs2	-0.12	1.00
Bzw2	-0.14	1.00
Yeats2	-0.14	1.00
Endov	0.12	1.00

Zdhhc2	-0.05	1.00
Rhbg	0.06	1.00
Slc25a44	0.10	1.00
Esyt2	-0.44	1.00
Wdfy3	-0.36	1.00
Tmem154	-0.04	1.00
Fahd1	0.00	1.00
Plekhg4	0.04	1.00
Bambi	0.22	1.00
Rnf114	0.16	1.00
Nlrx1	0.03	1.00
Klhdc1	-0.14	1.00
Tmem203	0.05	1.00
Phf12	0.09	1.00
Trub1	0.09	1.00
Srgap1	-1.24	1.00
Arhgap40	0.06	1.00
Tbck	0.06	1.00
Fgfr1	-0.11	1.00
Gata6	0.01	1.00
Gnpat	0.00	1.00
Cdkn1b	-0.02	1.00
Neo1	0.02	1.00
B4galt4	0.10	1.00
Nebl	0.20	1.00
Atp6v1d	-0.08	1.00
Nudt5	-0.26	1.00
Tra2a	-0.15	1.00
Ubap1	-0.15	1.00
Retreg1	-0.05	1.00
Commd9	0.14	1.00
Yy2	0.06	1.00
Rab3b	0.18	1.00
Nop2	0.01	1.00
Cep44	-0.08	1.00
Asns	0.08	1.00
Lta4h	0.09	1.00
Fam219a	0.01	1.00
Aldh1a2	0.00	1.00
Erc2	0.01	1.00
Nhlrc3	0.07	1.00
Fto	0.17	1.00
Timm8a2	0.02	1.00
Usp25	-0.09	1.00
B3glct	0.09	1.00
Wdr19	-0.02	1.00

Zfp704	-0.02	1.00
Rilpl1	0.02	1.00
Cilk1	-0.39	1.00
Syt16	-0.16	1.00
Gnb4	-0.15	1.00
Suox	0.15	1.00
Zfp503	0.13	1.00
Qars	0.13	1.00
Ccdc92	-0.17	1.00
Ndnf	-0.15	1.00
Trdmt1	-0.16	1.00
Pcif1	-0.04	1.00
Pkib	0.11	1.00
Tmem87a	-0.09	1.00
Atxn7	0.00	1.00
Ube2v2	-0.03	1.00
Sugt1	0.03	1.00
Hif1an	0.06	1.00
Clip4	0.04	1.00
Lin54	0.13	1.00
Hnrnpu	0.04	1.00
Ctu2	0.12	1.00
Col5a2	-0.48	1.00
Lzic	0.07	1.00
Cetn3	0.06	1.00
Lman2	-0.07	1.00
Neto2	0.02	1.00
Acot10	-0.40	1.00
Gata3	-0.46	1.00
Trps1	-0.38	1.00
Fyttd1	-0.02	1.00
Fam214a	-0.04	1.00
Ttll11	0.14	1.00
Esf1	-0.12	1.00
Nupl1	-0.04	1.00
Cldnd1	0.12	1.00
Rad54l2	0.19	1.00
Tsr1	0.03	1.00
Uhrf1	0.09	1.00
Prkrip1	0.11	1.00
Hadha	0.08	1.00
Psmd4	0.03	1.00
Fabp5	-0.12	1.00
Wsb2	0.05	1.00
Kbtbd8	0.00	1.00
Sgtb	0.07	1.00

Tmem258	-0.10	1.00
R3hdm1	-0.06	1.00
Tanc2	-0.04	1.00
Pigc	-0.03	1.00
Adamts17	-0.20	1.00
Cdc123	0.14	1.00
Slc36a4	0.19	1.00
Slc2a8	0.11	1.00
Tm2d1	0.17	1.00
Xpot	0.04	1.00
Gfus	0.11	1.00
Ptp4a2	-0.13	1.00
Lox	0.10	1.00
Rmc1	-0.04	1.00
Plekha7	0.12	1.00
Gtpbp6	0.07	1.00
Sec61a1	-0.10	1.00
Rb1	-0.52	1.00
Ythdf3	-0.34	1.00
Katna1	-0.15	1.00
Dnajc30	0.12	1.00
Cdk9	0.12	1.00
Fam53a	0.02	1.00
Glcci1	-0.51	1.00
Scamp3	-0.05	1.00
Rfc3	0.03	1.00
Gga3	0.00	1.00
Glod4	0.06	1.00
Gyg	-0.05	1.00
Psme3	-0.02	1.00
Wbp2nl	0.06	1.00
Rexo5	-0.21	1.00
Zcchc4	0.05	1.00
Lars	-0.44	1.00
Pop4	0.12	1.00
Slc4a4	0.42	1.00
Hddc2	0.03	1.00
Pm20d2	0.10	1.00
Fat4	0.18	1.00
Ttll10	0.24	1.00
Srp9	0.09	1.00
Cntrl	-0.68	1.00
Tex10	-0.14	1.00
Srpk2	-0.25	1.00
Yipf5	0.03	1.00
Plxnb1	-0.25	1.00

Zfyve26	-0.05	1.00
Tor4a	0.04	1.00
Itgb4	0.05	1.00
Galk1	0.15	1.00
Brd1	0.02	1.00
Pik3ca	-0.23	1.00
Tprkb	0.16	1.00
Pla2g4f	0.10	1.00
Ccnb2	0.17	1.00
Exoc3	0.09	1.00
Plod1	-0.12	1.00
Rlim	0.02	1.00
Ric8a	0.12	1.00
Fmn1	0.09	1.00
Fam161a	0.02	1.00
Snx31	0.02	1.00
Gpatch2	-0.01	1.00
Tcp11l2	-0.02	1.00
Gtf2h3	0.08	1.00
Apbb2	0.09	1.00
Cdk5rap2	-0.62	1.00
Snx6	-0.52	1.00
Nolc1	0.01	1.00
Cntf	0.07	1.00
Pum2	-0.17	1.00
Cdadc1	-0.09	1.00
Sbno2	0.02	1.00
Nf2	0.01	1.00
Btbd10	-0.24	1.00
Trim23	0.08	1.00
Ccdc127	0.12	1.00
Sesn1	-0.14	1.00
Wiz	-0.08	1.00
Ube2d1	0.16	1.00
Zcchc2	-0.17	1.00
Phlda1	0.09	1.00
Slc25a46	0.00	1.00
Fnbp1l	-0.48	1.00
Fbxl5	-0.10	1.00
Ttc9	-0.28	1.00
Dpf3	0.18	1.00
Ablim1	-0.36	1.00
Cacna1d	0.08	1.00
Castor2	0.00	1.00
Lyplal1	-0.17	1.00
Tarbp1	0.07	1.00

Iqgap2	-0.62	1.00
Ilk	0.17	1.00
Heatr5a	-0.16	1.00
Usb1	0.01	1.00
Zdhhc8	0.22	1.00
Pepd	0.01	1.00
Zfp407	0.01	1.00
Ccpg1	-0.42	1.00
Ddx47	0.19	1.00
Urm1	-0.16	1.00
Rpgr	-0.14	1.00
Rad51c	0.05	1.00
Xpo4	0.03	1.00
Rbm34	-0.13	1.00
Fam81a	0.00	1.00
B3gntl1	-0.03	1.00
Etv6	-0.11	1.00
Ibtk	0.01	1.00
Nrg4	-0.28	1.00
Csl	-0.31	1.00
Stoml3	0.10	1.00
Daam2	0.13	1.00
Runx2	-0.51	1.00
Noc2l	0.10	1.00
Naa16	-0.02	1.00
Slc25a29	0.13	1.00
Ubl3	-0.11	1.00
Jph1	-0.18	1.00
Pde6d	0.02	1.00
Samd8	0.17	1.00
Faf1	-0.04	1.00
Tssc4	0.04	1.00
Suz12	0.21	1.00
Tpm4	-0.23	1.00
Eif4ebp2	-0.11	1.00
Fam13b	-0.03	1.00
Gfm2	0.09	1.00
Tmem245	-0.68	1.00
Tmx1	-0.17	1.00
Chst14	0.11	1.00
Osbpl1a	-0.08	1.00
Tmf1	-0.13	1.00
Pot1b	0.12	1.00
Hcfc2	0.15	1.00
Bms1	-0.01	1.00
Lamb2	0.03	1.00

Ntn4	0.03	1.00
Nefl	-0.27	1.00
Esrp1	0.12	1.00
Zdhhc13	0.00	1.00
Casr	-0.10	1.00
Paqr7	0.11	1.00
Tnks	-0.08	1.00
Zfp131	-0.14	1.00
Klhl29	-0.43	1.00
Thoc5	0.16	1.00
Rwdd4a	-0.04	1.00
Vps13c	-0.60	1.00
Prmt3	0.06	1.00
Klhl42	0.07	1.00
Cald1	0.13	1.00
Asb7	0.12	1.00
Armc9	0.03	1.00
Fbn1	-0.19	1.00
Cwc25	0.07	1.00
Thumpd3	-0.05	1.00
Cotl1	-0.06	1.00
Nlk	0.07	1.00
Nr2c1	0.14	1.00
Lztr1	-0.31	1.00
Ampd3	0.01	1.00
Kitl	-0.34	1.00
Fam214b	0.07	1.00
Sirt1	0.01	1.00
Ufsp2	-0.01	1.00
Eef1d	-0.03	1.00
Golgb1	-0.55	1.00
Pik3cd	0.19	1.00
Ptges3l	0.11	1.00
Plekhm2	-0.01	1.00
Iqgap1	-0.51	1.00
Eps15l1	-0.30	1.00
Efnb2	0.18	1.00
Parg	-0.18	1.00
Cltc	-0.43	1.00
Frmpd4	-0.36	1.00
Tinag	-0.86	1.00
Pcgf6	0.09	1.00
Gstcd	0.03	1.00
Pmm2	-0.09	1.00
Trio	0.03	1.00
Cdkn1a	0.10	1.00

Rnf217	0.10	1.00
Wdr24	0.09	1.00
Nfat5	0.05	1.00
Pcgf5	-0.03	1.00
Gnpda1	0.10	1.00
Setd3	-0.14	1.00
Ppp1r16b	-0.21	1.00
Ncoa4	-0.56	1.00
Commd4	0.04	1.00
Nup88	0.07	1.00
Thap4	0.10	1.00
Papolg	0.07	1.00
Sec61a2	0.13	1.00
Grip1	-0.60	1.00
Tmem164	-0.15	1.00
Apool	-0.01	1.00
Gpn1	0.00	1.00
Ankrd46	0.02	1.00
Tbccd1	0.07	1.00
Cldn23	0.10	1.00
D5Ertd579e	-0.22	1.00
Tuft1	0.17	1.00
Arhgap45	0.05	1.00
Zfp622	-0.13	1.00
Rsf1	-0.13	1.00
Agpat4	0.00	1.00
St3gal5	-0.18	1.00
Nupl2	0.10	1.00
Sos1	-0.58	1.00
Pick1	-0.06	1.00
Rnpepl1	0.13	1.00
Reps2	-0.04	1.00
Ppp2r3a	0.10	1.00
Pabpc4	0.10	1.00
Kdm1a	-0.15	1.00
Nol8	-0.13	1.00
Rcn2	0.07	1.00
Rnps1	0.06	1.00
Fgfr2	-0.28	1.00
Timmdc1	0.08	1.00
Pitpnm2	-0.12	1.00
Fhl4	-0.04	1.00
Pde8a	-0.03	1.00
Phc2	0.00	1.00
Tmem30a	-0.03	1.00
Adora2a	-0.17	1.00

Gcnt4	-0.38	1.00
Emp1	-0.44	1.00
Sephs1	0.00	1.00
Trappc6b	0.04	1.00
Nampt	-0.27	1.00
Pgam5	0.06	1.00
Gale	0.00	1.00
Tmem35a	0.15	1.00
Ulk2	0.01	1.00
Hmgn3	-0.11	1.00
Aars2	0.02	1.00
Slc5a8	-0.44	1.00
P3h1	0.09	1.00
Ttc7	0.00	1.00
Stoml1	0.13	1.00
Ppm1g	0.11	1.00
Arfgap1	0.01	1.00
Rpa1	0.13	1.00
Arf1	0.03	1.00
Ndufa4	0.02	1.00
Fgd4	0.00	1.00
Pus10	-0.03	1.00
Cops7b	-0.46	1.00
Actr10	-0.01	1.00
Grhl1	0.08	1.00
Lrrc28	0.05	1.00
Rasa3	-0.47	1.00
Dapk2	-0.07	1.00
Lnx2	-0.01	1.00
Sccpdh	-0.09	1.00
Esrp2	-0.01	1.00
Fnbp1	0.08	1.00
Wdr81	0.14	1.00
Tab2	-0.18	1.00
Rbis	-0.11	1.00
Alg6	0.07	1.00
Lpar6	0.01	1.00
Capn15	-0.02	1.00
Cux2	0.10	1.00
Ephb1	-0.01	1.00
Smchd1	-0.19	1.00
Stk24	-0.04	1.00
Smarcal1	0.04	1.00
Emsy	0.14	1.00
Ubxn2b	0.15	1.00
Gnb1	-0.07	1.00

Hbs1l	-0.18	1.00
Slc35c2	0.15	1.00
Fuca1	0.22	1.00
Nck2	0.02	1.00
Lrp3	-0.12	1.00
Eea1	-0.49	1.00
Atg10	0.13	1.00
Pogz	-0.01	1.00
Evc	-0.03	1.00
Ebna1bp2	0.11	1.00
Kdm5b	0.00	1.00
Cdk12	-0.02	1.00
Lama5	-0.63	1.00
Pomgnt2	0.06	1.00
Acer3	-0.41	1.00
Slk	-0.12	1.00
Topbp1	0.13	1.00
Cfap99	0.07	1.00
Cops5	0.01	1.00
Prox1	-0.18	1.00
Ston2	0.14	1.00
Dock11	-0.81	1.00
Ndst4	-0.63	1.00
Chp1	-0.50	1.00
Vps51	0.13	1.00
Armc1	-0.08	1.00
Sirt3	0.11	1.00
Ezh2	0.13	1.00
Cdv3	-0.06	1.00
Tmem266	0.17	1.00
Cops8	0.11	1.00
Spock3	-0.49	1.00
Zfp703	-0.18	1.00
Txnl1	-0.07	1.00
Per2	-0.05	1.00
Lin9	0.12	1.00
Hes6	0.07	1.00
Thoc7	0.02	1.00
G3bp2	-0.14	1.00
Rbfox1	-0.15	1.00
Fam76a	-0.08	1.00
St8sia1	0.06	1.00
Arpp19	0.05	1.00
Galnt12	0.18	1.00
Rexo4	-0.05	1.00
Sun1	-0.45	1.00

Negr1	0.09	1.00
Trim24	-0.47	1.00
Chd7	-0.01	1.00
Galt	0.04	1.00
Cnppd1	0.00	1.00
Lrp6	0.08	1.00
Rab35	0.09	1.00
Kmt5b	-0.04	1.00
Fbxo21	-0.15	1.00
Hectd4	-0.41	1.00
Shroom2	-0.62	1.00
E2f6	0.03	1.00
Rab11fip1	-0.02	1.00
Grip2	0.09	1.00
Evc2	-0.05	1.00
Fbln5	-0.23	1.00
Epha4	-0.14	1.00
Carhsp1	-0.32	1.00
Has2	0.22	1.00
Prpf6	-0.08	1.00
Tgm2	-0.55	1.00
Tmem53	0.08	1.00
Zfyve21	0.11	1.00
Slc25a33	0.12	1.00
Ppp1cc	-0.12	1.00
Agk	0.15	1.00
Tspan6	0.05	1.00
Jade1	-0.09	1.00
Pls3	-0.46	1.00
Dpy30	-0.03	1.00
Sgsm1	-0.07	1.00
Fgd3	-0.07	1.00
Rxylt1	0.16	1.00
Rbm45	0.01	1.00
Hfm1	-0.45	1.00
Hdac11	-0.28	1.00
Ccdc117	0.12	1.00
Ap4s1	0.09	1.00
Gnl2	0.15	1.00
Ap3d1	-0.14	1.00
Cks2	0.13	1.00
Ikbkb	0.17	1.00
Rogdi	-0.12	1.00
Nip7	0.05	1.00
Exosc3	-0.39	1.00
Tbk1	-0.01	1.00

4930523C07Rik	0.10	1.00
Uba3	0.03	1.00
Fam169a	-0.23	1.00
Rassf9	-0.12	1.00
Inpp5b	-0.15	1.00
Pip5k1a	-0.12	1.00
Dnajc9	-0.09	1.00
Ahcyl1	0.01	1.00
Atxn7l2	0.06	1.00
Pcsk7	0.02	1.00
Tax1bp3	-0.55	1.00
Ghr	0.24	1.00
Ccser2	0.02	1.00
Dynll2	-0.10	1.00
Il6st	0.13	1.00
Ddx41	0.09	1.00
Hp1bp3	-0.37	1.00
Ccnl2	-0.53	1.00
Bspry	-0.01	1.00
Nr2c2	-0.13	1.00
Gtf2ird1	0.11	1.00
Frmpd1	-0.30	1.00
Fmnl2	-0.45	1.00
Tmem165	-0.15	1.00
Taf4	-0.17	1.00
4933400A11Rik	0.08	1.00
Wdr1	-0.01	1.00
Greb1l	0.02	1.00
Tmem185a	-0.03	1.00
Reck	-0.08	1.00
Llgl2	-0.46	1.00
Fign	0.08	1.00
Gfod2	0.08	1.00
Eipr1	0.14	1.00
Bcl2	-0.17	1.00
Hmgb3	0.02	1.00
Adat1	-0.07	1.00
Ahdc1	0.09	1.00
Trmt1l	0.00	1.00
Ofd1	-0.04	1.00
Nr3c2	0.14	1.00
Pik3r5	0.14	1.00
Parvg	-0.07	1.00
Strip1	-0.08	1.00
Eed	0.04	1.00
Cenpk	0.07	1.00

Ltbp2	0.13	1.00
Iffo1	0.11	1.00
Eif4e3	0.03	1.00
Sinhcaf	-0.12	1.00
Galnt11	0.02	1.00
Dicer1	-0.24	1.00
Bbs7	0.03	1.00
Tbc1d22a	-0.18	1.00
Vasn	0.11	1.00
Adam12	-0.53	1.00
Nae1	-0.05	1.00
Arhgap6	-1.07	1.00
Ppid	0.15	1.00
Slc22a15	-0.31	1.00
Arntl2	-0.12	1.00
Ankrd28	-0.10	1.00
Rtf1	-0.13	1.00
Atp1b1	0.00	1.00
Cebpg	0.02	1.00
Rgl1	0.16	1.00
Glyr1	-0.14	1.00
Angptl2	-0.29	1.00
Ehd3	0.06	1.00
Fbxo32	0.03	1.00
Cpsf7	0.11	1.00
Ndufa5	-0.19	1.00
Dalrd3	0.05	1.00
Zup1	0.00	1.00
Sumf2	0.10	1.00
Rab11fip4	-0.51	1.00
Add3	-0.27	1.00
Atg13	0.00	1.00
Exoc1	0.05	1.00
Thoc3	0.14	1.00
Prickle1	0.07	1.00
Plekhm3	-0.11	1.00
Ppat	-0.12	1.00
Vps4b	-0.14	1.00
Pnpla8	-0.06	1.00
Ptar1	-0.42	1.00
Nkiras2	0.00	1.00
Vti1a	-0.14	1.00
Rabl6	-0.03	1.00
Pcnx	0.00	1.00
Slc4a2	0.11	1.00
Cep57	-0.21	1.00

Adam10	-0.07	1.00
Unc50	0.02	1.00
Josd1	0.00	1.00
Tmem128	0.05	1.00
Pds5b	-0.03	1.00
Usp53	0.01	1.00
Irak1bp1	0.10	1.00
Hectd1	-0.03	1.00
Agpat5	-0.34	1.00
Gins2	0.07	1.00
Ddx1	-0.16	1.00
Sltm	-0.41	1.00
Pdlim4	-0.64	1.00
Arhgef37	0.10	1.00
Abat	0.02	1.00
Poglut1	0.11	1.00
Spata5l1	0.06	1.00
Ttc19	-0.28	1.00
Echdc1	0.12	1.00
Fam167b	0.10	1.00
Ago4	0.01	1.00
Cc2d2a	-0.36	1.00
Cep350	-0.51	1.00
Cntln	0.15	1.00
Flot2	-0.10	1.00
Nrp1	-0.42	1.00
Fbf1	0.03	1.00
Wdr43	-0.02	1.00
Ppara	0.08	1.00
Sdcbp2	0.19	1.00
Irf2bpl	0.02	1.00
Cbr4	0.10	1.00
Slc2a12	-0.15	1.00
Tusc1	0.08	1.00
Xbp1	0.04	1.00
Smad1	0.14	1.00
Sema4g	-0.13	1.00
Trpc1	-0.16	1.00
Syn2	0.06	1.00
Unc5c	-0.17	1.00
Ercc8	0.10	1.00
Far1	0.01	1.00
Adal	0.04	1.00
Nuak2	-0.04	1.00
Alkbh4	-0.02	1.00
Tstd2	0.09	1.00

Slco2a1	-0.72	1.00
Dbt	-0.42	1.00
Ipmk	-0.59	1.00
Fsd1l	0.06	1.00
Cpsf3	0.08	1.00
Tmem208	0.10	1.00
Gla	0.07	1.00
Pappa	0.17	1.00
Llgl1	-0.07	1.00
Nubpl	0.04	1.00
Sft2d2	-0.06	1.00
Tmem39b	0.07	1.00
Tspan13	0.11	1.00
Xpr1	-0.08	1.00
Ttpal	-0.05	1.00
Lekr1	-0.18	1.00
Ddx24	0.14	1.00
Wdr48	-0.05	1.00
Ccndbp1	0.10	1.00
Tbc1d15	-0.18	1.00
Cdc16	0.13	1.00
Tmem209	0.07	1.00
Kat7	0.13	1.00
Abcb6	-0.12	1.00
Capzb	-0.04	1.00
Caln1	-0.20	1.00
Sec62	-0.02	1.00
Naa60	-0.04	1.00
Styk1	0.04	1.00
Kdm5a	-0.60	1.00
Dnajc16	-0.05	1.00
Cradd	0.07	1.00
Stkld1	0.06	1.00
Impact	0.04	1.00
Ints4	0.03	1.00
Ppp2r2c	-0.26	1.00
Dyrk3	-0.24	1.00
Rsrp1	-0.01	1.00
Rarb	-0.01	1.00
Cnot6l	-0.29	1.00
Paip2	-0.06	1.00
Smim19	-0.13	1.00
Nagpa	-0.06	1.00
Scyl2	-0.61	1.00
Rrbp1	-0.41	1.00
Cers5	0.06	1.00

Ttf2	0.08	1.00
Dnajb14	-0.13	1.00
Sel1l	-0.49	1.00
Sugct	-0.72	1.00
Tial1	-0.02	1.00
Clcn7	0.09	1.00
Stk11	0.09	1.00
Top1	0.03	1.00
Rmnd1	-0.10	1.00
Blm	0.00	1.00
Tmtc3	-0.17	1.00
Nars2	0.02	1.00
Raver2	0.13	1.00
Pofut1	-0.02	1.00
Commd1b	-0.03	1.00
Lca5	-0.01	1.00
Utp6	0.03	1.00
Prpf38a	0.05	1.00
Nbn	-0.28	1.00
Adipor2	-0.31	1.00
Zfand6	-0.10	1.00
Dhx40	-0.02	1.00
Scnn1a	-0.43	1.00
Csrp1	-0.10	1.00
Stard5	0.15	1.00
Bicd2	-0.05	1.00
Spryd7	0.02	1.00
Rassf8	-0.25	1.00
Ascc1	-0.04	1.00
Evl	-0.56	1.00
Lmbrd1	-0.08	1.00
Daam1	-0.26	1.00
Adcy5	-0.73	1.00
Atl1	0.09	1.00
Tmem186	0.07	1.00
Dipk2a	-0.03	1.00
Pgm2l1	-0.20	1.00
Faap24	0.02	1.00
Gprc5c	0.10	1.00
Ino80	-0.19	1.00
Tex11	-0.08	1.00
Enoph1	0.04	1.00
Vps37a	0.05	1.00
Otud3	0.11	1.00
Trim33	-0.08	1.00
Ankrd17	-0.03	1.00

Ppp2ca	-0.18	1.00
Akap1	-0.22	1.00
Phf21a	-0.09	1.00
Stt3b	-0.09	1.00
Enc1	0.05	1.00
Aldh18a1	0.12	1.00
Gpsm2	-0.09	1.00
Fbxo25	-0.28	1.00
Skp1	-0.04	1.00
Zfp618	-0.08	1.00
Ppt1	0.08	1.00
Lrmda	0.03	1.00
Atf6	-0.09	1.00
Ythdf2	-0.07	1.00
Sestd1	-0.07	1.00
Cnih1	0.07	1.00
Plekhb2	-0.01	1.00
Rpp30	0.10	1.00
Emb	-0.25	1.00
Zfp687	0.08	1.00
Ikzf5	0.01	1.00
Abhd17b	0.11	1.00
Zbtb5	-0.04	1.00
Cab39l	-0.12	1.00
Cep85	0.07	1.00
Stxbp5	-0.05	1.00
Tcf4	-0.19	1.00
Anxa6	-0.39	1.00
Nup43	0.10	1.00
Cep85l	-0.29	1.00
Prmt9	0.01	1.00
Denr	0.01	1.00
Nol10	0.08	1.00
Ttll12	-0.11	1.00
Tyro3	-0.12	1.00
Dis3l	-0.06	1.00
Asah1	-0.05	1.00
Snx13	-0.10	1.00
Chmp2b	-0.09	1.00
Ece1	0.08	1.00
Hyou1	-0.15	1.00
Iqck	-0.15	1.00
Xpc	0.08	1.00
4833439L19Rik	-0.43	1.00
Tnfrsf13b	-0.15	1.00
Fh1	-0.08	1.00

Dtx4	0.09	1.00
Tmem184a	0.00	1.00
Ip6k1	0.03	1.00
Gatb	0.09	1.00
Smg5	-0.14	1.00
Oard1	0.17	1.00
Ppcdc	0.11	1.00
Tef	-0.09	1.00
Thap1	0.01	1.00
Abraxas2	0.00	1.00
Socs5	-0.30	1.00
Lrmp	-0.13	1.00
Tcp1	-0.13	1.00
Shroom3	0.01	1.00
Cnnm2	-0.10	1.00
Bend6	0.08	1.00
Cers2	0.16	1.00
Dpysl2	-0.28	1.00
Hdc	-0.54	1.00
Desi1	0.03	1.00
Tdp1	0.10	1.00
Dnajc3	-0.24	1.00
Ift172	-0.25	1.00
Dhx37	0.11	1.00
Zfp532	-0.03	1.00
Bop1	0.08	1.00
Txndc15	-0.33	1.00
Fam114a1	0.02	1.00
Zfp277	-0.10	1.00
Rsrc1	-0.03	1.00
Stk39	0.06	1.00
Ripk1	0.07	1.00
Larp4b	0.03	1.00
Pacsin2	0.04	1.00
Zfp821	-0.15	1.00
Sema4b	0.08	1.00
E2f5	0.00	1.00
Snx9	-0.11	1.00
Lifr	0.00	1.00
Gatad1	-0.01	1.00
Adprs	0.03	1.00
Tfcp2	-0.01	1.00
Tram2	-0.09	1.00
Pdk1	-0.12	1.00
Cep152	0.03	1.00
Cdca7l	-0.22	1.00

Dab1	-0.05	1.00
Cdh22	0.07	1.00
Rbm33	-0.11	1.00
Golga5	-0.46	1.00
Ckap5	-0.69	1.00
Srgap3	-0.29	1.00
Lpin1	-0.53	1.00
Traf7	0.05	1.00
Ripk4	-0.45	1.00
Jmy	-0.08	1.00
Stxbp4	-0.10	1.00
Fam204a	-0.07	1.00
Orc4	-0.15	1.00
Scaper	-0.13	1.00
Slc25a21	-0.43	1.00
Rbbp5	0.10	1.00
Abhd17a	-0.01	1.00
Supt6	-0.11	1.00
Alg2	0.04	1.00
Uxs1	-0.03	1.00
Ttbk2	0.14	1.00
Nudt7	-0.05	1.00
Trmt5	0.06	1.00
Gipc2	-0.06	1.00
Srrd	0.08	1.00
Drosha	-0.04	1.00
Galc	0.13	1.00
Eif2ak1	0.06	1.00
Drg2	0.12	1.00
Tmprss2	-0.43	1.00
Celf1	-0.03	1.00
Ing5	0.02	1.00
Adamtsl3	0.04	1.00
Rora	-0.51	1.00
Tulp4	-0.23	1.00
Dusp19	-0.09	1.00
Colgalt2	0.17	1.00
Enpp6	-0.27	1.00
Tenm4	0.33	1.00
Eif4enif1	-0.12	1.00
Naa15	-0.01	1.00
Gde1	-0.17	1.00
Vgll4	-0.31	1.00
Exosc2	0.00	1.00
Gins3	0.04	1.00
Dnajb6	-0.09	1.00

Akap8l	0.02	1.00
Tbc1d32	0.06	1.00
Pla2r1	0.08	1.00
Gata5	-0.16	1.00
Kif2a	-0.10	1.00
Rrp1b	-0.07	1.00
Dync2li1	0.03	1.00
Tmem60	-0.11	1.00
Ccdc93	-0.05	1.00
Btbd8	0.02	1.00
Ptpn9	0.08	1.00
Glt8d1	-0.01	1.00
Eif3l	-0.14	1.00
Cux1	-0.26	1.00
Atp11a	0.13	1.00
Ptchd3	-0.10	1.00
Ssbp1	-0.09	1.00
Blmh	0.03	1.00
Dlc1	0.11	1.00
Ptpn12	-0.28	1.00
Virma	-0.06	1.00
Cap1	-0.18	1.00
Ppwd1	0.08	1.00
Cdk17	-0.20	1.00
Acp6	0.12	1.00
Rfc2	0.11	1.00
Dennd6a	-0.02	1.00
Phf13	0.12	1.00
Lrch3	-0.05	1.00
Tlr3	-0.08	1.00
Spg21	0.09	1.00
Snx14	0.05	1.00
Ppp6r3	-0.22	1.00
Chsy3	-0.48	1.00
Nmnat2	0.08	1.00
Cgas	0.00	1.00
Rab7	-0.17	1.00
Zfp652	0.01	1.00
Satb1	-0.70	1.00
Zbtb44	-0.16	1.00
Cbx6	0.16	1.00
Selenoi	0.06	1.00
Serpinh1	-0.20	1.00
Grk3	-0.42	1.00
Deaf1	0.00	1.00
St3gal6	-0.02	1.00

Slc38a9	0.04	1.00
Nyx	-0.13	1.00
Cep68	0.06	1.00
Tcea1	0.05	1.00
Scoc	-0.04	1.00
Klhl18	0.02	1.00
Bloc1s4	0.05	1.00
Oxnad1	0.04	1.00
Prrc2c	-0.24	1.00
Hscb	-0.11	1.00
Pdia4	-0.12	1.00
Ube2a	-0.05	1.00
Pak2	-0.01	1.00
Senp5	-0.10	1.00
Wsb1	-0.04	1.00
Itpa	-0.01	1.00
Pars2	0.14	1.00
Stard6	0.10	1.00
Gpc3	0.12	1.00
Sorbs1	-0.42	1.00
Ipo7	-0.45	1.00
Fat3	-0.19	1.00
Ccdc126	-0.17	1.00
Snx18	0.08	1.00
Crkl	0.01	1.00
Luc7l3	0.03	1.00
Ei24	-0.12	1.00
Fbxo3	-0.47	1.00
Ndufaf3	-0.05	1.00
Dnajc5	-0.21	1.00
Eef1b2	-0.11	1.00
Zdhhc17	-0.07	1.00
Ercc6	-0.01	1.00
B9d1	0.02	1.00
Dennd5a	-0.16	1.00
Hip1r	-0.22	1.00
Gusb	-0.01	1.00
Slc25a20	0.13	1.00
Plcg2	-1.45	1.00
Asap2	-0.12	1.00
Gna12	-0.04	1.00
Tspan4	0.04	1.00
Zbtb49	0.03	1.00
B3galt6	0.08	1.00
Ttll7	-0.31	1.00
Taf5	-0.09	1.00

Agap3	0.09	1.00
Pqlc3	0.01	1.00
Farsb	-0.07	1.00
Nucks1	-0.34	1.00
Hmbox1	-0.42	1.00
Kpna3	-0.08	1.00
Ctsb	-0.14	1.00
Eif2a	0.04	1.00
Ercc3	0.00	1.00
Slain2	-0.10	1.00
Brca1	-0.05	1.00
Pdcl	-0.05	1.00
Zbtb7b	0.11	1.00
Ehbp1	0.04	1.00
Akt3	0.07	1.00
Clstn1	-0.20	1.00
Lgals8	-0.17	1.00
Pwwp2b	-0.25	1.00
Kcnq3	-0.66	1.00
Trappc8	-0.08	1.00
Dennd6b	0.01	1.00
Gab2	-0.13	1.00
Fbxl14	-0.01	1.00
Tfip11	0.08	1.00
Zbtb1	-0.26	1.00
Rasal2	-0.02	1.00
Snorc	0.09	1.00
Rad18	0.05	1.00
Pomt2	0.12	1.00
Sspn	-0.02	1.00
Kat2b	-0.18	1.00
Cpsf2	-0.17	1.00
Cdh11	-1.02	1.00
Dclre1a	0.05	1.00
Tada2b	-0.07	1.00
Rtn4	-0.12	1.00
Clic5	-0.05	1.00
Zmym3	-0.18	1.00
Vkorc1l1	-0.09	1.00
Anks6	0.09	1.00
Abhd10	0.09	1.00
Ptgfr	-0.29	1.00
Tmem183a	0.07	1.00
Pigq	0.00	1.00
Sgms2	-0.07	1.00
Epc2	-0.13	1.00

Ccnf	0.06	1.00
Pten	-0.12	1.00
Casp9	-0.02	1.00
Exoc5	-0.14	1.00
Pdia5	-0.16	1.00
Polr2l	-0.20	1.00
C77080	-0.04	1.00
Pcnx4	0.12	1.00
Sf3a2	-0.02	1.00
Dhrs13	0.13	1.00
Tbc1d14	-0.21	1.00
Pum1	-0.11	1.00
Dhtkd1	-0.01	1.00
Sybu	-0.12	1.00
Ints13	-0.13	1.00
Ints8	-0.20	1.00
Elovl1	-0.07	1.00
Wwtr1	0.05	1.00
Sipa1l1	-0.41	1.00
Cdc26	0.00	1.00
Tepsin	0.09	1.00
Tank	0.13	1.00
Crbn	-0.23	1.00
Dhrs7b	0.13	1.00
Dlg2	0.10	1.00
Dpp8	0.06	1.00
Pdk4	-0.10	1.00
Pde4b	-0.09	1.00
Ankrd44	-0.07	1.00
Snrnp48	0.00	1.00
Poldip2	0.13	1.00
Nde1	-0.30	1.00
Ttll9	-0.01	1.00
Fat1	-0.52	1.00
Srp72	-0.18	1.00
Clcn3	-0.16	1.00
Cdc37l1	0.02	1.00
Cdk14	-0.49	1.00
Slc39a11	-0.07	1.00
Lyar	-0.06	1.00
St6gal1	-0.41	1.00
Acss1	-0.11	1.00
Cemip2	-0.10	1.00
Usp15	-0.30	1.00
Hnrnph3	-0.48	1.00
Dctn5	0.07	1.00

Rmi2	0.02	1.00
Exoc2	-0.19	1.00
Ulk1	-0.41	1.00
Larp7	-0.08	1.00
Pnpt1	0.10	1.00
Spdl1	0.03	1.00
Ddx31	0.11	1.00
Lmtk2	-0.15	1.00
St13	-0.15	1.00
Fabp7	-0.04	1.00
Tead4	0.10	1.00
Lhfpl2	-0.21	1.00
Lrrc75a	0.03	1.00
Rif1	0.12	1.00
Atp5pb	-0.16	1.00
Zcchc8	-0.15	1.00
Ndfip1	-0.10	1.00
Clk4	-0.14	1.00
Nptn	-0.10	1.00
Gapvd1	-0.14	1.00
Zmat3	-0.06	1.00
Nufip1	-0.18	1.00
Pfdn1	-0.24	1.00
Klhl8	0.09	1.00
Amot	-0.39	1.00
Gpr85	-0.08	1.00
Dipk1a	-0.11	1.00
Sap130	0.00	1.00
Atf1	-0.11	1.00
P3h2	-0.04	1.00
Ubap2l	-0.05	1.00
Snapc1l	0.03	1.00
Fam136a	0.08	1.00
Rap2c	-0.02	1.00
Dym	-0.05	1.00
Abcd2	-0.11	1.00
Cpe	-0.58	1.00
Ybx3	-0.33	1.00
Tyw3	-0.17	1.00
Uckl1	-0.11	1.00
Coq7	0.08	1.00
Golph3l	0.02	1.00
Stox2	-0.36	1.00
Prxl2c	-0.09	1.00
Swap70	-0.11	1.00
Abcc5	-0.15	1.00

Slc44a1	-0.12	1.00
Ttc37	-0.18	1.00
Chgb	-0.39	1.00
Rpap2	0.04	1.00
Tox	-0.29	1.00
Dync2i1	0.01	1.00
Dock1	-0.36	1.00
Rwdd1	-0.05	1.00
Foxk2	-0.02	1.00
Rbm25	-0.12	1.00
Herc4	-0.38	1.00
Plekhj1	-0.05	1.00
Fry	-0.46	1.00
Dlg5	-0.28	1.00
Lmo4	0.02	1.00
Hspa4	0.08	1.00
Itgav	-0.26	1.00
Nphp1	-0.02	1.00
C2cd2	-0.53	1.00
Bclaf3	-0.30	1.00
Ppp2r2d	-0.10	1.00
Akap8	-0.19	1.00
Nprl2	-0.19	1.00
Zbtb33	-0.10	1.00
Tmem131	-0.11	1.00
Gpr158	-0.26	1.00
Kansl3	0.04	1.00
Cramp1	0.00	1.00
Dpp9	0.06	1.00
Prune1	0.01	1.00
Cdc42bpb	-0.30	1.00
Klf11	0.04	1.00
Col8a1	-0.62	1.00
Chst12	0.04	1.00
Ube2q1	-0.18	1.00
Zhx1	-0.21	1.00
Fyn	-0.22	1.00
Pacc1	0.04	1.00
Snupn	-0.12	1.00
Drg1	-0.05	1.00
Ahsa1	-0.05	1.00
Inpp5k	-0.39	1.00
Lsm11	0.14	1.00
Tex264	-0.06	1.00
Thyn1	-0.02	1.00
Nudcd1	0.14	1.00

Dyrk2	0.01	1.00
Zbtb8os	-0.20	1.00
Slc35a4	0.06	1.00
Afdn	-0.29	1.00
Spsb3	0.10	1.00
Rars2	0.05	1.00
Nsd1	0.02	1.00
Diaph2	0.03	1.00
Ncstn	0.05	1.00
Vps36	0.10	1.00
Taf1a	0.10	1.00
Stau2	0.00	1.00
Gan	-0.34	1.00
Tube1	-0.08	1.00
Cdk13	-0.16	1.00
Stau1	-0.06	1.00
Bnip3l	-0.54	1.00
Srpk1	-0.16	1.00
Kif21a	-0.16	1.00
Stx2	0.06	1.00
P4ha1	-0.64	1.00
Zc2hc1a	-0.12	1.00
Peak1	0.00	1.00
Acad11	0.03	1.00
Tedc1	-0.02	1.00
Tmem138	-0.11	1.00
Prdx6	0.05	1.00
Sema7a	-0.19	1.00
Zcchc14	0.03	1.00
Smarcad1	-0.05	1.00
Dusp14	-0.03	1.00
Nisch	0.04	1.00
Gpc1	-0.26	1.00
Zfp518a	0.06	1.00
Eepd1	-0.44	1.00
Edem3	-0.17	1.00
Hmbs	0.08	1.00
Pdia6	-0.15	1.00
Pde7b	-0.81	1.00
Tradd	-0.22	1.00
Rfc1	-0.02	1.00
Rab21	-0.06	1.00
Rpp14	0.09	1.00
Tc2n	-0.37	1.00
Usp31	0.05	1.00
Lysmd2	0.05	1.00

Trmt11	0.02	1.00
Ap3s2	-0.17	1.00
Gar1	0.02	1.00
Cul4b	-0.01	1.00
Orai1	-0.11	1.00
Wipf3	-0.01	1.00
Rpia	-0.02	1.00
Kctd10	-0.16	1.00
Ankrd50	-0.46	1.00
Pi4k2b	0.06	1.00
Slc37a1	0.04	1.00
Pitpna	0.06	1.00
Dqx1	0.08	1.00
Prune2	-0.20	1.00
Kif5c	-0.61	1.00
Aimp2	0.00	1.00
B4galt5	-0.19	1.00
Upf1	-0.01	1.00
Hnf4g	0.07	1.00
Brd3	0.04	1.00
Stk4	-0.04	1.00
Nudt14	0.06	1.00
Hs6st2	-0.32	1.00
Tnfaip8	-0.39	1.00
Ylpm1	-0.01	1.00
Adam22	-0.09	1.00
Wdr44	-0.12	1.00
Cd81	-0.28	1.00
Stk10	-0.14	1.00
Ccnt1	-0.26	1.00
Tmem67	0.03	1.00
Trit1	0.09	1.00
Lingo2	-0.43	1.00
Spsb4	-0.57	1.00
Rmdn1	-0.08	1.00
Chn1	-0.10	1.00
Chst11	-0.62	1.00
C9orf72	-0.10	1.00
Rnf220	-0.28	1.00
Itprid2	-0.13	1.00
Hsd17b4	-0.24	1.00
Usp16	-0.31	1.00
Skil	-0.09	1.00
Snx16	0.02	1.00
Eif3b	-0.51	1.00
Tti1	0.10	1.00

Epcam	-0.16	1.00
Acaa2	-0.40	1.00
Bub3	-0.03	1.00
Flot1	0.07	1.00
Yipf6	-0.23	1.00
Ehd4	0.10	1.00
Pecr	0.10	1.00
Decr1	-0.08	1.00
Exoc8	-0.09	1.00
Eri3	-0.08	1.00
Cog6	-0.07	1.00
Tbc1d13	0.08	1.00
Slc66a1	0.02	1.00
Nudt18	0.08	1.00
Nbeal2	-0.08	1.00
Trp53bp2	-0.58	1.00
Ciao3	0.06	1.00
Tmem223	0.13	1.00
Zfp414	0.03	1.00
Kdm7a	0.01	1.00
Vav2	0.03	1.00
Zdhhc18	0.12	1.00
Rc3h2	-0.08	1.00
Fam189a2	-0.05	1.00
Rprd1b	-0.02	1.00
Iqcb1	-0.14	1.00
Hikeshi	-0.07	1.00
Elp2	0.07	1.00
Naa50	-0.23	1.00
Stard13	0.02	1.00
Anxa7	-0.02	1.00
Setd6	-0.02	1.00
Rprd1a	-0.10	1.00
Ift46	-0.07	1.00
Spcs2	-0.52	1.00
Ndufs6	-0.09	1.00
Kmt2e	-0.13	1.00
Dnajc1	-0.42	1.00
Lxn	0.06	1.00
Tmtc2	0.06	1.00
Casp3	0.08	1.00
Rpp38	0.02	1.00
Asf1a	-0.01	1.00
Gpatch3	-0.05	1.00
Bag2	-0.01	1.00
Bmp2	0.00	1.00

Smpdl3b	0.08	1.00
Slf2	0.02	1.00
Ogdh	-0.12	1.00
Zc3hc1	-0.04	1.00
Vps8	0.02	1.00
Ipo9	0.05	1.00
Adap1	-0.34	1.00
Chpf2	0.02	1.00
Adam23	0.06	1.00
Prpf19	0.02	1.00
Slc25a47	0.05	1.00
Gkap1	-0.02	1.00
Slc3a1	0.05	1.00
Nup85	-0.04	1.00
Zfp462	0.06	1.00
Epb41	-0.31	1.00
Zbtb34	0.09	1.00
Dusp23	0.09	1.00
Smpd4	-0.05	1.00
Sfxn1	0.01	1.00
Smyd3	-0.43	1.00
Taok1	-0.09	1.00
Phactr4	0.00	1.00
Hps3	0.08	1.00
Plekha2	-0.20	1.00
Ciapin1	-0.28	1.00
Sugp1	-0.08	1.00
Iars	-0.01	1.00
Xkr6	0.06	1.00
Nfe2l1	-0.07	1.00
Rab30	0.02	1.00
Smurf1	-0.01	1.00
Tmem267	0.03	1.00
Gpatch11	-0.04	1.00
Antxr2	-0.34	1.00
Khdc4	-0.01	1.00
Anxa5	-0.08	1.00
Fam135a	-0.12	1.00
Rnmt	-0.21	1.00
Washc3	0.01	1.00
Scmh1	-0.02	1.00
Pgpep1	-0.05	1.00
Ndufv2	-0.13	1.00
Vat1	-0.13	1.00
Rsrc2	-0.13	1.00
Qser1	-0.01	1.00

Fam3c	-0.22	1.00
Gnl3	-0.05	1.00
Nln	-0.31	1.00
Tyms	0.06	1.00
Seh1l	0.04	1.00
Ddx6	-0.14	1.00
Sytl2	-0.11	1.00
Stxbp1	0.07	1.00
Exph5	-0.20	1.00
Rassf3	-0.36	1.00
Nab1	-0.09	1.00
Pdcd11	0.00	1.00
Ubiad1	0.03	1.00
Sin3a	0.01	1.00
Casd1	-0.32	1.00
Vps26c	0.11	1.00
Triqk	0.13	1.00
Hbegf	-0.32	1.00
Anapc13	-0.01	1.00
Tgfbr3	0.02	1.00
Anp32e	0.00	1.00
Rnf128	-0.13	1.00
Sema3d	-0.49	1.00
Per3	-0.44	1.00
Wnk2	0.06	1.00
Fam199x	-0.04	1.00
Sh3glb2	0.02	1.00
Fktn	-0.09	1.00
Zfp800	-0.03	1.00
Rbms1	-0.04	1.00
Kctd5	-0.20	1.00
Scaf4	-0.40	1.00
Septin12	0.00	1.00
Tmem65	-0.25	1.00
Iws1	-0.16	1.00
Gna11	-0.17	1.00
Grb2	-0.44	1.00
Slc4a7	-0.96	1.00
Dmxl1	-0.05	1.00
Xpo6	-0.52	1.00
Gdf11	-0.02	1.00
Aqp3	-0.24	1.00
Rc3h1	-0.30	1.00
Cd247	-0.59	1.00
Ap4e1	-0.23	1.00
Ugp2	0.06	1.00

Pdpk1	-0.05	1.00
Cuedc1	-0.23	1.00
Smim5	-0.07	1.00
Spag6l	-0.11	1.00
Ppard	-0.09	1.00
Pomgnt1	-0.01	1.00
Thnsl1	-0.18	1.00
Sla	-0.19	1.00
Odf2	-0.08	1.00
Trappc2l	0.02	1.00
Clec16a	-0.08	1.00
Chuk	-0.17	1.00
Prpf8	0.00	1.00
Zbtb21	-0.57	1.00
Tnrc18	-0.03	1.00
Ankrd52	0.11	1.00
Ehmt1	-0.09	1.00
Trap1	-0.01	1.00
Jag2	0.07	1.00
Qtrt2	-0.11	1.00
Pmpca	0.06	1.00
Pola1	-0.02	1.00
Erp29	-0.32	1.00
Ikzf1	-0.36	1.00
Itpripl2	-0.34	1.00
Rnf41	-0.07	1.00
Sav1	-0.13	1.00
Gpr180	-0.04	1.00
Ift52	0.04	1.00
Cln6	-0.14	1.00
Chchd1	0.00	1.00
Atad2b	-0.17	1.00
Gdnf	0.09	1.00
Vps35l	-0.10	1.00
Atg16l1	0.05	1.00
Zfyve19	0.08	1.00
Zfp513	0.11	1.00
Umad1	-0.04	1.00
Copg2	-0.17	1.00
Nup155	-0.01	1.00
Slc7a5	-0.12	1.00
Tln1	0.01	1.00
Zdhhc7	-0.17	1.00
Ssbp2	0.10	1.00
Leprotl1	0.03	1.00
Fubp3	-0.13	1.00

Efcab14	-0.33	1.00
Stx18	0.11	1.00
Golim4	-0.43	1.00
Nup133	0.06	1.00
Zfp644	-0.48	1.00
Taf6l	0.06	1.00
Rpusd4	0.02	1.00
Acsl3	-0.06	1.00
Rida	-0.03	1.00
Smap1	-0.11	1.00
Arhgap5	-0.03	1.00
Phyhd1	-0.03	1.00
Tada1	-0.14	1.00
Hacd3	-0.76	1.00
Dnase2b	-0.10	1.00
Cdpf1	0.13	1.00
Fam149b	0.11	1.00
Ptges2	-0.02	1.00
Slc9a7	-0.01	1.00
Trip4	-0.22	1.00
Ift122	0.02	1.00
Iqsec1	-0.50	1.00
Pigw	0.08	1.00
Gabpa	-0.42	1.00
Cep20	-0.05	1.00
Dcbld2	0.08	1.00
Lig3	-0.25	1.00
Eef2k	-0.11	1.00
Edc4	-0.05	1.00
Prpf3	0.04	1.00
Vps13b	-0.66	1.00
Kcnip4	-0.77	1.00
Kctd3	-0.01	1.00
Kpna4	-0.24	1.00
Wars2	0.10	1.00
Tead1	0.02	1.00
Slc6a6	-0.14	1.00
Sfxn5	-0.19	1.00
Prps2	-0.01	1.00
Nsd2	-0.17	1.00
Ift81	0.07	1.00
Cebpz	-0.13	1.00
Idh3a	-0.13	1.00
Eogt	-0.30	1.00
Lias	0.10	1.00
Ing4	-0.04	1.00

Triap1	0.05	1.00
Proser2	0.00	1.00
Alpk2	-0.24	1.00
Pgap4	0.11	1.00
Tppp3	-0.21	1.00
Pi4ka	-0.07	1.00
Gle1	-0.28	1.00
Zbtb2	0.05	1.00
Rgs9	-0.24	1.00
Dele1	0.00	1.00
Yme1l1	-0.44	1.00
Nars	-0.37	1.00
Tfdp1	0.04	1.00
Osbpl3	-0.66	1.00
Zfp609	-0.01	1.00
Camsap1	-0.17	1.00
Dip2b	-0.27	1.00
Arid1b	0.00	1.00
Cep162	-0.08	1.00
Lonp1	0.08	1.00
Pitrm1	0.09	1.00
Nelfa	-0.06	1.00
Fbxo46	-0.06	1.00
Stk25	0.07	1.00
Ube2cbp	-0.02	1.00
Sf3a3	-0.12	1.00
Serinc1	-0.36	1.00
Eif2b5	0.08	1.00
Apod	-0.10	1.00
Slc25a24	-0.68	1.00
Epb41l2	-0.02	1.00
Utp25	-0.06	1.00
Tbc1d8	-0.19	1.00
Bbx	-0.16	1.00
Nr1h3	0.03	1.00
Zc4h2	0.07	1.00
Rnf8	-0.04	1.00
Auts2	-0.04	1.00
Hint3	0.04	1.00
Stx1a	0.07	1.00
Pla2g15	-0.22	1.00
Lrrk2	-0.12	1.00
Dhx8	0.04	1.00
Bcl2l11	-0.04	1.00
Ythdf1	-0.18	1.00
Ttbk1	0.02	1.00

Tgs1	0.02	1.00
Tpr	-0.36	1.00
Zswim5	-0.08	1.00
Vps29	-0.46	1.00
Nudt9	-0.05	1.00
Dhcr7	-0.05	1.00
Lamc1	0.02	1.00
Xrcc6	0.06	1.00
Garre1	0.08	1.00
Atp23	0.05	1.00
Rab11fip3	-0.13	1.00
Snap29	-0.31	1.00
Amer1	0.04	1.00
Atg4c	-0.19	1.00
Api5	-0.16	1.00
Aig1	-0.17	1.00
C2cd3	-0.08	1.00
Sulf2	-0.09	1.00
Lpgat1	-0.08	1.00
Itm2b	-0.03	1.00
Tmem94	-0.02	1.00
Dnaaf5	-0.03	1.00
Tob1	-0.06	1.00
Nfrkb	-0.03	1.00
St3gal3	0.10	1.00
Ints10	-0.17	1.00
Cep192	0.02	1.00
Vps26b	0.11	1.00
Arhgef6	-0.08	1.00
Smad5	-0.11	1.00
Pdcd10	0.04	1.00
Necap1	0.15	1.00
Naf1	-0.04	1.00
Ninj1	-0.18	1.00
Nmt1	0.06	1.00
Plekhf1	0.02	1.00
Plekha8	-0.19	1.00
Lrif1	0.02	1.00
Cep97	0.09	1.00
Psmd10	0.00	1.00
Cactin	-0.04	1.00
Zbtb7a	-0.17	1.00
Ankrd13a	-0.10	1.00
Ywhae	-0.34	1.00
Acads	-0.10	1.00
Rhbdd1	-0.13	1.00

Pptc7	-0.53	1.00
Zbtb43	-0.26	1.00
Hmg20a	-0.05	1.00
Cep43	0.01	1.00
Zfp654	-0.09	1.00
Tubb4b	-0.15	1.00
Clk2	0.10	1.00
Parva	-0.51	1.00
Gss	0.04	1.00
Tmem260	0.01	1.00
Hspa4l	-0.06	1.00
Psme3ip1	-0.14	1.00
F2rl1	0.08	1.00
Smg1	-0.24	1.00
Kin	-0.03	1.00
Hunk	-0.02	1.00
Ddx23	0.02	1.00
Slc5a1	0.07	1.00
Aatf	0.03	1.00
Zranb3	-0.03	1.00
Atmin	0.00	1.00
Erlin2	-0.36	1.00
Fitm2	-0.27	1.00
Tmem251	-0.22	1.00
Ncald	-0.74	1.00
Igsf3	0.00	1.00
Amacr	-0.06	1.00
Fam161b	-0.02	1.00
Cep290	-0.14	1.00
Nox4	0.07	1.00
Cul3	-0.12	1.00
Dbf4	0.05	1.00
Prrg1	-0.22	1.00
St14	0.04	1.00
Gatd1	0.05	1.00
Sin3b	-0.10	1.00
Lactb2	-0.42	1.00
Ppp2cb	-0.08	1.00
Rnf34	-0.12	1.00
Trnt1	-0.08	1.00
Chac1	-0.02	1.00
Ahctf1	-0.42	1.00
Cpsf4	0.09	1.00
Lpcat1	-0.97	1.00
Srebf2	-0.21	1.00
Uso1	-0.08	1.00

Tut4	-0.21	1.00
Slc9a6	-0.18	1.00
Hivep3	-0.38	1.00
Fhod3	-0.30	1.00
Harbi1	0.08	1.00
Dscc1	-0.04	1.00
Ep300	-0.07	1.00
Pak1ip1	0.11	1.00
Aftph	-0.13	1.00
Bend5	-0.08	1.00
Tgfb3	-0.02	1.00
Abhd2	-0.30	1.00
Fubp1	-0.13	1.00
Fan1	0.06	1.00
Fam234a	0.02	1.00
Dnajc11	0.08	1.00
Pno1	-0.07	1.00
Eif2s2	-0.13	1.00
Stxbp3	0.01	1.00
Fbxo8	0.09	1.00
Rdx	-0.52	1.00
Clip2	-0.03	1.00
Wnk1	-0.28	1.00
Prdm2	-0.13	1.00
Zc3h18	-0.40	1.00
Acss3	0.05	1.00
Ulk4	-0.01	1.00
Crybg1	-0.09	1.00
Wdfy2	-0.07	1.00
Tet2	-0.55	1.00
Acot13	-0.13	1.00
Jpt1	0.08	1.00
Fbxw2	0.05	1.00
Dnajc2	-0.19	1.00
Itm2a	-0.10	1.00
Lmna	-0.21	1.00
Cep83	-0.11	1.00
Dsn1	-0.01	1.00
Cyth3	0.02	1.00
Clasp1	-0.11	1.00
Ppme1	-0.14	1.00
S1pr3	0.10	1.00
Slc25a5	-0.21	1.00
Qdpr	-0.60	1.00
Fbxo28	-0.06	1.00
Pgs1	0.17	1.00

Zmynd8	-0.31	1.00
Gphn	-0.52	1.00
Cwc15	-0.27	1.00
Acsf2	-0.15	1.00
Pex14	-0.24	1.00
Trib2	-0.14	1.00
Phc3	-0.08	1.00
Galnt5	0.00	1.00
Frs2	0.10	1.00
Pin1	-0.03	1.00
Zfp521	-0.50	1.00
Filip1	-0.33	1.00
Gfer	-0.10	1.00
Rcl1	0.02	1.00
Frmd7	-0.06	1.00
Etfdh	0.01	1.00
Rest	-0.12	1.00
Irf8	0.00	1.00
Lipc	0.08	1.00
Asb3	0.15	1.00
Lratd2	-0.09	1.00
Jak1	-0.20	1.00
Ywhab	-0.30	1.00
Slc7a6os	0.11	1.00
Fkbp14	-0.14	1.00
Pip4p2	-0.15	1.00
Dhx36	-0.05	1.00
Hs1bp3	-0.01	1.00
Eci3	0.06	1.00
Anapc1	-0.02	1.00
Bdh1	-0.32	1.00
Cacna1i	-0.13	1.00
Arhgef12	-0.31	1.00
Arid4b	-0.03	1.00
Ppp6c	-0.08	1.00
Exoc6	-0.08	1.00
Slc25a37	0.03	1.00
Efr3a	-0.17	1.00
Dock9	-0.27	1.00
Atp9b	-0.35	1.00
Tamm41	0.06	1.00
Arrdc1	0.04	1.00
Itch	-0.53	1.00
Jmjd6	-0.07	1.00
Ube3a	-0.12	1.00
Cacna1b	0.09	1.00

Prkd1	-0.55	1.00
Ubr2	-0.47	1.00
Pik3r6	-0.10	1.00
Fgf18	0.08	1.00
Ythdc1	-0.13	1.00
Lbh	-0.36	1.00
Anks3	-0.02	1.00
Fhl3	0.04	1.00
Gtf2h1	0.10	1.00
Ildr1	0.08	1.00
Ice2	-0.10	1.00
Coq8a	-0.05	1.00
Frs3	-0.02	1.00
Crtc1	-0.19	1.00
Smarca5	-0.18	1.00
Sdf4	-0.25	1.00
Ccdc59	-0.08	1.00
Eml1	-0.04	1.00
Qsox1	-0.24	1.00
Bdp1	-0.28	1.00
D630045J12Rik	-0.22	1.00
Ogfod3	-0.12	1.00
E4f1	-0.14	1.00
Cbx1	-0.25	1.00
Natd1	0.08	1.00
Zmym2	-0.06	1.00
Arhgef38	-0.87	1.00
Rheb	-0.20	1.00
Inpp4a	-0.29	1.00
Pikfyve	-0.13	1.00
Spry2	-0.23	1.00
Dhdds	-0.10	1.00
Arhgap42	-0.05	1.00
Hacd2	-0.24	1.00
Eif4g1	0.04	1.00
Lamb1	-0.23	1.00
Nup35	0.04	1.00
Ctnnbip1	0.04	1.00
Palb2	0.05	1.00
Gpatch1	-0.19	1.00
Dazap1	-0.31	1.00
Vcl	-0.33	1.00
Nelfb	-0.09	1.00
Atf4	-0.04	1.00
Sec23b	-0.01	1.00
Bcl11a	-0.42	1.00

Cyrib	-0.34	1.00
Lmo2	-0.13	1.00
Cmas	-0.10	1.00
Ywhag	-0.06	1.00
Atp8b1	-0.13	1.00
Ror2	-0.22	1.00
Thoc1	0.02	1.00
Cdip1	0.04	1.00
Dennd4a	-0.53	1.00
Pdap1	-0.18	1.00
Cul2	-0.26	1.00
4933427D14Rik	-0.06	1.00
Fth1	0.09	1.00
Snap23	-0.09	1.00
Cdhr1	-0.37	1.00
Prkcz	-0.04	1.00
Cnot7	-0.18	1.00
Nek1	0.05	1.00
Frem2	-0.58	1.00
Rock1	-0.06	1.00
Larp1b	-0.27	1.00
Xylt1	-1.05	1.00
Herc1	-0.42	1.00
Adgrg1	-0.10	1.00
Haus2	-0.05	1.00
Vamp4	0.03	1.00
Copb1	-0.01	1.00
Sirt7	-0.01	1.00
Upp1	-0.26	1.00
Naaa	-0.07	1.00
Elk3	-0.14	1.00
Utp11	0.10	1.00
Pop5	0.02	1.00
Pgrmc2	-0.32	1.00
Gpatch8	-0.26	1.00
Setx	-0.16	1.00
Rbl1	0.09	1.00
Dcaf13	0.00	1.00
Tmc7	-0.14	1.00
Qrich1	-0.03	1.00
Ncoa5	-0.20	1.00
Batf	-0.24	1.00
Cog1	0.05	1.00
Nipsnap1	-0.06	1.00
Aurkaip1	-0.02	1.00
Lrrc42	-0.08	1.00

Trim13	-0.07	1.00
Ptprk	-0.16	1.00
Rnf13	-0.16	1.00
Bin1	-0.02	1.00
Tnrc6a	-0.28	1.00
Bpnt1	-0.12	1.00
Creb1	-0.16	1.00
Xylb	0.02	1.00
Cdk10	0.01	1.00
Smarce1	-0.30	1.00
Fam162a	-0.23	1.00
Tango2	-0.05	1.00
Pomt1	-0.02	1.00
Ndufs1	-0.01	1.00
Ralgapb	-0.45	1.00
Slc7a11	-0.62	1.00
Polr1a	-0.33	1.00
Phka1	0.02	1.00
Zadh2	-0.18	1.00
Dock7	-0.20	1.00
Prpf40a	-0.13	1.00
Slc30a9	-0.19	1.00
Dpm2	-0.27	1.00
Atp11c	0.04	1.00
Cmtm3	-0.03	1.00
Dstyk	-0.10	1.00
Dnajc21	0.07	1.00
Gpbp1l1	-0.27	1.00
Hmox2	-0.14	1.00
Taf5l	0.06	1.00
Nf1	-0.15	1.00
Pcyt1a	-0.03	1.00
Nmd3	0.02	1.00
Gpatch4	0.05	1.00
Sbspon	0.07	1.00
Nub1	-0.31	1.00
Dennd2c	0.10	1.00
Stam2	-0.35	1.00
Ubxn7	-0.18	1.00
Orai2	-0.12	1.00
Galnt3	-0.16	1.00
Yes1	-0.43	1.00
Kbtbd2	-0.06	1.00
Lrch1	-0.08	1.00
Gnpda2	-0.27	1.00
Ttc39a	-0.35	1.00

Timp2	0.15	1.00
Trabd	-0.20	1.00
Pemt	-0.15	1.00
Brcc3	0.01	1.00
Tent4b	-0.33	1.00
Plxnb2	-0.03	1.00
Aamdc	-0.02	1.00
Palld	-0.93	1.00
Nubp1	0.06	1.00
Uprt	-0.07	1.00
Zfp326	-0.19	1.00
Ndufaf4	-0.06	1.00
Gstk1	-0.29	1.00
Glb1	0.04	1.00
Kdm3b	-0.08	1.00
Gas7	-0.20	1.00
Tmem43	-0.29	1.00
Swt1	-0.07	1.00
Dtwd1	0.05	1.00
Actl6a	-0.24	1.00
Pcnt	-0.33	1.00
Nectin1	0.01	1.00
Gabpb2	0.03	1.00
Itgb1	-0.44	1.00
Capn5	0.02	1.00
Smad4	-0.16	1.00
Rexo1	-0.13	1.00
Trim3	-0.03	1.00
Galns	-0.16	1.00
Brca2	-0.08	1.00
Plod2	-0.19	1.00
Itgae	-0.33	1.00
Proser1	0.07	1.00
Nat10	0.03	1.00
Stub1	-0.01	1.00
Asap1	-0.30	1.00
Upf2	-0.20	1.00
Pdzd2	0.19	1.00
Ube2o	-0.21	1.00
Leprot	-0.19	1.00
Zfp148	-0.22	1.00
Il16	-0.07	1.00
Slc24a2	0.01	1.00
Ppib	-0.05	1.00
Cep70	0.04	1.00
Thumpd2	-0.05	1.00

Nipal3	-0.22	1.00
Zfp292	-0.28	1.00
Rnf111	-0.23	1.00
Cul5	-0.25	1.00
Lsm6	-0.10	1.00
Cars2	0.06	1.00
Grk4	-0.03	1.00
Slc16a2	0.10	1.00
Acox2	-0.05	1.00
Abhd17c	-0.08	1.00
Wdr45b	0.06	1.00
Atp2c1	-0.25	1.00
Dpp10	-0.75	1.00
Lrrc31	-0.30	1.00
Arl6	-0.19	1.00
Pcna	-0.05	1.00
Ro60	-0.03	1.00
Xrn2	-0.26	1.00
Ppif	0.02	1.00
Dus3l	0.01	1.00
Fam98a	-0.17	1.00
Pigh	-0.04	1.00
Phospho1	-0.07	1.00
Pcgf3	-0.15	1.00
Clpx	-0.24	1.00
Adcy7	-0.69	1.00
Nr3c1	-0.26	1.00
Arl5a	0.04	1.00
Prdm5	-0.03	1.00
Kansl1l	-0.10	1.00
Slc30a4	-0.26	1.00
Alg8	0.08	1.00
Abi1	-0.40	1.00
Tbl3	0.06	1.00
Scube2	-0.09	1.00
Ccdc102a	-0.09	1.00
Sp3	-0.45	1.00
Ddx27	-0.08	1.00
Dapk3	-0.10	1.00
Pts	-0.10	1.00
Rab11fip2	-0.22	1.00
Bzw1	-0.38	1.00
Psmd1	-0.22	1.00
Pnpla2	-0.06	1.00
Bclaf1	-0.13	1.00
Ing1	0.00	1.00

Gorasp2	-0.28	1.00
Eri1	-0.03	1.00
Nmt2	-0.12	1.00
Fam210b	0.08	1.00
Zmpste24	-0.07	1.00
Dpy19l3	-0.13	1.00
Rnf214	0.09	1.00
Nprl3	-0.27	1.00
Pmp22	-0.59	1.00
Gfpt1	-0.20	1.00
Dlat	0.00	1.00
Ankmy2	-0.02	1.00
Slc31a1	-0.11	1.00
Zfand4	-0.10	1.00
Jpt2	-0.35	1.00
Bcl2l1	0.15	1.00
Pmpcb	-0.11	1.00
Heg1	-0.15	1.00
Rbm11	0.03	1.00
Gtse1	0.05	1.00
Pik3cb	-0.13	1.00
Rtcb	-0.03	1.00
Camta1	-0.24	1.00
Asrgl1	0.06	1.00
C1qtnf12	-0.30	1.00
Rnaseh2b	-0.19	1.00
Cbx8	-0.15	1.00
Bnip1	0.10	1.00
Actr6	-0.12	1.00
Gcc2	-0.55	1.00
Cops2	-0.28	1.00
Tmem231	0.03	1.00
Pigv	0.01	1.00
Rab8a	-0.06	1.00
Glce	-0.03	1.00
Sytl4	0.05	1.00
Commd6	-0.01	1.00
Nfxl1	-0.05	1.00
Baz1a	-0.29	1.00
Fzd7	-0.11	1.00
Gpx2	-0.27	1.00
Csnk2a2	-0.30	1.00
Snw1	-0.05	1.00
Pla2g1b	-0.01	1.00
Alg11	0.03	1.00
Alg13	-0.15	1.00

Trmt44	0.11	1.00
Rbbp8	-0.23	1.00
Socs4	0.08	1.00
Smim4	0.00	1.00
Bcl7a	-0.30	1.00
Usp34	-0.31	1.00
Slc25a16	-0.09	1.00
Pnrc1	0.01	1.00
Clasp2	-0.19	1.00
Selenoo	-0.46	1.00
Phtf2	-0.02	1.00
Caml	0.10	1.00
Tent4a	0.02	1.00
Tmem62	0.07	1.00
Cdc40	0.01	1.00
Eif5a2	-0.22	1.00
Tatdn3	-0.07	1.00
Elmod2	-0.02	1.00
Grk2	0.08	1.00
Rab6a	-0.41	1.00
Rap1b	-0.42	1.00
Znhit6	-0.09	1.00
Ano5	-0.22	1.00
Slc35a1	-0.04	1.00
Pnisr	-0.54	1.00
Rabep1	-0.35	1.00
Slc44a2	-0.24	1.00
Stk38l	-0.66	1.00
Bbox1	-0.09	1.00
Fyb	-0.20	1.00
Cobll1	-0.24	1.00
Thap12	-0.28	1.00
Kpna6	-0.30	1.00
Tmem214	-0.14	1.00
Gab3	-0.42	1.00
Gga1	-0.01	1.00
Kiz	0.03	1.00
Scrn3	-0.05	1.00
Rnf2	-0.07	1.00
Zzz3	-0.26	1.00
Ccdc82	0.03	1.00
Cdkl5	-0.16	1.00
Spg11	-0.25	1.00
Anxa4	-0.50	1.00
Lemd3	-0.38	1.00
Wasf2	-0.19	1.00

Ttc14	-0.07	1.00
Rhbdf1	0.01	1.00
Psmd12	-0.08	1.00
Dhx38	0.08	1.00
Limch1	-0.42	1.00
Asph	-0.55	1.00
Rrp7a	-0.02	1.00
Dusp8	0.00	1.00
Pphln1	0.06	1.00
Zc3h7a	-0.37	1.00
Anks1	-0.25	1.00
Tada2a	0.00	1.00
Cntnap2	-0.22	1.00
Fbxo38	-0.01	1.00
Erc1	-0.05	1.00
Asb6	-0.09	1.00
Tsc2	-0.18	1.00
Sec22b	0.00	1.00
Dynlt1a	0.02	1.00
Itgb8	0.07	1.00
Slc38a1	-0.47	1.00
Pdcl3	-0.17	1.00
Setd5	-0.03	1.00
Guf1	-0.14	1.00
Qrsl1	0.07	1.00
Chd9	-0.20	1.00
Gtf2a1	-0.06	1.00
Nfya	-0.06	1.00
Pigf	-0.18	1.00
Arih2	-0.11	1.00
Rbbp7	0.00	1.00
Tmem161a	-0.03	1.00
Hipk1	-0.35	1.00
P3h3	-0.02	1.00
Supv3l1	0.08	1.00
Ankle2	-0.29	1.00
Slc66a2	-0.30	1.00
Hat1	-0.26	1.00
Kcnab2	0.01	1.00
Plppr5	-0.17	1.00
Extl2	-0.16	1.00
Crk	-0.29	1.00
Cavin1	-0.28	1.00
Pbdc1	-0.21	1.00
Pms2	-0.11	1.00
Smarcb1	0.00	1.00

Nkrf	-0.10	1.00
Pcsk5	-0.04	1.00
Ascc2	-0.01	1.00
Traf6	-0.16	1.00
Sec31b	0.07	1.00
Kifbp	-0.20	1.00
Pgap1	-0.08	1.00
Tbc1d5	-0.38	1.00
Ormdl3	-0.10	1.00
Dusp11	0.02	1.00
Atrnl1	-0.75	1.00
Polr3f	0.04	1.00
Katnip	-0.04	1.00
Impa1	-0.12	1.00
Acat1	-0.20	1.00
Rab23	-0.24	1.00
Aida	-0.24	1.00
Aar2	0.08	1.00
Cenpf	-0.39	1.00
Ift80	-0.13	1.00
Ssh1	0.07	1.00
Plekhf2	-0.13	1.00
Nrbf2	-0.08	1.00
Tmco4	-0.31	1.00
Rsbn1	0.10	1.00
Tnks2	-0.39	1.00
Exosc10	-0.03	1.00
Ppp3cc	-0.15	1.00
Iqch	-0.18	1.00
Urb1	0.00	1.00
Pcbd1	-0.04	1.00
Apcdd1	-0.49	1.00
Ide	-0.25	1.00
Smap2	-0.10	1.00
Tubgcp4	-0.07	1.00
Hells	-0.17	1.00
Rragc	-0.01	1.00
Smim13	-0.20	1.00
Adprm	-0.15	1.00
Gorab	-0.15	1.00
Atf3	-0.50	1.00
Pms1	-0.20	1.00
Cnep1r1	0.04	1.00
Tmem70	-0.12	1.00
Zc3h12b	-0.11	1.00
Rpap3	-0.25	1.00

Trmt2a	-0.10	1.00
Zbtb38	-0.05	1.00
Trappc11	-0.18	1.00
Trp53inp2	-0.24	1.00
Srsf10	-0.29	1.00
Fam20b	-0.18	1.00
Rhot1	-0.04	1.00
Cers6	0.02	1.00
Cep128	0.04	1.00
Susd1	-0.07	1.00
Slc12a4	-0.22	1.00
Nceh1	-0.43	1.00
Lrrc45	-0.19	1.00
Nt5c3b	0.07	1.00
Slc25a25	-0.34	1.00
Tma16	-0.47	1.00
Gid8	-0.34	1.00
Rab10	-0.22	1.00
Nipsnap2	-0.22	1.00
Prepl	-0.12	1.00
Prpf39	-0.25	1.00
Hdgfl3	0.10	1.00
Rufy1	-0.14	1.00
Agl	-0.25	1.00
Lbr	-0.25	1.00
Trmt6	-0.07	1.00
Pias4	0.06	1.00
Rab9b	0.02	1.00
Dnaja3	-0.11	1.00
Casp8ap2	-0.20	1.00
Sgcb	-0.21	1.00
Strn	-0.36	1.00
Cab39	-0.23	1.00
Smim14	-0.25	1.00
Slc16a1	0.04	1.00
Nipal2	-0.10	1.00
Sox8	-0.01	1.00
Alg5	0.01	1.00
Usp48	-0.30	1.00
Pygl	-0.21	1.00
Bend3	-0.23	1.00
Lzts3	-0.07	1.00
Pdcd4	-0.10	1.00
Bmper	-0.39	1.00
Gnai3	-0.39	1.00
Lyrm2	0.12	1.00

Armt1	0.00	1.00
Wdr76	0.01	1.00
Rbm12b2	0.04	1.00
Suv39h2	-0.34	1.00
Poglut2	0.04	1.00
Pde9a	-0.12	1.00
Wfs1	-0.18	1.00
Nexmif	-0.07	1.00
Vrk1	0.02	1.00
Usp19	-0.23	1.00
Fbxl20	-0.34	1.00
Hibch	-0.12	1.00
Usp1	-0.22	1.00
Fbxo45	-0.25	1.00
Rwdd2b	0.03	1.00
Pheta2	-0.10	1.00
Nadsyn1	0.03	1.00
Wdr53	0.05	1.00
Agps	-0.26	1.00
Nvl	-0.37	1.00
Osbpl11	-0.07	1.00
Ubn1	-0.26	1.00
Foxo1	-0.82	1.00
Tbpl1	0.11	1.00
Riox2	0.01	1.00
Hmg20b	0.07	1.00
Atxn1l	-0.12	1.00
Kif3b	0.01	1.00
Fbxw8	-0.15	1.00
Dennd1b	-0.28	1.00
Aff4	-0.19	1.00
Caprin1	-0.19	1.00
Fam234b	-0.02	1.00
Gtpbp1	-0.02	1.00
Ppp4r3a	-0.18	1.00
Ago2	-0.65	1.00
Pi4kb	-0.08	1.00
Cdc14a	-0.35	1.00
Pank2	0.11	1.00
Csde1	-0.27	1.00
Tmem64	-0.30	1.00
Efl1	-0.11	1.00
Ift88	-0.03	1.00
Baz1b	-0.26	1.00
Cobl	0.04	1.00
Fam189a1	0.15	1.00

Klhl2	-0.26	1.00
Akap11	-0.25	1.00
Tmem131l	-0.38	1.00
P4htm	-0.07	1.00
Synj2	0.02	1.00
Slc17a5	-0.01	1.00
Dnajc28	-0.09	1.00
Ccp110	-0.22	1.00
Srbd1	-0.34	1.00
Gnptab	-0.36	1.00
Ints7	-0.07	1.00
Phrf1	-0.16	1.00
Katnbl1	-0.15	1.00
Csnk1g1	-0.16	1.00
Lgals3	-0.48	1.00
Erich1	-0.01	1.00
Ube2j2	-0.18	1.00
Csrnp1	-0.24	1.00
Inpp5a	-0.61	1.00
Narf	-0.24	1.00
Heatr3	-0.11	1.00
Cct5	-0.09	1.00
Ap1g1	-0.21	1.00
Sp4	0.00	1.00
Vegfa	0.15	1.00
Kifc3	-0.14	1.00
Gpn3	-0.06	1.00
Klhl36	0.10	1.00
Tmem237	-0.02	1.00
Zc3h14	-0.10	1.00
Azi2	-0.43	1.00
Tmx4	-0.15	1.00
Tnip3	0.15	1.00
Ubtd1	-0.06	1.00
S100a13	-0.37	1.00
Niban1	0.02	1.00
Pyroxd1	0.00	1.00
Gtf3c6	-0.13	1.00
Slc41a2	-0.10	1.00
Slc26a5	-0.12	1.00
Pik3r4	0.01	1.00
Dip2a	-0.09	1.00
Septin6	-0.26	1.00
Ccni	-0.39	1.00
Tmem222	0.06	1.00
Erbb2	0.04	1.00

Dcaf1	-0.13	1.00
Nif3l1	-0.16	1.00
Commd2	-0.27	1.00
Khdrbs1	-0.13	1.00
Anapc5	0.09	1.00
Syt14	-0.26	1.00
Aga	-0.08	1.00
Lrrc58	-0.15	1.00
Ankrd49	-0.17	1.00
Fndc3a	-0.01	1.00
B4galt3	0.06	1.00
Klhl12	0.03	1.00
Zbtb6	0.06	1.00
Uck1	0.01	1.00
Lrrcc1	0.00	1.00
Kidins220	-0.37	1.00
Spag9	-0.21	1.00
Stard3nl	0.02	1.00
Klhl7	-0.30	1.00
Sh3gl1	-0.01	1.00
St3gal1	-0.59	1.00
Enpp4	-0.15	1.00
Tm9sf4	-0.06	1.00
Rnf170	-0.19	1.00
Slc25a15	0.04	1.00
Cnst	0.04	1.00
Dph5	0.00	1.00
Wbp4	-0.22	1.00
Ubp1	-0.11	1.00
Impdh2	0.03	1.00
Rnf4	-0.45	1.00
Smc3	-0.11	1.00
Ift22	0.04	1.00
Timm22	0.07	1.00
Ccdc47	-0.29	1.00
Aqr	-0.15	1.00
Tubgcp5	-0.10	1.00
Sec22c	-0.07	1.00
Idh1	-0.16	1.00
Cds2	-0.24	1.00
Grb10	-0.06	1.00
Wdr77	0.02	1.00
Zbtb17	-0.03	1.00
Etaa1	-0.01	1.00
Apaf1	-0.02	1.00
Nin	0.03	1.00

Eml4	-0.23	1.00
Brms1l	-0.10	1.00
Dpysl3	-0.15	1.00
Sema6d	-0.09	1.00
Angel1	0.08	1.00
Fam114a2	-0.07	1.00
Tubd1	-0.29	1.00
Rrn3	-0.14	1.00
Prpf4	0.03	1.00
LTO1	-0.22	1.00
Slc25a12	-0.30	1.00
Atpaf1	-0.12	1.00
Arntl	-0.55	1.00
Wdr26	-0.35	1.00
Klhdc4	0.04	1.00
Zfp318	-0.30	1.00
Cldn12	-0.07	1.00
Gpd1l	-0.21	1.00
Rbbp6	-0.19	1.00
Rfxank	-0.06	1.00
Zbtb18	-0.13	1.00
Atm	-0.10	1.00
Cep131	0.00	1.00
Oaz2	-0.20	1.00
Gpr107	-0.29	1.00
Ube2q2	-0.33	1.00
Serinc2	0.00	1.00
Chaf1a	-0.15	1.00
Immp1l	-0.12	1.00
Zfp639	0.03	1.00
Cwf19l1	-0.09	1.00
Sdr16c5	-0.23	1.00
Aasdhppt	-0.09	1.00
Eaf1	-0.07	1.00
Nsmce1	-0.11	1.00
Ndrg4	-0.06	1.00
Prdm15	-0.03	1.00
Pbx4	-0.16	1.00
Fam174b	0.04	1.00
Prkar2a	-0.35	1.00
P4hb	-0.32	1.00
Cct8	-0.27	1.00
Tcaim	0.03	1.00
Tshr	0.05	1.00
Fanci	-0.03	1.00
Ireb2	-0.30	1.00

Lzts2	0.03	1.00
Zrsr1	-0.31	1.00
Abraxas1	-0.21	1.00
Fnta	-0.19	1.00
Raph1	-0.19	1.00
Ctps	-0.09	1.00
Pccb	-0.19	1.00
Gpt2	-0.20	1.00
Tnfaip1	0.03	1.00
Chmp5	-0.41	1.00
P4ha2	-0.13	1.00
Dus1l	-0.29	1.00
Coasy	0.03	1.00
Napg	-0.22	1.00
Ccnk	-0.39	1.00
Brip1	-0.01	1.00
Ttc1	-0.10	1.00
Rgma	-0.11	1.00
C2cd2l	-0.09	1.00
Coro1b	0.09	1.00
Tle3	-0.49	1.00
Smarcc1	-0.19	1.00
Lypla1	-0.17	1.00
Rnf144a	-0.41	1.00
Surf2	-0.10	1.00
Fam102a	-0.21	1.00
Evi5	-0.45	1.00
Alg12	0.09	1.00
Nek6	-0.46	1.00
Glul	-0.25	1.00
Ptprf	-0.25	1.00
Sipa1l2	-0.32	1.00
Hdac2	-0.38	1.00
Tm9sf3	-0.20	1.00
Utp23	-0.05	1.00
Gosr1	-0.40	1.00
Rfng	-0.28	1.00
Ggps1	-0.07	1.00
Kif3a	-0.02	1.00
Cluh	-0.18	1.00
Zfp410	-0.01	1.00
Crls1	-0.10	1.00
Nbas	-0.23	1.00
Bcar3	-0.04	1.00
Raly	-0.27	1.00
Vps35	-0.17	1.00

Pcm1	-0.28	1.00
Eif2ak3	-0.68	1.00
Cog2	-0.15	1.00
Prkd3	-0.08	1.00
Lima1	-0.19	1.00
Gxylt2	-0.15	1.00
Tmem106b	-0.17	1.00
Usp45	-0.15	1.00
Ppp2r3c	-0.17	1.00
Rab3gap2	-0.32	1.00
Rbm5	-0.32	1.00
Slit3	-0.27	1.00
Lrrfip2	-0.39	1.00
Plcd1	-0.17	1.00
Eif2d	-0.37	1.00
Gga2	0.07	1.00
Fbxo33	0.01	1.00
Rnf169	-0.02	1.00
Tm9sf2	-0.42	1.00
Dock5	-0.22	1.00
Klhl15	-0.16	1.00
Ap3m2	0.03	1.00
Pskh1	-0.09	1.00
Telo2	-0.02	1.00
Usp20	-0.09	1.00
Sap30l	-0.21	1.00
Dnttip2	-0.15	1.00
Ptpn11	-0.17	1.00
Pkp2	-0.31	1.00
Zmynd19	-0.09	1.00
Aco2	-0.32	1.00
Clic4	-0.07	1.00
Pkn2	-0.42	1.00
Dynlrb1	-0.23	1.00
Qk	0.00	1.00
Zbtb26	0.01	1.00
Ror1	-0.30	1.00
Zfp330	0.05	1.00
Setdb1	-0.14	1.00
Slx4	-0.08	1.00
Ralb	-0.50	1.00
Hpgds	-0.06	1.00
Pacsin3	-0.01	1.00
Adgra3	-0.39	1.00
Phka2	-0.07	1.00
Dnajc17	-0.04	1.00

Ddx55	0.05	1.00
Tubgcp3	-0.29	1.00
Il13ra1	-0.76	1.00
Tmem123	0.00	1.00
Pkd2	0.13	1.00
Insr	-0.46	1.00
Gprc5b	-0.53	1.00
Parp8	-0.34	1.00
Cks1brt	-0.16	1.00
Rpusd2	0.06	1.00
Coq10a	-0.07	1.00
Zfp384	0.06	1.00
Srsf4	-0.07	1.00
Ppil3	-0.22	1.00
Rnf141	-0.08	1.00
Gtf2e1	-0.47	1.00
Zfp827	-0.12	1.00
Fam83f	-0.01	1.00
Dync1li1	-0.05	1.00
B4galt2	-0.16	1.00
Smc4	-0.23	1.00
Spast	-0.20	1.00
Ptrhd1	0.06	1.00
Actr8	-0.09	1.00
Tmco3	-0.11	1.00
Ints9	0.11	1.00
Nectin3	-0.25	1.00
Ammecr1	-0.12	1.00
Trip11	-0.27	1.00
C2cd5	-0.38	1.00
Coq2	-0.07	1.00
Arnt2	-0.28	1.00
Uchl5	0.05	1.00
Sh3glb1	-0.03	1.00
Qsox2	0.13	1.00
Ppip5k1	0.03	1.00
Fam98b	-0.20	1.00
Vrk2	-0.15	1.00
Bloc1s6	-0.13	1.00
Arel1	-0.13	1.00
Epn2	-0.42	1.00
Srsf11	-0.11	1.00
Ppp1r12a	-0.14	1.00
Gbe1	-0.26	1.00
Hnrnpab	-0.24	1.00
Slc18b1	0.03	1.00

Rbm26	-0.24	1.00
Pfn2	-0.21	1.00
Celf5	-0.08	1.00
Arid2	-0.41	1.00
Nudt19	0.04	1.00
Intu	-0.07	1.00
Ncapd2	-0.02	1.00
Uap1l1	-0.05	1.00
Cfap20	0.09	1.00
Eif3a	-0.24	1.00
Zfp638	-0.19	1.00
Fam91a1	-0.25	1.00
Supt7l	0.04	1.00
Wapl	-0.24	1.00
Wdr47	-0.01	1.00
Fam110c	0.02	1.00
Sprtn	-0.08	1.00
Nup205	0.00	1.00
Spats2l	-0.47	1.00
Brwd3	-0.24	1.00
Spg7	0.05	1.00
Abce1	-0.19	1.00
Cfap57	0.00	1.00
Grik3	-0.07	1.00
Traf4	-0.05	1.00
Tmem168	-0.09	1.00
Atf2	-0.39	1.00
Opa1	-0.02	1.00
Cped1	-0.08	1.00
Prkx	-0.49	1.00
Abcc4	-0.56	1.00
Zfp711	-0.15	1.00
Rtn4r	0.09	1.00
Jade2	-0.21	1.00
Gpatch2l	-0.23	1.00
Spout1	0.05	1.00
Gemin5	-0.04	1.00
Dclk3	-0.25	1.00
Ogfod1	0.03	1.00
Gpam	-0.01	1.00
Pex6	-0.03	1.00
Cplane1	0.02	1.00
Atp6v0a2	-0.09	1.00
Gtpbp10	-0.42	1.00
Efcab7	-0.12	1.00
Zfp598	0.01	1.00

Nup107	-0.14	1.00
Acacb	-0.01	1.00
Vgll1	-0.26	1.00
Tpp2	-0.16	1.00
Pank1	-0.01	1.00
Atp7a	-0.20	1.00
Ktn1	-0.36	1.00
Vangl1	-0.07	1.00
Ppp1r13b	-0.41	1.00
Ndc1	0.04	1.00
Tecpr1	-0.11	1.00
Arfgap2	-0.13	1.00
Pgm3	0.07	1.00
Aadat	-0.04	1.00
Kat6b	-0.45	1.00
Ccdc84	-0.13	1.00
Eprs	-0.39	1.00
Dzip1l	-0.01	1.00
Clba1	0.04	1.00
Slc7a4	0.01	1.00
Helb	-0.03	1.00
Atg14	-0.02	1.00
Nol4l	-0.04	1.00
Chd1l	-0.14	1.00
Vps37b	-0.30	1.00
Noa1	-0.01	1.00
Ociad1	-0.08	1.00
Sos2	-0.02	1.00
Ccdc6	-0.19	1.00
Entpd1	-0.09	1.00
Pdlim7	0.10	1.00
Otud7b	0.00	1.00
Ggnbp2	-0.14	1.00
Vps18	-0.10	1.00
Ccdc174	-0.21	1.00
Fam120a	-0.24	1.00
Polr3d	0.06	1.00
Lrrc39	-0.82	1.00
Wipi1	-0.02	1.00
St6galnac4	0.05	1.00
Ldlrap1	0.01	1.00
Cggbp1	0.05	1.00
Rhbdd2	0.01	1.00
Sde2	-0.25	1.00
Slc35b2	0.00	1.00
Zbtb14	-0.09	1.00

Zfyve9	-0.08	1.00
Flrt3	-0.43	1.00
Hsd11b2	0.10	1.00
Lztfl1	-0.08	1.00
Vezf1	-0.36	1.00
Rbbp4	-0.37	1.00
Lrp12	-0.36	1.00
Atg2b	-0.34	1.00
Rnf146	-0.12	1.00
Tspan18	-0.38	1.00
Nfe2l2	-0.17	1.00
Arfgef2	-0.38	1.00
Sars	-0.09	1.00
Prkci	-0.12	1.00
Zfp362	-0.01	1.00
Ate1	-0.09	1.00
Il17rd	0.04	1.00
Slc2a9	-0.11	1.00
Uba5	0.00	1.00
Dglucy	-0.04	1.00
Fgf9	-0.28	1.00
Ankrd54	-0.02	1.00
Aagab	-0.10	1.00
Strada	-0.07	1.00
Cep164	-0.03	1.00
Capsl	-0.14	1.00
Smpdl3a	-0.18	1.00
Ccdc88a	-0.26	1.00
Ndrg1	-0.15	1.00
Wwox	-0.18	1.00
Taf1b	0.02	1.00
Ap1s3	-0.03	1.00
Lss	-0.08	1.00
Polr3b	-0.26	1.00
Apba1	-0.38	1.00
Smarcd1	-0.13	1.00
Smyd2	-0.07	1.00
Tmem144	-0.06	1.00
Samsn1	0.01	1.00
Lrrc34	-0.12	1.00
Apbb1	-0.06	1.00
Fermt1	-0.10	1.00
Cop1	-0.48	1.00
Abhd12	-0.06	1.00
Uhmk1	-0.05	1.00
Shld3	-0.06	1.00

Dnajb4	-0.28	1.00
Cnot10	0.02	1.00
Bmf	-0.08	1.00
Paxip1	-0.35	1.00
Tbl2	-0.05	1.00
Prr7	0.03	1.00
Bnip3	-0.19	1.00
Gpsm1	-0.02	1.00
Bfar	0.01	1.00
Jak2	-0.43	1.00
Eftud2	-0.06	1.00
Ttc26	-0.05	1.00
Gtf3c3	-0.09	1.00
Letm1	-0.14	1.00
Atp10a	-0.21	1.00
Arcn1	-0.24	1.00
Slc7a1	-0.06	1.00
Ralgapa1	-0.26	1.00
Vps50	-0.06	1.00
Fut8	-0.27	1.00
Cep63	-0.02	1.00
Scp2	-0.44	1.00
Sucla2	-0.02	1.00
Prex1	-0.25	1.00
Cep95	-0.16	1.00
Nt5c3	-0.16	1.00
Heatr5b	-0.03	1.00
Trp53bp1	-0.33	1.00
Epas1	-0.21	1.00
Slc25a19	-0.16	1.00
Prkdc	-0.30	1.00
Ccdc32	-0.11	1.00
Nhlrc2	0.04	1.00
Selenop	-0.49	1.00
Oma1	-0.06	1.00
Fam117a	-0.05	1.00
Blzf1	-0.09	1.00
Usf3	-0.03	1.00
Slc46a3	0.04	1.00
Phtf1	0.05	1.00
Ern2	0.00	1.00
Tarsl2	-0.14	1.00
Tlk1	-0.09	1.00
Czib	0.05	1.00
Clk1	-0.16	1.00
Atg4b	-0.22	1.00

Pknox1	-0.45	1.00
Ss18	-0.26	1.00
Sppl2b	-0.02	1.00
Tmem129	-0.10	1.00
Enpp2	-0.30	1.00
Erlin1	-0.06	1.00
Uggt2	-0.29	1.00
Crlf3	0.00	1.00
Cars	-0.20	1.00
Pus7l	-0.10	1.00
Adss	-0.14	1.00
Zswim8	-0.07	1.00
Recql	0.04	1.00
Rabgap1	-0.22	1.00
Scai	0.02	1.00
Gsk3b	-0.15	1.00
Lrrc40	0.05	1.00
Sppl2a	-0.21	1.00
Dmxl2	-0.20	1.00
Tbxas1	-0.17	1.00
Dcun1d4	-0.22	1.00
Washc4	-0.07	1.00
Pacs2	-0.18	1.00
Fryl	-0.34	1.00
Sypl2	-0.10	1.00
Rnf168	-0.24	1.00
Plekha3	-0.06	1.00
Syap1	-0.04	1.00
Rere	-0.01	1.00
Ncam1	-0.68	1.00
Pak3	-0.11	1.00
Kpnb1	-0.19	1.00
Timm44	-0.10	1.00
Aars	-0.02	1.00
Spata2	-0.02	1.00
Ambra1	-0.77	1.00
Wnt4	-0.07	1.00
Uhrf1bp1	-0.08	1.00
Pdk3	-0.40	1.00
Cnot11	-0.27	1.00
Rnf6	0.08	1.00
Dbr1	-0.11	1.00
Bace1	0.00	1.00
Golm1	-0.07	1.00
Baz2b	-0.18	1.00
Txndc16	-0.10	1.00

Dmap1	-0.13	1.00
Lnpk	0.01	1.00
Tmem19	-0.39	1.00
Ccnyl1	-0.16	1.00
Ypel5	-0.12	1.00
Rabggtb	-0.13	1.00
Pex11g	-0.05	1.00
Crb2	-0.30	1.00
Tmem47	-0.12	1.00
Pnpla7	-0.28	1.00
Siah2	-0.03	1.00
Rhobtb2	-0.04	1.00
Ccser1	-0.11	1.00
Cep104	-0.17	1.00
Rpp40	0.09	1.00
Nfx1	-0.09	1.00
Adpgk	-0.03	1.00
Rhof	-0.33	1.00
Daglb	-0.39	1.00
Fam210a	-0.02	1.00
Npepps	-0.23	1.00
Pik3c2a	-0.47	1.00
Obi1	-0.06	1.00
Pawr	-0.21	1.00
Serinc5	-0.47	1.00
Dip2c	-0.15	1.00
Tmem38b	0.01	1.00
Nedd4l	-0.25	1.00
Plcxd1	-0.05	1.00
Papola	-0.19	1.00
Bphl	-0.08	1.00
Ogt	-0.21	1.00
Nol11	-0.23	1.00
Eif2s1	-0.15	1.00
Wac	-0.30	1.00
Arih1	-0.25	1.00
Gpcpd1	-0.47	1.00
Slu7	-0.18	1.00
Ptcd3	-0.01	1.00
Wdr11	0.09	1.00
Sec14l1	-0.16	1.00
Senp6	-0.16	1.00
Smndc1	-0.08	1.00
Desi2	-0.02	1.00
Wdr35	0.04	1.00
Syde2	-0.08	1.00

Taf10	-0.07	1.00
Pwwp2a	-0.33	1.00
Rcan3	-0.22	1.00
Polr3k	-0.07	1.00
Eif3e	-0.32	1.00
Zfp236	-0.13	1.00
Smcr8	-0.01	1.00
Rad54b	-0.11	1.00
Camkmt	-0.07	1.00
Sec16a	-0.12	1.00
Ttc13	-0.07	1.00
Hyi	-0.03	1.00
Lsm14b	-0.07	1.00
Prrc2b	-0.18	1.00
Klhl28	-0.16	1.00
Gtf3c1	-0.21	1.00
Ccdc186	-0.40	1.00
Plekha1	-0.10	1.00
Pla2g7	-0.01	1.00
Ttc21b	-0.04	1.00
Slc16a3	-0.07	1.00
Prkca	-0.19	1.00
Hsf2	-0.03	1.00
Phc1	-0.14	1.00
Gzf1	-0.02	1.00
Cfl2	-0.05	1.00
Sdc1	0.05	1.00
Cep250	-0.02	1.00
Fam118b	-0.04	1.00
Ublcp1	-0.14	1.00
Fbxw11	-0.27	1.00
Ccna2	-0.14	1.00
Socs7	0.03	1.00
Nap1l1	-0.33	1.00
Cdk6	-0.17	1.00
Tns3	-0.27	1.00
Tacc1	-0.17	1.00
Trmt10a	-0.18	1.00
Vdac3	-0.22	1.00
Fgfr1op2	-0.12	1.00
Scaf11	-0.28	1.00
Ccdc43	-0.15	1.00
Stk3	-0.20	1.00
Ddx20	-0.05	1.00
Senp8	0.05	1.00
Zfp507	-0.17	1.00

Tbc1d9b	-0.03	1.00
Gpr161	-0.26	1.00
Dis3	0.00	1.00
Ippk	-0.10	1.00
Dapk1	-0.36	1.00
Fbxo9	-0.38	1.00
Crybg3	-0.35	1.00
Rae1	-0.32	1.00
Sesn3	-0.21	1.00
Echdc3	0.00	1.00
Uspl1	-0.03	1.00
Spata7	-0.27	1.00
Flnb	-0.07	1.00
Tgif2	-0.02	1.00
Rnft1	-0.09	1.00
Fpgt	0.06	1.00
Ctdspl2	-0.21	1.00
Cd9	-0.10	1.00
Acad9	-0.21	1.00
Uchl1	0.05	1.00
Rabif	-0.05	1.00
Osbpl5	-0.29	1.00
Trmu	-0.04	1.00
Tjp1	-0.21	1.00
Wnt3a	-0.09	1.00
Psmg2	-0.16	1.00
Zfp346	0.07	1.00
Phyh	-0.23	1.00
Pcmt1	-0.32	1.00
Cdk19	-0.28	1.00
Nuf2	-0.08	1.00
Rnh1	-0.15	1.00
Zdhhc16	0.02	1.00
Sar1a	-0.19	1.00
Ppm1f	-0.20	1.00
Dync1i1	0.00	1.00
Csnk1a1	-0.08	1.00
Sik2	-0.50	1.00
Zfp830	-0.25	1.00
Git2	-0.05	1.00
Tmem14a	-0.11	1.00
Yod1	-0.02	1.00
Ctsh	-0.28	1.00
Klhdc3	-0.01	1.00
Nfatc1	-0.54	1.00
Atp6v1c1	-0.36	1.00

Phf14	-0.25	1.00
Lrig2	-0.04	1.00
Vapb	-0.49	1.00
Nr6a1	-0.09	1.00
Hace1	-0.10	1.00
Lrsam1	0.04	1.00
Kif13b	-0.31	1.00
Nsdhl	-0.19	1.00
Zzef1	-0.43	1.00
Canx	-0.25	1.00
Tspan12	-0.27	1.00
Zfp512	-0.29	1.00
Cdr2	-0.07	1.00
Golm2	-0.30	1.00
Dhx57	0.04	1.00
Tpm3-rs7	-0.25	1.00
Nrbp1	-0.37	1.00
Ndufaf6	0.02	1.00
Pkp4	-0.10	1.00
Ube3b	-0.06	1.00
Wdr3	-0.08	1.00
Ankrd40	-0.03	1.00
Ndfip2	-0.05	1.00
Arglu1	-0.29	1.00
Plpbp	-0.12	1.00
Galnt2	-0.35	1.00
B4galnt3	-0.06	1.00
Rab29	-0.12	1.00
Trmt10c	-0.10	1.00
Eral1	0.01	1.00
Zfyve1	0.03	1.00
Ints12	0.03	1.00
Tbc1d1	-0.33	1.00
Snrpa1	-0.06	1.00
Uba2	-0.17	1.00
Dag1	-0.19	1.00
Atp11b	-0.27	1.00
Sclt1	-0.11	1.00
Bmt2	-0.25	1.00
Vdac2	-0.27	1.00
Vps39	-0.22	1.00
Otud4	-0.22	1.00
Bnc2	-0.15	1.00
Fen1	-0.13	1.00
Nsl1	0.08	1.00
Dut	-0.21	1.00

Bak1	-0.25	1.00
Dsg2	-0.05	1.00
Txlng	-0.21	1.00
Npb	0.01	1.00
Ccdc66	-0.20	1.00
Rfc4	-0.14	1.00
Nap1l4	-0.17	1.00
Dcaf17	0.02	1.00
Thrap3	-0.23	1.00
Usp33	-0.41	1.00
Rchy1	-0.02	1.00
Eif3d	-0.17	1.00
Septin7	-0.39	1.00
Bche	-0.66	1.00
Ubxn2a	0.04	1.00
Shank2	-0.15	1.00
Fam171a1	-0.32	1.00
Adcy9	-0.59	1.00
Srrm1	-0.27	1.00
Nod1	-0.05	1.00
Dop1a	-0.12	1.00
Rtkn	0.06	1.00
Taok3	-0.38	1.00
Gas8	0.02	1.00
Npnt	-0.44	1.00
Immt	-0.11	1.00
C1d	-0.03	1.00
Xpo5	0.02	1.00
Kbtbd4	-0.09	1.00
Txlna	-0.12	1.00
Xpnpep1	-0.10	1.00
Nedd9	-0.28	1.00
Tesk2	-0.13	1.00
Akr1a1	-0.04	1.00
Cutc	-0.03	1.00
Prdm16	-0.13	1.00
Cdc27	-0.31	1.00
Gnpnat1	0.03	1.00
Lap3	0.07	1.00
Ttc4	0.00	1.00
Haus8	-0.22	1.00
Lman1	-0.37	1.00
Fgd6	-0.09	1.00
Srsf6	-0.23	1.00
B3galnt2	-0.05	1.00
Epb41l1	-0.21	1.00

Terf1	0.00	1.00
Tmem184b	-0.17	1.00
Ccdc149	-0.15	1.00
Idh2	-0.09	1.00
Epm2a	-0.07	1.00
Nemp2	0.01	1.00
Rab40b	-0.05	1.00
Dhx15	-0.16	1.00
Fam185a	-0.10	1.00
Gorasp1	-0.11	1.00
Zc3h13	-0.24	1.00
Abcc1	-0.30	1.00
Usp24	-0.32	1.00
Rasa2	-0.64	1.00
Ddx17	-0.21	1.00
Usp4	-0.06	1.00
Cdc25a	-0.35	1.00
Rabl3	-0.06	1.00
Ppp2r5d	-0.09	1.00
Lyrm1	-0.09	1.00
Whamm	0.00	1.00
Cep170b	-0.29	1.00
St3gal4	0.00	1.00
Cfap36	-0.14	1.00
Slc35e4	-0.09	1.00
Hs6st3	0.02	1.00
Tom1	-0.08	1.00
Pex13	-0.20	1.00
Phf3	-0.09	1.00
Rbpms	-0.09	1.00
Secisbp2l	-0.17	1.00
Rnpc3	-0.10	1.00
Vps53	-0.10	1.00
Bahd1	-0.08	1.00
Pgbd5	-0.14	1.00
Hectd2	0.00	1.00
Sf3b1	-0.27	1.00
Btbd2	-0.11	1.00
Sepsecs	-0.36	1.00
Unk	-0.36	1.00
Ywhaz	-0.16	1.00
Psmd5	-0.05	1.00
Wdtc1	0.06	1.00
Gemin2	0.10	1.00
Plin2	-0.25	1.00
Spata6	0.03	1.00

Trim9	-0.06	1.00
Uri1	-0.25	1.00
Fam118a	-0.10	1.00
Dnajc24	0.04	1.00
Acat3	-0.52	1.00
Slc15a4	-0.44	1.00
Cish	-0.29	1.00
Epdr1	-0.10	1.00
Wdr17	-0.09	1.00
Brox	0.00	1.00
Srm	-0.07	1.00
Wdr55	-0.25	1.00
Ormdl1	-0.21	1.00
Rab5a	-0.43	1.00
Gramd3	-0.24	1.00
Gdap2	-0.07	1.00
Akap13	-0.21	1.00
Grsf1	-0.39	1.00
F3	-0.51	1.00
Cacnb2	0.07	1.00
Clcn4	0.01	1.00
Rmi1	-0.15	1.00
Eif3h	-0.23	1.00
Bcl2l13	-0.12	1.00
Aasdh	-0.05	1.00
Snrnp35	-0.25	1.00
Tdg	-0.20	1.00
Ttc28	-0.33	1.00
Pla2g4a	-0.85	1.00
Pafah1b1	-0.31	1.00
Snx21	0.03	1.00
Jmjd1c	-0.32	1.00
Crppa	-0.03	1.00
Terf2	-0.04	1.00
Irf1	-0.58	1.00
Atp2b1	-0.31	1.00
Ddx46	-0.23	1.00
Usp8	-0.25	1.00
Prickle2	-0.25	1.00
Heatr6	-0.03	1.00
Ccne1	0.03	1.00
Pus1	-0.03	1.00
Btbd3	-0.06	1.00
Kif26b	-0.04	1.00
Capn2	-0.32	1.00
Rbpj	-0.24	1.00

Phf20	-0.25	1.00
Srp54a	-0.18	1.00
Aspm	-0.01	1.00
Rai1	-0.23	1.00
Irf2	-0.38	1.00
Gng12	-0.02	1.00
Ccnt2	-0.16	1.00
Cisd1	-0.30	1.00
Tbc1d31	-0.10	1.00
Prss23	-0.44	1.00
Camk1d	-0.58	1.00
Otulin	-0.08	1.00
Hsdl1	-0.19	1.00
Parp4	-0.43	1.00
Btaf1	-0.22	1.00
Dpy19l4	-0.17	1.00
Cct3	-0.25	1.00
Rala	-0.29	1.00
Phf10	0.05	1.00
Ube2g2	-0.04	1.00
Tk2	-0.24	1.00
Ophn1	-0.08	1.00
Usp22	-0.34	1.00
Uimc1	-0.07	1.00
Wwc1	-0.29	1.00
Dyrk1a	-0.21	1.00
Ralgapa2	-0.41	1.00
Ak3	-0.22	1.00
Ubox5	0.06	1.00
Eif1b	-0.18	1.00
Zw10	-0.12	1.00
Cgrrf1	-0.13	1.00
Gemin4	-0.02	1.00
Cryz	-0.06	1.00
Fam102b	-0.76	1.00
Phldb2	-0.30	1.00
Brd7	-0.23	1.00
Trim59	-0.33	1.00
Eif3m	-0.34	1.00
Tiam1	-0.50	1.00
Amd2	-0.30	1.00
Sh2b3	-0.46	1.00
Ufl1	-0.13	1.00
Thap11	0.03	1.00
Tsc1	-0.22	1.00
Ints1	0.06	1.00

Ahcyl2	-0.06	1.00
Gse1	-0.27	1.00
Cpne1	-0.43	1.00
Prdm10	-0.03	1.00
Ddx59	0.03	1.00
Tk1	-0.05	1.00
Abca2	0.01	1.00
Gas6	-0.18	1.00
Lmcd1	-0.18	1.00
Slc2a1	-0.28	1.00
Zer1	-0.02	1.00
Cd276	-0.14	1.00
Sult4a1	-0.07	1.00
Snx1	0.05	1.00
Otud7a	-0.05	1.00
Orc3	-0.01	1.00
Kdm1b	-0.02	1.00
Btbd7	-0.06	1.00
Xkr8	0.03	1.00
Arl4a	-0.43	1.00
Ruvbl1	-0.08	1.00
Hgs	-0.07	1.00
Smg7	-0.26	1.00
Washc5	0.03	1.00
Pnrc2	-0.20	1.00
Scyl3	0.01	1.00
Galk2	-0.02	1.00
Ncdn	-0.11	1.00
Nit2	-0.06	1.00
Spg20	-0.08	1.00
Armc6	-0.05	1.00
Gcfc2	-0.16	1.00
Arfrp1	-0.05	1.00
Cdc23	-0.01	1.00
Rab14	-0.24	1.00
Zbed4	-0.16	1.00
Rlf	-0.54	1.00
Appl1	-0.27	1.00
Ttll1	-0.06	1.00
Klhdc2	0.04	1.00
Cdca4	0.12	1.00
Atp5j2	-0.31	1.00
Nbr1	-0.29	1.00
Ankrd10	-0.26	1.00
Dcun1d2	-0.25	1.00
Snx5	-0.09	1.00

Slc27a4	0.06	1.00
Naa30	-0.48	1.00
Uba6	-0.37	1.00
Pard6g	-0.09	1.00
Ctnna1	-0.14	1.00
Plce1	0.02	1.00
Sfmbt1	-0.07	1.00
Zbtb37	-0.18	1.00
Tec	-0.17	1.00
AF366264	-0.09	1.00
Bdh2	-0.08	1.00
Pibf1	0.03	1.00
Tlk2	-0.41	1.00
Zfp839	0.05	1.00
Tmed2	-0.42	1.00
Lonp2	-0.32	1.00
Sar1b	-0.15	1.00
Pdcd5	-0.09	1.00
Parp11	-0.03	1.00
Kctd7	-0.05	1.00
Cant1	-0.01	1.00
Ttc32	-0.07	1.00
Tcea2	-0.10	1.00
Rtca	-0.44	1.00
Prkar1b	0.05	1.00
Nup54	0.03	1.00
Pgpep1l	0.05	1.00
Chd5	0.05	1.00
Cipc	-0.10	1.00
Ubac2	-0.42	1.00
Plgrkt	-0.33	1.00
Eps15	-0.35	1.00
Boc	-0.21	1.00
Nfs1	-0.08	1.00
Nup50	-0.21	1.00
Nfatc3	-0.29	1.00
Ddx28	-0.01	1.00
Rasgrf2	0.39	1.00
Nadk2	-0.02	1.00
Hnrnpa3	-0.07	1.00
Fbxl18	-0.08	1.00
Eme1	-0.13	1.00
Zdhhc23	-0.25	1.00
Wnt16	-0.27	1.00
Taf12	-0.13	1.00
Pitpnm3	-0.25	1.00

Nup214	-0.11	1.00
Cisd2	-0.15	1.00
Cenph	-0.10	1.00
Poc1b	-0.22	1.00
Pan2	0.05	1.00
Usp14	-0.04	1.00
Fundc1	-0.15	1.00
Rbl2	-0.50	1.00
Gucd1	-0.28	1.00
Dnajb2	0.02	1.00
Fadd	0.04	1.00
Top3b	-0.20	1.00
Wee1	-0.33	1.00
Rab39b	-0.11	1.00
Dnaja2	-0.27	1.00
Arv1	0.02	1.00
Tubgcp6	-0.34	1.00
Polr3e	-0.12	1.00
Sgsm2	0.00	1.00
Ctps2	-0.04	1.00
Ndufaf5	0.07	1.00
Fem1b	-0.31	1.00
Lcmt1	-0.08	1.00
Tbc1d23	-0.07	1.00
Plrg1	-0.11	1.00
Glrx5	-0.29	1.00
Asb1	-0.02	1.00
Arfip1	-0.37	1.00
Rab33b	-0.24	1.00
Thsd4	-0.16	1.00
Trpm7	-0.49	1.00
Grpel1	-0.10	1.00
Trim37	0.04	1.00
Rufy2	-0.31	1.00
Napepld	-0.09	1.00
Polr3a	-0.11	1.00
Parp1	-0.28	1.00
Gfod1	-0.04	1.00
Cracr2a	0.00	1.00
Crym	-0.04	1.00
Rab11a	0.04	1.00
Sparc	-0.59	1.00
Rab12	-0.41	1.00
Ccdc167	-0.09	1.00
Cstf1	-0.06	1.00
Spring1	-0.06	1.00

Tmem38a	-0.10	1.00
Rnf123	0.06	1.00
Slc44a3	-0.16	1.00
Dtd2	-0.10	1.00
Zfp217	-0.30	1.00
Bag5	-0.22	1.00
Odr4	-0.16	1.00
Atp1b3	-0.29	1.00
Katnb1	0.03	1.00
St3gal2	-0.03	1.00
Gabpb1	-0.09	1.00
Tpcn1	-0.16	1.00
Copb2	-0.33	1.00
Ranbp10	-0.13	1.00
Yae1d1	0.09	1.00
Sema3c	-0.19	1.00
Wdr25	-0.02	1.00
Dync1li2	-0.27	1.00
Asxl1	-0.03	1.00
Rell1	-0.36	1.00
Dnm1l	-0.07	1.00
Adssl1	-0.05	1.00
Fig4	-0.06	1.00
Etf1	-0.17	1.00
Coq10b	0.04	1.00
Eif5b	-0.21	1.00
Cldn1	-0.28	1.00
Tnfrsf21	-0.17	1.00
Atxn2	-0.24	1.00
Htatsf1	-0.27	1.00
Pex7	-0.02	1.00
Hsf1	-0.09	1.00
Vezt	-0.12	1.00
Gpn2	-0.05	1.00
Chfr	-0.11	1.00
Prpf4b	-0.22	1.00
Chac2	-0.12	1.00
Pusl1	-0.04	1.00
Lyn	-0.44	1.00
Galnt18	-0.14	1.00
Ank3	-0.25	1.00
Usp6nl	-0.14	1.00
Cnot1	-0.13	1.00
Add1	-0.26	1.00
Chek2	-0.03	1.00
Dhcr24	-0.42	1.00

Tfam	-0.31	1.00
Sema3a	-0.13	1.00
Scnn1b	-0.18	1.00
Wdr7	-0.18	1.00
Hnrnpr	-0.17	1.00
Sugp2	-0.30	1.00
Crnkl1	-0.21	1.00
Sass6	-0.44	1.00
Eif2b1	-0.05	1.00
Tbc1d8b	-0.20	1.00
Ift27	-0.09	1.00
Golga4	-0.34	1.00
Armc2	-0.20	1.00
Nup160	-0.06	1.00
Rcc2	-0.05	1.00
Dgke	-0.03	1.00
Nsun2	-0.08	1.00
Ppp6r2	-0.25	1.00
Fnip2	-0.47	1.00
Clptm1l	-0.23	1.00
Dph7	-0.13	1.00
Tbc1d2b	-0.02	1.00
Rergl	-0.32	1.00
Cyld	-0.16	1.00
Gstz1	-0.12	1.00
Zdhhc12	-0.10	1.00
Bnip2	-0.38	1.00
Ctr9	-0.27	1.00
Umps	0.00	1.00
Wdr4	-0.33	1.00
Zbtb41	-0.06	1.00
Sec24b	-0.17	1.00
Fkbp4	-0.39	1.00
Vta1	-0.50	1.00
Cxadr	-0.30	1.00
Zbtb8a	-0.07	1.00
Itpr3	-0.11	1.00
Kat6a	-0.27	1.00
Nom1	-0.01	1.00
Ctdp1	-0.20	1.00
Ghitm	-0.27	1.00
Npepl1	-0.12	1.00
Fbxl22	-0.21	1.00
Pex1	-0.08	1.00
Golga1	-0.32	1.00
Pip4k2b	-0.06	1.00

Pex5	0.02	1.00
Pip4k2a	-0.62	1.00
Vps11	-0.14	1.00
Twf1	0.01	1.00
Dph3	-0.24	1.00
Ranbp1	-0.21	1.00
Wrn	-0.32	1.00
Serbp1	-0.13	1.00
Prkar1a	-0.17	1.00
Eloc	-0.38	1.00
Rnf121	-0.14	1.00
2510039O18Rik	-0.19	1.00
Fbxo30	-0.27	1.00
Rab9	-0.09	1.00
Ppcs	-0.05	1.00
Ifnar1	-0.71	1.00
Ovol2	-0.01	1.00
Golga3	-0.35	1.00
Aff2	-0.63	1.00
Fam83h	-0.22	1.00
Cnn3	-0.39	1.00
Smarca1	-0.11	1.00
Lig4	0.02	1.00
Crim1	-0.22	1.00
Rpusd1	0.03	1.00
Vdac1	-0.27	1.00
Svil	-0.26	1.00
Fam168b	-0.30	1.00
Dync1i2	-0.28	1.00
Rars	-0.20	1.00
Stx7	-0.16	1.00
Esco1	-0.25	1.00
Gps1	-0.11	1.00
Steep1	-0.36	1.00
Sh3pxd2a	-0.41	1.00
Elmo2	-0.15	1.00
Hdhd5	0.02	1.00
Eif4h	-0.27	1.00
Zeb2	-0.09	1.00
Akap10	-0.43	1.00
Vcpip1	-0.40	1.00
Stradb	-0.05	1.00
Birc6	-0.19	1.00
Depdc1b	-0.68	1.00
Stk17b	-0.48	1.00
Tor1b	-0.08	1.00

Pdgfc	-0.23	1.00
Aldh5a1	-0.11	1.00
Camsap2	-0.41	1.00
Dgkh	-0.30	1.00
Srp14	-0.27	1.00
Exog	-0.16	1.00
Bhlhe41	-0.07	1.00
Camk2g	-0.40	1.00
Osbpl8	-0.27	1.00
Klhl11	-0.01	1.00
Ankfy1	-0.47	1.00
Itfg2	-0.10	1.00
Bptf	-0.22	1.00
Thoc2	-0.22	1.00
Fam168a	-0.08	1.00
Fbxo7	-0.09	1.00
Ssbp3	-0.31	1.00
Ubac1	-0.04	1.00
Ipo11	-0.19	1.00
Sirt2	0.04	1.00
Cadm1	-0.49	1.00
Ero1b	-0.20	1.00
Kat14	-0.12	1.00
Kctd9	-0.17	1.00
Usp5	-0.02	1.00
Ptpn2	-0.35	1.00
Selenok	-0.19	1.00
Ap1b1	-0.01	1.00
Bloc1s2	-0.11	1.00
Tln2	-0.22	1.00
Usp30	0.01	1.00
Rnf126	-0.04	1.00
Hras	-0.09	1.00
Gtf2e2	-0.04	1.00
Nsmce4a	0.07	1.00
Ablim2	-0.07	1.00
Samd11	-0.11	1.00
Ipo5	-0.37	1.00
Stk38	-0.23	1.00
Fut11	-0.03	1.00
Klhdc10	-0.10	1.00
Stim1	-0.09	1.00
Ppp4r1	-0.27	1.00
Kyat3	0.00	1.00
Ankrd11	-0.14	1.00
Spen	-0.18	1.00

Emp2	-0.17	1.00
5730480H06Rik	0.03	1.00
Hspa5	-0.11	1.00
Traf1	-0.12	1.00
Sart3	-0.25	1.00
Snapc4	-0.10	1.00
Slc25a43	-0.12	1.00
Atad1	-0.11	1.00
Xrn1	-0.24	1.00
Tmem51	-0.01	1.00
Tbc1d4	-0.36	1.00
Lrrc47	-0.12	1.00
Rmnd5a	-0.29	1.00
Lin7c	-0.01	1.00
Cct4	-0.20	1.00
Gsn	-0.19	1.00
Traf5	-0.35	1.00
Ric8b	-0.01	1.00
Szrd1	0.04	1.00
Dusp22	-0.10	1.00
Lrrc59	-0.43	1.00
Nrde2	-0.22	1.00
Cblb	-1.23	1.00
Bod1l	-0.28	1.00
Chmp4c	-0.34	1.00
Chchd3	-0.39	1.00
Filip1l	-0.16	1.00
Dhx30	-0.11	1.00
Anapc7	-0.20	1.00
Poli	-0.06	1.00
Tacc3	-0.10	1.00
Pdgfa	-0.33	1.00
Reps1	-0.18	1.00
Efna4	0.03	1.00
Rap2a	0.01	1.00
Tiprl	-0.07	1.00
Tmem120b	0.03	1.00
Abi2	-0.25	1.00
Abcb7	-0.25	1.00
Btbd1	-0.33	1.00
Stambpl1	-0.06	1.00
Kansl2	-0.12	1.00
Itpr2	-0.27	1.00
Cracdl	-0.22	1.00
Clp1	-0.06	1.00
Gng10	-0.18	1.00

Rpap1	-0.01	1.00
Bach2	-0.39	1.00
Ppp1r21	-0.38	1.00
Wnt7a	-0.49	1.00
Wdsub1	-0.09	1.00
Ubn2	-0.12	1.00
Clock	-0.28	1.00
Ints11	-0.18	1.00
Arid5b	-0.04	1.00
Ncoa6	-0.43	1.00
Rab22a	-0.19	1.00
Ppil4	-0.27	1.00
Vps72	-0.01	1.00
Ppfia1	-0.20	1.00
Slc25a1	0.01	1.00
Gramd4	-0.09	1.00
Trip12	-0.26	1.00
Shoc2	-0.04	1.00
Rad17	-0.18	1.00
Habp4	-0.10	1.00
Zfp518b	-0.25	1.00
Trak2	-0.23	1.00
Hprt	-0.16	1.00
Chic1	-0.14	1.00
Tpd52l2	-0.22	1.00
Pbrm1	-0.26	1.00
Otud6b	-0.28	1.00
Ipo8	-0.15	1.00
Arl5b	-0.10	1.00
Chchd5	-0.13	1.00
Snx11	0.00	1.00
Ranbp3	-0.56	1.00
Ppp2r5a	-0.21	1.00
Yars2	-0.04	1.00
Rbfa	-0.12	1.00
Gnb5	-0.02	1.00
Zfp592	-0.01	1.00
Tcf3	-0.21	1.00
Hemk1	-0.14	1.00
Arnt	-0.22	1.00
Polr1c	-0.08	1.00
Arhgap1	-0.01	1.00
Rbsn	-0.12	1.00
Ing3	-0.32	1.00
Dot1l	-0.16	1.00
Appbp2	-0.18	1.00

Aqp4	-0.12	1.00
Tctn3	-0.01	1.00
Prdx4	-0.30	1.00
Cenpq	-0.09	1.00
Pank4	-0.14	1.00
Sbno1	-0.31	1.00
Grhpr	0.05	1.00
Nono	-0.20	1.00
Snx7	-0.21	1.00
Rab33a	5.40	0.00
Tpt1	5.03	0.00
Ezr	4.32	0.00
App	2.92	0.00
Phip	2.45	0.00
Cldn3	3.39	0.00
Lpp	2.19	0.00
Cnbp	1.97	0.00
Pcf11	1.97	0.00
Slmap	2.17	0.00
Atp5b	2.14	0.00
Txnip	2.00	0.00
Sdcbp	2.34	0.00
Sap18	1.82	0.00
Tent5a	2.54	0.00
Cmip	2.25	0.00
Dido1	1.94	0.00
Sgk1	3.64	0.00
Atp5g3	1.72	0.00
Arpc2	1.78	0.00
Rin2	1.71	0.00
Oga	1.61	0.00
Sytl3	1.83	0.00
Tollip	1.56	0.00
Slirp	1.28	0.00
Ankhd1	1.71	0.00
Slc12a7	1.69	0.00
Atp5k	1.47	0.00
U2surp	1.60	0.00
Plscr1	1.67	0.00
Ccz1	1.20	0.00
Trpc4ap	1.37	0.00
Adss	1.70	0.00
Cars	1.49	0.00
Adnp	1.34	0.00
Cdc37	1.25	0.00
Atf7ip	2.06	0.00

Ccdc25	1.36	0.00
Vcp	1.16	0.00
Tmbim1	1.22	0.00
Tiparp	1.80	0.00
Wtap	1.61	0.00
Eprs	1.25	0.00
Ndufa1	1.25	0.00
Nfib	2.04	0.00
Rgs2	2.44	0.00
Tardbp	1.37	0.00
Kdm3a	1.14	0.00
Adrb2	2.06	0.00
Rnf38	1.69	0.00
Jup	1.34	0.00
Enox2	1.07	0.00
Eif4b	1.17	0.00
Il1r1	1.46	0.00
Syncrip	1.35	0.00
Ccn1	2.74	0.00
Cacybp	1.11	0.00
Cklf	1.15	0.00
Bri3bp	1.07	0.00
Nasp	1.19	0.00
Tdpoz8	1.14	0.00
Pomp	0.96	0.00
Tgfbr1	1.26	0.00
Apmap	1.10	0.00
Cd2ap	1.61	0.00
Snrpd2	1.03	0.00
Pcnp	1.04	0.00
Rnf103	0.89	0.00
Il1rap	1.49	0.00
Golm2	0.84	0.00
Trrap	1.35	0.00
Spcs1	0.93	0.00
Kars	0.83	0.00
Zdhhc20	1.04	0.00
Pcdhgb4	1.20	0.00
Anapc15-ps	0.99	0.00
Atp5c1	0.79	0.00
Strbp	1.23	0.00
Nfic	0.80	0.00
Ncmap	1.12	0.00
Tppp	1.01	0.00
Rhob	1.85	0.00
Sdhc	0.77	0.00

LTO1	0.93	0.00
Inpp4b	1.31	0.00
Dars	1.00	0.00
C1qbp	0.88	0.00
Gadd45b	1.49	0.00
Bccip	0.68	0.00
Strap	0.75	0.00
Lnpep	1.09	0.00
Tars	1.33	0.00
Calu	0.68	0.00
Dsp	1.00	0.00
Tbp	0.90	0.00
Apip	0.75	0.00
Fbxl21	0.74	0.00
Blcap	0.77	0.00
Prlr	1.43	0.00
Pfkp	0.80	0.00
Ahsa2	0.74	0.00
Nars	0.68	0.00
Tut7	0.82	0.00
Banp	1.16	0.00
Brd8	0.64	0.00
Cul4a	0.96	0.00
Tapbpl	0.81	0.00
Wdpcp	2.28	0.00
Dtd1	0.71	0.00
Sec23ip	0.65	0.00
Kifbp	0.79	0.00
Crip2	0.62	0.00
Aktip	0.77	0.00
Csnk1d	0.78	0.00
Sarnp	0.56	0.00
Pfkfb1	0.68	0.00
Eya2	2.11	0.00
Ros1	1.49	0.00
Rad23b	0.99	0.00
Dpp4	0.79	0.00
Sfswap	0.67	0.00
Phax	0.65	0.00
Rtf2	0.67	0.00
Nanp	0.62	0.00
Ccsap	0.86	0.00
Psme3ip1	0.64	0.00
Dctn2	1.05	0.00
Dohh	0.46	0.00
Polr2i	0.70	0.00

Ank3	2.66	0.00
Limk2	1.61	0.00
Ripk4	1.26	0.00
Sorl1	1.17	0.00
Elf3	2.70	0.00
Ptpn13	1.26	0.00
Dtna	1.13	0.00
Cyria	1.38	0.00
Wtip	1.06	0.00
Dedd	0.52	0.00
Gcdh	0.54	0.00
Enpp4	0.65	0.00
Bcas1	1.16	0.00
Vdac1	0.98	0.00
Irf6	0.87	0.00
Tmem163	1.34	0.00
Cited2	1.51	0.00
Ubtf	0.51	0.00
Vim	0.86	0.00
Prep	0.83	0.00
Glrx3	0.51	0.00
Frk	1.70	0.00
Nhs	0.88	0.00
Sema6d	0.82	0.00
Pak1	1.15	0.00
Dagla	0.98	0.00
Sinhcaf	0.63	0.00
Fbh1	0.52	0.00
Haus6	0.84	0.00
Terf2ip	0.54	0.00
Rars	0.65	0.00
Crcp	0.56	0.00
Cnp	0.40	0.00
Gss	0.76	0.00
Ttc29	1.63	0.00
Hnf1b	0.55	0.00
Bace2	1.42	0.00
Esrp2	1.75	0.00
Clmn	0.92	0.00
Ppp4r4	1.60	0.00
Fam110c	0.70	0.00
Erbb2	0.72	0.00
Fmn1	1.04	0.00
Tead1	1.42	0.00
Cdh1	1.36	0.00
B3gnt7	1.19	0.00

Pip5k1b	1.26	0.00
Cobl	1.28	0.00
Aadat	0.53	0.00
Slc16a10	2.08	0.00
Polr1d	0.53	0.00
Rybp	0.73	0.00
Brap	0.49	0.00
Sap30bp	0.64	0.00
Slc25a29	1.32	0.00
C6	0.60	0.00
Kctd14	1.58	0.00
Fkbp5	1.65	0.00
Vopp1	1.35	0.00
Synj2	0.98	0.00
Bivm	0.56	0.00
Smox	1.26	0.00
Slc39a8	1.00	0.00
Dazl	0.61	0.00
Rasd1	2.10	0.00
Trpm6	1.21	0.00
Scd2	1.24	0.00
Plxnb2	2.47	0.00
Loxl1	0.55	0.00
Rbm47	1.50	0.00
Klf15	0.85	0.00
Dsg2	1.05	0.00
Sh3rf1	1.08	0.00
Sh3yl1	1.36	0.00
Lipg	0.84	0.00
Ift57	0.93	0.00
Cog3	0.48	0.00
Gnai1	0.66	0.00
Zfp777	0.35	0.00
Nop56	0.44	0.00
Fbp1	0.56	0.00
Naa20	0.48	0.00
Ivns1abp	0.43	0.00
Atp5j2	0.35	0.00
Asb8	0.34	0.00
Il1b	1.04	0.00
Areg	2.00	0.00
Ephx4	0.91	0.00
C77080	0.67	0.00
Ppl	1.35	0.00
Spag6l	1.65	0.00
Kctd1	0.76	0.00

Baiap2l1	1.75	0.00
Rasef	0.70	0.00
Ankrd6	1.44	0.00
Pawr	0.91	0.00
Dram1	1.09	0.00
Scnn1a	1.06	0.00
Rtn1	0.97	0.00
Uap1	0.75	0.00
Osbpl5	2.04	0.00
Fat1	1.34	0.00
Unc45a	0.59	0.00
Sntb1	1.41	0.00
Sec24d	0.47	0.00
Riok2	0.52	0.00
Spef2	2.22	0.00
Eapp	0.35	0.00
Porcn	0.34	0.00
AU040320	0.79	0.00
Plch2	0.85	0.00
Cdcp1	0.50	0.00
Tjp3	0.66	0.00
Golm1	1.10	0.00
Wwc1	0.95	0.00
Garem1	1.37	0.00
St14	0.82	0.00
Rnf128	1.32	0.00
Fam3b	0.82	0.00
Pde9a	0.61	0.00
Got2	0.59	0.00
Rab3b	1.23	0.00
Utp20	1.26	0.00
Cxadr	1.24	0.00
Chst9	1.86	0.00
Tc2n	0.96	0.00
Serinc5	0.86	0.00
Alpk1	1.72	0.00
Ech1	0.94	0.00
Rsph9	1.40	0.00
Alpk3	0.71	0.00
Epcam	1.65	0.00
Crtap	0.45	0.00
Vipr1	0.72	0.00
Ttl	0.56	0.00
Idh3b	0.31	0.00
Cdkn2aip	0.44	0.00
Gskip	0.33	0.00

Cp	1.73	0.00
Aars	0.37	0.00
Antkmt	0.32	0.00
Ifitm10	1.34	0.00
Efcab7	0.51	0.00
Ccdc85c	1.53	0.00
Zadh2	0.55	0.00
Hbegf	1.41	0.00
Ghrhr	1.66	0.00
Cpne8	0.97	0.00
Fcamr	1.34	0.00
Bckdhb	1.00	0.00
Itgb8	1.27	0.00
Ano6	1.14	0.00
Cln5	0.72	0.00
Lgi3	1.91	0.00
C3	2.46	0.00
Slc35f5	1.16	0.00
Ftcd	0.98	0.00
Shisa9	2.48	0.00
Efhd2	0.66	0.00
Chka	1.34	0.00
Cldn1	2.28	0.00
Tmem255b	0.64	0.00
Kcnk2	0.83	0.00
Ffar4	0.36	0.00
Abhd3	1.01	0.00
Arhgap39	1.24	0.00
Tdrp	0.30	0.00
Klf8	0.42	0.00
Lhpp	0.35	0.00
Arl2bp	0.30	0.00
Tirap	0.31	0.00
Hinfp	0.56	0.00
Yars	0.33	0.00
Rnpep	0.39	0.00
Khdc4	0.49	0.00
Armc3	1.50	0.00
Pip5k1c	0.70	0.00
Sh3bgrl2	1.60	0.00
Bdnf	1.86	0.00
Abl1	0.45	0.00
Nus1	0.61	0.00
Tpd52	0.73	0.00
Tmem41b	0.43	0.00
Kcnip4	1.57	0.00

Gpr137c	0.74	0.00
Jdp2	1.45	0.00
Gfpt2	1.14	0.00
Vash2	0.59	0.00
Tmprss2	0.71	0.00
Atp9a	0.99	0.00
Atp8b1	0.43	0.00
Nkx2-1	0.99	0.00
Ptprk	2.66	0.00
Lrp6	1.30	0.00
Cbx7	0.75	0.00
Hnf4g	0.98	0.00
Lrrk2	0.79	0.00
Gnpnat1	0.58	0.00
Golph3l	0.70	0.00
Arfgef3	0.97	0.00
Syt8	0.82	0.00
Spats2l	0.98	0.00
Itga8	0.44	0.00
C8a	0.75	0.00
Hagh	0.69	0.00
Frrs1l	0.31	0.00
Atp1b1	2.15	0.00
Eva1c	1.14	0.00
Sh3pxd2b	0.71	0.00
Slc9a7	1.04	0.00
Heph	0.69	0.00
Tert	1.41	0.00
Kcnma1	2.51	0.00
Plekha5	1.37	0.00
Slc38a1	0.79	0.00
Gpt2	0.58	0.00
Gpam	0.84	0.00
Sema5b	1.54	0.00
Necab1	0.45	0.00
Rsph1	1.12	0.00
Ccdc58	0.34	0.00
Tent4a	0.62	0.00
Eif2b4	0.27	0.00
Rbmx2	0.29	0.00
Itprip	0.37	0.00
Qars	0.22	0.00
Sntn	1.69	0.00
Trpv2	0.32	0.00
Psmc3ip	0.37	0.00
Tmem79	0.43	0.00

Irx5	0.66	0.00
Notch2	1.18	0.00
Nadk	0.97	0.00
Cfd	-5.41	0.00
Got1	0.46	0.00
Coro2a	0.63	0.00
Slc41a1	1.26	0.00
Ttll12	0.66	0.00
Fam210b	0.64	0.00
Aldh1a1	2.52	0.00
Cks2	0.79	0.00
Lmo4	0.95	0.00
Tor4a	1.00	0.00
Dusp16	1.00	0.00
Prkca	0.83	0.00
Sqle	1.18	0.00
Ugt8a	1.11	0.00
Vwa1	0.58	0.00
Aig1	0.89	0.00
Ccdc148	1.03	0.00
Kif6	2.32	0.00
Neurl1a	1.20	0.00
Galnt3	0.79	0.00
Hsf2	0.57	0.00
Bhlhe41	0.67	0.00
Fam83b	0.73	0.00
Sync	0.44	0.00
Ccnd2	0.78	0.00
Bspry	0.43	0.00
Ctbs	0.33	0.00
Eaf2	0.38	0.00
Tppp3	1.05	0.00
Flrt3	0.76	0.00
Tox3	0.92	0.00
Ptprf	1.91	0.00
Ift122	1.33	0.00
Zmynd12	0.68	0.00
Glis1	2.39	0.00
Pdgfa	0.87	0.00
Brdt	0.63	0.00
Ttc26	0.90	0.00
Gse1	0.85	0.00
Ugp2	1.07	0.00
Arg2	1.00	0.00
Npas2	0.79	0.00
Tbc1d8	0.72	0.00

Cds1	0.59	0.00
Srebf2	0.76	0.00
Snx27	0.63	0.00
Tenm2	0.59	0.00
Plce1	0.86	0.00
Agrn	0.96	0.00
Ppargc1a	2.66	0.00
Ddx60	1.26	0.00
Tub	0.96	0.00
Slc7a3	0.42	0.00
F3	1.50	0.00
Ikbip	0.31	0.00
Slc25a17	0.51	0.00
Gpat3	0.71	0.00
Nrap	0.58	0.00
Paip2	0.26	0.00
Sars	0.35	0.00
Stambp	0.26	0.00
Cherp	0.29	0.00
Trappc5	0.28	0.00
Itpr2	1.40	0.00
Pgap1	0.42	0.00
Hspb11	0.86	0.00
Kctd19	0.59	0.00
Rnf19b	0.60	0.00
Togaram1	1.24	0.00
Cep126	1.48	0.00
Wnt5a	0.68	0.00
Tmem229b	0.70	0.00
Tln2	1.12	0.00
Ptpn3	1.39	0.00
Ugdh	0.53	0.00
Dnah5	2.56	0.00
Rgma	0.42	0.00
Fat3	1.41	0.00
Anxa7	0.57	0.00
Plagl1	0.49	0.00
Tmem248	0.79	0.00
Sc5d	0.67	0.00
Irx2	0.55	0.00
Nlk	0.76	0.00
Taf15	0.43	0.00
Tbc1d19	0.55	0.00
Dag1	0.74	0.00
Etv1	0.90	0.00
Slc16a2	0.36	0.00

Rhobtb2	0.81	0.00
Sh3glb2	0.34	0.00
Ctsh	0.64	0.00
Slc25a22	0.40	0.00
Nipal2	0.48	0.00
Pard6b	0.99	0.00
Slc1a4	0.71	0.00
Pdlim4	0.44	0.00
Dhcr24	0.69	0.00
Gpr39	1.35	0.00
Patj	0.78	0.00
Rhov	0.74	0.00
Pcnx4	0.44	0.00
Gcat	0.35	0.00
Piezo2	1.54	0.00
Tecpr1	0.42	0.00
Bbs2	0.46	0.00
Psmd3	0.32	0.00
Retsat	0.52	0.00
Tsen2	0.70	0.00
Prdx3	0.66	0.00
Insig2	0.74	0.00
Anxa2	2.38	0.00
Tmem255a	0.61	0.00
Rab20	0.77	0.00
Ddah1	0.62	0.00
Wnt11	0.48	0.00
Bend5	2.20	0.00
Pdxk	1.01	0.00
Dthd1	1.68	0.00
Gpc4	0.77	0.00
Tom1l1	0.52	0.00
Ttc21b	0.53	0.00
Fbxo25	0.40	0.00
Carmil1	1.20	0.00
Rftn1	1.00	0.00
Il1rapl2	1.32	0.00
Phlda2	0.51	0.00
Fam3c	0.76	0.00
Lgr4	1.19	0.00
Tmem51	0.55	0.00
Acaca	0.91	0.00
Lin7a	0.59	0.00
Cfap221	1.28	0.00
Ppip5k2	0.71	0.00
Ptk2b	0.48	0.00

Prrg4	0.44	0.00
Ctso	0.36	0.00
Enkur	1.16	0.00
Naaladl2	1.88	0.00
Tmem67	0.55	0.00
Armc9	0.80	0.00
Plcxd1	0.51	0.00
Rab9	0.37	0.00
Rxylt1	0.26	0.00
Lrguk	1.11	0.00
Pip4p2	0.42	0.00
Polr1f	0.29	0.00
Agbl5	0.30	0.00
Utp25	0.73	0.00
Slc4a1ap	0.28	0.00
Aifm1	0.21	0.00
Aamp	0.33	0.00
Sfxn3	0.25	0.00
Dnmbp	0.26	0.00
Niban1	0.50	0.00
Dzip1l	0.58	0.00
Ilkap	0.22	0.00
Cntn4	0.38	0.00
Hars	0.20	0.00
Dab2ip	0.47	0.00
Trim28	0.28	0.00
Nr2c2ap	0.27	0.00
Tomm40l	0.31	0.00
Snx19	0.54	0.00
Cat	0.54	0.00
Spag16	1.04	0.00
Vsig10	0.94	0.00
Tnfrsf19	0.62	0.00
Rab36	0.92	0.00
Nefl	0.66	0.00
Crlf1	0.40	0.00
Igf1r	1.68	0.00
Kif21a	0.96	0.00
Klf5	1.32	0.00
Jag2	1.41	0.00
Il22ra1	0.70	0.00
Slc2a12	1.19	0.00
Tmem35b	0.51	0.00
Tmem63b	0.71	0.00
Rab25	0.35	0.00
Crebl2	0.84	0.00

Yes1	0.47	0.00
Cfap44	1.04	0.00
Lzts3	0.40	0.00
B4galt5	0.72	0.00
Ubac2	0.87	0.00
Galns	0.79	0.00
Pcbd1	0.34	0.00
Atp6v0d1	0.39	0.00
Adgrg2	1.36	0.00
Pag1	0.74	0.00
Bcat1	0.75	0.00
Clint1	1.11	0.00
Abhd17c	0.48	0.00
Tbc1d16	0.48	0.00
Slc25a43	0.38	0.00
Acsl3	0.54	0.00
Ptp4a3	0.59	0.00
Laptm4b	0.64	0.00
Hey2	0.39	0.00
Ephb3	0.53	0.00
Sod2	0.49	0.00
Gng4	0.63	0.00
Prox1	0.79	0.00
Aldh6a1	0.45	0.00
Ppil6	1.05	0.00
Adamts15	0.37	0.00
Prag1	0.54	0.00
Hexa	0.29	0.00
Wnt16	0.89	0.00
Trim9	0.33	0.00
Usf3	0.50	0.00
Lrrc56	0.75	0.00
Dzank1	1.12	0.00
Ccdc88c	1.59	0.00
Il18	0.63	0.00
Park7	0.39	0.00
Esd	0.46	0.00
Kank1	0.37	0.00
Npy1r	0.45	0.00
Foxp4	0.69	0.00
Cep164	0.82	0.00
Ctnnbip1	0.40	0.00
Tmem50b	0.63	0.00
Nhsl1	0.63	0.00
Rpia	0.66	0.00
Ric8b	0.61	0.00

Xdh	0.79	0.00
Nudt14	0.39	0.00
Ero1a	0.30	0.00
Gas7	1.13	0.00
Ccl20	2.30	0.00
Plek2	0.62	0.00
Zdhhc13	0.43	0.00
Tctn1	0.31	0.00
Dhrs7b	0.47	0.00
Lonrf3	1.39	0.00
Tfb1m	0.32	0.00
Rragd	0.48	0.00
Slc25a36	0.96	0.00
Abhd2	0.41	0.00
Aass	0.59	0.00
Ptprm	-3.07	0.00
Ribc2	2.06	0.00
Rfx2	1.00	0.00
Bpnt1	0.37	0.00
Ccdc191	0.60	0.00
Prkag2	0.46	0.00
Slc52a3	0.30	0.00
Sema4b	0.28	0.00
Borcs5	0.90	0.00
Ndufa8	0.29	0.00
9530077C05Rik	0.32	0.00
Wnt7b	0.69	0.00
Creld2	0.48	0.00
Eci1	0.47	0.00
Psen2	0.42	0.00
Cbr4	0.50	0.00
Herpud1	0.43	0.00
4932411N23Rik	-4.30	0.00
Snx30	0.45	0.00
Ift46	0.53	0.00
Tmtc4	0.52	0.00
Ypel2	0.62	0.00
Nek10	2.54	0.00
Dio2	0.73	0.00
Sdf2l1	0.49	0.00
Ccm2	0.20	0.00
Gars	0.20	0.00
Fzd3	0.61	0.00
Creb3	0.27	0.00
Lrr1	0.21	0.00
Dnpep	0.23	0.00

Pnpo	0.21	0.00
Prnp	0.40	0.00
Adam9	0.29	0.00
Stambpl1	0.38	0.00
Fbxo16	0.72	0.00
Ccdc78	0.78	0.00
Epas1	-2.72	0.00
Pcdh15	2.04	0.00
Eny2	0.33	0.00
Farsb	0.56	0.00
Cfap99	1.64	0.00
Saxo2	1.01	0.00
Stx7	0.62	0.00
Dnase2b	0.32	0.00
Kifap3	1.09	0.00
Tlcd1	1.28	0.00
Adcy6	0.48	0.00
Lzic	0.26	0.00
Ptgfrn	0.60	0.00
Hmox1	0.73	0.00
Pyroxd1	0.61	0.00
Tpst1	0.61	0.00
Kiz	0.55	0.00
Ergic2	0.58	0.00
Nbea	1.13	0.00
Abl2	0.89	0.00
Cfap36	0.61	0.00
Dock5	1.37	0.00
Erp44	0.49	0.00
Runx1	1.67	0.00
Pdxdc1	0.49	0.00
Frmd4b	0.92	0.00
Odc1	0.72	0.00
Gnptab	0.78	0.00
Cnksr1	0.33	0.00
Igfbp7	-3.09	0.00
Prpf8	0.35	0.00
Fbxo31	0.50	0.00
Idh1	0.57	0.00
Gch1	0.83	0.00
Capn7	0.60	0.00
Rnh1	0.61	0.00
Sorbs2	1.13	0.00
Cadm1	1.90	0.00
Esrp1	0.48	0.00
Selenoi	0.34	0.00

Dlg3	0.36	0.00
Plin2	0.75	0.00
Pcdh1	0.65	0.00
Lzts2	0.28	0.00
Lama3	0.69	0.00
Sh2d4a	0.52	0.00
Rnf182	1.10	0.00
Esyt3	1.15	0.00
Eno4	0.99	0.00
Rhpn1	0.42	0.00
Prkcz	0.61	0.00
Scnn1b	0.43	0.00
Lgals3	0.92	0.00
Raly	0.38	0.00
Atp12a	1.71	0.00
Zdhhc7	0.69	0.00
Shroom3	0.96	0.00
Cidea	1.12	0.00
Slc16a5	0.57	0.00
Fam214a	0.50	0.00
Nhp2	0.32	0.00
Pgam1	0.45	0.00
Slc44a5	0.69	0.00
Fuca2	0.24	0.00
Fbxo30	0.30	0.00
Sppl2a	0.37	0.00
Eci3	0.87	0.00
Itch	0.29	0.00
Ntng1	1.02	0.00
Tox2	0.93	0.00
Zdhhc9	0.33	0.00
Rxra	0.46	0.00
Pecam1	-3.68	0.00
Hmces	0.37	0.00
Ttbk2	1.24	0.00
Sox5	1.44	0.00
Abcd3	0.49	0.00
Tctn3	0.82	0.00
Uhrf1bp1l	0.59	0.00
Acox2	0.29	0.00
Tmem106b	0.47	0.00
Sdhaf2	0.32	0.00
Clic5	1.13	0.00
Tti1	0.20	0.00
Calcrl	-4.03	0.00
Sybu	0.51	0.00

Abr	0.35	0.00
Sdc1	0.75	0.00
Fam107a	0.24	0.00
Kcnt2	0.60	0.00
Ano1	0.99	0.00
Rgmb	0.38	0.00
Hspa4l	0.37	0.00
Wipi1	0.39	0.00
B3gnt5	0.46	0.00
Rab11fip4	0.75	0.00
Acsl5	0.37	0.00
Tmem116	0.65	0.00
Wee1	0.44	0.00
Taok3	0.29	0.00
Shtn1	1.15	0.00
Atp6v0b	0.27	0.00
Aspg	0.56	0.00
Sccpdh	0.26	0.00
Scp2	0.38	0.00
Abcc5	0.47	0.00
Tmem222	0.27	0.00
Pi4k2b	0.44	0.00
Noc4l	0.25	0.00
Slc40a1	0.87	0.00
Ccdc65	0.75	0.00
Rab23	0.27	0.00
Pop5	0.33	0.00
Kif9	0.73	0.00
Nfs1	0.14	0.00
Grb10	0.56	0.00
Aprt	0.47	0.00
Ssr2	0.21	0.00
Ppcs	0.36	0.00
Irf2bpl	1.27	0.00
Cc2d1b	0.50	0.00
Ctsz	0.74	0.00
Klf10	0.82	0.00
Ndufa13	0.38	0.00
Il13ra1	0.32	0.00
Galnt7	0.67	0.00
Aldh7a1	0.37	0.00
Nfe2l1	0.68	0.00
Pacrg	2.52	0.00
Lrmp	0.40	0.00
Tnpo3	0.23	0.00
Tmem170b	0.43	0.00

Clcf1	0.23	0.00
Cinp	0.21	0.00
Dapl1	0.16	0.00
Stk11ip	0.25	0.00
Etv5	0.17	0.00
Cdhr4	0.68	0.00
Apeh	0.31	0.00
Dnaaf1	0.47	0.00
Phykpl	0.21	0.00
Slc26a2	0.28	0.00
Edem2	0.25	0.00
Plcg1	0.19	0.00
Pole	0.24	0.00
Cd3g	0.30	0.00
Adprs	0.27	0.00
R3hcc1	0.19	0.00
Nalcn	1.09	0.00
Nfu1	0.34	0.00
Ptgr2	0.36	0.00
Epha1	0.38	0.00
Polr2f	0.36	0.00
Tenm3	2.35	0.00
Acsbg2	0.55	0.00
Lpcat3	0.30	0.00
Thsd4	1.15	0.00
Tprgl	0.28	0.00
Arl3	0.43	0.00
Samd4	0.88	0.00
Psmc2	0.11	0.00
Hid1	0.38	0.00
Nudt1	0.30	0.00
C1galt1	0.42	0.00
Foxp2	1.53	0.00
Uggt2	0.49	0.00
Psmd14	0.76	0.00
Ankrd49	0.31	0.00
Igfbp2	1.29	0.00
Yars2	0.30	0.00
Trappc4	0.38	0.00
P2rx4	0.40	0.00
Coq9	0.18	0.00
Slc5a9	0.39	0.00
Dtd2	0.36	0.00
Acss3	0.56	0.00
Thoc7	0.30	0.00
Sowaha	0.25	0.00

Galnt17	0.62	0.00
Nudt21	0.26	0.00
Rbx1	0.36	0.00
Hprt	0.32	0.00
Trak2	0.29	0.00
Sgms2	1.37	0.00
Arhgap40	0.28	0.00
Atl1	0.64	0.00
Six4	0.49	0.00
Dusp14	0.36	0.00
Anks1b	1.95	0.00
Hook1	0.42	0.00
Ppfibp2	0.44	0.00
Naa15	0.31	0.00
Vps26a	0.22	0.00
Sort1	0.25	0.00
Gabra4	0.82	0.00
Stxbp3	0.49	0.00
Dcbld2	0.73	0.00
Ncald	1.39	0.00
Ftsj3	0.29	0.00
Lonp2	0.39	0.00
Tfg	0.16	0.00
Psmd13	0.15	0.00
Crls1	0.37	0.00
Rasgef1c	0.35	0.00
Insig1	1.12	0.00
Csnk2a2	0.40	0.00
Sucla2	0.65	0.00
Dip2b	0.56	0.00
DPCD	0.43	0.00
Hmgcs1	0.75	0.00
Hps5	0.56	0.00
Nelfcd	0.62	0.00
Kirrel3	1.42	0.00
Rassf5	0.24	0.00
Ndufa10	0.44	0.00
Tmprss13	0.24	0.00
Daglb	0.59	0.00
Sik2	0.47	0.00
Ddhd1	2.02	0.00
Gsr	0.38	0.00
Sap30	0.35	0.00
Lrrc38	0.31	0.00
Dynlrb2	0.48	0.00
Cog7	0.37	0.00

Ophn1	0.37	0.00
Galnt13	0.40	0.00
Far1	1.39	0.00
Bbs7	0.24	0.00
Washc5	0.32	0.00
Kcnj16	0.46	0.00
Hmgb2	0.39	0.00
Nubp1	0.34	0.00
Pdp1	0.44	0.00
Tusc3	0.68	0.00
Tesk2	0.71	0.00
Commd4	0.24	0.00
Ndrg1	1.03	0.00
Adcy7	0.32	0.00
Kdf1	0.17	0.00
Tbc1d1	0.80	0.00
Acox1	0.20	0.00
Sbno2	0.53	0.00
Zfp512b	0.31	0.00
Eed	0.29	0.00
Stc1	0.66	0.00
Hdgfl3	0.39	0.00
Sestd1	0.77	0.00
Ldb2	-3.44	0.00
Dennd2d	0.21	0.00
Ptprd	1.26	0.00
Tmem245	0.36	0.00
Pgm2	0.41	0.00
Lmo7	1.66	0.00
C1galt1c1	0.15	0.00
Ptrh2	0.22	0.00
Lrrc8d	0.50	0.00
Tnks2	0.31	0.00
Sel1l3	0.33	0.00
Dcxr	0.32	0.00
Cndp2	0.19	0.00
Rapgef5	-2.83	0.00
Tpi1	0.33	0.00
Jazf1	0.63	0.00
Gabarapl1	0.23	0.00
Tiam1	1.15	0.00
Ipo5	0.48	0.00
Rhbdf2	0.53	0.00
Eif3l	0.45	0.00
Nupl1	0.46	0.00
Dpp7	0.37	0.00

Smpdl3a	0.25	0.00
Vps13c	0.88	0.00
Sec23b	0.41	0.00
Gatb	0.64	0.00
Efhc2	0.77	0.00
Lca5l	0.64	0.00
Jchain	0.17	0.00
Smc6	0.42	0.00
Traf3ip1	0.79	0.00
Ptgr1	0.45	0.00
Flvcr2	0.38	0.00
Ubxn10	0.56	0.00
Acsl1	0.60	0.00
Ncstn	0.15	0.00
Acbd5	0.40	0.00
Pgs1	0.46	0.00
Itga7	0.26	0.00
Noc3l	0.44	0.00
Prpsap2	0.27	0.00
Pcid2	0.15	0.00
Gart	0.21	0.00
Gsap	0.31	0.00
Eno2	0.48	0.00
Gfus	0.19	0.00
Acvr2b	0.31	0.00
Vbp1	0.16	0.00
Paics	0.20	0.00
Slc25a14	0.18	0.00
Inip	0.29	0.00
Pggt1b	0.27	0.00
Saxo1	0.34	0.00
Ptgdr	0.21	0.00
Atp13a4	0.88	0.00
Cfap100	1.01	0.00
Atrip	0.18	0.00
Rgs9bp	0.33	0.00
Hdac3	0.14	0.00
Ciao3	0.28	0.00
Iqcd	0.20	0.00
Spaca9	0.56	0.00
Scrn2	0.16	0.00
Sp1	0.28	0.00
Hnrnpa1	0.19	0.00
Clptm1	0.12	0.00
Chmp2a	0.24	0.00
Scnm1	0.18	0.00

Them4	0.22	0.00
Net1	1.11	0.00
Abcb9	0.35	0.00
Akap14	0.58	0.00
Barx2	0.42	0.00
Fermt1	0.25	0.00
Fam171a1	0.34	0.00
Gng12	0.31	0.00
Tox	1.36	0.00
Hecw2	-3.11	0.00
Arap2	0.83	0.00
Nsa2	0.39	0.00
Atg4b	0.45	0.00
Hint1	0.24	0.00
Lrrc58	0.17	0.00
Bcl6	0.54	0.00
B4galt1	0.45	0.00
Vps37b	0.39	0.00
Nbeal1	0.98	0.00
Ikzf2	1.26	0.00
Glce	0.43	0.00
Lrp1	0.15	0.00
Dsel	0.36	0.00
Cul5	0.27	0.00
Daw1	1.55	0.00
Smurf1	0.44	0.00
Eif2b2	0.16	0.00
Ddit4	-2.61	0.00
Nae1	0.31	0.00
Zcchc9	0.17	0.00
Etf1	0.45	0.00
Dusp10	1.30	0.00
Pigt	0.27	0.00
Efhd1	0.18	0.00
Slc39a11	0.43	0.00
Acat1	0.23	0.00
Itpka	0.60	0.00
Setd5	0.48	0.00
Pptc7	0.27	0.00
Tuft1	0.23	0.00
Lancl1	0.28	0.00
Plbd1	0.46	0.00
Eif3g	0.24	0.00
Gatd1	0.26	0.00
Tmco3	0.68	0.00
Cfap69	0.79	0.00

Eps8	1.48	0.00
Lclat1	0.90	0.00
Bcl2l14	0.38	0.00
Ppm1l	0.42	0.00
Taldo1	0.47	0.00
Ppil1	0.19	0.00
Pde5a	0.44	0.00
Nfkbie	0.34	0.00
Emc8	0.23	0.00
Pcyt1b	0.70	0.00
Usp20	0.28	0.00
Pdk2	0.20	0.00
Xpot	0.19	0.00
Tspan1	0.81	0.00
Sar1b	0.19	0.00
Copg1	0.25	0.00
Lca5	1.22	0.00
Btbd3	0.45	0.00
Prkd1	0.56	0.00
Slc4a4	1.64	0.00
Arid3b	0.62	0.00
Jagn1	0.19	0.00
Nectin3	0.79	0.00
Scamp2	0.24	0.00
Erlin1	0.22	0.00
Itgb6	0.98	0.00
Hlcs	0.69	0.00
Ppib	0.52	0.00
Atp7a	0.10	0.00
Adar	0.15	0.00
Ush1c	0.81	0.00
Lman2	0.47	0.00
Sfxn1	0.41	0.00
Dynlt1a	0.33	0.00
Pdcd4	0.46	0.00
Adgrf5	-3.60	0.00
Agpat2	0.21	0.00
Clcn3	0.33	0.00
Tmem64	0.50	0.00
Pigw	0.45	0.00
B4galt7	0.26	0.00
Zcrb1	0.20	0.00
Rida	0.21	0.00
Clstn3	0.39	0.00
Dlgap4	0.40	0.00
Cln6	0.23	0.00

Rnf168	0.05	0.00
Ap1s3	0.40	0.00
Nudt3	0.24	0.00
Egfr	1.01	0.00
Sik1	0.72	0.00
Cep104	0.30	0.00
Srm	0.31	0.00
Get4	0.27	0.00
Iqgap2	0.38	0.00
Fdps	0.31	0.00
Nipsnap2	0.41	0.00
Dnajc1	0.68	0.00
Tgfb2	0.62	0.00
Chrac1	0.17	0.00
Disc1	0.83	0.00
Pdk4	0.51	0.00
Plpp1	0.32	0.00
Ist1	0.23	0.00
Eif3m	0.47	0.00
Slc20a2	0.14	0.00
Txndc11	0.12	0.00
Aagab	0.35	0.00
Acss2	0.64	0.00
Gorasp2	0.27	0.00
Rasgrf1	0.23	0.00
Aak1	0.32	0.00
Fli1	-3.54	0.00
Glis3	1.20	0.00
Lss	0.28	0.00
Nup50	0.27	0.00
Ak5	0.59	0.00
Ppp1ca	0.18	0.00
Rassf7	0.34	0.00
Slc16a3	0.28	0.00
Cdon	0.19	0.00
Slc35b2	0.23	0.00
Ilf3	0.12	0.00
Lpcat1	1.06	0.00
Desi1	0.39	0.00
Decr2	0.21	0.00
Chmp2b	0.15	0.00
Tspan12	0.24	0.00
Col8a1	0.64	0.00
Crispld1	0.32	0.00
Cggbp1	0.25	0.00
Tex264	0.20	0.00

Brf2	0.14	0.00
Sh3bp2	0.64	0.00
Ovch2	1.18	0.00
Clstn1	0.57	0.00
Acox3	0.46	0.00
Farp1	1.25	0.00
Tmem181a	0.07	0.00
Kras	0.57	0.00
Il17ra	0.33	0.00
Npepps	0.31	0.00
Tspan6	0.29	0.00
Rnf216	0.93	0.00
Bcl9l	0.73	0.00
Tbc1d9	0.86	0.00
Sbds	0.15	0.00
Acp2	0.28	0.00
Snx4	0.29	0.00
Oat	0.28	0.00
Slc35f3	0.83	0.00
E2f6	0.02	0.00
Slc25a28	0.21	0.00
Rasal2	-2.47	0.00
Papolg	0.73	0.00
Dennd4c	0.37	0.00
Ndufv3	0.60	0.00
Tmsb4x	-1.38	0.00
Wdr4	0.26	0.00
Fbxl3	0.18	0.00
Adgrl4	-2.99	0.00
Alg5	0.29	0.00
Wsb2	0.28	0.00
Psmd9	0.23	0.00
Chl1	0.93	0.00
Hsdl1	0.23	0.00
Josd1	0.35	0.00
Cript	0.19	0.00
Ccdc181	0.28	0.00
Prdm16	0.50	0.00
Slitrk6	0.77	0.00
Nipal3	0.37	0.00
Hecw1	0.72	0.00
Emc6	0.13	0.00
Alkal2	0.31	0.00
Fam83f	0.31	0.00
Abca3	1.30	0.00
Brd3	0.12	0.00

Dnajc15	0.24	0.00
Dzip1	0.91	0.00
Zfp352	-2.78	0.00
Arl1	0.41	0.00
Pdia6	0.27	0.00
Fbxw11	0.27	0.00
Vwa3a	0.88	0.00
Nptn	0.63	0.00
Cct6b	0.10	0.00
Cep128	0.90	0.00
E2f5	0.16	0.00
Trib1	1.23	0.00
Gas8	0.52	0.00
Tkt	0.35	0.00
Fam118b	0.48	0.00
Afap1	0.24	0.00
Sf3a2	0.02	0.00
Zmynd8	0.31	0.00
Atp2c1	0.51	0.00
Aup1	0.30	0.00
Copg2	0.15	0.00
Tmem186	0.20	0.00
Psmb3	0.26	0.00
Fam20b	0.17	0.00
Cnpy3	0.21	0.00
Abcc4	1.10	0.00
Copb1	0.03	0.00
Stc2	0.69	0.00
Cfap97	0.58	0.00
Larp1	0.42	0.00
Wdr44	0.33	0.00
Utp3	0.25	0.00
Aebp2	0.43	0.00
Tbc1d12	0.21	0.00
Ctsc	0.26	0.00
Arhgap24	0.49	0.00
Bcl2	0.24	0.00
Sugct	1.52	0.00
Tead3	0.32	0.00
Comtd1	0.19	0.00
Pcmtd2	0.39	0.00
Npnt	0.42	0.00
Chgb	0.33	0.00
Ctnnal1	0.26	0.00
Cnih4	0.13	0.00
Nip7	0.13	0.00

Lrrc43	0.53	0.00
Eef1akmt1	0.19	0.00
Acad11	0.26	0.00
Pes1	0.27	0.00
Prkce	-2.38	0.00
Ripk2	0.51	0.00
Rdh10	0.39	0.00
Nmd3	0.35	0.00
Impg2	0.14	0.00
Ankrd42	0.37	0.00
Ccdc90b	0.19	0.00
Rrm1	0.12	0.00
Rsph3b	0.25	0.00
Polr1b	0.15	0.00
Pbk	0.14	0.00
Cxcl13	0.36	0.00
Arl14ep	0.19	0.00
Zc2hc1c	0.29	0.00
Cfap65	0.84	0.00
Tll2	0.18	0.00
Sumf1	0.14	0.00
Ddx25	0.33	0.00
Ccdc103	0.48	0.00
Atxn7l3	0.33	0.00
Arrdc2	0.49	0.00
Kptn	0.25	0.00
Llgl2	0.63	0.00
Cachd1	0.29	0.00
Itga6	0.68	0.00
Rnf139	0.23	0.00
Rhou	0.16	0.00
Kctd9	0.24	0.00
Dda1	0.27	0.00
Nudcd2	0.15	0.00
Ccdc170	1.03	0.00
Agps	0.54	0.00
Spidr	0.76	0.00
Rnd3	1.27	0.00
Yipf1	0.39	0.00
Yipf4	0.25	0.00
Rad21	0.27	0.00
Elavl1	0.07	0.00
Lnx2	0.32	0.00
Cab39l	0.41	0.00
Enpp2	1.31	0.00
Zyx	0.56	0.00

Slc25a16	0.07	0.00
Pecr	0.15	0.00
Rab28	0.61	0.00
Slc25a12	0.35	0.00
Wdr47	0.23	0.00
Ttc17	0.20	0.00
Htra2	0.43	0.00
Htr4	0.47	0.00
Lrrc59	0.26	0.00
Sufu	0.61	0.00
Pithd1	0.36	0.00
Dnajc7	0.20	0.00
Psmg2	0.15	0.00
Nova1	-3.23	0.00
Trank1	1.88	0.00
Sema4d	0.75	0.00
Pros1	0.43	0.00
Slc9a8	0.55	0.00
Plcxd2	0.23	0.00
Efna5	0.39	0.00
Lrrc1	0.65	0.00
Spg7	0.29	0.00
Cfap77	0.73	0.00
Cep162	0.43	0.00
Rgs6	0.66	0.00
Ptprs	0.36	0.00
Nfrkb	0.18	0.00
Suclg2	0.28	0.00
Klhl32	0.94	0.00
Pih1d2	0.28	0.00
Acly	0.42	0.00
Dnajb2	0.19	0.00
Gba2	0.18	0.00
Chd1l	0.42	0.00
Serpine2	0.39	0.00
Plekhh1	0.44	0.00
Pigyl	0.25	0.00
Ndufab1	0.22	0.00
Nek1	0.38	0.00
Pcgf3	0.29	0.00
Socs1	0.22	0.00
Tmem199	0.26	0.00
Cd9	1.34	0.00
Gramd1c	0.41	0.00
Ppic	0.30	0.00
Naxe	0.30	0.00

Tmco1	0.18	0.00
Primpol	0.51	0.00
Cdc34	0.23	0.00
Fam110b	0.32	0.00
Fam104a	0.23	0.00
Pcsk1	0.67	0.00
Sec11a	0.00	0.00
Psma5	0.25	0.00
Sqstm1	0.96	0.00
Gnpat	0.44	0.00
Kcnk1	0.53	0.00
Eif2s3y	0.28	0.00
Fgd4	0.80	0.00
Tgds	0.07	0.00
Sri	0.11	0.00
Nsl1	-0.01	0.00
Fbxo7	0.18	0.00
Drd3	0.17	0.00
Sptlc3	0.41	0.00
Ift27	0.51	0.00
Neil1	0.15	0.00
Srpx	0.28	0.00
Tmem100	-3.39	0.00
Nmnat3	0.19	0.00
Hebp1	0.18	0.00
Naf1	0.34	0.00
Eif2d	0.16	0.00
Rbbp7	0.21	0.00
Abca2	0.25	0.00
Gnl3	0.19	0.00
Aif1l	0.29	0.00
Ikbke	0.46	0.00
Nolc1	0.14	0.00
Ints13	0.16	0.00
Snx3	0.47	0.00
Acat3	0.23	0.00
Paqr7	0.15	0.00
Baiap2	0.66	0.00
Tmem59	0.15	0.00
Akap12	-3.58	0.00
Lztfl1	0.37	0.00
Clk2	0.12	0.00
Ncoa6	0.24	0.00
Hmbox1	0.44	0.00
Hadhb	0.31	0.00
Sparc	-3.39	0.00

Pde6d	0.21	0.00
Ciao1	0.12	0.00
Gns	1.03	0.00
Hs1bp3	0.35	0.00
Lrig3	0.30	0.00
Nsmaf	0.49	0.00
Ginm1	0.04	0.00
Eif3f	0.23	0.00
Pdhb	0.09	0.00
Ralgps1	0.43	0.00
Plbd2	0.42	0.00
Irak1bp1	0.44	0.00
Ints10	0.14	0.00
Fadd	0.11	0.00
Nos2	0.46	0.00
Psmf1	0.06	0.00
Cdc14b	0.21	0.00
Prelid3b	0.03	0.00
Pcyt2	0.20	0.00
Polr1e	0.14	0.00
Azi2	0.19	0.00
Tshz1	0.37	0.00
Hsd17b7	0.44	0.00
Sec13	0.06	0.00
Hbs1l	0.37	0.00
Nsun3	0.15	0.00
Sptbn1	-1.55	0.00
Slc22a3	0.40	0.00
Dhx38	0.25	0.00
Entpd6	0.29	0.00
Rbpj	0.55	0.00
Osbpl11	0.13	0.00
Fosl2	0.84	0.00
Emc3	0.09	0.00
Ubfd1	0.11	0.00
Tex9	0.53	0.00
Fam149a	0.77	0.00
Arhgef38	0.26	0.00
Atp10a	1.03	0.00
Crim1	-1.99	0.00
Entpd7	0.22	0.00
Acp1	0.43	0.00
Socs2	0.24	0.00
Sytl1	0.22	0.00
Hs3st1	0.82	0.00
Xpnpep1	0.33	0.00

Cfap58	0.55	0.00
Ndufa6	0.18	0.00
Slc44a3	0.88	0.00
Gna11	0.22	0.00
Ap3b1	0.50	0.00
Yeats4	0.20	0.00
Tfcp2l1	1.00	0.00
Eif2ak3	0.58	0.00
Tgif1	0.40	0.00
Ankib1	0.40	0.00
Slc9a3r1	0.27	0.00
Cenpc1	0.31	0.00
Klhl7	0.44	0.00
Tmtc1	-2.91	0.00
Ift43	0.45	0.00
Fggy	0.57	0.00
Dcdc2a	0.75	0.00
Capn11	0.20	0.00
Cog4	0.13	0.00
Itga1	0.03	0.00
Xbp1	0.75	0.00
Lrpap1	0.24	0.00
Rrp12	0.24	0.00
Dpm2	0.36	0.00
Rbpms	0.21	0.00
Ppm1a	0.13	0.00
Ppa1	0.03	0.00
Dnah10	1.21	0.00
Alkbh5	0.26	0.00
Tmed3	0.07	0.00
Timm21	0.07	0.00
Qrsl1	0.33	0.00
Ctsb	0.61	0.00
Erlec1	0.11	0.00
Ctu2	0.13	0.00
Gsk3b	-1.98	0.00
Cct8	0.08	0.00
Tmed2	0.10	0.00
Polr2l	0.22	0.00
Kdelr2	0.38	0.00
Setbp1	1.17	0.00
Rpn1	0.28	0.00
Poldip2	0.05	0.00
Exoc7	0.34	0.00
Zbtb44	0.17	0.00
Errfi1	0.57	0.00

Rarres2	0.25	0.00
Large2	0.20	0.00
Bmp4	0.62	0.00
Tmtc3	0.30	0.00
Pex11g	0.22	0.00
Prickle2	1.36	0.00
Lancl2	0.25	0.00
Guf1	0.38	0.00
Ift22	0.17	0.00
Zc3h12a	0.30	0.00
Slc39a6	0.21	0.00
Fdft1	0.16	0.00
Serpinf2	0.13	0.00
Dync2h1	1.66	0.00
Slc35f4	0.15	0.00
Slco4c1	0.31	0.00
Ccdc137	0.18	0.00
Ndufaf6	0.34	0.00
Atp2a2	0.86	0.00
Pik3ip1	0.23	0.00
Qsox1	0.38	0.00
Sh3d19	0.45	0.00
Dbt	0.18	0.00
Nab1	0.46	0.00
Ints9	0.34	0.00
Ap3m2	0.38	0.00
Commd7	0.09	0.00
Btbd6	0.25	0.00
Vps33b	0.31	0.00
Cog1	0.16	0.00
Pdcd5	0.11	0.00
Tmem132a	0.12	0.00
Tmem170	0.26	0.00
Tmem63a	0.37	0.00
H13	0.00	0.00
Uggt1	0.19	0.00
Ube4b	0.15	0.00
Pdrg1	0.09	0.00
Ap2b1	0.24	0.00
Zdhhc2	0.33	0.00
Gfra3	0.28	0.00
Pank3	0.51	0.00
Pdcd11	0.32	0.00
Slc35b1	0.17	0.00
Pik3cb	0.33	0.00
Tmem69	0.11	0.00

Trappc3	0.11	0.00
Nr4a2	0.65	0.00
Vps35	0.07	0.00
Utp4	0.23	0.00
Tbl2	0.19	0.00
Lama5	0.21	0.00
Pomt1	0.19	0.00
Aimp1	0.27	0.00
Nup98	0.13	0.00
Phlpp1	0.43	0.00
Wls	0.77	0.00
Dnai1	0.69	0.00
Nfyc	0.08	0.00
Setd3	0.27	0.00
Pcnx2	0.19	0.00
Gapvd1	0.29	0.00
Clybl	0.48	0.00
Pex5	0.43	0.00
Slc16a6	0.34	0.00
Trpm8	0.36	0.00
Slit3	0.66	0.00
Yrdc	0.22	0.00
Jakmip2	0.26	0.00
Gpsm2	0.54	0.00
Xpc	0.23	0.00
Tes	1.16	0.00
Pdcl3	0.01	0.00
Plekhm2	0.24	0.00
Uaca	0.78	0.00
Camsap1	0.31	0.00
Nans	0.05	0.00
Ormdl1	0.25	0.00
Klhdc1	0.25	0.00
Sbspon	0.51	0.00
Amd2	0.33	0.00
Ccdc186	0.40	0.00
Oit3	0.62	0.00
Vamp4	0.16	0.00
Nifk	0.27	0.00
Fbxo32	0.78	0.00
Ciz1	0.26	0.00
Plekhb2	0.21	0.00
Ckap4	0.24	0.00
Wbp2nl	0.08	0.00
Slc9a1	0.22	0.00
Ndufv2	0.27	0.00

Tbc1d4	0.78	0.00
Fabp3	2.47	0.00
P3h1	0.15	0.00
Fh1	0.32	0.00
Rbms3	-1.54	0.00
Pts	0.18	0.00
Shisa5	0.26	0.00
Dpy19l4	0.26	0.00
Ebag9	0.13	0.00
Nhlrc3	0.18	0.00
Cfi	0.21	0.00
Nfil3	0.48	0.00
Ddb1	-0.03	0.00
Tm9sf2	0.73	0.00
Zw10	0.07	0.00
Anapc2	0.18	0.00
Ttc7	1.00	0.00
Exosc3	0.18	0.00
Lrif1	0.17	0.00
Wbp11	0.28	0.00
Pcgf5	0.41	0.00
Atp6v1c1	0.05	0.00
Atp13a3	0.25	0.00
Sdhaf4	0.04	0.00
Plod2	0.48	0.00
Nr1h3	0.22	0.00
Foxk1	0.10	0.00
Pabpc4	0.09	0.00
Slc16a7	0.75	0.00
Ube2w	0.24	0.00
Grsf1	0.14	0.00
Cnih1	0.05	0.00
St7l	0.04	0.00
Cdc27	-0.01	0.00
Lamtor2	0.17	0.00
Ppfia1	0.60	0.00
Ccdc47	0.25	0.00
Dcun1d5	0.14	0.00
Cluh	0.27	0.00
Tax1bp3	0.10	0.00
Rwdd4a	0.01	0.00
Tmem230	0.17	0.00
Rfc2	0.25	0.00
Unc13b	0.69	0.00
Ppil3	0.09	0.00
Foxj1	0.64	0.00

Usp24	0.28	0.00
Gprc5c	0.13	0.00
Tmem184c	0.15	0.00
Cry2	0.06	0.00
Prdx1	2.09	0.00
Irak2	0.27	0.00
Tfap2a	0.39	0.00
Traf5	0.60	0.00
Nek5	0.49	0.00
Akap1	0.26	0.00
Nmi	0.11	0.00
Wdr48	0.33	0.00
Tsr3	0.09	0.00
Supt3	0.23	0.00
Cflar	0.07	0.00
Ankrd66	0.38	0.00
Cfap57	0.40	0.00
Gata6	0.66	0.00
Gemin8	0.39	0.00
Vav2	0.56	0.00
Cpsf4	0.25	0.00
Ndufs4	0.08	0.00
Sbf1	0.76	0.00
Ndufb9	0.02	0.00
Gtpbp1	0.02	0.00
Pak3	0.42	0.00
Esrrg	0.53	0.00
Ibtk	0.29	0.00
Cldn18	0.19	0.00
Fam83h	0.21	0.00
Hnrnpll	0.33	0.00
Nav2	0.80	0.00
Atp10b	1.44	0.00
Nsmce2	0.57	0.00
Btf3	0.08	0.00
Gtpbp8	0.07	0.00
Kif2a	0.46	0.00
Ift88	1.03	0.00
Stard4	0.20	0.00
Ruvbl1	0.34	0.00
Spata6	0.45	0.00
Rab3gap1	0.25	0.00
Cdc42ep4	0.15	0.00
Kcnk5	0.58	0.00
Chek2	0.26	0.00
Gemin4	0.05	0.00

Tma16	0.22	0.00
Foxa1	0.33	0.00
Gfm1	0.16	0.00
Pex5l	0.86	0.00
Snrnp25	0.28	0.00
Ppp1r7	0.01	0.00
Spata17	0.80	0.00
Pmm2	0.10	0.00
Ndufa5	0.08	0.00
Fbxw5	0.10	0.00
Gtpbp4	0.12	0.00
Ndufa2	0.37	0.00
Ptk7	0.24	0.00
Rasgef1b	0.69	0.00
Etfa	0.12	0.00
Ankrd40	0.14	0.00
Gcnt1	0.29	0.00
Sft2d1	-0.01	0.00
Rrbp1	0.29	0.00
Tsnax	0.28	0.00
Atp6v0e	0.52	0.00
Azin1	0.40	0.00
Nrcam	0.67	0.00
Atp2b1	0.07	0.00
Bcas2	0.17	0.00
Rab11a	0.13	0.00
Polr2d	0.02	0.00
Stat3	0.15	0.00
Naif1	0.34	0.00
Frem1	0.56	0.00
Tbc1d32	1.05	0.00
Prr15l	0.15	0.00
Fxn	0.19	0.00
Pebp1	0.06	0.00
Snap29	0.15	0.00
Ttc1	0.20	0.00
Sema4g	0.25	0.00
Pigk	0.11	0.00
Desi2	-0.01	0.00
Bcl9	0.19	0.00
Irf1	1.15	0.00
Rad51b	0.64	0.00
Serpinb5	0.16	0.00
Ogdh	0.28	0.00
Zcchc7	0.45	0.00
Ywhab	0.11	0.00

Pigb	0.13	0.00
Sox9	0.11	0.00
Anapc1	0.15	0.00
Dctn3	0.12	0.00
Casd1	0.22	0.00
Ltf	0.38	0.00
Dock3	0.39	0.00
Lonp1	0.07	0.00
Tmem237	0.38	0.00
Rad54l2	0.33	0.00
Ankrd13a	0.10	0.00
Ddost	0.15	0.00
Lamp3	2.21	0.00
Rnf145	0.35	0.00
Alad	0.02	0.00
Ash2l	0.19	0.00
Cryzl1	0.61	0.00
Sowahc	0.29	0.00
Ptp4a2	0.91	0.00
Soat1	0.66	0.00
Srp68	0.11	0.00
Ift172	0.35	0.00
Ldah	0.27	0.00
Tmem258	0.33	0.00
Ap1ar	0.13	0.00
Ralgapa1	0.60	0.00
Siah1b	0.28	0.00
Kctd3	0.55	0.00
Rnf4	0.24	0.00
Pkp2	0.31	0.00
Tmem192	0.30	0.00
Lyrm2	0.13	0.00
Slc22a18	0.00	0.00
Tctn2	0.33	0.00
Kdelr3	0.18	0.00
Akap7	0.39	0.00
Slc15a4	0.10	0.00
Dkc1	0.15	0.00
Psma7	-0.01	0.00
Rpa2	0.04	0.00
Zfp827	0.39	0.00
Dnaja3	-0.01	0.00
Ppp1cc	-0.07	0.00
Slc41a3	0.05	0.00
Usp10	0.35	0.00
Lcor	0.51	0.00

Sf3a3	-0.02	0.00
Rmdn3	0.19	0.00
Scrn3	0.03	0.00
Pcm1	0.69	0.00
Fsip1	0.84	0.00
Echdc2	0.46	0.00
Tmem33	0.13	0.00
Axin1	0.42	0.00
Glul	0.57	0.00
Ttbk1	0.79	0.00
Snrpd3	-0.04	0.00
Gtf2a2	0.04	0.00
Scaf11	0.69	0.00
Commd9	0.04	0.00
Pxk	0.21	0.00
Zfp654	0.19	0.00
Snx12	0.15	0.00
Zfp385b	1.23	0.00
Tmbim6	0.29	0.00
Lgmn	1.11	0.00
Cntfr	0.44	0.00
Kyat3	0.25	0.00
Slc27a1	0.15	0.00
Ptprb	-3.26	0.00
Dnajc3	0.32	0.00
Krr1	0.24	0.00
Letm1	0.35	0.00
Igsf3	0.34	0.00
Bcl11a	0.71	0.00
Ogt	0.39	0.00
Aco1	0.17	0.00
Chp1	0.65	0.00
Gpm6b	0.27	0.00
Slc7a6os	-0.03	0.00
Srpr	0.03	0.00
Rgcc	-3.34	0.00
Sec23a	0.19	0.00
Fgfr3	0.31	0.00
Arl8b	0.13	0.00
Rnf138	0.00	0.00
Actr10	0.18	0.00
Dnah1	0.57	0.00
Fam168b	1.37	0.00
Plekhf2	-0.12	0.00
Rab22a	0.05	0.00
Aldh4a1	0.08	0.00

Vil1	0.00	0.00
Ndufs1	-0.02	0.00
Plk3	1.06	0.00
Eno1b	0.51	0.00
Dlst	0.25	0.00
Chrm3	2.20	0.00
Ly75	0.49	0.00
Sema3e	1.06	0.00
Eml4	0.78	0.00
Cabin1	0.61	0.00
Cacna2d2	0.18	0.00
Ddr2	0.17	0.00
Uba6	0.37	0.00
Lnpk	0.24	0.00
Lmbrd1	0.35	0.00
Rexo2	0.10	0.00
Ccdc112	0.28	0.00
Adamts17	0.95	0.00
Klhl20	0.24	0.00
Nrbf2	0.18	0.00
Anapc5	0.19	0.00
Nop16	0.12	0.00
Fbxo21	0.13	0.00
Qtrt2	0.18	0.00
Lamtor3	0.17	0.00
Cpsf2	0.11	0.00
Pdcd2l	0.16	0.00
Odf2	0.37	0.00
Exoc1	0.10	0.00
Wdr17	0.62	0.00
Fig4	0.27	0.00
Atm	0.25	0.00
Sf3b5	0.05	0.00
Arhgef9	-3.15	0.00
Clp1	0.10	0.00
Cep57l1	0.60	0.00
Dffa	0.15	0.00
Clic3	0.23	0.00
Wdr12	0.16	0.00
Cstf1	-0.06	0.00
Caps2	0.42	0.00
Lamp1	1.14	0.00
Gpx8	0.11	0.00
Nrd1	0.09	0.00
Cntln	0.35	0.00
Sirt1	0.30	0.00

Acbd3	0.08	0.00
Eri3	0.09	0.00
Arpc1a	0.13	0.00
Bend7	0.41	0.00
Acvr1b	0.22	0.00
Pla2g4a	0.17	0.00
Rala	0.34	0.00
Sec31b	0.18	0.00
Ift81	0.40	0.00
Psma6	0.14	0.00
Ptges3	0.07	0.00
Erp29	0.70	0.00
Psma4	0.04	0.00
Smap1	0.35	0.00
Fnip2	0.28	0.00
Smc4	0.74	0.00
Ift52	0.44	0.00
Cdk12	0.02	0.00
Tom1	0.18	0.00
Zhx2	0.04	0.00
Aimp2	0.06	0.00
Rmdn1	0.14	0.00
Hacd2	0.15	0.00
Fam183b	0.70	0.00
Acsl4	0.60	0.00
Grhl3	0.42	0.00
Efcab14	0.03	0.00
Hadh	0.06	0.00
Prkcd	0.45	0.00
Ranbp10	0.36	0.00
B4galnt3	0.32	0.00
Ddx27	0.15	0.00
Hipk3	1.00	0.00
Ptpdc1	0.21	0.00
Chst11	0.85	0.00
Vldlr	0.25	0.00
Erich1	0.31	0.00
Abcf3	0.22	0.00
Hspa9	-0.14	0.00
Vsig1	0.13	0.00
Cep70	0.14	0.00
Ppm1m	0.15	0.00
Nsmf	0.15	0.00
Capn10	0.38	0.00
Ran	0.33	0.00
Acot8	0.10	0.00

Naa25	0.19	0.00
Pomgnt1	0.21	0.00
Pibf1	0.58	0.00
Sec16a	0.00	0.00
Pak1ip1	0.12	0.00
Usp1	0.24	0.00
Nup155	0.21	0.00
Noct	0.43	0.00
Angptl2	0.29	0.00
Lrrc49	0.19	0.00
Necap1	0.00	0.00
Arfgap2	0.07	0.00
Ttpa	0.25	0.00
Srp72	0.13	0.00
L2hgdh	0.10	0.00
Rif1	0.12	0.00
Fuca1	0.14	0.00
Trim2	0.24	0.00
Slc45a4	0.90	0.00
Pde8a	0.97	0.00
Ofd1	0.30	0.00
Prkci	0.19	0.00
Zdhhc12	0.17	0.00
Scfd1	0.42	0.00
Phb2	0.16	0.00
Aamdc	0.07	0.00
Gpsm1	0.53	0.00
Lrig1	0.68	0.00
Hsbp1	0.27	0.00
S100a16	0.15	0.00
Tmcc1	0.22	0.00
Acads	0.08	0.00
Akap9	0.94	0.00
Psmc6	0.11	0.00
Peak1	0.41	0.00
Clta	0.21	0.00
Cdk10	0.21	0.00
Etnk1	0.87	0.00
Hspa14	0.18	0.00
Sdk1	-2.29	0.00
Uri1	0.28	0.00
Gpat4	0.16	0.00
Ahcyl1	0.33	0.00
Slc25a34	0.13	0.00
Klhl2	0.15	0.00
Gosr2	0.03	0.00

Puf60	0.15	0.00
Tex10	0.31	0.00
Tbc1d23	0.03	0.00
Tceanc	0.16	0.00
Vta1	0.01	0.00
Tmem94	0.16	0.00
Dnajc18	0.15	0.00
Aldh18a1	0.21	0.00
Pank2	0.09	0.00
Pdgfc	0.39	0.00
Bnip3	0.25	0.00
Scel	0.87	0.00
Dpp9	0.07	0.00
Snupn	0.17	0.00
Cables1	0.05	0.00
Tmem141	0.21	0.00
Ikbkb	0.25	0.00
Impa2	0.05	0.00
Skap1	-2.84	0.00
Prkab1	0.02	0.00
Alg14	0.14	0.00
Ikzf5	0.07	0.00
Sema3d	1.30	0.00
Sanbr	0.35	0.00
Akap11	0.08	0.00
Chid1	0.16	0.00
Rab11fip2	0.16	0.00
Nkiras2	0.03	0.00
Atf2	0.19	0.00
Oaz1	0.69	0.00
Ube2ql1	0.15	0.00
Polr2e	-0.08	0.00
Slc41a2	0.25	0.00
Ahi1	0.95	0.00
Vps29	0.00	0.00
Ppp1r3a	0.25	0.00
Inppl1	0.18	0.00
Trappc2l	0.15	0.00
Cnot1	0.06	0.00
Vmp1	0.88	0.00
Pp2d1	0.39	0.00
Sema3a	0.83	0.00
Anln	0.26	0.00
Copb2	0.03	0.00
Ndufb1	0.21	0.00
C8g	0.10	0.00

Trim50	0.13	0.00
Chdh	0.10	0.00
Tmem164	0.57	0.00
Scfd2	0.28	0.00
Slc25a46	0.27	0.00
Ptprj	0.18	0.00
Zfp330	0.24	0.00
Derl1	0.00	0.00
Sec61g	0.42	0.00
Dgkq	0.54	0.00
Dnajb9	0.26	0.00
Gpr137b	0.42	0.00
Ttll4	0.21	0.00
Hmgcr	0.31	0.00
Tnfrsf21	0.14	0.00
Fkbp8	-0.05	0.00
Ptpn6	0.17	0.00
Stx17	-0.13	0.00
Nub1	0.19	0.00
Prpf3	-0.03	0.00
Zmat2	0.07	0.00
Dld	0.18	0.00
Rapgef1	0.10	0.00
Kdm4b	0.22	0.00
Appbp2	0.17	0.00
Slc25a24	0.01	0.00
Yme1l1	0.10	0.00
Rai2	0.11	0.00
Erh	-0.13	0.00
Fam13c	2.39	0.00
Srek1ip1	0.00	0.00
Agr3	0.74	0.00
Ipo7	0.05	0.00
Nudt12	0.24	0.00
Arhgap31	-2.90	0.00
Iars2	0.14	0.00
B9d1	0.23	0.00
Yod1	0.27	0.00
Podxl	-3.03	0.00
Ndc1	0.23	0.00
Pprc1	0.15	0.00
Kpnb1	0.13	0.00
Trappc11	0.24	0.00
Arpp19	0.12	0.00
Rragc	0.13	0.00
Ssbp1	0.12	0.00

Zcchc17	0.13	0.00
Ndufs3	-0.05	0.00
Triqk	0.30	0.00
Jph1	0.41	0.00
Fasn	0.69	0.00
Fem1a	-0.04	0.00
Slc25a5	0.27	0.00
Smarce1	0.11	0.00
Hsbp1l1	0.21	0.00
Psat1	0.15	0.00
Heatr3	0.31	0.00
Ahsa1	0.04	0.00
Gorasp1	0.23	0.00
Zeb2	-2.75	0.00
Selenof	0.05	0.00
Acadl	0.08	0.00
Rpf1	0.08	0.00
Rpn2	0.01	0.00
Pnpla8	0.14	0.00
Kdsr	0.35	0.00
Cwc15	-0.02	0.00
Idua	0.06	0.00
Pias2	0.06	0.00
Slc23a2	0.43	0.00
Lrrc47	0.19	0.00
Rbks	0.58	0.00
Ndufs2	0.09	0.00
Nipa2	0.21	0.00
Tank	-0.10	0.00
Foxn2	0.10	0.00
Swap70	0.82	0.00
Naa50	0.01	0.00
Tbc1d20	0.15	0.00
Phf13	0.17	0.00
Zc2hc1a	0.31	0.00
Ndufb3	0.01	0.00
Tmem38a	0.12	0.00
Spata18	1.19	0.00
Hsdl2	0.01	0.00
Cisd2	-0.13	0.00
Aldh1a3	0.91	0.00
Hikeshi	0.00	0.00
Ints4	0.15	0.00
Nf1	0.27	0.00
Elmo3	0.16	0.00
Cd99l2	0.15	0.00

Stat2	0.09	0.00
Slc31a1	0.15	0.00
Ugcg	0.18	0.00
Gxylt2	0.22	0.00
Khdrbs1	-0.01	0.00
Fdx1	0.07	0.00
Dusp6	0.46	0.00
Nsun2	0.15	0.00
Ei24	-0.06	0.00
Amfr	-0.05	0.00
Cstf3	0.07	0.00
Lactb2	0.18	0.00
Atl2	0.02	0.00
Slc26a9	0.12	0.00
Arid5b	1.00	0.00
Ptp4a1	-0.12	0.00
Actr6	0.16	0.00
Arfip1	0.59	0.00
Cnksr2	0.40	0.00
Dmd	0.62	0.00
Pofut1	0.08	0.00
Dhx29	0.12	0.00
Scn3b	0.19	0.00
Gxylt1	0.15	0.00
Chaf1a	0.08	0.00
Epb41l5	0.43	0.00
Tnfaip3	0.32	0.00
Pik3r1	0.33	0.00
Srebf1	0.01	0.00
Chn1	0.39	0.00
Rnpc3	-0.05	0.00
Naxd	0.27	0.00
Ccser2	0.57	0.00
Cttn	0.09	0.00
Nnt	0.03	0.00
Supt6	0.03	0.00
Hlf	0.90	0.00
Slc4a3	0.37	0.00
Kif7	0.07	0.00
Smyd4	0.11	0.00
Ik	0.16	0.00
Nt5dc2	0.01	0.00
Arf4	0.26	0.00
Sumo1	-0.07	0.00
Xpr1	0.19	0.00
Hax1	-0.06	0.00

Sat1	1.03	0.00
Rbm38	0.51	0.00
Hibadh	0.57	0.00
Pam	0.10	0.00
Dnajc28	0.16	0.00
Ciapin1	-0.02	0.00
Pstk	0.66	0.00
Ube2b	0.12	0.00
Yipf3	0.10	0.00
Amn1	0.33	0.00
Rreb1	0.26	0.00
Asah1	0.46	0.00
Psmb2	-0.10	0.00
Zfp518a	0.15	0.00
Pmvk	0.26	0.00
Rbm15b	-0.05	0.00
Rpap3	0.22	0.00
Nat9	0.21	0.00
Eif3d	-0.12	0.00
Synrg	0.21	0.00
Fam120b	0.31	0.00
Dntt	0.30	0.00
Snrpc	0.21	0.00
Frem2	0.43	0.00
Angel1	0.03	0.00
Ranbp9	0.47	0.00
Trmt44	0.19	0.00
Sumf2	0.12	0.00
Sytl4	0.21	0.00
Fam185a	0.13	0.00
Ndufs8	-0.02	0.00
Rnls	0.29	0.00
Cacna2d1	0.44	0.00
Dhrs3	0.11	0.00
Nsmce1	0.09	0.00
Socs4	0.23	0.00
Rspry1	0.02	0.00
Prim2	0.20	0.00
Hsph1	0.31	0.00
Cebpz	-0.03	0.00
Srsf5	0.18	0.00
Seh1l	0.23	0.00
Dnm1l	0.24	0.00
Rab29	0.15	0.00
Cdc123	0.39	0.00
Fam53b	0.37	0.00

B3galnt2	0.18	0.00
Cmas	0.09	0.00
Tab2	0.21	0.00
Ube2a	0.02	0.00
Hipk2	0.72	0.00
Ptges2	0.11	0.00
Pitpna	-0.03	0.00
Nagpa	0.13	0.00
Aspscr1	0.07	0.00
Prmt9	-0.03	0.00
Tcp1	-0.08	0.00
Rabl3	0.18	0.00
Stxbp1	0.21	0.00
Klhl15	0.19	0.00
Snrnp48	0.09	0.00
Nrxn3	1.68	0.00
Gpbp1l1	0.06	0.00
Nr2f2	0.51	0.00
Pgam5	0.13	0.00
Glg1	0.01	0.00
Suclg1	-0.06	0.00
Dusp5	0.15	0.00
Wdr20	0.25	0.00
Slc7a1	1.24	0.00
Sec11c	-0.04	0.00
Sec24b	0.45	0.00
Tbc1d13	0.07	0.00
Cnot8	-0.03	0.00
Ankmy2	0.14	0.00
Qk	-1.52	0.00
Zrsr1	0.25	0.00
Ilf2	0.09	0.00
Tbc1d8b	0.15	0.00
Reps1	0.12	0.00
Isca1	-0.12	0.00
Acaa2	0.13	0.00
Cetn3	0.26	0.00
Ttc4	0.00	0.00
Dffb	0.24	0.00
Plekha6	0.59	0.00
Rtf1	0.05	0.00
Psmg1	0.23	0.00
Gcn1	0.02	0.00
Rel	0.61	0.00
Sel1l	-0.03	0.00
Rab18	-0.13	0.00

Ep400	-0.08	0.00
Enoph1	0.12	0.00
Etfdh	0.10	0.00
Efcab5	0.59	0.00
Stard3nl	0.20	0.00
Ift20	0.03	0.00
Faim	0.20	0.00
Stoml2	0.07	0.00
Nt5c3	0.43	0.00
Bmp7	0.55	0.00
Ttpal	0.26	0.00
Btbd10	0.28	0.00
Ube2i	-0.04	0.00
Uspl1	0.08	0.00
Tcea1	-0.01	0.00
Swt1	0.01	0.00
Impa1	0.08	0.00
Snrpb2	-0.11	0.00
Timm13	0.01	0.00
Ppie	-0.04	0.00
Fnip1	0.23	0.00
Cnot6l	-0.04	0.00
Ssrp1	-0.10	0.00
Appl2	-0.05	0.00
Uba2	0.03	0.00
Nr2c2	-0.09	0.00
Unc50	0.12	0.00
Ccnh	0.04	0.00
Sdha	-0.06	0.00
Clcn7	0.08	0.00
Ttc14	0.61	0.00
Zfp704	0.11	0.00
Ntmt1	0.20	0.00
Wdtc1	0.05	0.00
Ippk	0.66	0.00
Psmc1	-0.12	0.00
Btf3l4	0.01	0.00
Anxa11	0.17	0.00
Brms1l	0.07	0.00
Nbr1	-0.04	0.00
Ankrd54	0.15	0.00
Nfat5	0.25	0.00
Lcmt2	0.35	0.00
Esyt2	0.07	0.00
Cc2d2a	1.57	0.00
Zbtb48	0.06	0.00

Rmnd5a	0.04	0.00
N4bp1	-0.02	0.00
Arhgap8	-0.10	0.00
Iqca	0.71	0.00
Xxylt1	0.27	0.00
Ubn2	-0.03	0.00
Eif4g1	0.08	0.00
Abhd11	-0.01	0.00
Rilpl2	-0.04	0.00
Irak4	0.27	0.00
Slc17a5	0.15	0.00
Ing1	-0.01	0.00
Prkar2a	0.11	0.00
Ovol2	0.20	0.00
Cbfa2t3	0.02	0.00
Usp16	-0.03	0.00
Rnf146	0.02	0.00
Fancd2	-0.14	0.00
Sys1	0.13	0.00
Iqub	0.49	0.00
Agfg1	0.12	0.00
Armc8	-0.15	0.00
Stau1	0.07	0.00
Nelfa	0.24	0.00
Sdad1	0.08	0.00
Ndufb5	0.12	0.00
Rrm2b	0.10	0.00
Zfp821	0.42	0.00
Zfp644	0.40	0.00
Bysl	0.32	0.00
Rbbp4	0.08	0.00
Sf3a1	0.22	0.00
Fmr1	0.03	0.00
Gdap2	0.15	0.00
Txndc12	-0.01	0.00
Reep5	-0.01	0.00
Trdmt1	0.37	0.00
Cln8	0.08	0.00
Cntn1	0.19	0.00
Pik3r5	0.55	0.00
Arpc5l	-0.01	0.00
Abhd17a	0.14	0.00
Rad51c	0.08	0.00
Faap100	0.07	0.00
Pgap2	0.28	0.00
Pcyox1l	0.09	0.00

Rbbp8	0.18	0.00
Srp9	0.04	0.00
G3bp1	0.04	0.00
Pcsk5	0.15	0.00
Usp53	0.21	0.00
Slc2a8	0.09	0.00
S1pr1	-2.81	0.00
Stard5	0.01	0.00
Zc4h2	0.12	0.00
Zfp511	0.18	0.00
Hexb	0.12	0.00
Utp11	0.09	0.00
Neto2	0.19	0.00
Ssr1	0.02	0.00
Parp6	0.20	0.00
Fbxl4	0.21	0.00
P4hb	0.11	0.00
Arfrp1	0.01	0.00
Srsf11	0.22	0.00
Nufip2	0.22	0.00
Zfand5	0.02	0.00
Igfbp4	0.09	0.00
Trp73	0.46	0.00
Pik3r6	0.12	0.00
Kansl2	0.10	0.00
Snx33	0.12	0.00
Cdkl2	0.12	0.00
Nadsyn1	0.09	0.00
Selenok	0.14	0.00
Adgrl3	0.49	0.00
Supt20	0.20	0.00
Rbm41	0.16	0.00
Cep78	0.37	0.00
Ahdc1	0.33	0.00
Sesn3	0.26	0.00
Nsun6	0.23	0.00
Rgs14	0.12	0.00
Klhdc8a	0.10	0.00
Prkx	0.38	0.00
Cnot7	0.02	0.00
Syf2	0.01	0.00
Snx9	0.08	0.00
Tmem242	0.01	0.00
Atp6ap2	-0.13	0.00
Abce1	-0.03	0.00
Wipi2	-0.10	0.00

Acap2	-0.06	0.00
Rpp30	0.21	0.00
Akap10	-0.04	0.00
Tdrd3	0.09	0.00
Nol10	0.29	0.00
Hmg20b	-0.01	0.00
Atp13a1	-0.04	0.00
Dbndd1	0.34	0.00
E2f1	0.03	0.00
Sec63	0.22	0.00
Dynlrb1	-0.15	0.00
Spire2	0.31	0.00
Acss1	0.14	0.00
Slc18b1	0.23	0.00
Fpgt	0.15	0.00
Hs6st2	0.19	0.00
Bbs9	0.61	0.00
Fbxo9	0.19	0.00
Bag3	0.33	0.00
Tnfaip8l1	0.27	0.00
Klhl12	0.08	0.00
Rad54l	0.08	0.00
Lta4h	0.12	0.00
Col4a1	-2.65	0.00
Cenpt	0.17	0.00
Suv39h2	0.06	0.00
Guk1	0.09	0.00
Tbc1d22b	0.09	0.00
Smco4	0.04	0.00
Gpn3	0.01	0.00
Cops3	-0.07	0.00
Ndufa12	0.13	0.00
Ccpg1	0.00	0.00
Lamp2	0.18	0.00
Akr1a1	0.53	0.00
Ndufb8	0.12	0.00
Ghitm	0.39	0.00
Urm1	0.05	0.00
Zbtb24	0.27	0.00
Rab10	0.11	0.00
Gpatch2	0.88	0.00
Srp14	0.01	0.00
Trio	-1.92	0.00
Lin54	0.21	0.00
Tmcc3	-2.77	0.00
Rabl6	0.20	0.00

Bbs4	0.18	0.00
Ddx42	0.03	0.00
Bach1	0.05	0.00
Plekhg1	-2.70	0.00
Pcif1	0.06	0.00
Eif3b	0.24	0.00
Dusp8	0.22	0.00
Tbck	0.40	0.00
Htra1	0.46	0.00
Lonrf1	0.13	0.00
Abtb1	-0.14	0.00
Derl3	0.16	0.00
Hmox2	0.32	0.00
Umps	0.09	0.00
Csrnp1	0.42	0.00
Lgi1	0.35	0.00
Vps4b	0.13	0.00
Vapb	0.45	0.00
Dbi	0.88	0.00
Adi1	0.11	0.00
Sdsl	0.21	0.00
Dtx2	0.00	0.00
Tmem203	0.01	0.00
Batf3	0.08	0.00
Fahd1	0.19	0.00
Leo1	0.05	0.00
Aldoc	0.18	0.00
Atp11b	-0.03	0.00
Zdhhc3	0.06	0.00
Gtpbp10	0.16	0.00
Col14a1	0.25	0.00
Acaa1b	0.00	0.00
Fam131b	0.15	0.00
Lsm1	-0.01	0.00
Fam189a1	-2.69	0.00
Ssbp3	-0.01	0.00
Csnk1e	-0.08	0.00
Heg1	-2.92	0.00
Ube2v1	-0.06	0.00
Rab27a	0.00	0.00
Yaf2	-0.09	0.00
Larp7	-0.04	0.00
Prelid1	0.13	0.00
Dnajc19	0.17	0.00
Fam234a	-0.05	0.00
Pdk1	0.10	0.00

Fgf9	0.28	0.00
Trmt13	0.09	0.00
Fam13a	0.34	0.00
Slc25a42	0.00	0.00
Ccdc171	0.72	0.00
Pdcd10	-0.21	0.00
Cyld	-0.01	0.00
Usp31	0.20	0.00
Elmo1	-1.79	0.00
Ssu72	0.15	0.00
Tasp1	0.19	0.00
Glt8d1	0.09	0.00
Cd83	0.14	0.00
Thoc5	0.07	0.00
Ola1	0.10	0.00
Hmbs	-0.15	0.00
Timm10	0.11	0.00
Spg11	0.04	0.00
Zdhhc16	0.08	0.00
Arl4a	-2.08	0.00
Fan1	0.18	0.00
Dusp19	0.11	0.00
Galnt12	0.05	0.00
Slc38a6	0.18	0.00
Use1	-0.02	0.00
Eif4a3l1	-0.08	0.00
Parg	-0.01	0.00
Braf	0.21	0.00
Sec22c	0.21	0.00
Grpel1	-0.07	0.00
Saraf	-0.18	0.00
Plag1	0.07	0.00
Coq5	0.07	0.00
Usp38	0.13	0.00
Ccdc91	0.11	0.00
Hacd3	0.15	0.00
Ppil2	-0.03	0.00
Ints11	-0.09	0.00
Creb3l2	0.31	0.00
Hcfc2	-0.03	0.00
Ubr2	0.27	0.00
Corin	-3.17	0.00
Unc119b	0.22	0.00
Prelid3a	0.31	0.00
Ccnd1	0.18	0.00
Tmem65	0.32	0.00

Phb	-0.08	0.00
Cdc40	-0.07	0.00
Cpd	-0.08	0.00
Adam22	0.77	0.00
Atp13a2	0.28	0.00
Sgcg	0.35	0.00
Psen1	-0.19	0.00
Pgm1	0.11	0.00
Hbp1	-0.04	0.00
Tmem60	0.00	0.00
Nkrf	-0.11	0.00
Wdfy1	0.01	0.00
Zcchc8	-0.19	0.00
Hsd17b4	-0.02	0.00
Actb	-1.02	0.00
Tmx1	-0.04	0.00
Grik4	0.43	0.00
Ddhd2	0.01	0.00
Rfng	0.15	0.00
C1qtnf12	0.00	0.00
Sirt6	0.10	0.00
Fkbp11	0.45	0.00
Wasf1	0.10	0.00
Rwdd1	0.10	0.00
Exosc7	0.08	0.00
Fbxw2	0.04	0.00
Ift74	0.40	0.00
Opa1	0.01	0.00
Zbtb49	0.33	0.00
Anks6	-0.01	0.00
Rasa2	0.23	0.00
Tdp1	0.25	0.00
Stx18	0.32	0.00
Zbtb33	0.05	0.00
Prkaa1	0.16	0.00
Ing5	-0.05	0.00
Tnrc6c	0.22	0.00
Qsox2	0.28	0.00
Tmem128	0.14	0.00
Fhl3	0.14	0.00
Tmem135	0.34	0.00
Dock9	-1.75	0.00
Tada3	0.04	0.00
Skil	0.32	0.00
Fbxl20	0.15	0.00
Ppfibp1	0.67	0.00

Arhgap1	0.04	0.00
Hpgds	0.04	0.00
Nbas	0.07	0.00
Ncbp1	0.02	0.00
Avl9	0.20	0.00
Klhl36	0.07	0.00
Polr2c	0.13	0.00
Hyou1	0.36	0.00
Bud31	-0.14	0.00
Washc3	0.00	0.00
Fam199x	-0.03	0.00
Rmdn2	0.22	0.00
Fkbp3	-0.08	0.00
Kdm5a	0.14	0.00
Alg3	0.14	0.00
Fgfr2	0.95	0.00
Dynlt3	-0.16	0.00
Gpx4	0.02	0.00
Bach2	0.47	0.00
Ctr9	0.13	0.00
Arcn1	-0.03	0.00
Tcerg1	-0.12	0.00
Arhgap12	0.47	0.00
Prpf6	-0.19	0.00
Rundc1	0.11	0.00
Abracl	-0.07	0.00
Psme4	0.24	0.00
Anapc7	0.10	0.00
Paip1	-0.06	0.00
Slc25a25	-0.04	0.00
Irf7	0.22	0.00
Raf1	0.09	0.00
Uhrf2	-0.01	0.00
Bst1	0.16	0.00
Tmx3	-0.23	0.00
Tomm70a	-0.24	0.00
Fbln1	0.89	0.00
Sulf2	0.48	0.00
Akt1	0.04	0.00
Ddx20	0.07	0.00
Atg101	0.02	0.00
Adgrl2	-1.96	0.00
Atxn10	0.57	0.00
Nabp1	0.05	0.00
Hp1bp3	-0.07	0.00
Psmc5	-0.25	0.00

Wdr82	-0.08	0.00
Spire1	0.52	0.00
Dqx1	0.10	0.00
Cdkl5	0.24	0.00
Exosc1	-0.05	0.00
Acer3	0.16	0.00
Tm9sf4	0.22	0.00
Eml1	0.60	0.00
Ankrd9	0.06	0.00
Ube2g2	-0.06	0.00
Pepd	0.15	0.00
Secisbp2	0.11	0.00
Slc12a2	0.42	0.00
Ndufs7	-0.08	0.00
Crtc1	0.22	0.00
Grip1	0.40	0.00
Ap1b1	0.13	0.00
Ranbp3	0.21	0.00
Gucd1	-0.01	0.00
Cntrl	0.28	0.00
Pus10	0.12	0.00
Cda	0.16	0.00
Smarcc1	0.08	0.00
Pig	0.10	0.00
Nsd3	0.00	0.00
Tubb6	0.99	0.00
Tmc6	0.09	0.00
Etfrf1	0.07	0.00
Plcb1	-1.84	0.00
Herpud2	0.13	0.00
Usp33	-0.04	0.00
Thada	0.12	0.00
Ythdf1	-0.15	0.00
Golga5	-0.04	0.00
Gale	0.05	0.00
Adamts5	0.25	0.00
Pcsk7	0.13	0.00
Rrp9	0.11	0.00
Gpr107	0.18	0.00
Chchd4	0.01	0.00
Afg3l2	0.03	0.00
Arl6	0.17	0.00
Tcirg1	0.09	0.00
Tubgcp4	0.10	0.00
Cib2	0.41	0.00
Stk38l	0.17	0.00

Casp7	-0.01	0.00
Sdr42e1	0.06	0.00
Klf11	-0.05	0.00
Ddx23	-0.03	0.00
Rpap2	0.19	0.00
Plekhm3	0.33	0.00
Grk4	0.03	0.00
Kdm2b	-0.05	0.00
Asf1a	0.10	0.00
Psmd7	-0.08	0.00
Ndufaf4	-0.04	0.00
Ccnk	-0.12	0.00
Alkbh8	0.22	0.00
Atad3a	0.07	0.00
Dcaf17	0.07	0.00
Nob1	0.05	0.00
Igfbp5	-0.09	0.00
Katna1	-0.03	0.00
Ylpm1	0.23	0.00
Ern1	-0.06	0.00
Iqck	0.48	0.00
Ccdc66	0.19	0.00
Selenoo	0.06	0.00
Smcr8	-0.01	0.00
Rapgef2	-2.07	0.00
Usp44	0.12	0.00
Trmt11	0.08	0.00
Nme5	0.30	0.00
Ssb	0.03	0.00
Rimbp2	0.44	0.00
Sema6a	-2.64	0.00
Commd2	0.11	0.00
G6pc2	0.25	0.00
Nmt2	0.00	0.00
Sgms1	0.58	0.00
Lrrc73	0.09	0.00
Ift80	0.54	0.00
Nup93	-0.03	0.00
Cdh5	-2.78	0.00
Capsl	0.37	0.00
Arfgap3	0.21	0.00
Rnft1	0.24	0.00
Scarb1	0.10	0.00
Cnnm4	0.08	0.00
Camkmt	1.15	0.00
Egf	0.24	0.00

Slc43a2	0.16	0.00
Copa	-0.10	0.00
Sf3b3	-0.17	0.00
Zfp462	0.59	0.00
Trim35	0.06	0.00
Eif3j2	0.00	0.00
Psmc3	-0.10	0.00
Naa30	0.02	0.00
Ccnc	0.27	0.00
Btbd1	0.03	0.00
Snx2	-0.03	0.00
Hadha	0.18	0.00
Tpcn1	0.36	0.00
Tcf25	-0.06	0.00
St7	0.11	0.00
Gpatch8	0.04	0.00
Nsf	0.15	0.00
Bcr	0.30	0.00
Trabd2b	0.35	0.00
Tmem165	-0.18	0.00
Wdr37	0.21	0.00
Ppp1r26	0.15	0.00
Gabarapl2	-0.07	0.00
Rrp7a	-0.02	0.00
Arntl	0.10	0.00
Psma1	-0.08	0.00
Pla2g12a	-0.04	0.00
Adipor1	-0.02	0.00
Spg21	0.03	0.00
Tm9sf3	0.53	0.00
Brd7	-0.17	0.00
Tsn	0.13	0.00
Pdcd6	-0.10	0.00
Brox	0.16	0.00
Polr2k	-0.09	0.00
Psmg3	-0.12	0.00
Slc38a2	0.93	0.00
Tpmt	0.08	0.00
Atp23	0.06	0.00
Ube2d2a	0.10	0.00
Csf1	0.12	0.00
Dnaja4	-0.14	0.00
Itgb5	0.21	0.00
Pds5a	-0.12	0.00
Sec61a2	0.21	0.00
Snrpe	0.04	0.00

Cltb	-0.18	0.00
Elovl1	0.03	0.00
Nudc	-0.07	0.00
Rarb	1.05	0.00
Sft2d2	0.04	0.00
Tmem39a	0.05	0.00
Ccng1	-0.08	0.00
Immt	0.06	0.00
Pdpk1	0.00	0.00
Ptpn2	0.23	0.00
Psmb4	-0.10	0.00
Actr8	0.00	0.00
Herc3	0.20	0.00
Atf3	1.82	0.00
Parp8	0.53	0.00
Cavin2	-2.67	0.00
Acad8	0.06	0.00
Nit2	0.06	0.00
Pkp4	0.25	0.00
Dusp23	-0.08	0.00
Tnfrsf1a	0.01	0.00
Ccdc149	0.27	0.00
Cast	-0.01	0.00
Nr4a3	1.34	0.00
Lcmt1	0.13	0.00
Scnn1g	0.25	0.00
Erlin2	0.02	0.00
Zfp598	-0.06	0.00
Numb	-1.99	0.00
Nhlrc1	0.12	0.00
Tlk1	-0.11	0.00
Oxr1	0.24	0.00
Dgat2	0.19	0.00
Decr1	0.00	0.00
Gtf3c3	0.12	0.00
Dnajc2	-0.09	0.00
Iah1	0.09	0.00
Ube2f	-0.04	0.00
Tmem138	0.04	0.00
Slc22a15	0.09	0.00
Plpp6	0.32	0.00
Foxk2	0.49	0.00
Kctd10	0.18	0.00
Srpk1	-0.05	0.00
Dars2	0.09	0.00
Cdk7	0.26	0.00

Sin3a	0.03	0.00
B3gnt2	-0.03	0.00
Pdzd11	0.05	0.00
Nrp2	1.13	0.00
Ush2a	1.17	0.00
Smim4	0.11	0.00
Rint1	0.07	0.00
Rabggtb	-0.02	0.00
Zzz3	-0.09	0.00
Slc35g2	0.11	0.00
Chst2	0.06	0.00
Wfdc1	0.02	0.00
Cops7a	-0.07	0.00
Pi4ka	-0.10	0.00
Eif4e	-0.04	0.00
Gtf3a	0.05	0.00
Zmynd10	0.20	0.00
Pin4	-0.15	0.00
Chordc1	-0.13	0.00
Psma2	-0.12	0.00
Surf6	-0.03	0.00
Nf2	0.14	0.00
Fndc3b	0.28	0.00
Plcb4	0.37	0.00
Cmtm4	0.10	0.00
Psip1	0.21	0.00
Gpc6	1.45	0.00
Tspan13	0.05	0.00
Vps26b	-0.03	0.00
Pdk3	0.03	0.00
Psme3	0.01	0.00
Tcea2	-0.03	0.00
Aar2	0.06	0.00
Gnas	0.76	0.00
Adsl	0.07	0.00
Clptm1l	-0.07	0.00
Tmed10	-0.01	0.00
Golga1	0.06	0.00
Serac1	0.04	0.00
Dis3l2	0.53	0.00
Rnf215	0.15	0.00
Chmp6	-0.09	0.00
Acvr2a	0.29	0.00
Arntl2	0.10	0.00
Sms	0.25	0.00
Tor1b	0.07	0.00

Eif2s2	-0.22	0.00
4933400A11Rik	0.04	0.00
Wrn	0.23	0.00
Lpl	-2.56	0.00
Zfp618	0.06	0.00
Setx	0.38	0.00
Stk10	-0.10	0.00
Ypel1	0.11	0.00
Ogfod3	0.03	0.00
Bmt2	0.09	0.00
Nfxl1	-0.06	0.00
Gnai2	-0.02	0.00
Tango6	-0.10	0.00
Cdc14a	0.47	0.00
Pten	-0.03	0.00
Rhbdf1	-0.06	0.00
Stk40	0.23	0.00
Hdac2	-0.02	0.00
Ddx31	0.02	0.00
Tmem184b	-0.07	0.00
Arpc5	-0.01	0.00
Hif1a	0.01	0.00
Rnf10	-0.20	0.00
Nr4a1	0.95	0.00
Trap1	-0.01	0.00
Lypd6	0.52	0.00
Add3	0.43	0.00
Arhgef16	0.20	0.00
Cd47	0.31	0.00
Idh2	-0.13	0.00
Ptdss1	0.13	0.00
Iqch	0.90	0.00
Chfr	-0.11	0.00
Ehd3	-0.21	0.00
Ostc	-0.17	0.00
Rnf126	0.09	0.00
Ctps2	-0.09	0.00
Prdx4	0.02	0.00
Dpysl3	0.11	0.00
Ankle2	-0.28	0.00
Smim14	0.61	0.00
Ublcp1	-0.14	0.00
Emc2	-0.08	0.00
Ddx6	-0.06	0.00
Tmem167	-0.13	0.00
Rbm19	-0.02	0.00

Dnaja1	-0.21	0.00
Ccdc113	0.12	0.00
Fam193b	0.05	0.00
Kcnb1	0.15	0.00
Wipf3	0.26	0.00
Atp11a	0.53	0.00
Samd12	0.82	0.00
Cnot9	0.07	0.00
Vezf1	-0.27	0.00
Bms1	-0.06	0.00
Vdr	0.15	0.00
Trmt10c	-0.04	0.00
Tob1	0.50	0.00
Clpx	-0.02	0.00
Nucb2	0.00	0.00
Exoc5	0.16	0.00
Hscb	0.00	0.00
Abcf2	-0.19	0.00
Nudcd3	-0.01	0.00
Dhcr7	0.09	0.00
Sptlc2	0.10	0.00
Timp3	-0.91	0.00
Armt1	0.10	0.00
Thyn1	0.04	0.00
Rbm12	-0.07	0.00
Pik3ap1	0.05	0.00
Ap3m1	-0.15	0.00
Usp19	0.01	0.00
Bzw1	-0.06	0.00
Gpi1	-0.20	0.00
Dbndd2	0.14	0.00
Whamm	-0.14	0.00
Usp30	0.14	0.00
Cdc42	0.05	0.00
Chchd1	0.10	0.00
Ddx24	0.01	0.00
Farp2	0.14	0.00
Pitpnb	-0.19	0.00
Polr3h	0.12	0.00
Drg1	-0.05	0.00
Gbe1	0.46	0.00
Kpna6	0.05	0.00
Rpgr	0.52	0.00
Sos2	-0.06	0.00
Rbm15	-0.02	0.00
Xpo5	-0.09	0.00

Samhd1	0.36	0.00
Arih2	0.07	0.00
Sik3	0.08	0.00
Drosha	0.15	0.00
Lypd6b	0.61	0.00
Arrdc1	0.10	0.00
Ddrgk1	-0.06	0.00
Nsdhl	0.17	0.00
Tmem42	-0.11	0.00
Nif3l1	0.04	0.00
Dpp6	0.69	0.00
Unc119	0.26	0.00
Dnal1	0.63	0.00
Ube2j1	0.09	0.00
Ostf1	-0.20	0.00
Parva	0.10	0.00
Rexo1	-0.02	0.00
Slc25a21	0.31	0.00
Arxes2	-0.14	0.00
Pex10	0.11	0.00
Ubn1	0.05	0.00
Sugt1	0.13	0.00
Npas3	-2.69	0.00
Prepl	0.27	0.00
Tmem177	0.17	0.00
Emc7	-0.22	0.00
Thap1	0.10	0.00
Abca5	0.15	0.00
Hhat	0.32	0.00
Dync2li1	0.04	0.00
Kif3a	-0.05	0.00
Prpf40a	0.04	0.00
Raph1	1.08	0.00
Wdr36	0.02	0.00
Fut4	0.11	0.00
Eif2b1	-0.04	0.00
Slc25a15	0.25	0.00
Ptpmt1	0.09	0.00
Baz1b	-0.04	0.00
Aasdhppt	0.24	0.00
Ss18	-0.18	0.00
Klhdc2	-0.03	0.00
Fam184a	0.19	0.00
Hectd4	0.00	0.00
Gpd1l	0.14	0.00
Fam120a	0.11	0.00

Fam81a	0.43	0.00
Ppp6r2	0.20	0.00
Gtf3c1	0.04	0.00
Cops2	0.06	0.00
Rab4a	-0.05	0.00
Ostm1	-0.15	0.00
Ptprz1	0.26	0.00
Six2	0.32	0.00
Arrdc4	0.01	0.00
Arsg	0.08	0.00
Ptafr	0.08	0.00
Uso1	0.03	0.00
Snx13	0.20	0.00
Ppm1b	-0.01	0.00
Tpo	0.45	0.00
Iqcg	0.21	0.00
Emg1	0.01	0.00
Rer1	-0.06	0.00
Rad9a	0.10	0.00
Gde1	0.08	0.00
Efcab2	0.35	0.00
Bag2	0.02	0.00
Pex16	0.09	0.00
Pthlh	0.36	0.00
Rgs9	0.11	0.00
Pemt	0.14	0.00
Ubr1	0.00	0.00
Itgb1bp1	0.20	0.00
Ilvbl	0.07	0.00
Acsf2	0.06	0.00
Lrrfip1	0.79	0.00
Styk1	0.28	0.00
Pdcd7	-0.08	0.00
Pinx1	0.08	0.00
Phf2	-0.20	0.00
Lxn	0.13	0.00
Afg1l	0.18	0.00
Pm20d2	-0.09	0.00
Fbxo48	0.00	0.00
Shroom2	0.29	0.00
Vps50	0.00	0.00
Arel1	0.21	0.00
Bsg	-2.14	0.00
Pfdn1	-0.18	0.00
Trnt1	0.15	0.00
Snn	0.02	0.00

Rab43	0.22	0.00
Smg1	-0.07	0.00
Fam8a1	0.04	0.00
Slc38a10	0.16	0.00
Tmem70	0.03	0.00
Bhlhe40	0.04	0.00
Parp1	-0.13	0.00
Jcad	-2.46	0.00
Swi5	-0.11	0.00
Dok7	0.79	0.00
Pnpt1	0.09	0.00
Htatsf1	0.00	0.00
Ergic3	0.02	0.00
Ccdc117	-0.12	0.00
Plek	0.26	0.00
Adgrg6	0.53	0.00
Rlim	-0.23	0.00
Cul4b	-0.15	0.00
Stx2	0.06	0.00
Fam204a	-0.11	0.00
Rprd1a	0.04	0.00
Zfp609	-0.01	0.00
Crk	-0.19	0.00
Adprm	0.12	0.00
Ptpn9	0.02	0.00
Dph6	0.57	0.00
Zswim7	0.14	0.00
Tdp2	-0.05	0.00
Pmepa1	0.12	0.00
Nsun7	0.51	0.00
Efr3a	0.04	0.00
Bap1	-0.13	0.00
Polg	0.10	0.00
Hddc2	0.11	0.00
Slc37a3	0.00	0.00
Ermp1	0.00	0.00
Nox4	0.24	0.00
Zfhx3	-0.01	0.00
Tial1	-0.15	0.00
Cdh2	0.45	0.00
Eif2ak1	0.15	0.00
Nisch	-0.14	0.00
Nfe2l2	0.09	0.00
Aco2	-0.10	0.00
Pgghg	0.10	0.00
Npr3	-2.82	0.00

Cwf19l2	0.26	0.00
Ddx17	0.08	0.00
Kcnj5	-2.63	0.00
Osbpl2	-0.15	0.00
Scaper	0.21	0.00
Atxn2	0.12	0.00
Vill	0.34	0.00
Kalrn	0.07	0.00
Pum2	0.08	0.00
Actl6a	0.33	0.00
Dll4	-2.60	0.00
Ankrd27	0.49	0.00
Txndc17	0.07	0.00
Ncoa7	0.31	0.00
Lrrc8b	0.19	0.00
Vezt	0.13	0.00
Tk2	0.01	0.00
Wdr61	0.07	0.00
Nipsnap1	0.11	0.00
Ino80d	-0.11	0.00
Rnmt	-0.18	0.00
Itfg1	0.00	0.00
Zfp507	0.01	0.00
Pard3	0.93	0.00
Zfp106	0.09	0.00
Satb1	0.28	0.00
Tgs1	0.05	0.00
Rnf19a	0.28	0.00
Pm20d1	0.21	0.00
Rbm26	0.09	0.00
Rwdd3	0.08	0.00
Ankfy1	-0.08	0.00
Ampd3	0.04	0.00
Slc26a5	0.22	0.00
Phf10	-0.09	0.00
Pwp1	-0.09	0.00
Kctd12	0.26	0.00
Bub3	-0.01	0.00
Plxna1	-0.16	0.00
Pgm2l1	-0.04	0.00
Tor2a	0.14	0.00
Btbd7	-0.14	0.00
Sfmbt1	0.13	0.00
Hps3	0.17	0.00
Ccdc43	0.11	0.00
Hibch	0.39	0.00

Prex2	-2.62	0.00
5730480H06Rik	0.12	0.00
Senp8	0.07	0.00
Arid5a	0.11	0.00
Tada2a	0.22	0.00
Zmynd19	0.12	0.00
Ero1b	-0.20	0.00
Hspbap1	0.33	0.00
Elmod2	-0.01	0.00
Fam161b	0.06	0.00
Ppara	-0.09	0.00
Rab5c	-0.11	0.00
Selenot	-0.12	0.00
Szrd1	0.09	0.00
Tmx4	0.09	0.00
AF366264	-0.02	0.00
Ccdc125	0.19	0.00
Oxnad1	0.12	0.00
Phpt1	0.05	0.00
Gpr157	0.11	0.00
Ppa2	-0.11	0.00
Ndufs6	-0.11	0.00
Lman2l	0.02	0.00
Champ1	-0.06	0.00
Smim5	0.14	0.00
Brpf3	0.02	0.00
Rap1gds1	0.03	0.00
Sdc2	-2.46	0.00
Ahr	0.52	0.00
Aff4	-0.20	0.00
Srp54a	-0.13	0.00
Ackr4	0.11	0.00
Inpp5a	-2.13	0.00
Fbxl2	0.24	0.00
Bcl2l13	-0.25	0.00
Bcorl1	0.07	0.00
Pfdn4	-0.21	0.00
Sgcb	0.07	0.00
Atg9a	0.00	0.00
Tbl3	0.00	0.00
Ctdspl	-1.19	0.00
Dera	-0.03	0.00
Nol9	-0.18	0.00
Kbtbd8	-0.04	0.00
Scyl2	0.04	0.00
Ppm1k	0.00	0.00

G2e3	0.00	0.00
Vapa	-0.27	0.00
Stxbp6	0.46	0.00
Ypel5	0.05	0.00
Fyttd1	0.07	0.00
Ndufb6	-0.08	0.00
Cct7	-0.04	0.00
Exoc3	0.02	0.00
Alas1	0.07	0.00
Surf4	-0.17	0.00
Eif4e2	-0.05	0.00
Ppid	0.08	0.00
Srsf10	0.11	0.00
Strn	0.18	0.00
Trak1	0.47	0.00
Reck	0.20	0.00
Efl1	-0.10	0.00
Tmem30a	0.01	0.00
Arl13b	0.43	0.00
Rsl1d1	-0.01	0.00
Snx20	0.31	0.00
Kif27	0.27	0.00
Nubpl	0.64	0.00
Zdhhc21	0.45	0.00
Uprt	-0.04	0.00
Galnt2	0.19	0.00
Ttc39b	0.16	0.00
Gne	0.00	0.00
Fstl4	0.26	0.00
Wwox	0.60	0.00
Taf2	0.17	0.00
Zfp711	-0.22	0.00
Fam53a	0.12	0.00
Dip2c	0.29	0.00
Taf13	-0.20	0.00
Slc5a8	0.04	0.00
Adpgk	0.00	0.00
Ube3b	0.16	0.00
Gstk1	0.09	0.00
Kcne3	-2.71	0.00
Rsf1	-0.28	0.00
Leprot	-0.25	0.00
Rnf13	-0.07	0.00
Glo1	-0.08	0.00
Snrpa1	0.06	0.00
Zfp503	0.03	0.00

Extl2	0.02	0.00
Npepl1	-0.03	0.00
Grhl1	0.22	0.00
Ssr3	-0.07	0.00
Ube3c	0.11	0.00
Thoc1	-0.06	0.00
Tmem144	0.11	0.00
Top3b	0.14	0.00
Als2cl	0.26	0.00
Tle1	-0.03	0.00
Pgd	0.09	0.00
Ubap1	-0.05	0.00
Pskh1	0.07	0.00
Usp8	0.18	0.00
Sox2	0.56	0.00
Zbtb38	-0.04	0.00
Kin	0.01	0.00
Rora	0.41	0.00
Alg2	-0.02	0.00
Angel2	0.19	0.00
Pik3cg	0.08	0.00
Cpne2	0.03	0.00
Sec61a1	0.11	0.00
Kctd20	-0.20	0.00
Ggnbp2	-0.10	0.00
Klhl42	0.16	0.00
Rab14	-0.01	0.00
Zbtb1	0.08	0.00
Abcb6	0.07	0.00
Ube4a	-0.10	0.00
Galc	0.11	0.00
Zfyve21	0.02	0.00
Zfp622	-0.05	0.00
Snx18	0.23	0.00
Gem	0.33	0.00
Angptl3	0.10	0.00
Rnf123	0.36	0.00
Dcaf1	-0.25	0.00
Rbm6	-0.11	0.00
Als2	0.12	0.00
Slc30a6	0.08	0.00
Zfp346	0.12	0.00
Ercc6l	0.11	0.00
Ptpru	0.11	0.00
Fubp3	0.01	0.00
Zcchc2	0.01	0.00

Phospho1	0.02	0.00
Prkdc	0.07	0.00
Add1	-0.03	0.00
Klhl25	0.08	0.00
Qser1	-0.20	0.00
Pld1	0.58	0.00
Chd9	0.08	0.00
Tfip11	-0.07	0.00
Ctnnb1	0.17	0.00
Asb14	0.69	0.00
Gabpa	-0.21	0.00
Rabep1	-0.20	0.00
Eif4ebp1	0.12	0.00
Slc20a1	0.04	0.00
Impdh2	0.19	0.00
Poc1a	0.36	0.00
Eif4enif1	-0.01	0.00
Tmem263	0.12	0.00
Taf11	0.02	0.00
Zfp800	-0.02	0.00
Psmd1	-0.16	0.00
Phf12	-0.33	0.00
Fem1b	0.06	0.00
Commd1b	-0.18	0.00
Atad1	-0.06	0.00
L3mbtl2	0.18	0.00
Stau2	0.10	0.00
Idh3a	0.03	0.00
Senp5	-0.10	0.00
Cct4	-0.13	0.00
Zfat	0.11	0.00
Tmem183a	-0.16	0.00
Eif3e	0.04	0.00
Slc2a9	0.22	0.00
Cep63	0.07	0.00
Tspan4	0.51	0.00
Galk2	0.25	0.00
Naa35	0.18	0.00
Sun2	0.03	0.00
Tmco4	0.49	0.00
Nsfl1c	-0.10	0.00
Lemd2	0.13	0.00
Zbtb5	0.09	0.00
Tmem87a	0.00	0.00
Rnf170	0.08	0.00
Riok1	0.22	0.00

Rabgap1	-0.35	0.00
Synm	-2.59	0.00
D2hgdh	0.06	0.00
Zhx3	-0.18	0.00
Lysmd3	-0.12	0.00
Trub1	0.05	0.00
Ptpra	0.05	0.00
Scin	0.25	0.00
Bcap29	-0.17	0.00
Arl6ip4	0.20	0.00
Tprkb	0.03	0.00
Gadd45g	0.09	0.00
Trerf1	-0.17	0.00
Casp6	-0.02	0.00
Nudt6	0.15	0.00
Herc1	0.22	0.00
Pigh	0.12	0.00
Nsun5	0.02	0.00
Aqp3	0.12	0.00
Basp1	1.17	0.00
Atg2b	0.27	0.00
Dennd1a	0.15	0.00
Tnfrsf8	0.13	0.00
Ubqln4	-0.08	0.00
Atp6v0a2	0.08	0.00
Crat	0.04	0.00
Psmd5	0.09	0.00
Rfx7	0.16	0.00
Ppp1r27	0.15	0.00
Ipo8	0.13	0.00
Prkcb	0.10	0.00
Tekt1	0.28	0.00
Capn15	0.13	0.00
Gatad2b	-0.01	0.00
Gpatch2l	0.09	0.00
Smchd1	0.23	0.00
Scmh1	0.12	0.00
Xrcc6	0.02	0.00
Usb1	0.06	0.00
Psmd2	0.06	0.00
Pdzd8	-0.04	0.00
Ndrg3	0.12	0.00
Wsb1	-0.15	0.00
Erbb4	0.23	0.00
Setdb1	-0.02	0.00
Ddx47	-0.10	0.00

Cacna1d	0.07	0.00
Cnep1r1	-0.04	0.00
Cebpb	0.09	0.00
Patz1	0.04	0.00
Ywhag	0.03	0.00
Nup205	0.03	0.00
Gemin6	-0.06	0.00
Thoc2	-0.10	0.00
Htt	0.03	0.00
Clasp1	0.09	0.00
Ehbp1	0.17	0.00
Dcp2	-0.15	0.00
Ino80c	0.02	0.00
Rsl24d1	-0.08	0.00
Haus2	-0.06	0.00
Pign	0.31	0.00
Rsrc1	0.12	0.00
Ttc27	0.09	0.00
Utp23	-0.03	0.00
Lrpprc	0.37	0.00
Cep350	0.04	0.00
Tmem204	-2.49	0.00
Atf6	-0.12	0.00
Creb1	0.03	0.00
Cep44	0.04	0.00
Fbxo28	-0.04	0.00
Lsm5	0.06	0.00
Usp14	0.06	0.00
Llph	-0.18	0.00
Rab3gap2	-0.12	0.00
Csnk2a1	-0.02	0.00
Rcor1	-0.24	0.00
Agpat3	0.07	0.00
Chmp7	0.08	0.00
Tob2	0.04	0.00
Prrc2b	-0.09	0.00
Nup54	0.07	0.00
Cul1	0.00	0.00
Lsm8	-0.18	0.00
Pafah1b2	-0.24	0.00
Sin3b	0.04	0.00
Ntan1	-0.02	0.00
Adam17	-0.10	0.00
Stam2	-0.07	0.00
Api5	-0.39	0.00
Rufy3	0.14	0.00

Arl6ip5	-0.07	0.00
Armc2	0.50	0.00
Caprin1	-0.09	0.00
Homer2	-0.08	0.00
Pitrm1	-0.05	0.00
Xrcc5	-0.05	0.00
Wrnip1	0.00	0.00
U2af1	-0.09	0.00
Epb41	0.06	0.00
Ipo13	0.23	0.00
Vps41	-0.05	0.00
Vps18	0.20	0.00
Nudt9	0.06	0.00
Ndufa4	0.34	0.00
Tgfb3	0.13	0.00
Atp8b3	0.12	0.00
Zc3h15	-0.16	0.00
Cnot11	0.03	0.00
Snx25	-0.15	0.00
Tdrd7	0.04	0.00
Gnpda2	-0.07	0.00
Pdia4	0.21	0.00
Trp53bp1	-0.02	0.00
Lsm10	0.07	0.00
Usp48	-0.23	0.00
Nap1l4	-0.20	0.00
Pcgf6	0.20	0.00
Lztr1	0.07	0.00
B2m	-0.59	0.00
Adamts1	0.37	0.00
Smarca5	-0.23	0.00
Capzb	-0.14	0.00
Nudt2	0.07	0.00
Aurkaip1	0.07	0.00
Zmat5	0.07	0.00
Psmb1	-0.22	0.00
Kif3b	0.10	0.00
Fam222b	0.02	0.00
Cnppd1	-0.14	0.00
Zranb3	0.01	0.00
Tns3	0.07	0.00
Vps37a	-0.03	0.00
Sdhd	-0.10	0.00
Ssh2	0.10	0.00
Aasdh	0.09	0.00
Nutf2	-0.02	0.00

Il6st	-0.08	0.00
Rsbn1	0.09	0.00
Trmt1l	-0.04	0.00
Isca2	0.00	0.00
Lrp12	-0.02	0.00
Usp5	-0.01	0.00
Heatr5b	0.12	0.00
Bclaf1	0.09	0.00
Ptprn2	-1.86	0.00
Nek9	-0.24	0.00
Pdia5	0.13	0.00
Eftud2	0.14	0.00
Prpf39	0.00	0.00
Tpd52l2	-0.28	0.00
Tmem208	-0.14	0.00
Vps8	0.01	0.00
Helq	0.07	0.00
Pop4	0.12	0.00
Gemin5	0.01	0.00
Stk3	0.02	0.00
Snrnp35	0.00	0.00
Uck2	0.07	0.00
Stk26	-0.08	0.00
Shank2	0.70	0.00
Ehhadh	0.05	0.00
Agtpbp1	0.12	0.00
Nfkb2	-0.06	0.00
Bmpr1a	-0.13	0.00
Supv3l1	0.06	0.00
Klhl18	0.08	0.00
Ppp1r42	0.67	0.00
Aqr	-0.20	0.00
Fbxo15	0.48	0.00
Katnbl1	-0.14	0.00
Smim8	0.09	0.00
Atg5	-0.21	0.00
Ccne1	0.04	0.00
Gps1	-0.03	0.00
Fam162a	-0.07	0.00
Stt3b	-0.29	0.00
Zc3h7a	-0.23	0.00
2510039O18Rik	-0.06	0.00
Oard1	-0.18	0.00
Elovl6	0.11	0.00
Dgkz	0.04	0.00
Kcnab1	0.12	0.00

Tfb2m	0.08	0.00
Arhgef18	0.31	0.00
Polr2b	-0.17	0.00
Chmp4b	0.10	0.00
Pogz	0.10	0.00
Lrp2	0.19	0.00
Epb41l1	0.02	0.00
P3h3	0.07	0.00
Tti2	0.12	0.00
Dpagt1	0.18	0.00
Ak3	0.13	0.00
Fam20c	0.25	0.00
Wnk2	0.15	0.00
Pigc	0.05	0.00
Hip1r	0.04	0.00
Rexo4	-0.04	0.00
Clk1	-0.01	0.00
Pknox1	0.07	0.00
Kdm5b	-0.17	0.00
Taf1a	0.06	0.00
Stx1a	0.16	0.00
Naaa	-0.01	0.00
Ubap2	-0.10	0.00
Ndufb10	-0.14	0.00
Zfp839	0.00	0.00
Asb13	0.03	0.00
Fastkd3	0.06	0.00
St6galnac4	0.02	0.00
Zfyve19	0.04	0.00
Zbtb7a	-0.03	0.00
Wdr24	0.07	0.00
Eif2a	-0.14	0.00
Ncoa1	0.08	0.00
Snip1	-0.27	0.00
Nek7	0.12	0.00
Epha5	0.14	0.00
Dtnbp1	0.07	0.00
Tm2d1	-0.07	0.00
Lipt2	0.04	0.00
Polr3f	0.10	0.00
Gla	0.05	0.00
Slc30a1	-0.36	0.00
Stx19	0.09	0.00
Sod1	0.00	0.00
Uba5	0.02	0.00
Rasgrp1	0.32	0.00

Cdkn1b	0.14	0.00
Ncln	-0.01	0.00
Snx17	-0.34	0.00
Tra2b	0.19	0.00
Blmh	-0.19	0.00
Fgl2	0.19	0.00
Ap4s1	0.12	0.00
Icosl	0.01	0.00
Iqcb1	0.06	0.00
Phtf2	0.11	0.00
Cwc27	-0.07	0.00
Nfkbiz	0.38	0.00
Socs7	0.04	0.00
Ufm1	-0.17	0.00
Tbk1	-0.17	0.00
Snhg4	0.14	0.00
Wdr26	0.03	0.00
Ing2	-0.07	0.00
Clk4	-0.20	0.00
Spopl	-0.37	0.00
Txn2	-0.05	0.00
Ski	-1.14	0.00
Lactb	-0.25	0.00
Dctn6	-0.22	0.00
Gclc	0.05	0.00
Nme6	0.02	0.00
Tab3	-0.15	0.00
Usp6nl	0.42	0.00
Pdlim5	0.18	0.00
Kctd6	0.07	0.00
Slc39a9	-0.07	0.00
Itga2	0.07	0.00
Dynll2	-0.04	0.00
Rufy1	-0.46	0.00
Rpa3	0.08	0.00
Dtx3l	-0.11	0.00
Slit2	0.62	0.00
Rsbn1l	0.43	0.00
Ulk1	-0.03	0.00
Tgfbrap1	-0.03	0.00
4933427D14Rik	0.04	0.00
Bsdc1	-0.19	0.00
Fam102a	0.17	0.00
Trmt6	-0.07	0.00
Wwp1	0.04	0.00
Phlpp2	-0.27	0.00

Dtwd2	0.17	0.00
Ube2j2	-0.17	0.00
Ppp1r2	-0.21	0.00
Itm2b	-1.57	0.00
Xpo4	0.02	0.00
Arih1	-0.02	0.00
Ythdf3	-0.12	0.00
Gyg	0.04	0.00
Ankrd39	-0.09	0.00
Brwd1	0.02	0.00
Xylt1	0.10	0.00
Nbn	0.05	0.00
Cops4	-0.06	0.00
Prkar2b	0.00	0.00
Inpp5f	0.16	0.00
Gpr180	-0.08	0.00
Sf3b6	-0.24	0.00
Larp4	-0.09	0.00
Sgpp1	-0.28	0.00
Aatf	0.02	0.00
Daam2	0.63	0.00
Hipk1	-0.08	0.00
Samm50	0.04	0.00
Slc25a3	-0.09	0.00
Nemf	-0.22	0.00
Sec24a	-0.19	0.00
Fam133b	-0.22	0.00
Arf6	0.09	0.00
Rnf213	0.21	0.00
Jag1	-0.04	0.00
Epb41l4a	0.26	0.00
Sav1	-0.10	0.00
Itpa	-0.04	0.00
Bcl7a	-0.10	0.00
Zzef1	-0.06	0.00
Slc45a3	-0.08	0.00
Pacsin2	0.37	0.00
Atxn1l	0.05	0.00
Cuedc1	-0.07	0.00
Ptcd3	-0.06	0.00
Gipc2	0.10	0.00
Zmynd11	0.33	0.00
Tcaim	0.07	0.00
Ewsr1	0.16	0.00
Nrbp1	-0.13	0.00
Elp4	0.19	0.00

Denr	-0.21	0.00
Sorbs1	-2.11	0.00
Ubtd2	0.00	0.00
Rsu1	-0.07	0.00
St6gal1	0.43	0.00
Setd2	-0.26	0.00
Abcb7	-0.09	0.00
Pkdcc	0.06	0.00
Rtcb	-0.02	0.00
Cnot10	-0.14	0.00
Atg4a-ps	0.04	0.00
Srf	0.00	0.00
Nucks1	-0.30	0.00
Srsf2	-0.14	0.00
Insr	0.06	0.00
Galm	0.25	0.00
Crkl	-0.24	0.00
Ascc3	0.02	0.00
Lsm14b	0.01	0.00
Tspan8	0.13	0.00
Spata7	0.13	0.00
Tmem104	-0.08	0.00
Atp6v1g1	-0.18	0.00
Lyrm7	0.06	0.00
Arid4a	0.14	0.00
Lias	0.09	0.00
Bcar1	-0.20	0.00
Syap1	-0.11	0.00
Dnajc13	-0.21	0.00
Washc4	0.04	0.00
Pus7l	0.01	0.00
Elmo2	0.01	0.00
Fem1c	0.06	0.00
Nfatc3	0.41	0.00
Nt5c3b	0.00	0.00
Xpo6	-0.31	0.00
Rfc1	-0.13	0.00
Cdc23	0.02	0.00
Sptlc1	-0.09	0.00
Pccb	0.00	0.00
2610507B11Rik	-0.05	0.00
Acyp1	-0.10	0.00
Pla2g6	0.01	0.00
Cyfip1	0.17	0.00
Sf3b1	-0.16	0.00
Acp6	-0.04	0.00

Ppwd1	0.01	0.00
Fam13b	0.00	0.00
Fbxl5	-0.35	0.00
Prmt3	-0.17	0.00
Zbtb26	-0.06	0.00
Ncdn	0.04	0.00
Fgd5	-2.32	0.00
Cpeb3	-0.05	0.00
Rab40c	0.12	0.00
Nme7	0.02	0.00
Eya4	0.68	0.00
Ttc3	0.43	0.00
Clip4	0.13	0.00
Lsg1	0.00	0.00
Tdg	-0.27	0.00
Tcf12	-1.49	0.00
Tmem260	0.05	0.00
Slc35g1	0.09	0.00
Rc3h2	-0.03	0.00
Serpinf1	0.09	0.00
Stat1	-0.23	0.00
Erbin	0.20	0.00
Nudt16l1	-0.14	0.00
Bmpr2	-2.34	0.00
Fbxw7	0.59	0.00
Rpusd2	0.03	0.00
Traf2	-0.16	0.00
Txndc9	-0.32	0.00
Bcar3	0.04	0.00
Actg1	-0.70	0.00
Ralgapb	-0.13	0.00
Dclk1	-2.64	0.00
Dcbld1	-0.02	0.00
Phf20l1	0.00	0.00
Blm	0.09	0.00
Pnrc2	0.61	0.00
Asns	0.01	0.00
Katnal2	0.23	0.00
Smad3	-0.15	0.00
Atg4c	-0.06	0.00
Veph1	0.42	0.00
Srsf1	-0.09	0.00
Dusp4	0.08	0.00
Stard8	-0.01	0.00
Pbrm1	-0.29	0.00
Zfp521	-2.37	0.00

Phka2	0.10	0.00
Srsf4	0.02	0.00
Nrsn1	0.05	0.00
Prdm5	0.30	0.00
Sult4a1	0.15	0.00
Arl4c	0.09	0.00
Tecpr2	-0.16	0.00
Ogfr	-0.05	0.00
Srp19	-0.31	0.00
Ankrd28	0.37	0.00
Slc44a1	0.13	0.00
Cps1	0.16	0.00
Scamp5	0.09	0.00
Egr4	0.08	0.00
Tshr	0.03	0.00
Ncor2	0.03	0.00
Git1	-0.06	0.00
Nemp2	0.18	0.00
Gusb	0.07	0.00
Rab9b	0.03	0.00
Sergef	0.25	0.00
Dscc1	-0.03	0.00
Exoc8	-0.11	0.00
Emp2	-0.01	0.00
Rbl1	-0.12	0.00
Nup35	0.12	0.00
Pafah1b1	-0.16	0.00
Rab21	-0.37	0.00
Snx5	-0.06	0.00
Slc35a1	-0.03	0.00
Kcnc1	-2.42	0.00
Asxl2	-0.03	0.00
Ep300	-0.24	0.00
Ube2d1	-0.12	0.00
Dck	-0.01	0.00
Chst10	0.00	0.00
Psph	0.12	0.00
Gcsh	0.04	0.00
Cerk	-0.16	0.00
Nup214	-0.07	0.00
Larp4b	-0.09	0.00
Alkbh1	0.05	0.00
Ccdc6	-0.11	0.00
Zfp366	-2.13	0.00
Hexim1	-0.21	0.00
Timeless	0.09	0.00

Dus11	0.05	0.00
Cope	-0.25	0.00
Gpatch4	0.04	0.00
Znrf3	-0.10	0.00
Polr1c	0.18	0.00
Iqsec1	0.48	0.00
B4galt2	-0.05	0.00
Ubxn2b	-0.02	0.00
Taf4	0.06	0.00
Strada	0.11	0.00
Wdr11	0.11	0.00
Recql5	0.04	0.00
Plekha8	0.07	0.00
Tulp3	0.37	0.00
Abhd18	0.01	0.00
Cfdp1	0.03	0.00
Rin3	-0.16	0.00
Poldip3	-0.21	0.00
Eral1	-0.01	0.00
Lyplal1	0.16	0.00
Zfand1	0.03	0.00
Setd7	-0.17	0.00
Arl8a	-0.12	0.00
Supt4a	-0.25	0.00
Trappc13	0.02	0.00
Pex13	-0.12	0.00
Plpbp	0.03	0.00
Vti1b	0.04	0.00
Oxct1	0.02	0.00
Ctnnbl1	0.02	0.00
Npc1	-0.05	0.00
Armc1	-0.29	0.00
Sec22a	0.02	0.00
Utrn	-1.11	0.00
Plaa	0.14	0.00
Ergic1	0.10	0.00
Papln	-0.11	0.00
Erg	-2.21	0.00
Pds5b	-0.29	0.00
Usp3	-0.10	0.00
Xylb	0.26	0.00
Ddx1	-0.19	0.00
Kansl1	-0.15	0.00
Xrn2	-0.29	0.00
Crot	-0.07	0.00
Nxpe3	0.15	0.00

Scaf4	-0.16	0.00
Topbp1	0.10	0.00
Nfyb	-0.09	0.00
Dbr1	-0.04	0.00
Far2	0.82	0.00
Slc25a37	-0.06	0.00
Dgkb	0.74	0.00
Gab1	-0.04	0.00
Rabepk	-0.15	0.01
Uchl5	-0.11	0.01
Tmem123	-0.05	0.01
Eef2	0.42	0.01
Dis3	-0.15	0.01
Cep112	0.11	0.01
Blvra	-0.04	0.01
Trim54	-0.01	0.01
Ttll5	0.87	0.01
Ube2q2	0.43	0.01
Vps11	-0.05	0.01
Dlg1	0.13	0.01
Coq7	-0.02	0.01
Otud3	0.16	0.01
Arglu1	-0.23	0.01
Nop14	-0.22	0.01
Ankrd46	-0.23	0.01
Pink1	0.09	0.01
Wipf1	0.18	0.01
Osbpl9	-0.12	0.01
Prr5	0.19	0.01
Rbm34	-0.31	0.01
Eif4h	-0.42	0.01
Taf3	-0.25	0.01
Klhl24	-0.02	0.01
Rnf149	-0.15	0.01
Cab39	-0.25	0.01
Wars2	0.39	0.01
Glcci1	0.37	0.01
Flvcr1	0.10	0.01
Cep135	0.45	0.01
Rbm45	0.06	0.01
Ap1g1	-0.36	0.01
Rfxank	-0.01	0.01
Cisd1	-0.21	0.01
Wwp2	0.11	0.01
Yap1	0.54	0.01
Tmem185a	-0.10	0.01

Slc16a9	0.33	0.01
Zfand2b	-0.01	0.01
Otud7b	0.00	0.01
Csde1	-0.18	0.01
Ass1	0.38	0.01
Arhgap18	0.00	0.01
Kansl1l	0.08	0.01
Sepsecs	0.08	0.01
Evc	0.22	0.01
Dnajc24	0.01	0.01
Tcp11l1	0.22	0.01
Zfp770	0.01	0.01
Rbm12b2	0.04	0.01
Rp2	0.01	0.01
Nadk2	-0.08	0.01
Lrch1	0.03	0.01
Sftpc	2.11	0.01
Eaf1	-0.15	0.01
Btbd11	0.59	0.01
Trappc2	-0.12	0.01
P4ha2	0.01	0.01
Ltn1	-0.25	0.01
Socs6	-0.13	0.01
Ngly1	-0.14	0.01
Pck1	0.24	0.01
Rnasel	0.12	0.01
Tigd5	-0.03	0.01
Ccng2	-0.04	0.01
Nudt5	0.05	0.01
Sacm1l	-0.25	0.01
Clk3	-0.21	0.01
Ppme1	-0.08	0.01
Dalrd3	0.14	0.01
Nop58	-0.37	0.01
Ercc3	-0.22	0.01
Nceh1	0.05	0.01
Ifrd1	0.48	0.01
Klhl14	0.07	0.01
Drg2	0.04	0.01
Tbc1d24	0.04	0.01
Wdfy2	0.13	0.01
Tab1	-0.04	0.01
Ptger4	0.15	0.01
Cog2	-0.22	0.01
Zfp367	-0.02	0.01
Lysmd4	0.06	0.01

Chaf1b	0.01	0.01
Cog6	-0.19	0.01
Pdap1	-0.30	0.01
Atp2b4	-0.27	0.01
Nedd4	0.04	0.01
Rrp1b	-0.25	0.01
Stard13	-0.84	0.01
Ak7	0.48	0.01
Tsen15	0.04	0.01
Tomm20	-0.26	0.01
Ip6k2	-0.16	0.01
Nin	-0.08	0.01
Tcof1	-0.35	0.01
Snapc3	0.13	0.01
Zfp148	-0.27	0.01
Acot13	-0.12	0.01
Nup85	0.01	0.01
Caml	0.08	0.01
Hdac11	0.06	0.01
Wbp2	-0.07	0.01
Igf2bp2	0.00	0.01
Cfap20	0.13	0.01
Endog	0.03	0.01
Zfp512	-0.07	0.01
Arl5b	0.02	0.01
Patl1	0.02	0.01
Cbx4	0.02	0.01
Dus3l	0.07	0.01
Pwwp2a	-0.15	0.01
Def6	0.13	0.01
Ctnna1	0.17	0.01
Hsd17b12	-1.24	0.01
Slc9a6	-0.03	0.01
Nqo2	0.07	0.01
Tnfaip6	0.11	0.01
Slc35c2	-0.37	0.01
Dennd3	-0.09	0.01
Adrm1	-0.23	0.01
Tbx3	-2.15	0.01
Trim25	0.05	0.01
Clns1a	-0.08	0.01
Eif2ak2	-0.02	0.01
Tcf20	-0.08	0.01
Qrich1	0.03	0.01
Rrn3	-0.16	0.01
Sap130	-0.24	0.01

Larp1b	-0.26	0.01
Cgrrf1	-0.20	0.01
Ttll1	-0.05	0.01
Cul3	-0.17	0.01
Trappc12	-0.22	0.01
Aida	-0.01	0.01
Trit1	-0.11	0.01
Ccndbp1	-0.13	0.01
Sun1	-0.28	0.01
Gtf2h1	-0.20	0.01
Pdhx	-0.11	0.01
Sec14l1	-2.02	0.01
Chmp4c	-0.16	0.01
Ybx3	0.10	0.01
Edem1	-0.18	0.01
Tmem50a	-0.36	0.01
Bzw2	0.06	0.01
Fam114a2	-0.06	0.01
Lrrc8a	-0.13	0.01
Ube2g1	-0.03	0.01
Hace1	0.09	0.01
Phc1	-0.06	0.01
Capza2	-0.02	0.01
Apcdd1	-2.15	0.01
Gramd4	0.05	0.01
Slc7a5	-0.04	0.01
Ttll7	0.50	0.01
Crebrf	-0.15	0.01
Ptgs2	0.48	0.02
Surf2	-0.06	0.02
Coq8a	0.24	0.02
Chuk	0.15	0.02
Upf2	-0.33	0.02
Zfand4	-0.05	0.02
Dync1i2	-0.50	0.02
Ncam1	0.59	0.02
Rab11fip1	0.05	0.02
Rtn4ip1	0.04	0.02
Eda	0.15	0.02
Sprtn	-0.05	0.02
Fam98b	0.08	0.02
Lrrc40	0.05	0.02
Zfp335	-0.16	0.02
Ddx46	-0.37	0.02
Urb1	-0.25	0.02
Tango2	0.00	0.02

Entpd4	0.05	0.02
Atp6v1a	0.00	0.02
Echdc1	0.02	0.02
Snx1	0.09	0.02
Dnajc5	0.00	0.02
Ubl3	0.36	0.02
Ccdc40	0.19	0.02
9030025P20Rik	0.02	0.02
Smpd4	0.06	0.02
Vrk2	0.00	0.02
Ccdc93	-0.06	0.02
Usp45	-0.17	0.02
Tmf1	-0.41	0.02
Efcab1	0.17	0.02
Agk	0.08	0.02
Cdk9	-0.10	0.02
Becn2	-0.01	0.02
Rab24	0.02	0.02
Epc2	-0.19	0.02
Immp1l	-0.06	0.02
Dnaja2	-0.35	0.02
Otulin	-0.23	0.02
Cd63	0.49	0.02
Rad18	0.01	0.02
Tia1	-0.09	0.02
Ide	-0.42	0.02
Hmgcl	-0.16	0.02
Nrip1	0.05	0.02
Gcc2	0.05	0.02
Tpr	-0.15	0.02
4833439L19Rik	-0.11	0.02
Socs3	0.02	0.02
Polr2h	-0.04	0.02
Cd81	-1.88	0.02
Adal	-0.04	0.02
Zfp652	-0.42	0.02
Kpna3	-0.34	0.02
Ccnyl1	-0.39	0.02
Ube2e3	0.03	0.02
Stom	0.37	0.02
Sash1	0.22	0.02
Tnfsf10	-0.28	0.02
Kdr	-2.06	0.02
Tbc1d9b	-0.04	0.02
Usp25	-0.12	0.02
Tfdp1	-0.15	0.02

Dnah12	1.45	0.02
Psmb7	-0.37	0.02
Sbno1	-0.22	0.02
Arl5a	-0.23	0.02
Spry2	-0.36	0.02
Pigl	0.24	0.02
Rfc4	-0.13	0.02
Rnf8	-0.25	0.02
Rffl	0.03	0.03
Nelfb	-0.05	0.03
Ncoa5	-0.13	0.03
Fbxo8	-0.21	0.03
Hdac1	0.01	0.03
Hspd1	-0.27	0.03
Fryl	-1.24	0.03
Pisd	-0.31	0.03
Nrde2	-0.08	0.03
Trpm7	-0.15	0.03
Atg3	-0.29	0.03
Smpd2	-0.02	0.03
Nup88	-0.04	0.03
Rpgrip1l	0.36	0.03
Cir1	-0.32	0.03
Rlf	-0.17	0.03
Trabd	-0.15	0.03
Cenpu	0.01	0.03
Eif5b	-0.38	0.03
Fam98a	-0.26	0.03
Top1mt	0.05	0.03
Trp53rka	0.02	0.03
Sec22b	0.01	0.03
Dok1	-0.10	0.03
Tyw3	0.06	0.03
Gins3	0.01	0.03
Lbr	-0.43	0.03
Ints1	-0.08	0.03
Gbf1	0.07	0.03
Ccdc14	0.01	0.03
Suco	-0.10	0.03
Cse1l	-0.05	0.03
Anapc13	-0.05	0.03
Gnal	0.07	0.03
Eefsec	-0.12	0.03
Pik3r4	-0.02	0.03
Actr2	-0.36	0.03
Fbxo42	0.23	0.03

Suds3	-0.38	0.03
Atg14	0.19	0.03
Sgpl1	-0.01	0.04
Sass6	0.32	0.04
Fbxl14	-0.25	0.04
Paxbp1	-0.44	0.04
Elovl7	-0.34	0.04
Uba3	-0.15	0.04
Trp53bp2	0.08	0.04
Tek	-1.95	0.04
Rab11fip3	-0.21	0.04
Bicd1	-1.60	0.04
Eif3k	-0.18	0.04
Ptpn4	0.86	0.04
Asb1	0.03	0.04
Nebl	0.92	0.04
Pnn	-0.41	0.04
Edem3	-0.16	0.04
Scn8a	0.08	0.04
Bud13	-0.06	0.04
Zfp706	0.19	0.04
Hpse	0.23	0.04
Ston2	0.08	0.04
Rimklb	0.13	0.04
Cct5	-0.45	0.04
Rnf6	0.02	0.04
Nln	0.33	0.04
Zmpste24	-0.12	0.04
Npc2	-0.31	0.04
Ell	0.00	0.04
Tatdn1	0.09	0.04
Polr3e	0.06	0.04
Gpatch11	-0.09	0.04
Camk1d	1.27	0.04
Dcaf13	-0.05	0.04
Tesc	-0.04	0.04
Nol4l	0.00	0.04
Zc3h7b	0.00	0.04
Spz1	-0.01	0.04
Snx16	-0.07	0.04
Pias4	0.09	0.04
Ndufaf2	-0.02	0.04
Eif6	-0.08	0.04
Cntnap2	0.11	0.04
Tapt1	-0.04	0.04
Slc35a3	-0.06	0.04

Crbn	-0.08	0.04
Rbm22	-0.21	0.04
Slc25a33	0.00	0.04
Lrrk1	-0.15	0.04
Eef1b2	1.01	0.05
Rbbp5	0.01	0.05
Gpalpp1	-0.46	0.05
Rab6a	-0.36	0.05
Sde2	-0.29	0.05
Sesn1	0.03	0.05
Kazn	0.57	0.05
1700034I23Rik	-0.05	0.05
Senp7	-0.50	0.05
Lyrm1	-0.12	0.05
Ufsp2	-0.11	0.05
Ppil4	-0.43	0.05
Pdpr	0.13	0.05
Asap2	-0.15	0.05
Rhoj	-2.06	0.05
Boc	-0.17	0.05
Chmp5	-0.30	0.05
Vdac2	-0.34	0.05
Ropn1l	0.15	0.05
Ifi30	0.06	0.05
Hspa4	-0.36	0.05
Serinc3	-0.22	0.06
Ephb1	-2.08	0.06
Elk4	-0.21	0.06
Rpf2	-0.28	0.06
Ddx49	-0.04	0.06
Dram2	-0.26	0.06
Dnajc11	-0.03	0.06
Pms1	0.21	0.06
Tex2	-2.11	0.06
Gpr155	-0.29	0.06
Zfp36l1	1.21	0.06
Syde2	0.41	0.06
Gtf2f2	-0.11	0.06
Plod1	-0.13	0.06
Itpkb	-2.09	0.06
Adamts9	-0.03	0.07
Vgll3	-0.04	0.07
Ascc2	0.06	0.07
Hspa13	-0.11	0.07
Slf2	-0.36	0.07
Ipo11	0.02	0.07

Retreg1	-0.28	0.07
Actr1a	-0.46	0.07
Utp6	-0.12	0.07
Sil1	-0.11	0.07
Fam32a	-0.24	0.07
Dach1	-1.83	0.07
Ints2	-0.11	0.07
Pbx1	0.03	0.07
Ccdc85a	-2.23	0.07
Szt2	0.08	0.08
Atxn7l1	0.09	0.08
Alg12	-0.09	0.08
Cav1	-2.05	0.08
Tbc1d5	0.07	0.08
Dhx35	0.00	0.08
Bop1	-0.12	0.08
Rbm17	-0.36	0.08
Sfxn4	0.10	0.08
Cep192	-0.08	0.08
Dhfr	-0.07	0.08
Prdm10	-0.08	0.08
Prpf4	0.03	0.08
Nmnat1	0.06	0.08
Abcg1	0.34	0.08
Vps39	-0.29	0.08
Zbtb2	-0.29	0.08
Xrn1	-0.55	0.08
Hectd2	0.41	0.08
Pycrl	-0.02	0.08
Traf7	0.08	0.08
Il22ra2	-0.11	0.09
Fer	0.04	0.09
Ascc1	-0.34	0.09
Dlat	-0.06	0.09
Cmtm8	-0.16	0.09
Stam	-0.21	0.09
Cpsf7	0.10	0.09
Txnl4b	-0.01	0.09
Tada1	-0.14	0.09
Chst15	0.00	0.09
Arsb	-0.03	0.09
Ndfip1	-0.22	0.09
Tepsin	-0.03	0.09
Strip1	0.01	0.09
Pigf	0.00	0.09
Lin9	0.05	0.09

Tle3	-0.43	0.09
Bnip1	-0.09	0.09
Ulk2	-0.01	0.09
Nek4	0.12	0.09
Cdh4	0.22	0.09
Blzf1	-0.23	0.09
Ccdc134	0.06	0.09
Pkd2	-0.06	0.09
Acvr1	-0.27	0.09
Hmgb3	-0.20	0.09
Clcn6	0.26	0.09
Fabp7	0.03	0.09
Galnt18	-1.69	0.09
Hmg20a	-0.03	0.10
Dapk1	0.14	0.10
Zmat3	-0.02	0.10
Cdk19	-0.36	0.10
Tbc1d2b	-0.34	0.10
Pum1	-0.18	0.10
Ddx18	-0.07	0.10
Wasl	-0.35	0.10
Phf6	-0.15	0.10
Exosc2	0.04	0.10
Zdhhc17	-0.27	0.10
Cltc	-0.18	0.10
Aftph	-0.46	0.10
Lats1	-0.36	0.10
Amdhd2	-0.29	0.10
Fubp1	-0.17	0.10
Txnrd3	-0.19	0.11
Stx8	0.19	0.11
Optn	-0.29	0.11
Coq2	-0.02	0.11
Rab5a	-0.31	0.11
Raver2	0.12	0.11
Nfx1	-0.04	0.11
Heca	-0.29	0.11
Heatr6	-0.12	0.11
Klhdc4	-0.13	0.11
Sub1	-0.40	0.11
Zdhhc6	-0.42	0.11
Timm22	-0.09	0.11
Abhd12	-0.14	0.12
Wdr91	-0.04	0.12
Cnn2	-0.10	0.12
Dym	-0.27	0.12

Pwwp2b	-0.55	0.12
Krit1	-0.04	0.12
Thrap3	-0.01	0.12
Ddx51	-0.05	0.12
Hspa8	-0.85	0.12
Kansl3	-0.11	0.12
Spryd7	0.18	0.13
Fcf1	-0.15	0.13
Adgra3	-0.03	0.13
Abcd2	-0.39	0.13
Trappc8	-0.24	0.13
Eif2b3	-0.08	0.13
Rnf34	-0.15	0.13
Gtf2h5	-0.44	0.13
Prdm2	-0.29	0.13
Nup37	0.13	0.13
Specc1l	-0.49	0.13
Rnaseh1	-0.30	0.13
Bet1	-0.11	0.14
Zfp292	-0.19	0.14
Traf3	-0.16	0.14
Zswim8	-0.26	0.14
Ncapd2	0.12	0.14
Bcl2l1	0.13	0.14
Trmt2a	-0.16	0.14
Fbxo22	0.02	0.14
Ndel1	-0.16	0.14
Zfp143	-0.07	0.14
5031439G07Rik	-0.18	0.15
Zfp516	-0.05	0.15
Dph7	0.13	0.15
Arpc3	0.01	0.15
Ythdf2	-0.34	0.15
Pnisr	-0.21	0.15
Ccnj	0.02	0.16
Cpsf3	-0.20	0.16
Fgfr1op2	-0.33	0.16
Pin1	-0.22	0.16
Fzd7	-0.03	0.16
Slc25a20	-0.17	0.16
Fam107b	-0.24	0.16
Tbpl1	-0.02	0.16
Pmp22	-2.16	0.16
Dvl3	-0.03	0.16
Vps25	-0.19	0.17
Bphl	-0.04	0.17

Clcc1	-0.12	0.17
Btrc	-0.19	0.17
Sike1	-0.30	0.17
Arl6ip1	-0.33	0.17
Psmd12	-0.57	0.17
Dazap1	-0.24	0.18
Gnai3	-0.19	0.18
Cntf	0.08	0.18
Arvcf	-2.22	0.18
Natd1	-0.05	0.19
S100a11	0.11	0.19
Ranbp1	-0.31	0.19
Atad2b	-0.09	0.19
Rictor	-0.20	0.19
Lap3	-0.07	0.19
Celf2	0.36	0.20
Slc35b3	-0.24	0.20
Tbc1d14	-0.44	0.20
Usp15	-0.27	0.20
Fam149b	0.19	0.20
Cactin	-0.27	0.20
B4galt4	0.02	0.20
R3hdm4	-0.20	0.20
Tatdn3	-0.08	0.21
Cutc	-0.09	0.21
Dlec1	0.17	0.21
Ankrd52	0.09	0.21
Tmem168	-0.12	0.21
Gramd2	0.05	0.21
Pde12	-0.07	0.21
Dyrk3	0.06	0.21
Socs5	-0.04	0.21
Gtf2a1	-0.37	0.21
Eea1	-0.07	0.21
Rbm48	0.01	0.21
Jmjd7	0.04	0.21
Btbd19	0.06	0.21
Abi2	-0.07	0.21
Snf8	-0.07	0.21
Luc7l	-0.36	0.21
Cdk5rap1	0.03	0.21
Dimt1	0.01	0.22
Nsrp1	0.20	0.22
Epha2	-0.28	0.22
Fancm	0.01	0.22
Crem	-1.37	0.22

Secisbp2l	-0.22	0.22
Id2	-0.11	0.22
Vegfa	-0.17	0.22
Eef1e1	-0.24	0.22
Usp7	-0.50	0.22
Cep131	0.03	0.22
Gpatch1	-0.22	0.23
Gstz1	0.01	0.23
Apool	-0.11	0.23
Helz2	-0.25	0.23
Dync1li1	-0.36	0.23
Zfp207	-0.32	0.23
Rfx3	0.70	0.23
Hdac10	0.01	0.24
Scpep1	-0.13	0.24
Agpat5	-0.12	0.24
Cbx3	-0.23	0.24
Tnks	0.04	0.24
Otud7a	0.09	0.24
Umad1	0.61	0.24
Rptor	-0.07	0.24
Ubqln1	-0.05	0.25
Atp1b3	-1.88	0.25
Fgfrl1	0.37	0.25
P4ha1	-0.21	0.25
Acvrl1	-1.83	0.25
Arnt	-0.23	0.25
Ctcf	-0.27	0.26
Zbtb41	-0.37	0.26
Ufl1	-0.06	0.27
Tead4	-2.04	0.27
Eif5a2	-0.23	0.27
Papss1	-0.29	0.27
Orc4	-0.05	0.28
Pgpep1	-0.15	0.28
Gpd2	0.05	0.28
Tnrc6a	-0.21	0.28
Sox6	-1.46	0.28
Necap2	-0.21	0.28
Hyi	0.02	0.29
Kif13b	-0.22	0.29
Dicer1	-0.10	0.29
Ddx59	-0.14	0.29
Qdpr	-0.04	0.29
C1d	-0.12	0.30
Upf3b	-0.19	0.30

Glyr1	-0.31	0.30
Fktn	-0.07	0.30
Smg7	-0.20	0.30
Psmd6	-0.13	0.30
Nipa1	-0.05	0.30
Cdo1	-0.03	0.30
Rpap1	-0.04	0.30
Ncoa4	-0.38	0.31
Anapc10	-0.08	0.31
Apoo	0.07	0.31
Yeats2	-0.15	0.31
Flii	-0.14	0.31
Zbed4	-0.38	0.31
Ash1l	-0.40	0.31
Ghdc	-0.13	0.31
Gtf2h3	-0.14	0.31
Ap1m1	-0.10	0.32
Uchl3	-0.18	0.32
Bbx	0.04	0.32
Tubgcp5	-0.09	0.32
Cmpk2	0.06	0.32
Ifih1	-0.01	0.33
Znhit6	-0.13	0.33
Gatad2a	-0.12	0.33
Tpm1	-0.19	0.33
Lonrf2	-0.24	0.33
Cnot2	-0.18	0.33
Fam219a	-0.25	0.33
Agap1	0.31	0.33
Pusl1	-0.11	0.34
Ptbp2	0.01	0.34
Tjp1	-0.74	0.34
Rap2c	-0.24	0.34
Ramp2	-1.77	0.34
Nav3	0.71	0.34
Cstf2	-0.07	0.35
Snrpd1	-0.37	0.35
Homer1	1.75	0.36
Slain2	0.02	0.37
C2cd5	-0.42	0.37
Tulp4	-0.06	0.37
Asrgl1	0.01	0.37
Igfbp3	-0.03	0.38
Pdss2	0.15	0.38
D630045J12Rik	0.10	0.38
Laptm4a	-1.68	0.38

Crnkl1	-0.12	0.38
Pikfyve	-0.34	0.39
Ppp2r2d	-0.37	0.39
Pou2f1	-0.05	0.39
Wdr70	-0.22	0.39
Dr1	-0.56	0.40
Dnmt3a	-0.09	0.40
Pkn2	-0.39	0.40
Telo2	0.00	0.40
Tlr3	-0.17	0.40
Snx29	-0.34	0.40
Snx10	-0.18	0.41
Gnl2	-0.16	0.41
Ppard	-0.15	0.41
Wdr25	0.11	0.42
Fyco1	-0.25	0.42
Napg	-0.37	0.43
Sec62	-0.30	0.43
Luc7l3	-0.09	0.43
Dennd2a	-1.97	0.43
Flnb	0.61	0.44
Cd109	-0.09	0.44
Smyd3	0.09	0.44
Celf1	-0.24	0.44
Ldb1	0.02	0.45
Asxl3	-1.85	0.45
Ppig	-0.16	0.45
Deaf1	0.01	0.46
Colgalt2	-0.24	0.46
Igsf6	0.02	0.46
Zbtb21	-0.45	0.47
Jund	-0.12	0.47
Cipc	-0.18	0.48
Arl16	0.05	0.48
Heatr5a	-0.24	0.48
Pdf	0.11	0.48
Ston1	0.07	0.49
Btaf1	-0.28	0.50
Pus1	-0.05	0.50
Arhgap6	0.10	0.50
Rsrp1	-0.32	0.50
Abcb8	-0.01	0.50
Ppp4r3a	-0.39	0.50
Kdm3b	-0.32	0.50
Rnf20	-0.24	0.50
Fam126a	-0.10	0.51

Prkar1b	-0.07	0.51
Zranb2	-0.29	0.51
Trim33	-0.14	0.51
Elac2	-0.06	0.52
Zfp830	-0.24	0.52
Aacs	-0.35	0.52
Terf2	0.04	0.53
Igf2r	-0.46	0.53
Ttc39a	0.16	0.53
Arhgef3	-2.03	0.53
Lyar	-0.37	0.53
Tspan2	-0.01	0.54
Prune1	-0.14	0.54
Caprin2	-0.01	0.54
Rhobtb1	0.22	0.54
Wdr3	-0.12	0.56
Ckap5	-0.37	0.56
Clock	-0.35	0.56
Eif1ad12	-0.48	0.57
Clec16a	-0.06	0.57
Eml6	0.26	0.57
Gna13	-0.12	0.58
Fkbp4	-0.24	0.58
Lbh	0.08	0.58
Skp1	-0.10	0.58
Atg13	-0.16	0.58
Osbpl10	0.27	0.60
Tiprl	-0.14	0.60
Rmnd1	0.01	0.60
Cnot4	-0.17	0.60
Helz	-0.15	0.61
Pdcl	-0.14	0.62
Oma1	-0.27	0.62
Ccp110	0.09	0.63
Prpf38a	-0.14	0.64
Dnajb12	-0.51	0.64
Zc3h13	-0.33	0.65
Rheb	-0.46	0.65
Ago2	-0.25	0.66
Sppl3	-0.29	0.69
Ank2	1.16	0.69
Bfar	-0.34	0.69
Bmp6	-1.78	0.70
Dek	0.01	0.70
Slc3a1	-0.19	0.70
Sos1	-0.42	0.71

Btg2	0.55	0.72
Cers5	-0.05	0.72
Pfn2	-0.03	0.73
Lsmem1	0.20	0.73
Edf1	0.39	0.74
Prkn	0.18	0.74
B3glct	-0.13	0.74
Kdm1b	0.00	0.74
Golim4	-0.15	0.75
Dvl1	-0.15	0.75
Stn1	0.12	0.75
Atp11c	-0.34	0.75
Klf3	-0.41	0.76
Lrrc28	0.00	0.76
Stxbp4	0.09	0.77
Dhx36	-0.43	0.78
Polk	0.05	0.78
Golt1b	-0.19	0.78
Ap4b1	-0.05	0.78
Ddx52	-0.12	0.78
Bloc1s2	-0.10	0.78
Kat14	-0.10	0.80
Dhx8	-0.20	0.80
Ptbp1	0.28	0.81
Lyrm9	-0.11	0.82
Senp6	-0.19	0.83
Cald1	-1.82	0.83
Fads2b	-0.21	0.84
Gid4	-0.11	0.85
B3gntl1	0.04	0.85
Pacsin3	0.13	0.87
Tmem161b	-0.09	0.87
Pex1	-0.01	0.87
Tmem131l	-0.03	0.88
Esco1	-0.06	0.88
Plpp2	-0.18	0.89
Cnot6	-0.35	0.89
Thumpd3	-0.36	0.89
Pxylp1	0.23	0.90
Rnf214	-0.31	0.91
Spata20	-0.11	0.91
Gatad1	-0.13	0.92
Wac	-0.53	0.94
Fundc1	-0.15	0.94
Elovl5	-0.35	0.94
Hdac7	-1.87	0.96

Zfp326	-0.39	0.97
Gtpbp6	0.11	0.98
Abhd13	-0.29	0.99
Itgav	-0.33	0.99
Arfgap1	-0.24	1.00
Tbcd	-0.08	1.00
Aqp1	-0.60	1.00
Hoxa3	-1.66	1.00
Zfp236	-0.06	1.00
Cap1	-0.10	1.00
Ccnt1	0.01	1.00
Ccdc28a	0.02	1.00
Syne3	0.03	1.00
Gan	0.02	1.00
Ttc33	-0.13	1.00
Traf6	-0.01	1.00
Nfkb1	0.18	1.00
Elp3	0.09	1.00
Filip1	0.25	1.00
Siah2	-0.01	1.00
Dcps	0.00	1.00
Lysmd2	-0.10	1.00
Rae1	0.06	1.00
Stx12	-0.26	1.00
Foxf1	-1.60	1.00
Snw1	-0.33	1.00
Rcor3	-0.34	1.00
Fbxw8	-0.28	1.00
Stk11	0.19	1.00
Pdgfrl	0.09	1.00
Bag1	-0.24	1.00
Cpne1	-0.41	1.00
Ppp6r3	-0.08	1.00
Ing4	-0.14	1.00
Rprd2	-0.31	1.00
Tarbp1	0.06	1.00
Klf12	0.12	1.00
Snapc4	-0.03	1.00
Dennd1b	0.04	1.00
4932438A13Rik	-0.26	1.00
Pms2	-0.04	1.00
Lmcd1	-0.05	1.00
Exosc9	-0.18	1.00
Gle1	0.02	1.00
Brf1	-0.33	1.00
Tlk2	-0.29	1.00

Adam23	-0.26	1.00
Ap2m1	-0.52	1.00
Kpna1	-0.46	1.00
Aga	-0.06	1.00
Bloc1s4	-0.05	1.00
Rnf114	-0.14	1.00
Hmgxb3	-0.06	1.00
Pacs2	-0.14	1.00
Ppp3cc	-0.10	1.00
Exosc8	-0.09	1.00
Gpr63	-0.03	1.00
Anapc4	0.06	1.00
Ankrd13d	-0.02	1.00
Rbm43	0.04	1.00
Taf12	-0.17	1.00
Cdc37l1	-0.16	1.00
Ncoa3	-0.36	1.00
Ttc13	-0.06	1.00
Ube2h	-0.10	1.00
Atp1a1	0.21	1.00
Tada2b	-0.07	1.00
Ubap2l	-0.03	1.00
Sdc4	-0.97	1.00
Rhoa	-1.04	1.00
Ttc19	-0.09	1.00
Itm2a	-1.13	1.00
Zfand6	-0.15	1.00
Ubac1	-0.21	1.00
Cpne3	-0.43	1.00
Ankrd10	0.56	1.00
Cct2	-0.40	1.00
Rhof	-0.07	1.00
Numa1	-0.12	1.00
Faf2	-0.32	1.00
Kif13a	-0.01	1.00
Lgals8	-0.31	1.00
Rbsn	-0.10	1.00
Spty2d1	-1.15	1.00
Vps45	-0.34	1.00
Txlng	-0.37	1.00
Prr14l	-0.37	1.00
Zc3h12b	-0.04	1.00
Acyp2	-0.14	1.00
R3hdm1	-0.16	1.00
Rab35	-0.44	1.00
Ak2	-0.46	1.00

Ambra1	-0.19	1.00
Zc3h18	-0.36	1.00
Fbxo3	-0.38	1.00
L3mbtl3	-0.49	1.00
Tpp2	-0.45	1.00
Vps53	-0.32	1.00
Pim1	-0.12	1.00
Ifnar2	-0.66	1.00
Ckb	-0.35	1.00
Fars2	-0.25	1.00
Ube2v2	-0.32	1.00
Ubp1	-0.33	1.00
Pak5	0.32	1.00
Cmtr2	0.08	1.00
Zbtb18	-0.31	1.00
Kpna4	-0.21	1.00
Vcpip1	-0.42	1.00
Frs3	0.11	1.00
Ints3	0.06	1.00
Ezh2	-0.34	1.00
Rc3h1	-0.21	1.00
Gosr1	-0.27	1.00
Hdx	0.05	1.00
Rab8a	-0.23	1.00
Pik3c2b	-0.07	1.00
Edc3	-0.21	1.00
Ttc32	-0.01	1.00
Tiam2	0.14	1.00
Hif1an	-0.07	1.00
Tceanc2	-0.08	1.00
Acot10	-0.02	1.00
Ndufaf7	-0.07	1.00
Dpy30	-0.15	1.00
Lmod3	0.08	1.00
Rdx	-0.25	1.00
Lmbrd2	-0.31	1.00
Rac1	-0.19	1.00
Srsf3	-0.33	1.00
Urod	-0.05	1.00
Smu1	-0.37	1.00
G3bp2	-0.08	1.00
Galnt11	-0.05	1.00
Galnt1	-0.33	1.00
Hook3	-0.42	1.00
Snx24	-0.19	1.00
Kdm8	0.00	1.00

Ptpn1	-0.26	1.00
Slc39a3	-0.11	1.00
Psma3	-0.38	1.00
Ube3a	-0.20	1.00
Cdc16	-0.04	1.00
Gata3	-1.81	1.00
Gtpbp2	0.04	1.00
Invs	0.03	1.00
Fabp6	-0.09	1.00
Fermt2	-1.72	1.00
Thap4	-0.02	1.00
Srrm1	-0.24	1.00
Ints8	-0.16	1.00
Rbl2	-0.16	1.00
Cdca4	-0.47	1.00
Phyh	-0.04	1.00
Ank	-0.02	1.00
Exog	-0.20	1.00
Ptpn11	-0.26	1.00
Trp53i11	-1.58	1.00
Dgkd	-1.59	1.00
Trim24	-0.24	1.00
Ywhah	-0.31	1.00
Spata5	-0.22	1.00
Tmem266	0.43	1.00
Virma	-0.31	1.00
Dock7	-0.12	1.00
Tnfaip8	0.03	1.00
Rit1	-0.16	1.00
Dapp1	0.15	1.00
Tmem131	-0.25	1.00
Zfp276	0.00	1.00
Vps36	-0.33	1.00
Upf1	-0.22	1.00
Noc2l	-0.02	1.00
Usp47	-0.40	1.00
D5Ertd579e	-0.23	1.00
Acad9	-0.24	1.00
Snap23	-0.51	1.00
Jade1	-0.30	1.00
Parn	-0.40	1.00
Ccnl2	-0.38	1.00
Kat2b	-0.36	1.00
Plekha1	0.01	1.00
Uckl1	-0.33	1.00
Cmss1	0.12	1.00

Irf2	-0.26	1.00
Nono	-0.29	1.00
Bloc1s6	-0.62	1.00
Zfp318	-0.45	1.00
Gria3	0.60	1.00
Cmtr1	-0.10	1.00
Jmjd1c	0.32	1.00
Colec10	-1.68	1.00
Dnajc17	-0.16	1.00
Arhgap21	-1.55	1.00
Wdr43	-0.08	1.00
Rwdd2b	0.16	1.00
Lin52	-0.34	1.00
Usp12	-0.51	1.00
Acap3	-0.19	1.00
Nop2	-0.11	1.00
Sugp1	0.18	1.00
Eif5	0.10	1.00
Ifngr1	-0.02	1.00
Plpp3	-0.24	1.00
Limd1	-0.27	1.00
Commd8	-0.19	1.00
Timmdc1	-0.17	1.00
Cdadc1	0.07	1.00
Orai1	-0.03	1.00
Commd6	-0.07	1.00
Tmem268	0.02	1.00
Ccdc82	0.02	1.00
Frs2	0.47	1.00
Phc3	-0.04	1.00
Snx8	-0.06	1.00
Lig3	-0.10	1.00
Ighmbp2	-0.07	1.00
Spcs2	-0.06	1.00
Pcbd2	-0.11	1.00
Osbpl8	-0.15	1.00
Thap12	-0.10	1.00
Ppp2r3c	-0.01	1.00
Ifngr2	-0.48	1.00
Tra2a	-0.14	1.00
Nrg4	-0.02	1.00
Rbm18	-0.13	1.00
Tanc2	-0.24	1.00
Phf5a	-0.14	1.00
Arhgdib	-0.11	1.00
Cwh43	-0.13	1.00

Ebna1bp2	-0.15	1.00
Tmem11	-0.20	1.00
Vwa8	-0.55	1.00
Prkar1a	-0.02	1.00
Ramp3	-1.59	1.00
Pex7	-0.04	1.00
Saal1	-0.15	1.00
Ryk	-0.19	1.00
Ehmt1	-0.14	1.00
Lrch2	-0.21	1.00
Ppp1r8	-0.26	1.00
Iws1	-0.41	1.00
Stk38	-0.08	1.00
Srpk2	0.17	1.00
Sap30l	-0.35	1.00
Kdm1a	-0.32	1.00
Elp2	0.11	1.00
Pcna	-0.35	1.00
Wdr1	-0.36	1.00
Dcn	-1.94	1.00
Gid8	-0.30	1.00
Ireb2	-0.45	1.00
Gkap1	-0.27	1.00
Cpeb4	-0.13	1.00
N4bp2l2	-0.20	1.00
Exosc10	-0.28	1.00
Bicc1	0.00	1.00
Zbtb6	-0.08	1.00
Pcnx	-0.19	1.00
Dusp15	-0.05	1.00
Inpp5d	-1.70	1.00
Polg2	-0.45	1.00
Ifi35	-0.53	1.00
Pcyt1a	-0.12	1.00
Tbc1d15	-0.56	1.00
Dhx37	-0.04	1.00
Srgap3	0.02	1.00
Scaf8	-0.22	1.00
Tamm41	0.06	1.00
Cd74	-0.17	1.00
Cdc42se2	-0.32	1.00
Pmpcb	-0.10	1.00
Hoxa5	-1.47	1.00
Zdhhc14	0.11	1.00
Ywhae	-0.52	1.00
Ppp2ca	-0.29	1.00

Ctdspl2	-0.11	1.00
Bnip3l	-0.52	1.00
Napepld	-0.35	1.00
Chst14	-0.23	1.00
Cfap45	-0.23	1.00
Cul2	-0.34	1.00
Nrg1	-0.05	1.00
Trip4	-0.24	1.00
Glmn	-0.14	1.00
Gstcd	0.02	1.00
Lasp1	-0.26	1.00
Ttc8	-0.02	1.00
Unk	-0.25	1.00
Tubgcp3	-0.14	1.00
Zranb1	-0.27	1.00
Ehd4	-1.65	1.00
Usp4	-0.37	1.00
Lig4	-0.10	1.00
Lrmda	-0.03	1.00
Arhgef10	0.15	1.00
Ccdc127	-0.03	1.00
Recql	-0.07	1.00
Epm2a	0.11	1.00
Slc37a4	-0.05	1.00
Sh3bgrl3	-0.50	1.00
Scyl3	0.08	1.00
Pola1	-0.14	1.00
Pals1	-0.36	1.00
Rhbdd1	-0.12	1.00
Eif2s1	-0.44	1.00
Vrk1	0.02	1.00
Fndc3a	-0.02	1.00
Casp8ap2	-0.42	1.00
Hyal2	-0.11	1.00
Xpa	-0.57	1.00
Plcg2	0.19	1.00
Fbxo38	-0.11	1.00
Arid2	-0.46	1.00
Pik3c2a	-0.64	1.00
Trafd1	-0.33	1.00
Sh3gl1	-0.61	1.00
Vangl1	-0.07	1.00
Lypla1	-0.30	1.00
Sdf4	-0.63	1.00
Pank1	-0.02	1.00
Sp3	-0.55	1.00

Hs6st1	-1.64	1.00
Cpsf4l	-0.40	1.00
Ttf1	-0.10	1.00
Ap2a1	-0.25	1.00
Dtwd1	-0.09	1.00
Btbd9	-0.05	1.00
Fer1l6	0.13	1.00
Slu7	-0.51	1.00
Cry1	0.05	1.00
Ip6k1	0.07	1.00
Cebpg	-0.23	1.00
Smap2	-0.23	1.00
Scube3	-1.48	1.00
Cbll1	-0.17	1.00
Tpk1	-0.37	1.00
Lyn	-0.21	1.00
Gpx3	-1.01	1.00
Smad5	-0.51	1.00
Col13a1	0.24	1.00
Nxt2	-0.14	1.00
Kif14	-0.09	1.00
Ppp1cb	-0.43	1.00
Ppm1g	-0.13	1.00
Ammecr1	-0.07	1.00
Cdk17	-1.62	1.00
Diablo	-0.04	1.00
Nt5dc1	-0.01	1.00
Tmem9b	-0.28	1.00
Ube2k	-0.41	1.00
Prpsap1	-0.10	1.00
Prdm15	-0.27	1.00
Enox1	-1.45	1.00
Cygb	-0.03	1.00
Grtp1	-0.12	1.00
Taok1	-0.42	1.00
Nhlrc2	-0.12	1.00
Ttc39c	-0.07	1.00
Gnptg	-0.31	1.00
Atxn1	-0.14	1.00
Lrrc45	0.09	1.00
Rasgrp3	-1.23	1.00
Slc39a13	-0.07	1.00
Xpo1	-0.29	1.00
Ghr	-0.32	1.00
Zc3h12c	0.04	1.00
Dmxl1	-0.36	1.00

Scarf2	-0.05	1.00
Atrx	-0.45	1.00
Cpeb2	-0.05	1.00
Zfp592	-0.21	1.00
Fbxo33	-0.31	1.00
Ndfip2	-0.33	1.00
Naglu	-0.04	1.00
Dgkh	-1.43	1.00
Sdf2	-0.02	1.00
Eif2ak4	-0.45	1.00
Tacc3	-0.36	1.00
Gtf2b	-0.60	1.00
Shq1	-0.14	1.00
Klhl22	-0.25	1.00
Lsm6	-0.48	1.00
2210408I21Rik	-0.10	1.00
Purg	-0.04	1.00
Sertad2	0.54	1.00
Lrch3	-0.44	1.00
Trim13	0.05	1.00
Aph1a	-0.04	1.00
Pla2g7	-0.53	1.00
Atg12	-0.18	1.00
Dpp8	-0.06	1.00
Ctbp2	-0.36	1.00
Rbms1	-0.13	1.00
Dscam	-0.03	1.00
Sart3	-0.23	1.00
Zfp523	-0.19	1.00
Srfbp1	-0.20	1.00
Dctn4	-0.15	1.00
Kat7	-0.47	1.00
Pspc1	-0.28	1.00
Fgf18	0.02	1.00
Neurl1b	-1.60	1.00
Tshz2	0.25	1.00
Rtn4	0.32	1.00
Slc25a13	-0.22	1.00
Fam110d	-0.31	1.00
Xrcc4	-0.17	1.00
Nup153	-0.51	1.00
Ice2	-0.34	1.00
Orc3	-0.15	1.00
Nlrc5	-0.22	1.00
Dync1li2	-0.53	1.00
Brcc3	0.01	1.00

Slc25a26	0.10	1.00
Ppp2r5d	-0.17	1.00
Ppp3r1	-0.34	1.00
Uvrag	-0.42	1.00
Jak1	-0.39	1.00
Rft1	0.06	1.00
Actn1	-0.14	1.00
Zbtb8a	0.00	1.00
Col12a1	0.25	1.00
Tbc1d22a	-0.48	1.00
Edn1	-1.29	1.00
Bnip2	-0.41	1.00
Lrrc8c	0.01	1.00
Wdsub1	-0.33	1.00
Rassf6	-0.16	1.00
Jmy	-0.09	1.00
Ctdp1	-0.37	1.00
Fat4	-1.35	1.00
Pick1	-0.14	1.00
Rabgap1l	0.04	1.00
Cacnb4	0.01	1.00
Axin2	-0.58	1.00
Ets1	-1.82	1.00
Pde7a	-0.20	1.00
Ncbp3	-0.30	1.00
Auh	-0.11	1.00
Dnm3	0.19	1.00
Appl1	-0.36	1.00
Adipor2	-0.39	1.00
Rcan2	0.00	1.00
Fam91a1	-0.26	1.00
Slc6a6	0.39	1.00
Slc35e3	-0.17	1.00
Rai14	-1.69	1.00
Dhx30	-0.23	1.00
Exd2	-0.11	1.00
Egfl7	-1.50	1.00
Rsrc2	-0.66	1.00
Cdv3	-0.33	1.00
Fkbp1a	-1.54	1.00
Slc39a10	-0.32	1.00
Phkb	-0.23	1.00
Utp18	-0.38	1.00
Tubd1	-0.07	1.00
Apba2	-1.40	1.00
Sspn	-0.12	1.00

Pef1	-0.30	1.00
Hsp90aa1	-0.36	1.00
Rnf141	-0.28	1.00
Ube2q1	-0.55	1.00
Nap1l1	-0.08	1.00
Znfx1	-0.47	1.00
Ppp2r2a	-0.56	1.00
Smndc1	-0.73	1.00
Btbd2	-0.19	1.00
Cep250	0.02	1.00
Cemip2	-0.05	1.00
Zcchc10	-0.12	1.00
F2r	-1.45	1.00
Nudcd1	-0.19	1.00
Chchd3	-0.05	1.00
Ric1	-0.59	1.00
Fam168a	-1.68	1.00
Ubtd1	-0.24	1.00
Esf1	-0.41	1.00
Arhgap42	-0.35	1.00
Lmo2	-1.25	1.00
Ptpn14	-0.03	1.00
Lin7c	-0.14	1.00
Fam172a	-0.15	1.00
Sox18	-1.42	1.00
Narf	-0.25	1.00
Fnbp1l	-1.20	1.00
Snx7	-0.21	1.00
Chic1	-0.07	1.00
Dnmt1	-0.21	1.00
Rnf144b	-0.04	1.00
Rnf150	-0.12	1.00
Vkorc1l1	-0.22	1.00
C2cd2l	-0.17	1.00
Ift140	0.54	1.00
Bin1	-0.54	1.00
Zbtb11	-0.46	1.00
Cep83	-1.81	1.00
Pcsk2	-1.57	1.00
Zmat4	-0.21	1.00
Apaf1	-0.35	1.00
Parp16	-0.18	1.00
Rnaset2b	-0.36	1.00
Thrb	0.13	1.00
Ddx54	-0.08	1.00
Cant1	-0.24	1.00

Reep1	-1.20	1.00
Fxr1	-0.61	1.00
Cradd	-0.22	1.00
Ppp2r5e	-0.20	1.00
Ano10	-0.31	1.00
Fam161a	-0.10	1.00
Nploc4	-0.07	1.00
Chchd5	-0.12	1.00
Derl2	-0.09	1.00
Slc2a13	-0.09	1.00
Ndufaf1	-0.06	1.00
Cldnd1	-0.02	1.00
Cryl1	-0.05	1.00
Wdr90	0.02	1.00
Ptpn12	-0.68	1.00
Aff2	-1.00	1.00
Supt7l	-0.12	1.00
Bdp1	-0.36	1.00
Ets2	0.07	1.00
Wdr7	-0.29	1.00
Tubgcp6	-0.15	1.00
Lrrc57	-0.25	1.00
Proser1	-0.31	1.00
Nudt19	-0.06	1.00
Prpf4b	-0.53	1.00
Tnip2	0.00	1.00
Helb	-0.22	1.00
Ankrd50	-0.39	1.00
Dcun1d4	-0.40	1.00
Ube2l3	-0.36	1.00
Zfp410	-0.12	1.00
Zfp518b	-0.41	1.00
Hyal1	-0.14	1.00
Aqp11	-0.17	1.00
Dut	-0.13	1.00
Ttc37	-0.45	1.00
Hnrnpm	-0.47	1.00
Shoc2	-0.27	1.00
Rars2	-0.15	1.00
Evc2	-0.18	1.00
Nr3c2	0.19	1.00
9130409I23Rik	-0.38	1.00
Plxnc1	-0.13	1.00
Phactr2	-1.78	1.00
Kbtbd2	-0.43	1.00
Faf1	-0.31	1.00

Cdc42bpb	-0.37	1.00
Cops5	-0.40	1.00
Rasgrf2	-1.19	1.00
Lpin1	-0.17	1.00
Rubcnl	0.28	1.00
Klhl5	-0.31	1.00
Ptch1	-0.04	1.00
Cpt1a	0.21	1.00
Ppm1d	-0.46	1.00
Gga3	-0.30	1.00
Chmp1a	-0.39	1.00
9930021J03Rik	-0.22	1.00
Ddx3y	-0.56	1.00
Grk5	0.06	1.00
Dll1	-1.18	1.00
Pdia3	-0.30	1.00
Zeb1	-1.51	1.00
Cdyl2	-0.35	1.00
Asap1	-0.44	1.00
Baz2b	-0.08	1.00
Itpr1	-0.45	1.00
Dok3	0.04	1.00
Galnt10	-0.09	1.00
Nfya	-0.07	1.00
Eng	-1.16	1.00
Anxa6	-0.33	1.00
Spata2	-0.12	1.00
Dctn1	-0.40	1.00
Fut11	-0.11	1.00
Pus7	-0.09	1.00
Slc30a5	-0.12	1.00
Per3	-0.17	1.00
Gnpda1	-0.14	1.00
Slc2a1	-0.08	1.00
Rilpl1	-0.32	1.00
Ggps1	-0.59	1.00
Tspan18	-1.39	1.00
Cd44	-0.07	1.00
Sfpq	-0.59	1.00
Limd2	-0.12	1.00
Bambi	-0.36	1.00
AW554918	-0.21	1.00
Ncoa2	0.23	1.00
Cracr2b	0.09	1.00
Sh3glb1	-0.59	1.00
Lrwd1	-0.11	1.00

Zfyve9	-0.23	1.00
Nktr	-0.44	1.00
Pabpc1	0.32	1.00
Flt4	-1.07	1.00
Eif1	0.25	1.00
Eri1	-0.24	1.00
Ggh	0.04	1.00
Fbxo34	-0.01	1.00
Sltm	-0.62	1.00
Serpind1	-1.47	1.00
Cwc25	-0.14	1.00
Fam76a	-0.09	1.00
Top1	-0.27	1.00
Pstpip1	0.02	1.00
Rock2	-0.47	1.00
Hectd1	-0.31	1.00
Dus2	-0.28	1.00
Ripk1	-0.55	1.00
Fam114a1	0.05	1.00
Khdrbs3	-0.06	1.00
Dytn	0.30	1.00
Txndc15	-0.29	1.00
Zer1	-0.18	1.00
Pbld2	-0.22	1.00
Spag9	-0.60	1.00
Tmem117	-1.07	1.00
Eif3h	-0.25	1.00
Itpk1	-0.35	1.00
Aplp2	-0.03	1.00
Wasf2	-0.24	1.00
Peli2	-0.10	1.00
Arap3	-1.11	1.00
Tgfbr2	-1.53	1.00
Sgsm2	-0.06	1.00
Rprd1b	-0.28	1.00
Cpe	0.00	1.00
Gar1	-0.14	1.00
Crip1	0.01	1.00
Ywhaq	-0.04	1.00
Nrf1	-0.39	1.00
Kidins220	-0.45	1.00
F8	-1.10	1.00
Ednrb	-1.11	1.00
Hells	-0.51	1.00
Thumpd2	-0.14	1.00
Rab7	-0.15	1.00

Adcy8	-1.21	1.00
Cd151	-0.19	1.00
Wdr35	0.14	1.00
Usp46	-0.23	1.00
Dcun1d1	-0.26	1.00
Tbl1xr1	-0.34	1.00
Rab2a	-0.40	1.00
Yipf5	-0.05	1.00
Iffo2	-0.05	1.00
Casp2	-0.28	1.00
Hoxb3	-1.06	1.00
Lama4	-1.22	1.00
Ccdc59	-0.05	1.00
Tspo	-0.29	1.00
Itfg2	-0.50	1.00
Plat	-0.04	1.00
Slc30a4	-0.13	1.00
Tns1	-1.86	1.00
Bmp2k	-0.43	1.00
Rnaseh2b	-0.50	1.00
Nckap1	-0.23	1.00
Arhgef28	-1.07	1.00
Pde10a	0.71	1.00
Cdc42ep3	-0.56	1.00
Adamts2	-1.47	1.00
Slc35a5	-0.11	1.00
Txlna	-0.01	1.00
Znrf1	-0.50	1.00
Tjp2	-0.29	1.00
Edrf1	-0.31	1.00
Lrba	0.02	1.00
Chpt1	0.02	1.00
Pxdc1	-1.27	1.00
Smyd2	-0.19	1.00
Ccdc18	-0.17	1.00
Cramp1	-0.68	1.00
Ppp3ca	-0.22	1.00
Vps54	-0.67	1.00
Rapgef3	-0.23	1.00
Flt1	-1.06	1.00
Rab40b	-0.19	1.00
Slc30a9	-0.50	1.00
Dnajb14	-0.29	1.00
Kif1b	0.03	1.00
Pum3	-0.18	1.00
Uhmk1	-0.18	1.00

Coq10b	-0.17	1.00
Cds2	-0.37	1.00
Zbtb37	-0.24	1.00
Syt11	-0.66	1.00
Ccdc92	-0.20	1.00
Gpx1	0.30	1.00
Rab8b	-0.55	1.00
Atp2a3	-0.33	1.00
Gabpb1	-0.17	1.00
Rassf9	-1.69	1.00
Slx4	-0.12	1.00
Neo1	-0.08	1.00
Klf7	-1.81	1.00
Sptan1	-1.30	1.00
Tef	-0.57	1.00
Pias1	-0.24	1.00
Slk	-0.04	1.00
Phkg1	-0.30	1.00
Gnb1l	0.05	1.00
Zc3h12d	0.08	1.00
Cbx6	-0.31	1.00
Plekha3	-0.52	1.00
Chic2	-0.43	1.00
Rere	0.05	1.00
Golga4	-0.50	1.00
Cep85l	-0.22	1.00
Dock11	-0.39	1.00
Hnrnpr	-0.33	1.00
Xpo7	-0.39	1.00
Sh2d1a	-1.63	1.00
Psmd4	-0.16	1.00
Zbtb43	-0.31	1.00
Slc15a1	-1.28	1.00
Steap4	0.32	1.00
Rrad	0.00	1.00
Ptdss2	-0.14	1.00
Tbrg4	-0.21	1.00
Fam135a	-0.38	1.00
Adam10	-0.41	1.00
Ssbp2	-1.04	1.00
Zfyve26	-0.27	1.00
Lmbr1	-0.42	1.00
Fto	0.05	1.00
Rcc1	-0.62	1.00
Gtf2e1	-0.19	1.00
Zfp319	-0.28	1.00

Rundc3b	-0.28	1.00
Cdca7l	-1.46	1.00
Ezh1	-0.03	1.00
Dock2	-0.02	1.00
St3gal1	-1.31	1.00
Amot	-0.61	1.00
Kat6a	-0.49	1.00
Atp6v1e2	-0.66	1.00
Twf1	-0.64	1.00
Exoc6	0.04	1.00
Ppm1f	-1.47	1.00
Dlg5	-0.40	1.00
Pde3b	-1.37	1.00
Parp9	-0.41	1.00
Plcb2	0.03	1.00
Rab12	-0.39	1.00
Dusp7	-1.16	1.00
Fez2	-0.76	1.00
Tanc1	-0.59	1.00
Ywhaz	-0.18	1.00
Ext2	-0.56	1.00
Slc22a23	0.41	1.00
Kat6b	-0.56	1.00
Hnrnpab	0.01	1.00
Ska2	-0.35	1.00
Nrxn1	0.22	1.00
Taf5	-0.05	1.00
Atp6v0a1	-0.67	1.00
Enah	-0.73	1.00
Gzf1	-0.10	1.00
Smad1	-0.44	1.00
Upk1b	-0.36	1.00
Kdm4c	-0.60	1.00
Spen	-0.46	1.00
Grb2	-0.42	1.00
Hivep1	-0.47	1.00
Bicd2	0.09	1.00
Adnp2	-0.57	1.00
Elf1	-0.04	1.00
Arhgef12	-0.71	1.00
Gal	-0.22	1.00
Rpa1	-0.85	1.00
Wdr89	-0.22	1.00
Phf21a	-0.57	1.00
Zfp384	-0.33	1.00
Bin3	-1.54	1.00

Zfp831	-0.19	1.00
Bicral	-0.46	1.00
Dnajb6	-0.35	1.00
Dennd6a	-0.29	1.00
Oxsr1	-0.32	1.00
Riok3	-0.60	1.00
Hoxa7	-0.92	1.00
Ust	0.17	1.00
Lars2	-0.12	1.00
Tnpo1	-0.73	1.00
Smad6	-1.07	1.00
Osbpl3	-0.68	1.00
Adrb1	-0.89	1.00
Tfpi	-0.91	1.00
Snapc1l	-0.22	1.00
Usp40	-0.19	1.00
Exoc4	-0.21	1.00
Rangap1	-0.39	1.00
Reps2	-0.32	1.00
Tmem19	-0.23	1.00
Tmem62	-0.29	1.00
Camsap2	-0.16	1.00
Rnf111	-0.39	1.00
Arv1	0.04	1.00
Arrdc3	-1.23	1.00
Rcn2	-0.60	1.00
Nfatc1	-1.43	1.00
Chst1	-0.87	1.00
Ildr2	-0.87	1.00
Stk39	0.06	1.00
Pex14	-0.30	1.00
Tfdp2	-0.54	1.00
Fgd6	-0.89	1.00
Ube2z	-0.18	1.00
Ppm1h	0.07	1.00
Dab1	-0.17	1.00
Timp2	-0.43	1.00
Kmt5b	-0.58	1.00
Ppp4r1	-0.51	1.00
Jak2	-0.62	1.00
Carhsp1	-0.48	1.00
Rb1cc1	-1.12	1.00
Prrc2c	-0.46	1.00
Jun	0.73	1.00
Pcmt1	-0.30	1.00
Amotl1	-0.25	1.00

Strn3	-0.68	1.00
Focad	-0.25	1.00
Fhit	-0.42	1.00
Agl	-0.15	1.00
Zfp423	-0.93	1.00
Aldh9a1	-0.36	1.00
St3gal3	-0.35	1.00
Tie1	-1.06	1.00
Ube2cbp	-0.20	1.00
Orc5	0.08	1.00
Dhx15	-0.48	1.00
Kifc3	-0.30	1.00
Sp4	-0.52	1.00
Nr2c1	-0.10	1.00
Stx16	-0.12	1.00
Rad17	-0.25	1.00
Zfyve1	-0.63	1.00
Nup210	-0.56	1.00
Neu2	-0.02	1.00
Fa2h	-0.20	1.00
Smim3	-0.18	1.00
Rhot1	-0.31	1.00
Adat1	-0.22	1.00
Ikzf3	-0.20	1.00
Reep2	-0.21	1.00
Slc13a1	-0.42	1.00
Zfp131	-0.32	1.00
Asb5	-0.27	1.00
Fam76b	-0.30	1.00
Lsm14a	-0.64	1.00
Trpc1	-0.29	1.00
Tmed5	-0.84	1.00
Prkrip1	-0.54	1.00
Lpgat1	-0.09	1.00
Kdm2a	-0.17	1.00
Vps13d	-0.56	1.00
Atp9b	-0.39	1.00
Ogfrl1	-0.45	1.00
Cdk8	-0.20	1.00
Ndufa9	-0.11	1.00
Ttyh3	-1.12	1.00
Zbtb20	0.29	1.00
Pard6g	-0.09	1.00
Ythdc2	0.06	1.00
Abca1	-0.24	1.00
Nufip1	-0.25	1.00

Sec31a	-0.58	1.00
Smc3	-0.67	1.00
Gcnt4	-0.26	1.00
Gpc5	0.37	1.00
Jmjd6	-0.24	1.00
Vgll4	-0.27	1.00
Syne1	1.25	1.00
Slc35c1	-0.34	1.00
Ccni	-0.39	1.00
Rnf11	-0.65	1.00
Abi1	-0.17	1.00
Rgl1	-0.35	1.00
Svil	-0.09	1.00
Atxn7	-0.47	1.00
Pde4b	-0.11	1.00
Zfp639	-0.58	1.00
Itgb1	-1.36	1.00
Ythdc1	-0.40	1.00
Tmpo	-0.75	1.00
Tnip1	-0.30	1.00
Pde7b	-0.52	1.00
Ercc4	-0.39	1.00
Rbm33	-0.29	1.00
Pls3	-1.45	1.00
Dph5	-0.20	1.00
Hnrnph3	-0.73	1.00
Slc48a1	-0.48	1.00
Haus3	-0.22	1.00
Efnb2	-1.37	1.00
Trib2	-1.62	1.00
Tnfrsf25	-0.97	1.00
Fos	-0.10	1.00
Picalm	0.11	1.00
Cask	-0.15	1.00
Elk3	-0.91	1.00
Chd2	-0.39	1.00
Elf2	-0.71	1.00
Sag	-0.92	1.00
Eif4g3	-0.48	1.00
Afap1l2	-0.53	1.00
Tnfrsf11b	-0.90	1.00
Adgrg1	-1.24	1.00
Akirin2	-0.49	1.00
Trip12	-0.38	1.00
Dmap1	-1.47	1.00
Zcchc14	-0.23	1.00

Kitl	-0.26	1.00
Txnrd1	-1.30	1.00
Nuak1	-0.19	1.00
Cep95	-0.05	1.00
Nenf	-0.31	1.00
Fbxl17	-0.32	1.00
Zfp608	-0.07	1.00
Brix1	-0.22	1.00
Rcc1l	-0.22	1.00
Pphln1	-0.14	1.00
Atmin	-0.28	1.00
Wwc2	-0.56	1.00
Sacs	-0.26	1.00
Ercc6	-0.30	1.00
Rnf144a	-0.76	1.00
Stxbp5	-0.30	1.00
Smg6	-0.50	1.00
Slc4a7	-0.33	1.00
Vti1a	-0.28	1.00
Ints6	-0.22	1.00
Dip2a	-0.10	1.00
Tmtc2	-0.15	1.00
Rev3l	-0.37	1.00
Urb2	-0.28	1.00
Gpcpd1	-0.32	1.00
Fbxo11	-0.55	1.00
Smarcb1	-0.30	1.00
Sulf1	-0.54	1.00
Rbm39	-0.35	1.00
Cyth3	-0.73	1.00
Atrn	-0.67	1.00
Fgd3	-0.53	1.00
Trim8	-0.68	1.00
Gulp1	-1.25	1.00
Sgip1	-0.90	1.00
Wnk1	-0.16	1.00
Gcc1	-0.21	1.00
Sh3pxd2a	-0.48	1.00
Nol8	-0.36	1.00
Eif3i	-0.51	1.00
Zfhx4	-0.14	1.00
Zfc3h1	-0.38	1.00
Uxs1	-0.62	1.00
Exoc6b	-0.68	1.00
Atp6v1h	-0.48	1.00
Polr3a	-0.70	1.00

Hat1	-0.22	1.00
Pank4	-0.27	1.00
Serbp1	-0.04	1.00
Nectin1	-0.23	1.00
Ate1	-0.44	1.00
Rbfox2	-1.20	1.00
Dap3	-0.17	1.00
Tpm3-rs7	-0.53	1.00
Zdhhc8	-0.13	1.00
Dock4	-1.53	1.00
Fchsd2	-0.26	1.00
Tec	-0.09	1.00
Trp53inp1	-0.41	1.00
Hvcn1	-0.23	1.00
Cd4	-0.24	1.00
Emp1	-0.63	1.00
Pard3b	0.31	1.00
Oser1	-0.36	1.00
Lrrfip2	-0.77	1.00
Tinagl1	-0.62	1.00
Tln1	-0.71	1.00
Proc	-0.48	1.00
Wbp4	-0.68	1.00
Cdc73	-0.78	1.00
Notch1	-0.75	1.00
Ptpre	-0.65	1.00
Iqgap1	-0.44	1.00
Cd247	-0.27	1.00
Lpar1	-0.32	1.00
Ubr5	-0.58	1.00
Plrg1	-0.47	1.00
Anks1	-1.03	1.00
Bcor	-0.36	1.00
Bmp2	-0.51	1.00
Ppp1r16b	-0.47	1.00
Dot1l	-0.23	1.00
Thbs1	-1.44	1.00
C2cd2	-0.42	1.00
Arid4b	-0.29	1.00
Ntn4	-1.39	1.00
Srgap1	-0.43	1.00
Shroom4	-0.94	1.00
Fxyd6	-0.76	1.00
Acbd6	-0.19	1.00
Adk	0.16	1.00
Nusap1	-0.55	1.00

Borcs8	-0.28	1.00
Epc1	-0.33	1.00
Usp34	-0.70	1.00
Ablim1	-1.18	1.00
Serinc1	-0.39	1.00
Pak2	-0.89	1.00
Cgnl1	-1.36	1.00
Runx1t1	-1.35	1.00
Lrrcc1	-0.33	1.00
Rnf217	-0.49	1.00
Ripor1	-1.20	1.00
Itga4	-0.32	1.00
Rock1	-0.38	1.00
Cbfb	-0.63	1.00
Nprl3	-0.19	1.00
Arpc1b	-1.49	1.00
Rev1	-0.54	1.00
Fgr	-0.84	1.00
Cnst	-0.33	1.00
Ipo9	-0.30	1.00
Stmn1	-0.44	1.00
Pla2r1	-0.59	1.00
Arhgap5	-0.63	1.00
Arl15	-1.13	1.00
Atad5	-0.30	1.00
Dcp1a	-0.34	1.00
Phldb2	-0.33	1.00
Itsn1	-0.51	1.00
Tcf3	-0.90	1.00
Ptprc	-0.17	1.00
Cldn12	-0.50	1.00
Gk5	-0.65	1.00
Poc1b	-0.40	1.00
Ddx5	-0.31	1.00
Tspan9	-1.00	1.00
Samd11	-0.25	1.00
Spred2	-1.43	1.00
Rnps1	-0.27	1.00
Timm44	-0.32	1.00
Ica1	-0.68	1.00
Pgm3	-0.27	1.00
Cish	-0.64	1.00
Pcolce2	-0.76	1.00
Cacul1	-0.91	1.00
Gnb4	-0.45	1.00
Extl3	-0.44	1.00

Nol11	-0.58	1.00
Lcp2	-0.32	1.00
Rasa3	-0.83	1.00
Csrp1	-0.70	1.00
Rassf3	-0.70	1.00
Cog5	-0.63	1.00
Hoxb5	-0.86	1.00
Atp6v1d	-0.50	1.00
Pip4k2a	0.33	1.00
Bend3	-0.27	1.00
Ppia	-0.02	1.00
Cotl1	-0.70	1.00
Cbl	-0.34	1.00
Hspa12a	-0.44	1.00
Lrig2	-0.33	1.00
Jarid2	-0.31	1.00
Dcaf5	-0.89	1.00
Ing3	-0.30	1.00
Atp8a1	-1.40	1.00
Nde1	-0.32	1.00
Creg1	-0.99	1.00
Top2b	-0.51	1.00
Nvl	-0.61	1.00
Jade2	-0.48	1.00
Tmem150c	-0.34	1.00
Vcl	-0.57	1.00
Aplf	-0.31	1.00
Arhgef6	-0.75	1.00
Pcmtd1	-0.34	1.00
Tnrc6b	-0.86	1.00
Cdk14	-0.43	1.00
Sema3c	0.27	1.00
Pip5k1a	-0.90	1.00
Sfi1	-0.43	1.00
Cops8	-0.72	1.00
E2f3	-0.29	1.00
Erc1	-0.98	1.00
Ankrd11	-0.29	1.00
Hnrnpa2b1	-0.61	1.00
Smtnl2	-0.34	1.00
Akt3	-0.92	1.00
Parvg	-0.10	1.00
Spi1	-0.09	1.00
Pik3c3	-0.44	1.00
Cnnm2	-0.89	1.00
Apoa1	-0.72	1.00

Susd6	-0.37	1.00
Sema7a	-0.51	1.00
Lfng	-1.16	1.00
Dusp1	0.36	1.00
Kcnq1	-0.40	1.00
Ccny	-1.53	1.00
Spry1	-0.83	1.00
Sh3kbp1	-0.21	1.00
Arhgef26	-0.40	1.00
Ppp1r13b	-1.13	1.00
Exoc2	-0.55	1.00
Oaz2	-0.75	1.00
Negr1	0.16	1.00
Hsp90b1	-0.58	1.00
Zbtb46	-0.64	1.00
Bcl11b	-0.20	1.00
Osbpl1a	-1.24	1.00
Ppt1	-0.60	1.00
Stim2	-1.11	1.00
Cfl2	-0.28	1.00
Rnf130	-0.51	1.00
Pparg	-0.37	1.00
Smarca2	-0.40	1.00
Dner	-0.26	1.00
Ap3s1	-0.67	1.00
Sh3bp5	-0.30	1.00
Daam1	-0.31	1.00
Rbbp6	-0.66	1.00
Epha4	-0.03	1.00
Adamts3	-0.39	1.00
Sh3bp4	-0.57	1.00
Commd10	-0.35	1.00
Ube2o	-0.17	1.00
Nr6a1	-0.13	1.00
Comt	-0.16	1.00
Nek6	-0.46	1.00
Unc5c	-0.11	1.00
Bag5	-0.73	1.00
Dnajc10	-1.51	1.00
Lrp4	-0.70	1.00
Cdk6	-1.37	1.00
Shprh	-0.76	1.00
Scoc	-0.66	1.00
Clic4	-1.30	1.00
Ext1	-0.84	1.00
Arhgap29	-1.57	1.00

Robo1	0.37	1.00
Ak1	-0.84	1.00
Slc36a4	-0.33	1.00
Spsb4	-0.69	1.00
P3h2	-0.43	1.00
Arhgap26	-0.46	1.00
Dnajc9	-0.41	1.00
Rbfox1	-0.34	1.00
Jakmip1	-0.89	1.00
Cxcr4	-0.18	1.00
Cep170	-1.21	1.00
Fry	-0.34	1.00
Ptpn21	-0.77	1.00
Usp28	-0.69	1.00
Jakmip3	-1.11	1.00
Sbf2	-0.54	1.00
Slc10a7	-0.69	1.00
Ppp3cb	-0.43	1.00
Adgra1	-0.35	1.00
Chsy1	-0.08	1.00
Fbrsl1	0.43	1.00
Paxip1	-0.35	1.00
Tspan14	-0.86	1.00
Lpin2	-1.39	1.00
Dst	-0.08	1.00
Ptk2	-0.45	1.00
Zfand3	-0.48	1.00
Dennd5a	-0.94	1.00
Wapl	-0.29	1.00
Ap1s2	-0.52	1.00
Rcan1	-1.33	1.00
Parvb	-0.07	1.00
Stag2	-0.28	1.00
Camk2d	-0.89	1.00
Ddx28	-0.38	1.00
Gnb1	-0.52	1.00
Creb5	-0.49	1.00
Abcb10	-0.99	1.00
Snx14	-1.06	1.00
Acer2	-1.08	1.00
Stk24	-1.06	1.00
Ttc7b	-0.41	1.00
Dlc1	-1.44	1.00
Zfp438	0.03	1.00
Hdac4	-0.19	1.00
Col22a1	-1.05	1.00

Foxn3	-0.67	1.00
Kmt2e	-0.68	1.00
Rgs1	0.05	1.00
Immp2l	-1.05	1.00
Ino80	-0.87	1.00
Auts2	-1.27	1.00
Gtdc1	-0.49	1.00
Atp6v0c	-0.54	1.00
Nkd1	-0.15	1.00
Lims2	-1.29	1.00
Tpm4	-0.87	1.00
Ccdc126	-0.34	1.00
Tet2	-0.49	1.00
Sipa1l1	-0.14	1.00
Ankrd44	-0.39	1.00
Phex	-0.24	1.00
Rap1b	-0.44	1.00
Usp54	-0.75	1.00
Ranbp17	-0.75	1.00
Itsn2	-0.77	1.00
Serpini1	-0.55	1.00
Abcc1	-0.63	1.00
Rhoh	-0.16	1.00
Tcf7l2	-0.19	1.00
Asb3	-0.62	1.00
Cdkal1	-0.33	1.00
Atp10d	-0.43	1.00
Fyn	-0.95	1.00
Rbm31y	-0.90	1.00
Ralbp1	-0.79	1.00
Rell1	-1.32	1.00
Zhx1	-0.84	1.00
Aven	-0.49	1.00
Dpysl2	-1.42	1.00
Fnbp1	-0.74	1.00
Itga9	-0.30	1.00
Gnb5	-1.24	1.00
Etl4	0.18	1.00
Slc46a3	-0.54	1.00
Ralgps2	-0.94	1.00
Tmc7	-0.58	1.00
Laptm5	-0.55	1.00
Epb41l3	0.24	1.00
Chac1	-0.96	1.00
Ccdc88a	-0.31	1.00
Lmo3	-1.23	1.00

Rac2	-0.22	1.00
Canx	-0.22	1.00
Nup133	-0.55	1.00
Smurf2	-0.71	1.00
Lamc1	-0.55	1.00
Lifr	-1.00	1.00
Foxo1	-1.08	1.00
Csnk1a1	-0.46	1.00
Tgfbr3	-0.83	1.00
Klhl3	-0.84	1.00
Ppp2r5c	-0.80	1.00
Lrp3	-1.00	1.00
Cttnbp2	-0.84	1.00
Lemd3	-0.75	1.00
Dcun1d2	-0.55	1.00
Gphn	-1.00	1.00
Slc4a11	-0.98	1.00
Fam177a	-1.01	1.00
Flot2	-0.43	1.00
Eef1d	-0.08	1.00
S100a13	-0.72	1.00
Ccdc50	-0.85	1.00
Samsn1	-1.11	1.00
Gjd4	-0.67	1.00
Amotl2	-0.97	1.00
Naca	-0.17	1.00
Endou	-1.07	1.00
Stab2	-0.04	1.00
Nfia	-0.18	1.00
Garnl3	-0.40	1.00
Inpp4a	-1.10	1.00
Ano5	-0.30	1.00
Tlnrd1	-0.98	1.00
Tax1bp1	-0.34	1.00
Camk2g	-1.18	1.00
Cpt2	-0.55	1.00
Adarb1	-0.43	1.00
Rb1	-1.07	1.00
Stox2	-0.76	1.00
Serpinh1	-0.84	1.00
Ybx1	-0.37	1.00
Vav3	-0.57	1.00
Nfatc2	-0.10	1.00
Evl	0.05	1.00
Ldlrad3	-0.87	1.00
Efemp1	-1.28	1.00

Synj1	-0.79	1.00
Slc12a4	-1.21	1.00
Sh3bgrl	-1.19	1.00
Tacc1	-0.46	1.00
Klf13	-0.73	1.00
Sipa1l2	-0.40	1.00
Polr3b	-0.75	1.00
Nup107	-0.87	1.00
Coro1c	-0.81	1.00
Plk2	-1.03	1.00
Chd7	-0.09	1.00
Hivep2	-0.24	1.00
Arid1b	-0.24	1.00
St3gal6	-1.21	1.00
Ipmk	-0.75	1.00
Arhgap10	-0.92	1.00
Wwtr1	-0.57	1.00
Ppp1r9a	-0.65	1.00
Ppp1r12a	-0.21	1.00
Birc6	-0.57	1.00
Rnf220	-1.10	1.00
Rcbtb1	-1.17	1.00
Tmc5	-0.16	1.00
Tnfrsf18	-0.50	1.00
Hspa5	-0.27	1.00
Hpgd	-0.67	1.00
Cnn3	-0.89	1.00
Plcd1	-0.63	1.00
Nedd9	-0.70	1.00
Kmt2c	-0.48	1.00
Kif26a	-0.66	1.00
Trip11	-0.51	1.00
Ccnl1	-0.23	1.00
Syne2	0.37	1.00
Frmd4a	-0.51	1.00
Fut8	-0.50	1.00
Cdc42bpa	-0.78	1.00
Adcy9	-0.99	1.00
F5	-0.51	1.00
Aoah	-0.70	1.00
Usp22	-0.50	1.00
Epn2	-0.89	1.00
Fzd4	-0.79	1.00
Rcsd1	-0.57	1.00
Clip1	-0.97	1.00
S100a10	-0.29	1.00

Slf1	-0.63	1.00
Arhgef7	-0.46	1.00
Ctbp1	-0.33	1.00
Dyrk1a	-0.81	1.00
Stk17b	-1.35	1.00
Fbxl18	-0.73	1.00
Pim3	-0.10	1.00
Afdn	-0.38	1.00
Cd82	-0.95	1.00
2900026A02Rik	-0.92	1.00
Brd1	-0.80	1.00
Cadps2	-0.59	1.00
Hnrnpu	-0.38	1.00
Rusc2	-0.54	1.00
Luzp1	-1.22	1.00
Phf20	-0.80	1.00
Atg16l1	-0.73	1.00
Prmt7	-0.91	1.00
Dock1	-0.73	1.00
Eif4a2	-0.10	1.00
Disp1	-0.87	1.00
Pfkfb3	-0.90	1.00
Usp37	-0.57	1.00
Cplx1	-0.59	1.00
Ripor3	-1.33	1.00
Klf6	0.41	1.00
Borcs6	-0.71	1.00
Eepd1	-0.93	1.00
Slc5a1	-0.64	1.00
Rgs3	-0.73	1.00
Grk3	-1.12	1.00
Il16	-0.63	1.00
Hnrnpa3	-0.50	1.00
Pan3	-0.95	1.00
Ldlrad4	-0.36	1.00
Sorcs2	0.25	1.00
Scube2	-0.49	1.00
Gfod1	-0.73	1.00
Snrk	-1.09	1.00
Phf3	-0.66	1.00
Caskin2	-1.01	1.00
Egr1	0.21	1.00
Clasp2	-0.49	1.00
Eif4g2	-0.22	1.00
Sla	-0.96	1.00
Scarb2	-1.10	1.00

Nr1d2	-0.67	1.00
Antxr2	-0.50	1.00
Rassf8	-0.52	1.00
Fth1	-0.16	1.00
Cttnbp2nl	-1.15	1.00
Btg1b	-0.16	1.00
Dpyd	-0.71	1.00
Ppp2r5a	-0.77	1.00
Tfpi2	-0.89	1.00
Slc30a7	-0.47	1.00
Spred1	-0.66	1.00
Fzd6	-0.71	1.00
Ccser1	-0.92	1.00
Kdm7a	-0.56	1.00
Anxa5	-0.89	1.00
Hpcal1	-0.94	1.00
Nr3c1	-0.91	1.00
Hmgb1	-0.62	1.00
Pdgfb	-1.14	1.00
Jade3	-1.05	1.00
Cobll1	-0.77	1.00
Aff1	-0.91	1.00
Atf4	-0.14	1.00
Prrg1	-1.23	1.00
Arhgap15	0.13	1.00
Ebf1	-0.45	1.00
Inpp5k	-0.28	1.00
Rbfa	-1.09	1.00
Ttc28	-1.11	1.00
Wdfy3	-0.65	1.00
Dync1h1	-0.57	1.00
Dstn	-0.35	1.00
Agbl1	-1.07	1.00
Slc9a3r2	-1.15	1.00
Pdlim1	-1.08	1.00
Smarcal1	-0.17	1.00
Gsn	-1.32	1.00
Tsc2	-0.69	1.00
Per2	-0.22	1.00
Zfp407	-0.63	1.00
Nrp1	-1.10	1.00
Robo2	1.17	1.00
Lcp1	-0.69	1.00
Tcf7	0.64	1.00
Trps1	-0.39	1.00
Fmnl2	-0.95	1.00

Pik3ca	-1.27	1.00
Prkd3	-0.86	1.00
Gigyf2	-0.79	1.00
Ankrd17	-0.61	1.00
Skap2	-0.71	1.00
Ikzf1	-0.25	1.00
Dennd4a	-0.96	1.00
Pde1c	-1.09	1.00
Anp32e	-1.13	1.00
Pde4d	-0.97	1.00
Dnajb4	-1.16	1.00
Tmem47	-0.75	1.00
Ralb	-1.00	1.00
Gpc1	-0.55	1.00
Hmcn1	-0.35	1.00
Tnik	-0.58	1.00
Diaph2	-0.13	1.00
Taf5l	-1.26	1.00
Tfrc	-0.82	1.00
Kif5b	-0.85	1.00
Hhex	-1.14	1.00
Zfp469	-0.58	1.00
Plcl2	-0.67	1.00
Rasgef1a	-0.42	1.00
Prex1	-1.09	1.00
Klhl8	-0.64	1.00
Chsy3	-0.71	1.00
Etv6	-0.19	1.00
Tspan3	-0.78	1.00
Large1	-0.72	1.00
Alcam	-0.80	1.00
Twsg1	-1.08	1.00
Ifi27l2a	4.58	0.00
Bst2	2.89	0.00
Ifitm3	3.37	0.00
Isg15	3.06	0.00
Zbtb16	-1.64	0.00
B2m	1.78	0.00
H2-K1	2.24	0.00
H2-D1	1.87	0.00
Ly6e	2.23	0.00
Xaf1	1.39	0.00
H2-T23	1.70	0.00
Ifi44	1.55	0.00
Irf7	1.55	0.00
Oasl2	1.46	0.01

Ifit1	1.99	0.01
Rtp4	1.54	0.01
Ddx60	1.34	0.02
Psmb8	1.47	0.03
Ube2l6	1.24	0.03
Usp18	1.30	0.03
Lgals3bp	1.22	0.05
Stat2	1.11	0.06
Zbp1	1.28	0.10
AY036118	1.17	0.12
Tapbp	1.27	0.13
Rsad2	1.94	0.18
Adssl1	-0.35	0.23
Tap2	1.11	0.28
Ly6a	1.59	0.36
Parp14	0.97	0.42
Chia1	-1.19	0.55
Slirp	1.07	0.70
Sp100	1.43	0.78
Ank3	-1.20	0.84
Ifit3	1.89	0.92
Phf11d	0.70	1.00
Ccnj	-0.47	1.00
Ifit3b	1.02	1.00
Osgep	-0.94	1.00
Nkd1	-1.02	1.00
Cth	-0.52	1.00
Notch1	-0.42	1.00
Lgals9	1.15	1.00
Rnf213	0.68	1.00
Irgm1	0.63	1.00
Gbp3	0.70	1.00
Tap1	0.87	1.00
Adar	0.74	1.00
Dapk1	-0.75	1.00
Trim25	0.61	1.00
Zbtb7a	0.87	1.00
Cyp39a1	-0.45	1.00
Rgcc	-1.30	1.00
Kcnj15	-0.83	1.00
Spats2l	0.93	1.00
Dtx3l	0.58	1.00
Ralgapa2	-1.62	1.00
Samd9l	0.85	1.00
Plac8	2.11	1.00
Kat6b	-0.72	1.00

Cxxc1	-0.34	1.00
Ddb1	-0.87	1.00
Slc1a3	-0.17	1.00
Uqcrq	0.82	1.00
Ddx58	0.76	1.00
Ifi35	0.71	1.00
Hspa13	-0.29	1.00
Ulk1	-0.90	1.00
Lifr	-0.69	1.00
Prag1	-0.32	1.00
St3gal1	-0.69	1.00
Hibadh	-0.62	1.00
Pafah1b3	-0.24	1.00
Ppp1r14c	-1.01	1.00
Ifi47	0.57	1.00
Prps2	-0.88	1.00
Prdm16	-0.83	1.00
Psme2	0.74	1.00
Cd36	-0.80	1.00
Parp9	1.20	1.00
Hgsnat	-0.33	1.00
Oas1a	0.43	1.00
9530026P05Rik	-0.95	1.00
Irf2bp2	-0.84	1.00
Herc6	1.22	1.00
Pafah2	-0.21	1.00
Trafd1	0.62	1.00
Fnbp1	-0.45	1.00
Camk1	-0.64	1.00
St6gal1	-0.33	1.00
Stk39	-0.83	1.00
Trim30a	0.90	1.00
Rad54l2	-0.33	1.00
Igtp	0.43	1.00
Ccl4	1.82	1.00
Tbc1d30	-0.51	1.00
Car8	-0.97	1.00
Cxcl10	1.24	1.00
Sh3pxd2a	-0.95	1.00
Pip5k1b	-0.93	1.00
Agk	-0.64	1.00
Alkal1	-0.53	1.00
Rnf185	-0.44	1.00
Cnnm2	-0.71	1.00
Ccrl2	0.87	1.00
Ccl2	0.94	1.00

Ly6c2	0.88	1.00
Fut8	-0.89	1.00
Nprl3	-0.60	1.00
Nnmt	-0.29	1.00
Smg8	-0.13	1.00
Prcd	-0.14	1.00
Slc34a2	-0.93	1.00
Tspan11	-0.85	1.00
Dbi	-0.71	1.00
Sgms1	-1.05	1.00
Tubb5	1.06	1.00
Gcc1	-0.13	1.00
Cpped1	-0.26	1.00
Etfb	-0.68	1.00
Dlst	0.69	1.00
Ddx42	0.74	1.00
C1galt1	-0.94	1.00
Rbmx	-0.27	1.00
Isca1	-0.28	1.00
Psme1	0.60	1.00
Rnase4	-1.10	1.00
Gbp7	0.85	1.00
Pigr	0.55	1.00
Ccl3	1.20	1.00
Cfb	0.90	1.00
Synm	-0.72	1.00
Gbp2b	0.92	1.00
Isg20	0.67	1.00
Tnfrsf1a	0.56	1.00
Atp1b1	-0.76	1.00
Rttn	-0.30	1.00
Ralb	0.71	1.00
Cpm	-0.61	1.00
Psmb9	1.07	1.00
Klhl29	-0.62	1.00
Gas6	-0.50	1.00
Tinag	-0.46	1.00
Sftpc	-0.77	1.00
Fabp5	-1.21	1.00
Rab27a	-0.83	1.00
Sgsm2	0.39	1.00
Srcap	-0.50	1.00
Hadha	-0.33	1.00
Auts2	-1.09	1.00
Parp12	0.69	1.00
Ces1d	-0.80	1.00

Nedd4l	-0.77	1.00
Tars2	-0.16	1.00
Grb14	-0.43	1.00
C330007P06Rik	-0.38	1.00
Clip2	-0.50	1.00
Igsf8	0.41	1.00
Lamb2	-0.29	1.00
Ifi30	-0.78	1.00
Gbp2	0.90	1.00
Irx1	-0.58	1.00
Dnajc17	0.66	1.00
Scnn1g	0.89	1.00
Oasl1	0.58	1.00
Lilr4b	0.92	1.00
Slc24a4	-0.52	1.00
Tbc1d8	-0.49	1.00
Slc12a2	-1.23	1.00
Pcgf2	-0.26	1.00
Ctss	1.06	1.00
Cmss1	0.51	1.00
Agmo	-0.61	1.00
Cxcl1	0.61	1.00
Ptbp1	0.56	1.00
Iigp1	1.02	1.00
Vps13d	-0.69	1.00
Gsk3b	-0.93	1.00
Aass	-0.10	1.00
Asxl3	-0.55	1.00
Tyro3	-0.12	1.00
4930403P22Rik	-0.15	1.00
Ccdc163	-0.12	1.00
Npc1	-0.55	1.00
Lamp3	-0.73	1.00
Eef2	-0.50	1.00
Deptor	-0.66	1.00
Arfgap3	0.29	1.00
Gbp6	0.50	1.00
Cxcl15	-0.72	1.00
Nfia	-0.44	1.00
Gna13	-0.79	1.00
Vps9d1	-0.20	1.00
Elfn1	-0.45	1.00
Spr	-0.31	1.00
Selenop	-0.54	1.00
Acot1	-0.25	1.00
Snx21	-0.03	1.00

Hnmt	-0.32	1.00
Atp7b	-0.35	1.00
Edar	-0.21	1.00
Pbx1	1.04	1.00
Rcn2	-0.24	1.00
Col23a1	-0.38	1.00
Tor3a	0.70	1.00
Stat1	0.65	1.00
Sdad1	0.16	1.00
3110082I17Rik	-0.44	1.00
Eif2s3x	-0.63	1.00
Oma1	-0.27	1.00
Rasgef1b	-0.89	1.00
Arhgap24	-2.06	1.00
Cd14	0.40	1.00
Tpcn2	-1.00	1.00
Psmb10	0.38	1.00
Krt18	0.97	1.00
Pla2g1b	-0.60	1.00
Shc1	-0.28	1.00
Atp5e	0.73	1.00
Zfp637	-0.14	1.00
Cd274	0.76	1.00
Slco4c1	-0.66	1.00
Sept7	-0.61	1.00
Sec61g	1.21	1.00
Yae1d1	-0.41	1.00
Pi4k2b	-0.67	1.00
Nebl	1.31	1.00
Tfcp2l1	-0.51	1.00
Pde4d	-0.72	1.00
Alkbh6	-0.15	1.00
Tceal1	-0.20	1.00
Fbxl20	-0.21	1.00
Abca17	-0.48	1.00
Fnip2	-0.19	1.00
Rheb	0.99	1.00
Ifit2	1.04	1.00
Leng8	0.65	1.00
Arfgef3	-0.51	1.00
Ndufa4	-0.37	1.00
Runx1	-0.86	1.00
Slf2	-0.44	1.00
Tspan12	-0.31	1.00
Adamtsl1	-0.42	1.00
Safb2	-0.24	1.00

Slc7a11	0.77	1.00
Irgm2	0.46	1.00
Aox3	-0.31	1.00
Rab27b	-0.58	1.00
Slc30a5	-0.30	1.00
Ano10	-0.31	1.00
Kank2	-0.32	1.00
Pygl	-0.37	1.00
Glul	-0.87	1.00
Dbp	0.44	1.00
Ddah2	-0.35	1.00
Fkbp3	-0.40	1.00
Ggcx	-0.24	1.00
Ndufb11	-0.19	1.00
Tbc1d4	-0.49	1.00
Ppard	0.87	1.00
Otulinl	-0.12	1.00
Abcb8	-0.18	1.00
3110009E18Rik	-0.75	1.00
Eif2ak4	-0.19	1.00
Ccdc77	-0.24	1.00
Cbr2	-0.85	1.00
Gjb6	-0.13	1.00
Pigq	0.62	1.00
Slc25a20	-0.41	1.00
Oas3	0.31	1.00
Shfl	0.24	1.00
Slfn4	1.05	1.00
Apol9a	0.35	1.00
H2-Q7	0.86	1.00
Hc	-0.67	1.00
Pradc1	-0.19	1.00
Ubr5	-0.33	1.00
Eef1d	-0.52	1.00
Ppargc1a	-0.15	1.00
Tmem184c	-0.24	1.00
Frg2f1	-0.37	1.00
Cd79a	-0.81	1.00
Zfp1	-0.63	1.00
Ndufa3	0.70	1.00
S100g	-0.63	1.00
Phf1	-0.25	1.00
Snx14	-0.80	1.00
Apc	-0.65	1.00
Rbm42	0.75	1.00
C1d	-0.43	1.00

Firre	-0.31	1.00
Ube2a	-0.34	1.00
Ccnd3	-0.41	1.00
Serac1	-0.13	1.00
Plp1	-1.12	1.00
AA986860	-0.41	1.00
Khdrbs1	0.71	1.00
Scd2	-0.82	1.00
Tle2	-0.26	1.00
Aldh1l1	-0.21	1.00
Galnt4	-0.52	1.00
Gpam	-0.45	1.00
Rapgef5	-0.81	1.00
Tmem135	-0.31	1.00
Cmtm8	-0.67	1.00
Dtd2	-0.27	1.00
Zfp654	-0.53	1.00
Srf	0.28	1.00
Ckap4	0.62	1.00
Adgrf5	-0.58	1.00
Epas1	-0.90	1.00
Hhat	-0.24	1.00
Thnsl2	-0.13	1.00
Rfc5	-0.15	1.00
Dolk	-0.11	1.00
Chml	-0.10	1.00
A430033K04Rik	-0.11	1.00
Smim31	-0.14	1.00
Tmem44	-0.14	1.00
Supv3l1	-0.16	1.00
Cradd	-0.86	1.00
Snta1	-0.73	1.00
Acaca	-0.47	1.00
Rap2a	-0.50	1.00
Il33	-0.85	1.00
Rsrp1	0.59	1.00
Pigyl	-0.30	1.00
Tacstd2	1.12	1.00
Pxmp2	-0.30	1.00
Zmpste24	-0.32	1.00
Fgd6	-0.65	1.00
Asah2	-1.29	1.00
Sort1	-0.63	1.00
Hspd1	-0.49	1.00
Zfp809	-0.04	1.00
Usp5	0.30	1.00

Aox1	-0.12	1.00
Il18bp	0.29	1.00
Tex11	-0.53	1.00
Dhrs3	0.03	1.00
Rab1b	0.49	1.00
Dcaf10	0.51	1.00
Csad	-0.31	1.00
Dcun1d3	-0.26	1.00
Tiam1	0.33	1.00
Asl	-0.18	1.00
BC031181	-0.45	1.00
Cat	-0.42	1.00
Rab43	-0.51	1.00
Tyk2	-0.34	1.00
Oat	-0.78	1.00
Scrn3	0.23	1.00
Itpripl2	-0.76	1.00
Zbtb8os	0.36	1.00
Tph2	-0.18	1.00
Amer1	-0.17	1.00
Plekhd1	-0.34	1.00
Strn4	0.42	1.00
Ubtd1	0.22	1.00
Nbea	-0.42	1.00
Tyrobp	0.75	1.00
Alyref	-0.45	1.00
Stam	-0.33	1.00
Rnf14	0.28	1.00
Nit1	-0.18	1.00
Fhl2	0.80	1.00
Ccdc122	0.18	1.00
Exosc9	0.19	1.00
Vcan	0.64	1.00
Dhx58	0.14	1.00
Gbp9	0.20	1.00
Clec2d	0.25	1.00
Zfp629	0.19	1.00
Plau	0.82	1.00
Ly6i	0.55	1.00
Scgb3a2	1.45	1.00
Plekhn1	0.17	1.00
Uba7	0.24	1.00
Hpn	-0.16	1.00
Epsti1	0.18	1.00
Ick	-0.24	1.00
4930599N23Rik	0.51	1.00

Impa1	-0.03	1.00
Sugct	-0.46	1.00
Ppig	0.67	1.00
Etaa1	0.13	1.00
Pdk2	-0.07	1.00
Txnl1	-0.37	1.00
Arih1	-0.71	1.00
Arhgef10l	-0.14	1.00
Scand1	0.76	1.00
Nrg1	2.04	1.00
Ing1	-0.23	1.00
Tuft1	0.41	1.00
Gss	-0.26	1.00
Il1b	0.44	1.00
Dnajc14	-0.19	1.00
Irf2bpl	0.50	1.00
Ccnd1	0.62	1.00
Tspan2	-0.58	1.00
Samhd1	1.15	1.00
Psap	0.31	1.00
Pdia3	0.67	1.00
Sidt2	-0.62	1.00
Srpk1	0.67	1.00
Dusp11	-0.26	1.00
Snx7	-0.73	1.00
Atl3	-0.53	1.00
Ube2i	-0.71	1.00
Cln5	-0.10	1.00
Wipf1	-0.89	1.00
Rnd3	-0.41	1.00
Tnfrsf1b	0.77	1.00
Slc16a1	-0.43	1.00
Slc25a24	-0.20	1.00
Aqp5	1.70	1.00
Lmbr1	-0.33	1.00
Iqcc	-0.67	1.00
Cftr	-0.42	1.00
Pank1	-0.24	1.00
Nob1	-0.17	1.00
Ppp1r9a	-0.43	1.00
Abca3	-0.58	1.00
Dip2c	-0.12	1.00
Atp5a1	0.77	1.00
Tnfrsf23	0.24	1.00
Nlk	-0.46	1.00
Tmtc2	-0.75	1.00

Dnajc30	-0.19	1.00
Eppk1	0.42	1.00
Rida	-0.29	1.00
Ndufa6	-0.69	1.00
Bsdc1	-0.26	1.00
Sowahc	-0.31	1.00
Sptssa	-0.71	1.00
Trim59	-0.18	1.00
Lrig2	0.03	1.00
Acadm	-0.29	1.00
Samd12	-1.14	1.00
Psmb2	0.55	1.00
Adam22	-0.27	1.00
Zmat5	-0.67	1.00
Eif3l	-0.16	1.00
Focad	-0.74	1.00
Hexim1	-0.67	1.00
Rpe	0.45	1.00
Gtf2ird2	-0.10	1.00
Tle1	-0.48	1.00
Amotl2	0.68	1.00
Cxcl2	1.77	1.00
Atp5md	0.59	1.00
Ndufs2	-0.22	1.00
Baiap2l1	-0.64	1.00
Stat3	-0.79	1.00
Hsph1	-0.07	1.00
Tmem163	-0.07	1.00
Znrf3	-0.44	1.00
Wdr81	-0.14	1.00
Vps13a	0.79	1.00
Dram1	-0.44	1.00
Inpp4b	-0.46	1.00
Arpc3	0.84	1.00
Atp8a1	-0.72	1.00
Ehd1	0.67	1.00
Atg4d	-0.18	1.00
Ssr1	-0.17	1.00
Tsc22d3	-0.31	1.00
Sfxn2	-1.03	1.00
Tex10	-0.38	1.00
Rmdn2	-0.48	1.00
Hdac3	-0.32	1.00
Stk4	-0.24	1.00
Hmg20b	0.50	1.00
Cmtr1	0.25	1.00

9930111J21Rik2	0.28	1.00
Fah	-0.62	1.00
Unc5d	-0.37	1.00
Thap4	-0.08	1.00
Ercc4	-0.15	1.00
Simc1	-0.41	1.00
Pdia6	0.75	1.00
Pygo2	0.15	1.00
Hspa1b	1.31	1.00
Bad	-0.17	1.00
Bod1l	-0.15	1.00
Bmp2k	-0.21	1.00
H3f3b	0.38	1.00
Helz	-0.59	1.00
S100a6	0.15	1.00
Osbpl11	-0.25	1.00
Sftpb	-0.73	1.00
Alg14	-0.45	1.00
Tgm2	1.03	1.00
Slc1a2	-0.09	1.00
Tsacc	-0.13	1.00
Fam126a	-0.73	1.00
Krt7	0.93	1.00
Fbp2	-0.48	1.00
Lpcat1	-0.73	1.00
Lactb2	-0.07	1.00
Cops8	0.39	1.00
Ablim1	-0.42	1.00
Iglc1	-0.15	1.00
Igip	-0.10	1.00
Tmf1	-0.29	1.00
Nup210l	-0.79	1.00
Prdx5	1.12	1.00
Enpp5	0.30	1.00
Btbd11	0.21	1.00
F10	0.64	1.00
L3mbtl3	0.20	1.00
Tbc1d2	0.39	1.00
Six1	0.14	1.00
Ybey	0.19	1.00
B3galt1	0.39	1.00
Pomgnt1	0.19	1.00
Il12rb2	0.55	1.00
Cdh13	0.73	1.00
Smim5	0.40	1.00
Dscaml1	0.30	1.00

Tmem79	0.14	1.00
E2f2	0.14	1.00
C130026I21Rik	0.20	1.00
Tmem185b	0.22	1.00
Ifi204	0.20	1.00
D730003I15Rik	0.13	1.00
Fcgr1	0.69	1.00
Cxcl3	0.35	1.00
Trim30c	0.40	1.00
Slfn1	0.40	1.00
Tmem106a	0.27	1.00
Tcp11	0.32	1.00
Tnf	0.47	1.00
H2-T24	0.46	1.00
Nudt22	0.13	1.00
Trap1	-0.29	1.00
Lonp1	-0.32	1.00
Aup1	0.47	1.00
Pura	-0.33	1.00
Slc26a9	-0.20	1.00
Sec22a	0.43	1.00
Hk2	-0.22	1.00
Hap1	0.28	1.00
Bmyc	-0.15	1.00
Zdhhc4	0.24	1.00
Agap1	-0.36	1.00
Rhoc	-0.12	1.00
Eif1b	-0.31	1.00
Ppp2r5d	-0.12	1.00
Klf15	-0.04	1.00
Cep290	0.20	1.00
Gabre	-0.16	1.00
Lrrc40	-0.12	1.00
Cables2	-0.10	1.00
Bcl7c	-0.52	1.00
Zfat	-0.76	1.00
Nfkbia	0.67	1.00
Agfg2	0.20	1.00
Ror1	-0.73	1.00
Eif2ak2	0.42	1.00
4833420G17Rik	-0.45	1.00
Cmpk2	0.62	1.00
Znfx1	0.41	1.00
Sema3e	1.37	1.00
Slc38a4	-0.15	1.00
Papolg	-0.14	1.00

Samd8	0.17	1.00
Emc9	-0.20	1.00
Alkbh7	-0.16	1.00
Exoc6	-0.66	1.00
Zfp804b	-0.54	1.00
Rp2	-0.50	1.00
Hdhd2	-0.12	1.00
Gnpat	-0.21	1.00
Akap8	-0.27	1.00
Kcne2	-0.32	1.00
Crlf1	0.37	1.00
Cp	0.64	1.00
Sfta2	-0.34	1.00
Vsig2	-0.44	1.00
Tifa	0.40	1.00
Tbc1d2b	-0.09	1.00
Setdb2	-0.07	1.00
Dpf3	-0.08	1.00
Suox	-0.09	1.00
Ang4	-0.09	1.00
Ctsf	-0.03	1.00
Dpp4	-0.39	1.00
Tmem265	-0.14	1.00
Zfx	-0.22	1.00
Edf1	-0.70	1.00
Ccdc141	-0.67	1.00
Kpna3	0.58	1.00
Wdr77	0.43	1.00
Tmem106c	-0.16	1.00
Stard3nl	-0.52	1.00
Spop	0.58	1.00
Amotl1	-0.08	1.00
Lsm2	-0.74	1.00
Cgn	0.62	1.00
Strip2	-0.61	1.00
Hdgf	0.18	1.00
Tmem8	-0.19	1.00
Snhg11	-0.27	1.00
Aqr	0.33	1.00
Cln3	-0.57	1.00
Alg10b	-0.14	1.00
Gpd2	-0.64	1.00
Gpx4	-0.34	1.00
Lgals3	0.77	1.00
Ramp1	-0.26	1.00
Cd52	1.32	1.00

Sgpp2	-0.30	1.00
Ier3	1.43	1.00
Cyth3	-1.07	1.00
Rdh11	-0.22	1.00
Fign	-0.51	1.00
Alkbh8	-0.16	1.00
Aifm2	-0.16	1.00
Trub2	-0.26	1.00
Ppm1a	-0.50	1.00
Spg11	-0.11	1.00
Pim3	-0.53	1.00
Ociad2	-0.39	1.00
Slc24a3	0.13	1.00
Scgb1c1	-0.67	1.00
Ndufb1-ps	0.80	1.00
Klhl24	-0.08	1.00
Tst	-0.22	1.00
Acss3	-0.33	1.00
Igsf3	-0.16	1.00
Rsph1	1.04	1.00
Csf1r	0.22	1.00
Ascc3	0.66	1.00
Smo	-0.15	1.00
Rbm19	-0.22	1.00
Sel1l	-0.43	1.00
Lnpep	-0.07	1.00
Zfp512	0.49	1.00
Zfp62	-0.13	1.00
Nradd	-0.13	1.00
Dhx29	-0.63	1.00
Prickle2	0.81	1.00
Plcg1	-0.25	1.00
Zswim4	0.32	1.00
Nrp1	-0.38	1.00
Faf2	-0.17	1.00
Pold3	-0.28	1.00
Slx4ip	-0.11	1.00
Topors	-0.72	1.00
Pigu	-0.12	1.00
Spast	-0.22	1.00
Rpa3	-0.51	1.00
Fhdc1	-0.32	1.00
Rflnb	-0.12	1.00
Ypel2	-0.16	1.00
Nmt2	-0.93	1.00
2310015A10Rik	-0.02	1.00

Fbxo45	0.32	1.00
Jpt2	0.12	1.00
Irf6	0.60	1.00
Sell	0.32	1.00
Cenpb	-0.28	1.00
Ier2	0.66	1.00
Secisbp2l	-0.64	1.00
Eif1	-0.32	1.00
Atp6v1c2	-0.23	1.00
Pik3c2g	-0.41	1.00
2610035D17Rik	-0.39	1.00
Slc22a5	-0.24	1.00
Pde7b	-0.58	1.00
Tipin	0.19	1.00
Hmgn2	-0.66	1.00
H2-T22	0.68	1.00
Igfbp7	1.86	1.00
Sorbs3	-0.24	1.00
Armcx1	-0.14	1.00
Pros1	-0.82	1.00
Foxj2	-0.60	1.00
Foxn2	0.38	1.00
Zc2hc1a	0.31	1.00
Tent4a	0.63	1.00
Tdrd3	-0.37	1.00
Polr1d	-0.65	1.00
Cyb5r1	-0.12	1.00
Tkt	-0.26	1.00
Oga	-0.48	1.00
Tmem68	-0.13	1.00
Tnfaip8l3	-0.14	1.00
Hspa1a	1.74	1.00
H6pd	-0.28	1.00
Ifih1	0.54	1.00
Prcp	-0.21	1.00
Gpr19	-0.11	1.00
BC029722	-0.28	1.00
Zfp951	-0.13	1.00
Ipo4	-0.16	1.00
Scd1	-0.42	1.00
Pign	-0.24	1.00
Elmod3	-0.35	1.00
Nat9	-0.20	1.00
Cox6b2	-0.17	1.00
Tmem263	-0.11	1.00
B3gnt5	-0.58	1.00

Uqcc1	0.23	1.00
Phldb1	0.16	1.00
Sertad1	0.74	1.00
Fitm2	-0.20	1.00
Tbxas1	0.12	1.00
Tmem117	0.41	1.00
Ptpro	0.15	1.00
Ttl	0.16	1.00
Trerf1	0.23	1.00
Ints10	0.37	1.00
Nhsl2	0.14	1.00
Trim14	0.31	1.00
Slc4a3	0.15	1.00
Gnb4	0.25	1.00
Sema3f	0.14	1.00
Arl2bp	0.16	1.00
Prkar1b	0.14	1.00
Surf2	0.24	1.00
Asb8	0.13	1.00
Jhy	0.20	1.00
Hinfp	0.11	1.00
Suv39h1	0.20	1.00
Ifi202b	0.48	1.00
Sertad3	0.20	1.00
Ifitm6	0.25	1.00
Ccr2	0.34	1.00
Olfr56	0.19	1.00
Ccnq	0.21	1.00
Slfn8	0.21	1.00
Cd300a	0.48	1.00
Cd300c2	0.48	1.00
Snhg16	0.11	1.00
Adck5	0.16	1.00
2700054A10Rik	0.35	1.00
Tcte2	0.21	1.00
Trem1	0.28	1.00
Ifit1bl2	0.15	1.00
Sbno2	-0.79	1.00
Tnk2	-0.14	1.00
Lnx2	-0.25	1.00
Actb	0.53	1.00
Cadps2	-0.47	1.00
Trrap	-0.33	1.00
Ier3ip1	0.56	1.00
Nin	0.33	1.00
Lcp1	-0.56	1.00

Tnfaip3	0.72	1.00
Zpr1	-0.63	1.00
Entpd7	0.24	1.00
Hprt	-0.22	1.00
Tent2	0.66	1.00
Glyr1	0.49	1.00
Tm7sf3	-0.21	1.00
Vps26c	-0.67	1.00
Ankrd16	-0.18	1.00
Tshz2	0.64	1.00
Phf11b	0.47	1.00
Nfatc1	-0.27	1.00
Nipal1	-0.18	1.00
Phlpp2	-0.45	1.00
Pdpn	1.13	1.00
Socs7	0.26	1.00
Taf1c	-0.11	1.00
Arl15	-0.40	1.00
Lrrk2	-0.80	1.00
Whamm	0.42	1.00
Rrbp1	0.45	1.00
Pik3c2b	-0.21	1.00
Pak6	-0.18	1.00
Psma6	0.73	1.00
Tmem132d	-0.50	1.00
Farsb	0.12	1.00
Syne2	-0.61	1.00
Fut4	-0.19	1.00
Ogfrl1	0.19	1.00
Cep112	-0.33	1.00
Ints6l	-0.34	1.00
Coq8b	-0.03	1.00
Polk	0.64	1.00
Tm9sf4	0.43	1.00
Stn1	-0.39	1.00
Dcaf13	-0.07	1.00
Gpx8	-0.34	1.00
Ttc28	-0.06	1.00
Dnajb11	0.46	1.00
Atp6v0a4	-0.31	1.00
Abcc3	-0.18	1.00
Prodh	-0.19	1.00
Rasgef1a	-0.34	1.00
Slbp	0.65	1.00
Sema3a	0.85	1.00
Rftn1	-0.66	1.00

Zbtb41	-0.13	1.00
Chmp7	-0.18	1.00
6820408C15Rik	0.27	1.00
Eif3m	-0.63	1.00
Cybb	0.84	1.00
Eif2b4	0.20	1.00
Ttc13	-0.18	1.00
Anxa1	0.81	1.00
Tln2	0.10	1.00
Necap2	0.53	1.00
Kifc3	0.27	1.00
Tomm34	-0.49	1.00
Pip4p1	0.35	1.00
Slc7a4	-0.07	1.00
Plxna4	-0.39	1.00
Lyz2	-0.41	1.00
Zfp944	0.11	1.00
Elf4	0.40	1.00
Gnas	-0.38	1.00
Stx4a	0.70	1.00
Cyb5a	-0.19	1.00
Fdps	-0.20	1.00
Jmy	-0.51	1.00
Sfmbt1	0.32	1.00
Pex11a	-0.17	1.00
Narf	-0.32	1.00
Gsta4	-0.52	1.00
Gnmt	-0.11	1.00
Itga9	-0.43	1.00
Tnfaip1	-0.32	1.00
Pfdn5	-0.13	1.00
Nmrk1	-0.10	1.00
Sema4c	0.19	1.00
Plpp2	-0.21	1.00
Fxr1	-0.25	1.00
5330417C22Rik	-0.36	1.00
Actg1	0.75	1.00
Pabpn1	0.21	1.00
Npw	-0.12	1.00
Fam199x	-0.11	1.00
Snhg12	0.17	1.00
Sdhaf1	0.19	1.00
Phkb	-0.34	1.00
Agpat4	0.69	1.00
Hat1	-0.20	1.00
Zcchc9	0.25	1.00

Sdf2l1	0.37	1.00
Cgnl1	-0.33	1.00
Cpne8	-0.87	1.00
Tarbp2	-0.08	1.00
Hint1	0.45	1.00
Capn15	-0.05	1.00
Klhl28	0.07	1.00
Dcp1b	-0.19	1.00
Poc1b	-0.14	1.00
Ndufa2	0.69	1.00
Bag4	-0.17	1.00
Trpm7	0.64	1.00
Pptc7	-0.60	1.00
Orai3	-0.10	1.00
Scaf8	-0.55	1.00
Ngly1	-0.05	1.00
Acsl3	-0.26	1.00
Hnrnpu	0.94	1.00
Dlat	0.57	1.00
Hdac4	-0.48	1.00
Baz2a	0.48	1.00
Rbpj	0.56	1.00
Lrp10	0.25	1.00
Ppp1r15b	-0.32	1.00
C87436	-0.54	1.00
Syde2	0.12	1.00
Krt19	0.95	1.00
Ahcyl2	-0.69	1.00
Psmd11	0.45	1.00
Rtkn2	0.86	1.00
Irf9	0.38	1.00
Cd55	-0.25	1.00
Trim26	0.31	1.00
Ccdc59	0.43	1.00
Ifi27	0.84	1.00
Afap1	-0.10	1.00
Slc12a9	-0.08	1.00
Tmed9	0.53	1.00
Lncpint	-0.72	1.00
Creb3l1	-0.21	1.00
Rpain	0.26	1.00
5033403H07Rik	-0.10	1.00
Bambi	-0.34	1.00
Tjp3	0.18	1.00
Stk40	0.67	1.00
Csgalnact2	-0.23	1.00

Hs6st1	0.47	1.00
Ccdc6	0.73	1.00
Nab1	0.55	1.00
Ptprc	0.92	1.00
Daxx	0.35	1.00
Gatd3a	-0.13	1.00
Aars	0.49	1.00
Psme4	0.53	1.00
Stk35	0.26	1.00
Syngr1	-0.33	1.00
Ncf1	0.19	1.00
Cers6	0.30	1.00
Itm2c	-0.35	1.00
Vps36	-0.12	1.00
Ppdpf	-0.14	1.00
Bbip1	0.18	1.00
Dbnl	0.47	1.00
2610001J05Rik	0.70	1.00
Degs1	-0.23	1.00
Syap1	0.31	1.00
Blmh	-0.03	1.00
2610507B11Rik	-0.58	1.00
Parp8	0.48	1.00
Bicd2	0.26	1.00
Nup205	-0.07	1.00
Stap2	-0.14	1.00
Fto	-0.24	1.00
Gstm1	-0.46	1.00
Sorcs2	0.41	1.00
Sdcbp	0.74	1.00
Taok2	-0.07	1.00
Wdr61	0.74	1.00
Gstt1	-0.48	1.00
Osgin2	0.22	1.00
Fbln5	0.75	1.00
Sema4b	0.11	1.00
Noxo1	0.48	1.00
Cstf1	0.22	1.00
Slc35e1	0.19	1.00
Ccdc162	0.22	1.00
Gas5	0.70	1.00
Pdcd4	0.84	1.00
Prdm11	-0.10	1.00
Slc22a17	-0.16	1.00
Tsta3	0.03	1.00
Cpox	-0.26	1.00

Srek1ip1	-0.31	1.00
St6galnac4	0.25	1.00
Srp19	0.31	1.00
Arpc4	-0.27	1.00
Naga	0.18	1.00
Arhgef26	0.25	1.00
Fabp12	-0.08	1.00
Styx	0.45	1.00
Napsa	-0.49	1.00
Hopx	1.58	1.00
Brap	-0.63	1.00
Trps1	0.61	1.00
Dzip1	0.20	1.00
Htra1	0.29	1.00
Irf8	0.67	1.00
Klhdc4	0.21	1.00
Sh2b3	0.41	1.00
Oscp1	0.36	1.00
9330188P03Rik	0.20	1.00
Rrp36	0.12	1.00
Gstm2	-0.24	1.00
Zeb1	-0.16	1.00
Uqcc3	-0.21	1.00
Smim10l1	-0.34	1.00
Rab11fip1	1.21	1.00
Gpr89	0.17	1.00
Plgrkt	0.39	1.00
Atp5j2	0.80	1.00
Bach2	-0.35	1.00
H2-Q6	0.45	1.00
Trip12	-0.04	1.00
Igf2bp3	-0.22	1.00
Oxsm	-0.11	1.00
Nkain2	-1.51	1.00
Ap5s1	-0.08	1.00
Rftn2	-0.14	1.00
Nckipsd	-0.12	1.00
Castor1	-0.11	1.00
Pnpla7	-0.24	1.00
6030407O03Rik	-0.16	1.00
Gatc	-0.08	1.00
Zfp763	-0.09	1.00
Wwc2	-0.39	1.00
Emb	-0.49	1.00
Eif4enif1	-0.17	1.00
Psmc1	0.43	1.00

Champ1	0.27	1.00
Adgrg6	0.56	1.00
Dimt1	0.17	1.00
AW011738	0.18	1.00
Oas2	0.26	1.00
Adgb	0.28	1.00
Setbp1	0.74	1.00
Ppp6r3	-0.47	1.00
Cfl1	0.53	1.00
Shisa5	0.52	1.00
Bloc1s6	-0.07	1.00
Taco1	-0.09	1.00
Znrd2	0.21	1.00
Rrp1	0.63	1.00
Lekr1	0.42	1.00
Zfp653	-0.10	1.00
Rbck1	0.19	1.00
Dnajc9	-0.47	1.00
Golt1b	0.28	1.00
Cyb5r4	-0.01	1.00
Cnpy2	-0.64	1.00
Trib1	0.68	1.00
Ppp1r2	-0.34	1.00
Ifitm2	0.84	1.00
Atp23	-0.09	1.00
Fam167a	-0.16	1.00
P2rx6	-0.23	1.00
A3galt2	-0.18	1.00
Trp53cor1	-0.09	1.00
Acss2	-0.23	1.00
Ppa1	0.61	1.00
Tfip11	0.12	1.00
Snhg20	0.15	1.00
Pcmtd2	-0.16	1.00
Tmem126a	0.29	1.00
Trim2	-0.32	1.00
Pik3ip1	0.31	1.00
0610009B22Rik	-0.23	1.00
Acod1	0.98	1.00
Ap2a1	0.24	1.00
Slc4a4	-0.61	1.00
Calr	0.63	1.00
Smc6	-0.88	1.00
Pxk	-0.53	1.00
Ro60	-0.10	1.00
Avl9	-0.13	1.00

Atp2a2	-0.26	1.00
Rab3gap2	0.38	1.00
Casp7	0.32	1.00
Abhd11	-0.15	1.00
2310033P09Rik	0.50	1.00
Atrnl1	-0.37	1.00
Zfp160	0.18	1.00
Nt5c3b	-0.12	1.00
Snrpd3	0.40	1.00
Polr3c	0.09	1.00
Hemk1	-0.08	1.00
Cldn5	-0.89	1.00
Aatk	-0.27	1.00
E130102H24Rik	-0.11	1.00
Rbak	-0.06	1.00
Trpv6	-0.07	1.00
AV356131	-0.10	1.00
D130040H23Rik	-0.11	1.00
Arf4os	-0.08	1.00
Rnf219	-0.09	1.00
Nckap5los	-0.07	1.00
C330013E15Rik	-0.07	1.00
Zmiz1	-0.21	1.00
Chst11	0.17	1.00
Sar1b	-0.25	1.00
Alad	-0.16	1.00
Kmt2e	-0.14	1.00
N4bp2	0.39	1.00
Piwil2	-1.02	1.00
Abr	-0.63	1.00
Pdlim5	0.83	1.00
Wdfy2	-0.16	1.00
Ehd4	0.60	1.00
Socs2	-0.37	1.00
Hps3	0.45	1.00
Def6	0.56	1.00
Sppl2b	0.18	1.00
B430010I23Rik	0.10	1.00
Unk	0.17	1.00
Npat	0.14	1.00
Slc9a3r1	0.48	1.00
Vegfb	-0.17	1.00
Ly6c1	0.72	1.00
Gabbr1	-0.56	1.00
Uhrf1bp1l	-0.39	1.00
Ptpmt1	0.35	1.00

Wdr83os	-0.53	1.00
CAAA01118383.1	0.33	1.00
Proz	-0.07	1.00
Tiam2	-0.10	1.00
Otop1	-0.05	1.00
Cdkl1	0.10	1.00
Sft2d3	-0.04	1.00
Tlnrd1	-0.05	1.00
Vdr	-0.10	1.00
Ppargc1b	0.15	1.00
Plekhh3	-0.02	1.00
Baz2b	-0.68	1.00
Ccn1	1.71	1.00
Fam114a2	-0.13	1.00
Naa20	0.42	1.00
Smarcd1	-0.13	1.00
Rasa3	-0.25	1.00
Arid5b	-0.57	1.00
Ppm1m	-0.12	1.00
Pgk1	0.34	1.00
Srebf1	-0.17	1.00
Klf4	-0.28	1.00
Tmem164	-0.48	1.00
Slc30a1	0.18	1.00
Gda	0.36	1.00
Bace1	0.20	1.00
Ogg1	0.12	1.00
Ddx5	0.28	1.00
Lmo4	0.38	1.00
Smpd1	-0.33	1.00
Cab39l	-0.44	1.00
Slc15a2	-0.20	1.00
Prxl2c	-0.05	1.00
Ahsa1	0.31	1.00
Jmjd6	0.40	1.00
Ostc	0.44	1.00
Saraf	0.40	1.00
Adam19	-0.39	1.00
Zbtb38	-0.27	1.00
Zfp385b	-0.27	1.00
Lcor	0.67	1.00
Lrrc4	0.83	1.00
Hnrnpul2	-0.28	1.00
Eif4h	-0.29	1.00
AW112010	1.36	1.00
Bcl6	-0.49	1.00

Hcar2	0.75	1.00
Ifitm1	1.04	1.00
Dram2	-0.31	1.00
Itprid2	0.47	1.00
Abcg1	-0.49	1.00
Fat4	-0.23	1.00
Ap1s2	0.10	1.00
Arpc2	0.54	1.00
Trim44	-0.54	1.00
Tes	0.37	1.00
Bmp3	-0.32	1.00
Ewsr1	-0.12	1.00
Arhgdig	0.39	1.00
Car2	-0.51	1.00
Strn3	0.49	1.00
Cdc42bpa	-0.50	1.00
Rpgr	0.55	1.00
Dus3l	0.28	1.00
Slfn5	0.56	1.00
Cd2bp2	0.10	1.00
Ppm1b	-0.32	1.00
Cbx7	0.09	1.00
Cfap65	0.16	1.00
Ncf2	-0.08	1.00
Psmb6	0.31	1.00
Fam174a	0.31	1.00
Fam110a	0.26	1.00
4921531C22Rik	-0.12	1.00
Zfp866	-0.17	1.00
Zfp729a	-0.13	1.00
Zfand5	0.41	1.00
Zfp395	-0.09	1.00
Cnih1	0.07	1.00
Ezh2	-0.35	1.00
Pakap.1	0.88	1.00
Itsn1	-0.20	1.00
Susd6	0.62	1.00
Neat1	-0.66	1.00
Gna14	-0.22	1.00
2900060B14Rik	0.17	1.00
Nagk	0.05	1.00
Ctps2	-0.60	1.00
9130230L23Rik	0.65	1.00
Slc25a18	-0.13	1.00
Heatr1	-0.14	1.00
Grk3	-0.26	1.00

Cebpa	-0.41	1.00
Fgfr1	-0.27	1.00
Zfp672	-0.13	1.00
Xpa	0.26	1.00
Sart1	-0.05	1.00
Cadm1	-0.21	1.00
Tead3	-0.11	1.00
Usf3	0.39	1.00
Patl2	0.29	1.00
2900097C17Rik	-0.18	1.00
Col1a2	-0.57	1.00
Lrrc47	-0.14	1.00
Appbp2	-0.36	1.00
Skil	-0.32	1.00
Sipa1l3	-0.40	1.00
Pcyt2	-0.32	1.00
Nelfb	-0.21	1.00
Gramd1c	-0.36	1.00
Echdc3	-0.10	1.00
Klhl26	-0.17	1.00
Sf3a3	0.25	1.00
Pla2g12a	-0.12	1.00
Timm44	0.17	1.00
Gcnt2	0.72	1.00
Rnf138	0.35	1.00
Wdr7	0.40	1.00
Zfp652	-0.21	1.00
Stimate	0.03	1.00
Etv5	-0.32	1.00
Erbin	-0.36	1.00
Trmu	-0.09	1.00
Daam2	-0.21	1.00
Umps	-0.74	1.00
Adam15	-0.09	1.00
Pram1	-0.08	1.00
Rbm47	-0.16	1.00
Zswim7	0.09	1.00
Rsph10b	0.36	1.00
2310010J17Rik	0.18	1.00
Tor1aip2	0.43	1.00
Tmem241	-0.66	1.00
Nelfa	-0.21	1.00
Coro2b	-0.19	1.00
Tmlhe	-0.25	1.00
Slc39a10	-0.52	1.00
Lipo3	0.24	1.00

Psma4	0.60	1.00
Plin3	-0.01	1.00
Col6a1	-0.08	1.00
Fyco1	0.80	1.00
Chrac1	0.17	1.00
Clmn	-0.35	1.00
Arpc1b	-0.29	1.00
Ralgapb	0.32	1.00
Setx	-0.78	1.00
Ttc1	-0.08	1.00
Zc3h12c	0.65	1.00
Las1l	-0.28	1.00
Pcbd1	-0.18	1.00
Hsd17b11	-0.26	1.00
Bphl	-0.20	1.00
Eif4a3	-0.52	1.00
Klhl42	-0.08	1.00
Gng2	-0.49	1.00
Tesk2	-0.26	1.00
Nacc2	-0.51	1.00
2810006K23Rik	-0.12	1.00
Hnf1b	0.32	1.00
Pcca	-0.34	1.00
Wdr70	-0.87	1.00
Dbt	-0.17	1.00
Grpel1	-0.33	1.00
Atxn7	-0.70	1.00
Fam187b	-0.21	1.00
Robo2	-0.32	1.00
Ppp4c	-0.75	1.00
Nipa2	0.41	1.00
Alas1	0.67	1.00
Nt5c3	0.36	1.00
Nfkb1	0.02	1.00
Hes1	0.65	1.00
Proca1	-0.10	1.00
Prmt5	-0.13	1.00
Chsy1	-0.19	1.00
Xpnpep3	0.34	1.00
Dapk3	0.18	1.00
Tlr2	0.75	1.00
Ikzf5	0.17	1.00
Prss12	-0.13	1.00
Pgap1	-0.10	1.00
Crtc1	-0.08	1.00
Zcchc4	-0.16	1.00

Lamb1	-0.21	1.00
Igkc	-0.63	1.00
Cdk19	-0.24	1.00
Krt23	-0.26	1.00
Gpaa1	-0.18	1.00
4931406C07Rik	-0.30	1.00
Atxn7l3b	-0.37	1.00
Gpr180	0.60	1.00
Tmem80	0.15	1.00
Utp3	0.42	1.00
Antxr1	-0.05	1.00
Polr2f	-0.22	1.00
Abcd3	-0.29	1.00
Commd4	0.34	1.00
Cog6	-0.17	1.00
Dcp2	0.36	1.00
Fam71a	-0.33	1.00
Gabarapl2	-0.20	1.00
Acot13	-0.06	1.00
Sntb2	-0.34	1.00
Nr4a1	0.52	1.00
Atpsckmt	-0.13	1.00
Tbc1d25	-0.08	1.00
Stx3	0.15	1.00
Lsr	0.30	1.00
Csgalnact1	-0.19	1.00
Cttnbp2	0.59	1.00
Tnfaip2	0.36	1.00
Cep250	0.37	1.00
5730455P16Rik	0.15	1.00
Hip1	0.51	1.00
Itgb7	0.29	1.00
Csf3r	0.26	1.00
Spag1	0.15	1.00
Usp50	0.25	1.00
Dalrd3	0.12	1.00
Ivd	-0.15	1.00
Parn	-0.16	1.00
Sirt7	-0.09	1.00
Casp9	-0.14	1.00
Trak1	-0.21	1.00
Schip1	0.48	1.00
Zfp276	-0.16	1.00
Tmem268	-0.08	1.00
Ism2	-0.05	1.00
Supt20	0.42	1.00

Col12a1	-0.44	1.00
Ppia	-0.33	1.00
Gadd45b	0.44	1.00
Tmem63a	0.01	1.00
Tmem97	-0.10	1.00
Cir1	-0.21	1.00
Zfp771	-0.31	1.00
Pde7a	-0.33	1.00
Veph1	-0.51	1.00
Snhg18	-0.07	1.00
Nfkbib	0.50	1.00
Haus6	-0.12	1.00
Pla2g4f	-0.07	1.00
Arhgap25	-0.21	1.00
Pja1	-0.13	1.00
Tmpo	-0.25	1.00
Zfp704	-0.56	1.00
Gapvd1	-0.20	1.00
Pitpna	0.46	1.00
Tacc1	-0.47	1.00
Relb	0.58	1.00
Cwc27	-0.63	1.00
Psmd9	-0.09	1.00
Ndufaf5	0.13	1.00
Tmco6	0.15	1.00
Rnf41	0.32	1.00
Ccdc180	0.48	1.00
Lilrb4a	0.70	1.00
Gdpd1	-0.20	1.00
Fam129a	-0.71	1.00
Tmem223	-0.19	1.00
Letm2	-0.03	1.00
Arhgef19	-0.06	1.00
Prdm16os	-0.04	1.00
Rfx1	-0.10	1.00
Jrkl	-0.08	1.00
Rfxap	-0.13	1.00
Spata2	0.22	1.00
Adk	-0.53	1.00
Sfta3-ps	-0.52	1.00
Tpm3	-0.03	1.00
Agap3	-0.03	1.00
Edem1	-0.33	1.00
Rreb1	-0.55	1.00
Zdhhc3	-0.27	1.00
Ppic	-0.18	1.00

Stard5	-0.26	1.00
Elmsan1	0.00	1.00
Tuba1a	0.37	1.00
F2rl1	-0.10	1.00
Ttbk2	-0.16	1.00
Ypel1	-0.10	1.00
Tango2	-0.15	1.00
Tmem220	-0.12	1.00
Pfkl	-0.50	1.00
St6galnac6	-0.33	1.00
Dmd	-1.42	1.00
Aldh9a1	-0.08	1.00
Ncoa2	-0.29	1.00
Dnah11	0.51	1.00
Asxl2	-0.65	1.00
Echs1	-0.03	1.00
Sh3rf1	-0.25	1.00
Ube2q2	0.63	1.00
Tmem209	0.31	1.00
Cacna1a	0.22	1.00
Fyn	-0.91	1.00
Aph1a	0.37	1.00
Aasdhppt	-0.53	1.00
Trim28	-0.18	1.00
Emc3	-0.22	1.00
Brd8	-0.14	1.00
Spata6	0.56	1.00
AC149090.1	0.13	1.00
Sgsh	-0.05	1.00
Akap13	-0.35	1.00
Samm50	0.60	1.00
Psph	0.09	1.00
Suco	-0.13	1.00
Yy1	-0.27	1.00
Chchd10	-0.11	1.00
Snx16	-0.16	1.00
Cep85l	-0.32	1.00
Slc15a4	-0.31	1.00
Cog8	-0.31	1.00
Prelid3b	0.24	1.00
Ncaph2	0.55	1.00
N6amt1	-0.12	1.00
Slc35g1	-0.16	1.00
C920021L13Rik	-0.03	1.00
Ptrh2	0.35	1.00
Zfp869	-0.17	1.00

Ang	-0.31	1.00
Plcl2	0.24	1.00
Srm	0.14	1.00
Bysl	0.15	1.00
Htatip2	0.10	1.00
Zfp943	0.25	1.00
Vav1	0.30	1.00
Gde1	-0.15	1.00
Agrn	0.82	1.00
Rab38	0.22	1.00
Psmg2	0.16	1.00
Ift122	0.26	1.00
Csf1	0.36	1.00
Serping1	0.41	1.00
Psd4	0.20	1.00
Pml	0.16	1.00
2310061I04Rik	0.25	1.00
Lrp2	-0.21	1.00
Tmeff1	0.12	1.00
Tyw1	-0.10	1.00
Skp1a	0.38	1.00
Tmcc1	0.45	1.00
Fam174b	0.35	1.00
Uqcrc2	0.49	1.00
Rnf186	0.34	1.00
Pop5	-0.37	1.00
Nudt18	0.27	1.00
Bmp4	0.76	1.00
Tgfbrap1	-0.09	1.00
Upp2	-0.18	1.00
N4bp1	-0.11	1.00
Pet100	0.42	1.00
Etv6	-0.34	1.00
Tmem109	0.08	1.00
Nrcam	-0.12	1.00
Recql	-0.05	1.00
Ranbp17	-0.11	1.00
Chkb	0.05	1.00
Ppp5c	0.42	1.00
Prkca	-0.12	1.00
Ugcg	0.57	1.00
Sar1a	0.57	1.00
Klc3	0.21	1.00
Sptlc2	-0.53	1.00
Fam185a	0.14	1.00
Rgs12	0.23	1.00

Zfp263	0.15	1.00
Zbtb43	0.18	1.00
Taf4b	0.07	1.00
2610020C07Rik	0.39	1.00
Zc3h13	-0.27	1.00
1110038F14Rik	0.29	1.00
Vwa8	-0.33	1.00
Oaz2	-0.28	1.00
Gemin2	-0.01	1.00
Timm8a1	0.04	1.00
Idi1	0.03	1.00
Rybp	-0.31	1.00
Abraxas2	-0.16	1.00
Anapc13	0.16	1.00
Thrb	-0.01	1.00
Pak1ip1	0.32	1.00
Egr1	0.81	1.00
Shld2	-0.05	1.00
AU020206	-0.12	1.00
Lrp2bp	-0.14	1.00
Cdyl2	0.00	1.00
Atg4a	-0.11	1.00
Gstk1	-0.05	1.00
Lsp1	0.73	1.00
Atxn3	0.23	1.00
Sec23ip	0.20	1.00
Eftud2	-0.77	1.00
Ccdc84	0.17	1.00
Uimc1	-0.18	1.00
Rarg	0.37	1.00
Jup	0.41	1.00
Plcxd2	0.27	1.00
Cbfa2t3	0.22	1.00
Ralgps2	0.16	1.00
Nkiras1	-0.12	1.00
Gyg	-0.13	1.00
Setd1b	0.39	1.00
Pcx	-0.34	1.00
Dgat2	-0.07	1.00
Nhlrc3	-0.06	1.00
Ptpn23	-0.08	1.00
Tpm4	0.25	1.00
Rsbn1l	-0.27	1.00
Sec23b	0.02	1.00
Galnt18	0.74	1.00
Junb	0.59	1.00

Gpn3	-0.34	1.00
Lrrfip2	-0.49	1.00
Irf1	0.40	1.00
Lrpap1	0.63	1.00
Uba2	0.52	1.00
Ddx56	0.00	1.00
Arhgef38	0.00	1.00
Ppm1g	0.32	1.00
Prkcz	0.25	1.00
C6	-0.23	1.00
Dipk2a	-0.21	1.00
Birc6	-0.28	1.00
Ubp1	0.33	1.00
Ttc38	-0.06	1.00
Tyms	-0.05	1.00
Creg1	-0.04	1.00
Grk4	0.52	1.00
Atic	0.12	1.00
Ethe1	0.17	1.00
Cd82	0.02	1.00
Lsm1	0.10	1.00
Nav2	-0.53	1.00
Scyl1	-0.13	1.00
Vipas39	0.14	1.00
Sox2	0.17	1.00
Tctex1d4	0.28	1.00
Rhog	0.15	1.00
Tmem213	-0.02	1.00
Unc93b1	0.44	1.00
Gramd1b	-0.21	1.00
Smurf1	-0.16	1.00
Rnf2	-0.19	1.00
Snap29	0.07	1.00
Rilp	-0.08	1.00
Rbm45	0.17	1.00
Zdhhc6	-0.07	1.00
Cldn7	0.60	1.00
Rdm1	0.23	1.00
Prdm1	-0.61	1.00
Cyfip1	0.74	1.00
Spock2	0.99	1.00
BC049715	0.39	1.00
Lipe	0.17	1.00
Pds5a	-0.39	1.00
Rdh14	-0.12	1.00
Il1rap	0.02	1.00

Stt3b	-0.74	1.00
Pank3	-0.19	1.00
Nfic	0.31	1.00
Ptpn12	0.05	1.00
Rab34	-0.19	1.00
Ifnar2	-0.07	1.00
Coq8a	0.08	1.00
Sema6d	-0.03	1.00
Bri3bp	0.05	1.00
Ikbke	0.09	1.00
Cnbd2	0.10	1.00
Anapc15	0.07	1.00
4930423D22Rik	0.07	1.00
Aatf	0.31	1.00
Hspa2	-0.42	1.00
Laptm4b	-0.08	1.00
Sec31a	-0.36	1.00
Gstt3	-0.33	1.00
Ociad1	-0.17	1.00
Nr2f2	-0.57	1.00
Ccl5	0.20	1.00
Gpc3	0.20	1.00
Parp3	0.37	1.00
Sugp1	0.15	1.00
Sftpa1	-0.38	1.00
Abhd10	-0.26	1.00
Spsb2	0.18	1.00
Stox2	-0.32	1.00
Shoc2	-0.10	1.00
Wdtc1	0.26	1.00
Ik	0.54	1.00
Itpr2	-0.49	1.00
Tma7	0.34	1.00
Wdr73	0.15	1.00
Txnrd3	-0.33	1.00
Galm	-0.10	1.00
Otud1	0.03	1.00
Gpd1	0.39	1.00
Sp110	0.22	1.00
Sec14l2	-0.09	1.00
Pum1	-0.40	1.00
Card19	0.43	1.00
Steap2	-0.17	1.00
Higd2a	-0.10	1.00
Iqcg	0.66	1.00
Ptma	0.29	1.00

Timm8b	-0.53	1.00
Shh	-0.05	1.00
Ghdc	-0.02	1.00
Ppid	-0.22	1.00
Ceacam1	0.29	1.00
Calcoco1	0.43	1.00
Hdac2	-0.16	1.00
Acaa2	-0.24	1.00
Txndc9	0.29	1.00
Cep70	-0.08	1.00
Patz1	-0.04	1.00
Ccdc127	-0.14	1.00
Ep300	0.16	1.00
Ccpg1	-0.42	1.00
Nudt3	-0.19	1.00
Timp2	0.68	1.00
Grb7	-0.15	1.00
Cebpd	-0.36	1.00
Tspan31	0.46	1.00
Egfl6	-0.25	1.00
Cnksr3	-0.03	1.00
Ankrd13c	-0.30	1.00
Kif1bp	-0.26	1.00
Ctnnal1	-0.33	1.00
Fam183b	0.86	1.00
Ano1	0.49	1.00
Nrbp2	0.28	1.00
Cdh1	0.75	1.00
Dock9	-0.27	1.00
Smpdl3a	0.47	1.00
Rab20	-0.41	1.00
Igf1r	0.13	1.00
Ubr3	-0.12	1.00
Rnaset2a	0.33	1.00
Brix1	-0.15	1.00
Socs3	-0.43	1.00
Actn1	0.94	1.00
Stx11	0.35	1.00
Zfp574	0.20	1.00
Dhrs7	-0.28	1.00
B3glct	-0.08	1.00
Vac14	0.30	1.00
Polr3gl	-0.07	1.00
Prpf40b	0.00	1.00
Dhx38	0.37	1.00
Ap3d1	-0.20	1.00

Pnpla8	-0.05	1.00
Sp2	0.06	1.00
Noc2l	0.17	1.00
Lrrc51	0.22	1.00
Fam129b	-0.18	1.00
Rnf24	0.19	1.00
Anp32a	0.12	1.00
Plagl1	-0.14	1.00
Fryl	-0.89	1.00
Ptprm	-0.20	1.00
Dmkn	0.75	1.00
Setd3	0.04	1.00
Cxadr	0.36	1.00
Zfp445	0.24	1.00
Kcnq1	-0.20	1.00
Rfc1	0.15	1.00
Ing5	-0.08	1.00
Gigyf1	0.11	1.00
Smpd4	-0.06	1.00
Slc22a23	-0.28	1.00
Nmi	0.33	1.00
Ttc33	-0.29	1.00
Slc38a1	0.07	1.00
Rab31	-0.07	1.00
Tm4sf1	0.57	1.00
Heatr5a	-0.70	1.00
Sec62	0.24	1.00
Acot7	-0.23	1.00
Ctcf	-0.15	1.00
Lca5	0.11	1.00
Tekt4	0.14	1.00
Hdac8	-0.23	1.00
Rab33b	-0.18	1.00
Herpud1	-0.27	1.00
Git2	-0.25	1.00
Nt5dc1	0.29	1.00
Ppp1r12c	-0.03	1.00
Lyz1	-0.11	1.00
Radil	-0.12	1.00
Nars2	-0.24	1.00
Usb1	0.12	1.00
Wdr91	-0.19	1.00
Ints13	-0.18	1.00
Dusp5	-0.23	1.00
Ints4	0.00	1.00
Cnih4	-0.16	1.00

Ahcyl1	-0.24	1.00
Prxl2a	-0.12	1.00
Pja2	0.30	1.00
Nipsnap1	-0.20	1.00
Hmgb1	-0.32	1.00
Hira	-0.19	1.00
Taf11	-0.14	1.00
Dusp3	-0.48	1.00
Ube2g2	-0.03	1.00
Cdk10	-0.04	1.00
Kdelr1	0.40	1.00
Slc23a2	-0.45	1.00
Usp24	-0.36	1.00
Immp2l	-0.58	1.00
Slc50a1	-0.22	1.00
Cox7a2	-0.46	1.00
Slit2	-0.05	1.00
Grhl2	-0.27	1.00
Gpx1	-0.38	1.00
Ppib	-0.20	1.00
S100a14	0.42	1.00
Alcam	-0.32	1.00
Frs2	-0.22	1.00
Snx12	-0.15	1.00
Ehbp1	-0.27	1.00
Daam1	-0.41	1.00
Scarf1	-0.01	1.00
Dcaf11	-0.23	1.00
Per1	-0.90	1.00
Hsd17b4	0.05	1.00
Cracr2b	-0.58	1.00
Prrg3	-0.18	1.00
Lgi3	-0.21	1.00
Gtpbp4	0.28	1.00
Alkbh5	-0.60	1.00
Lsm8	0.37	1.00
Sept9	0.49	1.00
Atg5	-0.52	1.00
Slco2a1	0.03	1.00
Per2	0.44	1.00
Pip4k2c	-0.08	1.00
Hspb1	0.66	1.00
Secisbp2	-0.25	1.00
Kif3a	0.50	1.00
Rhod	-0.11	1.00
H2-Q4	0.20	1.00

Bex4	-0.09	1.00
Brd9	-0.10	1.00
Bag6	0.21	1.00
Smim3	0.02	1.00
Ormdl2	-0.12	1.00
Ankrd28	-0.77	1.00
Lonp2	-0.59	1.00
Pdrg1	0.28	1.00
Smim11	-0.15	1.00
Btg1	0.32	1.00
Ubl4a	0.15	1.00
Cyb5b	-0.26	1.00
Elmo1	-0.83	1.00
Gnpnat1	-0.43	1.00
Stk11	-0.34	1.00
Adgre5	0.37	1.00
Alg1	0.18	1.00
Zfp397	-0.17	1.00
Crebrf	-0.43	1.00
Tubgcp3	0.56	1.00
Lama3	0.52	1.00
Gch1	0.43	1.00
Dgkz	-0.55	1.00
Polr3f	0.07	1.00
Dnah9	0.44	1.00
Aftph	0.38	1.00
Ube2e1	-0.23	1.00
Pnpt1	-0.02	1.00
Pprc1	0.11	1.00
Sema4f	-0.17	1.00
Exosc7	-0.29	1.00
Ddx39	0.15	1.00
Slc44a4	-0.18	1.00
Rims2	0.59	1.00
Pfkfb2	-0.13	1.00
Hadhb	0.13	1.00
Ndufa5	0.47	1.00
Fuca1	-0.10	1.00
Abi1	0.34	1.00
4732471J01Rik	-0.15	1.00
Comt	-0.10	1.00
Ptcd2	0.08	1.00
Spi1	0.29	1.00
Palmd	0.13	1.00
Ehhadh	0.17	1.00
Edc3	0.10	1.00

Spsb3	0.08	1.00
Tpst1	0.20	1.00
G0s2	0.22	1.00
Tusc1	0.34	1.00
Gad1os	0.14	1.00
5430416N02Rik	0.13	1.00
Ggct	0.10	1.00
Fiz1	0.23	1.00
Ajuba	0.12	1.00
C330018D20Rik	0.10	1.00
Acot2	-0.18	1.00
Arl4c	0.47	1.00
Gem	-0.18	1.00
Enpp4	0.35	1.00
Hnrnpa2b1	-0.28	1.00
Azi2	0.32	1.00
Virma	-0.54	1.00
Ralgps1	0.22	1.00
Ubald2	0.63	1.00
Pcf11	-0.28	1.00
Clec16a	-0.06	1.00
Erich2	0.29	1.00
Lrrc42	-0.21	1.00
Rasd1	-0.11	1.00
Rnf216	-0.25	1.00
Tspan9	-0.09	1.00
Net1	0.31	1.00
Tpt1	-0.24	1.00
Igf2bp2	0.38	1.00
Kctd10	-0.12	1.00
Hspe1	0.48	1.00
Emc8	-0.09	1.00
Emc10	0.54	1.00
Fasn	-0.26	1.00
Atp6v0d1	-0.24	1.00
Hsdl2	-0.14	1.00
Vps72	-0.15	1.00
Kdm6b	0.88	1.00
Cryzl1	-0.16	1.00
Scp2	-0.19	1.00
Ctdnep1	-0.13	1.00
A930037H05Rik	0.27	1.00
Ahnak2	0.60	1.00
Slc15a3	0.62	1.00
Cryab	0.11	1.00
Tbrg1	-0.26	1.00

Eif3e	-0.51	1.00
Vps41	-0.25	1.00
Psmb3	0.56	1.00
Kdm1b	0.16	1.00
Atrip	0.68	1.00
Ccl9	0.45	1.00
Eml3	0.09	1.00
Rfx5	0.08	1.00
Il1a	0.11	1.00
Cfap74	0.30	1.00
Tmub1	0.09	1.00
Peg10	0.31	1.00
Tagln2	-0.34	1.00
Rnf121	0.59	1.00
Rbm10	0.18	1.00
Lrch3	0.50	1.00
Cdpf1	-0.53	1.00
Nup214	-0.21	1.00
Trappc6a	-0.12	1.00
Scx	-0.16	1.00
Ddx47	0.21	1.00
Abi2	-0.09	1.00
Fam50a	-0.31	1.00
Nnat	0.63	1.00
Gal3st1	0.06	1.00
Tmed5	-0.09	1.00
Slc25a30	-0.20	1.00
Nexmif	-0.56	1.00
Sgms2	0.53	1.00
Cd63	-0.38	1.00
Rprd2	-0.17	1.00
Tspyl1	0.43	1.00
Hnrnpl	-0.28	1.00
Aprt	0.46	1.00
Dock1	-0.31	1.00
Cdc40	-0.32	1.00
Fam120a	-0.65	1.00
Gzma	0.87	1.00
Adck1	0.17	1.00
Smpd3	0.14	1.00
Clec4e	0.46	1.00
Frat1	0.08	1.00
Frmd6	-0.13	1.00
Smad2	-0.15	1.00
Ptprg	-0.50	1.00
Uba5	-0.25	1.00

Ywhaq	-0.36	1.00
Hspa14	0.10	1.00
Rab3c	-0.50	1.00
Stard4	-0.11	1.00
Crbn	0.26	1.00
Stbd1	-0.22	1.00
Cep63	0.09	1.00
Ubac2	-0.05	1.00
Arl8b	-0.59	1.00
Lmna	0.23	1.00
Brms1	0.12	1.00
Pepd	0.03	1.00
Nfatc3	-0.56	1.00
Vapa	0.29	1.00
Cobl	0.24	1.00
Ptprz1	0.49	1.00
Nrn1	-0.28	1.00
Pigb	0.18	1.00
Ercc1	0.14	1.00
Zfp251	0.14	1.00
1700109H08Rik	-0.10	1.00
Zfp84	-0.09	1.00
Fkbp1a	-0.39	1.00
Zfyve9	-0.18	1.00
Nubp1	0.24	1.00
Atp2b1	0.38	1.00
Icam1	0.70	1.00
Camk1d	0.44	1.00
Prkacb	-0.01	1.00
Npm3	-0.27	1.00
Sf3a1	-0.18	1.00
Hsd17b7	-0.31	1.00
Abcf2	-0.57	1.00
Fbxo42	-0.10	1.00
Zfp362	-0.07	1.00
Serpine1	0.11	1.00
Snrnp25	-0.20	1.00
Wnk4	-0.47	1.00
Cd151	0.30	1.00
Pfdn1	0.25	1.00
Tmem87a	0.43	1.00
S100a10	-0.27	1.00
Bspry	-0.13	1.00
Lrig3	0.09	1.00
Hs6st3	0.31	1.00
Rabl3	0.08	1.00

Arc	0.13	1.00
Arv1	0.15	1.00
Bbs5	0.17	1.00
Tex9	0.23	1.00
Pex11b	0.10	1.00
Eef1a1	-0.23	1.00
Senp7	0.07	1.00
Arsb	0.71	1.00
Habp4	0.47	1.00
Naxe	-0.09	1.00
Arl6ip5	0.66	1.00
Heg1	-0.52	1.00
Rhbdd2	0.17	1.00
Ext1	-0.63	1.00
Nfe2l2	-0.13	1.00
Vcl	0.32	1.00
Kitl	-0.63	1.00
Ppfia1	-0.61	1.00
Lamtor5	0.31	1.00
Tbcel	-0.20	1.00
Spata17	0.71	1.00
Nrep	-0.11	1.00
Armc5	0.17	1.00
Aph1c	0.20	1.00
Usp12	-0.02	1.00
Nrbp1	0.18	1.00
Serinc1	-0.11	1.00
Ehmt1	-0.21	1.00
Vps35l	-0.08	1.00
Epn3	-0.18	1.00
Serf1	-0.17	1.00
Ufc1	-0.28	1.00
Tmem70	-0.11	1.00
Cxcr4	-0.88	1.00
Alg13	-0.20	1.00
Ino80dos	-0.52	1.00
Ccdc125	-0.12	1.00
Peli2	-0.88	1.00
Amdhd2	-0.06	1.00
Ampd1	-0.17	1.00
Ergic2	0.29	1.00
Cavin1	-0.01	1.00
Ier5	0.98	1.00
Ubxn2a	0.41	1.00
Robo1	-0.09	1.00
Tbc1d9	0.52	1.00

Il31ra	0.24	1.00
Zkscan17	0.59	1.00
Nudt21	0.24	1.00
Pyroxd1	-0.14	1.00
Plagl2	-0.40	1.00
Hecw2	0.17	1.00
Ptprt	0.27	1.00
Taf6	0.08	1.00
Ankrd52	0.09	1.00
Tirap	0.07	1.00
Ttll6	0.14	1.00
Azin2	-0.20	1.00
Acin1	0.18	1.00
Inpp5b	-0.07	1.00
Tsc22d4	-0.15	1.00
Gtf2h2	0.36	1.00
Rnf146	-0.50	1.00
Fancl	0.54	1.00
Sec22b	-0.17	1.00
Stard7	-0.12	1.00
Ccdc189	0.44	1.00
Copz2	-0.09	1.00
Herpud2	0.21	1.00
Srgap1	0.43	1.00
Twsg1	-0.14	1.00
Pgm3	-0.07	1.00
Slf1	0.33	1.00
Klc1	0.22	1.00
Cdk5rap2	-0.76	1.00
1700025G04Rik	-0.31	1.00
Rapgef6	-0.66	1.00
Sacm1l	-0.52	1.00
Stk25	0.27	1.00
Tbcd	0.22	1.00
Pcmtd1	0.07	1.00
Tmem60	0.13	1.00
Acd	0.12	1.00
Anapc16	-0.13	1.00
Abl1	0.24	1.00
Ptar1	-0.10	1.00
Nup210	0.07	1.00
Wtap	0.05	1.00
Sec63	-0.17	1.00
Hypk	-0.25	1.00
Lap3	-0.35	1.00
Adgrl2	0.33	1.00

Ganc	-0.15	1.00
Grap	-0.08	1.00
Dolpp1	-0.10	1.00
Dffa	-0.11	1.00
Zbtb45	-0.06	1.00
Zfp692	-0.13	1.00
Tigar	-0.06	1.00
Ube2g1	-0.41	1.00
Rab3ip	-0.19	1.00
Bpifb5	-0.48	1.00
Ccnt1	-1.13	1.00
Tmem56	-0.16	1.00
Szt2	0.15	1.00
Acat1	-0.07	1.00
Ptges3	0.44	1.00
Klhl7	-0.31	1.00
Snx10	0.09	1.00
Cped1	0.93	1.00
Rcl1	0.18	1.00
Chchd6	-0.49	1.00
Bicd1	0.08	1.00
1700088E04Rik	0.16	1.00
Fam83b	0.09	1.00
Txnrd2	0.10	1.00
Dmxl2	0.11	1.00
Dusp2	0.16	1.00
Cptp	0.18	1.00
Lpcat2	0.12	1.00
Lrrc29	0.13	1.00
Ppp1r18	0.13	1.00
Tmx3	0.03	1.00
Ndufc2	-0.30	1.00
Il1r1	-0.42	1.00
Cnpy3	-0.31	1.00
Slc39a13	-0.12	1.00
Hells	-0.17	1.00
Tctn2	0.22	1.00
Bak1	0.17	1.00
Tmem38a	-0.11	1.00
Zbtb7b	0.29	1.00
Flywch1	0.13	1.00
Tbck	-0.09	1.00
Cd81	-0.02	1.00
Psmb1	0.15	1.00
2510002D24Rik	-0.34	1.00
Stard10	-0.18	1.00

Kif2a	-0.11	1.00
Coq3	-0.30	1.00
Ccdc137	0.31	1.00
Kmt2b	0.16	1.00
Optn	-0.41	1.00
Slk	0.04	1.00
Traf6	-0.18	1.00
Spcs3	0.47	1.00
Odr4	-0.11	1.00
Afg1l	-0.41	1.00
Yif1b	0.17	1.00
Scai	0.30	1.00
Sox5	-0.54	1.00
Eva1c	-0.05	1.00
Eif2b1	-0.10	1.00
Paqr6	-0.08	1.00
3222401L13Rik	-0.11	1.00
G6pdx	-0.08	1.00
Thap2	-0.19	1.00
Ubl3	-0.04	1.00
Orc4	0.08	1.00
Tmem19	-0.08	1.00
Kif15	-0.07	1.00
Cenpu	-0.11	1.00
Trim23	-0.59	1.00
Pold2	-0.06	1.00
E330011O21Rik	-0.12	1.00
Pon2	-0.16	1.00
Snhg8	-0.39	1.00
Anp32e	-0.19	1.00
Tatdn1	-0.59	1.00
Abhd14a	0.18	1.00
Tchh	-0.13	1.00
Zkscan6	0.11	1.00
Slc52a2	0.09	1.00
Timm17a	-0.05	1.00
Hnrnph3	-0.15	1.00
Sgsm3	-0.25	1.00
Senp1	-0.10	1.00
Polr3d	-0.10	1.00
6230400D17Rik	-0.11	1.00
Kdelr3	0.06	1.00
Rab36	0.05	1.00
Il1rn	-0.22	1.00
Zfp60	0.10	1.00
Zkscan7	0.07	1.00

Apex1	0.04	1.00
1110012L19Rik	0.06	1.00
Platr21	0.11	1.00
Nr6a1	0.47	1.00
Brd1	-0.47	1.00
Atf6	-0.44	1.00
Entpd1	-0.57	1.00
Nol7	-0.24	1.00
Slc10a6	-0.24	1.00
Slco3a1	0.73	1.00
Nlrc5	0.21	1.00
Arhgap27	0.17	1.00
Fopnl	0.11	1.00
Klc2	0.11	1.00
Bex2	-0.44	1.00
Psmg4	0.52	1.00
Exoc7	0.16	1.00
Prkch	-0.53	1.00
Rchy1	0.19	1.00
Bptf	-0.30	1.00
Iars2	-0.20	1.00
Depp1	0.00	1.00
Batf	-0.73	1.00
Rabggta	-0.09	1.00
Tns2	0.00	1.00
Emc7	0.55	1.00
Tom1	0.00	1.00
Foxn3	-0.44	1.00
BC004004	-0.14	1.00
Gja1	-0.52	1.00
Ghr	0.16	1.00
Tmem131l	-0.32	1.00
Hp1bp3	-0.37	1.00
Zfp36l2	-0.03	1.00
Ptprf	-0.17	1.00
Cspp1	0.44	1.00
Ptpre	0.46	1.00
Idh2	-0.18	1.00
Ppara	-0.17	1.00
Glb1l	-0.59	1.00
Cfap410	-0.12	1.00
Tomm7	0.35	1.00
Gtf2i	-0.31	1.00
Supt5	0.71	1.00
Klhl9	0.11	1.00
Cmtm4	-0.30	1.00

Cmtm7	0.12	1.00
Exosc10	0.15	1.00
Tbc1d22b	-0.31	1.00
Sf3a2	0.10	1.00
Tep1	-0.19	1.00
Vars2	-0.60	1.00
Ubfd1	-0.09	1.00
Crebl2	-0.12	1.00
Glra1	0.02	1.00
Foxj1	0.72	1.00
Slc43a3	-0.15	1.00
Insyn1	0.36	1.00
Daglb	-0.06	1.00
Acsf2	-0.09	1.00
Dhx34	-0.10	1.00
Homer3	0.02	1.00
Izumo4	-0.06	1.00
Phf7	-0.07	1.00
Retnlg	-1.21	1.00
Zbtb20	-0.51	1.00
Il6st	-0.28	1.00
Wars2	0.27	1.00
Dnpep	0.09	1.00
Spats2	0.07	1.00
Frat2	-0.02	1.00
1600020E01Rik	0.51	1.00
Nsa2	-0.31	1.00
Nampt	-0.11	1.00
Hnrnpul1	-0.13	1.00
Zmynd11	-0.19	1.00
Glrx	-0.25	1.00
Lpin1	-0.27	1.00
Serinc5	-0.40	1.00
Xrcc4	0.16	1.00
Thoc6	-0.60	1.00
Hax1	-0.04	1.00
Gsap	-0.33	1.00
Erap1	0.40	1.00
Ctdsp1	0.05	1.00
Gopc	-0.30	1.00
Dgcr2	-0.12	1.00
8030462N17Rik	-0.65	1.00
Rab8a	-0.19	1.00
Cmip	-0.39	1.00
Scly	-0.13	1.00
Nop16	-0.10	1.00

Neurl4	-0.13	1.00
Xrcc6	-0.09	1.00
Rab3a	-0.09	1.00
Pla2g6	-0.10	1.00
Dglucy	0.28	1.00
Hspa12a	-0.25	1.00
Caskin2	-0.09	1.00
Cadm4	-0.10	1.00
5330439K02Rik	-0.07	1.00
Kiz	0.02	1.00
Ywhaz	0.38	1.00
Ccdc30	0.63	1.00
Plrg1	-0.02	1.00
Ube2o	-0.61	1.00
Trim62	0.09	1.00
Gba	-0.08	1.00
2410004B18Rik	0.04	1.00
Gpatch4	0.13	1.00
Tsc22d2	-0.21	1.00
Tcf7l2	-0.26	1.00
Ctsa	0.64	1.00
Supt16	-0.28	1.00
St7l	-0.56	1.00
Pon3	-0.36	1.00
Slc40a1	-0.49	1.00
Dusp28	-0.04	1.00
Bbs4	-0.12	1.00
Ice2	-0.08	1.00
Cenpp	-0.23	1.00
Gpt2	-0.06	1.00
Cit	-0.15	1.00
Tor4a	-0.04	1.00
Pex12	-0.15	1.00
Zkscan5	-0.04	1.00
1700021F05Rik	0.03	1.00
Bloc1s5	-0.08	1.00
Pwwp3b	-0.10	1.00
Sptlc1	0.21	1.00
Prkar2a	0.38	1.00
Rbm7	0.52	1.00
Zfp608	-0.18	1.00
Phldb2	-0.29	1.00
Pdzd2	0.85	1.00
Tigd2	0.15	1.00
Crebzf	0.19	1.00
Pibf1	0.47	1.00

Usp25	0.35	1.00
Scarb2	0.44	1.00
Tspan1	0.33	1.00
Coa5	-0.14	1.00
Cfap97	-0.13	1.00
Pdcd2l	-0.51	1.00
Fam178b	-0.40	1.00
Hist1h1c	-0.10	1.00
Cwc15	-0.22	1.00
Gpx2	0.25	1.00
Cd44	0.30	1.00
2610301B20Rik	-0.11	1.00
Ammecr1	-0.13	1.00
Aldh3b1	0.52	1.00
Vapb	0.57	1.00
Ube2h	-0.39	1.00
Srp72	0.40	1.00
Spata18	0.46	1.00
Bbof1	0.16	1.00
Urod	-0.10	1.00
Dvl3	-0.06	1.00
Pig	0.09	1.00
Nectin4	0.11	1.00
Pcp4l1	0.48	1.00
1110017D15Rik	0.93	1.00
Zfp524	-0.06	1.00
Zfp667	0.12	1.00
Tm2d1	-0.18	1.00
Cox6a2	-0.10	1.00
Cdh5	-1.17	1.00
Rian	-0.18	1.00
Ccdc152	-0.07	1.00
A630089N07Rik	-0.26	1.00
1810037I17Rik	0.26	1.00
Hadh	-0.05	1.00
C77080	-0.33	1.00
Atp6v1h	-0.53	1.00
Fbxl17	-0.26	1.00
Sema3c	0.39	1.00
Zcchc2	0.26	1.00
Upf2	0.51	1.00
Anks1	-0.29	1.00
Spryd3	-0.55	1.00
Fam49a	0.62	1.00
Phlda1	0.72	1.00
Psmd2	0.36	1.00

Gne	-0.11	1.00
Pfkfb4	-0.12	1.00
Sh3bgrl	-0.43	1.00
Trmt112	0.11	1.00
Tpp2	-0.42	1.00
Rbbp5	-0.13	1.00
AW209491	-0.06	1.00
Xkr4	0.04	1.00
Tmem132a	-0.04	1.00
Dmac2l	-0.05	1.00
Ctu2	-0.05	1.00
Arl16	0.05	1.00
Tmem82	-0.05	1.00
Zfp628	-0.07	1.00
Cd164l2	-0.05	1.00
Zfp846	-0.02	1.00
Notch3	-0.08	1.00
Bgn	-0.06	1.00
Sf3b6	0.21	1.00
Calcrl	-0.34	1.00
Rxra	-0.01	1.00
Ncdn	-0.09	1.00
Sfxn3	-0.03	1.00
Thap11	-0.10	1.00
Tsfm	0.17	1.00
Fam241a	0.11	1.00
Kif24	0.23	1.00
Ube2j1	-0.15	1.00
Klhdc2	-0.12	1.00
Pebp1	0.22	1.00
Pof1b	-0.34	1.00
Bbs9	0.26	1.00
Tuba1b	0.52	1.00
Csrp2	-0.33	1.00
Fam126b	-0.11	1.00
Poglut3	-0.08	1.00
Tmem192	0.17	1.00
Gpr137c	-0.14	1.00
Pcbp4	-0.01	1.00
Ccdc17	0.11	1.00
Itgal	-0.79	1.00
Wdr27	0.12	1.00
Trappc5	0.29	1.00
Nrp2	-0.29	1.00
Cog2	-0.09	1.00
Il7r	-0.47	1.00

Ddx55	-0.08	1.00
Rab44	-0.11	1.00
Areg	-0.15	1.00
Nrd1	0.18	1.00
Sytl2	-0.16	1.00
Slc25a23	-0.11	1.00
Vti1b	0.58	1.00
Eif4ebp2	-0.08	1.00
Glo1	0.46	1.00
Pde4b	-0.36	1.00
Srp14	0.15	1.00
a	-0.53	1.00
Synrg	0.27	1.00
Lmtk2	0.46	1.00
Ppp3ca	0.30	1.00
Anapc7	-0.05	1.00
Heatr6	-0.08	1.00
Pnpo	-0.24	1.00
Gpt	-0.05	1.00
Rab5a	0.02	1.00
Cdkl2	0.22	1.00
Kansl2	-0.10	1.00
Cachd1	-0.38	1.00
Hsp90ab1	0.28	1.00
Dgkh	0.58	1.00
Atrx	-0.44	1.00
9330182L06Rik	-0.43	1.00
Rpap3	0.14	1.00
Enah	0.45	1.00
Tbcc	0.44	1.00
Slc7a7	0.22	1.00
Jcad	-0.09	1.00
Dock11	-0.19	1.00
Smagp	-0.06	1.00
Lurap1l	-0.09	1.00
Nr2c2	-0.30	1.00
Hbs1l	0.36	1.00
Slc25a15	-0.05	1.00
Rhoh	0.10	1.00
Rad1	0.02	1.00
Rfesd	-0.05	1.00
Ciao3	-0.02	1.00
Aifm3	-0.07	1.00
Fbxo10	-0.05	1.00
Drosha	-0.08	1.00
Sap30bp	0.14	1.00

Wnt4	0.49	1.00
Pick1	0.05	1.00
Khk	-0.07	1.00
Coa6	0.08	1.00
Chsy3	-0.23	1.00
Clcc1	0.02	1.00
Fbf1	0.15	1.00
Fkrp	-0.09	1.00
Actn4	0.25	1.00
Pcgf5	0.64	1.00
Ghitm	0.35	1.00
Cbfb	0.38	1.00
Ctsz	0.02	1.00
Anapc1	0.42	1.00
Cdkn2aip	0.13	1.00
Cope	0.36	1.00
Selenoo	-0.06	1.00
Poglut1	0.02	1.00
Nfatc2	-0.25	1.00
Gsta3	-0.31	1.00
Fuz	-0.08	1.00
Pdpk1	0.12	1.00
Parp2	-0.24	1.00
Pard3	-0.33	1.00
Glis3	0.43	1.00
Cdip1	0.18	1.00
Aff4	-0.03	1.00
Gramd3	-0.65	1.00
Rassf2	-0.12	1.00
Dcun1d2	0.36	1.00
9230114K14Rik	-0.07	1.00
Xrcc1	-0.05	1.00
Ccdc114	0.68	1.00
Tmem159	-0.11	1.00
Lrrc8c	-0.11	1.00
Pdk1	-0.27	1.00
Fyttd1	0.30	1.00
Tax1bp3	-0.26	1.00
Nectin1	-0.42	1.00
Cacfd1	-0.09	1.00
Cdyl	0.11	1.00
Lamb3	0.24	1.00
Cxcl16	0.55	1.00
Nt5c2	-0.35	1.00
Stard13	-0.39	1.00
Cfl2	0.20	1.00

Nphp1	0.07	1.00
Kif23	-0.34	1.00
Usp20	0.04	1.00
Pck2	-0.04	1.00
Smg9	-0.11	1.00
Ttc17	-0.60	1.00
Wbp1	-0.04	1.00
Nrbf2	-0.15	1.00
Tpmt	-0.05	1.00
L3hypdh	-0.01	1.00
Lims2	0.01	1.00
Lysmd4	-0.04	1.00
Dpagt1	-0.03	1.00
Cwf19l1	0.23	1.00
Vgll4	-0.43	1.00
Sdf4	-0.18	1.00
Cep19	-0.48	1.00
Phkg2	-0.06	1.00
Dst	-0.69	1.00
Sem1	0.09	1.00
Stk31	-0.13	1.00
Ttc39c	0.29	1.00
Snhg6	0.34	1.00
Clic5	1.04	1.00
Gpr39	-0.11	1.00
Svil	0.47	1.00
Nucb2	-0.23	1.00
Ago1	-0.16	1.00
Senp3	-0.14	1.00
Dnajc3	-0.05	1.00
Rab9	0.21	1.00
Igfbp6	-0.08	1.00
Cstf2	0.01	1.00
Gcdh	-0.09	1.00
Smim8	0.12	1.00
Kars	-0.11	1.00
Stk10	0.31	1.00
Tmem50b	-0.09	1.00
Igsf5	-0.23	1.00
Nos1ap	-0.19	1.00
Sepsecs	0.15	1.00
Ran	0.44	1.00
Kidins220	-0.08	1.00
Gfod2	0.25	1.00
Slain2	-0.09	1.00
Reep3	-0.14	1.00

Lrp6	-0.57	1.00
Col4a2	0.29	1.00
Fam107b	-0.30	1.00
Gid8	-0.39	1.00
Arf1	0.33	1.00
Eif4g2	0.37	1.00
Rabac1	-0.14	1.00
Gab1	0.35	1.00
Trappc2	-0.05	1.00
Sh3yl1	-0.07	1.00
Lef1	-0.21	1.00
Tgfbr3	-0.09	1.00
Zfp512b	-0.06	1.00
Cep57	-0.28	1.00
Cstb	0.55	1.00
Grb10	-0.11	1.00
Sbds	0.25	1.00
Atpif1	-0.39	1.00
Ykt6	0.20	1.00
Bex3	-0.10	1.00
Rbks	0.05	1.00
St3gal4	-0.15	1.00
Cenpv	0.30	1.00
Ccdc61	0.28	1.00
Cnksr1	-0.07	1.00
Dhrs1	-0.06	1.00
S1pr1	0.11	1.00
Zmynd19	-0.46	1.00
Foxo4	-0.03	1.00
2700097O09Rik	-0.07	1.00
Tnfsf9	0.07	1.00
Clcn5	-0.20	1.00
Nckap5	-0.17	1.00
Rbms3	-0.20	1.00
Ptpn3	0.30	1.00
Retreg1	0.35	1.00
Cox16	-0.20	1.00
Slc16a2	-0.04	1.00
Tfrc	-0.39	1.00
Srpk2	0.49	1.00
Kansl3	-0.23	1.00
Slc35b2	-0.06	1.00
Rexo4	0.07	1.00
Akap5	0.72	1.00
Spice1	0.24	1.00
Rbm27	-0.04	1.00

Slc25a1	-0.27	1.00
St6galnac2	-0.07	1.00
Arf6	0.42	1.00
Creb3l2	0.23	1.00
Abce1	0.29	1.00
Naa38	0.18	1.00
Vdac1	-0.26	1.00
Wdfy1	-0.13	1.00
Cldn4	1.72	1.00
Cox6a1	-0.16	1.00
Pip5k1a	-0.36	1.00
Insr	-0.21	1.00
Sltm	0.35	1.00
Spint1	0.37	1.00
Paxbp1	-0.06	1.00
Cyp4b1	-0.11	1.00
Bicral	0.25	1.00
Kdm6a	0.37	1.00
Rbm39	-0.36	1.00
Ret	-0.04	1.00
Ppp1r1b	-0.06	1.00
Synj2bp	-0.12	1.00
Gca	0.32	1.00
Lmnb1	0.74	1.00
Ccdc91	-0.01	1.00
Ctdp1	-0.34	1.00
Akr1b8	0.02	1.00
Arfgef1	0.47	1.00
Stag1	-0.35	1.00
Pdss1	-0.18	1.00
Pcsk7	0.10	1.00
Efhb	0.39	1.00
Zc3h15	0.20	1.00
Rasl11b	0.12	1.00
Crem	0.18	1.00
Riok3	0.33	1.00
Eps8l1	-0.01	1.00
Cct8	-0.11	1.00
Slc7a5	-0.24	1.00
Ndufa1	0.51	1.00
Rbms2	-0.09	1.00
Rbl2	-0.10	1.00
Paxx	-0.13	1.00
Rnh1	0.49	1.00
Fap	-0.37	1.00
Rtn4rl1	-0.06	1.00

Frmd8	0.05	1.00
Nop53	-0.56	1.00
Ccdc112	0.13	1.00
Zfp36	0.01	1.00
Ncoa3	0.16	1.00
Aspscr1	-0.07	1.00
Psma3	0.42	1.00
Ctdsp2	0.34	1.00
Basp1	1.28	1.00
Acvr2a	0.30	1.00
Srgn	0.31	1.00
Scyl2	0.40	1.00
Srsf5	0.01	1.00
Pnrc1	-0.37	1.00
Ppp2r3a	0.19	1.00
Pcgf3	-0.03	1.00
Ypel5	0.15	1.00
Rmnd5b	0.10	1.00
Cops4	-0.03	1.00
Snrpg	0.02	1.00
Llgl2	0.24	1.00
Tmem238	-0.53	1.00
Fkbp2	0.31	1.00
Fam168b	-0.05	1.00
Cotl1	0.45	1.00
Stmn1	-0.11	1.00
Zfp568	-0.16	1.00
Cdcp1	0.10	1.00
Rogdi	0.55	1.00
Phpt1	0.02	1.00
Tex15	0.25	1.00
Rnf144b	-0.46	1.00
Nek6	-0.39	1.00
Gatad2a	-0.10	1.00
Smim12	-0.06	1.00
Tmprss4	0.06	1.00
Tmem8b	-0.03	1.00
Amfr	-0.39	1.00
Wbp2	-0.12	1.00
Chmp2b	0.43	1.00
Nbdy	-0.07	1.00
Tubb4b	0.72	1.00
Rasef	-0.17	1.00
Iah1	-0.15	1.00
Ass1	-0.03	1.00
Pdgfb	0.54	1.00

Tnfrsf11a	0.29	1.00
Bpnt1	0.26	1.00
Sh3bgrl2	0.15	1.00
Chrna9	0.27	1.00
Ap4e1	0.22	1.00
Pmf1	0.18	1.00
Sult2b1	0.20	1.00
Nop9	0.11	1.00
Hikeshi	-0.70	1.00
Cd164	0.27	1.00
Prkag2	-0.13	1.00
Ints6	-0.57	1.00
Fis1	-0.35	1.00
Rabl6	-0.23	1.00
Wdr13	0.24	1.00
Trim16	0.27	1.00
Klc4	-0.04	1.00
Csde1	-0.23	1.00
Cul1	-0.28	1.00
Sec16a	0.20	1.00
Dnah6	0.29	1.00
Pex7	-0.21	1.00
Aff3	-0.68	1.00
Prss48	0.26	1.00
Preb	-0.13	1.00
Taf5l	0.15	1.00
Stub1	0.29	1.00
Ndfip1	-0.31	1.00
Ptgfrn	-0.22	1.00
Tdg	0.14	1.00
Klhdc3	-0.05	1.00
D230025D16Rik	-0.30	1.00
Bcl2l13	-0.55	1.00
Rph3al	-0.35	1.00
Specc1l	0.27	1.00
Cytip	-0.05	1.00
Pfn1	0.40	1.00
Trappc13	-0.33	1.00
Fbxo38	-0.12	1.00
Vcp	0.23	1.00
Pgap2	-0.19	1.00
Gcsh	0.03	1.00
Nck1	-0.28	1.00
Snrnp70	0.44	1.00
Ythdf1	-0.15	1.00
Lsm12	-0.07	1.00

Zrsr1	-0.13	1.00
Wdr78	0.14	1.00
Fam149b	0.23	1.00
Armc10	-0.05	1.00
Cd24a	0.87	1.00
Ipo7	0.43	1.00
Trpc1	0.26	1.00
Asb7	0.17	1.00
Slc25a26	0.29	1.00
Atpaf2	0.10	1.00
Rgs22	0.83	1.00
Snap23	-0.33	1.00
Lbr	-0.59	1.00
Gng10	0.24	1.00
Eef2kmt	-0.01	1.00
Trmt10c	0.09	1.00
Ppp2r2b	0.50	1.00
Znrd1	-0.04	1.00
Tns1	-0.01	1.00
Sec14l3	0.27	1.00
Trim12c	0.31	1.00
Slc11a2	-0.21	1.00
Atp5o	-0.41	1.00
R3hdm4	0.54	1.00
Tspan4	-0.04	1.00
Gstt2	0.20	1.00
Tax1bp1	0.08	1.00
Nucks1	-0.28	1.00
Smarcc1	-0.17	1.00
Letm1	0.53	1.00
Terf2ip	0.22	1.00
D8Ertd738e	-0.72	1.00
Zeb2	1.00	1.00
Cpsf2	-0.17	1.00
Purg	-0.23	1.00
Ncbp2	0.07	1.00
Ddx21	0.18	1.00
Slmap	-0.41	1.00
Plekhh2	-0.19	1.00
Rnft1	0.01	1.00
Cd74	0.59	1.00
Rad23b	-0.19	1.00
Ino80c	0.13	1.00
Steap4	0.42	1.00
Atmin	0.24	1.00
Jade2	-0.04	1.00

Ogfod2	0.15	1.00
Josd1	0.11	1.00
Sugp2	0.16	1.00
Krtap17-1	0.77	1.00
Ikbkg	0.18	1.00
Gk5	-0.35	1.00
Cnnm3	-0.09	1.00
Fntb	0.41	1.00
Snrnp48	-0.12	1.00
Hagh	-0.11	1.00
Wdr43	0.38	1.00
Fbxw2	0.27	1.00
Aldh1a7	-0.17	1.00
Capsl	0.50	1.00
Haus7	0.39	1.00
U2af1	-0.09	1.00
Ptov1	-0.20	1.00
Son	0.25	1.00
Bbx	-0.52	1.00
Parp1	0.02	1.00
Spry1	-0.07	1.00
Elf5	-0.10	1.00
Capg	-0.13	1.00
Rit1	0.15	1.00
Ap2m1	0.11	1.00
Ddi2	-0.52	1.00
Chchd1	0.28	1.00
Serf2	0.35	1.00
Helz2	-0.03	1.00
Psmc2	0.34	1.00
Psmg1	-0.48	1.00
Tars	-0.10	1.00
Laptm4a	0.25	1.00
Sqstm1	0.39	1.00
St7	-0.45	1.00
Wdr1	0.40	1.00
Zbtb4	0.05	1.00
Snx29	-0.56	1.00
Rfk	-0.17	1.00
Agps	-0.07	1.00
Usp6nl	0.46	1.00
Acp6	0.14	1.00
Cmas	0.31	1.00
Slc8a1	0.43	1.00
Ripor1	0.11	1.00
Gsdmd	0.36	1.00

Slc25a32	0.08	1.00
Nr1h3	-0.04	1.00
Tmem127	-0.11	1.00
Hbegf	0.85	1.00
Pou2f1	0.44	1.00
Nup107	-0.16	1.00
Rasgrf2	0.39	1.00
Hic2	0.12	1.00
Ano6	-0.42	1.00
Zkscan3	-0.01	1.00
Bola2	0.22	1.00
Fbxw7	0.35	1.00
Phf20	-0.39	1.00
Psmc5	0.26	1.00
Usp34	-0.07	1.00
Chd1	0.20	1.00
Col4a3bp	-0.20	1.00
Tmem176a	0.15	1.00
Scgb1a1	4.82	1.00
Dock10	-0.27	1.00
Adcy7	0.04	1.00
Taf1	0.24	1.00
Ccdc186	-0.30	1.00
Commd6	-0.56	1.00
Riiad1	0.04	1.00
Adi1	-0.07	1.00
Rprm	-0.10	1.00
Ttc39a	0.35	1.00
Sfxn1	-0.16	1.00
Hcfc1r1	0.36	1.00
Sfr1	0.78	1.00
Slc25a16	-0.10	1.00
Pdlim2	-0.30	1.00
Cep126	0.54	1.00
Cbwd1	0.23	1.00
Klhdc8b	-0.12	1.00
Pkp4	0.34	1.00
Kcnk5	0.21	1.00
Brf2	0.15	1.00
Kptn	0.09	1.00
Cfap43	0.64	1.00
Capn2	0.27	1.00
Slc25a33	0.16	1.00
Fam102b	-0.29	1.00
Arap2	-0.07	1.00
Arf2	0.21	1.00

Qrich1	-0.21	1.00
Twf1	-0.21	1.00
Tmc4	0.22	1.00
Abcf1	0.28	1.00
Pced1b	-0.09	1.00
Tmed4	0.10	1.00
H1f0	-0.23	1.00
Stam2	0.66	1.00
Ccdc124	-0.05	1.00
Txn1	-0.11	1.00
Smg7	-0.25	1.00
Ets1	-0.40	1.00
Gin1	0.09	1.00
Imp4	0.21	1.00
Haghl	0.18	1.00
Rhof	0.19	1.00
Dnajc16	0.13	1.00
Wdr49	0.21	1.00
Wfdc17	0.59	1.00
Golga3	0.11	1.00
Iscu	0.37	1.00
B3gnt2	0.04	1.00
Prpsap2	0.20	1.00
Idh3b	0.27	1.00
Rnaseh2a	0.02	1.00
Gart	0.24	1.00
Cox10	-0.14	1.00
Tspan3	0.28	1.00
Papss1	0.09	1.00
Csnk1d	-0.78	1.00
Ern1	0.47	1.00
Lrch1	0.57	1.00
Psmb4	0.32	1.00
Exoc5	0.42	1.00
Vkorc1	-0.22	1.00
Pds5b	-0.25	1.00
Zfp787	0.25	1.00
Eif4a1	0.34	1.00
Shtn1	-0.29	1.00
Ostm1	0.18	1.00
Stambp	0.14	1.00
Ccdc153	1.01	1.00
Ptpn9	0.44	1.00
Tmem245	0.04	1.00
Enpep	-0.09	1.00
Ulk2	-0.10	1.00

Psmb5	-0.20	1.00
Ifrd1	0.69	1.00
Sec61b	0.35	1.00
Tab2	-0.52	1.00
Zfp646	-0.12	1.00
Fam89b	-0.02	1.00
Rcbtb2	0.15	1.00
Fosl1	0.10	1.00
Ube2l3	0.51	1.00
Zfc3h1	-0.40	1.00
Lamc1	-0.03	1.00
Cuedc2	0.05	1.00
Calm2	-0.20	1.00
Tmprss2	-0.28	1.00
Ntn4	0.44	1.00
Edn3	0.20	1.00
Ccdc173	0.13	1.00
0610010K14Rik	0.10	1.00
Pou3f1	-0.96	1.00
Frmd4a	0.04	1.00
Ccdc174	0.15	1.00
Ccdc68	0.05	1.00
Grpel2	-0.10	1.00
Dhrs4	-0.13	1.00
Arhgap44	0.48	1.00
Zfp592	-0.26	1.00
Tapbpl	0.14	1.00
Spef2	0.45	1.00
Tubgcp4	-0.15	1.00
Evi5	-0.28	1.00
Ss18	0.51	1.00
Rbpjl	-0.12	1.00
Id2	-0.38	1.00
Npm1	-0.48	1.00
Irf2	-0.13	1.00
Adam17	0.08	1.00
Smim1	0.02	1.00
Ube2n	0.24	1.00
Trappc3	-0.45	1.00
Tank	-0.41	1.00
Tmem179b	-0.46	1.00
Tmed2	0.13	1.00
Rwdd4a	-0.12	1.00
Cops5	0.03	1.00
Smarce1	-0.08	1.00
Sun2	-0.06	1.00

Gpr107	0.00	1.00
Afg3l1	-0.44	1.00
Snx30	-0.32	1.00
Gemin7	-0.71	1.00
S100a11	0.74	1.00
Polr2c	0.16	1.00
Smpd2	0.15	1.00
1500015L24Rik	0.12	1.00
Osbpl8	-0.30	1.00
Rnf38	-0.23	1.00
Cars2	-0.08	1.00
Isca2	0.03	1.00
Dtx2	-0.13	1.00
Fkbp11	-0.15	1.00
Sprtn	0.10	1.00
Plpp1	0.40	1.00
Cdhr4	0.47	1.00
Bap1	0.12	1.00
5430405H02Rik	0.46	1.00
Akap17b	0.20	1.00
Ppp1r21	-0.15	1.00
Tmem62	-0.13	1.00
Pdhx	-0.09	1.00
Osbpl2	0.11	1.00
Plekhg2	0.11	1.00
Csrnp1	0.12	1.00
Usp33	-0.32	1.00
Cst3	0.62	1.00
Tmem106b	-0.12	1.00
St3gal5	-0.13	1.00
Cox7a2l	-0.36	1.00
Limk2	-0.14	1.00
Frg1	0.32	1.00
Scfd1	0.36	1.00
Osbpl10	-0.15	1.00
Zbtb18	0.15	1.00
Dnal1	0.29	1.00
Slc25a39	-0.13	1.00
Zhx3	-0.20	1.00
Commd1	0.12	1.00
Abca1	-0.31	1.00
Tfdp2	0.14	1.00
Rnf139	0.30	1.00
Hectd4	0.65	1.00
Vps4b	0.14	1.00
Fxyd5	0.12	1.00

Akt3	0.43	1.00
Tasor2	0.33	1.00
Rdx	-0.19	1.00
Xpo1	0.22	1.00
Atf7	-0.42	1.00
Sirt1	-0.33	1.00
Cdca7l	-0.54	1.00
Acads	-0.34	1.00
Pwwp2b	0.32	1.00
Colgalt1	-0.14	1.00
B230369F24Rik	0.14	1.00
Prdx4	-0.14	1.00
Kdm4c	-0.19	1.00
H2-Ab1	0.84	1.00
Ofd1	-0.08	1.00
Slc17a5	-0.01	1.00
Dpy19l4	-0.17	1.00
Chd9	0.33	1.00
Zdhhc23	0.04	1.00
Pkib	0.16	1.00
L2hgdh	-0.31	1.00
Hscb	0.14	1.00
Qsox2	0.15	1.00
Snhg5	0.18	1.00
1700024G13Rik	0.75	1.00
Anxa8	0.19	1.00
Use1	-0.12	1.00
Pdlim1	-0.23	1.00
Lpin2	-0.47	1.00
Ciao2b	0.20	1.00
Cpsf3	0.08	1.00
Gab2	0.05	1.00
Vps16	0.21	1.00
Pdcd11	0.18	1.00
Cables1	-0.23	1.00
Foxk1	-0.06	1.00
Brf1	0.27	1.00
Cnot9	0.08	1.00
Tmem203	-0.02	1.00
Nbr1	0.25	1.00
Nr2c1	-0.69	1.00
Asb3	-0.14	1.00
Pum3	0.33	1.00
Rptor	-0.43	1.00
Tgfbr2	0.33	1.00
Hectd1	0.50	1.00

Actr1a	0.26	1.00
Triap1	-0.30	1.00
Tgif1	0.40	1.00
Ndufa8	0.37	1.00
Psmb7	0.33	1.00
Cep83	0.76	1.00
Gpm6b	-0.33	1.00
Sord	-0.03	1.00
Lin54	-0.18	1.00
Hmces	0.12	1.00
Fam222a	-0.07	1.00
Tspyl4	-0.12	1.00
Bop1	-0.10	1.00
Lpar3	-0.21	1.00
Pank4	-0.64	1.00
Rnf135	-0.29	1.00
Fbxo8	-0.28	1.00
Wnk1	-0.09	1.00
Ltbp3	0.25	1.00
Ddx24	0.47	1.00
Tmem134	-0.30	1.00
Zfp810	-0.12	1.00
Ankle2	0.13	1.00
Pcnx4	0.48	1.00
Slc9a9	0.45	1.00
Slc9a8	0.09	1.00
Kctd5	0.01	1.00
Trim46	0.13	1.00
Fam166b	0.13	1.00
Adam28	0.12	1.00
Tmem65	-0.16	1.00
Nf2	-0.16	1.00
Ctdspl2	-0.17	1.00
Ubb	0.29	1.00
Slc7a6os	0.16	1.00
Rab6a	0.60	1.00
BC051019	0.34	1.00
Wdsub1	0.46	1.00
Stom	0.10	1.00
Arpin	0.13	1.00
Trim11	-0.04	1.00
Tdrd7	0.18	1.00
Pbxip1	0.50	1.00
Actr2	-0.09	1.00
Tead1	0.84	1.00
Cib1	0.26	1.00

Eny2	-0.29	1.00
Brpf3	0.10	1.00
Zdhhc14	-0.44	1.00
Atp6ap1	0.32	1.00
Cct3	-0.08	1.00
Sdc4	-0.20	1.00
Pphln1	0.08	1.00
Arhgef7	-0.21	1.00
Hyou1	0.47	1.00
Stk16	0.18	1.00
Atad2	-0.58	1.00
Rapgef2	-0.34	1.00
Cnot6	0.28	1.00
Osmr	-0.31	1.00
Arl6ip1	-0.32	1.00
Zcchc7	-0.41	1.00
Sp140	0.48	1.00
Cyp2f2	0.85	1.00
Ap2a2	0.32	1.00
Clasp2	-0.53	1.00
Usp19	0.17	1.00
Taok3	-0.23	1.00
Cx3cr1	-0.45	1.00
Pex6	-0.04	1.00
Prmt7	0.10	1.00
Scoc	0.47	1.00
Nsmce3	0.15	1.00
Lrrc59	0.22	1.00
Rhou	-0.14	1.00
Cant1	0.12	1.00
Cyp2s1	0.02	1.00
Slc1a4	0.11	1.00
Frmd5	0.17	1.00
Hck	-0.17	1.00
Inpp1	0.05	1.00
Tgfb1i1	0.08	1.00
Nop10	-0.27	1.00
Ehd2	0.56	1.00
Fkbp8	-0.04	1.00
Sra1	-0.01	1.00
Ddx17	-0.17	1.00
Spin1	-0.10	1.00
Polr3h	-0.08	1.00
Cd79b	-0.61	1.00
Acad8	-0.10	1.00
Pnkd	-0.34	1.00

Cwc25	0.14	1.00
Ccl6	-0.10	1.00
Fance	-0.12	1.00
Foxa2	0.31	1.00
Gramd2	0.58	1.00
Pafah1b2	-0.36	1.00
Nfat5	-0.18	1.00
Ccdc9	0.16	1.00
Wdr26	-0.36	1.00
Ap3s1	-0.33	1.00
Bscl2	0.47	1.00
Sinhcaf	0.27	1.00
Eif2b2	-0.45	1.00
Trmt1l	0.31	1.00
Elavl1	-0.37	1.00
Bnip3l	-0.27	1.00
Nop2	0.52	1.00
Ptk2b	0.61	1.00
B230307C23Rik	0.16	1.00
Rhoq	-0.19	1.00
Trio	0.18	1.00
Sri	0.42	1.00
Ccser1	0.40	1.00
Acsl4	-0.21	1.00
Nenf	0.38	1.00
Arhgap15	-0.38	1.00
Efemp1	0.25	1.00
Esyt2	-0.94	1.00
Atp5j	0.25	1.00
Prmt3	-0.11	1.00
Ppip5k1	-0.09	1.00
Jpx	-0.44	1.00
Sf3b5	0.61	1.00
Ube2z	-0.27	1.00
Ngef	0.21	1.00
Rp1	0.54	1.00
Cdkl3	0.16	1.00
Tmem165	0.05	1.00
Hsd17b12	-0.15	1.00
Aldoa	0.47	1.00
Dtymk	0.13	1.00
Ngrn	-0.03	1.00
Efcab1	0.46	1.00
Slc49a4	0.36	1.00
Tmem242	0.22	1.00
Igfbp5	0.16	1.00

Cnn3	-0.56	1.00
Ddhd2	-0.08	1.00
Sdr39u1	-0.24	1.00
Sec24d	0.50	1.00
Slc30a7	0.01	1.00
Cpsf7	-0.07	1.00
Dhps	0.15	1.00
BC003965	0.28	1.00
Gripap1	0.32	1.00
Ptgr1	-0.09	1.00
Prr12	-0.05	1.00
Wdr45b	-0.22	1.00
Adora2b	-0.75	1.00
Psma7	0.21	1.00
Dnajc10	-0.15	1.00
Ppp1r10	-0.17	1.00
Cep120	-0.09	1.00
Pcnp	-0.08	1.00
Rictor	0.25	1.00
Hsf4	-0.27	1.00
Wdr60	-0.07	1.00
Atp5b	0.35	1.00
Hnrnpdl	0.02	1.00
Cdc42	0.18	1.00
Rab24	-0.70	1.00
Prpsap1	0.34	1.00
Hacd1	-0.04	1.00
Tmem230	0.22	1.00
Itpkb	0.24	1.00
Tspan8	-0.27	1.00
Gtf2a2	0.28	1.00
Sertad2	0.43	1.00
Golm1	-0.06	1.00
Bod1	0.00	1.00
Wdyhv1	-0.31	1.00
Fkbp5	-0.17	1.00
Atp1b3	-0.31	1.00
Dnah7a	0.17	1.00
Ddx27	0.05	1.00
Rab2b	-0.11	1.00
Prkaa1	-0.09	1.00
Zfyve16	-0.05	1.00
Triobp	0.11	1.00
Bcorl1	0.00	1.00
Ttc3	0.40	1.00
Tmem128	0.11	1.00

2610037D02Rik	0.19	1.00
Rad51b	-0.02	1.00
Fgfr2	-0.30	1.00
Cwf19l2	-0.45	1.00
Ube3c	0.34	1.00
Plxnc1	0.03	1.00
Brip1	0.07	1.00
Braf	0.02	1.00
Tor1a	-0.02	1.00
Cdc73	-0.55	1.00
Tril	-0.12	1.00
Prx	0.00	1.00
Fbxl6	-0.11	1.00
Pcnx	-0.20	1.00
Ubtf	-0.13	1.00
Polr3k	-0.08	1.00
Ptpa	0.30	1.00
Jade3	-0.09	1.00
Leprot	-0.72	1.00
Amn1	0.24	1.00
Iqck	0.24	1.00
Phc2	0.31	1.00
Gaa	0.00	1.00
Chp1	-0.39	1.00
Ssrp1	0.15	1.00
P2rx4	-0.32	1.00
Cd2ap	-0.36	1.00
Zc3hc1	-0.61	1.00
Urah	-0.05	1.00
Traf1	-0.04	1.00
Kif1c	0.20	1.00
Wdr37	-0.15	1.00
Sema4d	0.64	1.00
Vps39	-0.08	1.00
Ikzf1	0.33	1.00
Bcar1	0.05	1.00
Kdf1	0.07	1.00
Srsf11	0.31	1.00
Crppa	0.15	1.00
Nom1	-0.08	1.00
Snx15	-0.37	1.00
Serpinb9	0.86	1.00
Chtop	-0.31	1.00
Spopl	-0.27	1.00
Oxct1	-0.06	1.00
Npepps	-0.19	1.00

Impact	0.47	1.00
Tnpo2	-0.05	1.00
Uqcrh	-0.14	1.00
Hspg2	0.46	1.00
Aebp2	-0.44	1.00
Rnf4	0.18	1.00
Edem2	0.28	1.00
Stx1a	0.03	1.00
Ube3a	0.52	1.00
Gon4l	-0.55	1.00
Bahd1	0.33	1.00
Ide	0.20	1.00
Rspry1	0.14	1.00
Rce1	0.10	1.00
Polr2e	0.01	1.00
Copg2	-0.27	1.00
Dhrs7b	0.13	1.00
Tcf12	-0.13	1.00
Dync2h1	0.26	1.00
Phf10	-0.06	1.00
Pdxdc1	0.29	1.00
Ptk2	-0.41	1.00
Gtf3c2	-0.13	1.00
Fhit	0.29	1.00
Zscan26	-0.10	1.00
Casp8ap2	-0.56	1.00
Tmem131	-0.06	1.00
Stk24	-0.13	1.00
Ell2	0.00	1.00
Crk	-0.01	1.00
Soat1	-0.13	1.00
Ift172	-0.19	1.00
Arhgef18	-0.03	1.00
Smndc1	0.04	1.00
Thg1l	-0.30	1.00
Nfix	-0.15	1.00
Usp38	0.14	1.00
Itfg1	0.12	1.00
Prkrip1	-0.28	1.00
Ercc6l2	-0.12	1.00
Nol10	0.32	1.00
Ubald1	-0.40	1.00
Ogfod3	0.07	1.00
Fam98c	0.06	1.00
Acoxl	0.27	1.00
Rpn1	0.02	1.00

Usp32	-0.08	1.00
Tpst2	0.16	1.00
Fbxo6	-0.04	1.00
Dph6	-0.12	1.00
Chd7	-0.06	1.00
Cep68	0.20	1.00
Tmem175	-0.13	1.00
Pgpep1	-0.06	1.00
Lrrc63	0.01	1.00
Agpat1	-0.06	1.00
Gna11	0.27	1.00
Fndc3b	-0.37	1.00
Cish	-0.12	1.00
Pam16	0.12	1.00
Flrt3	0.61	1.00
Junos	0.17	1.00
Rgl2	0.16	1.00
Brwd3	0.33	1.00
Pspc1	-0.37	1.00
Numa1	0.62	1.00
Eci1	-0.30	1.00
Ctsl	-0.33	1.00
Adss	0.18	1.00
Ubxn1	0.00	1.00
Bag1	0.23	1.00
Bcl3	0.55	1.00
Chmp4b	0.15	1.00
Ilvbl	0.01	1.00
Tnn	0.45	1.00
Nbl1	0.77	1.00
Atp5g2	-0.18	1.00
Isoc1	-0.06	1.00
Ccndbp1	0.23	1.00
Oaz1	-0.13	1.00
Fnip1	-0.27	1.00
Otud6b	-0.07	1.00
Uchl5	-0.11	1.00
Lrp5	0.01	1.00
Dusp10	0.15	1.00
Parva	0.83	1.00
Ube2r2	0.43	1.00
Afap1l1	0.10	1.00
Dcaf17	-0.05	1.00
Plpp5	-0.11	1.00
Itpr3	-0.09	1.00
Ino80	-0.28	1.00

Sec11a	0.32	1.00
Cul5	-0.36	1.00
Pgghg	-0.07	1.00
Vps8	-0.10	1.00
Klhl6	-0.59	1.00
Cep89	-0.12	1.00
Rtl8b	-0.52	1.00
Rft1	-0.07	1.00
Nsdhl	-0.11	1.00
Epb41l4a	0.31	1.00
Sik2	-0.58	1.00
Dock4	-0.17	1.00
Kdelr2	0.31	1.00
Aig1	-0.29	1.00
Arhgef12	-0.25	1.00
Clpb	-0.04	1.00
Ipo9	-0.04	1.00
Zbtb44	-0.12	1.00
Ptcd3	0.19	1.00
Tbc1d12	-0.39	1.00
Prune1	0.45	1.00
Aida	0.20	1.00
Caprin2	-0.18	1.00
Tnfaip8	0.28	1.00
Ric8a	0.15	1.00
Scamp2	-0.05	1.00
Prkar1a	0.49	1.00
Dctn2	0.19	1.00
Tcf4	-0.13	1.00
1600014C10Rik	0.60	1.00
Slfn2	0.40	1.00
Wdr6	0.18	1.00
Ap2b1	0.72	1.00
Jak1	-0.07	1.00
Paip2	-0.21	1.00
Spg20	0.00	1.00
Kif1a	-0.23	1.00
Zfp319	0.04	1.00
4833427G06Rik	-0.02	1.00
Cfap36	0.78	1.00
Shprh	0.10	1.00
Heatr5b	-0.15	1.00
Zfp219	0.07	1.00
Pkd2	-0.02	1.00
Zfp462	-0.07	1.00
Tbc1d17	-0.04	1.00

Upf1	0.06	1.00
Fam136a	-0.28	1.00
Eps8	-0.20	1.00
Xiap	-0.49	1.00
Psmd1	0.20	1.00
Epc2	-0.17	1.00
Cand1	0.29	1.00
Sec11c	-0.20	1.00
Lamp2	-0.36	1.00
Trappc6b	0.16	1.00
Arcn1	-0.10	1.00
Ahr	-0.48	1.00
Lrrk1	0.40	1.00
Sephs1	-0.07	1.00
Osbpl7	0.11	1.00
Sh2b1	0.23	1.00
Zbtb21	-0.55	1.00
Arhgef10	-0.43	1.00
Atp6v0e2	-0.12	1.00
Kcnj13	-0.19	1.00
Zfp143	0.19	1.00
Traf3ip1	0.81	1.00
Ei24	0.12	1.00
B4galnt3	-0.18	1.00
Ttc21b	-0.07	1.00
Elac2	-0.14	1.00
Cabin1	-0.16	1.00
Avpi1	-0.42	1.00
Pea15a	-0.58	1.00
Rbm15	-0.30	1.00
Anxa7	0.11	1.00
Desi1	-0.09	1.00
Slc26a2	-0.06	1.00
Tomm6	-0.11	1.00
Tubb6	0.07	1.00
Brip1os	0.14	1.00
Prkar2b	0.21	1.00
Iqce	0.26	1.00
1600002K03Rik	0.15	1.00
Fzd6	-0.13	1.00
Commd10	0.01	1.00
Chchd4	-0.01	1.00
Tcaf1	0.05	1.00
Tnfsf12	0.23	1.00
Lpcat3	0.26	1.00
Ddr1	0.14	1.00

Anxa2	0.24	1.00
Lrg1	1.05	1.00
Larp4	0.04	1.00
Trappc2l	0.22	1.00
Rev3l	0.45	1.00
Gclc	-0.50	1.00
Epb41l5	0.27	1.00
Tob2	-0.52	1.00
Smad7	-0.15	1.00
Fam173a	-0.26	1.00
Dnajb14	-0.45	1.00
Zfp326	-0.09	1.00
Dbf4	-0.03	1.00
Tmem212	-0.08	1.00
Smarca2	-0.62	1.00
Sbf2	-0.31	1.00
Hibch	-0.19	1.00
Sh2d4a	0.04	1.00
Klhl15	-0.05	1.00
Zfp651	-0.07	1.00
Ston1	0.00	1.00
Uck1	-0.06	1.00
Ttc9c	-0.08	1.00
Bin1	-0.41	1.00
Cd200	-0.29	1.00
Trnau1ap	0.18	1.00
Npr2	0.11	1.00
Rnd1	0.42	1.00
Ctnnbip1	0.13	1.00
Pak4	0.14	1.00
Pigp	-0.27	1.00
Gnb1	0.18	1.00
Clpp	-0.21	1.00
Sfi1	0.42	1.00
Chic2	-0.35	1.00
Nit2	-0.08	1.00
Crot	-0.07	1.00
Ros1	-0.11	1.00
Ppp2r3c	-0.04	1.00
Slc16a6	-0.12	1.00
2610008E11Rik	-0.04	1.00
Fads2	-0.09	1.00
Cebpb	0.10	1.00
Csnk1g1	0.04	1.00
Rrp7a	0.19	1.00
Riok1	-0.12	1.00

Prdx1	-0.30	1.00
Yeats4	0.17	1.00
Ndufaf8	-0.38	1.00
Eapp	-0.06	1.00
Faah	-0.02	1.00
Hsd17b10	0.16	1.00
Pstpip2	0.18	1.00
Scnn1b	0.26	1.00
Dda1	0.11	1.00
Tmem246	0.13	1.00
Dmac2	0.09	1.00
Glipr1	0.24	1.00
Tmtc4	-0.12	1.00
Gypc	-0.64	1.00
Slc2a8	-0.16	1.00
Rgs3	-0.15	1.00
Slc1a5	0.02	1.00
Rnf20	-0.07	1.00
Cyhr1	0.22	1.00
Nme7	0.61	1.00
Htt	0.11	1.00
Cdk2ap2	0.07	1.00
Acox1	0.09	1.00
Wasf2	0.04	1.00
Ttc5	0.54	1.00
Src	0.13	1.00
Spidr	-0.06	1.00
Prpf19	-0.10	1.00
Sdccag8	0.08	1.00
Pofut2	0.40	1.00
Ywhae	0.08	1.00
Ybx3	0.00	1.00
Dstn	0.58	1.00
Clic1	0.36	1.00
Lztfl1	0.12	1.00
Fundc2	-0.12	1.00
Clns1a	0.14	1.00
Cep104	0.19	1.00
Scn1b	0.24	1.00
2310039H08Rik	-0.13	1.00
Pqbp1	0.15	1.00
Krr1	-0.11	1.00
Prpf6	0.21	1.00
Slc16a11	-0.09	1.00
Ripor2	-0.44	1.00
Zfp7	-0.08	1.00

Rasgrp2	-0.09	1.00
Rcan1	-0.28	1.00
Iqcb1	0.17	1.00
Tmem243	-0.29	1.00
Vps50	0.13	1.00
Ckb	0.63	1.00
Dr1	-0.01	1.00
Asf1a	-0.32	1.00
Tgoln1	-0.21	1.00
Ubr1	0.14	1.00
Ddx52	-0.11	1.00
Cenpj	0.13	1.00
Runx2	0.51	1.00
Tlr3	0.84	1.00
Hdac11	0.12	1.00
Ankrd27	0.22	1.00
Fbxo44	0.12	1.00
Trp53bp2	0.53	1.00
Atp5g1	0.47	1.00
Rpf2	-0.29	1.00
Camsap3	-0.10	1.00
Slc35f3	0.43	1.00
Fra10ac1	0.13	1.00
Kazn	0.21	1.00
Tmem214	0.17	1.00
Spns1	-0.04	1.00
Jmjd1c	-0.25	1.00
Pgrmc1	0.24	1.00
Cyb5r3	0.15	1.00
Ssr4	0.64	1.00
Xpo7	-0.46	1.00
Urb1	-0.10	1.00
Grhl1	-0.08	1.00
Dlg3	-0.08	1.00
Efhd1	-0.26	1.00
Slc16a7	-0.08	1.00
Zfand2b	0.10	1.00
Agbl4	0.42	1.00
Fam207a	-0.40	1.00
Cbx4	-0.30	1.00
Aak1	-0.27	1.00
Zbtb10	-0.23	1.00
Immt	-0.10	1.00
Hebp1	0.43	1.00
Susd4	-0.05	1.00
Slc4a1ap	0.15	1.00

Npepl1	-0.08	1.00
Nr3c2	0.04	1.00
Snrpf	0.54	1.00
Plxdc2	-0.81	1.00
Ntmt1	0.15	1.00
Chpf	-0.10	1.00
Smim22	0.19	1.00
Sfpq	-0.11	1.00
Derl1	0.44	1.00
Nop58	-0.15	1.00
Usp53	0.27	1.00
Plekhb1	-0.14	1.00
Cox7b	0.14	1.00
Nudcd1	0.01	1.00
Uqcrc1	0.23	1.00
Asap2	-0.15	1.00
Rras2	-0.66	1.00
Pum2	-0.33	1.00
Dazap1	-0.08	1.00
Fads1	-0.26	1.00
Bmpr2	-0.29	1.00
Rarb	0.89	1.00
Paxip1	0.26	1.00
Ehmt2	0.21	1.00
Slc3a2	0.69	1.00
Wapl	-0.12	1.00
Rab3gap1	0.15	1.00
Vta1	0.28	1.00
Rraga	0.24	1.00
Gpd1l	-0.08	1.00
Rrs1	-0.09	1.00
Trim36	-0.05	1.00
Il17re	-0.06	1.00
Angptl8	-0.08	1.00
1700017B05Rik	-0.05	1.00
Cttn	0.17	1.00
Crtc3	0.11	1.00
Ptpn11	-0.22	1.00
Lsm14b	0.09	1.00
Cybc1	0.29	1.00
Asnsd1	0.07	1.00
Chd8	-0.44	1.00
Phf12	0.17	1.00
Ric8b	0.42	1.00
Eif4b	-0.29	1.00
Tmod1	0.10	1.00

Pgap3	0.46	1.00
Slc10a1	0.17	1.00
Smim27	0.06	1.00
Fam241b	0.11	1.00
2810402E24Rik	0.08	1.00
Bhlhb9	0.18	1.00
Setd2	-0.36	1.00
Zmym2	0.42	1.00
Gprc5a	0.55	1.00
Desi2	0.39	1.00
Fnta	-0.20	1.00
Xpr1	0.20	1.00
Tab3	-0.08	1.00
Ctsh	-0.29	1.00
Echdc1	0.18	1.00
Filip1	-0.45	1.00
Zfyve26	-0.18	1.00
Add3	-0.45	1.00
Ccdc12	0.07	1.00
Yipf5	0.19	1.00
Pgp	-0.53	1.00
Itga3	0.42	1.00
Tug1	-0.83	1.00
Hps5	0.03	1.00
Pdzrn3	0.29	1.00
Tbc1d16	-0.05	1.00
Usp39	-0.10	1.00
Ncl	-0.12	1.00
Aga	-0.03	1.00
Ndufs1	0.04	1.00
Ldb1	-0.06	1.00
Dpyd	-0.20	1.00
Nsun3	-0.11	1.00
Trpm3	-0.46	1.00
Ipp	-0.11	1.00
Ubac1	-0.06	1.00
Ikzf3	-0.49	1.00
Col18a1	-0.25	1.00
Glis2	-0.48	1.00
1700102P08Rik	-0.07	1.00
Tmem178	-0.09	1.00
Bub3	-0.12	1.00
Rnf145	-1.10	1.00
Ggh	-0.01	1.00
Pik3c3	0.00	1.00
Ppip5k2	-0.06	1.00

Fbxw5	0.03	1.00
Ccdc71l	0.01	1.00
Il17rc	0.01	1.00
Syvn1	-0.05	1.00
Cops2	0.17	1.00
Ubxn4	0.01	1.00
Agpat3	0.20	1.00
Bmi1	-0.48	1.00
Epm2aip1	0.16	1.00
Rmnd5a	-0.21	1.00
Slc38a9	-0.07	1.00
Rnf220	-0.22	1.00
2310011J03Rik	-0.13	1.00
Tut4	-0.34	1.00
Atf5	-0.04	1.00
Kif5b	0.35	1.00
Bcl10	0.03	1.00
Tango6	-0.31	1.00
Prom1	0.56	1.00
Zswim3	0.38	1.00
Dnah7b	0.30	1.00
Hilpda	0.44	1.00
Pcif1	-0.44	1.00
Ube4a	0.51	1.00
Faap100	-0.04	1.00
Cd101	-0.03	1.00
AI506816	-0.05	1.00
Hipk3	0.49	1.00
Trpc4ap	0.10	1.00
Usp22	0.12	1.00
Slc4a5	-0.05	1.00
Snu13	-0.26	1.00
Lbp	0.87	1.00
Seh1l	0.06	1.00
Npc2	-0.21	1.00
Eif3g	-0.05	1.00
Gps1	0.45	1.00
Gpat4	0.17	1.00
Rpgrip1l	0.47	1.00
Zfp532	0.34	1.00
Gadd45g	-0.01	1.00
Zbtb46	0.09	1.00
Klhdc7a	0.07	1.00
Ak8	0.53	1.00
Pf4	0.50	1.00
Snapc2	0.08	1.00

2010315B03Rik	0.10	1.00
Pgrmc2	-0.02	1.00
Eed	-0.05	1.00
Fam133b	0.06	1.00
Smc3	-0.47	1.00
Yme1l1	0.01	1.00
Sorl1	-0.41	1.00
Gkap1	-0.48	1.00
Sash1	-0.42	1.00
Pggt1b	-0.09	1.00
Tcp11l2	0.43	1.00
Irs1	0.26	1.00
Dnlz	0.22	1.00
Clcn4	-0.12	1.00
Siah2	0.13	1.00
Pabpc4	-0.30	1.00
Fam3a	0.08	1.00
Gpatch2	-0.04	1.00
Slc30a9	-0.08	1.00
Carhsp1	-0.43	1.00
Abhd17a	-0.15	1.00
Gar1	-0.11	1.00
Sgpl1	0.03	1.00
Ebna1bp2	0.16	1.00
Ezr	-0.08	1.00
Pawr	0.63	1.00
Rbm8a	0.43	1.00
Rwdd1	-0.36	1.00
Pgm1	-0.02	1.00
Rcsd1	-0.31	1.00
Ikbip	-0.10	1.00
Alg12	-0.05	1.00
Abcb10	-0.05	1.00
Fam149a	0.06	1.00
Fam114a1	-0.09	1.00
Nol6	-0.08	1.00
Nicn1	0.09	1.00
Ptprs	-0.09	1.00
3300002I08Rik	0.09	1.00
L3mbtl1	-0.04	1.00
Kti12	-0.10	1.00
Dctpp1	-0.06	1.00
Nfe2l1	-0.33	1.00
Hnrnpf	0.21	1.00
Usp47	0.23	1.00
Pank2	0.41	1.00

Rsf1	-0.18	1.00
Pam	0.26	1.00
Sec14l4	0.03	1.00
Dusp14	0.20	1.00
Lhfp	0.33	1.00
Itpka	0.21	1.00
Cib2	0.08	1.00
Emp3	0.37	1.00
Khnyn	0.07	1.00
Kcnc3	-0.44	1.00
Slc25a17	-0.01	1.00
Snx9	0.51	1.00
Gle1	-0.17	1.00
Hexa	-0.08	1.00
Phc1	-0.07	1.00
Fmo5	-0.09	1.00
Spc24	-0.04	1.00
Fdxr	0.04	1.00
1500015A07Rik	-0.02	1.00
Tpcn1	0.09	1.00
Pikfyve	0.10	1.00
Zhx1	-0.06	1.00
Atg14	0.07	1.00
Il6ra	-0.03	1.00
Serpinh1	0.45	1.00
Tmbim6	-0.15	1.00
Ift74	0.75	1.00
Arl13b	-0.28	1.00
Ccn2	0.42	1.00
Adamts6	0.57	1.00
Rgl1	0.59	1.00
Dzip1l	0.19	1.00
Lcp2	0.34	1.00
Trpv2	0.19	1.00
Otud3	0.17	1.00
1700030K09Rik	0.10	1.00
Parp11	0.06	1.00
Erich3	0.37	1.00
Trmt10b	0.07	1.00
Cfap97d2	0.55	1.00
Insig2	0.32	1.00
Sap30l	0.04	1.00
Eci2	-0.09	1.00
Oxsr1	-0.53	1.00
Tbc1d1	0.42	1.00
Ccdc117	-0.14	1.00

Eif4g1	-0.19	1.00
Ddx18	0.18	1.00
Ccdc82	-0.01	1.00
Ndufs6	-0.09	1.00
Abhd17b	0.00	1.00
Zfp638	-0.38	1.00
Tdp2	-0.12	1.00
Snx5	-0.13	1.00
Ankrd44	-0.13	1.00
Trappc8	0.22	1.00
Kras	0.57	1.00
Cyb561	0.03	1.00
Cirbp	-0.11	1.00
Hes6	-0.11	1.00
Snrpd2	-0.02	1.00
Rhobtb3	-0.13	1.00
Coa7	-0.07	1.00
Rnf123	0.33	1.00
Pan2	-0.08	1.00
Fmnl3	-0.13	1.00
Zfp408	-0.03	1.00
Cyp3a13	-0.03	1.00
BC024978	0.19	1.00
Zfp952	-0.05	1.00
Znrd1as	-0.05	1.00
Hdac6	-0.08	1.00
Nek5	0.58	1.00
Zfp639	-0.14	1.00
Lst1	-0.23	1.00
Prkx	0.19	1.00
Reps2	0.51	1.00
Zup1	-0.12	1.00
Tmem9b	0.00	1.00
Itpk1	0.18	1.00
Iqub	0.29	1.00
Lmf2	0.10	1.00
Bcl11b	0.31	1.00
Prkaa2	0.08	1.00
Cdk18	0.11	1.00
Spata24	0.07	1.00
Dnajc7	-0.63	1.00
Smg1	-0.11	1.00
Srsf4	-0.10	1.00
Dnmt3a	-0.28	1.00
Fam168a	-0.16	1.00
Tmem260	0.03	1.00

Aktip	-0.02	1.00
Wipi1	0.09	1.00
Cxcl17	0.13	1.00
Thsd4	-0.02	1.00
Ssr2	0.42	1.00
Zc3hav1	0.55	1.00
P4ha1	0.01	1.00
Ambra1	-0.01	1.00
Pigt	-0.20	1.00
Fam45a	-0.15	1.00
Grap2	0.01	1.00
Uhmk1	0.34	1.00
Dnaja2	0.20	1.00
Naaladl2	0.19	1.00
Fbxl4	0.31	1.00
Naaa	-0.03	1.00
Drg2	-0.05	1.00
Vps26b	-0.04	1.00
Cc2d1a	-0.06	1.00
Zmym6	0.16	1.00
Fkbp4	-0.06	1.00
Apbb1	0.08	1.00
Fam110b	-0.18	1.00
Cd53	0.55	1.00
Rprd1b	-0.40	1.00
Ufm1	0.21	1.00
Igbp1	-0.12	1.00
Casc3	0.24	1.00
Blvrb	0.01	1.00
Rora	-0.29	1.00
Cdc42ep1	0.08	1.00
Prpf3	-0.10	1.00
Cacna2d1	0.66	1.00
Tmem67	0.12	1.00
Usp40	0.07	1.00
Hspbap1	0.18	1.00
Tnfsf10	0.09	1.00
Arid3b	0.10	1.00
Cdk2	0.12	1.00
Lypd2	0.34	1.00
Zfp983	0.06	1.00
Gbf1	0.15	1.00
Rab15	-0.01	1.00
Fam160a2	0.20	1.00
Tmem171	0.51	1.00
Dnm2	-0.73	1.00

Pip4k2a	0.25	1.00
Fam221a	0.03	1.00
Tasp1	-0.06	1.00
Kctd3	0.66	1.00
1110059E24Rik	0.57	1.00
Abracl	0.36	1.00
Tbl1xr1	0.00	1.00
Uqcc2	-0.36	1.00
Ate1	0.22	1.00
Wdr82	0.18	1.00
Tcirg1	0.12	1.00
Smg5	0.13	1.00
Arpc5	0.25	1.00
Orai2	0.09	1.00
Chchd7	-0.07	1.00
Rragc	-0.04	1.00
Rev1	-0.11	1.00
Ten1	-0.67	1.00
B3galnt2	-0.09	1.00
Tcf20	0.02	1.00
Bmp5	-0.19	1.00
Rapgef4	0.78	1.00
2200002D01Rik	0.15	1.00
Pir	0.14	1.00
Nedd8	0.15	1.00
Cnot7	0.31	1.00
Rubcn	0.19	1.00
Hk1	-0.19	1.00
Amacr	-0.03	1.00
Gfpt2	0.31	1.00
Slc23a1	0.02	1.00
Ptpn22	-0.42	1.00
S1pr2	-0.01	1.00
Colca2	-0.04	1.00
Ccdc13	-0.07	1.00
Foxq1	0.17	1.00
Ppp2r1a	-0.13	1.00
Ivns1abp	-0.42	1.00
Ttc19	-0.21	1.00
Tnpo1	-0.65	1.00
Pmm1	0.18	1.00
Greb1	0.01	1.00
Zdhhc9	-0.01	1.00
Enoph1	-0.02	1.00
Lig3	-0.03	1.00
Ntf5	0.00	1.00

Tspan7	-0.01	1.00
Pbdc1	0.11	1.00
Szrd1	0.16	1.00
Hbp1	-0.04	1.00
Ephx1	-0.08	1.00
Hpf1	0.11	1.00
Ndufc1	-0.15	1.00
Sufu	-0.02	1.00
Dnajc24	0.18	1.00
Gpcpd1	0.27	1.00
Prkd2	-0.07	1.00
Arrdc1	-0.12	1.00
Smu1	0.11	1.00
Psmc6	0.26	1.00
Ero1l	-0.20	1.00
Ncoa1	-0.20	1.00
Ptn	0.24	1.00
Pitrm1	0.07	1.00
Cdt1	0.09	1.00
Slx4	0.07	1.00
Rbl1	0.06	1.00
Ccdc92	0.11	1.00
Abca5	0.24	1.00
Rsph4a	0.14	1.00
Armcx5	0.06	1.00
Sap18	0.03	1.00
Usp3	-0.08	1.00
Nipal3	0.09	1.00
Lztr1	-0.23	1.00
Zfp609	0.06	1.00
Gars	0.20	1.00
2210408I21Rik	0.22	1.00
Srsf9	0.13	1.00
Rbm26	0.08	1.00
Zfp131	-0.19	1.00
Fam120b	0.10	1.00
Usp31	0.18	1.00
Bicra	-0.16	1.00
Fam91a1	0.00	1.00
Klf12	0.18	1.00
Vsig10	0.14	1.00
Git1	0.16	1.00
Arhgef16	0.06	1.00
B9d2	0.15	1.00
Cct4	0.14	1.00
Aldh2	-0.23	1.00

Phactr4	0.08	1.00
Eml2	0.46	1.00
Fgd4	-0.06	1.00
Coro1c	0.16	1.00
Eif4a2	-0.17	1.00
Phtf1	-0.02	1.00
Cobll1	-0.12	1.00
Cryl1	-0.07	1.00
Ptk7	0.03	1.00
Fam160b1	-0.06	1.00
Pogz	0.12	1.00
Rmdn1	0.37	1.00
Osbpl3	0.26	1.00
Plcb4	0.41	1.00
Pkm	-0.23	1.00
Gdi2	0.23	1.00
Tbpl1	-0.35	1.00
Lcmt2	-0.07	1.00
Pon1	0.04	1.00
Nagpa	-0.04	1.00
Selenom	-0.04	1.00
Tlcd2	-0.04	1.00
9430015G10Rik	-0.03	1.00
Fli1	-0.05	1.00
Nr6a1os	-0.03	1.00
Zfp865	-0.45	1.00
Slc6a8	-0.46	1.00
Traf4	0.08	1.00
Ftl1	-0.07	1.00
Bhlhe40	-0.37	1.00
Esrp2	0.26	1.00
Unc13d	0.44	1.00
Nrf1	-0.05	1.00
Gnai3	-0.05	1.00
Fam98a	-0.03	1.00
Commd9	-0.05	1.00
Serinc2	-0.03	1.00
Nelfe	0.00	1.00
Snd1	-0.32	1.00
Spred2	0.05	1.00
Ube2q1	0.19	1.00
Snrpa1	0.09	1.00
Abhd14b	-0.07	1.00
Rab35	0.16	1.00
Fam192a	-0.01	1.00
Tecr	-0.09	1.00

Clu	0.44	1.00
Camkmt	-0.07	1.00
Ttc21a	0.35	1.00
Foxp1	-0.16	1.00
Ralgapa1	-0.01	1.00
Kmt2c	-0.15	1.00
Qars	-0.50	1.00
Hcfc2	0.01	1.00
Zfp438	0.11	1.00
A830018L16Rik	0.11	1.00
Lipa	0.17	1.00
Ccdc113	0.39	1.00
Nedd1	0.08	1.00
2410004P03Rik	0.25	1.00
B230354K17Rik	0.08	1.00
Ehbp1l1	0.07	1.00
Timm17b	-0.02	1.00
Prkd1	0.72	1.00
Ncoa4	-0.28	1.00
Psme3	-0.11	1.00
Cbr1	-0.06	1.00
Syf2	-0.35	1.00
Phactr1	0.57	1.00
Fgfr3	0.11	1.00
Prr14l	0.17	1.00
Otud5	0.43	1.00
Tfdp1	0.18	1.00
Bst1	0.18	1.00
Rin3	-0.03	1.00
Abca2	-0.15	1.00
Kat8	0.05	1.00
Fank1	0.24	1.00
Vamp8	0.62	1.00
Pcna	0.08	1.00
Nsmce4a	0.15	1.00
Eya3	-0.28	1.00
Slc2a1	0.18	1.00
Retreg3	-0.09	1.00
Cox6c	0.15	1.00
Gtf2h5	-0.24	1.00
Arl1	-0.13	1.00
Dlg1	0.39	1.00
Rb1cc1	-0.53	1.00
Sike1	-0.04	1.00
Gdpd3	0.11	1.00
BC005537	-0.28	1.00

Ssbp1	0.43	1.00
Baiap2	-0.13	1.00
Atp2a3	0.62	1.00
Slc25a36	-0.36	1.00
Plxna2	-0.20	1.00
Gpkow	-0.59	1.00
Abcc1	0.14	1.00
Swi5	0.60	1.00
Ncf4	-0.11	1.00
Exoc1	-0.01	1.00
Cfap70	0.01	1.00
Axin2	-0.02	1.00
Fam83d	-0.02	1.00
Elp3	0.02	1.00
Pias4	-0.01	1.00
Tbk1	0.03	1.00
Foxp2	-0.36	1.00
Kdm4a	0.45	1.00
Slc35b3	-0.17	1.00
Suclg2	0.09	1.00
Ccdc18	-0.04	1.00
Pms2	-0.02	1.00
Lrsam1	0.17	1.00
Atp13a2	0.17	1.00
Cd3e	0.05	1.00
Rbm44	-0.04	1.00
Dpy19l1	0.16	1.00
Cul7	-0.03	1.00
Slc37a1	0.09	1.00
C1galt1c1	-0.10	1.00
Wrn	-0.10	1.00
Abcf3	0.02	1.00
Zfp422	0.06	1.00
Pex5	-0.02	1.00
Lamtor3	-0.09	1.00
Lman1	0.29	1.00
Sftpd	0.35	1.00
Itgb6	0.57	1.00
Fam20b	0.25	1.00
Ssbp2	0.35	1.00
Ndufab1	-0.35	1.00
Rbm28	0.32	1.00
Camk2d	-0.16	1.00
Gfpt1	0.06	1.00
Ccz1	-0.13	1.00
Lrrc6	0.33	1.00

Dsg2	0.07	1.00
Stk26	0.04	1.00
Arrdc2	0.24	1.00
D3Ertd751e	0.11	1.00
Krba1	0.03	1.00
Itpkc	0.03	1.00
Ect2l	0.13	1.00
Trp53i13	0.04	1.00
Klf3	0.37	1.00
Nadk2	0.17	1.00
Sh3bp5	-0.13	1.00
Zbtb2	0.02	1.00
Cdca4	0.10	1.00
Casp4	0.16	1.00
Lrriq1	0.34	1.00
Epc1	-0.14	1.00
Col4a4	0.54	1.00
Erf	-0.12	1.00
Pcm1	0.10	1.00
Zfp868	0.17	1.00
Neo1	0.25	1.00
Fndc3a	0.13	1.00
Dleu2	0.51	1.00
Arl5a	0.26	1.00
Wtip	0.21	1.00
Gatad2b	0.33	1.00
Eml4	0.33	1.00
Pex16	0.13	1.00
Sox6	0.53	1.00
Serp1	0.41	1.00
Cdkl4	-0.21	1.00
Eef1g	0.06	1.00
Pccb	-0.07	1.00
Csk	0.05	1.00
E4f1	-0.14	1.00
Plekhg5	-0.06	1.00
Nfu1	0.06	1.00
Etfbkmt	-0.08	1.00
Gnai1	-0.16	1.00
Ugdh	-0.22	1.00
Clta	-0.14	1.00
Itgb1bp1	0.14	1.00
Pdzd11	0.00	1.00
Ddx19a	0.07	1.00
Vps4a	-0.01	1.00
Zcchc17	0.26	1.00

Rbfa	0.06	1.00
Tmub2	0.05	1.00
Ccnl2	0.05	1.00
Gtf3a	0.04	1.00
Zcchc8	-0.07	1.00
2610028H24Rik	-0.26	1.00
Sfswap	0.23	1.00
Cyth2	0.29	1.00
Iqgap2	0.38	1.00
Aldh1a1	0.05	1.00
Zfp68	0.24	1.00
Rnf43	0.33	1.00
Ndufs8	0.31	1.00
Zdhhc7	0.12	1.00
Cd2	0.12	1.00
Ppp2ca	0.23	1.00
Cept1	0.03	1.00
Dnajc19	-0.17	1.00
B4galt4	0.01	1.00
Pkd1	0.07	1.00
Rars	0.03	1.00
Armc8	-0.02	1.00
Tomm22	0.40	1.00
Efhc1	0.07	1.00
Stk11ip	0.06	1.00
Eno4	0.14	1.00
Hddc3	0.04	1.00
Usp43	0.04	1.00
BC052040	0.16	1.00
Zscan21	0.06	1.00
Trim68	0.11	1.00
Zfp738	0.11	1.00
Odf3b	0.11	1.00
Tnrc6b	-0.42	1.00
9030622O22Rik	0.07	1.00
Smyd3	0.45	1.00
Ankrd33b	0.49	1.00
Xpo5	0.13	1.00
Fam219a	0.36	1.00
Nanos1	0.15	1.00
Nr2c2ap	0.15	1.00
Ibtk	-0.05	1.00
Zfp703	-0.11	1.00
Gprin3	0.05	1.00
Fdx1	0.09	1.00
Papola	0.01	1.00

Psip1	0.04	1.00
Ttc32	-0.07	1.00
Coq2	0.26	1.00
Wdr41	-0.08	1.00
Id4	0.06	1.00
Zfp791	-0.06	1.00
3300002A11Rik	0.52	1.00
Ostf1	0.24	1.00
Tlk1	0.67	1.00
Ccnd2	0.42	1.00
Calm1	0.23	1.00
Ndufb10	-0.02	1.00
Acer3	0.13	1.00
Ubap1	0.19	1.00
Pitpnb	0.21	1.00
Fbrsl1	-0.08	1.00
Tmx1	0.02	1.00
Ice1	-0.19	1.00
Cplane1	-0.19	1.00
Bnip2	0.00	1.00
Elof1	0.40	1.00
Znhit6	0.11	1.00
Sptbn1	-0.18	1.00
Tex261	-0.01	1.00
Tmsb10	1.16	1.00
Stxbp6	-0.43	1.00
Ing2	-0.09	1.00
Far1	0.22	1.00
Galnt1	-0.46	1.00
Cse1l	-0.07	1.00
Tmem216	-0.50	1.00
Sephs2	-0.05	1.00
Arhgef11	-0.31	1.00
Creld2	0.02	1.00
Cmya5	0.03	1.00
Abhd13	-0.03	1.00
Liph	0.09	1.00
Asb11	-0.02	1.00
Lyrm4	0.00	1.00
Tbp	0.04	1.00
Hkdc1	0.21	1.00
Kctd6	0.09	1.00
Tmem104	0.03	1.00
Ap1g1	0.49	1.00
Ago3	-0.16	1.00
Pola1	0.13	1.00

Cfap45	0.41	1.00
Arhgdib	-0.27	1.00
Ranbp3	0.29	1.00
Trim69	-0.03	1.00
Ank	-0.10	1.00
Eya1	-0.28	1.00
Hexb	0.04	1.00
Efcab10	0.14	1.00
Hddc2	0.04	1.00
Col3a1	0.19	1.00
Gabarapl1	0.28	1.00
Ddost	-0.31	1.00
Dyrk2	0.26	1.00
Sec13	0.41	1.00
Gabarap	-0.05	1.00
Tex2	0.17	1.00
Ripk1	0.11	1.00
N4bp2l2	-0.35	1.00
Fosb	-0.19	1.00
Ptgr2	0.23	1.00
Kpnb1	-0.40	1.00
Nedd9	-0.06	1.00
Gnl3	-0.13	1.00
Vasn	-0.11	1.00
Rrp1b	0.10	1.00
Dph5	0.01	1.00
Leprotl1	0.12	1.00
Zfp827	-0.04	1.00
Ino80b	0.03	1.00
Atg13	-0.15	1.00
Nelfcd	-0.07	1.00
Usp37	0.51	1.00
Rab1a	0.49	1.00
Dnah5	-0.17	1.00
Thyn1	0.01	1.00
Ttc39b	0.01	1.00
Sucla2	-0.69	1.00
Traf3	0.26	1.00
Sema5a	0.13	1.00
Eml6	0.13	1.00
Ipo11	-0.16	1.00
Trnt1	0.14	1.00
3110056K07Rik	0.10	1.00
Ctnnb1	-0.03	1.00
Tmem141	0.27	1.00
Serpinf1	0.27	1.00

Capn1	0.45	1.00
Ddx54	0.16	1.00
Atp9b	-0.36	1.00
Atm	-0.06	1.00
Pygo1	-0.05	1.00
Tmem222	-0.42	1.00
Ppp2r1b	-0.47	1.00
Ggps1	-0.21	1.00
Pak1	0.14	1.00
Dhx30	0.27	1.00
Vamp3	-0.37	1.00
Cenpc1	-0.09	1.00
Adamts5	-0.06	1.00
Sorbs2	0.07	1.00
Psat1	0.02	1.00
Abcb9	0.13	1.00
Pifo	-0.01	1.00
Dnaic2	0.03	1.00
Sh3rf3	0.35	1.00
Tbc1d8b	-0.32	1.00
Smarcad1	0.08	1.00
Tmed7	-0.19	1.00
Acsl1	-0.14	1.00
Faf1	-0.01	1.00
Trim33	0.14	1.00
Ranbp1	0.20	1.00
Adgrg1	-0.51	1.00
Rlf	0.27	1.00
Smarcc2	-0.37	1.00
Vps37c	0.09	1.00
Fam98b	0.33	1.00
Snapin	0.22	1.00
Zfp398	0.00	1.00
Ccar2	-0.15	1.00
Thap3	-0.10	1.00
Pi4ka	0.10	1.00
Ythdf3	0.26	1.00
Trappc11	0.03	1.00
Fbxl15	0.17	1.00
Soga1	0.29	1.00
Ctsd	-0.01	1.00
Psmd14	0.21	1.00
Fam13b	-0.22	1.00
Slc45a4	0.02	1.00
Fzd8	-0.18	1.00
Polr2i	-0.34	1.00

Arhgap5	-0.11	1.00
Eif4e	0.72	1.00
Spag9	0.28	1.00
Arhgap35	-0.24	1.00
Syncrip	0.10	1.00
Bmp2	0.06	1.00
Rfwd3	0.09	1.00
Thbs3	0.33	1.00
Ppp1r11	0.12	1.00
Tmem140	0.14	1.00
Ndufaf2	-0.55	1.00
Bola3	0.26	1.00
Usf2	0.43	1.00
Rab10	-0.12	1.00
Cdk14	0.31	1.00
Adsl	0.52	1.00
Slc33a1	-0.07	1.00
Kcnk1	0.15	1.00
Hmox2	0.50	1.00
Atxn2	0.55	1.00
Plekhm2	-0.06	1.00
Rtraf	-0.15	1.00
Rer1	0.31	1.00
Lamc2	0.28	1.00
S100a1	0.00	1.00
Ccny	-0.40	1.00
Ptpn18	0.54	1.00
Pgls	-0.06	1.00
Tm9sf3	-0.05	1.00
Fundc1	0.12	1.00
Tmem64	-0.26	1.00
Lpl	-0.02	1.00
Sergef	-0.09	1.00
Sympk	0.00	1.00
Sec61a2	-0.10	1.00
Atg9a	-0.08	1.00
Atp6v1f	-0.14	1.00
Tubb2a	0.18	1.00
Suclg1	0.07	1.00
Ddo	0.28	1.00
Ap5m1	0.07	1.00
Sgpp1	0.05	1.00
Ints9	-0.06	1.00
Ndrg3	-0.07	1.00
Zw10	-0.08	1.00
Dedd	-0.05	1.00

Fbl	0.13	1.00
Ugp2	-0.33	1.00
Camk4	-0.09	1.00
Hook1	0.29	1.00
Ube2d3	0.00	1.00
Tmco3	0.26	1.00
Dipk1a	-0.27	1.00
Chmp1b	-0.08	1.00
Tef	0.33	1.00
Gk	0.09	1.00
Ptgs1	0.14	1.00
Nap1l1	-0.20	1.00
Ganab	0.64	1.00
Ffar4	0.09	1.00
Fam216a	0.21	1.00
Spata13	-0.06	1.00
Plcb3	0.42	1.00
Chpt1	-0.42	1.00
Hivep1	0.55	1.00
Dip2b	-0.02	1.00
Zhx2	-0.35	1.00
Siva1	-0.12	1.00
Gclm	0.49	1.00
Gsk3a	0.20	1.00
Dgkd	0.11	1.00
Dnase2a	-0.05	1.00
Tmem37	0.39	1.00
Hcfc1	0.15	1.00
Nfkbid	-0.11	1.00
Cav2	-0.08	1.00
Pak2	-0.60	1.00
Trmt6	0.28	1.00
Cfap299	0.97	1.00
Clec4d	0.04	1.00
Osbpl9	-0.20	1.00
Unc45a	0.00	1.00
Tesc	-0.24	1.00
Tgfa	-0.03	1.00
Cdk12	-0.33	1.00
Btf3l4	0.06	1.00
Rab10os	0.20	1.00
Eif3d	-0.05	1.00
Hnrnpa1	-0.32	1.00
Acadvl	-0.08	1.00
Os9	0.17	1.00
Ppp3r1	-0.10	1.00

Pfkfb3	0.15	1.00
6030458C11Rik	-0.19	1.00
Hsdl1	-0.15	1.00
Sept10	0.31	1.00
Ocln	0.30	1.00
Arhgap12	-0.21	1.00
Tcn2	0.31	1.00
Rnf214	0.44	1.00
R3hdm1	0.08	1.00
Cblb	-0.04	1.00
Efl1	0.18	1.00
Sae1	0.05	1.00
9530068E07Rik	0.08	1.00
Paqr8	0.14	1.00
Tle4	-0.18	1.00
Tmx4	-0.46	1.00
Angel2	-0.23	1.00
Setdb1	0.25	1.00
Rpp21	0.11	1.00
Nedd4	-0.05	1.00
Slu7	0.12	1.00
Brcc3	-0.02	1.00
Nln	0.13	1.00
Slc31a2	0.23	1.00
Zmym5	0.08	1.00
Caprin1	0.35	1.00
Nfyb	0.10	1.00
Copb1	-0.34	1.00
Sptan1	0.21	1.00
Dtd1	0.12	1.00
As3mt	0.41	1.00
Kansl1	0.15	1.00
Smox	0.30	1.00
Nuak1	-0.08	1.00
Tprkb	-0.10	1.00
Kctd1	-0.06	1.00
Pex1	-0.06	1.00
Srr	-0.33	1.00
Pym1	-0.11	1.00
Nfya	-0.09	1.00
Ascc1	-0.20	1.00
Rnf34	-0.09	1.00
Ncstn	-0.08	1.00
Vmp1	0.21	1.00
Erh	0.21	1.00
Fancm	-0.07	1.00

Fcgrt	-0.05	1.00
Vrk2	0.22	1.00
Wdr66	0.80	1.00
Camta1	0.11	1.00
Ppl	-0.09	1.00
Abhd12	0.36	1.00
Ppp1ca	-0.23	1.00
Cux1	0.00	1.00
Ube2w	-0.05	1.00
Atxn2l	0.08	1.00
Sub1	-0.21	1.00
Dusp6	0.01	1.00
Eef1e1	-0.18	1.00
Efnb2	-0.26	1.00
Gtf3c6	0.01	1.00
Trim24	-0.39	1.00
Fmc1	-0.31	1.00
Ubxn6	-0.42	1.00
Cdc42ep4	-0.13	1.00
Phrf1	0.31	1.00
Armcx3	0.25	1.00
Abhd2	0.12	1.00
Sdhd	-0.23	1.00
Pa2g4	-0.42	1.00
Arid4b	-0.06	1.00
Usp9x	-0.15	1.00
Hhex	-0.10	1.00
Zfp821	0.18	1.00
Ntn1	0.18	1.00
Pde2a	0.51	1.00
Ccser2	-0.16	1.00
Epha2	0.43	1.00
Cd302	0.36	1.00
H2afv	-0.22	1.00
Ahnak	0.44	1.00
Clasp1	0.04	1.00
Atp13a4	0.20	1.00
Tsc2	0.27	1.00
Ocel1	-0.04	1.00
Trmt1	-0.07	1.00
Klf9	-0.58	1.00
Pxmp4	0.18	1.00
Slc6a14	-0.03	1.00
Kat7	0.51	1.00
Rnf44	0.61	1.00
Prkn	-0.81	1.00

Rbm22	0.05	1.00
Rbbp7	0.01	1.00
Cuedc1	0.26	1.00
Itgb1	-0.26	1.00
Prdx3	0.31	1.00
Rae1	-0.04	1.00
Drg1	-0.04	1.00
Pdcl	-0.03	1.00
Bin3	0.13	1.00
Rhbdf1	-0.08	1.00
Tmem199	-0.08	1.00
Snx11	-0.03	1.00
Cutal	-0.06	1.00
Chfr	-0.04	1.00
Serpinb6a	0.20	1.00
Fmn1	0.24	1.00
Ogfod1	0.13	1.00
Vps11	0.14	1.00
Kctd8	-0.17	1.00
Fer	-0.06	1.00
Erlin1	-0.05	1.00
Tmem30b	-0.05	1.00
Phb2	0.07	1.00
Pkp2	0.32	1.00
Trp53inp1	0.51	1.00
Lsm5	-0.41	1.00
Clock	-0.10	1.00
Sf1	-0.16	1.00
Lmbr1l	0.04	1.00
Ccdc57	0.06	1.00
Fus	0.44	1.00
Gpbp1l1	0.35	1.00
Ccnt2	0.23	1.00
Ech1	-0.44	1.00
Paip1	-0.24	1.00
Reps1	-0.12	1.00
Rtf2	-0.52	1.00
Zmynd8	0.72	1.00
Ankmy2	-0.41	1.00
R3hcc1l	-0.02	1.00
Lcmt1	-0.47	1.00
Slc5a3	-0.12	1.00
Uba6	-0.20	1.00
Cerk	-0.65	1.00
Asrgl1	-0.11	1.00
Fbxo21	0.07	1.00

Slc25a37	0.12	1.00
Ankrd39	0.08	1.00
Wdr19	0.22	1.00
Zfp26	-0.11	1.00
Nlrp3	0.69	1.00
Dock2	-0.60	1.00
Nfkb2	0.27	1.00
Keap1	-0.09	1.00
Efna1	0.36	1.00
Gpr146	-0.55	1.00
Dld	0.05	1.00
Zfp281	-0.35	1.00
Atp5mpl	0.27	1.00
Ebag9	-0.07	1.00
Cdc25a	-0.08	1.00
G2e3	-0.05	1.00
Zdhhc8	-0.32	1.00
Zfp322a	0.14	1.00
Stag2	0.04	1.00
Dcaf6	0.09	1.00
Peak1	0.21	1.00
Ndufb6	0.24	1.00
D630045J12Rik	-0.08	1.00
Ttc37	-0.15	1.00
Dcakd	-0.05	1.00
Zfp106	-0.24	1.00
Ppp1r12b	-0.21	1.00
Pbrm1	-0.23	1.00
Grk2	0.13	1.00
Spred1	0.13	1.00
Dennd1b	0.02	1.00
Rasa2	0.15	1.00
Siah1a	-0.11	1.00
Dnm3	0.10	1.00
S100pbp	0.04	1.00
Zc3h12a	0.25	1.00
Usp10	0.26	1.00
Exoc4	0.54	1.00
Nt5e	1.02	1.00
Nudt16l1	0.45	1.00
Dnaaf5	0.22	1.00
Cers5	0.36	1.00
Tcf7l1	0.25	1.00
Selenoh	0.00	1.00
Cdc42se2	-0.07	1.00
Akirin2	-0.21	1.00

Ift27	0.12	1.00
Smim19	-0.03	1.00
Dusp8	0.04	1.00
Fuom	-0.06	1.00
Tox	-0.59	1.00
Ccnyl1	-0.06	1.00
Dennd4a	1.08	1.00
Tmem208	-0.10	1.00
Ppp6c	0.67	1.00
Stx6	0.19	1.00
Cnp	0.17	1.00
Frk	0.11	1.00
Satb1	-0.43	1.00
Exph5	0.25	1.00
Csnk2a1	0.19	1.00
Dennd6a	0.28	1.00
Serinc3	-0.36	1.00
Tnrc18	0.24	1.00
Il17ra	0.07	1.00
Exd2	0.41	1.00
Itga7	0.06	1.00
Unkl	0.28	1.00
Ptgs2	-0.19	1.00
Ndufs5	0.34	1.00
Pik3ca	-0.31	1.00
Emc6	0.38	1.00
Tpi1	-0.10	1.00
Sh3bgrl3	0.48	1.00
Lman2l	-0.03	1.00
Ifngr1	0.35	1.00
Tln1	0.22	1.00
Cystm1	0.10	1.00
Gga2	-0.03	1.00
1700001K19Rik	0.06	1.00
Stat5b	-0.14	1.00
Appl2	0.54	1.00
Stx7	0.16	1.00
2900089D17Rik	0.01	1.00
2210408F21Rik	0.07	1.00
Cecr2	-0.29	1.00
Rbm25	0.11	1.00
Tfeb	0.01	1.00
C330002G04Rik	-0.46	1.00
Atp5h	0.48	1.00
Smarca4	0.08	1.00
Rack1	-0.27	1.00

Gramd1a	0.52	1.00
Arf3	0.03	1.00
S100a13	0.29	1.00
Tnip1	0.53	1.00
Crip2	-0.12	1.00
Fam117b	-0.45	1.00
Wbp11	0.19	1.00
Pld3	0.10	1.00
Apbb2	-0.30	1.00
Rnf144a	0.01	1.00
Dusp4	-0.06	1.00
Gskip	-0.24	1.00
Creld1	-0.04	1.00
Hivep3	-0.24	1.00
Ikbkb	-0.03	1.00
Bbc3	0.04	1.00
Ilf3	-0.32	1.00
Jkamp	-0.10	1.00
Naa10	0.19	1.00
Tmco1	0.53	1.00
Fbxo32	-0.13	1.00
Stk3	-0.08	1.00
Api5	0.23	1.00
Ndufs3	0.47	1.00
Pik3c2a	-0.18	1.00
Lta4h	0.06	1.00
Fbxo31	0.15	1.00
Gins4	0.16	1.00
Cdk5	0.17	1.00
Cisd3	0.44	1.00
Prob1	0.10	1.00
Rtl8a	0.16	1.00
Ski	-0.17	1.00
Prpf8	-0.10	1.00
Inpp5d	-0.13	1.00
Retsat	-0.04	1.00
Kcmf1	0.14	1.00
Ylpm1	0.27	1.00
Rnasek	-0.14	1.00
Rab12	0.35	1.00
Pim1	0.26	1.00
Eif2b5	-0.14	1.00
St6galnac3	0.19	1.00
Adprm	0.38	1.00
Dusp1	0.36	1.00
Vma21	0.15	1.00

Aco1	0.07	1.00
Prpf18	-0.65	1.00
Neurl1a	-0.10	1.00
Bnip1	-0.21	1.00
1500011B03Rik	-0.01	1.00
Cetn2	0.13	1.00
Dcps	-0.03	1.00
Ciz1	0.21	1.00
Rbbp8	-0.31	1.00
Larp7	0.07	1.00
Nectin3	0.38	1.00
Ldhb	-0.15	1.00
Ldah	0.07	1.00
Cox20	-0.35	1.00
Rab4b	0.15	1.00
Coro1a	-0.13	1.00
Tspo	0.24	1.00
Atp6v1c1	-0.05	1.00
Sema4a	-0.04	1.00
Ap1s1	-0.06	1.00
Arhgap8	0.28	1.00
Got1	-0.02	1.00
Snrnp40	0.06	1.00
Uqcrb	0.20	1.00
Zfp292	-0.34	1.00
Galnt2	-0.04	1.00
Slc39a9	0.22	1.00
Otulin	0.10	1.00
Txndc11	0.02	1.00
Elob	0.32	1.00
Atox1	-0.37	1.00
Ctsb	0.12	1.00
Hdlbp	-0.23	1.00
Rangap1	-0.29	1.00
Nf1	-0.10	1.00
Actr10	0.02	1.00
Gigyf2	-0.08	1.00
Gprc5b	-0.20	1.00
Garnl3	-0.20	1.00
Strada	-0.14	1.00
Nbas	-0.19	1.00
Kremen1	0.19	1.00
Urm1	0.20	1.00
Prkcsh	0.10	1.00
Nr1h2	0.02	1.00
Ptpn4	-0.04	1.00

Ankib1	-0.22	1.00
4933434E20Rik	-0.10	1.00
Ermp1	-0.05	1.00
Rhobtb1	0.00	1.00
Akap1	0.09	1.00
Pacc1	0.06	1.00
Cetn4	0.47	1.00
Klhl8	-0.09	1.00
Vps18	-0.10	1.00
Adrb1	0.15	1.00
Ddx19b	0.15	1.00
Cmc2	-0.03	1.00
Lncppara	0.11	1.00
Trim39	-0.12	1.00
Sumo2	-0.01	1.00
Adnp	0.20	1.00
Sdc2	0.06	1.00
Ankrd46	0.39	1.00
Eif2d	0.01	1.00
Ctsk	0.17	1.00
Tmem219	0.40	1.00
B4galnt1	0.44	1.00
Phykpl	0.12	1.00
Stat5a	-0.13	1.00
Sod1	0.37	1.00
Polr2a	0.07	1.00
Kdsr	0.32	1.00
Rhno1	-0.09	1.00
Hmg20a	0.37	1.00
Tor2a	-0.04	1.00
Tada2a	-0.04	1.00
Dnaaf2	0.08	1.00
Lzic	0.14	1.00
Cdk7	0.10	1.00
Get4	-0.03	1.00
Nfrkb	-0.04	1.00
Vamp7	-0.07	1.00
Slc25a25	0.19	1.00
Limd1	-0.26	1.00
Dennd4c	-0.09	1.00
Zc3h14	-0.12	1.00
Abhd5	-0.12	1.00
Erbb3	0.18	1.00
Litaf	0.37	1.00
Pde4dip	0.36	1.00
Luzp1	0.13	1.00

Ecpas	-0.05	1.00
Cs	0.22	1.00
Dym	-0.14	1.00
Arhgap17	0.32	1.00
Arg2	-0.19	1.00
Washc2	-0.01	1.00
Sec24c	0.44	1.00
Ufsp2	0.30	1.00
Atg12	0.24	1.00
Acss1	-0.02	1.00
Nktr	-0.37	1.00
Rai14	-0.16	1.00
Gcc2	0.39	1.00
Eif2b3	-0.22	1.00
Lgmn	0.32	1.00
Cdh16	0.00	1.00
Tmem181a	0.18	1.00
Ythdc2	-0.32	1.00
Dus1l	0.12	1.00
Eva1b	0.19	1.00
Ccdc85b	0.01	1.00
Fubp1	0.19	1.00
Capza1	-0.20	1.00
Ptp4a2	-0.05	1.00
Fubp3	-0.57	1.00
Pgs1	-0.16	1.00
Nme1	0.76	1.00
Cmpk1	0.35	1.00
Sntb1	-0.36	1.00
Cdk13	-0.05	1.00
Rcor1	0.28	1.00
Ctbp1	0.26	1.00
Tmem161b	0.46	1.00
5530601H04Rik	0.46	1.00
Gabpb1	-0.57	1.00
Chpf2	0.07	1.00
Ccno	0.13	1.00
Spag16	0.45	1.00
Dnah10	0.41	1.00
Hid1	0.09	1.00
Ankfn1	0.26	1.00
Guca1a	-0.27	1.00
Trim72	0.14	1.00
Sh3bp1	0.06	1.00
Ythdc1	0.03	1.00
Zc3h6	0.37	1.00

Zfp217	0.09	1.00
Zdhhc16	-0.02	1.00
Rbmxl1	-0.20	1.00
Fam184a	0.06	1.00
Stx18	0.18	1.00
Huwe1	-0.24	1.00
Plk2	0.17	1.00
Tent4b	0.31	1.00
Actr3	0.46	1.00
Dhcr24	-0.24	1.00
Dennd2c	-0.23	1.00
Plod3	0.30	1.00
Col4a5	0.67	1.00
Hmgn1	-0.25	1.00
Bcl7b	-0.32	1.00
Dyrk1a	-0.31	1.00
Ergic3	-0.28	1.00
Slpi	-0.12	1.00
Pitpnc1	-0.76	1.00
Akt1	0.48	1.00
Rfx7	-0.15	1.00
Atp6v1b2	-0.18	1.00
Rnf114	0.43	1.00
Tmem14c	0.09	1.00
Nup153	0.24	1.00
Opa1	-0.01	1.00
Atp5d	0.35	1.00
Dph3	-0.33	1.00
Tra2a	-0.10	1.00
Acaa1a	-0.07	1.00
Rbm34	0.02	1.00
Dgka	0.08	1.00
Tnk1	-0.02	1.00
Arl4d	-0.02	1.00
Cyp4v3	-0.36	1.00
Supt6	0.01	1.00
Nhsl1	-0.03	1.00
Fos	0.20	1.00
Timp3	0.21	1.00
Cct6a	-0.06	1.00
Ctif	0.30	1.00
Fem1c	-0.15	1.00
Rbm14	0.07	1.00
Cdkn1c	-0.21	1.00
Tnrc6c	-0.15	1.00
2310009A05Rik	0.69	1.00

Ncapd3	0.05	1.00
Chchd5	0.28	1.00
Dgat1	0.09	1.00
Fam76a	0.19	1.00
Pdcd6ip	0.35	1.00
Pkn2	0.03	1.00
Cldn18	0.22	1.00
Pi4kb	-0.11	1.00
Agr3	0.34	1.00
Glipr2	0.17	1.00
Lrrc10b	0.16	1.00
Nprl2	-0.07	1.00
Ccdc66	-0.03	1.00
Ipo5	-0.05	1.00
Csnk2a2	0.12	1.00
Napa	0.27	1.00
Calm3	-0.28	1.00
Naprt	-0.02	1.00
1600012H06Rik	-0.13	1.00
Ttll4	-0.02	1.00
Ap3s2	-0.03	1.00
Cyc1	0.26	1.00
Dcun1d4	0.24	1.00
Zfp467	0.26	1.00
Swap70	0.13	1.00
Ndufb2	0.41	1.00
Sil1	-0.10	1.00
Prpf4	-0.08	1.00
Zfp612	0.24	1.00
Paip2b	0.11	1.00
Ngdn	-0.28	1.00
Cpeb4	0.14	1.00
Cbx5	0.17	1.00
Btbd7	0.03	1.00
Dpp8	0.17	1.00
Txnl4a	-0.04	1.00
Tiprl	0.08	1.00
Rad23a	0.11	1.00
Swt1	-0.04	1.00
Tmed1	-0.06	1.00
Zfpl1	-0.08	1.00
Ppm1l	0.49	1.00
Gga1	-0.33	1.00
Anpep	0.04	1.00
Zkscan1	0.02	1.00
Qdpr	0.08	1.00

Scamp3	0.09	1.00
Wdr33	0.13	1.00
Fbxo22	-0.03	1.00
Alpk1	-0.38	1.00
Ctbp2	0.12	1.00
Klf5	0.30	1.00
Arhgef17	0.12	1.00
Foxk2	0.08	1.00
Ttc6	-0.07	1.00
Fancc	0.13	1.00
Coasy	-0.01	1.00
Psd3	0.34	1.00
Lrmda	0.24	1.00
Pdhb	-0.07	1.00
Tusc2	-0.02	1.00
Wdr48	0.15	1.00
Bdp1	-0.10	1.00
Itga6	-0.18	1.00
Ddt	0.21	1.00
Cdc37	0.06	1.00
Ttc8	0.16	1.00
Fam210b	-0.06	1.00
Gltp	0.19	1.00
Tpgs1	0.16	1.00
Slc27a1	0.07	1.00
Lrrc36	0.50	1.00
Arl6ip4	0.18	1.00
Bzw2	-0.34	1.00
Ilkap	0.35	1.00
Cep78	-0.12	1.00
Gusb	0.16	1.00
Zfp414	0.05	1.00
Cfap52	0.16	1.00
Adgrv1	-0.17	1.00
Dnah7c	0.20	1.00
Prkra	-0.02	1.00
Cep295	-0.07	1.00
1700016K19Rik	0.22	1.00
1700037C18Rik	0.12	1.00
Ciita	-0.02	1.00
Armc4	0.25	1.00
Rabggtb	-0.04	1.00
Tmem42	0.10	1.00
Dars	0.02	1.00
Fbxw4	-0.84	1.00
Agfg1	0.14	1.00

Nufip2	-0.11	1.00
Btbd2	-0.13	1.00
Fgf1	0.27	1.00
Sept11	0.13	1.00
Atn1	-0.18	1.00
Ccdc88c	0.16	1.00
Arhgap18	0.25	1.00
Atp6v1g1	-0.25	1.00
Bsg	0.23	1.00
Ahsa2	-0.10	1.00
Glcci1	0.10	1.00
Ppp1cb	-0.25	1.00
Rap1a	-0.19	1.00
Slc7a2	-0.32	1.00
Hdac1	-0.11	1.00
Cyth1	-0.12	1.00
Timm10b	0.29	1.00
Cdc42ep3	0.36	1.00
Hspbp1	-0.38	1.00
Znrf2	0.32	1.00
Rab25	0.13	1.00
Art3	-0.46	1.00
Nckap5l	-0.21	1.00
Spire1	0.18	1.00
Ezh1	0.28	1.00
Sat1	-0.58	1.00
Fcho2	-0.08	1.00
Rhbdd1	-0.09	1.00
Srgap2	-0.38	1.00
Prorsd1	-0.18	1.00
Atp6v0c	0.30	1.00
Bclaf1	0.31	1.00
Creb5	-0.03	1.00
Plcb1	0.19	1.00
1700029J07Rik	-0.04	1.00
Ppil1	0.03	1.00
Lypla1	0.11	1.00
Ica1	-0.45	1.00
Ginm1	0.17	1.00
Adipor2	-0.28	1.00
Acap2	-0.09	1.00
Aamdc	-0.10	1.00
Dhx32	0.15	1.00
Lrrc8b	0.07	1.00
Dennd2d	0.00	1.00
Nol9	-0.07	1.00

Txnrd1	0.14	1.00
Orc2	0.02	1.00
Casc4	-0.07	1.00
Dnajb13	-0.10	1.00
Lrguk	0.05	1.00
Ccdc170	0.13	1.00
Phospho2	0.04	1.00
Lypd6b	0.04	1.00
Sntn	0.40	1.00
Afmid	0.03	1.00
Pmpca	0.04	1.00
Gosr1	-0.03	1.00
Rbbp9	0.08	1.00
Cfap206	0.17	1.00
Rab3d	0.10	1.00
D630024D03Rik	-0.19	1.00
AU040972	0.37	1.00
Zfp458	0.07	1.00
Slc7a8	0.11	1.00
C230072F16Rik	-0.18	1.00
Glg1	-0.29	1.00
Pom121	0.12	1.00
Hmgn5	0.24	1.00
Chka	-0.29	1.00
Tmem184b	0.14	1.00
Large1	-0.10	1.00
Dck	0.02	1.00
6720427I07Rik	0.12	1.00
Cox17	0.37	1.00
Arhgdia	-0.71	1.00
Cers2	0.17	1.00
Arl8a	0.16	1.00
Esam	0.35	1.00
Hspa9	-0.17	1.00
Dnajb4	0.40	1.00
Pno1	0.07	1.00
Fut11	0.03	1.00
Slc30a4	-0.16	1.00
Phf8	0.03	1.00
Nipa1	-0.05	1.00
Fbxo3	0.05	1.00
Cd320	0.11	1.00
Itfg2	0.05	1.00
Decr1	-0.14	1.00
C430049B03Rik	0.39	1.00
Gnl3l	0.11	1.00

Timm13	-0.17	1.00
Rab11b	0.25	1.00
Yipf4	-0.09	1.00
Picalm	-0.22	1.00
Rnf5	-0.20	1.00
Rasa1	-0.13	1.00
Plin2	0.43	1.00
Hnrnpll	0.65	1.00
Cdk2ap1	-0.02	1.00
Zc3h7a	-0.04	1.00
Ankhd1	-0.32	1.00
Rxylt1	0.03	1.00
Snapc1	0.56	1.00
Slc25a47	-0.06	1.00
Fam171b	-0.06	1.00
Plod1	-0.16	1.00
Eps8l2	0.15	1.00
Tex14	-0.62	1.00
Gnb2	0.19	1.00
Tle3	-0.39	1.00
Isyna1	0.01	1.00
Acly	0.05	1.00
Tbc1d5	0.21	1.00
Trappc9	-0.16	1.00
Usp46	0.13	1.00
Jdp2	0.11	1.00
Atad1	-0.10	1.00
Klf13	-0.46	1.00
Ndufaf7	0.21	1.00
Tmem251	0.11	1.00
Ttc4	-0.01	1.00
Tgif2	-0.01	1.00
Cog1	-0.04	1.00
Ercc5	-0.03	1.00
Zbed3	-0.06	1.00
Dnal4	-0.04	1.00
Abcd1	0.31	1.00
Ssbp3	0.08	1.00
Plekhf2	-0.01	1.00
Nipsnap2	0.05	1.00
Psma2	0.22	1.00
Acsl5	0.29	1.00
Lrrc58	0.24	1.00
Clstn1	0.03	1.00
Sin3b	0.18	1.00
Josd2	0.07	1.00

Bmp1	0.02	1.00
Trip11	-0.49	1.00
Slc25a51	0.00	1.00
Lsm14a	0.07	1.00
Lrrc23	0.11	1.00
Ankrd29	-0.02	1.00
Trappc12	-0.06	1.00
Rbm43	0.02	1.00
Rbsn	-0.07	1.00
Fhod1	0.03	1.00
Clcn7	0.02	1.00
Sh2d3c	0.08	1.00
Vps45	-0.03	1.00
C2cd3	0.04	1.00
Scml4	0.17	1.00
Hs3st3b1	-0.25	1.00
Arid1b	-0.30	1.00
Cited2	-0.01	1.00
Tardbp	-0.19	1.00
Nucb1	0.09	1.00
Limd2	0.15	1.00
Rragd	-0.11	1.00
Rbm5	0.40	1.00
Cdc26	-0.02	1.00
E130308A19Rik	0.10	1.00
Dnajc12	-0.15	1.00
Hist1h1e	0.00	1.00
Zfp410	0.19	1.00
Fam3c	-0.05	1.00
Lyrm2	0.07	1.00
Fsd1l	0.23	1.00
Ss18l2	0.17	1.00
Zxdb	-0.16	1.00
Nsfl1c	0.32	1.00
Slc31a1	-0.02	1.00
Atp5k	0.04	1.00
Ppp1r16a	0.34	1.00
Lrif1	0.10	1.00
Strn	0.48	1.00
Naa30	-0.07	1.00
Galnt3	0.27	1.00
Zfp598	-0.02	1.00
Ctnnbl1	-0.30	1.00
Slc41a1	-0.23	1.00
Arhgap10	0.07	1.00
Ak7	0.07	1.00

Ebpl	-0.02	1.00
Nck2	-0.05	1.00
Stim2	0.25	1.00
Ahctf1	0.49	1.00
Clip4	0.25	1.00
Copz1	0.15	1.00
Arl3	0.03	1.00
Tram1	0.39	1.00
Washc5	0.13	1.00
Ap1b1	-0.18	1.00
Lfng	-0.05	1.00
Fmn2	-0.04	1.00
Ulk3	-0.12	1.00
Peg13	-0.08	1.00
Agbl3	0.11	1.00
Nudt13	-0.03	1.00
Ttyh3	0.61	1.00
Chil3	0.47	1.00
H2afj	-0.33	1.00
Gdi1	0.25	1.00
Tet2	0.30	1.00
Zdhhc1	-0.23	1.00
Commd2	-0.27	1.00
Crat	0.00	1.00
Chchd3	0.30	1.00
Tomm5	0.31	1.00
D16Ertd472e	0.20	1.00
Rrp15	-0.40	1.00
Saxo2	0.08	1.00
Bcl7a	-0.05	1.00
Wdr18	-0.03	1.00
2410131K14Rik	-0.05	1.00
1110019D14Rik	-0.02	1.00
Stk33	0.15	1.00
Kri1	-0.05	1.00
Amz2	0.05	1.00
Gpr137b	-0.06	1.00
Psmd10	0.05	1.00
Slc1a1	0.03	1.00
Arhgef3	0.20	1.00
Sart3	0.08	1.00
Naa16	0.14	1.00
Taf12	0.01	1.00
Gabpa	0.02	1.00
Rnf6	0.08	1.00
Surf4	0.27	1.00

Cldn3	-0.08	1.00
Ptms	0.10	1.00
Kdm3a	0.09	1.00
Limch1	1.38	1.00
Smap2	0.29	1.00
Ramac	0.48	1.00
Dctn3	0.20	1.00
Utp18	-0.13	1.00
Dnmbp	-0.06	1.00
Lrrc1	0.28	1.00
Chst8	-0.07	1.00
Anapc2	0.01	1.00
Atp6ap2	0.15	1.00
Vti1a	-0.37	1.00
Pacsin3	0.00	1.00
Plekha3	-0.04	1.00
Ppie	0.16	1.00
Plekha5	0.28	1.00
Hnrnpa0	0.16	1.00
Rrm1	0.03	1.00
Trappc1	-0.06	1.00
Scamp1	0.10	1.00
Glrx3	-0.31	1.00
Gadd45a	0.14	1.00
Wdr36	0.02	1.00
Trim27	0.04	1.00
Gnl2	0.18	1.00
Ppp6r1	-0.41	1.00
Drap1	0.02	1.00
Snx17	0.17	1.00
Egfr	0.43	1.00
Isy1	-0.06	1.00
Hdc	0.73	1.00
Smoc2	0.13	1.00
Traf5	0.25	1.00
Prepl	0.05	1.00
Grhpr	0.05	1.00
Lmln	0.22	1.00
Clcn6	0.00	1.00
Cnot6l	0.11	1.00
1810013L24Rik	0.27	1.00
Idh3g	0.12	1.00
Rab14	-0.01	1.00
Dync2li1	0.42	1.00
Dicer1	0.34	1.00
Lpgat1	0.40	1.00

Apoe	0.47	1.00
Ocrl	0.11	1.00
Rnmt	0.09	1.00
Nup133	-0.01	1.00
P3h1	-0.09	1.00
Cdkn2d	-0.20	1.00
1810064F22Rik	-0.14	1.00
Luc7l2	-0.49	1.00
Spint2	-0.29	1.00
Canx	0.06	1.00
Terf1	-0.05	1.00
Inafm2	-0.38	1.00
Alkbh1	0.07	1.00
Ckmt1	0.21	1.00
Pgam1	0.46	1.00
0610040J01Rik	-0.16	1.00
Eif2ak3	0.32	1.00
Scaf4	0.16	1.00
Ep400	-0.15	1.00
Raly	-0.06	1.00
Pycrl	0.03	1.00
Hs6st2	0.06	1.00
Ccdc78	0.08	1.00
Rdh5	-0.08	1.00
Akip1	0.05	1.00
Dzip3	0.03	1.00
Slc25a4	0.02	1.00
Resf1	0.14	1.00
Ids	-0.06	1.00
Caml	0.27	1.00
Hmgcr	-0.30	1.00
Snhg3	0.24	1.00
Kdm5a	-0.14	1.00
Surf1	-0.37	1.00
Ndufa13	0.21	1.00
Crim1	1.01	1.00
Adamts9	-0.15	1.00
Pde4a	-0.03	1.00
Casd1	-0.01	1.00
Ankrd13a	0.09	1.00
Gzf1	0.06	1.00
Idh1	0.05	1.00
Fuca2	0.03	1.00
Ist1	0.04	1.00
Col4a3	0.68	1.00
Cry2	0.28	1.00

Eif3h	0.00	1.00
St14	0.23	1.00
Ing3	0.36	1.00
2210016F16Rik	-0.16	1.00
Ppp1cc	0.12	1.00
Cdc5l	0.21	1.00
Eif5b	-0.05	1.00
Arglu1	-0.25	1.00
Vps53	-0.01	1.00
Fbxl14	0.06	1.00
Antxr2	0.48	1.00
Cyp2b10	0.29	1.00
Snrpa	-0.07	1.00
Rtcb	0.28	1.00
Prkag1	0.02	1.00
Cltc	-0.02	1.00
Phlpp1	-0.26	1.00
Vezf1	0.00	1.00
Ccdc47	0.18	1.00
Sdhc	-0.07	1.00
Tmx2	0.11	1.00
Necap1	0.25	1.00
Tbc1d32	0.22	1.00
Znhit3	0.14	1.00
Abt1	-0.01	1.00
Cds2	0.22	1.00
Flna	0.25	1.00
Ddb2	0.09	1.00
Rffl	-0.18	1.00
Skap1	0.55	1.00
Pop7	0.30	1.00
Knop1	0.04	1.00
Slc44a2	0.31	1.00
Safb	0.24	1.00
Aff1	0.15	1.00
Dpysl2	0.34	1.00
Hp	0.93	1.00
Eif3a	0.09	1.00
Psen2	0.11	1.00
Fam234a	0.09	1.00
Yaf2	0.21	1.00
Abhd16a	0.30	1.00
Aamp	-0.06	1.00
Iyd	-0.08	1.00
Syne3	-0.06	1.00
Cfap57	-0.10	1.00

Pfn2	-0.12	1.00
Aimp2	-0.02	1.00
Naa40	-0.08	1.00
Epg5	-0.04	1.00
Arhgef40	-0.16	1.00
A930007I19Rik	-0.05	1.00
Coq5	0.18	1.00
Golga2	0.01	1.00
Ak3	0.09	1.00
Actl6a	-0.20	1.00
Rhpn2	0.07	1.00
Lbh	0.14	1.00
Cinp	-0.02	1.00
Tmem98	0.03	1.00
Slc35e2	-0.02	1.00
Trim32	0.04	1.00
Zbbx	0.06	1.00
Fam160b2	-0.02	1.00
Lrrc14	0.01	1.00
Celf1	0.29	1.00
Senp6	-0.03	1.00
Ppil4	0.31	1.00
Rufy3	0.03	1.00
Zbtb1	0.10	1.00
Kalrn	0.40	1.00
Bnip3	0.15	1.00
Nhlrc2	-0.35	1.00
Clic3	1.06	1.00
Phtf2	-0.43	1.00
Tbc1d20	0.13	1.00
Spcs2	0.40	1.00
Tob1	0.49	1.00
Cnbp	-0.64	1.00
Ciao2a	0.65	1.00
Ing4	-0.09	1.00
Pbx3	0.05	1.00
Dtnb	0.04	1.00
Flvcr1	-1.12	1.00
Osbpl5	0.30	1.00
Lama5	0.24	1.00
Dop1b	0.29	1.00
Samsn1	0.11	1.00
5031439G07Rik	0.10	1.00
Dclre1c	0.02	1.00
Gatd1	0.07	1.00
Pacs2	0.06	1.00

Iars	0.28	1.00
Coq9	0.24	1.00
Grk6	0.02	1.00
Tmem120b	0.01	1.00
Sh3glb2	0.06	1.00
Rrnad1	0.28	1.00
Dnajc4	0.17	1.00
Ckap5	-0.01	1.00
Lrig1	-0.04	1.00
Pdxk	-0.26	1.00
Tmem184a	0.23	1.00
Golph3l	0.08	1.00
Pih1d1	-0.08	1.00
Nomo1	-0.08	1.00
Carm1	0.06	1.00
Trip10	-0.07	1.00
Aifm1	0.09	1.00
Ptpn2	0.07	1.00
Clptm1l	-0.25	1.00
Efr3a	0.04	1.00
Bcas2	-0.31	1.00
Purb	0.29	1.00
Sos2	-0.18	1.00
Appl1	-0.09	1.00
Rhoa	0.17	1.00
Prdm2	0.00	1.00
Slc35a2	0.11	1.00
Uso1	0.01	1.00
Ttc7b	0.17	1.00
Rbm18	0.07	1.00
Nisch	0.22	1.00
Ccni	0.11	1.00
Epcam	0.51	1.00
Nkapd1	-0.04	1.00
Fbxw9	0.12	1.00
Cog5	0.05	1.00
Irs2	-0.07	1.00
Hsp90aa1	-0.05	1.00
Eif5a	0.10	1.00
Tmem123	-0.31	1.00
Crebbp	0.12	1.00
Gdap2	0.37	1.00
Pxylp1	-0.02	1.00
Ggt5	0.01	1.00
Ikzf4	0.08	1.00
Yju2	-0.03	1.00

Pot1a	-0.03	1.00
Pelp1	-0.04	1.00
Cdhr3	-0.02	1.00
Lmbrd2	-0.03	1.00
Blvra	0.11	1.00
Acvr2b	-0.09	1.00
Pqlc3	-0.11	1.00
Gla	-0.08	1.00
Hs3st1	0.04	1.00
Tmem129	-0.07	1.00
Dok1	-0.08	1.00
Tmprss7	-0.20	1.00
Lypla2	0.00	1.00
Ptprj	-0.15	1.00
Wipf2	0.32	1.00
Pdgfa	0.27	1.00
Trip6	0.02	1.00
Polr2g	0.19	1.00
Ndufv3	-0.11	1.00
Skap2	0.38	1.00
Ppp2r2d	0.22	1.00
Rnf130	0.34	1.00
Thbs1	-0.37	1.00
Tmem50a	0.15	1.00
Csnk1a1	0.03	1.00
Anxa3	0.42	1.00
Vamp2	0.07	1.00
Dera	0.08	1.00
Ercc6	0.13	1.00
Ccdc15	-0.16	1.00
Nudt5	0.11	1.00
Dcbld1	0.11	1.00
Vwa3a	0.32	1.00
Tmem94	0.19	1.00
Rhot2	0.14	1.00
Eef1akmt1	-0.53	1.00
Smn1	-0.07	1.00
Prrg2	-0.15	1.00
2610307P16Rik	0.46	1.00
Ubqln2	-0.01	1.00
Pag1	0.43	1.00
Aebp1	0.13	1.00
Zxdc	0.00	1.00
Rnf40	0.11	1.00
Tmem170b	0.02	1.00
Zdhhc5	0.14	1.00

Abtb2	0.64	1.00
Yap1	-0.16	1.00
Dek	0.03	1.00
Rnps1	-0.21	1.00
Gapdh	0.25	1.00
Slc12a6	0.01	1.00
Dennd1a	0.32	1.00
Hmgb3	0.11	1.00
Atg10	-0.14	1.00
Sp3os	-0.07	1.00
Gnl1	0.39	1.00
Eif4e2	0.11	1.00
Tom1l2	0.14	1.00
Syngr2	-0.13	1.00
Hipk1	0.40	1.00
2010001K21Rik	0.04	1.00
Snw1	0.02	1.00
Hars	0.48	1.00
Ubtd2	-0.20	1.00
Fzd5	0.00	1.00
Akap11	0.06	1.00
G3bp2	-0.43	1.00
Sec24a	0.18	1.00
Camk2n1	-0.22	1.00
Exosc5	-0.01	1.00
Rbbp6	0.36	1.00
Smarcb1	-0.03	1.00
Gsdme	0.01	1.00
Tma16	0.19	1.00
Echdc2	-0.01	1.00
Fzd2	0.00	1.00
Fbxl12	-0.05	1.00
9430038I01Rik	-0.01	1.00
Bcl2l2	0.01	1.00
B930095G15Rik	-0.02	1.00
Nasp	-0.12	1.00
Rerg	-0.01	1.00
Ust	-0.09	1.00
Ormdl1	-0.01	1.00
Polr3a	-0.04	1.00
Brdt	-0.07	1.00
Eif3j2	-0.02	1.00
Nup85	-0.04	1.00
Nol3	-0.08	1.00
Creb1	0.30	1.00
Snx6	-0.17	1.00

Socs6	-0.09	1.00
B4galt5	-0.12	1.00
Cnep1r1	-0.27	1.00
Nfe2l3	0.01	1.00
Slc38a10	0.13	1.00
Cul2	0.39	1.00
Tnfrsf9	0.59	1.00
Ppwd1	0.31	1.00
Acp2	0.06	1.00
Dgkg	0.06	1.00
Psmg3	0.16	1.00
Chd6	0.05	1.00
Arid4a	0.22	1.00
Plaa	0.12	1.00
Csrnp2	0.26	1.00
Dnaja4	-0.02	1.00
Tmem205	0.04	1.00
Psma5	0.54	1.00
Cpsf6	0.13	1.00
Tmed10	-0.01	1.00
Znrf1	0.17	1.00
Dhx36	0.31	1.00
Rap1gds1	-0.19	1.00
Pycard	0.44	1.00
Rnf149	0.30	1.00
Rnf187	0.11	1.00
Tcp1	-0.02	1.00
Smad4	0.18	1.00
Snrpb	0.08	1.00
Sgk1	-0.26	1.00
Cap1	0.28	1.00
Noct	0.38	1.00
Cox5b	0.39	1.00
H2-Aa	0.63	1.00
Ncor2	-0.13	1.00
Cycs	-0.01	1.00
Rbis	0.56	1.00
Rrm2b	0.21	1.00
Rab28	-0.20	1.00
Togaram1	0.60	1.00
Asna1	0.00	1.00
Stip1	-0.06	1.00
9530036O11Rik	0.35	1.00
Stk19	0.03	1.00
Cdipt	0.26	1.00
Zfp180	-0.09	1.00

Nr1d2	0.25	1.00
Prelid1	0.41	1.00
Srsf1	0.11	1.00
Selenos	0.28	1.00
Trmt11	0.01	1.00
Rbmx2	-0.02	1.00
Acer2	0.03	1.00
Abhd6	0.06	1.00
Dhrs11	0.00	1.00
Acvr1b	0.05	1.00
Dnali1	-0.06	1.00
Ccdc22	-0.02	1.00
Etfa	-0.23	1.00
Rab7	0.61	1.00
Rsph9	0.25	1.00
Sema3b	0.16	1.00
Cog7	-0.17	1.00
Bicc1	0.20	1.00
Epb41	0.15	1.00
Il10rb	0.07	1.00
Cnot11	-0.03	1.00
Klhl5	0.22	1.00
Flvcr2	-0.09	1.00
Aven	-0.01	1.00
Atg7	-0.37	1.00
Cacybp	0.08	1.00
Kdm7a	0.27	1.00
Tmem259	0.06	1.00
Lrpprc	-0.18	1.00
Slc20a2	-0.47	1.00
Blcap	0.11	1.00
Snf8	0.06	1.00
Cyp51	-0.31	1.00
Ubqln1	-0.43	1.00
4930523C07Rik	0.05	1.00
Rassf3	-0.40	1.00
Zfp800	0.14	1.00
Nek9	-0.19	1.00
Slc35e4	-0.04	1.00
Etv3	-0.08	1.00
Nek10	0.00	1.00
Rsph3b	-0.04	1.00
Ercc8	-0.02	1.00
Plch1	0.02	1.00
Limk1	0.00	1.00
Trim65	-0.02	1.00

Stk38l	0.63	1.00
Coq7	0.21	1.00
Brpf1	0.01	1.00
B9d1	0.12	1.00
Intu	0.07	1.00
Dmac1	0.09	1.00
Plb1	0.20	1.00
Itga5	-0.32	1.00
Ftx	0.09	1.00
Hmgcs1	0.25	1.00
Nectin2	0.13	1.00
Fam189a2	0.19	1.00
Zzef1	0.50	1.00
Ppp1r14b	0.41	1.00
Tecpr1	0.03	1.00
Sbno1	0.37	1.00
Syn3	0.20	1.00
Gxylt1	-0.02	1.00
Slc39a7	0.17	1.00
Pold4	0.25	1.00
Sys1	0.34	1.00
Cox4i1	0.25	1.00
Dnajc15	0.05	1.00
Bcas3	-0.48	1.00
Tmem237	-0.01	1.00
Lsmem1	-0.51	1.00
C3	0.56	1.00
Clpx	0.19	1.00
Pcnx3	-0.13	1.00
Acyp2	0.35	1.00
Ptbp2	0.12	1.00
Dock6	0.09	1.00
Iqank1	0.08	1.00
Natd1	-0.12	1.00
Leng1	-0.08	1.00
Ankrd17	-0.08	1.00
Ash1l	0.04	1.00
Bach1	0.05	1.00
Nceh1	-0.21	1.00
Bend7	0.17	1.00
Tpm3-rs7	0.27	1.00
Ubn2	0.38	1.00
Gnaq	0.08	1.00
Kif21a	-0.36	1.00
Dnajb12	-0.24	1.00
Odf2	0.24	1.00

Zyg11b	-0.01	1.00
A930015D03Rik	0.13	1.00
Nfib	0.30	1.00
Ndufv1	0.22	1.00
Babam2	-0.64	1.00
Ndufs4	-0.18	1.00
Gnpda2	0.50	1.00
Ncbp1	0.03	1.00
Polr2d	0.10	1.00
Pde12	-0.10	1.00
Zfp664	0.03	1.00
Elf3	0.47	1.00
Umad1	-0.21	1.00
4930402H24Rik	-0.09	1.00
1700123O20Rik	0.23	1.00
Fam118b	-0.02	1.00
Znhit2	0.03	1.00
Cbx6	-0.04	1.00
Smad5	-0.12	1.00
Hmgxb4	0.09	1.00
Setd1a	0.14	1.00
Spindoc	0.15	1.00
Utrn	-0.15	1.00
Ndel1	-0.23	1.00
Hmbs	0.44	1.00
Tec	-0.17	1.00
Snx27	-0.18	1.00
Kcnq1ot1	-0.16	1.00
Clybl	0.00	1.00
Invs	0.08	1.00
Slc18b1	-0.05	1.00
Uaca	-0.16	1.00
Llgl1	0.01	1.00
Il21r	0.03	1.00
Lrrc75a	-0.02	1.00
Zfp974	0.03	1.00
Zfp146	0.06	1.00
Plscr2	-0.02	1.00
Sept6	-0.05	1.00
Cdc14a	0.07	1.00
Luc7l3	0.34	1.00
Krtcap3	0.11	1.00
Usp14	0.13	1.00
Stx12	0.01	1.00
Furin	0.05	1.00
Kansl1l	-0.01	1.00

Diaph2	0.15	1.00
Coq10b	0.17	1.00
Cisd2	0.27	1.00
Nek1	-0.03	1.00
Ldha	0.10	1.00
Hsbp1	0.36	1.00
Aldh16a1	0.05	1.00
Tspan15	0.27	1.00
Btbd3	-0.17	1.00
Lgals1	0.72	1.00
Nifk	0.30	1.00
Ddit4	0.05	1.00
Dhx16	0.02	1.00
Bex1	0.25	1.00
Fam8a1	0.10	1.00
Casp3	0.15	1.00
Usp36	-0.08	1.00
Thrap3	0.02	1.00
Smco4	0.28	1.00
Atxn1	0.41	1.00
Slc7a1	0.01	1.00
Camk2g	-0.12	1.00
Vezt	0.04	1.00
Apip	-0.09	1.00
1810030O07Rik	-0.39	1.00
Reep5	0.65	1.00
Tecpr2	-0.07	1.00
Zfp64	-0.21	1.00
Zfand2a	0.08	1.00
Shkbp1	0.05	1.00
Ddx39b	-0.01	1.00
Ldlr	0.23	1.00
Wsb2	0.18	1.00
Tiparp	-0.21	1.00
F3	0.59	1.00
Taf10	-0.04	1.00
4930453N24Rik	0.12	1.00
Acbd5	-0.05	1.00
Ankrd10	0.01	1.00
Ncoa7	-0.05	1.00
Trp53inp2	-0.04	1.00
Zdhhc20	-0.12	1.00
Cdc14b	0.01	1.00
Rcc2	0.10	1.00
Dnttip2	0.38	1.00
Grip1	0.19	1.00

Parp10	0.08	1.00
Ift57	0.58	1.00
Lysmd3	-0.51	1.00
Dock5	0.20	1.00
Grasp	0.22	1.00
Tomm40	0.21	1.00
Tpd52l2	0.03	1.00
Srsf2	0.01	1.00
Brd2	0.06	1.00
Crip1	-0.13	1.00
Elp4	-0.19	1.00
Cast	0.04	1.00
Raf1	-0.10	1.00
Pttg1ip	-0.09	1.00
Cdc123	0.25	1.00
Lin52	-0.20	1.00
Tvp23b	-0.05	1.00
Rbm4b	-0.21	1.00
Idnk	0.32	1.00
Tmem38b	0.09	1.00
Gtf2h1	-0.03	1.00
Cep164	0.16	1.00
Fam117a	0.03	1.00
Ctnna1	-0.01	1.00
Washc3	0.15	1.00
Sult1a1	0.11	1.00
Pfdn2	0.25	1.00
Dsel	0.06	1.00
Rnaset2b	-0.01	1.00
Rassf6	-0.01	1.00
Jmjd7	-0.01	1.00
Nrip3	0.02	1.00
Gtf3c3	0.00	1.00
Zfp142	-0.04	1.00
Hykk	0.03	1.00
Nthl1	0.01	1.00
P2rx5	0.19	1.00
Rabep2	0.03	1.00
Eid3	-0.04	1.00
4430402I18Rik	0.00	1.00
Dap3	0.18	1.00
Endod1	-0.12	1.00
Fam171a1	-0.62	1.00
Orc3	-0.13	1.00
Cdc23	-0.08	1.00
1300002E11Rik	0.11	1.00

Tnks	0.20	1.00
Foxa1	-0.01	1.00
Slc25a28	0.15	1.00
A430005L14Rik	0.04	1.00
Alkbh3	-0.42	1.00
Prpf39	0.23	1.00
Pqlc1	0.26	1.00
Herc2	0.54	1.00
H13	-0.22	1.00
Brd4	-0.15	1.00
Hspa8	-0.18	1.00
Pmvk	0.11	1.00
Hsf1	0.13	1.00
Elp2	0.16	1.00
Cebpzos	0.06	1.00
D10Wsu102e	0.26	1.00
Atad2b	-0.16	1.00
Utp20	0.14	1.00
Sirt3	0.18	1.00
Nus1	-0.25	1.00
Akr7a5	-0.11	1.00
Naa50	0.22	1.00
Dock7	-0.24	1.00
Yipf3	0.34	1.00
Rxrb	0.20	1.00
4732440D04Rik	0.05	1.00
Spag17	-0.41	1.00
Hydin	-0.18	1.00
Aurkaip1	-0.03	1.00
Gtpbp8	0.03	1.00
Serpine3	0.19	1.00
Orc5	0.04	1.00
Asns	-0.17	1.00
Srfbp1	0.05	1.00
Socs4	0.23	1.00
Inpp5f	0.00	1.00
Cops7b	0.11	1.00
Leo1	0.04	1.00
Gpr132	-0.04	1.00
Zfp948	0.14	1.00
Esrra	-0.30	1.00
Ap1ar	0.32	1.00
Sec24b	-0.09	1.00
Pnn	0.07	1.00
Crnkl1	0.10	1.00
Txndc12	0.03	1.00

Uck2	-0.05	1.00
Chmp3	0.29	1.00
Elovl5	-0.18	1.00
Farp1	-0.18	1.00
Srek1	-0.23	1.00
Ppp4r2	-0.26	1.00
Adrm1	0.19	1.00
P3h2	0.12	1.00
Cd68	0.41	1.00
Plxna1	0.11	1.00
Hspa4	-0.38	1.00
Acad11	0.00	1.00
Klhl21	0.01	1.00
Fip1l1	-0.03	1.00
Uggt1	-0.17	1.00
Snx24	-0.04	1.00
Pnpla2	0.31	1.00
Sppl3	0.38	1.00
Itm2b	-0.09	1.00
Cox14	0.33	1.00
Serpinb8	0.00	1.00
Ccdc28a	-0.01	1.00
5730480H06Rik	0.07	1.00
Zfp367	0.02	1.00
Pigv	-0.01	1.00
Dmwd	0.00	1.00
Rbm4	-0.05	1.00
Ifngr2	0.25	1.00
Uhrf2	-0.07	1.00
Pdzd8	0.17	1.00
Usp48	0.08	1.00
Lrrc57	0.18	1.00
Lnpk	0.19	1.00
Clk4	-0.07	1.00
Klf7	0.20	1.00
Sin3a	-0.29	1.00
Tbca	-0.20	1.00
Rabgap1	0.04	1.00
Vps28	-0.32	1.00
Lsm4	0.18	1.00
Polr2l	0.18	1.00
Gnptg	0.36	1.00
Urgcp	0.15	1.00
Cep76	0.17	1.00
Tfb2m	0.12	1.00
Gpat3	0.18	1.00

Crybg1	0.20	1.00
Ap4m1	0.15	1.00
Uevld	0.05	1.00
BC065397	0.06	1.00
Smdt1	0.21	1.00
Ssh2	0.28	1.00
Cldnd1	0.22	1.00
Sft2d2	0.09	1.00
Rilpl2	0.07	1.00
Cd83	-0.57	1.00
Pdcd6	0.26	1.00
Ltn1	0.05	1.00
Ahi1	0.18	1.00
Nvl	0.04	1.00
Slc6a6	0.09	1.00
Set	0.03	1.00
Prr13	-0.43	1.00
Scfd2	-0.07	1.00
Ints7	-0.03	1.00
Tspan5	0.36	1.00
Atg16l1	-0.01	1.00
Nup88	0.09	1.00
Bola1	-0.01	1.00
Camkk2	0.18	1.00
Cops3	0.23	1.00
Cog4	-0.05	1.00
Pef1	0.09	1.00
Hdac5	0.29	1.00
Sumo1	-0.26	1.00
Eprs	-0.11	1.00
Psmd13	0.19	1.00
Cntrl	-0.02	1.00
Rtn3	0.01	1.00
Nsd3	-0.20	1.00
Arhgap39	0.03	1.00
Ccdc43	-0.51	1.00
Gpalpp1	-0.43	1.00
Tex30	0.06	1.00
Crnde	-0.02	1.00
Smc1a	0.21	1.00
Ip6k2	-0.06	1.00
Arfip2	0.11	1.00
Lgalsl	-0.03	1.00
Hivep2	0.04	1.00
Phf14	-0.04	1.00
Rnpepl1	0.13	1.00

Txndc5	0.08	1.00
Cript	0.07	1.00
Arhgef1	-0.13	1.00
Emd	-0.40	1.00
Nudt4	0.24	1.00
Ptpn14	0.15	1.00
Prpf38a	-0.02	1.00
Bet1l	0.15	1.00
Cr1l	-0.05	1.00
Kif1b	-0.39	1.00
Fmnl2	0.26	1.00
Tfe3	0.46	1.00
Selenbp1	-0.02	1.00
Tcf25	0.15	1.00
Lman2	-0.01	1.00
Tmem176b	0.32	1.00
Xrn2	-0.19	1.00
Rex1bd	-0.22	1.00
Anxa11	-0.19	1.00
Xpo4	0.06	1.00
Jag2	0.08	1.00
Shisa4	0.03	1.00
Ppp3cc	0.07	1.00
Fbxo46	0.00	1.00
Zcchc3	0.01	1.00
Apobec3	0.37	1.00
2410006H16Rik	0.05	1.00
Irx2	-0.05	1.00
Srsf6	-0.32	1.00
Ccng2	0.06	1.00
Pbx2	0.57	1.00
Rfc2	0.33	1.00
Pard6b	0.37	1.00
Vps13b	0.33	1.00
Dnajb6	0.13	1.00
Ggact	-0.10	1.00
Lyar	0.05	1.00
Dnmt1	0.05	1.00
Cox11	-0.07	1.00
Ppp2r5c	0.02	1.00
Pcnt	-0.03	1.00
Tns3	-0.25	1.00
Cuta	0.36	1.00
Tsga10	-0.02	1.00
Tmem154	0.03	1.00
Zfp687	0.05	1.00

Dand5	0.01	1.00
Cfap54	-0.02	1.00
Aplp2	-0.14	1.00
Stradb	0.06	1.00
Pcgf6	0.13	1.00
Arrdc4	0.11	1.00
Car4	0.85	1.00
Cyp4f13	0.20	1.00
Pdcd5	-0.20	1.00
Ift43	-0.10	1.00
Gnptab	0.31	1.00
Tasor	-0.14	1.00
Grn	0.05	1.00
Lamtor4	0.16	1.00
Ccdc97	0.10	1.00
Lyst	0.14	1.00
Ubn1	0.12	1.00
Arhgef2	0.18	1.00
Rnf169	0.11	1.00
Prkaca	-0.18	1.00
Phf2	-0.14	1.00
Galk2	0.10	1.00
Ube3b	-0.10	1.00
Polb	-0.27	1.00
Sugt1	-0.09	1.00
Ssu72	0.28	1.00
Kdm1a	0.18	1.00
Dnajb9	-0.27	1.00
Ppp1r9b	-0.37	1.00
Zfp511	-0.06	1.00
Wdpcp	0.28	1.00
Klhl2	-0.20	1.00
Pdzk1ip1	0.37	1.00
Lrrc41	-0.25	1.00
2810004N23Rik	-0.14	1.00
Senp2	-0.11	1.00
Nup50	-0.01	1.00
Taf4	0.15	1.00
Hgs	-0.02	1.00
Kdm3b	0.17	1.00
Dtnbp1	-0.11	1.00
Slc38a2	0.21	1.00
Slc25a12	0.16	1.00
Gpr108	0.07	1.00
Cmc1	0.02	1.00
Fkbp15	-0.05	1.00

Cdk5rap3	0.09	1.00
Kif13a	-0.01	1.00
Pdha1	0.05	1.00
Abl2	-0.20	1.00
AU040320	0.15	1.00
Kmt2d	0.27	1.00
Fnbp4	0.59	1.00
Cadm2	-0.40	1.00
Plekhm1	0.12	1.00
Pde4c	0.16	1.00
Il34	0.03	1.00
Atp11c	-0.04	1.00
Akap8l	-0.09	1.00
Ccdc107	0.13	1.00
Ccnc	0.20	1.00
Dedd2	0.04	1.00
Rhob	-0.01	1.00
Pknox1	-0.31	1.00
Bckdhb	0.01	1.00
Smarcal1	0.32	1.00
Pik3cd	0.04	1.00
4933408B17Rik	0.10	1.00
Wrnip1	-0.19	1.00
Wdr74	0.05	1.00
Pip5k1c	-0.36	1.00
Eya2	0.07	1.00
Gsr	0.73	1.00
Nipal2	0.24	1.00
Pik3ap1	0.83	1.00
Upf3a	0.03	1.00
Cic	0.19	1.00
Scpep1	-0.03	1.00
Akap7	-0.08	1.00
Fbh1	0.04	1.00
Rbm48	0.07	1.00
Pde5a	0.33	1.00
Itprip	0.07	1.00
0610043K17Rik	0.07	1.00
G930009F23Rik	0.11	1.00
Zranb1	0.03	1.00
Pacsin2	0.47	1.00
Rac3	-0.04	1.00
Timm22	0.01	1.00
Phax	0.03	1.00
Galnt13	-0.47	1.00
Hjurp	0.23	1.00

Crcp	-0.01	1.00
H2afz	-0.19	1.00
Csnk2b	0.19	1.00
Hist1h2bc	0.31	1.00
Dop1a	-0.03	1.00
Ogfr	0.34	1.00
B4galt3	0.05	1.00
Dcun1d1	-0.51	1.00
Slc25a11	-0.04	1.00
Rnf170	0.12	1.00
Ascc2	0.06	1.00
Gstp1	0.34	1.00
Eri3	0.11	1.00
Polr2k	0.09	1.00
Itsn2	0.00	1.00
Arfip1	-0.56	1.00
Usp15	-0.43	1.00
Dcaf8	0.20	1.00
1110004F10Rik	0.00	1.00
Lrrcc1	0.09	1.00
Pdcd7	-0.02	1.00
Rap1gap	0.18	1.00
Wiz	0.02	1.00
Tmem43	-0.16	1.00
Emp2	0.62	1.00
Snrpe	-0.11	1.00
Add1	-0.04	1.00
Psmd3	0.23	1.00
Tle5	-0.01	1.00
Ammecr1l	0.10	1.00
Chil1	0.62	1.00
Zcrb1	-0.14	1.00
Ubc	0.34	1.00
Dennd3	0.20	1.00
Nsrp1	0.09	1.00
Flot2	-0.03	1.00
Tnpo3	-0.08	1.00
1700094D03Rik	0.15	1.00
Dynlt1f	0.05	1.00
Sh2b2	0.00	1.00
Lzts2	0.08	1.00
Timm23	-0.44	1.00
Prps1	0.09	1.00
Fgf18	0.15	1.00
Ctse	0.41	1.00
Tmem29	0.04	1.00

Ttll5	0.57	1.00
Hmox1	-0.70	1.00
Sp1	-0.07	1.00
Nadk	0.19	1.00
Borcs5	-0.09	1.00
Topbp1	-0.09	1.00
Sh3d21	-0.14	1.00
Pcbp1	0.40	1.00
Tmbim4	0.19	1.00
Ube2d2a	0.30	1.00
Ruvbl1	0.15	1.00
Rpp14	0.01	1.00
Prex1	0.03	1.00
Nsun5	0.10	1.00
Phf13	0.03	1.00
Slc39a3	0.03	1.00
Fam151b	0.12	1.00
Sphk2	0.05	1.00
Zfp995	0.04	1.00
Brwd1	-0.25	1.00
Fh1	0.02	1.00
Ublcp1	0.10	1.00
Hdac7	0.18	1.00
Cdk17	-0.64	1.00
Cks2	0.07	1.00
Sp5	0.17	1.00
Il10	0.30	1.00
Prdx6	0.00	1.00
Ift46	-0.01	1.00
Cdk16	-0.05	1.00
Nt5c	0.08	1.00
Vps13c	0.15	1.00
Kpna6	0.08	1.00
Gpr155	-0.08	1.00
Endog	0.23	1.00
Iffo2	0.20	1.00
Socs1	0.32	1.00
Rab11a	-0.11	1.00
Gbe1	0.02	1.00
Ube4b	0.13	1.00
Pts	0.37	1.00
Selenow	0.13	1.00
Ube2f	-0.04	1.00
Insig1	0.33	1.00
Trim8	0.20	1.00
Tceal9	0.00	1.00

Ndrg1	-0.05	1.00
Zranb2	-0.80	1.00
Lrba	0.17	1.00
Khdrbs3	0.29	1.00
Chuk	0.14	1.00
Ppp1r13b	-0.19	1.00
Tbc1d19	0.27	1.00
Bloc1s2	0.09	1.00
P4ha2	0.34	1.00
Fam76b	-0.03	1.00
Lsg1	-0.30	1.00
Lactb	0.27	1.00
Pnpla6	-0.06	1.00
Clic6	0.08	1.00
Pdpr	-0.19	1.00
Selenon	0.00	1.00
Wbp4	0.03	1.00
Iqgap1	0.03	1.00
Arl6ip6	0.05	1.00
Tmem87b	-0.07	1.00
Txlna	0.18	1.00
Dstyk	0.11	1.00
Vasp	-0.06	1.00
Pias2	-0.05	1.00
Ager	0.95	1.00
Slc25a13	-0.09	1.00
Fas	-0.41	1.00
Vat1	-0.08	1.00
Tfcp2	0.07	1.00
Ruvbl2	0.04	1.00
Ppih	-0.05	1.00
Tbc1d10a	0.04	1.00
Cluap1	0.06	1.00
Tmem234	0.21	1.00
Irx5	0.00	1.00
Ppp1r15a	-0.30	1.00
Rad50	-0.05	1.00
Armh3	-0.20	1.00
Pcbd2	-0.03	1.00
Arl6	0.46	1.00
Atp7a	0.01	1.00
Smim20	0.00	1.00
Gfer	-0.25	1.00
Dnaja3	-0.16	1.00
Tmem147	0.08	1.00
Prmt8	0.05	1.00

Pxdc1	0.13	1.00
Tyw5	0.04	1.00
Ercc3	0.09	1.00
Zbtb37	0.04	1.00
Katnb1	0.14	1.00
Wdr59	0.04	1.00
Dyrk3	0.07	1.00
Pkp3	0.09	1.00
Ccdc115	0.04	1.00
Selplg	0.06	1.00
5930430L01Rik	0.03	1.00
Zfp474	0.09	1.00
Tspyl2	0.08	1.00
Ndufb3	-0.07	1.00
Dync1i2	0.18	1.00
Dcaf5	0.19	1.00
Rbm33	0.09	1.00
Sestd1	0.02	1.00
Opa3	0.17	1.00
Galc	0.09	1.00
Kdm2b	-0.32	1.00
Laptm5	0.13	1.00
Aarsd1	-0.01	1.00
Dync1li1	0.11	1.00
Atp6v0a1	-0.16	1.00
Ypel3	0.28	1.00
Tmem116	0.07	1.00
Prmt1	-0.27	1.00
Ap1s3	0.38	1.00
Gys1	0.21	1.00
Ddx46	-0.86	1.00
Ptbp3	0.24	1.00
Spen	0.09	1.00
Cebpz	0.09	1.00
Otub1	0.29	1.00
Yrdc	-0.05	1.00
Eea1	-0.22	1.00
Rnf11	0.14	1.00
Tmem9	0.22	1.00
Wdr45	0.30	1.00
Timmdc1	-0.21	1.00
U2surp	-0.10	1.00
Tsnax	-0.03	1.00
Top3b	0.03	1.00
Dnttip1	0.06	1.00
Cntln	0.03	1.00

Egr3	0.32	1.00
Btg3	0.14	1.00
Agtpbp1	-0.43	1.00
Dhx9	-0.26	1.00
Zfp407	-0.25	1.00
Ube2j2	-0.12	1.00
Caap1	-0.36	1.00
Rnaseh2c	-0.03	1.00
Uspl1	-0.01	1.00
Rmnd1	-0.44	1.00
Tfam	0.36	1.00
Rnf31	-0.01	1.00
Ttc14	0.16	1.00
Zfp706	-0.18	1.00
Slc44a1	-0.12	1.00
Wdr44	0.32	1.00
2300009A05Rik	0.01	1.00
Slc12a4	0.02	1.00
Ube2v2	0.18	1.00
Ccdc90b	-0.06	1.00
Wdr35	-0.11	1.00
Taldo1	0.18	1.00
Taf5	0.03	1.00
Rbp4	0.41	1.00
Usp28	0.12	1.00
Dpp3	0.11	1.00
App	0.09	1.00
Ints2	-0.17	1.00
Cbx3	-0.04	1.00
Wasl	0.14	1.00
Tnks2	-0.04	1.00
Jun	0.00	1.00
Hpcal1	-0.10	1.00
Fgl2	0.10	1.00
Six4	0.07	1.00
Exosc1	0.02	1.00
Rgs2	-0.01	1.00
Ippk	0.03	1.00
Cpne2	0.20	1.00
Smcr8	0.01	1.00
Whrn	0.04	1.00
Ska2	0.03	1.00
Bud13	-0.01	1.00
Thap7	0.01	1.00
Cherp	0.00	1.00
Tmbim1	0.11	1.00

Lars	-0.18	1.00
Flii	-0.32	1.00
Nr2f6	0.03	1.00
Ppp2r5e	-0.02	1.00
Ripk2	0.01	1.00
Hace1	0.08	1.00
Csrp1	0.06	1.00
Ilk	0.21	1.00
Oplah	0.06	1.00
Zfp280c	0.04	1.00
Snx2	0.42	1.00
Ptpn13	-0.34	1.00
Txndc17	-0.04	1.00
Brk1	0.16	1.00
Ankrd12	-0.01	1.00
Gatad1	-0.29	1.00
Tfg	0.27	1.00
Cdc42bpb	0.21	1.00
Hacd2	0.39	1.00
Sfn	0.29	1.00
Tmem30a	0.29	1.00
Sirt5	-0.34	1.00
Enkur	0.00	1.00
Slc35a3	0.06	1.00
Wdr63	0.09	1.00
Trmt61b	0.30	1.00
Arhgap31	0.12	1.00
Plaat3	0.03	1.00
Bag3	0.24	1.00
Etl4	0.28	1.00
Grb2	-0.02	1.00
Golga5	0.02	1.00
Copg1	0.05	1.00
Banp	0.01	1.00
Nsun4	0.10	1.00
D1Ertd622e	0.28	1.00
Anks1b	-0.09	1.00
Abcc4	-0.03	1.00
Ptp4a3	-0.04	1.00
Rusc2	-0.03	1.00
Zfyve27	-0.02	1.00
Dpf2	-0.48	1.00
Bcl2l11	0.25	1.00
Tuba1c	-0.13	1.00
Atp1a1	-0.10	1.00
Ggnbp2	-0.12	1.00

Cep162	-0.14	1.00
Atp6v0a2	0.06	1.00
Rras	0.27	1.00
Kcnk2	0.39	1.00
Atg2a	0.39	1.00
Cdk6	-0.11	1.00
Nudt1	-0.06	1.00
Ncln	-0.04	1.00
U2af1l4	-0.04	1.00
Ppp4r3b	-0.26	1.00
Gosr2	0.10	1.00
Ehf	0.07	1.00
Supt4a	0.10	1.00
Creb3	0.07	1.00
Asph	0.19	1.00
Dad1	0.27	1.00
Ppm1h	0.29	1.00
Dynlrb1	0.09	1.00
Cul4b	0.08	1.00
Ranbp10	0.21	1.00
Pacrg	0.20	1.00
Il18r1	0.04	1.00
Eif4g3	-0.06	1.00
Stx17	-0.26	1.00
Bcl2l1	-0.01	1.00
Hspb11	0.00	1.00
U2af2	0.34	1.00
Smc5	0.06	1.00
Fbxo34	0.04	1.00
Snrpd1	0.36	1.00
Cdv3	-0.15	1.00
P4hb	0.31	1.00
Cbll1	0.00	1.00
Cavin2	-0.13	1.00
Dlg5	0.04	1.00
Ppp2r3d	0.04	1.00
Tut7	-0.21	1.00
Spag6l	0.09	1.00
Zbtb24	0.02	1.00
Lrp4	0.04	1.00
Cln6	0.02	1.00
Cfap100	0.07	1.00
Apbb3	0.31	1.00
Hexdc	0.12	1.00
Proser2	0.13	1.00
Camta2	0.00	1.00

Ppp1r36	0.13	1.00
Rnf113a2	0.03	1.00
Ddx6	-0.35	1.00
Wsb1	-0.13	1.00
Eif3f	0.09	1.00
Stx5a	-0.36	1.00
Polr2m	0.47	1.00
Dynlt3	0.00	1.00
Wdr89	0.09	1.00
Nudcd2	0.07	1.00
E2f5	0.17	1.00
Fam53a	0.31	1.00
Czib	-0.02	1.00
D430042O09Rik	0.18	1.00
Arl4a	-0.03	1.00
Cox7c	-0.19	1.00
Prrc2a	0.00	1.00
Herc3	0.11	1.00
Polr2b	0.19	1.00
Arpc5l	0.43	1.00
Uchl3	0.14	1.00
Jag1	0.04	1.00
Zdhhc18	-0.30	1.00
Rac2	-0.37	1.00
Polr1a	0.09	1.00
Id1	0.31	1.00
Lyn	0.19	1.00
Dkc1	0.05	1.00
Fdx2	-0.24	1.00
Eda	0.11	1.00
Exoc3	0.10	1.00
Smim7	0.06	1.00
Epb41l4aos	0.09	1.00
1810058I24Rik	-0.12	1.00
Pgm2l1	0.10	1.00
Kctd12	0.04	1.00
Atp8b1	0.15	1.00
Clba1	0.14	1.00
Cfap77	0.37	1.00
Sarnp	0.10	1.00
Eif1a	-0.01	1.00
Eif2s1	-0.41	1.00
Dcaf1	-0.38	1.00
Lmo7	0.37	1.00
Cop1	-0.57	1.00
Zrsr2	0.12	1.00

Zwint	-0.21	1.00
Phf3	-0.04	1.00
Ttll7	-0.13	1.00
Golim4	0.02	1.00
Brd3	-0.07	1.00
Faim	-0.06	1.00
Hif1an	-0.08	1.00
Vpreb3	-0.25	1.00
Vps54	-0.29	1.00
Rabep1	-0.10	1.00
Lamp1	0.09	1.00
Dab2ip	0.28	1.00
Sparc	0.70	1.00
Ndnf	0.89	1.00
E330009J07Rik	0.00	1.00
Osbpl6	0.18	1.00
Tpd52	0.05	1.00
Pten	0.06	1.00
Aar2	0.02	1.00
Pnisr	0.23	1.00
Pmpcb	0.15	1.00
Tpp1	0.15	1.00
Zmym4	0.06	1.00
Tpr	0.26	1.00
Slc36a4	0.01	1.00
Cep44	-0.08	1.00
Osgepl1	0.06	1.00
Toe1	0.05	1.00
Zfp523	0.05	1.00
Rcn1	0.08	1.00
Acbd4	0.00	1.00
Nol12	0.00	1.00
Nfkbil1	0.03	1.00
Rin2	-0.32	1.00
Ppp1r8	0.11	1.00
2410002F23Rik	0.06	1.00
Ndufb8	0.04	1.00
Smurf2	0.05	1.00
Rab11fip2	-0.01	1.00
Eno1	-0.32	1.00
Kxd1	0.14	1.00
Xbp1	-0.11	1.00
Perp	0.16	1.00
Atat1	0.04	1.00
AW549877	0.00	1.00
Dcaf7	-0.12	1.00

Nabp2	-0.07	1.00
Wbp11	-0.24	1.00
Rnf181	0.14	1.00
Zfp53	0.00	1.00
Trim3	0.08	1.00
Sav1	-0.10	1.00
Commd7	0.03	1.00
Gorasp2	0.24	1.00
Rest	-0.09	1.00
Apoc1	-0.02	1.00
Svbp	0.04	1.00
Kdm4b	-0.03	1.00
Hist3h2a	0.07	1.00
Zfp148	0.06	1.00
Brms1l	0.04	1.00
Vrk1	0.24	1.00
Rnf168	0.07	1.00
Afdn	0.23	1.00
Scap	-0.01	1.00
Prpf31	-0.46	1.00
Pan3	-0.24	1.00
Tomm20	0.51	1.00
Arfgef2	0.29	1.00
Gprasp1	-0.06	1.00
Ncor1	0.06	1.00
Tuba4a	0.17	1.00
Cflar	0.28	1.00
B230219D22Rik	0.13	1.00
Ap2s1	-0.10	1.00
Ctso	-0.06	1.00
Nubp2	-0.06	1.00
Zfp335	-0.03	1.00
Zfp942	-0.03	1.00
Snx13	0.56	1.00
Rc3h2	-0.34	1.00
Casc1	-0.16	1.00
Sdhaf4	0.30	1.00
Taf7	0.04	1.00
Nr4a3	-0.42	1.00
Shroom4	0.28	1.00
Fbxo33	0.01	1.00
2610528J11Rik	-0.01	1.00
Vrk3	0.03	1.00
Atp6v1e1	-0.32	1.00
Rp9	0.17	1.00
Stau1	-0.31	1.00

Pcbp2	0.02	1.00
Nkx2-1	0.14	1.00
Slc9a3r2	-0.08	1.00
Psmd12	0.18	1.00
Prrc2c	-0.09	1.00
Katnbl1	0.04	1.00
Gtdc1	0.07	1.00
Arhgap26	0.34	1.00
Samd4b	0.09	1.00
Efhd2	-0.11	1.00
Pacs1	0.41	1.00
Faap20	0.00	1.00
Yipf2	0.03	1.00
Renbp	-0.10	1.00
Eif2a	-0.29	1.00
Pwwp2a	0.07	1.00
Chchd2	0.04	1.00
Znhit1	0.13	1.00
Senp5	-0.04	1.00
Pls3	0.39	1.00
Lonrf3	0.37	1.00
Scaf1	-0.14	1.00
Cyld	-0.23	1.00
Adh5	0.02	1.00
Nars	0.39	1.00
Cfap44	0.16	1.00
Zbtb14	0.01	1.00
Elovl6	0.09	1.00
Zfp507	-0.06	1.00
Lrrc71	0.08	1.00
Zfp426	0.02	1.00
Zfp472	-0.19	1.00
Sp3	0.16	1.00
Ubr7	0.09	1.00
Eloa	-0.12	1.00
Erlin2	0.03	1.00
Zfp212	0.03	1.00
Zfp260	-0.06	1.00
Rassf9	-0.01	1.00
Aacs	-0.02	1.00
Ints11	-0.01	1.00
Pgd	0.19	1.00
Ube2v1	0.39	1.00
Fastk	-0.45	1.00
Ctdspl	0.38	1.00
Ppat	0.05	1.00

Rbpms	0.15	1.00
Fmr1	-0.42	1.00
Flnb	-0.18	1.00
Nrip1	0.59	1.00
Hipk2	-0.12	1.00
Ppp2r2a	0.14	1.00
Stxbp5	0.16	1.00
Agpat2	0.32	1.00
Hltf	-0.01	1.00
Yipf1	0.08	1.00
Pole3	-0.39	1.00
Washc1	-0.41	1.00
Zfp384	0.01	1.00
Lsm7	-0.05	1.00
Rarres2	-0.15	1.00
Rpp25l	-0.02	1.00
Adcy6	-0.02	1.00
Gpr160	-0.04	1.00
Slc39a11	-0.05	1.00
Klhl12	0.07	1.00
Aen	-0.04	1.00
Erp44	0.08	1.00
Clptm1	0.16	1.00
Pik3cb	0.02	1.00
Ttn	-0.18	1.00
Lrrfip1	-0.06	1.00
Ift88	0.50	1.00
Vamp4	-0.20	1.00
Cluh	0.21	1.00
Rara	0.05	1.00
Upf3b	0.28	1.00
Higd1a	-0.02	1.00
Ston2	-0.25	1.00
Becn1	0.15	1.00
Pfdn6	-0.21	1.00
Xdh	0.08	1.00
Arih2	-0.25	1.00
Bcap29	-0.02	1.00
Dtx3	0.05	1.00
Sp4	0.15	1.00
Il13ra1	0.13	1.00
Dcaf12	0.29	1.00
Tmem189	0.05	1.00
Golph3	-0.40	1.00
Atp2c1	-0.02	1.00
Ctnnd2	0.17	1.00

Zc3h7b	0.10	1.00
Tmem167b	0.10	1.00
Rmc1	0.07	1.00
Ccdc167	0.01	1.00
Ier5l	-0.08	1.00
Hint2	-0.10	1.00
Slc41a2	-0.12	1.00
0610010F05Rik	0.10	1.00
Bcl2	0.07	1.00
Slc25a38	-0.02	1.00
Ankra2	-0.02	1.00
Rfx3	0.34	1.00
Fosl2	0.05	1.00
Elmod1	0.00	1.00
Ugt8a	0.15	1.00
Atp2b4	-0.45	1.00
Fzr1	-0.05	1.00
Prkcg	0.01	1.00
Stmnd1	0.03	1.00
Ppp1r12a	0.20	1.00
Ndufa9	0.11	1.00
Pmp22	0.06	1.00
Cacna1c	-0.07	1.00
Baz1b	0.03	1.00
Cry1	0.05	1.00
Dmtf1	0.08	1.00
Pdss2	-0.05	1.00
Dido1	0.18	1.00
Ppil2	0.25	1.00
Paf1	0.22	1.00
Washc4	0.61	1.00
Fam13a	0.24	1.00
Nop14	-0.10	1.00
Egr2	-0.03	1.00
Atg101	0.25	1.00
Rab21	0.08	1.00
Atf2	-0.23	1.00
Dnajb1	0.30	1.00
Dynlrb2	0.11	1.00
Eogt	0.05	1.00
Ets2	-0.07	1.00
Slc25a3	0.12	1.00
Tsn	-0.26	1.00
Gtpbp2	0.26	1.00
Rab40c	0.06	1.00
Cep135	0.02	1.00

Rbm3	-0.15	1.00
Prrc1	0.16	1.00
Abcd4	-0.03	1.00
Rgs11	-0.01	1.00
Atp13a1	-0.03	1.00
Tufm	0.02	1.00
Unc13b	0.00	1.00
Casz1	-0.16	1.00
Acbd3	0.13	1.00
Efnb1	-0.40	1.00
Nabp1	-0.39	1.00
Arl2	0.68	1.00
Rexo2	0.38	1.00
Ccdc50	0.03	1.00
Arhgef6	-0.13	1.00
Azin1	-0.07	1.00
Tmem33	0.01	1.00
C2cd4b	0.29	1.00
Ksr1	-0.16	1.00
T2	-0.16	1.00
H2afy	0.13	1.00
Etf1	0.01	1.00
Cnot2	0.08	1.00
Uqcr11	0.14	1.00
Xrcc5	0.19	1.00
Kank3	-0.03	1.00
Epb41l1	0.18	1.00
Vdac3	0.27	1.00
Rcbtb1	0.17	1.00
Acyp1	0.07	1.00
Ninj1	0.05	1.00
Fem1a	0.01	1.00
Ireb2	0.00	1.00
Tgs1	0.20	1.00
Usp54	0.08	1.00
Prkab1	0.05	1.00
Nsmce1	-0.08	1.00
Atp6v0e	-0.15	1.00
Blzf1	0.71	1.00
Kcnq5	0.70	1.00
Sept8	0.16	1.00
Arel1	0.06	1.00
Usp49	0.31	1.00
Akr1b10	0.23	1.00
Nsmce2	0.02	1.00
Lars2	0.27	1.00

Esrp1	0.05	1.00
Rprd1a	0.11	1.00
Fkbp9	0.29	1.00
Tbc1d22a	0.13	1.00
Cct5	0.36	1.00
Nras	-0.32	1.00
Zfand6	-0.05	1.00
Snapc3	-0.05	1.00
Ctnna3	-0.72	1.00
Nkiras2	-0.02	1.00
Ttc36	0.02	1.00
Ctla2a	-0.25	1.00
Nub1	0.23	1.00
Aco2	0.17	1.00
Gng12	0.12	1.00
Vcpip1	-0.02	1.00
Grcc10	0.35	1.00
Rexo1	0.00	1.00
Gls	-0.11	1.00
Lcorl	0.46	1.00
Hps4	0.06	1.00
Taf1d	0.24	1.00
Trib2	-0.02	1.00
Wdr12	-0.02	1.00
Dexi	-0.07	1.00
Paqr7	0.00	1.00
Pomp	-0.35	1.00
Plpbp	0.09	1.00
Ube2b	0.35	1.00
Cnot1	-0.25	1.00
Katna1	-0.02	1.00
Plekhb2	0.03	1.00
Cpd	0.41	1.00
Borcs8	0.03	1.00
Sod2	-0.12	1.00
Sh3d19	-0.35	1.00
Tbc1d23	0.04	1.00
Emc2	0.01	1.00
Akirin1	0.15	1.00
Wfdc2	-0.02	1.00
Hnrnpk	-0.20	1.00
Ccdc25	0.06	1.00
Cdkal1	0.30	1.00
Erp29	-0.15	1.00
Gse1	0.31	1.00
Slc4a7	0.07	1.00

Hmgb2	-0.21	1.00
Pcsk5	-0.10	1.00
Fxr2	-0.08	1.00
Rsrc1	0.24	1.00
Rsl1d1	-0.16	1.00
Rabif	0.01	1.00
Ino80e	0.04	1.00
Ccdc146	0.10	1.00
Kctd20	0.03	1.00
Arap1	0.03	1.00
Trappc10	0.04	1.00
Gpatch8	0.13	1.00
Hacd3	-0.03	1.00
C1qbp	0.24	1.00
Ilf2	0.02	1.00
Sco2	0.19	1.00
Itga1	0.08	1.00
Ubxn8	0.06	1.00
Syne1	0.27	1.00
Thumpd3	0.27	1.00
Cramp1l	0.15	1.00
Phactr2	-0.12	1.00
Sgce	0.21	1.00
Fcer1g	0.73	1.00
Pafah1b1	0.10	1.00
Efcab14	0.25	1.00
Sppl2a	-0.06	1.00
Phka1	0.07	1.00
Txlng	-0.07	1.00
Chordc1	-0.05	1.00
Slc39a1	0.03	1.00
Anapc5	-0.07	1.00
Ctbs	-0.03	1.00
Lrrc45	-0.03	1.00
Ash2l	0.05	1.00
Atp5g3	0.25	1.00
Hspa4l	0.38	1.00
Tsen34	0.24	1.00
Patj	0.06	1.00
Sypl	0.04	1.00
Cox5a	0.14	1.00
1110059G10Rik	-0.04	1.00
Cc2d1b	0.01	1.00
Fam131a	0.00	1.00
Emc1	0.07	1.00
Cavin3	-0.09	1.00

Park7	0.15	1.00
Odc1	0.08	1.00
Dnajc21	-0.01	1.00
Xist	-0.04	1.00
Ranbp9	0.19	1.00
Prr15l	-0.03	1.00
Ankrd54	0.34	1.00
Nans	-0.04	1.00
Sec23a	0.15	1.00
Plek	0.15	1.00
Ddx20	0.03	1.00
Thoc3	-0.04	1.00
Gabpb2	-0.05	1.00
Epb41l4b	0.20	1.00
Oxr1	0.37	1.00
Tc2n	-0.30	1.00
Inpp5a	-0.15	1.00
Wdr92	-0.12	1.00
Kif27	0.22	1.00
Nlgn2	-0.08	1.00
Thoc5	-0.04	1.00
Rufy1	-0.08	1.00
Tox4	-0.26	1.00
Plk3	0.19	1.00
Herc1	-0.33	1.00
Bccip	0.39	1.00
Emsy	-0.27	1.00
Synj2	-0.01	1.00
Scarf2	0.28	1.00
Tmtc3	0.09	1.00
Dusp12	0.12	1.00
Fbxl2	0.13	1.00
Dip2a	0.11	1.00
Tceanc2	0.07	1.00
Adpgk	0.05	1.00
Utp4	0.08	1.00
Tgfbi	0.09	1.00
Tsr2	0.01	1.00
Htatsf1	-0.02	1.00
Pin1	0.06	1.00
Paics	0.34	1.00
Glrx2	0.00	1.00
Wwtr1	0.11	1.00
Rab5if	0.23	1.00
Rnf167	-0.22	1.00
Slc10a7	-0.01	1.00

Axin1	0.01	1.00
Chd2	0.10	1.00
Atg2b	0.06	1.00
Pex19	0.05	1.00
Tia1	-0.11	1.00
Bri3	-0.09	1.00
Atrn	-0.27	1.00
Ankrd11	0.01	1.00
Id3	0.04	1.00
Phyh	0.04	1.00
Shld1	-0.48	1.00
Slc30a6	-0.06	1.00
Ogdh	-0.04	1.00
Ltv1	-0.41	1.00
Gspt1	0.45	1.00
Tusc3	0.39	1.00
Adipor1	0.04	1.00
Ccar1	0.06	1.00
Sh3kbp1	0.01	1.00
Gramd4	-0.02	1.00
Sigirr	0.04	1.00
Cyfip2	-0.11	1.00
Snrnp35	0.00	1.00
Nab2	0.00	1.00
Txk	0.16	1.00
Stpg4	0.09	1.00
Kpna4	-0.05	1.00
Kbtbd2	0.29	1.00
Rnf141	0.06	1.00
Smarcd2	-0.08	1.00
Dsp	0.32	1.00
Plekhm3	0.09	1.00
Fdft1	0.12	1.00
Nme2	-0.02	1.00
Numb	-0.21	1.00
Aggf1	0.07	1.00
Wwc1	-0.07	1.00
Stxbp3	0.22	1.00
Sesn1	-0.05	1.00
Lgals8	-0.11	1.00
Srxn1	0.71	1.00
Tmem250-ps	0.01	1.00
Ptges2	0.03	1.00
Cct2	0.39	1.00
Brd7	0.36	1.00
Rpn2	-0.03	1.00

Fbxl8	-0.10	1.00
B3gat3	-0.37	1.00
Suds3	0.14	1.00
Chm	-0.23	1.00
Foxo3	-0.02	1.00
Dapk2	0.13	1.00
Rabgef1	0.19	1.00
Ppp4r3a	0.03	1.00
Eps15	-0.20	1.00
Plec	0.13	1.00
Kmt5b	0.16	1.00
Ntpcr	-0.01	1.00
Chn1	-0.06	1.00
Gfra1	-0.77	1.00
Spout1	-0.54	1.00
Cd55b	-0.04	1.00
Xab2	-0.55	1.00
Apoo	0.15	1.00
Ppil6	0.23	1.00
Ephb3	0.07	1.00
Ccpg1os	0.13	1.00
Arl5c	0.09	1.00
Nsd1	-0.01	1.00
Glce	0.17	1.00
Ergic1	-0.09	1.00
Psmd5	-0.01	1.00
Phb	0.06	1.00
Eid1	0.53	1.00
Zbtb11	-0.07	1.00
Ucp2	0.04	1.00
Bcl9l	-0.12	1.00
Ciapin1	-0.09	1.00
Sumo3	0.01	1.00
Sh3gl1	-0.01	1.00
Srprb	-0.04	1.00
Imp3	0.11	1.00
Ubr2	-0.01	1.00
Taf15	0.78	1.00
Ubxn2b	0.10	1.00
Elf1	0.01	1.00
Fbxw11	0.26	1.00
Copb2	-0.24	1.00
Banf1	0.05	1.00
Utp6	0.12	1.00
Adgrl1	0.01	1.00
Fars2	0.16	1.00

Galnt11	-0.01	1.00
Ccnk	0.00	1.00
Impdh2	0.07	1.00
Fat1	-0.02	1.00
Naa15	0.04	1.00
Trp53	-0.34	1.00
Gtf2b	0.06	1.00
Trim35	0.02	1.00
Cox19	-0.15	1.00
Cfap126	0.04	1.00
Timm29	-0.01	1.00
Eif1ad	0.00	1.00
Zyx	0.01	1.00
Haus2	0.04	1.00
Coq10a	-0.16	1.00
Bbs2	-0.06	1.00
Npr1	-0.01	1.00
Ssx2ip	0.07	1.00
Spg7	-0.09	1.00
Actr1b	0.03	1.00
Cfap69	0.66	1.00
Sipa1l1	-0.01	1.00
Ip6k1	-0.17	1.00
Apool	0.16	1.00
Sf3b2	0.05	1.00
Dennd5a	0.07	1.00
Uqcrfs1	-0.09	1.00
Prep	0.48	1.00
Asap1	0.20	1.00
Entpd5	0.09	1.00
Egln2	0.17	1.00
Ap3m1	0.09	1.00
Atp6v1a	0.01	1.00
Lemd2	-0.17	1.00
Anxa6	0.07	1.00
Piezo1	0.34	1.00
Bcor	0.18	1.00
Pip4p2	0.15	1.00
Nkap	0.01	1.00
Tatdn2	-0.03	1.00
Cblc	0.03	1.00
Traf3ip2	-0.02	1.00
Elf2	-0.09	1.00
Trak2	0.03	1.00
Pitpnm2	0.04	1.00
Tmem168	0.05	1.00

Gas8	0.19	1.00
Dnajc11	0.18	1.00
Trit1	0.10	1.00
Zfp560	0.09	1.00
Carmil1	0.03	1.00
Vegfa	1.06	1.00
Rngtt	-0.66	1.00
Ankzf1	-0.02	1.00
Coro7	-0.06	1.00
Coq4	0.00	1.00
Fahd2a	0.01	1.00
Pdcd2	-0.02	1.00
Pclo	0.09	1.00
Ttll3	-0.24	1.00
Prr18	-0.01	1.00
Tnip2	-0.04	1.00
Tmco4	0.09	1.00
Rasal2	0.19	1.00
Stard8	0.13	1.00
Ndufa11	0.35	1.00
St3gal6	0.00	1.00
Tcof1	0.37	1.00
Clic4	-0.53	1.00
Inpp4a	-0.25	1.00
Pygb	0.03	1.00
Aip	0.19	1.00
Ccdc88a	0.18	1.00
Amd1	0.17	1.00
Dpm3	-0.12	1.00
Eif3k	-0.09	1.00
Uqcr10	0.07	1.00
Stx8	0.02	1.00
Traf7	0.12	1.00
Lat2	-0.06	1.00
Csf2	-0.10	1.00
Bik	-0.15	1.00
Psmc3	-0.15	1.00
Akap9	-0.20	1.00
Armc1	0.07	1.00
Plekha2	-0.01	1.00
Ttc23	0.01	1.00
Calu	0.13	1.00
Nudc	0.09	1.00
Ncoa6	-0.39	1.00
Pik3r3	0.06	1.00
Foxj3	0.05	1.00

Golgb1	0.13	1.00
Armc2	-0.24	1.00
Kif6	-0.20	1.00
Ccdc149	-0.01	1.00
Erbb2	0.29	1.00
Kat5	0.10	1.00
Ankfy1	-0.06	1.00
Arnt	0.05	1.00
Ndufv2	0.01	1.00
Acbd6	-0.08	1.00
Frmd4b	0.37	1.00
Irak1	0.42	1.00
Pfkp	0.05	1.00
Eipr1	-0.10	1.00
Etfdh	-0.01	1.00
Cgrrf1	0.13	1.00
Qsox1	0.04	1.00
Clk3	-0.25	1.00
Ap1m2	-0.01	1.00
Prr3	0.05	1.00
Ywhag	0.06	1.00
Larp4b	0.14	1.00
Atp5c1	0.14	1.00
Tpm1	0.09	1.00
Rnf115	0.04	1.00
Pin4	0.06	1.00
Pls1	-0.04	1.00
Rtf1	0.35	1.00
Nfkbiz	-0.08	1.00
Irak1bp1	0.06	1.00
Pmm2	0.02	1.00
Cux2	0.03	1.00
Ubash3b	0.14	1.00
Iqsec2	0.04	1.00
Ndufa12	0.25	1.00
Yes1	-0.25	1.00
Cnot4	-0.42	1.00
Prpf40a	-0.07	1.00
Enox2	-0.13	1.00
Cnppd1	0.26	1.00
Nol11	0.25	1.00
Ago4	0.05	1.00
Atf3	-0.11	1.00
Cpq	-0.18	1.00
Klf2	0.20	1.00
Ifnar1	0.29	1.00

Rbm17	0.00	1.00
Btrc	0.14	1.00
Adrb2	-0.29	1.00
Foxp4	0.17	1.00
Spry2	0.23	1.00
Itch	0.04	1.00
Fbxo25	-0.03	1.00
Taf1a	0.02	1.00
Cep128	-0.16	1.00
Pop4	-0.06	1.00
Slc8b1	0.02	1.00
Svip	-0.07	1.00
Iqcn	-0.04	1.00
Fam120c	0.06	1.00
Uri1	0.14	1.00
Baz1a	0.33	1.00
Bahcc1	-0.06	1.00
Wdr5	-0.39	1.00
Erg28	0.28	1.00
Ywhab	-0.59	1.00
Taf13	0.07	1.00
Fgfr1op2	0.31	1.00
1810026B05Rik	-0.15	1.00
Capza2	-0.11	1.00
Irf3	0.08	1.00
Dvl1	0.02	1.00
Tex264	0.18	1.00
Pex3	0.10	1.00
St5	0.02	1.00
Ppp2r5a	0.18	1.00
Smim15	-0.07	1.00
Rtn2	0.12	1.00
Arrdc3	-0.43	1.00
Piga	0.04	1.00
Il11ra1	0.13	1.00
Snrk	0.48	1.00
Ptprk	0.33	1.00
Shroom2	-0.07	1.00
Osbpl1a	0.09	1.00
Pdap1	0.09	1.00
Kdm5b	0.26	1.00
Cds1	0.25	1.00
Stard9	-0.10	1.00
Chd3	0.23	1.00
Slc12a7	-0.37	1.00
Agpat5	-0.02	1.00

Bfar	0.09	1.00
Pfdn4	-0.08	1.00
Diablo	0.01	1.00
Ndufb5	-0.12	1.00
Pdk3	0.06	1.00
Syt7	0.34	1.00
Greb1l	-0.36	1.00
Fam214a	0.08	1.00
Sgta	-0.21	1.00
Rsu1	-0.14	1.00
Cav1	0.37	1.00
Crkl	-0.04	1.00
Ndufaf3	0.22	1.00
Bag5	-0.58	1.00
Nuak2	-0.25	1.00
Gak	0.33	1.00
Zcchc14	0.01	1.00
Ntan1	-0.09	1.00
Atxn7l1	0.24	1.00
Spryd7	0.06	1.00
Phf6	-0.01	1.00
Plekhh1	0.13	1.00
Prorp	0.08	1.00
Cfap47	0.12	1.00
Dnajc18	0.07	1.00
Fam172a	-0.36	1.00
Ppfibp2	0.17	1.00
Fam204a	0.25	1.00
Vps26a	-0.03	1.00
Btbd1	0.00	1.00
Stk38	-0.15	1.00
Tnks1bp1	0.15	1.00
Thap12	0.26	1.00
Usp45	-0.04	1.00
Nudt14	-0.20	1.00
Orc6	-0.27	1.00
Plscr3	-0.02	1.00
Dlgap1	-0.49	1.00
Dnase1l1	-0.04	1.00
Tollip	-0.05	1.00
Kif13b	-0.15	1.00
Tom1l1	0.18	1.00
LTO1	-0.04	1.00
Arvcf	-0.06	1.00
Kctd2	-0.02	1.00
Pou6f1	-0.05	1.00

1700013F07Rik	0.01	1.00
Spa17	-0.10	1.00
4933406C10Rik	-0.01	1.00
Cfap298	0.22	1.00
Ptpra	0.38	1.00
Capns1	0.19	1.00
Airn	0.15	1.00
Nfx1	-0.03	1.00
C2cd2	0.04	1.00
S100a4	0.33	1.00
Abhd4	0.18	1.00
Vars	0.03	1.00
Unc50	0.19	1.00
Endov	0.00	1.00
Rad21	0.18	1.00
Hnrnph1	0.27	1.00
Nop56	0.14	1.00
Ophn1	0.24	1.00
Tm9sf2	0.47	1.00
Ece1	0.21	1.00
Sik1	0.04	1.00
Catspere2	0.05	1.00
Edrf1	-0.03	1.00
Nosip	-0.04	1.00
Kin	-0.33	1.00
Slc48a1	0.31	1.00
Ncoa5	0.00	1.00
Unc119b	0.08	1.00
Hs2st1	0.74	1.00
Arid1a	-0.11	1.00
Atf7ip	-0.07	1.00
Gns	0.23	1.00
Ola1	-0.07	1.00
Osbp	0.05	1.00
Eif3j1	-0.08	1.00
Srp9	0.12	1.00
Tmem256	0.24	1.00
Trim56	0.10	1.00
Akr1b3	0.15	1.00
Acadsb	0.14	1.00
Kifap3	0.28	1.00
Dbndd2	0.08	1.00
Srrt	0.14	1.00
Zswim6	-0.11	1.00
Snx25	-0.23	1.00
Kmt2a	0.19	1.00

Scaf11	0.05	1.00
Smc4	0.33	1.00
Srp68	0.08	1.00
A330023F24Rik	0.08	1.00
Rpf1	0.05	1.00
Ccdc171	0.04	1.00
Wdr11	0.26	1.00
Cbr3	0.00	1.00
Homer1	0.46	1.00
F2r	0.36	1.00
Gan	-0.05	1.00
Plekhj1	0.10	1.00
Uba1	0.09	1.00
AU021092	0.38	1.00
Psma1	0.13	1.00
Smad1	-0.06	1.00
Slc25a10	-0.04	1.00
Por	0.33	1.00
Srbd1	0.01	1.00
Slc9a6	-0.16	1.00
Fktn	0.02	1.00
Smim4	-0.26	1.00
Nae1	0.04	1.00
Ankrd37	-0.03	1.00
Dnah3	-0.32	1.00
Apopt1	-0.04	1.00
Wdr46	-0.05	1.00
Chmp2a	0.31	1.00
Top1	-0.12	1.00
Parl	-0.08	1.00
Selenof	0.25	1.00
Fbxo9	-0.04	1.00
Nipbl	0.29	1.00
Adam10	-0.14	1.00
Scmh1	0.21	1.00
Cracr2a	-0.01	1.00
0610030E20Rik	0.04	1.00
Epha4	-0.23	1.00
Pvr	-0.08	1.00
E130309D02Rik	-0.03	1.00
Slc25a44	0.04	1.00
Rgmb	0.03	1.00
Tapt1	0.11	1.00
Ctr9	0.59	1.00
Fzd3	0.13	1.00
Ccp110	0.20	1.00

Tgfb1	0.23	1.00
Nacc1	0.03	1.00
Rnf217	0.20	1.00
Ppm1d	0.00	1.00
Atp5f1	-0.09	1.00
Nup54	0.04	1.00
Tra2b	0.06	1.00
Snrnp27	0.06	1.00
Borcs6	0.11	1.00
Cars	0.09	1.00
Tox3	0.06	1.00
Rhbdf2	-0.50	1.00
Rnf19a	-0.12	1.00
Atp5l	0.07	1.00
Slc25a5	-0.13	1.00
Strbp	0.24	1.00
Galk1	-0.13	1.00
Khsrp	0.40	1.00
Dhx8	-0.09	1.00
Fgfr1op	0.08	1.00
Nfxl1	-0.45	1.00
Ppp1r37	0.04	1.00
Ndufb7	0.22	1.00
Larp1	0.34	1.00
Rab5c	0.03	1.00
Ybx1	-0.13	1.00
Itgb3bp	0.16	1.00
Pithd1	0.14	1.00
Tbc1d9b	-0.08	1.00
Dynll2	-0.01	1.00
Parp4	-0.35	1.00
Drc3	0.02	1.00
Tmc6	0.03	1.00
Rnf126	-0.01	1.00
Zfp36l1	0.14	1.00
Xylt1	-0.22	1.00
Sdc1	0.09	1.00
B4galt1	0.04	1.00
Klhl18	-0.01	1.00
Fbxl18	-0.16	1.00
Krt80	0.13	1.00
Dpy30	0.06	1.00
Smad3	-0.06	1.00
Cpne1	0.10	1.00
Exoc6b	0.11	1.00
Csnk1g3	0.04	1.00

Cnot10	0.45	1.00
Ints12	-0.02	1.00
Vps33a	0.02	1.00
Socs5	0.17	1.00
Dctn6	0.10	1.00
Tor1b	0.19	1.00
Pnp	0.29	1.00
Cpeb2	0.31	1.00
Ext2	0.10	1.00
Chd4	0.08	1.00
Fem1b	0.33	1.00
Cep170b	0.10	1.00
Prrc2b	-0.17	1.00
Btf3	0.02	1.00
Kdm2a	0.03	1.00
Prdm15	0.20	1.00
Fstl1	-0.08	1.00
Timm21	0.04	1.00
Itgb5	-0.05	1.00
Atpaf1	-0.48	1.00
Nek4	-0.02	1.00
Nudt19	0.16	1.00
Snhg4	0.00	1.00
Lsm10	0.01	1.00
Zmat4	0.02	1.00
Exoc2	0.18	1.00
Ssna1	0.04	1.00
Ube2m	0.16	1.00
Ppp6r2	0.12	1.00
Taf3	-0.37	1.00
AI661453	-0.16	1.00
Commd8	0.27	1.00
Snrpb2	0.09	1.00
Tmem183a	-0.37	1.00
Ubap2l	-0.22	1.00
Cipc	-0.03	1.00
Lsm6	-0.08	1.00
Zfhx3	0.11	1.00
Fam122a	0.29	1.00
Flot1	0.28	1.00
Zfp236	-0.11	1.00
5330438D12Rik	-0.10	1.00
St13	-0.23	1.00
Ogt	0.10	1.00
Asxl1	0.14	1.00
Coa3	-0.05	1.00

Cemip2	-0.71	1.00
Anp32b	-0.09	1.00
Gps2	0.06	1.00
Zfyve1	0.16	1.00
Borcs7	0.49	1.00
Fahd1	0.03	1.00
Scarb1	-0.10	1.00
Usp42	0.08	1.00
Auh	0.33	1.00
Elp5	0.27	1.00
Sdhb	0.05	1.00
D5Ertd579e	0.25	1.00
Arhgap1	0.06	1.00
Zfp318	-0.07	1.00
Rap2c	0.05	1.00
Spata1	-0.09	1.00
Arid2	-0.17	1.00
Ryk	0.14	1.00
Inppl1	0.26	1.00
Babam1	-0.41	1.00
Ndufa10	0.49	1.00
Cox8a	0.01	1.00
Fig4	0.06	1.00
Spp1	0.16	1.00
Dohh	0.08	1.00
Slc35a4	0.25	1.00
Ak9	-0.07	1.00
Cog3	-0.01	1.00
Ppil3	-0.16	1.00
Edem3	0.18	1.00
Nsun2	0.02	1.00
Exosc4	0.00	1.00
Stard3	0.17	1.00
Bcl2a1b	-0.30	1.00
Cwc22	0.04	1.00
Gtf2f2	0.21	1.00
Ric1	0.28	1.00
Hint3	-0.07	1.00
Ncbp3	0.07	1.00
Dcxr	-0.08	1.00
Commd3	-0.35	1.00
Srrm2	-0.03	1.00
Zfp277	-0.23	1.00
Sf3b3	0.00	1.00
Ddx10	0.03	1.00
Lage3	0.03	1.00

Tmem258	-0.05	1.00
Rab18	0.14	1.00
Atf1	0.25	1.00
Jade1	0.03	1.00
Oxa1l	0.35	1.00
Casp6	0.00	1.00
Evl	-0.09	1.00
Smim26	-0.16	1.00
Sdha	0.12	1.00
Rabepk	-0.06	1.00
Clk2	-0.13	1.00
Tcea1	0.07	1.00
Eloc	0.14	1.00
Hnrnpd	-0.35	1.00
Atraid	-0.17	1.00
Btbd9	0.05	1.00
Gata6	0.34	1.00
Dmxl1	0.31	1.00
Got2	0.13	1.00
Tbc1d15	-0.15	1.00
9930021J03Rik	-0.44	1.00
Kcnip4	0.00	1.00
Bicdl1	0.05	1.00
Ndufb4	0.24	1.00
Arsk	0.03	1.00
Abtb1	0.20	1.00
Actr8	0.01	1.00
Tsr3	-0.39	1.00
Slc52a3	0.00	1.00
Zfp444	0.06	1.00
H3f3a	-0.18	1.00
Fth1	0.03	1.00
Rtkn	0.04	1.00
Zmiz2	0.20	1.00
Zfp451	0.04	1.00
Rnpep	-0.32	1.00
Psmd4	0.19	1.00
Shb	-0.26	1.00
Gtpbp6	-0.06	1.00
Rnf166	-0.36	1.00
Sf3b4	0.07	1.00
Klhdc10	0.00	1.00
Gfm1	0.04	1.00
Arhgap32	0.13	1.00
Sipa1l2	0.19	1.00
Vwa5a	-0.36	1.00

Dnajc1	-0.07	1.00
Gtf2a1	-0.10	1.00
Zdhhc17	-0.41	1.00
Rpa1	0.06	1.00
Dnajc25	-0.02	1.00
Ktn1	-0.25	1.00
Naa25	-0.01	1.00
Cep95	-0.26	1.00
Btaf1	-0.06	1.00
Fam193a	0.09	1.00
Ccdc58	-0.49	1.00
Lrrc8a	0.00	1.00
Celsr1	-0.04	1.00
Nxf1	-0.35	1.00
Ralbp1	0.12	1.00
Serbp1	-0.08	1.00
Pabpc1	-0.07	1.00
Ttc7	0.08	1.00
Tmem248	0.06	1.00
Aldh6a1	0.05	1.00
H2-Ke6	0.16	1.00
Uap1	0.00	1.00
5031425E22Rik	0.01	1.00
Ago2	0.07	1.00
Asah1	-0.10	1.00
Kif16b	-0.22	1.00
Pik3r1	0.20	1.00
Ndufs7	0.00	1.00
Dnm1l	-0.36	1.00
Serpine2	0.20	1.00
Sorbs1	-0.32	1.00
Capzb	0.02	1.00
Ubxn7	0.26	1.00
Fam53b	0.24	1.00
Ppp2cb	0.07	1.00
Sec14l1	0.20	1.00
Ndufa7	0.07	1.00
Klf10	0.02	1.00
Dctn4	0.04	1.00
Rsbn1	0.35	1.00
Ints1	0.04	1.00
Ptpn1	-0.33	1.00
Bckdha	0.42	1.00
Ebp	-0.06	1.00
Tlk2	-0.11	1.00
Pygm	-0.26	1.00

Xpo6	0.20	1.00
Chd1l	0.00	1.00
Slc35a5	0.02	1.00
Adamts1	-0.02	1.00
Smyd2	0.11	1.00
Zfp518a	0.08	1.00
Icam5	0.00	1.00
Setd5	-0.11	1.00
2310030G06Rik	0.03	1.00
Hdgfl2	-0.01	1.00
Spty2d1	0.03	1.00
Cops6	0.24	1.00
Cby1	0.07	1.00
Pltp	0.28	1.00
Slc7a6	0.07	1.00
Trim41	0.08	1.00
Zdhhc2	-0.04	1.00
Lyrm1	-0.09	1.00
Pdk4	0.31	1.00
Stoml2	0.04	1.00
Hmgcs2	-0.03	1.00
Tada3	-0.22	1.00
Ring1	0.00	1.00
1110038B12Rik	0.20	1.00
AW554918	-0.12	1.00
Nr4a2	-0.95	1.00
Esyt1	0.07	1.00
Crb3	0.05	1.00
Bcar3	0.15	1.00
Sun1	-0.18	1.00
Nploc4	0.64	1.00
Cdc37l1	0.16	1.00
Dnajc8	-0.07	1.00
Usp16	0.10	1.00
Txn2	-0.47	1.00
Oser1	0.06	1.00
Orai1	0.11	1.00
Tsc22d1	-0.03	1.00
Tmem51	0.30	1.00
BC005561	-0.33	1.00
Rnf19b	-0.04	1.00
Prnp	0.21	1.00
Ptrhd1	-0.02	1.00
Adgra3	0.13	1.00
Twf2	0.39	1.00
Naa60	-0.03	1.00

Zer1	-0.02	1.00
Ccdc39	-0.16	1.00
Nap1l4	0.01	1.00
Dync1li2	-0.46	1.00
Tent5a	0.14	1.00
Glmp	0.10	1.00
Dnah12	0.67	1.00
Smap1	0.13	1.00
Wdr20	-0.08	1.00
Bud31	-0.01	1.00
0610012G03Rik	0.05	1.00
Atg3	0.42	1.00
Top2b	0.26	1.00
Slc35d1	-0.63	1.00
Dnaja1	-0.08	1.00
Sirt2	0.32	1.00
Araf	0.03	1.00
Bms1	0.06	1.00
Pcyox1	0.21	1.00
Pde8b	0.83	1.00
Tada2b	0.10	1.00
Poldip2	-0.15	1.00
Bax	0.07	1.00
Sars	0.22	1.00
Nmd3	-0.08	1.00
Cd47	0.17	1.00
Vamp1	-0.07	1.00
Gstp3	0.13	1.00
Ipo13	-0.04	1.00
Tnik	-0.52	1.00
Itgb4	-0.18	1.00
Ddx41	0.19	1.00
Golga1	0.00	1.00
Bckdk	0.06	1.00
Clip1	0.40	1.00
Slc37a3	0.13	1.00
Sharpin	0.05	1.00
Srd5a3	0.05	1.00
Rock2	0.04	1.00
Piwil4	-0.04	1.00
Aldh3a2	0.09	1.00
Ddit3	0.32	1.00
Ccnl1	-0.18	1.00
Tet3	0.33	1.00
Llph	-0.12	1.00
Cdk11b	0.21	1.00

Rbm12b2	0.01	1.00
Bmf	0.01	1.00
Rbm15b	0.00	1.00
Pard6a	0.01	1.00
Actr5	0.00	1.00
Scrn2	-0.01	1.00
Lrch4	0.06	1.00
Tbc1d10b	0.02	1.00
Zfp945	0.13	1.00
Fbxo28	-0.07	1.00
Naa35	-0.14	1.00
Tmem107	0.10	1.00
Phf21a	0.19	1.00
Thra	0.28	1.00
Synj1	0.26	1.00
Trim37	0.04	1.00
Glod4	0.20	1.00
Flcn	-0.02	1.00
Zdhhc21	0.17	1.00
2510009E07Rik	0.01	1.00
Porcn	-0.06	1.00
Ciao1	0.01	1.00
Iqsec1	-0.06	1.00
Gtf2ird1	0.13	1.00
Cfap20	0.06	1.00
Rint1	0.30	1.00
Cfdp1	0.02	1.00
Rtn4	-0.12	1.00
Arfgap2	-0.25	1.00
Plekha1	-0.47	1.00
Ttc27	-0.18	1.00
Ubl5	0.04	1.00
Rbfox2	0.09	1.00
Irx3	0.37	1.00
Bclaf3	0.17	1.00
Itgav	-0.01	1.00
Snrpc	-0.24	1.00
Tmsb4x	0.18	1.00
Slc4a2	0.04	1.00
Agtrap	0.02	1.00
Lima1	-0.04	1.00
Tspan14	0.00	1.00
Oard1	0.46	1.00
Rassf1	0.39	1.00
Rala	0.22	1.00
Selenoi	-0.02	1.00

Nup155	-0.32	1.00
Neto2	0.21	1.00
Denr	0.05	1.00
Tial1	0.07	1.00
Nfil3	0.37	1.00
Krtcap2	0.26	1.00
Eif3c	0.07	1.00
Gtf2e2	-0.03	1.00
Sel1l3	-0.01	1.00
Tstd2	0.15	1.00
Hk3	-0.20	1.00
Ppp1r35	-0.05	1.00
Proser1	0.35	1.00
Heatr3	0.03	1.00
Tlr4	0.10	1.00
Tlcd1	0.04	1.00
Arrb2	0.02	1.00
Arhgap21	-0.20	1.00
Gsto1	0.01	1.00
Polg	0.05	1.00
Hmgxb3	0.14	1.00
Gt(ROSA)26Sor	0.25	1.00
Anxa5	0.16	1.00
Ufl1	-0.03	1.00
Pkig	0.55	1.00
Fchsd2	-0.13	1.00
Xrn1	0.15	1.00
Psmd7	0.33	1.00
Rbms1	0.12	1.00
Fam162a	-0.08	1.00
Psmd6	0.05	1.00
Cnst	-0.03	1.00
Fibp	0.06	1.00
Gtpbp1	0.05	1.00
Ccdc126	0.11	1.00
Alg2	0.03	1.00
Hccs	0.14	1.00
Aagab	0.08	1.00
Edc4	0.04	1.00
Shmt1	0.02	1.00
Tk1	0.03	1.00
Stxbp1	0.04	1.00
Palld	0.34	1.00
D030028A08Rik	0.04	1.00
Taf2	0.37	1.00
Pnrc2	0.17	1.00

Tnfsf13	0.07	1.00
D030056L22Rik	0.05	1.00
Gng5	-0.16	1.00
Nt5dc3	-0.15	1.00
Usp8	0.19	1.00
Serhl	0.43	1.00
Als2	-0.03	1.00
Zfp24	-0.10	1.00
Ropn1l	0.03	1.00
Fbxo11	0.18	1.00
Zc3h4	0.02	1.00
Eif3i	-0.20	1.00
Trir	0.22	1.00
Selenot	-0.03	1.00
Scaper	0.05	1.00
Stau2	-0.35	1.00
Tm9sf1	0.02	1.00
Asb13	0.06	1.00
Fmo2	0.06	1.00
Cnot3	-0.01	1.00
Lats2	0.36	1.00
Acat2	0.04	1.00
Kpna1	-0.08	1.00
Fbxw8	0.39	1.00
Ctnnd1	-0.02	1.00
Gna12	0.10	1.00
Atf4	-0.20	1.00
Wipi2	0.05	1.00
Pecam1	-0.44	1.00
Dguok	0.07	1.00
1110008P14Rik	0.09	1.00
1110032A03Rik	0.09	1.00
Arid5a	0.14	1.00
Errfi1	0.13	1.00
Arpc1a	0.00	1.00
Lats1	-0.04	1.00
Nip7	0.18	1.00
Fry	0.39	1.00
Cdc16	0.07	1.00
Ovol1	-0.09	1.00
Krcc1	-0.21	1.00
Grsf1	-0.29	1.00
Rdh10	-0.08	1.00
Ttf1	-0.01	1.00
AI837181	0.01	1.00
Smchd1	0.39	1.00

Herc4	0.24	1.00
Ndufb9	-0.10	1.00
Plcg2	0.49	1.00
Zfp768	0.02	1.00
1110065P20Rik	-0.01	1.00
Tfpt	0.10	1.00
Zfp282	-0.02	1.00
Pla2g4a	-0.08	1.00
Suz12	-0.02	1.00
Acadl	0.12	1.00
Hnrnpc	-0.10	1.00
Sh3bp4	0.11	1.00
Lamtor1	0.20	1.00
Tm2d2	-0.13	1.00
Ift52	0.06	1.00
Dusp7	0.26	1.00
Prdm10	0.22	1.00
Sec61a1	0.24	1.00
Tomm70a	-0.08	1.00
Pdia4	0.16	1.00
Specc1	0.08	1.00
Strap	-0.34	1.00
Wars	-0.29	1.00
Sned1	0.11	1.00
Akr1e1	0.09	1.00
Pelo	0.06	1.00
D930016D06Rik	0.01	1.00
Kctd13	0.10	1.00
Snrnp200	0.02	1.00
Nsmaf	0.08	1.00
Apmap	0.35	1.00
Hsp90b1	0.09	1.00
Cdk8	0.30	1.00
Sms	0.27	1.00
Prkd3	0.23	1.00
Cbfa2t2	-0.01	1.00
Npnt	0.31	1.00
Polg2	0.33	1.00
Zfp710	-0.44	1.00
Srsf7	0.07	1.00
Cc2d2a	0.05	1.00
Kcnmb2	0.12	1.00
Epb41l2	0.11	1.00
Zfand3	-0.04	1.00
Alg5	0.22	1.00
Tppp3	0.31	1.00

Anapc11	0.19	1.00
Zfp516	0.04	1.00
Plbd1	-0.03	1.00
Rnf25	0.00	1.00
Kif3b	0.00	1.00
Tmem161a	0.04	1.00
Smarca5	0.09	1.00
Fnbp1l	0.66	1.00
Rad17	0.09	1.00
Ahdc1	0.03	1.00
Stxbp4	-0.07	1.00
Nup160	-0.06	1.00
Snapc5	0.00	1.00
Eef1b2	0.12	1.00
Hmbox1	0.13	1.00
Jazf1	-0.12	1.00
Tor1aip1	0.08	1.00
Rpia	0.09	1.00
Klf11	-0.32	1.00
Ufd1	0.25	1.00
Bloc1s1	-0.05	1.00
Nupr1	1.17	1.00
Bcl9	0.18	1.00
Exosc3	0.10	1.00
Uba3	0.08	1.00
Atxn7l3	0.32	1.00
Ddrgk1	0.31	1.00
Tmem160	0.07	1.00
Taok1	0.16	1.00
Trabd	0.18	1.00
Casp8	0.07	1.00
Timm50	-0.04	1.00
Rab13	0.07	1.00
Shroom3	0.24	1.00
Prkce	-0.31	1.00
Tmod3	0.11	1.00
Arhgap6	0.10	1.00
Gli3	0.03	1.00
Dpm2	0.06	1.00
Cd59a	-0.06	1.00
Jpt1	0.30	1.00
Nxt1	0.03	1.00
Adnp2	0.11	1.00
Fam161a	0.44	1.00
Ulk4	0.16	1.00
Pus7	-0.07	1.00

Crls1	0.02	1.00
Hnrnpr	0.21	1.00
Khdc4	0.10	1.00
Tmem59	0.05	1.00
Tgfbr1	0.12	1.00
Ythdf2	-0.01	1.00
Bet1	0.18	1.00
Ak2	0.30	1.00
Ap3b1	-0.40	1.00
Spg21	0.18	1.00
Dnajc5	0.16	1.00
Fhad1	0.04	1.00
Ints8	0.01	1.00
Nhp2	0.41	1.00
Cttnbp2nl	0.26	1.00
Yif1a	-0.01	1.00
Usf1	0.07	1.00
Zfp266	-0.12	1.00
Lmbrd1	-0.21	1.00
Eif5	-0.06	1.00
Elk3	0.01	1.00
Fam210a	0.05	1.00
Tesk1	0.01	1.00
Fyb	0.20	1.00
Pigx	0.46	1.00
Pde6d	0.01	1.00
Nme5	0.03	1.00
Tjap1	0.03	1.00
Efemp2	0.00	1.00
Rab11fip3	-0.14	1.00
Srebf2	0.13	1.00
Kat6a	-0.05	1.00
Epha7	0.33	1.00
Inpp5k	0.01	1.00
Spcs1	0.20	1.00
Clp1	0.23	1.00
Sumf1	0.01	1.00
Traf2	-0.03	1.00
Kctd9	-0.01	1.00
Scrib	0.05	1.00
1810019D21Rik	-0.04	1.00
Rab2a	0.06	1.00
Stim1	-0.10	1.00
Tmem41b	-0.14	1.00
Cstf3	0.21	1.00
Cers4	0.07	1.00

Ildr1	0.27	1.00
Sbf1	0.22	1.00
E2f4	0.13	1.00
Usp2	-0.10	1.00
Fam92a	0.14	1.00
Srpr	-0.14	1.00
Psmd8	0.19	1.00
Fbrs	0.01	1.00
Utp14a	0.07	1.00
Csnk1e	0.09	1.00
Srsf3	-0.25	1.00
Foxo1	0.23	1.00
Eefsec	0.10	1.00
4833439L19Rik	-0.03	1.00
Slain1	-0.05	1.00
Zfp110	0.06	1.00
Smim14	-0.05	1.00
Bmpr1a	0.21	1.00
Cbx1	0.06	1.00
Nudcd3	-0.12	1.00
Gphn	0.12	1.00
Zfp644	0.26	1.00
Copa	-0.15	1.00
Cdk9	0.17	1.00
Eif3b	0.17	1.00
Hmgn3	0.02	1.00
Fam135a	0.22	1.00
4921524J17Rik	0.19	1.00
Vav2	-0.42	1.00
Clcn3	0.06	1.00
Yipf6	-0.08	1.00
Luc7l	0.00	1.00
Phc3	0.31	1.00
Zfp740	-0.07	1.00
Jak2	0.45	1.00
Rere	-0.17	1.00
Nol8	-0.01	1.00
Anks3	0.18	1.00
Dhx57	0.02	1.00
Gadd45gip1	-0.01	1.00
Fam53c	0.00	1.00
Vamp5	0.52	1.00
Arf5	-0.12	1.00
Stxbp2	-0.34	1.00
Erc1	0.16	1.00
Raph1	0.05	1.00

Arfrp1	0.14	1.00
Akap10	-0.09	1.00
Pi4k2a	0.20	1.00
Birc2	0.39	1.00
Rufy2	0.35	1.00
Pogk	0.30	1.00
2810013P06Rik	0.04	1.00
Dpm1	-0.36	1.00
Anapc4	0.16	1.00
Ccnh	0.13	1.00
Dlgap4	0.05	1.00
Pcid2	-0.11	1.00
Nudt2	-0.03	1.00
Parp6	-0.05	1.00
Ldlrap1	-0.01	1.00
Cul9	-0.04	1.00
Gpatch2l	0.09	1.00
Gid4	0.15	1.00
Emg1	-0.08	1.00
Fam104a	-0.01	1.00
Fam102a	0.66	1.00
Sox4	0.23	1.00
Churc1	0.05	1.00
Hmgcl	0.00	1.00
Farsa	0.08	1.00
Atg16l2	0.14	1.00
Naxd	0.19	1.00
Rc3h1	0.11	1.00
Stt3a	0.07	1.00
Slc35a1	0.01	1.00
Usp4	-0.04	1.00
N4bp2l1	0.20	1.00
Nup98	-0.01	1.00
Bcap31	0.08	1.00
Tubg1	0.13	1.00
Dele1	0.07	1.00
E030030I06Rik	0.05	1.00
Txnip	0.06	1.00
Abcb7	0.02	1.00
Tjp2	0.08	1.00
Ankrd40	0.10	1.00
Ppfibp1	0.28	1.00
Pycr2	0.15	1.00
Spsb1	-0.22	1.00
Rbx1	0.21	1.00
Klf6	0.12	1.00

Ddx3x	0.18	1.00
Ipmk	-0.38	1.00
Rassf8	-0.11	1.00
Emp1	-0.36	1.00
Wee1	-0.01	1.00
Slc39a4	0.15	1.00
Rbbp4	-0.05	1.00
Acp1	0.20	1.00
Dgcr6	0.01	1.00
Osgin1	0.15	1.00
Bud23	-0.16	1.00
Rab5b	-0.27	1.00
Tmem63b	0.33	1.00
Camsap1	0.15	1.00
Taf8	0.04	1.00
Golga7	0.00	1.00
Rsrc2	0.02	1.00
Fermt2	0.05	1.00
Fam32a	0.18	1.00
Snhg1	0.00	1.00
S100a8	1.32	1.00
Cops9	0.40	1.00
Ptpn21	-0.26	1.00
Prkab2	-0.01	1.00
Spata5	-0.09	1.00
Dcbld2	-0.04	1.00
Tradd	-0.01	1.00
Wdr76	0.08	1.00
Icmt	0.02	1.00
Cbarp	0.08	1.00
Ino80d	0.10	1.00
Lasp1	-0.20	1.00
Atp13a3	0.16	1.00
Ell	0.09	1.00
Sdhaf2	0.09	1.00
Lancl1	0.05	1.00
Pmepa1	0.08	1.00
Rgs10	0.26	1.00
Rsl24d1	0.08	1.00
4632427E13Rik	0.24	1.00
Rnf8	-0.47	1.00
Afg3l2	-0.42	1.00
Jagn1	-0.01	1.00
Cfap53	-0.04	1.00
Hnrnpab	0.30	1.00
Cdc27	0.12	1.00

Slc20a1	0.14	1.00
Dap	-0.01	1.00
Irak2	0.22	1.00
Ssbp4	-0.06	1.00
Snx3	-0.01	1.00
Tacc2	0.09	1.00
Cct7	0.15	1.00
Pgm5	0.06	1.00
Cenpx	-0.23	1.00
Ift140	0.09	1.00
Clca3a1	0.08	1.00
Igf2r	0.10	1.00
Erlec1	0.03	1.00
Ap4s1	0.12	1.00
Fcf1	-0.04	1.00
Vdac2	-0.09	1.00
Ndst1	0.53	1.00
Eif2s2	-0.03	1.00
Rabgap1l	0.13	1.00
Prpf4b	-0.40	1.00
Dnajc2	0.03	1.00
Pisd	0.08	1.00
Dynll1	0.21	1.00
Tshz1	0.10	1.00
Ube2e2	0.13	1.00
Pcyt1a	0.07	1.00
Lims1	0.15	1.00
Zzz3	-0.09	1.00
Zfp207	0.12	1.00
Hmgcll1	-0.15	1.00
Trip4	0.25	1.00
Akt1s1	0.09	1.00
Esf1	0.17	1.00
Btbd10	0.06	1.00
Cdkl5	0.52	1.00
Plxnb2	0.25	1.00
Tmem11	0.03	1.00
Zfp593	-0.01	1.00
Peli1	0.29	1.00
Ost4	0.15	1.00
Lpp	-0.03	1.00
Vps37b	0.23	1.00
Gipc2	0.16	1.00
Poldip3	-0.06	1.00
Acvr1	0.04	1.00
Grk5	0.29	1.00

Rhot1	0.25	1.00
Zfp655	-0.03	1.00
Vps35	0.13	1.00
Eri1	0.14	1.00
Cdc42bpg	0.23	1.00
Hook3	-0.19	1.00
Lsm3	-0.25	1.00
Ankrd42	0.23	1.00
Aldh4a1	0.00	1.00
Tsc1	0.14	1.00
Rnf10	0.33	1.00
Cndp2	0.11	1.00
Wwp2	0.08	1.00
Nfs1	0.05	1.00
Ddx23	0.09	1.00
Rnf7	0.13	1.00
Sh3glb1	0.15	1.00
Slc35f5	-0.04	1.00
Ints14	0.31	1.00
Adam9	0.06	1.00
Pus1	0.01	1.00
Phlda3	0.01	1.00
Itgam	-0.06	1.00
Fhl1	-0.11	1.00
Dcun1d5	-0.13	1.00
Wdfy3	0.07	1.00
Ppa2	-0.03	1.00
Ralgds	0.04	1.00
Saysd1	0.06	1.00
Dazap2	-0.41	1.00
Rlim	0.10	1.00
Ipo8	0.17	1.00
Vbp1	-0.20	1.00
Ndfip2	0.06	1.00
Ift80	0.02	1.00
Atp11b	0.25	1.00
Alg11	0.18	1.00
Tnfrsf21	0.36	1.00
Anapc10	0.08	1.00
Itpa	0.15	1.00
Fxyd4	0.03	1.00
Camsap2	-0.02	1.00
F11r	-0.12	1.00
Tcf3	-0.37	1.00
Arntl	-0.01	1.00
St3gal3	-0.13	1.00

Grina	0.30	1.00
Trpm6	0.20	1.00
Anxa4	0.12	1.00
Cep350	0.17	1.00
Heca	0.19	1.00
Zfp280d	0.14	1.00
Gcn1	0.02	1.00
Arhgef5	0.01	1.00
Cdkn1a	0.28	1.00
Yars	0.25	1.00
Zmat2	-0.35	1.00
Rbm6	-0.04	1.00
Nr3c1	0.06	1.00
Impad1	0.06	1.00
Sesn3	0.32	1.00
Vldlr	0.33	1.00
2310009B15Rik	0.01	1.00
Qk	0.43	1.00
Patl1	0.25	1.00
Nup188	-0.10	1.00
Togaram2	0.38	1.00
Fam193b	0.24	1.00
Ppt1	0.15	1.00
Nfyc	-0.26	1.00
Pianp	0.06	1.00
Scin	-0.20	1.00
Sgf29	0.09	1.00
Pes1	0.13	1.00
Gstcd	0.01	1.00
Cox18	0.00	1.00
Xpot	0.18	1.00
Tcea3	0.10	1.00
Wwp1	1.02	1.00
Pdcd10	0.15	1.00
Larp1b	0.11	1.00
Immp1l	0.00	1.00
Filip1l	0.27	1.00
Dync1h1	0.04	1.00
Ssh1	-0.09	1.00
Atr	-0.06	1.00
Ppp3cb	0.13	1.00
Aptx	0.13	1.00
Prcc	0.05	1.00
9930014A18Rik	-0.01	1.00
4930562C15Rik	0.02	1.00
Golga4	0.07	1.00

Ywhah	0.20	1.00
Glrx5	0.03	1.00
Slc44a3	0.23	1.00
Hnrnpm	-0.10	1.00
Celf2	0.16	1.00
Snx18	-0.29	1.00
Samd4	0.17	1.00
Srsf10	0.09	1.00
Zc3h18	0.12	1.00
Gpbp1	0.04	1.00
Sik3	0.10	1.00
Zfp871	-0.09	1.00
Jund	0.08	1.00
Vps29	0.13	1.00
Clint1	-0.13	1.00
Naf1	0.04	1.00
Trp53bp1	0.04	1.00
Gas2l1	0.05	1.00
Prmt2	0.42	1.00
Kmt5a	0.00	1.00
Ccdc181	0.25	1.00
Ubxn10	0.11	1.00
Zgpat	0.05	1.00
2810403D21Rik	0.13	1.00
Plxnb1	0.05	1.00
2210016L21Rik	0.45	1.00
Cep170	0.09	1.00
Xpnpep1	0.09	1.00
Strip1	0.04	1.00
Bmt2	-0.14	1.00
Capn7	0.23	1.00
Npdc1	0.23	1.00
Nckap1	0.07	1.00
Ddhd1	-0.06	1.00
Elp1	0.09	1.00
Slc6a9	0.21	1.00
Gnb5	-0.78	1.00
Pde8a	0.03	1.00
Kcnj2	-0.02	1.00
Crybg3	-0.43	1.00
Prkdc	0.03	1.00
Hook2	0.08	1.00
Ube2e3	0.22	1.00
Atl2	0.07	1.00
Wls	-0.43	1.00
Fez2	0.04	1.00

Pus10	0.14	1.00
Ptdss2	0.23	1.00
Cyp20a1	-0.02	1.00
Armc9	-0.02	1.00
Eif4e3	-0.07	1.00
Cmtm6	0.04	1.00
Cetn3	-0.19	1.00
Tmem115	-0.03	1.00
Dcp1a	-0.03	1.00
Utp14b	0.03	1.00
Fbxo36	0.30	1.00
Thada	0.00	1.00
Prpf38b	-0.08	1.00
Ppp1r7	0.01	1.00
Cdc34	-0.01	1.00
Ltbr	0.05	1.00
Cdk4	0.29	1.00
Sos1	-0.10	1.00
Kdm5c	0.20	1.00
Ubl7	0.07	1.00
Tbl1x	0.27	1.00
Tmem167	-0.21	1.00
Guk1	0.48	1.00
Elmo2	-0.39	1.00
Pink1	0.33	1.00
4932438A13Rik	-0.01	1.00
Cdkn1b	-0.01	1.00
Glud1	0.10	1.00
Egln1	-0.07	1.00
Chmp6	0.08	1.00
Nbeal1	0.23	1.00
Ttc9	0.36	1.00
Emc4	0.33	1.00
Plet1	0.12	1.00
Dis3l2	-0.15	1.00
BC005624	0.61	1.00
Csnk1g2	0.07	1.00
Tubgcp5	0.09	1.00
Slc16a10	-0.04	1.00
Nim1k	0.25	1.00
Clk1	0.28	1.00
Rap1b	0.01	1.00
Utp11	-0.01	1.00
Selenok	0.12	1.00
Zdhhc13	0.09	1.00
Pcsk6	0.13	1.00

Klhl11	0.02	1.00
Ssr3	0.06	1.00
Wwox	-0.08	1.00
Abcc5	0.04	1.00
Dusp18	0.01	1.00
Farp2	0.05	1.00
Hras	0.26	1.00
Ccs	0.21	1.00
Tsix	-0.05	1.00
Arhgap28	-0.26	1.00
Rpa2	-0.07	1.00
Hsd3b7	-0.51	1.00
Zfp597	0.09	1.00
Nup35	-0.07	1.00
Eif2ak1	0.21	1.00
Plbd2	0.15	1.00
Ubqln4	0.05	1.00
Cnn2	-0.07	1.00
Ripk4	0.11	1.00
Zfp330	0.04	1.00
Cggbp1	0.08	1.00
Epn1	0.03	1.00
Nmt1	-0.24	1.00
R3hdm2	-0.13	1.00
Sema6a	-0.63	1.00
Nubpl	0.11	1.00
Dnah2	0.00	1.00
Parg	0.13	1.00
H2-Eb1	0.33	1.00
Cpne3	0.30	1.00
Prr14	0.13	1.00
Phf20l1	-0.02	1.00
Tspan13	0.35	1.00
Rfx2	0.01	1.00
Tpk1	0.12	1.00
Diaph1	0.07	1.00
Arhgap29	0.29	1.00
Krt8	0.73	1.00
Ptdss1	0.28	1.00
Bloc1s4	0.04	1.00
Eaf1	0.11	1.00
Prim2	0.11	1.00
Sccpdh	0.05	1.00
Smim13	0.00	1.00
Qtrt1	-0.26	1.00
Tspan17	-0.02	1.00

Zfyve21	-0.14	1.00
Pcmt1	-0.05	1.00
S100a16	0.26	1.00
Spag7	-0.05	1.00
Il4ra	0.22	1.00
Zfp622	0.03	1.00
Rnf103	0.04	1.00
Arhgef28	-0.04	1.00
Nsd2	0.13	1.00
Fads3	0.37	1.00
Uba52	-0.08	1.00
Prkcd	0.10	1.00
Polr1c	0.19	1.00
Eml5	-0.08	1.00
Zadh2	0.09	1.00
Ryr2	-0.10	1.00
Neu1	-0.02	1.00
Nptn	0.04	1.00
Zfand1	0.06	1.00
Arrb1	0.01	1.00
Thumpd1	0.11	1.00
Vps37a	0.20	1.00
Cyba	0.53	1.00
Hspb8	0.04	1.00
Elovl7	0.08	1.00
Slc9a1	0.54	1.00
Elk4	0.22	1.00
Slc35b1	0.30	1.00
Elovl1	0.50	1.00
Ssb	0.18	1.00
Cul3	0.29	1.00
Ube2k	0.24	1.00
C2cd5	0.41	1.00
Hlcs	0.01	1.00
Dag1	0.16	1.00
Alms1	0.33	1.00
Rasl11a	-0.01	1.00
Tanc2	0.04	1.00
Hspa5	0.09	1.00
Cpt1a	-0.08	1.00
Rnpc3	0.05	1.00
Eif1ax	0.14	1.00
Cd99l2	0.03	1.00
Srp54a	0.15	1.00
Slc38a6	0.33	1.00
Tcf7	0.01	1.00

Il15ra	0.07	1.00
Tanc1	0.11	1.00
Trappc4	0.36	1.00
Stat6	0.25	1.00
Slc22a15	0.01	1.00
Efcab8	-0.01	1.00
Aimp1	-0.26	1.00
Oip5os1	0.09	1.00
Sqle	0.08	1.00
Dot1l	0.05	1.00
Ddx1	-0.01	1.00
Nemf	0.25	1.00
Tmcc3	-0.39	1.00
Psenen	0.04	1.00
Puf60	0.15	1.00
Tjp1	-0.04	1.00
Gpi1	0.41	1.00
Prss8	0.02	1.00
Rabl2	0.05	1.00
Dgke	0.02	1.00
Srcin1	0.06	1.00
Zfp386	0.03	1.00
Jarid2	0.09	1.00
Pias1	0.09	1.00
Dnajb2	0.14	1.00
Pitpnm3	-0.01	1.00
Bzw1	-0.22	1.00
G3bp1	-0.03	1.00
Bcam	0.29	1.00
Thoc1	0.19	1.00
2700049A03Rik	0.10	1.00
Qser1	0.04	1.00
Rbm38	0.12	1.00
Pkn1	0.06	1.00
Pdcl3	0.28	1.00
Ppp4r1	-0.10	1.00
Nbn	0.15	1.00
Cpn1	0.13	1.00
Ccdc120	0.03	1.00
Thoc7	-0.18	1.00
Brox	0.28	1.00
Arl5b	-0.13	1.00
Eif6	0.13	1.00
Otud7b	-0.56	1.00
Cab39	-0.08	1.00
Gstz1	0.10	1.00

Cald1	0.38	1.00
Cep192	0.12	1.00
Lias	0.17	1.00
Neurl3	-0.34	1.00
3830406C13Rik	0.12	1.00
Cbl	-0.03	1.00
Etnk1	0.16	1.00
Prkci	-0.08	1.00
Ints3	0.05	1.00
Serpinb6b	0.47	1.00
Hnrnph2	-0.05	1.00
Hip1r	0.06	1.00
Sc5d	0.63	1.00
Zfp91	-0.05	1.00
Rel	0.18	1.00
Kcnrg	-0.04	1.00
Chmp4c	-0.03	1.00
Snap47	0.07	1.00
Rars2	0.03	1.00
Rnf125	-0.27	1.00
Tcerg1	0.03	1.00
Tbcb	0.02	1.00
Twistnb	0.03	1.00
Gsn	-0.23	1.00
Psmc4	-0.03	1.00
E130307A14Rik	0.48	1.00
Acox3	0.00	1.00
Syk	0.44	1.00
Rgs1	0.27	1.00
Cyb561d2	-0.06	1.00
Prkcb	0.11	1.00
Ak1	-0.01	1.00
Fpgs	0.15	1.00
Itk	0.29	1.00
Abcb1b	0.30	1.00
Epop	0.01	1.00
Trf	0.09	1.00
Dnajc13	0.45	1.00
Rela	-0.37	1.00
Iws1	0.28	1.00
Vkorc1l1	0.14	1.00
Sesn2	0.15	1.00
Yod1	0.01	1.00
Arhgap45	0.57	1.00
Ccdc138	0.16	1.00
Lemd3	-0.02	1.00

Phf5a	0.07	1.00
Gtf2f1	0.15	1.00
Plp2	0.12	1.00
Plod2	0.04	1.00
Snip1	0.05	1.00
Sqor	0.08	1.00
Espn	0.05	1.00
Ankrd26	0.05	1.00
Tmed3	0.18	1.00
Cd9	0.17	1.00
Ppme1	0.04	1.00
Taz	0.10	1.00
Terf2	0.04	1.00
Unc119	0.19	1.00
Nsf	0.07	1.00
Slc43a2	0.12	1.00
Rb1	0.03	1.00
Ppp1r16b	0.01	1.00
Reep4	0.04	1.00
Ttc23l	0.28	1.00
Ift20	-0.06	1.00
Atp9a	-0.01	1.00
Rab22a	-0.21	1.00
Lclat1	-0.34	1.00
Pde3b	-0.06	1.00
Tm2d3	0.03	1.00
Glt8d1	-0.14	1.00
Chrnb1	0.11	1.00
Ccng1	0.37	1.00
Atp6v1d	0.06	1.00
Polr2j	0.34	1.00
Otud4	0.17	1.00
Dhx40	-0.09	1.00
Fam49b	0.17	1.00
Stk17b	-0.39	1.00
Tprgl	0.15	1.00
Acot9	-0.85	1.00
Dctn1	-0.10	1.00
Itih4	-0.31	1.00
Ilrun	-0.16	1.00
B230118H07Rik	-0.21	1.00
Ak6	0.04	1.00
Cacul1	0.02	1.00
Tceal8	0.42	1.00
Tmem39a	0.25	1.00
Cdadc1	0.04	1.00

Jtb	0.23	1.00
Polr3b	0.05	1.00
Birc3	0.30	1.00
Napg	0.27	1.00
Usp1	-0.16	1.00
Nipsnap3b	-0.04	1.00
Snx4	-0.27	1.00
Fbxl3	-0.08	1.00
Prss23	0.00	1.00
Efna5	0.01	1.00
Gdnf	0.03	1.00
Fzd7	0.02	1.00
Pla2g12b	0.06	1.00
Wdr53	0.02	1.00
Sh3bp5l	0.06	1.00
Cyp4f16	-0.01	1.00
Dhx15	-0.21	1.00
Garem1	0.13	1.00
Btg2	0.10	1.00
Fgfbp1	0.37	1.00
Dock8	0.10	1.00
Gipc1	0.04	1.00
Elmod2	0.07	1.00
Wdr47	0.15	1.00
Ppcdc	0.08	1.00
Homer2	0.14	1.00
Pglyrp1	0.19	1.00
Setd7	0.30	1.00
Zfp503	0.14	1.00
Epn2	0.06	1.00
Gtf3c1	0.18	1.00
Hif1a	0.28	1.00
Aopep	0.15	1.00
Pex14	0.03	1.00
Pole4	0.05	1.00
Tinagl1	0.07	1.00
Derl2	-0.07	1.00
Il6	0.16	1.00
Polr2h	-0.06	1.00
Pik3r2	0.00	1.00
Lrrc8d	-0.07	1.00
Relch	0.24	1.00
Cltb	0.34	1.00
Tulp4	0.22	1.00
Arpp19	0.21	1.00
Slc39a8	0.36	1.00

Adcy9	-0.08	1.00
Rac1	0.19	1.00
Col4a1	0.35	1.00
Rnf13	-0.17	1.00
Spata7	0.28	1.00
Rab8b	0.39	1.00
Plaur	0.13	1.00
Zfr	-0.44	1.00
Rock1	-0.07	1.00
Dclk1	-0.26	1.00
Cfap61	-0.30	1.00
Sde2	0.16	1.00
Supt3	-0.06	1.00
Ldlrad3	-0.14	1.00
Esd	0.08	1.00
Cul4a	0.06	1.00
Krit1	0.41	1.00
Eps15l1	-0.17	1.00
Cdc42se1	0.10	1.00
Txndc15	-0.04	1.00
Plscr1	0.03	1.00
Tbce	0.32	1.00
Ormdl3	-0.47	1.00
2510039O18Rik	-0.32	1.00
Pxn	0.19	1.00
Fbxo7	-0.01	1.00
Adprh	0.02	1.00
Rfc3	0.00	1.00
Ndufaf4	0.01	1.00
2310022B05Rik	0.02	1.00
Sap30	0.20	1.00
Rmdn3	-0.03	1.00
Tgfb3	-0.17	1.00
Tysnd1	0.17	1.00
Ppif	0.19	1.00
Fam43a	-0.01	1.00
Top3a	-0.01	1.00
2900026A02Rik	0.00	1.00
Egfl7	-0.04	1.00
Rundc1	0.00	1.00
Xndc1	0.02	1.00
Samd1	0.06	1.00
Zfhx2	0.02	1.00
Nek7	0.25	1.00
Ubap2	0.00	1.00
Plekha7	-0.07	1.00

Glb1	0.05	1.00
Galt	0.02	1.00
Smad6	0.07	1.00
Ctns	0.00	1.00
Fam222b	0.08	1.00
Rgl3	0.01	1.00
Zbtb42	0.12	1.00
Dusp16	-0.17	1.00
Retreg2	0.22	1.00
Wac	0.08	1.00
Ctsc	0.06	1.00
Hnrnpa3	-0.31	1.00
Akr1a1	0.28	1.00
Vim	-1.02	1.00
Pkia	0.17	1.00
Ccdc148	-0.12	1.00
Chmp1a	0.13	1.00
Ccdc191	-0.11	1.00
Xpc	0.02	1.00
Dixdc1	0.00	1.00
Arhgap23	-0.01	1.00
Entr1	-0.05	1.00
Ankrd50	0.18	1.00
Rnf215	0.21	1.00
Per3	0.11	1.00
2010300C02Rik	0.30	1.00
Romo1	-0.12	1.00
Gpc4	-0.05	1.00
Fbxo4	0.00	1.00
Ift81	0.12	1.00
Naca	0.19	1.00
Slc25a46	0.07	1.00
Atg4b	0.23	1.00
Snx1	-0.08	1.00
Cops7a	0.14	1.00
Zswim8	0.26	1.00
Fbxo30	0.42	1.00
Ctps	0.09	1.00
Stx16	0.11	1.00
Fbxl7	0.06	1.00
Cpeb3	-0.07	1.00
Tsg101	-0.31	1.00
Lratd2	0.14	1.00
Fbxl5	0.02	1.00
4931406P16Rik	-0.53	1.00
Sap130	0.14	1.00

Spaca6	-0.03	1.00
Rif1	-0.07	1.00
Ldlrad4	0.17	1.00
Bcr	0.52	1.00
Lcn2	2.37	1.00
Notch2	0.25	1.00
Pard3b	0.22	1.00
Arf4	-0.01	1.00
Srrm1	0.33	1.00
Fau	0.01	1.00
Lss	0.38	1.00
Cxxc5	0.12	1.00
Cnot8	0.01	1.00
Depdc5	0.42	1.00
Pld1	0.19	1.00
Psen1	0.09	1.00
Carnmt1	-0.08	1.00
Usp7	0.00	1.00
Chmp5	0.21	1.00
Thoc2	-0.16	1.00
Gnai2	0.15	1.00
Riox2	-0.02	1.00
Epb41l3	0.14	1.00
Plcl1	-0.02	1.00
Acot8	-0.01	1.00
Gucd1	-0.05	1.00
Nek8	0.20	1.00
Naglu	0.15	1.00
Stk36	0.00	1.00
Dnaaf3	-0.08	1.00
Zfp579	0.16	1.00
Fxyd3	-0.20	1.00
Pld2	-0.38	1.00
Nufip1	0.06	1.00
Odf2l	0.13	1.00
Elmo3	0.08	1.00
Cdkn2b	0.38	1.00
Rrp8	0.00	1.00
Tcta	0.09	1.00
Cdkn2aipnl	0.01	1.00
Zmynd15	0.26	1.00
Kcnmb4	0.03	1.00
Ikzf2	-0.04	1.00
Ube2d1	0.01	1.00
Rilpl1	0.03	1.00
Uap1l1	0.22	1.00

Sclt1	-0.17	1.00
Rrn3	-0.23	1.00
Stx19	0.05	1.00
Rell1	-0.16	1.00
Nudt9	0.10	1.00
Irf2bp1	-0.15	1.00
Tbc1d14	-0.08	1.00
Rapgef1	-0.05	1.00
Tctex1d2	-0.08	1.00
Pigf	0.06	1.00
Idh3a	0.10	1.00
Dhdds	-0.09	1.00
Esco1	0.00	1.00
Nolc1	-0.08	1.00
Dpp9	0.37	1.00
Lin7c	-0.08	1.00
Crlf3	0.84	1.00
Kat2b	0.14	1.00
Cisd1	-0.22	1.00
Ensa	0.20	1.00
Cebpg	0.00	1.00
S100a9	0.94	1.00
Rai1	-0.26	1.00
Cask	-0.02	1.00
Akt2	-0.38	1.00
Dlc1	0.13	1.00
Nr1d1	-0.08	1.00
Ube2s	0.16	1.00
Phip	-0.10	1.00
Scnn1a	0.19	1.00
Tnrc6a	-0.06	1.00
Atp6v0b	0.32	1.00
Atxn10	0.04	1.00
Atp11a	-0.12	1.00
Uvrag	-0.05	1.00
Ccm2	-0.03	1.00
Dtna	-0.52	1.00
Pex2	0.04	1.00
Rcor3	-0.01	1.00
Pex13	0.18	1.00
Efcab2	-0.83	1.00
Nono	-0.05	1.00
Kank1	0.16	1.00
Aldh7a1	0.02	1.00
Rmi1	-0.02	1.00
Ptch1	0.07	1.00

Riok2	-0.03	1.00
Shank2	0.20	1.00
Sf3b1	0.18	1.00
Plce1	0.08	1.00
Pigk	0.27	1.00
Yeats2	-0.08	1.00
Abhd17c	0.26	1.00
Rnf111	-0.04	1.00
Dut	-0.11	1.00
Itpr1	0.01	1.00
Pskh1	0.01	1.00
Snx8	0.19	1.00
Psmf1	0.01	1.00
1810010H24Rik	0.11	1.00
Zfp618	-0.11	1.00
Lrwd1	-0.06	1.00
Smg6	-0.25	1.00
Sdf2	0.16	1.00
Skiv2l	0.09	1.00
Lamtor2	-0.24	1.00
Fgd2	-0.06	1.00
Zfas1	0.09	1.00
Ubr4	-0.31	1.00
Cox6b1	0.23	1.00
Prdx2	-0.09	1.00
Ranbp2	-0.11	1.00
Ddx50	0.00	1.00
Phf23	0.13	1.00
Ero1lb	-0.44	1.00
Tnfrsf12a	0.47	1.00
Saa3	4.45	0.00
Kcnk1	1.47	0.04
Sgms2	1.42	0.12
Elf3	1.58	0.13
Fam91a1	0.84	0.56
Ifi27	1.67	0.74
Ank	-2.04	0.82
Grb2	0.91	0.83
Hspa8	1.51	0.86
C1qbp	1.03	1.00
Snrpc	1.08	1.00
Rbm38	0.57	1.00
Frk	1.06	1.00
Apoe	1.63	1.00
Sdad1	0.74	1.00
Epb41l4b	1.11	1.00

Slc44a4	1.68	1.00
Tnip3	1.27	1.00
Uqcrc1	0.80	1.00
Golm1	1.03	1.00
Eif3b	0.65	1.00
Aco2	0.84	1.00
Tacstd2	0.71	1.00
Cir1	1.39	1.00
Hsph1	1.62	1.00
Shisa5	0.70	1.00
Ipo4	0.46	1.00
Tm2d1	0.79	1.00
Serpine1	2.25	1.00
Gdf15	0.85	1.00
B2m	1.50	1.00
Sel1l3	1.72	1.00
Sc5d	0.56	1.00
Cyba	1.15	1.00
Ctsc	1.54	1.00
Cdk12	0.97	1.00
Usp43	1.29	1.00
Naaladl2	1.80	1.00
Tspan1	1.56	1.00
Slc5a3	0.93	1.00
Abtb2	1.58	1.00
Rtkn2	-3.65	1.00
Prorp	1.11	1.00
Sik2	0.97	1.00
Stxbp5	-2.12	1.00
Zbtb18	0.57	1.00
Baiap2l1	1.58	1.00
Arl15	-1.89	1.00
Spns1	0.31	1.00
Rabep1	1.15	1.00
Gtpbp8	1.23	1.00
Lmf1	0.38	1.00
Eya3	0.71	1.00
Hsp90b1	1.21	1.00
Ibtk	0.69	1.00
Acsl3	1.19	1.00
Prkar2a	1.06	1.00
Pcbd1	0.53	1.00
F3	0.30	1.00
Cyp2j13	0.90	1.00
Pdcd10	0.83	1.00
Ehd4	0.59	1.00

Stk24	0.98	1.00
Traf3	-0.02	1.00
Bcl10	0.45	1.00
Slc3a2	0.60	1.00
Snx6	1.50	1.00
Btbd10	0.55	1.00
Slc38a10	0.45	1.00
Tiam1	1.44	1.00
Hltf	0.52	1.00
Lcor	1.25	1.00
Nol10	1.18	1.00
Nectin3	-2.31	1.00
Dnajc13	0.63	1.00
Cdc40	0.91	1.00
Wdr12	0.44	1.00
Reps2	0.40	1.00
Tiparp	1.16	1.00
Actb	-0.74	1.00
Clptm1l	0.63	1.00
Eva1c	1.14	1.00
Srebf2	0.77	1.00
Exoc2	0.89	1.00
Tmem131l	1.14	1.00
Hdac4	0.79	1.00
Ppid	0.90	1.00
Nek4	0.76	1.00
Nfs1	0.66	1.00
Daglb	0.72	1.00
Rcbtb2	0.65	1.00
Lgals3bp	0.58	1.00
Spcs1	0.63	1.00
Prom1	1.86	1.00
Galns	0.35	1.00
Sox9	0.84	1.00
Ccdc24	0.31	1.00
Arg1	1.24	1.00
Paqr8	0.84	1.00
Ckb	1.13	1.00
Nck2	1.26	1.00
Rpusd1	0.53	1.00
Vav2	0.52	1.00
Hmgcr	0.75	1.00
Bhlhe40	1.34	1.00
Atp13a3	0.89	1.00
Ccn2	-2.93	1.00
Pdcl3	0.39	1.00

Txnrd3	0.53	1.00
Ifi30	0.90	1.00
Slc39a11	1.16	1.00
Zfp366	1.37	1.00
Ankib1	0.88	1.00
Rpn1	0.83	1.00
Lgmn	1.49	1.00
Ints8	0.51	1.00
Nop53	0.24	1.00
Odc1	0.81	1.00
Stk39	1.34	1.00
Degs2	0.68	1.00
Gcnt4	0.36	1.00
Aspscr1	-0.13	1.00
1810037I17Rik	0.99	1.00
Copg2	0.59	1.00
Nudt22	0.92	1.00
Col12a1	-1.94	1.00
Cp	1.59	1.00
Luzp1	0.93	1.00
Hspd1	0.71	1.00
Snx17	1.36	1.00
Fadd	0.70	1.00
Gpt	0.71	1.00
Dock8	-2.03	1.00
Phc3	0.89	1.00
Prdx1	1.10	1.00
Slc35a1	0.34	1.00
Eya2	1.40	1.00
Jak1	0.94	1.00
Fbxo32	1.09	1.00
Lrrc56	0.69	1.00
Safb2	0.58	1.00
Tpi1	1.26	1.00
Dmxl2	1.53	1.00
Nasp	1.32	1.00
Azi2	0.63	1.00
Dnaja3	0.34	1.00
Ddx27	0.48	1.00
AI661453	0.50	1.00
Rab11fip4	0.47	1.00
Efna1	0.60	1.00
Nutf2	0.81	1.00
Tmf1	0.47	1.00
Ctnnb1	0.83	1.00
Cdk8	0.71	1.00

P3h1	0.91	1.00
Slc4a7	1.49	1.00
Rpf1	0.36	1.00
Ddx31	0.07	1.00
Sertad3	0.58	1.00
Ubb	0.78	1.00
Laptm4a	0.79	1.00
Cybb	1.18	1.00
Kctd17	0.42	1.00
Nsun2	0.24	1.00
Ackr2	0.27	1.00
Synpo2	0.61	1.00
Rad17	0.24	1.00
Ankzf1	0.30	1.00
Pwwp2b	0.18	1.00
Gnl3	0.31	1.00
Acad9	0.40	1.00
Capn15	0.23	1.00
Cd40	0.54	1.00
Dram2	0.44	1.00
Fdps	0.58	1.00
Plekhg6	0.37	1.00
Grb7	0.35	1.00
Eftud2	0.81	1.00
Xrra1	0.46	1.00
Ranbp6	1.02	1.00
Kcnma1	1.99	1.00
Fam193b	0.33	1.00
Selenof	0.94	1.00
Cct5	0.99	1.00
Hsp90aa1	1.24	1.00
Smarca4	0.61	1.00
Atp6v1c2	1.46	1.00
Wdr54	0.64	1.00
Ddx47	0.89	1.00
Fam20b	0.36	1.00
Nsmce2	1.03	1.00
Aco1	0.89	1.00
Agr2	2.83	1.00
Fus	0.60	1.00
Cnot1	0.57	1.00
Baz1a	1.38	1.00
Lrrcc1	0.48	1.00
Fbxo6	0.40	1.00
Papss1	1.03	1.00
Ubqln1	0.91	1.00

Rsrc2	0.75	1.00
Atf2	-1.26	1.00
Tmem268	0.85	1.00
Pls3	-1.66	1.00
Tmx4	1.44	1.00
Aacs	0.54	1.00
Slc35b2	0.25	1.00
Cd2bp2	0.56	1.00
Gabbr1	0.88	1.00
Piezo1	0.68	1.00
Abcf1	0.67	1.00
Fut8	0.62	1.00
Slc4a1ap	0.37	1.00
Suz12	0.56	1.00
Kdelr2	0.18	1.00
Cntn5	1.11	1.00
Acads	0.34	1.00
Scgb3a2	3.90	1.00
Cux1	-1.52	1.00
Vps53	0.25	1.00
Sec11c	0.77	1.00
Cldn13	0.86	1.00
Ppfia1	0.90	1.00
Asph	0.52	1.00
Kctd16	1.30	1.00
Derl2	0.34	1.00
Tmed10	0.89	1.00
Tmem131	1.03	1.00
Ptpn21	-1.41	1.00
Ndfip1	0.86	1.00
Gprc5c	0.64	1.00
Ttll1	0.15	1.00
Tnfsf13	0.15	1.00
Tubgcp2	0.62	1.00
Stx6	0.40	1.00
Rai1	0.42	1.00
Hspa5	1.36	1.00
Dnm3	-1.19	1.00
Leo1	0.94	1.00
Lman2	0.68	1.00
Ciao2b	0.34	1.00
Sinhcaf	0.88	1.00
Blnk	0.60	1.00
Ddx60	0.39	1.00
Sppl2a	0.47	1.00
Prrc2c	1.02	1.00

Egln3	0.84	1.00
Upf3b	0.49	1.00
Rcbtb1	0.60	1.00
Pin1	0.57	1.00
Vps41	0.57	1.00
Cdcp1	0.65	1.00
Siah2	0.24	1.00
Stpg2	0.76	1.00
Emc4	0.09	1.00
Hsf1	0.32	1.00
Pigr	1.39	1.00
Acss1	0.51	1.00
Fermt1	0.59	1.00
Dlg2	0.89	1.00
Bclaf3	0.42	1.00
Aifm1	0.34	1.00
Ugdh	0.67	1.00
Ccdc171	0.47	1.00
Smg5	0.44	1.00
Eipr1	0.34	1.00
Itpk1	0.81	1.00
Tubb2b	1.25	1.00
Ipo5	0.87	1.00
Fam174a	0.93	1.00
Nsun6	0.88	1.00
Paip1	0.46	1.00
Safb	0.92	1.00
Psmd2	0.36	1.00
Supt16	0.33	1.00
Ptov1	0.48	1.00
Arhgef38	1.07	1.00
Gsr	0.87	1.00
Efhc2	1.02	1.00
S100a2	2.48	1.00
Samm50	0.31	1.00
Tctn1	0.44	1.00
Ap1s1	0.27	1.00
Plgrkt	-0.25	1.00
Cnga3	0.42	1.00
Asb15	0.27	1.00
Fam3b	0.82	1.00
Klhl42	0.36	1.00
Galnt12	0.23	1.00
Smpdl3a	1.05	1.00
Cdc42se2	0.30	1.00
Negr1	0.77	1.00

D2hgdh	0.53	1.00
Capn10	0.24	1.00
Rnf166	0.21	1.00
Sh3pxd2b	1.35	1.00
Hvcn1	0.83	1.00
Kcnj16	0.71	1.00
Ier5l	1.44	1.00
Rabl6	0.26	1.00
Ormdl3	0.27	1.00
Pcsk7	0.52	1.00
Ppil1	0.22	1.00
Dohh	0.48	1.00
Shkbp1	0.58	1.00
Nsd2	0.27	1.00
Cebpa	0.55	1.00
Polr3d	0.19	1.00
Plod3	0.26	1.00
Exosc5	0.50	1.00
Trp53	0.27	1.00
Fam53c	0.28	1.00
Frmd8	0.81	1.00
Cdc42ep4	0.31	1.00
Dcaf1	0.68	1.00
Glyr1	0.41	1.00
Psmf1	0.15	1.00
Ppcs	0.31	1.00
Slc35a2	0.23	1.00
Vcl	-1.38	1.00
Abhd12	0.72	1.00
Ndrg1	1.11	1.00
Nbas	0.75	1.00
Dusp5	0.60	1.00
Ilf2	0.77	1.00
Ehf	1.23	1.00
Atp11c	1.01	1.00
Ppif	0.90	1.00
Dner	0.62	1.00
Ftsj3	0.59	1.00
Ero1a	0.39	1.00
Wdr20	0.31	1.00
Csnk1g2	0.32	1.00
Gpaa1	0.32	1.00
Net1	0.35	1.00
Pigt	1.06	1.00
Kbtbd2	0.46	1.00
P4ha2	0.69	1.00

Eps8	1.56	1.00
Slc39a8	0.85	1.00
Tmc5	0.60	1.00
Nectin4	0.89	1.00
Akap11	0.35	1.00
Ints14	0.79	1.00
Amn1	0.50	1.00
Hs6st1	0.53	1.00
Gpx2	0.97	1.00
Oaz2	0.06	1.00
Drap1	0.49	1.00
Ankrd11	0.58	1.00
Rhpn2	0.77	1.00
Hnrnpul1	0.25	1.00
Rab3gap2	0.58	1.00
Npas3	1.69	1.00
Lrrc27	0.45	1.00
Lrrc42	-0.13	1.00
Tor3a	0.34	1.00
Dmac2	0.48	1.00
Trub2	0.05	1.00
Utp11	0.19	1.00
Sema4b	0.97	1.00
Psmb4	0.51	1.00
Grsf1	0.43	1.00
Hepacam2	0.81	1.00
Tmem17	0.23	1.00
Ccdc32	0.39	1.00
Hax1	0.31	1.00
Lrrc51	0.23	1.00
Samhd1	0.90	1.00
Septin9	0.21	1.00
Ager	-1.82	1.00
Omd	-1.35	1.00
Krt8	1.39	1.00
Cd81	0.66	1.00
Yipf1	0.91	1.00
Ino80c	0.24	1.00
Pak1ip1	0.15	1.00
Bpnt1	0.19	1.00
Ltbp1	0.38	1.00
Nup133	0.28	1.00
2410004B18Rik	0.22	1.00
Brd4	0.78	1.00
Adcy8	-1.86	1.00
Denr	0.35	1.00

Fkbp8	0.55	1.00
Adamts9	-1.74	1.00
Higd1a	0.66	1.00
9530068E07Rik	0.57	1.00
Tspan31	0.46	1.00
B3gat3	0.40	1.00
Ddit4	0.90	1.00
Krt14	1.48	1.00
Tbc1d2	0.13	1.00
Nmi	0.16	1.00
Abhd2	-0.10	1.00
Golm2	1.06	1.00
Grk3	0.47	1.00
Kdm5c	0.21	1.00
Ankrd40	0.60	1.00
Exoc4	1.14	1.00
Clta	0.66	1.00
H2az2	0.79	1.00
Sap18	0.58	1.00
Psmb1	0.46	1.00
Atp1b1	1.58	1.00
Ttc1	1.15	1.00
Tnfaip8l1	0.44	1.00
Dlst	0.34	1.00
Cftr	1.63	1.00
4933400A11Rik	1.05	1.00
Psmd7	0.42	1.00
Arrdc1	0.32	1.00
Dlat	0.26	1.00
Polr2a	0.65	1.00
Csde1	-0.84	1.00
Llgl2	-0.17	1.00
Thyn1	0.84	1.00
Lrp10	0.36	1.00
Llph	0.55	1.00
Psmc4	0.26	1.00
Nr6a1	0.75	1.00
Dmxl1	0.37	1.00
Nol4l	0.01	1.00
Clic5	-1.82	1.00
Tbc1d31	0.83	1.00
Cct3	0.65	1.00
Timp1	1.02	1.00
Larp4	0.64	1.00
Rnf121	0.75	1.00
Fyn	1.08	1.00

Bag5	0.36	1.00
Dym	0.93	1.00
Sdf4	0.47	1.00
Polr1f	0.49	1.00
Specc1	0.56	1.00
Ptpn2	0.97	1.00
Dnai2	0.74	1.00
Sema3e	-1.68	1.00
Psma5	0.26	1.00
Fbxo15	0.64	1.00
Sgpp2	1.29	1.00
Odad4	0.65	1.00
Eif4g1	0.06	1.00
Tardbp	0.38	1.00
Spint1	1.10	1.00
Ganab	0.21	1.00
Chmp2b	0.44	1.00
Klf7	0.54	1.00
Zmat3	0.42	1.00
Arl4c	0.44	1.00
Kif17	0.35	1.00
Gspt1	0.62	1.00
Creld2	0.48	1.00
Phldb2	-2.43	1.00
C2cd5	0.37	1.00
Ptpn3	0.59	1.00
Fbxl2	0.77	1.00
Rbms3	-2.00	1.00
Zfp622	0.45	1.00
Pdlim5	1.06	1.00
Dnaaf4	0.54	1.00
P2ry14	0.53	1.00
Glb1l2	0.40	1.00
Vav1	0.96	1.00
Neu1	0.59	1.00
Psmd1	0.40	1.00
Csnk2a2	0.64	1.00
Hpcal1	1.29	1.00
Ddrgk1	0.20	1.00
Ptgr1	0.64	1.00
Eloc	0.76	1.00
Wipf2	0.39	1.00
Ruvbl2	0.39	1.00
Prkag2	0.20	1.00
Csnk1d	0.33	1.00
Eif6	0.28	1.00

Phf5a	0.31	1.00
Dnajc5	0.58	1.00
Kansl3	0.31	1.00
Abl2	0.77	1.00
Selenos	0.48	1.00
Golgb1	0.53	1.00
Eif3l	0.72	1.00
Psmc2	0.35	1.00
Eif4a1	0.82	1.00
Tgfbrap1	0.31	1.00
Emg1	0.35	1.00
Rnf32	0.49	1.00
Idh3b	0.41	1.00
Nat10	0.28	1.00
Derl3	0.24	1.00
Emc1	0.32	1.00
B3gntl1	0.35	1.00
Ccnt2	0.46	1.00
Irf6	0.47	1.00
Ldlr	0.79	1.00
Rarres1	-1.82	1.00
Trap1	0.82	1.00
Rab2a	0.55	1.00
Dmd	-1.27	1.00
Hs3st1	0.31	1.00
Zdhhc21	0.42	1.00
Tfrc	0.28	1.00
Pofut1	0.22	1.00
Kpna6	0.30	1.00
Wdr27	0.42	1.00
Ift20	0.25	1.00
Ubc	0.78	1.00
Arl1	0.49	1.00
Iqub	0.31	1.00
Irak4	0.41	1.00
Pcid2	0.21	1.00
Setdb2	0.42	1.00
Lrrc23	0.26	1.00
Serpinb11	0.90	1.00
Emilin2	0.80	1.00
Cenpq	0.26	1.00
Tnfrsf21	0.67	1.00
Pnpt1	0.15	1.00
Rmi1	0.25	1.00
Egr2	0.81	1.00
Abcf3	0.16	1.00

Ippk	0.18	1.00
Bco1	0.21	1.00
P2rx4	0.41	1.00
Emid1	0.21	1.00
Cep131	0.16	1.00
Pigl	0.51	1.00
Ptrh2	0.26	1.00
Srcin1	0.52	1.00
Slc66a1	0.19	1.00
C1qc	1.36	1.00
Lztr1	0.19	1.00
Prnp	0.15	1.00
Calr3	0.20	1.00
Dhrs13	0.15	1.00
Layn	0.17	1.00
Tmem212	0.73	1.00
Pih1d1	0.21	1.00
Zfp688	0.15	1.00
Hsd3b7	0.33	1.00
Ido1	0.76	1.00
Tspoap1	0.32	1.00
Osgep	0.17	1.00
Tssk4	0.18	1.00
Cpox	0.21	1.00
Fermt3	0.14	1.00
Slc6a14	1.07	1.00
B3gnt5	0.66	1.00
Eif2s2	0.68	1.00
Cds1	0.83	1.00
Plch1	0.84	1.00
Cars	-0.01	1.00
Hif1a	0.60	1.00
Tlk2	0.56	1.00
Rif1	0.58	1.00
Pkd2	-0.73	1.00
Zfp365	-1.02	1.00
Hspa12a	0.71	1.00
Ppp1r14bl	0.80	1.00
Pik3r1	0.79	1.00
Sharpin	0.21	1.00
Tlr3	0.24	1.00
Lrrc18	0.44	1.00
Commd2	0.60	1.00
Telo2	0.25	1.00
B3gnt4	0.31	1.00
Plpp5	0.42	1.00

Txnl4a	0.34	1.00
Kctd9	-1.36	1.00
Arhgap10	1.08	1.00
Wdr25	0.28	1.00
Lfng	0.37	1.00
Ncs1	0.34	1.00
Pop7	0.17	1.00
Arpc3	0.71	1.00
Ykt6	0.42	1.00
Ndufs6	0.10	1.00
Chfr	0.51	1.00
Clec16a	0.00	1.00
Arhgef26	-1.58	1.00
Aldh2	0.50	1.00
Hsp90ab1	0.72	1.00
Uba6	0.34	1.00
Psmc5	0.69	1.00
Kctd8	0.86	1.00
Spata7	0.59	1.00
Tdg	0.35	1.00
Dnajc27	0.26	1.00
Cog4	0.53	1.00
Thap7	0.21	1.00
Dop1b	0.77	1.00
Rap1b	0.90	1.00
Fam227a	0.63	1.00
Col4a3	-1.79	1.00
Katna1	0.47	1.00
Rcl1	0.10	1.00
Sec23ip	0.20	1.00
Iqch	0.53	1.00
Nxt1	0.26	1.00
H2aj	0.37	1.00
Optn	0.52	1.00
Uqcrfs1	-0.14	1.00
Lztfl1	0.60	1.00
Tmem263	0.62	1.00
Dpep2	0.74	1.00
Arhgap39	0.63	1.00
Jup	0.48	1.00
Atp2a3	0.49	1.00
Dyrk3	0.31	1.00
Prr15	0.18	1.00
Dhx16	0.33	1.00
Dynlt1a	0.41	1.00
Arih2	0.39	1.00

Inpp4a	-0.11	1.00
Dnah1	0.23	1.00
Ep400	0.13	1.00
Efl1	0.98	1.00
Sec61g	0.64	1.00
Trappc6b	0.11	1.00
Pcca	1.14	1.00
Gpr171	0.87	1.00
Zc3h12a	0.65	1.00
St14	0.58	1.00
Irak1bp1	0.42	1.00
Ppme1	0.36	1.00
Ranbp1	0.64	1.00
Coq2	0.25	1.00
Hypk	0.60	1.00
Ddost	0.22	1.00
Ulk4	0.96	1.00
Lmna	0.82	1.00
Psma3	0.43	1.00
Pdzd8	0.55	1.00
Ywhaz	0.62	1.00
Ephx2	0.34	1.00
Ints12	0.12	1.00
Zfp511	-0.18	1.00
Tmem125	0.14	1.00
Pomgnt1	0.16	1.00
Cep97	0.02	1.00
Wrap53	0.17	1.00
Usp11	0.07	1.00
Tmx1	0.31	1.00
Rapgef4	-1.57	1.00
4930579F01Rik	0.52	1.00
Txnrd1	0.53	1.00
Cfap97	0.87	1.00
Wdr61	0.38	1.00
Tango6	0.99	1.00
Usp7	0.37	1.00
Nck1	0.35	1.00
Syap1	0.37	1.00
Krt18	0.95	1.00
Fndc3a	0.65	1.00
Ago2	0.98	1.00
Rnpep	0.72	1.00
Selenot	0.69	1.00
Cops4	0.52	1.00
Taf15	0.26	1.00

Afdn	0.71	1.00
Lamp1	0.56	1.00
Arhgef10l	0.81	1.00
Wwc2	-1.49	1.00
Pnkd	0.84	1.00
Ireb2	0.15	1.00
Tmem98	-0.08	1.00
Ccpg1	0.57	1.00
Anxa11	0.42	1.00
Stk11	0.39	1.00
Dnali1	0.46	1.00
Rnf19a	0.82	1.00
Cyld	0.41	1.00
Dennd6b	0.42	1.00
Klf10	0.55	1.00
Iars	0.76	1.00
Capns1	0.77	1.00
Stat3	0.84	1.00
Fbxo39	0.82	1.00
Ccdc191	0.52	1.00
Cct7	0.47	1.00
Ric1	1.15	1.00
Cetn3	0.41	1.00
AA467197	1.57	1.00
Simc1	0.34	1.00
Dbndd2	0.37	1.00
Cfap410	0.46	1.00
Errfi1	0.87	1.00
Lamc2	1.46	1.00
Smim5	0.06	1.00
B4galt1	0.45	1.00
Slc22a23	0.97	1.00
Ifngr2	1.18	1.00
Aasdh	0.60	1.00
Samd4	-1.37	1.00
Drg1	0.59	1.00
Il6ra	0.46	1.00
Spp1	1.89	1.00
Tmem128	0.26	1.00
Pdk1	0.15	1.00
Prelid1	0.27	1.00
Bcl7c	0.44	1.00
Runx1	0.65	1.00
Rer1	0.38	1.00
Traf3ip2	0.77	1.00
Dync1li2	0.40	1.00

Rlf	0.59	1.00
Calr	0.87	1.00
Herpud1	0.80	1.00
Pip5k1a	0.60	1.00
Tubb5	0.83	1.00
Cand1	0.31	1.00
Hnrnpa0	0.51	1.00
Papola	0.72	1.00
Dcaf5	0.27	1.00
Sptlc2	0.78	1.00
Nrg1	-1.11	1.00
Cebpd	0.16	1.00
Galm	-0.22	1.00
Ppp3ca	-0.95	1.00
Akr1a1	0.84	1.00
Gpr160	0.54	1.00
Snx9	0.18	1.00
Clpx	0.01	1.00
Ppip5k2	0.49	1.00
Orc4	0.59	1.00
Ublcp1	0.54	1.00
Gpnmb	1.07	1.00
Gtf3c3	0.47	1.00
Bud23	0.79	1.00
Flnb	0.77	1.00
Exph5	0.60	1.00
Rfx3	1.37	1.00
Notch2	-2.20	1.00
Tnfaip8	0.68	1.00
Rnh1	0.61	1.00
Fnbp4	0.31	1.00
Plk3	0.03	1.00
Pdia4	0.86	1.00
Dennd1a	0.91	1.00
Chd1	0.58	1.00
Kremen1	0.65	1.00
Asb3	0.31	1.00
Cntln	0.59	1.00
Rnf114	0.27	1.00
Pip5k1b	0.65	1.00
P3h2	-1.59	1.00
Sertad1	0.94	1.00
Lhfpl2	0.67	1.00
Tspan14	0.16	1.00
Tom1l2	0.02	1.00
Fam222a	0.21	1.00

Ube2v2	0.39	1.00
C2cd3	0.81	1.00
Csrnp1	0.58	1.00
Sccpdh	0.95	1.00
Nadk2	0.54	1.00
Pparg	0.46	1.00
Rfxank	0.23	1.00
Tmprss4	0.57	1.00
Cpsf6	0.69	1.00
Psmb2	0.60	1.00
Ppm1d	-0.01	1.00
Cuedc1	0.24	1.00
Odf2l	0.40	1.00
Psd3	0.16	1.00
Rrbp1	0.89	1.00
Gpi1	0.64	1.00
Atp9a	0.39	1.00
Coro1b	0.55	1.00
Dennd2d	0.75	1.00
Zfp131	0.60	1.00
Akap6	0.21	1.00
Kif3a	0.54	1.00
Ric8b	0.52	1.00
Ndufa2	0.12	1.00
Rbm22	0.11	1.00
Naa50	0.65	1.00
Srfbp1	0.62	1.00
Glipr2	0.75	1.00
Rap1gds1	1.08	1.00
Wdr49	1.13	1.00
Pdzd11	0.32	1.00
Nudt13	0.25	1.00
2610507B11Rik	0.59	1.00
Vcpkmt	0.34	1.00
Tmem216	0.34	1.00
Zfp606	0.30	1.00
Tmem101	0.04	1.00
Cdkn1a	0.17	1.00
Pdia6	0.17	1.00
Cttn	0.23	1.00
Szrd1	0.49	1.00
Sec16a	0.52	1.00
Folh1	-0.61	1.00
Spata1	-0.45	1.00
Gid8	0.40	1.00
Plk4	0.98	1.00

Zc3h7a	0.78	1.00
Ift52	0.46	1.00
Upf1	0.44	1.00
Cox8a	0.27	1.00
Slc30a6	-0.13	1.00
Atp6ap1	-0.03	1.00
Ddx24	0.49	1.00
Ulk2	1.19	1.00
Acot13	0.20	1.00
Nhsl1	1.11	1.00
Chmp4b	0.20	1.00
Txndc5	0.27	1.00
Retsat	0.44	1.00
Xpo1	0.50	1.00
Camta1	0.54	1.00
Sod1	-0.01	1.00
Sod2	0.68	1.00
Khdrbs1	0.59	1.00
Tbc1d4	1.42	1.00
C4b	1.04	1.00
9430038I01Rik	0.29	1.00
Pkhd1	-1.07	1.00
Atp8b1	0.89	1.00
Usp6nl	0.99	1.00
Pus7	0.69	1.00
Tuba1b	0.53	1.00
Cnih1	0.22	1.00
Cep104	0.41	1.00
Erlin2	0.25	1.00
Rnf138	0.71	1.00
Cavin2	-1.26	1.00
Fgfr2	0.42	1.00
Taf1b	0.50	1.00
Serinc2	0.44	1.00
Wdr66	0.39	1.00
Klf4	1.21	1.00
Sin3a	0.40	1.00
Amd2	1.26	1.00
Ptgs2	-0.72	1.00
Bcas1	0.54	1.00
Tars	0.81	1.00
2610528J11Rik	0.42	1.00
Fam76b	0.11	1.00
Pknox1	0.16	1.00
Dctn6	0.20	1.00
Rrp12	0.30	1.00

Actl6a	0.31	1.00
Lrrc49	0.21	1.00
Adora2b	0.05	1.00
Atg101	0.51	1.00
Sppl2b	-0.11	1.00
Poc5	0.30	1.00
Agpat3	0.36	1.00
Sdhd	0.67	1.00
Gars	1.13	1.00
Srpr	0.57	1.00
Bmp2k	0.68	1.00
Kmt5a	0.31	1.00
Caprin2	0.43	1.00
Zfp830	0.55	1.00
Lpin1	0.52	1.00
C1qb	0.39	1.00
C77080	0.31	1.00
Prickle3	0.30	1.00
Cfl1	1.18	1.00
Usp48	0.11	1.00
Phactr2	-1.58	1.00
Ppm1g	-0.12	1.00
Vps37b	0.51	1.00
Cdk2ap2	0.34	1.00
Rsph3b	0.63	1.00
Cops5	0.41	1.00
Vdac3	0.45	1.00
Slc45a4	0.28	1.00
Cdyl	0.09	1.00
Itgb6	-0.83	1.00
Ezh2	0.20	1.00
Tmem63a	0.23	1.00
Zfyve21	0.43	1.00
Kctd10	0.07	1.00
Tnfrsf9	0.16	1.00
Ddah2	0.22	1.00
Atf3	1.00	1.00
Vrk2	0.66	1.00
Snrnp200	0.54	1.00
Pias1	-1.11	1.00
Klf5	0.78	1.00
Vezf1	0.34	1.00
Carnmt1	0.33	1.00
Taok2	0.21	1.00
F13a1	0.96	1.00
Fuca2	0.18	1.00

Slc16a2	0.22	1.00
Stk26	0.47	1.00
Lsg1	0.23	1.00
Ap4e1	0.55	1.00
Unc119	0.24	1.00
Thap11	0.21	1.00
Tmub2	0.19	1.00
Hnrnpk	0.51	1.00
Arrb1	0.50	1.00
Cmas	0.43	1.00
Hmgcs1	1.05	1.00
Cadm1	-0.98	1.00
Itgb1bp1	0.28	1.00
Hrh4	0.29	1.00
Il4ra	1.84	1.00
Rbm14	0.75	1.00
Per1	0.05	1.00
Srp68	0.37	1.00
Tekt1	0.89	1.00
Tent4a	0.25	1.00
Slc41a2	0.59	1.00
Recql	0.48	1.00
Cdc25a	0.34	1.00
Eya1	0.65	1.00
Csmd1	1.25	1.00
Exoc8	0.43	1.00
Pak3	0.49	1.00
Tmem192	0.13	1.00
Spata4	0.23	1.00
Il7r	0.73	1.00
Creb3l1	0.37	1.00
Ptdss2	0.14	1.00
Riox1	0.18	1.00
Scg5	0.32	1.00
Rasgrp1	0.33	1.00
Gstz1	0.14	1.00
Clp1	0.15	1.00
Tnfaip2	0.41	1.00
Kmo	1.01	1.00
Trub1	0.12	1.00
Hells	0.22	1.00
Thap4	0.09	1.00
Trpm2	0.77	1.00
Pfn2	0.13	1.00
Atrip	0.13	1.00
Pskh1	0.20	1.00

Grpel2	0.21	1.00
Csf1r	0.77	1.00
Nptx2	0.13	1.00
Slc29a4	0.12	1.00
Fscn1	0.62	1.00
Ccdc92	0.17	1.00
Adora2a	0.79	1.00
Snapc4	0.12	1.00
Zswim7	0.12	1.00
Nab2	0.22	1.00
Hmbs	0.10	1.00
Nlrx1	0.11	1.00
Dcaf15	0.13	1.00
Ppp1r12c	0.48	1.00
Slc9a4	0.87	1.00
Fgfbp1	0.39	1.00
Zfyve28	0.16	1.00
B4galnt4	0.15	1.00
Psd	0.15	1.00
Dele1	0.22	1.00
Smim26	0.13	1.00
Lbp	0.51	1.00
Slc39a1	0.20	1.00
Cdkn2b	0.35	1.00
Emilin1	0.13	1.00
Cxcl10	1.09	1.00
Repin1	0.20	1.00
Acrbp	0.10	1.00
Ceacam20	0.17	1.00
Zfp428	0.16	1.00
Ccdc97	0.14	1.00
Bcl2l12	0.20	1.00
Trim72	0.61	1.00
Nol3	0.17	1.00
Gna15	0.20	1.00
Zmiz2	0.21	1.00
Prss22	0.39	1.00
Ubd	0.10	1.00
Clpp	0.15	1.00
Slc15a3	0.27	1.00
Plekhs1	0.44	1.00
Fosb	1.19	1.00
Nfxl1	0.19	1.00
Ogfr	0.18	1.00
Dhrs11	0.27	1.00
Nipal3	0.20	1.00

Tigd4	0.40	1.00
Dtx3	0.42	1.00
Psmb7	0.11	1.00
Hnrnpd	0.71	1.00
Wrnip1	0.37	1.00
Bmper	0.83	1.00
Adrb2	-0.40	1.00
Gna11	0.08	1.00
Tbca	0.09	1.00
Scyl2	0.10	1.00
Tm9sf2	0.56	1.00
Srp14	0.01	1.00
Tarbp1	0.35	1.00
Foxa1	0.53	1.00
Lss	0.25	1.00
Becn1	0.29	1.00
Igf2bp3	0.87	1.00
Ap3b1	-1.10	1.00
Tasor2	0.27	1.00
Ppp2r2a	0.59	1.00
Nup107	0.42	1.00
Rdh14	0.19	1.00
Crisp3	2.74	1.00
Castor2	0.25	1.00
G6pc3	0.15	1.00
Letmd1	0.34	1.00
Prune1	0.16	1.00
Nav2	0.28	1.00
Pwp1	0.55	1.00
Otud4	0.37	1.00
Qsox2	0.12	1.00
Slpi	-0.85	1.00
Agrn	-1.22	1.00
Cic	-0.29	1.00
Bnip3	0.26	1.00
Epsti1	-0.59	1.00
Zfp462	-1.02	1.00
Bzw2	1.17	1.00
Drc7	0.53	1.00
Capg	0.55	1.00
Epb41	0.53	1.00
Chd8	0.41	1.00
Abl1	1.30	1.00
Gramd4	0.48	1.00
Armh1	0.61	1.00
Tmem184b	0.21	1.00

Slc4a2	0.22	1.00
Uprt	0.22	1.00
Tmem175	0.42	1.00
Shq1	0.27	1.00
Cyb561d2	0.15	1.00
Gid4	0.21	1.00
Pdcl	0.38	1.00
Rundc1	0.28	1.00
Ints11	0.20	1.00
Star	0.76	1.00
Timm44	0.20	1.00
Scamp3	0.19	1.00
Tmub1	0.13	1.00
Cmip	0.90	1.00
Insc	-1.05	1.00
Lrrc59	0.15	1.00
Zbtb40	0.03	1.00
Pafah1b3	-0.15	1.00
Gnl1	0.01	1.00
Prdm2	0.28	1.00
Atp6v0b	0.53	1.00
Acat3	0.22	1.00
Prrg4	0.44	1.00
Dazap1	0.77	1.00
Cmpk1	0.80	1.00
Prr29	0.23	1.00
1700025G04Rik	0.43	1.00
Adat1	0.26	1.00
Acer3	-1.07	1.00
Ssr4	0.82	1.00
Kat6b	0.45	1.00
Arhgef18	-1.29	1.00
Rfc1	0.72	1.00
N4bp2	0.32	1.00
Cyc1	0.35	1.00
Smad3	0.90	1.00
Prmt1	0.59	1.00
Clint1	0.69	1.00
Tanc2	0.55	1.00
Dcun1d5	0.08	1.00
Smc6	0.04	1.00
Usp31	0.21	1.00
Rprd2	0.44	1.00
Xrcc6	0.11	1.00
Cep76	0.16	1.00
Slc25a38	0.10	1.00

Agtpbp1	-0.17	1.00
Fa2h	0.10	1.00
Cant1	0.12	1.00
C1qa	0.16	1.00
Inava	0.15	1.00
Serpini2	0.13	1.00
Sphk2	0.01	1.00
Dusp18	0.13	1.00
Gclc	0.56	1.00
Rgcc	-0.91	1.00
Ostc	0.65	1.00
Trrap	0.69	1.00
Cwf19l2	-0.17	1.00
Gna13	0.48	1.00
Eprs	0.51	1.00
Ndufs3	-0.04	1.00
Actr10	0.17	1.00
Tmem14a	0.21	1.00
Armh4	0.18	1.00
Lrp2bp	-0.62	1.00
Usp37	0.33	1.00
Dusp16	0.76	1.00
Pcnx	0.28	1.00
Wwox	0.96	1.00
Cwc22	0.48	1.00
Nolc1	0.24	1.00
Auts2	0.78	1.00
Anxa1	1.00	1.00
Ahi1	0.42	1.00
Itprid2	0.80	1.00
Nudc	0.79	1.00
Echs1	0.33	1.00
Poc1b	0.28	1.00
Zdhhc5	-0.11	1.00
Tpd52l2	0.10	1.00
Atp2c2	0.85	1.00
Bcam	-1.27	1.00
Cst6	0.43	1.00
Sntn	0.66	1.00
Spcs2	0.59	1.00
S100a1	1.14	1.00
Wdr90	0.26	1.00
Gtf2h3	-0.03	1.00
Ilf3	0.44	1.00
Ccdc18	0.42	1.00
Supt6	0.58	1.00

Rtf2	0.26	1.00
Ubac1	0.19	1.00
Ammecr1l	0.11	1.00
Gak	-0.22	1.00
Efcab12	0.18	1.00
Epm2aip1	0.38	1.00
Gpx4	0.35	1.00
Spata5	-0.88	1.00
Ahsa1	0.43	1.00
Efemp1	-1.20	1.00
Srsf7	0.57	1.00
P4ha1	0.59	1.00
Psme2	1.16	1.00
Fyb2	0.65	1.00
Dynll2	0.33	1.00
Cc2d2a	0.20	1.00
Narf	0.36	1.00
Pfdn2	-0.03	1.00
Eepd1	0.66	1.00
Sema4d	0.39	1.00
Trim3	0.60	1.00
Gga1	0.40	1.00
Katnb1	0.21	1.00
Timm22	0.23	1.00
Smpd2	0.27	1.00
Foxo1	0.70	1.00
Tex9	0.74	1.00
Chordc1	0.47	1.00
Irf2bp2	0.49	1.00
Zranb1	0.56	1.00
Fhad1	0.71	1.00
Napepld	0.37	1.00
Kif3b	0.89	1.00
Aars	0.56	1.00
Pik3ap1	0.62	1.00
Akna	0.40	1.00
Ccdc88c	0.57	1.00
St13	0.33	1.00
Psmd14	0.31	1.00
Casd1	0.46	1.00
Ppp4r1	0.30	1.00
Insig1	0.60	1.00
Strap	0.11	1.00
Zcchc2	0.25	1.00
Fhod3	0.68	1.00
Wars	0.61	1.00

Psme3	0.39	1.00
Rtcb	0.31	1.00
Wls	0.66	1.00
Slc20a1	0.52	1.00
Bckdhb	0.38	1.00
Abhd18	0.30	1.00
Sh3glb2	0.46	1.00
Spats2	0.21	1.00
Tpgs1	0.25	1.00
Camk1d	0.68	1.00
Rab11a	0.51	1.00
Ppp2r5c	0.31	1.00
Ppp1r15a	0.91	1.00
Rfc2	0.51	1.00
Ier5	1.07	1.00
Arpc4	0.06	1.00
Spidr	0.51	1.00
Stk10	0.14	1.00
Sparc	-0.71	1.00
Pfdn4	0.17	1.00
Rab40b	0.24	1.00
Prpf8	0.12	1.00
Fam180a	-0.78	1.00
Zfp318	-0.17	1.00
Fn1	-0.43	1.00
Exo5	-0.55	1.00
Zfp768	-0.24	1.00
Stau1	0.08	1.00
Rack1	0.86	1.00
Ggnbp2	0.26	1.00
Herc2	0.07	1.00
Spock2	-1.52	1.00
Sds	-2.92	1.00
Fh1	0.14	1.00
Dlgap4	0.18	1.00
Fuca1	0.16	1.00
Brk1	0.14	1.00
Zmynd8	0.54	1.00
Fam193a	0.46	1.00
Klf12	0.46	1.00
Pde7a	-1.12	1.00
Nudt14	0.10	1.00
Ralgapb	0.47	1.00
Sec22b	0.14	1.00
Tsnaxip1	0.46	1.00
Wbp1	0.37	1.00

Srm	0.41	1.00
Dnajc16	0.31	1.00
Nuak2	0.57	1.00
Gipc1	0.53	1.00
Nsmce1	0.30	1.00
Trim37	0.71	1.00
Appl2	0.43	1.00
Pde3b	-2.10	1.00
Ndufv2	0.01	1.00
Plaa	0.41	1.00
Dzip1l	0.81	1.00
Uqcrc2	0.14	1.00
Khsrp	0.31	1.00
Samd12	-1.79	1.00
Pdpn	-0.90	1.00
Nrip1	0.44	1.00
Uba5	0.76	1.00
Ak6	0.15	1.00
Ccdc47	0.06	1.00
Phlpp2	0.12	1.00
Tmem208	-0.03	1.00
Ski	-0.06	1.00
Eif1	0.51	1.00
Ss18	1.19	1.00
Btf3l4	0.75	1.00
Usp9x	0.31	1.00
Rdx	-1.44	1.00
Txndc11	0.53	1.00
Rchy1	0.53	1.00
Ubxn11	0.69	1.00
Lsm2	0.37	1.00
Psmb10	0.59	1.00
Alg5	0.33	1.00
Lmln	0.34	1.00
Hectd2	0.57	1.00
Bloc1s6	0.18	1.00
Asb14	0.38	1.00
Rac3	0.19	1.00
Fam166c	0.53	1.00
Itgb4	0.13	1.00
Trim27	0.21	1.00
Pdlim1	0.13	1.00
Etfdh	-0.59	1.00
Esyt3	0.36	1.00
Gnpda1	0.25	1.00
Stau2	-0.62	1.00

Angel2	0.75	1.00
Tspo	0.72	1.00
Cln3	-0.05	1.00
Csnk1a1	0.53	1.00
Fli1	-1.22	1.00
Parn	0.23	1.00
Cltb	0.66	1.00
H2-Ea	0.79	1.00
Socs6	-0.05	1.00
Soat1	0.38	1.00
Dot1l	0.22	1.00
Ppp1r3b	-0.60	1.00
Cacng6	0.31	1.00
Fosl2	0.65	1.00
Snx29	0.07	1.00
Slamf7	0.87	1.00
Dnajc7	0.10	1.00
Cebpz	0.25	1.00
Iqcd	0.34	1.00
Vps28	0.11	1.00
Igf2r	0.33	1.00
Ift46	0.21	1.00
Kitl	-1.32	1.00
Slc7a1	0.35	1.00
Dkc1	0.38	1.00
Creb5	0.45	1.00
Iah1	0.06	1.00
Gatb	0.20	1.00
Htatsf1	0.21	1.00
Nploc4	0.13	1.00
Cdipt	0.29	1.00
Nr4a1	0.57	1.00
Stx8	0.25	1.00
Acvr1	0.49	1.00
Elovl6	0.42	1.00
Tm9sf4	0.47	1.00
Comt	0.09	1.00
Ppp1cc	0.15	1.00
Ap2s1	0.23	1.00
Ap2a2	0.10	1.00
Rhoq	-0.06	1.00
Chchd4	0.10	1.00
Ube2a	-0.09	1.00
Smad4	0.63	1.00
Sp110	0.53	1.00
Pter	-0.65	1.00

0610040J01Rik	0.75	1.00
Uox	0.41	1.00
Nrf1	0.26	1.00
Tbc1d2b	0.73	1.00
Psip1	0.41	1.00
Aplf	0.41	1.00
Cep170	0.78	1.00
Nfkbia	-0.06	1.00
Gsto1	0.58	1.00
Septin2	0.54	1.00
B4galt4	0.27	1.00
Pfkp	0.20	1.00
Rnf217	0.31	1.00
Flvcr1	0.20	1.00
Nrp2	-0.07	1.00
Tmem69	0.59	1.00
Tceanc2	0.29	1.00
Ergic3	0.25	1.00
Ube2o	0.22	1.00
Tmem222	0.21	1.00
Tnni3	0.19	1.00
Gramd3	1.36	1.00
Tmem123	0.47	1.00
Atp6v1h	0.15	1.00
Asb7	0.23	1.00
Nisch	0.21	1.00
Hmgxb3	0.21	1.00
Kdm4a	0.24	1.00
Ercc8	-0.04	1.00
Cep128	1.11	1.00
Sdhb	0.29	1.00
Dync1h1	0.53	1.00
Sun1	0.17	1.00
Ttc38	0.34	1.00
Dgkh	0.55	1.00
Sms	0.68	1.00
4933434E20Rik	0.40	1.00
Gcc2	0.77	1.00
Erh	0.57	1.00
Ptprj	0.70	1.00
Ccdc117	0.60	1.00
Srrm4	1.10	1.00
Rcsd1	-0.96	1.00
Daam2	-0.86	1.00
Nup214	0.66	1.00
Caprin1	0.09	1.00

Cep170b	0.43	1.00
Ttc23l	0.09	1.00
Gfm1	0.55	1.00
Ndufb2	0.29	1.00
Cbfb	0.25	1.00
Rad51b	-0.03	1.00
Hbb-bh2	-1.07	1.00
Rbm25	0.51	1.00
Etv6	0.75	1.00
Dnajb6	0.64	1.00
Cox5a	0.88	1.00
Smdt1	-0.36	1.00
Chchd1	-0.39	1.00
Bud31	0.02	1.00
Smg6	0.07	1.00
Scaper	-1.26	1.00
Ndufb1	0.40	1.00
Phf2	0.32	1.00
Exosc8	0.15	1.00
Zfand1	-0.22	1.00
Lrp12	0.23	1.00
Ccdc127	0.20	1.00
Npl	0.23	1.00
Fes	0.37	1.00
Aimp2	0.21	1.00
Npdc1	0.39	1.00
Ccdc142	0.29	1.00
Sel1l	0.30	1.00
Nudcd2	0.25	1.00
Pbxip1	0.38	1.00
Nampt	0.46	1.00
Hspe1-rs1	0.68	1.00
Usp12	0.44	1.00
Atp5pb	0.05	1.00
Atp5d	0.61	1.00
Capsl	0.79	1.00
Ppm1l	-1.61	1.00
Sugt1	0.48	1.00
Osbpl10	-1.05	1.00
Enox2	0.62	1.00
Epcam	0.48	1.00
Polr3b	1.42	1.00
Serpinb5	0.83	1.00
Asap2	0.78	1.00
Rbmx2	0.32	1.00
Acp2	0.17	1.00

Ap5z1	0.34	1.00
Lad1	0.24	1.00
Cd163	1.63	1.00
Cyp2e1	0.23	1.00
Slc31a1	0.70	1.00
Pgk1	0.62	1.00
Snx4	0.36	1.00
Cdkl5	0.37	1.00
Eif4a3l1	0.64	1.00
Zfp800	0.11	1.00
Got1	0.01	1.00
Ctsz	0.51	1.00
Pde4b	-1.11	1.00
Spint2	0.79	1.00
Nfyb	0.35	1.00
Esr1	0.07	1.00
2210016F16Rik	0.23	1.00
Grk2	0.18	1.00
Gnpnat1	0.07	1.00
Ogfod1	0.17	1.00
Src	0.13	1.00
Ecsit	0.19	1.00
Tmem213	0.25	1.00
Pls1	-1.09	1.00
Ncl	0.65	1.00
Hipk2	0.97	1.00
Kat2b	-1.46	1.00
Cers6	1.30	1.00
Sema3c	1.00	1.00
Cltc	0.32	1.00
Tgfbr1	0.54	1.00
Sec31a	0.19	1.00
Ube2z	0.34	1.00
Gbf1	0.53	1.00
Cracd	0.57	1.00
Cdkn2aip	0.37	1.00
Spg21	0.18	1.00
Ftl1-ps1	1.13	1.00
Uxs1	0.42	1.00
Dgki	2.13	1.00
Sytl5	0.58	1.00
Polr1b	0.31	1.00
Kit	0.38	1.00
Saal1	0.22	1.00
Cmtm3	0.20	1.00
Slc26a2	0.48	1.00

Atp13a1	0.20	1.00
Heatr1	0.17	1.00
Pfkfb4	0.58	1.00
Apex1	0.15	1.00
Ankfn1	1.32	1.00
Nudt16	0.50	1.00
Pgm2l1	0.16	1.00
Asb13	0.12	1.00
Hebp1	-0.04	1.00
Ska3	0.10	1.00
Ttc39b	0.09	1.00
Zdhhc13	0.10	1.00
Itgb5	0.09	1.00
Rexo1	0.12	1.00
Dcdc2b	0.08	1.00
Pdia3	1.10	1.00
Alcam	-0.60	1.00
Enpp4	0.43	1.00
Galnt3	0.56	1.00
Tfcp2	0.52	1.00
Cerkl	0.66	1.00
Dnttip1	-0.12	1.00
Etl4	0.71	1.00
Sfmbt1	-0.15	1.00
Cdh13	0.26	1.00
Rab22a	0.21	1.00
Emp2	-1.24	1.00
Npnt	-0.68	1.00
Psmd12	0.45	1.00
Spred2	0.17	1.00
Wfs1	0.11	1.00
Pacrg	1.53	1.00
Arpp21	-0.53	1.00
Nenf	0.30	1.00
Ccndbp1	0.33	1.00
Sgta	0.19	1.00
Ksr1	0.59	1.00
Tspan17	0.26	1.00
Zfp24	0.26	1.00
Ehbp1	-1.23	1.00
Dnah7b	0.96	1.00
Slc9a3r1	0.56	1.00
Lamtor2	-0.15	1.00
Abce1	-0.12	1.00
Zc3hc1	0.15	1.00
Sgsm3	0.14	1.00

Haus6	0.16	1.00
Idnk	0.25	1.00
Lsp1	0.23	1.00
Clmn	0.20	1.00
Trmt5	0.26	1.00
Smarcal1	0.22	1.00
Cybc1	0.12	1.00
Hlf	0.23	1.00
1700012P22Rik	0.51	1.00
2310030G06Rik	0.25	1.00
Ccdc189	0.20	1.00
Plscr3	0.13	1.00
Pip4p1	0.15	1.00
Tasp1	0.42	1.00
Sipa1l2	0.22	1.00
B3gnt7	-0.15	1.00
Orai1	0.11	1.00
Slc25a39	-0.19	1.00
Rarg	0.01	1.00
Adam9	0.87	1.00
Hsf2	0.46	1.00
Dcxr	0.31	1.00
Spata6	0.78	1.00
Higd2a	0.15	1.00
Ccnl2	0.18	1.00
Jhy	0.32	1.00
B9d2	0.38	1.00
Slc9a2	0.57	1.00
BC016579	0.19	1.00
Tafa2	0.16	1.00
Ccdc77	0.30	1.00
Rab20	0.45	1.00
Kcne3	0.79	1.00
Acod1	1.05	1.00
A4galt	0.15	1.00
Strip2	0.10	1.00
Slc35b4	0.13	1.00
Bcl2l14	0.20	1.00
Dmc1	0.25	1.00
Pdxp	0.73	1.00
Sacs	0.75	1.00
Asb9	0.19	1.00
Hgd	0.50	1.00
Ccn4	0.44	1.00
Als2cl	0.10	1.00
Plcd1	0.15	1.00

Tppp	0.08	1.00
Cyp7a1	0.51	1.00
Rrs1	0.37	1.00
Serpinb8	0.09	1.00
Dynlt2a3	0.10	1.00
Ehd3	0.11	1.00
Zfp512	0.13	1.00
Gemin6	0.10	1.00
Sobp	0.17	1.00
Vash2	0.12	1.00
Plekhh2	0.37	1.00
Cyp1b1	0.49	1.00
Slc35f3	0.59	1.00
Ap5s1	0.10	1.00
Radx	0.11	1.00
Vsig4	0.11	1.00
Neil3	0.44	1.00
Edil3	1.04	1.00
F2r	0.06	1.00
Lipg	0.17	1.00
Prdm11	0.18	1.00
Bahd1	0.32	1.00
Fancm	0.13	1.00
Cox16	0.11	1.00
Ush1c	0.28	1.00
Vipas39	0.29	1.00
Tesmin	0.11	1.00
Exoc3l4	0.46	1.00
Brf1	0.08	1.00
Stk11ip	0.11	1.00
Tmem198	0.14	1.00
Bcs1l	0.26	1.00
Nxph2	0.10	1.00
Hoga1	0.15	1.00
Hkdc1	0.32	1.00
Stambpl1	0.14	1.00
Clca2	0.19	1.00
Tmem61	0.20	1.00
Atp6v1g3	0.34	1.00
Tyw3	0.11	1.00
Pappa2	0.85	1.00
Gpc1	0.14	1.00
Ube2g2	0.10	1.00
Serpine2	0.26	1.00
Fam131a	0.07	1.00
Efhd1	0.76	1.00

P2ry1	0.60	1.00
Gpr87	0.10	1.00
Arnt2	0.16	1.00
Fah	0.24	1.00
Hapln3	0.20	1.00
Rgma	0.12	1.00
Blm	0.13	1.00
Ppcdc	0.15	1.00
Scamp5	0.75	1.00
Gcnt3	0.27	1.00
Usp4	0.06	1.00
1600014C10Rik	0.14	1.00
Tk2	0.16	1.00
Gabrp	0.73	1.00
Kcnip1	1.03	1.00
Sgcd	0.23	1.00
Ttyh3	0.14	1.00
Elmo2	0.09	1.00
Sulf2	0.12	1.00
Arvcf	0.12	1.00
Smpd4	0.08	1.00
Slc25a19	0.07	1.00
Glp2r	0.75	1.00
Fn3k	0.11	1.00
Slc16a6	0.19	1.00
Uap1l1	0.12	1.00
Alad	0.17	1.00
Vps37d	0.09	1.00
Spns3	0.08	1.00
Csf3	0.61	1.00
Zfp385c	0.09	1.00
Limd2	0.24	1.00
Trpt1	0.13	1.00
Phf13	0.12	1.00
Nmnat1	0.13	1.00
Fblim1	0.07	1.00
Clcn6	0.24	1.00
Rpusd4	0.16	1.00
Ctps	0.15	1.00
Strip1	0.09	1.00
Traf3ip3	0.15	1.00
Tspan2	0.12	1.00
Dmtn	0.06	1.00
Letm2	0.12	1.00
Arid5a	0.20	1.00
Creb3l3	0.11	1.00

Diras1	0.12	1.00
Gtpbp3	0.20	1.00
Ticam1	0.09	1.00
Angptl4	0.09	1.00
Tube1	0.16	1.00
Slc12a8	0.15	1.00
Cfap157	0.09	1.00
Bpifa1	2.68	1.00
Lexm	0.48	1.00
Ccdc158	0.30	1.00
Pvrig	0.22	1.00
A2m	0.44	1.00
Rdh13	0.12	1.00
Ppp1r13l	0.08	1.00
Tulp2	0.75	1.00
AI429214	0.43	1.00
Nacc1	0.11	1.00
Nod2	0.58	1.00
Piwil4	0.80	1.00
AB124611	0.59	1.00
Dock6	0.08	1.00
Vwa5a	0.19	1.00
Ccdc51	0.51	1.00
Eef1akmt3	0.11	1.00
Pla2g3	0.49	1.00
Trim11	0.11	1.00
Alox8	0.75	1.00
Stat5a	0.25	1.00
Aldoart2	0.13	1.00
Edaradd	0.12	1.00
Tep1	0.14	1.00
Scara3	0.12	1.00
Gsdmd	0.14	1.00
Iqank1	0.10	1.00
Sh3bgr	0.21	1.00
Adgrf1	1.36	1.00
Renbp	0.11	1.00
Arhgap26	0.73	1.00
Atp6v1f	0.49	1.00
Kpna4	0.54	1.00
Nudcd3	-0.28	1.00
Ccdc115	-0.09	1.00
Tanc1	-0.95	1.00
Crk	0.43	1.00
Tsc22d4	0.48	1.00
Carm1	0.30	1.00

Dnah12	0.39	1.00
Tet3	0.44	1.00
Crybg1	0.97	1.00
Casp3	0.18	1.00
Rab28	-0.02	1.00
Top2b	0.33	1.00
Ranbp2	0.14	1.00
Phtf2	0.72	1.00
Slc12a2	0.88	1.00
Nr4a2	-0.11	1.00
Ctdsp2	-0.13	1.00
Pcmtd1	0.55	1.00
Ip6k2	0.38	1.00
Col4a4	-0.72	1.00
Iws1	0.30	1.00
Ctr9	0.16	1.00
Ist1	0.12	1.00
Cav1	-1.89	1.00
Galnt11	0.31	1.00
Stam	0.16	1.00
Rnf4	0.19	1.00
Tead1	-1.02	1.00
Nsa2	0.22	1.00
Psmd13	0.19	1.00
Ptprz1	0.42	1.00
Anxa13	0.16	1.00
Grhl1	0.49	1.00
Ccng2	0.15	1.00
Coro2a	0.15	1.00
Ptch1	0.28	1.00
Prxl2c	0.19	1.00
LTO1	0.16	1.00
Pigq	0.14	1.00
Rab36	0.17	1.00
Slc16a5	1.20	1.00
Ccdc43	0.15	1.00
Trim7	0.16	1.00
Aen	0.14	1.00
Tspyl1	0.42	1.00
Thtpa	0.10	1.00
Rbbp7	0.27	1.00
Gnptab	0.61	1.00
Rnaseh2c	-0.32	1.00
Tfdp1	0.23	1.00
Traf3ip1	0.45	1.00
Slc1a2	-0.85	1.00

Rrm2b	0.33	1.00
Xpo5	0.16	1.00
Sar1a	0.01	1.00
Gnl2	0.27	1.00
Plekha6	0.67	1.00
Syngap1	0.19	1.00
Ap1m2	0.20	1.00
H1f4	1.00	1.00
Kmt5b	0.21	1.00
Fnip2	0.51	1.00
Ube2m	0.42	1.00
Gsn	-2.11	1.00
Dock4	0.09	1.00
Slc2a13	0.19	1.00
Gfus	0.19	1.00
Taf5l	0.22	1.00
Hrh1	0.38	1.00
Tmtc2	-1.39	1.00
Rbpms	-0.81	1.00
Phf20l1	-0.98	1.00
Ints6	0.58	1.00
Rfx2	0.74	1.00
Arrdc3	0.66	1.00
Pnrc2	0.78	1.00
Spag1	0.77	1.00
Ctps2	0.60	1.00
Sqstm1	0.35	1.00
Btbd6	0.31	1.00
Ppp1r11	0.09	1.00
Lrch1	-1.14	1.00
Uchl5	0.45	1.00
Gpr107	0.80	1.00
Lmnb1	-0.90	1.00
Dnajc10	0.31	1.00
Itpr1	0.34	1.00
Asxl1	0.33	1.00
Fbxl14	0.19	1.00
Golt1b	0.21	1.00
Nup205	0.09	1.00
Utp25	-0.10	1.00
Cd82	0.06	1.00
Top3a	0.11	1.00
Itpr3	0.22	1.00
Tmc4	0.07	1.00
Prss8	0.46	1.00
Gpc5	0.58	1.00

Prr5	0.00	1.00
Nudt15	0.13	1.00
Atpsckmt	0.03	1.00
2310033P09Rik	0.12	1.00
Coq8a	0.11	1.00
Elp3	0.12	1.00
Zup1	-0.03	1.00
Polr1c	0.07	1.00
2310022B05Rik	0.02	1.00
Pof1b	0.01	1.00
Unc93b1	-0.05	1.00
Slc35c1	0.03	1.00
Cracr2b	0.10	1.00
Slc40a1	0.42	1.00
Adgra1	-0.17	1.00
Echdc2	-0.05	1.00
Txndc15	0.09	1.00
Traf7	0.06	1.00
Coq5	-0.02	1.00
Unc13d	-0.07	1.00
Lig3	0.07	1.00
Csrnp2	0.10	1.00
Padi2	0.10	1.00
Vps26b	-0.11	1.00
Slc37a4	0.05	1.00
Tekt2	-0.17	1.00
Yju2	-0.04	1.00
Sugp2	0.14	1.00
Ccdc83	-0.11	1.00
Dbnl	0.04	1.00
Zfp78	-0.37	1.00
Sympk	0.07	1.00
Kif19a	-0.05	1.00
Kcnh3	0.13	1.00
Spats1	0.12	1.00
Dnph1	0.12	1.00
Elf4	0.18	1.00
Fam50a	0.09	1.00
Prcp	0.78	1.00
Ube3a	0.46	1.00
Lonrf1	-0.94	1.00
Tpp2	0.26	1.00
Stip1	-0.03	1.00
Cbr2	-1.04	1.00
Egfr	0.56	1.00
Saraf	0.63	1.00

Oxct1	0.57	1.00
Ctbp2	0.29	1.00
Ddx18	0.12	1.00
Zfp592	0.51	1.00
Derl1	0.32	1.00
H2-Ab1	0.81	1.00
Psmd3	-0.01	1.00
Xpo6	-1.32	1.00
Selenop	0.04	1.00
Edf1	-0.09	1.00
Klc2	-0.10	1.00
Lpgat1	-2.10	1.00
Jade1	0.31	1.00
Prex1	-1.56	1.00
Aldh1a1	1.27	1.00
Endod1	-0.17	1.00
Chst7	-0.25	1.00
Zfp467	-0.73	1.00
Dsel	-0.49	1.00
Wdr86	-0.20	1.00
Rmnd1	-0.27	1.00
Il1rapl2	-1.67	1.00
Adssl1	-0.59	1.00
Tmeff2	-0.43	1.00
Ror1	-1.12	1.00
Alg3	-0.65	1.00
Stab1	-0.33	1.00
Rbfox3	-0.83	1.00
Garnl3	-0.47	1.00
Ncdn	-0.63	1.00
Hdac9	-0.82	1.00
Adgrg3	-0.52	1.00
Ica1l	-0.72	1.00
Dpy30	0.23	1.00
Ccna1	0.24	1.00
Bin1	0.05	1.00
Fubp3	0.17	1.00
Sf3a3	0.20	1.00
Arl6ip6	0.14	1.00
Ttc12	0.09	1.00
Synj2bp	0.08	1.00
Kazn	0.94	1.00
Ccnc	-0.14	1.00
Slc35b1	0.25	1.00
Acadm	0.19	1.00
F2rl1	0.23	1.00

Prrc1	0.45	1.00
Cuedc2	0.04	1.00
Cfap20	0.46	1.00
Lmtk2	0.24	1.00
Nrg4	0.48	1.00
Rnf157	0.64	1.00
Yes1	0.62	1.00
Srpk1	-0.13	1.00
Entpd1	0.48	1.00
Dgke	0.57	1.00
Ndufs2	0.44	1.00
Serpinh1	-0.72	1.00
Dync1li1	0.72	1.00
Nme9	1.07	1.00
Cfap58	0.59	1.00
Ddr1	0.28	1.00
Dnmbp	1.09	1.00
Clca1	1.37	1.00
Ap2m1	0.51	1.00
Sf3b4	-0.29	1.00
Fam126a	-0.91	1.00
Pakap	-1.50	1.00
Sec11a	0.54	1.00
Bach1	0.09	1.00
Arpc1b	0.86	1.00
Smndc1	0.39	1.00
Zfyve9	0.32	1.00
Rasal2	0.71	1.00
Lcorl	0.74	1.00
Tbc1d23	0.25	1.00
Igsf3	0.41	1.00
Abhd17a	0.04	1.00
Osbpl6	-1.25	1.00
Ncoa1	-0.71	1.00
Eda	-0.24	1.00
Pllp	0.24	1.00
Dnajc14	0.15	1.00
Ttc29	0.72	1.00
Stx12	0.25	1.00
Znrf2	0.53	1.00
Oxsr1	0.04	1.00
Sdhc	0.25	1.00
Tcte1	0.33	1.00
Ggps1	0.14	1.00
Cfp	-0.15	1.00
Ube2d3	0.16	1.00

Ell	0.56	1.00
Atp9b	0.15	1.00
Eef2k	0.19	1.00
Timm8a2	0.26	1.00
Tmem126b	0.22	1.00
Cd80	1.13	1.00
Zfp236	-0.10	1.00
Pomt2	0.04	1.00
Trim8	0.04	1.00
Cdkl2	-0.65	1.00
Stub1	-0.12	1.00
Eif4ebp1	0.59	1.00
Ncald	1.77	1.00
Plekhj1	0.05	1.00
Klhl32	0.37	1.00
Klhl2	0.16	1.00
Gk5	0.20	1.00
Ccdc167	0.04	1.00
Nsdhl	0.13	1.00
Naa60	0.10	1.00
Cdk5rap2	0.04	1.00
Arhgef28	0.80	1.00
Nme5	0.52	1.00
Ak8	0.48	1.00
Coil	0.46	1.00
Phf8	0.24	1.00
Fanci	0.31	1.00
Taf4	0.29	1.00
Cped1	-1.29	1.00
Pbld2	-0.34	1.00
Esrp2	-0.72	1.00
Ffar4	0.31	1.00
Cep89	0.44	1.00
Pcdhga4	0.65	1.00
Cda	-2.72	1.00
Car8	0.85	1.00
Caps2	0.51	1.00
Api5	0.76	1.00
Cfap69	0.84	1.00
Vdac2	0.29	1.00
Tfb1m	0.44	1.00
Il27ra	0.83	1.00
Lgr4	0.75	1.00
Polr2m	0.52	1.00
Snx24	0.36	1.00
Slc16a11	0.03	1.00

Smad2	0.37	1.00
Tmem45b	0.79	1.00
Atp2b2	0.85	1.00
Usp10	0.11	1.00
Edn1	-0.78	1.00
Gin1	-0.33	1.00
Anpep	-0.28	1.00
Nedd1	-0.76	1.00
Atg2a	-0.52	1.00
Spag8	0.27	1.00
Iqgap2	0.43	1.00
Ywhaq	0.19	1.00
Cldnd1	0.37	1.00
1700088E04Rik	-0.06	1.00
Tsr3	-0.17	1.00
Stxbp1	-0.23	1.00
Tmem205	0.04	1.00
Ccdc126	0.66	1.00
Ap1s3	-0.90	1.00
Nfu1	0.44	1.00
Atp6v1b2	0.50	1.00
Acly	0.38	1.00
Pdcd4	0.28	1.00
Parva	-0.78	1.00
Nek9	0.37	1.00
Ccdc25	0.11	1.00
Tc2n	0.75	1.00
Ccny	0.43	1.00
Phactr1	-1.16	1.00
Tomm70a	-0.07	1.00
Cfap20dc	0.60	1.00
Ccdc14	-0.10	1.00
Sdccag8	0.16	1.00
Otulin	0.44	1.00
Upp1	0.79	1.00
Pacc1	0.64	1.00
Nudt12	0.31	1.00
Cib2	0.59	1.00
Nthl1	0.48	1.00
Nubp2	0.73	1.00
Eml2	0.91	1.00
Akt1s1	0.60	1.00
Zswim4	0.38	1.00
Atf6b	0.51	1.00
Slc44a1	0.56	1.00
Eif3a	0.77	1.00

Shld1	-0.76	1.00
Usp1	0.27	1.00
Odr4	0.32	1.00
Tmem160	0.28	1.00
Clu	1.17	1.00
Cachd1	0.80	1.00
Nfkbiz	0.52	1.00
Atp6ap2	0.43	1.00
Slc23a2	0.33	1.00
Eml1	0.26	1.00
Rhobtb1	-1.24	1.00
Gadd45b	1.06	1.00
Ptges3	0.50	1.00
Pced1b	-0.86	1.00
Scfd1	0.29	1.00
Dnajc15	-0.20	1.00
Slc9a7	-0.13	1.00
Dap	-0.24	1.00
Acot11	0.07	1.00
Nr2f2	0.24	1.00
Pmpca	0.43	1.00
Ccdc138	0.24	1.00
2510039O18Rik	0.07	1.00
Scamp4	0.03	1.00
Fbrs	0.07	1.00
Stim2	-0.31	1.00
Rab31	-1.58	1.00
Plpp1	-1.00	1.00
Slc22a15	-0.42	1.00
Ralgps1	0.35	1.00
Spa17	0.51	1.00
Eif4ebp2	-0.93	1.00
Ifit2	-2.04	1.00
Ccp110	0.03	1.00
Cav2	-1.07	1.00
Dync1i2	0.22	1.00
Pkn2	0.43	1.00
Ttc3	0.31	1.00
Zfp385b	-1.16	1.00
Hdac2	0.56	1.00
Vwa3a	0.66	1.00
Agbl2	0.49	1.00
Ppp2cb	0.43	1.00
Gpx1	0.40	1.00
Ndufb6	0.09	1.00
Tubgcp3	0.17	1.00

Cep41	0.23	1.00
Hint3	0.18	1.00
Bcl11a	0.25	1.00
Efnb1	0.43	1.00
Faim	0.30	1.00
Pex11a	0.87	1.00
Nt5dc2	0.50	1.00
Snx8	0.20	1.00
Sh2b3	0.94	1.00
Chmp6	0.16	1.00
4921504E06Rik	0.38	1.00
Klhl15	-0.11	1.00
Ccdc170	0.71	1.00
Npepps	0.01	1.00
Ppp2r1a	0.34	1.00
Rab9	1.02	1.00
Cyb5r4	0.19	1.00
Zfand2b	-0.18	1.00
Ppil2	0.13	1.00
Tbrg1	-0.12	1.00
Ripk2	-0.10	1.00
Hsdl2	0.05	1.00
Ap1m1	-0.24	1.00
Kif1c	-0.07	1.00
Ndufb11b	0.13	1.00
Txndc9	0.27	1.00
1700001L19Rik	0.09	1.00
Trmt9b	0.28	1.00
Rex1bd	0.38	1.00
Prxl2a	0.13	1.00
Npc2	-0.02	1.00
Nmd3	0.52	1.00
Tshr	-0.67	1.00
Glis3	0.79	1.00
Ndufa5	0.32	1.00
Chd7	0.07	1.00
D930020B18Rik	-0.78	1.00
Arhgap1	-0.29	1.00
Ptpn4	-0.17	1.00
Spef2	0.74	1.00
Iqca	0.81	1.00
Clhc1	0.57	1.00
Ugp2	0.71	1.00
Ddhd1	0.61	1.00
Ppp4c	0.48	1.00
Snrpd1	0.14	1.00

Psmd4	1.05	1.00
Cfap61	0.91	1.00
Il6st	0.44	1.00
Clba1	0.30	1.00
Chpf	0.19	1.00
Hbs1l	0.93	1.00
Rcor1	0.55	1.00
Siva1	0.21	1.00
Dync2i1	0.15	1.00
Imp3	0.10	1.00
Lrrc10b	0.15	1.00
Brms1	0.02	1.00
Sdk1	0.51	1.00
Prrc2b	0.35	1.00
Alkbh5	0.08	1.00
Tmem181a	0.23	1.00
Ciao2a	0.33	1.00
Chchd3	0.16	1.00
Gnal	0.16	1.00
Cpsf3	0.12	1.00
Cxxc4	0.25	1.00
Grk4	0.16	1.00
Polr2h	-0.07	1.00
Tmed3	0.07	1.00
Nob1	0.09	1.00
Whrn	0.12	1.00
U2af1l4	0.17	1.00
Atg4d	0.18	1.00
Pelp1	0.15	1.00
Tmem109	0.19	1.00
Pim2	0.08	1.00
Eif4e	0.65	1.00
Fermt2	-1.31	1.00
Ubald2	0.44	1.00
Numa1	0.01	1.00
Slc6a9	-0.08	1.00
Dtwd1	-0.08	1.00
Wwp2	-0.77	1.00
Snapc1l	0.19	1.00
Dcaf12	0.33	1.00
Enpp6	-0.81	1.00
Ppib	0.73	1.00
Prkcsh	0.27	1.00
Ilrun	0.29	1.00
Zranb3	0.44	1.00
Gpm6a	-1.01	1.00

Peli2	-1.37	1.00
Avl9	0.24	1.00
Pikfyve	0.33	1.00
Dctn3	-0.08	1.00
Vps13a	0.47	1.00
Zfand6	0.08	1.00
Brd2	0.60	1.00
Gcn1	0.53	1.00
Abcc3	0.44	1.00
Tdrp	0.12	1.00
Polr2b	0.37	1.00
Focad	-0.63	1.00
Bcl7a	0.19	1.00
Tfip11	0.29	1.00
Scpep1	0.42	1.00
Efcab5	0.96	1.00
Rwdd1	0.29	1.00
Lpar3	-0.03	1.00
Ubap1	0.21	1.00
Psma7	0.97	1.00
Parp8	-1.21	1.00
Efhc1	0.60	1.00
Bicd1	-1.03	1.00
Sh3bp5	-1.06	1.00
Stxbp6	-0.71	1.00
Rassf2	-0.94	1.00
Fam189a2	-0.91	1.00
Galnt18	0.59	1.00
Arhgef6	-1.28	1.00
Nphp4	-0.01	1.00
Dnajc8	0.83	1.00
Sap30	0.13	1.00
Rnasel	0.09	1.00
Atpaf2	0.16	1.00
Nup85	0.44	1.00
Kdf1	0.21	1.00
Rbbp5	0.25	1.00
Dstyk	0.42	1.00
Ccdc34	-0.04	1.00
Spef1	0.13	1.00
Zfpl1	0.12	1.00
Gpkow	0.18	1.00
Ift122	0.48	1.00
Oscp1	0.56	1.00
Dzank1	0.60	1.00
Tgfb1	0.38	1.00

Jarid2	0.25	1.00
Srsf11	0.28	1.00
Eml6	0.54	1.00
Jmy	0.52	1.00
Ncf1	-0.48	1.00
Arl13b	0.46	1.00
Pfdn1	0.24	1.00
Lap3	0.31	1.00
Pcnx4	0.24	1.00
Ctss	0.35	1.00
Tfcp2l1	0.68	1.00
Cd9	0.48	1.00
Nek5	0.28	1.00
Wdr35	0.14	1.00
Dpy19l3	-0.21	1.00
Tmem189	0.18	1.00
Itsn1	-1.26	1.00
Nrbp1	0.24	1.00
Tfdp2	0.67	1.00
Tekt3	0.21	1.00
Slc43a2	0.42	1.00
Ktn1	0.31	1.00
Ankrd44	-0.64	1.00
Uros	-0.34	1.00
Zbtb7a	0.36	1.00
Rgs12	0.05	1.00
Atg3	0.08	1.00
Zfp277	0.44	1.00
Pdcd11	0.18	1.00
Slc7a6os	0.28	1.00
Tmem134	0.23	1.00
Limch1	-1.16	1.00
Fnip1	0.37	1.00
Atp6v1d	0.32	1.00
Crtc3	0.30	1.00
Rab10	0.29	1.00
Tnfaip3	0.23	1.00
Fez2	0.12	1.00
Nifk	0.32	1.00
Zdhhc23	0.16	1.00
Tmem42	0.15	1.00
Elp1	0.10	1.00
Grhpr	0.14	1.00
Jkamp	0.10	1.00
Slc22a18	0.42	1.00
Eefsec	0.09	1.00

Zfp821	0.14	1.00
Cep20	0.14	1.00
Ints3	0.10	1.00
Timm50	0.14	1.00
Fam229b	0.12	1.00
Tomm7	0.20	1.00
Shank2	0.62	1.00
Clock	-0.14	1.00
Scmh1	0.07	1.00
Sil1	0.35	1.00
Arf6	0.05	1.00
Iars2	-0.08	1.00
St3gal1	0.67	1.00
Tusc3	0.67	1.00
Cald1	-0.56	1.00
Tbc1d19	0.28	1.00
Ghitm	0.24	1.00
Tecta	0.49	1.00
Anks1	0.68	1.00
Bms1	-0.23	1.00
Exosc10	0.00	1.00
Ssr3	0.34	1.00
Slc39a10	-0.91	1.00
D6Wsu163e	-0.22	1.00
Zc3h14	0.19	1.00
Xpr1	-0.03	1.00
Sft2d1	0.23	1.00
Fam3c	0.21	1.00
Psmg4	0.20	1.00
Rnf103	0.08	1.00
Gch1	0.06	1.00
Arhgef37	0.33	1.00
Rrad	0.43	1.00
Dgkz	0.42	1.00
Lrrc57	0.14	1.00
Stk25	0.34	1.00
Eif4e2	-0.03	1.00
Scarb1	0.69	1.00
Psmc3	0.03	1.00
Ube2k	0.40	1.00
Cdh1	0.55	1.00
Dhx15	0.29	1.00
Cd47	-1.06	1.00
Zfp146	0.24	1.00
Deup1	0.44	1.00
Aldh5a1	0.09	1.00

Ogfrl1	0.18	1.00
Cstf2	0.12	1.00
Umps	-0.26	1.00
Wdr72	0.10	1.00
Tnnc2	0.10	1.00
Vat1	0.07	1.00
Spryd3	0.08	1.00
Cd164l2	0.12	1.00
Ash2l	0.10	1.00
Ralgapa1	-0.22	1.00
Ntn4	-0.42	1.00
Wbp4	-0.25	1.00
Nkain3	-0.90	1.00
Slc25a37	0.37	1.00
Tmem129	0.50	1.00
Plpp2	0.26	1.00
Vegfc	0.79	1.00
Gale	0.87	1.00
Stxbp3	-0.42	1.00
Nipa2	0.09	1.00
Sec14l1	-0.16	1.00
Pign	0.37	1.00
Wsb2	0.28	1.00
Ankmy2	0.09	1.00
Slc19a1	0.40	1.00
Lrriq3	0.49	1.00
Copa	-0.13	1.00
Rock1	0.25	1.00
Secisbp2l	-1.51	1.00
Serinc5	-0.72	1.00
Senp5	-0.73	1.00
Lhfp	-0.25	1.00
Raly	0.41	1.00
Frmpd2	0.50	1.00
Cpm	-1.12	1.00
Pitpna	0.18	1.00
Ift74	0.51	1.00
Osbpl3	-1.28	1.00
Txnl1	0.59	1.00
Psmd8	0.53	1.00
Bri3	-0.37	1.00
Rlim	0.24	1.00
Ttll9	0.20	1.00
Hexb	0.44	1.00
Clptm1	0.52	1.00
Cep126	0.02	1.00

Ppfibp1	0.81	1.00
Pkp4	-1.28	1.00
Pip4k2a	1.03	1.00
1700028P14Rik	0.31	1.00
Relb	-0.16	1.00
Gorasp2	0.37	1.00
Dcaf11	-0.53	1.00
Ankrd49	0.04	1.00
Triqk	0.09	1.00
Ctdp1	0.03	1.00
Ly96	0.07	1.00
Trmt2b	0.13	1.00
Hdhd2	-0.04	1.00
Nrbf2	-0.01	1.00
Bcar3	-0.05	1.00
Senp8	0.08	1.00
Rfwd3	-0.07	1.00
Naa40	0.12	1.00
AI413582	-0.03	1.00
Fam151b	0.11	1.00
Rp9	-0.11	1.00
Sdc2	0.23	1.00
Katnal2	0.15	1.00
Trmt10a	0.03	1.00
Dock9	-0.93	1.00
Dsp	0.49	1.00
Slc20a2	0.44	1.00
Phlpp1	-0.77	1.00
Ptp4a1	0.25	1.00
Pds5a	0.25	1.00
Plec	-0.08	1.00
Pax9	1.00	1.00
Slc7a5	0.50	1.00
Phykpl	0.12	1.00
Ikbke	0.50	1.00
Arrdc2	0.51	1.00
Adm	0.79	1.00
Tmem54	0.56	1.00
Bpifb1	1.01	1.00
Ubqln2	0.50	1.00
Phlda2	0.95	1.00
Fmn1	0.97	1.00
Epb41l2	0.39	1.00
Chmp5	0.69	1.00
Dr1	0.38	1.00
Tax1bp3	0.41	1.00

Txlna	0.21	1.00
Plekha2	0.36	1.00
Ecm1	-0.04	1.00
Sf3b2	0.14	1.00
Iqcg	0.13	1.00
Dnaja4	0.18	1.00
A830018L16Rik	0.08	1.00
Tial1	0.21	1.00
Rhbdd1	-0.02	1.00
Dlc1	-1.08	1.00
Lipa	-0.68	1.00
Utp6	-0.01	1.00
Trim28	0.28	1.00
Calu	0.28	1.00
Ccnt1	0.03	1.00
Nap1l1	0.52	1.00
Evl	0.26	1.00
Banp	0.34	1.00
Uqcc3	-0.75	1.00
Gdi2	0.07	1.00
Tbcd	0.61	1.00
Pnpla7	-0.23	1.00
Dnajc11	0.10	1.00
Ranbp3	-0.12	1.00
Wdr73	-0.11	1.00
Ptpre	-1.47	1.00
Wfdc2	0.83	1.00
Dennd1l	-2.07	1.00
Rptor	-0.28	1.00
Zswim6	0.31	1.00
Qpct	0.45	1.00
Tsen15	0.46	1.00
Fkbp5	0.73	1.00
Vps36	0.18	1.00
Agps	-0.08	1.00
Xpnpep1	0.44	1.00
Tcf3	0.41	1.00
Trim24	-0.71	1.00
Jak2	0.64	1.00
Hes1	0.13	1.00
Phf6	0.55	1.00
Ccdc181	0.14	1.00
Nop56	0.07	1.00
Arl3	-0.27	1.00
Ndufa11	-0.04	1.00
Ccdc91	0.19	1.00

Fbxo22	-0.28	1.00
Adrm1	-0.76	1.00
Hspb1	0.16	1.00
Gpr183	0.33	1.00
Fgfr1	0.47	1.00
Boc	0.55	1.00
F7	0.13	1.00
Gpa33	0.19	1.00
Ndufaf6	0.32	1.00
Pitrm1	0.75	1.00
Nox1	0.15	1.00
Lyrm7	0.13	1.00
Arhgef39	0.11	1.00
Fbxo4	0.10	1.00
Spc25	0.15	1.00
Nhej1	1.15	1.00
Tysnd1	0.35	1.00
Tdrd5	0.16	1.00
Sema3b	0.49	1.00
Cacna2d3	0.88	1.00
Zfpm1	0.95	1.00
Tsc2	0.17	1.00
Ei24	0.16	1.00
Yrdc	0.18	1.00
Wdr18	0.12	1.00
Usf1	0.24	1.00
Clnk	1.30	1.00
Ccdc68	0.16	1.00
Gli2	0.61	1.00
Zfp12	0.18	1.00
Wdr62	0.28	1.00
Pex3	0.34	1.00
Tnk1	0.12	1.00
Snx32	0.28	1.00
Syvn1	0.20	1.00
Ttc9c	0.12	1.00
Foxa2	0.08	1.00
Ube2j2	0.11	1.00
Atf4	0.29	1.00
Sec23b	0.45	1.00
Cwc15	0.22	1.00
9130409I23Rik	0.49	1.00
Tmem59	0.43	1.00
Dctn2	0.47	1.00
Cavin1	-0.42	1.00
Commd9	-0.44	1.00

Ogn	-0.83	1.00
Pafah1b1	0.41	1.00
Pdxk	0.36	1.00
Slc24a3	-0.92	1.00
Sec24d	0.56	1.00
Spsb1	0.08	1.00
Rest	0.57	1.00
Lsm3	0.10	1.00
Rbm10	0.39	1.00
Slc18b1	0.15	1.00
Zbtb25	0.26	1.00
Acadl	0.19	1.00
Otub1	0.24	1.00
Slc8b1	0.15	1.00
Nfkbil1	0.07	1.00
Uqcr10	0.06	1.00
Cfap65	0.19	1.00
Spag16	1.18	1.00
Dnajb1	0.41	1.00
Znhit1	0.05	1.00
Irf3	-0.09	1.00
Ripk4	0.23	1.00
Rogdi	0.40	1.00
Kcnab3	0.34	1.00
Cfap45	0.63	1.00
Fam184a	-0.25	1.00
Cox7a2l	0.15	1.00
Tnrc6a	0.08	1.00
Rev3l	0.72	1.00
Tmed4	0.18	1.00
Lnx2	0.33	1.00
Pdxdc1	0.24	1.00
Dnah9	1.01	1.00
Ano10	-1.05	1.00
Fam117a	0.66	1.00
Fundc2	0.09	1.00
Arhgef3	0.76	1.00
Ttll6	0.74	1.00
Atg7	-0.70	1.00
Rnf182	0.16	1.00
Plxdc2	0.64	1.00
Lratd1	0.85	1.00
Fam167a	0.10	1.00
Slc25a14	0.07	1.00
Osbpl5	0.11	1.00
Kcnh7	0.51	1.00

Fam13c	0.61	1.00
Fra10ac1	0.09	1.00
Pacs2	0.26	1.00
Dock10	0.64	1.00
Plxnd1	0.45	1.00
Pop4	0.10	1.00
Cyp3a57	0.43	1.00
Nlrc3	0.10	1.00
Trp53inp2	0.37	1.00
Pus1	0.13	1.00
Jmjd6	0.12	1.00
Slc16a3	0.32	1.00
Slc13a5	0.44	1.00
Dhx58	0.43	1.00
Itgb3	0.28	1.00
Barx2	0.18	1.00
Tmem79	0.35	1.00
Tm2d2	0.38	1.00
Pias4	0.08	1.00
Ubqln4	0.20	1.00
Fbxl12	0.32	1.00
Ccn6	0.12	1.00
Agfg2	0.11	1.00
Ap4m1	0.09	1.00
Acp4	0.19	1.00
Dkkl1	0.15	1.00
Grwd1	0.11	1.00
Stx4a	0.09	1.00
Hus1	0.18	1.00
Gadd45a	0.44	1.00
Flrt3	-0.72	1.00
Gatad2b	0.40	1.00
Wdr26	0.52	1.00
Kansl1l	0.59	1.00
Usp45	0.09	1.00
Dhrs7b	0.21	1.00
Rbck1	-0.08	1.00
Zfp654	0.36	1.00
Jazf1	0.78	1.00
Srf	-0.67	1.00
Cyria	-1.20	1.00
Slc35d1	-0.44	1.00
Vsir	-1.13	1.00
Dcaf8	0.25	1.00
Hprt	0.13	1.00
Lrrc74b	0.19	1.00

Rtf1	0.29	1.00
Dnttip2	0.27	1.00
Cox17	0.37	1.00
Serbp1	0.29	1.00
Rbm39	0.23	1.00
Epdr1	0.07	1.00
Fam8a1	0.24	1.00
Klhl13	-0.19	1.00
Adal	-0.14	1.00
4921524J17Rik	0.23	1.00
Prkd1	-1.08	1.00
Sesn1	-1.04	1.00
Atrnl1	-0.99	1.00
Ndufa4	-0.14	1.00
Pgrmc1	0.51	1.00
Kdm3a	0.26	1.00
Pex14	0.12	1.00
Dcaf13	0.33	1.00
Rgs7	0.38	1.00
Ctsa	0.41	1.00
Sf3a1	0.38	1.00
Ttc34	0.30	1.00
Ciao1	-0.28	1.00
Cfap36	0.31	1.00
Dipk2a	-0.81	1.00
Bcl2l1	-0.31	1.00
Zmym2	-0.62	1.00
Rpgrip1l	0.35	1.00
Snx25	0.56	1.00
Tmem248	-0.03	1.00
Rasgef1b	0.13	1.00
Drc3	0.41	1.00
Glrx5	0.02	1.00
Plcl1	-1.30	1.00
Znhit6	0.23	1.00
Ccdc40	0.21	1.00
Arhgap42	-0.44	1.00
Slc12a7	0.09	1.00
Kdm1b	-0.17	1.00
Ehd1	0.01	1.00
Atad1	-0.67	1.00
Kifap3	0.42	1.00
Rnf145	-0.17	1.00
Perp	0.28	1.00
Nr3c1	-0.91	1.00
Fam114a1	-0.37	1.00

Pmfbp1	-0.56	1.00
Nkap	0.17	1.00
Acot10	0.06	1.00
P4hb	0.58	1.00
Cd36	-2.00	1.00
Dtx3l	-0.74	1.00
Tamalin	1.38	1.00
Cavin3	-1.13	1.00
Tmem97	-0.34	1.00
App	0.18	1.00
Twf1	0.15	1.00
Grip1	0.20	1.00
Prkdc	0.19	1.00
Zbtb2	0.12	1.00
Tnfaip6	-0.94	1.00
Comtd1	0.08	1.00
Elovl1	0.09	1.00
Pink1	0.09	1.00
Sypl2	-0.01	1.00
Fndc7	0.02	1.00
Accs	0.12	1.00
Lemd3	0.28	1.00
Bhlhe41	0.07	1.00
Prdm15	0.07	1.00
Adsl	-0.11	1.00
Rhno1	-0.02	1.00
Osgin2	-0.02	1.00
Alg2	0.10	1.00
Entpd6	0.02	1.00
Batf3	-0.05	1.00
Ndufaf7	-0.09	1.00
Fam53a	0.04	1.00
Inip	0.09	1.00
Cdin1	0.04	1.00
Coq6	0.09	1.00
Bicc1	-0.14	1.00
Ephb1	-0.27	1.00
Wdr53	0.06	1.00
Bap1	0.03	1.00
Hesx1	0.06	1.00
Klhdc4	0.09	1.00
Zfand2a	0.08	1.00
Aar2	0.05	1.00
Gucd1	0.05	1.00
Arsg	-0.17	1.00
Lrrc75a	0.00	1.00

Slc4a8	0.03	1.00
Gys1	0.07	1.00
Stat2	0.07	1.00
Elmod3	-0.07	1.00
Sin3b	0.12	1.00
Dpp9	-0.01	1.00
Poln	0.10	1.00
Stk16	0.06	1.00
Wiz	0.05	1.00
Cd84	0.11	1.00
Slc9b1	-0.41	1.00
1110017D15Rik	-0.24	1.00
Ube2u	0.11	1.00
Pon2	0.07	1.00
Catsperg1	0.00	1.00
Prr14	0.08	1.00
Zkscan6	0.09	1.00
Zscan12	0.02	1.00
BC003965	0.00	1.00
Tnxb	-0.02	1.00
Vwa7	0.04	1.00
Pja1	0.13	1.00
Derpc	-0.17	1.00
Uchl3	0.28	1.00
Scaf1	0.30	1.00
Armc10	-0.08	1.00
Lgals8	-0.11	1.00
Zfp106	0.25	1.00
Usp40	-0.04	1.00
Timm17b	-0.13	1.00
Lifr	0.25	1.00
Slc10a7	-0.01	1.00
Ppargc1a	0.68	1.00
Tmem254a	0.53	1.00
Nadk	0.50	1.00
Rufy2	-0.89	1.00
Polr2f	0.08	1.00
Eif4h	0.47	1.00
Vps26c	0.31	1.00
Ccdc160	0.02	1.00
Gan	0.62	1.00
Ankrd6	0.49	1.00
Lrrc58	0.01	1.00
Tex10	-0.03	1.00
Enpp3	-0.08	1.00
Tgif2	0.01	1.00

Dnmt1	0.12	1.00
Stk36	-0.38	1.00
Psmb3	0.05	1.00
Sh3gl1	-0.27	1.00
Lrrc46	0.04	1.00
Ndufs4	0.23	1.00
Tcp11l2	0.26	1.00
Rassf8	-0.48	1.00
Uba3	0.30	1.00
Armh3	-0.03	1.00
Drosha	-1.16	1.00
Wnt3a	-0.78	1.00
Gask1b	-1.27	1.00
Zdhhc2	-0.35	1.00
Arl9	-0.84	1.00
Adamts19	-0.67	1.00
Adam8	-0.78	1.00
BC055324	-0.44	1.00
Snx33	-0.15	1.00
Actr1b	-0.45	1.00
Zfp879	-0.65	1.00
Rnf20	0.09	1.00
Cbx4	0.28	1.00
Ddx21	0.09	1.00
Abcd4	0.16	1.00
Cnot11	-0.08	1.00
Pim1	0.18	1.00
Gbe1	0.94	1.00
Eif4g2	0.59	1.00
Itgb8	0.17	1.00
Fam102b	0.59	1.00
Zbbx	0.50	1.00
Tnpo3	0.20	1.00
Sec13	0.17	1.00
Cdc27	0.29	1.00
Arpc5l	0.23	1.00
Phc2	0.23	1.00
Trnau1ap	0.37	1.00
Cops8	-0.51	1.00
Ift27	0.03	1.00
Eno2	-0.27	1.00
Srp72	0.07	1.00
Cyb561a3	0.33	1.00
Uqcrq	-0.18	1.00
Slc19a2	-0.22	1.00
Pibf1	0.25	1.00

Atp6v1c1	0.39	1.00
Raf1	0.37	1.00
Arxes2	0.30	1.00
Enpp2	1.07	1.00
Fsd1l	0.27	1.00
Ak7	0.78	1.00
Rsph14	0.02	1.00
Ppp2r1b	0.22	1.00
Erp29	0.61	1.00
Rheb	0.23	1.00
Jph1	-0.83	1.00
Efna5	-1.06	1.00
Cacna2d1	-0.83	1.00
Rrp15	0.29	1.00
Tec	0.21	1.00
Ccdc61	-0.27	1.00
Iqgap1	0.30	1.00
Vta1	0.30	1.00
Tln2	0.47	1.00
Zfat	0.03	1.00
Lmo1	0.18	1.00
Nemp2	-0.30	1.00
Abcc5	0.37	1.00
Tle3	0.16	1.00
Rexo5	0.33	1.00
Ier3	0.90	1.00
Tpr	0.50	1.00
Amotl2	-0.91	1.00
Nbr1	0.29	1.00
Ap3d1	0.47	1.00
Sell	-0.81	1.00
AW554918	-0.09	1.00
Pik3ca	-0.16	1.00
Dclre1c	0.11	1.00
5730480H06Rik	-0.04	1.00
Rgl1	-0.65	1.00
Insyn1	0.01	1.00
Cmc1	-0.35	1.00
Vps52	0.09	1.00
Tmem70	-0.32	1.00
Wee1	0.30	1.00
Rgs1	1.43	1.00
Nit1	0.15	1.00
Stxbp4	-0.23	1.00
Krt7	1.27	1.00
Iffo2	0.00	1.00

Slc16a1	0.62	1.00
Rapgef2	0.44	1.00
Esd	-0.99	1.00
Eif3g	-0.30	1.00
Ipo7	0.18	1.00
Hnrnpll	0.04	1.00
Wdr70	0.59	1.00
Tbc1d22a	-0.03	1.00
Ankrd13a	-0.25	1.00
Togaram1	0.30	1.00
Ahr	0.02	1.00
Dnah5	0.92	1.00
Bag3	0.73	1.00
Dync2li1	0.34	1.00
Tgfb2	-0.98	1.00
Foxj1	0.21	1.00
Ccdc153	0.35	1.00
Tmprss2	-0.26	1.00
Alpk1	0.76	1.00
Cdkn2aipnl	-0.41	1.00
Tmem39a	0.35	1.00
Selenoo	-0.49	1.00
Tmtc1	-0.87	1.00
Tead4	-0.45	1.00
Zfp282	-0.51	1.00
Hps3	-0.52	1.00
Rhoc	-0.45	1.00
Lancl2	-0.26	1.00
Cdc73	0.42	1.00
Cul4a	0.14	1.00
Ndufa9	0.13	1.00
Gla	0.03	1.00
Tctn2	0.13	1.00
Yif1a	0.14	1.00
Sbno2	0.62	1.00
Nek7	-0.68	1.00
Rabgap1l	0.64	1.00
Wdr37	0.18	1.00
Cep63	0.10	1.00
Socs1	1.07	1.00
1700094D03Rik	0.50	1.00
Blvrb	0.19	1.00
Acadvl	0.07	1.00
Rock2	0.35	1.00
B4galt5	0.11	1.00
Esrrg	0.99	1.00

Flrt2	-0.75	1.00
Cdkn1c	0.89	1.00
Arel1	-0.35	1.00
Lman2l	-0.49	1.00
Pgpep1	-0.47	1.00
S100a6	1.64	1.00
Gemin5	0.40	1.00
Lrig1	0.20	1.00
Arhgap31	-0.02	1.00
Abhd3	-0.04	1.00
Tssc4	-0.09	1.00
Tom1l1	-0.91	1.00
Gpd2	-0.95	1.00
Hibch	-0.77	1.00
Gnai2	0.70	1.00
Ncf4	0.57	1.00
B3glct	0.19	1.00
Cyp39a1	0.22	1.00
Lrrc73	0.31	1.00
Slc7a11	0.79	1.00
Acaa2	0.13	1.00
Trim14	0.20	1.00
Irf8	1.12	1.00
Fam210b	0.75	1.00
Tor1a	0.25	1.00
Cdc42se1	0.17	1.00
1810030O07Rik	0.19	1.00
Ctnnd1	0.43	1.00
Neo1	0.43	1.00
Slc30a7	-0.07	1.00
AA986860	-0.34	1.00
Wwp1	-1.25	1.00
Lca5	0.08	1.00
Ect2l	0.54	1.00
Plaat3	0.12	1.00
Hnrnpdl	0.64	1.00
Ndufa1	0.23	1.00
Dars	0.28	1.00
Emc3	0.19	1.00
Ssb	0.27	1.00
Rictor	0.18	1.00
Brd8	-1.02	1.00
Lnpk	-0.07	1.00
Kif26b	0.60	1.00
Fbxl7	0.10	1.00
Lrrc1	-0.84	1.00

Tmem223	0.17	1.00
Nr2c1	0.16	1.00
Ss18l2	0.08	1.00
Pabpc4	0.37	1.00
Zfp2	0.15	1.00
Pam16	0.10	1.00
Fibp	-0.24	1.00
Bag4	0.33	1.00
Vcan	0.11	1.00
Camsap1	-0.27	1.00
Tbpl1	0.43	1.00
Trib1	0.24	1.00
Crls1	-0.22	1.00
Tmc7	-0.51	1.00
Blmh	-0.13	1.00
Klhdc9	-0.13	1.00
Csnk1g1	0.51	1.00
Colec12	-0.80	1.00
Cry2	0.41	1.00
Cog7	0.21	1.00
Fam166b	0.23	1.00
Slc4a4	0.35	1.00
Ptprc	-1.65	1.00
Tmem116	-0.59	1.00
Tnfrsf1b	-0.10	1.00
Aftph	0.09	1.00
Rhoh	0.55	1.00
Zeb2	0.53	1.00
Cacna1d	0.47	1.00
Slc31a2	0.22	1.00
Tada2a	0.15	1.00
Arl8b	0.62	1.00
Hspa4	-0.19	1.00
Rsph9	-0.39	1.00
Zbtb41	-0.50	1.00
Akap12	-0.69	1.00
Nudt6	-0.42	1.00
Atoh8	-0.36	1.00
Rbfox1	-0.95	1.00
Tjap1	-0.20	1.00
Wdfy2	0.38	1.00
Radil	-0.85	1.00
F11r	0.31	1.00
Smap1	-0.01	1.00
Dnah10	1.01	1.00
Gas8	-0.06	1.00

Kynu	0.20	1.00
Hpn	0.11	1.00
Prkaca	-0.24	1.00
Klf9	0.80	1.00
Dyrk2	0.44	1.00
Poglut1	-0.33	1.00
Synj2	0.18	1.00
Timm10	0.26	1.00
Ybey	0.05	1.00
Nelfe	0.16	1.00
Dyrk1b	0.17	1.00
1700029J07Rik	0.24	1.00
Pithd1	-0.37	1.00
Rtraf	-0.67	1.00
Cstb	0.47	1.00
Arhgap5	0.28	1.00
Atp5c1	0.12	1.00
Spire2	0.30	1.00
B9d1	0.33	1.00
Pcna	0.45	1.00
Srrm2	0.45	1.00
Chchd6	-0.03	1.00
Tmem243	0.01	1.00
Xylt1	0.39	1.00
Wnk1	0.55	1.00
Tmem135	0.14	1.00
Lsm6	0.20	1.00
Fbxo36	0.76	1.00
Hsd17b11	-0.12	1.00
Nhlrc2	0.02	1.00
Ptpn18	0.38	1.00
Pbx1	0.39	1.00
Canx	0.26	1.00
Chd3	0.26	1.00
Itga4	-1.30	1.00
Pigk	-0.71	1.00
Litaf	0.53	1.00
Hipk1	-0.11	1.00
Tmem241	0.19	1.00
Ptprn2	0.55	1.00
Zfp518b	0.20	1.00
Klhl41	0.25	1.00
Pask	0.33	1.00
Spg7	0.13	1.00
Hdac3	0.14	1.00
Sco1	0.25	1.00

Pea15a	0.26	1.00
Synpr	0.13	1.00
Akap3	0.38	1.00
Tomm40	0.96	1.00
Jrkl	0.15	1.00
Erlec1	0.55	1.00
Fgf14	0.41	1.00
Dld	0.21	1.00
Tcaim	0.51	1.00
Hgs	0.20	1.00
Dhrs1	0.00	1.00
Nhlrc3	-0.80	1.00
Ttc26	0.08	1.00
Chid1	-0.11	1.00
Cdca4	0.10	1.00
Arl8a	0.07	1.00
Wdr77	-0.20	1.00
Nebl	0.59	1.00
Gnb2	-0.04	1.00
Vwa8	0.21	1.00
Usp46	0.14	1.00
Prkci	0.63	1.00
Dhrs9	-0.43	1.00
Tmem38b	0.20	1.00
Sfswap	-0.12	1.00
Wdr1	-0.13	1.00
Rad23b	0.37	1.00
Tmed9	0.24	1.00
Dip2b	0.27	1.00
Ythdc2	-0.07	1.00
Casp7	0.56	1.00
Ralgps2	0.28	1.00
Tbc1d9b	0.02	1.00
Bbx	0.29	1.00
Zfp644	0.53	1.00
Arid4a	0.39	1.00
Ugcg	0.21	1.00
Kdm4c	0.22	1.00
Dera	0.42	1.00
Dlec1	0.06	1.00
Trappc13	0.01	1.00
Rab3gap1	-0.13	1.00
Ttll7	-0.90	1.00
Epb41l5	-1.27	1.00
Sars	0.43	1.00
Atxn7	-0.71	1.00

Fam189a1	0.46	1.00
Tsc1	-0.27	1.00
Ccng1	0.23	1.00
Stmnd1	0.04	1.00
Atp6v0a2	-0.04	1.00
Ndufb4	-0.26	1.00
L3hypdh	0.15	1.00
Nucks1	0.19	1.00
Dcun1d4	0.60	1.00
Polr2i	-0.19	1.00
Ttc21a	0.16	1.00
Lyar	0.09	1.00
Fastkd1	0.16	1.00
Rtca	0.17	1.00
Pcdh1	0.35	1.00
Psmd5	0.13	1.00
Thsd7a	0.30	1.00
Fbxl13	0.17	1.00
Ugt2b34	0.24	1.00
Tulp3	0.19	1.00
Kdsr	0.23	1.00
Pms1	0.39	1.00
Brwd1	-0.73	1.00
Tor1aip1	0.25	1.00
Fzd5	-0.15	1.00
Gtf3c1	0.32	1.00
Snrnp48	-0.42	1.00
Rassf5	-0.79	1.00
Tmem140	-0.10	1.00
Cdc123	-0.68	1.00
Zfp444	-0.15	1.00
Naga	0.19	1.00
Nprl3	0.15	1.00
Aqp5	-0.19	1.00
Calm1	0.13	1.00
Ssr1	0.08	1.00
Ptpra	0.21	1.00
Lamb3	0.46	1.00
Nus1	-0.05	1.00
Washc5	-0.22	1.00
Nkapd1	0.14	1.00
Tmem67	0.17	1.00
Ccdc58	0.04	1.00
Ap2a1	-0.18	1.00
Six4	-0.01	1.00
Dbi	0.38	1.00

Xrn2	0.21	1.00
Dram1	0.61	1.00
Ankrd33b	-1.21	1.00
Purg	-0.35	1.00
Gnb4	-0.56	1.00
Rsl24d1	0.37	1.00
Zmym6	0.76	1.00
Xpot	-0.07	1.00
Ccdc59	0.16	1.00
Ankrd66	-0.35	1.00
Ttc8	0.15	1.00
4833439L19Rik	0.02	1.00
Taf8	-0.03	1.00
Cox4i1	-0.07	1.00
Rnf13	-1.04	1.00
Vcp	-0.07	1.00
Tle5	0.09	1.00
Zmynd10	0.59	1.00
Rsph4a	0.27	1.00
Wdsub1	-0.36	1.00
E130308A19Rik	0.21	1.00
Ints6l	0.64	1.00
Cfap70	0.58	1.00
Nedd8	-0.06	1.00
Tm7sf3	0.06	1.00
Tspan9	0.15	1.00
Irf4	0.13	1.00
Ttc27	0.07	1.00
Sepsecs	0.08	1.00
Ppwd1	0.10	1.00
Tpgs2	0.22	1.00
Rnf170	0.05	1.00
Pex16	-0.34	1.00
Aip	0.09	1.00
Orc2	0.13	1.00
Dbt	0.11	1.00
Klf15	0.15	1.00
Hps4	0.31	1.00
Sirt7	-0.26	1.00
Rabepk	0.52	1.00
Ak1	0.65	1.00
Cct6b	0.40	1.00
Senp1	0.62	1.00
Gpatch4	0.34	1.00
Cad	0.06	1.00
Wasf3	0.19	1.00

Hspbp1	-0.02	1.00
Keap1	-0.17	1.00
Urgcp	0.40	1.00
AI837181	0.10	1.00
Tomm20	0.05	1.00
Tmem41b	0.16	1.00
Lonp2	0.45	1.00
Adgra3	0.80	1.00
1700037H04Rik	0.58	1.00
Dctpp1	-0.73	1.00
Col8a1	-1.24	1.00
Fbxo7	-0.05	1.00
Csrp2	-0.23	1.00
Il7	-0.06	1.00
Kank1	0.15	1.00
Tmem161b	0.25	1.00
Cnot9	0.00	1.00
Eloa	0.14	1.00
Rnf126	0.12	1.00
Ica1	0.20	1.00
Paxip1	0.34	1.00
Commd3	0.21	1.00
Hnf4g	0.29	1.00
Dnajb2	0.16	1.00
Frrs1	0.49	1.00
Col3a1	0.75	1.00
Fsip1	0.49	1.00
Spaca9	0.27	1.00
Kif27	0.68	1.00
Trp53inp1	0.50	1.00
Sorcs2	0.03	1.00
Ddb1	-0.09	1.00
Ccdc9	-0.14	1.00
Psme1	0.04	1.00
Cpeb2	0.25	1.00
Ddx5	0.35	1.00
Pak2	0.13	1.00
Dpy19l1	-0.04	1.00
Tnfrsf19	0.34	1.00
Ssbp4	-0.32	1.00
Ano6	-0.45	1.00
Ptp4a2	-0.05	1.00
Rnf146	-0.05	1.00
Emc7	0.02	1.00
Trappc5	0.08	1.00
Prmt9	-0.66	1.00

Inpp5a	-0.66	1.00
Rgs18	-0.64	1.00
Zdhhc4	-0.63	1.00
Gpn3	-0.46	1.00
Ndrg2	-0.46	1.00
Trem1	-1.15	1.00
Eef2	0.21	1.00
Sorbs2	0.11	1.00
Prpf40a	0.17	1.00
Ube2e2	-0.81	1.00
Cep83	-0.03	1.00
Slc35a3	0.28	1.00
Ccdc148	0.87	1.00
Bmt2	-0.84	1.00
Kpna1	0.26	1.00
Nek11	0.86	1.00
Sav1	-0.60	1.00
Bccip	-0.09	1.00
Tmem214	0.10	1.00
Ankra2	0.08	1.00
Dnajc17	0.09	1.00
Iqcb1	-0.05	1.00
Prr7	0.07	1.00
Gusb	0.07	1.00
Git1	0.07	1.00
Pip4k2b	0.04	1.00
Use1	0.07	1.00
Tsr1	0.07	1.00
Pigg	-0.09	1.00
Lkaaear1	-0.19	1.00
Nat14	-0.17	1.00
Cd3eap	-0.26	1.00
Ocel1	0.13	1.00
Cog5	0.15	1.00
Ankrd22	-0.01	1.00
Fam168b	0.10	1.00
Prpf6	0.11	1.00
Fbrsl1	0.02	1.00
Rnf214	0.00	1.00
Fam161a	0.12	1.00
Lonrf3	-1.27	1.00
Dstn	0.39	1.00
Ergic2	0.21	1.00
Virma	0.32	1.00
Yae1d1	0.19	1.00
Zfp143	0.01	1.00

Cers2	0.05	1.00
Gar1	0.69	1.00
Rit1	0.10	1.00
Thoc6	0.37	1.00
Wdr45	0.12	1.00
Uhrf2	-0.02	1.00
Wdr19	0.23	1.00
Rhobtb3	-0.50	1.00
DPCD	-0.10	1.00
Sema6d	-0.46	1.00
Trappc4	-0.35	1.00
Atl2	0.32	1.00
Rnf38	0.32	1.00
Ndufb7	0.16	1.00
Trmt112	0.01	1.00
Thrap3	0.33	1.00
Tbck	0.18	1.00
Fam162a	-0.09	1.00
Ift172	0.12	1.00
Ndufaf2	-0.08	1.00
Cnot4	0.39	1.00
Psmd11	0.37	1.00
Pdlim3	-0.78	1.00
Dnai1	0.75	1.00
Krt10	-0.63	1.00
BC048507	-0.67	1.00
Tent5a	0.56	1.00
Sh3bp4	-0.78	1.00
Pno1	0.03	1.00
Dhx40	0.09	1.00
Intu	0.09	1.00
Ttc22	0.11	1.00
Zfp407	0.19	1.00
Ifngr1	0.15	1.00
Trp53bp1	0.07	1.00
Srsf1	0.23	1.00
Pwwp2a	-0.28	1.00
Pan3	0.20	1.00
Podn	-0.11	1.00
Tbc1d9	0.29	1.00
Nqo2	-0.07	1.00
Ccdc85a	-0.08	1.00
Obsl1	0.08	1.00
Bcar1	-0.03	1.00
Dnaaf1	-0.01	1.00
Septin8	0.06	1.00

Pomt1	-0.01	1.00
Socs7	-0.74	1.00
Brca1	-0.14	1.00
Iqcc	0.04	1.00
Acsf3	-0.04	1.00
Sidt2	0.05	1.00
Enho	-0.01	1.00
Rnf183	-0.52	1.00
Usp42	0.03	1.00
Tex26	0.02	1.00
Ddit3	0.04	1.00
Trim41	0.01	1.00
Cdkl3	0.06	1.00
Gas2l2	-0.06	1.00
Gulp1	0.70	1.00
Ro60	0.42	1.00
Lgals3	0.22	1.00
Gfpt1	0.34	1.00
Nfe2l1	-0.01	1.00
Cep43	-0.07	1.00
Cxxc5	-0.09	1.00
Erc1	0.33	1.00
Ncbp1	-0.98	1.00
Paip2	0.33	1.00
Exog	0.09	1.00
Abcb10	0.14	1.00
Trim36	-0.05	1.00
Atpaf1	0.30	1.00
Fbxw5	0.40	1.00
Smg9	0.68	1.00
Emc9	0.12	1.00
Atp11a	0.53	1.00
Znfx1	-0.86	1.00
Slc13a1	0.88	1.00
Eri1	0.24	1.00
Pdia5	0.13	1.00
Cldn18	-0.69	1.00
Rbbp6	0.51	1.00
G3bp1	0.08	1.00
Pdpk1	0.22	1.00
Cul5	0.50	1.00
Fkbp4	0.43	1.00
Zbtb10	0.02	1.00
Lrrc34	0.22	1.00
Aspdh	0.27	1.00
Naa35	0.17	1.00

Kif1b	0.31	1.00
Cnpy3	-0.43	1.00
Ptar1	0.05	1.00
Idh1	0.25	1.00
Efhb	-0.03	1.00
Fam110c	0.03	1.00
Cxcr4	0.38	1.00
Ruvbl1	0.07	1.00
Scel	-0.55	1.00
Hyou1	0.26	1.00
Nup188	0.20	1.00
Hnrnpm	0.08	1.00
Hk2	0.04	1.00
Stk17b	-0.81	1.00
Ndufc2	0.00	1.00
Nans	0.53	1.00
Adam17	0.18	1.00
Stx7	-0.24	1.00
Sf3b6	0.18	1.00
Kif2a	-0.55	1.00
Nfe2l2	0.19	1.00
Rab30	-0.71	1.00
Fbxl18	-0.24	1.00
Lmbr1	0.31	1.00
Niban1	0.43	1.00
Thumpd1	-0.21	1.00
Luc7l3	0.14	1.00
Psap	0.32	1.00
Slc9a3r2	-0.78	1.00
Czib	0.03	1.00
Ldlrad1	-0.38	1.00
Nr1h2	-0.23	1.00
Vsig2	-0.47	1.00
Camk2g	-0.41	1.00
Pla2g6	0.14	1.00
Tlr2	0.39	1.00
Dlg3	0.05	1.00
Trpm6	0.39	1.00
Ckap5	-0.35	1.00
Tbc1d16	0.14	1.00
Shisa4	0.37	1.00
Ddx49	0.04	1.00
Dpf1	0.07	1.00
Tcn2	0.12	1.00
Slain2	-0.22	1.00
Ppard	-0.56	1.00

Hsbp1	0.25	1.00
Smarcc1	0.04	1.00
Npat	0.66	1.00
Sec63	-0.01	1.00
Usp25	-1.01	1.00
Ddx52	0.07	1.00
Eif1b	0.28	1.00
Itm2b	0.45	1.00
Entpd5	0.08	1.00
Ccdc65	-0.36	1.00
Rnf115	-0.32	1.00
Zfp639	-0.29	1.00
Lsm8	0.56	1.00
Abhd11	-0.36	1.00
Idi1	0.34	1.00
Commd1b	0.83	1.00
Cfap221	0.06	1.00
1110032A03Rik	0.08	1.00
Rab8b	-0.67	1.00
Tbc1d1	0.17	1.00
Tent2	0.43	1.00
Wdhd1	-0.37	1.00
Rfng	-0.25	1.00
Sult1a1	-0.55	1.00
Sipa1l1	0.72	1.00
Echdc1	-0.19	1.00
Gapvd1	0.21	1.00
Fam13a	0.59	1.00
Fzd6	-0.05	1.00
Rsl1d1	0.06	1.00
Arfgef2	0.12	1.00
Fam172a	0.10	1.00
Taf1d	0.21	1.00
Adss	0.30	1.00
Usf3	-0.17	1.00
Bag1	0.27	1.00
Ipo9	0.28	1.00
Ndnf	0.14	1.00
Clcn5	0.30	1.00
Spopl	0.08	1.00
Arfrp1	0.13	1.00
Ankle2	0.44	1.00
Rfc5	0.38	1.00
Wdr13	0.16	1.00
Tiprl	0.14	1.00
Nkiras1	0.12	1.00

Rpp38	0.12	1.00
Idua	-0.23	1.00
Ppp1ca	0.10	1.00
Cib1	-0.32	1.00
Tti2	0.08	1.00
Armc7	0.00	1.00
Ccar2	0.13	1.00
Spry1	0.44	1.00
Lrpprc	0.22	1.00
Stk33	0.56	1.00
Ambra1	0.45	1.00
Ssbp1	0.58	1.00
Dlg5	0.34	1.00
Rnf44	-0.15	1.00
Zcchc17	-0.22	1.00
Krt5	-0.43	1.00
Septin11	0.23	1.00
Hnrnpu	0.29	1.00
Nit2	-0.17	1.00
Rars2	-0.11	1.00
Stk40	0.35	1.00
Eef1g	0.29	1.00
Akt3	0.14	1.00
Tmem106c	0.18	1.00
Col19a1	0.62	1.00
Dek	-0.84	1.00
Chd9	0.41	1.00
Erp44	-0.13	1.00
Strbp	0.18	1.00
Csnk1g3	-0.78	1.00
Pag1	-2.12	1.00
Nr4a3	0.05	1.00
Elavl1	0.03	1.00
Timp3	-0.77	1.00
Dhx35	-0.51	1.00
Anln	0.47	1.00
Col1a2	0.70	1.00
Afap1	0.77	1.00
Rgs4	1.38	1.00
H2-Ob	0.52	1.00
Ecpas	0.07	1.00
Plekhf2	0.41	1.00
Fam133b	0.31	1.00
Crlf3	-0.78	1.00
Dcps	0.05	1.00
Tspan12	-0.49	1.00

Sun2	-0.10	1.00
Galc	0.07	1.00
Spata18	0.31	1.00
Etaa1	0.14	1.00
Tmem242	-0.40	1.00
Fbxl4	0.23	1.00
Xpa	0.02	1.00
Stn1	0.04	1.00
Prkab2	0.06	1.00
Cramp1	-0.02	1.00
Usp28	-0.35	1.00
Sae1	0.09	1.00
Erg28	0.11	1.00
Retreg2	0.02	1.00
Bckdk	0.04	1.00
Dusp6	0.48	1.00
Ccdc93	0.12	1.00
Cbfa2t2	0.26	1.00
Irak3	0.67	1.00
Pgap1	-0.05	1.00
Btbd19	0.47	1.00
Carmil1	-0.18	1.00
Eif3h	-0.12	1.00
Ep300	0.12	1.00
Ewsr1	0.29	1.00
Shprh	0.00	1.00
Armc8	0.24	1.00
Cndp2	0.24	1.00
Tapt1	0.04	1.00
Ppp6r3	0.25	1.00
Vgll4	-0.36	1.00
Flot1	-0.12	1.00
Frmpd4	1.10	1.00
Pgd	0.36	1.00
Necap2	-0.15	1.00
Ap2b1	0.47	1.00
Atg5	0.43	1.00
Etv3	0.70	1.00
Col4a5	-1.09	1.00
Zfp451	0.45	1.00
Fam183b	0.39	1.00
Cfap74	0.29	1.00
Snrpn	0.27	1.00
Vbp1	0.30	1.00
Fbxo33	0.00	1.00
Ttc14	-0.27	1.00

Lsm14a	0.03	1.00
Dnajc2	-0.36	1.00
Eci3	-0.19	1.00
Polr3k	-0.28	1.00
Hacd3	-0.58	1.00
Itfg1	0.26	1.00
Tmtc4	0.18	1.00
Lgals1	0.73	1.00
Pank1	0.21	1.00
Zfp248	0.18	1.00
Ubr2	-0.55	1.00
Tbc1d8	0.26	1.00
Tcf20	-1.38	1.00
Ino80	0.21	1.00
Xbp1	0.22	1.00
Pgm3	0.41	1.00
Cog2	0.16	1.00
Whamm	0.36	1.00
Zfp280c	0.09	1.00
Gpat4	-0.36	1.00
Hcfc2	-0.07	1.00
Yeats4	0.05	1.00
Agpat4	0.05	1.00
Tkfc	0.67	1.00
Ivd	-0.06	1.00
Agl	0.09	1.00
Eif2b5	0.08	1.00
Bnip2	0.10	1.00
Klhl36	-0.31	1.00
Zmpste24	0.00	1.00
Brd9	0.02	1.00
Vars	-0.07	1.00
Cdc42bpb	0.06	1.00
Hdac7	0.30	1.00
Etfa	-0.25	1.00
Oas2	-0.12	1.00
Lsmem1	0.09	1.00
Tmem117	0.30	1.00
Syt1	0.14	1.00
Fam234b	0.16	1.00
Cerk	0.17	1.00
Tubgcp5	0.12	1.00
F11	0.18	1.00
Sec16b	0.09	1.00
Cln6	0.22	1.00
Sord	0.29	1.00

Fbn1	0.46	1.00
Abhd6	0.12	1.00
Rab40c	0.17	1.00
Ciao3	0.12	1.00
Slco4a1	0.49	1.00
Lrrc8a	0.11	1.00
Galnt6	0.17	1.00
Acot7	0.15	1.00
Cd3e	0.78	1.00
Tinagl1	0.27	1.00
Tfeb	0.09	1.00
Tbc1d25	0.32	1.00
Sh2b2	0.07	1.00
Ifnlr1	0.43	1.00
Lin7b	0.15	1.00
Chrnb1	0.43	1.00
Trim15	0.19	1.00
Batf2	0.11	1.00
Trps1	0.35	1.00
Syne3	-0.06	1.00
Prr5l	-1.17	1.00
Supt20	0.19	1.00
Cdc14a	0.59	1.00
Abcg1	-0.49	1.00
Runx1t1	-1.40	1.00
Csmd3	-0.70	1.00
Vsnl1	-0.33	1.00
Sprtn	-0.58	1.00
Npffr2	-0.47	1.00
Adamts6	-0.41	1.00
Rftn2	-0.33	1.00
Ercc3	-0.53	1.00
Urod	-0.15	1.00
Dnajc6	-0.81	1.00
Rwdd3	-0.58	1.00
Sphkap	-0.72	1.00
Zfp276	-0.37	1.00
Ripor1	-0.57	1.00
Sh2d3c	-0.59	1.00
Slc26a9	-0.21	1.00
Zfp703	-0.31	1.00
Foxo4	-0.74	1.00
Adgrv1	-0.60	1.00
Prkra	-0.49	1.00
Zfp383	-0.26	1.00
Rnase6	-0.13	1.00

Dnase1	-0.65	1.00
Cul9	-0.55	1.00
Dut	0.62	1.00
Habp4	-0.35	1.00
Cpne3	0.19	1.00
Dnah11	0.66	1.00
Runx2	0.23	1.00
Cdh23	-0.97	1.00
Anapc5	-0.18	1.00
Kmt2e	-0.04	1.00
Ssrp1	0.15	1.00
C3	0.92	1.00
Ralgapa2	0.66	1.00
Prkaa1	-0.15	1.00
Rala	-0.18	1.00
Brd1	0.14	1.00
Isca2	-0.07	1.00
Slc25a29	0.15	1.00
Sh3pxd2a	0.41	1.00
Spns2	0.13	1.00
Wdr5	-0.24	1.00
Ubac2	0.19	1.00
Snrnp70	0.18	1.00
Trib2	-0.17	1.00
Ndst3	-0.80	1.00
Trmt10b	0.18	1.00
Psmd6	0.03	1.00
Afmid	0.30	1.00
Tex2	0.38	1.00
Slc25a25	0.17	1.00
Bscl2	0.16	1.00
Rrp1b	0.41	1.00
Polr2d	-0.11	1.00
Ccdc174	-0.02	1.00
Nlrc5	-0.12	1.00
Lsm14b	0.01	1.00
Fras1	0.11	1.00
Dab2	1.06	1.00
Odad2	0.54	1.00
Gxylt1	0.04	1.00
B3galnt2	-0.03	1.00
Dnaaf6b	-0.19	1.00
Ecd	0.06	1.00
Tmem53	0.06	1.00
Eif4e3	-0.11	1.00
Tamm41	0.00	1.00

Gde1	0.08	1.00
Tpcn1	0.07	1.00
Urm1	-0.14	1.00
Ifi35	-0.18	1.00
Eif5b	-0.95	1.00
Slc39a14	0.20	1.00
Atl3	0.14	1.00
Lypla1	0.20	1.00
Ift22	0.08	1.00
Adcy6	-0.68	1.00
Tgfbr2	0.41	1.00
Cabcoco1	0.23	1.00
Vps35l	-0.31	1.00
1700012B09Rik	-0.27	1.00
Htt	0.09	1.00
Fundc1	-0.82	1.00
Akap8l	-0.42	1.00
Cfap46	0.19	1.00
Sumo2	0.33	1.00
Usp47	-0.02	1.00
Adnp	0.07	1.00
Pes1	-0.01	1.00
Pafah2	0.14	1.00
Ppfia3	0.08	1.00
Pros1	0.23	1.00
Ctbp1	0.44	1.00
Il1a	0.65	1.00
Atpif1	-0.53	1.00
Kbtbd3	0.31	1.00
Tfec	0.78	1.00
Sh3rf3	0.73	1.00
Smo	0.08	1.00
Suv39h2	0.05	1.00
Pick1	0.10	1.00
Xk	0.08	1.00
Atp8a2	0.37	1.00
Pard6g	0.07	1.00
Tfap2a	0.06	1.00
Nrxn1	0.70	1.00
Tatdn3	0.20	1.00
Haao	0.10	1.00
Fam83b	0.09	1.00
Urb2	0.09	1.00
Dhcr7	0.12	1.00
Cacnb4	0.06	1.00
Klhl23	0.21	1.00

Npm3	0.15	1.00
Taf5	0.05	1.00
Ddah1	0.13	1.00
Cdcp2	0.65	1.00
Atg4b	0.07	1.00
Calml4	0.21	1.00
Frmd5	0.25	1.00
Enkd1	0.06	1.00
Gdf9	0.07	1.00
Dpysl3	0.35	1.00
Gprc5b	0.07	1.00
Kpna7	0.64	1.00
Soga1	0.14	1.00
Thop1	0.16	1.00
Tmem161a	0.07	1.00
Zfp687	0.05	1.00
Erich6	0.20	1.00
Adamtsl3	0.72	1.00
Sh3gl2	0.40	1.00
4930444P10Rik	0.16	1.00
Crocc2	0.08	1.00
Sh2d2a	0.12	1.00
Ccdc60	0.29	1.00
Qtrt1	0.12	1.00
Cd3d	0.13	1.00
Shmt2	0.39	1.00
Zkscan17	0.09	1.00
Cntrob	0.10	1.00
Hap1	0.09	1.00
Ppp1r36	0.14	1.00
Serf1	0.09	1.00
Dzip3	0.14	1.00
Tmem151b	0.07	1.00
Ltbp3	0.12	1.00
Xpo4	0.30	1.00
Sltm	0.17	1.00
Rnmt	0.51	1.00
Slirp	-0.16	1.00
Lims2	-0.34	1.00
Tm4sf4	-0.80	1.00
Osbpl2	-0.38	1.00
Pde1c	3.80	1.00
Kctd3	0.24	1.00
Ccdc39	0.27	1.00
Prdx2	0.30	1.00
Igfbp7	0.18	1.00

Dusp4	0.95	1.00
Chrac1	0.05	1.00
Dhtkd1	0.07	1.00
Letm1	-0.06	1.00
Scrn3	-0.05	1.00
Tcof1	-0.24	1.00
Plbd2	-0.54	1.00
Zbtb48	0.02	1.00
Gba	-0.06	1.00
Esyt2	0.38	1.00
Hmbox1	-0.23	1.00
Glg1	-1.30	1.00
Cfap99	0.35	1.00
Cnn2	-0.27	1.00
Zc3h12c	0.71	1.00
Cntn3	-0.69	1.00
Tuba4a	0.61	1.00
Tmem65	-0.21	1.00
Spred1	-0.23	1.00
Pde6d	-0.33	1.00
Il13ra1	0.01	1.00
Insig2	-0.09	1.00
Cyb561	-0.12	1.00
Suclg2	0.22	1.00
Kdm2a	0.08	1.00
Cpne1	0.13	1.00
Tmem50b	0.15	1.00
Trim33	0.33	1.00
Depdc1b	0.01	1.00
Dennd5b	-0.94	1.00
Tmcc1	-0.84	1.00
Rubcnl	-1.61	1.00
Spi1	-0.65	1.00
Colca2	-0.55	1.00
Gramd1a	0.67	1.00
Pdcd6	0.26	1.00
Fhdc1	0.07	1.00
Xkr6	-0.07	1.00
Slc36a4	0.09	1.00
Sash1	0.28	1.00
Ninj1	0.16	1.00
Axin1	-0.31	1.00
Tmco1	0.17	1.00
Ing1	0.08	1.00
Lca5l	0.17	1.00
Ust	-0.13	1.00

Tor1aip2	0.01	1.00
Dctn5	-0.03	1.00
Plxna2	0.20	1.00
Klhl9	0.02	1.00
Zfp94	0.19	1.00
Tmed7	0.16	1.00
Rab27a	0.86	1.00
Camta2	0.05	1.00
Yipf2	-0.26	1.00
Ropn1l	0.44	1.00
Ube2j1	0.12	1.00
Bub3	-0.23	1.00
Ift140	0.04	1.00
Ftsj1	0.16	1.00
Slc11a2	0.64	1.00
Pik3c3	-0.06	1.00
Pcsk6	0.11	1.00
Pxdn	-0.26	1.00
Pmp22	-0.74	1.00
Ncbp3	-0.16	1.00
Tmem107	-0.33	1.00
Dock7	0.33	1.00
Ntan1	-0.46	1.00
Auh	-0.82	1.00
Il1r1	-0.13	1.00
Ldb1	-0.04	1.00
Poldip2	-0.03	1.00
Gcfc2	-0.04	1.00
Wdr93	-0.01	1.00
Trpv5	0.50	1.00
Pdgfd	-0.64	1.00
Pdzrn4	-0.54	1.00
Arhgef33	-0.57	1.00
C5ar2	-0.46	1.00
Tmem45a	-0.37	1.00
Gdpd5	0.07	1.00
Serhl	-0.16	1.00
Cenpm	0.02	1.00
Clcn1	0.05	1.00
Anks6	0.04	1.00
Nbeal2	0.03	1.00
Acvr2b	0.03	1.00
Ly86	-0.04	1.00
Gatad1	0.04	1.00
Slc1a4	-0.19	1.00
Trmt6	0.03	1.00

Hddc2	0.05	1.00
Cd109	-0.65	1.00
Dll1	-0.21	1.00
Commd5	0.07	1.00
Sash3	0.05	1.00
Galntl6	-0.10	1.00
Dimt1	-0.09	1.00
Aptx	-0.03	1.00
Enc1	0.04	1.00
Nmrk1	0.01	1.00
Frmd3	0.01	1.00
Dnajb5	0.00	1.00
Tesk1	0.07	1.00
Sgpp1	-0.12	1.00
Wdr89	0.00	1.00
Ackr3	-0.40	1.00
Nexn	-0.27	1.00
Dmap1	0.04	1.00
Ddx59	-0.40	1.00
S1pr1	0.03	1.00
Hes6	0.06	1.00
Abtb1	0.07	1.00
Rrp9	0.03	1.00
Rbm15b	0.06	1.00
Wnk2	0.02	1.00
Ddx28	0.06	1.00
Jpt2	0.03	1.00
Ercc4	0.07	1.00
Kdm8	-0.10	1.00
Cit	-0.17	1.00
Fbxw8	-0.06	1.00
Atad5	-0.46	1.00
Ccdc137	-0.10	1.00
Ntmt1	0.02	1.00
Ptges2	0.03	1.00
Brip1	0.06	1.00
Ghdc	-0.15	1.00
Pou6f1	0.06	1.00
Zfp740	0.05	1.00
Plod1	-0.03	1.00
Zbtb45	-0.05	1.00
Tmem39b	0.05	1.00
Pex11g	0.06	1.00
Lonp1	0.07	1.00
Pmvk	0.07	1.00
Ppox	0.02	1.00

Fbxo46	0.01	1.00
Rgsl1	-0.03	1.00
Unc13c	0.15	1.00
Dnaaf5	0.01	1.00
Itgae	0.06	1.00
Faah	0.06	1.00
Crocc	-0.09	1.00
Oas1e	-0.03	1.00
Leng1	-0.02	1.00
Fiz1	0.00	1.00
Zfp784	0.04	1.00
Syt3	0.04	1.00
Klhl26	0.02	1.00
Bst2	-0.03	1.00
Ulk3	-0.10	1.00
Zfr2	0.04	1.00
Neurl4	0.04	1.00
Sdr39u1	0.03	1.00
Spaca6	-0.10	1.00
Scyl1	0.07	1.00
Frat2	0.03	1.00
Bex2	-0.21	1.00
Pir	0.03	1.00
Tex264	0.12	1.00
Hspa9	0.21	1.00
Veph1	-1.17	1.00
Ccbe1	-0.58	1.00
Hipk3	0.44	1.00
Rassf6	-0.35	1.00
Cnot2	-0.34	1.00
Gnai3	0.19	1.00
Nup35	-0.68	1.00
Ubxn8	-0.81	1.00
Galnt2	0.31	1.00
Klf2	-0.30	1.00
Avpi1	0.11	1.00
Ptpn1	0.68	1.00
Ttll12	0.22	1.00
Nfatc1	0.07	1.00
Dhx32	-0.07	1.00
Trp73	0.22	1.00
Prmt5	0.13	1.00
Acox2	0.31	1.00
Tob2	0.49	1.00
Lrrk2	-1.60	1.00
Galk2	0.62	1.00

Klf13	0.16	1.00
Cox7c	-0.31	1.00
Arhgap35	-0.90	1.00
Ndufa6	-0.01	1.00
Ptdss1	0.04	1.00
Grb10	-0.05	1.00
Tshz1	0.47	1.00
Ppp1r42	0.07	1.00
Pex1	0.01	1.00
Slco4c1	-0.03	1.00
Srsf5	0.00	1.00
Skap1	-0.64	1.00
Ubr3	-0.52	1.00
Tmem147	0.22	1.00
Prpf4b	0.17	1.00
Snx10	-0.72	1.00
Crebbp	-0.92	1.00
Sfpq	0.26	1.00
Ddx1	-0.12	1.00
Plcb4	0.33	1.00
Fggy	-0.13	1.00
Bnip3l	-0.05	1.00
Rxra	-0.43	1.00
Sh3d19	0.15	1.00
Celsr1	0.27	1.00
Faf1	-0.55	1.00
L3mbtl1	0.12	1.00
Ehmt1	0.22	1.00
Clip1	-0.63	1.00
Naa15	0.42	1.00
Nme6	0.21	1.00
Areg	0.99	1.00
Pigb	0.15	1.00
Slc25a26	0.38	1.00
Adgrg1	0.23	1.00
Traf1	0.95	1.00
Palld	0.99	1.00
Tnk2	0.15	1.00
H2bc14	0.63	1.00
Cox7a2	0.18	1.00
Nedd9	-0.68	1.00
Ndufs5	-0.10	1.00
Rel	0.75	1.00
Far2	0.11	1.00
Hace1	0.20	1.00
Eif2ak1	-0.24	1.00

Pcif1	-0.14	1.00
Atxn2l	0.12	1.00
Gtf2f1	0.84	1.00
Kat5	-0.07	1.00
Lars	-0.81	1.00
Dnmt3a	-0.16	1.00
Sox5	-0.75	1.00
Lias	-0.12	1.00
Bmp4	-0.24	1.00
Pla2g1b	-0.45	1.00
Tbc1d13	-0.53	1.00
Stac2	-0.23	1.00
Acbd4	-0.16	1.00
Slc25a23	-0.22	1.00
Prkn	-0.42	1.00
Itprid1	-0.65	1.00
Zfp518a	-0.09	1.00
Foxk2	0.08	1.00
Ccdc107	0.01	1.00
Cfap73	-0.17	1.00
Asgr2	-0.11	1.00
Isg20	0.13	1.00
Ifi44l	0.07	1.00
Etv1	0.83	1.00
Lhpp	-0.08	1.00
Ndufaf3	-0.03	1.00
Vamp4	0.09	1.00
Rab43	0.21	1.00
Nsf	0.15	1.00
Golga4	0.42	1.00
Ptprk	-0.46	1.00
Pdcl2	-0.24	1.00
Katnbl1	-0.17	1.00
Pitpnm3	0.08	1.00
Pcm1	0.13	1.00
Trp53bp2	0.14	1.00
Stom	-0.10	1.00
Nup88	0.03	1.00
Cfap43	-0.06	1.00
Plekha3	-0.70	1.00
Vps4b	0.00	1.00
Rrp36	-0.62	1.00
Asxl2	-0.85	1.00
Scai	-0.87	1.00
Bcl2a1d	-0.48	1.00
Ribc2	0.34	1.00

Tnrc18	0.15	1.00
Cyth4	-0.10	1.00
Ankrd50	-0.65	1.00
Scfd2	0.28	1.00
Rapgef1	0.01	1.00
Atf1	0.04	1.00
Alox5ap	0.08	1.00
Rassf7	0.07	1.00
Bcl9l	0.53	1.00
Ltn1	0.21	1.00
Ehd2	-0.46	1.00
Fam185a	-0.82	1.00
Wnt9a	-0.46	1.00
Arhgap28	-1.04	1.00
Actg1	0.51	1.00
Pxn	-1.43	1.00
Scin	0.09	1.00
Armc3	0.18	1.00
Papolg	0.13	1.00
Klhl20	-0.03	1.00
Yipf5	0.06	1.00
Rab35	-0.45	1.00
Pitpnm2	0.55	1.00
Hsd11b1	-0.08	1.00
Ring1	0.16	1.00
Serpinb1a	-0.25	1.00
Six1	-0.10	1.00
Calm3	0.33	1.00
Ldlrad3	-0.16	1.00
Chuk	-0.02	1.00
Cc2d1b	-0.08	1.00
Stk35	0.00	1.00
Usp2	0.03	1.00
Snrpa	-0.39	1.00
Tff3	-0.68	1.00
Dcn	0.19	1.00
Dusp10	0.36	1.00
Ccsap	0.13	1.00
Slc25a27	0.17	1.00
Il34	0.21	1.00
4930550C14Rik	0.20	1.00
Tjp3	-0.10	1.00
Kif13a	-0.34	1.00
Gosr1	-0.90	1.00
Fam76a	0.07	1.00
Snd1	-0.12	1.00

Slc50a1	0.39	1.00
Fmr1	0.51	1.00
Plin2	0.34	1.00
Irx5	-0.08	1.00
Klhdc8a	0.05	1.00
Ncaph	-0.08	1.00
Tmigd3	0.21	1.00
1110065P20Rik	0.13	1.00
Zfp185	-0.15	1.00
Ap4s1	-0.65	1.00
Traf6	-0.37	1.00
Vps50	0.21	1.00
Kat7	-0.72	1.00
Ncln	-0.46	1.00
Eif3d	0.07	1.00
Thrb	-0.90	1.00
Puf60	0.24	1.00
Nubpl	0.24	1.00
Zcchc14	0.22	1.00
Nsun7	0.56	1.00
Uso1	-0.32	1.00
Abhd10	0.08	1.00
Rab33b	0.01	1.00
Itgb2l	-0.16	1.00
Plxna1	0.30	1.00
Bfar	1.79	1.00
Chtop	0.34	1.00
Pqbp1	-0.37	1.00
Edem3	0.04	1.00
Tra2a	0.28	1.00
Snx14	-0.04	1.00
Zbtb16	0.39	1.00
Coq7	-0.14	1.00
Golga7	-0.20	1.00
Zswim2	0.75	1.00
Ccdc82	0.05	1.00
Dhx57	0.40	1.00
Arcn1	0.51	1.00
Ccm2	0.21	1.00
Galnt1	0.00	1.00
Cdc42	0.23	1.00
Ap1g1	-0.32	1.00
Dydc2	-0.28	1.00
Idh2	0.13	1.00
Lypla2	-0.71	1.00
C5ar1	-0.86	1.00

Gripap1	-0.59	1.00
Nupl2	-0.22	1.00
Slc39a6	-0.06	1.00
Bloc1s2	-0.28	1.00
Cldn3	-0.18	1.00
Flot2	0.05	1.00
Pde4dip	-0.23	1.00
Znrf3	-0.76	1.00
Syf2	0.35	1.00
Lrrc71	0.74	1.00
Lsr	0.13	1.00
Elp4	0.14	1.00
Hsd17b12	0.23	1.00
Hadh	0.10	1.00
Zzef1	0.04	1.00
Prkd3	-0.16	1.00
Babam2	0.48	1.00
Aldh3a2	-0.52	1.00
Krcc1	-0.55	1.00
Rbfox2	-1.17	1.00
Dtna	0.72	1.00
Ccdc112	0.21	1.00
Gemin2	0.13	1.00
Entpd7	0.18	1.00
Slit3	0.23	1.00
Bri3bp	0.10	1.00
Prpf4	0.09	1.00
Scrn2	0.08	1.00
Clspn	0.84	1.00
Plekho1	0.71	1.00
Sowahb	0.19	1.00
Igsf8	0.18	1.00
Ifi204	0.30	1.00
Acad12	0.19	1.00
Xab2	0.09	1.00
Ccl28	0.24	1.00
Iqsec2	0.23	1.00
Hexim1	0.71	1.00
Terb1	-0.70	1.00
Asap3	-0.84	1.00
Naa20	-0.04	1.00
Exoc5	-0.31	1.00
Nktr	-0.14	1.00
Gphn	-0.55	1.00
D630045J12Rik	0.16	1.00
Zdhhc3	0.10	1.00

Zfp827	-0.09	1.00
Ide	0.15	1.00
Pla2g4a	-0.82	1.00
Stk4	0.06	1.00
Gns	-0.37	1.00
Tspan5	-1.20	1.00
Vps13d	0.46	1.00
Plscr1	-0.13	1.00
Cox5b-ps	0.38	1.00
Fbxo3	-0.13	1.00
Crbn	-0.15	1.00
Zfp341	-0.03	1.00
Scand1	0.03	1.00
Id4	0.82	1.00
Eml5	-0.11	1.00
Lratd2	0.23	1.00
Stpg1	-0.22	1.00
Emc10	0.11	1.00
Acaca	-0.86	1.00
Dynlrb1	-0.57	1.00
Sesn3	-0.16	1.00
Tmem30a	0.17	1.00
Pygm	0.10	1.00
Arf1	0.08	1.00
Nudcd1	-0.19	1.00
Grn	0.39	1.00
Dagla	-0.03	1.00
Pnpo	-0.13	1.00
Def8	-0.07	1.00
Ak3	0.02	1.00
Ilkap	0.33	1.00
Cep295	-0.53	1.00
Lrriq1	0.04	1.00
Nf1	0.39	1.00
Sugct	-0.24	1.00
Cct8	-0.11	1.00
Zc3h7b	-0.75	1.00
Ankrd28	0.26	1.00
Psme4	-0.04	1.00
Steap4	-0.84	1.00
Pld1	-0.46	1.00
Nup98	0.09	1.00
Topbp1	0.57	1.00
Ndufb8	0.13	1.00
Cfap126	0.20	1.00
Cmtm7	-0.54	1.00

Cpsf2	-0.13	1.00
Srgap1	-0.72	1.00
Dhx30	-0.10	1.00
Tmem33	-0.59	1.00
Gne	0.53	1.00
Slc25a24	0.42	1.00
Akap8	-0.16	1.00
Helb	0.08	1.00
Lig4	0.17	1.00
Nsmaf	0.09	1.00
Pde7b	0.38	1.00
Nsl1	0.43	1.00
Rmdn3	0.10	1.00
Casp1	0.11	1.00
Dnajc12	0.31	1.00
Cutc	0.08	1.00
Pdp2	0.09	1.00
Dbn1	0.07	1.00
Acot8	0.11	1.00
Patz1	0.04	1.00
Zmat5	0.07	1.00
Recql5	0.23	1.00
Kirrel3	0.29	1.00
Reep4	0.19	1.00
Plin3	0.18	1.00
Atp8b2	0.15	1.00
Trim26	0.09	1.00
Pelo	0.09	1.00
B3galt4	0.10	1.00
Gng10	-0.62	1.00
Cnst	-0.13	1.00
Foxp2	-0.60	1.00
Dpyd	-0.52	1.00
Fem1c	-0.30	1.00
3110082I17Rik	0.07	1.00
Cab39l	0.25	1.00
Polb	0.25	1.00
Cstf3	0.16	1.00
Pepd	0.02	1.00
Cttnbp2nl	-0.33	1.00
Apbb2	-0.79	1.00
Uaca	-0.35	1.00
Hscb	-0.36	1.00
Lrrc47	-0.05	1.00
Fbln5	-0.41	1.00
Lpcat2	-0.36	1.00

Ptprb	-1.22	1.00
Erg	-0.67	1.00
Ldah	-0.18	1.00
Traf5	-0.39	1.00
Parp16	-0.80	1.00
Garre1	-0.30	1.00
Spsb3	-0.36	1.00
Galnt5	-0.38	1.00
Hemgn	-0.24	1.00
Pfkfb3	0.05	1.00
Edrf1	-0.90	1.00
Dusp1	-0.96	1.00
Lsm7	0.33	1.00
Atm	0.15	1.00
Fmnl1	-0.03	1.00
Trit1	0.03	1.00
Cfap52	-0.31	1.00
Cibar2	-0.09	1.00
Prdx5	0.29	1.00
Bmp3	-0.55	1.00
Hook3	-1.03	1.00
Serp1	-0.06	1.00
Herc6	-1.61	1.00
Sigirr	-0.36	1.00
Slc6a6	-0.23	1.00
Dpp7	-0.13	1.00
Rhod	0.23	1.00
Ppp1r35	-0.07	1.00
Ctdnep1	-0.43	1.00
Hnrnph3	0.00	1.00
Lyplal1	-0.08	1.00
Cipc	-0.06	1.00
Ccdc113	0.27	1.00
Sap30l	0.04	1.00
Tlr7	-0.03	1.00
Emp3	0.34	1.00
Cmtr1	-0.26	1.00
Jdp2	-0.23	1.00
Dnah6	0.52	1.00
Kpnb1	0.09	1.00
Vwa3b	0.69	1.00
Ubap2	0.03	1.00
Tenm2	0.73	1.00
Scgb3a1	-0.16	1.00
Ggh	0.10	1.00
Cnnm4	0.38	1.00

Rnf149	-1.39	1.00
Rac1	0.16	1.00
Laptm4b	0.28	1.00
Ptpn13	-0.28	1.00
Drc1	0.34	1.00
Cfap77	0.27	1.00
Iqck	0.42	1.00
Tpm1	-0.38	1.00
Yars2	0.10	1.00
Rabl3	0.06	1.00
Utp23	0.05	1.00
Rbm48	-0.01	1.00
Cgas	0.03	1.00
Atp7a	0.09	1.00
Las1l	-0.19	1.00
Sh3bp2	0.34	1.00
Fxn	0.04	1.00
AW549877	0.11	1.00
Ddb2	0.07	1.00
Txndc16	0.06	1.00
Tgfb3	0.19	1.00
Nif3l1	0.01	1.00
Lancl1	0.07	1.00
Rgs21	-0.10	1.00
Slco3a1	0.08	1.00
Snupn	0.00	1.00
Parp3	0.30	1.00
Zfp598	0.37	1.00
Pik3r6	0.18	1.00
Tmem199	-0.05	1.00
Asic2	0.08	1.00
Dvl1	0.13	1.00
Bud13	0.05	1.00
Cnksr1	0.21	1.00
Plpbp	0.06	1.00
Pitpnm1	0.10	1.00
Crtc2	0.19	1.00
Ssc5d	-0.01	1.00
Xrcc1	0.08	1.00
Rhbdd3	0.07	1.00
Cpped1	0.15	1.00
Stap2	-0.02	1.00
Pcnx3	0.05	1.00
Tfam	-0.19	1.00
Apoo	-0.39	1.00
Gss	-0.13	1.00

Ttc5	-0.17	1.00
Arpc2	0.51	1.00
Pard3b	-0.89	1.00
Lmbrd2	0.07	1.00
Pfkl	0.05	1.00
Eif3m	0.28	1.00
Dnai3	0.41	1.00
Trpc1	-0.31	1.00
Fnbp1l	-0.83	1.00
Osbpl8	0.03	1.00
Plag1	-0.40	1.00
Slc39a13	-0.27	1.00
Zswim5	-0.73	1.00
Smad6	-0.80	1.00
Stox1	-0.37	1.00
Lrrc36	-0.30	1.00
2810004N23Rik	-0.21	1.00
Gabpb1	-0.51	1.00
Cul2	-0.44	1.00
Tbc1d32	-0.36	1.00
Frmd6	-1.15	1.00
Cask	0.11	1.00
Nek10	0.37	1.00
Zfp410	0.17	1.00
Pptc7	0.17	1.00
Dscaml1	0.26	1.00
Camsap2	-0.25	1.00
Arl14ep	-0.17	1.00
Ptpa	0.04	1.00
Cdc26	-0.22	1.00
H6pd	0.05	1.00
Rab25	-0.02	1.00
Fam135a	-0.75	1.00
Ppp4r3b	0.21	1.00
Fam120b	0.00	1.00
Farp2	-0.71	1.00
Sdf2	0.01	1.00
Paf1	-0.10	1.00
Hexim2	-0.07	1.00
Hivep1	0.04	1.00
Dicer1	-0.10	1.00
Carhsp1	0.15	1.00
Gramd1b	-0.09	1.00
Braf	0.18	1.00
Crot	0.27	1.00
Ice2	0.23	1.00

Golph3l	-0.01	1.00
Cfap44	0.44	1.00
Ndufa8	-0.28	1.00
Xpo7	0.08	1.00
Fbl	0.73	1.00
Edem2	0.49	1.00
Tprgl	0.17	1.00
Gpatch2	0.47	1.00
Flacc1	-0.18	1.00
Casp6	0.18	1.00
Smc3	0.04	1.00
Gfod1	-0.79	1.00
Ajuba	0.01	1.00
Uvrag	0.35	1.00
Rhot1	-0.12	1.00
Hsd17b10	-0.17	1.00
Dennd5a	-0.37	1.00
Plekhb2	-0.17	1.00
Igfbp5	0.30	1.00
Olfml3	-0.21	1.00
Phf1	-0.63	1.00
Il1rapl1	-0.21	1.00
Crppa	0.08	1.00
Thnsl1	-0.05	1.00
Gpr137b	-0.05	1.00
Rab17	-0.40	1.00
Adarb1	-0.03	1.00
Pcgf6	-0.42	1.00
Zfyve27	0.04	1.00
Ssx2ip	-0.04	1.00
Cpt2	-0.01	1.00
Ccdc71	-0.01	1.00
Elmo3	0.00	1.00
Ddx55	0.01	1.00
Prdm10	0.03	1.00
Ano8	-0.06	1.00
Cabyr	-0.38	1.00
Arhgap23	0.08	1.00
Ankrd35	0.03	1.00
Zfp398	0.02	1.00
Grina	0.04	1.00
Rxrb	0.04	1.00
Agpat1	0.04	1.00
Taf9b	-0.04	1.00
Bicd2	0.06	1.00
Leng8	-0.07	1.00

Enpp5	-0.10	1.00
Irf7	-0.33	1.00
Bbs9	0.52	1.00
Slc35a5	-0.30	1.00
Pde12	-0.39	1.00
Decr2	-0.31	1.00
Itgam	-0.92	1.00
Cited1	-0.25	1.00
Trio	0.03	1.00
Arhgap44	-0.64	1.00
Zyx	0.28	1.00
Sgpl1	0.34	1.00
Dguok	-0.20	1.00
Inpp4b	0.43	1.00
Rpia	0.12	1.00
Gpr39	-0.05	1.00
Hlcs	-0.14	1.00
Chpt1	0.16	1.00
Rb1	0.42	1.00
Rpn2	0.10	1.00
Prkcg	-0.13	1.00
Ppp1cb	0.19	1.00
Svil	-0.03	1.00
Copb2	0.54	1.00
Cyth1	-0.41	1.00
Kras	0.15	1.00
Zfp207	0.07	1.00
Ppfibp2	-0.37	1.00
Ifnar1	-0.51	1.00
Taok3	-0.89	1.00
Setd5	0.26	1.00
Zbtb5	0.17	1.00
Acer2	0.11	1.00
Car9	0.30	1.00
Plek2	0.42	1.00
Commd4	0.05	1.00
Sfxn1	-0.06	1.00
Pkd1	-0.12	1.00
Rae1	0.64	1.00
Slc25a1	0.17	1.00
Usp18	0.18	1.00
Zfp90	0.08	1.00
Tsfm	0.21	1.00
Cpt1b	0.03	1.00
Zfp516	-0.64	1.00
Tmem63b	-0.02	1.00

Dnai4	0.15	1.00
Col23a1	0.21	1.00
Zhx1	-0.77	1.00
Bbs7	-0.58	1.00
Agfg1	0.02	1.00
Plekhg1	0.03	1.00
Ubl7	0.94	1.00
Edem1	-0.11	1.00
Shfl	-0.21	1.00
Stat6	-0.01	1.00
Lamc1	-0.45	1.00
Tdrd3	0.00	1.00
Sgms1	-0.57	1.00
Gabpa	0.02	1.00
Cops2	0.23	1.00
Sdcbp	-0.37	1.00
Btg1b	-0.31	1.00
Pik3cb	-0.59	1.00
Col5a2	-1.22	1.00
Tnip1	0.46	1.00
Eapp	0.31	1.00
Smad1	0.23	1.00
Arhgef7	-0.05	1.00
Xpnpep3	0.01	1.00
6030458C11Rik	-0.38	1.00
Smim8	-0.02	1.00
Gnpat	-0.01	1.00
Steep1	-0.18	1.00
Acvr1c	0.00	1.00
Upp2	-0.12	1.00
Asb1	-0.25	1.00
Atg16l1	-0.09	1.00
Hip1r	-0.07	1.00
Utp18	0.03	1.00
Tirap	-0.38	1.00
Iho1	-0.10	1.00
Trib3	0.03	1.00
Zkscan5	0.03	1.00
Gpr137	0.03	1.00
Uba1	0.01	1.00
Tmem165	0.10	1.00
Cep290	0.08	1.00
Camkmt	0.10	1.00
Arap2	-0.67	1.00
Stpg4	-0.27	1.00
Cuta	-0.10	1.00

Ccdc88a	0.52	1.00
Psmg3	0.15	1.00
Nf2	0.19	1.00
Hdac5	0.59	1.00
Ntrk2	-0.62	1.00
Leprot	-0.58	1.00
Col4a2	-0.62	1.00
Cyhr1	-0.28	1.00
Hcfc1r1	-0.20	1.00
Lrmda	-0.67	1.00
Rbm6	-0.61	1.00
Pih1d2	-0.19	1.00
Nfix	-0.38	1.00
Abca5	-0.30	1.00
Galnt7	0.04	1.00
Zer1	-0.08	1.00
Dusp14	-0.26	1.00
Dennd2c	0.27	1.00
Grhl2	0.03	1.00
Epb41l4a	0.27	1.00
Rsbn1	0.47	1.00
Clk1	-0.57	1.00
Sec23a	-0.73	1.00
Eps8l2	-0.33	1.00
Slc12a6	-0.40	1.00
Pomp	0.27	1.00
Pou2f1	0.22	1.00
Vav3	0.11	1.00
Tut7	-1.46	1.00
Tsc22d2	0.45	1.00
Hsd17b7	-0.02	1.00
Polr2c	-0.16	1.00
Rhbdd2	-0.14	1.00
N6amt1	0.09	1.00
Utp20	0.22	1.00
5031439G07Rik	0.17	1.00
Sucla2	-0.03	1.00
Ptpn6	0.13	1.00
Rap2c	-0.10	1.00
Smc2	0.15	1.00
Deaf1	0.05	1.00
Il17b	0.11	1.00
Neurl1b	0.07	1.00
Heatr6	0.27	1.00
Srprb	0.19	1.00
Baiap3	0.09	1.00

Oaz1	0.07	1.00
Nbea	-0.72	1.00
Irf2	-0.16	1.00
Lekr1	0.34	1.00
H1f3	0.06	1.00
Fmc1	0.30	1.00
Nelfcd	0.08	1.00
Cadps2	-0.54	1.00
Dnlz	-0.16	1.00
Ribc1	-0.43	1.00
Gpbp1	0.12	1.00
Arhgap12	-0.15	1.00
Nin	-1.00	1.00
Denn2b	-0.13	1.00
Epc2	-0.93	1.00
Tgfbr3	-0.43	1.00
Evi5	0.02	1.00
Rbm8a2	0.40	1.00
Ryr2	-0.35	1.00
Slc34a2	-0.13	1.00
Neto2	-0.10	1.00
Ascc1	-0.49	1.00
Glrx3	-0.09	1.00
Ubp1	-0.21	1.00
Arl5a	0.00	1.00
Ranbp17	-0.29	1.00
Smarcb1	0.22	1.00
Ccdc175	-0.03	1.00
Npc1	0.35	1.00
Ubfd1	0.22	1.00
Rnd1	1.23	1.00
Agk	-0.01	1.00
Tmem60	0.30	1.00
Rpf2	0.02	1.00
Pank2	0.22	1.00
Susd1	0.36	1.00
Ranbp10	0.03	1.00
Polr2g	0.16	1.00
S100a10	0.66	1.00
Zbtb44	-0.74	1.00
Ppp6r2	0.10	1.00
Nop58	0.05	1.00
Cenpu	-0.11	1.00
Rubcn	-0.16	1.00
S100a4	-0.56	1.00
Nfe2l3	-0.53	1.00

Snx15	-0.17	1.00
Gnl3l	-0.20	1.00
Stt3b	0.21	1.00
N4bp1	-0.49	1.00
Eif4enif1	-0.07	1.00
Cep164	0.01	1.00
Ccdc15	-0.26	1.00
Ppp1r10	0.06	1.00
Tmem184c	0.05	1.00
Sri	0.09	1.00
Chka	0.15	1.00
Kdm4b	0.68	1.00
Josd1	0.16	1.00
Fam149b	0.24	1.00
Fbxw7	0.57	1.00
Donson	0.62	1.00
Akt2	-0.07	1.00
Eny2	-0.28	1.00
Ube2e3	0.32	1.00
Eed	-0.16	1.00
Trpv4	-0.34	1.00
Gkap1	0.65	1.00
Aif1	0.22	1.00
Larp1	0.13	1.00
Napg	0.04	1.00
Tgs1	-0.11	1.00
Cbr4	0.05	1.00
Gba2	-0.04	1.00
Ppil3	0.28	1.00
Lins1	0.12	1.00
Rhbdf2	0.07	1.00
Alkbh6	0.01	1.00
Tbc1d17	-0.11	1.00
Zfp169	-0.31	1.00
Tspyl2	0.04	1.00
Fmo5	-1.34	1.00
Serinc3	0.39	1.00
Ikbip	-0.26	1.00
Ccnd2	-1.10	1.00
Sppl3	0.25	1.00
0610030E20Rik	-0.27	1.00
Cnot7	-0.04	1.00
Hibadh	-0.60	1.00
Abhd13	-0.01	1.00
Proser1	-0.03	1.00
Nars	0.02	1.00

Fam204a	-0.25	1.00
Lrba	0.29	1.00
Epb41l3	-0.68	1.00
Ptpn9	-0.64	1.00
Cdr2	-0.46	1.00
Gprc5a	-0.49	1.00
Flad1	-0.70	1.00
Brcc3	0.18	1.00
Brap	0.00	1.00
Ola1	0.05	1.00
Atp2c1	0.27	1.00
Zfp395	0.19	1.00
Sclt1	0.27	1.00
Unc13b	-0.07	1.00
Fbxo34	-0.25	1.00
Wdr7	-0.67	1.00
Rab6a	-0.64	1.00
Acaa1b	0.18	1.00
Prim2	0.29	1.00
Zfyve26	0.17	1.00
Pccb	0.13	1.00
Abca3	0.29	1.00
Rbm15	0.03	1.00
Pkig	-0.24	1.00
Luc7l	-0.02	1.00
Tbl1xr1	0.10	1.00
Cage1	-1.12	1.00
Dnajb13	0.16	1.00
Odf2	-0.25	1.00
Btrc	-0.09	1.00
Plekha5	-0.75	1.00
Pid1	0.00	1.00
Smarcd2	-0.56	1.00
Ptpdc1	-0.27	1.00
Pgm5	-0.37	1.00
Lzts2	-0.30	1.00
Lrrc31	-0.44	1.00
Ppp1r16b	-0.24	1.00
3110040N11Rik	0.40	1.00
Usp14	0.26	1.00
Hyal2	-0.16	1.00
Pinx1	-0.15	1.00
Sptlc1	-0.01	1.00
Wipi2	-0.13	1.00
Wipi1	-0.05	1.00
Pafah1b2	-0.10	1.00

Nfic	-0.71	1.00
Tacc2	0.23	1.00
Plagl2	0.12	1.00
Sypl	-0.41	1.00
Elp2	0.13	1.00
Arg2	-0.28	1.00
Gyg	-0.13	1.00
Thsd4	0.07	1.00
Zfp507	0.07	1.00
Snrnp35	0.04	1.00
Cdk5rap3	0.03	1.00
Zbtb7b	-0.02	1.00
Aste1	0.42	1.00
Araf	0.05	1.00
Setbp1	0.43	1.00
Prkce	-0.12	1.00
Ncapd2	-0.36	1.00
Cfap299	1.29	1.00
Ube2r2	0.17	1.00
Irs2	-0.18	1.00
Ttc13	-0.79	1.00
Fbxo21	-0.52	1.00
Ube2h	-0.56	1.00
Spag9	0.27	1.00
Rdh10	0.18	1.00
Ergic1	0.21	1.00
Ezh1	0.41	1.00
Fam3a	-0.02	1.00
Ubxn2a	-0.12	1.00
Tns1	-0.51	1.00
Ankrd12	0.13	1.00
Thoc2	-0.02	1.00
Smim7	-0.16	1.00
Sort1	-0.07	1.00
Reep5	0.46	1.00
Ift88	0.26	1.00
Elapor2	-0.18	1.00
Naa30	-0.23	1.00
Zdhhc6	0.30	1.00
Tctn3	0.12	1.00
Oma1	-0.04	1.00
Srebf1	0.31	1.00
Aatf	0.09	1.00
Rp1	0.11	1.00
Cox6a2	0.20	1.00
Stxbp2	-0.40	1.00

Cyp4b1	-0.24	1.00
Faf2	0.02	1.00
Coq4	-0.45	1.00
Serping1	0.59	1.00
Stoml2	0.04	1.00
Plcg2	-0.25	1.00
Hs6st3	-0.20	1.00
Ube2l6	-0.02	1.00
Ubn1	0.08	1.00
Garem1	-0.32	1.00
Pmepa1	0.80	1.00
Fbxo9	-0.46	1.00
Cpeb4	0.52	1.00
Tmem164	-0.10	1.00
Lrsam1	-0.17	1.00
Hmgxb4	-0.22	1.00
Tmem127	-0.38	1.00
Ttc7b	-0.19	1.00
Ablim3	-1.03	1.00
Pi4ka	-0.84	1.00
Kndc1	0.10	1.00
BC005537	0.05	1.00
Osbpl1a	0.20	1.00
Dennd4a	-0.18	1.00
Nptn	-0.28	1.00
Dnaja2	-0.10	1.00
Tnrc6b	0.26	1.00
Ppara	-0.35	1.00
Ano1	0.07	1.00
Ttc17	0.06	1.00
Hdgf	-0.16	1.00
Gna14	-0.56	1.00
Rpa3	-0.38	1.00
Ccn1	-1.83	1.00
Tstd2	-0.35	1.00
Itgav	-0.48	1.00
Xrn1	0.04	1.00
Il17ra	-0.17	1.00
Tmpo	-0.07	1.00
Rint1	-0.18	1.00
Bbs12	0.03	1.00
Inppl1	-0.56	1.00
Wdr36	0.05	1.00
Pdlim7	-0.03	1.00
Rilpl1	-0.76	1.00
Tbc1d22b	0.00	1.00

Tgfa	-0.34	1.00
1700102P08Rik	-0.14	1.00
Pkib	0.60	1.00
Prpf19	0.17	1.00
Elf1	0.19	1.00
Srgap3	-0.52	1.00
Cse1l	-0.06	1.00
Ttc28	-0.68	1.00
Wsb1	-0.09	1.00
Fcho2	-0.48	1.00
Nr2c2	-0.02	1.00
Kansl1	0.06	1.00
Eef1d	0.14	1.00
Stag2	-0.02	1.00
Skil	0.06	1.00
Ppil6	0.25	1.00
1700021F07Rik	-0.17	1.00
Cdk17	-0.07	1.00
Tppp3	-0.56	1.00
Vangl1	0.13	1.00
Rrm1	-0.01	1.00
Slc49a4	0.33	1.00
Dido1	0.10	1.00
Irx1	0.58	1.00
Uhrf1bp1l	-0.40	1.00
Lpin2	-0.96	1.00
Decr1	-0.04	1.00
Gpld1	-0.12	1.00
Cops3	-0.25	1.00
Rnf139	-0.75	1.00
Bcl2l13	-0.10	1.00
Spata17	0.20	1.00
Dapk1	0.29	1.00
Exoc7	-0.37	1.00
Kdm6a	0.22	1.00
Katnip	0.30	1.00
Tnrc6c	-0.76	1.00
Gtf2f2	-0.13	1.00
Abhd17b	0.23	1.00
Zc3hav1	-0.43	1.00
Vasp	-0.40	1.00
9930021J03Rik	-0.82	1.00
Ablim1	0.19	1.00
Chst11	-0.94	1.00
Zfp618	-0.02	1.00
Esrp1	-0.04	1.00

Ythdf3	-0.21	1.00
Ndufb3	0.40	1.00
1110004F10Rik	0.04	1.00
Stx17	-0.28	1.00
2210408I21Rik	0.98	1.00
Pygl	-0.11	1.00
Gsap	-0.54	1.00
Stk3	-0.41	1.00
Rars	-0.06	1.00
Prrc2a	0.34	1.00
Adar	-0.07	1.00
Ypel5	-0.08	1.00
Psmb8	0.47	1.00
Cdc42ep3	-0.50	1.00
Bdnf	-1.07	1.00
Zdbf2	-0.35	1.00
Septin7	0.29	1.00
Nme7	0.34	1.00
Gfod2	0.07	1.00
Nsfl1c	0.26	1.00
Kctd1	-0.06	1.00
Kat14	0.04	1.00
Ciapin1	-0.35	1.00
Tmem184a	-0.03	1.00
Akap10	-0.21	1.00
Atat1	-0.41	1.00
Rragb	-0.40	1.00
1700007K13Rik	-0.31	1.00
Ldlrad4	0.08	1.00
Cnot6	0.19	1.00
Usp39	0.01	1.00
Zfand5	-0.41	1.00
Lpcat1	0.05	1.00
Aup1	0.05	1.00
Tmem138	0.06	1.00
Exosc3	0.20	1.00
Adpgk	0.06	1.00
Nubp1	0.05	1.00
Tpst2	-0.13	1.00
Brpf3	0.11	1.00
Dip2c	-0.16	1.00
Ago4	-0.96	1.00
Cul1	-0.19	1.00
Fhit	0.42	1.00
Abcb7	-0.45	1.00
Abca1	-0.68	1.00

Epn2	-0.67	1.00
Efr3a	-0.10	1.00
Actn1	-0.30	1.00
Igf2bp2	-0.58	1.00
Snw1	-0.46	1.00
Klf3	-0.53	1.00
Smu1	0.11	1.00
Smg7	0.16	1.00
Phip	-0.01	1.00
Atp6v0a1	-0.65	1.00
Suclg1	0.48	1.00
Tmem50a	0.11	1.00
Ndfip2	-0.14	1.00
Tstd1	-0.55	1.00
Zdhhc20	0.07	1.00
Tmem238	-0.01	1.00
Phactr4	-0.73	1.00
Aig1	0.23	1.00
Katnal1	-0.10	1.00
Gipc2	0.47	1.00
Dnajc1	0.12	1.00
Zc3h8	0.24	1.00
Gbp2b	0.07	1.00
C2cd2	-0.02	1.00
Trip4	0.09	1.00
Cd44	-0.01	1.00
Pgls	-0.05	1.00
Lcp1	-0.75	1.00
Acp1	-0.06	1.00
Ift80	0.42	1.00
Aplp2	0.03	1.00
8030462N17Rik	0.12	1.00
Endov	-0.27	1.00
Ptges	0.10	1.00
Jmjd8	0.06	1.00
Lrrc9	0.10	1.00
Ufm1	-0.54	1.00
Zfp503	-0.41	1.00
Cryab	0.14	1.00
Sntb1	0.04	1.00
Phc1	-0.06	1.00
Zmynd12	-0.06	1.00
Ttll4	-0.78	1.00
Ska2	-0.57	1.00
Nabp1	-0.13	1.00
Ebna1bp2	-0.04	1.00

Trp53rka	-0.07	1.00
Ppp6c	-0.26	1.00
Ddx23	0.04	1.00
Stmn1	0.29	1.00
Gimap4	-0.04	1.00
Zfp445	0.03	1.00
Fstl1	-0.40	1.00
Aebp2	-0.56	1.00
Nt5dc1	-0.01	1.00
Rnf10	0.22	1.00
Nono	0.05	1.00
Qrich1	0.01	1.00
Tcp1	0.06	1.00
Eri3	0.02	1.00
Ssbp3	-0.01	1.00
U2af2	0.04	1.00
Arhgdib	-0.27	1.00
Tasor	0.05	1.00
Gemin4	-0.34	1.00
Psmb9	-0.02	1.00
Cdkal1	0.30	1.00
Fkbp15	0.45	1.00
Vkorc1l1	0.20	1.00
Fam126b	0.38	1.00
Dip2a	-0.64	1.00
Uri1	0.40	1.00
Pard6b	-0.72	1.00
Polh	-0.07	1.00
Immt	0.11	1.00
Os9	-0.53	1.00
Zfp704	-0.38	1.00
Utrn	-1.26	1.00
Cracdl	0.29	1.00
Cept1	0.07	1.00
Ndufa13	-0.12	1.00
Dpy19l4	0.09	1.00
Ppp1r14c	-0.07	1.00
Kif24	-0.06	1.00
Dtymk	0.06	1.00
Ufc1	-0.08	1.00
Prpf31	0.18	1.00
Ndufv1	0.00	1.00
Rc3h1	0.35	1.00
Srsf10	0.45	1.00
Ufl1	-0.33	1.00
H2ac20	0.58	1.00

Nfkb2	0.24	1.00
Trafd1	0.07	1.00
Sf3b1	0.14	1.00
Capzb	-0.04	1.00
Ccdc50	-0.33	1.00
Ppig	0.05	1.00
Usp53	-0.20	1.00
Gpr137c	-0.39	1.00
Gnb5	-0.53	1.00
Rspry1	-0.15	1.00
Zeb1	-1.14	1.00
Ube3c	-0.18	1.00
Cep135	0.28	1.00
Rnf34	0.20	1.00
Spag6l	0.40	1.00
Plekha7	-0.15	1.00
Smurf1	0.37	1.00
Selenow	-0.03	1.00
Noa1	-0.51	1.00
Lcp2	-0.07	1.00
Nat9	0.13	1.00
Wdr74	0.24	1.00
Rgs22	-0.78	1.00
Uevld	-0.09	1.00
Rgl2	0.00	1.00
Churc1	-0.11	1.00
Acp6	-0.51	1.00
Helz	-0.59	1.00
Ndufb9	-0.60	1.00
Kif5b	-0.19	1.00
Cdadc1	-0.41	1.00
Snf8	-0.23	1.00
Ccdc124	-0.02	1.00
Slf1	-0.45	1.00
Armc9	0.15	1.00
Tmem231	0.35	1.00
Hira	-0.64	1.00
Anxa4	-0.68	1.00
Ahctf1	-0.62	1.00
Ccdc180	-0.03	1.00
Cilk1	-0.70	1.00
Spg11	-0.34	1.00
Anapc15-ps	0.07	1.00
Gemin8	-0.14	1.00
Phf10	-0.17	1.00
Chsy1	-0.98	1.00

Fan1	-0.53	1.00
Phf14	-0.15	1.00
Klhl7	0.04	1.00
Fmo3	-0.08	1.00
Ntn1	0.05	1.00
Socs5	-0.77	1.00
Ubxn4	0.06	1.00
Tmem19	-0.41	1.00
Qk	-0.97	1.00
Dync2h1	0.34	1.00
4930453N24Rik	-0.06	1.00
Polr1d	0.48	1.00
Brwd3	-0.64	1.00
Nup50	-0.32	1.00
Bptf	-0.11	1.00
Rassf1	-0.04	1.00
Dad1	-0.04	1.00
Rbsn	-0.02	1.00
Nol9	-0.04	1.00
Unc5d	-0.52	1.00
Dop1a	-0.12	1.00
Krtcap2	0.17	1.00
Farp1	0.37	1.00
Rusc2	-0.01	1.00
Rbm42	0.35	1.00
Alg11	-0.25	1.00
Pkia	-0.43	1.00
Asf1a	-0.44	1.00
Atp6v0d1	-0.18	1.00
Nmral1	-0.45	1.00
Zbtb43	-0.35	1.00
Slc35e1	-0.11	1.00
Tpd52l1	-0.50	1.00
Tmem251	0.16	1.00
Wdr11	-0.39	1.00
Sbf2	0.09	1.00
Bcap31	0.04	1.00
Guf1	-0.52	1.00
Rwdd4a	-0.67	1.00
Kiz	-0.44	1.00
Phb	-0.28	1.00
Bbof1	-0.01	1.00
Ccdc66	0.16	1.00
Eea1	-0.38	1.00
Vegfa	0.26	1.00
Psmc6	-0.05	1.00

Plxna4	0.38	1.00
Cytip	-0.51	1.00
Arrdc4	0.07	1.00
Elapor1	-0.21	1.00
BC004004	0.31	1.00
Ankrd37	0.31	1.00
Ppp5c	-0.04	1.00
Tmco3	-0.03	1.00
Wars2	-0.10	1.00
Gtpbp4	-0.12	1.00
Rfk	-0.14	1.00
Tmed8	-0.16	1.00
Ung	-0.27	1.00
Bicra	-0.12	1.00
Ddx39a	-0.31	1.00
Wwtr1	-0.24	1.00
Aagab	-0.80	1.00
Vti1a	0.37	1.00
Ezr	0.09	1.00
Dock1	0.22	1.00
Rufy3	0.10	1.00
Gtpbp1	-0.12	1.00
Lrrc2	0.19	1.00
Tcf12	0.09	1.00
Wdr91	-0.47	1.00
Stx2	-0.75	1.00
Cisd3	-0.32	1.00
Bend7	-0.10	1.00
Cblb	-0.11	1.00
Lpcat3	-0.57	1.00
Acbd6	-0.27	1.00
Snx7	-0.99	1.00
Copg1	-0.17	1.00
Sacm1l	0.35	1.00
Sar1b	0.12	1.00
Ctdspl	-1.02	1.00
Ankfy1	0.04	1.00
Ptpn12	0.87	1.00
Plk2	0.76	1.00
Ppm1a	-0.86	1.00
Nap1l4	0.27	1.00
Wasf2	-0.51	1.00
Srsf4	0.15	1.00
Tom1	-0.16	1.00
Naxd	-0.05	1.00
Lnx1	-0.20	1.00

Aqp3	0.51	1.00
Pet100	-0.09	1.00
Agap3	0.11	1.00
Abhd5	-0.09	1.00
Heatr5b	0.34	1.00
Fbxo31	-0.53	1.00
Cdc34	0.22	1.00
Tmem232	0.49	1.00
Bid	-0.31	1.00
Clic6	-0.16	1.00
Ggt7	-0.17	1.00
Tbc1d5	-0.05	1.00
Acin1	0.21	1.00
Yap1	-0.21	1.00
Cep112	-0.33	1.00
Cdc37l1	-0.13	1.00
Plekhm3	-1.58	1.00
Adipor2	0.18	1.00
Rap2a	0.01	1.00
Guk1	-0.38	1.00
Hk3	-0.40	1.00
4930505A04Rik	-0.01	1.00
Gdi1	-0.03	1.00
Tsga10	-0.13	1.00
Pdgfa	-0.05	1.00
Cwc25	-0.18	1.00
Bsdc1	0.00	1.00
Tpm4	0.24	1.00
Vrk3	-0.24	1.00
Ptprf	-0.49	1.00
Atrx	-0.41	1.00
Reps1	-0.61	1.00
Strn3	-0.14	1.00
Itsn2	-0.42	1.00
Lurap1l	-0.21	1.00
Akirin1	-0.05	1.00
Nup153	-0.03	1.00
Cbl	0.26	1.00
Oga	0.06	1.00
Rassf9	-0.56	1.00
Dynlt3	-0.70	1.00
Isoc1	-0.23	1.00
Cryl1	-0.01	1.00
Ctsb	-0.15	1.00
Creb3	-0.08	1.00
Ncor2	0.23	1.00

Pard3	-0.22	1.00
Borcs5	0.48	1.00
Hc	-0.77	1.00
Gpr108	-0.53	1.00
Ppt1	-0.38	1.00
Jmjd1c	-0.28	1.00
Wasl	-0.21	1.00
Grcc10	0.08	1.00
Cdk7	-0.05	1.00
Zbtb37	-0.29	1.00
Serpinb6b	-0.24	1.00
Dnm2	0.01	1.00
Hk1	-0.05	1.00
Herpud2	-0.25	1.00
Arhgap15	-2.90	1.00
Pde10a	0.79	1.00
Usb1	0.13	1.00
Pglyrp1	2.42	1.00
Txlng	-0.07	1.00
Tab3	-0.08	1.00
Szt2	-0.13	1.00
Fam81a	-0.22	1.00
Chmp1a	-0.06	1.00
Pgam5	-0.23	1.00
Dus1l	-0.13	1.00
Ccnq	-0.06	1.00
Tcf25	-0.67	1.00
Tma7	-0.70	1.00
Ryk	0.10	1.00
Ptk2b	-0.55	1.00
Arfgef1	0.18	1.00
Rpp30	-0.06	1.00
Ece1	-0.79	1.00
Ets2	0.24	1.00
Glul	0.29	1.00
Scnn1b	-0.80	1.00
Hcls1	-0.26	1.00
Gtf2h1	-0.01	1.00
Lrig2	-0.60	1.00
Usp24	-0.62	1.00
Trerf1	0.16	1.00
Rasa2	-0.40	1.00
Vps29	-0.14	1.00
Zbtb7c	-0.05	1.00
Yipf6	0.01	1.00
Snrpa1	0.12	1.00

Ifit3	-0.45	1.00
Lrp5	-0.11	1.00
Pi4kb	-0.38	1.00
Leprotl1	-0.08	1.00
Osgepl1	0.00	1.00
Cacul1	-0.09	1.00
Samd8	-0.39	1.00
Unk	-0.15	1.00
Snx16	-0.24	1.00
Nt5c3	-0.57	1.00
Commd8	-0.18	1.00
Wdr75	-0.07	1.00
Zcchc7	0.15	1.00
Lta4h	-0.43	1.00
Pcgf5	-0.81	1.00
Blzf1	0.00	1.00
Ubald1	-0.04	1.00
Inpp5e	-0.08	1.00
Entpd4	0.14	1.00
Ncstn	-0.05	1.00
Strn4	-0.10	1.00
Smchd1	-0.10	1.00
Riok3	-0.31	1.00
Hmgb2	-0.41	1.00
Reck	-0.63	1.00
Rc3h2	0.22	1.00
Zdhhc17	-0.06	1.00
Sufu	-0.60	1.00
Arhgef12	-0.05	1.00
Pwwp3a	-0.08	1.00
Eno4	0.05	1.00
Atad2b	-0.28	1.00
Cct4	0.22	1.00
Herc4	-0.80	1.00
Rad21	-0.03	1.00
Trim44	0.10	1.00
Spry2	0.09	1.00
Slc48a1	0.14	1.00
Syn2	0.42	1.00
Ttc16	0.10	1.00
Appl1	-0.17	1.00
Rhob	-0.67	1.00
Gpcpd1	-1.14	1.00
Ubl5	-0.82	1.00
Kdm5b	-1.11	1.00
Dcaf10	-0.12	1.00

Hmox1	0.41	1.00
Taf1a	0.19	1.00
Fam20c	0.39	1.00
St6galnac4	0.21	1.00
Camkk2	0.28	1.00
Trak1	0.07	1.00
Wbp2	-0.13	1.00
Crip2	0.03	1.00
Slc25a3	-0.11	1.00
Nfatc3	-0.17	1.00
Cfdp1	-0.25	1.00
Chic2	0.11	1.00
Kcnj15	-0.39	1.00
Tmem8b	-0.47	1.00
Flii	0.01	1.00
Rela	-0.06	1.00
Spata6l	-0.32	1.00
Sorl1	-1.43	1.00
Akr1b3	-0.01	1.00
Osbp2	0.31	1.00
Tmco4	0.12	1.00
Ccnl1	0.09	1.00
Pxk	-0.04	1.00
Fam234a	-0.47	1.00
Frmd4b	0.40	1.00
Phf23	-0.01	1.00
Atg2b	-0.04	1.00
Elovl5	-0.12	1.00
Cnot10	0.06	1.00
L2hgdh	-0.42	1.00
Aprt	-0.06	1.00
Pim3	0.45	1.00
Ltbp2	-0.12	1.00
Noc3l	-0.32	1.00
Rcc2	-0.51	1.00
Pja2	-0.35	1.00
Prpf38a	0.16	1.00
Slc5a10	-0.08	1.00
Cers5	0.08	1.00
Asap1	-0.06	1.00
Fam102a	-0.14	1.00
Prep	-0.37	1.00
Ern1	-0.61	1.00
Atp6v0a4	-0.32	1.00
Nrcam	-0.60	1.00
Impg2	-0.12	1.00

Blvra	-0.09	1.00
Trappc11	-0.37	1.00
Thnsl2	-0.24	1.00
Zfyve19	-0.49	1.00
Nudt7	-0.28	1.00
Hsd17b2	-0.40	1.00
Crispld2	-1.15	1.00
St3gal2	-0.34	1.00
Aktip	-0.35	1.00
Sbk1	-0.18	1.00
Ctnnbip1	-0.23	1.00
Kcnn3	-0.24	1.00
Zfp532	-0.14	1.00
Tmed1	-0.28	1.00
Aph1b	-0.85	1.00
Otub2	-0.41	1.00
Ssr2	-0.27	1.00
Immp1l	0.00	1.00
Psmc1	0.04	1.00
Pml	0.02	1.00
Svopl	0.10	1.00
Coro1c	0.08	1.00
Tsc22d1	0.26	1.00
Smap2	-0.62	1.00
Cdk13	0.08	1.00
Terf1	0.52	1.00
Atp8a1	-0.23	1.00
Umad1	-0.12	1.00
Tspan15	-0.04	1.00
Epha2	-0.02	1.00
Rapgef5	0.01	1.00
Smc4	-0.51	1.00
Cradd	-0.63	1.00
Adh5	0.20	1.00
Bach2	-0.36	1.00
Dtx2	-0.34	1.00
Inpp1	0.65	1.00
Tox	0.27	1.00
Psen1	-0.34	1.00
Rac2	-0.28	1.00
Zfp36l2	-0.18	1.00
Robo2	1.16	1.00
St8sia1	0.21	1.00
Kctd12	0.40	1.00
Pde9a	0.51	1.00
Timm21	0.05	1.00

Hhat	0.17	1.00
Hnmt	0.78	1.00
Alg6	0.41	1.00
Hmcn1	0.50	1.00
Serpini1	0.11	1.00
Tox3	0.07	1.00
Ess2	0.07	1.00
Tor4a	0.12	1.00
Rab34	0.17	1.00
Rnf122	0.40	1.00
Nkx3-1	0.10	1.00
Ccdc62	0.15	1.00
Atp6v1b1	0.22	1.00
Stat1	0.29	1.00
Diaph1	0.00	1.00
Tbc1d15	-0.14	1.00
Dctn4	-0.49	1.00
Gcnt2	-0.42	1.00
Dhx36	0.73	1.00
Fam171a1	0.07	1.00
Ccdc141	-0.72	1.00
Fbxo38	-0.86	1.00
Fam222b	0.35	1.00
Tmx3	-0.39	1.00
Hpf1	-0.10	1.00
Ptprd	-0.48	1.00
Sp1	-0.07	1.00
Tsen34	-0.18	1.00
Creb3l2	-1.14	1.00
Tet2	-0.02	1.00
Txnip	-0.41	1.00
Tmem260	0.05	1.00
Hoatz	-0.11	1.00
Pdha1	-0.07	1.00
Twsg1	0.06	1.00
Slco2a1	-0.11	1.00
Dnase2a	0.63	1.00
Tmem87b	-0.94	1.00
Ing2	0.08	1.00
Phkg2	-0.01	1.00
Nsrp1	-0.49	1.00
Gadd45g	0.79	1.00
Iqsec1	-0.27	1.00
Acsl1	-0.86	1.00
Srp19	0.05	1.00
Acss3	0.24	1.00

Tspan3	0.04	1.00
Limk2	-1.11	1.00
Nsd3	-0.23	1.00
Wdr47	0.51	1.00
Prpf3	-0.51	1.00
Ypel2	0.16	1.00
Zfp36	-0.27	1.00
Lama3	-0.48	1.00
Crim1	-0.33	1.00
Eml4	-0.36	1.00
Hint1	0.09	1.00
Tef	-0.08	1.00
Plcl2	-0.22	1.00
9030025P20Rik	-0.18	1.00
St3gal6	-0.44	1.00
Prkx	-0.34	1.00
Ppic	-0.35	1.00
Pnpla2	-0.15	1.00
Zw10	-0.25	1.00
Rb1cc1	-0.55	1.00
Fam53b	-0.20	1.00
Dnajb9	0.25	1.00
Zc3h6	-0.15	1.00
Dusp2	-1.15	1.00
Ptbp2	-0.01	1.00
Shoc2	-0.15	1.00
Atp5a1	-0.02	1.00
Als2	0.23	1.00
Pde1a	0.44	1.00
Cpeb3	0.11	1.00
Kdm2b	0.38	1.00
Elf2	-0.24	1.00
Rassf3	0.47	1.00
Fxr1	-0.07	1.00
Glcci1	-0.60	1.00
Srsf9	0.23	1.00
Pnpla8	-0.36	1.00
Tln1	0.29	1.00
Stx18	-0.08	1.00
Dock5	-0.78	1.00
Rnf14	-0.73	1.00
Gatad2a	-0.25	1.00
Dusp11	-0.09	1.00
1600012H06Rik	-0.07	1.00
Gclm	-0.16	1.00
Dpf2	-0.28	1.00

Shtn1	-0.08	1.00
Bcat2	-0.61	1.00
Prox1	0.09	1.00
Ermp1	0.62	1.00
Trpm3	0.16	1.00
Cd14	0.17	1.00
Nlrp3	0.28	1.00
Slc9a8	-0.60	1.00
Ilk	-0.28	1.00
Fam98a	0.15	1.00
Cox20	-0.16	1.00
Galk1	0.34	1.00
Smarca5	-0.67	1.00
Tnik	0.50	1.00
Zfp512b	-0.18	1.00
Tcp11	-0.14	1.00
Bmx	0.34	1.00
Brms1l	0.02	1.00
Aldh18a1	0.12	1.00
Coro2b	0.22	1.00
Loxl1	0.14	1.00
Nudt19	0.67	1.00
Crym	0.60	1.00
Tesc	0.05	1.00
Zkscan14	0.02	1.00
Tspan11	0.39	1.00
H2-Oa	0.42	1.00
Ccdc22	0.02	1.00
Ccdc120	0.21	1.00
Taok1	-0.04	1.00
Kdm7a	0.06	1.00
Arfip1	0.03	1.00
Fbxl17	-0.11	1.00
Fbxo42	-0.95	1.00
Adcy9	-0.24	1.00
Rfx7	-0.12	1.00
Rsrc1	-0.76	1.00
Hectd4	-0.84	1.00
Plek	-0.78	1.00
Cd2ap	-0.10	1.00
Stox2	-0.06	1.00
Pla1a	0.24	1.00
Haus3	0.11	1.00
Prdx3	-0.10	1.00
Arhgap29	-0.25	1.00
Nme1	-0.53	1.00

Ago1	-0.42	1.00
Lrch3	-0.57	1.00
Ephx1	-0.05	1.00
Ubn2	-0.27	1.00
St8sia4	-0.95	1.00
Ncoa6	0.18	1.00
Kidins220	0.13	1.00
Eya4	-0.70	1.00
Cds2	0.22	1.00
Unc50	-0.20	1.00
Dcun1d2	-0.14	1.00
Nufip1	-0.46	1.00
Fancb	-0.55	1.00
Iba57	-0.29	1.00
Slc25a40	-0.35	1.00
Tram2	-0.22	1.00
Capn8	-0.25	1.00
Amot	-0.25	1.00
Cenpi	-0.20	1.00
Itga1	-0.31	1.00
Dnpep	-0.33	1.00
Col17a1	-0.36	1.00
Rnpepl1	-0.45	1.00
Ficd	-0.13	1.00
Tagln	-0.48	1.00
6820408C15Rik	-0.15	1.00
Dhrs4	-0.80	1.00
Idh3g	-0.22	1.00
Nrde2	0.27	1.00
Sbno1	-0.40	1.00
Saxo2	-0.91	1.00
Serf2	-0.09	1.00
Pde4d	0.19	1.00
Taf13	0.27	1.00
Tab1	0.07	1.00
Nelfa	0.07	1.00
Vps37c	-0.12	1.00
Cinp	0.12	1.00
Lactb	0.15	1.00
Qars	0.00	1.00
Inpp5b	0.07	1.00
Napa	0.01	1.00
Ovol2	0.16	1.00
St3gal5	0.04	1.00
Shld2	0.24	1.00
Gpsm2	0.63	1.00

Bdh1	0.06	1.00
Tlnrd1	0.04	1.00
Caml	0.14	1.00
Slc25a5	0.11	1.00
Brd7	-0.52	1.00
Otud6b	-0.12	1.00
Cfap100	-0.26	1.00
B230219D22Rik	0.35	1.00
Atxn7l1	-0.20	1.00
Cebpg	-0.35	1.00
Ythdf2	-0.35	1.00
Alms1	0.04	1.00
Rp2	-0.69	1.00
Clstn1	-0.25	1.00
Accsl	-0.88	1.00
Stard10	-0.05	1.00
Ubtd2	0.30	1.00
Ghsr	-0.03	1.00
Ddi2	-0.06	1.00
Disp1	-0.70	1.00
Snrpe	-0.16	1.00
Senp6	-0.05	1.00
Rxylt1	-0.13	1.00
B4galnt3	0.06	1.00
Tmem209	0.03	1.00
Itfg2	0.07	1.00
Nhs	0.13	1.00
Stk31	0.04	1.00
Armt1	0.33	1.00
Agbl5	0.03	1.00
Trim35	0.03	1.00
Ntpcr	0.16	1.00
Cdc25b	0.03	1.00
Sgcz	-0.05	1.00
Zc3h12b	-0.64	1.00
Fbxo8	0.35	1.00
Rad1	0.11	1.00
Rhov	-0.03	1.00
Sirt3	-0.22	1.00
Fam171b	0.59	1.00
Ak5	0.31	1.00
Ttc4	0.03	1.00
Tafa1	0.10	1.00
Fstl4	-0.09	1.00
Pdrg1	0.40	1.00
Ascc2	0.14	1.00

Ttyh2	0.04	1.00
Nek6	0.05	1.00
Pgap3	0.05	1.00
Ikzf3	-0.10	1.00
Otud3	0.05	1.00
Il18	-0.01	1.00
Runx3	0.31	1.00
Usp49	0.17	1.00
Fstl3	0.07	1.00
Rorc	-0.03	1.00
Slc44a2	0.17	1.00
Rtn4rl1	-0.18	1.00
Itpripl1	0.05	1.00
Cd2	0.13	1.00
Lrrc66	0.01	1.00
Ltbp4	0.21	1.00
Trpm4	0.50	1.00
Retn	0.06	1.00
Dvl2	0.14	1.00
Arrb2	0.21	1.00
Phf7	0.15	1.00
Cacna1e	-0.22	1.00
Fgd6	0.19	1.00
Arl6ip4	0.07	1.00
Ubash3b	0.17	1.00
Rasa1	-0.52	1.00
Pot1b	0.08	1.00
Angpt1	0.46	1.00
Atg13	-0.27	1.00
Fos	-0.42	1.00
Skp1	-0.12	1.00
Abhd17c	-0.05	1.00
Zmiz1	-0.11	1.00
Fbxw11	0.08	1.00
Prkcd	-0.32	1.00
Bicdl1	-0.78	1.00
Orai3	-0.38	1.00
Tmem168	0.47	1.00
Nupl1	-0.24	1.00
Ppp1r14a	-0.09	1.00
H1f0	-0.60	1.00
Creb1	-0.25	1.00
Fam13b	-0.28	1.00
Psma4	-0.23	1.00
Dapp1	-0.33	1.00
Vldlr	-0.44	1.00

Slc39a9	-0.07	1.00
Slc25a16	-0.53	1.00
Pcgf2	-0.08	1.00
Aph1a	-0.20	1.00
Zfp128	-0.10	1.00
Evpl	-0.19	1.00
Rpp21	-0.07	1.00
Trmt13	0.03	1.00
Slc5a1	0.35	1.00
Rnft1	-0.29	1.00
Sox13	0.02	1.00
Sugp1	0.33	1.00
Itm2c	-0.12	1.00
Efcab1	-0.53	1.00
Lmo7	-0.49	1.00
Rab18	-0.22	1.00
Zc2hc1a	-0.04	1.00
Scarb2	-0.17	1.00
Apaf1	-0.12	1.00
Paxx	-0.30	1.00
Desi1	0.07	1.00
Lamb1	0.01	1.00
Ikzf1	0.03	1.00
Rftn1	0.08	1.00
Gpat3	0.10	1.00
Camk4	0.20	1.00
Ptger4	0.14	1.00
Anxa6	0.09	1.00
Cep95	0.05	1.00
Atxn7l3	0.31	1.00
Zfp422	0.15	1.00
Avil	0.13	1.00
Ciita	0.36	1.00
Txndc17	-0.71	1.00
Amfr	-0.02	1.00
Sf3b3	-0.72	1.00
Srrt	-0.40	1.00
Rnf5	0.03	1.00
Scd2	-0.77	1.00
Jade3	-0.53	1.00
Rbmx	-0.25	1.00
Azin1	-0.48	1.00
Papss2	-0.15	1.00
Paqr3	-0.02	1.00
Lrrk1	-0.52	1.00
Kif21a	-0.11	1.00

Rsu1	-0.33	1.00
Nol7	-0.20	1.00
Gpm6b	-0.51	1.00
Vps39	-0.03	1.00
Cpt1a	0.13	1.00
Scly	0.58	1.00
Cyth3	0.13	1.00
Pecam1	0.02	1.00
Axdnd1	0.16	1.00
Sftpd	0.06	1.00
Slc39a7	0.08	1.00
Shroom4	0.44	1.00
Gpc4	0.16	1.00
Ovol1	0.25	1.00
Zfp28	-0.41	1.00
Fnbp1	0.08	1.00
Ptpn11	-0.15	1.00
Rprd1a	0.13	1.00
Fhod1	-0.21	1.00
Washc3	-0.28	1.00
Epas1	-0.41	1.00
Pawr	-0.44	1.00
Cadm2	-0.39	1.00
Smpd1	-0.13	1.00
Sox10	-0.52	1.00
Rtn4ip1	0.03	1.00
Napb	-0.08	1.00
Rsph6a	-0.17	1.00
Nop14	0.00	1.00
Lzts3	-0.16	1.00
Gda	-0.39	1.00
Galt	-0.06	1.00
Rad9a	-0.03	1.00
Eif2b2	0.01	1.00
Dpp10	-0.62	1.00
Lrp1b	-0.53	1.00
Sec31b	-0.01	1.00
Rnf220	-0.56	1.00
Uck2	0.03	1.00
Hccs	-0.21	1.00
Polg	0.02	1.00
Cyfip2	-0.01	1.00
Drg2	-0.17	1.00
Astn2	0.08	1.00
Akr7a5	-0.03	1.00
Ldlrap1	0.04	1.00

Rab29	0.04	1.00
Setdb1	0.02	1.00
Fcsk	0.02	1.00
Gamt	-0.06	1.00
Adam15	0.04	1.00
Aldh16a1	0.09	1.00
Ino80e	0.00	1.00
Tnfsf12	0.01	1.00
Thap3	-0.37	1.00
Tubb4b	-0.02	1.00
Cdkl1	-0.46	1.00
Cenpp	0.31	1.00
Cdkn1b	0.10	1.00
Sh3rf1	-0.41	1.00
Ppp4r3a	0.25	1.00
Thoc7	-0.86	1.00
Pum1	-0.21	1.00
Itga3	-0.34	1.00
Zfp292	-0.14	1.00
Dennd2a	0.06	1.00
Lrp11	0.06	1.00
Nipal1	0.58	1.00
Sag	-0.33	1.00
Irx3	-0.05	1.00
Etf1	-0.48	1.00
Nfyc	-0.53	1.00
Kin	0.05	1.00
Tnfsf13b	0.12	1.00
Utp4	-0.14	1.00
Cluh	-0.03	1.00
Fdx1	-0.53	1.00
Dennd6a	-0.20	1.00
Pkn1	0.24	1.00
Nr2f6	-0.46	1.00
Retreg1	-0.07	1.00
Clk4	-0.45	1.00
Trabd	0.20	1.00
Anks1b	0.00	1.00
E2f6	-0.07	1.00
Pigf	-0.02	1.00
Zc4h2	0.02	1.00
Rnf141	0.03	1.00
Nfatc2	-0.01	1.00
Rab14	0.36	1.00
Hfm1	-0.14	1.00
Preb	-0.03	1.00

Parm1	0.06	1.00
Dpp3	-0.01	1.00
Pogz	-0.28	1.00
Prkca	-0.06	1.00
Atp5k	-0.40	1.00
Rsbn1l	-0.09	1.00
Arhgap17	-0.37	1.00
BC005624	0.05	1.00
A130010J15Rik	0.06	1.00
Snrk	-0.50	1.00
Zbtb34	-0.06	1.00
Gck	0.13	1.00
Igf1r	-0.47	1.00
Cdk9	-0.44	1.00
Ophn1	-0.66	1.00
Dusp22	-0.36	1.00
Sema5a	0.18	1.00
Vps35	-0.31	1.00
Apobec4	-0.58	1.00
Rsf1	-0.88	1.00
Prex2	-0.93	1.00
Tubgcp4	-0.66	1.00
Frem2	-0.13	1.00
Ppp2r5d	-0.24	1.00
Arsb	-0.51	1.00
Selenbp2	-0.61	1.00
Ndufa10	-0.32	1.00
Rhoa	0.04	1.00
Sik3	-0.30	1.00
Slmap	-0.92	1.00
Fam81b	-0.72	1.00
Zbtb38	-0.24	1.00
Isg15	-0.05	1.00
Ctsh	0.79	1.00
Rpap3	-0.02	1.00
Brox	-0.33	1.00
Oser1	0.02	1.00
Tlr4	-0.08	1.00
Card19	-0.13	1.00
Vapb	-0.22	1.00
Cdk14	-0.84	1.00
Ildr1	0.22	1.00
Slitrk6	0.04	1.00
Usp5	-0.04	1.00
Cul4b	-0.20	1.00
Cox18	0.20	1.00

Ptcd3	0.05	1.00
Trp53i11	0.09	1.00
Fam161b	0.09	1.00
Dalrd3	0.02	1.00
Rbm19	0.61	1.00
Dapk3	0.04	1.00
D030056L22Rik	0.02	1.00
Pspc1	0.07	1.00
Prdm4	-0.50	1.00
Polr3h	-0.18	1.00
Nos3	-0.05	1.00
Zfp777	-0.25	1.00
Rpp40	-0.26	1.00
Eif2b4	-0.12	1.00
Bdh2	-0.10	1.00
Apool	-0.26	1.00
Stard4	-0.03	1.00
Thap1	-0.14	1.00
Tdp1	-0.16	1.00
Kcnj13	-0.08	1.00
Snapc5	-0.24	1.00
Kif23	-0.02	1.00
Ccdc102a	-0.16	1.00
Cox19	-0.23	1.00
Sdcbp2	-0.07	1.00
Zfp335	0.02	1.00
Oxld1	-0.36	1.00
Psph	0.01	1.00
Eml3	-0.11	1.00
Pogk	-0.06	1.00
Ptrh1	-0.30	1.00
Zfp661	-0.50	1.00
D3Ertd751e	-0.04	1.00
Tars2	-0.02	1.00
Gjb3	-0.01	1.00
Ndn	0.00	1.00
Sall2	0.01	1.00
Abhd4	-0.01	1.00
Eif1ad	-0.09	1.00
Hp1bp3	-0.26	1.00
Wtap	0.03	1.00
Nupr1	0.59	1.00
Fig4	0.09	1.00
Rcor3	-0.01	1.00
Rap1gap2	-0.71	1.00
Ucp2	-0.27	1.00

Srrm1	-0.12	1.00
Ufsp2	-0.41	1.00
Pyroxd1	-0.08	1.00
Ppp1r21	0.18	1.00
Trank1	-0.68	1.00
Smad5	-0.09	1.00
Pde3a	-0.15	1.00
Dcp1a	-0.37	1.00
Pfdn6	0.06	1.00
1700013F07Rik	0.24	1.00
Ints13	0.13	1.00
Sco2	0.02	1.00
Gtf3a	-0.08	1.00
Satb1	0.59	1.00
Dclk2	-0.10	1.00
Fgfr3	0.27	1.00
Nadsyn1	-0.03	1.00
Cdk1	0.54	1.00
Clcn7	-0.15	1.00
Usp30	-0.12	1.00
Pop5	-0.22	1.00
Traf2	-0.15	1.00
Stt3a	0.04	1.00
Fabp3	-0.29	1.00
Cactin	0.27	1.00
Ifrd2	0.36	1.00
Tle2	0.17	1.00
Dnaaf2	0.00	1.00
Fam111a	0.47	1.00
Rnf19b	0.27	1.00
Nipbl	0.16	1.00
Btaf1	-0.98	1.00
Smarce1	0.09	1.00
Parp12	-0.99	1.00
Ptprm	-0.71	1.00
Ogfod3	-0.26	1.00
Phkb	0.12	1.00
Slc37a1	-0.42	1.00
Thumpd2	-0.77	1.00
Itprip	-0.14	1.00
Slc9a9	-0.89	1.00
Enkur	-0.10	1.00
Acbd3	-0.49	1.00
Cat	-0.56	1.00
Ubr5	-0.05	1.00
Ankrd29	-0.41	1.00

Zdhhc9	-0.35	1.00
Atg14	-0.29	1.00
Aldh6a1	-0.16	1.00
Ppm1m	-0.14	1.00
Clasp2	-0.11	1.00
Tnks2	0.03	1.00
Pnp	0.73	1.00
Arglu1	0.16	1.00
Antxr1	0.27	1.00
Lrp6	0.10	1.00
Rragc	-0.55	1.00
Snrpf	-0.27	1.00
Homer1	0.56	1.00
Cdca7l	-0.04	1.00
Pycrl	-0.05	1.00
Btbd3	0.01	1.00
Fzd3	0.01	1.00
Ccnh	-0.01	1.00
Atg4c	0.07	1.00
Trim59	-0.28	1.00
Idh3a	0.04	1.00
Creld1	0.00	1.00
Htr4	-0.20	1.00
Smarcd1	0.07	1.00
Rmnd5b	0.02	1.00
Adam28	0.00	1.00
Tm7sf2	-0.01	1.00
Trpm7	-0.28	1.00
Cops7a	0.05	1.00
Ptk7	0.25	1.00
Psme3ip1	0.37	1.00
Pcyox1	-0.45	1.00
Tex14	0.29	1.00
Vps26a	0.03	1.00
Bad	-0.77	1.00
Kyat3	-0.36	1.00
Dusp28	-0.17	1.00
Epg5	-0.91	1.00
Oxnad1	0.04	1.00
Gpn1	-0.37	1.00
Adk	-0.18	1.00
Apbb3	-0.11	1.00
Lrrc45	-0.09	1.00
Fam227b	0.07	1.00
Ppp2r3a	0.18	1.00
Zfp330	-0.38	1.00

Swap70	-0.35	1.00
Slc30a5	-0.49	1.00
Paxbp1	-0.35	1.00
Picalm	-0.34	1.00
Suco	-0.13	1.00
Eif3e	-0.63	1.00
Trip12	-0.45	1.00
Pgap2	-0.33	1.00
Chmp4c	-0.05	1.00
Cgrrf1	0.01	1.00
Tmem86a	-0.01	1.00
Hmces	-0.04	1.00
Thg1l	-0.03	1.00
Pgp	-0.03	1.00
Bspry	-0.56	1.00
Kcnab2	-0.23	1.00
Ppp1r8	-0.03	1.00
Oard1	-0.18	1.00
Adap1	-0.04	1.00
Cd37	-0.21	1.00
Junb	-0.02	1.00
Zfhx2	-0.08	1.00
Stk19	-0.04	1.00
Znhit2	0.00	1.00
Kcmf1	-0.18	1.00
Glb1	-0.27	1.00
Spr	-0.04	1.00
Cfl2	-0.15	1.00
Insr	-0.41	1.00
Cacybp	-0.45	1.00
Pkp2	-0.02	1.00
Nphp1	-0.19	1.00
Prkg1	0.18	1.00
Etnk1	-0.02	1.00
Crebzf	0.37	1.00
Sulf1	-1.20	1.00
Casp8ap2	-0.21	1.00
Utp15	-0.35	1.00
Gpatch1	-0.45	1.00
Golga1	-0.81	1.00
Vnn1	-0.65	1.00
Arl6ip1	0.60	1.00
Chd1l	-0.46	1.00
Nfil3	-1.32	1.00
Ell2	-1.06	1.00
Smarca2	-0.27	1.00

Slu7	-0.16	1.00
Capn1	-0.49	1.00
Fam168a	-0.46	1.00
Btg3	0.27	1.00
Arhgef5	-0.42	1.00
Nlk	-0.15	1.00
Zmynd11	-0.52	1.00
Tmem68	-0.63	1.00
Pus10	0.05	1.00
Qdpr	-0.12	1.00
Ttc33	-0.55	1.00
Aamp	0.25	1.00
Wbp11	0.11	1.00
Dock2	-0.62	1.00
Hook2	0.43	1.00
Dgkd	-0.01	1.00
Spast	-0.50	1.00
Sorbs1	-0.76	1.00
Efcab7	-0.37	1.00
Smim15	-0.62	1.00
Actr6	-0.06	1.00
Dcx	0.00	1.00
Rgmb	-0.41	1.00
Tbl3	0.06	1.00
Tet1	-0.55	1.00
Kmt2b	0.04	1.00
Ccnb1ip1	-0.02	1.00
Ccdc85b	-0.03	1.00
Tsr2	0.01	1.00
Polr1h	-0.18	1.00
Dapk2	-0.62	1.00
F2rl2	-0.31	1.00
Rab5c	0.37	1.00
Arnt	0.02	1.00
Flvcr2	-0.63	1.00
Abr	-0.27	1.00
Rora	-0.27	1.00
Fank1	0.16	1.00
Slc25a4	-0.07	1.00
Pprc1	-0.10	1.00
Gca	0.35	1.00
Ifitm6	-0.16	1.00
Hadhb	-0.40	1.00
Filip1l	-0.53	1.00
Cers4	-0.48	1.00
Pcyt1a	-0.72	1.00

Bola1	0.16	1.00
Wdr38	-0.46	1.00
Efcab11	-0.16	1.00
Phf21a	-0.69	1.00
Cd247	0.59	1.00
Limd1	-0.91	1.00
Prepl	0.27	1.00
Cert1	0.01	1.00
Sntg1	-0.71	1.00
Fnta	-0.16	1.00
Nt5c3b	-0.31	1.00
Cxxc1	-0.20	1.00
Gpr161	-0.07	1.00
Plekha8	-0.12	1.00
Kng2	-0.06	1.00
Cdk10	-0.05	1.00
Vdr	-0.17	1.00
Brf2	-0.09	1.00
Ninl	-0.44	1.00
P2ry12	-0.12	1.00
Arhgap33	-0.08	1.00
Parp2	-0.35	1.00
Zfp213	-0.24	1.00
Bicdl2	-0.07	1.00
Slc25a12	0.17	1.00
Foxn3	0.12	1.00
Pcf11	-0.23	1.00
Rbpj	-0.52	1.00
Ube2w	-0.03	1.00
Ubr4	-0.68	1.00
Sqle	-0.34	1.00
Arl6	-0.11	1.00
Rida	-0.07	1.00
Pdss2	-0.14	1.00
Srbd1	-0.17	1.00
Ccdc190	-0.31	1.00
Trabd2b	0.04	1.00
Plau	0.15	1.00
Smurf2	-0.20	1.00
Bzw1	0.32	1.00
Panx1	-0.74	1.00
Got2	-0.21	1.00
Cibar1	-0.10	1.00
Dph6	0.09	1.00
Nmt1	0.13	1.00
Cwf19l1	0.16	1.00

Abcd3	0.18	1.00
Aqr	-0.41	1.00
Sec24b	-0.35	1.00
Top1	-0.29	1.00
Capn2	-0.04	1.00
Fgfr1op2	-0.66	1.00
Npr3	-0.28	1.00
Imp4	-0.11	1.00
Arhgap45	-0.25	1.00
Prmt2	-0.14	1.00
Tinf2	-0.24	1.00
Ap1b1	-0.28	1.00
Ddhd2	-0.38	1.00
Plxnc1	-0.90	1.00
Zc2hc1c	-0.43	1.00
Akr1c13	0.28	1.00
Fgl2	-0.23	1.00
Kcnh8	-0.41	1.00
Rundc3b	-0.09	1.00
Smyd2	-0.10	1.00
Nup155	-0.56	1.00
Slc44a3	0.00	1.00
Pura	0.00	1.00
Acss2	0.04	1.00
Gnb1l	0.00	1.00
Timm17a	0.01	1.00
Ppp1r37	0.24	1.00
Tcf4	-0.26	1.00
Ing3	-0.06	1.00
Fbxo25	-0.01	1.00
Exosc9	0.12	1.00
Setd3	0.17	1.00
Ncoa4	-0.22	1.00
Ube3b	-0.15	1.00
Bcl3	0.00	1.00
Prpsap1	-0.48	1.00
Ddx6	-0.13	1.00
G3bp2	0.18	1.00
Ndufb10	-0.43	1.00
Zdhhc18	-0.49	1.00
Gpatch8	-0.41	1.00
Eef1b2	-0.44	1.00
2700062C07Rik	-0.14	1.00
Dennd3	-0.87	1.00
Dnajb14	-0.27	1.00
Ccdc146	-0.24	1.00

Cdh16	-0.30	1.00
Anapc13	-0.02	1.00
Fkbp7	0.22	1.00
Akap9	-0.13	1.00
Snx13	0.05	1.00
Tsc22d3	-0.64	1.00
Tdpoz8	-0.18	1.00
Atxn2	-0.21	1.00
Ppp2ca	0.25	1.00
Slc38a2	0.53	1.00
Wapl	-0.06	1.00
Ccdc12	-0.64	1.00
Trip11	-0.11	1.00
Lrrfip2	0.06	1.00
Zdhhc14	0.24	1.00
Tes	0.47	1.00
Fasn	-0.37	1.00
Tagap1	-0.55	1.00
Setd7	-0.23	1.00
Irs1	-0.48	1.00
Cog6	-0.67	1.00
Arhgdia	-0.57	1.00
Cfap300	0.03	1.00
Hivep2	0.30	1.00
Cast	-0.43	1.00
Nudt21	-0.48	1.00
Dedd	-0.48	1.00
Fastk	-0.05	1.00
Abracl	-0.37	1.00
Cnih3	-0.05	1.00
Vps51	-0.37	1.00
Vps11	-0.12	1.00
Colgalt1	-0.54	1.00
Nipsnap3b	-0.03	1.00
Olr1	-0.62	1.00
Clpb	-0.01	1.00
1700123O20Rik	0.00	1.00
Pde6a	-0.32	1.00
Gtf2e2	-0.68	1.00
Resf1	-0.38	1.00
Abraxas2	-1.02	1.00
Btbd1	-0.32	1.00
Rilpl2	-0.70	1.00
Rbm26	-0.24	1.00
Ndufab1	-0.20	1.00
Iyd	0.24	1.00

Ulk1	0.08	1.00
Cyp51	0.15	1.00
Rab23	-0.09	1.00
Cacna1b	-0.37	1.00
Sike1	-0.34	1.00
Cep192	-0.10	1.00
Yipf4	-0.68	1.00
Slc5a6	-0.05	1.00
Adcy3	-0.15	1.00
Klhl8	-0.01	1.00
Tmem267	-0.25	1.00
Atmin	-0.18	1.00
Rcan3	-0.04	1.00
Gigyf1	0.06	1.00
Impa1	-0.01	1.00
1110059G10Rik	-0.02	1.00
Faxc	-0.06	1.00
Rbm45	-0.41	1.00
Rfc4	-0.13	1.00
Arl6ip5	-0.63	1.00
Trnt1	-0.13	1.00
Sec24a	-0.14	1.00
Ears2	-0.26	1.00
Golga3	-0.08	1.00
Surf1	-0.07	1.00
Tubg1	-0.14	1.00
Wrap73	0.01	1.00
Foxp4	-0.04	1.00
Arf5	-0.07	1.00
Adgb	0.28	1.00
Snx2	-0.37	1.00
Tnfrsf1a	-0.20	1.00
Csgalnact2	-0.32	1.00
Chchd5	-0.09	1.00
Ttc6	0.61	1.00
Usf2	-0.18	1.00
Pitpnb	-0.25	1.00
Spats2l	-0.08	1.00
Lrpap1	0.14	1.00
Lmbrd1	0.10	1.00
Obi1	-0.14	1.00
Nrp1	-0.13	1.00
Ankrd27	-0.46	1.00
Fmo2	-0.96	1.00
Rbis	0.17	1.00
Fdft1	-0.14	1.00

Nfkb1	0.06	1.00
Kdm5a	0.08	1.00
Pde5a	-0.47	1.00
Vgll3	-1.14	1.00
Psmd10	-0.16	1.00
Rrn3	-0.32	1.00
Ssna1	-0.41	1.00
Kcnrg	-0.19	1.00
Wdr44	0.04	1.00
Ggcx	-0.05	1.00
Parp14	-0.89	1.00
Ginm1	-0.33	1.00
Actr3	-0.85	1.00
Park7	-0.54	1.00
Thoc1	-0.25	1.00
Sh3yl1	0.16	1.00
Ocln	-0.01	1.00
Hsd17b4	-0.64	1.00
Grk5	-0.64	1.00
Vps13c	-0.59	1.00
Rnf111	-0.03	1.00
Zcrb1	-0.28	1.00
Afg1l	-0.48	1.00
Tmc1	-0.33	1.00
Txndc12	0.02	1.00
Selenoi	-0.03	1.00
Erbb4	-0.09	1.00
Parg	-0.20	1.00
Hspb11	-0.52	1.00
Furin	-0.26	1.00
Nop10	-0.25	1.00
Ube2q1	0.34	1.00
Senp7	-0.83	1.00
Commd10	-0.24	1.00
Ccdc173	-0.28	1.00
Kmt2c	0.03	1.00
Gsk3b	-0.53	1.00
Ift81	0.51	1.00
Hdgfl3	-0.40	1.00
Spdya	-0.08	1.00
Gpatch11	-0.20	1.00
Glmn	-0.07	1.00
Per2	-0.37	1.00
Cox11	-0.22	1.00
Gtf2ird1	-0.10	1.00
Uhrf1bp1	-0.04	1.00

Rnf40	-0.14	1.00
Nt5c	-0.85	1.00
Cmbl	-0.17	1.00
Snrnp25	-0.18	1.00
Emsy	-0.21	1.00
Lrrc8d	-0.55	1.00
Ahcyl1	-0.34	1.00
Fyttd1	-0.36	1.00
Nufip2	0.08	1.00
Tut4	-0.26	1.00
Lamp2	-0.47	1.00
Pggt1b	0.10	1.00
Atp2b1	-0.03	1.00
Ankhd1	0.05	1.00
Ebag9	-0.84	1.00
Prkab1	-0.46	1.00
Klhl21	0.04	1.00
Ralb	-0.18	1.00
Tnip2	-0.06	1.00
Thbs1	-0.90	1.00
Zhx3	-0.33	1.00
Usp8	-0.44	1.00
Tmem259	-0.29	1.00
Wac	-0.42	1.00
Ankrd46	-0.36	1.00
Ccdc125	-0.47	1.00
Edc3	-0.47	1.00
Ccdc69	-0.42	1.00
Arap1	-0.36	1.00
Ddx50	0.03	1.00
Cwc27	0.37	1.00
Phax	-0.18	1.00
Slc16a7	0.17	1.00
Srsf3	-0.15	1.00
Cfap161	-0.17	1.00
Plekhm1	-0.11	1.00
Kansl2	-0.41	1.00
Scamp1	0.01	1.00
Tmem43	-0.88	1.00
Rsph1	-0.24	1.00
Rsrp1	0.35	1.00
Slc25a33	-0.19	1.00
Clic4	0.26	1.00
Pik3r4	-0.12	1.00
Hnrnph2	0.10	1.00
Nde1	-0.16	1.00

Bcap29	-0.06	1.00
Skap2	-0.28	1.00
Jag1	0.09	1.00
Kcnq1	-0.25	1.00
Rbm28	-0.47	1.00
Itga2	-0.38	1.00
Fbxo30	0.20	1.00
Coq10b	-0.58	1.00
Ttc39a	-0.40	1.00
Sos1	-0.12	1.00
Susd6	-0.68	1.00
Pdk4	0.90	1.00
Dhx29	0.03	1.00
Tmed5	-0.17	1.00
Polr3f	-0.45	1.00
Wdr41	-0.49	1.00
Dmac2l	-0.44	1.00
Gorab	-0.21	1.00
Tbc1d20	-0.40	1.00
Eps15l1	-0.13	1.00
Pld3	-0.40	1.00
Sart1	-0.54	1.00
Spindoc	-0.08	1.00
Ankrd42	0.27	1.00
Btbd11	-0.46	1.00
Tpt1	-0.19	1.00
Eif3j2	-0.62	1.00
Spen	0.32	1.00
Ptpn14	0.55	1.00
Emb	-0.50	1.00
Rbm17	-0.02	1.00
Pknox2	-0.05	1.00
R3hdm4	-0.22	1.00
Copb1	0.25	1.00
Arhgap21	-0.12	1.00
Kif9	-0.39	1.00
Riok1	-0.16	1.00
Asprv1	-0.12	1.00
Syk	-0.45	1.00
Tmem62	-0.26	1.00
Arntl	-0.34	1.00
Gpr155	-0.22	1.00
Anapc7	-0.28	1.00
Serpinf1	-0.19	1.00
Cbx1	-0.28	1.00
Eno3	-0.49	1.00

Ttc37	0.13	1.00
Ctcf	-0.13	1.00
Gas6	0.13	1.00
Nmnat2	-0.22	1.00
Ppl	-0.40	1.00
Ddx17	-0.36	1.00
Fcer1g	0.03	1.00
Larp1b	-0.10	1.00
Thra	0.15	1.00
Arhgef11	0.02	1.00
Cxcl3	-1.63	1.00
Hck	0.48	1.00
Srek1ip1	-0.05	1.00
Rbx1	0.00	1.00
Bmpr2	-0.77	1.00
Tfg	-0.39	1.00
Sat1	-0.14	1.00
Pds5b	-0.06	1.00
Arl5b	-0.91	1.00
Itgb1	-0.39	1.00
Gorasp1	-0.42	1.00
Fam199x	-0.19	1.00
Traf4	-0.28	1.00
Rara	-0.22	1.00
Ccs	-0.11	1.00
A430005L14Rik	-0.28	1.00
Vps72	-0.43	1.00
Topaz1	-0.40	1.00
Ogdh	-0.60	1.00
Pik3r5	0.21	1.00
Rbm5	0.21	1.00
Nemf	-0.38	1.00
Tsen2	0.41	1.00
Plekha1	-0.50	1.00
Cfd	0.80	1.00
Bicral	-0.07	1.00
Rngtt	0.19	1.00
AI597479	-0.18	1.00
Slc46a3	-0.17	1.00
Angpt2	-0.10	1.00
Enoph1	-0.82	1.00
Dvl3	-0.33	1.00
Zfp787	-0.34	1.00
Lactb2	0.11	1.00
Gnpda2	-0.50	1.00
Rnf2	-0.64	1.00

Esco1	-0.21	1.00
Eif4b	-0.68	1.00
Prss55	0.20	1.00
Hadha	0.03	1.00
Pigu	-0.66	1.00
Btbd2	-0.58	1.00
Coro1a	-0.62	1.00
Supt3	-0.30	1.00
Lbr	-0.11	1.00
Ccdc28a	-0.57	1.00
Cryz	-0.01	1.00
Vps54	-0.42	1.00
Ascc3	-0.74	1.00
Ankef1	-0.59	1.00
Por	-0.35	1.00
Zbtb11	-0.27	1.00
H13	-0.42	1.00
Gga2	0.11	1.00
Syne2	-0.50	1.00
Zswim8	-0.09	1.00
Wdr59	-0.18	1.00
Snap29	-0.28	1.00
Nfia	0.01	1.00
Trim2	-0.07	1.00
Prpf39	0.15	1.00
Scnn1a	-0.25	1.00
Atp5md	-0.27	1.00
Cd164	0.09	1.00
Capn7	-0.63	1.00
Tspan6	-0.33	1.00
Cnot8	-0.19	1.00
Tcea2	-0.70	1.00
Synj1	-0.40	1.00
Ccser1	0.21	1.00
Chd6	-0.42	1.00
Cox6a1	-0.73	1.00
Aopep	-0.50	1.00
Adgrf5	0.31	1.00
Lin7c	-0.43	1.00
Trim75	0.55	1.00
Eif2ak4	-0.05	1.00
Dlg1	-0.04	1.00
Abcc1	-1.01	1.00
Hdac1	-0.56	1.00
Pole4	0.31	1.00
Nub1	0.16	1.00

Lysmd3	-0.12	1.00
Tax1bp1	-0.05	1.00
Dnajb4	-0.17	1.00
Opa1	-0.38	1.00
Phf11a	-0.94	1.00
Nvl	-0.33	1.00
Dazap2	-0.20	1.00
Foxj3	0.34	1.00
Arhgap18	-0.22	1.00
Zdhhc7	-0.92	1.00
Ets1	-0.10	1.00
Adgrg6	-0.07	1.00
Ikbkb	0.02	1.00
Cmss1	-0.08	1.00
Spryd7	-0.25	1.00
Rtn2	-0.24	1.00
Ube4b	-0.13	1.00
Zfp217	-0.21	1.00
Plagl1	0.00	1.00
Abi2	0.27	1.00
Trmt1l	-0.18	1.00
Tnfsf4	-0.38	1.00
Slc7a7	-0.62	1.00
Arpc5	-0.02	1.00
Dcdc2a	-0.39	1.00
Ift57	-0.32	1.00
Zfyve1	-0.23	1.00
Sftpb	-0.58	1.00
Parp4	-0.65	1.00
Rbl2	-0.83	1.00
Crem	-0.71	1.00
Atxn1	0.41	1.00
Taldo1	-0.57	1.00
Sptan1	-0.21	1.00
Cggbp1	-0.61	1.00
Dst	0.13	1.00
Get3	0.60	1.00
Naa25	-0.54	1.00
Trdmt1	-0.47	1.00
Ccnk	-0.16	1.00
Nras	-0.46	1.00
Zc3h4	-0.21	1.00
Yeats2	-0.24	1.00
Ywhab	-0.03	1.00
Asah1	-0.01	1.00
Hectd1	-0.10	1.00

Cplane1	0.21	1.00
Tlk1	-0.25	1.00
Ereg	-0.16	1.00
L3mbtl2	-0.13	1.00
Usp38	-0.18	1.00
Polr2e	-0.52	1.00
Fmnl2	0.23	1.00
Kdm1a	-0.46	1.00
Slc25a21	-0.56	1.00
Inpp5d	-0.72	1.00
R3hdm1	0.06	1.00
Sumf1	-0.20	1.00
Snrnp40	-0.58	1.00
Trappc3	-0.10	1.00
Pias2	0.31	1.00
Tm4sf1	0.46	1.00
Ikzf2	0.25	1.00
Dis3l2	0.07	1.00
Ylpm1	-0.39	1.00
Fryl	-0.30	1.00
Zfp148	-0.25	1.00
Sirt1	-0.05	1.00
Pdap1	0.02	1.00
Gpbp1l1	0.02	1.00
Btf3	-0.19	1.00
Srp9	-0.12	1.00
Ttc21b	-0.21	1.00
Sftpa1	0.86	1.00
Ssbp2	-0.49	1.00
Baiap2	0.30	1.00
Xpc	-0.40	1.00
Hspa1l	-0.20	1.00
Rcan2	0.05	1.00
Foxn2	-0.35	1.00
Pamr1	-0.49	1.00
Baz1b	-0.14	1.00
Diablo	-0.44	1.00
Vapa	-0.16	1.00
Ptbp3	-0.59	1.00
Lin54	-0.58	1.00
Ddx3x	-0.17	1.00
Armc2	0.15	1.00
Xiap	0.16	1.00
Arfgef3	-0.14	1.00
Ubtd1	0.03	1.00
Zbtb20	-0.34	1.00

Phyh	-0.41	1.00
Prickle2	-0.61	1.00
Yars	-0.05	1.00
Ppm1h	0.55	1.00
Prkcb	-0.70	1.00
Rbm18	-0.22	1.00
Phtf1	-0.41	1.00
Ppp1r15b	-0.85	1.00
Rnf6	0.05	1.00
Itpr2	0.53	1.00
Dennd4c	-0.65	1.00
Gna12	-0.52	1.00
Relch	-0.82	1.00
Smg1	0.13	1.00
Ogt	-0.29	1.00
Gtdc1	-0.23	1.00
Sp4	-0.20	1.00
Vti1b	-0.49	1.00
Peak1	-0.20	1.00
Hnrnpa3	0.05	1.00
Eif2s1	0.05	1.00
Tmtc3	-0.02	1.00
Scaf4	0.02	1.00
Lrrc41	-0.46	1.00
Gja1	0.16	1.00
Fubp1	-0.05	1.00
Gabarapl1	-0.05	1.00
1700030J22Rik	-0.29	1.00
Vim	-1.07	1.00
Usp33	-0.55	1.00
Exoc6	-0.43	1.00
Gab1	-0.38	1.00
Tnpo1	-0.43	1.00
Tm9sf3	-0.37	1.00
Slc30a9	-0.05	1.00
Pdhb	-0.24	1.00
Id1	0.93	1.00
Klc4	0.43	1.00
St7l	-0.44	1.00
Sergef	0.49	1.00
Pxylp1	0.02	1.00
Zfand3	-0.50	1.00
Tkt	-0.40	1.00
Cmtm4	-0.14	1.00
Cyb5b	-0.35	1.00
Otud5	-0.30	1.00

Pin4	-0.39	1.00
Osbp	-0.33	1.00
Hat1	-0.07	1.00
Reep3	-0.15	1.00
Ros1	0.20	1.00
Rab11fip2	-0.90	1.00
Kars	-0.41	1.00
Tcerg1	-0.26	1.00
Fam216b	-0.02	1.00
Aasdhppt	-0.36	1.00
Ubr1	-0.48	1.00
Taf10	-0.70	1.00
Smarcad1	-0.15	1.00
Slc1a5	-0.52	1.00
Pnn	-0.31	1.00
Arf4	-0.25	1.00
Dync2i2	-0.11	1.00
Ethe1	0.10	1.00
Zc3h13	-0.04	1.00
Ralbp1	0.15	1.00
Prdm16	0.14	1.00
Lats1	-0.44	1.00
Dthd1	-0.01	1.00
Eogt	-0.73	1.00
Kctd20	-0.63	1.00
Surf4	-0.02	1.00
Pam	-0.08	1.00
Hbegf	0.20	1.00
Osmr	0.31	1.00
Atf7ip	-0.31	1.00
Gata6	-0.21	1.00
Gps1	-0.20	1.00
Cd24a	0.08	1.00
Fbxw4	0.29	1.00
Flna	-0.31	1.00
4932438A13Rik	-0.74	1.00
Son	-0.14	1.00
Smim19	-0.35	1.00
Grtp1	-0.24	1.00
Prelid2	-0.37	1.00
Ppa2	0.03	1.00
Atg12	0.08	1.00
Dnajc24	-0.13	1.00
Herc1	-0.22	1.00
Dnajc19	-0.15	1.00
Lrrc43	-0.39	1.00

Ly75	-0.64	1.00
Nedd4l	-0.22	1.00
Ddx42	-0.25	1.00
Prkaa2	0.02	1.00
Pnisr	-0.24	1.00
Krr1	0.61	1.00
Nop2	0.08	1.00
Ampd3	0.50	1.00
Fam98c	0.50	1.00
Cmtm8	0.21	1.00
Eps15	-0.66	1.00
Cisd2	-0.23	1.00
Ddx20	0.05	1.00
Apmap	-0.03	1.00
Cttnbp2	0.55	1.00
Rabgap1	-0.57	1.00
Ext1	-0.46	1.00
Stam2	-0.57	1.00
Timm8b	-0.26	1.00
Tmem154	-1.88	1.00
Casp8	-0.39	1.00
Oxa1l	-0.23	1.00
Sh3bgrl2	-0.54	1.00
Dnal1	-0.02	1.00
Cep120	-0.81	1.00
Ap5m1	-0.36	1.00
Armcx3	-0.29	1.00
Ppil4	0.12	1.00
Napsa	-0.51	1.00
Arl4a	-0.25	1.00
Crnkl1	0.02	1.00
Spin1	-0.17	1.00
Nbn	-0.46	1.00
Stk38l	-0.40	1.00
Tbl2	-0.16	1.00
Supt4a	-0.70	1.00
Dhrs3	-0.33	1.00
Peli1	-0.57	1.00
Cflar	0.10	1.00
Arid2	-0.39	1.00
Cnih4	-0.03	1.00
Cd48	-0.36	1.00
Rtn4	-0.30	1.00
Nos2	0.09	1.00
Snap23	-0.50	1.00
Ctnnbl1	0.08	1.00

Ero1b	0.12	1.00
Tsnax	-0.35	1.00
Cpsf7	-0.12	1.00
Setd2	-0.45	1.00
Klf6	-0.66	1.00
Antxr2	-1.15	1.00
Rexo2	-0.29	1.00
Cap1	-0.17	1.00
Smc1a	-0.39	1.00
Emc2	-0.16	1.00
Rnf144b	-0.38	1.00
Zmym4	0.04	1.00
Phf12	-0.73	1.00
Apc	-0.33	1.00
Cacnb2	0.25	1.00
Pex7	0.32	1.00
Retreg3	0.10	1.00
Filip1	0.52	1.00
Alg14	0.40	1.00
Tsen54	0.51	1.00
Smyd3	-0.41	1.00
Hyi	-0.25	1.00
Brd3	-0.09	1.00
Prkcz	-0.77	1.00
Hnrnpr	-0.11	1.00
Acsl4	-0.35	1.00
Adam10	-0.43	1.00
Pabpc1	0.00	1.00
Git2	-0.90	1.00
Ppp1r13b	-0.18	1.00
Gtf2i	0.03	1.00
Eif3f	-0.26	1.00
Trim23	-0.45	1.00
Slc25a17	0.24	1.00
Rgs3	-0.11	1.00
Ptk2	-0.40	1.00
Arhgap32	-0.18	1.00
Ankrd17	-0.10	1.00
Sestd1	-0.14	1.00
Rpap2	-0.23	1.00
Rab1a	-0.36	1.00
Itch	-0.18	1.00
Enah	-0.50	1.00
1810013L24Rik	-0.27	1.00
Acvr2a	0.08	1.00
Dpysl2	0.15	1.00

Scgb1a1	-0.17	1.00
Pphln1	-0.32	1.00
Pgs1	-0.36	1.00
Rbm47	-0.08	1.00
Arhgef2	-0.53	1.00
Fchsd2	-0.49	1.00
Birc6	-0.16	1.00
Agpat5	-0.58	1.00
Patl1	-0.86	1.00
Ipmk	-0.72	1.00
Hmox2	-0.32	1.00
Spg20	0.39	1.00
Vcpip1	-0.09	1.00
Desi2	-0.04	1.00
Plekhh1	0.06	1.00
Tradd	-0.06	1.00
Gspt2	-0.27	1.00
Ripk1	-0.30	1.00
Pbrm1	-0.43	1.00
Tab2	-0.02	1.00
Agpat2	0.63	1.00
D8Ertd738e	0.46	1.00
Acbd5	-0.38	1.00
Hunk	-0.92	1.00
Tbc1d8b	-0.93	1.00
1110059E24Rik	-0.13	1.00
Nudt2	-0.27	1.00
Tada1	-0.22	1.00
Srxn1	-0.14	1.00
C1rl	-0.57	1.00
Fuom	-0.18	1.00
Cebpb	-1.16	1.00
Zc3h18	-0.37	1.00
Strada	-0.26	1.00
Zfp609	0.20	1.00
Rin2	0.38	1.00
R3hdm2	-0.06	1.00
Dab2ip	-0.20	1.00
Arfgap3	0.12	1.00
Ctdsp1	-0.18	1.00
Coq9	0.08	1.00
Fam110a	0.54	1.00
Zfp9	-0.08	1.00
Tfe3	-0.18	1.00
Capza2	0.04	1.00
Ddx54	-0.34	1.00

Bora	-0.24	1.00
Prdx4	-0.65	1.00
Qser1	-0.28	1.00
Kifbp	-0.58	1.00
Hsdl1	-0.48	1.00
Arhgap25	-0.75	1.00
Slc4a5	-0.73	1.00
Prpf40b	-0.46	1.00
Lars2	-0.13	1.00
Add3	0.07	1.00
St3gal3	-0.06	1.00
Tesk2	0.22	1.00
Thumpd3	-0.11	1.00
Naf1	-0.28	1.00
Dtnbp1	-0.68	1.00
Pcyt2	-0.07	1.00
Acad11	-0.43	1.00
Prcc	-0.65	1.00
Tbxas1	0.31	1.00
Vtcn1	0.25	1.00
Cndp1	0.31	1.00
Dusp19	0.00	1.00
Etv4	0.31	1.00
Cdk18	0.38	1.00
Dsn1	0.29	1.00
Nfatc2ip	0.40	1.00
4931414P19Rik	0.31	1.00
Trim13	-0.05	1.00
Btbd7	-0.10	1.00
Slc25a36	-0.38	1.00
Eif4g3	-0.05	1.00
Dcp2	-0.34	1.00
Bcr	0.26	1.00
Strn	-0.63	1.00
Pola1	-0.46	1.00
Usp34	-0.12	1.00
Icmt	-0.33	1.00
Tram1	0.00	1.00
Ppp3cb	-0.05	1.00
Twf2	-0.01	1.00
Ctnna1	-0.11	1.00
Syngr1	0.07	1.00
Cubn	-0.21	1.00
Orc3	-0.03	1.00
Arhgap24	-0.05	1.00
Kdm6b	0.03	1.00

Socs3	-0.50	1.00
Taf3	-0.32	1.00
Pcgf3	-0.13	1.00
Esf1	-0.55	1.00
Cabin1	-0.45	1.00
Secisbp2	-0.10	1.00
Ythdc1	-0.26	1.00
Cdc42bpg	-0.01	1.00
Zc3h15	0.00	1.00
Wdr33	0.11	1.00
Slc30a3	-0.44	1.00
Pex13	-0.59	1.00
Casq2	-0.30	1.00
Nup37	-0.16	1.00
Creg1	-0.31	1.00
E2f5	-0.34	1.00
Arv1	-0.32	1.00
Acadsb	-0.11	1.00
Nfam1	-0.36	1.00
S100a8	-0.41	1.00
Nosip	-0.61	1.00
Cdk16	-0.18	1.00
Cobll1	-0.35	1.00
Sft2d2	-0.02	1.00
Hras	-0.05	1.00
Bcl6	0.17	1.00
Zfp30	-0.03	1.00
Gls	-0.04	1.00
Tcf7l2	-0.02	1.00
Rcn2	0.47	1.00
Nipsnap2	-0.22	1.00
Nav3	0.11	1.00
Slc1a3	0.05	1.00
Sap30bp	-0.04	1.00
Rras	0.16	1.00
Tead2	-0.07	1.00
Akip1	-0.02	1.00
Ccdc90b	-0.37	1.00
Disc1	-0.40	1.00
Gstcd	-0.71	1.00
Syde2	-0.46	1.00
Ddx46	-0.50	1.00
Ap1s2	-0.45	1.00
Alkbh1	-0.29	1.00
Rhof	-0.27	1.00
Lat2	-0.44	1.00

Zxdc	-0.34	1.00
Zcchc8	0.02	1.00
Lasp1	-0.28	1.00
Sh3bgrl3	0.26	1.00
Ankrd13c	-0.17	1.00
Arid1b	-0.17	1.00
Atp5j	-0.03	1.00
Atp1a1	-0.18	1.00
Yipf3	-0.06	1.00
Stx3	0.31	1.00
Sgsm1	-0.65	1.00
Ccar1	-0.43	1.00
Tbrg4	0.02	1.00
Hacd4	0.07	1.00
Crat	-0.29	1.00
Anapc2	-0.08	1.00
Hnrnph1	-0.29	1.00
Tead3	-0.03	1.00
Anxa5	0.15	1.00
Tst	-0.33	1.00
Lyst	-0.52	1.00
B3gnt2	-0.40	1.00
Ttc9	0.45	1.00
Naxe	0.05	1.00
Huwe1	-0.13	1.00
Prkar1a	0.04	1.00
Gnb1	-0.59	1.00
Ppip5k1	-0.79	1.00
Crkl	-0.52	1.00
Heca	-0.17	1.00
Cop1	-0.39	1.00
Gstk1	0.06	1.00
Lzic	0.07	1.00
Taf12	-0.58	1.00
Rab6b	-0.04	1.00
Rnase4	-0.08	1.00
Dnajb12	-0.15	1.00
Vezt	0.52	1.00
Atf6	-0.06	1.00
Icam1	-0.35	1.00
Cst3	-0.56	1.00
Sp3	-0.97	1.00
Tpk1	-0.48	1.00
Zfr	-0.23	1.00
Crebl2	-0.04	1.00
Slc25a13	-0.11	1.00

Psca	-0.11	1.00
Ap3m1	-0.03	1.00
Pdcd7	-0.20	1.00
Dipk1a	0.39	1.00
Wnt7b	-0.50	1.00
Abraxas1	-0.28	1.00
Rab4a	-0.35	1.00
Laptm5	-0.83	1.00
Unkl	-0.23	1.00
U2surp	-0.19	1.00
Exoc1	-0.51	1.00
Pdhx	-0.22	1.00
Acsl5	-0.52	1.00
Acap2	-0.07	1.00
Etfrf1	0.32	1.00
Plcb1	0.62	1.00
Spic	-0.26	1.00
Tpp1	-0.29	1.00
Rsad2	-0.29	1.00
Tma16	-0.04	1.00
Haus1	-0.11	1.00
Btbd8	-0.22	1.00
Ints1	-0.09	1.00
Igsf6	-0.35	1.00
Ubxn2b	-0.16	1.00
Rad18	-0.25	1.00
Usp20	-0.16	1.00
Lpcat4	-0.30	1.00
Tmem178b	0.01	1.00
Tsku	0.03	1.00
Hspa13	0.02	1.00
Necap1	-0.09	1.00
Osr1	-0.16	1.00
Pum3	-0.17	1.00
Fam114a2	0.15	1.00
Ramp2	-0.88	1.00
Id3	-0.78	1.00
Zfp579	0.05	1.00
Heatr3	-0.29	1.00
Pmaip1	-0.16	1.00
Aak1	-0.55	1.00
Cpd	-0.59	1.00
Sbds	-0.29	1.00
Prr14l	-0.47	1.00
Vps13b	-0.38	1.00
Elob	0.00	1.00

Calcoco2	-0.24	1.00
Zfp326	-0.47	1.00
Ubap2l	-0.57	1.00
Fyco1	-0.45	1.00
Ino80d	-0.65	1.00
Ncoa3	-0.67	1.00
Cry1	0.01	1.00
Klhl24	-0.20	1.00
Rufy1	-0.39	1.00
Gramd1c	-0.27	1.00
Lamtor3	-0.26	1.00
Sema4a	-0.21	1.00
Deptor	-0.01	1.00
Nrbp2	0.01	1.00
Slc2a12	0.08	1.00
Ints9	0.02	1.00
Rnf128	-0.15	1.00
Slc1a1	0.04	1.00
4930447C04Rik	0.01	1.00
Zfp770	0.00	1.00
Vwc2l	0.05	1.00
Slc25a28	0.06	1.00
Fbxo45	0.04	1.00
Vps9d1	-0.15	1.00
Taf6	-0.40	1.00
Ddx51	0.02	1.00
Cog1	0.02	1.00
Alpl	0.04	1.00
Csf1	0.05	1.00
Tfpt	-0.01	1.00
Elp5	0.16	1.00
Alkbh7	-0.32	1.00
Dyrk1a	-0.26	1.00
Senp2	-0.51	1.00
Aida	-0.19	1.00
Prdm5	-0.16	1.00
Uhmk1	-0.23	1.00
Smad7	-0.49	1.00
Irx2	-0.11	1.00
Tyrobp	-0.05	1.00
Zranb2	-0.01	1.00
Crebrf	-0.54	1.00
Golga5	-0.65	1.00
Ndufs1	-0.28	1.00
Dis3	-0.06	1.00
Ttc7	-0.07	1.00

Klhdc1	-0.15	1.00
Tada3	0.01	1.00
Ubtf	-0.05	1.00
Patj	0.20	1.00
Ube2b	-0.21	1.00
Fbxo11	-0.26	1.00
Rfc3	-0.66	1.00
Gli3	-0.49	1.00
Fgd3	-0.63	1.00
Lyrm1	-0.90	1.00
Rab11fip3	-0.63	1.00
Bin3	-0.26	1.00
Tyk2	-0.17	1.00
Tbcb	-0.71	1.00
Spink5	-0.32	1.00
Scoc	-0.16	1.00
Rab5a	-0.26	1.00
Chmp2a	-0.44	1.00
Snrpb2	-0.48	1.00
Klhl28	-0.52	1.00
Appbp2	-0.19	1.00
Cd53	-0.15	1.00
Lemd2	-0.19	1.00
Ik	-0.01	1.00
Arid5b	-0.91	1.00
Cenpj	-0.24	1.00
Enox1	-1.06	1.00
Klhdc3	-0.30	1.00
Vps16	-0.17	1.00
Zfp329	-0.53	1.00
Tmem11	-0.94	1.00
Liph	-0.38	1.00
Elp6	-0.14	1.00
Dis3l	-0.09	1.00
Dpm2	0.01	1.00
Ciz1	0.07	1.00
Gle1	-0.09	1.00
Evi2	-0.13	1.00
Rev1	-0.07	1.00
Ctdspl2	-0.29	1.00
Adck2	-0.20	1.00
Lrig3	-0.23	1.00
Tmem170b	-0.08	1.00
Slc17a5	-0.13	1.00
Rab15	-0.04	1.00
Tmem51	-0.17	1.00

Slc41a1	-0.06	1.00
Nfya	-0.25	1.00
Pxmp2	-0.13	1.00
Rassf4	-0.11	1.00
Zfp105	-0.16	1.00
Amz2	-0.58	1.00
Aff1	-0.15	1.00
Snx3	-0.36	1.00
Tcea1	-0.13	1.00
Siah1b	-0.02	1.00
Cdv3	0.07	1.00
Nrd1	-0.05	1.00
Arid1a	-0.32	1.00
Acvr1b	0.54	1.00
Zfp36l1	-0.35	1.00
Scaf11	-0.12	1.00
Tgds	-0.38	1.00
Bphl	-0.24	1.00
Ncoa7	-0.54	1.00
Socs4	-0.27	1.00
Polr3a	0.09	1.00
Btg4	-0.05	1.00
Acot4	-0.38	1.00
Pnkp	-0.40	1.00
Sap130	-0.53	1.00
Sec61a1	-0.20	1.00
Tnks	-0.32	1.00
Apip	-0.23	1.00
Dynlrb2	-0.74	1.00
Pmpcb	-0.06	1.00
Sidt1	-0.15	1.00
Fbf1	-0.03	1.00
Ccdc150	-0.28	1.00
Atr	-0.51	1.00
Aff4	-0.17	1.00
Dcp1b	-0.01	1.00
Adprh	-0.01	1.00
Arhgap6	0.10	1.00
Nipal2	0.01	1.00
Invs	-0.43	1.00
Rarres2	0.14	1.00
Eaf1	0.01	1.00
Vill	-0.07	1.00
Sytl3	0.11	1.00
Gatd1	0.00	1.00
Tyro3	0.23	1.00

Zwilch	-0.46	1.00
Adgrg5	-0.22	1.00
Crcp	0.01	1.00
Gabpb2	0.00	1.00
Polr1a	-0.54	1.00
Zfp408	0.03	1.00
Ccdc9b	-0.05	1.00
Six5	0.58	1.00
Tufm	-0.08	1.00
Zfp672	0.26	1.00
Ctsf	0.04	1.00
Ehbp1l1	0.00	1.00
Plxna3	0.18	1.00
Sntb2	-0.33	1.00
Tcf7	0.07	1.00
Ybx3	-0.31	1.00
Tgif1	-0.17	1.00
Ccdc149	-0.29	1.00
Aga	-0.68	1.00
Adcy7	-0.18	1.00
Dcakd	-0.36	1.00
Dnajc3	-0.44	1.00
Usp16	-0.33	1.00
Zfp652	0.00	1.00
Ankrd39	-0.44	1.00
Scaf8	-0.51	1.00
Elk3	-0.23	1.00
Rbm41	-0.43	1.00
Slc9a6	-0.17	1.00
Suds3	-0.52	1.00
Pip5k1c	-0.32	1.00
Pram1	-0.24	1.00
Rnd3	-0.36	1.00
Fbxl3	-0.15	1.00
Zbtb8os	-0.21	1.00
Nbeal1	-0.14	1.00
Zfc3h1	-0.41	1.00
Erbin	-0.19	1.00
Actr2	-0.07	1.00
Txnl4b	-0.53	1.00
Cdpf1	-0.03	1.00
Sec22c	-0.05	1.00
Ttc39c	-0.06	1.00
Rtkn	-0.04	1.00
Bmf	0.00	1.00
Dph5	-0.57	1.00

Slc41a3	-0.03	1.00
E2f4	-0.01	1.00
AU021092	-0.09	1.00
Zfp64	-0.02	1.00
Isg20l2	-0.08	1.00
Alas1	0.31	1.00
Ppp1r12b	-0.04	1.00
Syn3	0.16	1.00
Pik3cg	-0.90	1.00
Zadh2	-0.51	1.00
Dock11	0.01	1.00
Aifm2	-0.16	1.00
Scamp2	-0.04	1.00
Nudt1	-0.55	1.00
Slc27a4	-0.50	1.00
Arid3a	-0.02	1.00
Vps45	-0.17	1.00
Pkp3	-0.07	1.00
Nostrin	0.12	1.00
Pdcd5	-0.15	1.00
Tbc1d12	-0.34	1.00
4932411N23Rik	-0.14	1.00
Adgrl1	-0.59	1.00
Wdfy3	-0.31	1.00
Ndel1	0.16	1.00
Tatdn1	-0.55	1.00
Cep85l	-0.05	1.00
Col6a1	-0.24	1.00
Carf	-0.13	1.00
Actr1a	-0.56	1.00
Cldn1	0.02	1.00
Rep15	0.33	1.00
Zfp13	-0.18	1.00
Arid3b	-0.17	1.00
Niban2	-0.29	1.00
Slc38a6	-0.28	1.00
Sec22a	-0.64	1.00
Acox1	-0.18	1.00
Bod1l	-0.40	1.00
Golim4	0.34	1.00
Nars2	-0.56	1.00
Cd151	-0.46	1.00
Elk4	-0.18	1.00
G0s2	-0.65	1.00
Fto	-0.42	1.00
Dag1	-0.01	1.00

Nagk	-0.40	1.00
Bag6	-0.07	1.00
Nr3c2	-0.49	1.00
Zfp638	-0.40	1.00
Rap1a	-0.97	1.00
Tra2b	-0.31	1.00
St6gal1	-0.05	1.00
Scyl3	-0.53	1.00
Sh3rf2	-0.16	1.00
Csf3r	-0.42	1.00
Rfx1	-0.71	1.00
Baz2b	-0.32	1.00
Upf2	-0.36	1.00
Poldip3	-0.41	1.00
Cpq	-0.42	1.00
Klhl18	-0.28	1.00
Stradb	0.13	1.00
Wdtc1	-0.19	1.00
Ptpn23	-0.52	1.00
Zfp664	-0.20	1.00
Acat1	-0.38	1.00
Tle1	0.22	1.00
Fbxl5	-0.28	1.00
Gap43	-0.46	1.00
Capn5	-0.04	1.00
Entpd3	-0.07	1.00
Aars2	-0.10	1.00
C9orf72	-0.01	1.00
Nrep	-0.11	1.00
Arfgap2	-0.04	1.00
Tcp11l1	-0.08	1.00
Cybrd1	-0.08	1.00
Ormdl1	-0.03	1.00
Zdhhc16	-0.04	1.00
Hif1an	-0.27	1.00
Usp19	-0.03	1.00
Jade2	-0.01	1.00
Pcmtd2	-0.11	1.00
2210016L21Rik	-0.07	1.00
Dhx8	-0.02	1.00
Prkag1	-0.03	1.00
Atad3a	0.00	1.00
Dpagt1	0.01	1.00
Skiv2l	-0.05	1.00
Sorbs3	0.00	1.00
Entr1	-0.06	1.00

Fuz	-0.28	1.00
Daxx	-0.07	1.00
Cd274	-0.12	1.00
Dnase1l1	-0.60	1.00
Usp15	-0.24	1.00
Frs2	-0.20	1.00
Yy1	-0.19	1.00
Atp6v1a	-0.54	1.00
Lyn	-0.47	1.00
Ostf1	-0.35	1.00
Cep68	-0.07	1.00
Snx18	-0.32	1.00
Pygo1	-0.12	1.00
Htatip2	-0.27	1.00
Atp8b4	-0.22	1.00
Noct	-0.66	1.00
Ccl5	-0.79	1.00
Pbx3	-0.02	1.00
Bmpr1a	-0.43	1.00
Scap	-0.03	1.00
Ccdc78	-0.44	1.00
Zfp473	0.04	1.00
Gadd45gip1	-0.46	1.00
Ing5	-0.41	1.00
Hars	-0.14	1.00
Angptl2	-0.09	1.00
Plekhm2	-0.04	1.00
Taf11	-0.05	1.00
Shc1	-0.07	1.00
Was	-0.21	1.00
Enpp1	-0.43	1.00
Poli	-0.23	1.00
Trim39	-0.20	1.00
Immp2l	-0.04	1.00
Ccnd3	0.06	1.00
Wdr48	-0.51	1.00
Slc10a1	-0.57	1.00
Rbm12b2	-0.34	1.00
Vps37a	-0.24	1.00
Gtf2b	0.13	1.00
Vps8	-0.01	1.00
Srpk2	0.04	1.00
Fbxl20	0.00	1.00
Cul3	-0.07	1.00
Cdc16	-0.34	1.00
Efnb2	-0.17	1.00

Urb1	-0.08	1.00
Ints7	-0.16	1.00
Zbtb17	0.03	1.00
Atg16l2	-0.26	1.00
Trak2	-0.02	1.00
Lig1	-0.09	1.00
Yod1	-0.06	1.00
Nckap5l	-0.10	1.00
Orc5	-0.75	1.00
Pbdc1	-0.07	1.00
Cox7a1	-0.17	1.00
Parp1	-0.08	1.00
Aldh7a1	-0.16	1.00
Cks2	-0.52	1.00
Cdk6	0.03	1.00
Trpc4ap	-0.20	1.00
Ankrd10	-0.07	1.00
Eif2ak3	0.38	1.00
Wnt5b	-0.85	1.00
Ssh1	-0.29	1.00
Gas7	-0.19	1.00
Lmntd1	0.10	1.00
Lman1	-0.81	1.00
Pgm2	-0.39	1.00
Pank3	0.05	1.00
Aurkaip1	-0.05	1.00
Clk2	-0.19	1.00
Nxf1	-0.14	1.00
Adgre5	-0.62	1.00
Zfhx3	-0.36	1.00
Srgn	-1.65	1.00
Large1	0.39	1.00
Sec62	-0.12	1.00
Nab1	-0.01	1.00
Ccdc186	-0.08	1.00
Frmd4a	0.18	1.00
Ltbr	-0.08	1.00
Trappc9	-0.38	1.00
Aimp1	0.23	1.00
Wdr82	-0.28	1.00
Stim1	-0.26	1.00
Nfx1	-0.40	1.00
Numb	0.11	1.00
AU040320	-0.22	1.00
Swt1	-0.61	1.00
Clybl	-0.40	1.00

Primpol	-0.55	1.00
Ghr	-0.13	1.00
Osbpl11	-0.57	1.00
Sftpc	-1.60	1.00
Prkd2	-0.31	1.00
Chpf2	-0.26	1.00
Gtpbp2	-0.25	1.00
Ppat	-0.06	1.00
Htra2	-0.12	1.00
Tollip	-0.05	1.00
Fjx1	-0.07	1.00
Dclre1a	-0.18	1.00
Sft2d3	-0.04	1.00
Pdpr	-0.17	1.00
Grk6	-0.38	1.00
Slc36a1	-0.27	1.00
Dipk1b	-0.10	1.00
Eif2d	-0.41	1.00
Crtc1	-0.15	1.00
Proser2	-0.30	1.00
Osgin1	-0.10	1.00
Tmem156	-0.08	1.00
Exosc4	-0.39	1.00
Slc6a8	-0.16	1.00
Fabp5	-0.31	1.00
Fam120a	-0.35	1.00
Il15	-1.02	1.00
Stx11	-0.51	1.00
Nol11	-0.40	1.00
Nckap1	-0.31	1.00
Nsd1	-0.17	1.00
Aqp4	-0.33	1.00
Zbtb1	0.26	1.00
Phrf1	-0.21	1.00
Btbd9	-0.20	1.00
Dhx9	0.02	1.00
Smim14	-0.03	1.00
Nsun3	-0.28	1.00
Clns1a	-0.13	1.00
Csk	-0.14	1.00
Kctd6	-0.10	1.00
Znhit3	-0.49	1.00
Ptpru	-0.09	1.00
Ces3a	-0.47	1.00
Egr1	-0.59	1.00
Septin10	0.14	1.00

Cars2	-0.13	1.00
Pik3ip1	-0.04	1.00
Bloc1s5	-0.88	1.00
Prdx6	-0.56	1.00
Hspa14	-0.48	1.00
Armc1	-0.58	1.00
Kdr	-0.35	1.00
Lxn	-0.29	1.00
Rbl1	-0.16	1.00
4930523C07Rik	-0.05	1.00
Serac1	-0.49	1.00
Trmt11	-0.41	1.00
Cep78	-0.36	1.00
Ston2	-0.18	1.00
Setx	-0.23	1.00
Galnt10	-0.43	1.00
Palmd	0.51	1.00
Lrrc28	0.07	1.00
Wdr76	0.46	1.00
Ptgs1	0.01	1.00
Clcn3	-0.22	1.00
Wwc1	-0.31	1.00
Id2	-0.38	1.00
Dynlt4	-0.43	1.00
Sptbn1	-0.43	1.00
Gdap2	0.16	1.00
Anapc1	-0.32	1.00
Cnn3	-0.15	1.00
Samd4b	-0.05	1.00
Topors	-0.19	1.00
Cbll1	-0.68	1.00
Ccser2	-0.43	1.00
Hmg20b	-0.55	1.00
Oxr1	-0.59	1.00
Cnot6l	-0.44	1.00
Ppp2r5e	-0.34	1.00
Ngly1	-0.41	1.00
Rai14	-0.43	1.00
Fam117b	-0.11	1.00
Pdgfc	-0.14	1.00
Ate1	-0.07	1.00
Atxn10	0.54	1.00
Ppp1r9a	-0.49	1.00
Rbbp8	-0.34	1.00
Cep162	-0.37	1.00
Trim25	-0.26	1.00

Zkscan1	-0.24	1.00
Asns	-0.09	1.00
Kif3c	-0.08	1.00
Arsk	0.02	1.00
Wtip	-0.12	1.00
Rpa1	-0.06	1.00
Cracr2a	-0.03	1.00
Gps2	-0.09	1.00
Rcan1	-0.27	1.00
Pm20d2	-0.17	1.00
Xrcc5	-0.19	1.00
D16Ertd472e	-0.27	1.00
Ctif	-1.06	1.00
Sh3glb1	0.13	1.00
Slc38a9	-0.01	1.00
Ikzf5	-0.02	1.00
Rprd1b	-0.21	1.00
Gart	-0.19	1.00
Apbb1ip	-0.35	1.00
Lima1	-0.40	1.00
Rrp1	-0.14	1.00
Thap2	-0.26	1.00
Sertad2	0.32	1.00
Fam177a	-0.67	1.00
Tle4	-0.20	1.00
Cdc42ep1	-0.11	1.00
Iftap	-0.19	1.00
Sult2b1	-0.53	1.00
Aard	-0.25	1.00
Wdr43	-0.21	1.00
Helq	-0.08	1.00
Utp3	-0.09	1.00
Erap1	-0.21	1.00
Ndufaf1	-0.10	1.00
Nrxn3	-0.89	1.00
Ptgfr	-0.16	1.00
Paqr9	-0.50	1.00
Kif7	-0.06	1.00
Cdyl2	-0.20	1.00
Bnip1	-0.05	1.00
Pms2	-0.02	1.00
Gltp	-0.69	1.00
Kcnj2	-0.07	1.00
Nacc2	-0.59	1.00
Cyb5d2	-0.12	1.00
Tpst1	-0.10	1.00

Ilvbl	-0.08	1.00
Chml	-0.17	1.00
Snai1	-0.57	1.00
Zkscan16	-0.12	1.00
Kctd13	-0.03	1.00
Khnyn	-0.16	1.00
Dennd10	-0.20	1.00
Ccdc6	-0.31	1.00
Nt5c2	-0.30	1.00
Slc35b3	-0.24	1.00
Dffa	-0.08	1.00
Tmem183a	-0.14	1.00
Bysl	-0.50	1.00
Arhgap30	-0.37	1.00
Scnm1	-0.24	1.00
Ywhah	-0.12	1.00
Sgf29	-0.14	1.00
Zhx2	0.41	1.00
Ostm1	0.02	1.00
Pde8a	-0.30	1.00
Ncoa5	-0.28	1.00
Fbh1	0.15	1.00
Serinc1	-0.25	1.00
Irf1	0.06	1.00
Zcchc9	-0.01	1.00
Tcta	-0.06	1.00
Plin5	-0.27	1.00
Fmo4	-0.44	1.00
Zmym5	-0.30	1.00
Ipo11	-0.18	1.00
Ifih1	-0.47	1.00
Nceh1	-0.45	1.00
Foxk1	-0.06	1.00
Arhgap27	0.11	1.00
Osbpl9	-0.11	1.00
Lpp	-0.27	1.00
Dpp8	-0.39	1.00
Ppp1r12a	-0.49	1.00
Rbks	-0.30	1.00
Rnf216	-0.32	1.00
Tmem9b	-0.15	1.00
Pigc	-0.11	1.00
Sgsm2	-0.11	1.00
Il10ra	-0.13	1.00
Ubxn1	-0.46	1.00
Washc4	-0.18	1.00

Rgs2	-0.06	1.00
Btd	-0.22	1.00
Slc66a2	-0.18	1.00
L3mbtl3	-0.08	1.00
Zdhhc15	-0.04	1.00
Cnppd1	-0.08	1.00
Prkrip1	0.01	1.00
Amh	-0.07	1.00
Tmem126a	-0.53	1.00
Cdk4	-0.77	1.00
Rmnd5a	-0.38	1.00
Zzz3	-0.31	1.00
Cenpc1	-0.07	1.00
Ubxn10	0.00	1.00
Inpp5f	0.06	1.00
Tlcd2	0.02	1.00
Klhdc10	-0.20	1.00
Sephs1	-0.87	1.00
Chm	-0.48	1.00
Spire1	-0.64	1.00
S100a11	0.02	1.00
Cspp1	-0.04	1.00
Nfib	-0.59	1.00
Fkbp3	-0.13	1.00
Cdc37	-0.17	1.00
Otud7b	-0.12	1.00
Nudt5	-0.13	1.00
Cables1	-0.46	1.00
Kcnk2	-1.05	1.00
Prmt3	-0.71	1.00
Fem1b	-0.42	1.00
Eral1	-0.31	1.00
Siae	-0.19	1.00
Fxr2	-0.13	1.00
Tbc1d7	-0.09	1.00
Uqcc1	0.00	1.00
Exoc6b	-0.08	1.00
Timmdc1	-0.67	1.00
Sdc1	-0.26	1.00
Klhdc2	-0.40	1.00
Acsf2	-0.14	1.00
Prkacb	-0.27	1.00
Slc10a3	-0.54	1.00
Folr1	-0.97	1.00
Bcl9	0.08	1.00
Pcmt1	-0.35	1.00

Cited2	-0.32	1.00
Eif2b3	-0.13	1.00
Zfp369	-0.19	1.00
Spring1	-0.61	1.00
Tspan13	-0.18	1.00
Clasp1	-0.09	1.00
Nfat5	-0.05	1.00
Ranbp9	-0.41	1.00
Cog3	-0.39	1.00
Cd200	0.04	1.00
Far1	0.10	1.00
Ivns1abp	-0.48	1.00
Slc35f5	-0.20	1.00
Tns3	-0.57	1.00
Fer	0.01	1.00
Epn1	-0.16	1.00
Tuft1	-0.54	1.00
Glt8d1	0.28	1.00
Usp3	-0.28	1.00
Gse1	-0.68	1.00
Add1	-0.73	1.00
Lamtor1	-0.01	1.00
Adgrl2	0.18	1.00
Hacd1	-0.53	1.00
Uggt1	-0.85	1.00
Stambp	-0.24	1.00
Ppp2r5a	-0.50	1.00
Dennd1b	-0.16	1.00
Atp2a2	0.40	1.00
Nuak1	0.02	1.00
Crtap	0.64	1.00
Wrn	-0.06	1.00
Snx12	-0.02	1.00
Greb1l	-0.17	1.00
Dcun1d3	0.16	1.00
Hspg2	-0.17	1.00
Pef1	-0.26	1.00
Cdhr3	0.06	1.00
Ypel3	-0.06	1.00
Itga9	-0.05	1.00
Rttn	-0.98	1.00
Cyb5a	-0.47	1.00
Sgk1	-0.71	1.00
Daam1	-0.39	1.00
Cnksr2	0.07	1.00
Gab2	-0.97	1.00

Pacsin2	0.08	1.00
Epc1	-0.52	1.00
Cops6	-0.45	1.00
Bex4	-0.52	1.00
Rph3al	-0.18	1.00
Akap7	0.42	1.00
Fam98b	0.08	1.00
Hnf1b	-0.08	1.00
Vps25	-0.11	1.00
Dusp23	0.05	1.00
Anxa7	-0.02	1.00
Yaf2	-0.64	1.00
Snx30	-0.46	1.00
Lclat1	-0.18	1.00
Cdc42bpa	-0.04	1.00
Bcas3	0.30	1.00
Tprkb	-0.13	1.00
Cgnl1	-0.68	1.00
Tjp1	-0.38	1.00
Tmod3	-0.10	1.00
Ifi44	-0.52	1.00
Ccdc33	-0.22	1.00
Emp1	-0.19	1.00
Ash1l	-0.25	1.00
Pnrc1	-0.07	1.00
Stag1	-0.19	1.00
Ppp2r3c	0.52	1.00
Rgs19	-0.66	1.00
Pacs1	0.07	1.00
Fndc3b	-0.36	1.00
Rad23a	0.02	1.00
Atrn	0.16	1.00
Rangap1	-0.29	1.00
Glud1	-0.35	1.00
Gtf2a1	0.97	1.00
Erbb2	0.03	1.00
Zmym1	0.15	1.00
Prdm1	-0.39	1.00
Krt80	-0.29	1.00
Pik3c2a	0.02	1.00
Vamp5	-0.23	1.00
Sh3kbp1	-0.91	1.00
Plce1	-0.62	1.00
Eif2a	-0.90	1.00
Exoc3	0.29	1.00
Nnt	0.36	1.00

2700049A03Rik	0.32	1.00
Chp1	-0.03	1.00
G2e3	0.00	1.00
Cnp	0.60	1.00
Polm	-0.05	1.00
Jchain	-0.91	1.00
Csrp1	-0.26	1.00
Eif2ak2	-0.42	1.00
Uap1	-0.29	1.00
Gigyf2	-0.49	1.00
Riox2	-0.47	1.00
Exosc7	-0.22	1.00
Snip1	-0.55	1.00
Cdk19	0.04	1.00
Agap1	0.17	1.00
Cecr2	0.01	1.00
Rin3	-0.04	1.00
Pcbp4	-0.10	1.00
Nfrkb	0.20	1.00
Septin6	0.36	1.00
G6pdx	0.72	1.00
Cab39	-0.19	1.00
Ncoa2	-0.18	1.00
Atp5g3	-0.62	1.00
Snap47	-0.37	1.00
Fyb	-0.34	1.00
Csf2rb2	-0.25	1.00
Ammecr1	-0.31	1.00
Clcc1	-0.70	1.00
Phf3	-0.33	1.00
Terf2ip	-0.49	1.00
Gpr176	-0.16	1.00
Ppa1	-0.11	1.00
Crybg3	0.02	1.00
Tmem245	-0.34	1.00
Usp54	-0.06	1.00
Efcab14	-0.55	1.00
C4bp	-1.00	1.00
Glce	0.01	1.00
Romo1	-0.56	1.00
Ccni	-0.12	1.00
Wdfy1	-0.61	1.00
Zbtb22	-0.38	1.00
Ddx39b	0.29	1.00
Ip6k1	-0.19	1.00
Ttbk2	-0.61	1.00

Kat8	-0.21	1.00
H2-Ke6	-0.79	1.00
Nek1	-0.22	1.00
Slk	-0.24	1.00
Bclaf1	-0.18	1.00
Rbms1	-0.27	1.00
Tmbim6	-0.16	1.00
Plaur	-1.18	1.00
Naa16	-0.54	1.00
Tmem108	0.14	1.00
Usp22	0.05	1.00
Scnn1g	0.09	1.00
Anp32e	-0.49	1.00
Ap1ar	-0.13	1.00
Kpna3	-0.58	1.00
Abi1	-0.33	1.00
Fam174b	0.05	1.00
Fam32a	0.02	1.00
Ipo8	0.04	1.00
Cemip2	-0.44	1.00
Nmnat3	-0.16	1.00
Hexdc	-0.34	1.00
Zfp438	-0.35	1.00
Dcbld1	0.41	1.00
Ccnd1	0.03	1.00
Ppm1e	-0.36	1.00
Tsn	0.21	1.00
Fbxo28	-0.46	1.00
Fmo1	-0.90	1.00
Synrg	-0.20	1.00
Il1rap	-0.15	1.00
Pi4k2b	-0.55	1.00
Snx27	0.02	1.00
Taf7	-0.28	1.00
Tmem87a	-0.06	1.00
Bpnt2	-0.51	1.00
Hacd2	-0.37	1.00
Rab7	0.27	1.00
Vmp1	-0.07	1.00
Asrgl1	-0.24	1.00
Taf4b	0.10	1.00
Abhd16a	-0.41	1.00
Dhx34	0.29	1.00
Sart3	-0.01	1.00
Adi1	-0.53	1.00
Atp2b4	-0.57	1.00

Fam214a	-0.07	1.00
Pcbp1	-0.21	1.00
Rnf169	-0.06	1.00
Ankrd54	-0.15	1.00
Hbp1	-0.18	1.00
Tmem106b	-0.22	1.00
Stard3nl	-0.32	1.00
Adnp2	-0.20	1.00
Pum2	-0.51	1.00
Arid4b	-0.14	1.00
Diaph2	-0.09	1.00
Polk	-0.27	1.00
Bdp1	-0.48	1.00
Lnpep	-0.07	1.00
Aven	0.67	1.00
Srp54a	-0.19	1.00
Hmg20a	-0.06	1.00
Rybp	-0.21	1.00
Prmt7	-0.23	1.00
Ufd1	-0.14	1.00
Casc3	-0.03	1.00
Stk38	-0.55	1.00
Tia1	-0.31	1.00
Snrpd2	-0.08	1.00
Dcaf6	-0.51	1.00
Nxn	0.14	1.00
Tap1	-0.37	1.00
Fam120c	0.05	1.00

pecies upon H5 HPAIV infection

host
Chicken
Chicken
Chicken
Chicken
Chicken
Chicken
Chicken
Chicken
Chicken
Chicken
Chicken
Chicken
Chicken
Chicken
Chicken
Chicken
Chicken
Chicken
Chicken
Chicken
Chicken
Chicken
Chicken
Chicken
Chicken
Chicken
Chicken
Chicken
Chicken
Chicken
Chicken
Chicken
Chicken
Chicken
Chicken
Chicken
Chicken
Chicken
Chicken
Chicken
Chicken

Chicken
Chicken
Chicken
Chicken
Chicken
Chicken
Chicken
Chicken
Chicken
Chicken
Chicken
Chicken
Chicken
Chicken
Chicken
Chicken
Chicken
Chicken
Chicken
Chicken
Chicken
Chicken
Chicken
Chicken
Chicken
Chicken
Chicken
Chicken
Chicken
Chicken
Chicken
Chicken
Chicken
Chicken
Chicken
Chicken
Chicken
Chicken
Chicken
Chicken
Chicken
Chicken
Chicken
Chicken
Chicken
Chicken

Chicken
Chicken
Chicken
Chicken
Chicken
Chicken
Chicken
Chicken
Chicken
Chicken
Chicken
Chicken
Chicken
Chicken
Chicken
Chicken
Chicken
Chicken
Chicken
Chicken
Chicken
Chicken
Chicken
Chicken
Chicken
Chicken
Chicken
Chicken
Chicken
Chicken
Chicken
Chicken
Chicken
Chicken
Chicken
Chicken
Chicken
Chicken
Chicken
Chicken
Chicken
Chicken
Chicken
Chicken
Chicken
Chicken

Chicken
Chicken
Chicken
Chicken
Chicken
Chicken
Chicken
Chicken
Chicken
Chicken
Chicken
Chicken
Chicken
Chicken
Chicken
Chicken
Chicken
Chicken
Chicken
Chicken
Chicken
Chicken
Chicken
Chicken
Chicken
Chicken
Chicken
Chicken
Chicken
Chicken
Chicken
Chicken
Chicken
Chicken
Chicken
Chicken
Chicken
Chicken
Chicken
Chicken
Chicken
Chicken
Chicken
Chicken
Chicken
Chicken

Chicken
Chicken
Chicken
Chicken
Chicken
Chicken
Chicken
Chicken
Chicken
Chicken
Chicken
Chicken
Chicken
Chicken
Chicken
Chicken
Chicken
Chicken
Chicken
Chicken
Chicken
Chicken
Chicken
Chicken
Chicken
Chicken
Chicken
Chicken
Chicken
Chicken
Chicken
Chicken
Chicken
Chicken
Chicken
Chicken
Chicken
Chicken
Chicken
Chicken
Chicken
Chicken
Chicken
Chicken
Chicken
Chicken

Chicken
Chicken
Chicken
Chicken
Chicken
Chicken
Chicken
Chicken
Chicken
Chicken
Chicken
Chicken
Chicken
Chicken
Chicken
Chicken
Chicken
Chicken
Chicken
Chicken
Chicken
Chicken
Chicken
Chicken
Chicken
Chicken
Chicken
Chicken
Chicken
Chicken
Chicken
Chicken
Chicken
Chicken
Chicken
Chicken
Chicken
Chicken
Chicken
Chicken
Chicken
Chicken
Chicken
Chicken
Chicken
Chicken

Chicken
Chicken
Chicken
Chicken
Chicken
Chicken
Chicken
Chicken
Chicken
Chicken
Chicken
Chicken
Chicken
Chicken
Chicken
Chicken
Chicken
Chicken
Chicken
Chicken
Chicken
Chicken
Chicken
Chicken
Chicken
Chicken
Chicken
Chicken
Chicken
Chicken
Chicken
Chicken
Chicken
Chicken
Chicken
Chicken
Chicken
Chicken
Chicken
Chicken
Chicken
Chicken
Chicken
Chicken
Chicken
Chicken

Chicken
Chicken
Chicken
Chicken
Chicken
Chicken
Chicken
Chicken
Chicken
Chicken
Chicken
Chicken
Chicken
Chicken
Chicken
Chicken
Chicken
Chicken
Chicken
Chicken
Chicken
Chicken
Chicken
Chicken
Chicken
Chicken
Chicken
Chicken
Chicken
Chicken
Chicken
Chicken
Chicken
Chicken
Chicken
Chicken
Chicken
Chicken
Chicken
Chicken
Chicken
Chicken
Chicken
Chicken
Chicken
Chicken

Chicken
Chicken
Chicken
Chicken
Chicken
Chicken
Chicken
Chicken
Chicken
Chicken
Chicken
Chicken
Chicken
Chicken
Chicken
Chicken
Chicken
Chicken
Chicken
Chicken
Chicken
Chicken
Chicken
Chicken
Chicken
Chicken
Chicken
Chicken
Chicken
Chicken
Chicken
Chicken
Chicken
Chicken
Chicken
Chicken
Chicken
Chicken
Chicken
Chicken
Chicken
Chicken
Chicken
Chicken
Chicken
Chicken

Chicken
Chicken
Chicken
Chicken
Chicken
Chicken
Chicken
Chicken
Chicken
Chicken
Chicken
Chicken
Chicken
Chicken
Chicken
Chicken
Chicken
Chicken
Chicken
Chicken
Chicken
Chicken
Chicken
Chicken
Chicken
Chicken
Chicken
Chicken
Chicken
Chicken
Chicken
Chicken
Chicken
Chicken
Chicken
Chicken
Chicken
Chicken
Chicken
Chicken
Chicken
Chicken
Chicken
Chicken
Chicken
Chicken

Chicken
Chicken
Chicken
Chicken
Chicken
Chicken
Chicken
Chicken
Chicken
Chicken
Chicken
Chicken
Chicken
Chicken
Chicken
Chicken
Chicken
Chicken
Chicken
Chicken
Chicken
Chicken
Chicken
Chicken
Chicken
Chicken
Chicken
Chicken
Chicken
Chicken
Chicken
Chicken
Chicken
Chicken
Chicken
Chicken
Chicken
Chicken
Chicken
Chicken
Chicken
Chicken
Chicken
Chicken
Chicken
Chicken

Chicken
Chicken
Chicken
Chicken
Chicken
Chicken
Chicken
Chicken
Chicken
Chicken
Chicken
Chicken
Chicken
Chicken
Chicken
Chicken
Chicken
Chicken
Chicken
Chicken
Chicken
Chicken
Chicken
Chicken
Chicken
Chicken
Chicken
Chicken
Chicken
Chicken
Chicken
Chicken
Chicken
Chicken
Chicken
Chicken
Chicken
Chicken
Chicken
Chicken
Chicken
Chicken
Chicken
Chicken
Chicken
Chicken

Chicken
Chicken
Chicken
Chicken
Chicken
Chicken
Chicken
Chicken
Chicken
Chicken
Chicken
Chicken
Chicken
Chicken
Chicken
Chicken
Chicken
Chicken
Chicken
Chicken
Chicken
Chicken
Chicken
Chicken
Chicken
Chicken
Chicken
Chicken
Chicken
Chicken
Chicken
Chicken
Chicken
Chicken
Chicken
Chicken
Chicken
Chicken
Chicken
Chicken
Chicken
Chicken
Chicken
Chicken
Chicken
Chicken

Chicken
Chicken
Chicken
Chicken
Chicken
Chicken
Chicken
Chicken
Chicken
Chicken
Chicken
Chicken
Chicken
Chicken
Chicken
Chicken
Chicken
Chicken
Chicken
Chicken
Chicken
Chicken
Chicken
Chicken
Chicken
Chicken
Chicken
Chicken
Chicken
Chicken
Chicken
Chicken
Chicken
Chicken
Chicken
Chicken
Chicken
Chicken
Chicken
Chicken
Chicken
Chicken
Chicken
Chicken
Chicken
Chicken

Chicken
Chicken
Chicken
Chicken
Chicken
Chicken
Chicken
Chicken
Chicken
Chicken
Chicken
Chicken
Chicken
Chicken
Chicken
Chicken
Chicken
Chicken
Chicken
Chicken
Chicken
Chicken
Chicken
Chicken
Chicken
Chicken
Chicken
Chicken
Chicken
Chicken
Chicken
Chicken
Chicken
Chicken
Chicken
Chicken
Chicken
Chicken
Chicken
Chicken
Chicken
Chicken
Chicken
Chicken
Chicken
Chicken

Chicken
Chicken
Chicken
Chicken
Chicken
Chicken
Chicken
Chicken
Chicken
Chicken
Chicken
Chicken
Chicken
Chicken
Chicken
Chicken
Chicken
Chicken
Chicken
Chicken
Chicken
Chicken
Chicken
Chicken
Chicken
Chicken
Chicken
Chicken
Chicken
Chicken
Chicken
Chicken
Chicken
Chicken
Chicken
Chicken
Chicken
Chicken
Chicken
Chicken
Chicken
Chicken
Chicken
Chicken
Chicken
Chicken

Chicken
Chicken
Chicken
Chicken
Chicken
Chicken
Chicken
Chicken
Chicken
Chicken
Chicken
Chicken
Chicken
Chicken
Chicken
Chicken
Chicken
Chicken
Chicken
Chicken
Chicken
Chicken
Chicken
Chicken
Chicken
Chicken
Chicken
Chicken
Chicken
Chicken
Chicken
Chicken
Chicken
Chicken
Chicken
Chicken
Chicken
Chicken
Chicken
Chicken
Chicken
Chicken
Chicken
Chicken
Chicken
Chicken

Chicken
Chicken
Chicken
Chicken
Chicken
Chicken
Chicken
Chicken
Chicken
Chicken
Chicken
Chicken
Chicken
Chicken
Chicken
Chicken
Chicken
Chicken
Chicken
Chicken
Chicken
Chicken
Chicken
Chicken
Chicken
Chicken
Chicken
Chicken
Chicken
Chicken
Chicken
Chicken
Chicken
Chicken
Chicken
Chicken
Chicken
Chicken
Chicken
Chicken
Chicken
Chicken
Chicken
Chicken
Chicken
Chicken

Chicken
Chicken
Chicken
Chicken
Chicken
Chicken
Chicken
Chicken
Chicken
Chicken
Chicken
Chicken
Chicken
Chicken
Chicken
Chicken
Chicken
Chicken
Chicken
Chicken
Chicken
Chicken
Chicken
Chicken
Chicken
Chicken
Chicken
Chicken
Chicken
Chicken
Chicken
Chicken
Chicken
Chicken
Chicken
Chicken
Chicken
Chicken
Chicken
Chicken
Chicken
Chicken
Chicken
Chicken
Chicken
Chicken

Chicken
Chicken
Chicken
Chicken
Chicken
Chicken
Chicken
Chicken
Chicken
Chicken
Chicken
Chicken
Chicken
Chicken
Chicken
Chicken
Chicken
Chicken
Chicken
Chicken
Chicken
Chicken
Chicken
Chicken
Chicken
Chicken
Chicken
Chicken
Chicken
Chicken
Chicken
Chicken
Chicken
Chicken
Chicken
Chicken
Chicken
Chicken
Chicken
Chicken
Chicken
Chicken
Chicken
Chicken
Chicken
Chicken

Chicken
Chicken
Chicken
Chicken
Chicken
Chicken
Chicken
Chicken
Chicken
Chicken
Chicken
Chicken
Chicken
Chicken
Chicken
Chicken
Chicken
Chicken
Chicken
Chicken
Chicken
Chicken
Chicken
Chicken
Chicken
Chicken
Chicken
Chicken
Chicken
Chicken
Chicken
Chicken
Chicken
Chicken
Chicken
Chicken
Chicken
Chicken
Chicken
Chicken
Chicken
Chicken
Chicken
Chicken
Chicken
Chicken

Chicken
Chicken
Chicken
Chicken
Chicken
Chicken
Chicken
Chicken
Chicken
Chicken
Chicken
Chicken
Chicken
Chicken
Chicken
Chicken
Chicken
Chicken
Chicken
Chicken
Chicken
Chicken
Chicken
Chicken
Chicken
Chicken
Chicken
Chicken
Chicken
Chicken
Chicken
Chicken
Chicken
Chicken
Chicken
Chicken
Chicken
Chicken
Chicken
Chicken
Chicken
Chicken
Chicken
Chicken
Chicken
Chicken

Chicken
Chicken
Chicken
Chicken
Chicken
Chicken
Chicken
Chicken
Chicken
Chicken
Chicken
Chicken
Chicken
Chicken
Chicken
Chicken
Chicken
Chicken
Chicken
Chicken
Chicken
Chicken
Chicken
Chicken
Chicken
Chicken
Chicken
Chicken
Chicken
Chicken
Chicken
Chicken
Chicken
Chicken
Chicken
Chicken
Chicken
Chicken
Chicken
Chicken
Chicken
Chicken
Chicken
Chicken
Chicken
Chicken

Chicken
Chicken
Chicken
Chicken
Chicken
Chicken
Chicken
Chicken
Chicken
Chicken
Chicken
Chicken
Chicken
Chicken
Chicken
Chicken
Chicken
Chicken
Chicken
Chicken
Chicken
Chicken
Chicken
Chicken
Chicken
Chicken
Chicken
Chicken
Chicken
Chicken
Chicken
Chicken
Chicken
Chicken
Chicken
Chicken
Chicken
Chicken
Chicken
Chicken
Chicken
Chicken
Chicken
Chicken
Chicken
Chicken

Chicken
Chicken
Chicken
Chicken
Chicken
Chicken
Chicken
Chicken
Chicken
Chicken
Chicken
Chicken
Chicken
Chicken
Chicken
Chicken
Chicken
Chicken
Chicken
Chicken
Chicken
Chicken
Chicken
Chicken
Chicken
Chicken
Chicken
Chicken
Chicken
Chicken
Chicken
Chicken
Chicken
Chicken
Chicken
Chicken
Chicken
Chicken
Chicken
Chicken
Chicken
Chicken
Chicken
Chicken
Chicken
Chicken

Chicken
Chicken
Chicken
Chicken
Chicken
Chicken
Chicken
Chicken
Chicken
Chicken
Chicken
Chicken
Chicken
Chicken
Chicken
Chicken
Chicken
Chicken
Chicken
Chicken
Chicken
Chicken
Chicken
Chicken
Chicken
Chicken
Chicken
Chicken
Chicken
Chicken
Chicken
Chicken
Chicken
Chicken
Chicken
Chicken
Chicken
Chicken
Chicken
Chicken
Chicken
Chicken
Chicken
Chicken
Chicken
Chicken

Chicken
Chicken
Chicken
Chicken
Chicken
Chicken
Chicken
Chicken
Chicken
Chicken
Chicken
Chicken
Chicken
Chicken
Chicken
Chicken
Chicken
Chicken
Chicken
Chicken
Chicken
Chicken
Chicken
Chicken
Chicken
Chicken
Chicken
Chicken
Chicken
Chicken
Chicken
Chicken
Chicken
Chicken
Chicken
Chicken
Chicken
Chicken
Chicken
Chicken
Chicken
Chicken
Chicken
Chicken
Chicken
Chicken

Chicken
Chicken
Chicken
Chicken
Chicken
Chicken
Chicken
Chicken
Chicken
Chicken
Chicken
Chicken
Chicken
Chicken
Chicken
Chicken
Chicken
Chicken
Chicken
Chicken
Chicken
Chicken
Chicken
Chicken
Chicken
Chicken
Chicken
Chicken
Chicken
Chicken
Chicken
Chicken
Chicken
Chicken
Chicken
Chicken
Chicken
Chicken
Chicken
Chicken
Chicken
Chicken
Chicken
Chicken
Chicken
Chicken

Chicken
Chicken
Chicken
Chicken
Chicken
Chicken
Chicken
Chicken
Chicken
Chicken
Chicken
Chicken
Chicken
Chicken
Chicken
Chicken
Chicken
Chicken
Chicken
Chicken
Chicken
Chicken
Chicken
Chicken
Chicken
Chicken
Chicken
Chicken
Chicken
Chicken
Chicken
Chicken
Chicken
Chicken
Chicken
Chicken
Chicken
Chicken
Chicken
Chicken
Chicken
Chicken
Chicken
Chicken
Chicken
Chicken

Chicken
Chicken
Chicken
Chicken
Chicken
Chicken
Chicken
Chicken
Chicken
Chicken
Chicken
Chicken
Chicken
Chicken
Chicken
Chicken
Chicken
Chicken
Chicken
Chicken
Chicken
Chicken
Chicken
Chicken
Chicken
Chicken
Chicken
Chicken
Chicken
Chicken
Chicken
Chicken
Chicken
Chicken
Chicken
Chicken
Chicken
Chicken
Chicken
Chicken
Chicken
Chicken
Chicken
Chicken
Chicken
Chicken

Chicken
Chicken
Chicken
Chicken
Chicken
Chicken
Chicken
Chicken
Chicken
Chicken
Chicken
Chicken
Chicken
Chicken
Chicken
Chicken
Chicken
Chicken
Chicken
Chicken
Chicken
Chicken
Chicken
Chicken
Chicken
Chicken
Chicken
Chicken
Chicken
Chicken
Chicken
Chicken
Chicken
Chicken
Chicken
Chicken
Chicken
Chicken
Chicken
Chicken
Chicken
Chicken
Chicken
Chicken
Chicken
Chicken

Chicken
Chicken
Chicken
Chicken
Chicken
Chicken
Chicken
Chicken
Chicken
Chicken
Chicken
Chicken
Chicken
Chicken
Chicken
Chicken
Chicken
Chicken
Chicken
Chicken
Chicken
Chicken
Chicken
Chicken
Chicken
Chicken
Chicken
Chicken
Chicken
Chicken
Chicken
Chicken
Chicken
Chicken
Chicken
Chicken
Chicken
Chicken
Chicken
Chicken
Chicken
Chicken
Chicken
Chicken
Chicken
Chicken

Chicken
Chicken
Chicken
Chicken
Chicken
Chicken
Chicken
Chicken
Chicken
Chicken
Chicken
Chicken
Chicken
Chicken
Chicken
Chicken
Chicken
Chicken
Chicken
Chicken
Chicken
Chicken
Chicken
Chicken
Chicken
Chicken
Chicken
Chicken
Chicken
Chicken
Chicken
Chicken
Chicken
Chicken
Chicken
Chicken
Chicken
Chicken
Chicken
Chicken
Chicken
Chicken
Chicken
Chicken
Chicken
Chicken

Chicken
Chicken
Chicken
Chicken
Chicken
Chicken
Chicken
Chicken
Chicken
Chicken
Chicken
Chicken
Chicken
Chicken
Chicken
Chicken
Chicken
Chicken
Chicken
Chicken
Chicken
Chicken
Chicken
Chicken
Chicken
Chicken
Chicken
Chicken
Chicken
Chicken
Chicken
Chicken
Chicken
Chicken
Chicken
Chicken
Chicken
Chicken
Chicken
Chicken
Chicken
Chicken
Chicken
Chicken
Chicken
Chicken

Chicken
Chicken
Chicken
Chicken
Chicken
Chicken
Chicken
Chicken
Chicken
Chicken
Chicken
Chicken
Chicken
Chicken
Chicken
Chicken
Chicken
Chicken
Chicken
Chicken
Chicken
Chicken
Chicken
Chicken
Chicken
Chicken
Chicken
Chicken
Chicken
Chicken
Chicken
Chicken
Chicken
Chicken
Chicken
Chicken
Chicken
Chicken
Chicken
Chicken
Chicken
Chicken
Chicken
Chicken
Chicken
Chicken

Chicken
Chicken
Chicken
Chicken
Chicken
Chicken
Chicken
Chicken
Chicken
Chicken
Chicken
Chicken
Chicken
Chicken
Chicken
Chicken
Chicken
Chicken
Chicken
Chicken
Chicken
Chicken
Chicken
Chicken
Chicken
Chicken
Chicken
Chicken
Chicken
Chicken
Chicken
Chicken
Chicken
Chicken
Chicken
Chicken
Chicken
Chicken
Chicken
Chicken
Chicken
Chicken
Chicken
Chicken
Chicken
Chicken

Chicken
Chicken
Chicken
Chicken
Chicken
Chicken
Chicken
Chicken
Chicken
Chicken
Chicken
Chicken
Chicken
Chicken
Chicken
Chicken
Chicken
Chicken
Chicken
Chicken
Chicken
Chicken
Chicken
Chicken
Chicken
Chicken
Chicken
Chicken
Chicken
Chicken
Chicken
Chicken
Chicken
Chicken
Chicken
Chicken
Chicken
Chicken
Chicken
Chicken
Chicken
Chicken
Chicken
Chicken
Chicken
Chicken

Chicken
Chicken
Chicken
Chicken
Chicken
Chicken
Chicken
Chicken
Chicken
Chicken
Chicken
Chicken
Chicken
Chicken
Chicken
Chicken
Chicken
Chicken
Chicken
Chicken
Chicken
Chicken
Chicken
Chicken
Chicken
Chicken
Chicken
Chicken
Chicken
Chicken
Chicken
Chicken
Chicken
Chicken
Chicken
Chicken
Chicken
Chicken
Chicken
Chicken
Chicken
Chicken
Chicken
Chicken
Chicken
Chicken

Chicken
Chicken
Chicken
Chicken
Chicken
Chicken
Chicken
Chicken
Chicken
Chicken
Chicken
Chicken
Chicken
Chicken
Chicken
Chicken
Chicken
Chicken
Chicken
Chicken
Chicken
Chicken
Chicken
Chicken
Chicken
Chicken
Chicken
Chicken
Chicken
Chicken
Chicken
Chicken
Chicken
Chicken
Chicken
Chicken
Chicken
Chicken
Chicken
Chicken
Chicken
Chicken
Chicken
Chicken
Chicken
Chicken

Chicken
Chicken
Chicken
Chicken
Chicken
Chicken
Chicken
Chicken
Chicken
Chicken
Chicken
Chicken
Chicken
Chicken
Chicken
Chicken
Chicken
Chicken
Chicken
Chicken
Chicken
Chicken
Chicken
Chicken
Chicken
Chicken
Chicken
Chicken
Chicken
Chicken
Chicken
Chicken
Chicken
Chicken
Chicken
Chicken
Chicken
Chicken
Chicken
Chicken
Chicken
Chicken
Chicken
Chicken
Chicken
Chicken

Chicken
Chicken
Chicken
Chicken
Chicken
Chicken
Chicken
Chicken
Chicken
Chicken
Chicken
Chicken
Chicken
Chicken
Chicken
Chicken
Chicken
Chicken
Chicken
Chicken
Chicken
Chicken
Chicken
Chicken
Chicken
Chicken
Chicken
Chicken
Chicken
Chicken
Chicken
Chicken
Chicken
Chicken
Chicken
Chicken
Chicken
Chicken
Chicken
Chicken
Chicken
Chicken
Chicken
Chicken
Chicken
Chicken

Chicken
Chicken
Chicken
Chicken
Chicken
Chicken
Chicken
Chicken
Chicken
Chicken
Chicken
Chicken
Chicken
Chicken
Chicken
Chicken
Chicken
Chicken
Chicken
Chicken
Chicken
Chicken
Chicken
Chicken
Chicken
Chicken
Chicken
Chicken
Chicken
Chicken
Chicken
Chicken
Chicken
Chicken
Chicken
Chicken
Chicken
Chicken
Chicken
Chicken
Chicken
Chicken
Chicken
Chicken
Chicken
Chicken

Chicken
Chicken
Chicken
Chicken
Chicken
Chicken
Chicken
Chicken
Chicken
Chicken
Chicken
Chicken
Chicken
Chicken
Chicken
Chicken
Chicken
Chicken
Chicken
Chicken
Chicken
Chicken
Chicken
Chicken
Chicken
Chicken
Chicken
Chicken
Chicken
Chicken
Chicken
Chicken
Chicken
Chicken
Chicken
Chicken
Chicken
Chicken
Chicken
Chicken
Chicken
Chicken
Chicken
Chicken
Chicken
Chicken

Chicken
Chicken
Chicken
Chicken
Chicken
Chicken
Chicken
Chicken
Chicken
Chicken
Chicken
Chicken
Chicken
Chicken
Chicken
Chicken
Chicken
Chicken
Chicken
Chicken
Chicken
Chicken
Chicken
Chicken
Chicken
Chicken
Chicken
Chicken
Chicken
Chicken
Chicken
Chicken
Chicken
Chicken
Chicken
Chicken
Chicken
Chicken
Chicken
Chicken
Chicken
Chicken
Chicken
Chicken
Chicken
Chicken

Chicken
Chicken
Chicken
Chicken
Chicken
Chicken
Chicken
Chicken
Chicken
Chicken
Chicken
Chicken
Chicken
Chicken
Chicken
Chicken
Chicken
Chicken
Chicken
Chicken
Chicken
Chicken
Chicken
Chicken
Chicken
Chicken
Chicken
Chicken
Chicken
Chicken
Chicken
Chicken
Chicken
Chicken
Chicken
Chicken
Chicken
Chicken
Chicken
Chicken
Chicken
Chicken
Chicken
Chicken
Chicken
Chicken

Chicken
Chicken
Chicken
Chicken
Chicken
Chicken
Chicken
Chicken
Chicken
Chicken
Chicken
Chicken
Chicken
Chicken
Chicken
Chicken
Chicken
Chicken
Chicken
Chicken
Chicken
Chicken
Chicken
Chicken
Chicken
Chicken
Chicken
Chicken
Chicken
Chicken
Chicken
Chicken
Chicken
Chicken
Chicken
Chicken
Chicken
Chicken
Chicken
Chicken
Chicken
Chicken
Chicken
Chicken
Chicken
Chicken

Chicken
Chicken
Chicken
Chicken
Chicken
Chicken
Chicken
Chicken
Chicken
Chicken
Chicken
Chicken
Chicken
Chicken
Chicken
Chicken
Chicken
Chicken
Chicken
Chicken
Chicken
Chicken
Chicken
Chicken
Chicken
Chicken
Chicken
Chicken
Chicken
Chicken
Chicken
Chicken
Chicken
Chicken
Chicken
Chicken
Chicken
Chicken
Chicken
Chicken
Chicken
Chicken
Chicken
Chicken
Chicken
Chicken

Chicken
Chicken
Chicken
Chicken
Chicken
Chicken
Chicken
Chicken
Chicken
Chicken
Chicken
Chicken
Chicken
Chicken
Chicken
Chicken
Chicken
Chicken
Chicken
Chicken
Chicken
Chicken
Chicken
Chicken
Chicken
Chicken
Chicken
Chicken
Chicken
Chicken
Chicken
Chicken
Chicken
Chicken
Chicken
Chicken
Chicken
Chicken
Chicken
Chicken
Chicken
Chicken
Chicken
Chicken
Chicken
Chicken

Chicken
Chicken
Chicken
Chicken
Chicken
Chicken
Chicken
Chicken
Chicken
Chicken
Chicken
Chicken
Chicken
Chicken
Chicken
Chicken
Chicken
Chicken
Chicken
Chicken
Chicken
Chicken
Chicken
Chicken
Chicken
Chicken
Chicken
Chicken
Chicken
Chicken
Chicken
Chicken
Chicken
Chicken
Chicken
Chicken
Chicken
Chicken
Chicken
Chicken
Chicken
Chicken
Chicken
Chicken
Chicken
Chicken

Chicken
Chicken
Chicken
Chicken
Chicken
Chicken
Chicken
Chicken
Chicken
Chicken
Chicken
Chicken
Chicken
Chicken
Chicken
Chicken
Chicken
Chicken
Chicken
Chicken
Chicken
Chicken
Chicken
Chicken
Chicken
Chicken
Chicken
Chicken
Chicken
Chicken
Chicken
Chicken
Chicken
Chicken
Chicken
Chicken
Chicken
Chicken
Chicken
Chicken
Chicken
Chicken
Chicken
Chicken
Chicken
Chicken

Chicken
Chicken
Chicken
Chicken
Chicken
Chicken
Chicken
Chicken
Chicken
Chicken
Chicken
Chicken
Chicken
Chicken
Chicken
Chicken
Chicken
Chicken
Chicken
Chicken
Chicken
Chicken
Chicken
Chicken
Chicken
Chicken
Chicken
Chicken
Chicken
Chicken
Chicken
Chicken
Chicken
Chicken
Chicken
Chicken
Chicken
Chicken
Chicken
Chicken
Chicken
Chicken
Chicken
Chicken
Chicken
Chicken

Chicken
Chicken
Chicken
Chicken
Chicken
Chicken
Chicken
Chicken
Chicken
Chicken
Chicken
Chicken
Chicken
Chicken
Chicken
Chicken
Chicken
Chicken
Chicken
Chicken
Chicken
Chicken
Chicken
Chicken
Chicken
Chicken
Chicken
Chicken
Chicken
Chicken
Chicken
Chicken
Chicken
Chicken
Chicken
Chicken
Chicken
Chicken
Chicken
Chicken
Chicken
Chicken
Chicken
Chicken
Chicken
Chicken

Chicken
Chicken
Chicken
Chicken
Chicken
Chicken
Chicken
Chicken
Chicken
Chicken
Chicken
Chicken
Chicken
Chicken
Chicken
Chicken
Chicken
Chicken
Chicken
Chicken
Chicken
Chicken
Chicken
Chicken
Chicken
Chicken
Chicken
Chicken
Chicken
Chicken
Chicken
Chicken
Chicken
Chicken
Chicken
Chicken
Chicken
Chicken
Chicken
Chicken
Chicken
Chicken
Chicken
Chicken
Chicken
Chicken

Chicken
Chicken
Chicken
Chicken
Chicken
Chicken
Chicken
Chicken
Chicken
Chicken
Chicken
Chicken
Chicken
Chicken
Chicken
Chicken
Chicken
Chicken
Chicken
Chicken
Chicken
Chicken
Chicken
Chicken
Chicken
Chicken
Chicken
Chicken
Chicken
Chicken
Chicken
Chicken
Chicken
Chicken
Chicken
Chicken
Chicken
Chicken
Chicken
Chicken
Chicken
Chicken
Chicken
Chicken
Chicken
Chicken

Chicken
Chicken
Chicken
Chicken
Chicken
Chicken
Chicken
Chicken
Chicken
Chicken
Chicken
Chicken
Chicken
Chicken
Chicken
Chicken
Chicken
Chicken
Chicken
Chicken
Chicken
Chicken
Chicken
Chicken
Chicken
Chicken
Chicken
Chicken
Chicken
Chicken
Chicken
Chicken
Chicken
Chicken
Chicken
Chicken
Chicken
Chicken
Chicken
Chicken
Chicken
Chicken
Chicken
Chicken
Chicken
Chicken

Chicken
Chicken
Chicken
Chicken
Chicken
Chicken
Chicken
Chicken
Chicken
Chicken
Chicken
Chicken
Chicken
Chicken
Chicken
Chicken
Chicken
Chicken
Chicken
Chicken
Chicken
Chicken
Chicken
Chicken
Chicken
Chicken
Chicken
Chicken
Chicken
Chicken
Chicken
Chicken
Chicken
Chicken
Chicken
Chicken
Chicken
Chicken
Chicken
Chicken
Chicken
Chicken
Chicken
Chicken
Chicken
Chicken

Chicken
Chicken
Chicken
Chicken
Chicken
Chicken
Chicken
Chicken
Chicken
Chicken
Chicken
Chicken
Chicken
Chicken
Chicken
Chicken
Chicken
Chicken
Chicken
Chicken
Chicken
Chicken
Chicken
Chicken
Chicken
Chicken
Chicken
Chicken
Chicken
Chicken
Chicken
Chicken
Chicken
Chicken
Chicken
Chicken
Chicken
Chicken
Chicken
Chicken
Chicken
Chicken
Chicken
Chicken
Chicken
Chicken

Chicken
Chicken
Chicken
Chicken
Chicken
Chicken
Chicken
Chicken
Chicken
Chicken
Chicken
Chicken
Chicken
Chicken
Chicken
Chicken
Chicken
Chicken
Chicken
Chicken
Chicken
Chicken
Chicken
Chicken
Chicken
Chicken
Chicken
Chicken
Chicken
Chicken
Chicken
Chicken
Chicken
Chicken
Chicken
Chicken
Chicken
Chicken
Chicken
Chicken
Chicken
Chicken
Chicken
Chicken
Chicken
Chicken

Chicken
Chicken
Chicken
Chicken
Chicken
Chicken
Chicken
Chicken
Chicken
Chicken
Chicken
Chicken
Chicken
Chicken
Chicken
Chicken
Chicken
Chicken
Chicken
Chicken
Chicken
Chicken
Chicken
Chicken
Chicken
Chicken
Chicken
Chicken
Chicken
Chicken
Chicken
Chicken
Chicken
Chicken
Chicken
Chicken
Chicken
Chicken
Chicken
Chicken
Chicken
Chicken
Chicken
Chicken
Chicken
Chicken

Chicken
Chicken
Chicken
Chicken
Chicken
Chicken
Chicken
Chicken
Chicken
Chicken
Chicken
Chicken
Chicken
Chicken
Chicken
Chicken
Chicken
Chicken
Chicken
Chicken
Chicken
Chicken
Chicken
Chicken
Chicken
Chicken
Chicken
Chicken
Chicken
Chicken
Chicken
Chicken
Chicken
Chicken
Chicken
Chicken
Chicken
Chicken
Chicken
Chicken
Chicken
Chicken
Chicken
Chicken
Chicken
Chicken

Chicken
Chicken
Chicken
Chicken
Chicken
Chicken
Chicken
Chicken
Chicken
Chicken
Chicken
Chicken
Chicken
Chicken
Chicken
Chicken
Chicken
Chicken
Chicken
Chicken
Chicken
Chicken
Chicken
Chicken
Chicken
Chicken
Chicken
Chicken
Chicken
Chicken
Chicken
Chicken
Chicken
Chicken
Chicken
Chicken
Chicken
Chicken
Chicken
Chicken
Chicken
Chicken
Chicken
Chicken
Chicken
Chicken

Chicken
Chicken
Chicken
Chicken
Chicken
Chicken
Chicken
Chicken
Chicken
Chicken
Chicken
Chicken
Chicken
Chicken
Chicken
Chicken
Chicken
Chicken
Chicken
Chicken
Chicken
Chicken
Chicken
Chicken
Chicken
Chicken
Chicken
Chicken
Chicken
Chicken
Chicken
Chicken
Chicken
Chicken
Chicken
Chicken
Chicken
Chicken
Chicken
Chicken
Chicken
Chicken
Chicken
Chicken
Chicken
Chicken

Chicken
Chicken
Chicken
Chicken
Chicken
Chicken
Chicken
Chicken
Chicken
Chicken
Chicken
Chicken
Chicken
Chicken
Chicken
Chicken
Chicken
Chicken
Chicken
Chicken
Chicken
Chicken
Chicken
Chicken
Chicken
Chicken
Chicken
Chicken
Chicken
Chicken
Chicken
Chicken
Chicken
Chicken
Chicken
Chicken
Chicken
Chicken
Chicken
Chicken
Chicken
Chicken
Chicken
Chicken
Chicken
Chicken

Chicken
Chicken
Chicken
Chicken
Chicken
Chicken
Chicken
Chicken
Chicken
Chicken
Chicken
Chicken
Chicken
Chicken
Chicken
Chicken
Chicken
Chicken
Chicken
Chicken
Chicken
Chicken
Chicken
Chicken
Chicken
Chicken
Chicken
Chicken
Chicken
Chicken
Chicken
Chicken
Chicken
Chicken
Chicken
Chicken
Chicken
Chicken
Chicken
Chicken
Chicken
Chicken
Chicken
Chicken
Chicken
Chicken

Chicken
Chicken
Chicken
Chicken
Chicken
Chicken
Chicken
Chicken
Chicken
Chicken
Chicken
Chicken
Chicken
Chicken
Chicken
Chicken
Chicken
Chicken
Chicken
Chicken
Chicken
Chicken
Chicken
Chicken
Chicken
Chicken
Chicken
Chicken
Chicken
Chicken
Chicken
Chicken
Chicken
Chicken
Chicken
Chicken
Chicken
Chicken
Chicken
Chicken
Chicken
Chicken
Chicken
Chicken
Chicken
Chicken

Chicken
Chicken
Chicken
Chicken
Chicken
Chicken
Chicken
Chicken
Chicken
Chicken
Chicken
Chicken
Chicken
Chicken
Chicken
Chicken
Chicken
Chicken
Chicken
Chicken
Chicken
Chicken
Chicken
Chicken
Chicken
Chicken
Chicken
Chicken
Chicken
Chicken
Chicken
Chicken
Chicken
Chicken
Chicken
Chicken
Chicken
Chicken
Chicken
Chicken
Chicken
Chicken
Chicken
Chicken
Chicken
Chicken

Chicken
Chicken
Chicken
Chicken
Chicken
Chicken
Chicken
Chicken
Chicken
Chicken
Chicken
Chicken
Chicken
Chicken
Chicken
Chicken
Chicken
Chicken
Chicken
Chicken
Chicken
Chicken
Chicken
Chicken
Chicken
Chicken
Chicken
Chicken
Chicken
Chicken
Chicken
Chicken
Chicken
Chicken
Chicken
Chicken
Chicken
Chicken
Chicken
Chicken
Chicken
Chicken
Chicken
Chicken
Chicken
Chicken

Chicken
Chicken
Chicken
Chicken
Chicken
Chicken
Chicken
Chicken
Chicken
Chicken
Chicken
Chicken
Chicken
Chicken
Chicken
Chicken
Chicken
Chicken
Chicken
Chicken
Chicken
Chicken
Chicken
Chicken
Chicken
Chicken
Chicken
Chicken
Chicken
Chicken
Chicken
Chicken
Chicken
Chicken
Chicken
Chicken
Chicken
Chicken
Chicken
Chicken
Chicken
Chicken
Chicken
Chicken
Chicken
Chicken

Chicken
Chicken
Chicken
Chicken
Chicken
Chicken
Chicken
Chicken
Chicken
Chicken
Chicken
Chicken
Chicken
Chicken
Chicken
Chicken
Chicken
Chicken
Chicken
Chicken
Chicken
Chicken
Chicken
Chicken
Chicken
Chicken
Chicken
Chicken
Chicken
Chicken
Chicken
Chicken
Chicken
Chicken
Chicken
Chicken
Chicken
Chicken
Chicken
Chicken
Chicken
Chicken
Chicken
Chicken
Chicken
Chicken

Chicken
Chicken
Chicken
Chicken
Chicken
Chicken
Chicken
Chicken
Chicken
Chicken
Chicken
Chicken
Chicken
Chicken
Chicken
Chicken
Chicken
Chicken
Chicken
Chicken
Chicken
Chicken
Chicken
Chicken
Chicken
Chicken
Chicken
Chicken
Chicken
Chicken
Chicken
Chicken
Chicken
Chicken
Chicken
Chicken
Chicken
Chicken
Chicken
Chicken
Chicken
Chicken
Chicken
Chicken
Chicken
Chicken

Chicken
Chicken
Chicken
Chicken
Chicken
Chicken
Chicken
Chicken
Chicken
Chicken
Chicken
Chicken
Chicken
Chicken
Chicken
Chicken
Chicken
Chicken
Chicken
Chicken
Chicken
Chicken
Chicken
Chicken
Chicken
Chicken
Chicken
Chicken
Chicken
Chicken
Chicken
Chicken
Chicken
Chicken
Chicken
Chicken
Chicken
Chicken
Chicken
Chicken
Chicken
Chicken
Chicken
Chicken
Chicken
Chicken

Chicken
Chicken
Chicken
Chicken
Chicken
Chicken
Chicken
Chicken
Chicken
Chicken
Chicken
Chicken
Chicken
Chicken
Chicken
Chicken
Chicken
Chicken
Chicken
Chicken
Chicken
Chicken
Chicken
Chicken
Chicken
Chicken
Chicken
Chicken
Chicken
Chicken
Chicken
Chicken
Chicken
Chicken
Chicken
Chicken
Chicken
Chicken
Chicken
Chicken
Chicken
Chicken
Chicken
Chicken
Chicken
Chicken

Chicken
Chicken
Chicken
Chicken
Chicken
Chicken
Chicken
Chicken
Chicken
Chicken
Chicken
Chicken
Chicken
Chicken
Chicken
Chicken
Chicken
Chicken
Chicken
Chicken
Chicken
Chicken
Chicken
Chicken
Chicken
Chicken
Chicken
Chicken
Chicken
Chicken
Chicken
Chicken
Chicken
Chicken
Chicken
Chicken
Chicken
Chicken
Chicken
Chicken
Chicken
Chicken
Chicken
Chicken
Chicken
Chicken

Chicken
Chicken
Chicken
Chicken
Chicken
Chicken
Chicken
Chicken
Chicken
Chicken
Chicken
Chicken
Chicken
Chicken
Chicken
Chicken
Chicken
Chicken
Chicken
Chicken
Chicken
Chicken
Chicken
Chicken
Chicken
Chicken
Chicken
Chicken
Chicken
Chicken
Chicken
Chicken
Chicken
Chicken
Chicken
Chicken
Chicken
Chicken
Chicken
Chicken
Chicken
Chicken
Chicken
Chicken
Chicken
Chicken

Chicken
Chicken
Chicken
Chicken
Chicken
Chicken
Chicken
Chicken
Chicken
Chicken
Chicken
Chicken
Chicken
Chicken
Chicken
Chicken
Chicken
Chicken
Chicken
Chicken
Chicken
Chicken
Chicken
Chicken
Chicken
Chicken
Chicken
Chicken
Chicken
Chicken
Chicken
Chicken
Chicken
Chicken
Chicken
Chicken
Chicken
Chicken
Chicken
Chicken
Chicken
Chicken
Chicken
Chicken
Chicken
Chicken

Chicken
Chicken
Chicken
Chicken
Chicken
Chicken
Chicken
Chicken
Chicken
Chicken
Chicken
Chicken
Chicken
Chicken
Chicken
Chicken
Chicken
Chicken
Chicken
Chicken
Chicken
Chicken
Chicken
Chicken
Chicken
Chicken
Chicken
Chicken
Chicken
Chicken
Chicken
Chicken
Chicken
Chicken
Chicken
Chicken
Chicken
Chicken
Chicken
Chicken
Chicken
Chicken
Chicken
Chicken
Chicken
Chicken

Chicken
Chicken
Chicken
Chicken
Chicken
Chicken
Chicken
Chicken
Chicken
Chicken
Chicken
Chicken
Chicken
Chicken
Chicken
Chicken
Chicken
Chicken
Chicken
Chicken
Chicken
Chicken
Chicken
Chicken
Chicken
Chicken
Chicken
Chicken
Chicken
Chicken
Chicken
Chicken
Chicken
Chicken
Chicken
Chicken
Chicken
Chicken
Chicken
Chicken
Chicken
Chicken
Chicken
Chicken
Chicken
Chicken

Chicken
Chicken
Chicken
Chicken
Chicken
Chicken
Chicken
Chicken
Chicken
Chicken
Chicken
Chicken
Chicken
Chicken
Chicken
Chicken
Chicken
Chicken
Chicken
Chicken
Chicken
Chicken
Chicken
Chicken
Chicken
Chicken
Chicken
Chicken
Chicken
Chicken
Chicken
Chicken
Chicken
Chicken
Chicken
Chicken
Chicken
Chicken
Chicken
Chicken
Chicken
Chicken
Chicken
Chicken
Chicken
Chicken

Chicken
Chicken
Chicken
Chicken
Chicken
Chicken
Chicken
Chicken
Chicken
Chicken
Chicken
Chicken
Chicken
Chicken
Chicken
Chicken
Chicken
Chicken
Chicken
Chicken
Chicken
Chicken
Chicken
Chicken
Chicken
Chicken
Chicken
Chicken
Chicken
Chicken
Chicken
Chicken
Chicken
Chicken
Chicken
Chicken
Chicken
Chicken
Chicken
Chicken
Chicken
Chicken
Chicken
Chicken
Chicken
Chicken

Chicken
Chicken
Chicken
Chicken
Chicken
Chicken
Chicken
Chicken
Chicken
Chicken
Chicken
Chicken
Chicken
Chicken
Chicken
Chicken
Chicken
Chicken
Chicken
Chicken
Chicken
Chicken
Chicken
Chicken
Chicken
Chicken
Chicken
Chicken
Chicken
Chicken
Chicken
Chicken
Chicken
Chicken
Chicken
Chicken
Chicken
Chicken
Chicken
Chicken
Chicken
Chicken
Chicken
Chicken
Chicken
Chicken

Chicken
Chicken
Chicken
Chicken
Chicken
Chicken
Chicken
Chicken
Chicken
Chicken
Chicken
Chicken
Chicken
Chicken
Chicken
Chicken
Chicken
Chicken
Chicken
Chicken
Chicken
Chicken
Chicken
Chicken
Chicken
Chicken
Chicken
Chicken
Chicken
Chicken
Chicken
Chicken
Chicken
Chicken
Chicken
Chicken
Chicken
Chicken
Chicken
Chicken
Chicken
Chicken
Chicken
Chicken
Chicken
Chicken

Chicken
Chicken
Chicken
Chicken
Chicken
Chicken
Chicken
Chicken
Chicken
Chicken
Chicken
Chicken
Chicken
Chicken
Chicken
Chicken
Chicken
Chicken
Chicken
Chicken
Chicken
Chicken
Chicken
Chicken
Chicken
Chicken
Chicken
Chicken
Chicken
Chicken
Chicken
Chicken
Chicken
Chicken
Chicken
Chicken
Chicken
Chicken
Chicken
Chicken
Chicken
Chicken
Chicken
Chicken
Chicken
Chicken

Chicken
Chicken
Chicken
Chicken
Chicken
Chicken
Chicken
Chicken
Chicken
Chicken
Chicken
Chicken
Chicken
Chicken
Chicken
Chicken
Chicken
Chicken
Chicken
Chicken
Chicken
Chicken
Chicken
Chicken
Chicken
Chicken
Chicken
Chicken
Chicken
Chicken
Chicken
Chicken
Chicken
Chicken
Chicken
Chicken
Chicken
Chicken
Chicken
Chicken
Chicken
Chicken
Chicken
Chicken
Chicken
Chicken

Chicken
Chicken
Chicken
Chicken
Chicken
Chicken
Chicken
Chicken
Chicken
Chicken
Chicken
Chicken
Chicken
Chicken
Chicken
Chicken
Chicken
Chicken
Chicken
Chicken
Chicken
Chicken
Chicken
Chicken
Chicken
Chicken
Chicken
Chicken
Chicken
Chicken
Chicken
Chicken
Chicken
Chicken
Chicken
Chicken
Chicken
Chicken
Chicken
Chicken
Chicken
Chicken
Chicken
Chicken
Chicken
Chicken

Chicken
Chicken
Chicken
Chicken
Chicken
Chicken
Chicken
Chicken
Chicken
Chicken
Chicken
Chicken
Chicken
Chicken
Chicken
Chicken
Chicken
Chicken
Chicken
Chicken
Chicken
Chicken
Chicken
Chicken
Chicken
Chicken
Chicken
Chicken
Chicken
Chicken
Chicken
Chicken
Chicken
Chicken
Chicken
Chicken
Chicken
Chicken
Chicken
Chicken
Chicken
Chicken
Chicken
Chicken
Chicken
Chicken

Chicken
Chicken
Chicken
Chicken
Chicken
Chicken
Chicken
Chicken
Chicken
Chicken
Chicken
Chicken
Chicken
Chicken
Chicken
Chicken
Chicken
Chicken
Chicken
Chicken
Chicken
Chicken
Chicken
Chicken
Chicken
Chicken
Chicken
Chicken
Chicken
Chicken
Chicken
Chicken
Chicken
Chicken
Chicken
Chicken
Chicken
Chicken
Chicken
Chicken
Chicken
Chicken
Chicken
Chicken
Chicken
Chicken

Chicken
Chicken
Chicken
Chicken
Chicken
Chicken
Chicken
Chicken
Chicken
Chicken
Chicken
Chicken
Chicken
Chicken
Chicken
Chicken
Chicken
Chicken
Chicken
Chicken
Chicken
Chicken
Chicken
Chicken
Chicken
Chicken
Chicken
Chicken
Chicken
Chicken
Chicken
Chicken
Chicken
Chicken
Chicken
Chicken
Chicken
Chicken
Chicken
Chicken
Chicken
Chicken
Chicken
Chicken
Chicken
Chicken

Chicken
Chicken
Chicken
Chicken
Chicken
Chicken
Chicken
Chicken
Chicken
Chicken
Chicken
Chicken
Chicken
Chicken
Chicken
Chicken
Chicken
Chicken
Chicken
Chicken
Chicken
Chicken
Chicken
Chicken
Chicken
Chicken
Chicken
Chicken
Chicken
Chicken
Chicken
Chicken
Chicken
Chicken
Chicken
Chicken
Chicken
Chicken
Chicken
Chicken
Chicken
Chicken
Chicken
Chicken
Chicken
Chicken

Chicken
Chicken
Chicken
Chicken
Chicken
Chicken
Chicken
Chicken
Chicken
Chicken
Chicken
Chicken
Chicken
Chicken
Chicken
Chicken
Chicken
Chicken
Chicken
Chicken
Chicken
Chicken
Chicken
Chicken
Chicken
Chicken
Chicken
Chicken
Chicken
Chicken
Chicken
Chicken
Chicken
Chicken
Chicken
Chicken
Chicken
Chicken
Chicken
Chicken
Chicken
Chicken
Chicken
Chicken
Chicken
Chicken

Chicken
Chicken
Chicken
Chicken
Chicken
Chicken
Chicken
Chicken
Chicken
Chicken
Chicken
Chicken
Chicken
Chicken
Chicken
Chicken
Chicken
Chicken
Chicken
Chicken
Chicken
Chicken
Chicken
Chicken
Chicken
Chicken
Chicken
Chicken
Chicken
Chicken
Chicken
Chicken
Chicken
Chicken
Chicken
Chicken
Chicken
Chicken
Chicken
Chicken
Chicken
Chicken
Chicken
Chicken
Chicken
Chicken

Chicken
Chicken
Chicken
Chicken
Chicken
Chicken
Chicken
Chicken
Chicken
Chicken
Chicken
Chicken
Chicken
Chicken
Chicken
Chicken
Chicken
Chicken
Chicken
Chicken
Chicken
Chicken
Chicken
Chicken
Chicken
Chicken
Chicken
Chicken
Chicken
Chicken
Chicken
Chicken
Chicken
Chicken
Chicken
Chicken
Chicken
Chicken
Chicken
Chicken
Chicken
Chicken
Chicken
Chicken
Chicken
Chicken

Chicken
Chicken
Chicken
Chicken
Chicken
Chicken
Chicken
Chicken
Chicken
Chicken
Chicken
Chicken
Chicken
Chicken
Chicken
Chicken
Chicken
Chicken
Chicken
Chicken
Chicken
Chicken
Chicken
Chicken
Chicken
Chicken
Chicken
Chicken
Chicken
Chicken
Chicken
Chicken
Chicken
Chicken
Chicken
Chicken
Chicken
Chicken
Chicken
Chicken
Chicken
Chicken
Chicken
Chicken
Chicken
Chicken

Chicken
Chicken
Chicken
Chicken
Chicken
Chicken
Chicken
Chicken
Chicken
Chicken
Chicken
Chicken
Chicken
Chicken
Chicken
Chicken
Chicken
Chicken
Chicken
Chicken
Chicken
Chicken
Chicken
Chicken
Chicken
Chicken
Chicken
Chicken
Chicken
Chicken
Chicken
Chicken
Chicken
Chicken
Chicken
Chicken
Chicken
Chicken
Chicken
Chicken
Chicken
Chicken
Chicken
Chicken
Chicken
Chicken

Chicken
Chicken
Chicken
Chicken
Chicken
Chicken
Chicken
Chicken
Chicken
Chicken
Chicken
Chicken
Chicken
Chicken
Chicken
Chicken
Chicken
Chicken
Chicken
Chicken
Chicken
Chicken
Chicken
Chicken
Chicken
Chicken
Chicken
Chicken
Chicken
Chicken
Chicken
Chicken
Chicken
Chicken
Chicken
Chicken
Chicken
Chicken
Chicken
Chicken
Chicken
Chicken
Chicken
Chicken
Chicken
Chicken

Chicken
Chicken
Chicken
Chicken
Chicken
Chicken
Chicken
Chicken
Chicken
Chicken
Chicken
Chicken
Chicken
Chicken
Chicken
Chicken
Chicken
Chicken
Chicken
Chicken
Chicken
Chicken
Chicken
Chicken
Chicken
Chicken
Chicken
Chicken
Chicken
Chicken
Chicken
Chicken
Chicken
Chicken
Chicken
Chicken
Chicken
Chicken
Chicken
Chicken
Chicken
Chicken
Chicken
Chicken
Chicken
Chicken

Chicken
Chicken
Chicken
Chicken
Chicken
Chicken
Chicken
Chicken
Chicken
Chicken
Chicken
Chicken
Chicken
Chicken
Chicken
Chicken
Chicken
Chicken
Chicken
Chicken
Chicken
Chicken
Chicken
Chicken
Chicken
Chicken
Chicken
Chicken
Chicken
Chicken
Chicken
Chicken
Chicken
Chicken
Chicken
Chicken
Chicken
Chicken
Chicken
Chicken
Chicken
Chicken
Chicken
Chicken
Chicken
Chicken

Chicken
Chicken
Chicken
Chicken
Chicken
Chicken
Chicken
Chicken
Chicken
Chicken
Chicken
Chicken
Chicken
Chicken
Chicken
Chicken
Chicken
Chicken
Chicken
Chicken
Chicken
Chicken
Chicken
Chicken
Chicken
Chicken
Chicken
Chicken
Chicken
Chicken
Chicken
Chicken
Chicken
Chicken
Chicken
Chicken
Chicken
Chicken
Chicken
Chicken
Chicken
Chicken
Chicken
Chicken
Chicken
Chicken

Chicken
Chicken
Chicken
Chicken
Chicken
Chicken
Chicken
Chicken
Chicken
Chicken
Chicken
Chicken
Chicken
Chicken
Chicken
Chicken
Chicken
Chicken
Chicken
Chicken
Chicken
Chicken
Chicken
Chicken
Chicken
Chicken
Chicken
Chicken
Chicken
Chicken
Chicken
Chicken
Chicken
Chicken
Chicken
Chicken
Chicken
Chicken
Chicken
Chicken
Chicken
Chicken
Chicken
Chicken
Chicken
Chicken

Chicken
Chicken
Chicken
Chicken
Chicken
Chicken
Chicken
Chicken
Chicken
Chicken
Chicken
Chicken
Chicken
Chicken
Chicken
Chicken
Chicken
Chicken
Chicken
Chicken
Chicken
Chicken
Chicken
Chicken
Chicken
Chicken
Chicken
Chicken
Chicken
Chicken
Chicken
Chicken
Chicken
Chicken
Chicken
Chicken
Chicken
Chicken
Chicken
Chicken
Chicken
Chicken
Chicken
Chicken
Chicken
Chicken

Chicken
Chicken
Chicken
Chicken
Chicken
Chicken
Chicken
Chicken
Chicken
Chicken
Chicken
Chicken
Chicken
Chicken
Chicken
Chicken
Chicken
Chicken
Chicken
Chicken
Chicken
Chicken
Chicken
Chicken
Chicken
Chicken
Chicken
Chicken
Chicken
Chicken
Chicken
Chicken
Chicken
Chicken
Chicken
Chicken
Chicken
Chicken
Chicken
Chicken
Chicken
Chicken
Chicken
Chicken
Chicken
Chicken

Chicken
Chicken
Chicken
Chicken
Chicken
Chicken
Chicken
Chicken
Chicken
Chicken
Chicken
Chicken
Chicken
Chicken
Chicken
Chicken
Chicken
Chicken
Chicken
Chicken
Chicken
Chicken
Chicken
Chicken
Chicken
Chicken
Chicken
Chicken
Chicken
Chicken
Chicken
Chicken
Chicken
Chicken
Chicken
Chicken
Chicken
Chicken
Chicken
Chicken
Chicken
Chicken
Chicken
Chicken
Chicken
Chicken

Chicken
Chicken
Chicken
Chicken
Chicken
Chicken
Chicken
Chicken
Chicken
Chicken
Chicken
Chicken
Chicken
Chicken
Chicken
Chicken
Chicken
Chicken
Chicken
Chicken
Chicken
Chicken
Chicken
Chicken
Chicken
Chicken
Chicken
Chicken
Chicken
Chicken
Chicken
Chicken
Chicken
Chicken
Chicken
Chicken
Chicken
Chicken
Chicken
Chicken
Chicken
Chicken
Chicken
Chicken
Chicken
Chicken

Chicken
Chicken
Chicken
Chicken
Chicken
Chicken
Chicken
Chicken
Chicken
Chicken
Chicken
Chicken
Chicken
Chicken
Chicken
Chicken
Chicken
Chicken
Chicken
Chicken
Chicken
Chicken
Chicken
Chicken
Chicken
Chicken
Chicken
Chicken
Chicken
Chicken
Chicken
Chicken
Chicken
Chicken
Chicken
Chicken
Chicken
Chicken
Chicken
Chicken
Chicken
Chicken
Chicken
Chicken
Chicken
Chicken

Chicken
Chicken
Chicken
Chicken
Chicken
Chicken
Chicken
Chicken
Chicken
Chicken
Chicken
Chicken
Chicken
Chicken
Chicken
Chicken
Chicken
Chicken
Chicken
Chicken
Chicken
Chicken
Chicken
Chicken
Chicken
Chicken
Chicken
Chicken
Chicken
Chicken
Chicken
Chicken
Chicken
Chicken
Chicken
Chicken
Chicken
Chicken
Chicken
Chicken
Chicken
Chicken
Chicken
Chicken
Chicken
Chicken

Chicken
Chicken
Chicken
Chicken
Chicken
Chicken
Chicken
Chicken
Chicken
Chicken
Chicken
Chicken
Chicken
Chicken
Chicken
Chicken
Chicken
Chicken
Chicken
Chicken
Chicken
Chicken
Chicken
Chicken
Chicken
Chicken
Chicken
Chicken
Chicken
Chicken
Chicken
Chicken
Chicken
Chicken
Chicken
Chicken
Chicken
Chicken
Chicken
Chicken
Chicken
Chicken
Chicken
Chicken
Chicken
Chicken

Chicken
Chicken
Chicken
Chicken
Chicken
Chicken
Chicken
Chicken
Chicken
Chicken
Chicken
Chicken
Chicken
Chicken
Chicken
Chicken
Chicken
Chicken
Chicken
Chicken
Chicken
Chicken
Chicken
Chicken
Chicken
Chicken
Chicken
Chicken
Chicken
Chicken
Chicken
Chicken
Chicken
Chicken
Chicken
Chicken
Chicken
Chicken
Chicken
Chicken
Chicken
Chicken
Chicken
Chicken
Chicken
Chicken

Chicken
Chicken
Chicken
Chicken
Chicken
Chicken
Chicken
Chicken
Chicken
Chicken
Chicken
Chicken
Chicken
Chicken
Chicken
Chicken
Chicken
Chicken
Chicken
Chicken
Chicken
Chicken
Chicken
Chicken
Chicken
Chicken
Chicken
Chicken
Chicken
Chicken
Chicken
Chicken
Chicken
Chicken
Chicken
Chicken
Chicken
Chicken
Chicken
Chicken
Chicken
Chicken
Chicken
Chicken
Chicken
Chicken

Chicken
Chicken
Chicken
Chicken
Chicken
Chicken
Chicken
Chicken
Chicken
Chicken
Chicken
Chicken
Chicken
Chicken
Chicken
Chicken
Chicken
Chicken
Chicken
Chicken
Chicken
Chicken
Chicken
Chicken
Chicken
Chicken
Chicken
Chicken
Chicken
Chicken
Chicken
Chicken
Chicken
Chicken
Chicken
Chicken
Chicken
Chicken
Chicken
Chicken
Chicken
Chicken
Chicken
Chicken
Chicken
Chicken

Chicken
Chicken
Chicken
Chicken
Chicken
Chicken
Chicken
Chicken
Chicken
Chicken
Chicken
Chicken
Chicken
Chicken
Chicken
Chicken
Chicken
Chicken
Chicken
Chicken
Chicken
Chicken
Chicken
Chicken
Chicken
Chicken
Chicken
Chicken
Chicken
Chicken
Chicken
Chicken
Chicken
Chicken
Chicken
Chicken
Chicken
Chicken
Chicken
Chicken
Chicken
Chicken
Chicken
Chicken
Chicken
Chicken

Chicken
Chicken
Chicken
Chicken
Chicken
Chicken
Chicken
Chicken
Chicken
Chicken
Chicken
Chicken
Chicken
Chicken
Chicken
Chicken
Chicken
Chicken
Chicken
Chicken
Chicken
Chicken
Chicken
Chicken
Chicken
Chicken
Chicken
Chicken
Chicken
Chicken
Chicken
Chicken
Chicken
Chicken
Chicken
Chicken
Chicken
Chicken
Chicken
Chicken
Chicken
Chicken
Chicken
Chicken
Chicken
Chicken

Chicken
Chicken
Chicken
Chicken
Chicken
Chicken
Chicken
Chicken
Chicken
Chicken
Chicken
Chicken
Chicken
Chicken
Chicken
Chicken
Chicken
Chicken
Chicken
Chicken
Chicken
Chicken
Chicken
Chicken
Chicken
Chicken
Chicken
Chicken
Chicken
Chicken
Chicken
Chicken
Chicken
Chicken
Chicken
Chicken
Chicken
Chicken
Chicken
Chicken
Chicken
Chicken
Chicken
Chicken
Chicken
Chicken

Chicken
Chicken
Chicken
Chicken
Chicken
Chicken
Chicken
Chicken
Chicken
Chicken
Chicken
Chicken
Chicken
Chicken
Chicken
Chicken
Chicken
Chicken
Chicken
Chicken
Chicken
Chicken
Chicken
Chicken
Chicken
Chicken
Chicken
Chicken
Chicken
Chicken
Chicken
Chicken
Chicken
Chicken
Chicken
Chicken
Chicken
Chicken
Chicken
Chicken
Chicken
Chicken
Chicken
Chicken
Chicken
Chicken

Chicken
Chicken
Chicken
Chicken
Chicken
Chicken
Chicken
Chicken
Chicken
Chicken
Chicken
Chicken
Chicken
Chicken
Chicken
Chicken
Chicken
Chicken
Chicken
Chicken
Chicken
Chicken
Chicken
Chicken
Chicken
Chicken
Chicken
Chicken
Chicken
Chicken
Chicken
Chicken
Chicken
Chicken
Chicken
Chicken
Chicken
Chicken
Chicken
Chicken
Chicken
Chicken
Chicken
Chicken
Chicken
Chicken

Chicken
Chicken
Chicken
Chicken
Chicken
Chicken
Chicken
Chicken
Chicken
Chicken
Chicken
Chicken
Chicken
Chicken
Chicken
Chicken
Chicken
Chicken
Chicken
Chicken
Chicken
Chicken
Chicken
Chicken
Chicken
Chicken
Chicken
Chicken
Chicken
Chicken
Chicken
Chicken
Chicken
Chicken
Chicken
Chicken
Chicken
Chicken
Chicken
Chicken
Chicken
Chicken
Chicken
Chicken
Chicken
Chicken

Chicken
Chicken
Chicken
Chicken
Chicken
Chicken
Chicken
Chicken
Chicken
Chicken
Chicken
Chicken
Chicken
Chicken
Chicken
Chicken
Chicken
Chicken
Chicken
Chicken
Chicken
Chicken
Chicken
Chicken
Chicken
Chicken
Chicken
Chicken
Chicken
Chicken
Chicken
Chicken
Chicken
Chicken
Chicken
Chicken
Chicken
Chicken
Chicken
Chicken
Chicken
Chicken
Chicken
Chicken
Chicken
Chicken

Chicken
Chicken
Chicken
Chicken
Chicken
Chicken
Chicken
Chicken
Chicken
Chicken
Chicken
Chicken
Chicken
Chicken
Chicken
Chicken
Chicken
Chicken
Chicken
Chicken
Chicken
Chicken
Chicken
Chicken
Chicken
Chicken
Chicken
Chicken
Chicken
Chicken
Chicken
Chicken
Chicken
Chicken
Chicken
Chicken
Chicken
Chicken
Chicken
Chicken
Chicken
Chicken
Chicken
Chicken
Chicken
Chicken

Chicken
Chicken
Chicken
Chicken
Chicken
Chicken
Chicken
Chicken
Chicken
Chicken
Chicken
Chicken
Chicken
Chicken
Chicken
Chicken
Chicken
Chicken
Chicken
Chicken
Chicken
Chicken
Chicken
Chicken
Chicken
Chicken
Chicken
Chicken
Chicken
Chicken
Chicken
Chicken
Chicken
Chicken
Chicken
Chicken
Chicken
Chicken
Chicken
Chicken
Chicken
Chicken
Chicken
Chicken
Duck
Duck

Duck
Duck
Duck
Duck
Duck
Duck
Duck
Duck
Duck
Duck
Duck
Duck
Duck
Duck
Duck
Duck
Duck
Duck
Duck
Duck
Duck
Duck
Duck
Duck
Duck
Duck
Duck
Duck
Duck
Duck
Duck
Duck
Duck
Duck
Duck
Duck
Duck
Duck
Duck
Duck
Duck
Duck
Duck
Duck
Duck
Duck

Duck
Duck
Duck
Duck
Duck
Duck
Duck
Duck
Duck
Duck
Duck
Duck
Duck
Duck
Duck
Duck
Duck
Duck
Duck
Duck
Duck
Duck
Duck
Duck
Duck
Duck
Duck
Duck
Duck
Duck
Duck
Duck
Duck
Duck
Duck
Duck
Duck
Duck
Duck
Duck
Duck
Duck
Duck
Duck
Duck
Duck

Duck
Duck
Duck
Duck
Duck
Duck
Duck
Duck
Duck
Duck
Duck
Duck
Duck
Duck
Duck
Duck
Duck
Duck
Duck
Duck
Duck
Duck
Duck
Duck
Duck
Duck
Duck
Duck
Duck
Duck
Duck
Duck
Duck
Duck
Duck
Duck
Duck
Duck
Duck
Duck
Duck
Duck
Duck
Duck
Duck
Duck

Duck
Duck
Duck
Duck
Duck
Duck
Duck
Duck
Duck
Duck
Duck
Duck
Duck
Duck
Duck
Duck
Duck
Duck
Duck
Duck
Duck
Duck
Duck
Duck
Duck
Duck
Duck
Duck
Duck
Duck
Duck
Duck
Duck
Duck
Duck
Duck
Duck
Duck
Duck
Duck
Duck
Duck
Duck
Duck
Duck
Duck

Duck
Duck
Duck
Duck
Duck
Duck
Duck
Duck
Duck
Duck
Duck
Duck
Duck
Duck
Duck
Duck
Duck
Duck
Duck
Duck
Duck
Duck
Duck
Duck
Duck
Duck
Duck
Duck
Duck
Duck
Duck
Duck
Duck
Duck
Duck
Duck
Duck
Duck
Duck
Duck
Duck
Duck
Duck
Duck
Duck
Duck

Duck
Duck
Duck
Duck
Duck
Duck
Duck
Duck
Duck
Duck
Duck
Duck
Duck
Duck
Duck
Duck
Duck
Duck
Duck
Duck
Duck
Duck
Duck
Duck
Duck
Duck
Duck
Duck
Duck
Duck
Duck
Duck
Duck
Duck
Duck
Duck
Duck
Duck
Duck
Duck
Duck
Duck
Duck
Duck
Duck
Duck

Duck
Duck
Duck
Duck
Duck
Duck
Duck
Duck
Duck
Duck
Duck
Duck
Duck
Duck
Duck
Duck
Duck
Duck
Duck
Duck
Duck
Duck
Duck
Duck
Duck
Duck
Duck
Duck
Duck
Duck
Duck
Duck
Duck
Duck
Duck
Duck
Duck
Duck
Duck
Duck
Duck
Duck
Duck
Duck
Duck
Duck

Duck
Duck
Duck
Duck
Duck
Duck
Duck
Duck
Duck
Duck
Duck
Duck
Duck
Duck
Duck
Duck
Duck
Duck
Duck
Duck
Duck
Duck
Duck
Duck
Duck
Duck
Duck
Duck
Duck
Duck
Duck
Duck
Duck
Duck
Duck
Duck
Duck
Duck
Duck
Duck
Duck
Duck
Duck
Duck
Duck
Duck

Duck
Duck
Duck
Duck
Duck
Duck
Duck
Duck
Duck
Duck
Duck
Duck
Duck
Duck
Duck
Duck
Duck
Duck
Duck
Duck
Duck
Duck
Duck
Duck
Duck
Duck
Duck
Duck
Duck
Duck
Duck
Duck
Duck
Duck
Duck
Duck
Duck
Duck
Duck
Duck
Duck
Duck
Duck
Duck
Duck
Duck

Duck
Duck
Duck
Duck
Duck
Duck
Duck
Duck
Duck
Duck
Duck
Duck
Duck
Duck
Duck
Duck
Duck
Duck
Duck
Duck
Duck
Duck
Duck
Duck
Duck
Duck
Duck
Duck
Duck
Duck
Duck
Duck
Duck
Duck
Duck
Duck
Duck
Duck
Duck
Duck
Duck
Duck
Duck
Duck
Duck
Duck

Duck
Duck
Duck
Duck
Duck
Duck
Duck
Duck
Duck
Duck
Duck
Duck
Duck
Duck
Duck
Duck
Duck
Duck
Duck
Duck
Duck
Duck
Duck
Duck
Duck
Duck
Duck
Duck
Duck
Duck
Duck
Duck
Duck
Duck
Duck
Duck
Duck
Duck
Duck
Duck
Duck
Duck
Duck
Duck
Duck
Duck

Duck
Duck
Duck
Duck
Duck
Duck
Duck
Duck
Duck
Duck
Duck
Duck
Duck
Duck
Duck
Duck
Duck
Duck
Duck
Duck
Duck
Duck
Duck
Duck
Duck
Duck
Duck
Duck
Duck
Duck
Duck
Duck
Duck
Duck
Duck
Duck
Duck
Duck
Duck
Duck
Duck
Duck
Duck
Duck
Duck
Duck

Duck
Duck
Duck
Duck
Duck
Duck
Duck
Duck
Duck
Duck
Duck
Duck
Duck
Duck
Duck
Duck
Duck
Duck
Duck
Duck
Duck
Duck
Duck
Duck
Duck
Duck
Duck
Duck
Duck
Duck
Duck
Duck
Duck
Duck
Duck
Duck
Duck
Duck
Duck
Duck
Duck
Duck
Duck
Duck
Duck
Duck

Duck
Duck
Duck
Duck
Duck
Duck
Duck
Duck
Duck
Duck
Duck
Duck
Duck
Duck
Duck
Duck
Duck
Duck
Duck
Duck
Duck
Duck
Duck
Duck
Duck
Duck
Duck
Duck
Duck
Duck
Duck
Duck
Duck
Duck
Duck
Duck
Duck
Duck
Duck
Duck
Duck
Duck
Duck
Duck
Duck
Duck

Duck
Duck
Duck
Duck
Duck
Duck
Duck
Duck
Duck
Duck
Duck
Duck
Duck
Duck
Duck
Duck
Duck
Duck
Duck
Duck
Duck
Duck
Duck
Duck
Duck
Duck
Duck
Duck
Duck
Duck
Duck
Duck
Duck
Duck
Duck
Duck
Duck
Duck
Duck
Duck
Duck
Duck
Duck
Duck
Duck
Duck

Duck
Duck
Duck
Duck
Duck
Duck
Duck
Duck
Duck
Duck
Duck
Duck
Duck
Duck
Duck
Duck
Duck
Duck
Duck
Duck
Duck
Duck
Duck
Duck
Duck
Duck
Duck
Duck
Duck
Duck
Duck
Duck
Duck
Duck
Duck
Duck
Duck
Duck
Duck
Duck
Duck
Duck
Duck
Duck
Duck
Duck

Duck
Duck
Duck
Duck
Duck
Duck
Duck
Duck
Duck
Duck
Duck
Duck
Duck
Duck
Duck
Duck
Duck
Duck
Duck
Duck
Duck
Duck
Duck
Duck
Duck
Duck
Duck
Duck
Duck
Duck
Duck
Duck
Duck
Duck
Duck
Duck
Duck
Duck
Duck
Duck
Duck
Duck
Duck
Duck
Duck
Duck

Duck
Duck
Duck
Duck
Duck
Duck
Duck
Duck
Duck
Duck
Duck
Duck
Duck
Duck
Duck
Duck
Duck
Duck
Duck
Duck
Duck
Duck
Duck
Duck
Duck
Duck
Duck
Duck
Duck
Duck
Duck
Duck
Duck
Duck
Duck
Duck
Duck
Duck
Duck
Duck
Duck
Duck
Duck
Duck
Duck
Duck

Duck
Duck
Duck
Duck
Duck
Duck
Duck
Duck
Duck
Duck
Duck
Duck
Duck
Duck
Duck
Duck
Duck
Duck
Duck
Duck
Duck
Duck
Duck
Duck
Duck
Duck
Duck
Duck
Duck
Duck
Duck
Duck
Duck
Duck
Duck
Duck
Duck
Duck
Duck
Duck
Duck
Duck
Duck
Duck
Duck
Duck

Duck
Duck
Duck
Duck
Duck
Duck
Duck
Duck
Duck
Duck
Duck
Duck
Duck
Duck
Duck
Duck
Duck
Duck
Duck
Duck
Duck
Duck
Duck
Duck
Duck
Duck
Duck
Duck
Duck
Duck
Duck
Duck
Duck
Duck
Duck
Duck
Duck
Duck
Duck
Duck
Duck
Duck
Duck
Duck
Duck
Duck

Duck
Duck
Duck
Duck
Duck
Duck
Duck
Duck
Duck
Duck
Duck
Duck
Duck
Duck
Duck
Duck
Duck
Duck
Duck
Duck
Duck
Duck
Duck
Duck
Duck
Duck
Duck
Duck
Duck
Duck
Duck
Duck
Duck
Duck
Duck
Duck
Duck
Duck
Duck
Duck
Duck
Duck
Duck
Duck
Duck
Duck

Duck
Duck
Duck
Duck
Duck
Duck
Duck
Duck
Duck
Duck
Duck
Duck
Duck
Duck
Duck
Duck
Duck
Duck
Duck
Duck
Duck
Duck
Duck
Duck
Duck
Duck
Duck
Duck
Duck
Duck
Duck
Duck
Duck
Duck
Duck
Duck
Duck
Duck
Duck
Duck
Duck
Duck
Duck
Duck
Duck
Duck

Duck
Duck
Duck
Duck
Duck
Duck
Duck
Duck
Duck
Duck
Duck
Duck
Duck
Duck
Duck
Duck
Duck
Duck
Duck
Duck
Duck
Duck
Duck
Duck
Duck
Duck
Duck
Duck
Duck
Duck
Duck
Duck
Duck
Duck
Duck
Duck
Duck
Duck
Duck
Duck
Duck
Duck
Duck
Duck
Duck
Duck

Duck
Duck
Duck
Duck
Duck
Duck
Duck
Duck
Duck
Duck
Duck
Duck
Duck
Duck
Duck
Duck
Duck
Duck
Duck
Duck
Duck
Duck
Duck
Duck
Duck
Duck
Duck
Duck
Duck
Duck
Duck
Duck
Duck
Duck
Duck
Duck
Duck
Duck
Duck
Duck
Duck
Duck
Duck
Duck
Duck
Duck

Duck
Duck
Duck
Duck
Duck
Duck
Duck
Duck
Duck
Duck
Duck
Duck
Duck
Duck
Duck
Duck
Duck
Duck
Duck
Duck
Duck
Duck
Duck
Duck
Duck
Duck
Duck
Duck
Duck
Duck
Duck
Duck
Duck
Duck
Duck
Duck
Duck
Duck
Duck
Duck
Duck
Duck
Duck
Duck
Duck
Duck

Duck
Duck
Duck
Duck
Duck
Duck
Duck
Duck
Duck
Duck
Duck
Duck
Duck
Duck
Duck
Duck
Duck
Duck
Duck
Duck
Duck
Duck
Duck
Duck
Duck
Duck
Duck
Duck
Duck
Duck
Duck
Duck
Duck
Duck
Duck
Duck
Duck
Duck
Duck
Duck
Duck
Duck
Duck
Duck
Duck
Duck

Duck
Duck
Duck
Duck
Duck
Duck
Duck
Duck
Duck
Duck
Duck
Duck
Duck
Duck
Duck
Duck
Duck
Duck
Duck
Duck
Duck
Duck
Duck
Duck
Duck
Duck
Duck
Duck
Duck
Duck
Duck
Duck
Duck
Duck
Duck
Duck
Duck
Duck
Duck
Duck
Duck
Duck
Duck
Duck
Duck
Duck

Duck
Duck
Duck
Duck
Duck
Duck
Duck
Duck
Duck
Duck
Duck
Duck
Duck
Duck
Duck
Duck
Duck
Duck
Duck
Duck
Duck
Duck
Duck
Duck
Duck
Duck
Duck
Duck
Duck
Duck
Duck
Duck
Duck
Duck
Duck
Duck
Duck
Duck
Duck
Duck
Duck
Duck
Duck
Duck
Duck
Duck

Duck
Duck
Duck
Duck
Duck
Duck
Duck
Duck
Duck
Duck
Duck
Duck
Duck
Duck
Duck
Duck
Duck
Duck
Duck
Duck
Duck
Duck
Duck
Duck
Duck
Duck
Duck
Duck
Duck
Duck
Duck
Duck
Duck
Duck
Duck
Duck
Duck
Duck
Duck
Duck
Duck
Duck
Duck
Duck
Duck
Duck

Duck
Duck
Duck
Duck
Duck
Duck
Duck
Duck
Duck
Duck
Duck
Duck
Duck
Duck
Duck
Duck
Duck
Duck
Duck
Duck
Duck
Duck
Duck
Duck
Duck
Duck
Duck
Duck
Duck
Duck
Duck
Duck
Duck
Duck
Duck
Duck
Duck
Duck
Duck
Duck
Duck
Duck
Duck
Duck
Duck
Duck

Duck
Duck
Duck
Duck
Duck
Duck
Duck
Duck
Duck
Duck
Duck
Duck
Duck
Duck
Duck
Duck
Duck
Duck
Duck
Duck
Duck
Duck
Duck
Duck
Duck
Duck
Duck
Duck
Duck
Duck
Duck
Duck
Duck
Duck
Duck
Duck
Duck
Duck
Duck
Duck
Duck
Duck
Duck
Duck
Duck
Duck

Duck
Duck
Duck
Duck
Duck
Duck
Duck
Duck
Duck
Duck
Duck
Duck
Duck
Duck
Duck
Duck
Duck
Duck
Duck
Duck
Duck
Duck
Duck
Duck
Duck
Duck
Duck
Duck
Duck
Duck
Duck
Duck
Duck
Duck
Duck
Duck
Duck
Duck
Duck
Duck
Duck
Duck
Duck
Duck
Duck
Duck

Duck
Duck
Duck
Duck
Duck
Duck
Duck
Duck
Duck
Duck
Duck
Duck
Duck
Duck
Duck
Duck
Duck
Duck
Duck
Duck
Duck
Duck
Duck
Duck
Duck
Duck
Duck
Duck
Duck
Duck
Duck
Duck
Duck
Duck
Duck
Duck
Duck
Duck
Duck
Duck
Duck
Duck
Duck
Duck
Duck
Duck

Duck
Duck
Duck
Duck
Duck
Duck
Duck
Duck
Duck
Duck
Duck
Duck
Duck
Duck
Duck
Duck
Duck
Duck
Duck
Duck
Duck
Duck
Duck
Duck
Duck
Duck
Duck
Duck
Duck
Duck
Duck
Duck
Duck
Duck
Duck
Duck
Duck
Duck
Duck
Duck
Duck
Duck
Duck
Duck
Duck
Duck

Duck
Duck
Duck
Duck
Duck
Duck
Duck
Duck
Duck
Duck
Duck
Duck
Duck
Duck
Duck
Duck
Duck
Duck
Duck
Duck
Duck
Duck
Duck
Duck
Duck
Duck
Duck
Duck
Duck
Duck
Duck
Duck
Duck
Duck
Duck
Duck
Duck
Duck
Duck
Duck
Duck
Duck
Duck
Duck
Duck
Duck

Duck
Duck
Duck
Duck
Duck
Duck
Duck
Duck
Duck
Duck
Duck
Duck
Duck
Duck
Duck
Duck
Duck
Duck
Duck
Duck
Duck
Duck
Duck
Duck
Duck
Duck
Duck
Duck
Duck
Duck
Duck
Duck
Duck
Duck
Duck
Duck
Duck
Duck
Duck
Duck
Duck
Duck
Duck
Duck
Duck
Duck

Duck
Duck
Duck
Duck
Duck
Duck
Duck
Duck
Duck
Duck
Duck
Duck
Duck
Duck
Duck
Duck
Duck
Duck
Duck
Duck
Duck
Duck
Duck
Duck
Duck
Duck
Duck
Duck
Duck
Duck
Duck
Duck
Duck
Duck
Duck
Duck
Duck
Duck
Duck
Duck
Duck
Duck
Duck
Duck
Duck
Duck

Duck
Duck
Duck
Duck
Duck
Duck
Duck
Duck
Duck
Duck
Duck
Duck
Duck
Duck
Duck
Duck
Duck
Duck
Duck
Duck
Duck
Duck
Duck
Duck
Duck
Duck
Duck
Duck
Duck
Duck
Duck
Duck
Duck
Duck
Duck
Duck
Duck
Duck
Duck
Duck
Duck
Duck
Duck
Duck
Duck
Duck

Duck
Duck
Duck
Duck
Duck
Duck
Duck
Duck
Duck
Duck
Duck
Duck
Duck
Duck
Duck
Duck
Duck
Duck
Duck
Duck
Duck
Duck
Duck
Duck
Duck
Duck
Duck
Duck
Duck
Duck
Duck
Duck
Duck
Duck
Duck
Duck
Duck
Duck
Duck
Duck
Duck
Duck
Duck
Duck
Duck
Duck

Duck
Duck
Duck
Duck
Duck
Duck
Duck
Duck
Duck
Duck
Duck
Duck
Duck
Duck
Duck
Duck
Duck
Duck
Duck
Duck
Duck
Duck
Duck
Duck
Duck
Duck
Duck
Duck
Duck
Duck
Duck
Duck
Duck
Duck
Duck
Duck
Duck
Duck
Duck
Duck
Duck
Duck
Duck
Duck
Duck
Duck

Duck
Duck
Duck
Duck
Duck
Duck
Duck
Duck
Duck
Duck
Duck
Duck
Duck
Duck
Duck
Duck
Duck
Duck
Duck
Duck
Duck
Duck
Duck
Duck
Duck
Duck
Duck
Duck
Duck
Duck
Duck
Duck
Duck
Duck
Duck
Duck
Duck
Duck
Duck
Duck
Duck
Duck
Duck
Duck
Duck
Duck

Duck
Duck
Duck
Duck
Duck
Duck
Duck
Duck
Duck
Duck
Duck
Duck
Duck
Duck
Duck
Duck
Duck
Duck
Duck
Duck
Duck
Duck
Duck
Duck
Duck
Duck
Duck
Duck
Duck
Duck
Duck
Duck
Duck
Duck
Duck
Duck
Duck
Duck
Duck
Duck
Duck
Duck
Duck
Duck
Duck
Duck

Duck
Duck
Duck
Duck
Duck
Duck
Duck
Duck
Duck
Duck
Duck
Duck
Duck
Duck
Duck
Duck
Duck
Duck
Duck
Duck
Duck
Duck
Duck
Duck
Duck
Duck
Duck
Duck
Duck
Duck
Duck
Duck
Duck
Duck
Duck
Duck
Duck
Duck
Duck
Duck
Duck
Duck
Duck
Duck
Duck
Duck

Duck
Duck
Duck
Duck
Duck
Duck
Duck
Duck
Duck
Duck
Duck
Duck
Duck
Duck
Duck
Duck
Duck
Duck
Duck
Duck
Duck
Duck
Duck
Duck
Duck
Duck
Duck
Duck
Duck
Duck
Duck
Duck
Duck
Duck
Duck
Duck
Duck
Duck
Duck
Duck
Duck
Duck
Duck
Duck
Duck
Duck

Duck
Duck
Duck
Duck
Duck
Duck
Duck
Duck
Duck
Duck
Duck
Duck
Duck
Duck
Duck
Duck
Duck
Duck
Duck
Duck
Duck
Duck
Duck
Duck
Duck
Duck
Duck
Duck
Duck
Duck
Duck
Duck
Duck
Duck
Duck
Duck
Duck
Duck
Duck
Duck
Duck
Duck
Duck
Duck
Duck
Duck

Duck
Duck
Duck
Duck
Duck
Duck
Duck
Duck
Duck
Duck
Duck
Duck
Duck
Duck
Duck
Duck
Duck
Duck
Duck
Duck
Duck
Duck
Duck
Duck
Duck
Duck
Duck
Duck
Duck
Duck
Duck
Duck
Duck
Duck
Duck
Duck
Duck
Duck
Duck
Duck
Duck
Duck
Duck
Duck
Duck
Duck

Duck
Duck
Duck
Duck
Duck
Duck
Duck
Duck
Duck
Duck
Duck
Duck
Duck
Duck
Duck
Duck
Duck
Duck
Duck
Duck
Duck
Duck
Duck
Duck
Duck
Duck
Duck
Duck
Duck
Duck
Duck
Duck
Duck
Duck
Duck
Duck
Duck
Duck
Duck
Duck
Duck
Duck
Duck
Duck
Duck
Duck

Duck
Duck
Duck
Duck
Duck
Duck
Duck
Duck
Duck
Duck
Duck
Duck
Duck
Duck
Duck
Duck
Duck
Duck
Duck
Duck
Duck
Duck
Duck
Duck
Duck
Duck
Duck
Duck
Duck
Duck
Duck
Duck
Duck
Duck
Duck
Duck
Duck
Duck
Duck
Duck
Duck
Duck
Duck
Duck
Duck
Duck

Duck
Duck
Duck
Duck
Duck
Duck
Duck
Duck
Duck
Duck
Duck
Duck
Duck
Duck
Duck
Duck
Duck
Duck
Duck
Duck
Duck
Duck
Duck
Duck
Duck
Duck
Duck
Duck
Duck
Duck
Duck
Duck
Duck
Duck
Duck
Duck
Duck
Duck
Duck
Duck
Duck
Duck
Duck
Duck
Duck
Duck

Duck
Duck
Duck
Duck
Duck
Duck
Duck
Duck
Duck
Duck
Duck
Duck
Duck
Duck
Duck
Duck
Duck
Duck
Duck
Duck
Duck
Duck
Duck
Duck
Duck
Duck
Duck
Duck
Duck
Duck
Duck
Duck
Duck
Duck
Duck
Duck
Duck
Duck
Duck
Duck
Duck
Duck
Duck
Duck
Duck
Duck

Duck
Duck
Duck
Duck
Duck
Duck
Duck
Duck
Duck
Duck
Duck
Duck
Duck
Duck
Duck
Duck
Duck
Duck
Duck
Duck
Duck
Duck
Duck
Duck
Duck
Duck
Duck
Duck
Duck
Duck
Duck
Duck
Duck
Duck
Duck
Duck
Duck
Duck
Duck
Duck
Duck
Duck
Duck
Duck
Duck
Duck

Duck
Duck
Duck
Duck
Duck
Duck
Duck
Duck
Duck
Duck
Duck
Duck
Duck
Duck
Duck
Duck
Duck
Duck
Duck
Duck
Duck
Duck
Duck
Duck
Duck
Duck
Duck
Duck
Duck
Duck
Duck
Duck
Duck
Duck
Duck
Duck
Duck
Duck
Duck
Duck
Duck
Duck
Duck
Duck
Duck
Duck

Duck
Duck
Duck
Duck
Duck
Duck
Duck
Duck
Duck
Duck
Duck
Duck
Duck
Duck
Duck
Duck
Duck
Duck
Duck
Duck
Duck
Duck
Duck
Duck
Duck
Duck
Duck
Duck
Duck
Duck
Duck
Duck
Duck
Duck
Duck
Duck
Duck
Duck
Duck
Duck
Duck
Duck
Duck
Duck
Duck
Duck

Duck
Duck
Duck
Duck
Duck
Duck
Duck
Duck
Duck
Duck
Duck
Duck
Duck
Duck
Duck
Duck
Duck
Duck
Duck
Duck
Duck
Duck
Duck
Duck
Duck
Duck
Duck
Duck
Duck
Duck
Duck
Duck
Duck
Duck
Duck
Duck
Duck
Duck
Duck
Duck
Duck
Duck
Duck
Duck
Duck
Duck

Duck
Duck
Duck
Duck
Duck
Duck
Duck
Duck
Duck
Duck
Duck
Duck
Duck
Duck
Duck
Duck
Duck
Duck
Duck
Duck
Duck
Duck
Duck
Duck
Duck
Duck
Duck
Duck
Duck
Duck
Duck
Duck
Duck
Duck
Duck
Duck
Duck
Duck
Duck
Duck
Duck
Duck
Duck
Duck
Duck
Duck

Duck
Duck
Duck
Duck
Duck
Duck
Duck
Duck
Duck
Duck
Duck
Duck
Duck
Duck
Duck
Duck
Duck
Duck
Duck
Duck
Duck
Duck
Duck
Duck
Duck
Duck
Duck
Duck
Duck
Duck
Duck
Duck
Duck
Duck
Duck
Duck
Duck
Duck
Duck
Duck
Duck
Duck
Duck
Duck
Duck
Duck

Duck
Duck
Duck
Duck
Duck
Duck
Duck
Duck
Duck
Duck
Duck
Duck
Duck
Duck
Duck
Duck
Duck
Duck
Duck
Duck
Duck
Duck
Duck
Duck
Duck
Duck
Duck
Duck
Duck
Duck
Duck
Duck
Duck
Duck
Duck
Duck
Duck
Duck
Duck
Duck
Duck
Duck
Duck
Duck
Duck
Duck

Duck
Duck
Duck
Duck
Duck
Duck
Duck
Duck
Duck
Duck
Duck
Duck
Duck
Duck
Duck
Duck
Duck
Duck
Duck
Duck
Duck
Duck
Duck
Duck
Duck
Duck
Duck
Duck
Duck
Duck
Duck
Duck
Duck
Duck
Duck
Duck
Duck
Duck
Duck
Duck
Duck
Duck
Duck
Duck
Duck
Duck

Duck
Duck
Duck
Duck
Duck
Duck
Duck
Duck
Duck
Duck
Duck
Duck
Duck
Duck
Duck
Duck
Duck
Duck
Duck
Duck
Duck
Duck
Duck
Duck
Duck
Duck
Duck
Duck
Duck
Duck
Duck
Duck
Duck
Duck
Duck
Duck
Duck
Duck
Duck
Duck
Duck
Duck
Duck
Duck
Duck
Duck

Duck
Duck
Duck
Duck
Duck
Duck
Duck
Duck
Duck
Duck
Duck
Duck
Duck
Duck
Duck
Duck
Duck
Duck
Duck
Duck
Duck
Duck
Duck
Duck
Duck
Duck
Duck
Duck
Duck
Duck
Duck
Duck
Duck
Duck
Duck
Duck
Duck
Duck
Duck
Duck
Duck
Duck
Duck
Duck
Duck
Duck

Duck
Duck
Duck
Duck
Duck
Duck
Duck
Duck
Duck
Duck
Duck
Duck
Duck
Duck
Duck
Duck
Duck
Duck
Duck
Duck
Duck
Duck
Duck
Duck
Duck
Duck
Duck
Duck
Duck
Duck
Duck
Duck
Duck
Duck
Duck
Duck
Duck
Duck
Duck
Duck
Duck
Duck
Duck
Duck
Duck
Duck

Duck
Duck
Duck
Duck
Duck
Duck
Duck
Duck
Duck
Duck
Duck
Duck
Duck
Duck
Duck
Duck
Duck
Duck
Duck
Duck
Duck
Duck
Duck
Duck
Duck
Duck
Duck
Duck
Duck
Duck
Duck
Duck
Duck
Duck
Duck
Duck
Duck
Duck
Duck
Duck
Duck
Duck
Duck
Duck
Duck
Duck

Duck
Duck
Duck
Duck
Duck
Duck
Duck
Duck
Duck
Duck
Duck
Duck
Duck
Duck
Duck
Duck
Duck
Duck
Duck
Duck
Duck
Duck
Duck
Duck
Duck
Duck
Duck
Duck
Duck
Duck
Duck
Duck
Duck
Duck
Duck
Duck
Duck
Duck
Duck
Duck
Duck
Duck
Duck
Duck
Duck
Duck

Duck
Duck
Duck
Duck
Duck
Duck
Duck
Duck
Duck
Duck
Duck
Duck
Duck
Duck
Duck
Duck
Duck
Duck
Duck
Duck
Duck
Duck
Duck
Duck
Duck
Duck
Duck
Duck
Duck
Duck
Duck
Duck
Duck
Duck
Duck
Duck
Duck
Duck
Duck
Duck
Duck
Duck
Duck
Duck
Duck
Duck

Duck
Duck
Duck
Duck
Duck
Duck
Duck
Duck
Duck
Duck
Duck
Duck
Duck
Duck
Duck
Duck
Duck
Duck
Duck
Duck
Duck
Duck
Duck
Duck
Duck
Duck
Duck
Duck
Duck
Duck
Duck
Duck
Duck
Duck
Duck
Duck
Duck
Duck
Duck
Duck
Duck
Duck
Duck
Duck
Duck
Duck

Duck
Duck
Duck
Duck
Duck
Duck
Duck
Duck
Duck
Duck
Duck
Duck
Duck
Duck
Duck
Duck
Duck
Duck
Duck
Duck
Duck
Duck
Duck
Duck
Duck
Duck
Duck
Duck
Duck
Duck
Duck
Duck
Duck
Duck
Duck
Duck
Duck
Duck
Duck
Duck
Duck
Duck
Duck
Duck
Duck
Duck

Duck
Duck
Duck
Duck
Duck
Duck
Duck
Duck
Duck
Duck
Duck
Duck
Duck
Duck
Duck
Duck
Duck
Duck
Duck
Duck
Duck
Duck
Duck
Duck
Duck
Duck
Duck
Duck
Duck
Duck
Duck
Duck
Duck
Duck
Duck
Duck
Duck
Duck
Duck
Duck
Duck
Duck
Duck
Duck
Duck
Duck

Duck
Duck
Duck
Duck
Duck
Duck
Duck
Duck
Duck
Duck
Duck
Duck
Duck
Duck
Duck
Duck
Duck
Duck
Duck
Duck
Duck
Duck
Duck
Duck
Duck
Duck
Duck
Duck
Duck
Duck
Duck
Duck
Duck
Duck
Duck
Duck
Duck
Duck
Duck
Duck
Duck
Duck
Duck
Duck
Duck
Duck

Duck
Duck
Duck
Duck
Duck
Duck
Duck
Duck
Duck
Duck
Duck
Duck
Duck
Duck
Duck
Duck
Duck
Duck
Duck
Duck
Duck
Duck
Duck
Duck
Duck
Duck
Duck
Duck
Duck
Duck
Duck
Duck
Duck
Duck
Duck
Duck
Duck
Duck
Duck
Duck
Duck
Duck
Duck
Duck
Duck
Duck

Duck
Duck
Duck
Duck
Duck
Duck
Duck
Duck
Duck
Duck
Duck
Duck
Duck
Duck
Duck
Duck
Duck
Duck
Duck
Duck
Duck
Duck
Duck
Duck
Duck
Duck
Duck
Duck
Duck
Duck
Duck
Duck
Duck
Duck
Duck
Duck
Duck
Duck
Duck
Duck
Duck
Duck
Duck
Duck
Duck
Duck

Duck
Duck
Duck
Duck
Duck
Duck
Duck
Duck
Duck
Duck
Duck
Duck
Duck
Duck
Duck
Duck
Duck
Duck
Duck
Duck
Duck
Duck
Duck
Duck
Duck
Duck
Duck
Duck
Duck
Duck
Duck
Duck
Duck
Duck
Duck
Duck
Duck
Duck
Duck
Duck
Duck
Duck
Duck
Duck
Duck
Duck

Duck
Duck
Duck
Duck
Duck
Duck
Duck
Duck
Duck
Duck
Duck
Duck
Duck
Duck
Duck
Duck
Duck
Duck
Duck
Duck
Duck
Duck
Duck
Duck
Duck
Duck
Duck
Duck
Duck
Duck
Duck
Duck
Duck
Duck
Duck
Duck
Duck
Duck
Duck
Duck
Duck
Duck
Duck
Duck
Duck
Duck

Duck
Duck
Duck
Duck
Duck
Duck
Duck
Duck
Duck
Duck
Duck
Duck
Duck
Duck
Duck
Duck
Duck
Duck
Duck
Duck
Duck
Duck
Duck
Duck
Duck
Duck
Duck
Duck
Duck
Duck
Duck
Duck
Duck
Duck
Duck
Duck
Duck
Duck
Duck
Duck
Duck
Duck
Duck
Duck
Duck
Duck

Duck
Duck
Duck
Duck
Duck
Duck
Duck
Duck
Duck
Duck
Duck
Duck
Duck
Duck
Duck
Duck
Duck
Duck
Duck
Duck
Duck
Duck
Duck
Duck
Duck
Duck
Duck
Duck
Duck
Duck
Duck
Duck
Duck
Duck
Duck
Duck
Duck
Duck
Duck
Duck
Duck
Duck
Duck
Duck
Duck
Duck

Duck
Duck
Duck
Duck
Duck
Duck
Duck
Duck
Duck
Duck
Duck
Duck
Duck
Duck
Duck
Duck
Duck
Duck
Duck
Duck
Duck
Duck
Duck
Duck
Duck
Duck
Duck
Duck
Duck
Duck
Duck
Duck
Duck
Duck
Duck
Duck
Duck
Duck
Duck
Duck
Duck
Duck
Duck
Duck
Duck
Duck

Duck
Duck
Duck
Duck
Duck
Duck
Duck
Duck
Duck
Duck
Duck
Duck
Duck
Duck
Duck
Duck
Duck
Duck
Duck
Duck
Duck
Duck
Duck
Duck
Duck
Duck
Duck
Duck
Duck
Duck
Duck
Duck
Duck
Duck
Duck
Duck
Duck
Duck
Duck
Duck
Duck
Duck
Duck
Duck
Duck
Duck

Duck
Duck
Duck
Duck
Duck
Duck
Duck
Duck
Duck
Duck
Duck
Duck
Duck
Duck
Duck
Duck
Duck
Duck
Duck
Duck
Duck
Duck
Duck
Duck
Duck
Duck
Duck
Duck
Duck
Duck
Duck
Duck
Duck
Duck
Duck
Duck
Duck
Duck
Duck
Duck
Duck
Duck
Duck
Duck
Duck
Duck

Duck
Duck
Duck
Duck
Duck
Duck
Duck
Duck
Duck
Duck
Duck
Duck
Duck
Duck
Duck
Duck
Duck
Duck
Duck
Duck
Duck
Duck
Duck
Duck
Duck
Duck
Duck
Duck
Duck
Duck
Duck
Duck
Duck
Duck
Duck
Duck
Duck
Duck
Duck
Duck
Duck
Duck
Duck
Duck
Duck
Duck

Duck
Duck
Duck
Duck
Duck
Duck
Duck
Duck
Duck
Duck
Duck
Duck
Duck
Duck
Duck
Duck
Duck
Duck
Duck
Duck
Duck
Duck
Duck
Duck
Duck
Duck
Duck
Duck
Duck
Duck
Duck
Duck
Duck
Duck
Duck
Duck
Duck
Duck
Duck
Duck
Duck
Duck
Duck
Duck
Duck
Duck

Duck
Duck
Duck
Duck
Duck
Duck
Duck
Duck
Duck
Duck
Duck
Duck
Duck
Duck
Duck
Duck
Duck
Duck
Duck
Duck
Duck
Duck
Duck
Duck
Duck
Duck
Duck
Duck
Duck
Duck
Duck
Duck
Duck
Duck
Duck
Duck
Duck
Duck
Duck
Duck
Duck
Duck
Duck
Duck
Duck
Duck

Duck
Duck
Duck
Duck
Duck
Duck
Duck
Duck
Duck
Duck
Duck
Duck
Duck
Duck
Duck
Duck
Duck
Duck
Duck
Duck
Duck
Duck
Duck
Duck
Duck
Duck
Duck
Duck
Duck
Duck
Duck
Duck
Duck
Duck
Duck
Duck
Duck
Duck
Duck
Duck
Duck
Duck
Duck
Duck
Duck
Duck

Duck
Duck
Duck
Duck
Duck
Duck
Duck
Duck
Duck
Duck
Duck
Duck
Duck
Duck
Duck
Duck
Duck
Duck
Duck
Duck
Duck
Duck
Duck
Duck
Duck
Duck
Duck
Duck
Duck
Duck
Duck
Duck
Duck
Duck
Duck
Duck
Duck
Duck
Duck
Duck
Duck
Duck
Duck
Duck
Duck
Duck

Duck
Duck
Duck
Duck
Duck
Duck
Duck
Duck
Duck
Duck
Duck
Duck
Duck
Duck
Duck
Duck
Duck
Duck
Duck
Duck
Duck
Duck
Duck
Duck
Duck
Duck
Duck
Duck
Duck
Duck
Duck
Duck
Duck
Duck
Duck
Duck
Duck
Duck
Duck
Duck
Duck
Duck
Duck
Duck
Duck
Duck

Duck
Duck
Duck
Duck
Duck
Duck
Duck
Duck
Duck
Duck
Duck
Duck
Duck
Duck
Duck
Duck
Duck
Duck
Duck
Duck
Duck
Duck
Duck
Duck
Duck
Duck
Duck
Duck
Duck
Duck
Duck
Duck
Duck
Duck
Duck
Duck
Duck
Duck
Duck
Duck
Duck
Duck
Duck
Duck
Duck
Duck

Duck
Duck
Duck
Duck
Duck
Duck
Duck
Duck
Duck
Duck
Duck
Duck
Duck
Duck
Duck
Duck
Duck
Duck
Duck
Duck
Duck
Duck
Duck
Duck
Duck
Duck
Duck
Duck
Duck
Duck
Duck
Duck
Duck
Duck
Duck
Duck
Duck
Duck
Duck
Duck
Duck
Duck
Duck
Duck
Duck
Duck

Duck
Duck
Duck
Duck
Duck
Duck
Duck
Duck
Duck
Duck
Duck
Duck
Duck
Duck
Duck
Duck
Duck
Duck
Duck
Duck
Duck
Duck
Duck
Duck
Duck
Duck
Duck
Duck
Duck
Duck
Duck
Duck
Duck
Duck
Duck
Duck
Duck
Duck
Duck
Duck
Duck
Duck
Duck
Duck
Duck
Duck

Duck
Duck
Duck
Duck
Duck
Duck
Duck
Duck
Duck
Duck
Duck
Duck
Duck
Duck
Duck
Duck
Duck
Duck
Duck
Duck
Duck
Duck
Duck
Duck
Duck
Duck
Duck
Duck
Duck
Duck
Duck
Duck
Duck
Duck
Duck
Duck
Duck
Duck
Duck
Duck
Duck
Duck
Duck
Duck
Duck
Duck

Duck
Duck
Duck
Duck
Duck
Duck
Duck
Duck
Duck
Duck
Duck
Duck
Duck
Duck
Duck
Duck
Duck
Duck
Duck
Duck
Duck
Duck
Duck
Duck
Duck
Duck
Duck
Duck
Duck
Duck
Duck
Duck
Duck
Duck
Duck
Duck
Duck
Duck
Duck
Duck
Duck
Duck
Duck
Duck
Duck
Duck

Duck
Duck
Duck
Duck
Duck
Duck
Duck
Duck
Duck
Duck
Duck
Duck
Duck
Duck
Duck
Duck
Duck
Duck
Duck
Duck
Duck
Duck
Duck
Duck
Duck
Duck
Duck
Duck
Duck
Duck
Duck
Duck
Duck
Duck
Duck
Duck
Duck
Duck
Duck
Duck
Duck
Duck
Duck
Duck
Duck
Duck

Duck
Duck
Duck
Duck
Duck
Duck
Duck
Duck
Duck
Duck
Duck
Duck
Duck
Duck
Duck
Duck
Duck
Duck
Duck
Duck
Duck
Duck
Duck
Duck
Duck
Duck
Duck
Duck
Duck
Duck
Duck
Duck
Duck
Duck
Duck
Duck
Duck
Duck
Duck
Duck
Duck
Duck
Duck
Duck
Duck
Duck

Duck
Duck
Duck
Duck
Duck
Duck
Duck
Duck
Duck
Duck
Duck
Duck
Duck
Duck
Duck
Duck
Duck
Duck
Duck
Duck
Duck
Duck
Duck
Duck
Duck
Duck
Duck
Duck
Duck
Duck
Duck
Duck
Duck
Duck
Duck
Duck
Duck
Duck
Duck
Duck
Duck
Duck
Duck
Duck
Duck
Duck

Duck
Duck
Duck
Duck
Duck
Duck
Duck
Duck
Duck
Duck
Duck
Duck
Duck
Duck
Duck
Duck
Duck
Duck
Duck
Duck
Duck
Duck
Duck
Duck
Duck
Duck
Duck
Duck
Duck
Duck
Duck
Duck
Duck
Duck
Duck
Duck
Duck
Duck
Duck
Duck
Duck
Duck
Duck
Duck
Duck
Duck

Duck
Duck
Duck
Duck
Duck
Duck
Duck
Duck
Duck
Duck
Duck
Duck
Duck
Duck
Duck
Duck
Duck
Duck
Duck
Duck
Duck
Duck
Duck
Duck
Duck
Duck
Duck
Duck
Duck
Duck
Duck
Duck
Duck
Duck
Duck
Duck
Duck
Duck
Duck
Duck
Duck
Duck
Duck
Duck
Duck
Duck

Duck
Duck
Duck
Duck
Duck
Duck
Duck
Duck
Duck
Duck
Duck
Duck
Duck
Duck
Duck
Duck
Duck
Duck
Duck
Duck
Duck
Duck
Duck
Duck
Duck
Duck
Duck
Duck
Duck
Duck
Duck
Duck
Duck
Duck
Duck
Duck
Duck
Duck
Duck
Duck
Duck
Duck
Duck
Duck
Duck
Duck

Duck
Duck
Duck
Duck
Duck
Duck
Duck
Duck
Duck
Duck
Duck
Duck
Duck
Duck
Duck
Duck
Duck
Duck
Duck
Duck
Duck
Duck
Duck
Duck
Duck
Duck
Duck
Duck
Duck
Duck
Duck
Duck
Duck
Duck
Duck
Duck
Duck
Duck
Duck
Duck
Duck
Duck
Duck
Duck
Duck
Duck

Duck
Duck
Duck
Duck
Duck
Duck
Duck
Duck
Duck
Duck
Duck
Duck
Duck
Duck
Duck
Duck
Duck
Duck
Duck
Duck
Duck
Duck
Duck
Duck
Duck
Duck
Duck
Duck
Duck
Duck
Duck
Duck
Duck
Duck
Duck
Duck
Duck
Duck
Duck
Duck
Duck
Duck
Duck
Duck
Duck
Duck

Duck
Duck
Duck
Duck
Duck
Duck
Duck
Duck
Duck
Duck
Duck
Duck
Duck
Duck
Duck
Duck
Duck
Duck
Duck
Duck
Duck
Duck
Duck
Duck
Duck
Duck
Duck
Duck
Duck
Duck
Duck
Duck
Duck
Duck
Duck
Duck
Duck
Duck
Duck
Duck
Duck
Duck
Duck
Duck
Duck
Duck

Duck
Duck
Duck
Duck
Duck
Duck
Duck
Duck
Duck
Duck
Duck
Duck
Duck
Duck
Duck
Duck
Duck
Duck
Duck
Duck
Duck
Duck
Duck
Duck
Duck
Duck
Duck
Duck
Duck
Duck
Duck
Duck
Duck
Duck
Duck
Duck
Duck
Duck
Duck
Duck
Duck
Duck
Duck
Duck
Duck
Duck

Duck
Duck
Duck
Duck
Duck
Duck
Duck
Duck
Duck
Duck
Duck
Duck
Duck
Duck
Duck
Duck
Duck
Duck
Duck
Duck
Duck
Duck
Duck
Duck
Duck
Duck
Duck
Duck
Duck
Duck
Duck
Duck
Duck
Duck
Duck
Duck
Duck
Duck
Duck
Duck
Duck
Duck
Duck
Duck
Duck
Duck

Duck
Duck
Duck
Duck
Duck
Duck
Duck
Duck
Duck
Duck
Duck
Duck
Duck
Duck
Duck
Duck
Duck
Duck
Duck
Duck
Duck
Duck
Duck
Duck
Duck
Duck
Duck
Duck
Duck
Duck
Duck
Duck
Duck
Duck
Duck
Duck
Duck
Duck
Duck
Duck
Duck
Duck
Duck
Duck
Duck
Duck

Duck
Duck
Duck
Duck
Duck
Duck
Duck
Duck
Duck
Duck
Duck
Duck
Duck
Duck
Duck
Duck
Duck
Duck
Duck
Duck
Duck
Duck
Duck
Duck
Duck
Duck
Duck
Duck
Duck
Duck
Duck
Duck
Duck
Duck
Duck
Duck
Duck
Duck
Duck
Duck
Duck
Duck
Duck
Duck
Duck
Duck

Duck
Duck
Duck
Duck
Duck
Duck
Duck
Duck
Duck
Duck
Duck
Duck
Duck
Duck
Duck
Duck
Duck
Duck
Duck
Duck
Duck
Duck
Duck
Duck
Duck
Duck
Duck
Duck
Duck
Duck
Duck
Duck
Duck
Duck
Duck
Duck
Duck
Duck
Duck
Duck
Duck
Duck
Duck
Duck
Duck
Duck

Duck
Duck
Duck
Duck
Duck
Duck
Duck
Duck
Duck
Duck
Duck
Duck
Duck
Duck
Duck
Duck
Duck
Duck
Duck
Duck
Duck
Duck
Duck
Duck
Duck
Duck
Duck
Duck
Duck
Duck
Duck
Duck
Duck
Duck
Duck
Duck
Duck
Duck
Duck
Duck
Duck
Duck
Duck
Duck
Duck
Duck

Duck
Duck
Duck
Duck
Duck
Duck
Duck
Duck
Duck
Duck
Duck
Duck
Duck
Duck
Duck
Duck
Duck
Duck
Duck
Duck
Duck
Duck
Duck
Duck
Duck
Duck
Duck
Duck
Duck
Duck
Duck
Duck
Duck
Duck
Duck
Duck
Duck
Duck
Duck
Duck
Duck
Duck
Duck
Duck
Duck
Duck

Duck
Duck
Duck
Duck
Duck
Duck
Duck
Duck
Duck
Duck
Duck
Duck
Duck
Duck
Duck
Duck
Duck
Duck
Duck
Duck
Duck
Duck
Duck
Duck
Duck
Duck
Duck
Duck
Duck
Duck
Duck
Duck
Duck
Duck
Duck
Duck
Duck
Duck
Duck
Duck
Duck
Duck
Duck
Duck
Duck
Duck

Duck
Duck
Duck
Duck
Duck
Duck
Duck
Duck
Duck
Duck
Duck
Duck
Duck
Duck
Duck
Duck
Duck
Duck
Duck
Duck
Duck
Duck
Duck
Duck
Duck
Duck
Duck
Duck
Duck
Duck
Duck
Duck
Duck
Duck
Duck
Duck
Duck
Duck
Duck
Duck
Duck
Duck
Duck
Duck
Duck
Duck

Duck
Duck
Duck
Duck
Duck
Duck
Duck
Duck
Duck
Duck
Duck
Duck
Duck
Duck
Duck
Duck
Duck
Duck
Duck
Duck
Duck
Duck
Duck
Duck
Duck
Duck
Duck
Duck
Duck
Duck
Duck
Duck
Duck
Duck
Duck
Duck
Duck
Duck
Duck
Duck
Duck
Duck
Duck
Duck
Duck
Duck

Duck
Duck
Duck
Duck
Duck
Duck
Duck
Duck
Duck
Duck
Duck
Duck
Duck
Duck
Duck
Duck
Duck
Duck
Duck
Duck
Duck
Duck
Duck
Duck
Duck
Duck
Duck
Duck
Duck
Duck
Duck
Duck
Duck
Duck
Duck
Duck
Duck
Duck
Duck
Duck
Duck
Duck
Duck
Duck
Duck
Duck

Duck
Duck
Duck
Duck
Duck
Duck
Duck
Duck
Duck
Duck
Duck
Duck
Duck
Duck
Duck
Duck
Duck
Duck
Duck
Duck
Duck
Duck
Duck
Duck
Duck
Duck
Duck
Duck
Duck
Duck
Duck
Duck
Duck
Duck
Duck
Duck
Duck
Duck
Duck
Duck
Duck
Duck
Duck
Duck
Duck
Duck

Duck
Duck
Duck
Duck
Duck
Duck
Duck
Duck
Duck
Duck
Duck
Duck
Duck
Duck
Duck
Duck
Duck
Duck
Duck
Duck
Duck
Duck
Duck
Duck
Duck
Duck
Duck
Duck
Duck
Duck
Duck
Duck
Duck
Duck
Duck
Duck
Duck
Duck
Duck
Duck
Duck
Duck
Duck
Duck
Duck
Duck

Duck
Duck
Duck
Duck
Duck
Duck
Duck
Duck
Duck
Duck
Duck
Duck
Duck
Duck
Duck
Duck
Duck
Duck
Duck
Duck
Duck
Duck
Duck
Duck
Duck
Duck
Duck
Duck
Duck
Duck
Duck
Duck
Duck
Duck
Duck
Duck
Duck
Duck
Duck
Duck
Duck
Duck
Duck
Duck
Duck
Duck

Duck
Duck
Duck
Duck
Duck
Duck
Duck
Duck
Duck
Duck
Duck
Duck
Duck
Duck
Duck
Duck
Duck
Duck
Duck
Duck
Duck
Duck
Duck
Duck
Duck
Duck
Duck
Duck
Duck
Duck
Duck
Duck
Duck
Duck
Duck
Duck
Duck
Duck
Duck
Duck
Duck
Duck
Duck
Duck
Duck
Duck

Duck
Duck
Duck
Duck
Duck
Duck
Duck
Duck
Duck
Duck
Duck
Duck
Duck
Duck
Duck
Duck
Duck
Duck
Duck
Duck
Duck
Duck
Duck
Duck
Duck
Duck
Duck
Duck
Duck
Duck
Duck
Duck
Duck
Duck
Duck
Duck
Duck
Duck
Duck
Duck
Duck
Duck
Duck
Duck
Duck
Duck

Duck
Duck
Duck
Duck
Duck
Duck
Duck
Duck
Duck
Duck
Duck
Duck
Duck
Duck
Duck
Duck
Duck
Duck
Duck
Duck
Duck
Duck
Duck
Duck
Duck
Duck
Duck
Duck
Duck
Duck
Duck
Duck
Duck
Duck
Duck
Duck
Duck
Duck
Duck
Duck
Duck
Duck
Duck
Duck
Duck
Duck

Duck
Duck
Duck
Duck
Duck
Duck
Duck
Duck
Duck
Duck
Duck
Duck
Duck
Duck
Duck
Duck
Duck
Duck
Duck
Duck
Duck
Duck
Duck
Duck
Duck
Duck
Duck
Duck
Duck
Duck
Duck
Duck
Duck
Duck
Duck
Duck
Duck
Duck
Duck
Duck
Duck
Duck
Duck
Duck
Duck
Duck

Duck
Duck
Duck
Duck
Duck
Duck
Duck
Duck
Duck
Duck
Duck
Duck
Duck
Duck
Duck
Duck
Duck
Duck
Duck
Duck
Duck
Duck
Duck
Duck
Duck
Duck
Duck
Duck
Duck
Duck
Duck
Duck
Duck
Duck
Duck
Duck
Duck
Duck
Duck
Duck
Duck
Duck
Duck
Duck
Duck
Duck

Duck
Duck
Duck
Duck
Duck
Duck
Duck
Duck
Duck
Duck
Duck
Duck
Duck
Duck
Duck
Duck
Duck
Duck
Duck
Duck
Duck
Duck
Duck
Duck
Duck
Duck
Duck
Duck
Duck
Duck
Duck
Duck
Duck
Duck
Duck
Duck
Duck
Duck
Duck
Duck
Duck
Duck
Duck
Duck
Duck
Duck

Duck
Duck
Duck
Duck
Duck
Duck
Duck
Duck
Duck
Duck
Duck
Duck
Duck
Duck
Duck
Duck
Duck
Duck
Duck
Duck
Duck
Duck
Duck
Duck
Duck
Duck
Duck
Duck
Duck
Duck
Duck
Duck
Duck
Duck
Duck
Duck
Duck
Duck
Duck
Duck
Duck
Duck
Duck
Duck
Duck
Duck

Duck
Duck
Duck
Duck
Duck
Duck
Duck
Duck
Duck
Duck
Duck
Duck
Duck
Duck
Duck
Duck
Duck
Duck
Duck
Duck
Duck
Duck
Duck
Duck
Duck
Duck
Duck
Duck
Duck
Duck
Duck
Duck
Duck
Duck
Duck
Duck
Duck
Duck
Duck
Duck
Duck
Duck
Duck
Duck
Duck
Duck

Duck
Duck
Duck
Duck
Duck
Duck
Duck
Duck
Duck
Duck
Duck
Duck
Duck
Duck
Duck
Duck
Duck
Duck
Duck
Duck
Duck
Duck
Duck
Duck
Duck
Duck
Duck
Duck
Duck
Duck
Duck
Duck
Duck
Duck
Duck
Duck
Duck
Duck
Duck
Duck
Duck
Duck
Duck
Duck
Duck
Duck

Duck
Duck
Duck
Duck
Duck
Duck
Duck
Duck
Duck
Duck
Duck
Duck
Duck
Duck
Duck
Duck
Duck
Duck
Duck
Duck
Duck
Duck
Duck
Duck
Duck
Duck
Duck
Duck
Duck
Duck
Duck
Duck
Duck
Duck
Duck
Duck
Duck
Duck
Duck
Duck
Duck
Duck
Duck
Duck
Duck
Duck

Duck
Duck
Duck
Duck
Duck
Duck
Duck
Duck
Duck
Duck
Duck
Duck
Duck
Duck
Duck
Duck
Duck
Duck
Duck
Duck
Duck
Duck
Duck
Duck
Duck
Duck
Duck
Duck
Duck
Duck
Duck
Duck
Duck
Duck
Duck
Duck
Duck
Duck
Duck
Duck
Duck
Duck
Duck
Duck
Duck
Duck

Duck
Duck
Duck
Duck
Duck
Duck
Duck
Duck
Duck
Duck
Duck
Duck
Duck
Duck
Duck
Duck
Duck
Duck
Duck
Duck
Duck
Duck
Duck
Duck
Duck
Duck
Duck
Duck
Duck
Duck
Duck
Duck
Duck
Duck
Duck
Duck
Duck
Duck
Duck
Duck
Duck
Duck
Duck
Duck
Duck
Duck

Duck
Duck
Duck
Duck
Duck
Duck
Duck
Duck
Duck
Duck
Duck
Duck
Duck
Duck
Duck
Duck
Duck
Duck
Duck
Duck
Duck
Duck
Duck
Duck
Duck
Duck
Duck
Duck
Duck
Duck
Duck
Duck
Duck
Duck
Duck
Duck
Duck
Duck
Duck
Duck
Duck
Duck
Duck
Duck
Duck
Duck

Duck
Duck
Duck
Duck
Duck
Duck
Duck
Duck
Duck
Duck
Duck
Duck
Duck
Duck
Duck
Duck
Duck
Duck
Duck
Duck
Duck
Duck
Duck
Duck
Duck
Duck
Duck
Duck
Duck
Duck
Duck
Duck
Duck
Duck
Duck
Duck
Duck
Duck
Duck
Duck
Duck
Duck
Duck
Duck
Duck
Duck

Duck
Duck
Duck
Duck
Duck
Duck
Duck
Duck
Duck
Duck
Duck
Duck
Duck
Duck
Duck
Duck
Duck
Duck
Duck
Duck
Duck
Duck
Duck
Duck
Duck
Duck
Duck
Duck
Duck
Duck
Duck
Duck
Duck
Duck
Duck
Duck
Duck
Duck
Duck
Duck
Duck
Duck
Duck
Duck
Duck
Duck

Duck
Duck
Duck
Duck
Duck
Duck
Duck
Duck
Duck
Duck
Duck
Duck
Duck
Duck
Duck
Duck
Duck
Duck
Duck
Duck
Duck
Duck
Duck
Duck
Duck
Duck
Duck
Duck
Duck
Duck
Duck
Duck
Duck
Duck
Duck
Duck
Duck
Duck
Duck
Duck
Duck
Duck
Duck
Duck
Duck
Duck

Duck
Duck
Duck
Duck
Duck
Duck
Duck
Duck
Duck
Duck
Duck
Duck
Duck
Duck
Duck
Duck
Duck
Duck
Duck
Duck
Duck
Duck
Duck
Duck
Duck
Duck
Duck
Duck
Duck
Duck
Duck
Duck
Duck
Duck
Duck
Duck
Duck
Duck
Duck
Duck
Duck
Duck
Duck
Duck
Duck
Duck

Duck
Duck
Duck
Duck
Duck
Duck
Duck
Duck
Duck
Duck
Duck
Duck
Duck
Duck
Duck
Duck
Duck
Duck
Duck
Duck
Duck
Duck
Duck
Duck
Duck
Duck
Duck
Duck
Duck
Duck
Duck
Duck
Duck
Duck
Duck
Duck
Duck
Duck
Duck
Duck
Duck
Duck
Duck
Duck
Duck
Duck

Duck
Duck
Duck
Duck
Duck
Duck
Duck
Duck
Duck
Duck
Duck
Duck
Duck
Duck
Duck
Duck
Duck
Duck
Duck
Duck
Duck
Duck
Duck
Duck
Duck
Duck
Duck
Duck
Duck
Duck
Duck
Duck
Duck
Duck
Duck
Duck
Duck
Duck
Duck
Duck
Duck
Duck
Duck
Duck
Duck
Duck

Duck
Duck
Duck
Duck
Duck
Duck
Duck
Duck
Duck
Duck
Duck
Duck
Duck
Duck
Duck
Duck
Duck
Duck
Duck
Duck
Duck
Duck
Duck
Duck
Duck
Duck
Duck
Duck
Duck
Duck
Duck
Duck
Duck
Duck
Duck
Duck
Duck
Duck
Duck
Duck
Duck
Duck
Duck
Duck
Duck
Duck

Duck
Duck
Duck
Duck
Duck
Duck
Duck
Duck
Duck
Duck
Duck
Duck
Duck
Duck
Duck
Duck
Duck
Duck
Duck
Duck
Duck
Duck
Duck
Duck
Duck
Duck
Duck
Duck
Duck
Duck
Duck
Duck
Duck
Duck
Duck
Duck
Duck
Duck
Duck
Duck
Duck
Duck
Duck
Duck
Duck
Duck

Duck
Duck
Duck
Duck
Duck
Duck
Duck
Duck
Duck
Duck
Duck
Duck
Duck
Duck
Duck
Duck
Duck
Duck
Duck
Duck
Duck
Duck
Duck
Duck
Duck
Duck
Duck
Duck
Duck
Duck
Duck
Duck
Duck
Duck
Duck
Duck
Duck
Duck
Duck
Duck
Duck
Duck
Duck
Duck
Duck
Duck

Duck
Duck
Duck
Duck
Duck
Duck
Duck
Duck
Duck
Duck
Duck
Duck
Duck
Duck
Duck
Duck
Duck
Duck
Duck
Duck
Duck
Duck
Duck
Duck
Duck
Duck
Duck
Duck
Duck
Duck
Duck
Duck
Duck
Duck
Duck
Duck
Duck
Duck
Duck
Duck
Duck
Duck
Duck
Duck
Duck
Duck

Duck
Duck
Duck
Duck
Duck
Duck
Duck
Duck
Duck
Duck
Duck
Duck
Duck
Duck
Duck
Duck
Duck
Duck
Duck
Duck
Duck
Duck
Duck
Duck
Duck
Duck
Duck
Duck
Duck
Duck
Duck
Duck
Duck
Duck
Duck
Duck
Duck
Duck
Duck
Duck
Duck
Duck
Duck
Duck
Duck
Duck

Duck
Duck
Duck
Duck
Duck
Duck
Duck
Duck
Duck
Duck
Duck
Duck
Duck
Duck
Duck
Duck
Duck
Duck
Duck
Duck
Duck
Duck
Duck
Duck
Duck
Duck
Duck
Duck
Duck
Duck
Duck
Duck
Duck
Duck
Duck
Duck
Duck
Duck
Duck
Duck
Duck
Duck
Duck
Duck
Duck
Duck

Duck
Duck
Duck
Duck
Duck
Duck
Duck
Duck
Duck
Duck
Duck
Duck
Duck
Duck
Duck
Duck
Duck
Duck
Duck
Duck
Duck
Duck
Duck
Duck
Duck
Duck
Duck
Duck
Duck
Duck
Duck
Duck
Duck
Duck
Duck
Duck
Duck
Duck
Duck
Duck
Duck
Duck
Duck
Duck
Duck
Duck

Duck
Duck
Duck
Duck
Duck
Duck
Duck
Duck
Duck
Duck
Duck
Duck
Duck
Duck
Duck
Duck
Duck
Duck
Duck
Duck
Duck
Duck
Duck
Duck
Duck
Duck
Duck
Duck
Duck
Duck
Duck
Duck
Duck
Duck
Duck
Duck
Duck
Duck
Duck
Duck
Duck
Duck
Duck
Duck
Duck
Duck

Duck
Duck
Duck
Duck
Duck
Duck
Duck
Duck
Duck
Duck
Duck
Duck
Duck
Duck
Duck
Duck
Duck
Duck
Duck
Duck
Duck
Duck
Duck
Duck
Duck
Duck
Duck
Duck
Duck
Duck
Duck
Duck
Duck
Duck
Duck
Duck
Duck
Duck
Duck
Duck
Duck
Duck
Duck
Duck
Duck
Duck

Duck
Duck
Duck
Duck
Duck
Duck
Duck
Duck
Duck
Duck
Duck
Duck
Duck
Duck
Duck
Duck
Duck
Duck
Duck
Duck
Duck
Duck
Duck
Duck
Duck
Duck
Duck
Pigeon
Pigeon
Pigeon
Pigeon
Pigeon
Pigeon
Pigeon
Pigeon
Pigeon
Pigeon
Pigeon
Pigeon
Pigeon
Pigeon
Pigeon
Pigeon
Pigeon
Pigeon
Pigeon

Pigeon
Pigeon
Pigeon
Pigeon
Pigeon
Pigeon
Pigeon
Pigeon
Pigeon
Pigeon
Pigeon
Pigeon
Pigeon
Pigeon
Pigeon
Pigeon
Pigeon
Pigeon
Pigeon
Pigeon
Pigeon
Pigeon
Pigeon
Pigeon
Pigeon
Pigeon
Pigeon
Pigeon
Pigeon
Pigeon
Pigeon
Pigeon
Pigeon
Pigeon
Pigeon
Pigeon
Pigeon
Pigeon
Pigeon
Pigeon
Pigeon
Pigeon
Pigeon
Pigeon
Pigeon
Pigeon

Pigeon
Pigeon
Pigeon
Pigeon
Pigeon
Pigeon
Pigeon
Pigeon
Pigeon
Pigeon
Pigeon
Pigeon
Pigeon
Pigeon
Pigeon
Pigeon
Pigeon
Pigeon
Pigeon
Pigeon
Pigeon
Pigeon
Pigeon
Pigeon
Pigeon
Pigeon
Pigeon
Pigeon
Pigeon
Pigeon
Pigeon
Pigeon
Pigeon
Pigeon
Pigeon
Pigeon
Pigeon
Pigeon
Pigeon
Pigeon
Pigeon
Pigeon
Pigeon
Pigeon
Pigeon
Pigeon

Pigeon
Pigeon
Pigeon
Pigeon
Pigeon
Pigeon
Pigeon
Pigeon
Pigeon
Pigeon
Pigeon
Pigeon
Pigeon
Pigeon
Pigeon
Pigeon
Pigeon
Pigeon
Pigeon
Pigeon
Pigeon
Pigeon
Pigeon
Pigeon
Pigeon
Pigeon
Pigeon
Pigeon
Pigeon
Pigeon
Pigeon
Pigeon
Pigeon
Pigeon
Pigeon
Pigeon
Pigeon
Pigeon
Pigeon
Pigeon
Pigeon
Pigeon
Pigeon
Pigeon
Pigeon
Pigeon

Pigeon
Pigeon
Pigeon
Pigeon
Pigeon
Pigeon
Pigeon
Pigeon
Pigeon
Pigeon
Pigeon
Pigeon
Pigeon
Pigeon
Pigeon
Pigeon
Pigeon
Pigeon
Pigeon
Pigeon
Pigeon
Pigeon
Pigeon
Pigeon
Pigeon
Pigeon
Pigeon
Pigeon
Pigeon
Pigeon
Pigeon
Pigeon
Pigeon
Pigeon
Pigeon
Pigeon
Pigeon
Pigeon
Pigeon
Pigeon
Pigeon
Pigeon
Pigeon
Pigeon
Pigeon
Pigeon

Pigeon
Pigeon
Pigeon
Pigeon
Pigeon
Pigeon
Pigeon
Pigeon
Pigeon
Pigeon
Pigeon
Pigeon
Pigeon
Pigeon
Pigeon
Pigeon
Pigeon
Pigeon
Pigeon
Pigeon
Pigeon
Pigeon
Pigeon
Pigeon
Pigeon
Pigeon
Pigeon
Pigeon
Pigeon
Pigeon
Pigeon
Pigeon
Pigeon
Pigeon
Pigeon
Pigeon
Pigeon
Pigeon
Pigeon
Pigeon
Pigeon
Pigeon
Pigeon
Pigeon
Pigeon
Pigeon

Pigeon
Pigeon
Pigeon
Pigeon
Pigeon
Pigeon
Pigeon
Pigeon
Pigeon
Pigeon
Pigeon
Pigeon
Pigeon
Pigeon
Pigeon
Pigeon
Pigeon
Pigeon
Pigeon
Pigeon
Pigeon
Pigeon
Pigeon
Pigeon
Pigeon
Pigeon
Pigeon
Pigeon
Pigeon
Pigeon
Pigeon
Pigeon
Pigeon
Pigeon
Pigeon
Pigeon
Pigeon
Pigeon
Pigeon
Pigeon
Pigeon
Pigeon
Pigeon
Pigeon
Pigeon
Pigeon

Pigeon
Pigeon
Pigeon
Pigeon
Pigeon
Pigeon
Pigeon
Pigeon
Pigeon
Pigeon
Pigeon
Pigeon
Pigeon
Pigeon
Pigeon
Pigeon
Pigeon
Pigeon
Pigeon
Pigeon
Pigeon
Pigeon
Pigeon
Pigeon
Pigeon
Pigeon
Pigeon
Pigeon
Pigeon
Pigeon
Pigeon
Pigeon
Pigeon
Pigeon
Pigeon
Pigeon
Pigeon
Pigeon
Pigeon
Pigeon
Pigeon
Pigeon
Pigeon
Pigeon
Pigeon
Pigeon

Pigeon
Pigeon
Pigeon
Pigeon
Pigeon
Pigeon
Pigeon
Pigeon
Pigeon
Pigeon
Pigeon
Pigeon
Pigeon
Pigeon
Pigeon
Pigeon
Pigeon
Pigeon
Pigeon
Pigeon
Pigeon
Pigeon
Pigeon
Pigeon
Pigeon
Pigeon
Pigeon
Pigeon
Pigeon
Pigeon
Pigeon
Pigeon
Pigeon
Pigeon
Pigeon
Pigeon
Pigeon
Pigeon
Pigeon
Pigeon
Pigeon
Pigeon
Pigeon
Pigeon
Pigeon
Pigeon

Pigeon
Pigeon
Pigeon
Pigeon
Pigeon
Pigeon
Pigeon
Pigeon
Pigeon
Pigeon
Pigeon
Pigeon
Pigeon
Pigeon
Pigeon
Pigeon
Pigeon
Pigeon
Pigeon
Pigeon
Pigeon
Pigeon
Pigeon
Pigeon
Pigeon
Pigeon
Pigeon
Pigeon
Pigeon
Pigeon
Pigeon
Pigeon
Pigeon
Pigeon
Pigeon
Pigeon
Pigeon
Pigeon
Pigeon
Pigeon
Pigeon
Pigeon
Pigeon
Pigeon
Pigeon
Pigeon

Pigeon
Pigeon
Pigeon
Pigeon
Pigeon
Pigeon
Pigeon
Pigeon
Pigeon
Pigeon
Pigeon
Pigeon
Pigeon
Pigeon
Pigeon
Pigeon
Pigeon
Pigeon
Pigeon
Pigeon
Pigeon
Pigeon
Pigeon
Pigeon
Pigeon
Pigeon
Pigeon
Pigeon
Pigeon
Pigeon
Pigeon
Pigeon
Pigeon
Pigeon
Pigeon
Pigeon
Pigeon
Pigeon
Pigeon
Pigeon
Pigeon
Pigeon
Pigeon
Pigeon
Pigeon
Pigeon

Pigeon
Pigeon
Pigeon
Pigeon
Pigeon
Pigeon
Pigeon
Pigeon
Pigeon
Pigeon
Pigeon
Pigeon
Pigeon
Pigeon
Pigeon
Pigeon
Pigeon
Pigeon
Pigeon
Pigeon
Pigeon
Pigeon
Pigeon
Pigeon
Pigeon
Pigeon
Pigeon
Pigeon
Pigeon
Pigeon
Pigeon
Pigeon
Pigeon
Pigeon
Pigeon
Pigeon
Pigeon
Pigeon
Pigeon
Pigeon
Pigeon
Pigeon
Pigeon
Pigeon
Pigeon
Pigeon

Pigeon
Pigeon
Pigeon
Pigeon
Pigeon
Pigeon
Pigeon
Pigeon
Pigeon
Pigeon
Pigeon
Pigeon
Pigeon
Pigeon
Pigeon
Pigeon
Pigeon
Pigeon
Pigeon
Pigeon
Pigeon
Pigeon
Pigeon
Pigeon
Pigeon
Pigeon
Pigeon
Pigeon
Pigeon
Pigeon
Pigeon
Pigeon
Pigeon
Pigeon
Pigeon
Pigeon
Pigeon
Pigeon
Pigeon
Pigeon
Pigeon
Pigeon
Pigeon
Pigeon
Pigeon
Pigeon

Pigeon
Pigeon
Pigeon
Pigeon
Pigeon
Pigeon
Pigeon
Pigeon
Pigeon
Pigeon
Pigeon
Pigeon
Pigeon
Pigeon
Pigeon
Pigeon
Pigeon
Pigeon
Pigeon
Pigeon
Pigeon
Pigeon
Pigeon
Pigeon
Pigeon
Pigeon
Pigeon
Pigeon
Pigeon
Pigeon
Pigeon
Pigeon
Pigeon
Pigeon
Pigeon
Pigeon
Pigeon
Pigeon
Pigeon
Pigeon
Pigeon
Pigeon
Pigeon
Pigeon
Pigeon
Pigeon

Pigeon
Pigeon
Pigeon
Pigeon
Pigeon
Pigeon
Pigeon
Pigeon
Pigeon
Pigeon
Pigeon
Pigeon
Pigeon
Pigeon
Pigeon
Pigeon
Pigeon
Pigeon
Pigeon
Pigeon
Pigeon
Pigeon
Pigeon
Pigeon
Pigeon
Pigeon
Pigeon
Pigeon
Pigeon
Pigeon
Pigeon
Pigeon
Pigeon
Pigeon
Pigeon
Pigeon
Pigeon
Pigeon
Pigeon
Pigeon
Pigeon
Pigeon
Pigeon
Pigeon
Pigeon
Pigeon

Pigeon
Pigeon
Pigeon
Pigeon
Pigeon
Pigeon
Pigeon
Pigeon
Pigeon
Pigeon
Pigeon
Pigeon
Pigeon
Pigeon
Pigeon
Pigeon
Pigeon
Pigeon
Pigeon
Pigeon
Pigeon
Pigeon
Pigeon
Pigeon
Pigeon
Pigeon
Pigeon
Pigeon
Pigeon
Pigeon
Pigeon
Pigeon
Pigeon
Pigeon
Pigeon
Pigeon
Pigeon
Pigeon
Pigeon
Pigeon
Pigeon
Pigeon
Pigeon
Pigeon
Pigeon
Pigeon

Pigeon
Pigeon
Pigeon
Pigeon
Pigeon
Pigeon
Pigeon
Pigeon
Pigeon
Pigeon
Pigeon
Pigeon
Pigeon
Pigeon
Pigeon
Pigeon
Pigeon
Pigeon
Pigeon
Pigeon
Pigeon
Pigeon
Pigeon
Pigeon
Pigeon
Pigeon
Pigeon
Pigeon
Pigeon
Pigeon
Pigeon
Pigeon
Pigeon
Pigeon
Pigeon
Pigeon
Pigeon
Pigeon
Pigeon
Pigeon
Pigeon
Pigeon
Pigeon
Pigeon
Pigeon
Pigeon

Pigeon
Pigeon
Pigeon
Pigeon
Pigeon
Pigeon
Pigeon
Pigeon
Pigeon
Pigeon
Pigeon
Pigeon
Pigeon
Pigeon
Pigeon
Pigeon
Pigeon
Pigeon
Pigeon
Pigeon
Pigeon
Pigeon
Pigeon
Pigeon
Pigeon
Pigeon
Pigeon
Pigeon
Pigeon
Pigeon
Pigeon
Pigeon
Pigeon
Pigeon
Pigeon
Pigeon
Pigeon
Pigeon
Pigeon
Pigeon
Pigeon
Pigeon
Pigeon
Pigeon
Pigeon
Pigeon

Pigeon
Pigeon
Pigeon
Pigeon
Pigeon
Pigeon
Pigeon
Pigeon
Pigeon
Pigeon
Pigeon
Pigeon
Pigeon
Pigeon
Pigeon
Pigeon
Pigeon
Pigeon
Pigeon
Pigeon
Pigeon
Pigeon
Pigeon
Pigeon
Pigeon
Pigeon
Pigeon
Pigeon
Pigeon
Pigeon
Pigeon
Pigeon
Pigeon
Pigeon
Pigeon
Pigeon
Pigeon
Pigeon
Pigeon
Pigeon
Pigeon
Pigeon
Pigeon
Pigeon
Pigeon
Pigeon

Pigeon
Pigeon
Pigeon
Pigeon
Pigeon
Pigeon
Pigeon
Pigeon
Pigeon
Pigeon
Pigeon
Pigeon
Pigeon
Pigeon
Pigeon
Pigeon
Pigeon
Pigeon
Pigeon
Pigeon
Pigeon
Pigeon
Pigeon
Pigeon
Pigeon
Pigeon
Pigeon
Pigeon
Pigeon
Pigeon
Pigeon
Pigeon
Pigeon
Pigeon
Pigeon
Pigeon
Pigeon
Pigeon
Pigeon
Pigeon
Pigeon
Pigeon
Pigeon
Pigeon
Pigeon
Pigeon

Pigeon
Pigeon
Pigeon
Pigeon
Pigeon
Pigeon
Pigeon
Pigeon
Pigeon
Pigeon
Pigeon
Pigeon
Pigeon
Pigeon
Pigeon
Pigeon
Pigeon
Pigeon
Pigeon
Pigeon
Pigeon
Pigeon
Pigeon
Pigeon
Pigeon
Pigeon
Pigeon
Pigeon
Pigeon
Pigeon
Pigeon
Pigeon
Pigeon
Pigeon
Pigeon
Pigeon
Pigeon
Pigeon
Pigeon
Pigeon
Pigeon
Pigeon
Pigeon
Pigeon
Pigeon
Pigeon

Pigeon
Pigeon
Pigeon
Pigeon
Pigeon
Pigeon
Pigeon
Pigeon
Pigeon
Pigeon
Pigeon
Pigeon
Pigeon
Pigeon
Pigeon
Pigeon
Pigeon
Pigeon
Pigeon
Pigeon
Pigeon
Pigeon
Pigeon
Pigeon
Pigeon
Pigeon
Pigeon
Pigeon
Pigeon
Pigeon
Pigeon
Pigeon
Pigeon
Pigeon
Pigeon
Pigeon
Pigeon
Pigeon
Pigeon
Pigeon
Pigeon
Pigeon
Pigeon
Pigeon
Pigeon
Pigeon

Pigeon
Pigeon
Pigeon
Pigeon
Pigeon
Pigeon
Pigeon
Pigeon
Pigeon
Pigeon
Pigeon
Pigeon
Pigeon
Pigeon
Pigeon
Pigeon
Pigeon
Pigeon
Pigeon
Pigeon
Pigeon
Pigeon
Pigeon
Pigeon
Pigeon
Pigeon
Pigeon
Pigeon
Pigeon
Pigeon
Pigeon
Pigeon
Pigeon
Pigeon
Pigeon
Pigeon
Pigeon
Pigeon
Pigeon
Pigeon
Pigeon
Pigeon
Pigeon
Pigeon
Pigeon
Pigeon

Pigeon
Pigeon
Pigeon
Pigeon
Pigeon
Pigeon
Pigeon
Pigeon
Pigeon
Pigeon
Pigeon
Pigeon
Pigeon
Pigeon
Pigeon
Pigeon
Pigeon
Pigeon
Pigeon
Pigeon
Pigeon
Pigeon
Pigeon
Pigeon
Pigeon
Pigeon
Pigeon
Pigeon
Pigeon
Pigeon
Pigeon
Pigeon
Pigeon
Pigeon
Pigeon
Pigeon
Pigeon
Pigeon
Pigeon
Pigeon
Pigeon
Pigeon
Pigeon
Pigeon
Pigeon
Pigeon

Pigeon
Pigeon
Pigeon
Pigeon
Pigeon
Pigeon
Pigeon
Pigeon
Pigeon
Pigeon
Pigeon
Pigeon
Pigeon
Pigeon
Pigeon
Pigeon
Pigeon
Pigeon
Pigeon
Pigeon
Pigeon
Pigeon
Pigeon
Pigeon
Pigeon
Pigeon
Pigeon
Pigeon
Pigeon
Pigeon
Pigeon
Pigeon
Pigeon
Pigeon
Pigeon
Pigeon
Pigeon
Pigeon
Pigeon
Pigeon
Pigeon
Pigeon
Pigeon
Pigeon
Pigeon
Pigeon

Pigeon
Pigeon
Pigeon
Pigeon
Pigeon
Pigeon
Pigeon
Pigeon
Pigeon
Pigeon
Pigeon
Pigeon
Pigeon
Pigeon
Pigeon
Pigeon
Pigeon
Pigeon
Pigeon
Pigeon
Pigeon
Pigeon
Pigeon
Pigeon
Pigeon
Pigeon
Pigeon
Pigeon
Pigeon
Pigeon
Pigeon
Pigeon
Pigeon
Pigeon
Pigeon
Pigeon
Pigeon
Pigeon
Pigeon
Pigeon
Pigeon
Pigeon
Pigeon
Pigeon
Pigeon
Pigeon

Pigeon
Pigeon
Pigeon
Pigeon
Pigeon
Pigeon
Pigeon
Pigeon
Pigeon
Pigeon
Pigeon
Pigeon
Pigeon
Pigeon
Pigeon
Pigeon
Pigeon
Pigeon
Pigeon
Pigeon
Pigeon
Pigeon
Pigeon
Pigeon
Pigeon
Pigeon
Pigeon
Pigeon
Pigeon
Pigeon
Pigeon
Pigeon
Pigeon
Pigeon
Pigeon
Pigeon
Pigeon
Pigeon
Pigeon
Pigeon
Pigeon
Pigeon
Pigeon
Pigeon
Pigeon
Pigeon

Pigeon
Pigeon
Pigeon
Pigeon
Pigeon
Pigeon
Pigeon
Pigeon
Pigeon
Pigeon
Pigeon
Pigeon
Pigeon
Pigeon
Pigeon
Pigeon
Pigeon
Pigeon
Pigeon
Pigeon
Pigeon
Pigeon
Pigeon
Pigeon
Pigeon
Pigeon
Pigeon
Pigeon
Pigeon
Pigeon
Pigeon
Pigeon
Pigeon
Pigeon
Pigeon
Pigeon
Pigeon
Pigeon
Pigeon
Pigeon
Pigeon
Pigeon
Pigeon
Pigeon
Pigeon
Pigeon

Pigeon
Pigeon
Pigeon
Pigeon
Pigeon
Pigeon
Pigeon
Pigeon
Pigeon
Pigeon
Pigeon
Pigeon
Pigeon
Pigeon
Pigeon
Pigeon
Pigeon
Pigeon
Pigeon
Pigeon
Pigeon
Pigeon
Pigeon
Pigeon
Pigeon
Pigeon
Pigeon
Pigeon
Pigeon
Pigeon
Pigeon
Pigeon
Pigeon
Pigeon
Pigeon
Pigeon
Pigeon
Pigeon
Pigeon
Pigeon
Pigeon
Pigeon
Pigeon
Pigeon
Pigeon
Pigeon

Pigeon
Pigeon
Pigeon
Pigeon
Pigeon
Pigeon
Pigeon
Pigeon
Pigeon
Pigeon
Pigeon
Pigeon
Pigeon
Pigeon
Pigeon
Pigeon
Pigeon
Pigeon
Pigeon
Pigeon
Pigeon
Pigeon
Pigeon
Pigeon
Pigeon
Pigeon
Pigeon
Pigeon
Pigeon
Pigeon
Pigeon
Pigeon
Pigeon
Pigeon
Pigeon
Pigeon
Pigeon
Pigeon
Pigeon
Pigeon
Pigeon
Pigeon
Pigeon
Pigeon
Pigeon
Pigeon

Pigeon
Pigeon
Pigeon
Pigeon
Pigeon
Pigeon
Pigeon
Pigeon
Pigeon
Pigeon
Pigeon
Pigeon
Pigeon
Pigeon
Pigeon
Pigeon
Pigeon
Pigeon
Pigeon
Pigeon
Pigeon
Pigeon
Pigeon
Pigeon
Pigeon
Pigeon
Pigeon
Pigeon
Pigeon
Pigeon
Pigeon
Pigeon
Pigeon
Pigeon
Pigeon
Pigeon
Pigeon
Pigeon
Pigeon
Pigeon
Pigeon
Pigeon
Pigeon
Pigeon
Pigeon
Pigeon

Pigeon
Pigeon
Pigeon
Pigeon
Pigeon
Pigeon
Pigeon
Pigeon
Pigeon
Pigeon
Pigeon
Pigeon
Pigeon
Pigeon
Pigeon
Pigeon
Pigeon
Pigeon
Pigeon
Pigeon
Pigeon
Pigeon
Pigeon
Pigeon
Pigeon
Pigeon
Pigeon
Pigeon
Pigeon
Pigeon
Pigeon
Pigeon
Pigeon
Pigeon
Pigeon
Pigeon
Pigeon
Pigeon
Pigeon
Pigeon
Pigeon
Pigeon
Pigeon
Pigeon
Pigeon
Pigeon

Pigeon
Pigeon
Pigeon
Pigeon
Pigeon
Pigeon
Pigeon
Pigeon
Pigeon
Pigeon
Pigeon
Pigeon
Pigeon
Pigeon
Pigeon
Pigeon
Pigeon
Pigeon
Pigeon
Pigeon
Pigeon
Pigeon
Pigeon
Pigeon
Pigeon
Pigeon
Pigeon
Pigeon
Pigeon
Pigeon
Pigeon
Pigeon
Pigeon
Pigeon
Pigeon
Pigeon
Pigeon
Pigeon
Pigeon
Pigeon
Pigeon
Pigeon
Pigeon
Pigeon
Pigeon
Pigeon

Pigeon
Pigeon
Pigeon
Pigeon
Pigeon
Pigeon
Pigeon
Pigeon
Pigeon
Pigeon
Pigeon
Pigeon
Pigeon
Pigeon
Pigeon
Pigeon
Pigeon
Pigeon
Pigeon
Pigeon
Pigeon
Pigeon
Pigeon
Pigeon
Pigeon
Pigeon
Pigeon
Pigeon
Pigeon
Pigeon
Pigeon
Pigeon
Pigeon
Pigeon
Pigeon
Pigeon
Pigeon
Pigeon
Pigeon
Pigeon
Pigeon
Pigeon
Pigeon
Pigeon
Pigeon
Pigeon

Pigeon
Pigeon
Pigeon
Pigeon
Pigeon
Pigeon
Pigeon
Pigeon
Pigeon
Pigeon
Pigeon
Pigeon
Pigeon
Pigeon
Pigeon
Pigeon
Pigeon
Pigeon
Pigeon
Pigeon
Pigeon
Pigeon
Pigeon
Pigeon
Pigeon
Pigeon
Pigeon
Pigeon
Pigeon
Pigeon
Pigeon
Pigeon
Pigeon
Pigeon
Pigeon
Pigeon
Pigeon
Pigeon
Pigeon
Pigeon
Pigeon
Pigeon
Pigeon
Pigeon
Pigeon
Pigeon

Pigeon
Pigeon
Pigeon
Pigeon
Pigeon
Pigeon
Pigeon
Pigeon
Pigeon
Pigeon
Pigeon
Pigeon
Pigeon
Pigeon
Pigeon
Pigeon
Pigeon
Pigeon
Pigeon
Pigeon
Pigeon
Pigeon
Pigeon
Pigeon
Pigeon
Pigeon
Pigeon
Pigeon
Pigeon
Pigeon
Pigeon
Pigeon
Pigeon
Pigeon
Pigeon
Pigeon
Pigeon
Pigeon
Pigeon
Pigeon
Pigeon
Pigeon
Pigeon
Pigeon
Pigeon
Pigeon

Pigeon
Pigeon
Pigeon
Pigeon
Pigeon
Pigeon
Pigeon
Pigeon
Pigeon
Pigeon
Pigeon
Pigeon
Pigeon
Pigeon
Pigeon
Pigeon
Pigeon
Pigeon
Pigeon
Pigeon
Pigeon
Pigeon
Pigeon
Pigeon
Pigeon
Pigeon
Pigeon
Pigeon
Pigeon
Pigeon
Pigeon
Pigeon
Pigeon
Pigeon
Pigeon
Pigeon
Pigeon
Pigeon
Pigeon
Pigeon
Pigeon
Pigeon
Pigeon
Pigeon
Pigeon
Pigeon

Pigeon
Pigeon
Pigeon
Pigeon
Pigeon
Pigeon
Pigeon
Pigeon
Pigeon
Pigeon
Pigeon
Pigeon
Pigeon
Pigeon
Pigeon
Pigeon
Pigeon
Pigeon
Pigeon
Pigeon
Pigeon
Pigeon
Pigeon
Pigeon
Pigeon
Pigeon
Pigeon
Pigeon
Pigeon
Pigeon
Pigeon
Pigeon
Pigeon
Pigeon
Pigeon
Pigeon
Pigeon
Pigeon
Pigeon
Pigeon
Pigeon
Pigeon
Pigeon
Pigeon
Pigeon
Pigeon

Pigeon
Pigeon
Pigeon
Pigeon
Pigeon
Pigeon
Pigeon
Pigeon
Pigeon
Pigeon
Pigeon
Pigeon
Pigeon
Pigeon
Pigeon
Pigeon
Pigeon
Pigeon
Pigeon
Pigeon
Pigeon
Pigeon
Pigeon
Pigeon
Pigeon
Pigeon
Pigeon
Pigeon
Pigeon
Pigeon
Pigeon
Pigeon
Pigeon
Pigeon
Pigeon
Pigeon
Pigeon
Pigeon
Pigeon
Pigeon
Pigeon
Pigeon
Pigeon
Pigeon
Pigeon
Pigeon

Pigeon
Pigeon
Pigeon
Pigeon
Pigeon
Pigeon
Pigeon
Pigeon
Pigeon
Pigeon
Pigeon
Pigeon
Pigeon
Pigeon
Pigeon
Pigeon
Pigeon
Pigeon
Pigeon
Pigeon
Pigeon
Pigeon
Pigeon
Pigeon
Pigeon
Pigeon
Pigeon
Pigeon
Pigeon
Pigeon
Pigeon
Pigeon
Pigeon
Pigeon
Pigeon
Pigeon
Pigeon
Pigeon
Pigeon
Pigeon
Pigeon
Pigeon
Pigeon
Pigeon
Pigeon
Pigeon

Pigeon
Pigeon
Pigeon
Pigeon
Pigeon
Pigeon
Pigeon
Pigeon
Pigeon
Pigeon
Pigeon
Pigeon
Pigeon
Pigeon
Pigeon
Pigeon
Pigeon
Pigeon
Pigeon
Pigeon
Pigeon
Pigeon
Pigeon
Pigeon
Pigeon
Pigeon
Pigeon
Pigeon
Pigeon
Pigeon
Pigeon
Pigeon
Pigeon
Pigeon
Pigeon
Pigeon
Pigeon
Pigeon
Pigeon
Pigeon
Pigeon
Pigeon
Pigeon
Pigeon
Pigeon
Pigeon

Pigeon
Pigeon
Pigeon
Pigeon
Pigeon
Pigeon
Pigeon
Pigeon
Pigeon
Pigeon
Pigeon
Pigeon
Pigeon
Pigeon
Pigeon
Pigeon
Pigeon
Pigeon
Pigeon
Pigeon
Pigeon
Pigeon
Pigeon
Pigeon
Pigeon
Pigeon
Pigeon
Pigeon
Pigeon
Pigeon
Pigeon
Pigeon
Pigeon
Pigeon
Pigeon
Pigeon
Pigeon
Pigeon
Pigeon
Pigeon
Pigeon
Pigeon
Pigeon
Pigeon
Pigeon
Pigeon

Pigeon
Pigeon
Pigeon
Pigeon
Pigeon
Pigeon
Pigeon
Pigeon
Pigeon
Pigeon
Pigeon
Pigeon
Pigeon
Pigeon
Pigeon
Pigeon
Pigeon
Pigeon
Pigeon
Pigeon
Pigeon
Pigeon
Pigeon
Pigeon
Pigeon
Pigeon
Pigeon
Pigeon
Pigeon
Pigeon
Pigeon
Pigeon
Pigeon
Pigeon
Pigeon
Pigeon
Pigeon
Pigeon
Pigeon
Pigeon
Pigeon
Pigeon
Pigeon
Pigeon
Pigeon
Pigeon

Pigeon
Pigeon
Pigeon
Pigeon
Pigeon
Pigeon
Pigeon
Pigeon
Pigeon
Pigeon
Pigeon
Pigeon
Pigeon
Pigeon
Pigeon
Pigeon
Pigeon
Pigeon
Pigeon
Pigeon
Pigeon
Pigeon
Pigeon
Pigeon
Pigeon
Pigeon
Pigeon
Pigeon
Pigeon
Pigeon
Pigeon
Pigeon
Pigeon
Pigeon
Pigeon
Pigeon
Pigeon
Pigeon
Pigeon
Pigeon
Pigeon
Pigeon
Pigeon
Pigeon
Pigeon
Pigeon

Pigeon
Pigeon
Pigeon
Pigeon
Pigeon
Pigeon
Pigeon
Pigeon
Pigeon
Pigeon
Pigeon
Pigeon
Pigeon
Pigeon
Pigeon
Pigeon
Pigeon
Pigeon
Pigeon
Pigeon
Pigeon
Pigeon
Pigeon
Pigeon
Pigeon
Pigeon
Pigeon
Pigeon
Pigeon
Pigeon
Pigeon
Pigeon
Pigeon
Pigeon
Pigeon
Pigeon
Pigeon
Pigeon
Pigeon
Pigeon
Pigeon
Pigeon
Pigeon
Pigeon
Pigeon
Pigeon

Pigeon
Pigeon
Pigeon
Pigeon
Pigeon
Pigeon
Pigeon
Pigeon
Pigeon
Pigeon
Pigeon
Pigeon
Pigeon
Pigeon
Pigeon
Pigeon
Pigeon
Pigeon
Pigeon
Pigeon
Pigeon
Pigeon
Pigeon
Pigeon
Pigeon
Pigeon
Pigeon
Pigeon
Pigeon
Pigeon
Pigeon
Pigeon
Pigeon
Pigeon
Pigeon
Pigeon
Pigeon
Pigeon
Pigeon
Pigeon
Pigeon
Pigeon
Pigeon
Pigeon
Pigeon
Pigeon

Pigeon
Pigeon
Pigeon
Pigeon
Pigeon
Pigeon
Pigeon
Pigeon
Pigeon
Pigeon
Pigeon
Pigeon
Pigeon
Pigeon
Pigeon
Pigeon
Pigeon
Pigeon
Pigeon
Pigeon
Pigeon
Pigeon
Pigeon
Pigeon
Pigeon
Pigeon
Pigeon
Pigeon
Pigeon
Pigeon
Pigeon
Pigeon
Pigeon
Pigeon
Pigeon
Pigeon
Pigeon
Pigeon
Pigeon
Pigeon
Pigeon
Pigeon
Pigeon
Pigeon
Pigeon
Pigeon

Pigeon
Pigeon
Pigeon
Pigeon
Pigeon
Pigeon
Pigeon
Pigeon
Pigeon
Pigeon
Pigeon
Pigeon
Pigeon
Pigeon
Pigeon
Pigeon
Pigeon
Pigeon
Pigeon
Pigeon
Pigeon
Pigeon
Pigeon
Pigeon
Pigeon
Pigeon
Pigeon
Pigeon
Pigeon
Pigeon
Pigeon
Pigeon
Pigeon
Pigeon
Pigeon
Pigeon
Pigeon
Pigeon
Pigeon
Pigeon
Pigeon
Pigeon
Pigeon
Pigeon
Pigeon
Pigeon

Pigeon
Pigeon
Pigeon
Pigeon
Pigeon
Pigeon
Pigeon
Pigeon
Pigeon
Pigeon
Pigeon
Pigeon
Pigeon
Pigeon
Pigeon
Pigeon
Pigeon
Pigeon
Pigeon
Pigeon
Pigeon
Pigeon
Pigeon
Pigeon
Pigeon
Pigeon
Pigeon
Pigeon
Pigeon
Pigeon
Pigeon
Pigeon
Pigeon
Pigeon
Pigeon
Pigeon
Pigeon
Pigeon
Pigeon
Pigeon
Pigeon
Pigeon
Pigeon
Pigeon
Pigeon
Pigeon

Pigeon
Pigeon
Pigeon
Pigeon
Pigeon
Pigeon
Pigeon
Pigeon
Pigeon
Pigeon
Pigeon
Pigeon
Pigeon
Pigeon
Pigeon
Pigeon
Pigeon
Pigeon
Pigeon
Pigeon
Pigeon
Pigeon
Pigeon
Pigeon
Pigeon
Pigeon
Pigeon
Pigeon
Pigeon
Pigeon
Pigeon
Pigeon
Pigeon
Pigeon
Pigeon
Pigeon
Pigeon
Pigeon
Pigeon
Pigeon
Pigeon
Pigeon
Pigeon
Pigeon
Pigeon
Pigeon

Pigeon
Pigeon
Pigeon
Pigeon
Pigeon
Pigeon
Pigeon
Pigeon
Pigeon
Pigeon
Pigeon
Pigeon
Pigeon
Pigeon
Pigeon
Pigeon
Pigeon
Pigeon
Pigeon
Pigeon
Pigeon
Pigeon
Pigeon
Pigeon
Pigeon
Pigeon
Pigeon
Pigeon
Pigeon
Pigeon
Pigeon
Pigeon
Pigeon
Pigeon
Pigeon
Pigeon
Pigeon
Pigeon
Pigeon
Pigeon
Pigeon
Pigeon
Pigeon
Pigeon
Pigeon
Pigeon

Pigeon
Pigeon
Pigeon
Pigeon
Pigeon
Pigeon
Pigeon
Pigeon
Pigeon
Pigeon
Pigeon
Pigeon
Pigeon
Pigeon
Pigeon
Pigeon
Pigeon
Pigeon
Pigeon
Pigeon
Pigeon
Pigeon
Pigeon
Pigeon
Pigeon
Pigeon
Pigeon
Pigeon
Pigeon
Pigeon
Pigeon
Pigeon
Pigeon
Pigeon
Pigeon
Pigeon
Pigeon
Pigeon
Pigeon
Pigeon
Pigeon
Pigeon
Pigeon
Pigeon
Pigeon
Pigeon

Pigeon
Pigeon
Pigeon
Pigeon
Pigeon
Pigeon
Pigeon
Pigeon
Pigeon
Pigeon
Pigeon
Pigeon
Pigeon
Pigeon
Pigeon
Pigeon
Pigeon
Pigeon
Pigeon
Pigeon
Pigeon
Pigeon
Pigeon
Pigeon
Pigeon
Pigeon
Pigeon
Pigeon
Pigeon
Pigeon
Pigeon
Pigeon
Pigeon
Pigeon
Pigeon
Pigeon
Pigeon
Pigeon
Pigeon
Pigeon
Pigeon
Pigeon
Pigeon
Pigeon
Pigeon
Pigeon

Pigeon
Pigeon
Pigeon
Pigeon
Pigeon
Pigeon
Pigeon
Pigeon
Pigeon
Pigeon
Pigeon
Pigeon
Pigeon
Pigeon
Pigeon
Pigeon
Pigeon
Pigeon
Pigeon
Pigeon
Pigeon
Pigeon
Pigeon
Pigeon
Pigeon
Pigeon
Pigeon
Pigeon
Pigeon
Pigeon
Pigeon
Pigeon
Pigeon
Pigeon
Pigeon
Pigeon
Pigeon
Pigeon
Pigeon
Pigeon
Pigeon
Pigeon
Pigeon
Pigeon
Pigeon
Pigeon

Pigeon
Pigeon
Pigeon
Pigeon
Pigeon
Pigeon
Pigeon
Pigeon
Pigeon
Pigeon
Pigeon
Pigeon
Pigeon
Pigeon
Pigeon
Pigeon
Pigeon
Pigeon
Pigeon
Pigeon
Pigeon
Pigeon
Pigeon
Pigeon
Pigeon
Pigeon
Pigeon
Pigeon
Pigeon
Pigeon
Pigeon
Pigeon
Pigeon
Pigeon
Pigeon
Pigeon
Pigeon
Pigeon
Pigeon
Pigeon
Pigeon
Pigeon
Pigeon
Pigeon
Pigeon
Pigeon

Pigeon
Pigeon
Pigeon
Pigeon
Pigeon
Pigeon
Pigeon
Pigeon
Pigeon
Pigeon
Pigeon
Pigeon
Pigeon
Pigeon
Pigeon
Pigeon
Pigeon
Pigeon
Pigeon
Pigeon
Pigeon
Pigeon
Pigeon
Pigeon
Pigeon
Pigeon
Pigeon
Pigeon
Pigeon
Pigeon
Pigeon
Pigeon
Pigeon
Pigeon
Pigeon
Pigeon
Pigeon
Pigeon
Pigeon
Pigeon
Pigeon
Pigeon
Pigeon
Pigeon
Pigeon
Pigeon

Pigeon
Pigeon
Pigeon
Pigeon
Pigeon
Pigeon
Pigeon
Pigeon
Pigeon
Pigeon
Pigeon
Pigeon
Pigeon
Pigeon
Pigeon
Pigeon
Pigeon
Pigeon
Pigeon
Pigeon
Pigeon
Pigeon
Pigeon
Pigeon
Pigeon
Pigeon
Pigeon
Pigeon
Pigeon
Pigeon
Pigeon
Pigeon
Pigeon
Pigeon
Pigeon
Pigeon
Pigeon
Pigeon
Pigeon
Pigeon
Pigeon
Pigeon
Pigeon
Pigeon
Pigeon
Pigeon

Pigeon
Pigeon
Pigeon
Pigeon
Pigeon
Pigeon
Pigeon
Pigeon
Pigeon
Pigeon
Pigeon
Pigeon
Pigeon
Pigeon
Pigeon
Pigeon
Pigeon
Pigeon
Pigeon
Pigeon
Pigeon
Pigeon
Pigeon
Pigeon
Pigeon
Pigeon
Pigeon
Pigeon
Pigeon
Pigeon
Pigeon
Pigeon
Pigeon
Pigeon
Pigeon
Pigeon
Pigeon
Pigeon
Pigeon
Pigeon
Pigeon
Pigeon
Pigeon
Pigeon
Pigeon
Pigeon

Pigeon
Pigeon
Pigeon
Pigeon
Pigeon
Pigeon
Pigeon
Pigeon
Pigeon
Pigeon
Pigeon
Pigeon
Pigeon
Pigeon
Pigeon
Pigeon
Pigeon
Pigeon
Pigeon
Pigeon
Pigeon
Pigeon
Pigeon
Pigeon
Pigeon
Pigeon
Pigeon
Pigeon
Pigeon
Pigeon
Pigeon
Pigeon
Pigeon
Pigeon
Pigeon
Pigeon
Pigeon
Pigeon
Pigeon
Pigeon
Pigeon
Pigeon
Pigeon
Pigeon
Pigeon
Pigeon

Pigeon
Pigeon
Pigeon
Pigeon
Pigeon
Pigeon
Pigeon
Pigeon
Pigeon
Pigeon
Pigeon
Pigeon
Pigeon
Pigeon
Pigeon
Pigeon
Pigeon
Pigeon
Pigeon
Pigeon
Pigeon
Pigeon
Pigeon
Pigeon
Pigeon
Pigeon
Pigeon
Pigeon
Pigeon
Pigeon
Pigeon
Pigeon
Pigeon
Pigeon
Pigeon
Pigeon
Pigeon
Pigeon
Pigeon
Pigeon
Pigeon
Pigeon
Pigeon
Pigeon
Pigeon
Pigeon

Pigeon
Pigeon
Pigeon
Pigeon
Pigeon
Pigeon
Pigeon
Pigeon
Pigeon
Pigeon
Pigeon
Pigeon
Pigeon
Pigeon
Pigeon
Pigeon
Pigeon
Pigeon
Pigeon
Pigeon
Pigeon
Pigeon
Pigeon
Pigeon
Pigeon
Pigeon
Pigeon
Pigeon
Pigeon
Pigeon
Pigeon
Pigeon
Pigeon
Pigeon
Pigeon
Pigeon
Pigeon
Pigeon
Pigeon
Pigeon
Pigeon
Pigeon
Pigeon
Pigeon
Pigeon
Pigeon

Pigeon
Pigeon
Pigeon
Pigeon
Pigeon
Pigeon
Pigeon
Pigeon
Pigeon
Pigeon
Pigeon
Pigeon
Pigeon
Pigeon
Pigeon
Pigeon
Pigeon
Pigeon
Pigeon
Pigeon
Pigeon
Pigeon
Pigeon
Pigeon
Pigeon
Pigeon
Pigeon
Pigeon
Pigeon
Pigeon
Pigeon
Pigeon
Pigeon
Pigeon
Pigeon
Pigeon
Pigeon
Pigeon
Pigeon
Pigeon
Pigeon
Pigeon
Pigeon
Pigeon
Pigeon
Pigeon

Pigeon
Pigeon
Pigeon
Pigeon
Pigeon
Pigeon
Pigeon
Pigeon
Pigeon
Pigeon
Pigeon
Pigeon
Pigeon
Pigeon
Pigeon
Pigeon
Pigeon
Pigeon
Pigeon
Pigeon
Pigeon
Pigeon
Pigeon
Pigeon
Pigeon
Pigeon
Pigeon
Pigeon
Pigeon
Pigeon
Pigeon
Pigeon
Pigeon
Pigeon
Pigeon
Pigeon
Pigeon
Pigeon
Pigeon
Pigeon
Pigeon
Pigeon
Pigeon
Pigeon
Pigeon
Pigeon

Pigeon
Pigeon
Pigeon
Pigeon
Pigeon
Pigeon
Pigeon
Pigeon
Pigeon
Pigeon
Pigeon
Pigeon
Pigeon
Pigeon
Pigeon
Pigeon
Pigeon
Pigeon
Pigeon
Pigeon
Pigeon
Pigeon
Pigeon
Pigeon
Pigeon
Pigeon
Pigeon
Pigeon
Pigeon
Pigeon
Pigeon
Pigeon
Pigeon
Pigeon
Pigeon
Pigeon
Pigeon
Pigeon
Pigeon
Pigeon
Pigeon
Pigeon
Pigeon
Pigeon
Pigeon
Pigeon

Pigeon
Pigeon
Pigeon
Pigeon
Pigeon
Pigeon
Pigeon
Pigeon
Pigeon
Pigeon
Pigeon
Pigeon
Pigeon
Pigeon
Pigeon
Pigeon
Pigeon
Pigeon
Pigeon
Pigeon
Pigeon
Pigeon
Pigeon
Pigeon
Pigeon
Pigeon
Pigeon
Pigeon
Pigeon
Pigeon
Pigeon
Pigeon
Pigeon
Pigeon
Pigeon
Pigeon
Pigeon
Pigeon
Pigeon
Pigeon
Pigeon
Pigeon
Pigeon
Pigeon
Pigeon
Pigeon

Pigeon
Pigeon
Pigeon
Pigeon
Pigeon
Pigeon
Pigeon
Pigeon
Pigeon
Pigeon
Pigeon
Pigeon
Pigeon
Pigeon
Pigeon
Pigeon
Pigeon
Pigeon
Pigeon
Pigeon
Pigeon
Pigeon
Pigeon
Pigeon
Pigeon
Pigeon
Pigeon
Pigeon
Pigeon
Pigeon
Pigeon
Pigeon
Pigeon
Pigeon
Pigeon
Pigeon
Pigeon
Pigeon
Pigeon
Pigeon
Pigeon
Pigeon
Pigeon
Pigeon
Pigeon
Pigeon

Pigeon
Pigeon
Pigeon
Pigeon
Pigeon
Pigeon
Pigeon
Pigeon
Pigeon
Pigeon
Pigeon
Pigeon
Pigeon
Pigeon
Pigeon
Pigeon
Pigeon
Pigeon
Pigeon
Pigeon
Pigeon
Pigeon
Pigeon
Pigeon
Pigeon
Pigeon
Pigeon
Pigeon
Pigeon
Pigeon
Pigeon
Pigeon
Pigeon
Pigeon
Pigeon
Pigeon
Pigeon
Pigeon
Pigeon
Pigeon
Pigeon
Pigeon
Pigeon
Pigeon
Pigeon
Pigeon

Pigeon
Pigeon
Pigeon
Pigeon
Pigeon
Pigeon
Pigeon
Pigeon
Pigeon
Pigeon
Pigeon
Pigeon
Pigeon
Pigeon
Pigeon
Pigeon
Pigeon
Pigeon
Pigeon
Pigeon
Pigeon
Pigeon
Pigeon
Pigeon
Pigeon
Pigeon
Pigeon
Pigeon
Pigeon
Pigeon
Pigeon
Pigeon
Pigeon
Pigeon
Pigeon
Pigeon
Pigeon
Pigeon
Pigeon
Pigeon
Pigeon
Pigeon
Pigeon
Pigeon
Pigeon
Pigeon

Pigeon
Pigeon
Pigeon
Pigeon
Pigeon
Pigeon
Pigeon
Pigeon
Pigeon
Pigeon
Pigeon
Pigeon
Pigeon
Pigeon
Pigeon
Pigeon
Pigeon
Pigeon
Pigeon
Pigeon
Pigeon
Pigeon
Pigeon
Pigeon
Pigeon
Pigeon
Pigeon
Pigeon
Pigeon
Pigeon
Pigeon
Pigeon
Pigeon
Pigeon
Pigeon
Pigeon
Pigeon
Pigeon
Pigeon
Pigeon
Pigeon
Pigeon
Pigeon
Pigeon
Pigeon
Pigeon

Pigeon
Pigeon
Pigeon
Pigeon
Pigeon
Pigeon
Pigeon
Pigeon
Pigeon
Pigeon
Pigeon
Pigeon
Pigeon
Pigeon
Pigeon
Pigeon
Pigeon
Pigeon
Pigeon
Pigeon
Pigeon
Pigeon
Pigeon
Pigeon
Pigeon
Pigeon
Pigeon
Pigeon
Pigeon
Pigeon
Pigeon
Pigeon
Pigeon
Pigeon
Pigeon
Pigeon
Pigeon
Pigeon
Pigeon
Pigeon
Pigeon
Pigeon
Pigeon
Pigeon
Pigeon
Pigeon

Pigeon
Pigeon
Pigeon
Pigeon
Pigeon
Pigeon
Pigeon
Pigeon
Pigeon
Pigeon
Pigeon
Pigeon
Pigeon
Pigeon
Pigeon
Pigeon
Pigeon
Pigeon
Pigeon
Pigeon
Pigeon
Pigeon
Pigeon
Pigeon
Pigeon
Pigeon
Pigeon
Pigeon
Pigeon
Pigeon
Pigeon
Pigeon
Pigeon
Pigeon
Pigeon
Pigeon
Pigeon
Pigeon
Pigeon
Pigeon
Pigeon
Pigeon
Pigeon
Pigeon
Pigeon
Pigeon

Pigeon
Pigeon
Pigeon
Pigeon
Pigeon
Pigeon
Pigeon
Pigeon
Pigeon
Pigeon
Pigeon
Pigeon
Pigeon
Pigeon
Pigeon
Pigeon
Pigeon
Pigeon
Pigeon
Pigeon
Pigeon
Pigeon
Pigeon
Pigeon
Pigeon
Pigeon
Pigeon
Pigeon
Pigeon
Pigeon
Pigeon
Pigeon
Pigeon
Pigeon
Pigeon
Pigeon
Pigeon
Pigeon
Pigeon
Pigeon
Pigeon
Pigeon
Pigeon
Pigeon
Pigeon
Pigeon

Pigeon
Pigeon
Pigeon
Pigeon
Pigeon
Pigeon
Pigeon
Pigeon
Pigeon
Pigeon
Pigeon
Pigeon
Pigeon
Pigeon
Pigeon
Pigeon
Pigeon
Pigeon
Pigeon
Pigeon
Pigeon
Pigeon
Pigeon
Pigeon
Pigeon
Pigeon
Pigeon
Pigeon
Pigeon
Pigeon
Pigeon
Pigeon
Pigeon
Pigeon
Pigeon
Pigeon
Pigeon
Pigeon
Pigeon
Pigeon
Pigeon
Pigeon
Pigeon
Pigeon
Pigeon
Pigeon

Pigeon
Pigeon
Pigeon
Pigeon
Pigeon
Pigeon
Pigeon
Pigeon
Pigeon
Pigeon
Pigeon
Pigeon
Pigeon
Pigeon
Pigeon
Pigeon
Pigeon
Pigeon
Pigeon
Pigeon
Pigeon
Pigeon
Pigeon
Pigeon
Pigeon
Pigeon
Pigeon
Pigeon
Pigeon
Pigeon
Pigeon
Pigeon
Pigeon
Pigeon
Pigeon
Pigeon
Pigeon
Pigeon
Pigeon
Pigeon
Pigeon
Pigeon
Pigeon
Pigeon
Pigeon
Pigeon

Pigeon
Pigeon
Pigeon
Pigeon
Pigeon
Pigeon
Pigeon
Pigeon
Pigeon
Pigeon
Pigeon
Pigeon
Pigeon
Pigeon
Pigeon
Pigeon
Pigeon
Pigeon
Pigeon
Pigeon
Pigeon
Pigeon
Pigeon
Pigeon
Pigeon
Pigeon
Pigeon
Pigeon
Pigeon
Pigeon
Pigeon
Pigeon
Pigeon
Pigeon
Pigeon
Pigeon
Pigeon
Pigeon
Pigeon
Pigeon
Pigeon
Pigeon
Pigeon
Pigeon
Pigeon
Pigeon

Pigeon
Pigeon
Pigeon
Pigeon
Pigeon
Pigeon
Pigeon
Pigeon
Pigeon
Pigeon
Pigeon
Pigeon
Pigeon
Pigeon
Pigeon
Pigeon
Pigeon
Pigeon
Pigeon
Pigeon
Pigeon
Pigeon
Pigeon
Pigeon
Pigeon
Pigeon
Pigeon
Pigeon
Pigeon
Pigeon
Pigeon
Pigeon
Pigeon
Pigeon
Pigeon
Pigeon
Pigeon
Pigeon
Pigeon
Pigeon
Pigeon
Pigeon
Pigeon
Pigeon
Pigeon
Pigeon

Pigeon
Pigeon
Pigeon
Pigeon
Pigeon
Pigeon
Pigeon
Pigeon
Pigeon
Pigeon
Pigeon
Pigeon
Pigeon
Pigeon
Pigeon
Pigeon
Pigeon
Pigeon
Pigeon
Pigeon
Pigeon
Pigeon
Pigeon
Pigeon
Pigeon
Pigeon
Pigeon
Pigeon
Pigeon
Pigeon
Pigeon
Pigeon
Pigeon
Pigeon
Pigeon
Pigeon
Pigeon
Pigeon
Pigeon
Pigeon
Pigeon
Pigeon
Pigeon
Pigeon
Pigeon
Pigeon

Pigeon
Pigeon
Pigeon
Pigeon
Pigeon
Pigeon
Pigeon
Pigeon
Pigeon
Pigeon
Pigeon
Pigeon
Pigeon
Pigeon
Pigeon
Pigeon
Pigeon
Pigeon
Pigeon
Pigeon
Pigeon
Pigeon
Pigeon
Pigeon
Pigeon
Pigeon
Pigeon
Pigeon
Pigeon
Pigeon
Pigeon
Pigeon
Pigeon
Pigeon
Pigeon
Pigeon
Pigeon
Pigeon
Pigeon
Pigeon
Pigeon
Pigeon
Pigeon
Pigeon
Pigeon
Pigeon

Pigeon
Pigeon
Pigeon
Pigeon
Pigeon
Pigeon
Pigeon
Pigeon
Pigeon
Pigeon
Pigeon
Pigeon
Pigeon
Pigeon
Pigeon
Pigeon
Pigeon
Pigeon
Pigeon
Pigeon
Pigeon
Pigeon
Pigeon
Pigeon
Pigeon
Pigeon
Pigeon
Pigeon
Pigeon
Pigeon
Pigeon
Pigeon
Pigeon
Pigeon
Pigeon
Pigeon
Pigeon
Pigeon
Pigeon
Pigeon
Pigeon
Pigeon
Pigeon
Pigeon
Pigeon
Pigeon

Pigeon
Pigeon
Pigeon
Pigeon
Pigeon
Pigeon
Pigeon
Pigeon
Pigeon
Pigeon
Pigeon
Pigeon
Pigeon
Pigeon
Pigeon
Pigeon
Pigeon
Pigeon
Pigeon
Pigeon
Pigeon
Pigeon
Pigeon
Pigeon
Pigeon
Pigeon
Pigeon
Pigeon
Pigeon
Pigeon
Pigeon
Pigeon
Pigeon
Pigeon
Pigeon
Pigeon
Pigeon
Pigeon
Pigeon
Pigeon
Pigeon
Pigeon
Pigeon
Pigeon
Pigeon
Pigeon

Pigeon
Pigeon
Pigeon
Pigeon
Pigeon
Pigeon
Pigeon
Pigeon
Pigeon
Pigeon
Pigeon
Pigeon
Pigeon
Pigeon
Pigeon
Pigeon
Pigeon
Pigeon
Pigeon
Pigeon
Pigeon
Pigeon
Pigeon
Pigeon
Pigeon
Pigeon
Pigeon
Pigeon
Pigeon
Pigeon
Pigeon
Pigeon
Pigeon
Pigeon
Pigeon
Pigeon
Pigeon
Pigeon
Pigeon
Pigeon
Pigeon
Pigeon
Pigeon
Pigeon
Pigeon
Pigeon

Pigeon
Pigeon
Pigeon
Pigeon
Pigeon
Pigeon
Pigeon
Pigeon
Pigeon
Pigeon
Pigeon
Pigeon
Pigeon
Pigeon
Pigeon
Pigeon
Pigeon
Pigeon
Pigeon
Pigeon
Pigeon
Pigeon
Pigeon
Pigeon
Pigeon
Pigeon
Pigeon
Pigeon
Pigeon
Pigeon
Pigeon
Pigeon
Pigeon
Pigeon
Pigeon
Pigeon
Pigeon
Pigeon
Pigeon
Pigeon
Pigeon
Pigeon
Pigeon
Pigeon
Pigeon
Pigeon

Pigeon
Pigeon
Pigeon
Pigeon
Pigeon
Pigeon
Pigeon
Pigeon
Pigeon
Pigeon
Pigeon
Pigeon
Pigeon
Pigeon
Pigeon
Pigeon
Pigeon
Pigeon
Pigeon
Pigeon
Pigeon
Pigeon
Pigeon
Pigeon
Pigeon
Pigeon
Pigeon
Pigeon
Pigeon
Pigeon
Pigeon
Pigeon
Pigeon
Pigeon
Pigeon
Pigeon
Pigeon
Pigeon
Pigeon
Pigeon
Pigeon
Pigeon
Pigeon
Pigeon
Pigeon
Pigeon

Pigeon
Pigeon
Pigeon
Pigeon
Pigeon
Pigeon
Pigeon
Pigeon
Pigeon
Pigeon
Pigeon
Pigeon
Pigeon
Pigeon
Pigeon
Pigeon
Pigeon
Pigeon
Pigeon
Pigeon
Pigeon
Pigeon
Pigeon
Pigeon
Pigeon
Pigeon
Pigeon
Pigeon
Pigeon
Pigeon
Pigeon
Pigeon
Pigeon
Pigeon
Pigeon
Pigeon
Pigeon
Pigeon
Pigeon
Pigeon
Pigeon
Pigeon
Pigeon
Pigeon
Pigeon
Pigeon

Pigeon
Pigeon
Pigeon
Pigeon
Pigeon
Pigeon
Pigeon
Pigeon
Pigeon
Pigeon
Pigeon
Pigeon
Pigeon
Pigeon
Pigeon
Pigeon
Pigeon
Pigeon
Pigeon
Pigeon
Pigeon
Pigeon
Pigeon
Pigeon
Pigeon
Pigeon
Pigeon
Pigeon
Pigeon
Pigeon
Pigeon
Pigeon
Pigeon
Pigeon
Pigeon
Pigeon
Pigeon
Pigeon
Pigeon
Pigeon
Pigeon
Pigeon
Pigeon
Pigeon
Pigeon
Pigeon

Pigeon
Pigeon
Pigeon
Pigeon
Pigeon
Pigeon
Pigeon
Pigeon
Pigeon
Pigeon
Pigeon
Pigeon
Pigeon
Pigeon
Pigeon
Pigeon
Pigeon
Pigeon
Pigeon
Pigeon
Pigeon
Pigeon
Pigeon
Pigeon
Pigeon
Pigeon
Pigeon
Pigeon
Pigeon
Pigeon
Pigeon
Pigeon
Pigeon
Pigeon
Pigeon
Pigeon
Pigeon
Pigeon
Pigeon
Pigeon
Pigeon
Pigeon
Pigeon
Pigeon
Pigeon
Pigeon

Pigeon
Pigeon
Pigeon
Pigeon
Pigeon
Pigeon
Pigeon
Pigeon
Pigeon
Pigeon
Pigeon
Pigeon
Pigeon
Pigeon
Pigeon
Pigeon
Pigeon
Pigeon
Pigeon
Pigeon
Pigeon
Pigeon
Pigeon
Pigeon
Pigeon
Pigeon
Pigeon
Pigeon
Pigeon
Pigeon
Pigeon
Pigeon
Pigeon
Pigeon
Pigeon
Pigeon
Pigeon
Pigeon
Pigeon
Pigeon
Pigeon
Pigeon
Pigeon
Pigeon
Pigeon
Pigeon

Pigeon
Pigeon
Pigeon
Pigeon
Pigeon
Pigeon
Pigeon
Pigeon
Pigeon
Pigeon
Pigeon
Pigeon
Pigeon
Pigeon
Pigeon
Pigeon
Pigeon
Pigeon
Pigeon
Pigeon
Pigeon
Pigeon
Pigeon
Pigeon
Pigeon
Pigeon
Pigeon
Pigeon
Pigeon
Pigeon
Pigeon
Pigeon
Pigeon
Pigeon
Pigeon
Pigeon
Pigeon
Pigeon
Pigeon
Pigeon
Pigeon
Pigeon
Pigeon
Pigeon
Pigeon
Pigeon

Pigeon
Pigeon
Pigeon
Pigeon
Pigeon
Pigeon
Pigeon
Pigeon
Pigeon
Pigeon
Pigeon
Pigeon
Pigeon
Pigeon
Pigeon
Pigeon
Pigeon
Pigeon
Pigeon
Pigeon
Pigeon
Pigeon
Pigeon
Pigeon
Pigeon
Pigeon
Pigeon
Pigeon
Pigeon
Pigeon
Pigeon
Pigeon
Pigeon
Pigeon
Pigeon
Pigeon
Pigeon
Pigeon
Pigeon
Pigeon
Pigeon
Pigeon
Pigeon
Pigeon
Pigeon
Pigeon

Pigeon
Pigeon
Pigeon
Pigeon
Pigeon
Pigeon
Pigeon
Pigeon
Pigeon
Pigeon
Pigeon
Pigeon
Pigeon
Pigeon
Pigeon
Pigeon
Pigeon
Pigeon
Pigeon
Pigeon
Pigeon
Pigeon
Pigeon
Pigeon
Pigeon
Pigeon
Pigeon
Pigeon
Pigeon
Pigeon
Pigeon
Pigeon
Pigeon
Pigeon
Pigeon
Pigeon
Pigeon
Pigeon
Pigeon
Pigeon
Pigeon
Pigeon
Pigeon
Pigeon
Pigeon
Pigeon

Pigeon
Pigeon
Pigeon
Pigeon
Pigeon
Pigeon
Pigeon
Pigeon
Pigeon
Pigeon
Pigeon
Pigeon
Pigeon
Pigeon
Pigeon
Pigeon
Pigeon
Pigeon
Pigeon
Pigeon
Pigeon
Pigeon
Pigeon
Pigeon
Pigeon
Pigeon
Pigeon
Pigeon
Pigeon
Pigeon
Pigeon
Pigeon
Pigeon
Pigeon
Pigeon
Pigeon
Pigeon
Pigeon
Pigeon
Pigeon
Pigeon
Pigeon
Pigeon
Pigeon
Pigeon
Pigeon

Pigeon
Pigeon
Pigeon
Pigeon
Pigeon
Pigeon
Pigeon
Pigeon
Pigeon
Pigeon
Pigeon
Pigeon
Pigeon
Pigeon
Pigeon
Pigeon
Pigeon
Pigeon
Pigeon
Pigeon
Pigeon
Pigeon
Pigeon
Pigeon
Pigeon
Pigeon
Pigeon
Pigeon
Pigeon
Pigeon
Pigeon
Pigeon
Pigeon
Pigeon
Pigeon
Pigeon
Pigeon
Pigeon
Pigeon
Pigeon
Pigeon
Pigeon
Pigeon
Pigeon
Pigeon
Pigeon

Pigeon
Pigeon
Pigeon
Pigeon
Pigeon
Pigeon
Pigeon
Pigeon
Pigeon
Pigeon
Pigeon
Pigeon
Pigeon
Pigeon
Pigeon
Pigeon
Pigeon
Pigeon
Pigeon
Pigeon
Pigeon
Pigeon
Pigeon
Pigeon
Pigeon
Pigeon
Pigeon
Pigeon
Pigeon
Pigeon
Pigeon
Pigeon
Pigeon
Pigeon
Pigeon
Pigeon
Pigeon
Pigeon
Pigeon
Pigeon
Pigeon
Pigeon
Pigeon
Pigeon
Pigeon
Pigeon

Pigeon
Pigeon
Pigeon
Pigeon
Pigeon
Pigeon
Pigeon
Pigeon
Pigeon
Pigeon
Pigeon
Pigeon
Pigeon
Pigeon
Pigeon
Pigeon
Pigeon
Pigeon
Pigeon
Pigeon
Pigeon
Pigeon
Pigeon
Pigeon
Pigeon
Pigeon
Pigeon
Pigeon
Pigeon
Pigeon
Pigeon
Pigeon
Pigeon
Pigeon
Pigeon
Pigeon
Pigeon
Pigeon
Pigeon
Pigeon
Pigeon
Pigeon
Pigeon
Pigeon
Pigeon
Pigeon

Pigeon
Pigeon
Pigeon
Pigeon
Pigeon
Pigeon
Pigeon
Pigeon
Pigeon
Pigeon
Pigeon
Pigeon
Pigeon
Pigeon
Pigeon
Pigeon
Pigeon
Pigeon
Pigeon
Pigeon
Pigeon
Pigeon
Pigeon
Pigeon
Pigeon
Pigeon
Pigeon
Pigeon
Pigeon
Pigeon
Pigeon
Pigeon
Pigeon
Pigeon
Pigeon
Pigeon
Pigeon
Pigeon
Pigeon
Pigeon
Pigeon
Pigeon
Pigeon
Pigeon
Pigeon
Pigeon

Pigeon
Pigeon
Pigeon
Pigeon
Pigeon
Pigeon
Pigeon
Pigeon
Pigeon
Pigeon
Pigeon
Pigeon
Pigeon
Pigeon
Pigeon
Pigeon
Pigeon
Pigeon
Pigeon
Pigeon
Pigeon
Pigeon
Pigeon
Pigeon
Pigeon
Pigeon
Pigeon
Pigeon
Pigeon
Pigeon
Pigeon
Pigeon
Pigeon
Pigeon
Pigeon
Pigeon
Pigeon
Pigeon
Pigeon
Pigeon
Pigeon
Pigeon
Pigeon
Pigeon
Pigeon
Pigeon

Pigeon
Pigeon
Pigeon
Pigeon
Pigeon
Pigeon
Pigeon
Pigeon
Pigeon
Pigeon
Pigeon
Pigeon
Pigeon
Pigeon
Pigeon
Pigeon
Pigeon
Pigeon
Pigeon
Pigeon
Pigeon
Pigeon
Pigeon
Pigeon
Pigeon
Pigeon
Pigeon
Pigeon
Pigeon
Pigeon
Pigeon
Pigeon
Pigeon
Pigeon
Pigeon
Pigeon
Pigeon
Pigeon
Pigeon
Pigeon
Pigeon
Pigeon
Pigeon
Pigeon
Pigeon
Pigeon

Pigeon
Pigeon
Pigeon
Pigeon
Pigeon
Pigeon
Pigeon
Pigeon
Pigeon
Pigeon
Pigeon
Pigeon
Pigeon
Pigeon
Pigeon
Pigeon
Pigeon
Pigeon
Pigeon
Pigeon
Pigeon
Pigeon
Pigeon
Pigeon
Pigeon
Pigeon
Pigeon
Pigeon
Pigeon
Pigeon
Pigeon
Pigeon
Pigeon
Pigeon
Pigeon
Pigeon
Pigeon
Pigeon
Pigeon
Pigeon
Pigeon
Pigeon
Pigeon
Pigeon
Pigeon
Pigeon

Pigeon
Pigeon
Pigeon
Pigeon
Pigeon
Pigeon
Pigeon
Pigeon
Pigeon
Pigeon
Pigeon
Pigeon
Pigeon
Pigeon
Pigeon
Pigeon
Pigeon
Pigeon
Pigeon
Pigeon
Pigeon
Pigeon
Pigeon
Pigeon
Pigeon
Pigeon
Pigeon
Pigeon
Pigeon
Pigeon
Pigeon
Pigeon
Pigeon
Pigeon
Pigeon
Pigeon
Pigeon
Pigeon
Pigeon
Pigeon
Pigeon
Pigeon
Pigeon
Pigeon
Pigeon
Pigeon

Pigeon
Pigeon
Pigeon
Pigeon
Pigeon
Pigeon
Pigeon
Pigeon
Pigeon
Pigeon
Pigeon
Pigeon
Pigeon
Pigeon
Pigeon
Pigeon
Pigeon
Pigeon
Pigeon
Pigeon
Pigeon
Pigeon
Pigeon
Pigeon
Pigeon
Pigeon
Pigeon
Pigeon
Pigeon
Pigeon
Pigeon
Pigeon
Pigeon
Pigeon
Pigeon
Pigeon
Pigeon
Pigeon
Pigeon
Pigeon
Pigeon
Pigeon
Pigeon
Pigeon
Pigeon
Pigeon

Pigeon
Pigeon
Pigeon
Pigeon
Pigeon
Pigeon
Pigeon
Pigeon
Pigeon
Pigeon
Pigeon
Pigeon
Pigeon
Pigeon
Pigeon
Pigeon
Pigeon
Pigeon
Pigeon
Pigeon
Pigeon
Pigeon
Pigeon
Pigeon
Pigeon
Pigeon
Pigeon
Pigeon
Pigeon
Pigeon
Pigeon
Pigeon
Pigeon
Pigeon
Pigeon
Pigeon
Pigeon
Pigeon
Pigeon
Pigeon
Pigeon
Pigeon
Pigeon
Pigeon
Pigeon
Pigeon

Pigeon
Pigeon
Pigeon
Pigeon
Pigeon
Pigeon
Pigeon
Pigeon
Pigeon
Pigeon
Pigeon
Pigeon
Pigeon
Pigeon
Pigeon
Pigeon
Pigeon
Pigeon
Pigeon
Pigeon
Pigeon
Pigeon
Pigeon
Pigeon
Pigeon
Pigeon
Pigeon
Pigeon
Pigeon
Pigeon
Pigeon
Pigeon
Pigeon
Pigeon
Pigeon
Pigeon
Pigeon
Pigeon
Pigeon
Pigeon
Pigeon
Pigeon
Pigeon
Pigeon
Pigeon
Pigeon

Pigeon
Pigeon
Pigeon
Pigeon
Pigeon
Pigeon
Pigeon
Pigeon
Pigeon
Pigeon
Pigeon
Pigeon
Pigeon
Pigeon
Pigeon
Pigeon
Pigeon
Pigeon
Pigeon
Pigeon
Pigeon
Pigeon
Pigeon
Pigeon
Pigeon
Pigeon
Pigeon
Pigeon
Pigeon
Pigeon
Pigeon
Pigeon
Pigeon
Pigeon
Pigeon
Pigeon
Pigeon
Pigeon
Pigeon
Pigeon
Pigeon
Pigeon
Pigeon
Pigeon
Pigeon
Pigeon

Pigeon
Pigeon
Pigeon
Pigeon
Pigeon
Pigeon
Pigeon
Pigeon
Pigeon
Pigeon
Pigeon
Pigeon
Pigeon
Pigeon
Pigeon
Pigeon
Pigeon
Pigeon
Pigeon
Pigeon
Pigeon
Pigeon
Pigeon
Pigeon
Pigeon
Pigeon
Pigeon
Pigeon
Pigeon
Pigeon
Pigeon
Pigeon
Pigeon
Pigeon
Pigeon
Pigeon
Pigeon
Pigeon
Pigeon
Pigeon
Pigeon
Pigeon
Pigeon
Pigeon
Pigeon
Pigeon

Pigeon
Pigeon
Pigeon
Pigeon
Pigeon
Pigeon
Pigeon
Pigeon
Pigeon
Pigeon
Pigeon
Pigeon
Pigeon
Pigeon
Pigeon
Pigeon
Pigeon
Pigeon
Pigeon
Pigeon
Pigeon
Pigeon
Pigeon
Pigeon
Pigeon
Pigeon
Pigeon
Pigeon
Pigeon
Pigeon
Pigeon
Pigeon
Pigeon
Pigeon
Pigeon
Pigeon
Pigeon
Pigeon
Pigeon
Pigeon
Pigeon
Pigeon
Pigeon
Pigeon
Pigeon
Pigeon

Pigeon
Pigeon
Pigeon
Pigeon
Pigeon
Pigeon
Pigeon
Pigeon
Pigeon
Pigeon
Pigeon
Pigeon
Pigeon
Pigeon
Pigeon
Pigeon
Pigeon
Pigeon
Pigeon
Pigeon
Pigeon
Pigeon
Pigeon
Pigeon
Pigeon
Pigeon
Pigeon
Pigeon
Pigeon
Pigeon
Pigeon
Pigeon
Pigeon
Pigeon
Pigeon
Pigeon
Pigeon
Pigeon
Pigeon
Pigeon
Pigeon
Pigeon
Pigeon
Pigeon
Pigeon
Pigeon

Pigeon
Pigeon
Pigeon
Pigeon
Pigeon
Pigeon
Pigeon
Pigeon
Pigeon
Pigeon
Pigeon
Pigeon
Pigeon
Pigeon
Pigeon
Pigeon
Pigeon
Pigeon
Pigeon
Pigeon
Pigeon
Pigeon
Pigeon
Pigeon
Pigeon
Pigeon
Pigeon
Pigeon
Pigeon
Pigeon
Pigeon
Pigeon
Pigeon
Pigeon
Pigeon
Pigeon
Pigeon
Pigeon
Pigeon
Pigeon
Pigeon
Pigeon
Pigeon
Pigeon
Pigeon
Pigeon

Pigeon
Pigeon
Pigeon
Pigeon
Pigeon
Pigeon
Pigeon
Pigeon
Pigeon
Pigeon
Pigeon
Pigeon
Pigeon
Pigeon
Pigeon
Pigeon
Pigeon
Pigeon
Pigeon
Pigeon
Pigeon
Pigeon
Pigeon
Pigeon
Pigeon
Pigeon
Pigeon
Pigeon
Pigeon
Pigeon
Pigeon
Pigeon
Pigeon
Pigeon
Pigeon
Pigeon
Pigeon
Pigeon
Pigeon
Pigeon
Pigeon
Pigeon
Pigeon
Pigeon
Pigeon
Pigeon

Pigeon
Pigeon
Pigeon
Pigeon
Pigeon
Pigeon
Pigeon
Pigeon
Pigeon
Pigeon
Pigeon
Pigeon
Pigeon
Pigeon
Pigeon
Pigeon
Pigeon
Pigeon
Pigeon
Pigeon
Pigeon
Pigeon
Pigeon
Pigeon
Pigeon
Pigeon
Pigeon
Pigeon
Pigeon
Pigeon
Pigeon
Pigeon
Pigeon
Pigeon
Pigeon
Pigeon
Pigeon
Pigeon
Pigeon
Pigeon
Pigeon
Pigeon
Pigeon
Pigeon
Pigeon
Pigeon

Pigeon
Pigeon
Pigeon
Pigeon
Pigeon
Pigeon
Pigeon
Pigeon
Pigeon
Pigeon
Pigeon
Pigeon
Pigeon
Pigeon
Pigeon
Pigeon
Pigeon
Pigeon
Pigeon
Pigeon
Pigeon
Pigeon
Pigeon
Pigeon
Pigeon
Pigeon
Pigeon
Pigeon
Pigeon
Pigeon
Pigeon
Pigeon
Pigeon
Pigeon
Pigeon
Pigeon
Pigeon
Pigeon
Pigeon
Pigeon
Pigeon
Pigeon
Pigeon
Pigeon
Pigeon
Pigeon

Pigeon
Pigeon
Pigeon
Pigeon
Pigeon
Pigeon
Pigeon
Pigeon
Pigeon
Pigeon
Pigeon
Pigeon
Pigeon
Pigeon
Pigeon
Pigeon
Pigeon
Pigeon
Pigeon
Pigeon
Pigeon
Pigeon
Pigeon
Pigeon
Pigeon
Pigeon
Pigeon
Pigeon
Pigeon
Pigeon
Pigeon
Pigeon
Pigeon
Pigeon
Pigeon
Pigeon
Pigeon
Pigeon
Pigeon
Pigeon
Pigeon
Pigeon
Pigeon
Pigeon
Pigeon
Pigeon

Pigeon
Pigeon
Pigeon
Pigeon
Pigeon
Pigeon
Pigeon
Pigeon
Pigeon
Pigeon
Pigeon
Pigeon
Pigeon
Pigeon
Pigeon
Pigeon
Pigeon
Pigeon
Pigeon
Pigeon
Pigeon
Pigeon
Pigeon
Pigeon
Pigeon
Pigeon
Pigeon
Pigeon
Pigeon
Pigeon
Pigeon
Pigeon
Pigeon
Pigeon
Pigeon
Pigeon
Pigeon
Pigeon
Pigeon
Pigeon
Pigeon
Pigeon
Pigeon
Pigeon
Pigeon
Pigeon

Pigeon
Pigeon
Pigeon
Pigeon
Pigeon
Pigeon
Pigeon
Pigeon
Pigeon
Pigeon
Pigeon
Pigeon
Pigeon
Pigeon
Pigeon
Pigeon
Pigeon
Pigeon
Pigeon
Pigeon
Pigeon
Pigeon
Pigeon
Pigeon
Pigeon
Pigeon
Pigeon
Pigeon
Pigeon
Pigeon
Pigeon
Pigeon
Pigeon
Pigeon
Pigeon
Pigeon
Pigeon
Pigeon
Pigeon
Pigeon
Pigeon
Pigeon
Pigeon
Pigeon
Pigeon
Pigeon

Pigeon
Pigeon
Pigeon
Pigeon
Pigeon
Pigeon
Pigeon
Pigeon
Pigeon
Pigeon
Pigeon
Pigeon
Pigeon
Pigeon
Pigeon
Pigeon
Pigeon
Pigeon
Pigeon
Pigeon
Pigeon
Pigeon
Pigeon
Pigeon
Pigeon
Pigeon
Pigeon
Pigeon
Pigeon
Pigeon
Pigeon
Pigeon
Pigeon
Pigeon
Pigeon
Pigeon
Pigeon
Pigeon
Pigeon
Pigeon
Pigeon
Pigeon
Pigeon
Pigeon
Pigeon
Pigeon

Pigeon
Pigeon
Pigeon
Pigeon
Pigeon
Pigeon
Pigeon
Pigeon
Pigeon
Pigeon
Pigeon
Pigeon
Pigeon
Pigeon
Pigeon
Pigeon
Pigeon
Pigeon
Pigeon
Pigeon
Pigeon
Pigeon
Pigeon
Pigeon
Pigeon
Pigeon
Pigeon
Pigeon
Pigeon
Pigeon
Pigeon
Pigeon
Pigeon
Pigeon
Pigeon
Pigeon
Pigeon
Pigeon
Pigeon
Pigeon
Pigeon
Pigeon
Pigeon
Pigeon
Pigeon
Pigeon

Pigeon
Pigeon
Pigeon
Pigeon
Pigeon
Pigeon
Pigeon
Pigeon
Pigeon
Pigeon
Pigeon
Pigeon
Pigeon
Pigeon
Pigeon
Pigeon
Pigeon
Pigeon
Pigeon
Pigeon
Pigeon
Pigeon
Pigeon
Pigeon
Pigeon
Pigeon
Pigeon
Pigeon
Pigeon
Pigeon
Pigeon
Pigeon
Pigeon
Pigeon
Pigeon
Pigeon
Pigeon
Pigeon
Pigeon
Pigeon
Pigeon
Pigeon
Pigeon
Pigeon
Pigeon
Pigeon

Pigeon
Pigeon
Pigeon
Pigeon
Pigeon
Pigeon
Pigeon
Pigeon
Pigeon
Pigeon
Pigeon
Pigeon
Pigeon
Pigeon
Pigeon
Pigeon
Pigeon
Pigeon
Pigeon
Pigeon
Pigeon
Pigeon
Pigeon
Pigeon
Pigeon
Pigeon
Pigeon
Pigeon
Pigeon
Pigeon
Pigeon
Pigeon
Pigeon
Pigeon
Pigeon
Pigeon
Pigeon
Pigeon
Pigeon
Pigeon
Pigeon
Pigeon
Pigeon
Pigeon
Pigeon
Pigeon

Pigeon
Pigeon
Pigeon
Pigeon
Pigeon
Pigeon
Pigeon
Pigeon
Pigeon
Pigeon
Pigeon
Pigeon
Pigeon
Pigeon
Pigeon
Pigeon
Pigeon
Pigeon
Pigeon
Pigeon
Pigeon
Pigeon
Pigeon
Pigeon
Pigeon
Pigeon
Pigeon
Pigeon
Pigeon
Pigeon
Pigeon
Pigeon
Pigeon
Pigeon
Pigeon
Pigeon
Pigeon
Pigeon
Pigeon
Pigeon
Pigeon
Pigeon
Pigeon
Pigeon
Pigeon
Pigeon

Pigeon
Pigeon
Pigeon
Pigeon
Pigeon
Pigeon
Pigeon
Pigeon
Pigeon
Pigeon
Pigeon
Pigeon
Pigeon
Pigeon
Pigeon
Pigeon
Pigeon
Pigeon
Pigeon
Pigeon
Pigeon
Pigeon
Pigeon
Pigeon
Pigeon
Pigeon
Pigeon
Pigeon
Pigeon
Pigeon
Pigeon
Pigeon
Pigeon
Pigeon
Pigeon
Pigeon
Pigeon
Pigeon
Pigeon
Pigeon
Pigeon
Pigeon
Pigeon
Pigeon
Pigeon
Pigeon

Pigeon
Pigeon
Pigeon
Pigeon
Pigeon
Pigeon
Pigeon
Pigeon
Pigeon
Pigeon
Pigeon
Pigeon
Pigeon
Pigeon
Pigeon
Pigeon
Pigeon
Pigeon
Pigeon
Pigeon
Pigeon
Pigeon
Pigeon
Pigeon
Pigeon
Pigeon
Pigeon
Pigeon
Pigeon
Pigeon
Pigeon
Pigeon
Pigeon
Pigeon
Pigeon
Pigeon
Pigeon
Pigeon
Pigeon
Pigeon
Pigeon
Pigeon
Pigeon
Pigeon
Pigeon
Pigeon

Pigeon
Pigeon
Pigeon
Pigeon
Pigeon
Pigeon
Pigeon
Pigeon
Pigeon
Pigeon
Pigeon
Pigeon
Pigeon
Pigeon
Pigeon
Pigeon
Pigeon
Pigeon
Pigeon
Pigeon
Pigeon
Pigeon
Pigeon
Pigeon
Pigeon
Pigeon
Pigeon
Pigeon
Pigeon
Pigeon
Pigeon
Pigeon
Pigeon
Pigeon
Pigeon
Pigeon
Pigeon
Pigeon
Pigeon
Pigeon
Pigeon
Pigeon
Pigeon
Pigeon
Pigeon
Pigeon

Pigeon
Pigeon
Pigeon
Pigeon
Pigeon
Pigeon
Pigeon
Pigeon
Pigeon
Pigeon
Pigeon
Pigeon
Pigeon
Pigeon
Pigeon
Pigeon
Pigeon
Pigeon
Pigeon
Pigeon
Pigeon
Pigeon
Pigeon
Pigeon
Pigeon
Pigeon
Pigeon
Pigeon
Pigeon
Pigeon
Pigeon
Pigeon
Pigeon
Pigeon
Pigeon
Pigeon
Pigeon
Pigeon
Pigeon
Pigeon
Pigeon
Pigeon
Pigeon
Pigeon
Pigeon
Pigeon

Pigeon
Pigeon
Pigeon
Pigeon
Pigeon
Pigeon
Pigeon
Pigeon
Pigeon
Pigeon
Pigeon
Pigeon
Pigeon
Pigeon
Pigeon
Pigeon
Pigeon
Pigeon
Pigeon
Pigeon
Pigeon
Pigeon
Pigeon
Pigeon
Pigeon
Pigeon
Pigeon
Pigeon
Pigeon
Pigeon
Pigeon
Pigeon
Pigeon
Pigeon
Pigeon
Pigeon
Pigeon
Pigeon
Pigeon
Pigeon
Pigeon
Pigeon
Pigeon
Pigeon
Pigeon
Pigeon

Pigeon
Pigeon
Pigeon
Pigeon
Pigeon
Pigeon
Pigeon
Pigeon
Pigeon
Pigeon
Pigeon
Pigeon
Pigeon
Pigeon
Pigeon
Pigeon
Pigeon
Pigeon
Pigeon
Pigeon
Pigeon
Pigeon
Pigeon
Pigeon
Pigeon
Pigeon
Pigeon
Pigeon
Pigeon
Pigeon
Pigeon
Pigeon
Pigeon
Pigeon
Pigeon
Pigeon
Pigeon
Pigeon
Pigeon
Pigeon
Pigeon
Pigeon
Pigeon
Pigeon
Pigeon
Pigeon

Pigeon
Pigeon
Pigeon
Pigeon
Pigeon
Pigeon
Pigeon
Pigeon
Pigeon
Pigeon
Pigeon
Pigeon
Pigeon
Pigeon
Pigeon
Pigeon
Pigeon
Pigeon
Pigeon
Pigeon
Pigeon
Pigeon
Pigeon
Pigeon
Pigeon
Pigeon
Pigeon
Pigeon
Pigeon
Pigeon
Pigeon
Pigeon
Pigeon
Pigeon
Pigeon
Pigeon
Pigeon
Pigeon
Pigeon
Pigeon
Pigeon
Pigeon
Pigeon
Pigeon
Pigeon
Pigeon

Pigeon
Pigeon
Pigeon
Pigeon
Pigeon
Pigeon
Pigeon
Pigeon
Pigeon
Pigeon
Pigeon
Pigeon
Pigeon
Pigeon
Pigeon
Pigeon
Pigeon
Pigeon
Pigeon
Pigeon
Pigeon
Pigeon
Pigeon
Pigeon
Pigeon
Pigeon
Pigeon
Pigeon
Pigeon
Pigeon
Pigeon
Pigeon
Pigeon
Pigeon
Pigeon
Pigeon
Pigeon
Pigeon
Pigeon
Pigeon
Pigeon
Pigeon
Pigeon
Pigeon
Pigeon
Pigeon

Pigeon
Pigeon
Pigeon
Pigeon
Pigeon
Pigeon
Pigeon
Pigeon
Pigeon
Pigeon
Pigeon
Pigeon
Pigeon
Pigeon
Pigeon
Pigeon
Pigeon
Pigeon
Pigeon
Pigeon
Pigeon
Pigeon
Pigeon
Pigeon
Pigeon
Pigeon
Pigeon
Pigeon
Pigeon
Pigeon
Pigeon
Pigeon
Pigeon
Pigeon
Pigeon
Pigeon
Pigeon
Pigeon
Pigeon
Pigeon
Pigeon
Pigeon
Pigeon
Pigeon
Pigeon
Pigeon

Pigeon
Pigeon
Pigeon
Pigeon
Pigeon
Pigeon
Pigeon
Pigeon
Pigeon
Pigeon
Pigeon
Pigeon
Pigeon
Pigeon
Pigeon
Pigeon
Pigeon
Pigeon
Pigeon
Pigeon
Pigeon
Pigeon
Pigeon
Pigeon
Pigeon
Pigeon
Pigeon
Pigeon
Pigeon
Pigeon
Pigeon
Pigeon
Pigeon
Pigeon
Pigeon
Pigeon
Pigeon
Pigeon
Pigeon
Pigeon
Pigeon
Pigeon
Pigeon
Pigeon
Pigeon
Pigeon

Pigeon
Pigeon
Pigeon
Pigeon
Pigeon
Pigeon
Pigeon
Pigeon
Pigeon
Pigeon
Pigeon
Pigeon
Pigeon
Pigeon
Pigeon
Pigeon
Pigeon
Pigeon
Pigeon
Pigeon
Pigeon
Pigeon
Pigeon
Pigeon
Pigeon
Pigeon
Pigeon
Pigeon
Pigeon
Pigeon
Pigeon
Pigeon
Pigeon
Pigeon
Pigeon
Pigeon
Pigeon
Pigeon
Pigeon
Pigeon
Pigeon
Pigeon
Pigeon
Pigeon
Pigeon
Pigeon

Pigeon
Pigeon
Pigeon
Pigeon
Pigeon
Pigeon
Pigeon
Pigeon
Pigeon
Pigeon
Pigeon
Pigeon
Pigeon
Pigeon
Pigeon
Pigeon
Pigeon
Pigeon
Pigeon
Pigeon
Pigeon
Pigeon
Pigeon
Pigeon
Pigeon
Pigeon
Pigeon
Pigeon
Pigeon
Pigeon
Pigeon
Pigeon
Pigeon
Pigeon
Pigeon
Pigeon
Pigeon
Pigeon
Pigeon
Pigeon
Pigeon
Pigeon
Pigeon
Pigeon
Pigeon
Pigeon

Pigeon
Pigeon
Pigeon
Pigeon
Mice
Mice
Mice
Mice
Mice
Mice
Mice
Mice
Mice
Mice
Mice
Mice
Mice
Mice
Mice
Mice
Mice
Mice
Mice
Mice
Mice
Mice
Mice
Mice
Mice
Mice
Mice
Mice
Mice
Mice
Mice
Mice
Mice
Mice
Mice
Mice
Mice
Mice
Mice
Mice
Mice
Mice

Mice
Mice
Mice
Mice
Mice
Mice
Mice
Mice
Mice
Mice
Mice
Mice
Mice
Mice
Mice
Mice
Mice
Mice
Mice
Mice
Mice
Mice
Mice
Mice
Mice
Mice
Mice
Mice
Mice
Mice
Mice
Mice
Mice
Mice
Mice
Mice
Mice
Mice
Mice
Mice
Mice
Mice
Mice
Mice
Mice
Mice

Mice
Mice
Mice
Mice
Mice
Mice
Mice
Mice
Mice
Mice
Mice
Mice
Mice
Mice
Mice
Mice
Mice
Mice
Mice
Mice
Mice
Mice
Mice
Mice
Mice
Mice
Mice
Mice
Mice
Mice
Mice
Mice
Mice
Mice
Mice
Mice
Mice
Mice
Mice
Mice
Mice
Mice
Mice
Mice
Mice
Mice

Mice
Mice
Mice
Mice
Mice
Mice
Mice
Mice
Mice
Mice
Mice
Mice
Mice
Mice
Mice
Mice
Mice
Mice
Mice
Mice
Mice
Mice
Mice
Mice
Mice
Mice
Mice
Mice
Mice
Mice
Mice
Mice
Mice
Mice
Mice
Mice
Mice
Mice
Mice
Mice
Mice
Mice
Mice
Mice
Mice
Mice

Mice
Mice
Mice
Mice
Mice
Mice
Mice
Mice
Mice
Mice
Mice
Mice
Mice
Mice
Mice
Mice
Mice
Mice
Mice
Mice
Mice
Mice
Mice
Mice
Mice
Mice
Mice
Mice
Mice
Mice
Mice
Mice
Mice
Mice
Mice
Mice
Mice
Mice
Mice
Mice
Mice
Mice
Mice
Mice
Mice
Mice

Mice
Mice
Mice
Mice
Mice
Mice
Mice
Mice
Mice
Mice
Mice
Mice
Mice
Mice
Mice
Mice
Mice
Mice
Mice
Mice
Mice
Mice
Mice
Mice
Mice
Mice
Mice
Mice
Mice
Mice
Mice
Mice
Mice
Mice
Mice
Mice
Mice
Mice
Mice
Mice
Mice
Mice
Mice
Mice
Mice
Mice

Mice
Mice
Mice
Mice
Mice
Mice
Mice
Mice
Mice
Mice
Mice
Mice
Mice
Mice
Mice
Mice
Mice
Mice
Mice
Mice
Mice
Mice
Mice
Mice
Mice
Mice
Mice
Mice
Mice
Mice
Mice
Mice
Mice
Mice
Mice
Mice
Mice
Mice
Mice
Mice
Mice
Mice
Mice
Mice
Mice
Mice

Mice
Mice
Mice
Mice
Mice
Mice
Mice
Mice
Mice
Mice
Mice
Mice
Mice
Mice
Mice
Mice
Mice
Mice
Mice
Mice
Mice
Mice
Mice
Mice
Mice
Mice
Mice
Mice
Mice
Mice
Mice
Mice
Mice
Mice
Mice
Mice
Mice
Mice
Mice
Mice
Mice
Mice
Mice
Mice
Mice
Mice

Mice
Mice
Mice
Mice
Mice
Mice
Mice
Mice
Mice
Mice
Mice
Mice
Mice
Mice
Mice
Mice
Mice
Mice
Mice
Mice
Mice
Mice
Mice
Mice
Mice
Mice
Mice
Mice
Mice
Mice
Mice
Mice
Mice
Mice
Mice
Mice
Mice
Mice
Mice
Mice
Mice
Mice
Mice
Mice
Mice
Mice

Mice
Mice
Mice
Mice
Mice
Mice
Mice
Mice
Mice
Mice
Mice
Mice
Mice
Mice
Mice
Mice
Mice
Mice
Mice
Mice
Mice
Mice
Mice
Mice
Mice
Mice
Mice
Mice
Mice
Mice
Mice
Mice
Mice
Mice
Mice
Mice
Mice
Mice
Mice
Mice
Mice
Mice
Mice
Mice
Mice
Mice

Mice
Mice
Mice
Mice
Mice
Mice
Mice
Mice
Mice
Mice
Mice
Mice
Mice
Mice
Mice
Mice
Mice
Mice
Mice
Mice
Mice
Mice
Mice
Mice
Mice
Mice
Mice
Mice
Mice
Mice
Mice
Mice
Mice
Mice
Mice
Mice
Mice
Mice
Mice
Mice
Mice
Mice
Mice
Mice
Mice
Mice

Mice
Mice
Mice
Mice
Mice
Mice
Mice
Mice
Mice
Mice
Mice
Mice
Mice
Mice
Mice
Mice
Mice
Mice
Mice
Mice
Mice
Mice
Mice
Mice
Mice
Mice
Mice
Mice
Mice
Mice
Mice
Mice
Mice
Mice
Mice
Mice
Mice
Mice
Mice
Mice
Mice
Mice
Mice
Mice
Mice
Mice

Mice
Mice
Mice
Mice
Mice
Mice
Mice
Mice
Mice
Mice
Mice
Mice
Mice
Mice
Mice
Mice
Mice
Mice
Mice
Mice
Mice
Mice
Mice
Mice
Mice
Mice
Mice
Mice
Mice
Mice
Mice
Mice
Mice
Mice
Mice
Mice
Mice
Mice
Mice
Mice
Mice
Mice
Mice
Mice
Mice
Mice

Mice
Mice
Mice
Mice
Mice
Mice
Mice
Mice
Mice
Mice
Mice
Mice
Mice
Mice
Mice
Mice
Mice
Mice
Mice
Mice
Mice
Mice
Mice
Mice
Mice
Mice
Mice
Mice
Mice
Mice
Mice
Mice
Mice
Mice
Mice
Mice
Mice
Mice
Mice
Mice
Mice
Mice
Mice
Mice
Mice
Mice

Mice
Mice
Mice
Mice
Mice
Mice
Mice
Mice
Mice
Mice
Mice
Mice
Mice
Mice
Mice
Mice
Mice
Mice
Mice
Mice
Mice
Mice
Mice
Mice
Mice
Mice
Mice
Mice
Mice
Mice
Mice
Mice
Mice
Mice
Mice
Mice
Mice
Mice
Mice
Mice
Mice
Mice
Mice
Mice
Mice
Mice

Mice
Mice
Mice
Mice
Mice
Mice
Mice
Mice
Mice
Mice
Mice
Mice
Mice
Mice
Mice
Mice
Mice
Mice
Mice
Mice
Mice
Mice
Mice
Mice
Mice
Mice
Mice
Mice
Mice
Mice
Mice
Mice
Mice
Mice
Mice
Mice
Mice
Mice
Mice
Mice
Mice
Mice
Mice
Mice
Mice
Mice

Mice
Mice
Mice
Mice
Mice
Mice
Mice
Mice
Mice
Mice
Mice
Mice
Mice
Mice
Mice
Mice
Mice
Mice
Mice
Mice
Mice
Mice
Mice
Mice
Mice
Mice
Mice
Mice
Mice
Mice
Mice
Mice
Mice
Mice
Mice
Mice
Mice
Mice
Mice
Mice
Mice
Mice
Mice
Mice
Mice
Mice

Mice
Mice
Mice
Mice
Mice
Mice
Mice
Mice
Mice
Mice
Mice
Mice
Mice
Mice
Mice
Mice
Mice
Mice
Mice
Mice
Mice
Mice
Mice
Mice
Mice
Mice
Mice
Mice
Mice
Mice
Mice
Mice
Mice
Mice
Mice
Mice
Mice
Mice
Mice
Mice
Mice
Mice
Mice
Mice
Mice
Mice

Mice
Mice
Mice
Mice
Mice
Mice
Mice
Mice
Mice
Mice
Mice
Mice
Mice
Mice
Mice
Mice
Mice
Mice
Mice
Mice
Mice
Mice
Mice
Mice
Mice
Mice
Mice
Mice
Mice
Mice
Mice
Mice
Mice
Mice
Mice
Mice
Mice
Mice
Mice
Mice
Mice
Mice
Mice
Mice
Mice
Mice

Mice
Mice
Mice
Mice
Mice
Mice
Mice
Mice
Mice
Mice
Mice
Mice
Mice
Mice
Mice
Mice
Mice
Mice
Mice
Mice
Mice
Mice
Mice
Mice
Mice
Mice
Mice
Mice
Mice
Mice
Mice
Mice
Mice
Mice
Mice
Mice
Mice
Mice
Mice
Mice
Mice
Mice
Mice
Mice
Mice
Mice

Mice
Mice
Mice
Mice
Mice
Mice
Mice
Mice
Mice
Mice
Mice
Mice
Mice
Mice
Mice
Mice
Mice
Mice
Mice
Mice
Mice
Mice
Mice
Mice
Mice
Mice
Mice
Mice
Mice
Mice
Mice
Mice
Mice
Mice
Mice
Mice
Mice
Mice
Mice
Mice
Mice
Mice
Mice
Mice
Mice
Mice

Mice
Mice
Mice
Mice
Mice
Mice
Mice
Mice
Mice
Mice
Mice
Mice
Mice
Mice
Mice
Mice
Mice
Mice
Mice
Mice
Mice
Mice
Mice
Mice
Mice
Mice
Mice
Mice
Mice
Mice
Mice
Mice
Mice
Mice
Mice
Mice
Mice
Mice
Mice
Mice
Mice
Mice
Mice
Mice
Mice
Mice

Mice
Mice
Mice
Mice
Mice
Mice
Mice
Mice
Mice
Mice
Mice
Mice
Mice
Mice
Mice
Mice
Mice
Mice
Mice
Mice
Mice
Mice
Mice
Mice
Mice
Mice
Mice
Mice
Mice
Mice
Mice
Mice
Mice
Mice
Mice
Mice
Mice
Mice
Mice
Mice
Mice
Mice
Mice
Mice
Mice
Mice

Mice
Mice
Mice
Mice
Mice
Mice
Mice
Mice
Mice
Mice
Mice
Mice
Mice
Mice
Mice
Mice
Mice
Mice
Mice
Mice
Mice
Mice
Mice
Mice
Mice
Mice
Mice
Mice
Mice
Mice
Mice
Mice
Mice
Mice
Mice
Mice
Mice
Mice
Mice
Mice
Mice
Mice
Mice
Mice
Mice
Mice

Mice
Mice
Mice
Mice
Mice
Mice
Mice
Mice
Mice
Mice
Mice
Mice
Mice
Mice
Mice
Mice
Mice
Mice
Mice
Mice
Mice
Mice
Mice
Mice
Mice
Mice
Mice
Mice
Mice
Mice
Mice
Mice
Mice
Mice
Mice
Mice
Mice
Mice
Mice
Mice
Mice
Mice
Mice
Mice
Mice
Mice

Mice
Mice
Mice
Mice
Mice
Mice
Mice
Mice
Mice
Mice
Mice
Mice
Mice
Mice
Mice
Mice
Mice
Mice
Mice
Mice
Mice
Mice
Mice
Mice
Mice
Mice
Mice
Mice
Mice
Mice
Mice
Mice
Mice
Mice
Mice
Mice
Mice
Mice
Mice
Mice
Mice
Mice
Mice
Mice
Mice
Mice

Mice
Mice
Mice
Mice
Mice
Mice
Mice
Mice
Mice
Mice
Mice
Mice
Mice
Mice
Mice
Mice
Mice
Mice
Mice
Mice
Mice
Mice
Mice
Mice
Mice
Mice
Mice
Mice
Mice
Mice
Mice
Mice
Mice
Mice
Mice
Mice
Mice
Mice
Mice
Mice
Mice
Mice
Mice
Mice
Mice
Mice

Mice
Mice
Mice
Mice
Mice
Mice
Mice
Mice
Mice
Mice
Mice
Mice
Mice
Mice
Mice
Mice
Mice
Mice
Mice
Mice
Mice
Mice
Mice
Mice
Mice
Mice
Mice
Mice
Mice
Mice
Mice
Mice
Mice
Mice
Mice
Mice
Mice
Mice
Mice
Mice
Mice
Mice
Mice
Mice
Mice
Mice

Mice
Mice
Mice
Mice
Mice
Mice
Mice
Mice
Mice
Mice
Mice
Mice
Mice
Mice
Mice
Mice
Mice
Mice
Mice
Mice
Mice
Mice
Mice
Mice
Mice
Mice
Mice
Mice
Mice
Mice
Mice
Mice
Mice
Mice
Mice
Mice
Mice
Mice
Mice
Mice
Mice
Mice
Mice
Mice
Mice
Mice

Mice
Mice
Mice
Mice
Mice
Mice
Mice
Mice
Mice
Mice
Mice
Mice
Mice
Mice
Mice
Mice
Mice
Mice
Mice
Mice
Mice
Mice
Mice
Mice
Mice
Mice
Mice
Mice
Mice
Mice
Mice
Mice
Mice
Mice
Mice
Mice
Mice
Mice
Mice
Mice
Mice
Mice
Mice
Mice
Mice
Mice

Mice
Mice
Mice
Mice
Mice
Mice
Mice
Mice
Mice
Mice
Mice
Mice
Mice
Mice
Mice
Mice
Mice
Mice
Mice
Mice
Mice
Mice
Mice
Mice
Mice
Mice
Mice
Mice
Mice
Mice
Mice
Mice
Mice
Mice
Mice
Mice
Mice
Mice
Mice
Mice
Mice
Mice
Mice
Mice
Mice
Mice

Mice
Mice
Mice
Mice
Mice
Mice
Mice
Mice
Mice
Mice
Mice
Mice
Mice
Mice
Mice
Mice
Mice
Mice
Mice
Mice
Mice
Mice
Mice
Mice
Mice
Mice
Mice
Mice
Mice
Mice
Mice
Mice
Mice
Mice
Mice
Mice
Mice
Mice
Mice
Mice
Mice
Mice
Mice
Mice
Mice
Mice

Mice
Mice
Mice
Mice
Mice
Mice
Mice
Mice
Mice
Mice
Mice
Mice
Mice
Mice
Mice
Mice
Mice
Mice
Mice
Mice
Mice
Mice
Mice
Mice
Mice
Mice
Mice
Mice
Mice
Mice
Mice
Mice
Mice
Mice
Mice
Mice
Mice
Mice
Mice
Mice
Mice
Mice
Mice
Mice
Mice
Mice

Mice
Mice
Mice
Mice
Mice
Mice
Mice
Mice
Mice
Mice
Mice
Mice
Mice
Mice
Mice
Mice
Mice
Mice
Mice
Mice
Mice
Mice
Mice
Mice
Mice
Mice
Mice
Mice
Mice
Mice
Mice
Mice
Mice
Mice
Mice
Mice
Mice
Mice
Mice
Mice
Mice
Mice
Mice
Mice
Mice
Mice

Mice
Mice
Mice
Mice
Mice
Mice
Mice
Mice
Mice
Mice
Mice
Mice
Mice
Mice
Mice
Mice
Mice
Mice
Mice
Mice
Mice
Mice
Mice
Mice
Mice
Mice
Mice
Mice
Mice
Mice
Mice
Mice
Mice
Mice
Mice
Mice
Mice
Mice
Mice
Mice
Mice
Mice
Mice
Mice
Mice
Mice

Mice
Mice
Mice
Mice
Mice
Mice
Mice
Mice
Mice
Mice
Mice
Mice
Mice
Mice
Mice
Mice
Mice
Mice
Mice
Mice
Mice
Mice
Mice
Mice
Mice
Mice
Mice
Mice
Mice
Mice
Mice
Mice
Mice
Mice
Mice
Mice
Mice
Mice
Mice
Mice
Mice
Mice
Mice
Mice
Mice
Mice

Mice
Mice
Mice
Mice
Mice
Mice
Mice
Mice
Mice
Mice
Mice
Mice
Mice
Mice
Mice
Mice
Mice
Mice
Mice
Mice
Mice
Mice
Mice
Mice
Mice
Mice
Mice
Mice
Mice
Mice
Mice
Mice
Mice
Mice
Mice
Mice
Mice
Mice
Mice
Mice
Mice
Mice
Mice
Mice
Mice
Mice

Mice
Mice
Mice
Mice
Mice
Mice
Mice
Mice
Mice
Mice
Mice
Mice
Mice
Mice
Mice
Mice
Mice
Mice
Mice
Mice
Mice
Mice
Mice
Mice
Mice
Mice
Mice
Mice
Mice
Mice
Mice
Mice
Mice
Mice
Mice
Mice
Mice
Mice
Mice
Mice
Mice
Mice
Mice
Mice
Mice
Mice

Mice
Mice
Mice
Mice
Mice
Mice
Mice
Mice
Mice
Mice
Mice
Mice
Mice
Mice
Mice
Mice
Mice
Mice
Mice
Mice
Mice
Mice
Mice
Mice
Mice
Mice
Mice
Mice
Mice
Mice
Mice
Mice
Mice
Mice
Mice
Mice
Mice
Mice
Mice
Mice
Mice
Mice
Mice
Mice
Mice
Mice

Mice
Mice
Mice
Mice
Mice
Mice
Mice
Mice
Mice
Mice
Mice
Mice
Mice
Mice
Mice
Mice
Mice
Mice
Mice
Mice
Mice
Mice
Mice
Mice
Mice
Mice
Mice
Mice
Mice
Mice
Mice
Mice
Mice
Mice
Mice
Mice
Mice
Mice
Mice
Mice
Mice
Mice
Mice
Mice
Mice
Mice

Mice
Mice
Mice
Mice
Mice
Mice
Mice
Mice
Mice
Mice
Mice
Mice
Mice
Mice
Mice
Mice
Mice
Mice
Mice
Mice
Mice
Mice
Mice
Mice
Mice
Mice
Mice
Mice
Mice
Mice
Mice
Mice
Mice
Mice
Mice
Mice
Mice
Mice
Mice
Mice
Mice
Mice
Mice
Mice
Mice
Mice

Mice
Mice
Mice
Mice
Mice
Mice
Mice
Mice
Mice
Mice
Mice
Mice
Mice
Mice
Mice
Mice
Mice
Mice
Mice
Mice
Mice
Mice
Mice
Mice
Mice
Mice
Mice
Mice
Mice
Mice
Mice
Mice
Mice
Mice
Mice
Mice
Mice
Mice
Mice
Mice
Mice
Mice
Mice
Mice
Mice
Mice

Mice
Mice
Mice
Mice
Mice
Mice
Mice
Mice
Mice
Mice
Mice
Mice
Mice
Mice
Mice
Mice
Mice
Mice
Mice
Mice
Mice
Mice
Mice
Mice
Mice
Mice
Mice
Mice
Mice
Mice
Mice
Mice
Mice
Mice
Mice
Mice
Mice
Mice
Mice
Mice
Mice
Mice
Mice
Mice
Mice
Mice

Mice
Mice
Mice
Mice
Mice
Mice
Mice
Mice
Mice
Mice
Mice
Mice
Mice
Mice
Mice
Mice
Mice
Mice
Mice
Mice
Mice
Mice
Mice
Mice
Mice
Mice
Mice
Mice
Mice
Mice
Mice
Mice
Mice
Mice
Mice
Mice
Mice
Mice
Mice
Mice
Mice
Mice
Mice
Mice
Mice
Mice

Mice
Mice
Mice
Mice
Mice
Mice
Mice
Mice
Mice
Mice
Mice
Mice
Mice
Mice
Mice
Mice
Mice
Mice
Mice
Mice
Mice
Mice
Mice
Mice
Mice
Mice
Mice
Mice
Mice
Mice
Mice
Mice
Mice
Mice
Mice
Mice
Mice
Mice
Mice
Mice
Mice
Mice
Mice
Mice
Mice
Mice

Mice
Mice
Mice
Mice
Mice
Mice
Mice
Mice
Mice
Mice
Mice
Mice
Mice
Mice
Mice
Mice
Mice
Mice
Mice
Mice
Mice
Mice
Mice
Mice
Mice
Mice
Mice
Mice
Mice
Mice
Mice
Mice
Mice
Mice
Mice
Mice
Mice
Mice
Mice
Mice
Mice
Mice
Mice
Mice
Mice
Mice

Mice
Mice
Mice
Mice
Mice
Mice
Mice
Mice
Mice
Mice
Mice
Mice
Mice
Mice
Mice
Mice
Mice
Mice
Mice
Mice
Mice
Mice
Mice
Mice
Mice
Mice
Mice
Mice
Mice
Mice
Mice
Mice
Mice
Mice
Mice
Mice
Mice
Mice
Mice
Mice
Mice
Mice
Mice
Mice
Mice
Mice

Mice
Mice
Mice
Mice
Mice
Mice
Mice
Mice
Mice
Mice
Mice
Mice
Mice
Mice
Mice
Mice
Mice
Mice
Mice
Mice
Mice
Mice
Mice
Mice
Mice
Mice
Mice
Mice
Mice
Mice
Mice
Mice
Mice
Mice
Mice
Mice
Mice
Mice
Mice
Mice
Mice
Mice
Mice
Mice
Mice
Mice

Mice
Mice
Mice
Mice
Mice
Mice
Mice
Mice
Mice
Mice
Mice
Mice
Mice
Mice
Mice
Mice
Mice
Mice
Mice
Mice
Mice
Mice
Mice
Mice
Mice
Mice
Mice
Mice
Mice
Mice
Mice
Mice
Mice
Mice
Mice
Mice
Mice
Mice
Mice
Mice
Mice
Mice
Mice
Mice
Mice
Mice

Mice
Mice
Mice
Mice
Mice
Mice
Mice
Mice
Mice
Mice
Mice
Mice
Mice
Mice
Mice
Mice
Mice
Mice
Mice
Mice
Mice
Mice
Mice
Mice
Mice
Mice
Mice
Mice
Mice
Mice
Mice
Mice
Mice
Mice
Mice
Mice
Mice
Mice
Mice
Mice
Mice
Mice
Mice
Mice
Mice
Mice

Mice
Mice
Mice
Mice
Mice
Mice
Mice
Mice
Mice
Mice
Mice
Mice
Mice
Mice
Mice
Mice
Mice
Mice
Mice
Mice
Mice
Mice
Mice
Mice
Mice
Mice
Mice
Mice
Mice
Mice
Mice
Mice
Mice
Mice
Mice
Mice
Mice
Mice
Mice
Mice
Mice
Mice
Mice
Mice
Mice
Mice

Mice
Mice
Mice
Mice
Mice
Mice
Mice
Mice
Mice
Mice
Mice
Mice
Mice
Mice
Mice
Mice
Mice
Mice
Mice
Mice
Mice
Mice
Mice
Mice
Mice
Mice
Mice
Mice
Mice
Mice
Mice
Mice
Mice
Mice
Mice
Mice
Mice
Mice
Mice
Mice
Mice
Mice
Mice
Mice
Mice
Mice

Mice
Mice
Mice
Mice
Mice
Mice
Mice
Mice
Mice
Mice
Mice
Mice
Mice
Mice
Mice
Mice
Mice
Mice
Mice
Mice
Mice
Mice
Mice
Mice
Mice
Mice
Mice
Mice
Mice
Mice
Mice
Mice
Mice
Mice
Mice
Mice
Mice
Mice
Mice
Mice
Mice
Mice
Mice
Mice
Mice
Mice

Mice
Mice
Mice
Mice
Mice
Mice
Mice
Mice
Mice
Mice
Mice
Mice
Mice
Mice
Mice
Mice
Mice
Mice
Mice
Mice
Mice
Mice
Mice
Mice
Mice
Mice
Mice
Mice
Mice
Mice
Mice
Mice
Mice
Mice
Mice
Mice
Mice
Mice
Mice
Mice
Mice
Mice
Mice
Mice
Mice
Mice

Mice
Mice
Mice
Mice
Mice
Mice
Mice
Mice
Mice
Mice
Mice
Mice
Mice
Mice
Mice
Mice
Mice
Mice
Mice
Mice
Mice
Mice
Mice
Mice
Mice
Mice
Mice
Mice
Mice
Mice
Mice
Mice
Mice
Mice
Mice
Mice
Mice
Mice
Mice
Mice
Mice
Mice
Mice
Mice
Mice
Mice

Mice
Mice
Mice
Mice
Mice
Mice
Mice
Mice
Mice
Mice
Mice
Mice
Mice
Mice
Mice
Mice
Mice
Mice
Mice
Mice
Mice
Mice
Mice
Mice
Mice
Mice
Mice
Mice
Mice
Mice
Mice
Mice
Mice
Mice
Mice
Mice
Mice
Mice
Mice
Mice
Mice
Mice
Mice
Mice
Mice
Mice

Mice
Mice
Mice
Mice
Mice
Mice
Mice
Mice
Mice
Mice
Mice
Mice
Mice
Mice
Mice
Mice
Mice
Mice
Mice
Mice
Mice
Mice
Mice
Mice
Mice
Mice
Mice
Mice
Mice
Mice
Mice
Mice
Mice
Mice
Mice
Mice
Mice
Mice
Mice
Mice
Mice
Mice
Mice
Mice
Mice
Mice

Mice
Mice
Mice
Mice
Mice
Mice
Mice
Mice
Mice
Mice
Mice
Mice
Mice
Mice
Mice
Mice
Mice
Mice
Mice
Mice
Mice
Mice
Mice
Mice
Mice
Mice
Mice
Mice
Mice
Mice
Mice
Mice
Mice
Mice
Mice
Mice
Mice
Mice
Mice
Mice
Mice
Mice
Mice
Mice
Mice
Mice

Mice
Mice
Mice
Mice
Mice
Mice
Mice
Mice
Mice
Mice
Mice
Mice
Mice
Mice
Mice
Mice
Mice
Mice
Mice
Mice
Mice
Mice
Mice
Mice
Mice
Mice
Mice
Mice
Mice
Mice
Mice
Mice
Mice
Mice
Mice
Mice
Mice
Mice
Mice
Mice
Mice
Mice
Mice
Mice
Mice
Mice

Mice
Mice
Mice
Mice
Mice
Mice
Mice
Mice
Mice
Mice
Mice
Mice
Mice
Mice
Mice
Mice
Mice
Mice
Mice
Mice
Mice
Mice
Mice
Mice
Mice
Mice
Mice
Mice
Mice
Mice
Mice
Mice
Mice
Mice
Mice
Mice
Mice
Mice
Mice
Mice
Mice
Mice
Mice
Mice
Mice
Mice

Mice
Mice
Mice
Mice
Mice
Mice
Mice
Mice
Mice
Mice
Mice
Mice
Mice
Mice
Mice
Mice
Mice
Mice
Mice
Mice
Mice
Mice
Mice
Mice
Mice
Mice
Mice
Mice
Mice
Mice
Mice
Mice
Mice
Mice
Mice
Mice
Mice
Mice
Mice
Mice
Mice
Mice
Mice
Mice
Mice
Mice

Mice
Mice
Mice
Mice
Mice
Mice
Mice
Mice
Mice
Mice
Mice
Mice
Mice
Mice
Mice
Mice
Mice
Mice
Mice
Mice
Mice
Mice
Mice
Mice
Mice
Mice
Mice
Mice
Mice
Mice
Mice
Mice
Mice
Mice
Mice
Mice
Mice
Mice
Mice
Mice
Mice
Mice
Mice
Mice
Mice
Mice

Mice
Mice
Mice
Mice
Mice
Mice
Mice
Mice
Mice
Mice
Mice
Mice
Mice
Mice
Mice
Mice
Mice
Mice
Mice
Mice
Mice
Mice
Mice
Mice
Mice
Mice
Mice
Mice
Mice
Mice
Mice
Mice
Mice
Mice
Mice
Mice
Mice
Mice
Mice
Mice
Mice
Mice
Mice
Mice
Mice
Mice

Mice
Mice
Mice
Mice
Mice
Mice
Mice
Mice
Mice
Mice
Mice
Mice
Mice
Mice
Mice
Mice
Mice
Mice
Mice
Mice
Mice
Mice
Mice
Mice
Mice
Mice
Mice
Mice
Mice
Mice
Mice
Mice
Mice
Mice
Mice
Mice
Mice
Mice
Mice
Mice
Mice
Mice
Mice
Mice
Mice
Mice

Mice
Mice
Mice
Mice
Mice
Mice
Mice
Mice
Mice
Mice
Mice
Mice
Mice
Mice
Mice
Mice
Mice
Mice
Mice
Mice
Mice
Mice
Mice
Mice
Mice
Mice
Mice
Mice
Mice
Mice
Mice
Mice
Mice
Mice
Mice
Mice
Mice
Mice
Mice
Mice
Mice
Mice
Mice
Mice
Mice
Mice

Mice
Mice
Mice
Mice
Mice
Mice
Mice
Mice
Mice
Mice
Mice
Mice
Mice
Mice
Mice
Mice
Mice
Mice
Mice
Mice
Mice
Mice
Mice
Mice
Mice
Mice
Mice
Mice
Mice
Mice
Mice
Mice
Mice
Mice
Mice
Mice
Mice
Mice
Mice
Mice
Mice
Mice
Mice
Mice
Mice
Mice

Mice
Mice
Mice
Mice
Mice
Mice
Mice
Mice
Mice
Mice
Mice
Mice
Mice
Mice
Mice
Mice
Mice
Mice
Mice
Mice
Mice
Mice
Mice
Mice
Mice
Mice
Mice
Mice
Mice
Mice
Mice
Mice
Mice
Mice
Mice
Mice
Mice
Mice
Mice
Mice
Mice
Mice
Mice
Mice
Mice
Mice

Mice
Mice
Mice
Mice
Mice
Mice
Mice
Mice
Mice
Mice
Mice
Mice
Mice
Mice
Mice
Mice
Mice
Mice
Mice
Mice
Mice
Mice
Mice
Mice
Mice
Mice
Mice
Mice
Mice
Mice
Mice
Mice
Mice
Mice
Mice
Mice
Mice
Mice
Mice
Mice
Mice
Mice
Mice
Mice
Mice
Mice

Mice
Mice
Mice
Mice
Mice
Mice
Mice
Mice
Mice
Mice
Mice
Mice
Mice
Mice
Mice
Mice
Mice
Mice
Mice
Mice
Mice
Mice
Mice
Mice
Mice
Mice
Mice
Mice
Mice
Mice
Mice
Mice
Mice
Mice
Mice
Mice
Mice
Mice
Mice
Mice
Mice
Mice
Mice
Mice
Mice
Mice

Mice
Mice
Mice
Mice
Mice
Mice
Mice
Mice
Mice
Mice
Mice
Mice
Mice
Mice
Mice
Mice
Mice
Mice
Mice
Mice
Mice
Mice
Mice
Mice
Mice
Mice
Mice
Mice
Mice
Mice
Mice
Mice
Mice
Mice
Mice
Mice
Mice
Mice
Mice
Mice
Mice
Mice
Mice
Mice
Mice
Mice

Mice
Mice
Mice
Mice
Mice
Mice
Mice
Mice
Mice
Mice
Mice
Mice
Mice
Mice
Mice
Mice
Mice
Mice
Mice
Mice
Mice
Mice
Mice
Mice
Mice
Mice
Mice
Mice
Mice
Mice
Mice
Mice
Mice
Mice
Mice
Mice
Mice
Mice
Mice
Mice
Mice
Mice
Mice
Mice
Mice
Mice

Mice
Mice
Mice
Mice
Mice
Mice
Mice
Mice
Mice
Mice
Mice
Mice
Mice
Mice
Mice
Mice
Mice
Mice
Mice
Mice
Mice
Mice
Mice
Mice
Mice
Mice
Mice
Mice
Mice
Mice
Mice
Mice
Mice
Mice
Mice
Mice
Mice
Mice
Mice
Mice
Mice
Mice
Mice
Mice
Mice
Mice

Mice
Mice
Mice
Mice
Mice
Mice
Mice
Mice
Mice
Mice
Mice
Mice
Mice
Mice
Mice
Mice
Mice
Mice
Mice
Mice
Mice
Mice
Mice
Mice
Mice
Mice
Mice
Mice
Mice
Mice
Mice
Mice
Mice
Mice
Mice
Mice
Mice
Mice
Mice
Mice
Mice
Mice
Mice
Mice
Mice
Mice

Mice
Mice
Mice
Mice
Mice
Mice
Mice
Mice
Mice
Mice
Mice
Mice
Mice
Mice
Mice
Mice
Mice
Mice
Mice
Mice
Mice
Mice
Mice
Mice
Mice
Mice
Mice
Mice
Mice
Mice
Mice
Mice
Mice
Mice
Mice
Mice
Mice
Mice
Mice
Mice
Mice
Mice
Mice
Mice
Mice
Mice

Mice
Mice
Mice
Mice
Mice
Mice
Mice
Mice
Mice
Mice
Mice
Mice
Mice
Mice
Mice
Mice
Mice
Mice
Mice
Mice
Mice
Mice
Mice
Mice
Mice
Mice
Mice
Mice
Mice
Mice
Mice
Mice
Mice
Mice
Mice
Mice
Mice
Mice
Mice
Mice
Mice
Mice
Mice
Mice
Mice
Mice

Mice
Mice
Mice
Mice
Mice
Mice
Mice
Mice
Mice
Mice
Mice
Mice
Mice
Mice
Mice
Mice
Mice
Mice
Mice
Mice
Mice
Mice
Mice
Mice
Mice
Mice
Mice
Mice
Mice
Mice
Mice
Mice
Mice
Mice
Mice
Mice
Mice
Mice
Mice
Mice
Mice
Mice
Mice
Mice
Mice
Mice

Mice
Mice
Mice
Mice
Mice
Mice
Mice
Mice
Mice
Mice
Mice
Mice
Mice
Mice
Mice
Mice
Mice
Mice
Mice
Mice
Mice
Mice
Mice
Mice
Mice
Mice
Mice
Mice
Mice
Mice
Mice
Mice
Mice
Mice
Mice
Mice
Mice
Mice
Mice
Mice
Mice
Mice
Mice
Mice
Mice
Mice

Mice
Mice
Mice
Mice
Mice
Mice
Mice
Mice
Mice
Mice
Mice
Mice
Mice
Mice
Mice
Mice
Mice
Mice
Mice
Mice
Mice
Mice
Mice
Mice
Mice
Mice
Mice
Mice
Mice
Mice
Mice
Mice
Mice
Mice
Mice
Mice
Mice
Mice
Mice
Mice
Mice
Mice
Mice
Mice
Mice
Mice

Mice
Mice
Mice
Mice
Mice
Mice
Mice
Mice
Mice
Mice
Mice
Mice
Mice
Mice
Mice
Mice
Mice
Mice
Mice
Mice
Mice
Mice
Mice
Mice
Mice
Mice
Mice
Mice
Mice
Mice
Mice
Mice
Mice
Mice
Mice
Mice
Mice
Mice
Mice
Mice
Mice
Mice
Mice
Mice
Mice
Mice

Mice
Mice
Mice
Mice
Mice
Mice
Mice
Mice
Mice
Mice
Mice
Mice
Mice
Mice
Mice
Mice
Mice
Mice
Mice
Mice
Mice
Mice
Mice
Mice
Mice
Mice
Mice
Mice
Mice
Mice
Mice
Mice
Mice
Mice
Mice
Mice
Mice
Mice
Mice
Mice
Mice
Mice
Mice
Mice
Mice
Mice

Mice
Mice
Mice
Mice
Mice
Mice
Mice
Mice
Mice
Mice
Mice
Mice
Mice
Mice
Mice
Mice
Mice
Mice
Mice
Mice
Mice
Mice
Mice
Mice
Mice
Mice
Mice
Mice
Mice
Mice
Mice
Mice
Mice
Mice
Mice
Mice
Mice
Mice
Mice
Mice
Mice
Mice
Mice
Mice
Mice
Mice

Mice
Mice
Mice
Mice
Mice
Mice
Mice
Mice
Mice
Mice
Mice
Mice
Mice
Mice
Mice
Mice
Mice
Mice
Mice
Mice
Mice
Mice
Mice
Mice
Mice
Mice
Mice
Mice
Mice
Mice
Mice
Mice
Mice
Mice
Mice
Mice
Mice
Mice
Mice
Mice
Mice
Mice
Mice
Mice
Mice
Mice

Mice
Mice
Mice
Mice
Mice
Mice
Mice
Mice
Mice
Mice
Mice
Mice
Mice
Mice
Mice
Mice
Mice
Mice
Mice
Mice
Mice
Mice
Mice
Mice
Mice
Mice
Mice
Mice
Mice
Mice
Mice
Mice
Mice
Mice
Mice
Mice
Mice
Mice
Mice
Mice
Mice
Mice
Mice
Mice
Mice
Mice

Mice
Mice
Mice
Mice
Mice
Mice
Mice
Mice
Mice
Mice
Mice
Mice
Mice
Mice
Mice
Mice
Mice
Mice
Mice
Mice
Mice
Mice
Mice
Mice
Mice
Mice
Mice
Mice
Mice
Mice
Mice
Mice
Mice
Mice
Mice
Mice
Mice
Mice
Mice
Mice
Mice
Mice
Mice
Mice
Mice
Mice

Mice
Mice
Mice
Mice
Mice
Mice
Mice
Mice
Mice
Mice
Mice
Mice
Mice
Mice
Mice
Mice
Mice
Mice
Mice
Mice
Mice
Mice
Mice
Mice
Mice
Mice
Mice
Mice
Mice
Mice
Mice
Mice
Mice
Mice
Mice
Mice
Mice
Mice
Mice
Mice
Mice
Mice
Mice
Mice
Mice
Mice

Mice
Mice
Mice
Mice
Mice
Mice
Mice
Mice
Mice
Mice
Mice
Mice
Mice
Mice
Mice
Mice
Mice
Mice
Mice
Mice
Mice
Mice
Mice
Mice
Mice
Mice
Mice
Mice
Mice
Mice
Mice
Mice
Mice
Mice
Mice
Mice
Mice
Mice
Mice
Mice
Mice
Mice
Mice
Mice
Mice
Mice

Mice
Mice
Mice
Mice
Mice
Mice
Mice
Mice
Mice
Mice
Mice
Mice
Mice
Mice
Mice
Mice
Mice
Mice
Mice
Mice
Mice
Mice
Mice
Mice
Mice
Mice
Mice
Mice
Mice
Mice
Mice
Mice
Mice
Mice
Mice
Mice
Mice
Mice
Mice
Mice
Mice
Mice
Mice
Mice
Mice
Mice

Mice
Mice
Mice
Mice
Mice
Mice
Mice
Mice
Mice
Mice
Mice
Mice
Mice
Mice
Mice
Mice
Mice
Mice
Mice
Mice
Mice
Mice
Mice
Mice
Mice
Mice
Mice
Mice
Mice
Mice
Mice
Mice
Mice
Mice
Mice
Mice
Mice
Mice
Mice
Mice
Mice
Mice
Mice
Mice
Mice
Mice

Mice
Mice
Mice
Mice
Mice
Mice
Mice
Mice
Mice
Mice
Mice
Mice
Mice
Mice
Mice
Mice
Mice
Mice
Mice
Mice
Mice
Mice
Mice
Mice
Mice
Mice
Mice
Mice
Mice
Mice
Mice
Mice
Mice
Mice
Mice
Mice
Mice
Mice
Mice
Mice
Mice
Mice
Mice
Mice
Mice
Mice

Mice
Mice
Mice
Mice
Mice
Mice
Mice
Mice
Mice
Mice
Mice
Mice
Mice
Mice
Mice
Mice
Mice
Mice
Mice
Mice
Mice
Mice
Mice
Mice
Mice
Mice
Mice
Mice
Mice
Mice
Mice
Mice
Mice
Mice
Mice
Mice
Mice
Mice
Mice
Mice
Mice
Mice
Mice
Mice
Mice
Mice

Mice
Mice
Mice
Mice
Mice
Mice
Mice
Mice
Mice
Mice
Mice
Mice
Mice
Mice
Mice
Mice
Mice
Mice
Mice
Mice
Mice
Mice
Mice
Mice
Mice
Mice
Mice
Mice
Mice
Mice
Mice
Mice
Mice
Mice
Mice
Mice
Mice
Mice
Mice
Mice
Mice
Mice
Mice
Mice
Mice
Mice

Mice
Mice
Mice
Mice
Mice
Mice
Mice
Mice
Mice
Mice
Mice
Mice
Mice
Mice
Mice
Mice
Mice
Mice
Mice
Mice
Mice
Mice
Mice
Mice
Mice
Mice
Mice
Mice
Mice
Mice
Mice
Mice
Mice
Mice
Mice
Mice
Mice
Mice
Mice
Mice
Mice
Mice
Mice
Mice
Mice
Mice

Mice
Mice
Mice
Mice
Mice
Mice
Mice
Mice
Mice
Mice
Mice
Mice
Mice
Mice
Mice
Mice
Mice
Mice
Mice
Mice
Mice
Mice
Mice
Mice
Mice
Mice
Mice
Mice
Mice
Mice
Mice
Mice
Mice
Mice
Mice
Mice
Mice
Mice
Mice
Mice
Mice
Mice
Mice
Mice
Mice
Mice

Mice
Mice
Mice
Mice
Mice
Mice
Mice
Mice
Mice
Mice
Mice
Mice
Mice
Mice
Mice
Mice
Mice
Mice
Mice
Mice
Mice
Mice
Mice
Mice
Mice
Mice
Mice
Mice
Mice
Mice
Mice
Mice
Mice
Mice
Mice
Mice
Mice
Mice
Mice
Mice
Mice
Mice
Mice
Mice
Mice
Mice

Mice
Mice
Mice
Mice
Mice
Mice
Mice
Mice
Mice
Mice
Mice
Mice
Mice
Mice
Mice
Mice
Mice
Mice
Mice
Mice
Mice
Mice
Mice
Mice
Mice
Mice
Mice
Mice
Mice
Mice
Mice
Mice
Mice
Mice
Mice
Mice
Mice
Mice
Mice
Mice
Mice
Mice
Mice
Mice
Mice
Mice

Mice
Mice
Mice
Mice
Mice
Mice
Mice
Mice
Mice
Mice
Mice
Mice
Mice
Mice
Mice
Mice
Mice
Mice
Mice
Mice
Mice
Mice
Mice
Mice
Mice
Mice
Mice
Mice
Mice
Mice
Mice
Mice
Mice
Mice
Mice
Mice
Mice
Mice
Mice
Mice
Mice
Mice
Mice
Mice
Mice
Mice

Mice
Mice
Mice
Mice
Mice
Mice
Mice
Mice
Mice
Mice
Mice
Mice
Mice
Mice
Mice
Mice
Mice
Mice
Mice
Mice
Mice
Mice
Mice
Mice
Mice
Mice
Mice
Mice
Mice
Mice
Mice
Mice
Mice
Mice
Mice
Mice
Mice
Mice
Mice
Mice
Mice
Mice
Mice
Mice
Mice
Mice

Mice
Mice
Mice
Mice
Mice
Mice
Mice
Mice
Mice
Mice
Mice
Mice
Mice
Mice
Mice
Mice
Mice
Mice
Mice
Mice
Mice
Mice
Mice
Mice
Mice
Mice
Mice
Mice
Mice
Mice
Mice
Mice
Mice
Mice
Mice
Mice
Mice
Mice
Mice
Mice
Mice
Mice
Mice
Mice
Mice
Mice

Mice
Mice
Mice
Mice
Mice
Mice
Mice
Mice
Mice
Mice
Mice
Mice
Mice
Mice
Mice
Mice
Mice
Mice
Mice
Mice
Mice
Mice
Mice
Mice
Mice
Mice
Mice
Mice
Mice
Mice
Mice
Mice
Mice
Mice
Mice
Mice
Mice
Mice
Mice
Mice
Mice
Mice
Mice
Mice
Mice
Mice

Mice
Mice
Mice
Mice
Mice
Mice
Mice
Mice
Mice
Mice
Mice
Mice
Mice
Mice
Mice
Mice
Mice
Mice
Mice
Mice
Mice
Mice
Mice
Mice
Mice
Mice
Mice
Mice
Mice
Mice
Mice
Mice
Mice
Mice
Mice
Mice
Mice
Mice
Mice
Mice
Mice
Mice
Mice
Mice
Mice
Mice

Mice
Mice
Mice
Mice
Mice
Mice
Mice
Mice
Mice
Mice
Mice
Mice
Mice
Mice
Mice
Mice
Mice
Mice
Mice
Mice
Mice
Mice
Mice
Mice
Mice
Mice
Mice
Mice
Mice
Mice
Mice
Mice
Mice
Mice
Mice
Mice
Mice
Mice
Mice
Mice
Mice
Mice
Mice
Mice
Mice
Mice

Mice
Mice
Mice
Mice
Mice
Mice
Mice
Mice
Mice
Mice
Mice
Mice
Mice
Mice
Mice
Mice
Mice
Mice
Mice
Mice
Mice
Mice
Mice
Mice
Mice
Mice
Mice
Mice
Mice
Mice
Mice
Mice
Mice
Mice
Mice
Mice
Mice
Mice
Mice
Mice
Mice
Mice
Mice
Mice
Mice
Mice

Mice
Mice
Mice
Mice
Mice
Mice
Mice
Mice
Mice
Mice
Mice
Mice
Mice
Mice
Mice
Mice
Mice
Mice
Mice
Mice
Mice
Mice
Mice
Mice
Mice
Mice
Mice
Mice
Mice
Mice
Mice
Mice
Mice
Mice
Mice
Mice
Mice
Mice
Mice
Mice
Mice
Mice
Mice
Mice
Mice
Mice

Mice
Mice
Mice
Mice
Mice
Mice
Mice
Mice
Mice
Mice
Mice
Mice
Mice
Mice
Mice
Mice
Mice
Mice
Mice
Mice
Mice
Mice
Mice
Mice
Mice
Mice
Mice
Mice
Mice
Mice
Mice
Mice
Mice
Mice
Mice
Mice
Mice
Mice
Mice
Mice
Mice
Mice
Mice
Mice
Mice
Mice

Mice
Mice
Mice
Mice
Mice
Mice
Mice
Mice
Mice
Mice
Mice
Mice
Mice
Mice
Mice
Mice
Mice
Mice
Mice
Mice
Mice
Mice
Mice
Mice
Mice
Mice
Mice
Mice
Mice
Mice
Mice
Mice
Mice
Mice
Mice
Mice
Mice
Mice
Mice
Mice
Mice
Mice
Mice
Mice
Mice
Mice

Mice
Mice
Mice
Mice
Mice
Mice
Mice
Mice
Mice
Mice
Mice
Mice
Mice
Mice
Mice
Mice
Mice
Mice
Mice
Mice
Mice
Mice
Mice
Mice
Mice
Mice
Mice
Mice
Mice
Mice
Mice
Mice
Mice
Mice
Mice
Mice
Mice
Mice
Mice
Mice
Mice
Mice
Mice
Mice
Mice
Mice

Mice
Mice
Mice
Mice
Mice
Mice
Mice
Mice
Mice
Mice
Mice
Mice
Mice
Mice
Mice
Mice
Mice
Mice
Mice
Mice
Mice
Mice
Mice
Mice
Mice
Mice
Mice
Mice
Mice
Mice
Mice
Mice
Mice
Mice
Mice
Mice
Mice
Mice
Mice
Mice
Mice
Mice
Mice
Mice
Mice
Mice

Mice
Mice
Mice
Mice
Mice
Mice
Mice
Mice
Mice
Mice
Mice
Mice
Mice
Mice
Mice
Mice
Mice
Mice
Mice
Mice
Mice
Mice
Mice
Mice
Mice
Mice
Mice
Mice
Mice
Mice
Mice
Mice
Mice
Mice
Mice
Mice
Mice
Mice
Mice
Mice
Mice
Mice
Mice
Mice
Mice
Mice

Mice
Mice
Mice
Mice
Mice
Mice
Mice
Mice
Mice
Mice
Mice
Mice
Mice
Mice
Mice
Mice
Mice
Mice
Mice
Mice
Mice
Mice
Mice
Mice
Mice
Mice
Mice
Mice
Mice
Mice
Mice
Mice
Mice
Mice
Mice
Mice
Mice
Mice
Mice
Mice
Mice
Mice
Mice
Mice
Mice
Mice

Mice
Mice
Mice
Mice
Mice
Mice
Mice
Mice
Mice
Mice
Mice
Mice
Mice
Mice
Mice
Mice
Mice
Mice
Mice
Mice
Mice
Mice
Mice
Mice
Mice
Mice
Mice
Mice
Mice
Mice
Mice
Mice
Mice
Mice
Mice
Mice
Mice
Mice
Mice
Mice
Mice
Mice
Mice
Mice
Mice
Mice

Mice
Mice
Mice
Mice
Mice
Mice
Mice
Mice
Mice
Mice
Mice
Mice
Mice
Mice
Mice
Mice
Mice
Mice
Mice
Mice
Mice
Mice
Mice
Mice
Mice
Mice
Mice
Mice
Mice
Mice
Mice
Mice
Mice
Mice
Mice
Mice
Mice
Mice
Mice
Mice
Mice
Mice
Mice
Mice
Mice
Mice

Mice
Mice
Mice
Mice
Mice
Mice
Mice
Mice
Mice
Mice
Mice
Mice
Mice
Mice
Mice
Mice
Mice
Mice
Mice
Mice
Mice
Mice
Mice
Mice
Mice
Mice
Mice
Mice
Mice
Mice
Mice
Mice
Mice
Mice
Mice
Mice
Mice
Mice
Mice
Mice
Mice
Mice
Mice
Mice
Mice
Mice

Mice
Mice
Mice
Mice
Mice
Mice
Mice
Mice
Mice
Mice
Mice
Mice
Mice
Mice
Mice
Mice
Mice
Mice
Mice
Mice
Mice
Mice
Mice
Mice
Mice
Mice
Mice
Mice
Mice
Mice
Mice
Mice
Mice
Mice
Mice
Mice
Mice
Mice
Mice
Mice
Mice
Mice
Mice
Mice
Mice
Mice

Mice
Mice
Mice
Mice
Mice
Mice
Mice
Mice
Mice
Mice
Mice
Mice
Mice
Mice
Mice
Mice
Mice
Mice
Mice
Mice
Mice
Mice
Mice
Mice
Mice
Mice
Mice
Mice
Mice
Mice
Mice
Mice
Mice
Mice
Mice
Mice
Mice
Mice
Mice
Mice
Mice
Mice
Mice
Mice
Mice
Mice

Mice
Mice
Mice
Mice
Mice
Mice
Mice
Mice
Mice
Mice
Mice
Mice
Mice
Mice
Mice
Mice
Mice
Mice
Mice
Mice
Mice
Mice
Mice
Mice
Mice
Mice
Mice
Mice
Mice
Mice
Mice
Mice
Mice
Mice
Mice
Mice
Mice
Mice
Mice
Mice
Mice
Mice
Mice
Mice
Mice
Mice

Mice
Mice
Mice
Mice
Mice
Mice
Mice
Mice
Mice
Mice
Mice
Mice
Mice
Mice
Mice
Mice
Mice
Mice
Mice
Mice
Mice
Mice
Mice
Mice
Mice
Mice
Mice
Mice
Mice
Mice
Mice
Mice
Mice
Mice
Mice
Mice
Mice
Mice
Mice
Mice
Mice
Mice
Mice
Mice
Mice
Mice

Mice
Mice
Mice
Mice
Mice
Mice
Mice
Mice
Mice
Mice
Mice
Mice
Mice
Mice
Mice
Mice
Mice
Mice
Mice
Mice
Mice
Mice
Mice
Mice
Mice
Mice
Mice
Mice
Mice
Mice
Mice
Mice
Mice
Mice
Mice
Mice
Mice
Mice
Mice
Mice
Mice
Mice
Mice
Mice
Mice
Mice

Mice
Mice
Mice
Mice
Mice
Mice
Mice
Mice
Mice
Mice
Mice
Mice
Mice
Mice
Mice
Mice
Mice
Mice
Mice
Mice
Mice
Mice
Mice
Mice
Mice
Mice
Mice
Mice
Mice
Mice
Mice
Mice
Mice
Mice
Mice
Mice
Mice
Mice
Mice
Mice
Mice
Mice
Mice
Mice
Mice
Mice

Mice
Mice
Mice
Mice
Mice
Mice
Mice
Mice
Mice
Mice
Mice
Mice
Mice
Mice
Mice
Mice
Mice
Mice
Mice
Mice
Mice
Mice
Mice
Mice
Mice
Mice
Mice
Mice
Mice
Mice
Mice
Mice
Mice
Mice
Mice
Mice
Mice
Mice
Mice
Mice
Mice
Mice
Mice
Mice
Mice
Mice

Mice
Mice
Mice
Mice
Mice
Mice
Mice
Mice
Mice
Mice
Mice
Mice
Mice
Mice
Mice
Mice
Mice
Mice
Mice
Mice
Mice
Mice
Mice
Mice
Mice
Mice
Mice
Mice
Mice
Mice
Mice
Mice
Mice
Mice
Mice
Mice
Mice
Mice
Mice
Mice
Mice
Mice
Mice
Mice
Mice
Mice

Mice
Mice
Mice
Mice
Mice
Mice
Mice
Mice
Mice
Mice
Mice
Mice
Mice
Mice
Mice
Mice
Mice
Mice
Mice
Mice
Mice
Mice
Mice
Mice
Mice
Mice
Mice
Mice
Mice
Mice
Mice
Mice
Mice
Mice
Mice
Mice
Mice
Mice
Mice
Mice
Mice
Mice
Mice
Mice
Mice
Mice

Mice
Mice
Mice
Mice
Mice
Mice
Mice
Mice
Mice
Mice
Mice
Mice
Mice
Mice
Mice
Mice
Mice
Mice
Mice
Mice
Mice
Mice
Mice
Mice
Mice
Mice
Mice
Mice
Mice
Mice
Mice
Mice
Mice
Mice
Mice
Mice
Mice
Mice
Mice
Mice
Mice
Mice
Mice
Mice
Mice
Mice

Mice
Mice
Mice
Mice
Mice
Mice
Mice
Mice
Mice
Mice
Mice
Mice
Mice
Mice
Mice
Mice
Mice
Mice
Mice
Mice
Mice
Mice
Mice
Mice
Mice
Mice
Mice
Mice
Mice
Mice
Mice
Mice
Mice
Mice
Mice
Mice
Mice
Mice
Mice
Mice
Mice
Mice
Mice
Mice
Mice
Mice

Mice
Mice
Mice
Mice
Mice
Mice
Mice
Mice
Mice
Mice
Mice
Mice
Mice
Mice
Mice
Mice
Mice
Mice
Mice
Mice
Mice
Mice
Mice
Mice
Mice
Mice
Mice
Mice
Mice
Mice
Mice
Mice
Mice
Mice
Mice
Mice
Mice
Mice
Mice
Mice
Mice
Mice
Mice
Mice
Mice
Mice

Mice
Mice
Mice
Mice
Mice
Mice
Mice
Mice
Mice
Mice
Mice
Mice
Mice
Mice
Mice
Mice
Mice
Mice
Mice
Mice
Mice
Mice
Mice
Mice
Mice
Mice
Mice
Mice
Mice
Mice
Mice
Mice
Mice
Mice
Mice
Mice
Mice
Mice
Mice
Mice
Mice
Mice
Mice
Mice
Mice
Mice

Mice
Mice
Mice
Mice
Mice
Mice
Mice
Mice
Mice
Mice
Mice
Mice
Mice
Mice
Mice
Mice
Mice
Mice
Mice
Mice
Mice
Mice
Mice
Mice
Mice
Mice
Mice
Mice
Mice
Mice
Mice
Mice
Mice
Mice
Mice
Mice
Mice
Mice
Mice
Mice
Mice
Mice
Mice
Mice
Mice
Mice

Mice
Mice
Mice
Mice
Mice
Mice
Mice
Mice
Mice
Mice
Mice
Mice
Mice
Mice
Mice
Mice
Mice
Mice
Mice
Mice
Mice
Mice
Mice
Mice
Mice
Mice
Mice
Mice
Mice
Mice
Mice
Mice
Mice
Mice
Mice
Mice
Mice
Mice
Mice
Mice
Mice
Mice
Mice
Mice
Mice
Mice

Mice
Mice
Mice
Mice
Mice
Mice
Mice
Mice
Mice
Mice
Mice
Mice
Mice
Mice
Mice
Mice
Mice
Mice
Mice
Mice
Mice
Mice
Mice
Mice
Mice
Mice
Mice
Mice
Mice
Mice
Mice
Mice
Mice
Mice
Mice
Mice
Mice
Mice
Mice
Mice
Mice
Mice
Mice
Mice
Mice
Mice

Mice
Mice
Mice
Mice
Mice
Mice
Mice
Mice
Mice
Mice
Mice
Mice
Mice
Mice
Mice
Mice
Mice
Mice
Mice
Mice
Mice
Mice
Mice
Mice
Mice
Mice
Mice
Mice
Mice
Mice
Mice
Mice
Mice
Mice
Mice
Mice
Mice
Mice
Mice
Mice
Mice
Mice
Mice
Mice
Mice
Mice

Mice
Mice
Mice
Mice
Mice
Mice
Mice
Mice
Mice
Mice
Mice
Mice
Mice
Mice
Mice
Mice
Mice
Mice
Mice
Mice
Mice
Mice
Mice
Mice
Mice
Mice
Mice
Mice
Mice
Mice
Mice
Mice
Mice
Mice
Mice
Mice
Mice
Mice
Mice
Mice
Mice
Mice
Mice
Mice
Mice
Mice

Mice
Mice
Mice
Mice
Mice
Mice
Mice
Mice
Mice
Mice
Mice
Mice
Mice
Mice
Mice
Mice
Mice
Mice
Mice
Mice
Mice
Mice
Mice
Mice
Mice
Mice
Mice
Mice
Mice
Mice
Mice
Mice
Mice
Mice
Mice
Mice
Mice
Mice
Mice
Mice
Mice
Mice
Mice
Mice
Mice
Mice

Mice
Mice
Mice
Mice
Mice
Mice
Mice
Mice
Mice
Mice
Mice
Mice
Mice
Mice
Mice
Mice
Mice
Mice
Mice
Mice
Mice
Mice
Mice
Mice
Mice
Mice
Mice
Mice
Mice
Mice
Mice
Mice
Mice
Mice
Mice
Mice
Mice
Mice
Mice
Mice
Mice
Mice
Mice
Mice
Mice
Mice

Mice
Mice
Mice
Mice
Mice
Mice
Mice
Mice
Mice
Mice
Mice
Mice
Mice
Mice
Mice
Mice
Mice
Mice
Mice
Mice
Mice
Mice
Mice
Mice
Mice
Mice
Mice
Mice
Mice
Mice
Mice
Mice
Mice
Mice
Mice
Mice
Mice
Mice
Mice
Mice
Mice
Mice
Mice
Mice
Mice
Mice

Mice
Mice
Mice
Mice
Mice
Mice
Mice
Mice
Mice
Mice
Mice
Mice
Mice
Mice
Mice
Mice
Mice
Mice
Mice
Mice
Mice
Mice
Mice
Mice
Mice
Mice
Mice
Mice
Mice
Mice
Mice
Mice
Mice
Mice
Mice
Mice
Mice
Mice
Mice
Mice
Mice
Mice
Mice
Mice
Mice
Mice

Mice
Mice
Mice
Mice
Mice
Mice
Mice
Mice
Mice
Mice
Mice
Mice
Mice
Mice
Mice
Mice
Mice
Mice
Mice
Mice
Mice
Mice
Mice
Mice
Mice
Mice
Mice
Mice
Mice
Mice
Mice
Mice
Mice
Mice
Mice
Mice
Mice
Mice
Mice
Mice
Mice
Mice
Mice
Mice
Mice
Mice

Mice
Mice
Mice
Mice
Mice
Mice
Mice
Mice
Mice
Mice
Mice
Mice
Mice
Mice
Mice
Mice
Mice
Mice
Mice
Mice
Mice
Mice
Mice
Mice
Mice
Mice
Mice
Mice
Mice
Mice
Mice
Mice
Mice
Mice
Mice
Mice
Mice
Mice
Mice
Mice
Mice
Mice
Mice
Mice
Mice
Mice

Mice
Mice
Mice
Mice
Mice
Mice
Mice
Mice
Mice
Mice
Mice
Mice
Mice
Mice
Mice
Mice
Mice
Mice
Mice
Mice
Mice
Mice
Mice
Mice
Mice
Mice
Mice
Mice
Mice
Mice
Mice
Mice
Mice
Mice
Mice
Mice
Mice
Mice
Mice
Mice
Mice
Mice
Mice
Mice
Mice
Mice

Mice
Mice
Mice
Mice
Mice
Mice
Mice
Mice
Mice
Mice
Mice
Mice
Mice
Mice
Mice
Mice
Mice
Mice
Mice
Mice
Mice
Mice
Mice
Mice
Mice
Mice
Mice
Mice
Mice
Mice
Mice
Mice
Mice
Mice
Mice
Mice
Mice
Mice
Mice
Mice
Mice
Mice
Mice
Mice
Mice
Mice

Mice
Mice
Mice
Mice
Mice
Mice
Mice
Mice
Mice
Mice
Mice
Mice
Mice
Mice
Mice
Mice
Mice
Mice
Mice
Mice
Mice
Mice
Mice
Mice
Mice
Mice
Mice
Mice
Mice
Mice
Mice
Mice
Mice
Mice
Mice
Mice
Mice
Mice
Mice
Mice
Mice
Mice
Mice
Mice
Mice
Mice

Mice
Mice
Mice
Mice
Mice
Mice
Mice
Mice
Mice
Mice
Mice
Mice
Mice
Mice
Mice
Mice
Mice
Mice
Mice
Mice
Mice
Mice
Mice
Mice
Mice
Mice
Mice
Mice
Mice
Mice
Mice
Mice
Mice
Mice
Mice
Mice
Mice
Mice
Mice
Mice
Mice
Mice
Mice
Mice
Mice
Mice

Mice
Mice
Mice
Mice
Mice
Mice
Mice
Mice
Mice
Mice
Mice
Mice
Mice
Mice
Mice
Mice
Mice
Mice
Mice
Mice
Mice
Mice
Mice
Mice
Mice
Mice
Mice
Mice
Mice
Mice
Mice
Mice
Mice
Mice
Mice
Mice
Mice
Mice
Mice
Mice
Mice
Mice
Mice
Mice
Mice
Mice

Mice
Mice
Mice
Mice
Mice
Mice
Mice
Mice
Mice
Mice
Mice
Mice
Mice
Mice
Mice
Mice
Mice
Mice
Mice
Mice
Mice
Mice
Mice
Mice
Mice
Mice
Mice
Mice
Mice
Mice
Mice
Mice
Mice
Mice
Mice
Mice
Mice
Mice
Mice
Mice
Mice
Mice
Mice
Mice
Mice
Mice

Mice
Mice
Mice
Mice
Mice
Mice
Mice
Mice
Mice
Mice
Mice
Mice
Mice
Mice
Mice
Mice
Mice
Mice
Mice
Mice
Mice
Mice
Mice
Mice
Mice
Mice
Mice
Mice
Mice
Mice
Mice
Mice
Mice
Mice
Mice
Mice
Mice
Mice
Mice
Mice
Mice
Mice
Mice
Mice
Mice
Mice

Mice
Mice
Mice
Mice
Mice
Mice
Mice
Mice
Mice
Mice
Mice
Mice
Mice
Mice
Mice
Mice
Mice
Mice
Mice
Mice
Mice
Mice
Mice
Mice
Mice
Mice
Mice
Mice
Mice
Mice
Mice
Mice
Mice
Mice
Mice
Mice
Mice
Mice
Mice
Mice
Mice
Mice
Mice
Mice
Mice
Mice

Mice
Mice
Mice
Mice
Mice
Mice
Mice
Mice
Mice
Mice
Mice
Mice
Mice
Mice
Mice
Mice
Mice
Mice
Mice
Mice
Mice
Mice
Mice
Mice
Mice
Mice
Mice
Mice
Mice
Mice
Mice
Mice
Mice
Mice
Mice
Mice
Mice
Mice
Mice
Mice
Mice
Mice
Mice
Mice
Mice
Mice

Mice
Mice
Mice
Mice
Mice
Mice
Mice
Mice
Mice
Mice
Mice
Mice
Mice
Mice
Mice
Mice
Mice
Mice
Mice
Mice
Mice
Mice
Mice
Mice
Mice
Mice
Mice
Mice
Mice
Mice
Mice
Mice
Mice
Mice
Mice
Mice
Mice
Mice
Mice
Mice
Mice
Mice
Mice
Mice
Mice
Mice

Mice
Mice
Mice
Mice
Mice
Mice
Mice
Mice
Mice
Mice
Mice
Mice
Mice
Mice
Mice
Mice
Mice
Mice
Mice
Mice
Mice
Mice
Mice
Mice
Mice
Mice
Mice
Mice
Mice
Mice
Mice
Mice
Mice
Mice
Mice
Mice
Mice
Mice
Mice
Mice
Mice
Mice
Mice
Mice
Mice
Mice

Mice
Mice
Mice
Mice
Mice
Mice
Mice
Mice
Mice
Mice
Mice
Mice
Mice
Mice
Mice
Mice
Mice
Mice
Mice
Mice
Mice
Mice
Mice
Mice
Mice
Mice
Mice
Mice
Mice
Mice
Mice
Mice
Mice
Mice
Mice
Mice
Mice
Mice
Mice
Mice
Mice
Mice
Mice
Mice
Mice
Mice

Mice
Mice
Mice
Mice
Mice
Mice
Mice
Mice
Mice
Mice
Mice
Mice
Mice
Mice
Mice
Mice
Mice
Mice
Mice
Mice
Mice
Mice
Mice
Mice
Mice
Mice
Mice
Mice
Mice
Mice
Mice
Mice
Mice
Mice
Mice
Mice
Mice
Mice
Mice
Mice
Mice
Mice
Mice
Mice
Mice
Mice

Mice
Mice
Mice
Mice
Mice
Mice
Mice
Mice
Mice
Mice
Mice
Mice
Mice
Mice
Mice
Mice
Mice
Mice
Mice
Mice
Mice
Mice
Mice
Mice
Mice
Mice
Mice
Mice
Mice
Mice
Mice
Mice
Mice
Mice
Mice
Mice
Mice
Mice
Mice
Mice
Mice
Mice
Mice
Mice
Mice
Mice

Mice
Mice
Mice
Mice
Mice
Mice
Mice
Mice
Mice
Mice
Mice
Mice
Mice
Mice
Mice
Mice
Mice
Mice
Mice
Mice
Mice
Mice
Mice
Mice
Mice
Mice
Mice
Mice
Mice
Mice
Mice
Mice
Mice
Mice
Mice
Mice
Mice
Mice
Mice
Mice
Mice
Mice
Mice
Mice
Mice
Mice

Mice
Mice
Mice
Mice
Mice
Mice
Mice
Mice
Mice
Mice
Mice
Mice
Mice
Mice
Mice
Mice
Mice
Mice
Mice
Mice
Mice
Mice
Mice
Mice
Mice
Mice
Mice
Mice
Mice
Mice
Mice
Mice
Mice
Mice
Mice
Mice
Mice
Mice
Mice
Mice
Mice
Mice
Mice
Mice
Mice
Mice

Mice
Mice
Mice
Mice
Mice
Mice
Mice
Mice
Mice
Mice
Mice
Mice
Mice
Mice
Mice
Mice
Mice
Mice
Mice
Mice
Mice
Mice
Mice
Mice
Mice
Mice
Mice
Mice
Mice
Mice
Mice
Mice
Mice
Mice
Mice
Mice
Mice
Mice
Mice
Mice
Mice
Mice
Mice
Mice
Mice
Mice

Mice
Mice
Mice
Mice
Mice
Mice
Mice
Mice
Mice
Mice
Mice
Mice
Mice
Mice
Mice
Mice
Mice
Mice
Mice
Mice
Mice
Mice
Mice
Mice
Mice
Mice
Mice
Mice
Mice
Mice
Mice
Mice
Mice
Mice
Mice
Mice
Mice
Mice
Mice
Mice
Mice
Mice
Mice
Mice
Mice
Mice

Mice
Mice
Mice
Mice
Mice
Mice
Mice
Mice
Mice
Mice
Mice
Mice
Mice
Mice
Mice
Mice
Mice
Mice
Mice
Mice
Mice
Mice
Mice
Mice
Mice
Mice
Mice
Mice
Mice
Mice
Mice
Mice
Mice
Mice
Mice
Mice
Mice
Mice
Mice
Mice
Mice
Mice
Mice
Mice
Mice
Mice

Mice
Mice
Mice
Mice
Mice
Mice
Mice
Mice
Mice
Mice
Mice
Mice
Mice
Mice
Mice
Mice
Mice
Mice
Mice
Mice
Mice
Mice
Mice
Mice
Mice
Mice
Mice
Mice
Mice
Mice
Mice
Mice
Mice
Mice
Mice
Mice
Mice
Mice
Mice
Mice
Mice
Mice
Mice
Mice
Mice
Mice

Mice
Mice
Mice
Mice
Mice
Mice
Mice
Mice
Mice
Mice
Mice
Mice
Mice
Mice
Mice
Mice
Mice
Mice
Mice
Mice
Mice
Mice
Mice
Mice
Mice
Mice
Mice
Mice
Mice
Mice
Mice
Mice
Mice
Mice
Mice
Mice
Mice
Mice
Mice
Mice
Mice
Mice
Mice
Mice
Mice
Mice

Mice
Mice
Mice
Mice
Mice
Mice
Mice
Mice
Mice
Mice
Mice
Mice
Mice
Mice
Mice
Mice
Mice
Mice
Mice
Mice
Mice
Mice
Mice
Mice
Mice
Mice
Mice
Mice
Mice
Mice
Mice
Mice
Mice
Mice
Mice
Mice
Mice
Mice
Mice
Mice
Mice
Mice
Mice
Mice
Mice
Mice

Mice
Mice
Mice
Mice
Mice
Mice
Mice
Mice
Mice
Mice
Mice
Mice
Mice
Mice
Mice
Mice
Mice
Mice
Mice
Mice
Mice
Mice
Mice
Mice
Mice
Mice
Mice
Mice
Mice
Mice
Mice
Mice
Mice
Mice
Mice
Mice
Mice
Mice
Mice
Mice
Mice
Mice
Mice
Mice
Mice
Mice

Mice
Mice
Mice
Mice
Mice
Mice
Mice
Mice
Mice
Mice
Mice
Mice
Mice
Mice
Mice
Mice
Mice
Mice
Mice
Mice
Mice
Mice
Mice
Mice
Mice
Mice
Mice
Mice
Mice
Mice
Mice
Mice
Mice
Mice
Mice
Mice
Mice
Mice
Mice
Mice
Mice
Mice
Mice
Mice
Mice
Mice

Mice
Mice
Mice
Mice
Mice
Mice
Mice
Mice
Mice
Mice
Mice
Mice
Mice
Mice
Mice
Mice
Mice
Mice
Mice
Mice
Mice
Mice
Mice
Mice
Mice
Mice
Mice
Mice
Mice
Mice
Mice
Mice
Mice
Mice
Mice
Mice
Mice
Mice
Mice
Mice
Mice
Mice
Mice
Mice
Mice
Mice

Mice
Mice
Mice
Mice
Mice
Mice
Mice
Mice
Mice
Mice
Mice
Mice
Mice
Mice
Mice
Mice
Mice
Mice
Mice
Mice
Mice
Mice
Mice
Mice
Mice
Mice
Mice
Mice
Mice
Mice
Mice
Mice
Mice
Mice
Mice
Mice
Mice
Mice
Mice
Mice
Mice
Mice
Mice
Mice
Mice
Mice

Mice
Mice
Mice
Mice
Mice
Mice
Mice
Mice
Mice
Mice
Mice
Mice
Mice
Mice
Mice
Mice
Mice
Mice
Mice
Mice
Mice
Mice
Mice
Mice
Mice
Mice
Mice
Mice
Mice
Mice
Mice
Mice
Mice
Mice
Mice
Mice
Mice
Mice
Mice
Mice
Mice
Mice
Mice
Mice
Mice
Mice

Mice
Mice
Mice
Mice
Mice
Mice
Mice
Mice
Mice
Mice
Mice
Mice
Mice
Mice
Mice
Mice
Mice
Mice
Mice
Mice
Mice
Mice
Mice
Mice
Mice
Mice
Mice
Mice
Mice
Mice
Mice
Mice
Mice
Mice
Mice
Mice
Mice
Mice
Mice
Mice
Mice
Mice
Mice
Mice
Mice
Mice

Mice
Mice
Mice
Mice
Mice
Mice
Mice
Mice
Mice
Mice
Mice
Mice
Mice
Mice
Mice
Mice
Mice
Mice
Mice
Mice
Mice
Mice
Mice
Mice
Mice
Mice
Mice
Mice
Mice
Mice
Mice
Mice
Mice
Mice
Mice
Mice
Mice
Mice
Mice
Mice
Mice
Mice
Mice
Mice
Mice
Mice

Mice
Mice
Mice
Mice
Mice
Mice
Mice
Mice
Mice
Mice
Mice
Mice
Mice
Mice
Mice
Mice
Mice
Mice
Mice
Mice
Mice
Mice
Mice
Mice
Mice
Mice
Mice
Mice
Mice
Mice
Mice
Mice
Mice
Mice
Mice
Mice
Mice
Mice
Mice
Mice
Mice
Mice
Mice
Mice
Mice
Mice

Mice
Mice
Mice
Mice
Mice
Mice
Mice
Mice
Mice
Mice
Mice
Mice
Mice
Mice
Mice
Mice
Mice
Mice
Mice
Mice
Mice
Mice
Mice
Mice
Mice
Mice
Mice
Mice
Mice
Mice
Mice
Mice
Mice
Mice
Mice
Mice
Mice
Mice
Mice
Mice
Mice
Mice
Mice
Mice
Mice
Mice

Mice
Mice
Mice
Mice
Mice
Mice
Mice
Mice
Mice
Mice
Mice
Mice
Mice
Mice
Mice
Mice
Mice
Mice
Mice
Mice
Mice
Mice
Mice
Mice
Mice
Mice
Mice
Mice
Mice
Mice
Mice
Mice
Mice
Mice
Mice
Mice
Mice
Mice
Mice
Mice
Mice
Mice
Mice
Mice
Mice
Mice

Mice
Mice
Mice
Mice
Mice
Mice
Mice
Mice
Mice
Mice
Mice
Mice
Mice
Mice
Mice
Mice
Mice
Mice
Mice
Mice
Mice
Mice
Mice
Mice
Mice
Mice
Mice
Mice
Mice
Mice
Mice
Mice
Mice
Mice
Mice
Mice
Mice
Mice
Mice
Mice
Mice
Mice
Mice
Mice
Mice
Mice

Mice
Mice
Mice
Mice
Mice
Mice
Mice
Mice
Mice
Mice
Mice
Mice
Mice
Mice
Mice
Mice
Mice
Mice
Mice
Mice
Mice
Mice
Mice
Mice
Mice
Mice
Mice
Mice
Mice
Mice
Mice
Mice
Mice
Mice
Mice
Mice
Mice
Mice
Mice
Mice
Mice
Mice
Mice
Mice
Mice
Mice

Mice
Mice
Mice
Mice
Mice
Mice
Mice
Mice
Mice
Mice
Mice
Mice
Mice
Mice
Mice
Mice
Mice
Mice
Mice
Mice
Mice
Mice
Mice
Mice
Mice
Mice
Mice
Mice
Mice
Mice
Mice
Mice
Mice
Mice
Mice
Mice
Mice
Mice
Mice
Mice
Mice
Mice
Mice
Mice
Mice
Mice

Mice
Mice
Mice
Mice
Mice
Mice
Mice
Mice
Mice
Mice
Mice
Mice
Mice
Mice
Mice
Mice
Mice
Mice
Mice
Mice
Mice
Mice
Mice
Mice
Mice
Mice
Mice
Mice
Mice
Mice
Mice
Mice
Mice
Mice
Mice
Mice
Mice
Mice
Mice
Mice
Mice
Mice
Mice
Mice
Mice
Mice

Mice
Mice
Mice
Mice
Mice
Mice
Mice
Mice
Mice
Mice
Mice
Mice
Mice
Mice
Mice
Mice
Mice
Mice
Mice
Mice
Mice
Mice
Mice
Mice
Mice
Mice
Mice
Mice
Mice
Mice
Mice
Mice
Mice
Mice
Mice
Mice
Mice
Mice
Mice
Mice
Mice
Mice
Mice
Mice
Mice
Mice

Mice
Mice
Mice
Mice
Mice
Mice
Mice
Mice
Mice
Mice
Mice
Mice
Mice
Mice
Mice
Mice
Mice
Mice
Mice
Mice
Mice
Mice
Mice
Mice
Mice
Mice
Mice
Mice
Mice
Mice
Mice
Mice
Mice
Mice
Mice
Mice
Mice
Mice
Mice
Mice
Mice
Mice
Mice
Mice
Mice
Mice

Mice
Mice
Mice
Mice
Mice
Mice
Mice
Mice
Mice
Mice
Mice
Mice
Mice
Mice
Mice
Mice
Mice
Mice
Mice
Mice
Mice
Mice
Mice
Mice
Mice
Mice
Mice
Mice
Mice
Mice
Mice
Mice
Mice
Mice
Mice
Mice
Mice
Mice
Mice
Mice
Mice
Mice
Mice
Mice
Mice
Mice

Mice
Mice
Mice
Mice
Mice
Mice
Mice
Mice
Mice
Mice
Mice
Mice
Mice
Mice
Mice
Mice
Mice
Mice
Mice
Mice
Mice
Mice
Mice
Mice
Mice
Mice
Mice
Mice
Mice
Mice
Mice
Mice
Mice
Mice
Mice
Mice
Mice
Mice
Mice
Mice
Mice
Mice
Mice
Mice
Mice
Mice

Mice
Mice
Mice
Mice
Mice
Mice
Mice
Mice
Mice
Mice
Mice
Mice
Mice
Mice
Mice
Mice
Mice
Mice
Mice
Mice
Mice
Mice
Mice
Mice
Mice
Mice
Mice
Mice
Mice
Mice
Mice
Mice
Mice
Mice
Mice
Mice
Mice
Mice
Mice
Mice
Mice
Mice
Mice
Mice
Mice
Mice

Mice
Mice
Mice
Mice
Mice
Mice
Mice
Mice
Mice
Mice
Mice
Mice
Mice
Mice
Mice
Mice
Mice
Mice
Mice
Mice
Mice
Mice
Mice
Mice
Mice
Mice
Mice
Mice
Mice
Mice
Mice
Mice
Mice
Mice
Mice
Mice
Mice
Mice
Mice
Mice
Mice
Mice
Mice
Mice
Mice
Mice

Mice
Mice
Mice
Mice
Mice
Mice
Mice
Mice
Mice
Mice
Mice
Mice
Mice
Mice
Mice
Mice
Mice
Mice
Mice
Mice
Mice
Mice
Mice
Mice
Mice
Mice
Mice
Mice
Mice
Mice
Mice
Mice
Mice
Mice
Mice
Mice
Mice
Mice
Mice
Mice
Mice
Mice
Mice
Mice
Mice
Mice

Mice
Mice
Mice
Mice
Mice
Mice
Mice
Mice
Mice
Mice
Mice
Mice
Mice
Mice
Mice
Mice
Mice
Mice
Mice
Mice
Mice
Mice
Mice
Mice
Mice
Mice
Mice
Mice
Mice
Mice
Mice
Mice
Mice
Mice
Mice
Mice
Mice
Mice
Mice
Mice
Mice
Mice
Mice
Mice
Mice
Mice

Mice
Mice
Mice
Mice
Mice
Mice
Mice
Mice
Mice
Mice
Mice
Mice
Mice
Mice
Mice
Mice
Mice
Mice
Mice
Mice
Mice
Mice
Mice
Mice
Mice
Mice
Mice
Mice
Mice
Mice
Mice
Mice
Mice
Mice
Mice
Mice
Mice
Mice
Mice
Mice
Mice
Mice
Mice
Mice
Mice
Mice

Mice
Mice
Mice
Mice
Mice
Mice
Mice
Mice
Mice
Mice
Mice
Mice
Mice
Mice
Mice
Mice
Mice
Mice
Mice
Mice
Mice
Mice
Mice
Mice
Mice
Mice
Mice
Mice
Mice
Mice
Mice
Mice
Mice
Mice
Mice
Mice
Mice
Mice
Mice
Mice
Mice
Mice
Mice
Mice
Mice
Mice

Mice
Mice
Mice
Mice
Mice
Mice
Mice
Mice
Mice
Mice
Mice
Mice
Mice
Mice
Mice
Mice
Mice
Mice
Mice
Mice
Mice
Mice
Mice
Mice
Mice
Mice
Mice
Mice
Mice
Mice
Mice
Mice
Mice
Mice
Mice
Mice
Mice
Mice
Mice
Mice
Mice
Mice
Mice
Mice
Mice
Mice

Mice
Mice
Mice
Mice
Mice
Mice
Mice
Mice
Mice
Mice
Mice
Mice
Mice
Mice
Mice
Mice
Mice
Mice
Mice
Mice
Mice
Mice
Mice
Mice
Mice
Mice
Mice
Mice
Mice
Mice
Mice
Mice
Mice
Mice
Mice
Mice
Mice
Mice
Mice
Mice
Mice
Mice
Mice
Mice
Mice
Mice

Mice
Mice
Mice
Mice
Mice
Mice
Mice
Mice
Mice
Mice
Mice
Mice
Mice
Mice
Mice
Mice
Mice
Mice
Mice
Mice
Mice
Mice
Mice
Mice
Mice
Mice
Mice
Mice
Mice
Mice
Mice
Mice
Mice
Mice
Mice
Mice
Mice
Mice
Mice
Mice
Mice
Mice
Mice
Mice
Mice
Mice

Mice
Mice
Mice
Mice
Mice
Mice
Mice
Mice
Mice
Mice
Mice
Mice
Mice
Mice
Mice
Mice
Mice
Mice
Mice
Mice
Mice
Mice
Mice
Mice
Mice
Mice
Mice
Mice
Mice
Mice
Mice
Mice
Mice
Mice
Mice
Mice
Mice
Mice
Mice
Mice
Mice
Mice
Mice
Mice
Mice
Mice

Mice
Mice
Mice
Mice
Mice
Mice
Mice
Mice
Mice
Mice
Mice
Mice
Mice
Mice
Mice
Mice
Mice
Mice
Mice
Mice
Mice
Mice
Mice
Mice
Mice
Mice
Mice
Mice
Mice
Mice
Mice
Mice
Mice
Mice
Mice
Mice
Mice
Mice
Mice
Mice
Mice
Mice
Mice
Mice
Mice
Mice

Mice
Mice
Mice
Mice
Mice
Mice
Mice
Mice
Mice
Mice
Mice
Mice
Mice
Mice
Mice
Mice
Mice
Mice
Mice
Mice
Mice
Mice
Mice
Mice
Mice
Mice
Mice
Mice
Mice
Mice
Mice
Mice
Mice
Mice
Mice
Mice
Mice
Mice
Mice
Mice
Mice
Mice
Mice
Mice
Mice
Mice

Mice
Mice
Mice
Mice
Mice
Mice
Mice
Mice
Mice
Mice
Mice
Mice
Mice
Mice
Mice
Mice
Mice
Mice
Mice
Mice
Mice
Mice
Mice
Mice
Mice
Mice
Mice
Mice
Mice
Mice
Mice
Mice
Mice
Mice
Mice
Mice
Mice
Mice
Mice
Mice
Mice
Mice
Mice
Mice
Mice
Mice

Mice
Mice
Pig
Pig
Pig
Pig
Pig
Pig
Pig
Pig
Pig
Pig
Pig
Pig
Pig
Pig
Pig
Pig
Pig
Pig
Pig
Pig
Pig
Pig
Pig
Pig
Pig
Pig
Pig
Pig
Pig
Pig
Pig
Pig
Pig
Pig
Pig
Pig
Pig
Pig
Pig
Pig
Pig
Pig
Pig
Pig

Pig
Pig
Pig
Pig
Pig
Pig
Pig
Pig
Pig
Pig
Pig
Pig
Pig
Pig
Pig
Pig
Pig
Pig
Pig
Pig
Pig
Pig
Pig
Pig
Pig
Pig
Pig
Pig
Pig
Pig
Pig
Pig
Pig
Pig
Pig
Pig
Pig
Pig
Pig
Pig
Pig
Pig
Pig
Pig
Pig
Pig

Pig
Pig
Pig
Pig
Pig
Pig
Pig
Pig
Pig
Pig
Pig
Pig
Pig
Pig
Pig
Pig
Pig
Pig
Pig
Pig
Pig
Pig
Pig
Pig
Pig
Pig
Pig
Pig
Pig
Pig
Pig
Pig
Pig
Pig
Pig
Pig
Pig
Pig
Pig
Pig
Pig
Pig
Pig
Pig
Pig
Pig

Pig
Pig
Pig
Pig
Pig
Pig
Pig
Pig
Pig
Pig
Pig
Pig
Pig
Pig
Pig
Pig
Pig
Pig
Pig
Pig
Pig
Pig
Pig
Pig
Pig
Pig
Pig
Pig
Pig
Pig
Pig
Pig
Pig
Pig
Pig
Pig
Pig
Pig
Pig
Pig
Pig
Pig
Pig
Pig
Pig
Pig

Pig
Pig
Pig
Pig
Pig
Pig
Pig
Pig
Pig
Pig
Pig
Pig
Pig
Pig
Pig
Pig
Pig
Pig
Pig
Pig
Pig
Pig
Pig
Pig
Pig
Pig
Pig
Pig
Pig
Pig
Pig
Pig
Pig
Pig
Pig
Pig
Pig
Pig
Pig
Pig
Pig
Pig
Pig
Pig
Pig
Pig

Pig
Pig
Pig
Pig
Pig
Pig
Pig
Pig
Pig
Pig
Pig
Pig
Pig
Pig
Pig
Pig
Pig
Pig
Pig
Pig
Pig
Pig
Pig
Pig
Pig
Pig
Pig
Pig
Pig
Pig
Pig
Pig
Pig
Pig
Pig
Pig
Pig
Pig
Pig
Pig
Pig
Pig
Pig
Pig
Pig
Pig

Pig
Pig
Pig
Pig
Pig
Pig
Pig
Pig
Pig
Pig
Pig
Pig
Pig
Pig
Pig
Pig
Pig
Pig
Pig
Pig
Pig
Pig
Pig
Pig
Pig
Pig
Pig
Pig
Pig
Pig
Pig
Pig
Pig
Pig
Pig
Pig
Pig
Pig
Pig
Pig
Pig
Pig
Pig
Pig
Pig
Pig

Pig
Pig
Pig
Pig
Pig
Pig
Pig
Pig
Pig
Pig
Pig
Pig
Pig
Pig
Pig
Pig
Pig
Pig
Pig
Pig
Pig
Pig
Pig
Pig
Pig
Pig
Pig
Pig
Pig
Pig
Pig
Pig
Pig
Pig
Pig
Pig
Pig
Pig
Pig
Pig
Pig
Pig
Pig
Pig
Pig
Pig

Pig
Pig
Pig
Pig
Pig
Pig
Pig
Pig
Pig
Pig
Pig
Pig
Pig
Pig
Pig
Pig
Pig
Pig
Pig
Pig
Pig
Pig
Pig
Pig
Pig
Pig
Pig
Pig
Pig
Pig
Pig
Pig
Pig
Pig
Pig
Pig
Pig
Pig
Pig
Pig
Pig
Pig
Pig
Pig
Pig
Pig

Pig
Pig
Pig
Pig
Pig
Pig
Pig
Pig
Pig
Pig
Pig
Pig
Pig
Pig
Pig
Pig
Pig
Pig
Pig
Pig
Pig
Pig
Pig
Pig
Pig
Pig
Pig
Pig
Pig
Pig
Pig
Pig
Pig
Pig
Pig
Pig
Pig
Pig
Pig
Pig
Pig
Pig
Pig
Pig
Pig
Pig

Pig
Pig
Pig
Pig
Pig
Pig
Pig
Pig
Pig
Pig
Pig
Pig
Pig
Pig
Pig
Pig
Pig
Pig
Pig
Pig
Pig
Pig
Pig
Pig
Pig
Pig
Pig
Pig
Pig
Pig
Pig
Pig
Pig
Pig
Pig
Pig
Pig
Pig
Pig
Pig
Pig
Pig
Pig
Pig
Pig
Pig

Pig
Pig
Pig
Pig
Pig
Pig
Pig
Pig
Pig
Pig
Pig
Pig
Pig
Pig
Pig
Pig
Pig
Pig
Pig
Pig
Pig
Pig
Pig
Pig
Pig
Pig
Pig
Pig
Pig
Pig
Pig
Pig
Pig
Pig
Pig
Pig
Pig
Pig
Pig
Pig
Pig
Pig
Pig
Pig
Pig
Pig

Pig
Pig
Pig
Pig
Pig
Pig
Pig
Pig
Pig
Pig
Pig
Pig
Pig
Pig
Pig
Pig
Pig
Pig
Pig
Pig
Pig
Pig
Pig
Pig
Pig
Pig
Pig
Pig
Pig
Pig
Pig
Pig
Pig
Pig
Pig
Pig
Pig
Pig
Pig
Pig
Pig
Pig
Pig
Pig
Pig
Pig

Pig
Pig
Pig
Pig
Pig
Pig
Pig
Pig
Pig
Pig
Pig
Pig
Pig
Pig
Pig
Pig
Pig
Pig
Pig
Pig
Pig
Pig
Pig
Pig
Pig
Pig
Pig
Pig
Pig
Pig
Pig
Pig
Pig
Pig
Pig
Pig
Pig
Pig
Pig
Pig
Pig
Pig
Pig
Pig
Pig
Pig

Pig
Pig
Pig
Pig
Pig
Pig
Pig
Pig
Pig
Pig
Pig
Pig
Pig
Pig
Pig
Pig
Pig
Pig
Pig
Pig
Pig
Pig
Pig
Pig
Pig
Pig
Pig
Pig
Pig
Pig
Pig
Pig
Pig
Pig
Pig
Pig
Pig
Pig
Pig
Pig
Pig
Pig
Pig
Pig
Pig
Pig

Pig
Pig
Pig
Pig
Pig
Pig
Pig
Pig
Pig
Pig
Pig
Pig
Pig
Pig
Pig
Pig
Pig
Pig
Pig
Pig
Pig
Pig
Pig
Pig
Pig
Pig
Pig
Pig
Pig
Pig
Pig
Pig
Pig
Pig
Pig
Pig
Pig
Pig
Pig
Pig
Pig
Pig
Pig
Pig
Pig
Pig

Pig
Pig
Pig
Pig
Pig
Pig
Pig
Pig
Pig
Pig
Pig
Pig
Pig
Pig
Pig
Pig
Pig
Pig
Pig
Pig
Pig
Pig
Pig
Pig
Pig
Pig
Pig
Pig
Pig
Pig
Pig
Pig
Pig
Pig
Pig
Pig
Pig
Pig
Pig
Pig
Pig
Pig
Pig
Pig
Pig
Pig

Pig
Pig
Pig
Pig
Pig
Pig
Pig
Pig
Pig
Pig
Pig
Pig
Pig
Pig
Pig
Pig
Pig
Pig
Pig
Pig
Pig
Pig
Pig
Pig
Pig
Pig
Pig
Pig
Pig
Pig
Pig
Pig
Pig
Pig
Pig
Pig
Pig
Pig
Pig
Pig
Pig
Pig
Pig
Pig
Pig
Pig

Pig
Pig
Pig
Pig
Pig
Pig
Pig
Pig
Pig
Pig
Pig
Pig
Pig
Pig
Pig
Pig
Pig
Pig
Pig
Pig
Pig
Pig
Pig
Pig
Pig
Pig
Pig
Pig
Pig
Pig
Pig
Pig
Pig
Pig
Pig
Pig
Pig
Pig
Pig
Pig
Pig
Pig
Pig
Pig
Pig
Pig

Pig
Pig
Pig
Pig
Pig
Pig
Pig
Pig
Pig
Pig
Pig
Pig
Pig
Pig
Pig
Pig
Pig
Pig
Pig
Pig
Pig
Pig
Pig
Pig
Pig
Pig
Pig
Pig
Pig
Pig
Pig
Pig
Pig
Pig
Pig
Pig
Pig
Pig
Pig
Pig
Pig
Pig
Pig
Pig
Pig
Pig

Pig
Pig
Pig
Pig
Pig
Pig
Pig
Pig
Pig
Pig
Pig
Pig
Pig
Pig
Pig
Pig
Pig
Pig
Pig
Pig
Pig
Pig
Pig
Pig
Pig
Pig
Pig
Pig
Pig
Pig
Pig
Pig
Pig
Pig
Pig
Pig
Pig
Pig
Pig
Pig
Pig
Pig
Pig
Pig
Pig
Pig

Pig
Pig
Pig
Pig
Pig
Pig
Pig
Pig
Pig
Pig
Pig
Pig
Pig
Pig
Pig
Pig
Pig
Pig
Pig
Pig
Pig
Pig
Pig
Pig
Pig
Pig
Pig
Pig
Pig
Pig
Pig
Pig
Pig
Pig
Pig
Pig
Pig
Pig
Pig
Pig
Pig
Pig
Pig
Pig
Pig
Pig

Pig
Pig
Pig
Pig
Pig
Pig
Pig
Pig
Pig
Pig
Pig
Pig
Pig
Pig
Pig
Pig
Pig
Pig
Pig
Pig
Pig
Pig
Pig
Pig
Pig
Pig
Pig
Pig
Pig
Pig
Pig
Pig
Pig
Pig
Pig
Pig
Pig
Pig
Pig
Pig
Pig
Pig
Pig
Pig
Pig
Pig

Pig
Pig
Pig
Pig
Pig
Pig
Pig
Pig
Pig
Pig
Pig
Pig
Pig
Pig
Pig
Pig
Pig
Pig
Pig
Pig
Pig
Pig
Pig
Pig
Pig
Pig
Pig
Pig
Pig
Pig
Pig
Pig
Pig
Pig
Pig
Pig
Pig
Pig
Pig
Pig
Pig
Pig
Pig
Pig
Pig
Pig

Pig
Pig
Pig
Pig
Pig
Pig
Pig
Pig
Pig
Pig
Pig
Pig
Pig
Pig
Pig
Pig
Pig
Pig
Pig
Pig
Pig
Pig
Pig
Pig
Pig
Pig
Pig
Pig
Pig
Pig
Pig
Pig
Pig
Pig
Pig
Pig
Pig
Pig
Pig
Pig
Pig
Pig
Pig
Pig
Pig
Pig

Pig
Pig
Pig
Pig
Pig
Pig
Pig
Pig
Pig
Pig
Pig
Pig
Pig
Pig
Pig
Pig
Pig
Pig
Pig
Pig
Pig
Pig
Pig
Pig
Pig
Pig
Pig
Pig
Pig
Pig
Pig
Pig
Pig
Pig
Pig
Pig
Pig
Pig
Pig
Pig
Pig
Pig
Pig
Pig
Pig
Pig

Pig
Pig
Pig
Pig
Pig
Pig
Pig
Pig
Pig
Pig
Pig
Pig
Pig
Pig
Pig
Pig
Pig
Pig
Pig
Pig
Pig
Pig
Pig
Pig
Pig
Pig
Pig
Pig
Pig
Pig
Pig
Pig
Pig
Pig
Pig
Pig
Pig
Pig
Pig
Pig
Pig
Pig
Pig
Pig
Pig
Pig

Pig
Pig
Pig
Pig
Pig
Pig
Pig
Pig
Pig
Pig
Pig
Pig
Pig
Pig
Pig
Pig
Pig
Pig
Pig
Pig
Pig
Pig
Pig
Pig
Pig
Pig
Pig
Pig
Pig
Pig
Pig
Pig
Pig
Pig
Pig
Pig
Pig
Pig
Pig
Pig
Pig
Pig
Pig
Pig
Pig
Pig

Pig
Pig
Pig
Pig
Pig
Pig
Pig
Pig
Pig
Pig
Pig
Pig
Pig
Pig
Pig
Pig
Pig
Pig
Pig
Pig
Pig
Pig
Pig
Pig
Pig
Pig
Pig
Pig
Pig
Pig
Pig
Pig
Pig
Pig
Pig
Pig
Pig
Pig
Pig
Pig
Pig
Pig
Pig
Pig
Pig
Pig

Pig
Pig
Pig
Pig
Pig
Pig
Pig
Pig
Pig
Pig
Pig
Pig
Pig
Pig
Pig
Pig
Pig
Pig
Pig
Pig
Pig
Pig
Pig
Pig
Pig
Pig
Pig
Pig
Pig
Pig
Pig
Pig
Pig
Pig
Pig
Pig
Pig
Pig
Pig
Pig
Pig
Pig
Pig
Pig
Pig
Pig

Pig
Pig
Pig
Pig
Pig
Pig
Pig
Pig
Pig
Pig
Pig
Pig
Pig
Pig
Pig
Pig
Pig
Pig
Pig
Pig
Pig
Pig
Pig
Pig
Pig
Pig
Pig
Pig
Pig
Pig
Pig
Pig
Pig
Pig
Pig
Pig
Pig
Pig
Pig
Pig
Pig
Pig
Pig
Pig
Pig
Pig

Pig
Pig
Pig
Pig
Pig
Pig
Pig
Pig
Pig
Pig
Pig
Pig
Pig
Pig
Pig
Pig
Pig
Pig
Pig
Pig
Pig
Pig
Pig
Pig
Pig
Pig
Pig
Pig
Pig
Pig
Pig
Pig
Pig
Pig
Pig
Pig
Pig
Pig
Pig
Pig
Pig
Pig
Pig
Pig
Pig
Pig

Pig
Pig
Pig
Pig
Pig
Pig
Pig
Pig
Pig
Pig
Pig
Pig
Pig
Pig
Pig
Pig
Pig
Pig
Pig
Pig
Pig
Pig
Pig
Pig
Pig
Pig
Pig
Pig
Pig
Pig
Pig
Pig
Pig
Pig
Pig
Pig
Pig
Pig
Pig
Pig
Pig
Pig
Pig
Pig
Pig
Pig

Pig
Pig
Pig
Pig
Pig
Pig
Pig
Pig
Pig
Pig
Pig
Pig
Pig
Pig
Pig
Pig
Pig
Pig
Pig
Pig
Pig
Pig
Pig
Pig
Pig
Pig
Pig
Pig
Pig
Pig
Pig
Pig
Pig
Pig
Pig
Pig
Pig
Pig
Pig
Pig
Pig
Pig
Pig
Pig
Pig
Pig

Pig
Pig
Pig
Pig
Pig
Pig
Pig
Pig
Pig
Pig
Pig
Pig
Pig
Pig
Pig
Pig
Pig
Pig
Pig
Pig
Pig
Pig
Pig
Pig
Pig
Pig
Pig
Pig
Pig
Pig
Pig
Pig
Pig
Pig
Pig
Pig
Pig
Pig
Pig
Pig
Pig
Pig
Pig
Pig
Pig
Pig

Pig
Pig
Pig
Pig
Pig
Pig
Pig
Pig
Pig
Pig
Pig
Pig
Pig
Pig
Pig
Pig
Pig
Pig
Pig
Pig
Pig
Pig
Pig
Pig
Pig
Pig
Pig
Pig
Pig
Pig
Pig
Pig
Pig
Pig
Pig
Pig
Pig
Pig
Pig
Pig
Pig
Pig
Pig
Pig
Pig
Pig

Pig
Pig
Pig
Pig
Pig
Pig
Pig
Pig
Pig
Pig
Pig
Pig
Pig
Pig
Pig
Pig
Pig
Pig
Pig
Pig
Pig
Pig
Pig
Pig
Pig
Pig
Pig
Pig
Pig
Pig
Pig
Pig
Pig
Pig
Pig
Pig
Pig
Pig
Pig
Pig
Pig
Pig
Pig
Pig
Pig
Pig

Pig
Pig
Pig
Pig
Pig
Pig
Pig
Pig
Pig
Pig
Pig
Pig
Pig
Pig
Pig
Pig
Pig
Pig
Pig
Pig
Pig
Pig
Pig
Pig
Pig
Pig
Pig
Pig
Pig
Pig
Pig
Pig
Pig
Pig
Pig
Pig
Pig
Pig
Pig
Pig
Pig
Pig
Pig
Pig
Pig
Pig

Pig
Pig
Pig
Pig
Pig
Pig
Pig
Pig
Pig
Pig
Pig
Pig
Pig
Pig
Pig
Pig
Pig
Pig
Pig
Pig
Pig
Pig
Pig
Pig
Pig
Pig
Pig
Pig
Pig
Pig
Pig
Pig
Pig
Pig
Pig
Pig
Pig
Pig
Pig
Pig
Pig
Pig
Pig
Pig
Pig
Pig

Pig
Pig
Pig
Pig
Pig
Pig
Pig
Pig
Pig
Pig
Pig
Pig
Pig
Pig
Pig
Pig
Pig
Pig
Pig
Pig
Pig
Pig
Pig
Pig
Pig
Pig
Pig
Pig
Pig
Pig
Pig
Pig
Pig
Pig
Pig
Pig
Pig
Pig
Pig
Pig
Pig
Pig
Pig
Pig
Pig
Pig

Pig
Pig
Pig
Pig
Pig
Pig
Pig
Pig
Pig
Pig
Pig
Pig
Pig
Pig
Pig
Pig
Pig
Pig
Pig
Pig
Pig
Pig
Pig
Pig
Pig
Pig
Pig
Pig
Pig
Pig
Pig
Pig
Pig
Pig
Pig
Pig
Pig
Pig
Pig
Pig
Pig
Pig
Pig
Pig
Pig
Pig

Pig
Pig
Pig
Pig
Pig
Pig
Pig
Pig
Pig
Pig
Pig
Pig
Pig
Pig
Pig
Pig
Pig
Pig
Pig
Pig
Pig
Pig
Pig
Pig
Pig
Pig
Pig
Pig
Pig
Pig
Pig
Pig
Pig
Pig
Pig
Pig
Pig
Pig
Pig
Pig
Pig
Pig
Pig
Pig
Pig
Pig

Pig
Pig
Pig
Pig
Pig
Pig
Pig
Pig
Pig
Pig
Pig
Pig
Pig
Pig
Pig
Pig
Pig
Pig
Pig
Pig
Pig
Pig
Pig
Pig
Pig
Pig
Pig
Pig
Pig
Pig
Pig
Pig
Pig
Pig
Pig
Pig
Pig
Pig
Pig
Pig
Pig
Pig
Pig
Pig
Pig
Pig

Pig
Pig
Pig
Pig
Pig
Pig
Pig
Pig
Pig
Pig
Pig
Pig
Pig
Pig
Pig
Pig
Pig
Pig
Pig
Pig
Pig
Pig
Pig
Pig
Pig
Pig
Pig
Pig
Pig
Pig
Pig
Pig
Pig
Pig
Pig
Pig
Pig
Pig
Pig
Pig
Pig
Pig
Pig
Pig
Pig
Pig

Pig
Pig
Pig
Pig
Pig
Pig
Pig
Pig
Pig
Pig
Pig
Pig
Pig
Pig
Pig
Pig
Pig
Pig
Pig
Pig
Pig
Pig
Pig
Pig
Pig
Pig
Pig
Pig
Pig
Pig
Pig
Pig
Pig
Pig
Pig
Pig
Pig
Pig
Pig
Pig
Pig
Pig
Pig
Pig
Pig
Pig

Pig
Pig
Pig
Pig
Pig
Pig
Pig
Pig
Pig
Pig
Pig
Pig
Pig
Pig
Pig
Pig
Pig
Pig
Pig
Pig
Pig
Pig
Pig
Pig
Pig
Pig
Pig
Pig
Pig
Pig
Pig
Pig
Pig
Pig
Pig
Pig
Pig
Pig
Pig
Pig
Pig
Pig
Pig
Pig
Pig
Pig

Pig
Pig
Pig
Pig
Pig
Pig
Pig
Pig
Pig
Pig
Pig
Pig
Pig
Pig
Pig
Pig
Pig
Pig
Pig
Pig
Pig
Pig
Pig
Pig
Pig
Pig
Pig
Pig
Pig
Pig
Pig
Pig
Pig
Pig
Pig
Pig
Pig
Pig
Pig
Pig
Pig
Pig
Pig
Pig
Pig
Pig

Pig
Pig
Pig
Pig
Pig
Pig
Pig
Pig
Pig
Pig
Pig
Pig
Pig
Pig
Pig
Pig
Pig
Pig
Pig
Pig
Pig
Pig
Pig
Pig
Pig
Pig
Pig
Pig
Pig
Pig
Pig
Pig
Pig
Pig
Pig
Pig
Pig
Pig
Pig
Pig
Pig
Pig
Pig
Pig
Pig
Pig

Pig
Pig
Pig
Pig
Pig
Pig
Pig
Pig
Pig
Pig
Pig
Pig
Pig
Pig
Pig
Pig
Pig
Pig
Pig
Pig
Pig
Pig
Pig
Pig
Pig
Pig
Pig
Pig
Pig
Pig
Pig
Pig
Pig
Pig
Pig
Pig
Pig
Pig
Pig
Pig
Pig
Pig
Pig
Pig
Pig
Pig

Pig
Pig
Pig
Pig
Pig
Pig
Pig
Pig
Pig
Pig
Pig
Pig
Pig
Pig
Pig
Pig
Pig
Pig
Pig
Pig
Pig
Pig
Pig
Pig
Pig
Pig
Pig
Pig
Pig
Pig
Pig
Pig
Pig
Pig
Pig
Pig
Pig
Pig
Pig
Pig
Pig
Pig
Pig
Pig
Pig
Pig

Pig
Pig
Pig
Pig
Pig
Pig
Pig
Pig
Pig
Pig
Pig
Pig
Pig
Pig
Pig
Pig
Pig
Pig
Pig
Pig
Pig
Pig
Pig
Pig
Pig
Pig
Pig
Pig
Pig
Pig
Pig
Pig
Pig
Pig
Pig
Pig
Pig
Pig
Pig
Pig
Pig
Pig
Pig
Pig
Pig
Pig

Pig
Pig
Pig
Pig
Pig
Pig
Pig
Pig
Pig
Pig
Pig
Pig
Pig
Pig
Pig
Pig
Pig
Pig
Pig
Pig
Pig
Pig
Pig
Pig
Pig
Pig
Pig
Pig
Pig
Pig
Pig
Pig
Pig
Pig
Pig
Pig
Pig
Pig
Pig
Pig
Pig
Pig
Pig
Pig
Pig
Pig

Pig
Pig
Pig
Pig
Pig
Pig
Pig
Pig
Pig
Pig
Pig
Pig
Pig
Pig
Pig
Pig
Pig
Pig
Pig
Pig
Pig
Pig
Pig
Pig
Pig
Pig
Pig
Pig
Pig
Pig
Pig
Pig
Pig
Pig
Pig
Pig
Pig
Pig
Pig
Pig
Pig
Pig
Pig
Pig
Pig
Pig

Pig
Pig
Pig
Pig
Pig
Pig
Pig
Pig
Pig
Pig
Pig
Pig
Pig
Pig
Pig
Pig
Pig
Pig
Pig
Pig
Pig
Pig
Pig
Pig
Pig
Pig
Pig
Pig
Pig
Pig
Pig
Pig
Pig
Pig
Pig
Pig
Pig
Pig
Pig
Pig
Pig
Pig
Pig
Pig
Pig
Pig

Pig
Pig
Pig
Pig
Pig
Pig
Pig
Pig
Pig
Pig
Pig
Pig
Pig
Pig
Pig
Pig
Pig
Pig
Pig
Pig
Pig
Pig
Pig
Pig
Pig
Pig
Pig
Pig
Pig
Pig
Pig
Pig
Pig
Pig
Pig
Pig
Pig
Pig
Pig
Pig
Pig
Pig
Pig
Pig
Pig
Pig

Pig
Pig
Pig
Pig
Pig
Pig
Pig
Pig
Pig
Pig
Pig
Pig
Pig
Pig
Pig
Pig
Pig
Pig
Pig
Pig
Pig
Pig
Pig
Pig
Pig
Pig
Pig
Pig
Pig
Pig
Pig
Pig
Pig
Pig
Pig
Pig
Pig
Pig
Pig
Pig
Pig
Pig
Pig
Pig
Pig
Pig

Pig
Pig
Pig
Pig
Pig
Pig
Pig
Pig
Pig
Pig
Pig
Pig
Pig
Pig
Pig
Pig
Pig
Pig
Pig
Pig
Pig
Pig
Pig
Pig
Pig
Pig
Pig
Pig
Pig
Pig
Pig
Pig
Pig
Pig
Pig
Pig
Pig
Pig
Pig
Pig
Pig
Pig
Pig
Pig
Pig
Pig

Pig
Pig
Pig
Pig
Pig
Pig
Pig
Pig
Pig
Pig
Pig
Pig
Pig
Pig
Pig
Pig
Pig
Pig
Pig
Pig
Pig
Pig
Pig
Pig
Pig
Pig
Pig
Pig
Pig
Pig
Pig
Pig
Pig
Pig
Pig
Pig
Pig
Pig
Pig
Pig
Pig
Pig
Pig
Pig
Pig
Pig

Pig
Pig
Pig
Pig
Pig
Pig
Pig
Pig
Pig
Pig
Pig
Pig
Pig
Pig
Pig
Pig
Pig
Pig
Pig
Pig
Pig
Pig
Pig
Pig
Pig
Pig
Pig
Pig
Pig
Pig
Pig
Pig
Pig
Pig
Pig
Pig
Pig
Pig
Pig
Pig
Pig
Pig
Pig
Pig
Pig
Pig

Pig
Pig
Pig
Pig
Pig
Pig
Pig
Pig
Pig
Pig
Pig
Pig
Pig
Pig
Pig
Pig
Pig
Pig
Pig
Pig
Pig
Pig
Pig
Pig
Pig
Pig
Pig
Pig
Pig
Pig
Pig
Pig
Pig
Pig
Pig
Pig
Pig
Pig
Pig
Pig
Pig
Pig
Pig
Pig
Pig
Pig

Pig
Pig
Pig
Pig
Pig
Pig
Pig
Pig
Pig
Pig
Pig
Pig
Pig
Pig
Pig
Pig
Pig
Pig
Pig
Pig
Pig
Pig
Pig
Pig
Pig
Pig
Pig
Pig
Pig
Pig
Pig
Pig
Pig
Pig
Pig
Pig
Pig
Pig
Pig
Pig
Pig
Pig
Pig
Pig
Pig
Pig

Pig
Pig
Pig
Pig
Pig
Pig
Pig
Pig
Pig
Pig
Pig
Pig
Pig
Pig
Pig
Pig
Pig
Pig
Pig
Pig
Pig
Pig
Pig
Pig
Pig
Pig
Pig
Pig
Pig
Pig
Pig
Pig
Pig
Pig
Pig
Pig
Pig
Pig
Pig
Pig
Pig
Pig
Pig
Pig
Pig
Pig

Pig
Pig
Pig
Pig
Pig
Pig
Pig
Pig
Pig
Pig
Pig
Pig
Pig
Pig
Pig
Pig
Pig
Pig
Pig
Pig
Pig
Pig
Pig
Pig
Pig
Pig
Pig
Pig
Pig
Pig
Pig
Pig
Pig
Pig
Pig
Pig
Pig
Pig
Pig
Pig
Pig
Pig
Pig
Pig
Pig
Pig

Pig
Pig
Pig
Pig
Pig
Pig
Pig
Pig
Pig
Pig
Pig
Pig
Pig
Pig
Pig
Pig
Pig
Pig
Pig
Pig
Pig
Pig
Pig
Pig
Pig
Pig
Pig
Pig
Pig
Pig
Pig
Pig
Pig
Pig
Pig
Pig
Pig
Pig
Pig
Pig
Pig
Pig
Pig
Pig
Pig
Pig

Pig
Pig
Pig
Pig
Pig
Pig
Pig
Pig
Pig
Pig
Pig
Pig
Pig
Pig
Pig
Pig
Pig
Pig
Pig
Pig
Pig
Pig
Pig
Pig
Pig
Pig
Pig
Pig
Pig
Pig
Pig
Pig
Pig
Pig
Pig
Pig
Pig
Pig
Pig
Pig
Pig
Pig
Pig
Pig
Pig
Pig

Pig
Pig
Pig
Pig
Pig
Pig
Pig
Pig
Pig
Pig
Pig
Pig
Pig
Pig
Pig
Pig
Pig
Pig
Pig
Pig
Pig
Pig
Pig
Pig
Pig
Pig
Pig
Pig
Pig
Pig
Pig
Pig
Pig
Pig
Pig
Pig
Pig
Pig
Pig
Pig
Pig
Pig
Pig
Pig
Pig
Pig

Pig
Pig
Pig
Pig
Pig
Pig
Pig
Pig
Pig
Pig
Pig
Pig
Pig
Pig
Pig
Pig
Pig
Pig
Pig
Pig
Pig
Pig
Pig
Pig
Pig
Pig
Pig
Pig
Pig
Pig
Pig
Pig
Pig
Pig
Pig
Pig
Pig
Pig
Pig
Pig
Pig
Pig
Pig
Pig
Pig
Pig

Pig
Pig
Pig
Pig
Pig
Pig
Pig
Pig
Pig
Pig
Pig
Pig
Pig
Pig
Pig
Pig
Pig
Pig
Pig
Pig
Pig
Pig
Pig
Pig
Pig
Pig
Pig
Pig
Pig
Pig
Pig
Pig
Pig
Pig
Pig
Pig
Pig
Pig
Pig
Pig
Pig
Pig
Pig
Pig
Pig
Pig

Pig
Pig
Pig
Pig
Pig
Pig
Pig
Pig
Pig
Pig
Pig
Pig
Pig
Pig
Pig
Pig
Pig
Pig
Pig
Pig
Pig
Pig
Pig
Pig
Pig
Pig
Pig
Pig
Pig
Pig
Pig
Pig
Pig
Pig
Pig
Pig
Pig
Pig
Pig
Pig
Pig
Pig
Pig
Pig
Pig
Pig

Pig
Pig
Pig
Pig
Pig
Pig
Pig
Pig
Pig
Pig
Pig
Pig
Pig
Pig
Pig
Pig
Pig
Pig
Pig
Pig
Pig
Pig
Pig
Pig
Pig
Pig
Pig
Pig
Pig
Pig
Pig
Pig
Pig
Pig
Pig
Pig
Pig
Pig
Pig
Pig
Pig
Pig
Pig
Pig
Pig
Pig

Pig
Pig
Pig
Pig
Pig
Pig
Pig
Pig
Pig
Pig
Pig
Pig
Pig
Pig
Pig
Pig
Pig
Pig
Pig
Pig
Pig
Pig
Pig
Pig
Pig
Pig
Pig
Pig
Pig
Pig
Pig
Pig
Pig
Pig
Pig
Pig
Pig
Pig
Pig
Pig
Pig
Pig
Pig
Pig
Pig
Pig

Pig
Pig
Pig
Pig
Pig
Pig
Pig
Pig
Pig
Pig
Pig
Pig
Pig
Pig
Pig
Pig
Pig
Pig
Pig
Pig
Pig
Pig
Pig
Pig
Pig
Pig
Pig
Pig
Pig
Pig
Pig
Pig
Pig
Pig
Pig
Pig
Pig
Pig
Pig
Pig
Pig
Pig
Pig
Pig
Pig
Pig

Pig
Pig
Pig
Pig
Pig
Pig
Pig
Pig
Pig
Pig
Pig
Pig
Pig
Pig
Pig
Pig
Pig
Pig
Pig
Pig
Pig
Pig
Pig
Pig
Pig
Pig
Pig
Pig
Pig
Pig
Pig
Pig
Pig
Pig
Pig
Pig
Pig
Pig
Pig
Pig
Pig
Pig
Pig
Pig
Pig
Pig

Pig
Pig
Pig
Pig
Pig
Pig
Pig
Pig
Pig
Pig
Pig
Pig
Pig
Pig
Pig
Pig
Pig
Pig
Pig
Pig
Pig
Pig
Pig
Pig
Pig
Pig
Pig
Pig
Pig
Pig
Pig
Pig
Pig
Pig
Pig
Pig
Pig
Pig
Pig
Pig
Pig
Pig
Pig
Pig
Pig
Pig

Pig
Pig
Pig
Pig
Pig
Pig
Pig
Pig
Pig
Pig
Pig
Pig
Pig
Pig
Pig
Pig
Pig
Pig
Pig
Pig
Pig
Pig
Pig
Pig
Pig
Pig
Pig
Pig
Pig
Pig
Pig
Pig
Pig
Pig
Pig
Pig
Pig
Pig
Pig
Pig
Pig
Pig
Pig
Pig
Pig
Pig

Pig
Pig
Pig
Pig
Pig
Pig
Pig
Pig
Pig
Pig
Pig
Pig
Pig
Pig
Pig
Pig
Pig
Pig
Pig
Pig
Pig
Pig
Pig
Pig
Pig
Pig
Pig
Pig
Pig
Pig
Pig
Pig
Pig
Pig
Pig
Pig
Pig
Pig
Pig
Pig
Pig
Pig
Pig
Pig
Pig
Pig

Pig
Pig
Pig
Pig
Pig
Pig
Pig
Pig
Pig
Pig
Pig
Pig
Pig
Pig
Pig
Pig
Pig
Pig
Pig
Pig
Pig
Pig
Pig
Pig
Pig
Pig
Pig
Pig
Pig
Pig
Pig
Pig
Pig
Pig
Pig
Pig
Pig
Pig
Pig
Pig
Pig
Pig
Pig
Pig
Pig
Pig

Pig
Pig
Pig
Pig
Pig
Pig
Pig
Pig
Pig
Pig
Pig
Pig
Pig
Pig
Pig
Pig
Pig
Pig
Pig
Pig
Pig
Pig
Pig
Pig
Pig
Pig
Pig
Pig
Pig
Pig
Pig
Pig
Pig
Pig
Pig
Pig
Pig
Pig
Pig
Pig
Pig
Pig
Pig
Pig
Pig
Pig

Pig
Pig
Pig
Pig
Pig
Pig
Pig
Pig
Pig
Pig
Pig
Pig
Pig
Pig
Pig
Pig
Pig
Pig
Pig
Pig
Pig
Pig
Pig
Pig
Pig
Pig
Pig
Pig
Pig
Pig
Pig
Pig
Pig
Pig
Pig
Pig
Pig
Pig
Pig
Pig
Pig
Pig
Pig
Pig
Pig
Pig

Pig
Pig
Pig
Pig
Pig
Pig
Pig
Pig
Pig
Pig
Pig
Pig
Pig
Pig
Pig
Pig
Pig
Pig
Pig
Pig
Pig
Pig
Pig
Pig
Pig
Pig
Pig
Pig
Pig
Pig
Pig
Pig
Pig
Pig
Pig
Pig
Pig
Pig
Pig
Pig
Pig
Pig
Pig
Pig
Pig
Pig

Pig
Pig
Pig
Pig
Pig
Pig
Pig
Pig
Pig
Pig
Pig
Pig
Pig
Pig
Pig
Pig
Pig
Pig
Pig
Pig
Pig
Pig
Pig
Pig
Pig
Pig
Pig
Pig
Pig
Pig
Pig
Pig
Pig
Pig
Pig
Pig
Pig
Pig
Pig
Pig
Pig
Pig
Pig
Pig
Pig
Pig

Pig
Pig
Pig
Pig
Pig
Pig
Pig
Pig
Pig
Pig
Pig
Pig
Pig
Pig
Pig
Pig
Pig
Pig
Pig
Pig
Pig
Pig
Pig
Pig
Pig
Pig
Pig
Pig
Pig
Pig
Pig
Pig
Pig
Pig
Pig
Pig
Pig
Pig
Pig
Pig
Pig
Pig
Pig
Pig
Pig
Pig

Pig
Pig
Pig
Pig
Pig
Pig
Pig
Pig
Pig
Pig
Pig
Pig
Pig
Pig
Pig
Pig
Pig
Pig
Pig
Pig
Pig
Pig
Pig
Pig
Pig
Pig
Pig
Pig
Pig
Pig
Pig
Pig
Pig
Pig
Pig
Pig
Pig
Pig
Pig
Pig
Pig
Pig
Pig
Pig
Pig
Pig

Pig
Pig
Pig
Pig
Pig
Pig
Pig
Pig
Pig
Pig
Pig
Pig
Pig
Pig
Pig
Pig
Pig
Pig
Pig
Pig
Pig
Pig
Pig
Pig
Pig
Pig
Pig
Pig
Pig
Pig
Pig
Pig
Pig
Pig
Pig
Pig
Pig
Pig
Pig
Pig
Pig
Pig
Pig
Pig
Pig
Pig

Pig
Pig
Pig
Pig
Pig
Pig
Pig
Pig
Pig
Pig
Pig
Pig
Pig
Pig
Pig
Pig
Pig
Pig
Pig
Pig
Pig
Pig
Pig
Pig
Pig
Pig
Pig
Pig
Pig
Pig
Pig
Pig
Pig
Pig
Pig
Pig
Pig
Pig
Pig
Pig
Pig
Pig
Pig
Pig
Pig
Pig

Pig
Pig
Pig
Pig
Pig
Pig
Pig
Pig
Pig
Pig
Pig
Pig
Pig
Pig
Pig
Pig
Pig
Pig
Pig
Pig
Pig
Pig
Pig
Pig
Pig
Pig
Pig
Pig
Pig
Pig
Pig
Pig
Pig
Pig
Pig
Pig
Pig
Pig
Pig
Pig
Pig
Pig
Pig
Pig
Pig
Pig

Pig
Pig
Pig
Pig
Pig
Pig
Pig
Pig
Pig
Pig
Pig
Pig
Pig
Pig
Pig
Pig
Pig
Pig
Pig
Pig
Pig
Pig
Pig
Pig
Pig
Pig
Pig
Pig
Pig
Pig
Pig
Pig
Pig
Pig
Pig
Pig
Pig
Pig
Pig
Pig
Pig
Pig
Pig
Pig
Pig
Pig

Pig
Pig
Pig
Pig
Pig
Pig
Pig
Pig
Pig
Pig
Pig
Pig
Pig
Pig
Pig
Pig
Pig
Pig
Pig
Pig
Pig
Pig
Pig
Pig
Pig
Pig
Pig
Pig
Pig
Pig
Pig
Pig
Pig
Pig
Pig
Pig
Pig
Pig
Pig
Pig
Pig
Pig
Pig
Pig
Pig
Pig

Pig
Pig
Pig
Pig
Pig
Pig
Pig
Pig
Pig
Pig
Pig
Pig
Pig
Pig
Pig
Pig
Pig
Pig
Pig
Pig
Pig
Pig
Pig
Pig
Pig
Pig
Pig
Pig
Pig
Pig
Pig
Pig
Pig
Pig
Pig
Pig
Pig
Pig
Pig
Pig
Pig
Pig
Pig
Pig
Pig
Pig

Pig
Pig
Pig
Pig
Pig
Pig
Pig
Pig
Pig
Pig
Pig
Pig
Pig
Pig
Pig
Pig
Pig
Pig
Pig
Pig
Pig
Pig
Pig
Pig
Pig
Pig
Pig
Pig
Pig
Pig
Pig
Pig
Pig
Pig
Pig
Pig
Pig
Pig
Pig
Pig
Pig
Pig
Pig
Pig
Pig
Pig

Pig
Pig
Pig
Pig
Pig
Pig
Pig
Pig
Pig
Pig
Pig
Pig
Pig
Pig
Pig
Pig
Pig
Pig
Pig
Pig
Pig
Pig
Pig
Pig
Pig
Pig
Pig
Pig
Pig
Pig
Pig
Pig
Pig
Pig
Pig
Pig
Pig
Pig
Pig
Pig
Pig
Pig
Pig
Pig
Pig
Pig

Pig
Pig
Pig
Pig
Pig
Pig
Pig
Pig
Pig
Pig
Pig
Pig
Pig
Pig
Pig
Pig
Pig
Pig
Pig
Pig
Pig
Pig
Pig
Pig
Pig
Pig
Pig
Pig
Pig
Pig
Pig
Pig
Pig
Pig
Pig
Pig
Pig
Pig
Pig
Pig
Pig
Pig
Pig
Pig
Pig
Pig

Pig
Pig
Pig
Pig
Pig
Pig
Pig
Pig
Pig
Pig
Pig
Pig
Pig
Pig
Pig
Pig
Pig
Pig
Pig
Pig
Pig
Pig
Pig
Pig
Pig
Pig
Pig
Pig
Pig
Pig
Pig
Pig
Pig
Pig
Pig
Pig
Pig
Pig
Pig
Pig
Pig
Pig
Pig
Pig
Pig
Pig

Pig
Pig
Pig
Pig
Pig
Pig
Pig
Pig
Pig
Pig
Pig
Pig
Pig
Pig
Pig
Pig
Pig
Pig
Pig
Pig
Pig
Pig
Pig
Pig
Pig
Pig
Pig
Pig
Pig
Pig
Pig
Pig
Pig
Pig
Pig
Pig
Pig
Pig
Pig
Pig
Pig
Pig
Pig
Pig
Pig
Pig

Pig
Pig
Pig
Pig
Pig
Pig
Pig
Pig
Pig
Pig
Pig
Pig
Pig
Pig
Pig
Pig
Pig
Pig
Pig
Pig
Pig
Pig
Pig
Pig
Pig
Pig
Pig
Pig
Pig
Pig
Pig
Pig
Pig
Pig
Pig
Pig
Pig
Pig
Pig
Pig
Pig
Pig
Pig
Pig
Pig
Pig

Pig
Pig
Pig
Pig
Pig
Pig
Pig
Pig
Pig
Pig
Pig
Pig
Pig
Pig
Pig
Pig
Pig
Pig
Pig
Pig
Pig
Pig
Pig
Pig
Pig
Pig
Pig
Pig
Pig
Pig
Pig
Pig
Pig
Pig
Pig
Pig
Pig
Pig
Pig
Pig
Pig
Pig
Pig
Pig
Pig
Pig

Pig
Pig
Pig
Pig
Pig
Pig
Pig
Pig
Pig
Pig
Pig
Pig
Pig
Pig
Pig
Pig
Pig
Pig
Pig
Pig
Pig
Pig
Pig
Pig
Pig
Pig
Pig
Pig
Pig
Pig
Pig
Pig
Pig
Pig
Pig
Pig
Pig
Pig
Pig
Pig
Pig
Pig
Pig
Pig
Pig
Pig

Pig
Pig
Pig
Pig
Pig
Pig
Pig
Pig
Pig
Pig
Pig
Pig
Pig
Pig
Pig
Pig
Pig
Pig
Pig
Pig
Pig
Pig
Pig
Pig
Pig
Pig
Pig
Pig
Pig
Pig
Pig
Pig
Pig
Pig
Pig
Pig
Pig
Pig
Pig
Pig
Pig
Pig
Pig
Pig
Pig
Pig

Pig
Pig
Pig
Pig
Pig
Pig
Pig
Pig
Pig
Pig
Pig
Pig
Pig
Pig
Pig
Pig
Pig
Pig
Pig
Pig
Pig
Pig
Pig
Pig
Pig
Pig
Pig
Pig
Pig
Pig
Pig
Pig
Pig
Pig
Pig
Pig
Pig
Pig
Pig
Pig
Pig
Pig
Pig
Pig
Pig
Pig

Pig
Pig
Pig
Pig
Pig
Pig
Pig
Pig
Pig
Pig
Pig
Pig
Pig
Pig
Pig
Pig
Pig
Pig
Pig
Pig
Pig
Pig
Pig
Pig
Pig
Pig
Pig
Pig
Pig
Pig
Pig
Pig
Pig
Pig
Pig
Pig
Pig
Pig
Pig
Pig
Pig
Pig
Pig
Pig
Pig
Pig

Pig
Pig
Pig
Pig
Pig
Pig
Pig
Pig
Pig
Pig
Pig
Pig
Pig
Pig
Pig
Pig
Pig
Pig
Pig
Pig
Pig
Pig
Pig
Pig
Pig
Pig
Pig
Pig
Pig
Pig
Pig
Pig
Pig
Pig
Pig
Pig
Pig
Pig
Pig
Pig
Pig
Pig
Pig
Pig
Pig
Pig

Pig
Pig
Pig
Pig
Pig
Pig
Pig
Pig
Pig
Pig
Pig
Pig
Pig
Pig
Pig
Pig
Pig
Pig
Pig
Pig
Pig
Pig
Pig
Pig
Pig
Pig
Pig
Pig
Pig
Pig
Pig
Pig
Pig
Pig
Pig
Pig
Pig
Pig
Pig
Pig
Pig
Pig
Pig
Pig
Pig
Pig

Pig
Pig
Pig
Pig
Pig
Pig
Pig
Pig
Pig
Pig
Pig
Pig
Pig
Pig
Pig
Pig
Pig
Pig
Pig
Pig
Pig
Pig
Pig
Pig
Pig
Pig
Pig
Pig
Pig
Pig
Pig
Pig
Pig
Pig
Pig
Pig
Pig
Pig
Pig
Pig
Pig
Pig
Pig
Pig
Pig
Pig

Pig
Pig
Pig
Pig
Pig
Pig
Pig
Pig
Pig
Pig
Pig
Pig
Pig
Pig
Pig
Pig
Pig
Pig
Pig
Pig
Pig
Pig
Pig
Pig
Pig
Pig
Pig
Pig
Pig
Pig
Pig
Pig
Pig
Pig
Pig
Pig
Pig
Pig
Pig
Pig
Pig
Pig
Pig
Pig
Pig
Pig

Pig
Pig
Pig
Pig
Pig
Pig
Pig
Pig
Pig
Pig
Pig
Pig
Pig
Pig
Pig
Pig
Pig
Pig
Pig
Pig
Pig
Pig
Pig
Pig
Pig
Pig
Pig
Pig
Pig
Pig
Pig
Pig
Pig
Pig
Pig
Pig
Pig
Pig
Pig
Pig
Pig
Pig
Pig
Pig
Pig
Pig

Pig
Pig
Pig
Pig
Pig
Pig
Pig
Pig
Pig
Pig
Pig
Pig
Pig
Pig
Pig
Pig
Pig
Pig
Pig
Pig
Pig
Pig
Pig
Pig
Pig
Pig
Pig
Pig
Pig
Pig
Pig
Pig
Pig
Pig
Pig
Pig
Pig
Pig
Pig
Pig
Pig
Pig
Pig
Pig
Pig
Pig

Pig
Pig
Pig
Pig
Pig
Pig
Pig
Pig
Pig
Pig
Pig
Pig
Pig
Pig
Pig
Pig
Pig
Pig
Pig
Pig
Pig
Pig
Pig
Pig
Pig
Pig
Pig
Pig
Pig
Pig
Pig
Pig
Pig
Pig
Pig
Pig
Pig
Pig
Pig
Pig
Pig
Pig
Pig
Pig
Pig
Pig

Pig
Pig
Pig
Pig
Pig
Pig
Pig
Pig
Pig
Pig
Pig
Pig
Pig
Pig
Pig
Pig
Pig
Pig
Pig
Pig
Pig
Pig
Pig
Pig
Pig
Pig
Pig
Pig
Pig
Pig
Pig
Pig
Pig
Pig
Pig
Pig
Pig
Pig
Pig
Pig
Pig
Pig
Pig
Pig
Pig
Pig

Pig
Pig
Pig
Pig
Pig
Pig
Pig
Pig
Pig
Pig
Pig
Pig
Pig
Pig
Pig
Pig
Pig
Pig
Pig
Pig
Pig
Pig
Pig
Pig
Pig
Pig
Pig
Pig
Pig
Pig
Pig
Pig
Pig
Pig
Pig
Pig
Pig
Pig
Pig
Pig
Pig
Pig
Pig
Pig
Pig
Pig

Pig
Pig
Pig
Pig
Pig
Pig
Pig
Pig
Pig
Pig
Pig
Pig
Pig
Pig
Pig
Pig
Pig
Pig
Pig
Pig
Pig
Pig
Pig
Pig
Pig
Pig
Pig
Pig
Pig
Pig
Pig
Pig
Pig
Pig
Pig
Pig
Pig
Pig
Pig
Pig
Pig
Pig
Pig
Pig
Pig
Pig

Pig
Pig
Pig
Pig
Pig
Pig
Pig
Pig
Pig
Pig
Pig
Pig
Pig
Pig
Pig
Pig
Pig
Pig
Pig
Pig
Pig
Pig
Pig
Pig
Pig
Pig
Pig
Pig
Pig
Pig
Pig
Pig
Pig
Pig
Pig
Pig
Pig
Pig
Pig
Pig
Pig
Pig
Pig
Pig
Pig
Pig

Pig
Pig
Pig
Pig
Pig
Pig
Pig
Pig
Pig
Pig
Pig
Pig
Pig
Pig
Pig
Pig
Pig
Pig
Pig
Pig
Pig
Pig
Pig
Pig
Pig
Pig
Pig
Pig
Pig
Pig
Pig
Pig
Pig
Pig
Pig
Pig
Pig
Pig
Pig
Pig
Pig
Pig
Pig
Pig
Pig
Pig

Pig
Pig
Pig
Pig
Pig
Pig
Pig
Pig
Pig
Pig
Pig
Pig
Pig
Pig
Pig
Pig
Pig
Pig
Pig
Pig
Pig
Pig
Pig
Pig
Pig
Pig
Pig
Pig
Pig
Pig
Pig
Pig
Pig
Pig
Pig
Pig
Pig
Pig
Pig
Pig
Pig
Pig
Pig
Pig
Pig
Pig

Pig
Pig
Pig
Pig
Pig
Pig
Pig
Pig
Pig
Pig
Pig
Pig
Pig
Pig
Pig
Pig
Pig
Pig
Pig
Pig
Pig
Pig
Pig
Pig
Pig
Pig
Pig
Pig
Pig
Pig
Pig
Pig
Pig
Pig
Pig
Pig
Pig
Pig
Pig
Pig
Pig
Pig
Pig
Pig
Pig
Pig

Pig
Pig
Pig
Pig
Pig
Pig
Pig
Pig
Pig
Pig
Pig
Pig
Pig
Pig
Pig
Pig
Pig
Pig
Pig
Pig
Pig
Pig
Pig
Pig
Pig
Pig
Pig
Pig
Pig
Pig
Pig
Pig
Pig
Pig
Pig
Pig
Pig
Pig
Pig
Pig
Pig
Pig
Pig
Pig
Pig
Pig

Pig
Pig
Pig
Pig
Pig
Pig
Pig
Pig
Pig
Pig
Pig
Pig
Pig
Pig
Pig
Pig
Pig
Pig
Pig
Pig
Pig
Pig
Pig
Pig
Pig
Pig
Pig
Pig
Pig
Pig
Pig
Pig
Pig
Pig
Pig
Pig
Pig
Pig
Pig
Pig
Pig
Pig
Pig
Pig
Pig
Pig

Pig
Pig
Pig
Pig
Pig
Pig
Pig
Pig
Pig
Pig
Pig
Pig
Pig
Pig
Pig
Pig
Pig
Pig
Pig
Pig
Pig
Pig
Pig
Pig
Pig
Pig
Pig
Pig
Pig
Pig
Pig
Pig
Pig
Pig
Pig
Pig
Pig
Pig
Pig
Pig
Pig
Pig
Pig
Pig
Pig
Pig

Pig
Pig
Pig
Pig
Pig
Pig
Pig
Pig
Pig
Pig
Pig
Pig
Pig
Pig
Pig
Pig
Pig
Pig
Pig
Pig
Pig
Pig
Pig
Pig
Pig
Pig
Pig
Pig
Pig
Pig
Pig
Pig
Pig
Pig
Pig
Pig
Pig
Pig
Pig
Pig
Pig
Pig
Pig
Pig
Pig
Pig

Pig
Pig
Pig
Pig
Pig
Pig
Pig
Pig
Pig
Pig
Pig
Pig
Pig
Pig
Pig
Pig
Pig
Pig
Pig
Pig
Pig
Pig
Pig
Pig
Pig
Pig
Pig
Pig
Pig
Pig
Pig
Pig
Pig
Pig
Pig
Pig
Pig
Pig
Pig
Pig
Pig
Pig
Pig
Pig
Pig
Pig

Pig
Pig
Pig
Pig
Pig
Pig
Pig
Pig
Pig
Pig
Pig
Pig
Pig
Pig
Pig
Pig
Pig
Pig
Pig
Pig
Pig
Pig
Pig
Pig
Pig
Pig
Pig
Pig
Pig
Pig
Pig
Pig
Pig
Pig
Pig
Pig
Pig
Pig
Pig
Pig
Pig
Pig
Pig
Pig
Pig
Pig

Pig
Pig
Pig
Pig
Pig
Pig
Pig
Pig
Pig
Pig
Pig
Pig
Pig
Pig
Pig
Pig
Pig
Pig
Pig
Pig
Pig
Pig
Pig
Pig
Pig
Pig
Pig
Pig
Pig
Pig
Pig
Pig
Pig
Pig
Pig
Pig
Pig
Pig
Pig
Pig
Pig
Pig
Pig
Pig
Pig
Pig

Pig
Pig
Pig
Pig
Pig
Pig
Pig
Pig
Pig
Pig
Pig
Pig
Pig
Pig
Pig
Pig
Pig
Pig
Pig
Pig
Pig
Pig
Pig
Pig
Pig
Pig
Pig
Pig
Pig
Pig
Pig
Pig
Pig
Pig
Pig
Pig
Pig
Pig
Pig
Pig
Pig
Pig
Pig
Pig
Pig
Pig

Pig
Pig
Pig
Pig
Pig
Pig
Pig
Pig
Pig
Pig
Pig
Pig
Pig
Pig
Pig
Pig
Pig
Pig
Pig
Pig
Pig
Pig
Pig
Pig
Pig
Pig
Pig
Pig
Pig
Pig
Pig
Pig
Pig
Pig
Pig
Pig
Pig
Pig
Pig
Pig
Pig
Pig
Pig
Pig
Pig
Pig

Pig
Pig
Pig
Pig
Pig
Pig
Pig
Pig
Pig
Pig
Pig
Pig
Pig
Pig
Pig
Pig
Pig
Pig
Pig
Pig
Pig
Pig
Pig
Pig
Pig
Pig
Pig
Pig
Pig
Pig
Pig
Pig
Pig
Pig
Pig
Pig
Pig
Pig
Pig
Pig
Pig
Pig
Pig
Pig
Pig
Pig

Pig
Pig
Pig
Pig
Pig
Pig
Pig
Pig
Pig
Pig
Pig
Pig
Pig
Pig
Pig
Pig
Pig
Pig
Pig
Pig
Pig
Pig
Pig
Pig
Pig
Pig
Pig
Pig
Pig
Pig
Pig
Pig
Pig
Pig
Pig
Pig
Pig
Pig
Pig
Pig
Pig
Pig
Pig
Pig
Pig
Pig

Pig
Pig
Pig
Pig
Pig
Pig
Pig
Pig
Pig
Pig
Pig
Pig
Pig
Pig
Pig
Pig
Pig
Pig
Pig
Pig
Pig
Pig
Pig
Pig
Pig
Pig
Pig
Pig
Pig
Pig
Pig
Pig
Pig
Pig
Pig
Pig
Pig
Pig
Pig
Pig
Pig
Pig
Pig
Pig
Pig
Pig

Pig
Pig
Pig
Pig
Pig
Pig
Pig
Pig
Pig
Pig
Pig
Pig
Pig
Pig
Pig
Pig
Pig
Pig
Pig
Pig
Pig
Pig
Pig
Pig
Pig
Pig
Pig
Pig
Pig
Pig
Pig
Pig
Pig
Pig
Pig
Pig
Pig
Pig
Pig
Pig
Pig
Pig
Pig
Pig
Pig
Pig

Pig
Pig
Pig
Pig
Pig
Pig
Pig
Pig
Pig
Pig
Pig
Pig
Pig
Pig
Pig
Pig
Pig
Pig
Pig
Pig
Pig
Pig
Pig
Pig
Pig
Pig
Pig
Pig
Pig
Pig
Pig
Pig
Pig
Pig
Pig
Pig
Pig
Pig
Pig
Pig
Pig
Pig
Pig
Pig
Pig
Pig

Pig
Pig
Pig
Pig
Pig
Pig
Pig
Pig
Pig
Pig
Pig
Pig
Pig
Pig
Pig
Pig
Pig
Pig
Pig
Pig
Pig
Pig
Pig
Pig
Pig
Pig
Pig
Pig
Pig
Pig
Pig
Pig
Pig
Pig
Pig
Pig
Pig
Pig
Pig
Pig
Pig
Pig
Pig
Pig
Pig
Pig

Pig
Pig
Pig
Pig
Pig
Pig
Pig
Pig
Pig
Pig
Pig
Pig
Pig
Pig
Pig
Pig
Pig
Pig
Pig
Pig
Pig
Pig
Pig
Pig
Pig
Pig
Pig
Pig
Pig
Pig
Pig
Pig
Pig
Pig
Pig
Pig
Pig
Pig
Pig
Pig
Pig
Pig
Pig
Pig
Pig
Pig

Pig
Pig
Pig
Pig
Pig
Pig
Pig
Pig
Pig
Pig
Pig
Pig
Pig
Pig
Pig
Pig
Pig
Pig
Pig
Pig
Pig
Pig
Pig
Pig
Pig
Pig
Pig
Pig
Pig
Pig
Pig
Pig
Pig
Pig
Pig
Pig
Pig
Pig
Pig
Pig
Pig
Pig
Pig
Pig
Pig
Pig

Pig
Pig
Pig
Pig
Pig
Pig
Pig
Pig
Pig
Pig
Pig
Pig
Pig
Pig
Pig
Pig
Pig
Pig
Pig
Pig
Pig
Pig
Pig
Pig
Pig
Pig
Pig
Pig
Pig
Pig
Pig
Pig
Pig
Pig
Pig
Pig
Pig
Pig
Pig
Pig
Pig
Pig
Pig
Pig
Pig
Pig

Pig
Pig
Pig
Pig
Pig
Pig
Pig
Pig
Pig
Pig
Pig
Pig
Pig
Pig
Pig
Pig
Pig
Pig
Pig
Pig
Pig
Pig
Pig
Pig
Pig
Pig
Pig
Pig
Pig
Pig
Pig
Pig
Pig
Pig
Pig
Pig
Pig
Pig
Pig
Pig
Pig
Pig
Pig
Pig
Pig
Pig

Pig
Pig
Pig
Pig
Pig
Pig
Pig
Pig
Pig
Pig
Pig
Pig
Pig
Pig
Pig
Pig
Pig
Pig
Pig
Pig
Pig
Pig
Pig
Pig
Pig
Pig
Pig
Pig
Pig
Pig
Pig
Pig
Pig
Pig
Pig
Pig
Pig
Pig
Pig
Pig
Pig
Pig
Pig
Pig
Pig
Pig

Pig
Pig
Pig
Pig
Pig
Pig
Pig
Pig
Pig
Pig
Pig
Pig
Pig
Pig
Pig
Pig
Pig
Pig
Pig
Pig
Pig
Pig
Pig
Pig
Pig
Pig
Pig
Pig
Pig
Pig
Pig
Pig
Pig
Pig
Pig
Pig
Pig
Pig
Pig
Pig
Pig
Pig
Pig
Pig
Pig
Pig

Pig
Pig
Pig
Pig
Pig
Pig
Pig
Pig
Pig
Pig
Pig
Pig
Pig
Pig
Pig
Pig
Pig
Pig
Pig
Pig
Pig
Pig
Pig
Pig
Pig
Pig
Pig
Pig
Pig
Pig
Pig
Pig
Pig
Pig
Pig
Pig
Pig
Pig
Pig
Pig
Pig
Pig
Pig
Pig
Pig
Pig

Pig
Pig
Pig
Pig
Pig
Pig
Pig
Pig
Pig
Pig
Pig
Pig
Pig
Pig
Pig
Pig
Pig
Pig
Pig
Pig
Pig
Pig
Pig
Pig
Pig
Pig
Pig
Pig
Pig
Pig
Pig
Pig
Pig
Pig
Pig
Pig
Pig
Pig
Pig
Pig
Pig
Pig
Pig
Pig
Pig
Pig

Pig
Pig
Pig
Pig
Pig
Pig
Pig
Pig
Pig
Pig
Pig
Pig
Pig
Pig
Pig
Pig
Pig
Pig
Pig
Pig
Pig
Pig
Pig
Pig
Pig
Pig
Pig
Pig
Pig
Pig
Pig
Pig
Pig
Pig
Pig
Pig
Pig
Pig
Pig
Pig
Pig
Pig
Pig
Pig
Pig
Pig

Pig
Pig
Pig
Pig
Pig
Pig
Pig
Pig
Pig
Pig
Pig
Pig
Pig
Pig
Pig
Pig
Pig
Pig
Pig
Pig
Pig
Pig
Pig
Pig
Pig
Pig
Pig
Pig
Pig
Pig
Pig
Pig
Pig
Pig
Pig
Pig
Pig
Pig
Pig
Pig
Pig
Pig
Pig
Pig
Pig
Pig

Pig
Pig
Pig
Pig
Pig
Pig
Pig
Pig
Pig
Pig
Pig
Pig
Pig
Pig
Pig
Pig
Pig
Pig
Pig
Pig
Pig
Pig
Pig
Pig
Pig
Pig
Pig
Pig
Pig
Pig
Pig
Pig
Pig
Pig
Pig
Pig
Pig
Pig
Pig
Pig
Pig
Pig
Pig
Pig
Pig
Pig

Pig
Pig
Pig
Pig
Pig
Pig
Pig
Pig
Pig
Pig
Pig
Pig
Pig
Pig
Pig
Pig
Pig
Pig
Pig
Pig
Pig
Pig
Pig
Pig
Pig
Pig
Pig
Pig
Pig
Pig
Pig
Pig
Pig
Pig
Pig
Pig
Pig
Pig
Pig
Pig
Pig
Pig
Pig
Pig
Pig
Pig

Pig
Pig
Pig
Pig
Pig
Pig
Pig
Pig
Pig
Pig
Pig
Pig
Pig
Pig
Pig
Pig
Pig
Pig
Pig
Pig
Pig
Pig
Pig
Pig
Pig
Pig
Pig
Pig
Pig
Pig
Pig
Pig
Pig
Pig
Pig
Pig
Pig
Pig
Pig
Pig
Pig
Pig
Pig
Pig
Pig
Pig

Pig
Pig
Pig
Pig
Pig
Pig
Pig
Pig
Pig
Pig
Pig
Pig
Pig
Pig
Pig
Pig
Pig
Pig
Pig
Pig
Pig
Pig
Pig
Pig
Pig
Pig
Pig
Pig
Pig
Pig
Pig
Pig
Pig
Pig
Pig
Pig
Pig
Pig
Pig
Pig
Pig
Pig
Pig
Pig
Pig
Pig

Pig
Pig
Pig
Pig
Pig
Pig
Pig
Pig
Pig
Pig
Pig
Pig
Pig
Pig
Pig
Pig
Pig
Pig
Pig
Pig
Pig
Pig
Pig
Pig
Pig
Pig
Pig
Pig
Pig
Pig
Pig
Pig
Pig
Pig
Pig
Pig
Pig
Pig
Pig
Pig
Pig
Pig
Pig
Pig
Pig
Pig

Pig
Pig
Pig
Pig
Pig
Pig
Pig
Pig
Pig
Pig
Pig
Pig
Pig
Pig
Pig
Pig
Pig
Pig
Pig
Pig
Pig
Pig
Pig
Pig
Pig
Pig
Pig
Pig
Pig
Pig
Pig
Pig
Pig
Pig
Pig
Pig
Pig
Pig
Pig
Pig
Pig
Pig
Pig
Pig
Pig
Pig

Pig
Pig
Pig
Pig
Pig
Pig
Pig
Pig
Pig
Pig
Pig
Pig
Pig
Pig
Pig
Pig
Pig
Pig
Pig
Pig
Pig
Pig
Pig
Pig
Pig
Pig
Pig
Pig
Pig
Pig
Pig
Pig
Pig
Pig
Pig
Pig
Pig
Pig
Pig
Pig
Pig
Pig
Pig
Pig
Pig
Pig

Pig
Pig
Pig
Pig
Pig
Pig
Pig
Pig
Pig
Pig
Pig
Pig
Pig
Pig
Pig
Pig
Pig
Pig
Pig
Pig
Pig
Pig
Pig
Pig
Pig
Pig
Pig
Pig
Pig
Pig
Pig
Pig
Pig
Pig
Pig
Pig
Pig
Pig
Pig
Pig
Pig
Pig
Pig
Pig
Pig
Pig

Pig
Pig
Pig
Pig
Pig
Pig
Pig
Pig
Pig
Pig
Pig
Pig
Pig
Pig
Pig
Pig
Pig
Pig
Pig
Pig
Pig
Pig
Pig
Pig
Pig
Pig
Pig
Pig
Pig
Pig
Pig
Pig
Pig
Pig
Pig
Pig
Pig
Pig
Pig
Pig
Pig
Pig
Pig
Pig
Pig
Pig

Pig
Pig
Pig
Pig
Pig
Pig
Pig
Pig
Pig
Pig
Pig
Pig
Pig
Pig
Pig
Pig
Pig
Pig
Pig
Pig
Pig
Pig
Pig
Pig
Pig
Pig
Pig
Pig
Pig
Pig
Pig
Pig
Pig
Pig
Pig
Pig
Pig
Pig
Pig
Pig
Pig
Pig
Pig
Pig
Pig
Pig

Pig
Pig
Pig
Pig
Pig
Pig
Pig
Pig
Pig
Pig
Pig
Pig
Pig
Pig
Pig
Pig
Pig
Pig
Pig
Pig
Pig
Pig
Pig
Pig
Pig
Pig
Pig
Pig
Pig
Pig
Pig
Pig
Pig
Pig
Pig
Pig
Pig
Pig
Pig
Pig
Pig
Pig
Pig
Pig
Pig
Pig

Pig
Pig
Pig
Pig
Pig
Pig
Pig
Pig
Pig
Pig
Pig
Pig
Pig
Pig
Pig
Pig
Pig
Pig
Pig
Pig
Pig
Pig
Pig
Pig
Pig
Pig
Pig
Pig
Pig
Pig
Pig
Pig
Pig
Pig
Pig
Pig
Pig
Pig
Pig
Pig
Pig
Pig
Pig
Pig
Pig
Pig

Pig
Pig
Pig
Pig
Pig
Pig
Pig
Pig
Pig
Pig
Pig
Pig
Pig
Pig
Pig
Pig
Pig
Pig
Pig
Pig
Pig
Pig
Pig
Pig
Pig
Pig
Pig
Pig
Pig
Pig
Pig
Pig
Pig
Pig
Pig
Pig
Pig
Pig
Pig
Pig
Pig
Pig
Pig
Pig
Pig
Pig

Pig
Pig
Pig
Pig
Pig
Pig
Pig
Pig
Pig
Pig
Pig
Pig
Pig
Pig
Pig
Pig
Pig
Pig
Pig
Pig
Pig
Pig
Pig
Pig
Pig
Pig
Pig
Pig
Pig
Pig
Pig
Pig
Pig
Pig
Pig
Pig
Pig
Pig
Pig
Pig
Pig
Pig
Pig
Pig
Pig
Pig

Pig
Pig
Pig
Pig
Pig
Pig
Pig
Pig
Pig
Pig
Pig
Pig
Pig
Pig
Pig
Pig
Pig
Pig
Pig
Pig
Pig
Pig
Pig
Pig
Pig
Pig
Pig
Pig
Pig
Pig
Pig
Pig
Pig
Pig
Pig
Pig
Pig
Pig
Pig
Pig
Pig
Pig
Pig
Pig
Pig
Pig

Pig
Pig
Pig
Pig
Pig
Pig
Pig
Pig
Pig
Pig
Pig
Pig
Pig
Pig
Pig
Pig
Pig
Pig
Pig
Pig
Pig
Pig
Pig
Pig
Pig
Pig
Pig
Pig
Pig
Pig
Pig
Pig
Pig
Pig
Pig
Pig
Pig
Pig
Pig
Pig
Pig
Pig
Pig
Pig
Pig
Pig

Pig
Pig
Pig
Pig
Pig
Pig
Pig
Pig
Pig
Pig
Pig
Pig
Pig
Pig
Pig
Pig
Pig
Pig
Pig
Pig
Pig
Pig
Pig
Pig
Pig
Pig
Pig
Pig
Pig
Pig
Pig
Pig
Pig
Pig
Pig
Pig
Pig
Pig
Pig
Pig
Pig
Pig
Pig
Pig
Pig
Pig

Pig
Pig
Pig
Pig
Pig
Pig
Pig
Pig
Pig
Pig
Pig
Pig
Pig
Pig
Pig
Pig
Pig
Pig
Pig
Pig
Pig
Pig
Pig
Pig
Pig
Pig
Pig
Pig
Pig
Pig
Pig
Pig
Pig
Pig
Pig
Pig
Pig
Pig
Pig
Pig
Pig
Pig
Pig
Pig
Pig
Pig

Pig
Pig
Pig
Pig
Pig
Pig
Pig
Pig
Pig
Pig
Pig
Pig
Pig
Pig
Pig
Pig
Pig
Pig
Pig
Pig
Pig
Pig
Pig
Pig
Pig
Pig
Pig
Pig
Pig
Pig
Pig
Pig
Pig
Pig
Pig
Pig
Pig
Pig
Pig
Pig
Pig
Pig
Pig
Pig
Pig
Pig

Pig
Pig
Pig
Pig
Pig
Pig
Pig
Pig
Pig
Pig
Pig
Pig
Pig
Pig
Pig
Pig
Pig
Pig
Pig
Pig
Pig
Pig
Pig
Pig
Pig
Pig
Pig
Pig
Pig
Pig
Pig
Pig
Pig
Pig
Pig
Pig
Pig
Pig
Pig
Pig
Pig
Pig
Pig
Pig
Pig
Pig

Pig
Pig
Pig
Pig
Pig
Pig
Pig
Pig
Pig
Pig
Pig
Pig
Pig
Pig
Pig
Pig
Pig
Pig
Pig
Pig
Pig
Pig
Pig
Pig
Pig
Pig
Pig
Pig
