# Supplementary material for: Heterogeneous viral-host response at single-cell resolution and viral adaptive mutational atlas in birds and mammals upon H5 influenza virus infection
Source: Natl Sci Rev. 2026 Jun 25;13(14):nwag380. doi: 10.1093/nsr/nwag380 (PMC13458601; doi:10.1093/nsr/nwag380)
Supplement: Supplementary file 1 [file nwag380_supplemental_files.zip › Supplementary Data 18.pdf]

lementary Data 18. Differentially expressed genes of fibriblasts across different species upon H5 HPAIV infe

gene	log2FoldChange	p value	host
Ifit2	5.40	0.00000000	chicken
Oasl2	3.89	0.00000000	chicken
Ly6e	3.73	0.00000000	chicken
Rnf213	3.05	0.00000000	chicken
AA467197	3.50	0.00000000	chicken
Obp2a	5.58	0.00000000	chicken
Plac8	2.70	0.00000000	chicken
Cmpk2	2.31	0.00000000	chicken
Fkbp5	2.71	0.00000000	chicken
Rsad2	2.70	0.00000000	chicken
Trank1	2.64	0.00000000	chicken
Ctss	2.15	0.00000000	chicken
B2m	1.86	0.00000000	chicken
Smchd1	2.54	0.00000000	chicken
C1s1	2.18	0.00000000	chicken
Ifih1	2.51	0.00000000	chicken
Tpt1	-1.71	0.00000000	chicken
Stat1	2.04	0.00000000	chicken
Pdxk	2.30	0.00000000	chicken
Plin2	2.48	0.00000000	chicken
Ggt5	2.71	0.00000000	chicken
Olfml1	2.07	0.00000000	chicken
Usp25	2.18	0.00000000	chicken
Smarcal1	-2.06	0.00000000	chicken
Sdc4	2.08	0.00000000	chicken
Dcn	-1.77	0.00000000	chicken
Ecrg4	-3.25	0.00000000	chicken
Lum	-2.64	0.00000000	chicken
Epb41l3	1.97	0.00000000	chicken
Fth1	-1.35	0.00000000	chicken
Birc2	2.06	0.00000000	chicken
Pdgfd	2.52	0.00000000	chicken
Serpine2	1.81	0.00000000	chicken
Hspb1	1.98	0.00000000	chicken
1600014C10Rik	1.79	0.00000000	chicken
Znfx1	1.73	0.00000000	chicken
Nrep	-2.40	0.00000000	chicken
Svbp	-1.88	0.00000000	chicken
Trim25	1.54	0.00000000	chicken
Zc3hav1l	1.66	0.00000000	chicken
Parp12	1.48	0.00000000	chicken

Slc9a9	-2.31	0.00000000	chicken
Sting1	1.56	0.00000000	chicken
Cmtr1	1.73	0.00000000	chicken
Cpne8	2.32	0.00000000	chicken
Nox4	-2.74	0.00000000	chicken
Rora	-2.10	0.00000000	chicken
Plscr1	1.63	0.00000000	chicken
Ptx3	2.48	0.00000000	chicken
Dot1l	1.63	0.00000000	chicken
Lama4	-2.24	0.00000000	chicken
Trafd1	1.34	0.00000000	chicken
Esrrg	1.74	0.00000000	chicken
Nmi	1.44	0.00000000	chicken
Clip2	1.48	0.00000000	chicken
Dab2	1.98	0.00000000	chicken
Rnh1	1.51	0.00000000	chicken
Snx10	1.44	0.00000000	chicken
Il6	3.23	0.00000000	chicken
Dram1	1.28	0.00000000	chicken
Col1a1	-2.30	0.00000000	chicken
Atp8a1	2.01	0.00000000	chicken
Tenm4	-1.99	0.00000000	chicken
Foxs1	1.76	0.00000000	chicken
Lamp5	1.78	0.00000000	chicken
Csnk1a1	1.51	0.00000000	chicken
Nme2	-1.76	0.00000000	chicken
Col5a2	-1.53	0.00000000	chicken
Eef1b2	-1.38	0.00000000	chicken
Cdh11	-1.88	0.00000000	chicken
Ppib	-1.52	0.00000000	chicken
Plod2	1.82	0.00000000	chicken
Hnrnpa1	-1.47	0.00000000	chicken
Cox6a1	-1.61	0.00000000	chicken
Tbc1d1	1.83	0.00000000	chicken
Crip1	-2.34	0.00000000	chicken
Kcnj5	1.42	0.00000000	chicken
Aqp1	2.86	0.00000000	chicken
Phf11a	1.30	0.00000000	chicken
Samhd1	1.52	0.00000000	chicken
Acsl1	2.10	0.00000000	chicken
Vmp1	1.42	0.00000000	chicken
Sqstm1	1.85	0.00000000	chicken
Wdfy2	1.54	0.00000000	chicken
Rnf24	1.37	0.00000000	chicken
Parp9	1.19	0.00000000	chicken
Gpx3	-2.01	0.00000000	chicken

Irf7	1.88	0.00000000	chicken
Ybx1	-1.22	0.00000000	chicken
Pard3b	1.57	0.00000000	chicken
Bcap29	1.43	0.00000000	chicken
Fau	-1.30	0.00000000	chicken
Thy1	1.56	0.00000000	chicken
Rack1	-1.46	0.00000000	chicken
Igfbp4	-1.84	0.00000000	chicken
Eef1d	-1.44	0.00000000	chicken
Angpt1	2.29	0.00000000	chicken
Dtx3l	1.04	0.00000000	chicken
Eps8	1.99	0.00000000	chicken
St7	1.65	0.00000000	chicken
Fat4	-1.92	0.00000000	chicken
Chac1	-2.16	0.00000000	chicken
Ntng2	1.14	0.00000000	chicken
Tlr3	0.99	0.00000000	chicken
Nampt	1.21	0.00000000	chicken
Cst3	-1.44	0.00000000	chicken
Ntm	2.22	0.00000000	chicken
Was	-1.40	0.00000000	chicken
Thsd4	2.09	0.00000000	chicken
Trerf1	1.53	0.00000000	chicken
H3f3a	-1.15	0.00000000	chicken
Ehd3	1.31	0.00000000	chicken
Stat2	1.01	0.00000000	chicken
Pde4b	1.29	0.00000000	chicken
H2az2	-1.39	0.00000000	chicken
Ttc3	1.65	0.00000000	chicken
Gns	1.23	0.00000000	chicken
Rhoq	0.98	0.00000000	chicken
Igf2bp3	1.48	0.00000000	chicken
BC048507	-1.55	0.00000000	chicken
Acot10	1.10	0.00000000	chicken
Rab40b	1.12	0.00000000	chicken
Rnf19b	1.02	0.00000000	chicken
Plxnb2	1.34	0.00000000	chicken
Col8a1	-2.14	0.00000000	chicken
Fli1	1.39	0.00000000	chicken
Lhfpl5	0.97	0.00000000	chicken
Col1a2	-1.82	0.00000000	chicken
Erap1	0.95	0.00000000	chicken
Ugdh	1.56	0.00000000	chicken
Nacc2	1.13	0.00000000	chicken
Hexim1	-1.30	0.00000000	chicken
Tcf21	-1.49	0.00000000	chicken

Flnb	1.69	0.00000000	chicken
Rarres2	-1.35	0.00000000	chicken
Tnip1	1.69	0.00000000	chicken
Tet2	1.54	0.00000000	chicken
Hlf	-1.42	0.00000000	chicken
Ebf1	1.66	0.00000000	chicken
Ptgs2	2.31	0.00000000	chicken
Bcl2l1	1.09	0.00000000	chicken
Fgl2	1.77	0.00000000	chicken
Rbms3	1.11	0.00000000	chicken
Cavin1	1.42	0.00000000	chicken
Ltbp1	-1.11	0.00000000	chicken
Alcam	2.01	0.00000000	chicken
4932438A13Rik	1.30	0.00000000	chicken
Ccnl1	-1.12	0.00000000	chicken
Tapbp	0.95	0.00000000	chicken
Farp1	1.54	0.00000000	chicken
Klf12	1.23	0.00000000	chicken
Hacd3	1.24	0.00000000	chicken
Pecam1	-1.90	0.00000000	chicken
Calhm6	0.87	0.00000000	chicken
Has2	2.04	0.00000000	chicken
Egfr	-1.09	0.00000000	chicken
C1ra	0.87	0.00000000	chicken
Bglap3	-2.57	0.00000000	chicken
Gfpt2	1.55	0.00000000	chicken
Tuba1a	-1.37	0.00000000	chicken
Vnn3	1.30	0.00000000	chicken
Tnfaip2	1.50	0.00000000	chicken
Tcim	1.55	0.00000000	chicken
Pparg	1.56	0.00000000	chicken
Filip1	-1.09	0.00000000	chicken
Ddx60	1.25	0.00000000	chicken
Creb5	1.64	0.00000000	chicken
Stmn2	-1.92	0.00000000	chicken
Pmp22	1.22	0.00000000	chicken
Gch1	0.75	0.00000000	chicken
Ccl9	1.61	0.00000000	chicken
Ell	1.03	0.00000000	chicken
Nt5c3b	0.75	0.00000000	chicken
Zdhhc8	1.27	0.00000000	chicken
Lrrfip1	1.26	0.00000000	chicken
Eif2ak2	0.89	0.00000000	chicken
Dpep2	0.87	0.00000000	chicken
Irf2	1.08	0.00000000	chicken
Hipk2	1.70	0.00000000	chicken

Tmem123	0.96	0.00000000	chicken
Calm1	-0.91	0.00000000	chicken
Traf3	1.20	0.00000000	chicken
Crot	0.97	0.00000000	chicken
Pxk	0.90	0.00000000	chicken
Camk2d	1.06	0.00000000	chicken
Art1	0.78	0.00000000	chicken
Gdpd5	1.33	0.00000000	chicken
Nav3	-1.31	0.00000000	chicken
Arfgap3	1.18	0.00000000	chicken
Ncor2	0.93	0.00000000	chicken
Txn1	1.85	0.00000000	chicken
Cnn3	0.87	0.00000000	chicken
Otud4	0.77	0.00000000	chicken
Fn1	1.16	0.00000000	chicken
Akap12	-1.20	0.00000000	chicken
Zfp593	-1.20	0.00000000	chicken
Ppia	-0.79	0.00000000	chicken
Ndufb1	-1.09	0.00000000	chicken
Igf1r	1.07	0.00000000	chicken
Adar	0.64	0.00000000	chicken
Atp10a	1.27	0.00000000	chicken
Cd47	0.84	0.00000000	chicken
Fgf7	-1.31	0.00000000	chicken
Prelid3b	0.86	0.00000000	chicken
Crispld2	1.08	0.00000000	chicken
Cept1	0.89	0.00000000	chicken
Dnajc13	1.05	0.00000000	chicken
B4galt5	0.86	0.00000000	chicken
Fndc3a	1.10	0.00000000	chicken
Ube2e2	1.54	0.00000000	chicken
Khdrbs3	-0.96	0.00000000	chicken
Tmsb4x	-0.75	0.00000000	chicken
Sbf2	1.13	0.00000000	chicken
Frem1	-1.09	0.00000000	chicken
Setx	0.99	0.00000000	chicken
H3f3b	-0.96	0.00000000	chicken
Tgfb1	-0.92	0.00000000	chicken
Slc1a2	1.03	0.00000000	chicken
Heatr5a	1.32	0.00000000	chicken
Zmiz1	1.03	0.00000000	chicken
Grk5	-1.32	0.00000000	chicken
Gbe1	1.46	0.00000000	chicken
Endov	0.65	0.00000000	chicken
Cast	0.93	0.00000000	chicken
Pik3ca	1.05	0.00000000	chicken

Tnfaip3	0.94	0.00000000	chicken
Commd8	0.78	0.00000000	chicken
Fgf10	1.46	0.00000000	chicken
Saraf	0.96	0.00000000	chicken
Fnip1	1.03	0.00000000	chicken
Rxra	1.02	0.00000000	chicken
Efhd1	1.02	0.00000000	chicken
Tnip2	1.90	0.00000000	chicken
Akr1e1	-1.13	0.00000000	chicken
Ecm2	-1.04	0.00000000	chicken
Txnrd1	1.71	0.00000000	chicken
Rad51b	-1.39	0.00000000	chicken
Ndufa4	-1.14	0.00000000	chicken
Nfix	-1.10	0.00000000	chicken
Cd34	-1.17	0.00000000	chicken
Ifi35	0.73	0.00000000	chicken
Atf4	-0.90	0.00000000	chicken
Niban1	-1.17	0.00000000	chicken
Errfi1	0.91	0.00000000	chicken
Ogfr	0.62	0.00000000	chicken
Col5a1	-0.91	0.00000000	chicken
Smoc2	-1.44	0.00000000	chicken
Usp16	0.78	0.00000000	chicken
Pde8a	1.32	0.00000000	chicken
Bcat1	1.25	0.00000000	chicken
Glo1	-1.02	0.00000000	chicken
Fmc1	-0.94	0.00000000	chicken
Gls	1.00	0.00000000	chicken
Ass1	1.01	0.00000000	chicken
Stk38l	1.05	0.00000000	chicken
Emp1	1.88	0.00000000	chicken
Romo1	-0.98	0.00000000	chicken
Acvr2a	1.04	0.00000000	chicken
Bach1	1.17	0.00000000	chicken
Oaf	1.01	0.00000000	chicken
Sorcs2	1.38	0.00000000	chicken
Tgfb3	-0.99	0.00000000	chicken
Dap	-1.11	0.00000000	chicken
Id4	-1.27	0.00000000	chicken
Hs6st1	1.22	0.00000000	chicken
Nbn	0.64	0.00000000	chicken
Sema3c	1.70	0.00000000	chicken
Acsl4	1.38	0.00000000	chicken
Zc3h12a	0.77	0.00000000	chicken
Trpv2	1.01	0.00000000	chicken
Rfc1	0.81	0.00000000	chicken

Ankrd39	-0.91	0.00000000	chicken
Ackr3	1.16	0.00000000	chicken
Helz2	1.17	0.00000000	chicken
Gsta4	-1.07	0.00000000	chicken
Cox4i1	-0.94	0.00000000	chicken
Xylt1	-1.18	0.00000000	chicken
Il6st	0.81	0.00000000	chicken
Batf3	0.67	0.00000000	chicken
Nfkb1	0.93	0.00000000	chicken
Cav1	0.87	0.00000000	chicken
Tapt1	0.86	0.00000000	chicken
Hbegf	0.95	0.00000000	chicken
Dusp1	0.92	0.00000000	chicken
Nhs	1.32	0.00000000	chicken
Papss2	0.77	0.00000000	chicken
Cpxm1	-0.93	0.00000000	chicken
Tnfrsf1a	0.78	0.00000000	chicken
Golgb1	-1.22	0.00000000	chicken
Wdfy3	0.76	0.00000000	chicken
Bcl6	0.71	0.00000000	chicken
Gnb1	0.72	0.00000000	chicken
Urm1	-0.98	0.00000000	chicken
Bbs2	0.75	0.00000000	chicken
Gas7	1.30	0.00000000	chicken
Cnp	0.64	0.00000000	chicken
Timp3	1.98	0.00000000	chicken
Eif4g3	0.73	0.00000000	chicken
Nub1	0.72	0.00000000	chicken
Gab2	1.18	0.00000000	chicken
Slc2a6	0.58	0.00000000	chicken
Ly75	0.85	0.00000000	chicken
Tmem255a	0.70	0.00000000	chicken
Brwd1	-1.20	0.00000000	chicken
Ago3	0.80	0.00000000	chicken
Uvrag	0.70	0.00000000	chicken
Adamts4	1.55	0.00000000	chicken
Vim	-0.71	0.00000000	chicken
Angptl4	1.76	0.00000000	chicken
Eef2	-0.71	0.00000000	chicken
Jarid2	-0.96	0.00000000	chicken
1810013L24Rik	-1.11	0.00000000	chicken
Arhgef28	0.81	0.00000000	chicken
Upf2	0.76	0.00000000	chicken
Calm2	-0.79	0.00000000	chicken
Atxn7l1	1.01	0.00000000	chicken
Grcc10	-0.91	0.00000000	chicken

Prex2	0.94	0.00000000	chicken
Col22a1	1.28	0.00000000	chicken
Lamb1	-1.13	0.00000000	chicken
Gabra5	0.97	0.00000000	chicken
Rasa3	1.41	0.00000000	chicken
Ulbp1	0.69	0.00000000	chicken
Adora2b	0.54	0.00000000	chicken
Gulp1	1.04	0.00000000	chicken
Id2	-1.09	0.00000000	chicken
Zfp36l2	0.70	0.00000000	chicken
Rb1cc1	0.80	0.00000000	chicken
Aacs	-0.99	0.00000000	chicken
Ndufb2	-0.90	0.00000000	chicken
Sod3	0.97	0.00000000	chicken
Hpgd	1.10	0.00000000	chicken
Abi2	-1.11	0.00000000	chicken
Zfp407	0.94	0.00000000	chicken
Slc25a25	0.80	0.00000000	chicken
Reps2	-1.16	0.00000000	chicken
Klhl24	-1.03	0.00000000	chicken
Smim4	-0.80	0.00000000	chicken
Churc1	-0.97	0.00000000	chicken
Prrc2c	0.64	0.00000000	chicken
Fkbp1b	-0.77	0.00000000	chicken
Aff1	0.82	0.00000000	chicken
Tns1	0.91	0.00000000	chicken
Hmcn1	-1.03	0.00000000	chicken
Glul	0.86	0.00000000	chicken
Icosl	0.71	0.00000000	chicken
2900026A02Rik	-1.01	0.00000000	chicken
Slc25a3	-1.13	0.00000000	chicken
Nfasc	-1.02	0.00000000	chicken
Blcap	0.63	0.00000000	chicken
Kdm7a	1.25	0.00000000	chicken
Dazap1	0.78	0.00000000	chicken
Grn	-0.95	0.00000000	chicken
Stxbp6	0.97	0.00000000	chicken
Htra3	1.04	0.00000000	chicken
Zeb1	0.95	0.00000000	chicken
Ptpn2	0.81	0.00000000	chicken
Srsf6	-0.93	0.00000000	chicken
Cox7b	-0.84	0.00000000	chicken
Rasgrp1	1.20	0.00000000	chicken
Dad1	-0.93	0.00000000	chicken
Spred1	0.91	0.00000000	chicken
Ranbp9	0.66	0.00000000	chicken

Zswim5	0.85	0.00000000	chicken
Sema3d	-1.31	0.00000000	chicken
Anxa2	0.91	0.00000000	chicken
Srgap1	1.13	0.00000000	chicken
Cxcl12	1.31	0.00000000	chicken
Ndufs5	-0.89	0.00000000	chicken
Vegfd	-1.01	0.00000000	chicken
Daam2	-0.79	0.00000000	chicken
Lipa	0.67	0.00000000	chicken
Pnrc1	-0.53	0.00000000	chicken
Lrrc1	0.96	0.00000000	chicken
Sat1	0.70	0.00000000	chicken
Fosl2	0.98	0.00000000	chicken
Ndufaf8	-0.75	0.00000000	chicken
Spata5	1.07	0.00000000	chicken
Sp1	0.63	0.00000000	chicken
Prkd1	1.01	0.00000000	chicken
Grem1	-1.37	0.00000000	chicken
Cygb	0.88	0.00000000	chicken
Cavin2	0.77	0.00000000	chicken
Smurf1	0.70	0.00000000	chicken
Itm2a	-0.85	0.00000000	chicken
Serpinb10	1.42	0.00000000	chicken
Cd109	0.75	0.00000000	chicken
Ptprd	-1.05	0.00000000	chicken
Zhx1	0.66	0.00000000	chicken
Eps15	0.79	0.00000000	chicken
Shisa5	0.54	0.00000000	chicken
Il1b	0.83	0.00000000	chicken
Jph3	0.65	0.00000000	chicken
Aspg	0.50	0.00000000	chicken
Fryl	-1.25	0.00000000	chicken
Ndufb4	-0.80	0.00000000	chicken
Adamts2	0.98	0.00000000	chicken
Tpst1	-0.92	0.00000000	chicken
C1qtnf7	-0.84	0.00000000	chicken
Vegfa	1.29	0.00000000	chicken
Rbbp8	0.68	0.00000000	chicken
Hmgb1	-0.83	0.00000000	chicken
Iffo2	0.66	0.00000000	chicken
Tead1	0.83	0.00000000	chicken
Il1rap	0.62	0.00000000	chicken
F2rl1	0.65	0.00000000	chicken
Btbd9	0.85	0.00000000	chicken
Vrk2	0.81	0.00000000	chicken
Uba2	0.60	0.00000000	chicken

Dlc1	-1.11	0.00000000	chicken
Chd2	0.83	0.00000000	chicken
Lyg2	1.11	0.00000000	chicken
Pebp1	-0.83	0.00000000	chicken
Bco1	0.44	0.00000000	chicken
Cercam	-0.82	0.00000000	chicken
Ndufc2	-0.84	0.00000000	chicken
Snx24	0.89	0.00000000	chicken
Ifitm5	0.50	0.00000000	chicken
Stoml1	0.54	0.00000000	chicken
Hsd17b12	0.77	0.00000000	chicken
Clec3b	-1.13	0.00000000	chicken
Tgfb2	-0.73	0.00000000	chicken
Rfx2	0.93	0.00000000	chicken
Aplp2	-0.79	0.00000000	chicken
Slc16a10	-1.06	0.00000000	chicken
P2rx7	0.48	0.00000000	chicken
Fmnl2	0.91	0.00000000	chicken
Lamtor2	-0.88	0.00000000	chicken
Agfg1	0.66	0.00000000	chicken
Klf5	0.75	0.00000000	chicken
Tasor2	0.58	0.00000000	chicken
Itpr1	1.10	0.00000000	chicken
Tpi1	-0.98	0.00000000	chicken
Fgfrl1	-0.82	0.00000000	chicken
Atp5d	-0.86	0.00000000	chicken
Saal1	0.82	0.00000000	chicken
Piga	0.43	0.00000000	chicken
Lrig3	-0.87	0.00000000	chicken
Hvcn1	-0.67	0.00000000	chicken
Cpne2	-1.38	0.00000000	chicken
Epn2	0.76	0.00000000	chicken
Irf1	0.96	0.00000000	chicken
Stam2	0.65	0.00000000	chicken
Bcr	0.71	0.00000000	chicken
Nek6	0.78	0.00000000	chicken
Pard3	0.79	0.00000000	chicken
Polg2	-0.79	0.00000000	chicken
Synm	-0.84	0.00000000	chicken
Wdr45b	0.58	0.00000000	chicken
Tll1	-1.07	0.00000000	chicken
Btg2	0.68	0.00000000	chicken
Ginm1	0.61	0.00000000	chicken
Arhgap1	0.61	0.00000000	chicken
Ssr2	-0.76	0.00000000	chicken
Nfatc2	1.17	0.00000000	chicken

Bcl2	0.79	0.00000000	chicken
Ccni	-0.83	0.00000000	chicken
Fmr1	0.59	0.00000000	chicken
Ppic	-0.94	0.00000000	chicken
Stard13	0.92	0.00000000	chicken
Tob2	-0.94	0.00000000	chicken
Cd3g	-0.89	0.00000000	chicken
Chchd10	-0.88	0.00000000	chicken
Syne1	-0.96	0.00000000	chicken
Cox7c	-0.85	0.00000000	chicken
Fam160a1	0.57	0.00000000	chicken
Slit2	-1.16	0.00000000	chicken
Cdk14	0.93	0.00000000	chicken
Apoa1	1.10	0.00000000	chicken
Arhgap12	1.13	0.00000000	chicken
Ythdf2	0.62	0.00000000	chicken
Cpne3	-0.83	0.00000000	chicken
Wwox	-0.98	0.00000000	chicken
Vps50	0.79	0.00000000	chicken
Gask1b	-0.75	0.00000000	chicken
Wdfy1	0.68	0.00000000	chicken
Prss23	-0.73	0.00000000	chicken
Creld1	-0.83	0.00000000	chicken
Pard6b	0.58	0.00000000	chicken
Plod1	0.93	0.00000000	chicken
Pfkp	1.02	0.00000000	chicken
Ankrd44	-1.04	0.00000000	chicken
Abhd14b	-0.67	0.00000000	chicken
Timp2	-0.75	0.00000000	chicken
Tjp2	0.72	0.00000000	chicken
Dlg1	0.86	0.00000000	chicken
Ncoa7	0.70	0.00000000	chicken
Eif2ak3	-0.85	0.00000000	chicken
Tubb2a	-1.02	0.00000000	chicken
Ube2d2a	0.62	0.00000000	chicken
Sipa1l1	0.90	0.00000000	chicken
Cited4	0.55	0.00000000	chicken
Cd44	1.52	0.00000000	chicken
Rpn1	-0.79	0.00000000	chicken
Rala	0.68	0.00000000	chicken
Ptgfrn	0.54	0.00000000	chicken
Tcf7	-1.06	0.00000000	chicken
Osbpl5	1.09	0.00000000	chicken
Csgalnact2	0.62	0.00000000	chicken
Arhgef18	0.59	0.00000000	chicken
Lsamp	1.13	0.00000000	chicken

Bnip3l	-0.83	0.00000000	chicken
Tmed10	0.64	0.00000000	chicken
Bmp5	-1.03	0.00000000	chicken
Rasgef1b	0.66	0.00000000	chicken
Lonrf1	-0.89	0.00000000	chicken
Ctsh	0.41	0.00000000	chicken
Eif4g1	0.58	0.00000000	chicken
Adamts17	-0.84	0.00000000	chicken
Elf1	0.68	0.00000000	chicken
Cdk17	0.98	0.00000000	chicken
Rspo2	-0.67	0.00000000	chicken
Ar	0.59	0.00000000	chicken
Cpt1a	0.80	0.00000000	chicken
Ywhaq	0.60	0.00000000	chicken
Hgf	-0.79	0.00000000	chicken
Rarb	-0.66	0.00000000	chicken
Col6a3	-0.68	0.00000000	chicken
Bbip1	-0.66	0.00000000	chicken
Rprm	0.99	0.00000000	chicken
Pacsin2	0.87	0.00000000	chicken
Chp1	0.76	0.00000000	chicken
Nr0b1	-0.91	0.00000000	chicken
Phlda1	0.56	0.00000000	chicken
Cox5a	-0.78	0.00000000	chicken
Rhob	-1.08	0.00000000	chicken
Brd2	-0.86	0.00000000	chicken
Gpc1	0.81	0.00000000	chicken
Ppfia1	-0.83	0.00000000	chicken
Nectin4	-0.56	0.00000000	chicken
Lclat1	0.82	0.00000000	chicken
Adcy6	-0.75	0.00000000	chicken
Atp7a	0.47	0.00000000	chicken
Wdr26	-1.11	0.00000000	chicken
Pkm	0.83	0.00000000	chicken
Rbm24	-0.87	0.00000000	chicken
Ctnna1	0.65	0.00000000	chicken
Slco4c1	0.39	0.00000000	chicken
Gapvd1	1.03	0.00000000	chicken
Gpr1	0.82	0.00000000	chicken
Glcci1	-0.87	0.00000000	chicken
Colec12	-0.93	0.00000000	chicken
Osr1	-0.98	0.00000000	chicken
Thrb	0.84	0.00000000	chicken
Ccdc85c	0.82	0.00000000	chicken
Tprgl	-0.79	0.00000000	chicken
Rab7b	0.39	0.00000000	chicken

Osbpl6	0.92	0.00000000	chicken
Ctsc	0.52	0.00000000	chicken
Sdcbp	0.63	0.00000000	chicken
Tor1aip2	0.41	0.00000000	chicken
Ext1	0.73	0.00000000	chicken
Fez1	-0.46	0.00000000	chicken
Adcy8	-0.77	0.00000000	chicken
Smc6	0.60	0.00000000	chicken
Spry2	-0.64	0.00000000	chicken
Fry	1.10	0.00000000	chicken
Ndufb7	-0.80	0.00000000	chicken
Ubl5b	-0.79	0.00000000	chicken
Foxk2	0.82	0.00000000	chicken
Smap2	0.53	0.00000000	chicken
Gata5	-0.75	0.00000000	chicken
Bend5	-1.37	0.00000000	chicken
Fhl4	-0.76	0.00000000	chicken
Gsk3b	0.72	0.00000000	chicken
Pisd	0.60	0.00000000	chicken
Zfand2a	0.58	0.00000000	chicken
Tlk2	0.55	0.00000000	chicken
Rffl	0.77	0.00000000	chicken
Adamts7	-0.50	0.00000000	chicken
Itga11	-0.60	0.00000000	chicken
Il13ra1	0.81	0.00000000	chicken
Csrnp1	0.62	0.00000000	chicken
Fap	-0.72	0.00000000	chicken
Fstl1	-0.65	0.00000000	chicken
Pdgfrl	-0.63	0.00000000	chicken
Arhgdib	-0.65	0.00000000	chicken
Tpp1	-0.80	0.00000000	chicken
Uaca	-0.88	0.00000000	chicken
Naxe	-0.60	0.00000000	chicken
Dcxr	-0.62	0.00000000	chicken
Hrh1	0.35	0.00000000	chicken
Ptk2	0.96	0.00000000	chicken
Atic	-0.73	0.00000000	chicken
Prdx6	0.70	0.00000000	chicken
Fer	0.65	0.00000000	chicken
Fbln5	-1.11	0.00000000	chicken
Lcat	0.63	0.00000000	chicken
Ackr4	0.64	0.00000000	chicken
Slbp	0.32	0.00000000	chicken
Cyp7b1	0.56	0.00000000	chicken
Smim3	-0.60	0.00000000	chicken
Nktr	0.66	0.00000000	chicken

Dgkd	0.83	0.00000000	chicken
Hspa8	0.58	0.00000000	chicken
Ddx27	0.55	0.00000000	chicken
Sgms2	0.52	0.00000000	chicken
Sema5b	-0.74	0.00000000	chicken
Rock2	0.59	0.00000000	chicken
Synpo2	-0.66	0.00000000	chicken
Otub1	-0.75	0.00000000	chicken
Klf6	0.69	0.00000000	chicken
Pnpt1	0.42	0.00000000	chicken
Itgbl1	-0.82	0.00000000	chicken
Trp53bp2	0.68	0.00000000	chicken
Timm13	-0.77	0.00000000	chicken
Sema6d	-1.07	0.00000000	chicken
Rab3gap2	0.61	0.00000000	chicken
Vav2	0.78	0.00000000	chicken
Hapln3	-0.66	0.00000000	chicken
Yap1	0.71	0.00000000	chicken
Gja6	-0.88	0.00000000	chicken
Cd3e	-0.67	0.00000000	chicken
Usp36	-0.75	0.00000000	chicken
Actg1	-0.51	0.00000000	chicken
Nfatc3	-0.80	0.00000000	chicken
Il1rapl1	-1.85	0.00000000	chicken
Fos	-0.73	0.00000000	chicken
Baz2b	0.76	0.00000000	chicken
Galk2	-0.68	0.00000000	chicken
Sobp	-0.66	0.00000000	chicken
Nfkb2	0.41	0.00000000	chicken
Cat	-0.76	0.00000000	chicken
Smarca5	0.62	0.00000000	chicken
Prex1	0.69	0.00000000	chicken
Gab1	0.89	0.00000000	chicken
Fhod3	-1.22	0.00000000	chicken
Zfp423	-0.76	0.00000000	chicken
Tns3	-0.87	0.00000000	chicken
Klf15	0.49	0.00000000	chicken
Smg1	0.60	0.00000000	chicken
Sec24d	0.54	0.00000000	chicken
Dennd4c	0.89	0.00000000	chicken
Ikbip	0.46	0.00000000	chicken
Eda2r	0.41	0.00000000	chicken
Tcp11l2	-0.77	0.00000000	chicken
Hmgn3	-0.73	0.00000000	chicken
Olfm3	-0.73	0.00000000	chicken
Atp6v1a	0.56	0.00000000	chicken

Lao1	-0.51	0.00000000	chicken
B3gnt7	0.31	0.00000000	chicken
Pcmtd1	-0.73	0.00000000	chicken
Tmem168	0.35	0.00000000	chicken
Tox	-1.07	0.00000000	chicken
Xpr1	0.57	0.00000000	chicken
Cd83	1.02	0.00000000	chicken
Rb1	0.63	0.00000000	chicken
Gab3	-0.91	0.00000000	chicken
Id3	-0.74	0.00000000	chicken
Ddx5	-0.53	0.00000000	chicken
Cacna2d1	-0.72	0.00000000	chicken
Slc25a29	0.32	0.00000000	chicken
Ugp2	0.66	0.00000000	chicken
Lrba	0.72	0.00000000	chicken
Fat3	-0.97	0.00000000	chicken
Tomm6	-0.74	0.00000000	chicken
BC016579	0.74	0.00000000	chicken
Tomm7	-0.65	0.00000000	chicken
Soat1	0.53	0.00000000	chicken
Cars	0.60	0.00000000	chicken
Dusp23	-0.66	0.00000000	chicken
Lrrfip2	0.86	0.00000000	chicken
Twf2	0.46	0.00000000	chicken
Pde3b	-1.08	0.00000000	chicken
Lrrc39	0.29	0.00000000	chicken
Eif3e	-0.67	0.00000000	chicken
P4ha2	0.65	0.00000000	chicken
Tut4	0.42	0.00000000	chicken
Fus	-0.65	0.00000000	chicken
Itgav	0.64	0.00000000	chicken
Dalrd3	0.32	0.00000000	chicken
Usp10	0.57	0.00000000	chicken
Tead3	0.47	0.00000000	chicken
Rbm43	0.32	0.00000000	chicken
Csf1	0.53	0.00000000	chicken
Jmjd1c	0.73	0.00000000	chicken
Hoxb5	-0.73	0.00000000	chicken
Serp1	-0.75	0.00000000	chicken
Tnfrsf21	0.52	0.00000000	chicken
Nrxn1	-1.53	0.00000000	chicken
Extl3	0.52	0.00000000	chicken
Kdelr3	-0.57	0.00000000	chicken
Bmpr2	-0.77	0.00000000	chicken
Aldh1a3	1.16	0.00000000	chicken
Llph	0.53	0.00000000	chicken

Ppm1d	0.52	0.00000000	chicken
Car2	0.77	0.00000000	chicken
Cntnap2	-0.74	0.00000000	chicken
Cav2	0.30	0.00000000	chicken
Ube2k	0.50	0.00000000	chicken
Sh3pxd2a	-0.57	0.00000000	chicken
Col6a2	-0.60	0.00000000	chicken
Ip6k2	0.60	0.00000000	chicken
Adgrl4	-1.01	0.00000000	chicken
Tmem258	-0.71	0.00000000	chicken
Gpx1	-0.50	0.00000000	chicken
Sesn1	-0.52	0.00000000	chicken
Creb3	0.49	0.00000000	chicken
Atp13a3	0.52	0.00000000	chicken
Arid4b	-0.68	0.00000000	chicken
Dock2	-1.07	0.00000000	chicken
Scin	-0.53	0.00000000	chicken
Vegfc	-0.61	0.00000000	chicken
Celf2	0.73	0.00000000	chicken
Larp1	0.50	0.00000000	chicken
Erp29	-0.61	0.00000000	chicken
Clasp2	0.76	0.00000000	chicken
Dhrs3	-0.86	0.00000000	chicken
Itga1	0.50	0.00000000	chicken
Sdhd	-0.68	0.00000000	chicken
Pdgfrb	0.65	0.00000000	chicken
Hnrnpr	0.53	0.00000000	chicken
Itga8	0.79	0.00000000	chicken
Ckap2l	0.36	0.00000000	chicken
Ramp2	-0.75	0.00000000	chicken
Tmem132b	-0.70	0.00000000	chicken
Hlcs	0.76	0.00000000	chicken
Psma7	0.56	0.00000000	chicken
Kank3	-0.62	0.00000000	chicken
Itga4	-0.76	0.00000000	chicken
Notch2	0.67	0.00000000	chicken
Rnf19a	0.61	0.00000000	chicken
Hectd1	0.63	0.00000000	chicken
Rnf13	0.54	0.00000000	chicken
Hivep3	0.57	0.00000000	chicken
Pigyl	-0.37	0.00000000	chicken
Rad23a	-0.68	0.00000000	chicken
Adamts5	0.59	0.00000000	chicken
Clstn1	0.55	0.00000000	chicken
Pgrmc1	0.55	0.00000000	chicken
Uqcrh	-0.65	0.00000000	chicken

Tomm20	-0.64	0.00000000	chicken
Ntf3	-0.87	0.00000000	chicken
Afdn	0.73	0.00000000	chicken
Bin1	-0.84	0.00000000	chicken
Denn2b	-0.57	0.00000000	chicken
Il1r1	0.55	0.00000000	chicken
Acvrl1	-0.61	0.00000000	chicken
Il10ra	0.41	0.00000000	chicken
Fbn2	-0.63	0.00000000	chicken
Optn	0.37	0.00000000	chicken
Kazn	0.47	0.00000000	chicken
Slc4a7	0.85	0.00000000	chicken
Atp5k	-0.63	0.00000000	chicken
Bcas3	-0.65	0.00000000	chicken
Nolc1	0.40	0.00000000	chicken
Il27ra	-0.58	0.00000000	chicken
Lama5	0.60	0.00000000	chicken
Camk4	-0.74	0.00000000	chicken
Col4a1	0.55	0.00000000	chicken
Prrx2	0.42	0.00000000	chicken
Ptgds	0.76	0.00000000	chicken
P3h4	-0.42	0.00000000	chicken
Ypel1	-0.67	0.00000000	chicken
Plxna1	0.55	0.00000000	chicken
Ubr5	0.51	0.00000000	chicken
Dnaaf9	-0.70	0.00000000	chicken
Srsf2	-0.70	0.00000000	chicken
Klhl18	-0.69	0.00000000	chicken
Parp16	-0.46	0.00000000	chicken
Hook1	0.45	0.00000000	chicken
Chd9	0.54	0.00000000	chicken
Fgfr2	-0.62	0.00000000	chicken
Ern1	-1.12	0.00000000	chicken
Kpna1	0.56	0.00000000	chicken
Pcsk5	-0.77	0.00000000	chicken
Ddx24	0.41	0.00000000	chicken
Robo1	1.40	0.00000000	chicken
Pdzrn4	-1.10	0.00000000	chicken
Adipor2	0.42	0.00000000	chicken
Lrig1	0.64	0.00000000	chicken
Dync1h1	-0.62	0.00000000	chicken
Dennd1a	0.49	0.00000000	chicken
Ikbke	0.35	0.00000000	chicken
Kmt2e	-0.60	0.00000000	chicken
Slc25a4	0.36	0.00000000	chicken
Noct	0.34	0.00000000	chicken

Cnbp	0.45	0.00000000	chicken
Rcor3	-0.53	0.00000000	chicken
Rab1a	0.48	0.00000000	chicken
Antkmt	-0.53	0.00000000	chicken
Pabpc1	-0.44	0.00000000	chicken
Ndufb10	-0.62	0.00000000	chicken
Itsn2	0.56	0.00000000	chicken
Scara5	0.49	0.00000000	chicken
Cenpu	0.75	0.00000000	chicken
Agap3	0.40	0.00000000	chicken
Csgalnact1	0.46	0.00000000	chicken
Ccng2	-0.71	0.00000000	chicken
Srf	-0.74	0.00000000	chicken
Ism1	0.50	0.00000000	chicken
Tjp1	-0.81	0.00000000	chicken
Hsp90ab1	-0.68	0.00000000	chicken
Rnf216	0.50	0.00000000	chicken
Pum1	0.51	0.00000000	chicken
Ndufb5	-0.56	0.00000000	chicken
Pid1	0.59	0.00000000	chicken
Gstm5	-0.66	0.00000000	chicken
Ttc19	0.38	0.00000000	chicken
Tnfaip8l3	-0.51	0.00000000	chicken
Tex2	-0.92	0.00000000	chicken
Etv6	0.35	0.00000000	chicken
H2az1	-0.79	0.00000000	chicken
Gng2	-0.48	0.00000000	chicken
G0s2	1.42	0.00000000	chicken
Pgk1	0.51	0.00000000	chicken
Negr1	0.55	0.00000000	chicken
Klf11	0.47	0.00000000	chicken
Hnrnpa3	0.40	0.00000000	chicken
Tmem164	0.63	0.00000000	chicken
Dnajb6	0.43	0.00000000	chicken
Rnf111	0.50	0.00000000	chicken
Helz	0.58	0.00000000	chicken
Gpatch1	0.44	0.00000000	chicken
Sh3bgrl	-0.64	0.00000000	chicken
Vstm4	-0.43	0.00000000	chicken
Rnf149	0.52	0.00000000	chicken
Zfpm1	0.52	0.00000000	chicken
Nubpl	0.60	0.00000000	chicken
C7	-1.55	0.00000000	chicken
Sgms1	0.63	0.00000000	chicken
Cdr2	-0.49	0.00000000	chicken
Fkbp1a	-0.65	0.00000000	chicken

Rrbp1	-0.61	0.00000000	chicken
Atp5j2	-0.58	0.00000000	chicken
Sptssa	-0.62	0.00000000	chicken
Babam2	0.50	0.00000000	chicken
Bambi	-0.61	0.00000000	chicken
Ccnd1	0.53	0.00000000	chicken
Cc2d2a	-0.43	0.00000000	chicken
Ebf3	0.73	0.00000000	chicken
Tnfsf15	0.58	0.00000000	chicken
Chrd	0.34	0.00000000	chicken
2510009E07Rik	-0.53	0.00000000	chicken
Rwdd3	-0.53	0.00000000	chicken
Fgf18	-0.48	0.00000000	chicken
Spz1	-0.69	0.00000000	chicken
Zfp706	-0.63	0.00000000	chicken
Dtna	0.73	0.00000000	chicken
Gtf2b	-0.64	0.00000000	chicken
Zc3h6	-0.59	0.00000000	chicken
Il13ra2	0.50	0.00000000	chicken
Nup50	0.37	0.00000000	chicken
Cyba	-0.61	0.00000000	chicken
Cracr2b	-0.61	0.00000000	chicken
Gpx7	-0.40	0.00000000	chicken
Pkd2	0.47	0.00000000	chicken
Chsy1	0.69	0.00000000	chicken
Rngtt	0.51	0.00000000	chicken
Pou3f2	0.35	0.00000000	chicken
Fign	-0.89	0.00000000	chicken
Txlna	0.39	0.00000000	chicken
Arxes2	-0.60	0.00000000	chicken
Spata18	-0.51	0.00000000	chicken
Ctps	0.30	0.00000000	chicken
Garem1	0.50	0.00000000	chicken
P4ha3	-0.40	0.00000000	chicken
Antxr2	-0.51	0.00000000	chicken
Sp3	-0.68	0.00000000	chicken
Cd81	0.41	0.00000000	chicken
Bsg	-0.62	0.00000000	chicken
Satb1	-0.59	0.00000000	chicken
Ngf	0.44	0.00000000	chicken
Gpx4	-0.57	0.00000000	chicken
Sec11c	0.36	0.00000000	chicken
Bnc2	1.01	0.00000000	chicken
Cdc26	-0.62	0.00000000	chicken
Glt8d2	-0.37	0.00000001	chicken
Larp6	0.28	0.00000001	chicken

Cab39l	-0.68	0.00000001	chicken
Tmem35b	-0.61	0.00000001	chicken
Ifi30	-0.67	0.00000001	chicken
Picalm	-0.76	0.00000001	chicken
Pdia4	-0.67	0.00000001	chicken
Stard5	0.30	0.00000001	chicken
Tmem65	-0.65	0.00000001	chicken
Znrf1	0.30	0.00000001	chicken
Stat3	0.41	0.00000001	chicken
Bzw2	-0.69	0.00000001	chicken
Oser1	0.29	0.00000001	chicken
Top1	0.47	0.00000001	chicken
Kif5c	0.46	0.00000001	chicken
Ankrd28	0.47	0.00000001	chicken
Ripor3	0.50	0.00000001	chicken
Abcc4	0.78	0.00000001	chicken
Rab20	0.55	0.00000001	chicken
Cdk6	0.54	0.00000001	chicken
Pdcd4	-0.59	0.00000001	chicken
Yeats2	-0.53	0.00000001	chicken
Eif4b	-0.68	0.00000001	chicken
Nap1l1	0.61	0.00000001	chicken
Pde4d	-0.87	0.00000001	chicken
Trim24	-0.60	0.00000001	chicken
Ptprc	-1.04	0.00000001	chicken
Tmem63b	0.43	0.00000001	chicken
Cdkal1	0.49	0.00000001	chicken
Dgkq	0.62	0.00000001	chicken
Etv1	-0.66	0.00000001	chicken
Helb	0.25	0.00000001	chicken
Pfdn5	-0.54	0.00000001	chicken
Casp7	0.30	0.00000001	chicken
Zfhx3	-0.78	0.00000001	chicken
Uqcr11	-0.51	0.00000001	chicken
Hagh	-0.48	0.00000001	chicken
Slc12a4	0.56	0.00000002	chicken
Ankrd11	0.47	0.00000002	chicken
Tuft1	0.33	0.00000002	chicken
Zcchc14	0.65	0.00000002	chicken
Chchd3	0.47	0.00000002	chicken
Rbms1	0.38	0.00000002	chicken
Secisbp2l	0.49	0.00000002	chicken
Agtr1a	0.33	0.00000002	chicken
Dram2	0.34	0.00000002	chicken
Ndufa6	-0.49	0.00000002	chicken
Osbpl10	0.56	0.00000002	chicken

Psmd1	0.43	0.00000002	chicken
Runx3	-0.67	0.00000002	chicken
St6galnac3	-0.43	0.00000002	chicken
Cdca4	0.34	0.00000003	chicken
Islr	0.38	0.00000003	chicken
Idh2	-0.45	0.00000003	chicken
Pgls	-0.56	0.00000003	chicken
Spen	0.49	0.00000003	chicken
Ndfip2	0.55	0.00000003	chicken
Crcp	-0.44	0.00000003	chicken
Cd63	0.43	0.00000003	chicken
Capn2	0.39	0.00000003	chicken
Tfdp2	-0.67	0.00000004	chicken
Trio	0.50	0.00000004	chicken
BC005537	0.45	0.00000004	chicken
Fbxl2	-0.44	0.00000004	chicken
Cidea	0.24	0.00000004	chicken
Plekhm2	0.27	0.00000005	chicken
Tbc1d8	-0.56	0.00000005	chicken
Ranbp10	0.44	0.00000006	chicken
Ndel1	0.39	0.00000006	chicken
Hsp90b1	-0.36	0.00000006	chicken
Dock7	0.42	0.00000006	chicken
Hdac7	0.37	0.00000006	chicken
Cirbp	-0.45	0.00000007	chicken
Sec11a	0.38	0.00000007	chicken
Zdhhc20	0.46	0.00000007	chicken
Fgfr1	0.54	0.00000008	chicken
Ipo5	0.34	0.00000008	chicken
Sdk1	-0.42	0.00000008	chicken
Zfand6	0.33	0.00000009	chicken
Commd1b	-0.49	0.00000009	chicken
Sec61g	-0.58	0.00000009	chicken
Pik3cb	0.65	0.00000010	chicken
Slc29a1	-0.34	0.00000010	chicken
Cdh13	1.56	0.00000010	chicken
Rrad	1.08	0.00000010	chicken
Znrf2	0.44	0.00000011	chicken
Prnp	0.41	0.00000011	chicken
Cog5	0.66	0.00000011	chicken
Eif3l	-0.56	0.00000011	chicken
Pi15	-0.51	0.00000012	chicken
Cntn1	0.50	0.00000012	chicken
Rasgef1a	0.49	0.00000012	chicken
Sugct	-0.76	0.00000012	chicken
Gadd45g	0.54	0.00000013	chicken

Ctsa	-0.43	0.00000013	chicken
Naif1	-0.46	0.00000013	chicken
Fam13a	0.53	0.00000013	chicken
Tsc22d3	-0.47	0.00000014	chicken
App	-0.52	0.00000014	chicken
Pcbd2	-0.44	0.00000014	chicken
Ndnf	-0.49	0.00000014	chicken
Adamtsl2	0.67	0.00000014	chicken
Olfml2b	-0.39	0.00000015	chicken
Engase	-0.42	0.00000015	chicken
Krt42	0.62	0.00000015	chicken
Smim20	-0.44	0.00000015	chicken
Hdgf	-0.60	0.00000015	chicken
Fndc1	-0.50	0.00000015	chicken
Wnt5a	-0.63	0.00000015	chicken
Lin54	0.41	0.00000016	chicken
Lcp2	-0.62	0.00000016	chicken
Aff2	-0.42	0.00000016	chicken
Nfatc1	0.53	0.00000016	chicken
Tcn2	-0.39	0.00000017	chicken
Aldh9a1	-0.41	0.00000018	chicken
Tmcc1	0.45	0.00000019	chicken
Camk1d	0.51	0.00000021	chicken
Arhgap26	0.39	0.00000022	chicken
Cops3	0.41	0.00000022	chicken
Traf7	0.26	0.00000023	chicken
Cct5	0.51	0.00000024	chicken
Cyfip1	0.42	0.00000025	chicken
F2rl2	0.31	0.00000025	chicken
Tor1b	0.34	0.00000026	chicken
Rbmxl1	-0.56	0.00000026	chicken
Abl1	0.45	0.00000026	chicken
Wbp1	-0.53	0.00000026	chicken
Galnt18	0.82	0.00000028	chicken
Syn3	0.49	0.00000029	chicken
Smc4	-0.44	0.00000029	chicken
Jdp2	-0.89	0.00000029	chicken
Bloc1s6	0.39	0.00000031	chicken
Elovl1	0.40	0.00000031	chicken
Srebf2	0.35	0.00000032	chicken
Tax1bp1	0.49	0.00000033	chicken
Npr3	-0.52	0.00000034	chicken
Col12a1	0.66	0.00000035	chicken
Faf2	0.36	0.00000035	chicken
Atp6v0a1	0.41	0.00000038	chicken
Nrp1	0.66	0.00000038	chicken

Frzb	-0.65	0.00000038	chicken
Ikzf1	-0.65	0.00000039	chicken
Psmd2	0.33	0.00000041	chicken
Arhgap17	0.36	0.00000041	chicken
Usp1	0.37	0.00000041	chicken
Rel	0.25	0.00000041	chicken
Slc40a1	0.56	0.00000041	chicken
Nebl	-0.50	0.00000042	chicken
Setbp1	-0.53	0.00000043	chicken
Pprc1	0.24	0.00000044	chicken
Wif1	-1.20	0.00000044	chicken
Ptprm	0.44	0.00000046	chicken
Disc1	-0.61	0.00000047	chicken
Etnk1	0.40	0.00000048	chicken
Ric1	0.36	0.00000049	chicken
Dtwd1	-0.35	0.00000049	chicken
Eif1	-0.39	0.00000049	chicken
Vps13a	0.66	0.00000050	chicken
Ebf2	0.69	0.00000050	chicken
Eml4	-0.68	0.00000052	chicken
Cacna1c	0.90	0.00000052	chicken
Dnm1l	0.37	0.00000053	chicken
Exoc3	0.38	0.00000054	chicken
Hnrnpm	0.36	0.00000056	chicken
Slc38a2	-0.48	0.00000057	chicken
Tmem229b	0.23	0.00000057	chicken
Itpripl2	0.30	0.00000058	chicken
Akr1d1	-0.65	0.00000059	chicken
Psmg4	-0.33	0.00000060	chicken
Vav3	-0.78	0.00000061	chicken
Plcb1	-0.11	0.00000063	chicken
Atoh8	-0.55	0.00000066	chicken
Rnf103	0.41	0.00000067	chicken
Cul4a	-0.79	0.00000067	chicken
Ank2	-0.40	0.00000071	chicken
Pm20d2	0.43	0.00000073	chicken
Sc5d	0.27	0.00000074	chicken
Ezr	0.55	0.00000074	chicken
Eif3m	-0.59	0.00000079	chicken
Rnf141	0.30	0.00000082	chicken
Ccdc141	-0.56	0.00000083	chicken
Vcp	0.43	0.00000086	chicken
Copz1	-0.57	0.00000086	chicken
Tacc1	0.42	0.00000087	chicken
Efnb1	-0.58	0.00000087	chicken
Runx1t1	-0.58	0.00000087	chicken

Dbi	-0.49	0.00000088	chicken
Npc2	-0.53	0.00000096	chicken
Sptan1	-0.56	0.00000096	chicken
Ppm1e	0.26	0.00000100	chicken
Kif13b	-0.72	0.00000100	chicken
Cib1	-0.44	0.00000104	chicken
Ncbp2	0.32	0.00000109	chicken
Zfc3h1	0.47	0.00000112	chicken
Lamp1	0.45	0.00000114	chicken
Laptm5	-0.45	0.00000119	chicken
Eif4ebp1	-0.53	0.00000120	chicken
Itih5	-0.64	0.00000121	chicken
Coro1c	0.32	0.00000122	chicken
Nlrc5	-1.13	0.00000125	chicken
Rasd1	0.58	0.00000128	chicken
Ctnnb1	-0.60	0.00000130	chicken
Cpq	-0.99	0.00000150	chicken
Rbm5	-0.59	0.00000152	chicken
Plcg1	-0.49	0.00000153	chicken
Dock4	-0.71	0.00000155	chicken
Ak3	-0.33	0.00000156	chicken
Tcirg1	0.30	0.00000171	chicken
Rpa3	0.27	0.00000175	chicken
Abhd12	0.36	0.00000175	chicken
Rfx3	-0.87	0.00000178	chicken
Prr16	0.52	0.00000179	chicken
Rrp12	0.23	0.00000185	chicken
Edc4	0.33	0.00000192	chicken
Psmd6	0.24	0.00000193	chicken
Serbp1	-0.32	0.00000199	chicken
Zfp462	-0.55	0.00000202	chicken
Tnik	-0.56	0.00000203	chicken
Kcnk5	0.67	0.00000204	chicken
Phaf1	0.51	0.00000208	chicken
Ppm1k	0.35	0.00000219	chicken
Nat8f7	-0.42	0.00000222	chicken
Nmrk1	0.46	0.00000222	chicken
Angptl2	0.61	0.00000223	chicken
Taok3	0.34	0.00000254	chicken
S100a4	0.36	0.00000262	chicken
Rassf8	0.60	0.00000266	chicken
Invs	0.48	0.00000267	chicken
Pgm5	-0.50	0.00000269	chicken
Pcnt	-0.68	0.00000271	chicken
Bmp4	-0.90	0.00000284	chicken
Kansl1l	0.64	0.00000310	chicken

Timp4	-0.94	0.00000326	chicken
Hivep1	0.56	0.00000348	chicken
Cnot6l	0.28	0.00000348	chicken
Cnnm2	-0.65	0.00000356	chicken
Usp22	0.32	0.00000357	chicken
Bcl11b	-0.65	0.00000390	chicken
Snx14	-0.76	0.00000404	chicken
Neo1	-0.58	0.00000426	chicken
Wsb2	-0.37	0.00000437	chicken
Eif2s3y	-0.58	0.00000456	chicken
Cobl	0.92	0.00000478	chicken
Rasgef1c	0.03	0.00000487	chicken
Prdx1	-0.49	0.00000489	chicken
Riok3	0.53	0.00000507	chicken
Rnaseh2b	-0.50	0.00000508	chicken
Sema5a	-0.69	0.00000514	chicken
Tgfbi	-0.45	0.00000545	chicken
Eif3f	-0.56	0.00000578	chicken
Lyar	0.26	0.00000583	chicken
Wdr3	0.39	0.00000592	chicken
Chd7	0.38	0.00000593	chicken
Serpinh1	-0.41	0.00000594	chicken
Usp47	0.41	0.00000616	chicken
Sinhcaf	0.32	0.00000625	chicken
Nabp1	0.31	0.00000631	chicken
Sla	-0.60	0.00000642	chicken
Ccdc88a	0.60	0.00000668	chicken
Socs1	0.59	0.00000672	chicken
Cemip2	0.34	0.00000677	chicken
Ammecr1	0.46	0.00000774	chicken
Itgb8	-0.66	0.00000798	chicken
Ppat	-0.47	0.00000837	chicken
Cdon	-0.52	0.00000837	chicken
Disp1	0.43	0.00000856	chicken
Rsu1	0.51	0.00000870	chicken
Ntrk2	-0.64	0.00000959	chicken
Tpk1	-0.73	0.00000974	chicken
Kif26a	-0.50	0.00000982	chicken
Ecpas	0.37	0.00000992	chicken
Fbxl17	0.71	0.00000999	chicken
Nuak1	0.35	0.00001006	chicken
Tiam2	0.46	0.00001012	chicken
Heph	0.34	0.00001035	chicken
Atp9b	0.41	0.00001046	chicken
Sf3b5	-0.46	0.00001067	chicken
Ror2	-0.54	0.00001101	chicken

Lsm12	0.33	0.00001153	chicken
S100a6	0.42	0.00001163	chicken
Lrp12	0.37	0.00001174	chicken
Pros1	-0.57	0.00001199	chicken
Calcb	-0.99	0.00001217	chicken
Postn	-1.51	0.00001222	chicken
Samd4	0.38	0.00001261	chicken
Hspa4l	0.40	0.00001280	chicken
Nin	0.20	0.00001288	chicken
Abcb8	-0.41	0.00001299	chicken
Gnl3	0.31	0.00001475	chicken
Zzef1	0.51	0.00001486	chicken
Cfap45	-0.43	0.00001516	chicken
Slc20a2	0.39	0.00001555	chicken
Snrnp25	-0.42	0.00001567	chicken
Zbtb46	0.28	0.00001586	chicken
Ergic1	0.34	0.00001592	chicken
Ablim1	-0.75	0.00001612	chicken
Arid5a	0.26	0.00001613	chicken
Zcchc2	0.36	0.00001623	chicken
Ccnl2	0.29	0.00001627	chicken
Edil3	-0.36	0.00001635	chicken
P4hb	-0.54	0.00001635	chicken
Stmn1	-0.43	0.00001642	chicken
Cd82	-0.55	0.00001683	chicken
Dennd4a	0.46	0.00001695	chicken
Spred2	0.44	0.00001707	chicken
Basp1	0.86	0.00001709	chicken
Plekhf1	0.39	0.00001752	chicken
Nckap1	0.34	0.00001754	chicken
Fto	-0.60	0.00001824	chicken
C2cd5	0.44	0.00001865	chicken
Sf3b3	0.37	0.00001922	chicken
Syf2	0.33	0.00001975	chicken
Crim1	-0.65	0.00002057	chicken
Lrrc3b	-0.48	0.00002057	chicken
Arf4	0.36	0.00002116	chicken
Slc24a2	0.49	0.00002131	chicken
Lpgat1	0.55	0.00002146	chicken
Pdk3	-0.57	0.00002165	chicken
Cdk9	0.24	0.00002184	chicken
Sntg1	1.61	0.00002187	chicken
Tmem141	-0.47	0.00002190	chicken
Patj	-0.62	0.00002202	chicken
Enox1	-0.63	0.00002297	chicken
Sash1	-0.52	0.00002342	chicken

Tmem104	-0.48	0.00002370	chicken
B3gnt2	-0.50	0.00002473	chicken
Tacc3	0.33	0.00002530	chicken
Thada	0.59	0.00002537	chicken
Sh3bp2	0.44	0.00002571	chicken
Chmp4b	0.34	0.00002583	chicken
Cldnd1	-0.43	0.00002587	chicken
Vapa	-0.55	0.00002617	chicken
Pin4	-0.49	0.00002748	chicken
Pik3r1	-0.50	0.00002794	chicken
Ltbp2	-0.79	0.00002827	chicken
Grid1	0.89	0.00002850	chicken
Ets1	0.38	0.00002887	chicken
Rangap1	0.28	0.00003051	chicken
Pmepa1	0.68	0.00003059	chicken
Dusp8	0.16	0.00003193	chicken
Kpna6	0.29	0.00003199	chicken
Stat4	-0.62	0.00003396	chicken
Tst	-0.35	0.00003478	chicken
Ankrd10	-0.42	0.00003623	chicken
Clpx	0.34	0.00003657	chicken
Fhod1	0.39	0.00003720	chicken
Kbtbd11	-0.41	0.00003731	chicken
Nr1h3	0.28	0.00003740	chicken
Lasp1	0.39	0.00003819	chicken
Commd7	-0.48	0.00003849	chicken
Snx25	-0.58	0.00003901	chicken
Rgs10	0.30	0.00003969	chicken
Kctd20	0.36	0.00003984	chicken
Arl5b	0.39	0.00004129	chicken
C1galt1c1	0.31	0.00004225	chicken
Smo	-0.44	0.00004445	chicken
Coro7	-0.42	0.00004580	chicken
Srpr	0.28	0.00004663	chicken
Uggt2	-0.40	0.00004671	chicken
Aebp1	-0.55	0.00004839	chicken
Tomm40l	-0.49	0.00004864	chicken
Tnfaip6	0.44	0.00004975	chicken
Ndufb6	-0.51	0.00005047	chicken
Lnx2	0.23	0.00005068	chicken
Erc1	0.50	0.00005284	chicken
Zfp64	-0.36	0.00005353	chicken
Atp5j	-0.47	0.00005668	chicken
Klf10	0.37	0.00005753	chicken
Tle5	-0.49	0.00005828	chicken
Ube2f	-0.46	0.00005913	chicken

Frs2	0.34	0.00006024	chicken
Smarca1	-0.54	0.00006027	chicken
Hmgcs1	0.27	0.00006060	chicken
Rrs1	0.26	0.00006148	chicken
Prickle1	-0.66	0.00006258	chicken
Dynlt3	-0.57	0.00006541	chicken
Ulk1	-0.34	0.00006597	chicken
Eif3h	-0.44	0.00006714	chicken
Bhlhe40	0.71	0.00006760	chicken
Hypk	-0.50	0.00006934	chicken
Tmed2	-0.53	0.00007178	chicken
Jund	0.29	0.00007445	chicken
Amd2	0.32	0.00007453	chicken
Ube2w	0.29	0.00007460	chicken
Rbm19	0.25	0.00007565	chicken
Fam174c	0.23	0.00007937	chicken
Kat6b	-0.23	0.00007981	chicken
Ahr	-0.52	0.00008363	chicken
Aff4	0.40	0.00008365	chicken
Ncln	0.19	0.00008487	chicken
Sik3	0.34	0.00008549	chicken
Flot2	0.33	0.00008724	chicken
Brix1	0.25	0.00008744	chicken
Serpini1	-0.49	0.00008796	chicken
Ubqln1	-0.61	0.00009058	chicken
Ckap4	-0.57	0.00009100	chicken
Fzd1	0.28	0.00009544	chicken
Prmt8	0.32	0.00009910	chicken
Spag16	-0.53	0.00009987	chicken
Pdia3	-0.47	0.00010055	chicken
Smim14	0.33	0.00010111	chicken
Ptch1	-0.70	0.00010118	chicken
Gpc4	0.50	0.00010384	chicken
Cyb5r2	0.30	0.00010575	chicken
Arl1	-0.47	0.00010732	chicken
Brsk2	-0.46	0.00010765	chicken
Smc3	-0.65	0.00010917	chicken
Nfe2l2	0.29	0.00011053	chicken
Dnajb9	0.36	0.00011312	chicken
Clta	-0.43	0.00011532	chicken
Rilpl2	-0.34	0.00011680	chicken
Irf8	0.31	0.00011764	chicken
Rdx	0.37	0.00011834	chicken
Egr2	0.21	0.00012594	chicken
Ttc27	0.29	0.00013304	chicken
Oxct1	-0.38	0.00013356	chicken

Dsel	-0.40	0.00013688	chicken
Prdx4	-0.51	0.00013819	chicken
Itpr2	0.59	0.00013844	chicken
Gkap1	0.39	0.00014196	chicken
Slc38a1	0.23	0.00014799	chicken
Fam122a	-0.43	0.00014928	chicken
St6gal1	-0.48	0.00015102	chicken
Esd	-0.45	0.00015249	chicken
Utp20	0.27	0.00015402	chicken
Dync1li1	0.23	0.00015901	chicken
Kazald1	-0.36	0.00017766	chicken
Batf	-0.41	0.00018045	chicken
Cap1	0.25	0.00018332	chicken
Ola1	0.33	0.00018612	chicken
Cops4	0.25	0.00018860	chicken
Park7	-0.43	0.00018870	chicken
Glt8d1	-0.36	0.00019008	chicken
Fgd3	-0.57	0.00019014	chicken
Ednra	-0.47	0.00019209	chicken
Ndufb9	-0.50	0.00019291	chicken
Fmo3	-0.43	0.00019671	chicken
Hspd1	0.37	0.00019823	chicken
Kpna4	0.34	0.00019979	chicken
Atp2a2	-0.54	0.00020507	chicken
Fbxo3	0.26	0.00020605	chicken
Tnfrsf19	-0.44	0.00020929	chicken
Hmgb2	-0.41	0.00021351	chicken
Cdcp1	0.22	0.00021665	chicken
Gabpb2	-0.23	0.00021793	chicken
Axin2	-0.49	0.00021874	chicken
Ndrg1	0.52	0.00021933	chicken
Rcn2	-0.51	0.00022114	chicken
Vldlr	-0.36	0.00023769	chicken
Kdelr2	-0.57	0.00023934	chicken
Rab10	0.42	0.00024752	chicken
Tbl1xr1	-0.45	0.00025312	chicken
Adam10	0.46	0.00026346	chicken
Ccn2	-0.41	0.00026596	chicken
Pdcd10	0.30	0.00026931	chicken
Ndufb8	-0.49	0.00027063	chicken
Sh3bgrl3	-0.45	0.00028080	chicken
Ppa2	-0.34	0.00028952	chicken
Adamts3	-0.42	0.00028966	chicken
Efna5	0.91	0.00029458	chicken
Tmem14a	-0.35	0.00031481	chicken
Fstl3	0.32	0.00032028	chicken

Cbx3	-0.59	0.00032264	chicken
Ythdc1	-0.43	0.00032433	chicken
Zfp618	0.43	0.00032722	chicken
Rrp1b	0.27	0.00032859	chicken
Strn	-0.48	0.00035815	chicken
Eif4e3	0.23	0.00035841	chicken
Nadk	0.34	0.00037035	chicken
Nav2	-0.45	0.00037108	chicken
Baz1b	0.47	0.00038376	chicken
Zfp821	-0.39	0.00038591	chicken
S100a11	-0.49	0.00038788	chicken
Hsbp1	-0.40	0.00038835	chicken
Ddx18	0.23	0.00039292	chicken
Zdhhc9	-0.37	0.00040582	chicken
Dnajb12	0.24	0.00041148	chicken
Twist1	0.34	0.00041713	chicken
Timm10	-0.45	0.00041753	chicken
Rsl1d1	0.26	0.00041970	chicken
Rin3	0.34	0.00042139	chicken
Akap9	0.37	0.00042276	chicken
Uhrf2	-0.61	0.00042708	chicken
Plekhf2	0.21	0.00043496	chicken
Synj1	0.20	0.00044931	chicken
Acsbg2	0.40	0.00046238	chicken
Frmd6	0.14	0.00046285	chicken
Fmnl1	-0.35	0.00046322	chicken
Flt1	0.62	0.00046384	chicken
Sdad1	0.20	0.00046575	chicken
Rtkn	-0.30	0.00049737	chicken
Uqcr10	-0.59	0.00051136	chicken
Zdhhc17	0.33	0.00053123	chicken
Afap1	-0.67	0.00054790	chicken
Abcf2	0.19	0.00055047	chicken
Prkca	-0.44	0.00055422	chicken
Runx1	-0.39	0.00055819	chicken
Smad2	-0.47	0.00056714	chicken
Amotl2	0.13	0.00057520	chicken
Cox7a2	-0.49	0.00058074	chicken
Paics	-0.56	0.00058688	chicken
Exoc6b	0.32	0.00062176	chicken
Lypla2	0.13	0.00063288	chicken
Tgfbr3	0.49	0.00063528	chicken
Slc10a7	0.50	0.00064458	chicken
Aass	0.26	0.00064784	chicken
Rbpj	0.25	0.00064934	chicken
Eny2	-0.31	0.00067833	chicken

Ets2	0.43	0.00068378	chicken
Txn2	-0.38	0.00068998	chicken
Igsf3	0.25	0.00070457	chicken
Eefsec	0.52	0.00073092	chicken
Abi1	0.35	0.00073097	chicken
Rgs3	0.24	0.00077778	chicken
Adamts15	0.46	0.00077788	chicken
Ptch2	-0.45	0.00077920	chicken
Gtpbp1	-0.37	0.00078223	chicken
Atrx	0.33	0.00080128	chicken
Eif2s2	0.35	0.00082389	chicken
Vps13c	-0.39	0.00082675	chicken
Dusp14	-0.29	0.00083260	chicken
Gnal	-0.52	0.00085251	chicken
Jade2	0.30	0.00085600	chicken
Inpp4b	-0.50	0.00087746	chicken
Ripk1	0.23	0.00087820	chicken
Rbm33	0.26	0.00090348	chicken
Foxp1	0.41	0.00095022	chicken
Nfib	-0.30	0.00095285	chicken
Arhgef7	-0.54	0.00096335	chicken
Cd164	0.27	0.00097902	chicken
Prkce	0.54	0.00098479	chicken
Ddx20	0.16	0.00098603	chicken
Ptpn9	0.20	0.00103135	chicken
Evl	-0.57	0.00103578	chicken
Dusp5	0.44	0.00107943	chicken
Sspn	-0.37	0.00108402	chicken
Ptpru	-0.53	0.00109747	chicken
Bcl9l	0.23	0.00113503	chicken
D630045J12Rik	0.52	0.00114121	chicken
Spopl	0.30	0.00115561	chicken
Snhg4	0.34	0.00117981	chicken
Plxnd1	-0.46	0.00118673	chicken
Gpatch2l	0.26	0.00119516	chicken
Dnaja1	-0.74	0.00119687	chicken
Usp28	-0.31	0.00119802	chicken
Specc1l	-0.52	0.00121318	chicken
Angptl1	-0.65	0.00121671	chicken
Lrrc8c	0.32	0.00124027	chicken
Spsb1	0.42	0.00124369	chicken
Galnt16	0.72	0.00125392	chicken
Hsp90aa1	0.32	0.00126794	chicken
Cd151	-0.49	0.00130038	chicken
Wdr11	0.16	0.00134154	chicken
Papola	0.40	0.00137255	chicken

Noc2l	0.25	0.00137730	chicken
Sgcd	-0.42	0.00141047	chicken
Lpin2	-0.38	0.00141136	chicken
Eif4ebp2	-0.36	0.00142719	chicken
Sox4	-0.50	0.00147578	chicken
Wnt2b	-0.56	0.00151463	chicken
Btbd10	0.23	0.00151516	chicken
Wasf2	0.18	0.00156455	chicken
Pcmtd2	-0.32	0.00157267	chicken
Chst5	-0.31	0.00158328	chicken
Creg1	0.34	0.00166414	chicken
Usb1	-0.26	0.00168777	chicken
Loxl1	0.37	0.00175254	chicken
Alpk1	0.38	0.00176669	chicken
Paqr7	-0.24	0.00191145	chicken
Naa40	-0.48	0.00198602	chicken
Ptgs1	0.25	0.00200213	chicken
Gclc	0.45	0.00202053	chicken
Gpi1	-0.55	0.00203037	chicken
Loxl2	-0.30	0.00204268	chicken
Ascc3	0.35	0.00214701	chicken
Ids	-0.33	0.00219677	chicken
Atp5h	-0.44	0.00220647	chicken
Psma3	0.30	0.00221662	chicken
Fam172a	0.46	0.00223632	chicken
Sec14l1	0.35	0.00226031	chicken
Cep350	0.34	0.00227910	chicken
Dync1li2	0.36	0.00234556	chicken
Fbxo22	0.32	0.00237550	chicken
Hbs1l	0.19	0.00237938	chicken
Fhl5	-0.55	0.00240783	chicken
Braf	0.22	0.00242358	chicken
Hsd17b10	-0.28	0.00257548	chicken
Psmd13	0.26	0.00260828	chicken
Slc25a5	-0.44	0.00261122	chicken
Actr2	0.17	0.00261504	chicken
Hyal1	-0.35	0.00262801	chicken
Tmtc1	-0.76	0.00265856	chicken
Plcb4	-0.41	0.00272827	chicken
Gpatch8	-0.49	0.00272853	chicken
Nkain3	-0.71	0.00275632	chicken
Psmd3	0.25	0.00280430	chicken
Syt11	-0.29	0.00281324	chicken
Lrmda	0.72	0.00289687	chicken
Psmc1	0.25	0.00305624	chicken
Tub	0.43	0.00312197	chicken

Smarca2	-0.41	0.00323559	chicken
Itsn1	-0.40	0.00325971	chicken
Ptk7	-0.39	0.00332545	chicken
Atp5c1	-0.31	0.00338620	chicken
Dnlz	-0.42	0.00339093	chicken
Gnai1	-0.56	0.00340761	chicken
Fgfr3	-0.61	0.00361843	chicken
Serpinf1	-0.73	0.00362454	chicken
Rab18	0.28	0.00363644	chicken
Ppard	0.31	0.00364852	chicken
Fat1	-0.45	0.00366941	chicken
Rhoc	0.19	0.00373592	chicken
Fuca1	-0.31	0.00373716	chicken
Jmjd6	-0.41	0.00375558	chicken
8030462N17Rik	0.23	0.00377817	chicken
Ehd2	0.28	0.00379805	chicken
Ndufb3	-0.43	0.00384321	chicken
Stk40	0.28	0.00389017	chicken
Ddx54	0.23	0.00414677	chicken
Cct2	0.28	0.00415011	chicken
Ncam1	-0.58	0.00417157	chicken
Jup	-0.45	0.00417367	chicken
Psmd7	0.32	0.00418992	chicken
Taf5	0.15	0.00426705	chicken
Sumo3	-0.49	0.00430411	chicken
Rorb	0.85	0.00432521	chicken
Trak1	0.51	0.00437647	chicken
Vps37a	0.21	0.00437974	chicken
Rab11a	0.20	0.00444117	chicken
Rarres1	1.56	0.00451947	chicken
Scg5	-0.38	0.00453685	chicken
Gtf2h1	0.25	0.00454152	chicken
Pak3	0.26	0.00464002	chicken
Kcnj8	-0.43	0.00470538	chicken
Tm2d2	-0.37	0.00474876	chicken
Hmox1	0.44	0.00479062	chicken
Septin14	-0.45	0.00480089	chicken
Lifr	-0.61	0.00488303	chicken
Eif4g2	-0.34	0.00496465	chicken
Ube3c	0.41	0.00498131	chicken
Pcna	-0.38	0.00505491	chicken
Dapk2	0.34	0.00512077	chicken
Fadd	0.22	0.00527868	chicken
Tcea2	-0.26	0.00568560	chicken
Arhgap10	-0.23	0.00586811	chicken
Immp2l	-0.46	0.00588888	chicken

Nrp2	0.42	0.00590540	chicken
1810058I24Rik	-0.31	0.00607932	chicken
Nras	-0.42	0.00624106	chicken
Iqsec1	0.06	0.00630663	chicken
Akt1	0.28	0.00632573	chicken
Selenoi	0.21	0.00641151	chicken
Nhsl1	0.57	0.00643661	chicken
Wapl	0.33	0.00645081	chicken
Fam98a	0.21	0.00649312	chicken
Arhgef26	0.31	0.00706435	chicken
Ypel2	-0.55	0.00707002	chicken
Rspo3	0.33	0.00720480	chicken
Selenbp2	0.21	0.00721280	chicken
Arf1	0.28	0.00727311	chicken
Cdin1	-0.50	0.00728309	chicken
Golim4	-0.55	0.00745273	chicken
Prrg1	-0.49	0.00758791	chicken
Piezo2	0.91	0.00785230	chicken
Igfbp7	-0.50	0.00788798	chicken
Pten	-0.40	0.00797980	chicken
Cilp	-0.36	0.00799050	chicken
Pgap1	0.20	0.00806676	chicken
Elmo1	-0.47	0.00816621	chicken
Ccn4	0.31	0.00821681	chicken
Atrnl1	0.61	0.00837429	chicken
Psmd14	0.28	0.00853669	chicken
Prdm6	-0.40	0.00864578	chicken
Ank3	0.28	0.00868016	chicken
2510002D24Rik	-0.21	0.00884471	chicken
AW554918	0.32	0.00898254	chicken
Tmbim6	-0.39	0.00912762	chicken
Tiam1	0.23	0.00918342	chicken
Acaa2	-0.28	0.00924384	chicken
Ndrg4	0.48	0.00930189	chicken
Itm2b	-0.39	0.00943180	chicken
Ptprs	0.35	0.00954060	chicken
Qk	0.20	0.00990195	chicken
Arhgef6	-0.39	0.00990907	chicken
Ninj1	0.20	0.01016052	chicken
Tmem144	-0.36	0.01020623	chicken
Dcun1d3	0.43	0.01026072	chicken
Trabd	0.15	0.01042092	chicken
Abcc5	-0.50	0.01046879	chicken
Apaf1	0.18	0.01046994	chicken
Plcl2	0.50	0.01047599	chicken
Prr5	-0.37	0.01074131	chicken

Hbp1	0.22	0.01079400	chicken
Pdgfa	-0.35	0.01082565	chicken
Brd1	-0.47	0.01092976	chicken
Acss3	-0.55	0.01100783	chicken
Sox5	0.64	0.01101847	chicken
Sipa1l2	-0.54	0.01102138	chicken
Tmem50b	0.17	0.01118088	chicken
Sfrp2	0.07	0.01123242	chicken
Hdgfl3	-0.44	0.01148194	chicken
Rbm12	0.21	0.01168179	chicken
Czib	-0.29	0.01193634	chicken
Peak1	0.28	0.01194421	chicken
Gxylt2	-0.46	0.01225391	chicken
Ube2l3	0.25	0.01226860	chicken
Gli3	0.67	0.01238404	chicken
Prrg4	-0.36	0.01253904	chicken
C3	0.34	0.01303152	chicken
Rere	0.32	0.01318588	chicken
Pim1	-0.59	0.01326345	chicken
Podn	0.40	0.01352750	chicken
L3mbtl3	0.09	0.01354365	chicken
Ndufv3	-0.49	0.01377288	chicken
Raph1	-0.55	0.01383096	chicken
Irs4	0.25	0.01392483	chicken
Pdlim5	0.37	0.01412979	chicken
Fbln1	-0.34	0.01417275	chicken
Tmem263	-0.46	0.01426466	chicken
Emg1	0.24	0.01457035	chicken
Cstb	-0.40	0.01468262	chicken
Tuba1b	-0.44	0.01480725	chicken
Hivep2	0.33	0.01503588	chicken
Cep170	0.30	0.01507759	chicken
Pde5a	-0.41	0.01511855	chicken
Supt3	-0.39	0.01516724	chicken
Tspan4	0.73	0.01523857	chicken
Cebpz	0.27	0.01525489	chicken
Crem	0.45	0.01608174	chicken
Traf2	0.20	0.01640155	chicken
Dock11	-0.40	0.01664685	chicken
Lpcat3	0.26	0.01678377	chicken
Hdac4	0.38	0.01716560	chicken
Ndufa13	-0.51	0.01761243	chicken
Kif21a	0.28	0.01767163	chicken
Sars	-0.34	0.01812216	chicken
Cdc42ep4	0.29	0.01821832	chicken
Appl2	0.33	0.01826422	chicken

Adamts9	-0.36	0.01947803	chicken
Oxr1	-0.44	0.01951110	chicken
Ubxn2b	-0.35	0.01952702	chicken
Ano5	0.51	0.01955423	chicken
Pgrmc2	-0.41	0.01959704	chicken
Smim12	-0.35	0.01964830	chicken
Ube2j1	0.14	0.01977032	chicken
Adprh	-0.36	0.02023210	chicken
Nfkbiz	-0.60	0.02042245	chicken
Lsmem1	0.54	0.02042754	chicken
Dpysl3	-0.46	0.02107705	chicken
Wdr43	0.23	0.02132986	chicken
Pde10a	-0.39	0.02144469	chicken
Dazap2	0.21	0.02162499	chicken
Idh1	-0.45	0.02174630	chicken
Hdx	0.35	0.02184415	chicken
Srsf5	-0.45	0.02186953	chicken
Ssb	0.26	0.02274488	chicken
Dgkh	0.51	0.02283487	chicken
Bahcc1	0.24	0.02309231	chicken
Ap3s1	-0.35	0.02478698	chicken
Kitl	-0.49	0.02513084	chicken
Ccdc91	-0.47	0.02554273	chicken
Naa15	0.27	0.02563651	chicken
Jade1	0.15	0.02640175	chicken
Rimklb	-0.42	0.02668598	chicken
Ncald	0.62	0.02724642	chicken
Jag1	-0.38	0.02769840	chicken
Acad9	0.33	0.02797584	chicken
Gtf2f2	0.26	0.02797616	chicken
Kif13a	-0.33	0.02812377	chicken
Naa25	0.22	0.02895321	chicken
Ift80	-0.30	0.02955473	chicken
Gatd3a	-0.36	0.02994090	chicken
Eya3	0.18	0.03027915	chicken
Ccnk	0.10	0.03135542	chicken
Actr3	0.29	0.03147999	chicken
Pdhx	0.17	0.03203804	chicken
Itpka	0.29	0.03222684	chicken
Cdo1	-0.35	0.03284978	chicken
Anxa6	-0.43	0.03332489	chicken
Jak2	0.23	0.03496399	chicken
Fzd7	-0.28	0.03507815	chicken
Cpe	-0.33	0.03602423	chicken
Fbxl5	0.19	0.03732205	chicken
Commd3	-0.25	0.03733676	chicken

Atg101	-0.37	0.03769553	chicken
Rnpep	-0.33	0.03875147	chicken
Apbb1ip	-0.39	0.03956243	chicken
Skil	0.26	0.03967222	chicken
Ogn	-0.57	0.03970318	chicken
Fermt2	0.38	0.03974099	chicken
Atl1	-0.27	0.04008795	chicken
Sgcb	-0.33	0.04019072	chicken
Rab11fip2	-0.44	0.04112893	chicken
Sema4g	-0.31	0.04139302	chicken
Fam98b	0.28	0.04153111	chicken
Ddx6	0.15	0.04170913	chicken
Hnrnpul1	-0.32	0.04182640	chicken
Scaf4	0.21	0.04187477	chicken
Tbc1d9	-0.48	0.04203175	chicken
Cecr2	-0.45	0.04248612	chicken
Slc6a6	-0.28	0.04284299	chicken
Cald1	-0.14	0.04408366	chicken
Slc35b2	0.12	0.04411908	chicken
Tex10	0.27	0.04499743	chicken
Pef1	0.22	0.04553802	chicken
Phip	-0.55	0.04571241	chicken
A830018L16Rik	0.61	0.04651013	chicken
Pcmt1	-0.27	0.04786148	chicken
Exog	-0.25	0.04843250	chicken
Ptbp1	-0.37	0.04873045	chicken
Per3	0.42	0.04897887	chicken
Twsg1	-0.29	0.04911058	chicken
Rpa2	0.18	0.04968594	chicken
Trip11	0.20	0.05026990	chicken
Reep5	-0.37	0.05037886	chicken
Foxp4	0.17	0.05047184	chicken
Clk1	-0.42	0.05167879	chicken
Pip5k1b	-0.26	0.05174826	chicken
Pfdn6	-0.43	0.05195583	chicken
Arhgap29	-0.43	0.05320872	chicken
Rnd3	-0.22	0.05366786	chicken
Hibadh	-0.40	0.05448994	chicken
Acadl	-0.43	0.05449865	chicken
Ccnyl1	0.16	0.05574813	chicken
Nt5dc2	-0.30	0.05635347	chicken
Sorbs1	-0.47	0.05772542	chicken
Baiap2l1	0.18	0.05839200	chicken
Sirt2	-0.35	0.05921618	chicken
Dek	-0.39	0.05954081	chicken
Wipf1	-0.22	0.06024120	chicken

Tshz3	-0.38	0.06040606	chicken
Garnl3	-0.44	0.06126511	chicken
Ehbp1	-0.42	0.06173493	chicken
Ankrd17	0.18	0.06388819	chicken
Sntb1	0.42	0.06509279	chicken
Sin3a	0.16	0.06667990	chicken
Zdhhc14	-0.53	0.06691447	chicken
Bmpr1a	0.27	0.06712098	chicken
Srp9	0.21	0.06737116	chicken
Nploc4	0.12	0.06845273	chicken
Prkar2b	0.08	0.06883090	chicken
Ulk2	-0.52	0.07021199	chicken
Nr2f2	0.28	0.07029966	chicken
Dapk1	-0.19	0.07051955	chicken
Sh3gl1	0.18	0.07080128	chicken
Lbh	-0.41	0.07088920	chicken
Cacna1d	-0.37	0.07170399	chicken
Tomm22	-0.29	0.07253793	chicken
Ssr1	0.29	0.07266795	chicken
St13	0.33	0.07290806	chicken
Larp4b	0.17	0.07320156	chicken
Pxn	0.31	0.07505503	chicken
Grb10	-0.16	0.07508617	chicken
Cpne1	-0.39	0.07680273	chicken
Rap1b	0.20	0.07686243	chicken
Eif4a2	-0.23	0.07787755	chicken
Nudc	0.17	0.07837186	chicken
Pakap	0.77	0.08006621	chicken
Ptges3l	-0.12	0.08039153	chicken
Cfap20	-0.34	0.08261240	chicken
Fam204a	-0.39	0.08299928	chicken
Ston1	-0.36	0.08345818	chicken
Vwa8	-0.37	0.08469716	chicken
Casp8ap2	-0.53	0.08482964	chicken
Tubb6	0.25	0.08524996	chicken
Gfod1	0.36	0.08551810	chicken
Prpf4b	0.22	0.08725701	chicken
Agtr2	-0.47	0.08755298	chicken
Bcl11a	-0.35	0.09036390	chicken
Rsbn1l	-0.43	0.09108266	chicken
Utp3	0.13	0.09189397	chicken
Slc25a13	0.31	0.09190500	chicken
Phkg1	-0.35	0.09275802	chicken
Dph6	0.19	0.09368328	chicken
Ociad1	-0.23	0.09563200	chicken
Itgb5	0.21	0.09576382	chicken

Caprin2	-0.37	0.09737095	chicken
Tgif1	0.32	0.09862941	chicken
Cyb5r4	0.13	0.10024045	chicken
Pdgfra	-0.44	0.10349875	chicken
Gatad2b	0.18	0.10377585	chicken
Arid3a	-0.33	0.10509306	chicken
Stxbp5	-0.68	0.10664578	chicken
Adam12	-0.28	0.10667461	chicken
Nbea	-0.43	0.10704064	chicken
Lonrf2	0.29	0.10805247	chicken
Slc25a22	0.14	0.10884429	chicken
Rev3l	0.37	0.10922554	chicken
Paip2	0.20	0.11026543	chicken
Dst	-0.18	0.11171468	chicken
Clasp1	0.27	0.11211527	chicken
Commd5	-0.28	0.11228729	chicken
Arid5b	0.31	0.11416921	chicken
Fcf1	0.18	0.11660914	chicken
Prrx1	0.24	0.11706983	chicken
Nudt3	0.17	0.11740906	chicken
Twnk	0.16	0.12011411	chicken
Pcdh19	0.27	0.12020466	chicken
Cacnb2	0.01	0.12036434	chicken
Nr4a3	0.37	0.12037933	chicken
Cyp46a1	0.14	0.12302938	chicken
Rabgap1	0.17	0.12786123	chicken
Cblb	-0.40	0.12814450	chicken
Dennd2a	-0.40	0.12839092	chicken
Htra2	-0.30	0.12911130	chicken
Galnt7	0.19	0.13088798	chicken
Eif4a3l1	0.18	0.13509106	chicken
Cep85l	0.44	0.13646557	chicken
Ruvbl1	0.02	0.13706153	chicken
Stx12	0.22	0.13883540	chicken
Grk3	-0.47	0.14303780	chicken
Arfgef1	0.36	0.14595405	chicken
Sertad2	-0.47	0.14950039	chicken
Clic4	0.24	0.15319686	chicken
Add1	-0.36	0.15456475	chicken
Ttf1	0.18	0.15660501	chicken
Zfp326	0.26	0.15832480	chicken
Atp5pb	-0.31	0.16085608	chicken
Atp1b1	-0.52	0.16429319	chicken
Asf1a	-0.31	0.16902236	chicken
Ctsk	-0.30	0.16985809	chicken
Lhfpl2	-0.35	0.17410821	chicken

Hp1bp3	-0.28	0.17580818	chicken
Larp4	0.24	0.17654788	chicken
Kcnq1	-0.69	0.18305105	chicken
Thoc2	0.20	0.18458757	chicken
Eif4e	0.19	0.18522804	chicken
Lima1	0.37	0.18972637	chicken
Pam	0.49	0.19438161	chicken
Bet1	-0.24	0.19860359	chicken
Tmtc2	-0.47	0.20493241	chicken
Hif1a	0.40	0.20680261	chicken
Zmat5	-0.28	0.21260922	chicken
Foxo6	0.14	0.21403750	chicken
Bcap31	-0.34	0.21639171	chicken
Iqgap2	0.04	0.21886203	chicken
Fbxw8	0.15	0.22202257	chicken
Erc2	-0.43	0.22303708	chicken
Lpcat1	-0.36	0.23313087	chicken
Slf2	0.12	0.23387296	chicken
Hps1	-0.41	0.24023373	chicken
Tmem106b	-0.37	0.24277931	chicken
Fbh1	0.19	0.24646181	chicken
Ubl7	-0.46	0.24734692	chicken
Nkap	0.14	0.24892270	chicken
Otud7b	0.19	0.25585594	chicken
Sephs1	-0.32	0.25677113	chicken
Cotl1	-0.34	0.25944191	chicken
Nop16	0.21	0.25980100	chicken
Nf1	0.37	0.26217270	chicken
Erg	0.32	0.26376516	chicken
Oxsr1	0.42	0.26394097	chicken
Lmbrd1	0.11	0.26599878	chicken
Slc43a3	0.13	0.26792491	chicken
Hsd17b7	-0.36	0.26891898	chicken
Prox1	0.23	0.27079845	chicken
Syt9	0.29	0.27132213	chicken
Ndufs4	-0.33	0.27576981	chicken
Ppp1ca	-0.40	0.27850316	chicken
Ahsa1	0.11	0.27919658	chicken
Dync2h1	-0.26	0.27947163	chicken
Ptprk	0.57	0.28436913	chicken
Ipo7	0.26	0.28453897	chicken
U2af1	0.20	0.28473537	chicken
Hibch	-0.34	0.29572804	chicken
Setd3	0.13	0.29725838	chicken
Arhgef12	0.29	0.30266438	chicken
Pfdn4	-0.30	0.30268109	chicken

Ankrd13d	-0.38	0.30854049	chicken
Tyro3	-0.37	0.30898294	chicken
Yy2	0.27	0.31732827	chicken
Ccdc50	0.21	0.31968408	chicken
Slk	-0.43	0.33011543	chicken
Baiap2	-0.35	0.33882002	chicken
Ppig	0.17	0.34208627	chicken
Tnks	0.26	0.34360559	chicken
Txndc5	-0.31	0.35151170	chicken
Scp2	-0.30	0.35271840	chicken
Serinc3	0.17	0.35382369	chicken
Sesn3	-0.30	0.35469042	chicken
Tle4	-0.53	0.36020419	chicken
Lhpp	-0.29	0.36170317	chicken
Plpp1	-0.44	0.36371980	chicken
Rnps1	0.14	0.36638598	chicken
Prmt3	0.20	0.36990449	chicken
Il17ra	0.18	0.37332240	chicken
Gtf2h5	-0.42	0.37703039	chicken
Foxn3	-0.44	0.38832030	chicken
Dnajb14	-0.32	0.39277708	chicken
E2f4	0.07	0.39464728	chicken
Zfp131	0.14	0.40130753	chicken
Prdx3	-0.37	0.40274054	chicken
Lpar1	-0.18	0.40579361	chicken
Ppm1a	0.16	0.40616439	chicken
Rnf145	0.28	0.41314739	chicken
Zfp36l1	0.24	0.42641144	chicken
Ngly1	0.24	0.43413682	chicken
Arid4a	-0.33	0.43492549	chicken
Dipk2a	-0.23	0.45225079	chicken
Golga3	0.14	0.45432310	chicken
Sun1	0.14	0.47241031	chicken
Tbl2	-0.28	0.47406860	chicken
Prkci	0.20	0.47541643	chicken
Bicc1	0.46	0.47831271	chicken
Wwc1	0.46	0.47994858	chicken
Akirin2	-0.32	0.48938223	chicken
Rsf1	-0.34	0.49419843	chicken
Slain2	0.16	0.50085356	chicken
Copg1	-0.32	0.50381992	chicken
Cdc42ep3	0.26	0.50385392	chicken
Unc5b	0.18	0.51033331	chicken
Sec24b	0.23	0.51655672	chicken
Coq9	-0.27	0.51858480	chicken
Sgk3	0.26	0.51998934	chicken

Aida	-0.26	0.52334236	chicken
Hoxa5	0.19	0.52535732	chicken
Lactb	0.22	0.52740254	chicken
Diaph2	-0.36	0.53090187	chicken
Dusp10	0.23	0.53391527	chicken
Gnptab	0.22	0.53620204	chicken
Gphn	0.41	0.53887474	chicken
Pxdc1	0.20	0.54705124	chicken
Isca1	0.22	0.55277268	chicken
Greb1l	0.46	0.55412751	chicken
Ubap1	0.07	0.57054803	chicken
Nkiras2	-0.25	0.57792262	chicken
Osbpl9	0.39	0.58064689	chicken
Rnf166	-0.42	0.58757542	chicken
Uqcrb	-0.35	0.58878190	chicken
Sh3bp5	0.42	0.59209530	chicken
Tagln2	0.20	0.59272347	chicken
Ppp2r5a	-0.50	0.59702771	chicken
Pcdh18	-0.45	0.60122131	chicken
Zmynd11	-0.29	0.60128017	chicken
Snu13	0.17	0.60249782	chicken
Nup107	0.15	0.60453832	chicken
Fam20c	0.54	0.61376044	chicken
Arhgef17	0.13	0.61412085	chicken
Zeb2	0.43	0.61718032	chicken
Gstm4	-0.35	0.62003373	chicken
Dpysl2	-0.29	0.63352404	chicken
Ippk	0.10	0.64160412	chicken
Vps33b	-0.31	0.64949164	chicken
Sf3a3	0.16	0.65649812	chicken
Ypel5	0.18	0.66900836	chicken
Pggt1b	0.13	0.67518419	chicken
Adrm1	0.13	0.69187286	chicken
Tmtc3	0.20	0.69946271	chicken
Stat5b	0.18	0.69991308	chicken
Rin2	0.08	0.71028514	chicken
Ncstn	0.21	0.71046554	chicken
Ttc13	0.15	0.71635389	chicken
Sub1	0.18	0.71973475	chicken
St3gal3	0.40	0.72139129	chicken
Ywhag	0.26	0.72644748	chicken
Ccny	-0.32	0.75440960	chicken
Dnah12	-0.51	0.77045881	chicken
Agpat5	-0.40	0.77131929	chicken
Ambra1	0.20	0.77837444	chicken
Fam13b	0.15	0.78807095	chicken

Nav1	-0.55	0.78931004	chicken
Nr4a1	0.18	0.79318293	chicken
Ripk2	0.15	0.79628288	chicken
Sulf2	0.18	0.79645304	chicken
Zfp438	0.25	0.79693722	chicken
Fxyd6	0.33	0.79929177	chicken
Lamtor3	-0.34	0.81357821	chicken
Samsn1	-0.63	0.81926424	chicken
Rap2b	0.17	0.82235822	chicken
Aldh18a1	-0.45	0.82609072	chicken
Atg7	0.25	0.82803093	chicken
Cdkn1b	0.29	0.82866964	chicken
Lemd3	0.27	0.83060973	chicken
Axin1	0.20	0.84194925	chicken
Herc2	-0.33	0.86217033	chicken
Fam107b	0.56	0.86559969	chicken
Usp54	-0.47	0.86753489	chicken
Gbf1	0.24	0.89038178	chicken
Pigx	-0.20	0.89187668	chicken
Wasl	-0.37	0.90771354	chicken
Esf1	0.12	0.90925169	chicken
Faf1	0.31	0.91674148	chicken
Yars	0.11	0.91737187	chicken
Tle1	-0.24	0.92119541	chicken
Tpr	-0.47	0.92411515	chicken
Rheb	0.23	0.93273281	chicken
Pex13	0.12	0.93397166	chicken
Pla2g6	0.12	0.94442486	chicken
Efl1	0.22	0.94456489	chicken
Cdc25b	-0.32	0.95192918	chicken
Rab2a	-0.35	0.96310320	chicken
Dnaja4	-0.40	0.96688461	chicken
Sgpl1	-0.33	0.98624855	chicken
Lpxn	0.12	0.99742610	chicken
Timmdc1	-0.26	1.00000000	chicken
Luc7l2	0.23	1.00000000	chicken
Fam114a1	-0.34	1.00000000	chicken
Pitpna	0.28	1.00000000	chicken
Dnajc1	0.25	1.00000000	chicken
Nhp2	0.13	1.00000000	chicken
Sod2	-0.32	1.00000000	chicken
Arhgap11a	0.21	1.00000000	chicken
Cnn1	0.36	1.00000000	chicken
Slc2a3	-0.23	1.00000000	chicken
Rsl24d1	0.14	1.00000000	chicken
Adamts1	0.10	1.00000000	chicken

Rnf146	0.16	1.00000000	chicken
Zfp521	0.34	1.00000000	chicken
Smg6	0.09	1.00000000	chicken
Poglut1	-0.28	1.00000000	chicken
Coa5	-0.27	1.00000000	chicken
Rapgef2	-0.38	1.00000000	chicken
Rad18	-0.29	1.00000000	chicken
Ei24	0.13	1.00000000	chicken
Lsp1	-0.43	1.00000000	chicken
Ago1	0.08	1.00000000	chicken
Gtf2a2	-0.18	1.00000000	chicken
Dut	-0.23	1.00000000	chicken
Susd6	0.15	1.00000000	chicken
Tmem19	-0.26	1.00000000	chicken
Lamb2	0.22	1.00000000	chicken
Leo1	0.10	1.00000000	chicken
Wdr44	0.23	1.00000000	chicken
Bri3	-0.31	1.00000000	chicken
Nars	0.27	1.00000000	chicken
Jam3	0.41	1.00000000	chicken
Anks1b	-0.38	1.00000000	chicken
Tnks1bp1	0.20	1.00000000	chicken
Uqcc1	0.11	1.00000000	chicken
Eif3k	-0.69	1.00000000	chicken
Nol9	-0.33	1.00000000	chicken
Pum2	0.21	1.00000000	chicken
Cd2ap	0.39	1.00000000	chicken
Lrch1	-0.53	1.00000000	chicken
C1d	0.21	1.00000000	chicken
Azin1	0.22	1.00000000	chicken
Dis3l2	0.12	1.00000000	chicken
Snx8	0.21	1.00000000	chicken
Asb7	-0.30	1.00000000	chicken
Cacnb4	0.19	1.00000000	chicken
Epas1	-1.17	1.00000000	chicken
Rbsn	0.17	1.00000000	chicken
Ssrp1	0.13	1.00000000	chicken
Gk5	-0.21	1.00000000	chicken
Gnl2	0.14	1.00000000	chicken
Ndufab1	-0.34	1.00000000	chicken
Ciao2a	-0.24	1.00000000	chicken
Vapb	-0.36	1.00000000	chicken
Cramp1	0.24	1.00000000	chicken
Tmem79	-0.24	1.00000000	chicken
Hnrnph2	-0.24	1.00000000	chicken
Ptpra	0.28	1.00000000	chicken

Wdr47	-0.34	1.00000000	chicken
Tead4	0.18	1.00000000	chicken
Sap18	-0.33	1.00000000	chicken
Il15	-0.41	1.00000000	chicken
Plin4	-0.33	1.00000000	chicken
St3gal6	0.41	1.00000000	chicken
Rida	-0.22	1.00000000	chicken
Psmg1	-0.25	1.00000000	chicken
Slc30a5	-0.30	1.00000000	chicken
Anxa7	0.19	1.00000000	chicken
Commd4	-0.25	1.00000000	chicken
Cdc42se1	0.14	1.00000000	chicken
Zzz3	0.15	1.00000000	chicken
Bmp2	0.15	1.00000000	chicken
Rfxap	-0.19	1.00000000	chicken
Lamc1	0.20	1.00000000	chicken
Fbxw7	0.31	1.00000000	chicken
Ndufa2	-0.38	1.00000000	chicken
Arid2	0.35	1.00000000	chicken
Wnk1	-0.37	1.00000000	chicken
Gabpb1	0.10	1.00000000	chicken
Gas2	0.19	1.00000000	chicken
Pfdn2	-0.37	1.00000000	chicken
Inpp5a	0.30	1.00000000	chicken
D5Ertd579e	0.28	1.00000000	chicken
Nova1	-0.36	1.00000000	chicken
Syne3	-0.32	1.00000000	chicken
Ctdspl	0.28	1.00000000	chicken
Snrpg	0.17	1.00000000	chicken
Usp14	0.16	1.00000000	chicken
Pogz	0.13	1.00000000	chicken
Wdr82	0.10	1.00000000	chicken
Nedd9	0.34	1.00000000	chicken
Scarb2	-0.22	1.00000000	chicken
Capn11	0.17	1.00000000	chicken
Arpc4	-0.33	1.00000000	chicken
Itch	0.12	1.00000000	chicken
H1f10	-0.33	1.00000000	chicken
Qrich1	0.11	1.00000000	chicken
Anapc15-ps	-0.33	1.00000000	chicken
Capzb	0.17	1.00000000	chicken
Utp25	0.11	1.00000000	chicken
Atf1	0.08	1.00000000	chicken
Klf13	0.33	1.00000000	chicken
Vdac2	-0.30	1.00000000	chicken
Ube2v1	0.09	1.00000000	chicken

Ogdh	0.14	1.00000000	chicken
Unc45a	0.12	1.00000000	chicken
Phtf2	0.33	1.00000000	chicken
Crebrf	0.21	1.00000000	chicken
Tef	-0.42	1.00000000	chicken
Hook3	-0.39	1.00000000	chicken
Aopep	-0.39	1.00000000	chicken
Tmem237	-0.25	1.00000000	chicken
Dnajc2	0.12	1.00000000	chicken
Vgll3	0.24	1.00000000	chicken
Rbm14	-0.38	1.00000000	chicken
Pls3	-0.32	1.00000000	chicken
Ptpn21	-0.24	1.00000000	chicken
Eif6	0.13	1.00000000	chicken
Yipf4	-0.37	1.00000000	chicken
Plagl1	0.12	1.00000000	chicken
Cadm1	0.18	1.00000000	chicken
Pdha1	-0.23	1.00000000	chicken
Htra1	0.39	1.00000000	chicken
Rasa2	0.11	1.00000000	chicken
Cox10	0.24	1.00000000	chicken
Antxr1	-0.33	1.00000000	chicken
Focad	0.13	1.00000000	chicken
Rbm34	-0.31	1.00000000	chicken
Cul2	0.09	1.00000000	chicken
Phf3	0.18	1.00000000	chicken
Pank2	0.10	1.00000000	chicken
Cdc14a	-0.46	1.00000000	chicken
Cep112	-0.29	1.00000000	chicken
Uggt1	0.08	1.00000000	chicken
Fbxo25	-0.23	1.00000000	chicken
Pdcl	0.15	1.00000000	chicken
Apmap	-0.23	1.00000000	chicken
Stim2	-0.41	1.00000000	chicken
Zcchc10	0.15	1.00000000	chicken
Cd247	-0.58	1.00000000	chicken
Bag3	-0.03	1.00000000	chicken
Elof1	-0.28	1.00000000	chicken
Hnrnpab	-0.30	1.00000000	chicken
Prkx	0.27	1.00000000	chicken
Cbr1	-0.18	1.00000000	chicken
Pinx1	0.13	1.00000000	chicken
Fgl1	-0.33	1.00000000	chicken
Snrk	-0.35	1.00000000	chicken
Eya4	-0.37	1.00000000	chicken
9130409I23Rik	-0.37	1.00000000	chicken

Lsm14a	-0.35	1.00000000	chicken
Ubl3	0.32	1.00000000	chicken
Slc9a1	-0.15	1.00000000	chicken
Arhgef11	0.08	1.00000000	chicken
Igf2bp2	0.18	1.00000000	chicken
Rab30	-0.35	1.00000000	chicken
Tmem131l	-0.29	1.00000000	chicken
Wdr75	0.11	1.00000000	chicken
Spdl1	-0.22	1.00000000	chicken
Pkdcc	0.38	1.00000000	chicken
Abcd2	-0.36	1.00000000	chicken
Polr2e	-0.24	1.00000000	chicken
Stk10	-0.45	1.00000000	chicken
Cyp2d22	0.33	1.00000000	chicken
Rnf220	0.20	1.00000000	chicken
Tshz1	-0.37	1.00000000	chicken
Insr	0.21	1.00000000	chicken
Stau2	0.32	1.00000000	chicken
Ckb	0.03	1.00000000	chicken
Rnf144a	-0.44	1.00000000	chicken
Rif1	0.16	1.00000000	chicken
Tspan5	0.30	1.00000000	chicken
Cnksr2	-0.46	1.00000000	chicken
Tanc1	0.20	1.00000000	chicken
Elovl5	0.15	1.00000000	chicken
Uxs1	-0.39	1.00000000	chicken
Crk	0.06	1.00000000	chicken
Tmem35a	-0.24	1.00000000	chicken
Kbtbd8	0.11	1.00000000	chicken
Ptpn12	0.28	1.00000000	chicken
Fbn1	-0.15	1.00000000	chicken
Lgals8	0.19	1.00000000	chicken
Sema3f	0.09	1.00000000	chicken
Fbxo45	0.08	1.00000000	chicken
Dgcr6	-0.22	1.00000000	chicken
Fasn	0.17	1.00000000	chicken
Armh3	0.20	1.00000000	chicken
Rap1gds1	0.28	1.00000000	chicken
Golga4	0.26	1.00000000	chicken
Ppp1r10	0.15	1.00000000	chicken
Spats2	-0.25	1.00000000	chicken
Hikeshi	-0.23	1.00000000	chicken
Reep2	-0.29	1.00000000	chicken
Scfd1	0.18	1.00000000	chicken
Psma2	0.18	1.00000000	chicken
Zfp292	-0.40	1.00000000	chicken

Hic2	-0.38	1.00000000	chicken
Zfp148	0.15	1.00000000	chicken
Poc1b	-0.37	1.00000000	chicken
Cdh5	-0.54	1.00000000	chicken
Sprtn	0.06	1.00000000	chicken
Chm	0.13	1.00000000	chicken
Emilin2	0.38	1.00000000	chicken
Vezt	-0.32	1.00000000	chicken
Ahdc1	-0.34	1.00000000	chicken
Vcl	-0.15	1.00000000	chicken
Olfml3	-0.39	1.00000000	chicken
Rhoa	0.15	1.00000000	chicken
Klhl36	-0.22	1.00000000	chicken
Idh3a	0.11	1.00000000	chicken
Champ1	-0.34	1.00000000	chicken
Qpctl	-0.20	1.00000000	chicken
Ktn1	0.17	1.00000000	chicken
Ran	-0.33	1.00000000	chicken
Arhgap5	0.24	1.00000000	chicken
Dars	-0.29	1.00000000	chicken
Ahi1	-0.31	1.00000000	chicken
Ddhd1	0.10	1.00000000	chicken
Phf2	0.24	1.00000000	chicken
Slc66a2	0.28	1.00000000	chicken
Kdm4b	-0.36	1.00000000	chicken
Fam171a1	-0.47	1.00000000	chicken
Hspa12a	0.21	1.00000000	chicken
Prkar2a	-0.40	1.00000000	chicken
Zdhhc6	0.14	1.00000000	chicken
Pthlh	-0.36	1.00000000	chicken
Ube2i	0.07	1.00000000	chicken
Lbr	-0.40	1.00000000	chicken
Nmt1	0.07	1.00000000	chicken
Camsap2	0.25	1.00000000	chicken
Tnrc6b	-0.38	1.00000000	chicken
Csnk2a1	0.13	1.00000000	chicken
Naa50	0.19	1.00000000	chicken
BC051665	0.23	1.00000000	chicken
Bub3	0.09	1.00000000	chicken
Zmynd8	-0.16	1.00000000	chicken
Spast	-0.21	1.00000000	chicken
Usp53	0.23	1.00000000	chicken
Eif5	0.15	1.00000000	chicken
Cttnbp2	-0.40	1.00000000	chicken
Btf3	-0.42	1.00000000	chicken
B3galnt2	-0.27	1.00000000	chicken

Tomm70a	0.11	1.00000000	chicken
Lmbr1	-0.34	1.00000000	chicken
Tubgcp5	-0.21	1.00000000	chicken
Ctbp1	-0.34	1.00000000	chicken
Tmem178b	-0.35	1.00000000	chicken
BC048403	-0.24	1.00000000	chicken
Rdh10	-0.22	1.00000000	chicken
Thrap3	0.21	1.00000000	chicken
Enpp6	-0.30	1.00000000	chicken
Pigf	0.11	1.00000000	chicken
Dmd	-0.48	1.00000000	chicken
Wac	0.18	1.00000000	chicken
Tkt	0.24	1.00000000	chicken
Tubb4b	0.14	1.00000000	chicken
Pip5k1a	0.11	1.00000000	chicken
Bach2	-0.38	1.00000000	chicken
Cdkl5	-0.20	1.00000000	chicken
Sclt1	0.21	1.00000000	chicken
3110082I17Rik	-0.44	1.00000000	chicken
Erlin1	-0.24	1.00000000	chicken
Stt3a	-0.34	1.00000000	chicken
Nfrkb	0.05	1.00000000	chicken
Tpp2	-0.38	1.00000000	chicken
Tdrd7	0.20	1.00000000	chicken
Ppil1	-0.23	1.00000000	chicken
Nrbf2	-0.30	1.00000000	chicken
Usp34	0.13	1.00000000	chicken
Pltp	-0.24	1.00000000	chicken
Tmem87a	0.21	1.00000000	chicken
Prpf39	0.04	1.00000000	chicken
Brf1	0.28	1.00000000	chicken
Tmeff2	0.32	1.00000000	chicken
Dok6	-0.25	1.00000000	chicken
Ssbp1	-0.28	1.00000000	chicken
Thbs1	1.35	1.00000000	chicken
Atp6v0e	-0.28	1.00000000	chicken
Dtnbp1	-0.28	1.00000000	chicken
Fam104a	-0.28	1.00000000	chicken
Exosc7	0.04	1.00000000	chicken
Cdc42	0.20	1.00000000	chicken
Cdca7l	-0.19	1.00000000	chicken
Sav1	-0.31	1.00000000	chicken
Usf3	0.22	1.00000000	chicken
Calr	-0.28	1.00000000	chicken
Dcbld2	0.16	1.00000000	chicken
Acap2	-0.32	1.00000000	chicken

Septin11	-0.38	1.00000000	chicken
Zfp318	-0.40	1.00000000	chicken
Epha7	-0.30	1.00000000	chicken
Ankrd46	0.10	1.00000000	chicken
Sap130	0.08	1.00000000	chicken
Asns	0.13	1.00000000	chicken
Snx6	0.14	1.00000000	chicken
Btbd6	-0.34	1.00000000	chicken
Ech1	-0.25	1.00000000	chicken
Txndc11	0.13	1.00000000	chicken
Dnajc18	-0.31	1.00000000	chicken
Erlin2	-0.33	1.00000000	chicken
Pdhb	0.28	1.00000000	chicken
Kank4	-0.44	1.00000000	chicken
Tor1a	0.12	1.00000000	chicken
Nfkbib	0.08	1.00000000	chicken
Enah	0.36	1.00000000	chicken
Ccpg1	0.12	1.00000000	chicken
Psme3ip1	-0.28	1.00000000	chicken
Fbxl21	-0.16	1.00000000	chicken
Gyg	0.22	1.00000000	chicken
Serpinf2	0.08	1.00000000	chicken
Epc1	0.11	1.00000000	chicken
Lgr4	-0.40	1.00000000	chicken
Qdpr	-0.20	1.00000000	chicken
Borcs5	0.21	1.00000000	chicken
AU040320	0.11	1.00000000	chicken
Exoc8	0.13	1.00000000	chicken
Vipr1	0.27	1.00000000	chicken
Derl1	0.11	1.00000000	chicken
Gtdc1	0.32	1.00000000	chicken
Ccng1	-0.22	1.00000000	chicken
Syne2	-0.37	1.00000000	chicken
Coq8a	-0.29	1.00000000	chicken
Exoc2	0.21	1.00000000	chicken
Pptc7	0.08	1.00000000	chicken
Nsun2	0.13	1.00000000	chicken
Col14a1	0.17	1.00000000	chicken
Rnf4	0.10	1.00000000	chicken
Pcca	0.18	1.00000000	chicken
Ncoa6	0.07	1.00000000	chicken
Desi2	0.14	1.00000000	chicken
Eloc	0.16	1.00000000	chicken
Tspan3	0.15	1.00000000	chicken
Nid1	-0.39	1.00000000	chicken
Usp24	0.20	1.00000000	chicken

Vps4b	0.10	1.00000000	chicken
Nop58	0.21	1.00000000	chicken
Dlgap4	-0.44	1.00000000	chicken
Cox19	-0.22	1.00000000	chicken
Adgrl2	0.54	1.00000000	chicken
Ppp1r12a	-0.21	1.00000000	chicken
Phf12	-0.39	1.00000000	chicken
Ssbp2	-0.29	1.00000000	chicken
Ube2e3	-0.31	1.00000000	chicken
Vps16	-0.20	1.00000000	chicken
Bfar	0.10	1.00000000	chicken
Zbtb20	0.15	1.00000000	chicken
Tab2	0.07	1.00000000	chicken
Srpk2	-0.32	1.00000000	chicken
Bag5	0.10	1.00000000	chicken
Bmi1	-0.41	1.00000000	chicken
Sparc	-0.13	1.00000000	chicken
Efemp1	-0.28	1.00000000	chicken
Ndufa10	-0.31	1.00000000	chicken
Nup98	0.09	1.00000000	chicken
Tulp4	0.22	1.00000000	chicken
Dcaf1	0.17	1.00000000	chicken
Fzd4	-0.30	1.00000000	chicken
Nat10	0.11	1.00000000	chicken
Trappc12	0.10	1.00000000	chicken
Ppp1r8	0.10	1.00000000	chicken
Sbno1	0.12	1.00000000	chicken
Gpbp1l1	0.12	1.00000000	chicken
Rac1	0.17	1.00000000	chicken
Rbl1	0.20	1.00000000	chicken
Cnih4	-0.18	1.00000000	chicken
Ftsj3	0.11	1.00000000	chicken
Ddx42	0.05	1.00000000	chicken
Fndc3b	0.12	1.00000000	chicken
Sh3d19	0.29	1.00000000	chicken
Atad2b	-0.30	1.00000000	chicken
Lyrm1	0.09	1.00000000	chicken
Sh3glb1	0.19	1.00000000	chicken
Top3b	0.06	1.00000000	chicken
Pwwp4a	0.11	1.00000000	chicken
Tiparp	-0.43	1.00000000	chicken
Atxn7	0.22	1.00000000	chicken
Atp5b	-0.23	1.00000000	chicken
Impdh2	0.14	1.00000000	chicken
Klhdc1	-0.26	1.00000000	chicken
Rexo2	-0.25	1.00000000	chicken

Gnpnat1	0.06	1.00000000	chicken
Tspan6	-0.15	1.00000000	chicken
Nol10	0.15	1.00000000	chicken
Rapgef5	0.05	1.00000000	chicken
Lrpprc	0.13	1.00000000	chicken
Tspan18	0.27	1.00000000	chicken
Atad1	0.13	1.00000000	chicken
Abhd2	0.07	1.00000000	chicken
Rabgap1l	-0.43	1.00000000	chicken
Gpatch2	0.18	1.00000000	chicken
Phf5a	0.07	1.00000000	chicken
Jmy	-0.23	1.00000000	chicken
Srp72	-0.33	1.00000000	chicken
Drosha	0.06	1.00000000	chicken
Dcp1a	0.07	1.00000000	chicken
U2surp	0.12	1.00000000	chicken
Pgd	-0.31	1.00000000	chicken
Usp33	0.09	1.00000000	chicken
Fundc1	-0.23	1.00000000	chicken
Sil1	-0.26	1.00000000	chicken
Eif2a	0.15	1.00000000	chicken
Ppp2r2a	0.11	1.00000000	chicken
Nedd8	-0.29	1.00000000	chicken
Wars	0.11	1.00000000	chicken
Tax1bp3	0.14	1.00000000	chicken
Hprt	0.11	1.00000000	chicken
Cebpd	0.13	1.00000000	chicken
Papln	-0.36	1.00000000	chicken
Pdia6	-0.38	1.00000000	chicken
Sdhc	-0.34	1.00000000	chicken
Ankrd50	-0.30	1.00000000	chicken
Ube4b	-0.27	1.00000000	chicken
Aurkaip1	0.14	1.00000000	chicken
Grk4	-0.27	1.00000000	chicken
Kat2a	0.08	1.00000000	chicken
Grem2	0.80	1.00000000	chicken
Eif2b2	0.10	1.00000000	chicken
Zdhhc3	-0.28	1.00000000	chicken
Tesk2	-0.21	1.00000000	chicken
Nrbp1	0.08	1.00000000	chicken
Fn3krp	0.09	1.00000000	chicken
Aak1	0.08	1.00000000	chicken
Zfat	0.22	1.00000000	chicken
Pla2g7	-0.23	1.00000000	chicken
Ggps1	-0.31	1.00000000	chicken
Snrpe	-0.25	1.00000000	chicken

Rab4a	-0.26	1.00000000	chicken
Wdr86	0.10	1.00000000	chicken
Rpia	-0.32	1.00000000	chicken
Abca12	-0.36	1.00000000	chicken
Anp32e	-0.31	1.00000000	chicken
Wdr1	0.20	1.00000000	chicken
Daam1	0.28	1.00000000	chicken
Pde1a	-0.30	1.00000000	chicken
Pphln1	0.24	1.00000000	chicken
Pnpla2	-0.22	1.00000000	chicken
Jpt2	-0.20	1.00000000	chicken
Swap70	0.15	1.00000000	chicken
Fahd2a	-0.20	1.00000000	chicken
Actr6	-0.32	1.00000000	chicken
Carhsp1	0.35	1.00000000	chicken
Csrp1	-0.02	1.00000000	chicken
Farp2	0.16	1.00000000	chicken
Nipsnap2	-0.31	1.00000000	chicken
Prmt7	0.34	1.00000000	chicken
Hmgb3	-0.26	1.00000000	chicken
Samd12	0.27	1.00000000	chicken
Ndst4	0.22	1.00000000	chicken
Fbxw5	0.08	1.00000000	chicken
Pspc1	-0.36	1.00000000	chicken
Ndufv2	-0.21	1.00000000	chicken
Capza2	0.12	1.00000000	chicken
Itgb6	-0.20	1.00000000	chicken
Arhgap31	0.22	1.00000000	chicken
Eif3j2	0.13	1.00000000	chicken
Zfp516	0.36	1.00000000	chicken
Klhdc4	0.17	1.00000000	chicken
Tspan14	0.14	1.00000000	chicken
Kras	0.30	1.00000000	chicken
Dennd5a	0.13	1.00000000	chicken
Nkd1	-0.28	1.00000000	chicken
Herpud1	-0.24	1.00000000	chicken
Hnrnpk	-0.35	1.00000000	chicken
Zc3h15	-0.43	1.00000000	chicken
Adam23	-0.26	1.00000000	chicken
Usp38	-0.28	1.00000000	chicken
Rai2	0.12	1.00000000	chicken
Adk	-0.58	1.00000000	chicken
Ppp3cb	0.11	1.00000000	chicken
Slmap	0.16	1.00000000	chicken
Lsm5	-0.19	1.00000000	chicken
Snx9	-0.36	1.00000000	chicken

Zfp703	-0.24	1.00000000	chicken
Ppif	-0.24	1.00000000	chicken
Chpt1	-0.16	1.00000000	chicken
Dnajc5	0.13	1.00000000	chicken
Spire1	0.11	1.00000000	chicken
Klf9	-0.28	1.00000000	chicken
Ddrgk1	-0.30	1.00000000	chicken
Rpf2	0.12	1.00000000	chicken
Zcchc7	0.20	1.00000000	chicken
Gcn1	-0.27	1.00000000	chicken
Ppp2cb	0.07	1.00000000	chicken
Dab2ip	-0.46	1.00000000	chicken
Igfbp5	-0.52	1.00000000	chicken
Fbxw11	0.18	1.00000000	chicken
Tmco1	0.11	1.00000000	chicken
B230219D22Rik	-0.30	1.00000000	chicken
Nek1	-0.24	1.00000000	chicken
Ttyh3	-0.20	1.00000000	chicken
Tmem254a	0.21	1.00000000	chicken
Nr2f1	-0.24	1.00000000	chicken
Cyria	-0.51	1.00000000	chicken
Phlda2	0.30	1.00000000	chicken
Socs4	0.03	1.00000000	chicken
Ahctf1	0.07	1.00000000	chicken
Clip1	-0.31	1.00000000	chicken
Klhl20	0.13	1.00000000	chicken
Tmed5	0.05	1.00000000	chicken
Arhgap6	1.13	1.00000000	chicken
Zyx	0.18	1.00000000	chicken
Gdi2	-0.36	1.00000000	chicken
Prkag2	0.27	1.00000000	chicken
Edf1	-0.37	1.00000000	chicken
Cfd	-0.04	1.00000000	chicken
Acsl3	-0.20	1.00000000	chicken
Cwc15	-0.33	1.00000000	chicken
Khdrbs1	-0.27	1.00000000	chicken
Rbis	0.16	1.00000000	chicken
Rpap2	0.10	1.00000000	chicken
Tmbim1	0.15	1.00000000	chicken
E2f5	0.12	1.00000000	chicken
Ppp1cc	-0.30	1.00000000	chicken
Pwp1	0.10	1.00000000	chicken
Gnai2	0.07	1.00000000	chicken
Rara	-0.20	1.00000000	chicken
Pawr	0.16	1.00000000	chicken
Tmem41b	0.14	1.00000000	chicken

Szt2	0.12	1.00000000	chicken
Trpc1	-0.21	1.00000000	chicken
Gpatch4	0.14	1.00000000	chicken
Adh5	-0.31	1.00000000	chicken
Pla2g4a	-0.21	1.00000000	chicken
Ciao2b	-0.22	1.00000000	chicken
Slc7a6os	0.10	1.00000000	chicken
Rnf214	-0.25	1.00000000	chicken
Rspry1	0.02	1.00000000	chicken
H2ac20	0.16	1.00000000	chicken
Smarcb1	-0.17	1.00000000	chicken
Phactr2	-0.33	1.00000000	chicken
Stx17	-0.06	1.00000000	chicken
Aldh7a1	-0.20	1.00000000	chicken
Tctn3	-0.22	1.00000000	chicken
Ntng1	-0.20	1.00000000	chicken
Atl2	0.11	1.00000000	chicken
Gramd4	0.21	1.00000000	chicken
G3bp2	0.15	1.00000000	chicken
Becn2	0.09	1.00000000	chicken
Tafa5	0.71	1.00000000	chicken
C77080	0.03	1.00000000	chicken
Psma6	0.18	1.00000000	chicken
Fkbp4	-0.41	1.00000000	chicken
Ndufa1	-0.25	1.00000000	chicken
Ryk	0.38	1.00000000	chicken
Tmem43	-0.20	1.00000000	chicken
Uhrf1bp1l	0.36	1.00000000	chicken
Tbx2	-0.31	1.00000000	chicken
Gabarapl1	0.21	1.00000000	chicken
Ccdc25	-0.28	1.00000000	chicken
Polk	0.10	1.00000000	chicken
Dnajc3	-0.31	1.00000000	chicken
Gucy1b1	-0.38	1.00000000	chicken
Fhl3	-0.31	1.00000000	chicken
Wls	-0.37	1.00000000	chicken
Supt4a	-0.26	1.00000000	chicken
Tpm3-rs7	-0.56	1.00000000	chicken
Wdr70	0.19	1.00000000	chicken
Snapin	-0.21	1.00000000	chicken
Jph1	0.23	1.00000000	chicken
Btbd11	-0.56	1.00000000	chicken
Ccdc186	-0.21	1.00000000	chicken
Dag1	-0.33	1.00000000	chicken
Ints6l	0.05	1.00000000	chicken
Lin7c	0.01	1.00000000	chicken

Reep3	0.08	1.00000000	chicken
Oaz2	-0.22	1.00000000	chicken
Egr1	0.14	1.00000000	chicken
Usp7	-0.35	1.00000000	chicken
Slc25a24	0.20	1.00000000	chicken
Dhx29	-0.25	1.00000000	chicken
Lsg1	0.13	1.00000000	chicken
Rybp	-0.27	1.00000000	chicken
Uqcrq	-0.26	1.00000000	chicken
Fkbp8	0.09	1.00000000	chicken
Zfand5	0.07	1.00000000	chicken
Eng	-0.26	1.00000000	chicken
Ifnar2	0.16	1.00000000	chicken
Taf3	0.19	1.00000000	chicken
Pofut1	0.09	1.00000000	chicken
Stk17b	-0.25	1.00000000	chicken
Ddost	-0.32	1.00000000	chicken
Ifrd1	0.33	1.00000000	chicken
Insig1	-0.07	1.00000000	chicken
Vcpip1	0.09	1.00000000	chicken
Csnk1d	0.09	1.00000000	chicken
Vps26c	-0.21	1.00000000	chicken
Gtpbp4	0.14	1.00000000	chicken
Srsf1	0.15	1.00000000	chicken
Tmem64	0.09	1.00000000	chicken
Ublcp1	0.09	1.00000000	chicken
Slc39a9	0.11	1.00000000	chicken
Lvrn	0.30	1.00000000	chicken
Decr1	0.06	1.00000000	chicken
Uhmk1	0.08	1.00000000	chicken
Cnot2	0.24	1.00000000	chicken
Xpa	0.11	1.00000000	chicken
Pds5a	-0.26	1.00000000	chicken
Npr2	0.10	1.00000000	chicken
Pou2f1	0.06	1.00000000	chicken
Iqgap1	0.19	1.00000000	chicken
Setd2	0.10	1.00000000	chicken
Aftph	-0.22	1.00000000	chicken
Wdr61	0.09	1.00000000	chicken
Klf3	0.17	1.00000000	chicken
Acbd5	-0.37	1.00000000	chicken
Fam168b	0.14	1.00000000	chicken
Ptrh2	-0.30	1.00000000	chicken
Dhx15	0.08	1.00000000	chicken
Trappc9	0.18	1.00000000	chicken
Naaladl2	-0.23	1.00000000	chicken

Armc1	0.12	1.00000000	chicken
Setd5	0.07	1.00000000	chicken
Seh1l	0.05	1.00000000	chicken
Xpot	-0.19	1.00000000	chicken
Rtf1	-0.40	1.00000000	chicken
Ppfibp2	0.28	1.00000000	chicken
Aprt	0.13	1.00000000	chicken
Zdhhc5	0.04	1.00000000	chicken
Hhip	-0.59	1.00000000	chicken
Srebf1	0.18	1.00000000	chicken
Crebbp	0.20	1.00000000	chicken
Camkmt	-0.38	1.00000000	chicken
Zfp512b	-0.26	1.00000000	chicken
Ufm1	0.09	1.00000000	chicken
Nbas	-0.29	1.00000000	chicken
Cntf	0.17	1.00000000	chicken
Rras2	0.14	1.00000000	chicken
Nudt14	0.21	1.00000000	chicken
Pnn	-0.37	1.00000000	chicken
Nucb2	0.22	1.00000000	chicken
Ggnbp2	0.10	1.00000000	chicken
Ccn3	1.13	1.00000000	chicken
Dlst	0.10	1.00000000	chicken
Cwc27	0.11	1.00000000	chicken
Nsa2	-0.37	1.00000000	chicken
Uri1	0.23	1.00000000	chicken
Ccdc32	-0.15	1.00000000	chicken
Atg9a	0.06	1.00000000	chicken
Psmc2	0.05	1.00000000	chicken
Eps15l1	0.15	1.00000000	chicken
Slc38a10	0.03	1.00000000	chicken
Upf1	0.08	1.00000000	chicken
Cog7	-0.22	1.00000000	chicken
Pde6d	-0.27	1.00000000	chicken
Surf4	-0.30	1.00000000	chicken
Ldb2	0.48	1.00000000	chicken
Cope	-0.30	1.00000000	chicken
Btrc	0.10	1.00000000	chicken
Psme4	0.45	1.00000000	chicken
Atp6ap2	-0.20	1.00000000	chicken
Dock9	-0.24	1.00000000	chicken
Pdxdc1	-0.36	1.00000000	chicken
G2e3	-0.22	1.00000000	chicken
Efnb2	-0.31	1.00000000	chicken
Znhit3	-0.21	1.00000000	chicken
Ccnd2	-0.28	1.00000000	chicken

Lonp1	0.09	1.00000000	chicken
Ppp2r2d	0.10	1.00000000	chicken
Ttl	-0.23	1.00000000	chicken
Atg2b	0.27	1.00000000	chicken
Uqcc2	0.10	1.00000000	chicken
Zbtb7a	0.07	1.00000000	chicken
Cops7a	-0.35	1.00000000	chicken
Cct4	0.06	1.00000000	chicken
Gsn	-0.49	1.00000000	chicken
Dnajc11	0.01	1.00000000	chicken
Boc	-0.31	1.00000000	chicken
Nr3c1	0.29	1.00000000	chicken
Sik2	0.23	1.00000000	chicken
Ncoa2	-0.32	1.00000000	chicken
Stx8	0.07	1.00000000	chicken
Ube2o	0.14	1.00000000	chicken
Erlec1	0.11	1.00000000	chicken
Tasor	-0.28	1.00000000	chicken
Crebl2	-0.13	1.00000000	chicken
Ccser2	0.35	1.00000000	chicken
Nfyb	-0.25	1.00000000	chicken
Ndfip1	-0.34	1.00000000	chicken
Folh1	-0.26	1.00000000	chicken
Fas	0.08	1.00000000	chicken
Dctn3	-0.25	1.00000000	chicken
Slc7a5	0.34	1.00000000	chicken
Ubp1	0.16	1.00000000	chicken
Txndc9	0.12	1.00000000	chicken
Cers5	0.07	1.00000000	chicken
Wwtr1	0.21	1.00000000	chicken
Akap8	-0.24	1.00000000	chicken
Nrip1	0.15	1.00000000	chicken
Ciapin1	0.08	1.00000000	chicken
Dkc1	0.13	1.00000000	chicken
Casp3	0.25	1.00000000	chicken
Cmtm3	-0.29	1.00000000	chicken
Acbd7	0.25	1.00000000	chicken
B4galt2	-0.16	1.00000000	chicken
Stau1	-0.28	1.00000000	chicken
Otud3	0.05	1.00000000	chicken
Alkbh1	0.09	1.00000000	chicken
5031439G07Rik	0.13	1.00000000	chicken
Zfpm2	0.29	1.00000000	chicken
Cct8	0.10	1.00000000	chicken
Fbxo9	-0.25	1.00000000	chicken
Nfe2l1	0.13	1.00000000	chicken

Vkorc1l1	-0.01	1.00000000	chicken
Nfs1	0.03	1.00000000	chicken
Epb41l5	-0.24	1.00000000	chicken
Atxn10	-0.20	1.00000000	chicken
Edem1	0.08	1.00000000	chicken
Agps	-0.19	1.00000000	chicken
Agl	-0.10	1.00000000	chicken
Birc6	-0.30	1.00000000	chicken
Atf7ip	0.23	1.00000000	chicken
Polr2l	-0.32	1.00000000	chicken
Gpd2	0.12	1.00000000	chicken
Zcrb1	-0.36	1.00000000	chicken
Krit1	-0.16	1.00000000	chicken
Dip2a	0.11	1.00000000	chicken
Gas6	-0.20	1.00000000	chicken
Polr2m	0.06	1.00000000	chicken
Nt5c3	0.16	1.00000000	chicken
Sdk2	-0.21	1.00000000	chicken
Skap2	0.33	1.00000000	chicken
Hmbox1	-0.24	1.00000000	chicken
Slc2a8	-0.34	1.00000000	chicken
S100a10	0.20	1.00000000	chicken
Zfyve1	0.08	1.00000000	chicken
Sptlc1	0.03	1.00000000	chicken
Tmem50a	-0.25	1.00000000	chicken
Lrrk1	0.24	1.00000000	chicken
Sorl1	-0.18	1.00000000	chicken
Dnajb1	0.03	1.00000000	chicken
Tnks2	0.12	1.00000000	chicken
Cntnap1	-0.19	1.00000000	chicken
Nelfe	-0.14	1.00000000	chicken
Tmem39a	0.02	1.00000000	chicken
Skp1	-0.35	1.00000000	chicken
Lrrc28	-0.21	1.00000000	chicken
Golga1	-0.23	1.00000000	chicken
Cln6	-0.17	1.00000000	chicken
Plag1	-0.27	1.00000000	chicken
Scfd2	-0.30	1.00000000	chicken
Smurf2	0.09	1.00000000	chicken
Fam133b	0.00	1.00000000	chicken
Crnkl1	0.05	1.00000000	chicken
Ski	-0.19	1.00000000	chicken
Hoxb2	0.00	1.00000000	chicken
Plxdc2	0.45	1.00000000	chicken
Cradd	-0.28	1.00000000	chicken
Epha4	0.31	1.00000000	chicken

Wdr13	-0.24	1.00000000	chicken
Exosc3	0.04	1.00000000	chicken
Hk1	-0.22	1.00000000	chicken
Sart3	0.12	1.00000000	chicken
Wdr20	-0.42	1.00000000	chicken
Lmbrd2	0.10	1.00000000	chicken
Slc44a1	0.20	1.00000000	chicken
Smdt1	0.09	1.00000000	chicken
As3mt	-0.14	1.00000000	chicken
Zfp652	-0.24	1.00000000	chicken
Xpnpep1	0.17	1.00000000	chicken
Poldip3	0.11	1.00000000	chicken
Sod1	0.19	1.00000000	chicken
Inpp4a	-0.36	1.00000000	chicken
Tmem181a	0.11	1.00000000	chicken
Fam20a	-0.22	1.00000000	chicken
Pmpcb	0.06	1.00000000	chicken
Trub1	0.10	1.00000000	chicken
Rftn2	-0.18	1.00000000	chicken
Srsf4	-0.23	1.00000000	chicken
Numb	0.26	1.00000000	chicken
Rabl6	0.09	1.00000000	chicken
Kcnq5	-0.25	1.00000000	chicken
Gars	0.00	1.00000000	chicken
Bbx	0.16	1.00000000	chicken
Rad23b	-0.32	1.00000000	chicken
Pigs	-0.28	1.00000000	chicken
Dhx38	-0.24	1.00000000	chicken
Car5b	-0.20	1.00000000	chicken
9930021J03Rik	-0.25	1.00000000	chicken
Slc9a8	0.13	1.00000000	chicken
Btbd7	0.10	1.00000000	chicken
Trrap	0.05	1.00000000	chicken
Nenf	-0.20	1.00000000	chicken
Aldh1a2	-0.23	1.00000000	chicken
Psen1	0.09	1.00000000	chicken
Adamts19	-0.37	1.00000000	chicken
Slc9a3r1	-0.25	1.00000000	chicken
Aven	0.20	1.00000000	chicken
Dlg5	-0.22	1.00000000	chicken
Hmces	-0.13	1.00000000	chicken
Setd1b	-0.20	1.00000000	chicken
Golt1b	0.04	1.00000000	chicken
Sdhb	-0.16	1.00000000	chicken
Lancl2	-0.13	1.00000000	chicken
Trappc8	0.08	1.00000000	chicken

Dld	0.04	1.00000000	chicken
Ssr3	-0.29	1.00000000	chicken
Vps39	-0.20	1.00000000	chicken
Eci1	-0.19	1.00000000	chicken
Ostf1	0.09	1.00000000	chicken
Nudcd2	0.09	1.00000000	chicken
Prickle2	-0.27	1.00000000	chicken
Tmem131	-0.23	1.00000000	chicken
Fbxo32	0.04	1.00000000	chicken
Cask	0.22	1.00000000	chicken
Arvcf	-0.14	1.00000000	chicken
Serinc5	-0.09	1.00000000	chicken
Fnbp1l	-0.16	1.00000000	chicken
Fyco1	-0.16	1.00000000	chicken
Vps29	0.02	1.00000000	chicken
Timm50	-0.25	1.00000000	chicken
Gtf2a1	-0.28	1.00000000	chicken
Fnbp1	-0.25	1.00000000	chicken
Pkd1	-0.35	1.00000000	chicken
Selenot	0.12	1.00000000	chicken
Ptges3	0.01	1.00000000	chicken
Kat6a	0.09	1.00000000	chicken
Cadm3	0.08	1.00000000	chicken
Arih1	0.22	1.00000000	chicken
Btf3l4	0.06	1.00000000	chicken
Eif3a	-0.24	1.00000000	chicken
Snx29	-0.33	1.00000000	chicken
Yaf2	-0.27	1.00000000	chicken
Rad21	-0.30	1.00000000	chicken
Lnpk	0.05	1.00000000	chicken
Rab27a	-0.30	1.00000000	chicken
Zfp592	0.04	1.00000000	chicken
Dnajc19	-0.22	1.00000000	chicken
Naa20	-0.18	1.00000000	chicken
Ogt	-0.28	1.00000000	chicken
Pik3r4	0.02	1.00000000	chicken
Ntrk3	-0.16	1.00000000	chicken
Trim8	-0.03	1.00000000	chicken
Ssbp3	-0.32	1.00000000	chicken
Brd8	0.04	1.00000000	chicken
Hadha	0.07	1.00000000	chicken
Irf2bp2	-0.26	1.00000000	chicken
Clcn3	0.11	1.00000000	chicken
Rab34	-0.22	1.00000000	chicken
Eloa	0.05	1.00000000	chicken
Ttbk1	-0.34	1.00000000	chicken

Rpa1	0.13	1.00000000	chicken
Fkbp14	-0.25	1.00000000	chicken
Ezh2	-0.38	1.00000000	chicken
Taz	-0.18	1.00000000	chicken
Chd1	0.12	1.00000000	chicken
Rev1	-0.16	1.00000000	chicken
Cnpy3	-0.15	1.00000000	chicken
Wdr81	-0.19	1.00000000	chicken
Ube2h	0.14	1.00000000	chicken
Cpeb2	-0.27	1.00000000	chicken
Cdc40	0.11	1.00000000	chicken
Sf3b1	-0.31	1.00000000	chicken
Pdcd5	0.08	1.00000000	chicken
Rusc2	0.19	1.00000000	chicken
C1qtnf2	-0.04	1.00000000	chicken
Szrd1	0.05	1.00000000	chicken
Hs6st2	0.23	1.00000000	chicken
Rpp30	-0.01	1.00000000	chicken
Usp45	0.07	1.00000000	chicken
Stt3b	0.02	1.00000000	chicken
Tlk1	-0.24	1.00000000	chicken
Epha3	-0.18	1.00000000	chicken
Eif2s1	0.02	1.00000000	chicken
Oit3	0.16	1.00000000	chicken
Dpyd	0.25	1.00000000	chicken
Ppm1b	-0.31	1.00000000	chicken
Ing3	0.01	1.00000000	chicken
Tbp	0.07	1.00000000	chicken
Sptlc2	0.03	1.00000000	chicken
Fut11	0.08	1.00000000	chicken
Fbxl3	0.09	1.00000000	chicken
Aebp2	-0.30	1.00000000	chicken
Nop2	0.07	1.00000000	chicken
Chst10	-0.22	1.00000000	chicken
Nufip1	0.00	1.00000000	chicken
Hddc2	-0.27	1.00000000	chicken
Soga1	-0.25	1.00000000	chicken
Cacybp	0.16	1.00000000	chicken
Zfp644	0.18	1.00000000	chicken
Crls1	-0.24	1.00000000	chicken
Spag9	-0.45	1.00000000	chicken
Glg1	-0.27	1.00000000	chicken
Git1	0.01	1.00000000	chicken
Lrrc8a	0.09	1.00000000	chicken
Snrpd1	-0.25	1.00000000	chicken
Lrwd1	-0.18	1.00000000	chicken

Zfp638	0.07	1.00000000	chicken
Tmem101	0.02	1.00000000	chicken
Trip12	0.14	1.00000000	chicken
Gnai3	0.11	1.00000000	chicken
Arl6ip1	-0.17	1.00000000	chicken
Cul1	0.01	1.00000000	chicken
Tmem184c	-0.26	1.00000000	chicken
Chmp6	-0.23	1.00000000	chicken
Nutf2	-0.20	1.00000000	chicken
Eif3i	-0.34	1.00000000	chicken
Ppara	-0.26	1.00000000	chicken
Atp6v1b2	0.01	1.00000000	chicken
Nr4a2	0.25	1.00000000	chicken
Luzp1	0.02	1.00000000	chicken
Pcgf3	0.07	1.00000000	chicken
Sgpp1	0.08	1.00000000	chicken
Ppm1g	0.00	1.00000000	chicken
Pak1	-0.22	1.00000000	chicken
Ppp3ca	-0.06	1.00000000	chicken
Nck2	0.21	1.00000000	chicken
Arglu1	-0.28	1.00000000	chicken
Ddr2	0.09	1.00000000	chicken
Rbfox2	-0.24	1.00000000	chicken
Nup153	0.06	1.00000000	chicken
Prss45	-0.38	1.00000000	chicken
Zrsr1	0.07	1.00000000	chicken
Arpp19	0.10	1.00000000	chicken
Pbxip1	-0.17	1.00000000	chicken
Pappa	-0.20	1.00000000	chicken
Khdc4	0.06	1.00000000	chicken
Slit3	0.86	1.00000000	chicken
Emc1	0.01	1.00000000	chicken
Ankrd13a	-0.24	1.00000000	chicken
Efr3a	-0.19	1.00000000	chicken
Iws1	-0.35	1.00000000	chicken
Sco1	0.03	1.00000000	chicken
St6galnac4	-0.17	1.00000000	chicken
Sos2	0.12	1.00000000	chicken
Tmem223	-0.14	1.00000000	chicken
Ash1l	-0.34	1.00000000	chicken
Carmil1	0.32	1.00000000	chicken
Rassf1	0.15	1.00000000	chicken
Hace1	0.06	1.00000000	chicken
P4ha1	-0.26	1.00000000	chicken
Gtf3c1	-0.02	1.00000000	chicken
Hspe1-rs1	-0.12	1.00000000	chicken

Snrpb2	0.10	1.00000000	chicken
Znhit6	0.03	1.00000000	chicken
Ext2	0.05	1.00000000	chicken
Lhfp	-0.09	1.00000000	chicken
Prpf40a	0.22	1.00000000	chicken
Btg1b	-0.17	1.00000000	chicken
Tbc1d15	-0.28	1.00000000	chicken
Cdc42se2	-0.23	1.00000000	chicken
Gpcpd1	0.12	1.00000000	chicken
Pik3cg	-0.21	1.00000000	chicken
Hk2	0.07	1.00000000	chicken
Zbtb1	0.05	1.00000000	chicken
Crispld1	-0.23	1.00000000	chicken
Cdk12	0.06	1.00000000	chicken
Fra10ac1	-0.22	1.00000000	chicken
Usp6nl	-0.25	1.00000000	chicken
Ttll12	-0.23	1.00000000	chicken
Galnt1	-0.15	1.00000000	chicken
Jak1	0.05	1.00000000	chicken
Ccdc47	0.07	1.00000000	chicken
Vdac1	-0.49	1.00000000	chicken
Fgd6	-0.13	1.00000000	chicken
Suds3	0.17	1.00000000	chicken
Glrx5	0.09	1.00000000	chicken
Scoc	0.06	1.00000000	chicken
Tent5a	-0.42	1.00000000	chicken
Nsmce2	-0.25	1.00000000	chicken
Napa	0.06	1.00000000	chicken
Pcid2	-0.20	1.00000000	chicken
Vcan	0.55	1.00000000	chicken
Fbxo30	0.00	1.00000000	chicken
Ier5	-0.23	1.00000000	chicken
Usp37	0.08	1.00000000	chicken
Rufy1	-0.25	1.00000000	chicken
Angel1	-0.21	1.00000000	chicken
Igf2r	0.10	1.00000000	chicken
Isca2	-0.23	1.00000000	chicken
Phldb2	0.21	1.00000000	chicken
Gatad2a	0.02	1.00000000	chicken
Dctn2	-0.20	1.00000000	chicken
Jpt1	0.10	1.00000000	chicken
Jagn1	0.07	1.00000000	chicken
Cyb5a	-0.30	1.00000000	chicken
Grsf1	0.05	1.00000000	chicken
Kansl1	0.06	1.00000000	chicken
Gadd45a	0.10	1.00000000	chicken

Glb1	-0.22	1.00000000	chicken
Scaper	0.08	1.00000000	chicken
Hscb	-0.25	1.00000000	chicken
Zfp143	0.04	1.00000000	chicken
Cd276	-0.21	1.00000000	chicken
Papss1	-0.22	1.00000000	chicken
Gid8	0.05	1.00000000	chicken
Itpk1	-0.21	1.00000000	chicken
Atg10	0.22	1.00000000	chicken
Qser1	-0.37	1.00000000	chicken
Cab39	0.08	1.00000000	chicken
Lzic	-0.18	1.00000000	chicken
Caprin1	-0.01	1.00000000	chicken
Htatsf1	-0.16	1.00000000	chicken
Slc25a16	0.04	1.00000000	chicken
Cpsf4l	0.16	1.00000000	chicken
Gadd45b	-0.08	1.00000000	chicken
Akap7	-0.23	1.00000000	chicken
Hoxb3	0.08	1.00000000	chicken
Dnajc7	0.06	1.00000000	chicken
Rab35	-0.26	1.00000000	chicken
Erp44	0.14	1.00000000	chicken
Cap2	-0.14	1.00000000	chicken
Ap2m1	-0.28	1.00000000	chicken
Cbfb	0.27	1.00000000	chicken
Cish	-0.32	1.00000000	chicken
Lpin1	-0.45	1.00000000	chicken
Zfp830	0.08	1.00000000	chicken
Tmem185a	0.12	1.00000000	chicken
Pomp	0.10	1.00000000	chicken
Vti1a	0.30	1.00000000	chicken
Kpnb1	0.08	1.00000000	chicken
Xpo5	-0.22	1.00000000	chicken
Hic1	-0.24	1.00000000	chicken
Yipf1	-0.13	1.00000000	chicken
Pdzd8	0.10	1.00000000	chicken
Dusp16	0.09	1.00000000	chicken
Rbm6	0.07	1.00000000	chicken
Ube2e1	0.09	1.00000000	chicken
Ube2g1	0.02	1.00000000	chicken
Ppip5k2	-0.27	1.00000000	chicken
Hlx	-0.02	1.00000000	chicken
Dbr1	0.02	1.00000000	chicken
Tenm2	0.18	1.00000000	chicken
Gpr137b	0.10	1.00000000	chicken
Zfp827	-0.14	1.00000000	chicken

Slc41a2	0.08	1.00000000	chicken
Apoo	0.05	1.00000000	chicken
Trp53rka	-0.14	1.00000000	chicken
Ylpm1	-0.27	1.00000000	chicken
Bcor	-0.35	1.00000000	chicken
Wbp4	-0.29	1.00000000	chicken
Hadh	-0.21	1.00000000	chicken
Ppil4	-0.29	1.00000000	chicken
Npas3	0.49	1.00000000	chicken
Rhot1	0.00	1.00000000	chicken
Herpud2	-0.17	1.00000000	chicken
Tnfsf10	0.13	1.00000000	chicken
Pgm1	0.07	1.00000000	chicken
Fancm	0.15	1.00000000	chicken
Nip7	0.09	1.00000000	chicken
Zbed4	0.15	1.00000000	chicken
Hoxa6	0.02	1.00000000	chicken
Rsrp1	-0.48	1.00000000	chicken
4933434E20Rik	0.04	1.00000000	chicken
Zbtb38	-0.26	1.00000000	chicken
Arhgef3	0.09	1.00000000	chicken
Txndc17	-0.28	1.00000000	chicken
Arhgap24	-0.03	1.00000000	chicken
Rtraf	-0.18	1.00000000	chicken
Eif3g	-0.28	1.00000000	chicken
Rgl1	-0.11	1.00000000	chicken
Rassf3	0.03	1.00000000	chicken
Eea1	0.05	1.00000000	chicken
Pink1	-0.21	1.00000000	chicken
Dpf3	0.27	1.00000000	chicken
Cluh	0.07	1.00000000	chicken
Uqcrfs1	-0.28	1.00000000	chicken
Irf2bpl	0.05	1.00000000	chicken
Tbc1d14	-0.25	1.00000000	chicken
Zfand2b	0.03	1.00000000	chicken
2700049A03Rik	-0.24	1.00000000	chicken
Leprot	-0.34	1.00000000	chicken
Dock1	0.10	1.00000000	chicken
Tob1	0.09	1.00000000	chicken
Kmt5b	-0.28	1.00000000	chicken
Osbpl8	0.19	1.00000000	chicken
4921524J17Rik	-0.33	1.00000000	chicken
Pus10	0.02	1.00000000	chicken
Rreb1	0.12	1.00000000	chicken
Itgb1	0.06	1.00000000	chicken
Adipor1	0.08	1.00000000	chicken

Immt	0.09	1.00000000	chicken
Sestd1	0.29	1.00000000	chicken
Arl13b	-0.23	1.00000000	chicken
Ago4	0.04	1.00000000	chicken
Ttc7	0.17	1.00000000	chicken
Ndufa5	-0.13	1.00000000	chicken
Wdr48	0.08	1.00000000	chicken
Slc2a1	0.02	1.00000000	chicken
Ppp1r13b	0.03	1.00000000	chicken
Esyt2	0.13	1.00000000	chicken
Zfp503	-0.19	1.00000000	chicken
Xbp1	0.00	1.00000000	chicken
Aldoc	-0.28	1.00000000	chicken
Hectd2	-0.33	1.00000000	chicken
Copb1	-0.05	1.00000000	chicken
Nol7	-0.19	1.00000000	chicken
Srsf10	0.03	1.00000000	chicken
Smu1	0.14	1.00000000	chicken
Surf6	0.05	1.00000000	chicken
4933400A11Rik	0.11	1.00000000	chicken
Gen1	0.06	1.00000000	chicken
Tcea1	0.02	1.00000000	chicken
Cops2	0.07	1.00000000	chicken
Ipo9	0.11	1.00000000	chicken
Tmem183a	-0.18	1.00000000	chicken
Ifngr1	0.08	1.00000000	chicken
Tln1	0.10	1.00000000	chicken
Cdc42bpa	-0.22	1.00000000	chicken
Fam241a	-0.23	1.00000000	chicken
Txlng	-0.31	1.00000000	chicken
Chd5	0.05	1.00000000	chicken
Slc35e2	-0.26	1.00000000	chicken
Svep1	0.00	1.00000000	chicken
Cog2	-0.24	1.00000000	chicken
Trim33	-0.02	1.00000000	chicken
Eif5b	-0.07	1.00000000	chicken
Setd7	-0.20	1.00000000	chicken
Etl4	0.34	1.00000000	chicken
Atf2	0.03	1.00000000	chicken
Eri3	0.03	1.00000000	chicken
Ilf2	-0.31	1.00000000	chicken
Tmed7	0.13	1.00000000	chicken
Flot1	-0.16	1.00000000	chicken
Ufl1	-0.21	1.00000000	chicken
Spg7	-0.27	1.00000000	chicken
Zfp512	0.06	1.00000000	chicken

Nfu1	-0.19	1.00000000	chicken
Fbln2	-0.16	1.00000000	chicken
Eml1	-0.28	1.00000000	chicken
Adgrd1	-0.08	1.00000000	chicken
Prpsap2	-0.13	1.00000000	chicken
Ralgapb	0.07	1.00000000	chicken
Ywhae	0.05	1.00000000	chicken
Atp1b3	0.20	1.00000000	chicken
Nt5dc1	-0.21	1.00000000	chicken
Ppp6r2	0.04	1.00000000	chicken
Auh	0.01	1.00000000	chicken
Dync1i2	0.00	1.00000000	chicken
Wrn	-0.17	1.00000000	chicken
Spg11	-0.21	1.00000000	chicken
Zcchc8	-0.21	1.00000000	chicken
Nfic	-0.22	1.00000000	chicken
Srsf11	-0.21	1.00000000	chicken
Ndufs2	-0.21	1.00000000	chicken
Sh3kbp1	-0.35	1.00000000	chicken
Camk2b	-0.17	1.00000000	chicken
Dcun1d4	-0.02	1.00000000	chicken
Elk3	0.00	1.00000000	chicken
Atp6v0c	-0.42	1.00000000	chicken
Wipi2	0.10	1.00000000	chicken
Fh1	-0.28	1.00000000	chicken
Paip1	0.03	1.00000000	chicken
Pnpla8	-0.37	1.00000000	chicken
Pkia	0.30	1.00000000	chicken
Usp46	0.06	1.00000000	chicken
Tcf25	0.03	1.00000000	chicken
Clk2	0.07	1.00000000	chicken
Chmp5	0.02	1.00000000	chicken
Kctd9	0.01	1.00000000	chicken
Smim7	-0.30	1.00000000	chicken
Stab1	-0.15	1.00000000	chicken
9530068E07Rik	-0.01	1.00000000	chicken
Spidr	0.34	1.00000000	chicken
Xpo4	0.17	1.00000000	chicken
Tsn	0.03	1.00000000	chicken
Gnas	0.18	1.00000000	chicken
Crtap	-0.18	1.00000000	chicken
Swt1	-0.14	1.00000000	chicken
Nop14	0.00	1.00000000	chicken
Armc8	0.07	1.00000000	chicken
Lsm8	0.10	1.00000000	chicken
Rpn2	-0.24	1.00000000	chicken

Sbno2	-0.58	1.00000000	chicken
Ckap5	0.07	1.00000000	chicken
Rbx1	-0.19	1.00000000	chicken
Tdrd3	0.18	1.00000000	chicken
Pno1	0.10	1.00000000	chicken
Dnmt3a	-0.24	1.00000000	chicken
Rhou	0.12	1.00000000	chicken
Fggy	-0.24	1.00000000	chicken
Sdhaf2	-0.18	1.00000000	chicken
Nsmf	-0.06	1.00000000	chicken
Clec16a	0.05	1.00000000	chicken
Ube3a	0.03	1.00000000	chicken
Imp4	0.06	1.00000000	chicken
Ythdf3	-0.28	1.00000000	chicken
Cir1	0.03	1.00000000	chicken
Acot13	0.05	1.00000000	chicken
Cul5	-0.04	1.00000000	chicken
Anapc10	-0.28	1.00000000	chicken
Nectin3	-0.09	1.00000000	chicken
Alg12	-0.19	1.00000000	chicken
Gsr	0.02	1.00000000	chicken
Slc25a1	-0.18	1.00000000	chicken
Cebpb	0.00	1.00000000	chicken
Tnrc6c	-0.28	1.00000000	chicken
Cul4b	0.01	1.00000000	chicken
Tardbp	0.07	1.00000000	chicken
Tmem259	0.01	1.00000000	chicken
Phlpp2	-0.16	1.00000000	chicken
Ak2	0.03	1.00000000	chicken
Lfng	0.11	1.00000000	chicken
Rapgef1	0.05	1.00000000	chicken
Slc30a9	-0.05	1.00000000	chicken
Bzw1	0.07	1.00000000	chicken
Sar1a	0.02	1.00000000	chicken
Ttc28	-0.20	1.00000000	chicken
Tnfrsf9	0.07	1.00000000	chicken
Yes1	-0.06	1.00000000	chicken
Sap30bp	-0.06	1.00000000	chicken
Dpp7	-0.16	1.00000000	chicken
Plpp3	-0.23	1.00000000	chicken
Hs1bp3	0.05	1.00000000	chicken
Scaf11	-0.25	1.00000000	chicken
Nop56	-0.06	1.00000000	chicken
Wwp1	0.22	1.00000000	chicken
Dph3	-0.33	1.00000000	chicken
Polr2f	-0.16	1.00000000	chicken

Cwc22	0.04	1.00000000	chicken
Rab31	0.51	1.00000000	chicken
Ppp1r2	0.05	1.00000000	chicken
Zfand4	-0.15	1.00000000	chicken
Fbxo33	-0.23	1.00000000	chicken
Sdhaf4	-0.17	1.00000000	chicken
Tgfbr2	-0.30	1.00000000	chicken
Xrcc5	0.06	1.00000000	chicken
Asxl2	0.00	1.00000000	chicken
Sufu	0.11	1.00000000	chicken
Lpar2	-0.20	1.00000000	chicken
Tbk1	-0.18	1.00000000	chicken
Sptbn1	0.18	1.00000000	chicken
Plcd1	0.23	1.00000000	chicken
Fbxw2	-0.15	1.00000000	chicken
Lta4h	-0.25	1.00000000	chicken
Htt	-0.29	1.00000000	chicken
Afap1l2	-0.13	1.00000000	chicken
Dyrk3	0.00	1.00000000	chicken
Uqcrc2	-0.21	1.00000000	chicken
Abca2	-0.19	1.00000000	chicken
Pex14	0.02	1.00000000	chicken
Ddx19a	0.03	1.00000000	chicken
Syngr2	0.06	1.00000000	chicken
Ppp2r5e	-0.20	1.00000000	chicken
Nup85	-0.22	1.00000000	chicken
Psma5	0.13	1.00000000	chicken
Slc43a2	0.06	1.00000000	chicken
Galnt11	-0.23	1.00000000	chicken
Dctn1	0.01	1.00000000	chicken
Rab14	-0.25	1.00000000	chicken
Csnk1e	-0.19	1.00000000	chicken
Far1	0.09	1.00000000	chicken
Ntan1	-0.21	1.00000000	chicken
N4bp1	-0.05	1.00000000	chicken
Thumpd3	-0.03	1.00000000	chicken
Pwwp2b	0.19	1.00000000	chicken
Rab6a	0.03	1.00000000	chicken
Cmtm4	-0.33	1.00000000	chicken
Ralb	-0.16	1.00000000	chicken
Tasp1	0.04	1.00000000	chicken
Klhl13	-0.25	1.00000000	chicken
Kalrn	1.10	1.00000000	chicken
Nono	0.03	1.00000000	chicken
Thoc1	0.03	1.00000000	chicken
Trnt1	0.07	1.00000000	chicken

Smim19	-0.12	1.00000000	chicken
Scaf8	0.02	1.00000000	chicken
Cnot8	0.00	1.00000000	chicken
Rtca	-0.09	1.00000000	chicken
Ssu72	0.05	1.00000000	chicken
Ndufa12	-0.17	1.00000000	chicken
Dip2b	0.04	1.00000000	chicken
Stx18	-0.20	1.00000000	chicken
Wdsub1	0.10	1.00000000	chicken
Stard3nl	0.02	1.00000000	chicken
Aco2	-0.19	1.00000000	chicken
Edem3	0.08	1.00000000	chicken
Cpsf3	0.02	1.00000000	chicken
Qpct	-0.18	1.00000000	chicken
Necap1	0.13	1.00000000	chicken
Sppl2a	0.12	1.00000000	chicken
Rmnd5a	-0.12	1.00000000	chicken
Bud31	-0.21	1.00000000	chicken
Kif5b	-0.34	1.00000000	chicken
Stxbp1	-0.18	1.00000000	chicken
Sec63	-0.19	1.00000000	chicken
Sirt1	-0.23	1.00000000	chicken
Rnmt	-0.21	1.00000000	chicken
Eif4e2	-0.18	1.00000000	chicken
Sdf4	-0.24	1.00000000	chicken
Celf1	0.03	1.00000000	chicken
Tm9sf3	0.08	1.00000000	chicken
Acaa1b	-0.19	1.00000000	chicken
Hipk3	-0.06	1.00000000	chicken
Psme3	0.01	1.00000000	chicken
Zfp384	0.05	1.00000000	chicken
Sgce	-0.15	1.00000000	chicken
Cops8	0.13	1.00000000	chicken
Ddx3y	-0.06	1.00000000	chicken
Pag1	-0.15	1.00000000	chicken
Add3	-0.03	1.00000000	chicken
Zc3h11a	-0.02	1.00000000	chicken
Senp6	-0.29	1.00000000	chicken
Ltf	0.04	1.00000000	chicken
Atp11c	0.08	1.00000000	chicken
Nek7	0.06	1.00000000	chicken
Fkbp3	-0.14	1.00000000	chicken
Rars	0.03	1.00000000	chicken
Pmf1	-0.27	1.00000000	chicken
Sirt5	0.04	1.00000000	chicken
Asap1	-0.18	1.00000000	chicken

Kif2a	-0.29	1.00000000	chicken
Cd74	-0.65	1.00000000	chicken
Rftn1	-0.27	1.00000000	chicken
Drg2	0.04	1.00000000	chicken
Ift20	-0.22	1.00000000	chicken
Eif2ak1	0.06	1.00000000	chicken
Asb5	-0.27	1.00000000	chicken
Atp6v1e2	0.02	1.00000000	chicken
Fzd2	0.00	1.00000000	chicken
Galnt17	0.12	1.00000000	chicken
Nus1	0.16	1.00000000	chicken
Sfpq	-0.22	1.00000000	chicken
Skp2	-0.27	1.00000000	chicken
Sdr16c5	0.05	1.00000000	chicken
Katnbl1	-0.06	1.00000000	chicken
Rab8b	0.04	1.00000000	chicken
Klhl7	-0.28	1.00000000	chicken
Emc7	0.06	1.00000000	chicken
Kif1b	-0.18	1.00000000	chicken
Ostc	-0.30	1.00000000	chicken
Eif1ad12	0.00	1.00000000	chicken
Ubr7	0.02	1.00000000	chicken
Fgf2	0.07	1.00000000	chicken
Ghitm	0.08	1.00000000	chicken
Dpt	-0.55	1.00000000	chicken
Slc9a3r2	-0.18	1.00000000	chicken
Eif5a2	-0.16	1.00000000	chicken
Tmx4	0.02	1.00000000	chicken
Ppp6c	0.02	1.00000000	chicken
Golm2	-0.19	1.00000000	chicken
Letm1	0.08	1.00000000	chicken
Arhgap21	0.14	1.00000000	chicken
Gfm1	-0.03	1.00000000	chicken
Cnot11	-0.19	1.00000000	chicken
Tcof1	0.00	1.00000000	chicken
Snap29	-0.19	1.00000000	chicken
Pcm1	-0.26	1.00000000	chicken
Ralbp1	-0.25	1.00000000	chicken
Ilrun	0.03	1.00000000	chicken
Tfdp1	-0.19	1.00000000	chicken
Iba57	0.01	1.00000000	chicken
Thoc5	-0.18	1.00000000	chicken
Ephb2	0.01	1.00000000	chicken
Tshz2	-0.09	1.00000000	chicken
Suclg2	0.10	1.00000000	chicken
Riok1	0.07	1.00000000	chicken

Vbp1	0.06	1.00000000	chicken
Ide	0.04	1.00000000	chicken
Sacm1l	0.02	1.00000000	chicken
Dennd1b	-0.43	1.00000000	chicken
Stx7	0.00	1.00000000	chicken
Prkn	-0.28	1.00000000	chicken
Ipmk	0.07	1.00000000	chicken
Sra1	-0.13	1.00000000	chicken
Sys1	0.04	1.00000000	chicken
Smad3	-0.27	1.00000000	chicken
Zfp207	-0.18	1.00000000	chicken
Tbc1d2b	0.17	1.00000000	chicken
Cox16	-0.02	1.00000000	chicken
Wtip	-0.33	1.00000000	chicken
Ralgps1	0.04	1.00000000	chicken
Exoc5	0.05	1.00000000	chicken
Rab8a	-0.21	1.00000000	chicken
Dhx37	0.07	1.00000000	chicken
Sae1	0.01	1.00000000	chicken
Sgf29	-0.17	1.00000000	chicken
Cpeb4	0.16	1.00000000	chicken
Nol11	-0.07	1.00000000	chicken
Zhx3	-0.01	1.00000000	chicken
Hdac2	-0.20	1.00000000	chicken
Ntmt1	-0.21	1.00000000	chicken
Ensa	-0.09	1.00000000	chicken
Zranb1	-0.12	1.00000000	chicken
Hsf2	-0.09	1.00000000	chicken
Gorab	0.02	1.00000000	chicken
Tut7	-0.21	1.00000000	chicken
Hsf1	-0.03	1.00000000	chicken
Cntrl	-0.25	1.00000000	chicken
Cstf3	-0.01	1.00000000	chicken
Psmb1	-0.25	1.00000000	chicken
Nup205	-0.14	1.00000000	chicken
Fam168a	0.13	1.00000000	chicken
Anks1	-0.01	1.00000000	chicken
Xkr4	0.16	1.00000000	chicken
Rbm18	0.08	1.00000000	chicken
Chpf2	0.07	1.00000000	chicken
Api5	0.06	1.00000000	chicken
Smyd3	-0.21	1.00000000	chicken
Ywhah	0.08	1.00000000	chicken
Cobll1	0.00	1.00000000	chicken
Psmd12	0.01	1.00000000	chicken
Osbpl2	-0.21	1.00000000	chicken

Chsy3	0.22	1.00000000	chicken
Agap1	0.10	1.00000000	chicken
Nucks1	-0.14	1.00000000	chicken
Strbp	-0.18	1.00000000	chicken
Prkcd	0.03	1.00000000	chicken
Pds5b	-0.22	1.00000000	chicken
Smpd1	-0.20	1.00000000	chicken
Phf10	-0.17	1.00000000	chicken
Dnajc10	0.00	1.00000000	chicken
Ints13	0.05	1.00000000	chicken
Ptpn14	0.23	1.00000000	chicken
Psmd9	-0.19	1.00000000	chicken
Arrdc2	0.00	1.00000000	chicken
Luc7l	-0.34	1.00000000	chicken
Ubac2	0.19	1.00000000	chicken
Jun	-0.16	1.00000000	chicken
Ppp3r1	0.08	1.00000000	chicken
Wbp1l	-0.18	1.00000000	chicken
Serpinb8	-0.18	1.00000000	chicken
Pde3a	0.19	1.00000000	chicken
Vps53	-0.18	1.00000000	chicken
Usp8	0.07	1.00000000	chicken
Tnfrsf11b	0.13	1.00000000	chicken
Tmem199	-0.08	1.00000000	chicken
Ss18l2	-0.21	1.00000000	chicken
Cdc42bpb	-0.18	1.00000000	chicken
Galnt15	0.00	1.00000000	chicken
Sdf2l1	0.01	1.00000000	chicken
Slc44a3	0.28	1.00000000	chicken
Jazf1	-0.17	1.00000000	chicken
Ubxn7	-0.04	1.00000000	chicken
Fam120a	0.08	1.00000000	chicken
Ythdf1	0.02	1.00000000	chicken
Brip1	-0.28	1.00000000	chicken
Pccb	-0.19	1.00000000	chicken
Rbm26	-0.32	1.00000000	chicken
Cyb5b	-0.14	1.00000000	chicken
Slc8a1	0.16	1.00000000	chicken
Elavl1	0.02	1.00000000	chicken
Fbxo34	-0.30	1.00000000	chicken
Lrch3	-0.04	1.00000000	chicken
Sgsm2	0.04	1.00000000	chicken
Spats2l	-0.18	1.00000000	chicken
Ttll5	-0.16	1.00000000	chicken
Raver2	-0.14	1.00000000	chicken
Tbc1d4	0.18	1.00000000	chicken

Zranb2	-0.20	1.00000000	chicken
Pibf1	0.18	1.00000000	chicken
Eef1e1	0.01	1.00000000	chicken
Kdm4c	-0.17	1.00000000	chicken
Cflar	0.03	1.00000000	chicken
Ercc3	0.04	1.00000000	chicken
Scamp2	-0.01	1.00000000	chicken
Chmp2b	0.01	1.00000000	chicken
Lats1	-0.24	1.00000000	chicken
Yipf3	-0.13	1.00000000	chicken
Jcad	0.03	1.00000000	chicken
Cln8	0.03	1.00000000	chicken
Polr1f	-0.21	1.00000000	chicken
Trim59	0.01	1.00000000	chicken
Pip4k2a	0.10	1.00000000	chicken
Dip2c	0.19	1.00000000	chicken
Arl8a	-0.10	1.00000000	chicken
Ankib1	-0.23	1.00000000	chicken
Serinc1	-0.22	1.00000000	chicken
Prkar1b	-0.20	1.00000000	chicken
Upf3b	-0.23	1.00000000	chicken
Ska2	-0.13	1.00000000	chicken
Notch1	-0.19	1.00000000	chicken
Tmf1	0.10	1.00000000	chicken
N6amt1	-0.12	1.00000000	chicken
Ppie	-0.22	1.00000000	chicken
Clint1	0.06	1.00000000	chicken
Angel2	-0.16	1.00000000	chicken
Zfp598	0.03	1.00000000	chicken
C1qtnf5	-0.27	1.00000000	chicken
Arl6ip5	0.09	1.00000000	chicken
Psmd5	-0.19	1.00000000	chicken
Rnf10	0.07	1.00000000	chicken
Hmgcr	-0.16	1.00000000	chicken
Tex264	-0.20	1.00000000	chicken
Arntl2	0.10	1.00000000	chicken
Spcs1	-0.23	1.00000000	chicken
Ptpa	0.06	1.00000000	chicken
Bccip	0.06	1.00000000	chicken
Selenok	-0.18	1.00000000	chicken
Evi5	-0.24	1.00000000	chicken
Tnrc6a	-0.03	1.00000000	chicken
Smad6	0.47	1.00000000	chicken
Trpc4ap	0.02	1.00000000	chicken
Tbc1d12	-0.12	1.00000000	chicken
Ctdp1	0.00	1.00000000	chicken

Jade3	0.06	1.00000000	chicken
Parp4	-0.26	1.00000000	chicken
Nfx1	-0.16	1.00000000	chicken
Commd2	-0.18	1.00000000	chicken
Ube2b	0.04	1.00000000	chicken
Tdg	0.03	1.00000000	chicken
Smad1	-0.05	1.00000000	chicken
Ptp4a1	0.00	1.00000000	chicken
Hnrnpd	-0.25	1.00000000	chicken
Emc8	0.09	1.00000000	chicken
Hyou1	0.01	1.00000000	chicken
Ift122	-0.16	1.00000000	chicken
Trim35	0.06	1.00000000	chicken
Rtcb	0.02	1.00000000	chicken
Adssl1	0.04	1.00000000	chicken
Npepps	-0.02	1.00000000	chicken
Aimp1	-0.09	1.00000000	chicken
Psmb2	0.10	1.00000000	chicken
Ik	0.01	1.00000000	chicken
Thyn1	0.01	1.00000000	chicken
Snw1	-0.03	1.00000000	chicken
Amfr	-0.03	1.00000000	chicken
Akap1	-0.01	1.00000000	chicken
Rundc1	0.00	1.00000000	chicken
Dgkz	-0.17	1.00000000	chicken
Cdc73	0.07	1.00000000	chicken
Tmpo	-0.17	1.00000000	chicken
Sf3a1	-0.17	1.00000000	chicken
Slc30a7	0.07	1.00000000	chicken
Syap1	-0.14	1.00000000	chicken
Cdk11b	0.03	1.00000000	chicken
Nbr1	-0.22	1.00000000	chicken
Atp2c1	0.01	1.00000000	chicken
Aco1	0.05	1.00000000	chicken
Smndc1	0.04	1.00000000	chicken
Rilp	-0.14	1.00000000	chicken
Lrrn4	0.01	1.00000000	chicken
Prkab1	0.06	1.00000000	chicken
Ttc17	0.02	1.00000000	chicken
Psmc6	0.06	1.00000000	chicken
Fam3c	-0.28	1.00000000	chicken
Ncbp3	-0.25	1.00000000	chicken
Rilpl1	-0.19	1.00000000	chicken
Rab3il1	-0.11	1.00000000	chicken
Cdk19	0.11	1.00000000	chicken
Tram1l1	-0.23	1.00000000	chicken

Plekhh2	0.07	1.00000000	chicken
H13	-0.30	1.00000000	chicken
Cstf2	0.01	1.00000000	chicken
St3gal2	-0.10	1.00000000	chicken
Sf3a2	0.00	1.00000000	chicken
Sdc2	0.15	1.00000000	chicken
Slc16a2	-0.19	1.00000000	chicken
Fam53a	0.05	1.00000000	chicken
Cyld	-0.01	1.00000000	chicken
Pcdh10	0.42	1.00000000	chicken
Snx4	0.04	1.00000000	chicken
Wdr37	-0.01	1.00000000	chicken
Prdm2	-0.07	1.00000000	chicken
Tspan9	-0.12	1.00000000	chicken
Ifnar1	0.01	1.00000000	chicken
Bap1	0.03	1.00000000	chicken
Ndufaf2	-0.03	1.00000000	chicken
Nubp1	0.10	1.00000000	chicken
Ranbp1	-0.19	1.00000000	chicken
Txnip	-0.27	1.00000000	chicken
Rae1	-0.02	1.00000000	chicken
Sdf2	0.02	1.00000000	chicken
Naxd	0.05	1.00000000	chicken
Ndufa9	0.02	1.00000000	chicken
Net1	-0.02	1.00000000	chicken
Cstf1	-0.14	1.00000000	chicken
Heatr5b	-0.16	1.00000000	chicken
Hsph1	-0.45	1.00000000	chicken
Pik3c2a	0.00	1.00000000	chicken
Trappc4	0.09	1.00000000	chicken
Clmp	0.17	1.00000000	chicken
Zc3h13	0.07	1.00000000	chicken
Polr3b	-0.09	1.00000000	chicken
Cplane2	-0.17	1.00000000	chicken
Tial1	-0.27	1.00000000	chicken
Zfp365	0.05	1.00000000	chicken
Tcf3	-0.19	1.00000000	chicken
Dnaja2	0.03	1.00000000	chicken
Pcgf6	-0.20	1.00000000	chicken
Ep400	-0.08	1.00000000	chicken
Atp6v1g1	-0.25	1.00000000	chicken
Smg7	-0.02	1.00000000	chicken
Fbxl7	0.17	1.00000000	chicken
Pim3	-0.48	1.00000000	chicken
Erh	-0.21	1.00000000	chicken
Arhgap15	-0.49	1.00000000	chicken

Cul3	0.13	1.00000000	chicken
Bud13	-0.16	1.00000000	chicken
Hnrnpll	-0.19	1.00000000	chicken
Cox5b-ps	-0.28	1.00000000	chicken
Nupl1	0.06	1.00000000	chicken
Poglut2	-0.20	1.00000000	chicken
Plrg1	-0.06	1.00000000	chicken
Srbd1	-0.10	1.00000000	chicken
Gcc2	-0.02	1.00000000	chicken
Stom	-0.03	1.00000000	chicken
Vps54	-0.15	1.00000000	chicken
Eipr1	-0.01	1.00000000	chicken
Plekho1	-0.11	1.00000000	chicken
Ofd1	-0.19	1.00000000	chicken
Tmem184b	0.10	1.00000000	chicken
Tbx3	-0.18	1.00000000	chicken
Ghdc	0.00	1.00000000	chicken
Trmt1l	-0.13	1.00000000	chicken
Bsdc1	0.14	1.00000000	chicken
Pdcd6	-0.01	1.00000000	chicken
Arhgdia	-0.22	1.00000000	chicken
Slc39a13	-0.08	1.00000000	chicken
Akt3	0.14	1.00000000	chicken
Adgra1	-0.06	1.00000000	chicken
Larp7	0.04	1.00000000	chicken
Kars	-0.20	1.00000000	chicken
Yipf5	-0.17	1.00000000	chicken
Nbeal1	-0.06	1.00000000	chicken
Pacs2	-0.08	1.00000000	chicken
Chn1	-0.22	1.00000000	chicken
Fkbp9	-0.11	1.00000000	chicken
Tmem30a	-0.04	1.00000000	chicken
Sp4	-0.19	1.00000000	chicken
Tenm3	-0.15	1.00000000	chicken
Ldah	0.05	1.00000000	chicken
Ctcf	-0.19	1.00000000	chicken
Tnc	1.93	1.00000000	chicken
Cep164	-0.06	1.00000000	chicken
Ric8b	-0.02	1.00000000	chicken
Ddx17	-0.22	1.00000000	chicken
Acp1	-0.05	1.00000000	chicken
Nvl	-0.07	1.00000000	chicken
Cnst	-0.03	1.00000000	chicken
Snrpd2	-0.13	1.00000000	chicken
Rdm1	0.01	1.00000000	chicken
Bdkrb1	0.08	1.00000000	chicken

Dcaf5	0.11	1.00000000	chicken
Rictor	0.04	1.00000000	chicken
Xpo7	0.02	1.00000000	chicken
Washc4	0.02	1.00000000	chicken
Ada	-0.19	1.00000000	chicken
Hint1	-0.21	1.00000000	chicken
Hras	-0.18	1.00000000	chicken
Kdm3a	-0.27	1.00000000	chicken
St14	0.18	1.00000000	chicken
Laptm4a	0.02	1.00000000	chicken
Tpm1	0.20	1.00000000	chicken
Apool	-0.18	1.00000000	chicken
Lnpep	0.01	1.00000000	chicken
Bclaf3	-0.04	1.00000000	chicken
Runx2	0.00	1.00000000	chicken
Rprd1a	-0.16	1.00000000	chicken
Lamtor5	-0.21	1.00000000	chicken
Klf7	-0.12	1.00000000	chicken
Tdp2	0.00	1.00000000	chicken
Rrp15	0.02	1.00000000	chicken
Colec10	-0.19	1.00000000	chicken
Sh3pxd2b	0.08	1.00000000	chicken
Csnk2a2	-0.16	1.00000000	chicken
Abitram	0.03	1.00000000	chicken
Herc3	-0.01	1.00000000	chicken
Acer3	-0.20	1.00000000	chicken
Arfip1	-0.09	1.00000000	chicken
Senp7	-0.20	1.00000000	chicken
Arl15	0.11	1.00000000	chicken
Acaca	-0.07	1.00000000	chicken
Vps35l	-0.16	1.00000000	chicken
Sri	0.06	1.00000000	chicken
Ubr2	-0.24	1.00000000	chicken
Pdia5	-0.20	1.00000000	chicken
Eif2ak4	-0.01	1.00000000	chicken
Abce1	0.05	1.00000000	chicken
Ghr	0.13	1.00000000	chicken
Por	0.08	1.00000000	chicken
2210016F16Rik	-0.15	1.00000000	chicken
Sccpdh	-0.12	1.00000000	chicken
Fam214a	0.12	1.00000000	chicken
Phf21a	0.17	1.00000000	chicken
Taf15	0.01	1.00000000	chicken
Ano10	-0.27	1.00000000	chicken
Slc4a4	0.33	1.00000000	chicken
Zc3h18	-0.01	1.00000000	chicken

Ndufa8	-0.12	1.00000000	chicken
Apip	-0.01	1.00000000	chicken
Zbtb16	0.04	1.00000000	chicken
Cyth1	-0.22	1.00000000	chicken
Samm50	-0.15	1.00000000	chicken
Haus2	-0.17	1.00000000	chicken
Gpalpp1	-0.01	1.00000000	chicken
Cbll1	-0.05	1.00000000	chicken
Kdm3b	-0.26	1.00000000	chicken
Bcl9	0.03	1.00000000	chicken
Cbx1	-0.24	1.00000000	chicken
Ppp1r9b	0.02	1.00000000	chicken
Umad1	0.06	1.00000000	chicken
Rragd	0.00	1.00000000	chicken
Pnrc2	-0.29	1.00000000	chicken
Sf3b6	-0.14	1.00000000	chicken
Spryd7	-0.08	1.00000000	chicken
Rcor1	-0.18	1.00000000	chicken
Aqr	0.12	1.00000000	chicken
Cert1	0.07	1.00000000	chicken
Rmc1	-0.22	1.00000000	chicken
Tfam	-0.16	1.00000000	chicken
Sos1	0.07	1.00000000	chicken
Oaz1	-0.03	1.00000000	chicken
Sec16a	-0.03	1.00000000	chicken
Eif2b3	-0.17	1.00000000	chicken
Slc3a1	-0.13	1.00000000	chicken
Rcan1	0.14	1.00000000	chicken
Lsm1	0.00	1.00000000	chicken
Kdm5b	-0.16	1.00000000	chicken
Foxo1	0.07	1.00000000	chicken
Hspa9	-0.05	1.00000000	chicken
Abhd6	0.04	1.00000000	chicken
Actr1a	0.00	1.00000000	chicken
Prkar1a	-0.05	1.00000000	chicken
Arhgef10	-0.12	1.00000000	chicken
Lemd2	0.08	1.00000000	chicken
Oga	-0.12	1.00000000	chicken
Arf6	-0.27	1.00000000	chicken
Bdp1	-0.04	1.00000000	chicken
Hsd17b4	-0.15	1.00000000	chicken
Trib2	0.05	1.00000000	chicken
Ltn1	-0.09	1.00000000	chicken
Pigt	0.05	1.00000000	chicken
Pbrm1	-0.21	1.00000000	chicken
Bin3	-0.01	1.00000000	chicken

Snrpf	-0.23	1.00000000	chicken
G3bp1	-0.18	1.00000000	chicken
Cmss1	0.11	1.00000000	chicken
Eftud2	-0.18	1.00000000	chicken
Parg	-0.14	1.00000000	chicken
Ndufs8	-0.20	1.00000000	chicken
Snx17	-0.16	1.00000000	chicken
Gna11	0.02	1.00000000	chicken
Cnot10	0.00	1.00000000	chicken
Rptor	-0.06	1.00000000	chicken
LTO1	-0.09	1.00000000	chicken
Timm17a	0.03	1.00000000	chicken
Rlf	0.03	1.00000000	chicken
F3	0.07	1.00000000	chicken
Lig3	-0.15	1.00000000	chicken
Creb3l2	0.06	1.00000000	chicken
Lrp6	-0.01	1.00000000	chicken
Iars	0.04	1.00000000	chicken
Smn1	0.02	1.00000000	chicken
Bckdhb	0.05	1.00000000	chicken
Top2b	0.12	1.00000000	chicken
Ranbp3	-0.14	1.00000000	chicken
Anxa5	-0.10	1.00000000	chicken
Kansl3	-0.03	1.00000000	chicken
Xiap	-0.19	1.00000000	chicken
Fbxo7	-0.19	1.00000000	chicken
Pdlim7	0.02	1.00000000	chicken
Lin52	0.07	1.00000000	chicken
Rab29	0.03	1.00000000	chicken
Ap1s2	0.39	1.00000000	chicken
Castor2	-0.22	1.00000000	chicken
Tvp23b	-0.03	1.00000000	chicken
Prep	0.02	1.00000000	chicken
Ston2	0.22	1.00000000	chicken
Foxk1	-0.04	1.00000000	chicken
Ccz1	0.04	1.00000000	chicken
D430041D05Rik	-0.07	1.00000000	chicken
Rnpc3	-0.24	1.00000000	chicken
Hdgfl2	-0.21	1.00000000	chicken
Chic2	0.03	1.00000000	chicken
Hira	0.03	1.00000000	chicken
Wdr4	-0.03	1.00000000	chicken
Synj2	-0.29	1.00000000	chicken
Cmip	0.10	1.00000000	chicken
Chmp7	-0.18	1.00000000	chicken
Srsf7	-0.21	1.00000000	chicken

Copa	-0.18	1.00000000	chicken
C1qbp	-0.11	1.00000000	chicken
Dock10	0.07	1.00000000	chicken
Zfp236	0.10	1.00000000	chicken
Rpp14	-0.11	1.00000000	chicken
Usp15	-0.02	1.00000000	chicken
Stox2	0.02	1.00000000	chicken
Hoxb4	-0.19	1.00000000	chicken
Zfyve9	-0.12	1.00000000	chicken
Tab3	-0.01	1.00000000	chicken
Phpt1	-0.03	1.00000000	chicken
Tbc1d5	-0.13	1.00000000	chicken
Ppp4r3a	0.04	1.00000000	chicken
Fosb	0.01	1.00000000	chicken
Cyrib	-0.46	1.00000000	chicken
Psmb6	0.08	1.00000000	chicken
Ube4a	-0.04	1.00000000	chicken
Tgfbrap1	-0.02	1.00000000	chicken
Sec61a1	-0.17	1.00000000	chicken
Radx	-0.12	1.00000000	chicken
Actr10	-0.21	1.00000000	chicken
Igfbp3	-0.18	1.00000000	chicken
2210408I21Rik	0.19	1.00000000	chicken
Cnot1	-0.22	1.00000000	chicken
Septin10	0.02	1.00000000	chicken
Hcfc2	-0.05	1.00000000	chicken
Fmn1	-0.15	1.00000000	chicken
Lman2	-0.02	1.00000000	chicken
Prelid1	0.11	1.00000000	chicken
Paxbp1	-0.20	1.00000000	chicken
Clic3	-0.05	1.00000000	chicken
Ythdc2	0.01	1.00000000	chicken
Nop53	-0.16	1.00000000	chicken
Wdr5b	0.02	1.00000000	chicken
Atp5a1	0.08	1.00000000	chicken
Rbm17	-0.08	1.00000000	chicken
Cisd1	-0.01	1.00000000	chicken
Srpk1	-0.04	1.00000000	chicken
Strap	-0.19	1.00000000	chicken
Igf2	0.08	1.00000000	chicken
Prpsap1	0.01	1.00000000	chicken
Fam102a	-0.11	1.00000000	chicken
Ate1	-0.14	1.00000000	chicken
Il1rl1	0.12	1.00000000	chicken
Sbds	0.08	1.00000000	chicken
Acp2	0.01	1.00000000	chicken

Osbpl3	0.19	1.00000000	chicken
Sumo1	-0.13	1.00000000	chicken
Adamts12	-0.09	1.00000000	chicken
Ywhab	-0.09	1.00000000	chicken
Gigyf2	0.20	1.00000000	chicken
Dcaf13	-0.09	1.00000000	chicken
St8sia4	0.13	1.00000000	chicken
Klhl2	0.03	1.00000000	chicken
Kifap3	-0.11	1.00000000	chicken
Kpna3	0.01	1.00000000	chicken
Shroom2	0.02	1.00000000	chicken
Senp5	-0.01	1.00000000	chicken
Nsun3	-0.13	1.00000000	chicken
Ncoa3	-0.04	1.00000000	chicken
Tmem245	-0.19	1.00000000	chicken
Rtn4	-0.10	1.00000000	chicken
Dr1	-0.22	1.00000000	chicken
Tia1	-0.09	1.00000000	chicken
Ppp1cb	0.02	1.00000000	chicken
Ago2	-0.05	1.00000000	chicken
Rnf130	-0.20	1.00000000	chicken
Tent4b	-0.27	1.00000000	chicken
Supt6	-0.05	1.00000000	chicken
Adsl	-0.15	1.00000000	chicken
Slu7	-0.15	1.00000000	chicken
Snrpd3	-0.12	1.00000000	chicken
Hacd2	-0.04	1.00000000	chicken
Rab7	-0.19	1.00000000	chicken
Adgrg2	-0.01	1.00000000	chicken
Cog6	0.10	1.00000000	chicken
Stag2	0.06	1.00000000	chicken
Nfat5	-0.05	1.00000000	chicken
Arcn1	-0.20	1.00000000	chicken
Fyn	-0.14	1.00000000	chicken
Fhit	0.18	1.00000000	chicken
Ints2	-0.17	1.00000000	chicken
Cop1	0.04	1.00000000	chicken
Polr2c	-0.13	1.00000000	chicken
Rab5c	0.06	1.00000000	chicken
Kat2b	-0.20	1.00000000	chicken
Pdss2	-0.04	1.00000000	chicken
Pdlim1	-0.12	1.00000000	chicken
Adam17	-0.07	1.00000000	chicken
Aig1	0.04	1.00000000	chicken
Krr1	-0.02	1.00000000	chicken
Slx4	0.02	1.00000000	chicken

Csl	-0.06	1.00000000	chicken
Tsc22d2	-0.11	1.00000000	chicken
Dkk3	0.06	1.00000000	chicken
Slc9a6	-0.16	1.00000000	chicken
Yme1l1	-0.01	1.00000000	chicken
Kctd2	0.01	1.00000000	chicken
Nfia	0.05	1.00000000	chicken
Ctnnd1	-0.13	1.00000000	chicken
Tbc1d25	0.03	1.00000000	chicken
Uqcrc1	-0.03	1.00000000	chicken
Utp6	0.04	1.00000000	chicken
Cabin1	-0.10	1.00000000	chicken
Tpd52	-0.15	1.00000000	chicken
Nasp	-0.23	1.00000000	chicken
Pcf11	-0.16	1.00000000	chicken
Ube2q1	-0.22	1.00000000	chicken
Rer1	-0.19	1.00000000	chicken
Hnrnpu	0.01	1.00000000	chicken
Btaf1	-0.04	1.00000000	chicken
Tmem241	-0.13	1.00000000	chicken
Vps11	-0.15	1.00000000	chicken
Prpf3	-0.05	1.00000000	chicken
Lars	-0.18	1.00000000	chicken
Chchd1	-0.18	1.00000000	chicken
Srp54a	-0.08	1.00000000	chicken
Gxylt1	-0.17	1.00000000	chicken
Eapp	0.04	1.00000000	chicken
Larp1b	0.00	1.00000000	chicken
Bnip2	-0.11	1.00000000	chicken
Asxl3	0.09	1.00000000	chicken
Slc7a3	-0.03	1.00000000	chicken
Bms1	-0.04	1.00000000	chicken
Syncrip	-0.05	1.00000000	chicken
E2f6	-0.04	1.00000000	chicken
Atg4a-ps	-0.04	1.00000000	chicken
Btbd1	0.01	1.00000000	chicken
Ddx41	0.01	1.00000000	chicken
Gdf6	0.09	1.00000000	chicken
Ikzf2	0.01	1.00000000	chicken
Ralgapa1	0.03	1.00000000	chicken
Anxa11	0.11	1.00000000	chicken
Stk26	-0.08	1.00000000	chicken
Grk2	-0.08	1.00000000	chicken
Utrn	-0.02	1.00000000	chicken
Ift22	-0.01	1.00000000	chicken
Sec31a	-0.17	1.00000000	chicken

Atf6	-0.03	1.00000000	chicken
C2cd2l	-0.18	1.00000000	chicken
Enpp2	-0.12	1.00000000	chicken
Phf6	-0.12	1.00000000	chicken
Prdm16	0.07	1.00000000	chicken
Uba3	-0.02	1.00000000	chicken
Etf1	-0.01	1.00000000	chicken
Cttnbp2nl	-0.01	1.00000000	chicken
Inpp5k	-0.15	1.00000000	chicken
Dpagt1	-0.09	1.00000000	chicken
Chmp2a	-0.01	1.00000000	chicken
Vps41	-0.18	1.00000000	chicken
Peli1	-0.01	1.00000000	chicken
Ubn2	0.01	1.00000000	chicken
Gnptg	0.04	1.00000000	chicken
Spty2d1	-0.25	1.00000000	chicken
Zfp451	-0.07	1.00000000	chicken
Abhd5	0.05	1.00000000	chicken
Arsj	0.15	1.00000000	chicken
Alas1	0.12	1.00000000	chicken
Rab5a	-0.10	1.00000000	chicken
Itfg1	0.01	1.00000000	chicken
Afg3l2	-0.02	1.00000000	chicken
Zfp704	0.16	1.00000000	chicken
Ppfibp1	0.31	1.00000000	chicken
Eno1b	-0.15	1.00000000	chicken
Tubg1	0.04	1.00000000	chicken
Desi1	-0.07	1.00000000	chicken
Fars2	0.10	1.00000000	chicken
Trappc2	-0.14	1.00000000	chicken
Pkn2	-0.01	1.00000000	chicken
Luc7l3	-0.19	1.00000000	chicken
Apba1	-0.19	1.00000000	chicken
Ddit4	-0.10	1.00000000	chicken
Polr2i	-0.15	1.00000000	chicken
Ptgr1	0.16	1.00000000	chicken
Hoxa3	-0.06	1.00000000	chicken
Fez2	-0.05	1.00000000	chicken
Zfp352	-0.14	1.00000000	chicken
Epm2a	-0.01	1.00000000	chicken
Tmem135	-0.02	1.00000000	chicken
Qars	-0.03	1.00000000	chicken
Emc3	0.01	1.00000000	chicken
Ireb2	-0.15	1.00000000	chicken
Hes1	-0.01	1.00000000	chicken
Pfdn1	-0.25	1.00000000	chicken

Rab3gap1	0.09	1.00000000	chicken
Homer1	0.09	1.00000000	chicken
Tm9sf2	0.00	1.00000000	chicken
Ubn1	-0.15	1.00000000	chicken
Ppp4r1	0.08	1.00000000	chicken
Heca	-0.19	1.00000000	chicken
Rlim	-0.01	1.00000000	chicken
Creb1	0.03	1.00000000	chicken
Tbc1d22a	0.04	1.00000000	chicken
Ppp1r7	0.05	1.00000000	chicken
Ergic2	-0.03	1.00000000	chicken
Vps35	0.05	1.00000000	chicken
Ube2q2	-0.10	1.00000000	chicken
Acbd3	-0.21	1.00000000	chicken
Hpcal1	-0.24	1.00000000	chicken
Psmb7	-0.19	1.00000000	chicken
Galnt2	-0.11	1.00000000	chicken
Drg1	0.02	1.00000000	chicken
Brpf3	-0.10	1.00000000	chicken
Rbm39	-0.16	1.00000000	chicken
Stk24	-0.08	1.00000000	chicken
Ybx3	-0.02	1.00000000	chicken
Atg3	0.02	1.00000000	chicken
Fam120b	-0.14	1.00000000	chicken
Cript	-0.06	1.00000000	chicken
Alkbh5	-0.23	1.00000000	chicken
Vamp3	-0.07	1.00000000	chicken
Ppp6r3	0.01	1.00000000	chicken
Elp4	0.08	1.00000000	chicken
Eif3d	-0.13	1.00000000	chicken
Arl6ip4	-0.05	1.00000000	chicken
Cttn	-0.19	1.00000000	chicken
Cndp2	-0.16	1.00000000	chicken
Rsbn1	-0.05	1.00000000	chicken
Ing1	-0.08	1.00000000	chicken
Cars2	-0.19	1.00000000	chicken
Ikbkb	0.01	1.00000000	chicken
Ergic3	-0.18	1.00000000	chicken
Atp6v0a2	-0.07	1.00000000	chicken
Tbcd	0.04	1.00000000	chicken
Inpp5f	-0.17	1.00000000	chicken
Phf20	0.04	1.00000000	chicken
Iars2	-0.14	1.00000000	chicken
Cped1	0.45	1.00000000	chicken
Tmem115	-0.18	1.00000000	chicken
Tcerg1	-0.05	1.00000000	chicken

Sfxn3	0.02	1.00000000	chicken
Pcgf5	-0.24	1.00000000	chicken
Sec62	-0.01	1.00000000	chicken
Blmh	0.00	1.00000000	chicken
Dnpep	-0.13	1.00000000	chicken
Bag1	-0.18	1.00000000	chicken
Pcnp	-0.03	1.00000000	chicken
Cactin	-0.01	1.00000000	chicken
Clk4	-0.09	1.00000000	chicken
Snx1	-0.08	1.00000000	chicken
Slc30a4	0.05	1.00000000	chicken
Fgfr1op2	-0.05	1.00000000	chicken
Camk2g	-0.02	1.00000000	chicken
Lman1	-0.17	1.00000000	chicken
Tln2	0.09	1.00000000	chicken
Elf2	-0.06	1.00000000	chicken
Fxr1	-0.04	1.00000000	chicken
Socs5	-0.04	1.00000000	chicken
Atp11a	0.15	1.00000000	chicken
Ank	-0.15	1.00000000	chicken
Vma21	-0.11	1.00000000	chicken
Tcf12	0.02	1.00000000	chicken
Acox1	0.06	1.00000000	chicken
Psap	-0.18	1.00000000	chicken
Phb	-0.13	1.00000000	chicken
Rc3h1	0.05	1.00000000	chicken
Sdha	-0.20	1.00000000	chicken
Gfpt1	0.03	1.00000000	chicken
Lamp2	-0.12	1.00000000	chicken
Vgll4	0.08	1.00000000	chicken
Dyrk2	-0.20	1.00000000	chicken
Slc25a36	0.02	1.00000000	chicken
Kidins220	-0.08	1.00000000	chicken
Smarcd2	-0.19	1.00000000	chicken
Arl4a	-0.23	1.00000000	chicken
Uso1	-0.04	1.00000000	chicken
Dym	0.08	1.00000000	chicken
Amot	-0.01	1.00000000	chicken
Wdr74	-0.08	1.00000000	chicken
Fbrsl1	-0.02	1.00000000	chicken
Vps37b	0.00	1.00000000	chicken
Rad54l2	-0.15	1.00000000	chicken
Gnpat	-0.03	1.00000000	chicken
Ewsr1	-0.25	1.00000000	chicken
Cerk	-0.04	1.00000000	chicken
Sec22b	0.06	1.00000000	chicken

Phlpp1	0.01	1.00000000	chicken
Hipk1	-0.08	1.00000000	chicken
Nelfa	-0.07	1.00000000	chicken
Tgs1	-0.05	1.00000000	chicken
Xpo6	-0.24	1.00000000	chicken
Pdzrn3	0.14	1.00000000	chicken
Kirrel	-0.28	1.00000000	chicken
Selenof	0.03	1.00000000	chicken
Slc49a4	-0.22	1.00000000	chicken
Timm44	-0.03	1.00000000	chicken
Bbs9	-0.05	1.00000000	chicken
Gstp3	-0.08	1.00000000	chicken
Chuk	-0.11	1.00000000	chicken
Tcf20	0.02	1.00000000	chicken
Tmem167	-0.14	1.00000000	chicken
Slc2a13	-0.12	1.00000000	chicken
Gna13	0.11	1.00000000	chicken
1700025G04Rik	0.14	1.00000000	chicken
Fchsd2	-0.12	1.00000000	chicken
Anapc16	0.00	1.00000000	chicken
Dhx30	-0.05	1.00000000	chicken
Ist1	-0.14	1.00000000	chicken
Cdc14b	-0.03	1.00000000	chicken
Lrrc59	-0.15	1.00000000	chicken
Socs3	0.12	1.00000000	chicken
Vps8	-0.13	1.00000000	chicken
Prpf6	-0.24	1.00000000	chicken
Rpf1	0.03	1.00000000	chicken
Pfkfb3	-0.16	1.00000000	chicken
Snap23	0.01	1.00000000	chicken
Bcas2	-0.07	1.00000000	chicken
Taldo1	-0.21	1.00000000	chicken
Tes	-0.06	1.00000000	chicken
Opa1	-0.11	1.00000000	chicken
Akr1c13	-0.09	1.00000000	chicken
Wtap	-0.17	1.00000000	chicken
Bclaf1	-0.17	1.00000000	chicken
Ccdc59	-0.02	1.00000000	chicken
Tcp11l1	-0.05	1.00000000	chicken
Hps3	-0.21	1.00000000	chicken
Tmem9b	-0.09	1.00000000	chicken
Copb2	-0.06	1.00000000	chicken
Bnip3	0.09	1.00000000	chicken
Smad5	-0.10	1.00000000	chicken
Unc5c	0.96	1.00000000	chicken
Siah1b	-0.06	1.00000000	chicken

Ankmy2	-0.15	1.00000000	chicken
Sar1b	-0.15	1.00000000	chicken
Pcolce2	-0.11	1.00000000	chicken
Azi2	-0.09	1.00000000	chicken
Psmc5	0.00	1.00000000	chicken
Sap30l	-0.17	1.00000000	chicken
Srrm1	-0.18	1.00000000	chicken
Ap2b1	-0.17	1.00000000	chicken
Abcd3	-0.14	1.00000000	chicken
Anxa1	0.43	1.00000000	chicken
Rab39b	-0.05	1.00000000	chicken
Adss	0.10	1.00000000	chicken
Psma4	0.03	1.00000000	chicken
Pde7b	-0.18	1.00000000	chicken
Pias1	0.04	1.00000000	chicken
Xpo1	0.11	1.00000000	chicken
Exd2	-0.03	1.00000000	chicken
Pcdhgb4	0.08	1.00000000	chicken
Sarnp	-0.10	1.00000000	chicken
Prpf4	-0.01	1.00000000	chicken
Ppp2ca	-0.21	1.00000000	chicken
Sde2	-0.21	1.00000000	chicken
Rasal2	0.12	1.00000000	chicken
Ppp2r5c	-0.11	1.00000000	chicken
Asah1	-0.06	1.00000000	chicken
Itprid2	-0.04	1.00000000	chicken
Gnb4	-0.18	1.00000000	chicken
Ttc7b	-0.08	1.00000000	chicken
Sel1l	-0.13	1.00000000	chicken
Ice2	-0.01	1.00000000	chicken
Ubr1	-0.19	1.00000000	chicken
Ctdspl2	-0.15	1.00000000	chicken
Tnfaip8	-0.02	1.00000000	chicken
Strn3	0.00	1.00000000	chicken
Crtc1	-0.09	1.00000000	chicken
Eml6	-0.03	1.00000000	chicken
Srp68	-0.07	1.00000000	chicken
Srp14	-0.12	1.00000000	chicken
Lyrm4	-0.04	1.00000000	chicken
Ecsit	0.15	1.00000000	chicken
Atg12	-0.11	1.00000000	chicken
Stx2	-0.13	1.00000000	chicken
Abhd18	-0.05	1.00000000	chicken
Reck	-0.16	1.00000000	chicken
Prkcsh	0.02	1.00000000	chicken
Ilk	-0.27	1.00000000	chicken

Zfp639	-0.12	1.00000000	chicken
Dusp6	-0.12	1.00000000	chicken
Rab22a	-0.03	1.00000000	chicken
Secisbp2	-0.16	1.00000000	chicken
Ep300	-0.17	1.00000000	chicken
Ehmt1	-0.18	1.00000000	chicken
Wdr12	0.01	1.00000000	chicken
Nedd4	0.00	1.00000000	chicken
Kat7	-0.01	1.00000000	chicken
Dstn	-0.03	1.00000000	chicken
Siva1	-0.05	1.00000000	chicken
Fam126a	0.04	1.00000000	chicken
Eif1b	-0.10	1.00000000	chicken
Rell1	-0.21	1.00000000	chicken
Zbtb2	0.01	1.00000000	chicken
Abca1	0.08	1.00000000	chicken
Pld1	0.02	1.00000000	chicken
Canx	-0.16	1.00000000	chicken
Vps26a	-0.03	1.00000000	chicken
Yeats4	-0.04	1.00000000	chicken
Plekha5	0.03	1.00000000	chicken
Cpeb3	-0.13	1.00000000	chicken
Sfmbt2	-0.14	1.00000000	chicken
Reps1	-0.14	1.00000000	chicken
D16Ertd472e	-0.08	1.00000000	chicken
Wbp2nl	-0.04	1.00000000	chicken
Itga9	0.08	1.00000000	chicken
Pudp	-0.06	1.00000000	chicken
Pak2	-0.08	1.00000000	chicken
Glrx3	-0.10	1.00000000	chicken
Brms1l	-0.12	1.00000000	chicken
Parp1	-0.04	1.00000000	chicken
BC005624	-0.03	1.00000000	chicken
Hspg2	-0.03	1.00000000	chicken
Relch	-0.12	1.00000000	chicken
Ube2g2	-0.10	1.00000000	chicken
Atrn	0.10	1.00000000	chicken
Taf2	-0.05	1.00000000	chicken
1110004F10Rik	-0.15	1.00000000	chicken
Ubqln4	-0.02	1.00000000	chicken
Rchy1	-0.11	1.00000000	chicken
Rtf2	-0.15	1.00000000	chicken
Ndufs6	-0.10	1.00000000	chicken
Adnp	-0.10	1.00000000	chicken
Tra2a	-0.11	1.00000000	chicken
Nifk	-0.07	1.00000000	chicken

Foxp2	0.24	1.00000000	chicken
Ptpn11	-0.06	1.00000000	chicken
Tbx4	0.02	1.00000000	chicken
Ap1ar	-0.12	1.00000000	chicken
Ephb1	-0.05	1.00000000	chicken
Cybrd1	0.03	1.00000000	chicken
Ift57	-0.12	1.00000000	chicken
Sh3rf1	0.00	1.00000000	chicken
Dicer1	-0.01	1.00000000	chicken
Get4	-0.14	1.00000000	chicken
Vps45	-0.05	1.00000000	chicken
Cdk7	-0.12	1.00000000	chicken
Tubb5	-0.19	1.00000000	chicken
Sec13	-0.11	1.00000000	chicken
Nptn	-0.11	1.00000000	chicken
Il18	0.00	1.00000000	chicken
Phf20l1	-0.15	1.00000000	chicken
Nipa2	-0.14	1.00000000	chicken
Rufy3	-0.05	1.00000000	chicken
Gabarapl2	-0.02	1.00000000	chicken
Rbm22	-0.04	1.00000000	chicken
Lmna	-0.07	1.00000000	chicken
Arap2	-0.24	1.00000000	chicken
Ube2d1	-0.04	1.00000000	chicken
Ephb3	-0.04	1.00000000	chicken
Thoc7	0.05	1.00000000	chicken
Cox7a2l	-0.14	1.00000000	chicken
Enox2	-0.01	1.00000000	chicken
Snx7	-0.05	1.00000000	chicken
Zfp654	-0.11	1.00000000	chicken
Cbx4	-0.26	1.00000000	chicken
Usp3	-0.10	1.00000000	chicken
Exoc6	-0.12	1.00000000	chicken
Ivns1abp	-0.14	1.00000000	chicken
Rit1	0.01	1.00000000	chicken
Hectd4	-0.16	1.00000000	chicken
Cwc25	-0.04	1.00000000	chicken
Cpm	0.02	1.00000000	chicken
Aldh3a2	-0.06	1.00000000	chicken
Hs2st1	-0.06	1.00000000	chicken
Dhx36	-0.12	1.00000000	chicken
Bend3	-0.13	1.00000000	chicken
Ttc1	-0.04	1.00000000	chicken
Acap3	0.00	1.00000000	chicken
Ncoa1	-0.13	1.00000000	chicken
Nmt2	-0.14	1.00000000	chicken

Gpc5	-0.03	1.00000000	chicken
Lrif1	0.03	1.00000000	chicken
Tecpr2	-0.07	1.00000000	chicken
Gng12	-0.12	1.00000000	chicken
Cnot7	-0.10	1.00000000	chicken
Leprotl1	-0.06	1.00000000	chicken
Gosr2	-0.04	1.00000000	chicken
Fubp1	-0.08	1.00000000	chicken
Ccdc88c	-0.16	1.00000000	chicken
Slc35c1	-0.05	1.00000000	chicken
Rbbp6	-0.23	1.00000000	chicken
Trps1	-0.01	1.00000000	chicken
Cnot6	-0.04	1.00000000	chicken
Kdm1a	-0.16	1.00000000	chicken
Rbm31y	-0.13	1.00000000	chicken
Txnrd3	-0.18	1.00000000	chicken
Ino80	0.02	1.00000000	chicken
Arfip2	-0.10	1.00000000	chicken
Napg	-0.03	1.00000000	chicken
Nudt16l1	-0.18	1.00000000	chicken
Pknox1	-0.09	1.00000000	chicken
Ahsa2	-0.05	1.00000000	chicken
Rcan3	-0.15	1.00000000	chicken
Phf14	-0.23	1.00000000	chicken
Cisd2	-0.06	1.00000000	chicken
Ibtk	-0.21	1.00000000	chicken
Cry1	-0.02	1.00000000	chicken
Hecw2	0.04	1.00000000	chicken
Lrch2	-0.08	1.00000000	chicken
Nisch	-0.02	1.00000000	chicken
Tfpi2	0.14	1.00000000	chicken
Adcy9	-0.07	1.00000000	chicken
Dmxl1	-0.16	1.00000000	chicken
Ldhb	0.04	1.00000000	chicken
Glipr2	0.12	1.00000000	chicken
Rasl11b	-0.11	1.00000000	chicken
Gipc2	0.04	1.00000000	chicken
Zfp711	-0.08	1.00000000	chicken
Sucla2	-0.06	1.00000000	chicken
Scyl2	-0.15	1.00000000	chicken
Csde1	-0.06	1.00000000	chicken
Ap3b1	-0.11	1.00000000	chicken
Plekhm3	-0.09	1.00000000	chicken
Ctsz	-0.20	1.00000000	chicken
Tnpo1	0.02	1.00000000	chicken
Plekha1	-0.15	1.00000000	chicken

Bicd2	-0.17	1.00000000	chicken
Arrdc3	-0.22	1.00000000	chicken
Plekhg1	0.05	1.00000000	chicken
Ddx1	-0.15	1.00000000	chicken
Fst	-0.21	1.00000000	chicken
Ankhd1	0.02	1.00000000	chicken
Gorasp2	-0.06	1.00000000	chicken
Cdk8	-0.05	1.00000000	chicken
Nemf	-0.13	1.00000000	chicken
Abracl	-0.19	1.00000000	chicken
Ppcs	-0.08	1.00000000	chicken
Tmem100	-0.18	1.00000000	chicken
Gtf3a	-0.08	1.00000000	chicken
Cherp	-0.13	1.00000000	chicken
Smad7	-0.04	1.00000000	chicken
Sugt1	-0.01	1.00000000	chicken
Nbl1	-0.12	1.00000000	chicken
Nudcd3	-0.01	1.00000000	chicken
Nab1	-0.08	1.00000000	chicken
Slc16a9	0.09	1.00000000	chicken
Ctr9	-0.09	1.00000000	chicken
Plekhb2	-0.06	1.00000000	chicken
Naa30	-0.06	1.00000000	chicken
Per2	0.01	1.00000000	chicken
Kctd3	-0.13	1.00000000	chicken
Noc3l	-0.02	1.00000000	chicken
Snx3	-0.12	1.00000000	chicken
Psmg3	-0.10	1.00000000	chicken
Denr	-0.16	1.00000000	chicken
Ubtf	-0.05	1.00000000	chicken
Ube2a	-0.13	1.00000000	chicken
Tmem59	0.01	1.00000000	chicken
Cnih1	-0.18	1.00000000	chicken
Atp5g3	0.07	1.00000000	chicken
Ino80d	-0.15	1.00000000	chicken
Odc1	-0.24	1.00000000	chicken
Slc25a20	-0.12	1.00000000	chicken
Rnf20	-0.18	1.00000000	chicken
Atp6v0b	-0.03	1.00000000	chicken
Polr2k	-0.05	1.00000000	chicken
Ywhaz	-0.08	1.00000000	chicken
Exoc4	0.01	1.00000000	chicken
Golga7	-0.06	1.00000000	chicken
Arfgap1	-0.01	1.00000000	chicken
Wmp	0.03	1.00000000	chicken
Lgmn	-0.04	1.00000000	chicken

Fem1b	-0.07	1.00000000	chicken
Arntl	-0.08	1.00000000	chicken
Osbpl1a	0.04	1.00000000	chicken
Thra	-0.12	1.00000000	chicken
Pdk4	0.25	1.00000000	chicken
Dcp1b	-0.08	1.00000000	chicken
Lrpap1	0.02	1.00000000	chicken
Hnrnph3	-0.04	1.00000000	chicken
Dact1	-0.07	1.00000000	chicken
Ncoa4	-0.04	1.00000000	chicken
Tfg	0.01	1.00000000	chicken
Ppid	0.00	1.00000000	chicken
Hnrnpa2b1	0.03	1.00000000	chicken
Exoc1	0.02	1.00000000	chicken
Ss18	-0.14	1.00000000	chicken
Pdcl3	0.09	1.00000000	chicken
Spg21	-0.11	1.00000000	chicken
Cbwd1	0.00	1.00000000	chicken
Cc2d1b	-0.13	1.00000000	chicken
Ccdc93	-0.05	1.00000000	chicken
Unc50	-0.04	1.00000000	chicken
Dedd	-0.21	1.00000000	chicken
Acat1	-0.13	1.00000000	chicken
Tra2b	-0.06	1.00000000	chicken
Clns1a	-0.14	1.00000000	chicken
Akr1a1	-0.05	1.00000000	chicken
Ormdl2	0.02	1.00000000	chicken
Usp4	-0.05	1.00000000	chicken
Snx33	-0.05	1.00000000	chicken
Gypc	0.07	1.00000000	chicken
Nxt2	-0.07	1.00000000	chicken
Cyth3	0.08	1.00000000	chicken
Lrp1	-0.13	1.00000000	chicken
Nelfcd	-0.01	1.00000000	chicken
Glyr1	-0.16	1.00000000	chicken
Tmem165	-0.01	1.00000000	chicken
Steep1	-0.09	1.00000000	chicken
Klhl15	-0.06	1.00000000	chicken
Epc2	-0.06	1.00000000	chicken
Hoxa4	-0.09	1.00000000	chicken
Uap1	-0.09	1.00000000	chicken
Ppil2	-0.15	1.00000000	chicken
Nr3c2	0.14	1.00000000	chicken
Etv5	-0.13	1.00000000	chicken
Colgalt1	-0.01	1.00000000	chicken
Cnot4	-0.15	1.00000000	chicken

Pcsk7	-0.16	1.00000000	chicken
Nrf1	0.00	1.00000000	chicken
Nckipsd	-0.07	1.00000000	chicken
Cebpg	-0.06	1.00000000	chicken
Chid1	0.01	1.00000000	chicken
Nfil3	0.04	1.00000000	chicken
Pdap1	-0.10	1.00000000	chicken
Tmod3	-0.02	1.00000000	chicken
Ccn1	-0.07	1.00000000	chicken
Clptm1l	-0.21	1.00000000	chicken
Sel1l3	-0.05	1.00000000	chicken
Ube2v2	-0.10	1.00000000	chicken
Parva	-0.24	1.00000000	chicken
Exosc10	-0.04	1.00000000	chicken
Kmt2c	-0.06	1.00000000	chicken
Far2	0.12	1.00000000	chicken
Pum3	0.01	1.00000000	chicken
Smarcc1	-0.08	1.00000000	chicken
Fbxo8	-0.08	1.00000000	chicken
Ddx46	-0.13	1.00000000	chicken
Zcchc17	-0.03	1.00000000	chicken
Rasl11a	0.01	1.00000000	chicken
Dido1	-0.16	1.00000000	chicken
Fam135a	-0.09	1.00000000	chicken
Cltc	-0.17	1.00000000	chicken
Bmp2k	-0.09	1.00000000	chicken
Commd10	-0.23	1.00000000	chicken
Ccdc171	-0.07	1.00000000	chicken
Vezf1	-0.05	1.00000000	chicken
Srfbp1	0.05	1.00000000	chicken
Nek9	-0.11	1.00000000	chicken
Appbp2	-0.08	1.00000000	chicken
Zfhx4	-0.06	1.00000000	chicken
Ebag9	-0.03	1.00000000	chicken
Washc2	-0.10	1.00000000	chicken
Lonp2	-0.10	1.00000000	chicken
Pfn2	-0.15	1.00000000	chicken
Banp	-0.04	1.00000000	chicken
Nsd3	-0.10	1.00000000	chicken
Sec23b	-0.22	1.00000000	chicken
Wwc2	-0.08	1.00000000	chicken
Pla2r1	-0.17	1.00000000	chicken
Wsb1	-0.07	1.00000000	chicken
Peli2	0.24	1.00000000	chicken
Sacs	-0.15	1.00000000	chicken
Emp2	-0.05	1.00000000	chicken

Vps25	-0.12	1.00000000	chicken
Pkp4	-0.15	1.00000000	chicken
Naca	-0.11	1.00000000	chicken
Bmt2	-0.04	1.00000000	chicken
Gosr1	-0.05	1.00000000	chicken
Zbtb8b	-0.08	1.00000000	chicken
Plekha3	0.00	1.00000000	chicken
Taok1	-0.08	1.00000000	chicken
Adam9	-0.10	1.00000000	chicken
Psmb3	-0.01	1.00000000	chicken
Rbbp4	-0.11	1.00000000	chicken
Nol8	-0.05	1.00000000	chicken
Parn	-0.02	1.00000000	chicken
Nup93	-0.10	1.00000000	chicken
Gabpa	-0.13	1.00000000	chicken
Wnt11	-0.04	1.00000000	chicken
Sik1	-0.03	1.00000000	chicken
Nr2c2	-0.09	1.00000000	chicken
Atp1a1	0.11	1.00000000	chicken
Txndc12	-0.09	1.00000000	chicken
Fam177a	-0.10	1.00000000	chicken
Tssc4	-0.09	1.00000000	chicken
Sppl3	-0.05	1.00000000	chicken
Tmx3	-0.11	1.00000000	chicken
Prr14l	-0.17	1.00000000	chicken
Prrc2b	-0.22	1.00000000	chicken
Esco1	-0.09	1.00000000	chicken
Traf6	-0.07	1.00000000	chicken
Tmem230	-0.07	1.00000000	chicken
Atp11b	-0.11	1.00000000	chicken
Ccndbp1	0.02	1.00000000	chicken
Rnf114	-0.08	1.00000000	chicken
Trabd2b	0.13	1.00000000	chicken
Tpd52l2	-0.07	1.00000000	chicken
Chrdl1	-0.14	1.00000000	chicken
Nap1l4	-0.18	1.00000000	chicken
Raf1	-0.18	1.00000000	chicken
Arpc1b	-0.08	1.00000000	chicken
Rsrc1	-0.13	1.00000000	chicken
Acbd6	-0.05	1.00000000	chicken
Slc48a1	-0.09	1.00000000	chicken
Dcun1d1	-0.06	1.00000000	chicken
Ube2j2	-0.12	1.00000000	chicken
Bcar3	-0.19	1.00000000	chicken
Snx2	-0.01	1.00000000	chicken
Rprd2	-0.10	1.00000000	chicken

Ube2r2	-0.14	1.00000000	chicken
Tulp3	-0.02	1.00000000	chicken
Srpx	-0.09	1.00000000	chicken
Tcp1	-0.09	1.00000000	chicken
Lmo4	-0.12	1.00000000	chicken
Rgs6	0.08	1.00000000	chicken
Rwdd1	-0.11	1.00000000	chicken
Abcc1	-0.03	1.00000000	chicken
Hmox2	-0.10	1.00000000	chicken
Acadsb	-0.10	1.00000000	chicken
Gpr107	-0.11	1.00000000	chicken
Cdc37	-0.15	1.00000000	chicken
Tcf7l2	-0.14	1.00000000	chicken
Stk3	-0.16	1.00000000	chicken
Suclg1	-0.10	1.00000000	chicken
Ctnnbl1	0.04	1.00000000	chicken
Sms	-0.07	1.00000000	chicken
Suco	-0.06	1.00000000	chicken
Septin8	-0.17	1.00000000	chicken
Pcbp2	-0.14	1.00000000	chicken
Ccdc82	-0.10	1.00000000	chicken
Ccnc	-0.04	1.00000000	chicken
Otud6b	-0.13	1.00000000	chicken
Irak2	-0.13	1.00000000	chicken
Sem1	-0.06	1.00000000	chicken
Slc35b1	-0.07	1.00000000	chicken
Ankle2	-0.03	1.00000000	chicken
Ralgps2	0.02	1.00000000	chicken
Hspa5	0.17	1.00000000	chicken
Ints6	0.00	1.00000000	chicken
Nnt	-0.05	1.00000000	chicken
Ldlrad3	-0.04	1.00000000	chicken
Tm9sf5	-0.05	1.00000000	chicken
Rbl2	-0.09	1.00000000	chicken
Prkd3	-0.04	1.00000000	chicken
Twist2	-0.03	1.00000000	chicken
Pde1c	0.06	1.00000000	chicken
Ccdc6	-0.11	1.00000000	chicken
Pals1	-0.13	1.00000000	chicken
Dnajb2	-0.13	1.00000000	chicken
Twf1	-0.04	1.00000000	chicken
Ctnna2	0.31	1.00000000	chicken
Atp2b1	-0.14	1.00000000	chicken
Numa1	-0.08	1.00000000	chicken
Rab5if	-0.05	1.00000000	chicken
Zfyve21	-0.17	1.00000000	chicken

Sike1	-0.07	1.00000000	chicken
Lin7a	-0.06	1.00000000	chicken
Rabggtb	-0.12	1.00000000	chicken
Cdc37l1	-0.10	1.00000000	chicken
Stim1	-0.05	1.00000000	chicken
Dynlrb1	-0.12	1.00000000	chicken
Sec24a	-0.06	1.00000000	chicken
Acvr1	-0.18	1.00000000	chicken
Ccdc43	-0.07	1.00000000	chicken
Nsrp1	-0.10	1.00000000	chicken
Phax	-0.03	1.00000000	chicken
Dyrk1a	-0.17	1.00000000	chicken
Ano3	0.04	1.00000000	chicken
Atxn2	-0.18	1.00000000	chicken
Vps13d	-0.14	1.00000000	chicken
Plk3	-0.20	1.00000000	chicken
Atm	-0.06	1.00000000	chicken
Fut8	-0.04	1.00000000	chicken
Smarce1	-0.11	1.00000000	chicken
Pafah1b1	-0.04	1.00000000	chicken
Gga3	-0.10	1.00000000	chicken
Ptpn13	-0.04	1.00000000	chicken
Col6a1	0.01	1.00000000	chicken
Arnt	-0.07	1.00000000	chicken
Prpf8	-0.11	1.00000000	chicken
Stub1	0.00	1.00000000	chicken
Bend7	-0.10	1.00000000	chicken
Ap2a1	-0.05	1.00000000	chicken
Snrpb	-0.09	1.00000000	chicken
Sft2d1	-0.11	1.00000000	chicken
Ap1g1	-0.14	1.00000000	chicken
Smap1	-0.07	1.00000000	chicken
Gata6	0.03	1.00000000	chicken
Wfdc1	0.07	1.00000000	chicken
Edc3	-0.09	1.00000000	chicken
Rabep1	-0.09	1.00000000	chicken
Auts2	-0.07	1.00000000	chicken
Ctsb	-0.07	1.00000000	chicken
Ust	0.10	1.00000000	chicken
Prdm11	-0.06	1.00000000	chicken
Pafah1b2	-0.09	1.00000000	chicken
Snrpc	-0.10	1.00000000	chicken
Aup1	-0.14	1.00000000	chicken
Tdpoz8	-0.08	1.00000000	chicken
Uspl1	-0.09	1.00000000	chicken
Tgfbr1	-0.07	1.00000000	chicken

Cfl2	-0.10	1.00000000	chicken
Brd7	-0.09	1.00000000	chicken
Clcn5	-0.09	1.00000000	chicken
Sltm	-0.02	1.00000000	chicken
Srsf3	-0.12	1.00000000	chicken
Arpc3	-0.11	1.00000000	chicken
Dvl1	-0.09	1.00000000	chicken
Ubap2	-0.02	1.00000000	chicken
Ptges	-0.01	1.00000000	chicken
Slc35e3	-0.01	1.00000000	chicken
Aars	-0.04	1.00000000	chicken
Shoc2	-0.06	1.00000000	chicken
Rsrc2	-0.09	1.00000000	chicken
Lmo2	0.04	1.00000000	chicken
Arpc2	-0.13	1.00000000	chicken
Dnajb4	-0.25	1.00000000	chicken
Gspt1	-0.04	1.00000000	chicken
Lonrf3	-0.10	1.00000000	chicken
Dusp7	-0.19	1.00000000	chicken
Fkbp11	-0.18	1.00000000	chicken
Lypla1	-0.07	1.00000000	chicken
Pcnx	-0.21	1.00000000	chicken
Ptp4a2	-0.10	1.00000000	chicken
Phkb	-0.05	1.00000000	chicken
Cdv3	-0.11	1.00000000	chicken
Rab21	-0.11	1.00000000	chicken
Ece1	-0.06	1.00000000	chicken
Trmt11	-0.06	1.00000000	chicken
Zmpste24	-0.05	1.00000000	chicken
Nrd1	-0.03	1.00000000	chicken
Rock1	-0.12	1.00000000	chicken
Psmd4	-0.01	1.00000000	chicken
Emc2	-0.09	1.00000000	chicken
Pdpk1	-0.17	1.00000000	chicken
Ccdc127	0.01	1.00000000	chicken
Itpkb	0.03	1.00000000	chicken
Gemin4	-0.12	1.00000000	chicken
Sgtb	-0.09	1.00000000	chicken
Snx13	-0.09	1.00000000	chicken
Eif3b	-0.10	1.00000000	chicken
Fubp3	-0.08	1.00000000	chicken
Appl1	-0.11	1.00000000	chicken
Rab12	-0.17	1.00000000	chicken
Sfswap	-0.12	1.00000000	chicken
Gzf1	-0.10	1.00000000	chicken
Cgnl1	0.07	1.00000000	chicken

Cltb	-0.16	1.00000000	chicken
Uchl5	-0.05	1.00000000	chicken
Lpcat2b	-0.13	1.00000000	chicken
Chka	-0.01	1.00000000	chicken
Lamtor1	-0.08	1.00000000	chicken
Trpm7	-0.13	1.00000000	chicken
Arl3	-0.20	1.00000000	chicken
Arpc1a	-0.17	1.00000000	chicken
Ercc6	-0.15	1.00000000	chicken
Atp6v1d	-0.18	1.00000000	chicken
Atxn1	-0.06	1.00000000	chicken
Zcchc24	0.21	1.00000000	chicken
Cct6b	-0.07	1.00000000	chicken
Ndufaf1	-0.09	1.00000000	chicken
Kbtbd2	-0.05	1.00000000	chicken
Scpep1	-0.01	1.00000000	chicken
Spg20	-0.15	1.00000000	chicken
Pepd	-0.04	1.00000000	chicken
Zc3h7a	-0.11	1.00000000	chicken
Cbfa2t3	-0.14	1.00000000	chicken
Prpf19	-0.10	1.00000000	chicken
Ipo8	-0.02	1.00000000	chicken
Erbin	-0.15	1.00000000	chicken
Rnf11	-0.07	1.00000000	chicken
Arhgap42	-0.01	1.00000000	chicken
Herc1	-0.11	1.00000000	chicken
Tsg101	-0.10	1.00000000	chicken
Prelp	-0.07	1.00000000	chicken
Septin9	-0.03	1.00000000	chicken
Ndufs7	-0.11	1.00000000	chicken
Atp2b4	0.00	1.00000000	chicken
Nr1d2	0.02	1.00000000	chicken
Akap8l	-0.06	1.00000000	chicken
Bbs7	-0.08	1.00000000	chicken
Psmc3	-0.09	1.00000000	chicken
Ufd1	-0.06	1.00000000	chicken
Snx12	-0.09	1.00000000	chicken
Eprs	0.04	1.00000000	chicken
Elmo2	-0.12	1.00000000	chicken
Casc3	-0.08	1.00000000	chicken
Sult4a1	-0.06	1.00000000	chicken
Ppt1	-0.03	1.00000000	chicken
Cdc27	-0.14	1.00000000	chicken
Carnmt1	-0.16	1.00000000	chicken
Psmb4	-0.15	1.00000000	chicken
Xrn1	-0.03	1.00000000	chicken

Ahcyl1	-0.14	1.00000000	chicken
Pigk	-0.08	1.00000000	chicken
Uchl3	-0.13	1.00000000	chicken
Nanp	-0.07	1.00000000	chicken
Znrf3	-0.05	1.00000000	chicken
Nfyc	-0.05	1.00000000	chicken
Polr2b	-0.08	1.00000000	chicken
Ankrd52	-0.07	1.00000000	chicken
Tspo	-0.12	1.00000000	chicken
Cops5	-0.10	1.00000000	chicken
Ebna1bp2	-0.13	1.00000000	chicken
Zmat2	-0.11	1.00000000	chicken
Cse1l	-0.06	1.00000000	chicken
Rexo1	-0.09	1.00000000	chicken
Ssh2	-0.18	1.00000000	chicken
Rnf34	-0.09	1.00000000	chicken
Pgam1	-0.06	1.00000000	chicken
Ctnnal1	-0.04	1.00000000	chicken
Pwwp2a	-0.06	1.00000000	chicken
Slc12a7	-0.08	1.00000000	chicken
Pbx1	0.00	1.00000000	chicken
Ifngr2	-0.11	1.00000000	chicken
Usp48	-0.10	1.00000000	chicken
Aamp	-0.06	1.00000000	chicken
R3hdm1	-0.15	1.00000000	chicken
Sgk1	0.01	1.00000000	chicken
Tmem33	-0.12	1.00000000	chicken
Large1	0.15	1.00000000	chicken
Ascc1	0.01	1.00000000	chicken
Tma16	0.00	1.00000000	chicken
Ddah1	-0.11	1.00000000	chicken
Arl8b	-0.05	1.00000000	chicken
Ddx23	-0.13	1.00000000	chicken
Ccser1	-0.01	1.00000000	chicken
Dctn6	-0.01	1.00000000	chicken
Kdm5a	-0.11	1.00000000	chicken
Hspa4	0.17	1.00000000	chicken
Xrn2	-0.12	1.00000000	chicken
Ost4	-0.11	1.00000000	chicken
Aktip	-0.14	1.00000000	chicken
Lpp	0.10	1.00000000	chicken
Spry1	-0.10	1.00000000	chicken
Ubtd2	-0.05	1.00000000	chicken
Ldlrad4	0.36	1.00000000	chicken
Hadhb	-0.08	1.00000000	chicken
Adarb1	0.17	1.00000000	chicken

Utp11	-0.07	1.00000000	chicken
Tmem208	-0.05	1.00000000	chicken
Ptbp2	-0.15	1.00000000	chicken
Fbxo11	-0.08	1.00000000	chicken
Rprd1b	-0.10	1.00000000	chicken
Oat	-0.04	1.00000000	chicken
Pitpnb	-0.07	1.00000000	chicken
Klhl5	-0.02	1.00000000	chicken
Kifbp	-0.04	1.00000000	chicken
Rubcnl	-0.05	1.00000000	chicken
Lcor	-0.11	1.00000000	chicken
Slirp	-0.07	1.00000000	chicken
Rnf150	0.14	1.00000000	chicken
Pi4ka	-0.11	1.00000000	chicken
Pdzd2	0.03	1.00000000	chicken
Prcc	-0.11	1.00000000	chicken
Ndufs3	-0.10	1.00000000	chicken
Zfand3	0.03	1.00000000	chicken
Pde7a	-0.07	1.00000000	chicken
Pan3	-0.09	1.00000000	chicken
Aagab	-0.01	1.00000000	chicken
Rab9	0.00	1.00000000	chicken
Washc3	0.00	1.00000000	chicken
Slc23a2	-0.10	1.00000000	chicken
Ap3m1	-0.12	1.00000000	chicken
Akap11	-0.14	1.00000000	chicken
Rpap3	-0.10	1.00000000	chicken
Ptpn1	-0.24	1.00000000	chicken
Ppp1r21	-0.10	1.00000000	chicken
Spon1	-0.30	1.00000000	chicken
Tollip	-0.09	1.00000000	chicken
Rrn3	-0.09	1.00000000	chicken
Ilf3	-0.10	1.00000000	chicken
Etfa	-0.06	1.00000000	chicken
Polr1d	-0.06	1.00000000	chicken
Dlgap1	-0.21	1.00000000	chicken
Exoc7	-0.09	1.00000000	chicken
Zfp609	-0.05	1.00000000	chicken
Foxn2	-0.10	1.00000000	chicken
Grb2	-0.21	1.00000000	chicken
Srgap3	-0.03	1.00000000	chicken
Arpc5	-0.13	1.00000000	chicken
Actb	0.22	1.00000000	chicken
Rnf217	-0.05	1.00000000	chicken
Pnisr	-0.09	1.00000000	chicken
Rasgrf2	0.26	1.00000000	chicken

Arid1b	-0.04	1.00000000	chicken
Atg5	-0.01	1.00000000	chicken
Zswim8	0.00	1.00000000	chicken
Pxylp1	-0.16	1.00000000	chicken
Pdp1	-0.12	1.00000000	chicken
Hsf3	-0.09	1.00000000	chicken
Npas2	0.22	1.00000000	chicken
Pank3	-0.07	1.00000000	chicken
Svil	0.12	1.00000000	chicken
Atf3	-0.23	1.00000000	chicken
Creld2	-0.14	1.00000000	chicken
Cct7	-0.09	1.00000000	chicken
Nde1	-0.12	1.00000000	chicken
Pola1	-0.12	1.00000000	chicken
Eif4h	-0.08	1.00000000	chicken
Fyttd1	-0.15	1.00000000	chicken
BC004004	-0.06	1.00000000	chicken
Rnf41	-0.08	1.00000000	chicken
Actn1	0.15	1.00000000	chicken
Smarca4	-0.08	1.00000000	chicken
Cfdp1	-0.19	1.00000000	chicken
Xrcc4	-0.09	1.00000000	chicken
Rhoj	0.17	1.00000000	chicken
Csnk1g1	-0.11	1.00000000	chicken
Wdr36	-0.08	1.00000000	chicken
Nfasc	-1.43	0.00000000	duck
Fscn1	-0.82	0.00000000	duck
Edil3	-2.34	0.00000000	duck
Cpe	-1.66	0.00000000	duck
Sema3d	-1.79	0.00000000	duck
Samhd1	2.36	0.00000000	duck
Pkp2	-1.13	0.00000000	duck
Itga11	-0.96	0.00000000	duck
Col1a1	-2.17	0.00000000	duck
Lvrn	-1.08	0.00000000	duck
Chdh	-0.69	0.00000000	duck
Itih5	-0.63	0.00000000	duck
Gfra4	-1.20	0.00000000	duck
Tll1	-1.30	0.00000000	duck
Nav2	-1.22	0.00000000	duck
Fn1	-1.77	0.00000000	duck
Selenop	-1.41	0.00000000	duck
Prss23	-1.31	0.00000000	duck
Kcne4	-0.76	0.00000000	duck
Igfbp7	-1.42	0.00000000	duck
Sh3pxd2a	-1.32	0.00000000	duck

Fgl2	-1.48	0.00000000	duck
C1qtnf2	-0.55	0.00000000	duck
Col1a2	-1.51	0.00000000	duck
Eva1b	-0.66	0.00000000	duck
Sparc	-1.10	0.00000000	duck
Dcn	-0.91	0.00000000	duck
Sox6	-3.10	0.00000000	duck
Sptan1	-1.14	0.00000000	duck
Tef	-1.17	0.00000000	duck
Ecrg4	-1.06	0.00000000	duck
Fkbp10	-0.51	0.00000000	duck
Postn	-1.71	0.00000000	duck
Clec3b	-1.24	0.00000000	duck
Lhfpl2	-0.93	0.00000000	duck
Fam180a	-0.85	0.00000000	duck
Bche	-0.98	0.00000000	duck
Colec10	-0.71	0.00000000	duck
Rspo2	-1.07	0.00000000	duck
Sulf2	-1.21	0.00000000	duck
Synm	-1.78	0.00000000	duck
Col8a1	-1.61	0.00000000	duck
Flrt2	-0.99	0.00000000	duck
Prkar2b	-0.54	0.00000000	duck
Scara5	-0.49	0.00000000	duck
Olfml1	-0.58	0.00000000	duck
Thbs1	-1.52	0.00000000	duck
Stmn2	-1.26	0.00000000	duck
Dock4	-0.86	0.00000000	duck
Boc	-0.65	0.00000000	duck
Epha4	-0.77	0.00000000	duck
Tnfaip2	-0.71	0.00000000	duck
Spon2	-1.87	0.00000000	duck
Sh3bp4	-0.48	0.00000000	duck
C1qtnf6	-0.29	0.00000000	duck
Srpx	-1.05	0.00000000	duck
Ecm2	-1.13	0.00000000	duck
Hic1	-0.52	0.00000000	duck
Gdf7	-0.36	0.00000000	duck
Dact1	-0.89	0.00000000	duck
Ephb2	-0.59	0.00000000	duck
Limch1	-0.50	0.00000000	duck
S1pr3	-0.54	0.00000000	duck
Adamts12	-0.59	0.00000000	duck
Stat1	1.18	0.00000000	duck
Pcdh18	-0.53	0.00000000	duck
Fkbp5	1.20	0.00000000	duck

Lamb2	-0.54	0.00000000	duck
Antxr1	-0.87	0.00000000	duck
Serpinh1	-0.79	0.00000000	duck
Dpysl3	-0.55	0.00000000	duck
Tcerg1l	-0.33	0.00000000	duck
Ckap4	-0.67	0.00000000	duck
Tsc22d3	1.42	0.00000000	duck
Jcad	-0.48	0.00000000	duck
Zbtb16	1.31	0.00000000	duck
Vegfd	-0.84	0.00000000	duck
Lmcd1	-0.65	0.00000000	duck
Ctsk	-0.57	0.00000000	duck
B2m	0.99	0.00000000	duck
Rfesd	-1.36	0.00000000	duck
Lum	-0.99	0.00000000	duck
Fbn1	-0.94	0.00000000	duck
Lima1	-0.51	0.00000000	duck
Pros1	-0.75	0.00000000	duck
Ramp3	-0.43	0.00000000	duck
Frmd4b	-0.60	0.00000000	duck
Atp10a	-0.74	0.00000000	duck
Ogn	-1.81	0.00000000	duck
Dclk1	-0.83	0.00000000	duck
Pmepa1	-0.94	0.00000000	duck
Emp1	-0.83	0.00000000	duck
Nid1	-0.97	0.00000000	duck
B3gnt9	-0.33	0.00000000	duck
Gsg1	-0.51	0.00000000	duck
Fbln1	-0.61	0.00000000	duck
Abca1	1.53	0.00000000	duck
Txndc5	-0.37	0.00000000	duck
Sgce	-0.60	0.00000000	duck
Poglut3	-0.58	0.00000000	duck
Cd99l2	-0.46	0.00000000	duck
Ctdspl	1.02	0.00000000	duck
C1s1	1.48	0.00000000	duck
P4ha2	-0.63	0.00000000	duck
Cln6	-0.30	0.00000000	duck
Arrdc3	1.01	0.00000000	duck
Gdf11	-0.24	0.00000000	duck
Hspa2	1.91	0.00000000	duck
Epsti1	1.52	0.00000000	duck
Tenm4	-1.14	0.00000000	duck
Gng10	-0.51	0.00000000	duck
Aif1l	-0.31	0.00000000	duck
Prrx1	-0.70	0.00000000	duck

Fam162a	-0.56	0.00000000	duck
Lama4	-0.82	0.00000000	duck
Parp1	-0.51	0.00000000	duck
Vash1	-0.80	0.00000000	duck
Cct3	-0.52	0.00000000	duck
Vgll3	-0.54	0.00000000	duck
Rpia	-0.51	0.00000000	duck
Cdr2	-0.45	0.00000000	duck
Adam12	-0.66	0.00000000	duck
Tmem200a	-0.29	0.00000000	duck
Etfrf1	-0.41	0.00000000	duck
Dok5	-0.26	0.00000000	duck
Lrrc17	-0.27	0.00000000	duck
Dennd2a	-0.36	0.00000000	duck
Crybg1	-0.50	0.00000000	duck
Acaa2	-0.34	0.00000000	duck
Plod2	-0.44	0.00000000	duck
Hvcn1	-0.50	0.00000000	duck
Osr1	-0.93	0.00000000	duck
Flt1	-0.79	0.00000000	duck
Tmem131	-0.91	0.00000000	duck
Trpc1	-0.27	0.00000000	duck
Rhoj	-0.74	0.00000000	duck
Pxdn	-0.51	0.00000000	duck
Col5a2	-0.85	0.00000000	duck
Tcaim	-0.48	0.00000000	duck
Fuca1	-0.39	0.00000000	duck
Ank2	-0.47	0.00000000	duck
Eogt	-0.45	0.00000000	duck
Palld	-1.46	0.00000000	duck
Vstm4	-0.25	0.00000000	duck
Setbp1	1.02	0.00000000	duck
Plac8	1.29	0.00000000	duck
Clip2	1.47	0.00000000	duck
Snx6	-0.51	0.00000000	duck
Kank4	-0.32	0.00000000	duck
Capn7	-0.64	0.00000000	duck
Plec	-0.68	0.00000000	duck
Tmtc1	-0.61	0.00000000	duck
Ldb2	-0.53	0.00000000	duck
Ntrk2	-1.13	0.00000000	duck
Cotl1	-0.39	0.00000000	duck
Psd3	-0.43	0.00000000	duck
Txndc9	-0.61	0.00000000	duck
Fmnl2	-0.66	0.00000000	duck
Creg1	-1.60	0.00000000	duck

Ifnar1	-0.71	0.00000000	duck
Fam114a1	-0.37	0.00000000	duck
Pi15	-0.57	0.00000000	duck
Fkbp9	-0.40	0.00000000	duck
Wfs1	-0.31	0.00000000	duck
Lrig3	-0.52	0.00000000	duck
Herc2	-0.84	0.00000000	duck
Wnt11	-0.59	0.00000000	duck
Aida	-0.41	0.00000000	duck
Ank1	-0.84	0.00000000	duck
Azi2	-0.50	0.00000000	duck
Ppib	-0.66	0.00000000	duck
Col6a3	-0.92	0.00000000	duck
Tacc2	-0.54	0.00000000	duck
Olfml3	-0.61	0.00000000	duck
Tgm2	-0.48	0.00000000	duck
Prickle1	-0.23	0.00000000	duck
Vdac2	-0.43	0.00000000	duck
Prkx	-0.65	0.00000000	duck
Bmf	-0.34	0.00000000	duck
Ahdc1	-0.37	0.00000000	duck
C1qtnf7	-0.38	0.00000000	duck
Rasd2	-0.32	0.00000000	duck
Bicc1	1.02	0.00000000	duck
Pcdh7	-0.86	0.00000000	duck
Pacs2	-0.29	0.00000000	duck
Tgfb3	-0.48	0.00000000	duck
Tmem200b	-0.20	0.00000000	duck
Nkd1	-0.56	0.00000000	duck
Golm2	-0.49	0.00000000	duck
Glt8d2	-0.18	0.00000000	duck
Cpz	-0.38	0.00000000	duck
Sec16a	-0.47	0.00000000	duck
Dpf3	-0.48	0.00000000	duck
Gramd3	-0.44	0.00000000	duck
Tmem47	-0.65	0.00000000	duck
Xylt1	-0.73	0.00000000	duck
Nup107	-0.41	0.00000000	duck
Sipa1l2	-0.82	0.00000000	duck
Nfil3	1.03	0.00000000	duck
Tpd52l2	-0.29	0.00000000	duck
Ncoa4	-0.52	0.00000000	duck
Luzp2	-0.58	0.00000000	duck
Extl2	-0.50	0.00000000	duck
Camk1d	-1.16	0.00000000	duck
Fkbp14	-0.32	0.00000000	duck

Ripor3	-1.87	0.00000000	duck
Adamts2	-0.75	0.00000000	duck
Galnt17	-0.68	0.00000000	duck
Cdh5	-0.34	0.00000000	duck
Arhgap17	-0.49	0.00000000	duck
Hspa5	-0.69	0.00000000	duck
Cab39l	-0.48	0.00000000	duck
Zfp651	-0.29	0.00000000	duck
Anxa6	-0.48	0.00000000	duck
Arsj	-0.22	0.00000000	duck
Rell1	-0.41	0.00000000	duck
Tpm1	-0.55	0.00000000	duck
Zmiz1	0.80	0.00000000	duck
Cish	-0.68	0.00000000	duck
Hectd2	-0.56	0.00000000	duck
Elk3	-0.26	0.00000000	duck
Sgcb	-0.38	0.00000000	duck
Lpin1	1.38	0.00000000	duck
Col18a1	-0.32	0.00000000	duck
Rflnb	-0.25	0.00000000	duck
Sytl2	-0.27	0.00000000	duck
Fst	1.70	0.00000000	duck
Cdh11	-0.64	0.00000000	duck
Arcn1	-0.51	0.00000000	duck
Septin7	-0.63	0.00000000	duck
Lrig1	-0.29	0.00000000	duck
Fat1	-0.57	0.00000000	duck
Ndufb9	-0.23	0.00000000	duck
Svep1	-0.51	0.00000000	duck
Slc16a7	-0.34	0.00000000	duck
Fzd1	-0.49	0.00000000	duck
Vrk2	-0.60	0.00000000	duck
Ybx1	-0.59	0.00000000	duck
Igsf10	-0.23	0.00000000	duck
Hacd3	-0.23	0.00000000	duck
Septin6	-0.41	0.00000000	duck
Adamts17	-0.44	0.00000000	duck
Dnm1	-0.16	0.00000000	duck
Cd82	-0.47	0.00000000	duck
Jag1	-0.24	0.00000000	duck
Adgrl3	-0.44	0.00000000	duck
Larp1b	-1.16	0.00000000	duck
Ephb1	-0.37	0.00000000	duck
Fbxo3	-0.70	0.00000000	duck
Efemp1	-0.81	0.00000000	duck
Pmp22	-0.55	0.00000000	duck

Abcc1	-0.93	0.00000000	duck
Gjc2	-0.25	0.00000000	duck
Glce	-0.23	0.00000000	duck
Epb41	-1.59	0.00000000	duck
Pak6	-0.39	0.00000000	duck
Adra2b	-0.20	0.00000000	duck
Serbp1	-0.35	0.00000000	duck
Klhl29	-0.51	0.00000000	duck
Lrrc59	-0.39	0.00000000	duck
Serpinf1	-0.75	0.00000000	duck
Acadsb	-0.35	0.00000000	duck
Cbfa2t2	-0.27	0.00000000	duck
Capn5	-0.25	0.00000000	duck
Cbx1	-0.35	0.00000000	duck
Prom1	-0.28	0.00000000	duck
Coro2b	-0.16	0.00000000	duck
Arfip1	-0.49	0.00000000	duck
Gstk1	-0.25	0.00000000	duck
Enah	-0.60	0.00000000	duck
Nectin3	-0.82	0.00000000	duck
Iqgap2	-0.30	0.00000000	duck
Acsf2	-0.16	0.00000000	duck
Clk4	-0.45	0.00000000	duck
Add1	-0.60	0.00000000	duck
Reck	-0.39	0.00000000	duck
Adamts7	-0.15	0.00000000	duck
Cfl2	-0.26	0.00000000	duck
Golga5	-0.39	0.00000000	duck
Slit2	-0.72	0.00000000	duck
Igf1	-0.46	0.00000000	duck
Neto2	-0.35	0.00000000	duck
Slc9a3r2	-0.13	0.00000000	duck
Gnptab	-0.35	0.00000000	duck
Nipsnap1	-0.13	0.00000000	duck
Anxa2	-0.65	0.00000000	duck
Loxl2	-0.26	0.00000000	duck
Plod1	-0.35	0.00000000	duck
Calm2	-0.57	0.00000000	duck
Samd12	-0.43	0.00000000	duck
Slc17a5	-0.37	0.00000000	duck
Inka1	-0.11	0.00000000	duck
Stox2	-0.42	0.00000000	duck
Ldah	-0.16	0.00000000	duck
Tmem116	-0.19	0.00000000	duck
Chst11	-0.40	0.00000000	duck
Otud6b	-0.32	0.00000000	duck

Eif3e	-0.45	0.00000000	duck
Pamr1	-0.41	0.00000000	duck
Trank1	1.21	0.00000000	duck
Thra	-0.15	0.00000000	duck
Ccndbp1	-0.25	0.00000000	duck
Septin8	-0.24	0.00000000	duck
Rnf213	1.17	0.00000000	duck
Fjx1	-0.26	0.00000000	duck
Atrx	-0.81	0.00000000	duck
Ubr2	-0.94	0.00000000	duck
S100a13	-0.32	0.00000000	duck
Uba5	-0.21	0.00000000	duck
Gxylt2	-0.40	0.00000000	duck
Gata5	-0.27	0.00000000	duck
Dhrs3	-0.78	0.00000000	duck
Aldh9a1	-0.15	0.00000000	duck
Papln	-0.39	0.00000000	duck
Gng7	0.67	0.00000000	duck
Carhsp1	-0.38	0.00000000	duck
Rfx2	-1.12	0.00000000	duck
Cct8	-0.34	0.00000000	duck
Tmem100	-0.40	0.00000000	duck
Smap1	-0.36	0.00000000	duck
Zbtb8a	-0.18	0.00000000	duck
Tanc2	-0.17	0.00000000	duck
Appl2	-0.25	0.00000000	duck
Cbfa2t3	-0.16	0.00000000	duck
Dll4	-0.14	0.00000000	duck
Pbx1	0.96	0.00000000	duck
Senp6	-0.36	0.00000000	duck
Pdcd4	-0.22	0.00000000	duck
Cltc	-0.45	0.00000000	duck
Ubap2	0.93	0.00000000	duck
Tbc1d12	-0.32	0.00000000	duck
Erp29	-0.43	0.00000000	duck
Copb1	-0.21	0.00000000	duck
Uso1	-0.43	0.00000000	duck
Copb2	-0.23	0.00000000	duck
Eif3d	-0.51	0.00000000	duck
Tradd	-0.21	0.00000000	duck
Fgf7	-0.28	0.00000000	duck
Ccn2	-0.99	0.00000000	duck
Fbln5	-0.57	0.00000000	duck
Tctn3	-0.11	0.00000000	duck
Akap12	-0.62	0.00000000	duck
Aco2	-0.30	0.00000000	duck

Tspan4	0.86	0.00000000	duck
Ppm1k	-0.24	0.00000000	duck
Alcam	-0.62	0.00000000	duck
Emilin2	-0.67	0.00000000	duck
Srgap1	-0.59	0.00000000	duck
Ric8b	-0.42	0.00000000	duck
Arl1	-0.18	0.00000000	duck
Igf1r	0.82	0.00000000	duck
Slc9a9	-0.34	0.00000000	duck
Socs5	-0.20	0.00000000	duck
Itgb1	-0.45	0.00000000	duck
Slc25a37	-0.80	0.00000000	duck
Gdi2	-0.30	0.00000000	duck
Zfp703	-0.10	0.00000000	duck
Fam3c	-0.51	0.00000000	duck
Chac1	-0.21	0.00000000	duck
Smad1	-0.23	0.00000000	duck
Pnrc2	-0.49	0.00000000	duck
Dbi	-0.27	0.00000000	duck
Elovl7	-0.35	0.00000000	duck
Sh3d19	-0.46	0.00000000	duck
Borcs6	1.15	0.00000000	duck
Actr6	-0.33	0.00000000	duck
Zc2hc1a	-0.27	0.00000000	duck
Thsd4	-0.47	0.00000000	duck
Pdlim7	-0.17	0.00000000	duck
Tpst1	-0.37	0.00000000	duck
Zfp207	-0.46	0.00000000	duck
Ccdc32	-0.19	0.00000000	duck
Zfpm2	-0.68	0.00000000	duck
Idh1	-0.10	0.00000000	duck
Cnep1r1	-0.17	0.00000000	duck
Cygb	-0.74	0.00000000	duck
Synpo2	-0.11	0.00000000	duck
Abcd2	-0.42	0.00000000	duck
Lman2	-0.29	0.00000000	duck
Fam185a	-0.22	0.00000000	duck
Cat	-0.30	0.00000000	duck
Gpc1	-0.74	0.00000000	duck
Gorab	-0.12	0.00000000	duck
Adcy9	-0.57	0.00000000	duck
Nars	-0.33	0.00000000	duck
Tppp3	-0.50	0.00000000	duck
Hivep3	-0.52	0.00000000	duck
Adgrd1	-0.28	0.00000000	duck
Ncam1	-0.12	0.00000000	duck

Pcm1	-0.50	0.00000000	duck
Cd276	-0.15	0.00000000	duck
Atp5pb	-0.24	0.00000000	duck
Hspa12a	-0.21	0.00000000	duck
Sh3bp5	-0.43	0.00000000	duck
Sgsm2	-0.33	0.00000000	duck
Lpar6	-0.40	0.00000000	duck
Tmem45a	-0.25	0.00000000	duck
Sertad4	-0.05	0.00000000	duck
Slc27a3	-0.13	0.00000000	duck
Lox	0.98	0.00000000	duck
Cerk	-0.25	0.00000000	duck
Prdx4	-0.32	0.00000000	duck
Golga1	-0.38	0.00000000	duck
Bnip3l	-0.44	0.00000000	duck
Aig1	-0.34	0.00000000	duck
Prss35	-0.40	0.00000000	duck
Calm1	-0.53	0.00000000	duck
Tomm20	-0.25	0.00000000	duck
Lrrc15	-0.15	0.00000000	duck
Faim	-0.09	0.00000000	duck
Erlin2	-0.39	0.00000000	duck
Golgb1	-0.60	0.00000000	duck
Fxr1	-0.78	0.00000000	duck
Nt5dc2	-0.31	0.00000000	duck
Tmf1	-0.34	0.00000000	duck
Rras2	-0.33	0.00000000	duck
Arhgap6	-0.58	0.00000000	duck
Angptl2	-0.46	0.00000000	duck
Acss3	-0.13	0.00000000	duck
Cfap36	-0.18	0.00000000	duck
Fhl4	-0.40	0.00000000	duck
Ptges	-0.23	0.00000000	duck
Gpx7	-0.18	0.00000000	duck
Zfp639	-0.29	0.00000000	duck
Asph	-0.34	0.00000000	duck
5730480H06Rik	-0.22	0.00000000	duck
Sbno2	-0.94	0.00000000	duck
Hsp90b1	-0.61	0.00000000	duck
P4hb	-0.35	0.00000000	duck
Hp1bp3	-0.25	0.00000000	duck
Gpx3	0.62	0.00000000	duck
Nsun2	-0.25	0.00000000	duck
Tecpr2	-0.31	0.00000000	duck
Slit3	-0.51	0.00000000	duck
Cald1	-0.44	0.00000000	duck

Tnrc18	-0.27	0.00000000	duck
Rala	-0.23	0.00000000	duck
Cdkl5	-0.26	0.00000000	duck
Cpeb3	-0.29	0.00000000	duck
Wsb2	-0.19	0.00000000	duck
Cdk17	-0.74	0.00000000	duck
Khdc4	-0.21	0.00000000	duck
Slc43a2	1.14	0.00000000	duck
Hnrnpab	-0.47	0.00000000	duck
Stk40	-0.20	0.00000000	duck
Hic2	-0.31	0.00000000	duck
Acat1	-0.19	0.00000000	duck
Anks1	-0.18	0.00000000	duck
Eda	-0.16	0.00000000	duck
Sdc2	-0.57	0.00000000	duck
Zdhhc2	-0.29	0.00000001	duck
Dipk2a	-0.35	0.00000001	duck
Sec23a	-0.41	0.00000001	duck
Cep170	-0.29	0.00000001	duck
Spats2l	-0.19	0.00000001	duck
Itgbl1	-0.28	0.00000001	duck
Frmd6	-0.13	0.00000001	duck
Srp72	-0.27	0.00000001	duck
Col6a2	-0.53	0.00000001	duck
Dcun1d2	-0.92	0.00000001	duck
Pcolce2	-0.27	0.00000001	duck
Pcdh19	-0.30	0.00000001	duck
Phldb2	-0.34	0.00000001	duck
Fam118b	-0.19	0.00000001	duck
Dnaja2	-0.22	0.00000001	duck
Shroom2	-0.33	0.00000001	duck
Cavin1	-0.37	0.00000001	duck
Fzd7	-0.13	0.00000001	duck
Agpat5	-0.22	0.00000001	duck
Slc45a1	-0.30	0.00000001	duck
Hrh1	-0.14	0.00000001	duck
Dip2c	-0.38	0.00000001	duck
Tbc1d8b	-0.25	0.00000001	duck
Gli3	-0.29	0.00000001	duck
Cercam	-0.11	0.00000001	duck
Pdgfrl	-0.28	0.00000001	duck
Wdr26	-0.71	0.00000001	duck
Eif4g1	-0.16	0.00000001	duck
Atg5	-0.33	0.00000001	duck
Iqgap1	-0.40	0.00000002	duck
Dock9	-0.43	0.00000002	duck

Clstn1	-0.13	0.00000002	duck
Sfxn1	-0.17	0.00000002	duck
Sphk1	-0.34	0.00000002	duck
Rfng	-0.13	0.00000002	duck
Hnrnph3	-0.38	0.00000002	duck
Dync1li1	-0.15	0.00000002	duck
Sinhcaf	-0.15	0.00000002	duck
P4ha1	-0.28	0.00000002	duck
Vat1	-0.24	0.00000002	duck
Lta4h	-0.11	0.00000003	duck
Acad9	-0.36	0.00000003	duck
Epb41l1	-0.14	0.00000003	duck
Sgcg	-0.22	0.00000003	duck
Tax1bp3	-0.34	0.00000003	duck
Oaz2	-0.18	0.00000003	duck
Chst2	-0.19	0.00000003	duck
Gorasp2	-0.25	0.00000003	duck
Nid2	-0.45	0.00000003	duck
Ddx5	-0.56	0.00000003	duck
Rrad	-0.66	0.00000003	duck
St6galnac3	-0.21	0.00000003	duck
Jpt2	-0.16	0.00000003	duck
Eif2b1	-0.13	0.00000003	duck
Dynlt1a	-0.25	0.00000004	duck
Tapt1	-0.57	0.00000004	duck
Ankrd40	-0.14	0.00000004	duck
Xrn1	-0.27	0.00000004	duck
Nxt2	-0.59	0.00000004	duck
Sema3c	-0.47	0.00000005	duck
Slc25a43	-0.14	0.00000005	duck
Pjvk	-0.22	0.00000005	duck
Snrk	-0.42	0.00000005	duck
Gpd1l	-0.09	0.00000005	duck
Pikfyve	-0.34	0.00000005	duck
Ehd3	-0.49	0.00000005	duck
Ro60	-0.09	0.00000006	duck
Larp6	-0.08	0.00000006	duck
Osbpl1a	-0.49	0.00000006	duck
Polr2b	-0.27	0.00000006	duck
Tubgcp5	-0.14	0.00000007	duck
Tmx4	-0.20	0.00000007	duck
Sos1	-0.34	0.00000007	duck
Edrf1	-0.50	0.00000007	duck
Plekhg4	-0.17	0.00000007	duck
Fam122b	-0.18	0.00000007	duck
Tcea1	-0.24	0.00000007	duck

Osbpl5	-0.20	0.00000008	duck
Vcpip1	-0.31	0.00000008	duck
Spdl1	-0.14	0.00000008	duck
Ociad1	-0.32	0.00000008	duck
Tbc1d2b	-0.31	0.00000008	duck
Galnt12	-0.21	0.00000008	duck
Rbsn	-0.15	0.00000008	duck
Atp5c1	-0.31	0.00000008	duck
Rprd1b	-0.28	0.00000009	duck
Fat4	-0.54	0.00000009	duck
Eml1	-0.30	0.00000009	duck
Dynlt3	-0.38	0.00000009	duck
Snx1	-0.18	0.00000009	duck
Gtf2e2	-0.12	0.00000009	duck
Efhd1	-0.19	0.00000010	duck
Zfp326	-0.42	0.00000010	duck
Gpatch11	-0.27	0.00000010	duck
Setd5	-0.38	0.00000010	duck
Plcb2	-0.39	0.00000010	duck
Uap1l1	-0.23	0.00000011	duck
Creld2	-0.24	0.00000011	duck
Rgs3	-0.11	0.00000011	duck
Tm9sf4	-0.18	0.00000011	duck
Gpr161	-0.19	0.00000012	duck
Chek2	-0.16	0.00000012	duck
Rhoq	-0.15	0.00000012	duck
Slc41a2	-0.08	0.00000012	duck
Rnf165	-0.13	0.00000012	duck
Washc3	-0.13	0.00000012	duck
Chuk	-0.40	0.00000013	duck
Dpy19l3	-0.13	0.00000013	duck
Zfand5	1.03	0.00000013	duck
Tfrc	-0.82	0.00000013	duck
Snap29	-0.35	0.00000013	duck
Slc38a1	-0.07	0.00000014	duck
Prrc2b	-0.35	0.00000015	duck
Itpk1	-1.11	0.00000015	duck
Thrb	0.00	0.00000015	duck
Cdon	-0.20	0.00000015	duck
Erlin1	-0.18	0.00000015	duck
Atp6v0a2	0.00	0.00000015	duck
Pank1	-0.12	0.00000016	duck
Fgfr2	-0.11	0.00000016	duck
Ddx27	-0.17	0.00000016	duck
Podn	-0.56	0.00000018	duck
Wwtr1	-0.36	0.00000018	duck

Pdia4	-0.30	0.00000018	duck
Dupd1	-0.08	0.00000018	duck
Usp48	-0.79	0.00000018	duck
Fam98a	-0.18	0.00000019	duck
Scoc	-0.08	0.00000020	duck
Nae1	-0.12	0.00000021	duck
Cep85l	-0.25	0.00000022	duck
Ywhaz	-0.42	0.00000022	duck
Pid1	1.11	0.00000023	duck
Cisd2	-0.15	0.00000023	duck
Gpatch1	-0.19	0.00000024	duck
Vangl1	-0.19	0.00000025	duck
Ssbp3	-0.34	0.00000026	duck
Anxa4	-0.12	0.00000026	duck
Pls3	-0.32	0.00000027	duck
Aco1	-0.21	0.00000027	duck
Pkd2	-0.28	0.00000029	duck
Cped1	-0.34	0.00000029	duck
Amotl2	-0.23	0.00000031	duck
Osmr	-0.10	0.00000031	duck
Dcaf1	-0.36	0.00000031	duck
Sh3glb2	-0.28	0.00000032	duck
Grb10	1.16	0.00000032	duck
Bcl6	-0.42	0.00000032	duck
Lars	-0.16	0.00000033	duck
Emp2	-0.24	0.00000034	duck
Rest	-0.30	0.00000034	duck
Fut8	-0.27	0.00000035	duck
Cnot7	-0.17	0.00000035	duck
Spty2d1	-0.49	0.00000036	duck
Itm2b	-0.45	0.00000038	duck
Zfyve9	-0.13	0.00000038	duck
Inhba	-1.17	0.00000038	duck
Usp14	-0.27	0.00000039	duck
Xpnpep1	-0.18	0.00000041	duck
Sh3glb1	-0.27	0.00000041	duck
Fkbp4	-0.14	0.00000042	duck
Adrm1	-0.21	0.00000045	duck
Vps37a	-0.29	0.00000046	duck
Hapln3	-0.80	0.00000047	duck
Fkbp7	-0.17	0.00000047	duck
Fermt2	-0.27	0.00000048	duck
Golim4	-0.46	0.00000048	duck
Ccdc102a	-0.12	0.00000051	duck
Rgs1	-0.30	0.00000051	duck
Scamp1	-0.34	0.00000052	duck

Golm1	-0.09	0.00000055	duck
Stk26	-0.51	0.00000055	duck
Pip5k1a	-0.21	0.00000058	duck
Hunk	-0.28	0.00000058	duck
St13	-0.10	0.00000058	duck
Cmtm3	-0.05	0.00000060	duck
Sec14l1	-0.22	0.00000062	duck
Slc31a1	-0.13	0.00000063	duck
Palm	0.01	0.00000066	duck
Large1	-0.25	0.00000067	duck
Tmc7	-0.14	0.00000069	duck
Uba3	-0.22	0.00000070	duck
Prpf4	-0.23	0.00000072	duck
Cep63	-0.13	0.00000077	duck
Btbd6	-0.40	0.00000078	duck
Ndrg4	-0.09	0.00000079	duck
Bcr	-0.41	0.00000080	duck
Nufip1	-0.26	0.00000082	duck
Dnmt1	-0.10	0.00000085	duck
C1ra	0.95	0.00000087	duck
Pdia5	-0.25	0.00000088	duck
Ndfip2	-0.31	0.00000088	duck
Ywhah	-0.39	0.00000091	duck
Ogdh	-0.18	0.00000096	duck
Psma4	-0.43	0.00000097	duck
Ank3	-0.32	0.00000101	duck
Ppt1	-0.06	0.00000102	duck
P3h1	-0.13	0.00000102	duck
Sec13	-0.01	0.00000102	duck
Hras	-0.18	0.00000103	duck
Snx9	-0.29	0.00000103	duck
Tial1	-0.38	0.00000104	duck
Abce1	-0.24	0.00000104	duck
Yes1	-0.05	0.00000104	duck
St6gal1	-0.65	0.00000108	duck
Rab9	-0.29	0.00000113	duck
Asah1	-0.43	0.00000120	duck
Uggt2	-0.27	0.00000121	duck
Sec22b	-0.09	0.00000125	duck
Stam2	-0.36	0.00000125	duck
Ilrun	-0.67	0.00000125	duck
Luzp1	-0.08	0.00000130	duck
Sde2	-0.37	0.00000136	duck
Celf2	0.73	0.00000141	duck
Phb	-0.15	0.00000141	duck
Pdia2	-0.11	0.00000141	duck

Commd2	-0.21	0.00000144	duck
Cdc14a	-0.76	0.00000147	duck
Ccdc167	-0.10	0.00000150	duck
Copa	-0.18	0.00000150	duck
Glrx5	-0.47	0.00000151	duck
Gask1a	-0.14	0.00000155	duck
Slc30a7	-0.43	0.00000157	duck
Gnpnat1	-0.18	0.00000163	duck
Clk2	-0.09	0.00000163	duck
Chst3	-0.11	0.00000166	duck
Rabif	-0.19	0.00000166	duck
Sdf2l1	-0.13	0.00000175	duck
Ltbp2	-0.19	0.00000183	duck
Cttn	-0.12	0.00000185	duck
Vps13b	-0.85	0.00000186	duck
Stk32c	-0.26	0.00000195	duck
Ngly1	-0.21	0.00000197	duck
Ubap2l	-0.23	0.00000202	duck
Dnajc1	-0.19	0.00000204	duck
Adprh	-0.09	0.00000208	duck
Kdelr3	-0.08	0.00000215	duck
Tcf3	-0.23	0.00000215	duck
Golga3	-0.20	0.00000218	duck
Disp1	-0.41	0.00000223	duck
Ptges3	-0.42	0.00000224	duck
Gsn	-0.58	0.00000235	duck
Fbrsl1	0.48	0.00000235	duck
Drg1	-0.12	0.00000238	duck
Wdr61	0.01	0.00000241	duck
Epb41l2	-0.35	0.00000245	duck
Hddc2	-0.11	0.00000253	duck
Nvl	-0.13	0.00000255	duck
Rimklb	-0.12	0.00000259	duck
Chst7	-0.06	0.00000262	duck
Ebna1bp2	-0.11	0.00000264	duck
Ccdc93	-0.18	0.00000266	duck
Crybg3	-0.54	0.00000268	duck
Glb1	-0.23	0.00000270	duck
Ppp2r5a	-0.10	0.00000270	duck
Tmem204	-0.13	0.00000272	duck
Pknox1	-0.73	0.00000274	duck
Ndufs1	-0.28	0.00000285	duck
Gadd45g	1.05	0.00000285	duck
Smndc1	-0.12	0.00000292	duck
Dlg2	-0.95	0.00000301	duck
Bace1	-0.06	0.00000304	duck

P3h4	-0.07	0.00000316	duck
Rnf141	-0.17	0.00000324	duck
Rab21	-0.20	0.00000328	duck
Commd9	-0.17	0.00000339	duck
Cpne3	-0.23	0.00000348	duck
Zfp532	-0.10	0.00000354	duck
Tpr	-0.30	0.00000354	duck
Nnt	0.00	0.00000359	duck
Phf6	-0.25	0.00000362	duck
Isca2	-0.25	0.00000366	duck
Exosc7	-0.22	0.00000389	duck
Snx4	-0.25	0.00000401	duck
Trmt1l	-0.30	0.00000403	duck
Cfap20	-0.08	0.00000406	duck
Sars	-0.05	0.00000408	duck
Herc4	-0.42	0.00000410	duck
Evi5	-0.42	0.00000421	duck
Nup98	-0.39	0.00000427	duck
Yipf4	-0.40	0.00000430	duck
Bok	-0.06	0.00000433	duck
Cep95	-0.14	0.00000446	duck
Prdm4	-0.02	0.00000448	duck
Zcchc8	-0.25	0.00000460	duck
Lrrc58	-0.18	0.00000461	duck
Slc25a5	-0.10	0.00000463	duck
Rasgef1c	-0.23	0.00000490	duck
Rap1b	-0.44	0.00000490	duck
Fnta	-0.19	0.00000507	duck
Nexn	-0.15	0.00000511	duck
Atp2b1	-0.24	0.00000512	duck
Tfam	-0.23	0.00000541	duck
Exoc3	-0.34	0.00000549	duck
Tfg	-0.16	0.00000555	duck
Agap3	-0.05	0.00000559	duck
D5Ertd579e	-0.95	0.00000565	duck
Slco3a1	-0.19	0.00000570	duck
C2cd2	-0.37	0.00000587	duck
Alg12	-0.11	0.00000590	duck
Ckap5	-0.23	0.00000598	duck
Sltm	-0.20	0.00000612	duck
Adk	-0.35	0.00000627	duck
Suz12	-0.14	0.00000629	duck
Rhbdf2	-0.17	0.00000635	duck
Noa1	-0.08	0.00000637	duck
Tuft1	-0.06	0.00000657	duck
Thoc7	-0.40	0.00000659	duck

Nfe2l2	-0.43	0.00000662	duck
Atl1	-0.06	0.00000691	duck
Rb1	-0.23	0.00000692	duck
Lims2	-0.12	0.00000695	duck
Eef1a1	-0.40	0.00000699	duck
Trp53inp2	-0.18	0.00000716	duck
Esyt2	-0.19	0.00000722	duck
Nemp2	-0.10	0.00000722	duck
Brinp3	-0.53	0.00000751	duck
Clk1	-0.40	0.00000754	duck
Cpeb4	-0.44	0.00000765	duck
Gclc	-0.25	0.00000778	duck
Dut	-0.02	0.00000785	duck
Ednra	-0.07	0.00000801	duck
Sar1b	-0.12	0.00000829	duck
Tjp1	-0.10	0.00000830	duck
Cwc15	-0.20	0.00000839	duck
Cndp2	-0.12	0.00000849	duck
Hoxb4	-0.04	0.00000860	duck
Dnm1l	0.05	0.00000877	duck
Ranbp1	-0.16	0.00000903	duck
Gse1	-0.16	0.00000925	duck
Colec12	-0.37	0.00000931	duck
Cilk1	-0.11	0.00000939	duck
Zfp277	-0.21	0.00000940	duck
Hmox2	0.00	0.00000944	duck
Sephs1	-0.14	0.00000981	duck
Timp2	-0.40	0.00001047	duck
Kremen1	-0.22	0.00001050	duck
Tnfsf10	-0.20	0.00001056	duck
Ankfy1	-0.16	0.00001059	duck
Ints13	-0.25	0.00001094	duck
Eif3b	-0.28	0.00001118	duck
Adcy7	-0.22	0.00001132	duck
Ptcd3	-0.03	0.00001143	duck
Txnrd3	-0.16	0.00001147	duck
Csl	-0.20	0.00001172	duck
Sin3a	-0.08	0.00001187	duck
Rhot1	-0.12	0.00001189	duck
Arl5b	-0.20	0.00001224	duck
Brox	-0.06	0.00001226	duck
Usp8	-0.39	0.00001227	duck
Rheb	-0.24	0.00001283	duck
Wasf2	-0.28	0.00001307	duck
Camsap1	-0.15	0.00001320	duck
Pdgfd	0.89	0.00001395	duck

Nbas	-0.16	0.00001399	duck
Asb9	-0.18	0.00001455	duck
Clint1	-0.32	0.00001459	duck
Bag5	-0.14	0.00001466	duck
Acbd3	-0.10	0.00001551	duck
Stk3	-0.31	0.00001583	duck
Trip12	-0.48	0.00001619	duck
Tube1	-0.38	0.00001630	duck
Fos	0.63	0.00001644	duck
Trim59	-0.08	0.00001666	duck
Tacc3	-0.58	0.00001752	duck
Pdcd6	-0.23	0.00001756	duck
Rad54l2	-0.06	0.00001760	duck
Yars2	-0.07	0.00001805	duck
Fkbp3	-0.21	0.00001848	duck
Poglut2	-0.09	0.00001894	duck
Setd3	0.00	0.00001916	duck
Jph1	-0.42	0.00001918	duck
Fyttd1	-0.12	0.00001921	duck
Nrbf2	-0.13	0.00001924	duck
Zc3h7a	-0.25	0.00001931	duck
Pdk3	-0.61	0.00001943	duck
Vcl	-0.43	0.00001949	duck
Tbc1d9b	-0.22	0.00001984	duck
Il21r	-0.18	0.00002034	duck
Tcf4	-0.26	0.00002108	duck
Ints10	-0.23	0.00002111	duck
Wdr86	-0.04	0.00002202	duck
Ak2	-0.73	0.00002211	duck
Nedd4	-0.10	0.00002256	duck
Eci3	-0.06	0.00002306	duck
Wnt4	-0.11	0.00002342	duck
Ccdc85a	-0.29	0.00002357	duck
Actr8	-0.04	0.00002387	duck
Cpq	-0.15	0.00002418	duck
Birc6	-0.46	0.00002441	duck
Pex10	-0.13	0.00002443	duck
Abcd3	-0.09	0.00002485	duck
Pxdc1	-0.38	0.00002517	duck
Ybx3	-0.44	0.00002528	duck
Dcaf17	-0.08	0.00002565	duck
Ndufa8	-0.23	0.00002643	duck
Poli	-0.11	0.00002646	duck
4933400A11Rik	-0.08	0.00002652	duck
Swap70	-0.17	0.00002661	duck
Aldh1a2	-0.02	0.00002723	duck

Tob1	-0.27	0.00002773	duck
Glipr2	-0.48	0.00002828	duck
Ttll12	-0.12	0.00002835	duck
Pgrmc2	-0.17	0.00002867	duck
Styk1	-0.49	0.00002940	duck
Gpc4	-0.29	0.00002975	duck
Tmem14a	-0.11	0.00003034	duck
Kdm3b	-0.22	0.00003048	duck
C6	-0.20	0.00003064	duck
Gpam	-0.09	0.00003065	duck
Tmem165	-0.18	0.00003087	duck
Yipf5	-0.14	0.00003095	duck
Parp4	-0.04	0.00003240	duck
Efcab10	-0.07	0.00003249	duck
Pbx4	-0.60	0.00003367	duck
Endod1	-0.11	0.00003507	duck
Ralgapa2	-0.54	0.00003558	duck
Arhgap18	-0.11	0.00003579	duck
Daam1	-0.20	0.00003633	duck
Trp53i11	-0.35	0.00003640	duck
Adgra2	0.00	0.00003644	duck
Nos2	1.16	0.00003686	duck
Lmo2	-0.28	0.00003778	duck
Smc4	-0.33	0.00003787	duck
Pik3r4	-0.12	0.00003841	duck
Plekhj1	-0.16	0.00003883	duck
Cops7a	-0.02	0.00004050	duck
Rad51b	-0.25	0.00004096	duck
Cttnbp2	-0.08	0.00004188	duck
Sesn1	1.04	0.00004268	duck
Tcf7l2	0.94	0.00004297	duck
Cited2	-0.11	0.00004345	duck
Plbd2	-0.06	0.00004356	duck
Anapc7	-0.10	0.00004406	duck
Gcfc2	-0.12	0.00004416	duck
Spsb4	-0.13	0.00004453	duck
Pheta2	-0.13	0.00004598	duck
Coq10b	-0.11	0.00004624	duck
Srpr	-0.14	0.00004651	duck
Psmd13	-0.14	0.00004652	duck
Gtpbp1	-0.04	0.00004733	duck
Pitpna	-0.04	0.00004794	duck
Serpind1	-0.39	0.00004799	duck
Arf4	-0.33	0.00004815	duck
G3bp2	-0.19	0.00004931	duck
Lin7c	-0.16	0.00005087	duck

Klf6	-0.40	0.00005261	duck
Ankrd12	-0.61	0.00005375	duck
Ankrd50	-0.19	0.00005450	duck
Purg	-0.41	0.00005463	duck
Cluh	-0.13	0.00005523	duck
Sema6a	-0.10	0.00005633	duck
Tlcd3a	-0.02	0.00005675	duck
Arih1	-0.59	0.00005741	duck
Arl5a	-0.27	0.00005750	duck
Ctsh	-0.12	0.00005793	duck
Rhou	-0.14	0.00005964	duck
BC034090	-0.06	0.00006062	duck
Ahcyl1	-0.16	0.00006082	duck
Slc2a13	-0.32	0.00006087	duck
Smarca1	-0.06	0.00006097	duck
Angel2	-0.35	0.00006284	duck
Aldh7a1	-0.04	0.00006433	duck
Tcp1	-0.07	0.00006469	duck
Ints8	-0.46	0.00006494	duck
Ift140	-0.06	0.00006587	duck
F3	-0.30	0.00006622	duck
Rabep1	-0.34	0.00006656	duck
Taf5l	-0.23	0.00006737	duck
Chmp2b	-0.13	0.00006950	duck
Rbpms	-0.11	0.00007111	duck
Psmg2	-0.10	0.00007397	duck
Serp1	-0.34	0.00007403	duck
Sdf2	-0.11	0.00007497	duck
Ccdc88a	-0.14	0.00007513	duck
Sec11a	-0.13	0.00007626	duck
Atxn1	-0.28	0.00007641	duck
Rcn2	-0.26	0.00008028	duck
Nipbl	0.59	0.00008043	duck
Leprot	-0.30	0.00008181	duck
Smyd2	-0.04	0.00008318	duck
Rcan3	-0.16	0.00008406	duck
Sspn	-0.11	0.00008501	duck
Ddx1	-0.14	0.00008508	duck
Naa25	-0.33	0.00008511	duck
Pi4ka	-0.28	0.00008538	duck
Plcb1	-0.31	0.00008591	duck
Axin1	-0.12	0.00008685	duck
Lonp1	-0.14	0.00009034	duck
Slco2a1	-0.09	0.00009271	duck
Iqsec1	-0.45	0.00009299	duck
Ccnk	-0.05	0.00009301	duck

Sucla2	-0.10	0.00009425	duck
Lysmd3	-0.09	0.00009519	duck
Dusp14	-0.08	0.00009561	duck
Pkp4	-0.16	0.00009607	duck
Zfp512	-0.15	0.00009676	duck
Rbbp6	-0.49	0.00009717	duck
Enox1	-0.35	0.00009722	duck
Tgfb2	-0.56	0.00010049	duck
Sowahc	-0.07	0.00010063	duck
Pccb	-0.08	0.00010077	duck
Pbdc1	-0.19	0.00010085	duck
Ctbs	-0.20	0.00010153	duck
Pak1ip1	-0.24	0.00010158	duck
Lrrc40	-0.16	0.00010445	duck
Ing5	-0.38	0.00010621	duck
Rlf	-0.61	0.00010645	duck
Acadl	-0.17	0.00010850	duck
Specc1	-0.25	0.00010907	duck
Cdk8	-0.49	0.00010948	duck
Eif5a2	-0.31	0.00010962	duck
Rars	-0.13	0.00011519	duck
Fbxo30	-0.23	0.00011575	duck
Prrg1	0.02	0.00011643	duck
Vdac3	-0.27	0.00012095	duck
Rgl1	-0.19	0.00012213	duck
Nup50	-0.11	0.00012334	duck
Ssr3	-0.16	0.00012335	duck
Snta1	-0.10	0.00012652	duck
Cers6	-0.19	0.00013086	duck
Phyh	-0.14	0.00013104	duck
Tagln3	-0.12	0.00013218	duck
Cpt1a	0.94	0.00013728	duck
Cdpf1	-0.13	0.00014005	duck
Pdik1l	-0.21	0.00014023	duck
Ank	-0.18	0.00014280	duck
Acvr1	-0.25	0.00014531	duck
Atp11c	-0.20	0.00014543	duck
Ube3a	-0.22	0.00014552	duck
Hcfc2	-0.08	0.00014785	duck
Rbbp4	-0.09	0.00015235	duck
Rab24	-0.03	0.00015383	duck
Tmem64	-0.04	0.00015420	duck
Nap1l4	-0.25	0.00015506	duck
Itga9	1.00	0.00015622	duck
Cd44	-0.82	0.00015963	duck
Osgin1	-0.50	0.00016146	duck

Fam98b	-0.14	0.00016177	duck
Noc2l	-0.14	0.00016473	duck
Cbx3	-0.22	0.00017052	duck
Cul3	-0.19	0.00017328	duck
Pde12	-0.08	0.00017386	duck
Igsf9	-0.37	0.00017575	duck
Hagh	-0.36	0.00017580	duck
Cbx6	-0.24	0.00017838	duck
Ctsb	0.72	0.00018145	duck
Aasdhppt	-0.20	0.00018367	duck
Ssr1	-0.14	0.00018630	duck
Cdc42bpb	-0.37	0.00018749	duck
Zmynd8	-0.16	0.00018797	duck
Idh2	-0.18	0.00019192	duck
Rhbdf1	-0.06	0.00019537	duck
Crlf1	-0.14	0.00019559	duck
Smchd1	-0.43	0.00019603	duck
Atxn10	1.09	0.00019641	duck
Rnf150	1.14	0.00019935	duck
Gpcpd1	-0.71	0.00020349	duck
Ypel5	-0.30	0.00020637	duck
Srrm1	-0.21	0.00021162	duck
Atp6v1c1	-0.26	0.00021537	duck
Erlec1	-0.17	0.00021883	duck
Iffo1	-0.03	0.00021981	duck
Tspan18	-0.10	0.00022198	duck
Lsm6	0.00	0.00022961	duck
Ssbp1	-0.10	0.00022986	duck
Osbpl2	-0.07	0.00023151	duck
Adgrv1	-0.30	0.00023414	duck
Iars2	-0.10	0.00023763	duck
Brms1l	-0.14	0.00024149	duck
Ube2e2	-0.22	0.00024158	duck
Plcl2	-0.22	0.00024332	duck
Nde1	-0.29	0.00024636	duck
Grb2	-0.33	0.00024661	duck
Btaf1	-0.34	0.00024790	duck
Smim14	-0.11	0.00024925	duck
Cct5	-0.11	0.00025998	duck
Uba2	-0.16	0.00026270	duck
Lactb2	-0.09	0.00026621	duck
Gtf2h1	-0.22	0.00026862	duck
Wiz	-0.27	0.00027241	duck
Raph1	-0.15	0.00027520	duck
Ulk1	-0.44	0.00027612	duck
Gk5	-0.21	0.00027968	duck

Chchd7	-0.13	0.00028091	duck
Enc1	-0.15	0.00029238	duck
Dync1li2	-0.32	0.00029320	duck
Trim37	-0.16	0.00029853	duck
Polr3e	-0.19	0.00030017	duck
Ncoa7	-0.03	0.00030370	duck
Ampd3	-0.08	0.00030630	duck
Ddx6	-0.31	0.00031245	duck
Net1	-0.06	0.00031544	duck
Eif2ak2	0.64	0.00031796	duck
Ahsa1	-0.08	0.00032965	duck
Ablim1	0.66	0.00032983	duck
Park7	-0.10	0.00033101	duck
Fzd2	0.04	0.00033131	duck
Esco1	-0.39	0.00033483	duck
Fgfr1	-0.07	0.00033904	duck
Ctr9	-0.03	0.00034491	duck
Paip2	-0.24	0.00034554	duck
Vasn	-0.07	0.00034638	duck
9330159F19Rik	-0.04	0.00034801	duck
Jam2	-0.12	0.00035754	duck
Eif4g2	-0.34	0.00035764	duck
Selenof	-0.16	0.00036228	duck
Dock11	-0.22	0.00036477	duck
Leo1	-0.17	0.00036598	duck
Rab8b	-0.21	0.00036615	duck
Pwwp2a	-0.05	0.00036621	duck
Scpep1	-0.11	0.00036909	duck
Cggbp1	-0.04	0.00037045	duck
Zfp236	-0.25	0.00038080	duck
Acot10	-0.15	0.00038388	duck
Acox1	-0.12	0.00038856	duck
Grk3	0.07	0.00039453	duck
Nfatc2	-0.55	0.00039466	duck
Ndufs3	-0.10	0.00039627	duck
Dhx38	-0.09	0.00040325	duck
Sgtb	-0.04	0.00040453	duck
Hoxa4	-0.12	0.00040785	duck
Wee1	-0.18	0.00040871	duck
Klf11	-0.09	0.00041375	duck
Eef2k	-0.16	0.00041553	duck
Bend6	-0.17	0.00042802	duck
Pcmt1	-0.03	0.00043963	duck
Prxl2c	0.03	0.00044178	duck
Wbp4	-0.43	0.00044900	duck
Kifap3	-0.01	0.00045882	duck

Idh3a	-0.23	0.00047062	duck
Wrn	-0.25	0.00048356	duck
Tspo	-0.04	0.00049133	duck
Nek1	-0.08	0.00049756	duck
Lman1	-0.17	0.00050720	duck
Panx1	0.00	0.00050921	duck
Cdc73	-0.14	0.00051087	duck
Prrx2	-0.11	0.00051203	duck
Gdap2	-0.03	0.00051476	duck
Klhl7	-0.05	0.00051550	duck
Dhx58	0.44	0.00052002	duck
Jph3	-0.04	0.00052459	duck
Tulp3	-0.01	0.00053589	duck
Vps35	-0.02	0.00053848	duck
Rilpl1	-0.06	0.00055664	duck
Iws1	-0.23	0.00056070	duck
Gemin2	-0.23	0.00056157	duck
Tada2a	-0.07	0.00056424	duck
Ythdf1	0.01	0.00059584	duck
Tnfaip8l3	-0.05	0.00059736	duck
Prelid2	-0.18	0.00059952	duck
Wdr44	-0.27	0.00061291	duck
Dock7	-0.06	0.00061821	duck
Ttc14	-0.26	0.00062808	duck
Kpna3	-0.08	0.00063026	duck
Rab11fip3	-0.17	0.00065274	duck
Fam168a	-0.14	0.00065331	duck
Brd7	-0.28	0.00065681	duck
Wwc1	-0.02	0.00065835	duck
Smim4	-0.25	0.00066007	duck
Tcf7	-0.04	0.00067341	duck
Cnot6l	-0.04	0.00068152	duck
Gabarapl1	0.91	0.00069215	duck
Usp15	-0.27	0.00070313	duck
Cfap97	-0.08	0.00070470	duck
Twsg1	-0.09	0.00071103	duck
Ptpn11	-0.05	0.00072387	duck
Hprt	-0.23	0.00072830	duck
Smc3	-0.08	0.00073412	duck
Septin11	-0.24	0.00074600	duck
Nav3	-0.25	0.00075723	duck
Ap3m1	0.02	0.00076027	duck
Zfp638	-0.25	0.00076647	duck
Dyrk3	-0.10	0.00077497	duck
Xbp1	-0.28	0.00077988	duck
Hipk1	-0.45	0.00078861	duck

Nrbp2	-0.51	0.00078890	duck
Kera	-0.37	0.00079224	duck
Psme3ip1	-0.10	0.00080489	duck
Svil	-0.06	0.00080674	duck
Cfd	0.95	0.00080908	duck
Fundc1	-0.17	0.00082649	duck
Septin9	-0.05	0.00083767	duck
Rnmt	-0.04	0.00083887	duck
Ap3d1	-0.25	0.00084693	duck
Tubb4b	-0.19	0.00085186	duck
Atp7a	-0.10	0.00085456	duck
Nt5c3b	0.00	0.00085551	duck
Bfar	-0.06	0.00087156	duck
Rpap3	-0.34	0.00087785	duck
Dmap1	-0.13	0.00088668	duck
Rab30	-0.15	0.00089239	duck
Serpini1	-0.38	0.00089488	duck
Abi1	-0.52	0.00089541	duck
Phlda2	-0.55	0.00089645	duck
Dnaja1	-0.18	0.00089936	duck
Sap30l	-0.27	0.00089968	duck
Aopep	0.87	0.00090528	duck
Kdm2b	-0.02	0.00092886	duck
Arntl	-0.09	0.00093312	duck
Kalrn	0.86	0.00093704	duck
Cope	-0.08	0.00093805	duck
Zfp652	-0.13	0.00094493	duck
Chst12	-0.04	0.00095434	duck
Shisa5	-0.26	0.00095495	duck
Etl4	-0.50	0.00095631	duck
Ripk1	-0.17	0.00096222	duck
Cstf1	0.04	0.00098216	duck
Ap3b1	-0.49	0.00098746	duck
Zbtb37	-0.25	0.00099570	duck
Cspg4	-0.11	0.00100974	duck
Paxip1	-0.16	0.00101795	duck
Add3	-0.33	0.00101924	duck
Atp2b4	-0.10	0.00102987	duck
Lmnb2	0.04	0.00104057	duck
Adam19	-0.06	0.00104674	duck
Cuedc1	-0.33	0.00105386	duck
Dnajc9	-0.17	0.00105537	duck
Daglb	-0.25	0.00106293	duck
Fbxo8	-0.17	0.00107888	duck
Vegfa	-0.17	0.00108215	duck
Nisch	-0.20	0.00108248	duck

Wdr11	-0.06	0.00108554	duck
Psmc1	-0.24	0.00111634	duck
Snrpa1	0.00	0.00112179	duck
Wdr3	-0.03	0.00113122	duck
Hebp1	-0.08	0.00114564	duck
Sash1	-0.33	0.00116180	duck
Tor2a	-0.11	0.00117823	duck
Gramd4	-0.38	0.00120517	duck
Vezf1	-0.30	0.00122664	duck
Fuca2	-0.13	0.00124673	duck
Dock10	-0.28	0.00125828	duck
Fbxo28	-0.18	0.00126130	duck
Aagab	-0.06	0.00126245	duck
Fbxo45	-0.14	0.00126424	duck
Pdzrn3	-0.18	0.00127162	duck
Zfp830	-0.29	0.00127203	duck
Ywhab	-0.16	0.00127629	duck
Ap1s2	-0.11	0.00128133	duck
Chgb	-0.24	0.00129144	duck
Kdm1a	-0.16	0.00130206	duck
Aars	-0.12	0.00132812	duck
Zfp518a	-0.15	0.00136332	duck
Cstf3	-0.25	0.00136655	duck
Stx2	-0.12	0.00139075	duck
Pkn2	-0.23	0.00139363	duck
Sema6d	0.11	0.00139615	duck
Nol4l	-0.01	0.00140217	duck
St3gal6	-0.13	0.00143735	duck
Pspc1	-0.42	0.00145957	duck
Ece1	-0.02	0.00148787	duck
Pik3cg	-0.03	0.00151689	duck
Rnf34	0.00	0.00151837	duck
Commd8	-0.04	0.00152071	duck
G2e3	-0.16	0.00152156	duck
Cdin1	1.36	0.00152239	duck
Psen1	-0.15	0.00155922	duck
Sclt1	-0.11	0.00155966	duck
Fam177a	-0.21	0.00156652	duck
Tshz2	-0.23	0.00160356	duck
Lsm14a	-0.38	0.00164307	duck
Thyn1	-0.07	0.00167087	duck
Ccnl1	-0.43	0.00167652	duck
Fgd4	-0.20	0.00167826	duck
Ube2q1	-0.12	0.00168733	duck
Ext2	0.00	0.00171075	duck
Lztr1	-0.12	0.00174475	duck

Ndufb1	-0.17	0.00175651	duck
Dusp11	-0.02	0.00176558	duck
Ppp6c	-0.09	0.00180322	duck
Ufl1	0.07	0.00181846	duck
Ubn1	-0.15	0.00184013	duck
Psmg4	-0.09	0.00184325	duck
Trappc8	-0.25	0.00184633	duck
Adam10	-0.24	0.00185599	duck
Pnpla7	-0.03	0.00186403	duck
Wnt2b	-0.02	0.00187299	duck
Btg1b	0.52	0.00187447	duck
Ate1	0.07	0.00192472	duck
Kat2b	-0.31	0.00192994	duck
Chchd5	-0.11	0.00193714	duck
Psme3	-0.20	0.00206248	duck
Cc2d2a	-0.14	0.00206330	duck
Hdac4	0.57	0.00210631	duck
Cdca4	-0.22	0.00215448	duck
Gps1	0.02	0.00218562	duck
Btbd10	-0.19	0.00220753	duck
Cav2	-0.10	0.00222635	duck
Cdc37l1	-0.15	0.00229793	duck
Ppp1r21	-0.13	0.00232229	duck
Tpm3-rs7	-0.16	0.00233453	duck
Psd	-0.08	0.00233647	duck
Ube2q2	-0.18	0.00235504	duck
Rtn4	-0.27	0.00240518	duck
Fubp1	-0.30	0.00240689	duck
Sgpl1	-0.10	0.00240878	duck
Eif1	0.36	0.00241011	duck
Prr5l	-0.26	0.00241480	duck
Clic4	-0.32	0.00243432	duck
Eif2s1	-0.13	0.00243663	duck
Guk1	-0.03	0.00249285	duck
Surf4	-0.16	0.00250041	duck
Nipal3	-0.18	0.00252302	duck
Grsf1	-0.11	0.00254661	duck
Ppil2	-0.34	0.00257096	duck
Sdr16c5	-0.04	0.00262085	duck
Rprd1a	-0.55	0.00262632	duck
Nans	-0.18	0.00264491	duck
Ing1	-0.09	0.00265767	duck
Opa1	-0.18	0.00265899	duck
Abi2	-0.23	0.00270266	duck
Stk24	-0.20	0.00272066	duck
Prrc2c	-0.45	0.00273059	duck

Txlng	-0.22	0.00274621	duck
Rac1	-0.23	0.00279549	duck
Dkk3	-0.08	0.00283055	duck
Odf2	-0.09	0.00283283	duck
Srsf11	-0.19	0.00285183	duck
Vps36	-0.01	0.00287523	duck
Ntpcr	-0.01	0.00290069	duck
Sertad2	-0.31	0.00292658	duck
Ndufs5	-0.15	0.00294342	duck
Stmn1	-0.14	0.00296181	duck
Kif3a	-0.01	0.00297012	duck
Bach1	-0.55	0.00300452	duck
Ciao2b	-0.11	0.00300696	duck
Rundc1	0.75	0.00301718	duck
Tent4a	-0.03	0.00302039	duck
Nus1	-0.01	0.00303953	duck
Rftn2	0.10	0.00311163	duck
Slc35a1	-0.05	0.00312135	duck
Garre1	-0.21	0.00317188	duck
Ggps1	-0.09	0.00322573	duck
Stx7	0.07	0.00323669	duck
Qdpr	-0.02	0.00328062	duck
Top2b	-0.25	0.00330097	duck
Gins3	-0.15	0.00334275	duck
Akap8l	-0.15	0.00334348	duck
Pdcd10	-0.17	0.00334655	duck
Ndufs7	-0.02	0.00335890	duck
Ift52	-0.03	0.00338726	duck
Bzw1	-0.24	0.00338728	duck
Rap1gds1	-0.16	0.00339978	duck
Crym	0.03	0.00341278	duck
Inpp4a	-0.36	0.00345006	duck
Slc44a2	0.04	0.00349044	duck
Ythdf3	-0.10	0.00350092	duck
Ppme1	-0.07	0.00350161	duck
Vkorc1l1	-0.10	0.00355875	duck
Pofut1	-0.01	0.00356657	duck
Adnp2	-0.19	0.00356894	duck
Cbl	-0.18	0.00357495	duck
Papola	-0.24	0.00359900	duck
Fam91a1	-0.08	0.00362318	duck
Washc4	-0.07	0.00362835	duck
Lgals8	-0.05	0.00364885	duck
Dcun1d3	-0.30	0.00365105	duck
Fam53b	-0.15	0.00366996	duck
Trmt2a	-0.22	0.00370137	duck

Sh2b3	-0.30	0.00379041	duck
Riok1	0.05	0.00382003	duck
Dok6	-0.13	0.00383214	duck
Ei24	-0.10	0.00384010	duck
Cebpa	-0.10	0.00385645	duck
Tnip2	-0.77	0.00397125	duck
Emc2	-0.04	0.00398006	duck
Errfi1	-0.42	0.00416745	duck
Khdrbs3	-0.03	0.00421518	duck
Srp54a	-0.15	0.00423309	duck
Crim1	-0.58	0.00423884	duck
Nfs1	-0.11	0.00428295	duck
Snx18	-0.16	0.00428624	duck
Cpne8	-0.05	0.00434703	duck
Plekho1	-0.14	0.00435129	duck
Fam8a1	-0.08	0.00436127	duck
Timp4	0.80	0.00441344	duck
Katnbl1	-0.02	0.00441555	duck
Filip1	-0.36	0.00446306	duck
Ywhag	-0.11	0.00455026	duck
Ppfibp2	-0.06	0.00456701	duck
Crnkl1	0.00	0.00458965	duck
Asxl1	0.00	0.00466549	duck
Fgl1	0.63	0.00466668	duck
Slc35a5	-0.26	0.00469640	duck
Poglut1	-0.04	0.00484250	duck
Lsg1	-0.06	0.00484548	duck
Gatad1	-0.05	0.00488386	duck
Slc44a3	0.03	0.00488959	duck
Spring1	-0.03	0.00490895	duck
Pacsin2	-0.32	0.00494432	duck
Gpn3	-0.08	0.00503469	duck
Esr1	-0.12	0.00503840	duck
Ccnl2	-0.23	0.00510623	duck
Noc3l	-0.09	0.00512505	duck
Papss1	-0.06	0.00516337	duck
Sccpdh	-0.06	0.00519744	duck
Dennd6a	-0.04	0.00521825	duck
Cibar1	-0.05	0.00525090	duck
Snx7	0.03	0.00527360	duck
Eloc	-0.16	0.00536132	duck
Zbtb41	-0.21	0.00541226	duck
Nptn	-0.11	0.00545187	duck
Rac2	0.98	0.00548294	duck
Ranbp9	0.78	0.00549728	duck
Rxra	0.86	0.00552896	duck

Cct2	-0.02	0.00556175	duck
Gcc2	-0.29	0.00559212	duck
Txn2	-0.01	0.00566286	duck
Nab1	-0.12	0.00577277	duck
Elmod2	-0.05	0.00579885	duck
B4galt7	-0.01	0.00581881	duck
Eef1b2	-0.32	0.00595210	duck
Qser1	0.00	0.00602949	duck
Urb1	0.00	0.00603303	duck
Gnal	-0.06	0.00604781	duck
Rint1	-0.05	0.00605504	duck
Amot	0.05	0.00612489	duck
Anapc5	-0.05	0.00614792	duck
Pdk1	-0.10	0.00627029	duck
Sprtn	-0.13	0.00636598	duck
Ctnna1	-0.14	0.00645966	duck
Bnip2	-0.04	0.00646030	duck
Nif3l1	-0.03	0.00647599	duck
Cops3	-0.19	0.00657055	duck
Tmx1	-0.04	0.00662558	duck
Osbpl8	-0.09	0.00663114	duck
Pitpnm2	-0.50	0.00664477	duck
Cpne1	-0.30	0.00671841	duck
Lmo4	-0.04	0.00676830	duck
Abcc4	-0.15	0.00687812	duck
Gfpt1	-0.08	0.00702721	duck
Bpnt2	-0.08	0.00703080	duck
Fut11	-0.05	0.00710201	duck
Cul5	-0.14	0.00720484	duck
Jazf1	-0.25	0.00721128	duck
Akr1a1	-0.06	0.00726948	duck
Hadha	-0.01	0.00756174	duck
Ywhaq	-0.23	0.00757658	duck
Uchl5	-0.03	0.00762707	duck
Ndrg3	-0.22	0.00770351	duck
Cd34	-0.09	0.00770404	duck
Pdap1	-0.15	0.00773010	duck
Sod2	-0.16	0.00774189	duck
Tcim	-0.09	0.00779551	duck
Gpn1	-0.11	0.00795130	duck
Rab22a	-0.19	0.00796718	duck
Sfrp2	-0.21	0.00797815	duck
Bdnf	-0.11	0.00805385	duck
Lamtor2	-0.16	0.00805565	duck
Pla2r1	-0.03	0.00806964	duck
Dysf	-0.07	0.00809496	duck

Slc6a6	-0.48	0.00812854	duck
Scai	0.04	0.00818039	duck
Ufm1	0.08	0.00819951	duck
Slc16a1	-0.03	0.00822935	duck
Zfp608	-0.19	0.00834992	duck
Rcbtb1	0.00	0.00842318	duck
Cdk6	-0.08	0.00870112	duck
Fam210a	-0.14	0.00893576	duck
Timm17a	-0.02	0.00900835	duck
Zmpste24	0.07	0.00907436	duck
Tmem258	-0.11	0.00931903	duck
Glod4	-0.06	0.00946372	duck
Ptpn9	-0.09	0.00946473	duck
Utp6	0.02	0.00948382	duck
Ywhae	-0.12	0.00962822	duck
Dhx36	-0.10	0.00963058	duck
Inpp5k	0.01	0.00970623	duck
Tpm4	-0.06	0.01017885	duck
Galk2	-0.06	0.01030505	duck
Ipo8	-0.06	0.01034293	duck
Ube2o	-0.12	0.01034483	duck
Gtf3c4	-0.15	0.01058169	duck
Pafah1b1	-0.28	0.01062737	duck
B4galt5	0.01	0.01069112	duck
Dpy30	-0.08	0.01081567	duck
Cep20	-0.12	0.01105843	duck
Emb	-0.09	0.01107272	duck
Tet1	-0.30	0.01119305	duck
Rexo5	-0.17	0.01120548	duck
Fstl1	-0.21	0.01121950	duck
Zfp592	-0.16	0.01122459	duck
Alas1	-0.04	0.01157604	duck
Nkain3	-0.17	0.01158885	duck
Trio	-0.30	0.01171590	duck
Tk2	-0.15	0.01176760	duck
Gga3	-0.04	0.01188946	duck
Rab35	-0.02	0.01192093	duck
Zfp821	-0.05	0.01196134	duck
Clip1	-0.25	0.01201086	duck
Sel1l	-0.32	0.01204185	duck
Timmdc1	-0.03	0.01216996	duck
Glrx	0.59	0.01217924	duck
Rbm22	-0.02	0.01225777	duck
Ibtk	-0.25	0.01233388	duck
Fxyd6	0.08	0.01242127	duck
Evc2	-0.07	0.01262351	duck

Uhrf1bp1	-0.28	0.01279743	duck
Ppfia1	-0.15	0.01302488	duck
Pitrm1	-0.22	0.01313042	duck
Rev1	-0.43	0.01323336	duck
Fhl3	0.05	0.01335830	duck
Cntln	-0.17	0.01366482	duck
Dazap1	-0.28	0.01382951	duck
Cry1	-0.08	0.01389296	duck
Vps29	0.02	0.01398300	duck
Prpf38a	-0.10	0.01399203	duck
Czib	-0.16	0.01400106	duck
Pithd1	0.06	0.01407393	duck
Ctnnb1	-0.13	0.01411085	duck
2510039O18Rik	-0.10	0.01412101	duck
Ltbp1	-0.33	0.01415196	duck
Rgma	0.07	0.01415990	duck
Prmt3	-0.06	0.01424164	duck
Tmem237	-0.11	0.01440920	duck
Gtf2a2	-0.05	0.01478413	duck
Ccni	-0.16	0.01506464	duck
Prdm6	-0.15	0.01506591	duck
Fbxo46	-0.16	0.01507095	duck
Pus1	-0.07	0.01530503	duck
Pot1b	-0.56	0.01544270	duck
Srsf6	-0.32	0.01564025	duck
Cep290	-0.38	0.01572041	duck
Bcl2l1	-0.14	0.01589016	duck
Cnih4	0.01	0.01591856	duck
Ccdc148	-0.08	0.01595286	duck
Selenok	0.01	0.01595631	duck
Tnfaip1	-0.01	0.01600354	duck
Dnajc16	0.03	0.01604085	duck
Zzef1	-0.31	0.01630302	duck
Jak2	-0.60	0.01639929	duck
Ext1	-0.33	0.01644643	duck
Eif5b	-0.25	0.01651034	duck
Tbc1d23	0.06	0.01682638	duck
Elovl1	0.01	0.01689441	duck
Galnt10	0.00	0.01702883	duck
Slc66a2	-0.06	0.01704512	duck
Akap10	-0.13	0.01711806	duck
Nip7	-0.02	0.01712489	duck
Cap1	-0.05	0.01729114	duck
Eif2s2	-0.36	0.01737173	duck
Ptch1	1.18	0.01743471	duck
Trappc11	-0.06	0.01751245	duck

Dnajb4	-0.02	0.01753354	duck
Vopp1	-0.10	0.01786959	duck
Usp22	-0.42	0.01799192	duck
Ppp4r1	-0.09	0.01803543	duck
Erich1	-0.10	0.01811508	duck
Senp5	-0.02	0.01812874	duck
Kif5b	-0.27	0.01812987	duck
Telo2	-0.22	0.01836673	duck
Pank3	-0.15	0.01866707	duck
Ghitm	-0.14	0.01912073	duck
Abhd17b	-0.15	0.01944948	duck
Cir1	-0.03	0.01946038	duck
Ppil3	-0.12	0.01947909	duck
Sh3kbp1	0.05	0.01948886	duck
Uggt1	-0.11	0.01955153	duck
Sult4a1	-0.08	0.01965426	duck
Ccdc28a	-0.06	0.01971118	duck
Spata7	-0.04	0.01971451	duck
Scfd1	-0.24	0.01991925	duck
Fgfr3	0.13	0.01998838	duck
Smarcd2	-0.10	0.02010240	duck
Itpr1	-0.37	0.02032328	duck
Rsrp1	-0.24	0.02044849	duck
Snx10	1.32	0.02056831	duck
Sntb2	0.00	0.02070319	duck
Arl13b	0.12	0.02071292	duck
Ccne1	0.05	0.02103994	duck
Zcchc24	-0.36	0.02111694	duck
Tmem70	-0.02	0.02114742	duck
Nfia	0.38	0.02132953	duck
Lias	-0.11	0.02149194	duck
Txnrd1	-0.50	0.02149992	duck
Cldn12	-0.02	0.02155044	duck
Ing2	-0.05	0.02205066	duck
Zfp36l1	-0.37	0.02251943	duck
Tnfrsf11b	-0.26	0.02268890	duck
Snapc4	-0.19	0.02294820	duck
Twist2	-0.02	0.02298067	duck
Atp11b	-0.28	0.02303432	duck
Dag1	-0.02	0.02305079	duck
Senp7	-0.37	0.02335669	duck
Ufd1	-0.16	0.02381005	duck
Cyld	-0.10	0.02434632	duck
Pla2g12a	-0.20	0.02438178	duck
Gata6	-0.12	0.02446572	duck
Fam120a	-0.08	0.02460719	duck

Parp9	0.56	0.02464480	duck
Dhdds	-0.12	0.02481269	duck
Bdh1	-0.15	0.02535229	duck
Rpap2	-0.11	0.02553062	duck
Dnajc24	-0.04	0.02577221	duck
Rnf214	-0.16	0.02581077	duck
Akap11	-0.10	0.02592501	duck
Phlda1	-0.14	0.02606297	duck
Ccdc174	-0.27	0.02609157	duck
Dync1i2	-0.15	0.02638059	duck
Lmna	-0.50	0.02665131	duck
Gtf2f2	-0.20	0.02666992	duck
Fmn1	-0.06	0.02681671	duck
Hapln1	-0.02	0.02685789	duck
Gga2	-0.06	0.02724639	duck
Dbf4	-0.15	0.02741299	duck
Golt1b	0.06	0.02774106	duck
Smad7	0.99	0.02777598	duck
Ankrd27	-0.15	0.02785833	duck
Phactr2	0.05	0.02810463	duck
Rpa2	-0.04	0.02846565	duck
Smarca5	-0.17	0.02898232	duck
C1d	0.05	0.02899545	duck
Rab11fip2	-0.25	0.02936880	duck
Stx18	0.07	0.02945982	duck
Ppp1r8	-0.02	0.02968826	duck
Oard1	-0.18	0.03019830	duck
Eea1	-0.04	0.03030053	duck
Immp1l	-0.41	0.03115351	duck
Pdxdc1	-0.02	0.03134217	duck
Sf3a3	-0.08	0.03154679	duck
Cd109	-0.18	0.03262276	duck
Tbl2	0.06	0.03262376	duck
Zfp518b	-0.03	0.03332714	duck
Colgalt2	-0.06	0.03345674	duck
Cdc42ep3	-0.12	0.03385068	duck
Napg	-0.15	0.03412119	duck
Lrrfip2	0.00	0.03413631	duck
Kidins220	-0.13	0.03423234	duck
Surf2	-0.11	0.03424001	duck
Tril	0.06	0.03426006	duck
Afap1l2	-0.13	0.03436215	duck
Txnl1	-0.08	0.03442671	duck
Rabgap1l	-0.32	0.03490550	duck
Zbtb1	-0.01	0.03493739	duck
Smarce1	0.00	0.03495837	duck

Fam151b	0.50	0.03501710	duck
Suox	-0.17	0.03592537	duck
Zfp503	-0.05	0.03620182	duck
Lpl	0.01	0.03652159	duck
Gtf3c1	-0.07	0.03677838	duck
Ncor2	0.45	0.03691569	duck
Stim1	-0.03	0.03692111	duck
Nfatc3	-0.18	0.03764895	duck
Nub1	-0.07	0.03794690	duck
Arih2	-0.09	0.03843703	duck
Smim19	-0.08	0.03884658	duck
Galnt1	-0.10	0.03946684	duck
Snx14	-0.04	0.03980873	duck
Psma5	-0.02	0.04033624	duck
Htatsf1	-0.19	0.04047196	duck
Eps15l1	-0.15	0.04069456	duck
Ercc3	0.11	0.04103842	duck
Btbd2	-0.30	0.04108333	duck
Drg2	-0.11	0.04110603	duck
Crls1	0.08	0.04114491	duck
Stau1	-0.01	0.04117616	duck
Fdps	-0.13	0.04165125	duck
Cnot10	-0.15	0.04169504	duck
Fbxo7	-0.12	0.04288776	duck
Jmy	0.88	0.04327531	duck
Sardh	0.04	0.04359026	duck
Pdgfa	0.00	0.04369623	duck
Slc25a25	-0.11	0.04376199	duck
Erg28	-0.12	0.04381425	duck
Rae1	-0.29	0.04425258	duck
Spg11	-0.14	0.04441193	duck
Klhl5	0.02	0.04445472	duck
Filip1l	-0.17	0.04486454	duck
Rchy1	-0.21	0.04570026	duck
Armc10	-0.03	0.04620878	duck
Vti1b	-0.06	0.04647595	duck
Extl3	-0.07	0.04683284	duck
Rabl6	0.00	0.04689073	duck
Nfrkb	-0.04	0.04783644	duck
Apod	-0.10	0.04828180	duck
Sfmbt2	0.01	0.04829552	duck
Ankrd33	0.03	0.04990151	duck
Phlpp1	0.12	0.05022928	duck
Dync1h1	-0.30	0.05039536	duck
Cep104	0.04	0.05049356	duck
Proser2	-0.13	0.05066551	duck

Spata2	-0.16	0.05117746	duck
Kctd20	-0.19	0.05155518	duck
Rtcb	-0.11	0.05172293	duck
Alg13	-0.07	0.05215793	duck
Tubgcp6	-0.03	0.05304072	duck
Cep112	0.09	0.05305805	duck
Vps39	-0.01	0.05347388	duck
Dnajc3	-0.07	0.05373477	duck
Szrd1	-0.07	0.05393156	duck
Crk	-0.02	0.05404115	duck
Lbh	-0.23	0.05466233	duck
Slu7	-0.26	0.05470466	duck
Srpk2	0.07	0.05490717	duck
Zc3h13	-0.03	0.05503836	duck
Sema3a	1.24	0.05544130	duck
Cipc	-0.19	0.05560898	duck
Fem1b	-0.08	0.05656365	duck
Hivep2	-0.30	0.05679512	duck
Tspan5	-0.04	0.05685452	duck
Tshz3	0.14	0.05703886	duck
Hif1an	-0.06	0.05725604	duck
Ruvbl1	0.07	0.05755831	duck
Sppl2b	-0.10	0.05778139	duck
Arhgdia	0.00	0.05805202	duck
Pi4kb	-0.02	0.05919501	duck
Usp12	-0.05	0.05954932	duck
Bud23	-0.06	0.05985471	duck
Tspan6	0.06	0.05987594	duck
Ebf1	-0.32	0.06070163	duck
Tnfaip6	-0.11	0.06137361	duck
Rufy1	-0.13	0.06166904	duck
Cgrrf1	-0.24	0.06209609	duck
Dhx40	-0.06	0.06251372	duck
Jpt1	-0.15	0.06266816	duck
Nol11	-0.15	0.06299939	duck
Gnl3	-0.05	0.06348755	duck
Gnpda2	-0.27	0.06364672	duck
Rrp15	-0.01	0.06472760	duck
Arhgap28	-0.20	0.06518367	duck
Arhgef6	-0.12	0.06531645	duck
Cdc25a	-0.04	0.06734017	duck
Plpbp	-0.01	0.06740381	duck
Gng12	-0.03	0.06770748	duck
Fabp3	0.44	0.06810315	duck
Sema5a	-0.31	0.06848732	duck
Slc25a24	-0.32	0.06868711	duck

Secisbp2l	-0.11	0.06951943	duck
Ficd	-0.06	0.07028276	duck
Sos2	-0.16	0.07041134	duck
Fam76b	-0.02	0.07092502	duck
Zeb2	-0.21	0.07166410	duck
Zfp362	0.06	0.07313568	duck
Ccser2	-0.04	0.07334275	duck
Lifr	-0.05	0.07423789	duck
Tbl3	-0.12	0.07439359	duck
Cnpy3	-0.12	0.07504989	duck
Wapl	-0.32	0.07506991	duck
Lingo1	-0.02	0.07552256	duck
Twf1	-0.05	0.07632988	duck
Clptm1l	0.04	0.07674061	duck
Tfdp1	-0.32	0.07708768	duck
Rbms3	0.46	0.07711354	duck
Prpf3	-0.07	0.07718621	duck
Tdrd7	0.64	0.07759035	duck
Srgn	0.72	0.07863901	duck
Lrrc49	0.00	0.07913256	duck
Nol9	0.00	0.08092427	duck
Dab1	-0.35	0.08136659	duck
Polr2e	-0.07	0.08198848	duck
Dtwd1	-0.02	0.08242863	duck
Smim5	-0.11	0.08244724	duck
Fam171a1	-0.16	0.08318412	duck
Lzic	-0.06	0.08322025	duck
Unc50	0.00	0.08322079	duck
Arhgap10	-0.02	0.08328853	duck
Npnt	-0.15	0.08389422	duck
Kdm5b	0.00	0.08445314	duck
Tnrc6a	-0.08	0.08473089	duck
Tmem184c	-0.01	0.08508415	duck
Txlnb	0.05	0.08578886	duck
Snrnp200	-0.19	0.08613960	duck
Nup214	-0.06	0.08635394	duck
Fam161a	-0.14	0.08687240	duck
Atf6	-0.12	0.08721146	duck
Pdzd8	-0.07	0.08729376	duck
Arxes2	0.02	0.08763057	duck
Pdcl	-0.01	0.08778430	duck
Smpdl3a	-0.05	0.08815097	duck
Ctdspl2	-0.19	0.08962113	duck
Bcl9l	0.09	0.08982093	duck
Rsrc2	-0.33	0.09124016	duck
Txn1	0.89	0.09128306	duck

Csnk1a1	-0.16	0.09130641	duck
Abhd10	0.01	0.09159649	duck
Klf5	-0.11	0.09250495	duck
Elf2	0.02	0.09271381	duck
Wdr20	-0.18	0.09278173	duck
Sec63	-0.09	0.09298466	duck
B9d1	0.00	0.09353033	duck
Rangap1	0.03	0.09421748	duck
Per2	-0.31	0.09481500	duck
Bst1	-0.18	0.09608431	duck
Arhgap12	-0.05	0.09903871	duck
Tm7sf3	0.02	0.09936909	duck
Pno1	-0.01	0.10031986	duck
Eif4enif1	-0.21	0.10086532	duck
Armt1	0.03	0.10092563	duck
Il17b	-0.01	0.10147217	duck
Ifngr2	0.05	0.10213852	duck
Rnf144a	0.54	0.10252178	duck
Efl1	-0.07	0.10262689	duck
Zfyve1	-0.06	0.10318845	duck
Herc1	-0.40	0.10393970	duck
Sav1	-0.07	0.10448972	duck
Slc12a4	-0.21	0.10462997	duck
Aimp2	-0.02	0.10510725	duck
Zc3h14	-0.17	0.10571456	duck
Ankle2	0.01	0.10635596	duck
Zdhhc7	-0.33	0.10658292	duck
Ralgps1	-0.16	0.10675363	duck
Glyr1	-0.09	0.10771980	duck
Ivd	-0.02	0.10773003	duck
Suclg1	-0.06	0.10782856	duck
Tsku	-0.13	0.10821406	duck
Snrnp48	0.01	0.10902782	duck
Dpm2	-0.05	0.11042193	duck
Prkar2a	-0.06	0.11102755	duck
Chd1l	-0.05	0.11105485	duck
Gde1	0.01	0.11184508	duck
Heatr5a	0.06	0.11208864	duck
Washc5	0.02	0.11313886	duck
Ccp110	-0.13	0.11365935	duck
Sufu	-0.36	0.11421010	duck
Dnajb2	-0.04	0.11422581	duck
Ap3m2	-0.03	0.11521342	duck
Calhm5	-0.12	0.11554735	duck
Taok3	-0.40	0.11592854	duck
Ppm1d	-0.04	0.11603228	duck

Anxa7	-0.07	0.11603914	duck
Tubb5	-0.18	0.11738752	duck
Frs2	-0.16	0.11826575	duck
Scarb1	-0.10	0.11890112	duck
Rnpc3	-0.28	0.11985822	duck
Ggnbp2	-0.09	0.11994155	duck
Ap1g1	-0.10	0.12161218	duck
Hint1	0.02	0.12194889	duck
Rbm5	-0.34	0.12214990	duck
Ormdl3	-0.02	0.12281395	duck
Npepps	0.06	0.12425500	duck
Dcbld2	0.01	0.12427213	duck
Tob2	0.01	0.12483854	duck
Tmem43	0.06	0.12493567	duck
Ufsp2	-0.08	0.12581214	duck
Pop4	-0.13	0.12716747	duck
Ints12	-0.06	0.12849838	duck
Klf15	0.75	0.12852021	duck
Nrbp1	-0.23	0.12876947	duck
Usp5	-0.05	0.12930674	duck
Bpnt1	-0.13	0.12983884	duck
Smim13	0.00	0.12999300	duck
Hikeshi	0.01	0.13002922	duck
Hook1	-0.10	0.13003932	duck
Dph7	0.02	0.13050268	duck
Heatr3	-0.10	0.13092102	duck
Trim14	-0.07	0.13110716	duck
Lig3	-0.08	0.13320391	duck
Ube2k	-0.04	0.13345253	duck
Txnrd2	0.01	0.13364459	duck
Rab7	-0.05	0.13528006	duck
Pank4	0.03	0.13681784	duck
Srebf2	0.03	0.13747724	duck
Psmd7	-0.06	0.13853388	duck
Pawr	-0.19	0.13861873	duck
Tcea2	-0.08	0.13967277	duck
Wdr4	0.00	0.14065996	duck
Tbc1d5	0.05	0.14207769	duck
Aimp1	-0.05	0.14282682	duck
Nckap1	-0.07	0.14339326	duck
Stat4	-0.19	0.14341456	duck
Naa30	-0.20	0.14392692	duck
Arglu1	-0.20	0.14479994	duck
Prdm2	-0.03	0.14550839	duck
Spg20	0.06	0.14556102	duck
Lrrk1	0.05	0.14573494	duck

Topbp1	-0.07	0.14750123	duck
Ciapin1	-0.08	0.14865440	duck
Sel1l3	0.13	0.15158112	duck
Dagla	-0.13	0.15302664	duck
Celf1	-0.30	0.15438300	duck
Pip4k2b	-0.01	0.15555216	duck
Osbp2	-0.02	0.15678321	duck
Ssbp2	-0.37	0.15860362	duck
Ttc4	-0.05	0.15895268	duck
Eif4h	0.05	0.16062185	duck
Angptl1	0.03	0.16071045	duck
Yipf6	-0.05	0.16092698	duck
Dach2	1.00	0.16165977	duck
Picalm	-0.27	0.16246251	duck
Cep350	-0.38	0.16254244	duck
Ythdc2	-0.21	0.16531776	duck
Plagl1	0.00	0.16671815	duck
Iffo2	-0.14	0.16694282	duck
Ccdc47	-0.12	0.17176790	duck
Cryz	-0.01	0.17235373	duck
Prex2	-0.28	0.17314218	duck
Tspan2	-0.05	0.17332426	duck
Timm22	0.01	0.17354796	duck
Ddx46	-0.18	0.17490092	duck
Prmt7	-0.06	0.17562539	duck
Nr4a1	-0.15	0.17645584	duck
Eef2	-0.27	0.17677523	duck
Nup88	-0.11	0.17912477	duck
Cog6	-0.02	0.17959104	duck
Camk4	0.03	0.17963489	duck
Slc16a2	0.04	0.18053531	duck
Ube2j2	-0.18	0.18054201	duck
Nono	-0.08	0.18192793	duck
Sntg1	-0.51	0.18228920	duck
Rab3gap2	-0.07	0.18319056	duck
Galnt18	0.00	0.18463267	duck
Wdr45b	-0.06	0.18578127	duck
Odc1	-0.36	0.18604403	duck
Prr5	-0.15	0.18822057	duck
Hs1bp3	0.04	0.18908204	duck
Sirt6	-0.08	0.19169211	duck
Kras	-0.26	0.19181105	duck
Gpatch8	-0.15	0.19456903	duck
Ndufb6	-0.10	0.19503912	duck
Anapc13	0.00	0.19517887	duck
Fam20b	-0.05	0.19691609	duck

Spg21	-0.03	0.19733190	duck
Sh3rf3	-0.20	0.19827168	duck
Taf10	-0.05	0.19864630	duck
Amotl1	0.01	0.20090776	duck
Polr2l	-0.08	0.20209780	duck
Nanos1	-0.03	0.20428468	duck
Stk25	-0.10	0.20436142	duck
Slc30a1	-0.04	0.20798581	duck
Nolc1	0.07	0.21000309	duck
Tmem97	0.02	0.21051256	duck
Pds5b	0.06	0.21156934	duck
Kdm4b	0.08	0.21306073	duck
Rragd	-0.11	0.21412416	duck
Heatr5b	-0.05	0.21612289	duck
Tm2d1	-0.04	0.21726743	duck
Stub1	-0.21	0.21926427	duck
Btbd7	-0.03	0.21972356	duck
Prdm11	-0.01	0.22335995	duck
Reep2	-0.01	0.22403817	duck
Ppig	-0.24	0.22469099	duck
Snw1	-0.03	0.22962693	duck
Acer3	-0.05	0.22986047	duck
Erap1	0.52	0.23076152	duck
Klhl24	0.63	0.23099551	duck
Dhx8	0.02	0.23102727	duck
Lonrf1	-0.39	0.23343695	duck
Cers2	0.02	0.23423821	duck
Nudt5	-0.01	0.23812568	duck
Snrpc	-0.04	0.23843754	duck
Afap1	-0.22	0.24153954	duck
Atp5g3	-0.04	0.24201184	duck
Tasor2	-0.04	0.24222053	duck
Heg1	-0.08	0.24384700	duck
Ppard	-0.06	0.24443753	duck
Ldlrad3	-0.07	0.24463344	duck
Tfdp2	-0.01	0.24645764	duck
Lysmd2	-0.11	0.24684169	duck
Atad1	-0.09	0.24765499	duck
Ttc1	-0.24	0.24880115	duck
Dnajb14	0.05	0.25035089	duck
Ddb1	-0.16	0.25096905	duck
Atic	-0.12	0.25156698	duck
Eed	-0.01	0.25503862	duck
Dapk3	-0.01	0.25574096	duck
Gpbp1	0.11	0.25596445	duck
Btrc	-0.20	0.25817342	duck

Snx16	-0.25	0.25833789	duck
Clcn3	-0.03	0.25862062	duck
Rnf220	-0.20	0.26329849	duck
Dstn	-0.06	0.26415835	duck
Ndufa10	-0.06	0.26603857	duck
Plrg1	0.03	0.26814379	duck
Ptprk	0.04	0.26866924	duck
DPCD	-0.20	0.27022540	duck
Castor2	-0.08	0.27170482	duck
Zfp148	-0.10	0.27528690	duck
Rftn1	-0.01	0.27743938	duck
Acap2	-0.06	0.27790681	duck
Rnf111	-0.29	0.28428345	duck
Tcerg1	-0.03	0.28590746	duck
Arv1	-0.22	0.28838729	duck
Pcyt2	-0.09	0.29232190	duck
Zfand6	-0.29	0.29516752	duck
Lrpap1	-0.09	0.29557788	duck
Slc16a10	-0.08	0.29926876	duck
Odr4	0.03	0.30062554	duck
Sap130	0.00	0.30192609	duck
Itgav	-0.14	0.30470157	duck
Rmnd5a	-0.07	0.30612730	duck
Ilk	0.02	0.30718355	duck
Pigw	-0.03	0.30967895	duck
Vps53	-0.07	0.31133294	duck
Zbtb33	0.03	0.31468782	duck
Ndufa6	-0.09	0.31475488	duck
Hmgn3	-0.03	0.31527059	duck
Gapvd1	0.00	0.31686552	duck
Fgd6	0.03	0.31722085	duck
Pou6f1	-0.07	0.31753083	duck
Nhsl1	-0.06	0.31944570	duck
Ackr3	-0.16	0.31968251	duck
Ppp2cb	-0.02	0.32115673	duck
Tma16	-0.02	0.32446700	duck
Smg1	-0.17	0.32447897	duck
Dop1a	-0.05	0.32835529	duck
Pcmtd2	-0.03	0.32874547	duck
Cnn3	-0.20	0.33066997	duck
B3galt1	-0.22	0.33184017	duck
Caprin1	-0.19	0.33219735	duck
Cep170b	0.16	0.33436129	duck
Prkci	-0.31	0.33465237	duck
Rhoa	-0.29	0.33617652	duck
Selenoo	-0.25	0.33675290	duck

Hacd2	0.01	0.33727037	duck
Ptp4a2	-0.01	0.34000754	duck
Tmem223	0.10	0.34161933	duck
Cav1	0.25	0.34270711	duck
Hace1	-0.02	0.35027343	duck
Ap1ar	0.00	0.35083261	duck
Slain2	-0.12	0.35087217	duck
Tsc22d2	-0.28	0.36061376	duck
Bloc1s2	-0.10	0.36523216	duck
Gid8	-0.08	0.36869855	duck
Lcat	0.07	0.36974489	duck
Htt	-0.21	0.37048453	duck
Psmd1	-0.21	0.37087697	duck
Slc4a2	-0.10	0.37333266	duck
Rbm33	-0.53	0.37767698	duck
Papss2	0.87	0.37881575	duck
Gnai3	-0.12	0.38452257	duck
Ncoa5	-0.01	0.38570742	duck
Cnih1	-0.14	0.38962125	duck
Prdx6	0.06	0.39002541	duck
Gyg	0.07	0.39731785	duck
Hnrnpa3	-0.29	0.39766241	duck
Ipo5	-0.04	0.40058371	duck
Trip11	-0.29	0.40255294	duck
Smarcc1	-0.34	0.40368072	duck
Pbrm1	-0.04	0.40535541	duck
Sulf1	-0.21	0.40579602	duck
Zbtb43	-0.31	0.40957233	duck
Tmem183a	-0.21	0.41035898	duck
Ipo7	-0.07	0.41089620	duck
Slc30a4	-0.06	0.41818700	duck
Rnf169	-0.01	0.42298646	duck
Poc1b	0.00	0.42456323	duck
Phf13	0.02	0.42495387	duck
Ciao2a	0.04	0.42679103	duck
Hacd4	0.90	0.42762860	duck
Sugp1	0.00	0.42845771	duck
Pus7l	-0.08	0.43029942	duck
Ppm1m	-0.09	0.43194795	duck
Ide	-0.21	0.43358016	duck
Fasn	0.21	0.43360374	duck
Srsf10	-0.10	0.43456067	duck
Ppic	0.01	0.44200836	duck
Klhl15	-0.29	0.44562486	duck
Ptgfrn	-0.15	0.44605744	duck
Rab14	0.05	0.44630320	duck

Dpy19l4	-0.10	0.44794797	duck
Btbd1	-0.05	0.45068805	duck
Zbed4	-0.03	0.45399290	duck
Ift46	-0.13	0.45472038	duck
Rpgrip1l	-0.05	0.45657942	duck
Nexmif	-0.03	0.45888848	duck
Polr2c	0.12	0.45914870	duck
Epha3	-0.20	0.45934359	duck
Wasl	-0.15	0.46114368	duck
Commd6	-0.13	0.46185105	duck
Vps37b	0.04	0.46352586	duck
Rab23	0.11	0.46815585	duck
Tmem119	0.12	0.46967953	duck
Pex16	0.05	0.47202462	duck
Ddx31	-0.16	0.47435887	duck
Fam126a	0.06	0.47678467	duck
Ciao3	-0.06	0.47684077	duck
Bloc1s6	0.02	0.47698260	duck
Rwdd1	-0.07	0.47707165	duck
Api5	-0.17	0.47919086	duck
Copg1	-0.01	0.48315563	duck
Parg	-0.17	0.48557428	duck
Dusp16	-0.03	0.48569209	duck
Psmg3	-0.08	0.48991632	duck
Nceh1	-0.01	0.49321962	duck
Bclaf3	0.05	0.49478768	duck
Prpf8	0.05	0.49559350	duck
Rab5a	-0.05	0.49596178	duck
Immt	0.03	0.49709163	duck
Ccdc91	-0.12	0.49831607	duck
Ptpn21	0.13	0.49917182	duck
Lpcat1	0.15	0.49932657	duck
Gosr1	-0.06	0.50719504	duck
Rcbtb2	0.10	0.50994929	duck
Sppl2a	-0.15	0.51100098	duck
Flot2	-0.10	0.51616817	duck
Exosc2	0.00	0.51853313	duck
Plat	-0.16	0.52477903	duck
Slc25a3	-0.15	0.53150598	duck
Utp18	0.03	0.53287698	duck
Ch25h	-0.05	0.53383210	duck
Plekha3	-0.15	0.53494331	duck
Itpripl2	0.00	0.53813636	duck
Sybu	0.60	0.54462581	duck
Relch	-0.12	0.55028912	duck
Pik3ip1	0.01	0.55135616	duck

Phf19	0.58	0.55401371	duck
Edc3	-0.09	0.55928958	duck
Fignl2	0.01	0.56057945	duck
Ugp2	0.03	0.56790571	duck
Hspa4	0.24	0.56814318	duck
Usp16	-0.03	0.57067803	duck
Tln2	-0.10	0.57387216	duck
Socs4	-0.03	0.57727851	duck
Eif3f	0.01	0.58143851	duck
Ppp4r3a	-0.26	0.58435779	duck
Ippk	-0.06	0.58566598	duck
Slc9a1	-0.02	0.58998147	duck
Chsy3	1.11	0.59344771	duck
Dync2li1	-0.02	0.59506658	duck
Shoc2	0.06	0.59734041	duck
Nt5c3	-0.15	0.60199984	duck
Ccnt1	-0.08	0.60412414	duck
Etf1	0.02	0.60703627	duck
Ndufa5	-0.09	0.60780969	duck
Ubxn2a	-0.10	0.60944018	duck
Ttll1	-0.08	0.61000420	duck
Bbs4	0.03	0.61026546	duck
Pigf	-0.01	0.61272710	duck
Syap1	-0.11	0.61297985	duck
Setd1b	-0.31	0.61342222	duck
Atl2	-0.02	0.62140193	duck
Gramd2	-0.04	0.62315438	duck
Rnft1	-0.05	0.62365446	duck
Actr10	0.11	0.63298070	duck
Ing3	-0.12	0.63626599	duck
Vapb	-0.01	0.63632648	duck
Lyrm2	0.02	0.64151840	duck
Eftud2	-0.01	0.64295805	duck
Usp45	0.02	0.64671003	duck
Clpx	-0.06	0.64926845	duck
Egflam	0.09	0.65060813	duck
Cebpb	0.54	0.65788966	duck
Ppp1cb	-0.36	0.66016811	duck
Smarcad1	-0.15	0.66033379	duck
Zmat3	-0.02	0.66172108	duck
Wdr43	-0.12	0.67106097	duck
Rsbn1	0.03	0.67570761	duck
Nedd4l	0.11	0.67579654	duck
Itgb6	-0.05	0.69001260	duck
Hscb	-0.01	0.69230334	duck
Tsc1	-0.26	0.69557991	duck

Cul2	0.06	0.69655782	duck
Dhx15	-0.16	0.69735339	duck
Fbxo22	-0.01	0.71188113	duck
Plpp1	0.83	0.71223140	duck
C1galt1	0.04	0.71517768	duck
Ern1	-0.09	0.72917769	duck
Adamts3	-0.07	0.73094618	duck
Creb1	0.02	0.73130923	duck
Zfp711	-0.27	0.73694086	duck
Setdb1	-0.09	0.73796839	duck
Kdsr	-0.06	0.73907180	duck
Cnot8	0.00	0.75075196	duck
Dock1	-0.11	0.75181595	duck
Fam168b	-0.21	0.76413464	duck
Znfx1	0.43	0.76743738	duck
Capzb	-0.10	0.76772140	duck
Scp2	-0.18	0.79248416	duck
Trim24	-0.19	0.79876452	duck
Hdgfl3	0.00	0.80010838	duck
Pcsk6	-0.10	0.80241875	duck
Tshz1	0.21	0.80287352	duck
Ehbp1	0.13	0.81341575	duck
Bcor	0.92	0.81504886	duck
Tpp2	0.00	0.81772917	duck
Pdcd5	-0.17	0.82154796	duck
Ric3	0.05	0.82168063	duck
Tlk1	-0.18	0.82322118	duck
Hspa4l	-0.16	0.83348870	duck
Fam20a	-0.08	0.83398174	duck
Wars	0.01	0.83619929	duck
Ascc1	-0.03	0.83644100	duck
Plekhh3	-0.02	0.84725793	duck
Fign	0.28	0.85037918	duck
Ndufb4	0.02	0.85593671	duck
Tpi1	-0.03	0.85887243	duck
Cblb	-0.14	0.86264159	duck
Pdhx	-0.18	0.86523531	duck
Hyou1	0.06	0.86668055	duck
Dlat	-0.02	0.86868127	duck
Adgra3	0.13	0.87630366	duck
Il6st	0.68	0.88776684	duck
Pcgf5	-0.08	0.89156571	duck
Lamc1	-0.28	0.91001770	duck
Tmem231	0.01	0.91263347	duck
Hyi	0.01	0.91734847	duck
Aurkaip1	0.02	0.91994622	duck

Fgfr1op2	-0.19	0.92240235	duck
Dsel	0.09	0.94137156	duck
Lrrk2	0.04	0.94502432	duck
Cct7	0.10	0.94602036	duck
Pias2	-0.14	0.94628558	duck
Synj2	-0.01	0.95581812	duck
C2cd3	-0.03	0.96142372	duck
Usp4	-0.08	0.97038999	duck
Hadh	-0.06	0.97388494	duck
Ift122	-0.01	0.97595817	duck
Gpsm2	-0.13	0.98280407	duck
Ttll7	-0.06	0.98666030	duck
Pin1	-0.04	0.98802847	duck
Tm9sf2	-0.05	0.99342306	duck
Plxna2	-0.13	0.99770982	duck
Nfkbiz	0.79	1.00000000	duck
Orc3	-0.08	1.00000000	duck
Amer1	0.03	1.00000000	duck
Npepl1	-0.01	1.00000000	duck
Jade3	0.03	1.00000000	duck
Caml	-0.03	1.00000000	duck
Sorbs1	0.32	1.00000000	duck
Ndrg1	0.49	1.00000000	duck
Ylpm1	-0.03	1.00000000	duck
Samm50	0.03	1.00000000	duck
Pgm2	0.06	1.00000000	duck
Fmr1	-0.03	1.00000000	duck
Samd11	0.09	1.00000000	duck
Pms2	-0.06	1.00000000	duck
Gnptg	0.02	1.00000000	duck
Cdc40	-0.20	1.00000000	duck
Nr4a3	-0.47	1.00000000	duck
Ddost	0.11	1.00000000	duck
Nup54	-0.05	1.00000000	duck
Ghr	0.81	1.00000000	duck
Zbtb34	-0.05	1.00000000	duck
Tex264	-0.01	1.00000000	duck
Nsl1	0.01	1.00000000	duck
Cdc26	0.05	1.00000000	duck
Spata5	1.03	1.00000000	duck
Nup85	0.02	1.00000000	duck
Plekha1	0.04	1.00000000	duck
Gucy1b1	0.09	1.00000000	duck
Prkdc	-0.05	1.00000000	duck
Eprs	-0.06	1.00000000	duck
Abcc9	-0.02	1.00000000	duck

Lap3	-0.03	1.00000000	duck
Tmem98	0.00	1.00000000	duck
Smurf1	-0.19	1.00000000	duck
Slc25a13	0.08	1.00000000	duck
Zmynd19	0.08	1.00000000	duck
Tm2d2	-0.06	1.00000000	duck
Tmem9	0.01	1.00000000	duck
Spout1	0.03	1.00000000	duck
Cct4	-0.09	1.00000000	duck
Trp53bp1	0.08	1.00000000	duck
Rxylt1	-0.06	1.00000000	duck
Hnrnpa0	0.03	1.00000000	duck
Smap2	0.17	1.00000000	duck
Uhrf2	0.64	1.00000000	duck
Bmp2	-0.08	1.00000000	duck
Noct	-0.07	1.00000000	duck
Naa60	0.08	1.00000000	duck
Prickle2	0.20	1.00000000	duck
Pard3b	0.56	1.00000000	duck
Pibf1	0.00	1.00000000	duck
Pgpep1	-0.02	1.00000000	duck
Chst14	0.07	1.00000000	duck
Tmem39a	0.05	1.00000000	duck
Snrpb2	0.06	1.00000000	duck
Zfyve21	-0.01	1.00000000	duck
N6amt1	-0.02	1.00000000	duck
Klhl36	0.04	1.00000000	duck
Hoxb5	-0.03	1.00000000	duck
Lypla1	0.03	1.00000000	duck
Rdx	-0.24	1.00000000	duck
Rabggtb	0.16	1.00000000	duck
Lasp1	-0.20	1.00000000	duck
Rpap1	-0.12	1.00000000	duck
Ino80	-0.11	1.00000000	duck
Limk2	-0.03	1.00000000	duck
Dapk2	-0.03	1.00000000	duck
Pwwp2b	0.00	1.00000000	duck
Slc37a4	-0.02	1.00000000	duck
Tnrc6b	-1.33	1.00000000	duck
Tmem208	0.00	1.00000000	duck
Tmtc2	0.84	1.00000000	duck
Ube2a	0.07	1.00000000	duck
Rbbp7	0.05	1.00000000	duck
Fibin	-0.10	1.00000000	duck
Arfrp1	-0.02	1.00000000	duck
Bco1	0.00	1.00000000	duck

Rasl11b	0.10	1.00000000	duck
Casd1	-0.14	1.00000000	duck
Chmp5	-0.06	1.00000000	duck
Stard4	0.04	1.00000000	duck
Recql	-0.05	1.00000000	duck
Spryd7	0.00	1.00000000	duck
Arhgap31	0.15	1.00000000	duck
Cnot4	-0.06	1.00000000	duck
Ccdc127	-0.08	1.00000000	duck
Zfhx4	0.02	1.00000000	duck
Crlf3	0.08	1.00000000	duck
Hps3	-0.02	1.00000000	duck
Sec61a1	0.08	1.00000000	duck
Gns	-0.17	1.00000000	duck
Bmp5	-0.02	1.00000000	duck
Bmpr1a	-0.05	1.00000000	duck
Zfp513	0.02	1.00000000	duck
Il17rd	0.10	1.00000000	duck
Rnf121	0.01	1.00000000	duck
Khdrbs1	0.03	1.00000000	duck
Pcgf6	-0.07	1.00000000	duck
Eif3g	-0.13	1.00000000	duck
Lmbrd1	-0.10	1.00000000	duck
Ero1b	0.07	1.00000000	duck
Zdhhc15	0.00	1.00000000	duck
Cmtm7	0.02	1.00000000	duck
Pex1	0.01	1.00000000	duck
Tnfaip8	-0.02	1.00000000	duck
Atmin	-0.10	1.00000000	duck
Atp6v1d	0.02	1.00000000	duck
Ntmt1	0.02	1.00000000	duck
Spsb1	0.67	1.00000000	duck
Prkca	-0.21	1.00000000	duck
Bnip3	-0.09	1.00000000	duck
Pank2	-0.02	1.00000000	duck
Rnf138	-0.03	1.00000000	duck
Usp33	-0.21	1.00000000	duck
Vps35l	-0.05	1.00000000	duck
Galns	0.00	1.00000000	duck
B4galt2	0.07	1.00000000	duck
Psmd4	-0.05	1.00000000	duck
Psmb4	-0.16	1.00000000	duck
Actl6a	-0.02	1.00000000	duck
Cntrl	-0.13	1.00000000	duck
Tesk2	-0.22	1.00000000	duck
Rexo2	0.05	1.00000000	duck

Fbxo38	-0.10	1.00000000	duck
Dctn3	0.65	1.00000000	duck
Nucb2	-0.16	1.00000000	duck
Rsl24d1	-0.04	1.00000000	duck
Lysmd4	0.03	1.00000000	duck
Aff4	-0.11	1.00000000	duck
Phkb	-0.04	1.00000000	duck
Zbtb6	-0.01	1.00000000	duck
Zcrb1	0.01	1.00000000	duck
Fam222b	-0.24	1.00000000	duck
Tmcc1	-0.57	1.00000000	duck
Prdx3	-0.02	1.00000000	duck
Fosb	0.41	1.00000000	duck
Ncoa6	-0.07	1.00000000	duck
Fgf2	0.74	1.00000000	duck
Arfip2	0.06	1.00000000	duck
Nap1l1	-0.19	1.00000000	duck
Arhgap5	-0.16	1.00000000	duck
Natd1	0.00	1.00000000	duck
Dennd2c	0.00	1.00000000	duck
Blmh	0.03	1.00000000	duck
Snx21	0.02	1.00000000	duck
Qsox2	0.10	1.00000000	duck
Ccnt2	-0.09	1.00000000	duck
Plekhf2	0.07	1.00000000	duck
Pde10a	0.45	1.00000000	duck
Fkbp15	0.05	1.00000000	duck
Zdhhc3	0.02	1.00000000	duck
Ptdss2	-0.03	1.00000000	duck
Gak	0.38	1.00000000	duck
Acsl3	-0.02	1.00000000	duck
Coq7	0.10	1.00000000	duck
Phf10	0.09	1.00000000	duck
Tm9sf3	-0.14	1.00000000	duck
Scyl3	-0.06	1.00000000	duck
Piezo2	-0.30	1.00000000	duck
G3bp1	-0.04	1.00000000	duck
Xkr8	0.02	1.00000000	duck
Rcc1l	-0.07	1.00000000	duck
Hdac2	-0.01	1.00000000	duck
Swt1	0.02	1.00000000	duck
Dvl1	0.06	1.00000000	duck
Ano10	0.05	1.00000000	duck
Rab12	0.11	1.00000000	duck
Pde8a	-0.77	1.00000000	duck
Wdr55	0.10	1.00000000	duck

Spi1	0.54	1.00000000	duck
Clcc1	0.09	1.00000000	duck
Lrig2	-0.02	1.00000000	duck
Zmat2	0.05	1.00000000	duck
Spire1	0.08	1.00000000	duck
Vsir	-0.11	1.00000000	duck
Cwc25	0.04	1.00000000	duck
Plek2	0.00	1.00000000	duck
Cep250	-0.09	1.00000000	duck
Neurl1b	0.04	1.00000000	duck
Pskh1	0.10	1.00000000	duck
Dram1	0.53	1.00000000	duck
Fancl	0.03	1.00000000	duck
Chchd3	0.12	1.00000000	duck
Rab33b	-0.04	1.00000000	duck
Scaf8	-0.15	1.00000000	duck
Lmtk2	-0.08	1.00000000	duck
Tbcd	0.05	1.00000000	duck
Pnpla2	-0.05	1.00000000	duck
Ccdc66	-0.06	1.00000000	duck
Kif15	0.55	1.00000000	duck
Pcnx	-0.02	1.00000000	duck
Zswim6	0.73	1.00000000	duck
Rtraf	-0.03	1.00000000	duck
Prkar1b	0.10	1.00000000	duck
Pafah1b2	0.05	1.00000000	duck
Hsdl1	-0.04	1.00000000	duck
Zfp330	-0.01	1.00000000	duck
Snx5	-0.05	1.00000000	duck
Pdcl3	-0.03	1.00000000	duck
Far1	0.04	1.00000000	duck
Snf8	0.00	1.00000000	duck
Desi2	0.04	1.00000000	duck
Smim20	0.13	1.00000000	duck
Rnf10	-0.02	1.00000000	duck
Ptprd	-0.03	1.00000000	duck
Zfp598	-0.08	1.00000000	duck
Ptafr	0.76	1.00000000	duck
Rab8a	0.01	1.00000000	duck
Slc4a4	1.21	1.00000000	duck
Irf7	0.60	1.00000000	duck
Ift81	0.09	1.00000000	duck
Zcchc2	-0.02	1.00000000	duck
Tnks2	-0.19	1.00000000	duck
Wdr36	0.06	1.00000000	duck
Oma1	0.11	1.00000000	duck

Rbm26	-0.28	1.00000000	duck
Fryl	-0.31	1.00000000	duck
Eaf1	0.05	1.00000000	duck
Plpp2	0.09	1.00000000	duck
Slc44a5	-0.13	1.00000000	duck
Tgs1	0.11	1.00000000	duck
Dpt	-0.31	1.00000000	duck
Tpk1	-0.03	1.00000000	duck
Tango2	-0.08	1.00000000	duck
Ndfip1	-0.11	1.00000000	duck
Nktr	0.04	1.00000000	duck
Sec23b	-0.02	1.00000000	duck
Ppat	-0.01	1.00000000	duck
Spg7	0.09	1.00000000	duck
Bmp4	-0.03	1.00000000	duck
Tram2	-0.01	1.00000000	duck
Cactin	-0.04	1.00000000	duck
Rad51c	0.00	1.00000000	duck
Ets1	0.07	1.00000000	duck
Piga	-0.05	1.00000000	duck
Cops8	0.02	1.00000000	duck
Bola3	-0.09	1.00000000	duck
Pdia3	-0.21	1.00000000	duck
Areg	-0.03	1.00000000	duck
Thap12	-0.01	1.00000000	duck
Srf	-0.06	1.00000000	duck
Nsd1	0.05	1.00000000	duck
Cstf2	0.01	1.00000000	duck
Tiprl	-0.11	1.00000000	duck
Dcaf13	0.03	1.00000000	duck
Pag1	-0.02	1.00000000	duck
Pptc7	-0.05	1.00000000	duck
Chd5	0.06	1.00000000	duck
Atg14	-0.08	1.00000000	duck
Ambra1	0.04	1.00000000	duck
Pik3ca	-0.15	1.00000000	duck
Ddx55	0.01	1.00000000	duck
Ndufv2	0.06	1.00000000	duck
Puf60	-0.04	1.00000000	duck
Tent5a	0.09	1.00000000	duck
Clta	0.00	1.00000000	duck
Asb7	-0.05	1.00000000	duck
Smarcb1	0.07	1.00000000	duck
Akt3	-0.13	1.00000000	duck
Psma3	-0.04	1.00000000	duck
Eipr1	-0.19	1.00000000	duck

Slc9a6	0.09	1.00000000	duck
Ahctf1	-0.08	1.00000000	duck
Trim25	0.58	1.00000000	duck
Acbd5	0.14	1.00000000	duck
Tmem185a	0.01	1.00000000	duck
Ppp2r5c	-0.43	1.00000000	duck
Septin2	0.02	1.00000000	duck
Smc6	-0.08	1.00000000	duck
Dennd1b	-0.05	1.00000000	duck
Drosha	0.05	1.00000000	duck
Epas1	-0.13	1.00000000	duck
Sec62	-0.07	1.00000000	duck
Fig4	-0.09	1.00000000	duck
Neo1	0.05	1.00000000	duck
Pgam5	0.02	1.00000000	duck
Ccdc50	0.18	1.00000000	duck
Isca1	0.00	1.00000000	duck
Ppp2r3a	0.01	1.00000000	duck
Golph3	-0.03	1.00000000	duck
Akap1	0.05	1.00000000	duck
Katna1	-0.01	1.00000000	duck
Fam114a2	0.05	1.00000000	duck
Hdac11	0.06	1.00000000	duck
Ptdss1	-0.04	1.00000000	duck
Ndufa4	0.05	1.00000000	duck
Kpna1	-0.08	1.00000000	duck
Arl4a	0.01	1.00000000	duck
Tedc1	0.13	1.00000000	duck
Tmbim6	-0.15	1.00000000	duck
Stat3	-0.05	1.00000000	duck
Pogz	-0.20	1.00000000	duck
Txlna	0.09	1.00000000	duck
Ubtd2	0.00	1.00000000	duck
Ttll11	0.06	1.00000000	duck
Gpbp1l1	-0.01	1.00000000	duck
Aldh5a1	0.02	1.00000000	duck
Tmed2	-0.03	1.00000000	duck
Ctso	0.01	1.00000000	duck
Ctbp2	0.10	1.00000000	duck
Scaf11	-0.11	1.00000000	duck
Gpi1	0.09	1.00000000	duck
Clcn4	-0.04	1.00000000	duck
Emc6	-0.01	1.00000000	duck
Chmp4b	-0.07	1.00000000	duck
Reps1	-0.13	1.00000000	duck
Ankrd44	-0.02	1.00000000	duck

Kifbp	-0.03	1.00000000	duck
Usp24	0.09	1.00000000	duck
Spag9	-0.01	1.00000000	duck
Sec11c	0.09	1.00000000	duck
Cpd	-0.08	1.00000000	duck
Ints6	-0.20	1.00000000	duck
Ppp1r26	-0.05	1.00000000	duck
Riox2	-0.04	1.00000000	duck
Ccdc186	-0.25	1.00000000	duck
Dtymk	0.09	1.00000000	duck
Bod1l	-0.32	1.00000000	duck
Uimc1	-0.13	1.00000000	duck
B4galt4	-0.01	1.00000000	duck
Ska2	-0.04	1.00000000	duck
Eefsec	-0.03	1.00000000	duck
Wsb1	-0.03	1.00000000	duck
Ptges3l	-0.07	1.00000000	duck
Nup133	-0.17	1.00000000	duck
Npas3	-0.31	1.00000000	duck
Arnt2	0.13	1.00000000	duck
Calcrl	0.16	1.00000000	duck
Ubac2	0.20	1.00000000	duck
Klhl21	-0.02	1.00000000	duck
Rida	-0.06	1.00000000	duck
Nubp1	-0.08	1.00000000	duck
Gpr180	-0.01	1.00000000	duck
Rab20	0.02	1.00000000	duck
Tle5	-0.05	1.00000000	duck
Tgif1	0.07	1.00000000	duck
Cdo1	0.09	1.00000000	duck
Scyl2	0.01	1.00000000	duck
Cul1	-0.13	1.00000000	duck
Csnk2a2	0.03	1.00000000	duck
Prdm16	0.75	1.00000000	duck
Tmem41b	-0.04	1.00000000	duck
Bend3	-0.04	1.00000000	duck
Rpusd2	-0.05	1.00000000	duck
Syt14	0.05	1.00000000	duck
Golga4	-0.26	1.00000000	duck
Ctbp1	-0.16	1.00000000	duck
Tada2b	-0.10	1.00000000	duck
Esf1	0.05	1.00000000	duck
Prkag1	-0.05	1.00000000	duck
Aldh18a1	0.02	1.00000000	duck
Sin3b	-0.03	1.00000000	duck
Sdf4	-0.12	1.00000000	duck

Gan	-0.04	1.00000000	duck
Emc3	0.03	1.00000000	duck
Prr16	0.13	1.00000000	duck
Scamp2	0.04	1.00000000	duck
Ostc	0.02	1.00000000	duck
Tmem63b	0.02	1.00000000	duck
Fam102a	0.06	1.00000000	duck
Wdr47	-0.10	1.00000000	duck
Ogt	0.03	1.00000000	duck
Utp11	0.00	1.00000000	duck
Rmdn3	-0.09	1.00000000	duck
Taf1a	-0.05	1.00000000	duck
Kctd5	0.07	1.00000000	duck
Clock	-0.15	1.00000000	duck
Phtf1	-0.23	1.00000000	duck
Surf6	-0.04	1.00000000	duck
Rogdi	-0.06	1.00000000	duck
Pemt	0.08	1.00000000	duck
Ikbkb	-0.03	1.00000000	duck
Kctd7	0.00	1.00000000	duck
Tmem104	-0.06	1.00000000	duck
Lonp2	0.10	1.00000000	duck
Phf20l1	-0.09	1.00000000	duck
Casp8ap2	-0.01	1.00000000	duck
Rbms1	0.38	1.00000000	duck
Aebp2	-0.03	1.00000000	duck
Fth1	-0.01	1.00000000	duck
Fam135a	0.12	1.00000000	duck
Irf2bpl	0.02	1.00000000	duck
Rnf8	-0.06	1.00000000	duck
Ralgapa1	-0.01	1.00000000	duck
Ankrd13a	0.00	1.00000000	duck
Syf2	-0.17	1.00000000	duck
Prpf4b	0.02	1.00000000	duck
Snap23	0.11	1.00000000	duck
Lyrm1	-0.27	1.00000000	duck
Zfp423	-0.03	1.00000000	duck
Nlk	-0.11	1.00000000	duck
Lsmem1	0.58	1.00000000	duck
Suco	-0.11	1.00000000	duck
Cars	-0.20	1.00000000	duck
Sirt7	-0.08	1.00000000	duck
Uri1	-0.12	1.00000000	duck
Ccdc6	-0.01	1.00000000	duck
Spcs2	0.19	1.00000000	duck
Unk	0.08	1.00000000	duck

Ppp2r2d	-0.19	1.00000000	duck
Atp5j2	-0.07	1.00000000	duck
Zfp654	0.02	1.00000000	duck
Zpr1	0.05	1.00000000	duck
Gnl2	-0.01	1.00000000	duck
Per3	-0.04	1.00000000	duck
Elmo2	-0.06	1.00000000	duck
Plxnd1	-0.04	1.00000000	duck
Zc3h15	-0.03	1.00000000	duck
Psmb7	-0.05	1.00000000	duck
Sirt1	-0.02	1.00000000	duck
Ubac1	-0.07	1.00000000	duck
Afg3l2	-0.03	1.00000000	duck
Cwf19l1	-0.08	1.00000000	duck
Cdc27	-0.03	1.00000000	duck
Slc35f5	0.06	1.00000000	duck
Prpf40a	-0.06	1.00000000	duck
Tmem251	-0.02	1.00000000	duck
Klhl18	-0.06	1.00000000	duck
Xrn2	-0.11	1.00000000	duck
Ptpru	0.04	1.00000000	duck
Akt1	0.13	1.00000000	duck
Ankrd13c	0.03	1.00000000	duck
Rbbp8	-0.09	1.00000000	duck
Leprotl1	0.05	1.00000000	duck
Exoc7	0.00	1.00000000	duck
Thoc3	-0.01	1.00000000	duck
Zbtb7a	0.01	1.00000000	duck
Pfn2	-0.05	1.00000000	duck
Slc45a4	-0.05	1.00000000	duck
Nmd3	0.02	1.00000000	duck
Agpat3	0.23	1.00000000	duck
Snx8	0.06	1.00000000	duck
Rdh10	-0.11	1.00000000	duck
Rab39b	-0.08	1.00000000	duck
Wac	-0.20	1.00000000	duck
Pgrmc1	0.01	1.00000000	duck
Jam3	-0.02	1.00000000	duck
Tbc1d13	-0.02	1.00000000	duck
Dstyk	0.04	1.00000000	duck
B4galt3	-0.13	1.00000000	duck
Anxa5	-0.17	1.00000000	duck
Vps13c	-0.11	1.00000000	duck
Pxn	0.02	1.00000000	duck
Trak2	-0.07	1.00000000	duck
Adamts8	0.15	1.00000000	duck

Ogfrl1	-0.09	1.00000000	duck
Rcor1	-0.04	1.00000000	duck
Ptpn12	-0.05	1.00000000	duck
Nle1	0.02	1.00000000	duck
Cwf19l2	0.02	1.00000000	duck
Orai2	0.01	1.00000000	duck
Lrrc75a	0.05	1.00000000	duck
Sf3b3	0.09	1.00000000	duck
Vps13d	-0.08	1.00000000	duck
Upf2	-0.27	1.00000000	duck
Plcxd3	-0.11	1.00000000	duck
Ythdc1	-0.05	1.00000000	duck
Trmt11	0.04	1.00000000	duck
Ddx28	-0.03	1.00000000	duck
Decr1	-0.13	1.00000000	duck
Dcun1d4	-0.07	1.00000000	duck
Bcl7a	-0.06	1.00000000	duck
Hibadh	-0.01	1.00000000	duck
Thoc5	0.04	1.00000000	duck
Rc3h1	-0.16	1.00000000	duck
Atf1	-0.01	1.00000000	duck
Prpf6	-0.06	1.00000000	duck
Phldb1	0.15	1.00000000	duck
Vps11	0.07	1.00000000	duck
Kif2a	0.05	1.00000000	duck
Gpx1	0.77	1.00000000	duck
Gas7	0.03	1.00000000	duck
Ilf2	0.00	1.00000000	duck
Wdr33	-0.17	1.00000000	duck
Syde2	0.06	1.00000000	duck
Sart3	0.01	1.00000000	duck
Psmc6	0.01	1.00000000	duck
Qars	0.10	1.00000000	duck
Thap11	-0.07	1.00000000	duck
Tgfbr2	-0.15	1.00000000	duck
Trappc2l	0.05	1.00000000	duck
Tmco1	-0.06	1.00000000	duck
Hdac1	0.08	1.00000000	duck
Ubp1	0.09	1.00000000	duck
Rabgap1	0.12	1.00000000	duck
Irf2	-0.07	1.00000000	duck
Nprl3	-0.23	1.00000000	duck
Yod1	-0.08	1.00000000	duck
Tmem199	0.07	1.00000000	duck
Eif3i	0.14	1.00000000	duck
Pnrc1	0.17	1.00000000	duck

Phf12	-0.12	1.00000000	duck
Kansl2	0.04	1.00000000	duck
Vdac1	-0.05	1.00000000	duck
Tpra1	-0.04	1.00000000	duck
Prkab1	0.06	1.00000000	duck
Slc30a9	-0.34	1.00000000	duck
Ppil1	-0.07	1.00000000	duck
Trmt44	-0.04	1.00000000	duck
Derl2	0.01	1.00000000	duck
Rasa3	0.03	1.00000000	duck
Appl1	0.22	1.00000000	duck
Supt4a	0.01	1.00000000	duck
Ice2	-0.13	1.00000000	duck
Iars	0.16	1.00000000	duck
Steep1	0.07	1.00000000	duck
Sugp2	-0.03	1.00000000	duck
Slc25a15	0.03	1.00000000	duck
Uhrf1bp1l	0.23	1.00000000	duck
Wdr77	0.12	1.00000000	duck
Tmem86a	0.72	1.00000000	duck
Ezh1	0.02	1.00000000	duck
Vps8	-0.02	1.00000000	duck
Irf1	-0.57	1.00000000	duck
Nbn	-0.10	1.00000000	duck
Oaz1	0.43	1.00000000	duck
Grk2	0.02	1.00000000	duck
Atf3	-0.66	1.00000000	duck
Als2	0.01	1.00000000	duck
Rasgef1b	0.12	1.00000000	duck
Pitpnm3	0.13	1.00000000	duck
Emc7	0.08	1.00000000	duck
2210408I21Rik	0.82	1.00000000	duck
Atp5b	0.03	1.00000000	duck
Rbl2	0.03	1.00000000	duck
Naa15	-0.17	1.00000000	duck
Zdhhc12	0.03	1.00000000	duck
Cttnbp2nl	0.07	1.00000000	duck
Atxn7l1	-0.16	1.00000000	duck
Gigyf2	-0.06	1.00000000	duck
Enoph1	0.05	1.00000000	duck
Ppid	0.10	1.00000000	duck
Adgrl2	-0.24	1.00000000	duck
Hoxa5	0.11	1.00000000	duck
Ss18	0.05	1.00000000	duck
Fam43a	0.07	1.00000000	duck
Cobll1	-0.23	1.00000000	duck

Gabpb1	-0.25	1.00000000	duck
Otud7b	-0.04	1.00000000	duck
Srp14	0.01	1.00000000	duck
Impdh2	0.02	1.00000000	duck
Hdx	0.05	1.00000000	duck
Lrrc45	0.01	1.00000000	duck
Rilpl2	-0.07	1.00000000	duck
Acvr1b	0.64	1.00000000	duck
Serinc5	0.12	1.00000000	duck
Pde3a	0.09	1.00000000	duck
Ormdl2	-0.04	1.00000000	duck
Cdk13	-0.25	1.00000000	duck
Anapc1	0.01	1.00000000	duck
Pigc	0.04	1.00000000	duck
Gar1	-0.04	1.00000000	duck
Chn1	-0.05	1.00000000	duck
Cnot11	-0.05	1.00000000	duck
Uck1	0.05	1.00000000	duck
Coq5	-0.19	1.00000000	duck
Cks2	-0.04	1.00000000	duck
Adal	-0.02	1.00000000	duck
Cpsf2	0.07	1.00000000	duck
Tlk2	0.14	1.00000000	duck
Ube2g1	-0.03	1.00000000	duck
Fdx1	-0.02	1.00000000	duck
Tead4	-0.14	1.00000000	duck
Hypk	0.10	1.00000000	duck
Slc27a4	0.00	1.00000000	duck
Tusc2	-0.07	1.00000000	duck
Git2	0.04	1.00000000	duck
Arhgef12	-0.16	1.00000000	duck
Kpna6	-0.16	1.00000000	duck
Hspa9	0.04	1.00000000	duck
Bcas2	0.15	1.00000000	duck
Tab2	-0.23	1.00000000	duck
Tmem245	0.09	1.00000000	duck
Rnf146	0.00	1.00000000	duck
Dnajb12	0.02	1.00000000	duck
Psmc2	0.08	1.00000000	duck
Taf12	0.09	1.00000000	duck
Bcl2	0.68	1.00000000	duck
Tbk1	-0.05	1.00000000	duck
Ccn1	-0.25	1.00000000	duck
Nsmce1	-0.09	1.00000000	duck
Rpn2	-0.04	1.00000000	duck
Klhdc2	0.00	1.00000000	duck

Ube4a	0.09	1.00000000	duck
Pphln1	-0.25	1.00000000	duck
Gfod1	-0.07	1.00000000	duck
Strn3	-0.07	1.00000000	duck
Kars	0.04	1.00000000	duck
Cacul1	-0.07	1.00000000	duck
Clba1	0.19	1.00000000	duck
Camta1	-0.09	1.00000000	duck
Creb3l2	0.54	1.00000000	duck
Lgr4	0.10	1.00000000	duck
Rrp1b	-0.04	1.00000000	duck
Ubl3	0.02	1.00000000	duck
Hmces	-0.08	1.00000000	duck
Wwc2	0.17	1.00000000	duck
Dars	0.06	1.00000000	duck
Tmem35a	0.08	1.00000000	duck
Galnt15	0.52	1.00000000	duck
Gpatch2	0.15	1.00000000	duck
Srp9	-0.02	1.00000000	duck
Nop58	0.13	1.00000000	duck
Sec22a	-0.01	1.00000000	duck
Dnaja4	-0.15	1.00000000	duck
Ankrd11	-0.19	1.00000000	duck
Rflna	0.04	1.00000000	duck
Decr2	0.15	1.00000000	duck
Isoc1	0.03	1.00000000	duck
Tti1	-0.09	1.00000000	duck
Stim2	-0.13	1.00000000	duck
Nol8	0.13	1.00000000	duck
Tgm4	0.45	1.00000000	duck
Kirrel	0.16	1.00000000	duck
Ppp2ca	-0.14	1.00000000	duck
Cnot6	-0.05	1.00000000	duck
Sptlc2	0.15	1.00000000	duck
Eps15	0.47	1.00000000	duck
Atp2a2	-0.11	1.00000000	duck
Hexim1	0.49	1.00000000	duck
Zfp827	0.05	1.00000000	duck
Wdr1	-0.01	1.00000000	duck
Loxl1	0.18	1.00000000	duck
Tnfrsf19	-0.08	1.00000000	duck
Ccnb2	0.16	1.00000000	duck
Cdk5rap2	-0.03	1.00000000	duck
Igf2r	-0.26	1.00000000	duck
Tmed10	-0.12	1.00000000	duck
Dda1	0.61	1.00000000	duck

Zcchc7	0.78	1.00000000	duck
Pigk	0.04	1.00000000	duck
Stt3a	0.14	1.00000000	duck
Arl6ip4	-0.05	1.00000000	duck
Bach2	0.29	1.00000000	duck
Cep97	0.01	1.00000000	duck
Ldlrap1	0.13	1.00000000	duck
Fgf18	0.21	1.00000000	duck
Copg2	-0.24	1.00000000	duck
Zrsr1	-0.08	1.00000000	duck
Trerf1	0.21	1.00000000	duck
Osbpl6	-0.28	1.00000000	duck
Rnf19a	0.48	1.00000000	duck
Fhod3	-0.11	1.00000000	duck
Actn4	0.15	1.00000000	duck
Cript	0.14	1.00000000	duck
Stxbp5	-0.08	1.00000000	duck
Plekha2	-0.14	1.00000000	duck
Lnx2	-0.11	1.00000000	duck
Eif2a	-0.06	1.00000000	duck
Bclaf1	-0.15	1.00000000	duck
Vgll4	0.16	1.00000000	duck
Atf4	-0.23	1.00000000	duck
Nagpa	-0.02	1.00000000	duck
Agps	0.03	1.00000000	duck
Ralgapb	-0.03	1.00000000	duck
Nipsnap2	0.01	1.00000000	duck
Slc25a46	0.00	1.00000000	duck
Rapgef1	0.02	1.00000000	duck
Ankrd54	0.05	1.00000000	duck
Adam17	0.09	1.00000000	duck
Rbx1	0.04	1.00000000	duck
Npc2	0.16	1.00000000	duck
Aamdc	-0.09	1.00000000	duck
Zdhhc9	-0.02	1.00000000	duck
Rit1	-0.03	1.00000000	duck
Dclk2	0.09	1.00000000	duck
Rap2c	-0.11	1.00000000	duck
Oat	0.00	1.00000000	duck
Wdr81	0.01	1.00000000	duck
Nkiras2	-0.03	1.00000000	duck
Hectd4	-0.18	1.00000000	duck
Sema3f	0.08	1.00000000	duck
Usp28	-0.04	1.00000000	duck
Rc3h2	-0.14	1.00000000	duck
Rnps1	0.00	1.00000000	duck

Bcl2l13	-0.15	1.00000000	duck
As3mt	0.08	1.00000000	duck
Xpa	0.61	1.00000000	duck
Prdx1	-0.07	1.00000000	duck
Csrnp1	-0.29	1.00000000	duck
Tmem248	0.18	1.00000000	duck
Lig4	-0.02	1.00000000	duck
Fam199x	0.09	1.00000000	duck
Rsl1d1	0.19	1.00000000	duck
Pcna	0.09	1.00000000	duck
Ptpn14	0.19	1.00000000	duck
Ktn1	0.06	1.00000000	duck
Phyhd1	0.03	1.00000000	duck
Samd8	-0.03	1.00000000	duck
Hpgd	0.61	1.00000000	duck
Atrnl1	0.60	1.00000000	duck
Znhit6	-0.01	1.00000000	duck
Fam219a	0.00	1.00000000	duck
Hsf2	0.06	1.00000000	duck
Phospho1	0.06	1.00000000	duck
Rbm19	0.16	1.00000000	duck
Ggcx	0.17	1.00000000	duck
Vcan	0.03	1.00000000	duck
Pole4	0.09	1.00000000	duck
Ipmk	0.75	1.00000000	duck
Ttc28	-0.03	1.00000000	duck
Agl	0.12	1.00000000	duck
Slc8a1	0.16	1.00000000	duck
Dis3	0.09	1.00000000	duck
Ppp2r5e	0.00	1.00000000	duck
Erc1	-0.15	1.00000000	duck
Ecd	0.01	1.00000000	duck
Tmem186	-0.03	1.00000000	duck
Usp30	-0.04	1.00000000	duck
Zfp36l2	0.49	1.00000000	duck
Rab11fip1	0.04	1.00000000	duck
Supt6	0.02	1.00000000	duck
Vps26c	0.04	1.00000000	duck
Stk17b	-0.13	1.00000000	duck
Trabd2b	-0.22	1.00000000	duck
Lrmp	-0.01	1.00000000	duck
Rreb1	0.06	1.00000000	duck
Triap1	-0.06	1.00000000	duck
Plxdc2	0.42	1.00000000	duck
Aplf	0.03	1.00000000	duck
Plekhm3	0.10	1.00000000	duck

Dusp6	0.07	1.00000000	duck
Csde1	-0.09	1.00000000	duck
Ap1b1	0.05	1.00000000	duck
Cdc16	0.05	1.00000000	duck
Nf1	-0.06	1.00000000	duck
Arid5b	-0.32	1.00000000	duck
Ube2v2	-0.01	1.00000000	duck
Dbr1	-0.02	1.00000000	duck
Alg5	0.05	1.00000000	duck
Acyp2	0.06	1.00000000	duck
Rbis	-0.08	1.00000000	duck
Atp13a1	-0.08	1.00000000	duck
Hoxb2	0.14	1.00000000	duck
Fbxo11	0.05	1.00000000	duck
Xrcc6	0.04	1.00000000	duck
Pds5a	0.00	1.00000000	duck
Siah1b	0.05	1.00000000	duck
Uxs1	0.11	1.00000000	duck
Ppp1r13b	-0.15	1.00000000	duck
Abl2	-0.04	1.00000000	duck
Edem3	0.11	1.00000000	duck
Pdss2	0.14	1.00000000	duck
Acadm	0.03	1.00000000	duck
Hs6st2	0.11	1.00000000	duck
Esd	0.15	1.00000000	duck
Dym	0.04	1.00000000	duck
Cops5	0.00	1.00000000	duck
Saal1	0.06	1.00000000	duck
Serinc3	0.01	1.00000000	duck
Stt3b	0.15	1.00000000	duck
Desi1	-0.05	1.00000000	duck
Eif4e	-0.01	1.00000000	duck
Hdac7	0.13	1.00000000	duck
Stx6	0.13	1.00000000	duck
Rarres1	1.03	1.00000000	duck
Tm2d3	0.05	1.00000000	duck
Lactb	0.11	1.00000000	duck
Wdsub1	-0.13	1.00000000	duck
Dnajc11	-0.11	1.00000000	duck
Vps4b	0.12	1.00000000	duck
Slc38a2	0.51	1.00000000	duck
Adora2b	0.49	1.00000000	duck
Wwp1	0.11	1.00000000	duck
Dnajb6	-0.02	1.00000000	duck
Ulk4	-0.05	1.00000000	duck
Heph	0.62	1.00000000	duck

Asf1a	0.05	1.00000000	duck
Tssc4	0.00	1.00000000	duck
Ctnnal1	0.12	1.00000000	duck
Sntb1	-0.32	1.00000000	duck
Tet2	-0.10	1.00000000	duck
Osbpl11	0.02	1.00000000	duck
Psmd6	-0.06	1.00000000	duck
Parva	0.00	1.00000000	duck
Tmem179b	0.05	1.00000000	duck
Pi4k2b	-0.14	1.00000000	duck
Ddx47	0.10	1.00000000	duck
Tmx3	0.08	1.00000000	duck
Swi5	-0.02	1.00000000	duck
Tyro3	0.03	1.00000000	duck
Foxf1	0.05	1.00000000	duck
Rab29	-0.01	1.00000000	duck
Lhfp	-0.10	1.00000000	duck
Rwdd2b	-0.01	1.00000000	duck
Zadh2	-0.02	1.00000000	duck
Psmd12	0.02	1.00000000	duck
Bmt2	-0.08	1.00000000	duck
Tmem167	-0.03	1.00000000	duck
Vav3	1.20	1.00000000	duck
Ergic1	0.05	1.00000000	duck
Tub	0.06	1.00000000	duck
Letm1	-0.10	1.00000000	duck
Rtf1	0.12	1.00000000	duck
Tfip11	0.04	1.00000000	duck
Ola1	0.12	1.00000000	duck
Eif4a3l1	0.07	1.00000000	duck
Gna11	0.12	1.00000000	duck
Tbc1d22a	0.13	1.00000000	duck
Rpgr	0.15	1.00000000	duck
Tor1b	-0.01	1.00000000	duck
Zdhhc8	0.03	1.00000000	duck
Nudcd2	0.07	1.00000000	duck
Fadd	0.08	1.00000000	duck
Pex2	0.01	1.00000000	duck
Sigmar1	-0.03	1.00000000	duck
Chrac1	0.05	1.00000000	duck
Pmm2	0.17	1.00000000	duck
Slc25a38	-0.13	1.00000000	duck
Tbc1d8	0.04	1.00000000	duck
Nectin1	0.00	1.00000000	duck
Ddah1	0.14	1.00000000	duck
Ephb3	0.22	1.00000000	duck

Dynll2	0.11	1.00000000	duck
Pip4k2a	-0.14	1.00000000	duck
Cdk7	0.03	1.00000000	duck
Klhdc4	0.04	1.00000000	duck
Dctn4	0.00	1.00000000	duck
Arl3	0.09	1.00000000	duck
Slc4a7	-0.25	1.00000000	duck
Exoc8	-0.05	1.00000000	duck
Etfdh	-0.10	1.00000000	duck
Acaca	0.04	1.00000000	duck
Dnttip2	0.05	1.00000000	duck
Otud4	0.09	1.00000000	duck
Cyth3	-0.18	1.00000000	duck
Adgrg2	-0.08	1.00000000	duck
Rnf180	0.01	1.00000000	duck
Crispld1	0.00	1.00000000	duck
Scmh1	0.08	1.00000000	duck
Tor3a	0.05	1.00000000	duck
Fnbp1	0.78	1.00000000	duck
Dnajc7	0.08	1.00000000	duck
Cgas	0.35	1.00000000	duck
Usf3	0.00	1.00000000	duck
Arhgef7	0.16	1.00000000	duck
Zfp609	0.04	1.00000000	duck
Camk2g	-0.06	1.00000000	duck
Pdzd2	0.15	1.00000000	duck
Cep57	-0.06	1.00000000	duck
Lpin2	-0.06	1.00000000	duck
Strn	0.53	1.00000000	duck
Traf6	-0.10	1.00000000	duck
Hlcs	0.16	1.00000000	duck
Ccnyl1	0.01	1.00000000	duck
Tbpl1	0.11	1.00000000	duck
Arg2	0.34	1.00000000	duck
Slc39a14	-0.02	1.00000000	duck
Tmem203	0.14	1.00000000	duck
Lin54	-0.04	1.00000000	duck
Arfgap2	-0.01	1.00000000	duck
Paxbp1	-0.11	1.00000000	duck
Pex7	0.04	1.00000000	duck
Disc1	0.33	1.00000000	duck
Llph	0.20	1.00000000	duck
Sri	-0.06	1.00000000	duck
Klhdc10	-0.05	1.00000000	duck
Rasal2	0.07	1.00000000	duck
Fgd5	0.10	1.00000000	duck

Bak1	0.09	1.00000000	duck
Rwdd4a	-0.05	1.00000000	duck
Fam241a	0.00	1.00000000	duck
Pknox2	0.12	1.00000000	duck
Fbxo32	0.55	1.00000000	duck
Dsn1	0.09	1.00000000	duck
Terf2	0.09	1.00000000	duck
Prelid1	0.00	1.00000000	duck
Dip2a	0.04	1.00000000	duck
Tspan12	0.19	1.00000000	duck
Atp1b3	-0.02	1.00000000	duck
Tmem60	-0.02	1.00000000	duck
Ankrd52	0.02	1.00000000	duck
Fam81a	0.00	1.00000000	duck
Il16	-0.24	1.00000000	duck
Polr1c	0.01	1.00000000	duck
Ldb1	-0.17	1.00000000	duck
Pdcd11	0.11	1.00000000	duck
Arf6	0.10	1.00000000	duck
Rapgef2	-0.04	1.00000000	duck
Rpa1	0.05	1.00000000	duck
Sms	-0.74	1.00000000	duck
Prlr	0.11	1.00000000	duck
Ezh2	0.13	1.00000000	duck
Slc16a3	-0.12	1.00000000	duck
Eif1b	0.01	1.00000000	duck
Pepd	0.20	1.00000000	duck
Suds3	0.00	1.00000000	duck
Cebpg	0.08	1.00000000	duck
Aak1	-0.03	1.00000000	duck
Heatr1	0.12	1.00000000	duck
Uchl3	-0.01	1.00000000	duck
Kdm7a	-0.04	1.00000000	duck
Tifa	0.08	1.00000000	duck
Ppm1a	0.00	1.00000000	duck
Uaca	0.68	1.00000000	duck
Cep44	-0.24	1.00000000	duck
Eif2ak3	0.01	1.00000000	duck
Dlg1	0.13	1.00000000	duck
Utp20	0.04	1.00000000	duck
Pnpla8	-0.09	1.00000000	duck
Gem	0.08	1.00000000	duck
Armc1	0.12	1.00000000	duck
Gtf2e1	-0.02	1.00000000	duck
Lztfl1	0.02	1.00000000	duck
Pxylp1	-0.10	1.00000000	duck

Kansl1l	-0.01	1.00000000	duck
Cldnd1	-0.03	1.00000000	duck
Adora2a	-0.16	1.00000000	duck
Slc35b3	0.07	1.00000000	duck
Rmdn1	0.05	1.00000000	duck
Ppp3cc	0.04	1.00000000	duck
Commd4	0.13	1.00000000	duck
Tnks	0.00	1.00000000	duck
Adat1	0.05	1.00000000	duck
Ndst4	0.14	1.00000000	duck
Sstr4	-0.03	1.00000000	duck
Zdhhc6	0.06	1.00000000	duck
Iqcb1	-0.01	1.00000000	duck
Insyn2a	0.03	1.00000000	duck
Pigt	0.02	1.00000000	duck
Sdad1	0.11	1.00000000	duck
Stom	0.59	1.00000000	duck
Adm	-0.30	1.00000000	duck
Zfand4	0.14	1.00000000	duck
Kpnb1	0.19	1.00000000	duck
Pcnt	-0.09	1.00000000	duck
Ptbp1	-0.08	1.00000000	duck
Adssl1	0.07	1.00000000	duck
Lsm11	0.07	1.00000000	duck
Ppm1l	-0.02	1.00000000	duck
Zfp384	0.02	1.00000000	duck
LTO1	0.00	1.00000000	duck
Wdr12	0.14	1.00000000	duck
Ppcs	-0.06	1.00000000	duck
Appbp2	0.11	1.00000000	duck
Lrmda	-0.20	1.00000000	duck
Synpo	-0.01	1.00000000	duck
Tmem19	0.19	1.00000000	duck
Tnpo1	0.47	1.00000000	duck
Prkrip1	0.04	1.00000000	duck
Slc39a6	0.16	1.00000000	duck
Abcb7	-0.14	1.00000000	duck
Sepsecs	0.01	1.00000000	duck
Eif3m	0.06	1.00000000	duck
Crkl	0.01	1.00000000	duck
Pdlim1	-0.03	1.00000000	duck
Lca5	-0.03	1.00000000	duck
Ttbk1	0.08	1.00000000	duck
Tax1bp1	-0.23	1.00000000	duck
P4htm	0.04	1.00000000	duck
Kbtbd2	-0.01	1.00000000	duck

Ogfr	0.59	1.00000000	duck
Il17ra	0.53	1.00000000	duck
Rpf2	0.05	1.00000000	duck
Ntan1	0.05	1.00000000	duck
Scaf4	0.06	1.00000000	duck
Acbd6	0.07	1.00000000	duck
Dicer1	-0.09	1.00000000	duck
Larp7	0.08	1.00000000	duck
Kif1b	-0.14	1.00000000	duck
Kansl3	0.03	1.00000000	duck
Hsbp1	0.04	1.00000000	duck
Tsc2	0.11	1.00000000	duck
Kat6a	-0.12	1.00000000	duck
Nfx1	0.16	1.00000000	duck
Trappc6b	0.04	1.00000000	duck
Galnt2	0.15	1.00000000	duck
Tbck	0.03	1.00000000	duck
Fbxl5	-0.14	1.00000000	duck
Gpr155	-0.29	1.00000000	duck
F2r	0.25	1.00000000	duck
Aasdh	-0.08	1.00000000	duck
Krr1	0.03	1.00000000	duck
Capg	0.06	1.00000000	duck
Rufy2	-0.19	1.00000000	duck
Abitram	0.02	1.00000000	duck
Rer1	0.09	1.00000000	duck
Flnb	0.13	1.00000000	duck
Dyrk1a	-0.05	1.00000000	duck
Bsg	-0.04	1.00000000	duck
Kif3b	0.08	1.00000000	duck
Osbpl9	0.08	1.00000000	duck
Slc16a9	0.07	1.00000000	duck
Crispld2	0.45	1.00000000	duck
Btbd8	-0.10	1.00000000	duck
Klf4	0.41	1.00000000	duck
Arf1	-0.07	1.00000000	duck
Taf5	0.08	1.00000000	duck
Hoxa3	0.24	1.00000000	duck
Susd6	-0.50	1.00000000	duck
Txndc17	-0.02	1.00000000	duck
Bicd2	0.13	1.00000000	duck
Ttc17	0.05	1.00000000	duck
Batf	0.00	1.00000000	duck
Cdk5rap3	0.10	1.00000000	duck
Rere	0.48	1.00000000	duck
Arfgap1	0.04	1.00000000	duck

N4bp1	-0.30	1.00000000	duck
Kdm2a	0.02	1.00000000	duck
Zfp704	0.01	1.00000000	duck
Adpgk	0.02	1.00000000	duck
Zfp644	-0.23	1.00000000	duck
Snrpb	0.12	1.00000000	duck
Purb	0.02	1.00000000	duck
Htra3	-0.02	1.00000000	duck
Zbtb2	0.04	1.00000000	duck
Ctss	0.33	1.00000000	duck
Rad21	0.08	1.00000000	duck
Hexb	0.07	1.00000000	duck
Gpd2	-0.10	1.00000000	duck
Osbpl3	-0.17	1.00000000	duck
Nop14	0.10	1.00000000	duck
Dclre1a	-0.01	1.00000000	duck
Igf2	0.31	1.00000000	duck
Gorasp1	0.08	1.00000000	duck
Atg13	0.11	1.00000000	duck
Ccdc126	-0.15	1.00000000	duck
Rad51ap1	-0.04	1.00000000	duck
Sned1	-0.07	1.00000000	duck
Rnf14	0.15	1.00000000	duck
Esrrg	0.78	1.00000000	duck
Fbxo4	0.11	1.00000000	duck
Dcun1d1	-0.07	1.00000000	duck
Btg2	-0.29	1.00000000	duck
Nrde2	-0.06	1.00000000	duck
Furin	0.00	1.00000000	duck
Rlim	0.16	1.00000000	duck
Got1	0.07	1.00000000	duck
Gpatch4	0.03	1.00000000	duck
Ctcf	0.06	1.00000000	duck
Erc2	-0.28	1.00000000	duck
Ppp6r3	0.01	1.00000000	duck
Rora	-0.17	1.00000000	duck
Sipa1l1	-0.20	1.00000000	duck
Hint3	-0.06	1.00000000	duck
Dyrk2	0.17	1.00000000	duck
Rsu1	0.07	1.00000000	duck
Get4	0.06	1.00000000	duck
Hsf1	0.05	1.00000000	duck
Ift172	0.05	1.00000000	duck
Sap30	0.09	1.00000000	duck
Tsn	0.06	1.00000000	duck
Ssr2	0.06	1.00000000	duck

Csnk1g1	0.13	1.00000000	duck
Slc40a1	0.40	1.00000000	duck
Usp7	0.00	1.00000000	duck
Cnst	0.24	1.00000000	duck
Bdp1	-0.07	1.00000000	duck
Snx12	0.16	1.00000000	duck
Atf2	0.04	1.00000000	duck
Slc25a16	-0.11	1.00000000	duck
Slc35c2	0.04	1.00000000	duck
Pla2g4a	0.20	1.00000000	duck
Yipf3	0.12	1.00000000	duck
Thap4	0.01	1.00000000	duck
Bnip1	0.03	1.00000000	duck
Arsb	0.25	1.00000000	duck
Nrp2	0.50	1.00000000	duck
Serac1	-0.08	1.00000000	duck
Insig1	0.69	1.00000000	duck
Irag1	0.07	1.00000000	duck
Anks6	0.04	1.00000000	duck
Stau2	0.10	1.00000000	duck
C2cd5	0.14	1.00000000	duck
Asxl2	-0.07	1.00000000	duck
Triqk	0.01	1.00000000	duck
Yme1l1	0.00	1.00000000	duck
Dennd4a	0.28	1.00000000	duck
Nsfl1c	0.14	1.00000000	duck
Nudcd1	0.04	1.00000000	duck
Sdc1	0.24	1.00000000	duck
Ndufb2	0.21	1.00000000	duck
Npc1	0.12	1.00000000	duck
Rrp7a	-0.02	1.00000000	duck
Wdr7	-0.07	1.00000000	duck
Ncbp3	0.11	1.00000000	duck
Fam234a	-0.04	1.00000000	duck
Nr2c1	0.07	1.00000000	duck
Slc35e1	0.07	1.00000000	duck
Vamp3	0.01	1.00000000	duck
Efnb1	0.19	1.00000000	duck
Tead1	0.09	1.00000000	duck
Lpcat2b	0.00	1.00000000	duck
Azin1	0.00	1.00000000	duck
Rmnd1	-0.16	1.00000000	duck
Fry	0.24	1.00000000	duck
Farp2	0.12	1.00000000	duck
Naf1	0.15	1.00000000	duck
Pcnx4	0.06	1.00000000	duck

Pdpk1	-0.21	1.00000000	duck
Tafa5	0.09	1.00000000	duck
Prpf39	-0.03	1.00000000	duck
Atg4c	-0.15	1.00000000	duck
Xrcc4	0.49	1.00000000	duck
Chaf1b	-0.03	1.00000000	duck
Atp1a1	0.03	1.00000000	duck
Rab3gap1	0.05	1.00000000	duck
Eny2	0.17	1.00000000	duck
Nhlrc2	-0.08	1.00000000	duck
Nfxl1	0.09	1.00000000	duck
Pip4p2	0.15	1.00000000	duck
Tbc1d15	0.04	1.00000000	duck
Uspl1	0.22	1.00000000	duck
Scamp4	0.09	1.00000000	duck
Bend5	-0.03	1.00000000	duck
Arnt	-0.06	1.00000000	duck
Vamp4	0.04	1.00000000	duck
Spred2	0.52	1.00000000	duck
Kdm5a	0.03	1.00000000	duck
Pms1	0.08	1.00000000	duck
Nup205	0.19	1.00000000	duck
Usp46	0.15	1.00000000	duck
Ror1	0.08	1.00000000	duck
Cebpz	0.07	1.00000000	duck
Tlnrd1	0.11	1.00000000	duck
Dpp9	-0.04	1.00000000	duck
Apool	0.11	1.00000000	duck
Herpud1	0.07	1.00000000	duck
Plce1	0.20	1.00000000	duck
Rab6a	-0.16	1.00000000	duck
Trim33	0.18	1.00000000	duck
Dph3	0.07	1.00000000	duck
Cpeb2	0.08	1.00000000	duck
Ophn1	0.16	1.00000000	duck
Col12a1	-0.23	1.00000000	duck
Sil1	0.00	1.00000000	duck
Pum3	0.14	1.00000000	duck
Jdp2	-0.02	1.00000000	duck
Ppl	-0.12	1.00000000	duck
Cgnl1	0.22	1.00000000	duck
Sp3	0.04	1.00000000	duck
Slc7a6	0.06	1.00000000	duck
Trip4	0.08	1.00000000	duck
Gxylt1	0.02	1.00000000	duck
Lmf2	0.15	1.00000000	duck

N4bp2	0.10	1.00000000	duck
Chst10	0.15	1.00000000	duck
Rab1a	-0.02	1.00000000	duck
Evc	0.09	1.00000000	duck
Usp38	-0.07	1.00000000	duck
Kdelr2	0.03	1.00000000	duck
Ubap1	0.08	1.00000000	duck
Thoc2	-0.11	1.00000000	duck
Col16a1	0.09	1.00000000	duck
Dhx57	-0.02	1.00000000	duck
Edc4	0.05	1.00000000	duck
Bms1	0.05	1.00000000	duck
Lrp12	0.09	1.00000000	duck
Galnt7	0.17	1.00000000	duck
Ing4	0.00	1.00000000	duck
Nmt2	0.20	1.00000000	duck
Ankrd46	0.17	1.00000000	duck
Tmtc3	0.04	1.00000000	duck
Ercc6	0.11	1.00000000	duck
Ubxn7	0.04	1.00000000	duck
Wdtc1	0.02	1.00000000	duck
Ptprf	0.00	1.00000000	duck
Cebpd	0.24	1.00000000	duck
Ireb2	0.13	1.00000000	duck
Deaf1	0.08	1.00000000	duck
Borcs5	0.09	1.00000000	duck
Txndc12	0.03	1.00000000	duck
Angptl4	0.14	1.00000000	duck
Lamtor5	0.15	1.00000000	duck
Pard3	0.37	1.00000000	duck
Stk38	0.07	1.00000000	duck
Scarb2	0.19	1.00000000	duck
Dele1	0.09	1.00000000	duck
Dpp7	0.20	1.00000000	duck
Ublcp1	0.02	1.00000000	duck
Pex13	0.16	1.00000000	duck
Ctps2	-0.13	1.00000000	duck
Exoc2	0.10	1.00000000	duck
Sf3a1	0.03	1.00000000	duck
Snx13	0.16	1.00000000	duck
Sugt1	-0.06	1.00000000	duck
Brwd3	-0.13	1.00000000	duck
Rnf145	0.13	1.00000000	duck
Gzf1	-0.01	1.00000000	duck
Nav1	0.09	1.00000000	duck
Pdlim4	0.06	1.00000000	duck

Chid1	0.06	1.00000000	duck
Bend7	0.04	1.00000000	duck
Sh3gl1	0.11	1.00000000	duck
Tbc1d31	0.03	1.00000000	duck
Ubfd1	-0.09	1.00000000	duck
Kcnk5	0.21	1.00000000	duck
Snx2	-0.06	1.00000000	duck
Fam126b	-0.15	1.00000000	duck
Flii	-0.09	1.00000000	duck
Lrrn4	0.24	1.00000000	duck
Zhx3	0.07	1.00000000	duck
Polr3a	-0.08	1.00000000	duck
Rock2	-0.02	1.00000000	duck
Slc39a11	-0.03	1.00000000	duck
Rnf216	0.18	1.00000000	duck
Rb1cc1	-0.19	1.00000000	duck
Psmc3	0.04	1.00000000	duck
Pabpc4	0.01	1.00000000	duck
Ddx20	-0.03	1.00000000	duck
Rbm6	0.06	1.00000000	duck
Cnot1	0.00	1.00000000	duck
Capza2	0.09	1.00000000	duck
Ddr2	0.14	1.00000000	duck
Tst	0.31	1.00000000	duck
Nudt19	0.15	1.00000000	duck
Ndufs6	0.06	1.00000000	duck
Xpo6	-0.17	1.00000000	duck
Syn3	0.12	1.00000000	duck
Nf2	0.19	1.00000000	duck
Ppp2r2a	0.10	1.00000000	duck
Rnf139	0.01	1.00000000	duck
Rai1	-0.03	1.00000000	duck
Arfgef3	-0.06	1.00000000	duck
Tes	-0.04	1.00000000	duck
Arl9	0.03	1.00000000	duck
Chpf2	0.07	1.00000000	duck
Sec61a2	0.13	1.00000000	duck
Srpk1	0.01	1.00000000	duck
Abl1	0.48	1.00000000	duck
Nadk2	0.13	1.00000000	duck
Yeats4	0.11	1.00000000	duck
Znrf3	0.19	1.00000000	duck
Tank	0.07	1.00000000	duck
Uhrf1	0.17	1.00000000	duck
Gtdc1	0.11	1.00000000	duck
Dcaf5	-0.33	1.00000000	duck

Luc7l3	0.00	1.00000000	duck
Sf3b1	0.01	1.00000000	duck
Pdss1	0.02	1.00000000	duck
Tyw3	0.10	1.00000000	duck
Naa16	0.02	1.00000000	duck
Ccdc12	0.04	1.00000000	duck
Ranbp10	-0.01	1.00000000	duck
Hgs	-0.09	1.00000000	duck
Rasgrf2	0.19	1.00000000	duck
Actr2	0.17	1.00000000	duck
Kif13a	-0.11	1.00000000	duck
Ergic2	0.09	1.00000000	duck
Zzz3	-0.06	1.00000000	duck
Scrib	0.17	1.00000000	duck
Ralb	0.22	1.00000000	duck
Lmnb1	0.40	1.00000000	duck
Cryl1	-0.02	1.00000000	duck
Raf1	0.03	1.00000000	duck
Cep120	0.09	1.00000000	duck
Gna13	0.00	1.00000000	duck
Rnf130	0.59	1.00000000	duck
Nek6	-0.06	1.00000000	duck
Top3b	0.05	1.00000000	duck
Celf5	-0.06	1.00000000	duck
Brca2	0.04	1.00000000	duck
Pus7	0.07	1.00000000	duck
Bin1	0.12	1.00000000	duck
Gpt2	0.09	1.00000000	duck
Golga7	-0.06	1.00000000	duck
Pdk4	-0.13	1.00000000	duck
Habp4	0.16	1.00000000	duck
Sugct	0.06	1.00000000	duck
Dph5	-0.01	1.00000000	duck
Dnaja3	0.00	1.00000000	duck
Pgd	0.17	1.00000000	duck
Capn2	0.03	1.00000000	duck
Cib1	-0.05	1.00000000	duck
Ccng1	0.03	1.00000000	duck
Fam76a	-0.04	1.00000000	duck
Fbxw7	0.10	1.00000000	duck
Lamb1	-0.06	1.00000000	duck
Gabpa	-0.03	1.00000000	duck
Arhgap26	-0.13	1.00000000	duck
Dennd5a	-0.16	1.00000000	duck
Hnrnpll	0.27	1.00000000	duck
Dnajc19	-0.06	1.00000000	duck

Fbf1	0.03	1.00000000	duck
Usp34	-0.04	1.00000000	duck
Plcd1	0.18	1.00000000	duck
Rpn1	-0.01	1.00000000	duck
Ttc39a	0.05	1.00000000	duck
Spats2	0.11	1.00000000	duck
Il13ra1	-0.02	1.00000000	duck
Thap1	0.03	1.00000000	duck
Arhgap1	0.12	1.00000000	duck
Ddx49	0.01	1.00000000	duck
Slc2a1	0.04	1.00000000	duck
Ttc32	0.02	1.00000000	duck
Id2	0.06	1.00000000	duck
Abtb2	1.21	1.00000000	duck
F2rl2	0.14	1.00000000	duck
Ptprs	-0.20	1.00000000	duck
Fzd8	0.11	1.00000000	duck
Foxk2	0.05	1.00000000	duck
Xpot	-0.04	1.00000000	duck
Pkm	0.03	1.00000000	duck
Cep164	0.15	1.00000000	duck
Cnnm2	0.25	1.00000000	duck
Ift22	0.03	1.00000000	duck
Nifk	0.06	1.00000000	duck
Gnpat	-0.02	1.00000000	duck
Nubpl	0.46	1.00000000	duck
Prelid3b	0.10	1.00000000	duck
Ptpn3	0.28	1.00000000	duck
Vwa8	0.79	1.00000000	duck
Ssb	0.04	1.00000000	duck
Akap8	0.04	1.00000000	duck
Aff1	0.00	1.00000000	duck
Nudc	0.16	1.00000000	duck
Lzts2	0.03	1.00000000	duck
4833439L19Rik	0.11	1.00000000	duck
Plekhm2	0.09	1.00000000	duck
Col4a6	0.09	1.00000000	duck
Btf3	0.55	1.00000000	duck
Nras	-0.03	1.00000000	duck
Rmi2	0.00	1.00000000	duck
Sgpp1	0.10	1.00000000	duck
Usp1	0.08	1.00000000	duck
Atg16l1	0.07	1.00000000	duck
Atm	-0.15	1.00000000	duck
Gtf2h5	-0.10	1.00000000	duck
Gfm1	0.08	1.00000000	duck

Eri1	0.06	1.00000000	duck
Zranb1	-0.06	1.00000000	duck
Arid3b	0.00	1.00000000	duck
Atad3a	0.06	1.00000000	duck
Pde6d	0.20	1.00000000	duck
Etaa1	-0.04	1.00000000	duck
Snrpg	0.18	1.00000000	duck
Kctd3	0.18	1.00000000	duck
Abhd17a	0.01	1.00000000	duck
Cdc23	0.03	1.00000000	duck
Dtx3l	0.56	1.00000000	duck
Fzr1	0.16	1.00000000	duck
Chchd1	0.03	1.00000000	duck
Glul	0.65	1.00000000	duck
Emc8	0.14	1.00000000	duck
Nrp1	0.02	1.00000000	duck
Rsf1	-0.09	1.00000000	duck
Slc23a2	-0.04	1.00000000	duck
Wnt5a	0.21	1.00000000	duck
Tmem242	0.02	1.00000000	duck
Hadhb	0.09	1.00000000	duck
Ccdc43	0.09	1.00000000	duck
Zswim8	-0.13	1.00000000	duck
Itpa	0.09	1.00000000	duck
Echs1	0.07	1.00000000	duck
Apaf1	-0.16	1.00000000	duck
Rabl3	-0.03	1.00000000	duck
Ost4	0.22	1.00000000	duck
Ptgs2	0.15	1.00000000	duck
Cdkal1	0.60	1.00000000	duck
Fgf10	0.21	1.00000000	duck
Kmt5b	-0.09	1.00000000	duck
Acads	0.08	1.00000000	duck
Vps26b	0.06	1.00000000	duck
Coq2	-0.02	1.00000000	duck
Slc48a1	0.08	1.00000000	duck
Ppara	0.16	1.00000000	duck
Bmp2k	0.08	1.00000000	duck
Foxs1	0.13	1.00000000	duck
Wdr91	0.14	1.00000000	duck
Fbxw11	0.03	1.00000000	duck
Taf3	-0.03	1.00000000	duck
Ssh2	0.04	1.00000000	duck
Dnajc5	0.02	1.00000000	duck
Rab10	-0.18	1.00000000	duck
Ankrd33b	0.13	1.00000000	duck

Cog5	0.19	1.00000000	duck
Bub3	0.06	1.00000000	duck
Lancl2	0.14	1.00000000	duck
Josd1	0.11	1.00000000	duck
Zfp438	-0.34	1.00000000	duck
Lcmt1	0.08	1.00000000	duck
Ier5	-0.03	1.00000000	duck
Ccng2	-0.04	1.00000000	duck
Polr1a	0.02	1.00000000	duck
Ndufb10	-0.01	1.00000000	duck
Cse1l	0.21	1.00000000	duck
4932411N23Rik	-0.05	1.00000000	duck
Snx17	0.05	1.00000000	duck
Fbxo9	0.05	1.00000000	duck
Aldh6a1	0.16	1.00000000	duck
AW554918	0.60	1.00000000	duck
Ugcg	0.12	1.00000000	duck
Pola1	0.36	1.00000000	duck
Ttc7	0.30	1.00000000	duck
Phf14	0.20	1.00000000	duck
Col15a1	-0.07	1.00000000	duck
Tubd1	-0.03	1.00000000	duck
Kcnq5	-0.22	1.00000000	duck
Scd2	-0.04	1.00000000	duck
Lsm5	0.13	1.00000000	duck
Cetn3	0.08	1.00000000	duck
Erp44	0.03	1.00000000	duck
Ppp3cb	0.10	1.00000000	duck
Kctd15	0.08	1.00000000	duck
Srfbp1	0.50	1.00000000	duck
Nutf2	0.26	1.00000000	duck
Runx1t1	0.65	1.00000000	duck
Ddx18	0.09	1.00000000	duck
Kctd10	-0.03	1.00000000	duck
Ugdh	0.25	1.00000000	duck
Ipo11	0.06	1.00000000	duck
Dlgap4	0.07	1.00000000	duck
Faap24	-0.10	1.00000000	duck
Tspan3	-0.08	1.00000000	duck
Psma7	0.04	1.00000000	duck
Ptprm	-0.21	1.00000000	duck
Cmas	0.06	1.00000000	duck
Hif1a	-0.05	1.00000000	duck
Vav2	0.19	1.00000000	duck
Gemin5	0.12	1.00000000	duck
Id3	0.12	1.00000000	duck

Wbp2nl	0.15	1.00000000	duck
Scrn3	0.04	1.00000000	duck
Efcab14	-0.13	1.00000000	duck
Arsk	-0.09	1.00000000	duck
Creb5	-0.44	1.00000000	duck
Numb	0.01	1.00000000	duck
Rbm12b2	0.10	1.00000000	duck
Igfbp3	1.25	1.00000000	duck
Tsr1	-0.09	1.00000000	duck
Foxk1	0.12	1.00000000	duck
Tia1	0.05	1.00000000	duck
Star	0.06	1.00000000	duck
Kitl	0.15	1.00000000	duck
Baz1a	-0.03	1.00000000	duck
Pmpcb	0.03	1.00000000	duck
Slc9a3r1	0.19	1.00000000	duck
Rarb	0.03	1.00000000	duck
Smg5	-0.03	1.00000000	duck
Ttc13	0.10	1.00000000	duck
Dvl3	0.12	1.00000000	duck
Agpat4	0.07	1.00000000	duck
Runx1	0.57	1.00000000	duck
Jmjd6	0.14	1.00000000	duck
Sbno1	-0.27	1.00000000	duck
Tmem87a	0.07	1.00000000	duck
Polr2f	0.13	1.00000000	duck
Lrrc8a	0.17	1.00000000	duck
Cds2	0.07	1.00000000	duck
Csnk1g2	0.09	1.00000000	duck
Ctsc	0.23	1.00000000	duck
Fancm	0.07	1.00000000	duck
Fbxo25	0.01	1.00000000	duck
Atg7	0.33	1.00000000	duck
Cdc123	0.00	1.00000000	duck
Lcor	0.18	1.00000000	duck
Wipi1	0.04	1.00000000	duck
Nelfa	0.07	1.00000000	duck
Nr3c1	0.08	1.00000000	duck
Ash2l	0.03	1.00000000	duck
Pm20d2	0.06	1.00000000	duck
Ptk7	-0.01	1.00000000	duck
Slc25a12	-0.19	1.00000000	duck
Grk4	-0.09	1.00000000	duck
Fbln2	-0.06	1.00000000	duck
Chic2	-0.11	1.00000000	duck
Sec24b	0.03	1.00000000	duck

Sesn3	0.42	1.00000000	duck
Larp1	0.08	1.00000000	duck
Polr3b	-0.05	1.00000000	duck
Rab11a	-0.06	1.00000000	duck
Fbxw2	-0.04	1.00000000	duck
Epb41l3	0.08	1.00000000	duck
Snrpf	0.20	1.00000000	duck
Smad3	0.58	1.00000000	duck
Tle4	0.61	1.00000000	duck
Phrf1	0.08	1.00000000	duck
Nfe2l1	0.14	1.00000000	duck
Nfatc1	0.52	1.00000000	duck
Tarsl2	-0.01	1.00000000	duck
Naxd	-0.04	1.00000000	duck
Bcar1	0.13	1.00000000	duck
Susd1	-0.41	1.00000000	duck
Lars2	0.10	1.00000000	duck
Bloc1s4	0.03	1.00000000	duck
Atp1b1	0.34	1.00000000	duck
Zbtb26	0.01	1.00000000	duck
Pex14	0.09	1.00000000	duck
Fbn2	0.05	1.00000000	duck
Gars	0.17	1.00000000	duck
Tdg	0.11	1.00000000	duck
Slc7a5	-0.04	1.00000000	duck
Ttc7b	0.06	1.00000000	duck
Ints1	0.08	1.00000000	duck
Immp2l	-0.07	1.00000000	duck
Lclat1	0.17	1.00000000	duck
Baz1b	-0.15	1.00000000	duck
Atp6v1b2	0.52	1.00000000	duck
Tprkb	0.06	1.00000000	duck
Hnrnpk	0.42	1.00000000	duck
Ak3	0.08	1.00000000	duck
Larp4b	-0.06	1.00000000	duck
Cers5	0.12	1.00000000	duck
Ints9	0.06	1.00000000	duck
Psmd10	-0.10	1.00000000	duck
Nbr1	0.03	1.00000000	duck
Dpyd	0.41	1.00000000	duck
Focad	0.16	1.00000000	duck
Vps72	0.10	1.00000000	duck
Zfp217	0.22	1.00000000	duck
Daam2	0.73	1.00000000	duck
Eif4e2	-0.03	1.00000000	duck
Eif3h	-0.10	1.00000000	duck

Vps54	0.07	1.00000000	duck
Hnrnpr	-0.11	1.00000000	duck
Itch	-0.30	1.00000000	duck
Xrcc5	0.01	1.00000000	duck
Hoxb3	0.36	1.00000000	duck
Naa50	0.19	1.00000000	duck
Tada1	-0.09	1.00000000	duck
Cd47	-0.02	1.00000000	duck
Drd4	-0.04	1.00000000	duck
Hibch	-0.05	1.00000000	duck
Ctnnbl1	0.12	1.00000000	duck
Tmem168	0.00	1.00000000	duck
Ppp1ca	0.08	1.00000000	duck
Ampd2	-0.11	1.00000000	duck
Braf	0.08	1.00000000	duck
Prpf19	0.11	1.00000000	duck
Atxn2	0.07	1.00000000	duck
Ddx23	0.17	1.00000000	duck
Chaf1a	0.03	1.00000000	duck
Trp53inp1	0.10	1.00000000	duck
Ncln	0.08	1.00000000	duck
Trnt1	0.11	1.00000000	duck
Denr	0.14	1.00000000	duck
Cyria	0.16	1.00000000	duck
Slk	-0.08	1.00000000	duck
Dennd4c	0.19	1.00000000	duck
Rel	0.13	1.00000000	duck
Socs1	0.06	1.00000000	duck
Romo1	0.11	1.00000000	duck
Dlgap1	0.17	1.00000000	duck
Fam13b	0.13	1.00000000	duck
Gch1	0.71	1.00000000	duck
Itga8	0.78	1.00000000	duck
Acp2	0.42	1.00000000	duck
Eif3l	0.06	1.00000000	duck
Auh	0.64	1.00000000	duck
Rnf13	-0.04	1.00000000	duck
Gpatch2l	-0.09	1.00000000	duck
Fbxo33	0.18	1.00000000	duck
Parp16	0.21	1.00000000	duck
Hipk2	0.33	1.00000000	duck
Rnf41	-0.01	1.00000000	duck
Fbxl2	0.18	1.00000000	duck
Aff2	0.47	1.00000000	duck
Arap2	0.31	1.00000000	duck
Tnip1	-0.05	1.00000000	duck

Atxn1l	0.05	1.00000000	duck
Txndc15	0.12	1.00000000	duck
Tbc1d9	0.07	1.00000000	duck
Entpd5	-0.15	1.00000000	duck
Nck2	0.13	1.00000000	duck
Bysl	-0.08	1.00000000	duck
Cd9	0.63	1.00000000	duck
Zfp507	-0.03	1.00000000	duck
Umps	-0.11	1.00000000	duck
Sft2d1	0.19	1.00000000	duck
Bzw2	0.13	1.00000000	duck
Radx	0.19	1.00000000	duck
Pisd	0.25	1.00000000	duck
Cul4b	0.14	1.00000000	duck
Tgfbr3	0.30	1.00000000	duck
Ralgps2	0.01	1.00000000	duck
Art4	0.08	1.00000000	duck
Vps26a	0.09	1.00000000	duck
Pfdn1	0.11	1.00000000	duck
Atp2c1	0.23	1.00000000	duck
Phb2	0.10	1.00000000	duck
Snrpd1	0.03	1.00000000	duck
Chd1	-0.10	1.00000000	duck
Adipor2	0.22	1.00000000	duck
Rngtt	0.76	1.00000000	duck
Gcn1	0.20	1.00000000	duck
Jade2	-0.04	1.00000000	duck
Ap3s1	-0.06	1.00000000	duck
Sik2	0.08	1.00000000	duck
Klf10	0.14	1.00000000	duck
Pigx	0.21	1.00000000	duck
Cep89	0.11	1.00000000	duck
Gdpd5	0.18	1.00000000	duck
Wipi2	-0.17	1.00000000	duck
Cacna2d1	0.69	1.00000000	duck
Klf3	0.11	1.00000000	duck
Zw10	0.06	1.00000000	duck
Rab3il1	0.08	1.00000000	duck
Tmem11	-0.07	1.00000000	duck
Adamtsl3	0.65	1.00000000	duck
Gulp1	0.09	1.00000000	duck
Snx24	0.17	1.00000000	duck
Psmb1	0.04	1.00000000	duck
Ifih1	-0.09	1.00000000	duck
Sf3b6	-0.06	1.00000000	duck
Clec16a	0.06	1.00000000	duck

St7l	0.13	1.00000000	duck
Slc25a26	0.37	1.00000000	duck
Nuak1	0.29	1.00000000	duck
Orai1	0.14	1.00000000	duck
Spast	-0.10	1.00000000	duck
Tenm3	0.02	1.00000000	duck
Adprm	0.07	1.00000000	duck
Aldh1a3	0.09	1.00000000	duck
Lpar1	1.23	1.00000000	duck
Ell	0.35	1.00000000	duck
Epn2	-0.12	1.00000000	duck
Slco2b1	0.74	1.00000000	duck
Lrrc28	0.12	1.00000000	duck
Zbtb21	0.17	1.00000000	duck
Kpna4	-0.34	1.00000000	duck
Lonrf2	0.07	1.00000000	duck
Eif2d	0.00	1.00000000	duck
Zbtb8os	0.07	1.00000000	duck
Lgmn	0.04	1.00000000	duck
Tsr3	0.17	1.00000000	duck
Epc1	0.06	1.00000000	duck
Slc7a6os	0.10	1.00000000	duck
Tarbp1	0.14	1.00000000	duck
Tiam2	0.03	1.00000000	duck
Gpalpp1	0.01	1.00000000	duck
Arl8b	0.22	1.00000000	duck
Gbe1	0.05	1.00000000	duck
Slc12a2	0.24	1.00000000	duck
Agfg1	0.06	1.00000000	duck
Kin	-0.20	1.00000000	duck
Eml4	0.03	1.00000000	duck
Ptpn2	-0.24	1.00000000	duck
Ppa2	0.16	1.00000000	duck
Anxa11	0.02	1.00000000	duck
Ccpg1	-0.26	1.00000000	duck
Sdhaf4	0.01	1.00000000	duck
Rnf6	0.07	1.00000000	duck
Cxxc5	0.06	1.00000000	duck
Fam117a	0.13	1.00000000	duck
Blzf1	0.11	1.00000000	duck
Ist1	-0.02	1.00000000	duck
Jmjd8	0.19	1.00000000	duck
Tkt	0.07	1.00000000	duck
Exoc1	0.18	1.00000000	duck
Prkg1	0.40	1.00000000	duck
Slc35b2	0.11	1.00000000	duck

Cdc42	-0.04	1.00000000	duck
Ccdc85c	-0.01	1.00000000	duck
Tex10	0.19	1.00000000	duck
Snu13	0.11	1.00000000	duck
Lfng	0.08	1.00000000	duck
Rufy3	0.18	1.00000000	duck
Cmtm6	0.11	1.00000000	duck
Derl1	0.07	1.00000000	duck
Nr6a1	-0.05	1.00000000	duck
Prrc1	0.10	1.00000000	duck
Ubr3	-0.06	1.00000000	duck
E4f1	0.00	1.00000000	duck
Phf21a	-0.02	1.00000000	duck
Ppp3ca	0.07	1.00000000	duck
Sox11	-0.02	1.00000000	duck
Fez2	0.20	1.00000000	duck
Angpt1	0.58	1.00000000	duck
Cks1brt	0.05	1.00000000	duck
Plag1	-0.09	1.00000000	duck
Rexo4	0.14	1.00000000	duck
Tmem62	0.11	1.00000000	duck
Lrp6	0.39	1.00000000	duck
C7	0.64	1.00000000	duck
Thada	0.68	1.00000000	duck
Clp1	0.04	1.00000000	duck
Slc15a4	0.22	1.00000000	duck
Anapc2	0.13	1.00000000	duck
Dync2i1	0.22	1.00000000	duck
Zmym3	0.12	1.00000000	duck
Traf3	0.65	1.00000000	duck
Lats1	0.08	1.00000000	duck
Hip1	0.06	1.00000000	duck
1110059G10Rik	-0.01	1.00000000	duck
Lsm14b	0.23	1.00000000	duck
Dek	0.19	1.00000000	duck
Pdia6	0.13	1.00000000	duck
Psmd14	0.16	1.00000000	duck
Zfr	0.51	1.00000000	duck
Ammecr1l	-0.01	1.00000000	duck
Anp32e	0.22	1.00000000	duck
Lyar	0.16	1.00000000	duck
Pak2	0.14	1.00000000	duck
Tmem50b	0.09	1.00000000	duck
Tab3	0.00	1.00000000	duck
Rrm2b	-0.01	1.00000000	duck
Trappc13	0.04	1.00000000	duck

B3gnt2	0.49	1.00000000	duck
Dnajc30	0.12	1.00000000	duck
Cog2	0.20	1.00000000	duck
Polr3k	0.10	1.00000000	duck
Polk	0.05	1.00000000	duck
Eif4ebp2	0.10	1.00000000	duck
Scn8a	0.15	1.00000000	duck
Arfgef2	0.05	1.00000000	duck
Ints2	0.12	1.00000000	duck
Lgals3	0.22	1.00000000	duck
Zfp511	0.05	1.00000000	duck
Ago4	0.08	1.00000000	duck
Slc26a5	-0.07	1.00000000	duck
Plekha8	-0.03	1.00000000	duck
Asb3	-0.06	1.00000000	duck
Rap2b	0.17	1.00000000	duck
Tent2	0.37	1.00000000	duck
Tmem50a	0.06	1.00000000	duck
Gpr107	0.05	1.00000000	duck
Dcaf6	0.22	1.00000000	duck
Psma6	0.07	1.00000000	duck
Agap1	0.17	1.00000000	duck
Coq8a	0.22	1.00000000	duck
Otud1	0.72	1.00000000	duck
Aup1	0.08	1.00000000	duck
Zmynd11	-0.02	1.00000000	duck
Ric1	0.49	1.00000000	duck
Tbc1d4	0.26	1.00000000	duck
Tmem33	0.21	1.00000000	duck
Ppip5k2	0.12	1.00000000	duck
Kank1	0.16	1.00000000	duck
Canx	0.06	1.00000000	duck
Thoc1	0.04	1.00000000	duck
Nol7	0.11	1.00000000	duck
Ahcyl2	0.11	1.00000000	duck
Helz	-0.01	1.00000000	duck
Slc39a13	0.23	1.00000000	duck
Ntrk3	0.04	1.00000000	duck
Trim23	0.10	1.00000000	duck
Vps18	0.13	1.00000000	duck
Spry2	0.20	1.00000000	duck
Ccny	0.89	1.00000000	duck
Atg4b	0.09	1.00000000	duck
Pomt2	0.18	1.00000000	duck
Sod1	0.04	1.00000000	duck
Prkcd	0.41	1.00000000	duck

Dnajb5	0.44	1.00000000	duck
Tex14	0.44	1.00000000	duck
Rab11fip4	0.45	1.00000000	duck
Rad18	0.76	1.00000000	duck
Gipc2	0.17	1.00000000	duck
Camsap2	-0.11	1.00000000	duck
Rnpepl1	0.11	1.00000000	duck
Cep162	0.13	1.00000000	duck
Slc35a3	-0.05	1.00000000	duck
Ube2g2	0.10	1.00000000	duck
Itpkb	0.14	1.00000000	duck
Pdgfrb	0.13	1.00000000	duck
Klf9	0.05	1.00000000	duck
Smarcd1	0.06	1.00000000	duck
Xpo4	0.18	1.00000000	duck
Commd1b	0.24	1.00000000	duck
Wdr48	0.01	1.00000000	duck
Wdr75	0.07	1.00000000	duck
Pik3r5	0.22	1.00000000	duck
Qrich1	0.23	1.00000000	duck
Ube2e3	0.08	1.00000000	duck
Exosc9	0.03	1.00000000	duck
Zfand2a	-0.20	1.00000000	duck
Ifngr1	0.09	1.00000000	duck
Kansl1	0.44	1.00000000	duck
Fam13a	0.11	1.00000000	duck
Erh	0.12	1.00000000	duck
Cass4	-0.01	1.00000000	duck
Rbbp5	0.06	1.00000000	duck
Gsr	0.05	1.00000000	duck
Taok1	0.10	1.00000000	duck
Xpc	0.14	1.00000000	duck
Prune1	0.02	1.00000000	duck
Enpp4	0.05	1.00000000	duck
Seh1l	-0.12	1.00000000	duck
Plekhb2	0.12	1.00000000	duck
Pcmtd1	-0.16	1.00000000	duck
Dlec1	0.10	1.00000000	duck
Slx4	-0.02	1.00000000	duck
Lrif1	0.11	1.00000000	duck
Uap1	0.26	1.00000000	duck
Rrn3	0.12	1.00000000	duck
Rap2a	0.11	1.00000000	duck
Sidt2	0.14	1.00000000	duck
Etnk1	0.08	1.00000000	duck
Ranbp3	0.10	1.00000000	duck

Prkar1a	0.01	1.00000000	duck
Dip2b	0.20	1.00000000	duck
Shprh	-0.02	1.00000000	duck
Exog	0.17	1.00000000	duck
Fbxo21	0.01	1.00000000	duck
Virma	0.07	1.00000000	duck
Pde7b	0.28	1.00000000	duck
Smad4	0.17	1.00000000	duck
Plcb4	0.28	1.00000000	duck
Faf1	-0.08	1.00000000	duck
Lrsam1	0.08	1.00000000	duck
Nelfb	0.02	1.00000000	duck
Ipo9	0.15	1.00000000	duck
Nmt1	0.08	1.00000000	duck
Trabd	0.16	1.00000000	duck
Hmox1	0.83	1.00000000	duck
Stk11	0.07	1.00000000	duck
Kctd19	-0.05	1.00000000	duck
Dtwd2	0.43	1.00000000	duck
Hnrnpu	0.32	1.00000000	duck
Dnajc2	-0.08	1.00000000	duck
Fchsd2	0.04	1.00000000	duck
Smdt1	0.06	1.00000000	duck
Srsf5	0.08	1.00000000	duck
Dcun1d5	0.11	1.00000000	duck
Pcsk5	0.08	1.00000000	duck
Dctn6	0.13	1.00000000	duck
Phlpp2	0.13	1.00000000	duck
Fam210b	0.03	1.00000000	duck
Bcl7b	0.10	1.00000000	duck
Usp6nl	-0.04	1.00000000	duck
Trap1	0.07	1.00000000	duck
Ap2m1	0.07	1.00000000	duck
Galnt11	0.23	1.00000000	duck
Klf13	-0.06	1.00000000	duck
Dpysl2	0.01	1.00000000	duck
Itfg1	0.07	1.00000000	duck
Ubqln1	0.06	1.00000000	duck
Zbtb38	0.03	1.00000000	duck
Pnisr	0.02	1.00000000	duck
Ip6k1	0.08	1.00000000	duck
Igf2bp3	0.16	1.00000000	duck
Cep70	0.08	1.00000000	duck
Hbs1l	0.07	1.00000000	duck
Fam166a	0.08	1.00000000	duck
Sorbs3	0.20	1.00000000	duck

Fam204a	0.12	1.00000000	duck
Limd2	0.33	1.00000000	duck
Rbm39	-0.12	1.00000000	duck
Pik3r1	0.32	1.00000000	duck
Wbp2	0.08	1.00000000	duck
Pcdh17	0.25	1.00000000	duck
Ap2b1	0.24	1.00000000	duck
Hpgds	0.22	1.00000000	duck
Afdn	0.13	1.00000000	duck
Crebl2	0.43	1.00000000	duck
Chd9	-0.03	1.00000000	duck
Inpp5b	0.08	1.00000000	duck
Prmt9	0.11	1.00000000	duck
Ncoa2	0.45	1.00000000	duck
Stag1	0.18	1.00000000	duck
Cplane1	0.08	1.00000000	duck
Psip1	0.34	1.00000000	duck
Pfkfb3	0.60	1.00000000	duck
Wdr35	0.44	1.00000000	duck
Hspa8	0.10	1.00000000	duck
Nin	0.12	1.00000000	duck
Rbl1	0.54	1.00000000	duck
Exoc5	0.01	1.00000000	duck
Abraxas1	0.02	1.00000000	duck
Pcif1	0.07	1.00000000	duck
Sec61g	0.15	1.00000000	duck
Usp25	0.23	1.00000000	duck
Acad11	0.13	1.00000000	duck
Lcp1	0.67	1.00000000	duck
Intu	0.15	1.00000000	duck
St6galnac4	0.03	1.00000000	duck
Ehmt1	0.14	1.00000000	duck
Stxbp3	0.14	1.00000000	duck
Lrp4	0.41	1.00000000	duck
Alox5	-0.69	1.00000000	duck
Sema5b	0.13	1.00000000	duck
Alpk1	0.01	1.00000000	duck
Kbtbd8	0.00	1.00000000	duck
Tmem138	-0.03	1.00000000	duck
Rasgef1a	0.33	1.00000000	duck
Rusc2	0.08	1.00000000	duck
Phyhipl	0.67	1.00000000	duck
Ddx17	-0.18	1.00000000	duck
Fcho2	-0.18	1.00000000	duck
Chsy1	0.26	1.00000000	duck
Tmem65	0.04	1.00000000	duck

Exd2	0.08	1.00000000	duck
Dlg5	0.13	1.00000000	duck
Pld1	0.25	1.00000000	duck
Enpp6	0.16	1.00000000	duck
Slc20a2	0.14	1.00000000	duck
P2rx4	0.07	1.00000000	duck
Eif3j2	-0.05	1.00000000	duck
Thsd1	0.07	1.00000000	duck
Nsrp1	0.09	1.00000000	duck
Irak2	0.01	1.00000000	duck
Dbnl	0.16	1.00000000	duck
Fam193a	0.07	1.00000000	duck
Cdk19	0.12	1.00000000	duck
Zcchc10	0.12	1.00000000	duck
Lnpk	0.19	1.00000000	duck
Olfml2b	-0.10	1.00000000	duck
Armh3	-0.34	1.00000000	duck
Acsbg2	0.32	1.00000000	duck
Pdcd2	0.09	1.00000000	duck
Polr2k	0.10	1.00000000	duck
Smad2	0.11	1.00000000	duck
Flot1	0.05	1.00000000	duck
Xpr1	0.13	1.00000000	duck
Vma21	0.17	1.00000000	duck
Ndufb3	0.08	1.00000000	duck
Hmgb3	0.20	1.00000000	duck
Atp5a1	0.17	1.00000000	duck
Dot1l	0.24	1.00000000	duck
Arid4a	-0.02	1.00000000	duck
Gabarapl2	0.07	1.00000000	duck
Asap1	0.25	1.00000000	duck
Basp1	0.04	1.00000000	duck
Prkcz	-0.08	1.00000000	duck
Ebf2	-0.16	1.00000000	duck
Skp1	-0.17	1.00000000	duck
Pgm1	0.07	1.00000000	duck
Efr3a	-0.06	1.00000000	duck
Hsd17b4	0.22	1.00000000	duck
Eya3	0.21	1.00000000	duck
Itpr2	0.05	1.00000000	duck
Stag2	-0.15	1.00000000	duck
Cisd1	0.20	1.00000000	duck
Ubxn2b	0.14	1.00000000	duck
Snx31	0.42	1.00000000	duck
Ppp1r12a	-0.08	1.00000000	duck
Sfmbt1	0.05	1.00000000	duck

Tbc1d14	0.16	1.00000000	duck
Rbm34	0.00	1.00000000	duck
Setx	0.15	1.00000000	duck
Ino80c	0.03	1.00000000	duck
Atp6ap2	0.19	1.00000000	duck
Rnh1	0.24	1.00000000	duck
Rpf1	0.08	1.00000000	duck
Tln1	0.24	1.00000000	duck
Btf3l4	0.13	1.00000000	duck
Tfpi2	0.07	1.00000000	duck
Snx29	0.20	1.00000000	duck
Supv3l1	0.14	1.00000000	duck
Bcap29	0.03	1.00000000	duck
Exoc6	0.00	1.00000000	duck
Pla2g15	0.23	1.00000000	duck
Cdk14	0.17	1.00000000	duck
Bbx	0.15	1.00000000	duck
Rnls	0.03	1.00000000	duck
Rnf114	0.14	1.00000000	duck
Timm13	0.13	1.00000000	duck
Ddx24	0.16	1.00000000	duck
Avl9	0.07	1.00000000	duck
Ythdf2	0.18	1.00000000	duck
Stxbp1	0.18	1.00000000	duck
Cdyl2	0.12	1.00000000	duck
Arvcf	0.23	1.00000000	duck
Oxr1	0.18	1.00000000	duck
Thumpd3	0.03	1.00000000	duck
D630045J12Rik	0.15	1.00000000	duck
Ap1m1	0.22	1.00000000	duck
Glcci1	0.13	1.00000000	duck
Ddx10	0.17	1.00000000	duck
Rtf2	0.01	1.00000000	duck
Aqr	-0.16	1.00000000	duck
Tom1l2	0.04	1.00000000	duck
Ncald	0.26	1.00000000	duck
Rpp14	0.11	1.00000000	duck
Stk38l	0.19	1.00000000	duck
Slc18b1	0.07	1.00000000	duck
Stxbp6	0.32	1.00000000	duck
Spen	-0.04	1.00000000	duck
Pofut2	0.05	1.00000000	duck
D430041D05Rik	0.07	1.00000000	duck
Serinc1	0.04	1.00000000	duck
Cux1	0.31	1.00000000	duck
Cxcl12	0.15	1.00000000	duck

Pdp1	0.20	1.00000000	duck
Eif3a	0.12	1.00000000	duck
Nek9	0.24	1.00000000	duck
Pak4	0.19	1.00000000	duck
Pkdcc	0.17	1.00000000	duck
Pgap1	0.03	1.00000000	duck
Trpm7	0.14	1.00000000	duck
Tnfaip3	0.59	1.00000000	duck
Nadk	0.56	1.00000000	duck
Ppif	0.12	1.00000000	duck
Prkaa1	0.43	1.00000000	duck
Enpp1	0.39	1.00000000	duck
Slc39a9	0.07	1.00000000	duck
Atad2b	-0.08	1.00000000	duck
Nsd2	0.19	1.00000000	duck
Vps50	0.07	1.00000000	duck
Has2	0.15	1.00000000	duck
Diaph2	-0.03	1.00000000	duck
Bcl2l11	0.27	1.00000000	duck
Spred1	0.16	1.00000000	duck
Ppargc1b	0.05	1.00000000	duck
Znrf2	0.16	1.00000000	duck
Atp6v0c	0.10	1.00000000	duck
Grem2	0.44	1.00000000	duck
Arsg	0.01	1.00000000	duck
Gle1	-0.02	1.00000000	duck
Nfyb	0.01	1.00000000	duck
Dcp2	0.02	1.00000000	duck
Whamm	0.04	1.00000000	duck
Ralbp1	0.05	1.00000000	duck
Zdhhc14	0.19	1.00000000	duck
Rexo1	0.12	1.00000000	duck
Nr3c2	0.38	1.00000000	duck
Gja6	0.24	1.00000000	duck
Lrba	0.14	1.00000000	duck
Nckipsd	0.12	1.00000000	duck
Abca2	0.01	1.00000000	duck
Exosc8	0.02	1.00000000	duck
Uvrag	0.10	1.00000000	duck
Tdrd3	0.07	1.00000000	duck
Capn15	0.09	1.00000000	duck
Nampt	0.27	1.00000000	duck
Zfp395	0.27	1.00000000	duck
Fars2	0.19	1.00000000	duck
Ccdc117	-0.09	1.00000000	duck
Trappc3	-0.02	1.00000000	duck

Dtnbp1	-0.04	1.00000000	duck
Optn	0.43	1.00000000	duck
Anxa1	0.31	1.00000000	duck
B3galnt2	0.11	1.00000000	duck
Reep5	0.40	1.00000000	duck
Acox3	0.24	1.00000000	duck
Poldip3	0.15	1.00000000	duck
Pfdn4	0.15	1.00000000	duck
Trib1	0.48	1.00000000	duck
Tars	0.22	1.00000000	duck
Rab31	0.55	1.00000000	duck
Fbxw8	0.13	1.00000000	duck
Asap2	0.18	1.00000000	duck
Tfcp2	0.32	1.00000000	duck
Cadm1	-0.04	1.00000000	duck
Bud31	0.17	1.00000000	duck
Hectd1	-0.05	1.00000000	duck
Kcnj8	0.13	1.00000000	duck
Ctdp1	0.22	1.00000000	duck
Ism1	0.32	1.00000000	duck
Cramp1	0.20	1.00000000	duck
Sec31a	0.28	1.00000000	duck
Scamp5	-0.01	1.00000000	duck
Trmt6	0.12	1.00000000	duck
Fyn	0.29	1.00000000	duck
Lmbr1	0.10	1.00000000	duck
Ripk2	0.06	1.00000000	duck
Cdk5r1	-0.02	1.00000000	duck
Fam110b	0.14	1.00000000	duck
Utp25	0.16	1.00000000	duck
Itprid2	0.16	1.00000000	duck
Ptpa	0.41	1.00000000	duck
Vmp1	0.55	1.00000000	duck
Slc44a1	0.19	1.00000000	duck
Scamp3	0.08	1.00000000	duck
Cep152	0.08	1.00000000	duck
Parn	0.21	1.00000000	duck
Robo1	0.34	1.00000000	duck
Ube2ql1	0.27	1.00000000	duck
Bambi	0.42	1.00000000	duck
Spopl	0.08	1.00000000	duck
Itm2a	-0.25	1.00000000	duck
Zfhx3	0.37	1.00000000	duck
Ttc21b	0.14	1.00000000	duck
Rnf168	0.33	1.00000000	duck
Got2	0.09	1.00000000	duck

Ust	0.43	1.00000000	duck
Hnrnpa2b1	-0.07	1.00000000	duck
Lrrc1	0.20	1.00000000	duck
Slc49a4	0.17	1.00000000	duck
Yap1	0.31	1.00000000	duck
Slc22a23	0.11	1.00000000	duck
Nob1	0.15	1.00000000	duck
St8sia4	0.27	1.00000000	duck
Fam107b	0.27	1.00000000	duck
Stam	0.16	1.00000000	duck
Ebag9	0.13	1.00000000	duck
Sacs	0.09	1.00000000	duck
Serinc2	0.23	1.00000000	duck
Rnd3	0.46	1.00000000	duck
Ccser1	0.33	1.00000000	duck
Tent4b	0.10	1.00000000	duck
Tpm2	0.63	1.00000000	duck
Slc25a33	0.11	1.00000000	duck
Rock1	0.13	1.00000000	duck
Kmt2e	-0.12	1.00000000	duck
Thrap3	-0.02	1.00000000	duck
Clasp1	0.27	1.00000000	duck
Stbd1	0.23	1.00000000	duck
Ano6	-0.04	1.00000000	duck
Glt8d1	0.08	1.00000000	duck
Chic1	0.14	1.00000000	duck
Vps13a	0.04	1.00000000	duck
Shc1	0.17	1.00000000	duck
Dlc1	0.41	1.00000000	duck
Dgke	0.18	1.00000000	duck
Bcl9	0.53	1.00000000	duck
Epg5	0.08	1.00000000	duck
Fhl5	0.21	1.00000000	duck
Igf2bp2	0.41	1.00000000	duck
Sf3a2	0.16	1.00000000	duck
Abhd12	0.06	1.00000000	duck
Rcc2	-0.05	1.00000000	duck
Efna1	0.13	1.00000000	duck
Grpel1	-0.06	1.00000000	duck
Plekhf1	0.37	1.00000000	duck
Atp6v0e	-0.03	1.00000000	duck
Gna12	0.20	1.00000000	duck
Abraxas2	0.16	1.00000000	duck
Zfp706	0.32	1.00000000	duck
Brix1	0.25	1.00000000	duck
Atg12	0.25	1.00000000	duck

Klf7	0.29	1.00000000	duck
Fam174a	0.42	1.00000000	duck
S100a11	0.57	1.00000000	duck
Gpc5	-0.03	1.00000000	duck
Nox4	-0.13	1.00000000	duck
Uprt	0.04	1.00000000	duck
Aven	0.03	1.00000000	duck
Adgrg6	0.14	1.00000000	duck
Cab39	-0.01	1.00000000	duck
Aptx	0.08	1.00000000	duck
Arhgap42	0.39	1.00000000	duck
Pten	-0.03	1.00000000	duck
Heatr6	0.06	1.00000000	duck
Timm44	0.13	1.00000000	duck
Dcps	0.07	1.00000000	duck
Ptar1	0.17	1.00000000	duck
Elovl5	0.08	1.00000000	duck
Phactr3	0.23	1.00000000	duck
Dctn5	0.09	1.00000000	duck
Nfyc	0.17	1.00000000	duck
Hecw2	0.18	1.00000000	duck
Pmvk	0.20	1.00000000	duck
Faf2	0.16	1.00000000	duck
Urm1	0.12	1.00000000	duck
Top1	0.00	1.00000000	duck
Tec	0.05	1.00000000	duck
Atp9b	0.29	1.00000000	duck
Phtf2	0.07	1.00000000	duck
Nup43	0.10	1.00000000	duck
Kat6b	0.12	1.00000000	duck
Greb1l	0.45	1.00000000	duck
Elk4	0.51	1.00000000	duck
Fgd3	0.20	1.00000000	duck
Tcp11l2	0.19	1.00000000	duck
Laptm4a	0.12	1.00000000	duck
Hmgb2	0.11	1.00000000	duck
Ccdc59	0.18	1.00000000	duck
Ankmy2	0.08	1.00000000	duck
Eif2ak1	0.12	1.00000000	duck
Rp2	0.02	1.00000000	duck
Rbm17	0.25	1.00000000	duck
Hsdl2	0.08	1.00000000	duck
Zfp318	0.11	1.00000000	duck
Antxr2	-0.10	1.00000000	duck
Epc2	0.01	1.00000000	duck
Scfd2	0.27	1.00000000	duck

Pcgf3	0.48	1.00000000	duck
Zmym2	0.07	1.00000000	duck
Arhgdib	0.56	1.00000000	duck
Pitpnb	0.13	1.00000000	duck
Strada	0.08	1.00000000	duck
Socs3	0.25	1.00000000	duck
Trp53bp2	0.19	1.00000000	duck
Nipa2	0.07	1.00000000	duck
Gatad2a	0.13	1.00000000	duck
Tacc1	0.14	1.00000000	duck
Adsl	0.05	1.00000000	duck
Herpud2	0.25	1.00000000	duck
Tmco4	-0.10	1.00000000	duck
Npr2	0.16	1.00000000	duck
Ccdc149	0.10	1.00000000	duck
Hnrnpd	0.13	1.00000000	duck
Ryk	0.27	1.00000000	duck
Rasa2	-0.10	1.00000000	duck
Dgkd	0.25	1.00000000	duck
Stk4	-0.09	1.00000000	duck
Cacna1c	0.10	1.00000000	duck
Fzd4	0.18	1.00000000	duck
Bmpr2	0.07	1.00000000	duck
Xdh	0.19	1.00000000	duck
Ube4b	0.15	1.00000000	duck
Sub1	0.41	1.00000000	duck
Sgms2	0.19	1.00000000	duck
Reep3	0.12	1.00000000	duck
Prex1	0.34	1.00000000	duck
Dusp8	0.46	1.00000000	duck
Sergef	0.59	1.00000000	duck
Fnbp1l	-0.29	1.00000000	duck
Crbn	0.02	1.00000000	duck
Ttc39b	0.42	1.00000000	duck
Cflar	0.04	1.00000000	duck
Dnm3	0.27	1.00000000	duck
Nr1h3	0.12	1.00000000	duck
Arid1b	0.25	1.00000000	duck
Efna2	0.29	1.00000000	duck
Jak1	0.14	1.00000000	duck
Pip5k1b	0.22	1.00000000	duck
Smad6	0.41	1.00000000	duck
Auts2	0.14	1.00000000	duck
Adamtsl2	0.53	1.00000000	duck
Haus8	0.08	1.00000000	duck
Poldip2	0.13	1.00000000	duck

Ttc27	0.41	1.00000000	duck
Klhdc1	0.47	1.00000000	duck
Ostm1	0.09	1.00000000	duck
Jade1	-0.15	1.00000000	duck
B3glct	0.25	1.00000000	duck
Sp4	0.16	1.00000000	duck
Hat1	-0.29	1.00000000	duck
Katnip	0.09	1.00000000	duck
Apbb2	0.35	1.00000000	duck
Ginm1	0.38	1.00000000	duck
Ulk2	0.40	1.00000000	duck
Rnf149	0.17	1.00000000	duck
Gucd1	-0.03	1.00000000	duck
Fam172a	0.61	1.00000000	duck
Actb	0.40	1.00000000	duck
Gab2	0.38	1.00000000	duck
Tcf25	0.28	1.00000000	duck
Cidea	0.17	1.00000000	duck
Foxn2	0.30	1.00000000	duck
Yeats2	0.28	1.00000000	duck
Zfp469	0.51	1.00000000	duck
Amacr	0.11	1.00000000	duck
Gphn	0.28	1.00000000	duck
Ube2i	0.20	1.00000000	duck
Cyrib	0.27	1.00000000	duck
Atp6v0d1	0.05	1.00000000	duck
Crot	0.18	1.00000000	duck
Erbin	-0.08	1.00000000	duck
Rmc1	0.18	1.00000000	duck
Zranb2	-0.10	1.00000000	duck
Alg2	0.21	1.00000000	duck
Sptlc1	0.15	1.00000000	duck
Ccdc71l	0.18	1.00000000	duck
Crtc1	0.13	1.00000000	duck
Dazap2	0.29	1.00000000	duck
Gpat4	0.11	1.00000000	duck
Trappc4	0.18	1.00000000	duck
Pgm2l1	0.56	1.00000000	duck
Phf20	0.04	1.00000000	duck
Adarb1	0.01	1.00000000	duck
Eif4a2	-0.03	1.00000000	duck
Abhd5	0.29	1.00000000	duck
Vti1a	0.22	1.00000000	duck
Rapgef5	0.64	1.00000000	duck
Jmjd1c	-0.14	1.00000000	duck
Gadl1	0.35	1.00000000	duck

Fam102b	0.09	1.00000000	duck
Ube2r2	0.15	1.00000000	duck
Sh3bp2	0.19	1.00000000	duck
Pim3	-0.03	1.00000000	duck
Dab2	0.41	1.00000000	duck
Vta1	0.08	1.00000000	duck
Otulin	0.06	1.00000000	duck
Gda	0.18	1.00000000	duck
Oga	0.01	1.00000000	duck
Elp1	-0.02	1.00000000	duck
Chrdl1	-0.14	1.00000000	duck
Tmed5	0.20	1.00000000	duck
Id4	0.10	1.00000000	duck
Unc119b	0.09	1.00000000	duck
Arl6ip5	0.25	1.00000000	duck
Impa1	0.07	1.00000000	duck
Atp11a	0.43	1.00000000	duck
Robo2	0.60	1.00000000	duck
Ppip5k1	0.03	1.00000000	duck
Cd81	0.15	1.00000000	duck
Cdk12	0.15	1.00000000	duck
Ccnh	0.39	1.00000000	duck
Rnf217	0.19	1.00000000	duck
Lonrf3	0.19	1.00000000	duck
Atp23	0.12	1.00000000	duck
Tra2b	0.13	1.00000000	duck
Hs6st1	0.19	1.00000000	duck
Rfc5	0.02	1.00000000	duck
Cops2	0.21	1.00000000	duck
Actr3	0.20	1.00000000	duck
Lemd2	0.29	1.00000000	duck
Fbxl20	-0.02	1.00000000	duck
Fam32a	0.08	1.00000000	duck
Cnot9	0.29	1.00000000	duck
Comt	0.38	1.00000000	duck
Nol10	0.15	1.00000000	duck
Lsm7	0.01	1.00000000	duck
Ubl7	0.11	1.00000000	duck
Tatdn3	0.07	1.00000000	duck
Foxj2	0.17	1.00000000	duck
Hlf	-0.03	1.00000000	duck
Apba1	0.26	1.00000000	duck
Plin2	0.37	1.00000000	duck
Elf1	0.19	1.00000000	duck
Als2cl	0.66	1.00000000	duck
Phf3	-0.23	1.00000000	duck

Ano5	0.22	1.00000000	duck
Plekha5	0.15	1.00000000	duck
Setd7	0.19	1.00000000	duck
Nfya	0.00	1.00000000	duck
C1qtnf1	0.16	1.00000000	duck
Notch2	0.38	1.00000000	duck
Sod3	0.01	1.00000000	duck
Agtpbp1	0.13	1.00000000	duck
Secisbp2	0.36	1.00000000	duck
Lrp5	0.34	1.00000000	duck
Tatdn1	0.12	1.00000000	duck
Samsn1	0.40	1.00000000	duck
Naaa	0.19	1.00000000	duck
Cables1	0.35	1.00000000	duck
Eef1d	-0.03	1.00000000	duck
Rbfox2	0.11	1.00000000	duck
Dipk1a	0.16	1.00000000	duck
Sorcs2	0.39	1.00000000	duck
Tmem131l	-0.09	1.00000000	duck
Bptf	-0.15	1.00000000	duck
Sdhb	0.11	1.00000000	duck
Cd151	0.23	1.00000000	duck
Trafd1	0.25	1.00000000	duck
Tmem35b	0.21	1.00000000	duck
Ttc33	0.29	1.00000000	duck
Arpp19	0.09	1.00000000	duck
Lmbrd2	0.12	1.00000000	duck
Aplp2	-0.07	1.00000000	duck
Taf2	0.22	1.00000000	duck
Chd7	0.31	1.00000000	duck
Ascc3	0.27	1.00000000	duck
Endov	0.16	1.00000000	duck
Fbxl17	0.52	1.00000000	duck
Ccdc134	0.13	1.00000000	duck
Plxnc1	0.10	1.00000000	duck
Wbp1l	0.42	1.00000000	duck
Exosc10	0.13	1.00000000	duck
Tle1	0.19	1.00000000	duck
Npas2	0.55	1.00000000	duck
Haus2	0.17	1.00000000	duck
9930021J03Rik	0.20	1.00000000	duck
Lipa	0.19	1.00000000	duck
Irak4	0.04	1.00000000	duck
Lamp2	0.00	1.00000000	duck
Prdm10	-0.15	1.00000000	duck
Ino80d	0.19	1.00000000	duck

Egfr	0.30	1.00000000	duck
Hmgxb4	0.23	1.00000000	duck
Soat1	0.44	1.00000000	duck
Hmgcr	0.17	1.00000000	duck
Poc1a	0.31	1.00000000	duck
Sh3bgrl3	0.29	1.00000000	duck
Zfp143	0.19	1.00000000	duck
Yae1d1	0.13	1.00000000	duck
Wdr82	0.25	1.00000000	duck
Slc35b1	0.23	1.00000000	duck
Ica1	0.38	1.00000000	duck
Flvcr2	-0.16	1.00000000	duck
Cmss1	0.31	1.00000000	duck
Aatf	0.09	1.00000000	duck
Pelo	0.21	1.00000000	duck
Rnf4	-0.01	1.00000000	duck
Fzd6	0.13	1.00000000	duck
Snrpd3	0.28	1.00000000	duck
Ttf1	0.17	1.00000000	duck
Ago3	0.17	1.00000000	duck
Kdm4c	0.54	1.00000000	duck
Tpcn1	0.21	1.00000000	duck
Rsrc1	0.22	1.00000000	duck
Ston2	0.36	1.00000000	duck
Eif4g3	-0.01	1.00000000	duck
Tns1	0.05	1.00000000	duck
Rassf8	0.26	1.00000000	duck
Ppwd1	0.18	1.00000000	duck
Arid2	-0.23	1.00000000	duck
Slc38a10	0.32	1.00000000	duck
Fnip1	0.61	1.00000000	duck
Pxk	0.27	1.00000000	duck
Rsbn1l	0.00	1.00000000	duck
Acss2	0.08	1.00000000	duck
Six4	0.13	1.00000000	duck
Smu1	0.20	1.00000000	duck
Naaladl2	0.32	1.00000000	duck
Gas6	0.64	1.00000000	duck
Carnmt1	0.14	1.00000000	duck
Fbxl14	0.15	1.00000000	duck
Supt5	0.50	1.00000000	duck
Rabepk	-0.03	1.00000000	duck
Srp68	0.18	1.00000000	duck
Insr	0.42	1.00000000	duck
Echdc2	0.19	1.00000000	duck
Prkd1	0.29	1.00000000	duck

St3gal2	0.15	1.00000000	duck
Pigg	0.23	1.00000000	duck
Fh1	0.13	1.00000000	duck
Sumo1	0.18	1.00000000	duck
Dcp1a	0.19	1.00000000	duck
Dennd1a	0.11	1.00000000	duck
Ptpn4	-0.22	1.00000000	duck
Gtf2a1	0.09	1.00000000	duck
Runx2	0.26	1.00000000	duck
Parp8	0.09	1.00000000	duck
Rnf185	0.07	1.00000000	duck
Tle3	0.48	1.00000000	duck
Baz2b	0.17	1.00000000	duck
Trmt13	0.12	1.00000000	duck
Bcat1	0.20	1.00000000	duck
Laptm5	0.61	1.00000000	duck
Aldoc	0.09	1.00000000	duck
Ppm1g	0.29	1.00000000	duck
Acsl1	0.45	1.00000000	duck
Rspry1	0.16	1.00000000	duck
Hk2	0.41	1.00000000	duck
Lrrc8c	0.16	1.00000000	duck
Stk16	0.10	1.00000000	duck
Arhgef18	-0.07	1.00000000	duck
H13	0.15	1.00000000	duck
Aox1	0.23	1.00000000	duck
Bnc2	0.31	1.00000000	duck
Bicral	0.30	1.00000000	duck
Slc25a47	0.23	1.00000000	duck
Ammecr1	0.12	1.00000000	duck
Osr2	0.11	1.00000000	duck
Wnt5b	0.14	1.00000000	duck
Ssh1	0.24	1.00000000	duck
Phf2	0.32	1.00000000	duck
Lmf1	0.32	1.00000000	duck
Ppm1f	0.08	1.00000000	duck
Tmem222	0.22	1.00000000	duck
Ada	0.40	1.00000000	duck
Cemip2	0.08	1.00000000	duck
Zc3h7b	0.21	1.00000000	duck
Pgs1	0.08	1.00000000	duck
Chfr	0.18	1.00000000	duck
Coro1c	0.12	1.00000000	duck
Lsm3	0.24	1.00000000	duck
Zfpm1	0.32	1.00000000	duck
Insig2	0.18	1.00000000	duck

Tiam1	0.32	1.00000000	duck
Ppargc1a	0.51	1.00000000	duck
Eps8	0.66	1.00000000	duck
Rnf166	0.12	1.00000000	duck
Strip1	0.04	1.00000000	duck
Yy2	0.36	1.00000000	duck
Zbtb20	-0.02	1.00000000	duck
Ppp1cc	0.25	1.00000000	duck
Exosc3	0.20	1.00000000	duck
Sdk1	0.40	1.00000000	duck
9130409I23Rik	0.20	1.00000000	duck
Foxp2	0.48	1.00000000	duck
Garem1	0.31	1.00000000	duck
Serpine2	0.04	1.00000000	duck
Etv1	0.40	1.00000000	duck
Dmxl1	0.07	1.00000000	duck
Psmb3	0.11	1.00000000	duck
Wdfy2	0.17	1.00000000	duck
Cdc34	0.17	1.00000000	duck
Nkrf	-0.12	1.00000000	duck
Fbxo34	0.47	1.00000000	duck
Tmem30a	0.17	1.00000000	duck
Tmem184b	0.33	1.00000000	duck
Smpd1	0.27	1.00000000	duck
Asns	0.19	1.00000000	duck
Dcp1b	0.15	1.00000000	duck
Supt7l	0.16	1.00000000	duck
Dgkq	0.22	1.00000000	duck
Fam20c	0.34	1.00000000	duck
Rab40c	0.14	1.00000000	duck
Ndufs4	0.50	1.00000000	duck
Pold2	0.11	1.00000000	duck
Slc30a6	0.19	1.00000000	duck
Cdc42bpa	0.24	1.00000000	duck
Arhgef26	0.45	1.00000000	duck
Ror2	0.43	1.00000000	duck
Pomgnt1	0.07	1.00000000	duck
Tut7	0.16	1.00000000	duck
Slc25a36	0.15	1.00000000	duck
Arhgap15	0.79	1.00000000	duck
Snrnp40	0.17	1.00000000	duck
Arpc3	0.28	1.00000000	duck
Arid1a	0.26	1.00000000	duck
Atrn	0.12	1.00000000	duck
Ep300	0.02	1.00000000	duck
Dimt1	0.11	1.00000000	duck

Tenm2	0.49	1.00000000	duck
Nhs	0.37	1.00000000	duck
Zdhhc21	0.31	1.00000000	duck
Brd3	0.21	1.00000000	duck
Nova1	0.48	1.00000000	duck
Nfkb1	0.30	1.00000000	duck
Crebrf	0.07	1.00000000	duck
Sf3b4	0.16	1.00000000	duck
Atp5j	0.15	1.00000000	duck
Itfg2	0.09	1.00000000	duck
Acsl4	-0.02	1.00000000	duck
Tasp1	0.27	1.00000000	duck
Gtf2b	0.39	1.00000000	duck
Nedd9	0.44	1.00000000	duck
Arhgap21	0.20	1.00000000	duck
Gnai2	0.31	1.00000000	duck
Rnf20	0.42	1.00000000	duck
Afg1l	0.47	1.00000000	duck
Ttbk2	0.21	1.00000000	duck
Slf2	0.19	1.00000000	duck
Commd10	0.35	1.00000000	duck
Hivep1	0.17	1.00000000	duck
Ankrd17	0.07	1.00000000	duck
Tra2a	0.12	1.00000000	duck
Zc3h18	0.33	1.00000000	duck
Sh3rf1	0.64	1.00000000	duck
Ube2d1	0.39	1.00000000	duck
Lin52	0.26	1.00000000	duck
Imp3	0.24	1.00000000	duck
Taf4	0.14	1.00000000	duck
Plaa	0.12	1.00000000	duck
Rnf170	0.25	1.00000000	duck
Thumpd2	0.19	1.00000000	duck
Kctd2	0.12	1.00000000	duck
Ago2	0.06	1.00000000	duck
Ankra2	0.41	1.00000000	duck
Cenpu	0.29	1.00000000	duck
Timp3	-0.09	1.00000000	duck
Pum1	0.19	1.00000000	duck
Fosl2	0.23	1.00000000	duck
Armc9	0.01	1.00000000	duck
Zfp407	0.10	1.00000000	duck
Lrch3	0.20	1.00000000	duck
Fubp3	0.22	1.00000000	duck
Nfat5	0.25	1.00000000	duck
Sestd1	0.31	1.00000000	duck

Peli1	0.45	1.00000000	duck
Skil	0.18	1.00000000	duck
Nfkbie	0.07	1.00000000	duck
Fam53a	0.27	1.00000000	duck
Tctn2	0.13	1.00000000	duck
Peli2	0.33	1.00000000	duck
Bin3	0.24	1.00000000	duck
Ar	0.20	1.00000000	duck
Kat7	0.27	1.00000000	duck
Taf15	0.30	1.00000000	duck
Abhd13	0.28	1.00000000	duck
Ssrp1	0.23	1.00000000	duck
Ap3s2	0.18	1.00000000	duck
Adss	0.03	1.00000000	duck
Zbtb44	0.30	1.00000000	duck
Phc2	0.25	1.00000000	duck
Pparg	-0.10	1.00000000	duck
Chst13	0.33	1.00000000	duck
Pde1c	0.35	1.00000000	duck
Pappa	0.36	1.00000000	duck
Bhlhe40	0.41	1.00000000	duck
Trps1	0.24	1.00000000	duck
Niban1	0.41	1.00000000	duck
Utp15	-0.03	1.00000000	duck
Wdr70	0.26	1.00000000	duck
Trappc9	0.29	1.00000000	duck
Gsk3b	0.11	1.00000000	duck
Ncoa3	0.26	1.00000000	duck
Rrbp1	0.20	1.00000000	duck
Foxo1	0.22	1.00000000	duck
Bsdc1	0.22	1.00000000	duck
Nelfcd	0.00	1.00000000	duck
Pcca	0.25	1.00000000	duck
Rfx3	0.13	1.00000000	duck
Cert1	0.42	1.00000000	duck
Nat10	0.14	1.00000000	duck
Stk10	0.31	1.00000000	duck
Hook3	0.25	1.00000000	duck
Pam	0.50	1.00000000	duck
Xpo5	0.17	1.00000000	duck
Fto	0.24	1.00000000	duck
Slc25a22	0.43	1.00000000	duck
Dhx30	0.25	1.00000000	duck
Oser1	0.29	1.00000000	duck
Larp4	0.19	1.00000000	duck
Carf	0.02	1.00000000	duck

Bahd1	0.08	1.00000000	duck
Dbt	0.39	1.00000000	duck
Dgkh	0.10	1.00000000	duck
Cwc27	0.39	1.00000000	duck
Lpcat3	0.34	1.00000000	duck
Zfp622	0.21	1.00000000	duck
Sirt5	0.27	1.00000000	duck
Zhx1	0.30	1.00000000	duck
Pdzrn4	0.46	1.00000000	duck
Tbc1d1	0.39	1.00000000	duck
Gramd1b	0.33	1.00000000	duck
Smg7	0.15	1.00000000	duck
Sun1	0.09	1.00000000	duck
Tasor	0.32	1.00000000	duck
AU040320	-0.06	1.00000000	duck
Xpo7	0.03	1.00000000	duck
Homer1	0.31	1.00000000	duck
Rbm25	0.18	1.00000000	duck
Cdc42se1	0.01	1.00000000	duck
Nsa2	0.19	1.00000000	duck
Farsb	0.16	1.00000000	duck
Bckdhb	0.43	1.00000000	duck
Pip5k1c	0.12	1.00000000	duck
Itpka	0.12	1.00000000	duck
Ptbp3	0.46	1.00000000	duck
Prkce	0.12	1.00000000	duck
Pde5a	0.40	1.00000000	duck
Slc30a5	0.13	1.00000000	duck
Tnfaip8l1	0.27	1.00000000	duck
Luc7l	0.28	1.00000000	duck
Pan3	0.03	1.00000000	duck
Zc3h12a	0.32	1.00000000	duck
Arl15	0.52	1.00000000	duck
Nup93	0.36	1.00000000	duck
Cep192	0.19	1.00000000	duck
Peak1	0.31	1.00000000	duck
Nt5dc1	0.24	1.00000000	duck
Gpn2	0.13	1.00000000	duck
Bcar3	0.06	1.00000000	duck
Akap13	-0.06	1.00000000	duck
Dhx29	0.18	1.00000000	duck
Lrch1	0.40	1.00000000	duck
Retreg1	0.28	1.00000000	duck
Kazn	0.25	1.00000000	duck
Nlrc5	0.33	1.00000000	duck
Chpt1	0.24	1.00000000	duck

Traf1	0.35	1.00000000	duck
Ncstn	0.09	1.00000000	duck
Eif4ebp1	0.07	1.00000000	duck
Polr2h	0.19	1.00000000	duck
Clasp2	0.07	1.00000000	duck
Nop2	0.09	1.00000000	duck
Fndc3a	0.10	1.00000000	duck
Cep83	0.12	1.00000000	duck
Vps41	0.13	1.00000000	duck
Acaa1b	0.09	1.00000000	duck
Zfp292	0.27	1.00000000	duck
Zfp521	0.22	1.00000000	duck
Snx25	0.11	1.00000000	duck
Dennd11	0.21	1.00000000	duck
Phf5a	0.16	1.00000000	duck
Slc25a44	0.17	1.00000000	duck
Zfp131	0.12	1.00000000	duck
Cbx8	0.03	1.00000000	duck
Adipor1	0.26	1.00000000	duck
Rbks	0.00	1.00000000	duck
Ninj1	0.18	1.00000000	duck
Bcas3	0.18	1.00000000	duck
Ran	0.21	1.00000000	duck
Parvg	0.25	1.00000000	duck
Srgap3	0.38	1.00000000	duck
Tomm7	0.27	1.00000000	duck
Zcchc17	0.26	1.00000000	duck
AF366264	0.15	1.00000000	duck
Ube2h	0.00	1.00000000	duck
Pik3c2a	0.08	1.00000000	duck
Tmem230	0.33	1.00000000	duck
Hspb8	0.57	1.00000000	duck
Slc10a7	0.09	1.00000000	duck
Camkmt	0.26	1.00000000	duck
Eri3	0.16	1.00000000	duck
Syn2	0.24	1.00000000	duck
Smyd3	0.03	1.00000000	duck
Adamts9	0.69	1.00000000	duck
Pex5	0.09	1.00000000	duck
Pradc1	0.18	1.00000000	duck
Lpgat1	0.38	1.00000000	duck
Lrrc39	0.76	1.00000000	duck
Arhgap29	0.04	1.00000000	duck
Foxo4	0.14	1.00000000	duck
Xxylt1	0.26	1.00000000	duck
Rassf9	0.62	1.00000000	duck

Elavl1	0.22	1.00000000	duck
Scaper	0.20	1.00000000	duck
Ints4	0.17	1.00000000	duck
Pef1	0.20	1.00000000	duck
Exoc6b	0.25	1.00000000	duck
Zfand3	0.23	1.00000000	duck
Csgalnact2	0.23	1.00000000	duck
Pou2f1	0.40	1.00000000	duck
Srek1ip1	0.29	1.00000000	duck
Saraf	0.20	1.00000000	duck
Trib2	0.30	1.00000000	duck
Gnb4	0.05	1.00000000	duck
Ppa1	0.24	1.00000000	duck
Riok3	0.28	1.00000000	duck
Bri3	0.24	1.00000000	duck
Usp53	0.24	1.00000000	duck
R3hdm1	0.16	1.00000000	duck
Reps2	0.18	1.00000000	duck
L3mbtl3	0.40	1.00000000	duck
Jund	0.05	1.00000000	duck
Wnk1	0.01	1.00000000	duck
Plekhh2	0.36	1.00000000	duck
Nr1d2	0.27	1.00000000	duck
Carmil1	0.47	1.00000000	duck
Gnb1	0.14	1.00000000	duck
Ift74	0.21	1.00000000	duck
Polg2	0.32	1.00000000	duck
Ttc39c	0.07	1.00000000	duck
Sppl3	0.31	1.00000000	duck
Cbfb	0.49	1.00000000	duck
Cdh13	0.28	1.00000000	duck
Invs	0.44	1.00000000	duck
Prdm15	0.23	1.00000000	duck
Polr1e	0.25	1.00000000	duck
Cnot2	0.20	1.00000000	duck
Brd1	0.26	1.00000000	duck
Adcy5	0.81	1.00000000	duck
Taldo1	0.20	1.00000000	duck
Zyx	0.33	1.00000000	duck
Yaf2	0.36	1.00000000	duck
Ephx1	0.18	1.00000000	duck
Lemd3	0.31	1.00000000	duck
Prkn	0.30	1.00000000	duck
Pcyox1	0.22	1.00000000	duck
Zswim5	-0.05	1.00000000	duck
Cdkn1a	0.22	1.00000000	duck

Fxn	0.33	1.00000000	duck
Oxtr	0.43	1.00000000	duck
Raver2	0.18	1.00000000	duck
Chordc1	0.29	1.00000000	duck
Abcc5	0.31	1.00000000	duck
Paip1	0.34	1.00000000	duck
Klf12	0.10	1.00000000	duck
Rif1	0.08	1.00000000	duck
Rragc	0.19	1.00000000	duck
Ptpn1	0.39	1.00000000	duck
Rbpj	0.37	1.00000000	duck
Tmem9b	0.19	1.00000000	duck
Tbl1xr1	0.07	1.00000000	duck
Spag1	0.09	1.00000000	duck
Rai2	0.40	1.00000000	duck
Arpc5	0.32	1.00000000	duck
Sla	-0.04	1.00000000	duck
Zeb1	0.42	1.00000000	duck
Rasa1	0.05	1.00000000	duck
Kif13b	0.37	1.00000000	duck
Kdm1b	-0.07	1.00000000	duck
Fbxl7	0.24	1.00000000	duck
Zdhhc17	0.40	1.00000000	duck
Ndufaf2	-0.02	1.00000000	duck
Gpx8	0.22	1.00000000	duck
Lrrcc1	0.16	1.00000000	duck
Pink1	0.19	1.00000000	duck
Pik3ap1	0.28	1.00000000	duck
Rictor	0.08	1.00000000	duck
Gab1	0.37	1.00000000	duck
Nr2c2	0.13	1.00000000	duck
Nrip1	0.60	1.00000000	duck
Ssu72	0.15	1.00000000	duck
Ell2	-0.21	1.00000000	duck
Tmem241	0.22	1.00000000	duck
St3gal3	-0.17	1.00000000	duck
Ube2b	0.29	1.00000000	duck
Tmem250-ps	0.25	1.00000000	duck
Zfat	0.24	1.00000000	duck
Pik3c3	0.33	1.00000000	duck
Trappc12	0.19	1.00000000	duck
Pdxk	0.31	1.00000000	duck
Prkd3	0.44	1.00000000	duck
Atp6v1h	0.19	1.00000000	duck
Cask	0.24	1.00000000	duck
Akirin2	0.05	1.00000000	duck

Emsy	0.38	1.00000000	duck
Rbm31y	0.07	1.00000000	duck
Etv3	0.25	1.00000000	duck
Rpain	0.25	1.00000000	duck
Arhgef3	0.38	1.00000000	duck
Hsd17b12	0.15	1.00000000	duck
Rpa3	0.26	1.00000000	duck
Tmem218	0.28	1.00000000	duck
Cd74	0.13	1.00000000	duck
Islr	0.34	1.00000000	duck
Hmbox1	0.36	1.00000000	duck
Taf13	0.29	1.00000000	duck
Timm8a2	0.21	1.00000000	duck
Cacnb2	0.67	1.00000000	duck
Usp20	0.29	1.00000000	duck
Traf7	0.13	1.00000000	duck
Zfp462	0.48	1.00000000	duck
Fam174b	0.52	1.00000000	duck
Icmt	0.13	1.00000000	duck
Pde4b	0.05	1.00000000	duck
Lsm8	0.27	1.00000000	duck
Cdc42se2	0.17	1.00000000	duck
Sat1	0.12	1.00000000	duck
Cacna1i	0.23	1.00000000	duck
Itgb5	0.26	1.00000000	duck
Thsd7a	0.44	1.00000000	duck
Dop1b	0.21	1.00000000	duck
Arid4b	0.23	1.00000000	duck
Rai14	0.87	1.00000000	duck
Cep126	0.43	1.00000000	duck
Trak1	0.37	1.00000000	duck
Lyrm7	0.19	1.00000000	duck
St3gal1	0.49	1.00000000	duck
Fer	0.22	1.00000000	duck
Fli1	0.24	1.00000000	duck
Emg1	0.19	1.00000000	duck
Elp3	0.37	1.00000000	duck
Btbd9	-0.27	1.00000000	duck
Fem1a	0.40	1.00000000	duck
Cdv3	0.11	1.00000000	duck
Psmb2	0.33	1.00000000	duck
Fgfrl1	0.05	1.00000000	duck
Ptbp2	0.31	1.00000000	duck
Tmem164	0.24	1.00000000	duck
Arhgap24	0.18	1.00000000	duck
Traf2	0.39	1.00000000	duck

Il6ra	0.14	1.00000000	duck
Ctps	0.14	1.00000000	duck
Ackr4	0.62	1.00000000	duck
Amd2	0.18	1.00000000	duck
Rfc1	0.44	1.00000000	duck
Ppp6r2	0.10	1.00000000	duck
Rnf122	0.40	1.00000000	duck
Tbx4	0.37	1.00000000	duck
Nsmaf	0.01	1.00000000	duck
Adamts6	0.20	1.00000000	duck
Cops4	0.39	1.00000000	duck
Zbtb10	0.30	1.00000000	duck
Spry1	0.30	1.00000000	duck
Chp1	0.34	1.00000000	duck
Ash1l	0.06	1.00000000	duck
Cop1	0.26	1.00000000	duck
Gfpt2	0.96	1.00000000	duck
Nup155	0.32	1.00000000	duck
Jun	0.15	1.00000000	duck
5031439G07Rik	0.40	1.00000000	duck
Trub1	0.25	1.00000000	duck
Itgae	0.34	1.00000000	duck
Pik3cb	0.41	1.00000000	duck
Hipk3	0.36	1.00000000	duck
C9	0.44	1.00000000	duck
Arntl2	0.33	1.00000000	duck
Fam117b	0.37	1.00000000	duck
Ip6k2	0.26	1.00000000	duck
Hfm1	0.48	1.00000000	duck
Btbd11	-0.06	1.00000000	duck
Usp47	0.22	1.00000000	duck
Samd4	-0.05	1.00000000	duck
Supt3	-0.02	1.00000000	duck
Inpp5a	0.04	1.00000000	duck
Ube2j1	0.26	1.00000000	duck
Pinx1	0.40	1.00000000	duck
Babam2	0.15	1.00000000	duck
Hbp1	0.24	1.00000000	duck
Lsp1	0.20	1.00000000	duck
Dcaf10	0.18	1.00000000	duck
Nabp1	0.38	1.00000000	duck
Actn1	0.37	1.00000000	duck
Rspo3	0.24	1.00000000	duck
Hbegf	0.17	1.00000000	duck
Sox5	0.30	1.00000000	duck
Pqlc3	0.37	1.00000000	duck

Adar	0.08	1.00000000	duck
1700034I23Rik	0.28	1.00000000	duck
Usp10	0.12	1.00000000	duck
Wdr37	0.43	1.00000000	duck
Casp3	0.21	1.00000000	duck
Gga1	0.20	1.00000000	duck
Nr4a2	0.27	1.00000000	duck
Polr2d	0.25	1.00000000	duck
Ednrb	0.46	1.00000000	duck
Etv6	0.31	1.00000000	duck
Cradd	0.17	1.00000000	duck
Tubgcp3	0.25	1.00000000	duck
Tulp4	0.38	1.00000000	duck
Gnas	0.29	1.00000000	duck
Apoo	0.19	1.00000000	duck
Stx17	0.41	1.00000000	duck
Hsph1	1.07	1.00000000	duck
Ubn2	0.35	1.00000000	duck
Arpc1b	0.45	1.00000000	duck
Dusp1	0.05	1.00000000	duck
Cops7b	0.28	1.00000000	duck
Cbx7	0.43	1.00000000	duck
Tut4	0.35	1.00000000	duck
Dnajb9	0.23	1.00000000	duck
Srp19	0.29	1.00000000	duck
Pdlim5	0.49	1.00000000	duck
Stard13	0.42	1.00000000	duck
Ereg	0.04	1.00000000	duck
Siva1	0.40	1.00000000	duck
Tor4a	0.13	1.00000000	duck
Pard6b	0.30	1.00000000	duck
Tmem140	0.27	1.00000000	duck
Foxc1	0.30	1.00000000	duck
Bbs12	0.21	1.00000000	duck
Tpmt	0.16	1.00000000	duck
Kif21a	0.26	1.00000000	duck
Ecpas	0.29	1.00000000	duck
Tanc1	0.18	1.00000000	duck
Syngr2	0.16	1.00000000	duck
Ostf1	0.11	1.00000000	duck
Sgms1	0.20	1.00000000	duck
Cbx4	0.31	1.00000000	duck
Gab3	0.32	1.00000000	duck
Ankrd10	0.19	1.00000000	duck
Susd3	0.17	1.00000000	duck
Pwp1	0.21	1.00000000	duck

Amfr	0.29	1.00000000	duck
Plk2	0.26	1.00000000	duck
Prrg3	0.19	1.00000000	duck
Il1rl1	0.35	1.00000000	duck
Baiap2l1	0.32	1.00000000	duck
Glis2	0.27	1.00000000	duck
Dusp10	0.42	1.00000000	duck
Rassf2	0.13	1.00000000	duck
Dcaf12	0.14	1.00000000	duck
Ptpre	0.28	1.00000000	duck
Ncbp1	0.13	1.00000000	duck
Pak3	0.44	1.00000000	duck
Abhd2	0.19	1.00000000	duck
Tomm22	0.34	1.00000000	duck
Ppm1e	0.23	1.00000000	duck
Rnf19b	0.45	1.00000000	duck
Spon1	0.45	1.00000000	duck
Upf1	0.27	1.00000000	duck
Efna5	0.18	1.00000000	duck
Dnajc21	-0.05	1.00000000	duck
Nek7	0.30	1.00000000	duck
Zcchc14	0.51	1.00000000	duck
Dhx35	0.16	1.00000000	duck
Zfp618	0.23	1.00000000	duck
Ranbp17	0.28	1.00000000	duck
Wwox	0.34	1.00000000	duck
Zfp516	0.46	1.00000000	duck
Nme7	0.37	1.00000000	duck
Dusp5	0.24	1.00000000	duck
Atxn7	0.19	1.00000000	duck
G0s2	0.22	1.00000000	duck
Hmg20a	0.23	1.00000000	duck
Nsmce2	0.38	1.00000000	duck
Ttc19	0.22	1.00000000	duck
Ctif	0.29	1.00000000	duck
Ttc37	0.12	1.00000000	duck
Psmf1	0.10	1.00000000	duck
Abtb1	0.13	1.00000000	duck
Ppie	0.23	1.00000000	duck
Ubqln4	0.27	1.00000000	duck
Fstl3	0.47	1.00000000	duck
Smurf2	0.35	1.00000000	duck
Qk	0.10	1.00000000	duck
Rnf11	0.16	1.00000000	duck
Pum2	0.10	1.00000000	duck
Wdfy3	0.18	1.00000000	duck

Srbd1	0.26	1.00000000	duck
Nudt21	0.27	1.00000000	duck
Crem	0.08	1.00000000	duck
Gkap1	0.20	1.00000000	duck
Hnrnpm	0.16	1.00000000	duck
Aftph	0.24	1.00000000	duck
Ptgfr	0.50	1.00000000	duck
Zfyve28	0.31	1.00000000	duck
Cdkn1b	0.25	1.00000000	duck
Ensa	0.38	1.00000000	duck
Irs2	0.26	1.00000000	duck
Dapp1	0.06	1.00000000	duck
Rfc4	0.16	1.00000000	duck
Skap2	0.32	1.00000000	duck
Diablo	0.22	1.00000000	duck
Cachd1	0.49	1.00000000	duck
Nucks1	0.14	1.00000000	duck
2700049A03Rik	0.07	1.00000000	duck
Syne3	0.32	1.00000000	duck
Ncoa1	0.48	1.00000000	duck
Tmem106b	0.42	1.00000000	duck
Slc9a7	0.34	1.00000000	duck
Jarid2	0.27	1.00000000	duck
Plxnb2	0.28	1.00000000	duck
Zfyve26	0.12	1.00000000	duck
Sacm1l	0.31	1.00000000	duck
Tns3	0.31	1.00000000	duck
Atp13a3	0.37	1.00000000	duck
Slc2a8	0.15	1.00000000	duck
Gpr137c	0.35	1.00000000	duck
Lbr	0.00	1.00000000	duck
Tent5b	0.28	1.00000000	duck
Lamp1	0.15	1.00000000	duck
Ankrd28	0.16	1.00000000	duck
Sh3bgrl	0.45	1.00000000	duck
Tnrc6c	0.22	1.00000000	duck
Itga1	0.23	1.00000000	duck
Epha7	0.33	1.00000000	duck
Gpr63	0.30	1.00000000	duck
Npr3	0.51	1.00000000	duck
Vapa	0.29	1.00000000	duck
Csrp1	0.45	1.00000000	duck
Htra1	0.07	1.00000000	duck
Trim8	0.33	1.00000000	duck
Atp2b2	0.36	1.00000000	duck
Ski	0.07	1.00000000	duck

E2f6	0.18	1.00000000	duck
Nupl1	0.26	1.00000000	duck
Pdrg1	0.12	1.00000000	duck
Tcf21	0.27	1.00000000	duck
Rnaseh2b	0.19	1.00000000	duck
B3gntl1	0.36	1.00000000	duck
Fndc3b	0.26	1.00000000	duck
Gpr137b	0.24	1.00000000	duck
Rab2a	0.20	1.00000000	duck
Stx8	0.23	1.00000000	duck
Acss1	0.23	1.00000000	duck
Fbxl4	0.14	1.00000000	duck
Otud7a	0.35	1.00000000	duck
Fam13c	0.40	1.00000000	duck
Edem1	0.42	1.00000000	duck
Ube3c	0.29	1.00000000	duck
Pigl	0.31	1.00000000	duck
Pdlim3	0.24	1.00000000	duck
Lrp3	-0.03	1.00000000	duck
Rab27a	0.22	1.00000000	duck
Eif5	-0.03	1.00000000	duck
Tmeff2	0.41	1.00000000	duck
Ero1a	0.26	1.00000000	duck
Naa35	0.25	1.00000000	duck
Abca3	0.36	1.00000000	duck
Pde1a	0.24	1.00000000	duck
Capn6	0.25	1.00000000	duck
Psmd5	0.25	1.00000000	duck
Slc1a2	0.45	1.00000000	duck
Dusp7	0.28	1.00000000	duck
Ccdc171	0.32	1.00000000	duck
Fam214a	0.48	1.00000000	duck
Phc3	0.24	1.00000000	duck
Dynlrb1	0.30	1.00000000	duck
Abcg1	0.56	1.00000000	duck
Ypel1	0.34	1.00000000	duck
Tmem123	0.13	1.00000000	duck
Slc25a29	0.26	1.00000000	duck
Slf1	0.25	1.00000000	duck
Nyx	0.41	1.00000000	duck
Lsm4	0.12	1.00000000	duck
Cmtr1	-0.09	1.00000000	duck
Atp6v1a	0.17	1.00000000	duck
Caap1	0.17	1.00000000	duck
B4galt1	0.41	1.00000000	duck
Suclg2	0.40	1.00000000	duck

Zc3h3	0.31	1.00000000	duck
Rassf3	0.30	1.00000000	duck
Psmd2	0.27	1.00000000	duck
Zc3h12b	0.29	1.00000000	duck
Wmp	0.32	1.00000000	duck
Chka	0.45	1.00000000	duck
Synrg	0.38	1.00000000	duck
Itsn1	0.43	1.00000000	duck
Foxc2	0.21	1.00000000	duck
Tspan14	0.36	1.00000000	duck
Prkag2	0.43	1.00000000	duck
Atg2b	0.37	1.00000000	duck
Nudt3	0.21	1.00000000	duck
Rtca	0.28	1.00000000	duck
Fem1c	0.28	1.00000000	duck
Efnb2	0.45	1.00000000	duck
Slc1a4	0.40	1.00000000	duck
Lyn	0.37	1.00000000	duck
Tmem161b	0.24	1.00000000	duck
Smad5	0.42	1.00000000	duck
Hells	0.18	1.00000000	duck
Usp44	0.16	1.00000000	duck
Cry2	0.29	1.00000000	duck
Paqr7	0.30	1.00000000	duck
Tmem59	0.26	1.00000000	duck
Ubl5b	0.28	1.00000000	duck
Epb41l5	0.38	1.00000000	duck
Smarca2	0.24	1.00000000	duck
Cept1	0.37	1.00000000	duck
Npl	0.26	1.00000000	duck
Trdmt1	0.15	1.00000000	duck
Dapk1	0.06	1.00000000	duck
Acvr2a	0.46	1.00000000	duck
App	2.95	0.00000000	pigeons
Tpt1	4.86	0.00000000	pigeons
Rhob	3.10	0.00000000	pigeons
Rab33a	5.23	0.00000000	pigeons
Ccn1	5.47	0.00000000	pigeons
Cnbp	2.44	0.00000000	pigeons
Pcdhgb4	2.73	0.00000000	pigeons
Txnip	4.35	0.00000000	pigeons
Prelp	2.49	0.00000000	pigeons
Sap18	2.10	0.00000000	pigeons
Pcf11	2.23	0.00000000	pigeons
Sgk1	2.53	0.00000000	pigeons
Gadd45b	2.81	0.00000000	pigeons

Sdcbp	1.90	0.00000000	pigeons
Atp5b	1.74	0.00000000	pigeons
Phip	2.19	0.00000000	pigeons
Atp5g3	1.72	0.00000000	pigeons
Tmbim1	1.64	0.00000000	pigeons
Arpc2	1.76	0.00000000	pigeons
U2surp	1.69	0.00000000	pigeons
Lpp	1.86	0.00000000	pigeons
Dido1	1.67	0.00000000	pigeons
Vim	2.37	0.00000000	pigeons
Eprs	1.58	0.00000000	pigeons
Adnp	1.48	0.00000000	pigeons
Oga	1.53	0.00000000	pigeons
Cilp	1.98	0.00000000	pigeons
Cdc37	1.42	0.00000000	pigeons
Wtap	1.52	0.00000000	pigeons
Syncrip	1.53	0.00000000	pigeons
Anapc15-ps	1.33	0.00000000	pigeons
Atf7ip	1.87	0.00000000	pigeons
Pomp	1.29	0.00000000	pigeons
Rtf2	1.19	0.00000000	pigeons
Ankhd1	1.30	0.00000000	pigeons
Trpc4ap	1.31	0.00000000	pigeons
Slirp	1.19	0.00000000	pigeons
Cyria	1.77	0.00000000	pigeons
Nasp	1.48	0.00000000	pigeons
Nfic	1.14	0.00000000	pigeons
Cmip	1.71	0.00000000	pigeons
Gnai1	1.39	0.00000000	pigeons
Pcnp	1.29	0.00000000	pigeons
Ahsa2	1.21	0.00000000	pigeons
Ccdc25	1.20	0.00000000	pigeons
Wtip	1.48	0.00000000	pigeons
Vcp	1.11	0.00000000	pigeons
Cd2ap	1.38	0.00000000	pigeons
Nfib	1.30	0.00000000	pigeons
Rnf103	1.20	0.00000000	pigeons
Cars	1.23	0.00000000	pigeons
Arrdc2	1.75	0.00000000	pigeons
Lsamp	1.91	0.00000000	pigeons
Crip2	1.12	0.00000000	pigeons
Tardbp	1.11	0.00000000	pigeons
Dab2ip	1.20	0.00000000	pigeons
Rnf38	1.51	0.00000000	pigeons
Cacybp	1.09	0.00000000	pigeons
Tgfbr1	1.16	0.00000000	pigeons

Ndufa1	1.13	0.00000000	pigeons
Strap	1.08	0.00000000	pigeons
Tollip	1.02	0.00000000	pigeons
Rin2	1.16	0.00000000	pigeons
Pfkp	1.09	0.00000000	pigeons
C1s1	1.65	0.00000000	pigeons
Enox2	0.99	0.00000000	pigeons
Fkbp5	1.75	0.00000000	pigeons
Brd8	0.97	0.00000000	pigeons
Rad23b	0.97	0.00000000	pigeons
C1qbp	0.92	0.00000000	pigeons
Pfkfb1	1.09	0.00000000	pigeons
Atp5k	0.96	0.00000000	pigeons
Galnt15	1.86	0.00000000	pigeons
Lnpep	1.18	0.00000000	pigeons
Plscr1	1.10	0.00000000	pigeons
Golm2	0.93	0.00000000	pigeons
Trrap	0.91	0.00000000	pigeons
Zdhhc20	1.02	0.00000000	pigeons
Sobp	1.00	0.00000000	pigeons
Sdhc	0.88	0.00000000	pigeons
Adss	1.17	0.00000000	pigeons
Crispld2	2.70	0.00000000	pigeons
Tent5a	1.11	0.00000000	pigeons
Slmap	0.89	0.00000000	pigeons
Tdpoz8	0.87	0.00000000	pigeons
Eif4b	0.88	0.00000000	pigeons
Apip	0.94	0.00000000	pigeons
Il1rap	1.22	0.00000000	pigeons
Il1r1	1.05	0.00000000	pigeons
Phax	0.84	0.00000000	pigeons
Ccz1	0.96	0.00000000	pigeons
Atp5c1	0.85	0.00000000	pigeons
Acvr2b	1.31	0.00000000	pigeons
Sarnp	0.93	0.00000000	pigeons
Fbxl21	0.85	0.00000000	pigeons
Bri3bp	0.83	0.00000000	pigeons
Crtap	0.84	0.00000000	pigeons
Rgs2	1.57	0.00000000	pigeons
Cklf	0.90	0.00000000	pigeons
Snrpd2	0.76	0.00000000	pigeons
Sfswap	0.77	0.00000000	pigeons
Trpv2	1.11	0.00000000	pigeons
Kifbp	0.76	0.00000000	pigeons
Ivns1abp	0.93	0.00000000	pigeons
Kars	0.86	0.00000000	pigeons

Prep	1.11	0.00000000	pigeons
LTO1	0.85	0.00000000	pigeons
Spcs1	0.75	0.00000000	pigeons
Ubtf	0.88	0.00000000	pigeons
Il1b	1.95	0.00000000	pigeons
Nars	0.79	0.00000000	pigeons
Prnp	0.71	0.00000000	pigeons
Cul4a	0.93	0.00000000	pigeons
Slc12a7	0.97	0.00000000	pigeons
Nop56	0.72	0.00000000	pigeons
Sec24d	0.77	0.00000000	pigeons
Blcap	0.70	0.00000000	pigeons
Lhfpl5	0.82	0.00000000	pigeons
Sap30bp	0.85	0.00000000	pigeons
Tmeff2	1.65	0.00000000	pigeons
Apoa1	2.78	0.00000000	pigeons
Calu	0.69	0.00000000	pigeons
Cbx7	1.18	0.00000000	pigeons
Sec23ip	0.70	0.00000000	pigeons
Tppp	0.83	0.00000000	pigeons
Antkmt	0.58	0.00000000	pigeons
Svep1	1.49	0.00000000	pigeons
Epas1	-2.04	0.00000000	pigeons
Aktip	0.66	0.00000000	pigeons
Csnk1d	0.62	0.00000000	pigeons
Nanp	0.73	0.00000000	pigeons
Gypc	0.81	0.00000000	pigeons
Sinhcaf	0.66	0.00000000	pigeons
Polr1f	0.68	0.00000000	pigeons
4932411N23Rik	-3.21	0.00000000	pigeons
Rybp	0.66	0.00000000	pigeons
Cdkn2aip	0.62	0.00000000	pigeons
Gcdh	0.55	0.00000000	pigeons
Pecam1	-2.36	0.00000000	pigeons
Tbp	0.54	0.00000000	pigeons
Plcg1	0.59	0.00000000	pigeons
Setbp1	1.60	0.00000000	pigeons
Rbmx2	0.65	0.00000000	pigeons
Tiparp	0.83	0.00000000	pigeons
Ptprm	-1.76	0.00000000	pigeons
Sertad4	1.00	0.00000000	pigeons
Gars	0.60	0.00000000	pigeons
Eapp	0.59	0.00000000	pigeons
Jup	0.65	0.00000000	pigeons
Tars	0.60	0.00000000	pigeons
Ptgdr	0.59	0.00000000	pigeons

Dars	0.73	0.00000000	pigeons
Apmap	0.62	0.00000000	pigeons
Rars	0.62	0.00000000	pigeons
C7	1.64	0.00000000	pigeons
Kdm3a	0.61	0.00000000	pigeons
Timp4	2.10	0.00000000	pigeons
Dapl1	0.62	0.00000000	pigeons
Bccip	0.54	0.00000000	pigeons
Hecw2	-3.18	0.00000000	pigeons
Ezr	0.71	0.00000000	pigeons
Terf2ip	0.57	0.00000000	pigeons
Glrx3	0.52	0.00000000	pigeons
Aamp	0.48	0.00000000	pigeons
Bcl6	1.11	0.00000000	pigeons
Rasal2	-2.45	0.00000000	pigeons
Calcrl	-2.61	0.00000000	pigeons
Naa20	0.54	0.00000000	pigeons
Gskip	0.51	0.00000000	pigeons
Cpne8	0.93	0.00000000	pigeons
Pdgfra	1.34	0.00000000	pigeons
Atp5j2	0.53	0.00000000	pigeons
Plxdc2	1.50	0.00000000	pigeons
Lrp6	1.14	0.00000000	pigeons
Banp	0.73	0.00000000	pigeons
Paip2	0.45	0.00000000	pigeons
Edem2	0.51	0.00000000	pigeons
Tnfaip2	1.17	0.00000000	pigeons
Rxylt1	0.51	0.00000000	pigeons
Idh3b	0.57	0.00000000	pigeons
Ginm1	0.92	0.00000000	pigeons
Rapgef5	-1.98	0.00000000	pigeons
Zfp777	0.51	0.00000000	pigeons
Tut7	0.48	0.00000000	pigeons
Porcn	0.42	0.00000000	pigeons
Asb8	0.44	0.00000000	pigeons
Dedd	0.49	0.00000000	pigeons
Dsel	0.91	0.00000000	pigeons
Dact1	1.09	0.00000000	pigeons
Cracr2b	1.22	0.00000000	pigeons
Igfbp4	1.02	0.00000000	pigeons
Trim28	0.51	0.00000000	pigeons
Adgrf5	-2.53	0.00000000	pigeons
Adgrl4	-2.41	0.00000000	pigeons
Polr2i	0.44	0.00000000	pigeons
Fli1	-2.42	0.00000000	pigeons
Galnt16	0.73	0.00000000	pigeons

Aldh1a3	0.98	0.00000000	pigeons
Prkce	-2.12	0.00000000	pigeons
Ada	0.78	0.00000000	pigeons
Fgf10	0.96	0.00000000	pigeons
Psme3ip1	0.34	0.00000000	pigeons
Cd3g	0.92	0.00000000	pigeons
Gas7	0.90	0.00000000	pigeons
Slit2	1.28	0.00000000	pigeons
Notch2	0.94	0.00000000	pigeons
Sqstm1	1.22	0.00000000	pigeons
Lpin1	0.82	0.00000000	pigeons
Ikbip	0.45	0.00000000	pigeons
Gpat3	0.55	0.00000000	pigeons
Polr1d	0.51	0.00000000	pigeons
Niban1	0.90	0.00000000	pigeons
Aars	0.37	0.00000000	pigeons
Plod2	0.80	0.00000000	pigeons
Ptpn13	0.98	0.00000000	pigeons
Htra1	1.24	0.00000000	pigeons
Etv1	0.78	0.00000000	pigeons
Ranbp9	1.14	0.00000000	pigeons
Slc43a2	0.95	0.00000000	pigeons
Il1rl1	1.04	0.00000000	pigeons
Vopp1	0.84	0.00000000	pigeons
Fbh1	0.45	0.00000000	pigeons
Aip	0.42	0.00000000	pigeons
Spry1	1.32	0.00000000	pigeons
Bicc1	1.02	0.00000000	pigeons
Pid1	0.93	0.00000000	pigeons
Cacnb2	1.21	0.00000000	pigeons
Fzd7	0.73	0.00000000	pigeons
Ldb2	-1.78	0.00000000	pigeons
Gsk3b	-1.92	0.00000000	pigeons
Ccdc90b	0.45	0.00000000	pigeons
Tent4a	0.51	0.00000000	pigeons
Riok2	0.39	0.00000000	pigeons
Daam1	1.14	0.00000000	pigeons
Sybu	0.94	0.00000000	pigeons
Kdelr3	0.61	0.00000000	pigeons
Ston1	0.78	0.00000000	pigeons
Plekhh2	0.79	0.00000000	pigeons
C1ra	0.94	0.00000000	pigeons
Ptprb	-2.28	0.00000000	pigeons
Sox6	-2.50	0.00000000	pigeons
Cenpp	0.63	0.00000000	pigeons
Vegfd	0.85	0.00000000	pigeons

Negr1	1.26	0.00000000	pigeons
Nr2f2	1.23	0.00000000	pigeons
Slc25a17	0.38	0.00000000	pigeons
Dtd1	0.43	0.00000000	pigeons
Inpp4b	1.03	0.00000000	pigeons
Sptlc2	0.76	0.00000000	pigeons
Glul	1.19	0.00000000	pigeons
Plxnb2	0.97	0.00000000	pigeons
Grb10	1.00	0.00000000	pigeons
Crim1	-1.52	0.00000000	pigeons
Cog3	0.32	0.00000000	pigeons
Utp25	0.37	0.00000000	pigeons
Slc27a6	0.45	0.00000000	pigeons
Cinp	0.45	0.00000000	pigeons
Dctn2	0.51	0.00000000	pigeons
Arhgef9	-2.63	0.00000000	pigeons
Ctnnal1	0.54	0.00000000	pigeons
Hbp1	0.59	0.00000000	pigeons
Sytl3	0.60	0.00000000	pigeons
Eif2b4	0.33	0.00000000	pigeons
Hhip	0.60	0.00000000	pigeons
Rxra	0.75	0.00000000	pigeons
Akap7	0.83	0.00000000	pigeons
Edil3	0.62	0.00000000	pigeons
Sun2	0.69	0.00000000	pigeons
Dap	0.53	0.00000000	pigeons
Ciao3	0.36	0.00000000	pigeons
Crcp	0.34	0.00000000	pigeons
Clmp	0.36	0.00000000	pigeons
Plekha5	0.94	0.00000000	pigeons
Ddr2	1.25	0.00000000	pigeons
Sdk1	-2.48	0.00000000	pigeons
Emilin2	1.10	0.00000000	pigeons
Zcchc24	1.08	0.00000000	pigeons
Fgfrl1	1.11	0.00000000	pigeons
Nfasc	0.67	0.00000000	pigeons
Ttpa	0.66	0.00000000	pigeons
Pdlim5	0.51	0.00000000	pigeons
Angptl1	0.94	0.00000000	pigeons
Khdc4	0.34	0.00000000	pigeons
Arl14ep	0.47	0.00000000	pigeons
Rnpep	0.32	0.00000000	pigeons
Podxl	-2.20	0.00000000	pigeons
Tjp1	-1.72	0.00000000	pigeons
Islr	0.68	0.00000000	pigeons
Adamts5	0.80	0.00000000	pigeons

Sema3c	1.05	0.00000000	pigeons
Pkd1	0.65	0.00000000	pigeons
Dock9	-2.15	0.00000000	pigeons
Cd74	-1.39	0.00000000	pigeons
Rgcc	-2.42	0.00000000	pigeons
Ank2	1.04	0.00000000	pigeons
Fbln5	0.97	0.00000000	pigeons
Greb1l	0.92	0.00000000	pigeons
Brap	0.38	0.00000000	pigeons
Scrn2	0.31	0.00000000	pigeons
Psmc3ip	0.40	0.00000000	pigeons
Tbx4	0.88	0.00000000	pigeons
Kcne3	-2.43	0.00000000	pigeons
Sox5	0.95	0.00000000	pigeons
Hgf	0.87	0.00000000	pigeons
Pdgfd	0.99	0.00000000	pigeons
Fbxl3	0.53	0.00000000	pigeons
Net1	1.04	0.00000000	pigeons
Lamp2	0.61	0.00000000	pigeons
Slc1a4	0.81	0.00000000	pigeons
Egfr	0.82	0.00000000	pigeons
Lonrf3	0.77	0.00000000	pigeons
Nhs	0.62	0.00000000	pigeons
Otud3	0.67	0.00000000	pigeons
Uap1	0.88	0.00000000	pigeons
Gpt2	0.45	0.00000000	pigeons
Klf15	0.67	0.00000000	pigeons
Arhgap31	-2.31	0.00000000	pigeons
Ndrg1	0.84	0.00000000	pigeons
Fat1	0.68	0.00000000	pigeons
Arl4a	-1.76	0.00000000	pigeons
Fgf7	0.86	0.00000000	pigeons
Cacna2d2	0.48	0.00000000	pigeons
Lama4	0.94	0.00000000	pigeons
Btg2	1.29	0.00000000	pigeons
Pdk4	0.71	0.00000000	pigeons
Nfil3	0.71	0.00000000	pigeons
Pla2g4a	0.54	0.00000000	pigeons
Dram1	0.52	0.00000000	pigeons
Plpp3	0.65	0.00000000	pigeons
Fam189a1	-2.38	0.00000000	pigeons
Lrrc49	0.61	0.00000000	pigeons
Usp44	0.49	0.00000000	pigeons
Rapgef2	-1.87	0.00000000	pigeons
Trank1	1.00	0.00000000	pigeons
Lrrk2	0.48	0.00000000	pigeons

Cebpb	0.52	0.00000000	pigeons
Abca1	0.73	0.00000000	pigeons
Slc25a28	0.41	0.00000000	pigeons
Fstl1	0.73	0.00000000	pigeons
Fgl1	0.69	0.00000000	pigeons
Zfhx4	0.64	0.00000000	pigeons
Cables1	0.66	0.00000000	pigeons
Tmcc3	-2.12	0.00000000	pigeons
Tmem100	-1.50	0.00000000	pigeons
Dhrs3	0.53	0.00000000	pigeons
Ccnd2	0.62	0.00000000	pigeons
Rbms3	-0.84	0.00000000	pigeons
Rnd3	0.73	0.00000000	pigeons
Prdm6	0.72	0.00000000	pigeons
Creb3l2	0.68	0.00000000	pigeons
Igf1r	0.77	0.00000000	pigeons
Tmtc1	-1.15	0.00000000	pigeons
Runx1	0.77	0.00000000	pigeons
Gem	0.60	0.00000000	pigeons
Nrp2	0.61	0.00000000	pigeons
Gadd45g	1.15	0.00000000	pigeons
Dok6	0.75	0.00000000	pigeons
Rsrp1	0.55	0.00000000	pigeons
Ahi1	0.59	0.00000000	pigeons
Cd99l2	0.60	0.00000000	pigeons
Osr1	0.74	0.00000000	pigeons
Gata5	0.68	0.00000000	pigeons
Cped1	0.82	0.00000000	pigeons
Cggbp1	0.61	0.00000000	pigeons
Thsd4	0.69	0.00000000	pigeons
Rspo2	1.34	0.00000000	pigeons
Rnf150	0.66	0.00000000	pigeons
Nanos1	0.68	0.00000000	pigeons
Carmil1	0.66	0.00000000	pigeons
Atf3	1.40	0.00000000	pigeons
Lrp1	0.63	0.00000000	pigeons
Zdhhc3	0.49	0.00000000	pigeons
Dkk3	0.54	0.00000000	pigeons
Chka	0.74	0.00000000	pigeons
Sema6a	-2.13	0.00000000	pigeons
Sgcb	0.55	0.00000000	pigeons
Pik3ip1	0.42	0.00000000	pigeons
Tmsb4x	-0.63	0.00000000	pigeons
Dpy19l4	0.52	0.00000000	pigeons
Bsg	-1.41	0.00000000	pigeons
Syt14	0.50	0.00000000	pigeons

Nfe2l1	0.53	0.00000000	pigeons
Tead3	0.56	0.00000000	pigeons
Jun	1.32	0.00000000	pigeons
Gns	0.34	0.00000000	pigeons
Rab30	0.43	0.00000000	pigeons
Bcl2	0.68	0.00000000	pigeons
Bnip3	0.41	0.00000000	pigeons
Sfmbt1	0.41	0.00000000	pigeons
Agrn	0.47	0.00000000	pigeons
Syne2	-1.54	0.00000000	pigeons
Deaf1	0.28	0.00000000	pigeons
Ctsz	0.45	0.00000000	pigeons
Heg1	-2.07	0.00000000	pigeons
Pdzrn3	0.53	0.00000000	pigeons
Inpp5a	-1.47	0.00000000	pigeons
Sccpdh	0.33	0.00000000	pigeons
Phldb1	0.40	0.00000000	pigeons
Lamb1	0.59	0.00000000	pigeons
Csf1	0.58	0.00000000	pigeons
Col5a2	0.55	0.00000000	pigeons
Fyn	0.41	0.00000000	pigeons
Lztfl1	0.36	0.00000000	pigeons
Cdh11	0.69	0.00000000	pigeons
Tbx5	0.40	0.00000000	pigeons
Atp2b1	0.41	0.00000000	pigeons
Kank4	0.53	0.00000000	pigeons
Fbln1	0.49	0.00000000	pigeons
Uaca	0.62	0.00000000	pigeons
Socs7	0.36	0.00000000	pigeons
Zfp352	-1.19	0.00000000	pigeons
Rab11fip2	0.42	0.00000000	pigeons
Kbtbd8	0.40	0.00000000	pigeons
Slc16a2	0.36	0.00000000	pigeons
Brd3	0.43	0.00000000	pigeons
Nt5c3b	0.35	0.00000000	pigeons
Sorbs1	-1.76	0.00000000	pigeons
Corin	-2.19	0.00000000	pigeons
Wnt11	0.49	0.00000000	pigeons
Snx9	0.29	0.00000000	pigeons
Pdlim3	0.85	0.00000000	pigeons
Gpc4	0.58	0.00000000	pigeons
Igfbp7	-0.68	0.00000000	pigeons
Pde5a	0.51	0.00000000	pigeons
S1pr1	-2.03	0.00000000	pigeons
Hmox1	0.52	0.00000000	pigeons
Bbs2	0.33	0.00000000	pigeons

Pde10a	-1.11	0.00000000	pigeons
Npr3	-2.20	0.00000000	pigeons
Il6st	0.50	0.00000000	pigeons
Grk5	0.62	0.00000000	pigeons
Axin2	0.51	0.00000000	pigeons
L3mbtl3	0.38	0.00000000	pigeons
Sparc	-0.97	0.00000000	pigeons
Col8a1	0.95	0.00000000	pigeons
Ppfibp1	0.67	0.00000000	pigeons
Pik3r1	0.48	0.00000000	pigeons
Tmem35b	0.32	0.00000000	pigeons
Rftn1	0.55	0.00000000	pigeons
Fam3c	0.42	0.00000000	pigeons
Gabrb2	0.64	0.00000000	pigeons
Ust	0.50	0.00000000	pigeons
Ccdc3	0.51	0.00000000	pigeons
Cdh5	-2.06	0.00000000	pigeons
Vldlr	0.38	0.00000000	pigeons
Ddx17	0.37	0.00000000	pigeons
Sema6d	0.53	0.00000000	pigeons
Gpx4	0.33	0.00000000	pigeons
Ulk2	0.39	0.00000000	pigeons
Gli3	0.53	0.00000000	pigeons
Ugdh	0.40	0.00000000	pigeons
Serpine2	0.78	0.00000000	pigeons
Colec12	0.65	0.00000000	pigeons
Hmces	0.39	0.00000000	pigeons
Tle1	0.47	0.00000000	pigeons
Prkar2b	0.38	0.00000000	pigeons
Ptprs	0.50	0.00000000	pigeons
Fryl	-1.60	0.00000000	pigeons
Fem1a	0.30	0.00000000	pigeons
Jade1	0.25	0.00000000	pigeons
Skap1	-1.81	0.00000000	pigeons
B4galt1	0.39	0.00000000	pigeons
Eif3k	0.83	0.00000000	pigeons
Atp9a	0.30	0.00000000	pigeons
Bag3	0.59	0.00000000	pigeons
Ptgfr	0.37	0.00000000	pigeons
Crls1	0.53	0.00000000	pigeons
Ints13	0.35	0.00000000	pigeons
Tnfrsf19	0.55	0.00000000	pigeons
Nox4	0.72	0.00000000	pigeons
Kcnj5	-2.04	0.00000000	pigeons
Hoxb3	0.40	0.00000000	pigeons
Rasgef1a	0.55	0.00000000	pigeons

Utp3	0.34	0.00000000	pigeons
Arpc5	0.31	0.00000000	pigeons
Larp6	0.32	0.00000000	pigeons
Zfpm2	0.68	0.00000000	pigeons
Wmp	0.46	0.00000000	pigeons
Hexim1	0.28	0.00000000	pigeons
Lrrfip1	0.81	0.00000000	pigeons
Bnc2	0.79	0.00000000	pigeons
Pmepa1	0.61	0.00000000	pigeons
Ltbp2	0.54	0.00000000	pigeons
Dll4	-1.92	0.00000000	pigeons
Pik3cb	0.45	0.00000000	pigeons
Gna11	0.27	0.00000000	pigeons
Nectin3	0.49	0.00000000	pigeons
Csnk1e	0.42	0.00000000	pigeons
Loxl1	0.56	0.00000000	pigeons
Prdx4	0.46	0.00000000	pigeons
Zbtb20	-0.85	0.00000000	pigeons
Adgrl2	-1.19	0.00000000	pigeons
Pnpla2	0.37	0.00000000	pigeons
Capn6	0.83	0.00000000	pigeons
Sftpc	-1.69	0.00000000	pigeons
Eda	0.44	0.00000000	pigeons
Vav2	0.43	0.00000000	pigeons
Arl6ip5	0.31	0.00000000	pigeons
Tmem106b	0.41	0.00000000	pigeons
Wdr47	0.33	0.00000000	pigeons
Hax1	0.23	0.00000000	pigeons
Clstn1	0.24	0.00000000	pigeons
Cygb	0.63	0.00000000	pigeons
Acbd3	0.34	0.00000000	pigeons
Eif3b	0.24	0.00000000	pigeons
Gpr137c	0.43	0.00000000	pigeons
Slc25a29	0.43	0.00000000	pigeons
Ppp1r27	0.33	0.00000000	pigeons
Sod2	0.29	0.00000000	pigeons
Celf2	0.79	0.00000000	pigeons
Aldh6a1	0.26	0.00000000	pigeons
Eya4	0.44	0.00000000	pigeons
Ash2l	0.35	0.00000000	pigeons
Nova1	-1.20	0.00000000	pigeons
Ppid	0.43	0.00000000	pigeons
Sh3pxd2b	0.25	0.00000000	pigeons
Igf2	0.59	0.00000000	pigeons
Nfat5	0.35	0.00000000	pigeons
Rasgef1c	0.60	0.00000000	pigeons

Dyrk2	0.32	0.00000000	pigeons
Ube2b	0.39	0.00000000	pigeons
Slc48a1	0.27	0.00000000	pigeons
Pde3a	0.60	0.00000000	pigeons
Pten	0.35	0.00000000	pigeons
Kdelr2	0.32	0.00000000	pigeons
Lonrf1	0.37	0.00000000	pigeons
Rassf3	-1.88	0.00000000	pigeons
Lonp2	0.25	0.00000000	pigeons
Slc9a9	0.52	0.00000000	pigeons
Ror1	0.61	0.00000000	pigeons
Sod3	0.54	0.00000000	pigeons
Zyx	0.39	0.00000000	pigeons
Ppm1k	0.31	0.00000000	pigeons
Syne3	0.34	0.00000000	pigeons
Npr2	0.36	0.00000000	pigeons
Fzd1	0.42	0.00000000	pigeons
Fam20c	0.45	0.00000000	pigeons
Podn	0.41	0.00000000	pigeons
Pcif1	0.12	0.00000000	pigeons
Whamm	0.13	0.00000000	pigeons
Rgl1	0.33	0.00000000	pigeons
Ddah1	0.40	0.00000000	pigeons
Ncoa7	0.45	0.00000000	pigeons
Smox	0.44	0.00000000	pigeons
Hsf2	0.39	0.00000000	pigeons
Fbxo25	0.46	0.00000000	pigeons
Sh3rf1	0.51	0.00000000	pigeons
Rgma	0.37	0.00000000	pigeons
Pros1	0.29	0.00000000	pigeons
Yeats2	0.44	0.00000000	pigeons
Pcgf5	0.47	0.00000000	pigeons
Kcnk5	0.87	0.00000000	pigeons
Trib1	0.88	0.00000000	pigeons
Lpl	-1.48	0.00000000	pigeons
Crebrf	0.17	0.00000000	pigeons
Clptm1l	0.19	0.00000000	pigeons
Vwa1	0.41	0.00000000	pigeons
Bmp4	0.63	0.00000000	pigeons
Setd7	0.28	0.00000000	pigeons
Arhgap42	0.63	0.00000000	pigeons
Pkdcc	0.76	0.00000000	pigeons
Tprgl	0.20	0.00000000	pigeons
Neto2	0.34	0.00000000	pigeons
Osbpl5	0.42	0.00000000	pigeons
Srsf11	0.25	0.00000000	pigeons

Tmem119	0.50	0.00000000	pigeons
Tmem63a	0.27	0.00000000	pigeons
Acvr1b	0.20	0.00000000	pigeons
Arvcf	-1.90	0.00000000	pigeons
Wasl	0.35	0.00000000	pigeons
Fos	1.02	0.00000000	pigeons
Slc35e4	0.25	0.00000000	pigeons
Angptl2	0.41	0.00000000	pigeons
Fbxo34	0.21	0.00000000	pigeons
Eva1b	0.32	0.00000000	pigeons
Cep170	0.33	0.00000000	pigeons
Gnptab	0.21	0.00000000	pigeons
Spon1	0.65	0.00000000	pigeons
Limd2	0.44	0.00000000	pigeons
Cln8	0.31	0.00000000	pigeons
Actg1	-0.52	0.00000000	pigeons
Ccng2	0.36	0.00000000	pigeons
Crbn	0.26	0.00000000	pigeons
Hadhb	0.33	0.00000000	pigeons
Fkbp3	0.34	0.00000000	pigeons
Nid1	0.42	0.00000000	pigeons
P4ha1	0.40	0.00000000	pigeons
Bbox1	1.02	0.00000000	pigeons
Atp6v0e	0.20	0.00000000	pigeons
Tcf7l1	0.26	0.00000000	pigeons
Scarf2	0.36	0.00000000	pigeons
Rbpms	0.36	0.00000000	pigeons
Klhdc1	0.23	0.00000000	pigeons
Tln2	0.43	0.00000000	pigeons
Cdkn1b	0.22	0.00000000	pigeons
Plekhg1	-1.64	0.00000000	pigeons
Eif3m	0.16	0.00000000	pigeons
Lpgat1	0.45	0.00000000	pigeons
Pcsk6	0.36	0.00000000	pigeons
Ube2w	0.20	0.00000000	pigeons
Htra3	0.63	0.00000000	pigeons
Aprt	0.24	0.00000000	pigeons
Pde3b	-1.60	0.00000000	pigeons
Hmcn1	0.49	0.00000000	pigeons
Arl3	0.29	0.00000000	pigeons
Arrdc4	0.29	0.00000000	pigeons
St7	0.43	0.00000000	pigeons
Tspan4	0.56	0.00000000	pigeons
Nr1h3	0.26	0.00000000	pigeons
Ech1	0.16	0.00000000	pigeons
Snx33	0.28	0.00000000	pigeons

Usp20	0.29	0.00000000	pigeons
Afap1	0.38	0.00000000	pigeons
Gsn	1.54	0.00000000	pigeons
Ncoa6	0.15	0.00000000	pigeons
Trabd2b	0.59	0.00000000	pigeons
Nbeal1	0.34	0.00000000	pigeons
Ttc21b	0.25	0.00000000	pigeons
Bicd1	-1.69	0.00000000	pigeons
Sesn1	0.46	0.00000000	pigeons
Cep57l1	0.30	0.00000000	pigeons
Fbxo7	0.26	0.00000000	pigeons
Get4	0.18	0.00000000	pigeons
Snrpe	0.28	0.00000000	pigeons
Ssrp1	0.12	0.00000000	pigeons
Pcdh19	0.45	0.00000000	pigeons
Rpn2	0.25	0.00000000	pigeons
Slc25a43	0.23	0.00000000	pigeons
Zmynd11	0.47	0.00000000	pigeons
Plpp1	0.49	0.00000000	pigeons
Glis1	0.55	0.00000000	pigeons
Fam214a	0.28	0.00000000	pigeons
Heyl	0.33	0.00000000	pigeons
Lzic	0.27	0.00000000	pigeons
Acadl	0.19	0.00000000	pigeons
Washc4	0.37	0.00000000	pigeons
Grem2	0.46	0.00000000	pigeons
Ephb2	0.40	0.00000000	pigeons
Cc2d2a	0.26	0.00000000	pigeons
Sh3d19	0.44	0.00000000	pigeons
Irak4	0.28	0.00000000	pigeons
Fbxo48	0.46	0.00000000	pigeons
Tshz3	0.31	0.00000000	pigeons
Sin3a	0.17	0.00000000	pigeons
Bhlhe41	0.24	0.00000000	pigeons
Tgfb2	0.50	0.00000000	pigeons
Abhd2	0.23	0.00000000	pigeons
Vegfa	0.28	0.00000000	pigeons
Parp16	0.39	0.00000000	pigeons
Srpr	0.27	0.00000000	pigeons
Adrm1	0.20	0.00000000	pigeons
Chst2	0.31	0.00000000	pigeons
Ckap4	0.33	0.00000000	pigeons
Kpnb1	0.17	0.00000000	pigeons
Irf2bpl	0.29	0.00000000	pigeons
Pdgfa	0.41	0.00000000	pigeons
Eif3g	0.21	0.00000000	pigeons

Trio	-1.22	0.00000000	pigeons
Pcdh18	0.70	0.00000000	pigeons
Fgd4	0.44	0.00000000	pigeons
Pdp1	0.22	0.00000000	pigeons
Gng12	0.31	0.00000000	pigeons
Bcat1	0.31	0.00000000	pigeons
Lactb	0.19	0.00000000	pigeons
Nop16	0.28	0.00000000	pigeons
Ccnt1	0.22	0.00000000	pigeons
Ankmy2	0.19	0.00000000	pigeons
Hoxb5	0.31	0.00000000	pigeons
Pcmtd2	0.30	0.00000000	pigeons
Spata5	0.55	0.00000000	pigeons
Kcnc1	-1.67	0.00000000	pigeons
Stx2	0.19	0.00000000	pigeons
Prim2	0.31	0.00000000	pigeons
Pes1	0.18	0.00000000	pigeons
Tef	0.15	0.00000000	pigeons
Alg5	0.26	0.00000000	pigeons
Arl13b	0.23	0.00000000	pigeons
Tmem9b	0.20	0.00000000	pigeons
Arpp19	0.23	0.00000000	pigeons
Fnip1	0.39	0.00000000	pigeons
Pkd2	0.37	0.00000000	pigeons
Ldb1	0.19	0.00000000	pigeons
P3h1	0.26	0.00000000	pigeons
Tpi1	0.22	0.00000000	pigeons
Cript	0.30	0.00000000	pigeons
Sf3a3	0.25	0.00000000	pigeons
Zbtb38	0.18	0.00000000	pigeons
Ptprn2	-1.44	0.00000000	pigeons
Abl1	0.33	0.00000000	pigeons
Sucla2	0.31	0.00000000	pigeons
Bag2	0.52	0.00000000	pigeons
Zfp366	-1.68	0.00000000	pigeons
Ubac2	0.17	0.00000000	pigeons
Suds3	0.08	0.00000000	pigeons
Tex2	-1.84	0.00000000	pigeons
Ppfia1	0.26	0.00000000	pigeons
Zdhhc2	0.32	0.00000000	pigeons
Agpat2	0.16	0.00000000	pigeons
Fgfr3	0.40	0.00000000	pigeons
Reck	0.22	0.00000000	pigeons
Znhit6	0.08	0.00000000	pigeons
Ppp1cc	0.14	0.00000000	pigeons
Ednra	0.62	0.00000000	pigeons

Htr6	0.36	0.00000000	pigeons
Robo1	0.78	0.00000000	pigeons
Olfml2b	0.32	0.00000000	pigeons
Zfp706	0.26	0.00000000	pigeons
Ccdc85a	-1.78	0.00000000	pigeons
Cdon	0.42	0.00000000	pigeons
Anxa7	0.19	0.00000000	pigeons
Agtr1a	0.55	0.00000000	pigeons
Sec14l1	-1.58	0.00000000	pigeons
Pcdh7	0.69	0.00000000	pigeons
Rasgef1b	0.37	0.00000000	pigeons
Zmat2	0.19	0.00000000	pigeons
Rhbdf2	0.12	0.00000000	pigeons
Chst10	0.20	0.00000000	pigeons
Dcun1d4	0.31	0.00000000	pigeons
Cdo1	0.54	0.00000000	pigeons
Fabp3	0.56	0.00000000	pigeons
Cops5	0.13	0.00000000	pigeons
Tbc1d12	0.21	0.00000000	pigeons
Dkc1	0.29	0.00000000	pigeons
Slc38a2	0.74	0.00000000	pigeons
Slc16a10	0.77	0.00000000	pigeons
Actb	-0.48	0.00000000	pigeons
Gpr63	0.38	0.00000000	pigeons
Klhl15	0.20	0.00000000	pigeons
Acvr2a	0.14	0.00000000	pigeons
Smim3	0.26	0.00000000	pigeons
Hnrnpll	0.15	0.00000000	pigeons
Ankib1	0.21	0.00000000	pigeons
Icosl	0.28	0.00000000	pigeons
Uvrag	0.16	0.00000000	pigeons
Rprd1b	0.16	0.00000000	pigeons
Jdp2	0.42	0.00000000	pigeons
Bambi	0.47	0.00000000	pigeons
Ei24	0.29	0.00000000	pigeons
Tril	0.35	0.00000000	pigeons
Tubgcp2	0.16	0.00000000	pigeons
Igsf3	0.22	0.00000000	pigeons
Cited2	0.57	0.00000000	pigeons
Dclk1	-1.69	0.00000000	pigeons
Cotl1	0.28	0.00000000	pigeons
Rpn1	0.11	0.00000000	pigeons
Dynlt1a	0.25	0.00000000	pigeons
Setd2	0.12	0.00000000	pigeons
Sbds	0.20	0.00000000	pigeons
Fam131b	0.22	0.00000000	pigeons

Adgra2	0.29	0.00000000	pigeons
Slc35a5	0.21	0.00000000	pigeons
Tmem43	0.25	0.00000000	pigeons
Klhl2	0.24	0.00000000	pigeons
Efr3a	0.16	0.00000000	pigeons
Rnasel	0.28	0.00000000	pigeons
Pde7b	0.58	0.00000000	pigeons
Amd2	0.13	0.00000000	pigeons
Xpo1	0.43	0.00000000	pigeons
Pdcd7	0.21	0.00000000	pigeons
Sat1	0.56	0.00000000	pigeons
Polr2l	0.17	0.00000000	pigeons
Ndufa2	0.07	0.00000000	pigeons
Peak1	0.29	0.00000000	pigeons
B4galt5	0.32	0.00000000	pigeons
Pdgfrb	0.36	0.00000000	pigeons
Acaa2	0.20	0.00000000	pigeons
Spidr	0.32	0.00000000	pigeons
Npepps	0.23	0.00000000	pigeons
Ctbp2	0.25	0.00000000	pigeons
Zfhx3	0.24	0.00000000	pigeons
Arap2	0.36	0.00000000	pigeons
Tsku	0.41	0.00000000	pigeons
Nr4a3	1.37	0.00000000	pigeons
St8sia1	0.34	0.00000000	pigeons
Ndufs2	0.10	0.00000000	pigeons
Tcf12	-1.27	0.00000000	pigeons
Fbn1	0.46	0.00000000	pigeons
Elmo1	-1.20	0.00000000	pigeons
Gabarapl1	0.26	0.00000000	pigeons
Tmem184c	0.31	0.00000000	pigeons
Csgalnact2	0.31	0.00000000	pigeons
Cpt2	0.25	0.00000000	pigeons
Fth1	-0.52	0.00000000	pigeons
Kifap3	0.33	0.00000000	pigeons
Fggy	0.33	0.00000000	pigeons
Pcnx4	0.20	0.00000000	pigeons
C1galt1c1	0.25	0.00000000	pigeons
Samd12	0.34	0.00000000	pigeons
Epha3	0.58	0.00000000	pigeons
Wipi2	0.12	0.00000000	pigeons
Sesn3	0.27	0.00000000	pigeons
Ebf1	0.83	0.00000000	pigeons
Cacna2d1	0.55	0.00000000	pigeons
Chp1	0.39	0.00000000	pigeons
Ndufa8	0.09	0.00000000	pigeons

Serpini1	0.29	0.00000000	pigeons
Ephb3	0.20	0.00000000	pigeons
Cwc15	0.16	0.00000000	pigeons
Ccpg1	0.15	0.00000000	pigeons
Ophn1	0.28	0.00000000	pigeons
Fam13a	0.30	0.00000000	pigeons
Dock3	0.47	0.00000000	pigeons
Stom	0.13	0.00000000	pigeons
Slc16a9	0.39	0.00000000	pigeons
Nsa2	0.21	0.00000000	pigeons
Gyg	0.13	0.00000000	pigeons
Tnks2	0.12	0.00000000	pigeons
Pcm1	0.22	0.00000000	pigeons
Ctsh	0.22	0.00000000	pigeons
Tor1b	0.14	0.00000000	pigeons
Tmx1	0.20	0.00000000	pigeons
Rit1	0.10	0.00000000	pigeons
Dhrs7b	0.23	0.00000000	pigeons
Nudc	0.17	0.00000000	pigeons
Ctr9	0.19	0.00000000	pigeons
Cep112	0.24	0.00000000	pigeons
Sppl3	0.15	0.00000000	pigeons
Lnpk	0.18	0.00000000	pigeons
Adgra3	0.26	0.00000000	pigeons
Seh1l	0.11	0.00000000	pigeons
Pfdn4	0.17	0.00000000	pigeons
Gorasp2	0.16	0.00000000	pigeons
Tns3	0.51	0.00000000	pigeons
Polr2d	0.16	0.00000000	pigeons
Prdm5	0.41	0.00000000	pigeons
Lrig1	0.44	0.00000000	pigeons
Anks6	0.22	0.00000000	pigeons
Abl2	0.33	0.00000000	pigeons
Ctsb	0.10	0.00000000	pigeons
Aldh7a1	0.21	0.00000000	pigeons
Clk4	0.11	0.00000000	pigeons
Grsf1	0.37	0.00000000	pigeons
Trmt11	0.18	0.00000000	pigeons
Hs1bp3	0.23	0.00000000	pigeons
Adra2a	1.00	0.00000000	pigeons
Phb2	0.13	0.00000000	pigeons
Nol9	0.21	0.00000000	pigeons
Dbndd2	0.19	0.00000000	pigeons
Nhp2	0.08	0.00000000	pigeons
Nkiras2	0.15	0.00000000	pigeons
Prss23	0.31	0.00000000	pigeons

Ube2i	0.13	0.00000000	pigeons
Cldnd1	0.21	0.00000000	pigeons
Aebp1	0.29	0.00000000	pigeons
Plcb1	-1.23	0.00000000	pigeons
Naaa	0.28	0.00000000	pigeons
Tcea1	0.22	0.00000000	pigeons
Pam	0.32	0.00000000	pigeons
Rab18	0.02	0.00000000	pigeons
Galnt18	-1.41	0.00000000	pigeons
Yipf4	0.11	0.00000000	pigeons
Togaram1	0.19	0.00000000	pigeons
Suclg1	0.23	0.00000000	pigeons
Eif4h	0.08	0.00000000	pigeons
Grem1	0.60	0.00000000	pigeons
Psma2	0.07	0.00000000	pigeons
Gxylt1	0.18	0.00000000	pigeons
Pgs1	0.31	0.00000000	pigeons
Ssr2	0.15	0.00000000	pigeons
Lrig2	0.20	0.00000000	pigeons
Prpf40a	0.09	0.00000000	pigeons
Ddhd1	0.09	0.00000000	pigeons
Prdx3	0.22	0.00000000	pigeons
Trpc1	0.31	0.00000000	pigeons
Tubb6	0.80	0.00000000	pigeons
Lig3	0.20	0.00000000	pigeons
Dst	-0.43	0.00000000	pigeons
Socs4	0.25	0.00000000	pigeons
Arl1	0.13	0.00000000	pigeons
Ing5	0.00	0.00000000	pigeons
Usp46	0.31	0.00000000	pigeons
Lca5	0.32	0.00000000	pigeons
Daam2	0.57	0.00000000	pigeons
Vps41	0.22	0.00000000	pigeons
Smc6	0.16	0.00000000	pigeons
Tmem50b	0.15	0.00000000	pigeons
Xpc	0.23	0.00000000	pigeons
Ptpn9	0.18	0.00000000	pigeons
Fam166a	0.18	0.00000000	pigeons
Bet1	0.23	0.00000000	pigeons
Sestd1	0.37	0.00000000	pigeons
Ptpru	0.23	0.00000000	pigeons
Neurl1b	-1.51	0.00000000	pigeons
Jarid2	-0.96	0.00000000	pigeons
Utp20	0.21	0.00000000	pigeons
Samd4	0.36	0.00000000	pigeons
Lgals3	0.14	0.00000000	pigeons

Gnl3	0.25	0.00000000	pigeons
Cct4	0.13	0.00000000	pigeons
Abhd3	0.20	0.00000000	pigeons
Commd9	0.33	0.00000000	pigeons
Emc7	0.06	0.00000000	pigeons
Fut11	0.20	0.00000000	pigeons
Naa50	0.13	0.00000000	pigeons
Rassf9	-1.63	0.00000000	pigeons
Cln5	0.21	0.00000000	pigeons
Galnt13	0.22	0.00000000	pigeons
Ezh1	0.30	0.00000000	pigeons
Stk38	0.12	0.00000000	pigeons
Ndufaf4	0.24	0.00000000	pigeons
Dusp16	0.35	0.00000000	pigeons
Tmed2	0.13	0.00000000	pigeons
Dach1	-1.37	0.00000000	pigeons
Arsb	0.15	0.00000000	pigeons
Sh3pxd2a	0.07	0.00000000	pigeons
Eva1c	0.22	0.00000000	pigeons
Eif4ebp1	0.20	0.00000000	pigeons
Glce	0.23	0.00000000	pigeons
Tspan6	0.26	0.00000000	pigeons
Cnot1	0.03	0.00000000	pigeons
Dpt	1.09	0.00000000	pigeons
Farp2	0.30	0.00000000	pigeons
Prkd1	0.17	0.00000000	pigeons
Nrbp1	0.12	0.00000000	pigeons
1700034I23Rik	0.15	0.00000000	pigeons
Pgf	0.29	0.00000000	pigeons
Fkbp4	0.20	0.00000000	pigeons
Atp8a1	-1.59	0.00000000	pigeons
Efnb2	-1.61	0.00000000	pigeons
Lamtor2	0.10	0.00000000	pigeons
Sppl2a	0.17	0.00000000	pigeons
Rasl11a	0.47	0.00000000	pigeons
Adamts9	0.44	0.00000000	pigeons
Prpsap2	0.31	0.00000000	pigeons
Creb1	0.18	0.00000000	pigeons
Cntn3	0.42	0.00000000	pigeons
Arl8b	0.14	0.00000000	pigeons
Adamts10	0.20	0.00000000	pigeons
Rae1	0.12	0.00000000	pigeons
Cntnap2	0.51	0.00000000	pigeons
Eaf1	0.28	0.00000000	pigeons
Pi4kb	0.16	0.00000000	pigeons
Rala	0.22	0.00000000	pigeons

Luc7l3	0.15	0.00000000	pigeons
Dpm2	0.19	0.00000000	pigeons
Foxp2	0.61	0.00000000	pigeons
Kctd20	0.19	0.00000000	pigeons
Slc25a33	0.21	0.00000000	pigeons
Nudt3	0.13	0.00000000	pigeons
Ntan1	0.06	0.00000000	pigeons
Ecd	0.23	0.00000000	pigeons
Wls	0.20	0.00000000	pigeons
Taldo1	0.11	0.00000000	pigeons
Tgfb3	0.32	0.00000000	pigeons
Bcas2	0.32	0.00000000	pigeons
Ypel1	0.07	0.00000000	pigeons
Rnmt	0.09	0.00000000	pigeons
Numb	-1.18	0.00000000	pigeons
Eif2b2	0.17	0.00000000	pigeons
Rabgap1	0.10	0.00000000	pigeons
Tek	-1.44	0.00000000	pigeons
Gtpbp4	0.14	0.00000000	pigeons
Dcbld2	0.15	0.00000000	pigeons
Serinc3	0.08	0.00000000	pigeons
Tbc1d8	0.25	0.00000000	pigeons
Fign	0.55	0.00000000	pigeons
Serinc1	0.17	0.00000000	pigeons
Eif2d	0.21	0.00000000	pigeons
Stat1	0.27	0.00000000	pigeons
Ttc17	0.06	0.00000000	pigeons
Eif2s2	0.16	0.00000000	pigeons
Filip1	0.28	0.00000000	pigeons
Nedd4	0.27	0.00000000	pigeons
Wipi1	0.19	0.00000000	pigeons
Nup85	0.18	0.00000000	pigeons
Ggt5	0.23	0.00000000	pigeons
Ank	0.19	0.00000000	pigeons
Eif4a3l1	0.11	0.00000000	pigeons
Ap3m1	0.13	0.00000000	pigeons
Erich1	0.15	0.00000000	pigeons
Atrnl1	0.50	0.00000000	pigeons
Ufl1	0.23	0.00000000	pigeons
Guk1	0.12	0.00000000	pigeons
Tmem230	0.12	0.00000000	pigeons
Tet2	-1.17	0.00000000	pigeons
Pdzd11	0.10	0.00000000	pigeons
Ints10	0.14	0.00000000	pigeons
Zhx3	0.28	0.00000000	pigeons
Psma7	0.17	0.00000000	pigeons

Ankle2	0.13	0.00000000	pigeons
Exoc3	0.13	0.00000000	pigeons
Rasa2	0.17	0.00000000	pigeons
Reps1	0.03	0.00000000	pigeons
Nfs1	0.10	0.00000000	pigeons
Pgam1	0.23	0.00000000	pigeons
Ahr	0.20	0.00000000	pigeons
Ebag9	0.15	0.00000000	pigeons
Nipa2	-0.02	0.00000000	pigeons
Dnajc3	0.10	0.00000000	pigeons
Plekhf2	0.15	0.00000000	pigeons
Npas3	-1.24	0.00000000	pigeons
Laptm4a	-1.21	0.00000000	pigeons
Gab2	0.06	0.00000000	pigeons
Ttbk2	0.25	0.00000000	pigeons
Sdhaf4	0.15	0.00000000	pigeons
Emp2	0.18	0.00000000	pigeons
Atm	0.32	0.00000000	pigeons
Lgmn	0.33	0.00000000	pigeons
Tfg	0.04	0.00000000	pigeons
Rad21	0.19	0.00000000	pigeons
Itgav	0.21	0.00000000	pigeons
Josd1	0.25	0.00000000	pigeons
Trim35	0.23	0.00000000	pigeons
Ripk2	0.21	0.00000000	pigeons
Rarb	0.77	0.00000000	pigeons
Hacd2	0.03	0.00000000	pigeons
Flt1	0.69	0.00000000	pigeons
Agpat5	0.12	0.00000000	pigeons
Zfp651	0.25	0.00000000	pigeons
Tlk1	0.02	0.00000000	pigeons
Gata6	0.29	0.00000000	pigeons
Dnajb9	0.31	0.00000000	pigeons
Txnl4b	0.12	0.00000000	pigeons
Hsd17b12	-0.69	0.00000000	pigeons
B3galnt2	0.19	0.00000000	pigeons
Syt11	0.05	0.00000000	pigeons
Rfc1	0.07	0.00000000	pigeons
Prpf8	0.09	0.00000000	pigeons
Casd1	0.21	0.00000000	pigeons
Gpatch8	0.14	0.00000000	pigeons
Ugp2	0.16	0.00000000	pigeons
Sulf1	0.28	0.00000000	pigeons
Oxr1	0.14	0.00000000	pigeons
Cep83	-1.54	0.00000000	pigeons
Ctso	0.25	0.00000000	pigeons

Ppp1ca	0.06	0.00000000	pigeons
Phb	0.04	0.00000000	pigeons
Nbea	0.45	0.00000000	pigeons
Unc5b	0.32	0.00000000	pigeons
Add1	0.12	0.00000000	pigeons
Penk	0.66	0.00000000	pigeons
Sdc3	0.29	0.00000000	pigeons
Kctd10	0.17	0.00000000	pigeons
Tob1	0.26	0.00000000	pigeons
Pde1c	0.56	0.00000000	pigeons
Egr1	0.98	0.00000000	pigeons
Ptprd	0.56	0.00000000	pigeons
Rtcb	0.18	0.00000000	pigeons
Thada	0.17	0.00000000	pigeons
Tulp4	0.21	0.00000000	pigeons
Eif2ak3	0.21	0.00000000	pigeons
Itpkb	-1.43	0.00000000	pigeons
Ccdc6	0.11	0.00000000	pigeons
Fst	0.58	0.00000000	pigeons
Slc29a1	0.17	0.00000000	pigeons
Lats1	0.09	0.00000000	pigeons
Exosc9	0.18	0.00000000	pigeons
Yes1	0.17	0.00000000	pigeons
Garem1	0.38	0.00000000	pigeons
Chfr	0.17	0.00000000	pigeons
Dhx38	0.11	0.00000000	pigeons
Fam171b	0.27	0.00000000	pigeons
Akap11	0.04	0.00000000	pigeons
Thbs2	0.44	0.00000000	pigeons
Rnf139	0.14	0.00000000	pigeons
Rsl1d1	0.23	0.00000000	pigeons
Usp16	0.10	0.00000000	pigeons
Yrdc	0.07	0.00000000	pigeons
Bdnf	0.44	0.00000000	pigeons
Tbc1d32	0.31	0.00000000	pigeons
Cdc42ep1	0.17	0.00000000	pigeons
Pole4	0.19	0.00000000	pigeons
Rtf1	0.09	0.00000000	pigeons
Tspan12	0.18	0.00000000	pigeons
Fbrsl1	-0.86	0.00000000	pigeons
Ip6k2	0.32	0.00000000	pigeons
Hic2	0.25	0.00000000	pigeons
Sspn	0.24	0.00000000	pigeons
Fndc3b	0.26	0.00000000	pigeons
Cebpz	0.24	0.00000000	pigeons
Taf15	0.10	0.00000000	pigeons

Foxp4	0.12	0.00000000	pigeons
Gab1	0.20	0.00000000	pigeons
Hhat	0.41	0.00000000	pigeons
G2e3	0.18	0.00000000	pigeons
Sgpp1	0.07	0.00000000	pigeons
Fem1b	0.08	0.00000000	pigeons
Nr2c2	0.17	0.00000000	pigeons
Uba3	0.26	0.00000000	pigeons
Synj2	0.19	0.00000000	pigeons
Igfbp3	0.37	0.00000000	pigeons
Btbd7	0.16	0.00000000	pigeons
Crot	0.10	0.00000000	pigeons
Wnt5b	0.21	0.00000000	pigeons
Ttc4	0.24	0.00000000	pigeons
Rhoa	-0.90	0.00000000	pigeons
Vezf1	0.14	0.00000000	pigeons
Oat	0.15	0.00000000	pigeons
Rnf13	0.04	0.00000000	pigeons
Sec61g	0.11	0.00000000	pigeons
Rai2	0.11	0.00000000	pigeons
Ybx3	0.12	0.00000000	pigeons
Aamdc	0.18	0.00000000	pigeons
Sec31b	0.08	0.00000000	pigeons
Sirt6	0.21	0.00000000	pigeons
Psip1	0.08	0.00000000	pigeons
Tgfbr3	0.38	0.00000000	pigeons
Capn7	0.14	0.00000000	pigeons
Thyn1	0.12	0.00000000	pigeons
Klhl24	0.24	0.00000000	pigeons
Erp29	0.01	0.00000000	pigeons
Hsp90aa1	0.63	0.00000000	pigeons
Ndufb1	0.10	0.00000000	pigeons
Fuca2	0.17	0.00000000	pigeons
Timm22	0.16	0.00000000	pigeons
Abr	0.14	0.00000000	pigeons
Tmem241	0.17	0.00000000	pigeons
Pdcd10	0.04	0.00000000	pigeons
Rpp30	0.13	0.00000000	pigeons
Raly	0.20	0.00000000	pigeons
Sap30	0.17	0.00000000	pigeons
Secisbp2l	0.04	0.00000000	pigeons
St8sia4	0.42	0.00000000	pigeons
Skil	0.12	0.00000000	pigeons
Cdc27	0.13	0.00000000	pigeons
Hadh	0.00	0.00000000	pigeons
Atad1	0.21	0.00000000	pigeons

Hs6st2	0.18	0.00000000	pigeons
Tomm70a	0.18	0.00000000	pigeons
Dnaja4	0.11	0.00000000	pigeons
Pdcd5	0.19	0.00000000	pigeons
Zbtb41	0.10	0.00000000	pigeons
Ddx5	-0.53	0.00000000	pigeons
Krr1	0.04	0.00000000	pigeons
Eps8	0.21	0.00000000	pigeons
Ptpn21	0.15	0.00000000	pigeons
Zrsr1	-0.01	0.00000000	pigeons
Ypel2	0.05	0.00000000	pigeons
Adipor1	0.06	0.00000000	pigeons
Ssb	0.17	0.00000000	pigeons
Suv39h2	0.15	0.00000000	pigeons
Slc39a6	0.22	0.00000000	pigeons
Ppm1a	0.05	0.00000000	pigeons
Rab29	0.16	0.00000000	pigeons
Col6a2	0.13	0.00000000	pigeons
Caprin1	0.01	0.00000000	pigeons
Sptbn1	-0.42	0.00000000	pigeons
Tmem42	0.17	0.00000000	pigeons
Fbxl4	0.15	0.00000000	pigeons
Ssbp2	-1.23	0.00000000	pigeons
Esf1	0.21	0.00000000	pigeons
Aimp1	0.17	0.00000000	pigeons
Lrch2	0.18	0.00000000	pigeons
Dusp1	0.91	0.00000001	pigeons
Gpatch2l	0.10	0.00000001	pigeons
Atp6v0b	0.05	0.00000001	pigeons
Lsm5	0.11	0.00000001	pigeons
Tbl2	0.18	0.00000001	pigeons
Inpp5k	-0.65	0.00000001	pigeons
Adam17	0.10	0.00000001	pigeons
Cdkl5	0.32	0.00000001	pigeons
Nkd1	0.14	0.00000001	pigeons
Cbx6	0.32	0.00000001	pigeons
Wwc1	0.24	0.00000001	pigeons
Tgs1	0.19	0.00000001	pigeons
Hsd17b7	0.29	0.00000001	pigeons
Oaz1	0.26	0.00000001	pigeons
Sel1l	-0.09	0.00000001	pigeons
Soga1	0.13	0.00000001	pigeons
Hivep2	-0.63	0.00000001	pigeons
Ndufa9	0.19	0.00000001	pigeons
Aifm2	0.15	0.00000001	pigeons
Hmg20b	0.12	0.00000001	pigeons

Eif1b	0.20	0.00000001	pigeons
Tmem30a	0.02	0.00000001	pigeons
Prkca	0.36	0.00000001	pigeons
Tmem87a	0.01	0.00000001	pigeons
Cab39l	0.22	0.00000001	pigeons
Tnfrsf1a	0.25	0.00000001	pigeons
Ralgps1	0.29	0.00000001	pigeons
Flot2	0.19	0.00000001	pigeons
Gnpda2	0.13	0.00000001	pigeons
Eif3f	0.09	0.00000001	pigeons
Fam8a1	0.06	0.00000001	pigeons
Ggnbp2	0.05	0.00000001	pigeons
Ankrd49	0.09	0.00000001	pigeons
Add3	0.29	0.00000001	pigeons
Cabin1	0.14	0.00000001	pigeons
Stam	0.10	0.00000001	pigeons
Ppp3r1	-0.04	0.00000001	pigeons
Lap3	0.19	0.00000001	pigeons
Upf2	0.00	0.00000001	pigeons
Psmg2	0.19	0.00000001	pigeons
Thoc7	0.16	0.00000001	pigeons
Sfrp2	0.31	0.00000001	pigeons
Angpt1	0.54	0.00000001	pigeons
Tmem192	0.25	0.00000001	pigeons
Wdr61	0.13	0.00000001	pigeons
Ift20	-0.02	0.00000001	pigeons
Sel1l3	0.21	0.00000001	pigeons
Rab23	0.19	0.00000001	pigeons
Pdap1	0.04	0.00000001	pigeons
Lrrc59	0.23	0.00000001	pigeons
Fancd2	0.09	0.00000001	pigeons
Cstf1	0.13	0.00000001	pigeons
Csrnp1	0.32	0.00000001	pigeons
Emc3	-0.10	0.00000001	pigeons
Txnrd1	-1.36	0.00000001	pigeons
Ndufv3	0.15	0.00000001	pigeons
Rexo2	0.13	0.00000001	pigeons
Nfatc3	0.17	0.00000001	pigeons
Ghr	0.25	0.00000001	pigeons
Pde6d	0.17	0.00000001	pigeons
Laptm5	0.16	0.00000001	pigeons
Scyl2	0.15	0.00000001	pigeons
Iffo1	0.16	0.00000001	pigeons
Fbln2	0.20	0.00000001	pigeons
Ddx27	0.08	0.00000001	pigeons
Slc40a1	0.12	0.00000002	pigeons

Pofut1	0.21	0.00000002	pigeons
Dennd4c	0.10	0.00000002	pigeons
Timp3	-0.26	0.00000002	pigeons
Npepl1	0.17	0.00000002	pigeons
Dcn	0.39	0.00000002	pigeons
Rnf19b	0.11	0.00000002	pigeons
Acly	0.12	0.00000002	pigeons
Gabpb1	0.04	0.00000002	pigeons
Ebf3	0.62	0.00000002	pigeons
Nuak1	0.20	0.00000002	pigeons
Taf3	0.15	0.00000002	pigeons
Rasgrf2	-1.49	0.00000002	pigeons
Slc35f5	0.06	0.00000002	pigeons
4833439L19Rik	0.00	0.00000002	pigeons
Zcchc14	0.10	0.00000002	pigeons
Ipo7	0.09	0.00000002	pigeons
Jund	0.21	0.00000002	pigeons
Ddx1	0.01	0.00000002	pigeons
Epb41l3	-0.30	0.00000002	pigeons
Elac2	0.11	0.00000002	pigeons
Got2	0.15	0.00000002	pigeons
Ube2e3	0.20	0.00000002	pigeons
Foxn2	0.18	0.00000002	pigeons
Krit1	0.07	0.00000002	pigeons
Psma3	-0.03	0.00000002	pigeons
Pim1	0.28	0.00000002	pigeons
Ercc4	0.14	0.00000002	pigeons
Zcchc10	0.16	0.00000002	pigeons
Tcf7	0.63	0.00000002	pigeons
Ndufb9	0.11	0.00000003	pigeons
Arid4a	0.21	0.00000003	pigeons
Klhl7	0.08	0.00000003	pigeons
Ran	0.15	0.00000003	pigeons
Pik3r4	0.14	0.00000003	pigeons
Rrn3	0.16	0.00000003	pigeons
Tmem60	0.11	0.00000003	pigeons
Smurf1	0.16	0.00000003	pigeons
Anxa11	0.05	0.00000003	pigeons
Thoc2	0.09	0.00000003	pigeons
Chic2	0.07	0.00000003	pigeons
Cfl2	0.23	0.00000003	pigeons
Ndel1	0.02	0.00000003	pigeons
Ube2v1	0.10	0.00000003	pigeons
Col12a1	0.28	0.00000003	pigeons
Sowahc	0.24	0.00000003	pigeons
Tdrd3	-0.09	0.00000004	pigeons

Traf3	0.34	0.00000004	pigeons
Nub1	0.04	0.00000004	pigeons
Pxdn	0.46	0.00000004	pigeons
Nek7	0.03	0.00000004	pigeons
Erh	-0.02	0.00000004	pigeons
Btf3l4	-0.03	0.00000004	pigeons
2900026A02Rik	-1.14	0.00000004	pigeons
Bcl9	0.03	0.00000004	pigeons
Ankrd6	0.23	0.00000004	pigeons
Tsnax	0.12	0.00000004	pigeons
Aspscr1	0.24	0.00000004	pigeons
Dok1	0.08	0.00000004	pigeons
Dus1l	0.14	0.00000005	pigeons
Ndnf	0.19	0.00000005	pigeons
Clint1	0.07	0.00000005	pigeons
Ruvbl1	0.15	0.00000005	pigeons
Ube2d1	0.04	0.00000005	pigeons
Plin2	0.24	0.00000005	pigeons
Tnfrsf11b	0.16	0.00000005	pigeons
Nupl1	0.19	0.00000005	pigeons
Hspd1	0.05	0.00000005	pigeons
Hoxa5	-0.02	0.00000005	pigeons
Sik2	0.22	0.00000005	pigeons
Klhl36	0.22	0.00000005	pigeons
Snrpa1	0.08	0.00000005	pigeons
Zbtb48	0.11	0.00000005	pigeons
Cdc40	-0.01	0.00000005	pigeons
Spg7	0.20	0.00000005	pigeons
Dock11	0.16	0.00000005	pigeons
Hdac2	0.14	0.00000005	pigeons
Socs1	0.24	0.00000005	pigeons
Cisd1	0.06	0.00000005	pigeons
Poldip3	-0.06	0.00000006	pigeons
Lamtor3	0.20	0.00000006	pigeons
Psmb3	0.28	0.00000006	pigeons
Tm9sf4	0.16	0.00000006	pigeons
Cnot6l	-0.09	0.00000006	pigeons
Pigt	0.08	0.00000006	pigeons
Tbx2	0.30	0.00000006	pigeons
Herpud1	0.22	0.00000006	pigeons
Paqr7	0.20	0.00000006	pigeons
Stard13	-0.56	0.00000006	pigeons
Slc41a1	0.14	0.00000006	pigeons
Serinc5	0.15	0.00000006	pigeons
Sugt1	-0.03	0.00000006	pigeons
Tial1	0.01	0.00000006	pigeons

Wdr26	-0.03	0.00000006	pigeons
Plce1	0.61	0.00000006	pigeons
Plagl1	0.22	0.00000006	pigeons
Pkia	0.09	0.00000006	pigeons
Cct5	0.02	0.00000007	pigeons
Fgd5	-1.38	0.00000007	pigeons
Tshz1	0.20	0.00000007	pigeons
Cwf19l2	0.12	0.00000007	pigeons
Plag1	0.22	0.00000007	pigeons
Eif2ak2	0.14	0.00000008	pigeons
Ypel5	-0.03	0.00000008	pigeons
Slc9a7	0.21	0.00000008	pigeons
Pop5	0.14	0.00000008	pigeons
Bach2	0.28	0.00000009	pigeons
Ccdc88a	0.14	0.00000009	pigeons
Extl3	0.17	0.00000009	pigeons
Sys1	0.17	0.00000009	pigeons
Dgkd	-1.38	0.00000009	pigeons
Cnih4	0.15	0.00000009	pigeons
Kcnq1	-1.18	0.00000009	pigeons
Fbxl14	-0.07	0.00000010	pigeons
Rilpl1	0.08	0.00000010	pigeons
Zfp704	0.05	0.00000010	pigeons
Zfand4	0.13	0.00000010	pigeons
Baz1b	0.00	0.00000010	pigeons
Ncam1	0.20	0.00000010	pigeons
Parva	0.13	0.00000011	pigeons
Gse1	0.09	0.00000011	pigeons
Ccdc47	0.01	0.00000011	pigeons
Ubl3	0.24	0.00000011	pigeons
Aebp2	0.10	0.00000011	pigeons
Lrp4	0.21	0.00000011	pigeons
Pebp1	0.10	0.00000011	pigeons
Csde1	-0.09	0.00000012	pigeons
Prmt3	0.15	0.00000012	pigeons
Eif5	0.32	0.00000012	pigeons
Xpo4	0.13	0.00000012	pigeons
Fkbp1a	-1.20	0.00000012	pigeons
Ik	0.08	0.00000013	pigeons
Crtc1	0.21	0.00000013	pigeons
Arl6ip1	0.04	0.00000013	pigeons
Cog1	0.20	0.00000013	pigeons
Dhx29	0.13	0.00000013	pigeons
Dbi	0.11	0.00000014	pigeons
Vgll3	0.21	0.00000014	pigeons
Gtf2h5	0.01	0.00000014	pigeons

Ppil3	0.03	0.00000014	pigeons
Slc25a21	0.29	0.00000014	pigeons
Zc3h12d	0.58	0.00000015	pigeons
Pak1ip1	0.25	0.00000015	pigeons
Gsr	0.12	0.00000015	pigeons
Fstl3	0.22	0.00000015	pigeons
Tk2	0.12	0.00000016	pigeons
Cmas	0.17	0.00000016	pigeons
Cavin2	-1.06	0.00000016	pigeons
Inpp5d	-1.36	0.00000016	pigeons
Rmnd5a	0.09	0.00000016	pigeons
Isca1	0.07	0.00000017	pigeons
Gorasp1	0.14	0.00000017	pigeons
Ghdc	0.19	0.00000017	pigeons
Pdxk	0.11	0.00000018	pigeons
Dusp8	0.27	0.00000018	pigeons
Pdgfrl	0.13	0.00000018	pigeons
Pbx1	0.19	0.00000019	pigeons
Lin54	-0.01	0.00000020	pigeons
Polr2f	0.03	0.00000021	pigeons
Scp2	0.10	0.00000021	pigeons
Il31ra	0.27	0.00000021	pigeons
Nrp1	0.34	0.00000021	pigeons
Stat2	0.10	0.00000021	pigeons
C1galt1	0.07	0.00000021	pigeons
Nup37	0.22	0.00000021	pigeons
Wbp1l	0.04	0.00000022	pigeons
Prickle1	0.01	0.00000022	pigeons
Cops2	0.08	0.00000022	pigeons
Helz	-0.12	0.00000022	pigeons
Nmt2	0.16	0.00000022	pigeons
Pigb	0.19	0.00000022	pigeons
Pde4d	-1.25	0.00000022	pigeons
Ddx42	0.00	0.00000023	pigeons
Slc39a13	0.11	0.00000023	pigeons
Btbd6	0.14	0.00000023	pigeons
Atp1b3	-1.31	0.00000023	pigeons
Cercam	0.25	0.00000023	pigeons
Tcerg1	-0.03	0.00000024	pigeons
Ptp4a2	-0.01	0.00000024	pigeons
Pdxdc1	0.12	0.00000025	pigeons
Luc7l	0.07	0.00000025	pigeons
Nrbf2	0.08	0.00000025	pigeons
Snrpb2	0.22	0.00000025	pigeons
Hipk1	-0.04	0.00000025	pigeons
Bub3	0.09	0.00000026	pigeons

Crebl2	0.07	0.00000026	pigeons
Gpx3	0.26	0.00000027	pigeons
Zfp654	0.03	0.00000027	pigeons
P4ha2	0.30	0.00000027	pigeons
Psma1	-0.06	0.00000027	pigeons
Psmd12	0.09	0.00000027	pigeons
Utp23	0.14	0.00000027	pigeons
Oxct1	0.15	0.00000027	pigeons
Rbm15	0.15	0.00000028	pigeons
Arpc5l	0.15	0.00000028	pigeons
Cdv3	0.07	0.00000028	pigeons
Acads	0.22	0.00000029	pigeons
Eed	0.11	0.00000029	pigeons
Pacs2	0.11	0.00000030	pigeons
Scrn3	0.07	0.00000030	pigeons
Bud31	0.04	0.00000030	pigeons
Plekhb2	-0.02	0.00000030	pigeons
Nup43	0.19	0.00000030	pigeons
Sorcs2	0.37	0.00000031	pigeons
Qk	-0.59	0.00000033	pigeons
Fdx1	0.09	0.00000034	pigeons
Rnf214	0.14	0.00000034	pigeons
Tbc1d8b	0.11	0.00000035	pigeons
Bmpr2	-1.32	0.00000035	pigeons
Gnl2	0.15	0.00000037	pigeons
Zbtb7a	0.04	0.00000037	pigeons
Dnajb2	0.16	0.00000038	pigeons
Cog2	0.09	0.00000039	pigeons
Slc25a15	0.14	0.00000040	pigeons
Spg21	0.12	0.00000040	pigeons
Nutf2	-0.05	0.00000040	pigeons
Hlf	0.28	0.00000041	pigeons
Nbn	0.13	0.00000043	pigeons
Ubxn2b	0.27	0.00000043	pigeons
Dip2b	0.07	0.00000043	pigeons
Lrrc1	0.26	0.00000043	pigeons
Psmc5	0.15	0.00000043	pigeons
Nudt16l1	0.08	0.00000044	pigeons
Kat7	0.04	0.00000044	pigeons
Sec22a	0.06	0.00000044	pigeons
Polr2k	0.11	0.00000044	pigeons
Glo1	0.09	0.00000045	pigeons
Osbpl1a	-1.46	0.00000045	pigeons
Surf4	0.07	0.00000045	pigeons
Psmb1	-0.13	0.00000045	pigeons
Acer3	0.20	0.00000046	pigeons

Cnot11	0.12	0.00000048	pigeons
Rnf4	0.10	0.00000051	pigeons
Insr	0.00	0.00000052	pigeons
Raf1	0.01	0.00000053	pigeons
Tasp1	0.18	0.00000053	pigeons
Lgi1	0.27	0.00000054	pigeons
Per3	0.18	0.00000054	pigeons
Cdk19	-0.08	0.00000055	pigeons
Clk3	-0.05	0.00000055	pigeons
Srrm1	0.06	0.00000056	pigeons
F3	0.23	0.00000057	pigeons
Nsl1	0.06	0.00000059	pigeons
2510039O18Rik	0.02	0.00000061	pigeons
Puf60	0.03	0.00000061	pigeons
Pex13	0.16	0.00000062	pigeons
Plcl1	0.27	0.00000062	pigeons
Hsdl1	0.19	0.00000062	pigeons
Acss3	0.33	0.00000063	pigeons
Sema3a	0.33	0.00000064	pigeons
Ptges	0.21	0.00000064	pigeons
Hspa9	-0.03	0.00000067	pigeons
Cnep1r1	0.12	0.00000068	pigeons
Cenpc1	0.01	0.00000069	pigeons
Caps2	0.04	0.00000069	pigeons
Nfe2l2	-0.03	0.00000069	pigeons
Eftud2	0.06	0.00000069	pigeons
Sf3a1	-0.05	0.00000070	pigeons
Pcnx2	0.09	0.00000071	pigeons
Tspo	0.13	0.00000071	pigeons
Secisbp2	-0.10	0.00000071	pigeons
Ahdc1	0.20	0.00000072	pigeons
B3gnt9	0.27	0.00000073	pigeons
Rab3gap1	0.13	0.00000074	pigeons
Eef2k	0.17	0.00000077	pigeons
Alkbh5	0.00	0.00000078	pigeons
Lum	0.54	0.00000078	pigeons
Pias1	0.09	0.00000079	pigeons
Xrcc5	0.07	0.00000080	pigeons
Nfyb	0.07	0.00000080	pigeons
Ap2b1	-0.03	0.00000082	pigeons
Prdm2	-0.01	0.00000083	pigeons
Ctu2	0.12	0.00000085	pigeons
Tmx3	-0.02	0.00000085	pigeons
Lrrc56	0.24	0.00000086	pigeons
Pard3b	0.36	0.00000086	pigeons
Adcy6	0.12	0.00000087	pigeons

Rwdd4a	0.08	0.00000088	pigeons
Ndufab1	0.04	0.00000089	pigeons
Ubn2	-0.03	0.00000091	pigeons
Ltn1	0.05	0.00000091	pigeons
Rgs4	0.36	0.00000092	pigeons
Wsb1	0.09	0.00000093	pigeons
Herc3	0.11	0.00000095	pigeons
Top1	0.05	0.00000096	pigeons
Itpa	0.15	0.00000098	pigeons
Cpsf4	0.11	0.00000098	pigeons
Ssbp1	0.03	0.00000101	pigeons
Rnf138	0.08	0.00000102	pigeons
Ap3b1	0.04	0.00000103	pigeons
Acsbg2	0.11	0.00000106	pigeons
G3bp2	0.10	0.00000107	pigeons
Lman2	0.02	0.00000108	pigeons
Ralgps2	-1.58	0.00000108	pigeons
Pank3	0.14	0.00000110	pigeons
Cct6b	-0.04	0.00000110	pigeons
Lrig3	0.36	0.00000112	pigeons
Srp68	0.04	0.00000112	pigeons
Atp1b1	0.10	0.00000120	pigeons
Rad54l2	0.02	0.00000120	pigeons
Stx7	0.16	0.00000120	pigeons
Appl1	0.07	0.00000121	pigeons
Dvl1	0.04	0.00000122	pigeons
Foxs1	0.27	0.00000126	pigeons
Rab9	0.17	0.00000128	pigeons
Srpk1	-0.01	0.00000131	pigeons
Smchd1	0.03	0.00000134	pigeons
Strn	0.14	0.00000136	pigeons
Copb2	0.08	0.00000137	pigeons
Tmf1	-0.02	0.00000138	pigeons
Washc3	-0.08	0.00000148	pigeons
Hspa4	0.00	0.00000148	pigeons
Gab3	0.03	0.00000148	pigeons
Specc1l	0.08	0.00000150	pigeons
Lrp12	-0.04	0.00000153	pigeons
Rcor3	0.09	0.00000154	pigeons
Hadha	0.13	0.00000157	pigeons
Txlng	0.03	0.00000159	pigeons
Cacna1g	0.21	0.00000161	pigeons
Cdk17	-1.42	0.00000166	pigeons
Ptges3	-0.05	0.00000168	pigeons
Becn2	0.09	0.00000175	pigeons
Ttc39b	0.16	0.00000181	pigeons

Itgb1	-0.97	0.00000182	pigeons
Fam20b	0.19	0.00000183	pigeons
Gramd4	0.11	0.00000187	pigeons
Pus10	0.04	0.00000191	pigeons
Commd2	0.13	0.00000192	pigeons
Naxe	0.15	0.00000196	pigeons
Kdm1b	0.15	0.00000196	pigeons
Azi2	0.02	0.00000196	pigeons
Psmc3	0.11	0.00000198	pigeons
Bmp5	-0.09	0.00000204	pigeons
Tes	0.12	0.00000207	pigeons
Invs	0.19	0.00000208	pigeons
Zhx2	0.17	0.00000219	pigeons
Zbtb2	0.07	0.00000219	pigeons
Usf3	0.09	0.00000222	pigeons
Sacs	0.24	0.00000223	pigeons
Gata3	-1.21	0.00000230	pigeons
Pcgf6	0.10	0.00000231	pigeons
Lpcat3	0.03	0.00000233	pigeons
Srsf4	0.03	0.00000237	pigeons
Dhx8	0.16	0.00000244	pigeons
Dctn3	-0.01	0.00000251	pigeons
Ssu72	0.01	0.00000251	pigeons
Cebpg	0.09	0.00000251	pigeons
Arfgap3	0.05	0.00000256	pigeons
Retsat	0.13	0.00000256	pigeons
Dnaja3	0.01	0.00000258	pigeons
Elmod2	0.11	0.00000259	pigeons
Fkbp11	0.00	0.00000261	pigeons
Polr1c	0.14	0.00000266	pigeons
Zfp503	-0.05	0.00000270	pigeons
Dicer1	-0.03	0.00000272	pigeons
Acsf2	0.20	0.00000273	pigeons
Zfp318	0.15	0.00000273	pigeons
Fgf18	0.17	0.00000273	pigeons
Srp72	0.14	0.00000277	pigeons
Ing1	0.01	0.00000297	pigeons
B3gnt2	0.10	0.00000300	pigeons
Naxd	0.00	0.00000300	pigeons
Uba6	0.04	0.00000302	pigeons
Jcad	-1.28	0.00000303	pigeons
Capn11	-0.03	0.00000306	pigeons
Rspry1	0.10	0.00000307	pigeons
Supt4a	-0.07	0.00000308	pigeons
Tbc1d23	-0.07	0.00000311	pigeons
Nol8	0.09	0.00000318	pigeons

Loxl3	0.22	0.00000320	pigeons
Rbbp4	0.11	0.00000324	pigeons
Fto	0.17	0.00000330	pigeons
Lancl1	0.14	0.00000334	pigeons
Uhrf2	0.09	0.00000334	pigeons
Dpysl2	0.08	0.00000334	pigeons
Ttc14	0.26	0.00000351	pigeons
Rif1	0.00	0.00000357	pigeons
Rrbp1	0.04	0.00000357	pigeons
Scamp2	-0.02	0.00000361	pigeons
Nsmaf	0.10	0.00000364	pigeons
Vps36	-0.05	0.00000364	pigeons
Atg9a	0.04	0.00000367	pigeons
Jam3	0.16	0.00000367	pigeons
Nexn	0.21	0.00000368	pigeons
Rbm26	0.01	0.00000370	pigeons
Adora2b	0.10	0.00000373	pigeons
Bap1	0.08	0.00000391	pigeons
Ndufb5	0.02	0.00000394	pigeons
Gcn1	0.04	0.00000404	pigeons
Eif3j2	0.01	0.00000404	pigeons
Saraf	0.01	0.00000408	pigeons
Bcap29	-0.05	0.00000414	pigeons
Gtf2a2	0.02	0.00000415	pigeons
Scfd2	0.26	0.00000416	pigeons
Sec11c	0.02	0.00000425	pigeons
Ttf1	0.12	0.00000426	pigeons
Dnajc15	0.03	0.00000430	pigeons
Ccnh	0.02	0.00000434	pigeons
Usp8	-0.13	0.00000451	pigeons
Chst14	0.03	0.00000453	pigeons
Supt6	-0.11	0.00000454	pigeons
Cdk14	0.30	0.00000460	pigeons
Gpbp1l1	-0.05	0.00000461	pigeons
Actr6	0.11	0.00000474	pigeons
Tpst1	0.10	0.00000475	pigeons
Cct7	-0.04	0.00000481	pigeons
Usp45	-0.16	0.00000486	pigeons
Zbtb1	0.03	0.00000497	pigeons
Fam168b	0.01	0.00000501	pigeons
Afg3l2	-0.06	0.00000501	pigeons
Cstf3	0.12	0.00000504	pigeons
Esd	0.00	0.00000518	pigeons
Ralgapb	0.04	0.00000547	pigeons
Snrnp48	0.05	0.00000550	pigeons
Nudt21	0.03	0.00000568	pigeons

Cbfa2t3	0.03	0.00000583	pigeons
Hipk3	0.05	0.00000601	pigeons
Rap1gds1	0.14	0.00000602	pigeons
Psmd4	0.09	0.00000615	pigeons
Idh1	0.05	0.00000622	pigeons
Asxl1	0.09	0.00000652	pigeons
Gss	0.13	0.00000666	pigeons
Tmtc3	0.08	0.00000669	pigeons
Rrp1b	0.05	0.00000670	pigeons
Nbas	0.13	0.00000679	pigeons
Ltbp1	0.96	0.00000682	pigeons
Ylpm1	-0.09	0.00000686	pigeons
Osbpl10	-1.36	0.00000710	pigeons
Tmco1	0.01	0.00000727	pigeons
Gfpt2	0.16	0.00000733	pigeons
Exoc8	0.02	0.00000739	pigeons
Acsl3	0.08	0.00000759	pigeons
Pag1	0.53	0.00000771	pigeons
Dlg3	0.15	0.00000774	pigeons
Gch1	0.55	0.00000776	pigeons
Galnt2	0.21	0.00000786	pigeons
Wdr82	-0.12	0.00000790	pigeons
Ndufa13	0.02	0.00000792	pigeons
Eri3	0.04	0.00000853	pigeons
Crk	-0.04	0.00000949	pigeons
Dbr1	0.07	0.00000972	pigeons
Senp6	0.01	0.00000982	pigeons
E2f6	-0.04	0.00001003	pigeons
Itgb1bp1	-0.01	0.00001029	pigeons
Atg12	0.13	0.00001031	pigeons
Amotl1	0.28	0.00001034	pigeons
Rab8a	0.06	0.00001049	pigeons
Dab2	0.28	0.00001059	pigeons
Uba2	0.00	0.00001092	pigeons
Ergic3	0.13	0.00001103	pigeons
Fhl3	0.14	0.00001109	pigeons
Atg3	0.15	0.00001117	pigeons
Clk1	-0.05	0.00001135	pigeons
Rab5c	0.09	0.00001175	pigeons
Commd8	0.08	0.00001183	pigeons
Reep1	-1.15	0.00001184	pigeons
Tgfbr2	0.30	0.00001190	pigeons
Dvl3	0.16	0.00001194	pigeons
Hif1a	0.07	0.00001212	pigeons
Rbpj	-0.04	0.00001231	pigeons
Npc2	0.07	0.00001231	pigeons

Hsdl2	0.13	0.00001248	pigeons
Nin	0.13	0.00001252	pigeons
Exosc8	0.13	0.00001259	pigeons
Rnf213	0.13	0.00001264	pigeons
Rsf1	-0.10	0.00001281	pigeons
Ctss	0.12	0.00001333	pigeons
Pcna	0.00	0.00001349	pigeons
Osbpl11	0.10	0.00001396	pigeons
Bach1	-0.21	0.00001406	pigeons
Arfip1	-0.01	0.00001420	pigeons
Qser1	-0.04	0.00001428	pigeons
Psmd1	-0.04	0.00001430	pigeons
B2m	-0.22	0.00001432	pigeons
Adamts15	0.19	0.00001435	pigeons
Dnajc19	0.02	0.00001447	pigeons
Ednrb	0.25	0.00001459	pigeons
Armc8	-0.04	0.00001485	pigeons
Cwh43	0.08	0.00001504	pigeons
Park7	-0.04	0.00001537	pigeons
Nolc1	0.10	0.00001539	pigeons
Pepd	0.13	0.00001546	pigeons
Chd9	-0.01	0.00001567	pigeons
Zfp236	-0.04	0.00001596	pigeons
Gfra3	0.06	0.00001606	pigeons
Lcmt2	0.04	0.00001664	pigeons
Pafah1b2	-0.04	0.00001711	pigeons
Cbx3	-0.09	0.00001729	pigeons
Arf4	-0.07	0.00001745	pigeons
Riok3	0.11	0.00001774	pigeons
Supt20	0.12	0.00001837	pigeons
Tgif1	-0.01	0.00001856	pigeons
Ltf	0.10	0.00001888	pigeons
Ercc3	-0.10	0.00001904	pigeons
Adgrg2	0.24	0.00001909	pigeons
Cadps2	-1.07	0.00002023	pigeons
Rnf34	-0.01	0.00002080	pigeons
Tpd52	0.10	0.00002088	pigeons
Pogz	0.12	0.00002108	pigeons
Btf3	-0.07	0.00002110	pigeons
Dffa	0.17	0.00002110	pigeons
Syf2	-0.11	0.00002116	pigeons
Tctn3	0.14	0.00002198	pigeons
Jmjd7	0.22	0.00002200	pigeons
Lonrf2	0.03	0.00002223	pigeons
Sec11a	0.05	0.00002277	pigeons
Ddx24	0.07	0.00002279	pigeons

Nup88	0.02	0.00002292	pigeons
Iqsec1	0.27	0.00002312	pigeons
Rnls	0.13	0.00002342	pigeons
Nceh1	0.18	0.00002367	pigeons
Zc2hc1a	0.07	0.00002386	pigeons
Sash1	0.58	0.00002387	pigeons
Itgb5	0.08	0.00002405	pigeons
Olfml1	0.20	0.00002441	pigeons
Ptprk	0.43	0.00002532	pigeons
Twist2	0.54	0.00002570	pigeons
Arpp21	0.44	0.00002581	pigeons
Smarcb1	-0.01	0.00002632	pigeons
Dip2a	0.11	0.00002654	pigeons
Rev3l	0.22	0.00002655	pigeons
Inpp5b	0.14	0.00002745	pigeons
Hmgb2	0.18	0.00002753	pigeons
Pcmtd1	0.13	0.00002760	pigeons
Pdcd4	-0.03	0.00002784	pigeons
Abhd12	0.12	0.00002795	pigeons
Chordc1	0.02	0.00002825	pigeons
Fbxo21	0.09	0.00002830	pigeons
Zer1	0.25	0.00002839	pigeons
Nadk	-0.03	0.00002866	pigeons
Amn1	0.18	0.00002931	pigeons
Aco2	-0.07	0.00002980	pigeons
Ppib	-0.02	0.00003010	pigeons
Ndufb6	-0.09	0.00003011	pigeons
Abcd3	0.06	0.00003032	pigeons
Psmc1	0.05	0.00003158	pigeons
Txndc11	-0.07	0.00003187	pigeons
Rpap3	0.13	0.00003196	pigeons
Lrpap1	0.08	0.00003231	pigeons
Vcan	0.20	0.00003371	pigeons
Ubfd1	0.02	0.00003430	pigeons
Phf2	-0.05	0.00003441	pigeons
Ctbs	0.11	0.00003539	pigeons
Txndc17	0.02	0.00003547	pigeons
Zdhhc9	0.09	0.00003572	pigeons
Bend3	0.19	0.00003630	pigeons
Wdr44	-0.04	0.00003637	pigeons
Chd7	-1.09	0.00003668	pigeons
Ascc1	-0.05	0.00003673	pigeons
Upf3b	-0.14	0.00003690	pigeons
Akt1	-0.13	0.00003700	pigeons
Agbl1	-1.04	0.00003700	pigeons
Dapk1	0.06	0.00003714	pigeons

Drg1	0.10	0.00003733	pigeons
Cnot8	0.01	0.00003746	pigeons
Rragd	0.14	0.00003811	pigeons
Spon2	0.55	0.00003883	pigeons
Ankrd46	0.01	0.00003933	pigeons
Pank2	0.09	0.00003969	pigeons
Zbtb6	0.08	0.00003983	pigeons
Faap100	0.18	0.00004035	pigeons
Iws1	-0.13	0.00004175	pigeons
Psma6	0.04	0.00004208	pigeons
Dync2li1	0.26	0.00004248	pigeons
Npc1	0.08	0.00004254	pigeons
Ccdc93	-0.05	0.00004420	pigeons
Pdgfb	-1.12	0.00004490	pigeons
Ifngr1	-0.04	0.00004555	pigeons
Pwwp2b	-0.03	0.00004633	pigeons
Drg2	-0.01	0.00004665	pigeons
Decr1	0.05	0.00004666	pigeons
Fam162a	-0.01	0.00004686	pigeons
Fbxw5	0.01	0.00004732	pigeons
Pak3	-0.05	0.00004819	pigeons
Nt5dc2	0.21	0.00004852	pigeons
Itpr2	0.05	0.00004870	pigeons
Pinx1	0.06	0.00004909	pigeons
Galnt17	0.31	0.00004951	pigeons
Adam19	0.10	0.00005072	pigeons
Fam110b	0.24	0.00005179	pigeons
Rabepk	-0.02	0.00005185	pigeons
Rab40c	-0.03	0.00005225	pigeons
Clp1	0.09	0.00005313	pigeons
Arhgap6	-0.02	0.00005334	pigeons
Gpam	0.15	0.00005528	pigeons
Etfa	-0.11	0.00005532	pigeons
Rasl11b	0.27	0.00005664	pigeons
Grid2	0.45	0.00005734	pigeons
Akap1	0.17	0.00005745	pigeons
Ptch1	0.14	0.00005777	pigeons
Tmem204	-1.24	0.00005805	pigeons
Arnt	-0.05	0.00005807	pigeons
Sec63	-0.14	0.00005993	pigeons
Atg13	0.02	0.00006001	pigeons
Vps50	-0.08	0.00006010	pigeons
Klhdc2	0.06	0.00006038	pigeons
Enc1	0.08	0.00006085	pigeons
Sin3b	0.11	0.00006124	pigeons
Timp2	0.19	0.00006183	pigeons

Cldn12	-0.02	0.00006209	pigeons
Dtx3l	-0.02	0.00006382	pigeons
Kat2b	-0.08	0.00006553	pigeons
N4bp1	0.11	0.00006633	pigeons
Sirt1	-0.07	0.00006656	pigeons
Tmtc2	0.39	0.00006670	pigeons
Sumo1	-0.13	0.00006749	pigeons
Socs6	0.08	0.00006972	pigeons
Golim4	0.08	0.00007024	pigeons
Gramd1c	0.31	0.00007071	pigeons
Phf6	0.02	0.00007075	pigeons
Cops7a	-0.01	0.00007123	pigeons
Stx8	0.02	0.00007204	pigeons
Exosc3	0.13	0.00007255	pigeons
Pgam5	0.16	0.00007271	pigeons
Vezt	0.03	0.00007400	pigeons
Ccdc186	-0.07	0.00007442	pigeons
Pcdh15	0.40	0.00007466	pigeons
Heatr6	0.09	0.00007506	pigeons
Osbpl2	0.16	0.00007627	pigeons
Eif3e	0.05	0.00007873	pigeons
Nucb2	-0.07	0.00007912	pigeons
Fyco1	0.03	0.00007926	pigeons
Lrrc8a	0.05	0.00007962	pigeons
Ndufa5	-0.21	0.00008205	pigeons
Syap1	-0.07	0.00008270	pigeons
Dusp10	0.27	0.00008296	pigeons
E4f1	0.11	0.00008379	pigeons
Dpp6	0.67	0.00008441	pigeons
Dis3	0.09	0.00008613	pigeons
Epb41l5	0.03	0.00008805	pigeons
Heph	0.13	0.00009009	pigeons
Zfp423	0.11	0.00009082	pigeons
Abce1	-0.11	0.00009190	pigeons
Btbd11	0.16	0.00009454	pigeons
Snrpd1	-0.17	0.00009577	pigeons
Sbno2	-0.02	0.00009675	pigeons
Rnf19a	-0.04	0.00009700	pigeons
Boc	0.13	0.00009793	pigeons
Appbp2	0.15	0.00009807	pigeons
Kansl3	0.08	0.00009813	pigeons
Fam126a	0.11	0.00009992	pigeons
Egfl7	-1.03	0.00010088	pigeons
Got1	0.09	0.00010278	pigeons
Cdc23	0.03	0.00010315	pigeons
Psmg3	-0.03	0.00010486	pigeons

Ski	-0.48	0.00010739	pigeons
Dtwd1	0.14	0.00010754	pigeons
Slc30a5	0.09	0.00010790	pigeons
Fam98a	-0.06	0.00010837	pigeons
Trap1	0.08	0.00010924	pigeons
Zhx1	-0.02	0.00011028	pigeons
Gtf2b	0.03	0.00011128	pigeons
Ift46	0.17	0.00011143	pigeons
Ddx59	0.02	0.00011228	pigeons
Ddx52	0.14	0.00011645	pigeons
Gps1	0.15	0.00011652	pigeons
Lamp1	0.09	0.00012137	pigeons
Srek1ip1	0.12	0.00012218	pigeons
Eif3l	-0.08	0.00012522	pigeons
Cdc14b	0.06	0.00012641	pigeons
Rundc1	0.14	0.00012704	pigeons
Stau2	-0.02	0.00012738	pigeons
Bcl2l13	-0.13	0.00012775	pigeons
Tmem135	0.00	0.00012817	pigeons
Aplp2	0.30	0.00012942	pigeons
Ss18	0.05	0.00013240	pigeons
Erlec1	-0.05	0.00013482	pigeons
Flrt2	0.29	0.00013995	pigeons
Tfpi2	0.23	0.00014211	pigeons
Atxn1l	0.10	0.00014428	pigeons
Ccser1	0.23	0.00014799	pigeons
Nufip2	0.00	0.00014996	pigeons
Rnf144a	0.10	0.00015022	pigeons
Exoc1	0.11	0.00015088	pigeons
Psma4	-0.07	0.00015121	pigeons
Epb41l1	0.04	0.00015128	pigeons
Arv1	0.13	0.00015141	pigeons
Hp1bp3	0.05	0.00015257	pigeons
Dnmt1	0.08	0.00015294	pigeons
Dgkh	-1.23	0.00015410	pigeons
Lingo1	0.23	0.00015566	pigeons
Atp23	0.05	0.00015611	pigeons
Slu7	-0.21	0.00015840	pigeons
Arhgap24	0.60	0.00016184	pigeons
Ube2g1	-0.04	0.00016745	pigeons
Sdha	0.06	0.00016749	pigeons
Sf3b5	0.00	0.00016829	pigeons
Pptc7	0.06	0.00016977	pigeons
Snw1	-0.03	0.00016999	pigeons
Sft2d1	-0.03	0.00017094	pigeons
Erlin2	0.04	0.00017095	pigeons

Rnh1	-0.01	0.00017210	pigeons
Rc3h2	0.12	0.00017350	pigeons
Vps26a	0.09	0.00017549	pigeons
Ctnnbl1	0.06	0.00017611	pigeons
Zfp106	0.18	0.00017684	pigeons
Tbk1	-0.13	0.00017703	pigeons
Rlf	-0.03	0.00018210	pigeons
Armt1	0.03	0.00018214	pigeons
Srp14	0.03	0.00018269	pigeons
Tmem39a	0.20	0.00018633	pigeons
Gas6	0.05	0.00018840	pigeons
Fam104a	0.08	0.00018919	pigeons
Gtpbp1	-0.03	0.00019104	pigeons
Dnajb4	0.15	0.00019425	pigeons
Nptn	-0.03	0.00019453	pigeons
Lmbrd1	0.00	0.00019506	pigeons
Vps37b	0.01	0.00019845	pigeons
Nkrf	0.00	0.00020160	pigeons
Brd7	-0.09	0.00020278	pigeons
Ripor3	-1.23	0.00020412	pigeons
Synpo2	0.15	0.00020573	pigeons
Uggt1	-0.03	0.00020718	pigeons
Tcf25	0.00	0.00020719	pigeons
Cttnbp2	0.00	0.00020789	pigeons
Nucks1	-0.14	0.00020917	pigeons
Tmem185a	-0.04	0.00020994	pigeons
Arid5b	0.19	0.00021230	pigeons
Zc3h15	-0.12	0.00021456	pigeons
Cep192	0.12	0.00021632	pigeons
Dnajc13	-0.03	0.00021646	pigeons
Ndufa10	0.07	0.00021735	pigeons
Hdx	0.06	0.00021753	pigeons
Ntrk3	0.33	0.00021813	pigeons
Rassf8	-0.05	0.00021843	pigeons
Nubpl	0.07	0.00021865	pigeons
Ccser2	0.26	0.00022098	pigeons
Tmem248	-0.06	0.00022142	pigeons
Blmh	0.06	0.00022391	pigeons
Wee1	0.10	0.00022447	pigeons
Zfp711	0.08	0.00022649	pigeons
Yipf1	0.00	0.00022649	pigeons
Atxn10	0.00	0.00023230	pigeons
Tpmt	0.14	0.00023250	pigeons
Fbxo31	0.01	0.00023488	pigeons
Oard1	-0.10	0.00024675	pigeons
Clpx	-0.04	0.00024839	pigeons

Vkorc1l1	0.01	0.00025061	pigeons
Rnf114	0.05	0.00025080	pigeons
Yipf5	0.02	0.00025380	pigeons
Ddb1	-0.03	0.00025900	pigeons
Cmtm8	-0.96	0.00025936	pigeons
Bzw1	-0.08	0.00026139	pigeons
Pum2	-0.16	0.00026372	pigeons
Kcnma1	0.18	0.00026552	pigeons
Cc2d1b	0.08	0.00026835	pigeons
Ap2a1	-0.10	0.00027120	pigeons
Rab20	0.17	0.00027321	pigeons
Pin4	0.04	0.00027915	pigeons
Zswim7	0.05	0.00028002	pigeons
Serpinh1	-0.06	0.00028026	pigeons
Gpatch1	-0.07	0.00028054	pigeons
Cfap97	0.07	0.00028361	pigeons
Ints8	-0.02	0.00028930	pigeons
Copg2	0.12	0.00029506	pigeons
Tecpr2	0.01	0.00029960	pigeons
Rufy1	-0.04	0.00030160	pigeons
Pgpep1	0.12	0.00031058	pigeons
Epm2a	0.06	0.00031910	pigeons
Atl2	-0.14	0.00032002	pigeons
Smad3	-0.06	0.00032008	pigeons
Acat1	0.01	0.00032033	pigeons
Igf2bp3	0.17	0.00032326	pigeons
Cpsf2	0.02	0.00033208	pigeons
Usp30	0.04	0.00033251	pigeons
Hcfc2	0.00	0.00033270	pigeons
Sumf2	0.09	0.00033559	pigeons
Nup93	0.09	0.00033929	pigeons
Trmt6	0.28	0.00033948	pigeons
Samm50	0.07	0.00034086	pigeons
Zfp207	-0.17	0.00034207	pigeons
Cep44	-0.07	0.00034365	pigeons
Eno1b	-0.12	0.00034494	pigeons
Samd11	0.12	0.00034526	pigeons
Ifnar2	-0.18	0.00034816	pigeons
Nxt2	0.07	0.00035334	pigeons
Smad5	0.00	0.00035352	pigeons
Yeats4	-0.01	0.00035468	pigeons
Zfp507	0.12	0.00035738	pigeons
Hhex	-1.05	0.00036471	pigeons
Atp6v0d1	0.05	0.00037142	pigeons
Luzp1	-1.28	0.00037682	pigeons
Tnrc6b	-1.16	0.00038010	pigeons

Smpd2	0.11	0.00038226	pigeons
4933400A11Rik	0.03	0.00039417	pigeons
Reep5	0.02	0.00039428	pigeons
Tcf20	0.02	0.00039774	pigeons
Cep85l	-0.03	0.00040541	pigeons
Rasgrp3	-1.00	0.00040560	pigeons
C2cd5	-0.04	0.00040755	pigeons
Atf2	-0.13	0.00041586	pigeons
Rictor	0.03	0.00041599	pigeons
Cul5	-0.18	0.00042113	pigeons
Snrpd3	0.07	0.00042708	pigeons
Rnf146	-0.06	0.00043032	pigeons
Coq7	0.12	0.00043720	pigeons
Xrn2	-0.14	0.00043742	pigeons
Rab21	-0.03	0.00044350	pigeons
Unc50	-0.01	0.00044763	pigeons
Eif2ak4	-0.18	0.00045075	pigeons
Dag1	0.05	0.00046488	pigeons
Ifi30	0.05	0.00047150	pigeons
Phtf2	0.05	0.00047535	pigeons
Sass6	0.08	0.00049854	pigeons
Fkbp8	0.00	0.00051153	pigeons
Ptp4a1	-0.24	0.00051249	pigeons
Ppil1	-0.07	0.00051918	pigeons
Bmpr1a	0.12	0.00052313	pigeons
Tead4	-1.25	0.00053663	pigeons
Sec62	0.10	0.00054421	pigeons
Eny2	-0.02	0.00055216	pigeons
Arcn1	-0.02	0.00057301	pigeons
Cmtm7	0.07	0.00058559	pigeons
Snap23	-0.04	0.00058719	pigeons
Dot1l	-0.93	0.00059514	pigeons
Osgepl1	0.07	0.00059539	pigeons
Nmd3	0.11	0.00060101	pigeons
Suclg2	0.12	0.00060271	pigeons
Sav1	0.02	0.00060587	pigeons
Nup50	-0.11	0.00060592	pigeons
H13	-0.11	0.00060927	pigeons
Aurkaip1	0.01	0.00060991	pigeons
Ncbp3	0.05	0.00061635	pigeons
Ywhae	-0.22	0.00062087	pigeons
Cttn	-0.04	0.00062232	pigeons
Enah	-1.13	0.00063546	pigeons
Emc6	-0.05	0.00063692	pigeons
Cdc42ep4	0.14	0.00064006	pigeons
G3bp1	0.03	0.00064588	pigeons

Atp6v1g1	-0.12	0.00064686	pigeons
Slc37a2	0.18	0.00065042	pigeons
Bbx	0.06	0.00065128	pigeons
Tmem104	0.11	0.00065656	pigeons
Adarb1	0.07	0.00065879	pigeons
Col22a1	0.16	0.00066108	pigeons
Cnih1	0.20	0.00066121	pigeons
Sox18	-1.02	0.00066382	pigeons
Ralgapa1	-0.07	0.00066523	pigeons
Gpc1	0.11	0.00066712	pigeons
Rcan1	-1.27	0.00066859	pigeons
Atf6	-0.08	0.00067241	pigeons
Zfp330	0.06	0.00067651	pigeons
Itga9	0.21	0.00067809	pigeons
Vps13c	0.00	0.00067896	pigeons
Zfp518a	0.07	0.00068154	pigeons
Nr2c1	-0.06	0.00068924	pigeons
Prpf6	-0.04	0.00068979	pigeons
Eif3d	-0.15	0.00071941	pigeons
Vta1	-0.05	0.00072343	pigeons
Tnip2	0.17	0.00073245	pigeons
Efl1	-0.03	0.00073721	pigeons
Noct	0.04	0.00074391	pigeons
Dalrd3	0.13	0.00074898	pigeons
Snx27	0.04	0.00075398	pigeons
Zmpste24	0.02	0.00075868	pigeons
Cacna1c	0.60	0.00076631	pigeons
Comtd1	0.08	0.00078061	pigeons
Kdm4b	-0.02	0.00078384	pigeons
Aimp2	0.05	0.00078626	pigeons
Vdac2	-0.07	0.00079692	pigeons
Pcmt1	-0.08	0.00081753	pigeons
Pias2	0.04	0.00081833	pigeons
Jade2	0.15	0.00083279	pigeons
Ift43	0.16	0.00086083	pigeons
Slc25a16	-0.01	0.00086356	pigeons
Ghitm	-0.09	0.00087228	pigeons
Ehd4	-1.00	0.00087827	pigeons
Rsl24d1	-0.17	0.00088095	pigeons
Ublcp1	0.00	0.00088541	pigeons
Slc35b1	0.01	0.00088884	pigeons
Iah1	0.07	0.00090055	pigeons
Ep300	-0.14	0.00090584	pigeons
Ogt	-0.11	0.00090658	pigeons
Acp1	-0.10	0.00092266	pigeons
Kalrn	0.19	0.00092575	pigeons

Ank3	0.29	0.00093688	pigeons
Ppic	0.02	0.00094045	pigeons
Selenof	-0.13	0.00094512	pigeons
Rnaseh1	0.01	0.00095114	pigeons
Cdk7	0.09	0.00095528	pigeons
Ccnd1	-0.05	0.00095539	pigeons
Rmdn2	0.10	0.00095829	pigeons
Helq	0.03	0.00096211	pigeons
Cfap36	-0.04	0.00096299	pigeons
Svil	0.12	0.00096945	pigeons
Tmem203	0.19	0.00097780	pigeons
Insig2	0.05	0.00098984	pigeons
Xpo5	0.03	0.00100272	pigeons
Nf1	-0.07	0.00100765	pigeons
Rab8b	-0.07	0.00100834	pigeons
Gtf2a1	0.00	0.00101744	pigeons
Wasf2	-0.18	0.00102107	pigeons
Casp2	0.00	0.00102231	pigeons
Gng4	0.17	0.00103099	pigeons
Hspa13	0.08	0.00103509	pigeons
Zzz3	-0.15	0.00105656	pigeons
Sdhd	-0.22	0.00107570	pigeons
Slco3a1	0.18	0.00111012	pigeons
Rab12	-0.14	0.00111699	pigeons
Thrap3	-0.04	0.00112413	pigeons
Snx2	0.02	0.00112906	pigeons
Hsph1	0.23	0.00113111	pigeons
Fam114a2	0.09	0.00113150	pigeons
Ssr3	-0.15	0.00115324	pigeons
Tax1bp3	-0.04	0.00116836	pigeons
Taok3	-0.12	0.00121505	pigeons
Pxk	0.01	0.00122568	pigeons
Fam102a	0.00	0.00125095	pigeons
Ints9	0.14	0.00126097	pigeons
Emc2	0.05	0.00127657	pigeons
Ikbkb	0.00	0.00128128	pigeons
Rabl6	-0.12	0.00128265	pigeons
Atg5	-0.13	0.00131919	pigeons
Zc3h18	-0.11	0.00133392	pigeons
Esyt2	-0.10	0.00134021	pigeons
Cdca4	-0.09	0.00134182	pigeons
Cog6	-0.17	0.00135475	pigeons
Steap4	0.41	0.00136426	pigeons
Slit3	0.18	0.00136847	pigeons
Rnf215	0.03	0.00141340	pigeons
Lbh	0.26	0.00141502	pigeons

Fmr1	-0.09	0.00142216	pigeons
Fbxl20	-0.07	0.00142268	pigeons
Tfb2m	0.09	0.00142433	pigeons
Dek	-0.17	0.00142446	pigeons
Ostm1	0.00	0.00142505	pigeons
Erc1	-1.03	0.00143682	pigeons
Nbr1	0.02	0.00143913	pigeons
Lyar	0.03	0.00144265	pigeons
Nelfa	0.00	0.00145770	pigeons
Prkag2	0.05	0.00149547	pigeons
Lsm14b	0.04	0.00150537	pigeons
Tbc1d15	-0.07	0.00151064	pigeons
Pnisr	-0.06	0.00151402	pigeons
Elavl1	-0.05	0.00152113	pigeons
Bsdc1	0.01	0.00152413	pigeons
Gpalpp1	-0.06	0.00152609	pigeons
Arl8a	0.07	0.00158467	pigeons
Far2	0.28	0.00160633	pigeons
Aldh4a1	0.07	0.00163015	pigeons
Vstm4	0.28	0.00164110	pigeons
Cse1l	-0.02	0.00165040	pigeons
Ahsa1	-0.04	0.00165120	pigeons
Tmed10	-0.04	0.00166786	pigeons
Tmem138	0.16	0.00168047	pigeons
Abcb7	0.06	0.00168943	pigeons
Clip4	0.00	0.00171416	pigeons
Tbcd	0.14	0.00171654	pigeons
Tob2	0.14	0.00177720	pigeons
Phka2	0.02	0.00179095	pigeons
Ssh2	-0.17	0.00179209	pigeons
Telo2	0.03	0.00180846	pigeons
Dcun1d5	-0.06	0.00181681	pigeons
Efcab7	0.12	0.00182744	pigeons
Derl1	-0.07	0.00184678	pigeons
Cops4	0.00	0.00186696	pigeons
Tacc1	-0.01	0.00186878	pigeons
Tkt	-0.08	0.00187487	pigeons
Fam234a	-0.10	0.00188790	pigeons
Eif2s3y	-0.09	0.00190205	pigeons
Selenot	-0.14	0.00191351	pigeons
Anxa6	-0.08	0.00194184	pigeons
Lgals8	-0.08	0.00196955	pigeons
Cpne3	-0.07	0.00198330	pigeons
Pi4k2b	0.00	0.00200343	pigeons
Haus2	-0.02	0.00211807	pigeons
Sike1	0.02	0.00213291	pigeons

Tmem59	-0.17	0.00213482	pigeons
Lztr1	0.09	0.00214589	pigeons
Srsf10	-0.04	0.00216421	pigeons
Sap130	-0.07	0.00218317	pigeons
Rpa2	0.00	0.00220296	pigeons
Rhbdf1	0.06	0.00221477	pigeons
Gpatch2	0.09	0.00222235	pigeons
Smc4	0.09	0.00229039	pigeons
Pnrc2	0.07	0.00229200	pigeons
Cipc	-0.02	0.00229251	pigeons
Oaf	0.21	0.00229341	pigeons
Ccdc117	-0.11	0.00231692	pigeons
Abcd2	0.00	0.00233205	pigeons
Traf3ip1	0.06	0.00236739	pigeons
Dynlrb1	-0.01	0.00236997	pigeons
Hvcn1	0.14	0.00240037	pigeons
Tmem242	-0.18	0.00244465	pigeons
Tdp2	0.02	0.00249509	pigeons
Cd151	-0.07	0.00252506	pigeons
Usp7	-0.14	0.00253883	pigeons
Nav3	0.35	0.00256297	pigeons
Paxbp1	-0.15	0.00256365	pigeons
Psmb2	-0.15	0.00258820	pigeons
Arl6ip4	0.08	0.00260881	pigeons
Ankrd44	0.19	0.00262622	pigeons
Bckdhb	0.09	0.00265795	pigeons
Gria3	0.27	0.00269478	pigeons
Eif1ad12	-0.05	0.00274671	pigeons
Foxn3	-0.90	0.00282367	pigeons
Sgce	0.01	0.00287274	pigeons
Slc38a6	0.09	0.00289744	pigeons
Rbm17	-0.11	0.00291351	pigeons
Fbxo22	-0.02	0.00294308	pigeons
Cdca7l	-1.15	0.00294930	pigeons
Slc25a3	-0.11	0.00301728	pigeons
Srsf2	-0.14	0.00303015	pigeons
Sulf2	-0.13	0.00306500	pigeons
Hoxa6	0.02	0.00311917	pigeons
Abca3	-0.15	0.00312713	pigeons
Dnajc1	0.01	0.00313695	pigeons
Srp19	-0.11	0.00315516	pigeons
Snx13	-0.08	0.00316868	pigeons
Tie1	-0.96	0.00321968	pigeons
Sec16a	-0.08	0.00322227	pigeons
Ndufs3	0.01	0.00323329	pigeons
Vps35	-0.04	0.00324454	pigeons

Glg1	-0.08	0.00330051	pigeons
Etfdh	0.02	0.00333152	pigeons
Dync2h1	0.00	0.00337893	pigeons
Bcl7a	-0.02	0.00339200	pigeons
Ston2	0.25	0.00341437	pigeons
B4galt2	0.04	0.00344227	pigeons
Lysmd3	0.05	0.00346733	pigeons
Sf3b1	-0.19	0.00348344	pigeons
Prpf39	-0.02	0.00348351	pigeons
Pdlim1	-1.09	0.00348575	pigeons
Wipf1	-0.07	0.00351736	pigeons
Snapc4	0.12	0.00357270	pigeons
Cttnbp2nl	-1.27	0.00357776	pigeons
Serac1	0.03	0.00358050	pigeons
Hacd3	-0.02	0.00362794	pigeons
Zw10	0.14	0.00365436	pigeons
Nr4a2	0.20	0.00367385	pigeons
Klf5	0.34	0.00369259	pigeons
Pnn	-0.11	0.00371516	pigeons
Usp24	-0.04	0.00375497	pigeons
Clip2	0.15	0.00379974	pigeons
Pick1	0.00	0.00383479	pigeons
Cntf	0.07	0.00388521	pigeons
Nek9	0.02	0.00394149	pigeons
Atp6v0a2	0.00	0.00394388	pigeons
Tbck	0.14	0.00394409	pigeons
Qrich1	-0.11	0.00396720	pigeons
Abhd18	0.06	0.00402058	pigeons
Paxip1	0.08	0.00404878	pigeons
Nek1	0.16	0.00409034	pigeons
Jagn1	0.04	0.00414339	pigeons
Sun1	-0.22	0.00419238	pigeons
Fam32a	-0.12	0.00420813	pigeons
Hecw1	0.32	0.00424239	pigeons
Psmd7	0.06	0.00431526	pigeons
St7l	0.01	0.00433688	pigeons
Chmp2b	-0.12	0.00444694	pigeons
Psmd13	-0.01	0.00452967	pigeons
Tead1	0.08	0.00455056	pigeons
Azin1	-0.16	0.00458599	pigeons
Srsf5	-0.03	0.00462258	pigeons
Fbxo11	0.13	0.00463170	pigeons
Rbx1	-0.11	0.00464085	pigeons
Aida	-0.05	0.00467428	pigeons
Pkp4	-0.17	0.00468642	pigeons
Vps11	-0.05	0.00479627	pigeons

Noc3l	-0.02	0.00493472	pigeons
Dtnbp1	0.06	0.00499662	pigeons
Prkaa1	-0.01	0.00503078	pigeons
Poc1a	0.05	0.00504781	pigeons
Alg2	0.10	0.00508489	pigeons
Tmem181a	0.04	0.00511247	pigeons
Phf5a	-0.03	0.00516043	pigeons
Col5a1	-0.05	0.00521527	pigeons
Tpcn1	0.09	0.00530323	pigeons
Jakmip3	-0.89	0.00530403	pigeons
Trabd	-0.03	0.00531276	pigeons
Xpnpep1	-0.09	0.00540437	pigeons
Erp44	0.00	0.00548763	pigeons
Wdr4	0.07	0.00557030	pigeons
Cnot7	-0.02	0.00557969	pigeons
St6galnac4	0.03	0.00563825	pigeons
Rcbtb2	0.03	0.00565711	pigeons
Gpn3	-0.07	0.00568011	pigeons
Capza2	-0.16	0.00574765	pigeons
Phf12	-0.14	0.00575294	pigeons
Lpcat1	-0.12	0.00579315	pigeons
Cant1	0.13	0.00582278	pigeons
Lmo4	-0.05	0.00585461	pigeons
Tgfbrap1	0.00	0.00586989	pigeons
Zeb2	-0.43	0.00587148	pigeons
Ubqln4	-0.07	0.00588152	pigeons
Bmt2	-0.04	0.00595435	pigeons
Rbm15b	-0.11	0.00595790	pigeons
Etv6	-0.96	0.00595961	pigeons
Lrwd1	0.09	0.00599177	pigeons
Nelfcd	0.04	0.00600755	pigeons
Fbxo8	-0.03	0.00603550	pigeons
Fam199x	0.02	0.00605779	pigeons
Lrif1	-0.05	0.00633086	pigeons
Nubp1	-0.11	0.00636835	pigeons
Nsd3	-0.30	0.00637923	pigeons
Lcat	0.12	0.00642261	pigeons
Isca2	0.04	0.00645707	pigeons
Cnpy3	-0.13	0.00648681	pigeons
Ippk	-0.09	0.00662850	pigeons
Rreb1	-0.21	0.00663868	pigeons
Ptgr2	0.11	0.00664630	pigeons
Aasdh	-0.01	0.00666620	pigeons
Ywhab	-0.08	0.00668064	pigeons
Ppt1	-0.14	0.00674128	pigeons
Prrx1	0.20	0.00682770	pigeons

Adamts1	0.37	0.00683255	pigeons
Fbxo32	-0.05	0.00685746	pigeons
Zfp462	0.06	0.00686429	pigeons
Psmd6	0.01	0.00691137	pigeons
Itch	-0.12	0.00706477	pigeons
Osbpl8	-0.01	0.00707681	pigeons
Nr3c2	-0.06	0.00709759	pigeons
Rbms1	-0.12	0.00711957	pigeons
Cd109	0.17	0.00713785	pigeons
Eif2ak1	0.05	0.00716942	pigeons
Pno1	0.02	0.00727238	pigeons
Hoxa3	-0.15	0.00730309	pigeons
Timm13	-0.05	0.00731003	pigeons
Atp11c	-0.21	0.00737833	pigeons
Scube3	-0.97	0.00741652	pigeons
Pds5a	-0.11	0.00746855	pigeons
Siah1b	0.08	0.00756470	pigeons
Acyp1	0.05	0.00766096	pigeons
Tab2	-0.17	0.00768287	pigeons
Xpa	-0.12	0.00773505	pigeons
Psmf1	-0.02	0.00785530	pigeons
Tnik	-0.06	0.00787163	pigeons
Rab22a	-0.02	0.00795624	pigeons
Entpd4	-0.01	0.00805463	pigeons
Gid8	-0.01	0.00806390	pigeons
Poc1b	-0.04	0.00811720	pigeons
Stat3	0.00	0.00815819	pigeons
Copb1	-0.16	0.00818928	pigeons
Adgrg1	-1.04	0.00819304	pigeons
Nufip1	0.03	0.00819465	pigeons
Nsmce2	0.11	0.00824033	pigeons
Ogfr	-0.01	0.00825170	pigeons
Ppil4	-0.13	0.00831289	pigeons
Rnft1	-0.09	0.00831845	pigeons
Stx17	-0.12	0.00831865	pigeons
Xpo7	-0.16	0.00838790	pigeons
Cers5	-0.01	0.00850521	pigeons
Cyth3	-0.18	0.00851017	pigeons
Nap1l1	0.09	0.00853053	pigeons
Napepld	-0.16	0.00854043	pigeons
Dnajc7	-0.05	0.00868540	pigeons
Rsbn1l	-0.16	0.00876358	pigeons
Ppme1	-0.07	0.00889358	pigeons
Pcsk5	0.34	0.00897537	pigeons
Khdrbs3	0.12	0.00930825	pigeons
N4bp2l2	-0.10	0.00934674	pigeons

Cgnl1	-0.08	0.00945503	pigeons
Fam53a	-0.01	0.00949705	pigeons
Dock10	0.02	0.00953433	pigeons
Tmcc1	-0.03	0.00954407	pigeons
Derl3	0.06	0.00958757	pigeons
Pgd	-0.06	0.00965383	pigeons
Dync1li1	-0.03	0.00978707	pigeons
Tmem63b	0.07	0.00982624	pigeons
Kbtbd11	0.07	0.00988686	pigeons
Letm1	-0.04	0.00989915	pigeons
Sf3b3	-0.15	0.00993984	pigeons
Xylt1	0.11	0.01005311	pigeons
Rer1	-0.08	0.01036081	pigeons
Pknox1	0.07	0.01039220	pigeons
Ubr1	-0.05	0.01039738	pigeons
Kdr	-0.99	0.01044473	pigeons
Ripk1	-0.11	0.01044931	pigeons
Agps	-0.14	0.01048585	pigeons
Fam185a	0.06	0.01057067	pigeons
Lypla1	-0.04	0.01077205	pigeons
Tjp2	-1.20	0.01078160	pigeons
Cachd1	0.01	0.01079906	pigeons
Smim4	0.03	0.01087628	pigeons
Fam135a	-0.06	0.01088906	pigeons
Surf2	0.08	0.01102310	pigeons
Tank	0.03	0.01112752	pigeons
Dync1li2	-0.19	0.01119534	pigeons
Hsd17b4	-0.20	0.01128174	pigeons
Lbr	-0.27	0.01130007	pigeons
Rilpl2	0.01	0.01133540	pigeons
Dnaja1	-0.15	0.01151713	pigeons
Usp10	-0.09	0.01153019	pigeons
Armc1	-0.08	0.01169038	pigeons
Borcs8	0.06	0.01181820	pigeons
Vps53	0.08	0.01197283	pigeons
Prelid3b	-0.09	0.01209825	pigeons
Fasn	-0.13	0.01213589	pigeons
Rpap2	0.04	0.01229449	pigeons
Hps5	0.07	0.01231972	pigeons
Tmem263	0.08	0.01237923	pigeons
Usp53	-0.07	0.01246223	pigeons
Hpgd	-1.07	0.01273892	pigeons
Rnf20	-0.18	0.01274591	pigeons
S1pr3	0.22	0.01288640	pigeons
Znfx1	-0.20	0.01293824	pigeons
Atg4c	-0.07	0.01306457	pigeons

Nop58	-0.13	0.01306874	pigeons
Rfng	0.07	0.01321278	pigeons
Zfp639	-0.10	0.01330722	pigeons
Rmdn3	-0.01	0.01335703	pigeons
Hagh	-0.01	0.01341100	pigeons
Cd47	-0.01	0.01355413	pigeons
Lhfpl2	0.04	0.01355518	pigeons
Vapa	-0.16	0.01394591	pigeons
Xpot	-0.11	0.01394934	pigeons
Papss1	-0.07	0.01411050	pigeons
Kitl	0.08	0.01415509	pigeons
Prdx1	-0.11	0.01419479	pigeons
Lims2	-1.12	0.01430954	pigeons
Zbtb46	-0.17	0.01450413	pigeons
Actr1a	-0.06	0.01460695	pigeons
Nrd1	-0.03	0.01470497	pigeons
Scn8a	0.00	0.01489609	pigeons
Cnppd1	0.00	0.01498003	pigeons
Fbxw2	-0.03	0.01499277	pigeons
Rab3il1	0.12	0.01500367	pigeons
Txn2	0.00	0.01513405	pigeons
Desi2	-0.01	0.01535028	pigeons
Edn1	-0.87	0.01554488	pigeons
Leprot	-0.12	0.01578188	pigeons
Zbtb33	-0.12	0.01592427	pigeons
Xpr1	-0.02	0.01607723	pigeons
Snapc5	0.04	0.01618809	pigeons
Dld	0.04	0.01621509	pigeons
Srf	0.08	0.01625389	pigeons
Rabep1	-0.19	0.01626253	pigeons
Ppcs	0.10	0.01637900	pigeons
Lamc1	0.21	0.01638338	pigeons
Ssbp3	-0.13	0.01659099	pigeons
Cflar	-0.23	0.01672235	pigeons
Etnk1	0.18	0.01690830	pigeons
Ldah	-0.01	0.01713201	pigeons
Rnpc3	-0.04	0.01726756	pigeons
Spen	-0.07	0.01754557	pigeons
Serpinf1	0.41	0.01759799	pigeons
Golga5	-0.12	0.01761565	pigeons
Aff1	-1.11	0.01780980	pigeons
Hmbs	-0.16	0.01782179	pigeons
Cast	-0.15	0.01784238	pigeons
Sik1	0.27	0.01805091	pigeons
Gkap1	-0.06	0.01808758	pigeons
Thoc5	0.09	0.01816306	pigeons

Nfu1	-0.02	0.01859664	pigeons
Thoc1	-0.01	0.01881302	pigeons
Abcc5	-0.05	0.01886343	pigeons
Cope	-0.08	0.01919925	pigeons
Klf11	-0.07	0.01941200	pigeons
Bcl11b	0.24	0.01948891	pigeons
Ranbp10	-0.03	0.01990046	pigeons
Tmem33	-0.03	0.01993809	pigeons
Ctnnb1	-0.10	0.02047361	pigeons
Zfp148	-0.09	0.02071069	pigeons
Tctn2	0.01	0.02085292	pigeons
Cd63	-0.09	0.02098859	pigeons
Btbd3	0.03	0.02126192	pigeons
Nup214	-0.12	0.02136070	pigeons
Clcc1	-0.03	0.02137622	pigeons
Srsf3	-0.06	0.02139478	pigeons
Tmem117	-0.70	0.02178662	pigeons
Tiprl	-0.11	0.02181453	pigeons
Necap1	0.09	0.02187079	pigeons
Ramp2	-1.05	0.02190392	pigeons
Nr4a1	0.45	0.02235175	pigeons
Ism1	0.31	0.02253765	pigeons
Prmt9	-0.07	0.02253913	pigeons
Tex10	0.01	0.02275204	pigeons
Aagab	0.05	0.02294747	pigeons
Pdia6	-0.11	0.02295835	pigeons
Kif26a	-0.82	0.02371677	pigeons
Gpr107	0.00	0.02388226	pigeons
Psme3	-0.06	0.02413641	pigeons
Lsm1	-0.06	0.02414249	pigeons
Slc15a1	-0.85	0.02453018	pigeons
Crip1	-0.94	0.02454229	pigeons
Atxn2	-0.21	0.02459481	pigeons
Anxa2	0.13	0.02479075	pigeons
Tspan9	-1.14	0.02487501	pigeons
Trim33	-0.16	0.02491855	pigeons
Txndc12	-0.11	0.02589283	pigeons
Snx14	-0.92	0.02615492	pigeons
Clta	0.06	0.02623397	pigeons
Psmc6	-0.04	0.02632022	pigeons
Usp12	-0.10	0.02636561	pigeons
Sdhaf2	0.11	0.02640913	pigeons
Gabpa	-0.11	0.02654646	pigeons
Sar1b	-0.10	0.02660813	pigeons
Ptp4a3	0.08	0.02671348	pigeons
Tmem64	0.00	0.02681450	pigeons

Rgs1	0.68	0.02682904	pigeons
Appl2	-0.15	0.02684212	pigeons
Ccdc102a	0.07	0.02684758	pigeons
Rnf217	0.07	0.02696298	pigeons
D630045J12Rik	0.10	0.02741798	pigeons
Usb1	0.02	0.02796654	pigeons
Pwwp2a	-0.09	0.02816428	pigeons
Galnt11	0.01	0.02823307	pigeons
Tpm1	-0.06	0.02839655	pigeons
Cwc25	0.08	0.02860709	pigeons
Ddx31	-0.02	0.02891090	pigeons
Dtx2	-0.16	0.02902867	pigeons
Snx19	-0.02	0.02923609	pigeons
Tle3	-0.14	0.02930756	pigeons
Trappc8	-0.11	0.02996535	pigeons
Bpnt1	0.12	0.03009089	pigeons
Wdfy3	0.13	0.03020512	pigeons
Denr	-0.14	0.03078365	pigeons
Tnpo1	-0.17	0.03082608	pigeons
Senp5	-0.09	0.03110780	pigeons
Auh	-0.01	0.03124129	pigeons
Psmg1	0.14	0.03135105	pigeons
Ppp3cb	-0.20	0.03162739	pigeons
Pdpk1	-0.20	0.03171140	pigeons
Zmynd8	-0.11	0.03191273	pigeons
Spred2	-1.25	0.03220369	pigeons
Chst3	0.05	0.03235081	pigeons
Pdia4	-0.20	0.03235669	pigeons
Gabarapl2	-0.20	0.03255088	pigeons
Paip1	-0.12	0.03260951	pigeons
Cd9	0.06	0.03342711	pigeons
Wdr37	-0.12	0.03447285	pigeons
Kansl1l	0.06	0.03481001	pigeons
Eif4e2	0.10	0.03580265	pigeons
Iars2	-0.02	0.03589380	pigeons
Usp3	-0.16	0.03686730	pigeons
Hook1	-0.01	0.03709018	pigeons
Stard3nl	-0.11	0.03732920	pigeons
Swap70	0.10	0.03777222	pigeons
Atp6v1c1	-0.05	0.03857213	pigeons
Jak1	-0.11	0.03884626	pigeons
Ndufs7	-0.07	0.03921835	pigeons
Lta4h	-0.01	0.03955728	pigeons
Stmn1	-0.06	0.03975917	pigeons
Fbxl2	0.07	0.03988649	pigeons
Tspan18	-0.13	0.04058512	pigeons

Nmi	-0.12	0.04080966	pigeons
Arsj	0.09	0.04105021	pigeons
Parg	-0.09	0.04110047	pigeons
Wbp2nl	0.00	0.04142889	pigeons
Smap1	-0.07	0.04142997	pigeons
Stt3b	-0.20	0.04147184	pigeons
Cct8	-0.18	0.04253557	pigeons
Zfp821	-0.02	0.04283900	pigeons
Pkn2	-0.30	0.04338209	pigeons
Rab27a	-0.24	0.04338683	pigeons
Hdac7	-1.38	0.04339753	pigeons
Brcc3	0.01	0.04398668	pigeons
Txndc9	-0.12	0.04408418	pigeons
Wdr91	-0.12	0.04409910	pigeons
Nap1l4	-0.19	0.04425973	pigeons
Rbm19	-0.12	0.04455758	pigeons
Pin1	-0.02	0.04463196	pigeons
Bloc1s4	-0.02	0.04468992	pigeons
Nipsnap2	-0.03	0.04526697	pigeons
Hdac11	0.06	0.04538516	pigeons
Hectd4	-0.14	0.04586093	pigeons
Arfgap1	-0.11	0.04612395	pigeons
Unc119b	-0.07	0.04688283	pigeons
Nus1	-0.08	0.04711576	pigeons
Abcf2	-0.08	0.04734044	pigeons
Urb1	-0.06	0.04736077	pigeons
Uchl3	0.04	0.04802209	pigeons
Nudt19	0.02	0.04813678	pigeons
Alad	0.06	0.04891054	pigeons
Agfg1	-0.13	0.04984206	pigeons
Tmem184b	-0.22	0.05076105	pigeons
Pafah1b1	-0.09	0.05230687	pigeons
Asb1	0.06	0.05253283	pigeons
Clasp1	-0.05	0.05272267	pigeons
Atg2b	-0.05	0.05273092	pigeons
Tfip11	-0.06	0.05300339	pigeons
Smtn	0.15	0.05366588	pigeons
Nek6	0.03	0.05366790	pigeons
Vps29	-0.10	0.05375112	pigeons
Golph3l	-0.11	0.05453849	pigeons
Vmp1	-0.16	0.05520754	pigeons
Plekha3	-0.08	0.05615846	pigeons
Brms1l	-0.03	0.05640852	pigeons
S100a11	-0.01	0.05707695	pigeons
Ndfip2	-0.07	0.05747866	pigeons
Zfp512b	-0.05	0.05788569	pigeons

Htatsf1	-0.10	0.05790577	pigeons
Cmtr1	-0.04	0.05808854	pigeons
Tmpo	-0.28	0.05856366	pigeons
Ep400	-0.27	0.05875499	pigeons
Pdzrn4	0.40	0.05877211	pigeons
Arih1	-0.16	0.05880237	pigeons
Smarcc1	-0.18	0.05902349	pigeons
Ythdf3	-0.28	0.05949932	pigeons
Tcea2	0.01	0.06016074	pigeons
Nsfl1c	-0.06	0.06018443	pigeons
Pnpla8	-0.05	0.06050866	pigeons
Zranb2	-0.18	0.06094219	pigeons
Tbc1d5	-0.03	0.06109093	pigeons
Kif13b	-0.19	0.06236471	pigeons
Tiam1	0.22	0.06288149	pigeons
Ankrd27	-0.06	0.06294247	pigeons
Pdcd11	0.01	0.06296556	pigeons
Fxyd6	-0.22	0.06344979	pigeons
Plrg1	-0.07	0.06363676	pigeons
Nt5dc1	0.23	0.06422574	pigeons
Spty2d1	-0.41	0.06440614	pigeons
Spata18	0.04	0.06480435	pigeons
Tra2a	-0.12	0.06536174	pigeons
Vil1	0.02	0.06582055	pigeons
Dpy30	-0.05	0.06600229	pigeons
Rpgrip1l	0.01	0.06600507	pigeons
Ttbk1	0.02	0.06603217	pigeons
Nudcd3	-0.03	0.06754077	pigeons
Slc39a9	0.03	0.06765977	pigeons
Rangap1	-0.05	0.06782480	pigeons
Emc8	-0.05	0.06831836	pigeons
Pcsk2	-1.02	0.06858849	pigeons
Tbc1d2b	-0.18	0.06890038	pigeons
Efhd1	0.00	0.06893424	pigeons
Ppie	0.08	0.06965459	pigeons
Dip2c	-0.19	0.07063384	pigeons
Adk	-0.98	0.07123284	pigeons
Ccny	-1.17	0.07132045	pigeons
Snrnp25	0.01	0.07145378	pigeons
Fam222b	-0.03	0.07196338	pigeons
Zc3h14	0.02	0.07215189	pigeons
Akap9	-0.03	0.07274459	pigeons
Sacm1l	-0.22	0.07284224	pigeons
Swi5	-0.14	0.07313041	pigeons
Cul1	-0.14	0.07450547	pigeons
Cry2	-0.05	0.07451085	pigeons

Galc	-0.06	0.07531552	pigeons
Ndufs8	-0.22	0.07573323	pigeons
Vegfc	0.10	0.07603844	pigeons
Zfyve21	-0.09	0.07605439	pigeons
Frs2	-0.07	0.07632371	pigeons
Rbm34	-0.05	0.07644474	pigeons
Shisa5	-0.11	0.07691569	pigeons
Timmdc1	-0.02	0.07794420	pigeons
Drosha	0.07	0.07834272	pigeons
Psen2	-0.10	0.07885919	pigeons
Fam76b	0.00	0.08016304	pigeons
Dio2	0.32	0.08016468	pigeons
Ankrd50	-0.02	0.08168331	pigeons
Dnaja2	-0.21	0.08242747	pigeons
Gapvd1	-0.01	0.08255796	pigeons
Atpaf1	0.10	0.08350241	pigeons
Naa25	0.01	0.08363015	pigeons
Itga11	0.04	0.08385680	pigeons
Commd1b	-0.11	0.08414677	pigeons
Yme1l1	-0.10	0.08481621	pigeons
Otud7a	0.13	0.08589885	pigeons
Rnf141	-0.05	0.08621093	pigeons
Nsun2	0.09	0.08731607	pigeons
Bnip1	-0.03	0.08864780	pigeons
Ddx51	-0.05	0.08944005	pigeons
Ndufa6	-0.09	0.09068372	pigeons
Ncald	0.10	0.09069320	pigeons
Actr8	-0.05	0.09085612	pigeons
Coq9	0.07	0.09220005	pigeons
Ppm1f	-1.13	0.09239510	pigeons
Ell	-0.08	0.09303764	pigeons
Psmd9	-0.14	0.09436849	pigeons
Tcof1	-0.10	0.09652842	pigeons
Ciapin1	-0.05	0.09815662	pigeons
Snx3	-0.08	0.09839732	pigeons
Zfp292	-0.20	0.09846056	pigeons
Chmp6	-0.11	0.09884944	pigeons
Llph	-0.09	0.09908787	pigeons
Hipk2	-0.15	0.09928650	pigeons
Wdr20	-0.23	0.09980620	pigeons
Lin52	-0.05	0.10106508	pigeons
Dpp9	0.09	0.10206629	pigeons
Nsf	0.06	0.10284267	pigeons
Trip4	-0.09	0.10532303	pigeons
Unk	-0.12	0.10566621	pigeons
Fn1	-0.06	0.10625085	pigeons

Fam184a	0.08	0.10666336	pigeons
Ndufs4	0.06	0.10804231	pigeons
Rfc4	0.02	0.10823612	pigeons
Cmtm4	-0.01	0.10882067	pigeons
Pwp1	-0.06	0.10943993	pigeons
Ibtk	-0.05	0.10995779	pigeons
Ttc3	0.00	0.11002964	pigeons
Il15	-0.18	0.11148121	pigeons
Zcrb1	0.06	0.11164918	pigeons
Txndc15	-0.04	0.11205845	pigeons
Arl5a	-0.10	0.11418692	pigeons
Rnf111	-0.11	0.11464153	pigeons
Spats2l	0.03	0.11558566	pigeons
Eif4a2	0.19	0.11671059	pigeons
Cnot2	-0.09	0.11777545	pigeons
Rbfox2	-1.06	0.11780460	pigeons
Peli2	0.03	0.11879782	pigeons
Chsy3	0.17	0.11890010	pigeons
Kdm1a	-0.05	0.12012128	pigeons
Elovl1	-0.05	0.12016073	pigeons
Ube2v2	-0.11	0.12377550	pigeons
Lpar1	0.06	0.12377788	pigeons
Kif3a	0.02	0.12464878	pigeons
Wdfy1	-0.05	0.12467945	pigeons
Dnajb12	-0.23	0.12603900	pigeons
Parn	-0.05	0.12781960	pigeons
Psmd2	-0.01	0.12949754	pigeons
Ddit4	-0.15	0.13082663	pigeons
Rab2a	-0.24	0.13291159	pigeons
Xrcc4	0.09	0.13410951	pigeons
Hmbox1	-0.12	0.13433598	pigeons
Apool	0.02	0.13559940	pigeons
Nup205	0.07	0.13562175	pigeons
Pum1	-0.19	0.13639897	pigeons
Irf1	-0.01	0.13826435	pigeons
Atl1	0.07	0.13980589	pigeons
Angpt2	0.10	0.14001678	pigeons
Phf10	-0.12	0.14154244	pigeons
Acot13	0.06	0.14179948	pigeons
AU040320	-0.09	0.14304547	pigeons
Washc5	-0.05	0.14337134	pigeons
Bst1	-0.02	0.14473119	pigeons
Usp25	-0.21	0.14555710	pigeons
Rapgef1	-0.16	0.14569084	pigeons
Fyttd1	-0.24	0.14724289	pigeons
Vti1b	-0.07	0.14734484	pigeons

Ero1b	-0.12	0.14755473	pigeons
Arid1b	-0.55	0.14859486	pigeons
Pxdc1	-0.21	0.15006111	pigeons
Snx18	0.03	0.15238685	pigeons
Psmc2	-0.05	0.15248947	pigeons
Atrx	-0.20	0.15278762	pigeons
Tab3	-0.12	0.15304334	pigeons
Stk38l	0.14	0.15355059	pigeons
Ccdc59	-0.03	0.15437719	pigeons
Rbl1	-0.17	0.15468207	pigeons
Osbpl6	0.06	0.15472306	pigeons
Trak1	-0.10	0.15605834	pigeons
Efemp1	-0.92	0.15983171	pigeons
Psmb7	-0.22	0.16033203	pigeons
Ppara	0.09	0.16472085	pigeons
Zfp523	-0.07	0.16479948	pigeons
Rpf1	0.06	0.16792380	pigeons
Ift172	0.08	0.16927294	pigeons
Nipa1	0.07	0.16964430	pigeons
Nod1	0.03	0.17329448	pigeons
Crem	-0.44	0.17376166	pigeons
Chst15	0.15	0.17381588	pigeons
Spsb1	0.04	0.17415723	pigeons
Six4	-0.04	0.17454219	pigeons
Itgb8	0.25	0.17586714	pigeons
Ncoa3	-0.22	0.17735596	pigeons
Leo1	-0.13	0.17843170	pigeons
Naa15	-0.06	0.18035393	pigeons
Chaf1a	0.16	0.18229265	pigeons
Sf3b6	-0.18	0.18292248	pigeons
Pm20d2	-0.12	0.18397333	pigeons
Dram2	-0.21	0.18517425	pigeons
Ints2	0.00	0.18697568	pigeons
Trappc4	0.13	0.18753804	pigeons
Cdk8	-0.11	0.18888900	pigeons
Fuca1	-0.03	0.18938497	pigeons
Slc25a36	0.17	0.19093256	pigeons
Ints4	0.04	0.19123450	pigeons
Katnbl1	-0.04	0.19455227	pigeons
Eif2b1	-0.04	0.19673271	pigeons
Haus3	0.11	0.19717248	pigeons
Rbm12b2	-0.04	0.20090581	pigeons
Rab10	-0.17	0.20224755	pigeons
Ppp6r3	-0.16	0.20363438	pigeons
Slc7a6os	-0.09	0.20414468	pigeons
Sptlc1	0.03	0.20428606	pigeons

Dynlt3	-0.07	0.20484731	pigeons
Urm1	-0.23	0.20610719	pigeons
Ftsj3	0.02	0.20719872	pigeons
Psma5	-0.16	0.20736620	pigeons
Eif5b	-0.21	0.21060627	pigeons
Heatr5b	-0.07	0.21183475	pigeons
Acvrl1	-1.16	0.21249288	pigeons
Cyld	-0.06	0.21372555	pigeons
Utp6	-0.08	0.21647076	pigeons
Tmem165	-0.14	0.21652028	pigeons
Zfp618	0.01	0.21706024	pigeons
Gne	0.03	0.21812274	pigeons
Arglu1	-0.25	0.21859666	pigeons
Slc31a1	-0.02	0.22039800	pigeons
Zdhhc8	-0.01	0.22042805	pigeons
Kdm3b	-0.23	0.22084620	pigeons
Ska2	-0.11	0.22218536	pigeons
Sema7a	-0.96	0.22449053	pigeons
Cask	0.09	0.22477657	pigeons
Tbpl1	-0.05	0.22493239	pigeons
Rsu1	-0.09	0.22923707	pigeons
Fbxo33	-0.23	0.23010113	pigeons
Stoml2	-0.03	0.23126589	pigeons
Zfp652	-0.19	0.23571097	pigeons
Ankrd13a	-0.12	0.23601250	pigeons
Prelid1	-0.19	0.24648478	pigeons
Arfgap2	-0.09	0.25014447	pigeons
Bmp2k	-0.13	0.25345705	pigeons
Akirin2	-0.17	0.25499418	pigeons
Nifk	-0.15	0.25598451	pigeons
Ube2h	-0.05	0.25756738	pigeons
Ugcg	0.21	0.25792129	pigeons
Eif5a2	-0.16	0.26506339	pigeons
Etl4	-1.06	0.26648560	pigeons
Exosc10	0.03	0.27856752	pigeons
Tatdn3	-0.03	0.28093610	pigeons
Ndufa12	-0.22	0.28315031	pigeons
Ntrk2	0.06	0.28887118	pigeons
Grk4	-0.05	0.29030976	pigeons
Ttll12	0.00	0.29495010	pigeons
Swt1	-0.06	0.30057911	pigeons
Tns1	0.13	0.31385977	pigeons
Tango6	-0.04	0.31413368	pigeons
Pou2f1	-0.08	0.31745306	pigeons
Oaz2	-0.11	0.31803662	pigeons
Tfrc	-0.87	0.32039257	pigeons

Slc4a11	-0.74	0.32090973	pigeons
Srebf1	-0.04	0.32454511	pigeons
Tmem258	-0.16	0.32503994	pigeons
Tmem50a	-0.17	0.32604968	pigeons
Ipo5	-0.03	0.32732277	pigeons
Arhgap10	0.03	0.32972240	pigeons
Ogn	-0.09	0.33185978	pigeons
Dync1i2	-0.31	0.33320295	pigeons
Dusp14	0.21	0.33539094	pigeons
Acbd5	-0.11	0.33731314	pigeons
Aplf	-0.11	0.33940672	pigeons
Aff4	-0.23	0.35156602	pigeons
Tmem200b	0.09	0.35306596	pigeons
Iffo2	0.09	0.35687619	pigeons
Igf2r	-0.27	0.35818946	pigeons
Ddx23	-0.11	0.36002177	pigeons
Atp6v1d	-0.16	0.36034087	pigeons
Cltc	-0.20	0.36088524	pigeons
Ppm1d	-0.26	0.36338177	pigeons
Tmem222	-0.13	0.36420450	pigeons
Tm9sf2	-0.31	0.36631903	pigeons
Rnf145	-0.20	0.36668550	pigeons
Wdr48	-0.02	0.36872390	pigeons
Camsap2	0.12	0.36957340	pigeons
Fbxl5	-0.18	0.37545622	pigeons
Ppp1r7	0.05	0.37883953	pigeons
Flnb	-1.06	0.38216255	pigeons
Ppp2r2d	-0.26	0.38327372	pigeons
Riok1	-0.04	0.38724561	pigeons
Hmgb3	-0.07	0.38980661	pigeons
Copa	-0.14	0.39293476	pigeons
Arhgef3	-1.09	0.39333010	pigeons
Saal1	0.10	0.39800038	pigeons
Vapb	-0.16	0.40247226	pigeons
Helb	-0.02	0.41173086	pigeons
Rarres2	-0.17	0.41491333	pigeons
Disc1	0.03	0.41944913	pigeons
Kpna4	-0.22	0.41958428	pigeons
Fbxo30	-0.07	0.42156697	pigeons
Zdhhc7	-0.03	0.42262146	pigeons
Ube3c	-0.06	0.42286408	pigeons
Uchl5	-0.10	0.42505744	pigeons
Ptpn6	-0.08	0.42512081	pigeons
Trdmt1	-0.04	0.42592025	pigeons
Smurf2	-1.02	0.42859412	pigeons
Adam23	-0.02	0.43766742	pigeons

Atp2a2	-0.11	0.43935509	pigeons
Cwc27	-0.07	0.44020565	pigeons
Ube2d2a	-0.20	0.44582953	pigeons
Irf2	-0.34	0.45306052	pigeons
Rhoc	0.10	0.45479350	pigeons
Ndufv2	-0.03	0.46050415	pigeons
Cmtm3	0.06	0.46069314	pigeons
Snx21	-0.02	0.46472699	pigeons
Csnk2a1	-0.04	0.46687764	pigeons
Agtpbp1	-0.05	0.47192491	pigeons
Ptgs2	0.33	0.47974186	pigeons
Bmp6	-0.86	0.47982403	pigeons
Slc4a7	0.17	0.48421928	pigeons
Fhl4	-0.07	0.48489499	pigeons
Gnas	-0.10	0.48547702	pigeons
Slc16a6	0.14	0.49159343	pigeons
Kdm5b	-0.10	0.49267832	pigeons
Ccnyl1	-0.27	0.49485658	pigeons
Dlgap4	-0.26	0.50412260	pigeons
Ptpn11	-0.11	0.50502158	pigeons
Zfand1	-0.01	0.50871058	pigeons
Ndufb10	-0.27	0.51033440	pigeons
Opa1	-0.06	0.51307314	pigeons
Galnt1	-0.23	0.51430505	pigeons
Aqp1	-0.02	0.51995644	pigeons
Rwdd1	-0.06	0.52173252	pigeons
Taf5	-0.07	0.52318020	pigeons
B9d1	-0.10	0.52391888	pigeons
Polg2	-0.22	0.52396014	pigeons
Wsb2	-0.05	0.52484948	pigeons
Rad51b	0.13	0.53011424	pigeons
Sdf2l1	-0.07	0.53189905	pigeons
Nisch	-0.39	0.53425672	pigeons
Srsf1	-0.14	0.54495051	pigeons
Lmod1	0.23	0.54645863	pigeons
Zfp319	-0.10	0.54684257	pigeons
Plod1	-0.02	0.54685410	pigeons
Rmdn1	-0.04	0.55374181	pigeons
Ly75	-0.25	0.55516884	pigeons
Itfg1	-0.20	0.56121784	pigeons
Rbsn	-0.09	0.56269685	pigeons
Prpf4b	-0.24	0.56491685	pigeons
Scaper	-0.11	0.57324797	pigeons
Zfat	-0.09	0.57440760	pigeons
Heatr3	-0.08	0.57771187	pigeons
Nelfb	0.03	0.59188290	pigeons

Cry1	0.11	0.59464476	pigeons
Esco1	-0.10	0.59475700	pigeons
Tc2n	-0.01	0.60143344	pigeons
Zfp335	-0.09	0.61429312	pigeons
Spg11	-0.05	0.61448097	pigeons
Tpr	-0.35	0.61778753	pigeons
Setd3	0.03	0.62130512	pigeons
Ptpn2	-0.28	0.62203843	pigeons
Cir1	-0.03	0.62957941	pigeons
Ephb1	-1.16	0.63643287	pigeons
Hsbp1	-0.24	0.63878398	pigeons
Tpd52l2	-0.22	0.64178807	pigeons
Acsl1	-0.08	0.64262705	pigeons
Ergic2	-0.11	0.64492092	pigeons
Htt	-0.35	0.64527980	pigeons
Tbc1d1	0.02	0.65800407	pigeons
Arpc1a	-0.17	0.67570127	pigeons
Pabpc4	-0.05	0.69133178	pigeons
Ppil2	0.01	0.69619679	pigeons
Thumpd3	-0.21	0.70262683	pigeons
St3gal3	-0.14	0.70658255	pigeons
Ric8b	-0.13	0.70819460	pigeons
Tmem116	-0.10	0.71385526	pigeons
Zfc3h1	-0.23	0.71960540	pigeons
Far1	-0.06	0.72149156	pigeons
Entpd6	0.16	0.72171622	pigeons
Phlpp1	-0.13	0.73604184	pigeons
Tmem19	-0.07	0.74269794	pigeons
Ddrgk1	-0.21	0.74853072	pigeons
Ppp3cc	-0.02	0.75213291	pigeons
Pibf1	-0.03	0.75453325	pigeons
Stk40	-0.07	0.75461488	pigeons
Ndufs1	-0.07	0.75466100	pigeons
Wdr3	-0.13	0.75618321	pigeons
Sprtn	-0.07	0.75627591	pigeons
Bfar	-0.14	0.75675974	pigeons
Bdp1	-0.03	0.75986345	pigeons
Lysmd2	-0.02	0.78092226	pigeons
Nup54	-0.09	0.78663569	pigeons
Rbm18	-0.08	0.79090076	pigeons
Hnrnph3	-0.20	0.79227760	pigeons
Larp1	-0.21	0.79747696	pigeons
Ccni	-0.16	0.80967724	pigeons
Tmem123	-0.11	0.81083101	pigeons
Xbp1	-0.15	0.81398690	pigeons
Ctdspl	0.20	0.81589499	pigeons

Erbin	-0.17	0.82332396	pigeons
Kin	-0.20	0.82458719	pigeons
Exoc6	0.09	0.83431521	pigeons
Klf7	-1.13	0.83640570	pigeons
Pard6g	0.04	0.83773013	pigeons
Klhl5	-0.09	0.83773272	pigeons
9130409I23Rik	-0.06	0.83991148	pigeons
U2af1	-0.18	0.85047679	pigeons
S100a13	-1.04	0.85319037	pigeons
Ahcyl1	0.03	0.87496962	pigeons
Pef1	-0.06	0.87773418	pigeons
Arid4b	-0.12	0.87788761	pigeons
Ranbp1	-0.31	0.88325293	pigeons
Cnot6	-0.28	0.89818737	pigeons
Dcun1d2	-1.02	0.90130938	pigeons
Zswim5	0.00	0.90953630	pigeons
Dis3l2	-0.17	0.91238934	pigeons
Rab14	-0.11	0.91483577	pigeons
Hdgfl3	-0.09	0.94576769	pigeons
Tcp1	-0.19	0.94935911	pigeons
Sod1	-0.26	0.97155337	pigeons
Nktr	-0.22	0.97597013	pigeons
Ncbp1	-0.14	1.00000000	pigeons
Bmp2	-0.03	1.00000000	pigeons
Ddost	-0.23	1.00000000	pigeons
Rab31	-0.15	1.00000000	pigeons
Trp53bp2	-0.20	1.00000000	pigeons
Dnajb14	-0.05	1.00000000	pigeons
Flt4	-0.73	1.00000000	pigeons
Pdzd8	-0.19	1.00000000	pigeons
Ola1	-0.26	1.00000000	pigeons
Uspl1	-0.09	1.00000000	pigeons
Nfkb1	-0.07	1.00000000	pigeons
Foxk2	-0.28	1.00000000	pigeons
Ift122	0.01	1.00000000	pigeons
Nfyc	-0.12	1.00000000	pigeons
Clk2	-0.09	1.00000000	pigeons
Ppp4r3a	-0.19	1.00000000	pigeons
Col6a3	-0.06	1.00000000	pigeons
Atp13a3	-0.16	1.00000000	pigeons
Dock1	-0.90	1.00000000	pigeons
Polk	-0.24	1.00000000	pigeons
Rab5a	-0.27	1.00000000	pigeons
Taf11	-0.11	1.00000000	pigeons
Zfp622	-0.07	1.00000000	pigeons
Tenm4	-0.11	1.00000000	pigeons

Tada1	-0.17	1.00000000	pigeons
Tlr3	0.00	1.00000000	pigeons
Ube2g2	-0.20	1.00000000	pigeons
Zfp518b	-0.12	1.00000000	pigeons
Hells	-0.20	1.00000000	pigeons
Wwp1	0.05	1.00000000	pigeons
Npnt	-0.01	1.00000000	pigeons
Uxs1	-0.26	1.00000000	pigeons
Oxsr1	-1.03	1.00000000	pigeons
Ist1	-0.04	1.00000000	pigeons
Rcc1	-0.04	1.00000000	pigeons
Polr1e	-0.01	1.00000000	pigeons
Atxn7l1	-0.21	1.00000000	pigeons
Zbtb18	-0.25	1.00000000	pigeons
Taok1	-0.10	1.00000000	pigeons
Pknox2	0.11	1.00000000	pigeons
Ube2j2	-0.21	1.00000000	pigeons
Grpel1	-0.01	1.00000000	pigeons
Chmp5	-0.25	1.00000000	pigeons
Taf4	0.03	1.00000000	pigeons
Eif4e	-0.12	1.00000000	pigeons
Snhg4	-0.12	1.00000000	pigeons
Ncoa2	-0.08	1.00000000	pigeons
Cisd2	-0.13	1.00000000	pigeons
Nectin1	-0.10	1.00000000	pigeons
Acox3	0.01	1.00000000	pigeons
Cpeb3	-0.07	1.00000000	pigeons
Brpf3	-0.04	1.00000000	pigeons
Slc15a4	-0.08	1.00000000	pigeons
Pikfyve	-0.30	1.00000000	pigeons
Sri	-0.19	1.00000000	pigeons
Itpr1	-0.02	1.00000000	pigeons
Ano5	0.20	1.00000000	pigeons
Lmbr1	-0.18	1.00000000	pigeons
Dnajc2	-0.19	1.00000000	pigeons
Nrf1	-0.12	1.00000000	pigeons
Trappc3	0.04	1.00000000	pigeons
Scaf11	-0.18	1.00000000	pigeons
Tmem11	-0.03	1.00000000	pigeons
Zfp830	-0.07	1.00000000	pigeons
Slf2	-0.25	1.00000000	pigeons
Errfi1	-0.04	1.00000000	pigeons
Dctn6	-0.17	1.00000000	pigeons
Ncoa5	-0.01	1.00000000	pigeons
Rdx	-0.24	1.00000000	pigeons
Borcs5	0.07	1.00000000	pigeons

Arrdc3	-0.58	1.00000000	pigeons
Itsn1	-0.19	1.00000000	pigeons
Rlim	-0.30	1.00000000	pigeons
Rpa3	0.04	1.00000000	pigeons
Jmy	-0.11	1.00000000	pigeons
Ywhaz	-0.03	1.00000000	pigeons
Pld1	-0.03	1.00000000	pigeons
Trmt13	-0.11	1.00000000	pigeons
Slc35b3	-0.07	1.00000000	pigeons
Zbtb37	0.05	1.00000000	pigeons
Gzf1	-0.25	1.00000000	pigeons
Atp11b	-0.19	1.00000000	pigeons
Fndc3a	-0.12	1.00000000	pigeons
Eif1	0.06	1.00000000	pigeons
Sh3bgrl	-0.22	1.00000000	pigeons
Canx	-0.06	1.00000000	pigeons
Ccdc91	0.10	1.00000000	pigeons
Golga1	0.00	1.00000000	pigeons
Dpysl3	0.11	1.00000000	pigeons
Rock2	-0.13	1.00000000	pigeons
Atp10a	0.04	1.00000000	pigeons
Timm21	0.07	1.00000000	pigeons
Synm	-0.98	1.00000000	pigeons
Prpf38a	-0.14	1.00000000	pigeons
Pdcd6	-0.22	1.00000000	pigeons
Osbpl9	-0.18	1.00000000	pigeons
Pla2g12a	-0.06	1.00000000	pigeons
Usp54	-1.11	1.00000000	pigeons
Gpi1	-0.21	1.00000000	pigeons
Tmco3	-0.16	1.00000000	pigeons
Rnf8	-0.14	1.00000000	pigeons
Rhot1	0.00	1.00000000	pigeons
Snip1	-0.15	1.00000000	pigeons
Smarca5	-0.28	1.00000000	pigeons
Pds5b	-0.28	1.00000000	pigeons
Klf13	-1.04	1.00000000	pigeons
Col6a1	-0.12	1.00000000	pigeons
Taf2	-0.21	1.00000000	pigeons
Kdm2b	-0.08	1.00000000	pigeons
Ergic1	-0.24	1.00000000	pigeons
Ankfy1	-0.17	1.00000000	pigeons
Ecm2	0.10	1.00000000	pigeons
Asf1a	0.02	1.00000000	pigeons
Vcpip1	-0.14	1.00000000	pigeons
Lyn	-0.80	1.00000000	pigeons
Snap29	-0.16	1.00000000	pigeons

Ifih1	-0.04	1.00000000	pigeons
Ino80	-1.02	1.00000000	pigeons
Dlc1	0.09	1.00000000	pigeons
Trmt10c	-0.02	1.00000000	pigeons
Fosl2	-0.01	1.00000000	pigeons
Pdk1	-0.19	1.00000000	pigeons
Slc2a13	0.02	1.00000000	pigeons
Tarbp1	0.02	1.00000000	pigeons
Tra2b	-0.21	1.00000000	pigeons
Ptges2	0.12	1.00000000	pigeons
Sap30l	-0.09	1.00000000	pigeons
Jazf1	-0.02	1.00000000	pigeons
Tssc4	0.08	1.00000000	pigeons
Lmcd1	0.00	1.00000000	pigeons
Xxylt1	-0.03	1.00000000	pigeons
Edrf1	-0.02	1.00000000	pigeons
Tacc3	-0.21	1.00000000	pigeons
Klf10	-0.15	1.00000000	pigeons
Foxo1	-0.26	1.00000000	pigeons
Chmp4b	-0.22	1.00000000	pigeons
R3hdm1	-0.28	1.00000000	pigeons
Fmn1	0.06	1.00000000	pigeons
Zbtb43	-0.03	1.00000000	pigeons
Fbxo9	-0.12	1.00000000	pigeons
Ryk	-0.08	1.00000000	pigeons
Rpap1	-0.14	1.00000000	pigeons
Stk24	-0.90	1.00000000	pigeons
Zfp407	-0.73	1.00000000	pigeons
Slc25a24	-0.20	1.00000000	pigeons
Larp4	-0.08	1.00000000	pigeons
Pdhb	-0.14	1.00000000	pigeons
Nusap1	-0.03	1.00000000	pigeons
Lonp1	-0.14	1.00000000	pigeons
Efcab14	-0.19	1.00000000	pigeons
Bclaf1	-0.36	1.00000000	pigeons
Picalm	0.25	1.00000000	pigeons
Sptan1	-0.84	1.00000000	pigeons
Dnajc5	-0.10	1.00000000	pigeons
Ndfip1	-0.22	1.00000000	pigeons
Jmjd1c	-0.09	1.00000000	pigeons
Senp7	-0.38	1.00000000	pigeons
Actn1	-0.07	1.00000000	pigeons
Cish	-1.04	1.00000000	pigeons
Homer2	0.00	1.00000000	pigeons
Polr2b	-0.15	1.00000000	pigeons
Tnip1	-0.11	1.00000000	pigeons

Afdn	-0.77	1.00000000	pigeons
Pdhx	-0.28	1.00000000	pigeons
Ccdc66	-0.02	1.00000000	pigeons
Thy1	-0.06	1.00000000	pigeons
Ift88	-0.13	1.00000000	pigeons
Dnm1l	-0.02	1.00000000	pigeons
Pex14	-0.13	1.00000000	pigeons
Ncstn	-0.11	1.00000000	pigeons
Eml6	-0.88	1.00000000	pigeons
Agl	-0.09	1.00000000	pigeons
Lrrcc1	-0.18	1.00000000	pigeons
Exosc1	0.10	1.00000000	pigeons
Vps39	-0.12	1.00000000	pigeons
Elp4	-0.11	1.00000000	pigeons
Cpeb2	-0.06	1.00000000	pigeons
Cenpt	-0.03	1.00000000	pigeons
Lrrc4c	0.29	1.00000000	pigeons
Stau1	0.00	1.00000000	pigeons
Nlrc5	0.08	1.00000000	pigeons
Snx17	-0.26	1.00000000	pigeons
Runx2	-0.01	1.00000000	pigeons
Cstf2	0.02	1.00000000	pigeons
Dcun1d1	-0.03	1.00000000	pigeons
Kctd3	-0.12	1.00000000	pigeons
Pik3c3	-0.02	1.00000000	pigeons
Crnkl1	-0.05	1.00000000	pigeons
Fermt2	-0.77	1.00000000	pigeons
Sp4	-0.02	1.00000000	pigeons
Cfd	-0.02	1.00000000	pigeons
Prkar2a	-0.11	1.00000000	pigeons
Nudcd1	-0.09	1.00000000	pigeons
Tmem167	-0.17	1.00000000	pigeons
Cdk12	-0.15	1.00000000	pigeons
Bnip2	-0.21	1.00000000	pigeons
Vps4b	-0.35	1.00000000	pigeons
Setx	-0.38	1.00000000	pigeons
Zfp384	-0.15	1.00000000	pigeons
Rheb	-0.28	1.00000000	pigeons
Slc35c1	-0.06	1.00000000	pigeons
Zc3h12b	-0.03	1.00000000	pigeons
Immt	-0.03	1.00000000	pigeons
Gnpat	-0.05	1.00000000	pigeons
Pde4b	0.10	1.00000000	pigeons
Ndufa4	-0.25	1.00000000	pigeons
Hdac1	-0.08	1.00000000	pigeons
Ube2a	-0.31	1.00000000	pigeons

Sec24a	-0.19	1.00000000	pigeons
Dcaf1	-0.20	1.00000000	pigeons
Tnfaip3	-0.03	1.00000000	pigeons
Ptpn12	-0.20	1.00000000	pigeons
Ccndbp1	-0.10	1.00000000	pigeons
Rprd2	-0.05	1.00000000	pigeons
Fmnl2	-0.06	1.00000000	pigeons
Cnot4	-0.25	1.00000000	pigeons
Rbm12	-0.16	1.00000000	pigeons
Uri1	-0.16	1.00000000	pigeons
Brox	-0.22	1.00000000	pigeons
Fnbp1	-0.32	1.00000000	pigeons
Slc20a2	0.01	1.00000000	pigeons
Ptprj	0.10	1.00000000	pigeons
Angptl4	0.08	1.00000000	pigeons
Ift80	-0.19	1.00000000	pigeons
Kras	-0.33	1.00000000	pigeons
Ctsc	-0.04	1.00000000	pigeons
Etf1	-0.14	1.00000000	pigeons
Cpsf3	-0.11	1.00000000	pigeons
Bin1	-0.94	1.00000000	pigeons
Fbxw8	-0.15	1.00000000	pigeons
Rgs3	-0.83	1.00000000	pigeons
Dach2	0.19	1.00000000	pigeons
Eif2s1	-0.18	1.00000000	pigeons
Ddx28	-0.05	1.00000000	pigeons
Ipo8	-0.13	1.00000000	pigeons
Nhsl2	-0.04	1.00000000	pigeons
Naf1	-0.12	1.00000000	pigeons
Tbc1d9	-0.02	1.00000000	pigeons
Exog	-0.01	1.00000000	pigeons
Ap1g1	-0.31	1.00000000	pigeons
Fez2	-0.33	1.00000000	pigeons
Ctdspl2	-0.02	1.00000000	pigeons
Pisd	-0.22	1.00000000	pigeons
Usp33	-0.18	1.00000000	pigeons
Prpf3	-0.16	1.00000000	pigeons
Ilf2	-0.17	1.00000000	pigeons
Tm9sf3	-0.25	1.00000000	pigeons
Rrad	0.04	1.00000000	pigeons
Fam133b	-0.17	1.00000000	pigeons
Lcp2	0.10	1.00000000	pigeons
Jade3	-0.98	1.00000000	pigeons
Ube2j1	-0.21	1.00000000	pigeons
Ube3a	-0.17	1.00000000	pigeons
Enpp6	-0.04	1.00000000	pigeons

Klf6	-0.25	1.00000000	pigeons
Ap3s1	-0.22	1.00000000	pigeons
Zc3h12a	-0.20	1.00000000	pigeons
Ap1ar	-0.06	1.00000000	pigeons
Gtf2f2	-0.14	1.00000000	pigeons
Nr1d2	0.10	1.00000000	pigeons
Rhoj	-1.06	1.00000000	pigeons
Wdfy2	-0.07	1.00000000	pigeons
Skp1	-0.25	1.00000000	pigeons
Dr1	-0.37	1.00000000	pigeons
Slc10a7	-0.17	1.00000000	pigeons
Rbm6	-0.20	1.00000000	pigeons
Gtdc1	-0.12	1.00000000	pigeons
Prrc2b	-0.33	1.00000000	pigeons
Tspan5	-0.16	1.00000000	pigeons
Rpf2	-0.12	1.00000000	pigeons
Nav2	-0.72	1.00000000	pigeons
Focad	-0.09	1.00000000	pigeons
Ets2	-0.10	1.00000000	pigeons
Plcl2	-0.95	1.00000000	pigeons
Hlcs	-0.10	1.00000000	pigeons
Adamts3	-0.11	1.00000000	pigeons
Fnbp1l	-0.61	1.00000000	pigeons
Bnip3l	-0.40	1.00000000	pigeons
Dusp7	-0.65	1.00000000	pigeons
Ndufb3	-0.30	1.00000000	pigeons
Traf2	-0.14	1.00000000	pigeons
Dlg5	-0.17	1.00000000	pigeons
Kif13a	-0.25	1.00000000	pigeons
Trp53bp1	-0.09	1.00000000	pigeons
Ppp2r5d	-0.12	1.00000000	pigeons
Tnfaip6	0.04	1.00000000	pigeons
Jag1	-0.18	1.00000000	pigeons
Qdpr	-0.05	1.00000000	pigeons
Atp2b4	-0.26	1.00000000	pigeons
Ddx6	-0.26	1.00000000	pigeons
Suco	-0.19	1.00000000	pigeons
Traf6	-0.08	1.00000000	pigeons
Aak1	-0.05	1.00000000	pigeons
Pard3	0.00	1.00000000	pigeons
Acaca	0.09	1.00000000	pigeons
Zc3h7a	-0.15	1.00000000	pigeons
Tulp3	-0.16	1.00000000	pigeons
Tubgcp3	-0.04	1.00000000	pigeons
Smg1	-0.30	1.00000000	pigeons
Trim24	-0.16	1.00000000	pigeons

Cpne2	-0.05	1.00000000	pigeons
Snx7	-0.03	1.00000000	pigeons
Antxr2	-0.03	1.00000000	pigeons
Rock1	0.03	1.00000000	pigeons
Bend5	0.12	1.00000000	pigeons
Hexa	0.10	1.00000000	pigeons
Ctcf	-0.25	1.00000000	pigeons
Cd81	0.10	1.00000000	pigeons
Dmap1	-1.08	1.00000000	pigeons
Ror2	0.14	1.00000000	pigeons
Bhlhe40	-0.11	1.00000000	pigeons
Dennd5a	-0.23	1.00000000	pigeons
Rcor1	-0.22	1.00000000	pigeons
Vps45	-0.10	1.00000000	pigeons
Heatr5a	-0.16	1.00000000	pigeons
Ythdf2	-0.28	1.00000000	pigeons
Cdh13	0.24	1.00000000	pigeons
Ube4b	-0.25	1.00000000	pigeons
Hikeshi	-0.15	1.00000000	pigeons
Zfand5	-0.27	1.00000000	pigeons
Rbm45	-0.06	1.00000000	pigeons
Adgrd1	0.03	1.00000000	pigeons
Bms1	-0.06	1.00000000	pigeons
Ppp2r5a	-0.11	1.00000000	pigeons
Gosr1	-0.17	1.00000000	pigeons
Dlst	-0.13	1.00000000	pigeons
Ube2cbp	-0.02	1.00000000	pigeons
Edem3	-0.24	1.00000000	pigeons
Creg1	-0.30	1.00000000	pigeons
Atp6ap2	-0.14	1.00000000	pigeons
Ctdp1	-0.20	1.00000000	pigeons
Slc35c2	-0.18	1.00000000	pigeons
Apba2	-0.75	1.00000000	pigeons
Cln6	-0.09	1.00000000	pigeons
Tm2d1	-0.15	1.00000000	pigeons
Trappc2	-0.09	1.00000000	pigeons
Kctd19	0.03	1.00000000	pigeons
Commd4	-0.05	1.00000000	pigeons
Esrrg	-0.06	1.00000000	pigeons
Arhgap5	-0.34	1.00000000	pigeons
Hnrnpm	-0.37	1.00000000	pigeons
Zfp592	-0.08	1.00000000	pigeons
Ppp2ca	-0.19	1.00000000	pigeons
Nfatc2	0.24	1.00000000	pigeons
Smarce1	-0.20	1.00000000	pigeons
Cdr2	0.22	1.00000000	pigeons

Sec23a	-0.30	1.00000000	pigeons
Prickle2	0.07	1.00000000	pigeons
Lsm14a	-0.30	1.00000000	pigeons
Api5	-0.27	1.00000000	pigeons
Ttc1	-0.07	1.00000000	pigeons
Htra2	-0.05	1.00000000	pigeons
Tfdp1	-0.15	1.00000000	pigeons
Pi4ka	-0.21	1.00000000	pigeons
Zfp644	-0.37	1.00000000	pigeons
Ccng1	-0.07	1.00000000	pigeons
Abtb1	-0.11	1.00000000	pigeons
Yipf3	-0.16	1.00000000	pigeons
Pfdn1	-0.33	1.00000000	pigeons
Prelid3a	-0.18	1.00000000	pigeons
Trib2	-1.08	1.00000000	pigeons
Nemf	-0.07	1.00000000	pigeons
Cmss1	0.04	1.00000000	pigeons
Rabggtb	-0.07	1.00000000	pigeons
Elp3	-0.20	1.00000000	pigeons
Prr14l	-0.22	1.00000000	pigeons
Zmynd19	-0.01	1.00000000	pigeons
Spopl	-0.32	1.00000000	pigeons
Sipa1l2	0.10	1.00000000	pigeons
Ufm1	-0.17	1.00000000	pigeons
Nans	-0.23	1.00000000	pigeons
Ctps2	-0.12	1.00000000	pigeons
Inpp4a	-1.11	1.00000000	pigeons
Ptpra	-0.26	1.00000000	pigeons
Amfr	-0.38	1.00000000	pigeons
Commd7	-0.09	1.00000000	pigeons
Clock	-0.19	1.00000000	pigeons
Pola1	-0.19	1.00000000	pigeons
Kdm7a	-0.43	1.00000000	pigeons
Gnai3	-0.40	1.00000000	pigeons
Rfx2	-0.07	1.00000000	pigeons
Ncor2	-0.17	1.00000000	pigeons
Pdcl3	-0.12	1.00000000	pigeons
Ptpn14	-1.07	1.00000000	pigeons
Hnrnpab	-0.14	1.00000000	pigeons
Fubp3	-0.23	1.00000000	pigeons
Chmp7	-0.22	1.00000000	pigeons
Fam204a	-0.34	1.00000000	pigeons
Phf20l1	-0.22	1.00000000	pigeons
Ehmt1	-0.16	1.00000000	pigeons
Tenm3	0.36	1.00000000	pigeons
Stx12	-0.20	1.00000000	pigeons

Col1a2	-0.23	1.00000000	pigeons
Nrsn1	0.07	1.00000000	pigeons
Usp1	-0.12	1.00000000	pigeons
Basp1	-0.01	1.00000000	pigeons
Smad6	-0.72	1.00000000	pigeons
Skap2	-0.18	1.00000000	pigeons
Dennd1a	0.09	1.00000000	pigeons
Nop14	-0.24	1.00000000	pigeons
Arxes2	-0.28	1.00000000	pigeons
Phkg1	-0.13	1.00000000	pigeons
D5Ertd579e	-0.07	1.00000000	pigeons
Epc2	-0.36	1.00000000	pigeons
Slain2	-0.23	1.00000000	pigeons
Srebf2	-0.11	1.00000000	pigeons
Impdh2	-0.03	1.00000000	pigeons
Bzw2	-0.38	1.00000000	pigeons
Nfkbie	-0.03	1.00000000	pigeons
Ip6k1	-0.12	1.00000000	pigeons
Scamp1	-0.05	1.00000000	pigeons
Zcchc8	-0.19	1.00000000	pigeons
Tfpi	-0.84	1.00000000	pigeons
Acap2	-0.23	1.00000000	pigeons
Foxk1	-0.09	1.00000000	pigeons
Sh3bgrl3	-0.32	1.00000000	pigeons
Dyrk1a	-0.86	1.00000000	pigeons
Eif2a	-0.06	1.00000000	pigeons
Sec13	-0.03	1.00000000	pigeons
Zcchc17	-0.14	1.00000000	pigeons
Slc30a6	-0.02	1.00000000	pigeons
Cnst	-0.13	1.00000000	pigeons
Ino80d	-0.31	1.00000000	pigeons
Hyou1	-0.19	1.00000000	pigeons
Lias	0.01	1.00000000	pigeons
Immp1l	-0.07	1.00000000	pigeons
Arhgap21	-0.75	1.00000000	pigeons
Herpud2	-0.19	1.00000000	pigeons
Gpc5	0.21	1.00000000	pigeons
Uhrf1bp1l	-0.18	1.00000000	pigeons
Spred1	-0.88	1.00000000	pigeons
Naa35	-0.09	1.00000000	pigeons
Fbxw7	-0.05	1.00000000	pigeons
Tmem65	-0.28	1.00000000	pigeons
Noc2l	-0.10	1.00000000	pigeons
Ube2q2	-0.23	1.00000000	pigeons
Prkrip1	-0.09	1.00000000	pigeons
Cbr4	-0.03	1.00000000	pigeons

Edem1	-0.28	1.00000000	pigeons
Thrb	0.00	1.00000000	pigeons
Ndufb8	-0.22	1.00000000	pigeons
Hbs1l	-0.28	1.00000000	pigeons
Homer1	0.57	1.00000000	pigeons
Tmem131l	-0.23	1.00000000	pigeons
Tubgcp5	-0.12	1.00000000	pigeons
Ipo11	-0.04	1.00000000	pigeons
Cobll1	-0.86	1.00000000	pigeons
Topbp1	-0.12	1.00000000	pigeons
Fam120a	-0.30	1.00000000	pigeons
Chd2	-0.22	1.00000000	pigeons
Rab4a	0.03	1.00000000	pigeons
C2cd2l	-0.13	1.00000000	pigeons
Zfp512	-0.02	1.00000000	pigeons
Arhgef10	-0.09	1.00000000	pigeons
Cbl	-0.13	1.00000000	pigeons
Zfyve9	-0.14	1.00000000	pigeons
Atp2c1	-0.24	1.00000000	pigeons
Rcsd1	-0.19	1.00000000	pigeons
Frmd4a	0.05	1.00000000	pigeons
Pfkfb3	-0.84	1.00000000	pigeons
Fubp1	-0.41	1.00000000	pigeons
Glcci1	-0.09	1.00000000	pigeons
Nol10	-0.16	1.00000000	pigeons
Ankrd11	-0.34	1.00000000	pigeons
Sertad2	-0.80	1.00000000	pigeons
Zc3h13	-0.19	1.00000000	pigeons
Ostc	-0.23	1.00000000	pigeons
Scaf4	-0.28	1.00000000	pigeons
Heca	-0.31	1.00000000	pigeons
Ercc6	-0.20	1.00000000	pigeons
Slc30a9	-0.36	1.00000000	pigeons
Tax1bp1	-0.02	1.00000000	pigeons
Sergef	-0.06	1.00000000	pigeons
Khdrbs1	-0.05	1.00000000	pigeons
Rnf216	-0.11	1.00000000	pigeons
Pmp22	-0.59	1.00000000	pigeons
Ubn1	-0.10	1.00000000	pigeons
Hnrnpa3	-0.60	1.00000000	pigeons
Cops8	-0.35	1.00000000	pigeons
Copg1	-0.07	1.00000000	pigeons
Zfp598	-0.14	1.00000000	pigeons
Srp9	-0.44	1.00000000	pigeons
Usp37	-0.02	1.00000000	pigeons
Cdc42	-0.39	1.00000000	pigeons

Ash1l	-0.44	1.00000000	pigeons
Fgfr1op2	-0.22	1.00000000	pigeons
Ddx46	-0.22	1.00000000	pigeons
Dennd4a	-1.05	1.00000000	pigeons
Gphn	-0.15	1.00000000	pigeons
Zranb1	-0.27	1.00000000	pigeons
Fkbp14	-0.22	1.00000000	pigeons
Spry2	-0.17	1.00000000	pigeons
Otulin	-0.34	1.00000000	pigeons
Col4a1	-0.46	1.00000000	pigeons
Fam107b	0.11	1.00000000	pigeons
Kat6a	-0.33	1.00000000	pigeons
Wif1	0.21	1.00000000	pigeons
Ptk7	-0.13	1.00000000	pigeons
Cramp1	-0.27	1.00000000	pigeons
Fh1	-0.13	1.00000000	pigeons
Necap2	-0.19	1.00000000	pigeons
Arhgap26	-0.86	1.00000000	pigeons
Fry	-0.04	1.00000000	pigeons
Slc23a2	-0.25	1.00000000	pigeons
Sms	-0.20	1.00000000	pigeons
Fgfr2	-0.89	1.00000000	pigeons
Stk26	-0.30	1.00000000	pigeons
Gtf2h1	-0.25	1.00000000	pigeons
Ggps1	-0.31	1.00000000	pigeons
Cetn3	-0.02	1.00000000	pigeons
Cul3	-0.30	1.00000000	pigeons
Cd44	-0.84	1.00000000	pigeons
Ppp1r8	-0.05	1.00000000	pigeons
Ext2	-0.29	1.00000000	pigeons
Srp54a	-0.21	1.00000000	pigeons
Polr3b	-0.75	1.00000000	pigeons
Alas1	-0.12	1.00000000	pigeons
Auts2	-0.59	1.00000000	pigeons
Crkl	-0.17	1.00000000	pigeons
Golt1b	0.00	1.00000000	pigeons
Atp6v1h	-0.16	1.00000000	pigeons
Vps25	-0.09	1.00000000	pigeons
Parp9	-0.08	1.00000000	pigeons
Eif3h	-0.17	1.00000000	pigeons
Pacsin2	-0.17	1.00000000	pigeons
Ube2q1	-0.23	1.00000000	pigeons
Fgl2	0.02	1.00000000	pigeons
Rabgap1l	-0.77	1.00000000	pigeons
Aldh9a1	-0.03	1.00000000	pigeons
Ube4a	-0.12	1.00000000	pigeons

Pphln1	-0.11	1.00000000	pigeons
Polr2e	-0.15	1.00000000	pigeons
Actr10	-0.10	1.00000000	pigeons
Fktn	-0.08	1.00000000	pigeons
Ninj1	-0.10	1.00000000	pigeons
Cfdp1	-0.30	1.00000000	pigeons
Trp53i11	-0.83	1.00000000	pigeons
Tmem266	-0.82	1.00000000	pigeons
Zfp516	-0.25	1.00000000	pigeons
Xpo6	-0.18	1.00000000	pigeons
Lsmem1	-0.09	1.00000000	pigeons
Tdg	-0.28	1.00000000	pigeons
Ide	-0.22	1.00000000	pigeons
Spz1	-0.40	1.00000000	pigeons
Pitpnb	-0.32	1.00000000	pigeons
Spag9	-0.35	1.00000000	pigeons
Nab1	-0.31	1.00000000	pigeons
Arf6	-0.31	1.00000000	pigeons
Aqr	-0.31	1.00000000	pigeons
Fam172a	-0.20	1.00000000	pigeons
Btbd1	-0.20	1.00000000	pigeons
Nfxl1	-0.19	1.00000000	pigeons
Srfbp1	-0.08	1.00000000	pigeons
Ckap5	-0.24	1.00000000	pigeons
Atp9b	-0.15	1.00000000	pigeons
Usp6nl	-0.22	1.00000000	pigeons
Fhit	-0.52	1.00000000	pigeons
Slc25a25	-0.26	1.00000000	pigeons
Lclat1	-0.27	1.00000000	pigeons
Hnrnpr	-0.33	1.00000000	pigeons
Arl5b	-0.12	1.00000000	pigeons
Camk2g	-0.99	1.00000000	pigeons
Strn3	-0.50	1.00000000	pigeons
Endou	-0.76	1.00000000	pigeons
Tmed5	-0.35	1.00000000	pigeons
Itfg2	-0.22	1.00000000	pigeons
Gigyf2	-0.80	1.00000000	pigeons
Tppp3	-0.05	1.00000000	pigeons
Dph6	0.01	1.00000000	pigeons
Zcchc2	-0.46	1.00000000	pigeons
Uso1	-0.33	1.00000000	pigeons
Cul4b	-0.27	1.00000000	pigeons
Larp7	-0.20	1.00000000	pigeons
Psme4	-0.25	1.00000000	pigeons
Usp4	-0.26	1.00000000	pigeons
Rnf11	-0.27	1.00000000	pigeons

Prkcd	-0.13	1.00000000	pigeons
Ak2	-0.36	1.00000000	pigeons
Ufsp2	-0.15	1.00000000	pigeons
Als2cl	-0.62	1.00000000	pigeons
Pik3cg	-0.20	1.00000000	pigeons
Atp7a	-0.26	1.00000000	pigeons
Elf2	-0.26	1.00000000	pigeons
Enox1	-0.58	1.00000000	pigeons
Lsm8	-0.25	1.00000000	pigeons
Pik3ca	-0.99	1.00000000	pigeons
Sart3	-0.18	1.00000000	pigeons
Plk3	-0.25	1.00000000	pigeons
Arhgef6	-0.33	1.00000000	pigeons
Rnf10	-0.21	1.00000000	pigeons
St3gal1	-0.86	1.00000000	pigeons
Ap1s2	-0.13	1.00000000	pigeons
Elovl6	-0.07	1.00000000	pigeons
Epn2	-0.32	1.00000000	pigeons
Virma	-0.01	1.00000000	pigeons
Ptpn4	-0.26	1.00000000	pigeons
Stat4	-0.02	1.00000000	pigeons
Gcc2	-0.26	1.00000000	pigeons
Hace1	-0.16	1.00000000	pigeons
Braf	-0.29	1.00000000	pigeons
Clec3b	-0.03	1.00000000	pigeons
Casp8ap2	-0.21	1.00000000	pigeons
Ythdc1	-0.31	1.00000000	pigeons
Fer	-0.27	1.00000000	pigeons
Cuedc1	-0.17	1.00000000	pigeons
Dcxr	-0.08	1.00000000	pigeons
Anapc10	-0.13	1.00000000	pigeons
Itm2a	-0.43	1.00000000	pigeons
Aftph	-0.50	1.00000000	pigeons
Eri1	-0.15	1.00000000	pigeons
Sgms2	-0.07	1.00000000	pigeons
Ap2m1	-0.29	1.00000000	pigeons
Kif2a	-0.25	1.00000000	pigeons
Eea1	-0.22	1.00000000	pigeons
Twsg1	-0.25	1.00000000	pigeons
Bicd2	0.00	1.00000000	pigeons
Rimklb	-0.06	1.00000000	pigeons
Itgb6	0.01	1.00000000	pigeons
Sec24b	-0.08	1.00000000	pigeons
Ebna1bp2	-0.06	1.00000000	pigeons
Aif1l	-0.07	1.00000000	pigeons
Camk1d	0.23	1.00000000	pigeons

Glyr1	-0.31	1.00000000	pigeons
Cds2	-0.23	1.00000000	pigeons
Asah1	-0.31	1.00000000	pigeons
Rsrc2	-0.24	1.00000000	pigeons
Lrpprc	-0.14	1.00000000	pigeons
Exoc7	-0.08	1.00000000	pigeons
Stim2	-0.84	1.00000000	pigeons
Patj	0.00	1.00000000	pigeons
Fsip1	0.10	1.00000000	pigeons
Arhgdib	-0.11	1.00000000	pigeons
Nsrp1	-0.21	1.00000000	pigeons
Lmo3	-0.74	1.00000000	pigeons
Tnfrsf18	0.11	1.00000000	pigeons
Dazap1	-0.38	1.00000000	pigeons
Cactin	-0.16	1.00000000	pigeons
Cacul1	-1.05	1.00000000	pigeons
Cap1	-0.26	1.00000000	pigeons
Rora	-0.19	1.00000000	pigeons
F5	-0.75	1.00000000	pigeons
Prkd3	-0.27	1.00000000	pigeons
Surf6	-0.12	1.00000000	pigeons
Tspan3	-0.12	1.00000000	pigeons
Hspb11	-0.21	1.00000000	pigeons
Ppp3ca	-0.28	1.00000000	pigeons
Pbrm1	-0.23	1.00000000	pigeons
Foxf1	-0.44	1.00000000	pigeons
Rac2	0.00	1.00000000	pigeons
Ubp1	-0.32	1.00000000	pigeons
Wdr7	-0.78	1.00000000	pigeons
Eif4enif1	-0.05	1.00000000	pigeons
Nfkbiz	-0.16	1.00000000	pigeons
Utp18	-0.09	1.00000000	pigeons
Rab11fip4	-0.01	1.00000000	pigeons
Gfod1	-0.36	1.00000000	pigeons
Hmgcs1	-0.10	1.00000000	pigeons
Dennd6a	-0.17	1.00000000	pigeons
Herc1	-0.41	1.00000000	pigeons
Eml4	-0.11	1.00000000	pigeons
Klhl8	-0.24	1.00000000	pigeons
Acsl4	-0.02	1.00000000	pigeons
Flii	-0.15	1.00000000	pigeons
Zdhhc14	-0.26	1.00000000	pigeons
Ppp1cb	-0.29	1.00000000	pigeons
Parp1	-0.13	1.00000000	pigeons
Abracl	-0.30	1.00000000	pigeons
Lrch1	-0.17	1.00000000	pigeons

Id2	-0.26	1.00000000	pigeons
Zfp326	-0.25	1.00000000	pigeons
Pgm2l1	-0.26	1.00000000	pigeons
Cdc42bpa	-0.82	1.00000000	pigeons
Kmt2c	-0.09	1.00000000	pigeons
Ate1	-0.22	1.00000000	pigeons
Nfkb2	-0.20	1.00000000	pigeons
Gclc	-0.31	1.00000000	pigeons
Acad9	-0.31	1.00000000	pigeons
Clip1	-0.37	1.00000000	pigeons
Optn	-0.33	1.00000000	pigeons
Wdr36	-0.22	1.00000000	pigeons
Ostf1	-0.37	1.00000000	pigeons
Osbpl3	-0.93	1.00000000	pigeons
Kbtbd2	-0.24	1.00000000	pigeons
Napg	-0.25	1.00000000	pigeons
Dnmt3a	-0.16	1.00000000	pigeons
G0s2	0.17	1.00000000	pigeons
Tmem131	-0.32	1.00000000	pigeons
Edf1	-0.21	1.00000000	pigeons
Amdhd2	-0.12	1.00000000	pigeons
Fchsd2	-0.38	1.00000000	pigeons
Cdc14a	-0.31	1.00000000	pigeons
Xrn1	-0.27	1.00000000	pigeons
Caskin2	-0.92	1.00000000	pigeons
Smap2	-0.20	1.00000000	pigeons
Scara5	-0.28	1.00000000	pigeons
Dpyd	-0.68	1.00000000	pigeons
Arl15	-0.86	1.00000000	pigeons
Rac1	-0.33	1.00000000	pigeons
St3gal6	-0.40	1.00000000	pigeons
Stx18	-0.16	1.00000000	pigeons
Hspg2	-0.17	1.00000000	pigeons
Trpm7	-0.14	1.00000000	pigeons
Shoc2	-0.28	1.00000000	pigeons
Zfp276	-0.15	1.00000000	pigeons
Kidins220	-0.33	1.00000000	pigeons
Hspa4l	-0.21	1.00000000	pigeons
Rnaseh2b	-0.32	1.00000000	pigeons
Sde2	-0.24	1.00000000	pigeons
Ppm1g	-0.16	1.00000000	pigeons
Upf1	-0.35	1.00000000	pigeons
Tpk1	-0.54	1.00000000	pigeons
Arhgap8	-0.07	1.00000000	pigeons
Utrn	-0.29	1.00000000	pigeons
Kif1b	-0.17	1.00000000	pigeons

Fbxw11	-0.43	1.00000000	pigeons
Ttyh3	-0.72	1.00000000	pigeons
Axin1	-0.17	1.00000000	pigeons
Dclre1a	-0.11	1.00000000	pigeons
Abcb10	-0.68	1.00000000	pigeons
Lrmda	-0.58	1.00000000	pigeons
Rab11a	-0.16	1.00000000	pigeons
Rp2	-0.25	1.00000000	pigeons
Sltm	-0.35	1.00000000	pigeons
Zeb1	-0.15	1.00000000	pigeons
Zfp36l1	0.35	1.00000000	pigeons
Ranbp3	-0.09	1.00000000	pigeons
Pde7a	-0.12	1.00000000	pigeons
Ywhah	-0.32	1.00000000	pigeons
Fxr1	-0.36	1.00000000	pigeons
Ubap1	-0.31	1.00000000	pigeons
Srpk2	-0.41	1.00000000	pigeons
Rtn4	-0.62	1.00000000	pigeons
Nrip1	-0.34	1.00000000	pigeons
Prkar1a	-0.35	1.00000000	pigeons
Epc1	-0.31	1.00000000	pigeons
Wapl	-0.70	1.00000000	pigeons
Dusp23	-0.14	1.00000000	pigeons
Zdhhc21	-0.17	1.00000000	pigeons
Fam13b	-0.36	1.00000000	pigeons
Lasp1	-0.22	1.00000000	pigeons
Rb1	-0.70	1.00000000	pigeons
Hscb	-0.10	1.00000000	pigeons
Disp1	-0.84	1.00000000	pigeons
Pdrg1	-0.23	1.00000000	pigeons
Ubap2	-0.26	1.00000000	pigeons
Hnrnpu	-0.18	1.00000000	pigeons
Proser1	-0.15	1.00000000	pigeons
Exoc2	-0.23	1.00000000	pigeons
Trerf1	-0.16	1.00000000	pigeons
Vps37a	-0.22	1.00000000	pigeons
Actr2	-0.39	1.00000000	pigeons
Oser1	-0.23	1.00000000	pigeons
Atrn	-0.32	1.00000000	pigeons
Scfd1	-0.28	1.00000000	pigeons
Cul2	-0.44	1.00000000	pigeons
Cltb	-0.38	1.00000000	pigeons
Dock7	-0.26	1.00000000	pigeons
Wdr70	-0.18	1.00000000	pigeons
Rdh10	-0.18	1.00000000	pigeons
Ccnk	-0.28	1.00000000	pigeons

Ing4	-0.08	1.00000000	pigeons
Ctnna1	-0.35	1.00000000	pigeons
Vash1	-0.21	1.00000000	pigeons
Pals1	-0.40	1.00000000	pigeons
Clec16a	-0.39	1.00000000	pigeons
Usp48	-0.32	1.00000000	pigeons
Ptpre	-0.55	1.00000000	pigeons
Taf12	-0.08	1.00000000	pigeons
Ica1	-0.73	1.00000000	pigeons
Aff2	-0.52	1.00000000	pigeons
Ttc37	-0.30	1.00000000	pigeons
Cnn3	-0.71	1.00000000	pigeons
Tpm3-rs7	-0.25	1.00000000	pigeons
Eci3	-0.06	1.00000000	pigeons
Ramp3	-0.32	1.00000000	pigeons
Cd82	-0.42	1.00000000	pigeons
Arhgef26	-0.12	1.00000000	pigeons
Ilf3	-0.18	1.00000000	pigeons
Ambra1	-0.32	1.00000000	pigeons
Supt3	-0.14	1.00000000	pigeons
Ezh2	-0.33	1.00000000	pigeons
Ppp1r2	-0.31	1.00000000	pigeons
Grb2	-0.16	1.00000000	pigeons
Rfx7	-0.12	1.00000000	pigeons
Fbxo3	-0.27	1.00000000	pigeons
Phkb	-0.28	1.00000000	pigeons
Usp14	-0.17	1.00000000	pigeons
Dmxl1	-0.38	1.00000000	pigeons
Arfgef3	-0.09	1.00000000	pigeons
Ccp110	-0.25	1.00000000	pigeons
Ing2	-0.14	1.00000000	pigeons
Nup98	-0.29	1.00000000	pigeons
Pip4k2a	-0.09	1.00000000	pigeons
Fam76a	-0.17	1.00000000	pigeons
Fundc1	-0.21	1.00000000	pigeons
Cpt1a	-0.36	1.00000000	pigeons
Nol11	-0.31	1.00000000	pigeons
Grk3	-0.54	1.00000000	pigeons
Pcgf3	-0.16	1.00000000	pigeons
Bin3	-0.97	1.00000000	pigeons
Sub1	-0.31	1.00000000	pigeons
Cct2	-0.23	1.00000000	pigeons
Arpc3	-0.40	1.00000000	pigeons
Pdk3	-0.36	1.00000000	pigeons
Scaf8	-0.46	1.00000000	pigeons
Tbl1xr1	-0.82	1.00000000	pigeons

Btrc	-0.38	1.00000000	pigeons
Chsy1	-0.21	1.00000000	pigeons
Lpin2	-0.92	1.00000000	pigeons
Csnk1a1	-0.15	1.00000000	pigeons
Btaf1	-0.43	1.00000000	pigeons
Rbm22	-0.16	1.00000000	pigeons
Alg12	-0.26	1.00000000	pigeons
Brf1	-0.30	1.00000000	pigeons
Cd34	-0.18	1.00000000	pigeons
Psmb4	-0.37	1.00000000	pigeons
Smu1	-0.29	1.00000000	pigeons
Rpia	-0.29	1.00000000	pigeons
Elovl5	-0.31	1.00000000	pigeons
Epb41	-0.86	1.00000000	pigeons
Pan3	-0.40	1.00000000	pigeons
Phactr2	-0.71	1.00000000	pigeons
Ammecr1	-0.26	1.00000000	pigeons
Bcr	-0.30	1.00000000	pigeons
Pla2g7	-0.28	1.00000000	pigeons
Dstn	-0.44	1.00000000	pigeons
Sdf4	-0.44	1.00000000	pigeons
Pank4	-0.15	1.00000000	pigeons
Cops3	-0.37	1.00000000	pigeons
Syne1	-0.09	1.00000000	pigeons
Tma16	-0.17	1.00000000	pigeons
Shroom2	-0.70	1.00000000	pigeons
5031439G07Rik	-0.15	1.00000000	pigeons
Ppard	-0.25	1.00000000	pigeons
Ikzf1	0.21	1.00000000	pigeons
Large1	-0.25	1.00000000	pigeons
Gpr155	-0.36	1.00000000	pigeons
Eef2	-0.28	1.00000000	pigeons
Zdhhc6	-0.55	1.00000000	pigeons
Hspa12a	-0.15	1.00000000	pigeons
Samhd1	-0.21	1.00000000	pigeons
Atad2b	-0.17	1.00000000	pigeons
Irak2	-0.25	1.00000000	pigeons
Ppig	-0.39	1.00000000	pigeons
Ubr2	-0.39	1.00000000	pigeons
Taf13	-0.25	1.00000000	pigeons
Cerk	-0.44	1.00000000	pigeons
Odf2	-0.23	1.00000000	pigeons
Dhx15	-0.36	1.00000000	pigeons
Rffl	-0.14	1.00000000	pigeons
Asb3	-0.26	1.00000000	pigeons
Scpep1	-0.19	1.00000000	pigeons

Adamts2	-0.63	1.00000000	pigeons
Sf3a2	-0.15	1.00000000	pigeons
Ppa1	-0.26	1.00000000	pigeons
Slc4a4	-0.72	1.00000000	pigeons
Sgms1	-0.34	1.00000000	pigeons
Snx24	-0.12	1.00000000	pigeons
Plbd2	-0.34	1.00000000	pigeons
Eif4g3	-0.90	1.00000000	pigeons
Lemd3	-0.46	1.00000000	pigeons
Kansl1	-0.25	1.00000000	pigeons
Nabp1	-0.31	1.00000000	pigeons
Scarb2	-0.53	1.00000000	pigeons
Ppm1b	-0.41	1.00000000	pigeons
Ptbp2	-0.41	1.00000000	pigeons
Rab7	-0.38	1.00000000	pigeons
Yaf2	-0.35	1.00000000	pigeons
Ablim1	-0.19	1.00000000	pigeons
Rab6a	-0.40	1.00000000	pigeons
Wwc2	-0.30	1.00000000	pigeons
Eepd1	-0.28	1.00000000	pigeons
Smim14	-0.39	1.00000000	pigeons
Ythdf1	-0.24	1.00000000	pigeons
Acvr1	-0.20	1.00000000	pigeons
Agpat3	-0.17	1.00000000	pigeons
Smarca2	-0.29	1.00000000	pigeons
Vps13d	-0.45	1.00000000	pigeons
Usp22	-0.67	1.00000000	pigeons
Slc2a8	-0.18	1.00000000	pigeons
Eif2b3	-0.28	1.00000000	pigeons
Acer2	-0.81	1.00000000	pigeons
Hmgb1	-0.14	1.00000000	pigeons
Cnnm2	-0.16	1.00000000	pigeons
Adipor2	-0.52	1.00000000	pigeons
Cnot10	-0.25	1.00000000	pigeons
Dcp2	-0.29	1.00000000	pigeons
Gulp1	-0.27	1.00000000	pigeons
Eefsec	-0.30	1.00000000	pigeons
Psmd14	-0.20	1.00000000	pigeons
Sbno1	-0.49	1.00000000	pigeons
Golga4	-0.44	1.00000000	pigeons
Sdc4	-0.22	1.00000000	pigeons
Ngly1	-0.31	1.00000000	pigeons
Csnk2a2	-0.31	1.00000000	pigeons
Ext1	-0.40	1.00000000	pigeons
Arhgap15	0.14	1.00000000	pigeons
Nr3c1	-0.36	1.00000000	pigeons

Hspa8	0.28	1.00000000	pigeons
Dym	-0.35	1.00000000	pigeons
Lrrc28	-0.35	1.00000000	pigeons
Exoc6b	-0.86	1.00000000	pigeons
Ripor1	-0.78	1.00000000	pigeons
Cab39	-0.51	1.00000000	pigeons
Aven	-0.27	1.00000000	pigeons
Pls3	-0.77	1.00000000	pigeons
Sos2	-0.44	1.00000000	pigeons
Plxna1	-0.27	1.00000000	pigeons
Kdm5a	-0.49	1.00000000	pigeons
Lrba	-0.68	1.00000000	pigeons
Utp11	-0.13	1.00000000	pigeons
Ifrd1	-0.01	1.00000000	pigeons
Usp38	-0.20	1.00000000	pigeons
Csmd1	0.46	1.00000000	pigeons
Ppp2r2a	-0.41	1.00000000	pigeons
Cdc42se2	-0.33	1.00000000	pigeons
Notch1	-0.74	1.00000000	pigeons
Ptpn1	-0.30	1.00000000	pigeons
Sbf2	-0.22	1.00000000	pigeons
Edc3	-0.27	1.00000000	pigeons
Fam219a	-0.28	1.00000000	pigeons
Nckap1	-0.87	1.00000000	pigeons
Shroom3	0.05	1.00000000	pigeons
Pabpc1	0.03	1.00000000	pigeons
Eef1e1	-0.39	1.00000000	pigeons
Cav1	-0.73	1.00000000	pigeons
Ppp6r2	-0.52	1.00000000	pigeons
Adgra1	-0.49	1.00000000	pigeons
Tmem70	-0.13	1.00000000	pigeons
Tmem141	-0.13	1.00000000	pigeons
Tcf7l2	-0.17	1.00000000	pigeons
Smad1	-0.33	1.00000000	pigeons
Fars2	-0.25	1.00000000	pigeons
Ralb	-0.46	1.00000000	pigeons
Faf2	-0.36	1.00000000	pigeons
Ankrd17	-0.25	1.00000000	pigeons
Stk17b	-0.73	1.00000000	pigeons
Gnptg	-0.21	1.00000000	pigeons
Hs6st1	-0.37	1.00000000	pigeons
Slc22a23	-0.51	1.00000000	pigeons
Nfatc1	0.10	1.00000000	pigeons
Ppa2	-0.41	1.00000000	pigeons
Vps54	-0.77	1.00000000	pigeons
Zfp143	-0.37	1.00000000	pigeons

Ago2	-0.30	1.00000000	pigeons
Lrrc58	-0.16	1.00000000	pigeons
Lama5	-0.23	1.00000000	pigeons
Plk2	-0.29	1.00000000	pigeons
Sdc2	-0.36	1.00000000	pigeons
Tmem208	-0.21	1.00000000	pigeons
Il16	-0.26	1.00000000	pigeons
Ntmt1	-0.19	1.00000000	pigeons
Arhgap12	-0.40	1.00000000	pigeons
Angel2	-0.32	1.00000000	pigeons
Immp2l	-0.41	1.00000000	pigeons
Nup153	-0.40	1.00000000	pigeons
Ehbp1	-0.30	1.00000000	pigeons
Cpne1	-0.43	1.00000000	pigeons
Ogdh	-0.31	1.00000000	pigeons
Bag5	-0.91	1.00000000	pigeons
Trip12	-0.37	1.00000000	pigeons
Atg101	-0.30	1.00000000	pigeons
Rap2c	-0.46	1.00000000	pigeons
Capzb	-0.42	1.00000000	pigeons
Ncoa1	-0.28	1.00000000	pigeons
Trappc12	-0.42	1.00000000	pigeons
Erg	-0.82	1.00000000	pigeons
Sh3glb1	-0.42	1.00000000	pigeons
Kmt5b	-0.47	1.00000000	pigeons
Fads2b	-0.23	1.00000000	pigeons
Fam168a	-0.79	1.00000000	pigeons
Prrg1	-0.49	1.00000000	pigeons
Rbl2	-0.25	1.00000000	pigeons
Dctn1	-0.30	1.00000000	pigeons
Hsp90b1	-0.32	1.00000000	pigeons
Zfyve1	-0.37	1.00000000	pigeons
Spsb4	-0.62	1.00000000	pigeons
Ets1	-0.71	1.00000000	pigeons
Tanc1	-0.31	1.00000000	pigeons
Smyd3	-0.10	1.00000000	pigeons
Helz2	-0.43	1.00000000	pigeons
Unc5c	-0.46	1.00000000	pigeons
Ube2l3	-0.34	1.00000000	pigeons
Nedd9	-0.27	1.00000000	pigeons
Rnf130	-0.77	1.00000000	pigeons
Dda1	-0.26	1.00000000	pigeons
S100a10	-0.54	1.00000000	pigeons
Tln1	-0.39	1.00000000	pigeons
Vill	-0.68	1.00000000	pigeons
Bcar1	-0.19	1.00000000	pigeons

Creb5	0.12	1.00000000	pigeons
Naa30	-0.30	1.00000000	pigeons
Creld2	-0.17	1.00000000	pigeons
Ccnl1	-0.28	1.00000000	pigeons
Stk3	-0.45	1.00000000	pigeons
Glipr2	0.23	1.00000000	pigeons
Itm2b	0.06	1.00000000	pigeons
Nono	-0.30	1.00000000	pigeons
Lsm6	-0.17	1.00000000	pigeons
Ctbp1	-0.10	1.00000000	pigeons
Rexo1	-0.36	1.00000000	pigeons
Ifngr2	-0.43	1.00000000	pigeons
Ptprc	-0.12	1.00000000	pigeons
Kpna3	-0.45	1.00000000	pigeons
Spire1	-0.01	1.00000000	pigeons
Comt	-0.50	1.00000000	pigeons
Jak2	-0.80	1.00000000	pigeons
Klf12	-0.43	1.00000000	pigeons
Sipa1l1	0.10	1.00000000	pigeons
Kpna1	-0.47	1.00000000	pigeons
Sec31a	-0.67	1.00000000	pigeons
Garnl3	-0.29	1.00000000	pigeons
Rnf220	-0.57	1.00000000	pigeons
Adnp2	-0.48	1.00000000	pigeons
Atp11a	-0.17	1.00000000	pigeons
Gbf1	-0.73	1.00000000	pigeons
Tsc2	-0.29	1.00000000	pigeons
Pcnx	-0.38	1.00000000	pigeons
Arhgap29	-0.96	1.00000000	pigeons
Rab11fip3	-0.60	1.00000000	pigeons
Rab35	-0.33	1.00000000	pigeons
Ern1	-0.66	1.00000000	pigeons
Tnrc6c	-0.38	1.00000000	pigeons
Exoc5	-0.23	1.00000000	pigeons
Cdc42bpb	-0.48	1.00000000	pigeons
Ybx1	-0.39	1.00000000	pigeons
Ehd3	-0.33	1.00000000	pigeons
Clasp2	-0.47	1.00000000	pigeons
Stox2	-0.74	1.00000000	pigeons
Lrrfip2	-0.42	1.00000000	pigeons
Rbfox1	0.05	1.00000000	pigeons
Tspan14	-0.81	1.00000000	pigeons
Kdm4c	-0.70	1.00000000	pigeons
Gipc2	-0.58	1.00000000	pigeons
Tomm20	-0.53	1.00000000	pigeons
Itsn2	-0.87	1.00000000	pigeons

Asap2	-0.18	1.00000000	pigeons
Hmgcr	-0.36	1.00000000	pigeons
2210408I21Rik	-0.43	1.00000000	pigeons
Wnk1	-0.28	1.00000000	pigeons
Dmd	0.28	1.00000000	pigeons
Tmem183a	-0.26	1.00000000	pigeons
Cxcr4	0.15	1.00000000	pigeons
Adcy9	-0.54	1.00000000	pigeons
Kmt2e	-0.41	1.00000000	pigeons
Tbc1d9b	-0.27	1.00000000	pigeons
Rftn2	-0.57	1.00000000	pigeons
Bicral	-0.45	1.00000000	pigeons
Asap1	-0.40	1.00000000	pigeons
Usp47	-0.45	1.00000000	pigeons
Hectd1	-0.39	1.00000000	pigeons
Pspc1	-0.45	1.00000000	pigeons
Runx1t1	-0.27	1.00000000	pigeons
Rere	-0.29	1.00000000	pigeons
Abi1	-0.74	1.00000000	pigeons
Rbfa	-0.73	1.00000000	pigeons
Usp28	-0.68	1.00000000	pigeons
Bcor	-0.84	1.00000000	pigeons
Ppp1r12a	-0.55	1.00000000	pigeons
Slc9a6	-0.33	1.00000000	pigeons
Lrp3	-0.70	1.00000000	pigeons
Hook3	-0.44	1.00000000	pigeons
Ulk1	-0.85	1.00000000	pigeons
Scube2	-0.26	1.00000000	pigeons
Has2	-0.32	1.00000000	pigeons
Per2	-0.43	1.00000000	pigeons
Lcor	-0.43	1.00000000	pigeons
Colec10	-0.54	1.00000000	pigeons
Smarcal1	-0.08	1.00000000	pigeons
Klf3	-0.50	1.00000000	pigeons
Fat4	-0.54	1.00000000	pigeons
Phf21a	-0.82	1.00000000	pigeons
Smg6	-0.48	1.00000000	pigeons
Diaph2	-0.15	1.00000000	pigeons
Gk5	-0.90	1.00000000	pigeons
Tmbim6	-0.49	1.00000000	pigeons
Tpm4	-0.84	1.00000000	pigeons
Prrc2c	-0.56	1.00000000	pigeons
Cgrrf1	-0.19	1.00000000	pigeons
Nup107	-0.79	1.00000000	pigeons
Rsrc1	-0.20	1.00000000	pigeons
Ralbp1	-0.79	1.00000000	pigeons

Plcd1	-0.47	1.00000000	pigeons
Ctsk	-0.91	1.00000000	pigeons
Zfand6	-0.48	1.00000000	pigeons
Trip11	-0.33	1.00000000	pigeons
Asxl2	-0.31	1.00000000	pigeons
Tcf3	-0.87	1.00000000	pigeons
Dnajb6	-0.44	1.00000000	pigeons
Psen1	-0.38	1.00000000	pigeons
Ssr1	-0.47	1.00000000	pigeons
Grip1	-0.22	1.00000000	pigeons
Trps1	-0.34	1.00000000	pigeons
Ppia	-0.10	1.00000000	pigeons
Dennd2a	-0.73	1.00000000	pigeons
Arntl	-0.29	1.00000000	pigeons
Arpc1b	-0.66	1.00000000	pigeons
Atg16l1	-0.53	1.00000000	pigeons
Ywhaq	-0.32	1.00000000	pigeons
Arid2	-0.36	1.00000000	pigeons
Sh2d1a	-0.45	1.00000000	pigeons
Gbe1	-0.27	1.00000000	pigeons
Farp1	-0.40	1.00000000	pigeons
Ntn4	-0.65	1.00000000	pigeons
Epha2	-0.59	1.00000000	pigeons
Apcdd1	-0.40	1.00000000	pigeons
Rbbp6	-0.40	1.00000000	pigeons
Tfdp2	-0.40	1.00000000	pigeons
Ascc3	-0.52	1.00000000	pigeons
Tbc1d22a	-0.48	1.00000000	pigeons
Slc9a3r2	-0.57	1.00000000	pigeons
Pitpna	-0.41	1.00000000	pigeons
Clcn3	-0.36	1.00000000	pigeons
Phldb2	-0.47	1.00000000	pigeons
Zbtb11	-0.51	1.00000000	pigeons
Apoo	-0.38	1.00000000	pigeons
Wac	-0.64	1.00000000	pigeons
Ccdc88c	-0.45	1.00000000	pigeons
Taf5l	-0.73	1.00000000	pigeons
Atp6v0c	-0.21	1.00000000	pigeons
Ttll5	-0.38	1.00000000	pigeons
Rcn2	-0.44	1.00000000	pigeons
Nme7	-0.46	1.00000000	pigeons
Gna13	-0.41	1.00000000	pigeons
Evl	-0.10	1.00000000	pigeons
Ranbp17	-0.61	1.00000000	pigeons
Tnrc6a	-0.50	1.00000000	pigeons
Tox	-0.31	1.00000000	pigeons

Tlnrd1	-0.76	1.00000000	pigeons
Trim8	-0.68	1.00000000	pigeons
Colgalt2	-0.49	1.00000000	pigeons
Rel	-0.34	1.00000000	pigeons
Dennd1b	-0.33	1.00000000	pigeons
Larp4b	-0.49	1.00000000	pigeons
Phf20	-0.47	1.00000000	pigeons
Tlk2	-0.37	1.00000000	pigeons
Sh3bp2	-0.33	1.00000000	pigeons
Slc30a1	-0.61	1.00000000	pigeons
Akt3	-0.47	1.00000000	pigeons
Rfx3	-0.10	1.00000000	pigeons
Fut8	-0.76	1.00000000	pigeons
Cntrl	-0.32	1.00000000	pigeons
Cpq	-0.32	1.00000000	pigeons
Reps2	-0.72	1.00000000	pigeons
Snx10	-0.43	1.00000000	pigeons
Tcn2	-0.36	1.00000000	pigeons
Bbs9	-0.12	1.00000000	pigeons
Nt5c3	-0.58	1.00000000	pigeons
Fgd3	-0.57	1.00000000	pigeons
Stxbp6	-0.29	1.00000000	pigeons
Cdkal1	-0.44	1.00000000	pigeons
Sgip1	-0.52	1.00000000	pigeons
Celf1	-0.48	1.00000000	pigeons
Plcg2	-0.06	1.00000000	pigeons
Sh3gl1	-0.53	1.00000000	pigeons
Fgd6	-0.68	1.00000000	pigeons
Plcb4	-0.35	1.00000000	pigeons
Vgll4	-0.45	1.00000000	pigeons
Scoc	-0.40	1.00000000	pigeons
Cytl1	-0.59	1.00000000	pigeons
Kat6b	-0.76	1.00000000	pigeons
F2r	-0.52	1.00000000	pigeons
Plekhm3	-0.19	1.00000000	pigeons
Ccnl2	-0.63	1.00000000	pigeons
Ube2f	-0.44	1.00000000	pigeons
Rpgr	-0.25	1.00000000	pigeons
Cpeb4	-0.88	1.00000000	pigeons
Camkmt	-0.13	1.00000000	pigeons
Hdac4	-0.32	1.00000000	pigeons
Pdia3	-0.55	1.00000000	pigeons
Smndc1	-0.51	1.00000000	pigeons
Tbc1d14	-0.39	1.00000000	pigeons
Brwd1	-0.31	1.00000000	pigeons
Ndufs6	-0.47	1.00000000	pigeons

Wwtr1	-0.31	1.00000000	pigeons
Camk2d	-0.25	1.00000000	pigeons
Bloc1s6	-0.46	1.00000000	pigeons
Faf1	-0.73	1.00000000	pigeons
Ckb	-0.66	1.00000000	pigeons
Ewsr1	-0.30	1.00000000	pigeons
Ppp2r5c	-0.63	1.00000000	pigeons
Ldlrad3	-0.53	1.00000000	pigeons
Phlpp2	-0.38	1.00000000	pigeons
Slc25a13	-0.43	1.00000000	pigeons
Zdhhc17	-0.59	1.00000000	pigeons
Wdr1	-0.45	1.00000000	pigeons
Tbc1d4	-0.38	1.00000000	pigeons
Atf4	-0.11	1.00000000	pigeons
Wdr35	-0.53	1.00000000	pigeons
Dnah12	-0.38	1.00000000	pigeons
Eml1	-0.36	1.00000000	pigeons
Chid1	-0.35	1.00000000	pigeons
Ice2	-0.29	1.00000000	pigeons
Cbfb	-0.40	1.00000000	pigeons
Baz2b	-0.74	1.00000000	pigeons
Dcaf5	-0.62	1.00000000	pigeons
Rpa1	-0.52	1.00000000	pigeons
Eef1b2	-0.13	1.00000000	pigeons
Prkn	-0.04	1.00000000	pigeons
Cep350	-0.58	1.00000000	pigeons
Ptk2	-0.71	1.00000000	pigeons
Usp15	-0.57	1.00000000	pigeons
Emp1	-0.40	1.00000000	pigeons
Tnfaip8	-0.35	1.00000000	pigeons
Coro1c	-0.42	1.00000000	pigeons
Dock2	-0.15	1.00000000	pigeons
Dusp6	-0.54	1.00000000	pigeons
Ankrd39	-0.39	1.00000000	pigeons
Tpp2	-0.41	1.00000000	pigeons
Abcc1	-0.65	1.00000000	pigeons
Tbx3	-0.54	1.00000000	pigeons
Fzd4	-0.32	1.00000000	pigeons
Ccdc50	-0.50	1.00000000	pigeons
Dock4	-0.19	1.00000000	pigeons
Naca	-0.41	1.00000000	pigeons
Rbm31y	-0.44	1.00000000	pigeons
Csrp1	-0.41	1.00000000	pigeons
Sufu	-0.26	1.00000000	pigeons
Clic4	-0.53	1.00000000	pigeons
Dnajc10	-0.77	1.00000000	pigeons

Larp1b	-0.54	1.00000000	pigeons
Hivep1	-0.36	1.00000000	pigeons
Lcp1	-0.45	1.00000000	pigeons
Ireb2	-0.43	1.00000000	pigeons
Plekha1	-0.57	1.00000000	pigeons
Elk4	-0.40	1.00000000	pigeons
Btbd10	-0.36	1.00000000	pigeons
Atp6v1e2	-0.42	1.00000000	pigeons
Slc12a2	-0.34	1.00000000	pigeons
Tspan13	-0.49	1.00000000	pigeons
Sfpq	-0.67	1.00000000	pigeons
Anp32e	-0.68	1.00000000	pigeons
Gnb4	-0.60	1.00000000	pigeons
Raph1	-0.53	1.00000000	pigeons
Agap1	-0.21	1.00000000	pigeons
Sh3bp4	-0.26	1.00000000	pigeons
Slc6a6	-0.43	1.00000000	pigeons
P4hb	-0.34	1.00000000	pigeons
Atp1a1	-0.49	1.00000000	pigeons
Lmo7	-0.33	1.00000000	pigeons
Znrf1	-0.44	1.00000000	pigeons
Synj1	-0.41	1.00000000	pigeons
Sh3kbp1	-0.53	1.00000000	pigeons
Lifr	-0.40	1.00000000	pigeons
Ldlrad4	-0.04	1.00000000	pigeons
Ints6	-0.36	1.00000000	pigeons
Cpsf4l	-0.52	1.00000000	pigeons
Gpatch11	-0.24	1.00000000	pigeons
Pak2	-0.57	1.00000000	pigeons
Cfap45	-0.48	1.00000000	pigeons
Slk	-0.46	1.00000000	pigeons
Lfng	-0.38	1.00000000	pigeons
Gatad2a	-0.39	1.00000000	pigeons
Zzef1	-0.37	1.00000000	pigeons
Gpcpd1	-0.58	1.00000000	pigeons
Amot	-0.64	1.00000000	pigeons
Cog5	-0.43	1.00000000	pigeons
Prex1	-0.84	1.00000000	pigeons
Ttc28	-0.65	1.00000000	pigeons
Itga4	-0.30	1.00000000	pigeons
Fam177a	-0.62	1.00000000	pigeons
Btbd9	-0.40	1.00000000	pigeons
Zfand3	-0.58	1.00000000	pigeons
Hspa5	-0.36	1.00000000	pigeons
Anxa5	-0.29	1.00000000	pigeons
Ppp2r5e	-0.66	1.00000000	pigeons

Aacs	-0.60	1.00000000	pigeons
Pim3	0.03	1.00000000	pigeons
Igfbp5	-0.09	1.00000000	pigeons
Gnb1	-0.59	1.00000000	pigeons
Eng	-0.53	1.00000000	pigeons
Ric1	-0.61	1.00000000	pigeons
Ncoa4	-0.36	1.00000000	pigeons
Rbm39	-0.54	1.00000000	pigeons
Dync1h1	-0.41	1.00000000	pigeons
Pparg	-0.05	1.00000000	pigeons
Ppargc1a	-0.19	1.00000000	pigeons
Gnal	-0.41	1.00000000	pigeons
Afap1l2	-0.45	1.00000000	pigeons
Rnaset2b	-0.49	1.00000000	pigeons
Dhx36	-0.58	1.00000000	pigeons
Akap12	-0.08	1.00000000	pigeons
Stag2	-0.55	1.00000000	pigeons
Rnf149	-0.24	1.00000000	pigeons
Zbed4	-0.31	1.00000000	pigeons
Enpp2	-0.28	1.00000000	pigeons
Serbp1	-0.33	1.00000000	pigeons
Atp6v0a1	-0.65	1.00000000	pigeons
Stk10	-0.44	1.00000000	pigeons
Snx25	-0.43	1.00000000	pigeons
Brd1	-0.40	1.00000000	pigeons
Itpk1	-0.59	1.00000000	pigeons
Zswim8	-0.50	1.00000000	pigeons
Retreg1	-0.50	1.00000000	pigeons
Usp34	-0.59	1.00000000	pigeons
Rell1	-0.55	1.00000000	pigeons
Sema3d	-0.19	1.00000000	pigeons
4932438A13Rik	-0.61	1.00000000	pigeons
Pla2r1	-0.67	1.00000000	pigeons
Vcl	-0.56	1.00000000	pigeons
Thbs1	0.28	1.00000000	pigeons
Arhgef7	-0.69	1.00000000	pigeons
Snrk	-0.61	1.00000000	pigeons
Pip5k1a	-0.75	1.00000000	pigeons
Tshz2	-0.41	1.00000000	pigeons
Hnrnpa2b1	-0.28	1.00000000	pigeons
Gpx1	-0.34	1.00000000	pigeons
Neo1	-0.47	1.00000000	pigeons
Twf1	-0.54	1.00000000	pigeons
Tmem47	-0.62	1.00000000	pigeons
Rai14	-0.73	1.00000000	pigeons
Zfp521	-0.47	1.00000000	pigeons

Ppp4r1	-0.68	1.00000000	pigeons
Prex2	-0.17	1.00000000	pigeons
Pdss2	-0.40	1.00000000	pigeons
Lmo2	-0.61	1.00000000	pigeons
Asxl3	-0.79	1.00000000	pigeons
Ift140	-0.45	1.00000000	pigeons
Tnks	-0.49	1.00000000	pigeons
Igfbp2	-0.16	1.00000000	pigeons
Dpf3	-0.34	1.00000000	pigeons
Rap1b	-0.49	1.00000000	pigeons
Slc39a10	-0.34	1.00000000	pigeons
Cd247	-0.17	1.00000000	pigeons
Ttc7b	-0.49	1.00000000	pigeons
Yap1	-0.56	1.00000000	pigeons
Samsn1	-0.31	1.00000000	pigeons
Rusc2	-0.58	1.00000000	pigeons
Shprh	-0.71	1.00000000	pigeons
Rb1cc1	-0.26	1.00000000	pigeons
Zbtb21	-0.70	1.00000000	pigeons
Casp7	-0.53	1.00000000	pigeons
Prkx	-0.38	1.00000000	pigeons
Vti1a	-0.45	1.00000000	pigeons
Smc3	-0.65	1.00000000	pigeons
Top2b	-0.44	1.00000000	pigeons
Ubr5	-0.52	1.00000000	pigeons
Odc1	-0.24	1.00000000	pigeons
Kif5b	-0.60	1.00000000	pigeons
Rev1	-0.58	1.00000000	pigeons
Gnb5	-0.74	1.00000000	pigeons
Ifi35	-0.54	1.00000000	pigeons
Adgrg6	-0.53	1.00000000	pigeons
Birc6	-0.58	1.00000000	pigeons
Adam10	-0.67	1.00000000	pigeons
Susd6	-0.69	1.00000000	pigeons
Nfx1	-0.49	1.00000000	pigeons
Fbxl17	-0.47	1.00000000	pigeons
Vwa8	-0.57	1.00000000	pigeons
Cald1	-0.20	1.00000000	pigeons
Ankrd28	-0.55	1.00000000	pigeons
Elf1	-0.60	1.00000000	pigeons
Iqgap1	-0.68	1.00000000	pigeons
Gjd4	-0.49	1.00000000	pigeons
Tub	-0.29	1.00000000	pigeons
Pik3c2a	-0.62	1.00000000	pigeons
Hpcal1	-0.53	1.00000000	pigeons
Cxcl12	-0.47	1.00000000	pigeons

Stxbp5	-0.57	1.00000000	pigeons
Cdc73	-0.52	1.00000000	pigeons
Rc3h1	-0.54	1.00000000	pigeons
Pcolce2	0.05	1.00000000	pigeons
Sos1	-0.71	1.00000000	pigeons
Tapt1	-0.78	1.00000000	pigeons
Cdk6	-0.27	1.00000000	pigeons
Rasa3	-0.40	1.00000000	pigeons
Btg1b	-0.13	1.00000000	pigeons
Sp3	-0.71	1.00000000	pigeons
Vav3	-0.34	1.00000000	pigeons
Ano10	-0.46	1.00000000	pigeons
Rbm33	-0.62	1.00000000	pigeons
Abcc4	-0.38	1.00000000	pigeons
Stam2	-0.64	1.00000000	pigeons
Amotl2	-0.67	1.00000000	pigeons
Wbp4	-0.63	1.00000000	pigeons
Zfp469	-0.39	1.00000000	pigeons
Ube2k	-0.57	1.00000000	pigeons
Fzd6	-0.49	1.00000000	pigeons
Eef1d	-0.16	1.00000000	pigeons
Eif3i	-0.55	1.00000000	pigeons
Snx29	-0.55	1.00000000	pigeons
Atxn7	-0.50	1.00000000	pigeons
Rcbtb1	-0.69	1.00000000	pigeons
Polr3a	-0.55	1.00000000	pigeons
Chac1	-0.29	1.00000000	pigeons
Alcam	-0.57	1.00000000	pigeons
Nfia	-0.10	1.00000000	pigeons
Ppp1r13b	-0.51	1.00000000	pigeons
Ppp1r9a	-0.39	1.00000000	pigeons
Exoc4	-0.47	1.00000000	pigeons
Pde8a	-0.29	1.00000000	pigeons
Ipmk	-0.62	1.00000000	pigeons
Tiam2	-0.47	1.00000000	pigeons
Serpind1	-0.34	1.00000000	pigeons
Arhgef12	-0.72	1.00000000	pigeons
Ddx3y	-0.06	1.00000000	pigeons
Ptbp1	-0.55	1.00000000	pigeons
Slc12a4	-0.66	1.00000000	pigeons
Nvl	-0.42	1.00000000	pigeons
Dlg1	-0.64	1.00000000	pigeons
Phf3	-0.53	1.00000000	pigeons
Setd5	-0.66	1.00000000	pigeons
Carhsp1	-0.66	1.00000000	pigeons
Shroom4	-0.47	1.00000000	pigeons

Ankrd10	0.06	1.00000000	pigeons
Commd10	-0.50	1.00000000	pigeons
Cyfip1	-0.63	1.00000000	pigeons
Lrch3	-0.44	1.00000000	pigeons
Anks1	-0.70	1.00000000	pigeons
Eif4g2	-0.21	1.00000000	pigeons
Robo2	0.09	1.00000000	pigeons
Sla	-0.23	1.00000000	pigeons
Limd1	-0.49	1.00000000	pigeons
B2m	2.48	0.00000000	mice
Ifitm3	2.25	0.00000000	mice
Ifi27l2a	3.84	0.00000000	mice
Bst2	3.29	0.00000000	mice
Zbtb16	-2.35	0.00000000	mice
Ifit3	2.68	0.00000000	mice
Ly6e	2.80	0.00000000	mice
Isg15	3.10	0.00000000	mice
Irf7	2.52	0.00000000	mice
Ifit1	2.64	0.00000000	mice
Oasl2	1.89	0.00000000	mice
H2-K1	2.28	0.00000000	mice
Lgals3bp	2.17	0.00000000	mice
Iigp1	2.73	0.00000000	mice
H2-D1	2.00	0.00000000	mice
Ifit3b	1.83	0.00000000	mice
Gbp3	1.63	0.00000000	mice
Xaf1	1.56	0.00000000	mice
Ifi44	2.02	0.00000000	mice
Cxcl10	3.46	0.00000000	mice
Zbp1	1.61	0.00000000	mice
Usp18	1.23	0.00000000	mice
H2-T23	1.68	0.00000000	mice
Gbp7	1.62	0.00000000	mice
Plac8	2.16	0.00000000	mice
Gbp6	1.27	0.00000000	mice
Rsad2	2.18	0.00000000	mice
Stat1	1.43	0.00000000	mice
Rtp4	1.12	0.00000000	mice
Ddx60	1.24	0.00000000	mice
Phf11d	1.36	0.00000000	mice
Ifih1	1.57	0.00000000	mice
Rnf213	1.68	0.00000000	mice
Cebpd	-1.26	0.00000000	mice
AY036118	1.98	0.00000000	mice
Klf9	-0.99	0.00000000	mice
Herc6	1.36	0.00000000	mice

Eif1	-0.64	0.00000000	mice
Tmsb10	1.47	0.00000000	mice
Trim30a	1.37	0.00000000	mice
Saa3	5.42	0.00000000	mice
Igtp	1.19	0.00000000	mice
Psmb8	1.63	0.00000000	mice
9530026P05Rik	-1.64	0.00000000	mice
Samd9l	1.11	0.00000000	mice
Tsc22d3	-1.46	0.00000000	mice
Eif2ak2	1.09	0.00000000	mice
Ifi35	1.09	0.00000000	mice
Parp9	1.01	0.00000000	mice
Jund	-0.83	0.00000000	mice
Ly6a	2.50	0.00000000	mice
Parp14	1.33	0.00000000	mice
Irgm1	1.08	0.00000000	mice
Ddx58	0.98	0.00000000	mice
Ifi47	1.09	0.00000000	mice
Tor3a	1.07	0.00000000	mice
Nup210l	-1.72	0.00000000	mice
Sp100	1.20	0.00000000	mice
Lgals9	1.07	0.00000000	mice
Ifi203	1.32	0.00000000	mice
Errfi1	-0.96	0.00000000	mice
Irgm2	0.78	0.00000000	mice
Cfb	2.18	0.00000000	mice
Ifi27	-0.61	0.00000000	mice
Tap1	0.86	0.00000000	mice
Gbp4	1.17	0.00000000	mice
Gbp2b	1.40	0.00000000	mice
Celf2	-0.89	0.00000000	mice
Ccl4	1.92	0.00000000	mice
Cmpk2	0.76	0.00000000	mice
Phf11b	0.88	0.00000000	mice
Gbp5	0.84	0.00000000	mice
Ifi202b	1.24	0.00000000	mice
Slfn8	0.56	0.00000000	mice
Ube2l6	0.81	0.00000000	mice
Gstm1	-0.61	0.00000000	mice
Psme1	1.18	0.00000000	mice
Stat2	0.77	0.00000000	mice
Gbp2	1.23	0.00000000	mice
Psmb10	1.09	0.00000000	mice
Apod	2.42	0.00000000	mice
F830016B08Rik	0.81	0.00000000	mice
Parp12	0.62	0.00000000	mice

Cdkn1a	-0.99	0.00000000	mice
Fkbp5	-0.69	0.00000000	mice
Psmb9	1.01	0.00000000	mice
Znfx1	0.68	0.00000000	mice
Dhx58	0.44	0.00000000	mice
Lcn2	2.89	0.00000000	mice
Oasl1	0.81	0.00000000	mice
H2-T24	0.45	0.00000000	mice
Tspo	1.01	0.00000000	mice
Il18bp	0.54	0.00000000	mice
C2	1.02	0.00000000	mice
Dtx3l	0.56	0.00000000	mice
Samhd1	0.94	0.00000000	mice
Shisa5	0.94	0.00000000	mice
A930037H05Rik	0.64	0.00000000	mice
Ccl7	2.18	0.00000000	mice
Cxcl13	2.60	0.00000000	mice
Pml	0.59	0.00000000	mice
Oas2	0.44	0.00000000	mice
Nop58	-0.91	0.00000000	mice
Tap2	0.86	0.00000000	mice
Prkca	-0.91	0.00000000	mice
H2-T22	0.91	0.00000000	mice
Gbp10	0.46	0.00000000	mice
Gbp8	0.52	0.00000000	mice
Oas1a	0.33	0.00000000	mice
Gbp9	0.45	0.00000000	mice
Trafd1	0.69	0.00000000	mice
Huwe1	-0.67	0.00000000	mice
Ifit2	0.98	0.00000000	mice
Peli2	-0.89	0.00000000	mice
Rbms3	-0.74	0.00000000	mice
Jmjd1c	-0.76	0.00000000	mice
Arih1	-0.69	0.00000000	mice
Glul	-0.70	0.00000000	mice
Cxcl2	1.24	0.00000000	mice
Adar	0.43	0.00000000	mice
Irs2	-0.59	0.00000000	mice
Ddx3x	-0.56	0.00000000	mice
Creb5	-0.97	0.00000000	mice
Btg2	-0.57	0.00000000	mice
Hdac4	-0.52	0.00000000	mice
Nmi	0.69	0.00000000	mice
Tapbp	0.85	0.00000000	mice
9930111J21Rik2	0.49	0.00000000	mice
Ifi44l	0.57	0.00000000	mice

Slfn5	0.95	0.00000000	mice
Auts2	-0.71	0.00000000	mice
Npc2	1.05	0.00000000	mice
Ifitm2	0.93	0.00000000	mice
Parp10	0.45	0.00000000	mice
Igfbp7	0.78	0.00000000	mice
Ripk1	-0.65	0.00000000	mice
Ptma	0.50	0.00000000	mice
Ifi211	0.70	0.00000000	mice
Slc10a6	-0.61	0.00000000	mice
Herpud1	-0.57	0.00000000	mice
Bach2	-0.66	0.00000000	mice
Scgb1a1	1.20	0.00000000	mice
Usp37	-0.53	0.00000000	mice
Psme2	0.84	0.00000000	mice
Parp11	0.31	0.00000000	mice
Ccl2	1.58	0.00000000	mice
Daxx	0.58	0.00000000	mice
Ifi204	0.89	0.00000000	mice
Coq10b	-0.48	0.00000000	mice
H2-Q6	0.45	0.00000000	mice
Uqcrh	-0.42	0.00000000	mice
Tpm3	-0.57	0.00000000	mice
Fosb	-0.62	0.00000000	mice
Cd274	0.78	0.00000000	mice
Slfn2	0.76	0.00000000	mice
Calr	0.79	0.00000000	mice
Rora	-0.59	0.00000000	mice
Socs3	-0.52	0.00000000	mice
Sgk1	-0.60	0.00000000	mice
Trim12c	0.41	0.00000000	mice
Junb	-0.53	0.00000000	mice
Tlr3	0.34	0.00000000	mice
Dgkb	-0.74	0.00000000	mice
Pqlc1	-0.57	0.00000000	mice
Tmem176a	0.87	0.00000000	mice
AW112010	1.06	0.00000000	mice
Tpm3-rs7	-0.52	0.00000000	mice
Egfr	-0.67	0.00000000	mice
Timp1	2.08	0.00000000	mice
Tgtp2	0.29	0.00000000	mice
Sp110	0.49	0.00000000	mice
Apol9a	0.29	0.00000000	mice
Gab2	-0.67	0.00000000	mice
Trim25	0.50	0.00000000	mice
Pde4b	-0.78	0.00000000	mice

Klf15	-0.30	0.00000000	mice
Oas3	0.32	0.00000000	mice
Il1b	1.19	0.00000000	mice
Rbm39	-0.44	0.00000000	mice
Aftph	0.64	0.00000000	mice
Klhl2	-0.50	0.00000000	mice
Tcp11l2	-0.47	0.00000000	mice
Ccnd3	-0.57	0.00000000	mice
Trak1	-0.50	0.00000000	mice
Olfr56	0.38	0.00000000	mice
Stat3	-0.55	0.00000000	mice
Galnt15	-0.42	0.00000000	mice
Hsp90aa1	-0.42	0.00000000	mice
Samd4	-0.75	0.00000000	mice
Sdc3	0.42	0.00000000	mice
Clec2d	0.56	0.00000000	mice
Nlrc5	0.52	0.00000000	mice
Nfkbiz	-0.41	0.00000000	mice
Slfn9	0.27	0.00000000	mice
Ly6c2	0.79	0.00000000	mice
Vcam1	1.08	0.00000000	mice
Slfn4	0.87	0.00000000	mice
Arhgef12	-0.43	0.00000000	mice
Enpp3	-0.86	0.00000000	mice
Ubr5	-0.47	0.00000000	mice
Klf13	-0.35	0.00000000	mice
Klf4	-0.67	0.00000000	mice
Ints6	-0.47	0.00000001	mice
Chd1	-0.44	0.00000001	mice
Bphl	-0.44	0.00000001	mice
Pdk4	-0.28	0.00000001	mice
Pde4d	-0.72	0.00000001	mice
Calhm6	0.52	0.00000001	mice
Lsmem1	-0.78	0.00000001	mice
Camta1	-0.49	0.00000001	mice
Fndc3b	-0.62	0.00000001	mice
Ltbp4	-0.64	0.00000001	mice
Dnaja1	-0.43	0.00000001	mice
Tprgl	-0.23	0.00000002	mice
Akap13	-0.49	0.00000002	mice
Ski	-0.44	0.00000002	mice
Elmo1	-0.66	0.00000002	mice
Cnot4	-0.46	0.00000002	mice
Prxl2c	-0.37	0.00000003	mice
Cmss1	0.86	0.00000003	mice
Gabarapl2	-0.28	0.00000003	mice

Spats2l	0.65	0.00000003	mice
Rock2	-0.48	0.00000003	mice
Cebpb	-0.43	0.00000004	mice
Large1	-0.75	0.00000004	mice
Svil	-0.45	0.00000004	mice
Serpinh1	0.81	0.00000004	mice
Tgfbr3	-0.73	0.00000005	mice
Adamts1	-0.47	0.00000005	mice
Gcc1	-0.41	0.00000005	mice
Rapgef6	-0.42	0.00000006	mice
Spry1	-0.42	0.00000006	mice
Zbtb38	-0.52	0.00000006	mice
Ust	-0.53	0.00000006	mice
Lyz2	0.18	0.00000007	mice
Irf2bp2	-0.39	0.00000007	mice
Gls	-0.38	0.00000007	mice
Tnfaip2	0.75	0.00000007	mice
Eif1a	-0.49	0.00000008	mice
Tpt1	-0.30	0.00000009	mice
Txnrd1	-0.57	0.00000010	mice
Jak1	-0.52	0.00000011	mice
Gpc6	-1.03	0.00000013	mice
Naca	-0.31	0.00000013	mice
Arid5b	-0.48	0.00000014	mice
Aff3	-0.72	0.00000014	mice
Atf4	-0.44	0.00000015	mice
Eif3h	-0.30	0.00000017	mice
Angptl2	-0.24	0.00000018	mice
4930523C07Rik	-0.30	0.00000021	mice
Sftpc	-1.06	0.00000023	mice
Sbno2	-0.83	0.00000023	mice
Kpna4	-0.46	0.00000026	mice
Selenow	0.72	0.00000027	mice
4930599N23Rik	0.37	0.00000027	mice
Bag3	-0.45	0.00000031	mice
Ckb	-0.47	0.00000031	mice
H2-Q7	0.61	0.00000032	mice
Dnajb9	-0.47	0.00000033	mice
Eln	-0.64	0.00000036	mice
Camk1d	0.92	0.00000036	mice
Eef1b2	-0.31	0.00000037	mice
Srek1	-0.38	0.00000038	mice
Rrbp1	0.64	0.00000038	mice
Nfat5	-0.53	0.00000039	mice
Atf3	-0.53	0.00000040	mice
Zhx3	-0.41	0.00000041	mice

Skil	-0.41	0.00000048	mice
Irf9	0.42	0.00000054	mice
Ppp1r15a	-0.38	0.00000058	mice
Igf1r	-0.46	0.00000059	mice
Fap	0.91	0.00000062	mice
Pcolce2	-0.58	0.00000062	mice
Ube2s	-0.34	0.00000064	mice
Spaca6	-0.41	0.00000070	mice
Tdrd7	0.28	0.00000076	mice
Sik3	-0.59	0.00000080	mice
Pcf11	-0.41	0.00000109	mice
C3	0.77	0.00000116	mice
Rnf19a	-0.33	0.00000129	mice
Pdzrn3	-0.57	0.00000138	mice
Eif4b	-0.26	0.00000147	mice
Setdb2	0.29	0.00000151	mice
Sun2	-0.27	0.00000155	mice
Hsp90b1	0.62	0.00000166	mice
Adamts17	-0.51	0.00000191	mice
Nfatc2	-0.35	0.00000214	mice
Lama2	-0.58	0.00000222	mice
Pfdn5	-0.26	0.00000231	mice
Dnajb4	-0.37	0.00000235	mice
Ranbp9	-0.58	0.00000243	mice
Arhgap10	-0.49	0.00000270	mice
Ccl3	0.91	0.00000270	mice
Rrm1	-0.24	0.00000298	mice
Ppa1	0.47	0.00000337	mice
Pdia3	0.67	0.00000356	mice
Aplp2	-0.34	0.00000408	mice
Rhob	-0.45	0.00000420	mice
Ccn2	-1.07	0.00000435	mice
Ctss	0.78	0.00000454	mice
Slc41a1	-0.36	0.00000483	mice
Pard3b	-0.46	0.00000485	mice
Kif1b	-0.33	0.00000497	mice
Esyt2	-0.44	0.00000519	mice
Rlim	-0.40	0.00000554	mice
Fbxl7	-0.58	0.00000589	mice
Atf6	-0.52	0.00000716	mice
Egr1	-0.43	0.00000776	mice
Arntl	-0.33	0.00000782	mice
Ksr1	-0.43	0.00000992	mice
Igfbp6	-0.86	0.00001004	mice
Arl13b	-0.40	0.00001025	mice
Kalrn	-0.59	0.00001073	mice

Rassf1	-0.48	0.00001124	mice
Cped1	-0.46	0.00001141	mice
Tacc2	-0.30	0.00001150	mice
Smc1a	-0.54	0.00001180	mice
Rtn4	-0.51	0.00001183	mice
Hspa2	-0.33	0.00001207	mice
Uba7	0.19	0.00001231	mice
Camk2n1	-0.35	0.00001262	mice
Chka	-0.46	0.00001276	mice
Col3a1	0.68	0.00001310	mice
Spsb1	-0.48	0.00001310	mice
Il1a	0.56	0.00001335	mice
Appbp2	-0.37	0.00001338	mice
Aida	0.60	0.00001426	mice
Peg3	-0.67	0.00001464	mice
Sec61g	0.62	0.00001541	mice
Ntrk3	-0.52	0.00001772	mice
Tor1aip2	0.46	0.00001823	mice
Snu13	-0.35	0.00001847	mice
Slirp	0.53	0.00002030	mice
Isg20	0.66	0.00002204	mice
Dst	-0.49	0.00002306	mice
Pdia4	0.51	0.00002371	mice
Bace2	-0.46	0.00002395	mice
Naaladl2	-0.72	0.00002397	mice
Elmod3	-0.39	0.00002500	mice
Ablim1	-0.40	0.00002665	mice
Gstk1	-0.26	0.00002710	mice
Top1	-0.41	0.00003137	mice
Foxn3	-0.36	0.00003319	mice
H2afv	-0.33	0.00003400	mice
Ttc28	-0.38	0.00003518	mice
Rb1cc1	-0.43	0.00003594	mice
Pnrc1	-0.35	0.00003827	mice
Tubb6	-0.33	0.00003853	mice
Elf2	-0.47	0.00003986	mice
Ldlrad4	-0.74	0.00004031	mice
Trib1	-0.57	0.00004035	mice
Sumo1	-0.34	0.00004096	mice
Pprc1	-0.31	0.00004239	mice
Ipo5	-0.50	0.00004327	mice
Ly6c1	1.54	0.00004398	mice
Polr1d	-0.28	0.00004611	mice
Wfdc17	0.41	0.00005106	mice
Dock9	-0.38	0.00005435	mice
Tgfbi	-0.64	0.00005562	mice

Dennd4a	-0.39	0.00005718	mice
Foxo1	-0.41	0.00005901	mice
Plk3	-0.28	0.00005996	mice
Aspn	-0.58	0.00006233	mice
Prkg1	-0.27	0.00006371	mice
Khdc4	-0.49	0.00006375	mice
Hipk3	-0.36	0.00006524	mice
Tmem106a	0.28	0.00006848	mice
Ldb2	-0.52	0.00007137	mice
Sppl2a	0.59	0.00007138	mice
Ccni	-0.27	0.00007168	mice
Vcl	-0.38	0.00007443	mice
Olfml2b	-0.38	0.00007471	mice
Atp5k	0.43	0.00007535	mice
Lbp	0.94	0.00007636	mice
Pde12	-0.34	0.00008304	mice
Tnrc6a	-0.29	0.00009061	mice
Clasp2	-0.41	0.00009407	mice
Il1rn	0.63	0.00009814	mice
Iyd	-0.48	0.00010002	mice
Tlr2	0.30	0.00010625	mice
Bmpr2	-0.50	0.00010739	mice
Ubtd2	-0.36	0.00010829	mice
Zbtb11	-0.45	0.00011426	mice
Asxl1	-0.49	0.00011527	mice
P4htm	-0.13	0.00011640	mice
Ccnl1	-0.33	0.00011912	mice
Phf8	-0.40	0.00013095	mice
Cox7a2l	-0.16	0.00014722	mice
Sertad1	-0.31	0.00015383	mice
Smad1	-0.40	0.00016495	mice
Hexim1	-0.34	0.00016553	mice
Pnpt1	0.34	0.00016670	mice
6030407O03Rik	-0.31	0.00016916	mice
Polr2a	-0.39	0.00017513	mice
Qpct	-0.34	0.00018066	mice
Zfp131	-0.34	0.00018121	mice
Gcnt2	-0.49	0.00018470	mice
Apol6	0.21	0.00018510	mice
Arih2	-0.34	0.00018760	mice
Bcl2l1	-0.35	0.00019214	mice
Ascc3	0.73	0.00019834	mice
Ahcyl1	-0.37	0.00019901	mice
Apobec3	0.19	0.00020495	mice
Ddx5	-0.25	0.00021883	mice
Kdm6b	-0.30	0.00021887	mice

Gsn	-0.88	0.00022056	mice
Grn	0.85	0.00022138	mice
Plcb4	-0.37	0.00022324	mice
Ikzf4	-0.28	0.00023343	mice
Rhoj	-0.64	0.00023377	mice
Grik4	-0.53	0.00026660	mice
Selenop	-0.26	0.00027589	mice
Cd47	0.56	0.00028019	mice
Sparcl1	-0.29	0.00030224	mice
Fbxo31	-0.22	0.00031459	mice
Ppm1n	-0.30	0.00031633	mice
Pam	-0.41	0.00036207	mice
Cblb	-0.34	0.00037118	mice
Adcy2	-0.55	0.00037770	mice
Socs1	0.60	0.00038594	mice
Foxp2	-0.38	0.00041741	mice
Ubr1	-0.40	0.00042020	mice
Lncpint	-0.56	0.00042804	mice
Lepr	-0.46	0.00043894	mice
Snhg8	-0.30	0.00046411	mice
Prkcg	-0.68	0.00047942	mice
Ybx3	-0.20	0.00049207	mice
Adarb1	-0.50	0.00051600	mice
Cul1	-0.22	0.00053220	mice
Tbc1d15	-0.25	0.00053442	mice
Pcsk5	-0.94	0.00055058	mice
Srsf5	-0.23	0.00056653	mice
Ogfr	0.36	0.00057431	mice
Dmd	-0.90	0.00057598	mice
Ythdc1	-0.33	0.00061704	mice
Pum1	-0.26	0.00063699	mice
AW011738	0.19	0.00064471	mice
Tnfrsf12a	-0.50	0.00071865	mice
Helz2	0.39	0.00074634	mice
Tmtc1	-0.34	0.00078995	mice
Ttc19	-0.28	0.00079648	mice
Ost4	-0.26	0.00080932	mice
Nktr	-0.30	0.00081040	mice
Aff4	-0.30	0.00081710	mice
D430041D05Rik	-0.29	0.00083360	mice
Tpm2	-0.84	0.00088028	mice
Ppp1r12a	-0.31	0.00092164	mice
Aff1	-0.31	0.00093572	mice
Arid5a	-0.34	0.00095240	mice
Batf2	0.21	0.00102402	mice
Ptprm	-0.45	0.00103184	mice

Litaf	-0.31	0.00105667	mice
Rabac1	-0.20	0.00115030	mice
Cmip	-0.40	0.00115547	mice
Rusc2	-0.23	0.00123603	mice
Eef1d	-0.18	0.00127305	mice
Usp25	0.48	0.00130633	mice
Timp3	-0.38	0.00133920	mice
Serping1	0.43	0.00134116	mice
Ankrd44	-0.84	0.00135492	mice
Pi15	-0.32	0.00154538	mice
Xist	-0.26	0.00154681	mice
Trp53inp1	-0.19	0.00156994	mice
Lmbr1l	-0.24	0.00159134	mice
Txn1	0.72	0.00159755	mice
Foxo3	-0.39	0.00159870	mice
Cav1	-0.10	0.00163805	mice
Adgrl1	-0.25	0.00172140	mice
Lgals1	0.59	0.00185252	mice
Nnmt	-0.35	0.00192782	mice
Nt5dc3	-0.33	0.00201175	mice
Atp1b3	-0.37	0.00201527	mice
Smim14	-0.20	0.00205702	mice
Etf1	-0.34	0.00206035	mice
Oser1	-0.30	0.00206280	mice
H2-Q4	0.36	0.00212711	mice
Npr3	-0.34	0.00217143	mice
Hnrnph1	-0.30	0.00218666	mice
Zmiz1	-0.35	0.00222231	mice
Clip1	-0.37	0.00238642	mice
Ankfy1	0.37	0.00240789	mice
Spats2	-0.50	0.00248710	mice
a	-0.36	0.00250475	mice
Btbd9	-0.37	0.00252528	mice
Stbd1	-0.24	0.00263828	mice
Pink1	-0.16	0.00270721	mice
Phf10	-0.23	0.00276506	mice
Ccl5	0.82	0.00280130	mice
Larp1b	-0.50	0.00285310	mice
Epha4	-0.19	0.00292585	mice
Tm2d2	-0.17	0.00294841	mice
Zbtb20	-0.25	0.00296367	mice
Clock	-0.32	0.00297177	mice
4930403P22Rik	-0.25	0.00302354	mice
Ntn4	-0.34	0.00304512	mice
Jcad	-0.31	0.00304695	mice
Arrdc3	-0.36	0.00313233	mice

Stat5b	-0.55	0.00323407	mice
Spock2	-0.24	0.00326589	mice
Arfgap3	-0.26	0.00331926	mice
Ppp1r10	-0.38	0.00333816	mice
Ndrg2	-0.30	0.00340424	mice
Ltbp3	-0.20	0.00341405	mice
Ahnak	-0.32	0.00347311	mice
Cd9	-0.32	0.00347386	mice
Plekhg2	-0.32	0.00363246	mice
Rdm1	-0.19	0.00366502	mice
Acer3	-0.50	0.00371740	mice
Plce1	-0.62	0.00387628	mice
Wsb1	-0.34	0.00389508	mice
Oaz1	-0.16	0.00390364	mice
Plekha4	0.31	0.00392879	mice
Smpdl3a	-0.11	0.00398820	mice
Pdia6	0.62	0.00399962	mice
Slc7a6	-0.32	0.00415659	mice
Atp2a2	-0.31	0.00433465	mice
Zswim6	-0.45	0.00438927	mice
2610035D17Rik	-0.33	0.00439650	mice
Fam107b	-0.42	0.00454968	mice
Dbp	0.20	0.00465722	mice
Abcc9	-0.37	0.00469126	mice
Fam129a	-0.61	0.00470492	mice
Cntn4	-0.54	0.00474287	mice
Adam33	-0.31	0.00476353	mice
Tgfb3	-0.21	0.00502380	mice
B830012L14Rik	-0.64	0.00503931	mice
Serpina3n	0.88	0.00505095	mice
Erc1	-0.41	0.00510857	mice
Filip1	-0.56	0.00514871	mice
Nf1	-0.32	0.00530977	mice
Sesn1	-0.39	0.00531645	mice
Fus	-0.26	0.00534600	mice
Pcdh7	-0.85	0.00537897	mice
Naf1	-0.15	0.00546330	mice
Actg1	-0.27	0.00549005	mice
Sp140	0.43	0.00557862	mice
Lims2	-0.24	0.00575947	mice
Ctnna1	-0.27	0.00586670	mice
Ube2j1	-0.25	0.00588001	mice
Fnip1	-0.30	0.00593310	mice
Guk1	-0.12	0.00594922	mice
Klf2	-0.46	0.00595275	mice
Tgfbr1	-0.37	0.00598505	mice

Samd4b	-0.32	0.00603773	mice
Acadm	-0.23	0.00608183	mice
Sult5a1	-0.14	0.00608486	mice
Kdm5c	-0.31	0.00649814	mice
Apoe	-0.40	0.00652173	mice
Ecm2	-0.26	0.00689259	mice
Zcchc2	0.27	0.00699594	mice
Frat2	-0.31	0.00703609	mice
Adamts12	-0.44	0.00742209	mice
Nipbl	-0.27	0.00748448	mice
Fgfr2	-0.27	0.00754782	mice
Hnrnpc	-0.24	0.00762042	mice
Amd1	-0.24	0.00800243	mice
Cox7c	0.57	0.00803551	mice
Nenf	-0.26	0.00813613	mice
Aqp1	-0.49	0.00829382	mice
Ifngr1	-0.42	0.00835202	mice
Cul3	-0.20	0.00839792	mice
Trim21	0.17	0.00867791	mice
Dph3	-0.27	0.00875669	mice
H2-Aa	0.50	0.00878901	mice
Siva1	-0.25	0.00880057	mice
Dcp2	0.27	0.00884283	mice
Sbf2	-0.34	0.00893667	mice
Uap1	-0.36	0.00905285	mice
Polg	-0.18	0.00984412	mice
Rian	-0.85	0.00999183	mice
Ctsf	-0.18	0.01007533	mice
Adamts2	-0.43	0.01020370	mice
Pabpc1	-0.08	0.01025241	mice
Hdx	-0.33	0.01031991	mice
Slc7a6os	-0.26	0.01088023	mice
Zup1	0.34	0.01099665	mice
Anxa5	-0.24	0.01125789	mice
Klhl24	-0.30	0.01134738	mice
Elavl1	-0.18	0.01156511	mice
Gria3	-0.55	0.01176263	mice
Sfpq	-0.26	0.01179193	mice
Ifitm1	1.11	0.01194314	mice
Rbl2	-0.17	0.01204787	mice
Snai1	0.17	0.01283131	mice
Tmem176b	0.67	0.01329940	mice
Slit3	-0.74	0.01332590	mice
Camk4	-0.18	0.01342520	mice
Stag1	-0.33	0.01387849	mice
Banp	-0.27	0.01403985	mice

Dnajc3	-0.26	0.01423099	mice
Ehbp1	-0.38	0.01426597	mice
Pygm	-0.26	0.01435049	mice
Tnxb	-0.35	0.01461386	mice
Cacna1c	-0.54	0.01465848	mice
Epc2	-0.26	0.01477780	mice
Smyd3	-0.36	0.01490547	mice
Adk	-0.32	0.01508678	mice
Peli1	-0.21	0.01537785	mice
Gpx4	-0.14	0.01540367	mice
Polr3b	-0.30	0.01575117	mice
Stpg4	-0.23	0.01584162	mice
Rbm18	-0.20	0.01595821	mice
Klhdc2	-0.15	0.01657943	mice
Slc4a4	-0.42	0.01738350	mice
Atp11c	-0.40	0.01753228	mice
Nfil3	-0.23	0.01764021	mice
Arnt	-0.30	0.01802874	mice
Tmem140	0.35	0.01847278	mice
Stau1	-0.29	0.01910785	mice
Tomm20	-0.15	0.01921054	mice
Smarca5	-0.14	0.01930892	mice
Arhgap17	-0.29	0.01959154	mice
Rhoq	-0.42	0.01962506	mice
Sema5a	-0.33	0.02012583	mice
Aldh2	-0.06	0.02033253	mice
Efcab8	-0.22	0.02036647	mice
Apex2	-0.34	0.02080034	mice
Tcirg1	0.25	0.02161889	mice
Ror1	-0.58	0.02214627	mice
Hmgcs2	-0.21	0.02229961	mice
Itga9	-0.31	0.02265109	mice
Plekhm2	-0.28	0.02289303	mice
Atp13a3	-0.29	0.02312339	mice
S100a9	0.20	0.02343784	mice
Thoc6	-0.26	0.02375718	mice
Ttll3	-0.10	0.02375824	mice
Id3	-0.35	0.02404077	mice
Sptssa	-0.09	0.02406132	mice
Abl1	-0.31	0.02416892	mice
Crispld2	-0.36	0.02477738	mice
Fam168a	-0.23	0.02480142	mice
Smc6	-0.19	0.02493511	mice
Fbrsl1	-0.29	0.02596314	mice
Tiparp	-0.32	0.02609805	mice
Kctd10	-0.20	0.02636533	mice

Arl4a	-0.39	0.02690635	mice
Nipa1	-0.08	0.02694087	mice
Piga	-0.20	0.02706438	mice
Ube2d2a	-0.22	0.02713155	mice
Rgs6	-0.29	0.02734904	mice
Itpr1	-0.39	0.02874282	mice
Lhfp	-0.15	0.02893498	mice
Dpep1	-0.67	0.02901702	mice
Acod1	0.74	0.02949970	mice
Rassf3	-0.29	0.02952503	mice
Ifi207	0.45	0.02963180	mice
Lamb2	-0.26	0.03012021	mice
Limk2	-0.20	0.03040523	mice
Wee1	-0.17	0.03050050	mice
Pim3	-0.20	0.03093523	mice
Akap12	-0.32	0.03126367	mice
Neat1	-0.30	0.03338827	mice
Fcgrt	-0.34	0.03461149	mice
Ankrd11	-0.30	0.03535963	mice
Sh3bgr	-0.32	0.03574516	mice
Tsc22d2	-0.36	0.03743117	mice
Fermt2	-0.13	0.03819080	mice
Igf1	0.73	0.03886249	mice
Wapl	-0.20	0.03895471	mice
Idh2	-0.31	0.03933276	mice
Ddr2	-0.39	0.03978652	mice
Gse1	-0.36	0.04167670	mice
Ube2h	-0.31	0.04186349	mice
Nufip2	-0.25	0.04209139	mice
Rela	-0.20	0.04244066	mice
Fstl3	-0.20	0.04294670	mice
Zfand5	-0.26	0.04377511	mice
Prkn	-0.42	0.04564678	mice
Dpyd	-0.36	0.04568559	mice
Atrnl1	-0.31	0.04589518	mice
Ptger4	-0.19	0.04663867	mice
Sptan1	-0.29	0.04829903	mice
Sdc2	-0.21	0.04842588	mice
Dusp8	-0.21	0.04899422	mice
Nup98	-0.26	0.05082457	mice
Phlda3	0.48	0.05133835	mice
Tinagl1	-0.43	0.05158273	mice
1810010H24Rik	-0.16	0.05185300	mice
Usp34	-0.26	0.05194382	mice
Pir	-0.25	0.05210203	mice
Ankrd42	-0.19	0.05254160	mice

Cstf3	-0.32	0.05314097	mice
Rras2	-0.37	0.05315943	mice
Pura	-0.24	0.05342000	mice
Itpkc	-0.31	0.05353939	mice
Ahctf1	-0.35	0.05512846	mice
Nupr1	0.61	0.05531455	mice
Rnf103	-0.29	0.05569764	mice
Eef2	-0.22	0.05687979	mice
Bop1	-0.18	0.05745136	mice
Cnot7	-0.21	0.05796755	mice
Atp5md	0.56	0.05895222	mice
Sorbs1	-0.37	0.05933088	mice
Nav2	-0.36	0.05947296	mice
Brwd1	-0.41	0.05996889	mice
Kat6b	-0.17	0.06115407	mice
Npepps	-0.32	0.06136222	mice
Wasf2	-0.18	0.06204699	mice
Hectd1	-0.27	0.06374824	mice
Atp5e	0.52	0.06470656	mice
Ndufb1-ps	0.50	0.06475851	mice
Fzd2	-0.25	0.06535055	mice
Rnf114	0.28	0.06555262	mice
Paqr6	-0.12	0.06580232	mice
Fcho2	-0.27	0.06840904	mice
Tenm4	-0.32	0.06852960	mice
Pgrmc1	-0.23	0.06966965	mice
Fgfr1	-0.30	0.06978440	mice
Kdm3a	-0.28	0.07028992	mice
Dennd5a	-0.21	0.07194651	mice
Prkx	-0.23	0.07225800	mice
Ndufa6	-0.01	0.07361777	mice
C1ra	0.64	0.07363202	mice
Hspa8	-0.25	0.07416420	mice
Txnip	-0.51	0.07616158	mice
Sdf2l1	0.37	0.07634507	mice
Pde10a	-1.49	0.07698849	mice
Nkain4	-0.19	0.07783405	mice
Nop53	-0.10	0.07840514	mice
Ago2	-0.29	0.07900717	mice
Gclc	-0.44	0.08001295	mice
Fads1	-0.15	0.08017653	mice
Oxnad1	-0.21	0.08389206	mice
Rbbp8	-0.25	0.08459137	mice
Kcnb1	-0.28	0.08497667	mice
Sorl1	-0.29	0.08677342	mice
Sntb2	-0.42	0.08822761	mice

Bnip3l	-0.04	0.08860383	mice
Ubxn4	-0.24	0.08932935	mice
Runx1	-0.52	0.09094483	mice
Syne1	-0.33	0.09131460	mice
Kdm6a	-0.22	0.09142568	mice
Vit	-0.20	0.09297773	mice
Hs6st3	-0.22	0.09316691	mice
H1fx	-0.19	0.09322901	mice
Ptprk	-0.32	0.09492027	mice
Fmo2	-0.20	0.09507067	mice
Dhrs3	-0.31	0.09515905	mice
Rnaseh2a	-0.21	0.09621898	mice
Fbxl18	-0.21	0.09707075	mice
Ppp4r2	-0.25	0.09900415	mice
Tom1	-0.31	0.10022320	mice
Speg	-0.35	0.10141458	mice
Hspa5	-0.33	0.10159120	mice
Sobp	-0.30	0.10266556	mice
Cyth3	-0.30	0.10420832	mice
Dyrk1a	-0.21	0.10443191	mice
Vasn	-0.26	0.10505653	mice
Exoc3l4	0.11	0.10640221	mice
Tuba1c	-0.11	0.10736695	mice
Pltp	-0.38	0.10833456	mice
Lpp	-0.33	0.10977920	mice
B230307C23Rik	0.15	0.11318842	mice
Gsk3a	-0.30	0.11701698	mice
Col14a1	-0.45	0.11779223	mice
Slc8a2	-0.19	0.12450789	mice
Fto	-0.25	0.12485082	mice
2010310C07Rik	-0.24	0.12632782	mice
Dync2h1	-0.26	0.12642260	mice
Tdp2	-0.19	0.12767058	mice
Crebrf	-0.30	0.12863633	mice
Dip2c	-0.22	0.13285299	mice
Pafah1b1	-0.14	0.13507282	mice
Dpysl2	-0.24	0.13659404	mice
Tnfsf9	-0.33	0.13770997	mice
Smim11	-0.06	0.13980289	mice
Bgn	0.40	0.14096259	mice
Rbbp6	-0.28	0.14533749	mice
Pikfyve	0.15	0.14733239	mice
Itm2a	-0.14	0.15064355	mice
Bzw1	-0.19	0.15511194	mice
Sgms1	-0.25	0.15831300	mice
Srsf10	-0.21	0.15926900	mice

Gria1	-0.63	0.15981645	mice
Tagln	-0.73	0.16040880	mice
Ctsc	0.63	0.16126443	mice
Capn15	-0.22	0.16212078	mice
Tasor	-0.32	0.16301053	mice
Rbm43	0.20	0.16437521	mice
Crim1	-0.18	0.16515078	mice
Ppp2r3a	-0.39	0.16670528	mice
Rsu1	-0.19	0.16740582	mice
Serpinb9	0.31	0.16876257	mice
H2afz	-0.07	0.16986809	mice
Suco	-0.28	0.17018738	mice
Gprasp1	-0.27	0.17326907	mice
Angpt2	-0.19	0.17329708	mice
Atp6v0e	-0.21	0.17424120	mice
Auh	-0.31	0.17530219	mice
Lrmda	0.53	0.17531991	mice
Ezh2	-0.41	0.17786052	mice
Gan	-0.20	0.17859851	mice
Pcdh9	-0.49	0.17868068	mice
Pitpna	-0.16	0.17993899	mice
Aven	-0.22	0.18156507	mice
Eef1a1	-0.17	0.18168903	mice
Ypel3	-0.10	0.18322225	mice
Tmem11	-0.13	0.18413612	mice
Tmtc2	-0.49	0.18626264	mice
Csnk1e	-0.27	0.18882483	mice
Purb	-0.19	0.18974973	mice
Dstn	-0.27	0.19256828	mice
Rem1	-0.24	0.19312805	mice
Gsdmd	0.28	0.19557114	mice
Naxe	-0.15	0.20028796	mice
Wwc2	-0.35	0.20142964	mice
Sec61b	0.69	0.21178117	mice
Hnrnpul1	-0.13	0.21297268	mice
H2-Ke6	-0.11	0.21653437	mice
Otulinl	-0.12	0.21729489	mice
Il15	0.22	0.21881508	mice
Sgcd	-0.68	0.21956741	mice
Ddx24	0.55	0.22110087	mice
Foxp1	-0.26	0.22194786	mice
Tent5a	0.36	0.22231724	mice
Bicd2	-0.17	0.22484317	mice
Pcbp3	-0.40	0.22605820	mice
Ank3	-0.62	0.22811742	mice
Fcer1g	0.59	0.22915845	mice

Cd52	0.65	0.23146553	mice
Desi1	-0.31	0.23386437	mice
Cyp1b1	0.38	0.23743908	mice
Per1	-0.22	0.24129363	mice
Azin1	-0.25	0.24298590	mice
Tns1	-0.27	0.24345973	mice
Elmsan1	-0.30	0.24351580	mice
Pid1	-0.51	0.24409908	mice
Ppp2r5a	-0.25	0.25168937	mice
Ccrl2	0.76	0.25390200	mice
Rbmxl1	-0.29	0.25569242	mice
Setbp1	-0.27	0.25597051	mice
Zyx	-0.16	0.25960725	mice
Atp1a1	-0.19	0.26166225	mice
Dennd5b	-0.34	0.26230564	mice
Actc1	-0.79	0.26323711	mice
Pea15a	-0.22	0.26378248	mice
Dnah12	-0.31	0.26929865	mice
Creb3l2	-0.18	0.27106888	mice
Ednrb	-0.17	0.27532788	mice
Zfp592	-0.19	0.27869962	mice
Pxdc1	-0.27	0.27990643	mice
Sat1	-0.22	0.28028273	mice
Thra	-0.22	0.28050654	mice
Zmynd11	-0.27	0.28437059	mice
Eloc	-0.24	0.28681292	mice
Fer1l5	-0.39	0.28890780	mice
Ism1	-0.38	0.29099175	mice
Fibin	-0.32	0.29233320	mice
Impdh2	-0.07	0.29333007	mice
Usp32	-0.30	0.29510549	mice
Nedd4	-0.20	0.29588918	mice
Btbd1	-0.25	0.29796329	mice
Atl2	-0.21	0.29985031	mice
Bambi	-0.22	0.30127552	mice
Endov	0.18	0.30396656	mice
Csf1	0.41	0.30425326	mice
Rnf150	0.38	0.30428409	mice
Cnn1	-0.40	0.31149696	mice
Rnft1	-0.16	0.31403317	mice
Armc9	-0.18	0.31489928	mice
Smurf2	-0.25	0.31810587	mice
Tnfsf13b	0.47	0.31948570	mice
Braf	-0.22	0.32308226	mice
Stk17b	-0.26	0.32387947	mice
Ccl8	0.46	0.32597757	mice

A530013C23Rik	-0.27	0.33034661	mice
Pdlim7	-0.20	0.33134722	mice
Htra3	-0.31	0.33440918	mice
Npy1r	-0.27	0.33993593	mice
Pknox1	-0.27	0.34728677	mice
Nsmce2	-0.29	0.34773499	mice
Sox6	-0.27	0.34796006	mice
Slc15a3	0.49	0.35230290	mice
Cbx6	-0.23	0.35709980	mice
Dner	-0.29	0.36770597	mice
Birc2	-0.26	0.36858882	mice
Prrc2b	-0.17	0.36957210	mice
Trio	-0.34	0.37893886	mice
Stim1	-0.24	0.37919436	mice
Id4	-0.25	0.38397044	mice
Kif5b	-0.19	0.38897042	mice
Gtf2b	-0.17	0.39121668	mice
Adcy5	-0.34	0.39375130	mice
Dact3	-0.20	0.39727859	mice
Srsf4	-0.12	0.39859573	mice
Arhgap15	-0.23	0.40246280	mice
Ccl6	0.33	0.40336354	mice
Pcna	-0.03	0.40576717	mice
Gspt1	-0.28	0.40803842	mice
Azi2	0.37	0.41078002	mice
Shb	-0.24	0.41667293	mice
Pramef8	-0.24	0.42021368	mice
Tnfsf12	-0.09	0.42141779	mice
Fam71a	-0.22	0.43227454	mice
Oxct1	-0.22	0.43404232	mice
Ankrd28	-0.49	0.43726061	mice
Dlg4	-0.32	0.43827616	mice
Ifi205	0.35	0.45081015	mice
Dennd2a	-0.35	0.45273936	mice
Cdh13	-0.68	0.45944986	mice
Cnbp	-0.04	0.46661941	mice
Ulk1	-0.25	0.47403473	mice
Rabgef1	-0.15	0.47445171	mice
Ino80d	-0.27	0.47489048	mice
Rbpms	-0.38	0.48206008	mice
Airn	-0.25	0.48683839	mice
Gas1	-0.35	0.49093355	mice
Ntn1	-0.25	0.49143088	mice
Sertad2	-0.30	0.49261918	mice
Ppp6r3	-0.23	0.49738631	mice
Luc7l2	-0.20	0.49955476	mice

Rptor	-0.19	0.50456397	mice
Ctif	-0.19	0.50652735	mice
Dpm1	-0.23	0.50744314	mice
Ets2	-0.20	0.51569163	mice
Dynll1	-0.15	0.51968493	mice
C4b	0.31	0.52044307	mice
Exoc6b	-0.25	0.52330078	mice
Ell2	-0.16	0.53003827	mice
Cnksr3	-0.28	0.53263766	mice
Psmg4	0.24	0.53543756	mice
Col16a1	-0.23	0.54095680	mice
Wfdc1	-0.07	0.54259116	mice
Galnt2	-0.14	0.54492160	mice
Sec16a	-0.25	0.55946044	mice
Usp2	-0.17	0.56348634	mice
Helz	-0.14	0.56509325	mice
Cmtr1	0.20	0.58208167	mice
Srrm2	-0.13	0.59114922	mice
Epas1	-0.17	0.59572489	mice
Ctsl	-0.24	0.59854694	mice
Pi16	-0.63	0.60118903	mice
Dcaf1	-0.28	0.60254337	mice
Rsrp1	0.44	0.61165833	mice
Pla2g12a	-0.13	0.61980332	mice
Jun	-0.33	0.62233934	mice
Park7	-0.09	0.62487708	mice
Fbln5	-0.24	0.63716511	mice
Ralgapa2	-0.26	0.65623701	mice
Ndufb10	-0.17	0.65633699	mice
Tmem173	0.18	0.66943027	mice
Cp	0.38	0.67879010	mice
Slc16a10	-0.27	0.67961229	mice
Sde2	-0.27	0.68302501	mice
Ces1d	0.65	0.69094503	mice
Smim3	-0.36	0.69154667	mice
Il11ra1	-0.22	0.69220592	mice
Safb2	-0.15	0.69280475	mice
Picalm	-0.21	0.69783838	mice
Psmd14	0.40	0.70342869	mice
Rack1	-0.14	0.70725812	mice
Dctn6	-0.13	0.71030240	mice
Desi2	-0.19	0.71209007	mice
Atxn7	-0.20	0.71429927	mice
Ddx6	-0.12	0.71702817	mice
Rhobtb3	-0.13	0.73403366	mice
Ubn1	-0.16	0.75936291	mice

Tle5	0.02	0.76562474	mice
Galnt17	-0.33	0.77683173	mice
Abcb1b	-0.54	0.77992322	mice
Tnrc6c	-0.19	0.80295041	mice
Fos	-0.33	0.81816580	mice
Ppp1cb	-0.20	0.82517352	mice
Serinc3	-0.17	0.83236260	mice
Rap1b	-0.20	0.83443432	mice
Dusp5	-0.32	0.83959070	mice
Stk10	-0.32	0.84162484	mice
Phyhd1	-0.13	0.85055369	mice
Lrat	-0.51	0.85154964	mice
Wipi2	-0.24	0.85754631	mice
Sp4	-0.24	0.86640801	mice
Nfix	-0.17	0.86815576	mice
Chd3	-0.16	0.86871031	mice
Nim1k	-0.32	0.87267216	mice
Clk1	-0.18	0.87298687	mice
Jade1	-0.16	0.87803982	mice
Eif3f	0.08	0.87809370	mice
Tecr	-0.11	0.87906524	mice
Cdc42ep2	0.27	0.88233321	mice
Gabarap	-0.11	0.88486230	mice
Pappa	-0.27	0.89428881	mice
Hsph1	-0.21	0.90968047	mice
Kcnq1ot1	-0.43	0.92297159	mice
Ephb2	-0.26	0.97296419	mice
Ndufa2	0.44	0.98021922	mice
Wdfy3	-0.23	0.98219359	mice
Gabbr1	-0.25	0.99474189	mice
Spata5	-0.26	1.00000000	mice
Taf4b	-0.37	1.00000000	mice
Tob1	-0.18	1.00000000	mice
Ginm1	0.01	1.00000000	mice
Fads2	-0.09	1.00000000	mice
Nup153	-0.13	1.00000000	mice
Glt8d2	-0.14	1.00000000	mice
Ctdsp1	-0.22	1.00000000	mice
Inpp5k	-0.15	1.00000000	mice
Ccnk	-0.16	1.00000000	mice
Cnn3	-0.21	1.00000000	mice
Actg2	-0.54	1.00000000	mice
Srp9	-0.11	1.00000000	mice
Ing1	-0.16	1.00000000	mice
Ppp1r12b	-0.22	1.00000000	mice
Ddah2	-0.25	1.00000000	mice

Sema3d	-0.35	1.00000000	mice
Spag9	-0.18	1.00000000	mice
Cntfr	-0.19	1.00000000	mice
Pttg1	0.23	1.00000000	mice
Puf60	-0.14	1.00000000	mice
1700001K19Rik	-0.17	1.00000000	mice
Cyth2	-0.19	1.00000000	mice
Ephx1	-0.11	1.00000000	mice
Hivep2	-0.32	1.00000000	mice
Rnf122	-0.23	1.00000000	mice
Jdp2	-0.28	1.00000000	mice
Grb10	-0.37	1.00000000	mice
Klhl15	-0.26	1.00000000	mice
Enpp4	0.16	1.00000000	mice
Lgals8	0.34	1.00000000	mice
Gfpt2	-0.36	1.00000000	mice
Hbp1	-0.15	1.00000000	mice
Tln2	-0.40	1.00000000	mice
Pigp	-0.12	1.00000000	mice
Fbxl17	-0.18	1.00000000	mice
Hnrnpa0	-0.09	1.00000000	mice
Clk4	-0.17	1.00000000	mice
Lmod1	-0.27	1.00000000	mice
Ubash3b	-0.33	1.00000000	mice
Nolc1	-0.23	1.00000000	mice
Fbxl22	-0.17	1.00000000	mice
Col12a1	-0.40	1.00000000	mice
Nbeal1	-0.24	1.00000000	mice
Kpna1	-0.30	1.00000000	mice
Cdk8	0.52	1.00000000	mice
Cd81	-0.04	1.00000000	mice
Zfp36	-0.29	1.00000000	mice
F2r	-0.10	1.00000000	mice
Tent4a	0.17	1.00000000	mice
Senp2	-0.26	1.00000000	mice
Pde3b	-0.32	1.00000000	mice
Ctnna3	-0.86	1.00000000	mice
Rapgef1	-0.20	1.00000000	mice
Bptf	-0.18	1.00000000	mice
Fam13a	-0.23	1.00000000	mice
Smad7	-0.34	1.00000000	mice
Dennd4b	-0.08	1.00000000	mice
Sf3b1	-0.20	1.00000000	mice
Sf1	-0.11	1.00000000	mice
Zhx2	-0.23	1.00000000	mice
Snx14	-0.21	1.00000000	mice

Crtc2	-0.14	1.00000000	mice
Ggnbp2	-0.18	1.00000000	mice
Cfap53	-0.55	1.00000000	mice
Smim10l1	-0.17	1.00000000	mice
Plscr2	0.20	1.00000000	mice
Dnajb2	-0.02	1.00000000	mice
Il1r1	-0.45	1.00000000	mice
2300009A05Rik	-0.17	1.00000000	mice
Snhg6	0.24	1.00000000	mice
Gabra3	-0.33	1.00000000	mice
Il6st	-0.12	1.00000000	mice
Slc29a1	-0.12	1.00000000	mice
Prrc2a	-0.23	1.00000000	mice
1810058I24Rik	-0.28	1.00000000	mice
Ist1	-0.15	1.00000000	mice
Dnttip2	-0.18	1.00000000	mice
Rab7	-0.16	1.00000000	mice
Vamp2	-0.18	1.00000000	mice
Cyth1	-0.22	1.00000000	mice
G3bp1	-0.09	1.00000000	mice
Rasa1	-0.25	1.00000000	mice
Igkc	-0.09	1.00000000	mice
Psme4	-0.22	1.00000000	mice
Vcan	0.43	1.00000000	mice
H3f3a	-0.17	1.00000000	mice
Btg1	-0.25	1.00000000	mice
1700025G04Rik	-0.25	1.00000000	mice
Unc45a	-0.22	1.00000000	mice
Angptl4	0.57	1.00000000	mice
Dennd1a	-0.19	1.00000000	mice
Laptm4a	0.35	1.00000000	mice
Nup107	-0.24	1.00000000	mice
Gstm5	-0.03	1.00000000	mice
Cep192	-0.17	1.00000000	mice
Cpeb2	-0.25	1.00000000	mice
Arhgef26	-0.27	1.00000000	mice
Pdlim3	-0.28	1.00000000	mice
Usp54	-0.17	1.00000000	mice
Taf1	-0.09	1.00000000	mice
Fubp1	-0.27	1.00000000	mice
Cyp2d22	-0.04	1.00000000	mice
Prkag2	-0.28	1.00000000	mice
Phf1	-0.15	1.00000000	mice
Cybb	0.16	1.00000000	mice
Eif3j1	-0.21	1.00000000	mice
Zfp704	-0.26	1.00000000	mice

Dcun1d3	-0.33	1.00000000	mice
Ifrd1	-0.14	1.00000000	mice
Agap3	-0.09	1.00000000	mice
Wdr26	-0.18	1.00000000	mice
Dact1	0.29	1.00000000	mice
Galnt16	-0.26	1.00000000	mice
Anapc16	-0.24	1.00000000	mice
Sik2	-0.29	1.00000000	mice
Nrbp1	0.36	1.00000000	mice
Slc41a2	-0.30	1.00000000	mice
Ttc7b	-0.10	1.00000000	mice
Acaa2	-0.17	1.00000000	mice
Pros1	-0.21	1.00000000	mice
Lats1	-0.23	1.00000000	mice
Snrpb2	0.30	1.00000000	mice
Wbp11	-0.19	1.00000000	mice
Dchs2	-0.43	1.00000000	mice
S100a11	0.39	1.00000000	mice
Limch1	-0.13	1.00000000	mice
Col20a1	-0.06	1.00000000	mice
Wbp2	-0.11	1.00000000	mice
Hnrnpl	-0.14	1.00000000	mice
Sbds	-0.05	1.00000000	mice
Abhd17b	-0.17	1.00000000	mice
Vat1	-0.39	1.00000000	mice
Grcc10	-0.09	1.00000000	mice
Gli1	-0.13	1.00000000	mice
Cd79a	-0.07	1.00000000	mice
Ptpn12	-0.34	1.00000000	mice
Slc2a1	-0.20	1.00000000	mice
Il6ra	-0.37	1.00000000	mice
Rnd3	-0.29	1.00000000	mice
Higd2a	-0.20	1.00000000	mice
Nudt18	-0.08	1.00000000	mice
Atat1	-0.13	1.00000000	mice
Arhgap24	-0.63	1.00000000	mice
Plcl1	-0.27	1.00000000	mice
Kirrel	-0.15	1.00000000	mice
Nod1	0.12	1.00000000	mice
Csrp2	-0.25	1.00000000	mice
Arel1	0.14	1.00000000	mice
Ltbp1	0.59	1.00000000	mice
Spsb2	0.09	1.00000000	mice
Gdf10	-0.43	1.00000000	mice
Slmap	-0.27	1.00000000	mice
Pbxip1	-0.20	1.00000000	mice

G530011O06Rik	0.06	1.00000000	mice
Rnf216	-0.18	1.00000000	mice
Itgbl1	-0.50	1.00000000	mice
Pet100	0.24	1.00000000	mice
Fnta	-0.11	1.00000000	mice
Psma4	0.48	1.00000000	mice
Atp8a1	-0.24	1.00000000	mice
Clip3	-0.11	1.00000000	mice
Enah	-0.34	1.00000000	mice
Rad51b	-0.26	1.00000000	mice
Slc16a7	-0.25	1.00000000	mice
Opa3	-0.22	1.00000000	mice
Pld1	-0.25	1.00000000	mice
Hoxa5	0.44	1.00000000	mice
Spp1	0.28	1.00000000	mice
Pde3a	-0.24	1.00000000	mice
Rap1gds1	-0.40	1.00000000	mice
Chd6	-0.12	1.00000000	mice
Ubb	-0.11	1.00000000	mice
Ptbp3	-0.18	1.00000000	mice
Sod3	0.53	1.00000000	mice
Prss23	-0.27	1.00000000	mice
Pon2	-0.07	1.00000000	mice
Pls3	-0.28	1.00000000	mice
Gle1	-0.15	1.00000000	mice
Dpy19l1	0.22	1.00000000	mice
Zfc3h1	-0.40	1.00000000	mice
Pak2	-0.20	1.00000000	mice
Fndc4	-0.08	1.00000000	mice
Chp1	-0.03	1.00000000	mice
H2-Ab1	0.30	1.00000000	mice
Dnm3	-0.19	1.00000000	mice
Fhl1	-0.06	1.00000000	mice
Tbrg1	0.46	1.00000000	mice
Dcaf10	-0.18	1.00000000	mice
Erh	-0.14	1.00000000	mice
Slc25a33	-0.16	1.00000000	mice
Agrn	0.17	1.00000000	mice
Layn	-0.22	1.00000000	mice
Uhrf1bp1l	-0.20	1.00000000	mice
Selenok	-0.08	1.00000000	mice
Spire1	-0.25	1.00000000	mice
Pfdn2	-0.01	1.00000000	mice
Nras	-0.17	1.00000000	mice
Syne2	-0.14	1.00000000	mice
S100a8	0.54	1.00000000	mice

Srsf11	-0.21	1.00000000	mice
Snrnp70	-0.15	1.00000000	mice
BC004004	0.01	1.00000000	mice
Entr1	-0.03	1.00000000	mice
Wdr47	-0.09	1.00000000	mice
Bsdc1	-0.19	1.00000000	mice
Arhgef17	0.28	1.00000000	mice
Kif1c	-0.18	1.00000000	mice
Steap4	0.51	1.00000000	mice
Rlf	-0.20	1.00000000	mice
Tex14	-0.14	1.00000000	mice
Prex2	0.02	1.00000000	mice
Gng10	-0.13	1.00000000	mice
Hnrnpf	-0.10	1.00000000	mice
Osmr	-0.29	1.00000000	mice
Pdgfd	-0.36	1.00000000	mice
Gorab	0.11	1.00000000	mice
Diaph2	-0.19	1.00000000	mice
Tmem158	-0.13	1.00000000	mice
Ash1l	-0.18	1.00000000	mice
Acox1	-0.35	1.00000000	mice
Kmt2c	-0.16	1.00000000	mice
Atp5d	-0.08	1.00000000	mice
Bbs9	-0.20	1.00000000	mice
Hnrnpm	-0.18	1.00000000	mice
Evl	-0.20	1.00000000	mice
1600020E01Rik	-0.27	1.00000000	mice
Pcsk7	-0.13	1.00000000	mice
Gphn	-0.19	1.00000000	mice
Sec24a	-0.20	1.00000000	mice
Nol10	-0.21	1.00000000	mice
Ldhb	-0.15	1.00000000	mice
Ccdc25	0.17	1.00000000	mice
Stk35	-0.17	1.00000000	mice
Dedd2	-0.10	1.00000000	mice
Rab11b	-0.03	1.00000000	mice
Kif13a	-0.20	1.00000000	mice
Plp2	-0.06	1.00000000	mice
Ubn2	-0.20	1.00000000	mice
Pfkp	-0.19	1.00000000	mice
Thbd	-0.37	1.00000000	mice
Selenom	-0.11	1.00000000	mice
Rock1	-0.26	1.00000000	mice
Ppp1cc	-0.12	1.00000000	mice
Ccdc138	-0.27	1.00000000	mice
Kmt2a	-0.22	1.00000000	mice

Tmbim6	0.41	1.00000000	mice
Gpx1	-0.11	1.00000000	mice
Rrm2b	-0.26	1.00000000	mice
Luzp1	-0.38	1.00000000	mice
Prr5l	0.29	1.00000000	mice
Lox	-0.28	1.00000000	mice
Higd1a	-0.06	1.00000000	mice
Rasl11a	-0.23	1.00000000	mice
Nr1h2	-0.08	1.00000000	mice
Cavin1	-0.12	1.00000000	mice
Brd2	-0.20	1.00000000	mice
Smarcd3	-0.16	1.00000000	mice
Gna11	-0.03	1.00000000	mice
Paip2	-0.14	1.00000000	mice
Hecw2	-0.27	1.00000000	mice
Plaur	-0.25	1.00000000	mice
Nop56	-0.14	1.00000000	mice
Eif3m	-0.09	1.00000000	mice
Fbxo11	-0.31	1.00000000	mice
Cyp27a1	-0.13	1.00000000	mice
B4galt1	-0.21	1.00000000	mice
Prmt7	-0.11	1.00000000	mice
Srsf2	-0.14	1.00000000	mice
Nos1ap	-0.17	1.00000000	mice
Plxna4	-0.45	1.00000000	mice
Sfrp1	0.59	1.00000000	mice
Eid1	-0.11	1.00000000	mice
Cdh4	-0.38	1.00000000	mice
Carhsp1	0.28	1.00000000	mice
Cirbp	-0.13	1.00000000	mice
Arl4d	-0.13	1.00000000	mice
Gipc2	-0.38	1.00000000	mice
Stk40	-0.21	1.00000000	mice
Cep112	-0.30	1.00000000	mice
Grb2	-0.22	1.00000000	mice
Ints13	-0.12	1.00000000	mice
Gpatch2	0.15	1.00000000	mice
Yipf4	0.00	1.00000000	mice
Agbl3	-0.22	1.00000000	mice
Flnc	-0.10	1.00000000	mice
Lemd3	-0.15	1.00000000	mice
Crtc3	-0.13	1.00000000	mice
Nasp	-0.11	1.00000000	mice
Snta1	-0.18	1.00000000	mice
C2cd2	-0.18	1.00000000	mice
Slc24a3	-0.36	1.00000000	mice

Nop10	-0.12	1.00000000	mice
Ogdh	-0.16	1.00000000	mice
Tspan7	-0.12	1.00000000	mice
Stag2	-0.16	1.00000000	mice
B230369F24Rik	-0.14	1.00000000	mice
Ndufb11	0.07	1.00000000	mice
Ppp1r2	-0.10	1.00000000	mice
Ppp2r2a	-0.24	1.00000000	mice
S100a6	-0.23	1.00000000	mice
Arl15	-0.37	1.00000000	mice
Slc38a2	-0.17	1.00000000	mice
Cep57	-0.06	1.00000000	mice
Klhl13	-0.18	1.00000000	mice
Smndc1	-0.10	1.00000000	mice
Fam193a	-0.20	1.00000000	mice
Lonrf3	-0.18	1.00000000	mice
Clk3	0.01	1.00000000	mice
Ssh2	-0.21	1.00000000	mice
Ube2k	-0.09	1.00000000	mice
Pik3r3	-0.39	1.00000000	mice
Casp8	0.12	1.00000000	mice
Cry2	-0.25	1.00000000	mice
Cavin2	-0.26	1.00000000	mice
Afap1	-0.29	1.00000000	mice
Bcl6	-0.20	1.00000000	mice
BC005537	-0.11	1.00000000	mice
Ccdc9	-0.09	1.00000000	mice
Dbn1	-0.18	1.00000000	mice
Ppp1r12c	-0.05	1.00000000	mice
Antxr1	-0.21	1.00000000	mice
Frmd6	-0.23	1.00000000	mice
Rasa2	-0.13	1.00000000	mice
Pkp4	-0.26	1.00000000	mice
Rc3h2	-0.21	1.00000000	mice
Cpne8	-0.55	1.00000000	mice
Nfs1	-0.09	1.00000000	mice
Bcas3	-0.20	1.00000000	mice
Zfp868	-0.13	1.00000000	mice
Axin1	-0.25	1.00000000	mice
Pgf	0.21	1.00000000	mice
Snx3	-0.05	1.00000000	mice
Thsd4	-0.67	1.00000000	mice
Tra2b	-0.14	1.00000000	mice
Plin2	0.55	1.00000000	mice
Nt5c2	-0.05	1.00000000	mice
Fli1	-0.19	1.00000000	mice

Sfswap	-0.24	1.00000000	mice
Inmt	-0.41	1.00000000	mice
Gatad2b	-0.24	1.00000000	mice
Il33	0.74	1.00000000	mice
Papss2	-0.20	1.00000000	mice
Ppdpf	-0.11	1.00000000	mice
Itgb8	0.14	1.00000000	mice
Eif4g1	-0.17	1.00000000	mice
Gapvd1	-0.19	1.00000000	mice
Snx29	-0.17	1.00000000	mice
Larp4b	-0.21	1.00000000	mice
Ercc6	-0.09	1.00000000	mice
Usp9x	-0.21	1.00000000	mice
Gclm	-0.13	1.00000000	mice
Atp5j2	0.36	1.00000000	mice
Trir	-0.08	1.00000000	mice
Dido1	-0.17	1.00000000	mice
Ube2j2	-0.10	1.00000000	mice
Twsg1	-0.07	1.00000000	mice
Hspb8	-0.19	1.00000000	mice
Brap	-0.22	1.00000000	mice
Glyr1	-0.19	1.00000000	mice
Wnt5a	0.27	1.00000000	mice
Atraid	0.02	1.00000000	mice
Spidr	-0.21	1.00000000	mice
Rnf7	-0.07	1.00000000	mice
Tada3	-0.05	1.00000000	mice
Usp47	-0.21	1.00000000	mice
Vapb	-0.12	1.00000000	mice
Lysmd3	-0.25	1.00000000	mice
Dgcr6	-0.14	1.00000000	mice
Sec24d	-0.25	1.00000000	mice
Abhd14a	-0.05	1.00000000	mice
Ube2b	-0.08	1.00000000	mice
E2f3	0.16	1.00000000	mice
Dusp3	-0.11	1.00000000	mice
Btaf1	-0.22	1.00000000	mice
Polr2c	-0.18	1.00000000	mice
Wwtr1	-0.20	1.00000000	mice
Hpse2	-0.25	1.00000000	mice
Rnf10	-0.13	1.00000000	mice
Dgka	-0.12	1.00000000	mice
Hbs1l	-0.13	1.00000000	mice
Ccng2	-0.04	1.00000000	mice
Txndc11	-0.14	1.00000000	mice
Akap8l	-0.13	1.00000000	mice

Dnah8	-0.09	1.00000000	mice
Acot9	-0.27	1.00000000	mice
Sparc	0.20	1.00000000	mice
Tspan3	-0.17	1.00000000	mice
Sec63	-0.10	1.00000000	mice
Nfkb1	-0.22	1.00000000	mice
Crebbp	-0.14	1.00000000	mice
Tor1aip1	0.45	1.00000000	mice
Lilrb4a	0.41	1.00000000	mice
Src	0.22	1.00000000	mice
Chchd7	-0.16	1.00000000	mice
Hsf1	-0.09	1.00000000	mice
Entpd2	-0.18	1.00000000	mice
Rap1a	-0.13	1.00000000	mice
5031439G07Rik	-0.13	1.00000000	mice
Iqsec2	-0.31	1.00000000	mice
Arpc1a	-0.10	1.00000000	mice
Phf3	-0.13	1.00000000	mice
Psma7	0.55	1.00000000	mice
Brd1	-0.22	1.00000000	mice
Ptbp2	-0.15	1.00000000	mice
Fxyd1	-0.08	1.00000000	mice
Dtnbp1	-0.17	1.00000000	mice
Fgf2	-0.38	1.00000000	mice
2310022B05Rik	-0.08	1.00000000	mice
Atf2	-0.20	1.00000000	mice
Ss18	-0.14	1.00000000	mice
Rasl12	-0.25	1.00000000	mice
Gapdh	-0.04	1.00000000	mice
Zfr	-0.11	1.00000000	mice
Anxa4	0.33	1.00000000	mice
Ulk2	-0.20	1.00000000	mice
Des	-0.38	1.00000000	mice
Dnajb1	-0.03	1.00000000	mice
Ubc	-0.17	1.00000000	mice
Inpp1	0.15	1.00000000	mice
Zfp36l1	-0.16	1.00000000	mice
Cxxc1	-0.24	1.00000000	mice
Naa15	-0.13	1.00000000	mice
Rrn3	-0.09	1.00000000	mice
Slc6a8	-0.11	1.00000000	mice
Itgav	-0.35	1.00000000	mice
Strap	-0.10	1.00000000	mice
Prnp	-0.26	1.00000000	mice
Epb41l2	-0.11	1.00000000	mice
Smtn	-0.28	1.00000000	mice

Vps37c	-0.14	1.00000000	mice
Samd14	-0.08	1.00000000	mice
Fbxw8	-0.17	1.00000000	mice
Eif2b1	-0.08	1.00000000	mice
Tmem223	-0.08	1.00000000	mice
Kidins220	-0.12	1.00000000	mice
Tspan2	-0.12	1.00000000	mice
Taok1	-0.19	1.00000000	mice
Tead3	-0.13	1.00000000	mice
Lrrc58	-0.04	1.00000000	mice
Ecscr	0.15	1.00000000	mice
Itga1	0.05	1.00000000	mice
Casp7	0.12	1.00000000	mice
Faim2	-0.06	1.00000000	mice
Slc6a6	-0.26	1.00000000	mice
Paf1	-0.17	1.00000000	mice
Slc7a1	-0.32	1.00000000	mice
Arf5	-0.08	1.00000000	mice
Erap1	0.19	1.00000000	mice
Ccpg1	-0.06	1.00000000	mice
Gid4	-0.16	1.00000000	mice
Asb13	0.18	1.00000000	mice
Ssbp3	-0.23	1.00000000	mice
Ddrgk1	-0.09	1.00000000	mice
Cxcl16	0.43	1.00000000	mice
D830025C05Rik	-0.09	1.00000000	mice
Tmem59	-0.06	1.00000000	mice
Cap2	-0.19	1.00000000	mice
Zdhhc8	-0.16	1.00000000	mice
Eif4g3	-0.16	1.00000000	mice
Ttc39b	0.19	1.00000000	mice
Usp36	-0.15	1.00000000	mice
Nfatc1	-0.18	1.00000000	mice
Arid1b	-0.11	1.00000000	mice
Btbd7	-0.16	1.00000000	mice
Pwwp3b	-0.29	1.00000000	mice
Nrf1	-0.11	1.00000000	mice
Ier5	-0.24	1.00000000	mice
Cdk12	-0.27	1.00000000	mice
Bbc3	-0.08	1.00000000	mice
Pik3ca	-0.12	1.00000000	mice
Tapt1	-0.11	1.00000000	mice
Eif4a1	-0.08	1.00000000	mice
Timp2	-0.27	1.00000000	mice
Bcl2	-0.20	1.00000000	mice
Acta2	-0.91	1.00000000	mice

Zwint	-0.13	1.00000000	mice
Atp2b1	-0.15	1.00000000	mice
Qrich1	-0.11	1.00000000	mice
Kdm5b	-0.13	1.00000000	mice
Rnf11	-0.19	1.00000000	mice
Rundc1	-0.06	1.00000000	mice
Eif4e	-0.16	1.00000000	mice
Cldnd1	-0.09	1.00000000	mice
Acss3	-0.38	1.00000000	mice
Ston1	-0.25	1.00000000	mice
Stox2	-0.27	1.00000000	mice
Ctdp1	-0.09	1.00000000	mice
Pygl	-0.07	1.00000000	mice
Ptov1	-0.17	1.00000000	mice
Gstt1	-0.19	1.00000000	mice
Tob2	-0.20	1.00000000	mice
Rab10	-0.26	1.00000000	mice
Slc35b1	0.18	1.00000000	mice
Rasgef1c	-0.22	1.00000000	mice
Zeb1	-0.40	1.00000000	mice
Klf7	-0.13	1.00000000	mice
Ntan1	-0.08	1.00000000	mice
Cherp	-0.15	1.00000000	mice
Rgs7bp	-0.26	1.00000000	mice
Arhgef10	-0.25	1.00000000	mice
Dlgap4	-0.12	1.00000000	mice
Kptn	-0.04	1.00000000	mice
Erbin	-0.20	1.00000000	mice
Trim8	0.34	1.00000000	mice
P4ha3	-0.34	1.00000000	mice
Acot13	-0.19	1.00000000	mice
Rsl1d1	-0.02	1.00000000	mice
Ddx17	-0.15	1.00000000	mice
Kansl1	-0.28	1.00000000	mice
Pan3	-0.18	1.00000000	mice
Nbea	-0.20	1.00000000	mice
Pde4c	-0.09	1.00000000	mice
BC005561	-0.16	1.00000000	mice
Nbdy	-0.04	1.00000000	mice
Gadd45b	-0.21	1.00000000	mice
Dock6	-0.14	1.00000000	mice
Nme2	0.39	1.00000000	mice
Pkd2	-0.16	1.00000000	mice
Nt5c3	0.17	1.00000000	mice
Inpp4b	0.60	1.00000000	mice
Cox4i1	-0.02	1.00000000	mice

Nampt	0.42	1.00000000	mice
Usp33	-0.18	1.00000000	mice
Gnpnat1	0.10	1.00000000	mice
Csde1	-0.07	1.00000000	mice
Brk1	-0.09	1.00000000	mice
Zfpm2	-0.16	1.00000000	mice
Kdm3b	-0.26	1.00000000	mice
Taf15	-0.01	1.00000000	mice
Ampd1	-0.10	1.00000000	mice
Ramp1	-0.17	1.00000000	mice
Dram1	0.28	1.00000000	mice
Phf20	-0.23	1.00000000	mice
Chd2	-0.14	1.00000000	mice
Lst1	0.41	1.00000000	mice
Calm2	-0.17	1.00000000	mice
Ube2n	-0.09	1.00000000	mice
Rab13	-0.22	1.00000000	mice
Trabd2b	-0.17	1.00000000	mice
Nptn	-0.19	1.00000000	mice
Rbm27	-0.28	1.00000000	mice
Zfp800	-0.19	1.00000000	mice
Sars	0.26	1.00000000	mice
Thbs2	-0.26	1.00000000	mice
Zdhhc2	-0.16	1.00000000	mice
Slc49a4	-0.23	1.00000000	mice
Itk	-0.23	1.00000000	mice
Kank4	-0.09	1.00000000	mice
Cab39	-0.24	1.00000000	mice
Pycard	0.35	1.00000000	mice
Tmem64	-0.18	1.00000000	mice
Ttl	-0.09	1.00000000	mice
Col4a1	0.40	1.00000000	mice
Rb1	-0.09	1.00000000	mice
Cdc42bpa	-0.09	1.00000000	mice
Ipo7	-0.23	1.00000000	mice
Nkiras1	-0.13	1.00000000	mice
Canx	0.31	1.00000000	mice
Nsd3	-0.13	1.00000000	mice
Ctnnb1	-0.18	1.00000000	mice
P3h1	0.20	1.00000000	mice
Ttll5	-0.11	1.00000000	mice
Nosip	0.16	1.00000000	mice
H2afx	-0.07	1.00000000	mice
Znrf1	-0.21	1.00000000	mice
Pde7b	-0.64	1.00000000	mice
4931406P16Rik	-0.08	1.00000000	mice

Col15a1	-0.26	1.00000000	mice
Use1	0.03	1.00000000	mice
Smchd1	-0.07	1.00000000	mice
Ebf1	-0.13	1.00000000	mice
Rcn1	0.43	1.00000000	mice
Igfbp3	-0.13	1.00000000	mice
Ell	-0.12	1.00000000	mice
Vapa	-0.13	1.00000000	mice
Lnx2	-0.18	1.00000000	mice
Atad2b	-0.12	1.00000000	mice
Klhl29	-0.39	1.00000000	mice
Atp5j	-0.14	1.00000000	mice
Atp6ap1	-0.16	1.00000000	mice
Bcl10	-0.07	1.00000000	mice
Synpo2	-0.51	1.00000000	mice
Utp4	-0.12	1.00000000	mice
Utp14b	-0.09	1.00000000	mice
Nedd9	-0.18	1.00000000	mice
Sema4a	-0.31	1.00000000	mice
F3	-0.16	1.00000000	mice
Cx3cr1	-0.27	1.00000000	mice
Unc50	-0.12	1.00000000	mice
Eif1b	-0.06	1.00000000	mice
Dazap2	-0.19	1.00000000	mice
Zfp655	-0.15	1.00000000	mice
Agpat3	-0.11	1.00000000	mice
Steap3	0.23	1.00000000	mice
Pdlim2	-0.07	1.00000000	mice
Patl2	0.13	1.00000000	mice
Agtrap	0.12	1.00000000	mice
Pdcl3	0.00	1.00000000	mice
Eid3	-0.12	1.00000000	mice
Tmem159	-0.12	1.00000000	mice
Insr	-0.14	1.00000000	mice
Erf	-0.20	1.00000000	mice
Srcap	-0.12	1.00000000	mice
Aoc3	-0.16	1.00000000	mice
Tmem88	-0.09	1.00000000	mice
Npm3	-0.04	1.00000000	mice
Smg9	-0.04	1.00000000	mice
Raf1	-0.10	1.00000000	mice
Trpm7	-0.11	1.00000000	mice
Ndufb9	-0.02	1.00000000	mice
Nfkbie	0.20	1.00000000	mice
Dock7	-0.23	1.00000000	mice
Clint1	-0.19	1.00000000	mice

Zbtb44	-0.12	1.00000000	mice
Adprh	-0.15	1.00000000	mice
Rpe	0.13	1.00000000	mice
Arhgef7	-0.13	1.00000000	mice
Inpp5a	-0.17	1.00000000	mice
D10Wsu102e	-0.18	1.00000000	mice
Slc25a47	-0.06	1.00000000	mice
Prrg3	-0.02	1.00000000	mice
Slc35b4	0.14	1.00000000	mice
Pmvk	-0.07	1.00000000	mice
Boc	-0.24	1.00000000	mice
Psd3	-0.25	1.00000000	mice
Adamtsl5	-0.12	1.00000000	mice
Rsrc2	-0.12	1.00000000	mice
Btbd10	-0.12	1.00000000	mice
Arl8b	-0.05	1.00000000	mice
Psmb3	0.53	1.00000000	mice
Ncoa2	-0.21	1.00000000	mice
Wdcp	-0.06	1.00000000	mice
Tnik	-0.13	1.00000000	mice
Snrpf	0.48	1.00000000	mice
Hcfc1r1	-0.19	1.00000000	mice
Dock1	-0.22	1.00000000	mice
Pdhb	-0.07	1.00000000	mice
Pias1	-0.27	1.00000000	mice
Calml4	-0.06	1.00000000	mice
Adrb2	-0.19	1.00000000	mice
Lars2	0.41	1.00000000	mice
Prdm11	-0.09	1.00000000	mice
Htatsf1	-0.04	1.00000000	mice
Anp32e	-0.05	1.00000000	mice
Chst12	-0.06	1.00000000	mice
Aldoa	0.02	1.00000000	mice
Anxa3	-0.32	1.00000000	mice
Eps8	0.05	1.00000000	mice
Far1	-0.13	1.00000000	mice
Thoc2	-0.12	1.00000000	mice
Isy1	-0.11	1.00000000	mice
Simc1	-0.31	1.00000000	mice
Bloc1s6	0.13	1.00000000	mice
Trim26	0.16	1.00000000	mice
Syde2	-0.15	1.00000000	mice
Pdgfa	-0.08	1.00000000	mice
Fam76a	-0.25	1.00000000	mice
Polg2	-0.15	1.00000000	mice
Rasl11b	-0.25	1.00000000	mice

Slc11a1	-0.02	1.00000000	mice
Bmyc	-0.13	1.00000000	mice
A930015D03Rik	0.17	1.00000000	mice
Rab20	-0.07	1.00000000	mice
Rnf38	-0.21	1.00000000	mice
Btbd2	-0.13	1.00000000	mice
Bzw2	-0.15	1.00000000	mice
Tgif1	0.34	1.00000000	mice
Dad1	0.02	1.00000000	mice
Prxl2b	-0.02	1.00000000	mice
Crebl2	-0.13	1.00000000	mice
Rapgef5	-0.36	1.00000000	mice
Ndrg1	-0.24	1.00000000	mice
Zfp207	-0.16	1.00000000	mice
Spen	-0.16	1.00000000	mice
Itm2b	0.25	1.00000000	mice
Zfp335os	-0.12	1.00000000	mice
Trappc6b	-0.08	1.00000000	mice
Piezo1	-0.14	1.00000000	mice
Foxf2	0.22	1.00000000	mice
Etfb	0.04	1.00000000	mice
Bcl7c	-0.14	1.00000000	mice
B3gnt2	-0.06	1.00000000	mice
Fry	-0.22	1.00000000	mice
Oat	0.00	1.00000000	mice
Zc3hav1	0.39	1.00000000	mice
Alpl	0.26	1.00000000	mice
Pik3c2a	-0.26	1.00000000	mice
Comt	-0.09	1.00000000	mice
Sar1a	-0.10	1.00000000	mice
Fgf7	0.29	1.00000000	mice
Appl1	-0.15	1.00000000	mice
Fbxo30	-0.19	1.00000000	mice
Baz2b	0.10	1.00000000	mice
Lrrc17	-0.10	1.00000000	mice
Rab2a	-0.08	1.00000000	mice
Zdhhc1	-0.16	1.00000000	mice
1810013L24Rik	-0.14	1.00000000	mice
Senp6	-0.20	1.00000000	mice
Thrb	-0.27	1.00000000	mice
Abtb2	-0.14	1.00000000	mice
B3glct	-0.11	1.00000000	mice
Fyn	-0.15	1.00000000	mice
Sox4	0.31	1.00000000	mice
Srgn	0.35	1.00000000	mice
Plxnb2	0.19	1.00000000	mice

Cdkn1c	-0.08	1.00000000	mice
Tmem184c	-0.16	1.00000000	mice
Hspa9	-0.11	1.00000000	mice
Paip2b	-0.17	1.00000000	mice
Homer1	0.20	1.00000000	mice
Ddx50	-0.09	1.00000000	mice
Slc4a7	-0.15	1.00000000	mice
Tppp3	-0.15	1.00000000	mice
Srgap2	-0.30	1.00000000	mice
Acox3	-0.04	1.00000000	mice
Vldlr	-0.09	1.00000000	mice
Psma2	0.46	1.00000000	mice
Pvt1	-0.34	1.00000000	mice
Arhgap23	-0.12	1.00000000	mice
Thada	-0.18	1.00000000	mice
Pecam1	-0.22	1.00000000	mice
Tax1bp1	-0.07	1.00000000	mice
Uimc1	-0.17	1.00000000	mice
Notch2	-0.10	1.00000000	mice
Pald1	0.16	1.00000000	mice
Arid4b	-0.03	1.00000000	mice
Grk4	-0.14	1.00000000	mice
Adnp	-0.17	1.00000000	mice
Nol9	-0.09	1.00000000	mice
Cdk13	-0.17	1.00000000	mice
Tfb2m	-0.11	1.00000000	mice
Tead2	-0.02	1.00000000	mice
Qk	-0.16	1.00000000	mice
Adgra2	0.11	1.00000000	mice
Ttc3	-0.11	1.00000000	mice
Smg1	-0.13	1.00000000	mice
Pmp22	-0.08	1.00000000	mice
Jpt1	-0.06	1.00000000	mice
Peak1	-0.16	1.00000000	mice
Cables1	-0.12	1.00000000	mice
Nrip1	-0.17	1.00000000	mice
Emb	-0.32	1.00000000	mice
Tfdp2	-0.12	1.00000000	mice
Akap6	-0.34	1.00000000	mice
Btg3	-0.09	1.00000000	mice
Tshz3	-0.09	1.00000000	mice
Ranbp2	-0.07	1.00000000	mice
Hnrnph3	-0.14	1.00000000	mice
Uqcc2	0.30	1.00000000	mice
Gcsh	-0.19	1.00000000	mice
Jmy	-0.09	1.00000000	mice

Atp5a1	0.04	1.00000000	mice
Pgm3	-0.02	1.00000000	mice
Stk11	-0.06	1.00000000	mice
Sspn	-0.11	1.00000000	mice
Dnm1	-0.08	1.00000000	mice
Spr	-0.13	1.00000000	mice
Rnf167	-0.12	1.00000000	mice
Tcf7l1	-0.17	1.00000000	mice
Gstm2	-0.08	1.00000000	mice
Ndufa3	0.38	1.00000000	mice
Itga5	-0.16	1.00000000	mice
Pcmtd2	-0.10	1.00000000	mice
Exoc5	-0.24	1.00000000	mice
N4bp2	-0.13	1.00000000	mice
Rreb1	0.20	1.00000000	mice
Ptges	0.35	1.00000000	mice
Wdr45	-0.06	1.00000000	mice
Sptbn1	-0.12	1.00000000	mice
Stard10	-0.09	1.00000000	mice
Unc93b1	0.19	1.00000000	mice
Tmem251	-0.14	1.00000000	mice
Ier2	-0.16	1.00000000	mice
Sphk1	-0.09	1.00000000	mice
Zer1	-0.06	1.00000000	mice
Fbln1	-0.49	1.00000000	mice
Actr1a	-0.25	1.00000000	mice
Surf4	0.29	1.00000000	mice
Fads3	-0.06	1.00000000	mice
Eny2	-0.06	1.00000000	mice
Kif13b	-0.10	1.00000000	mice
Pds5b	-0.15	1.00000000	mice
Zfp385a	-0.14	1.00000000	mice
Srebf2	-0.11	1.00000000	mice
Golim4	-0.21	1.00000000	mice
Rrp1	-0.07	1.00000000	mice
Ndst1	-0.11	1.00000000	mice
Dand5	-0.19	1.00000000	mice
Yap1	-0.22	1.00000000	mice
Ric1	-0.11	1.00000000	mice
Kxd1	-0.06	1.00000000	mice
Trpc1	-0.01	1.00000000	mice
Rcn2	-0.11	1.00000000	mice
Kcnk3	-0.15	1.00000000	mice
S100a10	-0.20	1.00000000	mice
Hist1h1c	-0.15	1.00000000	mice
Slc43a3	-0.21	1.00000000	mice

Sh3pxd2a	-0.21	1.00000000	mice
Bmp8a	-0.09	1.00000000	mice
Stam	-0.14	1.00000000	mice
Gulp1	-0.17	1.00000000	mice
Entpd1	-0.19	1.00000000	mice
Adipor1	-0.01	1.00000000	mice
Diaph1	-0.17	1.00000000	mice
C1rb	0.30	1.00000000	mice
Ube2f	-0.15	1.00000000	mice
Naaa	-0.05	1.00000000	mice
Ubald1	-0.07	1.00000000	mice
Capza1	-0.15	1.00000000	mice
Tmed5	-0.09	1.00000000	mice
Phf20l1	-0.20	1.00000000	mice
Smad4	-0.01	1.00000000	mice
Akap9	-0.23	1.00000000	mice
Dctn2	-0.14	1.00000000	mice
Reep3	-0.17	1.00000000	mice
Gypc	0.20	1.00000000	mice
Birc3	-0.23	1.00000000	mice
Gabpb1	-0.12	1.00000000	mice
Bclaf3	-0.05	1.00000000	mice
Ankhd1	-0.12	1.00000000	mice
Ctnnbl1	-0.01	1.00000000	mice
Ambra1	-0.14	1.00000000	mice
Ints8	-0.14	1.00000000	mice
Arf6	-0.15	1.00000000	mice
Bloc1s4	-0.11	1.00000000	mice
Tex30	-0.06	1.00000000	mice
Prpf8	-0.14	1.00000000	mice
Rgs10	-0.16	1.00000000	mice
Flt3l	-0.07	1.00000000	mice
Notch3	-0.18	1.00000000	mice
Ndufa11	0.30	1.00000000	mice
Ybx1	-0.03	1.00000000	mice
Ptprj	-0.14	1.00000000	mice
Timm17a	-0.03	1.00000000	mice
Tusc2	-0.13	1.00000000	mice
Dip2b	-0.18	1.00000000	mice
Pcp4l1	-0.13	1.00000000	mice
Ints2	-0.18	1.00000000	mice
Leng8	-0.04	1.00000000	mice
Negr1	-0.19	1.00000000	mice
Btf3l4	-0.05	1.00000000	mice
Lpin1	-0.17	1.00000000	mice
Trp53	0.20	1.00000000	mice

Anapc11	-0.09	1.00000000	mice
Ndel1	-0.04	1.00000000	mice
Stau2	-0.23	1.00000000	mice
Itgb1	-0.11	1.00000000	mice
Prkce	-0.17	1.00000000	mice
Bex3	-0.12	1.00000000	mice
Rnasel	0.13	1.00000000	mice
Irf8	0.35	1.00000000	mice
C1galt1	-0.11	1.00000000	mice
Tmem47	-0.15	1.00000000	mice
Wac	-0.18	1.00000000	mice
Nup50	-0.14	1.00000000	mice
Nbl1	0.17	1.00000000	mice
Cbx4	-0.19	1.00000000	mice
Fam117b	-0.16	1.00000000	mice
Cdipt	0.25	1.00000000	mice
Tspan13	-0.28	1.00000000	mice
Jak2	0.35	1.00000000	mice
Ptpn2	-0.26	1.00000000	mice
Kdm4b	-0.08	1.00000000	mice
Uqcrfs1	0.00	1.00000000	mice
Serf1	0.02	1.00000000	mice
Foxn2	-0.18	1.00000000	mice
Cacna2d1	-0.08	1.00000000	mice
Slc41a3	0.05	1.00000000	mice
Gem	-0.06	1.00000000	mice
Ssc5d	0.05	1.00000000	mice
Rpp30	-0.09	1.00000000	mice
Zfp644	-0.21	1.00000000	mice
Copb1	-0.17	1.00000000	mice
Qdpr	-0.06	1.00000000	mice
Chmp5	0.40	1.00000000	mice
Bcl2l11	-0.07	1.00000000	mice
Bach1	-0.25	1.00000000	mice
Sfxn2	0.07	1.00000000	mice
Fbrs	-0.10	1.00000000	mice
Acat2	-0.08	1.00000000	mice
Dtnb	-0.09	1.00000000	mice
Gns	0.23	1.00000000	mice
Tsix	0.21	1.00000000	mice
Snx24	-0.22	1.00000000	mice
Ubl3	-0.12	1.00000000	mice
Cytip	-0.11	1.00000000	mice
Klhl8	-0.10	1.00000000	mice
Zdhhc14	-0.26	1.00000000	mice
Ivns1abp	-0.19	1.00000000	mice

Il1rl2	-0.15	1.00000000	mice
Rtl8b	-0.08	1.00000000	mice
Fam168b	0.03	1.00000000	mice
Rasgrp2	-0.05	1.00000000	mice
Tanc2	-0.16	1.00000000	mice
Dgat1	-0.12	1.00000000	mice
Srp68	-0.08	1.00000000	mice
Cenpb	0.02	1.00000000	mice
Cd63	0.38	1.00000000	mice
Iqcn	-0.03	1.00000000	mice
Plekhg3	-0.17	1.00000000	mice
Ntf3	-0.11	1.00000000	mice
Gnb4	0.29	1.00000000	mice
Hyou1	0.20	1.00000000	mice
Thap2	-0.09	1.00000000	mice
Dusp16	-0.14	1.00000000	mice
Ctsb	0.34	1.00000000	mice
Zfyve9	-0.19	1.00000000	mice
Tpst1	-0.07	1.00000000	mice
Cdk14	-0.16	1.00000000	mice
Scaf8	-0.05	1.00000000	mice
Spint2	-0.13	1.00000000	mice
Csrnp2	-0.06	1.00000000	mice
Hexb	-0.11	1.00000000	mice
Ccm2	-0.06	1.00000000	mice
Gpc3	-0.29	1.00000000	mice
Hnrnpr	-0.02	1.00000000	mice
Tmem50a	-0.01	1.00000000	mice
Rnf130	0.06	1.00000000	mice
Ntrk2	-0.38	1.00000000	mice
Cnot10	-0.14	1.00000000	mice
Prdm16	-0.18	1.00000000	mice
Cox6c	0.30	1.00000000	mice
Ppp2r1a	0.01	1.00000000	mice
Kctd12	-0.15	1.00000000	mice
Kcnab1	-0.19	1.00000000	mice
Epc1	-0.09	1.00000000	mice
Nsmce4a	-0.14	1.00000000	mice
Pnpla8	-0.09	1.00000000	mice
Ahdc1	-0.17	1.00000000	mice
Whamm	0.08	1.00000000	mice
Pum2	-0.09	1.00000000	mice
Hmgxb3	-0.18	1.00000000	mice
Dlg2	-0.16	1.00000000	mice
Cpeb1	-0.08	1.00000000	mice
Igip	-0.05	1.00000000	mice

S1pr3	0.26	1.00000000	mice
Copz2	-0.12	1.00000000	mice
Pcdh17	-0.17	1.00000000	mice
Bclaf1	-0.07	1.00000000	mice
Nfe2l1	0.33	1.00000000	mice
Zc2hc1a	0.29	1.00000000	mice
Slc25a17	-0.08	1.00000000	mice
Gjc1	-0.13	1.00000000	mice
Cers5	-0.17	1.00000000	mice
Ubp1	-0.09	1.00000000	mice
Col4a3bp	-0.09	1.00000000	mice
Pebp1	-0.06	1.00000000	mice
Iscu	-0.04	1.00000000	mice
Iqcb1	-0.17	1.00000000	mice
Arid2	-0.12	1.00000000	mice
L3hypdh	-0.14	1.00000000	mice
Atg101	-0.20	1.00000000	mice
Ercc5	-0.05	1.00000000	mice
Hagh	-0.07	1.00000000	mice
Jag1	-0.03	1.00000000	mice
Tead1	-0.19	1.00000000	mice
Ckap4	0.41	1.00000000	mice
Ikbkg	0.01	1.00000000	mice
Atp5c1	-0.07	1.00000000	mice
Pdcd4	-0.11	1.00000000	mice
Clic4	0.22	1.00000000	mice
Zfhx3	-0.06	1.00000000	mice
Calm3	0.05	1.00000000	mice
Usp49	-0.19	1.00000000	mice
Rdh13	-0.03	1.00000000	mice
Gfra2	0.37	1.00000000	mice
Tubb5	0.43	1.00000000	mice
Nfkb2	-0.14	1.00000000	mice
Pmepa1	-0.17	1.00000000	mice
Slk	-0.01	1.00000000	mice
Ngdn	-0.09	1.00000000	mice
Casp4	0.29	1.00000000	mice
Tjp1	-0.20	1.00000000	mice
Fbxo45	-0.06	1.00000000	mice
Efnb2	-0.03	1.00000000	mice
Ncl	-0.03	1.00000000	mice
Arl8a	-0.13	1.00000000	mice
Prdm2	-0.07	1.00000000	mice
St7	-0.16	1.00000000	mice
Ndufa13	0.36	1.00000000	mice
Inhba	0.20	1.00000000	mice

Pbrm1	-0.11	1.00000000	mice
Igfbp5	-0.10	1.00000000	mice
Hdlbp	-0.12	1.00000000	mice
Trim59	-0.09	1.00000000	mice
Bak1	0.15	1.00000000	mice
Agfg2	-0.04	1.00000000	mice
Zfp280c	0.07	1.00000000	mice
Cdc42ep3	-0.17	1.00000000	mice
Upp2	-0.14	1.00000000	mice
Scmh1	-0.22	1.00000000	mice
Alas1	-0.23	1.00000000	mice
D8Ertd738e	-0.03	1.00000000	mice
Rab12	-0.18	1.00000000	mice
Crnkl1	0.12	1.00000000	mice
Slc48a1	-0.04	1.00000000	mice
Rabep1	-0.10	1.00000000	mice
Dnajb12	-0.02	1.00000000	mice
Tns2	-0.07	1.00000000	mice
Ccn1	-0.22	1.00000000	mice
Kcnb2	-0.22	1.00000000	mice
0610010F05Rik	-0.02	1.00000000	mice
Abca8a	-0.18	1.00000000	mice
Timm9	-0.08	1.00000000	mice
Ids	-0.14	1.00000000	mice
Rnf5	-0.05	1.00000000	mice
Tyrobp	0.15	1.00000000	mice
Tpi1	-0.05	1.00000000	mice
Stard8	0.02	1.00000000	mice
Tep1	0.05	1.00000000	mice
Nedd4l	-0.36	1.00000000	mice
Lmcd1	-0.40	1.00000000	mice
Setd7	-0.06	1.00000000	mice
Rpain	-0.13	1.00000000	mice
Gnl3	-0.22	1.00000000	mice
Tmem100	-0.32	1.00000000	mice
Rtn2	-0.10	1.00000000	mice
Pitpnb	-0.11	1.00000000	mice
Adam9	-0.19	1.00000000	mice
Tm2d1	-0.04	1.00000000	mice
Ccdc32	0.13	1.00000000	mice
Akt2	-0.14	1.00000000	mice
Spop	0.08	1.00000000	mice
Ikzf1	-0.02	1.00000000	mice
Abcd2	0.22	1.00000000	mice
Ilf2	-0.06	1.00000000	mice
Zfp330	-0.15	1.00000000	mice

Eif5	0.01	1.00000000	mice
Plxna2	-0.21	1.00000000	mice
Satb1	-0.03	1.00000000	mice
Has1	-0.42	1.00000000	mice
Bicral	-0.15	1.00000000	mice
Rbm6	-0.04	1.00000000	mice
Limd1	-0.05	1.00000000	mice
Btd	-0.05	1.00000000	mice
Rybp	-0.16	1.00000000	mice
1700109H08Rik	-0.15	1.00000000	mice
Coq3	-0.14	1.00000000	mice
Creld1	-0.10	1.00000000	mice
Itgb5	-0.09	1.00000000	mice
Daam1	-0.05	1.00000000	mice
Crk	-0.17	1.00000000	mice
Tagln2	0.30	1.00000000	mice
Zmym5	-0.20	1.00000000	mice
Scn7a	-0.28	1.00000000	mice
Brix1	-0.13	1.00000000	mice
Lats2	-0.10	1.00000000	mice
Tnpo2	-0.11	1.00000000	mice
Csnk1d	-0.13	1.00000000	mice
Ptp4a2	0.02	1.00000000	mice
Rapgef2	-0.10	1.00000000	mice
Fam172a	-0.12	1.00000000	mice
Parn	-0.06	1.00000000	mice
Ywhab	0.34	1.00000000	mice
Casp12	0.24	1.00000000	mice
Zfp945	0.15	1.00000000	mice
Gpd2	-0.13	1.00000000	mice
Atp6ap2	0.00	1.00000000	mice
Raly	0.01	1.00000000	mice
Acsl3	-0.18	1.00000000	mice
Tnip2	-0.15	1.00000000	mice
Fkbp4	-0.13	1.00000000	mice
Brwd3	-0.10	1.00000000	mice
Clpx	-0.16	1.00000000	mice
Tmed4	-0.04	1.00000000	mice
Cnot1	-0.17	1.00000000	mice
Eif3k	-0.02	1.00000000	mice
Fdx1	-0.22	1.00000000	mice
Ehd2	0.03	1.00000000	mice
Nes	-0.10	1.00000000	mice
Csf2rb	0.14	1.00000000	mice
Lrp6	-0.14	1.00000000	mice
Ccnt1	-0.11	1.00000000	mice

Slpi	0.03	1.00000000	mice
Pxk	0.21	1.00000000	mice
Fam53c	0.01	1.00000000	mice
Ghr	-0.10	1.00000000	mice
Trim56	0.13	1.00000000	mice
Cox8a	-0.05	1.00000000	mice
Sash1	-0.18	1.00000000	mice
Pmm1	-0.01	1.00000000	mice
Agmo	-0.17	1.00000000	mice
Prpf4b	-0.12	1.00000000	mice
Yars	-0.19	1.00000000	mice
Jam3	-0.07	1.00000000	mice
Itga4	-0.11	1.00000000	mice
Dnajb11	0.36	1.00000000	mice
Cdc73	-0.16	1.00000000	mice
Hsd11b1	0.39	1.00000000	mice
Smarcc2	0.01	1.00000000	mice
Zfp367	-0.16	1.00000000	mice
Slbp	-0.22	1.00000000	mice
Ddx39b	-0.09	1.00000000	mice
Trip11	-0.13	1.00000000	mice
Ndufv3	0.00	1.00000000	mice
Zfand6	0.00	1.00000000	mice
Fhit	-0.21	1.00000000	mice
Tgfb1i1	-0.18	1.00000000	mice
Prdx4	-0.02	1.00000000	mice
Safb	-0.09	1.00000000	mice
Itsn2	-0.12	1.00000000	mice
Gamt	0.01	1.00000000	mice
Aktip	-0.08	1.00000000	mice
Ssu72	0.30	1.00000000	mice
Rgs3	0.16	1.00000000	mice
Tapbpl	0.18	1.00000000	mice
Il17d	0.06	1.00000000	mice
Polr2m	-0.15	1.00000000	mice
Gabarapl1	-0.12	1.00000000	mice
Gng5	0.01	1.00000000	mice
Lxn	0.17	1.00000000	mice
Jtb	-0.06	1.00000000	mice
Insig2	-0.02	1.00000000	mice
Psmd2	0.39	1.00000000	mice
Usp16	-0.12	1.00000000	mice
Eif5b	0.30	1.00000000	mice
Cerk	0.01	1.00000000	mice
Sh3rf1	-0.11	1.00000000	mice
Dnmt3a	-0.09	1.00000000	mice

Hat1	-0.10	1.00000000	mice
2900026A02Rik	-0.15	1.00000000	mice
Gstp1	-0.17	1.00000000	mice
Chchd3	0.01	1.00000000	mice
Elavl3	-0.14	1.00000000	mice
Cnpy2	0.10	1.00000000	mice
Scd2	-0.15	1.00000000	mice
Larp4	-0.14	1.00000000	mice
Igbp1	-0.05	1.00000000	mice
Gars	0.20	1.00000000	mice
Scaf1	-0.06	1.00000000	mice
Sub1	0.05	1.00000000	mice
Clpb	-0.13	1.00000000	mice
1700017B05Rik	-0.12	1.00000000	mice
Prr14	-0.06	1.00000000	mice
Bcl7a	-0.05	1.00000000	mice
Timm21	-0.02	1.00000000	mice
Fkbp2	0.48	1.00000000	mice
St7l	-0.03	1.00000000	mice
Kdm5a	-0.11	1.00000000	mice
Rnf145	-0.19	1.00000000	mice
Etv6	-0.18	1.00000000	mice
Txndc5	0.35	1.00000000	mice
C330007P06Rik	-0.08	1.00000000	mice
Ing3	-0.11	1.00000000	mice
Rc3h1	-0.16	1.00000000	mice
Tmem87a	-0.18	1.00000000	mice
Atp6v1a	0.23	1.00000000	mice
Klhl33	-0.09	1.00000000	mice
Gtf2a2	0.00	1.00000000	mice
Anp32a	0.08	1.00000000	mice
Bmper	0.33	1.00000000	mice
Ppp1r9a	-0.15	1.00000000	mice
Tenm3	0.30	1.00000000	mice
Immp1l	-0.06	1.00000000	mice
Coq8b	-0.17	1.00000000	mice
Nab2	0.07	1.00000000	mice
Vgll4	-0.15	1.00000000	mice
Cd3e	-0.15	1.00000000	mice
Slc1a3	0.36	1.00000000	mice
Foxp4	-0.14	1.00000000	mice
Nop2	-0.17	1.00000000	mice
Nhs	-0.18	1.00000000	mice
Pard3	-0.08	1.00000000	mice
Aspa	-0.14	1.00000000	mice
Kcmf1	-0.08	1.00000000	mice

6030458C11Rik	-0.05	1.00000000	mice
Bri3	0.03	1.00000000	mice
Rad23a	-0.03	1.00000000	mice
Arhgap42	0.12	1.00000000	mice
Wnk1	-0.03	1.00000000	mice
Gpbp1	-0.02	1.00000000	mice
Trrap	-0.12	1.00000000	mice
Klhl12	-0.09	1.00000000	mice
Dlg1	-0.15	1.00000000	mice
Lamc3	-0.10	1.00000000	mice
Ttpal	-0.09	1.00000000	mice
Rcan1	-0.36	1.00000000	mice
Art3	0.10	1.00000000	mice
Ehd4	-0.30	1.00000000	mice
Snrpb	0.06	1.00000000	mice
Trim69	0.22	1.00000000	mice
Zbtb2	-0.18	1.00000000	mice
Taf1b	0.12	1.00000000	mice
Uchl5	-0.09	1.00000000	mice
Tcof1	0.18	1.00000000	mice
Sord	-0.15	1.00000000	mice
Eif2s2	0.48	1.00000000	mice
P2rx4	0.25	1.00000000	mice
Slc25a4	-0.12	1.00000000	mice
A4galt	-0.06	1.00000000	mice
Ptar1	-0.01	1.00000000	mice
Ptgis	-0.11	1.00000000	mice
Txlna	-0.19	1.00000000	mice
Ucp2	0.02	1.00000000	mice
Wasl	-0.12	1.00000000	mice
Eif4a2	-0.06	1.00000000	mice
Kazald1	-0.07	1.00000000	mice
Lsamp	-0.20	1.00000000	mice
Plekhj1	0.01	1.00000000	mice
Gpbp1l1	-0.17	1.00000000	mice
Hspbp1	-0.05	1.00000000	mice
Cd74	0.22	1.00000000	mice
Dolk	-0.03	1.00000000	mice
Agpat5	-0.08	1.00000000	mice
Pld3	-0.07	1.00000000	mice
Card19	-0.09	1.00000000	mice
Pbx1	-0.02	1.00000000	mice
Sirt1	-0.09	1.00000000	mice
Abi1	-0.08	1.00000000	mice
Strn3	-0.09	1.00000000	mice
Rbm25	-0.10	1.00000000	mice

Cast	-0.15	1.00000000	mice
Zfyve16	-0.14	1.00000000	mice
Arhgap20	-0.17	1.00000000	mice
Hoxb2	0.22	1.00000000	mice
Ubl7	-0.06	1.00000000	mice
Ywhaz	-0.08	1.00000000	mice
Fh1	-0.10	1.00000000	mice
Fat4	-0.14	1.00000000	mice
Snx5	-0.06	1.00000000	mice
Abhd2	-0.41	1.00000000	mice
Rab10os	-0.01	1.00000000	mice
Arhgap28	-0.21	1.00000000	mice
Pawr	-0.06	1.00000000	mice
Cystm1	-0.02	1.00000000	mice
Pitrm1	-0.16	1.00000000	mice
Ppm1k	0.07	1.00000000	mice
Tmem165	-0.06	1.00000000	mice
4933406I18Rik	-0.21	1.00000000	mice
Ankrd10	-0.09	1.00000000	mice
Pttg1ip	-0.06	1.00000000	mice
Tiam2	-0.15	1.00000000	mice
Efs	0.11	1.00000000	mice
Npas2	-0.16	1.00000000	mice
Sdccag8	-0.15	1.00000000	mice
Ssbp4	0.18	1.00000000	mice
Serpinf1	-0.67	1.00000000	mice
Supt4a	0.06	1.00000000	mice
Pnisr	-0.14	1.00000000	mice
Acbd5	-0.15	1.00000000	mice
Ptpn11	-0.13	1.00000000	mice
Iws1	-0.15	1.00000000	mice
Rgs2	-0.12	1.00000000	mice
Stk24	-0.08	1.00000000	mice
Ptcd3	0.10	1.00000000	mice
Cisd2	-0.10	1.00000000	mice
Cul9	-0.13	1.00000000	mice
Kcna2	-0.04	1.00000000	mice
Adhfe1	-0.04	1.00000000	mice
Zfp664	-0.13	1.00000000	mice
Pgls	-0.13	1.00000000	mice
Sdad1	-0.19	1.00000000	mice
Ankrd12	-0.09	1.00000000	mice
Irf2bpl	0.24	1.00000000	mice
Rarres2	0.44	1.00000000	mice
Rpn2	0.30	1.00000000	mice
Ralbp1	-0.04	1.00000000	mice

BC031181	-0.02	1.00000000	mice
Trappc2l	-0.10	1.00000000	mice
Scai	-0.05	1.00000000	mice
Ccn3	-0.33	1.00000000	mice
P3h4	-0.16	1.00000000	mice
Rtl8a	-0.04	1.00000000	mice
Thrap3	-0.05	1.00000000	mice
Clasp1	-0.13	1.00000000	mice
Fam102b	-0.09	1.00000000	mice
Eef2kmt	0.09	1.00000000	mice
Phactr2	-0.11	1.00000000	mice
Casc4	0.04	1.00000000	mice
Dusp7	0.11	1.00000000	mice
St8sia2	-0.02	1.00000000	mice
Ube3b	-0.12	1.00000000	mice
Creld2	0.20	1.00000000	mice
Kcnq4	-0.05	1.00000000	mice
Emilin1	0.34	1.00000000	mice
Tsc22d1	-0.05	1.00000000	mice
Strbp	-0.15	1.00000000	mice
Parp3	0.17	1.00000000	mice
Gnas	-0.07	1.00000000	mice
Zfp948	-0.07	1.00000000	mice
Gnpda2	-0.09	1.00000000	mice
Togaram1	-0.16	1.00000000	mice
Id2	-0.05	1.00000000	mice
Slco2b1	-0.13	1.00000000	mice
Phf13	-0.09	1.00000000	mice
Coa6	0.00	1.00000000	mice
Arhgap6	0.02	1.00000000	mice
Sppl3	-0.07	1.00000000	mice
Rnaseh2c	-0.12	1.00000000	mice
Supt6	-0.12	1.00000000	mice
Hint3	0.00	1.00000000	mice
Tes	-0.08	1.00000000	mice
Ppp1r8	-0.13	1.00000000	mice
Gpatch8	-0.20	1.00000000	mice
Ehbp1l1	-0.09	1.00000000	mice
Slc36a4	-0.04	1.00000000	mice
Luc7l	-0.04	1.00000000	mice
Tsr3	0.11	1.00000000	mice
Prkar2a	-0.15	1.00000000	mice
P4hb	0.29	1.00000000	mice
Rab11fip3	-0.23	1.00000000	mice
Smc5	-0.10	1.00000000	mice
Atp1a2	0.04	1.00000000	mice

Fxyd6	-0.11	1.00000000	mice
Tcf3	0.01	1.00000000	mice
Synpo	-0.13	1.00000000	mice
Ncor1	-0.03	1.00000000	mice
Ptbp1	-0.16	1.00000000	mice
Tpp1	-0.05	1.00000000	mice
Gnai3	-0.10	1.00000000	mice
Plpp1	-0.11	1.00000000	mice
Fkrp	-0.03	1.00000000	mice
Pcnx	-0.23	1.00000000	mice
Bnip2	-0.04	1.00000000	mice
Zc3h12a	-0.07	1.00000000	mice
Fscn1	0.10	1.00000000	mice
Agap1	-0.14	1.00000000	mice
Lsm1	-0.07	1.00000000	mice
Acap2	-0.15	1.00000000	mice
Ccdc107	-0.02	1.00000000	mice
Kcnj8	-0.01	1.00000000	mice
Atp5o	-0.01	1.00000000	mice
Adamts4	-0.42	1.00000000	mice
Plekha5	-0.06	1.00000000	mice
Syncrip	-0.07	1.00000000	mice
Ammecr1l	-0.14	1.00000000	mice
Cacul1	-0.14	1.00000000	mice
Urod	0.01	1.00000000	mice
Stx5a	0.00	1.00000000	mice
Ube2w	-0.11	1.00000000	mice
Wdfy2	-0.20	1.00000000	mice
Rfx2	-0.10	1.00000000	mice
Enc1	0.18	1.00000000	mice
Slc9a1	-0.11	1.00000000	mice
Kmt2e	-0.02	1.00000000	mice
Zranb1	-0.09	1.00000000	mice
Scn1b	0.01	1.00000000	mice
Ccdc85b	-0.09	1.00000000	mice
Fars2	-0.15	1.00000000	mice
Glipr2	0.20	1.00000000	mice
1200007C13Rik	-0.27	1.00000000	mice
Lgalsl	-0.01	1.00000000	mice
Akip1	-0.11	1.00000000	mice
Inpp5d	-0.08	1.00000000	mice
Edem3	-0.13	1.00000000	mice
Aifm1	0.11	1.00000000	mice
Tnpo1	-0.16	1.00000000	mice
Gak	-0.19	1.00000000	mice
Garem1	-0.13	1.00000000	mice

Ppp2r5e	-0.08	1.00000000	mice
Usp4	-0.07	1.00000000	mice
Lrrc28	-0.08	1.00000000	mice
Osgep	-0.09	1.00000000	mice
Ap4s1	-0.02	1.00000000	mice
Tmem250-ps	-0.12	1.00000000	mice
Alg11	0.14	1.00000000	mice
Sema3c	-0.10	1.00000000	mice
Cry1	-0.07	1.00000000	mice
Gpn3	-0.05	1.00000000	mice
Ppp2cb	-0.07	1.00000000	mice
Atp2b4	-0.22	1.00000000	mice
Bcam	-0.20	1.00000000	mice
Cd164	0.38	1.00000000	mice
Stat5a	-0.12	1.00000000	mice
B4gat1	-0.06	1.00000000	mice
Tmbim1	-0.09	1.00000000	mice
Fnip2	-0.18	1.00000000	mice
Arid4a	-0.06	1.00000000	mice
Arpc3	0.03	1.00000000	mice
Sv2c	-0.14	1.00000000	mice
Gigyf2	-0.14	1.00000000	mice
Osbpl11	-0.10	1.00000000	mice
Uhrf2	-0.12	1.00000000	mice
Siah1a	-0.18	1.00000000	mice
Nrep	0.11	1.00000000	mice
Pold4	0.00	1.00000000	mice
Tbl3	-0.04	1.00000000	mice
Hspa14	0.22	1.00000000	mice
Egfem1	-0.14	1.00000000	mice
Egln2	-0.22	1.00000000	mice
Dpp3	0.15	1.00000000	mice
Exoc4	-0.11	1.00000000	mice
Lmbrd1	-0.09	1.00000000	mice
Pomgnt1	-0.06	1.00000000	mice
Atp9b	-0.11	1.00000000	mice
Brms1l	-0.07	1.00000000	mice
Slc25a23	-0.05	1.00000000	mice
Ptprd	0.26	1.00000000	mice
Nradd	-0.06	1.00000000	mice
Sin3b	-0.13	1.00000000	mice
Gbe1	-0.31	1.00000000	mice
Dip2a	-0.09	1.00000000	mice
Podxl2	0.00	1.00000000	mice
B230219D22Rik	-0.01	1.00000000	mice
Glod4	-0.12	1.00000000	mice

Kremen1	-0.15	1.00000000	mice
C1s1	0.27	1.00000000	mice
Nfia	-0.12	1.00000000	mice
Scamp1	-0.11	1.00000000	mice
Immp2l	-0.17	1.00000000	mice
Zfp217	0.11	1.00000000	mice
Sertad3	0.08	1.00000000	mice
Net1	-0.15	1.00000000	mice
Rasd1	-0.12	1.00000000	mice
Ezh1	-0.15	1.00000000	mice
Synm	-0.04	1.00000000	mice
Gsta4	-0.04	1.00000000	mice
Heca	-0.07	1.00000000	mice
Nap1l1	-0.06	1.00000000	mice
Ryk	0.11	1.00000000	mice
Gdpd3	-0.07	1.00000000	mice
Tcf25	-0.03	1.00000000	mice
Cxcl14	0.65	1.00000000	mice
Lrrfip1	-0.19	1.00000000	mice
Scaper	-0.15	1.00000000	mice
C2cd3	-0.11	1.00000000	mice
Pank1	-0.11	1.00000000	mice
Anapc5	-0.01	1.00000000	mice
Nup188	-0.22	1.00000000	mice
Atxn2	-0.09	1.00000000	mice
Tmcc3	-0.09	1.00000000	mice
Rnf214	-0.09	1.00000000	mice
Ppp4r4	0.25	1.00000000	mice
Aamp	0.07	1.00000000	mice
Ythdf3	-0.09	1.00000000	mice
Hlf	-0.09	1.00000000	mice
Zeb2	0.28	1.00000000	mice
Rmnd5a	-0.06	1.00000000	mice
Tcp1	-0.06	1.00000000	mice
Cul2	-0.19	1.00000000	mice
Stk16	-0.04	1.00000000	mice
Herpud2	-0.12	1.00000000	mice
Cfl1	-0.04	1.00000000	mice
Asap2	-0.09	1.00000000	mice
Tmem106b	-0.13	1.00000000	mice
St6gal1	-0.07	1.00000000	mice
Dctn4	-0.11	1.00000000	mice
Josd2	-0.07	1.00000000	mice
Ap1s2	0.00	1.00000000	mice
Dlst	-0.03	1.00000000	mice
Cpox	-0.05	1.00000000	mice

Afg3l2	-0.09	1.00000000	mice
Phkg2	-0.04	1.00000000	mice
Pqbp1	-0.04	1.00000000	mice
Hic1	0.12	1.00000000	mice
Adcy3	-0.26	1.00000000	mice
Nlrp3	0.45	1.00000000	mice
Scara3	-0.18	1.00000000	mice
Nfkbia	0.03	1.00000000	mice
Tmem167	0.31	1.00000000	mice
Rasal2	-0.15	1.00000000	mice
Ppp1r18	-0.11	1.00000000	mice
Ewsr1	-0.05	1.00000000	mice
Nop16	0.03	1.00000000	mice
Gda	0.26	1.00000000	mice
Il4ra	-0.03	1.00000000	mice
Armcx2	0.14	1.00000000	mice
Ak3	-0.05	1.00000000	mice
Numbl	-0.05	1.00000000	mice
Nectin3	-0.11	1.00000000	mice
Thap3	-0.04	1.00000000	mice
Crip1	-0.07	1.00000000	mice
Cdkn2aip	-0.08	1.00000000	mice
Odc1	-0.35	1.00000000	mice
Tmem38a	-0.05	1.00000000	mice
Camk2d	-0.12	1.00000000	mice
Odr4	-0.07	1.00000000	mice
Ldah	-0.10	1.00000000	mice
Angpt1	0.54	1.00000000	mice
Zswim4	-0.16	1.00000000	mice
Prdx2	-0.02	1.00000000	mice
Spcs2	0.40	1.00000000	mice
Tle1	-0.16	1.00000000	mice
Krt10	-0.05	1.00000000	mice
Stom	-0.12	1.00000000	mice
Enpp5	-0.04	1.00000000	mice
Vps54	0.30	1.00000000	mice
Tsen34	-0.05	1.00000000	mice
Slc1a5	-0.14	1.00000000	mice
Thbs3	-0.12	1.00000000	mice
Tmem51	-0.08	1.00000000	mice
Ndfip2	-0.13	1.00000000	mice
Atp5mpl	0.34	1.00000000	mice
Tceal1	-0.04	1.00000000	mice
Cdo1	-0.04	1.00000000	mice
Klhl7	-0.08	1.00000000	mice
Sun1	-0.06	1.00000000	mice

Gsta3	0.07	1.00000000	mice
P4ha2	-0.11	1.00000000	mice
Eif3i	0.00	1.00000000	mice
Yrdc	-0.07	1.00000000	mice
Srm	0.21	1.00000000	mice
Gigyf1	-0.01	1.00000000	mice
Tubb2a	-0.23	1.00000000	mice
Gpam	0.14	1.00000000	mice
Ptpn13	-0.17	1.00000000	mice
Phtf2	-0.14	1.00000000	mice
Oaz2	0.02	1.00000000	mice
Casp3	0.10	1.00000000	mice
Sfr1	0.03	1.00000000	mice
Ralgapa1	-0.14	1.00000000	mice
Zbtb21	-0.14	1.00000000	mice
Pkd1	-0.09	1.00000000	mice
Iqgap1	-0.14	1.00000000	mice
Ilkap	0.00	1.00000000	mice
Rnasek	0.14	1.00000000	mice
Prpf40a	-0.09	1.00000000	mice
Rab2b	0.13	1.00000000	mice
Cyp7b1	0.47	1.00000000	mice
Ptgfr	-0.08	1.00000000	mice
C87436	-0.15	1.00000000	mice
Churc1	-0.03	1.00000000	mice
Spty2d1	-0.03	1.00000000	mice
Swi5	0.03	1.00000000	mice
Camsap2	-0.15	1.00000000	mice
Hs6st1	-0.12	1.00000000	mice
Plcg1	-0.03	1.00000000	mice
Tgfb2	-0.09	1.00000000	mice
Sumo2	-0.08	1.00000000	mice
Xylt1	-0.28	1.00000000	mice
Hook3	-0.06	1.00000000	mice
Smoc2	-0.29	1.00000000	mice
Socs6	0.12	1.00000000	mice
Spcs3	0.21	1.00000000	mice
Sh3glb1	0.44	1.00000000	mice
Pygb	-0.05	1.00000000	mice
Eif3e	-0.04	1.00000000	mice
Pibf1	-0.20	1.00000000	mice
Fbf1	-0.06	1.00000000	mice
Phlda1	0.38	1.00000000	mice
Scfd2	-0.21	1.00000000	mice
Zcchc14	-0.12	1.00000000	mice
Dbndd2	0.01	1.00000000	mice

2810013P06Rik	-0.12	1.00000000	mice
Zfp710	0.11	1.00000000	mice
Hipk1	-0.10	1.00000000	mice
Tjp2	-0.15	1.00000000	mice
Tdrd3	-0.10	1.00000000	mice
Gusb	0.00	1.00000000	mice
Pex1	-0.13	1.00000000	mice
Acbd4	-0.09	1.00000000	mice
Pvr	-0.13	1.00000000	mice
Dmxl1	-0.10	1.00000000	mice
Sh3bp5	-0.10	1.00000000	mice
Eif4enif1	-0.18	1.00000000	mice
Ddx27	0.03	1.00000000	mice
Rab21	-0.08	1.00000000	mice
Laptm4b	-0.10	1.00000000	mice
Zfp260	0.14	1.00000000	mice
Uck2	-0.21	1.00000000	mice
Egln1	0.00	1.00000000	mice
Golph3	-0.07	1.00000000	mice
Xpot	-0.03	1.00000000	mice
Naa50	0.00	1.00000000	mice
2210016F16Rik	-0.12	1.00000000	mice
Hilpda	0.41	1.00000000	mice
Dusp11	0.02	1.00000000	mice
Ssbp1	0.13	1.00000000	mice
Col5a2	0.19	1.00000000	mice
Sem1	0.35	1.00000000	mice
Pdlim4	-0.15	1.00000000	mice
Shfl	0.10	1.00000000	mice
Afap1l2	0.12	1.00000000	mice
Tspan5	-0.15	1.00000000	mice
Crot	0.13	1.00000000	mice
Gnaq	-0.07	1.00000000	mice
Smad2	-0.09	1.00000000	mice
Ccdc186	-0.06	1.00000000	mice
Lrp4	0.34	1.00000000	mice
Plscr4	-0.15	1.00000000	mice
Zfp451	-0.12	1.00000000	mice
Snrpn	-0.04	1.00000000	mice
Zfp955b	0.07	1.00000000	mice
Gba	0.12	1.00000000	mice
Slc25a28	0.15	1.00000000	mice
Timm23	-0.15	1.00000000	mice
Tubb4a	-0.07	1.00000000	mice
Znhit1	0.21	1.00000000	mice
Plagl1	-0.16	1.00000000	mice

Tma7	0.26	1.00000000	mice
Gas7	-0.03	1.00000000	mice
Amn1	0.22	1.00000000	mice
Il13ra1	0.05	1.00000000	mice
Knop1	0.06	1.00000000	mice
Setd5	-0.04	1.00000000	mice
Chmp2b	-0.08	1.00000000	mice
Patl1	-0.12	1.00000000	mice
Bcl7b	-0.10	1.00000000	mice
Slc9a6	-0.03	1.00000000	mice
Lsm8	0.06	1.00000000	mice
Phldb1	-0.08	1.00000000	mice
Hdgfl2	-0.10	1.00000000	mice
Surf1	-0.06	1.00000000	mice
Zcrb1	0.07	1.00000000	mice
Cnot2	-0.13	1.00000000	mice
Usp7	-0.13	1.00000000	mice
Ptk6	0.08	1.00000000	mice
Adgrg6	0.41	1.00000000	mice
Rai1	-0.10	1.00000000	mice
Tfg	-0.13	1.00000000	mice
Pacsin2	-0.09	1.00000000	mice
Tox4	-0.06	1.00000000	mice
Irak3	-0.13	1.00000000	mice
Tmem87b	-0.25	1.00000000	mice
Stub1	0.05	1.00000000	mice
Exoc7	0.12	1.00000000	mice
Crlf3	-0.03	1.00000000	mice
Afdn	-0.11	1.00000000	mice
Glo1	0.00	1.00000000	mice
Stard3nl	-0.06	1.00000000	mice
Cpe	-0.31	1.00000000	mice
Syf2	0.03	1.00000000	mice
Gtf2i	0.06	1.00000000	mice
Neo1	-0.09	1.00000000	mice
Gpatch2l	-0.10	1.00000000	mice
Lrrk1	-0.21	1.00000000	mice
Bcar1	-0.14	1.00000000	mice
Fam43a	-0.13	1.00000000	mice
1110038F14Rik	0.19	1.00000000	mice
Eif2a	0.07	1.00000000	mice
Rfx3	-0.15	1.00000000	mice
Cited2	-0.03	1.00000000	mice
Tmlhe	-0.04	1.00000000	mice
Ralgps1	-0.05	1.00000000	mice
Syt11	-0.12	1.00000000	mice

Ep400	-0.06	1.00000000	mice
Dapk1	-0.20	1.00000000	mice
Gnl1	0.19	1.00000000	mice
Ppib	0.29	1.00000000	mice
Bok	0.03	1.00000000	mice
Idi1	0.17	1.00000000	mice
Ift172	-0.20	1.00000000	mice
Slc25a3	-0.09	1.00000000	mice
Tubb4b	-0.07	1.00000000	mice
Tgfbr2	0.39	1.00000000	mice
Srpx	-0.16	1.00000000	mice
Fkbp9	0.30	1.00000000	mice
Eed	-0.04	1.00000000	mice
Pgm5	-0.17	1.00000000	mice
Ccdc86	-0.01	1.00000000	mice
Ran	0.34	1.00000000	mice
Ggct	0.15	1.00000000	mice
Cct4	0.04	1.00000000	mice
S100a13	0.01	1.00000000	mice
Cox6b2	0.09	1.00000000	mice
Rpap3	0.14	1.00000000	mice
Flna	-0.27	1.00000000	mice
Pcgf3	-0.17	1.00000000	mice
Gatad1	-0.10	1.00000000	mice
Hnrnpdl	0.29	1.00000000	mice
Asb7	-0.08	1.00000000	mice
Adam19	0.09	1.00000000	mice
Ctbp1	0.02	1.00000000	mice
Eif2s3x	0.02	1.00000000	mice
Psmd11	-0.04	1.00000000	mice
Smg5	-0.04	1.00000000	mice
Bckdhb	0.13	1.00000000	mice
Rufy1	-0.01	1.00000000	mice
Nsmf	-0.08	1.00000000	mice
Epb41l1	-0.06	1.00000000	mice
Rfk	0.07	1.00000000	mice
Dpy30	0.00	1.00000000	mice
Wwox	-0.31	1.00000000	mice
Chkb	-0.06	1.00000000	mice
Pgam1	-0.12	1.00000000	mice
Ift43	-0.04	1.00000000	mice
Nup214	-0.10	1.00000000	mice
Bag6	-0.08	1.00000000	mice
Abca5	-0.05	1.00000000	mice
Sart3	-0.03	1.00000000	mice
Ndufaf3	-0.03	1.00000000	mice

Fosl2	-0.04	1.00000000	mice
Slc11a2	0.13	1.00000000	mice
1810030O07Rik	-0.09	1.00000000	mice
Npm1	0.05	1.00000000	mice
Gch1	0.08	1.00000000	mice
Extl2	0.14	1.00000000	mice
Fbxo32	-0.13	1.00000000	mice
Tm9sf3	0.02	1.00000000	mice
Phlpp2	-0.08	1.00000000	mice
Tbca	0.08	1.00000000	mice
Pggt1b	-0.02	1.00000000	mice
Serpinb6a	0.05	1.00000000	mice
Bcl2l13	-0.12	1.00000000	mice
Ptdss1	-0.17	1.00000000	mice
Rbm48	-0.10	1.00000000	mice
Ccnl2	-0.11	1.00000000	mice
Rnf13	-0.07	1.00000000	mice
Eda	-0.17	1.00000000	mice
Nup160	-0.10	1.00000000	mice
Dcn	-0.50	1.00000000	mice
Wif1	0.01	1.00000000	mice
Srsf7	-0.10	1.00000000	mice
Gabpb2	-0.07	1.00000000	mice
Glud1	-0.08	1.00000000	mice
Vegfa	0.35	1.00000000	mice
Irf1	0.40	1.00000000	mice
Bag1	-0.02	1.00000000	mice
Aars	0.20	1.00000000	mice
Ncoa3	-0.22	1.00000000	mice
Pcsk6	-0.15	1.00000000	mice
Gas5	0.36	1.00000000	mice
Zbtb10	-0.12	1.00000000	mice
Gng11	0.44	1.00000000	mice
Hhip	0.23	1.00000000	mice
Pfdn1	0.25	1.00000000	mice
Acyp2	-0.08	1.00000000	mice
Ndufs2	0.09	1.00000000	mice
Syngr2	0.07	1.00000000	mice
Prrg1	0.18	1.00000000	mice
Cbl	0.05	1.00000000	mice
Dhx9	-0.08	1.00000000	mice
Csmd1	-0.10	1.00000000	mice
Depdc5	-0.20	1.00000000	mice
Pip4k2c	-0.05	1.00000000	mice
Utp18	-0.22	1.00000000	mice
Nek9	0.02	1.00000000	mice

Lzts2	0.10	1.00000000	mice
Zc3h18	-0.16	1.00000000	mice
Ier3	-0.05	1.00000000	mice
Blmh	-0.10	1.00000000	mice
Grasp	-0.04	1.00000000	mice
Kank1	-0.15	1.00000000	mice
Pdzd2	0.54	1.00000000	mice
Lama5	-0.02	1.00000000	mice
Irf2	0.30	1.00000000	mice
Anapc7	-0.12	1.00000000	mice
Rbm22	-0.06	1.00000000	mice
Slc35f5	-0.10	1.00000000	mice
Nudcd2	0.14	1.00000000	mice
Ube2g1	-0.17	1.00000000	mice
Atad2	-0.14	1.00000000	mice
Hk1	-0.13	1.00000000	mice
Phtf1	-0.11	1.00000000	mice
Pspc1	-0.02	1.00000000	mice
Glrx3	0.06	1.00000000	mice
Ccdc167	0.14	1.00000000	mice
Dnajc11	0.11	1.00000000	mice
Fam45a	0.10	1.00000000	mice
Arl6ip5	0.29	1.00000000	mice
Pcyox1	0.01	1.00000000	mice
Polr3e	-0.06	1.00000000	mice
Arvcf	-0.02	1.00000000	mice
Txnl1	0.01	1.00000000	mice
Cdk17	-0.09	1.00000000	mice
Eif3d	0.24	1.00000000	mice
Plod1	0.09	1.00000000	mice
Pde7a	-0.05	1.00000000	mice
Oxr1	-0.06	1.00000000	mice
Txnrd3	-0.08	1.00000000	mice
Sos2	-0.05	1.00000000	mice
Adgrd1	-0.22	1.00000000	mice
Rbpms2	-0.12	1.00000000	mice
Slc3a2	-0.05	1.00000000	mice
Ahsa1	0.02	1.00000000	mice
Heyl	0.07	1.00000000	mice
Taf13	-0.10	1.00000000	mice
Gja1	-0.34	1.00000000	mice
Aarsd1	-0.09	1.00000000	mice
Exoc1	0.10	1.00000000	mice
Fcf1	0.24	1.00000000	mice
Gucy1a2	0.00	1.00000000	mice
Lnpep	-0.03	1.00000000	mice

Fam3c	-0.05	1.00000000	mice
Rnaset2a	0.22	1.00000000	mice
Zfp706	-0.11	1.00000000	mice
Zfp182	-0.06	1.00000000	mice
Prkd2	-0.05	1.00000000	mice
Ppm1b	-0.10	1.00000000	mice
Selenbp1	-0.12	1.00000000	mice
Rev1	-0.11	1.00000000	mice
Cpt1a	0.15	1.00000000	mice
Fxr1	-0.07	1.00000000	mice
Ifnar2	0.26	1.00000000	mice
Snapc5	0.05	1.00000000	mice
Tet2	-0.17	1.00000000	mice
D430042O09Rik	-0.06	1.00000000	mice
Ddost	0.24	1.00000000	mice
Btf3	0.09	1.00000000	mice
Snx7	-0.08	1.00000000	mice
Ube2d3	-0.03	1.00000000	mice
Kpnb1	-0.06	1.00000000	mice
Eif2b4	0.11	1.00000000	mice
Dkc1	-0.07	1.00000000	mice
Slc20a1	-0.12	1.00000000	mice
Rbm4b	-0.16	1.00000000	mice
Tmem50b	0.03	1.00000000	mice
Pxn	-0.04	1.00000000	mice
Ylpm1	-0.04	1.00000000	mice
Ppip5k1	-0.14	1.00000000	mice
Trim37	-0.07	1.00000000	mice
Rcl1	-0.23	1.00000000	mice
Cmas	-0.11	1.00000000	mice
Kazn	-0.14	1.00000000	mice
Hmgcs1	0.35	1.00000000	mice
Tnrc6b	-0.12	1.00000000	mice
Ankrd26	-0.21	1.00000000	mice
Yipf3	-0.06	1.00000000	mice
Prps2	-0.04	1.00000000	mice
Ankrd40	-0.11	1.00000000	mice
Tmcc1	-0.17	1.00000000	mice
Csnk1g3	-0.02	1.00000000	mice
Prdm5	-0.11	1.00000000	mice
Atpif1	-0.01	1.00000000	mice
Jkamp	-0.14	1.00000000	mice
Ndufb5	-0.06	1.00000000	mice
Zfp521	-0.11	1.00000000	mice
Phf2	-0.06	1.00000000	mice
Sh3bp4	0.09	1.00000000	mice

Nhsl2	-0.09	1.00000000	mice
Snhg5	0.14	1.00000000	mice
Psip1	-0.12	1.00000000	mice
Phykpl	0.15	1.00000000	mice
Pias2	-0.19	1.00000000	mice
Abca1	-0.12	1.00000000	mice
Col5a1	-0.07	1.00000000	mice
Rbp4	-0.30	1.00000000	mice
Tmem230	-0.12	1.00000000	mice
Snrpa	0.10	1.00000000	mice
Tmem131	-0.09	1.00000000	mice
Rbbp7	-0.09	1.00000000	mice
Tcf12	-0.03	1.00000000	mice
Oard1	0.06	1.00000000	mice
Tbcel	-0.21	1.00000000	mice
Alkbh1	-0.07	1.00000000	mice
Ppp4c	-0.04	1.00000000	mice
Tnpo3	-0.14	1.00000000	mice
Hivep3	0.54	1.00000000	mice
Dynll2	-0.01	1.00000000	mice
Nfya	-0.05	1.00000000	mice
Uvrag	-0.10	1.00000000	mice
Zfp326	-0.05	1.00000000	mice
Adpgk	0.24	1.00000000	mice
Tpp2	-0.08	1.00000000	mice
Polr2l	0.21	1.00000000	mice
Vkorc1	-0.08	1.00000000	mice
Alkbh3	0.09	1.00000000	mice
Akr7a5	0.00	1.00000000	mice
Ppp3cc	-0.04	1.00000000	mice
Rbm19	-0.08	1.00000000	mice
Commd6	-0.06	1.00000000	mice
Thbs1	-0.37	1.00000000	mice
Wdr48	-0.13	1.00000000	mice
Lsm3	0.06	1.00000000	mice
Pabpn1	0.01	1.00000000	mice
Smco4	0.08	1.00000000	mice
Rnf185	-0.12	1.00000000	mice
Dcun1d4	-0.06	1.00000000	mice
Cox17	0.26	1.00000000	mice
Farp2	-0.17	1.00000000	mice
Gnptab	0.11	1.00000000	mice
Sema4c	-0.06	1.00000000	mice
Ttyh3	-0.10	1.00000000	mice
Fam89b	-0.09	1.00000000	mice
Hmgn3	-0.06	1.00000000	mice

St3gal2	-0.04	1.00000000	mice
Derl1	-0.03	1.00000000	mice
Acly	-0.12	1.00000000	mice
Zfp511	-0.04	1.00000000	mice
4921524J17Rik	0.20	1.00000000	mice
Ppm1a	-0.15	1.00000000	mice
Cacnb2	-0.02	1.00000000	mice
Fam13b	-0.02	1.00000000	mice
Rab3ip	-0.09	1.00000000	mice
Rmnd5b	0.10	1.00000000	mice
Cnih4	0.14	1.00000000	mice
Ythdc2	-0.07	1.00000000	mice
Rbm42	0.01	1.00000000	mice
Prim2	-0.04	1.00000000	mice
Anpep	-0.05	1.00000000	mice
Cndp2	0.16	1.00000000	mice
Usp48	-0.05	1.00000000	mice
Zfp142	-0.11	1.00000000	mice
Hnrnpab	0.01	1.00000000	mice
Ubac2	-0.01	1.00000000	mice
Tusc3	-0.07	1.00000000	mice
Ilk	-0.06	1.00000000	mice
Spin1	-0.09	1.00000000	mice
Firre	-0.09	1.00000000	mice
Aggf1	0.02	1.00000000	mice
Pdxdc1	-0.02	1.00000000	mice
Dleu2	-0.21	1.00000000	mice
2610301B20Rik	0.14	1.00000000	mice
Brpf3	-0.04	1.00000000	mice
Prdx6	0.08	1.00000000	mice
Nr4a2	0.01	1.00000000	mice
Ces2g	0.07	1.00000000	mice
Cd44	-0.12	1.00000000	mice
Capzb	0.01	1.00000000	mice
Uqcrb	0.37	1.00000000	mice
Dtd1	-0.09	1.00000000	mice
Plekho1	-0.10	1.00000000	mice
Sec24b	-0.13	1.00000000	mice
Rnf14	0.25	1.00000000	mice
Pear1	-0.04	1.00000000	mice
Flot2	-0.15	1.00000000	mice
Pdgfrb	0.16	1.00000000	mice
Cnpy4	-0.09	1.00000000	mice
Skiv2l	0.11	1.00000000	mice
Eif4g2	-0.04	1.00000000	mice
Csnk1a1	-0.07	1.00000000	mice

4932438A13Rik	-0.05	1.00000000	mice
Cgrrf1	0.14	1.00000000	mice
Id1	-0.10	1.00000000	mice
Pcbd2	-0.11	1.00000000	mice
Trip6	-0.15	1.00000000	mice
Tbc1d10a	-0.02	1.00000000	mice
Trim44	-0.09	1.00000000	mice
S100a16	-0.04	1.00000000	mice
Ftl1	-0.13	1.00000000	mice
Dtna	-0.35	1.00000000	mice
Col4a5	0.03	1.00000000	mice
Zfp609	-0.15	1.00000000	mice
Gkap1	-0.01	1.00000000	mice
Ranbp3	0.02	1.00000000	mice
Osbpl1a	-0.12	1.00000000	mice
Nedd8	0.05	1.00000000	mice
Phip	-0.16	1.00000000	mice
Dzip1	-0.09	1.00000000	mice
Tmx4	-0.03	1.00000000	mice
Adamtsl3	0.14	1.00000000	mice
Tspan18	-0.13	1.00000000	mice
Tra2a	-0.07	1.00000000	mice
Ube2o	-0.16	1.00000000	mice
Vcp	0.30	1.00000000	mice
Ctsz	0.31	1.00000000	mice
Tmem161a	-0.08	1.00000000	mice
Cep63	-0.04	1.00000000	mice
Arf4	-0.10	1.00000000	mice
Letm2	-0.09	1.00000000	mice
Polr2e	-0.11	1.00000000	mice
Serpine3	-0.09	1.00000000	mice
Edrf1	-0.03	1.00000000	mice
Ubxn2a	-0.03	1.00000000	mice
Zbtb4	-0.05	1.00000000	mice
Zswim8	0.13	1.00000000	mice
Vps13b	-0.09	1.00000000	mice
Ppme1	-0.11	1.00000000	mice
Abcc5	-0.06	1.00000000	mice
Oaf	-0.12	1.00000000	mice
Kmt2b	-0.05	1.00000000	mice
Tsc22d4	0.11	1.00000000	mice
Idh3b	-0.07	1.00000000	mice
Ermard	-0.14	1.00000000	mice
Ptpn1	-0.09	1.00000000	mice
Npdc1	-0.10	1.00000000	mice
Adamts5	-0.07	1.00000000	mice

Cpeb4	-0.02	1.00000000	mice
Uba2	-0.11	1.00000000	mice
Ilf3	-0.17	1.00000000	mice
Dnajc24	-0.04	1.00000000	mice
Lrrc41	-0.09	1.00000000	mice
Dynlrb1	0.10	1.00000000	mice
Dcps	-0.07	1.00000000	mice
Vamp5	-0.01	1.00000000	mice
Car13	0.12	1.00000000	mice
Hfe	0.21	1.00000000	mice
Hcfc2	-0.04	1.00000000	mice
Spryd3	0.17	1.00000000	mice
Capn7	-0.12	1.00000000	mice
Ap3m1	0.23	1.00000000	mice
2610020C07Rik	-0.01	1.00000000	mice
Cdc42se2	-0.18	1.00000000	mice
Hdac8	-0.10	1.00000000	mice
Rab11a	-0.04	1.00000000	mice
Nr2f2	0.30	1.00000000	mice
Psmc5	0.28	1.00000000	mice
Loxl1	-0.11	1.00000000	mice
Ptpn14	-0.11	1.00000000	mice
Slc7a10	0.31	1.00000000	mice
Prps1	0.01	1.00000000	mice
Zzz3	-0.08	1.00000000	mice
Ppp5c	-0.11	1.00000000	mice
A330023F24Rik	-0.09	1.00000000	mice
Fam49a	-0.20	1.00000000	mice
Rcor1	-0.22	1.00000000	mice
Slc35d1	-0.16	1.00000000	mice
Rsf1	-0.10	1.00000000	mice
Eng	-0.07	1.00000000	mice
Zfp462	-0.14	1.00000000	mice
Vcpip1	0.22	1.00000000	mice
Zfp568	-0.06	1.00000000	mice
Cxcl1	0.80	1.00000000	mice
Ctps2	-0.07	1.00000000	mice
Sdha	0.02	1.00000000	mice
Dguok	0.03	1.00000000	mice
Tbx3os1	-0.11	1.00000000	mice
Atp1b2	-0.14	1.00000000	mice
Smim15	0.36	1.00000000	mice
Pde2a	-0.08	1.00000000	mice
Suclg2	0.02	1.00000000	mice
Lgi2	0.13	1.00000000	mice
Ccdc43	0.12	1.00000000	mice

Riok2	-0.07	1.00000000	mice
Fbxw11	-0.05	1.00000000	mice
Acvr1b	-0.05	1.00000000	mice
Naa25	0.08	1.00000000	mice
Klhdc8b	-0.04	1.00000000	mice
Zfp622	0.01	1.00000000	mice
Dnajc21	-0.04	1.00000000	mice
Aatf	0.07	1.00000000	mice
Stn1	-0.07	1.00000000	mice
Acp2	0.08	1.00000000	mice
Rad50	0.15	1.00000000	mice
Herc4	-0.15	1.00000000	mice
Ugdh	-0.08	1.00000000	mice
Tbc1d22a	-0.17	1.00000000	mice
Coq5	0.04	1.00000000	mice
Cdk5rap2	0.12	1.00000000	mice
Fbxo7	0.07	1.00000000	mice
Adap2	0.07	1.00000000	mice
Abi2	0.02	1.00000000	mice
Pdlim5	-0.13	1.00000000	mice
Wdr44	-0.07	1.00000000	mice
Gaa	-0.03	1.00000000	mice
Plekha2	-0.03	1.00000000	mice
Cbr1	-0.11	1.00000000	mice
Tbc1d12	-0.15	1.00000000	mice
Echdc2	-0.01	1.00000000	mice
Gpsm1	0.00	1.00000000	mice
Smarca2	-0.04	1.00000000	mice
Abt1	0.00	1.00000000	mice
Ramac	-0.15	1.00000000	mice
Actn1	-0.13	1.00000000	mice
Tmem161b	0.06	1.00000000	mice
Trmt6	0.10	1.00000000	mice
Ppid	-0.06	1.00000000	mice
Zfp651	-0.06	1.00000000	mice
Drap1	0.20	1.00000000	mice
Setd2	-0.07	1.00000000	mice
Pphln1	-0.10	1.00000000	mice
Ppp2r1b	-0.08	1.00000000	mice
Trp53bp1	-0.13	1.00000000	mice
Hint1	0.11	1.00000000	mice
Klf6	-0.03	1.00000000	mice
Rnase4	-0.18	1.00000000	mice
Ccdc80	0.22	1.00000000	mice
Plcd3	-0.06	1.00000000	mice
Scube2	0.43	1.00000000	mice

Rab9	-0.12	1.00000000	mice
Ripk2	0.08	1.00000000	mice
Ddx23	-0.06	1.00000000	mice
Htt	-0.07	1.00000000	mice
Smdt1	0.36	1.00000000	mice
Aco2	-0.01	1.00000000	mice
Ppil4	-0.05	1.00000000	mice
Prdx5	0.44	1.00000000	mice
Inpp4a	-0.10	1.00000000	mice
Tulp4	-0.08	1.00000000	mice
Pi4ka	-0.21	1.00000000	mice
Rhoa	0.24	1.00000000	mice
Ncoa5	-0.11	1.00000000	mice
G2e3	-0.02	1.00000000	mice
Wdr82	-0.02	1.00000000	mice
Ncor2	-0.03	1.00000000	mice
Bod1	0.00	1.00000000	mice
Astn2	-0.04	1.00000000	mice
Ogt	-0.06	1.00000000	mice
Emc10	0.04	1.00000000	mice
Sipa1l2	-0.17	1.00000000	mice
Leprot	-0.08	1.00000000	mice
Pde9a	0.07	1.00000000	mice
Nup54	-0.15	1.00000000	mice
Zkscan3	-0.06	1.00000000	mice
Gtf2h1	-0.11	1.00000000	mice
Ppp2ca	0.01	1.00000000	mice
Stx18	-0.07	1.00000000	mice
Atp6v0b	0.00	1.00000000	mice
Ppp2r2d	0.07	1.00000000	mice
Twistnb	0.18	1.00000000	mice
Atp6v0c	0.12	1.00000000	mice
Rnf187	0.10	1.00000000	mice
Abca8b	-0.12	1.00000000	mice
Tango6	-0.12	1.00000000	mice
Atp6v1h	-0.11	1.00000000	mice
Ppard	-0.16	1.00000000	mice
Eif1ad	0.14	1.00000000	mice
H3f3b	0.19	1.00000000	mice
Sephs1	0.15	1.00000000	mice
Cbx5	-0.12	1.00000000	mice
Arhgef10l	-0.14	1.00000000	mice
H2afj	-0.01	1.00000000	mice
Ethe1	-0.04	1.00000000	mice
Dtd2	-0.13	1.00000000	mice
Scarf2	-0.02	1.00000000	mice

Hexa	0.04	1.00000000	mice
Taf3	-0.04	1.00000000	mice
Prkar1a	0.04	1.00000000	mice
Akap11	-0.09	1.00000000	mice
Vkorc1l1	-0.04	1.00000000	mice
Ric8b	-0.12	1.00000000	mice
Epb41l5	-0.02	1.00000000	mice
Vim	-0.18	1.00000000	mice
Clasrp	-0.05	1.00000000	mice
Selenoh	0.06	1.00000000	mice
Snrk	-0.11	1.00000000	mice
Pign	0.14	1.00000000	mice
Adgre5	-0.01	1.00000000	mice
Ctdspl2	-0.11	1.00000000	mice
Slc25a36	-0.14	1.00000000	mice
AI838599	-0.02	1.00000000	mice
Ccndbp1	0.01	1.00000000	mice
Ckap5	-0.02	1.00000000	mice
Heatr1	-0.02	1.00000000	mice
Preb	-0.02	1.00000000	mice
Slc35b2	0.10	1.00000000	mice
Rpia	0.02	1.00000000	mice
Adh1	-0.01	1.00000000	mice
Atn1	-0.09	1.00000000	mice
Dctpp1	-0.04	1.00000000	mice
Prelp	-0.04	1.00000000	mice
Sept8	0.04	1.00000000	mice
Dpysl3	-0.11	1.00000000	mice
Ttc21a	-0.06	1.00000000	mice
Srsf9	-0.05	1.00000000	mice
Flot1	0.18	1.00000000	mice
Cdr2	-0.10	1.00000000	mice
Tmem65	-0.01	1.00000000	mice
0610043K17Rik	-0.16	1.00000000	mice
Dnajc27	-0.05	1.00000000	mice
Taf2	-0.01	1.00000000	mice
Elp4	-0.16	1.00000000	mice
Ncoa1	-0.01	1.00000000	mice
Fas	-0.09	1.00000000	mice
Egflam	0.10	1.00000000	mice
Ywhae	0.06	1.00000000	mice
Armh4	-0.05	1.00000000	mice
Yy1	-0.03	1.00000000	mice
Fxr2	-0.12	1.00000000	mice
Snrnp40	-0.09	1.00000000	mice
Rab5c	0.35	1.00000000	mice

Pcbp2	0.00	1.00000000	mice
Ptp4a3	0.03	1.00000000	mice
Pdpn	0.22	1.00000000	mice
Atp10a	0.16	1.00000000	mice
Fzd5	0.06	1.00000000	mice
Ifi30	-0.06	1.00000000	mice
Adss	-0.06	1.00000000	mice
Vdac2	0.11	1.00000000	mice
Ppfibp1	-0.12	1.00000000	mice
Fam222a	-0.07	1.00000000	mice
Nectin1	-0.04	1.00000000	mice
Eepd1	0.06	1.00000000	mice
Pigt	-0.06	1.00000000	mice
Clstn1	-0.18	1.00000000	mice
Gtf2f1	-0.06	1.00000000	mice
Hmcn1	0.04	1.00000000	mice
Thoc1	-0.09	1.00000000	mice
Rad52	-0.15	1.00000000	mice
Dmxl2	-0.08	1.00000000	mice
Sqle	0.17	1.00000000	mice
Dcaf8	0.05	1.00000000	mice
Pex11b	0.00	1.00000000	mice
Lgr4	-0.10	1.00000000	mice
Nectin2	-0.07	1.00000000	mice
Klhl26	-0.12	1.00000000	mice
Gtpbp1	-0.10	1.00000000	mice
Vwa5a	0.13	1.00000000	mice
Qtrt1	-0.06	1.00000000	mice
Bnip3	0.04	1.00000000	mice
Saraf	-0.06	1.00000000	mice
Cdk9	-0.09	1.00000000	mice
Ndnf	0.02	1.00000000	mice
Add1	-0.03	1.00000000	mice
Pnkd	-0.05	1.00000000	mice
Tcea1	0.28	1.00000000	mice
Rangap1	-0.02	1.00000000	mice
Rbmx	0.00	1.00000000	mice
Tnks	-0.11	1.00000000	mice
Gramd3	0.01	1.00000000	mice
4930402H24Rik	-0.07	1.00000000	mice
Plec	-0.12	1.00000000	mice
Zkscan17	-0.07	1.00000000	mice
Fryl	-0.14	1.00000000	mice
Tex264	-0.08	1.00000000	mice
Arfgap2	-0.04	1.00000000	mice
Ap1s1	0.06	1.00000000	mice

Eif2ak3	-0.16	1.00000000	mice
Cd34	-0.22	1.00000000	mice
Dpt	-0.36	1.00000000	mice
Slc44a2	-0.05	1.00000000	mice
Cwf19l2	0.09	1.00000000	mice
Snx18	-0.04	1.00000000	mice
Kif16b	-0.07	1.00000000	mice
Dnaja4	-0.03	1.00000000	mice
Khdrbs1	-0.03	1.00000000	mice
Tmem179b	0.06	1.00000000	mice
Hnrnpu	-0.02	1.00000000	mice
Fam214a	-0.09	1.00000000	mice
Rabgap1l	-0.08	1.00000000	mice
Afg1l	-0.05	1.00000000	mice
Psma5	0.29	1.00000000	mice
Lrrc42	-0.04	1.00000000	mice
Kansl3	-0.12	1.00000000	mice
Unc5b	-0.06	1.00000000	mice
Iffo2	0.12	1.00000000	mice
Chid1	0.04	1.00000000	mice
Pdpk1	-0.15	1.00000000	mice
Tpm1	-0.44	1.00000000	mice
Carnmt1	-0.08	1.00000000	mice
Robo1	-0.21	1.00000000	mice
Lonrf1	-0.06	1.00000000	mice
Plxnc1	0.09	1.00000000	mice
Gadd45g	-0.11	1.00000000	mice
Etfa	-0.04	1.00000000	mice
Abr	0.19	1.00000000	mice
Rprd2	-0.02	1.00000000	mice
Fuca1	-0.07	1.00000000	mice
Rabif	-0.05	1.00000000	mice
Sypl	-0.02	1.00000000	mice
Slc25a11	-0.16	1.00000000	mice
Tmbim4	-0.02	1.00000000	mice
Rraga	0.24	1.00000000	mice
Cox7a2	0.00	1.00000000	mice
Paxx	-0.05	1.00000000	mice
Galnt18	-0.12	1.00000000	mice
Zadh2	-0.10	1.00000000	mice
Cald1	-0.15	1.00000000	mice
Eif1ax	0.10	1.00000000	mice
Prrc2c	-0.04	1.00000000	mice
Sorcs2	-0.25	1.00000000	mice
4933412E12Rik	0.10	1.00000000	mice
Npepl1	-0.14	1.00000000	mice

Atp5b	-0.08	1.00000000	mice
Lmo2	0.04	1.00000000	mice
Erg28	0.14	1.00000000	mice
Efna2	-0.05	1.00000000	mice
Dcun1d1	-0.05	1.00000000	mice
Ring1	-0.08	1.00000000	mice
Cops4	0.07	1.00000000	mice
Plcb1	-0.21	1.00000000	mice
Oga	-0.11	1.00000000	mice
Dnajc1	-0.10	1.00000000	mice
Spg11	-0.06	1.00000000	mice
Emsy	-0.08	1.00000000	mice
Rnf126	-0.10	1.00000000	mice
Synrg	0.07	1.00000000	mice
Xrcc6	0.00	1.00000000	mice
Epb41l4aos	-0.04	1.00000000	mice
Postn	0.74	1.00000000	mice
Slc31a1	-0.05	1.00000000	mice
Timm8b	-0.01	1.00000000	mice
E2f4	-0.04	1.00000000	mice
Sbno1	-0.08	1.00000000	mice
Lrp5	0.03	1.00000000	mice
Rbm38	-0.07	1.00000000	mice
Fndc3a	-0.08	1.00000000	mice
Nol3	0.34	1.00000000	mice
Ccdc47	0.00	1.00000000	mice
Sipa1l1	-0.03	1.00000000	mice
Srsf6	-0.01	1.00000000	mice
Hpcal1	-0.06	1.00000000	mice
Sh3glb2	-0.03	1.00000000	mice
Dync1i2	0.28	1.00000000	mice
Ap2b1	-0.22	1.00000000	mice
Sympk	0.07	1.00000000	mice
Spata1	-0.16	1.00000000	mice
Atg2b	-0.14	1.00000000	mice
Trappc10	-0.05	1.00000000	mice
Il6	-0.24	1.00000000	mice
Dnajc19	-0.07	1.00000000	mice
Pnpla2	0.00	1.00000000	mice
Aebp2	-0.03	1.00000000	mice
Bcar3	-0.10	1.00000000	mice
Vezf1	0.00	1.00000000	mice
Tial1	-0.15	1.00000000	mice
Hmgcl	0.11	1.00000000	mice
Wtip	-0.07	1.00000000	mice
Rpn1	0.13	1.00000000	mice

Zfp445	-0.06	1.00000000	mice
Fam98c	-0.01	1.00000000	mice
Ociad1	0.04	1.00000000	mice
Stx12	0.04	1.00000000	mice
Rnf31	0.14	1.00000000	mice
Hint2	0.07	1.00000000	mice
Drosha	-0.06	1.00000000	mice
Cadps2	0.07	1.00000000	mice
Nipa2	0.04	1.00000000	mice
Isoc1	-0.07	1.00000000	mice
Shc1	0.03	1.00000000	mice
Rab40c	0.00	1.00000000	mice
Agpat4	-0.01	1.00000000	mice
Ttll7	-0.01	1.00000000	mice
Pwwp2a	-0.10	1.00000000	mice
Dicer1	-0.11	1.00000000	mice
Rxra	0.11	1.00000000	mice
Ice1	-0.12	1.00000000	mice
Trap1	-0.05	1.00000000	mice
Dhx40	-0.14	1.00000000	mice
Yif1a	0.13	1.00000000	mice
Parp1	-0.08	1.00000000	mice
Setd3	-0.03	1.00000000	mice
Hnrnpa2b1	-0.05	1.00000000	mice
G0s2	0.31	1.00000000	mice
6330403K07Rik	-0.06	1.00000000	mice
Nfkbib	-0.07	1.00000000	mice
Usp1	0.15	1.00000000	mice
Cop1	-0.05	1.00000000	mice
Lsm12	0.01	1.00000000	mice
3110056K07Rik	-0.01	1.00000000	mice
Parva	-0.13	1.00000000	mice
Snx27	0.06	1.00000000	mice
Zfp618	0.06	1.00000000	mice
Dmwd	-0.05	1.00000000	mice
Supt5	-0.28	1.00000000	mice
Robo2	0.13	1.00000000	mice
Cars	-0.13	1.00000000	mice
Chmp4b	0.16	1.00000000	mice
Zfp280d	-0.04	1.00000000	mice
Spindoc	0.01	1.00000000	mice
S1pr2	0.09	1.00000000	mice
Kif1bp	-0.05	1.00000000	mice
Amotl1	-0.15	1.00000000	mice
Cpsf7	-0.09	1.00000000	mice
Rhbdf2	0.09	1.00000000	mice

Lbh	0.44	1.00000000	mice
Eif3g	-0.04	1.00000000	mice
Hpgd	0.14	1.00000000	mice
Nudt13	0.09	1.00000000	mice
Stx17	0.06	1.00000000	mice
Amph	0.21	1.00000000	mice
Ace	-0.07	1.00000000	mice
Nacc2	-0.07	1.00000000	mice
Lpar1	-0.01	1.00000000	mice
Lyst	-0.04	1.00000000	mice
R3hdm2	-0.04	1.00000000	mice
Setd1a	-0.03	1.00000000	mice
Tspan6	-0.04	1.00000000	mice
Usp31	-0.07	1.00000000	mice
Kank3	0.00	1.00000000	mice
Csgalnact2	-0.03	1.00000000	mice
Rere	-0.07	1.00000000	mice
Abhd8	-0.05	1.00000000	mice
Car2	0.25	1.00000000	mice
Ston2	0.00	1.00000000	mice
Lsm6	0.05	1.00000000	mice
Glcci1	-0.13	1.00000000	mice
B3galnt2	-0.06	1.00000000	mice
Kdelr2	0.38	1.00000000	mice
Hcfc1	0.02	1.00000000	mice
Hprt	-0.11	1.00000000	mice
Tspan14	-0.04	1.00000000	mice
Rnpep	0.01	1.00000000	mice
Lin7c	-0.12	1.00000000	mice
Kras	-0.03	1.00000000	mice
Top2b	-0.03	1.00000000	mice
Ezr	0.07	1.00000000	mice
Chic2	-0.05	1.00000000	mice
Pelp1	0.09	1.00000000	mice
Ccdc71l	-0.02	1.00000000	mice
Trip4	0.09	1.00000000	mice
Smim41	-0.02	1.00000000	mice
Hsd17b10	0.06	1.00000000	mice
Pogk	-0.08	1.00000000	mice
1110059E24Rik	0.07	1.00000000	mice
Ubl5	0.36	1.00000000	mice
Dym	-0.14	1.00000000	mice
Nfib	0.02	1.00000000	mice
Tecpr1	-0.06	1.00000000	mice
Acvrl1	-0.02	1.00000000	mice
Unkl	-0.03	1.00000000	mice

Rcn3	-0.02	1.00000000	mice
Kank2	-0.07	1.00000000	mice
Ube4b	-0.14	1.00000000	mice
9930021J03Rik	-0.09	1.00000000	mice
Zfp703	0.13	1.00000000	mice
Alyref	0.03	1.00000000	mice
Sdf2	0.11	1.00000000	mice
Tada2a	-0.05	1.00000000	mice
Cnot6	-0.12	1.00000000	mice
A430005L14Rik	0.14	1.00000000	mice
Dtx3	-0.03	1.00000000	mice
Wars	0.23	1.00000000	mice
Sh2b2	0.00	1.00000000	mice
Nudt21	-0.05	1.00000000	mice
Peg13	0.00	1.00000000	mice
Tuba1b	0.11	1.00000000	mice
Xkr4	-0.21	1.00000000	mice
Ro60	0.01	1.00000000	mice
Gpx8	0.13	1.00000000	mice
Dhx15	0.00	1.00000000	mice
Hmgb2	0.16	1.00000000	mice
Uqcrq	0.36	1.00000000	mice
Sf3b6	0.02	1.00000000	mice
Zfp362	0.03	1.00000000	mice
Pcolce	0.24	1.00000000	mice
Seh1l	0.05	1.00000000	mice
Pik3ap1	0.42	1.00000000	mice
Fip1l1	-0.03	1.00000000	mice
Arhgap18	-0.08	1.00000000	mice
Zfp91	0.21	1.00000000	mice
Fgfr4	0.38	1.00000000	mice
Fam222b	0.04	1.00000000	mice
Eri3	-0.07	1.00000000	mice
Oip5os1	-0.06	1.00000000	mice
Fblim1	-0.03	1.00000000	mice
Tmem131l	-0.08	1.00000000	mice
Ndfip1	0.11	1.00000000	mice
Ubxn6	0.04	1.00000000	mice
Kif3a	0.03	1.00000000	mice
Tank	0.12	1.00000000	mice
Zfp384	-0.09	1.00000000	mice
Dnajc10	0.08	1.00000000	mice
Enpep	0.36	1.00000000	mice
Chrdl1	-0.05	1.00000000	mice
Poc1b	-0.14	1.00000000	mice
Rad54l2	-0.11	1.00000000	mice

Akap8	0.01	1.00000000	mice
Tbx2	0.50	1.00000000	mice
Ptms	0.02	1.00000000	mice
Bbx	-0.02	1.00000000	mice
Asna1	0.11	1.00000000	mice
Ensa	-0.03	1.00000000	mice
Ing2	-0.01	1.00000000	mice
Tomm22	0.00	1.00000000	mice
Snw1	0.16	1.00000000	mice
H2afy	0.07	1.00000000	mice
C1s2	0.07	1.00000000	mice
Cacybp	0.06	1.00000000	mice
Zfas1	0.28	1.00000000	mice
Hectd4	-0.10	1.00000000	mice
Slc39a3	-0.01	1.00000000	mice
Ercc3	0.06	1.00000000	mice
Rhobtb1	-0.10	1.00000000	mice
Ubqln2	-0.06	1.00000000	mice
Ndufs7	-0.06	1.00000000	mice
Stk3	-0.04	1.00000000	mice
Grpel1	0.09	1.00000000	mice
Rce1	0.00	1.00000000	mice
Nckap1	0.02	1.00000000	mice
Herc2	-0.06	1.00000000	mice
Abce1	-0.03	1.00000000	mice
Leng1	-0.06	1.00000000	mice
Eif4e3	0.24	1.00000000	mice
Adam23	-0.03	1.00000000	mice
Gli2	-0.13	1.00000000	mice
Epb41	-0.16	1.00000000	mice
Ncam1	-0.13	1.00000000	mice
Slc25a5	0.04	1.00000000	mice
Resf1	0.37	1.00000000	mice
Phlpp1	-0.02	1.00000000	mice
Clmp	0.35	1.00000000	mice
Stt3b	-0.06	1.00000000	mice
Esam	-0.05	1.00000000	mice
Sf3a2	0.03	1.00000000	mice
Dhdds	-0.05	1.00000000	mice
Tcaf1	0.02	1.00000000	mice
Uba52	0.27	1.00000000	mice
Slc39a11	0.15	1.00000000	mice
Tulp3	0.02	1.00000000	mice
Aimp1	0.33	1.00000000	mice
Slc20a2	-0.05	1.00000000	mice
Ppie	-0.08	1.00000000	mice

1110038B12Rik	0.07	1.00000000	mice
Tmsb4x	-0.11	1.00000000	mice
Abhd4	-0.02	1.00000000	mice
2510002D24Rik	-0.05	1.00000000	mice
Tyw1	-0.07	1.00000000	mice
Arhgap29	0.12	1.00000000	mice
Zfp281	0.03	1.00000000	mice
Ndufs1	-0.09	1.00000000	mice
Igfbp4	-0.02	1.00000000	mice
Emg1	-0.05	1.00000000	mice
Calhm5	0.08	1.00000000	mice
Cachd1	-0.18	1.00000000	mice
Nr1d1	0.15	1.00000000	mice
Zdhhc5	-0.10	1.00000000	mice
Traf5	-0.01	1.00000000	mice
Soat1	-0.04	1.00000000	mice
Gemin5	0.07	1.00000000	mice
Ercc1	-0.09	1.00000000	mice
Herc1	-0.30	1.00000000	mice
Atf7	-0.12	1.00000000	mice
Crebzf	-0.09	1.00000000	mice
Eif3b	0.19	1.00000000	mice
Cdip1	-0.08	1.00000000	mice
Denr	0.29	1.00000000	mice
B230118H07Rik	-0.07	1.00000000	mice
Prdm4	-0.02	1.00000000	mice
Ecm1	-0.06	1.00000000	mice
Ctcf	-0.05	1.00000000	mice
Cbfa2t3	-0.05	1.00000000	mice
Fam104a	0.01	1.00000000	mice
Wbp1	0.00	1.00000000	mice
Dctn5	0.08	1.00000000	mice
Tmem237	-0.09	1.00000000	mice
Bmp5	0.36	1.00000000	mice
Rbbp9	0.01	1.00000000	mice
Coro1c	-0.12	1.00000000	mice
Smurf1	-0.10	1.00000000	mice
Slc30a7	-0.05	1.00000000	mice
4933421O10Rik	-0.06	1.00000000	mice
Tm9sf1	0.18	1.00000000	mice
Endog	-0.05	1.00000000	mice
Dync1li2	0.02	1.00000000	mice
Igsf8	0.16	1.00000000	mice
Ets1	0.04	1.00000000	mice
Tnfaip6	0.12	1.00000000	mice
Zc3h12c	-0.03	1.00000000	mice

Prorsd1	0.15	1.00000000	mice
Exosc3	0.09	1.00000000	mice
Nucb2	0.19	1.00000000	mice
Cish	-0.04	1.00000000	mice
Dennd4c	-0.03	1.00000000	mice
Vav3	-0.32	1.00000000	mice
Romo1	0.30	1.00000000	mice
1110065P20Rik	0.02	1.00000000	mice
Ccser2	0.03	1.00000000	mice
Lin52	-0.11	1.00000000	mice
Grik5	-0.08	1.00000000	mice
Col5a3	-0.03	1.00000000	mice
Cenpx	0.13	1.00000000	mice
Cers6	0.54	1.00000000	mice
Psmc2	0.29	1.00000000	mice
Dnajb6	-0.01	1.00000000	mice
Cap1	0.01	1.00000000	mice
Fbxo34	0.02	1.00000000	mice
Emp1	-0.20	1.00000000	mice
Kctd16	0.06	1.00000000	mice
Eif3l	-0.01	1.00000000	mice
Lrrc20	-0.02	1.00000000	mice
Bmpr1a	-0.05	1.00000000	mice
Itga8	-0.02	1.00000000	mice
Prrc1	0.07	1.00000000	mice
P3h3	-0.12	1.00000000	mice
Slc7a5	-0.03	1.00000000	mice
Rbm5	-0.07	1.00000000	mice
Ahnak2	-0.10	1.00000000	mice
Lcor	-0.05	1.00000000	mice
Baz1b	-0.06	1.00000000	mice
Tifa	0.03	1.00000000	mice
Bace1	0.04	1.00000000	mice
Bnip1	-0.08	1.00000000	mice
Zfp608	0.21	1.00000000	mice
Phc2	0.14	1.00000000	mice
Ncoa6	-0.11	1.00000000	mice
Apba1	-0.06	1.00000000	mice
Pim1	-0.04	1.00000000	mice
Fkbp3	0.02	1.00000000	mice
Nab1	0.03	1.00000000	mice
Drg1	-0.03	1.00000000	mice
2310033P09Rik	-0.07	1.00000000	mice
Atrx	0.08	1.00000000	mice
Adh5	0.02	1.00000000	mice
Isg20l2	-0.08	1.00000000	mice

Isyna1	0.19	1.00000000	mice
Utrn	-0.06	1.00000000	mice
Col6a2	-0.01	1.00000000	mice
Ech1	0.09	1.00000000	mice
Tnfrsf19	0.17	1.00000000	mice
Atp5f1	0.07	1.00000000	mice
C1qtnf2	0.04	1.00000000	mice
Idnk	0.10	1.00000000	mice
Upf3a	0.01	1.00000000	mice
Islr	-0.18	1.00000000	mice
Ebp	0.14	1.00000000	mice
Ndufa5	0.21	1.00000000	mice
Slc12a6	-0.20	1.00000000	mice
Gpi1	0.08	1.00000000	mice
Sh2b1	-0.03	1.00000000	mice
Kat6a	-0.11	1.00000000	mice
Zdhhc3	0.08	1.00000000	mice
Nip7	-0.02	1.00000000	mice
Cav2	0.01	1.00000000	mice
Nlgn2	0.14	1.00000000	mice
Sae1	0.05	1.00000000	mice
Aph1a	-0.05	1.00000000	mice
Hnrnpa1	0.09	1.00000000	mice
Kmt5b	-0.19	1.00000000	mice
Cspp1	0.05	1.00000000	mice
Ep300	-0.01	1.00000000	mice
Ccar1	-0.01	1.00000000	mice
Smtnl2	-0.05	1.00000000	mice
Fyco1	0.13	1.00000000	mice
Smim19	0.00	1.00000000	mice
Atp8b1	-0.14	1.00000000	mice
Dhps	0.00	1.00000000	mice
Hspg2	-0.06	1.00000000	mice
Ints3	0.05	1.00000000	mice
Kdm7a	0.01	1.00000000	mice
Set	0.33	1.00000000	mice
Fign	-0.10	1.00000000	mice
Cacna2d3	-0.17	1.00000000	mice
Palld	-0.06	1.00000000	mice
Csnk1g2	-0.02	1.00000000	mice
Armcx3	-0.15	1.00000000	mice
Yeats2	-0.04	1.00000000	mice
Gnl2	-0.02	1.00000000	mice
As3mt	0.00	1.00000000	mice
Nr1d2	-0.02	1.00000000	mice
Lsp1	0.12	1.00000000	mice

Rbm15	-0.03	1.00000000	mice
Pcdh18	0.23	1.00000000	mice
Fau	-0.03	1.00000000	mice
Nrbp2	-0.03	1.00000000	mice
Smarcd2	-0.07	1.00000000	mice
Alg14	-0.03	1.00000000	mice
Clk2	0.00	1.00000000	mice
Araf	-0.09	1.00000000	mice
Praf2	-0.05	1.00000000	mice
Cd40	-0.03	1.00000000	mice
Tax1bp3	-0.01	1.00000000	mice
Arpc2	0.03	1.00000000	mice
Smim20	0.16	1.00000000	mice
Gtf2h5	0.09	1.00000000	mice
Timm17b	0.00	1.00000000	mice
Hsd17b11	0.09	1.00000000	mice
Xpo7	-0.14	1.00000000	mice
Akt1	0.02	1.00000000	mice
Zfp830	0.00	1.00000000	mice
Tram2	0.13	1.00000000	mice
Lpin2	0.18	1.00000000	mice
Tmpo	0.01	1.00000000	mice
Exosc1	-0.06	1.00000000	mice
Cnnm2	-0.09	1.00000000	mice
Loxl2	-0.11	1.00000000	mice
Nabp2	0.06	1.00000000	mice
Ddit3	0.12	1.00000000	mice
Haus2	0.01	1.00000000	mice
Ndufa9	0.08	1.00000000	mice
Bmp2	0.14	1.00000000	mice
Dvl2	0.00	1.00000000	mice
Fndc1	-0.16	1.00000000	mice
Bmp2k	0.11	1.00000000	mice
Bmp3	-0.03	1.00000000	mice
Pdgfrl	-0.05	1.00000000	mice
Ubap1	-0.06	1.00000000	mice
Tmem175	-0.05	1.00000000	mice
Sidt2	-0.12	1.00000000	mice
Srgap1	0.06	1.00000000	mice
Grk2	0.02	1.00000000	mice
Snx16	-0.03	1.00000000	mice
Rheb	0.01	1.00000000	mice
Lrrc8b	0.05	1.00000000	mice
Cox7b	-0.01	1.00000000	mice
Enpp2	-0.32	1.00000000	mice
Las1l	-0.03	1.00000000	mice

Nsmce1	0.13	1.00000000	mice
Srp14	0.04	1.00000000	mice
Adsl	0.13	1.00000000	mice
Rp9	-0.09	1.00000000	mice
Fam173a	-0.03	1.00000000	mice
Calm1	0.30	1.00000000	mice
Dph6	0.11	1.00000000	mice
Cdkl2	-0.04	1.00000000	mice
Tsfm	0.08	1.00000000	mice
Snx2	0.21	1.00000000	mice
Tspan11	-0.12	1.00000000	mice
Hmg20a	-0.11	1.00000000	mice
Csrnp1	0.08	1.00000000	mice
Irf3	-0.10	1.00000000	mice
Rnf170	-0.08	1.00000000	mice
Tmem109	0.01	1.00000000	mice
Basp1	0.37	1.00000000	mice
Vav2	-0.07	1.00000000	mice
Dhx38	0.04	1.00000000	mice
Aldh9a1	-0.02	1.00000000	mice
Nkd1	-0.15	1.00000000	mice
Slc39a6	-0.05	1.00000000	mice
Vegfb	-0.04	1.00000000	mice
Emp3	0.34	1.00000000	mice
Pfn1	0.32	1.00000000	mice
Klhdc3	0.04	1.00000000	mice
Taz	0.01	1.00000000	mice
Blvrb	-0.08	1.00000000	mice
Slc36a2	0.26	1.00000000	mice
Rtn4rl1	-0.02	1.00000000	mice
Sec11a	0.05	1.00000000	mice
Chrac1	-0.04	1.00000000	mice
Il21r	-0.11	1.00000000	mice
Lamtor5	-0.01	1.00000000	mice
Sos1	-0.07	1.00000000	mice
Plod3	0.03	1.00000000	mice
Rab18	0.07	1.00000000	mice
Dse	0.14	1.00000000	mice
Ggh	0.00	1.00000000	mice
Kiz	0.01	1.00000000	mice
Synj1	0.01	1.00000000	mice
Sh3d21	-0.11	1.00000000	mice
Pamr1	-0.11	1.00000000	mice
Dnajc13	-0.06	1.00000000	mice
Ndufb7	0.34	1.00000000	mice
2210408F21Rik	-0.04	1.00000000	mice

Fbxo22	-0.03	1.00000000	mice
Inpp5f	-0.09	1.00000000	mice
Wnt11	-0.14	1.00000000	mice
Rab6a	-0.05	1.00000000	mice
Ccdc88a	-0.03	1.00000000	mice
Lonp2	-0.07	1.00000000	mice
Txlng	-0.08	1.00000000	mice
Esf1	-0.05	1.00000000	mice
Plpp3	-0.11	1.00000000	mice
AW554918	-0.13	1.00000000	mice
Clns1a	-0.09	1.00000000	mice
Pdcd10	-0.02	1.00000000	mice
Dvl1	-0.02	1.00000000	mice
Pum3	0.21	1.00000000	mice
Usp28	-0.02	1.00000000	mice
Hacd1	0.00	1.00000000	mice
Grk6	0.00	1.00000000	mice
Hnrnpll	-0.11	1.00000000	mice
Rbm14	-0.03	1.00000000	mice
Gsk3b	0.03	1.00000000	mice
Ppm1d	0.00	1.00000000	mice
Zfp354c	-0.07	1.00000000	mice
Snap29	0.07	1.00000000	mice
Lag3	0.06	1.00000000	mice
Wnt5b	0.02	1.00000000	mice
Gng2	0.20	1.00000000	mice
Reck	0.08	1.00000000	mice
Zfhx4	-0.05	1.00000000	mice
Coq7	0.17	1.00000000	mice
Prkd1	-0.15	1.00000000	mice
Psmd8	0.18	1.00000000	mice
H6pd	0.18	1.00000000	mice
Pin4	0.11	1.00000000	mice
C1qtnf7	-0.30	1.00000000	mice
Glt8d1	0.02	1.00000000	mice
Tesk1	-0.03	1.00000000	mice
Tnfaip1	-0.09	1.00000000	mice
Rsrc1	0.12	1.00000000	mice
Lpcat2	-0.10	1.00000000	mice
Snx12	0.03	1.00000000	mice
Adamts8	-0.09	1.00000000	mice
Prpf38a	-0.05	1.00000000	mice
Acadl	0.17	1.00000000	mice
Tatdn1	-0.01	1.00000000	mice
Tbc1d16	-0.05	1.00000000	mice
Rin2	0.20	1.00000000	mice

Asah2	0.09	1.00000000	mice
Nfyb	0.01	1.00000000	mice
Wls	-0.13	1.00000000	mice
Baiap2	0.03	1.00000000	mice
1110004F10Rik	0.31	1.00000000	mice
Psmd3	0.24	1.00000000	mice
Anxa1	-0.26	1.00000000	mice
Rbms2	-0.03	1.00000000	mice
Nsa2	-0.07	1.00000000	mice
Cyhr1	0.05	1.00000000	mice
Sox5	0.08	1.00000000	mice
Galk2	0.09	1.00000000	mice
Zfp106	0.04	1.00000000	mice
Timm29	0.02	1.00000000	mice
Hspa13	0.02	1.00000000	mice
Fyttd1	-0.10	1.00000000	mice
Polr2j	0.08	1.00000000	mice
Rcbtb1	0.04	1.00000000	mice
Rbm7	0.01	1.00000000	mice
Parp8	0.23	1.00000000	mice
Pgm2	-0.15	1.00000000	mice
Rngtt	0.20	1.00000000	mice
H2-Eb1	0.13	1.00000000	mice
Srpk1	-0.05	1.00000000	mice
Nvl	-0.08	1.00000000	mice
Tmem9	0.19	1.00000000	mice
Art4	0.07	1.00000000	mice
Senp1	-0.12	1.00000000	mice
Cttnbp2nl	-0.02	1.00000000	mice
Clcn3	-0.06	1.00000000	mice
Bdp1	0.02	1.00000000	mice
Itih5	0.04	1.00000000	mice
Sod2	0.38	1.00000000	mice
A730049H05Rik	0.01	1.00000000	mice
Glg1	-0.05	1.00000000	mice
Colgalt1	0.26	1.00000000	mice
Insig1	0.15	1.00000000	mice
Cdc42se1	0.13	1.00000000	mice
Slc16a1	0.20	1.00000000	mice
Pag1	0.28	1.00000000	mice
Ralb	0.25	1.00000000	mice
Prkch	0.05	1.00000000	mice
AI506816	-0.03	1.00000000	mice
Tug1	0.09	1.00000000	mice
Hmgn1	0.12	1.00000000	mice
Cdc42	0.11	1.00000000	mice

Atg16l2	0.00	1.00000000	mice
Spred2	-0.10	1.00000000	mice
Adamtsl2	-0.08	1.00000000	mice
Reep5	-0.10	1.00000000	mice
Ubald2	0.02	1.00000000	mice
0610010K14Rik	-0.18	1.00000000	mice
Pdpr	0.01	1.00000000	mice
Hspa4	0.02	1.00000000	mice
Tmem258	0.34	1.00000000	mice
Pgm2l1	-0.16	1.00000000	mice
Armcx1	-0.12	1.00000000	mice
Ube2i	0.01	1.00000000	mice
Pitpnc1	0.05	1.00000000	mice
Cops5	0.18	1.00000000	mice
Xpr1	-0.18	1.00000000	mice
Arpp19	0.26	1.00000000	mice
Rbsn	-0.01	1.00000000	mice
Cryab	0.00	1.00000000	mice
Actr3	0.03	1.00000000	mice
Pigk	0.07	1.00000000	mice
Trappc9	-0.12	1.00000000	mice
Caprin2	-0.24	1.00000000	mice
Gramd1a	0.04	1.00000000	mice
Rala	0.13	1.00000000	mice
Rras	-0.03	1.00000000	mice
Nup205	-0.07	1.00000000	mice
Hras	0.12	1.00000000	mice
Vps37a	0.02	1.00000000	mice
Tmem45a	0.13	1.00000000	mice
Chfr	-0.02	1.00000000	mice
Kcnma1	-0.03	1.00000000	mice
Fam174a	-0.01	1.00000000	mice
Cpxm1	0.30	1.00000000	mice
Lmna	-0.06	1.00000000	mice
Atxn2l	0.01	1.00000000	mice
Hadhb	0.02	1.00000000	mice
Gsto1	-0.07	1.00000000	mice
Atf1	-0.05	1.00000000	mice
Zfp536	-0.23	1.00000000	mice
Clcn4	-0.03	1.00000000	mice
Tnip1	0.10	1.00000000	mice
Efemp2	0.26	1.00000000	mice
Pus7	-0.07	1.00000000	mice
Prkra	0.11	1.00000000	mice
Acvr1	-0.12	1.00000000	mice
Psmd4	0.23	1.00000000	mice

Vps13d	0.02	1.00000000	mice
Hars	0.02	1.00000000	mice
Chmp7	0.00	1.00000000	mice
Ola1	0.20	1.00000000	mice
Tmem248	-0.11	1.00000000	mice
Cr1l	-0.11	1.00000000	mice
Cdc42bpg	-0.10	1.00000000	mice
Ebag9	0.09	1.00000000	mice
Fgf18	-0.03	1.00000000	mice
Pdlim1	-0.04	1.00000000	mice
Agk	-0.04	1.00000000	mice
Camkmt	-0.20	1.00000000	mice
Cct5	0.17	1.00000000	mice
Pds5a	-0.10	1.00000000	mice
Deaf1	-0.03	1.00000000	mice
Psme3	-0.05	1.00000000	mice
Yod1	-0.07	1.00000000	mice
Sec61a2	-0.07	1.00000000	mice
Tle4	-0.19	1.00000000	mice
Psen1	-0.02	1.00000000	mice
Fabp3	-0.09	1.00000000	mice
Icmt	-0.03	1.00000000	mice
Igsf3	-0.02	1.00000000	mice
Elob	0.32	1.00000000	mice
Foxk1	0.09	1.00000000	mice
Acin1	-0.06	1.00000000	mice
Fam162a	-0.13	1.00000000	mice
Otud5	0.14	1.00000000	mice
Polr1a	-0.01	1.00000000	mice
Bola1	0.09	1.00000000	mice
Kctd2	-0.09	1.00000000	mice
Trmt1	0.01	1.00000000	mice
Cdk19	0.01	1.00000000	mice
Snd1	0.02	1.00000000	mice
Gyg	0.64	1.00000000	mice
Ralgds	0.11	1.00000000	mice
Plxdc2	0.09	1.00000000	mice
Cbr3	0.02	1.00000000	mice
Uba3	0.15	1.00000000	mice
Itpripl2	0.19	1.00000000	mice
Izumo4	0.01	1.00000000	mice
Chchd2	0.07	1.00000000	mice
Dzip3	0.08	1.00000000	mice
Eef1g	0.07	1.00000000	mice
Cuedc2	0.00	1.00000000	mice
Arap1	-0.01	1.00000000	mice

Pdcd5	-0.07	1.00000000	mice
Sri	-0.04	1.00000000	mice
Ttc4	0.23	1.00000000	mice
Taf11	-0.07	1.00000000	mice
Chpf2	0.07	1.00000000	mice
Osbpl9	-0.10	1.00000000	mice
Pou2f2	-0.10	1.00000000	mice
Cox5b	0.28	1.00000000	mice
Xrn1	0.15	1.00000000	mice
Ints14	-0.10	1.00000000	mice
Chn1	0.19	1.00000000	mice
Ndufb6	0.06	1.00000000	mice
Esco1	-0.06	1.00000000	mice
Luc7l3	0.00	1.00000000	mice
Cdk10	-0.09	1.00000000	mice
Tram1	0.16	1.00000000	mice
Ap3s2	0.08	1.00000000	mice
Aldh7a1	-0.13	1.00000000	mice
Snx13	0.14	1.00000000	mice
Ccdc66	-0.01	1.00000000	mice
Aacs	-0.01	1.00000000	mice
Srp54a	0.05	1.00000000	mice
Rnf141	-0.01	1.00000000	mice
Nol7	0.02	1.00000000	mice
Gata6	-0.17	1.00000000	mice
Eno1	-0.02	1.00000000	mice
Csnk2b	0.16	1.00000000	mice
Csnk2a2	-0.01	1.00000000	mice
Sgms2	0.10	1.00000000	mice
Trabd	-0.02	1.00000000	mice
Trerf1	0.08	1.00000000	mice
Rbfox2	-0.09	1.00000000	mice
Grk5	0.13	1.00000000	mice
R3hdm4	0.05	1.00000000	mice
Rwdd1	0.25	1.00000000	mice
Eif2ak4	-0.05	1.00000000	mice
Pex13	-0.01	1.00000000	mice
Lgmn	0.33	1.00000000	mice
Aprt	0.18	1.00000000	mice
Aopep	0.31	1.00000000	mice
Ergic2	-0.05	1.00000000	mice
Sp1	-0.02	1.00000000	mice
Prpf31	-0.05	1.00000000	mice
Bmp1	-0.12	1.00000000	mice
Rasgef1b	-0.21	1.00000000	mice
Asnsd1	0.14	1.00000000	mice

Wdr83os	-0.09	1.00000000	mice
Wdr33	-0.09	1.00000000	mice
Nubp1	0.12	1.00000000	mice
Brpf1	-0.02	1.00000000	mice
Cep350	0.04	1.00000000	mice
Cd200	0.25	1.00000000	mice
Ss18l2	0.01	1.00000000	mice
Acvr2a	0.16	1.00000000	mice
Efna5	-0.12	1.00000000	mice
Arsk	0.01	1.00000000	mice
Vamp8	0.08	1.00000000	mice
Zdhhc17	-0.08	1.00000000	mice
Cst3	-0.25	1.00000000	mice
Kcne4	-0.25	1.00000000	mice
2610507B11Rik	0.21	1.00000000	mice
Tmem147	0.04	1.00000000	mice
Emc6	0.00	1.00000000	mice
Prr3	0.06	1.00000000	mice
Psmg2	-0.07	1.00000000	mice
Eif4ebp2	-0.12	1.00000000	mice
Trim47	0.12	1.00000000	mice
Plekhm3	0.12	1.00000000	mice
Ctnnd2	0.17	1.00000000	mice
Ick	-0.08	1.00000000	mice
Bsg	0.31	1.00000000	mice
Hadh	0.13	1.00000000	mice
Apbb1ip	0.18	1.00000000	mice
Wipf1	-0.03	1.00000000	mice
Ttc27	-0.04	1.00000000	mice
Wrnip1	-0.02	1.00000000	mice
Pcnt	-0.10	1.00000000	mice
Hspd1	0.16	1.00000000	mice
Tsn	0.00	1.00000000	mice
Rfc2	0.10	1.00000000	mice
Snx9	0.02	1.00000000	mice
Pdcd11	0.09	1.00000000	mice
Usp24	-0.03	1.00000000	mice
N4bp1	0.19	1.00000000	mice
Tbl1xr1	-0.14	1.00000000	mice
Actn4	0.06	1.00000000	mice
Ppfia1	0.02	1.00000000	mice
Usf3	-0.08	1.00000000	mice
Cmklr1	0.27	1.00000000	mice
Pole4	-0.02	1.00000000	mice
Pcid2	-0.14	1.00000000	mice
Slain2	-0.01	1.00000000	mice

Hdac2	-0.04	1.00000000	mice
Nfe2l2	0.16	1.00000000	mice
Tbx5	0.15	1.00000000	mice
Slc35a3	-0.03	1.00000000	mice
Poldip2	0.10	1.00000000	mice
Cse1l	-0.07	1.00000000	mice
Pus1	0.00	1.00000000	mice
Npat	-0.04	1.00000000	mice
Htra1	-0.05	1.00000000	mice
Psmb7	-0.06	1.00000000	mice
Palm	0.08	1.00000000	mice
Plekhh2	-0.08	1.00000000	mice
Cwc15	0.05	1.00000000	mice
Rara	0.11	1.00000000	mice
2610307P16Rik	0.03	1.00000000	mice
Psmc4	0.17	1.00000000	mice
Shroom3	-0.19	1.00000000	mice
Sltm	0.01	1.00000000	mice
Ints6l	-0.01	1.00000000	mice
Urgcp	-0.06	1.00000000	mice
Sugct	-0.19	1.00000000	mice
Fhod3	0.00	1.00000000	mice
Usp10	0.06	1.00000000	mice
Arfip1	0.10	1.00000000	mice
Abcc1	-0.06	1.00000000	mice
Tcn2	0.05	1.00000000	mice
Bet1	0.16	1.00000000	mice
Ddx56	0.01	1.00000000	mice
Syvn1	-0.03	1.00000000	mice
Arl4c	-0.16	1.00000000	mice
Cpped1	0.03	1.00000000	mice
Zfp503	0.15	1.00000000	mice
Supt3	-0.05	1.00000000	mice
Cacna1d	0.24	1.00000000	mice
Ebpl	-0.08	1.00000000	mice
AI837181	0.15	1.00000000	mice
Sra1	0.12	1.00000000	mice
Abca9	-0.07	1.00000000	mice
Cog7	0.02	1.00000000	mice
Rell1	-0.05	1.00000000	mice
Cep120	-0.06	1.00000000	mice
Dmtf1	0.07	1.00000000	mice
Ube2e3	0.00	1.00000000	mice
Tiprl	0.07	1.00000000	mice
Tmx3	-0.08	1.00000000	mice
Wdr20	-0.07	1.00000000	mice

Arhgap12	-0.12	1.00000000	mice
Gatad2a	-0.09	1.00000000	mice
Ahi1	-0.11	1.00000000	mice
Psmc6	-0.01	1.00000000	mice
2900097C17Rik	0.24	1.00000000	mice
Atxn7l3b	0.01	1.00000000	mice
Fam32a	0.01	1.00000000	mice
Ap3s1	0.07	1.00000000	mice
1110059G10Rik	0.08	1.00000000	mice
Rap2c	0.12	1.00000000	mice
Nfyc	-0.05	1.00000000	mice
Hipk2	-0.13	1.00000000	mice
Srf	0.00	1.00000000	mice
Nav1	-0.18	1.00000000	mice
Tmem9b	-0.01	1.00000000	mice
Socs5	0.04	1.00000000	mice
Ext1	-0.04	1.00000000	mice
Lrrc49	-0.14	1.00000000	mice
Uaca	0.16	1.00000000	mice
Chmp3	0.07	1.00000000	mice
Ralgapb	-0.03	1.00000000	mice
Styx	-0.11	1.00000000	mice
Npnt	0.42	1.00000000	mice
Zmpste24	0.14	1.00000000	mice
Ciao2a	-0.06	1.00000000	mice
C1qbp	0.06	1.00000000	mice
Ier5l	-0.12	1.00000000	mice
Psmd12	0.09	1.00000000	mice
Stk39	0.03	1.00000000	mice
Sfxn3	-0.07	1.00000000	mice
Dedd	0.04	1.00000000	mice
Kdm2b	-0.05	1.00000000	mice
Kcnq5	0.60	1.00000000	mice
Lmf2	0.04	1.00000000	mice
Paics	0.00	1.00000000	mice
Otud6b	0.00	1.00000000	mice
Dnajc5	-0.04	1.00000000	mice
Cdkn1b	-0.04	1.00000000	mice
Osbp	-0.07	1.00000000	mice
Ar	-0.05	1.00000000	mice
Ccl19	0.22	1.00000000	mice
Ogn	0.14	1.00000000	mice
Cdk2ap1	0.12	1.00000000	mice
Llgl1	0.02	1.00000000	mice
Cep295	0.02	1.00000000	mice
Ago3	-0.09	1.00000000	mice

Hspa4l	-0.05	1.00000000	mice
Cactin	0.04	1.00000000	mice
Wipi1	0.03	1.00000000	mice
Tmem19	0.01	1.00000000	mice
Usp53	-0.12	1.00000000	mice
Rab43	-0.14	1.00000000	mice
Cdc42ep4	-0.01	1.00000000	mice
Kansl1l	-0.12	1.00000000	mice
Rbm28	-0.01	1.00000000	mice
Phc1	0.01	1.00000000	mice
Ide	0.05	1.00000000	mice
Pdzd8	-0.09	1.00000000	mice
Ndufaf7	-0.02	1.00000000	mice
Apba3	-0.05	1.00000000	mice
Ssbp2	-0.03	1.00000000	mice
Ddx54	0.16	1.00000000	mice
Dcaf13	-0.09	1.00000000	mice
Fbxo33	-0.04	1.00000000	mice
Atxn1	-0.09	1.00000000	mice
Lmtk2	-0.12	1.00000000	mice
Dock5	0.00	1.00000000	mice
Ppp2r5d	0.04	1.00000000	mice
Tent2	0.12	1.00000000	mice
Wdr45b	-0.06	1.00000000	mice
B4galt5	0.23	1.00000000	mice
Tmem245	-0.01	1.00000000	mice
Pou2f1	-0.04	1.00000000	mice
Smad6	-0.12	1.00000000	mice
Aox1	0.06	1.00000000	mice
Psmd10	0.14	1.00000000	mice
R3hcc1l	-0.09	1.00000000	mice
Pbdc1	-0.03	1.00000000	mice
Srp19	0.16	1.00000000	mice
Dnttip1	0.00	1.00000000	mice
Opa1	0.02	1.00000000	mice
Zkscan1	0.10	1.00000000	mice
Fam129b	-0.02	1.00000000	mice
Clic1	0.14	1.00000000	mice
Gpsm3	0.17	1.00000000	mice
Relb	-0.03	1.00000000	mice
Polr1c	0.02	1.00000000	mice
Dap	-0.03	1.00000000	mice
Snx4	-0.04	1.00000000	mice
Orc3	0.12	1.00000000	mice
Arl1	0.00	1.00000000	mice
Galnt11	-0.03	1.00000000	mice

Swap70	-0.02	1.00000000	mice
Thumpd3	-0.08	1.00000000	mice
Nrbf2	-0.03	1.00000000	mice
Ofd1	0.02	1.00000000	mice
Bicc1	-0.04	1.00000000	mice
Lmo4	0.30	1.00000000	mice
Tmem132c	-0.02	1.00000000	mice
Cox6a1	-0.07	1.00000000	mice
Bod1l	-0.02	1.00000000	mice
Apip	0.20	1.00000000	mice
Fam118a	-0.02	1.00000000	mice
S100a1	-0.01	1.00000000	mice
Wdr74	-0.10	1.00000000	mice
Fam20a	0.02	1.00000000	mice
Gstz1	-0.01	1.00000000	mice
Adipor2	-0.05	1.00000000	mice
Dach1	0.12	1.00000000	mice
Pik3ip1	0.00	1.00000000	mice
Akt1s1	0.02	1.00000000	mice
Caap1	0.05	1.00000000	mice
Ift80	0.02	1.00000000	mice
Acd	0.00	1.00000000	mice
Tpcn1	-0.05	1.00000000	mice
Txnl4a	0.03	1.00000000	mice
Trmt11	0.09	1.00000000	mice
Tlcd2	-0.02	1.00000000	mice
Pde4a	0.07	1.00000000	mice
Cct2	0.04	1.00000000	mice
Cd2bp2	0.08	1.00000000	mice
Srbd1	0.03	1.00000000	mice
Trak2	-0.09	1.00000000	mice
Cdon	-0.01	1.00000000	mice
Vps13a	-0.12	1.00000000	mice
Nemf	-0.06	1.00000000	mice
Inafm2	0.05	1.00000000	mice
Cdyl2	-0.01	1.00000000	mice
Ywhah	0.06	1.00000000	mice
Ptpmt1	0.02	1.00000000	mice
Iars2	0.00	1.00000000	mice
Arpc5l	0.02	1.00000000	mice
Nexn	0.15	1.00000000	mice
Katna1	-0.04	1.00000000	mice
Prdx3	0.05	1.00000000	mice
Aldh6a1	0.01	1.00000000	mice
Wdr35	-0.01	1.00000000	mice
Slc4a2	-0.02	1.00000000	mice

Dchs1	-0.06	1.00000000	mice
Fem1b	-0.04	1.00000000	mice
Pex6	-0.08	1.00000000	mice
Bdnf	-0.04	1.00000000	mice
Fgfr3	0.04	1.00000000	mice
Letm1	0.11	1.00000000	mice
Tril	0.07	1.00000000	mice
Plat	0.05	1.00000000	mice
Elk3	0.01	1.00000000	mice
Gramd4	0.00	1.00000000	mice
Pinx1	0.12	1.00000000	mice
Pou3f1	-0.13	1.00000000	mice
Atp5h	0.11	1.00000000	mice
Sh3rf3	-0.03	1.00000000	mice
Zbtb7a	-0.09	1.00000000	mice
Fbxw7	-0.12	1.00000000	mice
Plvap	0.02	1.00000000	mice
Iqsec1	0.07	1.00000000	mice
Aar2	0.12	1.00000000	mice
Acadvl	0.09	1.00000000	mice
Ythdf2	0.13	1.00000000	mice
Lrch1	0.15	1.00000000	mice
Ahsa2	-0.01	1.00000000	mice
Cluh	-0.12	1.00000000	mice
Dock11	-0.06	1.00000000	mice
Hif1a	0.21	1.00000000	mice
E130307A14Rik	-0.03	1.00000000	mice
Pabpc4	-0.06	1.00000000	mice
Tceal9	0.22	1.00000000	mice
Nck1	0.16	1.00000000	mice
Xrcc4	-0.03	1.00000000	mice
Gcn1	0.03	1.00000000	mice
Ndufs5	0.33	1.00000000	mice
Specc1	0.05	1.00000000	mice
Tmem243	-0.09	1.00000000	mice
Ror2	-0.15	1.00000000	mice
Tgm2	0.32	1.00000000	mice
Kdm2a	-0.10	1.00000000	mice
Ptk7	0.23	1.00000000	mice
Narf	0.00	1.00000000	mice
Idh1	0.08	1.00000000	mice
Cdk4	0.07	1.00000000	mice
Dnajc7	0.06	1.00000000	mice
Bcr	-0.12	1.00000000	mice
Ubqln1	-0.06	1.00000000	mice
Ppp1r15b	-0.01	1.00000000	mice

Fuom	0.06	1.00000000	mice
Bet1l	-0.04	1.00000000	mice
Tmed9	0.31	1.00000000	mice
Col27a1	-0.06	1.00000000	mice
Lpcat1	0.03	1.00000000	mice
Etnk1	0.17	1.00000000	mice
Specc1l	0.11	1.00000000	mice
Sec13	0.24	1.00000000	mice
Fam98b	0.04	1.00000000	mice
Calcoco1	0.00	1.00000000	mice
Uba1	0.11	1.00000000	mice
Ubap2l	-0.03	1.00000000	mice
Jph1	-0.14	1.00000000	mice
Zkscan5	-0.03	1.00000000	mice
Foxk2	-0.06	1.00000000	mice
Ppig	0.04	1.00000000	mice
Ubap2	-0.05	1.00000000	mice
Tmod3	-0.05	1.00000000	mice
Add3	-0.08	1.00000000	mice
Serinc5	0.00	1.00000000	mice
Dctn3	-0.01	1.00000000	mice
Jph2	-0.06	1.00000000	mice
Ankrd50	-0.03	1.00000000	mice
Pcmtd1	0.00	1.00000000	mice
Impact	0.06	1.00000000	mice
Diablo	0.01	1.00000000	mice
Cers4	-0.01	1.00000000	mice
Il10rb	-0.02	1.00000000	mice
S100pbp	-0.12	1.00000000	mice
Polb	-0.05	1.00000000	mice
Pkm	0.06	1.00000000	mice
Tm7sf3	-0.06	1.00000000	mice
Plxnd1	0.15	1.00000000	mice
Nipsnap3b	-0.06	1.00000000	mice
Zfand2a	0.08	1.00000000	mice
Sin3a	-0.09	1.00000000	mice
Strn4	0.00	1.00000000	mice
Fxyd5	0.24	1.00000000	mice
Ptprg	-0.05	1.00000000	mice
Ppat	-0.02	1.00000000	mice
Bcor	0.08	1.00000000	mice
Slc7a2	0.23	1.00000000	mice
Efhd2	0.31	1.00000000	mice
Adamts9	-0.31	1.00000000	mice
G3bp2	0.25	1.00000000	mice
Rnf220	0.11	1.00000000	mice

Spry4	-0.07	1.00000000	mice
Plaat3	0.16	1.00000000	mice
Atg16l1	-0.12	1.00000000	mice
Pfn2	0.05	1.00000000	mice
Capns1	0.13	1.00000000	mice
Rel	0.05	1.00000000	mice
Nabp1	-0.02	1.00000000	mice
Jup	-0.01	1.00000000	mice
Scpep1	-0.05	1.00000000	mice
Icam1	-0.11	1.00000000	mice
Ndufs8	-0.01	1.00000000	mice
Eef1akmt1	-0.04	1.00000000	mice
Rbms1	0.04	1.00000000	mice
Dot1l	-0.03	1.00000000	mice
Gucy1b1	0.36	1.00000000	mice
Usf2	-0.06	1.00000000	mice
Kdsr	-0.02	1.00000000	mice
Rbm26	-0.10	1.00000000	mice
Sgce	0.04	1.00000000	mice
Slc38a9	-0.07	1.00000000	mice
2310039H08Rik	0.08	1.00000000	mice
Dynlt3	0.04	1.00000000	mice
Gfpt1	-0.06	1.00000000	mice
Lrif1	0.05	1.00000000	mice
Elovl5	-0.02	1.00000000	mice
Axin2	0.14	1.00000000	mice
Sec61a1	0.19	1.00000000	mice
Snrnp27	0.19	1.00000000	mice
1810055G02Rik	-0.02	1.00000000	mice
Kbtbd2	0.06	1.00000000	mice
Fat3	0.23	1.00000000	mice
Pptc7	-0.01	1.00000000	mice
Rsbn1	-0.04	1.00000000	mice
Dbnl	0.17	1.00000000	mice
Selenoi	0.02	1.00000000	mice
Ptgfrn	-0.10	1.00000000	mice
Hmg20b	0.21	1.00000000	mice
Zfp821	-0.02	1.00000000	mice
Rabepk	-0.11	1.00000000	mice
Snrpa1	0.16	1.00000000	mice
Ece1	-0.35	1.00000000	mice
Rnf166	0.04	1.00000000	mice
Sec14l1	0.09	1.00000000	mice
Vgll3	0.07	1.00000000	mice
Vamp3	0.02	1.00000000	mice
Gart	0.00	1.00000000	mice

Pik3r4	-0.05	1.00000000	mice
Tlr4	-0.06	1.00000000	mice
Ndufc2	0.11	1.00000000	mice
Prkci	-0.03	1.00000000	mice
Rbfox1	-0.09	1.00000000	mice
Arhgef2	-0.10	1.00000000	mice
Sirt2	0.08	1.00000000	mice
Vamp4	0.03	1.00000000	mice
Pom121	-0.04	1.00000000	mice
A230057D06Rik	0.27	1.00000000	mice
Elmod2	0.09	1.00000000	mice
Ramp2	0.15	1.00000000	mice
Rtraf	0.14	1.00000000	mice
Cyp20a1	0.08	1.00000000	mice
Ndufs6	0.11	1.00000000	mice
Ppfibp2	0.11	1.00000000	mice
Taf4	0.00	1.00000000	mice
Ippk	-0.04	1.00000000	mice
Cd14	-0.03	1.00000000	mice
Asb3	-0.11	1.00000000	mice
Psmb6	0.07	1.00000000	mice
Ndn	-0.07	1.00000000	mice
Phrf1	0.05	1.00000000	mice
Rrs1	-0.04	1.00000000	mice
Tfam	-0.02	1.00000000	mice
Ldha	0.09	1.00000000	mice
Asxl2	0.23	1.00000000	mice
Arpc4	0.03	1.00000000	mice
Cnih1	0.00	1.00000000	mice
Gnl3l	-0.04	1.00000000	mice
Slc25a51	-0.03	1.00000000	mice
Tmco3	-0.08	1.00000000	mice
Gemin7	0.10	1.00000000	mice
Pde1a	-0.01	1.00000000	mice
Pcnx3	-0.05	1.00000000	mice
Sdcbp	0.14	1.00000000	mice
Fam214b	0.02	1.00000000	mice
Psmg1	-0.11	1.00000000	mice
Polr2f	0.00	1.00000000	mice
Il16	0.05	1.00000000	mice
Cdk2ap2	0.03	1.00000000	mice
Enpp1	-0.02	1.00000000	mice
Klhl18	-0.01	1.00000000	mice
Suz12	-0.07	1.00000000	mice
Fam102a	0.05	1.00000000	mice
Lars	0.18	1.00000000	mice

Wars2	0.02	1.00000000	mice
Tmem234	-0.03	1.00000000	mice
Scara5	-0.25	1.00000000	mice
Farsb	0.01	1.00000000	mice
Ugp2	0.13	1.00000000	mice
Ciao2b	0.20	1.00000000	mice
Dync1li1	-0.06	1.00000000	mice
Dstyk	0.05	1.00000000	mice
Nars2	-0.09	1.00000000	mice
Tab3	-0.08	1.00000000	mice
Nin	-0.07	1.00000000	mice
Rabgap1	0.01	1.00000000	mice
Rhbdl3	-0.13	1.00000000	mice
Col6a1	-0.05	1.00000000	mice
Klf5	-0.03	1.00000000	mice
Rae1	0.14	1.00000000	mice
Aspscr1	0.12	1.00000000	mice
Kifap3	-0.09	1.00000000	mice
Cd109	-0.07	1.00000000	mice
Cebpg	-0.03	1.00000000	mice
Lamtor1	0.11	1.00000000	mice
Agpat1	0.05	1.00000000	mice
Plek	0.11	1.00000000	mice
Zbtb8os	0.00	1.00000000	mice
Ikzf5	0.02	1.00000000	mice
Cdk5rap3	0.16	1.00000000	mice
Smim7	0.04	1.00000000	mice
Nuak1	0.14	1.00000000	mice
Efl1	-0.03	1.00000000	mice
Fbxo6	-0.05	1.00000000	mice
Spg7	0.20	1.00000000	mice
Ctbp2	-0.05	1.00000000	mice
Sipa1	-0.06	1.00000000	mice
Pde4dip	-0.08	1.00000000	mice
Pdha1	-0.03	1.00000000	mice
Thoc7	0.02	1.00000000	mice
9530068E07Rik	0.27	1.00000000	mice
Hmces	0.05	1.00000000	mice
Znhit3	0.01	1.00000000	mice
Klhl5	0.16	1.00000000	mice
Rtf1	-0.05	1.00000000	mice
Tshz2	-0.18	1.00000000	mice
Epha3	-0.05	1.00000000	mice
Sar1b	0.02	1.00000000	mice
Cmpk1	0.07	1.00000000	mice
Itch	0.05	1.00000000	mice

Kif2a	0.00	1.00000000	mice
Ercc6l2	0.12	1.00000000	mice
Cyfip1	0.00	1.00000000	mice
Papola	0.02	1.00000000	mice
Ppm1m	0.12	1.00000000	mice
Rtcb	0.05	1.00000000	mice
Eogt	0.08	1.00000000	mice
Scoc	0.07	1.00000000	mice
Cfap298	0.01	1.00000000	mice
Gxylt2	0.00	1.00000000	mice
Cacfd1	0.02	1.00000000	mice
Lcorl	-0.17	1.00000000	mice
Pkig	0.06	1.00000000	mice
Fmnl2	-0.05	1.00000000	mice
Dnajc8	0.34	1.00000000	mice
Vps33a	-0.05	1.00000000	mice
Fance	-0.08	1.00000000	mice
Cdc26	0.16	1.00000000	mice
Nudcd1	-0.01	1.00000000	mice
Zbtb1	0.01	1.00000000	mice
Nmd3	-0.03	1.00000000	mice
Usp3	-0.04	1.00000000	mice
Cradd	-0.06	1.00000000	mice
Sec24c	0.06	1.00000000	mice
Nudt4	-0.03	1.00000000	mice
Pex2	0.14	1.00000000	mice
Ciao1	0.07	1.00000000	mice
Tradd	0.10	1.00000000	mice
Sumf1	0.16	1.00000000	mice
Fbxo3	0.11	1.00000000	mice
Gpatch11	-0.06	1.00000000	mice
Hsd17b4	-0.10	1.00000000	mice
Cdadc1	-0.04	1.00000000	mice
Hopx	0.07	1.00000000	mice
Antxr2	-0.06	1.00000000	mice
Vps28	0.02	1.00000000	mice
Ppp1r14a	0.08	1.00000000	mice
Pnkp	0.08	1.00000000	mice
Pitpnm2	-0.05	1.00000000	mice
Tcf21	0.06	1.00000000	mice
Ero1l	-0.02	1.00000000	mice
Actr8	-0.05	1.00000000	mice
Sacm1l	-0.01	1.00000000	mice
Orai3	0.07	1.00000000	mice
Psma3	0.26	1.00000000	mice
Lpgat1	0.13	1.00000000	mice

Hltf	-0.02	1.00000000	mice
Tmem126a	0.09	1.00000000	mice
Scap	-0.11	1.00000000	mice
Ube2q2	0.02	1.00000000	mice
Sfxn1	0.06	1.00000000	mice
Sntg2	-0.13	1.00000000	mice
Eif4e2	0.19	1.00000000	mice
Phax	-0.06	1.00000000	mice
Tm2d3	-0.03	1.00000000	mice
Nr1h3	0.30	1.00000000	mice
Slc27a3	0.17	1.00000000	mice
Lemd2	0.03	1.00000000	mice
Dclk1	-0.13	1.00000000	mice
Spata13	-0.14	1.00000000	mice
St3gal5	-0.05	1.00000000	mice
Dbi	-0.03	1.00000000	mice
Cd302	-0.04	1.00000000	mice
Zfp322a	0.04	1.00000000	mice
Dock2	-0.08	1.00000000	mice
Smim27	0.18	1.00000000	mice
Trim24	-0.14	1.00000000	mice
Sept11	0.26	1.00000000	mice
Hsd17b7	-0.04	1.00000000	mice
Selenof	0.39	1.00000000	mice
Ndufaf4	0.11	1.00000000	mice
Rcor3	0.04	1.00000000	mice
Eef2k	-0.01	1.00000000	mice
Arfgef1	-0.04	1.00000000	mice
Ppp3cb	0.08	1.00000000	mice
Cnot3	-0.03	1.00000000	mice
Secisbp2l	0.04	1.00000000	mice
Hdac5	-0.03	1.00000000	mice
Sf3b2	0.07	1.00000000	mice
Ndufa10	0.02	1.00000000	mice
AU020206	0.06	1.00000000	mice
Clta	0.15	1.00000000	mice
Lamtor4	0.08	1.00000000	mice
Fdps	0.26	1.00000000	mice
Cept1	0.14	1.00000000	mice
Cltb	0.04	1.00000000	mice
Git1	0.13	1.00000000	mice
Kat7	-0.05	1.00000000	mice
Vps35l	0.08	1.00000000	mice
Chsy3	0.05	1.00000000	mice
Dipk1a	0.15	1.00000000	mice
Erp44	0.23	1.00000000	mice

Eif2b3	0.05	1.00000000	mice
Commd10	0.20	1.00000000	mice
8430419K02Rik	-0.06	1.00000000	mice
Elf1	-0.01	1.00000000	mice
Upf1	0.04	1.00000000	mice
Sel1l	0.06	1.00000000	mice
Ednra	0.03	1.00000000	mice
Ltn1	0.03	1.00000000	mice
Optn	0.08	1.00000000	mice
Sh3kbp1	0.04	1.00000000	mice
Ddhd1	-0.07	1.00000000	mice
Ubr4	0.04	1.00000000	mice
Pja2	-0.04	1.00000000	mice
Plod2	0.18	1.00000000	mice
Hmox1	-0.20	1.00000000	mice
Pip4k2b	-0.10	1.00000000	mice
Zfp646	-0.01	1.00000000	mice
Plin3	-0.01	1.00000000	mice
Skap2	0.00	1.00000000	mice
Ctnnbip1	0.01	1.00000000	mice
Eea1	-0.07	1.00000000	mice
Ppl	-0.06	1.00000000	mice
Paip1	0.06	1.00000000	mice
Ddx10	-0.02	1.00000000	mice
Btbd3	0.07	1.00000000	mice
Relch	0.06	1.00000000	mice
Ano6	-0.06	1.00000000	mice
Timm44	0.13	1.00000000	mice
Scaf11	0.11	1.00000000	mice
Nit2	-0.05	1.00000000	mice
Thoc5	-0.02	1.00000000	mice
Nudt3	0.01	1.00000000	mice
Plekhf2	0.08	1.00000000	mice
Arpc5	0.23	1.00000000	mice
Zfyve21	0.02	1.00000000	mice
Tmem214	-0.03	1.00000000	mice
Gpcpd1	-0.11	1.00000000	mice
Rbm17	0.01	1.00000000	mice
Arhgef5	0.03	1.00000000	mice
Adgrl3	0.07	1.00000000	mice
Timmdc1	0.09	1.00000000	mice
Sec23b	-0.03	1.00000000	mice
Adcy6	-0.05	1.00000000	mice
Bloc1s1	-0.06	1.00000000	mice
Tmed10	0.29	1.00000000	mice
Slu7	0.10	1.00000000	mice

Pdk3	0.07	1.00000000	mice
Selenot	0.23	1.00000000	mice
Ndufaf2	-0.06	1.00000000	mice
Arsj	0.00	1.00000000	mice
Zfand3	0.03	1.00000000	mice
Coq2	0.10	1.00000000	mice
Faf1	-0.01	1.00000000	mice
Sp2	0.02	1.00000000	mice
Alms1	-0.04	1.00000000	mice
Exoc3	0.10	1.00000000	mice
Crtap	0.01	1.00000000	mice
2410006H16Rik	0.33	1.00000000	mice
Fer	-0.07	1.00000000	mice
Ftx	-0.02	1.00000000	mice
Rnf138	-0.03	1.00000000	mice
Abhd5	0.12	1.00000000	mice
Tlk2	0.07	1.00000000	mice
Shoc2	0.14	1.00000000	mice
Nbn	0.02	1.00000000	mice
Arl6ip4	0.02	1.00000000	mice
Sptlc1	0.03	1.00000000	mice
Fam120b	-0.09	1.00000000	mice
Stxbp3	0.11	1.00000000	mice
Tbcd	0.05	1.00000000	mice
Ipmk	-0.16	1.00000000	mice
Pla2g15	-0.02	1.00000000	mice
Ctdspl	0.02	1.00000000	mice
Tmem222	-0.01	1.00000000	mice
Slc22a5	-0.16	1.00000000	mice
Fendrr	0.07	1.00000000	mice
Tpst2	0.04	1.00000000	mice
Pcyt1a	-0.04	1.00000000	mice
Lancl1	-0.02	1.00000000	mice
Phf6	0.13	1.00000000	mice
Tmed2	0.07	1.00000000	mice
Rbm8a	0.05	1.00000000	mice
Nomo1	0.04	1.00000000	mice
Rit1	0.02	1.00000000	mice
Arl6ip6	0.02	1.00000000	mice
Znrf3	0.28	1.00000000	mice
Arhgap21	0.07	1.00000000	mice
Ptrhd1	0.02	1.00000000	mice
Ncbp1	-0.10	1.00000000	mice
Chd9	-0.07	1.00000000	mice
Sap30l	0.03	1.00000000	mice
Tmem119	0.03	1.00000000	mice

Arhgap5	0.00	1.00000000	mice
2810004N23Rik	0.06	1.00000000	mice
Dis3l2	-0.13	1.00000000	mice
Dnm2	-0.09	1.00000000	mice
Tgif2	0.04	1.00000000	mice
Atp6v0d1	0.05	1.00000000	mice
Polr2h	-0.04	1.00000000	mice
Cnst	-0.06	1.00000000	mice
B130024G19Rik	0.13	1.00000000	mice
Coro7	0.08	1.00000000	mice
Sf3a1	0.11	1.00000000	mice
Rad23b	0.04	1.00000000	mice
Nus1	0.17	1.00000000	mice
Bscl2	0.25	1.00000000	mice
Rad17	0.07	1.00000000	mice
Pgd	0.14	1.00000000	mice
Ppp2r3d	0.09	1.00000000	mice
Tctex1d2	0.16	1.00000000	mice
Ppp4r3a	-0.01	1.00000000	mice
Rnf144a	-0.03	1.00000000	mice
Fut11	-0.01	1.00000000	mice
Pcif1	0.09	1.00000000	mice
Brd9	0.09	1.00000000	mice
Sept4	0.22	1.00000000	mice
App	0.01	1.00000000	mice
Bud23	0.03	1.00000000	mice
Bola2	0.08	1.00000000	mice
Tnfsf13	-0.07	1.00000000	mice
Atp11b	-0.09	1.00000000	mice
Tor1b	0.12	1.00000000	mice
Avl9	0.04	1.00000000	mice
Dock4	0.47	1.00000000	mice
Smarcc1	-0.03	1.00000000	mice
Fam126b	-0.08	1.00000000	mice
Ehmt1	-0.06	1.00000000	mice
Ttc1	0.05	1.00000000	mice
Tm4sf1	0.12	1.00000000	mice
Fam3a	0.07	1.00000000	mice
Edf1	0.20	1.00000000	mice
Ddx18	0.10	1.00000000	mice
Esyt1	0.08	1.00000000	mice
Fam193b	0.00	1.00000000	mice
Traf3ip2	-0.05	1.00000000	mice
Rev3l	0.01	1.00000000	mice
Tcim	-0.03	1.00000000	mice
Snrpc	0.21	1.00000000	mice

Elp1	-0.06	1.00000000	mice
Atrn	-0.02	1.00000000	mice
Prickle2	-0.13	1.00000000	mice
Pygo2	-0.05	1.00000000	mice
Gid8	0.10	1.00000000	mice
Slc39a14	0.20	1.00000000	mice
Crem	0.05	1.00000000	mice
Ppm1g	0.00	1.00000000	mice
Khnyn	-0.01	1.00000000	mice
Alkbh5	-0.07	1.00000000	mice
Xpo6	0.02	1.00000000	mice
Dpy19l4	0.01	1.00000000	mice
Nrd1	0.05	1.00000000	mice
Lonp1	-0.08	1.00000000	mice
Slc25a20	0.08	1.00000000	mice
Cul4a	-0.01	1.00000000	mice
Srfbp1	0.05	1.00000000	mice
Gprc5b	0.02	1.00000000	mice
Scd1	0.00	1.00000000	mice
Hoxb5	0.03	1.00000000	mice
Vdac1	0.01	1.00000000	mice
Cnpy3	0.00	1.00000000	mice
Zc3h14	0.10	1.00000000	mice
Adamts15	0.00	1.00000000	mice
Nek7	0.02	1.00000000	mice
Fgfr1op2	0.00	1.00000000	mice
Tnrc18	-0.05	1.00000000	mice
2210016L21Rik	0.21	1.00000000	mice
Noc3l	-0.04	1.00000000	mice
Dyrk2	-0.04	1.00000000	mice
Tle3	-0.03	1.00000000	mice
Hgsnat	0.03	1.00000000	mice
Txn2	0.13	1.00000000	mice
Ubxn1	0.05	1.00000000	mice
Sipa1l3	-0.01	1.00000000	mice
Trmt112	0.04	1.00000000	mice
Vbp1	0.09	1.00000000	mice
Lysmd4	0.11	1.00000000	mice
Kdelr1	0.02	1.00000000	mice
Oxsr1	-0.02	1.00000000	mice
Atp11a	0.02	1.00000000	mice
Hikeshi	0.02	1.00000000	mice
Zbtb41	-0.05	1.00000000	mice
Epha1	0.04	1.00000000	mice
Nup62	-0.07	1.00000000	mice
Slco3a1	0.22	1.00000000	mice

Coa5	0.04	1.00000000	mice
Ubr3	0.03	1.00000000	mice
Prpf4	0.07	1.00000000	mice
Gtf2e2	-0.06	1.00000000	mice
Hk2	-0.25	1.00000000	mice
Pnn	0.04	1.00000000	mice
Chst3	0.18	1.00000000	mice
Rrp8	0.09	1.00000000	mice
Rad21	-0.16	1.00000000	mice
Rab31	-0.13	1.00000000	mice
Dnal4	0.03	1.00000000	mice
Cyb5b	0.07	1.00000000	mice
Stk38	-0.05	1.00000000	mice
Klc1	0.00	1.00000000	mice
Ldb1	-0.05	1.00000000	mice
Ppp6r2	-0.10	1.00000000	mice
Rffl	-0.04	1.00000000	mice
Pop5	-0.04	1.00000000	mice
Tspan9	-0.02	1.00000000	mice
Vps53	-0.01	1.00000000	mice
Dtx2	0.03	1.00000000	mice
Vrk1	0.07	1.00000000	mice
6720427I07Rik	-0.04	1.00000000	mice
Ano10	0.03	1.00000000	mice
Poglut2	-0.02	1.00000000	mice
Cpz	-0.02	1.00000000	mice
Samm50	0.00	1.00000000	mice
Cplane1	0.03	1.00000000	mice
Bcl9	0.05	1.00000000	mice
Zswim7	0.01	1.00000000	mice
Apopt1	-0.08	1.00000000	mice
Zfp809	0.01	1.00000000	mice
Cct7	0.07	1.00000000	mice
Zc3h7b	0.04	1.00000000	mice
Srp72	0.04	1.00000000	mice
Evi5	0.04	1.00000000	mice
Rap2a	0.06	1.00000000	mice
Cbll1	0.06	1.00000000	mice
Uqcr11	0.31	1.00000000	mice
Epn2	-0.04	1.00000000	mice
Stmn1	-0.01	1.00000000	mice
Qser1	0.04	1.00000000	mice
Rpa3	0.22	1.00000000	mice
Lhfpl2	0.03	1.00000000	mice
Pi4k2b	-0.06	1.00000000	mice
Srrm1	0.06	1.00000000	mice

Dlc1	0.05	1.00000000	mice
Rbpj	0.00	1.00000000	mice
Psmd13	0.15	1.00000000	mice
Ifnar1	-0.06	1.00000000	mice
Dync2li1	0.04	1.00000000	mice
Pip4p2	-0.01	1.00000000	mice
Cd55	0.12	1.00000000	mice
Efr3b	-0.08	1.00000000	mice
Aldh1a1	0.28	1.00000000	mice
Kif21a	-0.03	1.00000000	mice
Ndufv2	0.20	1.00000000	mice
Nans	-0.04	1.00000000	mice
Ublcp1	0.11	1.00000000	mice
Bin1	-0.06	1.00000000	mice
Nbas	0.01	1.00000000	mice
Traf6	-0.04	1.00000000	mice
Hr	-0.04	1.00000000	mice
Zfp787	0.02	1.00000000	mice
Lrrfip2	-0.03	1.00000000	mice
Avil	-0.06	1.00000000	mice
Arhgdia	0.28	1.00000000	mice
Sgpp1	0.05	1.00000000	mice
Nxf1	0.05	1.00000000	mice
Ctsk	-0.14	1.00000000	mice
Carmn	-0.07	1.00000000	mice
Cd151	0.14	1.00000000	mice
Sugt1	0.02	1.00000000	mice
Trappc6a	0.01	1.00000000	mice
Nudc	0.02	1.00000000	mice
Dhrs7	0.11	1.00000000	mice
Gna12	0.10	1.00000000	mice
Gcc2	0.16	1.00000000	mice
Cebpa	0.01	1.00000000	mice
Extl3	0.00	1.00000000	mice
Dpm2	0.02	1.00000000	mice
Rnf25	-0.08	1.00000000	mice
Igf2r	0.05	1.00000000	mice
Sned1	-0.15	1.00000000	mice
Agl	0.06	1.00000000	mice
Qars	-0.04	1.00000000	mice
Ccl11	0.60	1.00000000	mice
Tcerg1	0.08	1.00000000	mice
Zc3h12b	-0.08	1.00000000	mice
Rars	0.17	1.00000000	mice
Rrp15	0.02	1.00000000	mice
Fabp5	0.02	1.00000000	mice

Prpf6	0.15	1.00000000	mice
Cox18	0.12	1.00000000	mice
Tbc1d20	0.11	1.00000000	mice
Fam210b	0.17	1.00000000	mice
Vstm4	-0.03	1.00000000	mice
Rnf44	0.01	1.00000000	mice
Prickle1	0.04	1.00000000	mice
Raph1	-0.07	1.00000000	mice
Cog4	-0.04	1.00000000	mice
Ubtd1	0.04	1.00000000	mice
Ttc23	-0.12	1.00000000	mice
Usp22	0.02	1.00000000	mice
Lrrc32	0.06	1.00000000	mice
Heg1	0.23	1.00000000	mice
Lamp1	0.17	1.00000000	mice
Glrx	0.21	1.00000000	mice
Arhgap35	0.14	1.00000000	mice
Ctsa	0.02	1.00000000	mice
Supt20	-0.06	1.00000000	mice
Cryl1	-0.09	1.00000000	mice
Gpkow	-0.06	1.00000000	mice
Ufsp2	0.08	1.00000000	mice
Smarcb1	0.15	1.00000000	mice
Ykt6	0.09	1.00000000	mice
Nap1l4	0.14	1.00000000	mice
Tmf1	0.06	1.00000000	mice
Bcl9l	0.14	1.00000000	mice
Zzef1	-0.04	1.00000000	mice
Akap10	-0.05	1.00000000	mice
Thap12	0.05	1.00000000	mice
Plpp5	0.03	1.00000000	mice
Slc39a7	0.05	1.00000000	mice
Commd8	0.10	1.00000000	mice
Ttc14	0.01	1.00000000	mice
Zc3h4	-0.01	1.00000000	mice
Xpo4	0.05	1.00000000	mice
Sft2d2	-0.06	1.00000000	mice
Hdac1	0.02	1.00000000	mice
Dkk3	0.06	1.00000000	mice
Arfrp1	0.04	1.00000000	mice
Rab1a	0.10	1.00000000	mice
Zfp36l2	0.21	1.00000000	mice
Pi4k2a	-0.03	1.00000000	mice
Crip2	0.17	1.00000000	mice
Psph	0.05	1.00000000	mice
Atp6v1d	0.04	1.00000000	mice

Zfp516	-0.06	1.00000000	mice
Znhit6	0.02	1.00000000	mice
Atp5g3	0.06	1.00000000	mice
Cramp1l	-0.02	1.00000000	mice
Wdr70	-0.01	1.00000000	mice
Zyg11b	0.09	1.00000000	mice
Psap	0.04	1.00000000	mice
Cemip2	-0.12	1.00000000	mice
Lrrc4	0.18	1.00000000	mice
Rab28	0.08	1.00000000	mice
Ero1lb	-0.08	1.00000000	mice
Tom1l2	0.00	1.00000000	mice
Laptm5	0.01	1.00000000	mice
Snhg12	0.06	1.00000000	mice
Calu	0.20	1.00000000	mice
Ern1	0.00	1.00000000	mice
Smarca4	-0.07	1.00000000	mice
Galnt7	-0.01	1.00000000	mice
Dag1	0.01	1.00000000	mice
Vipr2	0.08	1.00000000	mice
Hdgfl3	-0.01	1.00000000	mice
Clu	0.37	1.00000000	mice
Tomm6	0.07	1.00000000	mice
Ip6k2	0.01	1.00000000	mice
Ten1	-0.16	1.00000000	mice
Tnk2	-0.02	1.00000000	mice
Ddx42	0.02	1.00000000	mice
Slc2a13	-0.04	1.00000000	mice
Psma6	0.37	1.00000000	mice
Fchsd2	-0.19	1.00000000	mice
Pakap.1	0.19	1.00000000	mice
Usp11	-0.10	1.00000000	mice
Plekho2	-0.05	1.00000000	mice
Phka1	-0.09	1.00000000	mice
Ccnt2	-0.01	1.00000000	mice
Tmem216	-0.08	1.00000000	mice
Hdac7	0.04	1.00000000	mice
Spns1	0.01	1.00000000	mice
Ppp1r21	-0.04	1.00000000	mice
Nae1	0.07	1.00000000	mice
Pdrg1	0.16	1.00000000	mice
Rnmt	-0.03	1.00000000	mice
Sinhcaf	-0.01	1.00000000	mice
Zfp638	0.03	1.00000000	mice
Atp6v1g1	0.02	1.00000000	mice
Rnh1	0.25	1.00000000	mice

Eps15	-0.03	1.00000000	mice
Chsy1	0.09	1.00000000	mice
Dcaf6	-0.10	1.00000000	mice
Tex10	-0.03	1.00000000	mice
Ap3b1	-0.13	1.00000000	mice
Zfp598	-0.03	1.00000000	mice
N6amt1	0.12	1.00000000	mice
Ndufa1	0.29	1.00000000	mice
Anapc4	0.07	1.00000000	mice
Nol8	-0.05	1.00000000	mice
Prr16	0.12	1.00000000	mice
Prpf19	-0.01	1.00000000	mice
Tmem29	-0.05	1.00000000	mice
Gdap2	-0.06	1.00000000	mice
N4bp2l2	-0.05	1.00000000	mice
Ddx41	0.01	1.00000000	mice
Tpgs1	0.15	1.00000000	mice
Spon2	0.11	1.00000000	mice
Csgalnact1	0.05	1.00000000	mice
Dhcr24	0.15	1.00000000	mice
Slc25a25	-0.04	1.00000000	mice
Ergic1	0.03	1.00000000	mice
Vps13c	0.04	1.00000000	mice
Cnep1r1	-0.03	1.00000000	mice
Snrnp48	0.09	1.00000000	mice
Sgf29	-0.01	1.00000000	mice
Arl6	0.06	1.00000000	mice
Asap1	0.08	1.00000000	mice
Anks1	0.01	1.00000000	mice
Rnf169	-0.03	1.00000000	mice
Trim2	-0.04	1.00000000	mice
Dnajc14	0.03	1.00000000	mice
Retreg3	-0.01	1.00000000	mice
Usp19	-0.12	1.00000000	mice
Tns3	0.10	1.00000000	mice
C1galt1c1	0.03	1.00000000	mice
Borcs6	0.08	1.00000000	mice
Taf12	-0.02	1.00000000	mice
Castor2	-0.02	1.00000000	mice
Zfpl1	0.07	1.00000000	mice
Emp2	0.13	1.00000000	mice
Sh3d19	-0.03	1.00000000	mice
Rftn1	0.08	1.00000000	mice
Ap2s1	0.24	1.00000000	mice
P2ry14	-0.04	1.00000000	mice
Ncaph2	0.01	1.00000000	mice

Slc30a4	-0.02	1.00000000	mice
Neu1	-0.03	1.00000000	mice
Ift74	0.06	1.00000000	mice
Nt5dc1	0.16	1.00000000	mice
Zranb2	0.03	1.00000000	mice
Naa38	0.10	1.00000000	mice
2610008E11Rik	-0.02	1.00000000	mice
Smim13	-0.03	1.00000000	mice
Arfgap1	0.15	1.00000000	mice
Spg20	-0.02	1.00000000	mice
9430038I01Rik	0.00	1.00000000	mice
Hsf2	-0.01	1.00000000	mice
Dusp22	-0.01	1.00000000	mice
Cand1	-0.01	1.00000000	mice
Atg5	0.11	1.00000000	mice
Zscan26	0.02	1.00000000	mice
Sqor	0.14	1.00000000	mice
Ccdc50	0.07	1.00000000	mice
Dr1	0.03	1.00000000	mice
Eif4h	0.08	1.00000000	mice
Lims1	0.09	1.00000000	mice
Usf1	0.04	1.00000000	mice
Prpsap2	-0.25	1.00000000	mice
Commd7	0.03	1.00000000	mice
Sgpl1	-0.01	1.00000000	mice
Slc10a7	-0.02	1.00000000	mice
Ppp1r7	-0.07	1.00000000	mice
Ube3c	0.13	1.00000000	mice
Washc2	0.11	1.00000000	mice
Adam10	-0.01	1.00000000	mice
Stx8	0.08	1.00000000	mice
Crybg3	0.08	1.00000000	mice
Etv1	-0.02	1.00000000	mice
Sgta	0.12	1.00000000	mice
Nfatc3	-0.08	1.00000000	mice
Ddx19a	0.11	1.00000000	mice
Capn2	-0.07	1.00000000	mice
Cdv3	-0.01	1.00000000	mice
Anapc10	-0.01	1.00000000	mice
Cct8	0.19	1.00000000	mice
Prkrip1	0.20	1.00000000	mice
Svep1	0.16	1.00000000	mice
Nsmce3	-0.04	1.00000000	mice
Pdcd7	-0.06	1.00000000	mice
Adamtsl1	-0.25	1.00000000	mice
Twist2	0.07	1.00000000	mice

Suclg1	0.16	1.00000000	mice
Washc4	0.07	1.00000000	mice
Dgkd	-0.04	1.00000000	mice
Snap23	0.19	1.00000000	mice
Phf21a	0.01	1.00000000	mice
Trpc4ap	0.06	1.00000000	mice
Eef1e1	-0.04	1.00000000	mice
Grina	0.17	1.00000000	mice
Lactb2	0.12	1.00000000	mice
Acyp1	0.05	1.00000000	mice
Ddx21	0.02	1.00000000	mice
Smap2	-0.01	1.00000000	mice
Brd4	0.05	1.00000000	mice
Pip5k1a	0.11	1.00000000	mice
Gk5	-0.25	1.00000000	mice
Slc33a1	0.08	1.00000000	mice
Tuba1a	0.05	1.00000000	mice
Fbl	0.03	1.00000000	mice
Rab7b	-0.13	1.00000000	mice
Ctsh	0.11	1.00000000	mice
Retreg1	-0.02	1.00000000	mice
Serinc1	0.12	1.00000000	mice
Dnajc30	0.02	1.00000000	mice
Tbc1d8b	0.00	1.00000000	mice
Ppp6c	0.09	1.00000000	mice
Ccdc124	0.18	1.00000000	mice
Zcchc8	-0.05	1.00000000	mice
Zmynd8	0.02	1.00000000	mice
Cdk16	0.06	1.00000000	mice
Cpsf3	0.09	1.00000000	mice
Arhgap1	0.03	1.00000000	mice
Epn1	0.07	1.00000000	mice
Atp6v1f	0.12	1.00000000	mice
Olfml1	0.09	1.00000000	mice
Anxa11	0.06	1.00000000	mice
Pdcd6ip	0.03	1.00000000	mice
Lipa	0.20	1.00000000	mice
Zfp950	-0.03	1.00000000	mice
Ube3a	0.00	1.00000000	mice
Ddi2	-0.02	1.00000000	mice
Eya3	0.13	1.00000000	mice
Vti1a	-0.09	1.00000000	mice
4833439L19Rik	-0.02	1.00000000	mice
Sdc4	0.17	1.00000000	mice
Ube2a	0.05	1.00000000	mice
Cycs	0.27	1.00000000	mice

Uxs1	0.08	1.00000000	mice
Cgnl1	-0.04	1.00000000	mice
Tex261	0.13	1.00000000	mice
Slc50a1	0.00	1.00000000	mice
Bax	0.23	1.00000000	mice
Tnc	-0.16	1.00000000	mice
Xbp1	0.11	1.00000000	mice
Ptpn21	-0.03	1.00000000	mice
Txndc17	0.15	1.00000000	mice
Erlin1	-0.05	1.00000000	mice
Ank2	-0.18	1.00000000	mice
Wdr36	-0.08	1.00000000	mice
Ypel2	0.00	1.00000000	mice
Cdk11b	-0.07	1.00000000	mice
Wnt2	0.25	1.00000000	mice
Pip4k2a	-0.02	1.00000000	mice
Hjurp	0.00	1.00000000	mice
Sec23ip	0.11	1.00000000	mice
Rpf1	0.04	1.00000000	mice
Bola3	0.20	1.00000000	mice
Polr3gl	0.02	1.00000000	mice
Cd248	0.06	1.00000000	mice
Cox6b1	0.30	1.00000000	mice
Sh3bgrl	0.30	1.00000000	mice
Pkia	-0.06	1.00000000	mice
Hoxb4	-0.03	1.00000000	mice
Ndufa4	0.33	1.00000000	mice
Aip	0.08	1.00000000	mice
Hsd17b12	0.06	1.00000000	mice
Aig1	0.08	1.00000000	mice
Zfp148	-0.03	1.00000000	mice
Por	-0.06	1.00000000	mice
Yaf2	-0.07	1.00000000	mice
Ywhaq	0.09	1.00000000	mice
Fnbp4	0.10	1.00000000	mice
Osbpl8	-0.09	1.00000000	mice
Srrt	0.00	1.00000000	mice
Hnrnpd	0.23	1.00000000	mice
Thoc3	0.08	1.00000000	mice
Stard9	-0.01	1.00000000	mice
Snrpd3	0.17	1.00000000	mice
Chn2	-0.07	1.00000000	mice
Pkn1	0.04	1.00000000	mice
Nsun4	-0.07	1.00000000	mice
St3gal3	0.04	1.00000000	mice
Rrp9	0.07	1.00000000	mice

Rab5a	0.01	1.00000000	mice
Stk25	0.07	1.00000000	mice
Blzf1	0.00	1.00000000	mice
Tbc1d19	0.06	1.00000000	mice
Sdhd	0.00	1.00000000	mice
Reps1	0.03	1.00000000	mice
Cggbp1	0.00	1.00000000	mice
Atxn7l1	-0.04	1.00000000	mice
Nars	0.20	1.00000000	mice
Elp5	0.08	1.00000000	mice
Tpd52	0.01	1.00000000	mice
Zfp385b	-0.19	1.00000000	mice
Kctd20	-0.01	1.00000000	mice
Ostf1	0.23	1.00000000	mice
Cep164	-0.03	1.00000000	mice
Gipc1	0.16	1.00000000	mice
Sclt1	0.07	1.00000000	mice
Pam16	0.04	1.00000000	mice
Nsrp1	0.16	1.00000000	mice
Atl3	0.12	1.00000000	mice
Ube2r2	0.03	1.00000000	mice
Cyba	0.15	1.00000000	mice
Actl6a	-0.04	1.00000000	mice
Rfx7	-0.04	1.00000000	mice
Zfp654	-0.17	1.00000000	mice
Elof1	0.21	1.00000000	mice
Cib1	0.04	1.00000000	mice
Cln3	0.02	1.00000000	mice
St3gal4	0.28	1.00000000	mice
Pde8a	-0.12	1.00000000	mice
Fkbp1a	0.24	1.00000000	mice
Larp7	0.00	1.00000000	mice
Keap1	0.08	1.00000000	mice
Ctsd	0.25	1.00000000	mice
Epm2aip1	0.05	1.00000000	mice
Trip10	0.08	1.00000000	mice
Rbbp4	0.00	1.00000000	mice
Dda1	0.17	1.00000000	mice
Psmb5	0.23	1.00000000	mice
Hsp90ab1	0.09	1.00000000	mice
Arhgef3	-0.02	1.00000000	mice
Rbfa	0.10	1.00000000	mice
Scamp3	0.09	1.00000000	mice
Tmem123	0.00	1.00000000	mice
Tbc1d17	0.01	1.00000000	mice
Ik	0.28	1.00000000	mice

Pde5a	0.58	1.00000000	mice
Phf5a	-0.03	1.00000000	mice
Smim12	-0.07	1.00000000	mice
Svbp	0.08	1.00000000	mice
Tmem43	0.05	1.00000000	mice
Slf1	0.09	1.00000000	mice
Ptpra	-0.04	1.00000000	mice
Tcf7l2	-0.06	1.00000000	mice
Klhl28	0.04	1.00000000	mice
Slc12a2	-0.12	1.00000000	mice
Wsb2	0.09	1.00000000	mice
Stk4	-0.02	1.00000000	mice
Tfdp1	-0.01	1.00000000	mice
Pofut2	-0.01	1.00000000	mice
5031425E22Rik	-0.02	1.00000000	mice
Dnajc18	0.05	1.00000000	mice
Crlf2	0.08	1.00000000	mice
Wdpcp	-0.07	1.00000000	mice
Cope	0.25	1.00000000	mice
Tmem205	0.08	1.00000000	mice
Lclat1	0.03	1.00000000	mice
Zfp512	0.02	1.00000000	mice
Pccb	-0.07	1.00000000	mice
Cyld	0.03	1.00000000	mice
Decr1	0.05	1.00000000	mice
Pnpla7	-0.09	1.00000000	mice
Atp8b2	-0.06	1.00000000	mice
Hdgf	0.29	1.00000000	mice
Tbc1d23	-0.02	1.00000000	mice
Tmed3	-0.04	1.00000000	mice
Cds2	0.03	1.00000000	mice
Cant1	0.13	1.00000000	mice
Lrp1	0.15	1.00000000	mice
Lbr	0.07	1.00000000	mice
Fst	0.19	1.00000000	mice
Tor2a	0.04	1.00000000	mice
Coro1a	-0.04	1.00000000	mice
Stoml2	0.06	1.00000000	mice
Usp6nl	0.03	1.00000000	mice
Smox	0.23	1.00000000	mice
Tshz1	-0.08	1.00000000	mice
Tmed7	-0.07	1.00000000	mice
Xdh	0.14	1.00000000	mice
Nol12	0.01	1.00000000	mice
Ankrd13c	0.00	1.00000000	mice
Cisd1	0.07	1.00000000	mice

Trim27	-0.06	1.00000000	mice
Znrf2	0.00	1.00000000	mice
Serpine2	0.08	1.00000000	mice
Cog5	-0.01	1.00000000	mice
Gnai2	0.19	1.00000000	mice
Lnpk	0.17	1.00000000	mice
Cnnm3	-0.01	1.00000000	mice
Zc3h15	0.13	1.00000000	mice
Pakap	0.12	1.00000000	mice
Ino80e	0.02	1.00000000	mice
Ptgr2	0.03	1.00000000	mice
Kdelr3	0.00	1.00000000	mice
Pbx3	-0.25	1.00000000	mice
Lcmt1	0.00	1.00000000	mice
Vps37b	0.08	1.00000000	mice
Casd1	-0.07	1.00000000	mice
Yipf5	0.16	1.00000000	mice
Rnf24	0.06	1.00000000	mice
Zhx1	0.06	1.00000000	mice
Cfh	-0.14	1.00000000	mice
Epdr1	-0.04	1.00000000	mice
Sav1	0.00	1.00000000	mice
Ergic3	0.16	1.00000000	mice
Utp11	-0.11	1.00000000	mice
Dtx4	0.06	1.00000000	mice
Pdgfra	0.15	1.00000000	mice
Tut7	0.17	1.00000000	mice
Smc3	0.18	1.00000000	mice
Rab22a	0.09	1.00000000	mice
Cryzl1	0.01	1.00000000	mice
Stambp	0.06	1.00000000	mice
Chst11	0.06	1.00000000	mice
Hsbp1	0.13	1.00000000	mice
Krr1	0.07	1.00000000	mice
Srxn1	-0.05	1.00000000	mice
Atm	0.05	1.00000000	mice
Tmem208	0.03	1.00000000	mice
Tacc1	0.07	1.00000000	mice
Tars	0.13	1.00000000	mice
Prpf39	-0.04	1.00000000	mice
Ypel5	-0.01	1.00000000	mice
Prcc	-0.08	1.00000000	mice
Fbxo9	0.08	1.00000000	mice
Psmc1	0.19	1.00000000	mice
Socs2	0.03	1.00000000	mice
Tmem63a	-0.06	1.00000000	mice

Adamts10	0.02	1.00000000	mice
Etl4	0.07	1.00000000	mice
Ptch1	0.19	1.00000000	mice
Rgl1	0.19	1.00000000	mice
Rtn3	0.10	1.00000000	mice
AC149090.1	-0.03	1.00000000	mice
Dusp10	0.00	1.00000000	mice
Farp1	0.03	1.00000000	mice
Asah1	-0.08	1.00000000	mice
Senp3	0.03	1.00000000	mice
Atp7a	0.00	1.00000000	mice
Galk1	-0.04	1.00000000	mice
Brd8	-0.04	1.00000000	mice
Ocrl	0.04	1.00000000	mice
Prpf3	-0.03	1.00000000	mice
Eftud2	-0.06	1.00000000	mice
Tomm5	0.13	1.00000000	mice
Cuedc1	-0.04	1.00000000	mice
Spast	-0.01	1.00000000	mice
Lifr	0.11	1.00000000	mice
Sh3pxd2b	-0.01	1.00000000	mice
Col1a1	-0.11	1.00000000	mice
Cks1b	0.05	1.00000000	mice
Tbpl1	0.09	1.00000000	mice
Parp4	0.20	1.00000000	mice
Pnrc2	0.00	1.00000000	mice
Ftsj1	-0.02	1.00000000	mice
Sc5d	0.15	1.00000000	mice
Fyb	-0.04	1.00000000	mice
Cdh11	0.40	1.00000000	mice
Snapin	0.02	1.00000000	mice
Icosl	0.00	1.00000000	mice
Sh3gl1	0.06	1.00000000	mice
Hgs	-0.09	1.00000000	mice
Abi3bp	0.12	1.00000000	mice
Col4a2	0.27	1.00000000	mice
Pdzd11	0.02	1.00000000	mice
Ubfd1	-0.02	1.00000000	mice
Camkk2	-0.04	1.00000000	mice
Caml	0.07	1.00000000	mice
Wdsub1	-0.01	1.00000000	mice
Gps1	0.00	1.00000000	mice
Atad1	0.10	1.00000000	mice
Zfp422	-0.04	1.00000000	mice
Dusp6	0.14	1.00000000	mice
Cuta	0.14	1.00000000	mice

Tln1	0.01	1.00000000	mice
Hist1h2bc	0.09	1.00000000	mice
Klf11	-0.03	1.00000000	mice
Rilpl2	-0.05	1.00000000	mice
Ppm1l	0.03	1.00000000	mice
Ino80dos	-0.06	1.00000000	mice
Emc3	0.28	1.00000000	mice
Ing5	-0.04	1.00000000	mice
Golga1	-0.04	1.00000000	mice
Fam135a	-0.05	1.00000000	mice
Pgrmc2	0.00	1.00000000	mice
Adcy7	-0.07	1.00000000	mice
Atp5g2	0.19	1.00000000	mice
Tek	-0.07	1.00000000	mice
Cdc42ep5	-0.06	1.00000000	mice
Uba5	0.00	1.00000000	mice
Scyl1	0.08	1.00000000	mice
Prdm6	0.14	1.00000000	mice
Cic	-0.05	1.00000000	mice
Fn1	0.44	1.00000000	mice
Vamp7	0.13	1.00000000	mice
Exosc10	-0.06	1.00000000	mice
Penk	-0.02	1.00000000	mice
Cul5	0.05	1.00000000	mice
Tipin	0.17	1.00000000	mice
Deptor	-0.15	1.00000000	mice
Rtf2	0.08	1.00000000	mice
Vasp	0.15	1.00000000	mice
Gde1	-0.01	1.00000000	mice
Kdm4a	-0.03	1.00000000	mice
Ankrd17	0.08	1.00000000	mice
Ikzf2	0.13	1.00000000	mice
Cmc2	-0.07	1.00000000	mice
Ino80	-0.02	1.00000000	mice
Ptgs2	0.54	1.00000000	mice
Zc3h13	-0.01	1.00000000	mice
Dgcr2	0.09	1.00000000	mice
Aak1	-0.04	1.00000000	mice
Rassf8	-0.01	1.00000000	mice
Terf2	0.04	1.00000000	mice
Abraxas2	0.09	1.00000000	mice
Utp20	-0.10	1.00000000	mice
Scp2	0.37	1.00000000	mice
Cdkn2d	0.09	1.00000000	mice
Fbxo8	0.02	1.00000000	mice
Chmp2a	0.32	1.00000000	mice

Pole3	0.11	1.00000000	mice
Etv3	0.01	1.00000000	mice
Gorasp2	0.07	1.00000000	mice
Glrx2	0.21	1.00000000	mice
Tmem167b	0.02	1.00000000	mice
2510039O18Rik	0.10	1.00000000	mice
Atp5g1	0.19	1.00000000	mice
Tbc1d9	0.04	1.00000000	mice
Nt5dc2	0.13	1.00000000	mice
Nrg2	0.26	1.00000000	mice
Snx6	0.08	1.00000000	mice
Fasn	0.01	1.00000000	mice
Wrn	0.03	1.00000000	mice
Acad8	-0.01	1.00000000	mice
Snhg18	0.12	1.00000000	mice
Itgb1bp1	0.05	1.00000000	mice
Trappc3	0.02	1.00000000	mice
Tma16	0.06	1.00000000	mice
Dram2	0.04	1.00000000	mice
Itm2c	0.30	1.00000000	mice
Trp53bp2	-0.06	1.00000000	mice
Exosc5	0.04	1.00000000	mice
Clcn5	-0.03	1.00000000	mice
Colec12	0.08	1.00000000	mice
Tnks1bp1	0.09	1.00000000	mice
Tmem204	0.12	1.00000000	mice
Tpm4	0.22	1.00000000	mice
Rnpepl1	0.01	1.00000000	mice
Adamts3	0.13	1.00000000	mice
Slc1a4	0.10	1.00000000	mice
Apool	0.00	1.00000000	mice
Hspa1b	0.37	1.00000000	mice
Dhx16	-0.01	1.00000000	mice
Txndc12	0.17	1.00000000	mice
Uggt1	0.07	1.00000000	mice
Igsf10	-0.12	1.00000000	mice
Hace1	-0.01	1.00000000	mice
Taco1	0.01	1.00000000	mice
Gins4	0.04	1.00000000	mice
Brip1os	-0.01	1.00000000	mice
Kctd5	-0.09	1.00000000	mice
Cbwd1	0.05	1.00000000	mice
Ssr3	0.12	1.00000000	mice
Vti1b	0.10	1.00000000	mice
Hps3	0.03	1.00000000	mice
Pdss1	-0.07	1.00000000	mice

Hdac3	-0.02	1.00000000	mice
Fmr1	0.14	1.00000000	mice
Arfgef2	0.00	1.00000000	mice
Pts	-0.06	1.00000000	mice
Ctdnep1	0.09	1.00000000	mice
Wdr43	-0.04	1.00000000	mice
Hivep1	0.12	1.00000000	mice
Riok1	0.14	1.00000000	mice
Cyp2j6	0.09	1.00000000	mice
Tsr1	-0.02	1.00000000	mice
Rchy1	0.02	1.00000000	mice
Itsn1	0.12	1.00000000	mice
Srebf1	0.02	1.00000000	mice
Tubgcp4	0.00	1.00000000	mice
BC003965	0.01	1.00000000	mice
Fam210a	0.00	1.00000000	mice
Cops7a	-0.04	1.00000000	mice
Slc38a10	0.07	1.00000000	mice
Xpnpep1	0.12	1.00000000	mice
Stk38l	0.14	1.00000000	mice
Olfml3	0.14	1.00000000	mice
Tmem127	0.13	1.00000000	mice
Ift46	0.13	1.00000000	mice
Arrb1	-0.01	1.00000000	mice
Zcchc9	0.04	1.00000000	mice
Endod1	0.06	1.00000000	mice
Stk19	0.09	1.00000000	mice
Ino80b	0.18	1.00000000	mice
Heatr6	-0.07	1.00000000	mice
Wdr5	0.02	1.00000000	mice
Ptpn23	-0.09	1.00000000	mice
Gtpbp4	0.09	1.00000000	mice
Tcf4	0.21	1.00000000	mice
Ap3d1	0.11	1.00000000	mice
Abcf3	-0.02	1.00000000	mice
Trip12	0.05	1.00000000	mice
Rxrb	-0.06	1.00000000	mice
Lyn	-0.04	1.00000000	mice
Sgsm2	0.07	1.00000000	mice
Gtdc1	-0.02	1.00000000	mice
Caprin1	0.05	1.00000000	mice
Psmc3	0.30	1.00000000	mice
Stard7	0.10	1.00000000	mice
Ccdc34	-0.03	1.00000000	mice
Carm1	-0.01	1.00000000	mice
Atxn7l3	0.12	1.00000000	mice

Aurkaip1	0.09	1.00000000	mice
Abcd3	0.06	1.00000000	mice
Nrp1	0.15	1.00000000	mice
Cux1	-0.02	1.00000000	mice
Osbpl5	0.04	1.00000000	mice
Tmem97	-0.02	1.00000000	mice
Ddx1	0.12	1.00000000	mice
Rhbdf1	-0.04	1.00000000	mice
Tpbg	-0.01	1.00000000	mice
Rbm15b	0.03	1.00000000	mice
1700016P03Rik	0.00	1.00000000	mice
Kitl	0.06	1.00000000	mice
1300002E11Rik	-0.03	1.00000000	mice
Tslp	0.17	1.00000000	mice
Serbp1	0.11	1.00000000	mice
Lypla1	-0.04	1.00000000	mice
Bhlhe40	0.11	1.00000000	mice
Nsun2	0.08	1.00000000	mice
Bmp4	0.02	1.00000000	mice
Stxbp5	-0.08	1.00000000	mice
Cxcr4	0.01	1.00000000	mice
Apex1	0.11	1.00000000	mice
Suds3	0.11	1.00000000	mice
Tmem63b	-0.07	1.00000000	mice
Hoxb3	0.08	1.00000000	mice
Erlin2	0.00	1.00000000	mice
Scn3a	0.18	1.00000000	mice
Nfxl1	0.00	1.00000000	mice
Chst8	0.04	1.00000000	mice
Gskip	0.15	1.00000000	mice
Capg	0.18	1.00000000	mice
Cwc22	0.03	1.00000000	mice
Psmb4	0.19	1.00000000	mice
Rnf217	0.26	1.00000000	mice
Lamc1	0.17	1.00000000	mice
Echs1	0.13	1.00000000	mice
Cc2d2a	-0.08	1.00000000	mice
Plk2	0.12	1.00000000	mice
Plcb3	-0.01	1.00000000	mice
Zmiz2	-0.06	1.00000000	mice
Emc2	0.10	1.00000000	mice
Proca1	-0.01	1.00000000	mice
Adamts6	0.13	1.00000000	mice
Cep128	0.08	1.00000000	mice
Jmjd6	-0.01	1.00000000	mice
Gucy1a1	0.58	1.00000000	mice

Prkab2	0.01	1.00000000	mice
Ruvbl1	0.03	1.00000000	mice
Sil1	0.00	1.00000000	mice
Sec22b	0.00	1.00000000	mice
Shisa4	0.04	1.00000000	mice
Gga1	0.15	1.00000000	mice
Fem1c	0.03	1.00000000	mice
Sfi1	0.08	1.00000000	mice
Ankle2	-0.02	1.00000000	mice
Dpp8	0.13	1.00000000	mice
Setdb1	0.04	1.00000000	mice
Gpr153	0.02	1.00000000	mice
Fgd5	-0.02	1.00000000	mice
Fancl	-0.03	1.00000000	mice
Imp3	0.10	1.00000000	mice
Eif6	0.15	1.00000000	mice
Ube2g2	-0.04	1.00000000	mice
Anxa6	0.09	1.00000000	mice
Rab3gap2	-0.07	1.00000000	mice
Rer1	0.17	1.00000000	mice
Ank	-0.04	1.00000000	mice
Cep85l	-0.03	1.00000000	mice
Zfp407	-0.03	1.00000000	mice
Glce	-0.03	1.00000000	mice
Zfp869	0.04	1.00000000	mice
Pja1	0.03	1.00000000	mice
Akt3	0.12	1.00000000	mice
Tm9sf4	0.13	1.00000000	mice
2700049A03Rik	-0.10	1.00000000	mice
Actr10	-0.09	1.00000000	mice
Becn1	-0.01	1.00000000	mice
Upf3b	0.07	1.00000000	mice
Chmp1b	0.11	1.00000000	mice
Fbxw4	0.04	1.00000000	mice
Chpt1	0.21	1.00000000	mice
Pxdn	-0.07	1.00000000	mice
Ttbk2	-0.05	1.00000000	mice
Vps29	0.26	1.00000000	mice
Ubxn7	0.04	1.00000000	mice
Fam92a	-0.03	1.00000000	mice
Ccdc6	-0.04	1.00000000	mice
Rex1bd	0.20	1.00000000	mice
Arl5a	-0.01	1.00000000	mice
Naa20	0.08	1.00000000	mice
Flnb	0.10	1.00000000	mice
Pigyl	0.22	1.00000000	mice

Trappc5	0.08	1.00000000	mice
Otud1	0.05	1.00000000	mice
Atp2c1	0.12	1.00000000	mice
Tpd52l2	0.11	1.00000000	mice
Dhrs7b	0.03	1.00000000	mice
Baz1a	-0.06	1.00000000	mice
Bcap29	0.10	1.00000000	mice
Phb	0.07	1.00000000	mice
Asap3	-0.02	1.00000000	mice
Slit2	0.11	1.00000000	mice
A330076H08Rik	0.07	1.00000000	mice
Fam189a2	0.11	1.00000000	mice
Tmx2	0.09	1.00000000	mice
D16Ertd472e	0.03	1.00000000	mice
Vsir	0.00	1.00000000	mice
Xpc	0.11	1.00000000	mice
Uqcr10	0.17	1.00000000	mice
Cops8	0.07	1.00000000	mice
Eipr1	-0.06	1.00000000	mice
Snrpd2	0.22	1.00000000	mice
Rdh14	0.06	1.00000000	mice
Zfp292	0.10	1.00000000	mice
Khdrbs3	0.16	1.00000000	mice
Hnrnph2	0.04	1.00000000	mice
Cd2ap	0.08	1.00000000	mice
Cdkn2c	0.31	1.00000000	mice
Rexo1	-0.04	1.00000000	mice
Arid1a	0.14	1.00000000	mice
Rassf5	-0.02	1.00000000	mice
Bud31	0.17	1.00000000	mice
Brd3	-0.13	1.00000000	mice
Acads	0.02	1.00000000	mice
Cspg4	0.05	1.00000000	mice
Nsd2	0.12	1.00000000	mice
Rprd1a	-0.08	1.00000000	mice
Fat1	0.01	1.00000000	mice
Gtpbp2	0.16	1.00000000	mice
Zfp236	0.03	1.00000000	mice
Ltbr	-0.08	1.00000000	mice
Atg9a	-0.04	1.00000000	mice
Fkbp14	0.06	1.00000000	mice
Arhgef40	0.05	1.00000000	mice
Arhgap32	0.06	1.00000000	mice
Cacnb3	-0.01	1.00000000	mice
Prkag1	0.06	1.00000000	mice
Naa16	0.05	1.00000000	mice

Grsf1	0.07	1.00000000	mice
Fktn	0.13	1.00000000	mice
Fam76b	-0.01	1.00000000	mice
Crkl	0.07	1.00000000	mice
2200002D01Rik	0.07	1.00000000	mice
Nxn	0.15	1.00000000	mice
Fbxo42	0.06	1.00000000	mice
H2afy2	0.01	1.00000000	mice
Slc39a13	-0.02	1.00000000	mice
Fkbp15	0.10	1.00000000	mice
Copz1	0.07	1.00000000	mice
Snx30	0.07	1.00000000	mice
P4ha1	0.03	1.00000000	mice
Pdap1	0.30	1.00000000	mice
Fmc1	-0.03	1.00000000	mice
Vegfd	0.24	1.00000000	mice
Ccny	0.07	1.00000000	mice
Ywhag	0.14	1.00000000	mice
Camsap1	-0.08	1.00000000	mice
Cers2	0.12	1.00000000	mice
Ap2m1	0.09	1.00000000	mice
Tmem115	0.12	1.00000000	mice
Atp9a	0.02	1.00000000	mice
Sart1	-0.03	1.00000000	mice
Kat2b	0.00	1.00000000	mice
Reps2	0.01	1.00000000	mice
Cab39l	0.01	1.00000000	mice
Epm2a	0.01	1.00000000	mice
Prkdc	-0.01	1.00000000	mice
Paxip1	-0.01	1.00000000	mice
Impa1	0.04	1.00000000	mice
Tm9sf2	-0.01	1.00000000	mice
Rspry1	-0.08	1.00000000	mice
Ciz1	-0.03	1.00000000	mice
Dnajc4	0.05	1.00000000	mice
Ninl	0.00	1.00000000	mice
Usp5	0.04	1.00000000	mice
Ddr1	0.02	1.00000000	mice
Adcy9	0.09	1.00000000	mice
Tgs1	0.01	1.00000000	mice
Eml2	0.09	1.00000000	mice
Blcap	-0.01	1.00000000	mice
Plekha3	-0.01	1.00000000	mice
Lamtor2	0.21	1.00000000	mice
Ap1g1	0.01	1.00000000	mice
Jagn1	0.04	1.00000000	mice

Lrch3	0.07	1.00000000	mice
Cfl2	-0.10	1.00000000	mice
Zc3h7a	0.07	1.00000000	mice
Tab2	0.05	1.00000000	mice
Rab8b	0.16	1.00000000	mice
Erlec1	-0.01	1.00000000	mice
Frg1	0.19	1.00000000	mice
Ptpn3	-0.03	1.00000000	mice
Lsm2	0.08	1.00000000	mice
Strada	-0.01	1.00000000	mice
Hebp1	0.00	1.00000000	mice
Atg7	0.01	1.00000000	mice
Srpr	0.03	1.00000000	mice
R3hdm1	0.04	1.00000000	mice
Slc37a3	-0.02	1.00000000	mice
Cstb	0.25	1.00000000	mice
Rcc2	-0.10	1.00000000	mice
Pacs1	0.00	1.00000000	mice
Lin54	0.04	1.00000000	mice
Ythdf1	-0.05	1.00000000	mice
Pced1b	-0.02	1.00000000	mice
Gxylt1	-0.05	1.00000000	mice
Gabpa	0.03	1.00000000	mice
Kpna3	0.18	1.00000000	mice
Cmtm3	-0.08	1.00000000	mice
Ube2v2	0.11	1.00000000	mice
Pes1	-0.01	1.00000000	mice
Agtpbp1	-0.07	1.00000000	mice
Numb	0.00	1.00000000	mice
Phldb2	0.09	1.00000000	mice
Dubr	0.00	1.00000000	mice
Xxylt1	-0.14	1.00000000	mice
Naa35	0.07	1.00000000	mice
St13	0.20	1.00000000	mice
Slf2	-0.04	1.00000000	mice
Rbp1	0.08	1.00000000	mice
Fam219a	0.00	1.00000000	mice
Txndc16	0.04	1.00000000	mice
Ganab	-0.09	1.00000000	mice
Rfc1	0.10	1.00000000	mice
Nudt9	0.00	1.00000000	mice
Otud4	-0.06	1.00000000	mice
Rnpc3	-0.01	1.00000000	mice
Armh3	0.08	1.00000000	mice
Commd4	0.05	1.00000000	mice
Zmym2	-0.04	1.00000000	mice

Rab3gap1	0.00	1.00000000	mice
Ripor1	-0.02	1.00000000	mice
Nr3c1	0.14	1.00000000	mice
Golga7	0.07	1.00000000	mice
Tmem164	0.05	1.00000000	mice
Fut8	0.05	1.00000000	mice
Ankib1	-0.06	1.00000000	mice
Cdc23	-0.05	1.00000000	mice
Zfp423	-0.03	1.00000000	mice
Hnrnpa3	0.20	1.00000000	mice
Spag7	0.00	1.00000000	mice
Usp12	-0.07	1.00000000	mice
Lrrcc1	0.00	1.00000000	mice
Fdx2	0.16	1.00000000	mice
Cbfa2t2	0.02	1.00000000	mice
Bad	0.00	1.00000000	mice
Rictor	0.07	1.00000000	mice
Dpm3	0.11	1.00000000	mice
Elp2	0.03	1.00000000	mice
Fopnl	0.04	1.00000000	mice
2610001J05Rik	0.18	1.00000000	mice
Ak1	0.05	1.00000000	mice
Pdhx	0.01	1.00000000	mice
Hmgcr	0.08	1.00000000	mice
Ankrd13a	0.01	1.00000000	mice
Pfdn6	0.11	1.00000000	mice
Pcm1	-0.02	1.00000000	mice
Nisch	0.17	1.00000000	mice
Washc5	0.00	1.00000000	mice
Lsm14a	0.03	1.00000000	mice
Ppp1ca	0.21	1.00000000	mice
Hadha	0.10	1.00000000	mice
Nbr1	0.06	1.00000000	mice
Degs1	0.06	1.00000000	mice
Chd7	0.12	1.00000000	mice
Fam114a1	-0.01	1.00000000	mice
Susd6	0.17	1.00000000	mice
Sesn2	0.00	1.00000000	mice
Lgr6	0.01	1.00000000	mice
Gnb1	0.06	1.00000000	mice
Gdi2	0.13	1.00000000	mice
Ldlrad3	0.03	1.00000000	mice
Lcp1	0.14	1.00000000	mice
Arhgap26	-0.24	1.00000000	mice
Akr1b8	-0.04	1.00000000	mice
Plaa	0.00	1.00000000	mice

Vps4b	0.02	1.00000000	mice
Aamdc	0.05	1.00000000	mice
Frs2	0.00	1.00000000	mice
Ahcyl2	-0.04	1.00000000	mice
Necap2	0.09	1.00000000	mice
Vps36	0.21	1.00000000	mice
Ddx52	0.01	1.00000000	mice
Nck2	0.18	1.00000000	mice
Fez2	0.06	1.00000000	mice
Dut	-0.03	1.00000000	mice
Thap11	-0.01	1.00000000	mice
Acsl5	0.12	1.00000000	mice
Tsnax	-0.03	1.00000000	mice
Fbxl3	0.11	1.00000000	mice
Vars	0.03	1.00000000	mice
Efcab14	0.07	1.00000000	mice
Cpne1	-0.10	1.00000000	mice
Aup1	0.10	1.00000000	mice
Hbegf	0.01	1.00000000	mice
Creb3l1	0.04	1.00000000	mice
Pwp1	0.00	1.00000000	mice
Gtf2f2	0.01	1.00000000	mice
Nploc4	0.04	1.00000000	mice
Ago1	0.03	1.00000000	mice
Nln	-0.02	1.00000000	mice
Parp2	0.02	1.00000000	mice
Dpp9	-0.02	1.00000000	mice
Zfyve26	-0.05	1.00000000	mice
Cox5a	0.25	1.00000000	mice
Apcdd1	-0.01	1.00000000	mice
Filip1l	-0.01	1.00000000	mice
Sema6d	-0.06	1.00000000	mice
Golga3	0.01	1.00000000	mice
Creg1	0.04	1.00000000	mice
Adam17	0.06	1.00000000	mice
Sf3b3	-0.03	1.00000000	mice
Clec3b	-0.29	1.00000000	mice
Abhd16a	0.11	1.00000000	mice
Cyb5r3	0.07	1.00000000	mice
Tbx4	0.30	1.00000000	mice
Cpsf2	0.08	1.00000000	mice
Eci1	-0.04	1.00000000	mice
Srpk2	0.09	1.00000000	mice
Prdx1	0.11	1.00000000	mice
Tmem219	-0.01	1.00000000	mice
Cct3	0.13	1.00000000	mice

Psma1	0.18	1.00000000	mice
Krcc1	0.00	1.00000000	mice
Plcl2	0.00	1.00000000	mice
Cetn3	0.07	1.00000000	mice
Ccnyl1	-0.05	1.00000000	mice
Foxj3	0.01	1.00000000	mice
Nol11	-0.02	1.00000000	mice
Zpr1	0.00	1.00000000	mice
Amz2	0.05	1.00000000	mice
Ptpn4	0.14	1.00000000	mice
Slc12a4	0.07	1.00000000	mice
Abcf1	-0.01	1.00000000	mice
Chchd1	0.19	1.00000000	mice
Traf3	0.15	1.00000000	mice
Rabggtb	0.09	1.00000000	mice
Usp45	0.01	1.00000000	mice
Lsg1	0.02	1.00000000	mice
Topors	0.15	1.00000000	mice
Gtf2a1	0.02	1.00000000	mice
Akr1b3	0.20	1.00000000	mice
Nudcd3	0.15	1.00000000	mice
Adprhl2	0.01	1.00000000	mice
Cep83	-0.03	1.00000000	mice
Sept9	0.10	1.00000000	mice
Eif5a	0.26	1.00000000	mice
Rab8a	0.19	1.00000000	mice
Uri1	0.09	1.00000000	mice
Ppt1	0.22	1.00000000	mice
Acadsb	0.03	1.00000000	mice
Trp53i13	0.00	1.00000000	mice
Tmem14c	0.02	1.00000000	mice
Cobll1	0.07	1.00000000	mice
1700123O20Rik	0.07	1.00000000	mice
Prrx2	0.16	1.00000000	mice
E2f5	-0.03	1.00000000	mice
Eva1b	0.28	1.00000000	mice
Git2	0.07	1.00000000	mice
Dnajc15	0.12	1.00000000	mice
Hacd2	-0.03	1.00000000	mice
Sh3bgrl3	-0.02	1.00000000	mice
Efr3a	0.05	1.00000000	mice
Fgd4	-0.07	1.00000000	mice
Rdx	0.06	1.00000000	mice
Efnb1	-0.01	1.00000000	mice
Gpr108	-0.02	1.00000000	mice
Tomm7	0.17	1.00000000	mice

Phkb	0.06	1.00000000	mice
Cnot6l	0.02	1.00000000	mice
Nob1	-0.11	1.00000000	mice
Tnfrsf1a	0.17	1.00000000	mice
Ephb4	-0.02	1.00000000	mice
Dgkh	-0.03	1.00000000	mice
Gosr2	0.07	1.00000000	mice
Hira	0.02	1.00000000	mice
Col23a1	0.39	1.00000000	mice
Ldlr	0.08	1.00000000	mice
Glmp	0.04	1.00000000	mice
Lgals3	0.28	1.00000000	mice
Siah2	-0.06	1.00000000	mice
Akirin1	-0.02	1.00000000	mice
Rnf139	0.00	1.00000000	mice
St3gal1	-0.06	1.00000000	mice
Eprs	-0.01	1.00000000	mice
Yif1b	0.21	1.00000000	mice
Zfp24	0.06	1.00000000	mice
Pef1	0.05	1.00000000	mice
Ccng1	0.02	1.00000000	mice
Ing4	0.06	1.00000000	mice
Smap1	0.15	1.00000000	mice
Gtf3c6	0.04	1.00000000	mice
Pisd	0.02	1.00000000	mice
Rab4b	0.16	1.00000000	mice
Nlgn1	-0.19	1.00000000	mice
Lsm7	0.14	1.00000000	mice
Naa10	0.00	1.00000000	mice
Atp6v1b2	0.12	1.00000000	mice
Vegfc	-0.04	1.00000000	mice
Gpc4	-0.04	1.00000000	mice
Zdhhc21	-0.09	1.00000000	mice
Rtca	-0.01	1.00000000	mice
Orc6	0.10	1.00000000	mice
Sdhb	-0.03	1.00000000	mice
Runx1t1	0.11	1.00000000	mice
Plekha1	0.05	1.00000000	mice
Bmi1	0.10	1.00000000	mice
Sulf1	-0.16	1.00000000	mice
Smg6	0.06	1.00000000	mice
Smarcad1	-0.05	1.00000000	mice
Cox19	0.05	1.00000000	mice
Ssh1	0.05	1.00000000	mice
Tia1	0.06	1.00000000	mice
Rest	0.07	1.00000000	mice

Uqcrc1	0.03	1.00000000	mice
Scyl2	-0.06	1.00000000	mice
8030462N17Rik	0.08	1.00000000	mice
Rnf20	-0.08	1.00000000	mice
Gna13	0.19	1.00000000	mice
Frem1	0.12	1.00000000	mice
Pin1	0.05	1.00000000	mice
Actr1b	0.03	1.00000000	mice
Egr2	0.01	1.00000000	mice
Tspan4	0.13	1.00000000	mice
Adgra3	0.08	1.00000000	mice
Pex5	-0.01	1.00000000	mice
Fstl1	0.10	1.00000000	mice
Nsun6	-0.05	1.00000000	mice
Nebl	0.19	1.00000000	mice
Dync1h1	0.23	1.00000000	mice
D5Ertd579e	-0.03	1.00000000	mice
Zmym4	-0.08	1.00000000	mice
Tmem41b	-0.01	1.00000000	mice
Prkar1b	0.05	1.00000000	mice
Cluap1	0.05	1.00000000	mice
Ilrun	0.16	1.00000000	mice
Stxbp4	0.00	1.00000000	mice
Ctbs	0.04	1.00000000	mice
Dnpep	0.13	1.00000000	mice
Sucla2	0.12	1.00000000	mice
Farsa	0.18	1.00000000	mice
Gdf15	0.05	1.00000000	mice
Yae1d1	0.05	1.00000000	mice
Gk	0.00	1.00000000	mice
Nrp2	0.00	1.00000000	mice
Pcbp1	0.17	1.00000000	mice
Taldo1	0.04	1.00000000	mice
Casp8ap2	0.03	1.00000000	mice
Zfx	-0.07	1.00000000	mice
Nckap5	0.00	1.00000000	mice
Bin3	0.00	1.00000000	mice
Agfg1	-0.05	1.00000000	mice
Ssrp1	0.10	1.00000000	mice
Ehd3	-0.04	1.00000000	mice
Gon4l	-0.03	1.00000000	mice
Brf1	-0.08	1.00000000	mice
Dnajb14	-0.04	1.00000000	mice
Armc1	-0.02	1.00000000	mice
Birc6	0.01	1.00000000	mice
Ndrg3	0.01	1.00000000	mice

Amfr	0.17	1.00000000	mice
Dock10	-0.10	1.00000000	mice
Cyb5a	0.24	1.00000000	mice
Sgk3	0.03	1.00000000	mice
Brcc3	0.10	1.00000000	mice
Fbln2	-0.07	1.00000000	mice
Timm22	-0.08	1.00000000	mice
Tbc1d1	-0.03	1.00000000	mice
Atg12	0.03	1.00000000	mice
Hypk	-0.02	1.00000000	mice
Rai14	-0.05	1.00000000	mice
Isca1	0.01	1.00000000	mice
4930453N24Rik	-0.08	1.00000000	mice
Rac1	0.16	1.00000000	mice
Saysd1	-0.03	1.00000000	mice
Gnb2	0.10	1.00000000	mice
Rnf168	0.08	1.00000000	mice
Sdf4	0.09	1.00000000	mice
Sqstm1	0.03	1.00000000	mice
Traf7	-0.01	1.00000000	mice
Rnf4	-0.11	1.00000000	mice
C9orf72	0.00	1.00000000	mice
Sod1	0.25	1.00000000	mice
Arhgef6	-0.08	1.00000000	mice
Fmnl3	0.08	1.00000000	mice
Eif4ebp1	0.22	1.00000000	mice
Ammecr1	-0.09	1.00000000	mice
U2af1l4	0.06	1.00000000	mice
Ndufc1	0.26	1.00000000	mice
Tor1a	0.09	1.00000000	mice
Cdc27	0.09	1.00000000	mice
Exosc7	0.11	1.00000000	mice
Plekhh3	0.02	1.00000000	mice
Emc7	0.13	1.00000000	mice
Chd4	0.03	1.00000000	mice
Klf10	0.07	1.00000000	mice
Kpna6	-0.05	1.00000000	mice
Soga1	0.07	1.00000000	mice
Csk	0.00	1.00000000	mice
Dhrs4	0.00	1.00000000	mice
Cdkl5	0.08	1.00000000	mice
Ncbp2	0.00	1.00000000	mice
Kmt2d	0.01	1.00000000	mice
Dapk3	0.06	1.00000000	mice
3110082I17Rik	0.06	1.00000000	mice
Rexo2	0.30	1.00000000	mice

Slc35e1	0.05	1.00000000	mice
Phf12	-0.03	1.00000000	mice
Dhrs1	-0.04	1.00000000	mice
1700021F05Rik	0.07	1.00000000	mice
Grem2	0.00	1.00000000	mice
Rarg	-0.05	1.00000000	mice
Gng12	0.12	1.00000000	mice
Ap2a1	-0.01	1.00000000	mice
Dctn1	0.01	1.00000000	mice
Usp15	-0.02	1.00000000	mice
Rftn2	0.00	1.00000000	mice
Tigd2	0.00	1.00000000	mice
Fam8a1	0.05	1.00000000	mice
Per2	0.04	1.00000000	mice
Cflar	0.25	1.00000000	mice
Prr13	0.30	1.00000000	mice
Zmat5	0.14	1.00000000	mice
Scamp2	0.03	1.00000000	mice
Wdr75	0.10	1.00000000	mice
Ubtf	0.14	1.00000000	mice
Loxl3	0.08	1.00000000	mice
Cnrip1	-0.06	1.00000000	mice
Golgb1	0.15	1.00000000	mice
S100a4	-0.10	1.00000000	mice
Utp15	-0.02	1.00000000	mice
Ube2l3	0.25	1.00000000	mice
Smn1	0.14	1.00000000	mice
Leo1	0.04	1.00000000	mice
Tanc1	0.18	1.00000000	mice
Sptlc2	0.18	1.00000000	mice
Cd99l2	0.02	1.00000000	mice
Rif1	0.18	1.00000000	mice
Dazap1	0.10	1.00000000	mice
Rrad	0.01	1.00000000	mice
Erp29	0.16	1.00000000	mice
Plscr3	0.08	1.00000000	mice
Invs	0.04	1.00000000	mice
Naxd	0.06	1.00000000	mice
Spata6	0.06	1.00000000	mice
Rhoc	0.41	1.00000000	mice
Trp53inp2	-0.04	1.00000000	mice
1810037I17Rik	0.18	1.00000000	mice
Cxcl12	0.16	1.00000000	mice
Poglut1	0.01	1.00000000	mice
Lpcat3	0.07	1.00000000	mice
Nub1	0.25	1.00000000	mice

Heatr5b	-0.02	1.00000000	mice
Pknox2	0.20	1.00000000	mice
Lynx1	0.00	1.00000000	mice
Prmt1	0.08	1.00000000	mice
Fam234a	-0.01	1.00000000	mice
Tfeb	-0.05	1.00000000	mice
Polr2i	0.20	1.00000000	mice
Tlnrd1	-0.03	1.00000000	mice
Eif3a	0.10	1.00000000	mice
Ctdsp2	0.18	1.00000000	mice
Cpsf6	-0.02	1.00000000	mice
U2af2	-0.03	1.00000000	mice
Tsg101	0.12	1.00000000	mice
Wdtc1	-0.08	1.00000000	mice
Acbd3	0.15	1.00000000	mice
Bckdha	-0.03	1.00000000	mice
Gpx3	0.52	1.00000000	mice
Sap30bp	0.03	1.00000000	mice
Ddx39	0.11	1.00000000	mice
Pus10	0.01	1.00000000	mice
Apmap	0.14	1.00000000	mice
Ehmt2	0.06	1.00000000	mice
Arhgef25	0.11	1.00000000	mice
Dgat2	0.01	1.00000000	mice
Triap1	0.05	1.00000000	mice
Psenen	0.16	1.00000000	mice
Ppp4r1	-0.07	1.00000000	mice
Tmem30a	0.12	1.00000000	mice
Glb1	-0.04	1.00000000	mice
Rbm3	0.12	1.00000000	mice
Zc3h6	0.03	1.00000000	mice
Rgcc	-0.16	1.00000000	mice
Ppia	0.12	1.00000000	mice
Chordc1	0.02	1.00000000	mice
3830406C13Rik	0.11	1.00000000	mice
Tmem184b	0.07	1.00000000	mice
Gar1	0.02	1.00000000	mice
Iqgap2	-0.17	1.00000000	mice
Arf1	0.11	1.00000000	mice
Celf1	0.10	1.00000000	mice
Pgap2	0.07	1.00000000	mice
Anp32b	0.11	1.00000000	mice
Arfip2	0.05	1.00000000	mice
Syn2	0.06	1.00000000	mice
Cox4i2	0.37	1.00000000	mice
Cyp51	0.07	1.00000000	mice

Smcr8	0.02	1.00000000	mice
Fam160b1	-0.04	1.00000000	mice
Babam2	0.00	1.00000000	mice
Atox1	0.27	1.00000000	mice
Phc3	-0.04	1.00000000	mice
Abracl	0.22	1.00000000	mice
2310009B15Rik	-0.01	1.00000000	mice
Polr2d	0.04	1.00000000	mice
Secisbp2	0.01	1.00000000	mice
Copg1	0.09	1.00000000	mice
Pik3r1	0.26	1.00000000	mice
Sdhaf1	0.17	1.00000000	mice
Hpf1	0.07	1.00000000	mice
Prkcd	0.02	1.00000000	mice
Fkbp8	0.23	1.00000000	mice
Zrsr2	0.10	1.00000000	mice
Dcakd	-0.01	1.00000000	mice
Slc35a2	0.07	1.00000000	mice
Rnf19b	0.08	1.00000000	mice
Stx4a	0.10	1.00000000	mice
Urm1	0.10	1.00000000	mice
Emd	0.13	1.00000000	mice
Hnrnpul2	0.00	1.00000000	mice
Synj2bp	0.01	1.00000000	mice
Garnl3	-0.01	1.00000000	mice
Cask	0.03	1.00000000	mice
Imp4	0.17	1.00000000	mice
Ccdc141	-0.07	1.00000000	mice
Ncstn	0.07	1.00000000	mice
Yes1	-0.13	1.00000000	mice
Srr	-0.02	1.00000000	mice
Rab14	0.04	1.00000000	mice
Gas6	0.43	1.00000000	mice
Kansl2	0.09	1.00000000	mice
Prkd3	0.04	1.00000000	mice
Usp40	0.06	1.00000000	mice
Arhgap31	0.08	1.00000000	mice
Kars	0.03	1.00000000	mice
Glis3	-0.13	1.00000000	mice
Vps16	0.06	1.00000000	mice
Bcas2	0.21	1.00000000	mice
Ttc37	0.01	1.00000000	mice
Golga5	0.04	1.00000000	mice
Usp46	0.00	1.00000000	mice
Dennd1b	0.14	1.00000000	mice
Rrp7a	0.14	1.00000000	mice

Idh3g	0.10	1.00000000	mice
Sbf1	-0.04	1.00000000	mice
Ddt	0.09	1.00000000	mice
Atp6v1e1	0.19	1.00000000	mice
Plgrkt	0.05	1.00000000	mice
Clptm1l	0.23	1.00000000	mice
Poldip3	-0.01	1.00000000	mice
Spopl	0.08	1.00000000	mice
Zfp532	-0.02	1.00000000	mice
Polr3d	0.02	1.00000000	mice
Cmtm6	0.06	1.00000000	mice
Swt1	0.04	1.00000000	mice
Capn1	0.06	1.00000000	mice
CAAA01118383.1	0.04	1.00000000	mice
Golga2	0.04	1.00000000	mice
Cyb5r1	-0.02	1.00000000	mice
Ric8a	0.00	1.00000000	mice
Ppp4r3b	0.11	1.00000000	mice
Ufl1	0.05	1.00000000	mice
Ngly1	-0.03	1.00000000	mice
Rsbn1l	0.11	1.00000000	mice
Lmbr1	0.14	1.00000000	mice
Fam117a	-0.01	1.00000000	mice
Tent4b	0.03	1.00000000	mice
Eci2	0.17	1.00000000	mice
Gopc	0.01	1.00000000	mice
Tlk1	-0.05	1.00000000	mice
Ipo4	-0.01	1.00000000	mice
Acat1	0.08	1.00000000	mice
Nr4a1	0.18	1.00000000	mice
Syn3	0.14	1.00000000	mice
Srprb	0.04	1.00000000	mice
Pank2	0.00	1.00000000	mice
Arf2	0.14	1.00000000	mice
Sirpa	0.09	1.00000000	mice
2410002F23Rik	0.08	1.00000000	mice
Slc8b1	0.09	1.00000000	mice
Cd53	0.11	1.00000000	mice
Rasa3	-0.14	1.00000000	mice
Rbx1	0.15	1.00000000	mice
Pafah1b2	0.04	1.00000000	mice
Slc26a2	0.07	1.00000000	mice
Arsb	-0.03	1.00000000	mice
Selenon	-0.04	1.00000000	mice
Fam13c	-0.01	1.00000000	mice
Tsc1	-0.03	1.00000000	mice

Fkbp10	0.14	1.00000000	mice
Exosc8	0.01	1.00000000	mice
Pdss2	-0.13	1.00000000	mice
Stim2	0.03	1.00000000	mice
Pxmp4	-0.01	1.00000000	mice
Zfp62	0.06	1.00000000	mice
Ldlrap1	0.02	1.00000000	mice
Hmgn5	-0.09	1.00000000	mice
Slc39a1	0.12	1.00000000	mice
Bag5	0.11	1.00000000	mice
Clec16a	-0.01	1.00000000	mice
Pten	0.16	1.00000000	mice
Wbp1l	0.02	1.00000000	mice
Pigu	-0.01	1.00000000	mice
Gla	0.10	1.00000000	mice
Ppp1r13b	-0.01	1.00000000	mice
Pepd	0.03	1.00000000	mice
Adm	0.43	1.00000000	mice
Gatd3a	-0.02	1.00000000	mice
Cygb	-0.03	1.00000000	mice
Vma21	0.05	1.00000000	mice
Got2	-0.04	1.00000000	mice
Tmem128	0.02	1.00000000	mice
Pigs	0.06	1.00000000	mice
Snrpe	0.33	1.00000000	mice
Hp1bp3	0.04	1.00000000	mice
Xpo1	0.09	1.00000000	mice
Krtcap2	0.22	1.00000000	mice
Camk1	0.16	1.00000000	mice
1500011B03Rik	0.04	1.00000000	mice
Tmem259	-0.07	1.00000000	mice
Efcc1	0.05	1.00000000	mice
Chd8	0.04	1.00000000	mice
Yipf1	0.13	1.00000000	mice
Orc2	-0.06	1.00000000	mice
Wdr13	0.02	1.00000000	mice
Usp8	0.02	1.00000000	mice
Creb3	0.24	1.00000000	mice
Bfar	0.08	1.00000000	mice
Nek1	0.00	1.00000000	mice
Bbip1	0.02	1.00000000	mice
Dmac1	0.08	1.00000000	mice
Dnmbp	0.02	1.00000000	mice
Pacs2	0.08	1.00000000	mice
Ssr1	0.10	1.00000000	mice
Utp3	0.13	1.00000000	mice

Kdm4c	0.02	1.00000000	mice
Virma	0.07	1.00000000	mice
Dis3	-0.06	1.00000000	mice
Twf1	0.07	1.00000000	mice
Prpsap1	0.16	1.00000000	mice
Pla2r1	0.17	1.00000000	mice
Gltp	0.10	1.00000000	mice
Katnbl1	0.09	1.00000000	mice
Bcl3	-0.07	1.00000000	mice
Brinp1	-0.21	1.00000000	mice
Snf8	0.06	1.00000000	mice
Tcp11l1	0.02	1.00000000	mice
Idh3a	0.03	1.00000000	mice
Anapc1	0.05	1.00000000	mice
Agps	-0.05	1.00000000	mice
Nr3c2	0.07	1.00000000	mice
Aldh3a2	0.06	1.00000000	mice
Ptprc	0.08	1.00000000	mice
Abcg2	0.00	1.00000000	mice
Golga4	-0.05	1.00000000	mice
Nr2f6	0.00	1.00000000	mice
Sirt7	-0.03	1.00000000	mice
Dmpk	-0.02	1.00000000	mice
Fnbp1l	0.01	1.00000000	mice
Larp1	0.05	1.00000000	mice
Renbp	0.02	1.00000000	mice
Ppp6r1	0.02	1.00000000	mice
Nox4	0.12	1.00000000	mice
Tkt	0.11	1.00000000	mice
4931406C07Rik	0.13	1.00000000	mice
Polr2b	0.01	1.00000000	mice
Flii	-0.01	1.00000000	mice
Ugcg	0.18	1.00000000	mice
Yme1l1	-0.02	1.00000000	mice
Calhm2	-0.05	1.00000000	mice
Lmbrd2	0.00	1.00000000	mice
Cwc25	-0.01	1.00000000	mice
Eif4a3	0.21	1.00000000	mice
Hspa1a	0.18	1.00000000	mice
Col13a1	0.33	1.00000000	mice
Cmtm7	0.05	1.00000000	mice
Tssc4	0.02	1.00000000	mice
Prmt2	0.00	1.00000000	mice
Slc31a2	0.08	1.00000000	mice
Pdcd6	0.19	1.00000000	mice
Slc5a3	0.12	1.00000000	mice

Isca2	0.09	1.00000000	mice
Fundc2	0.33	1.00000000	mice
Nifk	-0.01	1.00000000	mice
Alcam	0.08	1.00000000	mice
Slc45a4	-0.01	1.00000000	mice
Paxbp1	0.02	1.00000000	mice
Apbb1	0.00	1.00000000	mice
Irak2	0.21	1.00000000	mice
Nav3	-0.13	1.00000000	mice
Zfat	-0.06	1.00000000	mice
Ptpa	0.01	1.00000000	mice
Cdc42ep1	0.07	1.00000000	mice
Smim1	0.03	1.00000000	mice
Prr12	-0.06	1.00000000	mice
B3gat3	0.02	1.00000000	mice
Cops9	0.17	1.00000000	mice
Elovl6	0.02	1.00000000	mice
Atp13a1	-0.03	1.00000000	mice
Atic	0.03	1.00000000	mice
Crbn	-0.02	1.00000000	mice
Cenpc1	-0.08	1.00000000	mice
Ube2m	0.16	1.00000000	mice
Tef	0.07	1.00000000	mice
Pomp	0.25	1.00000000	mice
Pa2g4	0.24	1.00000000	mice
Sik1	0.04	1.00000000	mice
Rhot1	0.05	1.00000000	mice
Ocel1	0.10	1.00000000	mice
Pigx	0.07	1.00000000	mice
Rnf6	-0.08	1.00000000	mice
Otud7b	-0.01	1.00000000	mice
Rbm34	0.04	1.00000000	mice
Ak6	-0.06	1.00000000	mice
Gatd1	-0.02	1.00000000	mice
Csad	0.11	1.00000000	mice
Tbc1d2b	-0.02	1.00000000	mice
Slc23a2	0.03	1.00000000	mice
Pip4p1	-0.01	1.00000000	mice
Kcnip4	0.61	1.00000000	mice
Lrpap1	0.26	1.00000000	mice
Iah1	-0.09	1.00000000	mice
Cct6a	0.05	1.00000000	mice
Chtop	0.12	1.00000000	mice
Sms	0.12	1.00000000	mice
Rprd1b	0.06	1.00000000	mice
Lsm5	0.10	1.00000000	mice

Sec23a	-0.07	1.00000000	mice
Rrp36	0.06	1.00000000	mice
Ints7	-0.03	1.00000000	mice
Lrig2	-0.07	1.00000000	mice
Orc4	0.00	1.00000000	mice
Ppwd1	0.04	1.00000000	mice
Stard13	-0.02	1.00000000	mice
Fibp	0.22	1.00000000	mice
Nhp2	0.14	1.00000000	mice
Rilpl1	0.00	1.00000000	mice
Emc1	0.04	1.00000000	mice
Ppp3ca	0.00	1.00000000	mice
Cdk7	-0.04	1.00000000	mice
Tmco1	0.35	1.00000000	mice
Stam2	-0.03	1.00000000	mice
Ranbp10	0.04	1.00000000	mice
Pex7	0.00	1.00000000	mice
Dnmt1	0.06	1.00000000	mice
Snapc3	0.07	1.00000000	mice
Ltv1	0.02	1.00000000	mice
Cpeb3	0.06	1.00000000	mice
Psen2	0.10	1.00000000	mice
Prpf38b	0.15	1.00000000	mice
Nkap	0.09	1.00000000	mice
Exosc4	0.09	1.00000000	mice
Slc44a1	0.13	1.00000000	mice
Cog8	0.00	1.00000000	mice
Bicra	0.04	1.00000000	mice
Naga	0.00	1.00000000	mice
Ggcx	-0.03	1.00000000	mice
Arf3	0.03	1.00000000	mice
Ndufa8	0.16	1.00000000	mice
Lrrc8c	-0.01	1.00000000	mice
Ubl4a	0.18	1.00000000	mice
Cox16	0.16	1.00000000	mice
Lama4	0.08	1.00000000	mice
Prkaca	0.10	1.00000000	mice
Rnf41	0.00	1.00000000	mice
Atg10	0.00	1.00000000	mice
Vps50	0.00	1.00000000	mice
Pbx2	-0.04	1.00000000	mice
Acsl4	0.00	1.00000000	mice
Dab2	-0.11	1.00000000	mice
Top3b	-0.06	1.00000000	mice
Pfkl	0.02	1.00000000	mice
Ogfrl1	0.11	1.00000000	mice

Tmx1	-0.11	1.00000000	mice
Enox2	-0.07	1.00000000	mice
Ndufs3	0.10	1.00000000	mice
Zfp68	0.00	1.00000000	mice
Cntrl	-0.04	1.00000000	mice
Ptk2b	0.10	1.00000000	mice
Tmem60	0.07	1.00000000	mice
Prkg2	0.10	1.00000000	mice
Rfxap	-0.08	1.00000000	mice
Ciapin1	0.01	1.00000000	mice
Lrig1	-0.02	1.00000000	mice
Tmed1	0.19	1.00000000	mice
Pmm2	0.00	1.00000000	mice
Atf7ip	0.17	1.00000000	mice
Amotl2	0.00	1.00000000	mice
Lrig3	0.02	1.00000000	mice
Setd1b	-0.02	1.00000000	mice
Ivd	-0.07	1.00000000	mice
Atf5	0.15	1.00000000	mice
2410004B18Rik	0.05	1.00000000	mice
Arglu1	0.16	1.00000000	mice
Dlg5	0.00	1.00000000	mice
Sap18	0.16	1.00000000	mice
Wwp2	0.10	1.00000000	mice
Nucks1	0.17	1.00000000	mice
Ift81	-0.03	1.00000000	mice
Hacd3	-0.01	1.00000000	mice
Stx11	0.06	1.00000000	mice
Alg5	0.10	1.00000000	mice
Edem1	-0.07	1.00000000	mice
Smad3	0.09	1.00000000	mice
Tet3	0.08	1.00000000	mice
Cdc40	-0.03	1.00000000	mice
Exoc2	0.04	1.00000000	mice
Srd5a3	-0.06	1.00000000	mice
Olfm1	0.08	1.00000000	mice
Lamp2	0.07	1.00000000	mice
Ndufb8	0.14	1.00000000	mice
Rnf215	-0.06	1.00000000	mice
Spcs1	0.21	1.00000000	mice
Vps26c	0.04	1.00000000	mice
Tjap1	0.04	1.00000000	mice
Nadk2	0.05	1.00000000	mice
Nelfb	0.07	1.00000000	mice
Pkn2	0.09	1.00000000	mice
Cox20	0.15	1.00000000	mice

Col1a2	-0.03	1.00000000	mice
Zfp639	-0.03	1.00000000	mice
Hspe1	0.20	1.00000000	mice
Fam120a	0.04	1.00000000	mice
Psmd9	0.11	1.00000000	mice
Rxylt1	0.10	1.00000000	mice
Tomm34	-0.06	1.00000000	mice
Blvra	-0.02	1.00000000	mice
Frmd4a	-0.18	1.00000000	mice
Zfp266	-0.02	1.00000000	mice
Tmem38b	0.20	1.00000000	mice
Ecpas	0.03	1.00000000	mice
5330438D12Rik	0.06	1.00000000	mice
H1f0	0.12	1.00000000	mice
Snx10	0.04	1.00000000	mice
Lztfl1	0.10	1.00000000	mice
Zfp9	-0.04	1.00000000	mice
Cript	0.27	1.00000000	mice
Gab1	0.06	1.00000000	mice
Aasdhppt	0.01	1.00000000	mice
Nup88	-0.01	1.00000000	mice
Dhx30	-0.03	1.00000000	mice
Oxa1l	0.04	1.00000000	mice
Tbcb	0.18	1.00000000	mice
Elmo2	0.03	1.00000000	mice
Rab11fip2	0.00	1.00000000	mice
Pola1	-0.04	1.00000000	mice
Tpk1	-0.01	1.00000000	mice
Upf2	-0.05	1.00000000	mice
Arg2	0.12	1.00000000	mice
Hspb11	0.10	1.00000000	mice
Fam49b	-0.05	1.00000000	mice
Asph	-0.10	1.00000000	mice
Lrba	0.03	1.00000000	mice
Setx	0.05	1.00000000	mice
Nkapd1	0.06	1.00000000	mice
Slc25a16	0.01	1.00000000	mice
Ikbip	-0.01	1.00000000	mice
Ostc	0.21	1.00000000	mice
Sgip1	0.18	1.00000000	mice
Fdft1	0.04	1.00000000	mice
Nf2	-0.02	1.00000000	mice
Adnp2	0.04	1.00000000	mice
Chst2	0.07	1.00000000	mice
Sec31a	0.12	1.00000000	mice
Carmil1	-0.09	1.00000000	mice

Washc1	0.10	1.00000000	mice
Leprotl1	0.00	1.00000000	mice
Psmb1	0.15	1.00000000	mice
Nsfl1c	-0.01	1.00000000	mice
Psmd7	0.11	1.00000000	mice
Gtf3c2	0.01	1.00000000	mice
Irf2bp1	0.04	1.00000000	mice
Rbm10	0.06	1.00000000	mice
Dimt1	0.07	1.00000000	mice
Fsd1l	-0.01	1.00000000	mice
Atp6v0a1	0.03	1.00000000	mice
Nono	0.08	1.00000000	mice
B3galt1	0.14	1.00000000	mice
Arl5b	0.08	1.00000000	mice
Fgl2	-0.11	1.00000000	mice
Lrrc59	0.20	1.00000000	mice
Vezt	0.10	1.00000000	mice
Usp39	0.05	1.00000000	mice
Nme1	0.25	1.00000000	mice
Arpc1b	0.10	1.00000000	mice
Ncoa4	0.18	1.00000000	mice
Gosr1	0.03	1.00000000	mice
Fbxl5	-0.09	1.00000000	mice
Snhg4	0.02	1.00000000	mice
Yeats4	0.06	1.00000000	mice
Ndufv1	0.02	1.00000000	mice
Ssr4	0.36	1.00000000	mice
Ptprf	-0.03	1.00000000	mice
Got1	0.03	1.00000000	mice
Fam133b	0.08	1.00000000	mice
Parl	0.09	1.00000000	mice
Ddb1	0.04	1.00000000	mice
Golt1b	0.07	1.00000000	mice
Fis1	0.09	1.00000000	mice
Ralgps2	-0.01	1.00000000	mice
Ppih	0.02	1.00000000	mice
Ap2a2	0.03	1.00000000	mice
Slc25a46	0.07	1.00000000	mice
Dusp1	0.04	1.00000000	mice
Gbf1	-0.09	1.00000000	mice
Ophn1	-0.02	1.00000000	mice
Armc10	0.09	1.00000000	mice
Sulf2	-0.01	1.00000000	mice
Ints11	0.04	1.00000000	mice
Ndufa4l2	0.31	1.00000000	mice
Prkacb	-0.05	1.00000000	mice

Col6a3	0.01	1.00000000	mice
Plcd1	0.05	1.00000000	mice
Rpa1	0.04	1.00000000	mice
Tsen15	0.08	1.00000000	mice
Anks3	0.02	1.00000000	mice
Trnt1	0.02	1.00000000	mice
Lztr1	0.02	1.00000000	mice
Phf14	-0.03	1.00000000	mice
Slc35a1	0.05	1.00000000	mice
Lap3	0.13	1.00000000	mice
Spon1	-0.17	1.00000000	mice
Srsf3	0.17	1.00000000	mice
Shprh	-0.03	1.00000000	mice
Ddx47	-0.02	1.00000000	mice
Gpm6b	-0.04	1.00000000	mice
Triobp	0.03	1.00000000	mice
2700081O15Rik	0.07	1.00000000	mice
Naglu	0.00	1.00000000	mice
Tnfaip3	0.24	1.00000000	mice
Ctr9	0.07	1.00000000	mice
Trappc13	0.06	1.00000000	mice
Notch1	0.06	1.00000000	mice
Phb2	0.08	1.00000000	mice
Dek	0.06	1.00000000	mice
Rgmb	0.10	1.00000000	mice
Zdhhc20	-0.01	1.00000000	mice
Hectd2	0.01	1.00000000	mice
Atg3	0.01	1.00000000	mice
Csnk2a1	0.15	1.00000000	mice
Wtap	-0.13	1.00000000	mice
Ttc17	0.10	1.00000000	mice
Tcea3	0.05	1.00000000	mice
Adgrl2	-0.05	1.00000000	mice
Smad5	-0.03	1.00000000	mice
Borcs8	0.06	1.00000000	mice
Nelfe	0.01	1.00000000	mice
Ndufb3	0.30	1.00000000	mice
Eml4	0.10	1.00000000	mice
Wiz	0.00	1.00000000	mice
0610012G03Rik	0.23	1.00000000	mice
Nop14	0.11	1.00000000	mice
Ufd1	0.00	1.00000000	mice
Cotl1	-0.02	1.00000000	mice
D030056L22Rik	0.08	1.00000000	mice
Phpt1	0.22	1.00000000	mice
Ssr2	0.25	1.00000000	mice

Gli3	-0.05	1.00000000	mice
Angel2	0.13	1.00000000	mice
Nid1	-0.03	1.00000000	mice
Dexi	0.07	1.00000000	mice
Hspb2	0.24	1.00000000	mice
Pold3	-0.01	1.00000000	mice
Ccdc59	0.22	1.00000000	mice
Topbp1	0.08	1.00000000	mice
Snapc1	0.01	1.00000000	mice
Son	0.15	1.00000000	mice
Plbd2	0.08	1.00000000	mice
Ktn1	0.14	1.00000000	mice
Ranbp1	0.21	1.00000000	mice
Fam114a2	0.13	1.00000000	mice
Napa	-0.04	1.00000000	mice
Hmbox1	0.00	1.00000000	mice
Dnm1l	0.07	1.00000000	mice
Ppp1r37	0.03	1.00000000	mice
Dap3	-0.06	1.00000000	mice
Plxna1	0.03	1.00000000	mice
Rnf34	0.14	1.00000000	mice
B3gnt9	0.04	1.00000000	mice
Otub1	0.10	1.00000000	mice
Noc2l	-0.08	1.00000000	mice
Akap7	0.12	1.00000000	mice
Fbxl20	0.02	1.00000000	mice
Cs	0.11	1.00000000	mice
Elk4	0.02	1.00000000	mice
Gpatch1	0.02	1.00000000	mice
2310009A05Rik	0.12	1.00000000	mice
Atf6b	0.02	1.00000000	mice
Tollip	0.07	1.00000000	mice
Ireb2	0.07	1.00000000	mice
Scaf4	-0.01	1.00000000	mice
Pgs1	0.02	1.00000000	mice
Trim28	0.05	1.00000000	mice
Arl2	0.06	1.00000000	mice
Lman1	0.10	1.00000000	mice
Actr2	0.09	1.00000000	mice
Pex19	0.14	1.00000000	mice
U2surp	0.06	1.00000000	mice
Egr3	0.14	1.00000000	mice
Akr1a1	0.16	1.00000000	mice
Lpl	0.13	1.00000000	mice
Epg5	-0.01	1.00000000	mice
Cdc42bpb	0.01	1.00000000	mice

Ppp1r35	0.04	1.00000000	mice
Copg2	-0.02	1.00000000	mice
Ube4a	-0.06	1.00000000	mice
Tfe3	0.10	1.00000000	mice
Dnajc25	0.03	1.00000000	mice
Slc43a2	0.03	1.00000000	mice
Polr2g	0.14	1.00000000	mice
Lta4h	0.02	1.00000000	mice
Gpsm2	0.09	1.00000000	mice
Clptm1	0.09	1.00000000	mice
Rab5b	-0.10	1.00000000	mice
Pex14	0.02	1.00000000	mice
Taf7	0.01	1.00000000	mice
Rap2b	-0.03	1.00000000	mice
Prex1	0.08	1.00000000	mice
Ncoa7	0.05	1.00000000	mice
Prmt3	-0.01	1.00000000	mice
Slc30a9	0.03	1.00000000	mice
Aco1	-0.09	1.00000000	mice
Utp6	0.01	1.00000000	mice
Nat10	-0.02	1.00000000	mice
Tmem160	0.22	1.00000000	mice
Pank3	0.01	1.00000000	mice
Nipsnap2	0.03	1.00000000	mice
Sept7	0.11	1.00000000	mice
Nhlrc2	0.04	1.00000000	mice
Tardbp	-0.09	1.00000000	mice
Zxdc	-0.01	1.00000000	mice
Ccs	0.02	1.00000000	mice
Ddhd2	0.05	1.00000000	mice
Plpbp	-0.07	1.00000000	mice
Dcaf12	0.07	1.00000000	mice
Eif2ak1	0.08	1.00000000	mice
Tmem189	0.02	1.00000000	mice
Txndc9	0.11	1.00000000	mice
Smim4	0.17	1.00000000	mice
Tspan17	0.10	1.00000000	mice
Zfp397	0.10	1.00000000	mice
Pgk1	0.08	1.00000000	mice
Ifngr2	0.00	1.00000000	mice
Faf2	0.05	1.00000000	mice
Dhx36	0.04	1.00000000	mice
L3mbtl3	0.02	1.00000000	mice
Fam126a	0.02	1.00000000	mice
Dcbld2	-0.03	1.00000000	mice
Tmem134	0.07	1.00000000	mice

Rbck1	0.03	1.00000000	mice
Tbc1d5	0.02	1.00000000	mice
Rbm4	0.03	1.00000000	mice
Cbfb	0.01	1.00000000	mice
Timm50	0.03	1.00000000	mice
Ccdc174	0.05	1.00000000	mice
Nufip1	-0.02	1.00000000	mice
Nfu1	0.09	1.00000000	mice
Ccdc127	0.03	1.00000000	mice
Fmo1	0.22	1.00000000	mice
Atp5l	0.15	1.00000000	mice
Cmc1	0.05	1.00000000	mice
Ankrd27	0.02	1.00000000	mice
Rdh10	0.03	1.00000000	mice
Nr2c2	-0.12	1.00000000	mice
Hes1	0.09	1.00000000	mice
Dlat	0.12	1.00000000	mice
Apbb2	0.04	1.00000000	mice
Polr2k	0.06	1.00000000	mice
Stt3a	0.13	1.00000000	mice
Eml1	0.03	1.00000000	mice
Tspan31	0.12	1.00000000	mice
Tango2	0.07	1.00000000	mice
Ubr7	-0.04	1.00000000	mice
Ctnnd1	0.00	1.00000000	mice
Inppl1	0.03	1.00000000	mice
Ctso	0.11	1.00000000	mice
Lias	0.00	1.00000000	mice
Xpa	0.09	1.00000000	mice
Cat	0.18	1.00000000	mice
Frmd4b	0.27	1.00000000	mice
Rnf111	-0.03	1.00000000	mice
Ndufb2	0.09	1.00000000	mice
Terf1	0.00	1.00000000	mice
Xrcc5	0.04	1.00000000	mice
Numa1	0.01	1.00000000	mice
Sec62	0.18	1.00000000	mice
Jarid2	0.08	1.00000000	mice
Atxn10	0.14	1.00000000	mice
Rmdn1	0.08	1.00000000	mice
Sowahc	0.04	1.00000000	mice
Emc4	0.15	1.00000000	mice
Fam53a	0.04	1.00000000	mice
Zcchc17	0.03	1.00000000	mice
Cwc27	0.09	1.00000000	mice
Dohh	0.09	1.00000000	mice

Eloa	0.08	1.00000000	mice
Pip5k1c	0.10	1.00000000	mice
Ccn4	0.05	1.00000000	mice
Naa30	0.05	1.00000000	mice
Lsm4	0.17	1.00000000	mice
Nelfa	-0.03	1.00000000	mice
Schip1	0.07	1.00000000	mice
Osbpl2	0.03	1.00000000	mice
Sf3a3	-0.08	1.00000000	mice
Lncppara	0.00	1.00000000	mice
Lamb1	0.01	1.00000000	mice
Dgkz	0.17	1.00000000	mice
Pde6d	0.06	1.00000000	mice
Hdhd2	0.05	1.00000000	mice
Cep170	0.14	1.00000000	mice
Gas2l1	0.10	1.00000000	mice
Eif3j2	0.02	1.00000000	mice
Sp3	0.11	1.00000000	mice
Fosl1	-0.01	1.00000000	mice
Ppp1r14b	0.26	1.00000000	mice
Cops6	0.08	1.00000000	mice
Esd	0.20	1.00000000	mice
Gpat4	0.05	1.00000000	mice
Prelid1	0.10	1.00000000	mice
Rnf146	-0.04	1.00000000	mice
Fam192a	0.03	1.00000000	mice
Ift27	0.13	1.00000000	mice
Gfod1	0.04	1.00000000	mice
Casc3	-0.04	1.00000000	mice
Znrd1	0.00	1.00000000	mice
Acbd6	0.13	1.00000000	mice
Unc5c	0.14	1.00000000	mice
Pcmt1	0.15	1.00000000	mice
Nadk	0.17	1.00000000	mice
Abhd17a	-0.01	1.00000000	mice
5530601H04Rik	-0.02	1.00000000	mice
Tmem263	-0.04	1.00000000	mice
Nsmaf	0.03	1.00000000	mice
Slc8a1	0.02	1.00000000	mice
Hmox2	0.06	1.00000000	mice
4732471J01Rik	0.01	1.00000000	mice
Clip2	-0.01	1.00000000	mice
Tvp23b	0.11	1.00000000	mice
Ttll4	-0.03	1.00000000	mice
Tmem135	-0.04	1.00000000	mice
Cdc123	0.06	1.00000000	mice

Slc9a3r1	0.18	1.00000000	mice
Cebpz	0.05	1.00000000	mice
Anxa2	0.15	1.00000000	mice
Szrd1	0.03	1.00000000	mice
Qsox1	0.06	1.00000000	mice
Ackr3	0.10	1.00000000	mice
Lrrc75a	0.06	1.00000000	mice
Il1rap	0.09	1.00000000	mice
Lum	-0.04	1.00000000	mice
Pqlc3	0.01	1.00000000	mice
Flrt2	0.10	1.00000000	mice
Snx17	0.05	1.00000000	mice
Llph	0.06	1.00000000	mice
Ppp1r11	0.24	1.00000000	mice
Ctps	0.03	1.00000000	mice
Copa	0.07	1.00000000	mice
Ttc39c	0.02	1.00000000	mice
Hax1	0.00	1.00000000	mice
Ndufs4	0.16	1.00000000	mice
Ndufab1	0.11	1.00000000	mice
Zfp560	0.06	1.00000000	mice
Ttc33	0.00	1.00000000	mice
Ablim3	0.32	1.00000000	mice
Tnfrsf1b	0.03	1.00000000	mice
Tmem70	-0.04	1.00000000	mice
Ppic	0.15	1.00000000	mice
Ltbp2	-0.02	1.00000000	mice
Sergef	0.01	1.00000000	mice
Zdhhc9	-0.01	1.00000000	mice
Tiam1	-0.09	1.00000000	mice
Spg21	0.12	1.00000000	mice
Dop1a	-0.03	1.00000000	mice
Czib	0.10	1.00000000	mice
Pfkfb4	0.01	1.00000000	mice
Ccdc12	-0.07	1.00000000	mice
Gask1b	0.03	1.00000000	mice
Fancc	0.07	1.00000000	mice
Tomm40	0.06	1.00000000	mice
Khsrp	0.14	1.00000000	mice
Faap20	0.08	1.00000000	mice
Cox14	0.10	1.00000000	mice
Tgoln1	0.08	1.00000000	mice
Ccnc	0.03	1.00000000	mice
Nagk	0.03	1.00000000	mice
Smarcd1	0.05	1.00000000	mice
Ufm1	0.13	1.00000000	mice

AW549877	0.08	1.00000000	mice
Lrrc8a	0.01	1.00000000	mice
Lrrc8d	0.08	1.00000000	mice
Cavin3	0.14	1.00000000	mice
Dcp1a	0.00	1.00000000	mice
Crppa	0.01	1.00000000	mice
Jazf1	0.00	1.00000000	mice
Rab35	0.07	1.00000000	mice
Pianp	0.03	1.00000000	mice
Sufu	-0.02	1.00000000	mice
Sf3b5	0.22	1.00000000	mice
Rcbtb2	-0.01	1.00000000	mice
Sdhaf4	0.11	1.00000000	mice
Bcorl1	0.09	1.00000000	mice
Fam98a	0.00	1.00000000	mice
Hotairm1	0.16	1.00000000	mice
Casp6	-0.03	1.00000000	mice
Abhd12	0.07	1.00000000	mice
Rab24	-0.02	1.00000000	mice
Csrp1	0.06	1.00000000	mice
Hoxa3	0.08	1.00000000	mice
Dcaf11	0.03	1.00000000	mice
Slc9a3r2	0.04	1.00000000	mice
Samd8	0.04	1.00000000	mice
Stx7	0.05	1.00000000	mice
Csnk1g1	0.01	1.00000000	mice
Thumpd1	0.06	1.00000000	mice
Ppp1r9b	0.07	1.00000000	mice
Fzd4	0.06	1.00000000	mice
Gt(ROSA)26Sor	-0.04	1.00000000	mice
Ptges3	0.16	1.00000000	mice
Gnai1	-0.05	1.00000000	mice
5730455P16Rik	0.03	1.00000000	mice
Smarce1	0.13	1.00000000	mice
Klf12	0.06	1.00000000	mice
Foxj2	-0.02	1.00000000	mice
Rnps1	0.04	1.00000000	mice
Zfp277	0.09	1.00000000	mice
Nlk	-0.02	1.00000000	mice
Rnf121	0.04	1.00000000	mice
Afg3l1	0.00	1.00000000	mice
Acsl1	0.01	1.00000000	mice
Armc8	0.01	1.00000000	mice
Cfdp1	0.24	1.00000000	mice
Pck2	0.00	1.00000000	mice
Slc39a10	0.04	1.00000000	mice

Zfp740	0.08	1.00000000	mice
Hs2st1	0.05	1.00000000	mice
Zcchc7	0.09	1.00000000	mice
Stambpl1	0.00	1.00000000	mice
Rab34	0.13	1.00000000	mice
Arhgdib	0.13	1.00000000	mice
Ccdc58	0.00	1.00000000	mice
Cdkal1	0.03	1.00000000	mice
Ubr2	0.12	1.00000000	mice
Utp14a	0.14	1.00000000	mice
Bnc2	0.07	1.00000000	mice
Cyb5r4	0.05	1.00000000	mice
Gdi1	0.09	1.00000000	mice
Snrnp200	0.08	1.00000000	mice
Fbxo4	0.10	1.00000000	mice
1110008P14Rik	0.13	1.00000000	mice
Cep68	0.08	1.00000000	mice
Cdc14b	-0.09	1.00000000	mice
Get4	-0.03	1.00000000	mice
Emc8	0.01	1.00000000	mice
Rbis	0.17	1.00000000	mice
Gsr	-0.02	1.00000000	mice
Slc30a5	0.02	1.00000000	mice
Tst	0.08	1.00000000	mice
Chpf	0.04	1.00000000	mice
Tbl1x	0.06	1.00000000	mice
Ufc1	0.12	1.00000000	mice
Nfic	0.08	1.00000000	mice
Cabin1	0.00	1.00000000	mice
Slc4a1ap	0.01	1.00000000	mice
Slc7a11	0.12	1.00000000	mice
Snx15	0.04	1.00000000	mice
Ptk2	-0.09	1.00000000	mice
Tnks2	0.10	1.00000000	mice
Ptpn9	0.08	1.00000000	mice
Rufy3	0.00	1.00000000	mice
Usp14	0.09	1.00000000	mice
Heatr5a	-0.02	1.00000000	mice
Vrk2	0.03	1.00000000	mice
Ftsj3	0.04	1.00000000	mice
Irak1bp1	0.04	1.00000000	mice
Uso1	-0.03	1.00000000	mice
Iars	0.00	1.00000000	mice
Dsel	0.03	1.00000000	mice
Flywch1	0.00	1.00000000	mice
Cog3	0.01	1.00000000	mice

Lipo3	-0.03	1.00000000	mice
Edc4	0.05	1.00000000	mice
Dab2ip	0.01	1.00000000	mice
Pfkfb3	0.05	1.00000000	mice
Nfx1	0.06	1.00000000	mice
Ube2e1	0.11	1.00000000	mice
Nudt2	-0.02	1.00000000	mice
Pak1ip1	0.21	1.00000000	mice
Uxt	-0.01	1.00000000	mice
Aebp1	0.12	1.00000000	mice
Ppa2	0.05	1.00000000	mice
Usp42	0.01	1.00000000	mice
Sumo3	0.14	1.00000000	mice
Smpd1	0.06	1.00000000	mice
Nsd1	0.02	1.00000000	mice
Zdhhc6	0.02	1.00000000	mice
Senp7	0.06	1.00000000	mice
Kmt5a	0.07	1.00000000	mice
Vps26b	-0.02	1.00000000	mice
Trim3	0.05	1.00000000	mice
Col18a1	-0.07	1.00000000	mice
Fam50a	0.05	1.00000000	mice
Trnau1ap	0.09	1.00000000	mice
Lyar	0.09	1.00000000	mice
Psmd1	0.13	1.00000000	mice
Bcap31	0.20	1.00000000	mice
Aph1c	0.12	1.00000000	mice
Txndc15	0.04	1.00000000	mice
Taf10	0.10	1.00000000	mice
Scly	0.03	1.00000000	mice
Erich1	0.00	1.00000000	mice
Trappc1	0.04	1.00000000	mice
Ccbe1	0.05	1.00000000	mice
Atg13	0.02	1.00000000	mice
H13	0.14	1.00000000	mice
Fubp3	0.03	1.00000000	mice
Slc35a4	0.05	1.00000000	mice
Lman2	0.19	1.00000000	mice
Prep	0.02	1.00000000	mice
Klf3	0.08	1.00000000	mice
Ube2e2	-0.08	1.00000000	mice
Podn	0.02	1.00000000	mice
Commd2	0.00	1.00000000	mice
Ggps1	0.05	1.00000000	mice
Rabl6	0.03	1.00000000	mice
Cfap36	0.04	1.00000000	mice

Cpne3	-0.06	1.00000000	mice
Hacd4	0.15	1.00000000	mice
Smo	-0.04	1.00000000	mice
Rab3d	0.06	1.00000000	mice
Rnf8	0.00	1.00000000	mice
Scarb2	0.09	1.00000000	mice
Bub3	0.06	1.00000000	mice
Chm	-0.02	1.00000000	mice
Aga	0.05	1.00000000	mice
Gnptg	0.06	1.00000000	mice
Derl2	0.12	1.00000000	mice
Clec11a	0.10	1.00000000	mice
Tmem168	0.01	1.00000000	mice
Fam204a	0.03	1.00000000	mice
Vps41	-0.04	1.00000000	mice
Cybc1	0.02	1.00000000	mice
Acaca	-0.01	1.00000000	mice
Itpk1	-0.03	1.00000000	mice
Selenos	0.27	1.00000000	mice
Stip1	0.13	1.00000000	mice
Tgfb1	0.11	1.00000000	mice
Anapc13	0.11	1.00000000	mice
Rhbdd1	-0.04	1.00000000	mice
Junos	-0.07	1.00000000	mice
Tut4	0.16	1.00000000	mice
Alg1	0.02	1.00000000	mice
Pcnp	0.17	1.00000000	mice
Atxn3	0.08	1.00000000	mice
Pcca	0.11	1.00000000	mice
Slc25a39	0.04	1.00000000	mice
Trim35	0.19	1.00000000	mice
Efemp1	0.19	1.00000000	mice
Sike1	0.08	1.00000000	mice
Strn	0.08	1.00000000	mice
Sestd1	0.08	1.00000000	mice
Cd59a	0.13	1.00000000	mice
Ash2l	-0.01	1.00000000	mice
Bckdk	-0.01	1.00000000	mice
Prpf18	-0.02	1.00000000	mice
BC029722	0.05	1.00000000	mice
Ccn5	0.11	1.00000000	mice
Ormdl3	-0.03	1.00000000	mice
Ncln	0.08	1.00000000	mice
Heatr3	0.04	1.00000000	mice
Dpf2	0.08	1.00000000	mice
Cdc37l1	0.02	1.00000000	mice

Eapp	0.05	1.00000000	mice
Banf1	0.12	1.00000000	mice
Poglut3	0.10	1.00000000	mice
D230025D16Rik	-0.01	1.00000000	mice
Pla2g4a	0.07	1.00000000	mice
Rpp21	0.01	1.00000000	mice
Cul4b	0.00	1.00000000	mice
Bmt2	-0.01	1.00000000	mice
Papss1	0.13	1.00000000	mice
Lyrm2	0.12	1.00000000	mice
Scfd1	0.15	1.00000000	mice
Vps26a	0.07	1.00000000	mice
Slc25a1	0.18	1.00000000	mice
Clpp	0.06	1.00000000	mice
Slc38a5	0.25	1.00000000	mice
Scand1	0.15	1.00000000	mice
Fkbp7	-0.02	1.00000000	mice
Timm10	-0.07	1.00000000	mice
Tmem39a	-0.05	1.00000000	mice
Hnrnpk	0.15	1.00000000	mice
Stx6	0.11	1.00000000	mice
Krit1	0.01	1.00000000	mice
Ipo11	-0.03	1.00000000	mice
Sarnp	0.20	1.00000000	mice
Gpr180	0.11	1.00000000	mice
Arhgef11	0.02	1.00000000	mice
Babam1	0.06	1.00000000	mice
Fgf10	0.24	1.00000000	mice
Brdt	0.02	1.00000000	mice
Pym1	-0.04	1.00000000	mice
Cntln	-0.01	1.00000000	mice
Ccnh	-0.04	1.00000000	mice
Snap47	0.06	1.00000000	mice
Pcyt2	-0.01	1.00000000	mice
Noct	0.03	1.00000000	mice
Pnp	0.25	1.00000000	mice
Borcs7	-0.02	1.00000000	mice
Ap1m1	0.06	1.00000000	mice
Prrx1	0.12	1.00000000	mice
Dop1b	0.04	1.00000000	mice
Vps8	0.02	1.00000000	mice
Ube2q1	0.13	1.00000000	mice
Cfap20	0.07	1.00000000	mice
St6galnac6	0.11	1.00000000	mice
Tspyl1	0.05	1.00000000	mice
4933434E20Rik	0.01	1.00000000	mice

Pogz	0.03	1.00000000	mice
Ssna1	0.05	1.00000000	mice
Tomm70a	0.03	1.00000000	mice
Tbk1	-0.08	1.00000000	mice
Epha7	0.12	1.00000000	mice
Anapc2	-0.02	1.00000000	mice
Sept10	0.07	1.00000000	mice
E130308A19Rik	0.01	1.00000000	mice
Elovl1	0.08	1.00000000	mice
Pex3	0.07	1.00000000	mice
Cops2	0.04	1.00000000	mice
Jam2	-0.09	1.00000000	mice
Zfyve1	-0.05	1.00000000	mice
Cdyl	0.12	1.00000000	mice
Ift20	0.14	1.00000000	mice
Dclre1c	0.05	1.00000000	mice
Rab5if	0.19	1.00000000	mice
Fbxo38	0.03	1.00000000	mice
Rexo4	0.12	1.00000000	mice
Ndufa12	0.22	1.00000000	mice
Spry2	0.05	1.00000000	mice
Tmem241	0.02	1.00000000	mice
Immt	0.04	1.00000000	mice
Sys1	0.07	1.00000000	mice
Uqcrc2	0.03	1.00000000	mice
Stat6	0.11	1.00000000	mice
Lasp1	-0.05	1.00000000	mice
Uqcc3	0.08	1.00000000	mice
Eif2b2	0.09	1.00000000	mice
Syde1	0.00	1.00000000	mice
Dnaja2	0.06	1.00000000	mice
Gps2	0.07	1.00000000	mice
Twf2	0.02	1.00000000	mice
Uchl3	0.14	1.00000000	mice
Wdr37	-0.03	1.00000000	mice
Ormdl2	0.12	1.00000000	mice
Retreg2	-0.02	1.00000000	mice
Cdc16	0.07	1.00000000	mice
Smim26	0.06	1.00000000	mice
Trps1	0.03	1.00000000	mice
Clcc1	-0.01	1.00000000	mice
Tdg	-0.02	1.00000000	mice
Spred1	0.10	1.00000000	mice
Rragc	0.14	1.00000000	mice
Os9	0.10	1.00000000	mice
Ndufb4	0.16	1.00000000	mice

Ipo9	-0.04	1.00000000	mice
Hip1	-0.02	1.00000000	mice
1500009L16Rik	0.16	1.00000000	mice
Sec11c	0.15	1.00000000	mice
Irs1	-0.05	1.00000000	mice
Tmem256	0.25	1.00000000	mice
Sfmbt1	0.00	1.00000000	mice
Lrpprc	-0.01	1.00000000	mice
Dnlz	0.04	1.00000000	mice
Odf2	-0.03	1.00000000	mice
Wdr61	0.06	1.00000000	mice
Cetn2	-0.04	1.00000000	mice
Nrcam	0.20	1.00000000	mice
Snx19	0.00	1.00000000	mice
Rnf2	0.11	1.00000000	mice
Snhg1	0.24	1.00000000	mice
Cdc37	0.25	1.00000000	mice
Rpf2	0.05	1.00000000	mice
2610037D02Rik	-0.07	1.00000000	mice
Focad	0.07	1.00000000	mice
Wdr18	-0.05	1.00000000	mice
Gripap1	0.04	1.00000000	mice
Plekha6	0.04	1.00000000	mice
Bloc1s2	0.08	1.00000000	mice
Parg	0.03	1.00000000	mice
Brd7	0.06	1.00000000	mice
Akirin2	0.18	1.00000000	mice
Chchd6	0.05	1.00000000	mice
Itpkb	0.03	1.00000000	mice
Igf2bp2	0.04	1.00000000	mice
Zbed3	0.04	1.00000000	mice
Phyh	0.02	1.00000000	mice
Rab1b	0.05	1.00000000	mice
Arhgef1	0.05	1.00000000	mice
Trappc4	0.12	1.00000000	mice
Ibtk	0.05	1.00000000	mice
Hspb1	-0.04	1.00000000	mice
Polr3k	0.10	1.00000000	mice
Ppil3	-0.04	1.00000000	mice
Rab33b	-0.02	1.00000000	mice
Fam20c	0.03	1.00000000	mice
Tsr2	0.04	1.00000000	mice
Anxa7	0.07	1.00000000	mice
Cdc34	0.06	1.00000000	mice
Prkcsh	0.11	1.00000000	mice
Cdk6	0.19	1.00000000	mice

Taf1d	0.10	1.00000000	mice
Tmem183a	-0.03	1.00000000	mice
Ncald	0.16	1.00000000	mice
Socs7	-0.06	1.00000000	mice
Ssb	0.19	1.00000000	mice
Riok3	0.07	1.00000000	mice
Cks2	0.20	1.00000000	mice
Atp6v0a2	0.06	1.00000000	mice
Asf1a	0.04	1.00000000	mice
Appl2	0.01	1.00000000	mice
Srsf1	0.03	1.00000000	mice
Brox	0.09	1.00000000	mice
C7	0.23	1.00000000	mice
Pak1	0.06	1.00000000	mice
Furin	0.25	1.00000000	mice
Trim33	0.10	1.00000000	mice
Ikbkb	0.17	1.00000000	mice
Syk	0.02	1.00000000	mice
Fam20b	0.05	1.00000000	mice
Xiap	0.02	1.00000000	mice
Ndufaf8	0.06	1.00000000	mice
Wbp4	0.11	1.00000000	mice
Snrpd1	0.21	1.00000000	mice
Socs4	0.06	1.00000000	mice
U2af1	0.25	1.00000000	mice
Abcf2	0.04	1.00000000	mice
Abl2	0.03	1.00000000	mice
Uba6	-0.09	1.00000000	mice
Dvl3	-0.02	1.00000000	mice
Timm10b	0.10	1.00000000	mice
Ccz1	0.05	1.00000000	mice
Prr14l	-0.05	1.00000000	mice
Nom1	0.02	1.00000000	mice
Zdhhc18	0.12	1.00000000	mice
Cd82	0.18	1.00000000	mice
Smg7	0.09	1.00000000	mice
Nr6a1	-0.02	1.00000000	mice
Nfrkb	-0.01	1.00000000	mice
Glis2	0.05	1.00000000	mice
2210408I21Rik	-0.03	1.00000000	mice
Tmem98	0.10	1.00000000	mice
Ext2	0.02	1.00000000	mice
Foxf1	0.07	1.00000000	mice
Gpx7	0.02	1.00000000	mice
Nsg1	0.07	1.00000000	mice
Sdhaf2	0.04	1.00000000	mice

Nmt1	0.09	1.00000000	mice
Tubgcp3	-0.03	1.00000000	mice
Trappc8	-0.01	1.00000000	mice
Sdhc	0.17	1.00000000	mice
Dennd6a	0.09	1.00000000	mice
Ptprs	0.05	1.00000000	mice
Npr2	-0.02	1.00000000	mice
Abhd17c	0.04	1.00000000	mice
Zfp652	0.04	1.00000000	mice
Hmgb1	0.09	1.00000000	mice
Nup35	0.05	1.00000000	mice
Bcl2l2	-0.02	1.00000000	mice
Lysmd2	-0.07	1.00000000	mice
Kctd17	-0.03	1.00000000	mice
Tasor2	-0.04	1.00000000	mice
Dcun1d5	0.05	1.00000000	mice
Uhmk1	0.09	1.00000000	mice
Smc4	0.17	1.00000000	mice
Ado	0.02	1.00000000	mice
Acp1	0.10	1.00000000	mice
Lage3	0.13	1.00000000	mice
Pcnx4	0.02	1.00000000	mice
Uggt2	0.06	1.00000000	mice
Pi4kb	0.11	1.00000000	mice
Stxbp1	0.00	1.00000000	mice
Adam15	-0.02	1.00000000	mice
Rhou	-0.04	1.00000000	mice
Wdfy1	0.02	1.00000000	mice
AU040320	0.02	1.00000000	mice
Rwdd4a	0.03	1.00000000	mice
Gpr107	0.03	1.00000000	mice
Bms1	0.21	1.00000000	mice
Pno1	0.11	1.00000000	mice
St5	0.12	1.00000000	mice
Ube2z	0.06	1.00000000	mice
Lypla2	0.06	1.00000000	mice
Dcaf5	-0.03	1.00000000	mice
Pgp	0.05	1.00000000	mice
Hmgxb4	0.02	1.00000000	mice
Pgghg	0.03	1.00000000	mice
Kdm1a	0.12	1.00000000	mice
Nek6	0.01	1.00000000	mice
Senp5	0.02	1.00000000	mice
Phf23	0.10	1.00000000	mice
Rnf149	0.12	1.00000000	mice
Glrx5	0.12	1.00000000	mice

Prkaa1	0.08	1.00000000	mice
Cpd	0.07	1.00000000	mice
Slc17a5	0.02	1.00000000	mice
Cbx1	0.17	1.00000000	mice
Ttc5	-0.10	1.00000000	mice
Xrn2	0.03	1.00000000	mice
Cpq	0.09	1.00000000	mice
Sema6a	0.03	1.00000000	mice
Adra1a	0.04	1.00000000	mice
Gsap	0.10	1.00000000	mice
0610009B22Rik	-0.08	1.00000000	mice
Nt5c	0.14	1.00000000	mice
Axl	0.13	1.00000000	mice
Flvcr1	0.11	1.00000000	mice
Josd1	0.02	1.00000000	mice
Skp1a	0.19	1.00000000	mice
B9d1	-0.03	1.00000000	mice
Tpr	0.18	1.00000000	mice
Sort1	0.22	1.00000000	mice
Vps35	-0.05	1.00000000	mice
Dhx8	-0.01	1.00000000	mice
Rhog	-0.08	1.00000000	mice
Snrpg	0.27	1.00000000	mice
Ddx46	0.04	1.00000000	mice
Raver2	0.23	1.00000000	mice
Yipf6	0.05	1.00000000	mice
Taok3	0.08	1.00000000	mice
4833420G17Rik	0.11	1.00000000	mice
Vps72	0.02	1.00000000	mice
Fundc1	0.04	1.00000000	mice
Dld	0.08	1.00000000	mice
4632427E13Rik	-0.11	1.00000000	mice
Kin	0.05	1.00000000	mice
Pdcl	0.03	1.00000000	mice
Emilin2	-0.06	1.00000000	mice
Pon3	-0.03	1.00000000	mice
Eif2s1	0.12	1.00000000	mice
Snx8	0.03	1.00000000	mice
Stimate	0.02	1.00000000	mice
BC005624	0.05	1.00000000	mice
Purg	-0.04	1.00000000	mice
Ppp2r5c	0.18	1.00000000	mice
Arcn1	0.10	1.00000000	mice
Nucb1	0.07	1.00000000	mice
Snhg3	0.07	1.00000000	mice
Ago4	0.07	1.00000000	mice

Atg4a	0.13	1.00000000	mice
Rbm33	0.07	1.00000000	mice
Sgcb	0.07	1.00000000	mice
Aqr	0.04	1.00000000	mice
Umad1	-0.06	1.00000000	mice
Wwp1	0.00	1.00000000	mice
Cinp	0.00	1.00000000	mice
Cbx3	0.21	1.00000000	mice
Klhdc10	-0.04	1.00000000	mice
Ak2	0.12	1.00000000	mice
Plekhg1	0.02	1.00000000	mice
Tmem68	-0.03	1.00000000	mice
Stx3	0.00	1.00000000	mice
Otulin	0.07	1.00000000	mice
Fzd1	0.08	1.00000000	mice
Apc	0.08	1.00000000	mice
Synj2	-0.01	1.00000000	mice
Slc9a9	0.11	1.00000000	mice
Atg4b	-0.01	1.00000000	mice
Itpr2	0.05	1.00000000	mice
Trf	0.09	1.00000000	mice
Osr1	0.01	1.00000000	mice
Pcbp4	0.07	1.00000000	mice
Ipo8	0.10	1.00000000	mice
Fbxw2	0.02	1.00000000	mice
Gadd45a	0.07	1.00000000	mice
Rufy2	0.01	1.00000000	mice
Ppil2	0.05	1.00000000	mice
Ate1	-0.01	1.00000000	mice
Lrp10	0.06	1.00000000	mice
Fbn1	0.07	1.00000000	mice
Tcf20	0.02	1.00000000	mice
Phactr1	0.01	1.00000000	mice
Fzd7	0.02	1.00000000	mice
Hsdl2	0.08	1.00000000	mice
Eif2b5	0.06	1.00000000	mice
Crls1	0.01	1.00000000	mice
Cttn	0.01	1.00000000	mice
Vwa8	-0.05	1.00000000	mice
Cnot8	-0.01	1.00000000	mice
Cops3	0.10	1.00000000	mice
Lrrk2	-0.02	1.00000000	mice
Cir1	0.05	1.00000000	mice
Zfp771	0.05	1.00000000	mice
Pigf	0.13	1.00000000	mice
Sf3b4	0.03	1.00000000	mice

Rnf115	0.07	1.00000000	mice
Nup155	0.00	1.00000000	mice
Syap1	0.24	1.00000000	mice
Fbxl14	0.03	1.00000000	mice
Tceal8	0.13	1.00000000	mice
Nsf	-0.07	1.00000000	mice
Dipk2a	0.09	1.00000000	mice
Eaf1	-0.04	1.00000000	mice
Arl6ip1	0.22	1.00000000	mice
Sdk1	-0.05	1.00000000	mice
Galnt1	0.11	1.00000000	mice
Hibadh	-0.03	1.00000000	mice
Creb1	0.08	1.00000000	mice
Col4a6	-0.04	1.00000000	mice
Gtf3c1	0.13	1.00000000	mice
Zfp318	-0.05	1.00000000	mice
Ap1ar	0.06	1.00000000	mice
Pard6g	0.02	1.00000000	mice
Ehd1	0.20	1.00000000	mice
Arhgef18	0.03	1.00000000	mice
Bccip	0.08	1.00000000	mice
Vmp1	0.03	1.00000000	mice
Sh2b3	0.12	1.00000000	mice
Ebna1bp2	0.12	1.00000000	mice
Cln5	-0.07	1.00000000	mice
Wdr1	0.08	1.00000000	mice
Rnf181	-0.02	1.00000000	mice
Fth1	0.14	1.00000000	mice
Zcchc24	0.02	1.00000000	mice
Fastk	0.08	1.00000000	mice
Ttc8	-0.02	1.00000000	mice
Sap130	0.02	1.00000000	mice
Tmem150a	0.06	1.00000000	mice
Prdm10	0.02	1.00000000	mice
Arl3	0.14	1.00000000	mice
Vta1	0.04	1.00000000	mice
Coa3	0.09	1.00000000	mice
Agpat2	0.07	1.00000000	mice
Ghitm	0.08	1.00000000	mice
Nt5c3b	0.03	1.00000000	mice
Hsd3b7	-0.04	1.00000000	mice
Ccdc82	0.13	1.00000000	mice
Ier3ip1	0.19	1.00000000	mice
Npc1	0.07	1.00000000	mice
Pih1d1	0.11	1.00000000	mice
Nckap5l	0.00	1.00000000	mice

Ntmt1	0.03	1.00000000	mice
Timm13	0.12	1.00000000	mice
C1qtnf6	0.03	1.00000000	mice
Smu1	0.12	1.00000000	mice
Slc25a12	0.04	1.00000000	mice
Gramd1b	0.13	1.00000000	mice
Pofut1	0.08	1.00000000	mice
Itprid2	-0.04	1.00000000	mice
Cnppd1	0.07	1.00000000	mice
Apaf1	-0.04	1.00000000	mice
Fam91a1	0.02	1.00000000	mice
Tbce	-0.03	1.00000000	mice
Slc25a24	-0.09	1.00000000	mice
Tmtc3	0.00	1.00000000	mice
Rbm41	0.00	1.00000000	mice
Dnaja3	0.10	1.00000000	mice
Capza2	0.21	1.00000000	mice
Nr4a3	0.28	1.00000000	mice
Tex2	0.06	1.00000000	mice
Rp2	0.02	1.00000000	mice
Api5	0.05	1.00000000	mice
Rab23	0.05	1.00000000	mice
Taok2	0.04	1.00000000	mice
Il17ra	-0.06	1.00000000	mice
Zfp637	0.04	1.00000000	mice
Commd1	0.04	1.00000000	mice
Wipf2	0.06	1.00000000	mice
Pfdn4	0.15	1.00000000	mice
Lmnb1	0.15	1.00000000	mice
Eif3c	0.17	1.00000000	mice
Gtf2ird1	-0.03	1.00000000	mice
Irak1	0.05	1.00000000	mice
Tbck	0.00	1.00000000	mice
Ip6k1	0.10	1.00000000	mice
Klhl21	-0.04	1.00000000	mice
Stx16	-0.01	1.00000000	mice
Adamtsl4	0.01	1.00000000	mice
Serf2	0.09	1.00000000	mice
Tmem33	0.07	1.00000000	mice
Il17rc	0.03	1.00000000	mice
Adrm1	0.02	1.00000000	mice
Supt16	0.05	1.00000000	mice
Baz2a	0.01	1.00000000	mice
Itfg1	0.11	1.00000000	mice
Orai1	0.10	1.00000000	mice
Pcgf5	0.01	1.00000000	mice

Zfp871	0.00	1.00000000	mice
Cdc5l	0.15	1.00000000	mice
Slc16a9	-0.02	1.00000000	mice
Atg2a	0.00	1.00000000	mice
Alpk1	-0.15	1.00000000	mice
Dars	0.01	1.00000000	mice
Ccnd2	0.08	1.00000000	mice
Vdac3	0.18	1.00000000	mice
Camk2g	0.09	1.00000000	mice
Lix1l	0.08	1.00000000	mice
Tbx3	0.01	1.00000000	mice
Vps4a	0.00	1.00000000	mice
Fbh1	0.03	1.00000000	mice
Snx1	0.01	1.00000000	mice
Rfwd3	-0.05	1.00000000	mice
C1qtnf1	0.03	1.00000000	mice
Hmgn2	0.30	1.00000000	mice
Cyc1	0.04	1.00000000	mice
2310011J03Rik	0.11	1.00000000	mice
Ncbp3	0.06	1.00000000	mice
Lima1	0.11	1.00000000	mice
Napg	0.02	1.00000000	mice
Rsl24d1	0.17	1.00000000	mice
Lamtor3	-0.03	1.00000000	mice
Dtymk	0.09	1.00000000	mice
Btrc	-0.02	1.00000000	mice
Actb	0.11	1.00000000	mice
Htra2	0.03	1.00000000	mice
Nmt2	-0.08	1.00000000	mice
2510009E07Rik	-0.01	1.00000000	mice
Ndufa7	0.16	1.00000000	mice
Klhl9	0.11	1.00000000	mice
Sigmar1	0.08	1.00000000	mice
Fbxo28	0.07	1.00000000	mice
Ppp2r3c	0.00	1.00000000	mice
Gfm1	0.01	1.00000000	mice
Ift57	-0.04	1.00000000	mice
Copb2	0.10	1.00000000	mice
Wdr7	-0.06	1.00000000	mice
Tbc1d9b	0.05	1.00000000	mice
Impad1	0.01	1.00000000	mice
Serpine1	0.15	1.00000000	mice
1810026B05Rik	0.03	1.00000000	mice
Chuk	-0.01	1.00000000	mice
Cltc	0.13	1.00000000	mice
Sesn3	0.04	1.00000000	mice

Tasp1	-0.01	1.00000000	mice
Zmat2	0.23	1.00000000	mice
C1d	0.08	1.00000000	mice
Pkdcc	0.02	1.00000000	mice
Gpalpp1	0.14	1.00000000	mice
Commd3	0.21	1.00000000	mice
Prelid3b	0.09	1.00000000	mice
Ei24	0.05	1.00000000	mice
Fhod1	0.02	1.00000000	mice
Ube2v1	0.10	1.00000000	mice
Pmpcb	0.15	1.00000000	mice
Hgf	0.18	1.00000000	mice
Dcaf7	-0.03	1.00000000	mice
Tubgcp5	-0.07	1.00000000	mice
Uqcc1	0.06	1.00000000	mice
Phactr4	0.10	1.00000000	mice
Eri1	0.02	1.00000000	mice
Slc16a2	0.03	1.00000000	mice
Cercam	0.00	1.00000000	mice
Dele1	0.06	1.00000000	mice
Eps15l1	0.06	1.00000000	mice
Slc39a9	-0.02	1.00000000	mice
Usp38	-0.05	1.00000000	mice
Tnfaip8	0.05	1.00000000	mice
Arsa	0.01	1.00000000	mice
Psmd6	0.17	1.00000000	mice
Zfp410	0.03	1.00000000	mice
Arap2	0.11	1.00000000	mice
Gadd45gip1	0.09	1.00000000	mice
Dhx29	0.02	1.00000000	mice
Sec22a	0.04	1.00000000	mice
Cxxc5	0.02	1.00000000	mice
Znrd2	0.13	1.00000000	mice
Dnajc2	-0.02	1.00000000	mice
Cep290	0.07	1.00000000	mice
Ube2d1	0.10	1.00000000	mice
Ninj1	0.05	1.00000000	mice
Pycr2	0.07	1.00000000	mice
Elf4	0.04	1.00000000	mice
Acaa1a	0.02	1.00000000	mice
Abcb7	0.07	1.00000000	mice
Fam171a1	0.14	1.00000000	mice
Ccdc90b	0.06	1.00000000	mice
Tmem242	0.04	1.00000000	mice
Tfpi	0.09	1.00000000	mice
Psmb2	0.19	1.00000000	mice

Serp1	0.08	1.00000000	mice
Atp6v1c1	0.02	1.00000000	mice
Jade2	0.05	1.00000000	mice
Slc9a8	-0.04	1.00000000	mice
Cnn2	0.13	1.00000000	mice
Ppp3r1	-0.12	1.00000000	mice
Fnbp1	0.08	1.00000000	mice
Washc3	0.05	1.00000000	mice
Saa3	4.21	0.00000000	pigs
Timp1	2.66	0.00000000	pigs
Dpt	-2.08	0.00000000	pigs
Ifi27	2.23	0.00000000	pigs
B2m	1.21	0.00000000	pigs
Tnxb	-1.42	0.00000000	pigs
Ccl19	2.60	0.00000000	pigs
H2-Ea	2.07	0.00000000	pigs
Bst2	1.10	0.00000000	pigs
Sod2	1.87	0.00000000	pigs
Lepr	-1.89	0.00000000	pigs
Lgals3bp	1.29	0.00000000	pigs
Timp3	-1.30	0.00000000	pigs
Ptx3	1.77	0.00000000	pigs
Piezo1	0.82	0.00000000	pigs
Serpine2	1.64	0.00000000	pigs
Nfkbia	1.38	0.00000000	pigs
Setbp1	-1.42	0.00000000	pigs
Rack1	1.00	0.00000000	pigs
Il4ra	0.99	0.00000000	pigs
Tnfaip3	1.38	0.00000000	pigs
Akap12	-1.23	0.00000000	pigs
Cstb	1.29	0.00000000	pigs
Gpm6b	1.32	0.00000000	pigs
Cpxm1	0.51	0.00000000	pigs
Cpz	0.65	0.00000000	pigs
Egr1	-0.94	0.00000000	pigs
Plac9a	-1.03	0.00000000	pigs
Gsn	-1.22	0.00000000	pigs
Nnmt	0.47	0.00000000	pigs
Lrrc20	0.44	0.00000000	pigs
Snai2	0.98	0.00000000	pigs
Klf15	0.41	0.00000000	pigs
Tmem176a	0.65	0.00000000	pigs
Adamts4	0.91	0.00000000	pigs
Per1	0.88	0.00000000	pigs
Oas2	0.86	0.00000000	pigs
Cd34	-1.04	0.00000000	pigs

Aplf	0.38	0.00000000	pigs
Bhlhe40	1.09	0.00000000	pigs
Slc47a1	0.32	0.00000000	pigs
H2-Ab1	1.91	0.00000000	pigs
Zfy1	0.24	0.00000000	pigs
Sestd1	-0.99	0.00000000	pigs
Ifitm6	0.83	0.00000000	pigs
Tppp3	-1.48	0.00000000	pigs
Sema3c	-1.33	0.00000000	pigs
Anpep	0.54	0.00000000	pigs
Trib1	0.97	0.00000000	pigs
Prss35	0.61	0.00000000	pigs
Notch2	-1.10	0.00000000	pigs
Panx1	0.31	0.00000000	pigs
Ggh	0.53	0.00000000	pigs
Plxna4	0.66	0.00000000	pigs
Cyp2j13	0.34	0.00000000	pigs
Wdr86	0.33	0.00000000	pigs
Lamb1	0.71	0.00000000	pigs
Psmg3	0.42	0.00000000	pigs
Rnd1	0.56	0.00000000	pigs
Jak2	1.35	0.00000000	pigs
Cir1	0.32	0.00000000	pigs
Spp1	0.89	0.00000000	pigs
Aldh1a2	1.15	0.00000000	pigs
Arsk	0.32	0.00000000	pigs
Klf10	0.78	0.00000000	pigs
Phyhd1	0.21	0.00000000	pigs
Sfrp2	1.37	0.00000000	pigs
Atp11a	0.66	0.00000000	pigs
Cry2	0.69	0.00000000	pigs
Cst3	-0.33	0.00000000	pigs
Epas1	0.81	0.00000000	pigs
Lgmn	0.83	0.00000000	pigs
Hk2	0.92	0.00000000	pigs
Prdx5	0.68	0.00000000	pigs
Cxcl10	1.14	0.00000000	pigs
Ltbp4	-0.78	0.00000000	pigs
Dusp5	0.60	0.00000000	pigs
Adgrd1	-0.82	0.00000000	pigs
Clec11a	0.48	0.00000000	pigs
Btf3	0.84	0.00000000	pigs
Postn	1.10	0.00000000	pigs
Plcb1	-1.19	0.00000000	pigs
Pid1	-0.74	0.00000000	pigs
Rbms1	-0.81	0.00000000	pigs

Zfand5	0.78	0.00000000	pigs
Eef1b2	0.72	0.00000000	pigs
Smad7	0.36	0.00000000	pigs
Sptbn1	-0.62	0.00000000	pigs
Prelp	-0.78	0.00000000	pigs
Pros1	0.62	0.00000000	pigs
Grk4	0.36	0.00000000	pigs
Pdpn	1.04	0.00000000	pigs
C1qa	0.27	0.00000000	pigs
Eef1g	0.74	0.00000000	pigs
Lipg	0.26	0.00000000	pigs
Capg	0.80	0.00000000	pigs
Usp49	0.22	0.00000000	pigs
Ube2l6	0.57	0.00000000	pigs
Cavin2	-0.75	0.00000000	pigs
Arg1	1.10	0.00000000	pigs
Grid1	0.50	0.00000000	pigs
Nfib	-0.66	0.00000000	pigs
Angptl4	0.60	0.00000000	pigs
Runx1	1.10	0.00000000	pigs
Sned1	0.84	0.00000000	pigs
Il6	0.74	0.00000000	pigs
Gprc5b	0.55	0.00000000	pigs
Foxk1	0.33	0.00000000	pigs
Nupr1	1.31	0.00000000	pigs
Dst	-0.75	0.00000000	pigs
Pxdn	0.41	0.00000000	pigs
Chchd5	0.46	0.00000000	pigs
Carhsp1	0.35	0.00000000	pigs
Slamf7	1.42	0.00000000	pigs
Lamc1	-0.68	0.00000000	pigs
Gpc3	-0.87	0.00000000	pigs
Cd82	0.44	0.00000000	pigs
Cfd	-0.82	0.00000000	pigs
Zfpm2	-0.60	0.00000000	pigs
Cyba	0.79	0.00000000	pigs
Omd	-0.88	0.00000000	pigs
Col3a1	0.98	0.00000000	pigs
Cmah	0.12	0.00000000	pigs
Rnase4	-0.56	0.00000000	pigs
Relb	0.23	0.00000000	pigs
Nfkb2	0.41	0.00000000	pigs
Rnf122	0.36	0.00000000	pigs
Ssr4	0.84	0.00000000	pigs
Ybey	0.52	0.00000000	pigs
Agt	-1.41	0.00000000	pigs

Ghitm	0.47	0.00000000	pigs
Ndrg1	0.92	0.00000000	pigs
C4b	0.89	0.00000000	pigs
Fam71f2	0.19	0.00000000	pigs
Hsp90ab1	0.86	0.00000000	pigs
Grip1	0.46	0.00000000	pigs
Arl9	0.13	0.00000000	pigs
Clec3b	-1.02	0.00000000	pigs
Gadd45a	0.78	0.00000000	pigs
Fbn1	-1.05	0.00000000	pigs
Pdxk	0.54	0.00000000	pigs
Tsen2	0.54	0.00000000	pigs
Pam	-0.86	0.00000000	pigs
Itih5	-0.62	0.00000000	pigs
Htra1	0.75	0.00000000	pigs
Hexb	0.35	0.00000000	pigs
Sod3	0.32	0.00000000	pigs
E2f3	0.33	0.00000000	pigs
Ppp1r10	-0.70	0.00000000	pigs
Icam1	0.69	0.00000000	pigs
Zeb2	-0.59	0.00000000	pigs
Esd	0.53	0.00000000	pigs
Pik3r1	-0.66	0.00000000	pigs
Cxcl14	0.45	0.00000000	pigs
Vipr2	-1.15	0.00000000	pigs
Smc4	0.40	0.00000000	pigs
Tead4	0.26	0.00000000	pigs
Tmem26	0.23	0.00000000	pigs
Fosl2	0.32	0.00000001	pigs
Slc7a1	0.31	0.00000001	pigs
F13a1	0.72	0.00000001	pigs
Scara3	0.34	0.00000001	pigs
Cacna1e	0.55	0.00000001	pigs
Nudcd2	0.23	0.00000001	pigs
Tra2b	-0.58	0.00000001	pigs
Sox5	-0.68	0.00000001	pigs
Stag1	-0.59	0.00000001	pigs
Polr2g	0.47	0.00000001	pigs
Pim3	0.47	0.00000001	pigs
Klf12	-0.78	0.00000002	pigs
Tmem205	0.36	0.00000002	pigs
Abca1	0.89	0.00000002	pigs
Ubxn6	0.23	0.00000002	pigs
Ly96	0.30	0.00000002	pigs
Traf2	0.27	0.00000002	pigs
Asah1	0.27	0.00000002	pigs

Eif3l	0.65	0.00000002	pigs
Tpt1	0.48	0.00000002	pigs
Cd40	0.39	0.00000003	pigs
H1f3	0.43	0.00000003	pigs
Pcbd1	0.33	0.00000003	pigs
Urm1	0.28	0.00000003	pigs
Rad51b	0.77	0.00000003	pigs
Adam9	0.64	0.00000003	pigs
Plau	0.65	0.00000003	pigs
Psmb8	0.90	0.00000004	pigs
C1qc	0.51	0.00000004	pigs
Ifngr2	0.68	0.00000004	pigs
Slc39a14	0.96	0.00000004	pigs
Tagln	-0.38	0.00000005	pigs
Col14a1	-0.59	0.00000005	pigs
AA467197	1.11	0.00000006	pigs
Cyp1b1	0.94	0.00000006	pigs
Nfkbie	0.24	0.00000006	pigs
C1qb	0.20	0.00000007	pigs
Il33	0.55	0.00000009	pigs
Ptn	0.17	0.00000009	pigs
Slpi	0.75	0.00000010	pigs
Apod	0.77	0.00000010	pigs
Ftl1-ps1	0.96	0.00000010	pigs
Cd81	0.44	0.00000014	pigs
Baz1a	0.68	0.00000015	pigs
Batf3	0.41	0.00000016	pigs
Ubb	0.44	0.00000017	pigs
Fkbp10	0.39	0.00000017	pigs
Uap1	-0.77	0.00000019	pigs
Nkain3	0.44	0.00000020	pigs
Tmem14a	0.14	0.00000020	pigs
Sfpq	-0.43	0.00000025	pigs
Prkg1	-0.73	0.00000025	pigs
Acvr1b	0.20	0.00000026	pigs
Rwdd3	0.21	0.00000029	pigs
Hspe1-rs1	0.83	0.00000031	pigs
Plin3	0.33	0.00000032	pigs
Twf2	0.33	0.00000033	pigs
Zc3h12a	0.43	0.00000033	pigs
Ifi44l	0.18	0.00000035	pigs
Tifa	0.15	0.00000036	pigs
Fabp5	1.22	0.00000037	pigs
Hyi	0.25	0.00000037	pigs
Nfix	-0.60	0.00000040	pigs
Atrnl1	-0.79	0.00000045	pigs

Gem	0.59	0.00000048	pigs
Cyth1	0.67	0.00000053	pigs
Shisa5	0.47	0.00000055	pigs
Idua	0.17	0.00000058	pigs
Pon2	0.32	0.00000065	pigs
Dkk2	-1.14	0.00000065	pigs
Tspo	0.57	0.00000068	pigs
Specc1	0.20	0.00000069	pigs
Zeb1	-0.82	0.00000074	pigs
Nrxn1	0.67	0.00000075	pigs
Trabd2b	0.22	0.00000080	pigs
Susd6	0.31	0.00000081	pigs
Apoe	0.88	0.00000084	pigs
Spc24	0.40	0.00000085	pigs
Dhx58	0.19	0.00000094	pigs
Dhrs4	0.18	0.00000109	pigs
Rab29	0.18	0.00000121	pigs
Pdlim5	0.63	0.00000122	pigs
Gtpbp8	0.16	0.00000124	pigs
P4ha1	0.71	0.00000132	pigs
Arpc3	0.82	0.00000137	pigs
Parp3	0.12	0.00000139	pigs
Ostc	0.70	0.00000144	pigs
Pim1	0.30	0.00000149	pigs
Xbp1	-0.58	0.00000152	pigs
Stx11	0.45	0.00000155	pigs
Srxn1	0.11	0.00000163	pigs
Gsto1	0.43	0.00000164	pigs
Gab1	0.53	0.00000165	pigs
Lpar1	-0.76	0.00000166	pigs
Tnfsf13b	0.22	0.00000182	pigs
Phc2	0.26	0.00000206	pigs
Psmb9	0.65	0.00000210	pigs
Adcyap1r1	0.16	0.00000217	pigs
Ifi30	0.61	0.00000219	pigs
Pard3b	-0.68	0.00000224	pigs
Fos	-0.66	0.00000229	pigs
Tgif1	0.42	0.00000233	pigs
Gfra1	0.50	0.00000237	pigs
Plxdc2	-0.51	0.00000240	pigs
Slc43a2	0.43	0.00000250	pigs
Il17ra	0.28	0.00000259	pigs
Pfkfb3	0.43	0.00000269	pigs
Dot1l	0.13	0.00000282	pigs
Ctsb	0.67	0.00000311	pigs
Col12a1	-0.93	0.00000312	pigs

Nudt22	0.11	0.00000315	pigs
Irf1	0.45	0.00000326	pigs
Lyplal1	0.22	0.00000341	pigs
Rcl1	0.23	0.00000343	pigs
Ism1	-0.85	0.00000354	pigs
Socs3	0.57	0.00000356	pigs
Lum	0.62	0.00000356	pigs
Aard	0.06	0.00000361	pigs
Rpp25l	0.23	0.00000368	pigs
Vcam1	0.75	0.00000371	pigs
Atp5k	-0.49	0.00000383	pigs
Atp6ap2	0.42	0.00000403	pigs
Clip3	0.15	0.00000409	pigs
Isg15	0.28	0.00000427	pigs
Ddx5	-0.32	0.00000449	pigs
Ctss	0.64	0.00000486	pigs
Zranb1	0.47	0.00000499	pigs
Rell1	0.55	0.00000532	pigs
Irak3	0.47	0.00000541	pigs
Tmem45a	0.33	0.00000546	pigs
Irf8	0.44	0.00000571	pigs
Spsb1	0.60	0.00000593	pigs
Tspan14	0.33	0.00000618	pigs
Ttc28	-0.58	0.00000664	pigs
Pmaip1	0.65	0.00000668	pigs
Noct	0.66	0.00000735	pigs
Ppa1	0.63	0.00000780	pigs
Vmp1	0.62	0.00000809	pigs
Ciart	0.19	0.00000821	pigs
Slc4a7	0.50	0.00000824	pigs
Ackr4	0.66	0.00000858	pigs
Aak1	-0.73	0.00000861	pigs
Xpnpep2	0.33	0.00000863	pigs
Rdh10	0.63	0.00000937	pigs
Pdia5	0.23	0.00000948	pigs
Cyld	0.49	0.00000991	pigs
Sh3pxd2b	0.35	0.00001005	pigs
Dnpep	0.29	0.00001042	pigs
Lpl	0.37	0.00001058	pigs
Sqstm1	0.61	0.00001072	pigs
Cd163	0.37	0.00001130	pigs
Hectd1	-0.67	0.00001133	pigs
Prr16	0.58	0.00001140	pigs
Nfatc2	0.37	0.00001194	pigs
Ubl5	-0.40	0.00001204	pigs
Limch1	-0.85	0.00001212	pigs

Aldh3a2	0.39	0.00001314	pigs
Grhpr	0.10	0.00001370	pigs
Cinp	0.26	0.00001383	pigs
Nr1d1	0.08	0.00001420	pigs
Phldb2	-0.83	0.00001526	pigs
B4galt1	0.51	0.00001564	pigs
Narf	0.13	0.00001609	pigs
Zfp423	-0.73	0.00001637	pigs
Plce1	0.39	0.00001652	pigs
Ell	0.55	0.00001722	pigs
Rnf144a	0.13	0.00001789	pigs
Zbtb20	-0.35	0.00001817	pigs
Traf3	0.32	0.00001860	pigs
Cryz	0.04	0.00001941	pigs
Slc25a3	0.72	0.00002224	pigs
Dmap1	0.14	0.00002254	pigs
Atp1b1	-0.74	0.00002380	pigs
Il6ra	0.48	0.00002530	pigs
Klf5	0.40	0.00002714	pigs
Hnmt	0.39	0.00002860	pigs
Tef	0.09	0.00002927	pigs
Pdcd1lg2	0.30	0.00003061	pigs
Igfbp7	0.62	0.00003114	pigs
Uchl1	0.29	0.00003259	pigs
Arsa	0.11	0.00003312	pigs
Gpc6	0.65	0.00003334	pigs
B4galt2	0.14	0.00003365	pigs
Slc19a2	0.54	0.00003450	pigs
Dtx1	0.19	0.00003726	pigs
Ddx23	0.25	0.00003730	pigs
Tollip	0.37	0.00003736	pigs
Plgrkt	0.44	0.00003744	pigs
Cxcl3	0.41	0.00003832	pigs
Gulp1	-0.83	0.00003835	pigs
Lap3	0.57	0.00003860	pigs
Gpr183	0.62	0.00003949	pigs
Farp1	0.59	0.00004077	pigs
Fam114a1	-0.60	0.00004080	pigs
Scpep1	0.26	0.00004183	pigs
Uqcrq	0.52	0.00004482	pigs
Nynrin	0.13	0.00004499	pigs
Ptgs2	0.85	0.00004591	pigs
Dbp	0.14	0.00004640	pigs
Hscb	0.11	0.00004840	pigs
Gemin8	0.11	0.00004909	pigs
Dcn	-0.41	0.00004946	pigs

Rab9	0.31	0.00005019	pigs
Tom1l2	0.04	0.00005180	pigs
Cetn3	0.26	0.00005212	pigs
C1qtnf6	0.35	0.00005235	pigs
Gucy1a1	0.36	0.00005548	pigs
Smim14	-0.67	0.00005722	pigs
Crip2	-0.57	0.00005904	pigs
Krcc1	0.26	0.00006718	pigs
Tmed3	0.55	0.00006765	pigs
Tnip3	0.33	0.00006817	pigs
Gpx8	0.60	0.00007015	pigs
Chka	0.28	0.00007018	pigs
Aldh1a3	0.76	0.00007497	pigs
Ksr1	0.40	0.00008053	pigs
Septin9	0.48	0.00008108	pigs
Ogfr	0.07	0.00008919	pigs
Itpa	0.27	0.00008996	pigs
Zmiz1	-0.57	0.00009632	pigs
Dr1	0.17	0.00009670	pigs
Prkcg	0.02	0.00009824	pigs
Ern1	0.50	0.00010122	pigs
Pmm1	0.17	0.00010270	pigs
Dnase2a	0.32	0.00010803	pigs
Col4a2	0.53	0.00011050	pigs
Fam162a	0.36	0.00011139	pigs
Fmnl3	0.18	0.00011216	pigs
Klhl36	0.17	0.00011907	pigs
Ccn2	-1.08	0.00012391	pigs
Tnc	0.57	0.00012649	pigs
Slc5a3	0.04	0.00012820	pigs
Baz2b	-0.52	0.00013143	pigs
Clip1	-0.70	0.00013758	pigs
Ifi35	0.27	0.00013811	pigs
Col5a3	0.19	0.00013971	pigs
Dzip1l	0.08	0.00015162	pigs
Enpp1	-0.92	0.00015455	pigs
Tada3	0.19	0.00015460	pigs
Plekha5	-0.74	0.00015625	pigs
Gls	-0.53	0.00015926	pigs
Nxn	0.37	0.00016857	pigs
Thy1	0.39	0.00016913	pigs
Smad3	0.41	0.00016937	pigs
Ezh2	0.44	0.00017071	pigs
Ackr3	-0.73	0.00017732	pigs
Bcl2a1d	0.98	0.00017895	pigs
Tmem42	0.06	0.00018311	pigs

Niban2	0.19	0.00018874	pigs
Sec62	-0.47	0.00019564	pigs
Nfu1	0.37	0.00020069	pigs
Nufip2	-0.55	0.00020234	pigs
Rae1	0.15	0.00020591	pigs
Csnk1a1	0.58	0.00020854	pigs
Lpcat2	0.38	0.00020938	pigs
Ptprk	-0.77	0.00021259	pigs
Olfm2	0.66	0.00021888	pigs
Plekho1	0.34	0.00021931	pigs
Nfic	-0.52	0.00021942	pigs
Wnt5b	-0.43	0.00022147	pigs
Lyrm1	0.12	0.00022316	pigs
Fbxo39	0.37	0.00022362	pigs
Epsti1	0.37	0.00023616	pigs
Usp18	0.23	0.00024702	pigs
Pcid2	0.17	0.00025287	pigs
Ebpl	0.12	0.00027091	pigs
Pbx1	-0.59	0.00027295	pigs
Stat3	0.45	0.00028172	pigs
Pml	0.33	0.00028499	pigs
Kif13a	-0.71	0.00029633	pigs
Eva1b	0.31	0.00031754	pigs
Prdx3	0.40	0.00034642	pigs
Ell2	0.43	0.00035308	pigs
Thyn1	0.14	0.00035371	pigs
Prkcsh	0.26	0.00035472	pigs
Cipc	0.05	0.00035869	pigs
Chil1	0.24	0.00036851	pigs
Thsd1	0.22	0.00039024	pigs
Bcl3	0.46	0.00039040	pigs
Pigg	0.02	0.00040256	pigs
Arntl	-0.65	0.00044792	pigs
Rrad	0.26	0.00045026	pigs
Oas1e	0.18	0.00045046	pigs
Tomm20	0.43	0.00045311	pigs
Selenow	-0.42	0.00046785	pigs
Ifi44	0.27	0.00047672	pigs
Pappa	0.43	0.00049980	pigs
Fgfr2	0.07	0.00051662	pigs
Ctsa	0.32	0.00052402	pigs
Tmem35b	0.16	0.00053769	pigs
Glrx5	0.26	0.00053806	pigs
Klhl21	0.59	0.00055919	pigs
Rpusd4	0.06	0.00056259	pigs
Psmb10	0.25	0.00056712	pigs

Arf5	0.24	0.00058702	pigs
Fmod	0.23	0.00059957	pigs
Pop7	0.28	0.00062465	pigs
Fmc1	0.08	0.00063091	pigs
Casp7	0.12	0.00064143	pigs
Ppp1r12a	-0.52	0.00067550	pigs
Wnt5a	-0.49	0.00070397	pigs
Slc35f2	0.04	0.00071886	pigs
Hif3a	0.13	0.00072904	pigs
Clec14a	0.08	0.00073530	pigs
Pfdn4	0.36	0.00073791	pigs
Txndc5	0.71	0.00073835	pigs
Txndc17	0.71	0.00074979	pigs
Igfbp5	-1.37	0.00076051	pigs
Kctd16	0.61	0.00078089	pigs
Dusp23	0.01	0.00085044	pigs
4930579F01Rik	0.25	0.00092401	pigs
Trib3	0.31	0.00093571	pigs
Rnf114	0.14	0.00095599	pigs
Smim8	0.17	0.00096983	pigs
Ripk2	0.24	0.00102104	pigs
Il1r1	0.98	0.00105075	pigs
Ehbp1l1	0.30	0.00105911	pigs
Ptpru	0.08	0.00108096	pigs
Lpin1	0.25	0.00109333	pigs
Dbn1	-0.79	0.00111348	pigs
Tamalin	0.40	0.00115839	pigs
Rassf9	0.10	0.00121538	pigs
Gpi1	0.13	0.00125154	pigs
Efna1	0.07	0.00125594	pigs
Pitpna	0.35	0.00125634	pigs
Prdx1	0.70	0.00133723	pigs
Bmper	0.36	0.00140401	pigs
Grk3	0.32	0.00141119	pigs
Ier5	0.43	0.00141210	pigs
Nfkb1	0.49	0.00141391	pigs
Add3	-0.53	0.00154672	pigs
Osmr	0.45	0.00156759	pigs
Esyt2	0.37	0.00159189	pigs
Ciao2b	0.25	0.00159819	pigs
Serf1	0.05	0.00161685	pigs
Srpx2	0.14	0.00164656	pigs
Sorbs1	0.21	0.00172423	pigs
Plod3	0.14	0.00172922	pigs
Dut	0.68	0.00178867	pigs
Stx6	0.18	0.00183268	pigs

Cercam	0.25	0.00184265	pigs
Rhobtb3	0.32	0.00186681	pigs
Uvrag	0.59	0.00191831	pigs
Abtb2	0.39	0.00195259	pigs
Ppif	0.73	0.00196988	pigs
Son	-0.41	0.00197497	pigs
Trnau1ap	0.25	0.00204253	pigs
Dhrs7b	0.07	0.00207448	pigs
Unkl	0.09	0.00212845	pigs
Irf7	0.23	0.00219422	pigs
Pdcd4	0.56	0.00221116	pigs
Nol3	0.12	0.00223581	pigs
Epc2	-0.57	0.00225179	pigs
Sil1	0.14	0.00228156	pigs
Cdc42bpa	-0.39	0.00229780	pigs
Pgm2	0.18	0.00234425	pigs
Gpaa1	0.34	0.00236362	pigs
Denn2b	0.22	0.00238167	pigs
Ppm1m	0.18	0.00239137	pigs
Hexim2	0.22	0.00240582	pigs
Slc11a2	0.43	0.00249062	pigs
Skiv2l	0.21	0.00252790	pigs
Ckap4	0.11	0.00258420	pigs
Fbxl15	0.08	0.00266901	pigs
Icam5	0.06	0.00268932	pigs
Dram1	0.21	0.00274737	pigs
Zfp512b	0.33	0.00274970	pigs
Anxa5	0.46	0.00280079	pigs
Spats2	0.22	0.00281385	pigs
Rnf19b	0.72	0.00283321	pigs
Gpr35	0.22	0.00283506	pigs
Kirrel	0.40	0.00283586	pigs
Adamts9	0.45	0.00295426	pigs
Kdm6b	0.22	0.00298748	pigs
Ccdc39	0.03	0.00302180	pigs
Trappc4	0.15	0.00302976	pigs
Ehbp1	-0.58	0.00303176	pigs
Acss1	0.13	0.00306562	pigs
Fbxw5	0.28	0.00308956	pigs
Glmp	0.27	0.00309462	pigs
Rnase1	0.05	0.00316303	pigs
1700001L19Rik	0.13	0.00317019	pigs
Zfand2b	0.19	0.00317119	pigs
Vav2	0.06	0.00321838	pigs
Nipsnap3b	0.27	0.00338774	pigs
Fzd2	0.16	0.00348994	pigs

Csnk1d	0.12	0.00353160	pigs
Sema5a	-0.87	0.00366034	pigs
Accsl	0.94	0.00366615	pigs
Ndufb8	0.41	0.00373406	pigs
2700062C07Rik	0.27	0.00399520	pigs
Csrp2	0.65	0.00403065	pigs
Cdk17	0.36	0.00405384	pigs
Tnfsf14	0.07	0.00406118	pigs
Nox4	-0.81	0.00411548	pigs
Bloc1s5	0.03	0.00420668	pigs
Scarb1	0.20	0.00423292	pigs
Rpa3	0.38	0.00431425	pigs
Ccn3	-0.77	0.00442332	pigs
Xpo1	0.28	0.00444449	pigs
Rap2c	0.07	0.00450208	pigs
Unc45a	0.06	0.00467177	pigs
Slc41a1	0.08	0.00468101	pigs
Papln	0.05	0.00469274	pigs
Irf3	0.18	0.00472695	pigs
Acp2	0.21	0.00487153	pigs
St6galnac4	0.43	0.00492794	pigs
Snrpa1	0.28	0.00499290	pigs
Hnrnpdl	-0.30	0.00507574	pigs
Alas1	0.15	0.00521894	pigs
Selenop	0.49	0.00532249	pigs
Fah	0.16	0.00534780	pigs
Rel	0.51	0.00539142	pigs
Bach1	0.53	0.00552868	pigs
Ccdc32	0.03	0.00566715	pigs
Tmem60	0.09	0.00576521	pigs
Nudt5	0.20	0.00580172	pigs
S100a11	0.41	0.00581406	pigs
Ruvbl2	0.14	0.00595239	pigs
Il1a	1.03	0.00598041	pigs
C1qtnf1	0.43	0.00605965	pigs
Amz2	0.07	0.00614629	pigs
Ptdss1	0.19	0.00617830	pigs
Fam234a	0.10	0.00618836	pigs
Ddr2	-0.68	0.00627356	pigs
Sdhaf2	0.17	0.00632694	pigs
Itpr2	-0.57	0.00637907	pigs
Dpysl2	-0.66	0.00639433	pigs
Arrdc3	0.29	0.00658768	pigs
Nme6	0.10	0.00659740	pigs
Wee1	0.21	0.00677656	pigs
Slc35a2	0.17	0.00693210	pigs

Atrx	-0.56	0.00707995	pigs
Xrcc6	0.10	0.00723019	pigs
Ubfd1	0.24	0.00736913	pigs
Comt	0.41	0.00791732	pigs
Tm2d2	0.21	0.00801029	pigs
Stmn1	0.42	0.00832556	pigs
Fbln5	-0.55	0.00836225	pigs
Golim4	-0.54	0.00844376	pigs
Ndufaf5	0.28	0.00890167	pigs
Prkag2	0.21	0.00893680	pigs
Pafah1b3	0.13	0.00929898	pigs
Ammecr1	0.26	0.00955650	pigs
Epc1	-0.51	0.00958163	pigs
Tiam2	0.26	0.00967213	pigs
Klhdc4	0.07	0.00970791	pigs
Cebpd	0.36	0.00973682	pigs
Atp13a3	0.45	0.00983570	pigs
Cd99l2	0.01	0.00983794	pigs
Luzp1	0.16	0.00987576	pigs
Slit2	-0.57	0.01010772	pigs
Cdkn2c	0.13	0.01014880	pigs
Cacna2d3	-0.64	0.01032055	pigs
Rassf5	0.85	0.01059884	pigs
Prdx4	0.69	0.01081311	pigs
F2r	0.17	0.01093035	pigs
Thoc6	0.21	0.01097699	pigs
Cfap20dc	0.22	0.01099555	pigs
Agpat1	0.17	0.01102153	pigs
Lhfpl2	0.36	0.01107719	pigs
Bhlhe41	0.34	0.01117175	pigs
Nkg7	0.37	0.01123011	pigs
Pomgnt1	0.12	0.01129091	pigs
Prorp	0.50	0.01154081	pigs
Hmcn2	-0.55	0.01167331	pigs
Itga11	-0.66	0.01177426	pigs
Cdon	-0.71	0.01201245	pigs
Sorcs2	0.20	0.01222868	pigs
Hpse2	0.26	0.01225326	pigs
Klhl29	0.34	0.01228620	pigs
Arhgdib	0.81	0.01276484	pigs
Kcnj8	0.14	0.01297109	pigs
Peli1	0.32	0.01300565	pigs
Enc1	0.07	0.01321690	pigs
Enox1	0.23	0.01325791	pigs
Ccdc142	0.30	0.01349062	pigs
L3mbtl1	-0.40	0.01360264	pigs

Tspan15	0.14	0.01362758	pigs
Usp11	0.15	0.01381151	pigs
Actb	0.38	0.01413252	pigs
Ecm2	-0.39	0.01440112	pigs
Sord	0.44	0.01483686	pigs
Tmod3	-0.51	0.01486656	pigs
Dclk2	0.15	0.01496330	pigs
Ndfip1	0.70	0.01501158	pigs
Psme2	0.44	0.01503100	pigs
Angpt4	0.21	0.01523083	pigs
Lin54	0.12	0.01586910	pigs
Trap1	0.22	0.01590548	pigs
Aif1	0.80	0.01618396	pigs
Aco1	0.26	0.01623723	pigs
Rcvrn	0.07	0.01633041	pigs
Islr	-0.63	0.01641956	pigs
Lxn	0.25	0.01678711	pigs
Trmt10a	0.21	0.01686785	pigs
Psma5	0.13	0.01722298	pigs
Oser1	0.15	0.01757248	pigs
Sumf2	0.11	0.01759303	pigs
Ston2	-0.71	0.01787857	pigs
Cavin3	-0.39	0.01791884	pigs
Acta2	0.43	0.01797354	pigs
Cdh13	0.14	0.01815119	pigs
Peli2	-1.04	0.01842366	pigs
Rbfox2	-0.41	0.01909294	pigs
Sin3a	0.11	0.01914166	pigs
Akap9	-0.61	0.01929031	pigs
Rsrp1	0.08	0.01944883	pigs
Kitl	0.09	0.02018050	pigs
Fkbp5	0.56	0.02027947	pigs
Adh5	0.31	0.02029466	pigs
Cep192	0.24	0.02056231	pigs
Psmb4	0.49	0.02066523	pigs
Epb41l2	-0.41	0.02079399	pigs
Skap2	-0.32	0.02084421	pigs
Golph3l	0.13	0.02093063	pigs
Tnfaip8	0.77	0.02228002	pigs
Slc24a3	-0.40	0.02228822	pigs
Bgn	0.57	0.02239000	pigs
Cnot9	0.36	0.02257055	pigs
Ccdc146	0.05	0.02306906	pigs
Egr2	0.10	0.02352267	pigs
Ddr1	-0.02	0.02353027	pigs
Lgals1	-0.40	0.02383235	pigs

Commd8	0.15	0.02398323	pigs
Tmem11	0.35	0.02439234	pigs
Ptov1	0.03	0.02468592	pigs
Dhx9	-0.34	0.02469434	pigs
Tsc22d4	0.30	0.02470959	pigs
C2	0.23	0.02498004	pigs
Gpcpd1	0.39	0.02543319	pigs
Socs1	0.51	0.02571304	pigs
Efemp2	0.35	0.02590493	pigs
Clpx	0.27	0.02592733	pigs
Fam32a	0.32	0.02600323	pigs
Ip6k2	0.04	0.02686825	pigs
Creb3	0.07	0.02708134	pigs
Clic4	-0.50	0.02740113	pigs
Itgb1	-0.32	0.02768986	pigs
Fmo2	-0.78	0.02798185	pigs
Gpc4	0.16	0.02861001	pigs
Adam12	0.18	0.02862570	pigs
Arl14epl	-0.30	0.02906123	pigs
Ebf1	-0.44	0.02921871	pigs
Ppp1r21	0.19	0.02943869	pigs
Eef2	0.46	0.02962523	pigs
Prdm1	0.25	0.02967406	pigs
Npl	0.36	0.02985847	pigs
Adcy3	0.28	0.03027226	pigs
Dph5	0.06	0.03035540	pigs
Shisa4	0.07	0.03132121	pigs
Trabd	0.19	0.03149582	pigs
Cars2	0.02	0.03200665	pigs
Tmtc2	-0.77	0.03290831	pigs
Adora2b	0.17	0.03300586	pigs
Gatm	0.15	0.03309885	pigs
Cops4	0.23	0.03351172	pigs
Glg1	-0.46	0.03381477	pigs
Tnrc6a	-0.63	0.03389354	pigs
Eri3	0.17	0.03490042	pigs
Ddb2	-0.01	0.03536706	pigs
Slco2b1	0.07	0.03560097	pigs
Lrriq3	0.09	0.03590282	pigs
Atp6ap1	0.20	0.03591631	pigs
Blvrb	0.23	0.03621851	pigs
Erp29	0.43	0.03726709	pigs
Ptger1	0.04	0.03868470	pigs
Wtap	0.57	0.03998765	pigs
Ypel5	0.21	0.04002080	pigs
Syngap1	0.05	0.04058284	pigs

Sharpin	0.23	0.04072009	pigs
Traf7	0.07	0.04096285	pigs
Dbndd2	0.25	0.04276287	pigs
Tmem86a	0.09	0.04285946	pigs
F3	0.44	0.04288948	pigs
Clu	0.41	0.04483784	pigs
Adamts2	-0.41	0.04520541	pigs
Ccbe1	0.30	0.04597379	pigs
Cblb	-0.46	0.04614989	pigs
Agrn	0.03	0.04669600	pigs
Edem1	0.33	0.04682486	pigs
Dcx	0.18	0.04686542	pigs
Osbpl5	0.11	0.04750865	pigs
Lifr	0.31	0.04769826	pigs
Arpc1b	0.58	0.04800746	pigs
Galns	0.22	0.04920792	pigs
Anxa4	0.29	0.04938319	pigs
Eif3d	0.44	0.04941717	pigs
Wars	0.17	0.05197593	pigs
Bscl2	0.03	0.05206685	pigs
Tgfb3	0.40	0.05244132	pigs
Rpusd3	0.19	0.05264911	pigs
Rab11fip3	0.14	0.05323716	pigs
Wrnip1	0.05	0.05353211	pigs
Usf1	0.08	0.05432779	pigs
Sptan1	-0.45	0.05447517	pigs
Gtpbp1	0.25	0.05707768	pigs
Slc25a5	0.55	0.05764019	pigs
Slc16a9	0.13	0.05776385	pigs
Chrdl1	-0.47	0.05907544	pigs
Tbc1d22a	0.04	0.06100034	pigs
Igf1	0.44	0.06234111	pigs
Prmt1	0.60	0.06297681	pigs
Polr1d	0.44	0.06401798	pigs
5730480H06Rik	0.05	0.06537094	pigs
Rbck1	0.25	0.06575879	pigs
Lancl1	0.10	0.06621926	pigs
Nemp2	0.04	0.06698127	pigs
1810037I17Rik	0.37	0.06769913	pigs
Sdhc	0.23	0.06865207	pigs
Ap2m1	0.36	0.06878701	pigs
Kbtbd2	0.06	0.07036813	pigs
Nmt2	0.43	0.07048224	pigs
Bcl6	0.10	0.07137262	pigs
Ppt1	0.26	0.07140479	pigs
Zbtb37	0.08	0.07159708	pigs

Cyb561d2	0.03	0.07361137	pigs
Tmem88	0.11	0.07455581	pigs
Zbtb1	0.11	0.07456776	pigs
Dach1	-0.82	0.07458885	pigs
Timm50	0.28	0.07636100	pigs
Ntpcr	0.07	0.07688016	pigs
Ntmt1	0.08	0.07714165	pigs
Lrrc57	0.14	0.07768946	pigs
Tmem106c	0.26	0.07801959	pigs
Cacna2d1	-0.55	0.07832718	pigs
Ccng1	0.51	0.08043311	pigs
Nrf1	-0.40	0.08116842	pigs
Usp25	-0.58	0.08152569	pigs
Crot	0.09	0.08295077	pigs
Ccpg1	-0.35	0.08303581	pigs
Ankrd17	-0.44	0.08637607	pigs
C2cd2	0.23	0.08740934	pigs
Lrrk2	-0.69	0.08778700	pigs
Urod	0.05	0.08781484	pigs
Stk16	0.10	0.08895121	pigs
Hook3	-0.40	0.09015083	pigs
Sec11c	0.40	0.09111923	pigs
Ankrd28	0.23	0.09459649	pigs
Serpinf1	0.44	0.09576225	pigs
Pla2r1	0.03	0.09700674	pigs
Glt8d1	0.19	0.09897268	pigs
Zfp804a	0.29	0.09938763	pigs
Adcy2	0.32	0.10003917	pigs
Zfp516	0.02	0.10005759	pigs
Lta4h	0.33	0.10009117	pigs
Clk4	0.15	0.10030207	pigs
Podn	-0.17	0.10032815	pigs
Cenpu	-0.41	0.10154436	pigs
Dync2i1	-0.03	0.10155477	pigs
Slc26a2	0.14	0.10420905	pigs
Aspa	0.04	0.10486778	pigs
Hif1a	0.49	0.10489347	pigs
Tmem54	0.08	0.10534528	pigs
Ugp2	0.26	0.10632013	pigs
Hvcn1	0.12	0.10651763	pigs
Ints11	0.19	0.10681096	pigs
Supt5	0.27	0.10682306	pigs
Suclg2	0.38	0.10734570	pigs
Pakap	0.32	0.10884518	pigs
Ccn4	0.17	0.10928663	pigs
Sfmbt2	0.11	0.11055621	pigs

Oxa1l	0.20	0.11222044	pigs
Rtraf	0.49	0.11326307	pigs
Exoc3l4	0.06	0.11398718	pigs
Bri3	0.47	0.11483689	pigs
Sh3bp2	0.02	0.11650590	pigs
Rpp38	0.02	0.11725350	pigs
Rab11a	0.31	0.11731615	pigs
Atp5pb	0.51	0.11754652	pigs
Cish	0.09	0.11862612	pigs
Tubd1	0.10	0.12124725	pigs
Higd1a	0.32	0.12183424	pigs
Coq9	0.16	0.12274928	pigs
Timm22	0.02	0.12364029	pigs
Acat1	0.31	0.12568366	pigs
Poc1a	0.07	0.12636971	pigs
Dram2	0.17	0.12787574	pigs
Syn3	-0.49	0.12802544	pigs
Phex	-0.86	0.12825127	pigs
Sdk1	0.37	0.12858937	pigs
Cped1	-0.69	0.13406525	pigs
Vopp1	0.03	0.13414778	pigs
N4bp1	0.34	0.13535918	pigs
Rcc2	0.12	0.13626936	pigs
Rab33b	0.08	0.13639415	pigs
Rsu1	-0.39	0.13657376	pigs
Dpagt1	0.12	0.14024841	pigs
Ptpn2	0.32	0.14056299	pigs
Sec31a	-0.37	0.14291218	pigs
Tmub1	0.09	0.14329899	pigs
Cd9	0.71	0.14449715	pigs
Negr1	-0.94	0.14537868	pigs
Pepd	0.24	0.14778225	pigs
Col8a1	0.07	0.14784746	pigs
Churc1	0.37	0.14882704	pigs
Tceal1	0.08	0.14888512	pigs
Ap2a1	0.11	0.14952743	pigs
Palld	0.37	0.15017553	pigs
Axin1	0.04	0.15125571	pigs
Prickle1	-0.71	0.15140932	pigs
Sertad3	0.26	0.15336007	pigs
Hsd11b1	0.39	0.15394051	pigs
Ak3	0.07	0.15416278	pigs
Srgn	0.60	0.15419509	pigs
Tnfaip6	0.51	0.16023084	pigs
Deaf1	0.16	0.16091946	pigs
Dop1b	0.06	0.16186319	pigs

Camsap2	0.20	0.16285699	pigs
Chmp5	0.39	0.16330848	pigs
Washc3	0.17	0.16554773	pigs
Grn	0.29	0.16597826	pigs
Acaa2	0.42	0.16656248	pigs
Serpine1	0.50	0.16663251	pigs
Nfkbil1	0.00	0.16764897	pigs
Kdm4a	0.10	0.16785647	pigs
Lrig1	0.00	0.16896235	pigs
Slc38a10	0.11	0.17232372	pigs
Clasrp	0.09	0.17441451	pigs
Ccn1	-0.67	0.17460704	pigs
Tcf12	-0.35	0.17478803	pigs
Retsat	0.07	0.17483261	pigs
Acot8	0.28	0.17607753	pigs
Znhit6	0.01	0.17737242	pigs
Cbr3	0.13	0.17799308	pigs
Actn1	0.41	0.17803786	pigs
Plpp5	0.24	0.17949032	pigs
Sp4	-0.02	0.18250866	pigs
Dguok	0.08	0.18291500	pigs
Coro1b	0.19	0.18320280	pigs
Selenbp2	-0.49	0.18451107	pigs
Elob	-0.20	0.18660195	pigs
Pgrmc1	0.24	0.19036661	pigs
Anapc15-ps	0.19	0.19419217	pigs
Lamtor2	0.36	0.19831593	pigs
Ttc7	0.14	0.19943938	pigs
Tk1	0.47	0.19979547	pigs
Ube2d3	0.40	0.20034771	pigs
Nmral1	0.13	0.20182284	pigs
Sdf2	0.09	0.20222990	pigs
Dzip3	0.14	0.20234929	pigs
Jarid2	0.44	0.20542670	pigs
Vbp1	0.34	0.20840484	pigs
Ttyh2	0.03	0.21040930	pigs
Vps16	0.07	0.21072826	pigs
Nek9	0.08	0.21174640	pigs
Xkr6	0.04	0.21559377	pigs
Rab40b	0.21	0.21861902	pigs
Cdc34	0.17	0.21921716	pigs
Strap	0.40	0.21949722	pigs
Gas7	0.38	0.23023780	pigs
Epn2	0.03	0.23610455	pigs
Scrn3	0.02	0.24393635	pigs
Gan	0.21	0.24541448	pigs

Ccm2	0.22	0.24563861	pigs
Ap1s1	0.12	0.24716599	pigs
Tubg1	0.29	0.25395535	pigs
Ivd	0.12	0.25822295	pigs
Gemin4	0.31	0.25938041	pigs
Fign	-0.83	0.26059130	pigs
Rptor	-0.03	0.26121399	pigs
Slc15a3	0.25	0.26177357	pigs
Ebf2	-0.64	0.26482125	pigs
Hsd17b11	0.00	0.26584902	pigs
N6amt1	0.05	0.26694871	pigs
Cks2	0.29	0.27241442	pigs
Eif4e	0.38	0.27843219	pigs
Nek6	0.17	0.27888008	pigs
Ehd1	0.31	0.28130166	pigs
Ubash3b	0.17	0.28413851	pigs
Armcx1	0.03	0.28429436	pigs
Npepps	-0.40	0.28539352	pigs
Wdr7	-0.64	0.28689023	pigs
Angptl1	-0.54	0.28873485	pigs
Lpcat3	-0.03	0.29002181	pigs
Tmem100	-0.61	0.29005846	pigs
Rnd3	0.38	0.29440226	pigs
Col19a1	0.14	0.29560070	pigs
Egfr	0.23	0.29751886	pigs
Sgf29	0.15	0.30051856	pigs
Atf6b	0.18	0.30209327	pigs
Taf4b	0.29	0.30575164	pigs
Necap2	0.39	0.30947715	pigs
Gpr176	0.04	0.31046233	pigs
Psme1	0.30	0.31168816	pigs
Icmt	0.41	0.31733373	pigs
Tmem101	0.10	0.31818701	pigs
Ssbp1	0.27	0.32052031	pigs
Glyr1	0.10	0.32182203	pigs
Atp5j	0.52	0.33549746	pigs
Ptpn13	0.42	0.33949466	pigs
Tspan6	0.19	0.34029785	pigs
Cdk12	0.18	0.34654108	pigs
Bptf	-0.43	0.34662450	pigs
Tmem70	0.11	0.34718758	pigs
Slc50a1	0.04	0.35371524	pigs
Tlr3	0.07	0.35444756	pigs
Fgd6	0.12	0.35923364	pigs
Nuak1	0.05	0.36156886	pigs
Igf2r	0.34	0.36160243	pigs

Tep1	0.05	0.36308098	pigs
Ndel1	0.45	0.36497887	pigs
Ogt	0.38	0.36762269	pigs
Papss1	0.16	0.37425023	pigs
Atp1a1	0.40	0.37597567	pigs
Chst3	0.03	0.37622490	pigs
Ptgs1	0.09	0.37639366	pigs
Sf3b6	0.53	0.37775923	pigs
Septin7	-0.28	0.38102519	pigs
Tmem129	0.29	0.38138294	pigs
Pmpcb	0.11	0.38408434	pigs
Tpd52l2	0.15	0.38501914	pigs
Zfpl1	0.18	0.38792661	pigs
Sgce	0.26	0.39796559	pigs
Plekhh3	0.16	0.40768797	pigs
Ndufa9	0.30	0.40975733	pigs
St3gal1	0.39	0.41079831	pigs
Trappc5	0.32	0.41468629	pigs
Cops8	0.09	0.42006524	pigs
Scrn2	0.03	0.42105694	pigs
Ccdc127	0.23	0.42621095	pigs
Cmip	0.35	0.42787574	pigs
Btf3l4	0.44	0.43031459	pigs
Vps72	-0.06	0.43208302	pigs
Alad	-0.05	0.43437402	pigs
Echdc3	0.04	0.43691356	pigs
Hdac3	0.08	0.44186764	pigs
Fam210b	0.21	0.44338600	pigs
Ppp2r3a	-0.64	0.44597763	pigs
Prkca	0.24	0.44898632	pigs
Hnrnph1	-0.21	0.45354996	pigs
Acp1	0.47	0.45376943	pigs
Scly	0.06	0.45614226	pigs
Rimklb	0.05	0.46081012	pigs
Tmem98	0.07	0.46678382	pigs
Sav1	0.06	0.46754515	pigs
Pign	0.11	0.46946160	pigs
Cramp1	0.08	0.47011284	pigs
Nmi	0.09	0.47087461	pigs
Zfp64	0.08	0.47423215	pigs
Optn	0.23	0.48207170	pigs
Hspa5	-0.24	0.48639670	pigs
Bphl	-0.02	0.48801271	pigs
Parp9	0.26	0.48860151	pigs
Ctsc	0.60	0.48980075	pigs
Fh1	0.11	0.49091467	pigs

Pin1	0.15	0.49254782	pigs
Clstn1	-0.43	0.49318813	pigs
Uxt	0.21	0.49659652	pigs
Abcd4	0.03	0.49860706	pigs
Lman2	0.47	0.50370242	pigs
Psmb2	0.43	0.51411968	pigs
Antxr1	-0.36	0.51963097	pigs
Nudt21	0.05	0.52040495	pigs
Adamtsl3	-0.45	0.52065719	pigs
Bnc2	-0.26	0.52147905	pigs
Paxx	0.32	0.52838646	pigs
Plaat3	0.13	0.53313842	pigs
Tuba1b	-0.08	0.54638644	pigs
Tspan31	0.25	0.54694644	pigs
Polr1h	0.04	0.54914851	pigs
Tatdn3	0.05	0.55081589	pigs
Rhbdl1	0.01	0.55282491	pigs
Tmem131l	0.29	0.55287458	pigs
Btg3	0.28	0.55647619	pigs
Klhl26	0.00	0.56669122	pigs
Dhx32	-0.02	0.57769842	pigs
Dym	-0.43	0.57896306	pigs
Gemin6	0.13	0.58029186	pigs
Dera	0.08	0.59550303	pigs
Slc35c2	0.10	0.59655521	pigs
Git1	0.05	0.59890404	pigs
Psmd3	0.39	0.60108158	pigs
Slc45a4	0.06	0.60178261	pigs
Hprt	0.14	0.60392796	pigs
Nipbl	-0.23	0.60586313	pigs
Dnajc14	0.05	0.61139520	pigs
Golm1	0.13	0.61570753	pigs
Ndrg4	0.20	0.61688614	pigs
Ghsr	-0.46	0.61833947	pigs
Lratd1	0.12	0.61938364	pigs
Kctd9	-0.46	0.62069197	pigs
Ulk2	0.25	0.62959129	pigs
Haus2	0.18	0.63279175	pigs
Accs	0.09	0.63343054	pigs
Nat14	0.02	0.63365362	pigs
Slc8b1	0.14	0.63578642	pigs
Cd24a	-0.85	0.64017674	pigs
Tanc1	-0.16	0.64044591	pigs
Bicc1	-0.41	0.64140503	pigs
Piga	0.05	0.64645301	pigs
Ankrd12	-0.33	0.65072085	pigs

Rab43	0.05	0.66069030	pigs
Hpcal1	0.23	0.66172984	pigs
A430005L14Rik	0.01	0.66279216	pigs
5031439G07Rik	0.19	0.66405342	pigs
Fscn1	0.13	0.66600926	pigs
Vps25	0.22	0.67471698	pigs
Ccdc170	-0.55	0.68089652	pigs
Elk3	0.25	0.68191300	pigs
Rgcc	0.31	0.68601091	pigs
Ntrk2	-1.07	0.69141629	pigs
Tmem135	-0.49	0.69238801	pigs
Spcs1	0.55	0.69922970	pigs
Gle1	0.16	0.70395358	pigs
Tmem168	0.16	0.71900032	pigs
Tex22	0.03	0.72131168	pigs
Fndc3a	-0.44	0.72201672	pigs
Ube2v2	0.20	0.72255232	pigs
Pgap2	0.15	0.72849571	pigs
Pabpc1	-0.24	0.73571043	pigs
Emp1	-0.31	0.73799901	pigs
Pcf11	-0.44	0.74435922	pigs
Snrnp70	-0.37	0.74576932	pigs
Gfus	0.19	0.74678332	pigs
Alg5	0.09	0.74740422	pigs
Hsd17b12	0.17	0.76241778	pigs
Ywhaq	-0.31	0.76419098	pigs
Evl	0.02	0.76448744	pigs
Nbeal1	-0.33	0.76696679	pigs
Paqr9	-0.55	0.76776521	pigs
Cltc	-0.18	0.76998636	pigs
Gfod2	0.25	0.77123396	pigs
Ash1l	-0.40	0.77462711	pigs
Jade2	0.06	0.77747228	pigs
Gla	0.09	0.79303898	pigs
Gpnmb	0.16	0.79512743	pigs
Tbc1d4	0.26	0.79795034	pigs
Slc66a2	0.06	0.80025028	pigs
Siah1b	0.08	0.80145333	pigs
Col16a1	0.17	0.80206362	pigs
Ints7	0.09	0.80212456	pigs
Btg1b	0.58	0.80422578	pigs
Slc30a1	0.15	0.81405376	pigs
Snap29	0.37	0.82272210	pigs
Cox20	0.16	0.82612099	pigs
Pin4	0.13	0.83274674	pigs
Tbc1d1	0.35	0.83810739	pigs

Pcsk6	-0.44	0.84373165	pigs
Cuta	0.02	0.84969236	pigs
Csrnp1	0.27	0.85276714	pigs
Ift22	0.07	0.85526515	pigs
Nucks1	-0.21	0.85686984	pigs
Usp6nl	0.15	0.85806634	pigs
Impdh1	0.10	0.86012743	pigs
Oaz1	0.45	0.86144916	pigs
Agap3	0.02	0.86485731	pigs
Cep85l	-0.63	0.86828575	pigs
Armcx2	0.10	0.87150311	pigs
Tmx1	0.23	0.87535848	pigs
Tmem251	0.09	0.88356710	pigs
Crebzf	0.06	0.88640531	pigs
Pced1a	0.18	0.89180292	pigs
Ndufs3	0.27	0.89731441	pigs
Appl1	-0.50	0.89782453	pigs
Kcnq1	0.05	0.90727269	pigs
Cfap69	-0.45	0.91403494	pigs
Bmp1	0.15	0.91467443	pigs
Hoga1	0.00	0.92435752	pigs
Vps37c	0.08	0.92649887	pigs
Ift46	0.07	0.92689034	pigs
1700123O20Rik	0.24	0.92937563	pigs
Stk24	0.28	0.94017139	pigs
Ablim2	0.07	0.94024855	pigs
Nr4a3	0.23	0.95441783	pigs
Cox5a	0.35	0.95501461	pigs
Ptk7	0.00	0.95520951	pigs
Zbp1	0.11	0.95581021	pigs
Gyg	0.20	0.95825584	pigs
Nme1	0.60	0.96349071	pigs
Hoxb2	0.07	0.96356488	pigs
Tmem175	0.34	0.96493094	pigs
Wbp1l	0.29	0.96869316	pigs
Prkdc	-0.13	0.97635725	pigs
Taf5l	-0.01	0.97645122	pigs
Phactr2	-0.45	0.98630673	pigs
Coq5	-0.02	1.00000000	pigs
Aga	0.07	1.00000000	pigs
Ppp2r5c	0.19	1.00000000	pigs
Iqgap1	-0.34	1.00000000	pigs
Tfrc	0.08	1.00000000	pigs
Ptrh1	0.02	1.00000000	pigs
Dact1	0.12	1.00000000	pigs
Prxl2c	0.12	1.00000000	pigs

Tsr1	0.02	1.00000000	pigs
Atp6v0b	0.33	1.00000000	pigs
Akt1s1	0.09	1.00000000	pigs
Cpsf3	0.12	1.00000000	pigs
Slc43a3	-0.01	1.00000000	pigs
Sft2d1	0.19	1.00000000	pigs
Ripor1	0.05	1.00000000	pigs
Tmem183a	0.11	1.00000000	pigs
Ypel2	0.16	1.00000000	pigs
Tti2	0.04	1.00000000	pigs
Clns1a	0.07	1.00000000	pigs
Tprgl	0.03	1.00000000	pigs
Ralgps2	-0.65	1.00000000	pigs
Top3a	0.10	1.00000000	pigs
Obsl1	0.13	1.00000000	pigs
Rgs6	-0.58	1.00000000	pigs
Ppm1f	0.10	1.00000000	pigs
Gspt2	0.12	1.00000000	pigs
Epm2aip1	0.02	1.00000000	pigs
Nup98	0.16	1.00000000	pigs
Zer1	0.30	1.00000000	pigs
Nop53	0.18	1.00000000	pigs
Clmn	0.05	1.00000000	pigs
Csk	0.28	1.00000000	pigs
Ltbr	0.10	1.00000000	pigs
Pmpca	0.14	1.00000000	pigs
Polr1c	0.23	1.00000000	pigs
Fam171b	0.15	1.00000000	pigs
Tbcd	0.06	1.00000000	pigs
Sfmbt1	-0.04	1.00000000	pigs
Sh3d19	-0.39	1.00000000	pigs
Rragc	0.17	1.00000000	pigs
Ssbp4	0.21	1.00000000	pigs
Ror1	0.49	1.00000000	pigs
Polr2a	0.33	1.00000000	pigs
Net1	0.71	1.00000000	pigs
Ccnd3	0.36	1.00000000	pigs
Lipa	0.11	1.00000000	pigs
Ndufb4	0.31	1.00000000	pigs
Lmnb1	0.20	1.00000000	pigs
Sdf4	0.37	1.00000000	pigs
Isyna1	0.39	1.00000000	pigs
Dnajc19	0.14	1.00000000	pigs
Nqo2	-0.02	1.00000000	pigs
Nadsyn1	0.11	1.00000000	pigs
Plxnd1	-0.04	1.00000000	pigs

Sco2	0.16	1.00000000	pigs
Smarcd2	0.11	1.00000000	pigs
Fam110c	0.07	1.00000000	pigs
Endov	0.07	1.00000000	pigs
Fap	0.16	1.00000000	pigs
Eif2b5	0.10	1.00000000	pigs
Fam126a	-0.52	1.00000000	pigs
Rapgef5	0.31	1.00000000	pigs
Ing2	-0.02	1.00000000	pigs
Rnf121	0.19	1.00000000	pigs
Cybb	0.60	1.00000000	pigs
Hspa14	0.25	1.00000000	pigs
Tmem254a	-0.04	1.00000000	pigs
Fbxo32	0.00	1.00000000	pigs
Nono	0.26	1.00000000	pigs
Rnf6	0.13	1.00000000	pigs
Ddost	0.46	1.00000000	pigs
Nr3c1	-0.41	1.00000000	pigs
Oaf	0.13	1.00000000	pigs
Shmt2	0.12	1.00000000	pigs
Baiap2	0.13	1.00000000	pigs
Gart	0.07	1.00000000	pigs
Arhgap21	-0.47	1.00000000	pigs
Kcp	0.14	1.00000000	pigs
Ago2	0.40	1.00000000	pigs
Calml4	0.04	1.00000000	pigs
Pomp	0.48	1.00000000	pigs
Pnrc1	0.35	1.00000000	pigs
Acot4	0.11	1.00000000	pigs
Wac	-0.37	1.00000000	pigs
Axl	-0.55	1.00000000	pigs
Dtymk	0.04	1.00000000	pigs
Exosc7	0.13	1.00000000	pigs
Brwd1	-0.52	1.00000000	pigs
Gpr137b	0.07	1.00000000	pigs
Taf8	0.04	1.00000000	pigs
9930012K11Rik	0.03	1.00000000	pigs
Usp12	0.10	1.00000000	pigs
Cxcr4	0.53	1.00000000	pigs
Hmbox1	-0.58	1.00000000	pigs
Dimt1	-0.03	1.00000000	pigs
Rasa3	0.19	1.00000000	pigs
Dyrk1b	0.06	1.00000000	pigs
Fer	-0.55	1.00000000	pigs
Frrs1	0.23	1.00000000	pigs
Atp11c	0.12	1.00000000	pigs

Rac1	0.57	1.00000000	pigs
Trib2	0.09	1.00000000	pigs
Fnip1	0.35	1.00000000	pigs
Plcd1	0.10	1.00000000	pigs
Gbe1	-0.56	1.00000000	pigs
Nckap5l	0.11	1.00000000	pigs
1110065P20Rik	0.13	1.00000000	pigs
Zdhhc20	0.29	1.00000000	pigs
Arl3	0.16	1.00000000	pigs
Sspn	-0.44	1.00000000	pigs
Tm2d3	0.11	1.00000000	pigs
Becn1	0.07	1.00000000	pigs
Pomt1	-0.01	1.00000000	pigs
Resf1	0.09	1.00000000	pigs
Slc39a13	0.10	1.00000000	pigs
Ttc23	0.10	1.00000000	pigs
Ccdc24	0.09	1.00000000	pigs
Csad	0.02	1.00000000	pigs
Enoph1	0.03	1.00000000	pigs
Rabgap1l	-0.44	1.00000000	pigs
Dock9	0.36	1.00000000	pigs
Pcnx3	-0.01	1.00000000	pigs
Dek	-0.33	1.00000000	pigs
Clstn3	0.07	1.00000000	pigs
Smc2	0.07	1.00000000	pigs
Trp53bp2	0.03	1.00000000	pigs
Srsf3	-0.03	1.00000000	pigs
Umad1	0.10	1.00000000	pigs
Cd79a	0.26	1.00000000	pigs
Kat6b	-0.43	1.00000000	pigs
Zdhhc18	0.40	1.00000000	pigs
Eif2ak2	0.07	1.00000000	pigs
Taf10	0.27	1.00000000	pigs
Eif3f	0.47	1.00000000	pigs
Rhou	-0.45	1.00000000	pigs
Prmt2	0.00	1.00000000	pigs
Ldlrad4	0.20	1.00000000	pigs
Sypl	0.15	1.00000000	pigs
Dcxr	0.17	1.00000000	pigs
Aasdhppt	0.06	1.00000000	pigs
Wdtc1	0.08	1.00000000	pigs
Snx6	0.29	1.00000000	pigs
Smyd4	0.08	1.00000000	pigs
Gsdmd	0.04	1.00000000	pigs
Spring1	-0.64	1.00000000	pigs
Tbl1xr1	-0.39	1.00000000	pigs

Plcg2	0.23	1.00000000	pigs
Tex264	0.10	1.00000000	pigs
Bin3	0.32	1.00000000	pigs
Nr1h2	0.11	1.00000000	pigs
Zfp281	0.04	1.00000000	pigs
Slc25a25	0.12	1.00000000	pigs
B3gntl1	0.07	1.00000000	pigs
Usp47	-0.44	1.00000000	pigs
Chchd6	0.06	1.00000000	pigs
Fbxo38	0.00	1.00000000	pigs
Cacfd1	0.10	1.00000000	pigs
Clptm1l	0.30	1.00000000	pigs
Uaca	-0.38	1.00000000	pigs
Uba1	0.04	1.00000000	pigs
Smad6	0.04	1.00000000	pigs
Il18	0.19	1.00000000	pigs
Emg1	0.06	1.00000000	pigs
Ciao2a	0.09	1.00000000	pigs
Arhgap1	0.16	1.00000000	pigs
Atp6v1b2	0.12	1.00000000	pigs
Boc	-0.56	1.00000000	pigs
Bfar	0.21	1.00000000	pigs
Ror2	0.27	1.00000000	pigs
Ptdss2	0.08	1.00000000	pigs
Acss3	0.50	1.00000000	pigs
Ormdl1	0.09	1.00000000	pigs
Prex2	-0.68	1.00000000	pigs
Fbn2	0.01	1.00000000	pigs
Sec24d	-0.46	1.00000000	pigs
Cherp	0.00	1.00000000	pigs
Ccnb1ip1	0.07	1.00000000	pigs
Tsfm	0.07	1.00000000	pigs
Apip	0.18	1.00000000	pigs
Ablim1	-0.37	1.00000000	pigs
Dnajc5	0.04	1.00000000	pigs
Gns	0.19	1.00000000	pigs
Tbc1d15	0.34	1.00000000	pigs
Plxdc1	-0.64	1.00000000	pigs
Castor1	0.07	1.00000000	pigs
Dcun1d4	0.00	1.00000000	pigs
Rhbdd2	0.01	1.00000000	pigs
Dmxl2	0.31	1.00000000	pigs
Arhgef37	0.03	1.00000000	pigs
Pdzrn3	0.40	1.00000000	pigs
Bcl2l1	0.06	1.00000000	pigs
Srrm1	-0.34	1.00000000	pigs

Cul4b	-0.02	1.00000000	pigs
Dock4	-0.61	1.00000000	pigs
Spr	-0.05	1.00000000	pigs
Nova1	-0.80	1.00000000	pigs
Tgfbr2	-0.47	1.00000000	pigs
Gstk1	0.06	1.00000000	pigs
Ccdc167	0.10	1.00000000	pigs
Vash2	0.03	1.00000000	pigs
Commd3	0.13	1.00000000	pigs
Jpt2	0.31	1.00000000	pigs
Rufy1	-0.35	1.00000000	pigs
Usp53	0.13	1.00000000	pigs
Wdr37	0.04	1.00000000	pigs
Rbm25	-0.31	1.00000000	pigs
Ccdc102a	0.07	1.00000000	pigs
Dennd11	-0.54	1.00000000	pigs
Hsd17b10	0.17	1.00000000	pigs
Lvrn	-0.60	1.00000000	pigs
Nsl1	0.00	1.00000000	pigs
Aldh7a1	0.08	1.00000000	pigs
Ugcg	0.24	1.00000000	pigs
Gtf2a1l	-0.03	1.00000000	pigs
Bcl2l12	0.04	1.00000000	pigs
Snrpa	0.05	1.00000000	pigs
Dennd10	0.03	1.00000000	pigs
B3galnt1	0.14	1.00000000	pigs
Tjp1	-0.41	1.00000000	pigs
Acbd5	0.20	1.00000000	pigs
Gid8	0.26	1.00000000	pigs
Rgs10	0.05	1.00000000	pigs
Vrk3	0.04	1.00000000	pigs
Tap1	0.09	1.00000000	pigs
Afap1	0.15	1.00000000	pigs
Calm1	-0.08	1.00000000	pigs
Cda	-0.57	1.00000000	pigs
Tssk4	0.06	1.00000000	pigs
Jmjd8	0.04	1.00000000	pigs
Chpt1	0.11	1.00000000	pigs
Ugdh	-0.69	1.00000000	pigs
Cdkn1a	0.32	1.00000000	pigs
Nthl1	0.06	1.00000000	pigs
Abhd13	0.02	1.00000000	pigs
Cryab	0.31	1.00000000	pigs
Kif7	0.06	1.00000000	pigs
Zfp644	-0.41	1.00000000	pigs
Zfp511	0.17	1.00000000	pigs

Ppcs	0.07	1.00000000	pigs
Gpc1	0.08	1.00000000	pigs
Znhit1	0.16	1.00000000	pigs
Eef2k	0.01	1.00000000	pigs
Alg12	-0.04	1.00000000	pigs
Trim47	0.17	1.00000000	pigs
Apc2	-0.03	1.00000000	pigs
Cdh18	0.01	1.00000000	pigs
Arl15	-0.56	1.00000000	pigs
Adsl	-0.05	1.00000000	pigs
Rasal2	-0.62	1.00000000	pigs
Rida	0.11	1.00000000	pigs
Rspo2	0.03	1.00000000	pigs
Prelid1	0.13	1.00000000	pigs
Rbp2	-0.32	1.00000000	pigs
Klhdc8b	0.07	1.00000000	pigs
Klf6	0.42	1.00000000	pigs
Ormdl3	0.04	1.00000000	pigs
Adgb	0.19	1.00000000	pigs
Rp2	0.15	1.00000000	pigs
Gnpda1	0.12	1.00000000	pigs
Eif6	0.39	1.00000000	pigs
Phrf1	0.00	1.00000000	pigs
B3glct	0.04	1.00000000	pigs
Azin2	0.14	1.00000000	pigs
4933434E20Rik	0.15	1.00000000	pigs
Gch1	0.14	1.00000000	pigs
B3gnt2	0.29	1.00000000	pigs
Tsku	0.08	1.00000000	pigs
Gnl3l	-0.04	1.00000000	pigs
Nepro	0.00	1.00000000	pigs
Wwox	0.31	1.00000000	pigs
Ube3c	-0.03	1.00000000	pigs
Fam71e1	0.10	1.00000000	pigs
Numb	-0.63	1.00000000	pigs
Lonrf1	0.07	1.00000000	pigs
Vps52	0.08	1.00000000	pigs
Gys1	0.14	1.00000000	pigs
Fam102a	0.09	1.00000000	pigs
Ociad2	0.05	1.00000000	pigs
Bok	0.10	1.00000000	pigs
Ints6l	0.04	1.00000000	pigs
Bmpr1a	-0.50	1.00000000	pigs
Chst12	0.21	1.00000000	pigs
Dnajc1	-0.40	1.00000000	pigs
Klf2	0.48	1.00000000	pigs

Nelfa	0.01	1.00000000	pigs
Slc7a5	0.27	1.00000000	pigs
Zfp366	0.21	1.00000000	pigs
Dnajb12	0.14	1.00000000	pigs
Zdhhc13	0.04	1.00000000	pigs
Qsox1	0.00	1.00000000	pigs
Il34	0.16	1.00000000	pigs
Hmgxb3	0.13	1.00000000	pigs
Tubgcp2	0.08	1.00000000	pigs
Polr1b	0.01	1.00000000	pigs
Fmo4	0.07	1.00000000	pigs
Lsp1	0.24	1.00000000	pigs
Cpsf6	0.30	1.00000000	pigs
Nol4l	0.02	1.00000000	pigs
Pde3a	-0.69	1.00000000	pigs
Ywhab	-0.06	1.00000000	pigs
Pef1	0.12	1.00000000	pigs
Smim19	0.36	1.00000000	pigs
Cdyl	-0.03	1.00000000	pigs
Eci3	0.24	1.00000000	pigs
Cacnb2	-0.38	1.00000000	pigs
Anapc2	0.08	1.00000000	pigs
Snrpf	0.11	1.00000000	pigs
Sec11a	0.41	1.00000000	pigs
Adora2a	-0.10	1.00000000	pigs
Ldb1	0.13	1.00000000	pigs
Cep290	0.09	1.00000000	pigs
Fbxo25	0.11	1.00000000	pigs
Ctdnep1	0.18	1.00000000	pigs
Rrp1	0.04	1.00000000	pigs
Serpinb1a	0.09	1.00000000	pigs
Pmepa1	-0.51	1.00000000	pigs
Alkbh6	0.15	1.00000000	pigs
Ssrp1	0.13	1.00000000	pigs
Tmem178b	0.17	1.00000000	pigs
Cdk14	-0.34	1.00000000	pigs
App	0.40	1.00000000	pigs
Gnai1	0.07	1.00000000	pigs
Samd8	0.15	1.00000000	pigs
Vps11	0.15	1.00000000	pigs
Dhrs11	-0.02	1.00000000	pigs
Nfia	-0.22	1.00000000	pigs
Ust	-0.54	1.00000000	pigs
Abca6	-0.44	1.00000000	pigs
Ltbp1	-0.39	1.00000000	pigs
Apbb1	0.01	1.00000000	pigs

Pbrm1	-0.43	1.00000000	pigs
Kdsr	-0.51	1.00000000	pigs
Srrm2	-0.23	1.00000000	pigs
Lsm2	0.33	1.00000000	pigs
Art4	-0.61	1.00000000	pigs
Plscr4	0.39	1.00000000	pigs
Akirin1	0.04	1.00000000	pigs
A130010J15Rik	0.07	1.00000000	pigs
Ppil1	0.11	1.00000000	pigs
Tsc1	-0.06	1.00000000	pigs
Ift27	0.12	1.00000000	pigs
Plbd2	0.12	1.00000000	pigs
Ralgapa1	-0.41	1.00000000	pigs
Fmn1	0.00	1.00000000	pigs
Polr2m	0.12	1.00000000	pigs
Aff2	-0.40	1.00000000	pigs
Ier3	0.19	1.00000000	pigs
Cradd	0.20	1.00000000	pigs
Rasl11a	1.51	1.00000000	pigs
Ephb2	-0.48	1.00000000	pigs
Arv1	0.11	1.00000000	pigs
Cttn	0.17	1.00000000	pigs
Diablo	0.18	1.00000000	pigs
Adamtsl1	-0.50	1.00000000	pigs
Dpy19l3	-0.03	1.00000000	pigs
Cop1	-0.45	1.00000000	pigs
Snx12	0.18	1.00000000	pigs
Wdr26	0.06	1.00000000	pigs
Apobec3	0.05	1.00000000	pigs
Fam185a	0.09	1.00000000	pigs
Dynlt1a	0.32	1.00000000	pigs
Prrx2	0.32	1.00000000	pigs
Eif4g3	-0.25	1.00000000	pigs
Plcb3	0.16	1.00000000	pigs
Cdip1	0.32	1.00000000	pigs
Uck1	0.10	1.00000000	pigs
Ncl	-0.19	1.00000000	pigs
Chmp1a	0.06	1.00000000	pigs
Trnt1	-0.01	1.00000000	pigs
Cep162	-0.51	1.00000000	pigs
Arglu1	-0.31	1.00000000	pigs
Rnf145	0.24	1.00000000	pigs
Zfp628	0.09	1.00000000	pigs
Dusp22	0.05	1.00000000	pigs
Cbx6	-0.39	1.00000000	pigs
Sccpdh	0.02	1.00000000	pigs

Ccdc149	0.05	1.00000000	pigs
Jam2	0.09	1.00000000	pigs
Rgs1	0.79	1.00000000	pigs
Tmem189	0.41	1.00000000	pigs
Stat2	0.01	1.00000000	pigs
Dpm2	0.09	1.00000000	pigs
Slc26a11	0.10	1.00000000	pigs
Phf13	0.02	1.00000000	pigs
Sgsm3	0.17	1.00000000	pigs
Coq4	0.10	1.00000000	pigs
Unc93b1	0.10	1.00000000	pigs
Psmc4	0.05	1.00000000	pigs
Atp6v1f	0.34	1.00000000	pigs
Zmynd11	-0.34	1.00000000	pigs
Araf	-0.03	1.00000000	pigs
Wdr3	0.12	1.00000000	pigs
Prdx2	0.57	1.00000000	pigs
Smc1a	-0.46	1.00000000	pigs
Reep5	0.26	1.00000000	pigs
Pkdcc	0.13	1.00000000	pigs
Uso1	-0.46	1.00000000	pigs
Lig4	-0.02	1.00000000	pigs
Pqlc3	0.42	1.00000000	pigs
Eif1ad	0.20	1.00000000	pigs
Htra3	-1.09	1.00000000	pigs
Snapc1l	0.18	1.00000000	pigs
P3h1	0.37	1.00000000	pigs
Otud4	0.19	1.00000000	pigs
Farp2	-0.01	1.00000000	pigs
Rgs16	0.39	1.00000000	pigs
Rnf138	0.08	1.00000000	pigs
Acaa1b	0.23	1.00000000	pigs
Cttnbp2	0.10	1.00000000	pigs
Sap18	0.56	1.00000000	pigs
Xpnpep1	0.07	1.00000000	pigs
Ehmt2	0.22	1.00000000	pigs
Sema3b	-0.37	1.00000000	pigs
Tnrc6b	-0.32	1.00000000	pigs
Lrp5	-0.10	1.00000000	pigs
Zswim7	0.06	1.00000000	pigs
Txnl4a	0.09	1.00000000	pigs
Qtrt2	0.05	1.00000000	pigs
Zhx2	0.30	1.00000000	pigs
Tspan9	0.06	1.00000000	pigs
Klhdc3	0.06	1.00000000	pigs
Psmd5	0.15	1.00000000	pigs

Prmt5	0.02	1.00000000	pigs
Pink1	0.19	1.00000000	pigs
Cpsf2	0.07	1.00000000	pigs
Polb	0.33	1.00000000	pigs
Wdr1	0.17	1.00000000	pigs
Ube2a	0.37	1.00000000	pigs
Nfkbiz	0.41	1.00000000	pigs
Washc5	0.23	1.00000000	pigs
Nxph2	-0.44	1.00000000	pigs
Pcdh9	0.33	1.00000000	pigs
Cnn2	0.14	1.00000000	pigs
Snai1	0.34	1.00000000	pigs
G6pc3	0.15	1.00000000	pigs
Paxip1	0.02	1.00000000	pigs
Wdr45	0.27	1.00000000	pigs
Tma7	-0.06	1.00000000	pigs
Lpgat1	-0.70	1.00000000	pigs
Rph3a	0.21	1.00000000	pigs
Ldb2	-0.34	1.00000000	pigs
Cct3	0.35	1.00000000	pigs
Abhd12	0.09	1.00000000	pigs
Id3	0.47	1.00000000	pigs
Stxbp1	0.04	1.00000000	pigs
Anapc13	0.20	1.00000000	pigs
Romo1	-0.26	1.00000000	pigs
Ccs	0.09	1.00000000	pigs
Prune1	-0.06	1.00000000	pigs
Rnft2	-0.04	1.00000000	pigs
Cdk19	0.05	1.00000000	pigs
9030025P20Rik	-0.07	1.00000000	pigs
Kif2a	-0.45	1.00000000	pigs
Fgd5	0.04	1.00000000	pigs
Zfp1	0.20	1.00000000	pigs
Gusb	0.18	1.00000000	pigs
Fcsk	-0.02	1.00000000	pigs
Cxxc1	0.07	1.00000000	pigs
Ptges	0.25	1.00000000	pigs
Pcdh7	-0.57	1.00000000	pigs
Hsp90aa1	-0.17	1.00000000	pigs
Tut7	-0.34	1.00000000	pigs
Camkk1	0.01	1.00000000	pigs
Fbxo6	0.18	1.00000000	pigs
Rfxank	0.27	1.00000000	pigs
Pqbp1	0.11	1.00000000	pigs
Ndufb9	-0.39	1.00000000	pigs
Setd2	-0.41	1.00000000	pigs

Ccnc	0.17	1.00000000	pigs
Emilin1	0.23	1.00000000	pigs
Pigt	-0.12	1.00000000	pigs
Stat5a	0.25	1.00000000	pigs
Dmac2	0.16	1.00000000	pigs
Tmem33	0.08	1.00000000	pigs
Bmf	0.14	1.00000000	pigs
Smpd1	0.06	1.00000000	pigs
Filip1	-0.57	1.00000000	pigs
Ywhaz	-0.28	1.00000000	pigs
Tnfrsf21	0.22	1.00000000	pigs
Trim38	0.03	1.00000000	pigs
Vasp	0.41	1.00000000	pigs
Clec16a	0.02	1.00000000	pigs
Ik	-0.18	1.00000000	pigs
Riox2	0.18	1.00000000	pigs
Aig1	-0.48	1.00000000	pigs
Ubxn1	0.31	1.00000000	pigs
Pih1d1	0.17	1.00000000	pigs
Eif4b	0.38	1.00000000	pigs
Ppp4c	0.36	1.00000000	pigs
Akap6	-0.09	1.00000000	pigs
Scnm1	-0.01	1.00000000	pigs
Uox	0.46	1.00000000	pigs
Prpsap1	0.05	1.00000000	pigs
Fnbp1l	0.07	1.00000000	pigs
Aamp	0.31	1.00000000	pigs
Uxs1	0.10	1.00000000	pigs
Zfp638	-0.41	1.00000000	pigs
Smap1	-0.29	1.00000000	pigs
Rdx	-0.33	1.00000000	pigs
Epha7	-0.18	1.00000000	pigs
Il4i1	-0.02	1.00000000	pigs
Ykt6	0.43	1.00000000	pigs
Pyroxd1	0.15	1.00000000	pigs
Slc35b4	0.07	1.00000000	pigs
Prr3	0.11	1.00000000	pigs
Rbp1	-0.32	1.00000000	pigs
Snrpc	0.14	1.00000000	pigs
Rcbtb1	-0.02	1.00000000	pigs
Cept1	0.03	1.00000000	pigs
Dcaf6	-0.62	1.00000000	pigs
Gemin2	0.02	1.00000000	pigs
Ubr2	-0.43	1.00000000	pigs
Gpx3	-0.01	1.00000000	pigs
Lsm7	0.21	1.00000000	pigs

Ubn1	0.13	1.00000000	pigs
Camsap1	0.03	1.00000000	pigs
Usb1	0.10	1.00000000	pigs
Sde2	0.13	1.00000000	pigs
Zfyve1	0.05	1.00000000	pigs
Rit1	0.08	1.00000000	pigs
Ccdc191	0.04	1.00000000	pigs
Eif3m	0.34	1.00000000	pigs
Rbm10	0.18	1.00000000	pigs
Tmem132a	0.12	1.00000000	pigs
Pik3ap1	0.30	1.00000000	pigs
Tm7sf3	-0.01	1.00000000	pigs
Loxl1	0.45	1.00000000	pigs
Tax1bp3	0.06	1.00000000	pigs
Cd320	0.00	1.00000000	pigs
Srpx	0.34	1.00000000	pigs
Gpatch3	0.16	1.00000000	pigs
Ndn	0.14	1.00000000	pigs
Slc25a27	-0.02	1.00000000	pigs
Aup1	0.07	1.00000000	pigs
Ergic3	-0.06	1.00000000	pigs
Col5a1	-0.18	1.00000000	pigs
Tmem184c	0.24	1.00000000	pigs
Gstz1	0.08	1.00000000	pigs
Nit1	0.07	1.00000000	pigs
Snrpb2	0.28	1.00000000	pigs
Sh2b3	0.15	1.00000000	pigs
Fbxw8	0.14	1.00000000	pigs
Arl6ip5	0.13	1.00000000	pigs
Gphn	-0.40	1.00000000	pigs
Poldip2	-0.05	1.00000000	pigs
Rpa2	0.17	1.00000000	pigs
Fam117b	0.14	1.00000000	pigs
Ddx31	0.10	1.00000000	pigs
Dennd2c	0.55	1.00000000	pigs
Fcho2	-0.51	1.00000000	pigs
Kmt5a	0.02	1.00000000	pigs
Cog1	0.08	1.00000000	pigs
Ubqln4	-0.04	1.00000000	pigs
Fam3c	-0.25	1.00000000	pigs
Adap1	0.14	1.00000000	pigs
Ppp4r3a	0.29	1.00000000	pigs
Echs1	0.38	1.00000000	pigs
Ranbp1	-0.07	1.00000000	pigs
Denr	0.24	1.00000000	pigs
Abr	0.21	1.00000000	pigs

Lmntd1	-0.44	1.00000000	pigs
Zc3h12c	0.15	1.00000000	pigs
Tyrobp	0.47	1.00000000	pigs
Cdc26	0.01	1.00000000	pigs
Pawr	0.03	1.00000000	pigs
Etfrf1	0.13	1.00000000	pigs
Tmem147	0.34	1.00000000	pigs
Decr1	0.15	1.00000000	pigs
Bcl10	0.10	1.00000000	pigs
Plxnc1	0.12	1.00000000	pigs
Dcaf12	0.06	1.00000000	pigs
Isca2	0.17	1.00000000	pigs
Prkd2	0.28	1.00000000	pigs
Atxn2l	0.03	1.00000000	pigs
Wsb1	-0.30	1.00000000	pigs
Wrap53	-0.01	1.00000000	pigs
Tpm1	0.15	1.00000000	pigs
Bola1	0.12	1.00000000	pigs
Tlcd2	0.02	1.00000000	pigs
Idh2	0.28	1.00000000	pigs
Ints2	0.11	1.00000000	pigs
Hax1	0.09	1.00000000	pigs
Ddx56	0.02	1.00000000	pigs
Adamts10	0.04	1.00000000	pigs
Tpra1	0.09	1.00000000	pigs
Smarcd3	0.08	1.00000000	pigs
Arid5a	-0.03	1.00000000	pigs
Tpcn1	-0.04	1.00000000	pigs
Fam135a	-0.78	1.00000000	pigs
Lix1l	0.08	1.00000000	pigs
Nek4	0.10	1.00000000	pigs
2610507B11Rik	0.07	1.00000000	pigs
Ccdc137	-0.04	1.00000000	pigs
Rbm45	-0.07	1.00000000	pigs
Ndufa6	0.04	1.00000000	pigs
Etfa	0.35	1.00000000	pigs
Tbc1d7	0.05	1.00000000	pigs
Rab22a	0.15	1.00000000	pigs
Plekhg2	0.04	1.00000000	pigs
Sinhcaf	0.34	1.00000000	pigs
Crtc1	0.01	1.00000000	pigs
Bud13	0.01	1.00000000	pigs
Polr2h	0.02	1.00000000	pigs
Zfp654	-0.06	1.00000000	pigs
Tmem19	0.32	1.00000000	pigs
Birc6	-0.35	1.00000000	pigs

Jmjd4	-0.03	1.00000000	pigs
Hsf2	0.04	1.00000000	pigs
Cdkn3	0.04	1.00000000	pigs
Wdr59	-0.04	1.00000000	pigs
Plekha6	-0.02	1.00000000	pigs
Dctn2	0.18	1.00000000	pigs
Slc5a6	-0.04	1.00000000	pigs
Rnf2	0.10	1.00000000	pigs
Arntl2	-0.09	1.00000000	pigs
Hnrnpa0	0.26	1.00000000	pigs
Bdkrb2	-0.04	1.00000000	pigs
Sdhb	0.38	1.00000000	pigs
Hmox1	0.36	1.00000000	pigs
Pdzd11	-0.01	1.00000000	pigs
Nab2	0.03	1.00000000	pigs
Slc31a1	-0.12	1.00000000	pigs
Eif4ebp2	-0.31	1.00000000	pigs
Parp14	0.03	1.00000000	pigs
Ltbp2	0.21	1.00000000	pigs
4930453N24Rik	0.13	1.00000000	pigs
Tango2	0.15	1.00000000	pigs
Ccnl1	-0.35	1.00000000	pigs
Hspd1	0.38	1.00000000	pigs
Chrnb1	0.19	1.00000000	pigs
Eef1d	0.35	1.00000000	pigs
Ankrd39	0.08	1.00000000	pigs
Hnrnpul1	-0.29	1.00000000	pigs
B9d1	0.03	1.00000000	pigs
Gpr108	0.07	1.00000000	pigs
Szt2	0.14	1.00000000	pigs
Rftn2	0.19	1.00000000	pigs
Ada	0.13	1.00000000	pigs
Poglut3	-0.01	1.00000000	pigs
Ppfibp1	-0.31	1.00000000	pigs
Pdxdc1	0.26	1.00000000	pigs
Zmiz2	0.00	1.00000000	pigs
Sell	-0.42	1.00000000	pigs
Zfp24	-0.04	1.00000000	pigs
Nelfcd	0.03	1.00000000	pigs
Llph	0.17	1.00000000	pigs
Atg4a-ps	0.00	1.00000000	pigs
Slc39a1	0.09	1.00000000	pigs
Tenm3	-0.37	1.00000000	pigs
Adar	0.10	1.00000000	pigs
Pecr	0.02	1.00000000	pigs
Prkar2b	0.05	1.00000000	pigs

Fblim1	0.13	1.00000000	pigs
Plod1	-0.05	1.00000000	pigs
Psmb1	0.37	1.00000000	pigs
Chd1l	0.21	1.00000000	pigs
Sox13	0.08	1.00000000	pigs
Stam2	-0.53	1.00000000	pigs
Haus3	0.01	1.00000000	pigs
Api5	0.31	1.00000000	pigs
Gne	0.01	1.00000000	pigs
Spry4	0.25	1.00000000	pigs
Cfap20	0.28	1.00000000	pigs
Edem3	0.17	1.00000000	pigs
Tnk2	-0.05	1.00000000	pigs
Phf1	-0.05	1.00000000	pigs
Hsf1	0.03	1.00000000	pigs
C3	0.39	1.00000000	pigs
Pgap1	0.14	1.00000000	pigs
Eif4a1	0.35	1.00000000	pigs
Apbb2	0.23	1.00000000	pigs
Tm2d1	0.18	1.00000000	pigs
Kif5b	-0.29	1.00000000	pigs
Smg7	0.05	1.00000000	pigs
Gclm	0.07	1.00000000	pigs
Fbxl7	-0.26	1.00000000	pigs
Banp	-0.10	1.00000000	pigs
Tmem268	0.42	1.00000000	pigs
Tmem30a	0.29	1.00000000	pigs
Cars	0.10	1.00000000	pigs
Pxk	0.11	1.00000000	pigs
Akap8l	-0.10	1.00000000	pigs
Amfr	0.11	1.00000000	pigs
DPCD	0.12	1.00000000	pigs
Pbld2	-0.02	1.00000000	pigs
Rab5c	0.29	1.00000000	pigs
Prkag1	0.21	1.00000000	pigs
Cep20	-0.01	1.00000000	pigs
Sbno2	0.04	1.00000000	pigs
Scmh1	0.07	1.00000000	pigs
Abcc1	-0.31	1.00000000	pigs
Adam32	0.00	1.00000000	pigs
Tfe3	0.11	1.00000000	pigs
Lrrcc1	0.14	1.00000000	pigs
Zfp36l1	-0.34	1.00000000	pigs
Paics	0.01	1.00000000	pigs
Cc2d2a	-0.48	1.00000000	pigs
Ranbp10	0.13	1.00000000	pigs

Tom1	0.23	1.00000000	pigs
Rftn1	0.24	1.00000000	pigs
Amd2	-0.27	1.00000000	pigs
Osbpl2	-0.11	1.00000000	pigs
Ndst1	0.06	1.00000000	pigs
Tmem216	-0.03	1.00000000	pigs
Tmco3	0.02	1.00000000	pigs
Dusp11	-0.04	1.00000000	pigs
Snx2	0.20	1.00000000	pigs
2310009B15Rik	-0.05	1.00000000	pigs
Arl6ip6	0.12	1.00000000	pigs
Por	-0.05	1.00000000	pigs
Tnip1	0.29	1.00000000	pigs
Trim35	0.01	1.00000000	pigs
C9orf72	0.38	1.00000000	pigs
Nit2	0.06	1.00000000	pigs
Fbxo7	0.06	1.00000000	pigs
Dkk3	0.01	1.00000000	pigs
Sh3bp4	0.03	1.00000000	pigs
Zfp398	0.08	1.00000000	pigs
Cib2	-0.05	1.00000000	pigs
Tubgcp5	-0.02	1.00000000	pigs
Tuba8	0.00	1.00000000	pigs
Czib	0.07	1.00000000	pigs
Ccndbp1	0.23	1.00000000	pigs
Tbx2	0.11	1.00000000	pigs
Nrg1	-0.76	1.00000000	pigs
Pgm5	-0.61	1.00000000	pigs
Chd9	-0.35	1.00000000	pigs
Carm1	0.04	1.00000000	pigs
Rfwd3	0.15	1.00000000	pigs
Fastk	0.05	1.00000000	pigs
Ctso	0.10	1.00000000	pigs
Polr2f	-0.24	1.00000000	pigs
Osbpl6	0.19	1.00000000	pigs
Xrn1	-0.43	1.00000000	pigs
Gna13	0.42	1.00000000	pigs
Chm	0.11	1.00000000	pigs
Zfp704	-0.67	1.00000000	pigs
Rph3al	0.14	1.00000000	pigs
Exoc6b	-0.27	1.00000000	pigs
Spats2l	-0.53	1.00000000	pigs
G6pdx	0.19	1.00000000	pigs
Yeats2	-0.08	1.00000000	pigs
Dhtkd1	-0.03	1.00000000	pigs
Cavin1	-0.17	1.00000000	pigs

Fndc1	0.26	1.00000000	pigs
Cnp	0.03	1.00000000	pigs
Duoxa2	0.24	1.00000000	pigs
Fads3	0.10	1.00000000	pigs
Ppp2r5b	-0.02	1.00000000	pigs
Plpp1	0.30	1.00000000	pigs
Sgsh	-0.01	1.00000000	pigs
Rcbtb2	-0.03	1.00000000	pigs
Adm	0.22	1.00000000	pigs
Lurap1l	-0.42	1.00000000	pigs
Pfdn1	0.28	1.00000000	pigs
2510039O18Rik	-0.06	1.00000000	pigs
Casp3	0.10	1.00000000	pigs
Hsph1	0.24	1.00000000	pigs
Hspa9	0.31	1.00000000	pigs
Cisd2	0.18	1.00000000	pigs
Ttc17	-0.53	1.00000000	pigs
Rlim	0.10	1.00000000	pigs
Cox7a2l	0.29	1.00000000	pigs
Rarres2	-0.29	1.00000000	pigs
H2aj	0.28	1.00000000	pigs
Tradd	0.09	1.00000000	pigs
Usf2	0.12	1.00000000	pigs
Commd9	0.01	1.00000000	pigs
Srsf5	-0.26	1.00000000	pigs
Ndfip2	0.20	1.00000000	pigs
Katnal1	0.07	1.00000000	pigs
Entpd1	-0.62	1.00000000	pigs
Kcnab3	0.18	1.00000000	pigs
Clk3	0.13	1.00000000	pigs
Ak6	0.16	1.00000000	pigs
Pmvk	0.12	1.00000000	pigs
Col5a2	-0.21	1.00000000	pigs
Rgn	0.15	1.00000000	pigs
Zc3hc1	-0.03	1.00000000	pigs
Ube4b	0.10	1.00000000	pigs
Sorbs3	-0.08	1.00000000	pigs
Saysd1	0.08	1.00000000	pigs
Osbpl3	0.01	1.00000000	pigs
Anapc5	0.03	1.00000000	pigs
Fuca1	0.05	1.00000000	pigs
Vps35l	0.07	1.00000000	pigs
Dennd6b	0.03	1.00000000	pigs
Olfm1	0.01	1.00000000	pigs
Ptpn11	-0.43	1.00000000	pigs
Fam171a1	0.09	1.00000000	pigs

Nkap	-0.08	1.00000000	pigs
Oxsm	0.07	1.00000000	pigs
Cnot8	-0.03	1.00000000	pigs
Nprl3	-0.01	1.00000000	pigs
Il1rapl1	0.09	1.00000000	pigs
Catsperg1	0.05	1.00000000	pigs
Ipo5	0.20	1.00000000	pigs
Wsb2	-0.01	1.00000000	pigs
Dennd5a	0.07	1.00000000	pigs
Cct8	0.34	1.00000000	pigs
Ccdc107	0.16	1.00000000	pigs
Rmdn1	0.15	1.00000000	pigs
Sgta	0.04	1.00000000	pigs
Fhit	0.10	1.00000000	pigs
Limd1	-0.04	1.00000000	pigs
Nrp2	0.20	1.00000000	pigs
Creg1	0.09	1.00000000	pigs
Ddx39a	0.12	1.00000000	pigs
Foxred2	0.00	1.00000000	pigs
Vasn	0.08	1.00000000	pigs
Btaf1	-0.48	1.00000000	pigs
Vps9d1	0.01	1.00000000	pigs
F2rl2	-0.41	1.00000000	pigs
Slc35e1	0.05	1.00000000	pigs
Rpap2	0.06	1.00000000	pigs
Yipf5	0.04	1.00000000	pigs
Rfesd	-0.04	1.00000000	pigs
Fundc1	0.35	1.00000000	pigs
Cib1	0.00	1.00000000	pigs
Gask1b	0.14	1.00000000	pigs
Slc5a1	-0.35	1.00000000	pigs
Shfl	0.06	1.00000000	pigs
Slmap	-0.49	1.00000000	pigs
Desi2	0.09	1.00000000	pigs
Smpdl3a	0.11	1.00000000	pigs
Supt3	0.02	1.00000000	pigs
Kmt2c	-0.27	1.00000000	pigs
Tcfl5	0.00	1.00000000	pigs
Ddi2	-0.02	1.00000000	pigs
Recql	-0.23	1.00000000	pigs
Terb1	0.08	1.00000000	pigs
Abcg3	-0.01	1.00000000	pigs
Lmf1	-0.03	1.00000000	pigs
Rnpep	0.18	1.00000000	pigs
Gnl1	0.09	1.00000000	pigs
Cops7a	0.00	1.00000000	pigs

Cdk5rap3	-0.07	1.00000000	pigs
Kansl1	-0.36	1.00000000	pigs
Eps8	-0.51	1.00000000	pigs
Ipo4	0.35	1.00000000	pigs
Prpf31	0.05	1.00000000	pigs
Fmr1	0.13	1.00000000	pigs
Smim4	0.08	1.00000000	pigs
Acsl5	0.10	1.00000000	pigs
Ppox	0.02	1.00000000	pigs
Cntrob	0.12	1.00000000	pigs
Imp3	0.07	1.00000000	pigs
Setd7	-0.03	1.00000000	pigs
Pgs1	0.09	1.00000000	pigs
Gdf10	-0.39	1.00000000	pigs
Zfc3h1	-0.48	1.00000000	pigs
Ppp1r35	0.16	1.00000000	pigs
Dbnl	0.05	1.00000000	pigs
Bud31	0.14	1.00000000	pigs
Gramd3	0.42	1.00000000	pigs
Wfs1	0.04	1.00000000	pigs
1110032A03Rik	0.10	1.00000000	pigs
Arl6	0.07	1.00000000	pigs
Smarcc1	0.14	1.00000000	pigs
Tm9sf3	-0.13	1.00000000	pigs
Duox2	0.26	1.00000000	pigs
Ube2z	0.05	1.00000000	pigs
Napg	-0.05	1.00000000	pigs
Abca9	-0.41	1.00000000	pigs
Ncoa4	0.16	1.00000000	pigs
Cobll1	-0.33	1.00000000	pigs
Acsl3	-0.02	1.00000000	pigs
Timmdc1	0.08	1.00000000	pigs
Exoc5	0.09	1.00000000	pigs
Ppp5c	-0.01	1.00000000	pigs
Rer1	0.42	1.00000000	pigs
Adamts16	0.35	1.00000000	pigs
Commd4	0.05	1.00000000	pigs
Vta1	0.05	1.00000000	pigs
Synj2	-0.03	1.00000000	pigs
Siah2	0.06	1.00000000	pigs
Hdac4	0.22	1.00000000	pigs
S100a6	-0.22	1.00000000	pigs
Nsmce1	0.31	1.00000000	pigs
Aida	-0.06	1.00000000	pigs
Nup37	0.04	1.00000000	pigs
Ndufb3	0.36	1.00000000	pigs

Rab2a	-0.18	1.00000000	pigs
Pgm3	-0.10	1.00000000	pigs
Pdk4	0.20	1.00000000	pigs
Atg4b	0.05	1.00000000	pigs
Heatr3	-0.42	1.00000000	pigs
Ankrd27	0.12	1.00000000	pigs
Tirap	0.00	1.00000000	pigs
Rbmx2	0.02	1.00000000	pigs
Tigd2	-0.02	1.00000000	pigs
Smurf2	-0.47	1.00000000	pigs
Trim28	0.07	1.00000000	pigs
Lrrc4c	0.01	1.00000000	pigs
Cul4a	0.15	1.00000000	pigs
Fbh1	-0.01	1.00000000	pigs
Stx8	0.12	1.00000000	pigs
Trp53rka	0.03	1.00000000	pigs
Dvl3	0.04	1.00000000	pigs
B9d2	-0.03	1.00000000	pigs
Slc5a10	0.04	1.00000000	pigs
Hmcn1	-0.39	1.00000000	pigs
Arfgap3	0.06	1.00000000	pigs
Ndufs6	0.07	1.00000000	pigs
Stoml2	0.20	1.00000000	pigs
S100a10	-0.13	1.00000000	pigs
Spata5	-0.57	1.00000000	pigs
Klhl42	0.00	1.00000000	pigs
Ubp1	0.24	1.00000000	pigs
Ccdc58	0.05	1.00000000	pigs
Ppara	0.03	1.00000000	pigs
Cog7	-0.01	1.00000000	pigs
Plxna2	-0.01	1.00000000	pigs
Glrx3	0.11	1.00000000	pigs
Eny2	0.22	1.00000000	pigs
Ndufaf2	-0.06	1.00000000	pigs
Surf2	0.21	1.00000000	pigs
Kcnma1	-0.39	1.00000000	pigs
Foxo1	-0.26	1.00000000	pigs
Gars	0.19	1.00000000	pigs
Adipor2	0.07	1.00000000	pigs
Enah	0.15	1.00000000	pigs
H1f0	0.00	1.00000000	pigs
Eif2b2	0.01	1.00000000	pigs
Vps13a	-0.08	1.00000000	pigs
Runx2	0.50	1.00000000	pigs
Ctbp2	0.04	1.00000000	pigs
Pnkp	0.13	1.00000000	pigs

Plcxd3	0.16	1.00000000	pigs
Slc39a8	0.03	1.00000000	pigs
Adck2	0.09	1.00000000	pigs
Fam214a	-0.49	1.00000000	pigs
Nhsl1	-0.65	1.00000000	pigs
Cpeb2	0.24	1.00000000	pigs
Dusp10	0.10	1.00000000	pigs
Sass6	0.09	1.00000000	pigs
Gtpbp4	0.13	1.00000000	pigs
Itga2	-0.46	1.00000000	pigs
Scamp2	0.10	1.00000000	pigs
Csf1r	0.23	1.00000000	pigs
Cers5	0.06	1.00000000	pigs
Zfp219	0.03	1.00000000	pigs
Ninj1	0.28	1.00000000	pigs
Zfp2	-0.03	1.00000000	pigs
Vcan	0.38	1.00000000	pigs
Trim13	0.12	1.00000000	pigs
Prkn	-0.62	1.00000000	pigs
Tlr2	0.24	1.00000000	pigs
Ift52	0.01	1.00000000	pigs
Aimp2	0.00	1.00000000	pigs
Vars	0.01	1.00000000	pigs
Rap2a	-0.31	1.00000000	pigs
Cog4	0.06	1.00000000	pigs
Leprot	-0.08	1.00000000	pigs
Tcf4	-0.30	1.00000000	pigs
Tmed1	0.01	1.00000000	pigs
Git2	0.11	1.00000000	pigs
Asb1	0.00	1.00000000	pigs
Scaf11	-0.24	1.00000000	pigs
Idh3g	0.04	1.00000000	pigs
Cdipt	0.02	1.00000000	pigs
Rfc2	0.20	1.00000000	pigs
Dclre1c	-0.02	1.00000000	pigs
Smarca4	0.13	1.00000000	pigs
Dip2a	0.02	1.00000000	pigs
Nat9	0.06	1.00000000	pigs
Atp2b4	-0.24	1.00000000	pigs
Fdft1	-0.02	1.00000000	pigs
Zdhhc14	-0.49	1.00000000	pigs
Nin	-0.50	1.00000000	pigs
Fkbp3	0.26	1.00000000	pigs
Tlr7	0.14	1.00000000	pigs
Hars	-0.11	1.00000000	pigs
Ttf2	-0.02	1.00000000	pigs

Scyl3	0.07	1.00000000	pigs
Rnf180	-0.53	1.00000000	pigs
Erlin2	0.03	1.00000000	pigs
Trp53i11	0.24	1.00000000	pigs
Ndufs2	0.08	1.00000000	pigs
Suco	0.09	1.00000000	pigs
Larp1	0.22	1.00000000	pigs
Tusc3	-0.01	1.00000000	pigs
Atp10d	0.20	1.00000000	pigs
Kpna7	0.06	1.00000000	pigs
Fus	-0.25	1.00000000	pigs
Wwc2	0.16	1.00000000	pigs
Nt5dc1	-0.45	1.00000000	pigs
Akr1d1	0.00	1.00000000	pigs
Nip7	0.06	1.00000000	pigs
Slco3a1	-0.32	1.00000000	pigs
Bsdc1	0.01	1.00000000	pigs
Zfp622	0.08	1.00000000	pigs
Pdhx	0.01	1.00000000	pigs
Cdca7l	0.16	1.00000000	pigs
Paxbp1	0.06	1.00000000	pigs
Bin1	-0.41	1.00000000	pigs
Smarca2	-0.26	1.00000000	pigs
Fem1b	0.12	1.00000000	pigs
Scand1	0.42	1.00000000	pigs
Rnmt	0.16	1.00000000	pigs
Ppil4	0.03	1.00000000	pigs
Alcam	0.12	1.00000000	pigs
Tbck	0.11	1.00000000	pigs
Ttll11	-0.38	1.00000000	pigs
Cdc42ep2	-0.02	1.00000000	pigs
Psmc3	0.35	1.00000000	pigs
Hoxa7	-0.05	1.00000000	pigs
Nf1	-0.27	1.00000000	pigs
Golga5	-0.01	1.00000000	pigs
Nsfl1c	0.06	1.00000000	pigs
Clcn7	0.01	1.00000000	pigs
Arrb1	0.10	1.00000000	pigs
Zcchc14	0.01	1.00000000	pigs
Abce1	0.13	1.00000000	pigs
Sfxn4	0.21	1.00000000	pigs
Rest	0.08	1.00000000	pigs
Klf4	0.28	1.00000000	pigs
Tmem8b	-0.01	1.00000000	pigs
Echdc1	-0.09	1.00000000	pigs
Otulinl	0.32	1.00000000	pigs

Ift140	-0.02	1.00000000	pigs
Cdc42se1	0.02	1.00000000	pigs
Nrep	-0.35	1.00000000	pigs
Ripor3	0.00	1.00000000	pigs
Rarres1	0.21	1.00000000	pigs
Lonrf3	-0.60	1.00000000	pigs
Nudt7	0.16	1.00000000	pigs
Fam189b	-0.02	1.00000000	pigs
Edf1	0.22	1.00000000	pigs
Sdhd	0.09	1.00000000	pigs
Auts2	0.28	1.00000000	pigs
Hgf	-0.11	1.00000000	pigs
Rnf125	0.17	1.00000000	pigs
Gpt	0.03	1.00000000	pigs
Smtnl2	-0.25	1.00000000	pigs
Rab7b	0.12	1.00000000	pigs
Chmp4b	0.16	1.00000000	pigs
Tmem259	0.05	1.00000000	pigs
Fbln1	-0.23	1.00000000	pigs
Nod2	0.34	1.00000000	pigs
Flrt3	0.00	1.00000000	pigs
Psmc2	0.05	1.00000000	pigs
Calhm2	0.01	1.00000000	pigs
Bmerb1	0.03	1.00000000	pigs
Tial1	0.21	1.00000000	pigs
Gpr137	0.05	1.00000000	pigs
Fancd2	0.04	1.00000000	pigs
Nmnat2	0.06	1.00000000	pigs
Ppm1k	0.12	1.00000000	pigs
Itch	-0.43	1.00000000	pigs
Galt	0.08	1.00000000	pigs
Zfp598	0.01	1.00000000	pigs
Szrd1	0.11	1.00000000	pigs
Dbt	-0.02	1.00000000	pigs
Pdcd6	0.03	1.00000000	pigs
Pex16	0.04	1.00000000	pigs
Dctd	0.16	1.00000000	pigs
Erg28	0.08	1.00000000	pigs
Aff1	0.25	1.00000000	pigs
Rai1	-0.06	1.00000000	pigs
Gtpbp3	-0.01	1.00000000	pigs
Ilkap	0.10	1.00000000	pigs
Ppp2r3c	-0.02	1.00000000	pigs
Ptpn12	-0.24	1.00000000	pigs
Ptcd2	0.00	1.00000000	pigs
Evc2	-0.15	1.00000000	pigs

Arhgap15	-1.06	1.00000000	pigs
Ctsz	0.35	1.00000000	pigs
Cops3	0.23	1.00000000	pigs
Ola1	0.23	1.00000000	pigs
Scube1	-0.49	1.00000000	pigs
Rnf141	0.10	1.00000000	pigs
Tmem43	-0.40	1.00000000	pigs
Srp9	0.02	1.00000000	pigs
Casp6	0.10	1.00000000	pigs
Zadh2	0.08	1.00000000	pigs
Rab3gap2	0.06	1.00000000	pigs
Sfxn3	-0.01	1.00000000	pigs
Gnl2	-0.06	1.00000000	pigs
Ppp2ca	0.11	1.00000000	pigs
Immp2l	-0.59	1.00000000	pigs
Ralbp1	-0.22	1.00000000	pigs
Ifit2	-0.19	1.00000000	pigs
Cep78	0.10	1.00000000	pigs
Fndc3b	-0.28	1.00000000	pigs
Tmed4	-0.28	1.00000000	pigs
Smg9	0.04	1.00000000	pigs
Lsg1	0.02	1.00000000	pigs
Eif2d	0.01	1.00000000	pigs
AW554918	-0.47	1.00000000	pigs
Hypk	-0.15	1.00000000	pigs
Brox	0.09	1.00000000	pigs
Xpot	0.07	1.00000000	pigs
Acat3	-0.04	1.00000000	pigs
Col15a1	-0.41	1.00000000	pigs
Dctn5	0.02	1.00000000	pigs
Hibadh	0.02	1.00000000	pigs
Ndufa8	0.26	1.00000000	pigs
Supt4a	0.31	1.00000000	pigs
Lrrc8a	0.01	1.00000000	pigs
Plxna1	0.11	1.00000000	pigs
Prkce	0.15	1.00000000	pigs
Ccl5	0.95	1.00000000	pigs
Fam126b	0.01	1.00000000	pigs
Eif2ak3	-0.53	1.00000000	pigs
Utp6	0.12	1.00000000	pigs
Efcab14	0.00	1.00000000	pigs
Akip1	-0.05	1.00000000	pigs
Fkbp7	0.39	1.00000000	pigs
Bcap29	0.04	1.00000000	pigs
Bzw1	-0.16	1.00000000	pigs
Togaram1	-0.14	1.00000000	pigs

Ints13	0.06	1.00000000	pigs
Slc39a11	0.01	1.00000000	pigs
4833439L19Rik	0.17	1.00000000	pigs
Safb2	0.11	1.00000000	pigs
Hnrnpa3	-0.10	1.00000000	pigs
Top1	0.25	1.00000000	pigs
Ethe1	-0.03	1.00000000	pigs
Ftsj3	0.05	1.00000000	pigs
Dock10	0.28	1.00000000	pigs
Renbp	0.06	1.00000000	pigs
Bmp4	-0.40	1.00000000	pigs
Glis3	0.34	1.00000000	pigs
Phb	0.36	1.00000000	pigs
Tmco1	-0.36	1.00000000	pigs
Fam50a	0.07	1.00000000	pigs
Nhlrc3	0.06	1.00000000	pigs
Xylt1	0.11	1.00000000	pigs
Cdkal1	-0.30	1.00000000	pigs
Hspa12b	-0.56	1.00000000	pigs
Lmnb2	-0.03	1.00000000	pigs
Higd2a	0.11	1.00000000	pigs
Pkn1	0.14	1.00000000	pigs
Enkur	0.06	1.00000000	pigs
Hdgf	-0.18	1.00000000	pigs
Rfx5	-0.51	1.00000000	pigs
Oaz2	0.03	1.00000000	pigs
9130409I23Rik	0.14	1.00000000	pigs
Tmem63b	-0.03	1.00000000	pigs
Tgfb1	0.31	1.00000000	pigs
Alkbh7	0.09	1.00000000	pigs
Spag8	0.21	1.00000000	pigs
Jak1	0.20	1.00000000	pigs
Jmy	0.11	1.00000000	pigs
H2-Ke6	0.07	1.00000000	pigs
Ino80c	0.03	1.00000000	pigs
Arap2	0.23	1.00000000	pigs
A4galt	0.13	1.00000000	pigs
Ebag9	0.02	1.00000000	pigs
Vegfc	-0.47	1.00000000	pigs
Atp9a	-0.07	1.00000000	pigs
Zfp148	-0.38	1.00000000	pigs
Sema4c	0.03	1.00000000	pigs
Tpgs2	0.08	1.00000000	pigs
Ckap5	-0.07	1.00000000	pigs
Cd109	-0.43	1.00000000	pigs
Sri	0.37	1.00000000	pigs

Utp18	-0.04	1.00000000	pigs
Slc25a37	-0.03	1.00000000	pigs
Arl8a	0.07	1.00000000	pigs
9530068E07Rik	-0.05	1.00000000	pigs
Slc25a29	0.07	1.00000000	pigs
Tcea1	-0.32	1.00000000	pigs
Aspscr1	-0.05	1.00000000	pigs
Tbl3	0.01	1.00000000	pigs
Fbxo31	0.06	1.00000000	pigs
Dhx40	0.12	1.00000000	pigs
Rbks	0.04	1.00000000	pigs
Glul	-0.27	1.00000000	pigs
Cfap70	0.04	1.00000000	pigs
Bcar1	0.00	1.00000000	pigs
Fyn	-0.45	1.00000000	pigs
Lztfl1	-0.02	1.00000000	pigs
Nr6a1	-0.06	1.00000000	pigs
Slirp	0.05	1.00000000	pigs
Acadsb	0.07	1.00000000	pigs
Ube2g2	0.17	1.00000000	pigs
Ssbp2	-0.30	1.00000000	pigs
Ift57	-0.05	1.00000000	pigs
Psmd4	0.19	1.00000000	pigs
Ap5m1	-0.09	1.00000000	pigs
Zfp609	-0.38	1.00000000	pigs
Fbxo11	-0.32	1.00000000	pigs
Thop1	0.01	1.00000000	pigs
Pex7	0.06	1.00000000	pigs
Bnip3	0.26	1.00000000	pigs
Bysl	0.09	1.00000000	pigs
Spon1	0.35	1.00000000	pigs
Cops5	0.10	1.00000000	pigs
Nav1	-0.47	1.00000000	pigs
Ccny	0.21	1.00000000	pigs
Naa50	0.07	1.00000000	pigs
Slc3a2	0.18	1.00000000	pigs
Cth	0.01	1.00000000	pigs
Armcx3	0.06	1.00000000	pigs
Srgap1	-0.46	1.00000000	pigs
Keap1	-0.01	1.00000000	pigs
Cic	0.03	1.00000000	pigs
Ccnl2	-0.02	1.00000000	pigs
Rexo2	0.44	1.00000000	pigs
1700102P08Rik	-0.01	1.00000000	pigs
Dpp8	-0.50	1.00000000	pigs
Gtf3a	-0.09	1.00000000	pigs

Loxl3	-0.40	1.00000000	pigs
Npc2	0.26	1.00000000	pigs
Uqcc3	0.02	1.00000000	pigs
Scoc	0.09	1.00000000	pigs
Gpsm2	0.06	1.00000000	pigs
Rnf34	-0.05	1.00000000	pigs
Atp2b1	-0.17	1.00000000	pigs
Pbx4	-0.01	1.00000000	pigs
Rrp1b	0.12	1.00000000	pigs
Ifrd2	0.20	1.00000000	pigs
Tbx3	0.08	1.00000000	pigs
Gask1a	0.02	1.00000000	pigs
Got1	0.01	1.00000000	pigs
Gnb1l	0.03	1.00000000	pigs
Clp1	0.08	1.00000000	pigs
Zbtb22	0.18	1.00000000	pigs
Ptpn3	0.05	1.00000000	pigs
Polr1e	-0.02	1.00000000	pigs
Thoc1	-0.11	1.00000000	pigs
Psmd8	0.29	1.00000000	pigs
Btbd8	-0.05	1.00000000	pigs
Arl5b	0.19	1.00000000	pigs
Trim56	0.06	1.00000000	pigs
Ttyh3	-0.10	1.00000000	pigs
Dlgap4	-0.12	1.00000000	pigs
Id2	0.30	1.00000000	pigs
Rrs1	-0.04	1.00000000	pigs
Pccb	0.18	1.00000000	pigs
Sec61g	0.08	1.00000000	pigs
Zmat5	0.07	1.00000000	pigs
Ptp4a1	0.25	1.00000000	pigs
Gpr63	0.05	1.00000000	pigs
Cyhr1	0.01	1.00000000	pigs
Armc10	-0.37	1.00000000	pigs
Gak	0.00	1.00000000	pigs
Cct4	0.35	1.00000000	pigs
Aspdh	0.05	1.00000000	pigs
Akap11	0.02	1.00000000	pigs
Snx10	0.95	1.00000000	pigs
Lrrc1	0.13	1.00000000	pigs
Nek1	-0.18	1.00000000	pigs
Flad1	0.18	1.00000000	pigs
Tmem237	0.09	1.00000000	pigs
Tmem44	0.07	1.00000000	pigs
Pdhb	-0.02	1.00000000	pigs
Slc2a10	0.05	1.00000000	pigs

Exoc3	0.03	1.00000000	pigs
Oxsr1	0.08	1.00000000	pigs
Amn1	0.05	1.00000000	pigs
Sp110	0.02	1.00000000	pigs
Itpk1	0.26	1.00000000	pigs
Setx	0.02	1.00000000	pigs
Cyp39a1	0.11	1.00000000	pigs
Ermp1	0.22	1.00000000	pigs
Acvr2a	-0.58	1.00000000	pigs
Rab1a	0.32	1.00000000	pigs
Zfp335	0.10	1.00000000	pigs
Serp2	0.08	1.00000000	pigs
Calm3	0.20	1.00000000	pigs
Cmtm7	0.08	1.00000000	pigs
Zbtb18	0.01	1.00000000	pigs
Usp20	0.14	1.00000000	pigs
Cnnm3	-0.01	1.00000000	pigs
Ppp1r12c	0.08	1.00000000	pigs
Stk25	-0.01	1.00000000	pigs
Lsm3	0.04	1.00000000	pigs
Pnpla7	-0.03	1.00000000	pigs
Lrrc8c	0.27	1.00000000	pigs
Wbp1	0.01	1.00000000	pigs
Ctif	0.01	1.00000000	pigs
Dazap2	0.22	1.00000000	pigs
Rtf2	0.12	1.00000000	pigs
Zcchc7	-0.48	1.00000000	pigs
Commd2	-0.06	1.00000000	pigs
H13	0.02	1.00000000	pigs
Erbb2	0.00	1.00000000	pigs
Pdgfrb	0.15	1.00000000	pigs
Trp53i13	0.00	1.00000000	pigs
Lrsam1	-0.04	1.00000000	pigs
Arid5b	0.31	1.00000000	pigs
Golga7	0.04	1.00000000	pigs
Camk1	0.20	1.00000000	pigs
Runx1t1	-0.43	1.00000000	pigs
Galnt2	0.05	1.00000000	pigs
Sdcbp	0.31	1.00000000	pigs
Hbegf	0.15	1.00000000	pigs
Pex14	-0.12	1.00000000	pigs
Kdm1b	0.08	1.00000000	pigs
Ccnt1	-0.10	1.00000000	pigs
Nubp2	-0.06	1.00000000	pigs
Entr1	0.07	1.00000000	pigs
Parp1	0.05	1.00000000	pigs

Atp6v1c1	0.20	1.00000000	pigs
Rassf2	-0.30	1.00000000	pigs
Zbtb11	-0.05	1.00000000	pigs
Clic5	0.01	1.00000000	pigs
Ncf4	0.16	1.00000000	pigs
Flvcr1	0.15	1.00000000	pigs
Folr1	-0.19	1.00000000	pigs
Kdm3a	-0.05	1.00000000	pigs
Pdlim1	0.09	1.00000000	pigs
Lgals8	0.04	1.00000000	pigs
Supt20	-0.03	1.00000000	pigs
Hdgfl3	-0.29	1.00000000	pigs
Rabl3	-0.01	1.00000000	pigs
Nup107	-0.08	1.00000000	pigs
Dele1	0.00	1.00000000	pigs
Cdc25b	0.15	1.00000000	pigs
Tbrg4	0.10	1.00000000	pigs
Abl2	-0.05	1.00000000	pigs
Rhoh	0.60	1.00000000	pigs
Tubb4b	-0.13	1.00000000	pigs
Psmf1	0.04	1.00000000	pigs
Gatad2a	0.10	1.00000000	pigs
Entpd5	-0.01	1.00000000	pigs
Col1a1	0.31	1.00000000	pigs
Pard3	-0.20	1.00000000	pigs
Gatad2b	-0.34	1.00000000	pigs
Elk4	-0.03	1.00000000	pigs
Pacs1	0.05	1.00000000	pigs
Vps37a	-0.05	1.00000000	pigs
Cgrrf1	0.05	1.00000000	pigs
Gorasp1	-0.04	1.00000000	pigs
Saraf	0.20	1.00000000	pigs
Pde3b	-0.45	1.00000000	pigs
Dnajc15	-0.14	1.00000000	pigs
Sec23ip	-0.09	1.00000000	pigs
Rrp15	0.04	1.00000000	pigs
Plekhf2	0.13	1.00000000	pigs
Klhl20	-0.35	1.00000000	pigs
Etv3	0.07	1.00000000	pigs
Ogfod3	0.18	1.00000000	pigs
Ganab	0.22	1.00000000	pigs
Acad12	-0.09	1.00000000	pigs
P4hb	0.38	1.00000000	pigs
1810013L24Rik	0.17	1.00000000	pigs
Hnrnph3	0.22	1.00000000	pigs
Eif2b1	-0.10	1.00000000	pigs

Nubp1	-0.07	1.00000000	pigs
Tead2	0.01	1.00000000	pigs
Nup88	-0.01	1.00000000	pigs
Txndc11	-0.38	1.00000000	pigs
Atp8b1	-0.45	1.00000000	pigs
Cldnd1	0.12	1.00000000	pigs
Spata7	-0.10	1.00000000	pigs
Arf4	0.33	1.00000000	pigs
Itgae	0.03	1.00000000	pigs
Tle1	0.06	1.00000000	pigs
Dnajc18	-0.04	1.00000000	pigs
Triqk	0.01	1.00000000	pigs
Slc16a2	0.06	1.00000000	pigs
Klhdc10	-0.09	1.00000000	pigs
Cflar	0.23	1.00000000	pigs
Zfp217	-0.05	1.00000000	pigs
Taf1b	-0.01	1.00000000	pigs
Fhod1	-0.08	1.00000000	pigs
Samd4b	0.09	1.00000000	pigs
Plekha4	-0.07	1.00000000	pigs
Senp6	-0.29	1.00000000	pigs
Bloc1s2	-0.04	1.00000000	pigs
Pabpc4	-0.02	1.00000000	pigs
Snrnp40	0.07	1.00000000	pigs
Cdc27	0.01	1.00000000	pigs
Slc25a28	-0.15	1.00000000	pigs
Pias4	0.07	1.00000000	pigs
Dpf2	0.06	1.00000000	pigs
Efcc1	-0.17	1.00000000	pigs
Nr4a2	0.30	1.00000000	pigs
Phf23	0.41	1.00000000	pigs
Hsbp1	0.28	1.00000000	pigs
Tsc22d1	0.20	1.00000000	pigs
Ppl	-0.02	1.00000000	pigs
Tmem208	0.25	1.00000000	pigs
Gpbp1l1	0.19	1.00000000	pigs
Vps36	-0.32	1.00000000	pigs
Ttc33	-0.40	1.00000000	pigs
Ndufs5	0.32	1.00000000	pigs
Rars	0.16	1.00000000	pigs
Csnk2a2	0.03	1.00000000	pigs
Cmtm3	-0.06	1.00000000	pigs
Dlc1	0.00	1.00000000	pigs
Usp10	-0.02	1.00000000	pigs
Ccdc90b	0.24	1.00000000	pigs
Zswim8	0.05	1.00000000	pigs

Eif2a	0.10	1.00000000	pigs
Tmem64	0.07	1.00000000	pigs
Txnrd1	0.39	1.00000000	pigs
Fbln2	-0.01	1.00000000	pigs
Selenom	0.44	1.00000000	pigs
Zfp503	-0.44	1.00000000	pigs
Smarcad1	-0.07	1.00000000	pigs
Adamts1	0.32	1.00000000	pigs
Ubac2	0.09	1.00000000	pigs
Setdb2	-0.02	1.00000000	pigs
Ncstn	-0.02	1.00000000	pigs
Synj2bp	0.16	1.00000000	pigs
Znhit3	-0.04	1.00000000	pigs
Prex1	0.31	1.00000000	pigs
Prcp	-0.05	1.00000000	pigs
Smarca1	0.17	1.00000000	pigs
Ctu2	0.13	1.00000000	pigs
Entpd4	-0.03	1.00000000	pigs
Stk11	0.16	1.00000000	pigs
Naga	-0.07	1.00000000	pigs
Bahd1	0.11	1.00000000	pigs
Ppp1r14bl	0.12	1.00000000	pigs
Aip	0.04	1.00000000	pigs
Trafd1	0.08	1.00000000	pigs
Exosc1	-0.03	1.00000000	pigs
Abhd10	-0.28	1.00000000	pigs
Ppp1r8	0.03	1.00000000	pigs
Psmd10	-0.02	1.00000000	pigs
Bcat1	0.08	1.00000000	pigs
C5ar1	-0.73	1.00000000	pigs
Utp23	0.04	1.00000000	pigs
Arhgap12	-0.11	1.00000000	pigs
Cnep1r1	0.02	1.00000000	pigs
Cyth3	0.13	1.00000000	pigs
Lars2	-0.05	1.00000000	pigs
Rbm39	-0.23	1.00000000	pigs
Emp3	-0.57	1.00000000	pigs
Nhsl2	-0.50	1.00000000	pigs
Pmp22	-0.15	1.00000000	pigs
Fnbp4	0.29	1.00000000	pigs
Bnip3l	0.01	1.00000000	pigs
Stat1	0.09	1.00000000	pigs
8030462N17Rik	0.02	1.00000000	pigs
Cnot7	-0.09	1.00000000	pigs
Znrf3	0.08	1.00000000	pigs
Tbpl1	0.17	1.00000000	pigs

Snx18	0.15	1.00000000	pigs
Ets1	0.26	1.00000000	pigs
Epn1	-0.07	1.00000000	pigs
Cnot6	-0.13	1.00000000	pigs
Trappc12	0.08	1.00000000	pigs
Glipr2	-0.01	1.00000000	pigs
Wasf3	-0.07	1.00000000	pigs
Jkamp	-0.15	1.00000000	pigs
Ing1	-0.02	1.00000000	pigs
Arid3b	0.05	1.00000000	pigs
Pcsk5	-0.28	1.00000000	pigs
Serp1	0.11	1.00000000	pigs
Ccdc18	-0.03	1.00000000	pigs
Scyl1	-0.05	1.00000000	pigs
Tnik	-0.30	1.00000000	pigs
Atp23	0.00	1.00000000	pigs
Dipk1a	0.17	1.00000000	pigs
Hadhb	0.04	1.00000000	pigs
Slc4a4	-0.54	1.00000000	pigs
Ndufab1	0.30	1.00000000	pigs
Sugt1	0.17	1.00000000	pigs
Ufd1	0.06	1.00000000	pigs
Rin3	0.09	1.00000000	pigs
Rdh14	-0.02	1.00000000	pigs
Ifih1	-0.09	1.00000000	pigs
Slc29a1	-0.03	1.00000000	pigs
Ap5z1	-0.04	1.00000000	pigs
Gpbp1	0.18	1.00000000	pigs
Tent5a	-0.36	1.00000000	pigs
Hdac5	0.36	1.00000000	pigs
Nup93	-0.03	1.00000000	pigs
Usp42	-0.01	1.00000000	pigs
Dtwd2	-0.41	1.00000000	pigs
Thbs2	0.15	1.00000000	pigs
Rasgef1b	0.16	1.00000000	pigs
Socs5	0.16	1.00000000	pigs
Ubr1	-0.45	1.00000000	pigs
Lrrc8d	-0.31	1.00000000	pigs
Septin8	-0.40	1.00000000	pigs
Plcl1	-1.02	1.00000000	pigs
Ddah2	0.03	1.00000000	pigs
Traf6	0.07	1.00000000	pigs
Cfl1	0.37	1.00000000	pigs
Eml4	0.34	1.00000000	pigs
Tcea3	0.05	1.00000000	pigs
Dhrs1	-0.08	1.00000000	pigs

Vdac2	0.21	1.00000000	pigs
Elp5	0.03	1.00000000	pigs
Ncoa2	-0.34	1.00000000	pigs
Gipc1	-0.22	1.00000000	pigs
Nenf	-0.31	1.00000000	pigs
4933400A11Rik	0.30	1.00000000	pigs
Acadl	0.00	1.00000000	pigs
Gdf9	0.11	1.00000000	pigs
Gemin5	0.01	1.00000000	pigs
Zbtb2	-0.02	1.00000000	pigs
Gucy1b1	0.11	1.00000000	pigs
Zfp13	-0.03	1.00000000	pigs
Nol10	0.00	1.00000000	pigs
Gabpb1	0.16	1.00000000	pigs
Ppp3ca	-0.44	1.00000000	pigs
Insyn1	-0.04	1.00000000	pigs
Odc1	0.16	1.00000000	pigs
Ube2o	-0.01	1.00000000	pigs
Plekhm3	-0.55	1.00000000	pigs
Acvrl1	0.13	1.00000000	pigs
Cul9	0.08	1.00000000	pigs
Osbp	-0.07	1.00000000	pigs
Naf1	0.04	1.00000000	pigs
Lrrfip2	-0.32	1.00000000	pigs
Hyal2	-0.01	1.00000000	pigs
Tmem161a	0.04	1.00000000	pigs
Fchsd2	0.25	1.00000000	pigs
Patl1	0.17	1.00000000	pigs
Kat2b	-0.45	1.00000000	pigs
Atp6v1a	0.03	1.00000000	pigs
Ndufb6	0.00	1.00000000	pigs
B3gnt9	0.06	1.00000000	pigs
Nbr1	0.06	1.00000000	pigs
Cops7b	0.12	1.00000000	pigs
Cmklr1	-0.04	1.00000000	pigs
Wdr13	-0.08	1.00000000	pigs
Lrrc42	0.04	1.00000000	pigs
Urb1	-0.01	1.00000000	pigs
Zfp787	-0.05	1.00000000	pigs
Ndufb11b	0.23	1.00000000	pigs
Tgm2	0.24	1.00000000	pigs
Whamm	0.07	1.00000000	pigs
Eef1akmt3	0.03	1.00000000	pigs
Tmem115	0.09	1.00000000	pigs
Scfd1	-0.29	1.00000000	pigs
Ilf2	0.25	1.00000000	pigs

Nedd8	0.35	1.00000000	pigs
D6Wsu163e	0.04	1.00000000	pigs
Flrt2	-0.41	1.00000000	pigs
Ddx49	0.00	1.00000000	pigs
Irak2	0.48	1.00000000	pigs
Tsen15	-0.02	1.00000000	pigs
Fbrs	0.00	1.00000000	pigs
Pcgf2	-0.09	1.00000000	pigs
Ccdc28b	0.07	1.00000000	pigs
Ect2	-0.05	1.00000000	pigs
Emsy	-0.09	1.00000000	pigs
Sh3bgrl3	-0.01	1.00000000	pigs
Zc3hav1	0.16	1.00000000	pigs
Pcdhga4	-0.30	1.00000000	pigs
Fam98a	0.07	1.00000000	pigs
Pcna	0.35	1.00000000	pigs
Tbc1d2b	0.12	1.00000000	pigs
Eif4a3l1	0.24	1.00000000	pigs
Tnpo1	-0.38	1.00000000	pigs
Commd1b	0.08	1.00000000	pigs
Irak4	0.08	1.00000000	pigs
Itgb5	-0.33	1.00000000	pigs
Pcolce	0.28	1.00000000	pigs
Bpnt2	0.15	1.00000000	pigs
Bnip1	0.07	1.00000000	pigs
Ubxn4	-0.31	1.00000000	pigs
Fermt2	-0.45	1.00000000	pigs
Chsy1	-0.01	1.00000000	pigs
Spast	-0.03	1.00000000	pigs
Dvl2	0.02	1.00000000	pigs
Tecta	0.04	1.00000000	pigs
Uqcrc2	0.09	1.00000000	pigs
Sp3	-0.30	1.00000000	pigs
Dido1	-0.06	1.00000000	pigs
Gdi2	0.23	1.00000000	pigs
Usp22	-0.01	1.00000000	pigs
Camta1	0.14	1.00000000	pigs
Lekr1	-0.25	1.00000000	pigs
Coil	-0.05	1.00000000	pigs
Stx12	0.05	1.00000000	pigs
Wdr61	0.17	1.00000000	pigs
Ndufa4	0.43	1.00000000	pigs
Btbd19	-0.03	1.00000000	pigs
Aph1a	0.23	1.00000000	pigs
Ocel1	0.02	1.00000000	pigs
Kcnd3	0.02	1.00000000	pigs

Parva	-0.26	1.00000000	pigs
Ypel3	-0.27	1.00000000	pigs
Cdkl5	-0.05	1.00000000	pigs
Cox17	0.00	1.00000000	pigs
Prkra	0.07	1.00000000	pigs
Prr19	-0.03	1.00000000	pigs
C1qtnf7	-0.28	1.00000000	pigs
Cutc	0.07	1.00000000	pigs
Ndufa2	0.34	1.00000000	pigs
Tgfbr1	0.17	1.00000000	pigs
Scap	0.02	1.00000000	pigs
Safb	0.03	1.00000000	pigs
Foxj3	-0.31	1.00000000	pigs
Slc25a38	-0.02	1.00000000	pigs
Lama4	-0.18	1.00000000	pigs
Tpr	-0.22	1.00000000	pigs
Babam2	0.16	1.00000000	pigs
1110059G10Rik	0.04	1.00000000	pigs
Katnip	-0.10	1.00000000	pigs
Uchl3	0.15	1.00000000	pigs
Nfkbid	0.00	1.00000000	pigs
AI837181	-0.12	1.00000000	pigs
Herc2	-0.45	1.00000000	pigs
Zfand2a	-0.04	1.00000000	pigs
Fbxl3	0.03	1.00000000	pigs
Zdhhc7	-0.06	1.00000000	pigs
Ccdc9	-0.02	1.00000000	pigs
Hspb6	-0.31	1.00000000	pigs
Nrip1	-0.41	1.00000000	pigs
Gna11	0.00	1.00000000	pigs
Ddx59	-0.03	1.00000000	pigs
Sntb1	-0.29	1.00000000	pigs
Tasp1	-0.34	1.00000000	pigs
Clpb	-0.02	1.00000000	pigs
Dis3	-0.02	1.00000000	pigs
Fam3a	0.15	1.00000000	pigs
Creb3l2	-0.26	1.00000000	pigs
Pspc1	0.13	1.00000000	pigs
Fbxl17	-0.43	1.00000000	pigs
Agpat5	0.02	1.00000000	pigs
Chpf2	0.07	1.00000000	pigs
Hdhd2	-0.26	1.00000000	pigs
Akr1a1	-0.14	1.00000000	pigs
Slc7a2	0.29	1.00000000	pigs
Rnf170	0.12	1.00000000	pigs
Zyx	-0.33	1.00000000	pigs

Zpr1	0.28	1.00000000	pigs
Adss	-0.01	1.00000000	pigs
Wrap73	-0.01	1.00000000	pigs
Fermt3	0.15	1.00000000	pigs
Gramd1a	-0.05	1.00000000	pigs
Ddx47	0.01	1.00000000	pigs
Sec22c	0.00	1.00000000	pigs
Kdm8	-0.18	1.00000000	pigs
Rnf19a	0.34	1.00000000	pigs
Tst	-0.01	1.00000000	pigs
Exoc2	0.11	1.00000000	pigs
Ddb1	0.02	1.00000000	pigs
Arhgap24	0.05	1.00000000	pigs
Spg21	0.14	1.00000000	pigs
Vps26c	-0.09	1.00000000	pigs
Nploc4	0.17	1.00000000	pigs
Ackr1	-0.17	1.00000000	pigs
Kbtbd7	-0.09	1.00000000	pigs
Lrp10	0.08	1.00000000	pigs
Gramd1b	0.02	1.00000000	pigs
Coro2b	-0.56	1.00000000	pigs
Ppid	0.14	1.00000000	pigs
Crebrf	0.18	1.00000000	pigs
P2rx4	-0.04	1.00000000	pigs
Pradc1	0.03	1.00000000	pigs
Taf7	-0.03	1.00000000	pigs
Tmem243	0.13	1.00000000	pigs
Tmem222	-0.02	1.00000000	pigs
Hadha	-0.01	1.00000000	pigs
Csrnp2	0.02	1.00000000	pigs
Pitpnb	0.06	1.00000000	pigs
Ogfod1	0.01	1.00000000	pigs
Rhbdd1	0.02	1.00000000	pigs
Dlg4	-0.06	1.00000000	pigs
Zfp90	0.05	1.00000000	pigs
Zfp524	-0.05	1.00000000	pigs
Cluh	0.03	1.00000000	pigs
Tcp11l2	-0.04	1.00000000	pigs
Ahctf1	-0.28	1.00000000	pigs
Eed	-0.08	1.00000000	pigs
Neu1	0.16	1.00000000	pigs
Arl4d	0.00	1.00000000	pigs
Grb2	0.30	1.00000000	pigs
Foxk2	0.03	1.00000000	pigs
Ncln	0.04	1.00000000	pigs
Klhl23	-0.05	1.00000000	pigs

Rgma	0.03	1.00000000	pigs
P3h3	0.06	1.00000000	pigs
1600012H06Rik	-0.07	1.00000000	pigs
Vgll3	-0.41	1.00000000	pigs
Sat1	0.15	1.00000000	pigs
Dtx3	-0.04	1.00000000	pigs
Kctd3	0.01	1.00000000	pigs
Arfip1	-0.32	1.00000000	pigs
Kif1b	-0.32	1.00000000	pigs
Saal1	0.02	1.00000000	pigs
Psmc6	0.10	1.00000000	pigs
Ap3d1	-0.12	1.00000000	pigs
Fn1	0.34	1.00000000	pigs
Atp5c1	0.16	1.00000000	pigs
Rc3h2	0.10	1.00000000	pigs
Top2b	-0.16	1.00000000	pigs
BC048507	0.42	1.00000000	pigs
Tspan18	-0.04	1.00000000	pigs
Zcchc24	-0.32	1.00000000	pigs
Psmd2	0.18	1.00000000	pigs
Ccdc82	0.20	1.00000000	pigs
Sdc3	-0.09	1.00000000	pigs
Hat1	0.11	1.00000000	pigs
Spire1	0.00	1.00000000	pigs
Lamb2	-0.17	1.00000000	pigs
Kif18a	-0.01	1.00000000	pigs
Sar1b	0.20	1.00000000	pigs
Tgif2	-0.06	1.00000000	pigs
Sema3d	-0.27	1.00000000	pigs
Rcsd1	0.29	1.00000000	pigs
Pex13	0.03	1.00000000	pigs
Sds	-0.20	1.00000000	pigs
Ccdc62	0.05	1.00000000	pigs
Dennd1b	0.13	1.00000000	pigs
Umps	-0.05	1.00000000	pigs
Ap2s1	0.25	1.00000000	pigs
Slc1a3	0.01	1.00000000	pigs
Bud23	0.22	1.00000000	pigs
Creb1	0.10	1.00000000	pigs
Cdc37l1	-0.01	1.00000000	pigs
9930021J03Rik	-0.41	1.00000000	pigs
Arhgap29	0.04	1.00000000	pigs
Sgpp1	0.06	1.00000000	pigs
Cwc27	-0.56	1.00000000	pigs
Laptm4b	-0.35	1.00000000	pigs
Ctsf	0.13	1.00000000	pigs

Iqgap2	0.33	1.00000000	pigs
Slc7a7	0.06	1.00000000	pigs
Kcmf1	0.17	1.00000000	pigs
Eif2ak4	-0.44	1.00000000	pigs
Rasd1	-0.09	1.00000000	pigs
Bcat2	-0.10	1.00000000	pigs
Atg12	0.02	1.00000000	pigs
Dhx33	-0.02	1.00000000	pigs
Cdk4	0.13	1.00000000	pigs
Smchd1	-0.24	1.00000000	pigs
S100a8	-0.70	1.00000000	pigs
Tmem165	0.04	1.00000000	pigs
Hsd17b4	0.13	1.00000000	pigs
Elovl5	-0.07	1.00000000	pigs
Cnrip1	0.07	1.00000000	pigs
Usp30	0.05	1.00000000	pigs
Wwp1	-0.47	1.00000000	pigs
Gaa	0.09	1.00000000	pigs
Ranbp2	0.02	1.00000000	pigs
Cant1	0.01	1.00000000	pigs
Aifm2	-0.05	1.00000000	pigs
Lrrc14	0.07	1.00000000	pigs
Sirt7	0.15	1.00000000	pigs
Steep1	-0.01	1.00000000	pigs
Insig1	0.28	1.00000000	pigs
Cdc123	0.22	1.00000000	pigs
Cfl2	0.04	1.00000000	pigs
Pygm	0.01	1.00000000	pigs
Snx8	0.01	1.00000000	pigs
Igf1r	-0.31	1.00000000	pigs
Slc35e2	-0.07	1.00000000	pigs
Vwa5a	-0.09	1.00000000	pigs
Cd14	0.30	1.00000000	pigs
Fam13c	-0.13	1.00000000	pigs
Trmt13	0.01	1.00000000	pigs
Naxe	0.04	1.00000000	pigs
Apcdd1	0.09	1.00000000	pigs
Fibin	-0.44	1.00000000	pigs
Srpr	-0.19	1.00000000	pigs
Pik3c2b	0.00	1.00000000	pigs
Pbdc1	0.11	1.00000000	pigs
Aprt	0.08	1.00000000	pigs
Bid	0.00	1.00000000	pigs
Ntn1	0.01	1.00000000	pigs
Ccni	0.30	1.00000000	pigs
Agpat3	-0.05	1.00000000	pigs

Dkc1	-0.05	1.00000000	pigs
Alg3	-0.01	1.00000000	pigs
Sgcd	-0.12	1.00000000	pigs
Adgrl2	-0.23	1.00000000	pigs
Tsga10	0.02	1.00000000	pigs
Dipk2a	-0.39	1.00000000	pigs
Slc30a5	0.27	1.00000000	pigs
Ptbp2	-0.01	1.00000000	pigs
Zfp169	-0.07	1.00000000	pigs
Hhat	-0.08	1.00000000	pigs
Slc15a4	0.05	1.00000000	pigs
Kank1	-0.06	1.00000000	pigs
Sdc2	-0.27	1.00000000	pigs
Prickle2	-0.48	1.00000000	pigs
Zfp639	0.04	1.00000000	pigs
Rhot1	-0.36	1.00000000	pigs
Glud1	0.18	1.00000000	pigs
Uqcrc1	0.30	1.00000000	pigs
Lsm6	0.07	1.00000000	pigs
Rnf44	0.12	1.00000000	pigs
Atp6v1h	0.06	1.00000000	pigs
Cited2	-0.32	1.00000000	pigs
B4gat1	-0.03	1.00000000	pigs
Eif1	-0.05	1.00000000	pigs
Traf4	0.02	1.00000000	pigs
Slc22a3	-0.06	1.00000000	pigs
Psma3	0.34	1.00000000	pigs
Dnajc3	-0.14	1.00000000	pigs
Dnm1	-0.05	1.00000000	pigs
Gde1	-0.02	1.00000000	pigs
Samhd1	0.13	1.00000000	pigs
Taf1d	0.17	1.00000000	pigs
Cmbl	0.04	1.00000000	pigs
Cuedc2	0.10	1.00000000	pigs
Sgip1	0.07	1.00000000	pigs
Nt5dc2	0.23	1.00000000	pigs
Lrch1	0.21	1.00000000	pigs
Hrh4	0.00	1.00000000	pigs
Polr1a	-0.07	1.00000000	pigs
Fes	0.02	1.00000000	pigs
Supt6	-0.07	1.00000000	pigs
Dusp6	0.00	1.00000000	pigs
Pgghg	0.03	1.00000000	pigs
Pak2	-0.17	1.00000000	pigs
Zdhhc5	-0.03	1.00000000	pigs
Slc44a1	0.12	1.00000000	pigs

Vps39	-0.02	1.00000000	pigs
Sart1	0.02	1.00000000	pigs
Desi1	0.02	1.00000000	pigs
Wdr25	-0.01	1.00000000	pigs
Fnbp1	0.01	1.00000000	pigs
Ttc9	0.06	1.00000000	pigs
Ctnnbip1	0.07	1.00000000	pigs
Tomm7	0.40	1.00000000	pigs
Sar1a	0.12	1.00000000	pigs
Carnmt1	0.07	1.00000000	pigs
Tkt	0.21	1.00000000	pigs
D8Ertd738e	0.07	1.00000000	pigs
Dvl1	-0.12	1.00000000	pigs
Dtnbp1	-0.05	1.00000000	pigs
Sigmar1	-0.05	1.00000000	pigs
Rnls	-0.18	1.00000000	pigs
Setd5	-0.39	1.00000000	pigs
Nol11	0.41	1.00000000	pigs
Tspan11	-0.04	1.00000000	pigs
Zwilch	-0.04	1.00000000	pigs
Rcor1	0.17	1.00000000	pigs
Dcun1d2	-0.09	1.00000000	pigs
Gtf2h3	-0.11	1.00000000	pigs
Actr1a	0.02	1.00000000	pigs
Nck1	0.07	1.00000000	pigs
Psmd11	0.12	1.00000000	pigs
Abhd11	0.07	1.00000000	pigs
Tns1	-0.33	1.00000000	pigs
Wdr48	-0.04	1.00000000	pigs
Ascc3	-0.44	1.00000000	pigs
C1qbp	0.11	1.00000000	pigs
Eif2b3	-0.46	1.00000000	pigs
Pdk1	0.30	1.00000000	pigs
Stk19	0.10	1.00000000	pigs
Retreg2	0.10	1.00000000	pigs
Sdad1	-0.05	1.00000000	pigs
Npas2	-0.04	1.00000000	pigs
Serinc5	0.00	1.00000000	pigs
Slc38a6	-0.11	1.00000000	pigs
Pde12	0.10	1.00000000	pigs
Ticam1	0.05	1.00000000	pigs
Ski	-0.14	1.00000000	pigs
Rasl11b	-0.06	1.00000000	pigs
Cnih4	0.17	1.00000000	pigs
Syde2	-0.18	1.00000000	pigs
Zc3h7a	-0.07	1.00000000	pigs

Lpcat4	-0.04	1.00000000	pigs
Vps13b	-0.18	1.00000000	pigs
Ubqln1	0.05	1.00000000	pigs
Lbr	-0.41	1.00000000	pigs
Prkx	-0.03	1.00000000	pigs
Ahsa1	0.02	1.00000000	pigs
Hipk2	0.17	1.00000000	pigs
Fgd1	-0.14	1.00000000	pigs
Pak1ip1	0.11	1.00000000	pigs
Ipo7	0.20	1.00000000	pigs
Plaa	0.07	1.00000000	pigs
Wdfy3	-0.35	1.00000000	pigs
Tbc1d17	-0.02	1.00000000	pigs
Ccdc61	-0.08	1.00000000	pigs
Arrdc2	0.23	1.00000000	pigs
Bccip	0.15	1.00000000	pigs
Ilvbl	0.01	1.00000000	pigs
Tmem39b	-0.07	1.00000000	pigs
Tbc1d19	-0.11	1.00000000	pigs
Faxc	0.02	1.00000000	pigs
Cadm3	-0.49	1.00000000	pigs
Utp4	-0.02	1.00000000	pigs
Hsdl2	-0.09	1.00000000	pigs
Fam114a2	-0.07	1.00000000	pigs
Pacs2	-0.08	1.00000000	pigs
Oxld1	-0.10	1.00000000	pigs
Def8	-0.06	1.00000000	pigs
Trim27	0.04	1.00000000	pigs
Ddx55	-0.02	1.00000000	pigs
Fam13a	-0.34	1.00000000	pigs
Pi16	0.23	1.00000000	pigs
Homer1	-0.07	1.00000000	pigs
Rfc4	0.29	1.00000000	pigs
C2cd3	-0.03	1.00000000	pigs
Xab2	0.04	1.00000000	pigs
Trp53	0.30	1.00000000	pigs
Shc1	-0.01	1.00000000	pigs
Actr10	0.20	1.00000000	pigs
Ints6	-0.28	1.00000000	pigs
Tbca	0.20	1.00000000	pigs
Tlnrd1	0.03	1.00000000	pigs
Npm3	0.16	1.00000000	pigs
Ero1a	0.18	1.00000000	pigs
Rac3	0.11	1.00000000	pigs
Xiap	-0.01	1.00000000	pigs
Ccdc28a	0.02	1.00000000	pigs

Thap4	0.07	1.00000000	pigs
Fbf1	-0.01	1.00000000	pigs
Rnf217	-0.45	1.00000000	pigs
Chchd3	-0.05	1.00000000	pigs
Ascc2	0.07	1.00000000	pigs
Cops2	0.07	1.00000000	pigs
Arid1b	-0.20	1.00000000	pigs
Bzw2	0.05	1.00000000	pigs
Chd1	0.19	1.00000000	pigs
Rora	0.29	1.00000000	pigs
Gpr107	0.07	1.00000000	pigs
Nebl	0.16	1.00000000	pigs
Retreg1	-0.33	1.00000000	pigs
Rnf146	0.08	1.00000000	pigs
Acsf3	0.07	1.00000000	pigs
Diaph2	-0.10	1.00000000	pigs
1110004F10Rik	0.19	1.00000000	pigs
Trip10	0.02	1.00000000	pigs
Stk38	-0.45	1.00000000	pigs
Zdbf2	-0.02	1.00000000	pigs
Il17d	-0.01	1.00000000	pigs
Gltp	0.20	1.00000000	pigs
Cspp1	-0.36	1.00000000	pigs
Cct5	0.19	1.00000000	pigs
Rbx1	0.36	1.00000000	pigs
Wdr5	0.25	1.00000000	pigs
Vkorc1l1	0.00	1.00000000	pigs
Gata5	-0.02	1.00000000	pigs
Aplp2	-0.23	1.00000000	pigs
Cdkl1	-0.08	1.00000000	pigs
Snrpd1	0.42	1.00000000	pigs
Zfp143	-0.20	1.00000000	pigs
Bcas3	0.07	1.00000000	pigs
Vps26b	0.06	1.00000000	pigs
Slc2a12	0.02	1.00000000	pigs
Snap47	-0.39	1.00000000	pigs
Ccnq	-0.05	1.00000000	pigs
Scaf1	0.07	1.00000000	pigs
Srm	0.21	1.00000000	pigs
Eif4ebp1	0.16	1.00000000	pigs
Plec	-0.02	1.00000000	pigs
Galnt7	0.00	1.00000000	pigs
Adamts7	0.01	1.00000000	pigs
Smtn	-0.04	1.00000000	pigs
Gps2	-0.33	1.00000000	pigs
Pank2	-0.06	1.00000000	pigs

Rgmb	0.12	1.00000000	pigs
Yrdc	0.07	1.00000000	pigs
Yap1	-0.34	1.00000000	pigs
Frmd3	-0.24	1.00000000	pigs
Zcchc2	0.10	1.00000000	pigs
Pgls	0.09	1.00000000	pigs
Rbbp9	0.15	1.00000000	pigs
Rnf103	0.02	1.00000000	pigs
Wdr17	-0.06	1.00000000	pigs
Nob1	0.01	1.00000000	pigs
Slc66a1	-0.04	1.00000000	pigs
Bbx	-0.43	1.00000000	pigs
Utrn	-0.11	1.00000000	pigs
Stat6	-0.08	1.00000000	pigs
Cxxc5	0.05	1.00000000	pigs
Fbrsl1	-0.04	1.00000000	pigs
Atf3	-0.34	1.00000000	pigs
Hmg20a	-0.17	1.00000000	pigs
Fam53c	-0.03	1.00000000	pigs
Nagk	0.19	1.00000000	pigs
Cln3	-0.05	1.00000000	pigs
Ints8	-0.02	1.00000000	pigs
Nrbp2	-0.08	1.00000000	pigs
Ocrl	-0.01	1.00000000	pigs
Ikzf2	0.06	1.00000000	pigs
St3gal2	0.09	1.00000000	pigs
Septin6	0.07	1.00000000	pigs
Atxn7l3	0.10	1.00000000	pigs
D11Wsu47e	-0.04	1.00000000	pigs
Ubald2	0.26	1.00000000	pigs
Klf7	-0.38	1.00000000	pigs
Srpk1	0.09	1.00000000	pigs
Thumpd2	0.00	1.00000000	pigs
Dnlz	0.00	1.00000000	pigs
Trim37	0.00	1.00000000	pigs
Actr6	-0.03	1.00000000	pigs
Lnx2	-0.09	1.00000000	pigs
Cdca4	0.13	1.00000000	pigs
Eif2ak1	0.07	1.00000000	pigs
Jup	0.01	1.00000000	pigs
Card19	0.12	1.00000000	pigs
Ppfia2	0.04	1.00000000	pigs
Ikbkb	0.31	1.00000000	pigs
Tsen34	-0.06	1.00000000	pigs
Ubap1	-0.08	1.00000000	pigs
Krr1	-0.14	1.00000000	pigs

Clptm1	-0.06	1.00000000	pigs
Itga1	0.16	1.00000000	pigs
Castor2	0.03	1.00000000	pigs
Bdh2	-0.11	1.00000000	pigs
Adamts5	-0.84	1.00000000	pigs
Dnajb9	0.18	1.00000000	pigs
U2af1l4	0.07	1.00000000	pigs
Selenof	0.22	1.00000000	pigs
Dstyk	0.00	1.00000000	pigs
Chml	0.04	1.00000000	pigs
Etv1	-0.08	1.00000000	pigs
Hectd2	-0.51	1.00000000	pigs
Ndufv1	0.04	1.00000000	pigs
Parp16	0.05	1.00000000	pigs
Dnajc13	0.02	1.00000000	pigs
Hltf	-0.05	1.00000000	pigs
Hspb11	-0.02	1.00000000	pigs
LTO1	0.09	1.00000000	pigs
Cnot4	-0.29	1.00000000	pigs
Lonp1	0.05	1.00000000	pigs
Aass	-0.09	1.00000000	pigs
Zfyve9	-0.43	1.00000000	pigs
Cd302	-0.30	1.00000000	pigs
Ankrd44	0.23	1.00000000	pigs
Azi2	0.17	1.00000000	pigs
Ranbp9	0.12	1.00000000	pigs
Hmgcr	0.12	1.00000000	pigs
Lonp2	0.02	1.00000000	pigs
Abhd18	0.00	1.00000000	pigs
Ppp2r5a	0.16	1.00000000	pigs
Stn1	0.02	1.00000000	pigs
Gtdc1	-0.41	1.00000000	pigs
0610030E20Rik	0.11	1.00000000	pigs
Numa1	0.05	1.00000000	pigs
Sulf2	-0.24	1.00000000	pigs
Elmod3	0.03	1.00000000	pigs
Csnk1g2	-0.03	1.00000000	pigs
Lin7a	-0.35	1.00000000	pigs
Osr1	-0.08	1.00000000	pigs
Naxd	0.03	1.00000000	pigs
Abhd4	-0.03	1.00000000	pigs
Chodl	0.07	1.00000000	pigs
Ackr2	-0.40	1.00000000	pigs
Taf3	-0.38	1.00000000	pigs
Cbx4	0.04	1.00000000	pigs
Lrig2	0.00	1.00000000	pigs

Plscr1	0.09	1.00000000	pigs
Rbms3	-0.10	1.00000000	pigs
Pskh1	0.00	1.00000000	pigs
Ric8a	0.17	1.00000000	pigs
Dnal1	-0.07	1.00000000	pigs
Fbxo8	-0.01	1.00000000	pigs
Cox7a1	0.07	1.00000000	pigs
Cdk5rap2	-0.10	1.00000000	pigs
Nup35	-0.04	1.00000000	pigs
Fstl1	-0.17	1.00000000	pigs
Rgs2	0.24	1.00000000	pigs
Uqcc1	-0.09	1.00000000	pigs
Tns3	0.12	1.00000000	pigs
Dld	0.28	1.00000000	pigs
Chmp6	-0.02	1.00000000	pigs
Rpp30	-0.03	1.00000000	pigs
Atg101	-0.01	1.00000000	pigs
Xpo5	0.02	1.00000000	pigs
Ddx6	-0.17	1.00000000	pigs
Dennd5b	-0.04	1.00000000	pigs
Stk38l	-0.22	1.00000000	pigs
Lyar	0.06	1.00000000	pigs
Nampt	0.11	1.00000000	pigs
Snx17	-0.03	1.00000000	pigs
Smdt1	0.25	1.00000000	pigs
Rbmx	0.17	1.00000000	pigs
Taf9b	-0.04	1.00000000	pigs
Upf3b	-0.04	1.00000000	pigs
Pfn2	0.05	1.00000000	pigs
Psma7	0.02	1.00000000	pigs
Clta	0.49	1.00000000	pigs
Prpf19	0.17	1.00000000	pigs
Zfp239	-0.01	1.00000000	pigs
Cntn3	-0.29	1.00000000	pigs
3110082I17Rik	0.07	1.00000000	pigs
Arhgef10l	-0.32	1.00000000	pigs
Rock1	-0.34	1.00000000	pigs
Hdac2	-0.08	1.00000000	pigs
Dmd	-0.45	1.00000000	pigs
Snx21	0.01	1.00000000	pigs
Rwdd1	-0.18	1.00000000	pigs
Ccnk	0.01	1.00000000	pigs
Cuedc1	-0.28	1.00000000	pigs
S1pr1	-0.28	1.00000000	pigs
Slc41a2	-0.05	1.00000000	pigs
Helb	0.03	1.00000000	pigs

Nars	0.14	1.00000000	pigs
Dlg1	-0.41	1.00000000	pigs
Tmco4	-0.03	1.00000000	pigs
Cfap45	-0.04	1.00000000	pigs
Lrrc45	0.02	1.00000000	pigs
Nfya	-0.02	1.00000000	pigs
Faf1	-0.32	1.00000000	pigs
Dis3l	0.02	1.00000000	pigs
Trim25	-0.02	1.00000000	pigs
Ndufa5	0.18	1.00000000	pigs
Papolg	-0.02	1.00000000	pigs
Herpud1	-0.14	1.00000000	pigs
Ubald1	-0.04	1.00000000	pigs
Ppib	0.30	1.00000000	pigs
Elovl6	-0.43	1.00000000	pigs
Fadd	-0.09	1.00000000	pigs
Fbxo28	-0.05	1.00000000	pigs
Slc35b2	-0.03	1.00000000	pigs
Capn2	0.17	1.00000000	pigs
Atp5d	0.42	1.00000000	pigs
Sugp1	0.02	1.00000000	pigs
Fut10	-0.26	1.00000000	pigs
Chchd4	-0.06	1.00000000	pigs
Plekha1	0.18	1.00000000	pigs
Npdc1	-0.33	1.00000000	pigs
Slc4a2	-0.06	1.00000000	pigs
Ccser1	0.02	1.00000000	pigs
Epb41l3	0.11	1.00000000	pigs
St8sia4	0.12	1.00000000	pigs
Ppp2r1b	-0.12	1.00000000	pigs
Timm44	-0.02	1.00000000	pigs
Ndrg2	-0.02	1.00000000	pigs
Cdk9	0.08	1.00000000	pigs
Fam122b	0.04	1.00000000	pigs
Klhl3	0.04	1.00000000	pigs
Klf16	-0.05	1.00000000	pigs
Sult1a1	0.32	1.00000000	pigs
Prpf38a	-0.30	1.00000000	pigs
Zswim6	0.25	1.00000000	pigs
Chfr	0.14	1.00000000	pigs
Tcf3	-0.03	1.00000000	pigs
Plin2	0.08	1.00000000	pigs
Cfap36	0.03	1.00000000	pigs
Dnah9	-0.18	1.00000000	pigs
Gpr155	0.05	1.00000000	pigs
Otud5	-0.03	1.00000000	pigs

Llgl1	0.02	1.00000000	pigs
E2f6	0.01	1.00000000	pigs
Gpd2	0.16	1.00000000	pigs
Dcps	0.24	1.00000000	pigs
Ctdp1	0.23	1.00000000	pigs
Ptgis	-0.22	1.00000000	pigs
Cox6a2	-0.08	1.00000000	pigs
Ralgps1	0.06	1.00000000	pigs
Senp8	-0.02	1.00000000	pigs
Sugct	0.09	1.00000000	pigs
Fbxo34	-0.31	1.00000000	pigs
Rab34	0.03	1.00000000	pigs
Wls	0.01	1.00000000	pigs
Slc6a8	-0.07	1.00000000	pigs
Tomm70a	0.03	1.00000000	pigs
Kif27	0.01	1.00000000	pigs
Phldb1	-0.35	1.00000000	pigs
Arpc5l	0.24	1.00000000	pigs
Tmem63a	-0.10	1.00000000	pigs
Trim75	-0.13	1.00000000	pigs
Ndufc2	0.20	1.00000000	pigs
Spag9	-0.24	1.00000000	pigs
Cops6	-0.01	1.00000000	pigs
Rnf115	0.05	1.00000000	pigs
Nf2	-0.06	1.00000000	pigs
Cbll1	0.09	1.00000000	pigs
Usp40	-0.11	1.00000000	pigs
Pgam5	0.04	1.00000000	pigs
Atxn7l1	0.00	1.00000000	pigs
Daxx	-0.07	1.00000000	pigs
Palm	-0.33	1.00000000	pigs
Ier5l	0.22	1.00000000	pigs
Spata20	0.13	1.00000000	pigs
Pcdh19	-0.10	1.00000000	pigs
Lrrc17	-0.20	1.00000000	pigs
Dapk3	0.01	1.00000000	pigs
Lemd2	0.12	1.00000000	pigs
Ccdc22	0.03	1.00000000	pigs
D2hgdh	0.02	1.00000000	pigs
Polh	-0.13	1.00000000	pigs
Gnptab	0.10	1.00000000	pigs
Fam120b	-0.12	1.00000000	pigs
Soat1	0.01	1.00000000	pigs
Oga	-0.05	1.00000000	pigs
Hadh	-0.09	1.00000000	pigs
Ccar1	-0.05	1.00000000	pigs

Zdhhc3	-0.01	1.00000000	pigs
Armh4	-0.04	1.00000000	pigs
Acox1	0.06	1.00000000	pigs
Rp9	-0.44	1.00000000	pigs
Cdk6	0.05	1.00000000	pigs
Topors	0.06	1.00000000	pigs
Srprb	-0.06	1.00000000	pigs
Tead3	0.04	1.00000000	pigs
Crebbp	-0.25	1.00000000	pigs
Tmem120b	0.03	1.00000000	pigs
Pacc1	-0.05	1.00000000	pigs
Tfg	0.21	1.00000000	pigs
Pigb	-0.06	1.00000000	pigs
Dnajc7	0.08	1.00000000	pigs
Abhd15	0.07	1.00000000	pigs
Cog2	-0.04	1.00000000	pigs
Ubxn8	0.08	1.00000000	pigs
Stk35	0.13	1.00000000	pigs
Rab4a	-0.12	1.00000000	pigs
Itpr3	0.04	1.00000000	pigs
Vps29	0.18	1.00000000	pigs
Pamr1	-0.29	1.00000000	pigs
Dffa	-0.02	1.00000000	pigs
Fam151b	0.03	1.00000000	pigs
Slc25a1	-0.08	1.00000000	pigs
Tmem107	-0.01	1.00000000	pigs
D16Ertd472e	-0.32	1.00000000	pigs
Plekhb2	-0.03	1.00000000	pigs
Dennd4a	0.16	1.00000000	pigs
Hoxa5	0.05	1.00000000	pigs
Taf4	-0.12	1.00000000	pigs
Ddx18	0.09	1.00000000	pigs
Zdhhc9	-0.05	1.00000000	pigs
Rars2	-0.03	1.00000000	pigs
Rnf5	-0.07	1.00000000	pigs
Cemip2	-0.03	1.00000000	pigs
Zbtb8a	-0.05	1.00000000	pigs
Elp6	0.12	1.00000000	pigs
Rela	0.17	1.00000000	pigs
Calcrl	0.06	1.00000000	pigs
Asap2	0.04	1.00000000	pigs
Tpst1	-0.39	1.00000000	pigs
Foxj2	0.08	1.00000000	pigs
Rrp36	0.02	1.00000000	pigs
Tmem117	-0.46	1.00000000	pigs
Blmh	-0.01	1.00000000	pigs

Ppp1r13b	0.01	1.00000000	pigs
Appl2	-0.18	1.00000000	pigs
Actg1	0.22	1.00000000	pigs
Tasor2	0.06	1.00000000	pigs
Slc39a9	-0.01	1.00000000	pigs
Arhgap6	0.05	1.00000000	pigs
Abhd17b	-0.08	1.00000000	pigs
Tmem184b	-0.07	1.00000000	pigs
Anks3	-0.06	1.00000000	pigs
Stx4a	0.01	1.00000000	pigs
Prr14	-0.11	1.00000000	pigs
Slc9a3r2	-0.10	1.00000000	pigs
Stard3nl	-0.01	1.00000000	pigs
Tufm	-0.06	1.00000000	pigs
Psmb7	-0.09	1.00000000	pigs
Zfp384	0.10	1.00000000	pigs
Zfand1	0.07	1.00000000	pigs
Dnajb6	0.06	1.00000000	pigs
Bcap31	0.25	1.00000000	pigs
Arl6ip4	-0.02	1.00000000	pigs
Zmat3	0.00	1.00000000	pigs
Zbtb25	-0.07	1.00000000	pigs
Atp2c1	-0.46	1.00000000	pigs
Acot13	0.20	1.00000000	pigs
Dgkz	0.27	1.00000000	pigs
Trim59	0.02	1.00000000	pigs
Ttc39c	0.00	1.00000000	pigs
Stox2	-0.41	1.00000000	pigs
Akap8	0.20	1.00000000	pigs
Lyn	0.17	1.00000000	pigs
Sf3a3	0.01	1.00000000	pigs
Itgav	0.10	1.00000000	pigs
Adgrb3	-0.53	1.00000000	pigs
Prrt1	0.02	1.00000000	pigs
Pnpla2	-0.04	1.00000000	pigs
Rnaseh2b	-0.01	1.00000000	pigs
Nutf2	0.20	1.00000000	pigs
Klf13	0.18	1.00000000	pigs
Nsmf	-0.04	1.00000000	pigs
Psmd14	0.24	1.00000000	pigs
Sh3pxd2a	-0.01	1.00000000	pigs
Urb2	-0.02	1.00000000	pigs
Tspan3	0.07	1.00000000	pigs
Cenph	0.03	1.00000000	pigs
Pik3r5	0.37	1.00000000	pigs
Pole4	0.29	1.00000000	pigs

Fam20b	-0.15	1.00000000	pigs
Synpo2	-0.70	1.00000000	pigs
Ggps1	0.01	1.00000000	pigs
B3galnt2	-0.05	1.00000000	pigs
Ppa2	-0.01	1.00000000	pigs
Spen	0.02	1.00000000	pigs
Ppp1r11	0.01	1.00000000	pigs
Cep95	-0.01	1.00000000	pigs
Lars	-0.34	1.00000000	pigs
Rwdd4a	0.03	1.00000000	pigs
Igsf3	-0.07	1.00000000	pigs
Rsl1d1	-0.06	1.00000000	pigs
Tardbp	0.16	1.00000000	pigs
Gna12	0.12	1.00000000	pigs
Helq	-0.01	1.00000000	pigs
Slc35f5	-0.13	1.00000000	pigs
Poglut2	-0.08	1.00000000	pigs
Naa60	0.17	1.00000000	pigs
Ublcp1	-0.12	1.00000000	pigs
Nsun2	0.08	1.00000000	pigs
Rala	0.25	1.00000000	pigs
Litaf	0.14	1.00000000	pigs
Fzd7	-0.37	1.00000000	pigs
Nrp1	-0.30	1.00000000	pigs
Prpf8	0.03	1.00000000	pigs
Ficd	0.05	1.00000000	pigs
Ankrd11	0.08	1.00000000	pigs
2210016F16Rik	-0.03	1.00000000	pigs
Pxylp1	0.08	1.00000000	pigs
Zbtb38	0.15	1.00000000	pigs
Uros	0.00	1.00000000	pigs
Zdhhc15	-0.08	1.00000000	pigs
Vcpip1	-0.06	1.00000000	pigs
Itprid2	0.05	1.00000000	pigs
Tsc2	-0.06	1.00000000	pigs
Psmg4	0.19	1.00000000	pigs
Limk1	0.20	1.00000000	pigs
Dsel	-0.11	1.00000000	pigs
Pno1	-0.02	1.00000000	pigs
Avpi1	0.01	1.00000000	pigs
Arhgap20	-0.28	1.00000000	pigs
Otulin	-0.03	1.00000000	pigs
Nudt1	0.01	1.00000000	pigs
Ccdc14	-0.38	1.00000000	pigs
Tab1	0.05	1.00000000	pigs
Angptl2	-0.36	1.00000000	pigs

Tpi1	-0.27	1.00000000	pigs
Clasp2	-0.42	1.00000000	pigs
Capn7	-0.15	1.00000000	pigs
Chd3	0.07	1.00000000	pigs
Wasl	-0.34	1.00000000	pigs
St3gal5	-0.17	1.00000000	pigs
Spg7	0.12	1.00000000	pigs
Osgin2	0.01	1.00000000	pigs
Ppfia1	-0.17	1.00000000	pigs
Kdelr2	0.37	1.00000000	pigs
Otud6b	0.02	1.00000000	pigs
Acad9	0.00	1.00000000	pigs
Ppp1r15b	-0.05	1.00000000	pigs
Lhfp	0.28	1.00000000	pigs
Plekhm2	-0.01	1.00000000	pigs
Atp9b	-0.10	1.00000000	pigs
Niban1	0.04	1.00000000	pigs
Sobp	-0.44	1.00000000	pigs
Rfx3	-0.21	1.00000000	pigs
Slc7a10	-0.37	1.00000000	pigs
Rxra	-0.14	1.00000000	pigs
Ascc1	0.01	1.00000000	pigs
Txndc15	-0.02	1.00000000	pigs
Copb2	-0.14	1.00000000	pigs
Aspn	-0.22	1.00000000	pigs
Lrrc47	0.03	1.00000000	pigs
Elf4	0.10	1.00000000	pigs
Ptar1	-0.13	1.00000000	pigs
Kmt2b	-0.06	1.00000000	pigs
Zfp36l2	0.19	1.00000000	pigs
Csde1	-0.14	1.00000000	pigs
Ap1g1	-0.25	1.00000000	pigs
Slc27a4	-0.07	1.00000000	pigs
Fkbp8	-0.15	1.00000000	pigs
Dhx15	0.13	1.00000000	pigs
Chmp2a	0.32	1.00000000	pigs
Herc6	-0.06	1.00000000	pigs
Emc3	0.02	1.00000000	pigs
Btbd11	-0.20	1.00000000	pigs
Crispld2	0.19	1.00000000	pigs
Ptprd	-0.25	1.00000000	pigs
Ergic1	-0.18	1.00000000	pigs
Tmcc1	-0.39	1.00000000	pigs
Atpif1	0.45	1.00000000	pigs
Hus1	0.13	1.00000000	pigs
Lrba	-0.32	1.00000000	pigs

Copg2	-0.04	1.00000000	pigs
Ezr	0.11	1.00000000	pigs
Tmem181a	-0.02	1.00000000	pigs
Amotl2	-0.32	1.00000000	pigs
Pcbp4	-0.06	1.00000000	pigs
Rgl2	0.03	1.00000000	pigs
Alox15	0.06	1.00000000	pigs
Nsmaf	0.07	1.00000000	pigs
Cibar1	0.03	1.00000000	pigs
Papola	0.20	1.00000000	pigs
Asxl1	0.09	1.00000000	pigs
Elf1	0.20	1.00000000	pigs
Prdm6	-0.57	1.00000000	pigs
Dcaf10	-0.36	1.00000000	pigs
Pgd	0.03	1.00000000	pigs
Smad2	-0.13	1.00000000	pigs
Cald1	0.03	1.00000000	pigs
Atp13a1	-0.03	1.00000000	pigs
Dnajb4	-0.23	1.00000000	pigs
Tor1a	-0.05	1.00000000	pigs
Wdr55	0.03	1.00000000	pigs
Tent2	-0.17	1.00000000	pigs
Ivns1abp	-0.48	1.00000000	pigs
Rab28	-0.02	1.00000000	pigs
Ccdc85a	0.01	1.00000000	pigs
Ldlr	-0.09	1.00000000	pigs
Ecsit	-0.10	1.00000000	pigs
Efnb1	0.02	1.00000000	pigs
Slfn9	-0.06	1.00000000	pigs
Prkaa2	-0.30	1.00000000	pigs
Phf2	0.01	1.00000000	pigs
Celf1	-0.07	1.00000000	pigs
Pgap3	-0.04	1.00000000	pigs
Glce	-0.45	1.00000000	pigs
Aifm1	-0.04	1.00000000	pigs
Fpgs	-0.08	1.00000000	pigs
Khnyn	0.00	1.00000000	pigs
Dagla	-0.06	1.00000000	pigs
Slc44a2	-0.03	1.00000000	pigs
Nde1	-0.01	1.00000000	pigs
Uba3	-0.05	1.00000000	pigs
Emc2	0.03	1.00000000	pigs
Cmtm8	-0.24	1.00000000	pigs
Ccdc115	0.13	1.00000000	pigs
Crat	-0.05	1.00000000	pigs
Slc30a6	0.10	1.00000000	pigs

Bag2	-0.02	1.00000000	pigs
Zfand6	-0.14	1.00000000	pigs
Lrrc28	-0.50	1.00000000	pigs
Soga1	0.01	1.00000000	pigs
Enpp6	0.15	1.00000000	pigs
Ankrd42	-0.02	1.00000000	pigs
Neo1	-0.41	1.00000000	pigs
Iqsec2	-0.06	1.00000000	pigs
Eif4e3	-0.29	1.00000000	pigs
Pip4k2b	0.08	1.00000000	pigs
Ercc8	-0.05	1.00000000	pigs
Ucp2	-0.33	1.00000000	pigs
Slc16a7	0.09	1.00000000	pigs
Arrdc4	0.32	1.00000000	pigs
Cntn4	-0.30	1.00000000	pigs
Ptpn18	-0.07	1.00000000	pigs
Cfp	0.16	1.00000000	pigs
Nsdhl	-0.07	1.00000000	pigs
Phf20l1	-0.27	1.00000000	pigs
Atg14	-0.39	1.00000000	pigs
Fosb	0.05	1.00000000	pigs
Gpx4	-0.03	1.00000000	pigs
Psmb3	0.06	1.00000000	pigs
Col27a1	-0.03	1.00000000	pigs
Dock11	-0.09	1.00000000	pigs
Wdr27	-0.06	1.00000000	pigs
Flot1	0.03	1.00000000	pigs
Pigf	-0.09	1.00000000	pigs
Phlda2	-0.07	1.00000000	pigs
Zfp330	0.06	1.00000000	pigs
9430038I01Rik	-0.05	1.00000000	pigs
Nvl	-0.43	1.00000000	pigs
Calu	-0.06	1.00000000	pigs
Iho1	-0.22	1.00000000	pigs
Rgs12	-0.05	1.00000000	pigs
Xrcc1	0.07	1.00000000	pigs
Rhoq	0.04	1.00000000	pigs
Ptpa	0.14	1.00000000	pigs
Itgb2l	0.34	1.00000000	pigs
Galnt15	-0.41	1.00000000	pigs
Ccdc180	-0.04	1.00000000	pigs
Tbc1d13	0.00	1.00000000	pigs
Zc3h18	-0.13	1.00000000	pigs
Pfkfb4	-0.09	1.00000000	pigs
Slc24a4	-0.03	1.00000000	pigs
Dpp9	0.02	1.00000000	pigs

Acadvl	0.10	1.00000000	pigs
Pecam1	-0.21	1.00000000	pigs
Asrgl1	0.12	1.00000000	pigs
Tut4	-0.31	1.00000000	pigs
Caprin1	-0.16	1.00000000	pigs
Pitpnm2	-0.26	1.00000000	pigs
Letm1	0.02	1.00000000	pigs
Cfap410	-0.06	1.00000000	pigs
Usp16	-0.10	1.00000000	pigs
St3gal3	-0.38	1.00000000	pigs
Pet100	0.07	1.00000000	pigs
Cbr4	-0.06	1.00000000	pigs
Impa1	0.01	1.00000000	pigs
Arpc2	0.42	1.00000000	pigs
Sema6d	-0.07	1.00000000	pigs
Taf6	0.02	1.00000000	pigs
Pik3c2a	-0.31	1.00000000	pigs
Nectin3	-0.46	1.00000000	pigs
Abcd3	-0.19	1.00000000	pigs
Has1	0.40	1.00000000	pigs
Zfat	-0.03	1.00000000	pigs
Pycr1	-0.05	1.00000000	pigs
Taok2	0.08	1.00000000	pigs
Btbd3	-0.23	1.00000000	pigs
Fam189a1	0.03	1.00000000	pigs
Washc1	0.04	1.00000000	pigs
Disc1	-0.39	1.00000000	pigs
Clhc1	-0.04	1.00000000	pigs
Timm10	0.15	1.00000000	pigs
Strip1	-0.05	1.00000000	pigs
Dctn4	-0.06	1.00000000	pigs
BC003965	-0.02	1.00000000	pigs
L3mbtl3	-0.10	1.00000000	pigs
Dact2	-0.25	1.00000000	pigs
Adamtsl5	-0.12	1.00000000	pigs
Abi3bp	0.32	1.00000000	pigs
Ubr5	0.25	1.00000000	pigs
Flii	0.05	1.00000000	pigs
Has2	-0.36	1.00000000	pigs
Arhgef3	0.09	1.00000000	pigs
Ino80	-0.34	1.00000000	pigs
Sesn3	-0.07	1.00000000	pigs
Usp33	-0.10	1.00000000	pigs
Kpna6	-0.06	1.00000000	pigs
Vim	-0.10	1.00000000	pigs
Smg1	-0.11	1.00000000	pigs

Itgb8	-0.36	1.00000000	pigs
Abhd17a	0.11	1.00000000	pigs
Ssr2	0.25	1.00000000	pigs
Ccdc12	-0.25	1.00000000	pigs
Ttll7	-0.31	1.00000000	pigs
Polr3k	0.00	1.00000000	pigs
Lrp12	-0.23	1.00000000	pigs
Cyb5a	-0.04	1.00000000	pigs
Yipf2	0.03	1.00000000	pigs
Nxpe3	0.01	1.00000000	pigs
Fam131a	0.11	1.00000000	pigs
Kat5	-0.11	1.00000000	pigs
Rmnd5a	0.06	1.00000000	pigs
Zfp771	-0.06	1.00000000	pigs
Focad	-0.35	1.00000000	pigs
Necap1	0.12	1.00000000	pigs
Cep68	-0.04	1.00000000	pigs
Fam174b	-0.05	1.00000000	pigs
Adk	-0.46	1.00000000	pigs
Aars	-0.08	1.00000000	pigs
Hyou1	-0.32	1.00000000	pigs
Stxbp6	-0.30	1.00000000	pigs
Pacsin2	0.21	1.00000000	pigs
Laptm4a	0.14	1.00000000	pigs
Vti1a	-0.29	1.00000000	pigs
Urgcp	-0.05	1.00000000	pigs
Ogfrl1	-0.02	1.00000000	pigs
Cd151	-0.31	1.00000000	pigs
Atf1	0.03	1.00000000	pigs
Cd2ap	-0.02	1.00000000	pigs
Atf7ip	-0.42	1.00000000	pigs
Rab3gap1	-0.12	1.00000000	pigs
Frs2	-0.44	1.00000000	pigs
Inpp1	-0.06	1.00000000	pigs
Shq1	-0.10	1.00000000	pigs
Sars	-0.02	1.00000000	pigs
Tmem203	0.00	1.00000000	pigs
Dsn1	0.01	1.00000000	pigs
Osbpl9	-0.44	1.00000000	pigs
Elf2	0.24	1.00000000	pigs
Cap2	-0.36	1.00000000	pigs
Unc5c	-0.59	1.00000000	pigs
Dop1a	-0.40	1.00000000	pigs
Nexn	0.04	1.00000000	pigs
Stxbp3	0.02	1.00000000	pigs
Fyco1	-0.28	1.00000000	pigs

Rab11fip5	-0.03	1.00000000	pigs
Gtpbp2	-0.03	1.00000000	pigs
Thap2	-0.01	1.00000000	pigs
Emc9	-0.03	1.00000000	pigs
Slc9a3r1	0.09	1.00000000	pigs
9930104L06Rik	-0.01	1.00000000	pigs
Leprotl1	0.13	1.00000000	pigs
Tmtc1	-0.35	1.00000000	pigs
Derpc	-0.03	1.00000000	pigs
Chchd1	-0.09	1.00000000	pigs
Topbp1	-0.29	1.00000000	pigs
Hnrnpu	0.24	1.00000000	pigs
Cog6	-0.18	1.00000000	pigs
Lemd3	-0.10	1.00000000	pigs
Sun2	-0.34	1.00000000	pigs
Brd1	-0.11	1.00000000	pigs
Nadk2	-0.04	1.00000000	pigs
Acer3	-0.17	1.00000000	pigs
Tlr4	-0.01	1.00000000	pigs
Tc2n	0.04	1.00000000	pigs
Chtop	0.07	1.00000000	pigs
Zbtb41	0.01	1.00000000	pigs
Traf3ip1	0.01	1.00000000	pigs
Pacsin3	0.20	1.00000000	pigs
Slc20a1	0.13	1.00000000	pigs
Tcp1	0.32	1.00000000	pigs
Ptbp3	0.22	1.00000000	pigs
Dync2i2	-0.08	1.00000000	pigs
1810030O07Rik	-0.11	1.00000000	pigs
Usp8	-0.09	1.00000000	pigs
Tmem248	-0.21	1.00000000	pigs
Vps37b	0.39	1.00000000	pigs
Psma4	0.07	1.00000000	pigs
Bicra	-0.07	1.00000000	pigs
B4galt4	-0.02	1.00000000	pigs
Hmg20b	-0.10	1.00000000	pigs
Bmt2	-0.03	1.00000000	pigs
Fam177a	0.00	1.00000000	pigs
Zcrb1	-0.08	1.00000000	pigs
Igfbp4	0.03	1.00000000	pigs
Ikbip	0.00	1.00000000	pigs
Usp28	-0.01	1.00000000	pigs
Prdm15	-0.02	1.00000000	pigs
Fnta	0.06	1.00000000	pigs
Slf1	0.07	1.00000000	pigs
Drg1	0.02	1.00000000	pigs

Nck2	0.23	1.00000000	pigs
Hspa4	0.02	1.00000000	pigs
Gucd1	-0.12	1.00000000	pigs
Bcorl1	-0.05	1.00000000	pigs
Tmem131	-0.29	1.00000000	pigs
Chd6	-0.23	1.00000000	pigs
Atad2b	-0.38	1.00000000	pigs
Galnt1	-0.08	1.00000000	pigs
BC004004	-0.04	1.00000000	pigs
Vamp4	-0.30	1.00000000	pigs
Fam20c	0.03	1.00000000	pigs
Ccdc91	0.01	1.00000000	pigs
Herc1	-0.50	1.00000000	pigs
Apoo	-0.01	1.00000000	pigs
Akr7a5	0.02	1.00000000	pigs
Wdr91	-0.02	1.00000000	pigs
Kcnb1	-0.08	1.00000000	pigs
Ctdsp1	-0.33	1.00000000	pigs
Serpinb9	0.08	1.00000000	pigs
Chordc1	-0.08	1.00000000	pigs
Elp2	-0.05	1.00000000	pigs
Eaf1	0.06	1.00000000	pigs
Gpx1	0.31	1.00000000	pigs
Os9	-0.09	1.00000000	pigs
Dock6	-0.11	1.00000000	pigs
Gnb1	0.22	1.00000000	pigs
Hsd3b7	-0.07	1.00000000	pigs
Stag2	-0.18	1.00000000	pigs
Ccnd1	-0.15	1.00000000	pigs
Dpp7	-0.14	1.00000000	pigs
Pias2	-0.02	1.00000000	pigs
Zfp277	0.00	1.00000000	pigs
Abhd2	-0.54	1.00000000	pigs
Cnst	-0.16	1.00000000	pigs
Tet2	0.00	1.00000000	pigs
Etv5	-0.05	1.00000000	pigs
Cacna1h	-0.09	1.00000000	pigs
Tnfrsf1a	-0.10	1.00000000	pigs
Traf3ip2	0.05	1.00000000	pigs
Taf1c	-0.07	1.00000000	pigs
Phf6	-0.03	1.00000000	pigs
Dnah12	-0.63	1.00000000	pigs
Tmem41b	0.08	1.00000000	pigs
Dhrs3	-0.12	1.00000000	pigs
Tmtc3	-0.16	1.00000000	pigs
Brf1	-0.06	1.00000000	pigs

Il1rap	0.18	1.00000000	pigs
Zfp369	-0.05	1.00000000	pigs
Ctnnd1	-0.36	1.00000000	pigs
Cul2	-0.11	1.00000000	pigs
Errfi1	0.00	1.00000000	pigs
Ints3	-0.04	1.00000000	pigs
Ostf1	-0.38	1.00000000	pigs
Sparc	0.22	1.00000000	pigs
Unk	-0.09	1.00000000	pigs
Tmem260	-0.03	1.00000000	pigs
Pik3ip1	0.05	1.00000000	pigs
Septin2	-0.12	1.00000000	pigs
Emc4	0.00	1.00000000	pigs
Arl1	0.12	1.00000000	pigs
Trps1	-0.16	1.00000000	pigs
Txlng	-0.04	1.00000000	pigs
Cass4	-0.31	1.00000000	pigs
Golm2	0.02	1.00000000	pigs
Srp54a	-0.04	1.00000000	pigs
Nxt2	0.04	1.00000000	pigs
Unc50	-0.03	1.00000000	pigs
Cc2d1a	0.04	1.00000000	pigs
Grb10	0.04	1.00000000	pigs
Sun1	0.08	1.00000000	pigs
Eif3a	0.15	1.00000000	pigs
Camkk2	-0.33	1.00000000	pigs
Cybc1	0.00	1.00000000	pigs
P3h4	-0.15	1.00000000	pigs
Rxrb	-0.09	1.00000000	pigs
Zfr	0.20	1.00000000	pigs
Pak3	-0.15	1.00000000	pigs
Tnip2	0.07	1.00000000	pigs
N4bp2	-0.34	1.00000000	pigs
Atg2b	-0.02	1.00000000	pigs
Lman2l	0.09	1.00000000	pigs
Hdac1	-0.09	1.00000000	pigs
Bckdhb	-0.48	1.00000000	pigs
Tor1aip2	-0.10	1.00000000	pigs
Fam227b	-0.49	1.00000000	pigs
Pip5k1a	-0.08	1.00000000	pigs
Erc1	0.24	1.00000000	pigs
Nars2	-0.03	1.00000000	pigs
Rab40c	-0.08	1.00000000	pigs
Synrg	-0.24	1.00000000	pigs
Ddx46	-0.29	1.00000000	pigs
Acsl1	-0.31	1.00000000	pigs

Wdr74	0.19	1.00000000	pigs
Tnfaip2	0.04	1.00000000	pigs
Antxr2	0.21	1.00000000	pigs
Nsd1	-0.32	1.00000000	pigs
Zfp282	-0.12	1.00000000	pigs
Suds3	-0.07	1.00000000	pigs
Tcta	-0.11	1.00000000	pigs
Snrpd2	-0.04	1.00000000	pigs
Snrnp200	0.00	1.00000000	pigs
Fig4	-0.20	1.00000000	pigs
Galnt16	-0.25	1.00000000	pigs
Zfp428	-0.07	1.00000000	pigs
Drg2	0.04	1.00000000	pigs
Atg3	-0.02	1.00000000	pigs
Paqr8	-0.21	1.00000000	pigs
Tmem214	-0.25	1.00000000	pigs
Acads	-0.10	1.00000000	pigs
Tmem97	-0.06	1.00000000	pigs
Rnf4	-0.04	1.00000000	pigs
Pias1	-0.11	1.00000000	pigs
Tbc1d23	-0.06	1.00000000	pigs
Antkmt	0.11	1.00000000	pigs
Zfp606	-0.05	1.00000000	pigs
Pdpr	-0.04	1.00000000	pigs
Pcyt1a	-0.09	1.00000000	pigs
Akap7	-0.21	1.00000000	pigs
Tfam	-0.05	1.00000000	pigs
Irak1	0.02	1.00000000	pigs
Crybg3	0.11	1.00000000	pigs
Ak1	0.01	1.00000000	pigs
Etv6	0.13	1.00000000	pigs
Fam193a	0.03	1.00000000	pigs
Syvn1	-0.05	1.00000000	pigs
Fmnl1	0.10	1.00000000	pigs
Opa1	-0.21	1.00000000	pigs
Txnrd3	-0.01	1.00000000	pigs
Ppp1ca	0.03	1.00000000	pigs
Exoc8	-0.05	1.00000000	pigs
Pdp1	-0.04	1.00000000	pigs
1700025G04Rik	0.17	1.00000000	pigs
Noa1	-0.05	1.00000000	pigs
Apmap	-0.06	1.00000000	pigs
Ei24	0.14	1.00000000	pigs
Gatd1	0.27	1.00000000	pigs
Sft2d2	0.00	1.00000000	pigs
Kdm5b	-0.03	1.00000000	pigs

Rb1	-0.33	1.00000000	pigs
Ncam1	-0.33	1.00000000	pigs
Triobp	-0.25	1.00000000	pigs
Nkiras1	0.09	1.00000000	pigs
Pus7	-0.01	1.00000000	pigs
Flna	0.05	1.00000000	pigs
Casp8	-0.16	1.00000000	pigs
Fundc2	-0.02	1.00000000	pigs
Tmem132c	-0.17	1.00000000	pigs
Slc9a8	-0.02	1.00000000	pigs
Ankrd54	-0.08	1.00000000	pigs
Etnk1	-0.20	1.00000000	pigs
Psme3	0.05	1.00000000	pigs
Ncoa6	-0.11	1.00000000	pigs
Nup50	0.09	1.00000000	pigs
Id4	-0.07	1.00000000	pigs
Ppp2r1a	0.25	1.00000000	pigs
Grsf1	-0.04	1.00000000	pigs
Spg11	-0.08	1.00000000	pigs
Syne3	0.09	1.00000000	pigs
Ralgapb	-0.20	1.00000000	pigs
Taok1	-0.28	1.00000000	pigs
Angpt1	-0.40	1.00000000	pigs
Itm2b	0.12	1.00000000	pigs
Rasa1	-0.20	1.00000000	pigs
Txndc12	0.04	1.00000000	pigs
Ciz1	0.06	1.00000000	pigs
Ints1	-0.04	1.00000000	pigs
Golga4	0.21	1.00000000	pigs
Ccdc97	-0.06	1.00000000	pigs
Rnf150	-0.33	1.00000000	pigs
Ppp1cc	0.11	1.00000000	pigs
Gk5	-0.12	1.00000000	pigs
Yipf3	-0.03	1.00000000	pigs
Jam3	-0.08	1.00000000	pigs
Brms1l	-0.03	1.00000000	pigs
Synj1	-0.10	1.00000000	pigs
Hif1an	-0.04	1.00000000	pigs
Zmpste24	0.04	1.00000000	pigs
Pygo1	-0.08	1.00000000	pigs
Exoc1	-0.05	1.00000000	pigs
Nfatc4	-0.02	1.00000000	pigs
Ss18l2	0.06	1.00000000	pigs
Ktn1	-0.17	1.00000000	pigs
Qars	-0.09	1.00000000	pigs
Rchy1	-0.03	1.00000000	pigs

Ankle2	-0.06	1.00000000	pigs
Zhx1	-0.08	1.00000000	pigs
Arid4a	-0.02	1.00000000	pigs
Gsr	-0.04	1.00000000	pigs
Zfp407	-0.36	1.00000000	pigs
Gimap4	0.07	1.00000000	pigs
Golt1b	-0.03	1.00000000	pigs
Cpsf7	0.01	1.00000000	pigs
Pknox1	-0.13	1.00000000	pigs
Ccdc43	-0.15	1.00000000	pigs
Pdpk1	0.06	1.00000000	pigs
C1rl	-0.05	1.00000000	pigs
Foxn2	0.13	1.00000000	pigs
Sms	0.02	1.00000000	pigs
Nsrp1	-0.30	1.00000000	pigs
Zzz3	-0.39	1.00000000	pigs
Ift80	-0.14	1.00000000	pigs
Rock2	-0.21	1.00000000	pigs
Lzts2	-0.08	1.00000000	pigs
Lox	-0.16	1.00000000	pigs
Plekhm1	0.01	1.00000000	pigs
Nhs	-0.39	1.00000000	pigs
Setdb1	-0.01	1.00000000	pigs
Bag1	0.06	1.00000000	pigs
Atf4	-0.02	1.00000000	pigs
Rras	-0.25	1.00000000	pigs
Csgalnact2	-0.08	1.00000000	pigs
Dph1	0.11	1.00000000	pigs
Slc25a33	0.00	1.00000000	pigs
Exoc6	-0.27	1.00000000	pigs
Vstm4	0.01	1.00000000	pigs
Ubr3	-0.28	1.00000000	pigs
Rbpms	-0.24	1.00000000	pigs
R3hdm1	-0.26	1.00000000	pigs
Rspry1	0.07	1.00000000	pigs
Anxa6	-0.24	1.00000000	pigs
Cfdp1	-0.11	1.00000000	pigs
Vat1	-0.08	1.00000000	pigs
Chst11	-0.06	1.00000000	pigs
Pank3	0.07	1.00000000	pigs
Plscr3	-0.11	1.00000000	pigs
Gpr161	-0.29	1.00000000	pigs
Prpf3	-0.03	1.00000000	pigs
Cep126	-0.20	1.00000000	pigs
Kras	-0.26	1.00000000	pigs
Arsb	0.03	1.00000000	pigs

Ppig	0.05	1.00000000	pigs
Snx30	-0.13	1.00000000	pigs
Tmem242	-0.06	1.00000000	pigs
Prrc2a	-0.10	1.00000000	pigs
Zcchc9	-0.10	1.00000000	pigs
Iffo2	-0.08	1.00000000	pigs
Efs	-0.02	1.00000000	pigs
Zmym2	-0.01	1.00000000	pigs
Extl2	-0.01	1.00000000	pigs
Herpud2	0.06	1.00000000	pigs
Cttnbp2nl	-0.09	1.00000000	pigs
Dtna	-0.07	1.00000000	pigs
Sap30l	-0.08	1.00000000	pigs
Hivep1	0.07	1.00000000	pigs
Tmem154	-0.37	1.00000000	pigs
Psme3ip1	0.21	1.00000000	pigs
Ncoa5	-0.24	1.00000000	pigs
Esco1	-0.01	1.00000000	pigs
Usp48	-0.10	1.00000000	pigs
Dlat	-0.05	1.00000000	pigs
Trip11	-0.35	1.00000000	pigs
Uspl1	0.03	1.00000000	pigs
Tab2	-0.17	1.00000000	pigs
Tk2	-0.07	1.00000000	pigs
Cry1	0.14	1.00000000	pigs
Rsrc1	-0.35	1.00000000	pigs
Tiprl	0.12	1.00000000	pigs
Tbc1d10a	-0.02	1.00000000	pigs
Tprkb	0.02	1.00000000	pigs
Nup153	0.06	1.00000000	pigs
Agpat2	0.01	1.00000000	pigs
Arl8b	0.12	1.00000000	pigs
Cep89	-0.41	1.00000000	pigs
Cdc14a	0.09	1.00000000	pigs
Ccdc112	-0.08	1.00000000	pigs
Ubac1	0.19	1.00000000	pigs
Cmss1	-0.27	1.00000000	pigs
Psmc1	-0.10	1.00000000	pigs
Txnl1	0.17	1.00000000	pigs
Yaf2	-0.12	1.00000000	pigs
Cfap46	-0.06	1.00000000	pigs
Arih2	0.08	1.00000000	pigs
Ptch1	-0.50	1.00000000	pigs
Acap2	0.25	1.00000000	pigs
Trim24	-0.11	1.00000000	pigs
P4ha2	-0.01	1.00000000	pigs

AI413582	0.04	1.00000000	pigs
Junb	-0.23	1.00000000	pigs
Lat2	0.07	1.00000000	pigs
Slc48a1	-0.03	1.00000000	pigs
Pdlim4	-0.08	1.00000000	pigs
E130308A19Rik	-0.27	1.00000000	pigs
Dennd2a	-0.26	1.00000000	pigs
Bckdk	0.10	1.00000000	pigs
Taco1	-0.05	1.00000000	pigs
Vgll4	-0.12	1.00000000	pigs
Zfp830	-0.05	1.00000000	pigs
Cpne1	0.04	1.00000000	pigs
Rubcn	-0.09	1.00000000	pigs
Syk	0.12	1.00000000	pigs
Spindoc	0.05	1.00000000	pigs
Vrk2	-0.07	1.00000000	pigs
Hlx	-0.04	1.00000000	pigs
Fkbp14	-0.19	1.00000000	pigs
Hexim1	0.08	1.00000000	pigs
Spopl	0.00	1.00000000	pigs
Fez2	-0.06	1.00000000	pigs
Arxes2	0.00	1.00000000	pigs
Strn	-0.10	1.00000000	pigs
Ppp1r18	0.06	1.00000000	pigs
Rusc2	-0.23	1.00000000	pigs
Utp11	0.22	1.00000000	pigs
Wdr82	-0.21	1.00000000	pigs
Rcan1	-0.28	1.00000000	pigs
Pam16	-0.01	1.00000000	pigs
Uck2	-0.03	1.00000000	pigs
Tubb6	-0.32	1.00000000	pigs
Ppp6r3	0.03	1.00000000	pigs
Npc1	-0.09	1.00000000	pigs
Wtip	0.03	1.00000000	pigs
Tfpt	-0.04	1.00000000	pigs
Ap1ar	0.31	1.00000000	pigs
Ppp3cb	-0.09	1.00000000	pigs
Srsf4	-0.26	1.00000000	pigs
Bclaf1	-0.11	1.00000000	pigs
Dpy30	-0.02	1.00000000	pigs
Rhoj	-0.07	1.00000000	pigs
Qtrt1	-0.12	1.00000000	pigs
Eml1	-0.40	1.00000000	pigs
Nup188	0.07	1.00000000	pigs
Rrn3	-0.02	1.00000000	pigs
Cables1	-0.11	1.00000000	pigs

Evi5	-0.33	1.00000000	pigs
Fam98b	-0.08	1.00000000	pigs
Iws1	-0.04	1.00000000	pigs
Kyat3	-0.12	1.00000000	pigs
Ptprf	-0.08	1.00000000	pigs
Atmin	-0.06	1.00000000	pigs
Spns1	0.19	1.00000000	pigs
Surf1	0.05	1.00000000	pigs
Rc3h1	-0.42	1.00000000	pigs
Tlk1	0.02	1.00000000	pigs
Coq10b	0.12	1.00000000	pigs
Tgfbrap1	-0.14	1.00000000	pigs
Snrpe	0.42	1.00000000	pigs
Ctr9	-0.24	1.00000000	pigs
Slc4a1ap	-0.16	1.00000000	pigs
Olfml2b	0.02	1.00000000	pigs
Nt5e	-0.13	1.00000000	pigs
Cerk	-0.02	1.00000000	pigs
Lrrn2	-0.09	1.00000000	pigs
Fubp3	-0.12	1.00000000	pigs
Efna5	-0.31	1.00000000	pigs
Il13ra1	0.18	1.00000000	pigs
Cmc1	0.10	1.00000000	pigs
Ntn4	-0.17	1.00000000	pigs
Tgs1	-0.19	1.00000000	pigs
Tshz1	-0.33	1.00000000	pigs
Col6a3	-0.04	1.00000000	pigs
Thsd4	0.15	1.00000000	pigs
Cert1	0.01	1.00000000	pigs
Dnmt3a	-0.02	1.00000000	pigs
Rhob	0.23	1.00000000	pigs
Uqcrfs1	0.02	1.00000000	pigs
Commd10	-0.37	1.00000000	pigs
Srsf7	-0.18	1.00000000	pigs
Ncf1	0.06	1.00000000	pigs
Sacm1l	-0.28	1.00000000	pigs
Canx	-0.03	1.00000000	pigs
Pts	0.00	1.00000000	pigs
AI597479	-0.07	1.00000000	pigs
Pld5	-0.60	1.00000000	pigs
Aldh6a1	-0.03	1.00000000	pigs
Etfdh	-0.20	1.00000000	pigs
Scyl2	-0.06	1.00000000	pigs
Stradb	-0.04	1.00000000	pigs
Large1	0.13	1.00000000	pigs
Sin3b	-0.02	1.00000000	pigs

Fmo1	-0.16	1.00000000	pigs
Phax	0.11	1.00000000	pigs
Zbtb8os	0.09	1.00000000	pigs
Cd276	-0.27	1.00000000	pigs
Tenm1	-0.14	1.00000000	pigs
Dnajc2	-0.12	1.00000000	pigs
Cdc42bpb	-0.03	1.00000000	pigs
Slc35b1	0.19	1.00000000	pigs
Cep120	-0.46	1.00000000	pigs
Zdhhc4	-0.22	1.00000000	pigs
Cacybp	-0.10	1.00000000	pigs
Tada2a	-0.10	1.00000000	pigs
Ddx21	-0.20	1.00000000	pigs
Itm2c	-0.08	1.00000000	pigs
Cox4i2	0.27	1.00000000	pigs
Larp1b	-0.11	1.00000000	pigs
Rnf126	-0.03	1.00000000	pigs
Gpat3	0.07	1.00000000	pigs
Zfp438	-0.50	1.00000000	pigs
Ptpn9	0.04	1.00000000	pigs
Per2	-0.07	1.00000000	pigs
Tanc2	-0.17	1.00000000	pigs
Use1	-0.09	1.00000000	pigs
Ppp1r15a	-0.06	1.00000000	pigs
Cux1	-0.22	1.00000000	pigs
Selenot	0.08	1.00000000	pigs
Pls3	-0.06	1.00000000	pigs
Col6a6	-0.39	1.00000000	pigs
Zmym6	-0.08	1.00000000	pigs
Rrbp1	-0.07	1.00000000	pigs
Fyttd1	-0.06	1.00000000	pigs
Ccser2	-0.05	1.00000000	pigs
Zfp395	-0.06	1.00000000	pigs
Gigyf2	-0.34	1.00000000	pigs
Aco2	-0.09	1.00000000	pigs
Plk3	0.14	1.00000000	pigs
Col1a2	0.14	1.00000000	pigs
Dhx36	-0.22	1.00000000	pigs
Cpt1a	-0.10	1.00000000	pigs
BC005537	0.12	1.00000000	pigs
Eftud2	0.04	1.00000000	pigs
Bub3	0.11	1.00000000	pigs
Pik3r4	-0.17	1.00000000	pigs
Spidr	0.15	1.00000000	pigs
Cep170	-0.26	1.00000000	pigs
Scarf2	-0.08	1.00000000	pigs

Pkp4	-0.47	1.00000000	pigs
Anapc1	-0.15	1.00000000	pigs
Phc3	-0.40	1.00000000	pigs
Ccng2	-0.04	1.00000000	pigs
Cyria	-0.17	1.00000000	pigs
Gba	-0.14	1.00000000	pigs
Fkbp9	0.13	1.00000000	pigs
Hnrnpr	-0.20	1.00000000	pigs
Dcbld2	-0.04	1.00000000	pigs
Vdac3	0.03	1.00000000	pigs
Ubtd1	0.08	1.00000000	pigs
Cmtm4	-0.05	1.00000000	pigs
Gorab	-0.11	1.00000000	pigs
Qdpr	-0.28	1.00000000	pigs
Ipmk	-0.35	1.00000000	pigs
Dag1	-0.01	1.00000000	pigs
Ppic	0.18	1.00000000	pigs
Apex1	0.02	1.00000000	pigs
Dync2h1	-0.43	1.00000000	pigs
Enpep	-0.04	1.00000000	pigs
Dnajc8	-0.28	1.00000000	pigs
Pofut1	-0.02	1.00000000	pigs
Cep295	-0.09	1.00000000	pigs
Nop10	0.24	1.00000000	pigs
Lpp	-0.19	1.00000000	pigs
Sec24a	-0.18	1.00000000	pigs
Psmd13	-0.08	1.00000000	pigs
E2f4	0.13	1.00000000	pigs
Lsm14b	-0.04	1.00000000	pigs
Surf4	0.14	1.00000000	pigs
Slc20a2	-0.01	1.00000000	pigs
Ube2j2	-0.18	1.00000000	pigs
Ubap2l	-0.03	1.00000000	pigs
Usp34	-0.15	1.00000000	pigs
Ndufv2	0.08	1.00000000	pigs
Ndufa1	-0.02	1.00000000	pigs
Yars	0.07	1.00000000	pigs
Slu7	-0.01	1.00000000	pigs
Laptm5	0.15	1.00000000	pigs
Psen1	-0.15	1.00000000	pigs
Sertad2	-0.12	1.00000000	pigs
Erh	0.45	1.00000000	pigs
Wrn	-0.18	1.00000000	pigs
Gar1	0.03	1.00000000	pigs
Nedd4	-0.08	1.00000000	pigs
Sh3glb1	-0.08	1.00000000	pigs

Vsig10	-0.05	1.00000000	pigs
Vps13c	-0.13	1.00000000	pigs
Atg2a	-0.02	1.00000000	pigs
Zcchc17	-0.07	1.00000000	pigs
Tmem241	-0.04	1.00000000	pigs
Itga8	-0.15	1.00000000	pigs
Lasp1	0.16	1.00000000	pigs
Hunk	-0.05	1.00000000	pigs
Tpp2	-0.03	1.00000000	pigs
Pja1	-0.04	1.00000000	pigs
Zc4h2	-0.09	1.00000000	pigs
Ahcyl2	0.06	1.00000000	pigs
Robo2	-0.21	1.00000000	pigs
Fgl2	-0.06	1.00000000	pigs
St6gal1	-0.19	1.00000000	pigs
Srrt	-0.07	1.00000000	pigs
Sec23b	0.07	1.00000000	pigs
Ung	0.01	1.00000000	pigs
Lamtor1	-0.01	1.00000000	pigs
Cdkn2aip	0.17	1.00000000	pigs
Lmbr1l	-0.06	1.00000000	pigs
Ufsp2	0.07	1.00000000	pigs
Ptp4a2	-0.14	1.00000000	pigs
Kpna1	-0.03	1.00000000	pigs
Itga9	-0.37	1.00000000	pigs
Alkbh5	0.12	1.00000000	pigs
Cad	-0.07	1.00000000	pigs
Ikzf1	0.31	1.00000000	pigs
Isoc1	0.06	1.00000000	pigs
Ubl7	-0.17	1.00000000	pigs
Plod2	-0.14	1.00000000	pigs
Ube3a	-0.26	1.00000000	pigs
Cdk16	0.01	1.00000000	pigs
Vapa	-0.10	1.00000000	pigs
Spi1	0.36	1.00000000	pigs
Cbfb	-0.03	1.00000000	pigs
Serf2	0.16	1.00000000	pigs
Ttbk2	-0.41	1.00000000	pigs
Slc10a3	0.05	1.00000000	pigs
Ptcd3	-0.01	1.00000000	pigs
Timm8a2	-0.03	1.00000000	pigs
Dis3l2	-0.23	1.00000000	pigs
Ccdc122	-0.18	1.00000000	pigs
Vamp5	-0.03	1.00000000	pigs
Ap4m1	0.03	1.00000000	pigs
Nifk	-0.06	1.00000000	pigs

Oxct1	-0.18	1.00000000	pigs
Sbds	0.04	1.00000000	pigs
Spryd7	0.04	1.00000000	pigs
Gfod1	-0.01	1.00000000	pigs
Spry2	0.11	1.00000000	pigs
Ube2q1	-0.01	1.00000000	pigs
Emc7	-0.06	1.00000000	pigs
Klhl13	-0.26	1.00000000	pigs
Rbbp8	-0.17	1.00000000	pigs
Rbm8a2	0.31	1.00000000	pigs
Stxbp4	-0.39	1.00000000	pigs
Cyb5b	-0.07	1.00000000	pigs
H1f4	0.23	1.00000000	pigs
Gorasp2	-0.30	1.00000000	pigs
Cndp2	-0.04	1.00000000	pigs
Hnrnpll	-0.14	1.00000000	pigs
Gsap	0.22	1.00000000	pigs
Tpk1	0.10	1.00000000	pigs
Srp68	-0.05	1.00000000	pigs
Polr3a	0.00	1.00000000	pigs
Tgfbi	-0.11	1.00000000	pigs
Ankhd1	0.02	1.00000000	pigs
Camk1d	-0.33	1.00000000	pigs
Epb41l5	0.02	1.00000000	pigs
Agtr1a	-0.23	1.00000000	pigs
Lactb	0.18	1.00000000	pigs
Irak1bp1	-0.09	1.00000000	pigs
Was	0.11	1.00000000	pigs
Hs1bp3	0.06	1.00000000	pigs
Rbbp7	0.07	1.00000000	pigs
Ogdh	-0.01	1.00000000	pigs
Eif4e2	0.07	1.00000000	pigs
Heatr5b	-0.18	1.00000000	pigs
Ubap2	0.15	1.00000000	pigs
Puf60	-0.01	1.00000000	pigs
Tbc1d31	-0.31	1.00000000	pigs
Rtf1	-0.34	1.00000000	pigs
Casd1	-0.12	1.00000000	pigs
Cadps2	-0.11	1.00000000	pigs
Trip6	-0.06	1.00000000	pigs
Kdm7a	-0.13	1.00000000	pigs
Taf15	0.06	1.00000000	pigs
Spon2	-0.10	1.00000000	pigs
Pcmtd2	0.00	1.00000000	pigs
Arid2	-0.17	1.00000000	pigs
Got2	0.05	1.00000000	pigs

Wdfy2	0.00	1.00000000	pigs
Atxn10	-0.03	1.00000000	pigs
Ulk1	-0.05	1.00000000	pigs
Gnl3	0.01	1.00000000	pigs
Snrnp48	0.06	1.00000000	pigs
Exosc5	0.05	1.00000000	pigs
Fam168b	-0.11	1.00000000	pigs
Ddx42	-0.32	1.00000000	pigs
Ptpn1	0.34	1.00000000	pigs
Bap1	-0.08	1.00000000	pigs
Gcc2	-0.18	1.00000000	pigs
Yju2	-0.11	1.00000000	pigs
Akr1b3	0.29	1.00000000	pigs
Irs1	-0.06	1.00000000	pigs
Dync1i1	0.15	1.00000000	pigs
St3gal6	-0.12	1.00000000	pigs
Adrm1	0.13	1.00000000	pigs
Nphp1	-0.11	1.00000000	pigs
Nbea	-0.36	1.00000000	pigs
Crppa	-0.38	1.00000000	pigs
Acadm	0.07	1.00000000	pigs
Rtn4	-0.20	1.00000000	pigs
Ebna1bp2	-0.03	1.00000000	pigs
Kat8	-0.09	1.00000000	pigs
Heatr1	-0.09	1.00000000	pigs
Rfx1	-0.07	1.00000000	pigs
Pcyox1	0.05	1.00000000	pigs
Ppm1d	-0.10	1.00000000	pigs
Pik3cb	-0.35	1.00000000	pigs
Sike1	-0.05	1.00000000	pigs
Lrp4	0.05	1.00000000	pigs
Plekha8	-0.03	1.00000000	pigs
Abcf3	0.02	1.00000000	pigs
Clk1	0.12	1.00000000	pigs
Trerf1	-0.06	1.00000000	pigs
Fam199x	-0.08	1.00000000	pigs
Adal	-0.03	1.00000000	pigs
Cul5	-0.33	1.00000000	pigs
Znrf2	-0.11	1.00000000	pigs
Carmil1	-0.16	1.00000000	pigs
Stk39	-0.14	1.00000000	pigs
Rexo1	-0.08	1.00000000	pigs
Tmtc4	-0.13	1.00000000	pigs
Ampd2	-0.25	1.00000000	pigs
Rfx2	-0.31	1.00000000	pigs
Slc9a6	-0.12	1.00000000	pigs

Rnf169	-0.25	1.00000000	pigs
Ppil2	0.11	1.00000000	pigs
Srsf1	0.18	1.00000000	pigs
B4galt3	0.02	1.00000000	pigs
Aldh1a1	-0.27	1.00000000	pigs
Rab18	-0.04	1.00000000	pigs
Lrch2	-0.02	1.00000000	pigs
Cpt2	0.00	1.00000000	pigs
Wdr77	0.06	1.00000000	pigs
Sparcl1	0.23	1.00000000	pigs
Angel2	-0.07	1.00000000	pigs
Bmp2	-0.05	1.00000000	pigs
Pip5k1c	0.11	1.00000000	pigs
Cd3eap	0.03	1.00000000	pigs
Tpm4	0.21	1.00000000	pigs
Senp7	-0.33	1.00000000	pigs
Capn1	0.23	1.00000000	pigs
Smyd3	0.02	1.00000000	pigs
Tcf21	-0.05	1.00000000	pigs
Slc36a1	-0.10	1.00000000	pigs
Zfp410	0.00	1.00000000	pigs
Cep43	-0.08	1.00000000	pigs
Adgra3	-0.45	1.00000000	pigs
Phf5a	0.09	1.00000000	pigs
Prxl2a	-0.01	1.00000000	pigs
Ralgapa2	-0.48	1.00000000	pigs
Rpf1	0.05	1.00000000	pigs
Tmem134	-0.06	1.00000000	pigs
Tfcp2	-0.05	1.00000000	pigs
Rprd1a	-0.08	1.00000000	pigs
Hk1	-0.20	1.00000000	pigs
Csf2rb2	0.32	1.00000000	pigs
Tbcb	-0.13	1.00000000	pigs
Slc38a5	-0.12	1.00000000	pigs
Pnp	0.15	1.00000000	pigs
Dcbld1	-0.27	1.00000000	pigs
Piezo2	-0.33	1.00000000	pigs
Calcoco2	-0.07	1.00000000	pigs
Prdm2	0.08	1.00000000	pigs
Cyc1	0.15	1.00000000	pigs
Gpatch11	-0.22	1.00000000	pigs
Rap1a	-0.15	1.00000000	pigs
Pitpnm3	0.04	1.00000000	pigs
Las1l	-0.19	1.00000000	pigs
Trmt6	-0.07	1.00000000	pigs
Cep63	-0.18	1.00000000	pigs

B3gat3	-0.19	1.00000000	pigs
Arl13b	-0.05	1.00000000	pigs
Otub1	-0.08	1.00000000	pigs
Faap100	0.04	1.00000000	pigs
Snx7	-0.10	1.00000000	pigs
Pnkd	-0.08	1.00000000	pigs
Il17rd	-0.09	1.00000000	pigs
Arfrp1	-0.05	1.00000000	pigs
Asf1a	0.02	1.00000000	pigs
Tyk2	0.01	1.00000000	pigs
Odf2l	-0.12	1.00000000	pigs
Ddx54	-0.04	1.00000000	pigs
Nav3	-0.04	1.00000000	pigs
Abcc5	-0.02	1.00000000	pigs
Ttc23l	0.01	1.00000000	pigs
Plk2	0.01	1.00000000	pigs
Yipf4	-0.16	1.00000000	pigs
Ttc37	-0.13	1.00000000	pigs
H2az2	0.27	1.00000000	pigs
Serpinb8	-0.11	1.00000000	pigs
Gpsm1	0.01	1.00000000	pigs
Tpst2	0.10	1.00000000	pigs
Pex3	-0.10	1.00000000	pigs
Il16	0.07	1.00000000	pigs
Ddx3x	-0.08	1.00000000	pigs
Rad17	-0.07	1.00000000	pigs
Ap3b1	-0.26	1.00000000	pigs
Nceh1	-0.04	1.00000000	pigs
Pcif1	-0.10	1.00000000	pigs
Tbc1d8b	-0.09	1.00000000	pigs
Tipin	-0.28	1.00000000	pigs
Adam33	-0.14	1.00000000	pigs
Coq7	0.10	1.00000000	pigs
Pum1	-0.15	1.00000000	pigs
Parn	-0.05	1.00000000	pigs
Aldh18a1	0.00	1.00000000	pigs
Arhgap10	0.17	1.00000000	pigs
Gnpat	-0.10	1.00000000	pigs
Exoc4	0.12	1.00000000	pigs
Psd3	-0.23	1.00000000	pigs
Rprd2	-0.27	1.00000000	pigs
Pi4kb	-0.08	1.00000000	pigs
Rpgrip1l	-0.10	1.00000000	pigs
Glt8d2	0.00	1.00000000	pigs
Arcn1	-0.07	1.00000000	pigs
Fbxw11	-0.22	1.00000000	pigs

Chst10	0.08	1.00000000	pigs
Kifap3	-0.45	1.00000000	pigs
Kiz	-0.05	1.00000000	pigs
Faf2	0.01	1.00000000	pigs
Slc30a7	-0.07	1.00000000	pigs
Pura	-0.25	1.00000000	pigs
Rex1bd	0.06	1.00000000	pigs
Rnf20	-0.09	1.00000000	pigs
Pot1b	-0.14	1.00000000	pigs
Nfyb	0.03	1.00000000	pigs
Vegfd	-0.09	1.00000000	pigs
Ccdc59	-0.15	1.00000000	pigs
Ccdc80	0.11	1.00000000	pigs
Armt1	-0.06	1.00000000	pigs
Zfp879	-0.24	1.00000000	pigs
Tpgs1	-0.01	1.00000000	pigs
Ddrgk1	-0.17	1.00000000	pigs
Nfatc1	0.03	1.00000000	pigs
Zfp207	-0.02	1.00000000	pigs
Hira	-0.30	1.00000000	pigs
Asns	-0.02	1.00000000	pigs
Nupl2	-0.07	1.00000000	pigs
Slc25a21	-0.42	1.00000000	pigs
Srek1ip1	-0.12	1.00000000	pigs
Alg14	0.03	1.00000000	pigs
Reps2	-0.04	1.00000000	pigs
Gripap1	-0.04	1.00000000	pigs
Rev1	-0.17	1.00000000	pigs
Ska2	-0.16	1.00000000	pigs
Tatdn1	-0.02	1.00000000	pigs
Josd1	-0.04	1.00000000	pigs
Guk1	-0.02	1.00000000	pigs
Sgpl1	0.06	1.00000000	pigs
Foxs1	0.02	1.00000000	pigs
Klhl5	-0.01	1.00000000	pigs
Galc	-0.01	1.00000000	pigs
Eda	-0.45	1.00000000	pigs
Edrf1	0.02	1.00000000	pigs
Eng	-0.11	1.00000000	pigs
Nubpl	-0.16	1.00000000	pigs
3110040N11Rik	-0.24	1.00000000	pigs
Spsb3	0.05	1.00000000	pigs
Swap70	-0.09	1.00000000	pigs
Cdh11	0.22	1.00000000	pigs
Dnaja3	-0.03	1.00000000	pigs
Exoc7	-0.03	1.00000000	pigs

Ptrh2	0.04	1.00000000	pigs
Igsf10	0.12	1.00000000	pigs
Usp54	-0.01	1.00000000	pigs
Serping1	-0.14	1.00000000	pigs
Utp3	-0.10	1.00000000	pigs
Ifit3	-0.12	1.00000000	pigs
Aurkaip1	-0.24	1.00000000	pigs
Uqcr10	0.21	1.00000000	pigs
Sort1	0.01	1.00000000	pigs
Slc35a1	-0.05	1.00000000	pigs
Raf1	0.12	1.00000000	pigs
Blm	-0.10	1.00000000	pigs
Tesk2	-0.13	1.00000000	pigs
Pelp1	-0.08	1.00000000	pigs
Slc46a3	-0.09	1.00000000	pigs
Pprc1	-0.10	1.00000000	pigs
Upf1	-0.07	1.00000000	pigs
Noc3l	0.01	1.00000000	pigs
Zfp800	0.03	1.00000000	pigs
Kdm4c	-0.09	1.00000000	pigs
Fbxl14	-0.10	1.00000000	pigs
Lcp2	0.22	1.00000000	pigs
Dchs1	-0.15	1.00000000	pigs
Sel1l	0.00	1.00000000	pigs
Siva1	0.17	1.00000000	pigs
Lrpap1	0.18	1.00000000	pigs
Gse1	-0.24	1.00000000	pigs
Pfdn6	-0.02	1.00000000	pigs
Elmo2	-0.11	1.00000000	pigs
Plat	-0.16	1.00000000	pigs
Ube2m	0.02	1.00000000	pigs
Hdac6	-0.01	1.00000000	pigs
Psat1	-0.05	1.00000000	pigs
Fbxl18	-0.34	1.00000000	pigs
Sdc1	-0.08	1.00000000	pigs
Zdhhc6	-0.05	1.00000000	pigs
Acsf2	-0.11	1.00000000	pigs
Eif3g	0.22	1.00000000	pigs
Fads2	-0.26	1.00000000	pigs
Arhgef2	0.01	1.00000000	pigs
Tbc1d32	-0.34	1.00000000	pigs
Nktr	-0.33	1.00000000	pigs
Inpp5b	-0.08	1.00000000	pigs
Paip1	0.07	1.00000000	pigs
Tmem185a	0.00	1.00000000	pigs
Srp72	-0.10	1.00000000	pigs

Npat	0.05	1.00000000	pigs
Dhx16	0.00	1.00000000	pigs
Primpol	-0.13	1.00000000	pigs
Hdac7	-0.07	1.00000000	pigs
Pdlim7	-0.09	1.00000000	pigs
Capza2	0.17	1.00000000	pigs
Phactr1	-0.08	1.00000000	pigs
Fbxw4	-0.04	1.00000000	pigs
Pde6d	-0.15	1.00000000	pigs
Lsmem1	-0.28	1.00000000	pigs
Tubgcp3	-0.06	1.00000000	pigs
Akna	-0.05	1.00000000	pigs
Cdk10	-0.02	1.00000000	pigs
Txnip	0.22	1.00000000	pigs
Cyp7b1	0.17	1.00000000	pigs
Rlbp1	-0.13	1.00000000	pigs
Chrac1	-0.10	1.00000000	pigs
Cadm2	-0.13	1.00000000	pigs
Zkscan1	-0.03	1.00000000	pigs
Ptgfrn	-0.15	1.00000000	pigs
Foxn3	-0.17	1.00000000	pigs
Usp46	-0.07	1.00000000	pigs
Thbs3	-0.13	1.00000000	pigs
Ddit4	0.04	1.00000000	pigs
Gadd45g	0.20	1.00000000	pigs
Nt5c3	-0.02	1.00000000	pigs
Aopep	-0.31	1.00000000	pigs
Gtpbp10	-0.11	1.00000000	pigs
Nfrkb	-0.18	1.00000000	pigs
Sec14l1	0.08	1.00000000	pigs
Lamtor3	-0.03	1.00000000	pigs
Nkapd1	-0.16	1.00000000	pigs
Lypla1	-0.01	1.00000000	pigs
Slc49a4	-0.14	1.00000000	pigs
Rbm42	0.00	1.00000000	pigs
Evc	-0.11	1.00000000	pigs
Tex10	0.01	1.00000000	pigs
Gadd45b	0.04	1.00000000	pigs
Naaa	0.17	1.00000000	pigs
Kremen1	-0.07	1.00000000	pigs
Slc25a13	-0.31	1.00000000	pigs
Elp3	-0.09	1.00000000	pigs
Insr	-0.49	1.00000000	pigs
Aagab	-0.20	1.00000000	pigs
Pbxip1	0.00	1.00000000	pigs
Gpank1	0.05	1.00000000	pigs

Pfkl	0.17	1.00000000	pigs
Fibp	0.00	1.00000000	pigs
Fgf18	-0.12	1.00000000	pigs
Dpy19l4	0.00	1.00000000	pigs
Orai3	-0.05	1.00000000	pigs
Chmp7	-0.11	1.00000000	pigs
Ehd2	-0.26	1.00000000	pigs
R3hdm4	-0.09	1.00000000	pigs
Gdap2	-0.30	1.00000000	pigs
Ist1	-0.08	1.00000000	pigs
Vps35	-0.11	1.00000000	pigs
Tcea2	-0.04	1.00000000	pigs
C2cd5	-0.14	1.00000000	pigs
Spag1	0.02	1.00000000	pigs
Efemp1	-0.07	1.00000000	pigs
Peg10	-0.15	1.00000000	pigs
Rheb	0.20	1.00000000	pigs
Rbis	-0.03	1.00000000	pigs
Tgfb2	-0.06	1.00000000	pigs
Cdc16	-0.11	1.00000000	pigs
Ophn1	-0.26	1.00000000	pigs
Tax1bp1	0.08	1.00000000	pigs
Coro1a	0.40	1.00000000	pigs
Ptpn23	-0.11	1.00000000	pigs
Fam117a	0.04	1.00000000	pigs
Upf2	-0.40	1.00000000	pigs
Nmt1	0.09	1.00000000	pigs
Cwf19l1	-0.18	1.00000000	pigs
Cilk1	-0.29	1.00000000	pigs
Serbp1	0.23	1.00000000	pigs
Prmt9	-0.07	1.00000000	pigs
Pcbp1	0.33	1.00000000	pigs
Proser1	0.02	1.00000000	pigs
Plekhj1	-0.02	1.00000000	pigs
Pum2	-0.18	1.00000000	pigs
Pygl	-0.24	1.00000000	pigs
Syt11	0.00	1.00000000	pigs
Zfp12	-0.04	1.00000000	pigs
Snx33	-0.10	1.00000000	pigs
Usp24	-0.24	1.00000000	pigs
Nosip	-0.17	1.00000000	pigs
Stam	-0.11	1.00000000	pigs
Kctd20	-0.25	1.00000000	pigs
Rubcnl	-0.27	1.00000000	pigs
Cnpy3	-0.12	1.00000000	pigs
Gas6	-0.10	1.00000000	pigs

Prim2	-0.03	1.00000000	pigs
Tbc1d5	-0.09	1.00000000	pigs
Zc3h8	0.13	1.00000000	pigs
Idh1	0.14	1.00000000	pigs
Srsf11	0.04	1.00000000	pigs
Fgfr1op2	-0.12	1.00000000	pigs
Tcaf1	-0.15	1.00000000	pigs
Cct7	0.02	1.00000000	pigs
Vps54	0.11	1.00000000	pigs
Ftsj1	0.08	1.00000000	pigs
Rab3il1	-0.19	1.00000000	pigs
Zfp36	-0.20	1.00000000	pigs
Cstf1	0.07	1.00000000	pigs
Iars	-0.15	1.00000000	pigs
Ccdc25	-0.09	1.00000000	pigs
Pld1	-0.39	1.00000000	pigs
Wnt2	-0.25	1.00000000	pigs
Snx13	-0.32	1.00000000	pigs
Adgrl1	-0.02	1.00000000	pigs
Prkab2	-0.04	1.00000000	pigs
Pds5a	-0.13	1.00000000	pigs
Eif3j2	-0.13	1.00000000	pigs
Arap1	0.01	1.00000000	pigs
Plagl1	-0.38	1.00000000	pigs
Wdr12	-0.09	1.00000000	pigs
Jcad	0.01	1.00000000	pigs
Taok3	-0.40	1.00000000	pigs
Dtx3l	0.06	1.00000000	pigs
Phf14	-0.45	1.00000000	pigs
Phykpl	0.00	1.00000000	pigs
Trp53bp1	-0.17	1.00000000	pigs
Wbp4	-0.04	1.00000000	pigs
Dazap1	0.19	1.00000000	pigs
Dcaf13	0.00	1.00000000	pigs
Arf6	0.21	1.00000000	pigs
Terf2	-0.09	1.00000000	pigs
Dnmbp	-0.39	1.00000000	pigs
Ankrd46	-0.18	1.00000000	pigs
Ulk4	-0.16	1.00000000	pigs
Slc35a5	0.07	1.00000000	pigs
Ago4	-0.11	1.00000000	pigs
Samd12	-0.30	1.00000000	pigs
Prkci	-0.06	1.00000000	pigs
Cav1	-0.35	1.00000000	pigs
Xpc	-0.18	1.00000000	pigs
Trmt1l	0.04	1.00000000	pigs

Sh3rf3	-0.04	1.00000000	pigs
Zfyve21	0.09	1.00000000	pigs
Yars2	0.02	1.00000000	pigs
Hnrnpd	-0.04	1.00000000	pigs
Cap1	0.06	1.00000000	pigs
Sesn1	-0.39	1.00000000	pigs
Cdc73	-0.12	1.00000000	pigs
Sf3b1	-0.02	1.00000000	pigs
Ano10	-0.45	1.00000000	pigs
Kidins220	-0.08	1.00000000	pigs
Crls1	0.06	1.00000000	pigs
Gprc5a	0.22	1.00000000	pigs
Emp2	-0.02	1.00000000	pigs
Azin1	-0.14	1.00000000	pigs
Adat1	-0.10	1.00000000	pigs
G2e3	-0.06	1.00000000	pigs
Dab2	-0.08	1.00000000	pigs
Garem1	-0.06	1.00000000	pigs
Cstf3	0.04	1.00000000	pigs
Agk	-0.10	1.00000000	pigs
Pdcd11	-0.09	1.00000000	pigs
Peak1	0.17	1.00000000	pigs
Picalm	-0.34	1.00000000	pigs
Sgsm1	-0.24	1.00000000	pigs
Cd48	0.15	1.00000000	pigs
Slc39a6	-0.09	1.00000000	pigs
Thap1	0.00	1.00000000	pigs
Rhoa	0.01	1.00000000	pigs
Tfip11	0.10	1.00000000	pigs
Tlk2	-0.25	1.00000000	pigs
Ostm1	0.08	1.00000000	pigs
Rtca	0.08	1.00000000	pigs
Sbf2	-0.17	1.00000000	pigs
Dpyd	-0.13	1.00000000	pigs
Camta2	0.12	1.00000000	pigs
Ep300	-0.02	1.00000000	pigs
Bmpr2	0.07	1.00000000	pigs
Slc25a26	-0.11	1.00000000	pigs
Crlf3	0.24	1.00000000	pigs
Ggcx	-0.01	1.00000000	pigs
Phtf2	-0.40	1.00000000	pigs
B230219D22Rik	-0.36	1.00000000	pigs
Tmem126b	-0.08	1.00000000	pigs
Inpp5f	-0.13	1.00000000	pigs
Dock1	-0.10	1.00000000	pigs
Smurf1	-0.03	1.00000000	pigs

Tssc4	-0.14	1.00000000	pigs
Lman1	0.08	1.00000000	pigs
Arl5a	-0.02	1.00000000	pigs
Ints14	-0.04	1.00000000	pigs
Kifbp	0.07	1.00000000	pigs
Odf2	-0.09	1.00000000	pigs
Frmd6	-0.26	1.00000000	pigs
Sae1	-0.04	1.00000000	pigs
Bag6	-0.09	1.00000000	pigs
Ccdc93	-0.10	1.00000000	pigs
Dok6	-0.14	1.00000000	pigs
Tbl2	-0.27	1.00000000	pigs
Ddx24	-0.10	1.00000000	pigs
Aqr	-0.15	1.00000000	pigs
Atp6v1d	0.03	1.00000000	pigs
Cdc42	0.09	1.00000000	pigs
Atp13a2	-0.16	1.00000000	pigs
Pop4	-0.12	1.00000000	pigs
Arid4b	0.17	1.00000000	pigs
Prkaa1	-0.10	1.00000000	pigs
Acad11	-0.23	1.00000000	pigs
Nfil3	0.14	1.00000000	pigs
Sephs1	-0.06	1.00000000	pigs
Dnajb1	0.06	1.00000000	pigs
Ppp2r5d	-0.08	1.00000000	pigs
Inppl1	-0.07	1.00000000	pigs
Furin	0.11	1.00000000	pigs
Klhl8	0.02	1.00000000	pigs
Donson	-0.10	1.00000000	pigs
Gabarapl1	-0.04	1.00000000	pigs
Sgk1	0.00	1.00000000	pigs
Ahr	-0.23	1.00000000	pigs
Adcy9	-0.23	1.00000000	pigs
Fbxo9	-0.09	1.00000000	pigs
Slc25a12	-0.31	1.00000000	pigs
Immp1l	0.07	1.00000000	pigs
Polr2c	0.06	1.00000000	pigs
Yeats4	-0.05	1.00000000	pigs
Wipi2	0.01	1.00000000	pigs
Rnf139	0.03	1.00000000	pigs
Ergic2	0.00	1.00000000	pigs
Nfatc3	-0.21	1.00000000	pigs
Usp38	-0.03	1.00000000	pigs
Ssbp3	-0.02	1.00000000	pigs
Rabl6	-0.09	1.00000000	pigs
Plppr1	-0.15	1.00000000	pigs

Tomm40	0.09	1.00000000	pigs
Tsn	-0.03	1.00000000	pigs
Dnm2	0.05	1.00000000	pigs
Stom	0.04	1.00000000	pigs
Serac1	-0.02	1.00000000	pigs
Gfpt2	0.29	1.00000000	pigs
Rnf10	-0.17	1.00000000	pigs
Hspa8	0.37	1.00000000	pigs
Slc25a39	-0.05	1.00000000	pigs
Ehd4	-0.31	1.00000000	pigs
Naaladl2	-0.14	1.00000000	pigs
Sucla2	0.07	1.00000000	pigs
Prrx1	-0.05	1.00000000	pigs
Erp44	0.18	1.00000000	pigs
Tmem164	-0.01	1.00000000	pigs
Fbxl5	-0.20	1.00000000	pigs
Chd7	0.08	1.00000000	pigs
Cav2	-0.12	1.00000000	pigs
Plekhf1	-0.22	1.00000000	pigs
Nr2c1	-0.13	1.00000000	pigs
Pus7l	-0.02	1.00000000	pigs
Snupn	-0.06	1.00000000	pigs
Dcp1a	-0.34	1.00000000	pigs
Hcfc1r1	-0.12	1.00000000	pigs
Tmem150a	-0.04	1.00000000	pigs
Ankrd13c	-0.30	1.00000000	pigs
Gdi1	-0.03	1.00000000	pigs
2810004N23Rik	-0.27	1.00000000	pigs
Arhgap31	0.01	1.00000000	pigs
Nabp1	0.03	1.00000000	pigs
Hspa13	0.04	1.00000000	pigs
Cpeb4	-0.07	1.00000000	pigs
Kansl3	0.13	1.00000000	pigs
Dpp3	-0.06	1.00000000	pigs
Cd248	-0.31	1.00000000	pigs
Bag3	-0.19	1.00000000	pigs
Phf21a	-0.18	1.00000000	pigs
Prpf6	-0.22	1.00000000	pigs
Fam111a	0.03	1.00000000	pigs
Prr14l	-0.23	1.00000000	pigs
Vps41	-0.16	1.00000000	pigs
Foxf1	0.02	1.00000000	pigs
Vcp	-0.03	1.00000000	pigs
Arhgap39	-0.10	1.00000000	pigs
Tnpo3	0.01	1.00000000	pigs
Hgs	-0.16	1.00000000	pigs

Polr3e	0.07	1.00000000	pigs
Lyst	-0.41	1.00000000	pigs
Adnp	-0.12	1.00000000	pigs
Ythdf2	0.10	1.00000000	pigs
Ncoa7	-0.09	1.00000000	pigs
Chd8	-0.21	1.00000000	pigs
Wdr41	-0.09	1.00000000	pigs
Lrrc41	-0.08	1.00000000	pigs
Bcr	-0.05	1.00000000	pigs
Aebp2	-0.18	1.00000000	pigs
Shoc2	-0.04	1.00000000	pigs
Ecpas	-0.16	1.00000000	pigs
Colgalt1	0.01	1.00000000	pigs
Atpaf1	-0.12	1.00000000	pigs
Adamts6	-0.15	1.00000000	pigs
B4galt5	0.24	1.00000000	pigs
Zfp260	0.01	1.00000000	pigs
Zbtb44	-0.28	1.00000000	pigs
Gtf3c1	-0.16	1.00000000	pigs
Rab35	-0.08	1.00000000	pigs
Ap4e1	-0.07	1.00000000	pigs
Prdm10	0.01	1.00000000	pigs
Htt	0.01	1.00000000	pigs
Nptn	0.08	1.00000000	pigs
Pnpo	-0.22	1.00000000	pigs
Clcn6	-0.04	1.00000000	pigs
Acaca	-0.18	1.00000000	pigs
Sntb2	-0.10	1.00000000	pigs
Gja1	-0.20	1.00000000	pigs
Tns2	-0.18	1.00000000	pigs
Ppfibp2	-0.25	1.00000000	pigs
Ywhah	-0.06	1.00000000	pigs
Tnfsf12	-0.11	1.00000000	pigs
Cds2	-0.17	1.00000000	pigs
Ctnna1	0.12	1.00000000	pigs
Eefsec	-0.27	1.00000000	pigs
Cask	-0.15	1.00000000	pigs
Scamp3	-0.07	1.00000000	pigs
Srp19	-0.02	1.00000000	pigs
Rara	-0.02	1.00000000	pigs
Slc12a2	-0.28	1.00000000	pigs
Ahcyl1	-0.13	1.00000000	pigs
Chuk	-0.10	1.00000000	pigs
Zfp131	-0.10	1.00000000	pigs
Gna14	-0.42	1.00000000	pigs
Adpgk	-0.13	1.00000000	pigs

Surf6	0.04	1.00000000	pigs
Helz	0.11	1.00000000	pigs
Adamtsl4	-0.31	1.00000000	pigs
Tmem47	-0.18	1.00000000	pigs
Nudt6	-0.24	1.00000000	pigs
Sec61a1	0.26	1.00000000	pigs
Ireb2	-0.07	1.00000000	pigs
Smarcal1	-0.06	1.00000000	pigs
Vav3	-0.39	1.00000000	pigs
Crk	0.11	1.00000000	pigs
Rabep1	-0.14	1.00000000	pigs
Slc35b3	-0.04	1.00000000	pigs
Dctpp1	-0.07	1.00000000	pigs
Slc25a36	0.07	1.00000000	pigs
Ptpn14	-0.18	1.00000000	pigs
Cytip	0.18	1.00000000	pigs
Plekhg1	0.06	1.00000000	pigs
Polr3b	-0.06	1.00000000	pigs
Hsp90b1	0.33	1.00000000	pigs
Rab23	-0.33	1.00000000	pigs
Gpatch4	-0.07	1.00000000	pigs
Slc39a10	-0.11	1.00000000	pigs
Chst7	-0.18	1.00000000	pigs
Rbm18	0.01	1.00000000	pigs
Ss18	-0.21	1.00000000	pigs
Creb3l1	0.02	1.00000000	pigs
Disp1	-0.14	1.00000000	pigs
Nus1	-0.01	1.00000000	pigs
Sos1	-0.19	1.00000000	pigs
Rbbp6	0.13	1.00000000	pigs
Osgepl1	-0.36	1.00000000	pigs
Cox11	0.06	1.00000000	pigs
Folh1	-0.23	1.00000000	pigs
Zc2hc1a	-0.32	1.00000000	pigs
Tcp11l1	-0.16	1.00000000	pigs
Afdn	-0.44	1.00000000	pigs
Cast	-0.07	1.00000000	pigs
Tmf1	-0.08	1.00000000	pigs
Pxn	-0.39	1.00000000	pigs
Tob2	-0.23	1.00000000	pigs
Ylpm1	0.09	1.00000000	pigs
Ndnf	-0.06	1.00000000	pigs
Acp4	-0.09	1.00000000	pigs
Cxcl12	0.29	1.00000000	pigs
Eif5b	-0.16	1.00000000	pigs
Atp5md	-0.16	1.00000000	pigs

AW549877	-0.15	1.00000000	pigs
Ammecr1l	-0.15	1.00000000	pigs
Galk1	-0.09	1.00000000	pigs
Fbxo42	-0.21	1.00000000	pigs
Nudcd3	-0.15	1.00000000	pigs
Alg6	-0.10	1.00000000	pigs
Fggy	-0.14	1.00000000	pigs
Exosc9	-0.05	1.00000000	pigs
Cyb561a3	0.01	1.00000000	pigs
Ubtf	-0.29	1.00000000	pigs
Fbl	0.28	1.00000000	pigs
Brd8	-0.10	1.00000000	pigs
Rnf166	-0.01	1.00000000	pigs
Dipk1b	-0.15	1.00000000	pigs
Zfp74	-0.17	1.00000000	pigs
Nhlrc2	-0.27	1.00000000	pigs
Vps13d	-0.28	1.00000000	pigs
Frmd4a	0.09	1.00000000	pigs
Asap1	0.04	1.00000000	pigs
Tbc1d9	0.12	1.00000000	pigs
Uprt	0.02	1.00000000	pigs
Fbxo22	0.17	1.00000000	pigs
Daam2	-0.26	1.00000000	pigs
Nfe2l2	0.25	1.00000000	pigs
Zfp521	-0.27	1.00000000	pigs
Immt	-0.14	1.00000000	pigs
Pelo	0.01	1.00000000	pigs
Wipf2	-0.09	1.00000000	pigs
Tmem123	-0.08	1.00000000	pigs
Ppil3	-0.10	1.00000000	pigs
Plekha2	-0.04	1.00000000	pigs
Idi1	-0.31	1.00000000	pigs
Uri1	-0.04	1.00000000	pigs
Cpne3	0.06	1.00000000	pigs
Pus1	0.04	1.00000000	pigs
Ttc3	-0.07	1.00000000	pigs
Dctn6	-0.03	1.00000000	pigs
Ap5s1	-0.07	1.00000000	pigs
Tmem126a	0.03	1.00000000	pigs
Calr	0.42	1.00000000	pigs
Tubgcp4	-0.28	1.00000000	pigs
Yif1a	-0.13	1.00000000	pigs
Cdk13	-0.21	1.00000000	pigs
Invs	-0.28	1.00000000	pigs
Slc10a7	-0.10	1.00000000	pigs
Riok3	0.10	1.00000000	pigs

Cnot6l	0.00	1.00000000	pigs
Zfp322a	-0.07	1.00000000	pigs
Uchl5	0.11	1.00000000	pigs
Dscc1	-0.09	1.00000000	pigs
Gtf2a1	0.03	1.00000000	pigs
Cpped1	0.06	1.00000000	pigs
Ptprj	0.16	1.00000000	pigs
Smap2	-0.05	1.00000000	pigs
Xpo4	0.09	1.00000000	pigs
Sppl2a	0.15	1.00000000	pigs
Gnpnat1	-0.37	1.00000000	pigs
Alpk1	-0.24	1.00000000	pigs
Ankmy2	-0.11	1.00000000	pigs
Uba6	0.13	1.00000000	pigs
Kat7	-0.04	1.00000000	pigs
Foxp2	-0.12	1.00000000	pigs
Akt3	0.20	1.00000000	pigs
Ppp2r2a	-0.05	1.00000000	pigs
Nfe2l3	-0.12	1.00000000	pigs
Fbxl20	-0.13	1.00000000	pigs
Wwp2	-0.11	1.00000000	pigs
Siae	-0.04	1.00000000	pigs
Prkab1	-0.08	1.00000000	pigs
Lpin2	0.01	1.00000000	pigs
Bloc1s6	0.02	1.00000000	pigs
Ccnd2	0.02	1.00000000	pigs
Slc2a13	-0.09	1.00000000	pigs
Flnc	-0.20	1.00000000	pigs
Sec24b	-0.16	1.00000000	pigs
Elovl1	0.05	1.00000000	pigs
Pwwp3a	-0.22	1.00000000	pigs
Ero1b	-0.12	1.00000000	pigs
Rspo1	-0.23	1.00000000	pigs
Snf8	-0.01	1.00000000	pigs
Dync1li1	-0.28	1.00000000	pigs
Sfrp1	-0.13	1.00000000	pigs
Ufc1	-0.11	1.00000000	pigs
Rin2	-0.29	1.00000000	pigs
1700037H04Rik	0.15	1.00000000	pigs
Zfp518a	0.02	1.00000000	pigs
Hmces	-0.16	1.00000000	pigs
Pde5a	-0.40	1.00000000	pigs
Pms1	-0.15	1.00000000	pigs
Gbp2b	0.02	1.00000000	pigs
Cdk8	-0.10	1.00000000	pigs
Nelfb	-0.13	1.00000000	pigs

Arl4a	-0.12	1.00000000	pigs
Rnf14	-0.08	1.00000000	pigs
Echdc2	-0.16	1.00000000	pigs
Orc4	0.09	1.00000000	pigs
Cyp51	-0.26	1.00000000	pigs
Akt2	-0.16	1.00000000	pigs
Hint1	0.60	1.00000000	pigs
Ppp4r3b	0.05	1.00000000	pigs
Pbx3	-0.35	1.00000000	pigs
Txlna	-0.10	1.00000000	pigs
Ddx27	0.07	1.00000000	pigs
Polm	0.03	1.00000000	pigs
Idh3b	0.02	1.00000000	pigs
Lrch3	0.06	1.00000000	pigs
Terf1	0.05	1.00000000	pigs
Leng1	-0.10	1.00000000	pigs
Nipa2	-0.08	1.00000000	pigs
Aimp1	-0.10	1.00000000	pigs
Tarbp1	-0.30	1.00000000	pigs
Tnks2	-0.06	1.00000000	pigs
Itgb3	-0.05	1.00000000	pigs
Ccdc124	0.03	1.00000000	pigs
Rbbp5	-0.05	1.00000000	pigs
Pcca	-0.13	1.00000000	pigs
Actr1b	0.06	1.00000000	pigs
Fam120c	-0.14	1.00000000	pigs
Zfp358	-0.05	1.00000000	pigs
Parm1	-0.01	1.00000000	pigs
Ambra1	-0.07	1.00000000	pigs
Timm17b	-0.12	1.00000000	pigs
Zfp292	-0.01	1.00000000	pigs
Rfk	-0.19	1.00000000	pigs
Adcy7	-0.18	1.00000000	pigs
Nsd2	-0.10	1.00000000	pigs
Pds5b	-0.15	1.00000000	pigs
Slk	-0.05	1.00000000	pigs
Eif4h	0.01	1.00000000	pigs
Ptges3	0.00	1.00000000	pigs
Fstl3	-0.32	1.00000000	pigs
Dock7	-0.30	1.00000000	pigs
Gtf2e2	0.16	1.00000000	pigs
Cwc15	-0.11	1.00000000	pigs
Dcaf5	0.00	1.00000000	pigs
Smad4	-0.12	1.00000000	pigs
Chmp2b	-0.01	1.00000000	pigs
Hbp1	-0.06	1.00000000	pigs

Ccdc50	-0.28	1.00000000	pigs
Ptch2	-0.13	1.00000000	pigs
Wbp2	0.02	1.00000000	pigs
Ssr3	0.31	1.00000000	pigs
Cebpg	0.09	1.00000000	pigs
Timm8b	-0.04	1.00000000	pigs
Tcf24	-0.19	1.00000000	pigs
Pram1	-0.08	1.00000000	pigs
Flot2	-0.10	1.00000000	pigs
Col6a2	0.03	1.00000000	pigs
Rspo3	-0.20	1.00000000	pigs
Rad52	-0.28	1.00000000	pigs
Uhrf1bp1	-0.01	1.00000000	pigs
Dclk1	-0.08	1.00000000	pigs
Rpia	-0.09	1.00000000	pigs
Polr2e	0.09	1.00000000	pigs
Rcc1l	-0.03	1.00000000	pigs
Slc35f6	-0.12	1.00000000	pigs
Cdh23	0.02	1.00000000	pigs
Cstf2	-0.07	1.00000000	pigs
Sap30bp	-0.11	1.00000000	pigs
Dapk1	0.12	1.00000000	pigs
Bex3	-0.15	1.00000000	pigs
Tmem65	0.01	1.00000000	pigs
Csf3r	0.25	1.00000000	pigs
Ddx39b	0.36	1.00000000	pigs
Arpc4	0.30	1.00000000	pigs
Tmed5	-0.12	1.00000000	pigs
Stpg4	0.20	1.00000000	pigs
Thap3	-0.12	1.00000000	pigs
Htatsf1	-0.01	1.00000000	pigs
Zmynd8	-0.13	1.00000000	pigs
Nupl1	-0.06	1.00000000	pigs
Ttc7b	-0.02	1.00000000	pigs
Ginm1	0.02	1.00000000	pigs
Apc	-0.30	1.00000000	pigs
Ndufb2	0.22	1.00000000	pigs
Eif2s1	-0.15	1.00000000	pigs
Zfp592	-0.15	1.00000000	pigs
A830018L16Rik	-0.19	1.00000000	pigs
Psip1	-0.27	1.00000000	pigs
Parp12	0.07	1.00000000	pigs
Adamts15	0.00	1.00000000	pigs
Clmp	-0.13	1.00000000	pigs
Pnn	-0.12	1.00000000	pigs
Nudt13	-0.09	1.00000000	pigs

Usp14	0.03	1.00000000	pigs
Tdrd3	-0.43	1.00000000	pigs
Uhrf2	-0.05	1.00000000	pigs
Bcl7c	0.00	1.00000000	pigs
Adi1	-0.02	1.00000000	pigs
Fra10ac1	-0.09	1.00000000	pigs
Arhgef12	-0.26	1.00000000	pigs
Ptpmt1	0.08	1.00000000	pigs
Parp4	-0.34	1.00000000	pigs
Tspan4	-0.10	1.00000000	pigs
Rbm22	-0.03	1.00000000	pigs
Apbb1ip	-0.32	1.00000000	pigs
Pgpep1	0.06	1.00000000	pigs
Bad	-0.24	1.00000000	pigs
Trit1	-0.16	1.00000000	pigs
Dcaf8	-0.05	1.00000000	pigs
Fcer1g	-0.04	1.00000000	pigs
Reep3	-0.24	1.00000000	pigs
Olfml3	-0.05	1.00000000	pigs
Sh3rf1	-0.29	1.00000000	pigs
Zfp280c	-0.30	1.00000000	pigs
Zmym4	-0.17	1.00000000	pigs
Pip4k2a	0.08	1.00000000	pigs
Ciao1	-0.07	1.00000000	pigs
Tmem87b	-0.15	1.00000000	pigs
Thrb	0.08	1.00000000	pigs
Atp2a2	-0.22	1.00000000	pigs
Fbxo33	-0.13	1.00000000	pigs
Slc23a2	0.07	1.00000000	pigs
Cpm	-0.08	1.00000000	pigs
Eif2s2	0.08	1.00000000	pigs
Efcab11	-0.24	1.00000000	pigs
Nop58	-0.20	1.00000000	pigs
Exog	-0.03	1.00000000	pigs
Atp5g3	0.21	1.00000000	pigs
Ube2h	-0.05	1.00000000	pigs
C4bp	-0.93	1.00000000	pigs
Tmem50b	-0.17	1.00000000	pigs
Acbd3	-0.26	1.00000000	pigs
Acbd6	-0.37	1.00000000	pigs
Phyh	-0.07	1.00000000	pigs
Coro1c	-0.09	1.00000000	pigs
Gamt	-0.16	1.00000000	pigs
Glb1	-0.12	1.00000000	pigs
Katnbl1	-0.27	1.00000000	pigs
Dync1i2	-0.24	1.00000000	pigs

Galnt18	-0.15	1.00000000	pigs
Nbn	-0.18	1.00000000	pigs
Slc17a5	0.05	1.00000000	pigs
Prkd3	-0.19	1.00000000	pigs
Tgfbr3	-0.06	1.00000000	pigs
A2m	-0.50	1.00000000	pigs
Ilf3	0.07	1.00000000	pigs
Ptprs	-0.16	1.00000000	pigs
Itpr1	-0.02	1.00000000	pigs
Pdcd7	0.04	1.00000000	pigs
Wdsub1	-0.14	1.00000000	pigs
Ecm1	-0.40	1.00000000	pigs
Sf3b3	-0.25	1.00000000	pigs
Cpeb3	-0.34	1.00000000	pigs
Ccdc174	0.03	1.00000000	pigs
Sco1	-0.05	1.00000000	pigs
Hip1	-0.08	1.00000000	pigs
Lmcd1	-0.18	1.00000000	pigs
Sh3gl1	-0.09	1.00000000	pigs
Coq2	-0.05	1.00000000	pigs
Pde10a	-0.46	1.00000000	pigs
Ttc13	-0.29	1.00000000	pigs
Rasa2	-0.15	1.00000000	pigs
Ankib1	-0.27	1.00000000	pigs
Lrrc66	-0.09	1.00000000	pigs
Tmem127	-0.11	1.00000000	pigs
Zbtb7a	-0.08	1.00000000	pigs
Lnpk	-0.08	1.00000000	pigs
Snrpn	0.01	1.00000000	pigs
Tmbim6	0.17	1.00000000	pigs
Ctnnb1	0.10	1.00000000	pigs
Lss	-0.14	1.00000000	pigs
Smarcb1	-0.08	1.00000000	pigs
Nlrc5	-0.19	1.00000000	pigs
Rfc1	-0.41	1.00000000	pigs
Polr2i	0.00	1.00000000	pigs
Ankrd40	-0.06	1.00000000	pigs
Csf1	-0.25	1.00000000	pigs
Adam17	-0.03	1.00000000	pigs
Ptpra	-0.19	1.00000000	pigs
Fkbp4	-0.10	1.00000000	pigs
Pdk3	0.18	1.00000000	pigs
Aph1b	-0.10	1.00000000	pigs
Rad23b	-0.18	1.00000000	pigs
Cdin1	-0.28	1.00000000	pigs
Bmp2k	-0.09	1.00000000	pigs

Hps3	-0.16	1.00000000	pigs
Klhl15	0.01	1.00000000	pigs
Dpysl3	-0.13	1.00000000	pigs
Prdm5	-0.15	1.00000000	pigs
Kcnq5	-0.66	1.00000000	pigs
Gjc1	-0.02	1.00000000	pigs
Hhip	-0.26	1.00000000	pigs
Ccar2	-0.06	1.00000000	pigs
Ift20	0.05	1.00000000	pigs
Fgfr1	-0.14	1.00000000	pigs
Cebpb	0.36	1.00000000	pigs
Cep104	0.12	1.00000000	pigs
Dusp2	0.54	1.00000000	pigs
Orc5	-0.36	1.00000000	pigs
Gata6	-0.22	1.00000000	pigs
Smad1	-0.14	1.00000000	pigs
Ngly1	-0.06	1.00000000	pigs
Atf6	-0.08	1.00000000	pigs
2210408I21Rik	-0.12	1.00000000	pigs
Clcn5	-0.12	1.00000000	pigs
Tasor	-0.11	1.00000000	pigs
Zmym5	-0.11	1.00000000	pigs
Fjx1	-0.05	1.00000000	pigs
Cep83	-0.13	1.00000000	pigs
Trim8	0.08	1.00000000	pigs
Exosc4	-0.10	1.00000000	pigs
Impg2	-0.12	1.00000000	pigs
Zfyve26	-0.08	1.00000000	pigs
Dcun1d5	-0.31	1.00000000	pigs
Nup85	-0.16	1.00000000	pigs
Cmtr1	-0.09	1.00000000	pigs
Pi4ka	-0.05	1.00000000	pigs
Lgals3	0.25	1.00000000	pigs
Sytl4	-0.09	1.00000000	pigs
Pafah1b1	-0.08	1.00000000	pigs
Rbpj	-0.07	1.00000000	pigs
Dnajc11	-0.22	1.00000000	pigs
Bclaf3	0.02	1.00000000	pigs
Cdpf1	-0.07	1.00000000	pigs
Hnrnph2	0.00	1.00000000	pigs
Npas3	-0.05	1.00000000	pigs
Tnfaip1	0.14	1.00000000	pigs
Plcl2	-0.18	1.00000000	pigs
Atpaf2	-0.05	1.00000000	pigs
Krtcap2	0.09	1.00000000	pigs
Ephx2	-0.10	1.00000000	pigs

Ess2	0.03	1.00000000	pigs
Pibf1	-0.16	1.00000000	pigs
Filip1l	-0.36	1.00000000	pigs
Timm21	0.00	1.00000000	pigs
Nat10	-0.03	1.00000000	pigs
Naa20	0.07	1.00000000	pigs
Carf	0.03	1.00000000	pigs
Svep1	-0.39	1.00000000	pigs
Pnpla8	-0.42	1.00000000	pigs
Star	-0.11	1.00000000	pigs
Prmt7	-0.01	1.00000000	pigs
Brwd3	-0.06	1.00000000	pigs
Rtcb	0.04	1.00000000	pigs
Habp4	-0.09	1.00000000	pigs
Khdrbs1	-0.13	1.00000000	pigs
Daglb	-0.04	1.00000000	pigs
Tapt1	-0.24	1.00000000	pigs
Atr	-0.11	1.00000000	pigs
Gabpb2	-0.06	1.00000000	pigs
Iah1	-0.09	1.00000000	pigs
Phf8	-0.10	1.00000000	pigs
Osbpl1a	-0.13	1.00000000	pigs
Vsir	-0.09	1.00000000	pigs
Zfhx3	-0.21	1.00000000	pigs
Dars	-0.03	1.00000000	pigs
Uggt2	-0.27	1.00000000	pigs
Lzic	-0.12	1.00000000	pigs
Ldlrad3	-0.28	1.00000000	pigs
Ifnar1	-0.04	1.00000000	pigs
Nras	-0.04	1.00000000	pigs
Ecd	-0.11	1.00000000	pigs
Trub2	-0.03	1.00000000	pigs
Prickle3	0.01	1.00000000	pigs
Nup205	-0.10	1.00000000	pigs
Tubb2b	-0.12	1.00000000	pigs
Avil	-0.09	1.00000000	pigs
Tle3	-0.26	1.00000000	pigs
Steap4	-0.12	1.00000000	pigs
Kctd6	-0.04	1.00000000	pigs
Hsd17b7	-0.19	1.00000000	pigs
Ezh1	-0.17	1.00000000	pigs
Hspb1	0.22	1.00000000	pigs
Itgb1bp1	-0.30	1.00000000	pigs
Hrh1	-0.48	1.00000000	pigs
Nsun6	-0.09	1.00000000	pigs
Snx16	-0.12	1.00000000	pigs

Taldo1	0.09	1.00000000	pigs
Asb3	-0.21	1.00000000	pigs
Ppp1r9b	-0.01	1.00000000	pigs
Bach2	0.00	1.00000000	pigs
Ube2k	-0.05	1.00000000	pigs
Sppl3	-0.03	1.00000000	pigs
Rmnd5b	-0.05	1.00000000	pigs
Tfb1m	-0.14	1.00000000	pigs
Camkmt	-0.50	1.00000000	pigs
Trmt5	-0.10	1.00000000	pigs
Tbc1d8	-0.05	1.00000000	pigs
Dnttip2	0.05	1.00000000	pigs
Spata17	-0.29	1.00000000	pigs
Cdkn1b	-0.05	1.00000000	pigs
Tsc22d2	0.08	1.00000000	pigs
Ash2l	0.07	1.00000000	pigs
Atm	-0.20	1.00000000	pigs
Dohh	-0.11	1.00000000	pigs
Grk5	0.04	1.00000000	pigs
Pomt2	0.07	1.00000000	pigs
Eif3h	0.19	1.00000000	pigs
Patj	-0.07	1.00000000	pigs
Fbxo30	-0.44	1.00000000	pigs
Sorl1	-0.14	1.00000000	pigs
Abhd5	-0.48	1.00000000	pigs
Ptpn21	-0.23	1.00000000	pigs
Jag1	-0.21	1.00000000	pigs
Zswim4	-0.01	1.00000000	pigs
Zfp202	-0.02	1.00000000	pigs
Ankfy1	0.07	1.00000000	pigs
Tead1	0.04	1.00000000	pigs
Dennd1a	-0.24	1.00000000	pigs
Nup214	-0.14	1.00000000	pigs
Slc1a5	0.13	1.00000000	pigs
Zc3h6	-0.08	1.00000000	pigs
Tent4a	-0.09	1.00000000	pigs
Lrp6	-0.08	1.00000000	pigs
Inpp4a	-0.08	1.00000000	pigs
Prpf4b	-0.19	1.00000000	pigs
Sdccag8	-0.13	1.00000000	pigs
Csrp1	-0.06	1.00000000	pigs
Zfp106	-0.33	1.00000000	pigs
Npr1	-0.15	1.00000000	pigs
Ap2a2	-0.08	1.00000000	pigs
Naa16	0.01	1.00000000	pigs
Acvr1	-0.16	1.00000000	pigs

Dynlrb1	0.12	1.00000000	pigs
Lrp3	-0.16	1.00000000	pigs
Slc25a17	-0.17	1.00000000	pigs
Ripk1	0.01	1.00000000	pigs
Il6st	-0.04	1.00000000	pigs
Ice2	-0.20	1.00000000	pigs
Sash1	-0.07	1.00000000	pigs
Tnks	-0.31	1.00000000	pigs
Acss2	-0.09	1.00000000	pigs
Nckap1	-0.22	1.00000000	pigs
Polr1f	-0.13	1.00000000	pigs
Colec12	0.07	1.00000000	pigs
Nolc1	-0.06	1.00000000	pigs
Adgre5	-0.13	1.00000000	pigs
Ppp1r14a	-0.23	1.00000000	pigs
Tctn1	-0.07	1.00000000	pigs
Hipk1	-0.05	1.00000000	pigs
Ryk	-0.16	1.00000000	pigs
Sidt2	-0.08	1.00000000	pigs
Zfp652	-0.26	1.00000000	pigs
Tma16	-0.01	1.00000000	pigs
Cdr2l	-0.05	1.00000000	pigs
Zfp507	-0.15	1.00000000	pigs
St3gal4	-0.17	1.00000000	pigs
Zfp651	-0.09	1.00000000	pigs
Spred1	-0.20	1.00000000	pigs
Ercc4	-0.16	1.00000000	pigs
Pcmt1	-0.06	1.00000000	pigs
Aftph	-0.21	1.00000000	pigs
Tmem38b	-0.12	1.00000000	pigs
Heca	-0.05	1.00000000	pigs
Herc4	-0.20	1.00000000	pigs
Pm20d2	-0.11	1.00000000	pigs
Naa15	-0.09	1.00000000	pigs
Acox3	-0.01	1.00000000	pigs
Nbas	0.04	1.00000000	pigs
Fanci	-0.03	1.00000000	pigs
Cers4	0.14	1.00000000	pigs
Add1	-0.22	1.00000000	pigs
Emc10	-0.01	1.00000000	pigs
Irag1	-0.26	1.00000000	pigs
Ift88	-0.16	1.00000000	pigs
Snx29	-0.16	1.00000000	pigs
Brk1	0.14	1.00000000	pigs
Capns1	-0.01	1.00000000	pigs
Slc25a20	0.04	1.00000000	pigs

Srebf2	-0.02	1.00000000	pigs
Aar2	0.14	1.00000000	pigs
Pla2g4a	-0.32	1.00000000	pigs
Yy1	-0.16	1.00000000	pigs
Prkar2a	-0.32	1.00000000	pigs
Dhx57	-0.17	1.00000000	pigs
Cggbp1	-0.13	1.00000000	pigs
Srsf9	0.14	1.00000000	pigs
Secisbp2	-0.15	1.00000000	pigs
Shld2	-0.23	1.00000000	pigs
Ppp6r2	-0.25	1.00000000	pigs
Trappc6b	0.01	1.00000000	pigs
Hoxa3	-0.10	1.00000000	pigs
Ext1	-0.03	1.00000000	pigs
Snap23	-0.02	1.00000000	pigs
Srsf10	-0.18	1.00000000	pigs
Ccdc134	-0.05	1.00000000	pigs
Zfp583	-0.03	1.00000000	pigs
Emc1	0.02	1.00000000	pigs
Cdc42ep5	-0.18	1.00000000	pigs
Fli1	0.02	1.00000000	pigs
Rpn1	0.09	1.00000000	pigs
Btbd9	0.00	1.00000000	pigs
Fam222b	-0.36	1.00000000	pigs
Golgb1	-0.16	1.00000000	pigs
Hcfc2	-0.05	1.00000000	pigs
Dynlt3	-0.05	1.00000000	pigs
Klhl24	-0.14	1.00000000	pigs
Luc7l3	-0.06	1.00000000	pigs
Snx14	-0.27	1.00000000	pigs
Park7	0.07	1.00000000	pigs
Vps8	-0.29	1.00000000	pigs
Zfp422	-0.09	1.00000000	pigs
Kctd10	-0.05	1.00000000	pigs
Dlg2	-0.52	1.00000000	pigs
Sf3b4	-0.17	1.00000000	pigs
Kank2	-0.16	1.00000000	pigs
Fbxo3	-0.13	1.00000000	pigs
Sema6a	-0.19	1.00000000	pigs
Crtap	-0.05	1.00000000	pigs
Tdp2	-0.31	1.00000000	pigs
Pafah2	0.00	1.00000000	pigs
Gbf1	0.01	1.00000000	pigs
Cbfa2t2	-0.16	1.00000000	pigs
Spg20	-0.15	1.00000000	pigs
Zfp146	-0.04	1.00000000	pigs

Kdm6a	0.02	1.00000000	pigs
Jade3	-0.05	1.00000000	pigs
Zzef1	-0.08	1.00000000	pigs
Gga2	-0.06	1.00000000	pigs
Rb1cc1	-0.08	1.00000000	pigs
Trappc13	-0.01	1.00000000	pigs
Acsl4	-0.08	1.00000000	pigs
Fam102b	-0.29	1.00000000	pigs
Tia1	-0.23	1.00000000	pigs
Ogn	-0.03	1.00000000	pigs
Ppard	-0.19	1.00000000	pigs
Usp31	0.00	1.00000000	pigs
Pus10	-0.19	1.00000000	pigs
Tiparp	-0.14	1.00000000	pigs
Arhgap18	0.00	1.00000000	pigs
Serinc3	-0.08	1.00000000	pigs
Trmt11	-0.14	1.00000000	pigs
Iqcg	-0.16	1.00000000	pigs
G3bp2	-0.12	1.00000000	pigs
Plaur	-0.14	1.00000000	pigs
Grcc10	-0.13	1.00000000	pigs
Ube3b	-0.09	1.00000000	pigs
Arhgdia	0.27	1.00000000	pigs
Zranb2	-0.12	1.00000000	pigs
Slc25a40	-0.04	1.00000000	pigs
Prmt3	-0.14	1.00000000	pigs
Cox6a1	-0.02	1.00000000	pigs
Aven	-0.28	1.00000000	pigs
Tsc22d3	0.07	1.00000000	pigs
Blvra	-0.15	1.00000000	pigs
Ttc39b	-0.20	1.00000000	pigs
Ggnbp2	-0.14	1.00000000	pigs
Thra	-0.29	1.00000000	pigs
Nfxl1	0.00	1.00000000	pigs
Ubr4	0.02	1.00000000	pigs
Tsr3	0.09	1.00000000	pigs
Rybp	0.01	1.00000000	pigs
Pla2g7	-0.17	1.00000000	pigs
Usp9x	-0.22	1.00000000	pigs
Prkd1	-0.30	1.00000000	pigs
Pi15	-0.10	1.00000000	pigs
Pigu	-0.01	1.00000000	pigs
Rlf	-0.15	1.00000000	pigs
Comtd1	-0.03	1.00000000	pigs
Nfe2l1	-0.12	1.00000000	pigs
Ninl	-0.03	1.00000000	pigs

Prelid2	-0.05	1.00000000	pigs
Galk2	-0.22	1.00000000	pigs
Cryl1	-0.09	1.00000000	pigs
Cmpk1	0.10	1.00000000	pigs
Cpd	-0.19	1.00000000	pigs
Eif2b4	-0.07	1.00000000	pigs
Sumo2	0.11	1.00000000	pigs
Nedd1	-0.21	1.00000000	pigs
Wars2	-0.22	1.00000000	pigs
Fam91a1	-0.18	1.00000000	pigs
Snapc4	0.03	1.00000000	pigs
Tor3a	0.04	1.00000000	pigs
Pdgfc	-0.06	1.00000000	pigs
Slc25a24	-0.13	1.00000000	pigs
Taf1a	-0.18	1.00000000	pigs
Eif4enif1	-0.17	1.00000000	pigs
Ddx20	-0.11	1.00000000	pigs
Ipo9	-0.10	1.00000000	pigs
Tex2	-0.18	1.00000000	pigs
Cfap97	-0.13	1.00000000	pigs
Nup155	-0.12	1.00000000	pigs
Tmod2	0.00	1.00000000	pigs
Cwf19l2	-0.19	1.00000000	pigs
Tra2a	-0.11	1.00000000	pigs
Gxylt2	0.01	1.00000000	pigs
Tmem245	-0.27	1.00000000	pigs
Hes1	0.12	1.00000000	pigs
Xpnpep3	-0.05	1.00000000	pigs
Jmjd1c	-0.14	1.00000000	pigs
Zfp532	-0.10	1.00000000	pigs
Pear1	-0.26	1.00000000	pigs
Ap1b1	-0.20	1.00000000	pigs
Gabbr1	-0.13	1.00000000	pigs
Tbx4	-0.22	1.00000000	pigs
Kazn	-0.05	1.00000000	pigs
4921524J17Rik	-0.23	1.00000000	pigs
Ctdspl2	-0.22	1.00000000	pigs
Glis2	-0.15	1.00000000	pigs
Polk	-0.03	1.00000000	pigs
Bnip2	-0.03	1.00000000	pigs
Arhgef11	-0.26	1.00000000	pigs
Pdcd5	0.00	1.00000000	pigs
Igf2bp3	0.00	1.00000000	pigs
Bdp1	-0.32	1.00000000	pigs
Dlg5	-0.16	1.00000000	pigs
Usp39	0.08	1.00000000	pigs

Eps15l1	-0.20	1.00000000	pigs
Gps1	-0.14	1.00000000	pigs
Dcaf11	-0.11	1.00000000	pigs
Cpq	-0.07	1.00000000	pigs
Ube2w	-0.27	1.00000000	pigs
Ift122	-0.15	1.00000000	pigs
Dgke	-0.04	1.00000000	pigs
Wdr35	-0.03	1.00000000	pigs
Copa	-0.15	1.00000000	pigs
Brap	-0.20	1.00000000	pigs
Cab39l	0.16	1.00000000	pigs
Ldah	-0.31	1.00000000	pigs
Rilpl2	-0.23	1.00000000	pigs
Cadm1	-0.20	1.00000000	pigs
Prpf39	-0.12	1.00000000	pigs
Zc3h4	-0.10	1.00000000	pigs
Tfpi2	-0.10	1.00000000	pigs
Edem2	-0.25	1.00000000	pigs
Ing5	-0.11	1.00000000	pigs
Vps51	-0.04	1.00000000	pigs
Atp8b2	-0.09	1.00000000	pigs
Tesc	0.20	1.00000000	pigs
Ssx2ip	-0.02	1.00000000	pigs
Rapgef1	-0.07	1.00000000	pigs
Dhx29	-0.20	1.00000000	pigs
Ptk2b	0.19	1.00000000	pigs
Tmem223	-0.01	1.00000000	pigs
Tinf2	-0.12	1.00000000	pigs
Dcun1d3	-0.28	1.00000000	pigs
Utp25	-0.13	1.00000000	pigs
Arhgap35	-0.14	1.00000000	pigs
Cdc40	-0.04	1.00000000	pigs
Csnk1g1	-0.22	1.00000000	pigs
Gnai2	0.02	1.00000000	pigs
Tdpoz8	-0.12	1.00000000	pigs
Abca8b	-0.24	1.00000000	pigs
Tm9sf2	0.02	1.00000000	pigs
Kpnb1	-0.01	1.00000000	pigs
Pde7a	-0.21	1.00000000	pigs
Cd2bp2	-0.12	1.00000000	pigs
Purg	-0.32	1.00000000	pigs
Atl2	-0.14	1.00000000	pigs
Slc3a1	-0.09	1.00000000	pigs
Agpat4	-0.11	1.00000000	pigs
Klhl9	-0.08	1.00000000	pigs
Pop5	0.07	1.00000000	pigs

Copb1	-0.02	1.00000000	pigs
Odr4	-0.24	1.00000000	pigs
Ndufb1	-0.11	1.00000000	pigs
Ppm1l	0.01	1.00000000	pigs
Sgms2	-0.10	1.00000000	pigs
Ptpn4	-0.15	1.00000000	pigs
Cnih1	0.19	1.00000000	pigs
Ppp6c	-0.09	1.00000000	pigs
Ctdsp2	0.01	1.00000000	pigs
Lcp1	-0.29	1.00000000	pigs
Tmem150c	-0.26	1.00000000	pigs
Rnaseh2c	0.01	1.00000000	pigs
Cgnl1	-0.06	1.00000000	pigs
Tmem128	-0.08	1.00000000	pigs
Ctsh	0.19	1.00000000	pigs
Gsk3b	-0.10	1.00000000	pigs
Zbtb10	-0.23	1.00000000	pigs
Nudt19	0.09	1.00000000	pigs
Polr3d	0.00	1.00000000	pigs
Wdr33	-0.02	1.00000000	pigs
Rnf157	-0.11	1.00000000	pigs
Kmt2e	-0.02	1.00000000	pigs
Snx24	0.29	1.00000000	pigs
Cdkn2aipnl	0.09	1.00000000	pigs
Dcakd	0.02	1.00000000	pigs
Topaz1	-0.24	1.00000000	pigs
Kdm5c	-0.04	1.00000000	pigs
Usp3	-0.10	1.00000000	pigs
Prr5	-0.01	1.00000000	pigs
Gpn3	-0.15	1.00000000	pigs
Nsmce2	-0.31	1.00000000	pigs
Ift74	-0.15	1.00000000	pigs
Klhl28	-0.21	1.00000000	pigs
Phf10	-0.05	1.00000000	pigs
Wfdc1	-0.09	1.00000000	pigs
Phtf1	-0.08	1.00000000	pigs
Snrk	-0.23	1.00000000	pigs
Tnfrsf1b	0.07	1.00000000	pigs
Ctps2	-0.02	1.00000000	pigs
Ddx1	-0.04	1.00000000	pigs
Pla2g6	-0.08	1.00000000	pigs
Tsnax	-0.16	1.00000000	pigs
Tbc1d9b	-0.10	1.00000000	pigs
Eif3e	0.21	1.00000000	pigs
Parp8	-0.17	1.00000000	pigs
Igf2bp2	-0.19	1.00000000	pigs

Cog3	-0.25	1.00000000	pigs
Tpp1	-0.02	1.00000000	pigs
Limk2	-0.21	1.00000000	pigs
Sh3kbp1	-0.14	1.00000000	pigs
Kcnab1	-0.15	1.00000000	pigs
Hivep2	0.15	1.00000000	pigs
Gtf2b	-0.08	1.00000000	pigs
Dact3	-0.24	1.00000000	pigs
Trio	0.03	1.00000000	pigs
Golga3	-0.21	1.00000000	pigs
Anp32e	-0.11	1.00000000	pigs
Olfml2a	-0.10	1.00000000	pigs
Prkacb	-0.24	1.00000000	pigs
Lmx1a	-0.36	1.00000000	pigs
Tex14	-0.24	1.00000000	pigs
Ube2r2	-0.05	1.00000000	pigs
Efl1	-0.05	1.00000000	pigs
Ccdc186	-0.18	1.00000000	pigs
Stambp	-0.16	1.00000000	pigs
Lgr4	-0.21	1.00000000	pigs
Ufm1	-0.04	1.00000000	pigs
Ric8b	-0.29	1.00000000	pigs
Sulf1	-0.10	1.00000000	pigs
Agfg1	-0.11	1.00000000	pigs
Hemgn	-0.06	1.00000000	pigs
Fem1c	-0.28	1.00000000	pigs
Scamp1	-0.14	1.00000000	pigs
Crkl	-0.10	1.00000000	pigs
Zw10	0.01	1.00000000	pigs
Fam110b	-0.25	1.00000000	pigs
Rbm14	-0.15	1.00000000	pigs
Hras	-0.02	1.00000000	pigs
Tmc1	-0.03	1.00000000	pigs
Hp1bp3	-0.19	1.00000000	pigs
Tmem68	-0.15	1.00000000	pigs
Sipa1l1	-0.37	1.00000000	pigs
Snrnp35	0.15	1.00000000	pigs
Cdkl2	-0.19	1.00000000	pigs
Usp1	-0.17	1.00000000	pigs
Erap1	-0.17	1.00000000	pigs
Acot10	-0.23	1.00000000	pigs
Tmem238	0.06	1.00000000	pigs
Rnf111	-0.09	1.00000000	pigs
Wnt2b	-0.16	1.00000000	pigs
Itfg1	-0.30	1.00000000	pigs
Tubb5	0.16	1.00000000	pigs

Rab7	-0.06	1.00000000	pigs
Slain2	-0.22	1.00000000	pigs
Ppp4r1	-0.13	1.00000000	pigs
Klhl7	-0.13	1.00000000	pigs
Ranbp6	-0.16	1.00000000	pigs
R3hdm2	-0.28	1.00000000	pigs
Auh	-0.26	1.00000000	pigs
Tnrc6c	-0.25	1.00000000	pigs
Dmxl1	-0.28	1.00000000	pigs
Ctcf	-0.17	1.00000000	pigs
Dctn3	0.04	1.00000000	pigs
Sec63	-0.03	1.00000000	pigs
Smarcd1	-0.20	1.00000000	pigs
Ube2e2	-0.02	1.00000000	pigs
Nr2c2	-0.23	1.00000000	pigs
Abraxas2	-0.19	1.00000000	pigs
Src	-0.19	1.00000000	pigs
Ccdc15	-0.12	1.00000000	pigs
Edc3	-0.12	1.00000000	pigs
Tmed9	0.22	1.00000000	pigs
Sbno1	-0.35	1.00000000	pigs
Ndufaf3	-0.12	1.00000000	pigs
Casp8ap2	-0.06	1.00000000	pigs
Nans	0.17	1.00000000	pigs
Spag16	-0.01	1.00000000	pigs
Ring1	-0.17	1.00000000	pigs
Erlec1	-0.17	1.00000000	pigs
Lig3	-0.06	1.00000000	pigs
Hbs1l	-0.06	1.00000000	pigs
Tmem39a	-0.24	1.00000000	pigs
Abhd3	-0.01	1.00000000	pigs
Ptpdc1	-0.09	1.00000000	pigs
Spata6	-0.07	1.00000000	pigs
Plekha3	0.13	1.00000000	pigs
Stub1	-0.08	1.00000000	pigs
Gucy2c	-0.43	1.00000000	pigs
Prrc1	-0.17	1.00000000	pigs
Clcc1	-0.35	1.00000000	pigs
Crem	0.05	1.00000000	pigs
Spcs2	0.28	1.00000000	pigs
Pitrm1	0.00	1.00000000	pigs
Ccdc66	-0.15	1.00000000	pigs
Dmac2l	-0.15	1.00000000	pigs
Gcfc2	-0.20	1.00000000	pigs
Osbpl11	-0.01	1.00000000	pigs
Amh	-0.06	1.00000000	pigs

Ddx52	-0.18	1.00000000	pigs
Scml2	-0.18	1.00000000	pigs
Trappc9	0.01	1.00000000	pigs
Ltbp3	-0.09	1.00000000	pigs
Leo1	-0.21	1.00000000	pigs
Simc1	-0.22	1.00000000	pigs
Usp45	-0.20	1.00000000	pigs
Sap130	-0.31	1.00000000	pigs
Gucy1a2	-0.20	1.00000000	pigs
Htra2	-0.06	1.00000000	pigs
Pdap1	-0.05	1.00000000	pigs
Pip5k1b	-0.08	1.00000000	pigs
Atxn7	-0.04	1.00000000	pigs
Pde8a	0.09	1.00000000	pigs
Nisch	-0.15	1.00000000	pigs
Prrc2b	-0.06	1.00000000	pigs
Dnajc10	-0.06	1.00000000	pigs
Pnisr	-0.07	1.00000000	pigs
Fxn	0.03	1.00000000	pigs
Glmn	-0.13	1.00000000	pigs
Bace1	-0.02	1.00000000	pigs
Ccp110	-0.04	1.00000000	pigs
D630045J12Rik	-0.13	1.00000000	pigs
Slc9a9	-0.01	1.00000000	pigs
Cmas	-0.11	1.00000000	pigs
Pgk1	-0.03	1.00000000	pigs
Pcnx	-0.16	1.00000000	pigs
Stim2	-0.22	1.00000000	pigs
Ppp1r9a	-0.28	1.00000000	pigs
Dicer1	-0.45	1.00000000	pigs
Tor1aip1	-0.23	1.00000000	pigs
Cnih3	-0.07	1.00000000	pigs
Prpf40a	-0.06	1.00000000	pigs
Ube2j1	0.10	1.00000000	pigs
Supt16	0.00	1.00000000	pigs
Sptlc2	-0.41	1.00000000	pigs
Snx9	-0.06	1.00000000	pigs
Chpf	-0.19	1.00000000	pigs
Lrrc58	-0.22	1.00000000	pigs
Hibch	-0.23	1.00000000	pigs
Adgra2	-0.11	1.00000000	pigs
Wdr47	-0.01	1.00000000	pigs
Tmed7	-0.18	1.00000000	pigs
Phf11a	-0.03	1.00000000	pigs
Dpy19l1	-0.17	1.00000000	pigs
Nsd3	-0.03	1.00000000	pigs

Nav2	0.05	1.00000000	pigs
Cbr2	0.22	1.00000000	pigs
Tex26	-0.29	1.00000000	pigs
Zfp341	-0.05	1.00000000	pigs
Cd164	0.06	1.00000000	pigs
Slc36a4	-0.09	1.00000000	pigs
Cnot10	-0.19	1.00000000	pigs
Cdyl2	-0.03	1.00000000	pigs
Idh3a	-0.14	1.00000000	pigs
Drosha	-0.12	1.00000000	pigs
H2ac20	-0.01	1.00000000	pigs
Gtf2f2	-0.18	1.00000000	pigs
Prrc2c	0.11	1.00000000	pigs
Dap	-0.15	1.00000000	pigs
Emilin2	0.19	1.00000000	pigs
Itsn2	-0.15	1.00000000	pigs
Parg	-0.13	1.00000000	pigs
Scfd2	-0.05	1.00000000	pigs
Ift172	-0.13	1.00000000	pigs
Slc40a1	-0.25	1.00000000	pigs
Isg20l2	0.01	1.00000000	pigs
Spry1	-0.32	1.00000000	pigs
Csnk1g3	-0.27	1.00000000	pigs
Stx17	-0.25	1.00000000	pigs
C1qtnf2	-0.19	1.00000000	pigs
Sgca	-0.17	1.00000000	pigs
Caprin2	-0.01	1.00000000	pigs
Fbp1	0.01	1.00000000	pigs
Smndc1	-0.13	1.00000000	pigs
Lancl2	-0.09	1.00000000	pigs
Kdm2b	-0.10	1.00000000	pigs
Wdr11	-0.12	1.00000000	pigs
Trip4	-0.04	1.00000000	pigs
Gfm2	-0.23	1.00000000	pigs
Cbl	-0.11	1.00000000	pigs
Fhod3	-0.16	1.00000000	pigs
Dcaf1	-0.06	1.00000000	pigs
Abcb7	-0.35	1.00000000	pigs
Psmd7	0.00	1.00000000	pigs
Klhl18	-0.14	1.00000000	pigs
Axin2	-0.08	1.00000000	pigs
Npr2	-0.15	1.00000000	pigs
Ube2cbp	-0.16	1.00000000	pigs
Kdm5a	-0.12	1.00000000	pigs
Swt1	-0.22	1.00000000	pigs
Cd69	0.12	1.00000000	pigs

Crtc3	-0.17	1.00000000	pigs
Cd47	0.02	1.00000000	pigs
Pdha1	0.05	1.00000000	pigs
Gtf3c6	-0.02	1.00000000	pigs
Cplane1	-0.30	1.00000000	pigs
Kif3b	-0.19	1.00000000	pigs
Lrmda	0.06	1.00000000	pigs
Arhgap23	-0.09	1.00000000	pigs
Dmwd	-0.06	1.00000000	pigs
Cacna1d	-0.06	1.00000000	pigs
Osbpl10	-0.14	1.00000000	pigs
Slc35a3	-0.13	1.00000000	pigs
Ptk2	0.02	1.00000000	pigs
Alg2	-0.01	1.00000000	pigs
Creld2	0.00	1.00000000	pigs
Paip2	-0.02	1.00000000	pigs
Tram2	-0.31	1.00000000	pigs
Trip12	-0.03	1.00000000	pigs
Fam133b	-0.21	1.00000000	pigs
S100pbp	-0.11	1.00000000	pigs
Nid1	0.01	1.00000000	pigs
Poc1b	0.01	1.00000000	pigs
Pgp	0.15	1.00000000	pigs
Alg11	-0.10	1.00000000	pigs
Adamts12	-0.38	1.00000000	pigs
Dync1li2	-0.13	1.00000000	pigs
Utp15	-0.11	1.00000000	pigs
Bod1l	-0.11	1.00000000	pigs
Calhm5	-0.14	1.00000000	pigs
Prep	-0.19	1.00000000	pigs
Trim26	0.00	1.00000000	pigs
Rprd1b	-0.23	1.00000000	pigs
Hmgb2	-0.11	1.00000000	pigs
Fuca2	-0.10	1.00000000	pigs
Stt3b	-0.16	1.00000000	pigs
Stxbp5	-0.30	1.00000000	pigs
Ankrd10	-0.29	1.00000000	pigs
Nedd4l	0.05	1.00000000	pigs
Rufy3	-0.25	1.00000000	pigs
Fxr2	-0.12	1.00000000	pigs
Dnttip1	-0.16	1.00000000	pigs
Rpap3	-0.25	1.00000000	pigs
Fut11	-0.05	1.00000000	pigs
Ipo8	-0.14	1.00000000	pigs
Ro60	-0.17	1.00000000	pigs
Gnal	-0.17	1.00000000	pigs

Scai	-0.21	1.00000000	pigs
Gpatch8	-0.33	1.00000000	pigs
Ibtk	-0.23	1.00000000	pigs
Vti1b	-0.06	1.00000000	pigs
Dip2c	-0.05	1.00000000	pigs
Yipf1	-0.10	1.00000000	pigs
Zfp451	-0.23	1.00000000	pigs
Rbl2	-0.11	1.00000000	pigs
Fndc7	-0.01	1.00000000	pigs
Ccdc85b	-0.14	1.00000000	pigs
Agps	-0.15	1.00000000	pigs
Syngr1	-0.13	1.00000000	pigs
Prcc	-0.12	1.00000000	pigs
Cyb5r4	-0.11	1.00000000	pigs
Khsrp	-0.06	1.00000000	pigs
Plpbp	-0.12	1.00000000	pigs
Dhx30	-0.04	1.00000000	pigs
Gnb5	-0.08	1.00000000	pigs
Cdv3	-0.06	1.00000000	pigs
Rtn4rl2	-0.12	1.00000000	pigs
Smc3	-0.08	1.00000000	pigs
Rarg	-0.20	1.00000000	pigs
Derl2	-0.11	1.00000000	pigs
Arid1a	-0.19	1.00000000	pigs
Kars	0.06	1.00000000	pigs
Kif3a	-0.25	1.00000000	pigs
Clint1	-0.12	1.00000000	pigs
Dennd6a	-0.29	1.00000000	pigs
Fam168a	-0.32	1.00000000	pigs
Serinc1	-0.11	1.00000000	pigs
Armc8	-0.26	1.00000000	pigs
S1pr2	-0.14	1.00000000	pigs
Get3	-0.01	1.00000000	pigs
Lactb2	-0.11	1.00000000	pigs
Kif1c	0.03	1.00000000	pigs
Arfgef2	-0.11	1.00000000	pigs
Cecr2	-0.11	1.00000000	pigs
Ehmt1	-0.03	1.00000000	pigs
Rngtt	-0.20	1.00000000	pigs
Scamp4	-0.10	1.00000000	pigs
Ncbp1	-0.13	1.00000000	pigs
Wnk1	-0.19	1.00000000	pigs
Alox5ap	-0.25	1.00000000	pigs
Pola1	-0.14	1.00000000	pigs
Aldh1l2	-0.08	1.00000000	pigs
Poglut1	0.02	1.00000000	pigs

Exosc8	0.15	1.00000000	pigs
Kirrel3	-0.32	1.00000000	pigs
Dhx8	-0.16	1.00000000	pigs
Clybl	-0.17	1.00000000	pigs
Chsy3	0.08	1.00000000	pigs
Ppm1g	-0.19	1.00000000	pigs
Insig2	-0.04	1.00000000	pigs
Fbxw7	-0.13	1.00000000	pigs
Fkbp15	-0.08	1.00000000	pigs
Acin1	-0.04	1.00000000	pigs
Gas2l1	-0.14	1.00000000	pigs
Hacd3	-0.07	1.00000000	pigs
Fsd1l	-0.09	1.00000000	pigs
Sp1	-0.05	1.00000000	pigs
Kctd12	0.10	1.00000000	pigs
Igdcc4	-0.25	1.00000000	pigs
Itgb7	0.43	1.00000000	pigs
Ncbp3	-0.11	1.00000000	pigs
Slc25a22	0.04	1.00000000	pigs
Pithd1	-0.11	1.00000000	pigs
Stk10	-0.10	1.00000000	pigs
Dhx37	-0.13	1.00000000	pigs
Il17b	0.19	1.00000000	pigs
Rnf216	-0.06	1.00000000	pigs
Rsbn1l	-0.17	1.00000000	pigs
Ppp1r12b	-0.15	1.00000000	pigs
Amot	-0.10	1.00000000	pigs
Dock8	0.06	1.00000000	pigs
Ep400	-0.19	1.00000000	pigs
Syap1	-0.09	1.00000000	pigs
Tbx5	-0.50	1.00000000	pigs
Ssna1	0.10	1.00000000	pigs
Jchain	0.90	1.00000000	pigs
Trpm7	0.05	1.00000000	pigs
Rsf1	-0.13	1.00000000	pigs
Frmd4b	-0.10	1.00000000	pigs
Ccdc88a	-0.18	1.00000000	pigs
Ap1s2	-0.13	1.00000000	pigs
Slc22a15	-0.19	1.00000000	pigs
Pdia3	0.29	1.00000000	pigs
Gosr1	-0.20	1.00000000	pigs
Tbccd1	-0.09	1.00000000	pigs
Efr3a	-0.09	1.00000000	pigs
Nudc	-0.02	1.00000000	pigs
Eno2	-0.12	1.00000000	pigs
Ccnh	-0.05	1.00000000	pigs

Zfp236	-0.26	1.00000000	pigs
Ranbp17	-0.26	1.00000000	pigs
Nufip1	-0.13	1.00000000	pigs
Ehhadh	-0.03	1.00000000	pigs
Septin10	-0.05	1.00000000	pigs
Tmem9b	-0.04	1.00000000	pigs
Sec22b	-0.08	1.00000000	pigs
Rai2	-0.08	1.00000000	pigs
Rtn2	-0.10	1.00000000	pigs
Rab31	-0.32	1.00000000	pigs
Pcsk7	-0.15	1.00000000	pigs
Spred2	-0.42	1.00000000	pigs
Prdx6	0.31	1.00000000	pigs
Taf12	-0.04	1.00000000	pigs
BC034090	-0.04	1.00000000	pigs
Pkd1	-0.17	1.00000000	pigs
Sirt1	-0.15	1.00000000	pigs
Brms1	0.04	1.00000000	pigs
Yes1	-0.18	1.00000000	pigs
Ptprc	-0.23	1.00000000	pigs
Nt5c2	-0.06	1.00000000	pigs
Cox18	-0.04	1.00000000	pigs
Zfp383	-0.18	1.00000000	pigs
Kat14	-0.06	1.00000000	pigs
Btrc	-0.49	1.00000000	pigs
Nol7	-0.07	1.00000000	pigs
Pcdh18	0.07	1.00000000	pigs
Thap7	-0.13	1.00000000	pigs
Zfand3	-0.16	1.00000000	pigs
Cbx1	-0.20	1.00000000	pigs
Aldh16a1	-0.19	1.00000000	pigs
Arhgap17	-0.22	1.00000000	pigs
Eloc	0.03	1.00000000	pigs
Wdr70	-0.22	1.00000000	pigs
Pafah1b2	-0.20	1.00000000	pigs
Ctdspl	-0.08	1.00000000	pigs
Serpinb6b	-0.13	1.00000000	pigs
Dlst	-0.06	1.00000000	pigs
Nudcd1	-0.24	1.00000000	pigs
Bms1	-0.18	1.00000000	pigs
Tada1	-0.06	1.00000000	pigs
U2surp	-0.11	1.00000000	pigs
Atp6v0a2	-0.07	1.00000000	pigs
Skil	0.03	1.00000000	pigs
Lmna	0.22	1.00000000	pigs
Flnb	-0.25	1.00000000	pigs

Col6a1	-0.02	1.00000000	pigs
Cebpz	-0.26	1.00000000	pigs
Lsm8	0.13	1.00000000	pigs
Ewsr1	0.09	1.00000000	pigs
Aebp1	-0.21	1.00000000	pigs
Nadk	-0.04	1.00000000	pigs
Rictor	-0.19	1.00000000	pigs
Ilrun	-0.08	1.00000000	pigs
Crnkl1	-0.03	1.00000000	pigs
Crcp	-0.13	1.00000000	pigs
Asb9	-0.25	1.00000000	pigs
Thoc7	-0.18	1.00000000	pigs
Bmp5	-0.20	1.00000000	pigs
Zc3h15	0.03	1.00000000	pigs
Oxnad1	0.01	1.00000000	pigs
Dhx34	-0.04	1.00000000	pigs
Kdm1a	-0.09	1.00000000	pigs
Trp53inp1	-0.02	1.00000000	pigs
Nemf	-0.27	1.00000000	pigs
Ndufa11	-0.02	1.00000000	pigs
Cacul1	-0.02	1.00000000	pigs
Vegfa	0.01	1.00000000	pigs
Cep112	-0.26	1.00000000	pigs
Raly	-0.06	1.00000000	pigs
Ssr1	0.20	1.00000000	pigs
Tmem109	-0.13	1.00000000	pigs
Bcl2l13	-0.13	1.00000000	pigs
Abi2	-0.24	1.00000000	pigs
L2hgdh	0.02	1.00000000	pigs
Smo	-0.14	1.00000000	pigs
Pan3	-0.02	1.00000000	pigs
Tcf25	-0.03	1.00000000	pigs
Trim44	-0.29	1.00000000	pigs
Scaf4	0.03	1.00000000	pigs
Btbd6	-0.04	1.00000000	pigs
Tbc1d20	-0.20	1.00000000	pigs
Rpusd1	-0.11	1.00000000	pigs
Pde4d	-0.14	1.00000000	pigs
Atxn1	0.05	1.00000000	pigs
Hk3	0.06	1.00000000	pigs
Rassf8	0.00	1.00000000	pigs
Hspg2	-0.06	1.00000000	pigs
Cdk7	-0.20	1.00000000	pigs
Anks6	-0.19	1.00000000	pigs
Atp5a1	0.24	1.00000000	pigs
Klhl2	-0.01	1.00000000	pigs

Qk	0.03	1.00000000	pigs
Polr2b	-0.09	1.00000000	pigs
Zbtb40	-0.06	1.00000000	pigs
Bbs9	-0.06	1.00000000	pigs
Sik3	0.08	1.00000000	pigs
Rbm48	-0.13	1.00000000	pigs
Abl1	-0.19	1.00000000	pigs
Efhd1	-0.05	1.00000000	pigs
Pwwp3b	-0.14	1.00000000	pigs
Uba5	-0.26	1.00000000	pigs
Chic2	-0.13	1.00000000	pigs
Klc2	-0.09	1.00000000	pigs
Nup133	-0.12	1.00000000	pigs
Dnmt1	-0.17	1.00000000	pigs
Ddx50	-0.10	1.00000000	pigs
Rad21	-0.16	1.00000000	pigs
Tbc1d16	-0.14	1.00000000	pigs
Uevld	-0.18	1.00000000	pigs
Trpc1	-0.28	1.00000000	pigs
Ythdc1	-0.05	1.00000000	pigs
Wipi1	-0.23	1.00000000	pigs
Eipr1	-0.09	1.00000000	pigs
Lcorl	-0.10	1.00000000	pigs
Rsbn1	-0.19	1.00000000	pigs
Strn4	-0.12	1.00000000	pigs
Asprv1	-0.04	1.00000000	pigs
Slc35e3	-0.10	1.00000000	pigs
Dyrk1a	0.00	1.00000000	pigs
Phf12	0.02	1.00000000	pigs
Cnot1	-0.16	1.00000000	pigs
Zfp703	-0.07	1.00000000	pigs
Zbtb43	-0.05	1.00000000	pigs
Ttll12	0.01	1.00000000	pigs
Tfdp1	-0.11	1.00000000	pigs
Dcp2	-0.15	1.00000000	pigs
Snx27	-0.27	1.00000000	pigs
Slc10a1	-0.19	1.00000000	pigs
Pag1	0.22	1.00000000	pigs
Ankrd13a	-0.07	1.00000000	pigs
Slc12a6	-0.30	1.00000000	pigs
Pea15a	-0.14	1.00000000	pigs
Akap10	-0.06	1.00000000	pigs
Rbpms2	-0.09	1.00000000	pigs
Dpp4	-0.22	1.00000000	pigs
Ndufs1	-0.15	1.00000000	pigs
Tfeb	-0.13	1.00000000	pigs

Brd2	-0.10	1.00000000	pigs
Cep135	-0.17	1.00000000	pigs
Rapgef2	-0.12	1.00000000	pigs
Tulp3	-0.04	1.00000000	pigs
Crim1	-0.47	1.00000000	pigs
Gfpt1	-0.12	1.00000000	pigs
Sh3bp5	-0.10	1.00000000	pigs
Lmbrd2	-0.15	1.00000000	pigs
Aatf	-0.09	1.00000000	pigs
Fmnl2	0.04	1.00000000	pigs
C7	0.11	1.00000000	pigs
Galnt11	0.05	1.00000000	pigs
2210016L21Rik	0.03	1.00000000	pigs
Dusp28	-0.04	1.00000000	pigs
Pde4b	-0.40	1.00000000	pigs
Jdp2	-0.08	1.00000000	pigs
Psap	0.05	1.00000000	pigs
Rnf149	-0.09	1.00000000	pigs
Rgl1	-0.10	1.00000000	pigs
Ap1m1	-0.10	1.00000000	pigs
Sfxn1	-0.08	1.00000000	pigs
Psmd12	-0.01	1.00000000	pigs
Papss2	-0.09	1.00000000	pigs
2310022B05Rik	-0.15	1.00000000	pigs
Cers2	-0.02	1.00000000	pigs
Cul1	-0.20	1.00000000	pigs
Trim23	-0.16	1.00000000	pigs
Zcchc4	-0.09	1.00000000	pigs
Washc4	-0.22	1.00000000	pigs
Plekhg3	-0.09	1.00000000	pigs
Prepl	-0.05	1.00000000	pigs
Wiz	-0.15	1.00000000	pigs
Brd7	-0.26	1.00000000	pigs
Sclt1	-0.17	1.00000000	pigs
Pltp	-0.09	1.00000000	pigs
Gfm1	-0.11	1.00000000	pigs
Lcor	-0.10	1.00000000	pigs
Rassf1	-0.08	1.00000000	pigs
Cnn3	0.06	1.00000000	pigs
Wapl	-0.08	1.00000000	pigs
Rint1	-0.14	1.00000000	pigs
Plk4	-0.09	1.00000000	pigs
Nlk	-0.32	1.00000000	pigs
Armc1	-0.15	1.00000000	pigs
Nub1	0.07	1.00000000	pigs
Fam149b	-0.20	1.00000000	pigs

Slc37a1	0.12	1.00000000	pigs
Rif1	-0.22	1.00000000	pigs
Cdc42ep1	-0.17	1.00000000	pigs
Prnp	-0.05	1.00000000	pigs
Lysmd3	-0.10	1.00000000	pigs
Cabin1	-0.06	1.00000000	pigs
Irf2	-0.11	1.00000000	pigs
Zfp248	-0.17	1.00000000	pigs
Eprs	-0.15	1.00000000	pigs
Ptprm	0.11	1.00000000	pigs
Shmt1	-0.17	1.00000000	pigs
Krt10	-0.12	1.00000000	pigs
Terf2ip	0.07	1.00000000	pigs
Tmem263	0.01	1.00000000	pigs
Kank3	-0.15	1.00000000	pigs
Ctnnbl1	-0.09	1.00000000	pigs
Bcl9	-0.17	1.00000000	pigs
Brcc3	-0.14	1.00000000	pigs
Cox7a2	0.20	1.00000000	pigs
Zc3h13	-0.21	1.00000000	pigs
Nid2	-0.04	1.00000000	pigs
Usp5	-0.09	1.00000000	pigs
Inmt	-0.33	1.00000000	pigs
Slc25a4	0.05	1.00000000	pigs
Pigc	-0.07	1.00000000	pigs
Srpk2	-0.18	1.00000000	pigs
Luc7l	-0.23	1.00000000	pigs
Hcls1	-0.24	1.00000000	pigs
Pou2f1	-0.29	1.00000000	pigs
Pptc7	-0.18	1.00000000	pigs
Rad23a	-0.20	1.00000000	pigs
Ilk	0.05	1.00000000	pigs
Anxa11	0.05	1.00000000	pigs
Rfng	-0.20	1.00000000	pigs
Pdgfra	0.08	1.00000000	pigs
Telo2	-0.09	1.00000000	pigs
Ncs1	0.00	1.00000000	pigs
Atg4c	-0.26	1.00000000	pigs
Rbm17	-0.10	1.00000000	pigs
Atg5	-0.19	1.00000000	pigs
Armh3	-0.14	1.00000000	pigs
Eif1b	0.13	1.00000000	pigs
Abcf1	-0.15	1.00000000	pigs
Ctbp1	-0.14	1.00000000	pigs
Tmed10	-0.07	1.00000000	pigs
Paf1	-0.07	1.00000000	pigs

Hoxb6	-0.16	1.00000000	pigs
Stk40	-0.16	1.00000000	pigs
Ets2	0.13	1.00000000	pigs
Deptor	-0.20	1.00000000	pigs
Arhgef18	-0.21	1.00000000	pigs
Ccdc150	-0.13	1.00000000	pigs
Arhgef6	-0.17	1.00000000	pigs
Plekhh2	-0.41	1.00000000	pigs
Tshz3	-0.26	1.00000000	pigs
Wdr18	-0.08	1.00000000	pigs
Lmf2	-0.13	1.00000000	pigs
Kansl1l	-0.41	1.00000000	pigs
Epb41	-0.30	1.00000000	pigs
Acly	-0.21	1.00000000	pigs
Sik2	-0.23	1.00000000	pigs
Rpa1	-0.07	1.00000000	pigs
Pdia4	0.05	1.00000000	pigs
Rbm26	-0.12	1.00000000	pigs
Ccdc47	-0.21	1.00000000	pigs
Enox2	-0.20	1.00000000	pigs
Pheta2	-0.17	1.00000000	pigs
Ahi1	-0.12	1.00000000	pigs
Tmem50a	-0.02	1.00000000	pigs
Tcp11	0.04	1.00000000	pigs
Abhd16a	-0.13	1.00000000	pigs
Wdhd1	-0.05	1.00000000	pigs
Ipo11	-0.38	1.00000000	pigs
Ankrd33b	-0.15	1.00000000	pigs
Dync2li1	-0.15	1.00000000	pigs
Cep128	-0.32	1.00000000	pigs
Stau2	-0.22	1.00000000	pigs
Erg	-0.14	1.00000000	pigs
H6pd	-0.22	1.00000000	pigs
Tbc1d22b	-0.14	1.00000000	pigs
Syf2	-0.07	1.00000000	pigs
Samd4	-0.11	1.00000000	pigs
Atp6v0d1	0.09	1.00000000	pigs
Slc25a15	-0.17	1.00000000	pigs
Gdpd5	-0.10	1.00000000	pigs
Smarca5	0.08	1.00000000	pigs
Trappc3	-0.01	1.00000000	pigs
Amotl1	-0.17	1.00000000	pigs
Pcmtd1	-0.18	1.00000000	pigs
Sqle	-0.04	1.00000000	pigs
Pkia	-0.13	1.00000000	pigs
Hmox2	0.04	1.00000000	pigs

Zc3h14	0.11	1.00000000	pigs
Gtf2h1	-0.05	1.00000000	pigs
Rbm6	-0.02	1.00000000	pigs
Ap3m1	0.11	1.00000000	pigs
Bambi	-0.10	1.00000000	pigs
Srebf1	-0.11	1.00000000	pigs
Btbd1	-0.07	1.00000000	pigs
Arfgef1	0.02	1.00000000	pigs
Cltb	-0.06	1.00000000	pigs
Nxt1	0.06	1.00000000	pigs
Selenos	-0.08	1.00000000	pigs
Orc2	-0.14	1.00000000	pigs
Ubtd2	-0.09	1.00000000	pigs
Retreg3	-0.11	1.00000000	pigs
Pde1a	-0.27	1.00000000	pigs
Hpf1	-0.09	1.00000000	pigs
Hnrnpm	0.15	1.00000000	pigs
Cnppd1	-0.12	1.00000000	pigs
Gpn1	-0.04	1.00000000	pigs
Elavl1	-0.04	1.00000000	pigs
Crbn	-0.18	1.00000000	pigs
Pphln1	-0.11	1.00000000	pigs
Kansl2	-0.05	1.00000000	pigs
Rnft1	-0.13	1.00000000	pigs
2410004B18Rik	-0.06	1.00000000	pigs
Ccdc6	-0.23	1.00000000	pigs
Ephb3	-0.06	1.00000000	pigs
St7l	-0.18	1.00000000	pigs
Otud3	-0.07	1.00000000	pigs
Ephx1	-0.12	1.00000000	pigs
Nrbf2	-0.08	1.00000000	pigs
Nipal2	-0.22	1.00000000	pigs
Phkg2	-0.16	1.00000000	pigs
Tmem59	0.18	1.00000000	pigs
Lmtk2	-0.15	1.00000000	pigs
Trpc4ap	-0.03	1.00000000	pigs
Esf1	-0.18	1.00000000	pigs
Rbm5	-0.08	1.00000000	pigs
Zfp28	-0.19	1.00000000	pigs
Zfp9	-0.14	1.00000000	pigs
Dennd4c	-0.17	1.00000000	pigs
Abi1	-0.11	1.00000000	pigs
Prkcb	0.08	1.00000000	pigs
Pdia6	0.18	1.00000000	pigs
Thoc2	-0.10	1.00000000	pigs
Tle5	-0.08	1.00000000	pigs

Sipa1l2	0.14	1.00000000	pigs
2510009E07Rik	-0.32	1.00000000	pigs
Lats1	-0.21	1.00000000	pigs
Rrp8	-0.03	1.00000000	pigs
BC005624	-0.05	1.00000000	pigs
Fnip2	-0.22	1.00000000	pigs
Samm50	-0.01	1.00000000	pigs
Usf3	-0.29	1.00000000	pigs
Larp4	-0.15	1.00000000	pigs
Ing3	-0.08	1.00000000	pigs
Erbin	0.13	1.00000000	pigs
Hlcs	-0.04	1.00000000	pigs
Cnot2	-0.10	1.00000000	pigs
Fbxo4	-0.17	1.00000000	pigs
Radil	-0.24	1.00000000	pigs
Snx32	-0.05	1.00000000	pigs
Zmym1	-0.22	1.00000000	pigs
Slit3	0.02	1.00000000	pigs
Snx4	-0.16	1.00000000	pigs
Sltm	-0.09	1.00000000	pigs
Rfc3	-0.12	1.00000000	pigs
Hebp1	-0.04	1.00000000	pigs
Ndufb10	-0.01	1.00000000	pigs
Slc35d1	-0.05	1.00000000	pigs
Guf1	-0.19	1.00000000	pigs
Capzb	0.20	1.00000000	pigs
Entpd7	-0.05	1.00000000	pigs
Twsg1	-0.16	1.00000000	pigs
Chp1	-0.22	1.00000000	pigs
Ppp2cb	-0.28	1.00000000	pigs
Smc6	-0.08	1.00000000	pigs
Commd5	-0.15	1.00000000	pigs
Stk4	0.10	1.00000000	pigs
Ap4s1	-0.05	1.00000000	pigs
Zc3h7b	-0.21	1.00000000	pigs
Cdadc1	0.11	1.00000000	pigs
Smg6	-0.25	1.00000000	pigs
Sergef	-0.32	1.00000000	pigs
Cnot11	-0.03	1.00000000	pigs
Skp1	0.04	1.00000000	pigs
Slc38a2	0.03	1.00000000	pigs
Pcnx4	-0.23	1.00000000	pigs
Prkar1a	0.17	1.00000000	pigs
Pik3ca	-0.21	1.00000000	pigs
Rbm47	-0.04	1.00000000	pigs
Pnpt1	-0.13	1.00000000	pigs

Rexo5	-0.19	1.00000000	pigs
Socs6	0.03	1.00000000	pigs
Zfp362	-0.07	1.00000000	pigs
Nnt	-0.09	1.00000000	pigs
AU040320	-0.20	1.00000000	pigs
Psmd6	-0.21	1.00000000	pigs
Srbd1	-0.30	1.00000000	pigs
Arl14ep	-0.18	1.00000000	pigs
Clasp1	0.00	1.00000000	pigs
Xpo6	-0.45	1.00000000	pigs
Slc9a1	-0.02	1.00000000	pigs
Sema3a	-0.12	1.00000000	pigs
Fst	-0.22	1.00000000	pigs
Hipk3	-0.13	1.00000000	pigs
Lrrk1	-0.33	1.00000000	pigs
Gnb4	-0.15	1.00000000	pigs
Trim33	-0.23	1.00000000	pigs
Ruvbl1	-0.07	1.00000000	pigs
Dnaja2	0.01	1.00000000	pigs
Psmd1	0.02	1.00000000	pigs
Rnf214	-0.26	1.00000000	pigs
Rev3l	0.01	1.00000000	pigs
Tfdp2	-0.02	1.00000000	pigs
Dapp1	0.20	1.00000000	pigs
Rnf40	-0.10	1.00000000	pigs
Armc2	-0.24	1.00000000	pigs
Klhdc1	-0.26	1.00000000	pigs
Vcl	-0.39	1.00000000	pigs
Derl1	0.06	1.00000000	pigs
Oxr1	-0.10	1.00000000	pigs
Senp2	-0.22	1.00000000	pigs
Dusp1	-0.10	1.00000000	pigs
Trim72	-0.20	1.00000000	pigs
Golga1	-0.08	1.00000000	pigs
Deup1	-0.14	1.00000000	pigs
Thbs1	-0.43	1.00000000	pigs
Pcgf3	-0.23	1.00000000	pigs
Gpat4	-0.22	1.00000000	pigs
Actr2	0.18	1.00000000	pigs
Ccdc117	-0.18	1.00000000	pigs
Rab30	-0.10	1.00000000	pigs
Sec16a	-0.06	1.00000000	pigs
Dync1h1	-0.15	1.00000000	pigs
Fam193b	-0.07	1.00000000	pigs
Rfx7	-0.26	1.00000000	pigs
Bend7	-0.22	1.00000000	pigs

Ano6	-0.03	1.00000000	pigs
Vezt	-0.15	1.00000000	pigs
Xxylt1	-0.13	1.00000000	pigs
Senp5	-0.10	1.00000000	pigs
Sf3b2	0.11	1.00000000	pigs
Aff4	-0.02	1.00000000	pigs
Vps28	-0.03	1.00000000	pigs
Ybx3	-0.02	1.00000000	pigs
Sprtn	-0.18	1.00000000	pigs
Nab1	-0.11	1.00000000	pigs
Cox4i1	0.05	1.00000000	pigs
Ate1	-0.18	1.00000000	pigs
Xpr1	-0.27	1.00000000	pigs
Rac2	0.69	1.00000000	pigs
Syne2	0.03	1.00000000	pigs
Smim15	-0.16	1.00000000	pigs
Zfp770	-0.10	1.00000000	pigs
Ppan	-0.03	1.00000000	pigs
Rbsn	-0.03	1.00000000	pigs
Cenpc1	0.00	1.00000000	pigs
Grk6	-0.13	1.00000000	pigs
Tmpo	-0.06	1.00000000	pigs
Atad1	-0.25	1.00000000	pigs
Stk17b	-0.11	1.00000000	pigs
Anxa7	-0.04	1.00000000	pigs
Usp7	-0.11	1.00000000	pigs
Secisbp2l	-0.18	1.00000000	pigs
Trappc11	-0.15	1.00000000	pigs
Apaf1	-0.30	1.00000000	pigs
Ino80d	-0.21	1.00000000	pigs
Hook2	-0.04	1.00000000	pigs
Adam15	-0.17	1.00000000	pigs
Sec13	-0.10	1.00000000	pigs
Fasn	-0.02	1.00000000	pigs
Sart3	-0.02	1.00000000	pigs
Fam13b	-0.21	1.00000000	pigs
Dusp16	-0.21	1.00000000	pigs
Wasf2	0.03	1.00000000	pigs
Ikzf5	-0.13	1.00000000	pigs
Gnai3	0.08	1.00000000	pigs
Nxf1	-0.17	1.00000000	pigs
Alms1	-0.20	1.00000000	pigs
Crebl2	-0.13	1.00000000	pigs
Arhgap26	-0.14	1.00000000	pigs
Reps1	-0.09	1.00000000	pigs
Scaper	-0.18	1.00000000	pigs

Ccnt2	-0.26	1.00000000	pigs
Thap11	0.14	1.00000000	pigs
Nme7	-0.19	1.00000000	pigs
Kdelr3	0.18	1.00000000	pigs
Yif1b	0.09	1.00000000	pigs
Zfp664	-0.02	1.00000000	pigs
Slc39a7	-0.25	1.00000000	pigs
Gpatch2	-0.21	1.00000000	pigs
Trak2	-0.23	1.00000000	pigs
Nap1l4	-0.09	1.00000000	pigs
Btbd7	-0.16	1.00000000	pigs
Slc30a4	-0.16	1.00000000	pigs
Als2	0.02	1.00000000	pigs
Sfswap	-0.29	1.00000000	pigs
Nek7	-0.04	1.00000000	pigs
Foxp4	-0.13	1.00000000	pigs
Rai14	-0.29	1.00000000	pigs
Qrich1	0.04	1.00000000	pigs
Cep97	-0.03	1.00000000	pigs
Stau1	-0.11	1.00000000	pigs
Rpp21	0.06	1.00000000	pigs
Atp6v0a1	0.01	1.00000000	pigs
Btbd2	-0.09	1.00000000	pigs
AB124611	-0.18	1.00000000	pigs
Robo1	-0.57	1.00000000	pigs
Inpp5d	-0.20	1.00000000	pigs
Tcf20	-0.19	1.00000000	pigs
Ide	-0.19	1.00000000	pigs
Larp6	-0.22	1.00000000	pigs
Nsa2	0.00	1.00000000	pigs
Zdhhc17	-0.19	1.00000000	pigs
Lsm14a	-0.06	1.00000000	pigs
Cdr2	-0.19	1.00000000	pigs
Dip2b	-0.06	1.00000000	pigs
Tmem160	0.03	1.00000000	pigs
Ddhd1	-0.35	1.00000000	pigs
Tars	-0.24	1.00000000	pigs
Selenoo	-0.12	1.00000000	pigs
Sufu	-0.34	1.00000000	pigs
Socs4	-0.18	1.00000000	pigs
Arpc5	0.17	1.00000000	pigs
Ghr	-0.20	1.00000000	pigs
Suclg1	0.02	1.00000000	pigs
Spin1	-0.22	1.00000000	pigs
Emb	-0.18	1.00000000	pigs
Cox8a	0.32	1.00000000	pigs

Arf1	0.25	1.00000000	pigs
Rab8b	-0.08	1.00000000	pigs
Rab6a	-0.19	1.00000000	pigs
Rep15	-0.19	1.00000000	pigs
Hacd1	0.01	1.00000000	pigs
Nrd1	-0.26	1.00000000	pigs
Stx7	0.17	1.00000000	pigs
Lamp2	0.01	1.00000000	pigs
Pcgf5	-0.22	1.00000000	pigs
Syde1	-0.11	1.00000000	pigs
Tcerg1	-0.15	1.00000000	pigs
Atp7a	-0.09	1.00000000	pigs
Bbs7	-0.06	1.00000000	pigs
Sertad1	0.07	1.00000000	pigs
Tnrc18	-0.18	1.00000000	pigs
Vwa8	-0.25	1.00000000	pigs
Pggt1b	-0.34	1.00000000	pigs
Rabgap1	-0.11	1.00000000	pigs
Gli3	0.06	1.00000000	pigs
Cox5b-ps	0.21	1.00000000	pigs
Brpf3	-0.17	1.00000000	pigs
Stk3	-0.04	1.00000000	pigs
Nasp	0.14	1.00000000	pigs
Cwc22	-0.08	1.00000000	pigs
Tspan5	-0.16	1.00000000	pigs
Dalrd3	0.02	1.00000000	pigs
Lrpprc	-0.14	1.00000000	pigs
Drap1	0.03	1.00000000	pigs
Svil	-0.10	1.00000000	pigs
Eps15	-0.06	1.00000000	pigs
Nr3c2	-0.26	1.00000000	pigs
Arhgap42	-0.48	1.00000000	pigs
Rnf13	-0.10	1.00000000	pigs
Naa30	-0.15	1.00000000	pigs
Eapp	-0.15	1.00000000	pigs
Dock5	-0.20	1.00000000	pigs
Uggt1	-0.12	1.00000000	pigs
Virma	-0.11	1.00000000	pigs
Hace1	-0.17	1.00000000	pigs
Tbc1d12	-0.16	1.00000000	pigs
Nrbp1	-0.13	1.00000000	pigs
Cd53	0.12	1.00000000	pigs
Wdr44	-0.16	1.00000000	pigs
Arfgap2	-0.11	1.00000000	pigs
Atf2	-0.23	1.00000000	pigs
Leng8	-0.15	1.00000000	pigs

Blzf1	-0.08	1.00000000	pigs
Abraxas1	-0.04	1.00000000	pigs
Sec22a	-0.29	1.00000000	pigs
Irs2	-0.01	1.00000000	pigs
Hmgcs1	-0.09	1.00000000	pigs
U2af2	-0.01	1.00000000	pigs
Wdr75	-0.23	1.00000000	pigs
Pum3	-0.21	1.00000000	pigs
Clock	-0.03	1.00000000	pigs
Cse1l	-0.03	1.00000000	pigs
Nr4a1	-0.22	1.00000000	pigs
Rhbdf1	-0.12	1.00000000	pigs
Klf9	0.00	1.00000000	pigs
Vav1	0.08	1.00000000	pigs
Fbxl4	-0.01	1.00000000	pigs
Pld3	-0.09	1.00000000	pigs
Enho	-0.21	1.00000000	pigs
Brd9	-0.29	1.00000000	pigs
Sf3a1	-0.19	1.00000000	pigs
Avl9	-0.05	1.00000000	pigs
Hs6st1	-0.15	1.00000000	pigs
Dock2	-0.12	1.00000000	pigs
Thumpd1	-0.16	1.00000000	pigs
Ranbp3	-0.17	1.00000000	pigs
Afg1l	-0.27	1.00000000	pigs
Tex9	-0.13	1.00000000	pigs
Itsn1	0.10	1.00000000	pigs
Vps50	-0.24	1.00000000	pigs
Fxr1	0.00	1.00000000	pigs
Elp4	-0.33	1.00000000	pigs
Bag4	-0.19	1.00000000	pigs
Tln2	-0.11	1.00000000	pigs
Braf	0.15	1.00000000	pigs
Gapvd1	-0.25	1.00000000	pigs
Rap1b	0.04	1.00000000	pigs
Ttc27	-0.16	1.00000000	pigs
Ago1	-0.04	1.00000000	pigs
Daam1	-0.09	1.00000000	pigs
Tsr2	-0.05	1.00000000	pigs
Klf3	-0.19	1.00000000	pigs
Taf13	-0.21	1.00000000	pigs
Pdcl3	-0.20	1.00000000	pigs
Taf11	-0.05	1.00000000	pigs
Cpe	-0.07	1.00000000	pigs
Ptger4	-0.07	1.00000000	pigs
Arl6ip1	0.14	1.00000000	pigs

Nop2	-0.10	1.00000000	pigs
Trrap	-0.09	1.00000000	pigs
Piwil4	-0.13	1.00000000	pigs
Pdss2	-0.15	1.00000000	pigs
Xpa	0.00	1.00000000	pigs
Gadd45gip1	-0.04	1.00000000	pigs
Rpn2	0.06	1.00000000	pigs
Strn3	-0.16	1.00000000	pigs
Pik3c3	-0.27	1.00000000	pigs
Lin7c	-0.11	1.00000000	pigs
Fam53b	-0.12	1.00000000	pigs
Tgfb1i1	-0.17	1.00000000	pigs
Ltn1	-0.13	1.00000000	pigs
Tiam1	-0.03	1.00000000	pigs
Cdc37	-0.04	1.00000000	pigs
Ndufaf1	-0.05	1.00000000	pigs
Rab11fip2	-0.15	1.00000000	pigs
Ccdc173	-0.13	1.00000000	pigs
Dus1l	-0.09	1.00000000	pigs
Relch	-0.25	1.00000000	pigs
G3bp1	0.07	1.00000000	pigs
Pwwp2a	-0.25	1.00000000	pigs
Nt5c3b	-0.10	1.00000000	pigs
St13	0.08	1.00000000	pigs
Ndufa13	0.01	1.00000000	pigs
Nop56	-0.10	1.00000000	pigs
Ints9	-0.19	1.00000000	pigs
Bicd2	-0.13	1.00000000	pigs
Tbrg1	-0.16	1.00000000	pigs
Baz1b	-0.22	1.00000000	pigs
Hdac9	-0.15	1.00000000	pigs
Pkd2	-0.12	1.00000000	pigs
Znfx1	-0.29	1.00000000	pigs
Ikbkg	-0.15	1.00000000	pigs
Dph6	-0.34	1.00000000	pigs
Ttc14	-0.23	1.00000000	pigs
Atrn	-0.21	1.00000000	pigs
Vldlr	-0.13	1.00000000	pigs
Tcf7l2	0.00	1.00000000	pigs
Brd3	-0.23	1.00000000	pigs
Xrcc5	-0.29	1.00000000	pigs
Lamc2	-0.16	1.00000000	pigs
Grk2	0.34	1.00000000	pigs
Ube2b	0.02	1.00000000	pigs
Lclat1	-0.23	1.00000000	pigs
Qser1	-0.10	1.00000000	pigs

Dstn	0.13	1.00000000	pigs
Scarb2	-0.04	1.00000000	pigs
Rrm1	0.01	1.00000000	pigs
Ubc	-0.03	1.00000000	pigs
Haus6	-0.15	1.00000000	pigs
Fut8	-0.18	1.00000000	pigs
Gkap1	-0.21	1.00000000	pigs
Rbm12b2	-0.09	1.00000000	pigs
Cat	-0.03	1.00000000	pigs
Ttc21b	-0.21	1.00000000	pigs
Igsf8	-0.06	1.00000000	pigs
Armc9	-0.19	1.00000000	pigs
Eea1	-0.11	1.00000000	pigs
Lima1	-0.17	1.00000000	pigs
Zyg11b	-0.03	1.00000000	pigs
Ric1	-0.15	1.00000000	pigs
Fto	-0.11	1.00000000	pigs
Pogz	-0.16	1.00000000	pigs
Tet1	-0.33	1.00000000	pigs
Aasdh	-0.22	1.00000000	pigs
Lmbrd1	-0.16	1.00000000	pigs
Wdr36	-0.22	1.00000000	pigs
Nfx1	-0.22	1.00000000	pigs
Cog5	-0.19	1.00000000	pigs
Slc16a1	-0.12	1.00000000	pigs
Apbb3	-0.08	1.00000000	pigs
Ttc1	-0.16	1.00000000	pigs
Tmed8	-0.12	1.00000000	pigs
Adarb1	-0.11	1.00000000	pigs
Gng10	0.24	1.00000000	pigs
Riok1	-0.10	1.00000000	pigs
Stim1	-0.22	1.00000000	pigs
Pja2	-0.24	1.00000000	pigs
Otud7b	-0.27	1.00000000	pigs
Cntln	-0.29	1.00000000	pigs
Fam76a	-0.01	1.00000000	pigs
Tmx4	-0.03	1.00000000	pigs
Pgm2l1	-0.19	1.00000000	pigs
Slc22a17	-0.13	1.00000000	pigs
Pinx1	-0.31	1.00000000	pigs
Rrp12	-0.12	1.00000000	pigs
Dedd	0.00	1.00000000	pigs
Setd3	-0.11	1.00000000	pigs
Serpinh1	0.11	1.00000000	pigs
Jade1	-0.05	1.00000000	pigs
Ufl1	-0.20	1.00000000	pigs

Trmt112	0.22	1.00000000	pigs
Agl	-0.17	1.00000000	pigs
Caml	-0.11	1.00000000	pigs
Poldip3	-0.19	1.00000000	pigs
Dyrk2	-0.18	1.00000000	pigs
Ntan1	-0.20	1.00000000	pigs
Nrde2	-0.02	1.00000000	pigs
Thumpd3	-0.06	1.00000000	pigs
Agap1	-0.04	1.00000000	pigs
Sgsm2	-0.13	1.00000000	pigs
Camk2g	-0.24	1.00000000	pigs
Xpo7	-0.36	1.00000000	pigs
Dnajb14	-0.15	1.00000000	pigs
Shld1	-0.29	1.00000000	pigs
Gli2	-0.12	1.00000000	pigs
Phlpp2	-0.04	1.00000000	pigs
Nsf	-0.17	1.00000000	pigs
Fbxo21	-0.16	1.00000000	pigs
Bag5	0.06	1.00000000	pigs
Jazf1	0.11	1.00000000	pigs
Zfp618	-0.13	1.00000000	pigs
Uhrf1bp1l	-0.11	1.00000000	pigs
Rnh1	0.06	1.00000000	pigs
Chid1	-0.06	1.00000000	pigs
Imp4	-0.07	1.00000000	pigs
Pes1	-0.10	1.00000000	pigs
Trak1	-0.15	1.00000000	pigs
Zhx3	-0.16	1.00000000	pigs
Klhdc2	-0.12	1.00000000	pigs
Cab39	-0.02	1.00000000	pigs
Rcn2	-0.22	1.00000000	pigs
Usp15	-0.33	1.00000000	pigs
Nfat5	-0.01	1.00000000	pigs
Ndufa10	0.06	1.00000000	pigs
Nhej1	-0.05	1.00000000	pigs
Lnpep	-0.15	1.00000000	pigs
Ppip5k2	-0.37	1.00000000	pigs
Pdgfrl	-0.18	1.00000000	pigs
Ccdc126	-0.21	1.00000000	pigs
Pde7b	-0.04	1.00000000	pigs
Creb5	-0.03	1.00000000	pigs
Ap2b1	-0.14	1.00000000	pigs
Smu1	-0.02	1.00000000	pigs
Srp14	0.13	1.00000000	pigs
Gcn1	-0.11	1.00000000	pigs
Aldh2	0.01	1.00000000	pigs

Bcl9l	-0.06	1.00000000	pigs
Trim3	0.11	1.00000000	pigs
Gigyf1	-0.07	1.00000000	pigs
Nr2f2	-0.23	1.00000000	pigs
Wdfy1	0.15	1.00000000	pigs
Shprh	-0.26	1.00000000	pigs
Ifngr1	-0.12	1.00000000	pigs
Exosc10	-0.03	1.00000000	pigs
Loxl2	-0.01	1.00000000	pigs
Adamts3	-0.29	1.00000000	pigs
Nap1l1	0.14	1.00000000	pigs
Pth1r	-0.20	1.00000000	pigs
Slc38a9	-0.28	1.00000000	pigs
Anxa1	-0.08	1.00000000	pigs
Rpf2	-0.05	1.00000000	pigs
Zbtb16	0.06	1.00000000	pigs
Lrrc59	0.07	1.00000000	pigs
Borcs5	-0.20	1.00000000	pigs
Vps4b	-0.13	1.00000000	pigs
Arhgap32	-0.12	1.00000000	pigs
Heatr6	-0.21	1.00000000	pigs
Kdm2a	-0.14	1.00000000	pigs
Ubxn2b	-0.06	1.00000000	pigs
Ncoa1	-0.25	1.00000000	pigs
Nup54	-0.17	1.00000000	pigs
Uhmk1	-0.16	1.00000000	pigs
Tm4sf1	0.14	1.00000000	pigs
Smoc2	-0.17	1.00000000	pigs
Naa25	-0.16	1.00000000	pigs
Pdcd10	0.05	1.00000000	pigs
Prss55	-0.18	1.00000000	pigs
Cpne8	-0.20	1.00000000	pigs
Tab3	-0.15	1.00000000	pigs
Cd44	0.07	1.00000000	pigs
S100a4	0.32	1.00000000	pigs
Gap43	-0.27	1.00000000	pigs
Tm9sf4	-0.08	1.00000000	pigs
G0s2	0.26	1.00000000	pigs
Zfp827	-0.13	1.00000000	pigs
Cisd3	-0.04	1.00000000	pigs
Tagap1	0.40	1.00000000	pigs
Pikfyve	-0.34	1.00000000	pigs
Slc12a4	-0.25	1.00000000	pigs
Rhoc	0.00	1.00000000	pigs
Tram1	0.03	1.00000000	pigs
Pfdn2	0.14	1.00000000	pigs

Kpna4	0.00	1.00000000	pigs
Pdcl	-0.16	1.00000000	pigs
Ythdf3	-0.17	1.00000000	pigs
Adamts17	-0.36	1.00000000	pigs
Ift81	-0.23	1.00000000	pigs
Nedd9	-0.42	1.00000000	pigs
Smarce1	-0.06	1.00000000	pigs
Eif3b	-0.02	1.00000000	pigs
Tgds	-0.13	1.00000000	pigs
Prdm8	-0.22	1.00000000	pigs
Rilpl1	-0.19	1.00000000	pigs
Cul3	-0.06	1.00000000	pigs
Efcab7	-0.11	1.00000000	pigs
Slc30a9	-0.18	1.00000000	pigs
Cdc42ep4	-0.15	1.00000000	pigs
Arhgap5	-0.14	1.00000000	pigs
4932438A13Rik	-0.24	1.00000000	pigs
Zfp462	-0.19	1.00000000	pigs
Pwp1	0.06	1.00000000	pigs
Hlf	-0.17	1.00000000	pigs
Zfp672	-0.02	1.00000000	pigs
Ank	-0.19	1.00000000	pigs
Ndufs4	-0.05	1.00000000	pigs
Glcci1	-0.22	1.00000000	pigs
Fryl	0.03	1.00000000	pigs
Sema4a	-0.13	1.00000000	pigs
Stt3a	-0.16	1.00000000	pigs
Fam174a	-0.14	1.00000000	pigs
Adnp2	-0.03	1.00000000	pigs
Dtwd1	-0.19	1.00000000	pigs
Ssh1	-0.16	1.00000000	pigs
Txndc9	-0.06	1.00000000	pigs
Septin11	0.03	1.00000000	pigs
Rcan2	0.20	1.00000000	pigs
Cybrd1	0.09	1.00000000	pigs
Rcor3	-0.23	1.00000000	pigs
Rbm41	-0.23	1.00000000	pigs
Preb	0.00	1.00000000	pigs
Asap3	-0.27	1.00000000	pigs
Pde4dip	-0.19	1.00000000	pigs
Stx18	-0.19	1.00000000	pigs
Pde4a	-0.04	1.00000000	pigs
Zfp326	-0.26	1.00000000	pigs
Pkig	-0.02	1.00000000	pigs
Klf11	-0.21	1.00000000	pigs
Psme4	-0.20	1.00000000	pigs

Vps26a	-0.17	1.00000000	pigs
Utp20	-0.11	1.00000000	pigs
Osbpl8	-0.11	1.00000000	pigs
Thrap3	0.00	1.00000000	pigs
Atg13	-0.26	1.00000000	pigs
Vps53	-0.25	1.00000000	pigs
Snd1	-0.03	1.00000000	pigs
Pced1b	-0.10	1.00000000	pigs
Strbp	-0.12	1.00000000	pigs
Scaf8	-0.13	1.00000000	pigs
Dgkd	-0.02	1.00000000	pigs
Prkrip1	-0.11	1.00000000	pigs
Ndufb7	0.06	1.00000000	pigs
Fhl2	-0.20	1.00000000	pigs
Pigk	-0.20	1.00000000	pigs
Ubn2	-0.23	1.00000000	pigs
Gclc	-0.17	1.00000000	pigs
Cox7c	0.23	1.00000000	pigs
Irf2bp2	-0.15	1.00000000	pigs
Trim2	-0.22	1.00000000	pigs
Ddx17	0.03	1.00000000	pigs
Lrig3	-0.25	1.00000000	pigs
Pfkp	-0.07	1.00000000	pigs
Phf3	-0.07	1.00000000	pigs
Cep164	-0.17	1.00000000	pigs
Fubp1	-0.07	1.00000000	pigs
Dbi	0.13	1.00000000	pigs
Zfp667	-0.16	1.00000000	pigs
Gpatch1	-0.16	1.00000000	pigs
Ece1	-0.18	1.00000000	pigs
Phkb	-0.28	1.00000000	pigs
Cenpp	-0.27	1.00000000	pigs
Napa	-0.17	1.00000000	pigs
Nipsnap2	-0.22	1.00000000	pigs
Tmem87a	-0.16	1.00000000	pigs
Zfp30	-0.33	1.00000000	pigs
Trank1	-0.24	1.00000000	pigs
Zcchc8	-0.26	1.00000000	pigs
Prkaca	-0.11	1.00000000	pigs
Hacd2	-0.14	1.00000000	pigs
Kin	-0.09	1.00000000	pigs
Fan1	-0.18	1.00000000	pigs
Tln1	-0.11	1.00000000	pigs
Ppm1a	-0.03	1.00000000	pigs
Reck	-0.23	1.00000000	pigs
Psmc5	-0.06	1.00000000	pigs

Tstd2	-0.10	1.00000000	pigs
Lrp2bp	-0.22	1.00000000	pigs
Plcg1	-0.05	1.00000000	pigs
Asxl2	-0.33	1.00000000	pigs
Fdx1	-0.23	1.00000000	pigs
Plek	0.27	1.00000000	pigs
Kdm4b	-0.06	1.00000000	pigs
Iars2	-0.11	1.00000000	pigs
Cpxm2	-0.22	1.00000000	pigs
Snw1	-0.11	1.00000000	pigs
Ptgr1	-0.08	1.00000000	pigs
Orai1	-0.01	1.00000000	pigs
Xrn2	-0.06	1.00000000	pigs
Iqsec1	-0.09	1.00000000	pigs
Yae1d1	-0.03	1.00000000	pigs
Bpnt1	0.01	1.00000000	pigs
Ssb	-0.02	1.00000000	pigs
Vapb	-0.14	1.00000000	pigs
Kmt5b	-0.16	1.00000000	pigs
Nup160	-0.20	1.00000000	pigs
Rab5a	-0.08	1.00000000	pigs
Etf1	-0.07	1.00000000	pigs
Gtf2f1	-0.10	1.00000000	pigs
Gtf2i	-0.20	1.00000000	pigs
Twf1	-0.10	1.00000000	pigs
Dnase1l1	-0.18	1.00000000	pigs
Kpna3	-0.29	1.00000000	pigs
Rap1gds1	-0.06	1.00000000	pigs
Hbb-bh2	-0.35	1.00000000	pigs
Arnt	0.09	1.00000000	pigs
Gck	-0.12	1.00000000	pigs
Slc8a1	-0.05	1.00000000	pigs
Dab2ip	-0.15	1.00000000	pigs
Ip6k1	-0.32	1.00000000	pigs
Gpkow	-0.15	1.00000000	pigs
Gxylt1	-0.19	1.00000000	pigs
2700049A03Rik	-0.13	1.00000000	pigs
Hnrnpk	0.17	1.00000000	pigs
Atg7	-0.26	1.00000000	pigs
Lims2	-0.10	1.00000000	pigs
Far1	-0.21	1.00000000	pigs
Eloa	-0.10	1.00000000	pigs
Brd4	-0.02	1.00000000	pigs
Nol9	-0.12	1.00000000	pigs
Gpr180	-0.24	1.00000000	pigs
Pex1	-0.06	1.00000000	pigs

Tle4	-0.22	1.00000000	pigs
Cwc25	-0.17	1.00000000	pigs
Hmgxb4	-0.14	1.00000000	pigs
Rgs19	0.19	1.00000000	pigs
Suz12	-0.15	1.00000000	pigs
Dnajc24	-0.05	1.00000000	pigs
Cdkn1c	-0.15	1.00000000	pigs
Slc1a2	0.09	1.00000000	pigs
Esr1	-0.20	1.00000000	pigs
Sgms1	-0.28	1.00000000	pigs
Eogt	-0.26	1.00000000	pigs
Naa35	-0.22	1.00000000	pigs
Cand1	-0.03	1.00000000	pigs
Rsrc2	-0.04	1.00000000	pigs
Exo5	-0.13	1.00000000	pigs
Tacc2	-0.21	1.00000000	pigs
Copz2	-0.01	1.00000000	pigs
Snrnp25	0.04	1.00000000	pigs
Ythdc2	-0.25	1.00000000	pigs
Inpp5a	-0.26	1.00000000	pigs
Sptlc1	-0.02	1.00000000	pigs
Shroom4	-0.04	1.00000000	pigs
Dad1	-0.03	1.00000000	pigs
Btbd10	-0.25	1.00000000	pigs
Adam10	-0.16	1.00000000	pigs
Ecrg4	-0.19	1.00000000	pigs
Ankrd50	-0.01	1.00000000	pigs
Pkn2	0.01	1.00000000	pigs
Cubn	-0.18	1.00000000	pigs
Stx2	-0.21	1.00000000	pigs
Sod1	0.24	1.00000000	pigs
Timm17a	0.05	1.00000000	pigs
Pcm1	-0.10	1.00000000	pigs
Nr2f6	-0.18	1.00000000	pigs
Wdr19	-0.17	1.00000000	pigs
Procr	-0.23	1.00000000	pigs
4932411N23Rik	-0.05	1.00000000	pigs
Fam172a	-0.18	1.00000000	pigs
Galnt10	-0.14	1.00000000	pigs
Nmd3	-0.12	1.00000000	pigs
Prss23	0.06	1.00000000	pigs
Spryd3	-0.19	1.00000000	pigs
Hectd4	-0.25	1.00000000	pigs
Tmem161b	-0.12	1.00000000	pigs
Eif4g1	-0.15	1.00000000	pigs
Nfyc	-0.26	1.00000000	pigs

Bicral	-0.16	1.00000000	pigs
Phactr4	-0.35	1.00000000	pigs
Nkd1	-0.14	1.00000000	pigs
Polr2d	-0.05	1.00000000	pigs
Ppp1cb	0.11	1.00000000	pigs
Smad5	-0.18	1.00000000	pigs
Wdr20	-0.13	1.00000000	pigs
Sec23a	-0.27	1.00000000	pigs
Yipf6	-0.24	1.00000000	pigs
Ercc3	-0.10	1.00000000	pigs
Gab2	0.00	1.00000000	pigs
Gtf3c3	-0.14	1.00000000	pigs
Dhx35	-0.09	1.00000000	pigs
Dgkh	-0.13	1.00000000	pigs
Orc3	-0.09	1.00000000	pigs
Fam204a	-0.29	1.00000000	pigs
Wwtr1	-0.17	1.00000000	pigs
Ppp2r5e	0.00	1.00000000	pigs
Ssc5d	-0.16	1.00000000	pigs
Gabpa	-0.16	1.00000000	pigs
Anapc7	-0.22	1.00000000	pigs
Eln	-0.10	1.00000000	pigs
Casc3	-0.04	1.00000000	pigs
Lamp1	0.15	1.00000000	pigs
Appbp2	-0.32	1.00000000	pigs
Trmt2a	0.02	1.00000000	pigs
Wnt11	-0.22	1.00000000	pigs
Fam76b	-0.15	1.00000000	pigs
Ralb	0.06	1.00000000	pigs
Rbm28	-0.11	1.00000000	pigs
Tmem106b	-0.23	1.00000000	pigs
Rab10	0.13	1.00000000	pigs
Usp37	-0.15	1.00000000	pigs
Rbm19	-0.21	1.00000000	pigs
Arhgap28	-0.37	1.00000000	pigs
Ptger3	-0.46	1.00000000	pigs
Vezf1	-0.16	1.00000000	pigs
Diaph1	-0.19	1.00000000	pigs
Asph	-0.05	1.00000000	pigs
Fam120a	-0.18	1.00000000	pigs
Rnf38	-0.20	1.00000000	pigs
Ncoa3	-0.26	1.00000000	pigs
Gpx7	-0.18	1.00000000	pigs
Prdm11	-0.17	1.00000000	pigs
Obi1	-0.11	1.00000000	pigs
Ramp2	-0.17	1.00000000	pigs

Ube2e3	-0.17	1.00000000	pigs
Gspt1	-0.07	1.00000000	pigs
Hpgd	-0.25	1.00000000	pigs
Itgam	-0.01	1.00000000	pigs
Cdk2ap2	0.02	1.00000000	pigs
Copg1	-0.19	1.00000000	pigs
Rassf3	0.05	1.00000000	pigs
Cachd1	-0.32	1.00000000	pigs
Anks1	-0.19	1.00000000	pigs
Rufy2	-0.19	1.00000000	pigs
Tspyl2	-0.13	1.00000000	pigs
Tmx3	-0.09	1.00000000	pigs
Ednra	-0.18	1.00000000	pigs
Fam8a1	-0.11	1.00000000	pigs
Rnpepl1	-0.19	1.00000000	pigs
Rbm15	-0.17	1.00000000	pigs
Tdg	-0.10	1.00000000	pigs
Lmbr1	-0.21	1.00000000	pigs
Elp1	-0.12	1.00000000	pigs
Eya3	-0.11	1.00000000	pigs
Pnrc2	-0.05	1.00000000	pigs
Snx3	0.13	1.00000000	pigs
Phlpp1	-0.16	1.00000000	pigs
Ppme1	-0.25	1.00000000	pigs
Srfbp1	-0.28	1.00000000	pigs
Dynll2	0.01	1.00000000	pigs
Arhgef26	-0.24	1.00000000	pigs
Coq8a	-0.07	1.00000000	pigs
Snip1	-0.06	1.00000000	pigs
Ncor2	-0.17	1.00000000	pigs
Actr3	0.00	1.00000000	pigs
Smim7	-0.05	1.00000000	pigs
Nelfe	0.02	1.00000000	pigs
Rsl24d1	0.09	1.00000000	pigs
Tcf7	-0.16	1.00000000	pigs
Id1	-0.20	1.00000000	pigs
Epg5	-0.20	1.00000000	pigs
Khk	-0.08	1.00000000	pigs
Hes6	0.05	1.00000000	pigs
Huwe1	-0.13	1.00000000	pigs
Nr2f1	-0.21	1.00000000	pigs
Gnpda2	-0.16	1.00000000	pigs
Phip	-0.24	1.00000000	pigs
Pknox2	-0.23	1.00000000	pigs
Paqr3	-0.12	1.00000000	pigs
Ubxn2a	-0.05	1.00000000	pigs

Tesk1	-0.03	1.00000000	pigs
Trdmt1	-0.35	1.00000000	pigs
Sumf1	-0.21	1.00000000	pigs
Pdzd8	-0.22	1.00000000	pigs
Atxn2	-0.20	1.00000000	pigs
Gnb2	0.06	1.00000000	pigs
Ddhd2	-0.14	1.00000000	pigs
Pi4k2b	-0.15	1.00000000	pigs
Atl3	0.01	1.00000000	pigs
Pdgfd	-0.34	1.00000000	pigs
Katna1	-0.08	1.00000000	pigs
Eif4g2	0.10	1.00000000	pigs
Galm	-0.38	1.00000000	pigs
Tango6	-0.24	1.00000000	pigs
Extl3	-0.26	1.00000000	pigs
