# Supplementary material for: Heterogeneous viral-host response at single-cell resolution and viral adaptive mutational atlas in birds and mammals upon H5 influenza virus infection
Source: Natl Sci Rev. 2026 Jun 25;13(14):nwag380. doi: 10.1093/nsr/nwag380 (PMC13458601; doi:10.1093/nsr/nwag380)
Supplement: Supplementary file 1 [file nwag380_supplemental_files.zip › Supplementary Data 19.pdf]

Supplementary Data 19. Differentially expressed genes of T cells across different species upon H5 HPAIV infection.

gene	log2FoldChange	p value	host
Ifit2	2.26	0.00000000	chicken
Stat1	2.36	0.00000000	chicken
Trank1	2.46	0.00000000	chicken
Rfx2	2.45	0.00000000	chicken
Oasl2	1.73	0.00000000	chicken
Tox3	1.32	0.00000000	chicken
Rack1	-2.38	0.00000000	chicken
Smarcal1	-2.12	0.00000000	chicken
Jak2	1.94	0.00000000	chicken
Pde3b	1.23	0.00000000	chicken
Tpt1	-1.92	0.00000000	chicken
Cd44	1.27	0.00000000	chicken
Ybx1	-1.93	0.00000000	chicken
Ccl9	1.97	0.00000000	chicken
Eef2	-1.62	0.00000000	chicken
Pabpc1	-1.39	0.00000000	chicken
Eef1b2	-1.71	0.00000000	chicken
Ifih1	1.89	0.00000000	chicken
Nme2	-1.83	0.00000000	chicken
Rnf213	1.24	0.00000000	chicken
Fau	-1.68	0.00000000	chicken
Foxn3	1.53	0.00000000	chicken
Helz2	1.14	0.00000000	chicken
Ly6e	1.30	0.00000000	chicken
Rsad2	1.79	0.00000000	chicken
Rasgef1a	2.28	0.00000000	chicken
Trim25	1.06	0.00000000	chicken
H3f3a	-1.42	0.00000000	chicken
Eef1d	-1.47	0.00000000	chicken
Pcsk2	1.04	0.00000000	chicken
Cd3g	-1.24	0.00000000	chicken
Susd6	1.46	0.00000000	chicken
Plcl2	1.65	0.00000000	chicken
Rap1gds1	1.25	0.00000000	chicken
Slbp	0.75	0.00000000	chicken
Arid5b	1.13	0.00000000	chicken
Foxk1	1.35	0.00000000	chicken
Tmsb4x	-1.16	0.00000000	chicken
Itpkb	1.31	0.00000000	chicken
Irf1	1.41	0.00000000	chicken
Ddx60	1.08	0.00000000	chicken

Nr4a3	1.24	0.00000000	chicken
Actg1	-1.30	0.00000000	chicken
Ulbp1	-1.27	0.00000000	chicken
Hivep1	1.47	0.00000000	chicken
Dennd1b	0.98	0.00000000	chicken
Crlf2	1.26	0.00000000	chicken
Rbm39	-0.95	0.00000000	chicken
Tle4	1.46	0.00000000	chicken
Wwp1	1.53	0.00000000	chicken
Prdx1	-1.66	0.00000000	chicken
Gramd4	1.18	0.00000000	chicken
Lrmda	1.85	0.00000000	chicken
Etv6	1.45	0.00000000	chicken
Irf8	0.59	0.00000000	chicken
Nfatc1	1.37	0.00000000	chicken
Ankrd6	1.46	0.00000000	chicken
Chd7	1.49	0.00000000	chicken
Fbxl17	1.22	0.00000000	chicken
Il27ra	-1.11	0.00000000	chicken
Samhd1	1.08	0.00000000	chicken
Dgkh	1.48	0.00000000	chicken
Eif3f	-1.37	0.00000000	chicken
Plppr5	1.43	0.00000000	chicken
Serbp1	-0.97	0.00000000	chicken
Chsy1	1.21	0.00000000	chicken
Fndc3a	1.23	0.00000000	chicken
Rc3h1	1.12	0.00000000	chicken
Foxn2	1.05	0.00000000	chicken
Cmtr1	0.96	0.00000000	chicken
Glcci1	2.06	0.00000000	chicken
Fosb	0.67	0.00000000	chicken
Cox4i1	-1.23	0.00000000	chicken
Smchd1	1.08	0.00000000	chicken
Ppp6r2	0.95	0.00000000	chicken
Zfp593	-1.19	0.00000000	chicken
Stat2	0.47	0.00000000	chicken
Ppia	-1.07	0.00000000	chicken
Cblb	0.96	0.00000000	chicken
Rubcnl	1.10	0.00000000	chicken
Cmip	0.87	0.00000000	chicken
Osbpl3	1.25	0.00000000	chicken
Eif3m	-1.22	0.00000000	chicken
Utrn	0.95	0.00000000	chicken
Hivep2	0.81	0.00000000	chicken
Eif4a2	-0.75	0.00000000	chicken
Csnk1a1	0.87	0.00000000	chicken

Eif1	-0.92	0.00000000	chicken
Pstpip1	1.15	0.00000000	chicken
Elf1	0.77	0.00000000	chicken
Setx	1.01	0.00000000	chicken
Gnb1	0.83	0.00000000	chicken
Btbd11	1.27	0.00000000	chicken
Niban1	1.71	0.00000000	chicken
Hnrnpa1	-1.16	0.00000000	chicken
Ptpn2	0.95	0.00000000	chicken
Gls	1.02	0.00000000	chicken
Dusp1	1.09	0.00000000	chicken
Prr5	1.13	0.00000000	chicken
Cbfb	1.19	0.00000000	chicken
Zc3hav1l	0.90	0.00000000	chicken
Tmcc1	0.93	0.00000000	chicken
Tspan4	0.95	0.00000000	chicken
Foxp1	0.84	0.00000000	chicken
Dock10	1.01	0.00000000	chicken
Pip4k2a	1.13	0.00000000	chicken
Fyn	0.87	0.00000000	chicken
Eif3e	-1.10	0.00000000	chicken
P2rx7	0.78	0.00000000	chicken
Lnpep	1.06	0.00000000	chicken
Phf2	1.10	0.00000000	chicken
Ptprc	0.70	0.00000000	chicken
Rbms1	1.37	0.00000000	chicken
Gfpt2	1.26	0.00000000	chicken
Tut4	0.91	0.00000000	chicken
Fbxo11	0.92	0.00000000	chicken
Ncoa1	1.13	0.00000000	chicken
Smad1	0.96	0.00000000	chicken
Rfx3	1.53	0.00000000	chicken
Cox7b	-1.18	0.00000000	chicken
Per2	0.85	0.00000000	chicken
Nsa2	-1.09	0.00000000	chicken
Gab3	1.48	0.00000000	chicken
Calm2	-1.04	0.00000000	chicken
Gna13	0.77	0.00000000	chicken
Wbp1l	0.95	0.00000000	chicken
Ahr	1.70	0.00000000	chicken
Xylt1	1.21	0.00000000	chicken
Tomm20	-1.06	0.00000000	chicken
Igf2bp3	0.93	0.00000000	chicken
Eif3l	-1.09	0.00000000	chicken
Fnbp1	0.78	0.00000000	chicken
Fth1	-0.75	0.00000000	chicken

Cotl1	-1.10	0.00000000	chicken
Tsc22d3	1.09	0.00000000	chicken
Slc2a3	-0.88	0.00000000	chicken
Calcb	0.61	0.00000000	chicken
Sema3c	1.44	0.00000000	chicken
Dstn	-0.84	0.00000000	chicken
Cds2	0.54	0.00000000	chicken
Picalm	1.12	0.00000000	chicken
Usp25	0.94	0.00000000	chicken
Ext1	-0.87	0.00000000	chicken
Slco3a1	1.20	0.00000000	chicken
Hnrnpa3	-0.63	0.00000000	chicken
Acap3	0.75	0.00000000	chicken
Ppfibp1	1.00	0.00000000	chicken
Ctdp1	1.14	0.00000000	chicken
Cd3e	-0.71	0.00000000	chicken
Sbno2	0.80	0.00000000	chicken
Actb	-1.21	0.00000000	chicken
Atp2a2	1.26	0.00000000	chicken
Urm1	-1.07	0.00000000	chicken
Eif3d	-1.00	0.00000000	chicken
Apaf1	0.60	0.00000000	chicken
Fryl	1.08	0.00000000	chicken
Cox6a1	-1.02	0.00000000	chicken
Arpc1b	-1.08	0.00000000	chicken
Inpp5d	0.84	0.00000000	chicken
Esrrg	0.93	0.00000000	chicken
Wdfy2	0.78	0.00000000	chicken
Samsn1	0.79	0.00000000	chicken
Pde4b	0.83	0.00000000	chicken
Bcl11b	0.77	0.00000000	chicken
Chd2	0.87	0.00000000	chicken
Cnot2	0.88	0.00000000	chicken
Calm1	-0.74	0.00000000	chicken
Eif4g2	-0.81	0.00000000	chicken
Disp1	0.90	0.00000000	chicken
Cbl	0.60	0.00000000	chicken
Zfand6	0.82	0.00000000	chicken
Lrrc1	0.82	0.00000000	chicken
Tomm7	-0.98	0.00000000	chicken
Eno1b	-0.85	0.00000000	chicken
Gsk3b	0.70	0.00000000	chicken
D5Ertd579e	0.96	0.00000000	chicken
Hivep3	0.92	0.00000000	chicken
Arxes2	-0.76	0.00000000	chicken
Ctsc	0.34	0.00000000	chicken

Vps13a	0.96	0.00000000	chicken
Il1b	0.53	0.00000000	chicken
Nadk	0.71	0.00000000	chicken
Arhgap15	0.64	0.00000000	chicken
H2az2	-0.95	0.00000000	chicken
Lonrf3	0.59	0.00000000	chicken
Ppib	-0.87	0.00000000	chicken
Dpyd	1.07	0.00000000	chicken
Cdk17	0.71	0.00000000	chicken
Slc29a4	-1.04	0.00000000	chicken
Cpt1a	0.66	0.00000000	chicken
Eif5a2	-0.96	0.00000000	chicken
Braf	0.73	0.00000000	chicken
Ptafr	0.42	0.00000000	chicken
Ppp1r16b	0.81	0.00000000	chicken
Pde7b	1.03	0.00000000	chicken
Wwox	1.08	0.00000000	chicken
Arpc4	-0.95	0.00000000	chicken
Foxk2	0.68	0.00000000	chicken
Sgk1	0.64	0.00000000	chicken
Atp10a	1.05	0.00000000	chicken
Ptgs2	0.69	0.00000000	chicken
Ndfip2	0.98	0.00000000	chicken
Itm2a	-0.86	0.00000000	chicken
Runx3	0.80	0.00000000	chicken
Tbl1xr1	0.79	0.00000000	chicken
Prkd1	1.45	0.00000000	chicken
Znfx1	0.72	0.00000000	chicken
Clec16a	0.85	0.00000000	chicken
Ikzf1	0.75	0.00000000	chicken
Scin	-0.90	0.00000000	chicken
Pik3r1	1.10	0.00000000	chicken
Kmt2e	0.61	0.00000000	chicken
Sgms1	0.72	0.00000000	chicken
Ralgapa1	0.78	0.00000000	chicken
Eif4b	-0.90	0.00000000	chicken
Ago2	0.66	0.00000000	chicken
Ets2	0.81	0.00000000	chicken
Nbeal2	0.59	0.00000000	chicken
Syne1	0.90	0.00000000	chicken
Fam107b	1.24	0.00000000	chicken
Trerf1	0.84	0.00000000	chicken
Plagl1	0.78	0.00000000	chicken
Polk	0.43	0.00000000	chicken
Nav3	1.10	0.00000000	chicken
Cox5a	-0.93	0.00000000	chicken

Hnrnph2	-0.53	0.00000000	chicken
Slc25a3	-0.80	0.00000000	chicken
Hif1a	0.84	0.00000000	chicken
Obp2a	0.51	0.00000000	chicken
Atic	-0.86	0.00000000	chicken
Runx2	1.43	0.00000000	chicken
Eif3g	-0.80	0.00000000	chicken
Spsb1	0.63	0.00000000	chicken
Rgs1	0.96	0.00000000	chicken
Prkar2a	-0.47	0.00000000	chicken
Adipor2	0.72	0.00000000	chicken
Atp2b1	0.76	0.00000000	chicken
Spag16	-1.93	0.00000000	chicken
Nap1l1	-0.71	0.00000000	chicken
Ddx5	-0.44	0.00000000	chicken
Ergic1	0.84	0.00000000	chicken
Atf4	-0.61	0.00000000	chicken
Hnrnpa2b1	-0.56	0.00000000	chicken
Rere	0.74	0.00000000	chicken
Rhoa	-0.71	0.00000000	chicken
Snx10	0.81	0.00000000	chicken
Gclc	-0.88	0.00000000	chicken
Zdhhc20	0.72	0.00000000	chicken
Ust	1.34	0.00000000	chicken
Zfp36l1	0.59	0.00000000	chicken
Dennd5a	0.86	0.00000000	chicken
Ttc7b	0.65	0.00000000	chicken
Acvr2a	0.72	0.00000000	chicken
St7	1.32	0.00000000	chicken
Stxbp5	1.32	0.00000000	chicken
H3f3b	-0.59	0.00000000	chicken
Cep85l	0.80	0.00000000	chicken
Nfil3	0.83	0.00000000	chicken
Hdgf	-0.91	0.00000000	chicken
Grm8	-1.63	0.00000000	chicken
Fas	0.85	0.00000000	chicken
Gpr68	0.52	0.00000000	chicken
Itpka	0.71	0.00000000	chicken
Hnrnpr	-0.57	0.00000000	chicken
Cd7	1.09	0.00000000	chicken
Cox7a2	-0.85	0.00000000	chicken
Rock1	0.61	0.00000000	chicken
Ndufa4	-0.75	0.00000000	chicken
Fkbp5	0.61	0.00000000	chicken
Stat4	0.63	0.00000000	chicken
Arhgap17	0.66	0.00000000	chicken

Gfod1	0.75	0.00000000	chicken
Bms1	-0.90	0.00000000	chicken
Rev3l	0.82	0.00000000	chicken
Bend3	-0.87	0.00000000	chicken
Ssbp3	0.79	0.00000000	chicken
Ptpn22	0.74	0.00000000	chicken
Svbp	-0.79	0.00000000	chicken
Hao1	0.34	0.00000000	chicken
Isl2	0.48	0.00000000	chicken
Cd247	0.70	0.00000000	chicken
Il20ra	-0.59	0.00000000	chicken
Eif2s3y	-0.86	0.00000000	chicken
Tnfrsf21	-0.89	0.00000000	chicken
Epn2	0.76	0.00000000	chicken
Nktr	0.53	0.00000000	chicken
Gimap5	0.34	0.00000000	chicken
Csrnp1	0.52	0.00000000	chicken
Arsg	0.71	0.00000000	chicken
Pnrc1	-0.42	0.00000000	chicken
Tmem123	0.71	0.00000000	chicken
Garem1	1.08	0.00000000	chicken
Plk3	0.64	0.00000000	chicken
Tet2	0.72	0.00000000	chicken
Cert1	0.78	0.00000000	chicken
Ptprj	0.89	0.00000000	chicken
Bicd2	0.92	0.00000000	chicken
Tbc1d5	0.79	0.00000000	chicken
Slc7a5	-0.80	0.00000000	chicken
Slc16a10	1.16	0.00000000	chicken
Gas7	0.94	0.00000000	chicken
Pnn	-0.88	0.00000000	chicken
Ppm1d	0.52	0.00000000	chicken
Slc41a1	0.52	0.00000000	chicken
Cnot6	0.58	0.00000000	chicken
Gpx1	-0.84	0.00000000	chicken
Nedd9	0.71	0.00000000	chicken
Pou2f1	0.82	0.00000000	chicken
Snrpf	-0.74	0.00000000	chicken
Jak1	0.59	0.00000000	chicken
Nlrc5	0.63	0.00000000	chicken
Rab2a	0.50	0.00000000	chicken
Sptbn1	-0.53	0.00000000	chicken
Hsp90ab1	-1.01	0.00000000	chicken
Pdia3	-0.85	0.00000000	chicken
Iqgap2	0.89	0.00000000	chicken
Gabrr2	0.44	0.00000000	chicken

Eif3a	-0.62	0.00000000	chicken
Eya3	0.58	0.00000000	chicken
Gatad2a	0.53	0.00000000	chicken
Ccni	-0.82	0.00000000	chicken
Csnk2a2	-0.64	0.00000000	chicken
Gnas	0.79	0.00000000	chicken
Itpr2	-0.94	0.00000000	chicken
Abi1	0.65	0.00000000	chicken
Zfp438	0.45	0.00000000	chicken
Il2rb	1.01	0.00000000	chicken
Tmed2	-0.63	0.00000000	chicken
Ttyh3	-0.60	0.00000000	chicken
Arhgap12	0.58	0.00000000	chicken
AA467197	0.64	0.00000000	chicken
Slc43a2	0.52	0.00000000	chicken
Rbmxl1	-0.64	0.00000000	chicken
Socs2	-0.90	0.00000000	chicken
Rnf130	-0.69	0.00000000	chicken
Fto	0.72	0.00000000	chicken
Bcas3	0.81	0.00000000	chicken
Pds5a	0.68	0.00000000	chicken
Antxr2	0.89	0.00000000	chicken
Plpp1	0.56	0.00000000	chicken
Ddx3y	-0.43	0.00000000	chicken
Ubac2	1.06	0.00000000	chicken
Ddx19a	-0.71	0.00000000	chicken
Cox7c	-0.74	0.00000000	chicken
Fbrsl1	-0.77	0.00000000	chicken
Ndufb5	-0.64	0.00000000	chicken
Gnl3	-0.69	0.00000000	chicken
Cyba	-0.79	0.00000000	chicken
Ssbp1	-0.68	0.00000000	chicken
Pudp	0.72	0.00000000	chicken
Ctnna1	0.54	0.00000000	chicken
Sap18	-0.72	0.00000000	chicken
Atad2b	0.62	0.00000000	chicken
Trdmt1	0.61	0.00000000	chicken
Hmgb1	-0.67	0.00000000	chicken
Lasp1	0.42	0.00000000	chicken
Sertad2	0.51	0.00000000	chicken
Rassf5	0.31	0.00000000	chicken
Lrrc8c	0.32	0.00000000	chicken
Trio	0.95	0.00000000	chicken
Sytl3	0.67	0.00000000	chicken
Zeb2	1.07	0.00000000	chicken
Dusp7	-0.99	0.00000000	chicken

Gab1	0.58	0.00000000	chicken
Lao1	-0.72	0.00000000	chicken
Pde7a	0.77	0.00000000	chicken
Tnik	0.67	0.00000000	chicken
C1qbp	-0.74	0.00000000	chicken
S100a11	-0.92	0.00000000	chicken
Btf3	-0.70	0.00000000	chicken
St8sia4	0.78	0.00000000	chicken
Rbm33	0.56	0.00000000	chicken
Gpr65	-0.79	0.00000000	chicken
Ikbke	0.38	0.00000000	chicken
Fbxo34	0.60	0.00000001	chicken
Ifi30	-0.72	0.00000001	chicken
Rnf19b	0.31	0.00000001	chicken
Erbin	0.68	0.00000001	chicken
Pfkp	0.63	0.00000001	chicken
Adsl	-0.83	0.00000001	chicken
Grk4	-0.55	0.00000001	chicken
Irf7	0.46	0.00000001	chicken
Stoml1	0.33	0.00000001	chicken
Oit3	0.41	0.00000001	chicken
Tsc22d2	0.35	0.00000001	chicken
Tanc1	0.51	0.00000001	chicken
Atp5g3	-0.61	0.00000001	chicken
Vav3	0.53	0.00000001	chicken
Helb	0.41	0.00000001	chicken
Glo1	-0.67	0.00000002	chicken
Eif2ak3	-0.59	0.00000002	chicken
Zfp423	0.28	0.00000002	chicken
Nt5c3b	0.37	0.00000002	chicken
Phf11a	0.43	0.00000002	chicken
Grap2	-0.44	0.00000002	chicken
Nr3c2	0.82	0.00000002	chicken
Tnrc6c	0.63	0.00000002	chicken
Pgls	-0.71	0.00000002	chicken
Ccnyl1	0.37	0.00000002	chicken
Rbm31y	-0.49	0.00000003	chicken
Add3	-0.59	0.00000003	chicken
Exoc4	0.69	0.00000003	chicken
Babam2	0.68	0.00000004	chicken
Snrpd1	-0.63	0.00000005	chicken
Arrdc1	0.31	0.00000005	chicken
Golgb1	-0.89	0.00000005	chicken
Dennd4a	0.70	0.00000006	chicken
Arpc5	-0.70	0.00000006	chicken
Aopep	0.65	0.00000006	chicken

St8sia1	-0.68	0.00000006	chicken
Ccdc88c	0.70	0.00000006	chicken
Disc1	0.78	0.00000006	chicken
Hnrnph3	-0.51	0.00000006	chicken
Pik3cb	0.85	0.00000007	chicken
Serp1	-0.68	0.00000007	chicken
Kansl1l	0.50	0.00000007	chicken
Ube2h	0.50	0.00000008	chicken
Bmp2k	0.89	0.00000008	chicken
Prtg	0.61	0.00000008	chicken
Dek	-0.66	0.00000008	chicken
Ndufb1	-0.66	0.00000008	chicken
Apoa1	0.40	0.00000009	chicken
Cdkal1	0.60	0.00000010	chicken
Tiam2	0.63	0.00000011	chicken
Atp5j	-0.55	0.00000011	chicken
Dok6	0.71	0.00000012	chicken
Cstf3	0.61	0.00000012	chicken
Prelid1	-0.59	0.00000012	chicken
Brf1	-0.74	0.00000012	chicken
Atp2a3	0.68	0.00000012	chicken
Ctbp1	0.72	0.00000013	chicken
Rin3	0.92	0.00000015	chicken
Ndufb4	-0.61	0.00000015	chicken
Tnks	0.57	0.00000019	chicken
Dync1li2	0.48	0.00000022	chicken
Clcn3	0.67	0.00000022	chicken
Lsm14a	-0.64	0.00000023	chicken
Arhgdia	-0.62	0.00000024	chicken
Card11	0.58	0.00000026	chicken
BC005624	-0.62	0.00000031	chicken
Exoc6	0.57	0.00000031	chicken
Naca	-0.61	0.00000038	chicken
Arid2	0.61	0.00000040	chicken
Ppp2r5c	0.52	0.00000042	chicken
Timm44	-0.54	0.00000048	chicken
Prex1	0.60	0.00000060	chicken
Zfp706	-0.63	0.00000067	chicken
Stmn1	-0.71	0.00000074	chicken
Nedd4	0.53	0.00000079	chicken
Cltc	0.44	0.00000090	chicken
Parp12	0.32	0.00000093	chicken
Itch	0.44	0.00000097	chicken
Igsf3	1.07	0.00000111	chicken
G3bp1	-0.50	0.00000111	chicken
Litaf	0.31	0.00000114	chicken

Ccnd1	-0.67	0.00000115	chicken
Bach1	0.45	0.00000117	chicken
Iqgap1	0.48	0.00000123	chicken
Hexim1	-0.56	0.00000145	chicken
Runx1	-0.39	0.00000147	chicken
Abce1	-0.64	0.00000148	chicken
Pdss2	-0.42	0.00000168	chicken
Grb10	0.87	0.00000172	chicken
Fos	0.58	0.00000173	chicken
Pomp	-0.68	0.00000175	chicken
Trip12	0.48	0.00000178	chicken
Ube2q2	0.71	0.00000182	chicken
Dusp5	0.42	0.00000183	chicken
Rragd	0.35	0.00000191	chicken
Rad23a	-0.63	0.00000207	chicken
Trappc9	0.55	0.00000230	chicken
Tln1	-0.59	0.00000238	chicken
Lamtor2	-0.52	0.00000253	chicken
Fhit	1.06	0.00000253	chicken
Ifnar2	0.56	0.00000259	chicken
2900026A02Rik	0.64	0.00000262	chicken
Galc	-0.61	0.00000278	chicken
Pla2g4a	0.40	0.00000283	chicken
Znrf2	0.73	0.00000300	chicken
Sdhd	-0.52	0.00000325	chicken
Auh	0.43	0.00000337	chicken
Tpm1	-0.60	0.00000339	chicken
Erh	-0.60	0.00000339	chicken
Sik3	0.45	0.00000347	chicken
Upf2	0.52	0.00000358	chicken
Vdac2	-0.58	0.00000365	chicken
Tcf7	-0.06	0.00000418	chicken
Slc16a6	0.26	0.00000422	chicken
Ppp6r3	0.52	0.00000454	chicken
Drd4	-0.62	0.00000460	chicken
Actn1	-0.50	0.00000511	chicken
Ndufc2	-0.54	0.00000547	chicken
Nr1d2	-0.60	0.00000594	chicken
Smad3	1.30	0.00000599	chicken
Il6st	0.47	0.00000604	chicken
Etnk1	0.60	0.00000607	chicken
Lpin2	-0.49	0.00000674	chicken
Atf2	0.49	0.00000710	chicken
Nup210	0.69	0.00000739	chicken
Kcnq3	-1.56	0.00000778	chicken
Sclt1	-0.78	0.00000840	chicken

Trps1	0.41	0.00000849	chicken
Snrpe	-0.55	0.00000866	chicken
Slc24a4	-0.99	0.00000870	chicken
Arid4b	0.49	0.00000888	chicken
Ptprf	0.98	0.00000896	chicken
Zer1	0.34	0.00000897	chicken
Hsbp1	-0.54	0.00000917	chicken
Rrs1	-0.58	0.00000931	chicken
Ptpra	-0.61	0.00001012	chicken
Api5	-0.60	0.00001026	chicken
Socs1	0.43	0.00001034	chicken
Psmb1	-0.57	0.00001074	chicken
Prr5l	-0.74	0.00001085	chicken
Ehd3	0.46	0.00001108	chicken
Nfat5	0.40	0.00001126	chicken
Cul4b	-0.63	0.00001163	chicken
Dot1l	0.53	0.00001335	chicken
Sqstm1	0.37	0.00001405	chicken
Gnai1	0.62	0.00001523	chicken
Hspe1-rs1	-0.48	0.00001538	chicken
Ankrd13a	-0.73	0.00001645	chicken
Ptk2	0.47	0.00001956	chicken
Ndufb2	-0.58	0.00001960	chicken
Pcmtd1	0.47	0.00002000	chicken
Nek6	0.54	0.00002061	chicken
Limd1	0.38	0.00002095	chicken
Nf1	0.48	0.00002138	chicken
Ecpas	0.46	0.00002145	chicken
Sbno1	0.42	0.00002303	chicken
Emp1	0.39	0.00002306	chicken
Zcchc24	0.82	0.00002311	chicken
Plac8	0.52	0.00002433	chicken
Nasp	-0.64	0.00002639	chicken
Tasp1	0.48	0.00002846	chicken
Phactr2	0.56	0.00002871	chicken
Ddx24	-0.66	0.00002908	chicken
Snrpd3	-0.55	0.00002980	chicken
Herpud1	0.49	0.00002998	chicken
Kmt5b	-0.52	0.00003106	chicken
Asb5	-0.68	0.00003126	chicken
Rybp	0.44	0.00003190	chicken
Yeats2	-0.51	0.00003778	chicken
Nudt16l1	-0.56	0.00004003	chicken
Polr1f	-0.61	0.00004020	chicken
Sntb1	0.98	0.00004169	chicken
Ube2j1	0.50	0.00004241	chicken

Hemk1	-0.74	0.00004285	chicken
Btg1b	-0.26	0.00004340	chicken
Ahi1	0.62	0.00004459	chicken
Psme3	-0.57	0.00004581	chicken
Slc25a24	0.60	0.00004640	chicken
Cnot6l	0.48	0.00004805	chicken
Ppp3cb	0.44	0.00004948	chicken
Atp5j2	-0.56	0.00005087	chicken
Atp1b1	0.50	0.00005261	chicken
Rcor1	0.43	0.00005291	chicken
Stau2	0.55	0.00005548	chicken
Rffl	0.54	0.00005618	chicken
Ran	-0.58	0.00005758	chicken
Smarce1	-0.62	0.00005785	chicken
Ppp1r12a	0.28	0.00005957	chicken
Pdia4	-0.53	0.00006038	chicken
Ncoa3	0.60	0.00006145	chicken
Zbtb21	0.28	0.00006157	chicken
Dpf3	0.64	0.00006967	chicken
Uqcrh	-0.49	0.00007159	chicken
Rsbn1l	0.53	0.00007886	chicken
Diaph2	0.52	0.00007931	chicken
Phc3	0.44	0.00008783	chicken
Slf2	0.34	0.00008848	chicken
Glipr1l1	0.44	0.00009308	chicken
Gtpbp4	-0.62	0.00009395	chicken
B3gnt5	0.48	0.00009591	chicken
Ilf3	-0.48	0.00010088	chicken
Klf12	0.67	0.00010107	chicken
Oxr1	0.61	0.00010197	chicken
Spty2d1	-0.41	0.00010453	chicken
Bsg	-0.60	0.00010578	chicken
Atp5pb	-0.58	0.00011182	chicken
Ankrd39	-0.53	0.00011768	chicken
Git2	-0.63	0.00011914	chicken
Arpc2	-0.53	0.00012009	chicken
Itga1	0.91	0.00012189	chicken
Zbtb16	0.66	0.00012196	chicken
Wac	0.41	0.00012207	chicken
Eif4ebp1	-0.45	0.00012259	chicken
Tgfb1	-0.53	0.00012406	chicken
Atf3	0.67	0.00013478	chicken
Snx14	-0.55	0.00013554	chicken
Lmna	-0.49	0.00013598	chicken
Gpd2	0.33	0.00015257	chicken
Eif3b	-0.56	0.00015364	chicken

Pfdn4	-0.53	0.00015947	chicken
Casp8	-0.45	0.00017407	chicken
Nampt	0.35	0.00017801	chicken
Ikzf2	1.41	0.00018276	chicken
Nop58	-0.53	0.00019246	chicken
Atp5a1	-0.46	0.00019437	chicken
Itga4	-0.29	0.00019685	chicken
Zfp608	-0.75	0.00019966	chicken
Atxn7	0.61	0.00025406	chicken
Trafd1	0.27	0.00028342	chicken
Tnfsf11	-0.34	0.00028554	chicken
Ago3	-0.33	0.00028953	chicken
Ankrd11	0.34	0.00029660	chicken
Cmtm3	-0.52	0.00031274	chicken
Srebf2	-0.51	0.00031502	chicken
Zswim5	0.70	0.00032078	chicken
Tub	0.45	0.00033303	chicken
Atp5d	-0.53	0.00033698	chicken
Rb1	0.57	0.00033783	chicken
Tmed5	-0.53	0.00034652	chicken
Atrn	-0.65	0.00036372	chicken
Ywhab	-0.49	0.00036487	chicken
Cst3	-0.68	0.00036528	chicken
Erc2	-0.12	0.00038245	chicken
Otud7a	0.86	0.00039299	chicken
Ndufb10	-0.50	0.00039309	chicken
Tcof1	-0.60	0.00039940	chicken
Synj1	0.42	0.00041386	chicken
Wdr20	0.49	0.00042131	chicken
Snrk	0.48	0.00044659	chicken
Tspan5	1.24	0.00045058	chicken
Atf7ip	0.54	0.00045105	chicken
Cxcr4	0.52	0.00051493	chicken
Rpn1	-0.54	0.00054299	chicken
B2m	-0.19	0.00054320	chicken
Chd9	0.57	0.00054323	chicken
Ubl5b	-0.58	0.00054826	chicken
Ppm1h	0.65	0.00056291	chicken
Orc1	0.39	0.00057057	chicken
Prdm2	0.37	0.00057542	chicken
Tcf20	0.47	0.00059617	chicken
Cep164	-0.64	0.00060684	chicken
Arhgdib	-0.59	0.00060818	chicken
Crim1	0.71	0.00062908	chicken
Inpp4a	0.66	0.00062955	chicken
Arl3	0.32	0.00065834	chicken

Usp34	0.50	0.00070056	chicken
Cdc25b	-0.47	0.00070676	chicken
Dedd	-0.46	0.00071405	chicken
Cry1	-0.32	0.00073818	chicken
Trib2	0.52	0.00074185	chicken
Pag1	-0.43	0.00076257	chicken
Hmgb3	-0.51	0.00076618	chicken
Fyco1	0.47	0.00080831	chicken
Stk24	0.60	0.00090624	chicken
Fbxw11	0.44	0.00092264	chicken
Oat	-0.46	0.00092426	chicken
Ndufa5	-0.48	0.00093370	chicken
Hnrnpll	0.58	0.00096080	chicken
Acsl4	0.61	0.00096192	chicken
Phf20	0.38	0.00098185	chicken
Ppard	0.38	0.00098306	chicken
Pspc1	0.36	0.00099971	chicken
Tiparp	0.66	0.00103096	chicken
Vapb	0.39	0.00107264	chicken
Ndufa13	-0.46	0.00109094	chicken
Rictor	0.50	0.00110021	chicken
Gigyf2	0.45	0.00111151	chicken
Ptpn14	-0.58	0.00119140	chicken
Slc25a36	0.35	0.00121168	chicken
Fbxl5	0.37	0.00121496	chicken
Sumo3	-0.50	0.00127384	chicken
Cdc73	0.52	0.00134651	chicken
Ola1	-0.54	0.00135468	chicken
Cd81	-0.49	0.00136355	chicken
Lrba	0.37	0.00138204	chicken
Aff4	0.33	0.00140975	chicken
Rel	0.55	0.00142267	chicken
Tef	-0.53	0.00143496	chicken
Extl3	-0.41	0.00145717	chicken
Tgfbr1	0.34	0.00146113	chicken
Coro7	-0.46	0.00148172	chicken
Trim59	0.19	0.00151258	chicken
Cdv3	-0.48	0.00152247	chicken
Clint1	0.48	0.00152317	chicken
Tnrc6b	0.43	0.00155522	chicken
Abhd2	0.38	0.00156103	chicken
Eif1b	-0.56	0.00158341	chicken
Bcat1	-0.30	0.00159610	chicken
Sec14l1	0.40	0.00165108	chicken
Tgfbr2	0.62	0.00169943	chicken
Pi4ka	-0.44	0.00190279	chicken

Il10ra	0.19	0.00193852	chicken
Snrpb	-0.39	0.00195883	chicken
Jarid2	0.31	0.00196116	chicken
Ldb2	-0.64	0.00199906	chicken
Smad6	0.52	0.00208666	chicken
Nras	-0.39	0.00221309	chicken
Zc3h15	-0.47	0.00222536	chicken
Kpna1	0.34	0.00229243	chicken
Kdm7a	0.46	0.00229567	chicken
Fbxo25	-0.63	0.00231865	chicken
Hdx	0.40	0.00245760	chicken
Eny2	-0.39	0.00245817	chicken
Ankrd28	0.70	0.00247706	chicken
Skil	0.34	0.00250175	chicken
Ell	0.34	0.00256426	chicken
Far1	0.43	0.00258727	chicken
Il16	-0.48	0.00276625	chicken
B3gnt4	0.17	0.00291837	chicken
Exoc2	0.43	0.00303986	chicken
Bnip3	0.35	0.00305405	chicken
Camk1d	0.47	0.00310577	chicken
Ptger4	0.38	0.00316358	chicken
Ipmk	-0.56	0.00325428	chicken
Gch1	0.28	0.00334493	chicken
Lrrfip1	0.73	0.00345796	chicken
Psmc3	-0.48	0.00347718	chicken
Tcf12	0.48	0.00364928	chicken
Cdin1	0.37	0.00375321	chicken
Ldhb	-0.47	0.00376668	chicken
Psma5	-0.43	0.00380190	chicken
Ptbp2	0.38	0.00387970	chicken
Kcnk1	0.35	0.00393156	chicken
Crebbp	0.33	0.00419178	chicken
Klf13	-0.34	0.00420816	chicken
Syt14	1.12	0.00442414	chicken
Aven	0.58	0.00443631	chicken
Nop14	-0.24	0.00466438	chicken
Cenpc1	0.25	0.00499286	chicken
Rilpl1	0.29	0.00507026	chicken
Umad1	0.79	0.00509112	chicken
Cib1	-0.28	0.00519211	chicken
Gabpa	-0.41	0.00549442	chicken
Tomm6	-0.44	0.00559185	chicken
Setbp1	0.69	0.00566178	chicken
Sergef	0.46	0.00567038	chicken
Fchsd2	0.34	0.00570887	chicken

Eif5b	-0.51	0.00573281	chicken
Ppp2r5e	0.68	0.00589220	chicken
Atxn7l1	0.49	0.00591879	chicken
Traf3	0.53	0.00625459	chicken
Ptrh2	-0.51	0.00627250	chicken
Ankrd44	0.46	0.00642932	chicken
Abracl	-0.49	0.00677289	chicken
Ranbp10	0.42	0.00680563	chicken
Dhx37	-0.42	0.00709433	chicken
Tlk2	0.36	0.00712610	chicken
Skap2	0.62	0.00717802	chicken
Sumo1	-0.46	0.00723331	chicken
Rab5if	-0.36	0.00753913	chicken
Bcor	0.47	0.00765065	chicken
Pdap1	-0.48	0.00785910	chicken
Arrdc3	-0.36	0.00801063	chicken
Sf3b5	-0.47	0.00806411	chicken
Csnk1d	0.36	0.00809597	chicken
Errfi1	0.40	0.00813608	chicken
Rbis	-0.46	0.00813781	chicken
Tbc1d4	1.33	0.00830307	chicken
Akt1	0.40	0.00833095	chicken
Ostc	-0.46	0.00906209	chicken
Rnf103	0.44	0.00909500	chicken
Ttf1	-0.52	0.00924645	chicken
Tcp1	-0.50	0.00925986	chicken
Ppp1r21	0.42	0.00943172	chicken
Ankrd52	0.22	0.00950620	chicken
Rxra	0.32	0.00956940	chicken
Rsrc2	0.29	0.01066294	chicken
Dnajc5	0.29	0.01086386	chicken
Prelid3b	0.35	0.01093208	chicken
Armh1	-0.47	0.01098161	chicken
Anapc16	-0.46	0.01107176	chicken
Prkag2	0.28	0.01128239	chicken
Parp8	0.60	0.01189630	chicken
Arf1	-0.47	0.01195541	chicken
Pde8a	1.23	0.01200746	chicken
Dock2	-0.29	0.01204971	chicken
Dnajc2	-0.42	0.01253724	chicken
Dapk1	0.69	0.01282918	chicken
Tmem115	-0.33	0.01317911	chicken
Cdk19	0.59	0.01320385	chicken
Lrch1	0.50	0.01381407	chicken
Fbxl2	-0.44	0.01382972	chicken
Lpp	0.36	0.01409593	chicken

Nol9	-0.41	0.01428521	chicken
L3mbtl3	0.41	0.01437946	chicken
Srsf3	-0.51	0.01466296	chicken
Inpp5f	0.35	0.01512076	chicken
Sfxn1	0.32	0.01521438	chicken
Pkd1	-0.49	0.01535423	chicken
Arhgap29	-0.46	0.01554511	chicken
Slc9a9	-0.46	0.01579573	chicken
Nek7	0.25	0.01592097	chicken
Ssbp2	0.55	0.01603311	chicken
Timm13	-0.42	0.01846221	chicken
Sema4d	0.51	0.01915467	chicken
Ryk	0.44	0.01936725	chicken
Foxo1	0.55	0.01944434	chicken
Arfip1	0.43	0.01945434	chicken
Rreb1	0.38	0.01992871	chicken
Zdhhc17	0.41	0.02044390	chicken
Stk38l	0.42	0.02059961	chicken
Slc23a2	-0.64	0.02061627	chicken
Plcg2	0.39	0.02109608	chicken
Ttbk1	0.58	0.02136203	chicken
Rhoh	0.39	0.02137139	chicken
Inpp5a	0.36	0.02213826	chicken
Polr2l	-0.43	0.02296863	chicken
Bcl6	0.41	0.02312466	chicken
Tia1	0.36	0.02337849	chicken
Arfgap3	0.44	0.02394058	chicken
Ap3s1	-0.29	0.02399599	chicken
Tmem168	0.27	0.02406520	chicken
9030025P20Rik	-0.56	0.02436982	chicken
Cct2	-0.48	0.02459004	chicken
Abca1	0.76	0.02529203	chicken
Cytip	0.36	0.02542762	chicken
Fli1	0.65	0.02609784	chicken
AU040320	0.35	0.02632498	chicken
Sptan1	-0.52	0.02698321	chicken
Nrbp1	0.32	0.02704645	chicken
Dcaf13	-0.40	0.02805492	chicken
Ostf1	-0.53	0.02849355	chicken
Psma6	-0.48	0.02941743	chicken
Paics	-0.44	0.02959287	chicken
Zmynd8	-0.46	0.02962148	chicken
Gpc1	1.26	0.02992382	chicken
Srgap3	0.41	0.02998208	chicken
Rsl24d1	-0.43	0.03035119	chicken
Celf2	0.32	0.03105215	chicken

Dcn	-0.42	0.03112170	chicken
Ccndbp1	-0.46	0.03206671	chicken
Syncrip	-0.42	0.03214460	chicken
Als2	0.44	0.03527160	chicken
Exoc3	0.43	0.03568940	chicken
Pik3r6	0.56	0.03594887	chicken
Psmb4	-0.42	0.03653141	chicken
Sdc4	0.61	0.03834914	chicken
Sesn1	0.42	0.03846646	chicken
Cd28	-0.25	0.03913191	chicken
Snrpg	-0.39	0.03944438	chicken
Nptn	0.26	0.04102381	chicken
Ccser2	0.66	0.04171625	chicken
Wsb1	0.27	0.04245878	chicken
Uqcrfs1	-0.32	0.04297267	chicken
Greb1l	0.64	0.04512592	chicken
Immt	-0.39	0.04584949	chicken
Siva1	-0.39	0.04848124	chicken
Slc9a7	-0.49	0.04896503	chicken
Ifnar1	0.50	0.04992935	chicken
Unc13d	-0.47	0.05053657	chicken
Rora	-0.05	0.05285137	chicken
Wapl	-0.34	0.05370696	chicken
Bhlhe41	-0.50	0.05400796	chicken
Yap1	0.42	0.05413211	chicken
Ubap1	0.31	0.05537680	chicken
Eif6	-0.34	0.05675256	chicken
Ilf2	-0.48	0.05676080	chicken
Pum1	0.32	0.05691791	chicken
Pitpna	0.38	0.05721167	chicken
Eepd1	-0.34	0.05739989	chicken
Dr1	-0.34	0.05853536	chicken
Tpi1	-0.41	0.05948553	chicken
Ptpn12	0.36	0.06050801	chicken
Hnrnpm	-0.35	0.06067541	chicken
Ptp4a2	-0.48	0.06202361	chicken
Cab39l	-0.52	0.06353636	chicken
Usp3	0.30	0.06524654	chicken
Tcerg1	-0.32	0.06537292	chicken
Atxn2	0.26	0.06582863	chicken
Far2	0.52	0.06692591	chicken
Ndufaf8	-0.39	0.06732678	chicken
Unk	0.28	0.06997057	chicken
Pdpk1	-0.39	0.07117221	chicken
Ubash3a	-0.44	0.07470926	chicken
Vps35l	-0.56	0.07518851	chicken

Rpp14	-0.44	0.07622651	chicken
Efhd1	-0.44	0.07764964	chicken
Wbp1	-0.35	0.07835616	chicken
Sfpq	-0.38	0.08138091	chicken
Ikzf3	0.33	0.08281243	chicken
Phtf2	0.37	0.08690774	chicken
Erp29	-0.37	0.08771172	chicken
Smarca4	-0.45	0.08860546	chicken
Lemd3	0.41	0.09021797	chicken
4932411N23Rik	0.23	0.09050964	chicken
Zfp831	0.20	0.09214960	chicken
Ube2e2	0.85	0.09722158	chicken
Pxk	0.26	0.09761115	chicken
Arhgap30	-0.49	0.09761992	chicken
Copb2	-0.45	0.09768224	chicken
Evi2a	0.33	0.10326096	chicken
Kcnq5	0.40	0.10400887	chicken
Rab29	0.29	0.10472154	chicken
Hsf1	-0.66	0.10477431	chicken
Nkrf	-0.43	0.10522135	chicken
Iars	-0.53	0.10545455	chicken
Impa2	0.43	0.10621242	chicken
Actr2	-0.50	0.10716668	chicken
Wdr3	-0.48	0.10749445	chicken
Itgb1	0.68	0.10814133	chicken
Prkn	0.62	0.10977542	chicken
Borcs5	0.54	0.10980693	chicken
Psmb2	-0.42	0.11145791	chicken
Fnip1	0.50	0.11661486	chicken
Exog	-0.32	0.11860819	chicken
Snu13	-0.45	0.11895391	chicken
Btf3l4	-0.42	0.11973598	chicken
Qpctl	-0.32	0.12022165	chicken
Tomm22	-0.42	0.12052027	chicken
Ankhd1	0.29	0.12086226	chicken
Rfc1	0.33	0.12181186	chicken
Cers5	0.30	0.12235809	chicken
Usp6nl	0.52	0.12451812	chicken
Psma4	-0.44	0.12842217	chicken
Nipa1	0.25	0.12879867	chicken
Qsox1	-0.44	0.13180914	chicken
Dkc1	-0.47	0.13199445	chicken
Cat	-0.44	0.13228639	chicken
Brsk2	-0.29	0.13392838	chicken
Fam53a	0.42	0.13510351	chicken
Bivm	-0.45	0.13716079	chicken

Rsu1	0.40	0.13848211	chicken
4921524J17Rik	-0.52	0.14217019	chicken
Tacc3	0.23	0.14667369	chicken
Cd63	-0.51	0.14758259	chicken
Saraf	0.18	0.15363115	chicken
Ncor2	0.30	0.15455230	chicken
Ncapd2	-0.44	0.15503785	chicken
Plin2	0.47	0.15826771	chicken
Eipr1	-0.28	0.15889931	chicken
Setd5	0.23	0.16296079	chicken
Ubn1	-0.46	0.16437240	chicken
Sla	0.31	0.16598809	chicken
Dnajc19	-0.35	0.16675529	chicken
Ppil2	-0.56	0.17077452	chicken
Hopx	0.20	0.17318883	chicken
Ppp3ca	0.72	0.17346654	chicken
Itm2c	0.30	0.17401859	chicken
Eif3h	-0.23	0.17426429	chicken
Hectd4	0.46	0.17907519	chicken
Psmb7	-0.40	0.18021175	chicken
Dtx3l	0.27	0.18117164	chicken
St3gal2	-0.42	0.18963189	chicken
Arl5a	-0.49	0.19015301	chicken
Tomm40l	-0.43	0.19086008	chicken
Gstm5	-0.37	0.19373222	chicken
Naif1	-0.32	0.19458342	chicken
Cdk6	-0.13	0.19677374	chicken
Tob2	-0.51	0.19714317	chicken
Rheb	0.26	0.20192149	chicken
Blk	0.71	0.20253979	chicken
Fam168b	-0.33	0.20275629	chicken
Rnf216	0.23	0.20860533	chicken
Prdx4	-0.32	0.20894806	chicken
Arl15	0.45	0.20919515	chicken
Klhl6	-0.39	0.21090611	chicken
Naa15	-0.38	0.21231938	chicken
Hint1	-0.35	0.21609072	chicken
Irf2	0.29	0.22405515	chicken
Sp3	-0.50	0.22409822	chicken
Zfp704	0.52	0.23193300	chicken
Gcnt4	0.28	0.23430380	chicken
Cdyl2	0.51	0.23876297	chicken
Elavl1	0.25	0.23877367	chicken
Pphln1	0.49	0.24513151	chicken
Tor1a	-0.36	0.24966031	chicken
Cish	0.26	0.25569527	chicken

Ctnnb1	0.27	0.26053742	chicken
Lsg1	-0.32	0.26104519	chicken
Spen	0.28	0.26269249	chicken
Cpeb4	0.39	0.26600557	chicken
Ndufb7	-0.43	0.27280348	chicken
Stard4	-0.37	0.28266556	chicken
Tmem39a	-0.50	0.28511743	chicken
Nhp2	-0.28	0.28760966	chicken
Elp6	-0.39	0.28953355	chicken
Wasf2	0.21	0.28966702	chicken
Zfp207	-0.54	0.28974148	chicken
Zmat2	-0.40	0.29261451	chicken
Tmem131	0.44	0.30162711	chicken
Ccdc25	-0.32	0.30254731	chicken
Pef1	-0.29	0.31096778	chicken
Chchd1	-0.39	0.31375121	chicken
Trappc12	0.35	0.31417050	chicken
Slc20a2	-0.63	0.31509012	chicken
N4bp1	-0.34	0.31535084	chicken
Phaf1	0.39	0.31820450	chicken
Romo1	-0.44	0.32368075	chicken
Sde2	-0.44	0.32541185	chicken
Lims2	0.21	0.32631782	chicken
Dhx38	-0.39	0.32948875	chicken
Ccdc137	-0.31	0.33288487	chicken
Usp38	-0.36	0.33423006	chicken
Wdr43	-0.39	0.33950161	chicken
Ndufab1	-0.39	0.34096639	chicken
Adss	0.39	0.34260995	chicken
Uxs1	0.55	0.34297861	chicken
Dnah12	-0.44	0.34334365	chicken
Rfx7	0.24	0.34366978	chicken
Dvl1	0.20	0.34983950	chicken
Sobp	-0.49	0.34985481	chicken
Dock11	0.35	0.35342545	chicken
Slk	-0.15	0.35722575	chicken
Eif2ak4	0.36	0.35740569	chicken
Bmpr2	0.68	0.35837739	chicken
Strn3	0.36	0.37289140	chicken
Cars	0.35	0.37701848	chicken
Frs2	0.28	0.37802866	chicken
Usp16	0.32	0.38551846	chicken
Cul2	0.31	0.38564498	chicken
Uhrf2	0.30	0.39778989	chicken
Atxn1	0.51	0.39785768	chicken
Osgin2	-0.34	0.39992882	chicken

Acsl3	-0.17	0.40291940	chicken
Sh2b3	0.24	0.40747537	chicken
Ldb1	0.25	0.41028085	chicken
Rsf1	0.40	0.42330523	chicken
Fkbp4	-0.46	0.44706562	chicken
Def6	0.17	0.44738273	chicken
Pfdn2	-0.38	0.45023520	chicken
Prpf39	0.24	0.45595662	chicken
Oaz1	-0.37	0.45853430	chicken
Ankib1	-0.40	0.46476992	chicken
Rgs9	-0.44	0.46736845	chicken
Kcnq1	-0.05	0.48189741	chicken
Id2	-0.45	0.48307341	chicken
Dad1	-0.44	0.48476578	chicken
Aftph	0.60	0.48861633	chicken
Ints13	-0.37	0.49077621	chicken
Tox2	0.86	0.49272539	chicken
Grk3	0.34	0.49635291	chicken
Hlcs	0.50	0.49827472	chicken
Atp5k	-0.32	0.50205146	chicken
Dbi	-0.37	0.50682434	chicken
Ndrg3	0.35	0.51422858	chicken
Ube2j2	-0.35	0.51448328	chicken
Tapbp	-0.44	0.51504322	chicken
Septin9	0.35	0.52839959	chicken
Brd2	-0.38	0.53368345	chicken
Cyth1	-0.39	0.53841632	chicken
Ndufs5	-0.34	0.53920623	chicken
Hsp90b1	-0.44	0.53926927	chicken
Rspry1	0.30	0.54123388	chicken
Ube2w	0.41	0.54308430	chicken
Baz1b	0.33	0.55556377	chicken
Cirbp	-0.35	0.56390763	chicken
Cd38	0.45	0.56588447	chicken
Arpp19	-0.43	0.58228760	chicken
Rbbp4	-0.41	0.58844330	chicken
Rpn2	-0.33	0.59667371	chicken
Ccdc59	-0.39	0.60752594	chicken
Usp22	0.35	0.61005529	chicken
Nfkb1	0.31	0.62287051	chicken
Ldah	-0.42	0.63059167	chicken
Champ1	-0.30	0.64318270	chicken
Xbp1	-0.40	0.64582080	chicken
Hnrnpab	-0.35	0.64605706	chicken
Gnal	0.74	0.66887721	chicken
Cdca4	0.23	0.66970793	chicken

Lactb	-0.35	0.68630648	chicken
Gnb4	0.26	0.70020228	chicken
Eif5	-0.38	0.70243424	chicken
Fbxw2	-0.43	0.71793260	chicken
Xxylt1	0.26	0.72390339	chicken
Psmc2	-0.39	0.73737616	chicken
Pgap1	0.32	0.74134244	chicken
Usp1	-0.35	0.74752389	chicken
Rbsn	-0.38	0.74801322	chicken
Ndufaf6	0.19	0.74964064	chicken
Taf3	0.53	0.75336240	chicken
Sar1a	-0.40	0.75739685	chicken
Smg7	0.26	0.75908721	chicken
Tbc1d1	0.21	0.76371939	chicken
Exosc2	-0.30	0.76829490	chicken
Timm50	-0.33	0.78315099	chicken
Reep3	0.33	0.79485453	chicken
Pik3cd	0.24	0.79612763	chicken
Ddx20	-0.38	0.82658870	chicken
Ap1g1	0.28	0.82904159	chicken
Polr2i	-0.32	0.83187637	chicken
Nmi	0.21	0.83365757	chicken
Dennd4c	0.50	0.83394805	chicken
Sf3b6	-0.39	0.85500063	chicken
Rftn1	0.66	0.89014983	chicken
Lzic	-0.32	0.89100569	chicken
Insc	0.44	0.90087582	chicken
Skp1	-0.38	0.91483748	chicken
Ermn	0.23	0.91643767	chicken
Parn	-0.36	0.92283348	chicken
Ppp1ca	-0.36	0.92555499	chicken
Zzz3	0.37	0.95695021	chicken
Tax1bp1	0.71	0.96878668	chicken
Eef1e1	-0.51	0.97916121	chicken
Traf3ip3	-0.42	0.98100186	chicken
Smndc1	-0.43	0.98112329	chicken
Lsp1	-0.39	0.98359967	chicken
Pias1	0.26	0.98789586	chicken
Glrx5	-0.33	1.00000000	chicken
Dmxl1	-0.36	1.00000000	chicken
Lman2	-0.31	1.00000000	chicken
Lap3	-0.39	1.00000000	chicken
Polr2m	-0.31	1.00000000	chicken
Stk17b	-0.39	1.00000000	chicken
Cope	-0.29	1.00000000	chicken
Dhx15	-0.28	1.00000000	chicken

Smim19	-0.33	1.00000000	chicken
Il2ra	0.74	1.00000000	chicken
Triap1	-0.32	1.00000000	chicken
Clptm1l	-0.51	1.00000000	chicken
Fam102a	-0.23	1.00000000	chicken
Smg1	0.32	1.00000000	chicken
Tubb2a	-0.32	1.00000000	chicken
Pum2	0.36	1.00000000	chicken
Pik3ap1	0.41	1.00000000	chicken
Rab1a	0.27	1.00000000	chicken
Ebag9	-0.38	1.00000000	chicken
Dyrk1a	0.33	1.00000000	chicken
Srp19	-0.32	1.00000000	chicken
Hyou1	-0.42	1.00000000	chicken
1110004F10Rik	-0.36	1.00000000	chicken
Svil	0.58	1.00000000	chicken
Cops7a	-0.29	1.00000000	chicken
Nip7	-0.30	1.00000000	chicken
Hspa9	-0.39	1.00000000	chicken
Srp72	-0.32	1.00000000	chicken
Casp3	0.20	1.00000000	chicken
Snx25	-0.40	1.00000000	chicken
Ubxn2a	0.18	1.00000000	chicken
Acbd5	0.28	1.00000000	chicken
Parp1	-0.35	1.00000000	chicken
Ttc4	-0.33	1.00000000	chicken
Bcap31	-0.38	1.00000000	chicken
Dido1	0.24	1.00000000	chicken
Phf21a	0.34	1.00000000	chicken
Impdh2	-0.30	1.00000000	chicken
Cox7a2l	-0.33	1.00000000	chicken
Hk2	0.23	1.00000000	chicken
Psmc5	-0.38	1.00000000	chicken
Abhd5	0.32	1.00000000	chicken
Anp32e	-0.37	1.00000000	chicken
Fam204a	-0.38	1.00000000	chicken
Ncoa7	0.24	1.00000000	chicken
Ints8	-0.35	1.00000000	chicken
Ftsj3	-0.38	1.00000000	chicken
Traf1	-0.44	1.00000000	chicken
Herc1	0.29	1.00000000	chicken
Cbx3	-0.41	1.00000000	chicken
Abca2	-0.35	1.00000000	chicken
Cebpz	-0.38	1.00000000	chicken
Nedd8	-0.37	1.00000000	chicken
Pcgf5	0.24	1.00000000	chicken

Sync	0.11	1.00000000	chicken
Apbb1ip	0.37	1.00000000	chicken
Zyx	-0.35	1.00000000	chicken
Pcid2	-0.37	1.00000000	chicken
Snx20	-0.45	1.00000000	chicken
Nolc1	-0.30	1.00000000	chicken
Tmem59	-0.42	1.00000000	chicken
Snap29	-0.30	1.00000000	chicken
Selenot	-0.44	1.00000000	chicken
Psma7	-0.39	1.00000000	chicken
Sult4a1	0.21	1.00000000	chicken
Lcp1	-0.42	1.00000000	chicken
B3gntl1	-0.21	1.00000000	chicken
Kdm4b	0.30	1.00000000	chicken
Ociad1	-0.34	1.00000000	chicken
Supt3	0.49	1.00000000	chicken
Paxbp1	-0.35	1.00000000	chicken
Pfdn6	-0.39	1.00000000	chicken
Cdca7l	-0.34	1.00000000	chicken
Hmgcr	-0.43	1.00000000	chicken
Nsmce2	0.19	1.00000000	chicken
Gramd1b	0.21	1.00000000	chicken
Aff2	0.39	1.00000000	chicken
Arid1b	0.31	1.00000000	chicken
Zfp644	0.27	1.00000000	chicken
Znhit6	0.23	1.00000000	chicken
Bri3	-0.31	1.00000000	chicken
Psme4	-0.37	1.00000000	chicken
Rbm19	-0.38	1.00000000	chicken
Abcb7	0.25	1.00000000	chicken
Snrnp25	-0.29	1.00000000	chicken
Itpr1	-0.21	1.00000000	chicken
Utp6	-0.33	1.00000000	chicken
Golga7	-0.30	1.00000000	chicken
1810058I24Rik	-0.25	1.00000000	chicken
Polr2b	-0.41	1.00000000	chicken
Orai1	0.27	1.00000000	chicken
Otub1	-0.35	1.00000000	chicken
Susd3	0.35	1.00000000	chicken
Skap1	0.46	1.00000000	chicken
Prpf40a	-0.33	1.00000000	chicken
Plxnd1	-0.32	1.00000000	chicken
Ranbp3	0.26	1.00000000	chicken
Epc2	-0.38	1.00000000	chicken
Prdx6	-0.32	1.00000000	chicken
Fhod1	0.33	1.00000000	chicken

Dipk1a	-0.39	1.00000000	chicken
Ttc28	0.21	1.00000000	chicken
Vps33b	-0.26	1.00000000	chicken
Ssu72	0.36	1.00000000	chicken
Nisch	0.27	1.00000000	chicken
Nufip1	-0.24	1.00000000	chicken
Csde1	-0.34	1.00000000	chicken
Fam83f	-0.34	1.00000000	chicken
Rtn4	0.30	1.00000000	chicken
Usp24	0.35	1.00000000	chicken
Smarca5	-0.38	1.00000000	chicken
Eif4g3	0.19	1.00000000	chicken
Sec63	0.29	1.00000000	chicken
Slc44a1	-0.34	1.00000000	chicken
Suclg1	-0.31	1.00000000	chicken
Osbpl10	0.32	1.00000000	chicken
Slc3a1	0.31	1.00000000	chicken
Klhl2	-0.29	1.00000000	chicken
Lrrc28	-0.49	1.00000000	chicken
Ap3m2	-0.42	1.00000000	chicken
Uqcrc1	-0.31	1.00000000	chicken
Rnf166	-0.33	1.00000000	chicken
Ei24	-0.27	1.00000000	chicken
Wnk1	0.49	1.00000000	chicken
Htra2	-0.33	1.00000000	chicken
Ppp3r1	0.34	1.00000000	chicken
Pdhb	-0.34	1.00000000	chicken
Helz	0.36	1.00000000	chicken
Fahd2a	-0.32	1.00000000	chicken
Tmed7	-0.39	1.00000000	chicken
Zmynd11	0.41	1.00000000	chicken
Uqcrb	-0.31	1.00000000	chicken
Ppp1cb	-0.32	1.00000000	chicken
Stt3b	0.46	1.00000000	chicken
Usp44	0.14	1.00000000	chicken
Cyrib	0.34	1.00000000	chicken
Bnip3l	-0.41	1.00000000	chicken
Kif5b	-0.41	1.00000000	chicken
Pcm1	0.23	1.00000000	chicken
Nudcd1	-0.33	1.00000000	chicken
Serpini1	0.15	1.00000000	chicken
St13	-0.31	1.00000000	chicken
Esd	-0.29	1.00000000	chicken
Rac2	-0.41	1.00000000	chicken
Nop56	-0.34	1.00000000	chicken
Sytl1	-0.38	1.00000000	chicken

Cdkn1b	0.33	1.00000000	chicken
Taok1	0.24	1.00000000	chicken
Ddx27	-0.41	1.00000000	chicken
Rabgap1	0.26	1.00000000	chicken
Thoc7	-0.32	1.00000000	chicken
Rassf2	0.15	1.00000000	chicken
Zcchc17	-0.31	1.00000000	chicken
Fam126a	0.24	1.00000000	chicken
Kansl1	0.18	1.00000000	chicken
Hcls1	-0.27	1.00000000	chicken
Rassf3	-0.36	1.00000000	chicken
Zfp318	-0.38	1.00000000	chicken
Ndufb8	-0.32	1.00000000	chicken
Clk4	-0.44	1.00000000	chicken
Slmap	0.35	1.00000000	chicken
Foxi1	-0.36	1.00000000	chicken
Atp5c1	-0.30	1.00000000	chicken
Iws1	-0.34	1.00000000	chicken
Pdcd5	-0.31	1.00000000	chicken
Dalrd3	0.19	1.00000000	chicken
Rbpj	0.28	1.00000000	chicken
Tshz2	0.60	1.00000000	chicken
Cnot7	-0.36	1.00000000	chicken
Anapc7	0.23	1.00000000	chicken
Snw1	-0.30	1.00000000	chicken
Cd164	-0.34	1.00000000	chicken
Ip6k1	0.22	1.00000000	chicken
Tent4b	0.40	1.00000000	chicken
Usp47	0.33	1.00000000	chicken
Dip2b	-0.30	1.00000000	chicken
Ddx42	0.22	1.00000000	chicken
Stt3a	-0.31	1.00000000	chicken
Orc3	0.20	1.00000000	chicken
Efhd2	0.15	1.00000000	chicken
Lrpprc	-0.27	1.00000000	chicken
Atp5b	-0.30	1.00000000	chicken
Sec31a	-0.34	1.00000000	chicken
Usp49	0.31	1.00000000	chicken
Hnrnpu	-0.35	1.00000000	chicken
Spag9	0.35	1.00000000	chicken
Nop2	-0.31	1.00000000	chicken
Ctdspl	0.46	1.00000000	chicken
Cdc42bpb	0.31	1.00000000	chicken
Ewsr1	-0.41	1.00000000	chicken
Chmp5	-0.44	1.00000000	chicken
Gtdc1	0.15	1.00000000	chicken

Slc25a5	-0.28	1.00000000	chicken
Birc2	0.50	1.00000000	chicken
Ppp2r5a	0.31	1.00000000	chicken
Dcp2	-0.43	1.00000000	chicken
Rlf	-0.40	1.00000000	chicken
Ghitm	-0.41	1.00000000	chicken
Ndufs7	-0.27	1.00000000	chicken
Psmd2	-0.30	1.00000000	chicken
Pank3	-0.42	1.00000000	chicken
Fkbp3	-0.29	1.00000000	chicken
Zdhhc14	0.46	1.00000000	chicken
Psmd6	-0.26	1.00000000	chicken
Srrm1	-0.31	1.00000000	chicken
Rcc2	-0.24	1.00000000	chicken
Klhl24	-0.42	1.00000000	chicken
C2cd2l	-0.39	1.00000000	chicken
Jade1	0.37	1.00000000	chicken
Chic2	-0.32	1.00000000	chicken
Ern1	0.27	1.00000000	chicken
Syf2	-0.40	1.00000000	chicken
Rbm45	-0.30	1.00000000	chicken
Jpt1	-0.40	1.00000000	chicken
Acap2	0.33	1.00000000	chicken
Yaf2	-0.43	1.00000000	chicken
Atp11b	0.31	1.00000000	chicken
Tmem223	-0.23	1.00000000	chicken
Nt5c3	-0.26	1.00000000	chicken
Htra1	0.12	1.00000000	chicken
Wdr70	-0.34	1.00000000	chicken
Kif1b	0.46	1.00000000	chicken
Inpp4b	0.82	1.00000000	chicken
Nol10	-0.30	1.00000000	chicken
Appl1	-0.43	1.00000000	chicken
Cct5	-0.32	1.00000000	chicken
Eif4g1	-0.37	1.00000000	chicken
Evl	0.53	1.00000000	chicken
Taf13	-0.26	1.00000000	chicken
Atp5h	-0.22	1.00000000	chicken
Lars	-0.32	1.00000000	chicken
Hvcn1	0.28	1.00000000	chicken
Rfxap	-0.26	1.00000000	chicken
Rab8b	-0.16	1.00000000	chicken
Ide	0.27	1.00000000	chicken
Nucks1	-0.29	1.00000000	chicken
Yy2	0.23	1.00000000	chicken
Bok	-0.26	1.00000000	chicken

Gxylt1	-0.39	1.00000000	chicken
Kctd20	0.15	1.00000000	chicken
Bcr	0.21	1.00000000	chicken
Abcb8	-0.28	1.00000000	chicken
Tuba1b	-0.40	1.00000000	chicken
Gtf2a1	0.11	1.00000000	chicken
Septin14	0.15	1.00000000	chicken
Anapc10	0.46	1.00000000	chicken
Rhot1	0.13	1.00000000	chicken
Cd4	-0.30	1.00000000	chicken
Khdc4	0.19	1.00000000	chicken
Rap1b	0.33	1.00000000	chicken
Lta4h	-0.24	1.00000000	chicken
Zfp652	0.18	1.00000000	chicken
Cul5	-0.33	1.00000000	chicken
Secisbp2	0.19	1.00000000	chicken
Eif3i	-0.31	1.00000000	chicken
Sun1	0.16	1.00000000	chicken
Pigl	0.15	1.00000000	chicken
Lmbrd2	0.27	1.00000000	chicken
Pcnx	0.31	1.00000000	chicken
Park7	-0.34	1.00000000	chicken
Cdk14	-0.08	1.00000000	chicken
Slc9a6	-0.32	1.00000000	chicken
Psmd14	-0.32	1.00000000	chicken
Trnt1	-0.26	1.00000000	chicken
Ndufb9	-0.32	1.00000000	chicken
Prpsap2	-0.24	1.00000000	chicken
Creb3	-0.23	1.00000000	chicken
Zc3h11a	-0.38	1.00000000	chicken
Epcam	0.30	1.00000000	chicken
Per3	0.24	1.00000000	chicken
Prrc2c	-0.19	1.00000000	chicken
Phka2	-0.35	1.00000000	chicken
C3	0.22	1.00000000	chicken
Rbm26	0.20	1.00000000	chicken
Sox4	-0.30	1.00000000	chicken
Thoc5	-0.27	1.00000000	chicken
Hells	-0.32	1.00000000	chicken
Scaf11	-0.30	1.00000000	chicken
Bud31	-0.27	1.00000000	chicken
Fut8	0.35	1.00000000	chicken
Ttc7	0.44	1.00000000	chicken
Slirp	-0.22	1.00000000	chicken
Atl2	0.25	1.00000000	chicken
Atxn3	-0.41	1.00000000	chicken

Zc3h13	-0.28	1.00000000	chicken
Uba2	0.17	1.00000000	chicken
Dnajb6	0.32	1.00000000	chicken
Nrp2	-0.04	1.00000000	chicken
Traf5	0.24	1.00000000	chicken
Vps11	-0.37	1.00000000	chicken
Txlng	-0.35	1.00000000	chicken
Ppp4r3a	0.29	1.00000000	chicken
Kpnb1	-0.31	1.00000000	chicken
Zap70	0.28	1.00000000	chicken
Golga4	0.35	1.00000000	chicken
Sec11a	-0.29	1.00000000	chicken
Ntng1	-0.48	1.00000000	chicken
Stk40	-0.20	1.00000000	chicken
Dlgap4	0.23	1.00000000	chicken
Sox13	0.38	1.00000000	chicken
Lpxn	0.11	1.00000000	chicken
Dlgap1	-0.27	1.00000000	chicken
Lrch3	0.28	1.00000000	chicken
Ndufa10	-0.29	1.00000000	chicken
Arhgef7	-0.40	1.00000000	chicken
Coro1c	-0.28	1.00000000	chicken
Ttc27	-0.37	1.00000000	chicken
Vezt	0.35	1.00000000	chicken
Atad1	-0.35	1.00000000	chicken
Ddx46	-0.37	1.00000000	chicken
8030462N17Rik	0.22	1.00000000	chicken
Rcsd1	-0.29	1.00000000	chicken
Ppp2r2a	0.26	1.00000000	chicken
Bclaf1	-0.19	1.00000000	chicken
Spidr	0.44	1.00000000	chicken
Dennd1a	0.21	1.00000000	chicken
Plekha1	0.27	1.00000000	chicken
Atp8a1	-0.43	1.00000000	chicken
Uqcr10	-0.36	1.00000000	chicken
Acap1	-0.30	1.00000000	chicken
Tmem183a	-0.29	1.00000000	chicken
Shld2	0.28	1.00000000	chicken
Ankrd13d	-0.27	1.00000000	chicken
Setd1b	-0.40	1.00000000	chicken
Dusp23	-0.28	1.00000000	chicken
Tram1l1	-0.34	1.00000000	chicken
Csnk1e	0.05	1.00000000	chicken
Tfdp2	-0.23	1.00000000	chicken
Gpsm2	-0.30	1.00000000	chicken
Ptpn6	0.13	1.00000000	chicken

Ndufv2	-0.25	1.00000000	chicken
Dusp16	0.31	1.00000000	chicken
Ric1	0.10	1.00000000	chicken
Snrpc	-0.32	1.00000000	chicken
Nup153	0.26	1.00000000	chicken
Rnf13	0.22	1.00000000	chicken
Tfrc	-0.27	1.00000000	chicken
Rb1cc1	0.38	1.00000000	chicken
Nfya	-0.32	1.00000000	chicken
Trat1	-0.32	1.00000000	chicken
Riok3	-0.48	1.00000000	chicken
Pbxip1	-0.23	1.00000000	chicken
Carhsp1	-0.30	1.00000000	chicken
Ndfip1	-0.23	1.00000000	chicken
Pmf1	-0.20	1.00000000	chicken
Dusp10	0.34	1.00000000	chicken
Azin1	0.14	1.00000000	chicken
Sem1	-0.22	1.00000000	chicken
D16Ertd472e	-0.34	1.00000000	chicken
Abcf2	-0.36	1.00000000	chicken
Acsbg2	0.27	1.00000000	chicken
Rnf149	-0.26	1.00000000	chicken
Cmtm7	0.16	1.00000000	chicken
Setdb2	-0.39	1.00000000	chicken
Tbxas1	-0.14	1.00000000	chicken
Derl1	-0.31	1.00000000	chicken
Prkd3	0.37	1.00000000	chicken
Arhgef3	0.38	1.00000000	chicken
Dhx36	-0.21	1.00000000	chicken
Tcf25	-0.37	1.00000000	chicken
Cmss1	0.48	1.00000000	chicken
Usp8	0.15	1.00000000	chicken
Tmem192	-0.32	1.00000000	chicken
Cabin1	0.32	1.00000000	chicken
Nrp1	-0.15	1.00000000	chicken
Fbxw7	-0.36	1.00000000	chicken
Nckap1l	-0.23	1.00000000	chicken
Ndrg1	0.23	1.00000000	chicken
Lamp2	0.18	1.00000000	chicken
Zfp451	0.28	1.00000000	chicken
Phax	-0.27	1.00000000	chicken
Tpr	-0.30	1.00000000	chicken
Ppip5k2	0.23	1.00000000	chicken
Tmem135	0.29	1.00000000	chicken
Gnai2	-0.25	1.00000000	chicken
Eif2a	-0.28	1.00000000	chicken

Golim4	-0.30	1.00000000	chicken
Arl1	-0.28	1.00000000	chicken
Fez2	0.24	1.00000000	chicken
Dhx30	-0.25	1.00000000	chicken
Gpr137b	-0.31	1.00000000	chicken
Naa50	-0.31	1.00000000	chicken
Dennd6a	0.30	1.00000000	chicken
Rras2	0.25	1.00000000	chicken
Idh1	-0.32	1.00000000	chicken
Vps29	-0.34	1.00000000	chicken
Ralbp1	-0.15	1.00000000	chicken
Spz1	-0.34	1.00000000	chicken
Nr3c1	0.17	1.00000000	chicken
Esf1	-0.35	1.00000000	chicken
Ndufa12	-0.28	1.00000000	chicken
Psma3	-0.23	1.00000000	chicken
Lmo2	0.30	1.00000000	chicken
Sh3kbp1	-0.09	1.00000000	chicken
Scaf8	0.19	1.00000000	chicken
Anxa6	-0.27	1.00000000	chicken
Eml4	0.47	1.00000000	chicken
Nlk	0.16	1.00000000	chicken
Smg6	-0.37	1.00000000	chicken
Igfbp3	0.19	1.00000000	chicken
Dnaja1	0.18	1.00000000	chicken
Edem1	-0.29	1.00000000	chicken
Asap1	-0.10	1.00000000	chicken
Fkbp8	-0.23	1.00000000	chicken
Ogfod3	0.57	1.00000000	chicken
Zfp352	-0.29	1.00000000	chicken
Nifk	-0.30	1.00000000	chicken
Vps50	0.30	1.00000000	chicken
Bhlhe40	0.13	1.00000000	chicken
Ywhaq	-0.23	1.00000000	chicken
Tmx3	-0.35	1.00000000	chicken
Cwc22	-0.19	1.00000000	chicken
Exosc10	-0.30	1.00000000	chicken
Rer1	-0.28	1.00000000	chicken
Itgav	0.41	1.00000000	chicken
Reep2	-0.21	1.00000000	chicken
Ccdc43	-0.33	1.00000000	chicken
Afg3l2	0.18	1.00000000	chicken
Emc3	-0.25	1.00000000	chicken
Wipf1	0.27	1.00000000	chicken
Sqle	-0.28	1.00000000	chicken
Pes1	-0.22	1.00000000	chicken

Ubr2	0.21	1.00000000	chicken
Rnf4	0.13	1.00000000	chicken
Slc2a8	-0.26	1.00000000	chicken
Gorab	-0.26	1.00000000	chicken
Prkca	0.25	1.00000000	chicken
Cd83	0.72	1.00000000	chicken
Kif13a	0.56	1.00000000	chicken
Dph3	-0.37	1.00000000	chicken
Cnn2	-0.25	1.00000000	chicken
Psme3ip1	-0.34	1.00000000	chicken
Sft2d1	0.23	1.00000000	chicken
Srpr	-0.28	1.00000000	chicken
Ypel1	0.19	1.00000000	chicken
Ddx1	-0.34	1.00000000	chicken
Armc8	0.28	1.00000000	chicken
Lrp12	0.30	1.00000000	chicken
Crip1	0.32	1.00000000	chicken
Arhgap10	0.55	1.00000000	chicken
Necap1	-0.27	1.00000000	chicken
Gtf2h5	-0.21	1.00000000	chicken
Tbl3	-0.29	1.00000000	chicken
Grap	-0.26	1.00000000	chicken
Dnajc10	-0.25	1.00000000	chicken
Tcp11l2	0.06	1.00000000	chicken
Spata5	0.47	1.00000000	chicken
Sec13	-0.32	1.00000000	chicken
Pptc7	0.16	1.00000000	chicken
Ldlrad4	0.44	1.00000000	chicken
Pde4d	0.34	1.00000000	chicken
Tmem167	0.08	1.00000000	chicken
Pggt1b	0.22	1.00000000	chicken
Rad21	-0.23	1.00000000	chicken
Ing3	-0.25	1.00000000	chicken
Ssr2	-0.34	1.00000000	chicken
Rpap2	0.11	1.00000000	chicken
Parp4	-0.25	1.00000000	chicken
Abcc1	0.24	1.00000000	chicken
Gpatch2l	0.09	1.00000000	chicken
Shoc2	0.32	1.00000000	chicken
Vps41	-0.41	1.00000000	chicken
Pogz	-0.18	1.00000000	chicken
Zbtb46	0.33	1.00000000	chicken
Pex13	0.06	1.00000000	chicken
Pdlim5	0.49	1.00000000	chicken
Lamtor5	-0.24	1.00000000	chicken
Phip	0.36	1.00000000	chicken

Cdc14b	0.24	1.00000000	chicken
Wrn	-0.27	1.00000000	chicken
Rrp15	-0.23	1.00000000	chicken
Vim	0.58	1.00000000	chicken
Chchd10	0.23	1.00000000	chicken
Scai	-0.29	1.00000000	chicken
Thumpd3	-0.34	1.00000000	chicken
Raf1	0.16	1.00000000	chicken
C1d	-0.32	1.00000000	chicken
D630045J12Rik	0.28	1.00000000	chicken
Tec	0.31	1.00000000	chicken
Vma21	-0.30	1.00000000	chicken
Gosr2	-0.34	1.00000000	chicken
Ddx54	-0.22	1.00000000	chicken
Nob1	-0.27	1.00000000	chicken
Opa1	-0.30	1.00000000	chicken
Canx	-0.33	1.00000000	chicken
Sec61g	-0.38	1.00000000	chicken
Dis3	0.13	1.00000000	chicken
Hras	-0.26	1.00000000	chicken
Ssr3	-0.26	1.00000000	chicken
Rcor3	-0.13	1.00000000	chicken
P2rx5	0.15	1.00000000	chicken
Snapin	-0.25	1.00000000	chicken
Cops2	-0.33	1.00000000	chicken
Uso1	-0.30	1.00000000	chicken
Cwc15	-0.30	1.00000000	chicken
Ppp1r10	0.11	1.00000000	chicken
Cc2d1b	0.14	1.00000000	chicken
Atp1a1	-0.27	1.00000000	chicken
Nfe2l2	0.18	1.00000000	chicken
Vapa	-0.23	1.00000000	chicken
Aldh18a1	-0.30	1.00000000	chicken
Traf2	0.14	1.00000000	chicken
Tbrg4	-0.16	1.00000000	chicken
Crebrf	0.21	1.00000000	chicken
Kras	0.23	1.00000000	chicken
Anxa11	-0.31	1.00000000	chicken
Lrrfip2	-0.13	1.00000000	chicken
Irak2	-0.33	1.00000000	chicken
Asf1a	-0.30	1.00000000	chicken
Sdhaf2	-0.19	1.00000000	chicken
Ppfia1	0.28	1.00000000	chicken
Slc39a9	-0.29	1.00000000	chicken
Dazap1	0.16	1.00000000	chicken
Nop53	-0.27	1.00000000	chicken

St6gal1	0.43	1.00000000	chicken
Cramp1	0.31	1.00000000	chicken
Sesn3	0.27	1.00000000	chicken
Rufy3	-0.28	1.00000000	chicken
Tmtc4	0.24	1.00000000	chicken
Gbe1	0.29	1.00000000	chicken
Snx8	-0.31	1.00000000	chicken
Pcna	-0.29	1.00000000	chicken
Ythdf3	0.18	1.00000000	chicken
Polr2f	-0.26	1.00000000	chicken
Slc1a4	-0.22	1.00000000	chicken
Cplane2	-0.20	1.00000000	chicken
Cfdp1	-0.35	1.00000000	chicken
Cir1	-0.32	1.00000000	chicken
Ndufaf2	0.26	1.00000000	chicken
Arcn1	-0.31	1.00000000	chicken
Hp1bp3	-0.26	1.00000000	chicken
Ncoa6	0.21	1.00000000	chicken
Pwp1	-0.34	1.00000000	chicken
Hipk1	0.12	1.00000000	chicken
Vps4b	-0.24	1.00000000	chicken
Cct7	-0.25	1.00000000	chicken
Prpf6	-0.22	1.00000000	chicken
Plekhf2	-0.34	1.00000000	chicken
Nr2c2	0.23	1.00000000	chicken
Pds5b	-0.20	1.00000000	chicken
Polg2	-0.37	1.00000000	chicken
Banp	0.12	1.00000000	chicken
Slc9a3r1	-0.29	1.00000000	chicken
Luc7l2	-0.26	1.00000000	chicken
Slc25a16	-0.34	1.00000000	chicken
Drosha	-0.32	1.00000000	chicken
Pin4	-0.26	1.00000000	chicken
Akap7	-0.14	1.00000000	chicken
Noc2l	-0.35	1.00000000	chicken
Aup1	-0.29	1.00000000	chicken
Ankrd17	-0.19	1.00000000	chicken
Rwdd1	-0.22	1.00000000	chicken
Arid4a	0.24	1.00000000	chicken
Ube2q1	-0.28	1.00000000	chicken
Ncbp2	-0.28	1.00000000	chicken
Tbck	0.25	1.00000000	chicken
Ikbkb	-0.30	1.00000000	chicken
Hspa4	-0.28	1.00000000	chicken
Eftud2	-0.27	1.00000000	chicken
Wbp4	-0.31	1.00000000	chicken

4930453N24Rik	0.10	1.00000000	chicken
Hspa8	-0.27	1.00000000	chicken
Galnt9	0.39	1.00000000	chicken
Cyld	0.09	1.00000000	chicken
Rae1	-0.19	1.00000000	chicken
Sf3a1	-0.25	1.00000000	chicken
Fmr1	0.19	1.00000000	chicken
Nrip1	0.07	1.00000000	chicken
Ahsa1	-0.24	1.00000000	chicken
Ly96	0.30	1.00000000	chicken
Asxl2	0.28	1.00000000	chicken
Churc1	-0.24	1.00000000	chicken
Rc3h2	0.08	1.00000000	chicken
Creld2	-0.20	1.00000000	chicken
Alcam	0.49	1.00000000	chicken
C2cd5	-0.27	1.00000000	chicken
Hcfc2	0.07	1.00000000	chicken
Laptm4a	-0.29	1.00000000	chicken
Il21r	0.12	1.00000000	chicken
Zfp326	-0.23	1.00000000	chicken
Psmd7	-0.27	1.00000000	chicken
Stard3nl	0.27	1.00000000	chicken
Zcchc8	-0.32	1.00000000	chicken
Fancm	-0.28	1.00000000	chicken
Tuba1a	-0.32	1.00000000	chicken
Tmem165	-0.28	1.00000000	chicken
Ythdf1	-0.26	1.00000000	chicken
Pan3	0.20	1.00000000	chicken
Pdzd8	0.33	1.00000000	chicken
Soat1	-0.27	1.00000000	chicken
Usf3	-0.27	1.00000000	chicken
Adam10	0.19	1.00000000	chicken
Tfdp1	0.37	1.00000000	chicken
Fam13b	0.35	1.00000000	chicken
Icos	-0.13	1.00000000	chicken
Ddrgk1	-0.29	1.00000000	chicken
Ireb2	-0.32	1.00000000	chicken
Acot7	-0.25	1.00000000	chicken
Ddx23	-0.20	1.00000000	chicken
Xpr1	0.26	1.00000000	chicken
4933434E20Rik	-0.26	1.00000000	chicken
Prpf3	-0.27	1.00000000	chicken
Cnpy3	-0.26	1.00000000	chicken
Ssr1	-0.26	1.00000000	chicken
Dnlz	-0.22	1.00000000	chicken
Appl2	-0.33	1.00000000	chicken

Rtf1	-0.30	1.00000000	chicken
Nuak1	-0.23	1.00000000	chicken
Ccdc88a	-0.04	1.00000000	chicken
Tex2	-0.25	1.00000000	chicken
Dolpp1	0.11	1.00000000	chicken
Abtb1	-0.24	1.00000000	chicken
Smim7	-0.30	1.00000000	chicken
Cdc37	-0.28	1.00000000	chicken
Cnih1	-0.19	1.00000000	chicken
Rab6a	0.08	1.00000000	chicken
Gng2	-0.19	1.00000000	chicken
5031439G07Rik	0.19	1.00000000	chicken
B3gnt3	-0.24	1.00000000	chicken
Hook1	0.18	1.00000000	chicken
Agpat4	0.19	1.00000000	chicken
Immp2l	0.45	1.00000000	chicken
Abcg1	-0.04	1.00000000	chicken
Zfc3h1	0.12	1.00000000	chicken
Hbs1l	0.19	1.00000000	chicken
Dnajc15	-0.26	1.00000000	chicken
Ik	-0.27	1.00000000	chicken
App	0.17	1.00000000	chicken
Selenok	-0.19	1.00000000	chicken
Gtf3a	-0.25	1.00000000	chicken
Pdxk	-0.27	1.00000000	chicken
Tmem258	-0.24	1.00000000	chicken
Hk1	-0.16	1.00000000	chicken
Gpalpp1	-0.32	1.00000000	chicken
Calr	-0.25	1.00000000	chicken
Stag2	0.16	1.00000000	chicken
Glipr2	0.20	1.00000000	chicken
Rabl6	0.15	1.00000000	chicken
Nipal3	0.12	1.00000000	chicken
Pfdn5	-0.20	1.00000000	chicken
Cyp2r1	0.12	1.00000000	chicken
2210408I21Rik	0.16	1.00000000	chicken
Cnot4	0.26	1.00000000	chicken
Wdr4	-0.19	1.00000000	chicken
Smurf2	0.28	1.00000000	chicken
Copa	-0.30	1.00000000	chicken
Spg11	-0.28	1.00000000	chicken
Lclat1	0.25	1.00000000	chicken
Poll	0.25	1.00000000	chicken
Tmem245	0.31	1.00000000	chicken
Gpatch8	0.08	1.00000000	chicken
Itgb2l	-0.20	1.00000000	chicken

Trip11	-0.34	1.00000000	chicken
Yme1l1	-0.27	1.00000000	chicken
Ciao1	-0.18	1.00000000	chicken
Txn1	-0.26	1.00000000	chicken
Pcf11	-0.22	1.00000000	chicken
Naa35	0.10	1.00000000	chicken
Exoc6b	0.11	1.00000000	chicken
Spopl	0.16	1.00000000	chicken
Gid8	-0.21	1.00000000	chicken
Sugt1	0.21	1.00000000	chicken
Ccdc93	-0.20	1.00000000	chicken
Zcchc7	-0.41	1.00000000	chicken
Zfp36l2	-0.36	1.00000000	chicken
Golm2	-0.12	1.00000000	chicken
Commd2	-0.26	1.00000000	chicken
Nop16	-0.28	1.00000000	chicken
Smu1	-0.28	1.00000000	chicken
Smad2	-0.12	1.00000000	chicken
Rsbn1	0.07	1.00000000	chicken
Lrrc34	0.13	1.00000000	chicken
Itsn2	0.12	1.00000000	chicken
Srsf10	0.14	1.00000000	chicken
Edf1	-0.20	1.00000000	chicken
Zbtb1	0.09	1.00000000	chicken
Spcs1	-0.26	1.00000000	chicken
Pcnx2	0.17	1.00000000	chicken
Vac14	-0.27	1.00000000	chicken
Pbrm1	-0.26	1.00000000	chicken
Rev1	0.16	1.00000000	chicken
Ehmt1	-0.28	1.00000000	chicken
Osbpl2	0.18	1.00000000	chicken
Stard8	-0.24	1.00000000	chicken
Nudt14	0.34	1.00000000	chicken
Hibch	-0.14	1.00000000	chicken
Asns	-0.26	1.00000000	chicken
St3gal6	-0.03	1.00000000	chicken
Wdr81	-0.29	1.00000000	chicken
Gtf3c6	-0.30	1.00000000	chicken
Prkcd	-0.15	1.00000000	chicken
Nsmaf	0.23	1.00000000	chicken
Ambra1	0.23	1.00000000	chicken
Htt	-0.21	1.00000000	chicken
Ppp1cc	0.18	1.00000000	chicken
Osbpl1a	0.18	1.00000000	chicken
Zfp521	-0.05	1.00000000	chicken
Smarca2	0.27	1.00000000	chicken

Insig1	-0.23	1.00000000	chicken
Dok1	0.09	1.00000000	chicken
Rab5c	-0.19	1.00000000	chicken
Ing4	0.10	1.00000000	chicken
Stard5	0.09	1.00000000	chicken
Srf	0.07	1.00000000	chicken
Cd226	0.40	1.00000000	chicken
Fdft1	-0.23	1.00000000	chicken
Washc3	0.08	1.00000000	chicken
Polr2c	-0.19	1.00000000	chicken
Denr	-0.20	1.00000000	chicken
Cep162	-0.29	1.00000000	chicken
Nbeal1	0.22	1.00000000	chicken
Klf10	0.07	1.00000000	chicken
Ssb	-0.32	1.00000000	chicken
Ly75	0.28	1.00000000	chicken
Gsdme	0.19	1.00000000	chicken
Tmem164	0.35	1.00000000	chicken
Tnfsf8	0.30	1.00000000	chicken
Rock2	0.32	1.00000000	chicken
Zcchc2	0.26	1.00000000	chicken
Bin1	0.22	1.00000000	chicken
Atg16l1	0.09	1.00000000	chicken
Bop1	-0.20	1.00000000	chicken
Sat1	0.51	1.00000000	chicken
Srsf7	-0.30	1.00000000	chicken
Ythdf2	-0.22	1.00000000	chicken
Arf6	-0.24	1.00000000	chicken
R3hdm4	0.13	1.00000000	chicken
Vps8	0.19	1.00000000	chicken
Rtraf	-0.22	1.00000000	chicken
Pgam1	-0.27	1.00000000	chicken
Stx8	0.16	1.00000000	chicken
Gpatch1	0.22	1.00000000	chicken
Ero1b	0.27	1.00000000	chicken
Scyl2	-0.26	1.00000000	chicken
Hif1an	-0.19	1.00000000	chicken
Glrx3	-0.21	1.00000000	chicken
Commd10	0.31	1.00000000	chicken
Tnrc6a	0.13	1.00000000	chicken
Nnt	0.24	1.00000000	chicken
Rnf141	0.30	1.00000000	chicken
Ube2a	-0.31	1.00000000	chicken
Ahcyl1	0.07	1.00000000	chicken
Thyn1	-0.26	1.00000000	chicken
Pim3	0.15	1.00000000	chicken

Tm9sf2	0.15	1.00000000	chicken
Zfyve21	-0.28	1.00000000	chicken
Creg1	-0.33	1.00000000	chicken
Sp4	-0.30	1.00000000	chicken
Pus7	-0.23	1.00000000	chicken
Zcchc10	0.08	1.00000000	chicken
Ppm1b	0.18	1.00000000	chicken
Zbtb2	0.16	1.00000000	chicken
Ufl1	-0.19	1.00000000	chicken
Agap1	0.33	1.00000000	chicken
Alkbh1	-0.28	1.00000000	chicken
Atp6v1a	-0.29	1.00000000	chicken
Ezh2	-0.25	1.00000000	chicken
Jazf1	0.11	1.00000000	chicken
Psip1	-0.15	1.00000000	chicken
Ndufa1	-0.25	1.00000000	chicken
Cd47	0.20	1.00000000	chicken
Nck2	0.32	1.00000000	chicken
Prmt3	-0.30	1.00000000	chicken
Tnks2	0.12	1.00000000	chicken
Sf3b1	-0.30	1.00000000	chicken
Hsf2	-0.27	1.00000000	chicken
Ccnk	-0.29	1.00000000	chicken
Sik1	0.20	1.00000000	chicken
Cyth3	0.28	1.00000000	chicken
Akap9	0.23	1.00000000	chicken
Sf3a2	-0.23	1.00000000	chicken
Tmf1	-0.33	1.00000000	chicken
Tpp1	-0.22	1.00000000	chicken
Iars2	-0.20	1.00000000	chicken
Dcun1d4	0.25	1.00000000	chicken
Rnf126	-0.19	1.00000000	chicken
Wdr13	-0.20	1.00000000	chicken
Kpna4	0.22	1.00000000	chicken
Tspan2	-0.30	1.00000000	chicken
Ss18l2	-0.28	1.00000000	chicken
Tfip11	-0.22	1.00000000	chicken
Ythdc2	0.12	1.00000000	chicken
Fam133b	-0.27	1.00000000	chicken
Xpo5	-0.20	1.00000000	chicken
Nr4a1	0.08	1.00000000	chicken
Copz1	-0.18	1.00000000	chicken
Ttc1	-0.27	1.00000000	chicken
Hpcal1	0.37	1.00000000	chicken
Ip6k2	0.09	1.00000000	chicken
Rnf220	0.17	1.00000000	chicken

Riok1	-0.23	1.00000000	chicken
Epb41	-0.22	1.00000000	chicken
Klf6	0.41	1.00000000	chicken
Rnmt	-0.30	1.00000000	chicken
Zc3h12d	0.26	1.00000000	chicken
Tom1l2	0.37	1.00000000	chicken
Sulf2	-0.31	1.00000000	chicken
Il18	-0.37	1.00000000	chicken
Cd8a	0.12	1.00000000	chicken
Bmt2	0.10	1.00000000	chicken
Usp33	0.17	1.00000000	chicken
Sh3bp5	-0.23	1.00000000	chicken
Sppl3	0.15	1.00000000	chicken
Dtd1	-0.16	1.00000000	chicken
Rab12	-0.24	1.00000000	chicken
Pigk	-0.19	1.00000000	chicken
Rnaseh1	-0.20	1.00000000	chicken
Rad18	-0.24	1.00000000	chicken
Ccdc186	-0.24	1.00000000	chicken
Ppp1r2	-0.23	1.00000000	chicken
Ap1ar	-0.25	1.00000000	chicken
Ddx31	0.13	1.00000000	chicken
Slc12a2	-0.40	1.00000000	chicken
Stab1	-0.30	1.00000000	chicken
Fus	0.09	1.00000000	chicken
Rilpl2	-0.27	1.00000000	chicken
B4galt1	0.07	1.00000000	chicken
Syap1	-0.26	1.00000000	chicken
Zfp654	0.26	1.00000000	chicken
Zcchc14	0.21	1.00000000	chicken
Ppp1r13b	0.20	1.00000000	chicken
Stat3	0.15	1.00000000	chicken
Tra2a	0.10	1.00000000	chicken
Herpud2	-0.29	1.00000000	chicken
Polr1c	-0.24	1.00000000	chicken
Foxj2	0.24	1.00000000	chicken
Reck	0.06	1.00000000	chicken
Gnl2	-0.27	1.00000000	chicken
Uspl1	0.09	1.00000000	chicken
Vcp	-0.24	1.00000000	chicken
Actr6	-0.25	1.00000000	chicken
Vti1a	0.45	1.00000000	chicken
Akirin2	0.16	1.00000000	chicken
Supt4a	0.12	1.00000000	chicken
Vps37a	0.07	1.00000000	chicken
Lpin1	0.15	1.00000000	chicken

Pcgf6	-0.26	1.00000000	chicken
Rprd1b	-0.25	1.00000000	chicken
Pim1	0.04	1.00000000	chicken
Tmbim6	-0.25	1.00000000	chicken
Nucb2	0.18	1.00000000	chicken
Zfat	0.11	1.00000000	chicken
Cpeb2	0.16	1.00000000	chicken
Smarcc1	-0.09	1.00000000	chicken
Vps26a	0.19	1.00000000	chicken
Get4	-0.22	1.00000000	chicken
Lcor	0.21	1.00000000	chicken
Tnfrsf9	0.16	1.00000000	chicken
Batf	0.43	1.00000000	chicken
Efr3a	0.16	1.00000000	chicken
Zfp827	0.31	1.00000000	chicken
Bcap29	-0.25	1.00000000	chicken
Stn1	-0.30	1.00000000	chicken
Ndel1	0.11	1.00000000	chicken
Eif2s2	-0.24	1.00000000	chicken
Togaram1	0.20	1.00000000	chicken
Cask	0.22	1.00000000	chicken
Snx6	-0.27	1.00000000	chicken
Rmnd5a	0.14	1.00000000	chicken
Smc4	0.34	1.00000000	chicken
Jund	0.09	1.00000000	chicken
Nono	-0.25	1.00000000	chicken
Klf7	-0.21	1.00000000	chicken
Copb1	-0.25	1.00000000	chicken
Ash1l	0.11	1.00000000	chicken
Zbed4	-0.24	1.00000000	chicken
Rab11a	-0.25	1.00000000	chicken
Uqcrq	-0.25	1.00000000	chicken
Slc35b2	-0.27	1.00000000	chicken
Nipa2	0.10	1.00000000	chicken
Ndst4	-0.43	1.00000000	chicken
Bak1	-0.20	1.00000000	chicken
E2f4	-0.26	1.00000000	chicken
Otud4	0.14	1.00000000	chicken
Kdm5a	0.22	1.00000000	chicken
Tdrd3	0.23	1.00000000	chicken
Atxn10	-0.29	1.00000000	chicken
Elf2	0.07	1.00000000	chicken
Homer1	-0.15	1.00000000	chicken
Smc6	0.16	1.00000000	chicken
Ubl3	0.18	1.00000000	chicken
Slu7	-0.29	1.00000000	chicken

Rbm5	0.12	1.00000000	chicken
Nat10	-0.23	1.00000000	chicken
Cebpg	0.11	1.00000000	chicken
Rexo2	-0.14	1.00000000	chicken
Tnfaip3	0.38	1.00000000	chicken
Wdr37	0.14	1.00000000	chicken
Tubb4b	0.19	1.00000000	chicken
Becn2	-0.14	1.00000000	chicken
Notch2	0.22	1.00000000	chicken
Add1	0.26	1.00000000	chicken
Fam214a	-0.18	1.00000000	chicken
Srsf1	-0.28	1.00000000	chicken
Fyb	0.33	1.00000000	chicken
Tpd52l2	0.04	1.00000000	chicken
Ppm1a	0.17	1.00000000	chicken
Washc4	-0.27	1.00000000	chicken
Ckap5	-0.20	1.00000000	chicken
Naf1	-0.19	1.00000000	chicken
Ebna1bp2	-0.12	1.00000000	chicken
Gpatch4	-0.17	1.00000000	chicken
Ppcs	-0.17	1.00000000	chicken
Fars2	0.28	1.00000000	chicken
Pcmt1	0.10	1.00000000	chicken
Hhex	0.16	1.00000000	chicken
Gosr1	0.16	1.00000000	chicken
Cyfip1	-0.32	1.00000000	chicken
Uba6	-0.24	1.00000000	chicken
Acbd6	-0.20	1.00000000	chicken
Eif2s1	-0.16	1.00000000	chicken
Srsf6	-0.16	1.00000000	chicken
Ifngr1	-0.16	1.00000000	chicken
Dnajb14	0.13	1.00000000	chicken
Rtf2	-0.29	1.00000000	chicken
Arhgef18	0.06	1.00000000	chicken
Vrk2	0.21	1.00000000	chicken
Ulk2	0.17	1.00000000	chicken
H13	-0.26	1.00000000	chicken
Ppil4	-0.04	1.00000000	chicken
Ccdc126	-0.21	1.00000000	chicken
Nsf	-0.22	1.00000000	chicken
Eps15l1	-0.28	1.00000000	chicken
Nqo2	0.14	1.00000000	chicken
Armh3	0.15	1.00000000	chicken
Rchy1	-0.27	1.00000000	chicken
Gpr107	0.23	1.00000000	chicken
Klhl15	-0.09	1.00000000	chicken

Aco2	-0.13	1.00000000	chicken
Themis	-0.30	1.00000000	chicken
Zfp292	0.17	1.00000000	chicken
Fyttd1	0.06	1.00000000	chicken
Ddost	-0.21	1.00000000	chicken
Kat6a	0.10	1.00000000	chicken
Ist1	-0.19	1.00000000	chicken
9930021J03Rik	-0.27	1.00000000	chicken
Nmt2	0.17	1.00000000	chicken
Atp6v0a2	-0.10	1.00000000	chicken
Kars	-0.21	1.00000000	chicken
Sh2d1a	-0.23	1.00000000	chicken
Eif3j2	-0.19	1.00000000	chicken
Uchl5	-0.18	1.00000000	chicken
Abraxas2	0.06	1.00000000	chicken
Smap2	-0.20	1.00000000	chicken
Psen1	-0.34	1.00000000	chicken
Sbf1	0.05	1.00000000	chicken
Rell1	-0.19	1.00000000	chicken
Stau1	-0.24	1.00000000	chicken
Prpf4	-0.20	1.00000000	chicken
Nemf	-0.11	1.00000000	chicken
Pcnp	-0.17	1.00000000	chicken
Ypel2	0.16	1.00000000	chicken
Suclg2	0.12	1.00000000	chicken
Ube2l3	-0.19	1.00000000	chicken
Nfkbib	-0.21	1.00000000	chicken
Arap2	0.06	1.00000000	chicken
Aldoc	-0.19	1.00000000	chicken
Phf3	-0.23	1.00000000	chicken
Tesk2	-0.22	1.00000000	chicken
Nbas	-0.06	1.00000000	chicken
Ubqln1	0.18	1.00000000	chicken
Pacs2	-0.27	1.00000000	chicken
Capzb	-0.16	1.00000000	chicken
Cct6b	-0.26	1.00000000	chicken
Dus3l	-0.23	1.00000000	chicken
Tm7sf3	-0.17	1.00000000	chicken
Zdhhc6	-0.15	1.00000000	chicken
Vamp4	-0.16	1.00000000	chicken
Slc12a4	0.14	1.00000000	chicken
Xrcc4	0.22	1.00000000	chicken
Atg2b	-0.28	1.00000000	chicken
Ino80c	0.14	1.00000000	chicken
Eif4ebp2	-0.21	1.00000000	chicken
Dcun1d1	-0.33	1.00000000	chicken

Psmb6	-0.18	1.00000000	chicken
Tsn	-0.14	1.00000000	chicken
Cnbp	-0.16	1.00000000	chicken
Sec62	0.09	1.00000000	chicken
Kdm3b	0.07	1.00000000	chicken
Usp20	0.09	1.00000000	chicken
Cdc42	-0.09	1.00000000	chicken
Ttc13	-0.20	1.00000000	chicken
Nol8	-0.19	1.00000000	chicken
Taf5l	-0.12	1.00000000	chicken
Supt6	0.04	1.00000000	chicken
Ibtk	-0.27	1.00000000	chicken
Khdrbs1	-0.20	1.00000000	chicken
Pum3	-0.26	1.00000000	chicken
Sod1	-0.17	1.00000000	chicken
Slc6a6	0.27	1.00000000	chicken
Cnot11	-0.20	1.00000000	chicken
Ubtf	-0.30	1.00000000	chicken
Ranbp9	0.13	1.00000000	chicken
Clns1a	-0.16	1.00000000	chicken
Hnrnpk	-0.29	1.00000000	chicken
Tnip1	-0.17	1.00000000	chicken
Ifi35	0.10	1.00000000	chicken
Crnkl1	-0.32	1.00000000	chicken
Ranbp1	-0.21	1.00000000	chicken
Chic1	-0.21	1.00000000	chicken
Prep	-0.16	1.00000000	chicken
Ascc1	-0.09	1.00000000	chicken
Lpcat1	-0.27	1.00000000	chicken
Btrc	0.18	1.00000000	chicken
Ric8b	0.19	1.00000000	chicken
Bcl2	0.48	1.00000000	chicken
Txlna	-0.21	1.00000000	chicken
Ptdss1	-0.01	1.00000000	chicken
Rbm14	-0.26	1.00000000	chicken
Hdgfl3	-0.15	1.00000000	chicken
Dock8	-0.10	1.00000000	chicken
Wdr41	0.06	1.00000000	chicken
Aak1	0.16	1.00000000	chicken
Pak2	-0.25	1.00000000	chicken
Tmem106b	-0.29	1.00000000	chicken
Sppl2a	0.10	1.00000000	chicken
Chd1	0.03	1.00000000	chicken
Kdm1a	0.14	1.00000000	chicken
Agpat5	-0.27	1.00000000	chicken
Hspa4l	-0.21	1.00000000	chicken

Raph1	0.35	1.00000000	chicken
Seh1l	-0.23	1.00000000	chicken
Fh1	-0.22	1.00000000	chicken
Tnip2	-0.31	1.00000000	chicken
Smarcd1	0.04	1.00000000	chicken
Pten	-0.05	1.00000000	chicken
Eif2ak2	0.12	1.00000000	chicken
Htatsf1	-0.13	1.00000000	chicken
Dtwd2	-0.25	1.00000000	chicken
Bend7	0.13	1.00000000	chicken
Sp1	0.09	1.00000000	chicken
Srebf1	-0.23	1.00000000	chicken
Ralgapb	0.09	1.00000000	chicken
Tdpoz8	0.10	1.00000000	chicken
Sgpl1	0.21	1.00000000	chicken
Parg	-0.24	1.00000000	chicken
Sirt1	-0.28	1.00000000	chicken
Sh3glb1	0.07	1.00000000	chicken
Rbm6	0.06	1.00000000	chicken
Taldo1	-0.22	1.00000000	chicken
Tasor2	0.15	1.00000000	chicken
Ifngr2	-0.18	1.00000000	chicken
Hdac4	0.07	1.00000000	chicken
Heatr5b	-0.25	1.00000000	chicken
N4bp2l2	0.11	1.00000000	chicken
Ahsa2	-0.18	1.00000000	chicken
Capza2	0.22	1.00000000	chicken
Qrich1	0.11	1.00000000	chicken
Sdk1	0.50	1.00000000	chicken
Gspt1	-0.20	1.00000000	chicken
Itpk1	0.19	1.00000000	chicken
Tnfaip8	0.14	1.00000000	chicken
H2az1	-0.21	1.00000000	chicken
Rsl1d1	-0.17	1.00000000	chicken
AI597479	0.09	1.00000000	chicken
Sdhc	-0.17	1.00000000	chicken
Rbm22	-0.29	1.00000000	chicken
Sdf4	-0.23	1.00000000	chicken
Jun	0.56	1.00000000	chicken
Slc25a13	0.19	1.00000000	chicken
Oxsr1	0.08	1.00000000	chicken
Rabggtb	0.04	1.00000000	chicken
Yipf5	-0.11	1.00000000	chicken
Srp9	0.11	1.00000000	chicken
Ttll5	-0.15	1.00000000	chicken
Wbp2nl	-0.20	1.00000000	chicken

Slc30a4	-0.07	1.00000000	chicken
Rsrp1	0.03	1.00000000	chicken
Crem	0.18	1.00000000	chicken
Cltb	-0.23	1.00000000	chicken
Edem3	0.18	1.00000000	chicken
Cyb5a	0.09	1.00000000	chicken
Lsm12	0.10	1.00000000	chicken
Tubgcp5	-0.13	1.00000000	chicken
Grk2	-0.21	1.00000000	chicken
Lrrc8d	0.03	1.00000000	chicken
Sh3bgrl3	-0.09	1.00000000	chicken
Glb1	0.10	1.00000000	chicken
Akap8l	-0.27	1.00000000	chicken
Cnot1	-0.10	1.00000000	chicken
Lonrf1	0.17	1.00000000	chicken
Psmc1	-0.20	1.00000000	chicken
Stk10	0.05	1.00000000	chicken
Zranb1	0.07	1.00000000	chicken
Zmiz1	-0.14	1.00000000	chicken
Taok3	0.08	1.00000000	chicken
Lemd2	-0.21	1.00000000	chicken
Ssh2	0.23	1.00000000	chicken
Cdk11b	-0.24	1.00000000	chicken
Nek9	0.11	1.00000000	chicken
Secisbp2l	0.03	1.00000000	chicken
Ift20	-0.17	1.00000000	chicken
Wdr74	-0.11	1.00000000	chicken
Xpo6	0.17	1.00000000	chicken
Rab27a	-0.13	1.00000000	chicken
Eea1	0.13	1.00000000	chicken
Cep170	0.23	1.00000000	chicken
Siah1b	-0.15	1.00000000	chicken
Tmem263	-0.11	1.00000000	chicken
Smad5	0.04	1.00000000	chicken
Nrbf2	-0.19	1.00000000	chicken
Cdc14a	0.05	1.00000000	chicken
Hmbs	-0.25	1.00000000	chicken
Ubxn7	0.09	1.00000000	chicken
Taz	-0.19	1.00000000	chicken
Ap3m1	-0.20	1.00000000	chicken
Snrpb2	-0.14	1.00000000	chicken
Xpa	0.06	1.00000000	chicken
Atp6v1e2	-0.15	1.00000000	chicken
Arih1	0.13	1.00000000	chicken
Etf1	-0.25	1.00000000	chicken
Cnnm2	0.16	1.00000000	chicken

Kmt2c	0.23	1.00000000	chicken
Adarb1	-0.13	1.00000000	chicken
Gapvd1	0.09	1.00000000	chicken
Cox5b-ps	-0.19	1.00000000	chicken
Hectd1	0.06	1.00000000	chicken
Bzw1	-0.24	1.00000000	chicken
Actr3	-0.20	1.00000000	chicken
Psmd4	-0.07	1.00000000	chicken
Pfkfb3	0.07	1.00000000	chicken
Hs6st2	0.46	1.00000000	chicken
Arl6ip4	0.08	1.00000000	chicken
Capn7	0.18	1.00000000	chicken
Pias2	-0.15	1.00000000	chicken
Rbl2	-0.25	1.00000000	chicken
Ppp1r12b	0.01	1.00000000	chicken
Scfd1	0.09	1.00000000	chicken
Rbx1	-0.11	1.00000000	chicken
Garnl3	0.14	1.00000000	chicken
Sav1	-0.22	1.00000000	chicken
Vcpip1	0.07	1.00000000	chicken
Ino80d	-0.24	1.00000000	chicken
Tgfbrap1	0.13	1.00000000	chicken
Ints6l	0.15	1.00000000	chicken
Sgk3	-0.25	1.00000000	chicken
Cd82	0.17	1.00000000	chicken
Xiap	-0.26	1.00000000	chicken
Tcf3	0.15	1.00000000	chicken
Tmco1	0.04	1.00000000	chicken
Erc1	0.56	1.00000000	chicken
Stxbp1	-0.21	1.00000000	chicken
Pwwp2b	0.14	1.00000000	chicken
Slain2	-0.21	1.00000000	chicken
Kdm4c	0.19	1.00000000	chicken
Vcl	-0.18	1.00000000	chicken
Cox10	0.21	1.00000000	chicken
Wdr36	-0.18	1.00000000	chicken
Sptssa	-0.14	1.00000000	chicken
Rara	0.12	1.00000000	chicken
Uhmk1	-0.20	1.00000000	chicken
Traf6	0.14	1.00000000	chicken
Slc10a7	-0.19	1.00000000	chicken
Parvg	0.06	1.00000000	chicken
Tpp2	-0.11	1.00000000	chicken
Prdm15	0.04	1.00000000	chicken
Bbs7	-0.25	1.00000000	chicken
Gphn	0.22	1.00000000	chicken

Papss1	-0.18	1.00000000	chicken
Pcsk7	-0.09	1.00000000	chicken
Faf1	0.01	1.00000000	chicken
Taf4b	-0.14	1.00000000	chicken
Kif2a	0.14	1.00000000	chicken
Wasl	0.21	1.00000000	chicken
Ccng2	-0.22	1.00000000	chicken
Acbd3	-0.20	1.00000000	chicken
Qdpr	0.21	1.00000000	chicken
Washc1	0.24	1.00000000	chicken
Wtap	0.11	1.00000000	chicken
Birc6	-0.02	1.00000000	chicken
Rapsn	0.11	1.00000000	chicken
Tnpo1	0.14	1.00000000	chicken
Sdha	0.11	1.00000000	chicken
Camk2d	0.31	1.00000000	chicken
Ppid	0.06	1.00000000	chicken
Ano10	-0.54	1.00000000	chicken
Slc25a32	-0.21	1.00000000	chicken
Tomm70a	-0.23	1.00000000	chicken
Exoc1	0.05	1.00000000	chicken
Pign	0.11	1.00000000	chicken
Atp1b3	-0.19	1.00000000	chicken
Cstf1	-0.21	1.00000000	chicken
Dcun1d3	0.07	1.00000000	chicken
Yipf3	-0.19	1.00000000	chicken
Dazap2	-0.25	1.00000000	chicken
Nrf1	0.15	1.00000000	chicken
Ccdc91	0.09	1.00000000	chicken
Abcc4	-0.21	1.00000000	chicken
Trmt10c	-0.16	1.00000000	chicken
Chmp4b	-0.17	1.00000000	chicken
Fggy	-0.05	1.00000000	chicken
Numb	-0.12	1.00000000	chicken
Esyt2	0.20	1.00000000	chicken
Phlpp1	0.29	1.00000000	chicken
Plrg1	0.17	1.00000000	chicken
Cop1	-0.24	1.00000000	chicken
Trim24	-0.22	1.00000000	chicken
Slc35b3	-0.14	1.00000000	chicken
Lct	-0.23	1.00000000	chicken
Trim33	0.08	1.00000000	chicken
Hdac11	0.17	1.00000000	chicken
Fkbp1a	-0.19	1.00000000	chicken
Cpne1	0.08	1.00000000	chicken
Osbpl9	-0.16	1.00000000	chicken

Ubap2l	-0.20	1.00000000	chicken
Xpo4	-0.26	1.00000000	chicken
Xrn2	-0.16	1.00000000	chicken
Pdk3	0.11	1.00000000	chicken
Btbd7	0.09	1.00000000	chicken
Pacsin2	0.23	1.00000000	chicken
Strada	0.13	1.00000000	chicken
Xpo7	-0.24	1.00000000	chicken
Ppp1r7	-0.16	1.00000000	chicken
Pola1	0.23	1.00000000	chicken
Sub1	-0.22	1.00000000	chicken
Virma	-0.18	1.00000000	chicken
Lnpk	0.12	1.00000000	chicken
Atp6v0e	-0.16	1.00000000	chicken
Prr14l	-0.20	1.00000000	chicken
Ubap2	-0.25	1.00000000	chicken
Was	-0.07	1.00000000	chicken
Oga	-0.12	1.00000000	chicken
Prkce	0.16	1.00000000	chicken
Zfp830	-0.23	1.00000000	chicken
Dld	-0.14	1.00000000	chicken
Arih2	0.00	1.00000000	chicken
Dctn5	-0.13	1.00000000	chicken
Axin1	0.09	1.00000000	chicken
Lyrm4	0.25	1.00000000	chicken
Pik3cg	-0.09	1.00000000	chicken
Ube2d1	-0.20	1.00000000	chicken
Zfp131	-0.26	1.00000000	chicken
Ctcf	-0.09	1.00000000	chicken
Zzef1	0.14	1.00000000	chicken
Setd2	-0.01	1.00000000	chicken
Tmem185a	-0.17	1.00000000	chicken
Atf1	0.08	1.00000000	chicken
Kifbp	-0.04	1.00000000	chicken
Cacul1	0.24	1.00000000	chicken
Tmem9b	-0.12	1.00000000	chicken
Caprin1	0.05	1.00000000	chicken
Aig1	0.11	1.00000000	chicken
Napa	-0.19	1.00000000	chicken
Dnmt3a	-0.15	1.00000000	chicken
Ndufa9	-0.21	1.00000000	chicken
Clk1	-0.18	1.00000000	chicken
Numa1	0.06	1.00000000	chicken
Rpf2	-0.16	1.00000000	chicken
Stk4	0.07	1.00000000	chicken
Ddx6	0.11	1.00000000	chicken

Chid1	0.15	1.00000000	chicken
Trappc8	0.13	1.00000000	chicken
Il17ra	-0.15	1.00000000	chicken
Adcy7	0.01	1.00000000	chicken
Ywhae	0.05	1.00000000	chicken
Paip2	-0.22	1.00000000	chicken
Ehbp1	0.08	1.00000000	chicken
Tlk1	0.27	1.00000000	chicken
Ifrd1	-0.16	1.00000000	chicken
Csl	0.06	1.00000000	chicken
Grb2	-0.37	1.00000000	chicken
Actr1a	-0.16	1.00000000	chicken
Rnps1	-0.19	1.00000000	chicken
Vmp1	0.29	1.00000000	chicken
Tvp23b	-0.17	1.00000000	chicken
Ss18	-0.16	1.00000000	chicken
Phf5a	-0.14	1.00000000	chicken
Ubash3b	0.11	1.00000000	chicken
Trrap	0.25	1.00000000	chicken
Cd2ap	-0.02	1.00000000	chicken
Rrp1b	-0.21	1.00000000	chicken
Ywhag	0.10	1.00000000	chicken
Psmd12	-0.19	1.00000000	chicken
Inpp5k	0.02	1.00000000	chicken
Prpf19	-0.20	1.00000000	chicken
Hypk	-0.19	1.00000000	chicken
Nkiras2	-0.15	1.00000000	chicken
Llph	-0.18	1.00000000	chicken
Pcca	0.07	1.00000000	chicken
Stat5b	0.04	1.00000000	chicken
Epc1	0.12	1.00000000	chicken
Tdg	-0.25	1.00000000	chicken
Kansl3	0.03	1.00000000	chicken
Lpar5	-0.19	1.00000000	chicken
Abraxas1	-0.14	1.00000000	chicken
Srp14	-0.09	1.00000000	chicken
Elmo1	0.09	1.00000000	chicken
Kat2b	0.05	1.00000000	chicken
Ercc6	-0.23	1.00000000	chicken
1600014C10Rik	0.04	1.00000000	chicken
Aoah	-0.42	1.00000000	chicken
Clpx	0.10	1.00000000	chicken
Prdm10	-0.13	1.00000000	chicken
Tor4a	0.11	1.00000000	chicken
Tex10	0.09	1.00000000	chicken
Ttc17	0.06	1.00000000	chicken

Cdc42se1	-0.19	1.00000000	chicken
Itga6	-0.06	1.00000000	chicken
Dis3l2	-0.18	1.00000000	chicken
Ttc39b	-0.10	1.00000000	chicken
Fubp1	-0.10	1.00000000	chicken
Rasgrp1	0.10	1.00000000	chicken
Cerk	0.06	1.00000000	chicken
Cd74	-0.12	1.00000000	chicken
Hook3	0.09	1.00000000	chicken
Ubr7	-0.15	1.00000000	chicken
Zc3h18	-0.26	1.00000000	chicken
Rpf1	-0.17	1.00000000	chicken
Xrn1	0.16	1.00000000	chicken
Cast	0.24	1.00000000	chicken
G3bp2	-0.17	1.00000000	chicken
Pkm	-0.10	1.00000000	chicken
Gtf2a2	-0.17	1.00000000	chicken
Top1	0.11	1.00000000	chicken
Rad51b	-0.16	1.00000000	chicken
Dlg1	0.17	1.00000000	chicken
Kdm1b	-0.23	1.00000000	chicken
Krit1	-0.04	1.00000000	chicken
Tmem186	-0.17	1.00000000	chicken
BC048403	-0.14	1.00000000	chicken
Tsg101	-0.18	1.00000000	chicken
Fubp3	0.07	1.00000000	chicken
Pafah1b2	-0.15	1.00000000	chicken
Herc2	-0.10	1.00000000	chicken
Rangap1	0.04	1.00000000	chicken
Lamp1	0.07	1.00000000	chicken
Slc30a5	-0.12	1.00000000	chicken
Washc2	-0.25	1.00000000	chicken
Tes	0.09	1.00000000	chicken
Rnf139	0.16	1.00000000	chicken
Rnf20	-0.18	1.00000000	chicken
Hbp1	0.04	1.00000000	chicken
Rnf144a	-0.20	1.00000000	chicken
Chchd2	-0.12	1.00000000	chicken
Sec16a	0.04	1.00000000	chicken
Gfi1	0.12	1.00000000	chicken
Elp3	-0.04	1.00000000	chicken
Bin3	-0.21	1.00000000	chicken
Rbl1	0.14	1.00000000	chicken
Klhl5	0.02	1.00000000	chicken
Atp13a3	-0.08	1.00000000	chicken
Nup50	0.01	1.00000000	chicken

Btg2	0.09	1.00000000	chicken
Tagap1	0.15	1.00000000	chicken
Ipo8	-0.09	1.00000000	chicken
Ptp4a1	-0.18	1.00000000	chicken
Rnf11	0.09	1.00000000	chicken
Ube2i	-0.13	1.00000000	chicken
Ep300	0.11	1.00000000	chicken
B3gnt2	-0.03	1.00000000	chicken
Auts2	0.01	1.00000000	chicken
Brip1	0.03	1.00000000	chicken
Srfbp1	-0.10	1.00000000	chicken
Gfpt1	-0.21	1.00000000	chicken
Dph6	-0.54	1.00000000	chicken
Nfkb2	-0.21	1.00000000	chicken
Eif1ad12	-0.19	1.00000000	chicken
Lrch2	-0.26	1.00000000	chicken
Zfp407	-0.02	1.00000000	chicken
Ltf	-0.13	1.00000000	chicken
Nipsnap2	0.04	1.00000000	chicken
Ccdc6	0.26	1.00000000	chicken
Btbd1	-0.17	1.00000000	chicken
Rnf111	0.13	1.00000000	chicken
Stx17	0.03	1.00000000	chicken
Ptcd3	-0.17	1.00000000	chicken
Tubb5	-0.23	1.00000000	chicken
Ppif	-0.08	1.00000000	chicken
Vps37b	0.14	1.00000000	chicken
Sass6	-0.14	1.00000000	chicken
Sec24a	0.07	1.00000000	chicken
Ube2cbp	0.06	1.00000000	chicken
Ube2e3	-0.10	1.00000000	chicken
Igf2r	-0.16	1.00000000	chicken
Psmd9	-0.16	1.00000000	chicken
Kbtbd8	-0.15	1.00000000	chicken
Rasa3	0.29	1.00000000	chicken
Lef1	-0.03	1.00000000	chicken
Ahrr	0.50	1.00000000	chicken
Npepps	0.06	1.00000000	chicken
Sipa1l1	0.29	1.00000000	chicken
Tpk1	0.06	1.00000000	chicken
Emc2	0.14	1.00000000	chicken
Gata3	-0.02	1.00000000	chicken
Rnf217	0.24	1.00000000	chicken
Nfx1	0.13	1.00000000	chicken
Ube2o	-0.16	1.00000000	chicken
Cse1l	-0.15	1.00000000	chicken

Rapgef2	-0.01	1.00000000	chicken
Sdhb	-0.17	1.00000000	chicken
Tmem101	-0.15	1.00000000	chicken
Rac1	-0.19	1.00000000	chicken
Cradd	-0.20	1.00000000	chicken
Dnaja2	-0.06	1.00000000	chicken
Txnrd1	-0.09	1.00000000	chicken
Trp53bp2	0.03	1.00000000	chicken
Rnf144b	-0.19	1.00000000	chicken
Cwc27	0.14	1.00000000	chicken
Dync1h1	0.16	1.00000000	chicken
Gpr155	-0.12	1.00000000	chicken
Zfand3	0.23	1.00000000	chicken
Lman1	-0.12	1.00000000	chicken
Timm17a	-0.06	1.00000000	chicken
Slc5a1	0.12	1.00000000	chicken
Ppat	0.05	1.00000000	chicken
Ctr9	-0.26	1.00000000	chicken
Galnt7	-0.23	1.00000000	chicken
Bzw2	-0.02	1.00000000	chicken
Peli2	0.21	1.00000000	chicken
Poc1b	0.20	1.00000000	chicken
Rab7	-0.19	1.00000000	chicken
Tox	0.48	1.00000000	chicken
Flnb	0.17	1.00000000	chicken
Rsrc1	0.19	1.00000000	chicken
Ogt	0.02	1.00000000	chicken
Ccnl2	0.01	1.00000000	chicken
Ptpn11	-0.11	1.00000000	chicken
Dip2c	0.48	1.00000000	chicken
Brd1	0.11	1.00000000	chicken
Wdr44	-0.18	1.00000000	chicken
Rab35	-0.21	1.00000000	chicken
Fam168a	0.07	1.00000000	chicken
Ess2	0.07	1.00000000	chicken
R3hdm1	0.15	1.00000000	chicken
Pde6d	0.08	1.00000000	chicken
Elovl1	0.06	1.00000000	chicken
4932438A13Rik	0.11	1.00000000	chicken
Crlf3	-0.15	1.00000000	chicken
Bcar3	-0.07	1.00000000	chicken
Pdha1	-0.20	1.00000000	chicken
Tmem181a	0.09	1.00000000	chicken
Dtnbp1	0.18	1.00000000	chicken
Scaper	0.06	1.00000000	chicken
Gga3	-0.02	1.00000000	chicken

Adrm1	-0.16	1.00000000	chicken
Ap2m1	-0.12	1.00000000	chicken
Slc25a20	0.07	1.00000000	chicken
Qser1	-0.12	1.00000000	chicken
Lrrc8b	0.05	1.00000000	chicken
Setd3	0.03	1.00000000	chicken
Tns3	0.08	1.00000000	chicken
Ythdc1	-0.09	1.00000000	chicken
Fam104a	-0.13	1.00000000	chicken
Tent2	-0.16	1.00000000	chicken
Pgk1	-0.26	1.00000000	chicken
Arpc1a	-0.07	1.00000000	chicken
Eif2ak1	0.06	1.00000000	chicken
Rnf214	-0.11	1.00000000	chicken
Sh3pxd2b	-0.30	1.00000000	chicken
Txndc11	0.02	1.00000000	chicken
Strap	-0.12	1.00000000	chicken
Nxt2	-0.19	1.00000000	chicken
Brd7	-0.20	1.00000000	chicken
Adrb2	0.10	1.00000000	chicken
Atp6v0c	-0.22	1.00000000	chicken
Zfp638	0.02	1.00000000	chicken
Cnot8	-0.21	1.00000000	chicken
Slc7a6os	-0.13	1.00000000	chicken
Slc9a8	0.01	1.00000000	chicken
Rprd2	-0.04	1.00000000	chicken
Cops5	-0.10	1.00000000	chicken
Naxd	-0.03	1.00000000	chicken
Rapgef1	-0.18	1.00000000	chicken
Rab39b	0.06	1.00000000	chicken
Larp7	-0.11	1.00000000	chicken
Sarnp	-0.21	1.00000000	chicken
Hnrnpul1	-0.19	1.00000000	chicken
Uqcrc2	-0.06	1.00000000	chicken
Stam2	-0.19	1.00000000	chicken
Slc36a4	0.12	1.00000000	chicken
Cab39	0.10	1.00000000	chicken
Angel2	-0.03	1.00000000	chicken
Lbr	-0.12	1.00000000	chicken
Scaf4	0.06	1.00000000	chicken
Crb1	-0.21	1.00000000	chicken
Abi2	0.08	1.00000000	chicken
Nanp	-0.02	1.00000000	chicken
Tab2	0.05	1.00000000	chicken
Arhgap11a	0.19	1.00000000	chicken
Pbx1	0.16	1.00000000	chicken

Erap1	0.04	1.00000000	chicken
Upb1	-0.03	1.00000000	chicken
Zfyve9	0.18	1.00000000	chicken
Ntan1	-0.19	1.00000000	chicken
Gsr	-0.11	1.00000000	chicken
Ggps1	-0.13	1.00000000	chicken
Bnip2	-0.12	1.00000000	chicken
Tmem259	0.07	1.00000000	chicken
Rab8a	-0.10	1.00000000	chicken
Pms1	-0.16	1.00000000	chicken
Sptlc1	-0.04	1.00000000	chicken
Dapk2	0.04	1.00000000	chicken
Atp6v1b2	-0.14	1.00000000	chicken
Utp15	-0.14	1.00000000	chicken
Zcrb1	-0.19	1.00000000	chicken
Aqr	-0.17	1.00000000	chicken
Tbc1d15	-0.13	1.00000000	chicken
Ints4	0.06	1.00000000	chicken
Rarg	-0.03	1.00000000	chicken
Rexo1	0.02	1.00000000	chicken
Rcan1	-0.01	1.00000000	chicken
Ipo5	-0.04	1.00000000	chicken
Bach2	0.18	1.00000000	chicken
Cep350	-0.18	1.00000000	chicken
Nfatc2	0.17	1.00000000	chicken
Fam172a	0.15	1.00000000	chicken
Pcbp2	-0.17	1.00000000	chicken
Tmem184b	-0.14	1.00000000	chicken
Hps5	-0.05	1.00000000	chicken
Gnai3	0.12	1.00000000	chicken
Klhdc4	-0.17	1.00000000	chicken
Ubr1	0.14	1.00000000	chicken
Ptpre	0.07	1.00000000	chicken
Tial1	0.03	1.00000000	chicken
Arntl	0.37	1.00000000	chicken
Rmdn3	-0.18	1.00000000	chicken
Upf3b	-0.19	1.00000000	chicken
Nup98	-0.21	1.00000000	chicken
Eif4h	-0.17	1.00000000	chicken
Uvrag	-0.04	1.00000000	chicken
Atp6v0a1	0.05	1.00000000	chicken
Anapc15-ps	-0.14	1.00000000	chicken
Etfa	0.09	1.00000000	chicken
Gtf2h1	0.07	1.00000000	chicken
Laptm5	0.20	1.00000000	chicken
Rab5a	-0.17	1.00000000	chicken

Pdxdc1	-0.18	1.00000000	chicken
Paip1	0.03	1.00000000	chicken
Smap1	0.23	1.00000000	chicken
Whamm	0.08	1.00000000	chicken
Srp68	-0.18	1.00000000	chicken
Ints6	0.17	1.00000000	chicken
Gpcpd1	0.03	1.00000000	chicken
Nfyc	-0.08	1.00000000	chicken
Pmm2	-0.16	1.00000000	chicken
Ppp2cb	0.05	1.00000000	chicken
Ddx17	0.00	1.00000000	chicken
Phkb	0.03	1.00000000	chicken
Ccz1	-0.08	1.00000000	chicken
Enox2	0.04	1.00000000	chicken
Dnajc13	0.05	1.00000000	chicken
Vgll4	0.08	1.00000000	chicken
Fer	0.23	1.00000000	chicken
Reep5	-0.13	1.00000000	chicken
Rab3gap1	-0.21	1.00000000	chicken
Dgkq	-0.11	1.00000000	chicken
Arhgef6	-0.09	1.00000000	chicken
Tmem104	0.17	1.00000000	chicken
Fam120a	0.08	1.00000000	chicken
Btbd10	-0.08	1.00000000	chicken
Klhl13	0.10	1.00000000	chicken
Heca	0.08	1.00000000	chicken
Zbtb38	0.37	1.00000000	chicken
Rmnd1	-0.20	1.00000000	chicken
Thoc1	-0.13	1.00000000	chicken
Smc3	-0.02	1.00000000	chicken
Klhdc1	0.06	1.00000000	chicken
Kif5c	-0.14	1.00000000	chicken
Eri3	-0.19	1.00000000	chicken
Peli1	-0.03	1.00000000	chicken
Hipk3	0.09	1.00000000	chicken
Stk11	-0.02	1.00000000	chicken
Sel1l3	-0.14	1.00000000	chicken
Phf20l1	-0.12	1.00000000	chicken
Arfgef2	0.01	1.00000000	chicken
Pkn2	-0.18	1.00000000	chicken
Nsun2	-0.12	1.00000000	chicken
Tmpo	-0.19	1.00000000	chicken
Papola	0.03	1.00000000	chicken
Rbm34	-0.12	1.00000000	chicken
Pnpt1	-0.11	1.00000000	chicken
Rcn2	-0.12	1.00000000	chicken

Pcgf3	0.12	1.00000000	chicken
Tollip	-0.03	1.00000000	chicken
Btbd9	0.17	1.00000000	chicken
Fndc3b	0.08	1.00000000	chicken
Hsf3	-0.13	1.00000000	chicken
Vps54	-0.09	1.00000000	chicken
Ilrun	0.06	1.00000000	chicken
Aldh5a1	-0.15	1.00000000	chicken
Dnajc7	-0.13	1.00000000	chicken
Surf4	-0.14	1.00000000	chicken
Chuk	0.02	1.00000000	chicken
Il1rap	-0.13	1.00000000	chicken
Nr2c1	0.04	1.00000000	chicken
Zc3h6	-0.26	1.00000000	chicken
Nupl1	-0.04	1.00000000	chicken
Klf3	-0.08	1.00000000	chicken
Lipa	-0.04	1.00000000	chicken
Sri	-0.12	1.00000000	chicken
Kdm5b	0.00	1.00000000	chicken
Psma2	-0.09	1.00000000	chicken
Wdr75	-0.13	1.00000000	chicken
Hspa5	0.15	1.00000000	chicken
Cyfip2	0.23	1.00000000	chicken
Daglb	0.01	1.00000000	chicken
Eps15	0.00	1.00000000	chicken
BC005537	-0.14	1.00000000	chicken
Arhgap45	0.01	1.00000000	chicken
Galnt1	0.35	1.00000000	chicken
Elovl5	-0.04	1.00000000	chicken
Ecd	-0.19	1.00000000	chicken
Pdcd10	-0.20	1.00000000	chicken
Zfyve26	-0.06	1.00000000	chicken
Gpr183	-0.09	1.00000000	chicken
Luc7l	-0.25	1.00000000	chicken
Senp1	-0.12	1.00000000	chicken
Snx29	0.16	1.00000000	chicken
Tgs1	-0.17	1.00000000	chicken
Ndufs2	-0.11	1.00000000	chicken
Gcc2	-0.14	1.00000000	chicken
Chchd3	-0.24	1.00000000	chicken
Larp4b	-0.12	1.00000000	chicken
Sh3bgrl	0.14	1.00000000	chicken
Rab43	0.01	1.00000000	chicken
Brix1	0.05	1.00000000	chicken
Scamp1	-0.16	1.00000000	chicken
Znrf3	0.13	1.00000000	chicken

Cdk8	-0.07	1.00000000	chicken
Naa25	-0.12	1.00000000	chicken
Uchl3	0.09	1.00000000	chicken
Dnaja4	-0.14	1.00000000	chicken
Cct8	-0.07	1.00000000	chicken
Ap3b1	-0.10	1.00000000	chicken
Btaf1	0.13	1.00000000	chicken
Tmem87a	-0.15	1.00000000	chicken
Ccr7	-0.17	1.00000000	chicken
Ascc3	-0.04	1.00000000	chicken
Tent5a	0.03	1.00000000	chicken
9130409I23Rik	-0.05	1.00000000	chicken
Rimbp2	0.16	1.00000000	chicken
1810013L24Rik	-0.10	1.00000000	chicken
Usp37	0.09	1.00000000	chicken
Eif2b5	-0.20	1.00000000	chicken
Aacs	-0.19	1.00000000	chicken
Rabgap1l	0.13	1.00000000	chicken
Fxr1	0.07	1.00000000	chicken
Rif1	0.04	1.00000000	chicken
Cog7	-0.14	1.00000000	chicken
Usp15	0.06	1.00000000	chicken
Lcp2	0.07	1.00000000	chicken
Lig3	-0.06	1.00000000	chicken
Rpa3	-0.08	1.00000000	chicken
Akap11	-0.12	1.00000000	chicken
Top2b	0.13	1.00000000	chicken
Utp11	-0.11	1.00000000	chicken
Basp1	-0.09	1.00000000	chicken
Utp3	-0.14	1.00000000	chicken
Fam117a	-0.14	1.00000000	chicken
Odc1	0.00	1.00000000	chicken
Gdi2	-0.11	1.00000000	chicken
Pikfyve	-0.19	1.00000000	chicken
Stx2	0.02	1.00000000	chicken
Prkdc	0.07	1.00000000	chicken
Tmod3	0.15	1.00000000	chicken
Bud13	0.00	1.00000000	chicken
Rars	-0.08	1.00000000	chicken
Rab18	-0.08	1.00000000	chicken
Fam160b1	0.04	1.00000000	chicken
Plxnc1	0.00	1.00000000	chicken
Zbtb37	-0.05	1.00000000	chicken
Nr4a2	-0.15	1.00000000	chicken
Rnf10	0.03	1.00000000	chicken
Eif2b3	-0.18	1.00000000	chicken

Cemip2	0.03	1.00000000	chicken
Kpna3	-0.24	1.00000000	chicken
Hadh	0.24	1.00000000	chicken
Dync1li1	0.05	1.00000000	chicken
Ghdc	-0.13	1.00000000	chicken
Tctn3	-0.23	1.00000000	chicken
Agfg1	0.18	1.00000000	chicken
Nup214	0.03	1.00000000	chicken
Trpm6	0.06	1.00000000	chicken
Sf3b3	-0.12	1.00000000	chicken
Dcp1a	0.13	1.00000000	chicken
Sik2	-0.04	1.00000000	chicken
Nploc4	-0.10	1.00000000	chicken
Elk3	-0.16	1.00000000	chicken
Tmtc3	0.08	1.00000000	chicken
Abcc5	0.04	1.00000000	chicken
Gns	-0.19	1.00000000	chicken
Pitpnb	-0.16	1.00000000	chicken
Trak2	0.02	1.00000000	chicken
Slc30a9	-0.14	1.00000000	chicken
Cnot9	-0.12	1.00000000	chicken
Cul1	0.11	1.00000000	chicken
Hnrnpd	-0.10	1.00000000	chicken
Nbr1	-0.01	1.00000000	chicken
Rcl1	-0.06	1.00000000	chicken
Dgkd	-0.03	1.00000000	chicken
Ing1	-0.01	1.00000000	chicken
Clip1	-0.17	1.00000000	chicken
Fhl3	0.12	1.00000000	chicken
Dip2a	-0.08	1.00000000	chicken
Cops8	-0.16	1.00000000	chicken
Szrd1	0.13	1.00000000	chicken
Fasn	-0.10	1.00000000	chicken
Slc12a7	-0.06	1.00000000	chicken
Eloc	0.02	1.00000000	chicken
Psma1	-0.16	1.00000000	chicken
Rasal2	0.20	1.00000000	chicken
Sms	-0.07	1.00000000	chicken
Thoc2	-0.11	1.00000000	chicken
Glg1	-0.13	1.00000000	chicken
Wdr7	-0.06	1.00000000	chicken
Clcn5	0.06	1.00000000	chicken
Pebp1	-0.12	1.00000000	chicken
Edc3	-0.02	1.00000000	chicken
Relch	-0.02	1.00000000	chicken
Nbn	0.01	1.00000000	chicken

Cfap20	-0.15	1.00000000	chicken
Tm9sf5	-0.07	1.00000000	chicken
Rptor	-0.22	1.00000000	chicken
Ldha	-0.10	1.00000000	chicken
Ctla4	0.19	1.00000000	chicken
Trmt1l	-0.12	1.00000000	chicken
Adam17	-0.09	1.00000000	chicken
Acaca	-0.16	1.00000000	chicken
Cbwd1	-0.12	1.00000000	chicken
Tmed10	-0.09	1.00000000	chicken
Tmem120b	-0.01	1.00000000	chicken
Amd2	-0.01	1.00000000	chicken
Radx	-0.24	1.00000000	chicken
Lgmn	0.18	1.00000000	chicken
Pak1	-0.16	1.00000000	chicken
Bckdhb	0.18	1.00000000	chicken
Il18r1	-0.15	1.00000000	chicken
Sfswap	-0.08	1.00000000	chicken
Ccdc171	0.01	1.00000000	chicken
Arhgap24	0.20	1.00000000	chicken
Flot2	-0.14	1.00000000	chicken
Slc38a1	-0.04	1.00000000	chicken
Usp14	0.02	1.00000000	chicken
Clasp1	-0.20	1.00000000	chicken
Atrx	0.06	1.00000000	chicken
Nap1l4	0.05	1.00000000	chicken
Chm	0.01	1.00000000	chicken
Dars	0.09	1.00000000	chicken
Ybx3	-0.03	1.00000000	chicken
4933400A11Rik	0.03	1.00000000	chicken
Ppa2	0.20	1.00000000	chicken
Spred2	-0.22	1.00000000	chicken
Specc1l	0.18	1.00000000	chicken
Tasor	0.16	1.00000000	chicken
Ubp1	-0.13	1.00000000	chicken
Psmc6	-0.07	1.00000000	chicken
Kdm3a	0.04	1.00000000	chicken
Slc39a10	-0.14	1.00000000	chicken
Gzf1	-0.06	1.00000000	chicken
Trmt13	0.01	1.00000000	chicken
Fry	-0.34	1.00000000	chicken
Zfhx3	-0.06	1.00000000	chicken
Hdac7	0.07	1.00000000	chicken
Chmp2a	-0.17	1.00000000	chicken
Zfp609	-0.11	1.00000000	chicken
Arfip2	-0.14	1.00000000	chicken

Ndufb6	-0.05	1.00000000	chicken
Xpo1	0.02	1.00000000	chicken
Terf2	0.01	1.00000000	chicken
Ube2g1	-0.03	1.00000000	chicken
Leo1	-0.12	1.00000000	chicken
Nab1	-0.08	1.00000000	chicken
Atp9b	-0.07	1.00000000	chicken
Fmnl1	0.08	1.00000000	chicken
Commd5	-0.05	1.00000000	chicken
Ndufs4	-0.07	1.00000000	chicken
Irs4	-0.11	1.00000000	chicken
Ercc3	-0.01	1.00000000	chicken
Lsmem1	0.11	1.00000000	chicken
Lin54	-0.13	1.00000000	chicken
Pak3	-0.19	1.00000000	chicken
Atp2c1	0.07	1.00000000	chicken
Lars2	-0.21	1.00000000	chicken
Arfgef3	0.08	1.00000000	chicken
Gabarapl1	-0.11	1.00000000	chicken
Tmem131l	0.03	1.00000000	chicken
Dhx29	-0.09	1.00000000	chicken
Pnisr	-0.03	1.00000000	chicken
Ice2	0.02	1.00000000	chicken
Limd2	0.00	1.00000000	chicken
Csnk2a1	-0.11	1.00000000	chicken
Tbcd	0.08	1.00000000	chicken
Rab10	-0.05	1.00000000	chicken
Klf11	0.04	1.00000000	chicken
Cdk12	-0.03	1.00000000	chicken
Ezr	0.06	1.00000000	chicken
Arfgap2	-0.08	1.00000000	chicken
Cpsf4l	-0.01	1.00000000	chicken
Bclaf3	-0.04	1.00000000	chicken
Ino80	-0.04	1.00000000	chicken
Pafah1b1	0.12	1.00000000	chicken
Nr1h3	0.06	1.00000000	chicken
Ccdc174	-0.13	1.00000000	chicken
Ap2b1	-0.05	1.00000000	chicken
Drg1	-0.12	1.00000000	chicken
Ift22	-0.01	1.00000000	chicken
Ube3a	0.01	1.00000000	chicken
Satb1	0.46	1.00000000	chicken
Rbbp6	-0.05	1.00000000	chicken
Arhgef12	-0.09	1.00000000	chicken
Ppp2ca	0.01	1.00000000	chicken
Tardbp	0.06	1.00000000	chicken

Scfd2	-0.23	1.00000000	chicken
Slc4a7	0.15	1.00000000	chicken
Nol11	-0.13	1.00000000	chicken
Ensa	-0.03	1.00000000	chicken
Pdhx	0.10	1.00000000	chicken
Eefsec	0.08	1.00000000	chicken
Scap	-0.12	1.00000000	chicken
Zmpste24	-0.11	1.00000000	chicken
Creb1	-0.03	1.00000000	chicken
Trabd	-0.04	1.00000000	chicken
Cops3	0.02	1.00000000	chicken
Pccb	-0.02	1.00000000	chicken
Rbm12b2	-0.05	1.00000000	chicken
Slc38a6	-0.05	1.00000000	chicken
Pnpla8	-0.13	1.00000000	chicken
Wdr77	-0.10	1.00000000	chicken
Exoc5	-0.10	1.00000000	chicken
Optn	0.03	1.00000000	chicken
Taf2	0.01	1.00000000	chicken
Fadd	-0.09	1.00000000	chicken
Cars2	0.01	1.00000000	chicken
Arhgap26	0.05	1.00000000	chicken
Pinx1	0.13	1.00000000	chicken
Napg	-0.17	1.00000000	chicken
Rnf19a	-0.10	1.00000000	chicken
Akap8	0.00	1.00000000	chicken
Steep1	-0.18	1.00000000	chicken
Atf6	0.07	1.00000000	chicken
Pfdn1	-0.04	1.00000000	chicken
Ripk1	-0.06	1.00000000	chicken
Nfatc3	0.02	1.00000000	chicken
Cyria	0.23	1.00000000	chicken
Arl8b	0.05	1.00000000	chicken
Adam19	-0.03	1.00000000	chicken
Ano5	0.02	1.00000000	chicken
Arnt	0.01	1.00000000	chicken
Lpcat2b	0.12	1.00000000	chicken
Copg1	-0.08	1.00000000	chicken
Tapt1	-0.05	1.00000000	chicken
Polr3a	-0.12	1.00000000	chicken
Nsrp1	0.09	1.00000000	chicken
Trpm7	0.05	1.00000000	chicken
Tubgcp3	-0.15	1.00000000	chicken
Tspan14	-0.05	1.00000000	chicken
Clip4	-0.09	1.00000000	chicken
Rit1	0.01	1.00000000	chicken

Fbxo22	-0.10	1.00000000	chicken
Utp25	-0.12	1.00000000	chicken
Erlin2	-0.13	1.00000000	chicken
Cass4	-0.04	1.00000000	chicken
Rnf146	-0.15	1.00000000	chicken
Zrsr1	0.03	1.00000000	chicken
Phf12	-0.14	1.00000000	chicken
Upf1	0.04	1.00000000	chicken
Ipo11	0.05	1.00000000	chicken
Taf6	-0.19	1.00000000	chicken
Gpi1	-0.09	1.00000000	chicken
Dync1i2	-0.07	1.00000000	chicken
C1galt1	-0.12	1.00000000	chicken
Usp19	-0.01	1.00000000	chicken
Ube2e1	0.04	1.00000000	chicken
Tle1	0.00	1.00000000	chicken
Strn	-0.12	1.00000000	chicken
Ago4	-0.08	1.00000000	chicken
Ost4	0.03	1.00000000	chicken
Anxa2	0.15	1.00000000	chicken
Stk3	-0.13	1.00000000	chicken
Dhx8	-0.09	1.00000000	chicken
Celf1	0.07	1.00000000	chicken
Phf10	0.01	1.00000000	chicken
Rmc1	0.00	1.00000000	chicken
Slamf8	-0.18	1.00000000	chicken
Crtc1	-0.05	1.00000000	chicken
Qk	0.10	1.00000000	chicken
Ski	0.09	1.00000000	chicken
Ube2v2	-0.12	1.00000000	chicken
Cactin	-0.14	1.00000000	chicken
Galnt2	0.18	1.00000000	chicken
Snx13	0.12	1.00000000	chicken
Notch1	-0.15	1.00000000	chicken
Ncald	0.26	1.00000000	chicken
Hdac3	-0.08	1.00000000	chicken
Lmbrd1	0.15	1.00000000	chicken
Nol7	-0.12	1.00000000	chicken
Jmjd1c	0.01	1.00000000	chicken
Prpf8	-0.15	1.00000000	chicken
Tmem50a	-0.05	1.00000000	chicken
Snx12	0.08	1.00000000	chicken
Nelfa	-0.08	1.00000000	chicken
Sap130	0.02	1.00000000	chicken
Cops4	-0.12	1.00000000	chicken
Chd5	-0.12	1.00000000	chicken

Sec24b	-0.04	1.00000000	chicken
Brwd1	-0.10	1.00000000	chicken
Fgfr1op2	-0.07	1.00000000	chicken
Amfr	-0.15	1.00000000	chicken
Grcc10	-0.11	1.00000000	chicken
Cdc27	-0.05	1.00000000	chicken
Sec61a1	-0.14	1.00000000	chicken
Phf14	-0.02	1.00000000	chicken
Trappc11	-0.11	1.00000000	chicken
Gars	-0.11	1.00000000	chicken
Luc7l3	-0.11	1.00000000	chicken
Gne	-0.17	1.00000000	chicken
Ugt8a	-0.10	1.00000000	chicken
Sirt2	-0.08	1.00000000	chicken
Acvr1	0.06	1.00000000	chicken
Paxip1	-0.11	1.00000000	chicken
Tspan18	0.18	1.00000000	chicken
Sptlc2	0.14	1.00000000	chicken
Cyb5r4	-0.10	1.00000000	chicken
Nfkbiz	0.07	1.00000000	chicken
Bfar	0.02	1.00000000	chicken
Dnajb12	-0.11	1.00000000	chicken
Gpr171	0.00	1.00000000	chicken
Sinhcaf	0.03	1.00000000	chicken
Gtf2f2	0.14	1.00000000	chicken
Cflar	0.00	1.00000000	chicken
Ube2r2	0.00	1.00000000	chicken
Kctd6	-0.02	1.00000000	chicken
Ctnnbl1	-0.14	1.00000000	chicken
LTO1	-0.15	1.00000000	chicken
Otulinl	-0.14	1.00000000	chicken
Erich1	0.06	1.00000000	chicken
Ivns1abp	-0.21	1.00000000	chicken
Tab3	-0.02	1.00000000	chicken
Nup155	-0.07	1.00000000	chicken
Tkt	-0.09	1.00000000	chicken
Acad9	0.06	1.00000000	chicken
Efl1	0.05	1.00000000	chicken
Aimp1	0.08	1.00000000	chicken
Fem1b	-0.01	1.00000000	chicken
Srpk1	-0.10	1.00000000	chicken
Eprs	-0.11	1.00000000	chicken
Rprd1a	-0.11	1.00000000	chicken
Azi2	0.02	1.00000000	chicken
Psmd1	0.07	1.00000000	chicken
Dcaf5	0.00	1.00000000	chicken

Sltm	0.01	1.00000000	chicken
Ptch1	0.14	1.00000000	chicken
U2surp	-0.04	1.00000000	chicken
Nup107	-0.12	1.00000000	chicken
Prpf4b	-0.11	1.00000000	chicken
Usp10	0.07	1.00000000	chicken
Tbc1d14	-0.07	1.00000000	chicken
Abhd17c	-0.01	1.00000000	chicken
Igf2bp2	-0.06	1.00000000	chicken
Arid3b	-0.09	1.00000000	chicken
Ypel5	-0.07	1.00000000	chicken
Abcd2	-0.14	1.00000000	chicken
Zfp148	-0.08	1.00000000	chicken
Yipf4	-0.12	1.00000000	chicken
Katnbl1	-0.12	1.00000000	chicken
Camk2g	0.07	1.00000000	chicken
Rngtt	-0.05	1.00000000	chicken
Pcnt	-0.18	1.00000000	chicken
Pecam1	-0.22	1.00000000	chicken
Sec23b	0.15	1.00000000	chicken
Nrd1	-0.07	1.00000000	chicken
Ubr5	-0.05	1.00000000	chicken
Ogfr	-0.10	1.00000000	chicken
Stk26	-0.05	1.00000000	chicken
Slc35e2	0.03	1.00000000	chicken
Nsd3	-0.11	1.00000000	chicken
Atg12	-0.06	1.00000000	chicken
Vdac1	-0.15	1.00000000	chicken
Gtf2e1	0.01	1.00000000	chicken
Fan1	0.03	1.00000000	chicken
Fbxo32	0.02	1.00000000	chicken
Gpatch2	0.10	1.00000000	chicken
Nup205	0.00	1.00000000	chicken
Rdx	0.02	1.00000000	chicken
Vta1	0.10	1.00000000	chicken
Csgalnact2	-0.01	1.00000000	chicken
Ilkap	0.09	1.00000000	chicken
Wipi2	-0.08	1.00000000	chicken
Wdr1	-0.05	1.00000000	chicken
Trpc4ap	-0.09	1.00000000	chicken
Casc3	0.00	1.00000000	chicken
Erp44	0.08	1.00000000	chicken
Smarcd2	-0.07	1.00000000	chicken
Gadd45b	0.06	1.00000000	chicken
Clasp2	0.01	1.00000000	chicken
Elk4	0.02	1.00000000	chicken

Adar	-0.05	1.00000000	chicken
Ets1	0.00	1.00000000	chicken
Ubqln4	-0.05	1.00000000	chicken
Ttc33	-0.02	1.00000000	chicken
AW554918	0.03	1.00000000	chicken
Dnajb4	0.00	1.00000000	chicken
Ufd1	-0.01	1.00000000	chicken
Dbr1	-0.10	1.00000000	chicken
Commd1b	-0.13	1.00000000	chicken
Sfmbt2	0.03	1.00000000	chicken
Kat6b	0.04	1.00000000	chicken
Prmt7	-0.14	1.00000000	chicken
Rab11fip1	-0.11	1.00000000	chicken
Senp6	0.15	1.00000000	chicken
Polr3b	-0.09	1.00000000	chicken
Sel1l	-0.07	1.00000000	chicken
Zfp236	-0.11	1.00000000	chicken
Ccpg1	-0.09	1.00000000	chicken
Xpot	-0.02	1.00000000	chicken
Aktip	-0.08	1.00000000	chicken
Igf1r	0.37	1.00000000	chicken
Rad17	-0.03	1.00000000	chicken
Nars	-0.15	1.00000000	chicken
Sf3a3	-0.08	1.00000000	chicken
Fgd3	0.12	1.00000000	chicken
Nkap	-0.11	1.00000000	chicken
Suds3	-0.17	1.00000000	chicken
Smarca1	-0.10	1.00000000	chicken
Ipo7	-0.05	1.00000000	chicken
Kdelr2	-0.04	1.00000000	chicken
Snip1	-0.09	1.00000000	chicken
Ahctf1	0.11	1.00000000	chicken
Pno1	-0.12	1.00000000	chicken
Ube2f	0.16	1.00000000	chicken
Dap	0.10	1.00000000	chicken
Dnajc1	0.06	1.00000000	chicken
Ppp4r1	0.06	1.00000000	chicken
Cnp	-0.03	1.00000000	chicken
Rab9	-0.09	1.00000000	chicken
Zc3h7a	0.03	1.00000000	chicken
Spast	0.02	1.00000000	chicken
Faf2	-0.09	1.00000000	chicken
E2f6	-0.09	1.00000000	chicken
Pgrmc2	-0.03	1.00000000	chicken
Vbp1	-0.07	1.00000000	chicken
Irf2bp2	-0.10	1.00000000	chicken

Fbxl22	-0.02	1.00000000	chicken
Nabp1	-0.12	1.00000000	chicken
Brd8	-0.07	1.00000000	chicken
Isca1	0.02	1.00000000	chicken
Arf4	-0.06	1.00000000	chicken
Clk2	-0.11	1.00000000	chicken
Commd8	0.00	1.00000000	chicken
Irf4	0.04	1.00000000	chicken
Nhlrc2	-0.06	1.00000000	chicken
Cnot10	0.03	1.00000000	chicken
S100a10	-0.13	1.00000000	chicken
Lbh	0.02	1.00000000	chicken
Zfp384	-0.12	1.00000000	chicken
Evi5	0.05	1.00000000	chicken
Arid3a	-0.06	1.00000000	chicken
Ep400	0.04	1.00000000	chicken
Apoo	-0.06	1.00000000	chicken
Appbp2	-0.01	1.00000000	chicken
Kat7	-0.14	1.00000000	chicken
Zbtb41	-0.08	1.00000000	chicken
Carnmt1	-0.14	1.00000000	chicken
Arhgap27	-0.05	1.00000000	chicken
Rundc1	-0.05	1.00000000	chicken
Bin2	-0.06	1.00000000	chicken
Slc39a11	-0.15	1.00000000	chicken
Ccnd2	-0.05	1.00000000	chicken
Hsp90aa1	-0.48	1.00000000	chicken
Il7r	-0.02	1.00000000	chicken
Arpc3	-0.10	1.00000000	chicken
Camsap1	-0.08	1.00000000	chicken
Ppig	-0.13	1.00000000	chicken
Tut7	-0.12	1.00000000	chicken
Rab22a	-0.09	1.00000000	chicken
Ube3c	0.06	1.00000000	chicken
Esco1	0.02	1.00000000	chicken
Camsap2	0.04	1.00000000	chicken
Taf5	-0.13	1.00000000	chicken
Nup93	-0.04	1.00000000	chicken
Prkx	0.08	1.00000000	chicken
Ywhah	-0.05	1.00000000	chicken
Adck1	-0.11	1.00000000	chicken
Ube2b	-0.03	1.00000000	chicken
Tacc1	-0.06	1.00000000	chicken
Stim2	0.02	1.00000000	chicken
Rnf38	-0.09	1.00000000	chicken
Srsf5	-0.06	1.00000000	chicken

Abhd18	-0.06	1.00000000	chicken
Gtf2b	0.02	1.00000000	chicken
Serpine2	-0.19	1.00000000	chicken
Pdcd4	-0.03	1.00000000	chicken
Hexb	-0.01	1.00000000	chicken
Slc49a4	0.12	1.00000000	chicken
Rnf145	-0.02	1.00000000	chicken
Zfand5	0.00	1.00000000	chicken
Ap2a1	0.14	1.00000000	chicken
Ltn1	-0.12	1.00000000	chicken
Sdcbp	-0.06	1.00000000	chicken
Gtf3c1	-0.05	1.00000000	chicken
Cdc42se2	0.08	1.00000000	chicken
Slc30a7	-0.07	1.00000000	chicken
Tmem161b	-0.05	1.00000000	chicken
Serinc1	-0.04	1.00000000	chicken
Rasa2	-0.02	1.00000000	chicken
Lin7c	0.05	1.00000000	chicken
Otud7b	-0.01	1.00000000	chicken
Trim8	-0.13	1.00000000	chicken
Ogdh	-0.04	1.00000000	chicken
Slc66a2	-0.04	1.00000000	chicken
Thrap3	-0.10	1.00000000	chicken
Slc2a13	-0.11	1.00000000	chicken
Ints7	-0.03	1.00000000	chicken
Itfg1	-0.05	1.00000000	chicken
Prrc2b	0.05	1.00000000	chicken
Tshz1	0.10	1.00000000	chicken
Uggt1	-0.10	1.00000000	chicken
Eif4a3l1	-0.04	1.00000000	chicken
Sart3	-0.07	1.00000000	chicken
Zfp365	0.05	1.00000000	chicken
Adk	0.23	1.00000000	chicken
Rbm17	-0.09	1.00000000	chicken
Dnajc3	-0.05	1.00000000	chicken
Ptpn1	0.01	1.00000000	chicken
Gstm4	-0.07	1.00000000	chicken
Crk	-0.02	1.00000000	chicken
Fosl2	-0.06	1.00000000	chicken
Rnf114	-0.04	1.00000000	chicken
Zeb1	0.00	1.00000000	chicken
Ncoa2	0.15	1.00000000	chicken
Tgif1	-0.04	1.00000000	chicken
Atg7	0.04	1.00000000	chicken
Jade2	-0.05	1.00000000	chicken
Cacybp	0.07	1.00000000	chicken

Gcn1	-0.05	1.00000000	chicken
Srpk2	0.03	1.00000000	chicken
Bag1	-0.08	1.00000000	chicken
Lpcat3	-0.03	1.00000000	chicken
Sos1	-0.03	1.00000000	chicken
Cog2	-0.13	1.00000000	chicken
Srp54a	-0.03	1.00000000	chicken
Zswim8	0.06	1.00000000	chicken
Disp3	0.00	1.00000000	chicken
Pik3r5	0.09	1.00000000	chicken
Slc22a3	0.12	1.00000000	chicken
Wdr82	-0.11	1.00000000	chicken
Ralb	-0.05	1.00000000	chicken
Smyd3	0.22	1.00000000	chicken
Sgsm2	-0.03	1.00000000	chicken
Hdac2	-0.16	1.00000000	chicken
Rab21	-0.17	1.00000000	chicken
Ube2g2	-0.08	1.00000000	chicken
Rab3gap2	-0.07	1.00000000	chicken
Eif4e	0.00	1.00000000	chicken
P4hb	-0.06	1.00000000	chicken
Tmx4	-0.05	1.00000000	chicken
Ttc3	0.06	1.00000000	chicken
Angel1	0.01	1.00000000	chicken
Pdcd6	-0.09	1.00000000	chicken
Dcp1b	0.02	1.00000000	chicken
Stim1	-0.07	1.00000000	chicken
Ptprs	-0.16	1.00000000	chicken
Fbxw8	0.03	1.00000000	chicken
Arglu1	-0.05	1.00000000	chicken
Ubn2	-0.13	1.00000000	chicken
Hmg20a	-0.01	1.00000000	chicken
Desi2	0.02	1.00000000	chicken
Slc38a2	0.04	1.00000000	chicken
Ank	0.23	1.00000000	chicken
Ssrp1	-0.08	1.00000000	chicken
Phlpp2	-0.09	1.00000000	chicken
Gnptab	0.14	1.00000000	chicken
Proser1	-0.01	1.00000000	chicken
Ankrd10	-0.04	1.00000000	chicken
Ktn1	-0.08	1.00000000	chicken
Dym	0.02	1.00000000	chicken
Nup85	0.00	1.00000000	chicken
Plscr1	0.03	1.00000000	chicken
Ywhaz	0.07	1.00000000	chicken
Plcg1	0.01	1.00000000	chicken

Eml3	-0.03	1.00000000	chicken
Cul3	0.05	1.00000000	chicken
Senp7	-0.05	1.00000000	chicken
Slx4	-0.08	1.00000000	chicken
2700049A03Rik	-0.09	1.00000000	chicken
Heatr5a	-0.07	1.00000000	chicken
Rhof	-0.02	1.00000000	chicken
Acp1	-0.03	1.00000000	chicken
Tubg1	-0.02	1.00000000	chicken
Ccnc	0.03	1.00000000	chicken
Tbc1d12	-0.08	1.00000000	chicken
Gorasp2	-0.05	1.00000000	chicken
Usp45	0.01	1.00000000	chicken
Trmt11	-0.03	1.00000000	chicken
Rnaseh2b	0.10	1.00000000	chicken
Ate1	0.05	1.00000000	chicken
Agps	-0.02	1.00000000	chicken
Vrk1	-0.01	1.00000000	chicken
Vps13d	0.06	1.00000000	chicken
Rab31	0.05	1.00000000	chicken
Uri1	-0.15	1.00000000	chicken
Tcea1	-0.07	1.00000000	chicken
Oxnad1	-0.10	1.00000000	chicken
Cog3	-0.01	1.00000000	chicken
Fbxo33	-0.07	1.00000000	chicken
Dock9	0.19	1.00000000	chicken
Glyr1	-0.17	1.00000000	chicken
Ctdspl2	-0.09	1.00000000	chicken
Prkar1a	-0.05	1.00000000	chicken
Rpap3	0.04	1.00000000	chicken
Ppp2r2d	-0.06	1.00000000	chicken
Pus10	0.07	1.00000000	chicken
Aurkaip1	-0.02	1.00000000	chicken
Ago1	-0.02	1.00000000	chicken
Usp48	-0.02	1.00000000	chicken
Agl	-0.05	1.00000000	chicken
Srsf2	-0.09	1.00000000	chicken
Osbpl8	0.13	1.00000000	chicken
Aagab	-0.01	1.00000000	chicken
Wdr48	-0.10	1.00000000	chicken
Arl4a	-0.01	1.00000000	chicken
Suco	-0.15	1.00000000	chicken
Cdc40	0.01	1.00000000	chicken
Vps13c	0.04	1.00000000	chicken
Strbp	-0.04	1.00000000	chicken
Gbf1	0.14	1.00000000	chicken

Letm2	-0.12	1.00000000	chicken
Usp7	0.00	1.00000000	chicken
Blcap	-0.09	1.00000000	chicken
Rassf1	-0.06	1.00000000	chicken
Tbk1	0.04	1.00000000	chicken
Snhg4	-0.09	1.00000000	chicken
Adora2a	0.04	1.00000000	chicken
Krr1	-0.01	1.00000000	chicken
Jmy	0.05	1.00000000	chicken
Oxct1	0.25	1.00000000	chicken
Snx9	-0.02	1.00000000	chicken
Sin3a	0.00	1.00000000	chicken
Nde1	-0.10	1.00000000	chicken
Ncbp3	-0.05	1.00000000	chicken
Fbxo8	-0.06	1.00000000	chicken
Arhgap6	0.11	1.00000000	chicken
Dnajc18	-0.05	1.00000000	chicken
Taf15	-0.03	1.00000000	chicken
Cntrl	-0.05	1.00000000	chicken
Ylpm1	-0.10	1.00000000	chicken
Dicer1	-0.04	1.00000000	chicken
Large1	0.07	1.00000000	chicken
Ube4a	-0.08	1.00000000	chicken
Dnm1l	-0.01	1.00000000	chicken
Dgkz	-0.06	1.00000000	chicken
Ccdc82	-0.05	1.00000000	chicken
Yars	-0.07	1.00000000	chicken
Herc3	-0.10	1.00000000	chicken
Ints11	-0.03	1.00000000	chicken
Lonrf2	-0.08	1.00000000	chicken
Reps1	0.02	1.00000000	chicken
Bub3	-0.06	1.00000000	chicken
Fam3c	-0.06	1.00000000	chicken
Cul4a	-0.13	1.00000000	chicken
Tm9sf3	0.04	1.00000000	chicken
Dcaf1	0.01	1.00000000	chicken
Zfp143	-0.03	1.00000000	chicken
Letm1	0.02	1.00000000	chicken
Rad23b	0.10	1.00000000	chicken
Adnp	-0.01	1.00000000	chicken
9530068E07Rik	-0.03	1.00000000	chicken
Arl6ip5	0.00	1.00000000	chicken
Slc25a25	-0.06	1.00000000	chicken
Ulk1	-0.08	1.00000000	chicken
Senp5	0.02	1.00000000	chicken
Naa30	-0.01	1.00000000	chicken

Pla2g7	0.00	1.00000000	chicken
Ercc8	-0.02	1.00000000	chicken
Nus1	0.02	1.00000000	chicken
Chka	-0.04	1.00000000	chicken
Thada	-0.05	1.00000000	chicken
Rbm12	-0.08	1.00000000	chicken
Prmt9	-0.07	1.00000000	chicken
Ints2	0.08	1.00000000	chicken
Noc3l	-0.08	1.00000000	chicken
Tbc1d22a	-0.06	1.00000000	chicken
Aebp2	0.14	1.00000000	chicken
Zfp800	-0.03	1.00000000	chicken
Klhl7	-0.03	1.00000000	chicken
Gnb1l	-0.05	1.00000000	chicken
Pik3c2a	0.22	1.00000000	chicken
Lrif1	0.03	1.00000000	chicken
Ube2d2a	-0.02	1.00000000	chicken
Larp1	-0.10	1.00000000	chicken
Ttc39c	-0.02	1.00000000	chicken
Sap30bp	-0.06	1.00000000	chicken
B230219D22Rik	-0.08	1.00000000	chicken
Rlim	-0.07	1.00000000	chicken
Ncapg	-0.02	1.00000000	chicken
Tatdn1	0.01	1.00000000	chicken
Hps3	0.00	1.00000000	chicken
Ciapin1	-0.05	1.00000000	chicken
Uhrf1bp1l	-0.03	1.00000000	chicken
Itm2b	-0.06	1.00000000	chicken
Gpbp1l1	-0.05	1.00000000	chicken
Poldip3	-0.09	1.00000000	chicken
Pnrc2	-0.07	1.00000000	chicken
Ccny	0.04	1.00000000	chicken
Smn1	-0.08	1.00000000	chicken
Wdr26	-0.11	1.00000000	chicken
Itpripl2	-0.13	1.00000000	chicken
Bdp1	0.01	1.00000000	chicken
Gyg	0.03	1.00000000	chicken
Casp8ap2	-0.17	1.00000000	chicken
Rgs19	-0.08	1.00000000	chicken
Xpnpep1	-0.01	1.00000000	chicken
Srsf11	-0.06	1.00000000	chicken
Rala	-0.07	1.00000000	chicken
Rnpc3	-0.03	1.00000000	chicken
Ctss	-0.08	1.00000000	chicken
Golga1	0.01	1.00000000	chicken
Zfp711	0.04	1.00000000	chicken

Brox	0.00	1.00000000	chicken
Ptpn4	0.03	1.00000000	chicken
Ptbp1	0.04	1.00000000	chicken
Kidins220	-0.03	1.00000000	chicken
Cwc25	-0.13	1.00000000	chicken
Sos2	0.04	1.00000000	chicken
Rab14	-0.07	1.00000000	chicken
Sufu	-0.09	1.00000000	chicken
Akap1	-0.10	1.00000000	chicken
Ccnl1	0.07	1.00000000	chicken
Ammecr1	0.01	1.00000000	chicken
Usp4	0.00	1.00000000	chicken
Tra2b	-0.05	1.00000000	chicken
Cog5	0.07	1.00000000	chicken
Tmem33	-0.06	1.00000000	chicken
Ube2k	0.02	1.00000000	chicken
Larp4	-0.09	1.00000000	chicken
Hipk2	0.24	1.00000000	chicken
Hs6st1	0.11	1.00000000	chicken
Tfg	-0.07	1.00000000	chicken
Uprt	-0.09	1.00000000	chicken
Prkcb	-0.19	1.00000000	chicken
Nub1	-0.08	1.00000000	chicken
Rpap1	-0.02	1.00000000	chicken
Atg5	-0.14	1.00000000	chicken
Arfgef1	0.02	1.00000000	chicken
Pxylp1	0.11	1.00000000	chicken
Bbx	0.13	1.00000000	chicken
Pik3ca	-0.12	1.00000000	chicken
Eif4e2	-0.13	1.00000000	chicken
Zfp598	-0.04	1.00000000	chicken
Lats1	-0.06	1.00000000	chicken
Tspan3	-0.06	1.00000000	chicken
Fbxl3	-0.02	1.00000000	chicken
Sap30l	0.09	1.00000000	chicken
U2af1	-0.01	1.00000000	chicken
Phb2	-0.02	1.00000000	chicken
Pik3c3	0.04	1.00000000	chicken
Wdr11	-0.09	1.00000000	chicken
Dpep2	-0.16	1.00000000	chicken
Plac8	2.38	0.00000000	duck
Srgn	2.01	0.00000000	duck
Capn7	-4.27	0.00000000	duck
Nfil3	1.84	0.00000000	duck
Casd1	-2.13	0.00000000	duck
Itm2c	-1.87	0.00000000	duck

Epsti1	1.07	0.00000000	duck
Glcci1	-2.18	0.00000000	duck
Abcc1	-1.71	0.00000000	duck
Traf3	1.50	0.00000000	duck
Rac2	1.77	0.00000000	duck
Anxa1	2.69	0.00000000	duck
Fabp3	1.21	0.00000000	duck
Nfkbiz	1.44	0.00000000	duck
Rundc1	1.38	0.00000000	duck
Ikzf2	1.78	0.00000000	duck
Klf13	-1.41	0.00000000	duck
Pik3c2a	-1.59	0.00000000	duck
Atf4	1.24	0.00000000	duck
S100a11	2.49	0.00000000	duck
Ptpn4	-1.61	0.00000000	duck
Itga4	-1.95	0.00000000	duck
Trib1	1.47	0.00000000	duck
Serpine2	1.68	0.00000000	duck
Tcf7	-1.56	0.00000000	duck
Fnip1	1.84	0.00000000	duck
Nfam1	1.51	0.00000000	duck
F13a1	1.07	0.00000000	duck
Rhog	0.95	0.00000000	duck
Bhlhe40	1.37	0.00000000	duck
Plcg2	1.24	0.00000000	duck
Txn1	1.47	0.00000000	duck
Inpp5d	-1.65	0.00000000	duck
Synm	-3.08	0.00000000	duck
Samhd1	1.44	0.00000000	duck
Plek	1.33	0.00000000	duck
Stat1	1.14	0.00000000	duck
Prok2	1.02	0.00000000	duck
Sox6	-2.58	0.00000000	duck
Btbd11	1.30	0.00000000	duck
Wnt5b	1.27	0.00000000	duck
Tor4a	1.02	0.00000000	duck
Trim24	-1.50	0.00000000	duck
Ncf1	0.88	0.00000000	duck
Npc2	1.15	0.00000000	duck
Efhd2	1.16	0.00000000	duck
Fyn	1.19	0.00000000	duck
Ptgs2	0.93	0.00000000	duck
Pim3	1.70	0.00000000	duck
Dach2	2.13	0.00000000	duck
Snrk	-1.16	0.00000000	duck
Slc39a13	1.05	0.00000000	duck

Cebpb	1.75	0.00000000	duck
Hs6st1	-1.57	0.00000000	duck
Arpc1b	1.53	0.00000000	duck
Abtb2	2.34	0.00000000	duck
Glrx	1.94	0.00000000	duck
Arhgap10	-1.63	0.00000000	duck
Nucb2	-1.26	0.00000000	duck
Prkd1	-1.59	0.00000000	duck
Dusp1	1.30	0.00000000	duck
Pnrc1	1.04	0.00000000	duck
Blnk	1.42	0.00000000	duck
Baz1b	-1.14	0.00000000	duck
Lmnb1	1.63	0.00000000	duck
Sptan1	-1.62	0.00000000	duck
Ctsb	1.15	0.00000000	duck
Epb41	-1.39	0.00000000	duck
Spi1	1.41	0.00000000	duck
Rasa3	-1.60	0.00000000	duck
Hmox1	0.84	0.00000000	duck
Hpgds	1.41	0.00000000	duck
Cpb2	0.99	0.00000000	duck
Il20ra	-1.41	0.00000000	duck
Eif1	0.87	0.00000000	duck
Ptchd3	0.91	0.00000000	duck
Angpt1	1.38	0.00000000	duck
Ly96	0.97	0.00000000	duck
Pank3	-1.13	0.00000000	duck
Ski	1.06	0.00000000	duck
Lrba	-1.39	0.00000000	duck
Klf6	1.51	0.00000000	duck
Fosb	1.02	0.00000000	duck
Rasa2	-1.03	0.00000000	duck
Sat1	1.23	0.00000000	duck
Zc3h12a	1.13	0.00000000	duck
G0s2	1.69	0.00000000	duck
Per2	-1.24	0.00000000	duck
Pgd	1.37	0.00000000	duck
Gpcpd1	-1.23	0.00000000	duck
Jade1	-1.19	0.00000000	duck
Smad7	1.30	0.00000000	duck
Atp1b1	1.06	0.00000000	duck
Cd9	1.35	0.00000000	duck
Csrp1	1.00	0.00000000	duck
Chd7	1.38	0.00000000	duck
Xpo6	-1.12	0.00000000	duck
Sh3bgrl3	1.66	0.00000000	duck

Atf3	1.64	0.00000000	duck
Fam241a	-1.05	0.00000000	duck
Abcc4	-1.72	0.00000000	duck
Nos2	1.33	0.00000000	duck
Tnrc6b	-1.91	0.00000000	duck
Anxa11	1.05	0.00000000	duck
Erbin	-1.18	0.00000000	duck
Arhgdib	1.68	0.00000000	duck
Ptafr	1.44	0.00000000	duck
Smpd1	0.84	0.00000000	duck
Acsbg2	0.97	0.00000000	duck
Ubl5b	0.97	0.00000000	duck
Rnf144a	-1.21	0.00000000	duck
Atxn1	1.01	0.00000000	duck
Foxo1	-0.98	0.00000000	duck
Pid1	1.19	0.00000000	duck
Adora2b	1.19	0.00000000	duck
Osbpl6	0.78	0.00000000	duck
Bri3	1.14	0.00000000	duck
Snx10	1.74	0.00000000	duck
Cfd	0.75	0.00000000	duck
Ntng1	1.19	0.00000000	duck
Rel	1.01	0.00000000	duck
Upp1	0.60	0.00000000	duck
Jund	1.07	0.00000000	duck
Tbc1d2	0.64	0.00000000	duck
Aff2	-1.29	0.00000000	duck
Cnot6l	-1.10	0.00000000	duck
Ssbp2	-1.47	0.00000000	duck
Osbpl1a	-1.09	0.00000000	duck
Smad2	-1.45	0.00000000	duck
Nrip1	1.05	0.00000000	duck
Atp6v1g3	0.61	0.00000000	duck
Ier5	1.19	0.00000000	duck
Lyn	1.16	0.00000000	duck
Id2	1.39	0.00000000	duck
Alox5	0.66	0.00000000	duck
Arg2	0.75	0.00000000	duck
Cebpa	0.61	0.00000000	duck
Pkm	1.06	0.00000000	duck
Ralgapa2	-1.33	0.00000000	duck
Pds5b	-1.01	0.00000000	duck
Dynlt1a	0.89	0.00000000	duck
Aspg	1.05	0.00000000	duck
Tnfaip3	0.97	0.00000000	duck
Zfp516	1.51	0.00000000	duck

Tanc1	0.67	0.00000000	duck
Tbc1d16	0.48	0.00000000	duck
Sh2d1a	-1.26	0.00000000	duck
Ssh2	-0.83	0.00000000	duck
Slc38a2	0.90	0.00000000	duck
Dctn3	0.86	0.00000000	duck
Fam107b	1.03	0.00000000	duck
Tst	0.78	0.00000000	duck
Zswim6	1.00	0.00000000	duck
Zbtb16	1.23	0.00000000	duck
Plin2	1.35	0.00000000	duck
Nfkbie	0.89	0.00000000	duck
Rrp1b	-1.24	0.00000000	duck
Ugt8a	1.09	0.00000000	duck
Laptm5	1.27	0.00000000	duck
Cdk14	-1.36	0.00000000	duck
Prps2	-1.34	0.00000000	duck
Dock11	-1.24	0.00000000	duck
Prr5	-1.13	0.00000000	duck
Dcaf1	-0.86	0.00000000	duck
Lrmda	1.34	0.00000000	duck
Hdx	-0.94	0.00000000	duck
Adm	1.02	0.00000000	duck
Nt5e	0.42	0.00000000	duck
Anxa4	0.48	0.00000000	duck
Pigf	-1.26	0.00000000	duck
Pstpip2	0.80	0.00000000	duck
Nabp1	1.05	0.00000000	duck
Slc16a6	-0.92	0.00000000	duck
Palld	-2.63	0.00000000	duck
Pla2g4a	1.23	0.00000000	duck
Vps13b	-1.33	0.00000000	duck
Aldoc	1.01	0.00000000	duck
Atrx	-1.15	0.00000000	duck
Exoc6b	-1.15	0.00000000	duck
Acod1	0.65	0.00000000	duck
Rxra	1.20	0.00000000	duck
Slc9a9	-1.20	0.00000000	duck
Btf3	1.05	0.00000000	duck
Hras	0.67	0.00000000	duck
Slc4a11	-1.26	0.00000000	duck
Spred2	0.91	0.00000000	duck
Lekr1	0.84	0.00000000	duck
Znrf2	-0.91	0.00000000	duck
Calm2	0.65	0.00000000	duck
Plekhb2	0.67	0.00000000	duck

Ngf	0.54	0.00000000	duck
Tomm7	1.00	0.00000000	duck
Dicer1	-1.06	0.00000000	duck
Ulk2	-1.43	0.00000000	duck
Atp6ap2	0.77	0.00000000	duck
Thap12	-0.80	0.00000000	duck
Sacm1l	0.94	0.00000000	duck
Fth1	1.15	0.00000000	duck
Vdr	0.85	0.00000000	duck
Ephx1	0.73	0.00000000	duck
Uhrf1bp1	-0.79	0.00000000	duck
Crispld2	0.55	0.00000000	duck
Gpr137b	0.64	0.00000000	duck
Tm6sf1	0.56	0.00000000	duck
Cd247	-0.84	0.00000000	duck
Slc37a1	-0.94	0.00000000	duck
Itgav	0.72	0.00000000	duck
Fam102b	0.65	0.00000000	duck
Upf2	-0.80	0.00000000	duck
Tfrc	-1.00	0.00000000	duck
Stt3b	-0.93	0.00000000	duck
Rfc1	-1.20	0.00000000	duck
Naa15	-0.87	0.00000000	duck
Hnrnpll	1.15	0.00000000	duck
Smad3	1.02	0.00000000	duck
Gadd45g	0.93	0.00000000	duck
B3gnt2	0.81	0.00000000	duck
Pam	1.14	0.00000000	duck
Nab1	0.85	0.00000000	duck
Sesn1	-0.74	0.00000000	duck
Cmtm7	-0.81	0.00000000	duck
Csf3r	0.87	0.00000000	duck
B2m	0.71	0.00000000	duck
Eif3a	-0.87	0.00000000	duck
Nxt2	-0.99	0.00000000	duck
Slc9a3r1	1.17	0.00000000	duck
Rasgef1b	0.73	0.00000000	duck
Hormad1	0.75	0.00000000	duck
Echdc2	0.75	0.00000000	duck
Prpf4b	-0.91	0.00000000	duck
Gab3	0.78	0.00000000	duck
Vdac1	0.83	0.00000000	duck
Itk	-1.13	0.00000000	duck
Pparg	1.03	0.00000000	duck
Adgrg3	0.37	0.00000000	duck
Hnrnpr	-0.61	0.00000000	duck

Rhoa	0.71	0.00000000	duck
Rab20	0.70	0.00000000	duck
Enpp4	0.68	0.00000000	duck
Cenpu	0.97	0.00000000	duck
Dock9	-1.09	0.00000000	duck
Aqp3	-0.82	0.00000000	duck
Cables1	0.61	0.00000000	duck
Elf2	-0.86	0.00000001	duck
Slc7a11	1.00	0.00000001	duck
Oat	1.12	0.00000001	duck
Larp1b	-1.48	0.00000001	duck
Fgd3	-0.89	0.00000001	duck
Rc3h1	-0.98	0.00000001	duck
Cerk	-0.80	0.00000001	duck
Neurl1b	0.57	0.00000001	duck
Fli1	0.86	0.00000001	duck
Usp48	-1.18	0.00000001	duck
Cldn18	1.30	0.00000001	duck
Tcirg1	0.66	0.00000001	duck
Actr2	0.95	0.00000001	duck
Ndufs4	0.76	0.00000001	duck
Ankrd22	0.75	0.00000001	duck
Zfp207	-0.99	0.00000001	duck
Actb	1.44	0.00000001	duck
Unc119	-0.72	0.00000001	duck
Chst12	0.49	0.00000001	duck
Trip12	-1.16	0.00000002	duck
Tnip3	1.04	0.00000002	duck
Ppp2r5e	-1.04	0.00000002	duck
Rhoq	-0.71	0.00000002	duck
Pik3ca	-1.22	0.00000002	duck
Tubb5	0.87	0.00000002	duck
Klf10	0.76	0.00000002	duck
Kif21a	0.50	0.00000002	duck
Sgce	-1.53	0.00000003	duck
Taldo1	1.01	0.00000003	duck
Agtpbp1	0.67	0.00000003	duck
Cdc34	0.80	0.00000004	duck
Ptger4	0.92	0.00000004	duck
Dnah10	0.70	0.00000004	duck
Ston2	0.72	0.00000004	duck
Zfp644	-0.87	0.00000004	duck
Jun	1.26	0.00000005	duck
Psma6	0.78	0.00000005	duck
Arpc3	1.04	0.00000005	duck
Crot	0.82	0.00000006	duck

Batf	0.95	0.00000006	duck
Atp6v1d	0.90	0.00000006	duck
Wdfy2	-1.25	0.00000006	duck
Inpp5b	-0.70	0.00000007	duck
Patz1	-0.51	0.00000007	duck
Rfesd	-2.07	0.00000007	duck
Prkar2a	-1.05	0.00000007	duck
Bod1l	-0.99	0.00000007	duck
Sestd1	0.53	0.00000008	duck
Nup210	-0.71	0.00000008	duck
Herc2	-1.47	0.00000008	duck
Ninj1	0.81	0.00000009	duck
Arid4b	-0.50	0.00000010	duck
Dtx2	0.68	0.00000012	duck
Gnpda1	0.45	0.00000012	duck
Peli1	-0.65	0.00000012	duck
Pef1	0.50	0.00000013	duck
Tnip2	0.57	0.00000015	duck
Ppat	-0.58	0.00000015	duck
Arl6ip5	0.88	0.00000016	duck
Tmem250-ps	0.57	0.00000017	duck
F3	0.80	0.00000018	duck
Frmd4b	0.61	0.00000019	duck
Zfp706	0.77	0.00000020	duck
Skap1	-0.74	0.00000021	duck
Epn2	0.73	0.00000022	duck
Lpar4	-0.65	0.00000023	duck
Dapk2	-1.17	0.00000025	duck
Pik3cg	-0.84	0.00000025	duck
Igsf9	1.00	0.00000026	duck
Panx2	0.59	0.00000026	duck
Klf15	0.59	0.00000028	duck
Vcl	-0.74	0.00000036	duck
Rspry1	-0.91	0.00000040	duck
Tkt	0.81	0.00000040	duck
Dcun1d4	-0.86	0.00000041	duck
Ptprm	0.79	0.00000042	duck
Ogt	-0.74	0.00000046	duck
Pard3	0.75	0.00000048	duck
Scd2	-0.84	0.00000052	duck
Fryl	-0.83	0.00000062	duck
Rnf111	-0.87	0.00000063	duck
Numb	1.24	0.00000065	duck
Sp4	-0.58	0.00000069	duck
Irak2	1.34	0.00000070	duck
Atp5g3	0.87	0.00000070	duck

Pik3ip1	-0.59	0.00000071	duck
Eif5a2	-0.74	0.00000072	duck
Itsn1	1.80	0.00000091	duck
Ccdc88a	-0.97	0.00000091	duck
Tet1	-0.94	0.00000094	duck
Bclaf1	-0.59	0.00000100	duck
5031439G07Rik	0.75	0.00000108	duck
Psmb2	0.87	0.00000109	duck
Slc41a3	-0.92	0.00000111	duck
Bmp2k	0.79	0.00000118	duck
Trappc3	0.75	0.00000121	duck
Sec14l1	-1.10	0.00000131	duck
Ncald	-0.69	0.00000131	duck
Rab11fip2	0.70	0.00000144	duck
Evl	-0.97	0.00000144	duck
Snx8	0.52	0.00000162	duck
Akirin2	0.70	0.00000165	duck
Plcl1	-1.27	0.00000178	duck
Rgs5	-0.68	0.00000178	duck
Atp6v1b2	0.75	0.00000184	duck
Timp3	1.11	0.00000188	duck
Vsir	0.83	0.00000198	duck
Osgin2	0.64	0.00000204	duck
Fam53a	-0.87	0.00000216	duck
Drd4	1.24	0.00000225	duck
Inpp5a	-1.33	0.00000225	duck
Ash1l	-0.72	0.00000231	duck
Tet2	0.66	0.00000249	duck
Pik3r5	0.78	0.00000268	duck
Ttc39b	1.13	0.00000299	duck
Smdt1	0.71	0.00000308	duck
Ehd3	0.75	0.00000315	duck
Cpt1a	0.81	0.00000333	duck
Sec23b	-0.52	0.00000362	duck
Vangl1	0.49	0.00000373	duck
Pwwp2b	-0.58	0.00000420	duck
Traf2	0.75	0.00000449	duck
Ahctf1	-0.78	0.00000469	duck
Fzd6	0.62	0.00000469	duck
Ctss	0.59	0.00000479	duck
Tfpi2	0.61	0.00000483	duck
Atp5b	0.84	0.00000490	duck
Tnfaip8l1	0.69	0.00000499	duck
Creb5	0.84	0.00000563	duck
Gnb4	-0.64	0.00000564	duck
Thrap3	-0.67	0.00000580	duck

Stk3	-1.03	0.00000590	duck
Kras	0.71	0.00000617	duck
Ahrr	0.99	0.00000626	duck
Prkcb	-0.89	0.00000631	duck
Tpi1	0.82	0.00000657	duck
Trim25	0.54	0.00000720	duck
Tpmt	0.68	0.00000724	duck
Rcsd1	-0.84	0.00000730	duck
Eif2ak2	0.83	0.00000771	duck
Sh3bp2	0.61	0.00000807	duck
Itgb6	0.60	0.00000815	duck
Bambi	0.44	0.00000838	duck
Asb2	0.53	0.00000872	duck
Acer2	-0.62	0.00000885	duck
Swap70	0.65	0.00000911	duck
Cdk5rap2	-1.19	0.00000914	duck
Vmp1	0.77	0.00000923	duck
Iqgap2	-0.41	0.00000925	duck
Dennd1b	-0.60	0.00001010	duck
Insr	-1.18	0.00001035	duck
Sh3bgrl	0.86	0.00001054	duck
Oaz1	0.64	0.00001055	duck
Rbm31y	0.69	0.00001103	duck
Txlng	-0.87	0.00001117	duck
Hbegf	1.01	0.00001170	duck
Grb2	0.66	0.00001194	duck
Iars	-0.73	0.00001234	duck
Grid1	0.64	0.00001238	duck
Sstr4	0.49	0.00001290	duck
Pxylp1	0.64	0.00001373	duck
Sp3	-0.76	0.00001379	duck
Prkcd	-1.10	0.00001386	duck
Optn	0.69	0.00001470	duck
Chst13	1.01	0.00001510	duck
Gnal	-0.88	0.00001723	duck
Phf20	-0.62	0.00001989	duck
Klf4	0.67	0.00002030	duck
Kcnk5	0.65	0.00002389	duck
Rsu1	0.81	0.00002400	duck
Dtwd1	0.49	0.00002639	duck
Ubr2	-1.03	0.00002675	duck
Cebpd	0.71	0.00002892	duck
Zeb2	0.63	0.00002927	duck
Dhx15	-0.58	0.00002953	duck
Clock	-0.91	0.00002954	duck
Ppp6r3	-0.61	0.00003043	duck

Galnt1	0.79	0.00003182	duck
Rnf19b	0.86	0.00003292	duck
Slc35a5	0.45	0.00003721	duck
Rbm25	-0.52	0.00003908	duck
Hk2	0.73	0.00004047	duck
Parp1	-0.76	0.00004094	duck
Ino80	-0.66	0.00004164	duck
Reps2	0.92	0.00004243	duck
Tspan4	1.08	0.00004315	duck
Slc16a10	-0.75	0.00004861	duck
Ehd4	0.42	0.00005487	duck
Prrc2b	-0.59	0.00005515	duck
Arid1a	-0.69	0.00005814	duck
Ilk	0.57	0.00006021	duck
Zdhhc6	0.69	0.00006159	duck
Ythdc2	-0.88	0.00006425	duck
Elmo2	-0.65	0.00006971	duck
Esd	0.44	0.00007091	duck
Rai1	-0.59	0.00007102	duck
Nr4a1	0.87	0.00007111	duck
Tspan8	0.46	0.00007389	duck
Gch1	0.57	0.00007501	duck
Trmt1l	-0.54	0.00007559	duck
Wdfy3	0.66	0.00007562	duck
Abca1	1.07	0.00007574	duck
9130409I23Rik	0.67	0.00007810	duck
Capn10	-0.67	0.00008050	duck
Tnfrsf11a	0.44	0.00008457	duck
Nktr	-0.98	0.00008886	duck
Taok3	-0.88	0.00009010	duck
Serp1	0.68	0.00009430	duck
Ythdc1	-0.58	0.00009452	duck
Gpx1	1.74	0.00009460	duck
Ndfip2	0.50	0.00009752	duck
Inpp4a	0.65	0.00010511	duck
Vash1	-1.18	0.00010572	duck
Tspo	0.37	0.00010757	duck
Pde7b	-1.26	0.00012733	duck
Rab12	-0.59	0.00012782	duck
Chmp1a	0.41	0.00012801	duck
Trak1	0.80	0.00012809	duck
Yipf3	0.63	0.00013213	duck
Sgpp1	0.76	0.00013259	duck
Pcare	-0.57	0.00013466	duck
Laptm4a	0.75	0.00014133	duck
Pmepa1	0.95	0.00014360	duck

Rabl6	-0.78	0.00014973	duck
Park7	-0.63	0.00015058	duck
Ppp1ca	0.64	0.00015332	duck
Atp5a1	0.77	0.00016389	duck
Dcun1d2	-1.39	0.00016429	duck
Fbxo3	-1.37	0.00016753	duck
Tmem131	-0.70	0.00017100	duck
Tiam1	0.48	0.00017416	duck
Ap1ar	-0.53	0.00018318	duck
Zc3h7a	-0.85	0.00018321	duck
Sema3d	-1.85	0.00018924	duck
Herpud1	0.60	0.00020592	duck
Pfdn1	0.71	0.00020958	duck
Grk3	-0.62	0.00020968	duck
Rbm5	-0.65	0.00021578	duck
Stag2	-0.75	0.00021911	duck
Oxr1	-0.68	0.00022404	duck
Pde4b	-0.67	0.00022470	duck
Clip4	-0.57	0.00023911	duck
Abracl	0.66	0.00024611	duck
Pdxdc1	-0.42	0.00024824	duck
Zfp521	-0.82	0.00025747	duck
Cnot6	-0.65	0.00027263	duck
Armc2	-0.85	0.00027798	duck
Irf2bpl	0.57	0.00029497	duck
Tmem104	0.42	0.00029886	duck
Polr2h	0.66	0.00029998	duck
Unc50	0.42	0.00030076	duck
Dop1b	0.31	0.00030309	duck
Itch	-0.76	0.00030337	duck
Lif	0.53	0.00032228	duck
Rab7	0.62	0.00033098	duck
Paqr7	0.43	0.00033731	duck
Gxylt1	0.57	0.00035353	duck
Arsb	1.00	0.00037352	duck
Eif3g	0.65	0.00037379	duck
Sdc2	0.62	0.00037904	duck
Prmt3	-0.38	0.00038353	duck
Sri	0.81	0.00040027	duck
Ran	0.70	0.00040407	duck
Cdc73	-0.67	0.00040445	duck
Sec16a	-0.77	0.00042452	duck
Rbx1	0.73	0.00044327	duck
Irf7	0.54	0.00045321	duck
Nfkb2	0.58	0.00045804	duck
Actr3	0.89	0.00047316	duck

Herc1	-0.70	0.00047506	duck
Polr1a	-0.49	0.00047680	duck
Arv1	-0.73	0.00050065	duck
Tspan18	-0.46	0.00050896	duck
Hivep2	-0.43	0.00051885	duck
Ddx10	-0.74	0.00053321	duck
Garem1	0.44	0.00054968	duck
Hpse	0.83	0.00055238	duck
Zmynd11	-0.56	0.00056656	duck
Dram1	0.33	0.00056919	duck
Ccnl2	-0.56	0.00059120	duck
Clta	0.47	0.00059749	duck
Wrn	-1.01	0.00060056	duck
Stx7	0.44	0.00060187	duck
Alpk2	-0.35	0.00062327	duck
Rras2	-0.59	0.00062781	duck
Anapc2	0.51	0.00063164	duck
Napg	-0.72	0.00066911	duck
Nudt12	0.46	0.00070856	duck
Txk	-0.50	0.00073319	duck
Trib2	-0.43	0.00074006	duck
Prdx1	0.53	0.00076068	duck
Smarcc1	-0.59	0.00077944	duck
Gpn1	-0.58	0.00079146	duck
Brd1	-0.63	0.00080126	duck
Creg1	-1.46	0.00080411	duck
Cblb	-0.56	0.00083168	duck
Sirt5	0.33	0.00086286	duck
Bcl11b	-0.49	0.00090925	duck
Lrig2	-0.51	0.00091348	duck
Arid1b	-0.52	0.00092147	duck
Gkn2	1.59	0.00094311	duck
Kat6b	-0.64	0.00098521	duck
Lmna	0.81	0.00098926	duck
Atp5j	0.51	0.00099472	duck
Nol8	-0.54	0.00100880	duck
Osbpl11	-0.62	0.00101532	duck
Esyt2	0.94	0.00101889	duck
Aoah	0.63	0.00102773	duck
Ctdp1	-0.69	0.00103118	duck
Ccr4	-0.53	0.00106291	duck
Sucnr1	0.43	0.00106441	duck
Cog5	-0.82	0.00108352	duck
Psmb1	0.75	0.00112363	duck
Ccdc71l	0.38	0.00112779	duck
Cntrl	-0.68	0.00114911	duck

Sec61g	0.81	0.00118129	duck
Ppp1r12a	-0.42	0.00127567	duck
Ide	-0.80	0.00127692	duck
Arpc5	0.86	0.00127898	duck
Sulf2	0.76	0.00130494	duck
Cul4b	-0.43	0.00133220	duck
Prkdc	-0.60	0.00133531	duck
Lpgat1	0.75	0.00134288	duck
Axin1	-0.77	0.00136176	duck
Pold2	0.38	0.00136494	duck
Cep170	-0.77	0.00137815	duck
Pstpip1	0.16	0.00137935	duck
Azi2	-0.96	0.00138040	duck
Tob1	-0.47	0.00140129	duck
Dazap1	-0.65	0.00141392	duck
Psma3	0.75	0.00142344	duck
Rsrp1	-0.82	0.00145667	duck
Fuca2	0.86	0.00147201	duck
Slc45a1	-0.42	0.00150883	duck
Fbxo11	-0.69	0.00150972	duck
Atp6v0c	0.51	0.00153537	duck
Cyrib	0.72	0.00158057	duck
Akap1	-0.38	0.00159322	duck
Tbc1d5	-0.64	0.00165652	duck
Diablo	0.47	0.00176368	duck
Ube2h	-0.73	0.00181177	duck
Reps1	-0.59	0.00202705	duck
Pik3cb	0.57	0.00203278	duck
Dgkq	-0.75	0.00205646	duck
Lhfpl2	0.63	0.00206185	duck
Dnm1l	-0.62	0.00206273	duck
Ctr9	-0.51	0.00208389	duck
Azin1	0.53	0.00210514	duck
Slc25a3	0.58	0.00211587	duck
Slco3a1	-0.53	0.00218774	duck
Cdkn1a	0.41	0.00223387	duck
Dapp1	0.58	0.00228685	duck
Kdm1a	-0.69	0.00229590	duck
Emsy	-0.68	0.00231624	duck
Naga	0.39	0.00232128	duck
Dtl	0.36	0.00233358	duck
Septin6	-0.47	0.00236545	duck
Cd44	0.36	0.00238347	duck
Spen	-0.60	0.00251096	duck
Smchd1	-0.71	0.00254652	duck
Akap13	0.65	0.00256171	duck

Ero1a	0.51	0.00259696	duck
Ncf2	0.43	0.00262298	duck
Lrpap1	0.34	0.00265618	duck
Nqo2	0.56	0.00265907	duck
Il2rb	0.39	0.00266446	duck
Htra1	0.52	0.00266875	duck
Prdm1	0.51	0.00272902	duck
Derl3	0.41	0.00275823	duck
Suco	-0.79	0.00275999	duck
Tnfsf11	0.75	0.00279424	duck
Nmnat2	0.41	0.00282311	duck
Odc1	0.68	0.00286986	duck
Haus8	0.40	0.00292167	duck
Pdk3	-1.08	0.00300074	duck
Reep5	0.60	0.00305942	duck
Ccndbp1	-0.58	0.00313841	duck
Ripor3	-1.63	0.00327577	duck
Sla	-0.55	0.00336377	duck
Kif15	0.82	0.00338392	duck
Spsb1	0.44	0.00343752	duck
Dusp5	0.76	0.00347708	duck
Jak1	-0.38	0.00354209	duck
Strn	-0.59	0.00356761	duck
Itpkb	0.57	0.00367358	duck
Rsl1d1	-0.41	0.00371426	duck
Zfp638	-0.73	0.00378137	duck
Snx2	0.06	0.00378157	duck
Ube2j1	0.50	0.00378472	duck
Tlr4	0.34	0.00379035	duck
Slc25a24	0.32	0.00382088	duck
Copb1	-0.41	0.00385143	duck
Tmem199	0.36	0.00396080	duck
Tmem38a	-0.40	0.00399746	duck
Sun1	-1.02	0.00416672	duck
Ptpre	1.00	0.00421452	duck
Psmd2	0.57	0.00426541	duck
Zfp704	-0.52	0.00434424	duck
Ica1	0.65	0.00475925	duck
Atp6v0e	0.58	0.00488025	duck
Pdrg1	0.41	0.00491871	duck
Rab11fip4	0.57	0.00494162	duck
Slc12a4	-1.07	0.00501975	duck
Gphn	-0.80	0.00514588	duck
R3hdm1	-0.60	0.00521456	duck
Atp23	0.34	0.00527203	duck
Hspa4l	-0.62	0.00568369	duck

Camkmt	-0.72	0.00572309	duck
Ext2	-0.76	0.00580583	duck
Ddx27	-0.40	0.00587419	duck
Prrc2c	-0.46	0.00587839	duck
Edem3	0.44	0.00600484	duck
Eif4ebp1	0.76	0.00653022	duck
Polr2d	0.61	0.00667174	duck
Scaf8	-0.65	0.00689022	duck
Ppargc1a	0.50	0.00693701	duck
Rmdn3	-0.45	0.00731070	duck
Cyth4	0.48	0.00750717	duck
Ubxn2a	-0.49	0.00755889	duck
Taf13	0.52	0.00766468	duck
Nsmce1	-0.55	0.00771570	duck
Ptx3	0.87	0.00777114	duck
Ptpn2	-0.70	0.00800611	duck
Calm1	0.49	0.00848648	duck
Ablim1	-0.78	0.00855586	duck
Chka	0.77	0.00900970	duck
Enpp6	0.49	0.00903318	duck
Cdca7l	-0.25	0.00904412	duck
Cldn10	-0.62	0.00915271	duck
Fxr1	-0.96	0.00917238	duck
Top1	0.74	0.00952264	duck
Scube2	0.42	0.00985123	duck
Septin7	-0.51	0.00989954	duck
Clasp2	-0.67	0.00991151	duck
Obi1	-0.25	0.01011094	duck
Ifnar1	-0.83	0.01036597	duck
Zmiz1	1.10	0.01048334	duck
Amacr	0.31	0.01055908	duck
Fosl2	0.46	0.01070841	duck
Romo1	0.69	0.01077296	duck
Ercc6	-0.60	0.01078167	duck
Ccdc82	-0.40	0.01079175	duck
Sytl2	-0.54	0.01202126	duck
Pbrm1	-0.64	0.01219325	duck
Leo1	-0.38	0.01228836	duck
Ebag9	0.56	0.01234665	duck
Ints6	-0.54	0.01255472	duck
Eif2d	-0.26	0.01273138	duck
Dgkh	0.87	0.01315867	duck
Fnip2	-0.26	0.01323042	duck
Rnf11	0.53	0.01333876	duck
Ssu72	-0.40	0.01337914	duck
Supt5	0.53	0.01345994	duck

Nudcd1	-0.48	0.01361900	duck
Ppil2	-0.60	0.01394167	duck
Nup93	-0.43	0.01424258	duck
Wdr7	-0.78	0.01426360	duck
Wac	-0.72	0.01472836	duck
Oser1	0.32	0.01534786	duck
Ap1s2	0.46	0.01539343	duck
Lamp1	0.70	0.01563982	duck
Tnrc6a	-0.63	0.01591114	duck
Cask	0.86	0.01624682	duck
Psma7	0.64	0.01628154	duck
Tapt1	-0.97	0.01659299	duck
Ereg	0.60	0.01686226	duck
Fubp3	-0.45	0.01695678	duck
Amfr	0.57	0.01711816	duck
Fam120a	-0.53	0.01747355	duck
Polr3e	-0.47	0.01758013	duck
Btbd9	-0.60	0.01779168	duck
Atxn2	-0.47	0.01784634	duck
Cdk13	-0.51	0.01798792	duck
Esf1	-0.60	0.01904256	duck
Znfx1	0.54	0.01920657	duck
Inpp5k	-0.59	0.01947261	duck
Arhgef38	0.44	0.02023007	duck
Dok5	-0.34	0.02036085	duck
Vcpip1	-0.69	0.02047648	duck
Pcnx	-0.68	0.02091487	duck
Rnf146	-0.48	0.02108245	duck
Tab3	-0.59	0.02174592	duck
Ankrd27	-0.55	0.02219428	duck
Arrdc4	0.35	0.02238001	duck
Cks1brt	0.58	0.02244906	duck
Sipa1l1	0.56	0.02273676	duck
Tent5a	0.80	0.02282804	duck
Snrpg	0.73	0.02297474	duck
Xrn2	-0.59	0.02327436	duck
Prelid3b	0.43	0.02357933	duck
Pitpna	-0.52	0.02406248	duck
Csnk1g1	-0.47	0.02475308	duck
Tmem35b	0.51	0.02477348	duck
Disp1	-1.10	0.02495723	duck
Zbed4	-0.47	0.02605126	duck
Srpk1	-0.45	0.02629954	duck
Rab27a	0.68	0.02675937	duck
Tbc1d23	-0.38	0.02731609	duck
Cmtm6	0.60	0.02831934	duck

Gigyf2	-0.39	0.02837912	duck
Btbd7	-0.38	0.02843517	duck
Pcnt	-0.80	0.02882949	duck
Vav3	1.17	0.02893726	duck
Rbms3	1.59	0.02972082	duck
Chd9	-0.62	0.02989019	duck
Desi2	-0.27	0.03067024	duck
Dctn6	0.60	0.03104937	duck
Edrf1	-0.55	0.03110734	duck
Adcy7	-0.86	0.03132355	duck
Lag3	0.31	0.03133851	duck
Cep350	-0.64	0.03200934	duck
Tcerg1	-0.39	0.03222542	duck
Arap2	-0.41	0.03348205	duck
Tcf7l2	0.68	0.03482756	duck
Shld2	0.74	0.03499131	duck
Gatad2a	-0.57	0.03556599	duck
Foxk2	-0.53	0.03592935	duck
Acsl4	-0.50	0.03739403	duck
Ssr1	0.60	0.03818230	duck
Hnrnpab	-0.59	0.03966219	duck
Stambpl1	0.38	0.03983224	duck
Nsfl1c	0.46	0.04023720	duck
1700034I23Rik	0.79	0.04110661	duck
Ensa	0.51	0.04133873	duck
Cd3e	-0.29	0.04152331	duck
Psmb7	0.46	0.04325034	duck
Ndufb3	0.40	0.04332080	duck
Setbp1	-0.47	0.04342617	duck
Ndufb2	0.71	0.04440724	duck
Bst1	0.79	0.04494946	duck
Nbas	-0.70	0.04607411	duck
Jdp2	0.54	0.04724908	duck
Trp53i11	-0.22	0.04775926	duck
Ncoa5	-0.49	0.04778073	duck
Pdp1	-0.39	0.04906400	duck
Kdm4b	-0.47	0.04944269	duck
Dync1h1	-0.50	0.04971714	duck
Plk2	0.43	0.05068197	duck
Bdh1	-0.48	0.05071755	duck
Swt1	-0.64	0.05144366	duck
Atp2b1	-0.60	0.05306567	duck
Rdx	-0.47	0.05326803	duck
H13	0.55	0.05332855	duck
Eif4a2	0.36	0.05332857	duck
Zfand2a	0.27	0.05343180	duck

Tmem59	-0.34	0.05402303	duck
Sub1	0.58	0.05437734	duck
Usp45	-0.72	0.05573956	duck
Lsp1	0.87	0.05654220	duck
Dusp6	0.66	0.05709335	duck
Pigx	0.51	0.05753117	duck
Add1	-0.87	0.05954120	duck
Trak2	-0.39	0.06481373	duck
Kcnq1	1.59	0.06710141	duck
Csde1	-0.47	0.06731590	duck
Phrf1	-0.41	0.06773603	duck
Nbeal2	-0.28	0.06783234	duck
Scaf11	-0.43	0.06939518	duck
Rgma	-0.32	0.07042013	duck
Phf12	-0.57	0.07310741	duck
Tpr	-0.60	0.07626675	duck
Ccdc18	-0.63	0.07662997	duck
Hspa8	0.66	0.07666573	duck
Ipo7	-0.37	0.07733345	duck
Akt3	-0.71	0.07755166	duck
Khdc4	-0.42	0.07891258	duck
Nnt	-0.39	0.08093086	duck
Pip4k2a	-0.45	0.08153656	duck
Dna2	-0.57	0.08243748	duck
Klhl24	-0.56	0.08246236	duck
Slc9a4	0.40	0.08388381	duck
Snrpd1	0.74	0.08401947	duck
Phf14	-0.50	0.08910437	duck
St8sia6	-0.42	0.08939888	duck
Dek	-0.31	0.09442988	duck
Fut8	-0.79	0.09450267	duck
Foxp2	0.76	0.09493588	duck
Fa2h	0.25	0.09682082	duck
Heatr3	-0.30	0.09684947	duck
Wdr20	-0.79	0.09712785	duck
Clec16a	-0.46	0.09803070	duck
Plekhf2	-0.45	0.09911210	duck
Tax1bp1	0.74	0.10002250	duck
Slc4a7	-0.33	0.10014227	duck
Sh2b3	-0.74	0.10049591	duck
Anxa5	0.50	0.10085338	duck
Dram2	0.46	0.10126875	duck
Gzf1	-0.33	0.10138545	duck
Gtf2b	0.53	0.10158025	duck
Irs2	0.43	0.10197025	duck
Atp9b	-0.54	0.10210085	duck

Polr2f	0.49	0.10219302	duck
Rgs9	-0.45	0.10424824	duck
Kank4	-0.27	0.10443458	duck
Arih1	-0.71	0.10449583	duck
Tbcd	-0.77	0.10719524	duck
Noc2l	-0.34	0.11089800	duck
Sap30l	-0.37	0.11276164	duck
Vma21	0.61	0.11469935	duck
Ssr2	0.56	0.11571602	duck
Efnb2	0.64	0.11619950	duck
Elovl5	0.26	0.11624062	duck
Fry	-1.01	0.11630014	duck
Rev1	-0.73	0.11698988	duck
Ctbs	0.36	0.11986238	duck
Ube2q2	-0.55	0.11987630	duck
Wdr43	-0.44	0.12102904	duck
Abce1	-0.39	0.12393293	duck
Slc3a1	0.49	0.12510284	duck
Cdc14a	-1.02	0.12883765	duck
Ulk1	-0.72	0.13002316	duck
Slain2	-0.68	0.13197602	duck
Dennd6a	-0.61	0.13286728	duck
Ppp1r13b	-0.48	0.13371948	duck
Fam126b	-0.79	0.13385834	duck
Efl1	-0.35	0.13546743	duck
Nadk2	-0.51	0.13827462	duck
Nprl3	-0.79	0.13917556	duck
Nlrc3	-0.30	0.13942139	duck
Plekha7	0.55	0.14213565	duck
Srrm1	-0.40	0.15068912	duck
Adcy9	-0.89	0.15075465	duck
Pak3	0.68	0.15099400	duck
Nr4a3	0.71	0.15128509	duck
Ost4	0.83	0.15728228	duck
Pcm1	-0.63	0.15936445	duck
Gramd1c	0.30	0.16137078	duck
Lonp2	-0.54	0.16465586	duck
Far2	-0.53	0.16635066	duck
Ank1	-0.85	0.16662698	duck
Tdrd3	-0.72	0.16861499	duck
Parp10	0.29	0.17531519	duck
Vps50	-0.35	0.17571641	duck
Sf3b4	0.53	0.17946047	duck
Fgl2	0.58	0.18122656	duck
Plcl2	-0.94	0.18729655	duck
Zmym2	-0.70	0.18793135	duck

Pdss2	-0.27	0.19354126	duck
Ercc3	-0.32	0.19489708	duck
Opa1	-0.51	0.19628765	duck
Eif4enif1	-0.58	0.19850217	duck
Pgm1	0.51	0.20016217	duck
Dmxl2	-0.42	0.20141337	duck
Klhl5	-0.54	0.20357506	duck
Tspan14	0.41	0.20777901	duck
Serinc1	-0.44	0.21099582	duck
Jak2	-0.73	0.21323517	duck
Riok3	0.60	0.21649512	duck
Tgds	0.33	0.21724381	duck
Ndufs5	0.65	0.22095182	duck
Parva	0.28	0.23055344	duck
Itpka	0.21	0.23124026	duck
Crem	0.62	0.23552024	duck
Sult4a1	-0.40	0.23594207	duck
Ppp6r2	-0.43	0.23607959	duck
Ciao3	-0.47	0.23930203	duck
Ptcd3	-0.32	0.24093158	duck
Parvg	-0.37	0.24872330	duck
Ccdc171	-0.56	0.25898552	duck
Ubr3	-0.63	0.26296776	duck
Tob2	0.50	0.26910484	duck
Ttc39c	0.38	0.27104146	duck
Cul5	-0.54	0.27705862	duck
Arhgap5	-0.57	0.27877957	duck
Ppp2r2a	-0.59	0.28220347	duck
Arih2	-0.50	0.28317294	duck
Eif2b5	-0.19	0.28500733	duck
Chn1	0.35	0.28884772	duck
Il16	-0.31	0.30014050	duck
Zmynd8	-0.51	0.30282350	duck
Dmxl1	-0.99	0.30441331	duck
Rbfox2	0.74	0.30942993	duck
Nck2	0.24	0.31383197	duck
Dazap2	0.37	0.33254881	duck
Fbxl22	-0.41	0.33484764	duck
Phf10	-0.43	0.33915077	duck
Ldlrap1	-0.41	0.33931324	duck
Tfdp1	-0.58	0.34538586	duck
Dapk1	-0.36	0.34681673	duck
Brsk2	-0.14	0.34747092	duck
Pgrmc2	-0.44	0.34924598	duck
Fam174a	0.40	0.35091208	duck
Lig3	-0.45	0.35171330	duck

Nf1	-0.44	0.35650589	duck
Lrrc42	-0.27	0.36002285	duck
P2ry13	0.33	0.36079223	duck
Ttbk1	-0.36	0.36554637	duck
Cdkal1	-0.57	0.37024416	duck
Rmnd5a	-0.59	0.37083317	duck
Nek6	0.54	0.37191176	duck
Adss	-0.35	0.37213278	duck
Rp2	0.55	0.37558600	duck
Asap1	-1.12	0.37791860	duck
Zfhx3	0.66	0.38139695	duck
Arf6	0.63	0.38219913	duck
Traf1	-0.25	0.38261624	duck
Ttc39a	0.43	0.38906203	duck
Rbm26	-0.51	0.39303700	duck
Pdia6	0.45	0.39837446	duck
Rhbdf2	0.24	0.39838008	duck
Nudt21	0.55	0.39954830	duck
Zbtb7b	0.30	0.40225533	duck
Sdf4	-0.45	0.40456443	duck
Srsf4	0.29	0.40514915	duck
Tnfrsf25	0.68	0.40547670	duck
Xpo4	-0.45	0.41128014	duck
Cobll1	0.17	0.41318288	duck
Nceh1	0.32	0.41776599	duck
Slc35f5	0.30	0.42231236	duck
Nadk	0.60	0.42326416	duck
Coro1c	0.55	0.43509842	duck
Kat2b	0.27	0.44366847	duck
Htr2a	0.35	0.44681488	duck
Ostm1	0.53	0.45152566	duck
Kcnk13	0.91	0.45299478	duck
Wdr70	-0.91	0.45681087	duck
Ascc3	-0.63	0.47281278	duck
Card11	-0.47	0.48074142	duck
Gabarapl2	0.51	0.48110440	duck
Psd	0.37	0.48494609	duck
Cemip2	0.55	0.49810546	duck
Cib1	0.38	0.50021455	duck
Ilrun	-1.04	0.50170458	duck
Heatr5b	-0.42	0.50580104	duck
Unc13d	0.63	0.50686346	duck
Acsl1	1.17	0.51561459	duck
Ufl1	-0.48	0.51657200	duck
Thoc2	-0.50	0.51836494	duck
Tmem251	0.49	0.52993287	duck

Phc2	0.39	0.55341886	duck
Ppp3cb	-0.54	0.55973320	duck
Dusp22	-0.90	0.56274774	duck
Usp22	-0.59	0.56282412	duck
Clasp1	-0.56	0.57528962	duck
Zfp652	-0.51	0.57687834	duck
Gtf2h1	-0.61	0.58566981	duck
Ormdl2	0.55	0.59380737	duck
Ipo5	-0.42	0.59816445	duck
Steep1	-0.32	0.59819416	duck
Tubb4b	0.58	0.59999600	duck
Rbl2	-0.54	0.60205554	duck
Taf12	0.44	0.60405918	duck
Anapc7	-0.35	0.60452949	duck
Clic4	0.60	0.60982329	duck
Jade2	-0.48	0.61357895	duck
Eif4g2	0.31	0.61381356	duck
Zfand5	0.57	0.61603855	duck
Lman2	0.41	0.61882834	duck
Lasp1	0.72	0.61997950	duck
Zbtb20	1.58	0.63243106	duck
Arid2	-0.63	0.63322320	duck
Cdc42se1	0.32	0.63523052	duck
Wnk1	0.50	0.64763415	duck
Phactr2	0.52	0.64928507	duck
Bbs12	0.42	0.65907852	duck
Dhx37	-0.32	0.66911305	duck
Nfatc1	0.84	0.67083908	duck
Il1rl1	0.38	0.67278829	duck
Samd4	0.62	0.67359868	duck
Usp16	-0.60	0.69100130	duck
Ostf1	0.56	0.69149055	duck
Stom	0.77	0.69716091	duck
Cep290	-0.61	0.69878576	duck
Ppm1a	-0.52	0.69885681	duck
Phf20l1	-0.51	0.73194001	duck
Guk1	0.42	0.74065441	duck
Nrde2	-0.38	0.75118442	duck
Sdhb	0.49	0.75801131	duck
Asah1	0.82	0.76360593	duck
Aqr	-0.36	0.76810814	duck
Golga3	-0.60	0.77229566	duck
Ddx5	-0.30	0.77467034	duck
Prkce	-0.41	0.77700390	duck
Plcd1	0.34	0.79442807	duck
Atad1	-0.48	0.80256365	duck

Znhit6	-0.34	0.82267872	duck
Socs5	-0.38	0.84997221	duck
Sltm	-0.20	0.85430732	duck
Cdk5r1	0.46	0.85584469	duck
Ppme1	-0.30	0.87597049	duck
Zzef1	-0.65	0.87952915	duck
Zbtb41	-0.50	0.88430867	duck
Rap1b	0.46	0.88563444	duck
B3gnt4	0.45	0.89599365	duck
Vps37a	-0.30	0.95248694	duck
Ctsc	0.51	0.95571277	duck
Cilk1	-0.31	0.96439494	duck
Cd82	-0.35	0.96800832	duck
Supt4a	0.42	0.98007196	duck
Tmx1	-0.43	0.98761633	duck
Cluh	-0.45	1.00000000	duck
Ppig	-0.52	1.00000000	duck
Als2cl	1.42	1.00000000	duck
Wdr89	0.22	1.00000000	duck
Srpr	-0.33	1.00000000	duck
Cebpz	-0.29	1.00000000	duck
Ciao2b	0.49	1.00000000	duck
Prdm10	-0.35	1.00000000	duck
Cog6	-0.54	1.00000000	duck
Uspl1	-0.31	1.00000000	duck
Hbs1l	-0.47	1.00000000	duck
Irf1	-0.32	1.00000000	duck
Pus7l	-0.50	1.00000000	duck
Sel1l	-0.56	1.00000000	duck
Cops7a	0.30	1.00000000	duck
Srsf10	-0.27	1.00000000	duck
Pptc7	-0.39	1.00000000	duck
Atxn7	-0.47	1.00000000	duck
Snx18	0.39	1.00000000	duck
Anks1	-0.69	1.00000000	duck
Eml4	0.55	1.00000000	duck
Xpnpep1	-0.35	1.00000000	duck
Satb1	-0.95	1.00000000	duck
Golgb1	-0.64	1.00000000	duck
Baz1a	0.33	1.00000000	duck
Hipk1	-0.63	1.00000000	duck
Vti1a	-0.71	1.00000000	duck
Syngr2	0.29	1.00000000	duck
Rnf41	0.31	1.00000000	duck
Naa30	-0.39	1.00000000	duck
Eefsec	-0.70	1.00000000	duck

Arhgap45	-0.39	1.00000000	duck
Gne	0.39	1.00000000	duck
Ppp1r21	-0.71	1.00000000	duck
Tmem138	0.19	1.00000000	duck
Gtf3c1	-0.35	1.00000000	duck
Tbc1d14	0.34	1.00000000	duck
Ago2	-0.66	1.00000000	duck
Tacc1	-0.27	1.00000000	duck
St3gal6	-0.43	1.00000000	duck
Kansl3	-0.27	1.00000000	duck
Kctd5	-0.53	1.00000000	duck
Chst10	-0.34	1.00000000	duck
Gpatch8	-0.31	1.00000000	duck
Gfod2	-0.35	1.00000000	duck
Ncoa4	-0.45	1.00000000	duck
Ano5	0.83	1.00000000	duck
Xbp1	0.40	1.00000000	duck
Luc7l3	-0.39	1.00000000	duck
Ngly1	-0.52	1.00000000	duck
Wdr45b	0.22	1.00000000	duck
Lpxn	0.62	1.00000000	duck
Gemin5	-0.19	1.00000000	duck
Ybx1	0.49	1.00000000	duck
Prpf39	-0.45	1.00000000	duck
Samsn1	0.41	1.00000000	duck
Angel2	-0.28	1.00000000	duck
Btrc	-0.39	1.00000000	duck
Nyx	0.41	1.00000000	duck
Aff4	-0.41	1.00000000	duck
Cyth1	-0.11	1.00000000	duck
Npr3	-0.33	1.00000000	duck
Zeb1	-0.79	1.00000000	duck
Dip2b	-0.34	1.00000000	duck
Slc25a13	-0.61	1.00000000	duck
Dnaja4	-0.40	1.00000000	duck
Hs6st2	-0.84	1.00000000	duck
Arsg	-0.44	1.00000000	duck
Dnajc2	-0.48	1.00000000	duck
Rrbp1	0.58	1.00000000	duck
Lcp1	0.73	1.00000000	duck
Xpa	0.57	1.00000000	duck
Ddx46	-0.36	1.00000000	duck
Ndufb10	0.47	1.00000000	duck
Tra2a	-0.42	1.00000000	duck
Rb1	-0.51	1.00000000	duck
Ubp1	-0.53	1.00000000	duck

Strn3	-0.54	1.00000000	duck
Wdr1	0.50	1.00000000	duck
Arf1	0.38	1.00000000	duck
Rnf213	0.61	1.00000000	duck
Usp44	0.53	1.00000000	duck
Paxbp1	-0.50	1.00000000	duck
Tsc1	-0.73	1.00000000	duck
Ctps2	-0.61	1.00000000	duck
Wapl	-0.48	1.00000000	duck
Abi1	0.38	1.00000000	duck
Prelid3a	0.21	1.00000000	duck
Usp24	-0.59	1.00000000	duck
Plekha1	-0.17	1.00000000	duck
Ssbp3	-0.43	1.00000000	duck
Skap2	0.51	1.00000000	duck
Sox13	-0.22	1.00000000	duck
Slc2a13	0.27	1.00000000	duck
C2cd3	-0.43	1.00000000	duck
Pnisr	-0.59	1.00000000	duck
Ccng2	0.27	1.00000000	duck
Taf3	-0.42	1.00000000	duck
Zfp592	-0.40	1.00000000	duck
Ric8b	-0.56	1.00000000	duck
Bcl6	0.60	1.00000000	duck
Lgmn	-0.55	1.00000000	duck
Bms1	-0.25	1.00000000	duck
Smad6	0.62	1.00000000	duck
Smarcd2	0.36	1.00000000	duck
Phc3	-0.31	1.00000000	duck
Acss1	0.29	1.00000000	duck
Itgb1	0.23	1.00000000	duck
Rsf1	-0.53	1.00000000	duck
Wdr11	-0.34	1.00000000	duck
Esrrg	-0.92	1.00000000	duck
Cds2	-0.33	1.00000000	duck
Xrn1	-0.46	1.00000000	duck
Dstn	0.44	1.00000000	duck
Rnpc3	-0.33	1.00000000	duck
Tmem131l	-0.70	1.00000000	duck
Vcpkmt	0.34	1.00000000	duck
Pxn	0.18	1.00000000	duck
Rsrc2	-0.46	1.00000000	duck
Fam168b	-0.47	1.00000000	duck
Nsun2	-0.39	1.00000000	duck
Ahsa1	0.47	1.00000000	duck
Nfia	0.33	1.00000000	duck

Eif3e	-0.38	1.00000000	duck
Ndufaf3	0.27	1.00000000	duck
Pum2	-0.45	1.00000000	duck
Nmt2	-0.36	1.00000000	duck
Tiam2	0.59	1.00000000	duck
Gnl3	-0.34	1.00000000	duck
Zranb2	-0.61	1.00000000	duck
Top2b	-0.60	1.00000000	duck
Pgm2	0.43	1.00000000	duck
Pbx4	-0.89	1.00000000	duck
Ccdc12	0.44	1.00000000	duck
Cnot7	-0.56	1.00000000	duck
Tial1	-0.39	1.00000000	duck
Emc6	0.41	1.00000000	duck
Srp19	0.40	1.00000000	duck
Rrn3	-0.26	1.00000000	duck
Smad4	-0.53	1.00000000	duck
Zbtb34	-0.60	1.00000000	duck
Rpap2	-0.44	1.00000000	duck
Rufy3	0.66	1.00000000	duck
Slc25a37	-0.75	1.00000000	duck
Gdpd5	0.59	1.00000000	duck
Gpx3	0.61	1.00000000	duck
Adgrl2	0.47	1.00000000	duck
Atm	-0.44	1.00000000	duck
Ric1	0.60	1.00000000	duck
Septin2	-0.26	1.00000000	duck
Ptprs	0.30	1.00000000	duck
Raver2	0.41	1.00000000	duck
Gsk3b	-0.28	1.00000000	duck
Gpatch1	-0.41	1.00000000	duck
Zc3h12d	-0.25	1.00000000	duck
Bloc1s4	0.30	1.00000000	duck
E4f1	-0.47	1.00000000	duck
Zfp148	-0.70	1.00000000	duck
Parp4	-0.34	1.00000000	duck
Kansl1l	-0.52	1.00000000	duck
Ccdc134	0.38	1.00000000	duck
Rasa1	-0.39	1.00000000	duck
Rbsn	-0.43	1.00000000	duck
Arsk	-0.56	1.00000000	duck
Cwf19l1	-0.31	1.00000000	duck
Ctla4	-0.50	1.00000000	duck
Larp1	-0.26	1.00000000	duck
Diaph2	0.48	1.00000000	duck
Ap4e1	-0.40	1.00000000	duck

Chst11	-0.47	1.00000000	duck
Rabep1	-0.53	1.00000000	duck
Slc66a2	-0.36	1.00000000	duck
Atad2b	-0.54	1.00000000	duck
Lrmp	0.16	1.00000000	duck
Ubap2l	-0.23	1.00000000	duck
Tmem164	-0.14	1.00000000	duck
Slc24a2	0.10	1.00000000	duck
Ppp2r2d	-0.54	1.00000000	duck
Zbtb43	-0.58	1.00000000	duck
Stk26	-0.60	1.00000000	duck
Pnpla7	-0.50	1.00000000	duck
Sesn3	-0.42	1.00000000	duck
Pdcd11	-0.30	1.00000000	duck
Psd3	0.36	1.00000000	duck
Kdm4c	-0.57	1.00000000	duck
Jpt1	0.55	1.00000000	duck
Specc1	-0.43	1.00000000	duck
Rcan2	-0.45	1.00000000	duck
Gpr55	-0.13	1.00000000	duck
Arhgap26	0.18	1.00000000	duck
Tecpr2	-0.32	1.00000000	duck
Cnot9	0.57	1.00000000	duck
Fkbp4	-0.21	1.00000000	duck
Ndrg1	0.28	1.00000000	duck
Ddx60	0.48	1.00000000	duck
Wdr4	-0.24	1.00000000	duck
Tec	-0.85	1.00000000	duck
Dot1l	0.81	1.00000000	duck
Itga1	-0.27	1.00000000	duck
Dennd1a	-0.57	1.00000000	duck
Nfatc2	-0.30	1.00000000	duck
Sccpdh	0.42	1.00000000	duck
Tmem64	0.49	1.00000000	duck
Prune1	0.12	1.00000000	duck
Snx29	-0.41	1.00000000	duck
Ppil4	-0.22	1.00000000	duck
Adnp2	-0.28	1.00000000	duck
Rprd1b	-0.37	1.00000000	duck
Lmbrd1	-0.31	1.00000000	duck
Hlf	0.58	1.00000000	duck
Stam2	-0.29	1.00000000	duck
Ankrd52	-0.30	1.00000000	duck
Erp44	0.54	1.00000000	duck
Eya3	-0.48	1.00000000	duck
Dym	-0.81	1.00000000	duck

Apaf1	-0.49	1.00000000	duck
Lztr1	-0.41	1.00000000	duck
Xxylt1	0.44	1.00000000	duck
Sptlc2	-0.51	1.00000000	duck
Armc1	-0.69	1.00000000	duck
Ckap5	-0.34	1.00000000	duck
Dars	-0.01	1.00000000	duck
Arrdc3	0.55	1.00000000	duck
Ptdss2	-0.25	1.00000000	duck
Rfng	-0.27	1.00000000	duck
Rnf166	0.43	1.00000000	duck
Papln	0.25	1.00000000	duck
St3gal1	-0.43	1.00000000	duck
Cdk17	0.27	1.00000000	duck
Extl3	-0.30	1.00000000	duck
Traf7	0.38	1.00000000	duck
LTO1	-0.35	1.00000000	duck
Il21r	-0.47	1.00000000	duck
Pdcd4	-0.17	1.00000000	duck
Ola1	-0.49	1.00000000	duck
Runx3	0.68	1.00000000	duck
Impdh2	-0.23	1.00000000	duck
Rnmt	-0.22	1.00000000	duck
Ddx17	-0.62	1.00000000	duck
Tbc1d15	-0.63	1.00000000	duck
Plcb1	0.57	1.00000000	duck
Ptdss1	-0.33	1.00000000	duck
Pcgf6	-0.32	1.00000000	duck
Cul1	-0.40	1.00000000	duck
Tent4a	-0.31	1.00000000	duck
Sbno1	-0.51	1.00000000	duck
Guf1	-0.33	1.00000000	duck
Ube2o	-0.41	1.00000000	duck
Ptbp3	0.52	1.00000000	duck
Lin7c	-0.27	1.00000000	duck
Acsl3	-0.40	1.00000000	duck
Hbp1	-0.44	1.00000000	duck
Hnrnpa3	-0.22	1.00000000	duck
Heatr1	-0.11	1.00000000	duck
Tab2	-0.53	1.00000000	duck
Parg	-0.66	1.00000000	duck
Fam214b	0.39	1.00000000	duck
Zfp236	-0.52	1.00000000	duck
Zfp512	-0.30	1.00000000	duck
Klf3	-0.58	1.00000000	duck
Sirt1	-0.36	1.00000000	duck

Fbxo21	-0.02	1.00000000	duck
Xrcc5	-0.24	1.00000000	duck
Bcat1	0.38	1.00000000	duck
Zdhhc14	-0.67	1.00000000	duck
Ss18	-0.27	1.00000000	duck
Ahcyl2	0.22	1.00000000	duck
Psme3ip1	-0.23	1.00000000	duck
Eipr1	-0.59	1.00000000	duck
Pitpnb	-0.32	1.00000000	duck
Fkbp15	-0.11	1.00000000	duck
Zw10	-0.17	1.00000000	duck
Ppip5k1	-0.40	1.00000000	duck
Srebf2	0.38	1.00000000	duck
Golm2	-0.33	1.00000000	duck
Commd4	0.44	1.00000000	duck
Retreg1	-0.48	1.00000000	duck
Vps13a	-0.59	1.00000000	duck
Emp1	0.67	1.00000000	duck
Dync2i1	0.60	1.00000000	duck
Slc39a9	-0.44	1.00000000	duck
Agpat4	0.13	1.00000000	duck
Senp7	-0.63	1.00000000	duck
Uprt	-0.26	1.00000000	duck
Nop58	-0.15	1.00000000	duck
G3bp2	-0.35	1.00000000	duck
Gsr	0.39	1.00000000	duck
Glb1	-0.19	1.00000000	duck
Gpc1	0.89	1.00000000	duck
Rbm33	-0.52	1.00000000	duck
Rrp15	-0.25	1.00000000	duck
Fbxo28	-0.42	1.00000000	duck
Kdm5b	-0.35	1.00000000	duck
Rock1	-0.28	1.00000000	duck
Pdap1	-0.34	1.00000000	duck
Cep57	-0.21	1.00000000	duck
Slc25a16	-0.61	1.00000000	duck
Pds5a	-0.35	1.00000000	duck
Slc9a7	0.65	1.00000000	duck
Eif4g1	-0.19	1.00000000	duck
Larp4	-0.17	1.00000000	duck
Aptx	0.25	1.00000000	duck
Zbtb26	-0.24	1.00000000	duck
Atp10a	0.82	1.00000000	duck
Wdr81	0.32	1.00000000	duck
Gpc5	0.73	1.00000000	duck
Ywhab	-0.17	1.00000000	duck

Soat1	0.21	1.00000000	duck
Gcn1	-0.29	1.00000000	duck
Pik3r4	-0.20	1.00000000	duck
Notch2	0.42	1.00000000	duck
Yeats2	-0.23	1.00000000	duck
Pan3	-0.30	1.00000000	duck
Niban1	0.22	1.00000000	duck
Pi4k2b	-0.24	1.00000000	duck
Luzp1	-0.28	1.00000000	duck
Golga4	-0.59	1.00000000	duck
Cdc16	-0.30	1.00000000	duck
Vezt	-0.51	1.00000000	duck
Sema4a	0.32	1.00000000	duck
Ddx28	-0.29	1.00000000	duck
Crybg3	-0.78	1.00000000	duck
Cggbp1	-0.34	1.00000000	duck
Trps1	-0.41	1.00000000	duck
Dusp11	-0.21	1.00000000	duck
Tecpr1	-0.19	1.00000000	duck
Slc16a12	0.37	1.00000000	duck
Parp9	0.14	1.00000000	duck
Lipa	0.28	1.00000000	duck
Bdp1	-0.57	1.00000000	duck
Tia1	-0.47	1.00000000	duck
Parn	-0.26	1.00000000	duck
Gpr155	0.11	1.00000000	duck
Zfyve9	-0.63	1.00000000	duck
Tm2d2	0.31	1.00000000	duck
Pknox1	-1.05	1.00000000	duck
Fam98a	-0.19	1.00000000	duck
Cers2	0.30	1.00000000	duck
Sf3b6	0.33	1.00000000	duck
Gabpa	-0.39	1.00000000	duck
Lypla1	-0.28	1.00000000	duck
Lpcat2b	0.37	1.00000000	duck
Arhgef18	-0.36	1.00000000	duck
Crlf2	0.32	1.00000000	duck
Fbxl5	-0.43	1.00000000	duck
Rc3h2	-0.29	1.00000000	duck
Elf1	0.30	1.00000000	duck
Ywhah	-0.32	1.00000000	duck
Abca3	0.34	1.00000000	duck
Gtf2f2	-0.49	1.00000000	duck
Atp5j2	0.48	1.00000000	duck
Phf2	-0.43	1.00000000	duck
Zzz3	-0.39	1.00000000	duck

Fam32a	0.42	1.00000000	duck
Chp1	0.59	1.00000000	duck
Vapa	0.39	1.00000000	duck
Cdk19	-0.51	1.00000000	duck
Ranbp10	-0.20	1.00000000	duck
Acap2	0.49	1.00000000	duck
Kcnmb4	-0.16	1.00000000	duck
Cbx7	-0.20	1.00000000	duck
Dyrk1a	-0.41	1.00000000	duck
Ppp3cc	-0.27	1.00000000	duck
Pars2	0.32	1.00000000	duck
1110059G10Rik	0.47	1.00000000	duck
Atp2a3	-0.09	1.00000000	duck
Nfx1	-0.53	1.00000000	duck
Klhl15	-0.32	1.00000000	duck
Cog2	-0.43	1.00000000	duck
Serbp1	-0.31	1.00000000	duck
Tlk1	-0.46	1.00000000	duck
Klhl2	-0.29	1.00000000	duck
Cwf19l2	-0.59	1.00000000	duck
Sms	-0.48	1.00000000	duck
Furin	0.24	1.00000000	duck
Etfdh	-0.35	1.00000000	duck
Pde8a	0.51	1.00000000	duck
Dop1a	-0.34	1.00000000	duck
Ints8	-0.46	1.00000000	duck
Fasn	-0.01	1.00000000	duck
Ythdf2	0.42	1.00000000	duck
Clk2	-0.25	1.00000000	duck
Tomm22	0.47	1.00000000	duck
Dcun1d5	0.37	1.00000000	duck
Ccnyl1	-0.40	1.00000000	duck
Tcf3	-0.47	1.00000000	duck
Fam122b	-0.21	1.00000000	duck
Cnot2	-0.38	1.00000000	duck
Smarca5	-0.38	1.00000000	duck
Btaf1	-0.58	1.00000000	duck
Igf1r	0.64	1.00000000	duck
Acvr2a	-0.23	1.00000000	duck
Clcn3	-0.53	1.00000000	duck
Ip6k2	-0.32	1.00000000	duck
Fam76b	-0.31	1.00000000	duck
Utp25	-0.13	1.00000000	duck
Sync	-0.14	1.00000000	duck
Arglu1	-0.36	1.00000000	duck
Gnai2	0.64	1.00000000	duck

Rexo5	-0.30	1.00000000	duck
Lars	-0.39	1.00000000	duck
Gpr171	-0.18	1.00000000	duck
Suds3	0.49	1.00000000	duck
4932411N23Rik	-0.25	1.00000000	duck
Sparc	0.45	1.00000000	duck
Lonrf2	-0.34	1.00000000	duck
Fam78a	-0.33	1.00000000	duck
Prkag1	0.20	1.00000000	duck
Impa1	0.41	1.00000000	duck
Itpr2	-0.26	1.00000000	duck
Rpap3	-0.42	1.00000000	duck
Slc17a5	0.13	1.00000000	duck
Crtc1	-0.28	1.00000000	duck
Txn2	0.28	1.00000000	duck
Faf1	-0.22	1.00000000	duck
Hmgxb4	-0.29	1.00000000	duck
Iqsec1	0.28	1.00000000	duck
Tbc1d2b	-0.41	1.00000000	duck
Epas1	-0.78	1.00000000	duck
Kit	0.48	1.00000000	duck
Fbxo38	-0.31	1.00000000	duck
Cdc27	-0.41	1.00000000	duck
Adap1	0.34	1.00000000	duck
Acp2	0.35	1.00000000	duck
Flnb	-0.24	1.00000000	duck
Ndfip1	0.58	1.00000000	duck
Sh3bp5	-0.07	1.00000000	duck
Ambra1	-0.37	1.00000000	duck
Ndufb6	0.53	1.00000000	duck
Rif1	-0.35	1.00000000	duck
Oga	-0.38	1.00000000	duck
Tnip1	0.39	1.00000000	duck
Pdk1	-0.43	1.00000000	duck
Bnc2	-0.49	1.00000000	duck
Arhgdia	0.42	1.00000000	duck
Raf1	-0.33	1.00000000	duck
Gtf2e1	-0.50	1.00000000	duck
Abcg1	0.45	1.00000000	duck
Ifngr2	0.50	1.00000000	duck
Fmnl1	-0.24	1.00000000	duck
Echs1	0.38	1.00000000	duck
Ppp2ca	0.23	1.00000000	duck
C2cd5	-0.57	1.00000000	duck
Hook1	-0.40	1.00000000	duck
Trim33	-0.53	1.00000000	duck

Sepsecs	-0.16	1.00000000	duck
Sirt6	0.33	1.00000000	duck
Gnpda2	-0.43	1.00000000	duck
Fam98b	-0.25	1.00000000	duck
Fxn	0.21	1.00000000	duck
Ube2q1	0.36	1.00000000	duck
Ostc	0.38	1.00000000	duck
Yaf2	0.28	1.00000000	duck
Stat3	0.38	1.00000000	duck
Snrpf	0.61	1.00000000	duck
Wbp2nl	0.33	1.00000000	duck
Otud6b	-0.24	1.00000000	duck
Erlec1	0.18	1.00000000	duck
Usp31	-0.26	1.00000000	duck
Crebrf	-0.66	1.00000000	duck
Daglb	-0.19	1.00000000	duck
Ust	-0.14	1.00000000	duck
Tmem184b	-0.70	1.00000000	duck
Vegfc	0.30	1.00000000	duck
Agpat5	-0.34	1.00000000	duck
Pde1c	-1.27	1.00000000	duck
Ywhaq	0.25	1.00000000	duck
Elavl1	-0.27	1.00000000	duck
Hnrnpk	0.42	1.00000000	duck
Ptpn22	-0.08	1.00000000	duck
Acadsb	-0.31	1.00000000	duck
Zfp711	-0.19	1.00000000	duck
Dcaf6	0.61	1.00000000	duck
Kansl2	-0.19	1.00000000	duck
Clptm1l	-0.16	1.00000000	duck
Lats1	-0.16	1.00000000	duck
Nfrkb	-0.35	1.00000000	duck
Lfng	0.51	1.00000000	duck
Atp6v1c1	0.28	1.00000000	duck
Tubd1	0.20	1.00000000	duck
Gtdc1	0.54	1.00000000	duck
Sugp2	-0.20	1.00000000	duck
Trappc11	-0.52	1.00000000	duck
Kctd7	0.35	1.00000000	duck
Dbi	-0.10	1.00000000	duck
Atf2	-0.49	1.00000000	duck
Ctps	-0.04	1.00000000	duck
Rapsn	0.18	1.00000000	duck
Asf1a	-0.26	1.00000000	duck
Tsc2	-0.26	1.00000000	duck
Rexo2	0.32	1.00000000	duck

Bcr	0.52	1.00000000	duck
Poldip3	0.43	1.00000000	duck
Prdx6	0.36	1.00000000	duck
Tbl1xr1	0.43	1.00000000	duck
Rnf138	0.50	1.00000000	duck
Tasor2	-0.14	1.00000000	duck
Fam20b	-0.46	1.00000000	duck
Abcb7	-0.26	1.00000000	duck
Coq5	0.22	1.00000000	duck
Eif5	0.08	1.00000000	duck
Nod1	-0.13	1.00000000	duck
Tmem154	0.43	1.00000000	duck
Ireb2	-0.46	1.00000000	duck
Ap1g1	-0.55	1.00000000	duck
Zfp384	-0.38	1.00000000	duck
Trmt10a	0.26	1.00000000	duck
Nolc1	-0.11	1.00000000	duck
Tgif1	-0.27	1.00000000	duck
Atg7	0.28	1.00000000	duck
Sugp1	-0.25	1.00000000	duck
Thap1	0.24	1.00000000	duck
Usp25	0.36	1.00000000	duck
Naa50	-0.26	1.00000000	duck
Suclg2	-0.29	1.00000000	duck
Kpna1	-0.27	1.00000000	duck
Ctdspl	-0.62	1.00000000	duck
Blk	-0.41	1.00000000	duck
Errfi1	-0.23	1.00000000	duck
Tgfbr2	0.45	1.00000000	duck
Psmb4	0.56	1.00000000	duck
Stk40	-0.29	1.00000000	duck
Emc8	0.32	1.00000000	duck
G3bp1	-0.26	1.00000000	duck
Tyw3	-0.35	1.00000000	duck
Zbtb49	-0.30	1.00000000	duck
Nol10	-0.29	1.00000000	duck
Cisd2	0.40	1.00000000	duck
Smu1	0.41	1.00000000	duck
Zranb1	-0.41	1.00000000	duck
Washc5	0.21	1.00000000	duck
Tpp2	-0.54	1.00000000	duck
Ndufa6	0.46	1.00000000	duck
Akap11	-0.27	1.00000000	duck
Deptor	0.41	1.00000000	duck
Nampt	0.53	1.00000000	duck
Trmt44	-0.17	1.00000000	duck

Pik3r1	0.04	1.00000000	duck
Dda1	0.26	1.00000000	duck
Kidins220	-0.25	1.00000000	duck
Kbtbd2	-0.36	1.00000000	duck
Prkci	-0.46	1.00000000	duck
Chuk	-0.26	1.00000000	duck
Kdm1b	-0.39	1.00000000	duck
Ddx24	-0.09	1.00000000	duck
Eif3h	-0.31	1.00000000	duck
Spag1	0.33	1.00000000	duck
Rgs19	0.48	1.00000000	duck
Eea1	-0.30	1.00000000	duck
Fkbp5	0.33	1.00000000	duck
Dock10	0.57	1.00000000	duck
Gps1	-0.16	1.00000000	duck
Grap2	-0.14	1.00000000	duck
Htt	-0.55	1.00000000	duck
Zbtb1	-0.19	1.00000000	duck
Cxcr4	-0.25	1.00000000	duck
Anxa6	-0.23	1.00000000	duck
Arhgap25	-0.31	1.00000000	duck
Taf4	-0.32	1.00000000	duck
Ankrd12	-0.53	1.00000000	duck
Ate1	-0.58	1.00000000	duck
Aplp2	0.16	1.00000000	duck
Erc1	-0.48	1.00000000	duck
Zfp217	0.47	1.00000000	duck
Lrrk2	0.38	1.00000000	duck
Letm1	-0.29	1.00000000	duck
Hdac4	-0.19	1.00000000	duck
Ogfrl1	0.43	1.00000000	duck
Dhrs7c	-0.27	1.00000000	duck
Ssr3	0.54	1.00000000	duck
Ncbp3	-0.13	1.00000000	duck
Usp6nl	0.25	1.00000000	duck
Lbh	-0.23	1.00000000	duck
Ergic1	0.36	1.00000000	duck
Phf6	-0.21	1.00000000	duck
Acvr1	-0.37	1.00000000	duck
Hexb	0.22	1.00000000	duck
Slc10a7	-0.43	1.00000000	duck
Pspc1	-0.30	1.00000000	duck
Nle1	-0.15	1.00000000	duck
Npl	0.29	1.00000000	duck
Plec	-0.30	1.00000000	duck
Ptbp2	-0.29	1.00000000	duck

Entpd1	0.17	1.00000000	duck
Anxa2	0.82	1.00000000	duck
Phf3	-0.53	1.00000000	duck
Eny2	0.18	1.00000000	duck
Ano10	0.45	1.00000000	duck
Hipk2	-0.18	1.00000000	duck
Nfat5	-0.27	1.00000000	duck
Rpa3	0.32	1.00000000	duck
Rhoh	0.65	1.00000000	duck
Fto	-0.49	1.00000000	duck
Tox2	0.61	1.00000000	duck
Jmjd4	0.32	1.00000000	duck
Lpcat3	0.28	1.00000000	duck
Tnfrsf19	0.52	1.00000000	duck
Hdac1	0.40	1.00000000	duck
Hmgcr	0.41	1.00000000	duck
Zbtb33	-0.19	1.00000000	duck
Srsf11	-0.28	1.00000000	duck
Pcca	-0.17	1.00000000	duck
Nvl	-0.23	1.00000000	duck
Pskh1	-0.29	1.00000000	duck
Pex1	-0.28	1.00000000	duck
Aopep	0.32	1.00000000	duck
Acaa2	-0.22	1.00000000	duck
Xpo7	-0.18	1.00000000	duck
Setd5	-0.23	1.00000000	duck
Bfar	-0.12	1.00000000	duck
Smg7	-0.33	1.00000000	duck
Tbc1d22a	-0.32	1.00000000	duck
Rab11a	0.21	1.00000000	duck
Dbnl	0.43	1.00000000	duck
Vkorc1l1	-0.22	1.00000000	duck
Smg1	-0.21	1.00000000	duck
Immt	-0.17	1.00000000	duck
Prdm2	-0.32	1.00000000	duck
Tmem50b	-0.28	1.00000000	duck
Zswim8	-0.41	1.00000000	duck
Wdr75	-0.23	1.00000000	duck
Hipk3	0.44	1.00000000	duck
Camsap2	-0.90	1.00000000	duck
Gas7	0.55	1.00000000	duck
Seh1l	-0.27	1.00000000	duck
Ankrd28	0.31	1.00000000	duck
Tmem165	0.42	1.00000000	duck
Serinc3	0.29	1.00000000	duck
Eif3b	-0.26	1.00000000	duck

Uhrf2	-0.57	1.00000000	duck
Atmin	-0.34	1.00000000	duck
Rab22a	-0.49	1.00000000	duck
Phtf1	-0.23	1.00000000	duck
Ehbp1	-0.31	1.00000000	duck
Rsbn1	-0.21	1.00000000	duck
Kcnab2	0.38	1.00000000	duck
Wdr33	-0.33	1.00000000	duck
Bicral	-0.42	1.00000000	duck
Fgfr1op2	-0.26	1.00000000	duck
Abhd6	0.33	1.00000000	duck
Lrif1	-0.28	1.00000000	duck
Rint1	0.24	1.00000000	duck
Prkca	-0.37	1.00000000	duck
Chic1	-0.17	1.00000000	duck
Rad54l2	-0.03	1.00000000	duck
Rreb1	-0.58	1.00000000	duck
Wdr44	-0.31	1.00000000	duck
Dnttip2	-0.38	1.00000000	duck
Kremen1	0.66	1.00000000	duck
Bach2	-0.07	1.00000000	duck
Sar1a	-0.23	1.00000000	duck
Smg5	0.24	1.00000000	duck
Lzic	0.17	1.00000000	duck
Casp3	0.22	1.00000000	duck
Psmb3	0.24	1.00000000	duck
Capg	0.42	1.00000000	duck
Ccnt2	-0.26	1.00000000	duck
Ufm1	0.36	1.00000000	duck
Ints2	-0.28	1.00000000	duck
Zmynd19	-0.10	1.00000000	duck
Cops2	-0.32	1.00000000	duck
Efcab14	0.29	1.00000000	duck
Fyb	-0.22	1.00000000	duck
Grsf1	-0.07	1.00000000	duck
Il7r	0.54	1.00000000	duck
Pank4	-0.12	1.00000000	duck
Zfp598	-0.09	1.00000000	duck
Nipbl	0.22	1.00000000	duck
Btbd10	-0.43	1.00000000	duck
Ralgapa1	-0.55	1.00000000	duck
Slc6a6	-0.43	1.00000000	duck
Wdr82	0.47	1.00000000	duck
Rassf2	0.40	1.00000000	duck
Elk3	0.28	1.00000000	duck
Zfp36l1	0.20	1.00000000	duck

Tmem167	0.33	1.00000000	duck
Dgke	-0.27	1.00000000	duck
Plxnc1	0.37	1.00000000	duck
Srfbp1	-0.30	1.00000000	duck
Cysltr1	0.60	1.00000000	duck
Septin9	-0.18	1.00000000	duck
Crim1	-0.60	1.00000000	duck
Gfm2	-0.16	1.00000000	duck
Clk1	-0.39	1.00000000	duck
Ctdspl2	-0.32	1.00000000	duck
Gfi1	0.39	1.00000000	duck
Cse1l	-0.16	1.00000000	duck
Fam167b	0.24	1.00000000	duck
Caprin1	-0.38	1.00000000	duck
Zfand3	0.50	1.00000000	duck
Scaf4	-0.31	1.00000000	duck
Psmd7	0.28	1.00000000	duck
Ranbp9	-0.49	1.00000000	duck
Qdpr	-0.32	1.00000000	duck
Stard13	0.28	1.00000000	duck
Tex10	-0.24	1.00000000	duck
Chchd3	-0.41	1.00000000	duck
Ctcf	-0.27	1.00000000	duck
Zfp407	-0.33	1.00000000	duck
Ikbke	0.25	1.00000000	duck
Dock1	0.40	1.00000000	duck
Pdpk1	-0.33	1.00000000	duck
Siva1	0.53	1.00000000	duck
Imp3	0.32	1.00000000	duck
Thoc1	-0.31	1.00000000	duck
Efr3a	-0.41	1.00000000	duck
Ammecr1	-0.23	1.00000000	duck
Plekha5	0.35	1.00000000	duck
Golph3	0.27	1.00000000	duck
Lonrf1	-0.64	1.00000000	duck
Setd1b	-0.34	1.00000000	duck
Ndst4	-1.06	1.00000000	duck
Chfr	0.19	1.00000000	duck
Timm44	-0.08	1.00000000	duck
Fan1	-0.14	1.00000000	duck
Tmem258	0.52	1.00000000	duck
Rexo1	-0.37	1.00000000	duck
Hsd11b2	0.59	1.00000000	duck
Hmbox1	-0.29	1.00000000	duck
Ikzf1	-0.21	1.00000000	duck
Arhgef12	-0.58	1.00000000	duck

Nufip1	-0.32	1.00000000	duck
St8sia1	0.26	1.00000000	duck
Whamm	-0.26	1.00000000	duck
Fnta	-0.15	1.00000000	duck
Actr10	-0.32	1.00000000	duck
Slc4a4	-0.67	1.00000000	duck
Plagl1	-0.20	1.00000000	duck
Zfyve28	0.22	1.00000000	duck
Alg2	0.30	1.00000000	duck
Emb	0.65	1.00000000	duck
Serinc5	-0.19	1.00000000	duck
Rnh1	0.27	1.00000000	duck
Tmem123	-0.18	1.00000000	duck
Asph	0.06	1.00000000	duck
Ttc13	-0.45	1.00000000	duck
Atp11b	-0.14	1.00000000	duck
Slc25a12	-0.58	1.00000000	duck
St8sia4	0.60	1.00000000	duck
Usp38	-0.42	1.00000000	duck
Pip5k1a	-0.23	1.00000000	duck
Usp12	-0.26	1.00000000	duck
Prelid1	0.48	1.00000000	duck
Ncoa3	-0.50	1.00000000	duck
Snap29	-0.28	1.00000000	duck
Gpr180	-0.51	1.00000000	duck
Fam117b	-0.25	1.00000000	duck
Ccr7	-0.23	1.00000000	duck
Evi5	-0.93	1.00000000	duck
Nuak1	0.53	1.00000000	duck
Noc3l	-0.34	1.00000000	duck
Bnip3l	-0.44	1.00000000	duck
Itpr1	-0.29	1.00000000	duck
Ankmy2	-0.27	1.00000000	duck
Cat	0.24	1.00000000	duck
Zyx	0.56	1.00000000	duck
Nipa2	-0.40	1.00000000	duck
Eif2ak3	0.00	1.00000000	duck
Plekha2	-0.08	1.00000000	duck
Cert1	0.32	1.00000000	duck
Ppif	0.26	1.00000000	duck
Prdm15	-0.30	1.00000000	duck
Pip5k1b	0.35	1.00000000	duck
Gak	-0.28	1.00000000	duck
Cacul1	-0.49	1.00000000	duck
Git2	-0.23	1.00000000	duck
Cept1	0.19	1.00000000	duck

Phka2	-0.12	1.00000000	duck
Cul3	-0.39	1.00000000	duck
Tef	-0.32	1.00000000	duck
Reep2	0.28	1.00000000	duck
Ipmk	-0.74	1.00000000	duck
Ypel5	0.39	1.00000000	duck
Bud31	0.34	1.00000000	duck
Cript	-0.29	1.00000000	duck
Hdgfl3	-0.06	1.00000000	duck
Agk	-0.20	1.00000000	duck
Tbck	-0.38	1.00000000	duck
Dcun1d3	-0.41	1.00000000	duck
Lclat1	-0.25	1.00000000	duck
DPCD	0.13	1.00000000	duck
Sec24b	-0.30	1.00000000	duck
Tbk1	-0.28	1.00000000	duck
Hmg20a	-0.20	1.00000000	duck
Rufy1	0.10	1.00000000	duck
Depdc1b	0.84	1.00000000	duck
Osbpl8	-0.37	1.00000000	duck
Gtf2h5	0.38	1.00000000	duck
Ndufa8	0.41	1.00000000	duck
Ing1	-0.18	1.00000000	duck
Dynlrb1	0.40	1.00000000	duck
Itfg1	0.36	1.00000000	duck
Hsf1	-0.16	1.00000000	duck
Slc25a36	0.34	1.00000000	duck
Gfm1	-0.07	1.00000000	duck
Sgpl1	0.42	1.00000000	duck
Eef1a1	0.19	1.00000000	duck
Secisbp2l	-0.52	1.00000000	duck
Gosr1	-0.41	1.00000000	duck
Lrrc45	-0.34	1.00000000	duck
Trp53bp1	-0.15	1.00000000	duck
Gpn3	-0.16	1.00000000	duck
Rab8b	0.10	1.00000000	duck
Dipk2a	-0.39	1.00000000	duck
Cep112	0.19	1.00000000	duck
Pgpep1	0.26	1.00000000	duck
Exoc2	-0.38	1.00000000	duck
Entpd5	-0.48	1.00000000	duck
Nol11	-0.29	1.00000000	duck
Wdr12	-0.21	1.00000000	duck
Cipc	-0.25	1.00000000	duck
Nisch	-0.47	1.00000000	duck
Atp6v1a	0.16	1.00000000	duck

Pde12	-0.05	1.00000000	duck
Tmc7	-0.23	1.00000000	duck
Cadm1	0.49	1.00000000	duck
Sf3a2	0.15	1.00000000	duck
L3mbtl3	-0.73	1.00000000	duck
Rell1	-0.19	1.00000000	duck
Rbms1	0.20	1.00000000	duck
Dnajb9	0.09	1.00000000	duck
Zfp622	-0.22	1.00000000	duck
Eepd1	-0.08	1.00000000	duck
Brix1	0.36	1.00000000	duck
Dip2c	0.40	1.00000000	duck
Slc31a1	-0.17	1.00000000	duck
Rap2a	0.43	1.00000000	duck
Dusp10	0.49	1.00000000	duck
Pphln1	-0.49	1.00000000	duck
Prkd3	-0.12	1.00000000	duck
Dnajb4	-0.47	1.00000000	duck
Abca2	-0.15	1.00000000	duck
Rbm39	0.14	1.00000000	duck
Rnaseh2b	-0.14	1.00000000	duck
Skil	-0.22	1.00000000	duck
Lias	-0.26	1.00000000	duck
Il17ra	0.34	1.00000000	duck
Hlcs	-0.40	1.00000000	duck
Ralgps1	-0.39	1.00000000	duck
Csl	-0.09	1.00000000	duck
Exoc7	-0.14	1.00000000	duck
Tm9sf3	0.36	1.00000000	duck
Ern1	0.06	1.00000000	duck
Lpar6	0.21	1.00000000	duck
Gtpbp1	-0.17	1.00000000	duck
Farsb	-0.12	1.00000000	duck
Wdr61	0.27	1.00000000	duck
Taf5l	-0.25	1.00000000	duck
Ptprk	0.14	1.00000000	duck
Lonrf3	0.19	1.00000000	duck
Tmem19	-0.14	1.00000000	duck
Rpap1	-0.33	1.00000000	duck
Atg2b	-0.25	1.00000000	duck
Immp1l	-0.14	1.00000000	duck
Psma5	0.50	1.00000000	duck
Plekha3	-0.11	1.00000000	duck
Erlin2	-0.32	1.00000000	duck
Dnajc1	-0.19	1.00000000	duck
Hgs	-0.25	1.00000000	duck

Dhdds	-0.30	1.00000000	duck
Rab3gap1	-0.14	1.00000000	duck
Gar1	-0.10	1.00000000	duck
Pld1	-0.54	1.00000000	duck
Dgkd	0.42	1.00000000	duck
Smc4	-0.23	1.00000000	duck
Zfp318	-0.25	1.00000000	duck
Polg2	0.43	1.00000000	duck
Naa60	0.30	1.00000000	duck
Chsy1	0.48	1.00000000	duck
Dync1i2	-0.35	1.00000000	duck
Hmox2	0.21	1.00000000	duck
Virma	-0.02	1.00000000	duck
Nfs1	-0.18	1.00000000	duck
Slc45a4	-0.31	1.00000000	duck
Vezf1	-0.45	1.00000000	duck
Stx11	0.50	1.00000000	duck
Wiz	-0.34	1.00000000	duck
Dclre1a	-0.28	1.00000000	duck
Nbr1	-0.31	1.00000000	duck
Eif4h	-0.24	1.00000000	duck
Fer	-0.38	1.00000000	duck
Pdcl3	-0.10	1.00000000	duck
Cep85l	-0.43	1.00000000	duck
Slc51a	-0.14	1.00000000	duck
Bcl7b	-0.24	1.00000000	duck
Eif4a3l1	0.28	1.00000000	duck
Hspa4	-0.21	1.00000000	duck
Trafd1	0.18	1.00000000	duck
Smap1	-0.20	1.00000000	duck
Rtn4	0.31	1.00000000	duck
Naxd	-0.21	1.00000000	duck
Glrx5	-0.33	1.00000000	duck
Smurf2	-0.33	1.00000000	duck
Ginm1	0.46	1.00000000	duck
Tor2a	-0.10	1.00000000	duck
Ints1	-0.12	1.00000000	duck
Ube3a	-0.21	1.00000000	duck
Ndufb1	0.44	1.00000000	duck
Skp1	0.00	1.00000000	duck
Ccr6	0.34	1.00000000	duck
Sos1	-0.59	1.00000000	duck
Papola	-0.38	1.00000000	duck
Snrpc	0.37	1.00000000	duck
Tle3	0.44	1.00000000	duck
Ppm1g	-0.09	1.00000000	duck

Cap1	0.33	1.00000000	duck
Kansl1	-0.37	1.00000000	duck
Arfrp1	-0.18	1.00000000	duck
Tmem65	-0.38	1.00000000	duck
Zbtb18	-0.09	1.00000000	duck
Nupl1	-0.27	1.00000000	duck
Fam102a	-0.12	1.00000000	duck
Ube4a	-0.14	1.00000000	duck
Fes	-0.15	1.00000000	duck
Add3	-0.23	1.00000000	duck
Rabgap1l	-0.28	1.00000000	duck
Tnrc6c	-0.20	1.00000000	duck
Dnajb2	0.29	1.00000000	duck
Aco1	0.29	1.00000000	duck
Cux1	-0.73	1.00000000	duck
Rars	-0.11	1.00000000	duck
Slc25a38	-0.18	1.00000000	duck
Ecpas	-0.53	1.00000000	duck
Edc4	-0.12	1.00000000	duck
Uba2	-0.19	1.00000000	duck
Hexim1	0.27	1.00000000	duck
Psmc2	0.41	1.00000000	duck
Cracr2a	-0.37	1.00000000	duck
Shprh	0.20	1.00000000	duck
Sgsm3	0.31	1.00000000	duck
Stk4	-0.31	1.00000000	duck
Serac1	0.37	1.00000000	duck
Bcas3	0.39	1.00000000	duck
Tti1	-0.21	1.00000000	duck
Atl2	-0.24	1.00000000	duck
Chek2	-0.24	1.00000000	duck
Phb	0.47	1.00000000	duck
Tnfaip8	-0.22	1.00000000	duck
Fmr1	-0.34	1.00000000	duck
Exosc2	-0.13	1.00000000	duck
Lactb	0.42	1.00000000	duck
Pak4	0.23	1.00000000	duck
Ppp2r5c	-0.51	1.00000000	duck
Arid4a	0.34	1.00000000	duck
Phlpp2	0.43	1.00000000	duck
Sugt1	-0.47	1.00000000	duck
Dynll2	0.09	1.00000000	duck
Dnmt1	-0.10	1.00000000	duck
Nat10	-0.18	1.00000000	duck
Fbxl4	-0.06	1.00000000	duck
Rasgrp1	-0.07	1.00000000	duck

Paip1	0.42	1.00000000	duck
Snrpd3	0.45	1.00000000	duck
Dmap1	-0.22	1.00000000	duck
Ctbp2	0.57	1.00000000	duck
Sin3a	-0.13	1.00000000	duck
Dennd4a	0.13	1.00000000	duck
Abhd17a	0.24	1.00000000	duck
Rufy2	-0.57	1.00000000	duck
Psip1	-0.17	1.00000000	duck
Hif1a	0.19	1.00000000	duck
Agps	-0.20	1.00000000	duck
Cpsf4	-0.07	1.00000000	duck
Nsa2	0.44	1.00000000	duck
Sdad1	-0.07	1.00000000	duck
Smarcad1	-0.32	1.00000000	duck
Atg16l1	-0.24	1.00000000	duck
Tubgcp5	-0.16	1.00000000	duck
Rlim	0.22	1.00000000	duck
Adipor2	0.31	1.00000000	duck
Ppm1d	0.17	1.00000000	duck
Gabarapl1	0.46	1.00000000	duck
Cacna1i	0.31	1.00000000	duck
Prmt9	-0.36	1.00000000	duck
Tcea1	-0.09	1.00000000	duck
Asb7	0.08	1.00000000	duck
Fam3c	0.23	1.00000000	duck
Iqcb1	-0.14	1.00000000	duck
Vps35l	-0.28	1.00000000	duck
Ylpm1	-0.36	1.00000000	duck
Zfp609	-0.29	1.00000000	duck
Srm	0.02	1.00000000	duck
Cramp1	-0.21	1.00000000	duck
Slc38a10	-0.09	1.00000000	duck
Dus1l	-0.25	1.00000000	duck
Sdk1	0.22	1.00000000	duck
Lcmt1	-0.13	1.00000000	duck
Znrf3	-0.27	1.00000000	duck
Rassf5	0.37	1.00000000	duck
Wdr3	-0.26	1.00000000	duck
Copa	-0.10	1.00000000	duck
Chpf2	0.20	1.00000000	duck
Tgs1	-0.15	1.00000000	duck
Usf3	-0.24	1.00000000	duck
Gng7	0.30	1.00000000	duck
Rock2	0.40	1.00000000	duck
Cnnm2	-0.55	1.00000000	duck

Tut4	-0.30	1.00000000	duck
Polr2e	0.35	1.00000000	duck
Arhgap17	-0.24	1.00000000	duck
Utp18	-0.14	1.00000000	duck
Krr1	0.25	1.00000000	duck
Acbd5	-0.22	1.00000000	duck
Uso1	-0.29	1.00000000	duck
Impa2	-0.05	1.00000000	duck
Otud4	-0.17	1.00000000	duck
Tnfsf13b	0.23	1.00000000	duck
Hes6	0.36	1.00000000	duck
Tox	0.41	1.00000000	duck
Vamp4	0.17	1.00000000	duck
Fam214a	0.43	1.00000000	duck
Lemd3	-0.14	1.00000000	duck
C2cd2	-0.58	1.00000000	duck
Etv3	0.30	1.00000000	duck
Ubqln1	0.06	1.00000000	duck
Fam168a	-0.47	1.00000000	duck
Ptp4a2	0.31	1.00000000	duck
Pnpt1	-0.08	1.00000000	duck
Grpel1	0.31	1.00000000	duck
Pdlim5	1.04	1.00000000	duck
Crbn	-0.34	1.00000000	duck
Slc7a3	0.41	1.00000000	duck
Bank1	0.53	1.00000000	duck
Nucks1	-0.12	1.00000000	duck
Glipr2	0.24	1.00000000	duck
Cpne1	-0.34	1.00000000	duck
Gbe1	0.12	1.00000000	duck
Scai	-0.32	1.00000000	duck
Gtf3c3	-0.19	1.00000000	duck
Hsf2	-0.17	1.00000000	duck
B3gnt5	-0.01	1.00000000	duck
Riok1	-0.24	1.00000000	duck
Pcmtd1	-0.61	1.00000000	duck
Fbxo22	-0.04	1.00000000	duck
Ehmt1	-0.17	1.00000000	duck
Hadha	-0.15	1.00000000	duck
Dlgap4	-0.15	1.00000000	duck
Smarca1	-0.14	1.00000000	duck
Nfatc3	-0.19	1.00000000	duck
Ttc17	-0.30	1.00000000	duck
Snorc	0.12	1.00000000	duck
Sf3b1	-0.27	1.00000000	duck
Chordc1	0.27	1.00000000	duck

Helq	0.01	1.00000000	duck
Lbr	-0.32	1.00000000	duck
Nhlrc2	-0.23	1.00000000	duck
Rest	0.16	1.00000000	duck
Dpm2	0.28	1.00000000	duck
Cope	0.37	1.00000000	duck
Tpm3-rs7	0.50	1.00000000	duck
Tmem203	0.31	1.00000000	duck
Slc40a1	0.17	1.00000000	duck
Nek7	-0.15	1.00000000	duck
Kdm3b	-0.32	1.00000000	duck
Rnf13	0.26	1.00000000	duck
Zfp326	-0.25	1.00000000	duck
Prpf8	-0.15	1.00000000	duck
Tpra1	0.18	1.00000000	duck
Ndufb4	0.36	1.00000000	duck
Stk17b	-0.24	1.00000000	duck
Spata7	-0.10	1.00000000	duck
Adat1	0.36	1.00000000	duck
Stau1	-0.16	1.00000000	duck
Eif3l	0.32	1.00000000	duck
Uba5	-0.09	1.00000000	duck
Zfp513	0.18	1.00000000	duck
Akap8	-0.06	1.00000000	duck
Ndufa10	-0.05	1.00000000	duck
Epc2	-0.36	1.00000000	duck
Rab5a	-0.22	1.00000000	duck
Ciapin1	-0.38	1.00000000	duck
Rdh10	0.31	1.00000000	duck
Aup1	0.34	1.00000000	duck
Gda	0.28	1.00000000	duck
Prr5l	0.46	1.00000000	duck
Slc35a1	0.34	1.00000000	duck
Eps15l1	-0.26	1.00000000	duck
Zfp292	-0.41	1.00000000	duck
Rfx2	0.39	1.00000000	duck
Hspa14	-0.13	1.00000000	duck
Dnajc11	-0.33	1.00000000	duck
Dync1li1	-0.15	1.00000000	duck
Parp8	0.26	1.00000000	duck
Ubn1	-0.25	1.00000000	duck
Rlf	-0.52	1.00000000	duck
Rtf1	-0.25	1.00000000	duck
Npc1	0.12	1.00000000	duck
Fchsd2	-0.16	1.00000000	duck
Acaca	-0.13	1.00000000	duck

Nubp1	-0.13	1.00000000	duck
Rbm19	-0.03	1.00000000	duck
Acat1	-0.14	1.00000000	duck
Tubgcp3	-0.08	1.00000000	duck
Armh3	-0.51	1.00000000	duck
Dyrk3	0.16	1.00000000	duck
Psmg4	0.26	1.00000000	duck
Narf	-0.44	1.00000000	duck
Khdrbs1	-0.09	1.00000000	duck
Lgals3	0.36	1.00000000	duck
Ppp4r3a	-0.29	1.00000000	duck
Arl3	0.20	1.00000000	duck
Zfp507	-0.26	1.00000000	duck
Bmpr2	-0.54	1.00000000	duck
Stim2	-0.06	1.00000000	duck
Cst7	0.41	1.00000000	duck
Eri3	-0.18	1.00000000	duck
Pigg	-0.25	1.00000000	duck
Uimc1	-0.34	1.00000000	duck
Adipor1	0.07	1.00000000	duck
Atxn10	-0.15	1.00000000	duck
Adsl	-0.17	1.00000000	duck
Rora	0.30	1.00000000	duck
Rala	-0.29	1.00000000	duck
Pex14	0.27	1.00000000	duck
Wars	-0.24	1.00000000	duck
Nfyc	-0.25	1.00000000	duck
Rubcnl	0.27	1.00000000	duck
Taf2	-0.25	1.00000000	duck
Arid5b	-0.24	1.00000000	duck
Arhgap6	0.74	1.00000000	duck
Zdhhc3	0.34	1.00000000	duck
Nup50	-0.30	1.00000000	duck
Prkag2	0.03	1.00000000	duck
Sh3gl3	-0.07	1.00000000	duck
Dennd5a	0.04	1.00000000	duck
Nde1	-0.06	1.00000000	duck
Bcl11a	-0.01	1.00000000	duck
Yeats4	0.24	1.00000000	duck
Eef2k	-0.11	1.00000000	duck
Antxr2	0.35	1.00000000	duck
Chmp4b	0.27	1.00000000	duck
Ubac1	-0.23	1.00000000	duck
Elp3	-0.09	1.00000000	duck
Slc25a46	-0.14	1.00000000	duck
Slc25a5	0.35	1.00000000	duck

Api5	-0.30	1.00000000	duck
Cnot1	-0.12	1.00000000	duck
Nr3c1	-0.01	1.00000000	duck
Cry2	0.25	1.00000000	duck
Ddx18	-0.11	1.00000000	duck
Psmf1	0.16	1.00000000	duck
Stim1	0.25	1.00000000	duck
Herc4	-0.32	1.00000000	duck
Yme1l1	0.58	1.00000000	duck
Proser1	-0.24	1.00000000	duck
Ube2g1	-0.33	1.00000000	duck
Gucd1	0.11	1.00000000	duck
Ippk	-0.10	1.00000000	duck
Katnbl1	-0.13	1.00000000	duck
Arntl2	0.04	1.00000000	duck
Zbtb38	0.30	1.00000000	duck
Dvl1	-0.15	1.00000000	duck
Lmnb2	0.55	1.00000000	duck
Fcho2	-0.07	1.00000000	duck
Runx2	0.53	1.00000000	duck
Supt3	-0.60	1.00000000	duck
Arl5a	-0.07	1.00000000	duck
Sod2	0.40	1.00000000	duck
Nup43	0.05	1.00000000	duck
Tnks2	-0.32	1.00000000	duck
Uvrag	-0.23	1.00000000	duck
Lonp1	-0.28	1.00000000	duck
Uxs1	-0.60	1.00000000	duck
Isca2	0.09	1.00000000	duck
Scrn3	-0.16	1.00000000	duck
Pgap1	0.18	1.00000000	duck
Taf5	-0.08	1.00000000	duck
Dph5	-0.26	1.00000000	duck
Tsn	-0.24	1.00000000	duck
Ncoa2	-0.40	1.00000000	duck
Pithd1	-0.17	1.00000000	duck
Nap1l4	-0.16	1.00000000	duck
Dock3	0.48	1.00000000	duck
Iffo2	0.35	1.00000000	duck
Lrch3	0.38	1.00000000	duck
Hsp90b1	-0.21	1.00000000	duck
Zdhhc9	0.24	1.00000000	duck
Bnip3	0.25	1.00000000	duck
Ankfy1	-0.31	1.00000000	duck
Tax1bp3	0.09	1.00000000	duck
Ctnnbl1	-0.11	1.00000000	duck

Psma4	-0.24	1.00000000	duck
Ppib	0.68	1.00000000	duck
Txnrd1	0.48	1.00000000	duck
Luc7l	-0.16	1.00000000	duck
Ldb1	-0.19	1.00000000	duck
Qars	-0.13	1.00000000	duck
Tube1	-0.29	1.00000000	duck
Pdcl	0.18	1.00000000	duck
Abraxas2	0.29	1.00000000	duck
Ufd1	-0.05	1.00000000	duck
Lpar5	0.31	1.00000000	duck
Spag9	-0.04	1.00000000	duck
Ipo9	-0.28	1.00000000	duck
Adprh	-0.14	1.00000000	duck
Fos	0.82	1.00000000	duck
Fam204a	0.33	1.00000000	duck
Gcc2	-0.26	1.00000000	duck
Iars2	-0.23	1.00000000	duck
Odr4	-0.10	1.00000000	duck
Ahcyl1	-0.11	1.00000000	duck
Acadl	0.38	1.00000000	duck
Zfyve26	-0.01	1.00000000	duck
Lin54	-0.35	1.00000000	duck
Scfd1	-0.33	1.00000000	duck
Cnot8	0.18	1.00000000	duck
Dis3	-0.14	1.00000000	duck
Psmd13	0.33	1.00000000	duck
Hace1	-0.14	1.00000000	duck
Ago3	-0.09	1.00000000	duck
Brwd3	-0.52	1.00000000	duck
Dnajc7	0.21	1.00000000	duck
Pcyt2	0.03	1.00000000	duck
Hdac7	0.51	1.00000000	duck
Atg14	-0.10	1.00000000	duck
Sort1	0.25	1.00000000	duck
Wipi2	-0.38	1.00000000	duck
Ralgapb	-0.18	1.00000000	duck
Abcc8	-0.03	1.00000000	duck
Zcchc2	-0.10	1.00000000	duck
Apoo	-0.10	1.00000000	duck
Ptpa	0.18	1.00000000	duck
Clstn1	0.14	1.00000000	duck
Hdac2	-0.17	1.00000000	duck
Dync1li2	-0.27	1.00000000	duck
Fem1c	0.15	1.00000000	duck
Etf1	-0.06	1.00000000	duck

Bnip2	-0.41	1.00000000	duck
Irf4	0.47	1.00000000	duck
Alk	-0.10	1.00000000	duck
Ube2b	0.20	1.00000000	duck
Herpud2	-0.19	1.00000000	duck
Sec61a1	0.18	1.00000000	duck
Acvr1b	0.34	1.00000000	duck
Kif2a	-0.07	1.00000000	duck
Srf	0.21	1.00000000	duck
Rpusd2	-0.03	1.00000000	duck
Ndufs1	-0.25	1.00000000	duck
Ythdf1	0.01	1.00000000	duck
Arl8b	0.17	1.00000000	duck
Immp2l	0.39	1.00000000	duck
Ing3	-0.23	1.00000000	duck
Rae1	-0.47	1.00000000	duck
Caml	-0.11	1.00000000	duck
Bak1	-0.03	1.00000000	duck
Papss1	0.48	1.00000000	duck
Sgsm2	-0.17	1.00000000	duck
Esco1	-0.41	1.00000000	duck
Nrf1	-0.18	1.00000000	duck
Cep83	-0.18	1.00000000	duck
Ywhag	0.22	1.00000000	duck
Ythdf3	-0.38	1.00000000	duck
Tpk1	-0.25	1.00000000	duck
Stxbp5	-0.56	1.00000000	duck
Pacsin2	0.20	1.00000000	duck
Ube2d1	-0.07	1.00000000	duck
Etfrf1	-0.14	1.00000000	duck
Nup155	-0.09	1.00000000	duck
Ctbp1	-0.20	1.00000000	duck
Ash2l	-0.15	1.00000000	duck
Pi4kb	-0.27	1.00000000	duck
Hacd2	-0.24	1.00000000	duck
Lcor	-0.20	1.00000000	duck
Copg2	-0.34	1.00000000	duck
Nlk	-0.48	1.00000000	duck
Washc4	-0.13	1.00000000	duck
Smim20	0.24	1.00000000	duck
Adam17	-0.14	1.00000000	duck
Plrg1	0.10	1.00000000	duck
Unk	-0.17	1.00000000	duck
Dcp2	0.26	1.00000000	duck
Rabepk	-0.12	1.00000000	duck
Usp14	-0.19	1.00000000	duck

Ptger3	0.18	1.00000000	duck
Cmss1	-0.11	1.00000000	duck
Ywhaz	0.18	1.00000000	duck
Usp4	-0.16	1.00000000	duck
Aatf	-0.05	1.00000000	duck
Ddx31	-0.23	1.00000000	duck
Pmvk	0.30	1.00000000	duck
Trappc8	-0.23	1.00000000	duck
Snu13	0.30	1.00000000	duck
P4ha1	-0.31	1.00000000	duck
Sec11a	0.38	1.00000000	duck
Sf3a3	-0.18	1.00000000	duck
Aff1	-0.25	1.00000000	duck
Tent4b	-0.06	1.00000000	duck
Sacs	-0.13	1.00000000	duck
Cflar	-0.38	1.00000000	duck
Osbpl9	0.14	1.00000000	duck
Ncor2	0.72	1.00000000	duck
Rasgef1a	0.14	1.00000000	duck
Emc2	-0.08	1.00000000	duck
Ipo8	-0.19	1.00000000	duck
Ddx1	-0.13	1.00000000	duck
Bzw1	-0.23	1.00000000	duck
Czib	-0.20	1.00000000	duck
Per3	-0.25	1.00000000	duck
Cul2	-0.15	1.00000000	duck
Lemd2	-0.10	1.00000000	duck
Actn4	0.20	1.00000000	duck
Rabgap1	0.01	1.00000000	duck
Pdcd5	-0.30	1.00000000	duck
Slu7	-0.29	1.00000000	duck
Tmem222	0.22	1.00000000	duck
Fam76a	0.22	1.00000000	duck
Bsg	0.28	1.00000000	duck
Snrpa1	0.02	1.00000000	duck
Rhobtb2	-0.04	1.00000000	duck
Fancm	0.05	1.00000000	duck
Tmed5	0.63	1.00000000	duck
Smcr8	-0.02	1.00000000	duck
Uggt1	-0.30	1.00000000	duck
Ubl7	0.17	1.00000000	duck
Zc3h7b	-0.19	1.00000000	duck
Bptf	-0.32	1.00000000	duck
Fbxo32	-0.31	1.00000000	duck
Uhrf1	-0.09	1.00000000	duck
Recql	0.18	1.00000000	duck

Trdmt1	-0.22	1.00000000	duck
Fh1	-0.13	1.00000000	duck
Ebna1bp2	-0.09	1.00000000	duck
Grk2	0.04	1.00000000	duck
Ppil3	0.19	1.00000000	duck
Naa16	-0.38	1.00000000	duck
Syap1	0.18	1.00000000	duck
Thoc7	0.13	1.00000000	duck
Larp7	-0.06	1.00000000	duck
Areg	0.49	1.00000000	duck
Tbpl1	-0.16	1.00000000	duck
Susd1	-0.41	1.00000000	duck
Ankrd11	0.17	1.00000000	duck
Utp6	-0.10	1.00000000	duck
Ldah	0.26	1.00000000	duck
Rai2	0.28	1.00000000	duck
Timp2	0.38	1.00000000	duck
Rgs1	0.37	1.00000000	duck
Polr1c	-0.08	1.00000000	duck
Hibadh	0.26	1.00000000	duck
Nt5c3	-0.07	1.00000000	duck
Otud7a	-0.03	1.00000000	duck
Zc3h14	-0.18	1.00000000	duck
Ptpn12	0.11	1.00000000	duck
Fem1a	0.06	1.00000000	duck
Dapk3	0.41	1.00000000	duck
Wasl	0.31	1.00000000	duck
Dele1	-0.03	1.00000000	duck
Commd8	0.22	1.00000000	duck
Psmd6	0.19	1.00000000	duck
Hyou1	0.00	1.00000000	duck
Nelfcd	0.20	1.00000000	duck
Prepl	0.16	1.00000000	duck
Disc1	0.36	1.00000000	duck
Exosc9	-0.04	1.00000000	duck
Cish	-0.41	1.00000000	duck
Btf3l4	0.33	1.00000000	duck
Basp1	0.07	1.00000000	duck
Trnt1	-0.19	1.00000000	duck
Homer1	0.30	1.00000000	duck
Cpq	-0.08	1.00000000	duck
Vps53	-0.06	1.00000000	duck
Grk4	-0.22	1.00000000	duck
Sin3b	-0.15	1.00000000	duck
Fbxw2	-0.12	1.00000000	duck
Pitrm1	-0.09	1.00000000	duck

Ip6k1	-0.17	1.00000000	duck
Dip2a	-0.30	1.00000000	duck
Klf9	0.12	1.00000000	duck
Bud23	0.04	1.00000000	duck
Eif2a	-0.20	1.00000000	duck
Acot7	0.24	1.00000000	duck
Ppp2cb	-0.10	1.00000000	duck
Slc35b1	0.16	1.00000000	duck
Jade3	-0.24	1.00000000	duck
Rbm6	-0.13	1.00000000	duck
Rbbp7	-0.10	1.00000000	duck
Itm2b	0.49	1.00000000	duck
Atg5	-0.48	1.00000000	duck
Spast	-0.34	1.00000000	duck
Wbp4	-0.64	1.00000000	duck
Slc2a8	-0.03	1.00000000	duck
Ptpn11	-0.18	1.00000000	duck
Ppara	0.25	1.00000000	duck
Tent2	-0.13	1.00000000	duck
Ssbp1	0.28	1.00000000	duck
Snrpb	0.41	1.00000000	duck
Sdf2l1	0.26	1.00000000	duck
Rab2a	0.26	1.00000000	duck
Hnrnpd	0.25	1.00000000	duck
Unc119b	0.19	1.00000000	duck
Wdtc1	-0.32	1.00000000	duck
Eftud2	-0.06	1.00000000	duck
Stk38l	-0.11	1.00000000	duck
Ccser2	0.01	1.00000000	duck
Desi1	-0.03	1.00000000	duck
Eif2s1	-0.12	1.00000000	duck
Pigk	-0.06	1.00000000	duck
Lsm8	0.19	1.00000000	duck
Pisd	-0.08	1.00000000	duck
Htatsf1	-0.36	1.00000000	duck
Plpbp	-0.11	1.00000000	duck
Tmem242	0.33	1.00000000	duck
Otulinl	-0.14	1.00000000	duck
Cotl1	0.44	1.00000000	duck
Cdc37l1	-0.44	1.00000000	duck
Cwc15	0.03	1.00000000	duck
Trappc9	-0.33	1.00000000	duck
Pfdn4	0.24	1.00000000	duck
Ankrd17	-0.17	1.00000000	duck
Tmem183a	0.03	1.00000000	duck
Spopl	0.11	1.00000000	duck

Slf2	-0.26	1.00000000	duck
Nr1d2	0.35	1.00000000	duck
Kdm7a	-0.09	1.00000000	duck
Nlrc5	-0.11	1.00000000	duck
Carhsp1	-0.16	1.00000000	duck
Gnb1	0.24	1.00000000	duck
Ncoa6	-0.17	1.00000000	duck
Katnip	0.17	1.00000000	duck
Tacc3	-0.45	1.00000000	duck
Gramd4	0.34	1.00000000	duck
Gan	-0.19	1.00000000	duck
Lmbr1	-0.28	1.00000000	duck
Rbbp5	-0.20	1.00000000	duck
Otud7b	-0.22	1.00000000	duck
Cog7	0.25	1.00000000	duck
Pccb	-0.08	1.00000000	duck
Pbdc1	-0.14	1.00000000	duck
Hivep3	0.45	1.00000000	duck
Rpia	0.15	1.00000000	duck
Zbtb37	-0.33	1.00000000	duck
Tpm1	0.03	1.00000000	duck
Dnaja1	0.34	1.00000000	duck
Rmc1	-0.21	1.00000000	duck
Atp6v1h	0.16	1.00000000	duck
Sash3	0.20	1.00000000	duck
Wdr37	0.54	1.00000000	duck
Tmem161b	-0.29	1.00000000	duck
Acadm	0.23	1.00000000	duck
Hif1an	-0.10	1.00000000	duck
Acbd3	0.34	1.00000000	duck
Slc1a4	0.24	1.00000000	duck
Bin2	0.37	1.00000000	duck
Ankrd54	-0.13	1.00000000	duck
Nr2c1	-0.18	1.00000000	duck
Vps45	-0.05	1.00000000	duck
Atic	-0.07	1.00000000	duck
Pinx1	-0.23	1.00000000	duck
Tusc2	0.22	1.00000000	duck
Il6st	-0.05	1.00000000	duck
Bbx	-0.23	1.00000000	duck
Sft2d2	-0.06	1.00000000	duck
Parp11	0.31	1.00000000	duck
Xylt1	-0.67	1.00000000	duck
Smc3	-0.03	1.00000000	duck
Agfg1	0.20	1.00000000	duck
Slc38a1	0.05	1.00000000	duck

Cdk12	-0.09	1.00000000	duck
Ankrd13c	0.02	1.00000000	duck
Ssrp1	0.31	1.00000000	duck
Jarid2	0.29	1.00000000	duck
Snrnp200	0.04	1.00000000	duck
Epb41l2	0.43	1.00000000	duck
Brd3	-0.21	1.00000000	duck
Glyr1	-0.14	1.00000000	duck
Nek9	-0.06	1.00000000	duck
Pex13	0.19	1.00000000	duck
Nrp2	0.43	1.00000000	duck
Caap1	0.13	1.00000000	duck
Sbno2	0.39	1.00000000	duck
Cdc26	-0.01	1.00000000	duck
Tdg	0.13	1.00000000	duck
Selenop	0.30	1.00000000	duck
Wipi1	0.05	1.00000000	duck
Chaf1a	-0.05	1.00000000	duck
Lamtor2	0.23	1.00000000	duck
Entpd4	-0.16	1.00000000	duck
Crlf3	-0.16	1.00000000	duck
Zcchc7	0.25	1.00000000	duck
Tk2	-0.12	1.00000000	duck
Plpp1	0.26	1.00000000	duck
Rnpepl1	-0.12	1.00000000	duck
Gnai3	-0.21	1.00000000	duck
Flii	0.30	1.00000000	duck
Anapc5	0.19	1.00000000	duck
Hemk1	-0.21	1.00000000	duck
Fbxo25	-0.35	1.00000000	duck
Ube2a	0.20	1.00000000	duck
Tshz2	0.31	1.00000000	duck
Eif3d	0.28	1.00000000	duck
Nup205	-0.11	1.00000000	duck
Nop2	-0.01	1.00000000	duck
Gdi2	0.28	1.00000000	duck
Bend6	0.40	1.00000000	duck
Ttc19	0.35	1.00000000	duck
Srgap3	-0.34	1.00000000	duck
Upb1	-0.15	1.00000000	duck
Exosc10	0.00	1.00000000	duck
Selenoo	-0.62	1.00000000	duck
Supt6	-0.12	1.00000000	duck
Pdcd10	0.18	1.00000000	duck
Smyd3	-0.32	1.00000000	duck
Npepps	-0.18	1.00000000	duck

Utp20	-0.15	1.00000000	duck
Commd1b	0.17	1.00000000	duck
Lmo2	-0.57	1.00000000	duck
Hypk	0.28	1.00000000	duck
Bcl2	0.49	1.00000000	duck
Gid8	-0.26	1.00000000	duck
Osbpl2	-0.20	1.00000000	duck
Phkb	-0.43	1.00000000	duck
Pdzd8	-0.28	1.00000000	duck
Nptn	0.26	1.00000000	duck
Prkx	0.22	1.00000000	duck
Tnpo1	0.14	1.00000000	duck
Cdc42se2	0.43	1.00000000	duck
Smarcb1	-0.15	1.00000000	duck
Elp2	-0.06	1.00000000	duck
Ttc27	-0.13	1.00000000	duck
Ccnt1	-0.06	1.00000000	duck
Cfap36	-0.10	1.00000000	duck
Commd6	-0.11	1.00000000	duck
Lsm7	0.35	1.00000000	duck
Kctd10	0.15	1.00000000	duck
Gng2	0.01	1.00000000	duck
Tnfrsf21	-0.21	1.00000000	duck
Rab11fip1	0.18	1.00000000	duck
Rnf217	0.15	1.00000000	duck
B4galt1	0.25	1.00000000	duck
Srsf5	-0.21	1.00000000	duck
Cep120	-0.08	1.00000000	duck
Trappc4	0.20	1.00000000	duck
Fam91a1	-0.20	1.00000000	duck
Pum1	-0.14	1.00000000	duck
Ugcg	0.17	1.00000000	duck
Fnbp1l	0.32	1.00000000	duck
Vps13d	-0.30	1.00000000	duck
Ppp2r5d	-0.08	1.00000000	duck
Trim8	0.37	1.00000000	duck
Btbd6	0.08	1.00000000	duck
Psmd14	-0.16	1.00000000	duck
Capn2	0.39	1.00000000	duck
Kdelr2	0.28	1.00000000	duck
Rangap1	0.32	1.00000000	duck
Pigt	0.18	1.00000000	duck
Nup133	-0.34	1.00000000	duck
Nrbp1	-0.30	1.00000000	duck
Nedd4l	-0.13	1.00000000	duck
Appl1	-0.05	1.00000000	duck

Dag1	0.00	1.00000000	duck
Zdhhc17	-0.27	1.00000000	duck
Lmtk2	-0.46	1.00000000	duck
Ints10	-0.03	1.00000000	duck
Gpatch2	-0.22	1.00000000	duck
Ecd	-0.31	1.00000000	duck
Tbl3	-0.13	1.00000000	duck
Cactin	-0.04	1.00000000	duck
Tmem41b	0.10	1.00000000	duck
Spata2	-0.15	1.00000000	duck
Polr2b	-0.07	1.00000000	duck
Lrp3	0.11	1.00000000	duck
Natd1	-0.06	1.00000000	duck
Ext1	-0.34	1.00000000	duck
Stxbp1	0.33	1.00000000	duck
Taok1	0.16	1.00000000	duck
Adarb1	0.17	1.00000000	duck
Nop14	0.37	1.00000000	duck
Bcas2	0.31	1.00000000	duck
Lrrcc1	0.01	1.00000000	duck
Hectd4	-0.24	1.00000000	duck
Reck	-0.08	1.00000000	duck
Paxip1	-0.17	1.00000000	duck
Cdk6	-0.04	1.00000000	duck
Dcp1b	0.09	1.00000000	duck
Sde2	-0.40	1.00000000	duck
Rab1a	0.25	1.00000000	duck
Ppp1cb	0.07	1.00000000	duck
Ndufs7	0.27	1.00000000	duck
Ap3m1	-0.06	1.00000000	duck
Dhx36	-0.13	1.00000000	duck
Ttc7	0.31	1.00000000	duck
Selenok	0.12	1.00000000	duck
Gpr107	-0.12	1.00000000	duck
Rnf10	-0.22	1.00000000	duck
Nutf2	0.07	1.00000000	duck
Canx	-0.19	1.00000000	duck
Trip11	-0.57	1.00000000	duck
Ddx20	0.11	1.00000000	duck
Pitpnm1	0.14	1.00000000	duck
Tmem86a	0.18	1.00000000	duck
Pcnx4	-0.12	1.00000000	duck
Snx17	0.21	1.00000000	duck
Ubqln4	0.22	1.00000000	duck
Slc39a6	0.08	1.00000000	duck
Klhdc4	-0.08	1.00000000	duck

Tsr1	0.17	1.00000000	duck
Pcmt1	-0.05	1.00000000	duck
Cop1	0.19	1.00000000	duck
Ei24	0.33	1.00000000	duck
Gper1	-0.17	1.00000000	duck
Triap1	0.24	1.00000000	duck
Ppp3ca	-0.01	1.00000000	duck
Uri1	-0.10	1.00000000	duck
Scamp1	-0.02	1.00000000	duck
Lyrm7	0.04	1.00000000	duck
Vps8	0.04	1.00000000	duck
Bcl2l13	-0.23	1.00000000	duck
Tmem245	0.23	1.00000000	duck
Cbx1	-0.04	1.00000000	duck
Dnajb6	0.17	1.00000000	duck
Lrrc8d	0.29	1.00000000	duck
Fundc1	-0.24	1.00000000	duck
Leprot	0.26	1.00000000	duck
Scaper	-0.17	1.00000000	duck
Snx12	0.24	1.00000000	duck
Kmt2e	-0.08	1.00000000	duck
Cdpf1	-0.16	1.00000000	duck
Sh3kbp1	0.09	1.00000000	duck
Slc44a2	-0.04	1.00000000	duck
Ccnh	0.14	1.00000000	duck
Stk10	0.25	1.00000000	duck
Usp8	0.10	1.00000000	duck
Ssb	0.07	1.00000000	duck
Cers5	0.10	1.00000000	duck
Tmem11	-0.10	1.00000000	duck
Crkl	0.20	1.00000000	duck
Ankrd44	-0.02	1.00000000	duck
Setdb1	-0.13	1.00000000	duck
Tada2b	0.23	1.00000000	duck
Igf2bp2	0.00	1.00000000	duck
Fam13b	0.35	1.00000000	duck
Wasf2	-0.06	1.00000000	duck
Zbtb10	0.22	1.00000000	duck
Stub1	-0.27	1.00000000	duck
Cbfb	0.33	1.00000000	duck
Hnrnpm	-0.17	1.00000000	duck
Snx9	-0.02	1.00000000	duck
Tm9sf4	0.05	1.00000000	duck
Sufu	-0.42	1.00000000	duck
Haus2	0.11	1.00000000	duck
Suz12	-0.19	1.00000000	duck

Gtf2a1	-0.44	1.00000000	duck
Rassf3	0.19	1.00000000	duck
Ubfd1	-0.33	1.00000000	duck
Vps11	-0.07	1.00000000	duck
Cpeb2	-0.49	1.00000000	duck
Chd1	0.14	1.00000000	duck
Tomm20	-0.12	1.00000000	duck
Golga5	-0.22	1.00000000	duck
Lims2	0.24	1.00000000	duck
Celf1	-0.20	1.00000000	duck
Ipo11	-0.30	1.00000000	duck
Hsd17b12	0.16	1.00000000	duck
Senp6	-0.16	1.00000000	duck
Nelfb	0.01	1.00000000	duck
P4hb	-0.08	1.00000000	duck
Nub1	-0.02	1.00000000	duck
Atrn	0.21	1.00000000	duck
Nr4a2	0.71	1.00000000	duck
Ppp6c	0.23	1.00000000	duck
Exosc3	0.17	1.00000000	duck
Pcif1	-0.05	1.00000000	duck
Upf1	0.24	1.00000000	duck
Saraf	0.16	1.00000000	duck
Slc7a6os	-0.16	1.00000000	duck
Cct3	-0.17	1.00000000	duck
Ube2v2	-0.28	1.00000000	duck
Ergic2	-0.15	1.00000000	duck
Bmpr1a	0.31	1.00000000	duck
4933400A11Rik	0.03	1.00000000	duck
Pak2	0.23	1.00000000	duck
Hprt	-0.37	1.00000000	duck
Ghitm	-0.31	1.00000000	duck
Txnrd3	0.30	1.00000000	duck
Rab21	-0.26	1.00000000	duck
Klhl12	0.15	1.00000000	duck
Aig1	0.12	1.00000000	duck
Xpot	-0.12	1.00000000	duck
Atp11a	-0.27	1.00000000	duck
Timm17a	-0.07	1.00000000	duck
Tmf1	-0.20	1.00000000	duck
Chmp2b	0.19	1.00000000	duck
Hmgb2	0.36	1.00000000	duck
Gpbp1l1	-0.18	1.00000000	duck
Runx1	-0.10	1.00000000	duck
Ube2i	0.24	1.00000000	duck
Ncln	0.15	1.00000000	duck

Dtx3l	0.08	1.00000000	duck
Klf12	-0.10	1.00000000	duck
Klhdc1	0.27	1.00000000	duck
Deaf1	-0.19	1.00000000	duck
Cct4	0.14	1.00000000	duck
Saal1	-0.04	1.00000000	duck
Atf1	-0.12	1.00000000	duck
Hp1bp3	-0.10	1.00000000	duck
Dbf4	-0.10	1.00000000	duck
Pgam5	-0.05	1.00000000	duck
Ruvbl1	0.02	1.00000000	duck
Stat4	0.00	1.00000000	duck
Slc30a4	0.25	1.00000000	duck
Fbxw8	-0.01	1.00000000	duck
Drg1	-0.13	1.00000000	duck
Atp13a3	0.28	1.00000000	duck
Synrg	-0.02	1.00000000	duck
Atp1b3	0.23	1.00000000	duck
Sf3b3	0.05	1.00000000	duck
Bach1	0.20	1.00000000	duck
Arxes2	0.18	1.00000000	duck
Cnst	0.53	1.00000000	duck
Ypel1	0.44	1.00000000	duck
Zfr	-0.23	1.00000000	duck
Supv3l1	-0.01	1.00000000	duck
Rit1	-0.28	1.00000000	duck
Ap2b1	-0.03	1.00000000	duck
Braf	-0.33	1.00000000	duck
Dcp1a	0.16	1.00000000	duck
Creb1	-0.12	1.00000000	duck
Ankra2	0.01	1.00000000	duck
Cdc40	-0.21	1.00000000	duck
Dnajb14	0.00	1.00000000	duck
D5Ertd579e	-0.59	1.00000000	duck
Kctd20	-0.41	1.00000000	duck
Lars2	-0.05	1.00000000	duck
Zcrb1	-0.04	1.00000000	duck
Ubash3a	0.05	1.00000000	duck
Psmd10	-0.08	1.00000000	duck
Tns3	0.09	1.00000000	duck
Ddb1	-0.29	1.00000000	duck
Pten	0.11	1.00000000	duck
Dhx29	0.13	1.00000000	duck
Wsb2	0.22	1.00000000	duck
Zer1	-0.09	1.00000000	duck
Chic2	-0.23	1.00000000	duck

Yars	0.19	1.00000000	duck
Zcchc10	0.04	1.00000000	duck
Capzb	0.27	1.00000000	duck
Camta1	-0.09	1.00000000	duck
Clip1	0.19	1.00000000	duck
Bin3	0.34	1.00000000	duck
Dennd4c	0.25	1.00000000	duck
Rnps1	0.11	1.00000000	duck
St6galnac4	0.23	1.00000000	duck
Cwc25	0.30	1.00000000	duck
Slc7a5	0.15	1.00000000	duck
Abhd2	-0.02	1.00000000	duck
Birc6	-0.05	1.00000000	duck
Fbxo33	-0.12	1.00000000	duck
Tra2b	0.28	1.00000000	duck
Cops7b	-0.07	1.00000000	duck
Klhl7	-0.64	1.00000000	duck
Atp1a1	0.04	1.00000000	duck
Pex7	-0.02	1.00000000	duck
Ccdc186	-0.13	1.00000000	duck
Fam114a2	-0.12	1.00000000	duck
Smim19	0.32	1.00000000	duck
Ube4b	-0.30	1.00000000	duck
Irf2	-0.12	1.00000000	duck
Adora2a	-0.18	1.00000000	duck
Poli	0.11	1.00000000	duck
Ubxn7	-0.13	1.00000000	duck
Usp1	-0.11	1.00000000	duck
Slc27a1	-0.09	1.00000000	duck
Sumo1	0.21	1.00000000	duck
Rab3gap2	-0.20	1.00000000	duck
Ptgfrn	0.34	1.00000000	duck
Zfand4	-0.31	1.00000000	duck
Ndufs6	0.14	1.00000000	duck
Rcor1	-0.24	1.00000000	duck
Zfand6	-0.37	1.00000000	duck
Atxn7l2	-0.01	1.00000000	duck
Dhx38	-0.12	1.00000000	duck
Ahdc1	0.10	1.00000000	duck
Aco2	-0.06	1.00000000	duck
Rmnd1	-0.15	1.00000000	duck
Syf2	0.19	1.00000000	duck
Hspa5	-0.15	1.00000000	duck
Abi2	0.32	1.00000000	duck
Ddx47	0.03	1.00000000	duck
Fndc3a	-0.15	1.00000000	duck

Pitpnm2	0.18	1.00000000	duck
Fars2	0.01	1.00000000	duck
Foxo4	-0.22	1.00000000	duck
Erap1	0.13	1.00000000	duck
Ldlrad3	0.21	1.00000000	duck
Kdm2a	-0.10	1.00000000	duck
Eprs	-0.12	1.00000000	duck
Hells	-0.03	1.00000000	duck
Ccnk	-0.11	1.00000000	duck
Rnf6	-0.23	1.00000000	duck
Gab1	0.11	1.00000000	duck
Ugp2	0.17	1.00000000	duck
Dok2	0.11	1.00000000	duck
Tfcp2	0.03	1.00000000	duck
Camk2g	-0.09	1.00000000	duck
Gtf2a2	0.24	1.00000000	duck
Rab35	-0.05	1.00000000	duck
Nek1	-0.22	1.00000000	duck
Cab39	-0.04	1.00000000	duck
Fam126a	-0.35	1.00000000	duck
Nom1	-0.14	1.00000000	duck
Aebp2	0.12	1.00000000	duck
Impact	0.34	1.00000000	duck
Nsmce2	0.06	1.00000000	duck
Swi5	0.17	1.00000000	duck
Tada1	-0.15	1.00000000	duck
Nt5dc1	-0.28	1.00000000	duck
Lin52	0.14	1.00000000	duck
Vps36	0.05	1.00000000	duck
Fam117a	0.03	1.00000000	duck
Taf10	0.23	1.00000000	duck
Tspan2	0.03	1.00000000	duck
Tfip11	-0.20	1.00000000	duck
Jmjd8	0.21	1.00000000	duck
Galc	-0.01	1.00000000	duck
Slc15a4	0.13	1.00000000	duck
Csnk1a1	0.09	1.00000000	duck
Zfp438	-0.63	1.00000000	duck
Bicd2	0.27	1.00000000	duck
Vapb	0.04	1.00000000	duck
Dcaf5	-0.34	1.00000000	duck
Usp10	0.05	1.00000000	duck
Derl1	0.20	1.00000000	duck
Ubap2	0.36	1.00000000	duck
Dcps	0.03	1.00000000	duck
Hook3	-0.15	1.00000000	duck

Snx20	0.32	1.00000000	duck
Gnpnat1	0.23	1.00000000	duck
Uap1l1	0.11	1.00000000	duck
Siah1b	-0.36	1.00000000	duck
Rnf130	0.00	1.00000000	duck
St6gal1	-0.12	1.00000000	duck
Selenoi	-0.01	1.00000000	duck
Fubp1	-0.16	1.00000000	duck
Psmc1	0.16	1.00000000	duck
Trio	0.46	1.00000000	duck
Ppp2r3c	-0.11	1.00000000	duck
Borcs6	0.35	1.00000000	duck
Atp6v0a2	0.27	1.00000000	duck
Adk	-0.51	1.00000000	duck
Ints11	0.15	1.00000000	duck
B4galt5	0.15	1.00000000	duck
Eif4e	-0.14	1.00000000	duck
Rb1cc1	-0.38	1.00000000	duck
Setd3	-0.31	1.00000000	duck
Dpy30	0.21	1.00000000	duck
Gpr65	0.12	1.00000000	duck
Kat7	0.03	1.00000000	duck
Ubl3	0.51	1.00000000	duck
Dnajc5	0.05	1.00000000	duck
Ppm1f	-0.16	1.00000000	duck
Actr6	0.14	1.00000000	duck
Tspan12	-0.04	1.00000000	duck
Rabl3	-0.02	1.00000000	duck
Arl1	0.17	1.00000000	duck
Nudt3	0.15	1.00000000	duck
Tcp1	-0.05	1.00000000	duck
Tpm4	0.11	1.00000000	duck
Prkaa1	0.08	1.00000000	duck
Rwdd1	-0.09	1.00000000	duck
Scamp2	0.15	1.00000000	duck
Pou2f1	-0.29	1.00000000	duck
Rbbp6	-0.29	1.00000000	duck
Tmem268	0.04	1.00000000	duck
Pcnx2	0.18	1.00000000	duck
Phb2	0.21	1.00000000	duck
Clk4	0.25	1.00000000	duck
Dtnbp1	-0.41	1.00000000	duck
Qk	-0.03	1.00000000	duck
Rtraf	0.05	1.00000000	duck
Dcun1d1	-0.27	1.00000000	duck
Tmco1	0.30	1.00000000	duck

2700049A03Rik	0.12	1.00000000	duck
Snrpb2	-0.06	1.00000000	duck
Farp2	0.07	1.00000000	duck
Zfp654	-0.23	1.00000000	duck
Tfdp2	-0.21	1.00000000	duck
Arntl	0.20	1.00000000	duck
Radx	0.10	1.00000000	duck
Scoc	-0.10	1.00000000	duck
Adgrv1	0.13	1.00000000	duck
Ap1m1	-0.15	1.00000000	duck
Ep300	-0.01	1.00000000	duck
Gpr68	0.30	1.00000000	duck
Pelo	-0.03	1.00000000	duck
Hsbp1	0.17	1.00000000	duck
Atad3a	-0.01	1.00000000	duck
Alcam	0.83	1.00000000	duck
Sft2d1	0.25	1.00000000	duck
Erh	0.31	1.00000000	duck
Polr1e	0.19	1.00000000	duck
Lman1	0.04	1.00000000	duck
Ankle2	-0.03	1.00000000	duck
Tcf4	0.27	1.00000000	duck
Acad9	0.11	1.00000000	duck
Zcchc8	-0.20	1.00000000	duck
Cyria	0.09	1.00000000	duck
Prex1	0.04	1.00000000	duck
Usp15	-0.15	1.00000000	duck
Kat6a	-0.17	1.00000000	duck
Ndrg3	-0.17	1.00000000	duck
Nf2	-0.07	1.00000000	duck
Acbd6	-0.15	1.00000000	duck
Puf60	0.14	1.00000000	duck
Tlk2	-0.02	1.00000000	duck
Phf5a	0.20	1.00000000	duck
Xpo5	0.05	1.00000000	duck
Fam219a	0.07	1.00000000	duck
Ripk1	0.18	1.00000000	duck
Arf4	-0.07	1.00000000	duck
Zfyve1	-0.26	1.00000000	duck
Pik3ap1	0.36	1.00000000	duck
E2f6	-0.10	1.00000000	duck
Ints4	-0.12	1.00000000	duck
Tspan5	0.20	1.00000000	duck
Ccdc174	-0.02	1.00000000	duck
Zfat	-0.27	1.00000000	duck
Tiprl	-0.02	1.00000000	duck

Agl	-0.26	1.00000000	duck
Gpr137c	0.03	1.00000000	duck
Eif3j2	0.13	1.00000000	duck
Polr3h	0.14	1.00000000	duck
Ubtd2	0.05	1.00000000	duck
Ibtk	-0.27	1.00000000	duck
Rasal2	0.36	1.00000000	duck
Cbx3	0.01	1.00000000	duck
Lrrc59	0.04	1.00000000	duck
Pno1	0.07	1.00000000	duck
Atp6v0d1	0.18	1.00000000	duck
Cnot11	-0.11	1.00000000	duck
Zfp831	-0.18	1.00000000	duck
Cstf3	0.04	1.00000000	duck
Arhgef7	-0.22	1.00000000	duck
Nol9	0.35	1.00000000	duck
Ubac2	0.45	1.00000000	duck
Smc6	-0.25	1.00000000	duck
Usp47	-0.12	1.00000000	duck
Pogz	-0.20	1.00000000	duck
Fkbp3	-0.07	1.00000000	duck
Sf3a1	-0.03	1.00000000	duck
Ptbp1	-0.01	1.00000000	duck
Cyld	-0.23	1.00000000	duck
Naa35	-0.09	1.00000000	duck
Psmd12	0.08	1.00000000	duck
Foxk1	-0.14	1.00000000	duck
Fbxo34	-0.02	1.00000000	duck
Zfp639	-0.01	1.00000000	duck
Acox1	0.28	1.00000000	duck
Ctnnb1	0.34	1.00000000	duck
Pik3c3	0.02	1.00000000	duck
Trappc12	-0.25	1.00000000	duck
Rbpj	-0.04	1.00000000	duck
Lamtor5	0.35	1.00000000	duck
Derl2	0.00	1.00000000	duck
Sec23a	-0.12	1.00000000	duck
Ndufa4	0.35	1.00000000	duck
Scp2	-0.13	1.00000000	duck
Ift74	0.35	1.00000000	duck
Stx18	-0.26	1.00000000	duck
Pla2g15	0.27	1.00000000	duck
Akap10	-0.13	1.00000000	duck
Rftn1	0.00	1.00000000	duck
Nfyb	0.25	1.00000000	duck
Ppp1cc	0.00	1.00000000	duck

Cmtr1	-0.11	1.00000000	duck
Nsrp1	-0.10	1.00000000	duck
Fam114a1	0.22	1.00000000	duck
Pard3b	0.34	1.00000000	duck
Stx6	0.25	1.00000000	duck
Hnrnpa2b1	-0.15	1.00000000	duck
Fnbp1	-0.05	1.00000000	duck
Txlna	-0.09	1.00000000	duck
Vps72	0.21	1.00000000	duck
Carnmt1	0.09	1.00000000	duck
Csnk1g2	-0.08	1.00000000	duck
Ndufb9	0.20	1.00000000	duck
Trank1	0.02	1.00000000	duck
Dut	0.19	1.00000000	duck
Cwc27	-0.37	1.00000000	duck
Pag1	0.18	1.00000000	duck
Gfod1	0.28	1.00000000	duck
Kif5b	-0.30	1.00000000	duck
Psmc6	-0.06	1.00000000	duck
Wbp2	0.05	1.00000000	duck
Sod1	0.27	1.00000000	duck
Ktn1	0.18	1.00000000	duck
Litaf	0.21	1.00000000	duck
Fam162a	0.13	1.00000000	duck
As3mt	0.32	1.00000000	duck
Rsbn1l	0.04	1.00000000	duck
Xpr1	-0.42	1.00000000	duck
Zdhhc7	-0.25	1.00000000	duck
Bsdc1	0.06	1.00000000	duck
Tmed2	0.22	1.00000000	duck
Ttc28	0.34	1.00000000	duck
Zbtb8os	0.12	1.00000000	duck
Ppp1r8	-0.02	1.00000000	duck
Tln2	-0.05	1.00000000	duck
Pin1	0.18	1.00000000	duck
Zbtb44	-0.42	1.00000000	duck
Ascc2	0.02	1.00000000	duck
Sel1l3	0.42	1.00000000	duck
Cnot10	0.00	1.00000000	duck
Babam2	0.05	1.00000000	duck
Lrch1	-0.19	1.00000000	duck
Ubap1	0.13	1.00000000	duck
Casp8ap2	-0.11	1.00000000	duck
Appl2	-0.05	1.00000000	duck
Rnf214	-0.12	1.00000000	duck
Cpm	0.17	1.00000000	duck

Cpeb3	-0.52	1.00000000	duck
Prkar1a	0.12	1.00000000	duck
Cep20	-0.12	1.00000000	duck
Ncbp1	-0.07	1.00000000	duck
Nup214	0.02	1.00000000	duck
Vps41	-0.09	1.00000000	duck
Arfgap2	-0.05	1.00000000	duck
Samm50	-0.16	1.00000000	duck
Vps35	-0.12	1.00000000	duck
Ctsh	0.02	1.00000000	duck
Tes	0.14	1.00000000	duck
Tatdn1	0.11	1.00000000	duck
Srp72	0.02	1.00000000	duck
Zbtb21	-0.03	1.00000000	duck
Rnf168	0.06	1.00000000	duck
Vps18	0.13	1.00000000	duck
Poldip2	0.07	1.00000000	duck
Fam199x	-0.12	1.00000000	duck
Golt1b	0.05	1.00000000	duck
Kdsr	-0.04	1.00000000	duck
Zc3h18	-0.02	1.00000000	duck
Rnf220	0.13	1.00000000	duck
Tmem70	-0.13	1.00000000	duck
Lct	-0.09	1.00000000	duck
Sart3	-0.09	1.00000000	duck
Eif4e2	0.06	1.00000000	duck
Gpi1	0.26	1.00000000	duck
Thumpd3	0.23	1.00000000	duck
Thyn1	0.38	1.00000000	duck
Tmem241	0.09	1.00000000	duck
Zrsr1	-0.28	1.00000000	duck
Klf7	0.41	1.00000000	duck
Camsap1	-0.05	1.00000000	duck
Cdca4	0.13	1.00000000	duck
Aven	-0.18	1.00000000	duck
Tango2	0.04	1.00000000	duck
Cops5	-0.20	1.00000000	duck
Slc12a2	-0.16	1.00000000	duck
Ndufv2	0.15	1.00000000	duck
Ttc37	-0.30	1.00000000	duck
Arid3b	-0.07	1.00000000	duck
Vta1	-0.06	1.00000000	duck
Ogfr	0.23	1.00000000	duck
Psmg3	0.01	1.00000000	duck
Arhgef6	0.39	1.00000000	duck
Tmx4	-0.09	1.00000000	duck

Scrib	0.20	1.00000000	duck
Lpin2	-0.29	1.00000000	duck
Exoc6	0.20	1.00000000	duck
Shisa5	0.21	1.00000000	duck
Surf6	0.23	1.00000000	duck
Ccdc47	-0.13	1.00000000	duck
Dpysl2	0.18	1.00000000	duck
Bcap29	-0.22	1.00000000	duck
Atg12	0.19	1.00000000	duck
Akap8l	-0.11	1.00000000	duck
Atf6	0.02	1.00000000	duck
Cir1	-0.41	1.00000000	duck
Etnk1	-0.11	1.00000000	duck
Tln1	0.08	1.00000000	duck
C2cd2l	0.17	1.00000000	duck
Acot10	-0.20	1.00000000	duck
Leprotl1	0.02	1.00000000	duck
Klhdc2	0.09	1.00000000	duck
Kdm5a	-0.15	1.00000000	duck
Ap3m2	0.05	1.00000000	duck
St13	0.00	1.00000000	duck
Tmcc1	-0.48	1.00000000	duck
Scyl2	0.08	1.00000000	duck
Tut7	0.02	1.00000000	duck
Kcnq5	0.10	1.00000000	duck
Btbd1	-0.07	1.00000000	duck
Lsm14a	0.02	1.00000000	duck
Slc44a1	0.10	1.00000000	duck
Slc35e1	0.14	1.00000000	duck
Papss2	0.11	1.00000000	duck
Eef1d	0.14	1.00000000	duck
Themis	-0.16	1.00000000	duck
Sec22a	0.05	1.00000000	duck
Psen1	-0.09	1.00000000	duck
Emc3	0.32	1.00000000	duck
Zpr1	-0.01	1.00000000	duck
Bcl7a	-0.09	1.00000000	duck
Nmd3	-0.01	1.00000000	duck
Ppard	0.22	1.00000000	duck
Brms1l	-0.02	1.00000000	duck
Nol7	0.08	1.00000000	duck
Snx4	-0.06	1.00000000	duck
Kif1b	-0.02	1.00000000	duck
Rilpl2	0.41	1.00000000	duck
Lrp8	-0.52	1.00000000	duck
Prpf4	-0.06	1.00000000	duck

Pdia4	0.15	1.00000000	duck
Cltc	-0.08	1.00000000	duck
Rictor	0.08	1.00000000	duck
Gsn	0.20	1.00000000	duck
Smarca2	0.03	1.00000000	duck
Hmgb3	0.12	1.00000000	duck
Ankrd13a	0.01	1.00000000	duck
Hnrnph3	-0.06	1.00000000	duck
Usp34	-0.17	1.00000000	duck
Spats2	0.26	1.00000000	duck
Gns	-0.15	1.00000000	duck
Abcd2	-0.09	1.00000000	duck
Bola3	0.11	1.00000000	duck
Sap130	-0.06	1.00000000	duck
Hadh	0.15	1.00000000	duck
Tmtc3	-0.27	1.00000000	duck
Prpf40a	-0.14	1.00000000	duck
Lsg1	0.06	1.00000000	duck
Eri1	0.05	1.00000000	duck
Limd2	0.16	1.00000000	duck
Nav3	0.06	1.00000000	duck
Smad5	0.17	1.00000000	duck
Sppl3	0.28	1.00000000	duck
Chmp5	0.05	1.00000000	duck
Eif3m	-0.08	1.00000000	duck
Hecw2	0.31	1.00000000	duck
Tfam	0.12	1.00000000	duck
Spg11	-0.09	1.00000000	duck
Sec11c	0.11	1.00000000	duck
Nfya	-0.29	1.00000000	duck
Lnpk	0.01	1.00000000	duck
Pola1	0.25	1.00000000	duck
Reep3	-0.13	1.00000000	duck
Cxcr6	0.16	1.00000000	duck
Jmy	0.07	1.00000000	duck
Purb	0.29	1.00000000	duck
Ncoa1	0.24	1.00000000	duck
Polr2c	0.14	1.00000000	duck
Tbc1d1	0.02	1.00000000	duck
Srp9	0.06	1.00000000	duck
Ppp1r16b	0.16	1.00000000	duck
Fzr1	-0.11	1.00000000	duck
Bzw2	-0.06	1.00000000	duck
Rragc	0.13	1.00000000	duck
Fbxl20	-0.02	1.00000000	duck
Ywhae	0.20	1.00000000	duck

Usp20	0.02	1.00000000	duck
Ing4	-0.08	1.00000000	duck
Plcb2	-0.10	1.00000000	duck
Susd3	-0.06	1.00000000	duck
Lmbrd2	0.18	1.00000000	duck
Phactr4	0.25	1.00000000	duck
Ccdc93	-0.08	1.00000000	duck
Senp5	-0.01	1.00000000	duck
Atp5pb	0.14	1.00000000	duck
Commd2	0.03	1.00000000	duck
Pafah1b1	-0.15	1.00000000	duck
Ccng1	0.33	1.00000000	duck
Hspa2	-0.15	1.00000000	duck
Itgb2l	-0.04	1.00000000	duck
Dgkz	0.11	1.00000000	duck
Cd74	0.65	1.00000000	duck
Wdr77	0.14	1.00000000	duck
Snap23	-0.04	1.00000000	duck
Smap2	0.03	1.00000000	duck
Yap1	0.06	1.00000000	duck
Yipf5	0.18	1.00000000	duck
Kif13a	0.22	1.00000000	duck
Lpin1	0.06	1.00000000	duck
Hectd1	0.15	1.00000000	duck
Rap2c	0.10	1.00000000	duck
Tsc22d3	0.13	1.00000000	duck
Rpa2	-0.01	1.00000000	duck
Palb2	-0.09	1.00000000	duck
Trabd	0.18	1.00000000	duck
Usp33	-0.30	1.00000000	duck
Rad51ap1	0.16	1.00000000	duck
Sox5	0.25	1.00000000	duck
Sntb1	0.30	1.00000000	duck
Ankrd46	0.05	1.00000000	duck
Sap30	0.10	1.00000000	duck
Ube2j2	-0.09	1.00000000	duck
Atg4c	-0.28	1.00000000	duck
Rfx3	0.11	1.00000000	duck
Elp1	0.01	1.00000000	duck
Llph	0.09	1.00000000	duck
Stx8	-0.15	1.00000000	duck
Polk	-0.36	1.00000000	duck
Frs2	-0.07	1.00000000	duck
Ccdc127	0.10	1.00000000	duck
Thoc5	0.28	1.00000000	duck
Pnrc2	0.07	1.00000000	duck

Tesk2	-0.11	1.00000000	duck
Sec63	0.00	1.00000000	duck
Gabpb1	-0.18	1.00000000	duck
Gnas	-0.29	1.00000000	duck
Cdkn1b	0.14	1.00000000	duck
Arfgef2	0.05	1.00000000	duck
Psmg2	-0.12	1.00000000	duck
Tmed10	0.13	1.00000000	duck
Vdac2	0.16	1.00000000	duck
Abat	0.26	1.00000000	duck
Btg1b	0.17	1.00000000	duck
Adam19	0.06	1.00000000	duck
Denr	0.22	1.00000000	duck
Ociad1	-0.03	1.00000000	duck
Suox	-0.37	1.00000000	duck
Spg21	0.08	1.00000000	duck
Hnrnpa0	0.16	1.00000000	duck
Tnfsf8	0.10	1.00000000	duck
Nedd4	0.21	1.00000000	duck
Rab10	-0.04	1.00000000	duck
Npepl1	-0.07	1.00000000	duck
Arhgap29	-0.23	1.00000000	duck
Kin	-0.17	1.00000000	duck
Ell	-0.14	1.00000000	duck
Pepd	-0.04	1.00000000	duck
Traf3ip3	-0.33	1.00000000	duck
Ino80d	-0.06	1.00000000	duck
Slc48a1	-0.07	1.00000000	duck
Smndc1	0.28	1.00000000	duck
Naa25	0.04	1.00000000	duck
Rbm22	0.07	1.00000000	duck
Pde6d	0.07	1.00000000	duck
Rpf2	-0.09	1.00000000	duck
Smurf1	0.02	1.00000000	duck
Abcc5	-0.01	1.00000000	duck
Cbl	0.20	1.00000000	duck
Wwp1	-0.08	1.00000000	duck
Tmem208	0.12	1.00000000	duck
Zbtb2	0.05	1.00000000	duck
Snx1	-0.17	1.00000000	duck
Ndufs3	0.24	1.00000000	duck
Akt1	0.13	1.00000000	duck
Cct8	0.20	1.00000000	duck
Igf2bp3	0.06	1.00000000	duck
Gnl2	0.10	1.00000000	duck
Gpd2	-0.19	1.00000000	duck

Bcor	0.10	1.00000000	duck
Gyg	-0.11	1.00000000	duck
Surf4	-0.06	1.00000000	duck
Bub3	0.09	1.00000000	duck
Fdx1	-0.07	1.00000000	duck
Ets1	0.03	1.00000000	duck
Itpk1	-0.08	1.00000000	duck
Fyttd1	-0.20	1.00000000	duck
Ubash3b	0.54	1.00000000	duck
Fig4	0.04	1.00000000	duck
Slc9a5	-0.03	1.00000000	duck
Emc7	-0.13	1.00000000	duck
Slc39a11	-0.02	1.00000000	duck
Nfkb1	-0.04	1.00000000	duck
Uchl5	0.13	1.00000000	duck
Cdc42bpb	-0.03	1.00000000	duck
Ptpn1	-0.02	1.00000000	duck
Rapgef2	0.44	1.00000000	duck
Cd151	0.14	1.00000000	duck
Tle1	-0.07	1.00000000	duck
Decr2	0.12	1.00000000	duck
Dhx30	0.10	1.00000000	duck
Gpbp1	-0.02	1.00000000	duck
Sirt7	-0.02	1.00000000	duck
N4bp2	0.11	1.00000000	duck
Cfl2	-0.20	1.00000000	duck
Snx13	-0.01	1.00000000	duck
Vti1b	0.13	1.00000000	duck
Snw1	0.10	1.00000000	duck
Ccni	0.21	1.00000000	duck
Pikfyve	-0.35	1.00000000	duck
Rbm34	0.03	1.00000000	duck
Vamp3	0.07	1.00000000	duck
Pik3cd	0.19	1.00000000	duck
Urm1	0.23	1.00000000	duck
Dnajc3	0.12	1.00000000	duck
Nkrf	0.10	1.00000000	duck
Ppip5k2	-0.04	1.00000000	duck
Hddc2	0.29	1.00000000	duck
Kif13b	0.09	1.00000000	duck
Nr3c2	0.55	1.00000000	duck
Cgrrf1	0.02	1.00000000	duck
Vps26a	0.06	1.00000000	duck
Cyfip2	-0.08	1.00000000	duck
Zfp131	0.09	1.00000000	duck
Fbxl14	0.14	1.00000000	duck

Pkn2	-0.08	1.00000000	duck
Als2	0.10	1.00000000	duck
Strada	0.05	1.00000000	duck
Stag1	-0.02	1.00000000	duck
Nup107	-0.07	1.00000000	duck
Cisd1	0.06	1.00000000	duck
Slc9a8	-0.02	1.00000000	duck
Ralbp1	-0.05	1.00000000	duck
Larp4b	-0.27	1.00000000	duck
Setd7	-0.07	1.00000000	duck
Rnf185	0.05	1.00000000	duck
Cc2d2a	0.01	1.00000000	duck
Kmt5b	-0.18	1.00000000	duck
Cstf2	0.16	1.00000000	duck
Ccdc117	-0.47	1.00000000	duck
Pdcd6	-0.03	1.00000000	duck
Rad51b	-0.02	1.00000000	duck
Pop4	0.07	1.00000000	duck
Dtymk	0.14	1.00000000	duck
Fez2	-0.09	1.00000000	duck
Txndc17	0.13	1.00000000	duck
Glod4	0.18	1.00000000	duck
Txnl1	-0.17	1.00000000	duck
Katna1	0.19	1.00000000	duck
Pik3r6	0.08	1.00000000	duck
Cd47	0.14	1.00000000	duck
Dctn4	0.00	1.00000000	duck
Zbtb7a	0.17	1.00000000	duck
Gclc	-0.22	1.00000000	duck
Cops4	-0.05	1.00000000	duck
Stt3a	0.25	1.00000000	duck
Abhd17c	-0.13	1.00000000	duck
Zcchc14	0.04	1.00000000	duck
Pwp1	0.11	1.00000000	duck
Exog	0.10	1.00000000	duck
Cpne3	-0.16	1.00000000	duck
Dpp9	-0.06	1.00000000	duck
Plekhj1	0.16	1.00000000	duck
Prpf38a	0.02	1.00000000	duck
Slc30a9	-0.35	1.00000000	duck
Uck2	0.07	1.00000000	duck
Arhgef3	0.05	1.00000000	duck
Trpm7	-0.05	1.00000000	duck
Sppl2a	0.01	1.00000000	duck
Lcp2	0.09	1.00000000	duck
Eif2s2	0.11	1.00000000	duck

Ddx6	-0.09	1.00000000	duck
Gfpt1	0.07	1.00000000	duck
Fndc3b	0.13	1.00000000	duck
Ddost	0.20	1.00000000	duck
Arl6ip4	0.15	1.00000000	duck
Stk38	-0.01	1.00000000	duck
Gapvd1	-0.35	1.00000000	duck
Ciao2a	0.07	1.00000000	duck
Ccne1	0.23	1.00000000	duck
Jmjd1c	-0.04	1.00000000	duck
2510039O18Rik	-0.04	1.00000000	duck
Cnih4	0.02	1.00000000	duck
Spg7	-0.24	1.00000000	duck
Garre1	-0.04	1.00000000	duck
Gse1	0.08	1.00000000	duck
Tbc1d12	-0.22	1.00000000	duck
Trerf1	0.02	1.00000000	duck
Gars	0.17	1.00000000	duck
Atxn1l	0.10	1.00000000	duck
Prdx4	0.04	1.00000000	duck
Scmh1	0.04	1.00000000	duck
Pbx1	0.33	1.00000000	duck
Tbc1d9b	0.03	1.00000000	duck
Gorasp2	0.05	1.00000000	duck
Wsb1	-0.07	1.00000000	duck
Ero1b	0.01	1.00000000	duck
Ryk	0.07	1.00000000	duck
Pdss1	0.10	1.00000000	duck
Qrich1	0.00	1.00000000	duck
Prpf6	-0.22	1.00000000	duck
Ddx23	0.00	1.00000000	duck
Fhod1	0.21	1.00000000	duck
Arhgap15	0.13	1.00000000	duck
Ccnl1	0.06	1.00000000	duck
Rbm17	-0.24	1.00000000	duck
Nelfa	-0.15	1.00000000	duck
Hmgn3	0.36	1.00000000	duck
Ppwd1	0.06	1.00000000	duck
Eif3i	0.24	1.00000000	duck
Uhrf1bp1l	0.22	1.00000000	duck
Zfp36l2	0.16	1.00000000	duck
Ppa1	-0.10	1.00000000	duck
Bcl2l1	0.01	1.00000000	duck
Slc49a4	0.21	1.00000000	duck
Adar	0.13	1.00000000	duck
Nus1	0.24	1.00000000	duck

Slc16a3	0.32	1.00000000	duck
Srp68	0.03	1.00000000	duck
Lpcat1	0.01	1.00000000	duck
Rassf8	-0.05	1.00000000	duck
Erich6	0.00	1.00000000	duck
Haus3	0.07	1.00000000	duck
Pqlc3	0.13	1.00000000	duck
Tasp1	0.06	1.00000000	duck
Cops8	-0.07	1.00000000	duck
Appbp2	-0.05	1.00000000	duck
Clint1	-0.01	1.00000000	duck
Ube2r2	0.00	1.00000000	duck
Cars	0.00	1.00000000	duck
Exosc7	-0.06	1.00000000	duck
Prkcq	0.47	1.00000000	duck
Rfc5	0.19	1.00000000	duck
Zswim5	0.36	1.00000000	duck
Tspan3	-0.07	1.00000000	duck
Smyd2	-0.02	1.00000000	duck
Erlin1	-0.06	1.00000000	duck
Sertad2	-0.01	1.00000000	duck
Coq2	-0.04	1.00000000	duck
Svil	0.31	1.00000000	duck
Tcf25	0.24	1.00000000	duck
Pdxk	0.11	1.00000000	duck
Sos2	-0.24	1.00000000	duck
Copb2	-0.04	1.00000000	duck
Ttc33	0.14	1.00000000	duck
Osbpl3	-0.03	1.00000000	duck
Spcs2	0.17	1.00000000	duck
Selenof	0.02	1.00000000	duck
Gnptg	0.21	1.00000000	duck
Dctn5	0.06	1.00000000	duck
Crls1	0.09	1.00000000	duck
Erp29	0.20	1.00000000	duck
Nr1h3	-0.08	1.00000000	duck
Snx14	-0.08	1.00000000	duck
Fem1b	0.04	1.00000000	duck
Ranbp1	0.13	1.00000000	duck
Vps4b	-0.08	1.00000000	duck
Iqgap1	0.14	1.00000000	duck
Zmat2	0.16	1.00000000	duck
Ddx55	0.15	1.00000000	duck
Rnf20	0.17	1.00000000	duck
Brd7	0.01	1.00000000	duck
Cct5	0.05	1.00000000	duck

Cass4	0.10	1.00000000	duck
Asns	0.28	1.00000000	duck
Hint1	0.31	1.00000000	duck
Lyar	0.22	1.00000000	duck
Tmem50a	0.13	1.00000000	duck
Arcn1	0.04	1.00000000	duck
Ybx3	0.07	1.00000000	duck
Kars	0.19	1.00000000	duck
Hps3	-0.03	1.00000000	duck
Get4	0.09	1.00000000	duck
Stk11	0.17	1.00000000	duck
Zfp618	-0.35	1.00000000	duck
Cbx6	0.12	1.00000000	duck
Polr2k	0.07	1.00000000	duck
Eif5b	0.14	1.00000000	duck
Tmem30a	-0.09	1.00000000	duck
Abtb1	-0.16	1.00000000	duck
Pdia3	0.15	1.00000000	duck
Kpna3	0.18	1.00000000	duck
Stx17	0.26	1.00000000	duck
Pmm2	0.15	1.00000000	duck
Hip1	0.10	1.00000000	duck
Ubn2	0.02	1.00000000	duck
Ptpn9	-0.06	1.00000000	duck
Uchl3	-0.04	1.00000000	duck
Otulin	0.04	1.00000000	duck
Cnep1r1	-0.14	1.00000000	duck
Tmx3	0.07	1.00000000	duck
Fbxo30	-0.23	1.00000000	duck
Hs6st3	-0.46	1.00000000	duck
Kctd2	-0.02	1.00000000	duck
Slc7a6	0.34	1.00000000	duck
Tasor	0.14	1.00000000	duck
Rnf145	0.04	1.00000000	duck
Pank2	0.03	1.00000000	duck
Lrp12	0.06	1.00000000	duck
Tmem33	0.09	1.00000000	duck
Pde10a	0.52	1.00000000	duck
Chst7	0.24	1.00000000	duck
Tfg	0.04	1.00000000	duck
Ccdc6	0.12	1.00000000	duck
Zfp143	0.22	1.00000000	duck
Creb3l2	-0.03	1.00000000	duck
Thumpd2	0.06	1.00000000	duck
Plekhm2	0.24	1.00000000	duck
Sfmbt1	0.17	1.00000000	duck

Rnf4	-0.09	1.00000000	duck
Nars	0.09	1.00000000	duck
Abcd3	0.13	1.00000000	duck
Lrrc8a	0.04	1.00000000	duck
Ube3c	0.10	1.00000000	duck
Siah2	0.08	1.00000000	duck
Rrm2b	0.07	1.00000000	duck
Ikbkb	0.15	1.00000000	duck
Elk4	0.06	1.00000000	duck
Sema4d	0.37	1.00000000	duck
Ube2e2	-0.01	1.00000000	duck
Tmem231	-0.01	1.00000000	duck
Wdr48	0.16	1.00000000	duck
Cry1	0.52	1.00000000	duck
Idh3a	0.20	1.00000000	duck
Rcc2	0.09	1.00000000	duck
Ino80c	0.15	1.00000000	duck
Btg2	0.05	1.00000000	duck
Pgs1	0.22	1.00000000	duck
Foxj2	-0.03	1.00000000	duck
Pnpla8	-0.40	1.00000000	duck
Txndc9	-0.39	1.00000000	duck
Ppm1l	0.10	1.00000000	duck
Hsph1	0.12	1.00000000	duck
Fam193a	-0.22	1.00000000	duck
Lsm3	0.17	1.00000000	duck
Rexo4	0.08	1.00000000	duck
Phtf2	0.16	1.00000000	duck
Arl5b	-0.05	1.00000000	duck
Amd2	0.24	1.00000000	duck
Dhx8	0.22	1.00000000	duck
Isca1	0.03	1.00000000	duck
Abraxas1	-0.06	1.00000000	duck
Ice2	-0.18	1.00000000	duck
Nono	0.23	1.00000000	duck
Asxl1	-0.12	1.00000000	duck
Rrp7a	0.03	1.00000000	duck
Zc3h15	0.02	1.00000000	duck
Actn1	0.28	1.00000000	duck
Spata5	0.01	1.00000000	duck
Dnajc16	-0.01	1.00000000	duck
Tpd52l2	0.05	1.00000000	duck
Cdk8	-0.19	1.00000000	duck
Cfap20	0.13	1.00000000	duck
Ap3d1	-0.14	1.00000000	duck
Galnt7	0.13	1.00000000	duck

Kdm2b	0.09	1.00000000	duck
Tmem230	0.20	1.00000000	duck
Rsl24d1	0.11	1.00000000	duck
Rpf1	-0.05	1.00000000	duck
Pdhx	0.03	1.00000000	duck
Ankrd10	-0.15	1.00000000	duck
Aurkaip1	0.16	1.00000000	duck
Rbm12b2	-0.20	1.00000000	duck
Rchy1	0.03	1.00000000	duck
Gpalpp1	0.03	1.00000000	duck
Dnaja3	0.12	1.00000000	duck
Stau2	-0.06	1.00000000	duck
Asxl2	-0.18	1.00000000	duck
Relch	-0.23	1.00000000	duck
Helz	-0.25	1.00000000	duck
Nedd9	-0.07	1.00000000	duck
Wdr91	-0.03	1.00000000	duck
Suclg1	0.11	1.00000000	duck
Psmd1	-0.19	1.00000000	duck
Cpsf2	0.07	1.00000000	duck
Klhdc10	0.02	1.00000000	duck
Crk	-0.05	1.00000000	duck
Nob1	-0.04	1.00000000	duck
Dlg1	-0.36	1.00000000	duck
Susd6	-0.02	1.00000000	duck
Tsr3	0.02	1.00000000	duck
Omg	0.10	1.00000000	duck
Vps54	0.12	1.00000000	duck
Sephs1	0.10	1.00000000	duck
Rad21	0.03	1.00000000	duck
Tmbim6	0.09	1.00000000	duck
Zcchc17	0.20	1.00000000	duck
Utp3	0.18	1.00000000	duck
Cnih1	0.20	1.00000000	duck
Cdyl2	-0.02	1.00000000	duck
Zc3h12b	0.08	1.00000000	duck
Arl4a	0.36	1.00000000	duck
Bclaf3	0.04	1.00000000	duck
Crnkl1	0.02	1.00000000	duck
Aftph	-0.25	1.00000000	duck
Fbxo9	-0.05	1.00000000	duck
Rab39b	0.05	1.00000000	duck
Eef1b2	0.02	1.00000000	duck
St3gal3	0.10	1.00000000	duck
Phf21a	0.01	1.00000000	duck
B4galt4	0.05	1.00000000	duck

Zmpste24	-0.11	1.00000000	duck
Cpeb4	-0.33	1.00000000	duck
Tmem185a	0.08	1.00000000	duck
Iws1	0.01	1.00000000	duck
Rprd1a	-0.05	1.00000000	duck
Rbis	0.18	1.00000000	duck
Nip7	0.05	1.00000000	duck
Pdcd2	-0.01	1.00000000	duck
Eif4g3	-0.14	1.00000000	duck
Syne3	0.13	1.00000000	duck
Stx2	-0.14	1.00000000	duck
Cdv3	-0.04	1.00000000	duck
Sergef	0.34	1.00000000	duck
Sec62	0.03	1.00000000	duck
Ranbp3	-0.20	1.00000000	duck
Pi4ka	-0.01	1.00000000	duck
Ell2	0.14	1.00000000	duck
Ccdc59	-0.06	1.00000000	duck
Usp7	-0.07	1.00000000	duck
Kpnb1	0.12	1.00000000	duck
Nsmaf	-0.03	1.00000000	duck
Ogdh	0.08	1.00000000	duck
Ints12	0.07	1.00000000	duck
Clcc1	0.02	1.00000000	duck
Hscb	0.07	1.00000000	duck
Cops3	-0.30	1.00000000	duck
Vdac3	0.03	1.00000000	duck
Hsd17b4	-0.04	1.00000000	duck
Sec31a	0.07	1.00000000	duck
Cdk7	0.06	1.00000000	duck
Ccpg1	-0.26	1.00000000	duck
Rap1gds1	-0.01	1.00000000	duck
Rpn2	0.08	1.00000000	duck
Agpat3	0.27	1.00000000	duck
Rhot1	0.16	1.00000000	duck
Ikzf5	-0.09	1.00000000	duck
Dcaf12	0.00	1.00000000	duck
Rad18	0.10	1.00000000	duck
Angptl2	0.24	1.00000000	duck
Tmem248	0.10	1.00000000	duck
Slc23a2	-0.21	1.00000000	duck
Rcn2	0.10	1.00000000	duck
Nup98	-0.21	1.00000000	duck
Cdc42	0.09	1.00000000	duck
Gna13	0.11	1.00000000	duck
Hikeshi	0.06	1.00000000	duck

Tmem9b	0.01	1.00000000	duck
Eaf1	-0.03	1.00000000	duck
Far1	0.10	1.00000000	duck
Bend7	0.04	1.00000000	duck
Rpa1	0.12	1.00000000	duck
Noct	0.06	1.00000000	duck
Ints13	-0.07	1.00000000	duck
Abl1	0.03	1.00000000	duck
Ptges3	-0.16	1.00000000	duck
Ap2m1	0.15	1.00000000	duck
Rheb	-0.19	1.00000000	duck
Rpn1	0.25	1.00000000	duck
Cebpg	-0.08	1.00000000	duck
Cmtm3	0.24	1.00000000	duck
Edc3	0.12	1.00000000	duck
Srp54a	-0.09	1.00000000	duck
Tsc22d2	0.00	1.00000000	duck
Focad	0.24	1.00000000	duck
Lamp2	-0.10	1.00000000	duck
Atp5c1	0.10	1.00000000	duck
Ppp2r5a	-0.11	1.00000000	duck
Ccny	0.15	1.00000000	duck
Arl15	-0.30	1.00000000	duck
Heg1	0.22	1.00000000	duck
St7l	-0.04	1.00000000	duck
Tulp4	-0.34	1.00000000	duck
Vps29	0.04	1.00000000	duck
Csrnp1	0.15	1.00000000	duck
Pfkfb3	-0.09	1.00000000	duck
Ezh2	0.15	1.00000000	duck
Trim23	-0.08	1.00000000	duck
Ampd2	0.18	1.00000000	duck
Dlat	-0.05	1.00000000	duck
Nap1l1	-0.08	1.00000000	duck
4833439L19Rik	0.13	1.00000000	duck
Rer1	0.05	1.00000000	duck
Polr2l	0.23	1.00000000	duck
Yy2	-0.07	1.00000000	duck
Zdhhc15	0.17	1.00000000	duck
Aida	0.17	1.00000000	duck
Wdr26	0.26	1.00000000	duck
Fbxw11	0.02	1.00000000	duck
Tmem223	0.14	1.00000000	duck
Rabggtb	-0.15	1.00000000	duck
Srp14	0.11	1.00000000	duck
Wdr36	0.19	1.00000000	duck

Eef2	-0.05	1.00000000	duck
Shoc2	0.23	1.00000000	duck
Slc35b3	0.06	1.00000000	duck
9930021J03Rik	0.04	1.00000000	duck
Rab14	0.02	1.00000000	duck
Zc3h3	0.08	1.00000000	duck
Heatr5a	0.20	1.00000000	duck
Fbxw7	0.10	1.00000000	duck
Ube2e3	-0.19	1.00000000	duck
Rnf149	0.00	1.00000000	duck
Pcna	0.27	1.00000000	duck
Anapc1	0.01	1.00000000	duck
Timm13	0.22	1.00000000	duck
Strip1	0.13	1.00000000	duck
Trp53bp2	0.20	1.00000000	duck
Hspa9	-0.05	1.00000000	duck
Tle4	0.50	1.00000000	duck
Zhx1	-0.12	1.00000000	duck
Bcl2l11	0.12	1.00000000	duck
Borcs5	-0.04	1.00000000	duck
Dph3	0.10	1.00000000	duck
Secisbp2	-0.01	1.00000000	duck
Traf6	0.09	1.00000000	duck
Atxn7l1	0.18	1.00000000	duck
Naf1	0.04	1.00000000	duck
Dhx40	-0.06	1.00000000	duck
Csnk2a2	0.09	1.00000000	duck
Capza2	0.03	1.00000000	duck
Btbd2	-0.10	1.00000000	duck
Rnf169	0.02	1.00000000	duck
Taf15	0.13	1.00000000	duck
Tcea2	0.04	1.00000000	duck
Rab6a	0.10	1.00000000	duck
Ctnna1	0.22	1.00000000	duck
Rngtt	0.05	1.00000000	duck
Rnf114	0.02	1.00000000	duck
Avl9	0.05	1.00000000	duck
Wbp11	-0.10	1.00000000	duck
Eif3f	0.09	1.00000000	duck
Slc43a2	0.04	1.00000000	duck
Hivep1	-0.17	1.00000000	duck
Tm9sf2	-0.11	1.00000000	duck
Nsd1	-0.11	1.00000000	duck
Psme3	0.10	1.00000000	duck
Ralb	0.06	1.00000000	duck
Eps15	-0.13	1.00000000	duck

Tmem87a	0.04	1.00000000	duck
Srek1ip1	-0.02	1.00000000	duck
Dnajb12	0.04	1.00000000	duck
Gtf2e2	0.10	1.00000000	duck
Lancl2	0.02	1.00000000	duck
Gorab	0.06	1.00000000	duck
Atp11c	0.35	1.00000000	duck
Bmf	0.19	1.00000000	duck
Smarce1	0.05	1.00000000	duck
Dsn1	0.07	1.00000000	duck
Srpk2	0.13	1.00000000	duck
Aplf	0.19	1.00000000	duck
Sinhcaf	0.02	1.00000000	duck
Ppp4r1	0.13	1.00000000	duck
AF366264	-0.05	1.00000000	duck
Kif3a	0.08	1.00000000	duck
Yipf4	-0.14	1.00000000	duck
Casp9	0.03	1.00000000	duck
Cstf1	-0.04	1.00000000	duck
Setx	0.13	1.00000000	duck
Spry1	0.23	1.00000000	duck
Cct7	0.25	1.00000000	duck
Ist1	-0.04	1.00000000	duck
Rap2b	-0.09	1.00000000	duck
Plekhm3	-0.06	1.00000000	duck
Rab9	0.10	1.00000000	duck
Ska2	0.02	1.00000000	duck
Trp53inp2	0.13	1.00000000	duck
Golga1	-0.06	1.00000000	duck
Adrm1	0.02	1.00000000	duck
Fbxo8	0.00	1.00000000	duck
Arpp19	-0.04	1.00000000	duck
Clpx	0.07	1.00000000	duck
Ccnb2	0.20	1.00000000	duck
Tlnrd1	0.13	1.00000000	duck
Faf2	0.00	1.00000000	duck
Csgalnact2	-0.04	1.00000000	duck
Arnt	0.01	1.00000000	duck
Commd9	0.13	1.00000000	duck
Slc30a7	-0.05	1.00000000	duck
Terf2	-0.06	1.00000000	duck
Kpna4	-0.30	1.00000000	duck
Etv6	-0.02	1.00000000	duck
Rbbp4	0.11	1.00000000	duck
Dennd2d	0.14	1.00000000	duck
Fbrsl1	0.12	1.00000000	duck

Gprin3	0.16	1.00000000	duck
Ercc8	0.01	1.00000000	duck
Nr2c2	-0.21	1.00000000	duck
Slc25a25	0.14	1.00000000	duck
Ifngr1	-0.03	1.00000000	duck
Arfip1	0.01	1.00000000	duck
Fam172a	0.26	1.00000000	duck
Lrrfip2	0.17	1.00000000	duck
Epb41l5	0.09	1.00000000	duck
Yae1d1	0.02	1.00000000	duck
Pafah1b2	0.12	1.00000000	duck
Fbxl2	-0.04	1.00000000	duck
Sucla2	-0.12	1.00000000	duck
Igf2r	-0.35	1.00000000	duck
Zc3h13	-0.18	1.00000000	duck
Tars	0.12	1.00000000	duck
Gna12	0.08	1.00000000	duck
Lsm5	0.14	1.00000000	duck
Vps37b	0.21	1.00000000	duck
Prdx3	0.06	1.00000000	duck
Dbr1	0.04	1.00000000	duck
Rcl1	0.19	1.00000000	duck
Prpf19	0.08	1.00000000	duck
Ak2	-0.45	1.00000000	duck
Dennd6b	0.03	1.00000000	duck
Nrbf2	-0.04	1.00000000	duck
Cd81	0.18	1.00000000	duck
Gnptab	0.35	1.00000000	duck
Ggnbp2	-0.15	1.00000000	duck
Dusp7	0.30	1.00000000	duck
Uhmk1	0.01	1.00000000	duck
Rere	0.16	1.00000000	duck
Stmn1	0.11	1.00000000	duck
Ndufa5	0.06	1.00000000	duck
Slk	-0.01	1.00000000	duck
Rac1	0.23	1.00000000	duck
Hnrnpu	0.01	1.00000000	duck
Ilf2	0.07	1.00000000	duck
Rnf216	0.06	1.00000000	duck
Traf5	-0.06	1.00000000	duck
Vwa8	0.10	1.00000000	duck
Adam10	-0.08	1.00000000	duck
Psmd4	0.00	1.00000000	duck
AW554918	0.09	1.00000000	duck
Ccdc126	-0.19	1.00000000	duck
Rragd	-0.11	1.00000000	duck

Trmt10c	0.02	1.00000000	duck
Picalm	-0.19	1.00000000	duck
Prpf3	0.15	1.00000000	duck
Fbxo45	-0.06	1.00000000	duck
Snx6	0.14	1.00000000	duck
Eloc	0.08	1.00000000	duck
Wwox	-0.16	1.00000000	duck
Dynlt3	-0.13	1.00000000	duck
Gna11	-0.12	1.00000000	duck
Dnajc19	-0.09	1.00000000	duck
Oard1	-0.15	1.00000000	duck
Yod1	0.06	1.00000000	duck
Plaa	0.01	1.00000000	duck
Nifk	0.22	1.00000000	duck
Sypl2	0.17	1.00000000	duck
Abhd5	0.13	1.00000000	duck
Ppid	0.22	1.00000000	duck
Acsf2	0.12	1.00000000	duck
Ago4	0.02	1.00000000	duck
Paip2	0.13	1.00000000	duck
Tcp11l2	-0.04	1.00000000	duck
Atp2a2	-0.17	1.00000000	duck
Tnks	-0.06	1.00000000	duck
Cdc123	0.06	1.00000000	duck
Rnf19a	-0.01	1.00000000	duck
Utp15	0.11	1.00000000	duck
Dcaf13	0.04	1.00000000	duck
Pcgf3	0.01	1.00000000	duck
Sec13	0.01	1.00000000	duck
Capn15	-0.08	1.00000000	duck
Nras	-0.07	1.00000000	duck
Fam222b	-0.07	1.00000000	duck
Sik2	0.28	1.00000000	duck
Pias2	0.17	1.00000000	duck
Gga3	0.13	1.00000000	duck
Gata3	0.30	1.00000000	duck
Sgms1	-0.10	1.00000000	duck
Prmt7	0.06	1.00000000	duck
Ttf1	0.06	1.00000000	duck
Rtf2	-0.09	1.00000000	duck
Anp32e	0.10	1.00000000	duck
Snrnp40	0.21	1.00000000	duck
Vgll4	0.09	1.00000000	duck
Atp2c1	-0.19	1.00000000	duck
Lck	0.17	1.00000000	duck
Nfe2l2	-0.47	1.00000000	duck

Golim4	0.02	1.00000000	duck
Fbxl17	0.03	1.00000000	duck
Scn8a	-0.01	1.00000000	duck
Epc1	-0.12	1.00000000	duck
Aprt	0.08	1.00000000	duck
Cpd	0.06	1.00000000	duck
Rhof	0.21	1.00000000	duck
Pcgf5	0.12	1.00000000	duck
Sppl2b	0.06	1.00000000	duck
Gpatch2l	-0.17	1.00000000	duck
C1d	0.15	1.00000000	duck
Fam8a1	-0.11	1.00000000	duck
Ppa2	0.05	1.00000000	duck
Celf2	0.19	1.00000000	duck
Lamc1	0.14	1.00000000	duck
Rnf34	-0.07	1.00000000	duck
Golga7	0.06	1.00000000	duck
Ube2k	-0.08	1.00000000	duck
Cnot4	-0.15	1.00000000	duck
Qser1	0.03	1.00000000	duck
Aak1	-0.02	1.00000000	duck
Zfp462	0.12	1.00000000	duck
Dnaja2	-0.08	1.00000000	duck
Itm2a	0.01	1.00000000	duck
Vps39	0.08	1.00000000	duck
Psmc3	0.04	1.00000000	duck
Eif2b1	0.11	1.00000000	duck
Pwwp2a	0.04	1.00000000	duck
Edem1	0.12	1.00000000	duck
Ap3b1	0.15	1.00000000	duck
Pum3	0.17	1.00000000	duck
Ccdc66	0.09	1.00000000	duck
Fhl3	0.09	1.00000000	duck
Gga2	0.25	1.00000000	duck
Nudc	0.21	1.00000000	duck
Sh3glb1	-0.01	1.00000000	duck
Rapgef1	-0.04	1.00000000	duck
Ppfia1	-0.14	1.00000000	duck
Elovl1	0.07	1.00000000	duck
Ap3s1	-0.13	1.00000000	duck
Pxk	-0.05	1.00000000	duck
Ppil1	0.16	1.00000000	duck
Hat1	0.13	1.00000000	duck
Foxn2	0.22	1.00000000	duck
Cct2	0.21	1.00000000	duck
Ttc1	0.12	1.00000000	duck

Lysmd2	0.09	1.00000000	duck
Srsf6	0.02	1.00000000	duck
Spty2d1	0.06	1.00000000	duck
Lsm14b	0.15	1.00000000	duck
Ptpn6	0.23	1.00000000	duck
Aimp1	0.08	1.00000000	duck
Creld2	0.20	1.00000000	duck
Peak1	0.06	1.00000000	duck
Chchd5	0.00	1.00000000	duck
Camk1d	-0.07	1.00000000	duck
Slc30a5	0.05	1.00000000	duck
Lsm4	0.12	1.00000000	duck
Prkrip1	0.01	1.00000000	duck
Atp2b4	0.22	1.00000000	duck
Lgals8	-0.16	1.00000000	duck
Rab8a	0.04	1.00000000	duck
Abhd13	-0.15	1.00000000	duck
AU040320	-0.18	1.00000000	duck
Thada	0.09	1.00000000	duck
Afg3l2	0.09	1.00000000	duck
Tssc4	0.09	1.00000000	duck
Kifbp	0.09	1.00000000	duck
N4bp1	-0.06	1.00000000	duck
Ptch1	0.15	1.00000000	duck
Auh	0.19	1.00000000	duck
Lsm6	0.14	1.00000000	duck
Ammecr1l	-0.01	1.00000000	duck
Blmh	0.02	1.00000000	duck
Tpt1	5.77	0.00000000	mice
Adss	3.74	0.00000000	mice
Rab33a	4.52	0.00000000	mice
Cmip	4.44	0.00000000	mice
Cd3g	3.58	0.00000000	mice
Ptprm	-4.58	0.00000000	mice
Epas1	-4.01	0.00000000	mice
Cd28	3.64	0.00000000	mice
Cfd	-4.64	0.00000000	mice
Dars	3.13	0.00000000	mice
Arpc2	2.81	0.00000000	mice
Aqp1	-4.61	0.00000000	mice
Igfbp7	-3.84	0.00000000	mice
Timp3	-3.86	0.00000000	mice
Fyb	2.92	0.00000000	mice
Rbms3	-3.71	0.00000000	mice
Pecam1	-3.74	0.00000000	mice
Zc3h12d	2.04	0.00000000	mice

Calcrl	-3.59	0.00000000	mice
Rgs1	1.82	0.00000000	mice
Atp5b	2.33	0.00000000	mice
Adgrf5	-3.28	0.00000000	mice
Ldb2	-3.62	0.00000000	mice
Rgs2	3.84	0.00000000	mice
Adgrl2	-3.35	0.00000000	mice
Cxcr4	2.11	0.00000000	mice
Oga	2.28	0.00000000	mice
Tmtc1	-3.51	0.00000000	mice
Tjp1	-3.36	0.00000000	mice
U2surp	2.33	0.00000000	mice
Cnbp	2.14	0.00000000	mice
Atp5g3	2.26	0.00000000	mice
Rapgef5	-3.54	0.00000000	mice
Dido1	2.26	0.00000000	mice
Wtap	2.17	0.00000000	mice
Sparc	-2.76	0.00000000	mice
Syne2	-3.01	0.00000000	mice
Atf7ip	2.52	0.00000000	mice
Phip	2.39	0.00000000	mice
Sap18	2.04	0.00000000	mice
Ankhd1	2.09	0.00000000	mice
Vim	2.42	0.00000000	mice
Tcf7	2.06	0.00000000	mice
Egr1	-2.97	0.00000000	mice
Cyria	2.50	0.00000000	mice
Adgrl4	-3.00	0.00000000	mice
Cd247	1.49	0.00000000	mice
Ptprb	-2.87	0.00000000	mice
Tmem100	-2.93	0.00000000	mice
Fos	-2.52	0.00000000	mice
Cavin2	-2.81	0.00000000	mice
4932411N23Rik	-3.48	0.00000000	mice
Akap12	-2.85	0.00000000	mice
Fam189a1	-2.66	0.00000000	mice
Sdcbp	1.92	0.00000000	mice
Nova1	-2.87	0.00000000	mice
Lpp	2.14	0.00000000	mice
Pfkp	2.23	0.00000000	mice
Pcf11	1.96	0.00000000	mice
Ptprc	1.31	0.00000000	mice
Sox6	-3.17	0.00000000	mice
Arhgap31	-2.46	0.00000000	mice
Txnip	1.89	0.00000000	mice
Gpr171	1.46	0.00000000	mice

Anapc15-ps	1.74	0.00000000	mice
Gpx3	-2.54	0.00000000	mice
Apbb1ip	1.96	0.00000000	mice
Arid5b	1.39	0.00000000	mice
Podxl	-2.42	0.00000000	mice
Plcb1	-2.26	0.00000000	mice
Tmcc3	-2.43	0.00000000	mice
App	2.17	0.00000000	mice
Evl	1.17	0.00000000	mice
Bcl11b	1.53	0.00000000	mice
Plekhg1	-2.40	0.00000000	mice
Col4a1	-2.30	0.00000000	mice
Rgcc	-2.50	0.00000000	mice
Tiparp	2.04	0.00000000	mice
Trpc4ap	1.71	0.00000000	mice
Dll4	-2.38	0.00000000	mice
Sla	1.61	0.00000000	mice
Syncrip	1.75	0.00000000	mice
Sema6a	-2.37	0.00000000	mice
Lnpep	1.83	0.00000000	mice
Rasal2	-2.99	0.00000000	mice
Arhgef9	-2.49	0.00000000	mice
Arhgap15	1.08	0.00000000	mice
Kars	1.66	0.00000000	mice
Pmp22	-2.16	0.00000000	mice
Cklf	1.60	0.00000000	mice
Npr3	-2.35	0.00000000	mice
Niban1	2.33	0.00000000	mice
Prep	1.83	0.00000000	mice
Fermt2	-2.28	0.00000000	mice
Synm	-2.22	0.00000000	mice
Zbtb20	-2.45	0.00000000	mice
Ddit4	-2.25	0.00000000	mice
Afdn	-2.43	0.00000000	mice
Nfia	-2.37	0.00000000	mice
Cdh5	-2.14	0.00000000	mice
Sp4	1.52	0.00000000	mice
Heg1	-2.05	0.00000000	mice
Pde4b	1.08	0.00000000	mice
Sdk1	-2.36	0.00000000	mice
Sptbn1	-1.87	0.00000000	mice
Fth1	-1.06	0.00000000	mice
Smad6	-2.21	0.00000000	mice
Bmpr2	-2.42	0.00000000	mice
Pde10a	-2.55	0.00000000	mice
Adnp	1.53	0.00000000	mice

Strap	1.46	0.00000000	mice
Jcad	-2.02	0.00000000	mice
Pomp	1.44	0.00000000	mice
Ccser2	1.29	0.00000000	mice
Lpl	-2.03	0.00000000	mice
Corin	-2.40	0.00000000	mice
Sytl3	2.65	0.00000000	mice
Ptger4	1.45	0.00000000	mice
Rnf38	1.64	0.00000000	mice
Arvcf	-2.16	0.00000000	mice
Ndufa1	1.37	0.00000000	mice
Rffl	1.25	0.00000000	mice
Gna13	1.04	0.00000000	mice
Prr5	1.25	0.00000000	mice
Zfp36l1	0.94	0.00000000	mice
Dock1	-2.00	0.00000000	mice
Runx1	1.37	0.00000000	mice
Ankrd10	1.07	0.00000000	mice
Brd8	1.40	0.00000000	mice
Evi2a	1.30	0.00000000	mice
Auts2	-1.94	0.00000000	mice
Tead4	-1.85	0.00000000	mice
Csnk1d	1.38	0.00000000	mice
Samsn1	1.14	0.00000000	mice
Sdhc	1.28	0.00000000	mice
Ccz1	1.34	0.00000000	mice
Gnai1	1.49	0.00000000	mice
Sorbs1	-2.09	0.00000000	mice
Atp5k	1.34	0.00000000	mice
Sdc4	-1.60	0.00000000	mice
Cav1	-1.85	0.00000000	mice
Slc16a6	1.29	0.00000000	mice
Prex2	-1.98	0.00000000	mice
Snrpd2	1.29	0.00000000	mice
Gsap	1.71	0.00000000	mice
Fam107b	1.28	0.00000000	mice
Ahsa2	1.49	0.00000000	mice
Kcnj5	-2.10	0.00000000	mice
Phax	1.30	0.00000000	mice
Crim1	-2.18	0.00000000	mice
Tmem204	-1.84	0.00000000	mice
Galnt18	-1.79	0.00000000	mice
Vcp	1.23	0.00000000	mice
Dock2	1.16	0.00000000	mice
Rassf9	-1.80	0.00000000	mice
Eif4b	1.22	0.00000000	mice

Tnfaip3	1.12	0.00000000	mice
Tgfbr1	1.39	0.00000000	mice
Ccdc25	1.22	0.00000000	mice
Jun	-1.56	0.00000000	mice
Sec24d	1.46	0.00000000	mice
Cdc37	1.23	0.00000000	mice
Tek	-1.68	0.00000000	mice
Tdpoz8	1.28	0.00000000	mice
Spred2	-1.99	0.00000000	mice
Sinhcaf	1.68	0.00000000	mice
Sdc2	-1.71	0.00000000	mice
Rai14	-1.71	0.00000000	mice
Kifbp	1.26	0.00000000	mice
Eprs	1.35	0.00000000	mice
Hecw2	-2.23	0.00000000	mice
Slc9a3r2	-1.90	0.00000000	mice
Cnn3	-1.73	0.00000000	mice
Dach1	-1.80	0.00000000	mice
Runx1t1	-1.64	0.00000000	mice
Neo1	-1.64	0.00000000	mice
Prkce	-1.73	0.00000000	mice
Atp5c1	1.15	0.00000000	mice
Nop56	1.19	0.00000000	mice
Ptpn14	-1.57	0.00000000	mice
Cacybp	1.21	0.00000000	mice
Xpo6	0.98	0.00000000	mice
Spcs1	1.12	0.00000000	mice
Efnb2	-1.93	0.00000000	mice
C1qbp	1.14	0.00000000	mice
Trim24	1.36	0.00000000	mice
Btg1b	0.74	0.00000000	mice
Arap2	1.29	0.00000000	mice
Slirp	1.17	0.00000000	mice
Rtf2	1.19	0.00000000	mice
Kcnc1	-1.73	0.00000000	mice
Dclk1	-2.04	0.00000000	mice
Trrap	1.21	0.00000000	mice
Tbx3	-1.63	0.00000000	mice
Apcdd1	-1.59	0.00000000	mice
Cd47	0.86	0.00000000	mice
Zfp352	-1.97	0.00000000	mice
Polr1f	1.17	0.00000000	mice
Creb5	-1.87	0.00000000	mice
Golm2	1.17	0.00000000	mice
Ephb1	-1.56	0.00000000	mice
Sipa1l1	0.81	0.00000000	mice

S100a13	-1.65	0.00000000	mice
Bsg	-2.07	0.00000000	mice
Ptprn2	-1.74	0.00000000	mice
Tex2	-2.02	0.00000000	mice
Plcg1	1.19	0.00000000	mice
Pcnp	1.13	0.00000000	mice
Tspan9	-1.86	0.00000000	mice
Ablim1	-1.82	0.00000000	mice
Arl4a	-2.25	0.00000000	mice
Tnfaip8	1.05	0.00000000	mice
Tnfrsf18	1.42	0.00000000	mice
Tns1	-1.74	0.00000000	mice
Spred1	-1.62	0.00000000	mice
Fgd5	-1.71	0.00000000	mice
Rad23b	1.20	0.00000000	mice
Stk17b	0.99	0.00000000	mice
Ccdc85a	-1.72	0.00000000	mice
Fgfr2	-1.54	0.00000000	mice
Tgfbr3	-1.70	0.00000000	mice
Ndst4	1.20	0.00000000	mice
Stat4	1.26	0.00000000	mice
Usp3	1.00	0.00000000	mice
Hpgd	-1.53	0.00000000	mice
Cytip	1.07	0.00000000	mice
B2m	-0.58	0.00000000	mice
Kcne3	-1.87	0.00000000	mice
Cobll1	-1.38	0.00000000	mice
Cdk5r1	1.08	0.00000000	mice
Baz2b	-1.61	0.00000000	mice
Pde3b	0.79	0.00000000	mice
Tjp2	-1.56	0.00000000	mice
Zhx2	1.13	0.00000000	mice
Erg	-1.41	0.00000000	mice
Rasgrf2	-1.33	0.00000000	mice
Dock9	-2.11	0.00000000	mice
Zfp366	-1.56	0.00000000	mice
Gpd1l	1.17	0.00000000	mice
Dennd2a	-1.47	0.00000000	mice
Tardbp	1.08	0.00000000	mice
Lcp2	0.93	0.00000000	mice
Cd2ap	1.23	0.00000000	mice
Dgkh	-1.77	0.00000000	mice
Zeb2	-1.11	0.00000000	mice
Cars	1.15	0.00000000	mice
Nckap1	-1.59	0.00000000	mice
Ankrd44	0.92	0.00000000	mice

Rhoj	-1.44	0.00000000	mice
Stat1	1.04	0.00000000	mice
Kdr	-1.42	0.00000000	mice
Gsk3b	-1.18	0.00000000	mice
Dlc1	-1.46	0.00000000	mice
Slc12a7	1.22	0.00000000	mice
Atp10a	1.22	0.00000000	mice
Npas3	-1.82	0.00000000	mice
Stard13	-1.46	0.00000000	mice
Acvrl1	-1.42	0.00000000	mice
Rhob	1.25	0.00000000	mice
Rybp	1.22	0.00000000	mice
Crtc1	1.00	0.00000000	mice
Cgnl1	-1.34	0.00000000	mice
Itm2b	-1.69	0.00000000	mice
Efemp1	-1.60	0.00000000	mice
Ezr	1.30	0.00000000	mice
Lrmp	1.17	0.00000000	mice
Enox2	0.98	0.00000000	mice
Sfswap	0.97	0.00000000	mice
Rars	0.93	0.00000000	mice
Lifr	-1.20	0.00000000	mice
Sat1	0.68	0.00000000	mice
Tcf7l2	-1.58	0.00000000	mice
Tollip	0.97	0.00000000	mice
Ramp2	-1.15	0.00000000	mice
Zfp521	-1.37	0.00000000	mice
Slc38a2	0.85	0.00000000	mice
Ddx28	0.81	0.00000000	mice
Fli1	-1.72	0.00000000	mice
Gpcpd1	0.81	0.00000000	mice
Adcy7	1.01	0.00000000	mice
Apip	0.94	0.00000000	mice
Colec10	-1.20	0.00000000	mice
Tut7	0.98	0.00000000	mice
Itga4	0.98	0.00000000	mice
Rel	0.76	0.00000000	mice
Dpyd	-1.44	0.00000000	mice
Slmap	0.98	0.00000000	mice
Abca1	0.89	0.00000000	mice
Rbfox2	-1.74	0.00000000	mice
Nfil3	1.11	0.00000000	mice
Col22a1	-1.18	0.00000000	mice
Dst	-0.61	0.00000000	mice
Pim3	-1.86	0.00000000	mice
Sesn1	0.82	0.00000000	mice

Jmjd1c	0.86	0.00000000	mice
Tnik	0.84	0.00000000	mice
Pls3	-1.66	0.00000000	mice
Ergic1	0.75	0.00000000	mice
Cd74	-1.46	0.00000000	mice
Cdc42bpa	-1.52	0.00000000	mice
Adamts2	-1.60	0.00000000	mice
Apba2	-1.16	0.00000000	mice
Foxf1	-1.15	0.00000000	mice
Hoxa3	-1.32	0.00000000	mice
Txnrd1	-1.68	0.00000000	mice
Ets1	0.67	0.00000000	mice
Agbl1	-1.24	0.00000000	mice
Zdhhc20	1.07	0.00000000	mice
Snrk	0.75	0.00000000	mice
Egfl7	-0.97	0.00000000	mice
Wipf1	0.87	0.00000000	mice
Dennd1b	0.77	0.00000000	mice
Rpf2	0.81	0.00000000	mice
Klhl5	0.88	0.00000000	mice
Actb	-0.64	0.00000000	mice
Rasgef1a	0.93	0.00000000	mice
Gulp1	-1.38	0.00000000	mice
Abcg1	1.03	0.00000000	mice
Ikzf1	0.93	0.00000000	mice
Dcn	-1.73	0.00000000	mice
Trp53i11	-1.06	0.00000000	mice
Cd3e	0.68	0.00000000	mice
Gdpd5	1.25	0.00000000	mice
Kcnk5	1.18	0.00000000	mice
Pggt1b	0.92	0.00000000	mice
Cd4	0.82	0.00000000	mice
Tox2	0.75	0.00000000	mice
Naa20	0.81	0.00000000	mice
Ripor3	-1.50	0.00000000	mice
Psip1	0.68	0.00000000	mice
Tmem123	0.73	0.00000000	mice
Fnbp1l	-1.71	0.00000000	mice
Igf1r	0.95	0.00000000	mice
Atp11b	0.91	0.00000000	mice
Prrg1	-1.05	0.00000000	mice
Tmbim1	0.92	0.00000000	mice
Card11	1.07	0.00000000	mice
Atp5j2	0.79	0.00000000	mice
Dock11	0.73	0.00000000	mice
Thbs1	-1.40	0.00000000	mice

Sap30bp	0.80	0.00000000	mice
Bmp6	-1.17	0.00000000	mice
Pcsk2	-1.11	0.00000000	mice
Arhgap21	-1.83	0.00000000	mice
Il1b	1.00	0.00000000	mice
Glrx3	0.77	0.00000000	mice
Rassf3	-1.63	0.00000000	mice
Chfr	0.77	0.00000000	mice
Psme3ip1	0.74	0.00000000	mice
Bri3bp	0.81	0.00000000	mice
Ramp3	-1.16	0.00000000	mice
Lpar4	1.26	0.00000000	mice
Il1rap	1.09	0.00000000	mice
Vav3	0.91	0.00000000	mice
Blvra	0.87	0.00000000	mice
Cttnbp2nl	-1.32	0.00000000	mice
Fbxl21	0.80	0.00000000	mice
Tars	0.77	0.00000000	mice
Nars	0.74	0.00000000	mice
Sarnp	0.76	0.00000000	mice
Pdlim1	-1.04	0.00000000	mice
Pik3cd	0.88	0.00000000	mice
Osbpl10	-1.37	0.00000000	mice
Dock10	0.78	0.00000000	mice
Reps2	-1.09	0.00000000	mice
Cdkn2aip	0.77	0.00000000	mice
Bin1	-1.20	0.00000000	mice
Inpp5k	-0.91	0.00000000	mice
Atf3	-1.78	0.00000000	mice
Fat4	-0.99	0.00000000	mice
Atxn7l1	0.71	0.00000000	mice
Pdgfb	-0.99	0.00000000	mice
Fyn	0.69	0.00000000	mice
Bcat1	0.79	0.00000000	mice
Stox2	-1.30	0.00000000	mice
Laptm4a	-1.47	0.00000000	mice
Cadps2	-1.24	0.00000000	mice
Wwc2	-1.08	0.00000000	mice
Nfatc3	0.63	0.00000000	mice
Tmem63a	0.95	0.00000000	mice
Foxo1	0.68	0.00000000	mice
Serpind1	-1.17	0.00000000	mice
Ehbp1	0.69	0.00000000	mice
Cbx7	0.75	0.00000000	mice
Ythdc2	0.89	0.00000000	mice
Bccip	0.69	0.00000000	mice

Rac2	0.59	0.00000000	mice
Qk	-1.29	0.00000000	mice
Spata5	0.71	0.00000000	mice
Banp	0.83	0.00000000	mice
Adgrg1	-0.98	0.00000000	mice
Cald1	-1.02	0.00000000	mice
Dennd4a	-1.68	0.00000000	mice
Sox18	-1.02	0.00000000	mice
Hif1a	0.65	0.00000000	mice
Ssbp2	-1.11	0.00000000	mice
Hsd17b12	-1.61	0.00000000	mice
Caskin2	-0.98	0.00000000	mice
Lrch3	0.70	0.00000000	mice
Dock4	-1.24	0.00000000	mice
Unc5c	-1.13	0.00000000	mice
Ski	-0.92	0.00000000	mice
Farp1	-1.05	0.00000000	mice
Enah	-1.06	0.00000000	mice
Scoc	0.70	0.00000000	mice
Fkbp5	0.81	0.00000000	mice
Scube3	-0.91	0.00000000	mice
Slc9a9	0.86	0.00000000	mice
Ccng2	0.71	0.00000000	mice
Sik1	0.60	0.00000000	mice
Pusl1	0.79	0.00000000	mice
Shroom2	-0.81	0.00000000	mice
Rbmx2	0.68	0.00000000	mice
Kdm3a	0.71	0.00000000	mice
Atad1	0.67	0.00000000	mice
Tcf12	-1.65	0.00000000	mice
Polr2i	0.65	0.00000000	mice
Amotl2	-0.92	0.00000000	mice
Traf3ip3	0.66	0.00000000	mice
Rgs4	1.25	0.00000000	mice
Nfkb1	0.58	0.00000000	mice
Slc22a23	-0.97	0.00000000	mice
Rbm26	0.48	0.00000000	mice
Pim1	0.82	0.00000000	mice
Cttnbp2	-0.99	0.00000000	mice
Tmem135	0.58	0.00000000	mice
Terf2ip	0.65	0.00000000	mice
Cep170	0.60	0.00000000	mice
Gphn	-1.27	0.00000000	mice
Syne1	0.62	0.00000000	mice
Dnajc1	0.92	0.00000000	mice
Sh3kbp1	0.65	0.00000000	mice

Bicd1	-1.35	0.00000000	mice
Ndufv3	0.50	0.00000000	mice
Foxn2	0.70	0.00000000	mice
S1pr1	-1.40	0.00000000	mice
St8sia4	0.96	0.00000000	mice
Idh3b	0.64	0.00000000	mice
Il22ra2	0.83	0.00000000	mice
Coro1c	0.51	0.00000000	mice
Ifnar1	0.67	0.00000000	mice
Blcap	0.62	0.00000000	mice
Tbp	0.64	0.00000000	mice
Pex13	0.68	0.00000000	mice
Nrp2	-1.08	0.00000000	mice
Ripor1	-0.96	0.00000000	mice
Wdr37	0.58	0.00000000	mice
Orai1	0.69	0.00000000	mice
Sec23ip	0.65	0.00000000	mice
LTO1	0.69	0.00000000	mice
Wdfy3	-1.52	0.00000000	mice
Ctdspl	-1.30	0.00000000	mice
Cbx6	0.77	0.00000000	mice
Fbh1	0.72	0.00000000	mice
Zfp777	0.61	0.00000000	mice
Anxa5	-1.41	0.00000000	mice
Etnk1	0.56	0.00000000	mice
Dtd1	0.65	0.00000000	mice
Pxdc1	-1.12	0.00000000	mice
Trim28	0.58	0.00000000	mice
Sftpc	-2.81	0.00000000	mice
Cd44	0.65	0.00000000	mice
Clic4	-1.40	0.00000000	mice
Antxr2	0.60	0.00000000	mice
Gadd45b	0.81	0.00000000	mice
Celf2	0.53	0.00000000	mice
F2r	-0.97	0.00000000	mice
Smad1	-0.95	0.00000000	mice
Spry1	0.81	0.00000000	mice
Aff2	0.65	0.00000000	mice
Smc4	0.66	0.00000000	mice
Psmc3ip	0.61	0.00000000	mice
Pten	0.48	0.00000000	mice
Cul1	0.57	0.00000000	mice
Arhgap30	0.57	0.00000000	mice
Ubac2	0.77	0.00000000	mice
Hoxa5	-0.87	0.00000000	mice
Trank1	0.83	0.00000000	mice

Laptm5	0.50	0.00000000	mice
Tapbpl	0.60	0.00000000	mice
Pstpip1	0.89	0.00000000	mice
Gnb5	-1.03	0.00000000	mice
Rin2	0.66	0.00000000	mice
Cnot1	0.51	0.00000000	mice
Cd81	-1.32	0.00000000	mice
Tagap1	0.93	0.00000000	mice
Dedd	0.56	0.00000000	mice
Dock8	0.56	0.00000000	mice
Colgalt2	-0.80	0.00000000	mice
Rnf19b	0.61	0.00000000	mice
Rgl1	-0.89	0.00000000	mice
Fgl2	0.81	0.00000000	mice
Rhoh	0.69	0.00000000	mice
Ctla4	1.05	0.00000000	mice
Eif2b4	0.55	0.00000000	mice
Tspan18	-0.94	0.00000000	mice
Cog3	0.59	0.00000000	mice
Traf3	0.72	0.00000000	mice
Polr1d	0.58	0.00000000	mice
Crem	0.46	0.00000000	mice
Rasa3	0.77	0.00000000	mice
Ubtf	0.59	0.00000000	mice
Atp2a3	0.52	0.00000000	mice
Kdelr3	0.48	0.00000000	mice
Rnf213	0.69	0.00000000	mice
Dgkq	0.69	0.00000000	mice
Lpgat1	0.66	0.00000000	mice
Khdc4	0.58	0.00000000	mice
Spag6l	0.79	0.00000000	mice
Hnrnpll	0.48	0.00000000	mice
Slc6a15	0.91	0.00000000	mice
Nasp	0.64	0.00000000	mice
Rxylt1	0.59	0.00000000	mice
Phldb2	-0.96	0.00000000	mice
Csnk1a1	0.47	0.00000000	mice
Diaph2	0.43	0.00000000	mice
Numb	-1.24	0.00000000	mice
Nr2f2	-0.85	0.00000000	mice
Dpysl2	-1.16	0.00000000	mice
Rock1	0.56	0.00000000	mice
Ttl	0.52	0.00000000	mice
Phf12	0.46	0.00000000	mice
Lfng	-1.47	0.00000000	mice
Anxa2	0.81	0.00000000	mice

Glcci1	0.79	0.00000000	mice
Smchd1	0.44	0.00000000	mice
Cab39	0.49	0.00000000	mice
Ifngr1	0.65	0.00000000	mice
Kcnq5	0.77	0.00000000	mice
Yars	0.53	0.00000000	mice
Gars	0.52	0.00000000	mice
Itprip	0.57	0.00000000	mice
Rad51b	0.84	0.00000000	mice
Dgkd	-1.16	0.00000000	mice
Ccdc88a	0.78	0.00000000	mice
Vmp1	0.49	0.00000000	mice
Trerf1	0.85	0.00000000	mice
Ntn4	-0.83	0.00000000	mice
Tbk1	0.55	0.00000000	mice
Arrdc3	0.65	0.00000000	mice
Utp25	0.53	0.00000000	mice
Chd2	0.52	0.00000000	mice
Ptpn22	0.66	0.00000000	mice
Vta1	0.48	0.00000000	mice
Trpv2	0.62	0.00000000	mice
Cyfip2	0.53	0.00000000	mice
Jak1	0.42	0.00000000	mice
Agap1	-1.34	0.00000000	mice
Pcid2	0.53	0.00000000	mice
Pde8a	-0.89	0.00000000	mice
Skap1	-0.70	0.00000000	mice
Pik3cg	0.48	0.00000000	mice
Pde7b	0.68	0.00000000	mice
Gcdh	0.49	0.00000000	mice
Cerk	0.50	0.00000000	mice
Eif2ak3	0.45	0.00000000	mice
Csnk2a2	0.47	0.00000000	mice
Apmap	0.52	0.00000000	mice
Stk38	0.46	0.00000000	mice
Cmtm8	-0.87	0.00000000	mice
Elf1	0.48	0.00000000	mice
Rnf34	0.64	0.00000000	mice
Rfx7	0.72	0.00000000	mice
Eapp	0.51	0.00000000	mice
Rictor	0.51	0.00000000	mice
Rnasel	0.70	0.00000000	mice
Aars	0.49	0.00000000	mice
Rassf5	0.58	0.00000000	mice
Dus2	0.57	0.00000000	mice
Vps13d	0.52	0.00000000	mice

Zfpm1	0.65	0.00000000	mice
S100a10	-1.03	0.00000000	mice
Spry2	-0.89	0.00000000	mice
Phtf2	0.72	0.00000000	mice
Sars	0.47	0.00000000	mice
Eml6	-1.00	0.00000000	mice
Braf	0.46	0.00000000	mice
Zfp706	0.30	0.00000000	mice
Asxl1	0.71	0.00000000	mice
Ccm2	0.51	0.00000000	mice
Pebp1	0.38	0.00000000	mice
Ano5	0.64	0.00000000	mice
Stx17	0.54	0.00000000	mice
Ccdc6	0.55	0.00000000	mice
Fam53a	0.64	0.00000000	mice
Kdm7a	0.61	0.00000000	mice
Cherp	0.49	0.00000000	mice
Tspan2	0.59	0.00000000	mice
Runx3	0.54	0.00000000	mice
Frs2	0.58	0.00000000	mice
Stk11ip	0.47	0.00000000	mice
Dapk1	0.59	0.00000000	mice
Gnal	0.90	0.00000000	mice
Ppm1k	0.62	0.00000000	mice
Atp1b3	-1.45	0.00000000	mice
Ppp6r3	0.45	0.00000000	mice
D630045J12Rik	0.90	0.00000000	mice
Strbp	0.55	0.00000000	mice
Crcp	0.48	0.00000000	mice
Aip	0.51	0.00000000	mice
Fmnl2	-1.15	0.00000000	mice
Gramd1c	0.80	0.00000000	mice
Letm1	0.46	0.00000000	mice
Pus10	0.55	0.00000000	mice
Rbpj	0.49	0.00000000	mice
Nrp1	-0.96	0.00000000	mice
Gk5	-1.15	0.00000000	mice
Lag3	0.55	0.00000000	mice
Neurl1b	-1.25	0.00000000	mice
Cotl1	0.45	0.00000000	mice
Serinc5	0.70	0.00000000	mice
Aktip	0.48	0.00000000	mice
Suv39h2	0.60	0.00000000	mice
Pnisr	0.42	0.00000000	mice
Ncoa3	0.49	0.00000000	mice
Cyld	0.43	0.00000000	mice

Flnb	-1.21	0.00000000	mice
Rsbn1l	0.38	0.00000000	mice
Nfkbie	0.44	0.00000000	mice
Scarb2	-1.10	0.00000000	mice
Lhfpl5	0.59	0.00000000	mice
Riok2	0.47	0.00000000	mice
Tnrc6c	0.47	0.00000000	mice
Tfpi2	-1.29	0.00000000	mice
Snrnp35	0.65	0.00000000	mice
Hook1	0.46	0.00000000	mice
Lrp3	0.48	0.00000000	mice
Ice2	0.64	0.00000000	mice
Srpk1	0.42	0.00000000	mice
Paip2	0.44	0.00000000	mice
Twsg1	-0.98	0.00000000	mice
Sgms2	0.71	0.00000000	mice
Sdf2l1	0.44	0.00000000	mice
Far1	0.45	0.00000000	mice
Irf4	0.66	0.00000000	mice
Kcnq1	-0.27	0.00000000	mice
Sec62	0.34	0.00000000	mice
Kpnb1	0.43	0.00000000	mice
Ppia	0.42	0.00000000	mice
Dnpep	0.42	0.00000000	mice
Il7r	0.45	0.00000000	mice
Hbp1	0.33	0.00000000	mice
Qars	0.47	0.00000000	mice
Fbp1	0.44	0.00000000	mice
Ogt	0.31	0.00000000	mice
Cnot2	0.43	0.00000000	mice
Ttc21b	0.53	0.00000000	mice
Dok1	0.55	0.00000000	mice
Zbtb38	0.47	0.00000000	mice
Asxl3	-1.03	0.00000000	mice
Sec14l1	-0.99	0.00000000	mice
Rbm34	0.38	0.00000000	mice
Kansl1	0.34	0.00000000	mice
Ube2j1	0.41	0.00000000	mice
Lhpp	0.45	0.00000000	mice
Usp10	0.34	0.00000000	mice
Satb1	0.85	0.00000000	mice
Rgs3	-0.84	0.00000000	mice
Sanbr	0.68	0.00000000	mice
Lrmda	-0.84	0.00000000	mice
Cdk14	0.61	0.00000000	mice
Lrpprc	0.51	0.00000000	mice

Ralgps2	-1.35	0.00000000	mice
Fryl	-1.17	0.00000000	mice
Crip2	0.42	0.00000000	mice
Pacsin2	0.39	0.00000000	mice
Tcf20	0.38	0.00000000	mice
Igf2bp3	0.57	0.00000000	mice
Sema7a	-0.99	0.00000000	mice
Eif3m	0.36	0.00000000	mice
Gtpbp4	0.43	0.00000000	mice
Rbm12	0.39	0.00000000	mice
Ndufa4	0.29	0.00000000	mice
Lbr	0.26	0.00000000	mice
Vcl	-1.16	0.00000000	mice
Socs1	0.54	0.00000000	mice
Tent4a	0.48	0.00000000	mice
Stambp	0.41	0.00000000	mice
Nek6	0.40	0.00000000	mice
Nanp	0.43	0.00000000	mice
Hdac3	0.38	0.00000000	mice
Traf5	0.58	0.00000000	mice
Ncoa6	0.32	0.00000000	mice
Hbs1l	0.34	0.00000000	mice
Znfx1	0.36	0.00000000	mice
Acvr1	0.47	0.00000000	mice
Lrba	0.47	0.00000000	mice
Cmtm7	0.51	0.00000000	mice
Spidr	0.54	0.00000000	mice
Klhl15	0.47	0.00000000	mice
Slc4a7	0.63	0.00000000	mice
Creg1	-1.51	0.00000000	mice
Pdhx	0.40	0.00000000	mice
Bzw1	0.35	0.00000000	mice
Trmt11	0.48	0.00000000	mice
Pde1c	-1.29	0.00000000	mice
Klf12	0.58	0.00000000	mice
Rab8b	0.35	0.00000000	mice
Arid4a	0.32	0.00000000	mice
Themis	0.59	0.00000000	mice
Eif3g	0.41	0.00000000	mice
Oaz1	0.29	0.00000000	mice
Nebl	0.72	0.00000000	mice
Dffb	0.42	0.00000000	mice
Tlk1	0.37	0.00000000	mice
Caprin1	0.29	0.00000000	mice
Rhoa	-0.54	0.00000000	mice
Ckb	-0.87	0.00000000	mice

Lef1	0.95	0.00000000	mice
Ep400	0.28	0.00000000	mice
Fam8a1	0.37	0.00000000	mice
Efl1	0.37	0.00000000	mice
Rpa2	0.42	0.00000000	mice
Rc3h1	0.38	0.00000000	mice
Ing1	0.46	0.00000000	mice
Ctbp1	0.53	0.00000000	mice
Pcmtd1	0.32	0.00000000	mice
Dhx15	0.31	0.00000000	mice
Eif3b	0.31	0.00000000	mice
Lrrc31	0.58	0.00000000	mice
Ptpn9	0.41	0.00000000	mice
Trmt10c	0.36	0.00000000	mice
Prkd3	0.31	0.00000000	mice
Ctdp1	0.35	0.00000000	mice
Rfc1	0.35	0.00000000	mice
Siah2	0.49	0.00000000	mice
Kif5c	0.87	0.00000000	mice
Ptk2	-1.09	0.00000000	mice
Tcea1	0.36	0.00000000	mice
Gcnt4	0.44	0.00000000	mice
Nol9	0.24	0.00000000	mice
Thyn1	0.28	0.00000000	mice
Nr4a3	0.41	0.00000000	mice
Atxn2	0.23	0.00000000	mice
Oxr1	0.31	0.00000000	mice
Lamtor2	0.27	0.00000000	mice
Ndufa13	0.38	0.00000000	mice
Rcsd1	0.45	0.00000000	mice
Ssrp1	0.36	0.00000000	mice
Auh	0.54	0.00000000	mice
Jarid2	0.55	0.00000000	mice
Usp44	0.45	0.00000000	mice
Tox	0.71	0.00000000	mice
Btbd9	0.41	0.00000000	mice
Rnf216	0.46	0.00000000	mice
Cep83	-1.10	0.00000000	mice
Atp2a2	0.49	0.00000000	mice
Xpo4	0.42	0.00000000	mice
Rasa2	0.38	0.00000000	mice
Lrrc8d	0.46	0.00000000	mice
Epb41l3	-0.45	0.00000000	mice
Rbm33	0.18	0.00000000	mice
Adam19	0.46	0.00000000	mice
Ttbk1	0.46	0.00000000	mice

Itm2a	-0.68	0.00000000	mice
Rilpl2	0.31	0.00000000	mice
Usp7	0.15	0.00000000	mice
Pms2	0.38	0.00000000	mice
Snrpe	0.28	0.00000000	mice
Ccr7	0.57	0.00000000	mice
Idua	0.36	0.00000000	mice
Sash3	0.43	0.00000000	mice
Wdr44	0.26	0.00000000	mice
Cabin1	0.37	0.00000000	mice
Clk1	0.28	0.00000000	mice
Vps37b	0.35	0.00000000	mice
Tial1	0.26	0.00000000	mice
Sms	0.39	0.00000000	mice
Ssb	0.38	0.00000000	mice
Gnl3	0.45	0.00000000	mice
Ppib	0.31	0.00000000	mice
Alcam	-0.80	0.00000000	mice
Fry	-0.75	0.00000000	mice
Ppp4r3a	0.20	0.00000000	mice
Ptges3	0.29	0.00000000	mice
Borcs5	0.51	0.00000000	mice
Smurf2	-0.99	0.00000000	mice
Psmg2	0.29	0.00000000	mice
Tab2	0.33	0.00000000	mice
Aff1	0.74	0.00000000	mice
Ankib1	0.48	0.00000000	mice
Rnf138	0.50	0.00000000	mice
Usp54	-1.12	0.00000000	mice
Nf1	0.32	0.00000000	mice
Tmem131l	0.35	0.00000000	mice
Capn7	0.37	0.00000000	mice
Chic2	0.35	0.00000000	mice
Bag5	-1.18	0.00000000	mice
Xpot	0.36	0.00000000	mice
Rpa1	0.29	0.00000000	mice
Nr4a1	0.21	0.00000000	mice
Rrp1b	0.30	0.00000000	mice
Cdca7l	-1.09	0.00000000	mice
Tspan4	-0.81	0.00000000	mice
Grk3	0.29	0.00000000	mice
Herc1	0.09	0.00000000	mice
Ebf1	-1.15	0.00000000	mice
Gnai2	0.40	0.00000000	mice
Grk4	0.43	0.00000000	mice
Arl3	0.28	0.00000000	mice

Cul3	0.20	0.00000000	mice
Rpn2	0.29	0.00000000	mice
Patj	0.55	0.00000000	mice
Dnajc2	0.32	0.00000000	mice
Nlk	0.32	0.00000000	mice
Slu7	0.16	0.00000000	mice
Arl6ip5	0.29	0.00000000	mice
Parp1	0.40	0.00000000	mice
Ptdss1	0.43	0.00000000	mice
Pip4k2a	-0.35	0.00000000	mice
Hps3	0.30	0.00000000	mice
Nab1	0.40	0.00000000	mice
Lpxn	0.47	0.00000000	mice
Amd2	0.33	0.00000000	mice
Pspc1	0.26	0.00000000	mice
Slc9a7	0.52	0.00000000	mice
Hsdl2	0.45	0.00000000	mice
Ssu72	0.25	0.00000000	mice
Arf6	0.27	0.00000000	mice
Iqgap2	0.39	0.00000000	mice
Aqr	0.28	0.00000000	mice
Cyfip1	-0.88	0.00000000	mice
Oxsr1	-1.02	0.00000000	mice
Rhof	0.33	0.00000000	mice
Snrpa1	0.35	0.00000000	mice
Thada	0.39	0.00000000	mice
Srsf11	0.27	0.00000000	mice
Atad5	0.55	0.00000000	mice
Btf3l4	0.29	0.00000000	mice
Pabpc1	0.31	0.00000000	mice
Rftn1	0.55	0.00000000	mice
Rab11fip4	0.43	0.00000000	mice
Tns3	-0.66	0.00000000	mice
Akap9	0.34	0.00000000	mice
Itpk1	-1.40	0.00000000	mice
Urb1	0.45	0.00000000	mice
Btaf1	0.24	0.00000000	mice
Tcerg1	0.29	0.00000000	mice
Slc43a2	0.49	0.00000000	mice
Rpgr	0.45	0.00000000	mice
Eif2s3y	0.32	0.00000000	mice
Ddx17	0.01	0.00000000	mice
Tasp1	0.45	0.00000000	mice
Scyl2	0.45	0.00000000	mice
Il10ra	0.39	0.00000000	mice
Bbx	0.27	0.00000000	mice

Sf3a3	0.30	0.00000000	mice
Bcl2	0.55	0.00000000	mice
Naa15	0.32	0.00000000	mice
Ep300	0.23	0.00000000	mice
Nfatc2	0.51	0.00000000	mice
Heatr5b	0.49	0.00000000	mice
Sun2	0.31	0.00000000	mice
Lipg	0.41	0.00000000	mice
Kctd20	0.33	0.00000000	mice
Tmc7	0.36	0.00000000	mice
Lyn	-0.94	0.00000000	mice
Acsl3	0.45	0.00000000	mice
Tulp3	0.33	0.00000000	mice
Smap2	0.27	0.00000000	mice
Nbn	0.31	0.00000000	mice
Atp6v0e	0.25	0.00000000	mice
Kdm2b	0.36	0.00000000	mice
Lmo2	-0.75	0.00000000	mice
Bmt2	0.33	0.00000000	mice
Cep112	0.36	0.00000000	mice
Tbl1xr1	0.38	0.00000000	mice
Sacs	0.40	0.00000000	mice
Ran	0.21	0.00000000	mice
Trio	-1.26	0.00000000	mice
Psme3	0.33	0.00000000	mice
Rad51c	0.39	0.00000000	mice
Gpr155	0.38	0.00000000	mice
Ifnar2	0.28	0.00000000	mice
Chd9	0.29	0.00000000	mice
F13a1	-0.57	0.00000000	mice
Helz	0.22	0.00000000	mice
Fkbp3	0.31	0.00000000	mice
Rfx3	0.67	0.00000000	mice
Utp20	0.27	0.00000000	mice
Elovl6	0.65	0.00000000	mice
Dpf3	0.75	0.00000000	mice
Celf1	0.23	0.00000000	mice
Cstf3	0.22	0.00000000	mice
Rbx1	0.24	0.00000000	mice
Klf13	0.38	0.00000000	mice
Snrpd1	0.23	0.00000000	mice
Il27ra	0.32	0.00000000	mice
Srsf3	0.23	0.00000000	mice
Arhgap5	-1.00	0.00000000	mice
Cep85l	0.43	0.00000000	mice
Thoc2	0.18	0.00000000	mice

Erc1	-0.94	0.00000000	mice
Emc2	0.35	0.00000000	mice
Cdv3	0.20	0.00000000	mice
Cntrl	0.28	0.00000000	mice
Hook3	0.20	0.00000000	mice
Ints13	0.28	0.00000000	mice
Nhp2	0.25	0.00000000	mice
Eif2a	0.43	0.00000000	mice
Ipo5	0.28	0.00000000	mice
Ccdc82	0.39	0.00000000	mice
Anapc1	0.30	0.00000000	mice
Dnajc13	0.32	0.00000000	mice
Eif2b2	0.37	0.00000000	mice
Smim14	-0.87	0.00000000	mice
Fbxo32	0.43	0.00000000	mice
Fbxl22	0.35	0.00000000	mice
Scaf8	0.23	0.00000000	mice
Ikzf3	0.38	0.00000000	mice
Foxk1	0.35	0.00000000	mice
Hs6st1	-0.95	0.00000000	mice
Stk26	0.25	0.00000000	mice
Arhgap25	0.39	0.00000000	mice
Hipk1	0.17	0.00000000	mice
Lck	0.30	0.00000000	mice
Itpr2	0.34	0.00000000	mice
Fbxl5	0.25	0.00000000	mice
Ahi1	0.37	0.00000000	mice
Ints6	-1.14	0.00000000	mice
Ptpn6	0.33	0.00000000	mice
Sugt1	0.40	0.00000000	mice
Dicer1	0.18	0.00000000	mice
Ddx6	0.20	0.00000000	mice
Bach2	0.55	0.00000000	mice
Rpn1	0.21	0.00000000	mice
Shoc2	0.40	0.00000000	mice
Ppig	0.21	0.00000000	mice
Scaper	0.32	0.00000000	mice
Gtdc1	0.57	0.00000000	mice
Gpbp1l1	0.17	0.00000000	mice
Aimp1	0.21	0.00000000	mice
Xrn2	0.26	0.00000000	mice
N4bp1	0.29	0.00000000	mice
Acsl5	0.51	0.00000000	mice
Slc39a6	0.33	0.00000000	mice
Fndc3a	0.35	0.00000000	mice
Zfp711	0.29	0.00000000	mice

Pcnx2	0.40	0.00000000	mice
Aldh18a1	0.29	0.00000000	mice
Pld1	0.32	0.00000000	mice
Ube3a	0.22	0.00000000	mice
Naa25	0.34	0.00000000	mice
Ndufb1	0.23	0.00000000	mice
Arglu1	0.08	0.00000000	mice
Tmem258	0.22	0.00000000	mice
Sptan1	-1.11	0.00000000	mice
Ibtk	0.17	0.00000000	mice
Sike1	0.29	0.00000000	mice
Zfp326	0.27	0.00000000	mice
Osbpl1a	-1.13	0.00000000	mice
Srpr	0.23	0.00000000	mice
Cyth3	-0.89	0.00000000	mice
Gramd4	0.48	0.00000000	mice
Edc3	0.30	0.00000000	mice
Sytl1	0.38	0.00000000	mice
Sf3b5	0.27	0.00000000	mice
Ppp1r27	0.35	0.00000000	mice
Notch2	0.47	0.00000000	mice
Pnldc1	0.38	0.00000000	mice
Stx11	0.61	0.00000000	mice
Anks1	-0.88	0.00000000	mice
Elavl1	0.17	0.00000000	mice
Atp2b1	0.38	0.00000000	mice
Nfatc1	0.92	0.00000000	mice
Ubash3a	0.50	0.00000000	mice
Acer2	-0.80	0.00000000	mice
St6gal1	0.48	0.00000000	mice
U2af1	0.20	0.00000000	mice
Sod2	0.37	0.00000000	mice
L3mbtl3	0.28	0.00000000	mice
Kdm5b	0.27	0.00000000	mice
Prkd1	0.48	0.00000000	mice
Prkcz	0.39	0.00000000	mice
Naxd	0.22	0.00000000	mice
Zc3h15	0.22	0.00000000	mice
Tnpo1	0.15	0.00000000	mice
Sipa1l2	-0.44	0.00000000	mice
Dcp2	0.31	0.00000000	mice
Epha5	0.43	0.00000000	mice
Grap2	0.55	0.00000000	mice
Pcnx	0.19	0.00000000	mice
Edem1	0.27	0.00000000	mice
Sgms1	0.29	0.00000000	mice

Etl4	-0.80	0.00000000	mice
Rap1gds1	0.35	0.00000000	mice
Dok2	0.36	0.00000000	mice
Acp1	0.15	0.00000000	mice
Atp1b1	0.24	0.00000000	mice
Cnot4	0.29	0.00000000	mice
Arid5a	0.34	0.00000000	mice
Hspa8	-0.54	0.00000000	mice
Odc1	0.21	0.00000000	mice
Xpnpep1	0.20	0.00000000	mice
Luzp1	-1.01	0.00000000	mice
Sulf2	0.87	0.00000000	mice
Lcp1	0.22	0.00000000	mice
Csgalnact2	0.30	0.00000000	mice
Pgam1	0.32	0.00000000	mice
Tmem11	0.30	0.00000000	mice
Rnmt	0.14	0.00000000	mice
Abcb10	-0.81	0.00000000	mice
Tsnax	0.33	0.00000000	mice
Supt3	0.42	0.00000000	mice
Cep104	0.29	0.00000000	mice
Yap1	-0.96	0.00000000	mice
Ccndbp1	0.26	0.00000000	mice
Eif4h	0.16	0.00000000	mice
Farsb	0.31	0.00000000	mice
Aagab	0.33	0.00000000	mice
Yeats4	0.34	0.00000001	mice
Lims2	-1.03	0.00000001	mice
Luc7l3	0.13	0.00000001	mice
Kidins220	0.11	0.00000001	mice
Hs6st2	0.27	0.00000001	mice
Ptbp2	0.14	0.00000001	mice
Fdx1	0.19	0.00000001	mice
Skil	0.19	0.00000001	mice
Ppp2r2a	0.08	0.00000001	mice
Ppp1r8	0.26	0.00000001	mice
Ankrd28	0.28	0.00000001	mice
Dhx29	0.37	0.00000001	mice
Prrc2b	0.16	0.00000001	mice
Dennd2d	0.44	0.00000001	mice
Def6	0.35	0.00000001	mice
Znhit6	0.24	0.00000001	mice
Aplp2	-1.04	0.00000001	mice
Bcl7a	0.30	0.00000001	mice
Camk1d	0.37	0.00000001	mice
Gsn	-1.09	0.00000001	mice

Wnk1	0.17	0.00000001	mice
Agps	0.23	0.00000001	mice
4933400A11Rik	0.34	0.00000001	mice
Srsf5	0.14	0.00000001	mice
Jade2	0.17	0.00000001	mice
Galnt9	0.36	0.00000001	mice
Rpap2	0.30	0.00000001	mice
Dtwd2	0.39	0.00000001	mice
Stk3	0.30	0.00000001	mice
Rcan1	-0.81	0.00000001	mice
Tk2	0.19	0.00000001	mice
Nr2c2	0.20	0.00000001	mice
Trp53rka	0.30	0.00000001	mice
Zbtb43	0.24	0.00000001	mice
Prpf40a	0.17	0.00000001	mice
Traf6	0.29	0.00000001	mice
Pcif1	0.28	0.00000001	mice
Slc41a1	0.23	0.00000001	mice
Sdhaf4	0.23	0.00000001	mice
Zbtb1	0.33	0.00000001	mice
Rapgef2	-0.78	0.00000002	mice
Bambi	-0.52	0.00000002	mice
Ccnd3	0.32	0.00000002	mice
Arfip1	0.26	0.00000002	mice
Enpp4	0.48	0.00000002	mice
Nbas	0.17	0.00000002	mice
Pdxk	0.30	0.00000002	mice
Klhl7	0.18	0.00000002	mice
Surf4	0.23	0.00000002	mice
Grsf1	0.23	0.00000002	mice
Spty2d1	-0.70	0.00000003	mice
Ppil1	0.31	0.00000003	mice
Hlf	-0.80	0.00000003	mice
Dusp5	0.31	0.00000003	mice
Ncoa2	0.33	0.00000003	mice
Gyg	0.18	0.00000003	mice
Irak4	0.19	0.00000004	mice
Asap2	0.52	0.00000004	mice
Stat2	0.26	0.00000004	mice
Fam168a	-0.91	0.00000004	mice
Ndufa5	0.25	0.00000004	mice
Bend5	-0.70	0.00000004	mice
Snapc1l	0.29	0.00000004	mice
Hnrnph3	0.10	0.00000005	mice
Zfp384	0.20	0.00000005	mice
Sav1	0.26	0.00000005	mice

Strn	0.30	0.00000005	mice
Yrdc	0.28	0.00000006	mice
Usp15	0.10	0.00000006	mice
Zmynd8	0.14	0.00000006	mice
Lmo3	-0.86	0.00000006	mice
Sowahc	0.40	0.00000006	mice
Ddx1	0.16	0.00000006	mice
Lats1	0.20	0.00000006	mice
Denr	0.25	0.00000006	mice
Acaca	0.34	0.00000006	mice
Setx	0.12	0.00000007	mice
G3bp1	0.14	0.00000007	mice
Yme1l1	0.20	0.00000007	mice
Svil	-0.85	0.00000007	mice
Tacc3	0.29	0.00000007	mice
Pigb	0.28	0.00000008	mice
Bivm	0.35	0.00000008	mice
Zyx	0.27	0.00000008	mice
Azi2	0.22	0.00000008	mice
Usp28	-0.81	0.00000009	mice
Inpp5a	-0.61	0.00000009	mice
Zbtb21	0.12	0.00000009	mice
Nup214	0.34	0.00000009	mice
Ubr5	0.16	0.00000009	mice
Senp6	0.33	0.00000010	mice
Prrc2c	0.11	0.00000010	mice
Ppp2r5c	0.12	0.00000010	mice
Pdk1	0.28	0.00000010	mice
Hax1	0.18	0.00000011	mice
Dynlt1a	0.21	0.00000011	mice
Ptpn2	0.16	0.00000011	mice
Actg1	-0.25	0.00000012	mice
Sec61g	0.16	0.00000012	mice
Sft2d2	0.25	0.00000013	mice
Slc16a10	0.58	0.00000013	mice
Pi4k2b	0.18	0.00000013	mice
Tnrc6a	0.15	0.00000014	mice
Nlrc3	0.29	0.00000014	mice
Dhx36	0.08	0.00000015	mice
Brd7	0.16	0.00000015	mice
Fam222b	0.25	0.00000015	mice
Pds5a	0.07	0.00000016	mice
Zfp831	0.34	0.00000016	mice
Tpr	0.12	0.00000016	mice
Zfat	0.01	0.00000018	mice
Snrpc	0.31	0.00000019	mice

Polr2l	0.21	0.00000019	mice
Zfand1	0.28	0.00000019	mice
Ranbp17	0.47	0.00000019	mice
Nav3	0.55	0.00000019	mice
Lmbrd1	0.24	0.00000019	mice
Anapc7	0.29	0.00000019	mice
Arhgap45	0.30	0.00000019	mice
Atm	0.34	0.00000019	mice
Rbl2	0.25	0.00000020	mice
Galc	0.35	0.00000020	mice
Kat2b	-0.87	0.00000020	mice
Ets2	-0.88	0.00000021	mice
Ppid	0.29	0.00000021	mice
Kdm3b	0.09	0.00000021	mice
Ndufb9	0.32	0.00000022	mice
Tdg	0.30	0.00000022	mice
Nup210	0.16	0.00000024	mice
Sntb1	0.35	0.00000024	mice
Btrc	0.15	0.00000024	mice
Rpp40	0.25	0.00000025	mice
Itpkb	0.23	0.00000025	mice
Tmx1	0.21	0.00000026	mice
Fbxo33	0.10	0.00000027	mice
Plcg2	0.45	0.00000028	mice
Srp14	0.26	0.00000029	mice
Gata3	0.53	0.00000029	mice
Gnptab	0.32	0.00000029	mice
Setd5	0.16	0.00000029	mice
Snx20	0.32	0.00000029	mice
Smc6	0.29	0.00000029	mice
Stom	0.10	0.00000030	mice
Dcaf1	0.11	0.00000031	mice
Dazap1	0.12	0.00000032	mice
Sbno2	0.21	0.00000032	mice
Ankrd13a	0.21	0.00000033	mice
Cog6	0.10	0.00000035	mice
Tada1	0.25	0.00000035	mice
Actr6	0.31	0.00000035	mice
Zcchc2	0.09	0.00000036	mice
Ankrd49	0.23	0.00000036	mice
Foxn3	-0.77	0.00000036	mice
Pphln1	0.26	0.00000038	mice
Dnmt1	0.22	0.00000038	mice
Raf1	0.16	0.00000038	mice
Eif4g1	0.22	0.00000039	mice
Tmem167	0.19	0.00000039	mice

Tfip11	0.16	0.00000039	mice
Dnaja4	0.66	0.00000040	mice
Baz1b	0.18	0.00000040	mice
Kin	0.14	0.00000041	mice
Ndufa8	0.15	0.00000042	mice
Glyr1	0.17	0.00000042	mice
Erp29	0.21	0.00000043	mice
Ankrd39	-0.54	0.00000045	mice
Ttll12	0.22	0.00000045	mice
Nmt2	0.30	0.00000047	mice
Exosc9	0.22	0.00000048	mice
Slain2	0.15	0.00000050	mice
Suclg2	0.43	0.00000052	mice
Stx8	0.23	0.00000052	mice
Psmd13	0.23	0.00000053	mice
Asxl2	0.22	0.00000053	mice
Eif1	0.19	0.00000055	mice
Ilf3	0.19	0.00000056	mice
Zbtb49	0.27	0.00000057	mice
Trappc8	0.26	0.00000057	mice
Papss1	0.12	0.00000057	mice
Utp23	0.30	0.00000059	mice
Cnst	0.29	0.00000059	mice
Kpna1	0.02	0.00000061	mice
Nudc	0.26	0.00000063	mice
Fbxo11	0.18	0.00000064	mice
Eny2	0.14	0.00000064	mice
Wdr77	0.27	0.00000066	mice
B3gntl1	0.42	0.00000066	mice
Bclaf1	0.07	0.00000069	mice
Iars2	0.15	0.00000069	mice
Rrbp1	0.23	0.00000069	mice
Atg16l1	0.20	0.00000071	mice
Arcn1	0.17	0.00000072	mice
Cdc42se2	0.52	0.00000074	mice
Fas	0.31	0.00000074	mice
Zcchc8	0.14	0.00000075	mice
Dcun1d2	-1.05	0.00000076	mice
Pum1	0.11	0.00000082	mice
Tecpr2	0.31	0.00000082	mice
Rdx	0.13	0.00000083	mice
Srp72	0.23	0.00000084	mice
Rbm15	0.28	0.00000085	mice
Aacs	-0.72	0.00000089	mice
Pyroxd1	0.33	0.00000097	mice
Uhrf1bp1l	0.24	0.00000100	mice

Gins2	0.30	0.00000101	mice
Qrich1	0.12	0.00000103	mice
Ndufa2	0.10	0.00000104	mice
Sync	0.26	0.00000105	mice
Fkbp4	0.20	0.00000105	mice
Eef1e1	0.20	0.00000107	mice
Hdx	0.11	0.00000112	mice
Gnpda2	0.18	0.00000114	mice
Tpi1	0.38	0.00000116	mice
Kcnk1	0.30	0.00000116	mice
Polr2e	0.11	0.00000117	mice
Ddx46	0.13	0.00000118	mice
Taf2	0.11	0.00000130	mice
Lrrfip1	0.23	0.00000134	mice
Smg1	0.16	0.00000135	mice
Cc2d2a	0.26	0.00000136	mice
Fam177a	-0.87	0.00000142	mice
Rpap3	0.22	0.00000145	mice
Pip5k1a	-0.77	0.00000147	mice
Cbl	0.22	0.00000160	mice
Tfg	0.22	0.00000164	mice
Prelid1	0.17	0.00000168	mice
Arv1	0.25	0.00000170	mice
Uxs1	0.31	0.00000177	mice
Osbpl11	0.20	0.00000178	mice
Btbd3	0.33	0.00000185	mice
Dusp10	0.23	0.00000185	mice
Srsf10	0.20	0.00000202	mice
Csde1	0.02	0.00000204	mice
Pign	0.26	0.00000214	mice
Fbxl3	0.15	0.00000217	mice
Snhg4	0.09	0.00000227	mice
Tmed10	0.12	0.00000229	mice
Ube2b	0.13	0.00000236	mice
St3gal1	-0.80	0.00000240	mice
Tnip3	0.33	0.00000241	mice
Rexo2	0.13	0.00000242	mice
Jak2	0.13	0.00000243	mice
Armt1	0.33	0.00000246	mice
Ptpn7	0.27	0.00000252	mice
Psma1	0.18	0.00000258	mice
Acvr2a	0.18	0.00000266	mice
Htra2	0.26	0.00000292	mice
Pard3b	-0.97	0.00000298	mice
Adipor1	0.14	0.00000299	mice
Champ1	0.30	0.00000326	mice

Pnpla8	-0.08	0.00000327	mice
Ogfr	0.20	0.00000330	mice
Pxk	0.24	0.00000344	mice
Rad21	0.13	0.00000350	mice
Prkca	0.27	0.00000356	mice
Xpa	0.10	0.00000367	mice
Tiam1	-0.55	0.00000370	mice
Pes1	0.24	0.00000373	mice
Atp23	0.17	0.00000380	mice
Rwdd2b	0.26	0.00000381	mice
Arpc5	0.10	0.00000397	mice
Ccnd2	0.31	0.00000397	mice
Prmt9	0.17	0.00000401	mice
Ddx23	0.24	0.00000414	mice
Tnfrsf26	0.26	0.00000421	mice
Zfp644	0.14	0.00000427	mice
Atf6	0.13	0.00000443	mice
Asap1	0.23	0.00000445	mice
Wdr82	0.12	0.00000446	mice
Sesn3	0.18	0.00000460	mice
Cpsf2	0.14	0.00000461	mice
Smarce1	0.15	0.00000484	mice
Pdpk1	0.15	0.00000503	mice
Clec16a	0.19	0.00000511	mice
Tcp1	0.12	0.00000521	mice
Ss18	0.17	0.00000523	mice
Sbno1	-0.01	0.00000527	mice
Ckap5	0.16	0.00000534	mice
Pnn	0.08	0.00000545	mice
Ppil4	0.16	0.00000550	mice
Uap1	0.20	0.00000556	mice
Ppp1r7	0.22	0.00000558	mice
Hdac2	0.21	0.00000568	mice
Vps39	0.19	0.00000571	mice
Krit1	0.22	0.00000583	mice
Eif4a3l1	0.15	0.00000588	mice
Dusp7	-0.90	0.00000607	mice
Gzf1	0.16	0.00000614	mice
Atl2	0.12	0.00000616	mice
Appl2	0.20	0.00000648	mice
Nsrp1	0.09	0.00000650	mice
Pofut1	0.24	0.00000714	mice
Plcb2	0.28	0.00000731	mice
Anapc5	0.23	0.00000772	mice
Sccpdh	0.23	0.00000775	mice
Nol11	0.21	0.00000777	mice

Rtn4	-0.83	0.00000788	mice
Thrap3	0.17	0.00000793	mice
Zbtb6	0.25	0.00000798	mice
Hsph1	0.32	0.00000802	mice
Tacc1	0.18	0.00000814	mice
Ghitm	0.04	0.00000929	mice
Gpr68	0.48	0.00000938	mice
Supt20	0.13	0.00000938	mice
Arih2	0.25	0.00000960	mice
Asns	0.30	0.00000962	mice
G3bp2	0.19	0.00000982	mice
Ppp1r9a	0.39	0.00000995	mice
Eif2ak2	0.24	0.00000995	mice
Ttc28	-0.83	0.00001009	mice
Tmsb4x	0.15	0.00001035	mice
Dnaja3	0.22	0.00001040	mice
Unc119b	0.16	0.00001072	mice
Uchl3	0.32	0.00001089	mice
Paqr3	0.23	0.00001123	mice
Ikbkb	0.28	0.00001130	mice
Zw10	0.26	0.00001185	mice
Prkx	0.31	0.00001224	mice
Acadl	0.10	0.00001259	mice
Snip1	0.14	0.00001300	mice
Kdm1b	0.25	0.00001322	mice
Hace1	0.19	0.00001336	mice
Lcor	0.11	0.00001336	mice
Akap1	0.24	0.00001361	mice
Lasp1	0.15	0.00001410	mice
Galnt7	0.26	0.00001454	mice
Ell	0.10	0.00001467	mice
Trmt2a	0.19	0.00001495	mice
Cript	0.12	0.00001498	mice
Usp22	-1.00	0.00001535	mice
Nop14	0.15	0.00001554	mice
Dhx38	0.16	0.00001576	mice
Oat	-0.57	0.00001624	mice
Nsl1	0.21	0.00001647	mice
Klhl36	0.21	0.00001677	mice
Setd2	0.12	0.00001690	mice
F5	-0.98	0.00001797	mice
Otud7a	0.44	0.00001814	mice
Clp1	0.28	0.00001858	mice
Atg2b	-0.01	0.00001865	mice
Limd2	0.23	0.00001938	mice
Fam32a	0.01	0.00001951	mice

Snw1	0.10	0.00002017	mice
Traf1	0.31	0.00002068	mice
Nfrkb	0.21	0.00002096	mice
Ifi35	0.05	0.00002161	mice
Slc6a6	-0.87	0.00002161	mice
Slc25a16	0.19	0.00002229	mice
Gcn1	0.12	0.00002289	mice
Dnajc7	0.10	0.00002319	mice
Hspa14	0.15	0.00002418	mice
Ubn2	0.11	0.00002479	mice
Sfmbt1	0.39	0.00002508	mice
Kif21b	0.23	0.00002542	mice
Primpol	0.21	0.00002574	mice
Ercc6	0.24	0.00002584	mice
Ipo11	0.20	0.00002599	mice
Tmem87a	0.11	0.00002660	mice
Gtf3c1	0.10	0.00002896	mice
Cwc27	0.24	0.00002899	mice
Hacd2	0.09	0.00002917	mice
Rfx2	0.17	0.00003041	mice
Fnbp1	0.14	0.00003051	mice
Trak1	-0.74	0.00003055	mice
Xpc	0.21	0.00003085	mice
Akap11	0.17	0.00003150	mice
Prim2	0.29	0.00003236	mice
Ostf1	0.13	0.00003282	mice
Ubr1	0.12	0.00003286	mice
Nsfl1c	0.20	0.00003414	mice
Ipo7	0.11	0.00003611	mice
Tbc1d15	0.15	0.00003628	mice
Hmgcs1	0.15	0.00003708	mice
Rasgef1b	0.37	0.00003729	mice
Prpf3	0.17	0.00003768	mice
Nbeal2	0.23	0.00003899	mice
Zcchc7	0.17	0.00004042	mice
Klf7	-0.73	0.00004074	mice
Dda1	0.15	0.00004093	mice
Tnfrsf21	0.34	0.00004145	mice
Osgin2	0.35	0.00004360	mice
Pibf1	0.28	0.00004406	mice
Ttyh3	0.07	0.00004413	mice
Lrig1	0.49	0.00004426	mice
Cnn2	0.24	0.00004642	mice
Tet2	-0.65	0.00004719	mice
Sf3a1	0.05	0.00004854	mice
Slc39a9	0.18	0.00004904	mice

Zfp207	0.07	0.00004967	mice
Rbfa	-0.67	0.00005082	mice
Rnf114	0.21	0.00005084	mice
Chordc1	0.22	0.00005132	mice
Trmt6	0.26	0.00005244	mice
Utp6	0.18	0.00005248	mice
Nipa1	0.22	0.00005335	mice
Cnot6	0.09	0.00005431	mice
Rit2	0.10	0.00005466	mice
Ric8b	0.20	0.00005544	mice
Luc7l	0.11	0.00005576	mice
Ap1g1	0.05	0.00005662	mice
Kdm4c	0.20	0.00005883	mice
Erich1	0.26	0.00005911	mice
Tgif1	0.21	0.00005924	mice
Gnai3	0.12	0.00005937	mice
Nmi	0.17	0.00006324	mice
Ccdc88c	0.10	0.00006522	mice
Dis3	0.22	0.00006532	mice
Trappc4	0.17	0.00006538	mice
Esf1	0.16	0.00006570	mice
Dek	0.06	0.00006885	mice
Exoc2	0.13	0.00007057	mice
Eml4	0.38	0.00007381	mice
Exosc7	0.21	0.00007591	mice
Eif3e	0.09	0.00007792	mice
Polr2k	0.10	0.00007826	mice
Panx1	0.28	0.00008008	mice
Rnaseh2b	0.06	0.00008010	mice
Dkc1	0.30	0.00008092	mice
Rmnd1	0.13	0.00008628	mice
Ywhaz	0.07	0.00008744	mice
Ankrd17	0.11	0.00008895	mice
Twf1	-0.63	0.00008946	mice
Nsd3	0.03	0.00008966	mice
Pou2f1	0.20	0.00009128	mice
Ptk2b	0.24	0.00009185	mice
Sec23b	0.18	0.00009254	mice
Hadh	0.15	0.00009331	mice
Serbp1	0.19	0.00009357	mice
Rer1	0.19	0.00009521	mice
Gnat2	0.30	0.00009761	mice
Ddx52	0.18	0.00009918	mice
Birc6	0.17	0.00010180	mice
Vps13c	0.22	0.00010347	mice
Sbds	0.16	0.00010444	mice

Emc6	0.15	0.00010491	mice
Syf2	0.08	0.00011039	mice
Ywhae	-0.02	0.00011219	mice
Cnot7	-0.03	0.00011533	mice
Heatr6	0.21	0.00011625	mice
Lrrc47	0.20	0.00011688	mice
Cdk6	0.31	0.00011715	mice
Eif5a2	0.14	0.00011946	mice
Nek7	0.20	0.00012072	mice
Wdr20	0.12	0.00012073	mice
Ing4	0.12	0.00012117	mice
Get4	0.14	0.00012443	mice
Slc22a15	0.24	0.00012454	mice
Atp13a3	0.28	0.00012454	mice
Aspm	0.29	0.00012499	mice
Cpeb4	-0.92	0.00012660	mice
Uso1	-0.02	0.00013037	mice
Drg1	0.19	0.00013517	mice
Txnrd3	0.18	0.00013678	mice
Psmd1	-0.01	0.00013995	mice
Bcas2	0.17	0.00014146	mice
Ralgapb	0.11	0.00014413	mice
Sec16a	-0.03	0.00014972	mice
Uhmk1	0.15	0.00015179	mice
Rsl1d1	0.23	0.00015415	mice
Taf15	0.21	0.00015431	mice
Arhgef18	0.14	0.00015453	mice
Nup85	0.18	0.00015707	mice
Gnl2	0.13	0.00015948	mice
Hsp90aa1	0.40	0.00016488	mice
Pin4	0.19	0.00017144	mice
Nap1l1	0.16	0.00017642	mice
Rasgrp1	0.27	0.00017901	mice
Xbp1	0.12	0.00017956	mice
Rapgef1	0.19	0.00018142	mice
Arhgef12	-0.99	0.00018851	mice
Eif2b1	0.17	0.00019024	mice
Dnajb6	0.11	0.00019121	mice
Ndufs4	0.11	0.00019394	mice
Vps54	-0.68	0.00020546	mice
Abce1	0.09	0.00020590	mice
Armc8	0.08	0.00020891	mice
Tcof1	0.13	0.00021039	mice
Katnbl1	0.26	0.00021127	mice
B3gnt5	0.25	0.00021253	mice
Chmp5	-0.06	0.00021831	mice

Ccnk	0.09	0.00021918	mice
Ppt1	-0.52	0.00022043	mice
Vdac2	0.08	0.00022261	mice
Dck	0.24	0.00022660	mice
Washc4	0.17	0.00023091	mice
Parn	0.06	0.00023341	mice
Scaf4	0.07	0.00023540	mice
Kat6a	0.00	0.00023816	mice
Eif2s2	0.07	0.00023829	mice
Cdk17	-0.85	0.00023959	mice
Fbxw11	0.11	0.00024006	mice
Slc25a13	0.09	0.00024160	mice
Deaf1	0.19	0.00024303	mice
Atp9b	0.06	0.00024556	mice
Cul2	0.04	0.00024927	mice
Tiprl	0.18	0.00025116	mice
Sec61a2	0.11	0.00025275	mice
Emp1	0.24	0.00025308	mice
Eif3i	-0.55	0.00025342	mice
Tab3	0.19	0.00025374	mice
Gosr1	0.14	0.00025518	mice
Thoc7	0.16	0.00025594	mice
Psme4	0.13	0.00026083	mice
Gpatch11	0.17	0.00026145	mice
Ctsc	0.18	0.00026356	mice
Lpcat3	0.13	0.00026505	mice
Hivep1	0.15	0.00027181	mice
Zc3h18	0.16	0.00028211	mice
Dnajc15	0.16	0.00028418	mice
Lrwd1	0.22	0.00029080	mice
Larp1	0.15	0.00029484	mice
Nceh1	0.36	0.00030068	mice
Atp6v0b	-0.05	0.00030574	mice
Hyou1	0.17	0.00031460	mice
Wdr25	0.18	0.00031612	mice
Uri1	0.12	0.00031658	mice
Ercc3	0.05	0.00033036	mice
Hs1bp3	0.14	0.00033474	mice
Zcchc10	0.16	0.00033492	mice
Eif3d	0.14	0.00033740	mice
Pnrc2	-0.05	0.00034050	mice
Tgfbr2	0.24	0.00034576	mice
Nub1	0.02	0.00035671	mice
Exoc6	0.17	0.00036138	mice
Iffo2	0.43	0.00036308	mice
Ik	0.03	0.00037549	mice

Fbxl4	0.09	0.00037587	mice
Ppp3cc	0.16	0.00037714	mice
Eepd1	0.27	0.00039138	mice
Fam168b	0.05	0.00039182	mice
Atp6v0c	-0.87	0.00039524	mice
Pisd	0.15	0.00040218	mice
Tsr3	0.22	0.00040691	mice
Fam120a	0.02	0.00041792	mice
Pkn2	0.04	0.00042250	mice
Ehd4	-0.87	0.00042616	mice
Hspa13	0.19	0.00042659	mice
Appbp2	0.21	0.00042670	mice
Pcm1	0.20	0.00043119	mice
Cct2	0.11	0.00043186	mice
Herpud2	-0.01	0.00043582	mice
Ubap1	0.08	0.00044972	mice
Npepps	0.07	0.00045698	mice
Zbtb46	0.10	0.00046742	mice
Ppp2r2d	0.09	0.00047489	mice
Nudt21	0.19	0.00048424	mice
Tnrc6b	-0.83	0.00048754	mice
Polr2f	0.06	0.00051313	mice
Srsf1	-0.03	0.00052067	mice
Tbpl1	0.20	0.00052167	mice
Psma4	0.02	0.00052231	mice
Btbd7	0.12	0.00052493	mice
Ttc17	0.08	0.00052957	mice
Khdrbs1	0.13	0.00054888	mice
Cops7a	0.14	0.00055000	mice
Foxk2	0.07	0.00055033	mice
Rnf145	0.01	0.00055225	mice
Tlnrd1	-0.73	0.00056951	mice
Abracl	0.08	0.00057451	mice
Sehl1	0.14	0.00057632	mice
Kpna6	0.10	0.00058312	mice
Eif2b3	0.13	0.00059431	mice
Kctd3	0.09	0.00060153	mice
Pdap1	0.05	0.00060476	mice
Agfg1	0.06	0.00060715	mice
Guk1	0.21	0.00061099	mice
Tbc1d5	0.23	0.00062581	mice
Fer	0.15	0.00062759	mice
Ube2g2	0.13	0.00063320	mice
Plagl2	0.26	0.00064159	mice
Gemin4	0.18	0.00065584	mice
Pik3r1	0.27	0.00066014	mice

Esyt2	0.22	0.00066608	mice
Ndufa9	0.23	0.00067216	mice
Tpk1	-0.67	0.00067690	mice
Ndufs3	0.09	0.00068280	mice
Tmem19	0.09	0.00069125	mice
St7l	0.12	0.00069330	mice
Btf3	0.12	0.00075135	mice
Tomm70a	0.13	0.00075181	mice
C2cd5	0.09	0.00075611	mice
Nr1d2	-0.72	0.00075838	mice
Prpf38a	0.16	0.00075901	mice
Rbm48	0.19	0.00076804	mice
Cep350	-0.09	0.00078048	mice
Suds3	0.06	0.00078196	mice
Arsg	0.22	0.00079045	mice
Prelid3b	0.08	0.00083532	mice
Galnt10	0.22	0.00084618	mice
Sec11a	0.21	0.00085718	mice
Ndufaf4	0.22	0.00086243	mice
Klhdc2	0.12	0.00086286	mice
Nup93	0.17	0.00086910	mice
Cct4	0.14	0.00087378	mice
Rit1	0.03	0.00088490	mice
Tnks2	0.07	0.00088636	mice
Arel1	0.11	0.00090629	mice
Txndc11	0.02	0.00092169	mice
Tia1	0.19	0.00092539	mice
Ccny	0.17	0.00092774	mice
Nop16	0.19	0.00096069	mice
Tmx3	0.04	0.00097582	mice
Wdr43	0.18	0.00098715	mice
Cwc25	0.11	0.00101757	mice
Capza2	0.03	0.00102066	mice
Apoa1	-1.31	0.00102259	mice
Fkbp1a	-0.88	0.00103097	mice
Ccdc59	0.18	0.00103610	mice
Aup1	0.24	0.00106828	mice
Kif3b	0.08	0.00108058	mice
Il20ra	0.36	0.00108728	mice
Tle1	0.38	0.00109046	mice
Nptn	-0.01	0.00110848	mice
Uba6	0.13	0.00112308	mice
Gpn3	0.11	0.00113214	mice
Prpf8	0.06	0.00114116	mice
Golga4	-0.85	0.00115312	mice
Fggy	0.21	0.00115430	mice

Uchl5	0.15	0.00115787	mice
Klhl2	0.20	0.00116469	mice
Pcgf5	0.10	0.00117433	mice
Selenot	0.08	0.00117659	mice
Ppm1a	-0.01	0.00122908	mice
Cipc	0.10	0.00123098	mice
Usp47	-0.03	0.00123245	mice
Hspa4	0.09	0.00123743	mice
Angel1	0.24	0.00131340	mice
Nek9	0.08	0.00137970	mice
Zfp335	0.03	0.00142523	mice
Ncoa5	0.12	0.00143107	mice
Sqstm1	0.07	0.00144758	mice
Kmt5b	-0.13	0.00148254	mice
Cdc42	-0.03	0.00148704	mice
Zranb2	0.09	0.00150074	mice
Selenof	0.02	0.00151944	mice
Gtf2a1	-0.04	0.00154219	mice
Git2	0.17	0.00160329	mice
Rbm39	0.01	0.00161356	mice
Rae1	0.13	0.00162065	mice
Srsf2	-0.01	0.00168892	mice
Fam104a	0.11	0.00169436	mice
Aco2	0.06	0.00173608	mice
Lonp1	0.15	0.00175248	mice
Rc3h2	0.19	0.00177571	mice
Fkbp11	0.15	0.00178247	mice
Erbin	0.11	0.00180340	mice
Ybx1	-0.38	0.00180472	mice
Isca2	0.17	0.00184942	mice
Ssh2	0.08	0.00187329	mice
Txndc17	0.00	0.00187702	mice
Upf1	0.02	0.00192311	mice
Wdr26	-0.01	0.00196716	mice
Stt3b	0.03	0.00198200	mice
Tgfbrap1	0.15	0.00201465	mice
Tbc1d1	0.20	0.00206380	mice
Exoc3	0.07	0.00207594	mice
Bmp2k	0.11	0.00210351	mice
Clk4	-0.04	0.00212943	mice
Fktn	0.07	0.00222587	mice
Arpc3	0.15	0.00222880	mice
Ola1	0.12	0.00233917	mice
Serinc1	0.10	0.00234103	mice
Ctcf	0.00	0.00236738	mice
Nol8	0.11	0.00241229	mice

Jmjd7	0.19	0.00241390	mice
Lrp12	0.02	0.00250607	mice
Pitpna	0.07	0.00250707	mice
Aprt	0.17	0.00253709	mice
Btbd11	0.26	0.00265756	mice
Pfkfb3	-0.88	0.00282086	mice
Nuak1	-0.53	0.00288669	mice
Sec24b	0.20	0.00298256	mice
Hagh	0.18	0.00301815	mice
Rprd2	0.12	0.00304324	mice
Lnpk	0.11	0.00310710	mice
Slc35b1	0.17	0.00312810	mice
Tnfaip8l1	0.17	0.00313074	mice
Zfp318	0.12	0.00314875	mice
Smarcb1	0.08	0.00316531	mice
Dgkz	0.23	0.00321735	mice
Ilf2	0.12	0.00322902	mice
Xrcc5	0.20	0.00324112	mice
Ap2a1	-0.02	0.00324867	mice
Spg11	0.18	0.00325854	mice
Ruvbl1	0.17	0.00327793	mice
Rabggtb	0.15	0.00330087	mice
Alad	0.18	0.00343158	mice
Arrdc1	0.19	0.00344574	mice
Topbp1	0.16	0.00347007	mice
Bub3	0.19	0.00348614	mice
Cd82	0.00	0.00350875	mice
Rad54l2	0.06	0.00360204	mice
Pias1	0.07	0.00371297	mice
Shq1	0.22	0.00378164	mice
Zc3h7a	0.11	0.00390362	mice
Immt	0.06	0.00395339	mice
Gpd2	0.24	0.00403868	mice
Slc30a9	0.10	0.00407606	mice
Dennd1a	0.09	0.00409460	mice
Zdhhc3	0.14	0.00412737	mice
Ctnnb1	-0.81	0.00413332	mice
Pik3ca	-0.75	0.00420440	mice
Ndufs8	0.07	0.00432021	mice
Pak1ip1	0.24	0.00434487	mice
Crlf3	0.13	0.00439609	mice
Rlf	0.07	0.00441678	mice
Rrn3	0.11	0.00450200	mice
Rab8a	0.00	0.00456768	mice
Ypel1	-0.83	0.00465917	mice
Nfxl1	0.06	0.00466193	mice

Prpf4b	-0.04	0.00467399	mice
Ube2v2	0.07	0.00471774	mice
Rtcb	0.13	0.00482831	mice
Cnnm2	-0.66	0.00483903	mice
Oxct1	0.05	0.00484167	mice
Stk10	0.06	0.00484969	mice
Ptges2	0.22	0.00490336	mice
Smarcc1	0.08	0.00495765	mice
Smarcal1	0.14	0.00497458	mice
Smim4	0.21	0.00501237	mice
Ankle2	0.03	0.00507197	mice
Ndel1	0.00	0.00507496	mice
Tmem140	0.09	0.00508236	mice
Ssbp1	0.19	0.00520191	mice
Gosr2	0.13	0.00522673	mice
Ncbp1	0.11	0.00525722	mice
Jagn1	0.17	0.00526097	mice
Lrig2	0.08	0.00526937	mice
Ech1	0.08	0.00527152	mice
Ppp1r13b	-0.82	0.00530994	mice
Prpsap2	0.18	0.00533528	mice
Ndufs2	0.08	0.00535540	mice
Gng2	0.13	0.00551208	mice
Hipk2	0.11	0.00556253	mice
Taf3	0.06	0.00561647	mice
Cct6b	0.02	0.00562167	mice
Cwc15	0.02	0.00567989	mice
Zmynd19	0.08	0.00569798	mice
Nup88	0.16	0.00590331	mice
Cpt1a	0.07	0.00591134	mice
Tank	0.07	0.00593956	mice
Usf3	0.12	0.00599537	mice
Ddx18	0.21	0.00624493	mice
Rundc3b	0.17	0.00639815	mice
Rabepk	-0.01	0.00641676	mice
Il2rb	0.32	0.00642246	mice
Abcd2	0.10	0.00643598	mice
Garnl3	-0.45	0.00649551	mice
Bnip2	0.00	0.00653543	mice
Dusp1	-0.34	0.00672602	mice
Bicral	-0.04	0.00677879	mice
Nucb2	0.02	0.00678711	mice
Hspa4l	0.10	0.00692576	mice
Il21r	0.21	0.00696766	mice
4833439L19Rik	0.12	0.00697741	mice
Usb1	0.19	0.00703778	mice

Cmtm3	0.21	0.00718061	mice
Casp3	0.14	0.00726422	mice
Gse1	0.03	0.00736284	mice
Stim2	-0.76	0.00741525	mice
Stx18	0.26	0.00743158	mice
Ralgps1	0.12	0.00748666	mice
Hectd4	0.07	0.00749996	mice
Epc1	0.00	0.00756612	mice
Nup205	0.09	0.00758149	mice
Ndufaf2	0.12	0.00766569	mice
Ift20	0.10	0.00774459	mice
Adat1	0.12	0.00779305	mice
Golga1	0.11	0.00788693	mice
Sin3a	-0.01	0.00805329	mice
Cltb	0.02	0.00807241	mice
Phf20l1	-0.04	0.00811409	mice
Gtpbp1	0.17	0.00822067	mice
Rnf130	0.09	0.00823783	mice
Eif3l	0.12	0.00851528	mice
Nipal3	0.15	0.00891512	mice
Ext1	-0.65	0.00892784	mice
Fbxo48	0.14	0.00892885	mice
Smyd2	0.16	0.00899854	mice
Zfp292	0.11	0.00933312	mice
Wdr36	0.22	0.00938973	mice
Smarca5	-0.04	0.00942908	mice
Arpc1a	-0.01	0.00945589	mice
Spen	-0.08	0.00947153	mice
Itgb2l	0.07	0.00951500	mice
Lct	0.26	0.00980453	mice
Pdia4	0.06	0.00991721	mice
Xpo5	0.08	0.01001979	mice
Naa35	0.11	0.01039115	mice
Serac1	0.24	0.01070227	mice
Lipt2	0.22	0.01073640	mice
Aatf	0.20	0.01081422	mice
Ccnh	0.14	0.01104227	mice
Nubpl	0.11	0.01107666	mice
Fam98a	0.07	0.01117007	mice
Spopl	0.05	0.01136347	mice
Gpatch8	0.06	0.01173142	mice
Chp1	-0.75	0.01217886	mice
Adk	-0.93	0.01222265	mice
Zbtb37	0.08	0.01223418	mice
Csrnp1	-0.02	0.01235540	mice
Nup98	0.01	0.01238799	mice

Dtx3l	0.07	0.01244560	mice
Psmb7	0.01	0.01247458	mice
Zfp654	-0.03	0.01247707	mice
Psmd14	0.16	0.01252780	mice
Crbn	0.01	0.01264962	mice
Ash2l	0.07	0.01274246	mice
Fig4	0.12	0.01274394	mice
Hnrnpm	0.00	0.01281654	mice
Zmynd11	0.05	0.01308922	mice
Dnajc10	0.08	0.01311454	mice
Ctdspl2	0.19	0.01322998	mice
Eea1	0.08	0.01366734	mice
Fam3c	0.13	0.01373628	mice
Tmed2	0.00	0.01396774	mice
Arhgap26	-0.63	0.01421184	mice
Fbxl14	0.11	0.01428048	mice
Surf6	0.20	0.01444552	mice
Jade1	0.04	0.01446921	mice
Psmc2	0.18	0.01451526	mice
Wwc1	0.18	0.01489856	mice
Dph6	0.27	0.01493614	mice
Rpap1	0.03	0.01502934	mice
Ttf1	0.22	0.01526366	mice
Stk39	0.15	0.01538285	mice
Whamm	0.11	0.01542785	mice
Commd1b	0.11	0.01547724	mice
Lonp2	0.04	0.01597618	mice
Puf60	0.14	0.01603422	mice
Cul5	0.02	0.01618102	mice
Acap2	0.05	0.01646353	mice
Utp3	0.11	0.01657805	mice
Fubp1	-0.05	0.01666185	mice
Rnpc3	0.20	0.01686680	mice
Vps4b	-0.01	0.01700304	mice
Gfod1	0.09	0.01707153	mice
Prdx3	0.15	0.01730214	mice
Plekhb2	0.07	0.01755255	mice
Nfu1	0.15	0.01764335	mice
Zcrb1	0.07	0.01766570	mice
Vps29	0.10	0.01792974	mice
Pdcd10	-0.03	0.01804335	mice
Atp11a	-0.48	0.01807492	mice
Cog2	0.12	0.01821832	mice
Ctr9	0.02	0.01836525	mice
Zdhhc13	0.11	0.01855100	mice
Xpr1	0.08	0.01871245	mice

Cct8	-0.04	0.01921120	mice
Abhd17c	0.02	0.01945189	mice
Tmem30a	-0.04	0.01958709	mice
Pdzd8	0.18	0.01961316	mice
Fbxw7	0.21	0.01973328	mice
Gps1	0.05	0.02028826	mice
Lrrc28	0.01	0.02103772	mice
Rnf146	-0.01	0.02114321	mice
Qser1	0.06	0.02121606	mice
Vkorc1l1	0.10	0.02149887	mice
9130409I23Rik	-0.79	0.02159069	mice
Atp8a1	-0.78	0.02166420	mice
C1galt1c1	0.07	0.02192626	mice
Spg7	0.15	0.02207780	mice
Upf2	0.00	0.02216570	mice
Pik3r4	0.05	0.02225384	mice
Slc25a3	-0.02	0.02237685	mice
Nelfb	0.08	0.02251360	mice
Ddx27	0.12	0.02280686	mice
Phb2	0.09	0.02307914	mice
Npc2	-0.77	0.02320687	mice
Dennd4c	-0.03	0.02341107	mice
Prrc1	0.15	0.02365741	mice
Ints2	0.09	0.02400861	mice
Rlim	-0.06	0.02427245	mice
Slc2a13	0.10	0.02444497	mice
Iqgap1	-0.01	0.02475872	mice
Tmem266	-0.82	0.02498897	mice
Paip1	0.06	0.02534454	mice
Nup107	-0.79	0.02552211	mice
Ndufb10	0.02	0.02558962	mice
Pabpc4	0.13	0.02566203	mice
Bdp1	0.11	0.02575115	mice
Rbms1	0.08	0.02624887	mice
Ftsj3	0.17	0.02652274	mice
Inpp5d	0.17	0.02661099	mice
Cnot6l	0.02	0.02757660	mice
Tmem229b	0.24	0.02758028	mice
Ppil3	0.04	0.02844194	mice
Add3	0.15	0.02852406	mice
Sft2d1	-0.03	0.02883922	mice
Sucla2	-0.08	0.02897743	mice
Dis3l2	0.05	0.02929210	mice
Slc35b3	0.19	0.02943492	mice
Rbm15b	0.11	0.02985961	mice
Prkrip1	0.04	0.03004055	mice

Rcc1	0.13	0.03008894	mice
Copb2	0.04	0.03050452	mice
Ndufab1	0.06	0.03070015	mice
Atf2	-0.08	0.03074774	mice
Itch	0.02	0.03122516	mice
Hp1bp3	0.02	0.03144918	mice
Usp16	0.06	0.03213979	mice
Slc12a4	0.10	0.03221790	mice
Lamc1	-0.80	0.03264206	mice
Oard1	-0.09	0.03282354	mice
Cct7	0.04	0.03369756	mice
Itsn1	0.05	0.03399464	mice
Riok3	0.00	0.03405917	mice
Slc25a28	0.15	0.03434777	mice
Sin3b	0.13	0.03439153	mice
Sgip1	0.01	0.03448856	mice
Sf3b3	-0.01	0.03497105	mice
Zfp830	0.07	0.03538694	mice
Bud13	0.19	0.03556321	mice
Dpm2	-0.06	0.03581853	mice
Tmem181a	0.17	0.03704165	mice
Otud4	0.16	0.03719063	mice
Dlst	0.16	0.03747691	mice
Pbrm1	-0.10	0.03754884	mice
E2f6	0.10	0.03811789	mice
Atg12	0.12	0.03821764	mice
Fbxw8	0.04	0.03832199	mice
Gpr65	0.21	0.03889389	mice
Immp1l	0.09	0.03920381	mice
Psmd12	0.00	0.03947143	mice
Ica1	-0.62	0.03949322	mice
Fam162a	0.11	0.03967263	mice
Adar	0.07	0.04275282	mice
Usp24	0.08	0.04303200	mice
Wasl	0.07	0.04375957	mice
Gpatch2	0.08	0.04388842	mice
Immp2l	-0.60	0.04409899	mice
Ppa2	-0.04	0.04450653	mice
Cog1	0.15	0.04576470	mice
Hmbs	-0.04	0.04612196	mice
Pigk	0.12	0.04685469	mice
Tprgl	0.13	0.04697601	mice
Nol10	0.02	0.04776456	mice
Dym	-0.12	0.04787196	mice
Ltn1	0.14	0.04836451	mice
Cbx3	-0.05	0.04876261	mice

Smg7	-0.03	0.05014733	mice
Tra2a	-0.10	0.05090624	mice
Nsa2	0.09	0.05121569	mice
Clk2	0.01	0.05125280	mice
Vps35	0.04	0.05177864	mice
Tbcd	0.16	0.05197096	mice
Ndufa10	0.12	0.05321449	mice
Slc30a5	0.16	0.05393862	mice
Sart3	-0.04	0.05443519	mice
Pin1	0.16	0.05500734	mice
Vapb	-0.06	0.05601907	mice
Actr8	0.07	0.05637537	mice
Carhsp1	0.02	0.05771498	mice
Rabl6	0.05	0.05851168	mice
Acsbg2	0.06	0.05910694	mice
Ing5	-0.04	0.06016871	mice
Snx9	0.02	0.06145058	mice
Tnfsf11	0.52	0.06161179	mice
Afg3l2	-0.07	0.06170578	mice
Ap3s1	-0.68	0.06182149	mice
Reps1	-0.04	0.06195495	mice
Cnih1	0.09	0.06274506	mice
Sri	0.02	0.06276706	mice
Kifap3	0.10	0.06338775	mice
Ube2l3	0.09	0.06436177	mice
Sec13	0.07	0.06473616	mice
Fkbp8	0.11	0.06594188	mice
Pum2	-0.01	0.06653490	mice
Phf21a	0.09	0.06706545	mice
Rab4a	0.06	0.06724089	mice
Top1	-0.10	0.06897260	mice
Cdc40	0.12	0.06934153	mice
Naa50	0.03	0.07007434	mice
Fam185a	0.16	0.07095330	mice
Ddx20	0.18	0.07101014	mice
Fbxo42	0.07	0.07179129	mice
Larp7	0.05	0.07253427	mice
Slc36a4	0.26	0.07388272	mice
Gne	0.16	0.07417817	mice
Nubp1	0.02	0.07476308	mice
Zbtb48	0.14	0.07593665	mice
Eri1	0.09	0.07797211	mice
Rbm17	0.05	0.07822457	mice
Nfe2l2	-0.91	0.07829180	mice
Zzz3	0.03	0.07900766	mice
Hdgfl3	0.05	0.08083194	mice

Nipsnap2	0.07	0.08273988	mice
Lemd3	0.00	0.08354041	mice
Nudt16l1	0.09	0.08378800	mice
Ankmy2	0.06	0.08402069	mice
Homer1	-0.58	0.08459234	mice
Setbp1	0.34	0.08855742	mice
Pdcd4	0.11	0.08859030	mice
Fhl3	0.17	0.08896520	mice
Rsl24d1	-0.01	0.08929377	mice
Snrpd3	0.03	0.09024759	mice
Ncor2	-0.05	0.09038436	mice
Umad1	0.11	0.09164419	mice
Nifk	0.01	0.09266188	mice
Nfkb2	0.07	0.09296490	mice
Aftph	-0.01	0.09376089	mice
Rangap1	0.02	0.09576086	mice
Pcmt1	0.06	0.09603443	mice
Snx24	-0.34	0.09637948	mice
Kras	0.09	0.09639673	mice
Golga5	0.11	0.09975730	mice
Pex14	0.00	0.10041451	mice
Noc3l	0.16	0.10287417	mice
Ttc1	0.09	0.10553957	mice
Atp6v0a2	0.09	0.10640049	mice
Rexo1	0.02	0.10660026	mice
C1galt1	0.14	0.10797995	mice
Usp46	0.18	0.11012575	mice
Crkl	0.06	0.11128106	mice
Sfpq	0.00	0.11188093	mice
Rnaseh1	0.11	0.11204382	mice
Gigyf2	0.13	0.11229825	mice
Tbc1d23	0.00	0.11268389	mice
Dnm1l	0.06	0.11283487	mice
Gtf3a	0.18	0.11299115	mice
Adnp2	-0.13	0.11323978	mice
Upf3b	0.01	0.11394167	mice
Limd1	0.12	0.11415770	mice
Ift122	0.07	0.11495765	mice
Tra2b	0.01	0.11517147	mice
Slc23a2	0.06	0.11789638	mice
Ntan1	-0.05	0.11832110	mice
Brd3	0.07	0.11979810	mice
Fbxw2	0.03	0.12037239	mice
Rabgap1	-0.02	0.12070230	mice
Psmb1	-0.05	0.12073200	mice
Yeats2	-0.02	0.12215282	mice

Cct5	-0.04	0.12327000	mice
Plk3	-0.60	0.12345846	mice
Nup43	0.18	0.12399448	mice
Zfp618	0.06	0.12415508	mice
Ddx42	-0.05	0.12518552	mice
Psmg3	0.08	0.12579334	mice
Supt4a	0.00	0.12677654	mice
Rspry1	0.12	0.12830800	mice
Anxa11	-0.07	0.13126879	mice
Pdia6	-0.03	0.13231202	mice
Cebpg	0.09	0.13333065	mice
Tmem154	0.06	0.13385036	mice
Ube2d2a	-0.03	0.13475641	mice
Pafah1b1	0.05	0.13687421	mice
Atp2b4	-0.51	0.13740877	mice
Supt6	0.02	0.14016864	mice
Copa	0.05	0.14036475	mice
Psmc1	0.10	0.14218608	mice
Sec24a	-0.05	0.14228570	mice
Hdac4	-0.73	0.14260222	mice
Brf1	0.01	0.14489933	mice
Ormdl1	0.14	0.14908365	mice
Cdk19	0.00	0.14995233	mice
Stk4	0.12	0.15096047	mice
Aak1	-0.01	0.15189067	mice
Srfbp1	0.13	0.15385889	mice
Ppp1cc	-0.02	0.15486705	mice
Fam204a	-0.11	0.15687298	mice
Tmem131	-0.13	0.16344354	mice
Taf11	0.06	0.16410624	mice
Dlgap4	0.03	0.16488237	mice
Sumo1	-0.08	0.16590038	mice
Ccng1	-0.05	0.16619903	mice
Cep44	0.03	0.16642398	mice
Tm9sf2	-0.07	0.16721045	mice
Picalm	-0.91	0.16813674	mice
Nrbp1	0.03	0.17254550	mice
Pias2	0.10	0.17469387	mice
Taf5	0.01	0.18146841	mice
Ylpm1	-0.03	0.18167480	mice
Csnk1e	0.01	0.18174308	mice
Cish	-0.05	0.18200603	mice
Tmem168	0.04	0.18960522	mice
Sertad2	-0.01	0.18964743	mice
Tmem60	0.08	0.18999880	mice
Atg13	0.09	0.19226112	mice

Tkt	0.12	0.19275339	mice
Poldip2	0.19	0.19275579	mice
Ewsr1	-0.02	0.19318364	mice
Hcrt	0.21	0.19439464	mice
Ptbp1	0.05	0.19498959	mice
Snap23	-0.11	0.19580660	mice
Ddx51	0.06	0.19854135	mice
Cpsf3	-0.01	0.19880914	mice
Eif2ak1	0.16	0.20235536	mice
Ube2d1	-0.03	0.20295204	mice
Ccne1	0.15	0.20604848	mice
Surf2	0.10	0.20697851	mice
Alkbh5	0.07	0.21068796	mice
Nupl1	0.11	0.21098728	mice
Gtf2h5	-0.01	0.21444657	mice
C2cd2l	0.05	0.21568972	mice
Etv6	-0.17	0.21660751	mice
Dag1	0.14	0.21856361	mice
Wwox	0.25	0.22325615	mice
Ppard	0.03	0.22366094	mice
Senp7	-0.14	0.22414898	mice
Slf2	0.00	0.22457225	mice
Gkap1	0.11	0.22617646	mice
Lap3	0.12	0.22824848	mice
Nop58	0.06	0.22890399	mice
Gpi1	0.12	0.23224183	mice
Pigt	0.11	0.23423482	mice
Rcor3	0.08	0.24357896	mice
Ddx3y	0.06	0.24850374	mice
Nelfcd	0.06	0.25277365	mice
Eif3f	0.02	0.25735364	mice
Ctu2	0.11	0.26186636	mice
Rab3gap1	0.05	0.26519727	mice
Gid8	0.01	0.26938873	mice
Srp9	-0.05	0.27021616	mice
Kdm2a	0.03	0.27127320	mice
Xxylt1	0.07	0.27212069	mice
Park7	0.01	0.27366964	mice
Arhgdib	0.08	0.27460141	mice
Tm9sf3	-0.07	0.27507685	mice
Zfp512b	0.14	0.28267624	mice
H13	0.08	0.28588861	mice
Kdm1a	0.06	0.28630326	mice
Vezt	0.16	0.28824246	mice
Ube2a	0.03	0.28840004	mice
Zcchc24	-0.44	0.28999172	mice

Cpsf4	0.12	0.29201740	mice
Ebag9	0.06	0.29290027	mice
Zbtb41	-0.08	0.29657215	mice
Slf1	0.10	0.29736452	mice
Tubgcp3	0.18	0.29913407	mice
Chsy1	0.19	0.30022479	mice
Cog5	0.14	0.30246848	mice
Iws1	-0.14	0.30382252	mice
Wdr61	0.05	0.30667778	mice
Ipo8	0.04	0.30762709	mice
Plxnc1	0.14	0.31374556	mice
Exosc2	0.13	0.31552264	mice
Tmem208	0.13	0.31701602	mice
Bin3	-0.81	0.31825703	mice
Nrf1	0.13	0.32341959	mice
Sirt1	-0.01	0.32462736	mice
Smarca2	0.03	0.32504920	mice
Ccnl2	-0.04	0.32602649	mice
Pikfyve	-0.03	0.32799727	mice
Pfdn4	-0.02	0.33053829	mice
Ei24	0.05	0.33077553	mice
Lrch1	-0.06	0.33113987	mice
Osbpl3	0.09	0.33267535	mice
Ptpra	0.04	0.33731785	mice
Arfgap2	0.10	0.34033732	mice
Atrn	-0.48	0.34195186	mice
Fosl2	0.07	0.34548864	mice
Tmcc1	-0.08	0.34688921	mice
Esd	0.08	0.34804954	mice
Scfd1	-0.01	0.34886566	mice
Tpra1	0.11	0.34919468	mice
Ttc7	0.14	0.34976187	mice
Exosc10	0.03	0.35070691	mice
Rab35	-0.07	0.35832284	mice
Eif4g2	-0.53	0.36115085	mice
Ndufb6	0.00	0.36130625	mice
Pkp4	0.07	0.36562874	mice
Cacna2d2	0.11	0.37082335	mice
Ddx24	0.14	0.37269373	mice
Tmem222	0.11	0.37540998	mice
Rnf111	-0.03	0.38059472	mice
Btbd6	0.11	0.38117413	mice
Ciapin1	0.04	0.38406648	mice
Phf20	-0.13	0.38780281	mice
Sltm	-0.03	0.38828528	mice
Setd3	0.06	0.38898401	mice

Ap2b1	0.04	0.39163662	mice
Cdk7	0.01	0.39495399	mice
Aurkaip1	0.10	0.39780304	mice
Zfp143	-0.03	0.39878026	mice
Aven	0.22	0.40079625	mice
Ints11	0.11	0.40349199	mice
Zcchc17	-0.02	0.40431583	mice
Polr2d	-0.02	0.41436092	mice
Commd6	0.07	0.41730948	mice
Tnip2	0.13	0.42241788	mice
Clk3	0.07	0.42266095	mice
Cflar	-0.07	0.42533510	mice
Aig1	0.17	0.42869287	mice
Xrn1	-0.03	0.42992384	mice
Igf2r	0.02	0.43201015	mice
Prdm15	0.00	0.43275084	mice
Psmd3	0.10	0.44011057	mice
Rsrc2	-0.05	0.44020642	mice
Dld	0.09	0.44081629	mice
Hat1	0.09	0.45062541	mice
Rilpl1	0.08	0.45562630	mice
Gdap2	0.10	0.45956772	mice
Zc3h14	0.08	0.46107846	mice
Zdhhc17	0.01	0.47251116	mice
Tgs1	0.12	0.47498217	mice
Chd1l	0.16	0.47875420	mice
Ifngr2	-0.62	0.48080996	mice
Chchd4	0.18	0.48853817	mice
Nudcd3	0.04	0.49212063	mice
Grip1	0.21	0.49267024	mice
Ccnd1	0.05	0.49364631	mice
Slc10a7	0.00	0.49887411	mice
Usp12	-0.57	0.51010305	mice
R3hdm1	0.01	0.51396693	mice
Glg1	-0.05	0.51491780	mice
Tshz2	0.49	0.52185351	mice
Cramp1	-0.13	0.52657555	mice
Wipi2	0.02	0.52969136	mice
Ccdc117	-0.05	0.53167993	mice
Rev3l	0.12	0.53228933	mice
Plrg1	0.09	0.53684932	mice
Bcr	0.06	0.53686271	mice
Traf2	-0.03	0.53945632	mice
Cdc42bpb	-0.77	0.54347622	mice
Abcb7	0.05	0.55498845	mice
Sdha	0.10	0.55539172	mice

Cenpt	0.16	0.56034977	mice
Ascc1	-0.02	0.57053088	mice
Ythdf3	-0.08	0.57256313	mice
Tex10	0.09	0.57532828	mice
Gpalpp1	-0.03	0.57763270	mice
Creb1	0.07	0.58078881	mice
Zbed4	0.01	0.58449281	mice
Spg21	0.07	0.58707502	mice
Lmbr1	0.05	0.58716994	mice
Borcs7	0.16	0.58767193	mice
Tango6	0.10	0.58838540	mice
Atp13a1	0.07	0.59135689	mice
Ncoa7	0.14	0.60092295	mice
Cnpy3	0.00	0.60413285	mice
Alg12	-0.06	0.61026876	mice
Dpy30	0.08	0.62005326	mice
Erp44	0.03	0.62943413	mice
Ddrgk1	-0.04	0.63083514	mice
Itfg1	-0.08	0.63115038	mice
Cebpz	0.12	0.63820413	mice
Faf1	-0.87	0.64625134	mice
Ldb1	0.08	0.64894199	mice
Ccni	-0.04	0.64934237	mice
Tm9sf4	0.13	0.65113936	mice
Eif6	0.15	0.65183103	mice
Uggt1	0.03	0.65325854	mice
Lbh	0.09	0.65896035	mice
Dstn	-0.25	0.66198869	mice
Arnt	0.01	0.66356350	mice
Ppp1ca	0.05	0.67514452	mice
Jade3	-0.83	0.68597057	mice
Stx12	0.08	0.68615692	mice
Brix1	0.17	0.68999836	mice
Samm50	0.08	0.69885127	mice
Ube2j2	-0.05	0.71119430	mice
Gpr107	0.07	0.71242069	mice
Derl1	0.00	0.71331048	mice
Gcsh	0.15	0.73386534	mice
Itpka	0.00	0.74617162	mice
Bcl2l13	-0.05	0.75319065	mice
Pgs1	0.02	0.75495477	mice
Txndc12	0.04	0.75608171	mice
Pla2g7	0.06	0.76184528	mice
Itgb1bp1	0.15	0.78375744	mice
Commd7	0.05	0.78508547	mice
Api5	-0.01	0.80653406	mice

Fh1	0.07	0.81879255	mice
Naf1	0.07	0.82004658	mice
Rpp30	0.09	0.82692793	mice
Adam23	-0.01	0.82698337	mice
Ptp4a2	-0.05	0.84570353	mice
Fuca2	0.19	0.85206084	mice
Arhgef6	-0.06	0.85477161	mice
Nfat5	-0.06	0.85814531	mice
Ambra1	-0.12	0.85904991	mice
Ap3b1	0.06	0.87032487	mice
Asf1a	0.10	0.87377474	mice
Cnot8	0.07	0.87733798	mice
Trp53bp1	0.13	0.88434558	mice
Ufl1	0.00	0.90027629	mice
Dhx40	0.10	0.90120269	mice
Hspd1	0.06	0.91061444	mice
Ndufb3	0.00	0.93039587	mice
Sap130	0.08	0.94271428	mice
Ttc14	0.05	0.94607490	mice
Epc2	0.02	0.94651391	mice
Nedd4	0.30	0.94756768	mice
Rnps1	0.11	0.95514565	mice
Rbbp4	0.08	0.97778474	mice
Pank2	0.08	1.00000000	mice
Comtd1	0.03	1.00000000	mice
Tnks	-0.03	1.00000000	mice
Ttc37	-0.02	1.00000000	mice
Bcor	-0.83	1.00000000	mice
Ublcp1	-0.02	1.00000000	mice
Pdcd11	0.06	1.00000000	mice
Prdm2	-0.01	1.00000000	mice
Spz1	-0.17	1.00000000	mice
Gnas	-0.02	1.00000000	mice
Nup54	0.02	1.00000000	mice
Ly75	-0.73	1.00000000	mice
Rbm12b2	0.09	1.00000000	mice
Kat7	0.00	1.00000000	mice
Fars2	0.11	1.00000000	mice
Pdxdc1	0.04	1.00000000	mice
Pno1	0.08	1.00000000	mice
Gpatch1	-0.04	1.00000000	mice
Lrif1	0.04	1.00000000	mice
Zfp276	0.09	1.00000000	mice
Ythdf2	0.03	1.00000000	mice
Vapa	-0.10	1.00000000	mice
Fam76a	0.04	1.00000000	mice

Desi2	0.07	1.00000000	mice
Arhgap10	0.11	1.00000000	mice
Ago2	-0.13	1.00000000	mice
Creb3l2	-0.48	1.00000000	mice
Arid2	-0.02	1.00000000	mice
Camsap2	-0.85	1.00000000	mice
Srrm1	-0.05	1.00000000	mice
Cdk12	-0.03	1.00000000	mice
Aspscr1	0.10	1.00000000	mice
Uba2	-0.09	1.00000000	mice
Clock	-0.08	1.00000000	mice
Cers5	0.04	1.00000000	mice
Dip2b	0.04	1.00000000	mice
Ino80d	-0.02	1.00000000	mice
Lsm8	-0.04	1.00000000	mice
Wdr1	-0.16	1.00000000	mice
Tspan3	-0.81	1.00000000	mice
Zfp131	0.06	1.00000000	mice
Sf3b1	-0.12	1.00000000	mice
Vps53	-0.03	1.00000000	mice
Osbpl9	-0.07	1.00000000	mice
Rab11a	-0.01	1.00000000	mice
Kpna4	-0.10	1.00000000	mice
Tbc1d12	0.00	1.00000000	mice
Wac	-0.07	1.00000000	mice
Eefsec	-0.06	1.00000000	mice
Ypel2	0.00	1.00000000	mice
Sprtn	0.09	1.00000000	mice
Ndufb8	0.01	1.00000000	mice
Creld2	0.08	1.00000000	mice
Taf5l	-0.76	1.00000000	mice
Pwwp2a	0.00	1.00000000	mice
Rnf214	0.05	1.00000000	mice
Csnk2a1	-0.02	1.00000000	mice
Ythdf1	-0.03	1.00000000	mice
Ppp2ca	0.05	1.00000000	mice
Kansl1l	-0.14	1.00000000	mice
Hmgb3	-0.02	1.00000000	mice
Usp34	-0.08	1.00000000	mice
Tbc1d22b	0.10	1.00000000	mice
Rbm6	-0.01	1.00000000	mice
Impdh2	0.07	1.00000000	mice
Bet1	0.02	1.00000000	mice
Trdmt1	-0.03	1.00000000	mice
Susd6	-0.04	1.00000000	mice
Txlng	0.05	1.00000000	mice

Zbtb2	-0.07	1.00000000	mice
Cc2d1b	0.14	1.00000000	mice
Htt	-0.21	1.00000000	mice
Cdkn1b	-0.07	1.00000000	mice
Ust	0.07	1.00000000	mice
Thoc1	-0.01	1.00000000	mice
Ttc33	0.17	1.00000000	mice
Ide	-0.07	1.00000000	mice
Ube2k	-0.01	1.00000000	mice
Rcbtb2	0.12	1.00000000	mice
Pi4ka	-0.11	1.00000000	mice
Riok1	0.07	1.00000000	mice
Sdf4	-0.08	1.00000000	mice
Fbxl20	0.04	1.00000000	mice
Slc25a36	-0.07	1.00000000	mice
Dcaf5	-0.33	1.00000000	mice
Cacul1	-0.73	1.00000000	mice
Pinx1	-0.02	1.00000000	mice
Rfc5	0.12	1.00000000	mice
Nfyc	0.07	1.00000000	mice
Aoah	0.15	1.00000000	mice
Zrsr1	-0.15	1.00000000	mice
Dynlrb1	-0.08	1.00000000	mice
Ralbp1	-0.66	1.00000000	mice
Ppwd1	0.07	1.00000000	mice
Phlpp2	-0.05	1.00000000	mice
Scaf11	-0.01	1.00000000	mice
Acsl4	0.17	1.00000000	mice
P4ha1	-0.69	1.00000000	mice
Sacm1l	-0.03	1.00000000	mice
Ttc27	0.01	1.00000000	mice
Eif3j2	-0.04	1.00000000	mice
Osbpl8	-0.02	1.00000000	mice
Lsm6	-0.59	1.00000000	mice
Ctnna1	-0.17	1.00000000	mice
Hip1r	0.16	1.00000000	mice
Nt5dc1	0.27	1.00000000	mice
Rabgap1l	0.06	1.00000000	mice
Esco1	-0.02	1.00000000	mice
Tfdp1	-0.11	1.00000000	mice
Zfp622	0.04	1.00000000	mice
Rpa3	0.12	1.00000000	mice
Shprh	-0.56	1.00000000	mice
Ip6k1	0.04	1.00000000	mice
Rnf19a	-0.11	1.00000000	mice
Sergef	0.13	1.00000000	mice

Ints8	-0.03	1.00000000	mice
Usp45	-0.13	1.00000000	mice
Usp33	0.06	1.00000000	mice
Senp5	0.09	1.00000000	mice
Fem1a	-0.03	1.00000000	mice
Paxbp1	-0.14	1.00000000	mice
Dusp15	0.14	1.00000000	mice
Gcc2	-0.03	1.00000000	mice
Casd1	0.04	1.00000000	mice
Yipf4	0.00	1.00000000	mice
Gpatch2l	-0.10	1.00000000	mice
Secisbp2l	-0.14	1.00000000	mice
Ubp1	-0.22	1.00000000	mice
Faf2	-0.07	1.00000000	mice
Hsf2	0.01	1.00000000	mice
Zfp148	-0.05	1.00000000	mice
Ankrd13d	0.08	1.00000000	mice
Ash1l	-0.13	1.00000000	mice
Dnajc5	0.08	1.00000000	mice
Wdr48	0.02	1.00000000	mice
Emc3	-0.05	1.00000000	mice
Ncbp3	-0.02	1.00000000	mice
Smpd2	0.09	1.00000000	mice
Rnf11	-0.78	1.00000000	mice
Ehd3	-0.03	1.00000000	mice
Gtf2f2	0.03	1.00000000	mice
Tfb2m	0.17	1.00000000	mice
Kmt2e	0.04	1.00000000	mice
Akt1	-0.07	1.00000000	mice
Abr	0.04	1.00000000	mice
St3gal6	-0.65	1.00000000	mice
Abcc5	0.10	1.00000000	mice
Acox3	0.07	1.00000000	mice
Sp3	-0.13	1.00000000	mice
Hells	-0.02	1.00000000	mice
Trpm7	-0.06	1.00000000	mice
Sub1	-0.03	1.00000000	mice
Lpcat1	-0.02	1.00000000	mice
Rbm45	0.00	1.00000000	mice
Cul4b	-0.13	1.00000000	mice
Wapl	0.01	1.00000000	mice
Copg2	0.02	1.00000000	mice
Pms1	0.18	1.00000000	mice
Hmgb2	0.26	1.00000000	mice
Grpel1	0.16	1.00000000	mice
Tmem70	0.04	1.00000000	mice

Plin2	-0.01	1.00000000	mice
Dcun1d4	0.05	1.00000000	mice
Abhd13	0.00	1.00000000	mice
Arfgap1	0.03	1.00000000	mice
Pdcd7	-0.07	1.00000000	mice
Pdcd5	0.08	1.00000000	mice
Sde2	-0.08	1.00000000	mice
Taok3	-0.16	1.00000000	mice
Ube3c	-0.12	1.00000000	mice
Togaram1	0.03	1.00000000	mice
Zfc3h1	-0.19	1.00000000	mice
Nipa2	-0.07	1.00000000	mice
Usp14	0.05	1.00000000	mice
Capn11	0.00	1.00000000	mice
Kdm4b	-0.01	1.00000000	mice
Bcap29	-0.06	1.00000000	mice
Ralgapa1	-0.10	1.00000000	mice
Abcf2	-0.02	1.00000000	mice
Ppp6r2	-0.06	1.00000000	mice
Sh3bp2	0.09	1.00000000	mice
Pptc7	-0.01	1.00000000	mice
Ric1	-0.05	1.00000000	mice
Nemf	0.05	1.00000000	mice
Wdr12	0.15	1.00000000	mice
Ipmk	-0.68	1.00000000	mice
Dip2a	0.00	1.00000000	mice
Cdc16	0.03	1.00000000	mice
Dpy19l4	-0.03	1.00000000	mice
Paxip1	0.05	1.00000000	mice
Zfp236	0.04	1.00000000	mice
Kif2a	-0.01	1.00000000	mice
Stag2	-0.05	1.00000000	mice
Zbtb33	0.10	1.00000000	mice
Tmem64	0.07	1.00000000	mice
Ints10	0.19	1.00000000	mice
Pfdn1	-0.09	1.00000000	mice
Vrk1	0.04	1.00000000	mice
Nkrf	-0.02	1.00000000	mice
Nfx1	-0.08	1.00000000	mice
Rnls	0.21	1.00000000	mice
Acat1	0.00	1.00000000	mice
Zfp592	-0.02	1.00000000	mice
Arhgap8	0.04	1.00000000	mice
Ube2g1	-0.20	1.00000000	mice
Fam13b	-0.12	1.00000000	mice
Nus1	0.03	1.00000000	mice

Nabp1	0.00	1.00000000	mice
Esrrg	0.13	1.00000000	mice
Elmod2	0.08	1.00000000	mice
Utrn	-0.18	1.00000000	mice
Scamp2	0.00	1.00000000	mice
Axin2	-0.51	1.00000000	mice
Sbf2	-0.54	1.00000000	mice
Ezh2	0.04	1.00000000	mice
Psma7	-0.08	1.00000000	mice
Bzw2	-0.07	1.00000000	mice
Ttc3	-0.06	1.00000000	mice
Vti1b	0.01	1.00000000	mice
Ino80	-0.62	1.00000000	mice
Tnfrsf25	-0.48	1.00000000	mice
Klhdc1	-0.01	1.00000000	mice
Hikeshi	0.00	1.00000000	mice
Pds5b	-0.10	1.00000000	mice
Crk	-0.12	1.00000000	mice
Eif4e	-0.07	1.00000000	mice
Tdp2	-0.06	1.00000000	mice
Ippk	-0.07	1.00000000	mice
Pde4d	0.17	1.00000000	mice
Tdrd3	-0.17	1.00000000	mice
Tec	0.33	1.00000000	mice
Cggbp1	0.06	1.00000000	mice
Ppm1b	-0.18	1.00000000	mice
Xrcc4	0.04	1.00000000	mice
Zfp330	0.05	1.00000000	mice
Virma	-0.01	1.00000000	mice
9930021J03Rik	-0.04	1.00000000	mice
Fbxo30	0.04	1.00000000	mice
Rbm19	0.06	1.00000000	mice
Trip12	-0.18	1.00000000	mice
Slc35c1	0.07	1.00000000	mice
Ubap2l	0.02	1.00000000	mice
Utp11	0.06	1.00000000	mice
Napepld	-0.16	1.00000000	mice
Pcmtd2	0.00	1.00000000	mice
Timmdc1	0.11	1.00000000	mice
Bsdc1	0.00	1.00000000	mice
Zfp598	0.02	1.00000000	mice
Tfb1m	-0.02	1.00000000	mice
Runx2	0.16	1.00000000	mice
Rsf1	-0.11	1.00000000	mice
Add1	-0.10	1.00000000	mice
Hsbp1	-0.03	1.00000000	mice

Ccdc66	0.04	1.00000000	mice
Dhx8	0.00	1.00000000	mice
Dnajc9	-0.54	1.00000000	mice
Ing3	0.08	1.00000000	mice
Srpk2	0.06	1.00000000	mice
Nbr1	-0.10	1.00000000	mice
Zeb1	-0.01	1.00000000	mice
Stxbp6	0.26	1.00000000	mice
2510039O18Rik	0.10	1.00000000	mice
Atp6v1c1	-0.48	1.00000000	mice
Helb	-0.03	1.00000000	mice
Helz2	-0.10	1.00000000	mice
Hnrnpu	-0.08	1.00000000	mice
Orc3	0.10	1.00000000	mice
Nufip2	-0.01	1.00000000	mice
Tspan13	0.08	1.00000000	mice
Cops2	0.02	1.00000000	mice
Atf1	0.03	1.00000000	mice
Sap30l	0.03	1.00000000	mice
Pik3c3	-0.06	1.00000000	mice
Hexim1	-0.07	1.00000000	mice
Hdac1	0.10	1.00000000	mice
Drosha	0.02	1.00000000	mice
Itm2c	0.17	1.00000000	mice
Cap1	-0.06	1.00000000	mice
Zcchc4	0.01	1.00000000	mice
Ankrd11	0.04	1.00000000	mice
Slc25a26	0.11	1.00000000	mice
Cst7	0.07	1.00000000	mice
Irf1	-0.02	1.00000000	mice
Ctnnbip1	0.00	1.00000000	mice
Ugcg	0.25	1.00000000	mice
Lman2	-0.01	1.00000000	mice
Gtf2b	-0.07	1.00000000	mice
Chd7	-0.60	1.00000000	mice
Ncstn	-0.02	1.00000000	mice
Pik3r5	-0.17	1.00000000	mice
Tmem184b	-0.09	1.00000000	mice
Trappc2	0.00	1.00000000	mice
Trp53bp2	-0.53	1.00000000	mice
Hmgcr	-0.09	1.00000000	mice
Fbxo7	-0.03	1.00000000	mice
Ccdc91	0.10	1.00000000	mice
Unk	-0.06	1.00000000	mice
Scfd2	0.10	1.00000000	mice
Plekha1	-0.08	1.00000000	mice

Lig3	-0.01	1.00000000	mice
Sptlc1	0.02	1.00000000	mice
Pdhb	0.04	1.00000000	mice
Rb1cc1	-0.50	1.00000000	mice
Cwf19l2	-0.02	1.00000000	mice
Abcc4	-0.05	1.00000000	mice
Ctbp2	0.05	1.00000000	mice
Ubl3	-0.17	1.00000000	mice
Vcpip1	-0.10	1.00000000	mice
Psma2	-0.09	1.00000000	mice
Xpo7	-0.16	1.00000000	mice
Suclg1	0.12	1.00000000	mice
Srek1ip1	0.00	1.00000000	mice
Timm13	-0.02	1.00000000	mice
Haus3	-0.01	1.00000000	mice
Arf4	-0.15	1.00000000	mice
Tiam2	-0.02	1.00000000	mice
Wdr4	0.08	1.00000000	mice
Slc35a1	0.11	1.00000000	mice
Htatsf1	0.01	1.00000000	mice
Bms1	0.02	1.00000000	mice
Lemd2	0.08	1.00000000	mice
Cnot11	0.02	1.00000000	mice
Atxn7	-0.04	1.00000000	mice
Akt3	-0.39	1.00000000	mice
Zfp516	-0.53	1.00000000	mice
Ext2	-0.02	1.00000000	mice
Vezf1	-0.16	1.00000000	mice
Dmap1	-0.79	1.00000000	mice
Sf3a2	0.00	1.00000000	mice
Polr1c	0.11	1.00000000	mice
Rora	0.08	1.00000000	mice
Zmpste24	0.02	1.00000000	mice
Nxt2	0.10	1.00000000	mice
Uba3	0.00	1.00000000	mice
Tmem230	-0.07	1.00000000	mice
Clcc1	-0.11	1.00000000	mice
Clint1	0.03	1.00000000	mice
Ttc32	0.08	1.00000000	mice
Susd3	0.05	1.00000000	mice
Nap1l4	-0.08	1.00000000	mice
Ppie	0.02	1.00000000	mice
Arid1b	0.08	1.00000000	mice
Ptcd3	0.07	1.00000000	mice
Abi2	0.05	1.00000000	mice
Golt1b	0.06	1.00000000	mice

Stk11	0.05	1.00000000	mice
Timm44	0.05	1.00000000	mice
Rnf10	-0.09	1.00000000	mice
Rap2c	-0.19	1.00000000	mice
Hdac7	-0.80	1.00000000	mice
Kdm5a	-0.07	1.00000000	mice
Cmtr1	-0.03	1.00000000	mice
Dnajb14	0.03	1.00000000	mice
Dcaf13	0.00	1.00000000	mice
Fto	0.03	1.00000000	mice
Ccdc50	-0.70	1.00000000	mice
Lmbrd2	-0.45	1.00000000	mice
Prkn	0.12	1.00000000	mice
Ccdc93	0.01	1.00000000	mice
Dync1li1	-0.10	1.00000000	mice
Pde12	0.06	1.00000000	mice
Ap3m1	-0.04	1.00000000	mice
Bnip3l	-0.72	1.00000000	mice
Gabpa	-0.11	1.00000000	mice
Rnf20	-0.04	1.00000000	mice
Gorasp2	0.03	1.00000000	mice
Ammecr1	-0.11	1.00000000	mice
Pak2	-0.10	1.00000000	mice
Tmem35b	-0.14	1.00000000	mice
Tmem33	0.10	1.00000000	mice
Tma16	0.01	1.00000000	mice
Psmc6	-0.06	1.00000000	mice
Cope	-0.02	1.00000000	mice
Dnajb4	-0.11	1.00000000	mice
Commd9	-0.02	1.00000000	mice
Supv3l1	0.02	1.00000000	mice
Pdss2	-0.06	1.00000000	mice
Tpd52l2	-0.06	1.00000000	mice
Psmc5	-0.03	1.00000000	mice
Sppl2a	-0.03	1.00000000	mice
Rab3gap2	0.01	1.00000000	mice
Tbc1d14	-0.08	1.00000000	mice
Aebp2	-0.16	1.00000000	mice
Nek1	0.00	1.00000000	mice
Pex7	0.03	1.00000000	mice
Washc5	0.06	1.00000000	mice
Ythdc1	-0.06	1.00000000	mice
Ciao1	0.04	1.00000000	mice
Cdc23	0.07	1.00000000	mice
Usp37	-0.08	1.00000000	mice
Csrp1	-0.65	1.00000000	mice

Psma6	-0.11	1.00000000	mice
Snap29	-0.02	1.00000000	mice
Commd10	0.05	1.00000000	mice
Zc3h13	-0.12	1.00000000	mice
Nktr	-0.15	1.00000000	mice
Trip4	0.05	1.00000000	mice
Cast	-0.08	1.00000000	mice
Sec63	-0.14	1.00000000	mice
Fbxo21	-0.04	1.00000000	mice
Zranb3	0.09	1.00000000	mice
Stam	-0.01	1.00000000	mice
Elf2	-0.15	1.00000000	mice
Parg	-0.21	1.00000000	mice
Tomm20	-0.07	1.00000000	mice
Rexo5	0.02	1.00000000	mice
Slc35f5	-0.07	1.00000000	mice
Rab27a	-0.04	1.00000000	mice
Fmr1	-0.19	1.00000000	mice
Sos2	-0.12	1.00000000	mice
Ahsa1	-0.07	1.00000000	mice
Brpf3	0.07	1.00000000	mice
Lta4h	0.06	1.00000000	mice
Nrd1	-0.03	1.00000000	mice
Ctnnbl1	0.04	1.00000000	mice
Rpgrip1l	-0.05	1.00000000	mice
Gtf2a2	-0.08	1.00000000	mice
Clasp2	-0.78	1.00000000	mice
Leo1	-0.01	1.00000000	mice
Rheb	-0.25	1.00000000	mice
Dusp23	0.01	1.00000000	mice
Ppp2r5e	-0.10	1.00000000	mice
Ghdc	0.04	1.00000000	mice
Necap2	0.03	1.00000000	mice
Kctd10	-0.07	1.00000000	mice
Ranbp1	-0.15	1.00000000	mice
Taok1	-0.06	1.00000000	mice
Pknox1	-0.17	1.00000000	mice
Rnf13	-0.08	1.00000000	mice
Ubqln4	-0.05	1.00000000	mice
Opa1	-0.12	1.00000000	mice
Smc3	-0.12	1.00000000	mice
Slc15a4	-0.05	1.00000000	mice
Adcy9	-0.71	1.00000000	mice
Rhot1	-0.03	1.00000000	mice
Brcc3	0.03	1.00000000	mice
Prdx4	-0.02	1.00000000	mice

Rab2a	-0.14	1.00000000	mice
Psmb2	-0.08	1.00000000	mice
Actr1a	-0.07	1.00000000	mice
Cog7	0.07	1.00000000	mice
Secisbp2	-0.19	1.00000000	mice
Tanc1	-0.03	1.00000000	mice
Polk	-0.08	1.00000000	mice
Dffa	0.01	1.00000000	mice
Atp2c1	-0.08	1.00000000	mice
Ncoa1	-0.02	1.00000000	mice
Ehmt1	-0.06	1.00000000	mice
Parp8	0.04	1.00000000	mice
Strada	0.11	1.00000000	mice
Oma1	-0.04	1.00000000	mice
Itga6	0.02	1.00000000	mice
Atp6v0d1	-0.10	1.00000000	mice
Pmm2	-0.02	1.00000000	mice
Nrde2	0.08	1.00000000	mice
Brd1	-0.70	1.00000000	mice
Zzef1	-0.06	1.00000000	mice
Il18r1	0.14	1.00000000	mice
Use1	0.01	1.00000000	mice
Snapc4	0.06	1.00000000	mice
Fxyd6	-0.48	1.00000000	mice
Gid4	0.06	1.00000000	mice
Lonrf1	-0.57	1.00000000	mice
Hspa9	-0.14	1.00000000	mice
Eif4e2	0.05	1.00000000	mice
Adal	0.08	1.00000000	mice
Wdr7	-0.59	1.00000000	mice
Pals1	-0.51	1.00000000	mice
Timm21	0.05	1.00000000	mice
Zfand5	0.00	1.00000000	mice
Chst10	0.02	1.00000000	mice
Bach1	-0.14	1.00000000	mice
Prkaa1	0.03	1.00000000	mice
Phc3	0.00	1.00000000	mice
Washc3	-0.01	1.00000000	mice
Kpna3	-0.17	1.00000000	mice
Srebf1	-0.09	1.00000000	mice
Ascc3	0.04	1.00000000	mice
Gnb4	-0.08	1.00000000	mice
Synj1	-0.14	1.00000000	mice
Nup155	0.11	1.00000000	mice
Arpp19	-0.14	1.00000000	mice
Phf6	-0.06	1.00000000	mice

Brwd1	-0.16	1.00000000	mice
Nr2c1	-0.07	1.00000000	mice
Txnl4b	0.03	1.00000000	mice
Fbxo25	0.05	1.00000000	mice
Optn	-0.10	1.00000000	mice
Ngly1	-0.19	1.00000000	mice
Fancd2	-0.06	1.00000000	mice
Tmem242	-0.06	1.00000000	mice
Clptm1l	-0.12	1.00000000	mice
Cops3	-0.19	1.00000000	mice
Otulin	-0.07	1.00000000	mice
Arid4b	-0.04	1.00000000	mice
Tapt1	-0.25	1.00000000	mice
Elp4	-0.06	1.00000000	mice
Fchsd2	-0.08	1.00000000	mice
Eif4a2	0.06	1.00000000	mice
Ap1ar	-0.01	1.00000000	mice
Stk38l	0.15	1.00000000	mice
Wsb1	-0.01	1.00000000	mice
Galnt1	-0.06	1.00000000	mice
Crot	0.02	1.00000000	mice
Cops5	-0.03	1.00000000	mice
Ripk1	0.02	1.00000000	mice
Ppm1h	0.04	1.00000000	mice
Rprd1a	-0.09	1.00000000	mice
Pik3c2a	-0.21	1.00000000	mice
Btbd2	-0.07	1.00000000	mice
Tfdp2	0.00	1.00000000	mice
Etfa	-0.14	1.00000000	mice
Ppil2	-0.03	1.00000000	mice
Ranbp3	-0.05	1.00000000	mice
Cisd1	-0.13	1.00000000	mice
Prkar2a	0.03	1.00000000	mice
Zfhx3	-0.10	1.00000000	mice
Nfs1	-0.02	1.00000000	mice
Drg2	0.07	1.00000000	mice
Samd4	-0.03	1.00000000	mice
Rtf1	-0.11	1.00000000	mice
Gabarapl2	-0.17	1.00000000	mice
Diablo	0.11	1.00000000	mice
Uspl1	-0.09	1.00000000	mice
Ptp4a1	-0.18	1.00000000	mice
Abi1	-0.18	1.00000000	mice
Ctso	0.07	1.00000000	mice
Slc30a4	-0.08	1.00000000	mice
Tm2d1	0.04	1.00000000	mice

Gsr	-0.05	1.00000000	mice
Zfand4	0.02	1.00000000	mice
Polr1e	0.09	1.00000000	mice
Extl3	0.01	1.00000000	mice
Ywhab	-0.10	1.00000000	mice
2210408I21Rik	0.07	1.00000000	mice
Tcn2	-0.05	1.00000000	mice
Scyl3	0.11	1.00000000	mice
Usp4	-0.17	1.00000000	mice
Psmb3	-0.07	1.00000000	mice
Fyco1	-0.15	1.00000000	mice
Rere	-0.12	1.00000000	mice
Fam149b	0.08	1.00000000	mice
Cpne2	0.04	1.00000000	mice
Serinc3	-0.64	1.00000000	mice
Dcp1a	-0.03	1.00000000	mice
Rcn2	-0.57	1.00000000	mice
Dctn3	0.01	1.00000000	mice
Lin7c	0.03	1.00000000	mice
B3gnt2	0.01	1.00000000	mice
Ostc	-0.01	1.00000000	mice
Rmdn3	-0.06	1.00000000	mice
Nutf2	-0.11	1.00000000	mice
Sik3	0.01	1.00000000	mice
Dcbld2	0.10	1.00000000	mice
Wwp1	-0.01	1.00000000	mice
Ip6k2	-0.24	1.00000000	mice
Ireb2	-0.14	1.00000000	mice
Lpin2	-0.17	1.00000000	mice
Tmco3	-0.60	1.00000000	mice
Atp10d	-0.08	1.00000000	mice
Bud31	-0.10	1.00000000	mice
Rufy1	-0.21	1.00000000	mice
Fam91a1	0.01	1.00000000	mice
Arl5a	-0.10	1.00000000	mice
Cfap97	-0.09	1.00000000	mice
Thumpd3	-0.12	1.00000000	mice
Zdhhc9	-0.01	1.00000000	mice
Ypel5	-0.09	1.00000000	mice
Commd2	-0.02	1.00000000	mice
Zer1	-0.01	1.00000000	mice
Ddhd1	-0.11	1.00000000	mice
Trps1	0.29	1.00000000	mice
Heatr5a	0.02	1.00000000	mice
Rab10	-0.05	1.00000000	mice
Nip7	0.04	1.00000000	mice

Ndrg3	-0.01	1.00000000	mice
Ldlrad4	0.18	1.00000000	mice
Syap1	-0.14	1.00000000	mice
Larp4	-0.02	1.00000000	mice
Pccb	0.07	1.00000000	mice
Psen1	-0.50	1.00000000	mice
Clcn3	-0.09	1.00000000	mice
Wdr70	-0.06	1.00000000	mice
Sdhd	-0.11	1.00000000	mice
Arxes2	-0.17	1.00000000	mice
Ryk	-0.07	1.00000000	mice
Angel2	-0.03	1.00000000	mice
Chgb	-0.03	1.00000000	mice
Decr1	-0.01	1.00000000	mice
Atp6v1e2	-0.17	1.00000000	mice
Ints1	-0.06	1.00000000	mice
Camk2g	-0.74	1.00000000	mice
Borcs8	0.04	1.00000000	mice
Tatdn1	0.00	1.00000000	mice
Trnt1	0.06	1.00000000	mice
Fgfr1op2	-0.13	1.00000000	mice
Pafah1b2	-0.06	1.00000000	mice
Rbm31y	-0.33	1.00000000	mice
Hcfc2	-0.10	1.00000000	mice
Sdhaf2	0.05	1.00000000	mice
Pola1	0.01	1.00000000	mice
Kat6b	-0.73	1.00000000	mice
Lias	0.05	1.00000000	mice
Smndc1	-0.10	1.00000000	mice
Lactb	-0.13	1.00000000	mice
Arfgap3	-0.10	1.00000000	mice
Txlna	-0.01	1.00000000	mice
Fbxo9	-0.13	1.00000000	mice
Pcna	-0.23	1.00000000	mice
Stat3	0.08	1.00000000	mice
Rif1	0.02	1.00000000	mice
Ptpn1	-0.08	1.00000000	mice
Pank3	-0.12	1.00000000	mice
Srp68	-0.03	1.00000000	mice
Copb1	-0.06	1.00000000	mice
Agtpbp1	0.02	1.00000000	mice
Uhrf2	-0.07	1.00000000	mice
Eef2	-0.01	1.00000000	mice
Zswim8	-0.49	1.00000000	mice
Gjd4	-0.03	1.00000000	mice
Cdc73	-0.20	1.00000000	mice

Edem3	-0.15	1.00000000	mice
Cbll1	0.03	1.00000000	mice
Ulk2	-0.57	1.00000000	mice
Slc2a8	-0.05	1.00000000	mice
Sos1	-0.19	1.00000000	mice
Atg3	-0.09	1.00000000	mice
Atg5	-0.19	1.00000000	mice
Snx12	-0.06	1.00000000	mice
Rwdd4a	-0.07	1.00000000	mice
Capzb	-0.07	1.00000000	mice
Erh	-0.13	1.00000000	mice
Ndfip2	-0.08	1.00000000	mice
Emc7	-0.10	1.00000000	mice
Epn2	-0.19	1.00000000	mice
Bloc1s2	0.09	1.00000000	mice
Adipor2	-0.26	1.00000000	mice
Smurf1	0.03	1.00000000	mice
Fbxo34	-0.08	1.00000000	mice
Polr2c	0.11	1.00000000	mice
Casp7	-0.14	1.00000000	mice
Dnajc3	-0.15	1.00000000	mice
Nedd9	-0.03	1.00000000	mice
Tmem245	0.02	1.00000000	mice
Ndufaf7	0.00	1.00000000	mice
Slc30a6	-0.09	1.00000000	mice
Camkmt	0.10	1.00000000	mice
Pank4	-0.13	1.00000000	mice
Hscb	0.05	1.00000000	mice
Urm1	-0.14	1.00000000	mice
Zfp518a	0.01	1.00000000	mice
Tmem106b	-0.06	1.00000000	mice
Alox5	0.25	1.00000000	mice
Arpc1b	-0.38	1.00000000	mice
Ecd	0.06	1.00000000	mice
Sh3gl1	-0.47	1.00000000	mice
Pbx1	-0.68	1.00000000	mice
Acaa2	-0.05	1.00000000	mice
Cfdp1	-0.14	1.00000000	mice
Reep5	-0.13	1.00000000	mice
Alg2	-0.01	1.00000000	mice
Nolc1	-0.04	1.00000000	mice
Casp2	0.00	1.00000000	mice
Clns1a	-0.02	1.00000000	mice
Efr3a	-0.17	1.00000000	mice
Ccdc167	0.08	1.00000000	mice
Ist1	0.02	1.00000000	mice

Prmt3	0.03	1.00000000	mice
Gbf1	-0.08	1.00000000	mice
Tmem248	-0.09	1.00000000	mice
Rab22a	-0.04	1.00000000	mice
Naaa	0.06	1.00000000	mice
Dbr1	0.01	1.00000000	mice
Ginm1	-0.48	1.00000000	mice
Ctsh	-0.01	1.00000000	mice
Nr4a2	-0.06	1.00000000	mice
Exosc1	0.00	1.00000000	mice
Rbm22	-0.07	1.00000000	mice
Peak1	-0.05	1.00000000	mice
Snx29	-0.63	1.00000000	mice
Fam172a	-0.13	1.00000000	mice
Cdc14a	-0.50	1.00000000	mice
Rab5a	-0.09	1.00000000	mice
Stard3nl	-0.14	1.00000000	mice
Aimp2	-0.03	1.00000000	mice
Heatr3	-0.05	1.00000000	mice
Bag1	-0.04	1.00000000	mice
Nfe2l1	-0.18	1.00000000	mice
Tmem65	0.05	1.00000000	mice
Hnrnpab	-0.11	1.00000000	mice
Psmd9	-0.17	1.00000000	mice
Vps36	-0.04	1.00000000	mice
Fbxo8	-0.13	1.00000000	mice
Lrrc34	0.12	1.00000000	mice
Llph	-0.20	1.00000000	mice
Ssbp3	-0.15	1.00000000	mice
Rprd1b	-0.17	1.00000000	mice
Eed	-0.05	1.00000000	mice
Eif1ad12	-0.09	1.00000000	mice
Hdac11	-0.01	1.00000000	mice
Gns	-0.24	1.00000000	mice
Kansl3	0.02	1.00000000	mice
Rnf4	-0.01	1.00000000	mice
Bckdhb	-0.04	1.00000000	mice
Impa1	-0.01	1.00000000	mice
Cstf1	-0.06	1.00000000	mice
Chmp1a	-0.15	1.00000000	mice
Dennd5a	-0.61	1.00000000	mice
Tcf25	-0.11	1.00000000	mice
Snx3	-0.48	1.00000000	mice
Adrm1	-0.14	1.00000000	mice
Lsm14a	-0.33	1.00000000	mice
Cnih4	-0.06	1.00000000	mice

Fcf1	0.01	1.00000000	mice
Snrnp25	-0.04	1.00000000	mice
Ppp2r5d	-0.06	1.00000000	mice
Agpat5	-0.15	1.00000000	mice
Fubp3	-0.04	1.00000000	mice
Prmt7	-0.41	1.00000000	mice
Cops4	-0.12	1.00000000	mice
Klhdc4	0.03	1.00000000	mice
Eif2s1	-0.13	1.00000000	mice
Cactin	-0.09	1.00000000	mice
Strn3	-0.20	1.00000000	mice
Prr14l	-0.19	1.00000000	mice
Casp8ap2	-0.08	1.00000000	mice
Snrpb2	-0.07	1.00000000	mice
Sfi1	-0.17	1.00000000	mice
Exoc1	-0.08	1.00000000	mice
Clasp1	-0.12	1.00000000	mice
Prex1	-0.08	1.00000000	mice
Cask	0.02	1.00000000	mice
Sec11c	-0.13	1.00000000	mice
Fxr1	-0.41	1.00000000	mice
Plek	-0.05	1.00000000	mice
N4bp2l2	-0.08	1.00000000	mice
Eif4g3	-0.61	1.00000000	mice
Ube2h	-0.59	1.00000000	mice
Naxe	-0.03	1.00000000	mice
Ankra2	0.04	1.00000000	mice
Phf2	-0.18	1.00000000	mice
Sh3bgrl	-0.08	1.00000000	mice
Slc35c2	0.01	1.00000000	mice
Trip11	-0.75	1.00000000	mice
Adam17	-0.14	1.00000000	mice
Phf5a	-0.03	1.00000000	mice
Ube2w	-0.18	1.00000000	mice
Rnh1	0.02	1.00000000	mice
Dnajc17	-0.02	1.00000000	mice
Insr	-0.21	1.00000000	mice
Psma3	-0.12	1.00000000	mice
Acvr1b	0.00	1.00000000	mice
Znrf1	-0.42	1.00000000	mice
Samhd1	-0.26	1.00000000	mice
Nudcd1	-0.08	1.00000000	mice
Polr2b	-0.11	1.00000000	mice
Usp25	-0.12	1.00000000	mice
Exoc7	-0.03	1.00000000	mice
Rab12	-0.44	1.00000000	mice

Socs6	-0.07	1.00000000	mice
Caps2	0.00	1.00000000	mice
Ikzf2	0.07	1.00000000	mice
Nadk	-0.07	1.00000000	mice
Akirin2	-0.23	1.00000000	mice
Bop1	0.05	1.00000000	mice
Eif4enif1	-0.04	1.00000000	mice
Pald1	-0.46	1.00000000	mice
Ggps1	-0.53	1.00000000	mice
Plaa	-0.09	1.00000000	mice
Snx13	-0.14	1.00000000	mice
Arl6ip1	-0.18	1.00000000	mice
Zfp106	-0.09	1.00000000	mice
Cd99l2	-0.13	1.00000000	mice
Rab14	-0.09	1.00000000	mice
Gnptg	-0.11	1.00000000	mice
Psmd2	-0.01	1.00000000	mice
Slc25a5	0.07	1.00000000	mice
Hlcs	-0.01	1.00000000	mice
Sh2d1a	-0.08	1.00000000	mice
Cdc123	0.09	1.00000000	mice
Noc2l	-0.11	1.00000000	mice
Eif2ak4	-0.10	1.00000000	mice
Usp48	-0.15	1.00000000	mice
Cpeb2	0.12	1.00000000	mice
Snx4	-0.02	1.00000000	mice
Efcab14	-0.22	1.00000000	mice
Cdkal1	0.04	1.00000000	mice
Ccnyl1	-0.18	1.00000000	mice
Gbe1	-0.09	1.00000000	mice
Ak2	-0.59	1.00000000	mice
Nufip1	0.08	1.00000000	mice
Zfp512	0.03	1.00000000	mice
Pak3	0.04	1.00000000	mice
Ppme1	-0.10	1.00000000	mice
Glo1	-0.06	1.00000000	mice
Emc8	0.07	1.00000000	mice
Gtf2h1	-0.23	1.00000000	mice
Telo2	0.03	1.00000000	mice
Aasdh	-0.08	1.00000000	mice
Smap1	-0.14	1.00000000	mice
Nr1h3	-0.20	1.00000000	mice
Vps11	-0.13	1.00000000	mice
Anxa6	-0.09	1.00000000	mice
Dcp1b	0.12	1.00000000	mice
Trappc11	-0.07	1.00000000	mice

Tbc1d22a	-0.19	1.00000000	mice
Usp6nl	-0.20	1.00000000	mice
Lrrfip2	-0.13	1.00000000	mice
Timm10	0.03	1.00000000	mice
Tle3	-0.17	1.00000000	mice
Cds2	-0.13	1.00000000	mice
Zdhhc6	-0.23	1.00000000	mice
Fgd3	-0.50	1.00000000	mice
Vgll4	0.00	1.00000000	mice
Gpx4	-0.10	1.00000000	mice
Ercc4	-0.16	1.00000000	mice
Ndufs7	-0.09	1.00000000	mice
Tmem9b	-0.09	1.00000000	mice
Clpx	-0.19	1.00000000	mice
Ppp3cb	-0.16	1.00000000	mice
Ap1s2	-0.09	1.00000000	mice
Smyd3	0.09	1.00000000	mice
Ascc2	-0.18	1.00000000	mice
Ldlrad3	-0.24	1.00000000	mice
Actr10	-0.07	1.00000000	mice
Usp38	-0.20	1.00000000	mice
Nup50	-0.22	1.00000000	mice
Nhlrc2	-0.15	1.00000000	mice
Ulk1	-0.69	1.00000000	mice
Ddb1	-0.01	1.00000000	mice
Naca	-0.20	1.00000000	mice
Ggnbp2	-0.10	1.00000000	mice
Utp18	-0.11	1.00000000	mice
Klhl6	0.01	1.00000000	mice
Stau2	-0.09	1.00000000	mice
Dync1i2	-0.27	1.00000000	mice
Ube2i	-0.14	1.00000000	mice
Lgmn	-0.10	1.00000000	mice
Smg6	-0.18	1.00000000	mice
Ppm1g	-0.12	1.00000000	mice
Cstf2	0.01	1.00000000	mice
Btbd10	-0.12	1.00000000	mice
Slc25a24	-0.18	1.00000000	mice
Lgals8	-0.17	1.00000000	mice
Tpm1	-0.15	1.00000000	mice
Krr1	-0.01	1.00000000	mice
Tmco1	-0.15	1.00000000	mice
Rcor1	-0.15	1.00000000	mice
Trappc3	-0.01	1.00000000	mice
Klf6	-0.32	1.00000000	mice
Tbc1d2b	-0.17	1.00000000	mice

Poldip3	-0.17	1.00000000	mice
Syne3	-0.18	1.00000000	mice
Aff4	-0.16	1.00000000	mice
Rbl1	-0.07	1.00000000	mice
Arhgef7	-0.60	1.00000000	mice
Pum3	-0.06	1.00000000	mice
Dync1h1	-0.21	1.00000000	mice
Hddc2	-0.01	1.00000000	mice
Pdk3	-0.10	1.00000000	mice
Slc7a5	0.02	1.00000000	mice
Tor1b	-0.06	1.00000000	mice
Sf3b6	-0.12	1.00000000	mice
Sh3bgrl3	-0.14	1.00000000	mice
Ube2v1	-0.14	1.00000000	mice
Saraf	-0.16	1.00000000	mice
Atp6v0a1	-0.52	1.00000000	mice
Acad9	-0.14	1.00000000	mice
Ppp1r12a	-0.05	1.00000000	mice
Cd63	-0.18	1.00000000	mice
Nsun2	-0.07	1.00000000	mice
Eva1c	0.16	1.00000000	mice
Vps50	-0.33	1.00000000	mice
Gramd1b	0.02	1.00000000	mice
Nsmce1	0.01	1.00000000	mice
Rac1	-0.55	1.00000000	mice
Irf2	-0.30	1.00000000	mice
Ranbp9	-0.39	1.00000000	mice
Cenpc1	-0.08	1.00000000	mice
Tcf3	-0.55	1.00000000	mice
Acox1	-0.07	1.00000000	mice
Elovl5	-0.15	1.00000000	mice
Ssr2	-0.20	1.00000000	mice
Fam53b	-0.02	1.00000000	mice
Ubr2	-0.28	1.00000000	mice
Tulp4	-0.07	1.00000000	mice
Tob2	-0.24	1.00000000	mice
Tmem141	-0.13	1.00000000	mice
Rsu1	-0.12	1.00000000	mice
Szrd1	0.01	1.00000000	mice
Ranbp10	-0.12	1.00000000	mice
Parp9	-0.05	1.00000000	mice
Slk	-0.20	1.00000000	mice
Zfp639	-0.17	1.00000000	mice
Gatad2a	-0.10	1.00000000	mice
Chmp2b	-0.07	1.00000000	mice
Specc1l	-0.47	1.00000000	mice

Ltf	-0.04	1.00000000	mice
Srebf2	-0.06	1.00000000	mice
Phf10	-0.18	1.00000000	mice
Cry1	-0.43	1.00000000	mice
Cpsf4l	-0.13	1.00000000	mice
Rab6a	-0.25	1.00000000	mice
4932438A13Rik	-0.39	1.00000000	mice
Taldo1	-0.13	1.00000000	mice
Dram2	-0.16	1.00000000	mice
Nono	-0.14	1.00000000	mice
Ube2f	-0.08	1.00000000	mice
Top2b	-0.31	1.00000000	mice
Eftud2	-0.04	1.00000000	mice
Fbxo3	-0.50	1.00000000	mice
Wdr3	-0.04	1.00000000	mice
Eif3h	-0.10	1.00000000	mice
Suco	-0.20	1.00000000	mice
Phactr2	-0.18	1.00000000	mice
Zfyve1	-0.49	1.00000000	mice
Snx14	-0.55	1.00000000	mice
Slc25a25	-0.23	1.00000000	mice
Cdc27	-0.13	1.00000000	mice
Ube2o	-0.09	1.00000000	mice
Rab5c	-0.11	1.00000000	mice
Zc2hc1a	-0.11	1.00000000	mice
Fndc3b	-0.11	1.00000000	mice
Prpf6	-0.16	1.00000000	mice
Plcl2	0.20	1.00000000	mice
Disp1	-0.56	1.00000000	mice
Phf3	-0.12	1.00000000	mice
Ebna1bp2	-0.06	1.00000000	mice
Ube4a	-0.07	1.00000000	mice
Rrp15	0.06	1.00000000	mice
Armc1	-0.23	1.00000000	mice
Odf2	-0.11	1.00000000	mice
Ubap2	-0.22	1.00000000	mice
Slx4	-0.04	1.00000000	mice
Klhl22	-0.10	1.00000000	mice
Rgs9	-0.10	1.00000000	mice
Btbd1	-0.03	1.00000000	mice
Elp3	-0.06	1.00000000	mice
Zbtb7a	-0.19	1.00000000	mice
Wasf2	-0.16	1.00000000	mice
Gnb1	-0.32	1.00000000	mice
Tob1	-0.51	1.00000000	mice
Fnip1	-0.33	1.00000000	mice

Tmbim6	-0.22	1.00000000	mice
Ndfip1	-0.46	1.00000000	mice
Zmat2	-0.13	1.00000000	mice
Uprt	-0.09	1.00000000	mice
Polr3a	-0.24	1.00000000	mice
Copg1	-0.08	1.00000000	mice
Rell1	-0.18	1.00000000	mice
Grb2	-0.20	1.00000000	mice
Nelfa	-0.15	1.00000000	mice
Slc39a8	-0.01	1.00000000	mice
Kdelr2	-0.25	1.00000000	mice
Actr2	-0.26	1.00000000	mice
Ralb	-0.53	1.00000000	mice
Tmem183a	-0.16	1.00000000	mice
Focad	-0.26	1.00000000	mice
Gpx1	-0.55	1.00000000	mice
Gtf2e1	-0.08	1.00000000	mice
Slco3a1	-0.01	1.00000000	mice
Wdr59	-0.10	1.00000000	mice
Phb	0.01	1.00000000	mice
Heca	-0.19	1.00000000	mice
Tbc1d4	-0.05	1.00000000	mice
Nkiras2	-0.01	1.00000000	mice
Fbrsl1	-0.12	1.00000000	mice
Tmem50a	-0.51	1.00000000	mice
Tpm3-rs7	-0.39	1.00000000	mice
Jmy	-0.20	1.00000000	mice
St8sia1	0.01	1.00000000	mice
Fyttd1	-0.23	1.00000000	mice
Tmem59	-0.27	1.00000000	mice
Nup153	-0.20	1.00000000	mice
Hsp90b1	-0.08	1.00000000	mice
Pan3	-0.55	1.00000000	mice
Uimc1	-0.01	1.00000000	mice
Exoc4	-0.20	1.00000000	mice
Dot1l	-0.64	1.00000000	mice
Polg2	-0.22	1.00000000	mice
Akap7	-0.07	1.00000000	mice
Bnip1	-0.11	1.00000000	mice
Snx17	-0.30	1.00000000	mice
Ovch2	0.09	1.00000000	mice
Skp1	-0.26	1.00000000	mice
Elp2	0.03	1.00000000	mice
Ppp1r2	-0.26	1.00000000	mice
Ino80c	-0.05	1.00000000	mice
Atxn10	0.15	1.00000000	mice

Pcgf6	-0.08	1.00000000	mice
Gapvd1	-0.07	1.00000000	mice
Ska2	-0.15	1.00000000	mice
Rabep1	-0.15	1.00000000	mice
Bbs9	-0.03	1.00000000	mice
Pik3cb	-0.17	1.00000000	mice
Chid1	-0.06	1.00000000	mice
Tubgcp5	-0.04	1.00000000	mice
Bap1	-0.04	1.00000000	mice
Pwwp2b	-0.45	1.00000000	mice
Xylt1	-0.18	1.00000000	mice
Dnajb12	-0.18	1.00000000	mice
Ccpg1	-0.21	1.00000000	mice
Sec31a	-0.66	1.00000000	mice
Rab21	-0.24	1.00000000	mice
Flii	-0.15	1.00000000	mice
Eif5	-0.26	1.00000000	mice
Rsrc1	-0.25	1.00000000	mice
Dcun1d5	-0.16	1.00000000	mice
Gnpat	-0.10	1.00000000	mice
Tmem185a	-0.11	1.00000000	mice
Tecpr1	-0.12	1.00000000	mice
Srp54a	-0.18	1.00000000	mice
Pitpnb	-0.19	1.00000000	mice
Zbtb11	-0.33	1.00000000	mice
Rab40c	-0.16	1.00000000	mice
Trim8	-0.29	1.00000000	mice
Yaf2	-0.21	1.00000000	mice
Hpcal1	-0.35	1.00000000	mice
Cdk8	-0.16	1.00000000	mice
Lyar	-0.11	1.00000000	mice
Trim33	-0.13	1.00000000	mice
Sun1	-0.34	1.00000000	mice
Klf3	-0.35	1.00000000	mice
Nudt3	-0.10	1.00000000	mice
Hipk3	0.25	1.00000000	mice
Plag1	0.04	1.00000000	mice
Yod1	-0.08	1.00000000	mice
Pdrg1	-0.18	1.00000000	mice
Slc7a6os	-0.14	1.00000000	mice
Ldah	-0.06	1.00000000	mice
Crnkl1	-0.04	1.00000000	mice
Agl	-0.12	1.00000000	mice
Rcbtb1	-0.54	1.00000000	mice
Nvl	-0.45	1.00000000	mice
Ifih1	-0.04	1.00000000	mice

Atad2b	-0.30	1.00000000	mice
Sod1	-0.23	1.00000000	mice
Sel1l	-0.31	1.00000000	mice
Ndufv2	-0.09	1.00000000	mice
Gclc	-0.41	1.00000000	mice
Ahr	0.02	1.00000000	mice
Fut8	-0.45	1.00000000	mice
Ctsb	-0.58	1.00000000	mice
Pdia3	-0.23	1.00000000	mice
Prkar1a	-0.22	1.00000000	mice
Adam10	-0.26	1.00000000	mice
Ubfd1	-0.04	1.00000000	mice
Snx10	-0.46	1.00000000	mice
Cdc14b	-0.08	1.00000000	mice
Ing2	-0.03	1.00000000	mice
Il16	-0.15	1.00000000	mice
Tspan14	-0.48	1.00000000	mice
Apaf1	-0.23	1.00000000	mice
Ssr3	-0.39	1.00000000	mice
Eif4ebp1	-0.46	1.00000000	mice
Sptlc2	-0.30	1.00000000	mice
Ywhah	-0.13	1.00000000	mice
Taf13	-0.35	1.00000000	mice
Trib2	-0.13	1.00000000	mice
Pik3ip1	-0.04	1.00000000	mice
Atp6ap2	-0.42	1.00000000	mice
Dlg1	-0.34	1.00000000	mice
Elmo1	0.02	1.00000000	mice
Nrip1	-0.03	1.00000000	mice
Ppp3r1	-0.38	1.00000000	mice
Fbxo22	-0.14	1.00000000	mice
Rad17	-0.16	1.00000000	mice
Fundc1	0.04	1.00000000	mice
Nfkbiz	-0.23	1.00000000	mice
Snx19	-0.10	1.00000000	mice
Ppm1d	-0.39	1.00000000	mice
Glipr2	-0.40	1.00000000	mice
Lrrc57	-0.11	1.00000000	mice
Ppfibp1	-0.42	1.00000000	mice
Dtnbp1	-0.03	1.00000000	mice
Zfp652	-0.46	1.00000000	mice
Hivep2	-0.15	1.00000000	mice
Ccnl1	-0.06	1.00000000	mice
Phkg1	-0.06	1.00000000	mice
Tnip1	-0.20	1.00000000	mice
Rbsn	-0.12	1.00000000	mice

Ate1	-0.14	1.00000000	mice
Tfrc	-0.75	1.00000000	mice
Hnrnpa2b1	-0.10	1.00000000	mice
Cops8	-0.45	1.00000000	mice
Brms1l	-0.13	1.00000000	mice
Hhex	-0.44	1.00000000	mice
Taf12	-0.14	1.00000000	mice
Erlin2	-0.32	1.00000000	mice
Eno1b	-0.23	1.00000000	mice
Ppp1cb	-0.20	1.00000000	mice
Nans	-0.17	1.00000000	mice
Phkb	-0.15	1.00000000	mice
Atp6v1d	-0.18	1.00000000	mice
Tln1	-0.46	1.00000000	mice
Ppa1	-0.35	1.00000000	mice
Cbfb	-0.20	1.00000000	mice
Rnaset2b	-0.41	1.00000000	mice
Hsd17b4	-0.19	1.00000000	mice
Hnrnpr	-0.18	1.00000000	mice
Atxn1	-0.05	1.00000000	mice
Psmd7	-0.17	1.00000000	mice
Sil1	-0.23	1.00000000	mice
Lin54	-0.47	1.00000000	mice
Wdr47	-0.08	1.00000000	mice
Ufm1	-0.20	1.00000000	mice
Napg	-0.17	1.00000000	mice
Rnf220	-0.30	1.00000000	mice
Ppm1f	-0.62	1.00000000	mice
Pdcd6	-0.24	1.00000000	mice
Elk4	-0.39	1.00000000	mice
Dmxl1	-0.32	1.00000000	mice
Dcaf17	-0.11	1.00000000	mice
Lin52	-0.28	1.00000000	mice
Axin1	-0.19	1.00000000	mice
Fam133b	-0.17	1.00000000	mice
Trappc12	-0.21	1.00000000	mice
Dctn1	-0.31	1.00000000	mice
Zdhhc14	-0.20	1.00000000	mice
Tmpo	-0.34	1.00000000	mice
Acbd3	-0.20	1.00000000	mice
Zranb1	-0.18	1.00000000	mice
Zdhhc7	-0.11	1.00000000	mice
Invs	-0.25	1.00000000	mice
Chmp4b	-0.37	1.00000000	mice
Plbd2	-0.36	1.00000000	mice
Hmgb1	-0.14	1.00000000	mice

Dr1	-0.25	1.00000000	mice
Ppp2r5a	-0.66	1.00000000	mice
Ndufb5	-0.21	1.00000000	mice
Eci3	-0.09	1.00000000	mice
Idh3a	-0.03	1.00000000	mice
Eef1b2	-0.03	1.00000000	mice
Vps41	-0.20	1.00000000	mice
Setd7	-0.30	1.00000000	mice
Clstn1	-0.20	1.00000000	mice
Zfp407	-0.32	1.00000000	mice
Atf4	-0.34	1.00000000	mice
Tmf1	-0.26	1.00000000	mice
Lrpap1	-0.43	1.00000000	mice
Nr3c1	-0.34	1.00000000	mice
Usp8	-0.35	1.00000000	mice
Rab18	-0.26	1.00000000	mice
Kbtbd2	-0.27	1.00000000	mice
Atp7a	-0.22	1.00000000	mice
S100a11	-0.36	1.00000000	mice
Sash1	0.34	1.00000000	mice
Trap1	-0.01	1.00000000	mice
Dnaja2	-0.24	1.00000000	mice
Aplf	-0.16	1.00000000	mice
Hnrnpa3	0.02	1.00000000	mice
Smu1	-0.24	1.00000000	mice
Rwdd1	-0.23	1.00000000	mice
Prkcd	-0.14	1.00000000	mice
Cmss1	-0.17	1.00000000	mice
Setdb1	-0.17	1.00000000	mice
Reck	-0.15	1.00000000	mice
Ddx5	-0.10	1.00000000	mice
Chmp6	-0.22	1.00000000	mice
Ankfy1	-0.21	1.00000000	mice
Fasn	-0.30	1.00000000	mice
Zbtb18	-0.33	1.00000000	mice
Fem1b	-0.02	1.00000000	mice
Dnajb2	-0.11	1.00000000	mice
Snx25	-0.16	1.00000000	mice
Yipf5	-0.14	1.00000000	mice
Dync2h1	-0.19	1.00000000	mice
Vps37a	-0.21	1.00000000	mice
Prpf39	-0.12	1.00000000	mice
Phlpp1	-0.08	1.00000000	mice
Erlec1	-0.22	1.00000000	mice
Oser1	-0.14	1.00000000	mice
Kif5b	-0.48	1.00000000	mice

Kif1b	-0.25	1.00000000	mice
Wbp2nl	-0.16	1.00000000	mice
Swi5	-0.41	1.00000000	mice
Blmh	-0.10	1.00000000	mice
Gab3	0.30	1.00000000	mice
Cpne3	-0.25	1.00000000	mice
Hectd1	-0.22	1.00000000	mice
Pde7a	-0.07	1.00000000	mice
D5Ertd579e	-0.48	1.00000000	mice
Arl15	-0.19	1.00000000	mice
Qdpr	-0.05	1.00000000	mice
Prelid3a	-0.30	1.00000000	mice
Srp19	-0.12	1.00000000	mice
Azin1	-0.26	1.00000000	mice
Edrf1	-0.12	1.00000000	mice
Large1	-0.18	1.00000000	mice
Wbp4	-0.82	1.00000000	mice
Ppfia1	-0.34	1.00000000	mice
Zcchc14	-0.14	1.00000000	mice
Lcmt2	-0.27	1.00000000	mice
Atp11c	-0.19	1.00000000	mice
Itsn2	-0.49	1.00000000	mice
Itfg2	-0.22	1.00000000	mice
Anapc10	-0.38	1.00000000	mice
Etf1	-0.24	1.00000000	mice
Dph5	-0.08	1.00000000	mice
Ybx3	-0.26	1.00000000	mice
Cir1	-0.23	1.00000000	mice
Tbc1d9	-0.08	1.00000000	mice
Lrch2	-0.10	1.00000000	mice
Akap10	-0.20	1.00000000	mice
Rnf144a	-0.20	1.00000000	mice
Disc1	-0.29	1.00000000	mice
Abcc1	-0.31	1.00000000	mice
Jakmip1	-0.32	1.00000000	mice
Aida	-0.22	1.00000000	mice
Sec23a	-0.38	1.00000000	mice
Zhx1	-0.61	1.00000000	mice
Tmem165	-0.12	1.00000000	mice
Nop2	-0.11	1.00000000	mice
Brox	-0.20	1.00000000	mice
Nbeal1	-0.44	1.00000000	mice
Ccdc47	-0.14	1.00000000	mice
Bcar3	-0.14	1.00000000	mice
Tpp2	-0.35	1.00000000	mice
Lamp1	-0.56	1.00000000	mice

Rreb1	-0.48	1.00000000	mice
Ero1b	-0.21	1.00000000	mice
Ifrd1	-0.56	1.00000000	mice
Dnaja1	-0.40	1.00000000	mice
Sppl3	-0.21	1.00000000	mice
Rala	-0.32	1.00000000	mice
Slc4a11	-0.29	1.00000000	mice
Kif13b	-0.29	1.00000000	mice
Stx2	-0.22	1.00000000	mice
Ube2q1	-0.31	1.00000000	mice
Dctn6	-0.27	1.00000000	mice
Lrrcc1	-0.17	1.00000000	mice
Fez2	-0.31	1.00000000	mice
Spag9	-0.48	1.00000000	mice
Gata2	-0.24	1.00000000	mice
Ube4b	-0.25	1.00000000	mice
Abtb1	-0.27	1.00000000	mice
Zfand6	-0.31	1.00000000	mice
Fhit	0.01	1.00000000	mice
Atrx	-0.34	1.00000000	mice
Nisch	-0.37	1.00000000	mice
Appl1	-0.32	1.00000000	mice
Rnf8	-0.22	1.00000000	mice
Rbbp6	-0.24	1.00000000	mice
Scp2	-0.32	1.00000000	mice
Psma5	-0.16	1.00000000	mice
Usp53	-0.26	1.00000000	mice
Vps25	-0.29	1.00000000	mice
Cry2	-0.20	1.00000000	mice
Apoo	-0.34	1.00000000	mice
Nusap1	-0.37	1.00000000	mice
Isca1	-0.26	1.00000000	mice
Pef1	-0.20	1.00000000	mice
Wbp11	-0.31	1.00000000	mice
Camk2d	-0.19	1.00000000	mice
Stam2	-0.46	1.00000000	mice
Nme7	-0.24	1.00000000	mice
Epb41	-0.19	1.00000000	mice
Atp1a1	-0.65	1.00000000	mice
Kmt2c	-0.27	1.00000000	mice
Naa30	-0.22	1.00000000	mice
Eef1d	-0.08	1.00000000	mice
Tes	-0.40	1.00000000	mice
Polr3b	-0.36	1.00000000	mice
Pla2g12a	-0.17	1.00000000	mice
Notch1	-0.51	1.00000000	mice

Slc4a4	-0.31	1.00000000	mice
Rnf141	-0.17	1.00000000	mice
Crip1	-0.27	1.00000000	mice
Smad3	-0.22	1.00000000	mice
Tpm4	-0.43	1.00000000	mice
Nucks1	-0.41	1.00000000	mice
Cltc	-0.43	1.00000000	mice
Id2	-0.13	1.00000000	mice
Ptpn4	-0.35	1.00000000	mice
Rsrp1	-0.61	1.00000000	mice
Poc1b	-0.15	1.00000000	mice
Ptpre	-0.37	1.00000000	mice
Ank	0.02	1.00000000	mice
Asah1	-0.48	1.00000000	mice
Atp6v1g1	-0.35	1.00000000	mice
Anp32e	-0.29	1.00000000	mice
Ppp1r16b	-0.26	1.00000000	mice
Tef	-0.36	1.00000000	mice
Itpr1	-0.21	1.00000000	mice
Slc39a10	-0.41	1.00000000	mice
Atp6v1h	-0.41	1.00000000	mice
Bloc1s6	-0.38	1.00000000	mice
Amfr	-0.47	1.00000000	mice
Stxbp5	-0.30	1.00000000	mice
Slc30a7	-0.24	1.00000000	mice
Tax1bp1	-0.41	1.00000000	mice
Dcun1d1	-0.27	1.00000000	mice
Ppp3ca	-0.34	1.00000000	mice
Dtx2	-0.29	1.00000000	mice
Ndufa6	-0.36	1.00000000	mice
Smad5	-0.45	1.00000000	mice
Shisa5	-0.36	1.00000000	mice
Proser1	-0.06	1.00000000	mice
Adgrg6	0.10	1.00000000	mice
Cmtm4	-0.20	1.00000000	mice
Cdca4	-0.32	1.00000000	mice
Syt11	-0.23	1.00000000	mice
Inpp4a	-0.09	1.00000000	mice
Tmem164	-0.06	1.00000000	mice
Rft1	-0.17	1.00000000	mice
Hspa5	-0.33	1.00000000	mice
Commd4	-0.13	1.00000000	mice
Pdcl3	-0.20	1.00000000	mice
Btg2	-0.49	1.00000000	mice
Fbxl17	-0.13	1.00000000	mice
Clip1	-0.51	1.00000000	mice

Stoml2	-0.13	1.00000000	mice
Ndufa12	-0.23	1.00000000	mice
Tmed5	-0.41	1.00000000	mice
Ptpn12	-0.21	1.00000000	mice
Xpo1	-0.33	1.00000000	mice
Ube2q2	-0.43	1.00000000	mice
Lsg1	-0.04	1.00000000	mice
Ywhaq	-0.31	1.00000000	mice
Per2	-0.32	1.00000000	mice
Bfar	-0.38	1.00000000	mice
Itgb1	-0.05	1.00000000	mice
Rpia	-0.21	1.00000000	mice
Dyrk1a	-0.30	1.00000000	mice
Crebrf	-0.40	1.00000000	mice
Nt5c3	-0.55	1.00000000	mice
Tlk2	-0.28	1.00000000	mice
Unc13b	-0.17	1.00000000	mice
Per3	-0.04	1.00000000	mice
Nsmce2	-0.23	1.00000000	mice
Kif13a	-0.46	1.00000000	mice
Cnot10	-0.25	1.00000000	mice
Pard3	-0.32	1.00000000	mice
Rptor	-0.13	1.00000000	mice
Ttc7b	-0.27	1.00000000	mice
Txndc9	-0.43	1.00000000	mice
Ndufs1	-0.20	1.00000000	mice
Psmd6	-0.17	1.00000000	mice
Irf2bpl	-0.32	1.00000000	mice
Inpp5b	-0.03	1.00000000	mice
Zfand3	-0.25	1.00000000	mice
Sgpp1	-0.30	1.00000000	mice
Exoc5	-0.24	1.00000000	mice
Tbc1d9b	-0.18	1.00000000	mice
Slc48a1	-0.24	1.00000000	mice
Tspan5	-0.99	1.00000000	mice
Rab7	-0.41	1.00000000	mice
Parvg	-0.32	1.00000000	mice
Arhgap29	-0.42	1.00000000	mice
Il6st	-0.32	1.00000000	mice
Ap2m1	-0.42	1.00000000	mice
Bicd2	-0.58	1.00000000	mice
Cpne1	-0.28	1.00000000	mice
Coq9	-0.12	1.00000000	mice
Lamp2	-0.37	1.00000000	mice
Nr3c2	-0.32	1.00000000	mice
Exoc6b	-0.50	1.00000000	mice

Oaz2	-0.36	1.00000000	mice
Arl8b	-0.26	1.00000000	mice
Rev1	-0.40	1.00000000	mice
Sulf1	-0.04	1.00000000	mice
Fbxl18	-0.46	1.00000000	mice
Ssr1	-0.33	1.00000000	mice
Actn1	-0.37	1.00000000	mice
Arl1	-0.31	1.00000000	mice
Vwa8	-0.40	1.00000000	mice
Gabpb1	-0.21	1.00000000	mice
Sh3glb1	-0.49	1.00000000	mice
Ano10	-0.24	1.00000000	mice
Arntl	-0.37	1.00000000	mice
Plcb4	-0.29	1.00000000	mice
Wdsub1	-0.23	1.00000000	mice
Slc12a2	-0.40	1.00000000	mice
Pank1	-0.22	1.00000000	mice
2900026A02Rik	-0.10	1.00000000	mice
Lonrf2	-0.29	1.00000000	mice
Canx	-0.25	1.00000000	mice
Rap1b	-0.40	1.00000000	mice
Stk24	-0.41	1.00000000	mice
Edf1	-0.25	1.00000000	mice
Psmc3	-0.28	1.00000000	mice
Wdfy1	-0.26	1.00000000	mice
Hmbox1	-0.30	1.00000000	mice
Prdx1	-0.43	1.00000000	mice
Stk40	-0.22	1.00000000	mice
Pwp1	-0.14	1.00000000	mice
Larp4b	-0.34	1.00000000	mice
Vti1a	-0.23	1.00000000	mice
Raph1	-0.25	1.00000000	mice
Irak2	-0.35	1.00000000	mice
Ndufs6	-0.20	1.00000000	mice
Fzd6	-0.37	1.00000000	mice
Psmb4	-0.31	1.00000000	mice
Ccdc186	-0.44	1.00000000	mice
Larp1b	-0.47	1.00000000	mice
Amot	-0.34	1.00000000	mice
Cisd2	-0.14	1.00000000	mice
Plekha3	-0.31	1.00000000	mice
Lclat1	-0.40	1.00000000	mice
Uvrag	-0.39	1.00000000	mice
Rock2	-0.46	1.00000000	mice
Yipf3	-0.21	1.00000000	mice
Rb1	-0.27	1.00000000	mice

Scpep1	-0.15	1.00000000	mice
Acbd5	-0.36	1.00000000	mice
Dbi	-0.39	1.00000000	mice
Eif5b	-0.42	1.00000000	mice
Serpini1	-0.25	1.00000000	mice
Ddost	-0.19	1.00000000	mice
Ppp4r1	-0.35	1.00000000	mice
Pam	-0.32	1.00000000	mice
Arhgef3	-0.50	1.00000000	mice
Atg101	-0.13	1.00000000	mice
Dync1li2	-0.45	1.00000000	mice
Zc3h12a	-0.42	1.00000000	mice
Ergic2	-0.24	1.00000000	mice
Psmd4	-0.26	1.00000000	mice
Fam135a	-0.23	1.00000000	mice
Arhgap12	-0.43	1.00000000	mice
Arih1	-0.43	1.00000000	mice
Saa3	2.13	0.00000000	pigs
Ago2	1.48	0.00000000	pigs
S100a6	-2.05	0.00000000	pigs
S100a4	-2.22	0.00000000	pigs
Nol11	1.11	0.00000000	pigs
Aldh3a2	1.08	0.00000000	pigs
Timp1	1.67	0.00000000	pigs
Lgmn	1.37	0.00000000	pigs
Abtb2	1.87	0.00000000	pigs
Runx2	0.86	0.00000000	pigs
Furin	1.51	0.00000000	pigs
Hif1a	0.91	0.00000000	pigs
Gramd3	1.29	0.00000000	pigs
Tsen2	0.72	0.00000000	pigs
Cyth1	0.82	0.00000000	pigs
Clasp1	1.06	0.00000000	pigs
Hsph1	1.09	0.00000000	pigs
Foxo1	0.97	0.00000000	pigs
Antxr2	0.84	0.00000000	pigs
Ccnl1	0.73	0.00000000	pigs
Sik3	0.85	0.00000000	pigs
Ndfip1	0.69	0.00000000	pigs
Hivep1	0.95	0.00000000	pigs
Zswim6	0.91	0.00000000	pigs
Nme8	0.72	0.00000000	pigs
Prkca	0.92	0.00000000	pigs
Card11	0.91	0.00000000	pigs
Gna13	0.77	0.00000000	pigs
Arid4b	0.70	0.00000000	pigs

Stat5a	0.68	0.00000000	pigs
Gfi1	0.73	0.00000000	pigs
Nebl	-1.34	0.00000000	pigs
Gpx1	-1.45	0.00000000	pigs
Jmjd1c	0.78	0.00000000	pigs
Il21r	0.73	0.00000000	pigs
H2-Ob	0.71	0.00000000	pigs
1700025G04Rik	0.79	0.00000000	pigs
Atp2b4	0.65	0.00000000	pigs
Dennd3	0.62	0.00000000	pigs
Jmy	0.82	0.00000000	pigs
Csnk1a1	0.56	0.00000000	pigs
Bhlhe40	0.86	0.00000000	pigs
Frmd4b	0.76	0.00000000	pigs
Serf2	-0.57	0.00000000	pigs
Ybey	0.46	0.00000000	pigs
Relb	0.60	0.00000000	pigs
Wnk1	0.72	0.00000000	pigs
Dusp4	0.90	0.00000000	pigs
Niban1	0.99	0.00000000	pigs
Nck2	0.69	0.00000000	pigs
Ppp1r12a	0.65	0.00000000	pigs
Ftl1-ps1	0.71	0.00000000	pigs
Traf3	0.70	0.00000000	pigs
Lrrc1	0.74	0.00000000	pigs
Hdac4	0.68	0.00000000	pigs
Prdm2	0.77	0.00000000	pigs
Ubl5	-0.87	0.00000000	pigs
Actb	-0.44	0.00000000	pigs
Pnp	0.75	0.00000000	pigs
Stat1	0.60	0.00000000	pigs
Rybp	0.69	0.00000000	pigs
Hivep2	0.65	0.00000000	pigs
Cd28	0.83	0.00000000	pigs
Spock2	0.57	0.00000000	pigs
Ctsb	0.62	0.00000000	pigs
Eif4a1	0.56	0.00000000	pigs
Rnf19a	0.98	0.00000000	pigs
Ranbp9	0.58	0.00000000	pigs
Cry2	0.60	0.00000000	pigs
Cdkn1a	0.54	0.00000000	pigs
Tifa	0.51	0.00000000	pigs
Isg20	0.55	0.00000000	pigs
Socs3	0.70	0.00000000	pigs
Prkce	0.66	0.00000000	pigs
Wwox	0.68	0.00000000	pigs

Lpin2	0.58	0.00000000	pigs
Rbpj	0.48	0.00000000	pigs
Tnip2	0.47	0.00000000	pigs
Hint3	0.51	0.00000000	pigs
Lcor	0.58	0.00000000	pigs
Jak2	0.67	0.00000000	pigs
Dennd5a	0.45	0.00000000	pigs
Atp2b1	0.77	0.00000000	pigs
Tnfrsf4	1.10	0.00000000	pigs
B4galt1	0.73	0.00000000	pigs
Dyrk1a	0.64	0.00000000	pigs
Il4ra	0.55	0.00000000	pigs
S100a10	-0.73	0.00000000	pigs
Braf	0.53	0.00000000	pigs
Stat3	0.60	0.00000000	pigs
Caprin1	0.57	0.00000000	pigs
Atp1a1	0.60	0.00000000	pigs
Prkcq	0.56	0.00000000	pigs
Rfx2	0.91	0.00000000	pigs
Susd6	0.52	0.00000000	pigs
Usp18	0.52	0.00000000	pigs
Ubr5	0.58	0.00000000	pigs
Gpr183	0.46	0.00000000	pigs
Usp15	0.55	0.00000000	pigs
Rora	0.75	0.00000000	pigs
Gtf3c1	0.50	0.00000000	pigs
Snapc1l	0.52	0.00000000	pigs
F2r	0.50	0.00000000	pigs
Pan3	0.49	0.00000000	pigs
Kdm6a	0.54	0.00000000	pigs
Zeb1	0.64	0.00000000	pigs
Ldlrad4	0.61	0.00000000	pigs
Pspc1	0.52	0.00000000	pigs
Per1	0.54	0.00000000	pigs
Ikzf1	0.56	0.00000000	pigs
Cavin3	-0.76	0.00000000	pigs
Zranb1	0.51	0.00000000	pigs
Atp2a2	0.62	0.00000000	pigs
Xylt1	0.61	0.00000000	pigs
Irf2bp2	0.52	0.00000000	pigs
Ddx3x	0.45	0.00000000	pigs
Cyth4	0.51	0.00000000	pigs
Szrd1	0.44	0.00000000	pigs
Ncoa7	0.64	0.00000000	pigs
Lpin1	0.71	0.00000000	pigs
Chordc1	0.43	0.00000000	pigs

Cab39	0.49	0.00000000	pigs
Slamf1	0.80	0.00000000	pigs
Eps15	0.56	0.00000000	pigs
Tet2	0.54	0.00000000	pigs
Ywhab	-0.67	0.00000000	pigs
Pde7a	0.69	0.00000000	pigs
Ecpas	0.43	0.00000000	pigs
Cytip	0.38	0.00000000	pigs
Ubash3a	0.56	0.00000000	pigs
Stx11	0.43	0.00000000	pigs
Rpap2	0.45	0.00000000	pigs
Pum1	0.41	0.00000000	pigs
Galnt7	0.73	0.00000000	pigs
Rasa3	-1.04	0.00000000	pigs
Zc3hav1	0.50	0.00000000	pigs
Rcor1	0.51	0.00000000	pigs
Traf1	0.48	0.00000000	pigs
Stambpl1	0.69	0.00000000	pigs
Phc3	0.49	0.00000000	pigs
Cflar	0.55	0.00000000	pigs
Evl	0.49	0.00000000	pigs
Zcchc14	0.47	0.00000000	pigs
Brd4	0.37	0.00000000	pigs
Ell2	0.33	0.00000000	pigs
Peli1	0.42	0.00000000	pigs
Dusp16	0.89	0.00000000	pigs
Safb	0.35	0.00000000	pigs
Arap2	0.54	0.00000000	pigs
Lgals1	-1.99	0.00000000	pigs
Ttc39c	0.49	0.00000000	pigs
Ppp4r3a	0.41	0.00000000	pigs
Cxcr6	0.55	0.00000000	pigs
Trerf1	0.83	0.00000000	pigs
Fam102a	0.44	0.00000000	pigs
Tmem131l	0.50	0.00000000	pigs
Eef2	0.41	0.00000000	pigs
Piezo1	0.39	0.00000000	pigs
Icos	0.70	0.00000000	pigs
Dpyd	0.45	0.00000000	pigs
Ndfip2	0.53	0.00000000	pigs
Gpbp1	0.38	0.00000000	pigs
Elf1	0.39	0.00000000	pigs
Usp9x	0.44	0.00000000	pigs
Spp1	0.43	0.00000000	pigs
Cnot6l	0.48	0.00000000	pigs
Rab27a	0.44	0.00000000	pigs

Rnf2	1.02	0.00000000	pigs
Ube2d3	0.36	0.00000000	pigs
Nfkbia	0.64	0.00000000	pigs
Nfkb2	0.36	0.00000000	pigs
Sh3bgrl3	-0.64	0.00000000	pigs
Qk	0.47	0.00000000	pigs
Kansl1	0.42	0.00000000	pigs
Zfp644	0.41	0.00000000	pigs
Ptprj	0.78	0.00000000	pigs
Plagl1	0.50	0.00000000	pigs
Vav1	0.42	0.00000000	pigs
Odc1	0.51	0.00000000	pigs
Lag3	0.85	0.00000000	pigs
Ptbp2	0.42	0.00000000	pigs
Lrrc8c	0.55	0.00000000	pigs
Efr3a	0.43	0.00000000	pigs
Satb1	0.60	0.00000000	pigs
Hspa8	0.46	0.00000000	pigs
Vmp1	0.48	0.00000000	pigs
Tab2	0.45	0.00000000	pigs
Apmap	0.37	0.00000000	pigs
Rasa2	0.29	0.00000000	pigs
Ccnd2	0.50	0.00000000	pigs
Arhgap25	0.56	0.00000000	pigs
Stk24	0.41	0.00000000	pigs
Narf	0.34	0.00000000	pigs
Tpi1	0.46	0.00000000	pigs
Nsd3	0.54	0.00000000	pigs
C1qa	0.49	0.00000000	pigs
Rnf145	0.38	0.00000000	pigs
Ext1	0.54	0.00000000	pigs
Trps1	0.58	0.00000000	pigs
Ppp2r5c	0.46	0.00000000	pigs
Rnf139	0.38	0.00000000	pigs
Tmem129	0.44	0.00000000	pigs
Nup50	0.36	0.00000000	pigs
Poc1b	0.51	0.00000000	pigs
Ranbp2	0.33	0.00000000	pigs
Smurf2	0.48	0.00000000	pigs
Trat1	-0.85	0.00000000	pigs
Jarid2	0.42	0.00000000	pigs
Stim1	0.48	0.00000000	pigs
Slc30a5	0.45	0.00000000	pigs
Slc7a1	0.40	0.00000000	pigs
Phtf1	0.42	0.00000000	pigs
Cfd	-1.06	0.00000000	pigs

Zc3h12d	0.46	0.00000000	pigs
Pxk	0.38	0.00000000	pigs
Edem3	0.46	0.00000000	pigs
Ezh2	0.39	0.00000000	pigs
Spen	0.40	0.00000000	pigs
Snrpd2	-0.60	0.00000000	pigs
Smndc1	0.36	0.00000000	pigs
Tnip3	0.65	0.00000000	pigs
Atp11a	0.40	0.00000000	pigs
Snrnp48	0.31	0.00000000	pigs
Nfkb1	0.23	0.00000000	pigs
Cir1	0.34	0.00000000	pigs
Ppp2r5d	0.33	0.00000000	pigs
Phc2	0.36	0.00000000	pigs
Lrp12	0.45	0.00000000	pigs
Pmepa1	0.39	0.00000000	pigs
Baz1a	0.43	0.00000000	pigs
Ahi1	0.56	0.00000000	pigs
Top3a	0.36	0.00000000	pigs
Tbc1d15	0.37	0.00000000	pigs
Slc11a2	0.44	0.00000000	pigs
Rel	0.53	0.00000000	pigs
Dmxl1	0.45	0.00000000	pigs
Fosl2	0.38	0.00000000	pigs
Plagl2	0.41	0.00000000	pigs
Rabep1	0.60	0.00000000	pigs
Zcchc2	0.43	0.00000000	pigs
Irf4	0.44	0.00000000	pigs
Tma7	-0.59	0.00000000	pigs
Ppp1r16b	0.48	0.00000000	pigs
Syne3	0.50	0.00000000	pigs
Tnip1	0.38	0.00000000	pigs
Oas2	0.33	0.00000000	pigs
Smad4	0.34	0.00000000	pigs
Plcg1	0.34	0.00000000	pigs
Wac	0.34	0.00000000	pigs
Fbxw11	0.33	0.00000000	pigs
Gpi1	0.35	0.00000000	pigs
Raf1	0.40	0.00000000	pigs
Rbm26	0.37	0.00000000	pigs
Ctnnb1	0.36	0.00000000	pigs
Ankrd11	0.34	0.00000000	pigs
Ppp6r3	0.31	0.00000000	pigs
Nfkbie	0.33	0.00000000	pigs
Nek9	0.30	0.00000000	pigs
Bnip3	0.44	0.00000000	pigs

Sat1	0.36	0.00000000	pigs
Vps54	0.44	0.00000000	pigs
Sik2	0.47	0.00000000	pigs
Stt3b	0.38	0.00000000	pigs
Etv6	0.49	0.00000000	pigs
Rp2	0.47	0.00000000	pigs
Traf5	0.43	0.00000000	pigs
Ewsr1	0.32	0.00000000	pigs
Arid5b	0.47	0.00000000	pigs
Clock	0.28	0.00000000	pigs
Smad3	0.54	0.00000000	pigs
Dhx15	0.30	0.00000000	pigs
Arhgap18	0.52	0.00000000	pigs
A4galt	0.33	0.00000000	pigs
Chd1	0.34	0.00000000	pigs
Cd53	0.33	0.00000000	pigs
Prdm1	0.51	0.00000000	pigs
Slc9a9	0.50	0.00000000	pigs
Scml4	0.46	0.00000000	pigs
Grk3	0.56	0.00000000	pigs
Kpnb1	0.27	0.00000000	pigs
Cnot9	0.37	0.00000000	pigs
Inpp5d	0.51	0.00000000	pigs
Rftn1	0.42	0.00000000	pigs
Etnk1	0.40	0.00000000	pigs
Pml	0.35	0.00000000	pigs
Rae1	0.59	0.00000000	pigs
P4ha1	0.46	0.00000000	pigs
Eif3a	0.32	0.00000000	pigs
Tcf20	0.37	0.00000000	pigs
Ctla4	0.70	0.00000000	pigs
Eml4	0.40	0.00000000	pigs
Ddx5	0.30	0.00000000	pigs
Ogt	0.34	0.00000000	pigs
Dnm2	0.30	0.00000000	pigs
Luc7l3	0.31	0.00000000	pigs
Tubb4b	0.29	0.00000000	pigs
Cyp2j13	0.25	0.00000000	pigs
B3gnt2	0.35	0.00000000	pigs
Slc20a1	0.30	0.00000000	pigs
Ptpn1	0.37	0.00000000	pigs
Rab3gap2	0.33	0.00000000	pigs
Helz	0.35	0.00000000	pigs
Plekha1	0.36	0.00000000	pigs
Klhl42	0.30	0.00000000	pigs
Ppm1k	0.37	0.00000000	pigs

Zmym2	0.41	0.00000000	pigs
Arg1	0.45	0.00000000	pigs
Smc4	0.37	0.00000000	pigs
Pgs1	0.30	0.00000000	pigs
Bclaf1	0.35	0.00000000	pigs
Ern1	0.35	0.00000000	pigs
Safb2	0.31	0.00000000	pigs
Hsp90aa1	0.45	0.00000000	pigs
Dctn4	0.33	0.00000000	pigs
Snd1	0.48	0.00000000	pigs
L3mbtl1	0.34	0.00000000	pigs
Asxl1	0.43	0.00000000	pigs
Rgcc	0.59	0.00000000	pigs
Top1	0.34	0.00000000	pigs
Pag1	0.38	0.00000000	pigs
Slc25a36	0.34	0.00000000	pigs
Ulk2	0.29	0.00000000	pigs
Bcl6	0.35	0.00000000	pigs
Cdv3	0.32	0.00000000	pigs
Ppp2r5e	0.43	0.00000000	pigs
Picalm	0.24	0.00000000	pigs
Anxa6	0.37	0.00000000	pigs
Daam1	0.42	0.00000000	pigs
Sf3b3	0.32	0.00000000	pigs
Tfrc	0.31	0.00000000	pigs
Cyfip2	0.44	0.00000000	pigs
Acss1	0.28	0.00000000	pigs
Adgrg1	-0.85	0.00000000	pigs
Klf2	-0.75	0.00000000	pigs
Tcerg1	0.33	0.00000000	pigs
Ccdc6	0.35	0.00000000	pigs
Arid5a	0.33	0.00000000	pigs
Rsf1	0.38	0.00000000	pigs
Pmaip1	0.51	0.00000000	pigs
Gls	0.28	0.00000000	pigs
Hnrnpu	0.32	0.00000000	pigs
Lonrf1	0.37	0.00000000	pigs
Irf8	0.45	0.00000000	pigs
Bud23	0.29	0.00000000	pigs
Tram1	0.26	0.00000000	pigs
Tax1bp1	0.33	0.00000000	pigs
Nr3c1	0.54	0.00000000	pigs
Zc3h13	0.33	0.00000000	pigs
Elob	-0.63	0.00000000	pigs
Phip	0.36	0.00000000	pigs
Ubc	0.38	0.00000000	pigs

Usp25	0.37	0.00000000	pigs
Nab1	0.29	0.00000000	pigs
Vta1	0.24	0.00000000	pigs
Ubp1	0.25	0.00000000	pigs
Vcp	0.29	0.00000000	pigs
Kcmf1	0.30	0.00000000	pigs
Slc23a2	0.42	0.00000000	pigs
Uvrag	0.41	0.00000000	pigs
Cdyl	0.28	0.00000000	pigs
Xpo7	0.26	0.00000000	pigs
Fndc3a	0.40	0.00000000	pigs
Hnrnpll	0.38	0.00000000	pigs
Ptpn12	0.47	0.00000000	pigs
Cpm	-0.93	0.00000000	pigs
Slc4a7	0.44	0.00000000	pigs
Ssr1	0.24	0.00000000	pigs
Hspb1	0.33	0.00000000	pigs
Zdhhc20	0.45	0.00000000	pigs
Bag3	0.56	0.00000000	pigs
Dennd11	-0.80	0.00000000	pigs
Ubr3	0.30	0.00000000	pigs
Nfat5	0.36	0.00000000	pigs
Prex1	0.33	0.00000000	pigs
Jak3	0.25	0.00000000	pigs
Nampt	0.33	0.00000000	pigs
Rnf19b	0.27	0.00000000	pigs
Bcl11b	0.39	0.00000000	pigs
Ghitm	0.19	0.00000000	pigs
Rictor	0.31	0.00000000	pigs
Smarca2	0.41	0.00000000	pigs
Snx25	0.35	0.00000000	pigs
Itpr2	0.39	0.00000000	pigs
Fyn	0.41	0.00000000	pigs
Nedd4l	0.57	0.00000000	pigs
D16Ertd472e	0.34	0.00000000	pigs
Herpud1	0.29	0.00000000	pigs
Lsm14a	0.25	0.00000000	pigs
Gapvd1	0.27	0.00000000	pigs
Arhgap17	0.28	0.00000000	pigs
Otud4	0.25	0.00000000	pigs
Tmem65	0.45	0.00000000	pigs
Ier5	0.38	0.00000000	pigs
Nup153	0.35	0.00000000	pigs
Smg7	0.27	0.00000000	pigs
Gtpbp1	0.20	0.00000000	pigs
1810013L24Rik	0.28	0.00000000	pigs

Csrnp1	0.31	0.00000000	pigs
Tnfaip3	0.37	0.00000000	pigs
Trappc9	0.28	0.00000000	pigs
Kmt2e	0.28	0.00000000	pigs
Ranbp3	0.29	0.00000000	pigs
Kif3b	0.43	0.00000000	pigs
Ccdc50	0.40	0.00000000	pigs
Ubr4	0.24	0.00000000	pigs
Rsbn1	0.29	0.00000000	pigs
AA467197	0.57	0.00000000	pigs
Luc7l	0.29	0.00000000	pigs
Ncoa3	0.36	0.00000000	pigs
Rala	0.41	0.00000000	pigs
Apoe	0.29	0.00000000	pigs
Rab1a	0.29	0.00000000	pigs
Sbno1	0.28	0.00000000	pigs
Gpnmb	0.23	0.00000000	pigs
Orc4	0.34	0.00000000	pigs
Flvcr1	0.28	0.00000000	pigs
Cpd	0.25	0.00000000	pigs
Rsrp1	0.35	0.00000000	pigs
Cyld	0.27	0.00000000	pigs
Ksr1	0.29	0.00000000	pigs
Fez2	0.39	0.00000000	pigs
Sod2	0.43	0.00000000	pigs
Gan	0.29	0.00000000	pigs
Hipk3	0.30	0.00000000	pigs
Tiparp	0.31	0.00000000	pigs
C4bp	-1.02	0.00000000	pigs
Sptlc2	0.35	0.00000000	pigs
Dnajb1	0.43	0.00000000	pigs
Suco	0.33	0.00000000	pigs
Rdx	-0.71	0.00000000	pigs
Pou2f1	0.44	0.00000000	pigs
Dgkd	0.37	0.00000000	pigs
Gnb1	0.29	0.00000000	pigs
Elovl5	0.24	0.00000000	pigs
Pdpk1	0.30	0.00000000	pigs
Nfkbiz	0.41	0.00000000	pigs
Gtf2h1	0.29	0.00000000	pigs
Abhd2	0.28	0.00000000	pigs
Smg1	0.23	0.00000000	pigs
Ly96	0.25	0.00000000	pigs
Aftph	0.24	0.00000000	pigs
Fam185a	0.31	0.00000000	pigs
Eif2ak2	0.32	0.00000000	pigs

Fam91a1	0.26	0.00000000	pigs
Pptc7	0.21	0.00000000	pigs
Uqcrc1	0.25	0.00000000	pigs
Il7r	0.35	0.00000000	pigs
C1qb	0.39	0.00000000	pigs
Vgll4	0.29	0.00000000	pigs
Rif1	0.30	0.00000000	pigs
Cox7c	-0.48	0.00000000	pigs
Ankhd1	0.34	0.00000000	pigs
Nrf1	0.35	0.00000000	pigs
Sox13	0.76	0.00000000	pigs
Ppp2r1b	0.25	0.00000000	pigs
Sdf4	0.33	0.00000000	pigs
Cndp2	0.30	0.00000000	pigs
Phf12	0.27	0.00000000	pigs
Api5	0.31	0.00000000	pigs
Llph	0.27	0.00000000	pigs
Sbno2	0.31	0.00000000	pigs
Dnajc2	0.20	0.00000000	pigs
Plgrkt	0.29	0.00000000	pigs
Rapgef2	0.37	0.00000000	pigs
Rab22a	0.38	0.00000000	pigs
Rnf10	0.25	0.00000000	pigs
Sin3a	0.24	0.00000000	pigs
Plekho1	0.26	0.00000000	pigs
Xiap	0.31	0.00000000	pigs
Epsti1	0.32	0.00000000	pigs
Psme4	0.32	0.00000000	pigs
Sap30l	0.27	0.00000000	pigs
Ppp2r5a	0.28	0.00000000	pigs
Kif5b	0.17	0.00000000	pigs
Klf10	0.28	0.00000000	pigs
Rrp15	0.28	0.00000000	pigs
Hmgcr	0.20	0.00000000	pigs
Zc3h12a	0.26	0.00000000	pigs
Dtnbp1	0.25	0.00000000	pigs
Zfp207	0.23	0.00000000	pigs
Usp7	0.23	0.00000000	pigs
Ubqln1	0.20	0.00000000	pigs
Cct8	0.18	0.00000000	pigs
Stat4	0.36	0.00000000	pigs
Emb	0.41	0.00000000	pigs
Usp3	0.34	0.00000000	pigs
Rabgap1l	0.39	0.00000000	pigs
Cx3cr1	-0.75	0.00000000	pigs
Sptan1	0.26	0.00000000	pigs

Ppp1r9b	0.27	0.00000000	pigs
Wdfy1	0.29	0.00000000	pigs
B4galt5	0.25	0.00000000	pigs
Dhrs3	0.37	0.00000000	pigs
Tuba1b	0.30	0.00000000	pigs
Apbb1ip	-0.60	0.00000000	pigs
Fes	0.40	0.00000000	pigs
Pde8a	0.43	0.00000000	pigs
Ccr4	0.39	0.00000000	pigs
Sec14l1	0.28	0.00000000	pigs
Panx1	0.21	0.00000000	pigs
Arl5b	0.32	0.00000000	pigs
Ostf1	-0.62	0.00000000	pigs
Zdhhc17	0.29	0.00000000	pigs
Usf1	0.21	0.00000000	pigs
Rassf1	0.27	0.00000000	pigs
Fosb	0.12	0.00000000	pigs
Il6ra	0.34	0.00000000	pigs
Thoc2	0.25	0.00000000	pigs
Itk	0.33	0.00000000	pigs
Ripk1	0.25	0.00000000	pigs
Cacna1e	0.21	0.00000000	pigs
Gpr107	0.20	0.00000000	pigs
Setd5	0.29	0.00000000	pigs
Foxk2	0.22	0.00000000	pigs
Tmem181a	0.27	0.00000000	pigs
Vdac3	0.22	0.00000000	pigs
Ambra1	0.25	0.00000000	pigs
Rc3h1	0.24	0.00000000	pigs
Eif1	-0.35	0.00000000	pigs
Ogdh	0.24	0.00000000	pigs
Ust	0.44	0.00000000	pigs
Spsb1	0.27	0.00000000	pigs
Ctsc	0.32	0.00000000	pigs
Sec23b	0.22	0.00000000	pigs
Shisa5	0.24	0.00000000	pigs
Rpia	0.27	0.00000000	pigs
Polh	0.24	0.00000000	pigs
Anks1	0.29	0.00000000	pigs
Ahr	0.26	0.00000000	pigs
Slamf7	0.64	0.00000000	pigs
Klrg1	-0.80	0.00000000	pigs
Trim33	0.28	0.00000000	pigs
Pcnx4	0.29	0.00000000	pigs
Lpcat3	0.27	0.00000000	pigs
P2ry10	0.23	0.00000000	pigs

Sh3kbp1	0.33	0.00000000	pigs
Zbtb10	0.38	0.00000000	pigs
Lgals3bp	0.27	0.00000000	pigs
Rnf115	0.28	0.00000000	pigs
Eif5b	0.25	0.00000000	pigs
Edem1	0.27	0.00000000	pigs
Clk4	0.20	0.00000000	pigs
Aff1	0.26	0.00000000	pigs
Emp3	-0.59	0.00000000	pigs
Atp6v1b2	0.24	0.00000000	pigs
Wdr37	0.21	0.00000000	pigs
Birc6	0.26	0.00000000	pigs
Psmc6	0.19	0.00000000	pigs
Eif2ak3	0.32	0.00000000	pigs
Eif3m	0.25	0.00000000	pigs
Ehd1	0.26	0.00000000	pigs
Zfp217	0.18	0.00000000	pigs
Ticam1	0.23	0.00000000	pigs
Wapl	0.27	0.00000000	pigs
Arf6	0.27	0.00000000	pigs
Eed	0.25	0.00000000	pigs
Sqle	0.21	0.00000000	pigs
Prkar2a	0.24	0.00000000	pigs
Arntl	0.30	0.00000000	pigs
Cpeb2	0.35	0.00000000	pigs
Sun2	-0.74	0.00000000	pigs
Ric1	0.26	0.00000000	pigs
Dr1	0.17	0.00000000	pigs
Pip5k1a	0.25	0.00000000	pigs
Ero1a	0.31	0.00000000	pigs
Thsd7b	-1.06	0.00000000	pigs
Rtf2	0.29	0.00000000	pigs
Scpep1	0.26	0.00000000	pigs
Lman2	0.19	0.00000000	pigs
Trim44	-0.51	0.00000000	pigs
Gnl1	0.21	0.00000000	pigs
Lmtk2	0.23	0.00000000	pigs
Csrnp2	0.22	0.00000000	pigs
Gbp2b	0.21	0.00000000	pigs
Hspa9	0.17	0.00000000	pigs
Ipo7	0.25	0.00000000	pigs
Serinc5	0.31	0.00000000	pigs
Rhoh	0.03	0.00000000	pigs
Bst2	0.27	0.00000000	pigs
Cda	-1.06	0.00000000	pigs
Ankrd17	0.24	0.00000000	pigs

Usp37	0.25	0.00000000	pigs
Rap1b	-0.60	0.00000000	pigs
Slc38a2	0.32	0.00000000	pigs
Pdxk	0.27	0.00000000	pigs
Zc3h7a	0.19	0.00000000	pigs
Ube2k	0.21	0.00000000	pigs
Tmem135	0.39	0.00000000	pigs
Lbr	-0.68	0.00000000	pigs
Sec31a	0.24	0.00000000	pigs
Vapb	0.22	0.00000000	pigs
Vopp1	0.23	0.00000000	pigs
Lipa	0.22	0.00000000	pigs
Lrch3	0.23	0.00000000	pigs
Npepps	0.16	0.00000000	pigs
Tm9sf2	0.27	0.00000000	pigs
Ugp2	0.16	0.00000000	pigs
Usp12	0.28	0.00000000	pigs
Aplf	0.26	0.00000000	pigs
Sema4d	0.24	0.00000000	pigs
Stk26	0.24	0.00000000	pigs
Tom1	0.21	0.00000000	pigs
Rock1	0.22	0.00000000	pigs
Kdm4c	0.28	0.00000000	pigs
Fdft1	0.20	0.00000000	pigs
Cox8a	-0.49	0.00000000	pigs
Surf4	0.22	0.00000000	pigs
Npl	0.24	0.00000000	pigs
Kpna4	0.25	0.00000000	pigs
H13	0.18	0.00000000	pigs
Srpk2	0.40	0.00000000	pigs
Stim2	0.31	0.00000000	pigs
Cul3	0.22	0.00000000	pigs
Klf13	0.30	0.00000000	pigs
Gpr35	0.22	0.00000000	pigs
Arid1b	0.32	0.00000000	pigs
B2m	0.21	0.00000000	pigs
Zfp326	0.17	0.00000000	pigs
Hsd17b4	0.25	0.00000000	pigs
Lax1	0.28	0.00000000	pigs
Tbc1d4	0.32	0.00000000	pigs
Pcdhga4	0.19	0.00000000	pigs
Xpo1	0.21	0.00000000	pigs
Hnrnph3	0.27	0.00000000	pigs
Zfp1	0.26	0.00000000	pigs
Pon2	0.20	0.00000000	pigs
Plaat3	0.21	0.00000000	pigs

Slc36a4	0.24	0.00000000	pigs
Brd2	0.17	0.00000000	pigs
Pik3ap1	0.70	0.00000000	pigs
Cdadc1	0.33	0.00000000	pigs
Gpbp1l1	0.19	0.00000000	pigs
Hs3st3b1	0.26	0.00000000	pigs
Rngtt	0.23	0.00000000	pigs
Ptbp3	0.26	0.00000000	pigs
Ppp1cb	0.20	0.00000000	pigs
Poldip3	0.19	0.00000000	pigs
Dbi	-0.65	0.00000000	pigs
Csnk1d	0.14	0.00000000	pigs
Ctdspl2	0.21	0.00000000	pigs
Kpna6	0.22	0.00000000	pigs
St8sia4	0.31	0.00000000	pigs
Bmp2k	0.31	0.00000000	pigs
Xpr1	0.25	0.00000000	pigs
Gcnt1	0.30	0.00000000	pigs
Fbxo11	0.29	0.00000000	pigs
Hpcal1	0.23	0.00000000	pigs
Hat1	0.18	0.00000000	pigs
Tent4a	0.23	0.00000000	pigs
Rap1a	0.31	0.00000000	pigs
Nfe2l2	0.30	0.00000000	pigs
Klrk1	-0.77	0.00000000	pigs
Usp48	0.21	0.00000000	pigs
Btbd9	0.37	0.00000000	pigs
Tspan31	0.19	0.00000000	pigs
Kdm3a	0.23	0.00000000	pigs
Psmd12	0.16	0.00000000	pigs
Setx	0.32	0.00000000	pigs
Kdm1b	0.24	0.00000000	pigs
Gatad2b	0.24	0.00000000	pigs
Sec24d	0.31	0.00000000	pigs
Atf4	0.24	0.00000000	pigs
Abcg1	0.25	0.00000000	pigs
Il1rl1	0.50	0.00000000	pigs
Ifngr2	0.45	0.00000000	pigs
Cox4i1	-0.37	0.00000000	pigs
Hsf1	0.25	0.00000000	pigs
Npat	0.23	0.00000000	pigs
Parp14	0.23	0.00000000	pigs
Pafah2	0.21	0.00000000	pigs
Psmd2	0.17	0.00000000	pigs
Cbfb	0.30	0.00000000	pigs
Sec24b	0.22	0.00000000	pigs

Tmem165	0.21	0.00000000	pigs
Naf1	0.29	0.00000000	pigs
Nop56	0.20	0.00000000	pigs
Abraxas2	0.21	0.00000000	pigs
Idh2	0.18	0.00000000	pigs
Kdm2a	0.19	0.00000000	pigs
Icam1	0.19	0.00000000	pigs
Prpf39	0.17	0.00000000	pigs
Nfatc2	0.31	0.00000000	pigs
Papola	0.22	0.00000000	pigs
Prrg4	0.17	0.00000000	pigs
Klhl15	0.39	0.00000000	pigs
Pop7	0.20	0.00000000	pigs
Ppp1cc	0.22	0.00000000	pigs
Hmgxb4	0.17	0.00000000	pigs
Irf3	0.20	0.00000000	pigs
Rev1	0.36	0.00000000	pigs
Zmiz1	0.26	0.00000000	pigs
Trim27	0.24	0.00000000	pigs
Hbp1	0.18	0.00000000	pigs
Zap70	0.18	0.00000000	pigs
Psmg3	0.13	0.00000000	pigs
Gns	0.21	0.00000000	pigs
Uggt1	0.19	0.00000000	pigs
Atad2b	0.22	0.00000000	pigs
Tiprl	0.12	0.00000000	pigs
Ap1ar	0.34	0.00000000	pigs
Tlk2	0.21	0.00000000	pigs
Akirin1	0.12	0.00000000	pigs
Csde1	0.22	0.00000000	pigs
Rapgef1	0.14	0.00000000	pigs
Dusp5	0.31	0.00000000	pigs
Tsc22d2	0.27	0.00000000	pigs
Cdc42se2	0.23	0.00000000	pigs
Cacybp	0.23	0.00000000	pigs
Sumo2	-0.45	0.00000000	pigs
Kif21b	0.26	0.00000000	pigs
Blk	0.58	0.00000000	pigs
Nsf	0.35	0.00000000	pigs
Ugcg	0.20	0.00000000	pigs
Atp13a3	0.23	0.00000000	pigs
Rfk	0.26	0.00000000	pigs
Oga	0.20	0.00000000	pigs
Ufl1	0.22	0.00000000	pigs
Derl1	0.21	0.00000000	pigs
Fnbp1	0.28	0.00000000	pigs

Nipbl	0.25	0.00000000	pigs
Nono	0.23	0.00000000	pigs
Chuk	0.15	0.00000000	pigs
Notch1	0.26	0.00000000	pigs
Arid4a	0.25	0.00000000	pigs
Pomp	0.12	0.00000000	pigs
Ube2z	0.12	0.00000000	pigs
Scmh1	0.25	0.00000000	pigs
Tmem140	0.22	0.00000000	pigs
Atp5k	-0.66	0.00000000	pigs
Vti1b	0.19	0.00000000	pigs
Etf1	0.16	0.00000000	pigs
N4bp1	0.29	0.00000000	pigs
Gna15	0.27	0.00000000	pigs
Fam210b	0.16	0.00000000	pigs
Tomm20	0.19	0.00000000	pigs
Crlf3	0.29	0.00000000	pigs
Gosr1	0.16	0.00000000	pigs
Esyt2	0.38	0.00000000	pigs
Cdk9	0.20	0.00000000	pigs
Ripk2	0.25	0.00000000	pigs
Crk	0.20	0.00000000	pigs
Klhl5	0.30	0.00000000	pigs
Pfkfb3	0.22	0.00000000	pigs
Rac1	0.27	0.00000000	pigs
Itgb1bp1	0.19	0.00000000	pigs
Rnf38	0.33	0.00000000	pigs
Pim1	0.16	0.00000000	pigs
8030462N17Rik	0.18	0.00000000	pigs
Rap1gap2	-0.73	0.00000000	pigs
Spag9	0.16	0.00000000	pigs
Ncapg	0.35	0.00000000	pigs
Larp4	0.25	0.00000000	pigs
Dhx9	0.22	0.00000000	pigs
Aebp2	0.22	0.00000000	pigs
Cep85l	0.18	0.00000000	pigs
Iffo2	0.28	0.00000000	pigs
Calm3	0.14	0.00000000	pigs
Ralgapa2	0.28	0.00000000	pigs
Foxk1	0.19	0.00000000	pigs
Ythdf2	0.19	0.00000000	pigs
Crebzf	0.15	0.00000000	pigs
Reps1	0.25	0.00000000	pigs
Faf2	0.16	0.00000000	pigs
Plekha8	0.26	0.00000000	pigs
Uox	-0.52	0.00000000	pigs

Prrc2c	0.25	0.00000000	pigs
H1f3	0.17	0.00000000	pigs
Lasp1	0.21	0.00000000	pigs
Agfg1	0.14	0.00000000	pigs
Stau1	0.16	0.00000000	pigs
Zfp277	0.16	0.00000000	pigs
Kdm6b	0.24	0.00000000	pigs
F2rl1	0.18	0.00000000	pigs
Zbtb17	0.19	0.00000000	pigs
Baz1b	0.23	0.00000000	pigs
Il6st	0.47	0.00000000	pigs
Lrp10	0.19	0.00000000	pigs
Arih2	0.18	0.00000000	pigs
Riok3	0.17	0.00000000	pigs
Gpr171	0.31	0.00000000	pigs
Abl2	0.21	0.00000000	pigs
4933400A11Rik	0.23	0.00000000	pigs
Klhdc10	0.43	0.00000000	pigs
Bptf	0.24	0.00000000	pigs
Zcchc8	0.16	0.00000001	pigs
Cltc	0.20	0.00000001	pigs
Comt	0.13	0.00000001	pigs
Dcaf5	0.20	0.00000001	pigs
Plin2	0.09	0.00000001	pigs
Ptpn13	0.47	0.00000001	pigs
Pacsin2	0.33	0.00000001	pigs
Wsb1	0.23	0.00000001	pigs
Ankfy1	0.27	0.00000001	pigs
Eif3l	0.16	0.00000001	pigs
Reep3	0.23	0.00000001	pigs
Donson	0.14	0.00000001	pigs
Fmnl3	0.18	0.00000001	pigs
Far1	0.16	0.00000001	pigs
Il2ra	0.60	0.00000001	pigs
Ttc23l	0.20	0.00000001	pigs
Acap2	0.19	0.00000001	pigs
Ccser2	0.32	0.00000001	pigs
Eif4g3	0.28	0.00000001	pigs
Tox	0.65	0.00000001	pigs
Sar1b	0.19	0.00000001	pigs
Glmn	0.21	0.00000001	pigs
Ccdc186	0.15	0.00000001	pigs
Peak1	0.23	0.00000001	pigs
Ube3c	0.13	0.00000001	pigs
Fbxl20	0.22	0.00000001	pigs
Ubap2l	0.18	0.00000001	pigs

Ywhaz	0.22	0.00000001	pigs
Ubr2	0.27	0.00000001	pigs
Bzw1	-0.44	0.00000001	pigs
Abi1	0.27	0.00000001	pigs
Phf11a	0.21	0.00000001	pigs
Icmt	-0.34	0.00000001	pigs
Atp10d	0.29	0.00000001	pigs
Ccnt2	0.16	0.00000001	pigs
Plek	-0.85	0.00000001	pigs
Zfp830	0.10	0.00000001	pigs
Pum2	0.19	0.00000001	pigs
Ptger4	0.12	0.00000001	pigs
Chst11	0.28	0.00000001	pigs
Stk17b	0.27	0.00000001	pigs
Rela	0.13	0.00000001	pigs
Eif4e	0.13	0.00000001	pigs
Rbbp6	0.19	0.00000001	pigs
Nemf	0.19	0.00000001	pigs
Strn3	0.16	0.00000001	pigs
Zfp654	0.26	0.00000001	pigs
St6gal1	0.30	0.00000001	pigs
Cd80	0.32	0.00000001	pigs
Kdelr2	0.13	0.00000001	pigs
Git2	0.16	0.00000001	pigs
Traf4	0.20	0.00000001	pigs
Nupr1	0.29	0.00000001	pigs
Elmo2	0.15	0.00000001	pigs
Jak1	0.18	0.00000002	pigs
Kdm5c	0.20	0.00000002	pigs
Ttc3	0.24	0.00000002	pigs
Tpm4	0.18	0.00000002	pigs
Ipo9	0.17	0.00000002	pigs
Zfp609	0.23	0.00000002	pigs
Khdrbs1	0.16	0.00000002	pigs
Arrdc2	0.20	0.00000002	pigs
Brd1	0.19	0.00000002	pigs
Calr	0.27	0.00000002	pigs
Clptm1l	0.15	0.00000002	pigs
Hnrnph1	0.19	0.00000002	pigs
Fam168b	0.13	0.00000002	pigs
Rnf138	0.19	0.00000002	pigs
Fam120a	0.21	0.00000002	pigs
Tial1	0.10	0.00000002	pigs
Foxn2	0.27	0.00000002	pigs
Rabepk	0.15	0.00000002	pigs
Ppp2r2a	0.20	0.00000002	pigs

Sec63	0.15	0.00000002	pigs
Ap3d1	0.18	0.00000002	pigs
Lypla1	0.13	0.00000002	pigs
Ptcd3	0.13	0.00000002	pigs
Cd2	-0.47	0.00000003	pigs
Usp10	0.13	0.00000003	pigs
Ermp1	0.17	0.00000003	pigs
Smap1	0.26	0.00000003	pigs
Dnajc3	0.18	0.00000003	pigs
Fam71e1	-0.42	0.00000003	pigs
Huwe1	0.24	0.00000003	pigs
Rlim	0.10	0.00000003	pigs
Kidins220	0.18	0.00000003	pigs
Wrnip1	0.17	0.00000003	pigs
Pdcd1	0.15	0.00000003	pigs
Trim75	0.20	0.00000003	pigs
Zfp280c	0.19	0.00000003	pigs
Usp14	0.15	0.00000004	pigs
Dnttip2	0.15	0.00000004	pigs
Ints6	0.27	0.00000004	pigs
Ttbk2	0.21	0.00000004	pigs
Srebf2	0.16	0.00000004	pigs
Psap	0.20	0.00000004	pigs
Ddx17	0.21	0.00000004	pigs
Nabp1	0.22	0.00000004	pigs
Brox	0.11	0.00000004	pigs
Alas1	0.10	0.00000004	pigs
Agpat4	0.25	0.00000005	pigs
Dip2b	0.30	0.00000005	pigs
Dusp11	0.18	0.00000005	pigs
Pgk1	0.22	0.00000005	pigs
Esco1	0.16	0.00000005	pigs
Tmem11	0.12	0.00000005	pigs
Pim2	0.22	0.00000005	pigs
Hexb	0.19	0.00000005	pigs
Rassf5	0.22	0.00000005	pigs
Exoc2	0.18	0.00000006	pigs
Akna	0.14	0.00000006	pigs
Smyd3	0.27	0.00000006	pigs
Dld	0.12	0.00000006	pigs
Casp3	0.20	0.00000006	pigs
Fbxl5	0.20	0.00000006	pigs
Trib2	0.25	0.00000006	pigs
Tgfbr2	0.33	0.00000006	pigs
Ube3a	0.22	0.00000006	pigs
Igf2r	0.18	0.00000007	pigs

Lrch1	0.22	0.00000007	pigs
Yipf5	0.16	0.00000007	pigs
Zfc3h1	0.14	0.00000007	pigs
Pdxdc1	0.13	0.00000007	pigs
Pi4ka	0.22	0.00000007	pigs
Zdhhc14	0.50	0.00000007	pigs
Tmem106b	0.15	0.00000007	pigs
Pde7b	0.53	0.00000007	pigs
Cnot2	0.27	0.00000007	pigs
Atg3	0.12	0.00000007	pigs
Sv2c	0.60	0.00000007	pigs
Brwd3	0.20	0.00000007	pigs
Nol10	0.17	0.00000008	pigs
Amfr	0.15	0.00000008	pigs
Hnrnpa0	0.17	0.00000008	pigs
Trip12	0.20	0.00000008	pigs
Sell	-0.68	0.00000008	pigs
Lix1l	0.19	0.00000008	pigs
Polr2a	0.17	0.00000009	pigs
Rab3gap1	0.18	0.00000009	pigs
Galnt2	0.21	0.00000009	pigs
Cnot1	0.16	0.00000009	pigs
Kdm5a	0.19	0.00000009	pigs
Dot1l	0.18	0.00000010	pigs
Ncoa6	0.12	0.00000010	pigs
Bms1	0.17	0.00000010	pigs
Aco2	0.10	0.00000010	pigs
Ncor2	0.16	0.00000010	pigs
Tg	0.25	0.00000010	pigs
Hexim1	0.17	0.00000010	pigs
9930021J03Rik	0.51	0.00000010	pigs
Ppil4	0.15	0.00000010	pigs
Arhgap15	0.21	0.00000010	pigs
Fabp5	0.14	0.00000010	pigs
Znhit6	0.17	0.00000010	pigs
Rheb	0.11	0.00000011	pigs
Tnpo3	0.14	0.00000011	pigs
Cds2	0.09	0.00000011	pigs
Tmem19	0.19	0.00000011	pigs
Rai1	0.16	0.00000011	pigs
Bloc1s5	0.19	0.00000011	pigs
Eif4b	0.17	0.00000012	pigs
Ap2m1	0.17	0.00000012	pigs
Tcf4	0.34	0.00000013	pigs
Cstf3	0.18	0.00000013	pigs
Zfr	0.14	0.00000013	pigs

Ndel1	0.14	0.00000013	pigs
Kcna3	0.23	0.00000013	pigs
Lactb	0.18	0.00000014	pigs
Tia1	0.13	0.00000014	pigs
Tbc1d22a	0.17	0.00000014	pigs
Larp1	0.15	0.00000015	pigs
Smarca4	0.14	0.00000016	pigs
Ankrd28	0.31	0.00000017	pigs
Chka	0.19	0.00000017	pigs
2510039O18Rik	0.17	0.00000018	pigs
Apobec3	0.15	0.00000019	pigs
Zfp292	0.26	0.00000019	pigs
Cops8	0.15	0.00000019	pigs
Tcf12	0.36	0.00000020	pigs
Cblb	0.32	0.00000020	pigs
Tasor2	0.15	0.00000021	pigs
Gnl2	0.11	0.00000021	pigs
Aspscr1	0.18	0.00000021	pigs
Ehmt1	0.12	0.00000021	pigs
Prpf40a	0.16	0.00000022	pigs
Tm7sf3	0.21	0.00000023	pigs
Tmem156	0.22	0.00000025	pigs
Ddi2	0.17	0.00000026	pigs
Gbf1	0.16	0.00000026	pigs
Rnf103	0.12	0.00000026	pigs
Cdc27	0.19	0.00000026	pigs
Itpr1	0.23	0.00000026	pigs
Pdzd8	0.17	0.00000027	pigs
Ddx54	0.22	0.00000027	pigs
Wdr59	0.16	0.00000029	pigs
Fam204a	0.06	0.00000030	pigs
Cramp1	0.17	0.00000031	pigs
Dusp10	0.18	0.00000032	pigs
Akap8l	0.16	0.00000033	pigs
Cdr2	0.31	0.00000033	pigs
Atp23	0.20	0.00000034	pigs
Dennd2d	0.17	0.00000034	pigs
Upf1	0.13	0.00000034	pigs
Ahsa1	0.20	0.00000035	pigs
Sema4a	0.21	0.00000035	pigs
Tent2	0.07	0.00000035	pigs
Tmem131	0.21	0.00000036	pigs
Cilk1	0.33	0.00000037	pigs
Ppp2ca	0.07	0.00000037	pigs
Nrbp1	0.16	0.00000037	pigs
Wdr20	0.10	0.00000039	pigs

Unc13d	0.16	0.00000039	pigs
Sh3bgrl2	0.40	0.00000040	pigs
Ibtk	0.15	0.00000040	pigs
Sypl	0.09	0.00000040	pigs
Anxa11	0.14	0.00000041	pigs
Tcp11l2	0.21	0.00000041	pigs
Uap1	0.22	0.00000042	pigs
Tspan14	0.15	0.00000042	pigs
Hpse	0.20	0.00000042	pigs
Cmip	0.20	0.00000044	pigs
Fam214a	0.23	0.00000046	pigs
Ankrd42	0.21	0.00000048	pigs
Klf4	0.29	0.00000048	pigs
Kat6b	0.29	0.00000048	pigs
Slc41a2	0.21	0.00000049	pigs
Zc3h18	0.14	0.00000049	pigs
Fam102b	0.18	0.00000050	pigs
Slc16a1	0.17	0.00000051	pigs
Lxn	0.12	0.00000051	pigs
Tpst2	0.17	0.00000052	pigs
Phf8	0.15	0.00000052	pigs
Atxn7	0.11	0.00000052	pigs
Lrrk2	-1.08	0.00000053	pigs
Zhx2	0.38	0.00000053	pigs
Tnks	0.12	0.00000054	pigs
Ilrun	0.13	0.00000054	pigs
Zfp831	0.19	0.00000055	pigs
Nop53	0.16	0.00000057	pigs
Brwd1	0.15	0.00000057	pigs
Rhoq	-0.68	0.00000058	pigs
Cubn	-0.51	0.00000059	pigs
Rbm22	0.17	0.00000060	pigs
Dazap2	0.08	0.00000061	pigs
Dnajb14	0.15	0.00000065	pigs
Tardbp	0.15	0.00000068	pigs
Rnf169	0.16	0.00000068	pigs
Polr2b	0.20	0.00000069	pigs
Ablim3	0.25	0.00000069	pigs
Arid2	0.19	0.00000071	pigs
Klf9	0.22	0.00000072	pigs
Gsk3b	0.16	0.00000074	pigs
Nek7	0.14	0.00000074	pigs
Flii	0.09	0.00000076	pigs
Tnfsf8	0.33	0.00000076	pigs
Ncoa4	0.11	0.00000077	pigs
Lats1	0.12	0.00000078	pigs

Rab9	0.12	0.00000080	pigs
Cnot4	0.19	0.00000080	pigs
Cdc123	0.19	0.00000081	pigs
Rnf149	-0.03	0.00000083	pigs
Ptdss1	0.24	0.00000084	pigs
Eif4h	0.13	0.00000085	pigs
Sdhb	0.14	0.00000085	pigs
Exoc5	0.13	0.00000088	pigs
Cct4	0.11	0.00000090	pigs
Psmd4	0.09	0.00000091	pigs
Rgs19	-0.60	0.00000091	pigs
Chst12	0.23	0.00000091	pigs
Scaf8	0.14	0.00000092	pigs
Nup88	0.15	0.00000094	pigs
Xpnpep1	0.19	0.00000094	pigs
Usp47	0.16	0.00000096	pigs
Zbtb43	0.15	0.00000098	pigs
Rtn4	0.19	0.00000099	pigs
Oxsr1	0.08	0.00000100	pigs
Topors	0.13	0.00000104	pigs
Tut4	-0.63	0.00000108	pigs
Cd164	0.15	0.00000112	pigs
Kpna1	0.22	0.00000113	pigs
Adnp	0.14	0.00000119	pigs
Clint1	0.14	0.00000122	pigs
Kcnab2	0.19	0.00000123	pigs
Fut8	0.26	0.00000124	pigs
Lancl2	0.13	0.00000125	pigs
Tnfaip8	0.31	0.00000126	pigs
Eif3d	0.07	0.00000127	pigs
Ube2a	0.09	0.00000130	pigs
Adora2b	0.14	0.00000131	pigs
Ctsz	0.19	0.00000131	pigs
Sec61a1	0.16	0.00000132	pigs
Glrx3	0.10	0.00000135	pigs
Sec24a	0.11	0.00000139	pigs
Setd3	0.15	0.00000139	pigs
Ccl5	-0.99	0.00000139	pigs
Ube2r2	0.14	0.00000140	pigs
Septin2	0.11	0.00000141	pigs
Ilk	0.14	0.00000143	pigs
Sf3a3	0.09	0.00000143	pigs
Gars	0.16	0.00000144	pigs
Numa1	0.10	0.00000147	pigs
Crem	0.22	0.00000148	pigs
Pus7	0.22	0.00000150	pigs

Ell	0.12	0.00000157	pigs
Ktn1	0.17	0.00000157	pigs
Anapc1	0.13	0.00000160	pigs
Tbc1d1	0.18	0.00000161	pigs
Psmc2	0.10	0.00000166	pigs
Inpp1	0.14	0.00000170	pigs
Rc3h2	0.12	0.00000172	pigs
Fmr1	0.06	0.00000173	pigs
Slc38a6	0.23	0.00000182	pigs
Cmas	0.13	0.00000183	pigs
Oaz1	-0.40	0.00000185	pigs
Snx27	0.18	0.00000185	pigs
Arsa	0.14	0.00000191	pigs
Cd86	0.13	0.00000193	pigs
Rab18	0.13	0.00000194	pigs
Gemin5	0.19	0.00000194	pigs
Smcr8	0.13	0.00000198	pigs
Gatad2a	0.14	0.00000200	pigs
Srrt	0.15	0.00000203	pigs
Rad21	0.14	0.00000208	pigs
Fubp1	0.14	0.00000212	pigs
Prrc2b	0.15	0.00000212	pigs
Ccny	0.13	0.00000217	pigs
Ss18	0.18	0.00000217	pigs
Zkscan1	0.12	0.00000219	pigs
Ralgapa1	0.25	0.00000221	pigs
Tcp1	0.12	0.00000222	pigs
Asap2	0.23	0.00000227	pigs
Csgalnact2	0.11	0.00000230	pigs
Anxa5	0.16	0.00000231	pigs
Pfdn2	0.11	0.00000242	pigs
Rap2c	0.11	0.00000244	pigs
Top2b	0.16	0.00000244	pigs
Sart3	0.12	0.00000263	pigs
Fbxo21	0.19	0.00000264	pigs
Srebf1	0.17	0.00000284	pigs
Irak2	0.17	0.00000295	pigs
Nr4a3	0.04	0.00000297	pigs
Prdm4	0.13	0.00000297	pigs
Ppp1r15b	0.11	0.00000302	pigs
Cdk6	0.25	0.00000302	pigs
Rhbdd1	0.13	0.00000305	pigs
Pcf11	0.10	0.00000307	pigs
Slc5a10	0.12	0.00000313	pigs
Ap2a2	0.16	0.00000313	pigs
Smg6	0.11	0.00000320	pigs

Smurf1	0.22	0.00000331	pigs
Bub3	0.04	0.00000349	pigs
Samhd1	0.25	0.00000353	pigs
Cnn2	0.17	0.00000356	pigs
Ssrp1	0.15	0.00000358	pigs
Ppp6r2	0.12	0.00000361	pigs
Rchy1	0.14	0.00000376	pigs
Gadd45g	0.13	0.00000379	pigs
Entpd4	0.15	0.00000402	pigs
Kbtbd2	0.07	0.00000405	pigs
Nelfa	0.17	0.00000409	pigs
Ikzf3	0.50	0.00000417	pigs
Lysmd3	0.13	0.00000425	pigs
Akap9	0.17	0.00000435	pigs
Fh1	0.10	0.00000436	pigs
Whamm	0.11	0.00000439	pigs
Plcl1	-0.57	0.00000442	pigs
Aplp2	0.25	0.00000447	pigs
Nktr	0.20	0.00000464	pigs
Tnfrsf9	0.25	0.00000477	pigs
Pde4d	0.15	0.00000486	pigs
Fxr1	0.13	0.00000489	pigs
Ppard	0.15	0.00000490	pigs
Rnf144b	-0.73	0.00000502	pigs
Casp8	0.13	0.00000505	pigs
Zbtb25	0.19	0.00000509	pigs
Rbm14	0.10	0.00000526	pigs
Cep120	0.19	0.00000579	pigs
Gabpb1	0.19	0.00000580	pigs
Pank3	0.12	0.00000594	pigs
Tesc	-0.75	0.00000596	pigs
Sos1	0.23	0.00000609	pigs
Rnf216	0.17	0.00000614	pigs
Fbxw4	0.16	0.00000625	pigs
Cd24a	-0.84	0.00000627	pigs
Taok1	0.15	0.00000652	pigs
Sugt1	0.09	0.00000661	pigs
Nemp2	0.20	0.00000674	pigs
Taf3	0.19	0.00000678	pigs
Atg5	0.17	0.00000727	pigs
Ssr4	-0.49	0.00000749	pigs
Ccar1	0.16	0.00000761	pigs
Tap1	0.15	0.00000775	pigs
Krr1	0.08	0.00000775	pigs
Rnf122	0.17	0.00000780	pigs
Cep83	0.22	0.00000785	pigs

Ptger2	0.24	0.00000800	pigs
Wdr91	0.11	0.00000807	pigs
Stxbp3	0.11	0.00000825	pigs
Fnbp4	0.17	0.00000826	pigs
Xab2	0.13	0.00000846	pigs
Rpa3	0.11	0.00000849	pigs
Sms	0.13	0.00000860	pigs
Pgap1	0.14	0.00000865	pigs
Axin1	0.14	0.00000886	pigs
Dnajc5	0.08	0.00000886	pigs
Itga2	-1.00	0.00000894	pigs
Ptk2b	0.17	0.00000924	pigs
Ing2	0.08	0.00000925	pigs
Tex2	0.18	0.00000957	pigs
Ctss	0.16	0.00000959	pigs
Lemd3	0.12	0.00000977	pigs
Exoc4	0.17	0.00001001	pigs
Abcc1	0.20	0.00001008	pigs
Crim1	0.53	0.00001037	pigs
Col3a1	0.15	0.00001063	pigs
Dcaf10	0.13	0.00001066	pigs
Rab28	0.10	0.00001069	pigs
Tgif2	0.13	0.00001086	pigs
Tmtc3	0.13	0.00001091	pigs
Rab35	0.08	0.00001108	pigs
Gnptab	0.18	0.00001119	pigs
Llgl1	0.12	0.00001128	pigs
Laptm4a	0.06	0.00001143	pigs
Psma5	0.07	0.00001155	pigs
Abca1	0.27	0.00001174	pigs
Cog6	0.10	0.00001186	pigs
Rrn3	0.15	0.00001202	pigs
Tmem101	0.07	0.00001244	pigs
Cert1	0.14	0.00001263	pigs
Psmd11	0.06	0.00001269	pigs
Rassf2	0.13	0.00001312	pigs
Snap29	0.08	0.00001316	pigs
Ino80d	0.16	0.00001336	pigs
Laptm5	0.20	0.00001338	pigs
Pam	0.18	0.00001344	pigs
Ptpra	0.15	0.00001359	pigs
Actr1a	0.17	0.00001368	pigs
Srp54a	0.11	0.00001371	pigs
Nus1	0.11	0.00001385	pigs
Rrp1b	0.13	0.00001387	pigs
Selenos	0.09	0.00001394	pigs

Daglb	0.18	0.00001408	pigs
Cul5	0.08	0.00001435	pigs
Chsy1	0.18	0.00001439	pigs
Rb1cc1	0.08	0.00001478	pigs
Serinc1	0.07	0.00001506	pigs
Ndufb9	-0.69	0.00001513	pigs
Ctr9	0.10	0.00001534	pigs
Sfmbt2	0.12	0.00001559	pigs
Anxa7	0.11	0.00001560	pigs
Uhrf2	0.15	0.00001561	pigs
Tnpo1	0.16	0.00001565	pigs
Psen1	0.09	0.00001641	pigs
Rab4a	-0.58	0.00001648	pigs
Orc6	0.10	0.00001652	pigs
Atxn1	0.26	0.00001662	pigs
Dedd	0.13	0.00001703	pigs
Trim25	0.15	0.00001709	pigs
Tgfb1	0.19	0.00001717	pigs
Fnip1	0.16	0.00001726	pigs
Tmem50b	0.25	0.00001750	pigs
Hspe1-rs1	0.17	0.00001783	pigs
U2surp	0.12	0.00001784	pigs
Serinc3	0.09	0.00001789	pigs
Arfgef1	0.07	0.00001851	pigs
Emsy	0.07	0.00001891	pigs
Pafah1b1	0.15	0.00001976	pigs
Ptpn23	0.14	0.00001977	pigs
Uba6	0.12	0.00002035	pigs
Dlg1	0.24	0.00002096	pigs
Babam2	0.22	0.00002116	pigs
Ptpn22	0.21	0.00002147	pigs
BC048507	0.09	0.00002151	pigs
Ifih1	0.12	0.00002181	pigs
Appbp2	0.09	0.00002191	pigs
Carhsp1	0.09	0.00002192	pigs
Vapa	0.13	0.00002207	pigs
Pfdn4	0.11	0.00002225	pigs
Rbm6	0.13	0.00002336	pigs
Rab5a	0.13	0.00002382	pigs
Lrrc41	0.10	0.00002427	pigs
Rbm10	0.15	0.00002427	pigs
Hdac2	0.11	0.00002432	pigs
Sdcbp	0.09	0.00002439	pigs
Txndc16	0.19	0.00002446	pigs
2610507B11Rik	0.11	0.00002465	pigs
Drg1	0.11	0.00002496	pigs

Tubb2b	0.08	0.00002551	pigs
Thrap3	0.17	0.00002558	pigs
Fam193a	0.16	0.00002574	pigs
Swap70	0.36	0.00002612	pigs
Frs2	0.11	0.00002616	pigs
Fus	0.13	0.00002623	pigs
Ccr5	0.17	0.00002678	pigs
Pias1	0.18	0.00002703	pigs
Galnt3	0.11	0.00002704	pigs
Nsmce1	0.13	0.00002718	pigs
Arl8b	0.11	0.00002748	pigs
Pofut1	0.12	0.00002803	pigs
Aldh18a1	0.16	0.00002845	pigs
Srsf11	0.11	0.00002881	pigs
Pdia4	0.05	0.00002895	pigs
Tmf1	0.13	0.00002931	pigs
Clpx	0.08	0.00002967	pigs
Ptp4a2	0.10	0.00002975	pigs
Arcn1	0.09	0.00003000	pigs
Ubap1	0.13	0.00003039	pigs
Coq4	0.10	0.00003061	pigs
Prpf3	0.12	0.00003226	pigs
Agtpbp1	0.06	0.00003271	pigs
Polr2g	0.12	0.00003289	pigs
Prkar1a	0.08	0.00003345	pigs
Tubb5	0.09	0.00003379	pigs
Tpp2	0.12	0.00003385	pigs
Nufip2	0.17	0.00003394	pigs
Echdc1	0.10	0.00003403	pigs
Golm1	0.19	0.00003430	pigs
Psme1	-0.60	0.00003547	pigs
Inpp4a	0.23	0.00003549	pigs
Ptpn9	0.19	0.00003603	pigs
Hmgxb3	0.13	0.00003622	pigs
Ube2o	0.15	0.00003653	pigs
Itgav	0.22	0.00003725	pigs
Acaca	0.11	0.00003737	pigs
Ppp2r1a	0.05	0.00003853	pigs
Rap1gds1	0.20	0.00003857	pigs
Rbm15	0.17	0.00003887	pigs
Rab7	0.14	0.00004032	pigs
Ifi30	0.26	0.00004039	pigs
Rrp1	0.11	0.00004058	pigs
Pygm	0.18	0.00004086	pigs
Ndrg1	0.19	0.00004130	pigs
Sec61g	-0.46	0.00004174	pigs

Wasl	0.04	0.00004249	pigs
Snx2	0.06	0.00004250	pigs
Ppcs	0.12	0.00004334	pigs
Smc3	0.12	0.00004385	pigs
Actr2	0.13	0.00004487	pigs
Atg16l1	0.12	0.00004586	pigs
Kansl2	0.12	0.00004590	pigs
Akt3	0.30	0.00004653	pigs
Eif3e	0.17	0.00004653	pigs
Gch1	0.11	0.00004700	pigs
Cnst	0.14	0.00004721	pigs
Pdlim5	0.23	0.00004784	pigs
Ascc3	0.15	0.00004811	pigs
Phkg2	0.15	0.00004873	pigs
Fkbp8	0.11	0.00004892	pigs
Ppif	0.24	0.00005056	pigs
Zbtb11	0.14	0.00005169	pigs
Cox7a2	-0.48	0.00005309	pigs
Trnt1	0.12	0.00005310	pigs
Scaf4	0.09	0.00005345	pigs
Isg15	-0.17	0.00005346	pigs
Ykt6	0.06	0.00005353	pigs
Naa50	0.14	0.00005423	pigs
Lsg1	0.10	0.00005437	pigs
Ifit2	-0.61	0.00005507	pigs
Cand1	0.11	0.00005836	pigs
Wdr26	0.10	0.00005943	pigs
Gdi2	0.13	0.00006098	pigs
Naa15	0.07	0.00006229	pigs
Trnau1ap	0.11	0.00006255	pigs
Cd81	0.19	0.00006258	pigs
Hbs1l	0.14	0.00006329	pigs
Rrbp1	0.05	0.00006422	pigs
Phtf2	0.20	0.00006577	pigs
Nifk	0.11	0.00006578	pigs
Ice2	0.16	0.00006708	pigs
Fam126b	0.10	0.00006878	pigs
Atp5pb	0.08	0.00007022	pigs
Gigyf2	0.08	0.00007031	pigs
Immp2l	-0.55	0.00007165	pigs
Inpp5f	0.14	0.00007190	pigs
Adam10	0.16	0.00007207	pigs
Chtop	0.11	0.00007293	pigs
Acin1	0.14	0.00007403	pigs
Ankrd12	0.20	0.00007416	pigs
Chd7	0.08	0.00007499	pigs

Hook3	0.09	0.00007531	pigs
Trim2	-0.56	0.00007532	pigs
Zfp622	0.09	0.00007554	pigs
Cog1	0.12	0.00007576	pigs
Pitpnm3	0.13	0.00007593	pigs
Hnrnpa3	0.11	0.00007603	pigs
Katnbl1	0.14	0.00008050	pigs
Xpo5	0.13	0.00008112	pigs
Akt2	0.10	0.00008510	pigs
Tmx3	0.05	0.00008526	pigs
Smarcd2	0.13	0.00008604	pigs
Erbin	0.21	0.00008694	pigs
Cdk16	0.12	0.00008862	pigs
Gab2	-0.10	0.00008891	pigs
Nudt21	0.13	0.00008940	pigs
Btbd7	0.12	0.00008979	pigs
Pnrc2	0.08	0.00009057	pigs
Btbd1	0.10	0.00009058	pigs
Klhl28	0.10	0.00009202	pigs
Tbc1d2b	0.14	0.00009285	pigs
Sit1	0.08	0.00009480	pigs
Tomm70a	0.12	0.00009519	pigs
Ctsa	0.09	0.00009549	pigs
Rnft1	-0.02	0.00009646	pigs
Polr3e	0.08	0.00009668	pigs
Clic4	0.22	0.00009677	pigs
Cldnd1	0.09	0.00009687	pigs
Pqbp1	0.13	0.00009729	pigs
Rgs10	0.10	0.00009775	pigs
Zbtb38	0.12	0.00009967	pigs
Ubap2	0.13	0.00010054	pigs
Atf7ip	0.13	0.00010381	pigs
Ube2h	0.10	0.00010665	pigs
Ikzf5	0.14	0.00010674	pigs
Ppp4r3b	0.08	0.00010889	pigs
Smarca5	0.19	0.00011174	pigs
Bbx	0.19	0.00011202	pigs
Stradb	0.10	0.00011209	pigs
Psmf1	0.14	0.00011300	pigs
Lmna	0.20	0.00011323	pigs
Patl1	0.08	0.00011389	pigs
Fbxo28	0.08	0.00011435	pigs
Gpr108	0.11	0.00011510	pigs
Cdk17	0.19	0.00011960	pigs
Sec23ip	0.08	0.00012036	pigs
Puf60	0.11	0.00012245	pigs

Atf2	0.14	0.00012254	pigs
Rnf4	0.07	0.00012302	pigs
Cyria	-0.70	0.00012722	pigs
Pfkp	0.10	0.00012737	pigs
Tmed7	0.06	0.00012842	pigs
Cyth3	0.53	0.00012946	pigs
Actn1	0.09	0.00013008	pigs
Yeats2	0.09	0.00014121	pigs
Tpk1	0.22	0.00014451	pigs
Higd1a	0.06	0.00014572	pigs
Tmem248	0.11	0.00014775	pigs
Ngly1	0.14	0.00014856	pigs
Nfatc1	0.14	0.00015102	pigs
Cdyl2	0.18	0.00015324	pigs
Supt4a	0.09	0.00015567	pigs
Itch	0.13	0.00015712	pigs
Zbtb16	0.24	0.00015733	pigs
Syt3	0.18	0.00015791	pigs
Fam135a	-0.52	0.00015895	pigs
Tnfrsf1b	0.15	0.00016288	pigs
Inpp5a	0.18	0.00016581	pigs
Fam3c	0.10	0.00016811	pigs
Twf2	0.06	0.00016846	pigs
Dnajb12	0.07	0.00016929	pigs
Ide	0.17	0.00017035	pigs
Cep95	0.10	0.00017816	pigs
Runx1	0.26	0.00017912	pigs
Kctd3	0.16	0.00017922	pigs
Naa30	0.07	0.00018197	pigs
Ubr1	-0.11	0.00018220	pigs
Senp6	0.15	0.00018283	pigs
Stx7	0.11	0.00018466	pigs
Pcbd1	0.05	0.00018795	pigs
Wipi2	0.04	0.00018827	pigs
Clip1	0.11	0.00018930	pigs
Sgpp1	0.20	0.00019083	pigs
Ash1l	0.13	0.00019430	pigs
Rnf114	0.07	0.00019475	pigs
Cct5	0.15	0.00019653	pigs
Herc4	0.11	0.00019898	pigs
Csnk1g1	0.16	0.00020148	pigs
Psmd3	0.09	0.00021067	pigs
Nub1	0.20	0.00021573	pigs
Ftsj1	0.13	0.00021591	pigs
Pds5b	0.13	0.00021854	pigs
Yaf2	0.12	0.00021870	pigs

Gcfc2	0.06	0.00021907	pigs
Stx17	0.15	0.00022066	pigs
Btbd11	0.39	0.00022087	pigs
Efcab14	0.13	0.00022199	pigs
Pkn2	0.12	0.00022203	pigs
Cse1l	0.14	0.00022885	pigs
Pola1	0.25	0.00022997	pigs
Ints6l	0.08	0.00023079	pigs
Arnt	0.14	0.00023581	pigs
Acat3	0.11	0.00024205	pigs
Trrap	0.13	0.00024527	pigs
Edc3	0.09	0.00024641	pigs
Dcaf1	0.10	0.00024684	pigs
Fyco1	0.04	0.00024708	pigs
Ccdc69	0.10	0.00024778	pigs
Chchd5	0.10	0.00025524	pigs
Hcfc2	0.09	0.00025675	pigs
Vps37b	0.12	0.00025677	pigs
Zfp236	0.13	0.00025809	pigs
Uhmk1	0.09	0.00025811	pigs
Ifi35	0.13	0.00026093	pigs
Btg3	0.09	0.00026153	pigs
Zfp90	0.14	0.00027066	pigs
Dram2	0.13	0.00027253	pigs
Ubash3b	0.13	0.00027569	pigs
Tbl1xr1	0.12	0.00028043	pigs
Usp6nl	0.18	0.00028410	pigs
Cpsf7	0.11	0.00028443	pigs
Rnf111	0.09	0.00028838	pigs
Htt	0.11	0.00028892	pigs
Ythdc2	0.11	0.00029178	pigs
Sart1	0.13	0.00029232	pigs
Ube2j2	0.03	0.00029296	pigs
Adal	0.11	0.00029364	pigs
Ifnar1	0.10	0.00030231	pigs
Crnkl1	0.10	0.00030301	pigs
Sclt1	0.26	0.00030358	pigs
Ip6k1	0.06	0.00030756	pigs
Ccdc32	0.10	0.00030958	pigs
Otulin	0.18	0.00030960	pigs
Tlr7	0.07	0.00031025	pigs
Nptn	0.10	0.00031568	pigs
Clec16a	0.08	0.00031837	pigs
Ccr7	0.27	0.00031847	pigs
Eloa	0.08	0.00032116	pigs
Wtap	0.11	0.00032479	pigs

Ube2v2	0.10	0.00033456	pigs
Trim28	0.12	0.00033561	pigs
Aida	0.08	0.00033561	pigs
Cd3d	0.13	0.00034427	pigs
Nasp	0.08	0.00034956	pigs
Eef1b2	0.14	0.00035610	pigs
Sde2	0.04	0.00035928	pigs
Brpf3	0.11	0.00036029	pigs
Tfg	0.07	0.00036090	pigs
Alkbh5	0.16	0.00037026	pigs
Plekhb2	0.05	0.00038499	pigs
Ccs	0.07	0.00039676	pigs
Epb41	0.22	0.00039919	pigs
Armc8	0.13	0.00040318	pigs
Glyr1	0.12	0.00040720	pigs
Rabl6	0.11	0.00040900	pigs
Sigirr	0.10	0.00042018	pigs
4930523C07Rik	0.05	0.00042430	pigs
Dhx33	0.11	0.00042530	pigs
Pik3ca	0.03	0.00043738	pigs
Srpk1	0.07	0.00044825	pigs
Zfp800	0.09	0.00045121	pigs
Gpatch3	0.13	0.00045214	pigs
Ifi44l	0.10	0.00045341	pigs
Gla	0.12	0.00045758	pigs
Pggt1b	0.12	0.00045792	pigs
Cd4	0.19	0.00046908	pigs
Uqcrfs1	0.07	0.00047140	pigs
B3glct	0.25	0.00048421	pigs
Ap4e1	0.12	0.00049653	pigs
Rack1	0.18	0.00050159	pigs
Vps13b	0.10	0.00050949	pigs
Usp11	0.10	0.00051819	pigs
Isoc1	0.11	0.00053342	pigs
Eef1d	0.12	0.00054459	pigs
Nup214	0.11	0.00054514	pigs
Ptpn2	0.14	0.00056332	pigs
Rpap3	0.13	0.00056426	pigs
Chd1l	0.11	0.00057006	pigs
Eef1g	0.16	0.00057089	pigs
Ifngr1	0.10	0.00058331	pigs
Slc7a11	0.27	0.00059450	pigs
Armcx3	0.03	0.00059653	pigs
Utp6	0.05	0.00059666	pigs
Pim3	0.09	0.00059670	pigs
Elf4	0.13	0.00060700	pigs

4933434E20Rik	0.06	0.00060940	pigs
Foxs1	0.13	0.00062462	pigs
Avpi1	0.10	0.00062657	pigs
Cops2	0.09	0.00063265	pigs
Ptpn18	-0.47	0.00063530	pigs
Il27ra	0.14	0.00064123	pigs
Twf1	0.13	0.00065402	pigs
Hectd1	0.11	0.00065447	pigs
Gabpa	0.11	0.00066056	pigs
Xrn2	0.06	0.00066411	pigs
Psmd7	0.03	0.00066766	pigs
Slc43a2	0.17	0.00067178	pigs
Ski	0.13	0.00067844	pigs
Sesn1	0.20	0.00067869	pigs
Calm1	-0.32	0.00070509	pigs
Eif3j2	0.08	0.00070948	pigs
Gclc	0.08	0.00072034	pigs
Crebbp	0.09	0.00072331	pigs
Npc2	0.00	0.00074429	pigs
Oser1	0.06	0.00074696	pigs
Tox2	0.26	0.00075986	pigs
Spata2	0.10	0.00076979	pigs
Hnrnpr	0.08	0.00077505	pigs
Fbxo3	0.13	0.00077545	pigs
Slain1	0.22	0.00078236	pigs
Gclm	0.06	0.00078261	pigs
Tsn	0.04	0.00078264	pigs
Eif2b4	0.08	0.00080908	pigs
Stk10	0.17	0.00081386	pigs
Efl1	0.04	0.00081537	pigs
Rad23b	0.03	0.00081843	pigs
Nipa2	0.04	0.00081904	pigs
Chic2	0.09	0.00082523	pigs
Cd96	0.26	0.00083139	pigs
Unkl	0.06	0.00083252	pigs
Ddost	0.06	0.00085003	pigs
Dync1h1	0.12	0.00087489	pigs
Usp16	0.10	0.00088431	pigs
Hectd4	0.10	0.00089996	pigs
Cinp	0.12	0.00090489	pigs
Slc38a10	0.11	0.00091672	pigs
Taf1a	0.04	0.00092293	pigs
Rbm5	0.10	0.00092862	pigs
Rgs2	0.13	0.00094084	pigs
Dcps	0.05	0.00094311	pigs
Hspa4	0.05	0.00094993	pigs

Trit1	0.11	0.00096233	pigs
Cd82	0.08	0.00099691	pigs
Hipk1	0.06	0.00100117	pigs
Scfd1	0.08	0.00100217	pigs
Endov	0.12	0.00100272	pigs
Ccng1	0.09	0.00100670	pigs
Tfcp2	0.17	0.00103702	pigs
Usp42	0.13	0.00103849	pigs
Scyl2	0.08	0.00105807	pigs
Snx9	0.33	0.00106707	pigs
Pmpcb	0.08	0.00107514	pigs
Saraf	0.16	0.00111534	pigs
Dpf3	0.18	0.00112899	pigs
Dbr1	0.07	0.00115677	pigs
Bloc1s2	0.12	0.00116368	pigs
Cpeb4	0.11	0.00117950	pigs
Uxs1	0.10	0.00119445	pigs
Ep400	0.03	0.00120012	pigs
Rubcnl	0.05	0.00121184	pigs
Ptpn11	0.07	0.00121495	pigs
Paxip1	0.08	0.00123419	pigs
Csnk1g2	0.09	0.00123895	pigs
Ppp5c	0.12	0.00124251	pigs
BC005537	0.07	0.00124332	pigs
Acsl3	0.14	0.00124549	pigs
Tle3	0.07	0.00125388	pigs
Dhrs1	0.05	0.00126776	pigs
Nlrc5	0.05	0.00127187	pigs
Pdhx	0.14	0.00131115	pigs
Plpp1	0.13	0.00131736	pigs
Hyi	0.04	0.00135471	pigs
Tet3	0.12	0.00135493	pigs
Polr1c	0.12	0.00137660	pigs
Ndufa4	-0.49	0.00139111	pigs
Dcp1a	0.06	0.00140577	pigs
Pacc1	0.10	0.00141993	pigs
Septin11	0.00	0.00142498	pigs
Snx12	0.09	0.00148649	pigs
Rab40b	0.16	0.00149046	pigs
Psme3	0.04	0.00149282	pigs
Ralgapb	0.15	0.00150736	pigs
Eif2a	0.07	0.00151567	pigs
Exoc6b	0.16	0.00151812	pigs
Stx12	0.01	0.00152817	pigs
Fos	-0.34	0.00154730	pigs
Esd	0.06	0.00156953	pigs

Tmx1	0.03	0.00158570	pigs
Tmem106c	0.13	0.00158913	pigs
Bap1	0.08	0.00159528	pigs
Adnp2	0.07	0.00161205	pigs
Usp38	0.11	0.00161809	pigs
Dusp1	-0.53	0.00162237	pigs
Cry1	0.22	0.00162440	pigs
Gpatch8	0.05	0.00163105	pigs
Rpn1	0.14	0.00165742	pigs
Sp1	0.14	0.00168579	pigs
Gpaa1	0.07	0.00170963	pigs
Rprd2	0.13	0.00172460	pigs
Rev3l	0.08	0.00174525	pigs
Pcna	0.06	0.00178166	pigs
Sirt1	0.02	0.00178350	pigs
Zfp148	0.11	0.00183335	pigs
Rell1	0.17	0.00183557	pigs
Akap11	0.11	0.00184015	pigs
Dennd4a	0.14	0.00188052	pigs
Cherp	0.12	0.00188543	pigs
Tpt1	0.12	0.00190335	pigs
Anapc7	0.06	0.00190344	pigs
Actr10	0.06	0.00190497	pigs
Klf12	0.16	0.00191770	pigs
Ganab	0.03	0.00192182	pigs
Fbxo42	0.08	0.00196143	pigs
Tbl2	0.09	0.00198651	pigs
Tnrc6a	0.14	0.00198764	pigs
Hnrnpd	0.10	0.00204450	pigs
Arrb1	0.09	0.00215858	pigs
App	0.18	0.00217047	pigs
Eif4g1	0.07	0.00222929	pigs
Usp34	0.09	0.00223730	pigs
Plaa	0.10	0.00229317	pigs
Psmd1	0.04	0.00232889	pigs
Shq1	0.12	0.00233832	pigs
Atp5md	-0.59	0.00237910	pigs
Uimc1	0.10	0.00242226	pigs
Nsd2	0.12	0.00242600	pigs
Cct7	0.03	0.00243011	pigs
Lamtor2	0.03	0.00243561	pigs
Kcnk5	0.11	0.00248423	pigs
Hyou1	0.08	0.00249806	pigs
Zfp592	0.11	0.00250422	pigs
Gpat4	-0.02	0.00253892	pigs
Lrrc47	0.08	0.00254150	pigs

Dmap1	0.05	0.00254505	pigs
Guf1	0.15	0.00255055	pigs
Tbc1d23	0.09	0.00255308	pigs
Ptges3	0.06	0.00255819	pigs
Lrba	0.16	0.00255831	pigs
Pik3cd	0.12	0.00257580	pigs
Sdhc	0.07	0.00259586	pigs
Sdad1	0.05	0.00260330	pigs
Rab10	0.16	0.00260594	pigs
Pik3c3	0.04	0.00261446	pigs
Prkcsh	0.10	0.00263378	pigs
Rprd1a	0.07	0.00266989	pigs
Hus1	0.06	0.00267661	pigs
Tra2a	0.18	0.00269936	pigs
Bag6	0.05	0.00270330	pigs
Trmt11	0.10	0.00271889	pigs
Zzz3	0.13	0.00275358	pigs
Kifbp	0.09	0.00280105	pigs
Atg12	0.10	0.00281284	pigs
Dcun1d4	0.10	0.00284625	pigs
Hltf	0.09	0.00292299	pigs
Spg7	0.06	0.00292529	pigs
Cysltr2	0.14	0.00302098	pigs
Pitpnb	0.08	0.00303626	pigs
Rprd1b	0.06	0.00304078	pigs
Ypel5	0.05	0.00310610	pigs
Stag2	0.13	0.00311374	pigs
Cdc42	0.15	0.00313034	pigs
Kmt2c	0.10	0.00315765	pigs
Txnrd1	0.12	0.00318548	pigs
Slain2	0.08	0.00323354	pigs
Emilin2	0.20	0.00324808	pigs
Srsf10	0.09	0.00326985	pigs
Naa25	0.10	0.00327637	pigs
Odf2	0.09	0.00327637	pigs
Glt8d1	0.07	0.00327894	pigs
Ptp4a1	0.07	0.00334107	pigs
Sds	-0.76	0.00343606	pigs
Rbl2	0.07	0.00345717	pigs
Nup54	0.08	0.00345986	pigs
Gorasp2	0.03	0.00351940	pigs
Secisbp2l	0.07	0.00353029	pigs
Sec13	0.08	0.00353362	pigs
Osbpl9	0.10	0.00354814	pigs
Nat10	0.12	0.00356502	pigs
Azin1	0.07	0.00368312	pigs

Arrdc3	0.26	0.00373784	pigs
Yy1	0.06	0.00378987	pigs
Scap	0.11	0.00386994	pigs
Slc6a9	0.15	0.00389204	pigs
Ddx47	0.08	0.00391172	pigs
Dock9	0.02	0.00393825	pigs
Ghsr	0.12	0.00395773	pigs
Acsl5	-0.07	0.00407760	pigs
Cep44	0.07	0.00409163	pigs
Tmem120b	0.14	0.00412026	pigs
Kdm1a	0.06	0.00422921	pigs
Zdhhc3	0.06	0.00430220	pigs
Ncbp1	0.02	0.00434109	pigs
Csf1	0.13	0.00434468	pigs
Sipa1	0.01	0.00435358	pigs
Polr1b	0.07	0.00435509	pigs
Sf3b1	0.12	0.00441800	pigs
Eif2b5	0.07	0.00441804	pigs
Atl2	0.12	0.00450090	pigs
Frmd6	0.17	0.00455197	pigs
Cpsf3	0.08	0.00456473	pigs
Ppp4c	0.02	0.00459777	pigs
Ints8	0.05	0.00460254	pigs
Ankib1	0.09	0.00460835	pigs
Cept1	0.11	0.00468852	pigs
Crkl	0.04	0.00480463	pigs
Usp22	0.06	0.00483920	pigs
Strap	0.09	0.00488555	pigs
Lcp2	0.06	0.00500828	pigs
Dcp2	0.03	0.00515855	pigs
Pknox1	0.07	0.00516758	pigs
Eif4ebp2	0.08	0.00520965	pigs
Tdrd3	0.07	0.00524306	pigs
Acap1	0.12	0.00533585	pigs
Tm9sf3	0.13	0.00542214	pigs
Slc36a1	0.13	0.00542419	pigs
Foxj2	0.10	0.00547348	pigs
Bach1	0.06	0.00548773	pigs
Exog	0.11	0.00551474	pigs
Tcaim	0.13	0.00555767	pigs
Lamp1	0.02	0.00557543	pigs
Arglu1	0.11	0.00562279	pigs
Cd247	0.18	0.00562929	pigs
Tmem59	-0.02	0.00567957	pigs
Ep300	0.11	0.00576301	pigs
Taf4	0.09	0.00584565	pigs

Arhgap10	0.13	0.00595611	pigs
Ing1	0.13	0.00595761	pigs
Fgl2	-0.56	0.00596677	pigs
Srsf5	0.13	0.00597071	pigs
Grn	0.17	0.00598248	pigs
Hbb-bh2	-0.65	0.00601389	pigs
Pip5k1c	0.11	0.00611905	pigs
Psmd8	0.03	0.00614460	pigs
Uhrf1bp1l	0.04	0.00617973	pigs
Nxt1	0.04	0.00643673	pigs
Fbxo38	0.03	0.00644476	pigs
Fam222a	0.10	0.00648191	pigs
Otud5	0.03	0.00652216	pigs
Kat14	0.02	0.00655116	pigs
Srsf3	0.14	0.00657495	pigs
Ccnc	0.02	0.00665351	pigs
Prdm10	0.10	0.00671132	pigs
Ncoa2	0.18	0.00674091	pigs
Klhdc4	0.04	0.00677980	pigs
Ddx31	0.07	0.00682618	pigs
Ppp3ca	-0.56	0.00683021	pigs
Tbc1d31	0.15	0.00690647	pigs
Cars	0.08	0.00691149	pigs
Pprc1	0.07	0.00697080	pigs
Supt20	0.07	0.00697566	pigs
Runx3	0.04	0.00701001	pigs
Rest	0.06	0.00707286	pigs
Ostm1	0.08	0.00712605	pigs
Os9	0.07	0.00718056	pigs
Socs4	0.10	0.00719912	pigs
Virma	0.02	0.00719918	pigs
Cers5	0.03	0.00721047	pigs
Ccar2	0.07	0.00722477	pigs
Desi1	0.10	0.00749955	pigs
Socs7	0.08	0.00757619	pigs
Fyttd1	0.05	0.00763153	pigs
Tmem184c	0.06	0.00771207	pigs
Gfpt1	0.03	0.00782272	pigs
Insig1	0.15	0.00790829	pigs
Agap2	0.12	0.00798787	pigs
Ankrd27	0.01	0.00799841	pigs
Krt10	-0.51	0.00810395	pigs
Yars	0.04	0.00824908	pigs
Yipf4	0.06	0.00832382	pigs
Fbxo7	0.01	0.00834321	pigs
Rasgrp1	0.02	0.00839440	pigs

Ddx18	0.02	0.00850540	pigs
Usp8	-0.01	0.00857462	pigs
Csnk1g3	0.12	0.00870351	pigs
Trpv2	0.11	0.00915374	pigs
Ddb2	0.09	0.00916307	pigs
Abce1	0.07	0.00923457	pigs
Dpy19l1	0.19	0.00934437	pigs
Ifi204	0.11	0.00955158	pigs
Zmym5	0.06	0.00963574	pigs
Atg2a	0.08	0.01009094	pigs
Agpat5	0.06	0.01010186	pigs
Cd8b1	-0.50	0.01012274	pigs
Slc39a9	0.10	0.01018822	pigs
Eif4a3l1	0.02	0.01021909	pigs
Ttc39b	0.16	0.01070188	pigs
Atxn2	0.10	0.01071840	pigs
Pnpla8	0.02	0.01078902	pigs
Heatr1	0.12	0.01087811	pigs
Hmgcs1	0.09	0.01094787	pigs
Pds5a	0.09	0.01100114	pigs
Ahcyl1	0.10	0.01103905	pigs
Sfswap	0.01	0.01104780	pigs
Pkn1	0.03	0.01113191	pigs
Cox17	-0.52	0.01117063	pigs
Snx13	0.08	0.01138341	pigs
Zbtb44	0.13	0.01179859	pigs
Camta1	0.05	0.01198052	pigs
Epg5	0.07	0.01214261	pigs
Slc10a3	-0.03	0.01241657	pigs
Canx	0.07	0.01271892	pigs
Senp2	0.05	0.01278230	pigs
Fbxo39	0.09	0.01286730	pigs
Sft2d2	0.09	0.01306290	pigs
Slc16a3	0.09	0.01319084	pigs
Bod1l	0.00	0.01330868	pigs
Rlf	0.02	0.01331159	pigs
Nup98	0.02	0.01331928	pigs
Fbh1	-0.01	0.01339372	pigs
Tmem87b	0.08	0.01353273	pigs
Dennd1a	0.06	0.01359446	pigs
Slc3a2	0.00	0.01361749	pigs
Cstb	0.07	0.01380454	pigs
Aspdh	0.09	0.01390358	pigs
Tspan5	0.21	0.01416799	pigs
Rpn2	0.05	0.01427499	pigs
Uprt	0.03	0.01439482	pigs

Pcid2	0.01	0.01462850	pigs
Dap	0.10	0.01483450	pigs
Tmub1	0.04	0.01495067	pigs
Ndufb8	0.05	0.01507580	pigs
Ruvbl2	0.09	0.01520788	pigs
Tanc2	0.17	0.01532202	pigs
Plxnc1	-0.01	0.01537333	pigs
Rasgef1b	0.18	0.01551423	pigs
Prmt1	-0.01	0.01587439	pigs
Sdhd	0.04	0.01587543	pigs
Sergef	0.18	0.01596743	pigs
Eif4enif1	0.09	0.01617478	pigs
Ets1	0.14	0.01621928	pigs
Polb	0.04	0.01649459	pigs
Grsf1	0.04	0.01655365	pigs
Sucla2	0.09	0.01656586	pigs
Eprs	0.01	0.01663100	pigs
Anxa1	-0.48	0.01692536	pigs
Cnep1r1	0.04	0.01707226	pigs
Iqgap1	0.12	0.01748806	pigs
Srsf7	0.09	0.01750886	pigs
Arhgap12	0.06	0.01767561	pigs
Ccdc22	0.06	0.01769444	pigs
Psmb7	0.05	0.01773858	pigs
Tmem41b	0.04	0.01782692	pigs
Ppp4r1	0.11	0.01783065	pigs
Gart	0.09	0.01799034	pigs
Fcho1	0.13	0.01810618	pigs
Cacul1	0.06	0.01816822	pigs
Lrmda	-0.01	0.01825184	pigs
Ppig	0.02	0.01828447	pigs
Kansl3	0.07	0.01865067	pigs
Lrrc57	0.06	0.01884946	pigs
Naa16	0.02	0.01888522	pigs
Papolg	0.07	0.01901598	pigs
Cfap20	0.02	0.01938874	pigs
Slc17a5	0.10	0.01956119	pigs
Prorp	0.06	0.01979096	pigs
Ypel3	-0.58	0.02005490	pigs
Xkr6	0.08	0.02024157	pigs
Zfp618	0.31	0.02026586	pigs
Znfx1	-0.06	0.02089535	pigs
Pafah1b2	0.03	0.02094652	pigs
Nop58	0.04	0.02098759	pigs
P4hb	0.05	0.02105272	pigs
Dnah11	-0.50	0.02145765	pigs

Blmh	0.09	0.02198231	pigs
Sun1	0.07	0.02214563	pigs
Actr1b	0.09	0.02219024	pigs
C5ar1	-0.64	0.02235457	pigs
Katnal1	0.08	0.02245866	pigs
Rrs1	0.09	0.02253470	pigs
Amh	0.06	0.02269071	pigs
Aco1	0.13	0.02322589	pigs
Zfp131	0.07	0.02361057	pigs
Phf20l1	-0.45	0.02361167	pigs
Alox5ap	-0.65	0.02364368	pigs
Csk	0.03	0.02382249	pigs
Wasf2	0.08	0.02387022	pigs
Camkk2	0.07	0.02393654	pigs
Pdcd1lg2	0.09	0.02427212	pigs
Vcpip1	0.07	0.02467926	pigs
Ppp1r8	0.09	0.02468026	pigs
Kctd12	0.18	0.02486253	pigs
Cdc73	0.05	0.02488006	pigs
1810037I17Rik	0.06	0.02488704	pigs
Slu7	0.01	0.02494138	pigs
Tlnrd1	0.04	0.02517665	pigs
Hspa13	0.05	0.02519442	pigs
Ddx23	0.07	0.02524475	pigs
Ino80c	0.08	0.02563991	pigs
Exosc7	0.04	0.02615912	pigs
Fam53b	0.04	0.02681073	pigs
Uso1	0.10	0.02682412	pigs
Bscl2	0.06	0.02733633	pigs
Prpf8	0.03	0.02738429	pigs
Tmem189	0.01	0.02766504	pigs
Cdc37l1	0.08	0.02771621	pigs
Spast	0.02	0.02780808	pigs
Ate1	0.05	0.02782306	pigs
Setdb2	0.10	0.02788341	pigs
Dpf2	0.03	0.02830186	pigs
Plekhm3	-0.58	0.02835105	pigs
Ankrd13a	-0.01	0.02849728	pigs
Ppan	0.02	0.02926600	pigs
Prpf31	0.10	0.02942977	pigs
Topbp1	0.00	0.02945080	pigs
Sf3b6	0.02	0.02959726	pigs
Rexo1	0.10	0.02973392	pigs
Arl6ip4	0.03	0.03007332	pigs
Hnrnpdl	-0.31	0.03031398	pigs
Pcm1	0.05	0.03044592	pigs

Itsn2	0.08	0.03078034	pigs
Txnip	-0.56	0.03078742	pigs
Rab2a	0.06	0.03089064	pigs
Slc9a6	0.07	0.03103351	pigs
Tm2d3	0.10	0.03109442	pigs
Ctdp1	0.04	0.03117933	pigs
Exoc3	0.00	0.03198534	pigs
Bfar	0.04	0.03220455	pigs
Ddhd1	0.12	0.03300728	pigs
Umps	0.05	0.03311256	pigs
Tollip	0.03	0.03314364	pigs
Fbxo33	0.12	0.03326279	pigs
Cd274	0.15	0.03359665	pigs
Trim26	0.03	0.03396818	pigs
Slmap	0.09	0.03397916	pigs
Txlna	0.08	0.03421970	pigs
Washc5	0.06	0.03439893	pigs
Fkbp5	0.13	0.03450425	pigs
Lman1	0.01	0.03475021	pigs
Traf7	0.06	0.03490556	pigs
Qser1	0.12	0.03505891	pigs
Armc7	0.08	0.03525352	pigs
Srsf1	0.01	0.03536867	pigs
Dync1li2	0.05	0.03544804	pigs
Trpm7	0.07	0.03562800	pigs
Rubcn	0.05	0.03566511	pigs
Rhot1	0.10	0.03569305	pigs
Ddb1	0.08	0.03616846	pigs
Ccm2	0.06	0.03620680	pigs
Txnl1	0.00	0.03628366	pigs
Cenpc1	0.12	0.03680676	pigs
Pias2	0.08	0.03699482	pigs
Trim47	0.12	0.03727607	pigs
Emc4	0.02	0.03777477	pigs
Golgb1	0.03	0.03779787	pigs
Samd8	0.07	0.03817880	pigs
Nkapd1	0.02	0.03880607	pigs
Nup85	0.08	0.03891066	pigs
Adss	-0.10	0.03963242	pigs
Rsrc2	0.07	0.03975430	pigs
Dyrk3	0.16	0.03994488	pigs
Atp6ap2	-0.02	0.04004338	pigs
Calu	0.06	0.04016984	pigs
Arhgap35	0.04	0.04041464	pigs
Kif2a	0.04	0.04042480	pigs
Herc2	0.05	0.04084673	pigs

Psmd13	0.06	0.04110964	pigs
Kdm5b	0.03	0.04149705	pigs
Ankrd13c	0.17	0.04196872	pigs
Capza2	0.05	0.04201284	pigs
Psmb10	0.00	0.04272059	pigs
Rbm45	0.03	0.04306624	pigs
Wbp4	0.01	0.04339535	pigs
Pcnx	-0.19	0.04361319	pigs
Vps26c	0.04	0.04368543	pigs
Cdk13	0.08	0.04400781	pigs
Slc25a26	0.10	0.04485815	pigs
Tmem87a	0.06	0.04531220	pigs
Wdr82	0.04	0.04572128	pigs
Prdx6	0.04	0.04579805	pigs
Tmem33	0.09	0.04644211	pigs
Vps13a	0.04	0.04715500	pigs
Scaf11	0.07	0.04719901	pigs
Polk	0.13	0.04755580	pigs
Slc45a4	0.11	0.04815612	pigs
Cdk5rap3	0.07	0.04840429	pigs
Cdc40	0.00	0.04859154	pigs
Ssbp2	0.19	0.04904202	pigs
Ly9	0.02	0.04950934	pigs
Atxn7l1	-0.51	0.04966569	pigs
Iars2	0.09	0.04971394	pigs
Dnajb6	0.07	0.04998615	pigs
Ero1b	0.12	0.05005565	pigs
Vps52	0.05	0.05023864	pigs
Pign	0.08	0.05141562	pigs
Wipf2	0.10	0.05157030	pigs
Ccdc82	0.00	0.05177010	pigs
Ipo5	-0.06	0.05364551	pigs
Ltn1	0.09	0.05369005	pigs
Cat	0.04	0.05493613	pigs
Nagk	0.08	0.05539606	pigs
Pfdn1	0.00	0.05548895	pigs
Cops4	-0.01	0.05569745	pigs
Rasgef1a	0.01	0.05579358	pigs
Sp110	0.04	0.05621960	pigs
Pstpip1	0.00	0.05636455	pigs
Smg9	0.09	0.05683559	pigs
Scyl3	0.02	0.05694983	pigs
Psmb4	-0.01	0.05705956	pigs
Wars2	0.04	0.05758612	pigs
Cdkal1	0.11	0.05784996	pigs
Xcl1	-0.74	0.05884789	pigs

Amz2	0.03	0.05913764	pigs
Wsb2	0.05	0.05939134	pigs
Btaf1	0.05	0.05962673	pigs
Ctdsp1	-0.42	0.05963293	pigs
Washc4	0.07	0.05966810	pigs
Ppme1	0.05	0.06020284	pigs
Chid1	0.02	0.06077537	pigs
Gpd2	0.11	0.06090056	pigs
Slc6a6	0.04	0.06159656	pigs
Bloc1s6	0.04	0.06320558	pigs
Tbc1d10a	0.11	0.06375730	pigs
Srsf9	0.07	0.06409752	pigs
Cul1	0.07	0.06421539	pigs
Pgls	0.01	0.06483407	pigs
Prcc	0.05	0.06501976	pigs
Gfm1	0.03	0.06526925	pigs
Ipo8	0.07	0.06569804	pigs
Strn	-0.03	0.06649000	pigs
Ehbp1l1	0.04	0.06801160	pigs
Lrrc58	0.12	0.06862900	pigs
Bud13	0.03	0.06866931	pigs
Plk3	0.03	0.06896372	pigs
Dennd2c	0.18	0.06974331	pigs
Ccni	0.01	0.06977837	pigs
Pip4k2a	0.15	0.06989173	pigs
Rab5c	-0.02	0.07048935	pigs
Ints7	0.09	0.07075946	pigs
Naa35	0.01	0.07087351	pigs
Nfyc	0.04	0.07089487	pigs
Pak1ip1	0.05	0.07127594	pigs
Dis3l2	0.04	0.07280156	pigs
Polr2i	0.01	0.07317276	pigs
Dnajc18	0.05	0.07368292	pigs
Wbp2	0.04	0.07399247	pigs
Plekhm2	0.06	0.07426549	pigs
Crebrf	0.11	0.07443849	pigs
Oxa1l	0.02	0.07506667	pigs
Bicra	0.04	0.07610764	pigs
Card19	0.01	0.07611013	pigs
Lnpep	0.11	0.07659530	pigs
Wdr61	0.04	0.07689649	pigs
Rcc2	0.06	0.07691093	pigs
Zdhhc9	0.06	0.07694586	pigs
Uchl3	0.06	0.07714673	pigs
Kmt5b	0.00	0.07774783	pigs
Slc35a1	-0.02	0.08076989	pigs

Acox1	0.04	0.08098637	pigs
Wdr70	0.04	0.08109823	pigs
Slc37a1	0.10	0.08210785	pigs
Araf	0.08	0.08233060	pigs
Bcl10	-0.09	0.08263629	pigs
Srp72	0.05	0.08331573	pigs
Stx6	0.10	0.08366060	pigs
Qars	0.02	0.08444962	pigs
Cox11	0.06	0.08519038	pigs
Prepl	0.10	0.08979536	pigs
Aak1	-0.47	0.09152970	pigs
Prrc2a	0.05	0.09248027	pigs
Egr1	-0.51	0.09312500	pigs
Elp2	0.06	0.09411240	pigs
4932411N23Rik	0.02	0.09446164	pigs
Zfp39	0.07	0.09457475	pigs
Selenoi	0.10	0.09531951	pigs
Tmem123	0.11	0.09569903	pigs
Crppa	0.15	0.09670568	pigs
Phrf1	0.08	0.09742353	pigs
Fnta	0.01	0.09746340	pigs
Inpp5b	0.09	0.09905901	pigs
Phf2	0.09	0.10002322	pigs
Nhlrc2	0.04	0.10054133	pigs
Dcaf6	0.02	0.10060987	pigs
Nap1l4	0.03	0.10270436	pigs
Wdr44	0.10	0.10318956	pigs
Gpn1	0.06	0.10330177	pigs
Dut	0.09	0.10365866	pigs
Rab11a	-0.05	0.10373080	pigs
Triobp	-0.72	0.10460775	pigs
Txndc5	0.12	0.10465857	pigs
9930104L06Rik	0.09	0.10519554	pigs
Orai1	0.09	0.10543179	pigs
Otub1	0.03	0.10573835	pigs
Ccdc91	0.16	0.10640584	pigs
Zmynd8	0.06	0.10641912	pigs
Tmed9	0.05	0.10685589	pigs
Ralgps2	0.22	0.10701689	pigs
Fkbp15	0.08	0.10704045	pigs
Dynlt1a	0.02	0.10737566	pigs
S100a11	-0.60	0.10790558	pigs
Copa	0.00	0.10848864	pigs
Adam17	-0.02	0.10881562	pigs
Fbxo22	0.02	0.10884377	pigs
Nt5c2	-0.21	0.10894050	pigs

Psmc5	-0.07	0.10907090	pigs
Gdi1	0.05	0.10948262	pigs
Slc35f2	0.08	0.11060988	pigs
Elavl1	-0.03	0.11363749	pigs
Vbp1	0.06	0.11447955	pigs
Samd4b	0.02	0.11602753	pigs
Edrf1	0.01	0.11629431	pigs
Zfp335	0.03	0.11758794	pigs
Phax	-0.06	0.11866697	pigs
Pik3c2b	0.18	0.12012971	pigs
G2e3	0.03	0.12016171	pigs
Ccdc9	0.09	0.12323887	pigs
Arl14ep	0.05	0.12348931	pigs
Tmed10	0.00	0.12384286	pigs
Tom1l2	0.09	0.12492398	pigs
Ip6k2	0.03	0.12572265	pigs
Pitpnm1	0.03	0.12596848	pigs
Nup93	0.10	0.12628691	pigs
Aff4	0.12	0.12806537	pigs
Slc44a2	0.07	0.13046895	pigs
Haus6	0.02	0.13097806	pigs
Lig3	0.02	0.13141469	pigs
Dusp2	-0.51	0.13241558	pigs
Sppl3	0.07	0.13249580	pigs
Aqr	0.04	0.13303590	pigs
Trabd	-0.01	0.13360256	pigs
Plekha3	0.07	0.13508482	pigs
Spag1	0.02	0.13762431	pigs
Clptm1	0.06	0.13763773	pigs
Tmem39a	0.03	0.13767976	pigs
Nt5c3	0.05	0.13820783	pigs
Cyp51	0.04	0.13897081	pigs
Zswim8	0.11	0.13912159	pigs
Cspp1	0.07	0.13936169	pigs
Atp6v1d	-0.02	0.13942327	pigs
Snx14	0.07	0.14049732	pigs
Alg12	0.10	0.14066183	pigs
Zbtb8os	0.02	0.14126870	pigs
Srsf4	-0.01	0.14188289	pigs
Trp53	0.04	0.14283130	pigs
Zbtb2	0.09	0.14642492	pigs
Ube2j1	-0.04	0.14761907	pigs
Ctps2	0.07	0.15181511	pigs
Tor1aip1	0.07	0.15191891	pigs
Klhl2	0.01	0.15217427	pigs
Sprtn	0.05	0.15300335	pigs

Chd8	0.03	0.15340771	pigs
Hsbp1	0.00	0.15478120	pigs
Pafah1b3	-0.05	0.15492559	pigs
Naga	0.04	0.15495806	pigs
Paxbp1	-0.04	0.15519109	pigs
Brap	0.01	0.15553127	pigs
Dhx36	0.03	0.15558820	pigs
Crtc3	-0.09	0.15722029	pigs
Rnf146	-0.04	0.15928486	pigs
Tmem128	0.00	0.16053341	pigs
Cop1	0.02	0.16120919	pigs
Slc25a5	-0.06	0.16149868	pigs
Ndufa9	0.01	0.16158229	pigs
Ncbp3	0.08	0.16158960	pigs
Ago1	0.02	0.16279008	pigs
Golm2	0.01	0.16450367	pigs
Nlk	0.11	0.16483832	pigs
Dmac2	0.03	0.16754654	pigs
Eipr1	0.03	0.17155209	pigs
Dele1	0.05	0.17224524	pigs
Ormdl1	0.04	0.17387294	pigs
Dip2a	0.12	0.17669253	pigs
Wbp11	0.01	0.18278646	pigs
Lamp2	-0.07	0.18325752	pigs
Hdac7	0.04	0.18340195	pigs
Pde4b	0.08	0.18621350	pigs
Ankrd44	0.13	0.18986699	pigs
Snrpa1	-0.05	0.19050594	pigs
Cep295	0.06	0.19145414	pigs
Galnt10	0.06	0.19155532	pigs
Khsrp	0.06	0.19162431	pigs
Hdac3	0.07	0.19169098	pigs
Lrrfip2	0.07	0.19169345	pigs
Ctdsp2	0.03	0.19307407	pigs
Grb2	0.08	0.19421535	pigs
Dhx35	0.07	0.19509211	pigs
Ap1g1	0.03	0.19650171	pigs
Slk	0.01	0.19846757	pigs
Pex14	0.06	0.19891872	pigs
Fndc7	0.00	0.19952991	pigs
Eif3g	0.05	0.20216174	pigs
Rsbn1l	0.07	0.20430193	pigs
Bdp1	0.04	0.20486207	pigs
Nsfl1c	0.04	0.20489967	pigs
Urm1	0.06	0.20654805	pigs
Ndufs2	-0.06	0.20950529	pigs

Eya3	0.06	0.21114135	pigs
Erp44	0.05	0.21209863	pigs
Irf5	0.05	0.21302258	pigs
Trp53bp1	0.06	0.21318263	pigs
Gnl3	0.09	0.21491004	pigs
Slc12a2	0.10	0.21614088	pigs
Nsmaf	0.04	0.22173766	pigs
Cul4a	0.04	0.22342456	pigs
Sgk1	-0.58	0.22342702	pigs
Txlng	0.02	0.22688611	pigs
Fmnl1	0.01	0.22690338	pigs
Zcrb1	-0.01	0.22880847	pigs
Ssbp3	0.03	0.23040679	pigs
Arhgap9	0.06	0.23589695	pigs
Lrrcc1	0.09	0.23905477	pigs
Dgke	0.03	0.24348930	pigs
Tgfbr3	0.17	0.24686693	pigs
Trp53inp1	0.06	0.25220923	pigs
Cetn3	0.01	0.25468890	pigs
Pabpc1	0.07	0.25555972	pigs
Ddx52	0.05	0.25728935	pigs
Hddc2	0.08	0.25762405	pigs
Nphp4	0.10	0.25879989	pigs
Cdin1	0.07	0.26013500	pigs
Tpd52l2	0.01	0.26321310	pigs
Cep290	0.10	0.26468900	pigs
Cebpg	0.00	0.26527355	pigs
Bnip3l	-0.04	0.26624526	pigs
Dhx38	0.05	0.26865504	pigs
Rbck1	-0.03	0.26967431	pigs
Tmem14a	0.02	0.27113188	pigs
Tbcd	0.08	0.27169087	pigs
Srp68	0.05	0.27412375	pigs
Txndc15	0.02	0.27515314	pigs
Kif3c	0.07	0.27591719	pigs
Cox6a1	-0.38	0.28121690	pigs
Phf6	0.01	0.28196819	pigs
Selenow	-0.55	0.28732522	pigs
Olfml3	0.08	0.28749520	pigs
Atrn	0.09	0.29007504	pigs
H2az2	-0.31	0.29141383	pigs
Cmpk2	0.05	0.29283639	pigs
Lrpprc	0.07	0.29284000	pigs
Utp18	0.01	0.29284841	pigs
Ttc7	0.07	0.29301985	pigs
Tmem183a	-0.03	0.29359813	pigs

Gsap	0.21	0.29436488	pigs
Snw1	-0.05	0.29534250	pigs
Proser1	0.08	0.29631779	pigs
Tpr	0.04	0.29805805	pigs
Ndufa13	-0.43	0.29939173	pigs
Ercc3	0.05	0.30225291	pigs
Tob2	-0.02	0.30733597	pigs
Themis	0.12	0.31027260	pigs
Tubgcp3	0.08	0.31036262	pigs
Umad1	0.03	0.31380497	pigs
9530068E07Rik	0.06	0.31537312	pigs
Zfp143	0.00	0.31580436	pigs
Pgm2	-0.03	0.31593628	pigs
Ccnl2	-0.03	0.31661228	pigs
Eefsec	0.01	0.31861888	pigs
Sirt7	0.00	0.32003416	pigs
Timmdc1	0.08	0.32034677	pigs
Sidt2	0.05	0.32046918	pigs
Adsl	0.02	0.32061196	pigs
Larp1b	0.04	0.32134922	pigs
Cd69	-0.03	0.32364635	pigs
Atp6v1a	0.10	0.32695851	pigs
Rtraf	0.01	0.32884756	pigs
Xpc	-0.01	0.33190645	pigs
Elf2	0.10	0.33500015	pigs
Klhl9	0.09	0.33543567	pigs
2410004B18Rik	0.00	0.33859421	pigs
Arid1a	-0.02	0.34804257	pigs
Nfya	0.05	0.35060970	pigs
Snx6	-0.01	0.35315825	pigs
Rrm1	0.02	0.35355558	pigs
Kif1b	0.06	0.36334874	pigs
Npc1	0.09	0.36350198	pigs
Wdr41	0.01	0.36493463	pigs
Yrdc	-0.03	0.36675677	pigs
Taf12	-0.02	0.36705510	pigs
Atl3	0.07	0.36823579	pigs
Tmem168	0.02	0.36876156	pigs
Taf5	0.07	0.36884210	pigs
Golga7	0.01	0.36919162	pigs
Exoc6	0.01	0.36952150	pigs
Tcf3	0.06	0.37118815	pigs
Btg1b	0.14	0.37217714	pigs
Rnf40	0.07	0.37360731	pigs
Clk1	0.04	0.37708660	pigs
Gna12	0.17	0.37834238	pigs

Pik3r5	0.13	0.38311594	pigs
Zzef1	0.01	0.38627946	pigs
Ggnbp2	0.03	0.38696475	pigs
Gtdc1	0.00	0.38877854	pigs
Selenot	0.00	0.39236944	pigs
Ift20	0.03	0.39378551	pigs
Tsc22d4	-0.03	0.39458765	pigs
Tigit	0.05	0.39603035	pigs
Eif3b	0.05	0.39609323	pigs
Ttc17	0.08	0.40389885	pigs
Ryk	0.09	0.40615786	pigs
Tlcd2	-0.02	0.40707016	pigs
Taok2	0.00	0.41020096	pigs
Fbxo4	0.07	0.41428319	pigs
Tsr2	0.05	0.41719205	pigs
Cep152	0.10	0.41772918	pigs
Rpf2	0.01	0.42061269	pigs
Rtca	0.03	0.42214384	pigs
Lrig2	0.09	0.42276101	pigs
Phf3	0.04	0.42542877	pigs
Casc3	-0.05	0.42851198	pigs
Gzma	0.48	0.43331641	pigs
Rbm19	0.03	0.43354992	pigs
Zbtb24	0.06	0.43631049	pigs
Hnrnpul1	-0.01	0.43800117	pigs
Pbdc1	-0.09	0.43806417	pigs
Tmem30a	-0.02	0.44087632	pigs
Uri1	0.04	0.44298626	pigs
E2f3	0.03	0.44458091	pigs
Fam98a	0.00	0.45903281	pigs
Zfp330	0.04	0.46132803	pigs
Cd47	0.03	0.46533168	pigs
Ddx39b	0.04	0.46619642	pigs
Atxn2l	-0.03	0.46682475	pigs
Tcp11	0.10	0.46742875	pigs
Rab6a	-0.05	0.47052531	pigs
Suclg2	0.04	0.47133485	pigs
Fbxo30	0.05	0.47266839	pigs
Tssc4	0.07	0.47287180	pigs
Zfp13	-0.36	0.47640045	pigs
Katna1	0.04	0.48121919	pigs
Zfp451	0.06	0.48423741	pigs
Pik3r4	0.00	0.48577429	pigs
Atp2a3	0.05	0.49233314	pigs
Stat6	0.03	0.49325339	pigs
Pabpc4	0.05	0.49697132	pigs

Bcl2l1	0.03	0.49911205	pigs
Pja2	-0.01	0.50805767	pigs
Gspt1	-0.01	0.50942983	pigs
Ilkap	-0.03	0.51740059	pigs
Prr5l	-0.30	0.51858970	pigs
Atad1	-0.07	0.52185322	pigs
Hells	0.03	0.52454029	pigs
Crls1	0.08	0.52607634	pigs
Cbll1	-0.04	0.52737947	pigs
Ddx49	0.07	0.52786198	pigs
Chmp1a	0.03	0.53003756	pigs
Tigar	0.02	0.53036115	pigs
Aatf	-0.01	0.53269274	pigs
Otud6b	0.06	0.54127331	pigs
Tdpoz8	-0.02	0.54188768	pigs
Dicer1	0.01	0.54191287	pigs
Zbtb22	0.05	0.54666886	pigs
Sorl1	-0.21	0.54770541	pigs
Ppil2	0.01	0.55119542	pigs
Exosc10	0.08	0.55185483	pigs
Txndc12	0.05	0.55423925	pigs
Pdcd4	-0.04	0.55685767	pigs
Ppid	0.08	0.55899917	pigs
Nfu1	-0.02	0.55984325	pigs
Nf1	0.15	0.56003880	pigs
Itgal	-0.60	0.56027525	pigs
Ddx39a	0.03	0.56048064	pigs
Irak1	0.10	0.56881854	pigs
Lpxn	0.00	0.57063141	pigs
Itm2a	0.15	0.57123529	pigs
Tnks2	-0.02	0.57266156	pigs
Lin54	0.05	0.57834461	pigs
Gcnt2	0.14	0.58171724	pigs
Clk3	0.05	0.58345348	pigs
Ttc1	0.03	0.58690963	pigs
Dnajc7	0.01	0.59310757	pigs
Tmod3	0.00	0.59586329	pigs
Dnajc10	0.01	0.60227676	pigs
Pcgf5	0.08	0.60284856	pigs
Fam222b	-0.05	0.60326511	pigs
Pi4k2b	0.01	0.60881326	pigs
Tmem60	-0.02	0.61380655	pigs
Gcnt4	-0.34	0.61858275	pigs
Creb1	0.04	0.62009978	pigs
Slc25a17	0.00	0.62118852	pigs
Trmt11	-0.04	0.62189830	pigs

Ric8b	0.23	0.62276024	pigs
Dcaf8	0.01	0.63093324	pigs
Trim24	0.03	0.63119725	pigs
Cog5	0.06	0.63182897	pigs
Pgrmc1	-0.03	0.63218108	pigs
Ldlr	0.07	0.63670853	pigs
Avl9	0.03	0.63791006	pigs
Creb3	-0.01	0.64051478	pigs
Hace1	0.08	0.64498888	pigs
Limd1	0.09	0.65247733	pigs
Dennd10	0.03	0.65572700	pigs
Dennd4c	0.01	0.66043932	pigs
Cep192	0.00	0.66407894	pigs
Ndufs1	0.01	0.66868103	pigs
Cic	0.04	0.66963788	pigs
Sft2d1	-0.06	0.67287492	pigs
Rnf5	0.02	0.67690158	pigs
Adam9	0.10	0.68108148	pigs
Taf1d	0.09	0.69254927	pigs
Vps53	0.05	0.69500549	pigs
Parvb	-0.31	0.72256674	pigs
Erg28	0.01	0.72493078	pigs
Ppfia1	-0.02	0.72552481	pigs
Hk1	0.01	0.73311584	pigs
Pard6b	0.06	0.74000430	pigs
Lamtor3	0.01	0.74317286	pigs
Adgre5	-0.47	0.75125737	pigs
Acly	0.01	0.75752102	pigs
Gramd1b	0.11	0.75842814	pigs
Pex13	0.09	0.77823768	pigs
Zc3h14	0.00	0.78308993	pigs
Rfx7	0.02	0.78382989	pigs
Grcc10	-0.54	0.78437242	pigs
Prss55	0.14	0.79194198	pigs
Rnf126	0.03	0.79307105	pigs
Atp6ap1	0.05	0.79325237	pigs
Fam234a	0.06	0.79518073	pigs
Ash2l	0.04	0.79793140	pigs
Dctn6	-0.02	0.80803851	pigs
Hes6	0.01	0.81157536	pigs
Itga1	-0.56	0.81387055	pigs
Agpat1	0.07	0.81821406	pigs
Elk4	0.04	0.82220521	pigs
Slc9b2	0.11	0.82394039	pigs
Eif4e2	0.05	0.82398838	pigs
Hadhb	0.03	0.82638536	pigs

Dtwd2	0.11	0.82852021	pigs
Nup188	0.00	0.82976790	pigs
Relch	0.04	0.83232501	pigs
Synrg	-0.05	0.83248523	pigs
G3bp1	0.02	0.83645578	pigs
Sec22b	0.07	0.83931839	pigs
Stag1	0.07	0.84591452	pigs
Akr1a1	0.01	0.84877022	pigs
Dync1li1	0.02	0.84936649	pigs
Prkag1	0.01	0.85030021	pigs
Cog2	0.04	0.86018737	pigs
Coro1c	0.05	0.86054716	pigs
Cyp2e1	0.04	0.86361486	pigs
Stam2	0.01	0.86430456	pigs
Tada3	0.03	0.86910581	pigs
Cd40lg	0.19	0.86982821	pigs
Dctn2	0.00	0.87245024	pigs
Fam98b	0.04	0.88007794	pigs
Rrp36	-0.09	0.88340392	pigs
Gata3	0.07	0.88769786	pigs
Zpr1	-0.03	0.88821863	pigs
Xpo4	0.01	0.88961514	pigs
Smarcc1	0.08	0.90050870	pigs
Rbm18	-0.05	0.90248519	pigs
Nsun2	0.01	0.90889000	pigs
Bicd2	0.06	0.91206254	pigs
Copb1	-0.02	0.91242101	pigs
5730455P16Rik	0.01	0.92564764	pigs
Rbm28	-0.01	0.92570751	pigs
Rbm42	0.03	0.92827830	pigs
Lmbrd1	0.03	0.93755016	pigs
Ccdc88c	-0.01	0.93924928	pigs
Dnajc13	0.02	0.94740911	pigs
Ikbkg	0.05	0.95143493	pigs
St6galnac4	-0.06	0.95417906	pigs
Tmem43	0.02	0.95497036	pigs
Cwc15	-0.04	0.95557808	pigs
Nop2	0.07	0.96053641	pigs
Pelp1	0.04	0.96235757	pigs
Zbtb41	0.07	0.96544206	pigs
Eny2	-0.09	0.97289379	pigs
Pik3c2a	0.07	0.97617477	pigs
Vim	0.07	0.98494452	pigs
Eloc	-0.04	0.99213027	pigs
Ptar1	0.04	0.99767937	pigs
Cox18	0.06	1.00000000	pigs

Ugdh	0.01	1.00000000	pigs
Nisch	0.05	1.00000000	pigs
Fli1	0.17	1.00000000	pigs
Ddx6	0.08	1.00000000	pigs
Rtcb	0.00	1.00000000	pigs
Zfp36	0.10	1.00000000	pigs
Fbxl17	0.13	1.00000000	pigs
Cops6	-0.04	1.00000000	pigs
Slc9a8	-0.04	1.00000000	pigs
Sf3a1	-0.04	1.00000000	pigs
Naxd	0.02	1.00000000	pigs
Fcmr	0.07	1.00000000	pigs
Rad51b	0.10	1.00000000	pigs
Smarcb1	0.05	1.00000000	pigs
Shoc2	-0.01	1.00000000	pigs
Pfkl	0.04	1.00000000	pigs
Pin4	-0.01	1.00000000	pigs
Osbpl11	0.04	1.00000000	pigs
Chmp2b	0.00	1.00000000	pigs
Rfwd3	0.05	1.00000000	pigs
Paxx	-0.10	1.00000000	pigs
Hnrnpm	0.05	1.00000000	pigs
Gemin4	-0.04	1.00000000	pigs
Copg1	0.03	1.00000000	pigs
Dnajb9	0.10	1.00000000	pigs
Taf5l	0.01	1.00000000	pigs
Ubn1	0.03	1.00000000	pigs
Atrx	0.09	1.00000000	pigs
Fam32a	-0.04	1.00000000	pigs
Sh2d2a	0.07	1.00000000	pigs
Srp9	0.00	1.00000000	pigs
Smu1	-0.03	1.00000000	pigs
Ankrd46	0.04	1.00000000	pigs
Ccnq	-0.04	1.00000000	pigs
Uba5	0.04	1.00000000	pigs
Cbx4	0.05	1.00000000	pigs
Pogz	0.04	1.00000000	pigs
Arhgap4	0.05	1.00000000	pigs
Gtf2f1	-0.03	1.00000000	pigs
Nploc4	0.08	1.00000000	pigs
Zc3h15	0.00	1.00000000	pigs
Got1	0.01	1.00000000	pigs
Sod1	0.00	1.00000000	pigs
Rida	-0.03	1.00000000	pigs
Riok1	0.00	1.00000000	pigs
Apbb3	-0.01	1.00000000	pigs

Wdr48	-0.02	1.00000000	pigs
Skap1	0.11	1.00000000	pigs
Gabarapl1	0.03	1.00000000	pigs
Lancl1	0.05	1.00000000	pigs
Nob1	0.06	1.00000000	pigs
Nmi	-0.03	1.00000000	pigs
Gnpat	0.02	1.00000000	pigs
Coq2	0.07	1.00000000	pigs
Acsf2	0.05	1.00000000	pigs
Ldb1	0.06	1.00000000	pigs
Fam114a2	0.02	1.00000000	pigs
Arpc5	0.10	1.00000000	pigs
Tmem107	-0.48	1.00000000	pigs
Desi2	0.04	1.00000000	pigs
Fam172a	0.09	1.00000000	pigs
Il17ra	-0.08	1.00000000	pigs
Ucp2	-0.46	1.00000000	pigs
Cd48	-0.01	1.00000000	pigs
Stk40	-0.02	1.00000000	pigs
Rbm38	0.06	1.00000000	pigs
Atp5c1	-0.03	1.00000000	pigs
Bcat1	0.04	1.00000000	pigs
Shkbp1	-0.04	1.00000000	pigs
Ccdc127	-0.02	1.00000000	pigs
Rcbtb1	0.04	1.00000000	pigs
Prdx5	-0.08	1.00000000	pigs
Tsc22d3	0.12	1.00000000	pigs
Eif2b2	-0.03	1.00000000	pigs
Pex16	-0.01	1.00000000	pigs
Epn1	0.06	1.00000000	pigs
Trappc11	-0.01	1.00000000	pigs
Dync1i2	-0.05	1.00000000	pigs
Rasgrp2	-0.35	1.00000000	pigs
Papss1	-0.01	1.00000000	pigs
Prep	-0.02	1.00000000	pigs
Trafd1	0.02	1.00000000	pigs
Zfand6	0.06	1.00000000	pigs
Tuba4a	0.03	1.00000000	pigs
Snrpc	-0.12	1.00000000	pigs
Skil	-0.04	1.00000000	pigs
Trpc4ap	-0.01	1.00000000	pigs
Sbds	-0.02	1.00000000	pigs
Gne	0.02	1.00000000	pigs
Kif18a	-0.01	1.00000000	pigs
Son	-0.25	1.00000000	pigs
Kpna3	0.00	1.00000000	pigs

Setd2	0.00	1.00000000	pigs
Arid3b	-0.06	1.00000000	pigs
1700123O20Rik	0.04	1.00000000	pigs
Sco1	0.05	1.00000000	pigs
Nosip	0.01	1.00000000	pigs
Diaph1	0.03	1.00000000	pigs
Ncoa1	0.03	1.00000000	pigs
Ckap5	-0.01	1.00000000	pigs
Ddit4	0.00	1.00000000	pigs
Aif1	-0.05	1.00000000	pigs
Leprotl1	-0.09	1.00000000	pigs
Cga	0.08	1.00000000	pigs
Opa1	0.00	1.00000000	pigs
Nars	-0.01	1.00000000	pigs
Cnpy3	0.06	1.00000000	pigs
Osbpl3	0.16	1.00000000	pigs
Ywhah	-0.04	1.00000000	pigs
Lpp	0.00	1.00000000	pigs
Fbxw7	0.03	1.00000000	pigs
U2af2	0.02	1.00000000	pigs
Usp46	0.00	1.00000000	pigs
Cnot10	0.03	1.00000000	pigs
Stpg4	-0.25	1.00000000	pigs
Dynlrb1	0.01	1.00000000	pigs
Clasp2	0.08	1.00000000	pigs
Znhit3	-0.01	1.00000000	pigs
Lpcat2	-0.42	1.00000000	pigs
Rara	0.03	1.00000000	pigs
Hsf2	0.02	1.00000000	pigs
Pip4p1	0.05	1.00000000	pigs
Fkbp4	-0.01	1.00000000	pigs
Ctsw	-0.54	1.00000000	pigs
Cers4	0.00	1.00000000	pigs
Vasp	-0.08	1.00000000	pigs
Glipr1	0.05	1.00000000	pigs
Sgf29	0.03	1.00000000	pigs
Usf3	0.01	1.00000000	pigs
Parp12	0.03	1.00000000	pigs
Colgalt1	0.08	1.00000000	pigs
Slco3a1	0.06	1.00000000	pigs
Arf1	0.02	1.00000000	pigs
Ndufs6	-0.01	1.00000000	pigs
Srgn	0.08	1.00000000	pigs
Flna	-0.47	1.00000000	pigs
Ccng2	-0.37	1.00000000	pigs
Kdm2b	-0.04	1.00000000	pigs

Cstf1	-0.01	1.00000000	pigs
Rock2	-0.02	1.00000000	pigs
Acaa1b	0.04	1.00000000	pigs
Kcnab3	-0.06	1.00000000	pigs
Pef1	0.02	1.00000000	pigs
Sesn3	0.06	1.00000000	pigs
Thyn1	-0.04	1.00000000	pigs
Sccpdh	0.00	1.00000000	pigs
Gadd45gip1	-0.02	1.00000000	pigs
Upf2	0.02	1.00000000	pigs
Vkorc1l1	0.05	1.00000000	pigs
Cwf19l1	-0.02	1.00000000	pigs
Ap1s1	-0.04	1.00000000	pigs
Slc25a13	0.03	1.00000000	pigs
Rfxank	-0.09	1.00000000	pigs
Psmb3	-0.02	1.00000000	pigs
Kdm4a	0.04	1.00000000	pigs
Id2	0.17	1.00000000	pigs
Exosc8	-0.02	1.00000000	pigs
Ammecr1	0.01	1.00000000	pigs
Reep5	-0.01	1.00000000	pigs
Xrcc6	0.04	1.00000000	pigs
Vamp4	0.04	1.00000000	pigs
Elp1	0.07	1.00000000	pigs
Stk11	0.02	1.00000000	pigs
Tbrg4	-0.04	1.00000000	pigs
Casd1	0.04	1.00000000	pigs
Rfc1	-0.01	1.00000000	pigs
Zfp341	0.00	1.00000000	pigs
Ahrr	0.03	1.00000000	pigs
Bag4	0.06	1.00000000	pigs
Bccip	-0.09	1.00000000	pigs
Iws1	-0.01	1.00000000	pigs
Tbl3	0.04	1.00000000	pigs
Apip	0.02	1.00000000	pigs
Rfc2	0.01	1.00000000	pigs
Tmem127	0.01	1.00000000	pigs
Ablim1	0.00	1.00000000	pigs
Zdhhc7	0.02	1.00000000	pigs
Gbe1	0.03	1.00000000	pigs
Lsm14b	0.03	1.00000000	pigs
Golga5	0.02	1.00000000	pigs
Zcchc7	0.04	1.00000000	pigs
Ehbp1	0.00	1.00000000	pigs
E2f4	0.02	1.00000000	pigs
Commd10	0.02	1.00000000	pigs

Tnfsf9	0.09	1.00000000	pigs
Nmt1	-0.02	1.00000000	pigs
Cnot7	-0.01	1.00000000	pigs
Ola1	-0.04	1.00000000	pigs
Arrb2	-0.01	1.00000000	pigs
Lhpp	0.04	1.00000000	pigs
Clcn3	0.03	1.00000000	pigs
Otulinl	-0.45	1.00000000	pigs
Prelid1	-0.03	1.00000000	pigs
Ctnnbl1	0.00	1.00000000	pigs
Stk39	0.05	1.00000000	pigs
Cep63	-0.03	1.00000000	pigs
Acsl1	-0.41	1.00000000	pigs
Zfand3	-0.07	1.00000000	pigs
Igf2bp3	-0.04	1.00000000	pigs
Kmt5a	0.02	1.00000000	pigs
Pdhb	-0.01	1.00000000	pigs
Ppp1r11	-0.01	1.00000000	pigs
Polr1d	0.04	1.00000000	pigs
Ippk	0.07	1.00000000	pigs
Tfdp1	0.00	1.00000000	pigs
Cyb561a3	0.06	1.00000000	pigs
Ctdnep1	-0.01	1.00000000	pigs
Pcca	0.11	1.00000000	pigs
Dynll2	-0.03	1.00000000	pigs
Osgin2	0.00	1.00000000	pigs
St3gal2	0.06	1.00000000	pigs
Nelfb	0.03	1.00000000	pigs
Tbc1d9b	0.01	1.00000000	pigs
Crtc1	0.06	1.00000000	pigs
Stk3	0.08	1.00000000	pigs
Zfand5	0.00	1.00000000	pigs
Utp25	0.00	1.00000000	pigs
Kin	-0.07	1.00000000	pigs
Keap1	0.05	1.00000000	pigs
Pced1b	0.12	1.00000000	pigs
Lmbrd2	0.04	1.00000000	pigs
Jakmip1	-0.04	1.00000000	pigs
Polr2m	0.03	1.00000000	pigs
Vcl	-0.42	1.00000000	pigs
Psmb1	-0.05	1.00000000	pigs
Cep250	0.04	1.00000000	pigs
4930453N24Rik	-0.01	1.00000000	pigs
Cc2d1b	0.05	1.00000000	pigs
Aurkaip1	-0.36	1.00000000	pigs
Cxxc1	-0.02	1.00000000	pigs

Irak4	0.01	1.00000000	pigs
Nudc	-0.03	1.00000000	pigs
Prkcb	0.03	1.00000000	pigs
Rbm39	0.05	1.00000000	pigs
Sugp1	0.00	1.00000000	pigs
Supt3	0.04	1.00000000	pigs
Yipf2	0.03	1.00000000	pigs
Snrk	0.00	1.00000000	pigs
Rbm41	0.04	1.00000000	pigs
Fbxo31	0.03	1.00000000	pigs
Tgif1	0.00	1.00000000	pigs
Dapl1	-0.54	1.00000000	pigs
Nudcd3	0.07	1.00000000	pigs
Ergic2	-0.01	1.00000000	pigs
Irf7	0.14	1.00000000	pigs
Prdx3	-0.01	1.00000000	pigs
Ube2l6	-0.02	1.00000000	pigs
4833439L19Rik	-0.03	1.00000000	pigs
Pikfyve	0.05	1.00000000	pigs
Znrf2	0.09	1.00000000	pigs
Fem1b	0.03	1.00000000	pigs
Pih1d1	0.04	1.00000000	pigs
Ammecr1l	0.02	1.00000000	pigs
Cenpp	0.05	1.00000000	pigs
Pbxip1	0.02	1.00000000	pigs
Phlda2	-0.59	1.00000000	pigs
Cep78	0.04	1.00000000	pigs
Dop1a	0.04	1.00000000	pigs
Suz12	-0.06	1.00000000	pigs
Sh2d3c	0.06	1.00000000	pigs
Esf1	-0.01	1.00000000	pigs
Rnf20	0.05	1.00000000	pigs
Rpf1	0.01	1.00000000	pigs
Ap1b1	-0.03	1.00000000	pigs
Dars	-0.02	1.00000000	pigs
Rexo2	-0.03	1.00000000	pigs
Heatr3	0.00	1.00000000	pigs
Alms1	0.00	1.00000000	pigs
Kifap3	0.00	1.00000000	pigs
Cops3	-0.01	1.00000000	pigs
Retreg3	0.02	1.00000000	pigs
Nsmce2	-0.01	1.00000000	pigs
Asap1	0.08	1.00000000	pigs
Emc2	0.03	1.00000000	pigs
Klc2	-0.03	1.00000000	pigs
Bcl3	-0.03	1.00000000	pigs

Ppa1	-0.02	1.00000000	pigs
Cog3	0.04	1.00000000	pigs
Agps	-0.03	1.00000000	pigs
Unk	0.07	1.00000000	pigs
LTO1	-0.04	1.00000000	pigs
Vps39	0.04	1.00000000	pigs
Odr4	0.07	1.00000000	pigs
Emg1	0.05	1.00000000	pigs
Creld1	0.02	1.00000000	pigs
Tgfbr1	-0.01	1.00000000	pigs
Oxr1	-0.04	1.00000000	pigs
Ciao2b	0.02	1.00000000	pigs
Rassf3	0.06	1.00000000	pigs
Btf3l4	-0.06	1.00000000	pigs
Spats2	-0.05	1.00000000	pigs
Osbpl8	0.01	1.00000000	pigs
Nedd9	0.04	1.00000000	pigs
Serp1	-0.11	1.00000000	pigs
Gak	0.01	1.00000000	pigs
Rbm12b2	-0.01	1.00000000	pigs
Kat2b	0.06	1.00000000	pigs
Borcs5	-0.05	1.00000000	pigs
Bsdc1	0.06	1.00000000	pigs
Ncstn	0.00	1.00000000	pigs
Usp39	-0.01	1.00000000	pigs
Ufc1	-0.01	1.00000000	pigs
Pnisr	0.01	1.00000000	pigs
Chp1	0.03	1.00000000	pigs
Kat7	-0.06	1.00000000	pigs
Asb3	-0.01	1.00000000	pigs
Tmem259	0.06	1.00000000	pigs
Eps15l1	0.00	1.00000000	pigs
Slc7a6os	0.05	1.00000000	pigs
Cops5	-0.06	1.00000000	pigs
Cwf19l2	0.00	1.00000000	pigs
Eogt	-0.03	1.00000000	pigs
Fryl	0.15	1.00000000	pigs
Sertad2	0.07	1.00000000	pigs
Gng10	-0.12	1.00000000	pigs
Ppm1l	0.05	1.00000000	pigs
Fyb	0.01	1.00000000	pigs
Atp11c	-0.01	1.00000000	pigs
Cpsf2	0.01	1.00000000	pigs
Sfxn1	0.01	1.00000000	pigs
Tmem70	0.04	1.00000000	pigs
Ppm1a	-0.03	1.00000000	pigs

Osbp	0.05	1.00000000	pigs
Stx4a	0.06	1.00000000	pigs
Fbrsl1	-0.02	1.00000000	pigs
Ifi44	-0.01	1.00000000	pigs
Trip4	0.01	1.00000000	pigs
Wrap53	0.04	1.00000000	pigs
Sltm	-0.10	1.00000000	pigs
Atp6v1h	0.04	1.00000000	pigs
Sec16a	0.03	1.00000000	pigs
Pwwp2a	-0.05	1.00000000	pigs
Rsl1d1	-0.12	1.00000000	pigs
Cnp	0.02	1.00000000	pigs
Scnm1	0.08	1.00000000	pigs
Chd6	0.01	1.00000000	pigs
Nras	-0.06	1.00000000	pigs
Arl5a	-0.01	1.00000000	pigs
Bach2	0.04	1.00000000	pigs
Fer	0.05	1.00000000	pigs
Trmt10a	0.01	1.00000000	pigs
Zfp64	0.07	1.00000000	pigs
Ivd	0.00	1.00000000	pigs
Hdac1	-0.05	1.00000000	pigs
Dlgap4	0.03	1.00000000	pigs
Slc4a4	0.44	1.00000000	pigs
Cct3	0.03	1.00000000	pigs
Rfx3	0.18	1.00000000	pigs
9430038I01Rik	-0.04	1.00000000	pigs
Ezh1	-0.03	1.00000000	pigs
Saysd1	0.04	1.00000000	pigs
Hsd17b12	-0.02	1.00000000	pigs
Dhrs11	-0.01	1.00000000	pigs
Glrx5	-0.03	1.00000000	pigs
Nme6	0.02	1.00000000	pigs
Naxe	-0.07	1.00000000	pigs
Tkt	0.01	1.00000000	pigs
Bicdl1	0.15	1.00000000	pigs
Sdf2	-0.04	1.00000000	pigs
Ist1	-0.05	1.00000000	pigs
Dcaf13	-0.01	1.00000000	pigs
Qrich1	0.04	1.00000000	pigs
Nrbf2	-0.04	1.00000000	pigs
Bzw2	-0.04	1.00000000	pigs
Atp6v0b	-0.12	1.00000000	pigs
Carm1	0.01	1.00000000	pigs
AU040320	0.03	1.00000000	pigs
Heatr6	0.01	1.00000000	pigs

Susd1	-0.51	1.00000000	pigs
Arl6ip6	-0.04	1.00000000	pigs
Nbas	0.00	1.00000000	pigs
Fchsd2	-0.15	1.00000000	pigs
Ubxn6	0.01	1.00000000	pigs
Abhd18	0.02	1.00000000	pigs
Ubxn4	-0.06	1.00000000	pigs
Vps72	0.03	1.00000000	pigs
Nudt1	-0.38	1.00000000	pigs
Fgfr1op2	0.01	1.00000000	pigs
Utp15	0.01	1.00000000	pigs
Nufip1	0.00	1.00000000	pigs
Taf7	0.00	1.00000000	pigs
Emc10	-0.05	1.00000000	pigs
Yipf3	0.02	1.00000000	pigs
Glce	0.06	1.00000000	pigs
Helq	0.04	1.00000000	pigs
Nkg7	-0.89	1.00000000	pigs
Ikbkb	0.01	1.00000000	pigs
Fermt3	-0.04	1.00000000	pigs
Psip1	-0.02	1.00000000	pigs
Abhd12	0.03	1.00000000	pigs
Abcb7	0.04	1.00000000	pigs
Hyal2	-0.04	1.00000000	pigs
Ap5m1	-0.01	1.00000000	pigs
Ube2m	-0.04	1.00000000	pigs
Lysmd2	-0.03	1.00000000	pigs
Ube2e3	-0.06	1.00000000	pigs
Togaram1	0.04	1.00000000	pigs
AI597479	0.03	1.00000000	pigs
Bpnt1	-0.03	1.00000000	pigs
Aamp	-0.03	1.00000000	pigs
Dipk1a	0.02	1.00000000	pigs
Dcakd	0.04	1.00000000	pigs
Fcer1g	-1.07	1.00000000	pigs
Snip1	0.01	1.00000000	pigs
Setd7	0.04	1.00000000	pigs
Drap1	-0.06	1.00000000	pigs
Psmd14	-0.04	1.00000000	pigs
Stk25	-0.01	1.00000000	pigs
Zfat	0.03	1.00000000	pigs
Wdr1	0.03	1.00000000	pigs
Epc1	0.09	1.00000000	pigs
Arl6ip5	0.00	1.00000000	pigs
Senp1	0.01	1.00000000	pigs
Fmnl2	0.20	1.00000000	pigs

Cab39l	-0.02	1.00000000	pigs
Capn2	-0.02	1.00000000	pigs
Amd2	-0.05	1.00000000	pigs
Slc66a2	-0.02	1.00000000	pigs
Dynlrb2	-0.36	1.00000000	pigs
Serac1	0.03	1.00000000	pigs
Rep15	-0.01	1.00000000	pigs
Prkacb	0.09	1.00000000	pigs
Ccdc174	-0.03	1.00000000	pigs
Gnpda2	-0.04	1.00000000	pigs
Impdh1	0.04	1.00000000	pigs
Commd4	-0.12	1.00000000	pigs
Raly	-0.01	1.00000000	pigs
Ubfd1	0.00	1.00000000	pigs
Polr2e	-0.08	1.00000000	pigs
Klhl18	0.01	1.00000000	pigs
Otud7b	0.02	1.00000000	pigs
Nenf	-0.01	1.00000000	pigs
Tnik	0.11	1.00000000	pigs
Apc	0.01	1.00000000	pigs
Nup107	-0.11	1.00000000	pigs
Ctsh	0.05	1.00000000	pigs
Siah2	-0.09	1.00000000	pigs
Arel1	0.00	1.00000000	pigs
Tmem223	-0.56	1.00000000	pigs
Cdk8	0.00	1.00000000	pigs
Uros	0.00	1.00000000	pigs
Adipor2	-0.01	1.00000000	pigs
Vps37a	-0.03	1.00000000	pigs
Zfp24	-0.01	1.00000000	pigs
Slc25a3	0.01	1.00000000	pigs
Vps51	-0.03	1.00000000	pigs
Ppp2cb	0.04	1.00000000	pigs
Svil	-0.05	1.00000000	pigs
Prkdc	0.00	1.00000000	pigs
Sh3glb1	0.02	1.00000000	pigs
Txndc11	-0.01	1.00000000	pigs
Ireb2	-0.02	1.00000000	pigs
Bag5	-0.03	1.00000000	pigs
Eif2ak1	0.05	1.00000000	pigs
Cox7a2l	0.01	1.00000000	pigs
Trio	-0.29	1.00000000	pigs
Dnmt3a	0.01	1.00000000	pigs
Ifrd2	0.04	1.00000000	pigs
Tubgcp2	0.04	1.00000000	pigs
Rptor	0.08	1.00000000	pigs

Ylpm1	0.04	1.00000000	pigs
Rac2	-0.22	1.00000000	pigs
Polr2f	-0.45	1.00000000	pigs
Bin3	0.03	1.00000000	pigs
Ppm1d	-0.04	1.00000000	pigs
Tmem268	-0.34	1.00000000	pigs
L2hgdh	0.02	1.00000000	pigs
Pitpna	-0.06	1.00000000	pigs
Nnt	-0.03	1.00000000	pigs
Hira	0.00	1.00000000	pigs
Prdx1	-0.02	1.00000000	pigs
Ndufv2	-0.06	1.00000000	pigs
Fam149b	-0.02	1.00000000	pigs
Vps35l	-0.04	1.00000000	pigs
Synj1	-0.03	1.00000000	pigs
3110040N11Rik	-0.32	1.00000000	pigs
Rfc5	0.01	1.00000000	pigs
Taf9b	-0.01	1.00000000	pigs
Phf10	0.02	1.00000000	pigs
Spred2	0.09	1.00000000	pigs
Xpot	-0.02	1.00000000	pigs
Taf4b	-0.01	1.00000000	pigs
Cul2	-0.05	1.00000000	pigs
Cbl	-0.03	1.00000000	pigs
Zdhhc5	-0.04	1.00000000	pigs
Arfgef2	-0.01	1.00000000	pigs
Fundc1	-0.05	1.00000000	pigs
Ppp1r15a	-0.05	1.00000000	pigs
Klhl24	0.00	1.00000000	pigs
Dnmt1	-0.09	1.00000000	pigs
Rhof	-0.10	1.00000000	pigs
Gpn3	-0.02	1.00000000	pigs
Snrnp40	-0.03	1.00000000	pigs
Sqstm1	-0.09	1.00000000	pigs
Utp4	0.03	1.00000000	pigs
Eif1b	-0.49	1.00000000	pigs
Gcn1	0.04	1.00000000	pigs
Kctd10	-0.01	1.00000000	pigs
Atp6v0d1	0.01	1.00000000	pigs
Zfp30	0.09	1.00000000	pigs
Cnot8	0.02	1.00000000	pigs
Castor1	0.03	1.00000000	pigs
Abhd16a	-0.03	1.00000000	pigs
Utp11	-0.07	1.00000000	pigs
Ncald	0.08	1.00000000	pigs
Slc16a6	-0.03	1.00000000	pigs

Cd44	0.04	1.00000000	pigs
Hsdl2	0.00	1.00000000	pigs
Angel2	-0.06	1.00000000	pigs
Ddx60	0.01	1.00000000	pigs
Exosc1	0.02	1.00000000	pigs
Adar	0.03	1.00000000	pigs
Zbtb7a	-0.04	1.00000000	pigs
Nipal3	-0.03	1.00000000	pigs
Tspan3	-0.03	1.00000000	pigs
Ahctf1	-0.17	1.00000000	pigs
R3hdm2	-0.02	1.00000000	pigs
Gtf2b	-0.04	1.00000000	pigs
Arap1	0.01	1.00000000	pigs
Zfp827	0.03	1.00000000	pigs
S1pr1	-0.29	1.00000000	pigs
Rnf13	-0.14	1.00000000	pigs
Dnttip1	0.02	1.00000000	pigs
Slc38a9	0.03	1.00000000	pigs
Fto	0.08	1.00000000	pigs
Rfx1	-0.01	1.00000000	pigs
Arpc1b	0.05	1.00000000	pigs
Ndufaf2	-0.04	1.00000000	pigs
Smchd1	0.07	1.00000000	pigs
Csnk2a2	-0.07	1.00000000	pigs
Scamp2	-0.02	1.00000000	pigs
Supt16	-0.09	1.00000000	pigs
Dffa	-0.02	1.00000000	pigs
Zmpste24	0.01	1.00000000	pigs
Acbd3	-0.10	1.00000000	pigs
Txnl4a	-0.07	1.00000000	pigs
Cd3e	0.08	1.00000000	pigs
Nek6	0.03	1.00000000	pigs
Abcf1	-0.10	1.00000000	pigs
Kctd13	0.01	1.00000000	pigs
Fkbp3	-0.07	1.00000000	pigs
Zfp36l1	0.04	1.00000000	pigs
Sec23a	-0.01	1.00000000	pigs
Chchd3	0.01	1.00000000	pigs
Ss18l2	-0.05	1.00000000	pigs
Gabpb2	0.03	1.00000000	pigs
Deaf1	0.04	1.00000000	pigs
Csrp1	0.03	1.00000000	pigs
Smc6	-0.02	1.00000000	pigs
Dctn3	-0.05	1.00000000	pigs
Ak3	-0.06	1.00000000	pigs
Dis3	-0.03	1.00000000	pigs

Zxdc	0.07	1.00000000	pigs
Cfl1	0.12	1.00000000	pigs
R3hdm4	-0.09	1.00000000	pigs
Ccdc47	0.05	1.00000000	pigs
Wdr7	-0.01	1.00000000	pigs
Dcaf12	0.00	1.00000000	pigs
Spring1	-0.02	1.00000000	pigs
Clk2	0.02	1.00000000	pigs
Slc35e1	0.03	1.00000000	pigs
Atf6	-0.08	1.00000000	pigs
Stoml2	-0.04	1.00000000	pigs
Trmt2a	0.04	1.00000000	pigs
Lclat1	-0.03	1.00000000	pigs
Eif2s1	-0.05	1.00000000	pigs
Nbr1	0.03	1.00000000	pigs
Nat9	0.01	1.00000000	pigs
Rnmt	-0.09	1.00000000	pigs
Chmp5	0.05	1.00000000	pigs
Abcd3	0.01	1.00000000	pigs
Ndufb2	-0.03	1.00000000	pigs
Xxylt1	0.01	1.00000000	pigs
Itgae	0.04	1.00000000	pigs
Rbbp8	0.09	1.00000000	pigs
Zfp369	-0.01	1.00000000	pigs
Bin2	0.07	1.00000000	pigs
Trappc6b	-0.05	1.00000000	pigs
Pmpca	0.01	1.00000000	pigs
Arl1	-0.06	1.00000000	pigs
Slc1a5	0.03	1.00000000	pigs
C3	-0.03	1.00000000	pigs
Hspd1	-0.03	1.00000000	pigs
Tmco3	0.02	1.00000000	pigs
Pdha1	-0.03	1.00000000	pigs
Idh3g	0.00	1.00000000	pigs
Agk	-0.07	1.00000000	pigs
Fam174a	0.05	1.00000000	pigs
Smim15	-0.11	1.00000000	pigs
Lrrc8d	0.08	1.00000000	pigs
Tradd	0.04	1.00000000	pigs
Rbx1	-0.02	1.00000000	pigs
Golga4	-0.01	1.00000000	pigs
Wwp1	0.03	1.00000000	pigs
H2aj	-0.13	1.00000000	pigs
Faf1	-0.05	1.00000000	pigs
Nolc1	-0.06	1.00000000	pigs
Lmbr1l	-0.02	1.00000000	pigs

Obi1	0.02	1.00000000	pigs
Crybg1	-0.08	1.00000000	pigs
Ufd1	-0.14	1.00000000	pigs
Inpp4b	0.03	1.00000000	pigs
Slc30a9	0.03	1.00000000	pigs
Arhgap30	-0.04	1.00000000	pigs
Ssr3	-0.02	1.00000000	pigs
Osgep	0.06	1.00000000	pigs
Hspa14	-0.06	1.00000000	pigs
Atf6b	0.01	1.00000000	pigs
Trmt13	0.07	1.00000000	pigs
Scg5	-0.05	1.00000000	pigs
Bpnt2	-0.06	1.00000000	pigs
Dnajc14	-0.02	1.00000000	pigs
Glg1	0.04	1.00000000	pigs
Gusb	-0.04	1.00000000	pigs
Nr1h2	0.00	1.00000000	pigs
Tgfbrap1	0.04	1.00000000	pigs
Abracl	-0.03	1.00000000	pigs
Slc12a6	-0.06	1.00000000	pigs
Hadh	-0.06	1.00000000	pigs
G3bp2	-0.04	1.00000000	pigs
Zbtb37	0.03	1.00000000	pigs
Cltb	-0.07	1.00000000	pigs
Smyd2	0.00	1.00000000	pigs
Dnajc11	-0.04	1.00000000	pigs
Wdfy2	0.01	1.00000000	pigs
Ddx20	-0.08	1.00000000	pigs
Cage1	-0.30	1.00000000	pigs
Hadha	-0.06	1.00000000	pigs
Rbms1	-0.07	1.00000000	pigs
Cd5	0.01	1.00000000	pigs
Ap2b1	-0.08	1.00000000	pigs
Slc4a1ap	-0.06	1.00000000	pigs
Acot7	-0.02	1.00000000	pigs
Fam168a	-0.02	1.00000000	pigs
Wdr47	-0.01	1.00000000	pigs
Utp20	-0.01	1.00000000	pigs
Srpr	-0.11	1.00000000	pigs
Thap7	0.00	1.00000000	pigs
Kif3a	-0.02	1.00000000	pigs
Zfp36l2	-0.17	1.00000000	pigs
Zfp146	-0.06	1.00000000	pigs
Nubp2	-0.07	1.00000000	pigs
Rasa1	-0.02	1.00000000	pigs
Shld2	0.07	1.00000000	pigs

0610030E20Rik	-0.02	1.00000000	pigs
Il16	-0.11	1.00000000	pigs
Rspry1	0.03	1.00000000	pigs
Rilpl2	-0.08	1.00000000	pigs
Wdr33	-0.01	1.00000000	pigs
Chmp4b	-0.11	1.00000000	pigs
Cdc42ep3	-0.33	1.00000000	pigs
Cmc1	-0.45	1.00000000	pigs
Copg2	0.00	1.00000000	pigs
Chmp2a	-0.05	1.00000000	pigs
Cpne1	-0.08	1.00000000	pigs
Strn4	-0.02	1.00000000	pigs
Cfp	0.02	1.00000000	pigs
Cwc22	-0.06	1.00000000	pigs
Atp5d	-0.11	1.00000000	pigs
Prr14l	-0.02	1.00000000	pigs
Ccdc58	0.01	1.00000000	pigs
Parp8	0.06	1.00000000	pigs
Ggcx	0.02	1.00000000	pigs
Dag1	-0.03	1.00000000	pigs
Vps13d	-0.05	1.00000000	pigs
Pik3r1	-0.02	1.00000000	pigs
Hnrnpk	0.03	1.00000000	pigs
Cul4b	0.02	1.00000000	pigs
Nutf2	-0.12	1.00000000	pigs
Bcl7c	-0.09	1.00000000	pigs
Hdac5	0.01	1.00000000	pigs
Pnkp	0.02	1.00000000	pigs
Tprgl	-0.05	1.00000000	pigs
Gnpda1	-0.04	1.00000000	pigs
Chm	0.03	1.00000000	pigs
Isg20l2	-0.01	1.00000000	pigs
Ttc27	0.05	1.00000000	pigs
Rcbtb2	-0.04	1.00000000	pigs
Apex1	-0.01	1.00000000	pigs
Abhd3	-0.04	1.00000000	pigs
Slc30a7	-0.06	1.00000000	pigs
Cmah	-0.05	1.00000000	pigs
Nars2	0.01	1.00000000	pigs
Ncoa5	-0.02	1.00000000	pigs
Zbtb1	0.05	1.00000000	pigs
Wbp1	0.02	1.00000000	pigs
Uba3	0.00	1.00000000	pigs
Pex1	0.01	1.00000000	pigs
Clns1a	-0.02	1.00000000	pigs
Cd9	-0.31	1.00000000	pigs

Dad1	-0.39	1.00000000	pigs
Trim37	0.01	1.00000000	pigs
Slc9a7	0.11	1.00000000	pigs
Ddx1	-0.04	1.00000000	pigs
Kynu	0.12	1.00000000	pigs
Josd1	-0.05	1.00000000	pigs
Thoc1	0.02	1.00000000	pigs
Vrk2	-0.04	1.00000000	pigs
Tab3	0.01	1.00000000	pigs
Irf1	-0.11	1.00000000	pigs
Ubac1	-0.03	1.00000000	pigs
Emc7	-0.08	1.00000000	pigs
Fbxl3	-0.01	1.00000000	pigs
Rsu1	-0.10	1.00000000	pigs
Bad	-0.03	1.00000000	pigs
Zfp410	0.04	1.00000000	pigs
Sap30bp	0.00	1.00000000	pigs
Usp24	-0.07	1.00000000	pigs
Ndufb1	-0.42	1.00000000	pigs
Ccdc59	-0.10	1.00000000	pigs
Spopl	0.04	1.00000000	pigs
Net1	-0.06	1.00000000	pigs
Anapc15-ps	-0.04	1.00000000	pigs
Ebna1bp2	0.01	1.00000000	pigs
Phactr2	-0.39	1.00000000	pigs
Flot2	-0.01	1.00000000	pigs
Zfp407	0.04	1.00000000	pigs
Herc6	0.05	1.00000000	pigs
Pphln1	-0.15	1.00000000	pigs
Golt1b	-0.06	1.00000000	pigs
Snrpa	-0.02	1.00000000	pigs
Glud1	-0.06	1.00000000	pigs
Ccdc66	-0.10	1.00000000	pigs
Prmt5	0.01	1.00000000	pigs
Tasor	0.03	1.00000000	pigs
Sacm1l	-0.10	1.00000000	pigs
Trim8	0.02	1.00000000	pigs
Sp3	-0.19	1.00000000	pigs
Rfc4	-0.02	1.00000000	pigs
Aars	-0.03	1.00000000	pigs
H2ac20	-0.11	1.00000000	pigs
Cdc14a	0.09	1.00000000	pigs
Ubald1	-0.02	1.00000000	pigs
Ap2a1	0.02	1.00000000	pigs
Atg101	-0.07	1.00000000	pigs
Gimap4	-0.05	1.00000000	pigs

Fam117b	-0.01	1.00000000	pigs
Aasdhppt	-0.02	1.00000000	pigs
Samm50	-0.04	1.00000000	pigs
Pdia6	-0.04	1.00000000	pigs
Zmynd11	-0.01	1.00000000	pigs
Tmx4	0.02	1.00000000	pigs
Ndufs3	-0.05	1.00000000	pigs
Dph6	-0.01	1.00000000	pigs
Chchd6	-0.02	1.00000000	pigs
Katnip	0.01	1.00000000	pigs
Zfp638	-0.07	1.00000000	pigs
Edem2	0.00	1.00000000	pigs
Tex10	0.01	1.00000000	pigs
Arl8a	-0.05	1.00000000	pigs
Nedd1	0.00	1.00000000	pigs
Rpp21	-0.32	1.00000000	pigs
Hmgb2	-0.03	1.00000000	pigs
Tsc22d1	0.03	1.00000000	pigs
Uspl1	0.01	1.00000000	pigs
Bri3bp	-0.05	1.00000000	pigs
Ivns1abp	-0.04	1.00000000	pigs
Bcl2l13	0.03	1.00000000	pigs
Polr1f	-0.08	1.00000000	pigs
Unc45a	-0.01	1.00000000	pigs
Zhx1	-0.04	1.00000000	pigs
Cmss1	0.08	1.00000000	pigs
Azi2	-0.02	1.00000000	pigs
Tfip11	-0.05	1.00000000	pigs
Plcl2	0.09	1.00000000	pigs
Arhgef18	-0.39	1.00000000	pigs
Sar1a	-0.07	1.00000000	pigs
Skp1	-0.02	1.00000000	pigs
Tmem97	-0.31	1.00000000	pigs
Spi1	-0.26	1.00000000	pigs
Pcif1	-0.01	1.00000000	pigs
Tsc1	0.02	1.00000000	pigs
Nipsnap3b	-0.04	1.00000000	pigs
Cdk12	-0.10	1.00000000	pigs
Fbxo34	-0.07	1.00000000	pigs
Rhoc	0.00	1.00000000	pigs
Dctn5	0.00	1.00000000	pigs
Tut7	-0.13	1.00000000	pigs
Psmc3	-0.08	1.00000000	pigs
Tmem243	-0.01	1.00000000	pigs
Sike1	0.01	1.00000000	pigs
Zfp2	0.00	1.00000000	pigs

Atg7	-0.09	1.00000000	pigs
Ghrl	-0.17	1.00000000	pigs
Zc3h7b	0.00	1.00000000	pigs
Itpa	-0.03	1.00000000	pigs
Gpank1	0.04	1.00000000	pigs
Cabin1	0.04	1.00000000	pigs
Usp20	-0.09	1.00000000	pigs
Aup1	-0.07	1.00000000	pigs
Cebpb	-0.45	1.00000000	pigs
Ruvbl1	0.02	1.00000000	pigs
Add1	-0.04	1.00000000	pigs
Acot4	-0.02	1.00000000	pigs
Usf2	-0.08	1.00000000	pigs
Kmt2b	-0.03	1.00000000	pigs
Zeb2	-0.52	1.00000000	pigs
Ddhd2	-0.02	1.00000000	pigs
Fam126a	-0.02	1.00000000	pigs
Ccdc90b	0.01	1.00000000	pigs
St3gal4	-0.04	1.00000000	pigs
Herc1	-0.05	1.00000000	pigs
Atf3	-0.03	1.00000000	pigs
Slc25a24	-0.04	1.00000000	pigs
Hmox2	-0.11	1.00000000	pigs
Dnaja2	-0.11	1.00000000	pigs
Prkab1	0.02	1.00000000	pigs
Spns1	-0.06	1.00000000	pigs
Rab43	0.02	1.00000000	pigs
Dusp28	-0.04	1.00000000	pigs
Ap3b1	-0.02	1.00000000	pigs
Il12rb1	0.03	1.00000000	pigs
C2cd5	-0.02	1.00000000	pigs
Supt6	-0.13	1.00000000	pigs
Terb1	-0.04	1.00000000	pigs
U2af1l4	-0.14	1.00000000	pigs
Fam78a	-0.05	1.00000000	pigs
Tspyl2	0.05	1.00000000	pigs
Nlrc3	0.04	1.00000000	pigs
Pdcl3	-0.02	1.00000000	pigs
Cnih4	-0.13	1.00000000	pigs
Rangap1	0.00	1.00000000	pigs
Ecd	0.01	1.00000000	pigs
Ddx42	-0.07	1.00000000	pigs
Coil	-0.05	1.00000000	pigs
Atp9b	-0.01	1.00000000	pigs
Letm1	0.00	1.00000000	pigs
Trak1	-0.05	1.00000000	pigs

Actg1	0.21	1.00000000	pigs
Arl4c	0.01	1.00000000	pigs
Lyrm1	0.00	1.00000000	pigs
Arpc2	-0.17	1.00000000	pigs
Aga	-0.04	1.00000000	pigs
Trank1	-0.05	1.00000000	pigs
Kdm8	-0.17	1.00000000	pigs
Prkag2	0.06	1.00000000	pigs
Cox6a2	-0.02	1.00000000	pigs
Fam120b	-0.01	1.00000000	pigs
Foxj3	-0.02	1.00000000	pigs
Dhx16	-0.02	1.00000000	pigs
Tmc1	-0.28	1.00000000	pigs
Ifitm6	-0.29	1.00000000	pigs
Capzb	-0.02	1.00000000	pigs
Ptgr1	0.00	1.00000000	pigs
Immt	-0.08	1.00000000	pigs
Ppp1r13b	-0.01	1.00000000	pigs
Rmnd5a	-0.05	1.00000000	pigs
Arhgef2	0.02	1.00000000	pigs
Pygl	-0.15	1.00000000	pigs
Poglut1	-0.10	1.00000000	pigs
Arf5	-0.04	1.00000000	pigs
Atmin	-0.06	1.00000000	pigs
Lss	-0.02	1.00000000	pigs
Tfam	-0.09	1.00000000	pigs
Cybc1	-0.02	1.00000000	pigs
Sf3b4	-0.07	1.00000000	pigs
Camk1d	-0.08	1.00000000	pigs
Arhgef6	-0.09	1.00000000	pigs
Nsun6	0.02	1.00000000	pigs
Clcn7	0.01	1.00000000	pigs
Pcyt1a	-0.12	1.00000000	pigs
P3h1	-0.07	1.00000000	pigs
Lemd2	-0.06	1.00000000	pigs
Psme3ip1	-0.04	1.00000000	pigs
Epm2aip1	0.00	1.00000000	pigs
Dcun1d5	-0.05	1.00000000	pigs
Ilf3	-0.09	1.00000000	pigs
Traf6	-0.12	1.00000000	pigs
Cep135	-0.04	1.00000000	pigs
Nup160	-0.04	1.00000000	pigs
Cd37	-0.06	1.00000000	pigs
Stxbp2	-0.08	1.00000000	pigs
Ttc14	0.00	1.00000000	pigs
Cd8a	-0.56	1.00000000	pigs

Rbmx	-0.09	1.00000000	pigs
Zdhhc18	0.01	1.00000000	pigs
Hsd17b10	-0.08	1.00000000	pigs
Zfp639	-0.03	1.00000000	pigs
Cnot6	-0.07	1.00000000	pigs
Hypk	-0.14	1.00000000	pigs
Xbp1	-0.05	1.00000000	pigs
Eif4g2	-0.02	1.00000000	pigs
Npm3	0.06	1.00000000	pigs
Slc41a1	0.03	1.00000000	pigs
Bcas3	0.08	1.00000000	pigs
Rbm25	-0.01	1.00000000	pigs
Atp6v1c1	-0.10	1.00000000	pigs
B230219D22Rik	-0.07	1.00000000	pigs
Pepd	-0.01	1.00000000	pigs
Focad	-0.05	1.00000000	pigs
Ccndbp1	-0.01	1.00000000	pigs
Ube2e2	0.12	1.00000000	pigs
Emc3	-0.10	1.00000000	pigs
Acat1	-0.09	1.00000000	pigs
Stub1	-0.11	1.00000000	pigs
G6pdx	0.02	1.00000000	pigs
Sil1	0.02	1.00000000	pigs
Pcmtd1	-0.03	1.00000000	pigs
Ctsf	-0.05	1.00000000	pigs
Ints13	-0.04	1.00000000	pigs
Prpf19	-0.03	1.00000000	pigs
Etv3	-0.04	1.00000000	pigs
Sel1l	-0.06	1.00000000	pigs
Ubtd2	-0.04	1.00000000	pigs
Atp6v0a2	-0.06	1.00000000	pigs
Sinhcaf	-0.06	1.00000000	pigs
Ubxn11	-0.07	1.00000000	pigs
Sae1	-0.01	1.00000000	pigs
Rnf166	-0.02	1.00000000	pigs
Cdk14	-0.62	1.00000000	pigs
Anp32e	-0.08	1.00000000	pigs
Aimp2	-0.05	1.00000000	pigs
Tnrc6b	0.07	1.00000000	pigs
Wdtc1	-0.02	1.00000000	pigs
Phactr1	-0.76	1.00000000	pigs
Bend7	-0.07	1.00000000	pigs
Taf15	-0.07	1.00000000	pigs
Itgb1	-0.43	1.00000000	pigs
Brcc3	-0.06	1.00000000	pigs
Iars	-0.02	1.00000000	pigs

Tmc6	0.00	1.00000000	pigs
Armh3	-0.04	1.00000000	pigs
Commd1b	-0.08	1.00000000	pigs
Cox5b-ps	-0.29	1.00000000	pigs
Jade2	-0.03	1.00000000	pigs
Gdap2	0.00	1.00000000	pigs
Rnaseh2b	-0.07	1.00000000	pigs
Nmd3	-0.07	1.00000000	pigs
Tfb1m	0.02	1.00000000	pigs
Tnrc18	-0.02	1.00000000	pigs
Ddx21	-0.04	1.00000000	pigs
Atg4c	-0.06	1.00000000	pigs
Slc22a15	-0.07	1.00000000	pigs
Dnajc15	-0.11	1.00000000	pigs
Galnt6	-0.01	1.00000000	pigs
Coq10b	-0.30	1.00000000	pigs
Heca	-0.01	1.00000000	pigs
Rbm8a2	-0.05	1.00000000	pigs
1810030O07Rik	-0.05	1.00000000	pigs
Appl1	-0.06	1.00000000	pigs
Cnot11	-0.05	1.00000000	pigs
Rsrc1	-0.02	1.00000000	pigs
Ubac2	-0.44	1.00000000	pigs
Srm	-0.06	1.00000000	pigs
Nup205	0.00	1.00000000	pigs
Xrn1	-0.05	1.00000000	pigs
Lrpap1	-0.09	1.00000000	pigs
Hsp90b1	0.01	1.00000000	pigs
Copb2	-0.09	1.00000000	pigs
Ccnk	-0.07	1.00000000	pigs
Telo2	-0.04	1.00000000	pigs
Abhd5	0.04	1.00000000	pigs
Lef1	0.45	1.00000000	pigs
Rad23a	-0.12	1.00000000	pigs
Uqcr10	-0.08	1.00000000	pigs
Add3	-0.29	1.00000000	pigs
Ogfod3	-0.04	1.00000000	pigs
Nlrp3	-0.18	1.00000000	pigs
Ppm1g	-0.10	1.00000000	pigs
Acadm	-0.05	1.00000000	pigs
Dtx3	-0.06	1.00000000	pigs
Ube3b	-0.05	1.00000000	pigs
Cpped1	-0.10	1.00000000	pigs
Ddx27	-0.11	1.00000000	pigs
Brk1	-0.42	1.00000000	pigs
Rgs16	0.12	1.00000000	pigs

Arf4	0.03	1.00000000	pigs
Acads	0.01	1.00000000	pigs
Smarcd1	-0.31	1.00000000	pigs
Bud31	-0.08	1.00000000	pigs
Xpo6	-0.31	1.00000000	pigs
Slc39a6	-0.10	1.00000000	pigs
Yju2	-0.05	1.00000000	pigs
H2-Ab1	-0.19	1.00000000	pigs
Tbx21	-0.06	1.00000000	pigs
Ap5z1	-0.03	1.00000000	pigs
Thumpd1	-0.06	1.00000000	pigs
Rwdd4a	0.06	1.00000000	pigs
Lin7c	-0.10	1.00000000	pigs
Capn15	-0.01	1.00000000	pigs
Rpa1	-0.01	1.00000000	pigs
Zfp106	-0.07	1.00000000	pigs
Atxn10	-0.04	1.00000000	pigs
Igfbp7	-0.08	1.00000000	pigs
Adcy7	-0.01	1.00000000	pigs
Bcat2	0.00	1.00000000	pigs
Usp1	-0.11	1.00000000	pigs
Ythdf3	-0.08	1.00000000	pigs
Fnip2	-0.06	1.00000000	pigs
Fam177a	-0.06	1.00000000	pigs
Cerk	-0.15	1.00000000	pigs
Specc1	0.04	1.00000000	pigs
Etfdh	-0.06	1.00000000	pigs
Btbd2	0.00	1.00000000	pigs
Senp5	0.00	1.00000000	pigs
Dhrs7b	-0.03	1.00000000	pigs
Ubxn2b	-0.02	1.00000000	pigs
Bid	-0.07	1.00000000	pigs
Rnf217	0.00	1.00000000	pigs
Ccnb1ip1	-0.09	1.00000000	pigs
Tmem63a	-0.05	1.00000000	pigs
Ranbp17	-0.09	1.00000000	pigs
Pdcd7	-0.07	1.00000000	pigs
Cdc34	-0.03	1.00000000	pigs
Nans	-0.06	1.00000000	pigs
Slamf6	-0.05	1.00000000	pigs
Cdkn2aip	-0.04	1.00000000	pigs
Cebpd	-0.01	1.00000000	pigs
Adh5	-0.12	1.00000000	pigs
Lrrc59	-0.04	1.00000000	pigs
Nde1	-0.05	1.00000000	pigs
Ogfod1	-0.03	1.00000000	pigs

Yipf6	-0.09	1.00000000	pigs
Abhd13	-0.08	1.00000000	pigs
Chrac1	-0.40	1.00000000	pigs
Socs1	-0.09	1.00000000	pigs
Psmc1	-0.03	1.00000000	pigs
Asns	0.01	1.00000000	pigs
Oxct1	-0.46	1.00000000	pigs
Dkc1	0.00	1.00000000	pigs
Ppt1	-0.03	1.00000000	pigs
Nfkbil1	-0.03	1.00000000	pigs
Polr1e	-0.01	1.00000000	pigs
Ikzf2	0.25	1.00000000	pigs
Galk2	-0.06	1.00000000	pigs
Agpat3	-0.03	1.00000000	pigs
Tatdn1	-0.04	1.00000000	pigs
Ublcp1	-0.08	1.00000000	pigs
Pno1	-0.03	1.00000000	pigs
Fam50a	-0.08	1.00000000	pigs
Atf1	-0.06	1.00000000	pigs
Sh3gl1	-0.01	1.00000000	pigs
Phf21a	-0.18	1.00000000	pigs
Rsad2	0.00	1.00000000	pigs
Ipo4	-0.07	1.00000000	pigs
Tmem164	-0.38	1.00000000	pigs
Jade1	-0.03	1.00000000	pigs
Clpb	0.00	1.00000000	pigs
Pygb	-0.03	1.00000000	pigs
Pgam5	-0.07	1.00000000	pigs
Sbf2	0.11	1.00000000	pigs
Lypla2	-0.07	1.00000000	pigs
Ppp2r3c	-0.04	1.00000000	pigs
Nudt5	-0.05	1.00000000	pigs
Ints2	0.05	1.00000000	pigs
Pank2	-0.04	1.00000000	pigs
Dennd1b	0.07	1.00000000	pigs
Pnrc1	0.05	1.00000000	pigs
Tshz1	0.04	1.00000000	pigs
Lamtor1	-0.05	1.00000000	pigs
Hprt	-0.03	1.00000000	pigs
Gadd45b	0.10	1.00000000	pigs
Capns1	-0.17	1.00000000	pigs
Gramd1a	0.01	1.00000000	pigs
Gtpbp8	-0.02	1.00000000	pigs
Ccdc117	-0.03	1.00000000	pigs
Cish	-0.02	1.00000000	pigs
Plekha2	-0.13	1.00000000	pigs

Pigt	-0.01	1.00000000	pigs
Ecsit	-0.01	1.00000000	pigs
Plscr3	-0.16	1.00000000	pigs
Erh	-0.34	1.00000000	pigs
Sumf1	-0.03	1.00000000	pigs
Acbd5	-0.03	1.00000000	pigs
Slc20a2	0.08	1.00000000	pigs
Ctcf	-0.09	1.00000000	pigs
Zranb2	-0.04	1.00000000	pigs
Tex14	-0.06	1.00000000	pigs
Plxnd1	-0.06	1.00000000	pigs
Serpini1	-0.07	1.00000000	pigs
Eif4ebp1	-0.08	1.00000000	pigs
Lgals3	0.01	1.00000000	pigs
Tmem38b	-0.05	1.00000000	pigs
Lsm2	-0.10	1.00000000	pigs
Ulk4	-0.26	1.00000000	pigs
Tdp2	-0.03	1.00000000	pigs
Cdk2ap2	-0.38	1.00000000	pigs
Spg21	-0.09	1.00000000	pigs
Smpd2	0.00	1.00000000	pigs
Chchd1	-0.09	1.00000000	pigs
Banp	-0.03	1.00000000	pigs
Ythdc1	-0.05	1.00000000	pigs
Hmces	-0.07	1.00000000	pigs
Rmc1	-0.08	1.00000000	pigs
Ggps1	-0.04	1.00000000	pigs
Smarcad1	0.00	1.00000000	pigs
Sptlc1	-0.06	1.00000000	pigs
Ccnh	0.00	1.00000000	pigs
Nrip1	0.04	1.00000000	pigs
Rbpms	-0.32	1.00000000	pigs
Rbl1	-0.03	1.00000000	pigs
Ube2w	-0.05	1.00000000	pigs
Tiam1	-0.06	1.00000000	pigs
Cops7a	-0.05	1.00000000	pigs
Wdr45	-0.08	1.00000000	pigs
Tra2b	0.03	1.00000000	pigs
Atg2b	-0.02	1.00000000	pigs
Dpy19l4	0.00	1.00000000	pigs
Armc10	-0.09	1.00000000	pigs
Anapc13	-0.08	1.00000000	pigs
Crbn	-0.06	1.00000000	pigs
Ilf2	-0.11	1.00000000	pigs
Alg5	-0.06	1.00000000	pigs
Golga1	0.00	1.00000000	pigs

Abhd6	-0.09	1.00000000	pigs
Wwp2	-0.03	1.00000000	pigs
Vezf1	-0.09	1.00000000	pigs
Rbm17	-0.07	1.00000000	pigs
Uqcrc2	-0.17	1.00000000	pigs
Nrd1	-0.12	1.00000000	pigs
Rab29	-0.01	1.00000000	pigs
Ciao1	-0.01	1.00000000	pigs
Kyat3	0.00	1.00000000	pigs
Ogfrl1	0.01	1.00000000	pigs
Dclre1c	-0.05	1.00000000	pigs
Ninl	-0.07	1.00000000	pigs
Ppa2	-0.17	1.00000000	pigs
Slc9a3r1	-0.39	1.00000000	pigs
Ubxn8	-0.09	1.00000000	pigs
Stk19	0.00	1.00000000	pigs
Lcorl	0.05	1.00000000	pigs
Usp45	0.02	1.00000000	pigs
Poldip2	-0.02	1.00000000	pigs
Ppp6c	-0.11	1.00000000	pigs
Ncf1	-0.38	1.00000000	pigs
Pcbp4	-0.06	1.00000000	pigs
Brms1l	-0.02	1.00000000	pigs
Fem1c	-0.06	1.00000000	pigs
Pde6d	-0.04	1.00000000	pigs
Gtf2e2	-0.12	1.00000000	pigs
Herpud2	-0.06	1.00000000	pigs
Pfdn6	-0.07	1.00000000	pigs
Slc30a1	0.02	1.00000000	pigs
Ephb6	-0.24	1.00000000	pigs
Utp3	-0.09	1.00000000	pigs
Ptpn7	-0.03	1.00000000	pigs
Spsb3	-0.14	1.00000000	pigs
Serpinb9	-0.01	1.00000000	pigs
Zc3h6	-0.08	1.00000000	pigs
Shfl	-0.08	1.00000000	pigs
Brd3	-0.01	1.00000000	pigs
Rufy1	-0.41	1.00000000	pigs
Rnf6	-0.42	1.00000000	pigs
Gtf2i	-0.06	1.00000000	pigs
Erp29	-0.05	1.00000000	pigs
Sec22a	-0.03	1.00000000	pigs
Eef2k	0.04	1.00000000	pigs
1110004F10Rik	0.01	1.00000000	pigs
Osbpl1a	-0.04	1.00000000	pigs
Itfg1	-0.10	1.00000000	pigs

Stx18	-0.07	1.00000000	pigs
Exosc5	-0.11	1.00000000	pigs
Kctd20	-0.16	1.00000000	pigs
Hmg20a	-0.04	1.00000000	pigs
Slc7a7	-0.04	1.00000000	pigs
Il10	0.09	1.00000000	pigs
Plekhf2	-0.09	1.00000000	pigs
Exoc7	0.00	1.00000000	pigs
Ubxn1	-0.11	1.00000000	pigs
Ssb	-0.04	1.00000000	pigs
Clcc1	-0.05	1.00000000	pigs
Nrg1	-0.38	1.00000000	pigs
Aqp3	-0.03	1.00000000	pigs
Dhx8	-0.13	1.00000000	pigs
Smim4	-0.24	1.00000000	pigs
Cass4	0.02	1.00000000	pigs
Klhl36	0.04	1.00000000	pigs
Clasrp	0.02	1.00000000	pigs
Entpd1	0.01	1.00000000	pigs
Fam162a	-0.04	1.00000000	pigs
Srrm2	-0.04	1.00000000	pigs
Fhl2	-0.22	1.00000000	pigs
Nxf1	-0.01	1.00000000	pigs
Synj2bp	0.02	1.00000000	pigs
Cyb561d2	-0.06	1.00000000	pigs
Paqr8	-0.03	1.00000000	pigs
Polr2h	-0.10	1.00000000	pigs
H2-Ea	-0.42	1.00000000	pigs
Psma3	-0.12	1.00000000	pigs
Cd2bp2	-0.05	1.00000000	pigs
Clcn5	-0.01	1.00000000	pigs
Nelfe	-0.10	1.00000000	pigs
Ndufab1	-0.37	1.00000000	pigs
Rnf44	-0.07	1.00000000	pigs
Asxl2	-0.01	1.00000000	pigs
Gca	-0.07	1.00000000	pigs
Ebag9	-0.07	1.00000000	pigs
Cdc37	-0.15	1.00000000	pigs
Dennd1c	-0.03	1.00000000	pigs
Smim19	-0.18	1.00000000	pigs
Irf2	-0.04	1.00000000	pigs
Ap2s1	-0.14	1.00000000	pigs
Arrdc4	0.03	1.00000000	pigs
Prkrip1	-0.01	1.00000000	pigs
Ik	-0.38	1.00000000	pigs
Fmo4	-0.04	1.00000000	pigs

Septin9	-0.09	1.00000000	pigs
Resf1	-0.07	1.00000000	pigs
Cd6	-0.39	1.00000000	pigs
Gpr174	-0.10	1.00000000	pigs
Tnfsf13b	-0.26	1.00000000	pigs
Zfp422	-0.08	1.00000000	pigs
Atp2c1	-0.09	1.00000000	pigs
Commd9	-0.06	1.00000000	pigs
Lsp1	-0.42	1.00000000	pigs
St3gal5	-0.11	1.00000000	pigs
Nfil3	-0.23	1.00000000	pigs
Plaur	-0.54	1.00000000	pigs
Imp4	-0.10	1.00000000	pigs
Tstd2	-0.04	1.00000000	pigs
Zbp1	0.00	1.00000000	pigs
Ints14	-0.06	1.00000000	pigs
Bcl9l	-0.20	1.00000000	pigs
Exoc1	-0.12	1.00000000	pigs
Stard3nl	-0.10	1.00000000	pigs
Acoxl	0.01	1.00000000	pigs
Phf1	-0.10	1.00000000	pigs
Snrpb2	-0.17	1.00000000	pigs
Hvcn1	-0.05	1.00000000	pigs
Cuedc2	-0.04	1.00000000	pigs
Apaf1	-0.07	1.00000000	pigs
Nubpl	-0.13	1.00000000	pigs
Egfl8	-0.09	1.00000000	pigs
Pxn	-0.28	1.00000000	pigs
Taf11	-0.11	1.00000000	pigs
Tnrc6c	-0.07	1.00000000	pigs
Cers2	-0.09	1.00000000	pigs
Gk5	-0.04	1.00000000	pigs
Arhgap45	-0.10	1.00000000	pigs
Ptprcap	-0.27	1.00000000	pigs
Cant1	-0.09	1.00000000	pigs
Elk3	-0.11	1.00000000	pigs
Nek1	0.01	1.00000000	pigs
Tcf25	-0.11	1.00000000	pigs
Rgs1	-0.01	1.00000000	pigs
Tmem251	-0.09	1.00000000	pigs
Ccsap	-0.24	1.00000000	pigs
Prim2	-0.02	1.00000000	pigs
Pin1	-0.08	1.00000000	pigs
L3mbtl2	-0.01	1.00000000	pigs
Aifm1	-0.03	1.00000000	pigs
Dhx40	-0.06	1.00000000	pigs

Cdkn1b	-0.10	1.00000000	pigs
Ddrgk1	-0.15	1.00000000	pigs
Tec	-0.32	1.00000000	pigs
Ciz1	0.02	1.00000000	pigs
Uba1	-0.05	1.00000000	pigs
Cst3	0.73	1.00000000	pigs
Brms1	-0.10	1.00000000	pigs
Higd2a	-0.10	1.00000000	pigs
Nupl1	-0.05	1.00000000	pigs
Tm2d1	-0.15	1.00000000	pigs
Bri3	-0.10	1.00000000	pigs
Srek1ip1	-0.15	1.00000000	pigs
Aagab	-0.05	1.00000000	pigs
Dbnl	-0.05	1.00000000	pigs
Pea15a	-0.12	1.00000000	pigs
Gcc2	-0.15	1.00000000	pigs
Lmnb1	-0.16	1.00000000	pigs
Caml	-0.15	1.00000000	pigs
Lfng	-0.10	1.00000000	pigs
Gnal	-0.03	1.00000000	pigs
Otud3	-0.04	1.00000000	pigs
Polr3d	-0.04	1.00000000	pigs
Rad17	-0.10	1.00000000	pigs
Trappc13	-0.04	1.00000000	pigs
Sppl2a	-0.09	1.00000000	pigs
Dhx30	-0.07	1.00000000	pigs
Dph1	-0.09	1.00000000	pigs
Pcmtd2	-0.03	1.00000000	pigs
Ppp1r18	-0.11	1.00000000	pigs
Dnmbp	0.00	1.00000000	pigs
Cisd2	-0.19	1.00000000	pigs
Kdm7a	-0.14	1.00000000	pigs
Cebpz	-0.11	1.00000000	pigs
Chfr	-0.13	1.00000000	pigs
Zbtb20	0.08	1.00000000	pigs
Slc39a14	-0.09	1.00000000	pigs
Hrh4	-0.26	1.00000000	pigs
Ro60	-0.16	1.00000000	pigs
Ppp1r35	-0.04	1.00000000	pigs
Bmt2	-0.07	1.00000000	pigs
Zcchc17	-0.11	1.00000000	pigs
Ube2b	-0.05	1.00000000	pigs
Snap23	-0.09	1.00000000	pigs
Ubald2	-0.09	1.00000000	pigs
Stau2	-0.06	1.00000000	pigs
AW554918	0.08	1.00000000	pigs

Tada2a	-0.11	1.00000000	pigs
Ssbp4	-0.17	1.00000000	pigs
Jkamp	-0.15	1.00000000	pigs
Ring1	-0.02	1.00000000	pigs
Vps13c	-0.23	1.00000000	pigs
Hsp90ab1	0.03	1.00000000	pigs
Gripap1	-0.05	1.00000000	pigs
Btbd10	-0.06	1.00000000	pigs
Ccdc126	-0.09	1.00000000	pigs
Nbeal1	-0.09	1.00000000	pigs
Arhgdia	-0.08	1.00000000	pigs
Ppp1ca	-0.09	1.00000000	pigs
Pinx1	-0.05	1.00000000	pigs
Capn1	-0.03	1.00000000	pigs
Slc35c2	-0.11	1.00000000	pigs
Kdm4b	-0.15	1.00000000	pigs
Hipk2	0.08	1.00000000	pigs
Dpy30	-0.15	1.00000000	pigs
Dock8	-0.21	1.00000000	pigs
Gpatch2	-0.02	1.00000000	pigs
Dgkz	-0.12	1.00000000	pigs
Sfpq	-0.03	1.00000000	pigs
Ndufa6	-0.08	1.00000000	pigs
Glipr2	-0.31	1.00000000	pigs
Tagap1	-0.41	1.00000000	pigs
Filip1l	-0.08	1.00000000	pigs
Serpinb6b	-0.39	1.00000000	pigs
Sh3bp2	-0.03	1.00000000	pigs
Aven	-0.02	1.00000000	pigs
Taf8	-0.02	1.00000000	pigs
Vps41	-0.19	1.00000000	pigs
Nudcd2	-0.05	1.00000000	pigs
Psmc4	-0.15	1.00000000	pigs
Acsl4	-0.43	1.00000000	pigs
Atf7ip2	-0.07	1.00000000	pigs
Tcof1	0.01	1.00000000	pigs
Cnih1	-0.13	1.00000000	pigs
Cdipt	-0.02	1.00000000	pigs
Pms1	0.04	1.00000000	pigs
Tle4	-0.11	1.00000000	pigs
Aip	-0.09	1.00000000	pigs
Ift80	0.03	1.00000000	pigs
Oaz2	-0.03	1.00000000	pigs
Leng8	-0.13	1.00000000	pigs
Cxcr3	-0.04	1.00000000	pigs
9130409I23Rik	-0.14	1.00000000	pigs

N4bp2	0.00	1.00000000	pigs
Bcap31	-0.15	1.00000000	pigs
Cyb5a	-0.09	1.00000000	pigs
Parp1	-0.11	1.00000000	pigs
Tgds	-0.09	1.00000000	pigs
Primpol	-0.04	1.00000000	pigs
Wdr74	-0.05	1.00000000	pigs
Phactr4	0.01	1.00000000	pigs
Pithd1	-0.05	1.00000000	pigs
Nr4a2	-0.27	1.00000000	pigs
Tcf7	-0.02	1.00000000	pigs
Rufy3	0.01	1.00000000	pigs
Khk	-0.06	1.00000000	pigs
Arl6ip1	-0.13	1.00000000	pigs
Wdr5	-0.10	1.00000000	pigs
Fbxo8	-0.10	1.00000000	pigs
Rer1	-0.43	1.00000000	pigs
Zc3h4	-0.04	1.00000000	pigs
Pdk3	-0.02	1.00000000	pigs
Ldlrad3	-0.20	1.00000000	pigs
Dek	-0.29	1.00000000	pigs
Ndufb7	-0.37	1.00000000	pigs
Ak6	-0.10	1.00000000	pigs
Bcap29	-0.43	1.00000000	pigs
Itga4	-0.27	1.00000000	pigs
Cwc27	-0.08	1.00000000	pigs
Tm9sf4	-0.05	1.00000000	pigs
Txnl4b	-0.03	1.00000000	pigs
Znhit1	-0.06	1.00000000	pigs
Get3	-0.10	1.00000000	pigs
Rab31	-0.14	1.00000000	pigs
Akap8	-0.09	1.00000000	pigs
Snrnp70	-0.10	1.00000000	pigs
Ccdc28b	-0.05	1.00000000	pigs
Commd3	-0.07	1.00000000	pigs
Acot10	-0.14	1.00000000	pigs
Gnpnat1	-0.10	1.00000000	pigs
Acot13	-0.16	1.00000000	pigs
Steep1	-0.07	1.00000000	pigs
Col19a1	-0.27	1.00000000	pigs
Ets2	0.03	1.00000000	pigs
Ndufs4	-0.10	1.00000000	pigs
Tango6	-0.03	1.00000000	pigs
Lpgat1	-0.36	1.00000000	pigs
Hacd2	-0.13	1.00000000	pigs
Ywhaq	-0.15	1.00000000	pigs

Accsl	-0.05	1.00000000	pigs
Dpm2	-0.07	1.00000000	pigs
Tmem263	-0.16	1.00000000	pigs
Scly	-0.15	1.00000000	pigs
Cactin	-0.08	1.00000000	pigs
Pnpt1	-0.04	1.00000000	pigs
Dazap1	-0.31	1.00000000	pigs
Ccnt1	-0.11	1.00000000	pigs
Sec11c	-0.16	1.00000000	pigs
Ppp3cb	-0.08	1.00000000	pigs
Orc3	-0.06	1.00000000	pigs
Ncf2	0.00	1.00000000	pigs
Cemip2	-0.31	1.00000000	pigs
Ikbke	-0.06	1.00000000	pigs
Slc35d1	-0.04	1.00000000	pigs
Ufsp2	-0.02	1.00000000	pigs
Pet100	-0.31	1.00000000	pigs
Cmtr1	-0.13	1.00000000	pigs
Rragc	-0.18	1.00000000	pigs
Nupl2	-0.11	1.00000000	pigs
Ccdc115	-0.08	1.00000000	pigs
Becn1	-0.11	1.00000000	pigs
Krtcap2	-0.42	1.00000000	pigs
Dbndd2	-0.01	1.00000000	pigs
Pole4	-0.16	1.00000000	pigs
Gzmk	-0.48	1.00000000	pigs
Ntan1	-0.34	1.00000000	pigs
Vrk3	-0.04	1.00000000	pigs
Dbt	-0.07	1.00000000	pigs
Ralbp1	-0.14	1.00000000	pigs
Nvl	-0.07	1.00000000	pigs
Snrnp200	-0.15	1.00000000	pigs
Vps26a	-0.27	1.00000000	pigs
Pi4kb	-0.03	1.00000000	pigs
Thoc6	-0.06	1.00000000	pigs
Trak2	-0.08	1.00000000	pigs
Tbpl1	-0.05	1.00000000	pigs
Wdr36	-0.09	1.00000000	pigs
Eif2b3	-0.22	1.00000000	pigs
Gnai2	-0.12	1.00000000	pigs
Slc1a2	-0.39	1.00000000	pigs
Cd226	-0.35	1.00000000	pigs
Pot1b	-0.14	1.00000000	pigs
Snrpd1	-0.08	1.00000000	pigs
Hars	-0.06	1.00000000	pigs
Khnyn	-0.03	1.00000000	pigs

Tmem115	-0.07	1.00000000	pigs
Dnajc24	-0.05	1.00000000	pigs
Pip5k1b	-0.32	1.00000000	pigs
Bcl2a1d	-0.17	1.00000000	pigs
Prpf38a	-0.13	1.00000000	pigs
G6pc3	-0.06	1.00000000	pigs
Nfkbid	-0.03	1.00000000	pigs
Dus1l	-0.08	1.00000000	pigs
Faxc	-0.06	1.00000000	pigs
Slc30a6	-0.12	1.00000000	pigs
Rad52	-0.17	1.00000000	pigs
Fam189a1	-0.07	1.00000000	pigs
Diaph2	-0.04	1.00000000	pigs
Scaper	-0.04	1.00000000	pigs
Vsir	-0.11	1.00000000	pigs
Pop4	-0.14	1.00000000	pigs
Dguok	-0.09	1.00000000	pigs
Churc1	-0.06	1.00000000	pigs
Atg4b	-0.10	1.00000000	pigs
Abr	-0.07	1.00000000	pigs
Hdgfl3	-0.28	1.00000000	pigs
Atp5g3	-0.40	1.00000000	pigs
Got2	-0.09	1.00000000	pigs
Fibp	-0.14	1.00000000	pigs
Wdr75	-0.03	1.00000000	pigs
Sgsm2	-0.07	1.00000000	pigs
Tbrg1	-0.14	1.00000000	pigs
Itm2c	-0.39	1.00000000	pigs
Ncl	-0.05	1.00000000	pigs
Ndufv1	-0.07	1.00000000	pigs
Abhd17a	-0.15	1.00000000	pigs
Hsd17b11	-0.06	1.00000000	pigs
Capg	-0.15	1.00000000	pigs
Upf3b	-0.06	1.00000000	pigs
B3gntl1	-0.10	1.00000000	pigs
Rnh1	-0.12	1.00000000	pigs
Aprt	-0.14	1.00000000	pigs
Trappc5	-0.13	1.00000000	pigs
Htatsf1	-0.09	1.00000000	pigs
2210408I21Rik	-0.34	1.00000000	pigs
Rtf1	-0.15	1.00000000	pigs
Rab8b	-0.13	1.00000000	pigs
Nrp1	-0.27	1.00000000	pigs
Gnai3	-0.11	1.00000000	pigs
Ppp1r21	-0.06	1.00000000	pigs
Las1l	-0.06	1.00000000	pigs

Tmem192	-0.02	1.00000000	pigs
Pla2g4a	-0.03	1.00000000	pigs
Parn	-0.12	1.00000000	pigs
Ing3	-0.11	1.00000000	pigs
Chd9	-0.08	1.00000000	pigs
Eaf1	-0.17	1.00000000	pigs
Nfia	-0.18	1.00000000	pigs
Tmem160	-0.39	1.00000000	pigs
Sptbn1	-0.06	1.00000000	pigs
Selenof	-0.10	1.00000000	pigs
Exosc9	-0.10	1.00000000	pigs
Pcbp1	-0.14	1.00000000	pigs
Gsr	-0.07	1.00000000	pigs
Cep170	-0.14	1.00000000	pigs
Dipk2a	0.02	1.00000000	pigs
Tapt1	-0.05	1.00000000	pigs
Emc9	-0.09	1.00000000	pigs
Gsn	-0.11	1.00000000	pigs
Spire1	-0.01	1.00000000	pigs
Trdmt1	-0.06	1.00000000	pigs
Camta2	-0.07	1.00000000	pigs
Nfrkb	-0.04	1.00000000	pigs
Kdsr	-0.14	1.00000000	pigs
Tinf2	-0.03	1.00000000	pigs
Pbrm1	-0.13	1.00000000	pigs
Tmem68	-0.14	1.00000000	pigs
Acer3	0.10	1.00000000	pigs
Pacs1	-0.10	1.00000000	pigs
Trmt6	-0.12	1.00000000	pigs
Tada1	-0.04	1.00000000	pigs
Chchd4	-0.06	1.00000000	pigs
Kiz	-0.05	1.00000000	pigs
Spg11	-0.02	1.00000000	pigs
Cxcr4	0.00	1.00000000	pigs
Uxt	-0.08	1.00000000	pigs
Fam133b	-0.20	1.00000000	pigs
Prkd2	-0.08	1.00000000	pigs
Tbca	-0.09	1.00000000	pigs
Agl	-0.02	1.00000000	pigs
Chml	-0.04	1.00000000	pigs
Idh3b	-0.08	1.00000000	pigs
Selenop	0.02	1.00000000	pigs
Hmbox1	-0.09	1.00000000	pigs
Pdia3	-0.21	1.00000000	pigs
Kars	-0.10	1.00000000	pigs
Zmym4	-0.07	1.00000000	pigs

Lmbr1	-0.09	1.00000000	pigs
Gnl3l	-0.05	1.00000000	pigs
Nbn	-0.15	1.00000000	pigs
Necap2	-0.15	1.00000000	pigs
Zfp511	-0.09	1.00000000	pigs
Plec	-0.12	1.00000000	pigs
Sap130	-0.17	1.00000000	pigs
Slf1	-0.13	1.00000000	pigs
Sec62	-0.43	1.00000000	pigs
Timm17b	-0.20	1.00000000	pigs
Akr1b3	-0.09	1.00000000	pigs
Trip11	-0.05	1.00000000	pigs
Bmpr2	-0.07	1.00000000	pigs
Limd2	-0.39	1.00000000	pigs
Ndufa10	-0.28	1.00000000	pigs
Ubxn2a	-0.16	1.00000000	pigs
Nmral1	-0.10	1.00000000	pigs
Prkaca	-0.06	1.00000000	pigs
Hras	-0.11	1.00000000	pigs
Dtx3l	-0.25	1.00000000	pigs
Sass6	-0.01	1.00000000	pigs
Carnmt1	-0.13	1.00000000	pigs
Atg13	-0.13	1.00000000	pigs
Ccdc28a	-0.14	1.00000000	pigs
Tmem134	-0.13	1.00000000	pigs
Atpif1	-0.38	1.00000000	pigs
Psmb8	0.00	1.00000000	pigs
Ndufb10	-0.13	1.00000000	pigs
Arl15	-0.35	1.00000000	pigs
Ints12	-0.07	1.00000000	pigs
Inppl1	0.02	1.00000000	pigs
Glcci1	-0.01	1.00000000	pigs
Skap2	-0.35	1.00000000	pigs
Swt1	-0.15	1.00000000	pigs
Nol9	-0.15	1.00000000	pigs
Slc35a3	-0.12	1.00000000	pigs
Stxbp5	-0.60	1.00000000	pigs
Denr	-0.11	1.00000000	pigs
Ccnd3	0.04	1.00000000	pigs
Casp6	-0.11	1.00000000	pigs
Cdk4	-0.08	1.00000000	pigs
Tmem208	-0.17	1.00000000	pigs
Rap2a	-0.13	1.00000000	pigs
Ocel1	-0.09	1.00000000	pigs
Ankle2	-0.06	1.00000000	pigs
Klhdc2	-0.16	1.00000000	pigs

Paip2	-0.27	1.00000000	pigs
Leo1	-0.16	1.00000000	pigs
Hk3	-0.11	1.00000000	pigs
3110082I17Rik	-0.17	1.00000000	pigs
Gltp	-0.07	1.00000000	pigs
Sec11a	-0.16	1.00000000	pigs
Slc39a10	-0.35	1.00000000	pigs
Cfap36	-0.09	1.00000000	pigs
Pcmt1	-0.16	1.00000000	pigs
Optn	-0.02	1.00000000	pigs
Spata5	-0.08	1.00000000	pigs
Ndufa8	-0.15	1.00000000	pigs
Dcaf11	-0.07	1.00000000	pigs
Thap11	-0.06	1.00000000	pigs
Nup133	-0.09	1.00000000	pigs
Gse1	-0.33	1.00000000	pigs
Pak2	0.01	1.00000000	pigs
Capn10	-0.08	1.00000000	pigs
Ginm1	-0.32	1.00000000	pigs
Ankrd33b	-0.39	1.00000000	pigs
Ranbp6	-0.11	1.00000000	pigs
Fbxo6	-0.20	1.00000000	pigs
Gtf2h3	-0.10	1.00000000	pigs
Cep164	-0.11	1.00000000	pigs
Lmf1	-0.08	1.00000000	pigs
Galnt1	-0.11	1.00000000	pigs
Flnb	0.06	1.00000000	pigs
2810004N23Rik	-0.17	1.00000000	pigs
Cdk19	-0.09	1.00000000	pigs
Clstn1	-0.04	1.00000000	pigs
Preb	-0.10	1.00000000	pigs
Itprid2	-0.13	1.00000000	pigs
Arfgap3	-0.11	1.00000000	pigs
Trappc3	-0.10	1.00000000	pigs
Snx8	-0.08	1.00000000	pigs
Cyhr1	-0.03	1.00000000	pigs
Clta	-0.13	1.00000000	pigs
Alcam	0.07	1.00000000	pigs
Ndufs5	-0.11	1.00000000	pigs
Folr1	-0.43	1.00000000	pigs
Trim23	-0.04	1.00000000	pigs
Tln1	-0.30	1.00000000	pigs
Gps1	-0.12	1.00000000	pigs
Cuta	-0.16	1.00000000	pigs
Urb2	-0.06	1.00000000	pigs
Akap10	-0.09	1.00000000	pigs

BC003965	-0.10	1.00000000	pigs
Pitpnm2	-0.10	1.00000000	pigs
Ldlrap1	0.01	1.00000000	pigs
Lyn	-0.26	1.00000000	pigs
Polr1a	-0.08	1.00000000	pigs
Tesk2	-0.42	1.00000000	pigs
Rhoa	-0.07	1.00000000	pigs
Atpaf2	-0.20	1.00000000	pigs
Gtf2a1	-0.13	1.00000000	pigs
Abhd17b	-0.09	1.00000000	pigs
Klhl20	-0.33	1.00000000	pigs
Klf11	-0.06	1.00000000	pigs
Ifit3	-0.52	1.00000000	pigs
Prkd3	-0.10	1.00000000	pigs
Nr4a1	-0.41	1.00000000	pigs
Smap2	-0.24	1.00000000	pigs
Ubtf	-0.17	1.00000000	pigs
Brd8	-0.16	1.00000000	pigs
Ppip5k2	-0.10	1.00000000	pigs
Tfb2m	-0.08	1.00000000	pigs
Atpaf1	-0.07	1.00000000	pigs
Ptpre	-0.40	1.00000000	pigs
Slc39a11	-0.10	1.00000000	pigs
Taldo1	-0.17	1.00000000	pigs
Akip1	-0.09	1.00000000	pigs
Prpf4b	-0.15	1.00000000	pigs
Dock5	-0.38	1.00000000	pigs
Ppil3	-0.15	1.00000000	pigs
Ankrd49	-0.15	1.00000000	pigs
Nuak2	-0.04	1.00000000	pigs
Spidr	-0.08	1.00000000	pigs
Nol4l	-0.19	1.00000000	pigs
Gorab	-0.04	1.00000000	pigs
Stambp	-0.08	1.00000000	pigs
R3hdm1	-0.11	1.00000000	pigs
Taf10	-0.17	1.00000000	pigs
Brd9	-0.20	1.00000000	pigs
Krcc1	-0.28	1.00000000	pigs
Psmg4	-0.33	1.00000000	pigs
Fndc3b	-0.67	1.00000000	pigs
Xk	-0.06	1.00000000	pigs
Bicral	-0.09	1.00000000	pigs
Prkaa1	-0.15	1.00000000	pigs
Pik3ip1	-0.08	1.00000000	pigs
Pum3	-0.08	1.00000000	pigs
Timm17a	-0.09	1.00000000	pigs

Nr2c2	-0.09	1.00000000	pigs
Gga2	-0.07	1.00000000	pigs
Etfrf1	-0.29	1.00000000	pigs
Rars	-0.06	1.00000000	pigs
Tbc1d7	-0.05	1.00000000	pigs
Gpr18	-0.14	1.00000000	pigs
Pam16	-0.11	1.00000000	pigs
Sp4	-0.17	1.00000000	pigs
Tmem147	-0.14	1.00000000	pigs
Rin3	-0.10	1.00000000	pigs
St8sia1	-0.33	1.00000000	pigs
Cmpk1	-0.19	1.00000000	pigs
Phf23	-0.12	1.00000000	pigs
Cradd	-0.16	1.00000000	pigs
Srp19	-0.18	1.00000000	pigs
Snx16	-0.05	1.00000000	pigs
BC004004	-0.13	1.00000000	pigs
Ethe1	-0.15	1.00000000	pigs
Fam13b	-0.10	1.00000000	pigs
Prkar2b	0.02	1.00000000	pigs
Rufy2	-0.19	1.00000000	pigs
Adk	-0.06	1.00000000	pigs
Arfip1	-0.14	1.00000000	pigs
Cpne3	-0.18	1.00000000	pigs
Socs5	-0.21	1.00000000	pigs
Slc9a1	-0.04	1.00000000	pigs
Shprh	-0.05	1.00000000	pigs
Smarce1	-0.16	1.00000000	pigs
Cdc25b	-0.26	1.00000000	pigs
Ndufa1	-0.34	1.00000000	pigs
Syne2	-0.08	1.00000000	pigs
Cdc16	-0.06	1.00000000	pigs
Upp1	-0.16	1.00000000	pigs
Tars	-0.11	1.00000000	pigs
Nfx1	-0.12	1.00000000	pigs
Rgs14	-0.04	1.00000000	pigs
Agpat2	-0.14	1.00000000	pigs
Ccdc85b	-0.17	1.00000000	pigs
Atp5a1	-0.14	1.00000000	pigs
Bola1	-0.15	1.00000000	pigs
Itgb7	-0.17	1.00000000	pigs
Dhx29	-0.11	1.00000000	pigs
Chmp6	-0.09	1.00000000	pigs
Necap1	-0.24	1.00000000	pigs
Ddx50	-0.18	1.00000000	pigs
Acadvl	-0.09	1.00000000	pigs

Fhit	-0.22	1.00000000	pigs
Phb	-0.17	1.00000000	pigs
Arhgap26	-0.25	1.00000000	pigs
Atxn7l3	-0.06	1.00000000	pigs
Terf2	-0.07	1.00000000	pigs
Impa1	-0.11	1.00000000	pigs
Grk2	-0.13	1.00000000	pigs
Cfdp1	-0.27	1.00000000	pigs
Ano6	-0.14	1.00000000	pigs
Sytl3	0.14	1.00000000	pigs
Fdx1	-0.12	1.00000000	pigs
Ube2q1	-0.34	1.00000000	pigs
Timm8b	-0.19	1.00000000	pigs
Cdc26	-0.11	1.00000000	pigs
Eif3f	-0.06	1.00000000	pigs
Eftud2	-0.15	1.00000000	pigs
Ptpn6	-0.06	1.00000000	pigs
Rcan3	-0.27	1.00000000	pigs
Coro1a	0.00	1.00000000	pigs
Zcchc9	-0.18	1.00000000	pigs
Polr1h	-0.26	1.00000000	pigs
Rbks	-0.16	1.00000000	pigs
Golga3	-0.09	1.00000000	pigs
Naa20	-0.18	1.00000000	pigs
Ubb	-0.07	1.00000000	pigs
Glul	-0.11	1.00000000	pigs
Gtpbp4	-0.14	1.00000000	pigs
Fam117a	-0.24	1.00000000	pigs
Acadl	-0.09	1.00000000	pigs
C1qbp	-0.05	1.00000000	pigs
Snx29	-0.19	1.00000000	pigs
Cap1	-0.10	1.00000000	pigs
Bckdhb	-0.08	1.00000000	pigs
Ttc37	-0.09	1.00000000	pigs
Lsm3	-0.17	1.00000000	pigs
Snx4	-0.13	1.00000000	pigs
Tmem242	-0.04	1.00000000	pigs
Tmed5	-0.19	1.00000000	pigs
Ap1s2	-0.38	1.00000000	pigs
Sdhaf2	-0.07	1.00000000	pigs
Tipin	-0.10	1.00000000	pigs
Serbp1	-0.18	1.00000000	pigs
Nme1	-0.01	1.00000000	pigs
Tbck	-0.14	1.00000000	pigs
Wrap73	-0.09	1.00000000	pigs
Lnpk	-0.14	1.00000000	pigs

Atr	-0.07	1.00000000	pigs
Tspo	-0.31	1.00000000	pigs
Cmtm3	-0.06	1.00000000	pigs
Adgrg5	-0.21	1.00000000	pigs
Trap1	-0.11	1.00000000	pigs
Prpf6	-0.18	1.00000000	pigs
Plscr1	-0.12	1.00000000	pigs
Ago4	-0.10	1.00000000	pigs
Slc10a1	-0.27	1.00000000	pigs
Eif3h	-0.24	1.00000000	pigs
Sri	-0.17	1.00000000	pigs
Psmd6	-0.17	1.00000000	pigs
Snrpf	-0.29	1.00000000	pigs
Tmem154	-0.41	1.00000000	pigs
Sipa1l1	-0.26	1.00000000	pigs
Ung	-0.21	1.00000000	pigs
Il10ra	-0.23	1.00000000	pigs
Abcg3	-0.10	1.00000000	pigs
Podn	-0.12	1.00000000	pigs
Dcun1d3	-0.07	1.00000000	pigs
Rex1bd	-0.32	1.00000000	pigs
Lsm7	-0.15	1.00000000	pigs
Snrpn	-0.19	1.00000000	pigs
Cib1	-0.23	1.00000000	pigs
Ino80	-0.14	1.00000000	pigs
Trp53i11	-0.17	1.00000000	pigs
Recql	-0.17	1.00000000	pigs
Dtymk	-0.13	1.00000000	pigs
Ppil1	-0.08	1.00000000	pigs
Ranbp1	-0.12	1.00000000	pigs
Nfe2l3	-0.09	1.00000000	pigs
Adpgk	-0.04	1.00000000	pigs
Tdg	-0.10	1.00000000	pigs
Dusp14	-0.14	1.00000000	pigs
Cwc25	-0.15	1.00000000	pigs
Phf14	-0.10	1.00000000	pigs
Lars	-0.23	1.00000000	pigs
Rcl1	-0.11	1.00000000	pigs
Psmb9	-0.25	1.00000000	pigs
Rnf14	-0.21	1.00000000	pigs
Ubl7	-0.10	1.00000000	pigs
Ndufb6	-0.15	1.00000000	pigs
Dnlz	-0.23	1.00000000	pigs
St3gal1	-0.10	1.00000000	pigs
Ap3m1	-0.16	1.00000000	pigs
Insyn2b	-0.31	1.00000000	pigs

Uck2	0.03	1.00000000	pigs
Dhx37	-0.04	1.00000000	pigs
Nfatc3	-0.10	1.00000000	pigs
Utp23	-0.13	1.00000000	pigs
Ap1m1	-0.08	1.00000000	pigs
Lyst	-0.35	1.00000000	pigs
Syf2	-0.20	1.00000000	pigs
Dnaja3	-0.10	1.00000000	pigs
Lcp1	-0.12	1.00000000	pigs
Scfd2	-0.02	1.00000000	pigs
Tomm7	-0.33	1.00000000	pigs
Dalrd3	-0.14	1.00000000	pigs
Wipi1	-0.11	1.00000000	pigs
Dnajb4	-0.11	1.00000000	pigs
Sgta	-0.12	1.00000000	pigs
Osgepl1	-0.12	1.00000000	pigs
Actr6	-0.12	1.00000000	pigs
Acp5	-0.29	1.00000000	pigs
Tnni2	-0.30	1.00000000	pigs
Cyc1	-0.12	1.00000000	pigs
Hnrnph2	-0.27	1.00000000	pigs
Arpc4	-0.22	1.00000000	pigs
Dpp7	-0.07	1.00000000	pigs
Zer1	-0.15	1.00000000	pigs
Parg	-0.07	1.00000000	pigs
Cdca7l	-0.14	1.00000000	pigs
Cdk5rap2	-0.05	1.00000000	pigs
Prmt3	-0.10	1.00000000	pigs
Por	-0.09	1.00000000	pigs
Pdk1	-0.27	1.00000000	pigs
Sting1	-0.11	1.00000000	pigs
Junb	-0.05	1.00000000	pigs
Tsen34	-0.13	1.00000000	pigs
Lsm6	-0.17	1.00000000	pigs
St7l	-0.10	1.00000000	pigs
Nsrp1	-0.28	1.00000000	pigs
Psmb2	-0.15	1.00000000	pigs
Retreg1	0.06	1.00000000	pigs
Fam171b	-0.02	1.00000000	pigs
Eif2b1	-0.09	1.00000000	pigs
Nck1	-0.26	1.00000000	pigs
Plpbp	-0.03	1.00000000	pigs
Sertad3	-0.27	1.00000000	pigs
Anapc5	-0.09	1.00000000	pigs
Snx17	-0.11	1.00000000	pigs
Bysl	-0.20	1.00000000	pigs

Rgs3	-0.11	1.00000000	pigs
Ndufa2	-0.35	1.00000000	pigs
Prmt7	-0.12	1.00000000	pigs
Psme2	-0.27	1.00000000	pigs
Nsd1	-0.12	1.00000000	pigs
Golim4	0.03	1.00000000	pigs
Smim7	-0.23	1.00000000	pigs
Sntb1	-0.18	1.00000000	pigs
Dera	-0.17	1.00000000	pigs
Fig4	-0.10	1.00000000	pigs
Pdap1	-0.30	1.00000000	pigs
Entr1	-0.04	1.00000000	pigs
Sash3	-0.13	1.00000000	pigs
Spcs1	-0.32	1.00000000	pigs
Septin1	-0.20	1.00000000	pigs
Acadsb	-0.09	1.00000000	pigs
Arxes2	-0.21	1.00000000	pigs
Itga6	-0.02	1.00000000	pigs
1600012H06Rik	-0.16	1.00000000	pigs
Fbl	-0.22	1.00000000	pigs
Pigb	-0.05	1.00000000	pigs
Polr2c	-0.08	1.00000000	pigs
H1f4	-0.19	1.00000000	pigs
Il12rb2	-0.12	1.00000000	pigs
Zfp512b	-0.05	1.00000000	pigs
Prdx4	-0.20	1.00000000	pigs
Grk6	-0.09	1.00000000	pigs
Hint1	-0.12	1.00000000	pigs
Slc10a7	-0.06	1.00000000	pigs
Hsd17b7	-0.13	1.00000000	pigs
Tfdp2	-0.24	1.00000000	pigs
Tmem161b	-0.15	1.00000000	pigs
Ccpg1	-0.10	1.00000000	pigs
Cyb5b	-0.16	1.00000000	pigs
Terf1	-0.18	1.00000000	pigs
Gtf2f2	-0.15	1.00000000	pigs
Auh	-0.15	1.00000000	pigs
Yipf1	-0.26	1.00000000	pigs
Rbmx2	-0.13	1.00000000	pigs
Ssr2	-0.21	1.00000000	pigs
Hgs	-0.10	1.00000000	pigs
Pqlc3	-0.17	1.00000000	pigs
Pex3	-0.10	1.00000000	pigs
Czib	-0.13	1.00000000	pigs
Chd3	-0.11	1.00000000	pigs
Yeats4	-0.17	1.00000000	pigs

Ppp1r14bl	-0.26	1.00000000	pigs
Dyrk2	-0.10	1.00000000	pigs
Ccdc107	-0.19	1.00000000	pigs
9030025P20Rik	-0.16	1.00000000	pigs
Disp1	-0.11	1.00000000	pigs
Sars	-0.22	1.00000000	pigs
Tmem71	-0.35	1.00000000	pigs
Pdcd10	-0.30	1.00000000	pigs
Pop5	-0.26	1.00000000	pigs
Ttf2	-0.13	1.00000000	pigs
Qdpr	-0.25	1.00000000	pigs
Smdt1	-0.25	1.00000000	pigs
Yif1a	-0.09	1.00000000	pigs
Noct	-0.10	1.00000000	pigs
Commd2	-0.06	1.00000000	pigs
Stk38	-0.12	1.00000000	pigs
Dyrk1b	-0.09	1.00000000	pigs
Ufm1	-0.19	1.00000000	pigs
Was	-0.21	1.00000000	pigs
Rbm47	-0.46	1.00000000	pigs
Nop10	-0.30	1.00000000	pigs
Rwdd1	-0.18	1.00000000	pigs
Poc1a	-0.04	1.00000000	pigs
L3mbtl3	-0.11	1.00000000	pigs
Calcoco2	-0.17	1.00000000	pigs
Nmur1	-0.18	1.00000000	pigs
Epc2	-0.16	1.00000000	pigs
Nucks1	-0.17	1.00000000	pigs
Stx8	-0.08	1.00000000	pigs
Ankrd39	-0.12	1.00000000	pigs
Rcor3	-0.08	1.00000000	pigs
Ssbp1	-0.18	1.00000000	pigs
Itm2b	-0.17	1.00000000	pigs
Ube4b	-0.11	1.00000000	pigs
Kras	-0.20	1.00000000	pigs
Nav3	0.15	1.00000000	pigs
Actr3	-0.14	1.00000000	pigs
Nipsnap2	-0.07	1.00000000	pigs
Tmem203	-0.24	1.00000000	pigs
Rnpepl1	-0.19	1.00000000	pigs
Snap47	-0.05	1.00000000	pigs
Vps50	-0.15	1.00000000	pigs
Slc25a39	-0.13	1.00000000	pigs
Commd8	-0.12	1.00000000	pigs
Atg14	-0.17	1.00000000	pigs
Irs2	-0.14	1.00000000	pigs

Vps25	-0.15	1.00000000	pigs
Gabbr1	-0.08	1.00000000	pigs
Hdac9	0.10	1.00000000	pigs
Pigc	-0.17	1.00000000	pigs
Tcfl5	-0.02	1.00000000	pigs
4932438A13Rik	-0.23	1.00000000	pigs
Rasal2	-0.06	1.00000000	pigs
1110059G10Rik	-0.10	1.00000000	pigs
Dnajc8	-0.21	1.00000000	pigs
Sf3b2	-0.20	1.00000000	pigs
Trmt112	-0.28	1.00000000	pigs
Pde3b	-0.01	1.00000000	pigs
Jchain	-0.09	1.00000000	pigs
Nfic	-0.24	1.00000000	pigs
Dock10	-0.08	1.00000000	pigs
Tm2d2	-0.17	1.00000000	pigs
Rnf214	-0.10	1.00000000	pigs
Septin3	-0.14	1.00000000	pigs
Uchl5	-0.17	1.00000000	pigs
Pnn	-0.28	1.00000000	pigs
Plekhj1	-0.30	1.00000000	pigs
Hp1bp3	-0.21	1.00000000	pigs
Ap4s1	-0.21	1.00000000	pigs
Btd	-0.20	1.00000000	pigs
Zfp395	-0.12	1.00000000	pigs
Nkap	-0.22	1.00000000	pigs
Tyrobp	-0.50	1.00000000	pigs
Vav3	-0.14	1.00000000	pigs
Ahcyl2	-0.25	1.00000000	pigs
Ogfr	-0.08	1.00000000	pigs
Prdx2	-0.11	1.00000000	pigs
Rb1	-0.27	1.00000000	pigs
Derl2	-0.22	1.00000000	pigs
Tmem50a	-0.30	1.00000000	pigs
Ndufaf3	-0.23	1.00000000	pigs
Washc3	-0.23	1.00000000	pigs
Snf8	-0.22	1.00000000	pigs
Star	-0.19	1.00000000	pigs
Noa1	-0.21	1.00000000	pigs
Ankrd10	-0.10	1.00000000	pigs
Wdr12	-0.22	1.00000000	pigs
Iah1	-0.26	1.00000000	pigs
Tcea1	-0.34	1.00000000	pigs
Ostc	-0.17	1.00000000	pigs
Numb	-0.29	1.00000000	pigs
Smad2	-0.13	1.00000000	pigs

Gtf3a	-0.13	1.00000000	pigs
Adi1	-0.15	1.00000000	pigs
Smim14	-0.25	1.00000000	pigs
Arhgef3	-0.18	1.00000000	pigs
Sap30	-0.24	1.00000000	pigs
Coq9	-0.08	1.00000000	pigs
Tubgcp4	-0.08	1.00000000	pigs
Eapp	-0.19	1.00000000	pigs
Afg1l	-0.18	1.00000000	pigs
Terf2ip	-0.25	1.00000000	pigs
Vps36	-0.29	1.00000000	pigs
Smad7	-0.16	1.00000000	pigs
Plcg2	-0.21	1.00000000	pigs
Dlst	-0.21	1.00000000	pigs
Cdk7	-0.17	1.00000000	pigs
Erc1	-0.10	1.00000000	pigs
Pdcd5	-0.38	1.00000000	pigs
Dapp1	-0.21	1.00000000	pigs
Gfus	-0.14	1.00000000	pigs
Fam3a	-0.06	1.00000000	pigs
Tlk1	-0.12	1.00000000	pigs
Ptprc	-0.05	1.00000000	pigs
Abraxas1	-0.23	1.00000000	pigs
Nup210	-0.09	1.00000000	pigs
Etfa	-0.14	1.00000000	pigs
Rnf125	-0.14	1.00000000	pigs
Txndc17	-0.16	1.00000000	pigs
Arpc5l	-0.31	1.00000000	pigs
Ercc8	-0.10	1.00000000	pigs
Ccdc150	-0.12	1.00000000	pigs
Nubp1	-0.24	1.00000000	pigs
Eif2s2	-0.16	1.00000000	pigs
Sntb2	-0.37	1.00000000	pigs
Fbxl14	-0.04	1.00000000	pigs
Elovl6	-0.27	1.00000000	pigs
Park7	-0.19	1.00000000	pigs
Ccdc43	-0.19	1.00000000	pigs
Fggy	-0.10	1.00000000	pigs
Gtf3c6	-0.13	1.00000000	pigs
Ascc1	-0.18	1.00000000	pigs
Zfyve26	-0.05	1.00000000	pigs
Arhgdib	0.02	1.00000000	pigs
Aph1a	-0.17	1.00000000	pigs
Klf3	-0.27	1.00000000	pigs
Nfe2l1	-0.12	1.00000000	pigs
Vps29	-0.21	1.00000000	pigs

Pik3cg	-0.13	1.00000000	pigs
Echs1	-0.19	1.00000000	pigs
Crtam	0.17	1.00000000	pigs
Lat2	-0.39	1.00000000	pigs
Ccdc12	-0.23	1.00000000	pigs
Slc35b3	-0.13	1.00000000	pigs
Tmem245	-0.15	1.00000000	pigs
Ndufa11	-0.27	1.00000000	pigs
Nup155	-0.25	1.00000000	pigs
Pms2	-0.15	1.00000000	pigs
Hibch	-0.10	1.00000000	pigs
Tmed4	-0.19	1.00000000	pigs
Pigu	-0.12	1.00000000	pigs
Cryl1	-0.19	1.00000000	pigs
Aopep	-0.14	1.00000000	pigs
Prr7	-0.21	1.00000000	pigs
Vamp5	-0.32	1.00000000	pigs
Thoc7	-0.33	1.00000000	pigs
Cox5a	-0.11	1.00000000	pigs
Orai3	-0.07	1.00000000	pigs
Phf5a	-0.34	1.00000000	pigs
Utrn	-0.11	1.00000000	pigs
Gpsm3	-0.21	1.00000000	pigs
Erap1	-0.17	1.00000000	pigs
Tle5	-0.15	1.00000000	pigs
Slc25a4	-0.20	1.00000000	pigs
Vti1a	-0.16	1.00000000	pigs
Dcaf15	-0.09	1.00000000	pigs
Suclg1	-0.16	1.00000000	pigs
Idi1	-0.19	1.00000000	pigs
Gpx4	-0.16	1.00000000	pigs
Casp7	-0.05	1.00000000	pigs
Diablo	-0.21	1.00000000	pigs
Anapc16	-0.09	1.00000000	pigs
Hlcs	-0.14	1.00000000	pigs
Srp14	-0.21	1.00000000	pigs
Imp3	-0.24	1.00000000	pigs
Ppib	-0.19	1.00000000	pigs
Fcho2	-0.13	1.00000000	pigs
Ccdc124	-0.13	1.00000000	pigs
Ndufa5	-0.16	1.00000000	pigs
Ankra2	-0.18	1.00000000	pigs
Hps3	-0.07	1.00000000	pigs
Baz2b	-0.11	1.00000000	pigs
Tmem9b	-0.28	1.00000000	pigs
Ptpdc1	-0.22	1.00000000	pigs

Tnf	-0.17	1.00000000	pigs
Unc119	-0.18	1.00000000	pigs
Fam89b	-0.10	1.00000000	pigs
Vps28	-0.22	1.00000000	pigs
Hcls1	-0.24	1.00000000	pigs
Gar1	-0.06	1.00000000	pigs
Serpinb1a	-0.10	1.00000000	pigs
Gemin2	-0.11	1.00000000	pigs
Sufu	-0.07	1.00000000	pigs
Cxcl3	-0.59	1.00000000	pigs
BC005624	-0.19	1.00000000	pigs
Rhob	-0.16	1.00000000	pigs
Dpp8	-0.15	1.00000000	pigs
Rp9	-0.23	1.00000000	pigs
2300009A05Rik	-0.15	1.00000000	pigs
Hpf1	-0.18	1.00000000	pigs
Tk2	-0.16	1.00000000	pigs
Flot1	-0.29	1.00000000	pigs
Far2	-0.30	1.00000000	pigs
Thumpd3	-0.16	1.00000000	pigs
Atp8a1	-0.06	1.00000000	pigs
Ttc13	-0.17	1.00000000	pigs
Smc1a	-0.25	1.00000000	pigs
Cdc42se1	-0.26	1.00000000	pigs
Edf1	-0.23	1.00000000	pigs
Tbc1d5	-0.07	1.00000000	pigs
Lrrc42	-0.16	1.00000000	pigs
Traf3ip3	-0.13	1.00000000	pigs
Lzic	-0.18	1.00000000	pigs
Smc2	-0.16	1.00000000	pigs
Ubd	-0.12	1.00000000	pigs
Ssna1	-0.23	1.00000000	pigs
Camkmt	-0.05	1.00000000	pigs
Cenph	-0.20	1.00000000	pigs
Polr2d	-0.18	1.00000000	pigs
Pdcd6	-0.22	1.00000000	pigs
Lonp2	-0.18	1.00000000	pigs
Pbx3	-0.22	1.00000000	pigs
B3gat3	-0.19	1.00000000	pigs
Enpp4	-0.19	1.00000000	pigs
Arrdc1	-0.13	1.00000000	pigs
Tbc1d20	-0.24	1.00000000	pigs
Dnajc1	-0.26	1.00000000	pigs
Stmn1	-0.29	1.00000000	pigs
Gps2	-0.26	1.00000000	pigs
Prf1	-0.07	1.00000000	pigs

Ndufb3	-0.21	1.00000000	pigs
Bag1	-0.19	1.00000000	pigs
S100pbp	-0.08	1.00000000	pigs
Naa60	-0.21	1.00000000	pigs
Camk2g	-0.17	1.00000000	pigs
Scand1	-0.29	1.00000000	pigs
Flad1	-0.12	1.00000000	pigs
Usp33	-0.09	1.00000000	pigs
Cux1	-0.18	1.00000000	pigs
Sdccag8	-0.16	1.00000000	pigs
2700062C07Rik	-0.27	1.00000000	pigs
Pus10	-0.12	1.00000000	pigs
Tes	-0.23	1.00000000	pigs
Romo1	-0.22	1.00000000	pigs
Txndc9	-0.22	1.00000000	pigs
Klhl7	-0.09	1.00000000	pigs
Taf13	-0.15	1.00000000	pigs
Litaf	-0.18	1.00000000	pigs
Ubn2	-0.19	1.00000000	pigs
Iqgap2	-0.17	1.00000000	pigs
Chst7	-0.09	1.00000000	pigs
Vrk1	-0.11	1.00000000	pigs
Nt5c	-0.17	1.00000000	pigs
Pgp	-0.15	1.00000000	pigs
Sc5d	-0.14	1.00000000	pigs
Bnip2	-0.16	1.00000000	pigs
Creld2	-0.22	1.00000000	pigs
Polm	-0.21	1.00000000	pigs
Pibf1	-0.10	1.00000000	pigs
Lap3	-0.17	1.00000000	pigs
Ddx24	-0.22	1.00000000	pigs
Vwa8	-0.17	1.00000000	pigs
Cpt1a	-0.11	1.00000000	pigs
Srbd1	-0.17	1.00000000	pigs
Ppp1r10	-0.19	1.00000000	pigs
Cst7	-0.30	1.00000000	pigs
Btf3	-0.06	1.00000000	pigs
Dennd6a	-0.18	1.00000000	pigs
Cdkn2aipnl	-0.25	1.00000000	pigs
Gfod1	-0.23	1.00000000	pigs
Lsm8	-0.36	1.00000000	pigs
Dusp22	-0.25	1.00000000	pigs
Aasdh	-0.23	1.00000000	pigs
Srrm1	-0.09	1.00000000	pigs
Ybx3	-0.01	1.00000000	pigs
Oxnad1	-0.08	1.00000000	pigs

Siva1	-0.16	1.00000000	pigs
Cggbp1	-0.26	1.00000000	pigs
H2-Ke6	-0.14	1.00000000	pigs
Vps8	-0.17	1.00000000	pigs
Antkmt	-0.15	1.00000000	pigs
Sertad1	-0.39	1.00000000	pigs
Pwwp3a	-0.09	1.00000000	pigs
Tsr3	-0.23	1.00000000	pigs
Eif4e3	-0.14	1.00000000	pigs
Cyba	-0.17	1.00000000	pigs
Atp13a2	-0.14	1.00000000	pigs
Cd38	-0.17	1.00000000	pigs
Surf1	-0.16	1.00000000	pigs
Smpd1	-0.18	1.00000000	pigs
Rbm48	-0.07	1.00000000	pigs
Sgms1	-0.10	1.00000000	pigs
Timm10	-0.14	1.00000000	pigs
Lrrc28	-0.10	1.00000000	pigs
Adrm1	-0.13	1.00000000	pigs
Prkx	-0.17	1.00000000	pigs
Pes1	-0.15	1.00000000	pigs
Itgb2l	-0.36	1.00000000	pigs
Atp8b4	-0.24	1.00000000	pigs
Txk	-0.10	1.00000000	pigs
1700037H04Rik	-0.19	1.00000000	pigs
Pdcl	-0.14	1.00000000	pigs
Cep104	-0.25	1.00000000	pigs
Vps35	-0.21	1.00000000	pigs
Rasal3	-0.26	1.00000000	pigs
D8Ertd738e	-0.18	1.00000000	pigs
Cmtm7	-0.32	1.00000000	pigs
Snrpe	-0.25	1.00000000	pigs
Fmc1	-0.18	1.00000000	pigs
Ndufc2	-0.29	1.00000000	pigs
Cited2	-0.15	1.00000000	pigs
Slc35b1	-0.18	1.00000000	pigs
Tex264	-0.22	1.00000000	pigs
Stk4	-0.06	1.00000000	pigs
Pcgf3	-0.08	1.00000000	pigs
Grk5	-0.16	1.00000000	pigs
Septin6	-0.06	1.00000000	pigs
Phkb	-0.17	1.00000000	pigs
Taok3	-0.23	1.00000000	pigs
Tnfsf4	-0.10	1.00000000	pigs
Ttc33	-0.16	1.00000000	pigs
Snx3	-0.25	1.00000000	pigs

Fam76b	-0.13	1.00000000	pigs
Cox20	-0.20	1.00000000	pigs
Atp6v1f	-0.28	1.00000000	pigs
Eif6	-0.27	1.00000000	pigs
Tmem260	-0.16	1.00000000	pigs
Tmem238	-0.22	1.00000000	pigs
Gadd45a	-0.06	1.00000000	pigs
Dock2	-0.09	1.00000000	pigs
Paip1	-0.20	1.00000000	pigs
Spag16	-0.31	1.00000000	pigs
Pram1	-0.14	1.00000000	pigs
Hook2	-0.13	1.00000000	pigs
Zmym1	-0.23	1.00000000	pigs
Hdgf	-0.19	1.00000000	pigs
Cast	-0.21	1.00000000	pigs
Sap18	-0.15	1.00000000	pigs
Limk2	-0.35	1.00000000	pigs
Klf6	-0.01	1.00000000	pigs
Dynlt3	-0.29	1.00000000	pigs
Ctbp1	-0.24	1.00000000	pigs
Alkbh7	-0.18	1.00000000	pigs
Nol7	-0.30	1.00000000	pigs
Xrcc5	-0.17	1.00000000	pigs
Rnaseh2c	-0.24	1.00000000	pigs
Gid8	-0.23	1.00000000	pigs
Dnajc19	-0.21	1.00000000	pigs
Hspa5	-0.17	1.00000000	pigs
Usp40	-0.12	1.00000000	pigs
Pnkd	-0.25	1.00000000	pigs
Septin7	-0.05	1.00000000	pigs
Dock11	-0.16	1.00000000	pigs
Invs	-0.16	1.00000000	pigs
Rsl24d1	-0.29	1.00000000	pigs
Ipmk	-0.28	1.00000000	pigs
Btrc	-0.10	1.00000000	pigs
Stk38l	-0.31	1.00000000	pigs
Tbc1d10c	-0.24	1.00000000	pigs
Ddx55	-0.09	1.00000000	pigs
Tmcc1	-0.29	1.00000000	pigs
Fbxo9	-0.23	1.00000000	pigs
C2cd3	-0.17	1.00000000	pigs
Ddx46	-0.28	1.00000000	pigs
Cd84	-0.27	1.00000000	pigs
Sh2d1a	-0.16	1.00000000	pigs
Fundc2	-0.25	1.00000000	pigs
Tmco1	-0.29	1.00000000	pigs

Guk1	-0.26	1.00000000	pigs
Trappc4	-0.23	1.00000000	pigs
Gnb2	-0.26	1.00000000	pigs
Nrde2	-0.15	1.00000000	pigs
Rbis	-0.22	1.00000000	pigs
Il1rap	-0.43	1.00000000	pigs
Ralb	-0.08	1.00000000	pigs
Cks2	-0.21	1.00000000	pigs
Pvrig	-0.16	1.00000000	pigs
Akap7	-0.18	1.00000000	pigs
AI413582	-0.19	1.00000000	pigs
Snrnp25	-0.20	1.00000000	pigs
Atp5j	-0.22	1.00000000	pigs
Zdhhc15	-0.16	1.00000000	pigs
Rcsd1	-0.22	1.00000000	pigs
Slc25a12	-0.20	1.00000000	pigs
Ndufb11b	-0.23	1.00000000	pigs
Pdlim7	-0.23	1.00000000	pigs
Brd7	-0.21	1.00000000	pigs
Pecam1	-0.17	1.00000000	pigs
Spag8	-0.30	1.00000000	pigs
St13	-0.16	1.00000000	pigs
Rcn2	-0.22	1.00000000	pigs
Pip5kl1	-0.13	1.00000000	pigs
Prr5	-0.23	1.00000000	pigs
Arpc3	-0.09	1.00000000	pigs
Bdh2	-0.20	1.00000000	pigs
Lck	-0.15	1.00000000	pigs
Htatip2	-0.24	1.00000000	pigs
Bin1	-0.26	1.00000000	pigs
Thumpd2	-0.16	1.00000000	pigs
Vps4b	-0.24	1.00000000	pigs
Nin	-0.30	1.00000000	pigs
Tasp1	-0.22	1.00000000	pigs
Rabgap1	-0.15	1.00000000	pigs
Nit2	-0.16	1.00000000	pigs
Ccdc25	-0.26	1.00000000	pigs
Cd79a	-0.23	1.00000000	pigs
Nsa2	-0.24	1.00000000	pigs
Spcs2	-0.26	1.00000000	pigs
Elp4	-0.17	1.00000000	pigs
Tarbp1	-0.10	1.00000000	pigs
Ssh1	-0.21	1.00000000	pigs
Ipo11	-0.18	1.00000000	pigs
Ptpn4	-0.14	1.00000000	pigs
Foxn3	-0.16	1.00000000	pigs

Nap1l1	-0.14	1.00000000	pigs
Fgd3	-0.19	1.00000000	pigs
Cgas	-0.24	1.00000000	pigs
Hemgn	-0.17	1.00000000	pigs
Eif2d	-0.23	1.00000000	pigs
Dctpp1	-0.25	1.00000000	pigs
Psma7	-0.22	1.00000000	pigs
Gspt2	-0.23	1.00000000	pigs
Zyx	-0.23	1.00000000	pigs
Uqcc3	-0.20	1.00000000	pigs
Ezr	-0.16	1.00000000	pigs
Gyg	-0.25	1.00000000	pigs
Aimp1	-0.20	1.00000000	pigs
Napg	-0.15	1.00000000	pigs
Heatr5b	-0.14	1.00000000	pigs
Nedd8	-0.24	1.00000000	pigs
Dym	-0.13	1.00000000	pigs
Jazf1	-0.08	1.00000000	pigs
Pgd	-0.09	1.00000000	pigs
Atm	-0.16	1.00000000	pigs
4921524J17Rik	-0.10	1.00000000	pigs
Klhl8	-0.59	1.00000000	pigs
Erlec1	-0.26	1.00000000	pigs
Ccdc93	-0.21	1.00000000	pigs
Eif2ak4	-0.20	1.00000000	pigs
Vdac2	-0.23	1.00000000	pigs
Psma4	-0.22	1.00000000	pigs
Btbd19	-0.21	1.00000000	pigs
Camk4	-0.08	1.00000000	pigs
Evi2	-0.16	1.00000000	pigs
Hacd3	-0.13	1.00000000	pigs
Nedd4	-0.09	1.00000000	pigs
Senp7	-0.16	1.00000000	pigs
Nsl1	-0.12	1.00000000	pigs
Tgs1	-0.16	1.00000000	pigs
Pwp1	-0.19	1.00000000	pigs
Acbd6	-0.22	1.00000000	pigs
Notch2	-0.55	1.00000000	pigs
Ndufb4	-0.30	1.00000000	pigs
Cep128	-0.08	1.00000000	pigs
Vezt	-0.13	1.00000000	pigs
Fam76a	-0.25	1.00000000	pigs
Snx10	-0.12	1.00000000	pigs
Acp1	-0.24	1.00000000	pigs
Rbbp7	-0.22	1.00000000	pigs
Rnpep	-0.25	1.00000000	pigs

Galnt11	-0.14	1.00000000	pigs
Tmbim6	-0.11	1.00000000	pigs
Abhd15	-0.14	1.00000000	pigs
Kcnq5	-0.09	1.00000000	pigs
Vcan	-0.14	1.00000000	pigs
Zfp438	-0.19	1.00000000	pigs
Secisbp2	-0.21	1.00000000	pigs
Ankmy2	-0.07	1.00000000	pigs
Gpcpd1	-0.28	1.00000000	pigs
Dpysl2	-0.15	1.00000000	pigs
Prmt2	-0.11	1.00000000	pigs
Immp1l	-0.23	1.00000000	pigs
Cyb5r4	-0.20	1.00000000	pigs
Syap1	-0.24	1.00000000	pigs
Uqcrq	-0.23	1.00000000	pigs
