# Supplementary material for: Heterogeneous viral-host response at single-cell resolution and viral adaptive mutational atlas in birds and mammals upon H5 influenza virus infection
Source: Natl Sci Rev. 2026 Jun 25;13(14):nwag380. doi: 10.1093/nsr/nwag380 (PMC13458601; doi:10.1093/nsr/nwag380)
Supplement: Supplementary file 1 [file nwag380_supplemental_files.zip › Supplementary Data 2.pdf]

Supplementary Data 2. Gene list of ISG, cytokines-, and inflammatory-related genes used in this study.

Interferon-stimulated genes	Cytokine-related genes	Inflammatory-related genes
Nfkbia	*Tgfb3*	*Has2*
Ifi44	*Il23r*	*Itga5*
Psmb9	*Fgf9*	*Ptger4*
Rsad2	*Tslp*	*Ifngr2*
Ifit3	*Il12a*	*Nfkbia*
Ifit3b	*Il12rb2*	*Myc*
Epsti1	*Ifng*	*Il7r*
Mx1	*Pdgfc*	*Edn1*
Mx2	*Tpo*	*Rela*
Apol6	*Scyl1*	*Ifnar1*
Hif1a	*Ebi3*	*Osmr*
Eif2ak2	*Il22*	*Hbegf*
Psme2	*Il22b*	*F3*
Psme2b	*Hbegf*	*Tlr2*
Stat1	*Tnfsf8*	*Ly6e*
Trim75	*Fgf23*	*Cd14*
Ifi35	*Bmp6*	*C3ar1*
B2m	*Lta*	*Tlr3*
Parp14	*Il15*	*Dcbld2*
Oas3	*Fgf15*	*Gpr183*
Ddx60	*Fgf2*	*Nampt*
Ifih1	*Tnf*	*Ebi3*
Herc6	*Il18*	*Rnf144b*
Vamp5	*Txlna*	*Lck*
Bank1	*Tnfsf9*	*P2rx4*
Lgals3bp	*Igf1*	*Sri*
Dhx58	*Spp1*	*Tnfrsf9*
Irf9	*Cd70*	*Acvr1b*
Pml	*Vegfc*	*Best1*
Stat3	*Ccl20*	*Csf3*
Oas2	*Il16*	*Sema4d*
Isg15	*Il2*	*Tacr1*
Adar	*Ltb*	*Adgre1*
Parp9	*Tnfsf14*	*Nlrp3*
Samhd1	*Fgf13*	*Cd70*
Nmi	*Scyl2*	*Slc31a2*

Trim25	*Il21*	*Ccl20*
Znfx1	*Tnfsf11*	*Atp2a2*
Ptpn1	*Cd40lg*	*Klf6*
Irf7	*Tgfb2*	*Tnfsf9*
Xaf1	*Il13*	*Met*
Ube2l6	*Gdf7*	*Ccr7*
	Cxcl11	*Gpc3*
	Xcl1	*Itgb8*
	Clcf1	*Il15*
	Cxcl5	*Stab1*
	Bmp3	*P2rx7*
	Il23a	*Bdkrb1*
	Fasl	*Il18*
	Eda	*Rgs16*
	Ppbp	*Kif1b*
	Ccl12	*Hif1a*
	Ccl28	*Slc31a1*
	Gdf9	*Mefv*
	Tgfa	*Lta*
	Il12rb1	*Scarf1*
	Il20	*Axl*
	Tsc1	*Psen1*
	Ifnb1	*Ahr*
	Mif	*Tpbg*
	Ccl7	*Rtp4*
	Tnfsf4	*Slc11a2*
	Vegfb	*Selenos*
	Gdf11	*Cdkn1a*
	Csf1	*Il2rb*
	Inhbe	*Ptger2*
	Il27	*Kcna3*
	Fgf18	*Fpr1*
	Il4	*Hrh1*
	Cxcl9	*Clec5a*
	Hgf	*Npffr2*
	Vegfa	*Chst2*
	Ifna15	*Gnai3*
	Ifna14	*Csf1*
	Ifna9	*Gabbr1*
	Ifna12	*Ccl7*
	Ifna13	*Mxd1*
	Ifna16	*Icam4*
	Ifna2	*Pde4b*
	Ifnab	*Vip*

Ifna7	*Slc4a4*
Ifna11	*Cmklr1*
Ifna6	*Tapbp*
Ifna5	*Eif2ak2*
Ifna4	*Il10ra*
Ifna1	*Adm*
Inhba	*Pcdh7*
Ifna15	*Gch1*
Ifna14	*Bst2*
Ifna9	*Cx3cl1*
Ifna12	*Btg2*
Ifna13	*Adora2b*
Ifna16	*Cxcl5*
Ifna2	*Raf1*
Ifnab	*Icam1*
Ifna7	*Csf3r*
Ifna11	*Tlr1*
Ifna6	*Cxcl11*
Ifna5	*Pdpn*
Ifna4	*Cd55b*
Ifna1	*Cd55*
Il24	*Tnfsf10*
Il17c	*Adrm1*
Il10	*Gm9774*
Bmp4	*Irf1*
Il6	*Ccl22*
Il5	*Tacr3*
Pdgfb	*Cxcl10*
Gdf15	*Slc7a1*
Scyl3	*Cybb*
Tnfsf13b	*Ptgir*
Ccl11	*Nfkb1*
Xcl1	*Il15ra*
Tnfsf10	*Ptafr*
Csf2	*Mmp14*
Gdf10	*Slc7a2*
Cxcl10	*Il12b*
Bmp7	*Aqp9*
Il18r1	*Nod2*
Ccl3	*Pvr*
Ccl4	*Ndp*
Fgf7	*Il6*
Pdgfa	*Sgms2*
Ccl9	*Irak2*

Ccl6	*Il10*
Lif	*Plaur*
Fgf11	*Pik3r5*
Osm	*Olr1*
Il1a	*Atp2b1*
Il1rn	*Lcp2*
Il1b	*Cd69*
Cxcl16	*Sphk1*
Tnfsf13	*Atp2c1*
Pdgfd	*Ereg*
Ccl3	*Sele*
Ccl19	*Sell*
Fgf22	*Kcnmb2*
Ccl4	*Ros1*
Amh	*Cxcl9*
Gh	*Ccl17*
Tnfsf12	*Timp1*
	Tnfrsf1b
	Ccrl2
	Msr1
	Inhba
	Nmur1
	Prok2
	Abca1
	Slc28a2
	Slc28a2b
	Cxcr6
	Il1a
	Oprk1
	Lyn
	Ccl24
	Cd82
	Osm
	Lif
	Abi1
	Acvr2a
	Il18rap
	Kcnj2
	Il4ra
	Emp3
	P2ry2
	Tnfsf15
	Rgs1
	Ptpre

Cd40
Lpar1
Cd48
Calcrl
Irf7
C5ar1
Rasgrp1
Ripk2
Il18r1
Itgb3
Il1r1
Ifitm6
Slamf1
Serpine1
Aplnr
Scn1b
Nmi
Icosl
Slc1a2
Gp1ba
Fzd5
Ffar2
Rhog
Gna15
Tnfaip6
Hpn
Il1b
Ldlr
