# Supplementary material for: Heterogeneous viral-host response at single-cell resolution and viral adaptive mutational atlas in birds and mammals upon H5 influenza virus infection
Source: Natl Sci Rev. 2026 Jun 25;13(14):nwag380. doi: 10.1093/nsr/nwag380 (PMC13458601; doi:10.1093/nsr/nwag380)
Supplement: Supplementary file 1 [file nwag380_supplemental_files.zip › Supplementary Data 3.pdf]

Supplementary Data 3. The MiP-seq probe sequences of host genes in chicken, duck, pigeon, mice, and pig, viral genes of eight segments, viral genes with specific adaptive mutations used in this study.

Species	Gene	Probe	Pad sequences	Amp sequences
Mouse	*Ifit1*	**Ifit1-1**	CACCATCAGCATTATGAGATCGTCATCGTATGATCTTCCGATCTAATCCATGACCTGGT	GCTGAAGCAGATTGTTAGATCGGAAGAT
		**Ifit1-2**	TGCTCAGCTGCTCATGAGATCGTCATCGTATGATCTTCCGATCTAAGCCAGGCTTCTCT	AGTTGCCCCAGGTGTTAGATCGGAAGAT
		**Ifit1-3**	CAGGCCATGGCTTATGAGATCGTCATCGTATGATCTTCCGATCTAAAGCCTTCGCAAAG	GGCTCCACTTTCACTTAGATCGGAAGAT
		**Ifit1-4**	TGTGTGCTTCTGCATGAGATCGTCATCGTATGATCTTCCGATCTAAAGGGCTTCTTCAA	AAGATGTGCTGCTCTTAGATCGGAAGAT
		**Ifit1-5**	AGGCCCTGTTTAGATGAGATCGTCATCGTATGATCTTCCGATCTAAGGTGATGCCTGCA	AGTAGCCAGAGGATTTAGATCGGAAGAT
		**Ifit1-6**	CTCAGTTGGGAGAATGAGATCGTCATCGTATGATCTTCCGATCTAAGTTTCGGGATGTC	CTTCTGGGCTGCCATTAGATCGGAAGAT
		**Ifit1-7**	TGTGGGCCTCAGTATGAGATCGTCATCGTATGATCTTCCGATCTAAAGGCCATCTCAAA	AGCCATGCAAACAATTAGATCGGAAGAT
		**Ifit1-8**	TCAGTGCCTTCTGATGAGATCGTCATCGTATGATCTTCCGATCTAAAGGGTCTTGTTGT	CTATGTGAGCCACCTTAGATCGGAAGAT
		**Ifit1-9**	AGTGCCTTCTCATATGAGATCGTCATCGTATGATCTTCCGATCTAACCCAGTGAGCCTC	GCAGGGTTCATTTGTTAGATCGGAAGAT
		**Ifit1-10**	CTTCCATGTGAAGATGAGATCGTCATCGTATGATCTTCCGATCTAATTTCAAATAGCAG	AGGTATGTCATTAATTAGATCGGAAGAT
		**Ifit1-11**	TGCTCTGAGATTCATGAGATCGTCATCGTATGATCTTCCGATCTAAAAGGAACTGGACC	GGGTTCTTGATGTGTTAGATCGGAAGAT
		**Ifit1-12**	TCACGTAGGCCAGATGAGATCGTCATCGTATGATCTTCCGATCTAACCTTTCAGGTGCC	CTTCGTCCTGCTGCTTAGATCGGAAGAT
	Ifit2	**Ifit2-1**	ATTGCTCTCCAGTATGAGATCGTCATCGTACAGATAGGACTAGTCATTAGCTGTCGCAG	GGTGAAATGGCATATGACTAGTCCTATC
		**Ifit2-2**	AGACCTCTGCAGTATGAGATCGTCATCGTACAGATAGGACTAGTCATGCTGCCTCATTT	CCTAAGCATTGCACTGACTAGTCCTATC
		**Ifit2-3**	TAGTTTCCCCAGGATGAGATCGTCATCGTACAGATAGGACTAGTCAGTAGACCCAAGCA	TGGCCCATGTGATTTGACTAGTCCTATC
		**Ifit2-4**	GTCAAGAGCAGGAATGAGATCGTCATCGTACAGATAGGACTAGTCAACCCCTCCTCACA	CTTTAATCGGGCCGTGACTAGTCCTATC
		**Ifit2-5**	CACACCTTCACTCATGAGATCGTCATCGTACAGATAGGACTAGTCAAGCCTTCTGGAAA	GGGTCCTTTTCCACTGACTAGTCCTATC
		**Ifit2-6**	TGGCCCATCCAGAATGAGATCGTCATCGTACAGATAGGACTAGTCACGGTAGTTCGCAA	GCCAGTCATCCAGTTGACTAGTCCTATC
		**Ifit2-7**	CAAGCTTCAGTGCATGAGATCGTCATCGTACAGATAGGACTAGTCATTATGTACCGCGT	CCATTGCTTGGTTATGACTAGTCCTATC
		**Ifit2-8**	TCCCCTCTCAGAGATGAGATCGTCATCGTACAGATAGGACTAGTCATTGCAGAGTCCAC	TGAAGGTACCTGGATGACTAGTCCTATC
		**Ifit2-9**	CAAGGACTCATCTATGAGATCGTCATCGTACAGATAGGACTAGTCACCTCAAACTCATC	GTTAAACACCCTGATGACTAGTCCTATC
		**Ifit2-10**	CTCACTGTTCTGAATGAGATCGTCATCGTACAGATAGGACTAGTCATGGTGGCTTTAAA	CAGTATGTTGCACTTGACTAGTCCTATC
		**Ifit2-11**	GATGAAGCCCTCAATGAGATCGTCATCGTACAGATAGGACTAGTCAGATGCTGTTGCTG	TTCTACTTGGTCACTGACTAGTCCTATC
		**Ifit2-12**	ATCCAGATAAGCCATGAGATCGTCATCGTACAGATAGGACTAGTCACCTGTTTCACTTT	AAACTTCTTGCAGTTGACTAGTCCTATC
	Ifit3	**Ifit3-1**	TCCCGGTTGACCTTGATCTTCCGATCTAAACAGATAGGACTAGTCACGCTTCCAGAGAT	AGCTGTGGAAGGAATGACTAGTCCTATC
		**Ifit3-2**	CAGTCGACGAATTTGATCTTCCGATCTAAACAGATAGGACTAGTCATTCCCCAGGTGAC	GATCCAGGCGTAGATGACTAGTCCTATC

		**Ifit3-3**	AAGCCTGAGCTTCTGATCTTCCGATCTAAACAGATAGGACTAGTCAACCTTGTCAACGT	GGCAAACTTGTCTGTGACTAGTCCTATC
		**Ifit3-4**	TGGGTCCTTTGGCTGATCTTCCGATCTAAACAGATAGGACTAGTCACAGAGGAGCACTC	GGCGATGGCCATCGTGACTAGTCCTATC
		**Ifit3-5**	TGCTTCTCAGGTTTGATCTTCCGATCTAAACAGATAGGACTAGTCAATCCACGGAGAAC	GCCTGCTTCAGAGGTGACTAGTCCTATC
		**Ifit3-6**	AGGGCCAGGAGAATGATCTTCCGATCTAAACAGATAGGACTAGTCACCTCAGCAGTTTC	GCTTCTTCTCCCAATGACTAGTCCTATC
		**Ifit3-7**	AGCGCTCATAGAGTGATCTTCCGATCTAAACAGATAGGACTAGTCATGGAGGTTGCAAT	TCCTGTGGTACTCGTGACTAGTCCTATC
	Isg15	**Isg15-1**	CTGGGCAATCTGCTATGTGACCGGGTACAATATGTGACCGGGTACAGCACACCAATCTT	CTGCTGGAAAGCCCTGTACCCGGTCACA
		**Isg15-2**	TTTGGTGGGCCAGTATGTGACCGGGTACAATATGTGACCGGGTACATGGAGCACTGCAG	GGGTAAGACCGTCGTGTACCCGGTCACA
		**Isg15-3**	CTCAGAGGCTCGCTATGTGACCGGGTACAATATGTGACCGGGTACACCTCACCAGGATG	TGGCCCCTTTCGTATGTACCCGGTCACA
		**Isg15-4**	CTCCATGGGCCTTTATGTGACCGGGTACAATATGTGACCGGGTACAGCAGCTCCTTGTC	GCCATACTCCCCCTTGTACCCGGTCACA
		**Isg15-5**	TCACTGTGCACTGTATGTGACCGGGTACAATATGTGACCGGGTACACGCAAATGCTTGA	CCCCACCCCTCAGCTGTACCCGGTCACA
		**Isg15-6**	TAGTCACGGACACTATGTGACCGGGTACAATATGTGACCGGGTACAACCGTCATGGAGT	TCTTCAGTTCTGAGTGTACCCGGTCACA
		**Isg15-7**	TTTAGGTCCCAGGTATGTGACCGGGTACAATATGTGACCGGGTACACAGCATCTTCACC	AAATCGTTACCCCGTGTACCCGGTCACA
		**Isg15-8**	TCAGAGGCTCGCTTATGTGACCGGGTACAATATGTGACCGGGTACACTCACCAGGATGC	GGCCCCTTTCGTTGTGTACCCGGTCACA
		**Isg15-9**	GGACCCAGACCCATATGTGACCGGGTACAATATGTGACCGGGTACACACTGTGCTGCTG	TGTACCACTAGCAATGTACCCGGTCACA
		**Isg15-10**	AGTCTGCGTCAGATATGTGACCGGGTACAATATGTGACCGGGTACATAAGCGTGTCTAC	GGACACCTTCTTCATGTACCCGGTCACA
	Rsad2	**Rsad2-1**	TCAGAAAGCGCATGTCTATTAGTGGAGCCAGTCTATTAGTGGAGCCCCACCGGTACAGT	AAGGGTCCTTCCGCGGCTCCACTAATAG
		**Rsad2-2**	CAGCTAGAGCAGTGTCTATTAGTGGAGCCAGTCTATTAGTGGAGCCCTCAGCAGCCGAG	GCTGCTGGAACAGCGGCTCCACTAATAG
		**Rsad2-3**	GAGGGAACCCAGCGTCTATTAGTGGAGCCAGTCTATTAGTGGAGCCCCAGGCCACTCCA	GCAGAACAGGATGCGGCTCCACTAATAG
		**Rsad2-4**	CACCTCCTCAGCTGTCTATTAGTGGAGCCAGTCTATTAGTGGAGCCGTAATCCCTGCAC	TTGAAAGCCACCTAGGCTCCACTAATAG
		**Rsad2-5**	AGCCACCCTAATGGTCTATTAGTGGAGCCAGTCTATTAGTGGAGCCCTTCCCGGGATCT	ACCTGTGGCTGTTGGGCTCCACTAATAG
		**Rsad2-6**	AGTGCTGTTCCCAGTCTATTAGTGGAGCCAGTCTATTAGTGGAGCCGGGTTGTGGGCTG	GTTGACACTCACGCGGCTCCACTAATAG
		**Rsad2-7**	AGCAGAAGCCACAGTCTATTAGTGGAGCCAGTCTATTAGTGGAGCCTTGGCTGTGTGGA	GCACGAAGGATGTGGGCTCCACTAATAG
		**Rsad2-8**	TGGCCTCCTCCAGGTCTATTAGTGGAGCCAGTCTATTAGTGGAGCCAGCAGTCCTCGCT	CCTGTTTGAGCAGTGGCTCCACTAATAG
		**Rsad2-9**	GCCAACATCCAGAGTCTATTAGTGGAGCCAGTCTATTAGTGGAGCCTTGCTTCTTCCAC	TCCACTGAACTTTTGGCTCCACTAATAG
		**Rsad2-10**	ACATACTTTCCGCGTCTATTAGTGGAGCCAGTCTATTAGTGGAGCCAGCTTTACTCCAC	TCCAGCTTCAGGTGGGCTCCACTAATAG
		**Rsad2-11**	AGGCACTGGAAAAGTCTATTAGTGGAGCCAGTCTATTAGTGGAGCCACCCTCAATTAGG	TCTCCTGAGTTCTGGGCTCCACTAATAG
		**Rsad2-12**	AGAAATCTTTCTGGTCTATTAGTGGAGCCAGTCTATTAGTGGAGCCTTCATTGCTTATA	AATGTTTCAAATTGGGCTCCACTAATAG
	Ddx60	**Ddx60-1**	CAGCCCGACCAGAATAACGATCCCTCACAAATAACGATCCCTCACATGCCCTCGTCTTC	CACCCAACAGGTCATGTGAGGGATCGTT
		**Ddx60-2**	AATCAGGGCTCAGATAACGATCCCTCACAAATAACGATCCCTCACAGAGTGCTCATGGT	CCAGCATCCTCCCATGTGAGGGATCGTT
		**Ddx60-3**	TCGCCATCAATCAATAACGATCCCTCACAAATAACGATCCCTCACAGATGAGCAAGGAG	TCACACGCACACGATGTGAGGGATCGTT
		**Ddx60-4**	TTCTGCCAACCTCATAACGATCCCTCACAAATAACGATCCCTCACAAGCCAATGCCCCT	GGAACTGTGGTGGATGTGAGGGATCGTT
		**Ddx60-5**	TCGCTCTCTTTCAATAACGATCCCTCACAAATAACGATCCCTCACAAACCACGCCTTCA	GGTGCAACATACAGTGTGAGGGATCGTT

		**Ddx60-6**	AGCCAGCAACATGATAACGATCCCTCACAAATAACGATCCCTCACACATCCGCCTTGGA	GCCATCCTCTAGGATGTGAGGGATCGTT
		**Ddx60-7**	CAAGCAAGCCTGTATAACGATCCCTCACAAATAACGATCCCTCACACCTTCCAGGCTTT	AGCCTGGCAATGTATGTGAGGGATCGTT
		**Ddx60-8**	TAGCGCTCAACCAATAACGATCCCTCACAAATAACGATCCCTCACAAACATCCAACAGG	CCTCCTTTGCTAAATGTGAGGGATCGTT
		**Ddx60-9**	CGTTCAGGCCTTCATAACGATCCCTCACAAATAACGATCCCTCACATGTGTCTGCAGGT	CAAAGTTGAAAAGTTGTGAGGGATCGTT
		**Ddx60-10**	AGAGCTTTGCATAATAACGATCCCTCACAAATAACGATCCCTCACATCTGCATTCCATG	CTCCTTGAAGAACTTGTGAGGGATCGTT
		**Ddx60-11**	TGTCCTGAGAAGAATAACGATCCCTCACAAATAACGATCCCTCACAGATGTCAGATGTC	GCATAAAGACGAACTGTGAGGGATCGTT
		**Ddx60-12**	TGTGTATTTTTTATAACGATCCCTCACAAATAACGATCCCTCACATTTTTAAAAAACC	TAAATGTGACATTTTGTGAGGGATCGTT
	Znfx1	**Znfx1-1**	AAACAGGGACACCTACCCGAGATAGAGTCATACCCGAGATAGAGTCTTACCAGCCGTTC	GGGGATGTTCACTAGACTCTATCTCGGG
		**Znfx1-2**	TAGCCAGCTCCTGTACCCGAGATAGAGTCATACCCGAGATAGAGTCGCCAACAGCATTT	GGTCCAGTCTCATCGACTCTATCTCGGG
		**Znfx1-3**	TGTTCCCAATGCATACCCGAGATAGAGTCATACCCGAGATAGAGTCGCAAGCATCTGCA	ACAAGGGCACCTTCGACTCTATCTCGGG
		**Znfx1-4**	TGACATGTTCGCTTACCCGAGATAGAGTCATACCCGAGATAGAGTCGACACAGCGGTTC	TTGCACTGGCTGTCGACTCTATCTCGGG
		**Znfx1-5**	CACTGGTAGTGCTTACCCGAGATAGAGTCATACCCGAGATAGAGTCGCAGAGTTTGGTG	TTGCAGGGCTCTGTGACTCTATCTCGGG
		**Znfx1-6**	TCAAAGATGTGGCTACCCGAGATAGAGTCATACCCGAGATAGAGTCCAAGGCTTGCACT	TTCATGAAGCGGTGGACTCTATCTCGGG
		**Znfx1-7**	AGATGCCCAAGCCTACCCGAGATAGAGTCATACCCGAGATAGAGTCCGTTCCTCATCTG	TGACAATCTGCACAGACTCTATCTCGGG
		**Znfx1-8**	CACAAAGAAAAGGTACCCGAGATAGAGTCATACCCGAGATAGAGTCGAAAGTTGTGCTC	GATCTCCTGCTCACGACTCTATCTCGGG
		**Znfx1-9**	ACGTGTGCACACATACCCGAGATAGAGTCATACCCGAGATAGAGTCATGGATGGCAGGC	GTGGGAGGAGTCAAGACTCTATCTCGGG
		**Znfx1-10**	ATGAAGTTGTTGGTACCCGAGATAGAGTCATACCCGAGATAGAGTCCTCCTCAAAGTTC	ATCATCCTGTGGAAGACTCTATCTCGGG
		**Znfx1-11**	TACTTGGCAGCACTACCCGAGATAGAGTCATACCCGAGATAGAGTCAAGGATCTGGCGG	GGCTCCACTTGCTCGACTCTATCTCGGG
		**Znfx1-12**	ATCTGCAGCCACATACCCGAGATAGAGTCATACCCGAGATAGAGTCGTCGGCTTGGTAC	ATCCTCCGGCGAGAGACTCTATCTCGGG
	Dhx58	**Dhx58-1**	CAATCAGCGAGAATATGTGACCGGGTACAAGTCTATTAGTGGAGCCCACTCGTCCACCA	TGTGGGTGTGGTGTGGCTCCACTAATAG
		**Dhx58-2**	CTGCTAGCTGGCCTATGTGACCGGGTACAAGTCTATTAGTGGAGCCTCAGGACCCCGAG	CCAACTTCGGGTTGGGCTCCACTAATAG
		**Dhx58-3**	TGCCCTCTGTAGCTATGTGACCGGGTACAAGTCTATTAGTGGAGCCTTCATCTCCCGAC	TGGTTAGCTCCCGGGGCTCCACTAATAG
		**Dhx58-4**	TGCCACACTTGTGTATGTGACCGGGTACAAGTCTATTAGTGGAGCCCCAGCCCCTCCTC	GCACTGAGCGATAAGGCTCCACTAATAG
		**Dhx58-5**	AGCCTTCTCCATCTATGTGACCGGGTACAAGTCTATTAGTGGAGCCGTACAGCAGCCAC	AGGGTCCATCTTCAGGCTCCACTAATAG
		**Dhx58-6**	ATGGAGAAAGGCATATGTGACCGGGTACAAGTCTATTAGTGGAGCCATCGAAGACTGGT	CAGTCCTGCAGGAAGGCTCCACTAATAG
		**Dhx58-7**	CAGTCTGACGGGTTATGTGACCGGGTACAAGTCTATTAGTGGAGCCAGGAGGGAGGAAG	GCCGAAGCCAGAGGGGCTCCACTAATAG
		**Dhx58-8**	ACAACTCTGCCGTTATGTGACCGGGTACAAGTCTATTAGTGGAGCCAGTGCCAACTGTA	CCTCAGAGCTGTTCGGCTCCACTAATAG
		**Dhx58-9**	AGTATGCTTCCGATATGTGACCGGGTACAAGTCTATTAGTGGAGCCAGGTGTTTCCAGT	TGGATCTTCCCTCCGGCTCCACTAATAG
		**Dhx58-10**	GTCTCCAGTCCTTTATGTGACCGGGTACAAGTCTATTAGTGGAGCCATGGTTCCTCCAG	AGTTACTGCACCTCGGCTCCACTAATAG
		**Dhx58-11**	CACGGCCACCATATATGTGACCGGGTACAAGTCTATTAGTGGAGCCCACTCCCGTAGCC	CACTTTCCGCAGGAGGCTCCACTAATAG
		**Dhx58-12**	GATCACTTCCTGCTATGTGACCGGGTACAAGTCTATTAGTGGAGCCCCCTGAACTCCTG	GCTCAGGATACCAAGGCTCCACTAATAG
	Ifi30	**Ifi30-1**	TGGCTTCTCCGTTTATGTGACCGGGTACAAATAACGATCCCTCACAAGGAGCAGATGTC	GGAGTCGGCAATGCTGTGAGGGATCGTT

		**Ifi30-2**	ACCTCAGGAGCATTATGTGACCGGGTACAAATAACGATCCCTCACAACTCTCTGGTGAC	GCGCACTCCATGAATGTGAGGGATCGTT
		**Ifi30-3**	TGCCCCGTTTTCCTATGTGACCGGGTACAAATAACGATCCCTCACATGCATGAGCTGTG	GCTGGGCATTCTCTTGTGAGGGATCGTT
		**Ifi30-4**	CTCGAGTGGGAACTATGTGACCGGGTACAAATAACGATCCCTCACACTGCTCTGGGAAC	AAGGGACGCAGTGCTGTGAGGGATCGTT
		**Ifi30-5**	TGAAGAAGCTGGGTATGTGACCGGGTACAAATAACGATCCCTCACACAGGTTGGCAATT	GGCAGCAGCTCTTCTGTGAGGGATCGTT
		**Ifi30-6**	AATTTAAATTTAGTATGTGACCGGGTACAAATAACGATCCCTCACAGATTTATTCTATG	ACAGGAGTATAGAATGTGAGGGATCGTT
		**Ifi30-7**	TTTGTGGGACATGTATGTGACCGGGTACAAATAACGATCCCTCACACTGGAGTCTGGGC	TGTCTCTGAGAAGATGTGAGGGATCGTT
		**Ifi30-8**	ACTTATAGCAGACTATGTGACCGGGTACAAATAACGATCCCTCACAAGGTTATGCCTTT	CTGGGACGAGTTTCTGTGAGGGATCGTT
		**Ifi30-9**	GCTGGGGTCCTTCTATGTGACCGGGTACAAATAACGATCCCTCACATGCTCAGGAGCTC	CAGCTGACAGACTTTGTGAGGGATCGTT
		**Ifi30-10**	GGGGTGGGTGTAGTATGTGACCGGGTACAAATAACGATCCCTCACAGGCACATACTCGT	TGACCAGCACCCACTGTGAGGGATCGTT
		**Ifi30-11**	GCTTATCCAGCAGTATGTGACCGGGTACAAATAACGATCCCTCACAGCCTCCTTTTCCA	TGGTTAGGAACGCATGTGAGGGATCGTT
		**Ifi30-12**	TCTCCTCCATACATATGTGACCGGGTACAAATAACGATCCCTCACATCCATATCATCCA	GTCCCAGTTTCTTGTGTGAGGGATCGTT
	Ifi35	**Ifi35-1**	TAAGTGCTGGGCCTATGTGACCGGGTACAATACCCGAGATAGAGTCGGCCAATCTGGCA	TGGGACTCTGAACAGACTCTATCTCGGG
		**Ifi35-2**	GTGGTTATCTGGCTATGTGACCGGGTACAATACCCGAGATAGAGTCTAACGAGCACCCT	AGCAGGAAAACCAGGACTCTATCTCGGG
		**Ifi35-3**	TTGGCCTTGGAATTATGTGACCGGGTACAATACCCGAGATAGAGTCTGCCCTGCTTAGT	CTTGGGCACTTGCGGACTCTATCTCGGG
		**Ifi35-4**	CAGGAGGACCTGCTATGTGACCGGGTACAATACCCGAGATAGAGTCAGGGAGAGACCCT	CTCACCACTCACAAGACTCTATCTCGGG
		**Ifi35-5**	AGGCAGAGGGCAGTATGTGACCGGGTACAATACCCGAGATAGAGTCGAGCAGAACCTTC	CTCAAAGGTGACCTGACTCTATCTCGGG
		**Ifi35-6**	ATCCCCACCTCCATATGTGACCGGGTACAATACCCGAGATAGAGTCCCCGGGTCTCTAC	CCCTTGCAGCATCAGACTCTATCTCGGG
		**Ifi35-7**	CAACTGTCAGGGCTATGTGACCGGGTACAATACCCGAGATAGAGTCTGCCCTGAAGGCA	TAGCCAGGCCCTGGGACTCTATCTCGGG
		**Ifi35-8**	AGACTGTAGAGGATATGTGACCGGGTACAATACCCGAGATAGAGTCTTGCTCCTCCTGA	ATCTTGAGCCTGGGGACTCTATCTCGGG
		**Ifi35-9**	TGAGCTGCTGCAGTATGTGACCGGGTACAATACCCGAGATAGAGTCGTGCGCTCCCTTT	CTCCGGGAGAGCCAGACTCTATCTCGGG
		**Ifi35-10**	CAACCGATCAACCTATGTGACCGGGTACAATACCCGAGATAGAGTCCCTTTTGTTGTAG	GTTAACTCTGTGTAGACTCTATCTCGGG
		**Ifi35-11**	CTGGACCTGCACCTATGTGACCGGGTACAATACCCGAGATAGAGTCGCAGCTCCAAGGG	GTTGGTCACCACACGACTCTATCTCGGG
		**Ifi35-12**	CCAACAGTTCCTCTATGTGACCGGGTACAATACCCGAGATAGAGTCATCTCCAGCTTGT	CCTTGCCAAAGAACGACTCTATCTCGGG
	Ifih1	**Ifih1-1**	GTGACCAGGACATGTCTATTAGTGGAGCCAATAACGATCCCTCACATGAGCCACTGCTG	CGTTCGGTAACTCGTGTGAGGGATCGTT
		**Ifih1-2**	TGACTCATCGACAGTCTATTAGTGGAGCCAATAACGATCCCTCACACTGTCTCCAGACT	TACGTTCCAGGCCATGTGAGGGATCGTT
		**Ifih1-3**	AGGCCATAACGAAGTCTATTAGTGGAGCCAATAACGATCCCTCACACTCGTTCGTGACA	TGGACCATGGCTAATGTGAGGGATCGTT
		**Ifih1-4**	GTATCTGCGGCAGGTCTATTAGTGGAGCCAATAACGATCCCTCACAGCTGTCAGTCCTA	CAACACCAGGTGATTGTGAGGGATCGTT
		**Ifih1-5**	CATTGAGGACTGCGTCTATTAGTGGAGCCAATAACGATCCCTCACACTCTTGTCGCTGT	TGGCCTCGTCATCTTGTGAGGGATCGTT
		**Ifih1-6**	CAGCAGTTCTGCCGTCTATTAGTGGAGCCAATAACGATCCCTCACACCAAGGTGCTCAG	CCATTGTCCCTGCATGTGAGGGATCGTT
		**Ifih1-7**	CACGAACATCTGCGTCTATTAGTGGAGCCAATAACGATCCCTCACAGCTCTAGGGCCTC	GGGATTGCCACTGATGTGAGGGATCGTT
		**Ifih1-8**	TGGGCAGTCTCAGGTCTATTAGTGGAGCCAATAACGATCCCTCACAGAGACACTCGTCA	AGGGTCAGCAAGTCTGTGAGGGATCGTT
		**Ifih1-9**	ACCATCATTGTTCGTCTATTAGTGGAGCCAATAACGATCCCTCACATAAACCTTTGTGC	AGACAAGGCAAATGTGTGAGGGATCGTT

		**Ifih1-10**	TGAAATTGACTACGTCTATTAGTGGAGCCAATAACGATCCCTCACAGGTGAGTTATTTT	TGTACTGTTTCTTGTGTGAGGGATCGTT
		**Ifih1-11**	AGATCAGGAAATCGTCTATTAGTGGAGCCAATAACGATCCCTCACATTCTGAGTAGTCA	CTATACAAGCAGTTTGTGAGGGATCGTT
		**Ifih1-12**	TGCTTTGTTTTCTGTCTATTAGTGGAGCCAATAACGATCCCTCACAATTTCTTTTGCAG	CTGATAATCAGCATTGTGAGGGATCGTT
	Samhd1	**Samhd1-1**	GAGCCGGCGATTTGTCTATTAGTGGAGCCATACCCGAGATAGAGTCAAAGGGCAGAAAC	CGATCCTCATCCATGACTCTATCTCGGG
		**Samhd1-2**	GTTTTTCGGCAAGGTCTATTAGTGGAGCCATACCCGAGATAGAGTCTGTAGCTCTGGCT	CTCGCTCACTGATGGACTCTATCTCGGG
		**Samhd1-3**	AATGCGGAACTTCGTCTATTAGTGGAGCCATACCCGAGATAGAGTCCAATGGCTGTGGA	GGCTTCCATGTCAAGACTCTATCTCGGG
		**Samhd1-4**	ATAGCGAAGTCGCGTCTATTAGTGGAGCCATACCCGAGATAGAGTCCCAGCTGCTTGAT	ATAGTAGCCGCCCGGACTCTATCTCGGG
		**Samhd1-5**	ACAAAGTGCTTCCGTCTATTAGTGGAGCCATACCCGAGATAGAGTCCGCACACCACTGA	GTGAAGTCCCTGACGACTCTATCTCGGG
		**Samhd1-6**	GAGGAGTGCTCGGGTCTATTAGTGGAGCCATACCCGAGATAGAGTCTTAGCTGTTGCAG	CGTCTGCAGACAGTGACTCTATCTCGGG
		**Samhd1-7**	TCGGAAACCACGAGTCTATTAGTGGAGCCATACCCGAGATAGAGTCGCACTTTCTTCTC	TCTGAAGATGTCCTGACTCTATCTCGGG
		**Samhd1-8**	AGCTATAATGTCAGTCTATTAGTGGAGCCATACCCGAGATAGAGTCGTGTTATGAGTGG	ATTCCATTTCAGACGACTCTATCTCGGG
		**Samhd1-9**	TTCTTGGAGGCAAGTCTATTAGTGGAGCCATACCCGAGATAGAGTCTTACTTTGGATAC	TTTTAGACATGTTAGACTCTATCTCGGG
		**Samhd1-10**	TTTCTCTGGCAGCGTCTATTAGTGGAGCCATACCCGAGATAGAGTCGCTGCTCTGCAAA	GTACACCCGAATGTGACTCTATCTCGGG
		**Samhd1-11**	CCACATTGATAACGTCTATTAGTGGAGCCATACCCGAGATAGAGTCTCCATCCCATAAT	TTGGATTCTTGTCAGACTCTATCTCGGG
		**Samhd1-12**	CCACATTGATAACGTCTATTAGTGGAGCCATACCCGAGATAGAGTCTCCATCCCATAAT	TTGGATTCTTGTCAGACTCTATCTCGGG
	Stat1	**Stat1-1**	CTACCACGAAGGAATAACGATCCCTCACAATACCCGAGATAGAGTCCACGGCTGTCGTT	GGTGAGTGGGCATCGACTCTATCTCGGG
		**Stat1-2**	GAAGCTCGAACCAATAACGATCCCTCACAATACCCGAGATAGAGTCGAGTCCAGCTGCT	GCTCCAGGAACTTCGACTCTATCTCGGG
		**Stat1-3**	GAGATCACCACGAATAACGATCCCTCACAATACCCGAGATAGAGTCCTGGCTGACGTTG	CAGCCACTGGGGACGACTCTATCTCGGG
		**Stat1-4**	TTTTTGGTCGCTCATAACGATCCCTCACAATACCCGAGATAGAGTCGCAGCTGTTCCTG	CATCTTGTGGAGCTGACTCTATCTCGGG
		**Stat1-5**	CTGGGACCACCACATAACGATCCCTCACAATACCCGAGATAGAGTCACACCTCTGAGAG	AAACTGCCAACTCTGACTCTATCTCGGG
		**Stat1-6**	TGGGTTTCAAAGCATAACGATCCCTCACAATACCCGAGATAGAGTCTGGCTGGCACAAC	TCAATCACCAAGCGGACTCTATCTCGGG
		**Stat1-7**	TGTGATGGCCCCTATAACGATCCCTCACAATACCCGAGATAGAGTCCCACCCATGTGAA	GTTCTGGGACCGTAGACTCTATCTCGGG
		**Stat1-8**	CTGCGTTCAGACCATAACGATCCCTCACAATACCCGAGATAGAGTCATGCTCAGCTGGT	GCTTCTCTCCCAGGGACTCTATCTCGGG
		**Stat1-9**	TCTGACAACACCTATAACGATCCCTCACAATACCCGAGATAGAGTCGAGGAAGGTTCGA	AGCTGCTGGAAGACGACTCTATCTCGGG
		**Stat1-10**	TCATAGGCAGCGTATAACGATCCCTCACAATACCCGAGATAGAGTCCGCAAACGAGACA	TGGAAGCGGATGGAGACTCTATCTCGGG
		**Stat1-11**	CCAGCTGTGAGAGATAACGATCCCTCACAATACCCGAGATAGAGTCCTGTACTGGTCGT	CCAGAGAAAAGCGCGACTCTATCTCGGG
		**Stat1-12**	GATGATCATGGACATAACGATCCCTCACAATACCCGAGATAGAGTCTCAGACAGTTGTA	CTTCCTTTCTTCCAGACTCTATCTCGGG
	Usp18	**Usp18-1**	AACCCCTTGCCCAACCATGCTGGATCTAGAACCATGCTGGATCTAGCCTCAGGAGCCCA	GACTGGCAGGGTTACTAGATCCAGCATG
		**Usp18-2**	ACACTGGACATCCACCATGCTGGATCTAGAACCATGCTGGATCTAGGGTTCCCATAGGT	CCAGCGGTATCTGACTAGATCCAGCATG
		**Usp18-3**	TCTTTGGGCTGGAACCATGCTGGATCTAGAACCATGCTGGATCTAGGGAGCTTGCTAAC	TCGCAGCACATGTGCTAGATCCAGCATG
		**Usp18-4**	CTGCTGTACAGCCACCATGCTGGATCTAGAACCATGCTGGATCTAGCTCCTGCTGCTCT	TGAGCAGTTTGCTGCTAGATCCAGCATG
		**Usp18-5**	TCCGGATGTAGGCACCATGCTGGATCTAGAACCATGCTGGATCTAGCCATCCACGGGAT	AGCAGAACCACTTACTAGATCCAGCATG

		**Usp18-6**	AACGAGCTCCGTGACCATGCTGGATCTAGAACCATGCTGGATCTAGTCTGCAGGCACTG	CGGCACGTTGTATACTAGATCCAGCATG
		**Usp18-7**	TTCATGGTCCTCTACCATGCTGGATCTAGAACCATGCTGGATCTAGCACTCTCTTCCTC	AGGTCTCTAGACACCTAGATCCAGCATG
		**Usp18-8**	GAGGGAAATTAACACCATGCTGGATCTAGAACCATGCTGGATCTAGAAATCCAAGCTCT	GCAGAACCTGACTCCTAGATCCAGCATG
		**Usp18-9**	GTGTAAACCAACCACCATGCTGGATCTAGAACCATGCTGGATCTAGTCTGTCCGATGTT	GTTAAGGCAACACCCTAGATCCAGCATG
		**Usp18-10**	TTCATCATGAACAACCATGCTGGATCTAGAACCATGCTGGATCTAGTCTGAAGTCCATG	CTCTTCAATATCAACTAGATCCAGCATG
		**Usp18-11**	TCCTCTCTTCTGCACCATGCTGGATCTAGAACCATGCTGGATCTAGGGGACACTTCTCT	AAAGCAGCTGGAACCTAGATCCAGCATG
		**Usp18-12**	TAGAGTTGAGCAGACCATGCTGGATCTAGAACCATGCTGGATCTAGCCAGATTGTAAGG	TCCTTAGTCAGGTACTAGATCCAGCATG
	Cd79a	**Cd79a-1**	ATTTTGCCACCGTTACTAGCTCGATTCAAATACTAGCTCGATTCAACCCCAAACTTCTC	ATCTGGCATGTCCTTTGAATCGAGCTAG
		**Cd79a-2**	TCTACCCGCAGGGTACTAGCTCGATTCAAATACTAGCTCGATTCAATGGTGGACCCCCT	TTCACCGTCAGGGTTTGAATCGAGCTAG
		**Cd79a-3**	TGCGGTTCTTGGTTACTAGCTCGATTCAAATACTAGCTCGATTCAATCTGCTGTGATGA	GCAAGATGATCCCATTGAATCGAGCTAG
		**Cd79a-4**	GTAGGTGCCCTGGTACTAGCTCGATTCAAATACTAGCTCGATTCAATGCCCACATCCTG	TCCAATGTGGAGGATTGAATCGAGCTAG
		**Cd79a-5**	AACCACCATGTGATACTAGCTCGATTCAAATACTAGCTCGATTCAAAGACTGAAGGCTG	GGCCATGTGATGTATTGAATCGAGCTAG
		**Cd79a-6**	GTACCCTGGCCAGTACTAGCTCGATTCAAATACTAGCTCGATTCAACAGCTGGCCTGTG	ACTTCGGGGAAGATTTGAATCGAGCTAG
		**Cd79a-7**	AGACCCCCTGGCATACTAGCTCGATTCAAATACTAGCTCGATTCAATCTGAGGGCTTCT	AGGAGAGGCAGGGGTTGAATCGAGCTAG
		**Cd79a-8**	GTTGTTTTCTATCTACTAGCTCGATTCAAATACTAGCTCGATTCAAAGCGTTTTAATAT	GTAAGTACCACAGCTTGAATCGAGCTAG
		**Cd79a-9**	ACTGCACAGAACATACTAGCTCGATTCAAATACTAGCTCGATTCAACGTCCCTGGCACC	CTGAATAGCAGCACTTGAATCGAGCTAG
		**Cd79a-10**	ATCAAGGTTCAGGTACTAGCTCGATTCAAATACTAGCTCGATTCAAACATAGAACAGTC	GGAGATGTCCTCAATTGAATCGAGCTAG
		**Cd79a-11**	GGCCTCCTCGCCCTACTAGCTCGATTCAAATACTAGCTCGATTCAACACAGGTGAGGCG	CCTGCCATTGTTTATTGAATCGAGCTAG
	Agbl1	**Agbl1-1**	TGGACAGGGTGGATAGGCATGAACGTCGAATAGGCATGAACGTCGAAGGACTTGGAGGA	AAAGCAGATCGCCCTCGACGTTCATGCC
		**Agbl1-2**	TGGCTCCCATTTCTAGGCATGAACGTCGAATAGGCATGAACGTCGACCCAAGCAGTACA	CCAAGATGAGCAGATCGACGTTCATGCC
		**Agbl1-3**	GTGCTGGGAGCTATAGGCATGAACGTCGAATAGGCATGAACGTCGAAGAACCACTGCTG	GCTCACCTTGAAGATCGACGTTCATGCC
		**Agbl1-4**	TGCAGAAGGGCCTTAGGCATGAACGTCGAATAGGCATGAACGTCGAGTCCACCAGAGTC	GATGACCTTGCTGATCGACGTTCATGCC
		**Agbl1-5**	TGGCAGAGAACATTAGGCATGAACGTCGAATAGGCATGAACGTCGACCCTCCCAACGTT	AGCGGACATGGATATCGACGTTCATGCC
		**Agbl1-6**	CTCGCAGGGTAACTAGGCATGAACGTCGAATAGGCATGAACGTCGACAGGGGAAGCCGA	CTGCTGGCTGTGAGTCGACGTTCATGCC
		**Agbl1-7**	GAGGATGTCCCAGTAGGCATGAACGTCGAATAGGCATGAACGTCGATGAAGAGAGGCAG	TTTAGCCAGCAGCGTCGACGTTCATGCC
		**Agbl1-8**	GAGTGGACTGTGTTAGGCATGAACGTCGAATAGGCATGAACGTCGAGTGCAGAATGGAC	CCTGTCTTCACCAATCGACGTTCATGCC
		**Agbl1-9**	TCTTGTTCAGCCATAGGCATGAACGTCGAATAGGCATGAACGTCGACAGGCCACTGCCT	TGCAGCAGCATCTCTCGACGTTCATGCC
		**Agbl1-10**	GCCAAAATCAAGGTAGGCATGAACGTCGAATAGGCATGAACGTCGACAGGTTTTTCCTG	TTCTGGCTGTGAGTTCGACGTTCATGCC
		**Agbl1-11**	TAAGGAGAAAGGATAGGCATGAACGTCGAATAGGCATGAACGTCGAGTGCTTTCGGGTG	CTGATTGTCCGGGATCGACGTTCATGCC
		**Agbl1-12**	GCCTTGGGCTGCCTAGGCATGAACGTCGAATAGGCATGAACGTCGAAGAGAGTCTCCAT	CTGTGCAATGCTGTTCGACGTTCATGCC
	Bank1	**Bank1-1**	AGCCACAGGAAGCCGTAGCTTACCTGCAAACGTAGCTTACCTGCAACCCTAGTGCCAGA	ATCCTGGGTGCTAGTTGCAGGTAAGCTA
		**Bank1-2**	TCTGTTGGAATGCCGTAGCTTACCTGCAAACGTAGCTTACCTGCAACTTGGCTCTTGAG	GTGTCCTCAGGATTTTGCAGGTAAGCTA

		**Bank1-3**	TGTCTGTGTTCCGCGTAGCTTACCTGCAAACGTAGCTTACCTGCAAGGCTCCTCAGATC	CCACAGACCTTTCTTGCAGGTAAGCTA
		**Bank1-4**	CAGATGAAGAGCCCGTAGCTTACCTGCAAACGTAGCTTACCTGCAACTGAACACTGCAG	TGCCCTGGTTGCTGTTGCAGGTAAGCTA
		**Bank1-5**	CTGCTTCTTCCTCCGTAGCTTACCTGCAAACGTAGCTTACCTGCAACTTGCCTCAGCAC	CTGACAGGCTGCGATTGCAGGTAAGCTA
		**Bank1-6**	AGTTGCCTTCGGTCGTAGCTTACCTGCAAACGTAGCTTACCTGCAAAGAGGCTCTTCAG	GCCTGCAGTGTGGCTTGCAGGTAAGCTA
		**Bank1-7**	TTCGAGCAGGAAACGTAGCTTACCTGCAAACGTAGCTTACCTGCAATTTTCAACTTGGA	AGAAACCAAACTCGTTGCAGGTAAGCTA
		**Bank1-8**	TGGGCAGTATTACCGTAGCTTACCTGCAAACGTAGCTTACCTGCAACATATTTTCATTG	GGACTGTTGTACATTTGCAGGTAAGCTA
		**Bank1-9**	GCCGTAGCTTTTCCGTAGCTTACCTGCAAACGTAGCTTACCTGCAATTGTCTCGTAGTT	TTTTCCCAATAATCTTGCAGGTAAGCTA
		**Bank1-10**	ATCCGAGTTTTGTCGTAGCTTACCTGCAAACGTAGCTTACCTGCAAGGTTGGGCCCTCC	CTTGTACTAGGGATTTGCAGGTAAGCTA
		**Bank1-11**	TTTTGTTGGAACACGTAGCTTACCTGCAAACGTAGCTTACCTGCAATCTTCGGGCTGCC	TCACCATCAGATTCTTGCAGGTAAGCTA
		**Bank1-12**	TCCAGTACTTTTCCGTAGCTTACCTGCAAACGTAGCTTACCTGCAATTCCATTTGGTCT	CAGTTTTGACTTCATTGCAGGTAAGCTA
		**Pkib-1**	CTGAGGACGCAAAACCATGCTGGATCTAGATACTAGCTCGATTCAACGGCCTGCCCTTG	GTAAGGCATTGCGCTTGAATCGAGCTAG
		**Pkib-2**	AGAGGATCCACTTACCATGCTGGATCTAGATACTAGCTCGATTCAATCAGTGGAAGATC	CAACGCTTCCAGTATTGAATCGAGCTAG
	Pkib	**Pkib-3**	TCAGTGGAAGATCACCATGCTGGATCTAGATACTAGCTCGATTCAAAACGCTTCCAGTT	CTTCCTTCACGGCGTTGAATCGAGCTAG
		**Pkib-4**	CCTCATCACAGAAACCATGCTGGATCTAGATACTAGCTCGATTCAACTGATGAATCTGT	CACATCAGTCATCATTGAATCGAGCTAG
		**Pkib-5**	GGCCTTGGTCTTTACCATGCTGGATCTAGATACTAGCTCGATTCAAGGTGTTTTTGGTT	TTCCTTCATTTAGCTTGAATCGAGCTAG
		**Ccr9-1**	ACTGCCTGACATTACCATGCTGGATCTAGATAGGCATGAACGTCGAAAATGGCTTGCAA	ACAGAGGTGGAAGTTCGACGTTCATGCC
		**Ccr9-2**	ATGAACACAAGCCACCATGCTGGATCTAGATAGGCATGAACGTCGACAAGGTGCCCACA	ACCAGGCTGTTGCGTCGACGTTCATGCC
		**Ccr9-3**	AGAGCAGATCAGCACCATGCTGGATCTAGATAGGCATGAACGTCGAAGAGTGGCAAGAA	TGGCCCAGAAGGGTTCGACGTTCATGCC
		**Ccr9-4**	TGGCCTGTACAATACCATGCTGGATCTAGATAGGCATGAACGTCGAACCTGAGCCTTCA	TTTTCTGCCTCCACTCGACGTTCATGCC
		**Ccr9-5**	CTTCTTGGCCTGTACCATGCTGGATCTAGATAGGCATGAACGTCGATGTGCTTGGATGA	CACCTTGAGGGCCATCGACGTTCATGCC
	Ccr9	**Ccr9-6**	CTCGCCAACAAAAACCATGCTGGATCTAGATAGGCATGAACGTCGACCCTTCGGAATCT	GGTCTTCACCAGAATCGACGTTCATGCC
		**Ccr9-7**	CCAGGAATAAGGCACCATGCTGGATCTAGATAGGCATGAACGTCGAGTCATCAAACATG	GAGTCATAGCTGATTCGACGTTCATGCC
		**Ccr9-8**	GTAGAAGTTCATCACCATGCTGGATCTAGATAGGCATGAACGTCGAGAAGCACACAGCT	GATGCACATGATGTTCGACGTTCATGCC
		**Ccr9-9**	CATGTGGCAATACACCATGCTGGATCTAGATAGGCATGAACGTCGAGTAGACCATGGTA	TTCTTATCCTTAGCTCGACGTTCATGCC
		**Ccr9-10**	CAGTAGCATAGAAACCATGCTGGATCTAGATAGGCATGAACGTCGACCGAAGTTGTCTC	TAGGGAGAGAGCCGTCGACGTTCATGCC
		**Ccr9-11**	CCACTGGGCCTGGACCATGCTGGATCTAGATAGGCATGAACGTCGATTGTGAATGAAAC	GCTACCCTCTCTCGTCGACGTTCATGCC
		**Ccr9-12**	AAGCGTCAACAGCACCATGCTGGATCTAGATAGGCATGAACGTCGAATGAACATGGCAT	TAGTGCAGTTGGACTCGACGTTCATGCC
		**Prickle2-1**	CACTCGTCTGCAAACCATGCTGGATCTAGACGTAGCTTACCTGCAACTCAGCCTCTGTG	TGCCAGTGTCGTCGTTGCAGGTAAGCTA
		**Prickle2-2**	AGGGGACTTTCTCACCATGCTGGATCTAGACGTAGCTTACCTGCAAGCACTGTTGACAT	GCAACTTCTCTCCTTTGCAGGTAAGCTA
	Prickle2	**Prickle2-3**	GTTGCTCAGCTTTACCATGCTGGATCTAGACGTAGCTTACCTGCAATGAGAGTGCCCAT	CTGCATTGAGGAGATTGCAGGTAAGCTA
		**Prickle2-4**	TCGTCATCTGAGGACCATGCTGGATCTAGACGTAGCTTACCTGCAAAGCACAGCCAGAG	GCGTACTCTTCCATTTGCAGGTAAGCTA
		**Prickle2-5**	ACTGTTGTGCGGAACCATGCTGGATCTAGACGTAGCTTACCTGCAATCCATCTCCTGGT	GGTGAAGGTTCCCGTTGCAGGTAAGCTA

		**Prickle2-6**	TTGCCGATTTTGGACCATGCTGGATCTAGACGTAGCTTACCTGCAACTTGCCCTTGTTC	ATGGCTTCCTCTGATTGCAGGTAAGCTA
		**Prickle2-7**	TCAAAGCTCTGGTACCATGCTGGATCTAGACGTAGCTTACCTGCAAGCCACCATCAAAA	TTGCTGCTCGCTAATTGCAGGTAAGCTA
		**Prickle2-8**	AGCCGGTTAGAACACCATGCTGGATCTAGACGTAGCTTACCTGCAACACATCAGCTGAG	ACTGACAGGGGGTGTTGCAGGTAAGCTA
		**Prickle2-9**	ACCGAGCGATGACACCATGCTGGATCTAGACGTAGCTTACCTGCAAGGCCTTTCCTTCA	TGGCTCTCAGAGGCTTGCAGGTAAGCTA
		**Prickle2-10**	TGCAAAGCCAGGTACCATGCTGGATCTAGACGTAGCTTACCTGCAACCCAAAGGCATTC	GGTCCCCATCGTTGTTGCAGGTAAGCTA
		**Prickle2-11**	ACCGTAAGAGCTGACCATGCTGGATCTAGACGTAGCTTACCTGCAAAGGATTTGGGGAC	GCCACCTAAGGTGCTTGCAGGTAAGCTA
		**Prickle2-12**	TCAGCAGACAGGCACCATGCTGGATCTAGACGTAGCTTACCTGCAACTTGGCACCACCT	AGGTGCTCCTGGCCTTGCAGGTAAGCTA
	Cldn5	**Cldn5-1**	TTCCAACGCTGCATACTAGCTCGATTCAAATAGGCATGAACGTCGACCAGACCCAGAAT	TACCAGACACAGCTTCGACGTTCATGCC
		**Cldn5-2**	AGCCCCTTCCAAGTACTAGCTCGATTCAAATAGGCATGAACGTCGAGCACGACATCCAC	GTACTCTGCACCAGTCGACGTTCATGCC
		**Cldn5-3**	ACCCGTGCCTTAATACTAGCTCGATTCAAATAGGCATGAACGTCGATCCCGTGAGTGCT	GCGTAAAGCGCTCGTCGACGTTCATGCC
		**Cldn5-4**	TTCTGTGACACCGTACTAGCTCGATTCAAATAGGCATGAACGTCGAGCCCAGCTCGTAC	ATGTACAGCGCCGGTCGACGTTCATGCC
		**Cldn5-5**	GAACTCAGGGCGCTACTAGCTCGATTCAAATAGGCATGAACGTCGATGACCGGGAAGCT	CGGCGCAGAGTACATCGACGTTCATGCC
		**Cldn5-6**	CTGCCACATGGGCTACTAGCTCGATTCAAATAGGCATGAACGTCGAGGAAGGCAGTCAC	GATGTTGTGGTCCTTCGACGTTCATGCC
		**Cldn5-7**	CAAAGAGTGCCACTACTAGCTCGATTCAAATAGGCATGAACGTCGACCGGTCAAGGTAA	TGGTGCACTGAGCCTCGACGTTCATGCC
		**Cldn5-8**	GATGTTGGCGAACTACTAGCTCGATTCAAATAGGCATGAACGTCGAACTCGCGGACAAC	CGTCGGATCATAGTTCGACGTTCATGCC
		**Cldn5-9**	ATACACCTTGCACTACTAGCTCGATTCAAATAGGCATGAACGTCGACCAGCACAGATTC	CTCCGCACTCAGCCTCGACGTTCATGCC
		**Cldn5-10**	ATTGGCCGTGGGCTACTAGCTCGATTCAAATAGGCATGAACGTCGATGTCGTAATCGCC	GACATAGTTCTTCATCGACGTTCATGCC
		**Cldn5-11**	GTGCGGAGGCCGCTACTAGCTCGATTCAAATAGGCATGAACGTCGACCGCACATGAGCA	ACACGAGGCCGCCTTCGACGTTCATGCC
	St8sia6	**St8sia6-1**	TCCCTCCATCAACTACTAGCTCGATTCAAACGTAGCTTACCTGCAATGTCCTCTCTGCT	TGAGGTACCACTGCTTGCAGGTAAGCTA
		**St8sia6-2**	CAGCATCAGGCTGTACTAGCTCGATTCAAACGTAGCTTACCTGCAAGCAGGAGGAGCAG	GCACCAGAGCATAGTTGCAGGTAAGCTA
		**St8sia6-3**	TCGGGCTCCAGAGTACTAGCTCGATTCAAACGTAGCTTACCTGCAAGGTACCGGGGTTG	TGTTCCTGGTGCGATTGCAGGTAAGCTA
		**St8sia6-4**	GAGGAGGAATGCATACTAGCTCGATTCAAACGTAGCTTACCTGCAAAAAATGCTGGCAG	GTTGGCCCGATAGCTTGCAGGTAAGCTA
		**St8sia6-5**	CAGTCACCCCTTTTACTAGCTCGATTCAAACGTAGCTTACCTGCAAGACAAGCGGTATG	TCATCAAGCCTGTCTTGCAGGTAAGCTA
		**St8sia6-6**	TCACACAGTTCCATACTAGCTCGATTCAAACGTAGCTTACCTGCAAGAGCTTCACGTTT	GGCCAGAATCCGTTTTGCAGGTAAGCTA
		**St8sia6-7**	ATGCTGGCAGGAGTACTAGCTCGATTCAAACGTAGCTTACCTGCAAGCCCGATAGGAAA	AGATGCCTGTGTTCTTGCAGGTAAGCTA
		**St8sia6-8**	CAAATTCTGGTACTACTAGCTCGATTCAAACGTAGCTTACCTGCAACTTTCTTCTCCTT	CTCCAAAAACTGTCTTGCAGGTAAGCTA
		**St8sia6-9**	ATAGTAGTGGTGATACTAGCTCGATTCAAACGTAGCTTACCTGCAAGTAACATGTTATC	GAAACCATGCTTACTTGCAGGTAAGCTA
		**St8sia6-10**	TGAGGATTCCTCTTACTAGCTCGATTCAAACGTAGCTTACCTGCAACTGAATTGCAGTT	CCGTTTCACATTTCTTGCAGGTAAGCTA
		**St8sia6-11**	GGGGAGGTTACACTACTAGCTCGATTCAAACGTAGCTTACCTGCAATCCCTGTGATTGG	ATCTTTACTAGCGGTTGCAGGTAAGCTA
		**St8sia6-12**	CAGGAAGCCAGTTTACTAGCTCGATTCAAACGTAGCTTACCTGCAAAATGGCATCGCAA	ACCACGAAGTCTTCTTGCAGGTAAGCTA
	Sftpa1	**Sftpa1-1**	TCCAGGTAAGCTGTAGGCATGAACGTCGAACGTAGCTTACCTGCAACTGAAGCTCCTCA	TCGTAGAGTGCAGATTGCAGGTAAGCTA
		**Sftpa1-2**	CTGGCATTCCTCCTAGGCATGAACGTCGAACGTAGCTTACCTGCAACGCCCAGGAAGAC	CAGGCAGCCCATCCTTGCAGGTAAGCTA

		**Sftpa1-3**	GATGCCAGCAACATAGGCATGAACGTCGAACGTAGCTTACCTGCAATCCCATTGCACTT	GGCACAAACTTCTCTTGCAGGTAAGCTA
		**Sftpa1-4**	GTTCTCCAGGTGCTAGGCATGAACGTCGAACGTAGCTTACCTGCAACCCTTGTCTCCAT	TTTCTCCAGGCTCATTGCAGGTAAGCTA
		**Sftpa1-5**	CAATGTGGCCACCTAGGCATGAACGTCGAACGTAGCTTACCTGCAATTCCTCGGGGCAG	CGTTCTCCTCAGGTTTGCAGGTAAGCTA
		**Sftpa1-6**	TGGCCTTCAATCATAGGCATGAACGTCGAACGTAGCTTACCTGCAAGTCTCCAGGAGTC	TCCAGGTAGTGGATTTGCAGGTAAGCTA
		**Sftpa1-7**	CTGGGTACCAGTTTAGGCATGAACGTCGAACGTAGCTTACCTGCAACCCCTGGGCTCTC	TCTCTTTGCCCCGTTTGCAGGTAAGCTA
		**Sftpa1-8**	GCCAAAGAGCCTATAGGCATGAACGTCGAACGTAGCTTACCTGCAAGAAGAGGGTGAAG	GCAACAACAGTCATTTGCAGGTAAGCTA
		**Sftpa1-9**	AGTGCCAGGGATCTAGGCATGAACGTCGAACGTAGCTTACCTGCAACATGGTTTCCGGG	TCTGCCGGGCAAAGTTGCAGGTAAGCTA
		**Sftpa1-10**	CATCTCTCCCATCTAGGCATGAACGTCGAACGTAGCTTACCTGCAATCTCCTTTGATAC	CTGGAGGTCCAGGCTTGCAGGTAAGCTA
		**Sftpa1-11**	CTTGCAAGCTGAGTAGGCATGAACGTCGAACGTAGCTTACCTGCAAGACAGCATGGATC	CTTTATCCCCCACATTGCAGGTAAGCTA
		**Sftpa1-12**	AAGTTGACTGACTTAGGCATGAACGTCGAACGTAGCTTACCTGCAATCTAATGGTATCA	CTGGTACACATCTGTTGCAGGTAAGCTA
	Sftpb	**Sftpb-1**	ATGCCACCCACCAGGCACTTGAGCTAGAAAGGCACTTGAGCTAGAACAGGCACTGGCAG	GTGTAGCGCTCAGGTTCTAGCTCAAGTG
		**Sftpb-2**	CAGCCACAGCCAGGGCACTTGAGCTAGAAAGGCACTTGAGCTAGAACACACCTGGGACA	GGGGTACCACGTGCTTCTAGCTCAAGTG
		**Sftpb-3**	TGGGGAGCTGTTGGGCACTTGAGCTAGAAAGGCACTTGAGCTAGAAAAGGGCAGGGGAA	TGCAAAGCCAGCACTTCTAGCTCAAGTG
		**Sftpb-4**	CACACTCCAGGGAGGCACTTGAGCTAGAAAGGCACTTGAGCTAGAATGAGGGCCTTGTG	TTTGGCACCAGAAATTCTAGCTCAAGTG
		**Sftpb-5**	CAGAAGGGCAGGGGGCACTTGAGCTAGAAAGGCACTTGAGCTAGAATCTGCAAAGCCAG	CGCTTGATCAGAGATTCTAGCTCAAGTG
		**Sftpb-6**	ACAGACTTGCAGAGGCACTTGAGCTAGAAAGGCACTTGAGCTAGAAGGCCTGGTTGATC	TCACTGGTGTTCCTTTCTAGCTCAAGTG
		**Sftpb-7**	AGGCCACAGACTAGGCACTTGAGCTAGAAAGGCACTTGAGCTAGAAACATCGGAGGACA	GCATCCTCAGTGGTTTCTAGCTCAAGTG
		**Sftpb-8**	GAACGGCATCCGGGGCACTTGAGCTAGAAAGGCACTTGAGCTAGAAAGCAGAGGGTTTG	GGACCAGCTTGTCGTTCTAGCTCAAGTG
		**Sftpb-9**	GTAGGTGCGACTTGGCACTTGAGCTAGAAAGGCACTTGAGCTAGAAAGTAGCCACTGCA	GGGTAGGAAGCAGGTTCTAGCTCAAGTG
		**Sftpb-10**	CTGCACTGCATGCGGCACTTGAGCTAGAAAGGCACTTGAGCTAGAACCAGGGCTCTGCA	CTGCAGGCAGTGCGTTCTAGCTCAAGTG
		**Sftpb-11**	GTGGACAATATCCGGCACTTGAGCTAGAAAGGCACTTGAGCTAGAATCTTTGTGAGGAG	ATCTTCCTTGGTCTTTCTAGCTCAAGTG
		**Sftpb-12**	GAACTTCCGGATTGGCACTTGAGCTAGAAAGGCACTTGAGCTAGAAATTCTTGTTCCAG	GGGAAGGATATCAGTTCTAGCTCAAGTG
	Col3a1	**Col3a1-1**	TTGCCTCGAGCACATGCTGCTGTAGTACTAATGCTGCTGTAGTACTCCGAGCACCATCA	TGCCCATCACTGCGAGTACTACAGCAGC
		**Col3a1-2**	TTTCTCCATTGCGATGCTGCTGTAGTACTAATGCTGCTGTAGTACTCCTGTTTCTCCCT	TCAGTCCAGGAGCTAGTACTACAGCAGC
		**Col3a1-3**	TTAGCCCTGCAATATGCTGCTGTAGTACTAATGCTGCTGTAGTACTCCTCGTGCTCCAG	GTGGTCCAGCAAGTAGTACTACAGCAGC
		**Col3a1-4**	TCTTCGCCCTTAGATGCTGCTGTAGTACTAATGCTGCTGTAGTACTTCCATCTTTGCCA	GGTTCTCCAGGTGTAGTACTACAGCAGC
		**Col3a1-5**	TTGTCCTGGTGAGATGCTGCTGTAGTACTAATGCTGCTGTAGTACTCAGGCTCCCCTCT	ATGTCCCTGCGGTGAGTACTACAGCAGC
		**Col3a1-6**	GTCTGAGGAATGCATGCTGCTGTAGTACTAATGCTGCTGTAGTACTCGGCTGGAAAGAA	TGTTCTGGGAGGCGAGTACTACAGCAGC
		**Col3a1-7**	CAGCAATGGCAGCATGCTGCTGTAGTACTAATGCTGCTGTAGTACTTCACCTCCAACTC	AGCCACCAGACTTAAGTACTACAGCAGC
		**Col3a1-8**	CTTTTGGTCCAGCATGCTGCTGTAGTACTAATGCTGCTGTAGTACTTGTCCAGCATCAC	CCTTCTCTCCAGGGAGTACTACAGCAGC
		**Col3a1-9**	TGCTTACGTGGGAATGCTGCTGTAGTACTAATGCTGCTGTAGTACTATCTGTCCACCAG	TTCTCTGCACCAGTAGTACTACAGCAGC
		**Col3a1-10**	TTCCGAGCAGGGTATGCTGCTGTAGTACTAATGCTGCTGTAGTACTTAGGTCTCTGCAG	GGGTGGCAGAATTAAGTACTACAGCAGC

		**Col3a1-11**	CACAAAGCTCATCATGCTGCTGTAGTACTAATGCTGCTGTAGTACTAGGTTCCACTTTG	GGTGAGAAGAAACGAGTACTACAGCAGC
		**Col3a1-12**	CTAGACTCATAGGATGCTGCTGTAGTACTAATGCTGCTGTAGTACTCTTCCAGACATCT	TGACATGGTTCTGCAGTACTACAGCAGC
	Col1a2	**Col1a2-1**	TTGCCAGGAGGACGGAGACTGATTACACGAGGAGACTGATTACACGGGTTGAACCACGA	ATTCCAGCAGGGCGCGTGTAATCAGTCT
		**Col1a2-2**	GTCCCGGGGAAACGGAGACTGATTACACGAGGAGACTGATTACACGAGGAAGTCCAGGA	ACGCCTTTGAAGCGCGTGTAATCAGTCT
		**Col1a2-3**	ACCCTGAGGACCTGGAGACTGATTACACGAGGAGACTGATTACACGGAGCACCAAGAAG	ACCCAGAATACCGCCGTGTAATCAGTCT
		**Col1a2-4**	CTGCAAGCAGCAAGGAGACTGATTACACGAGGAGACTGATTACACGAGGCACGAAGTTA	ATTGGCATGTTGCACGTGTAATCAGTCT
		**Col1a2-5**	AGGACCAGAAGGGGGAGACTGATTACACGAGGAGACTGATTACACGCTCGGCTTCCAAT	GGCTCCACTGGGAGCGTGTAATCAGTCT
		**Col1a2-6**	TTCACCACGGGGAGGAGACTGATTACACGAGGAGACTGATTACACGCTGGAAGACCCAC	AGGGCCAGAAAGAGCGTGTAATCAGTCT
		**Col1a2-7**	TGTTGCCAGCTTCGGAGACTGATTACACGAGGAGACTGATTACACGCCAGGGAATCCGA	AGGGGCCTTTGGGACGTGTAATCAGTCT
		**Col1a2-8**	GACCCACAGAACCGGAGACTGATTACACGAGGAGACTGATTACACGTGTTTCCCAGCAG	CACCTCGGTTTCCTCGTGTAATCAGTCT
		**Col1a2-9**	TCCAGTTCGGCCAGGAGACTGATTACACGAGGAGACTGATTACACGATGCACCTGTTTC	TCCAGGGGGTCCACCGTGTAATCAGTCT
		**Col1a2-10**	CTTGCAGTGGTAGGGAGACTGATTACACGAGGAGACTGATTACACGACGCAATGCTGTT	CTCCTCATCCAGGACGTGTAATCAGTCT
		**Col1a2-11**	TTCTTCGCTGGGGGGAGACTGATTACACGAGGAGACTGATTACACGGCGGCTGTATGAG	TTGTTGGCCTGGGGCGTGTAATCAGTCT
		**Col1a2-12**	TGTTCAAGCTGCCGGAGACTGATTACACGAGGAGACTGATTACACGAGAAGCACAGCCT	CGTTGGAACCCTGGCGTGTAATCAGTCT
	Kcnip4	**Kcnip4-1**	TGTTCTGCGCGTACCTCGATTAACATCGCACCTCGATTAACATCGCCTGCGCTTGGTGT	GCCGCTCTTTAATCGCGATGTTAATCGA
		**Kcnip4-2**	TAGCCATCTCCAGCCTCGATTAACATCGCACCTCGATTAACATCGCCGATGCCTGACAG	CCAGGGCTTCAGGGGCGATGTTAATCGA
		**Kcnip4-3**	GTCGAATGCATTGCCTCGATTAACATCGCACCTCGATTAACATCGCCATTGTGGTCCGT	AAAGCTCACAGCTGGCGATGTTAATCGA
		**Kcnip4-4**	TGAGGACCGGGTACCTCGATTAACATCGCACCTCGATTAACATCGCGGAGCATCTTCCT	CCACATGCTGTCGCGCGATGTTAATCGA
		**Kcnip4-5**	AGCAGGGCAAGAGCCTCGATTAACATCGCACCTCGATTAACATCGCGTTTTGGCAGCTG	TAGCAGGAGACGAGGCGATGTTAATCGA
		**Kcnip4-6**	AGAGACGCACATACCTCGATTAACATCGCACCTCGATTAACATCGCATGAAGCAGCTTC	ATCAGTCCCAGCATGCGATGTTAATCGA
		**Kcnip4-7**	CGCATTATGTTTTCCTCGATTAACATCGCACCTCGATTAACATCGCGAGCTGCATGGAG	ATCACATTTTCAATGCGATGTTAATCGA
		**Kcnip4-8**	ATGGTAACGACACCCTCGATTAACATCGCACCTCGATTAACATCGCAATGAACTCATCT	TTTTGGCAACTTTGGCGATGTTAATCGA
		**Kcnip4-9**	CATTATGTCCAGCCCTCGATTAACATCGCACCTCGATTAACATCGCCGTAGATTGCTTT	TTTCCCCATCATGAGCGATGTTAATCGA
		**Kcnip4-10**	GAAAGACCTTTGACCTCGATTAACATCGCACCTCGATTAACATCGCTCGAAGCAAAATG	TCTTGTACTGTCCGGCGATGTTAATCGA
		**Kcnip4-11**	TGTCATACAAATTCCTCGATTAACATCGCACCTCGATTAACATCGCCCATCTTTGTTTA	CTTTAGTGATGTACGCGATGTTAATCGA
		**Kcnip4-12**	TCATTAACAACACCCTCGATTAACATCGCACCTCGATTAACATCGCCTTGAAAGTTTCT	TGTGAGTAAATCTGGCGATGTTAATCGA
	Chil3	**Chil3-1**	GTGGCTCCTTCATGGCACTTGAGCTAGAAAATGCTGCTGTAGTACTATCCCAGACCTCA	ACTTCCTGGGGGGGAGTACTACAGCAGC
		**Chil3-2**	AGGCCATCAAAGTGGCACTTGAGCTAGAAAATGCTGCTGTAGTACTCCAGTCCAGGTTG	GACCCAGGGTACTCAGTACTACAGCAGC
		**Chil3-3**	TGAGCCACTGAGCGGCACTTGAGCTAGAAAATGCTGCTGTAGTACTAAATTGTTGTCCT	CCACGGCACCTCCAAGTACTACAGCAGC
		**Chil3-4**	GAACCACTGAAGTGGCACTTGAGCTAGAAAATGCTGCTGTAGTACTCTGGTGACAGAAA	AGAGGGAAATGTCAAGTACTACAGCAGC
		**Chil3-5**	GTAGGGGCACCAAGGCACTTGAGCTAGAAAATGCTGCTGTAGTACTGCCAGTACTAATT	TACTTTCCTGGTGCAGTACTACAGCAGC
		**Chil3-6**	TAAAGTAGATGTCGGCACTTGAGCTAGAAAATGCTGCTGTAGTACTTGAGATCTCCCTT	TGCACTGTGTATAAAGTACTACAGCAGC

		**Chil3-7**	CACTCATTACCCTGGCACTTGAGCTAGAAAATGCTGCTGTAGTACTGTCATAACCAACC	AAGCTCCTGACATAAGTACTACAGCAGC
		**Chil3-8**	CAGTAGGAAATGAGGCACTTGAGCTAGAAAATGCTGCTGTAGTACTTCCATGGTCCTTC	TTCTCAGAAGCTGGAGTACTACAGCAGC
		**Chil3-9**	ATGCTGGAAATCCGGCACTTGAGCTAGAAAATGCTGCTGTAGTACTAAGGTATGCCCAT	GGTCACTCAGGATTAGTACTACAGCAGC
		**Chil3-10**	ATAGTCAAGAGACGGCACTTGAGCTAGAAAATGCTGCTGTAGTACTTCATGACCTGAAT	ATGGAGATCATATCAGTACTACAGCAGC
		**Chil3-11**	CTATTTTCTCCAGGGCACTTGAGCTAGAAAATGCTGCTGTAGTACTTTTATAGAGGGGA	ATGTCATATGGAGTAGTACTACAGCAGC
		**Chil3-12**	CTTTACGCATTTCGGCACTTGAGCTAGAAAATGCTGCTGTAGTACTTCTTCCTCAAAAG	CTTTCTCCACAGAAAGTACTACAGCAGC
	S100a9	**S100a9-1**	AGGTGCTTTGTTGGGCACTTGAGCTAGAAAGGAGACTGATTACACGGCTCCATCTGAGA	GGTGGTTATGCTGGCGTGTAATCAGTCT
		**S100a9-2**	CATGACAGGCAAAGGCACTTGAGCTAGAAAGGAGACTGATTACACGTCATGCAGCTTCT	CACGTGGGTTGTTGCGTGTAATCAGTCT
		**S100a9-3**	CATTTATGAGGGCGGCACTTGAGCTAGAAAGGAGACTGATTACACGTCCTCCATGATGT	GGTTTGTGTCCAGCCGTGTAATCAGTCT
		**S100a9-4**	CAAAGCTCAGCTGGGCACTTGAGCTAGAAAGGAGACTGATTACACGATCATACACTCCT	ACTTTGCCATCAGGCGTGTAATCAGTCT
		**S100a9-5**	TATTGATGGAAGGGGCACTTGAGCTAGAAAGGAGACTGATTACACGTTCCTTCCTAGAG	GTGTCAGGGTGTCGCGTGTAATCAGTCT
		**S100a9-6**	CCATTTGTCTGAAGGCACTTGAGCTAGAAAGGAGACTGATTACACGAACTGTGCTTCCA	TCATAAAGGTTGCGCGTGTAATCAGTCT
	Retnlg	**Retnlg-1**	ACCAGTGACAGTCATGCTGCTGTAGTACTAGGAGACTGATTACACGAGCCACAAGCACA	AGATCCACAGCCAACGTGTAATCAGTCT
		**Retnlg-2**	AAGCCGTGATACTATGCTGCTGTAGTACTAGGAGACTGATTACACGGCCAGTCTGCCTG	CAGAAGGACAGGACCGTGTAATCAGTCT
		**Retnlg-3**	TATTCACTGGGACATGCTGCTGTAGTACTAGGAGACTGATTACACGAAGGTCCCCTCAG	CCACAATAGATTCACGTGTAATCAGTCT
		**Retnlg-4**	AGAAGGGAACAAGATGCTGCTGTAGTACTAGGAGACTGATTACACGGGAGATGCAGATG	ATCAGCTGGAGAACCGTGTAATCAGTCT
		**Retnlg-5**	GGAGGGACAGTCAATGCTGCTGTAGTACTAGGAGACTGATTACACGTCTTTGTTACAGT	AGTACAGGAGAAACCGTGTAATCAGTCT
		**Retnlg-6**	TCCCGGATATCCCATGCTGCTGTAGTACTAGGAGACTGATTACACGGCAAGTATTTCCA	GAGCACTGACAGTCCGTGTAATCAGTCT
		**Retnlg-7**	CCAGTCCATTGTTATGCTGCTGTAGTACTAGGAGACTGATTACACGAACGGGCGGTGGC	GGCCAGTTGGCAGGCGTGTAATCAGTCT
	Ccl5	**Ccl5-1**	GTGTCCGAGCCATATGCTGCTGTAGTACTACCTCGATTAACATCGCGCAGCAGGGAGTG	GAGAGGTAGGCAATGCGATGTTAATCGA
		**Ccl5-2**	ACGTGGGCACGAGATGCTGCTGTAGTACTACCTCGATTAACATCGCGAAATACTCCTTG	TTGCTGCTGGTGTTGCGATGTTAATCGA
		**Ccl5-3**	TGAACCCACTTCTATGCTGCTGTAGTACTACCTCGATTAACATCGCGTTGATGTATTCT	CTCATCTCCAAATTGCGATGTTAATCGA
		**Ccl5-4**	CAGAGATCTTCATATGCTGCTGTAGTACTACCTCGATTAACATCGCGTGAGGGCAGCTG	CAGTGAGGATGATCGCGATGTTAATCGA
	Gzma	**Gzma-1**	CTTCTCTCCACCAGGAGACTGATTACACGACCTCGATTAACATCGCGGCGATCTCCACA	GACACCAGGCCATGGCGATGTTAATCGA
		**Gzma-2**	TCCTGGTTTCACAGGAGACTGATTACACGACCTCGATTAACATCGCCTCGGCATCTGGT	CCCCCATCCTGCTTGCGATGTTAATCGA
		**Gzma-3**	GTCCAATTACAGGGGAGACTGATTACACGACCTCGATTAACATCGCCAAATCATGTTTA	GGAGGTCCCCTGCTGCGATGTTAATCGA
		**Gzma-4**	CTATGACAGTGATGGAGACTGATTACACGACCTCGATTAACATCGCCAGATTTTTCTGT	GTTTTTCATCATTCGCGATGTTAATCGA
		**Gzma-5**	AGCTGACTTATTGGGAGACTGATTACACGACCTCGATTAACATCGCGAGTTTCAGAGGG	GTTGACTTCTCTCTGCGATGTTAATCGA
		**Gzma-6**	TGCCAGAATCCCCGGAGACTGATTACACGACCTCGATTAACATCGCCATAGCAGAGGGC	GCAAAATACCATCTGCGATGTTAATCGA
		**Gzma-7**	ATTGAGGTGTTTAGGAGACTGATTACACGACCTCGATTAACATCGCTCTTCTTTATCCA	AGAACCCTTCATATGCGATGTTAATCGA
		**Gzma-8**	CCACATTTCTGTTGGAGACTGATTACACGACCTCGATTAACATCGCAGGTGAAGGATAG	CTCCCTTTTTAGGAGCGATGTTAATCGA
		**Gzma-9**	GTTCTTTTCAATCGGAGACTGATTACACGACCTCGATTAACATCGCCAGTCAACACCCA	GTTACAGTGGGCACGCGATGTTAATCGA

		**Gzma-10**	TCATCATAGCATGGGAGACTGATTACACGACCTCGATTAACATCGCCTCACGTGTATAT	AGTTGTAGATCCCGGCGATGTTAATCGA
		**Gzma-11**	GCTCCTTATTGATGGAGACTGATTACACGACCTCGATTAACATCGCATCTGTTGTTCTG	TCTTAACAGTCAAAGCGATGTTAATCGA
		**Gzma-12**	TAGAGCCATATACGGAGACTGATTACACGACCTCGATTAACATCGCAACTAAGTTTAAG	ACAGATGGTATTTCGCGATGTTAATCGA
		**Angptl4-1**	ACTGTCCAGCCTCGTGCTTGAACTGGTCTAGTGCTTGAACTGGTCTGCGTCTCTGAATC	ACAGAGCCGTTCACAGACCAGTTCAAGC
		**Angptl4-2**	CTTTTCCAGGCCCGTGCTTGAACTGGTCTAGTGCTTGAACTGGTCTTGATGCTGTGCAT	TCCTCGGTTCCCTCAGACCAGTTCAAGC
		**Angptl4-3**	TCCCCTCGAAGTCGTGCTTGAACTGGTCTAGTGCTTGAACTGGTCTTGGGAAGCCTCTT	GAGCTGGGTCATCAAGACCAGTTCAAGC
		**Angptl4-4**	TCCTGGAGCTGCAGTGCTTGAACTGGTCTAGTGCTTGAACTGGTCTATTGCCATCCCAG	TGGAGCAATTTGGGAGACCAGTTCAAGC
		**Angptl4-5**	GATGCAAAGCGCGGTGCTTGAACTGGTCTAGTGCTTGAACTGGTCTCATCTCGTCCCAG	TGAGCCAGCAAGTAAGACCAGTTCAAGC
	Angptl4	**Angptl4-6**	CTCACCCCCCAAAGTGCTTGAACTGGTCTAGTGCTTGAACTGGTCTTGTAGGCTGTGTC	AGTGAGCTGCAGGGAGACCAGTTCAAGC
		**Angptl4-7**	TCAGCAGGGTGGTGTGCTTGAACTGGTCTAGTGCTTGAACTGGTCTTCCATGGGCTGGA	AGGCTGCTGTAGCGAGACCAGTTCAAGC
		**Angptl4-8**	ATTGGTGGCACCCGTGCTTGAACTGGTCTAGTGCTTGAACTGGTCTCATTGGGGGAAAC	GGGCAGGGAAAGGGAGACCAGTTCAAGC
		**Angptl4-9**	TGGTCCCAAGTAGGTGCTTGAACTGGTCTAGTGCTTGAACTGGTCTGAGGTCATGGTCT	TTAAGGTCCCCACCAGACCAGTTCAAGC
		**Angptl4-10**	TTTTCCCTTGGGCGTGCTTGAACTGGTCTAGTGCTTGAACTGGTCTTGAAGGGTGCATC	ATCCTCGGAGTCTAAGACCAGTTCAAGC
		**Angptl4-11**	TTGAAGGGTGCATGTGCTTGAACTGGTCTAGTGCTTGAACTGGTCTATCCTCGGAGTCT	CCTTCAGGGACTCAAGACCAGTTCAAGC
		**Angptl4-12**	AACCACCAGCCACGTGCTTGAACTGGTCTAGTGCTTGAACTGGTCTGCTACAGGTACCA	TTGAGATTGGAATCAGACCAGTTCAAGC
		**Sdc4-1**	TATCCAGTGGCACCTGACTAACGACACCAACTGACTAACGACACCATCAGGGATGTGGT	CAGGCTGTGCATTGTGGTGTCGTTAGTC
		**Sdc4-2**	TGACCTCTGTCTCCTGACTAACGACACCAACTGACTAACGACACCATCCTGGGGGTCGA	TGCCTTCCAGGAGCTGGTGTCGTTAGTC
		**Sdc4-3**	GATGCCTACCACGCTGACTAACGACACCAACTGACTAACGACACCAAAACGGCAAAGAG	CAGCAGGATCAGGTTGGTGTCGTTAGTC
		**Sdc4-4**	TCTTCTTCATGCGCTGACTAACGACACCAACTGACTAACGACACCACTGCCTTCGTCCT	TGCCCAAGTCGTATTGGTGTCGTTAGTC
		**Sdc4-5**	TGTAGATGGGTTTCTGACTAACGACACCAACTGACTAACGACACCAGTGGGGGCTTTTT	CGTAGAACTCATTCTGGTGTCGTTAGTC
	Sdc4	**Sdc4-6**	CTTGGGCTCTGAGCTGACTAACGACACCAACTGACTAACGACACCATCTCTTCCAGTTC	AGGAATGACCTCAATGGTGTCGTTAGTC
		**Sdc4-7**	CATCCCCCACGTCCTGACTAACGACACCAACTGACTAACGACACCATTGTTGGACATGT	TGGACATGGATACATGGTGTCGTTAGTC
		**Sdc4-8**	GTTGCTGCCCTGGCTGACTAACGACACCAACTGACTAACGACACCATTCTCTCAAAGAT	TGCCAAGACCTCACTGGTGTCGTTAGTC
		**Sdc4-9**	TGGGCTCCTCCGTCTGACTAACGACACCAACTGACTAACGACACCATCAGGGAAGGGCC	AGGGCTCAATCACATGGTGTCGTTAGTC
		**Sdc4-10**	AGGGCTCCAGAGACTGACTAACGACACCAACTGACTAACGACACCATTCGTCGTCGGGG	GAGCCGCCAGCATGTGGTGTCGTTAGTC
		**Sdc4-11**	AGGCAGGCGCCATCTGACTAACGACACCAACTGACTAACGACACCAGGCGCAAGCAGGC	GCAGCAGCAGCAGGTGGTGTCGTTAGTC
		**Sdc2-1**	CAGGATCCACGCGACTTGGATCCACTCGTAACTTGGATCCACTCGTAGCCCAAGGTGAG	CACACAGGCCATCTACGAGTGGATCCAA
		**Sdc2-2**	AGCAGGATGAGGAACTTGGATCCACTCGTAACTTGGATCCACTCGTGCGGTACACCAAT	TCTTTCTTCCGCAAACGAGTGGATCCAA
	Sdc2	**Sdc2-3**	GTGGGATTCTTGGACTTGGATCCACTCGTAACTTGGATCCACTCGTGCAGCACTAGTGA	CCACTTTGGGGGATACGAGTGGATCCAA
		**Sdc2-4**	TCCAAGGTCGTAGACTTGGATCCACTCGTAACTTGGATCCACTCGTATGGTTTGCGTTC	GTAAGCTGCGCTGCACGAGTGGATCCAA
		**Sdc2-5**	AAGGTACATATCCACTTGGATCCACTCGTAACTTGGATCCACTCGTTGGAGCTATTGTC	TGAAGCTTCCTCATACGAGTGGATCCAA
		**Sdc2-6**	ATCATCAATAGGAACTTGGATCCACTCGTAACTTGGATCCACTCGTAAGAATAGTCATC	TGAGCCTGAGGCACACGAGTGGATCCAA

		**Sdc2-7**	TGTCTTCAACGTCACTTGGATCCACTCGTAACTTGGATCCACTCGTGTGTAATGCTTTG	CTCAGTCTGAGCACACGAGTGGATCCAA
		**Sdc2-8**	AGTTTCTTCAGGTACTTGGATCCACTCGTAACTTGGATCCACTCGTCTTCCTCCTTGTC	CTCAGAAATGTCATACGAGTGGATCCAA
		**Sdc2-9**	CCCAGCTTCTCTTACTTGGATCCACTCGTAACTTGGATCCACTCGTTTTTATAGCAGGG	TACACATCTGTGCAACGAGTGGATCCAA
		**Sdc2-10**	GATTGTCTGAATGACTTGGATCCACTCGTAACTTGGATCCACTCGTGTCCGTTTAAACA	CTGCTAGAACTTCAACGAGTGGATCCAA
		**Sdc2-11**	CACCAGCAATGACACTTGGATCCACTCGTAACTTGGATCCACTCGTAAGCCGATCACAC	AAATGGCAAAGAGTACGAGTGGATCCAA
	Tlr4	**Tlr4-1**	TGCCTTGTCTTCAGATCTCGAGAACGTCAAGATCTCGAGAACGTCAGTAAGCCATGCCA	GTTTGAGAGGTGGATGACGTTCTCGAGA
		**Tlr4-2**	AGGCAGGAGAAGAGATCTCGAGAACGTCAAGATCTCGAGAACGTCAGCTTCCTGGTGTC	ATGCAGGGATTCATTGACGTTCTCGAGA
		**Tlr4-3**	AAGTGTCTCAGGCGATCTCGAGAACGTCAAGATCTCGAGAACGTCAGAAGCTGAGGTCT	ATGATGGCACCATATGACGTTCTCGAGA
		**Tlr4-4**	TGGCTACACCAGGGATCTCGAGAACGTCAAGATCTCGAGAACGTCAATGTTGGCAGCAA	AGCCTTCCTGGATCTGACGTTCTCGAGA
		**Tlr4-5**	CTGCCATGTTTGAGATCTCGAGAACGTCAAGATCTCGAGAACGTCAGGCTGCTCAGAAA	GATGATGCCAGAGGTGACGTTCTCGAGA
		**Tlr4-6**	ATTCCACCTGCTGGATCTCGAGAACGTCAAGATCTCGAGAACGTCAAGAAGGCGATACA	AGGTGTTTCTGCTTTGACGTTCTCGAGA
		**Tlr4-7**	TGCTCAGGATTCGGATCTCGAGAACGTCAAGATCTCGAGAACGTCATTCCTCTGCTGTT	GCCGTTTCTTGTTGTGACGTTCTCGAGA
		**Tlr4-8**	AAGATCTTCAAGGGATCTCGAGAACGTCAAGATCTCGAGAACGTCAGCTATAGCTTTTT	GAAAAATTGGAGATTGACGTTCTCGAGA
		**Tlr4-9**	AGTTTCTTTAAGGGATCTCGAGAACGTCAAGATCTCGAGAACGTCAGTGAGCCACATTG	GAATGTATAAAATATGACGTTCTCGAGA
		**Tlr4-10**	TGAATGAAGTCAAGATCTCGAGAACGTCAAGATCTCGAGAACGTCAAAAGGCTTGGTCT	AGCTTAATTCCCTCTGACGTTCTCGAGA
		**Tlr4-11**	CCCAAGATCAACCGATCTCGAGAACGTCAAGATCTCGAGAACGTCAATCTTTAAATTCT	TCCAGATTCCTTTGTGACGTTCTCGAGA
		**Tlr4-12**	GATTGCCATTTGAGATCTCGAGAACGTCAAGATCTCGAGAACGTCAAATGATTGATAAG	TTAAGTTGACATCATGACGTTCTCGAGA
	Cd226	**Cd226-1**	TGCAGACAGGTAGGTGCTTGAACTGGTCTACTGACTAACGACACCACTTGTCCAGGTTC	AGTGAGTGTGACAATGGTGTCGTTAGTC
		**Cd226-2**	TGCTCCTTGTTTGGTGCTTGAACTGGTCTACTGACTAACGACACCACCCTGGCTACAGT	TGCTCTTCATGCTGTGGTGTCGTTAGTC
		**Cd226-3**	GTAACATAAGCCAGTGCTTGAACTGGTCTACTGACTAACGACACCAAGCCAAAAGCCAA	TGCACATGAAGAAATGGTGTCGTTAGTC
		**Cd226-4**	CTGGCCAAGTCCTGTGCTTGAACTGGTCTACTGACTAACGACACCAATGACTTGTTGCA	GGACTTTTTCCCAATGGTGTCGTTAGTC
		**Cd226-5**	AAGCTAAGATGTCGTGCTTGAACTGGTCTACTGACTAACGACACCAGATAGGTTACAGG	ATCTTGTCTCTTGTTGGTGTCGTTAGTC
		**Cd226-6**	GGCCATGGCATTTGTGCTTGAACTGGTCTACTGACTAACGACACCAGTCCTGAGTCAGC	GCGACATCTGTAATTGGTGTCGTTAGTC
		**Cd226-7**	TGTAATGGCCTCTGTGCTTGAACTGGTCTACTGACTAACGACACCAACTTGTTTTTTCC	CCTTATGACAAAGCTGGTGTCGTTAGTC
		**Cd226-8**	AATGTTTATTGGTGTGCTTGAACTGGTCTACTGACTAACGACACCAATGGGAAGGATAA	CTAACCCTCCAACCTGGTGTCGTTAGTC
		**Cd226-9**	ATTAGGATGACAAGTGCTTGAACTGGTCTACTGACTAACGACACCAGAAAATGATGATA	CTGTTATATAAAAATGGTGTCGTTAGTC
		**Cd226-10**	GTCTCCGTCTCTTGTGCTTGAACTGGTCTACTGACTAACGACACCAGGAATTCTCACCT	TGGGCTCTTTAAGATGGTGTCGTTAGTC
		**Cd226-11**	CAGTTGGTGGCTAGTGCTTGAACTGGTCTACTGACTAACGACACCAAGTAGGACTTCTG	GACTGGATGGGAGTTGGTGTCGTTAGTC
		**Cd226-12**	CTTTTTCATCATCGTGCTTGAACTGGTCTACTGACTAACGACACCAACATAAATGTCCT	AAGTTGGATAGTTATGGTGTCGTTAGTC
	Nectin2	**Nectin2-1**	CAGCGTGGTTCTCGTGCTTGAACTGGTCTAACTTGGATCCACTCGTACCTCCTGGGCTT	GGGGGCCAATTGTCACGAGTGGATCCAA
		**Nectin2-2**	CATCACAGGTCAGGTGCTTGAACTGGTCTAACTTGGATCCACTCGTGGGTTGCTTCGTA	AGTCTGTGGGCTCAACGAGTGGATCCAA
		**Nectin2-3**	CTCCCCCTCAGCAGTGCTTGAACTGGTCTAACTTGGATCCACTCGTCTTCCGCCTCCTC	CCTCCTCCAGGGCAACGAGTGGATCCAA

		**Nectin2-4**	CAACTGTGCCATCGTGCTTGAACTGGTCTAACTTGGATCCACTCGTTGGAAAGCAGCCA	CTCCGAAGGATGGCACGAGTGGATCCAA
		**Nectin2-5**	TGCTGAACTGAGAGTGCTTGAACTGGTCTAACTTGGATCCACTCGTGACAGACGGTCCT	TCGCTCTGACAAACACGAGTGGATCCAA
		**Nectin2-6**	AAGACAGGACCCCGTGCTTGAACTGGTCTAACTTGGATCCACTCGTTGCTGACCTCCAG	ATGGGCTCAGGGGTACGAGTGGATCCAA
		**Nectin2-7**	CTCGTGTTCCACTGTGCTTGAACTGGTCTAACTTGGATCCACTCGTGCTCTTCGAAGCT	TGGCAGCAGGATCCACGAGTGGATCCAA
		**Nectin2-8**	CTACCCTCAGTCCGTGCTTGAACTGGTCTAACTTGGATCCACTCGTTTGCCCTCGTCCT	ACTCGCAGGTGTATACGAGTGGATCCAA
		**Nectin2-9**	ACTCGTGTGGAGGGTGCTTGAACTGGTCTAACTTGGATCCACTCGTTCGTCCTTAGGTG	GATGGGACTCCATCACGAGTGGATCCAA
		**Nectin2-10**	AGGCCTGGGGGGTGTGCTTGAACTGGTCTAACTTGGATCCACTCGTCCCACATCTCGGG	CCCACACCAGCGGTACGAGTGGATCCAA
		**Nectin2-11**	GCACCAGCAATGTGTGCTTGAACTGGTCTAACTTGGATCCACTCGTCCAGCCAGGAGTA	AGGCCAGGAACCCGACGAGTGGATCCAA
		**Nectin2-12**	AAGACGCCCGAGGGTGCTTGAACTGGTCTAACTTGGATCCACTCGTTGCAGAGGCTGGG	GAGCCCTGGGCCAGACGAGTGGATCCAA
	Col6a3	**Col6a3-1**	CTCCCAATTCACCGTGCTTGAACTGGTCTAGATCTCGAGAACGTCATCCAAGCCGATTT	CACCATCCAGACCTTGACGTTCTCGAGA
		**Col6a3-2**	TGTCTCCCCTCTCGTGCTTGAACTGGTCTAGATCTCGAGAACGTCACCAATGGGCCCTC	TTGGCCCAATGCTCTGACGTTCTCGAGA
		**Col6a3-3**	ATCGCCCACAATGGTGCTTGAACTGGTCTAGATCTCGAGAACGTCACTGCAATGGTCAG	CGGACAGTTGCTCATGACGTTCTCGAGA
		**Col6a3-4**	AGTGGGCATTCAGGTGCTTGAACTGGTCTAGATCTCGAGAACGTCATCATCCTTGCTGA	CAGCGTTCTGTACATGACGTTCTCGAGA
		**Col6a3-5**	ACCGGATCTCTGTGTGCTTGAACTGGTCTAGATCTCGAGAACGTCATTGGAGTCAGCAA	GGGCAGACTTCTTGTGACGTTCTCGAGA
		**Col6a3-6**	TGATTCCGCAGCAGTGCTTGAACTGGTCTAGATCTCGAGAACGTCACTCCGGCACAAAG	AGGCGGCTACCAGGTGACGTTCTCGAGA
		**Col6a3-7**	TCCTCACACGTTTGTGCTTGAACTGGTCTAGATCTCGAGAACGTCAATCAGGAAGCCAC	CAGCCACTTTCCTGTGACGTTCTCGAGA
		**Col6a3-8**	CACTGACTTCCCTGTGCTTGAACTGGTCTAGATCTCGAGAACGTCACCTGAGCATCCTC	AGCCAGGCTCACAATGACGTTCTCGAGA
		**Col6a3-9**	ACCTTGACACCTTGTGCTTGAACTGGTCTAGATCTCGAGAACGTCAGCCAACGGCAAAC	TCGATGTTCCTCAGTGACGTTCTCGAGA
		**Col6a3-10**	ATCTTGCCCACCTGTGCTTGAACTGGTCTAGATCTCGAGAACGTCAACTGTTGGAGGCT	CGGAAGGCTGTGGGTGACGTTCTCGAGA
		**Col6a3-11**	TGACCCTCTCTTCGTGCTTGAACTGGTCTAGATCTCGAGAACGTCACCAAAGGAAAGCA	TAGCCCCAGAGGGATGACGTTCTCGAGA
		**Col6a3-12**	AGGCTCCTCGTTGGTGCTTGAACTGGTCTAGATCTCGAGAACGTCACAAAGCGCATCAG	TGGGAGCAGCCTCGTGACGTTCTCGAGA
	Col6a1	**Col6a1-1**	TTCCTCGCTCCCCCTGACTAACGACACCAAACTTGGATCCACTCGTGGAAGACCTGGCT	CTCCCTTCTCTCCGACGAGTGGATCCAA
		**Col6a1-2**	TCGAGCACGAAGACTGACTAACGACACCAAACTTGGATCCACTCGTACTCTCCGAGGTG	AGCCTCAAGGCCAGACGAGTGGATCCAA
		**Col6a1-3**	TGTAGTGCAGCGCCTGACTAACGACACCAAACTTGGATCCACTCGTTCCACCTCGTCAC	GCCCTCGGATGATGACGAGTGGATCCAA
		**Col6a1-4**	CTTGTAGCCCTCTCTGACTAACGACACCAAACTTGGATCCACTCGTCCCCGCATGGTTC	TGCATCTTCCAGAGACGAGTGGATCCAA
		**Col6a1-5**	CTTGCGATAAGCCCTGACTAACGACACCAAACTTGGATCCACTCGTATACCTGGCCGAC	CCTTCACCAGGGACACGAGTGGATCCAA
		**Col6a1-6**	AATGACCAGGGCACTGACTAACGACACCAAACTTGGATCCACTCGTAACGTCCATCCGT	CCGTTGAGTGTCACACGAGTGGATCCAA
		**Col6a1-7**	AGTACTTGACCGCCTGACTAACGACACCAAACTTGGATCCACTCGTGTGCCTTTCCCGA	CGCAGTCGGTGTACACGAGTGGATCCAA
		**Col6a1-8**	CTTGGTGGCACCTCTGACTAACGACACCAAACTTGGATCCACTCGTACAGCACTCTCTT	GCCGTCTGAAAACTACGAGTGGATCCAA
		**Col6a1-9**	TCTCAATGCCTGCCTGACTAACGACACCAAACTTGGATCCACTCGTACCACCACAAAGA	CCTGGGGTCCCACGACGAGTGGATCCAA
		**Col6a1-10**	GTAGAGTACGCCACTGACTAACGACACCAAACTTGGATCCACTCGTTGGAGACTGTCTG	CAGTGCCACCTTCGACGAGTGGATCCAA
		**Col6a1-11**	CGGGGAGGAGAATCTGACTAACGACACCAAACTTGGATCCACTCGTTGGTGATGTCAGC	GCTGTCTAGCAGGTACGAGTGGATCCAA

		**Col6a1-12**	AGTTGTGGCTGCCCTGACTAACGACACCAAACTTGGATCCACTCGTTTGGTGGTTTCGA	GCTTGGCGAAGACGACGAGTGGATCCAA
	Cd44	**Cd44-1**	TTTCTTCTGCCCACTGACTAACGACACCAAGATCTCGAGAACGTCATGATCACCAGCTT	CCCATTGCCACCGATGACGTTCTCGAGA
		**Cd44-2**	GTGCCACCAAAACCTGACTAACGACACCAAGATCTCGAGAACGTCAGTCCCCAAGCTGT	CTGCAAGAGGCAATTGACGTTCTCGAGA
		**Cd44-3**	GAGAGATGCCAAGCTGACTAACGACACCAAGATCTCGAGAACGTCAGAGCCAGTGCCAG	GACGGCAAGAATCTTGACGTTCTCGAGA
		**Cd44-4**	CTGTAGCGGCCATCTGACTAACGACACCAAGATCTCGAGAACGTCAAGTCCGGGAGATA	AGGTCAGCTGCCTGTGACGTTCTCGAGA
		**Cd44-5**	ACTGGGTTTCCTGCTGACTAACGACACCAAGATCTCGAGAACGTCACCCCGTTGAGCTC	AGACTTGCTGGCCATGACGTTCTCGAGA
		**Cd44-6**	GTTCCTTGTTCACCTGACTAACGACACCAAGATCTCGAGAACGTCAGGGGTCTCTGATG	TCATACACTGGTCGTGACGTTCTCGAGA
		**Cd44-7**	AGATTCCGGGTCTCTGACTAACGACACCAAGATCTCGAGAACGTCAGTCCACACTCTGC	ACCCCAATCTTCAATGACGTTCTCGAGA
		**Cd44-8**	GCACTTGAGTGTCCTGACTAACGACACCAAGATCTCGAGAACGTCAACTGTCCTGGTTC	GTTGTGGTCACTCGTGACGTTCTCGAGA
		**Cd44-9**	GAGTCTCGATCTCCTGACTAACGACACCAAGATCTCGAGAACGTCAGGAGTCCTTGGAT	CGGGAACTCCCCCATGACGTTCTCGAGA
		**Cd44-10**	CGGTTGACAATAGCTGACTAACGACACCAAGATCTCGAGAACGTCAGCGAGTACCATCA	CCCTTCTTGCTGTTTGACGTTCTCGAGA
		**Cd44-11**	TGAAGCATCGATGCTGACTAACGACACCAAGATCTCGAGAACGTCACATCTATAATGTT	GCTGCTGACATCGATGACGTTCTCGAGA
		**Cd44-12**	GTGCTCTTCTCGACTGACTAACGACACCAAGATCTCGAGAACGTCAGTAGCTTTCTGGG	TAGGTGTGCAAAAATGACGTTCTCGAGA
	Col4a1	**Col4a1-1**	CACCAGGATCTCCACTTGGATCCACTCGTAGATCTCGAGAACGTCATGGCCGAGAATTT	GTGTTCCGGGAACCTGACGTTCTCGAGA
		**Col4a1-2**	ACCCATTTCACCTACTTGGATCCACTCGTAGATCTCGAGAACGTCAGGGTTCCCATGAC	GCCTGGTTGTCCCCTGACGTTCTCGAGA
		**Col4a1-3**	CTGGAGGGCCATCACTTGGATCCACTCGTAGATCTCGAGAACGTCAATACCTGGGGGAC	CGTTGCATCCTGGCTGACGTTCTCGAGA
		**Col4a1-4**	CTCCCTTTGAACCACTTGGATCCACTCGTAGATCTCGAGAACGTCAGGCAGTCCCATAT	ATCCTGGAACTCCCTGACGTTCTCGAGA
		**Col4a1-5**	TCTCTCCCGGGATACTTGGATCCACTCGTAGATCTCGAGAACGTCACCCACAGCACCTT	CAATTCCTGGCTGCTGACGTTCTCGAGA
		**Col4a1-6**	TCCACGACTACCAACTTGGATCCACTCGTAGATCTCGAGAACGTCAGAGGGCCGATGTC	TCCTGGGGGTCCACTGACGTTCTCGAGA
		**Col4a1-7**	ACCGTTAGGGCACACTTGGATCCACTCGTAGATCTCGAGAACGTCAACAGTGAGGACCA	GGAATAGCCGATCGTGACGTTCTCGAGA
		**Col4a1-8**	TCCTTTGGCTCCCACTTGGATCCACTCGTAGATCTCGAGAACGTCAACTGCCCTTTCTC	GCCAGGTAGACCTCTGACGTTCTCGAGA
		**Col4a1-9**	CACCTTTGAGTCGACTTGGATCCACTCGTAGATCTCGAGAACGTCAGGGTCTCCTTTGT	GTCCCGGAAAACCATGACGTTCTCGAGA
		**Col4a1-10**	TCCAGGAAGGCCAACTTGGATCCACTCGTAGATCTCGAGAACGTCAGTGTCCCCTGCTG	CCCTGGAACTCCACTGACGTTCTCGAGA
		**Col4a1-11**	AGCACCATGTTGTACTTGGATCCACTCGTAGATCTCGAGAACGTCAAAGGGGGGGGAGT	AGGGAGAAGAGGAGTGACGTTCTCGAGA
		**Col4a1-12**	AAGCATTTGCGTAACTTGGATCCACTCGTAGATCTCGAGAACGTCAAGCCAAAAGCTGT	TCTCTATGGTGGCCTGACGTTCTCGAGA
	Col6a2	**Col6a2-1**	CACAGCGCTTCTCAGACGTAATGACAGCGATCCAGTACGTCGACTAACATCCAGGGCAC	CGATGACGAAGACGTAGTCGACGTACTG
		**Col6a2-2**	GTGTCCAACACGAAGACGTAATGACAGCGATCCAGTACGTCGACTACACGCTCTCTGAG	GGGGACTGCATGGGTAGTCGACGTACTG
		**Col6a2-3**	TCCCCCAATCAGGAGACGTAATGACAGCGATCCAGTACGTCGACTAGGACCCCCAAGAG	CTGCTGGGCATGGTTAGTCGACGTACTG
		**Col6a2-4**	GAGATGGCTTCCTAGACGTAATGACAGCGATCCAGTACGTCGACTACTGCTCCTGGGGT	GGTGAGACAGCTTGTAGTCGACGTACTG
		**Col6a2-5**	AGACCACCGTAGCAGACGTAATGACAGCGATCCAGTACGTCGACTAGTCCGAGAAGTGT	AACACCTCCACTTCTAGTCGACGTACTG
		**Col6a2-6**	TCTGGCTGCCATAAGACGTAATGACAGCGATCCAGTACGTCGACTAACCTGTTGCTGAT	TCAGTGGGAAGGCGTAGTCGACGTACTG
		**Col6a2-7**	GACCACTGCGAACAGACGTAATGACAGCGATCCAGTACGTCGACTAGCCCATCCGTGAT	TCGGGGGTCATGGGTAGTCGACGTACTG

		**Col6a2-8**	ACACAGTGCCCGAAGACGTAATGACAGCGATCCAGTACGTCGACTATGACATCTCGGTC	AATGGCTGTCACACTAGTCGACGTACTG
		**Col6a2-9**	CTGCCGGATCTGCAGACGTAATGACAGCGATCCAGTACGTCGACTACCTTGCCTACGTG	GAAGTTGACCACCGTAGTCGACGTACTG
		**Col6a2-10**	TTGATGCCCCCACAGACGTAATGACAGCGATCCAGTACGTCGACTACTCAGCCTGCATC	TCTTCACGGGCACCTAGTCGACGTACTG
		**Col6a2-11**	TGAACTTCTCGGCAGACGTAATGACAGCGATCCAGTACGTCGACTATCCATGTCATCGA	GACAAAGGACGTCATAGTCGACGTACTG
		**Col6a2-12**	GCCACATAGAGCTAGACGTAATGACAGCGATCCAGTACGTCGACTATTGTGTGCACTGG	ATGTCCACGGGCCCTAGTCGACGTACTG
**Chicken**	***IFIT5***	**IFIT5-1**	ACGAGACTTCTCCTATGTGACCGGGTACAATATGTGACCGGGTACAGTTCCCCCAGGTA	TAGATCCAAGCGTTTGTACCCGGTCACA
		**IFIT5-2**	CAGGGATGAGTCCTATGTGACCGGGTACAATATGTGACCGGGTACAGCTTCAGAGGCTC	CAGTTTCAGTGCAGTGTACCCGGTCACA
		**IFIT5-3**	AGGAATTCTTGGATATGTGACCGGGTACAATATGTGACCGGGTACAAGGGAGTTCTTCA	ATTCTAGCTGCAGGTGTACCCGGTCACA
		**IFIT5-4**	GCAAAGTCCATGTTATGTGACCGGGTACAATATGTGACCGGGTACAACATCCTCCTTCA	GACTGTCAAGATCATGTACCCGGTCACA
		**IFIT5-5**	TCTGATCCTCTATTATGTGACCGGGTACAATATGTGACCGGGTACAATGAAAAACTCAA	AAATGTTGGGTTTATGTACCCGGTCACA
		**IFIT5-6**	CATAAGATAGTAGTATGTGACCGGGTACAATATGTGACCGGGTACATTTAGGTGGTATA	CTTCATCTGAGAGGTGTACCCGGTCACA
		**IFIT5-7**	CTTTTTGGAGATATATGTGACCGGGTACAATATGTGACCGGGTACAATTTCTTCTTCAG	GATAGTATTTTTTTGTACCCGGTCACA
		**IFIT5-8**	CTTCTTCATATCTTATGTGACCGGGTACAATATGTGACCGGGTACAATATACACTTGAG	TTTCCACTTTATTATGTACCCGGTCACA
		**IFIT5-9**	TTGAAAGCTTTTTTATGTGACCGGGTACAATATGTGACCGGGTACAAAATGAGCAGTAT	GAAGTTGAATCTTGTGTACCCGGTCACA
		**IFIT5-10**	AATCCTTGCTCAGTATGTGACCGGGTACAATATGTGACCGGGTACATTTTAATAGTGCA	TACTTTCCTCCAATTGTACCCGGTCACA
		**IFIT5-11**	GATACACTGCTATTATGTGACCGGGTACAATATGTGACCGGGTACAAATTCTTCCAAAC	ATCTTCTGTAAGATTGTACCCGGTCACA
		**IFIT5-12**	GATGCATTTCTCTTATGTGACCGGGTACAATATGTGACCGGGTACATCATTCCTTCTTC	ATAAGGGGTTTTCATGTACCCGGTCACA
	RSAD2	**RSAD2-1**	TCGTCCTTGTCCTGTCTATTAGTGGAGCCAGTCTATTAGTGGAGCCAGTCGGAACTGTC	TTGACGCTGGTGGCGGCTCCACTAATAG
		**RSAD2-2**	AACTGGACCAGCTGTCTATTAGTGGAGCCAGTCTATTAGTGGAGCCCTCCTCTTTGCAG	CTTGGCAGTTTCACGGCTCCACTAATAG
		**RSAD2-3**	AGCAGAAACCACAGTCTATTAGTGGAGCCAGTCTATTAGTGGAGCCTTGGCCGTGTGGA	GCACGAAGGAGGTGGGCTCCACTAATAG
		**RSAD2-4**	TGCTCACAATGCTGTCTATTAGTGGAGCCAGTCTATTAGTGGAGCCATCAGGCTGCCAT	ACCACCGTTCCCTCGGCTCCACTAATAG
		**RSAD2-5**	GCCAGCGGCAGGTGTCTATTAGTGGAGCCAGTCTATTAGTGGAGCCCGCCCGCGCCAGG	AGCGCTGCCAGCAGGGCTCCACTAATAG
		**RSAD2-6**	ACAGCGCGCTCAGGTCTATTAGTGGAGCCAGTCTATTAGTGGAGCCGTCAGCCCCCAGC	GCAGAACGAGGGGGGGCTCCACTAATAG
		**RSAD2-7**	ACCGCCGCCTCCAGTCTATTAGTGGAGCCAGTCTATTAGTGGAGCCGTGTCGGGGCCCG	GGGGTGCCGCGGGCGGCTCCACTAATAG
		**RSAD2-8**	GCTTGGCTTCCTCGTCTATTAGTGGAGCCAGTCTATTAGTGGAGCCATTGCCAGCCCCC	CCGCCTCCTTAAGGGGCTCCACTAATAG
		**RSAD2-9**	CCAGAATGTCTAAGTCTATTAGTGGAGCCAGTCTATTAGTGGAGCCTCACATGAAATTG	CCTCATTAAAACTTGGCTCCACTAATAG
		**RSAD2-10**	CACGACCAATTAAGTCTATTAGTGGAGCCAGTCTATTAGTGGAGCCTTCCTTCCTTGAC	TTTCCACATGGTTGGGCTCCACTAATAG
		**RSAD2-11**	ATCTTCCTCAACAGTCTATTAGTGGAGCCAGTCTATTAGTGGAGCCTCTGCTCATTCAT	ATTGAGTGCCTTGTGGCTCCACTAATAG
		**RSAD2-12**	CGCTGATAACAAAGTCTATTAGTGGAGCCAGTCTATTAGTGGAGCCTCAAAGTCTTCAT	GTTCCAGGAATTGGGGCTCCACTAATAG
	DDX60	**DDX60-1**	TTCACCACCAAGAATAACGATCCCTCACAAATAACGATCCCTCACACCTCTGCTCCAAT	AAGATGCTCCCAATTGTGAGGGATCGTT
		**DDX60-2**	ACGAAGCTCATCAATAACGATCCCTCACAAATAACGATCCCTCACACAGAATGCCAGTG	AGACAGTGGTCTGGTGTGAGGGATCGTT
		**DDX60-3**	ACACCACCAATCCATAACGATCCCTCACAAATAACGATCCCTCACAGTGAAGACTCCAC	GGCGATAATCTCGATGTGAGGGATCGTT

		**DDX60-4**	TGTGCACTTTCCAATAACGATCCCTCACAAATAACGATCCCTCACATCCCAAGGAAGCA	TGCTCTGGTTTTGGTGTGAGGGATCGTT
		**DDX60-5**	TACCCAATGCCCCATAACGATCCCTCACAAATAACGATCCCTCACAAGCTCCGTGATGA	TCCTTCATGGCCAATGTGAGGGATCGTT
		**DDX60-6**	CACTCAGTGATGTATAACGATCCCTCACAAATAACGATCCCTCACATCATCGCAGAGCT	CATTATCGTTTGGTTGTGAGGGATCGTT
		**DDX60-7**	TCTCCCAGTTTTTATAACGATCCCTCACAAATAACGATCCCTCACAACTTTGGTAGACT	ACTGCATTCAGTTATGTGAGGGATCGTT
		**DDX60-8**	AGGTAGTATGCATATAACGATCCCTCACAAATAACGATCCCTCACAAAAATCCTTCAGG	TGAATGAGGAGTAGTGTGAGGGATCGTT
		**DDX60-9**	AGCGTAGTACAGCATAACGATCCCTCACAAATAACGATCCCTCACATTAATTCCAAGGG	GGCAGTTTGTGACTTGTGAGGGATCGTT
		**DDX60-10**	GCCATTTTTCAGAATAACGATCCCTCACAAATAACGATCCCTCACAAGTTCAGAGCCAT	TCAAGTAGGATGCATGTGAGGGATCGTT
		**DDX60-11**	GCAGATCTTCTAGATAACGATCCCTCACAAATAACGATCCCTCACAGCAAAGTCTTCTG	CATTTACAGCTTCATGTGAGGGATCGTT
		**DDX60-12**	GCACCAGCTTTTCATAACGATCCCTCACAAATAACGATCCCTCACATTTGCCAGAATGA	TTCTCCCAAACAGTTGTGAGGGATCGTT
	ZNFX1	**ZNFX1-1**	CACAGTCCAACCTTACCCGAGATAGAGTCATACCCGAGATAGAGTCGTGCAAACATGTC	GGTGGCATGCTCTAGACTCTATCTCGGG
		**ZNFX1-2**	CATCCTCAAAGCCTACCCGAGATAGAGTCATACCCGAGATAGAGTCCGTGCATCCTCAT	CAAGCTGTACGAAAGACTCTATCTCGGG
		**ZNFX1-3**	AAAAGCTGCCCAGTACCCGAGATAGAGTCATACCCGAGATAGAGTCCTTGCGCAAACAG	TTGGCTGGCATGACGACTCTATCTCGGG
		**ZNFX1-4**	TTTGTCCCTTCCATACCCGAGATAGAGTCATACCCGAGATAGAGTCCTCCGTGAACCTT	GCAGCCTCTTCCTAGACTCTATCTCGGG
		**ZNFX1-5**	TGATCCCCAGCCCTACCCGAGATAGAGTCATACCCGAGATAGAGTCCGCTCAGCTTCAG	TGACAATCTGCACAGACTCTATCTCGGG
		**ZNFX1-6**	AGCATTTGTTGCATACCCGAGATAGAGTCATACCCGAGATAGAGTCCATTCCTGCCCAC	GACACCGAGTAGTTGACTCTATCTCGGG
		**ZNFX1-7**	TCGAAGCACCTGATACCCGAGATAGAGTCATACCCGAGATAGAGTCAACCGCATGCTTT	TCTGTCCATTCGGCGACTCTATCTCGGG
		**ZNFX1-8**	TGAGCCTTTGCTGTACCCGAGATAGAGTCATACCCGAGATAGAGTCCTCCTTCCTCCTC	GCCCTGTGTTCTTGGACTCTATCTCGGG
		**ZNFX1-9**	GTCTGAAGGCTGATACCCGAGATAGAGTCATACCCGAGATAGAGTCCCATGAGGAAGGA	GGCAGTTGTCTCTTGACTCTATCTCGGG
		**ZNFX1-10**	GTTCTGCACATGGTACCCGAGATAGAGTCATACCCGAGATAGAGTCCGCCACTCACATG	GGTAGTGCTTGCAGGACTCTATCTCGGG
		**ZNFX1-11**	TGGTGAGTGCAAGTACCCGAGATAGAGTCATACCCGAGATAGAGTCATAGCCAGCTCCT	GAGGTCCTTGGATTGACTCTATCTCGGG
		**ZNFX1-12**	ATAGCAACCGTCTTACCCGAGATAGAGTCATACCCGAGATAGAGTCACCAGGGATCCAT	CTCTGGACATGCACGACTCTATCTCGGG
	DHX58	**DHX58-1**	TCTTCTTCAGCCGTATGTGACCGGGTACAAGTCTATTAGTGGAGCCATCTGTGCCATGA	CCATGTGCTCCTGCGGCTCCACTAATAG
		**DHX58-2**	GATGGGGAACGTCTATGTGACCGGGTACAAGTCTATTAGTGGAGCCAGCTGAACTCCTT	GCAGTACTCCAGGAGGCTCCACTAATAG
		**DHX58-3**	TGATCACGTCCTGTATGTGACCGGGTACAAGTCTATTAGTGGAGCCTAGCGGAACAGTG	GGTTGAGCTCTCCCGGCTCCACTAATAG
		**DHX58-4**	GTTTGCTCAGCCGTATGTGACCGGGTACAAGTCTATTAGTGGAGCCAGGATCTCCTCCA	GGAAGTGCTCCTGGGGCTCCACTAATAG
		**DHX58-5**	GACGTTTCCCCCCTATGTGACCGGGTACAAGTCTATTAGTGGAGCCAGCCCCTTCAAAG	AGGATGTGCTCCAGGGCTCCACTAATAG
		**DHX58-6**	ATCAGCAGTGCATTATGTGACCGGGTACAAGTCTATTAGTGGAGCCCACGGTGTCGTTG	GCGTCCATCATGCCGGCTCCACTAATAG
		**DHX58-7**	CTTGCCAGCTGAGTATGTGACCGGGTACAAGTCTATTAGTGGAGCCCTGGCTTCCTTCA	GCCTCTCACTGCTGGGCTCCACTAATAG
		**DHX58-8**	GATGGCCCTCTCCTATGTGACCGGGTACAAGTCTATTAGTGGAGCCCTTGCACTGCTCT	CCGCTCAGGCATGCGGCTCCACTAATAG
		**DHX58-9**	GTGTCCTGCAGCCTATGTGACCGGGTACAAGTCTATTAGTGGAGCCACACAGCCCAGCT	CTGATGTGCTGCCGGGCTCCACTAATAG
		**DHX58-10**	TGGGGTCCTTTGTTATGTGACCGGGTACAAGTCTATTAGTGGAGCCAGGAAGCGCTCAG	CAAAGGTGGTGGCGGGCTCCACTAATAG
		**DHX58-11**	ATGACCACATCGCTATGTGACCGGGTACAAGTCTATTAGTGGAGCCCTGTGCTGTGCAG	GCGTTCTGCAGGAAGGCTCCACTAATAG

		**DHX58-12**	AGCGCACCACAATTATGTGACCGGGTACAAGTCTATTAGTGGAGCCGTCATCAGCCCGT	TGGCAATCTCGTTCGGCTCCACTAATAG
	IFI30	**IFI30-1**	TGTTCAGGACCTCTATGTGACCGGGTACAAATAACGATCCCTCACAGGCACCAGAGTGA	GAGCGTTGCCGTAGTGTGAGGGATCGTT
		**IFI30-2**	ACTGCCACTTCCCTATGTGACCGGGTACAAATAACGATCCCTCACATGCTGGCACTGGA	ACTCCTCCGAGCCCTGTGAGGGATCGTT
		**IFI30-3**	TGCGGTGCATCAGTATGTGACCGGGTACAAATAACGATCCCTCACAGTCAGCTGAGCGT	GGTCGAGAGCGTCGTGTGAGGGATCGTT
		**IFI30-4**	CACCAGGAAGCCCTATGTGACCGGGTACAAATAACGATCCCTCACATGAAGAGCTGCTG	CAGCAGCCAGGTGCTGTGAGGGATCGTT
		**IFI30-5**	CGGGTTTGTCCCCTATGTGACCGGGTACAAATAACGATCCCTCACATCCTCGCAGGCCT	TCGGGCTCTCCGTCTGTGAGGGATCGTT
		**IFI30-6**	CGCCTGCAGCTCATATGTGACCGGGTACAAATAACGATCCCTCACAAGCTCTGGGCCTG	CAGCCTCAGCAGCCTGTGAGGGATCGTT
		**IFI30-7**	GCGTACAACTGCATATGTGACCGGGTACAAATAACGATCCCTCACAATCCAGCTGCGGG	GTGATGCGGCCTGGTGTGAGGGATCGTT
		**IFI30-8**	GTGCATCAGGCAGTATGTGACCGGGTACAAATAACGATCCCTCACAAGTTCTGGGCCTC	AAAATAGGTGGTGTTGTGAGGGATCGTT
		**IFI30-9**	CCATGCAGAAGATTATGTGACCGGGTACAAATAACGATCCCTCACAGAGGAGCCCGACT	GATTCTTGGTGGCCTGTGAGGGATCGTT
		**IFI30-10**	ACTGCCACTTCCCTATGTGACCGGGTACAAATAACGATCCCTCACATGCTGGCACTGGA	ACTCCTCCGAGCCCTGTGAGGGATCGTT
		**IFI30-11**	AGTGGCTCTCTGCTATGTGACCGGGTACAAATAACGATCCCTCACAGCGAGGTTGGCAC	CGGCTGTCGGGTGGTGTGAGGGATCGTT
		**IFI30-12**	CCCCAGCCCCACGTATGTGACCGGGTACAAATAACGATCCCTCACAGGGCCAGCCCCAG	GTCACAGCTGGGCTTGTGAGGGATCGTT
	IFI35	**IFI35-1**	TTCTTCGTCTTGCTATGTGACCGGGTACAATACCCGAGATAGAGTCCTCACTGCCTCCA	TCCCTGCTCTCCAGGACTCTATCTCGGG
		**IFI35-2**	TTTGCAGCATTGCTATGTGACCGGGTACAATACCCGAGATAGAGTCGCTTCCTTGTCCT	ACCTCTGCTCTACTGACTCTATCTCGGG
		**IFI35-3**	GAGCCCCCTGACATATGTGACCGGGTACAATACCCGAGATAGAGTCGGTGATGAGAGCT	TCTGCCTTCTCAATGACTCTATCTCGGG
		**IFI35-4**	TTCAGGGTCCTCCTATGTGACCGGGTACAATACCCGAGATAGAGTCACCGGTCAAGGTC	CACTCGCACCTGGGGACTCTATCTCGGG
		**IFI35-5**	CAGCTTGTCCATGTATGTGACCGGGTACAATACCCGAGATAGAGTCGAGGGGTGAGCAC	AGGATAGCGGACCTGACTCTATCTCGGG
		**IFI35-6**	CTGGCCACAGTCATATGTGACCGGGTACAATACCCGAGATAGAGTCAGGTCAGCACCAC	TCCATCCTGCGTGTGACTCTATCTCGGG
		**IFI35-7**	GTGATCAGCTGCTTATGTGACCGGGTACAATACCCGAGATAGAGTCGACCTGCCCTCTA	CCAATAGGCACCTCGACTCTATCTCGGG
		**IFI35-8**	CTCTCCACCACCATATGTGACCGGGTACAATACCCGAGATAGAGTCCGAGGACGTCCAC	AGCTGGGACATAGGGACTCTATCTCGGG
		**IFI35-9**	CTGGTGCAGTGGGTATGTGACCGGGTACAATACCCGAGATAGAGTCGAGCAGAACGGTC	TCTGGGATCCCTGTGACTCTATCTCGGG
		**IFI35-10**	CCGCACGAAGGAGTATGTGACCGGGTACAATACCCGAGATAGAGTCGGGGGCTGGCTAG	ATCCTCCCGCTGCAGACTCTATCTCGGG
		**IFI35-11**	ATCTCCGGGCTGCTATGTGACCGGGTACAATACCCGAGATAGAGTCTCGCTCGGGGCTC	ATCTCCTGCCGGGGGACTCTATCTCGGG
		**IFI35-12**	TTGCTTGAGGTTTTATGTGACCGGGTACAATACCCGAGATAGAGTCCTTTTGGAGGCTT	GGACACCTTTTCAAGACTCTATCTCGGG
	IFIH1	**IFIH1-1**	TCTTGCAACGTCCGTCTATTAGTGGAGCCAATAACGATCCCTCACAATCAGCATGCTTG	ACGTTTGTCTGGTTTGTGAGGGATCGTT
		**IFIH1-2**	CAGTGAACCATCCGTCTATTAGTGGAGCCAATAACGATCCCTCACAGCCAGCAGGAATT	CGCCTTTCTTCAGGTGTGAGGGATCGTT
		**IFIH1-3**	GTTCCCACGACTCGTCTATTAGTGGAGCCAATAACGATCCCTCACATTGCACCGTCTCT	GCTCAACAGCTCCGTGTGAGGGATCGTT
		**IFIH1-4**	CAGACACTTCTCGGTCTATTAGTGGAGCCAATAACGATCCCTCACAAGATGCCCAGCTC	GTCCTCCTCCTGGTTGTGAGGGATCGTT
		**IFIH1-5**	CTCATCAGCTCGAGTCTATTAGTGGAGCCAATAACGATCCCTCACAGTGCATAAGTGCT	ACTTGAAGCCACATTGTGAGGGATCGTT
		**IFIH1-6**	CTCCGACATCGCTGTCTATTAGTGGAGCCAATAACGATCCCTCACACGTCTCGGCACTC	GTAGAGGAAGCGCATGTGAGGGATCGTT
		**IFIH1-7**	AGGCTGTAAGTCCGTCTATTAGTGGAGCCAATAACGATCCCTCACACCAACTCCAGGTG	TGGATCTTGCACCATGTGAGGGATCGTT

		**IFIH1-8**	TCGCTTGCTGTTTGTCTATTAGTGGAGCCAATAACGATCCCTCACAGCGGCTTTCGGTT	ACATGAACCCCGCATGTGAGGGATCGTT
		**IFIH1-9**	GTGGTCGTCCTCCGTCTATTAGTGGAGCCAATAACGATCCCTCACAGCACACAGAGATC	CAGCTGCACCAAGATGTGAGGGATCGTT
		**IFIH1-10**	TGCCCTCTGCTTCGTCTATTAGTGGAGCCAATAACGATCCCTCACATCAGGTGAAGCTC	GGGTGAGATCTGGGTGTGAGGGATCGTT
		**IFIH1-11**	TGCCTGTAGGGAGGTCTATTAGTGGAGCCAATAACGATCCCTCACACTGGTTTTGCCAC	TGTAAACAGCCACATGTGAGGGATCGTT
		**IFIH1-12**	GGCACGGCGTCAGGTCTATTAGTGGAGCCAATAACGATCCCTCACAAGCCGGAGTCGCC	GTGGCGGCCGAAGCTGTGAGGGATCGTT
	SAMHD1	**SAMHD1-1**	GACATCCTGCAAGGTCTATTAGTGGAGCCATACCCGAGATAGAGTCAGTTCACGAACCA	GTTGCCGCTCCTTGGACTCTATCTCGGG
		**SAMHD1-2**	AAGGTCCCTGGTAGTCTATTAGTGGAGCCATACCCGAGATAGAGTCAACCTGCTCTGAA	CAGTAAACCCGGAAGACTCTATCTCGGG
		**SAMHD1-3**	TCCGAGTGTGGAAGTCTATTAGTGGAGCCATACCCGAGATAGAGTCCGGTGCAGGCAAT	GCTGGTACGCTCGGGACTCTATCTCGGG
		**SAMHD1-4**	TGTCTCGTTGGGTGTCTATTAGTGGAGCCATACCCGAGATAGAGTCTCCACACAGAGGA	AAAGCCCAGCAATAGACTCTATCTCGGG
		**SAMHD1-5**	TGAAGCTGTCGTGGTCTATTAGTGGAGCCATACCCGAGATAGAGTCTGCAGCTGGGTTC	AGAGAGCTTCTGCAGACTCTATCTCGGG
		**SAMHD1-6**	TGATGCGAACAAGGTCTATTAGTGGAGCCATACCCGAGATAGAGTCTGAGGCGTGTCAA	GCAGGCGCTGAAAGGACTCTATCTCGGG
		**SAMHD1-7**	ACCACATCACCATGTCTATTAGTGGAGCCATACCCGAGATAGAGTCTAGATGAGGGGCA	TGTTTCAGTGGAGAGACTCTATCTCGGG
		**SAMHD1-8**	CAAAATACTGCTTGTCTATTAGTGGAGCCATACCCGAGATAGAGTCATACACCACTGAA	TGAAATCCCTCTTAGACTCTATCTCGGG
		**SAMHD1-9**	ATCCATATTGACAGTCTATTAGTGGAGCCATACCCGAGATAGAGTCCTTTCATTCCATA	AATAGGGTTCTGAAGACTCTATCTCGGG
		**SAMHD1-10**	CCAGGCCATCGTAGTCTATTAGTGGAGCCATACCCGAGATAGAGTCGCAATGTCCCCTG	CTGGCTTGGAGTTTGACTCTATCTCGGG
		**SAMHD1-11**	GCAGAATTTCCAGGTCTATTAGTGGAGCCATACCCGAGATAGAGTCGGACGACTTGAGT	CCTCACTCAGTTCAGACTCTATCTCGGG
		**SAMHD1-12**	TTATTCTGGATCCGTCTATTAGTGGAGCCATACCCGAGATAGAGTCCCTATAATCAAAA	AATTTAATTAAGCCGACTCTATCTCGGG
	STAT1	**STAT1-1**	CTGCCTGATTTCCATAACGATCCCTCACAATACCCGAGATAGAGTCACTGTGCCAAGTA	CTGGTTTTCCAGCGGACTCTATCTCGGG
		**STAT1-2**	CAGTCTGTTGCCTATAACGATCCCTCACAATACCCGAGATAGAGTCCCACCAATGCATG	AGGCATTGGGTGGCGACTCTATCTCGGG
		**STAT1-3**	CAGGCTTTCAGCAATAACGATCCCTCACAATACCCGAGATAGAGTCGACGAACTTGCTG	CTTTTTGAGCTGCAGACTCTATCTCGGG
		**STAT1-4**	AGGCTGCCTCTCTATAACGATCCCTCACAATACCCGAGATAGAGTCGTGTTGGCATGCA	TGGCCTCTGTGGAAGACTCTATCTCGGG
		**STAT1-5**	GTGTGCTCTGTGGATAACGATCCCTCACAATACCCGAGATAGAGTCAAGAGCACTCTGT	AGCTCCTCGTTAAAGACTCTATCTCGGG
		**STAT1-6**	AGATTGGAGCCTGATAACGATCCCTCACAATACCCGAGATAGAGTCGCAGATTTTCTGG	AGGAGACATAGGCTGACTCTATCTCGGG
		**STAT1-7**	TGTATCCATAGGCATAACGATCCCTCACAATACCCGAGATAGAGTCCCTTGGGAGTATC	AATGTAGCCGTTTGGACTCTATCTCGGG
		**STAT1-8**	ACTGAGTGGAACTATAACGATCCCTCACAATACCCGAGATAGAGTCGGTGTATGGTTCT	GAGAGCTCCTTCTAGACTCTATCTCGGG
		**STAT1-9**	GTTGTCTTTGGGAATAACGATCCCTCACAATACCCGAGATAGAGTCATTTGCCAAAGGC	AGGCCTGGAGTAGAGACTCTATCTCGGG
		**STAT1-10**	TACTGATGAAACCATAACGATCCCTCACAATACCCGAGATAGAGTCCGTTCTTTGTCTT	CCTTCAATAAAGCTGACTCTATCTCGGG
		**STAT1-11**	CCAAAAAGGGAAAATAACGATCCCTCACAATACCCGAGATAGAGTCCCTCTATCCACAG	AAGTTCCAGGATTGGACTCTATCTCGGG
		**STAT1-12**	AGATCCTCCACTTATAACGATCCCTCACAATACCCGAGATAGAGTCTAAGGCCATCATG	TCTTGTCCAAGGATGACTCTATCTCGGG
	DENND5B	**DENND5B-1**	AACGCCCTTTCCCACCATGCTGGATCTAGAACCATGCTGGATCTAGAGCTGCCATCGTC	AAGGATTCGCTCCTCTAGATCCAGCATG
		**DENND5B-2**	ACACAAATCCACGACCATGCTGGATCTAGAACCATGCTGGATCTAGAAGCTCTCCCGAA	ACCCCCGTGTCTCGCTAGATCCAGCATG
		**DENND5B-3**	GAAGACTTCCCGCACCATGCTGGATCTAGAACCATGCTGGATCTAGGCCCCAACAGCTC	GACCAGGTTCTCCTCTAGATCCAGCATG

		**DENND5B-4**	CTGCACGTTCAGTACCATGCTGGATCTAGAACCATGCTGGATCTAGACACCTCACGGAG	GAAGCGGTTGGCATCTAGATCCAGCATG
		**DENND5B-5**	TGCTGCAACCAAGACCATGCTGGATCTAGAACCATGCTGGATCTAGAGACCTGCTCCAG	GAATCCGTGGTGGTCTAGATCCAGCATG
		**DENND5B-6**	CTCGTGACCTCCTACCATGCTGGATCTAGAACCATGCTGGATCTAGGCAGATCTGCTTG	GTCTGCATGGCTGACTAGATCCAGCATG
		**DENND5B-7**	CTCCCGAAACACAACCATGCTGGATCTAGAACCATGCTGGATCTAGGTGTCTCCAAGCT	TCTGCATGACCCCGCTAGATCCAGCATG
		**DENND5B-8**	TCCCGAAACACAAACCATGCTGGATCTAGAACCATGCTGGATCTAGTGTCTCCAAGCTC	CTGCATGACCCCCCCTAGATCCAGCATG
		**DENND5B-9**	GTGGCCCATTTTCACCATGCTGGATCTAGAACCATGCTGGATCTAGGCTCAACTGCTTC	TTCTCCATGTCCCTCTAGATCCAGCATG
		**DENND5B-10**	GAGTTGTACCGCTACCATGCTGGATCTAGAACCATGCTGGATCTAGGCTGATGTCGTAG	TACAGCGTGTCCCACTAGATCCAGCATG
		**DENND5B-11**	GTAGGGAACCGGAACCATGCTGGATCTAGAACCATGCTGGATCTAGGCAGCCCCATCAG	TCCCTCCTTGGACACTAGATCCAGCATG
		**DENND5B-12**	GAGAGAGGTCCGAACCATGCTGGATCTAGAACCATGCTGGATCTAGGCAATCACAGCTG	TCGAGTTGGTCTGTCTAGATCCAGCATG
	PAX5	**PAX5-1**	GTCGTTGTACGTTTACTAGCTCGATTCAAATACTAGCTCGATTCAAGAAACCTCCAGGA	AAGTCCGGGATTGCTTGAATCGAGCTAG
		**PAX5-2**	CATCCAGTCCTCCTACTAGCTCGATTCAAATACTAGCTCGATTCAATTGGCCTTCATGT	TGGGGCTGGTTATTTTGAATCGAGCTAG
		**PAX5-3**	TTGGCGTTTGTACTACTAGCTCGATTCAAATACTAGCTCGATTCAAACATGGTGGGATT	GATCTCCCAGGCATTTGAATCGAGCTAG
		**PAX5-4**	ACTGCTGCTGGGTTACTAGCTCGATTCAAATACTAGCTCGATTCAATCCAACACTTCCA	TCTCGAAGACTCGTTTGAATCGAGCTAG
		**PAX5-5**	TGGGTACTGGAGGTACTAGCTCGATTCAAATACTAGCTCGATTCAAAGGGAATGGCCAT	AATCTCTACCCGGCTTGAATCGAGCTAG
		**PAX5-6**	AACGGAGCCTGTGTACTAGCTCGATTCAAATACTAGCTCGATTCAAAGGACACCTGGGT	GTCAGTGCTGACACTTGAATCGAGCTAG
		**PAX5-7**	ATCGCACACTCGCTACTAGCTCGATTCAAATACTAGCTCGATTCAAGCACGGTGTCGTT	TGAACTGACGCTGCTTGAATCGAGCTAG
		**PAX5-8**	ATGGAGTAGGACGTACTAGCTCGATTCAAATACTAGCTCGATTCAACAGGATCCCACTG	GGAGAGGCGATGCGTTGAATCGAGCTAG
		**PAX5-9**	CAGTGTATTTCCATACTAGCTCGATTCAAATACTAGCTCGATTCAACGGGTCGTGCTTA	TGCATTGCTGCAATTTGAATCGAGCTAG
		**PAX5-10**	GCTGGTTCACACCTACTAGCTCGATTCAAATACTAGCTCGATTCAAAAAACGCCCCCCA	GGCGGCCATTCACTTTGAATCGAGCTAG
		**PAX5-11**	CACCCCCTGGTGCTACTAGCTCGATTCAAATACTAGCTCGATTCAATGTCGCACGGCCT	GAGCTGCCTGGAGTTTGAATCGAGCTAG
		**PAX5-12**	CCCGGTTTCATAATACTAGCTCGATTCAAATACTAGCTCGATTCAACAGGCTTAATGCT	TCCTCCAATCACTGTTGAATCGAGCTAG
	TIAM1	**TIAM1-1**	AGGAAAACAGCACTAGGCATGAACGTCGAATAGGCATGAACGTCGAAAGGATCCACCCA	CGGCGTAGTAAAGCTCGACGTTCATGCC
		**TIAM1-2**	ACCGGACTCCCCTTAGGCATGAACGTCGAATAGGCATGAACGTCGATCCTTCTCCTCAC	GGACATGGCTTGCCTCGACGTTCATGCC
		**TIAM1-3**	ATCCAGCTGGTGTTAGGCATGAACGTCGAATAGGCATGAACGTCGAAGTGAGCAGAGAG	CTTAAGACGCAGGATCGACGTTCATGCC
		**TIAM1-4**	TGTCCTCCTGCTCTAGGCATGAACGTCGAATAGGCATGAACGTCGATCCGTTTCCTTGA	CATCGCTGAGGATCTCGACGTTCATGCC
		**TIAM1-5**	TGCTTCCAAAGGTTAGGCATGAACGTCGAATAGGCATGAACGTCGACTCGAACCTGCAG	TGCACTTGCCAATCTCGACGTTCATGCC
		**TIAM1-6**	AGTGAACGTGATGTAGGCATGAACGTCGAATAGGCATGAACGTCGAATTGGAGAGGCGA	CGTCGAGAAGCTTATCGACGTTCATGCC
		**TIAM1-7**	ACGGCATCTGAGTTAGGCATGAACGTCGAATAGGCATGAACGTCGAGCTGCTTGTGAAG	GGCTCTTTTTCGGCTCGACGTTCATGCC
		**TIAM1-8**	ATGGCCTCACAGTTAGGCATGAACGTCGAATAGGCATGAACGTCGACACGGAACCCCTC	AGGTCTCGGTCGGGTCGACGTTCATGCC
		**TIAM1-9**	TCAGAGCAGGCTGTAGGCATGAACGTCGAATAGGCATGAACGTCGAGGGCGGATGACTG	GAGTGGTGTCCCCGTCGACGTTCATGCC
		**TIAM1-10**	TGACCTTTCGCAGTAGGCATGAACGTCGAATAGGCATGAACGTCGAAGAAGTTCGCAGA	TGCGCTCTGTTTCCTCGACGTTCATGCC
		**TIAM1-11**	CAGAGCTGTCCGGTAGGCATGAACGTCGAATAGGCATGAACGTCGAGGTGCTGTCTCAA	CTTCTGCCTCTTCATCGACGTTCATGCC

		**TIAM1-12**	TGAAAGCAGTGTCTAGGCATGAACGTCGAATAGGCATGAACGTCGATCCAGAAATGCCT	TGGGATTCTGAGCTTCGACGTTCATGCC
	NPR3	**NPR3-1**	CAGCCAACATGATCGTAGCTTACCTGCAAACGTAGCTTACCTGCAACCTTGCCGATGAG	CCCCATTGGTCATATTGCAGGTAAGCTA
		**NPR3-2**	TCCTCCTCCTCCTCGTAGCTTACCTGCAAACGTAGCTTACCTGCAACCGGATCAGCTTC	AACAGCACCAGCAGTTGCAGGTAAGCTA
		**NPR3-3**	TGTCTCCTCTCCTCGTAGCTTACCTGCAAACGTAGCTTACCTGCAATCAAGGTCATGCT	ATGCCTTCTTTGCATTGCAGGTAAGCTA
		**NPR3-4**	ATGGACGCCCTCCCGTAGCTTACCTGCAAACGTAGCTTACCTGCAACCTGGAAAACCAC	ATGGAAGGCCTCAATTGCAGGTAAGCTA
		**NPR3-5**	ATTCTCAGCCTGCCGTAGCTTACCTGCAAACGTAGCTTACCTGCAATCGGCTTTCATCT	GTGTGCTCCGAAAGTTGCAGGTAAGCTA
		**NPR3-6**	AGCAGCCAACATGCGTAGCTTACCTGCAAACGTAGCTTACCTGCAATTCCTTGCCGATG	GTCCCCATTGGTCTTTGCAGGTAAGCTA
		**NPR3-7**	GAAGCTGTGCACACGTAGCTTACCTGCAAACGTAGCTTACCTGCAAGGCTGGTCTCGTC	GATGTCGAGGTGGGTTGCAGGTAAGCTA
		**NPR3-8**	CAGCATCTCGCCCCGTAGCTTACCTGCAAACGTAGCTTACCTGCAAGGAAGAGGGCCAG	CCACTGGTGGTGGGTTGCAGGTAAGCTA
		**NPR3-9**	TCGCTGTAGAGCACGTAGCTTACCTGCAAACGTAGCTTACCTGCAACTCGGGGTTGCTG	AAGTAGCAGTTGCCTTGCAGGTAAGCTA
		**NPR3-10**	ATTCTGTACTTCTCGTAGCTTACCTGCAAACGTAGCTTACCTGCAATCTCTCAATAGTT	TCCTGTTGAGTTCCTTGCAGGTAAGCTA
		**NPR3-11**	TCTTCGCGTAACTCGTAGCTTACCTGCAAACGTAGCTTACCTGCAAAGATCTAATGGAG	GCAGCAGAAAAATCTTGCAGGTAAGCTA
		**NPR3-12**	CAAACATGTTCACCGTAGCTTACCTGCAAACGTAGCTTACCTGCAATGGAATCCTTCCA	GAAGAATAGCATCCTTGCAGGTAAGCTA
	VWF	**VWF-1**	AGGAGGCAAATCAACCATGCTGGATCTAGATACTAGCTCGATTCAACACAGTCAGGGAC	CTGCAGGCCAAACATTGAATCGAGCTAG
		**VWF-2**	ACTGCACACACTGACCATGCTGGATCTAGATACTAGCTCGATTCAACTTGACACCTCCA	CTTCCATGTGGGAATTGAATCGAGCTAG
		**VWF-3**	ACCATCAAGGAGCACCATGCTGGATCTAGATACTAGCTCGATTCAACAACACAACGTTC	ACAGTCAGAGCTTATTGAATCGAGCTAG
		**VWF-4**	TCATCTTGCTGCAACCATGCTGGATCTAGATACTAGCTCGATTCAAAAGGCCAAGTCCA	AGCCATCCACTAGTTTGAATCGAGCTAG
		**VWF-5**	GAGACTTGCTTGCACCATGCTGGATCTAGATACTAGCTCGATTCAACGGACTTTTCCTG	CTGGGATGATGGTCTTGAATCGAGCTAG
		**VWF-6**	TTTGGCATCCTCTACCATGCTGGATCTAGATACTAGCTCGATTCAAGTGACACACAGGT	GCAGGAAGATTGCATTGAATCGAGCTAG
		**VWF-7**	TCCTCTTCCACACACCATGCTGGATCTAGATACTAGCTCGATTCAATACTGGTCGGCAT	TGCAGTCTGGTACATTGAATCGAGCTAG
		**VWF-8**	TGGGATCGCTACTACCATGCTGGATCTAGATACTAGCTCGATTCAATGGGACACTGGAG	TACAACTGTGCCAATTGAATCGAGCTAG
		**VWF-9**	TCAGCTCTTGGGTACCATGCTGGATCTAGATACTAGCTCGATTCAAACACTCCAACCCT	TGGCAAGTCTTGGGTTGAATCGAGCTAG
		**VWF-10**	CAATGTCCCACACACCATGCTGGATCTAGATACTAGCTCGATTCAAATGTCTGGTTGCA	GGACAGAGATCGGGTTGAATCGAGCTAG
		**VWF-11**	TGCAGGCCCAAGAACCATGCTGGATCTAGATACTAGCTCGATTCAACAGGAGAGCTTTC	TGTTGGAGTGGGCATTGAATCGAGCTAG
		**VWF-12**	TAGAGGGCACTTCACCATGCTGGATCTAGATACTAGCTCGATTCAACTTCTACGCACTG	GTGAACATGGCATGTTGAATCGAGCTAG
	SFTPC	**SFTPC-1**	CTGCGCTCCATCCACCATGCTGGATCTAGATAGGCATGAACGTCGACGGCTCTTTGGTG	GGCTCCTCCAGCACTCGACGTTCATGCC
		**SFTPC-2**	AATGGAGCGGTCAACCATGCTGGATCTAGATAGGCATGAACGTCGATGGTGGTGCCCAA	GCAGAGGATGTTGTTCGACGTTCATGCC
		**SFTPC-3**	TGATGGTGGTGCCACCATGCTGGATCTAGATAGGCATGAACGTCGACTGCAGAGGATGT	ATATGGGGACGGTCTCGACGTTCATGCC
		**SFTPC-4**	AGCGATGGGCTGAACCATGCTGGATCTAGATAGGCATGAACGTCGAAAATGGAGCGGTC	GATGGTGGTGCCCTTCGACGTTCATGCC
		**SFTPC-5**	TTGATGGTGGTGCACCATGCTGGATCTAGATAGGCATGAACGTCGAGCTGCAGAGGATG	AATATGGGGACGGATCGACGTTCATGCC
		**SFTPC-6**	GAGCTGCAATGGTACCATGCTGGATCTAGATAGGCATGAACGTCGATCAGCGATGGGCT	CCAAAATGGAGCGCTCGACGTTCATGCC
		**SFTPC-7**	AAATGGAGCGGTCACCATGCTGGATCTAGATAGGCATGAACGTCGAATGGTGGTGCCCA	TGCAGAGGATGTTCTCGACGTTCATGCC

		**SFTPC-8**	CAGCGATGGGCTGACCATGCTGGATCTAGATAGGCATGAACGTCGAAAAATGGAGCGGT	TGATGGTGGTGCCGTCGACGTTCATGCC
		**SFTPC-9**	GATGGTGGTGCCCACCATGCTGGATCTAGATAGGCATGAACGTCGATGCAGAGGATGTT	TATGGGGACGGTGGTCGACGTTCATGCC
	RALYL	**RALYL-1**	ATCACTGCACGAGACCATGCTGGATCTAGACGTAGCTTACCTGCAAACGCTTCAATGGT	ACAGCCACACGAGCTTGCAGGTAAGCTA
		**RALYL-2**	TGCTCACTGACAGACCATGCTGGATCTAGACGTAGCTTACCTGCAAGCAGCGAGACAGT	CGTGCTTCTGCTTATTGCAGGTAAGCTA
		**RALYL-3**	AGGTGGATCGTGAACCATGCTGGATCTAGACGTAGCTTACCTGCAAGAAGTCCCACTTG	TGGATCCTGAGGATTTGCAGGTAAGCTA
		**RALYL-4**	CAGGGTAATACCAACCATGCTGGATCTAGACGTAGCTTACCTGCAATCTCAGAGAAAGG	GGTAACCAGCAATGTTGCAGGTAAGCTA
		**RALYL-5**	CACTTTCTAAGAAACCATGCTGGATCTAGACGTAGCTTACCTGCAATCTGAAGAAATTC	TTGTACATAGTCAATTGCAGGTAAGCTA
		**RALYL-6**	TTTGGTTCTCCTGACCATGCTGGATCTAGACGTAGCTTACCTGCAATGGTCTGTATGGT	TTGTTGGCAGGTTATTGCAGGTAAGCTA
		**RALYL-7**	AATCAAAGACATAACCATGCTGGATCTAGACGTAGCTTACCTGCAACTGTAGTAATCAT	TGTAGAAATCATCGTTGCAGGTAAGCTA
		**RALYL-8**	TCTGCGAGTTGTTACCATGCTGGATCTAGACGTAGCTTACCTGCAAAAACCCCTTTGCC	TCCTTTCATGGAGTTTGCAGGTAAGCTA
		**RALYL-9**	CTCATCTGATTTCACCATGCTGGATCTAGACGTAGCTTACCTGCAATGATTGTTTGCAA	GGTTAATTCCTTCATTGCAGGTAAGCTA
		**RALYL-10**	TCTTTTCCAGTCTACCATGCTGGATCTAGACGTAGCTTACCTGCAATGCTGTTTTTCAA	CTGCTTCAGCTTTATTGCAGGTAAGCTA
		**RALYL-11**	TTTCTTTTGAGCTACCATGCTGGATCTAGACGTAGCTTACCTGCAATATCTTCCATTTG	AATCAAATCAGCAATTGCAGGTAAGCTA
		**RALYL-12**	GATCTTCTGTAGAACCATGCTGGATCTAGACGTAGCTTACCTGCAACCTCCTTCAATTG	CATCTCCATCTGGGTTGCAGGTAAGCTA
	COL1A1	**COL1A1-1**	ACGTGCTTCTTCTTACTAGCTCGATTCAAATAGGCATGAACGTCGACTCGCCGAACCAG	CCGTCGCTCATCGATCGACGTTCATGCC
		**COL1A1-2**	GTTCACCGGTCTCTACTAGCTCGATTCAAATAGGCATGAACGTCGACCTCTGTCTCCCT	TGTGGCCCTTCATCTCGACGTTCATGCC
		**COL1A1-3**	TGACCTCTGGCTCTACTAGCTCGATTCAAATAGGCATGAACGTCGACATCACACCAGCT	GGACCGGGGAAACGTCGACGTTCATGCC
		**COL1A1-4**	AGCGGCTGTTTCCTACTAGCTCGATTCAAATAGGCATGAACGTCGAACCCCATAGGTGA	AGCCATCCTCGGTCTCGACGTTCATGCC
		**COL1A1-5**	TTAGCACCAGGCTTACTAGCTCGATTCAAATAGGCATGAACGTCGACCCAGTTTCACCT	TTGGCTCCAGCATGTCGACGTTCATGCC
		**COL1A1-6**	CAGGCGCAGGAAGTACTAGCTCGATTCAAATAGGCATGAACGTCGACCTCGGTGGACAT	GACGTTCTGGGTGCTCGACGTTCATGCC
		**COL1A1-7**	AGGTTGCCGGTGTTACTAGCTCGATTCAAATAGGCATGAACGTCGACAGAGCCTTCTTC	GCTCCCTGGAGCACTCGACGTTCATGCC
		**COL1A1-8**	TCCTTACCGCTGGTACTAGCTCGATTCAAATAGGCATGAACGTCGATCCTTTGCCACCT	GTCTCACCACGGGCTCGACGTTCATGCC
		**COL1A1-9**	GACCGACGACACCTACTAGCTCGATTCAAATAGGCATGAACGTCGACTCTGTCCGGGGA	AGCCTCTCTCGCCATCGACGTTCATGCC
		**COL1A1-10**	TTGAGGAGCGAAGTACTAGCTCGATTCAAATAGGCATGAACGTCGAAGCCGTAAGACAT	CGATTTCTCGTCGATCGACGTTCATGCC
		**COL1A1-11**	GTTGCAGCCCTGGTACTAGCTCGATTCAAATAGGCATGAACGTCGATGATGGCATCCAG	GTTGCAGTAGACCATCGACGTTCATGCC
		**COL1A1-12**	AAGTCGAAGCCGCTACTAGCTCGATTCAAATAGGCATGAACGTCGAAGGCAGGAAGCTG	TCTTGGGGCGGTTCTCGACGTTCATGCC
	APOA1	**APOA1-1**	AGTAGGGAGCCATTACTAGCTCGATTCAAACGTAGCTTACCTGCAACGCACCTCCTTGT	TCAGCCACATCTCCTTGCAGGTAAGCTA
		**APOA1-2**	CTCAGCACGCAGATACTAGCTCGATTCAAACGTAGCTTACCTGCAAGGTCCTTGGTCAG	CTTCACCTCCTCCTTTGCAGGTAAGCTA
		**APOA1-3**	TATCCCGAATGCGTACTAGCTCGATTCAAACGTAGCTTACCTGCAATAGACGTCCACCA	TCACCGTCTCCAGCTTGCAGGTAAGCTA
		**APOA1-4**	CTGGTCCAGGAAGTACTAGCTCGATTCAAACGTAGCTTACCTGCAAACTTGGCGGAGAA	CAGCTCCTCCGTCGTTGCAGGTAAGCTA
		**APOA1-5**	CTCCTGAGCCACGTACTAGCTCGATTCAAACGTAGCTTACCTGCAATGAGCTCCTTCAG	CACCTTCTGCTTGCTTGCAGGTAAGCTA
		**APOA1-6**	CAGCACGCCTCTCTACTAGCTCGATTCAAACGTAGCTTACCTGCAACAGCGAGGGTCAC	CGTCAGGAAGAGCTTTGCAGGTAAGCTA

		**APOA1-7**	CTTCTGGCTCAGCTACTAGCTCGATTCAAACGTAGCTTACCTGCAAGGATCTCCTCCAG	GATGCCCTTCTCGGTTGCAGGTAAGCTA
		**APOA1-8**	TGGCCTGGTACTCTACTAGCTCGATTCAAACGTAGCTTACCTGCAATGCTCCATCACCT	GCAGGTTGCTGAGGTTGCAGGTAAGCTA
		**APOA1-9**	AGGTTCTCAGCATTACTAGCTCGATTCAAACGTAGCTTACCTGCAACAAGCGGTTCTTG	TCCAGGAAGGAGAATTGCAGGTAAGCTA
		**APOA1-10**	AGCCAGCTTCAGGTACTAGCTCGATTCAAACGTAGCTTACCTGCAATGTCCAGGTTGTC	CGCGGCACTCAGCCTTGCAGGTAAGCTA
		**APOA1-11**	AGGAGCGGGCCTGTACTAGCTCGATTCAAACGTAGCTTACCTGCAATCGTGCTGCCAGA	GCGTCTGGGGCTCTTTGCAGGTAAGCTA
		**APOA1-12**	CGAACTGGGCGATTACTAGCTCGATTCAAACGTAGCTTACCTGCAAACAGCAGAGGACT	CAAGCTGTTTGCCGTTGCAGGTAAGCTA
	IL1R2	**IL1R2-1**	TTCCACGTCAGTATAGGCATGAACGTCGAACGTAGCTTACCTGCAACATTCGCTAACCA	ATCGATCGTGGTAATTGCAGGTAAGCTA
		**IL1R2-2**	TGCGACTGGAATTTAGGCATGAACGTCGAACGTAGCTTACCTGCAATGACACAGGCCAA	TATTCCTGCCAGGTTTGCAGGTAAGCTA
		**IL1R2-3**	CTTTCCCAGAGGATAGGCATGAACGTCGAACGTAGCTTACCTGCAAGAACCCATGCATT	ACCAGGGCAAAGATTTGCAGGTAAGCTA
		**IL1R2-4**	GACCGTGAGCTGGTAGGCATGAACGTCGAACGTAGCTTACCTGCAACTGCCTTCTGCAC	GATGTCCCGAGCACTTGCAGGTAAGCTA
		**IL1R2-5**	CACATCGTGCATGTAGGCATGAACGTCGAACGTAGCTTACCTGCAAGAAAGCAGAGGCA	CCTTGCTGGAGCTATTGCAGGTAAGCTA
		**IL1R2-6**	ATCTGGGGATAGGTAGGCATGAACGTCGAACGTAGCTTACCTGCAAGAAAGTGAAGAGG	ATCTTTCCAGAAGATTGCAGGTAAGCTA
		**IL1R2-7**	CCTGTTTATTGTATAGGCATGAACGTCGAACGTAGCTTACCTGCAAGCTCCATGTTTGT	CTTGTACCACTGGTTTGCAGGTAAGCTA
		**IL1R2-8**	CATCTTCCAAAAGTAGGCATGAACGTCGAACGTAGCTTACCTGCAAAATTTTTCACTGT	CTTTCAGAAATACTTTGCAGGTAAGCTA
		**IL1R2-9**	GAAGTGAAGAACATAGGCATGAACGTCGAACGTAGCTTACCTGCAATGTCGGTAATACA	TAGTAGCCGGAGTGTTGCAGGTAAGCTA
		**IL1R2-10**	TCATATGTGACACTAGGCATGAACGTCGAACGTAGCTTACCTGCAATGTTCTGGTGATT	GTCTGCAGTTTAAATTGCAGGTAAGCTA
		**IL1R2-11**	TTCTCTTTTCTTGTAGGCATGAACGTCGAACGTAGCTTACCTGCAAATTATGGGGGTAA	GAGTTGGATATACTTTGCAGGTAAGCTA
		**IL1R2-12**	AGTCATCTTGGAGTAGGCATGAACGTCGAACGTAGCTTACCTGCAACTTTACATGGGAG	CGGTCCAGCAAATTTTGCAGGTAAGCTA
	IL2RB	**IL2RB-1**	ACCTCTGCAGATGGGCACTTGAGCTAGAAAGGCACTTGAGCTAGAAAGTCACTCCACAC	TACCGTCTTGCTCGTTCTAGCTCAAGTG
		**IL2RB-2**	CTGTTCACACACAGGCACTTGAGCTAGAAAGGCACTTGAGCTAGAATTCTGGGGCTGTA	TCCAGCATTGAGAATTCTAGCTCAAGTG
		**IL2RB-3**	TGATTCCTCGTCGGGCACTTGAGCTAGAAAGGCACTTGAGCTAGAACCTTGGAAACCTG	AGGTGCCAGGGTTGTTCTAGCTCAAGTG
		**IL2RB-4**	AGGCAGTGAGGAGGGCACTTGAGCTAGAAAGGCACTTGAGCTAGAAAGAGGTGGTGCAA	CAGGCTAAGCAGCGTTCTAGCTCAAGTG
		**IL2RB-5**	CTGAGGGAGGATCGGCACTTGAGCTAGAAAGGCACTTGAGCTAGAATGCCCACTGGTTT	AGCTGGACACAGACTTCTAGCTCAAGTG
		**IL2RB-6**	CTCCTGGGTGAAAGGCACTTGAGCTAGAAAGGCACTTGAGCTAGAATGCTGCCATTGCT	AGTCTCACTGTCGGTTCTAGCTCAAGTG
		**IL2RB-7**	TGTTTTGGAAGCCGGCACTTGAGCTAGAAAGGCACTTGAGCTAGAAGCCACAAAGGAGA	CACCTTGTGTCTCATTCTAGCTCAAGTG
		**IL2RB-8**	TTCATATTGCCGCGGCACTTGAGCTAGAAAGGCACTTGAGCTAGAATTCGGTACCGCAC	CGACTGACTTGTGCTTCTAGCTCAAGTG
		**IL2RB-9**	TATGTCCACCTGGGGCACTTGAGCTAGAAAGGCACTTGAGCTAGAAAGTCAGGGAAGTC	ATGCAGGTCTGGTCTTCTAGCTCAAGTG
		**IL2RB-10**	AATTGGAGGGCCTGGCACTTGAGCTAGAAAGGCACTTGAGCTAGAAGGGCACTGGGATC	AGCATGTGCTGCTCTTCTAGCTCAAGTG
		**IL2RB-11**	AGCTGCCTCATACGGCACTTGAGCTAGAAAGGCACTTGAGCTAGAAGCTTTGCACGGAC	GCTTGAGGCACCTCTTCTAGCTCAAGTG
		**IL2RB-12**	GATCCTCGTCCTGGGCACTTGAGCTAGAAAGGCACTTGAGCTAGAAGGTGTTCTGCAAA	CTTGGCTGGAGAACTTCTAGCTCAAGTG
	CD7	**CD7-1**	CAATGACCCCACTATGCTGCTGTAGTACTAATGCTGCTGTAGTACTAGTGGTGACTGTT	GAGCATGGACAGAGAGTACTACAGCAGC
		**CD7-2**	ATACACAAATGGGATGCTGCTGTAGTACTAATGCTGCTGTAGTACTGCAGAAATAAGAG	GAAGAATGGGAGATAGTACTACAGCAGC

		**CD7-3**	TTTATTTTCTTCAATGCTGCTGTAGTACTAATGCTGCTGTAGTACTACCAATCAGTAAT	GATAATGTCTGTTCAGTACTACAGCAGC
		**CD7-4**	AATTAATGGTGTCATGCTGCTGTAGTACTAATGCTGCTGTAGTACTTTTGAACAAGTTA	TTTCTGAACTGTTGAGTACTACAGCAGC
		**CD7-5**	AATTGTGTCCCCAATGCTGCTGTAGTACTAATGCTGCTGTAGTACTCGCTCTAATCCTC	ATATTGGTTGGCGCAGTACTACAGCAGC
		**CD7-6**	TGGAGCATACTTGATGCTGCTGTAGTACTAATGCTGCTGTAGTACTCCGGGTCTATATA	GCGATTAGCAAAACAGTACTACAGCAGC
		**CD7-7**	AGATTTGTTCCTTATGCTGCTGTAGTACTAATGCTGCTGTAGTACTCACAGTGATTGTG	TTCTGTACGTTGTCAGTACTACAGCAGC
		**CD7-8**	TGCATAGGTAGATATGCTGCTGTAGTACTAATGCTGCTGTAGTACTACAGAATAGTGGA	GACGCTCATTTACAAGTACTACAGCAGC
		**CD7-9**	GGTAATGTTGATAATGCTGCTGTAGTACTAATGCTGCTGTAGTACTTTTTCAACACACA	ATCTCCATTCGAGGAGTACTACAGCAGC
		**CD7-10**	CGCTGACTGAGAAATGCTGCTGTAGTACTAATGCTGCTGTAGTACTATGGTGCCACTTT	CTTTAACCAGCACGAGTACTACAGCAGC
		**CD7-11**	TGTCTGCTTCTCAATGCTGCTGTAGTACTAATGCTGCTGTAGTACTTGATACTGCATGC	GATGGCCAAGGAAGAGTACTACAGCAGC
		**CD7-12**	CAGTGCAGATAACATGCTGCTGTAGTACTAATGCTGCTGTAGTACTAGATGAAGCATAT	ATTGATACGGTTCTAGTACTACAGCAGC
	CD28	**CD28-1**	CTCCTCCTCCCTGGGAGACTGATTACACGAGGAGACTGATTACACGTCACACAGCTTCT	GCACCATCACCAGGCGTGTAATCAGTCT
		**CD28-2**	CTTTGTGCAGCGAGGAGACTGATTACACGAGGAGACTGATTACACGGCACTGTCTGTTC	AGCAGACTTCGACACGTGTAATCAGTCT
		**CD28-3**	GATGGTGGGCCTGGGAGACTGATTACACGAGGAGACTGATTACACGCCAGCATGGTGAC	CACGACGAGGATCGCGTGTAATCAGTCT
		**CD28-4**	TTGGTGCATAGGAGGAGACTGATTACACGAGGAGACTGATTACACGGTGTAGTCTCGTG	AGGAGCGATATGCTCGTGTAATCAGTCT
		**CD28-5**	CATATTCATGTAGGGAGACTGATTACACGAGGAGACTGATTACACGGATGCCGGGGGGT	CTTCTGGTGAGGTCCGTGTAATCAGTCT
		**CD28-6**	TGGATGGGTGTCTGGAGACTGATTACACGAGGAGACTGATTACACGAGGTTCTTGTGTT	GAAGTTGCAGATTGCGTGTAATCAGTCT
		**CD28-7**	AAACCAAGAAGTCGGAGACTGATTACACGAGGAGACTGATTACACGAAGCATACTGTAG	AAGACTGCAGTTAACGTGTAATCAGTCT
		**CD28-8**	AGAATCTTGTTTTGGAGACTGATTACACGAGGAGACTGATTACACGACGCTGAGCCACT	ACAATGAGCAAAGCCGTGTAATCAGTCT
		**CD28-9**	ACTAGAGTTGCTGGGAGACTGATTACACGAGGAGACTGATTACACGTGTGTAATTGCAG	CCTGTTCCATTGTTCGTGTAATCAGTCT
		**CD28-10**	AATTTTGGTCATGGGAGACTGATTACACGAGGAGACTGATTACACGTTGAATTACTGTT	GTTGAATTCCTTAACGTGTAATCAGTCT
		**CD28-11**	TCCTTATCATGAAGGAGACTGATTACACGAGGAGACTGATTACACGGAAGATTACTTTG	ATATTCCAAAGATACGTGTAATCAGTCT
		**CD28-12**	AGATGTCAGTTTGGGAGACTGATTACACGAGGAGACTGATTACACGATTTTGCAGAAGT	GATACATGGCCTCCCGTGTAATCAGTCT
	G0S2	**G0S2-1**	GTGCATGGTTTCCCCTCGATTAACATCGCACCTCGATTAACATCGCAGGGGATCAGCTC	CATCTCTTTGGCGTGCGATGTTAATCGA
		**G0S2-2**	TCCTGTTGGGCTTCCTCGATTAACATCGCACCTCGATTAACATCGCAGCTTCACCATCT	TGCCCAGCACGTAGGCGATGTTAATCGA
		**G0S2-3**	CACGCCGAAGAACCCTCGATTAACATCGCACCTCGATTAACATCGCCCAGACCGATGAC	GCACACTGCCTCCTGCGATGTTAATCGA
		**G0S2-4**	ATATTCCCTTCGGCCTCGATTAACATCGCACCTCGATTAACATCGCCTTCTCCTCCTCT	GATGGGCTCGGTCAGCGATGTTAATCGA
		**G0S2-5**	CTTCCGAGGAAGCCCTCGATTAACATCGCACCTCGATTAACATCGCCCAAATCCTCCCG	GCTCTGCTCCAACTGCGATGTTAATCGA
		**G0S2-6**	CTGCACCGCTGACCCTCGATTAACATCGCACCTCGATTAACATCGCTCACCACCCCCCG	TGCATGCTGCCTGCGCGATGTTAATCGA
	ANGPTL4	**ANGPTL4-1**	TCTTCTCCAAGCCGGCACTTGAGCTAGAAAATGCTGCTGTAGTACTACCAGGTGATGGA	ACCTTCCCTCCTGCAGTACTACAGCAGC
		**ANGPTL4-2**	GTCCACATGCTCCGGCACTTGAGCTAGAAAATGCTGCTGTAGTACTGCCCCTTGGTCCT	GAGCTCCCGCATCAAGTACTACAGCAGC
		**ANGPTL4-3**	TCCAGCTCAATCAGGCACTTGAGCTAGAAAATGCTGCTGTAGTACTTCCCTCCCAGTCT	ATCTCCTGGGAGTAAGTACTACAGCAGC
		**ANGPTL4-4**	GACACCGCTGCTTGGCACTTGAGCTAGAAAATGCTGCTGTAGTACTGCTGCACCTGGAA	CTGAGACCCCGAGCAGTACTACAGCAGC

		**ANGPTL4-5**	TGCTCTCCCCACCGGCACTTGAGCTAGAAAATGCTGCTGTAGTACTAGTGTGTAGGCTG	GTCCCAGCAGGTTCAGTACTACAGCAGC
		**ANGPTL4-6**	TCCAGTTCTCCAGGGCACTTGAGCTAGAAAATGCTGCTGTAGTACTCCCAATGGCATTT	AGCTGCCTGAAGTGAGTACTACAGCAGC
		**ANGPTL4-7**	TGGATCACCGTCCGGCACTTGAGCTAGAAAATGCTGCTGTAGTACTATCCGTACGCCTC	AAGTCCACAGAGCGAGTACTACAGCAGC
		**ANGPTL4-8**	TGTCCTTCGGATGGGCACTTGAGCTAGAAAATGCTGCTGTAGTACTGACTGCGTTTTGT	TCTTCCACTTCGGTAGTACTACAGCAGC
		**ANGPTL4-9**	TCCCGAGTGGAGAGGCACTTGAGCTAGAAAATGCTGCTGTAGTACTGTCGTGGTCACGA	GTGTCCGCTTTGACAGTACTACAGCAGC
		**ANGPTL4-10**	CTGGGCGTCCATCGGCACTTGAGCTAGAAAATGCTGCTGTAGTACTCGATTCGCGAGTT	CTGCAGTAGCTCCAAGTACTACAGCAGC
		**ANGPTL4-11**	CTGCTGCTGCTTGGGCACTTGAGCTAGAAAATGCTGCTGTAGTACTTGTCCAGCTTGTA	CTGCAGATTCTGCAAGTACTACAGCAGC
		**ANGPTL4-12**	AACCACCACCCGCGGCACTTGAGCTAGAAAATGCTGCTGTAGTACTGCCGCAGGTGCTG	TTGAGGTTGGCGTCAGTACTACAGCAGC
	SDC4	**SDC4-1**	TGTTGGACAGGTTGGCACTTGAGCTAGAAAGGAGACTGATTACACGGCCATGGAGATCT	TGTTGGCTGTGCTACGTGTAATCAGTCT
		**SDC4-2**	CTCCTGCAATGAGGGCACTTGAGCTAGAAAGGAGACTGATTACACGAGGCCAACTGCTC	AGACAGCAAACAGCCGTGTAATCAGTCT
		**SDC4-3**	TCCATGGTCTCTGGGCACTTGAGCTAGAAAGGAGACTGATTACACGAAGCCATCGGGCA	GAGCCCACGTTGTGCGTGTAATCAGTCT
		**SDC4-4**	CGGGGCAGCGGCAGGCACTTGAGCTAGAAAGGAGACTGATTACACGCAGGAACGCGGCG	AGCAGGAGGCCGACCGTGTAATCAGTCT
		**SDC4-5**	TCTTCATCATCTGGGCACTTGAGCTAGAAAGGAGACTGATTACACGGAATTCACCAATG	GTTAAGTGAGGTGACGTGTAATCAGTCT
		**SDC4-6**	CAAACTCGTCAGAGGCACTTGAGCTAGAAAGGAGACTGATTACACGGTGTCATCTATAT	CTCCGGAGCCAGAACGTGTAATCAGTCT
		**SDC4-7**	TGGCATCATCATAGGCACTTGAGCTAGAAAGGAGACTGATTACACGGTGGTCAGGTATA	CAGGAGTATCCACTCGTGTAATCAGTCT
		**SDC4-8**	ATAGTTGTCAGATGGCACTTGAGCTAGAAAGGAGACTGATTACACGTATCTCCAGGGAT	CATCTTTCTCTCTCCGTGTAATCAGTCT
		**SDC4-9**	CATGGTGTTTTTCGGCACTTGAGCTAGAAAGGAGACTGATTACACGTTTCATTGTCCAG	TTTGTCTGGAATGTCGTGTAATCAGTCT
		**SDC4-10**	GACTAAGAGGAGGGGCACTTGAGCTAGAAAGGAGACTGATTACACGTCTTCATGCGATA	GCCCTCGTCCTTTACGTGTAATCAGTCT
		**SDC4-11**	CTTGTAGATGGGTGGCACTTGAGCTAGAAAGGAGACTGATTACACGTTGTAGGGGCTTT	AGCGTAGAACTCAACGTGTAATCAGTCT
	SDC2	**SDC2-1**	ACAGAGCTGTTGTGGCACTTGAGCTAGAAACCTCGATTAACATCGCCGATGCCTCTTCA	ATGGGATAGACGCGGCGATGTTAATCGA
		**SDC2-2**	CTTTCTCATGCGAGGCACTTGAGCTAGAAACCTCGATTAACATCGCTGCCTTCATCCTT	ACCAAGGTCGTAAGGCGATGTTAATCGA
		**SDC2-3**	CACTCGTTGTTGGGGCACTTGAGCTAGAAACCTCGATTAACATCGCGCTCTAGACGCAT	CTGTGGTGGTCTCTGCGATGTTAATCGA
		**SDC2-4**	AGACGGTTTACGTGGCACTTGAGCTAGAAACCTCGATTAACATCGCGATAGGCAGCACT	AGTAGGTGCCTTCAGCGATGTTAATCGA
		**SDC2-5**	GTCAAATCTGCTCGGCACTTGAGCTAGAAACCTCGATTAACATCGCATCTTTATCAGAG	TTGTCCAGGTATATGCGATGTTAATCGA
		**SDC2-6**	ATCTTCCTCAGCAGGCACTTGAGCTAGAAACCTCGATTAACATCGCCTGTGCTATCCTC	GGACGTTGTTGTCTGCGATGTTAATCGA
		**SDC2-7**	ATCGATTTCTTCAGGCACTTGAGCTAGAAACCTCGATTAACATCGCGTCTTTCCTCTTT	GGAATCCACCTCGCGCGATGTTAATCGA
		**SDC2-8**	CTGGCTCATTGCTGGCACTTGAGCTAGAAACCTCGATTAACATCGCTCAGTGTCATCCC	TTTCAGTGAACACTGCGATGTTAATCGA
		**SDC2-9**	GGTTTTCTGAATGGGCACTTGAGCTAGAAACCTCGATTAACATCGCGTCCTCTGGAAGA	CTGCCAGAACTTCAGCGATGTTAATCGA
		**SDC2-10**	GCCAGCAATAACAGGCACTTGAGCTAGAAACCTCGATTAACATCGCAACCAATAACTCC	GATTGCAAACAGATGCGATGTTAATCGA
	TLR4	**TLR4-1**	GAGTGAATTGGCAATGCTGCTGTAGTACTAGGAGACTGATTACACGGACCCAGGTGCTG	ATGGAAGGCTGCTTCGTGTAATCAGTCT
		**TLR4-2**	AGGTCAGACAGAGATGCTGCTGTAGTACTAGGAGACTGATTACACGGTGTCCGATGGGT	TGCAGGGTATTCATCGTGTAATCAGTCT
		**TLR4-3**	CAGGTTGGCAAAAATGCTGCTGTAGTACTAGGAGACTGATTACACGGCCTGAGAGAGGT	GGAAAAGCTCAGGACGTGTAATCAGTCT

		**TLR4-4**	TTCCCTCAGGGCAATGCTGCTGTAGTACTAGGAGACTGATTACACGTGAGCCTGTTTGT	TACCAACGTGAGGACGTGTAATCAGTCT
		**TLR4-5**	GAGTGCATCGCATATGCTGCTGTAGTACTAGGAGACTGATTACACGGCCCTGAAGAGAA	TGTAAACCTGCCACCGTGTAATCAGTCT
		**TLR4-6**	CTGAAATGCCGTTATGCTGCTGTAGTACTAGGAGACTGATTACACGTCAAGAGTCCGTT	CTGACACAGTCCAGCGTGTAATCAGTCT
		**TLR4-7**	TGCCTATGCAGTCATGCTGCTGTAGTACTAGGAGACTGATTACACGATAGTGGTGACGT	GGTCCACCAACCGTCGTGTAATCAGTCT
		**TLR4-8**	TTCAGCAGGAACTATGCTGCTGTAGTACTAGGAGACTGATTACACGTGGTGTTTGGGAT	GTCCAAGTCCAGGCCGTGTAATCAGTCT
		**TLR4-9**	GTTCAAGCCATGAATGCTGCTGTAGTACTAGGAGACTGATTACACGGCACTTGCAAAGA	GCCTGCCATCTTGTCGTGTAATCAGTCT
		**TLR4-10**	AGGGCTTGGAGTGATGCTGCTGTAGTACTAGGAGACTGATTACACGATCCAGAGCTGTG	TGGTTGTTGCTGATCGTGTAATCAGTCT
		**TLR4-11**	CTGAGCATGCTACATGCTGCTGTAGTACTAGGAGACTGATTACACGAGCAACACAGTCA	ACACTCCTGCAGCTCGTGTAATCAGTCT
		**TLR4-12**	CAGCACCAATGAGATGCTGCTGTAGTACTAGGAGACTGATTACACGTACACCCACTGAG	TTCTGCAGAGTGTACGTGTAATCAGTCT
		**CD226-1**	AGCAATCCACCAGATGCTGCTGTAGTACTACCTCGATTAACATCGCCTTGCCTGACCAG	TGACAACTGTGGACGCGATGTTAATCGA
		**CD226-2**	TCCAAAGTGCTCTATGCTGCTGTAGTACTACCTCGATTAACATCGCAAGACCAACGTCT	ATGGAGCAACAGTTGCGATGTTAATCGA
		**CD226-3**	GTAGTCAGAGGCTATGCTGCTGTAGTACTACCTCGATTAACATCGCAGCGGTACGTTGT	TCCAGTAGCCACGGGCGATGTTAATCGA
		**CD226-4**	GAAAGCTGCATGGATGCTGCTGTAGTACTACCTCGATTAACATCGCGAACAGCAACAAG	TCTACAAACTTGGTGCGATGTTAATCGA
		**CD226-5**	CACATTGTCTAAAATGCTGCTGTAGTACTACCTCGATTAACATCGCCAAACCTTCCTTC	TATAGTTGAATCCTGCGATGTTAATCGA
	CD226	**CD226-6**	GCTTCATCTTCTCATGCTGCTGTAGTACTACCTCGATTAACATCGCTACATACATTCCA	CAGCTTTTTTTGGTGCGATGTTAATCGA
		**CD226-7**	TATGCTTCATCCAATGCTGCTGTAGTACTACCTCGATTAACATCGCTTATGACTTCCAT	CAGCAACATTTTCAGCGATGTTAATCGA
		**CD226-8**	ATGCCATAGATAGATGCTGCTGTAGTACTACCTCGATTAACATCGCGTCTTCAATATGT	CTTCCTCTGTATTAGCGATGTTAATCGA
		**CD226-9**	GGAAGTATTTTCAATGCTGCTGTAGTACTACCTCGATTAACATCGCATTGGTCTTCCCT	ATTGAAGGATAAGCGCGATGTTAATCGA
		**CD226-10**	AGAGACTCCATCTATGCTGCTGTAGTACTACCTCGATTAACATCGCCTATTACCTTCTC	TGGATGAATAACTAGCGATGTTAATCGA
		**CD226-11**	TAATTGTTACCAGATGCTGCTGTAGTACTACCTCGATTAACATCGCATTGTGTTCTGCA	CATGTGGTATGATTGCGATGTTAATCGA
		**CD226-12**	GGCCTGAGTGATGATGCTGCTGTAGTACTACCTCGATTAACATCGCAATCTCCTGCTCC	ATGCAGTTCACGTTGCGATGTTAATCGA
		**NECTIN3-1**	CACTGCTGTCACAGGAGACTGATTACACGACCTCGATTAACATCGCCCTTCTTCCCCCA	GCATTTCAGTGCATGCGATGTTAATCGA
		**NECTIN3-2**	TGATTGGCACCATGGAGACTGATTACACGACCTCGATTAACATCGCGGCTGCTATTGTT	GCTGCTGTACAAAAGCGATGTTAATCGA
		**NECTIN3-3**	TCTCTGCAGCAGGGGAGACTGATTACACGACCTCGATTAACATCGCCCTTCCCAGTCAA	TCTCACCAAGACCAGCGATGTTAATCGA
		**NECTIN3-4**	CAGCACTGTCACAGGAGACTGATTACACGACCTCGATTAACATCGCTCCTTGGCGTCAG	GGATGGCCTTTTCAGCGATGTTAATCGA
		**NECTIN3-5**	CCGATCGCTGCCAGGAGACTGATTACACGACCTCGATTAACATCGCGCGAGAAACGGCT	GGAGCCGGCGAGAGGCGATGTTAATCGA
	NECTIN3	**NECTIN3-6**	CGGCCGCTGGAAAGGAGACTGATTACACGACCTCGATTAACATCGCCGCGGGGAGGCAC	GCCACCACCACCAGGCGATGTTAATCGA
		**NECTIN3-7**	CGCCGCCGCTGTTGGAGACTGATTACACGACCTCGATTAACATCGCCGAGCCCGCCCGG	CCCGGGGGCAGCGGGCGATGTTAATCGA
		**NECTIN3-8**	CCAGCAGCCCCAGGGAGACTGATTACACGACCTCGATTAACATCGCGAGAACACCAGTC	TGCAGCAGAAGCGCGCGATGTTAATCGA
		**NECTIN3-9**	TCTCCCAAGAAACGGAGACTGATTACACGACCTCGATTAACATCGCTTACCATGGATCT	TAGTCTCTGAAGTAGCGATGTTAATCGA
		**NECTIN3-10**	ATATTCAGGATGAGGAGACTGATTACACGACCTCGATTAACATCGCGAATAGAAATTCC	TTGGTATTTTTCTAGCGATGTTAATCGA
		**NECTIN3-11**	GTGAATAGTTTTTGGAGACTGATTACACGACCTCGATTAACATCGCGTTGCATCAGTAA	TTTTTAGGATGATAGCGATGTTAATCGA

		**NECTIN3-12**	TATTCTCCTGCATGGAGACTGATTACACGACCTCGATTAACATCGCTGCCTTACAGATA	AGAGGGAATGTAAGGCGATGTTAATCGA
	COL6A3	**COL6A3-1**	CTGTGCAACTTCCGTGCTTGAACTGGTCTAGTGCTTGAACTGGTCTCCTTGACACCCTT	ACCAACTGCAAACTAGACCAGTTCAAGC
		**COL6A3-2**	TCCTACACCCGGCGTGCTTGAACTGGTCTAGTGCTTGAACTGGTCTACCCCTTCCTCCA	CCAGCATGGGGAGGAGACCAGTTCAAGC
		**COL6A3-3**	TTGTCCGTCCGTTGTGCTTGAACTGGTCTAGTGCTTGAACTGGTCTCATCATCCTGGGA	TGATGGCAGAGCCTAGACCAGTTCAAGC
		**COL6A3-4**	CTCCAACAGCAAAGTGCTTGAACTGGTCTAGTGCTTGAACTGGTCTTCCGCATTCCTAA	CAAGCTCTGCCTTTAGACCAGTTCAAGC
		**COL6A3-5**	ACACATCGAAGCCGTGCTTGAACTGGTCTAGTGCTTGAACTGGTCTGCTCCGACATCTG	AGATGTTTTGGCCCAGACCAGTTCAAGC
		**COL6A3-6**	GTGGTGTCAAAGCGTGCTTGAACTGGTCTAGTGCTTGAACTGGTCTCACCGAAATCCGA	TCGCTGAACTGGAGAGACCAGTTCAAGC
		**COL6A3-7**	CTTTGTTTGGCCCGTGCTTGAACTGGTCTAGTGCTTGAACTGGTCTACTCCAATCCGCA	TGCTGAATTGCACGAGACCAGTTCAAGC
		**COL6A3-8**	GTCCCCTCTCTGCGTGCTTGAACTGGTCTAGTGCTTGAACTGGTCTCGGGAAGACCTCT	TGGTCCTATGGGGGAGACCAGTTCAAGC
		**COL6A3-9**	AGAGGGGTACGTAGTGCTTGAACTGGTCTAGTGCTTGAACTGGTCTGCACATCTTGGTT	CGCGATATGGGAGTAGACCAGTTCAAGC
		**COL6A3-10**	GACGATTTTGGCAGTGCTTGAACTGGTCTAGTGCTTGAACTGGTCTCCTCCAGCCTTTG	CAGGTCAGGTCCATAGACCAGTTCAAGC
		**COL6A3-11**	GTCCCCTCCTCCTGTGCTTGAACTGGTCTAGTGCTTGAACTGGTCTAAAGTCCCTGCAG	TGCCACTTGAGCAGAGACCAGTTCAAGC
		**COL6A3-12**	GTGCTCCTGGTTGGTGCTTGAACTGGTCTAGTGCTTGAACTGGTCTCCCCTTTCTCCAG	GTGGGCCAATCCTCAGACCAGTTCAAGC
	COL6A1	**COL6A1-1**	ATGTCTGCCGGATCTGACTAACGACACCAACTGACTAACGACACCACACCAACAGCATG	CTGGTGGAGCTGTGTGGTGTCGTTAGTC
		**COL6A1-2**	AGCCGCTTCACAACTGACTAACGACACCAACTGACTAACGACACCAGAACCGCTCTGCA	TTGCTGGCCTCAACTGGTGTCGTTAGTC
		**COL6A1-3**	ACGCGCACAGAATCTGACTAACGACACCAACTGACTAACGACACCACTGGACCACCGAG	TTTCTGCCGCTGTTTGGTGTCGTTAGTC
		**COL6A1-4**	TTCGACCTTCTGCCTGACTAACGACACCAACTGACTAACGACACCAGCTGAAATGGGAC	GACCGTGTAGTTGGTGGTGTCGTTAGTC
		**COL6A1-5**	GTACTGCAGAGCGCTGACTAACGACACCAACTGACTAACGACACCAACAGCCCCATGAT	AGAGGACCGCTGGATGGTGTCGTTAGTC
		**COL6A1-6**	CTGTAGCCCTTTGCTGACTAACGACACCAACTGACTAACGACACCAGCCATCATCTCCT	TCATTGCCACGGGCTGGTGTCGTTAGTC
		**COL6A1-7**	GATGGCCCTTGCACTGACTAACGACACCAACTGACTAACGACACCAGCACAGTCCTCTC	CTGCTGAACTTCCATGGTGTCGTTAGTC
		**COL6A1-8**	TGATCCCACTGGGCTGACTAACGACACCAACTGACTAACGACACCACCCTTTGCTCCTT	CAGGACCCCTGTGCTGGTGTCGTTAGTC
		**COL6A1-9**	CTGGCTTTCCTCTCTGACTAACGACACCAACTGACTAACGACACCATGACCAGGAAGAC	CAGCATCTCCCTTGTGGTGTCGTTAGTC
		**COL6A1-10**	ACACGAAGAGGAGCTGACTAACGACACCAACTGACTAACGACACCATCTGAGCTGTCCA	GCAGGCCAATACTGTGGTGTCGTTAGTC
		**COL6A1-11**	CTGCCAGCACGTACTGACTAACGACACCAACTGACTAACGACACCAACTTGGCTGCCCA	CATTGGGCTCGTTCTGGTGTCGTTAGTC
		**COL6A1-12**	TTGTGCTCTTCCCCTGACTAACGACACCAACTGACTAACGACACCAGCAACGTCATAGT	GACGCTCCCCATACTGGTGTCGTTAGTC
	CD44	**CD44-1**	AGCCGTCCTCATTACTTGGATCCACTCGTAACTTGGATCCACTCGTGCATTGTAACGTG	GACGTGTGCCATCAACGAGTGGATCCAA
		**CD44-2**	AATCAGCAGCTGTACTTGGATCCACTCGTAACTTGGATCCACTCGTTCTTCTCTGGGGA	GATGCTGCACATCTACGAGTGGATCCAA
		**CD44-3**	GTAGGTTCACCGTACTTGGATCCACTCGTAACTTGGATCCACTCGTTCCAGGGGAAGAA	GTTGTTGTGGACATACGAGTGGATCCAA
		**CD44-4**	AGGATCAATGCCAACTTGGATCCACTCGTAACTTGGATCCACTCGTGATGCACACGGCA	CGGCTGTTAACCGGACGAGTGGATCCAA
		**CD44-5**	TAGAAGTTGGCCAACTTGGATCCACTCGTAACTTGGATCCACTCGTAGTTGCCAATAAG	AAGCATAATCCAATACGAGTGGATCCAA
		**CD44-6**	CGTATCTGCAAGTACTTGGATCCACTCGTAACTTGGATCCACTCGTTGAAACACTCCTC	CATTTTTCTCCACTACGAGTGGATCCAA
		**CD44-7**	AGCTCGTGTGAGAACTTGGATCCACTCGTAACTTGGATCCACTCGTGCTCAATTGCTTC	GAGAGCTCTACAGTACGAGTGGATCCAA

		**CD44-8**	GCCAGTTGTATTTACTTGGATCCACTCGTAACTTGGATCCACTCGTATGCATCATACCG	TGCATTGTAACAAAACGAGTGGATCCAA
		**CD44-9**	CTATATTGGACGTACTTGGATCCACTCGTAACTTGGATCCACTCGTCCCTGATCACTGT	CTCCCTTGTGTGGAACGAGTGGATCCAA
		**CD44-10**	TTTTCTTCTGCCCACTTGGATCCACTCGTAACTTGGATCCACTCGTTTAATGACTAGCT	CACCTTTGCCATTTACGAGTGGATCCAA
		**CD44-11**	TTCTCTTGTCTTCACTTGGATCCACTCGTAACTTGGATCCACTCGTCATCTCCGTTTAA	TTGTGACTTGCTGCACGAGTGGATCCAA
		**CD44-12**	TCTTTGTGAACCAACTTGGATCCACTCGTAACTTGGATCCACTCGTGTCATTTGACTGT	CATGCTCCTGTTCCACGAGTGGATCCAA
	COL6A2	**COL6A2-1**	TCCTTTTGGTCCAGATCTCGAGAACGTCAAGATCTCGAGAACGTCATTGTCCCTTGTGT	CCCCTTCTCTCCTATGACGTTCTCGAGA
		**COL6A2-2**	TCTCTCACTGCCGGATCTCGAGAACGTCAAGATCTCGAGAACGTCACCACATTCTGCTC	GGTCAGTGGGAAGTTGACGTTCTCGAGA
		**COL6A2-3**	ACTTCACATCCCGGATCTCGAGAACGTCAAGATCTCGAGAACGTCAATGACCACTGCAA	CATGGCCATCAGTCTGACGTTCTCGAGA
		**COL6A2-4**	TCCAGGCGCTTTAGATCTCGAGAACGTCAAGATCTCGAGAACGTCATCCAGCAATCCAC	GGTGTCCAGGTGCGTGACGTTCTCGAGA
		**COL6A2-5**	ACTTGGGCTTTCTGATCTCGAGAACGTCAAGATCTCGAGAACGTCACACCACAGCAAAC	CGTCCATCTGTGAATGACGTTCTCGAGA
		**COL6A2-6**	GATCTCCACGTCGGATCTCGAGAACGTCAAGATCTCGAGAACGTCACCCTTCGTCCCTG	GACCGCCTTTGCTCTGACGTTCTCGAGA
		**COL6A2-7**	TTCGGTCAGGTTGGATCTCGAGAACGTCAAGATCTCGAGAACGTCAGGGCATTGGAGAT	CTTGATCTGAGCCTTGACGTTCTCGAGA
		**COL6A2-8**	GTTTCCCTTTGCAGATCTCGAGAACGTCAAGATCTCGAGAACGTCACTGGTTCACCCAT	CAGTCCAGGGGAGGTGACGTTCTCGAGA
		**COL6A2-9**	TGTCATCATCCCGGATCTCGAGAACGTCAAGATCTCGAGAACGTCAGCCCCTAGGTTCT	CTCTGCCACAAAGTTGACGTTCTCGAGA
		**COL6A2-10**	AGGTCATGTGGTGGATCTCGAGAACGTCAAGATCTCGAGAACGTCAGTTACTGCGGTAC	GGTGTGATGGTGTTTGACGTTCTCGAGA
		**COL6A2-11**	CTTGGGCCCAAGTGATCTCGAGAACGTCAAGATCTCGAGAACGTCATCCAGCATCACCT	TTTGGTCCCGGTGCTGACGTTCTCGAGA
		**COL6A2-12**	GTCATCACATCGCGATCTCGAGAACGTCAAGATCTCGAGAACGTCACTCTCGCACGTAG	CAGCATCCACATGATGACGTTCTCGAGA
	COL4A1	**COL4A1-1**	CAACAGATGCTGCGTGCTTGAACTGGTCTACTGACTAACGACACCAAGGAAGCCATGAG	TGTGTCTGGTGACTTGGTGTCGTTAGTC
		**COL4A1-2**	CACCTCCTGGCAAGTGCTTGAACTGGTCTACTGACTAACGACACCACCCTTTTCACCAG	GAGGCCCTAGATCTTGGTGTCGTTAGTC
		**COL4A1-3**	AGCTCCCTTCAGAGTGCTTGAACTGGTCTACTGACTAACGACACCACCTGCTCTCCTTT	CCAGCCATCGCTTGTGGTGTCGTTAGTC
		**COL4A1-4**	TACTCCCCACGTAGTGCTTGAACTGGTCTACTGACTAACGACACCATCCTGGTTCTCCA	TCCCTTCCTGGAAATGGTGTCGTTAGTC
		**COL4A1-5**	GACCAGGAACACCGTGCTTGAACTGGTCTACTGACTAACGACACCAATGGAACCAGCAG	CACCCTTCTGACCTTGGTGTCGTTAGTC
		**COL4A1-6**	TCACACACAGAGCGTGCTTGAACTGGTCTACTGACTAACGACACCACATAGCAGGAGCT	TGAACGGCGATTAGTGGTGTCGTTAGTC
		**COL4A1-7**	TGCCTGTTCTTCCGTGCTTGAACTGGTCTACTGACTAACGACACCAGACAAACCCGGAC	CACCTTTGTCTCCTTGGTGTCGTTAGTC
		**COL4A1-8**	AACCCTGGAGTTCGTGCTTGAACTGGTCTACTGACTAACGACACCACATTGGTCCCTGA	ATCCCTGGTGGCCGTGGTGTCGTTAGTC
		**COL4A1-9**	TCACCCTTTGCACGTGCTTGAACTGGTCTACTGACTAACGACACCATGGGAAACCCACT	ATTCCTGGGGATCGTGGTGTCGTTAGTC
		**COL4A1-10**	AGTTGACGGAGTCGTGCTTGAACTGGTCTACTGACTAACGACACCACCCCTGCTTTCAA	GTTGGAGCGCAGAATGGTGTCGTTAGTC
		**COL4A1-11**	TGTCCAGGAATGCGTGCTTGAACTGGTCTACTGACTAACGACACCAAGGAGGGCCATCT	ATGCCAGGTGGACGTGGTGTCGTTAGTC
		**COL4A1-12**	CAATGAAGGGAGCGTGCTTGAACTGGTCTACTGACTAACGACACCACGGCCATGGCATT	AATTGCAGGTGCCGTGGTGTCGTTAGTC
	COL4A2	**COL4A2-1**	TTCTCCCTTGCTTGTGCTTGAACTGGTCTAACTTGGATCCACTCGTCTGCTCGTCCACT	TTGTCCGCTCACAGACGAGTGGATCCAA
		**COL4A2-2**	TTCTCCCCGTAGTGTGCTTGAACTGGTCTAACTTGGATCCACTCGTCCTGCTCTCCTTG	CAGCCCACGATCAGACGAGTGGATCCAA
		**COL4A2-3**	TGTGTCTGCCGATGTGCTTGAACTGGTCTAACTTGGATCCACTCGTGTCCTGCCTTGAG	GTGGCTTCGGATGTACGAGTGGATCCAA

		**COL4A2-4**	TTGAGGCCTGGCAGTGCTTGAACTGGTCTAACTTGGATCCACTCGTACCAACTTCCCCT	GTAAGACCCGGCAGACGAGTGGATCCAA
		**COL4A2-5**	CTCCCAGAACGTCGTGCTTGAACTGGTCTAACTTGGATCCACTCGTGGACCTGCTGCTG	CATCATCCCCTCGTACGAGTGGATCCAA
		**COL4A2-6**	TACCTGGAAACCCGTGCTTGAACTGGTCTAACTTGGATCCACTCGTGGTTCACCGTGTT	GACCACGGATTCCAACGAGTGGATCCAA
		**COL4A2-7**	CTGGATCACCTCGGTGCTTGAACTGGTCTAACTTGGATCCACTCGTTATCCTTCCCGAC	CTGGAGGACCTGGTACGAGTGGATCCAA
		**COL4A2-8**	TGGCTGTGCTTCAGTGCTTGAACTGGTCTAACTTGGATCCACTCGTCTCTTGGTCGGAC	ATCGGGCACATTGCACGAGTGGATCCAA
		**COL4A2-9**	CTTTAGGGCCTTGGTGCTTGAACTGGTCTAACTTGGATCCACTCGTTCACCCTTGTGAC	CTGGATGTCCTCTAACGAGTGGATCCAA
		**COL4A2-10**	GACTCCTCTTTGCGTGCTTGAACTGGTCTAACTTGGATCCACTCGTCTGGGAAACCAGT	AACGCCATCTGCCGACGAGTGGATCCAA
		**COL4A2-11**	TGAGAGCCAGTAGGTGCTTGAACTGGTCTAACTTGGATCCACTCGTGAGGTGCTGTAGT	AGGCATCATTGGATACGAGTGGATCCAA
		**COL4A2-12**	CTCACCCTGCTCCGTGCTTGAACTGGTCTAACTTGGATCCACTCGTCTGGAGGACCCAT	ATAGGCAGGTGGTGACGAGTGGATCCAA
	FN1	**FN1-1**	AGTGTCCCCAATTGTGCTTGAACTGGTCTAGATCTCGAGAACGTCACCTTCTTGCTCCA	GTTTCCCCGGTTAATGACGTTCTCGAGA
		**FN1-2**	CTGCTCTCCAACTGTGCTTGAACTGGTCTAGATCTCGAGAACGTCAACTCCTTCTGCCA	GATGGCCCCGAAGATGACGTTCTCGAGA
		**FN1-3**	CTCCTCGCCAATGGTGCTTGAACTGGTCTAGATCTCGAGAACGTCAATAATCGCTCCCA	AAAGCCCGTTTCACTGACGTTCTCGAGA
		**FN1-4**	TGAGACGAACGTTGTGCTTGAACTGGTCTAGATCTCGAGAACGTCAACCCTGTAGCCAG	TGGGGTTCACTCTCTGACGTTCTCGAGA
		**FN1-5**	CACAAACTGCAGGGTGCTTGAACTGGTCTAGATCTCGAGAACGTCACGTCAGAAACCTC	GATGGTGACCTTGTTGACGTTCTCGAGA
		**FN1-6**	TGCTCCTCGAAGAGTGCTTGAACTGGTCTAGATCTCGAGAACGTCACCTCCTGTAGCCA	GTCGTGGGTACAGCTGACGTTCTCGAGA
		**FN1-7**	TAAGGAAGCGCAGGTGCTTGAACTGGTCTAGATCTCGAGAACGTCATTGGTTGTGGTTG	TAGCCAGGAGGGACTGACGTTCTCGAGA
		**FN1-8**	TTCACAGGTGAGTGTGCTTGAACTGGTCTAGATCTCGAGAACGTCAGTCCTCCTCCTTC	GTCAGCTCTGCAAGTGACGTTCTCGAGA
		**FN1-9**	GATGCTGATGCTGGTGCTTGAACTGGTCTAGATCTCGAGAACGTCAAAGGGAGCCATCT	GACTGGGGATGATCTGACGTTCTCGAGA
		**FN1-10**	CTGGCCATTCTCCGTGCTTGAACTGGTCTAGATCTCGAGAACGTCATGCAGCTCATCAT	ATTCCCGAGGCAGCTGACGTTCTCGAGA
		**FN1-11**	GTGACTTGCCCCTGTGCTTGAACTGGTCTAGATCTCGAGAACGTCACACCCTGTACCTG	GGGCTTGAGTAGGATGACGTTCTCGAGA
		**FN1-12**	CAGTGGACAGTGAGTGCTTGAACTGGTCTAGATCTCGAGAACGTCAACGAGGAGCAATC	CCCGTAATGGTGGGTGACGTTCTCGAGA
	LAMC1	**LAMC1-1**	TTGCTCTCCTCCACTGACTAACGACACCAAACTTGGATCCACTCGTGCACTTTCTGCTG	GCAAGCACTCGTTCACGAGTGGATCCAA
		**LAMC1-2**	CAGCTCACACCTCCTGACTAACGACACCAAACTTGGATCCACTCGTAGGCATCATCACA	TGGGTCTCCAAAAAACGAGTGGATCCAA
		**LAMC1-3**	TGCCCCTCGTAGCCTGACTAACGACACCAAACTTGGATCCACTCGTGCGCTCACAGAAC	TACCCAGGGGAGCTACGAGTGGATCCAA
		**LAMC1-4**	ATGCACTCGCTCGCTGACTAACGACACCAAACTTGGATCCACTCGTAAGCTCGTTCCTT	CAGACCAGCTTCCGACGAGTGGATCCAA
		**LAMC1-5**	GTTGCAGCTCGTCCTGACTAACGACACCAAACTTGGATCCACTCGTGCCCCGTCACTTG	GAGGCACTCGCACAACGAGTGGATCCAA
		**LAMC1-6**	GAGAAAGCCACGTCTGACTAACGACACCAAACTTGGATCCACTCGTCCCCTCCAACGTT	TAGGCGCTGGGACCACGAGTGGATCCAA
		**LAMC1-7**	GTCACATTGGCCACTGACTAACGACACCAAACTTGGATCCACTCGTGCCCTGTCCATAT	CTGGCACTCACACAACGAGTGGATCCAA
		**LAMC1-8**	AGTGCTGTTGATGCTGACTAACGACACCAAACTTGGATCCACTCGTGCTGACTTGTCAA	CCTCAGCCTGCTGTACGAGTGGATCCAA
		**LAMC1-9**	TGCCTCCTCAAGCCTGACTAACGACACCAAACTTGGATCCACTCGTTTCCAGCTTCCTC	CTGGAGGGCCTTAAACGAGTGGATCCAA
		**LAMC1-10**	CTGGTAGGGCACCCTGACTAACGACACCAAACTTGGATCCACTCGTACCCGCTGTAATA	GGTGCTCTCACACCACGAGTGGATCCAA
		**LAMC1-11**	AGCCAATGGCATTCTGACTAACGACACCAAACTTGGATCCACTCGTCTGTACACGCTGT	AGCTGGAGGTGATCACGAGTGGATCCAA

		**LAMC1-12**	TGCAAGTACACCGCTGACTAACGACACCAAACTTGGATCCACTCGTTCAACGTTGTCCT	CACAGTGGAAGCCGACGAGTGGATCCAA
**Pigeon**	***TPT1***	**TPT1-1**	CTTCAGCAGAGGCCAGATAGGACTAGTCAACAGATAGGACTAGTCATCTCCCTCAGGAC	TGGCTTCTGTTCCTTGACTAGTCCTATC
		**TPT1-2**	CTCCTACAAAGAACAGATAGGACTAGTCAACAGATAGGACTAGTCAGGATTCATGTTCT	CCACCATACCATCATGACTAGTCCTATC
		**TPT1-3**	ATCATATATGGGGCAGATAGGACTAGTCAACAGATAGGACTAGTCAGTCCTTAAAGAAA	TCAATTTCTAAGCGTGACTAGTCCTATC
		**TPT1-4**	TCTGGCTTTAATTCAGATAGGACTAGTCAACAGATAGGACTAGTCATGTGTTCCTCAAG	TACTCTCTCTGGCATGACTAGTCCTATC
		**TPT1-5**	ATTTGTTCTGCAGCAGATAGGACTAGTCAACAGATAGGACTAGTCAAAGGATGTGTTTG	TTTTTGAAGTTGGGTGACTAGTCCTATC
		**TPT1-6**	TCTGTCCTGGTGACAGATAGGACTAGTCAACAGATAGGACTAGTCAATCAATTTGACCC	CCAATTAGAGAGTGTGACTAGTCCTATC
		**TPT1-7**	ATATCAACACCAGCAGATAGGACTAGTCAACAGATAGGACTAGTCAGTGGTTTATTACT	GTTTCCTGAAGATCTGACTAGTCCTATC
		**TPT1-8**	TGTAGGATTCTTTCAGATAGGACTAGTCAACAGATAGGACTAGTCATTGATGTACTTCT	CTTTCATGTAGTCGTGACTAGTCCTATC
	TXNIP	**TXNIP-1**	CATCACCTCGAAGTAGTCGCAACTCTGAAAATGAGATCGTCATCGTCGTCGACAGGATC	TTCCGGAGTGTTCTACGATGACGATCTC
		**TXNIP-2**	GAAGCGCAGATACTAGTCGCAACTCTGAAAATGAGATCGTCATCGTCCAGCACGTCCTC	CTGCTCCTCCAGGCACGATGACGATCTC
		**TXNIP-3**	ACGTGGTACCCAGTAGTCGCAACTCTGAAAATGAGATCGTCATCGTTACTTGCCCTTGA	AATCCACGCAGCCCACGATGACGATCTC
		**TXNIP-4**	AGCCTTCACCCAATAGTCGCAACTCTGAAAATGAGATCGTCATCGTGGCGCTCCAGAAA	GGTGGGGAAGGACCACGATGACGATCTC
		**TXNIP-5**	TGCAGATGTCGTCTAGTCGCAACTCTGAAAATGAGATCGTCATCGTAAGTCGGCGTTGA	AGCACGTGTTCTCCACGATGACGATCTC
		**TXNIP-6**	GAGCTTCTTGACGTAGTCGCAACTCTGAAAATGAGATCGTCATCGTGGATGGAGGGTTT	GATGTTGCAGCCCTACGATGACGATCTC
		**TXNIP-7**	CATGCCCGAGATGTAGTCGCAACTCTGAAAATGAGATCGTCATCGTGCCAGGACTCGGA	GATGGTTTTGCCGGACGATGACGATCTC
		**TXNIP-8**	CTTGGTCTGGCCGTAGTCGCAACTCTGAAAATGAGATCGTCATCGTTCTGGGTGAAGAC	GACGCAGGAGAGTAACGATGACGATCTC
		**TXNIP-9**	CAGGTAACACGGGTAGTCGCAACTCTGAAAATGAGATCGTCATCGTCGGGGACGATGTC	CAGGCGATGGTCTAACGATGACGATCTC
		**TXNIP-10**	TGGGCACCACGATTAGTCGCAACTCTGAAAATGAGATCGTCATCGTATGATGGCGGCTT	AGGTGTGCTTGGCCACGATGACGATCTC
	SGK1	**SGK1-1**	AAAGCCTCAGCAGTAGTCGCAACTCTGAAATGATCTTCCGATCTAATGAGAAGCCCAAA	ACAGGTGGGGCATTTTAGATCGGAAGAT
		**SGK1-2**	TGCAAGAGGCCTTTAGTCGCAACTCTGAAATGATCTTCCGATCTAATGTCCTGTCCTTC	GCACCAAGCCTCTATTAGATCGGAAGAT
		**SGK1-3**	TGGCCGATGGAACTAGTCGCAACTCTGAAATGATCTTCCGATCTAAGCTGTCTGGGGAC	GCAGTGATGAGGAATTAGATCGGAAGAT
		**SGK1-4**	TGGCGATTTCAGCTAGTCGCAACTCTGAAATGATCTTCCGATCTAATAGCCCAGTGCAC	TCAGGGAGTGCAGCTTAGATCGGAAGAT
		**SGK1-5**	AACTTCAGGAGCATAGTCGCAACTCTGAAATGATCTTCCGATCTAAGCTGCTTATGGAG	AGTCCGGTCATAGCTTAGATCGGAAGAT
		**SGK1-6**	CACTGGGGCCACTTAGTCGCAACTCTGAAATGATCTTCCGATCTAAAAGTGTCGCAGGT	TAAACTCCGGATCTTTAGATCGGAAGAT
		**SGK1-7**	GAAGAAGATGTGATAGTCGCAACTCTGAAATGATCTTCCGATCTAAAGTTAATTGGGGA	AATGAGATCATCCGTTAGATCGGAAGAT
		**SGK1-8**	GTTCCTGCTGTAGTAGTCGCAACTCTGAAATGATCTTCCGATCTAAACATTTCTGCCGT	CAAGATGTTGTCGATTAGATCGGAAGAT
		**SGK1-9**	TATTTGGCTTCAGTAGTCGCAACTCTGAAATGATCTTCCGATCTAAGCAGAGTTGGTAA	CCAGGAGATGTCTTTTAGATCGGAAGAT
		**SGK1-10**	TCCAAGGCACCACTAGTCGCAACTCTGAAATGATCTTCCGATCTAACATACAGGACTGC	GCCATAAAGCATCATTAGATCGGAAGAT
	RAB33A	**RAB33A-1**	CTCCTTAGGGTCCTATGTGACCGGGTACAATATGTGACCGGGTACACCACGTTCTGGCT	CATGAAAATGGCGATGTACCCGGTCACA
		**RAB33A-2**	AGTCCACGCCGATTATGTGACCGGGTACAATATGTGACCGGGTACAGTCTTCTCGCGGA	CCTCGATCTCCACGTGTACCCGGTCACA
		**RAB33A-3**	CGTGTCCCACACCTATGTGACCGGGTACAATATGTGACCGGGTACATCTCCTGGCCGGC	GCTCTTGCGAAACGTGTACCCGGTCACA

		**RAB33A-4**	AGTAGTGCTCCACTATGTGACCGGGTACAATATGTGACCGGGTACATGCACGTTGCGGT	CGAAGACGACGGCCTGTACCCGGTCACA
		**RAB33A-5**	GAGGTCATCTTGGTATGTGACCGGGTACAATATGTGACCGGGTACAGAGGTTGGTGAAG	TCGATCCACATCTATGTACCCGGTCACA
		**RAB33A-6**	GGCACGGCGTGCCTATGTGACCGGGTACAATATGTGACCGGGTACAGGGGACCATGGCG	CCAACCAGCACCCATGTACCCGGTCACA
		**RAB33A-7**	GTCGGCGAACTTCTATGTGACCGGGTACAATATGTGACCGGGTACAGCATGTTGTGAGC	GGTCTCAAACAAATTGTACCCGGTCACA
		**RAB33A-8**	CTTTCAAGTCACATATGTGACCGGGTACAATATGTGACCGGGTACAACCTGGATCAGGT	CCATGCTGGATGGGTGTACCCGGTCACA
		**RAB33A-9**	GCTGCGCCTTCAGTATGTGACCGGGTACAATATGTGACCGGGTACACAGAGCAGCGAGC	CCTCCAGGTCCCGCTGTACCCGGTCACA
		**RAB33A-10**	CCTGCGGGCCTGGTATGTGACCGGGTACAATATGTGACCGGGTACAGCGCTGGCTCCAG	ACCGCTGGCGTCCCTGTACCCGGTCACA
	RSAD2	**RSAD2-1**	CAAATTCGCCTCTGTCTATTAGTGGAGCCAGTCTATTAGTGGAGCCACCAGTTTGCCTA	GCTTGCAAAACTGCGGCTCCACTAATAG
		**RSAD2-2**	CAGGCATTGGAACGTCTATTAGTGGAGCCAGTCTATTAGTGGAGCCCCCCTTCGATGAG	CTCACCACTGTTCAGGCTCCACTAATAG
		**RSAD2-3**	GAAGCAGAAGCCGGTCTATTAGTGGAGCCAGTCTATTAGTGGAGCCTTTTGGCCGTATG	CAGCACGAAGGAGCGGCTCCACTAATAG
		**RSAD2-4**	GCGCCCAGGGACAGTCTATTAGTGGAGCCAGTCTATTAGTGGAGCCCAGGCGGCACAGC	GCCACCGCCAGCGCGGCTCCACTAATAG
		**RSAD2-5**	ACAGCGCGGCCAGGTCTATTAGTGGAGCCAGTCTATTAGTGGAGCCCCCAGCTGTCCGC	TCCAGCACAGCGCTGGCTCCACTAATAG
		**RSAD2-6**	TATTTTCTCCATTGTCTATTAGTGGAGCCAGTCTATTAGTGGAGCCCTCCTGAGAAATT	AAGAAAGGGTTCTGGGCTCCACTAATAG
		**RSAD2-7**	AGAGGGGCGCCAGGTCTATTAGTGGAGCCAGTCTATTAGTGGAGCCGGCAGGGACAGGG	GCCAGCCAGGCAGCGGCTCCACTAATAG
		**RSAD2-8**	CTTCCTCCAGGGGGTCTATTAGTGGAGCCAGTCTATTAGTGGAGCCAGCCCCCTCTTGG	CCTTGAGCATCGCGGGCTCCACTAATAG
		**RSAD2-9**	ATGCTGACACTTGGTCTATTAGTGGAGCCAGTCTATTAGTGGAGCCGCCGTTACTAACA	TCTCTAATCAGGCAGGCTCCACTAATAG
		**RSAD2-10**	CCAGAATGTCTAAGTCTATTAGTGGAGCCAGTCTATTAGTGGAGCCTCACATGAAATTG	CCTCATCAAAACTTGGCTCCACTAATAG
	DDX60	**DDX60-1**	TTTCACTGCCAAGATAACGATCCCTCACAAATAACGATCCCTCACAACCTCTGCTCCAA	GAAGATGCTCCCATTGTGAGGGATCGTT
		**DDX60-2**	TGGTGTCATCCTCATAACGATCCCTCACAAATAACGATCCCTCACATCTGCTTCTTCGT	GCATAACAGCTCCATGTGAGGGATCGTT
		**DDX60-3**	CTCCTGCTCTTTCATAACGATCCCTCACAAATAACGATCCCTCACAAGGCTTCCCACTG	AGTTGGTATGCGCTTGTGAGGGATCGTT
		**DDX60-4**	GACGGAGCTCATGATAACGATCCCTCACAAATAACGATCCCTCACACCAGAATGCCAGT	CAGTCAAAGGTCGCTGTGAGGGATCGTT
		**DDX60-5**	GTCAACAGCATGAATAACGATCCCTCACAAATAACGATCCCTCACAATCGGCCTTTGAG	GCGTCTGCTTTGTGTGTGAGGGATCGTT
		**DDX60-6**	CACGCCGAATGTCATAACGATCCCTCACAAATAACGATCCCTCACAAGAACACAGACCA	CCACAGCACAAGATTGTGAGGGATCGTT
		**DDX60-7**	TCTGCAGAATCCAATAACGATCCCTCACAAATAACGATCCCTCACACTTGAAATTATCT	TTATGAATTACTTATGTGAGGGATCGTT
		**DDX60-8**	TTTTCTCACTGTTATAACGATCCCTCACAAATAACGATCCCTCACAGGAATGAAACCTA	CATTTTCCTTTGTATGTGAGGGATCGTT
		**DDX60-9**	GTAATGTTGATCTATAACGATCCCTCACAAATAACGATCCCTCACATGTAAGTGTTTCT	AGTGGTCAAGATAGTGTGAGGGATCGTT
		**DDX60-10**	ACTCTCAAATCCTATAACGATCCCTCACAAATAACGATCCCTCACATTTGTGCTGGAGA	CTTTGGGAACCACCTGTGAGGGATCGTT
	ZNFX1	**ZNFX1-1**	CAACCTGCATTCATACCCGAGATAGAGTCATACCCGAGATAGAGTCCATGCCCACAACT	TGCCCTTGTGCAATGACTCTATCTCGGG
		**ZNFX1-2**	GACAAGGCTTCAGTACCCGAGATAGAGTCATACCCGAGATAGAGTCCAGAGCACCTTCT	GGTGCCCATCTGCTGACTCTATCTCGGG
		**ZNFX1-3**	CTCCCCCGTGAATTACCCGAGATAGAGTCATACCCGAGATAGAGTCCTGCAGCCTCATC	CAAGGCCTTCTTCTGACTCTATCTCGGG
		**ZNFX1-4**	TTCCCCATCAGTGTACCCGAGATAGAGTCATACCCGAGATAGAGTCCTGCAGAACAGCA	ACGGGTAGATTCCAGACTCTATCTCGGG
		**ZNFX1-5**	CTCCAGCGTAGCATACCCGAGATAGAGTCATACCCGAGATAGAGTCACACATGACCACA	ACAGGAACCTGAGGGACTCTATCTCGGG

		**ZNFX1-6**	CATCAGCCTTTCCTACCCGAGATAGAGTCATACCCGAGATAGAGTCGCAGCCTCAGTTC	GCAGAGATCCTCCAGACTCTATCTCGGG
		**ZNFX1-7**	TGACATGGCAGCTTACCCGAGATAGAGTCATACCCGAGATAGAGTCGACGCAGTGGTTC	TGACACCTGCTGTCGACTCTATCTCGGG
		**ZNFX1-8**	CACGCACCATTCCTACCCGAGATAGAGTCATACCCGAGATAGAGTCCTTCTGCCTCCAA	GGACCTCACTGCTTGACTCTATCTCGGG
		**ZNFX1-9**	TTTCTGGCAAGGTTACCCGAGATAGAGTCATACCCGAGATAGAGTCCGCAGTTCAACTT	AGTGCATGTGTGCGGACTCTATCTCGGG
		**ZNFX1-10**	TCGACAAGAGGCTTACCCGAGATAGAGTCATACCCGAGATAGAGTCGCACCGTTGCATC	TGATCCTCGCACTGGACTCTATCTCGGG
	DHX58	**DHX58-1**	AAGCGTCGTTGTATATGTGACCGGGTACAAGTCTATTAGTGGAGCCTCGTTGATCAGCA	TCATCCGCACCGTCGGCTCCACTAATAG
		**DHX58-2**	TCTCCATTCCCCATATGTGACCGGGTACAAGTCTATTAGTGGAGCCTGCCGGTAGATCA	TGGGCAGCTTCACGGGCTCCACTAATAG
		**DHX58-3**	TCCACCACAAAGTTATGTGACCGGGTACAAGTCTATTAGTGGAGCCCTCAGCTGGCGTC	TTGTACCGCCTCTAGGCTCCACTAATAG
		**DHX58-4**	ATCACCAGGAGCGTATGTGACCGGGTACAAGTCTATTAGTGGAGCCGTGGCACTCGTCT	TCCTTGTGCGTGTCGGCTCCACTAATAG
		**DHX58-5**	CAGTGGGGTCCTTTATGTGACCGGGTACAAGTCTATTAGTGGAGCCGCGAGGAACTGTT	CCTCAAATGTGGCCGGCTCCACTAATAG
		**DHX58-6**	CTGGAGGCACTGGTATGTGACCGGGTACAAGTCTATTAGTGGAGCCTGGCGTAGAACTG	TGTGTCCCTTTCGCGGCTCCACTAATAG
		**DHX58-7**	ATCACATCCTGCTTATGTGACCGGGTACAAGTCTATTAGTGGAGCCTCGGAACAGCTTG	TTGAGGGCTCCCTGGGCTCCACTAATAG
		**DHX58-8**	CTCCAGCTTGCCCTATGTGACCGGGTACAAGTCTATTAGTGGAGCCCCTGCAAGATCTG	GGGCTGGAAGTGCAGGCTCCACTAATAG
		**DHX58-9**	AGCGGACCACGATTATGTGACCGGGTACAAGTCTATTAGTGGAGCCGTCATCAGCCCAT	TGGCGATCTCATTCGGCTCCACTAATAG
		**DHX58-10**	GTTAGGGCCCGTCTATGTGACCGGGTACAAGTCTATTAGTGGAGCCTGCTCCTGCTGCT	CCGCAGCTCCATTGGGCTCCACTAATAG
	IFI30	**IFI30-1**	CTGCACCAGGAACTATGTGACCGGGTACAAATAACGATCCCTCACACGGTGAAGAGCTC	CAGCACCAGCCACCTGTGAGGGATCGTT
		**IFI30-2**	GTGCATCAGGCAGTATGTGACCGGGTACAAATAACGATCCCTCACAAGTCCTTGGCCTC	GAAGTAGGTGCTGTTGTGAGGGATCGTT
		**IFI30-3**	GAGCGGAGGATGCTATGTGACCGGGTACAAATAACGATCCCTCACACCGAGGGGAGTGA	CACCACCAAGACCGTGTGAGGGATCGTT
		**IFI30-4**	AGACCTGCAGGCATATGTGACCGGGTACAAATAACGATCCCTCACAAGCTGCGGGGCGT	TGCGGCCGCTGTCGTGTGAGGGATCGTT
		**IFI30-5**	ACGTCCCCCTGCATATGTGACCGGGTACAAATAACGATCCCTCACACAGGGCGGCCCCC	GCGTTGCGGTGCAATGTGAGGGATCGTT
		**IFI30-6**	GTCGAGCGCCTCGTATGTGACCGGGTACAAATAACGATCCCTCACAATTGGTGCGGGGG	GATCCAGGGCACGATGTGAGGGATCGTT
		**IFI30-7**	CCCGACTCCAGGCTATGTGACCGGGTACAAATAACGATCCCTCACAGGTGACAGAGCTG	GCCTCCAGGTTCTATGTGAGGGATCGTT
		**IFI30-8**	AAGCGCCACTTCCTATGTGACCGGGTACAAATAACGATCCCTCACAGTGCTGGCACTGG	CACTCCTCGGGGCGTGTGAGGGATCGTT
		**IFI30-9**	CAGCGGCGCTCGGTATGTGACCGGGTACAAATAACGATCCCTCACACGAGCGGTTGGCG	GCTGCCGCTGGCTCTGTGAGGGATCGTT
		**IFI30-10**	ACGCTGAGGACGTTATGTGACCGGGTACAAATAACGATCCCTCACAGGGGACCAGGGTG	TGCGCGTTGCCATTTGTGAGGGATCGTT
	IFI35	**IFI35-1**	CTTCCCCTCCCACTATGTGACCGGGTACAATACCCGAGATAGAGTCCAGAAAATCGCCT	GCTGCAGTCCCAGCGACTCTATCTCGGG
		**IFI35-2**	TCGTCTTGCTGAATATGTGACCGGGTACAATACCCGAGATAGAGTCCCGCCTCCATTCT	TGCTCTCCACCTCCGACTCTATCTCGGG
		**IFI35-3**	CTCACCCAGCACGTATGTGACCGGGTACAATACCCGAGATAGAGTCCCCTCATGGCCTC	GATCTCCAGGGTGAGACTCTATCTCGGG
		**IFI35-4**	CAAGCTCCGTCAGTATGTGACCGGGTACAATACCCGAGATAGAGTCACCTTGCAGCAGT	CTGCCTGCACTCGGGACTCTATCTCGGG
		**IFI35-5**	TGCTCTACAGCTTTATGTGACCGGGTACAATACCCGAGATAGAGTCATCTCGTGTCCTT	TCCTCTTCCCTCACGACTCTATCTCGGG
		**IFI35-6**	GTCCACCTCTCCCTATGTGACCGGGTACAATACCCGAGATAGAGTCCATAGGCCAGCAC	GTGTCCTGCTGGGTGACTCTATCTCGGG
		**IFI35-7**	GAGTTGTCCAGGATATGTGACCGGGTACAATACCCGAGATAGAGTCCACCACCTGGCTG	TGCACGAAGGTCACGACTCTATCTCGGG

		**IFI35-8**	CGGGAGGGCTGGATATGTGACCGGGTACAATACCCGAGATAGAGTCGGTCCGGGGGCAG	CCCGAGAGCAGGAGGACTCTATCTCGGG
		**IFI35-9**	TGTCATATTTCCCTATGTGACCGGGTACAATACCCGAGATAGAGTCGGTGATATTTTGA	CTCCGCTCATATAGGACTCTATCTCGGG
		**IFI35-10**	CAATGAGCGGCTCTATGTGACCGGGTACAATACCCGAGATAGAGTCATGCGTCCTCTCG	CAATAAGCACCTGGGACTCTATCTCGGG
	IFIH1	**IFIH1-1**	TATCCACCAGCGTGTCTATTAGTGGAGCCAATAACGATCCCTCACAACGGTCTGCATTT	TCTCGGCCACCTGGTGTGAGGGATCGTT
		**IFIH1-2**	TCATGTTTCCCCAGTCTATTAGTGGAGCCAATAACGATCCCTCACACCTCTGTGAACCA	AAGGCAGGTCAAGTTGTGAGGGATCGTT
		**IFIH1-3**	CTCGACCACGAGCGTCTATTAGTGGAGCCAATAACGATCCCTCACATCATCAGCACGAG	GTGCATAGGTGCTGTGTGAGGGATCGTT
		**IFIH1-4**	TCACGTAGAGCAAGTCTATTAGTGGAGCCAATAACGATCCCTCACATCCATGTCGGGTT	TCATCTGCAAGGTGTGTGAGGGATCGTT
		**IFIH1-5**	ACGTTGAATGGCCGTCTATTAGTGGAGCCAATAACGATCCCTCACAGCATCTTCTGGAC	ATACTCTTCCCGCCTGTGAGGGATCGTT
		**IFIH1-6**	GTGAGGCTGTAAGGTCTATTAGTGGAGCCAATAACGATCCCTCACACCTCCTACACCAG	AGTTGGATGTTGCTTGTGAGGGATCGTT
		**IFIH1-7**	ATGGTCGGCCTCCGTCTATTAGTGGAGCCAATAACGATCCCTCACAGCACGCAGAGGTC	CAGCTGCACCAAGATGTGAGGGATCGTT
		**IFIH1-8**	GTCTCGGGACTCTGTCTATTAGTGGAGCCAATAACGATCCCTCACAAGAGGAAGCGTTC	GCAGGAGATCATGATGTGAGGGATCGTT
		**IFIH1-9**	CCACAAAATTTCTGTCTATTAGTGGAGCCAATAACGATCCCTCACATCTTCAAACACAA	TTGTTGTTTTCTTCTGTGAGGGATCGTT
		**IFIH1-10**	ATCAAAATTAGGGGTCTATTAGTGGAGCCAATAACGATCCCTCACAAATGAGCTGCATA	ATCACTTGAAGGGGTGTGAGGGATCGTT
	SAMHD1	**SAMHD1-1**	CAGGCATCCTGCCGTCTATTAGTGGAGCCATACCCGAGATAGAGTCTCAGCTCACGAAC	TGGTTGTCTCTCCAGACTCTATCTCGGG
		**SAMHD1-2**	TGATGCGGACAAGGTCTATTAGTGGAGCCATACCCGAGATAGAGTCTGAGGTGTGTCGA	GGAGGCGCTGAAAGGACTCTATCTCGGG
		**SAMHD1-3**	CTGCCAGGTAACCGTCTATTAGTGGAGCCATACCCGAGATAGAGTCCGAACCAGGCATC	TCTCCTTCAGCTCTGACTCTATCTCGGG
		**SAMHD1-4**	TCCTGTGCTCATCGTCTATTAGTGGAGCCATACCCGAGATAGAGTCGAGCGCTCAGGTG	GCTTTTGTCTGCAAGACTCTATCTCGGG
		**SAMHD1-5**	CCACATCACCATCGTCTATTAGTGGAGCCATACCCGAGATAGAGTCAGATGAGGGGCAA	TCTTCATTGGAGTGGACTCTATCTCGGG
		**SAMHD1-6**	AAATCCTTGAAACGTCTATTAGTGGAGCCATACCCGAGATAGAGTCAACGTCTTGGGTA	TAACCTGCTCTGATGACTCTATCTCGGG
		**SAMHD1-7**	GCCAAAAAAACATGTCTATTAGTGGAGCCATACCCGAGATAGAGTCATACTGTTTCGCA	CACCACTGAACAATGACTCTATCTCGGG
		**SAMHD1-8**	CATTCCATAATCCGTCTATTAGTGGAGCCATACCCGAGATAGAGTCGATTCTGGTCTTT	AACTTTGTCAATGCGACTCTATCTCGGG
		**SAMHD1-9**	AAACTCTCCTTCAGTCTATTAGTGGAGCCATACCCGAGATAGAGTCCTTGGATTTAGCA	GGATTCACTTTTGCGACTCTATCTCGGG
		**SAMHD1-10**	CCAGGTAGATGTTGTCTATTAGTGGAGCCATACCCGAGATAGAGTCGAGTGCAGAATCT	GTTCTGGACTAGTGGACTCTATCTCGGG
	STAT1	**STAT1-1**	TAGAAGCCCATCCATAACGATCCCTCACAATACCCGAGATAGAGTCTTGAACCACAGGA	CAGCAGACAGCATCGACTCTATCTCGGG
		**STAT1-2**	CTGTACGCTTCCCATAACGATCCCTCACAATACCCGAGATAGAGTCCGATCACAGTGTT	TTTGTCCAGCATCGGACTCTATCTCGGG
		**STAT1-3**	GACTTTCGGCAACATAACGATCCCTCACAATACCCGAGATAGAGTCCGAACTTGCTGTA	TTTTGAGCTGCTGTGACTCTATCTCGGG
		**STAT1-4**	ATCCATCGGCTCTATAACGATCCCTCACAATACCCGAGATAGAGTCTGGGACCATCCAA	GTAGCCATTTCCCAGACTCTATCTCGGG
		**STAT1-5**	ACCGAGTGGAACTATAACGATCCCTCACAATACCCGAGATAGAGTCGGTGTACGGCTCT	GAGAGCTCCTTCTAGACTCTATCTCGGG
		**STAT1-6**	GCTGATACCACTGATAACGATCCCTCACAATACCCGAGATAGAGTCTCAAGTTGCTGGA	CCAAGAACTTGGACGACTCTATCTCGGG
		**STAT1-7**	AGGTACTGCCTGAATAACGATCCCTCACAATACCCGAGATAGAGTCTAGCCATTGTGCC	CAGTCCTGGTTTTGGACTCTATCTCGGG
		**STAT1-8**	AAGACACGTTGCTATAACGATCCCTCACAATACCCGAGATAGAGTCAACACTGTAGCAA	GCAGGTCATGGAAAGACTCTATCTCGGG
		**STAT1-9**	TTGCAGCAAAAAGATAACGATCCCTCACAATACCCGAGATAGAGTCTCCTTATGTTGTG	ATTACGTTTGCTTAGACTCTATCTCGGG

		**STAT1-10**	CTTCCTGGAAGTTATAACGATCCCTCACAATACCCGAGATAGAGTCATGTGTACGGGGT	GGATGATCATTGCGGACTCTATCTCGGG
	DSCAM	**DSCAM-1**	CTGCCGAAATCTGACCATGCTGGATCTAGAACCATGCTGGATCTAGGACCTGTAACCGT	GTTCTCGATCAGCTCTAGATCCAGCATG
		**DSCAM-2**	AGACCCCGACCAAACCATGCTGGATCTAGAACCATGCTGGATCTAGACAAAGAGCAGCA	CGACCAGCAGCATGCTAGATCCAGCATG
		**DSCAM-3**	GTATTCGCCCTCGACCATGCTGGATCTAGAACCATGCTGGATCTAGGTACATTGCACGT	CTGCGGTTGAACATCTAGATCCAGCATG
		**DSCAM-4**	TGCTGACCTTGCAACCATGCTGGATCTAGAACCATGCTGGATCTAGGCTCCCACATCAT	ACTTGCTGACGTCACTAGATCCAGCATG
		**DSCAM-5**	ACTGTTGTCCCAAACCATGCTGGATCTAGAACCATGCTGGATCTAGAGCAGTTGTCCAC	AAGGATGTCCGCTCCTAGATCCAGCATG
		**DSCAM-6**	AGCCAACGATACTACCATGCTGGATCTAGAACCATGCTGGATCTAGAACGTTGTCCTTC	TCAGGCTCCCAGGCCTAGATCCAGCATG
		**DSCAM-7**	GTCCTGCCATGGCACCATGCTGGATCTAGAACCATGCTGGATCTAGAGCTCCACTCGCT	ATGCTTTGCAAGGGCTAGATCCAGCATG
		**DSCAM-8**	CAGGACAAGGACAACCATGCTGGATCTAGAACCATGCTGGATCTAGTCCCGTCACACTG	TCCTGGTCCTCAGACTAGATCCAGCATG
		**DSCAM-9**	ACCATGCCATACTACCATGCTGGATCTAGAACCATGCTGGATCTAGGCAAGCCTGGACT	ATTCCAGCCCGGTACTAGATCCAGCATG
		**DSCAM-10**	CAGCCTATCAGGTACCATGCTGGATCTAGAACCATGCTGGATCTAGGCCACCGTCATTC	GAGGTGATGGGGCTCTAGATCCAGCATG
	HVCN1	**HVCN1-1**	AGTCATTGCTCCATACTAGCTCGATTCAAATACTAGCTCGATTCAATCCCATTTCCGAT	CCTCCTCCTCGTCGTTGAATCGAGCTAG
		**HVCN1-2**	GATCCCGTCCAGCTACTAGCTCGATTCAAATACTAGCTCGATTCAAACACGATGACGAC	GTCGAGGATGAAGCTTGAATCGAGCTAG
		**HVCN1-3**	GTGCTCCCGGAAGTACTAGCTCGATTCAAATACTAGCTCGATTCAACGGCTTCAAACTC	AATCAGGAGCCCGTTTGAATCGAGCTAG
		**HVCN1-4**	TGCTTCAGGTACCTACTAGCTCGATTCAAATACTAGCTCGATTCAACACCACCGTGAAG	ATGGGGTCGTCCCGTTGAATCGAGCTAG
		**HVCN1-5**	TCTCCGAATCCGGTACTAGCTCGATTCAAATACTAGCTCGATTCAAGGCTCCAGCTTGA	CATGGCTCCTGGCCTTGAATCGAGCTAG
		**HVCN1-6**	CATCATGATCTGGTACTAGCTCGATTCAAATACTAGCTCGATTCAATGAAGAGCCTTTT	AAACCTGTGGGAGGTTGAATCGAGCTAG
		**HVCN1-7**	CAGACAACCAGGATACTAGCTCGATTCAAATACTAGCTCGATTCAATAGGATGACCAAG	ACCAACAAGGCATGTTGAATCGAGCTAG
		**HVCN1-8**	AGTCCATAAGCAATACTAGCTCGATTCAAATACTAGCTCGATTCAATGGATGATTTTCA	TATATTTGTCCGGTTTGAATCGAGCTAG
		**HVCN1-9**	GAGATAGTGGAAATACTAGCTCGATTCAAATACTAGCTCGATTCAAAAATGGACAGGGA	CAGGAAGATGGTTTTTGAATCGAGCTAG
		**HVCN1-10**	AGACTTTGAACCCTACTAGCTCGATTCAAATACTAGCTCGATTCAACAGCGGTAGATGA	GGTGGAAGAATTCGTTGAATCGAGCTAG
	SHTN1	**SHTN1-1**	GTTGATCAGGAGCTAGGCATGAACGTCGAATAGGCATGAACGTCGAGCCTTCAAAAGCT	CGTTGTCATCCAATTCGACGTTCATGCC
		**SHTN1-2**	ACTGCTGTCTGTTTAGGCATGAACGTCGAATAGGCATGAACGTCGACCAGCTCCGTTTC	CCGCAGAACTCGTATCGACGTTCATGCC
		**SHTN1-3**	TCTTGCTCACGTTTAGGCATGAACGTCGAATAGGCATGAACGTCGAGGAGGTTTCTCCT	CTGACTCCTGCTGTTCGACGTTCATGCC
		**SHTN1-4**	CTGGCTCCCGAACTAGGCATGAACGTCGAATAGGCATGAACGTCGATGTTTGTGCGGCA	TTCTTGTTCGGTGGTCGACGTTCATGCC
		**SHTN1-5**	TCCTCAATAACCATAGGCATGAACGTCGAATAGGCATGAACGTCGAAATGCAGTTTACT	TCAAGATGGTTCTCTCGACGTTCATGCC
		**SHTN1-6**	GCACGTCTTCTCATAGGCATGAACGTCGAATAGGCATGAACGTCGACAGCACTTTCACG	AGAAGCCAAAGCCATCGACGTTCATGCC
		**SHTN1-7**	TTTTCTTTGTTCATAGGCATGAACGTCGAATAGGCATGAACGTCGATTTCAAGGTCTTA	AGCATGCTAATTCATCGACGTTCATGCC
		**SHTN1-8**	ATCTGTGGATGAATAGGCATGAACGTCGAATAGGCATGAACGTCGAAGTTAGCTTCTGT	ACTGCATGATCCACTCGACGTTCATGCC
		**SHTN1-9**	AGATTTGATCTCGTAGGCATGAACGTCGAATAGGCATGAACGTCGATCATGAACAGATA	AGGTCTTCTTTTCGTCGACGTTCATGCC
		**SHTN1-10**	CCTTTTCTTCTTTTAGGCATGAACGTCGAATAGGCATGAACGTCGAGTTGTCAAAACAG	GCTTATTAACTTCATCGACGTTCATGCC
	IRF8	**IRF8-1**	CTGCCTGACACACCGTAGCTTACCTGCAAACGTAGCTTACCTGCAACCTCCTCCACCAC	GCATGTCTTTCGACTTGCAGGTAAGCTA

		**IRF8-2**	CACCTCCTCGAAGCGTAGCTTACCTGCAAACGTAGCTTACCTGCAAGGGACCTGTCTGT	AGAGATGTCCAGCATTGCAGGTAAGCTA
		**IRF8-3**	GAAGATGCCCTGCCGTAGCTTACCTGCAAACGTAGCTTACCTGCAAACAGCCTCTTGAT	GACCCTCCCCTGGGTTGCAGGTAAGCTA
		**IRF8-4**	TCTGGATGGCGTCCGTAGCTTACCTGCAAACGTAGCTTACCTGCAATTCTGACGGTCGT	TCCTGGTGATCTGGTTGCAGGTAAGCTA
		**IRF8-5**	TCACCATGGCTGGCGTAGCTTACCTGCAAACGTAGCTTACCTGCAAGGCATAGAGCTTC	TCCAGGGCTTCTGCTTGCAGGTAAGCTA
		**IRF8-6**	CCGTTACGGTCACCGTAGCTTACCTGCAAACGTAGCTTACCTGCAAGAGCCGCCGTCCA	ATCAGCCACTGCCCTTGCAGGTAAGCTA
		**IRF8-7**	TCACTGTCGATCTCGTAGCTTACCTGCAAACGTAGCTTACCTGCAAGCCAGGGTACAAG	TTCTCCCAGATGACTTGCAGGTAAGCTA
		**IRF8-8**	ATACGGAACATGGCGTAGCTTACCTGCAAACGTAGCTTACCTGCAAGTGTTTCCAGGGG	TCCTGCTTCCCAGGTTGCAGGTAAGCTA
		**IRF8-9**	TTTGAAAACAGCCCGTAGCTTACCTGCAAACGTAGCTTACCTGCAACTTTGAATTTGCC	CGCTTTATCACCTATTGCAGGTAAGCTA
		**IRF8-10**	GACCTTGTAGGGCCGTAGCTTACCTGCAAACGTAGCTTACCTGCAAGCACGATCCTGTA	CTGTTCCTCCTCTCTTGCAGGTAAGCTA
		**PROX1-1**	TGCTCTTTCCTCGACCATGCTGGATCTAGATACTAGCTCGATTCAACTCCTCTCGCTTC	TTCAGCTGTCTGCCTTGAATCGAGCTAG
		**PROX1-2**	CTTGGGACCTTCTACCATGCTGGATCTAGATACTAGCTCGATTCAACCTGCTCCTTCTC	GAAGGCATTTGGCGTTGAATCGAGCTAG
		**PROX1-3**	ACTCGACATCCTGACCATGCTGGATCTAGATACTAGCTCGATTCAATGCACCACTGAAT	CCCCATCTGCATGGTTGAATCGAGCTAG
		**PROX1-4**	CACTGAAGGCGCTACCATGCTGGATCTAGATACTAGCTCGATTCAAGACGTCCCCAAAG	GGGTTGGGAATGAATTGAATCGAGCTAG
	PROX1	**PROX1-5**	TCCACAACGGCACACCATGCTGGATCTAGATACTAGCTCGATTCAACAGCAAGCAGCAG	CGAGAGCGTTCCGCTTGAATCGAGCTAG
		**PROX1-6**	TCCCTACCGTCCTACCATGCTGGATCTAGATACTAGCTCGATTCAAAATGCAGATGCTG	TTGCCTTTGCAAATTTGAATCGAGCTAG
		**PROX1-7**	GACAAGAGGCAGCACCATGCTGGATCTAGATACTAGCTCGATTCAAAGTTTTTGCGGAC	GGACTGGTCGGAGCTTGAATCGAGCTAG
		**PROX1-8**	AGGTTGCCATCTTACCATGCTGGATCTAGATACTAGCTCGATTCAAGCTGTCTTCAGAC	GTTTCGGAGCGCAATTGAATCGAGCTAG
		**PROX1-9**	TGTCTCAGCCAAAACCATGCTGGATCTAGATACTAGCTCGATTCAAGCTCCTGCTTCAG	CATGGCGGTGTTGTTTGAATCGAGCTAG
		**PROX1-10**	CTCGTTGTCTGACACCATGCTGGATCTAGATACTAGCTCGATTCAACCAGCTCGCACAT	GAACTGTCCCGGGATTGAATCGAGCTAG
		**EBF3-1**	TGGTTGTGGTTGCACCATGCTGGATCTAGATAGGCATGAACGTCGAGAGAGTGGGGATC	GCTGGAGTGTTGGGTCGACGTTCATGCC
		**EBF3-2**	TTGCGACTGTAACACCATGCTGGATCTAGATAGGCATGAACGTCGAAACACTGCTTGTG	TATCCTCGCGGGGTTCGACGTTCATGCC
		**EBF3-3**	GTTTCACTGCAGAACCATGCTGGATCTAGATAGGCATGAACGTCGAAAGGCGCTCTTTT	TGACAACAGGAGCTTCGACGTTCATGCC
		**EBF3-4**	TCATCCCGGTGTGACCATGCTGGATCTAGATAGGCATGAACGTCGAGAGTTCACCCCCA	GCTGGCTGCTGAACTCGACGTTCATGCC
		**EBF3-5**	TTCTTGTCACAGCACCATGCTGGATCTAGATAGGCATGAACGTCGAGTTGCCACAACTT	GGCGTCTCATTTCATCGACGTTCATGCC
	EBF3	**EBF3-6**	CAAGATGGTGGAGACCATGCTGGATCTAGATAGGCATGAACGTCGAATTTGCACTGGTG	TGAAGTCCATTTCGTCGACGTTCATGCC
		**EBF3-7**	CTGAGGAGTACTGACCATGCTGGATCTAGATAGGCATGAACGTCGATGTAATTGGACTG	ATTGCTGACTGTGATCGACGTTCATGCC
		**EBF3-8**	AAATCCAGGGGAAACCATGCTGGATCTAGATAGGCATGAACGTCGAAGGAGCCATTAAG	AGGAGAGTTAGCTCTCGACGTTCATGCC
		**EBF3-9**	TTCGACAAGGTCTACCATGCTGGATCTAGATAGGCATGAACGTCGATCCCATATAGGGC	GTTGTTATGGGGCTTCGACGTTCATGCC
		**EBF3-10**	ATCTATGGTGGGTACCATGCTGGATCTAGATAGGCATGAACGTCGATCTGAAAGCCATA	AACTTTCTGCAACGTCGACGTTCATGCC
		**FABP3-1**	CTTGAAGGTGCTGACCATGCTGGATCTAGACGTAGCTTACCTGCAATGATCTCCGTGTT	GCCCAGCTTGAAGGTTGCAGGTAAGCTA
	FABP3	**FABP3-2**	CAGCGATGTCTCCACCATGCTGGATCTAGACGTAGCTTACCTGCAATCAGCTCCCGGAC	TAATTTGCCGTCCATTGCAGGTAAGCTA
		**FABP3-3**	TGCCCATGGTCAGACCATGCTGGATCTAGACGTAGCTTACCTGCAAGTGGAGACGACGT	TCTCGTAGGTGCGCTTGCAGGTAAGCTA

		**FABP3-4**	ACGGTGGTGGGTTACCATGCTGGATCTAGACGTAGCTTACCTGCAAGTCCACCTCGATG	GTGACCTTGTCGCGTTGCAGGTAAGCTA
		**FABP3-5**	TCCAGCTTGACCAACCATGCTGGATCTAGACGTAGCTTACCTGCAAGAGCTTCCCTCCA	TTCTGCACGTGGAGTTGCAGGTAAGCTA
		**FABP3-6**	GCAGGATGTGGCCACCATGCTGGATCTAGACGTAGCTTACCTGCAAGGGGTGTAGGGGG	TTGGGGGCGCTGACTTGCAGGTAAGCTA
		**FABP3-7**	CCACGCCTGGGGAACCATGCTGGATCTAGACGTAGCTTACCTGCAACGCGTGGCGAAGC	GCCCGGCCATCTGGTTGCAGGTAAGCTA
	THBS1	**THBS1-1**	CACCCACTTCATCTACTAGCTCGATTCAAATAGGCATGAACGTCGATTATCACAGCGGT	GATTGTAGGGGCACTCGACGTTCATGCC
		**THBS1-2**	AGGTCACAGAACATACTAGCTCGATTCAAATAGGCATGAACGTCGAATCCCATTCCCAC	GGCCTCTCTGCTGTTCGACGTTCATGCC
		**THBS1-3**	CATTCTGCAGCAGTACTAGCTCGATTCAAATAGGCATGAACGTCGAAACACAAACCGCA	CCAGAGTTGTTCCTTCGACGTTCATGCC
		**THBS1-4**	TCCAGGAGCCATCTACTAGCTCGATTCAAATAGGCATGAACGTCGACAGTCACCACACT	GGTAGCCAGCAGGTTCGACGTTCATGCC
		**THBS1-5**	CATGGTCCCCAGTTACTAGCTCGATTCAAATAGGCATGAACGTCGAATCCCACGGAGAC	GTCACTGTGCAAGGTCGACGTTCATGCC
		**THBS1-6**	TGCATAGTCGCTATACTAGCTCGATTCAAATAGGCATGAACGTCGAACCATCCGTCATC	TTCAGACCAGGGACTCGACGTTCATGCC
		**THBS1-7**	GTTCTTTGCCCTGTACTAGCTCGATTCAAATAGGCATGAACGTCGAACTGTCTGAACCA	CAGGGTCACAGTTCTCGACGTTCATGCC
		**THBS1-8**	GTTGTACCCAGGATACTAGCTCGATTCAAATAGGCATGAACGTCGAGACAAGGCAGACA	CGTGAAACGTGGTCTCGACGTTCATGCC
		**THBS1-9**	CTCCACAGACAGCTACTAGCTCGATTCAAATAGGCATGAACGTCGAAGCCATCCTTCTG	AAAGACATGGCCACTCGACGTTCATGCC
		**THBS1-10**	AGGTCCAGGGTGCTACTAGCTCGATTCAAATAGGCATGAACGTCGAACCAGAAAGGCTC	TGCTGCTTGCCATGTCGACGTTCATGCC
	DCN	**DCN-1**	CATCGGCATCAATTACTAGCTCGATTCAAACGTAGCTTACCTGCAAAGTCCAGACAGAG	TTGCCAAGTTGGTATTGCAGGTAAGCTA
		**DCN-2**	TTGGCACCTTTTCTACTAGCTCGATTCAAACGTAGCTTACCTGCAAGGAGGAAGGTCTC	GCAAAGTTGTGTCTTTGCAGGTAAGCTA
		**DCN-3**	CTGGCAGCGAAAGTACTAGCTCGATTCAAACGTAGCTTACCTGCAACGCGAAGATGGCA	AGAGCACTGTACCTTTGCAGGTAAGCTA
		**DCN-4**	CTTCATCCCCTGATACTAGCTCGATTCAAACGTAGCTTACCTGCAATGTAGGAGAGCCT	GTCTGCGATACGGTTTGCAGGTAAGCTA
		**DCN-5**	GTACTGCACGGGGTACTAGCTCGATTCAAACGTAGCTTACCTGCAAGCTGGATTTCCCA	TCGGAAAGCAGAGCTTGCAGGTAAGCTA
		**DCN-6**	AGCCTCTTCATCCTACTAGCTCGATTCAAACGTAGCTTACCTGCAAACGGATGTAGGAG	TTGGTGTCTGCGAATTGCAGGTAAGCTA
		**DCN-7**	AGAGAAGAGCCAGTACTAGCTCGATTCAAACGTAGCTTACCTGCAAAGCAGTAGGACGA	CCAAGCATGCTGGGTTGCAGGTAAGCTA
		**DCN-8**	TGAAGATAAACCATACTAGCTCGATTCAAACGTAGCTTACCTGCAAAATTTTGTTGTTG	ATACCGATGGAAGGTTGCAGGTAAGCTA
		**DCN-9**	GCTAAGACCTAATTACTAGCTCGATTCAAACGTAGCTTACCTGCAAAAATACTGTTGAA	ATTTTCAACTGAACTTGCAGGTAAGCTA
		**DCN-10**	TTCAAATGGAGCTTACTAGCTCGATTCAAACGTAGCTTACCTGCAAAAGTTCATTGTTG	CCAGGTACTCTAACTTGCAGGTAAGCTA
	ISM1	**ISM1-1**	CATCTCTCACAGCTAGGCATGAACGTCGAACGTAGCTTACCTGCAATTTGCAGCTCATC	TTCAAGAACTCGCATTGCAGGTAAGCTA
		**ISM1-2**	GTGCAGTGGGCTTTAGGCATGAACGTCGAACGTAGCTTACCTGCAACGGATGCAGTATC	CGAAGAGCATGGACTTGCAGGTAAGCTA
		**ISM1-3**	CATCCCACCCACTTAGGCATGAACGTCGAACGTAGCTTACCTGCAACTGGGTGTGTGAC	CAAATGTCCGGTGTTTGCAGGTAAGCTA
		**ISM1-4**	GAGGACCTCTGCCTAGGCATGAACGTCGAACGTAGCTTACCTGCAAGCGGGTCAAGGTG	TCACCGCTGCTCAATTGCAGGTAAGCTA
		**ISM1-5**	AATCGCTGCTTCGTAGGCATGAACGTCGAACGTAGCTTACCTGCAAGTTTTCCTGCCTG	AGGGACGTATGCCGTTGCAGGTAAGCTA
		**ISM1-6**	AGTCCAGACCAACTAGGCATGAACGTCGAACGTAGCTTACCTGCAAGCTCCTGCTGCTG	CGGCTCTCCTCCTCTTGCAGGTAAGCTA
		**ISM1-7**	CTCTCCTGCCACCTAGGCATGAACGTCGAACGTAGCTTACCTGCAAGCAGCTCCGTACT	TAGGGCTGGCAGGCTTGCAGGTAAGCTA
		**ISM1-8**	TTGACCACTTTGTTAGGCATGAACGTCGAACGTAGCTTACCTGCAAGCTGGGAAGATCA	TAGGAGCATGGGCTTTGCAGGTAAGCTA

		**ISM1-9**	TAGATTTCAGCAGTAGGCATGAACGTCGAACGTAGCTTACCTGCAACTTAATTCGATCA	CGAAAGTTTTCCATTGCAGGTAAGCTA
		**ISM1-10**	AGGTTTGGCGTACTAGGCATGAACGTCGAACGTAGCTTACCTGCAATTCGATGCTGATC	AGTTCTGCTGAGATTTGCAGGTAAGCTA
	EFEMP1	**EFEMP1-1**	TTCTCTTGGTCCCGGCACTTGAGCTAGAAAGGCACTTGAGCTAGAAGATCCACGATGTG	TGTGAGCATCTCCTTTCTAGCTCAAGTG
		**EFEMP1-2**	TTGATGAGTGGGGGGCACTTGAGCTAGAAAGGCACTTGAGCTAGAACTGCACACTGGAT	TTGCTCATACCCACTTCTAGCTCAAGTG
		**EFEMP1-3**	TAGCCATCTGTGCGGCACTTGAGCTAGAAAGGCACTTGAGCTAGAAGGGATCCCATTCA	CACCGTTGCCTAAGTTCTAGCTCAAGTG
		**EFEMP1-4**	CTTACGGCGCTTGGGCACTTGAGCTAGAAAGGCACTTGAGCTAGAACACAAGCATCGCA	AGCGATTTCACCACTTCTAGCTCAAGTG
		**EFEMP1-5**	TACTTGTGCACCAGGCACTTGAGCTAGAAAGGCACTTGAGCTAGAAGCGGATGCTCATG	ACCGTTCTGTCGGTTTCTAGCTCAAGTG
		**EFEMP1-6**	TGACGTTGTTTCGGGCACTTGAGCTAGAAAGGCACTTGAGCTAGAAGGAGTCCGTCGAA	GCTGAGGATCAGCATTCTAGCTCAAGTG
		**EFEMP1-7**	AGCCAAGAACATAGGCACTTGAGCTAGAAAGGCACTTGAGCTAGAATAAGAATGAGAGC	CGAGTGTACTGTATTTCTAGCTCAAGTG
		**EFEMP1-8**	TCACATTCATCAAGGCACTTGAGCTAGAAAGGCACTTGAGCTAGAAATCTGGGACTATT	CCACCTTTACATGGTTCTAGCTCAAGTG
		**EFEMP1-9**	TAGTGGTTAACACGGCACTTGAGCTAGAAAGGCACTTGAGCTAGAAGAGGTATCCTCCA	GTTCTGGGTAGACTTTCTAGCTCAAGTG
		**EFEMP1-10**	TGCACATTCATCTGGCACTTGAGCTAGAAAGGCACTTGAGCTAGAATATGGGCTCCAGT	GTCGGCTCTACAAATTCTAGCTCAAGTG
	STEAP4	**STEAP4-1**	TCCACCGTAAACCATGCTGCTGTAGTACTAATGCTGCTGTAGTACTTCAGGAACCGGTT	GTACGATGACGGGGAGTACTACAGCAGC
		**STEAP4-2**	GTCTGTGTGTTCCATGCTGCTGTAGTACTAATGCTGCTGTAGTACTAATCAGGCTGGGT	TCTGCATCCTTGGCAGTACTACAGCAGC
		**STEAP4-3**	AGTTTATGCCCCAATGCTGCTGTAGTACTAATGCTGCTGTAGTACTGCCGGACTGAATC	AAGACAACAGGGTTAGTACTACAGCAGC
		**STEAP4-4**	AGGCAAGGAAGTGATGCTGCTGTAGTACTAATGCTGCTGTAGTACTTGTTGCTGACTGA	TCGCCAGTTGACAAAGTACTACAGCAGC
		**STEAP4-5**	AGCGAGGTACTCTATGCTGCTGTAGTACTAATGCTGCTGTAGTACTCAGGCACCAGCTG	CACAACCTTTGCGGAGTACTACAGCAGC
		**STEAP4-6**	TGTTGTAAGAATTATGCTGCTGTAGTACTAATGCTGCTGTAGTACTGTCAAAGCCATTA	TAGAAGTGTTAGGCAGTACTACAGCAGC
		**STEAP4-7**	ACTGTCTCTCTTTATGCTGCTGTAGTACTAATGCTGCTGTAGTACTTCCAAATATGCAC	CCAAAATCTCCAGAAGTACTACAGCAGC
		**STEAP4-8**	TAATGTTGCCTCTATGCTGCTGTAGTACTAATGCTGCTGTAGTACTTGTAAGGAAGCTG	ATTTCTGCTAGTGTAGTACTACAGCAGC
		**STEAP4-9**	TGCAGGGCCCAGGATGCTGCTGTAGTACTAATGCTGCTGTAGTACTCAGCGTGCCCGAC	TGCCGGCTGGCATGAGTACTACAGCAGC
		**STEAP4-10**	TCATTTTTCAGTGATGCTGCTGTAGTACTAATGCTGCTGTAGTACTGAGTGGTTCTGTT	GCATTAGTCGTGTAAGTACTACAGCAGC
	G0S2	**G0S2-1**	ACCACCCCAAAGAGGAGACTGATTACACGAGGAGACTGATTACACGCACCAGACCGATA	CTGCACACTGTCTGCGTGTAATCAGTCT
		**G0S2-2**	CAGCTCGTGCATGGGAGACTGATTACACGAGGAGACTGATTACACGTGGCAAAGGGGAT	GCTGAGCATTTCTACGTGTAATCAGTCT
		**G0S2-3**	TCCTGTTGGGCTTGGAGACTGATTACACGAGGAGACTGATTACACGAGCTTGACCATTT	TGCCCAGCATGTAGCGTGTAATCAGTCT
	PDE1C	**PDE1C-1**	TGCTGCACCACCTCCTCGATTAACATCGCACCTCGATTAACATCGCTTTCTCCCGGTTC	GCTTGTGCTTTCCTGCGATGTTAATCGA
		**PDE1C-2**	GTGCTCTTGACACCCTCGATTAACATCGCACCTCGATTAACATCGCTCCTTCGGACCCA	TTGAGTGAGGCACAGCGATGTTAATCGA
		**PDE1C-3**	GTTTGTTCACCCCCCTCGATTAACATCGCACCTCGATTAACATCGCTGAGAGGTTTCGT	CTGCGTTTTTCCCAGCGATGTTAATCGA
		**PDE1C-4**	GAGCGGGAAGCTTCCTCGATTAACATCGCACCTCGATTAACATCGCCCCAGACATGCCA	CTGGAACGTCTGATGCGATGTTAATCGA
		**PDE1C-5**	CACCATCTCGATCCCTCGATTAACATCGCACCTCGATTAACATCGCTATCAGTGGCCAA	GAAGTGACAGGACTGCGATGTTAATCGA
		**PDE1C-6**	CTGAGGGCACAGACCTCGATTAACATCGCACCTCGATTAACATCGCCAGTCCCGGACCT	AGGTGGAAGCCAAGGCGATGTTAATCGA
		**PDE1C-7**	TTTTCAAGCACGGCCTCGATTAACATCGCACCTCGATTAACATCGCACTAACGTGGTGA	AGACGATATGCCGGGCGATGTTAATCGA

		**PDE1C-8**	GAAAGGAAGCCCTCCTCGATTAACATCGCACCTCGATTAACATCGCCACACAGTGGAGA	AGTAGACTTGCGAAGCGATGTTAATCGA
		**PDE1C-9**	CTGCTTTACCAAGCCTCGATTAACATCGCACCTCGATTAACATCGCCCCCTCTCTCTAA	TACAACTGAAGCTAGCGATGTTAATCGA
		**PDE1C-10**	AGAACAGTTGCAGCCTCGATTAACATCGCACCTCGATTAACATCGCGTATACTGACTCA	CTGGTTTCGTCAAAGCGATGTTAATCGA
	TCF7	**TCF7-1**	CACCTGGTTGTCAGGCACTTGAGCTAGAAAATGCTGCTGTAGTACTGGGCGCAGAAGAG	GTGGGGGACTCTGGAGTACTACAGCAGC
		**TCF7-2**	CTCCACTGGGAGGGGCACTTGAGCTAGAAAATGCTGCTGTAGTACTGTGATCTGTCCGA	ATCCCATCGACGGGAGTACTACAGCAGC
		**TCF7-3**	CACTCGGCAATGAGGCACTTGAGCTAGAAAATGCTGCTGTAGTACTCTCCTTCAGGGTG	TTGATGGCAGCGCAAGTACTACAGCAGC
		**TCF7-4**	GTACGGGATGAGCGGCACTTGAGCTAGAAAATGCTGCTGTAGTACTAATGGTCGTTGCT	GGAGCCGTGACTGTAGTACTACAGCAGC
		**TCF7-5**	AGGACGGTGCACTGGCACTTGAGCTAGAAAATGCTGCTGTAGTACTCTGAGGTCTGGGA	GAAACCGGGGATGAAGTACTACAGCAGC
		**TCF7-6**	CTGTGTGCGGAGTGGCACTTGAGCTAGAAAATGCTGCTGTAGTACTGTCGTGCTGTTGT	GAGTCGGGGAAGGCAGTACTACAGCAGC
		**TCF7-7**	CTGCATGTGGAGCGGCACTTGAGCTAGAAAATGCTGCTGTAGTACTAGCCGGGGTAGAG	GTCCCTGGCAGACGAGTACTACAGCAGC
		**TCF7-8**	GTGGATGAGGGAAGGCACTTGAGCTAGAAAATGCTGCTGTAGTACTGAGCCCAGCATCA	TGGTGTGCATGCCCAGTACTACAGCAGC
		**TCF7-9**	ACTTCTTTTCCTGGCACTTGAGCTAGAAAATGCTGCTGTAGTACTAAGTACCGAATGC	GTCCTTCTCCTTGAAGTACTACAGCAGC
		**TCF7-10**	GAAGAAACTGGGCGGCACTTGAGCTAGAAAATGCTGCTGTAGTACTAGCACTTCCATCG	GGAGGGGAGTCTAAAGTACTACAGCAGC
	COL6A3	**COL6A3-1**	ACTCCAACAGCAAGGCACTTGAGCTAGAAACCTCGATTAACATCGCATCCGCACTCCTA	TCAAGTTCTGCCTAGCGATGTTAATCGA
		**COL6A3-2**	CACCCAGCAGCACGGCACTTGAGCTAGAAACCTCGATTAACATCGCTTCCTCCATCCTG	ATGGGGAGCACCCGGCGATGTTAATCGA
		**COL6A3-3**	TCCAGCTGCATCAGGCACTTGAGCTAGAAACCTCGATTAACATCGCCCCACGACCGAAC	CTGTTGTAAGCGATGCGATGTTAATCGA
		**COL6A3-4**	CTCACACTGACAGGGCACTTGAGCTAGAAACCTCGATTAACATCGCAACGGGCTTGATG	GACTTGGTGCTCAGGCGATGTTAATCGA
		**COL6A3-5**	GTTCTCCTCGTTCGGCACTTGAGCTAGAAACCTCGATTAACATCGCCCACGGTCACCTC	GGTCTCCACGAAGGGCGATGTTAATCGA
		**COL6A3-6**	AGGCATGACCCCAGGCACTTGAGCTAGAAACCTCGATTAACATCGCTGGTGAGGAACAC	CGGGTCCTGACTGGGCGATGTTAATCGA
		**COL6A3-7**	AATGGCTTGCATGGGCACTTGAGCTAGAAACCTCGATTAACATCGCGCACTTCAGGAAG	CCGGATAGGTGACTGCGATGTTAATCGA
		**COL6A3-8**	TCTGCACAAGAGAGGCACTTGAGCTAGAAACCTCGATTAACATCGCGTCACGTTTTGCT	CATGCTCAGTGCCCGCGATGTTAATCGA
		**COL6A3-9**	AGGAGGCAGAACAGGCACTTGAGCTAGAAACCTCGATTAACATCGCAAAGCCTGAGAGT	GCATGAACTGAGCTGCGATGTTAATCGA
		**COL6A3-10**	CATGATGAAGGCGGGCACTTGAGCTAGAAACCTCGATTAACATCGCACTCCGAGCTATC	CAGAGGGGTAGTGCGCGATGTTAATCGA
	COL6A1	**COL6A1-1**	TGTTGGGCTCATTATGCTGCTGTAGTACTAGGAGACTGATTACACGACCAGGACACGGA	CACTCTTCCCCGTCCGTGTAATCAGTCT
		**COL6A1-2**	CACTGGTGGAACTATGCTGCTGTAGTACTAGGAGACTGATTACACGTTCTTGCTTCCCA	TGGTGGTCTCAAACCGTGTAATCAGTCT
		**COL6A1-3**	GTAGGTGCCCCCAATGCTGCTGTAGTACTAGGAGACTGATTACACGAGGCTTCTCCAGT	CCTGGTGAACTGCTCGTGTAATCAGTCT
		**COL6A1-4**	AGGGCTTCACTCTATGCTGCTGTAGTACTAGGAGACTGATTACACGACCAGGTCCCCAA	CTTTCACTTGGGCTCGTGTAATCAGTCT
		**COL6A1-5**	CAGCCCTCTCAATATGCTGCTGTAGTACTAGGAGACTGATTACACGTGAGCTTCCTGCA	CAATGCCAGCCTGGCGTGTAATCAGTCT
		**COL6A1-6**	GACGGGTCCACAGATGCTGCTGTAGTACTAGGAGACTGATTACACGCGAAGAGGAGGTC	CGAGCTGTCCAACTCGTGTAATCAGTCT
		**COL6A1-7**	ACGATGGCAACACATGCTGCTGTAGTACTAGGAGACTGATTACACGTCCGTCCGTGATG	AGCGTGTCAGAGCCCGTGTAATCAGTCT
		**COL6A1-8**	GACCGATGCTCTCATGCTGCTGTAGTACTAGGAGACTGATTACACGTGGAAGTTCTGCA	AGTCCTTGGCAATACGTGTAATCAGTCT
		**COL6A1-9**	CTCCCCCCTAGGAATGCTGCTGTAGTACTAGGAGACTGATTACACGCTGAGCCCATGAG	GTGATTACTGGGCCCGTGTAATCAGTCT

		**COL6A1-10**	GTAGTTCACAGCCATGCTGCTGTAGTACTAGGAGACTGATTACACGTGCCCTTCCCGAT	GCAGTCAGTGTAGCCGTGTAATCAGTCT
	CD44	**CD44-1**	CTCTCCGCTATGAATGCTGCTGTAGTACTACCTCGATTAACATCGCCTGAGGTTGAGGA	TTCATCATCCGCTGGCGATGTTAATCGA
		**CD44-2**	TGAGAGTGTAGCGATGCTGCTGTAGTACTACCTCGATTAACATCGCGCTTCAGATCGTG	TGCAGAGTTCAACAGCGATGTTAATCGA
		**CD44-3**	AATACGAGGGGCTATGCTGCTGTAGTACTACCTCGATTAACATCGCATAGCACTTGCGT	GCTCTGAGACTGGTGCGATGTTAATCGA
		**CD44-4**	ATCCAAAAGTTGCATGCTGCTGTAGTACTACCTCGATTAACATCGCTTCAGCAAACATA	CTGTTAGACAAAGGGCGATGTTAATCGA
		**CD44-5**	TGCAACTTACATTATGCTGCTGTAGTACTACCTCGATTAACATCGCACTCCTCCATATC	TCTCAACATGAAATGCGATGTTAATCGA
		**CD44-6**	GTTGACAAAGTACATGCTGCTGTAGTACTACCTCGATTAACATCGCCAACTGTTCTAGT	TCATGAGCTTTCTAGCGATGTTAATCGA
		**CD44-7**	ACTACAAAACCATATGCTGCTGTAGTACTACCTCGATTAACATCGCAACAATATTCCCC	TTGATTCGTGGAAAGCGATGTTAATCGA
		**CD44-8**	TATGATTTGCTGCATGCTGCTGTAGTACTACCTCGATTAACATCGCTTATAAATGCCAG	TGTTTGCTGAAAGAGCGATGTTAATCGA
		**CD44-9**	CTTTCAATTGGCTATGCTGCTGTAGTACTACCTCGATTAACATCGCAGAGGTATCTAGC	TGATGATTGAGGATGCGATGTTAATCGA
		**CD44-10**	TCTGCATTGTCAAATGCTGCTGTAGTACTACCTCGATTAACATCGCATAACGTGAGCCA	GTGCCATCTGCATGGCGATGTTAATCGA
	COL6A2	**COL6A2-1**	CTTGGGCTTTCTCGGAGACTGATTACACGACCTCGATTAACATCGCACCACAGCAAACA	GCCCATCTGTGATAGCGATGTTAATCGA
		**COL6A2-2**	CACCTTCATCCCCGGAGACTGATTACACGACCTCGATTAACATCGCCTCTTTCAGCCTG	CCCCATGTCTCGTCGCGATGTTAATCGA
		**COL6A2-3**	AAGTTCACACCCGGGAGACTGATTACACGACCTCGATTAACATCGCGATGACCACCGCA	ACATGGCCATCGGAGCGATGTTAATCGA
		**COL6A2-4**	AGCTCACCTCTTCGGAGACTGATTACACGACCTCGATTAACATCGCGCCTTTTGGTCCA	TTGGTTCCTTGCGGGCGATGTTAATCGA
		**COL6A2-5**	TCTGCCGCACAGAGGAGACTGATTACACGACCTCGATTAACATCGCTGACGACCACGTC	AATGCCAATGGTGAGCGATGTTAATCGA
		**COL6A2-6**	GTCCCTGGATCTCGGAGACTGATTACACGACCTCGATTAACATCGCTTTGCTGCCTTTC	GCTCCAGGAGTGCGGCGATGTTAATCGA
		**COL6A2-7**	CTGCAGAGCAATGGGAGACTGATTACACGACCTCGATTAACATCGCGGATAGGCACAGT	ATCCACGAGGCTCAGCGATGTTAATCGA
		**COL6A2-8**	CTGCCTCTGTCACGGAGACTGATTACACGACCTCGATTAACATCGCGCTAAAGCCAGGT	CTGGGTCCAGGGTTGCGATGTTAATCGA
		**COL6A2-9**	GTGGACCTGCAATGGAGACTGATTACACGACCTCGATTAACATCGCCCCTTGAGACCAG	TCTGCCCTCGGTAAGCGATGTTAATCGA
		**COL6A2-10**	ATGGAGACCAGGGGGAGACTGATTACACGACCTCGATTAACATCGCTTCATTGCAGGCA	TGGACTCGCTGGGCGCGATGTTAATCGA
	ANGPTL4	**ANGPTL4-1**	CAGCTCAATCAGGGTGCTTGAACTGGTCTAGTGCTTGAACTGGTCTCCTCCCAGTCTTC	CACCTGGGAATTCGAGACCAGTTCAAGC
		**ANGPTL4-2**	CTGCAAGAGCTCCGTGCTTGAACTGGTCTAGTGCTTGAACTGGTCTGCTGCTTGATCTT	CAGCTTGTACTGCAAGACCAGTTCAAGC
		**ANGPTL4-3**	GAAGGGCAGCTGCGTGCTTGAACTGGTCTAGTGCTTGAACTGGTCTGATCCCGGGTGGA	GAGGTCGTGGTCGGAGACCAGTTCAAGC
		**ANGPTL4-4**	ATCCAGCTGTGCCGTGCTTGAACTGGTCTAGTGCTTGAACTGGTCTCAGCCCCAGGAAG	CCAGGTGAACCCCTAGACCAGTTCAAGC
		**ANGPTL4-5**	TGCGGAGGAAAGAGTGCTTGAACTGGTCTAGTGCTTGAACTGGTCTATCCATCAGGGAC	CGCAAGTTCTGTGGAGACCAGTTCAAGC
		**ANGPTL4-6**	GATCAGTAGATTGGTGCTTGAACTGGTCTAGTGCTTGAACTGGTCTTTAGGTGTAAGGG	CGTTTTGTTGTCTAAGACCAGTTCAAGC
		**ANGPTL4-7**	GTTTATCTTCCACGTGCTTGAACTGGTCTAGTGCTTGAACTGGTCTGGCTTTTCTTCAG	GTTGGTGAGACTCTAGACCAGTTCAAGC
		**ANGPTL4-8**	ATCCTCCGGGAGCGTGCTTGAACTGGTCTAGTGCTTGAACTGGTCTAGAGCTGGTGGCA	CTGCCCGGCCAGGTAGACCAGTTCAAGC
		**ANGPTL4-9**	TCAGAACCCGCGGGTGCTTGAACTGGTCTAGTGCTTGAACTGGTCTGACTTTGAAGGGC	GTCATGTCGCAGTTAGACCAGTTCAAGC
		**ANGPTL4-10**	CGTCCAGCCACCTGTGCTTGAACTGGTCTAGTGCTTGAACTGGTCTGCCTCTGGATCAC	AGAGCCATCTGTGGAGACCAGTTCAAGC
	SDC4	**SDC4-1**	ATCGTCTGGCAAGCTGACTAACGACACCAACTGACTAACGACACCACGCCAATGTCTTC	GTGAGGCCTGAATGTGGTGTCGTTAGTC

		**SDC4-2**	TGTTGGACACGTCCTGACTAACGACACCAACTGACTAACGACACCAGCCATGGAGATCT	TGTTGGCTGTGCTATGGTGTCGTTAGTC
		**SDC4-3**	CGTCCATGGTCTCCTGACTAACGACACCAACTGACTAACGACACCATCAAGCCATCGGG	CGGAGGCAGGATACTGGTGTCGTTAGTC
		**SDC4-4**	GCATCTTCAGAATCTGACTAACGACACCAACTGACTAACGACACCAAGTCAGATACACG	GGAGTATCCATGGATGGTGTCGTTAGTC
		**SDC4-5**	ATAGTTGTCAGATCTGACTAACGACACCAACTGACTAACGACACCATATCTCCAGGGAT	AATCTTTCTCTCACTGGTGTCGTTAGTC
		**SDC4-6**	CAGTGTGTTTTTCCTGACTAACGACACCAACTGACTAACGACACCATTTCATTGTCCAC	TTTGTCTGGAATTTTGGTGTCGTTAGTC
		**SDC4-7**	AAGAGGCCCACTGCTGACTAACGACACCAACTGACTAACGACACCAGAAGACAGCAAAC	AGGAGGAGGATCACTGGTGTCGTTAGTC
		**SDC4-8**	CGTCCTTTTTCTTCTGACTAACGACACCAACTGACTAACGACACCATCGTAACTGCCTT	GTTTCTTCCCAAGCTGGTGTCGTTAGTC
	SDC2	**SDC2-1**	CTTTCTCATGCGAACTTGGATCCACTCGTAACTTGGATCCACTCGTTGCCTTCATCCTT	ACCAAGGTCGTAAGACGAGTGGATCCAA
		**SDC2-2**	CCAGCAATAACAGACTTGGATCCACTCGTAACTTGGATCCACTCGTACCGATAACTCCG	ATTGCGAACAGAATACGAGTGGATCCAA
		**SDC2-3**	CACAGGATGGTTTACTTGGATCCACTCGTAACTTGGATCCACTCGTTTTTGATAGGCAG	CCTTAGTAGGTGCGACGAGTGGATCCAA
		**SDC2-4**	TCAATTTCTTCAGACTTGGATCCACTCGTAACTTGGATCCACTCGTCCTTTCCTCTTTA	GCATCTGTCTCAGCACGAGTGGATCCAA
		**SDC2-5**	TCCCCTGGCTCATACTTGGATCCACTCGTAACTTGGATCCACTCGTTACATCGGTGTCG	TGTTTCTCAGTGATACGAGTGGATCCAA
		**SDC2-6**	ATCTTCTTCAGCAACTTGGATCCACTCGTAACTTGGATCCACTCGTCTGCACTATCCTC	GGATGTTGTTATCTACGAGTGGATCCAA
		**SDC2-7**	GTTGGTAACTTTGACTTGGATCCACTCGTAACTTGGATCCACTCGTTGCATCACTAGTT	GTCTCAGCCCTAGTACGAGTGGATCCAA
		**SDC2-8**	TCAAATCTGCTCTACTTGGATCCACTCGTAACTTGGATCCACTCGTTCTTTATCAGAGG	TGTCAAGGTATAATACGAGTGGATCCAA
		**SDC2-9**	CTGATGCTTCTTCACTTGGATCCACTCGTAACTTGGATCCACTCGTATTGGATAGACGC	AATCATCATCATCTACGAGTGGATCCAA
		**SDC2-10**	TGACTTGCAGTTGACTTGGATCCACTCGTAACTTGGATCCACTCGTTGGAATAAGAGTC	GAGGTAAATTCCATACGAGTGGATCCAA
	TLR4	**TLR4-1**	AGTTCAAGCCACAGATCTCGAGAACGTCAAGATCTCGAGAACGTCAAGCACTTGCAAAG	TGCCAGCCATCTTCTGACGTTCTCGAGA
		**TLR4-2**	ATGTGCCTGCTGAGATCTCGAGAACGTCAAGATCTCGAGAACGTCACTGCCTCCAGAAG	AGGACTCCTGTCACTGACGTTCTCGAGA
		**TLR4-3**	CACCGAGGATGCCGATCTCGAGAACGTCAAGATCTCGAGAACGTCACGAGCAACATGAT	GAACACCACTGCACTGACGTTCTCGAGA
		**TLR4-4**	GAGAGGCGATTCTGATCTCGAGAACGTCAAGATCTCGAGAACGTCAGCAACCAGTGAAG	AACTGCCGGGAACTTGACGTTCTCGAGA
		**TLR4-5**	CAGTTCCTGCAGGGATCTCGAGAACGTCAAGATCTCGAGAACGTCATGTGGCCCAAATT	TGAGGCAATGTTGATGACGTTCTCGAGA
		**TLR4-6**	TCCCTTCCCTCAGGATCTCGAGAACGTCAAGATCTCGAGAACGTCAAGGTTGAGCCTGT	AAAGCACCAGCGTCTGACGTTCTCGAGA
		**TLR4-7**	ACCAGGACCAAATGATCTCGAGAACGTCAAGATCTCGAGAACGTCAAGACAGGGTAGGA	CTGAAGGGACAGATTGACGTTCTCGAGA
		**TLR4-8**	GTGGTGGGACAAGGATCTCGAGAACGTCAAGATCTCGAGAACGTCACAGTTTGCCAGCT	AGGGGTTGGGGAGCTGACGTTCTCGAGA
		**TLR4-9**	TGGGCAATGTCAAGATCTCGAGAACGTCAAGATCTCGAGAACGTCAAAGCTGCCAGGAC	GCCTTTCCTTCGAGTGACGTTCTCGAGA
		**TLR4-10**	CTTTGCTGGAGTGGATCTCGAGAACGTCAAGATCTCGAGAACGTCAACCCATTCTTGGT	CCAGCTCCTTCATGTGACGTTCTCGAGA
	COL4A1	**COL4A1-1**	CTTTGGCCCCTTTGTGCTTGAACTGGTCTAACTTGGATCCACTCGTCTCCCCTGCTCTC	CAGGCCAGCCATTCACGAGTGGATCCAA
		**COL4A1-2**	TGCCTTCTCTTCCGTGCTTGAACTGGTCTAACTTGGATCCACTCGTTCTTTCCCTGGTC	GACCTGGCAATCCTACGAGTGGATCCAA
		**COL4A1-3**	ACCCTTTTCACCTGTGCTTGAACTGGTCTAACTTGGATCCACTCGTCGAATCCTGGAGA	GGGTCCAGGTAAACACGAGTGGATCCAA
		**COL4A1-4**	CACCCTTGAAACCGTGCTTGAACTGGTCTAACTTGGATCCACTCGTGGTAAGCCTCTGT	GGCCATCAAGTCCTACGAGTGGATCCAA
		**COL4A1-5**	TGTCCATCGTTCCGTGCTTGAACTGGTCTAACTTGGATCCACTCGTACCATGTCCATGT	GGCCTTTGAGACTTACGAGTGGATCCAA

		**COL4A1-6**	ACCCAATCACACCGTGCTTGAACTGGTCTAACTTGGATCCACTCGTTGCATTCCAGGAA	GACCTTCAGGTCCAACGAGTGGATCCAA
		**COL4A1-7**	TGCTCCCTGAAATGTGCTTGAACTGGTCTAACTTGGATCCACTCGTCTTGTGGCCCCAT	TTCTCCTGGAGGCGACGAGTGGATCCAA
		**COL4A1-8**	ACTCCAGGGAATCGTGCTTGAACTGGTCTAACTTGGATCCACTCGTTCTTTCACCACGT	GGGCCAGGAAAACGACGAGTGGATCCAA
		**COL4A1-9**	TTCCACAACCGGAGTGCTTGAACTGGTCTAACTTGGATCCACTCGTCTGCAGTCACATT	GCCCTTTTACTCCTACGAGTGGATCCAA
		**COL4A1-10**	TCCTACCCTGTCTGTGCTTGAACTGGTCTAACTTGGATCCACTCGTGAACACCAGGTGA	ACCCCTCTCTCCACACGAGTGGATCCAA
	COL4A2	**COL4A2-1**	TCCTGTGCCTCCTGTGCTTGAACTGGTCTAGATCTCGAGAACGTCACTTCGCCTTTTTG	ACCAGGATCACCAGTGACGTTCTCGAGA
		**COL4A2-2**	ACCCACTGCACCTGTGCTTGAACTGGTCTAGATCTCGAGAACGTCACGTCCCCTTTGTC	GCTCGGCTCTCCTCTGACGTTCTCGAGA
		**COL4A2-3**	GTCCCCTTTTGCAGTGCTTGAACTGGTCTAGATCTCGAGAACGTCACTTTCGCACCAAC	CAGACCTGCCAGCGTGACGTTCTCGAGA
		**COL4A2-4**	CTGGTTGTCCACTGTGCTTGAACTGGTCTAGATCTCGAGAACGTCATGTCCACTCACAC	CTTTTGGGCCGGTATGACGTTCTCGAGA
		**COL4A2-5**	AGCCTTGGAGGAAGTGCTTGAACTGGTCTAGATCTCGAGAACGTCATTGCTCAGCAACA	GGACCTGGTGAACGTGACGTTCTCGAGA
		**COL4A2-6**	CTTCTCCCCTAGCGTGCTTGAACTGGTCTAGATCTCGAGAACGTCACCTTGCTCACCAT	TGGGGCCAGGAACTTGACGTTCTCGAGA
		**COL4A2-7**	TGTTCACCTCTGGGTGCTTGAACTGGTCTAGATCTCGAGAACGTCATCCTGTTGCTCCT	ACTGGACCCTGAATTGACGTTCTCGAGA
		**COL4A2-8**	TGAGGCCTTCAATGTGCTTGAACTGGTCTAGATCTCGAGAACGTCACCCTGTGTGCCCT	CAGAGCCAGGGATTTGACGTTCTCGAGA
		**COL4A2-9**	AGCCCATGTTCACGTGCTTGAACTGGTCTAGATCTCGAGAACGTCATTCACCAGCAGGT	CAGACTGGCTGTGGTGACGTTCTCGAGA
		**COL4A2-10**	GAGCGTATCTGCCGTGCTTGAACTGGTCTAGATCTCGAGAACGTCATGAGTCCTGCCTT	GATGTGGCTTCGGTTGACGTTCTCGAGA
	FN1	**FN1-1**	TTGTCCCTCCGTCCTGACTAACGACACCAAACTTGGATCCACTCGTACACCACCTCATG	TTTGCAGTGGTTCGACGAGTGGATCCAA
		**FN1-2**	TCTCCACGGCCATCTGACTAACGACACCAAACTTGGATCCACTCGTAACGCAAGTCCAT	AGCTGGGAATAGGGACGAGTGGATCCAA
		**FN1-3**	GTACACCACTCCGCTGACTAACGACACCAAACTTGGATCCACTCGTGCATACCCACAGA	TGTCTTGAGCCACAACGAGTGGATCCAA
		**FN1-4**	TCCCCTACTCGGTCTGACTAACGACACCAAACTTGGATCCACTCGTGCGCTCATATGTC	ATGGAGTCCTTGGCACGAGTGGATCCAA
		**FN1-5**	CAGCTCCAATGCACTGACTAACGACACCAAACTTGGATCCACTCGTATTCTCCCACGTC	CAATGGTGCAGCTCACGAGTGGATCCAA
		**FN1-6**	AGCAAGTTTCCACCTGACTAACGACACCAAACTTGGATCCACTCGTACAGACGCACTGA	CTGCCGTTACCAGAACGAGTGGATCCAA
		**FN1-7**	TCTCCCCCTGACGCTGACTAACGACACCAAACTTGGATCCACTCGTATCATCTGGCCAT	GGCAGGTACAGCTGACGAGTGGATCCAA
		**FN1-8**	ACTCAATTGGGCACTGACTAACGACACCAAACTTGGATCCACTCGTAGGGGCATGAAAC	CTGCCTGTACATCAACGAGTGGATCCAA
		**FN1-9**	GTGACTTGCCCCTCTGACTAACGACACCAAACTTGGATCCACTCGTCACCCTGTACCTG	GGGCTTGAGTAGGAACGAGTGGATCCAA
		**FN1-10**	CATCATGACGCTTCTGACTAACGACACCAAACTTGGATCCACTCGTAGCATGTGCCCCT	AGCATGTGCAGTTGACGAGTGGATCCAA
	LAMC1	**LAMC1-1**	CAGGTCCTCTGCACTGACTAACGACACCAAGATCTCGAGAACGTCACCCCTTCCAACAC	CACCCTCAGCCCACTGACGTTCTCGAGA
		**LAMC1-2**	CTTCTGCTTCAGCCTGACTAACGACACCAAGATCTCGAGAACGTCACAGCATCATCCTG	CATCATGTCCTGCATGACGTTCTCGAGA
		**LAMC1-3**	TGCTTCAGCCTCTCTGACTAACGACACCAAGATCTCGAGAACGTCAATCTCTTTCCGCT	AGCAGCTCCGTAAGTGACGTTCTCGAGA
		**LAMC1-4**	TCCTCCACCCTCACTGACTAACGACACCAAGATCTCGAGAACGTCACAGGTCCTCCGTG	GAAGCCAAGCTGAATGACGTTCTCGAGA
		**LAMC1-5**	TCCTTGCATGCACCTGACTAACGACACCAAGATCTCGAGAACGTCAGCCTTCGACATTG	CTCTCGCAGTGGATTGACGTTCTCGAGA
		**LAMC1-6**	TGTCTTCTGCCTCCTGACTAACGACACCAAGATCTCGAGAACGTCAGCTTTTCTTGCGT	TCACGGAGTTCTTATGACGTTCTCGAGA
		**LAMC1-7**	CTCTTCTGCTTCCCTGACTAACGACACCAAGATCTCGAGAACGTCATCAGCTGATCCAA	CAGCTTTCTGGCATTGACGTTCTCGAGA

		**LAMC1-8**	TTGCAACTCGTCTCTGACTAACGACACCAAGATCTCGAGAACGTCACCCCGTCACTTGA	AGGCACTCGCACTCTGACGTTCTCGAGA
		**LAMC1-9**	CAGCCTCTTCAGCCTGACTAACGACACCAAGATCTCGAGAACGTCATTGCCCTTCCGAG	CCTGGAGAGTCCCCTGACGTTCTCGAGA
		**LAMC1-10**	TCTCGCCCATCACCTGACTAACGACACCAAGATCTCGAGAACGTCACAGCGGTCACACT	GGAAGCCTGGCTGCTGACGTTCTCGAGA
**Pig**	***IFIT1***	**IFIT1-1**	AACCAGCCTTCTCATGAGATCGTCATCGTATGATCTTCCGATCTAAAGTTGCCCCAGGT	ATACAGCCAGGCAATTAGATCGGAAGAT
		**IFIT1-2**	GTCCTTCACCTTGATGAGATCGTCATCGTATGATCTTCCGATCTAAGCTTCCTGCAAGT	GGAGGGATTTGCATTTAGATCGGAAGAT
		**IFIT1-3**	CTGAGGACACTCCATGAGATCGTCATCGTATGATCTTCCGATCTAACCTCACAGTCCAT	CAAGGCCCATCCTATTAGATCGGAAGAT
		**IFIT1-4**	TACCCAGTGCTGAATGAGATCGTCATCGTATGATCTTCCGATCTAAGATGGTGATTGCA	TGGTCTAGGCGATTTTAGATCGGAAGAT
		**IFIT1-5**	AATACTCCAGGGCATGAGATCGTCATCGTATGATCTTCCGATCTAAAGGGCCTGCTCAT	TAGAAGCCAGCCTGTTAGATCGGAAGAT
		**IFIT1-6**	TTCACATAGGCCAATGAGATCGTCATCGTATGATCTTCCGATCTAAGCCTTTCAGGTGT	GCTCCCTCATTCTCTTAGATCGGAAGAT
		**IFIT1-7**	ATTCTTGAAGCTTATGAGATCGTCATCGTATGATCTTCCGATCTAATCTTCTTGTCCTA	ACTTTTCTCCTTCTTTAGATCGGAAGAT
		**IFIT1-8**	AAGTATCTTCTGCATGAGATCGTCATCGTATGATCTTCCGATCTAAAACACTCTTTGAA	GTGATTCCATACAGTTAGATCGGAAGAT
		**IFIT1-9**	ATCTTCAGCATCTATGAGATCGTCATCGTATGATCTTCCGATCTAACTTGTTGGGTTAA	TTGGTCAGTATGTATTAGATCGGAAGAT
		**IFIT1-10**	GTTTTCTTTGTCCATGAGATCGTCATCGTATGATCTTCCGATCTAATTAGTCTGCTGAT	TGTGGCTAATTTTTTTAGATCGGAAGAT
		**IFIT1-11**	GCATTCAGACAAAATGAGATCGTCATCGTATGATCTTCCGATCTAAAGCCTGTTTTAGA	GGATTTAGCCTGAGTTAGATCGGAAGAT
		**IFIT1-12**	TCTTTCCTCCACAATGAGATCGTCATCGTATGATCTTCCGATCTAAGCCCGTTCATAAT	TGAAGCAGACCTTCTTAGATCGGAAGAT
	IFIT2	**IFIT2-1**	GAGCAGACAAGGAATGAGATCGTCATCGTACAGATAGGACTAGTCAGCAGGCGAGATAG	GCTTGCACACAGACTGACTAGTCCTATC
		**IFIT2-2**	TGGCCAGACCAGAATGAGATCGTCATCGTACAGATAGGACTAGTCACGGTAGCTTGCGA	GCCAGTTATCCAGTTGACTAGTCCTATC
		**IFIT2-3**	ATAGCAGGACCCAATGAGATCGTCATCGTACAGATAGGACTAGTCAGGACTTTGGCCCT	CTTCTGCGTCTCGTTGACTAGTCCTATC
		**IFIT2-4**	TGGCTACAGGAGGATGAGATCGTCATCGTACAGATAGGACTAGTCATGGAGCACTTGTT	TGCCGTATCGCAGTTGACTAGTCCTATC
		**IFIT2-5**	AGTAGACCCAGGCATGAGATCGTCATCGTACAGATAGGACTAGTCACTTCCCAGGTGAT	GAGCTTCTGCAAGATGACTAGTCCTATC
		**IFIT2-6**	CTCTTCAGCTTGCATGAGATCGTCATCGTACAGATAGGACTAGTCACTCGCCGGATGAA	CTGGCTAGCGTGTATGACTAGTCCTATC
		**IFIT2-7**	TACAGCTTTGCTGATGAGATCGTCATCGTACAGATAGGACTAGTCAACCTTTACTGCGA	GCTTGGTCCAGGTGTGACTAGTCCTATC
		**IFIT2-8**	ATTCCGAGGAGGTATGAGATCGTCATCGTACAGATAGGACTAGTCAGAGGGTCAATGGA	AACAGCTTGCCTCTTGACTAGTCCTATC
		**IFIT2-9**	TTTCATAGCCAAGATGAGATCGTCATCGTACAGATAGGACTAGTCATCATCTTTCGAAG	TTCATTTGCTTCAATGACTAGTCCTATC
		**IFIT2-10**	GTGAAATGGCATTATGAGATCGTCATCGTACAGATAGGACTAGTCATACCAAGTTCCAG	GAACTTTCTCCCTGTGACTAGTCCTATC
		**IFIT2-11**	TTTTCCTCTGCTGATGAGATCGTCATCGTACAGATAGGACTAGTCAATCCCTCTCAGAG	TTTCCAGAGTCCATTGACTAGTCCTATC
		**IFIT2-12**	CTCTGTCTTCAAAATGAGATCGTCATCGTACAGATAGGACTAGTCAGTCTTGTTACACA	TGTTCTGAAACTCTTGACTAGTCCTATC
	RASD2	**RASD2-1**	ACATCTCGTCCACGTCTATTAGTGGAGCCAGTCTATTAGTGGAGCCAAGAGCACGTAGA	GCTTGGCCATGCTCGGCTCCACTAATAG
		**RASD2-2**	TCGCGGTTATCCAGTCTATTAGTGGAGCCAGTCTATTAGTGGAGCCCTCGTCGAAGGAC	TGGAGGCGCTTGAGGGCTCCACTAATAG
		**RASD2-3**	CTCTTGCCCACCCGTCTATTAGTGGAGCCAGTCTATTAGTGGAGCCAGAGACGATGGAG	CCGTTGAGGAAGCCGGCTCCACTAATAG
		**RASD2-4**	GACTTGACCTCCAGTCTATTAGTGGAGCCAGTCTATTAGTGGAGCCGTTCTTCAGGCAG	GCCTCCTTGGTCTAGGCTCCACTAATAG
		**RASD2-5**	TCCCGCTGGACAAGTCTATTAGTGGAGCCAGTCTATTAGTGGAGCCCTGAGCGTGCAGT	TCTTGGCTGGCACCGGCTCCACTAATAG

		**RASD2-6**	ACTGGTCCTCGAAGTCTATTAGTGGAGCCAGTCTATTAGTGGAGCCATGGTGGGCGTGT	GGTGGAAGTCCTCCGGCTCCACTAATAG
		**RASD2-7**	TCCAGCTGGTACAGTCTATTAGTGGAGCCAGTCTATTAGTGGAGCCCGTGTCCAGGATG	GGGTGGTTGCCAGTGGCTCCACTAATAG
		**RASD2-8**	CCCGTACTGCACGGTCTATTAGTGGAGCCAGTCTATTAGTGGAGCCGGTGGAAGGCGTC	GCAGAAGGGCCTGCGGCTCCACTAATAG
		**RASD2-9**	CCTGGCGGCACAGGTCTATTAGTGGAGCCAGTCTATTAGTGGAGCCTCGGTGGTGGGCA	CCAGCAGCTCCGCGGGCTCCACTAATAG
		**RASD2-10**	ACCATGGGCAGCTGTCTATTAGTGGAGCCAGTCTATTAGTGGAGCCGTTGCCGCAGATG	CCGTGGTCGTTCTAGGCTCCACTAATAG
		**RASD2-11**	GTCCTTGACACGGGTCTATTAGTGGAGCCAGTCTATTAGTGGAGCCCGTAGGCGTCCAT	GGGCGAGACCATGGGGCTCCACTAATAG
		**RASD2-12**	AGTTCCCGTCGCCGTCTATTAGTGGAGCCAGTCTATTAGTGGAGCCTCGAAGTAGGCGC	TCTTGGCCGACACGGGCTCCACTAATAG
	DDX60	**DDX60-1**	TGCAGCTCATCAAATAACGATCCCTCACAAATAACGATCCCTCACAACTCTCCCCTCCT	AGGGAGAGATGGCCTGTGAGGGATCGTT
		**DDX60-2**	CAGCCCCTAACTTATAACGATCCCTCACAAATAACGATCCCTCACAGCCCTTTCCTCGA	TTTTCACACTCCGCTGTGAGGGATCGTT
		**DDX60-3**	TCTTGTCCACCACATAACGATCCCTCACAAATAACGATCCCTCACAACTGCAGACTCGT	TGGGGGCCACAATCTGTGAGGGATCGTT
		**DDX60-4**	AACCCCACCATTTATAACGATCCCTCACAAATAACGATCCCTCACACAGGCAAGCAGTT	TCTTTCCACGCTTATGTGAGGGATCGTT
		**DDX60-5**	CAGCTCTGCCTTCATAACGATCCCTCACAAATAACGATCCCTCACATCACCCAGTCGGT	GACGCAGCCACTCATGTGAGGGATCGTT
		**DDX60-6**	AGCTCCGCACCTTATAACGATCCCTCACAAATAACGATCCCTCACATGCCAACGCCTTC	CCAACGCCCCTTGGTGTGAGGGATCGTT
		**DDX60-7**	GAGACAGTGCAGTATAACGATCCCTCACAAATAACGATCCCTCACAGAAAGGCCACGCT	AGGTAAGCAGAGGTTGTGAGGGATCGTT
		**DDX60-8**	ACAGGCACCCAGAATAACGATCCCTCACAAATAACGATCCCTCACATCCCACTCTTGTG	CTTCCAGGAAAGCATGTGAGGGATCGTT
		**DDX60-9**	GAGCATGAGCCTCATAACGATCCCTCACAAATAACGATCCCTCACACCTTGGAAGCCAG	TCCGGGGTCATCTGTGTGAGGGATCGTT
		**DDX60-10**	CAGCCCGTCCAGAATAACGATCCCTCACAAATAACGATCCCTCACATGACCTCGACGTC	CTCCCAGCAGGTCATGTGAGGGATCGTT
		**DDX60-11**	GTCTCCATCGAGCATAACGATCCCTCACAAATAACGATCCCTCACATGATCAGCAAGGA	CTCACAGACGCACTTGTGAGGGATCGTT
		**DDX60-12**	CAAAGAACTCGCCATAACGATCCCTCACAAATAACGATCCCTCACAGCAGGAAGTGACA	CGCAAAGACAATGTTGTGAGGGATCGTT
	ZNFX1	**ZNFX1-1**	CTCCTCTTCCCCTTACCCGAGATAGAGTCATACCCGAGATAGAGTCCAATCAGCGAGCT	CTCCTCTGCAATCAGACTCTATCTCGGG
		**ZNFX1-2**	ATTCTCCCCTGCCTACCCGAGATAGAGTCATACCCGAGATAGAGTCCGCTGCCTTTCAC	GACCTCTCCTGCTTGACTCTATCTCGGG
		**ZNFX1-3**	ATTTCACTGGCCTTACCCGAGATAGAGTCATACCCGAGATAGAGTCCCAAACGTCCTGA	TTCAGGTCCAGCTCGACTCTATCTCGGG
		**ZNFX1-4**	CACCACAAAACGATACCCGAGATAGAGTCATACCCGAGATAGAGTCACTTGCACAGCTC	CTGGCACAGGAAGAGACTCTATCTCGGG
		**ZNFX1-5**	CACAGTCCTCACCTACCCGAGATAGAGTCATACCCGAGATAGAGTCGAACAGAACCGCA	CGGTGACCATTTCAGACTCTATCTCGGG
		**ZNFX1-6**	ACAAACCGATGCATACCCGAGATAGAGTCATACCCGAGATAGAGTCCATGGTTCCCCAC	GGCATTTCTTGGGCGACTCTATCTCGGG
		**ZNFX1-7**	TCCGAAGAACCTGTACCCGAGATAGAGTCATACCCGAGATAGAGTCGAACTGCAAGCCT	GGCGGTCCATTTTCGACTCTATCTCGGG
		**ZNFX1-8**	CTTCCTTCTTCCGTACCCGAGATAGAGTCATACCCGAGATAGAGTCATTGCCCCATTCT	GAGCCAACTCCTGCGACTCTATCTCGGG
		**ZNFX1-9**	GTTGCCGACACAATACCCGAGATAGAGTCATACCCGAGATAGAGTCCCAACATCTGCAT	CAAGGGCACCTTGCGACTCTATCTCGGG
		**ZNFX1-10**	TTGGCAGGCATGATACCCGAGATAGAGTCATACCCGAGATAGAGTCACCAGCAAATGTC	ACATGGACCCTGAGGACTCTATCTCGGG
		**ZNFX1-11**	TTCCACGATGACGTACCCGAGATAGAGTCATACCCGAGATAGAGTCCTTCAGCAGCCTC	GTGGGCCTCAAGGTGACTCTATCTCGGG
		**ZNFX1-12**	TCCTGGAAGCAAATACCCGAGATAGAGTCATACCCGAGATAGAGTCACAAGGCTGGCAC	GGCACCTTCACCTCGACTCTATCTCGGG
	DHX58	**DHX58-1**	TGCTGCCGACTCTTATGTGACCGGGTACAAGTCTATTAGTGGAGCCTGCCAGGAACTTC	AGCTGCACTTGCTGGGCTCCACTAATAG

		**DHX58-2**	GAAGTCAGGCACCTATGTGACCGGGTACAAGTCTATTAGTGGAGCCACTGCGTGTAATC	GCTCTCGGTGCAAAGGCTCCACTAATAG
		**DHX58-3**	AGCGCCTTCTGTATATGTGACCGGGTACAAGTCTATTAGTGGAGCCCTCCTCGCTGGTC	ACGTGCTCCTCCTGGGCTCCACTAATAG
		**DHX58-4**	CACCAGCAGGGAGTATGTGACCGGGTACAAGTCTATTAGTGGAGCCGACACTCATCCAC	TTTGTGTGTGTGGAGGCTCCACTAATAG
		**DHX58-5**	CAGTTGATGCACATATGTGACCGGGTACAAGTCTATTAGTGGAGCCCATGGCCACCATG	TCACTCCCGTAGCGGGCTCCACTAATAG
		**DHX58-6**	TCTCCAGATGCCTTATGTGACCGGGTACAAGTCTATTAGTGGAGCCGCTCCATCCACAG	GCACAACCACCTTCGGCTCCACTAATAG
		**DHX58-7**	CACTGGGGGATGTTATGTGACCGGGTACAAGTCTATTAGTGGAGCCCACCACCACGTTG	AGGAGCCCATAGCCGGCTCCACTAATAG
		**DHX58-8**	TCCTCACAATGCTTATGTGACCGGGTACAAGTCTATTAGTGGAGCCCATGCGGCTGAAC	CAGCGTCTGTCCACGGCTCCACTAATAG
		**DHX58-9**	TCTCCAGATGGCGTATGTGACCGGGTACAAGTCTATTAGTGGAGCCCTCAACTCCGGCA	TCCCAAAGTCCCGAGGCTCCACTAATAG
		**DHX58-10**	TCCACAGTCTGCATATGTGACCGGGTACAAGTCTATTAGTGGAGCCCTGGGCCCTGATG	GCCCCAATCAACACGGCTCCACTAATAG
		**DHX58-11**	CTGGCTGCTGTTCTATGTGACCGGGTACAAGTCTATTAGTGGAGCCTCATCGGGGTGCT	CTGGGTCATCTGGCGGCTCCACTAATAG
	IFI30	**IFI30-1**	CTCTGCAGGCATGTATGTGACCGGGTACAAATAACGATCCCTCACACTTCCCCACGGGT	GGTGGAGGGTCAGTTGTGAGGGATCGTT
		**IFI30-2**	GACATCCGGCTTCTATGTGACCGGGTACAAATAACGATCCCTCACACCAAGATTTGGCA	CTTGTGGGAGCTGCTGTGAGGGATCGTT
		**IFI30-3**	TCCTGAAAGGCAATATGTGACCGGGTACAAATAACGATCCCTCACAGCTGACATTTCGT	AATTCCCACCTGCGTGTGAGGGATCGTT
		**IFI30-4**	GACGGCAGAAGCATATGTGACCGGGTACAAATAACGATCCCTCACAGGGGCCAACGTAC	GCCATCACGACGGTTGTGAGGGATCGTT
		**IFI30-5**	TTCATGGGGTGGCTATGTGACCGGGTACAAATAACGATCCCTCACACCCAGGGCACATA	CCCATTGACGACGTTGTGAGGGATCGTT
		**IFI30-6**	TCCAAAAGGCAGGTATGTGACCGGGTACAAATAACGATCCCTCACACTTCTCCAGCTTG	AGAAAGGCCATGTATGTGAGGGATCGTT
		**IFI30-7**	CAGGAACGGCAGGTATGTGACCGGGTACAAATAACGATCCCTCACAGCAGCAGCAGAGG	CTCCAGCAGCAGCTTGTGAGGGATCGTT
		**IFI30-8**	ATTTCCCAGACCATATGTGACCGGGTACAAATAACGATCCCTCACATGTGACATTGAGG	CCATAGGGTACCACTGTGAGGGATCGTT
		**IFI30-9**	AGCCATTGCACAGTATGTGACCGGGTACAAATAACGATCCCTCACAAGGAAGTACCGGC	AGAGCTCTCGGACATGTGAGGGATCGTT
		**IFI30-10**	GCTGGTCCTTCTCTATGTGACCGGGTACAAATAACGATCCCTCACAACGAGGCGTAAGA	CGTACAACTGGCACTGTGAGGGATCGTT
		**IFI30-11**	GAGCATAGATCTGTATGTGACCGGGTACAAATAACGATCCCTCACAGGGGATACTTTTG	CCATGATAGAGTCATGTGAGGGATCGTT
		**IFI30-12**	GCAGACAATGGTCTATGTGACCGGGTACAAATAACGATCCCTCACACCAGTTCCTCTAT	TTTCTCCATGTCAATGTGAGGGATCGTT
	IFI35	**IFI35-1**	TGTAGCAGTTCCCTATGTGACCGGGTACAATACCCGAGATAGAGTCCACAACACCCCCT	TTAGTGAAGCCCACGACTCTATCTCGGG
		**IFI35-2**	TGGTCTTGCCAAATATGTGACCGGGTACAATACCCGAGATAGAGTCCCACCCCCGTTCT	GTGTCTCCACATCTGACTCTATCTCGGG
		**IFI35-3**	TGGCCATTTCCCTTATGTGACCGGGTACAATACCCGAGATAGAGTCGAGACCACAGACT	AGATCTGCAGGTTCGACTCTATCTCGGG
		**IFI35-4**	CTGACCCTTCTGGTATGTGACCGGGTACAATACCCGAGATAGAGTCGTGGCACTGAGAA	CTGCTGCTTACCCTGACTCTATCTCGGG
		**IFI35-5**	CAGGAATCCCACTTATGTGACCGGGTACAATACCCGAGATAGAGTCAGCTTGAGCCCAG	GCTCCTCTTCACTGGACTCTATCTCGGG
		**IFI35-6**	GAGCCTCCAGGCATATGTGACCGGGTACAATACCCGAGATAGAGTCGGTGACCAGAGCA	TTGGGGTCATCAATGACTCTATCTCGGG
		**IFI35-7**	CATTTTCAGCTGCTATGTGACCGGGTACAATACCCGAGATAGAGTCTGTCCCTGAGCTT	TGGGAAGCTTTCTGGACTCTATCTCGGG
		**IFI35-8**	CTCAGGCATTGGGTATGTGACCGGGTACAATACCCGAGATAGAGTCATACCAGGGGGAC	AATGTGTCCTCGGTGACTCTATCTCGGG
		**IFI35-9**	GCTGGGACCTGACTATGTGACCGGGTACAATACCCGAGATAGAGTCGACTGGGGCACAG	TGAGCACCAGCACGGACTCTATCTCGGG
		**IFI35-10**	GCAGCCGGAACTCTATGTGACCGGGTACAATACCCGAGATAGAGTCTGGACCTGCACCC	GCAGCTCCAAGGGGGACTCTATCTCGGG

		**IFI35-11**	AGACTGTCAGAGGTATGTGACCGGGTACAATACCCGAGATAGAGTCCTCACGTAGGGAG	TCTGGATCTCCCCCGACTCTATCTCGGG
		**IFI35-12**	CCCCACGGGTAGGTATGTGACCGGGTACAATACCCGAGATAGAGTCTCCACCTCCCCAC	CAACTGTCACGGCAGACTCTATCTCGGG
	IFIH1	**IFIH1-1**	GAGCCTGCACAAAGTCTATTAGTGGAGCCAATAACGATCCCTCACACCTGTTTCCCGGA	CCGCTAAGGAGTTCTGTGAGGGATCGTT
		**IFIH1-2**	GTTCAGCTGCGTTGTCTATTAGTGGAGCCAATAACGATCCCTCACAGTGTTCAGAAGCA	CCCCCTTCTCCAATTGTGAGGGATCGTT
		**IFIH1-3**	TCTGGTTTTCCCAGTCTATTAGTGGAGCCAATAACGATCCCTCACATGTACACGGCCAC	GTGATCCTTGGCATTGTGAGGGATCGTT
		**IFIH1-4**	GTAAGGCCTGAGAGTCTATTAGTGGAGCCAATAACGATCCCTCACACCACTTCCAGTTG	CAGAGCTGGCTGGCTGTGAGGGATCGTT
		**IFIH1-5**	CTTTGGGCAACCAGTCTATTAGTGGAGCCAATAACGATCCCTCACAAACTCCAGAGCCA	GTCTCACGTTCAAATGTGAGGGATCGTT
		**IFIH1-6**	TCTGCTCCTTCACGTCTATTAGTGGAGCCAATAACGATCCCTCACAACCGCCCTCTGAA	TCCCAGTGGTGGCCTGTGAGGGATCGTT
		**IFIH1-7**	TTCCGTCTCTTGCGTCTATTAGTGGAGCCAATAACGATCCCTCACACGATCTTTGCGAT	GTTCTGCACAAACTTGTGAGGGATCGTT
		**IFIH1-8**	AGGCTGAAGAAGGGTCTATTAGTGGAGCCAATAACGATCCCTCACATGTCCACCACTGT	GGTAACCAGAAGCATGTGAGGGATCGTT
		**IFIH1-9**	TGCAGGCAGAAAGGTCTATTAGTGGAGCCAATAACGATCCCTCACAGCTCCTTCACCTC	CGCCCTCTGAATCATGTGAGGGATCGTT
		**IFIH1-10**	GTCCAGCTCCAATGTCTATTAGTGGAGCCAATAACGATCCCTCACAAACTCACTGCTGT	GTGTCATGGGCTTCTGTGAGGGATCGTT
		**IFIH1-11**	GTTAATTCGCGGGGTCTATTAGTGGAGCCAATAACGATCCCTCACAGCAATCGGTGCCT	GCATTGCTTTCACTTGTGAGGGATCGTT
		**IFIH1-12**	AGGCTGTTAGTCCGTCTATTAGTGGAGCCAATAACGATCCCTCACACCAACACCTGGTG	GCTTTTTGGCTCCGTGTGAGGGATCGTT
	SAMHD1	**SAMHD1-1**	TAGGAAGCCGCACGTCTATTAGTGGAGCCATACCCGAGATAGAGTCAGCCACTGCGATT	TTCAGAATCGCCGTGACTCTATCTCGGG
		**SAMHD1-2**	AGAGATTACGGCAGTCTATTAGTGGAGCCATACCCGAGATAGAGTCCCCACAAACTTGT	CTGGTTGGGTCTCTGACTCTATCTCGGG
		**SAMHD1-3**	CTGGGCTGAATGCGTCTATTAGTGGAGCCATACCCGAGATAGAGTCTGCGAGCTTGACT	GGATGCTTCTCGGCGACTCTATCTCGGG
		**SAMHD1-4**	CAGCAAACAGACTGTCTATTAGTGGAGCCATACCCGAGATAGAGTCAAATGCTGTCGTG	AGCACCACTGAACTGACTCTATCTCGGG
		**SAMHD1-5**	TGTGACCATGGATGTCTATTAGTGGAGCCATACCCGAGATAGAGTCGGGTGGAGTTCAA	TTCGGATGAGGAGTGACTCTATCTCGGG
		**SAMHD1-6**	TTTGGCGGGGTTGGTCTATTAGTGGAGCCATACCCGAGATAGAGTCTGCTGGAGGGGTG	GACCTCTCTGCCGGGACTCTATCTCGGG
		**SAMHD1-7**	TGCTCAAACATATGTCTATTAGTGGAGCCATACCCGAGATAGAGTCAGAATTAACCAGG	TCTATGAGTCCATAGACTCTATCTCGGG
		**SAMHD1-8**	CCTCTTTTCATAGGTCTATTAGTGGAGCCATACCCGAGATAGAGTCTAAGGAGTTTCAT	TCGTTGGATATAAAGACTCTATCTCGGG
		**SAMHD1-9**	CCCAGCTGTTTAAGTCTATTAGTGGAGCCATACCCGAGATAGAGTCATAGTAACCGCCT	GCTCCTGGAAAAAGGACTCTATCTCGGG
		**SAMHD1-10**	CTCAGGTTGTTTTGTCTATTAGTGGAGCCATACCCGAGATAGAGTCCACTTATCTGCAG	AAGCATATCTCGTAGACTCTATCTCGGG
		**SAMHD1-11**	ATGTTACTGGGCTGTCTATTAGTGGAGCCATACCCGAGATAGAGTCAGCTTCTAGCTGT	TCAGCCTTCAGTTGGACTCTATCTCGGG
		**SAMHD1-12**	CAGATACCCCACAGTCTATTAGTGGAGCCATACCCGAGATAGAGTCCAAGACATCCTGC	ACTCAGTTCACGATGACTCTATCTCGGG
	STAT1	**STAT1-1**	TGAGTGCCCAAAAATAACGATCCCTCACAATACCCGAGATAGAGTCCATCACCTTCGTG	GACTCCTCCATGTAGACTCTATCTCGGG
		**STAT1-2**	TTCCACCAGTTCAATAACGATCCCTCACAATACCCGAGATAGAGTCGTCTCCGCTTCCA	ACAGGCACTCTGCAGACTCTATCTCGGG
		**STAT1-3**	TCCTCAGGAGACAATAACGATCCCTCACAATACCCGAGATAGAGTCCACCTCGTCAAAC	CCCACCATTCGAGTGACTCTATCTCGGG
		**STAT1-4**	TCGTGCACAAGGTATAACGATCCCTCACAATACCCGAGATAGAGTCAAAGCTGAGACCA	ACTCAGCACCTCTCGACTCTATCTCGGG
		**STAT1-5**	TGAACCGCAACAGATAACGATCCCTCACAATACCCGAGATAGAGTCCGGCAGCTCTCAC	TGATGGCCCCTTCGGACTCTATCTCGGG
		**STAT1-6**	TCATTGGCAGCGTATAACGATCCCTCACAATACCCGAGATAGAGTCGGCAAACGAAACA	TGAAAACGGATGGAGACTCTATCTCGGG

		**STAT1-7**	CAGGGCTGTCTTTATAACGATCCCTCACAATACCCGAGATAGAGTCATGAGTTGGCATG	AATGGCCTCTGAGCGACTCTATCTCGGG
		**STAT1-8**	GACGGGCAGAGAGATAACGATCCCTCACAATACCCGAGATAGAGTCTGGAGATCACCAC	GAGCTGGCTGACGAGACTCTATCTCGGG
		**STAT1-9**	GTCAAGCTCCTTAATAACGATCCCTCACAATACCCGAGATAGAGTCTTCTGACTTTGCT	TTTGTCCTTCACAAGACTCTATCTCGGG
		**STAT1-10**	AGAGCTGATGAACATAACGATCCCTCACAATACCCGAGATAGAGTCAAACTGTCATCGT	TGATTTCCATGGGTGACTCTATCTCGGG
		**STAT1-11**	CTTTCAATCCAAAATAACGATCCCTCACAATACCCGAGATAGAGTCGAGTTCAAGGATA	AGGTGTTTCTTAAAGACTCTATCTCGGG
		**STAT1-12**	TCTTCGTGTAAGGATAACGATCCCTCACAATACCCGAGATAGAGTCGCAGAGAGTTCTT	CAGGGAAGGTAACTGACTCTATCTCGGG
	USP18	**USP18-1**	CAGAAGCACTTGTACCATGCTGGATCTAGAACCATGCTGGATCTAGGCCACAGTTCTCA	CAACAGGTCTTCCACTAGATCCAGCATG
		**USP18-2**	CTCAGCTCCCCTTACCATGCTGGATCTAGAACCATGCTGGATCTAGTTCTCCTCTGCTC	CTGGAAGGGGACAACTAGATCCAGCATG
		**USP18-3**	AGAGCTCGTACCGACCATGCTGGATCTAGAACCATGCTGGATCTAGGCGATCACAGCGA	CCAGCCCCATGTGCCTAGATCCAGCATG
		**USP18-4**	TACAGTGAGTGGCACCATGCTGGATCTAGAACCATGCTGGATCTAGGCTCTGGGGGAAA	GGGTTCAGATCCATCTAGATCCAGCATG
		**USP18-5**	AAGGGGGAGGGTCACCATGCTGGATCTAGAACCATGCTGGATCTAGTGTCAAACAGCGA	GGGCTTTGAGTCCTCTAGATCCAGCATG
		**USP18-6**	TTGTGCAAACCGAACCATGCTGGATCTAGAACCATGCTGGATCTAGGGTCTGTCCAATG	GAGTTAAGGCAGCTCTAGATCCAGCATG
		**USP18-7**	ATAGAGGGCCTGCACCATGCTGGATCTAGAACCATGCTGGATCTAGTCACGCGGATGGT	GACGAAGGTCTCCACTAGATCCAGCATG
		**USP18-8**	CAGTGCAGGGCATACCATGCTGGATCTAGAACCATGCTGGATCTAGGGGCTGGAAGAAG	CCGGACAACTCCCACTAGATCCAGCATG
		**USP18-9**	CATCGTGCTGGACACCATGCTGGATCTAGAACCATGCTGGATCTAGTAGAGCTGGGCAG	TCCAGACGGTGAGCCTAGATCCAGCATG
		**USP18-10**	CCATTTTCCATCCACCATGCTGGATCTAGAACCATGCTGGATCTAGCATTGAAGCAGAA	ACAGACATTGGAGACTAGATCCAGCATG
		**USP18-11**	AAGGTCTGGGGCAACCATGCTGGATCTAGAACCATGCTGGATCTAGGAGGTGCAGGGTC	ATGGAGAACCGCAACTAGATCCAGCATG
		**USP18-12**	ATAATCAGCACCTACCATGCTGGATCTAGAACCATGCTGGATCTAGTAATCCCACATTC	TTCAGTATCTTGGACTAGATCCAGCATG
	PAX5	**PAX5-1**	TCCGCAAGCAGTCTAGGCATGAACGTCGAATAGGCATGAACGTCGAGTCACACACCCGT	GGCACTGTGTCATATCGACGTTCATGCC
		**PAX5-2**	TTTTGTCCGGATGTAGGCATGAACGTCGAATAGGCATGAACGTCGAGTGGCTGCTGTAC	GACCGGCTGGTTGCTCGACGTTCATGCC
		**PAX5-3**	TTCGGCACAGGAGTAGGCATGAACGTCGAATAGGCATGAACGTCGACAGTGAGTGACCA	AAATCTCTGCCCGCTCGACGTTCATGCC
		**PAX5-4**	AGGGTGGCTGTAGTAGGCATGAACGTCGAATAGGCATGAACGTCGAACGAGGGATACTG	CCAGGAATCGTTGATCGACGTTCATGCC
		**PAX5-5**	GAGTAGGAGGAGCTAGGCATGAACGTCGAATAGGCATGAACGTCGAGATGCCGCTGATG	GAGGTGATGCCCACTCGACGTTCATGCC
		**PAX5-6**	CTGCCCTGTCCAGTAGGCATGAACGTCGAATAGGCATGAACGTCGACGGCGCTGAGTAG	ATCCCTGTCAGCGATCGACGTTCATGCC
		**PAX5-7**	CTCCCCGCATCTGTAGGCATGAACGTCGAATAGGCATGAACGTCGATGTGTGAACAGGT	CCTCCAGCTGCTGGTCGACGTTCATGCC
		**PAX5-8**	GGATTTTGGCGTTTAGGCATGAACGTCGAATAGGCATGAACGTCGAGGCAAACATGGTG	TCCCTGATCTCCCTTCGACGTTCATGCC
		**PAX5-9**	GGGGCTGGTCAGGTAGGCATGAACGTCGAATAGGCATGAACGTCGATGTCGGCAGGGGT	CACGCTGCTCCCATTCGACGTTCATGCC
		**PAX5-10**	CCTCTCAAACACGTAGGCATGAACGTCGAATAGGCATGAACGTCGACTGAGTAGTGCTG	GGTGGTGAAGATGATCGACGTTCATGCC
	DYNC1I1	**DYNC1I1-1**	GACCGACATCACGCGTAGCTTACCTGCAAACGTAGCTTACCTGCAAAACGAGCGAAGCA	CAAGTTGGGATGGTTTGCAGGTAAGCTA
		**DYNC1I1-2**	TTCCCCGATGCCTCGTAGCTTACCTGCAAACGTAGCTTACCTGCAAGACCTTCGAAGAC	CACTGGTCCTTGGATTGCAGGTAAGCTA
		**DYNC1I1-3**	TTTCTTCTGCTGCCGTAGCTTACCTGCAAACGTAGCTTACCTGCAACCTGAACTGGCTC	CAGGTCAGAGTCAATTGCAGGTAAGCTA
		**DYNC1I1-4**	CTTTGACACGCCCCGTAGCTTACCTGCAAACGTAGCTTACCTGCAACCACTTGGGTAAC	CCTGGGCAGGAAAATTGCAGGTAAGCTA

		**DYNC1I1-5**	CAGCTTCACTGGGCGTAGCTTACCTGCAAACGTAGCTTACCTGCAAGAGTCTTGGCTTC	GTCCCAGGTCACCATTGCAGGTAAGCTA
		**DYNC1I1-6**	TCCCGCCAACTACCGTAGCTTACCTGCAAACGTAGCTTACCTGCAATGGCCCGAGTAAG	CCCACAGGACGATGTTGCAGGTAAGCTA
		**DYNC1I1-7**	AGCCCAACGAACACGTAGCTTACCTGCAAACGTAGCTTACCTGCAACTTTGCCAGCTTG	CCCAACGGCAACTATTGCAGGTAAGCTA
		**DYNC1I1-8**	AGCTCTGTTGGCGCGTAGCTTACCTGCAAACGTAGCTTACCTGCAACCTCCTCGCTATC	CTCAACAGTGCCTATTGCAGGTAAGCTA
		**DYNC1I1-9**	AACAGCCACAGGCCGTAGCTTACCTGCAAACGTAGCTTACCTGCAAAAGCCATTCCGGT	GTCTCCCGTTGGGTTTGCAGGTAAGCTA
		**DYNC1I1-10**	TGCCAACCACGAACGTAGCTTACCTGCAAACGTAGCTTACCTGCAAGTGCCCTCCTCAC	AGGCTGTGTAGACATTGCAGGTAAGCTA
	RTKN2	**RTKN2-1**	TCCAAGCTCAGGGTACTAGCTCGATTCAAATAGGCATGAACGTCGAATCCTCTGCACTT	TGGGTCTTGAAGCATCGACGTTCATGCC
		**RTKN2-2**	TCGGCAGCACATGTACTAGCTCGATTCAAATAGGCATGAACGTCGAGTTGGGCGACTAA	AGCCATACAAGCTCTCGACGTTCATGCC
		**RTKN2-3**	CATGCTGACCAATTACTAGCTCGATTCAAATAGGCATGAACGTCGAGGGGAGAATTCCT	TGGCCCAAGGAGGATCGACGTTCATGCC
		**RTKN2-4**	TGTAACTGCTTGCTACTAGCTCGATTCAAATAGGCATGAACGTCGACCCTTTCCCATCA	GCTCGTCTCTTTTATCGACGTTCATGCC
		**RTKN2-5**	CACATTAGTGGTATACTAGCTCGATTCAAATAGGCATGAACGTCGAATCAGAGTCTTTC	TTATTGCTGAAGTCTCGACGTTCATGCC
		**RTKN2-6**	AGTAATCATACCTTACTAGCTCGATTCAAATAGGCATGAACGTCGAATAATCTTCTCCA	TCTTAAAACACAAATCGACGTTCATGCC
		**RTKN2-7**	GTGTGTTTGTTATTACTAGCTCGATTCAAATAGGCATGAACGTCGAGCTAGTTTTTTTG	TCTTCAGTTTCTTTTCGACGTTCATGCC
		**RTKN2-8**	GAATGGAGCTTTATACTAGCTCGATTCAAATAGGCATGAACGTCGAGTGTCTTTAAGCT	ATCTGTATTGCTCATCGACGTTCATGCC
		**RTKN2-9**	AAAATCCACATCATACTAGCTCGATTCAAATAGGCATGAACGTCGAGAGCTTTATGTTC	TTTACATGCTGTTGTCGACGTTCATGCC
		**RTKN2-10**	AGAACAAAGGTGGTACTAGCTCGATTCAAATAGGCATGAACGTCGAGCCTCTTTTGTCA	GGTAGACTGAGGTATCGACGTTCATGCC
	DCN	**DCN-1**	AGCCTTTTTGGTGTACTAGCTCGATTCAAACGTAGCTTACCTGCAACCCCCGAATAAGA	GCTGAAAAGGCTCTTTGCAGGTAAGCTA
		**DCN-2**	CACATGGGTCCCATACTAGCTCGATTCAAACGTAGCTTACCTGCAAACAGCGGAAGGGA	CGAAGATGGCATTCTTGCAGGTAAGCTA
		**DCN-3**	ATCTTTGGGCACTTACTAGCTCGATTCAAACGTAGCTTACCTGCAATGTCAGGTGGAAG	ATCCAGCAGGGCACTTGCAGGTAAGCTA
		**DCN-4**	GTACTGGACTGGGTACTAGCTCGATTCAAACGTAGCTTACCTGCAAGCTGGATCTCCCA	TCGGAAGGTGGATCTTGCAGGTAAGCTA
		**DCN-5**	GTTATTTTGTTGTTACTAGCTCGATTCAAACGTAGCTTACCTGCAAATCTTTGATTTCA	TTCTTAAAGTCTCGTTGCAGGTAAGCTA
		**DCN-6**	CTCATTCTCATGGTACTAGCTCGATTCAAACGTAGCTTACCTGCAAGCACTTTGGTGAT	GAACACAGCCTTTGTTGCAGGTAAGCTA
		**DCN-7**	CAATTTCACCAAATACTAGCTCGATTCAAACGTAGCTTACCTGCAAGATAAAGTCGTTC	TTGATTCTTGGATTTTGCAGGTAAGCTA
		**DCN-8**	TTCCCTCAAATGATACTAGCTCGATTCAAACGTAGCTTACCTGCAATGTTCAGATGAAG	GTTAAGCTTGTTGATTGCAGGTAAGCTA
		**DCN-9**	TCTTCAGCGGGTTTACTAGCTCGATTCAAACGTAGCTTACCTGCAATCAATGCCTGAGC	GGAAAGCTCCGTTATTGCAGGTAAGCTA
		**DCN-10**	ATAGTTGCCTTCATACTAGCTCGATTCAAACGTAGCTTACCTGCAACAAGAGGAAGATG	GAAACTTGTGCAACTTGCAGGTAAGCTA
	COL1A2	**COL1A2-1**	ATACCTTGTGGGCTAGGCATGAACGTCGAACGTAGCTTACCTGCAACTTCTCACCTCGA	TCACCAGGCTCTCGTTGCAGGTAAGCTA
		**COL1A2-2**	ATTTCCACGGGCATAGGCATGAACGTCGAACGTAGCTTACCTGCAACCACACTTCCATC	AGGACCCACAGGAGTTGCAGGTAAGCTA
		**COL1A2-3**	CACACCAGGTTCGTAGGCATGAACGTCGAACGTAGCTTACCTGCAACTGGAGCACCAAG	TGGACCAGCAGTGGTTGCAGGTAAGCTA
		**COL1A2-4**	AGCACCAGGAGCATAGGCATGAACGTCGAACGTAGCTTACCTGCAACAGGGGCACCTAC	GGCTCCAGCAGGAGTTGCAGGTAAGCTA
		**COL1A2-5**	GAGGACCATTTGGTAGGCATGAACGTCGAACGTAGCTTACCTGCAACCAGCAGGACCAG	CATCACCACGACTCTTGCAGGTAAGCTA
		**COL1A2-6**	GAGCACCAGCAGGTAGGCATGAACGTCGAACGTAGCTTACCTGCAACCAGGTGGACCAC	CCTTGCCAGGAGGCTTGCAGGTAAGCTA

		**COL1A2-7**	ACAGGTCCAAGTTTAGGCATGAACGTCGAACGTAGCTTACCTGCAAACCAGGGTTACCA	GCAGGACCTGCAGCTTGCAGGTAAGCTA
		**COL1A2-8**	CTCTGAGACCAGTTAGGCATGAACGTCGAACGTAGCTTACCTGCAACTACCAACGTCAC	CATCTCTGCCAGGCTTGCAGGTAAGCTA
		**COL1A2-9**	TCCATCAGGACCTTAGGCATGAACGTCGAACGTAGCTTACCTGCAAGAGCACCATTGTT	TCCAGGAGGTCCCATTGCAGGTAAGCTA
		**COL1A2-10**	ACCACGAGAACCATAGGCATGAACGTCGAACGTAGCTTACCTGCAACAGCTCCAGGGAG	ACCAGCTCTGCCAATTGCAGGTAAGCTA
	PI16	**PI16-1**	GTCTTGGCCCAGAGGCACTTGAGCTAGAAAGGCACTTGAGCTAGAAGCCAATCCTCTCT	AAATGGGCACCACTTTCTAGCTCAAGTG
		**PI16-2**	TGGCAAACAGGTTGGCACTTGAGCTAGAAAGGCACTTGAGCTAGAACCCTTATCCGTGA	GTGGCACGTCCAGCTTCTAGCTCAAGTG
		**PI16-3**	GATGCCAGGCCTTGGCACTTGAGCTAGAAAGGCACTTGAGCTAGAATCAACCCTGACTC	CCTAGGGCTTGAGATTCTAGCTCAAGTG
		**PI16-4**	CTGGTAGGGCCTCGGCACTTGAGCTAGAAAGGCACTTGAGCTAGAAATGGAGTCCCCTC	AGGACATTGGGAGGTTCTAGCTCAAGTG
		**PI16-5**	ACCAAGTAAGGCAGGCACTTGAGCTAGAAAGGCACTTGAGCTAGAATGGGGCCTCATAT	GTTGTCAGGGAAGTTTCTAGCTCAAGTG
		**PI16-6**	TCCTCCATGGCCAGGCACTTGAGCTAGAAAGGCACTTGAGCTAGAACTCGTGGTACCAC	TTGTAGTGCTCCCTTTCTAGCTCAAGTG
		**PI16-7**	TGCCCTGGAGCTTGGCACTTGAGCTAGAAAGGCACTTGAGCTAGAATTGGCCTCCTCAA	CCAGCAGATGGATCTTCTAGCTCAAGTG
		**PI16-8**	TCCCAGGAAGAGGGGCACTTGAGCTAGAAAGGCACTTGAGCTAGAAGCAGTAGCAGCAG	CAACACCAGGGCACTTCTAGCTCAAGTG
		**PI16-9**	GAGCCTGAGGCTTGGCACTTGAGCTAGAAAGGCACTTGAGCTAGAAGCCCTTTTCCCTA	AGGGATGAAGTGGATTCTAGCTCAAGTG
		**PI16-10**	AAAGAAAGGGGCCGGCACTTGAGCTAGAAAGGCACTTGAGCTAGAATGTGAGTTGCCAA	CAAGGAGAGCAGGATTCTAGCTCAAGTG
	C1QB	**C1QB-1**	GAGAAGGGCCTTCGGAGACTGATTACACGAGGAGACTGATTACACGTCCCCAGACAAAC	AGCGGAAGTTCCAACGTGTAATCAGTCT
		**C1QB-2**	GAAGCAGGTTCAGGGAGACTGATTACACGAGGAGACTGATTACACGGCCTGGGAGACAT	TGCAGAAGACCTGCCGTGTAATCAGTCT
		**C1QB-3**	TCTCATTGGCGTTGGAGACTGATTACACGAGGAGACTGATTACACGCGAGACTCGTAGT	TGAACTTGCCACTACGTGTAATCAGTCT
		**C1QB-4**	GAGAGCTGGCATGGGAGACTGATTACACGAGGAGACTGATTACACGCACAGGTTCCCTC	GCATGAGGTTCACCCGTGTAATCAGTCT
		**C1QB-5**	GTTCTGGACCTGGGGAGACTGATTACACGAGGAGACTGATTACACGTGACCTGGAAGGT	AATGCTGCCCGTGCCGTGTAATCAGTCT
		**C1QB-6**	CTCCCTTCTCTCCGGAGACTGATTACACGAGGAGACTGATTACACGGGAATCCCTGGAT	TTCCTGGAATCCCTCGTGTAATCAGTCT
		**C1QB-7**	CTAGCTTGAGCACGGAGACTGATTACACGAGGAGACTGATTACACGGTCTCCTCCACCT	CCTGCAGGAAGACCCGTGTAATCAGTCT
		**C1QB-8**	CCGGTATCCCTGGGGAGACTGATTACACGAGGAGACTGATTACACGATGCCATCAGGGC	CCGGGGTCCCAGGACGTGTAATCAGTCT
		**C1QB-9**	GCAACCCTGGAAAGGAGACTGATTACACGAGGAGACTGATTACACGCTTACTTGCCCTG	CCTCATTGTGGTCACGTGTAATCAGTCT
		**C1QB-10**	CTCTTCTCGGTGGGGAGACTGATTACACGAGGAGACTGATTACACGGCCCAGCAGGGAG	TTGGCGCCGTCGAACGTGTAATCAGTCT
	C1QC	**C1QC-1**	TCCCAGGGATAGCCCTCGATTAACATCGCACCTCGATTAACATCGCTTGGGTCCACGTG	CTCCCTTCTCACCGGCGATGTTAATCGA
		**C1QC-2**	AGCAGCAGTAGGTCCTCGATTAACATCGCACCTCGATTAACATCGCCAGTGCCAGTAGA	TGGCCTTCAAGTGCGCGATGTTAATCGA
		**C1QC-3**	TCCCATGGGACCACCTCGATTAACATCGCACCTCGATTAACATCGCGAATCCCAGGGGT	TCCGGGAGTCCCTCGCGATGTTAATCGA
		**C1QC-4**	CAGCACACCGCCCCCTCGATTAACATCGCACCTCGATTAACATCGCCCTGCAGCCGCAA	CACCTGCTCGCCCAGCGATGTTAATCGA
		**C1QC-5**	GAACTTGCCAGTGCCTCGATTAACATCGCACCTCGATTAACATCGCGGACTTTGCAGGT	GTAGTAGAGGCCGCGCGATGTTAATCGA
		**C1QC-6**	GTCTGCCGTGTGACCTCGATTAACATCGCACCTCGATTAACATCGCTGGGTACTGGGCC	AAGCTGTTGGCCGGGCGATGTTAATCGA
		**C1QC-7**	ATCTTCCCTCCTCCCTCGATTAACATCGCACCTCGATTAACATCGCTGCTTCTGCTTAT	AGAACACTGACTGCGCGATGTTAATCGA
		**C1QC-8**	ATCTTAAGTTGTCCCTCGATTAACATCGCACCTCGATTAACATCGCGGATCCCATGGC	GGCCCGGTATCCCTGCGATGTTAATCGA

		**C1QC-9**	GTTGAACTTGACCCCTCGATTAACATCGCACCTCGATTAACATCGCTGGTGATGACGTT	ATCCCCCTGCGGGAGCGATGTTAATCGA
		**C1QC-10**	GATCACCTTGAAGCCTCGATTAACATCGCACCTCGATTAACATCGCGGTCACAGAAGGT	GCTATTAGACATGAGCGATGTTAATCGA
	ITGA2	**ITGA2-1**	GTGAACCAACCAGGGCACTTGAGCTAGAAAATGCTGCTGTAGTACTAAGCCACTCCATG	TTCGGTTCTCAGGTAGTACTACAGCAGC
		**ITGA2-2**	TCGGATCCCAAGAGGCACTTGAGCTAGAAAATGCTGCTGTAGTACTCCTAAAGGCTCCA	TACTGGAGACGGCAAGTACTACAGCAGC
		**ITGA2-3**	CAGCAGCTGCTGAGGCACTTGAGCTAGAAAATGCTGCTGTAGTACTCCTGGTCGTCCAC	TGACCTTGGTAGCTAGTACTACAGCAGC
		**ITGA2-4**	CTGCTGTGCCCACGGCACTTGAGCTAGAAAATGCTGCTGTAGTACTACTGGCTTCCACA	CCCGGTTGTGTAAAAGTACTACAGCAGC
		**ITGA2-5**	AAGCTCCTACTGCGGCACTTGAGCTAGAAAATGCTGCTGTAGTACTCCACTCCAGTCAT	GCTGAACAACAGTGAGTACTACAGCAGC
		**ITGA2-6**	GAAAGTCGACTGCGGCACTTGAGCTAGAAAATGCTGCTGTAGTACTGCTGTACTGTCTG	TGCAGCTGCTGTCTAGTACTACAGCAGC
		**ITGA2-7**	TGCTCACAGATGCGGCACTTGAGCTAGAAAATGCTGCTGTAGTACTTGAGGGATGTGGA	TGCCCTTGGTGTAAAGTACTACAGCAGC
		**ITGA2-8**	TCCACACCCTCAGGGCACTTGAGCTAGAAAATGCTGCTGTAGTACTAAACCGCGCATTT	GCAATCGCAGAGCGAGTACTACAGCAGC
		**ITGA2-9**	TGTCAAGCCAAGGGGCACTTGAGCTAGAAAATGCTGCTGTAGTACTCGTTCCTTGTCAG	TCCTCCCGTTCCCCAGTACTACAGCAGC
		**ITGA2-10**	ATCAGCGGGTTCTGGCACTTGAGCTAGAAAATGCTGCTGTAGTACTCCCAGTCACGTAC	TGGTCTGTGTGCAGAGTACTACAGCAGC
	IL12RB2	**IL12RB2-1**	CTCTTGCACACATGGCACTTGAGCTAGAAAGGAGACTGATTACACGCGTCACATCCCCT	TGGGAAGGCTGCAGCGTGTAATCAGTCT
		**IL12RB2-2**	ATGCTTGGTGCTAGGCACTTGAGCTAGAAAGGAGACTGATTACACGGACAGCAATGCAA	ACGCCCACTGTGAACGTGTAATCAGTCT
		**IL12RB2-3**	TGTCCGAGCCCTCGGCACTTGAGCTAGAAAGGAGACTGATTACACGTTCACCAGGAGGT	CCGGAGACGTCCAGCGTGTAATCAGTCT
		**IL12RB2-4**	ACGGAGGCATTTAGGCACTTGAGCTAGAAAGGAGACTGATTACACGGGTACATGTGCTC	TCTCTCCATTGCACCGTGTAATCAGTCT
		**IL12RB2-5**	ACTCTGGGCTGCAGGCACTTGAGCTAGAAAGGAGACTGATTACACGCAGGACGTATGTC	AGAGCTGTCATCCTCGTGTAATCAGTCT
		**IL12RB2-6**	TCGTCCCATGAAAGGCACTTGAGCTAGAAAGGAGACTGATTACACGCTGGGCTGGAATT	CAGCCCATTTGCTGCGTGTAATCAGTCT
		**IL12RB2-7**	GTCCAGGAGTTGCGGCACTTGAGCTAGAAAGGAGACTGATTACACGGGGTATGGTCCAG	CAGATGCCAGTTCACGTGTAATCAGTCT
		**IL12RB2-8**	CACTCCACCACATGGCACTTGAGCTAGAAAGGAGACTGATTACACGATGGAGCTCCCTC	ATGCCACCCCTCACCGTGTAATCAGTCT
		**IL12RB2-9**	TTGGGATGATGGGGGCACTTGAGCTAGAAAGGAGACTGATTACACGCTTTCCTGGCCAG	TGGAGCCTTTGTCGCGTGTAATCAGTCT
		**IL12RB2-10**	ACCACGCTGAAAGGGCACTTGAGCTAGAAAGGAGACTGATTACACGAACTGAGGGAGTG	ACCTGTGACTTGACCGTGTAATCAGTCT
		**IL12RB2-11**	CTTCCTCAGAGGCGGCACTTGAGCTAGAAAGGAGACTGATTACACGCTGGACCCTGTGT	GTGGACTTGAGGCTCGTGTAATCAGTCT
		**IL12RB2-12**	GTACCCAGGGGAAGGCACTTGAGCTAGAAAGGAGACTGATTACACGGACGAACAGGGTG	CAGGCCAGTTTGCTCGTGTAATCAGTCT
	IL7R	**IL7R-1**	CTGCGTCGTCAAAATGCTGCTGTAGTACTAGGAGACTGATTACACGTAGTCGTCCGGCT	AGCACGAGAAGGACCGTGTAATCAGTCT
		**IL7R-2**	AGTCCTCGACCTGATGCTGCTGTAGTACTAGGAGACTGATTACACGCTCTGCCTCTCAG	TACCCGCTCCGAGGCGTGTAATCAGTCT
		**IL7R-3**	TCCAGCTGACTGTATGCTGCTGTAGTACTAGGAGACTGATTACACGAGGTCCATCCACT	GTCAGCAAGTGCTCCGTGTAATCAGTCT
		**IL7R-4**	TGCCGTCCACTTTATGCTGCTGTAGTACTAGGAGACTGATTACACGTCCCTCGCTTGAA	AGCTTTCCGCTTCTCGTGTAATCAGTCT
		**IL7R-5**	ACGGTGACTCTCCATGCTGCTGTAGTACTAGGAGACTGATTACACGGCCAGGCATCTGA	CACTGACATTCCCTCGTGTAATCAGTCT
		**IL7R-6**	CTGGAGGAGGGGGATGCTGCTGTAGTACTAGGAGACTGATTACACGGCAGTTTGGGGAC	TTGCTACCTTCCCACGTGTAATCAGTCT
		**IL7R-7**	TGGTTCCAGGGCCATGCTGCTGTAGTACTAGGAGACTGATTACACGAGGGTGCTGTCTG	GAAACGGAGGGGCGCGTGTAATCAGTCT
		**IL7R-8**	AACGGCAGGATTCATGCTGCTGTAGTACTAGGAGACTGATTACACGGCCAGAGGGGTTC	ATGGGCTGTCCCTCCGTGTAATCAGTCT

		**IL7R-9**	GGATTGAAACTCAATGCTGCTGTAGTACTAGGAGACTGATTACACGCAGGAAACTTTCA	TGAATCTGACAGTGCGTGTAATCAGTCT
		**IL7R-10**	TAGAAAGCCATACATGCTGCTGTAGTACTAGGAGACTGATTACACGAGCTTGAAGTAAA	CTTTCTCCAGAAAGCGTGTAATCAGTCT
	LAMC1	**LAMC1-1**	TCCTGAGCAGCCTGGAGACTGATTACACGACCTCGATTAACATCGCATTGATCTCGGCT	TTGGCCTTTCTGGGGCGATGTTAATCGA
		**LAMC1-2**	CTCTGGCTAGGAGGGAGACTGATTACACGACCTCGATTAACATCGCTTGGCAGCATCAG	CTTCCGCAAGAGCGGCGATGTTAATCGA
		**LAMC1-3**	CATTGGTGGAACCGGAGACTGATTACACGACCTCGATTAACATCGCACGTCACACTGTC	ACTGGCCAGTGTGCGCGATGTTAATCGA
		**LAMC1-4**	TGCTTTACGGTCCGGAGACTGATTACACGACCTCGATTAACATCGCGCAGCTGCTCTGT	CCGGTCACAGGGTAGCGATGTTAATCGA
		**LAMC1-5**	TTGAGGCGCACATGGAGACTGATTACACGACCTCGATTAACATCGCGCTGGTGTGGAAC	AAGCTCTCTGGGCCGCGATGTTAATCGA
		**LAMC1-6**	CTGCAGCCGTCTTGGAGACTGATTACACGACCTCGATTAACATCGCCTTAGGGCCTCCT	TGGCAGGGATTCTGGCGATGTTAATCGA
		**LAMC1-7**	ATGCTTCAGAGCCGGAGACTGATTACACGACCTCGATTAACATCGCGACCACTCGAGTG	CCTGCCTCTCAGACGCGATGTTAATCGA
		**LAMC1-8**	CTGGCTTACAGCTGGAGACTGATTACACGACCTCGATTAACATCGCTCCCCCATCACTC	AACGGTCACACTTCGCGATGTTAATCGA
		**LAMC1-9**	GTGCAACCCCTAGGGAGACTGATTACACGACCTCGATTAACATCGCGCAGAAGCAGGGT	GAGGAATGCCCAATGCGATGTTAATCGA
		**LAMC1-10**	TCCAAGGTTTGCAGGAGACTGATTACACGACCTCGATTAACATCGCTCTCATCCCCAGT	TTGGTCCGTCACCTGCGATGTTAATCGA
		**LAMC1-11**	TCACAGCGACCATGGAGACTGATTACACGACCTCGATTAACATCGCGCCTTCTCTGCAT	CGGTTCCCCACAATGCGATGTTAATCGA
		**LAMC1-12**	AGGGCCATTCCTAGGAGACTGATTACACGACCTCGATTAACATCGCGGCAGAGCCTCAC	GCACTGGCACAGGGGCGATGTTAATCGA
	ANGPTL4	**ANGPTL4-1**	ACAGTCCAGCCTCGTGCTTGAACTGGTCTAGTGCTTGAACTGGTCTGCGCCTCTGAATT	ACCGAGCCATCCTCAGACCAGTTCAAGC
		**ANGPTL4-2**	CAGGTGCCAAACCGTGCTTGAACTGGTCTAGTGCTTGAACTGGTCTGTTGGAGTGGCTG	TACTGGCCATTGACAGACCAGTTCAAGC
		**ANGPTL4-3**	TGCAGCTGCACAGGTGCTTGAACTGGTCTAGTGCTTGAACTGGTCTGCCCTCCCAGTCT	AGGGACTCGGCATAAGACCAGTTCAAGC
		**ANGPTL4-4**	GACGGGGAACTGCGTGCTTGAACTGGTCTAGTGCTTGAACTGGTCTCACCACCCAGGTG	GTAGGCAGTGTCCAAGACCAGTTCAAGC
		**ANGPTL4-5**	CTAGCCACAGGTGGTGCTTGAACTGGTCTAGTGCTTGAACTGGTCTGGCACCCAGTTTG	GGTGTGTCGATGGAAGACCAGTTCAAGC
		**ANGPTL4-6**	AGGGCAGGGAGAGGTGCTTGAACTGGTCTAGTGCTTGAACTGGTCTTCCCAAGTGGAGA	GGTCGTGGTCCTGTAGACCAGTTCAAGC
		**ANGPTL4-7**	CGGTGAGAGGAGGGTGCTTGAACTGGTCTAGTGCTTGAACTGGTCTACCAGATTCCCCG	CGCTTTGGGAAGGCAGACCAGTTCAAGC
		**ANGPTL4-8**	GCCCAGGTGCATGGTGCTTGAACTGGTCTAGTGCTTGAACTGGTCTTGGCCACCCCATG	TCTCCTGGCAGCCAAGACCAGTTCAAGC
		**ANGPTL4-9**	GGCGGCCCCGCACGTGCTTGAACTGGTCTAGTGCTTGAACTGGTCTCCGGGTCTTTGCA	CACGGCGGACCCCAAGACCAGTTCAAGC
		**ANGPTL4-10**	GTTCCGGGCTGCCGTGCTTGAACTGGTCTAGTGCTTGAACTGGTCTGGCGCCTCAGGAG	AGGACGCGAAGCGGAGACCAGTTCAAGC
	TLR4	**TLR4-1**	GAGAAAGGCCATGGATCTCGAGAACGTCAAGATCTCGAGAACGTCAATCTCAGGCAGGA	GTCCCAGCTCTCACTGACGTTCTCGAGA
		**TLR4-2**	CAGTTCCTCCAGGGATCTCGAGAACGTCAAGATCTCGAGAACGTCAGGGTCAAATTGCA	CCGGAACTGTTCATTGACGTTCTCGAGA
		**TLR4-3**	ATGCCACGGAAGAGATCTCGAGAACGTCAAGATCTCGAGAACGTCACAAGCCAGCAAAA	GTTTGGAGGCTGAGTGACGTTCTCGAGA
		**TLR4-4**	ACCTGTTCCAGTTGATCTCGAGAACGTCAAGATCTCGAGAACGTCATGCCCTCTGGGAT	GGGAGGGAATGAATTGACGTTCTCGAGA
		**TLR4-5**	ACGCACAAGAAAAGATCTCGAGAACGTCAAGATCTCGAGAACGTCAGTCTGGTGTTCAC	CCCACTGCAAGAATTGACGTTCTCGAGA
		**TLR4-6**	CTGCTTCTGGTCCGATCTCGAGAACGTCAAGATCTCGAGAACGTCACTCCCACAAGGAG	CACCATTTGCTCACTGACGTTCTCGAGA
		**TLR4-7**	TAATGACGGCCTCGATCTCGAGAACGTCAAGATCTCGAGAACGTCAAGAACTGACGCAC	CCAGGAGGAAGGTCTGACGTTCTCGAGA
		**TLR4-8**	AACAAAGGCGTCAGATCTCGAGAACGTCAAGATCTCGAGAACGTCAGGCTGGAGTAGAT	CCAGTCTTCGTCCATGACGTTCTCGAGA

		**TLR4-9**	ATGTTAGCAGCGAGATCTCGAGAACGTCAAGATCTCGAGAACGTCAACCTTCCTGGATG	CGACTTTTGTGGATTGACGTTCTCGAGA
		**TLR4-10**	AAGATGTGCCGCCGATCTCGAGAACGTCAAGATCTCGAGAACGTCAGAGTCGTCTCCAG	AGCAAGGCTTTCTATGACGTTCTCGAGA
	CD226	**CD226-1**	GTGAGCGTGACATGTGCTTGAACTGGTCTACTGACTAACGACACCATTGAGGCTGACAG	AACTGCCACTTCAATGGTGTCGTTAGTC
		**CD226-2**	ATGAGGCTGGATCGTGCTTGAACTGGTCTACTGACTAACGACACCAGGAGGTCGATCTG	GTTGCAGCTAGTTTTGGTGTCGTTAGTC
		**CD226-3**	AGCCCTGAGTCAGGTGCTTGAACTGGTCTACTGACTAACGACACCAACAACAGCGGTAG	GTGCTGGCTTTGATTGGTGTCGTTAGTC
		**CD226-4**	CCTCCAACCATGAGTGCTTGAACTGGTCTACTGACTAACGACACCACAACAAAGCTGCT	AGAACCAACAGCATTGGTGTCGTTAGTC
		**CD226-5**	CACACCGTAAGTAGTGCTTGAACTGGTCTACTGACTAACGACACCAGCTTTCTCACAAT	CCTGTCAGCATAGCTGGTGTCGTTAGTC
		**CD226-6**	TTCCCTGGGACAAGTGCTTGAACTGGTCTACTGACTAACGACACCAGAAGTGTAACTAC	GTCTTGGGTACTTATGGTGTCGTTAGTC
		**CD226-7**	AAGTATGAAGAGAGTGCTTGAACTGGTCTACTGACTAACGACACCAGGTCCGTGTGGGA	TCATCTTTTTCCACTGGTGTCGTTAGTC
		**CD226-8**	GTTAAATTTGAAAGTGCTTGAACTGGTCTACTGACTAACGACACCAATTAGAGGCATTG	GATAGGCTCATATGTGGTGTCGTTAGTC
		**CD226-9**	AGAGGCATTATGAGTGCTTGAACTGGTCTACTGACTAACGACACCACAATATCTGCCTC	GCAGGAATAGAAGGTGGTGTCGTTAGTC
		**CD226-10**	GTTTCAATTTCGCGTGCTTGAACTGGTCTACTGACTAACGACACCATCTCATCACGAAA	TCAGTTACAGTCACTGGTGTCGTTAGTC
		**CD226-11**	CAGCTACCTCAAAGTGCTTGAACTGGTCTACTGACTAACGACACCACTAGTTGATGGCA	CTGAGACTGTCTGCTGGTGTCGTTAGTC
		**CD226-12**	CAGCAAGTTTAACGTGCTTGAACTGGTCTACTGACTAACGACACCAAGGGTCATATTCT	GATACACACATTCATGGTGTCGTTAGTC
		**CD226-13**	CTGTGTCATCTGAGTGCTTGAACTGGTCTACTGACTAACGACACCATAAACATCCTCCC	TTGGGTAGTTGACTTGGTGTCGTTAGTC
		**CD226-14**	GTGTTCATTGATGGTGCTTGAACTGGTCTACTGACTAACGACACCACATTTGGGTTAAA	ATCTTGAACCACTGTGGTGTCGTTAGTC
		**CD226-15**	TTCTGTCTCCTTCGTGCTTGAACTGGTCTACTGACTAACGACACCAAAATAGGCCTCTT	TCCCAGTACTCTTATGGTGTCGTTAGTC
	COL6A3	**COL6A3-1**	ACACCCCGTCTCTGTGCTTGAACTGGTCTAGATCTCGAGAACGTCACCAGGACTTCCAG	TTTTTCCGGGCTCATGACGTTCTCGAGA
		**COL6A3-2**	AGAAACACCACGTGTGCTTGAACTGGTCTAGATCTCGAGAACGTCAGGAACCGTCAATC	CTCCTGACGCCCTGTGACGTTCTCGAGA
		**COL6A3-3**	TGCCCGGAGCATTGTGCTTGAACTGGTCTAGATCTCGAGAACGTCATCGGTCACCCCTC	CTGCCAATGGGCCGTGACGTTCTCGAGA
		**COL6A3-4**	ACTCCAGCTGCATGTGCTTGAACTGGTCTAGATCTCGAGAACGTCAAACCCTCGCCCAA	GCCGGTTGTACGTCTGACGTTCTCGAGA
		**COL6A3-5**	GAAGCCCTTCTGAGTGCTTGAACTGGTCTAGATCTCGAGAACGTCACCACTTTGGACTC	GTTCAGGATGGTGATGACGTTCTCGAGA
		**COL6A3-6**	TGCTTGGAAGTTGGTGCTTGAACTGGTCTAGATCTCGAGAACGTCATGAAGTCACGGAT	GATGACCTTGGCATTGACGTTCTCGAGA
		**COL6A3-7**	TGTCCACGTCGCTGTGCTTGAACTGGTCTAGATCTCGAGAACGTCAAAAGCCATGTCGA	AGCTGTCCACGATCTGACGTTCTCGAGA
		**COL6A3-8**	TCCGGATCACAAAGTGCTTGAACTGGTCTAGATCTCGAGAACGTCAAGTCTACGCACAA	TGGGGCCGATGTTCTGACGTTCTCGAGA
		**COL6A3-9**	TCCACCAGCCTCTGTGCTTGAACTGGTCTAGATCTCGAGAACGTCACACATCCAGGGAG	CTCGTGTCGAAGCGTGACGTTCTCGAGA
		**COL6A3-10**	ACCGTGTCCACAAGTGCTTGAACTGGTCTAGATCTCGAGAACGTCAGCCCCCTTCGTAA	ACCTGGATGGAGTGTGACGTTCTCGAGA
		**COL6A3-11**	TGTCTCCTTGTCCGTGCTTGAACTGGTCTAGATCTCGAGAACGTCATCCCCACTCGCTG	TCTGACGCATGCTATGACGTTCTCGAGA
		**COL6A3-12**	GTCCTGTCCGATAGTGCTTGAACTGGTCTAGATCTCGAGAACGTCACCACCTGGATGAG	ATACTGAGCCACGCTGACGTTCTCGAGA
	COL6A2	**COL6A2-1**	TGACGTCGCACTCACTTGGATCCACTCGTAGATCTCGAGAACGTCACTCACGTAGGTCA	ATCCACAGGTCTCGTGACGTTCTCGAGA
		**COL6A2-2**	TCACCTCTGCCCCACTTGGATCCACTCGTAGATCTCGAGAACGTCATTTGGAGCCAAAG	GTCCCCGGTCTTCGTGACGTTCTCGAGA
		**COL6A2-3**	AGGGGGCAGGAAAACTTGGATCCACTCGTAGATCTCGAGAACGTCACCTGAGGGAGAAG	GCGCCCTAACTTGGTGACGTTCTCGAGA

		**COL6A2-4**	GTGGCTGTACTGCACTTGGATCCACTCGTAGATCTCGAGAACGTCACGAAGGTGCCCTC	CAGCTGGATGGCTATGACGTTCTCGAGA
		**COL6A2-5**	GACCACGTTGATGACTTGGATCCACTCGTAGATCTCGAGAACGTCACCCCCAGCCTGTT	GTCCTTGGCGATGCTGACGTTCTCGAGA
		**COL6A2-6**	ACGAAGGCCAGCTACTTGGATCCACTCGTAGATCTCGAGAACGTCAGTCCGTGAGGAAC	TTGCCCGTGATGCGTGACGTTCTCGAGA
		**COL6A2-7**	TCGGTGAAGGTGCACTTGGATCCACTCGTAGATCTCGAGAACGTCAGGACAGCATGCAG	TCCTGGGTCATGTATGACGTTCTCGAGA
		**COL6A2-8**	ATGTCCCCGATGCACTTGGATCCACTCGTAGATCTCGAGAACGTCACTTCTCGTGGAAC	TTCTCGCTCTCGTCTGACGTTCTCGAGA
		**COL6A2-9**	TTCGCACCTGCTGACTTGGATCCACTCGTAGATCTCGAGAACGTCAAAGAGCGTCATGT	CCACCAGGTCGGACTGACGTTCTCGAGA
		**COL6A2-10**	GCCCGCTCGGCCTACTTGGATCCACTCGTAGATCTCGAGAACGTCAGCCCTCCTCCCGG	GCGAAGAGCCGGAATGACGTTCTCGAGA
		**COL6A2-11**	ATGAACTTCTCCGACTTGGATCCACTCGTAGATCTCGAGAACGTCACTCCATGTCGTCG	GGGCACAGGACGTGTGACGTTCTCGAGA
		**COL6A2-12**	AGTCGATGCGCTCACTTGGATCCACTCGTAGATCTCGAGAACGTCAAAGCTGGACAGCG	TGACGGCCTCCTTCTGACGTTCTCGAGA
	COL4A2	**COL4A2-1**	ACGCCTCTTGCTCAGACGTAATGACAGCGATCCAGTACGTCGACTACGGGAATCCAGAG	ATTCCATCGGCCCGTAGTCGACGTACTG
		**COL4A2-2**	CAAAGCAGCCTCGAGACGTAATGACAGCGATCCAGTACGTCGACTATGCGGATGGGGAG	GTCCCACGTCACCGTAGTCGACGTACTG
		**COL4A2-3**	CAGGCCTCTGCTTAGACGTAATGACAGCGATCCAGTACGTCGACTACTTCGTAGCCGTC	GTATCCGTCAGGGGTAGTCGACGTACTG
		**COL4A2-4**	GAGAACCTCTGGTAGACGTAATGACAGCGATCCAGTACGTCGACTACCGTGCATCCCAG	GCATGCCTTGGAACTAGTCGACGTACTG
		**COL4A2-5**	TTACAGTCGCCGGAGACGTAATGACAGCGATCCAGTACGTCGACTAATCCTCTGCGCAT	CCGACGAATTGGTGTAGTCGACGTACTG
		**COL4A2-6**	TCCCTGGGGATCCAGACGTAATGACAGCGATCCAGTACGTCGACTATAAAGGCCTGCTC	CTCCGATCTCACCCTAGTCGACGTACTG
		**COL4A2-7**	CAGGAAGGGCATGAGACGTAATGACAGCGATCCAGTACGTCGACTACGGGGTTACAGTA	GTGGCAGATGTCGGTAGTCGACGTACTG
		**COL4A2-8**	TGCCTGGAAAGCCAGACGTAATGACAGCGATCCAGTACGTCGACTAGAAGGGGGGGTGA	ACCCAGAGACGTTCTAGTCGACGTACTG
		**COL4A2-9**	CACCCCTGTCCCCAGACGTAATGACAGCGATCCAGTACGTCGACTAGCCTCTCCTGGGT	CTGGAAGGGTGTTCTAGTCGACGTACTG
		**COL4A2-10**	CAAGGCCCTTCGTAGACGTAATGACAGCGATCCAGTACGTCGACTACCCCGATCTCCTG	CCAGTCCTGGGGGCTAGTCGACGTACTG
		**COL4A2-11**	AGGCCCTTCATGCAGACGTAATGACAGCGATCCAGTACGTCGACTATCTGTCCCCGGAG	AGGCCAGGGTCACGTAGTCGACGTACTG
		**COL4A2-12**	CTGGATCTCCCGGAGACGTAATGACAGCGATCCAGTACGTCGACTAAATCCCTCGCGAC	CTGGGGGTCCTGGCTAGTCGACGTACTG
	FN1	**FN1-1**	TGAAGGAGCCAGAAGACGTAATGACAGCGAGCAATTAGCGATGGCAGTTCGGACATCTG	GCTGGTAAATGGCATGCCATCGCTAATT
		**FN1-2**	TTCCAGGAACTCGAGACGTAATGACAGCGAGCAATTAGCGATGGCAGCACTAGCAGAAG	GACCCGTCAAGGTCTGCCATCGCTAATT
		**FN1-3**	TGTTGGTGGAGACAGACGTAATGACAGCGAGCAATTAGCGATGGCACCAGGTGCAAGTT	GTCAGGGTTTGCCATGCCATCGCTAATT
		**FN1-4**	TTGGATGGTGCATAGACGTAATGACAGCGAGCAATTAGCGATGGCACAGGAAACGCAAG	TTGGGTGTGGTGGGTGCCATCGCTAATT
		**FN1-5**	CAACGGTTTGGAGAGACGTAATGACAGCGAGCAATTAGCGATGGCAGGTGGCTTCCTTC	AAGTGGCCTGGAAATGCCATCGCTAATT
		**FN1-6**	TGCTGACCACATAAGACGTAATGACAGCGAGCAATTAGCGATGGCATTAACAGCGACGA	TTTCCTCTCTGCCTTGCCATCGCTAATT
		**FN1-7**	GTCCTTGGGAGGGAGACGTAATGACAGCGAGCAATTAGCGATGGCACCACAAACTGCAG	CACGTCCGTCACTATGCCATCGCTAATT
		**FN1-8**	TTCCACCTCGAGTAGACGTAATGACAGCGAGCAATTAGCGATGGCAGCACCATTGGAAT	GAAAGTGGCACAATTGCCATCGCTAATT
		**FN1-9**	AGTAGGGTCAGGAAGACGTAATGACAGCGAGCAATTAGCGATGGCACAACTTGGTCCAC	AATCGAGGTGTCAATGCCATCGCTAATT
		**FN1-10**	TGGAAGGGTTACCAGACGTAATGACAGCGAGCAATTAGCGATGGCAGAAGATTGGGGTG	GATCTCTGGTCCAATGCCATCGCTAATT
		**FN1-11**	CACTTGCATCTGGAGACGTAATGACAGCGAGCAATTAGCGATGGCACCTGGACATCAGT	ACTGATGCTGTTGATGCCATCGCTAATT

		**FN1-12**	CATGACTCCTTCGAGACGTAATGACAGCGAGCAATTAGCGATGGCACTCCGATGCGATA	TTTGTCCCACTGAATGCCATCGCTAATT
	ISG15	**ISG15-1**	GTCAGCCAGAACTTATGTGACCGGGTACAATATGTGACCGGGTACACTTCCCCTCGAAA	TCATCCTCCATGGCTGTACCCGGTCACA
		**ISG15-2**	CTCACCAGGATGCTATGTGACCGGGTACAATATGTGACCGGGTACAACCCTTGTCGTTC	TAGGCGTTGCTGCCTGTACCCGGTCACA
		**ISG15-3**	TGCAGTCCCTCAATATGTGACCGGGTACAATATGTGACCGGGTACATCCGATGCCATCA	TCTGCTGCTTCAGCTGTACCCGGTCACA
		**ISG15-4**	ACCTTCAGTTCCCTATGTGACCGGGTACAATATGTGACCGGGTACATCCCAGCATCTTC	AGGATCTCCTTGCGTGTACCCGGTCACA
		**ISG15-5**	ACACCTGCTGCTTTATGTGACCGGGTACAATATGTGACCGGGTACACCCTCCTGCTGAC	GGTCAGCTTGCACCTGTACCCGGTCACA
		**ISG15-6**	AAGCACATTGCCGTATGTGACCGGGTACAATATGTGACCGGGTACAGCACCCCATCCTG	CTGGTTGACCAGGCTGTACCCGGTCACA
		**ISG15-7**	TCAACAGGACTGTTATGTGACCGGGTACAATATGTGACCGGGTACAAAGCTTTGCACCA	TCAGTGGGTCTCTCTGTACCCGGTCACA
		**ISG15-8**	TCATACTCCCCCATATGTGACCGGGTACAATATGTGACCGGGTACACATAGGCTTGAGG	ATATACACGGTGCTTGTACCCGGTCACA
		**ISG15-9**	CCGATCTCCCGGGTATGTGACCGGGTACAATATGTGACCGGGTACAGAAGGCGGGCACA	GCCAGACGCTGCTCTGTACCCGGTCACA
		**ISG15-10**	CGCAGGCGCAGATTATGTGACCGGGTACAATATGTGACCGGGTACATGTCCCGCCCCCC	TGCTCCCCTGGCCGTGTACCCGGTCACA
	CD79B	**CD79B-1**	CAGCACCGTCTCATACTAGCTCGATTCAAATACTAGCTCGATTCAATTGTTTCGGCCTC	ATCCCGGAGTTCCATTGAATCGAGCTAG
		**CD79B-2**	CTGCCAGATCCGGTACTAGCTCGATTCAAATACTAGCTCGATTCAACGAAACGTGGGTG	CCGTTTCCTGGCCTTTGAATCGAGCTAG
		**CD79B-3**	CAGTTGCTGAGCCTACTAGCTCGATTCAAATACTAGCTCGATTCAACAGCAGCACCAAC	GAGAGGAGCAGCACTTGAATCGAGCTAG
		**CD79B-4**	TCAGCGTGTTCCGTACTAGCTCGATTCAAATACTAGCTCGATTCAAATGATGCCATCCT	GGGTCTGGATCATCTTGAATCGAGCTAG
		**CD79B-5**	GTCCGCAGTGTCATACTAGCTCGATTCAAATACTAGCTCGATTCAACTTCACCTCCCCT	TCACCCACCGACCTTTGAATCGAGCTAG
		**CD79B-6**	CTTACGATGCCGGTACTAGCTCGATTCAAATACTAGCTCGATTCAACTTCCGGAGCCAG	AAGTCCGCCTCCTCTTGAATCGAGCTAG
		**CD79B-7**	TATCTTGGGCTCCTACTAGCTCGATTCAAATACTAGCTCGATTCAACTTCGGGCGGAAA	GATGATGCGGCCCATTGAATCGAGCTAG
		**CD79B-8**	GAGTGGCCTCGGATACTAGCTCGATTCAAATACTAGCTCGATTCAACCCTGGATGATGA	CCTGAAACTGGACTTTGAATCGAGCTAG
		**CD79B-9**	TAGACACCGTTGTTACTAGCTCGATTCAAATACTAGCTCGATTCAACTGCTTGCAGTAA	TCCTTGGAGCACTGTTGAATCGAGCTAG
		**CD79B-10**	TGTCCTCAGGGGCTACTAGCTCGATTCAAATACTAGCTCGATTCAACACAGCCCTGCTC	CCGAAGCTCAGTGGTTGAATCGAGCTAG
	SLPI	**SLPI-1**	AGGCCACTGGACTACCATGCTGGATCTAGATACTAGCTCGATTCAACACGAAGGGCAAG	CCCAGGGCAAGAACTTGAATCGAGCTAG
		**SLPI-2**	TTCCCAGGCTTCAACCATGCTGGATCTAGATACTAGCTCGATTCAAGACCACTGGACAC	ATACACTGGCCATTTTGAATCGAGCTAG
		**SLPI-3**	GTGATTGGGGGGGACCATGCTGGATCTAGATACTAGCTCGATTCAATGTCTGTCTTGCA	ACCCAGGCACTGGGTTGAATCGAGCTAG
		**SLPI-4**	TGCCAGTCACTTGACCATGCTGGATCTAGATACTAGCTCGATTCAACTTGTCTGGACAC	CGGCAACATTTCTATTGAATCGAGCTAG
		**SLPI-5**	CTCTTGCAGCATTACCATGCTGGATCTAGATACTAGCTCGATTCAATTTCCCGCACATG	GGGGTGAGGCAGAGTTGAATCGAGCTAG
		**SLPI-6**	GATTCCGCAAGTAACCATGCTGGATCTAGATACTAGCTCGATTCAAGGTTCAGGCATTT	CGTGATAGCAACACTTGAATCGAGCTAG
		**SLPI-7**	TGGACAATTTTTCACCATGCTGGATCTAGATACTAGCTCGATTCAAATATCTAAGGCAC	CACTTGGGTTTCTGTTGAATCGAGCTAG
	CCL21	**CCL21-1**	CAGTGACTGAGCCACCATGCTGGATCTAGATAGGCATGAACGTCGAGGATGCGCAGAAC	CAGGACCAGGACATTCGACGTTCATGCC
		**CCL21-2**	TAGCTCTGGCTGAACCATGCTGGATCTAGATAGGCATGAACGTCGAGGGGGTCTGCACA	CACCCAGGCCTTTATCGACGTTCATGCC
		**CCL21-3**	CAACGTGAGTAGGACCATGCTGGATCTAGATAGGCATGAACGTCGACGGTAGCTGCGTA	TTGGCTCCTGCTTGTCGACGTTCATGCC
		**CCL21-4**	TGGCGCATCAGTTACCATGCTGGATCTAGATAGGCATGAACGTCGATGGCTTGTCCAGA	GTCCGTGGGACTGCTCGACGTTCATGCC

		**CCL21-5**	TTTGGAGGCCCTCACCATGCTGGATCTAGATAGGCATGAACGTCGACCTTCTTGCCAGA	TTTGGAGCCCTTCGTCGACGTTCATGCC
		**CCL21-6**	CAGTCCTGGGCTCACCATGCTGGATCTAGATAGGCATGAACGTCGAGTATTTGAGGCAA	ATCTTCCTTAGGCATCGACGTTCATGCC
	SAMD12	**SAMD12-1**	AAACCGCAGTGGAACCATGCTGGATCTAGACGTAGCTTACCTGCAAACCCCGTGGGTTC	GCAGGGTGATCAAATTGCAGGTAAGCTA
		**SAMD12-2**	AGCCACTGGTTTTACCATGCTGGATCTAGACGTAGCTTACCTGCAAGCTGAGTCCACAG	CTTGCAGACATCCATTGCAGGTAAGCTA
		**SAMD12-3**	TCGCTCGAGCTTTACCATGCTGGATCTAGACGTAGCTTACCTGCAAGGGCAATCCCCAT	CCGGAGGTTCTCCATTGCAGGTAAGCTA
		**SAMD12-4**	GTAGGATGTGCTGACCATGCTGGATCTAGACGTAGCTTACCTGCAATGGAGCACCTGTT	CTCGCACCTTCAGGTTGCAGGTAAGCTA
		**SAMD12-5**	TTTGGTCAAGCACACCATGCTGGATCTAGACGTAGCTTACCTGCAATTGGGGGTTCCTT	CTGCCTGCAGTCGGTTGCAGGTAAGCTA
		**SAMD12-6**	GCTGTAGATCTGAACCATGCTGGATCTAGACGTAGCTTACCTGCAAGTTTGAACGACTC	AGTTATGTCATGCATTGCAGGTAAGCTA
		**SAMD12-7**	GCAGTTTAATACCACCATGCTGGATCTAGACGTAGCTTACCTGCAATCACCTTCAATTT	GAGATTCAATGCCATTGCAGGTAAGCTA
	KLRK1	**KLRK1-1**	AGCACCACGGTTTGGCACTTGAGCTAGAAACCTCGATTAACATCGCGCCGCGACTTAAA	CTGCGTTCTCTTCAGCGATGTTAATCGA
		**KLRK1-2**	TGCCAGGATCTGTGGCACTTGAGCTAGAAACCTCGATTAACATCGCACCATCTTCCCAC	GGTGAGAGGATGGTGCGATGTTAATCGA
		**KLRK1-3**	TGCATTTCAACCAGGCACTTGAGCTAGAAACCTCGATTAACATCGCACAGCTTCCATTC	GAGCCATAGACTGGGCGATGTTAATCGA
		**KLRK1-4**	CTCTGGAGAAAAAGGCACTTGAGCTAGAAACCTCGATTAACATCGCGCAATTCCCTTAT	CACATCACGATTAAGCGATGTTAATCGA
		**KLRK1-5**	CCAGTCCCATCTTGGCACTTGAGCTAGAAACCTCGATTAACATCGCCACCGATCTCGGA	TGCTGGAAGGAGCGGCGATGTTAATCGA
		**KLRK1-6**	CAGTTTTTAGGACGGCACTTGAGCTAGAAACCTCGATTAACATCGCTCGATAGCATATC	TGGTAGCAGCTATAGCGATGTTAATCGA
		**KLRK1-7**	AATGATATGACTTGGCACTTGAGCTAGAAACCTCGATTAACATCGCACTAATCCCATCC	TTGTTGGAATTTGAGCGATGTTAATCGA
		**KLRK1-8**	CCGTATAACCTTTGGCACTTGAGCTAGAAACCTCGATTAACATCGCGTTAAGCAGTTTT	TGTATGTGTTCAGTGCGATGTTAATCGA
		**KLRK1-9**	TTGAGATCTACAAGGCACTTGAGCTAGAAACCTCGATTAACATCGCGAAGACTGGAATT	GCTGTATATCTTCTGCGATGTTAATCGA
	SDC2	**SDC2-1**	TACGTCCTCATCGACTTGGATCCACTCGTAACTTGGATCCACTCGTGCTCTGGACTCTC	TCGAGATGTGGTCTACGAGTGGATCCAA
		**SDC2-2**	GATCCAAACGCGCACTTGGATCCACTCGTAACTTGGATCCACTCGTCCAAGGTGAGCAG	GCAGGCCACCAAGGACGAGTGGATCCAA
		**SDC2-3**	CACTCTCGGAGCGACTTGGATCCACTCGTAACTTGGATCCACTCGTGCGTCGTGGTTTC	CTGCATCTTGTCCTACGAGTGGATCCAA
		**SDC2-4**	GATGGTTTGCGTTACTTGGATCCACTCGTAACTTGGATCCACTCGTGTAAGCAGCGCTG	GTCGGTGCCTTCTCACGAGTGGATCCAA
		**SDC2-5**	AAAGCCGATAACTACTTGGATCCACTCGTAACTTGGATCCACTCGTAAATGGCAAAGAG	CAGCAGAATAAGGTACGAGTGGATCCAA
		**SDC2-6**	GAAGCTTCTTCAAACTTGGATCCACTCGTAACTTGGATCCACTCGTAGGATACACTCCT	TCGTCATCATCAAAACGAGTGGATCCAA
		**SDC2-7**	TGCGATACACCAAACTTGGATCCACTCGTAACTTGGATCCACTCGTTCCTTCTTTCTCA	CATAACTTCCTTCTACGAGTGGATCCAA
		**SDC2-8**	AGTGCTTCTCCGTACTTGGATCCACTCGTAACTTGGATCCACTCGTAACAGATTGTCTG	CTTCTGTTCTTTTTACGAGTGGATCCAA
		**SDC2-9**	TCCTTATCAATTTACTTGGATCCACTCGTAACTTGGATCCACTCGTGAGGTGAACTTTC	CTTTCTGAGTCAGTACGAGTGGATCCAA
	COL6A1	**COL6A1-1**	CAGCACGAAGAGGCTGACTAACGACACCAAACTTGGATCCACTCGTTCTCAGAGCTGTC	CTGCAGGCCGATGGACGAGTGGATCCAA
	CD44	**CD44-1**	TGGTGCCACTTCTCTGACTAACGACACCAAGATCTCGAGAACGTCATCTCCATGAGCAC	CCCAGGTCCAGTTGTGACGTTCTCGAGA
		**CD44-2**	AACCCCCGGAAGTCTGACTAACGACACCAAGATCTCGAGAACGTCATGGAAGATGCTGT	TCGGAAGGTGGGTCTGACGTTCTCGAGA
	IFIT3	**IFIT3-1**	GTGTGTTCTTGCTTGATCTTCCGATCTAAACAGATAGGACTAGTCATTCTGCCTGGTCA	ACCAGGCTTCTGAATGACTAGTCCTATC
		**IFIT3-2**	ATGGCCAGTCCAGTGATCTTCCGATCTAAACAGATAGGACTAGTCAAAAGTACATCGCG	GGTTTGCCATTCACTGACTAGTCCTATC

		**IFIT3-3**	ACCAACTCTTCCCTGATCTTCCGATCTAAACAGATAGGACTAGTCACAGGGCCTCTACA	CGAGGAGCTTTTTGTGACTAGTCCTATC
		**IFIT3-4**	CTAGGGAGCTCCTTGATCTTCCGATCTAAACAGATAGGACTAGTCATTGCGCCTCAGTG	CGCTGATGAAGTTCTGACTAGTCCTATC
		**IFIT3-5**	GGGCTGTTCCTTATGATCTTCCGATCTAAACAGATAGGACTAGTCAGCCTATGCCTGAA	GGCTGGAAAATGCATGACTAGTCCTATC
		**IFIT3-6**	CAGCTCAGATGCTTGATCTTCCGATCTAAACAGATAGGACTAGTCACAGTGCCTTCTTC	CTGGTCCATTTCCATGACTAGTCCTATC
		**IFIT3-7**	AGAGCCATCTGCATGATCTTCCGATCTAAACAGATAGGACTAGTCAGCTCCCTGAGAGT	CCCAGGGTCAGGATTGACTAGTCCTATC
	C1QA	**C1QA-1**	CTGAAGACGCTGTATGCTGCTGTAGTACTAATGCTGCTGTAGTACTGATGAGGAAGCCG	CAGGTGGAGGGGATAGTACTACAGCAGC
		**C1QA-2**	ATGACCACGTTGCATGCTGCTGTAGTACTAATGCTGCTGTAGTACTGACCTCATCGAAG	TCCTGGTTGGTGAAAGTACTACAGCAGC
		**C1QA-3**	CCTTCAACCCTGGATGCTGCTGTAGTACTAATGCTGCTGTAGTACTTTGCCCTTGATGC	TGATGTTTCCCGGCAGTACTACAGCAGC
		**C1QA-4**	CCTTGCTGTTGAAATGCTGCTGTAGTACTAATGCTGCTGTAGTACTACCTGGAAGAGTC	TGCCCCCAGACACGAGTACTACAGCAGC
		**C1QA-5**	TCCTTTAAGGCCGATGCTGCTGTAGTACTAATGCTGCTGTAGTACTACTCCCCCTGGTC	TCCAGGGGACCCACAGTACTACAGCAGC
		**C1QA-6**	GGCTCTTGTACGCATGCTGCTGTAGTACTAATGCTGCTGTAGTACTAACTGGCCCAGCT	GCACATCGCAGATCAGTACTACAGCAGC
		**C1QA-7**	ATGATCACCAGCCATGCTGCTGTAGTACTAATGCTGCTGTAGTACTCAGCACACTGATC	GCCAGGGATACGGGAGTACTACAGCAGC
		**C1QA-8**	TCTCCCTTGGGGCATGCTGCTGTAGTACTAATGCTGCTGTAGTACTCGGCTCTCCTCGT	AAGGCAGGGGCTCGAGTACTACAGCAGC
		**C1QA-9**	ACCTGGAAGGTGAATGCTGCTGTAGTACTAATGCTGCTGTAGTACTCCACTTGGACACC	TACAGGCAGAGGTGAGTACTACAGCAGC
	COL4A1	**COL4A1-1**	CCCATGTCCACTTATGCTGCTGTAGTACTACCTCGATTAACATCGCGCCCTTCATGCTG	TGGTCTCCCTTCTCGCGATGTTAATCGA
		**COL4A1-2**	CTCCTTTTGCTCCATGCTGCTGTAGTACTACCTCGATTAACATCGCGCCTGTCCTTTCT	TGCCAGGTAGGCCGGCGATGTTAATCGA
		**COL4A1-3**	TGGAACTCCTGGAATGCTGCTGTAGTACTACCTCGATTAACATCGCGACCTTGAAACCC	AGGGAGGCCCTTCAGCGATGTTAATCGA
		**COL4A1-4**	ATCGCCCTTGAAGATGCTGCTGTAGTACTACCTCGATTAACATCGCCCACATCTCCCTT	CTTGCCGGGGAGAGGCGATGTTAATCGA
		**COL4A1-5**	GACCCTGGGATCCATGCTGCTGTAGTACTACCTCGATTAACATCGCCTTCTCTCCTGCC	AGACCTGGTTCACGGCGATGTTAATCGA
		**COL4A1-6**	CTGGCAACCCTGGATGCTGCTGTAGTACTACCTCGATTAACATCGCTCCCCCTTCATGC	GTATTTCACCGGGCGCGATGTTAATCGA
		**COL4A1-7**	GGCCAGTCTGTCCATGCTGCTGTAGTACTACCTCGATTAACATCGCTTGTCACCGGTTG	CTCCCCGGGATCCAGCGATGTTAATCGA
		**COL4A1-8**	GTCACCAATGGGAATGCTGCTGTAGTACTACCTCGATTAACATCGCCCGCCTGTCCTTT	GTTGCCCGGGAAGGGCGATGTTAATCGA
		**COL4A1-9**	TTTCGGTCCTTGGATGCTGCTGTAGTACTACCTCGATTAACATCGCCACCCTTGTCACC	ACTGACCCCTTGAAGCGATGTTAATCGA
		**COL4A1-10**	GTCGCCTTTTTCAATGCTGCTGTAGTACTACCTCGATTAACATCGCCTGGGAAGCCGTG	GGGTCCCGAGGAGGGCGATGTTAATCGA
		**COL4A1-11**	GCACCGTCAAACCATGCTGCTGTAGTACTACCTCGATTAACATCGCTTTCTGGCCAGGG	GGTCCCGTCTCTCGGCGATGTTAATCGA
		**COL4A1-12**	TTCACTCCGTGGCATGCTGCTGTAGTACTACCTCGATTAACATCGCTCCCTTCTGTCCC	GGGAGGCCTCTTTGGCGATGTTAATCGA
	NECTIN2	**NECTIN2-1**	TCGTGCTCCACTCGTGCTTGAACTGGTCTAACTTGGATCCACTCGTCTCCTTGAAGCTC	GGCAGCAGGACAGCACGAGTGGATCCAA
		**NECTIN2-2**	TCCTCCACCGTCAGTGCTTGAACTGGTCTAACTTGGATCCACTCGTGTAATTGCCCTCG	GCAAACTCGCAGGAACGAGTGGATCCAA
		**NECTIN2-3**	TCCTCACCCTCAGGTGCTTGAACTGGTCTAACTTGGATCCACTCGTCTCTTCCTCCTCC	CTCCTCCTCCAGGCACGAGTGGATCCAA
		**NECTIN2-4**	CGTGTTGAACAGCGTGCTTGAACTGGTCTAACTTGGATCCACTCGTTGCAGATGAAGGT	GGCGTTGGTGACCCACGAGTGGATCCAA
		**NECTIN2-5**	TGGTGACGGTGACGTGCTTGAACTGGTCTAACTTGGATCCACTCGTAAGGTGAAGCGGC	GGCCCAAGGGTACGACGAGTGGATCCAA
		**NECTIN2-6**	ACATCTTGGGCTCGTGCTTGAACTGGTCTAACTTGGATCCACTCGTCACCCGAACTCGC	CGCACCTCAGCTGGACGAGTGGATCCAA

		**NECTIN2-7**	ACAGCCGCTCCTTGTGCTTGAACTGGTCTAACTTGGATCCACTCGTGGTTTGACGAAGG	CAGTGCTCTGCCCCACGAGTGGATCCAA
		**NECTIN2-8**	GGAAGATGGCCACGTGCTTGAACTGGTCTAACTTGGATCCACTCGTCCGTACTGAGGGT	TAGGGAAGCTGGGTACGAGTGGATCCAA
		**NECTIN2-9**	CGTGGTTCTGGGGGTGCTTGAACTGGTCTAACTTGGATCCACTCGTTCCTGGGTCTCGG	GGCTGAGGGTGACGACGAGTGGATCCAA
		**NECTIN2-10**	TCAGAGTGGCCTCGTGCTTGAACTGGTCTAACTTGGATCCACTCGTCGAACGTCACAGT	GCTCTGGTTTGCTCACGAGTGGATCCAA
	SDC4	**SDC4-1**	CCAGAGCCAGACCCTGACTAACGACACCAACTGACTAACGACACCAACCCTCCAGATCT	TGCTTGTCCTCTGCTGGTGTCGTTAGTC
		**SDC4-2**	GGGGTGTGTCACTCTGACTAACGACACCAACTGACTAACGACACCACTAGAGGCACCAA	AGGGATGTGGTTGATGGTGTCGTTAGTC
		**SDC4-3**	GCTGACAGAACCTCTGACTAACGACACCAACTGACTAACGACACCAGCCCACAATCAGA	ATGCCCACGATGCGTGGTGTCGTTAGTC
		**SDC4-4**	AAGTATCGGCCTTCTGACTAACGACACCAACTGACTAACGACACCATAGGTCTCCGGAG	TCCTCGTCATCCGCTGGTGTCGTTAGTC
		**SDC4-5**	CTCGGATCGACTCCTGACTAACGACACCAACTGACTAACGACACCAATGACCTCGGTCT	GGTCCTGGGGGTTCTGGTGTCGTTAGTC
		**SDC4-6**	CGAGCAGGCGAGGCTGACTAACGACACCAACTGACTAACGACACCAAGCAGCAGCAGCG	AGGCCCCCACGAGCTGGTGTCGTTAGTC
**Duck**	***HSPA2***	**HSPA2-1**	AGACGCCCACACATGATCTTCCGATCTAAACAGATAGGACTAGTCATTGCCATGCTGGA	CGATGATCTCCACATGACTAGTCCTATC
		**HSPA2-2**	TACTCCACCTGCATGATCTTCCGATCTAAACAGATAGGACTAGTCACATCTCGCCCTTG	GGGAAGAAGGTCTATGACTAGTCCTATC
		**HSPA2-3**	TGCGCCCCAGATATGATCTTCCGATCTAAACAGATAGGACTAGTCAGCGTTCTGCACCT	GCACCGTGATGACTTGACTAGTCCTATC
		**HSPA2-4**	ATACGCATCACGTTGATCTTCCGATCTAAACAGATAGGACTAGTCAGGGCTCGTTGATA	ATCGCAGCTGCTGATGACTAGTCCTATC
		**HSPA2-5**	CTTGTCCAAGCCGTGATCTTCCGATCTAAACAGATAGGACTAGTCACCCGAGTACCTTT	GTTCTTCTCGCCACTGACTAGTCCTATC
		**HSPA2-6**	TGTGCTTACGCTTTGATCTTCCGATCTAAACAGATAGGACTAGTCAGCAATGTCACGCT	CACGCTTATTGCCTTGACTAGTCCTATC
		**HSPA2-7**	ATGCTAGCTTGCGTGATCTTCCGATCTAAACAGATAGGACTAGTCAGGAGTCGATCTCA	ATGCCCTCGTACACTGACTAGTCCTATC
		**HSPA2-8**	CTAGCTTGGCATCTGATCTTCCGATCTAAACAGATAGGACTAGTCAATCTGGCCCTTGT	GCACGATCTCCTGCTGACTAGTCCTATC
		**HSPA2-9**	TAGCCGCTTGCACTGATCTTCCGATCTAAACAGATAGGACTAGTCATCTCCCATGAGGA	CGTTCTCGGACTTCTGACTAGTCCTATC
		**HSPA2-10**	ACCTGGACCAGGATGATCTTCCGATCTAAACAGATAGGACTAGTCACTCACCCTCGTAC	TTGGTCATGGCCCATGACTAGTCCTATC
	RSAD2	**RSAD2-1**	CTCCTTGCAGAACGTCTATTAGTGGAGCCAGTCTATTAGTGGAGCCGCAGCTTCAGCTC	GATGCTGACACTCCGGCTCCACTAATAG
		**RSAD2-2**	ATGAGCAGGCACTGTCTATTAGTGGAGCCAGTCTATTAGTGGAGCCGTTCTCCCCTTCA	GCATCATCACCACAGGCTCCACTAATAG
		**RSAD2-3**	AAGCCGCACTTATGTCTATTAGTGGAGCCAGTCTATTAGTGGAGCCGGTGTGGAAGCAG	AAGGAGGTCTTGGGGGCTCCACTAATAG
		**RSAD2-4**	GCGCCCAGATGCAGTCTATTAGTGGAGCCAGTCTATTAGTGGAGCCCAGGTGGAGCAGC	GACAGCGCCAGCGCGGCTCCACTAATAG
		**RSAD2-5**	GTCCTCGGAGCGCGTCTATTAGTGGAGCCAGTCTATTAGTGGAGCCAGGGCGCCCAGTC	CCAGGCTCCAGCAGGGCTCCACTAATAG
		**RSAD2-6**	GGGGCAGGTGCAGGTCTATTAGTGGAGCCAGTCTATTAGTGGAGCCCCTGGAAGGGGCA	GTCCCCGTCGCCACGGCTCCACTAATAG
		**RSAD2-7**	GGGGCTGAGGGGCGTCTATTAGTGGAGCCAGTCTATTAGTGGAGCCGTGGGGGGCCGGT	TGCTCCCGCTGCCCGGCTCCACTAATAG
		**RSAD2-8**	GCTTGGCCTCCTCGTCTATTAGTGGAGCCAGTCTATTAGTGGAGCCATCTCCAGCCCCC	CCGCCTCCTTGAGGGGCTCCACTAATAG
		**RSAD2-9**	GATTTTCTCCATTGTCTATTAGTGGAGCCAGTCTATTAGTGGAGCCCTCCTGAGAAATT	AAGAAACGGTTCTGGGCTCCACTAATAG
		**RSAD2-10**	CTGCCATTGCTAAGTCTATTAGTGGAGCCAGTCTATTAGTGGAGCCCTCTCTAATCAGG	TTCTTGAACCACCCGGCTCCACTAATAG
	DDX60	**DDX60-1**	TTTCGCCACCAAGATAACGATCCCTCACAAATAACGATCCCTCACAACCTCTGCTCCAA	GAAGATGCTCCCATTGTGAGGGATCGTT
		**DDX60-2**	CTACGCGCCGAATATAACGATCCCTCACAAATAACGATCCCTCACAGCAAGAACACAGA	ACCCCACTGCACATTGTGAGGGATCGTT

		**DDX60-3**	TCGCAGCCAACAGATAACGATCCCTCACAAATAACGATCCCTCACATTGTCATCAGCCT	CACTGGCATCTGCATGTGAGGGATCGTT
		**DDX60-4**	TCCATCTGGAAGCATAACGATCCCTCACAAATAACGATCCCTCACACGCACACCACCAA	TCGTGTGAAGACTGTGTGAGGGATCGTT
		**DDX60-5**	TCTGCCCAAACAGATAACGATCCCTCACAAATAACGATCCCTCACAGCCGGAAGATACT	ATGTCATTGAGCGCTGTGAGGGATCGTT
		**DDX60-6**	TTGGTGTTGTCCAATAACGATCCCTCACAAATAACGATCCCTCACAAGCAGATATGCGT	GCAAAGGGTGAGAATGTGAGGGATCGTT
		**DDX60-7**	TTATCTTCTGCAGATAACGATCCCTCACAAATAACGATCCCTCACAAACCTTCTTGAAA	GTGGTATCACAAAGTGTGAGGGATCGTT
		**DDX60-8**	ATGCTTTCTGATTATAACGATCCCTCACAAATAACGATCCCTCACATCAGAAATTCAAG	TAGCTTCAAAATCATGTGAGGGATCGTT
		**DDX60-9**	ACAAACTGACATAATAACGATCCCTCACAAATAACGATCCCTCACAACGAAGTCTTTTA	AGAATTCAGACTCTTGTGAGGGATCGTT
		**DDX60-10**	TCTTTGAGTGAAGATAACGATCCCTCACAAATAACGATCCCTCACATATATCTTGCTCC	GAAGAAAACAACATTGTGAGGGATCGTT
	ZNFX1	**ZNFX1-1**	AAAAGCTGCCCAGTACCCGAGATAGAGTCATACCCGAGATAGAGTCCTTGCGCAAACAG	TTGGCTGGCATGACGACTCTATCTCGGG
		**ZNFX1-2**	AATGAGCTGAACGTACCCGAGATAGAGTCATACCCGAGATAGAGTCGAAGCACCTGACA	GCTGCATGCTTTTGGACTCTATCTCGGG
		**ZNFX1-3**	GTACCATCCCGTGTACCCGAGATAGAGTCATACCCGAGATAGAGTCCTCCCCCCAACAC	CCTCACTGCTACTAGACTCTATCTCGGG
		**ZNFX1-4**	ATGCAGCACTCCATACCCGAGATAGAGTCATACCCGAGATAGAGTCCCAAGTGACGTTC	GGCAATGCATGCTAGACTCTATCTCGGG
		**ZNFX1-5**	TGGTGAGTGCAAGTACCCGAGATAGAGTCATACCCGAGATAGAGTCATAGCCAGCTCCT	GGGGTCCTTGGATTGACTCTATCTCGGG
		**ZNFX1-6**	CATTGAAGGCCACTACCCGAGATAGAGTCATACCCGAGATAGAGTCGTCCCAAGGTTTC	CTGGGACTCATCTTGACTCTATCTCGGG
		**ZNFX1-7**	GAGTTCTGCCATCTACCCGAGATAGAGTCATACCCGAGATAGAGTCTAGCAAACGTCTG	ACCAGGGATCCATTGACTCTATCTCGGG
		**ZNFX1-8**	CATGCCCGTCCACTACCCGAGATAGAGTCATACCCGAGATAGAGTCCCCGGCCTTCTCA	ATGTTCCAGGGTCGGACTCTATCTCGGG
		**ZNFX1-9**	CTTCTCTTGCAGGTACCCGAGATAGAGTCATACCCGAGATAGAGTCTGCCTTCACGTCT	ATCAGCCCTCAGTCGACTCTATCTCGGG
		**ZNFX1-10**	AATCCGTGTGTCATACCCGAGATAGAGTCATACCCGAGATAGAGTCAGAGTGGGGCAAT	ACCAGTTGGGGAGGGACTCTATCTCGGG
	DHX58	**DHX58-1**	ACGTCCTGCTGCTTATGTGACCGGGTACAAGTCTATTAGTGGAGCCGAACTGCCTGATC	AGGTCTCCATTGCCGGCTCCACTAATAG
		**DHX58-2**	ACCAACAGGGAGATATGTGACCGGGTACAAGTCTATTAGTGGAGCCGCACTCGTCAATC	TTGTGCGTGTGGTCGGCTCCACTAATAG
		**DHX58-3**	CTTGCTCAGCCGATATGTGACCGGGTACAAGTCTATTAGTGGAGCCGGATCTCCTCCAG	GAAGTTCTCCCGCTGGCTCCACTAATAG
		**DHX58-4**	CTTCAGCCGCTGGTATGTGACCGGGTACAAGTCTATTAGTGGAGCCGGGCCATGATCTT	GTGCTGCTGGATCAGGCTCCACTAATAG
		**DHX58-5**	TGGGGTCCTTCATTATGTGACCGGGTACAAGTCTATTAGTGGAGCCAGGAAGCGCTCAG	CAAAGGTGGTGGCGGGCTCCACTAATAG
		**DHX58-6**	TAAACGTCGTCCGTATGTGACCGGGTACAAGTCTATTAGTGGAGCCGACGCAGTAGAAG	GCCACGTTGCAGTAGGCTCCACTAATAG
		**DHX58-7**	TGCACTTGGGGATTATGTGACCGGGTACAAGTCTATTAGTGGAGCCCGCACCACGATGT	TCATCAGCCCGTACGGCTCCACTAATAG
		**DHX58-8**	CTCTGCAGGTTCTTATGTGACCGGGTACAAGTCTATTAGTGGAGCCCTGGGGCACTTGT	TACTGCTTCCTGGCGGCTCCACTAATAG
		**DHX58-9**	AGGCGTCGACCATTATGTGACCGGGTACAAGTCTATTAGTGGAGCCTGCAGGTGCTGGA	TGGCGTAGAATTGGGGCTCCACTAATAG
		**DHX58-10**	CAGGACGTGGAACTATGTGACCGGGTACAAGTCTATTAGTGGAGCCTGAAGGTGCCCTG	GATGGCCGTCACCGGGCTCCACTAATAG
	IFI30	**IFI30-1**	CTCCACCAGGAAGTATGTGACCGGGTACAAATAACGATCCCTCACATGGTGAAGAGCTG	CAGCAGCAGCCAGCTGTGAGGGATCGTT
		**IFI30-2**	ACTGCCACTTGCCTATGTGACCGGGTACAAATAACGATCCCTCACATGCTGGCACTGGA	ACTCCTCCGAGCCCTGTGAGGGATCGTT
		**IFI30-3**	GCTCTGGCAGCCCTATGTGACCGGGTACAAATAACGATCCCTCACATCGGTGCCTTGGC	GACCGCCCTGGGTATGTGAGGGATCGTT
		**IFI30-4**	GCAGCTCGTCCGTTATGTGACCGGGTACAAATAACGATCCCTCACATGCGCCTGCTCCT	TCAGCAGCGAGCGGTGTGAGGGATCGTT

		**IFI30-5**	CCAGCTGCGGGGCTATGTGACCGGGTACAAATAACGATCCCTCACAATGCGGCCCGCGT	GCACGCAGGCGGCCTGTGAGGGATCGTT
		**IFI30-6**	CCCCAGGTCCCCGTATGTGACCGGGTACAAATAACGATCCCTCACAGCATCAGCGCGGT	CTGGGCGTTGCGGATGTGAGGGATCGTT
		**IFI30-7**	GTCGAGCGCCTCGTATGTGACCGGGTACAAATAACGATCCCTCACAATTGGTGCGGCGG	GATCCAGGGGACGATGTGAGGGATCGTT
		**IFI30-8**	CTCGTGCATCAGGTATGTGACCGGGTACAAATAACGATCCCTCACATGAAGTTCTCGCC	AGGGAAGTAGGTGCTGTGAGGGATCGTT
		**IFI30-9**	GCAGCGGCTCTCCTATGTGACCGGGTACAAATAACGATCCCTCACAGCGAGAGGTTGGC	AGGAGCCGTGGGGATGTGAGGGATCGTT
		**IFI30-10**	ATGCGGGGGCCATTATGTGACCGGGTACAAATAACGATCCCTCACAGCCAGCAGGGCCA	CCGGGGCCAGCAGCTGTGAGGGATCGTT
	IFI35	**IFI35-1**	TCGTCTTGCTGAATATGTGACCGGGTACAATACCCGAGATAGAGTCCTGCCCCCATTCT	TGCTCTCCACCTCCGACTCTATCTCGGG
		**IFI35-2**	CTCCTCCTTTGCTTATGTGACCGGGTACAATACCCGAGATAGAGTCTCAGTCCGCTCTT	CTCCTTCTCCTGCAGACTCTATCTCGGG
		**IFI35-3**	TCACCCTCTGGGCTATGTGACCGGGTACAATACCCGAGATAGAGTCTCCTTCACCTCGA	GCTCCACCGTGTGGGACTCTATCTCGGG
		**IFI35-4**	AGCTGCTCAGCCATATGTGACCGGGTACAATACCCGAGATAGAGTCCCCTCTAGCGATG	GGCACCTGGACTTCGACTCTATCTCGGG
		**IFI35-5**	AGCATCAGCTTGTTATGTGACCGGGTACAATACCCGAGATAGAGTCGATCAGCGGGGTG	TGCAGAGGGTAGCCGACTCTATCTCGGG
		**IFI35-6**	TCGTCCAGGAACTTATGTGACCGGGTACAATACCCGAGATAGAGTCCACCTGGCCACAG	ACGAAGGTCAGCAGGACTCTATCTCGGG
		**IFI35-7**	CGGGAGGGCTGGATATGTGACCGGGTACAATACCCGAGATAGAGTCGGTCCTGGCGCAG	CCCGACAGCAGGAGGACTCTATCTCGGG
		**IFI35-8**	TCATGGGCTCCTCTATGTGACCGGGTACAATACCCGAGATAGAGTCTCCAGAGCATCCC	TCTGGAAGTGGATGGACTCTATCTCGGG
		**IFI35-9**	CCACGGCTCTCCTTATGTGACCGGGTACAATACCCGAGATAGAGTCCACCTCTCCCCCG	TAGCCCAAGGTGTGGACTCTATCTCGGG
		**IFI35-10**	AGGGCGATATTTTTATGTGACCGGGTACAATACCCGAGATAGAGTCTCTCCTCTCATGT	GGATGTTAGTGACGGACTCTATCTCGGG
	IFIH1	**IFIH1-1**	AGGAAACCAACCTGTCTATTAGTGGAGCCAATAACGATCCCTCACACCTGCACGAACTC	GCCGTACTCCAGCCTGTGAGGGATCGTT
		**IFIH1-2**	GTCGTGGTCAGCCGTCTATTAGTGGAGCCAATAACGATCCCTCACAATTGCACACAGAG	GAGAAGCTGCACCTTGTGAGGGATCGTT
		**IFIH1-3**	AGCCACTCGAAGCGTCTATTAGTGGAGCCAATAACGATCCCTCACATCAACAGCTCCTG	TTACGTCTTCACGATGTGAGGGATCGTT
		**IFIH1-4**	ACTCCAGGTGATGGTCTATTAGTGGAGCCAATAACGATCCCTCACAATTTGCACCTCCT	GCTTTTTGGTTGGTTGTGAGGGATCGTT
		**IFIH1-5**	GTCTCGGCACTCCGTCTATTAGTGGAGCCAATAACGATCCCTCACAAGAGGAAGCACTC	GCAGGAGATCATGATGTGAGGGATCGTT
		**IFIH1-6**	GATGGGCAGTTCTGTCTATTAGTGGAGCCAATAACGATCCCTCACAAACTGGGGAACCT	AGCTGCATAGTCATTGTGAGGGATCGTT
		**IFIH1-7**	TATTTCCCCAAGCGTCTATTAGTGGAGCCAATAACGATCCCTCACACGGTGAACCATCA	GCAGGTCAAGACCATGTGAGGGATCGTT
		**IFIH1-8**	ACCACAAAATTTCGTCTATTAGTGGAGCCAATAACGATCCCTCACAGTCTTCAAAAACA	TTTGTTGCTTTCTATGTGAGGGATCGTT
		**IFIH1-9**	TATGGTAAAGATGGTCTATTAGTGGAGCCAATAACGATCCCTCACATTATTTTCTCTTG	TATCTTGAAGTGTGTGTGAGGGATCGTT
		**IFIH1-10**	GGAAGCTCTCAATGTCTATTAGTGGAGCCAATAACGATCCCTCACAATACTCTGCAACT	TTTGTTTTTCCATATGTGAGGGATCGTT
	SAMHD1	**SAMHD1-1**	CTAGGTACCCCACGTCTATTAGTGGAGCCATACCCGAGATAGAGTCACCAGGCATCCTG	CCTTCAGTGCACGAGACTCTATCTCGGG
		**SAMHD1-2**	CAATCACATCACTGTCTATTAGTGGAGCCATACCCGAGATAGAGTCGTTAGATGAGGGG	TTTGTTTCATTGGTGACTCTATCTCGGG
		**SAMHD1-3**	CGAAGCTGTATTGGTCTATTAGTGGAGCCATACCCGAGATAGAGTCTGCAACTTGGTTC	AGAAAGCCTTTGCAGACTCTATCTCGGG
		**SAMHD1-4**	AAAAGCCTTGAAAGTCTATTAGTGGAGCCATACCCGAGATAGAGTCAAATATCCTGGGT	ATAAGCTGTTCTGGGACTCTATCTCGGG
		**SAMHD1-5**	TTGGATCCAGATTGTCTATTAGTGGAGCCATACCCGAGATAGAGTCGCTGAAACAATAT	AATACTGTTTTGCTGACTCTATCTCGGG
		**SAMHD1-6**	CCATAATCCATACGTCTATTAGTGGAGCCATACCCGAGATAGAGTCCTGATCTTTCATT	TTATCAATGGGATAGACTCTATCTCGGG

		**SAMHD1-7**	GGCCATCATATTCGTCTATTAGTGGAGCCATACCCGAGATAGAGTCATTTCATCTGCCA	GCTTGGAATTAGCCGACTCTATCTCGGG
		**SAMHD1-8**	GCTTGAGTGCAGAGTCTATTAGTGGAGCCATACCCGAGATAGAGTCTCAGTTCTGGACG	TTCTCGTGCCTCAAGACTCTATCTCGGG
		**SAMHD1-9**	AGTCTCTCCTAGAGTCTATTAGTGGAGCCATACCCGAGATAGAGTCCTTTTTCAGGTTG	TGGAATTTCATTTTGACTCTATCTCGGG
		**SAMHD1-10**	TCCGTGTGTGGAAGTCTATTAGTGGAGCCATACCCGAGATAGAGTCCGATGCAGGCAAT	GCTGGTATGCTCGAGACTCTATCTCGGG
	STAT1	**STAT1-1**	CTCTCTACCACGAATAACGATCCCTCACAATACCCGAGATAGAGTCCATGCAAGGCTGT	TGAGGGTGGGTTGCGACTCTATCTCGGG
		**STAT1-2**	TGTTGGCCGCATGATAACGATCCCTCACAATACCCGAGATAGAGTCGCAAAAGAGACGT	GGAACAGCACCGTTGACTCTATCTCGGG
		**STAT1-3**	ACGCTCCCCACTTATAACGATCCCTCACAATACCCGAGATAGAGTCCACGGTGTTCTGT	TCCAGCATCCCAAAGACTCTATCTCGGG
		**STAT1-4**	AGCAACAATGGTGATAACGATCCCTCACAATACCCGAGATAGAGTCGCTGCAGGCTTTC	CTGCTGCCGAACTAGACTCTATCTCGGG
		**STAT1-5**	ACATGCAGTCTGTATAACGATCCCTCACAATACCCGAGATAGAGTCGTGGGCCACCAAT	AAGGCAGGCATTGCGACTCTATCTCGGG
		**STAT1-6**	TCCATAGGCTCTGATAACGATCCCTCACAATACCCGAGATAGAGTCGGGACCGTCCAAA	TAGCCGTTTCCCTAGACTCTATCTCGGG
		**STAT1-7**	AGCTGGTGGACTTATAACGATCCCTCACAATACCCGAGATAGAGTCGCTGTCATCGTAC	ATCTCCATGGGGATGACTCTATCTCGGG
		**STAT1-8**	CCTGGAGGGGTGGATAACGATCCCTCACAATACCCGAGATAGAGTCCTGGAGACTGGAG	GGGCAGCAGATTTAGACTCTATCTCGGG
		**STAT1-9**	CCTGGAGGGGTGGATAACGATCCCTCACAATACCCGAGATAGAGTCCTGGAGACTGGAG	GGGCAGCAGATTTAGACTCTATCTCGGG
		**STAT1-10**	GTGTACGGTTCTAATAACGATCCCTCACAATACCCGAGATAGAGTCGAGCTCCTTCTTG	AAAGTAACAGCAGTGACTCTATCTCGGG
	NOS2	**NOS2-1**	TGCCCCTCACCAAACCATGCTGGATCTAGAACCATGCTGGATCTAGGGCCCATCTCTAG	CTGCCTGAGGAACACTAGATCCAGCATG
		**NOS2-2**	TCTTGCCCATTGAACCATGCTGGATCTAGAACCATGCTGGATCTAGGCGAAAGGCTTCT	CTGACTGCCCATGACTAGATCCAGCATG
		**NOS2-3**	ATCATGCTTCCCAACCATGCTGGATCTAGAACCATGCTGGATCTAGTCCAAACACGGAA	GATGAGCTGGCTGACTAGATCCAGCATG
		**NOS2-4**	TACCAGCCATTGAACCATGCTGGATCTAGAACCATGCTGGATCTAGCTCTGTCCCCATG	TCTCGCACTCCTAACTAGATCCAGCATG
		**NOS2-5**	TTTGGCAGCAAAGACCATGCTGGATCTAGAACCATGCTGGATCTAGTCCTCCAGGCCTG	ACATCTCGGAGCAACTAGATCCAGCATG
		**NOS2-6**	AAGGCCCTTGTCCACCATGCTGGATCTAGAACCATGCTGGATCTAGACTCCATGATGCA	GCCATGTGCTGCATCTAGATCCAGCATG
		**NOS2-7**	TGTTGGCTAGTGTACCATGCTGGATCTAGAACCATGCTGGATCTAGAAGCTGCACAGAT	AGGCACAGCTGAAGCTAGATCCAGCATG
		**NOS2-8**	GTCAATGGCATGAACCATGCTGGATCTAGAACCATGCTGGATCTAGGGGCCAGTTTTTG	TGAAGCCCCTAGTACTAGATCCAGCATG
		**NOS2-9**	CATGTTGGCAACAACCATGCTGGATCTAGAACCATGCTGGATCTAGCCACCTCAAGGAG	AAATTCCAGGCCTGCTAGATCCAGCATG
		**NOS2-10**	AGTAACGTTTTGTACCATGCTGGATCTAGAACCATGCTGGATCTAGGGTGATCCATGAT	TTCAGCAGCTGAGACTAGATCCAGCATG
	ACOD1	**ACOD1-1**	CTGCCTTGCACCTTACTAGCTCGATTCAAATACTAGCTCGATTCAAAAAGCGTAGCAAC	CTGGCTTCGTTGGTTTGAATCGAGCTAG
		**ACOD1-2**	AAGCCCCACTCCCTACTAGCTCGATTCAAATACTAGCTCGATTCAATGCTGGTCCCCAG	GTGGTAGACCTCACTTGAATCGAGCTAG
		**ACOD1-3**	TCCAAGTGGCCCCTACTAGCTCGATTCAAATACTAGCTCGATTCAAAGGCAGTCGGAAA	GCTGCATACAGAGCTTGAATCGAGCTAG
		**ACOD1-4**	TGCCACGTGTCATTACTAGCTCGATTCAAATACTAGCTCGATTCAAGTGTGTGGCTGGA	ACAGCCCCAGAGGCTTGAATCGAGCTAG
		**ACOD1-5**	TTTCTTCTGAGGATACTAGCTCGATTCAAATACTAGCTCGATTCAAGACCTGAGATTTT	TAAGAGCAGGTCATTTGAATCGAGCTAG
		**ACOD1-6**	TTCCTGTAACTGTTACTAGCTCGATTCAAATACTAGCTCGATTCAAACATTGGCAAAGT	TCAAACCATGAATGTTGAATCGAGCTAG
		**ACOD1-7**	ACTTGATCTGTCATACTAGCTCGATTCAAATACTAGCTCGATTCAAGCTTCTCTGAATG	AGAATCATTCGCTATTGAATCGAGCTAG
	WNT5B	**WNT5B-1**	AGCTCGCACCCATTAGGCATGAACGTCGAATAGGCATGAACGTCGATCCGCAGCACATC	TGGTCGTAGCCCCCTCGACGTTCATGCC

		**WNT5B-2**	CTTCTCCCGCTCCTAGGCATGAACGTCGAATAGGCATGAACGTCGACCCTCACGTAGTT	CTGCTCCTCCGAGGTCGACGTTCATGCC
		**WNT5B-3**	CACGTTATCCCCGTAGGCATGAACGTCGAATAGGCATGAACGTCGAGGTACCCGTACTC	CTCCTTGGCGAAGGTCGACGTTCATGCC
		**WNT5B-4**	ACCAGCTCCAGCTTAGGCATGAACGTCGAATAGGCATGAACGTCGAGAAGCGGTTGTTC	TGCGTAGGCATGTATCGACGTTCATGCC
		**WNT5B-5**	GTTGTCCACCGTGTAGGCATGAACGTCGAATAGGCATGAACGTCGACGAAGACGGAGGT	CTTCATGACCCGGGTCGACGTTCATGCC
		**WNT5B-6**	GTAGAGCTGGCACTAGGCATGAACGTCGAATAGGCATGAACGTCGACCATGTGCTCCTG	CTCGCCGATGAACTTCGACGTTCATGCC
		**WNT5B-7**	TCATGGCCAAAGATAGGCATGAACGTCGAATAGGCATGAACGTCGACTCTGGATGGGGT	TGTACATCTCGGGGTCGACGTTCATGCC
		**WNT5B-8**	ACTGGCACTCCTTTAGGCATGAACGTCGAATAGGCATGAACGTCGATGCCGAAACTGGT	AGTTCCACCTGCGGTCGACGTTCATGCC
		**WNT5B-9**	TTGAGGCTGCAGGTAGGCATGAACGTCGAATAGGCATGAACGTCGACAGCCAGCAGGTC	AAGTCGGCCAGCTCTCGACGTTCATGCC
		**WNT5B-10**	CGGGACGGGGCGCTAGGCATGAACGTCGAATAGGCATGAACGTCGACGGAGCCTGCCGC	TGGGGCAGCCCCCCTCGACGTTCATGCC
		**PAX5-1**	TGGCGAACGACATCGTAGCTTACCTGCAAACGTAGCTTACCTGCAACTCCACGATCCTT	CCTTGGTGGGCAACTTGCAGGTAAGCTA
		**PAX5-2**	GATGTGCCACACTCGTAGCTTACCTGCAAACGTAGCTTACCTGCAAATTCCACCCACAG	AAAGGCTCAGCCAGTTGCAGGTAAGCTA
		**PAX5-3**	TCTAACTGCTGCTCGTAGCTTACCTGCAAACGTAGCTTACCTGCAATCGATCCAACACT	TGCCTCTCGAAGAGTTGCAGGTAAGCTA
		**PAX5-4**	CTGGGTGACAGACCGTAGCTTACCTGCAAACGTAGCTTACCTGCAATCACAGAGGACAC	GGCGGAGTCTGTAGTTGCAGGTAAGCTA
	PAX5	**PAX5-5**	ATCAGCATGGAGCCGTAGCTTACCTGCAAACGTAGCTTACCTGCAACACGTACTGTCTC	GCCTGCGGTTGTAGTTGCAGGTAAGCTA
		**PAX5-6**	CTGCACCTTTGTTCGTAGCTTACCTGCAAACGTAGCTTACCTGCAAGATTGGGTGGCTG	GGCTGGGACTTGCATTGCAGGTAAGCTA
		**PAX5-7**	TGCCAGCTCACGACGTAGCTTACCTGCAAACGTAGCTTACCTGCAAGGAGGGTTGTGCT	TGGAGGGTATCCTCTTGCAGGTAAGCTA
		**PAX5-8**	GGGCTGCAGCGCTCGTAGCTTACCTGCAAACGTAGCTTACCTGCAAGGGGCGGCTCCAC	CAGCCGCTGCAGGATTGCAGGTAAGCTA
		**PAX5-9**	GAAAACTCACTCCCGTAGCTTACCTGCAAACGTAGCTTACCTGCAAGTATGGGCTCCCA	TATTGTGGATGGCATTGCAGGTAAGCTA
		**PAX5-10**	TTGTGCTTGCCAGCGTAGCTTACCTGCAAACGTAGCTTACCTGCAATATCCTGGGAGGG	GGACATGTGGAGGCTTGCAGGTAAGCTA
		**TMEM163-1**	TCGCTGATGACAAACCATGCTGGATCTAGATACTAGCTCGATTCAACGCCACAGAACTA	CTGCGTTGCTGTATTTGAATCGAGCTAG
		**TMEM163-2**	AAAACGGAGACGCACCATGCTGGATCTAGATACTAGCTCGATTCAAAAGGATGCCACTT	GCCAAAACAGTGCTTTGAATCGAGCTAG
		**TMEM163-3**	GGGGAACCATGTCACCATGCTGGATCTAGATACTAGCTCGATTCAAGTTTGCCTTACGC	TTTCGTAGTGGCGGTTGAATCGAGCTAG
		**TMEM163-4**	AGAGAGTTGAAACACCATGCTGGATCTAGATACTAGCTCGATTCAATATTCCACCTACA	ATGGAAAATCCCAATTGAATCGAGCTAG
		**TMEM163-5**	TAAACACTTCTGCACCATGCTGGATCTAGATACTAGCTCGATTCAAGAAGAATTGTGTT	CCAGAAACCAGACTTTGAATCGAGCTAG
	TMEM163	**TMEM163-6**	AAACTCCTATACTACCATGCTGGATCTAGATACTAGCTCGATTCAAGTTAGTCCAATTA	CATAGGCAAATATATTGAATCGAGCTAG
		**TMEM163-7**	CTTTCCCAGCATAACCATGCTGGATCTAGATACTAGCTCGATTCAATGCTTGTAAGGAC	AGTTATCAGCGCTGTTGAATCGAGCTAG
		**TMEM163-8**	GATAACACAAGCTACCATGCTGGATCTAGATACTAGCTCGATTCAAATATGACACCCAA	AGATGATAGGAGGTTTGAATCGAGCTAG
		**TMEM163-9**	TGGATGGCTTTGAACCATGCTGGATCTAGATACTAGCTCGATTCAACTTCGCTAGATCG	TCTGGAAGCACCTATTGAATCGAGCTAG
		**TMEM163-10**	CTCATTACGGAGAACCATGCTGGATCTAGATACTAGCTCGATTCAATGATGCACTGTAT	GCAAATCCAAATGTTTGAATCGAGCTAG
		**LY86-1**	GTCTTGCTGGGGAACCATGCTGGATCTAGATAGGCATGAACGTCGATGCTGAAAGCAAA	AGAACAACGGTCATTCGACGTTCATGCC
	LY86	**LY86-2**	CTGTGCTGGCACTACCATGCTGGATCTAGATAGGCATGAACGTCGATGCGTAGGCCATT	CTTTGCAGACTGTCTCGACGTTCATGCC
		**LY86-3**	GCATTTGATGTCTACCATGCTGGATCTAGATAGGCATGAACGTCGAGATGAGAACGAGA	GAAAGCAGGACTATTCGACGTTCATGCC

		**LY86-4**	AGGTGTGGGTTGCACCATGCTGGATCTAGATAGGCATGAACGTCGAGCTCTGACGTTAA	TTAGGACCATGGCTTCGACGTTCATGCC
		**LY86-5**	GTGTCTGAGTAGCACCATGCTGGATCTAGATAGGCATGAACGTCGAAGGCTCGCAAAGT	AGCTGAGAATGACGTCGACGTTCATGCC
		**LY86-6**	TGACGTACAGTTCACCATGCTGGATCTAGATAGGCATGAACGTCGAATCAGATCAGCCT	TCTTCCCACCTATTTCGACGTTCATGCC
		**LY86-7**	GACAGGTCCCTCAACCATGCTGGATCTAGATAGGCATGAACGTCGATGATCCCCAGTGT	CTGTGGGATTTCTATCGACGTTCATGCC
		**LY86-8**	CAACAGTGACATGACCATGCTGGATCTAGATAGGCATGAACGTCGAAAATCAGCACAAG	AATTTTTCACAGTTTCGACGTTCATGCC
	DNM3	**DNM3-1**	CAGCTATCCACCTACCATGCTGGATCTAGACGTAGCTTACCTGCAACAGAGATGCCTTC	ACACCGGCTCGTACTTGCAGGTAAGCTA
		**DNM3-2**	TTGGAGGAGGAGAACCATGCTGGATCTAGACGTAGCTTACCTGCAATGAGTTGAGGGAC	ACGTAGGCCTCCTGTTGCAGGTAAGCTA
		**DNM3-3**	ATCGCCAATGATCACCATGCTGGATCTAGACGTAGCTTACCTGCAATGCTAGTGCTGAT	AGGGGTTGACACGCTTGCAGGTAAGCTA
		**DNM3-4**	ATGGGGGCAATGGACCATGCTGGATCTAGACGTAGCTTACCTGCAACCACCGCTTGGAA	CTCCATGGGCTTCCTTGCAGGTAAGCTA
		**DNM3-5**	GTGCCTGTATGCTACCATGCTGGATCTAGACGTAGCTTACCTGCAATCCGATCTGCCAT	ATACGGTGTTCCCTTTGCAGGTAAGCTA
		**DNM3-6**	TTCCCGGCAAGTCACCATGCTGGATCTAGACGTAGCTTACCTGCAAGGCACTTTTGTGA	GCTGATCCCCTACATTGCAGGTAAGCTA
		**DNM3-7**	CTGGGGGTCCATTACCATGCTGGATCTAGACGTAGCTTACCTGCAACCTGCCTCTCCAG	TCGAATCGTCTCCTTTGCAGGTAAGCTA
		**DNM3-8**	CTGAAGGCAGGCAACCATGCTGGATCTAGACGTAGCTTACCTGCAACTGGAGTTTGCTC	GAAAGCAGCTGACATTGCAGGTAAGCTA
		**DNM3-9**	GAGCCATCGGTATACCATGCTGGATCTAGACGTAGCTTACCTGCAAGTCTGGACGGGAG	AAGAGTGGAGGACATTGCAGGTAAGCTA
		**DNM3-10**	TAAGCACGGTCCTACCATGCTGGATCTAGACGTAGCTTACCTGCAAGATCCACGCTTTT	CTCTTGCATCACCTTTGCAGGTAAGCTA
	RHAG	**RHAG-1**	AGGCTGCTATGAGTACTAGCTCGATTCAAATAGGCATGAACGTCGACACTGAAGACCGA	GCATCAGGGTGCCGTCGACGTTCATGCC
		**RHAG-2**	TCCTCCTGCCAGGTACTAGCTCGATTCAAATAGGCATGAACGTCGATACCCACTGCTAC	CAGATCTGCACAGCTCGACGTTCATGCC
		**RHAG-3**	CTCCCAGTACTCCTACTAGCTCGATTCAAATAGGCATGAACGTCGAATGCCTGCAATAC	TCGCGGTTACTACTTCGACGTTCATGCC
		**RHAG-4**	AGGGCAAACGTTATACTAGCTCGATTCAAATAGGCATGAACGTCGACACCAGGCTGGAG	TTGCCTGTTCGATGTCGACGTTCATGCC
		**RHAG-5**	TGCAGGGCACTTTTACTAGCTCGATTCAAATAGGCATGAACGTCGACATGCCAGGGGTA	AGGGCAGCAGCTTCTCGACGTTCATGCC
		**RHAG-6**	GAAGGGCAGCTTTTACTAGCTCGATTCAAATAGGCATGAACGTCGAATGCTTGTCCCAG	GCAGTTCTTGTCACTCGACGTTCATGCC
		**RHAG-7**	TCTTCTTCTGGAATACTAGCTCGATTCAAATAGGCATGAACGTCGATACATGCAGTTTC	TTGTTGGAGTGACGTCGACGTTCATGCC
		**RHAG-8**	GATGCCAAAACAGTACTAGCTCGATTCAAATAGGCATGAACGTCGAAATGTTCAGCTTG	CCACAAGTATCATCTCGACGTTCATGCC
		**RHAG-9**	CCCAATGCACATATACTAGCTCGATTCAAATAGGCATGAACGTCGATTCCAGCAATTGC	GAGGACAGAGATGTTCGACGTTCATGCC
		**RHAG-10**	TGCATTTATCATGTACTAGCTCGATTCAAATAGGCATGAACGTCGACTGTACTGAAGTC	AATCAAAGCAGTTCTCGACGTTCATGCC
	TUBB1	**TUBB1-1**	CAAGCCACAGTCATACTAGCTCGATTCAAACGTAGCTTACCTGCAAGCCTCTGAAGATG	CTGGTAGACATGCCTTGCAGGTAAGCTA
		**TUBB1-2**	CATTCCTGACCACTACTAGCTCGATTCAAACGTAGCTTACCTGCAACAGCTCTCACACT	CCTGCAGGCAGTCTTTGCAGGTAAGCTA
		**TUBB1-3**	AGCTGCCATCTTCTACTAGCTCGATTCAAACGTAGCTTACCTGCAATGCCAATGAAGGT	GATGGCCGTGTTAATTGCAGGTAAGCTA
		**TUBB1-4**	TAGTTTCCAGCGATACTAGCTCGATTCAAACGTAGCTTACCTGCAATGCATCCCCACGA	TCAAGCTGCAGTGTTTGCAGGTAAGCTA
		**TUBB1-5**	CAGCGCCTGAATTTACTAGCTCGATTCAAACGTAGCTTACCTGCAAGCCCAGTTGTTTC	TGTAATGGCCCTTTTTGCAGGTAAGCTA
		**TUBB1-6**	TTAACAGCCAGCTTACTAGCTCGATTCAAACGTAGCTTACCTGCAAAAAGGGCACCATG	AAATGCAGGCGAGCTTGCAGGTAAGCTA
		**TUBB1-7**	TTGGCTACCTCGATACTAGCTCGATTCAAACGTAGCTTACCTGCAAGGGCTCTGTACTG	CTCTGGCACCGAGTTTGCAGGTAAGCTA

		**TUBB1-8**	AGAACGGGGTACGTACTAGCTCGATTCAAACGTAGCTTACCTGCAAGGTCCACCAAGAT	TGTACCAGGTTCCTTTGCAGGTAAGCTA
		**TUBB1-9**	GTGACTCCACTCATACTAGCTCGATTCAAACGTAGCTTACCTGCAAACGGAGCGAGGTT	AGCTGGCCAGGAATTTGCAGGTAAGCTA
		**TUBB1-10**	TGCGCGCATCAAATACTAGCTCGATTCAAACGTAGCTTACCTGCAAGCTGCCATCATGT	GACGGGGGTCACACTTGCAGGTAAGCTA
		**ROBO2-1**	TGTCTCCCCACTTTAGGCATGAACGTCGAACGTAGCTTACCTGCAACAGCACTCCATGA	TGTCACCTCCAGCTTTGCAGGTAAGCTA
		**ROBO2-2**	GATTGACAGCTCCTAGGCATGAACGTCGAACGTAGCTTACCTGCAAGGCCTCTGATTCT	TTTTAGTGGGCCTATTGCAGGTAAGCTA
		**ROBO2-3**	TAACTGGGGTCACTAGGCATGAACGTCGAACGTAGCTTACCTGCAATGCAAGCCAAGGA	GCAGGCCAAGAGTGTTGCAGGTAAGCTA
		**ROBO2-4**	CACACGGTACTGATAGGCATGAACGTCGAACGTAGCTTACCTGCAATTGCAGCAACTTC	ACCTGCACTGGTGGTTGCAGGTAAGCTA
	ROBO2	**ROBO2-5**	ACAACCAGATGCTTAGGCATGAACGTCGAACGTAGCTTACCTGCAATTTCTGCGCCAGT	GCCCCTTCCTCTTGTTGCAGGTAAGCTA
		**ROBO2-6**	CAGGGGATGGGAATAGGCATGAACGTCGAACGTAGCTTACCTGCAAGAGCTGTGTCCAC	AAGCTGTTCCTGATTTGCAGGTAAGCTA
		**ROBO2-7**	TTCCAGCTCTGTGTAGGCATGAACGTCGAACGTAGCTTACCTGCAACCACCCCGTAGTG	TGCTTCTTGCTGAATTGCAGGTAAGCTA
		**ROBO2-8**	ACCATCACTGTCATAGGCATGAACGTCGAACGTAGCTTACCTGCAAAAGGCGGACACCA	GGTCTGAACGGGCTTTGCAGGTAAGCTA
		**ROBO2-9**	ATTTGGCTGGAACTAGGCATGAACGTCGAACGTAGCTTACCTGCAATGTAGCTTGCGTT	GTGGTGGCATATGCTTGCAGGTAAGCTA
		**ROBO2-10**	GAGCACAGGGGAATAGGCATGAACGTCGAACGTAGCTTACCTGCAAGCACATCTGTGGT	TGGAACTGGTGGCTTTGCAGGTAAGCTA
		**TENM2-1**	GACAGACCGGAACGGCACTTGAGCTAGAAAGGCACTTGAGCTAGAAGTTTCGCGACTT	AGTGTGAGAGCCGTTTCTAGCTCAAGTG
		**TENM2-2**	CACCCTTCTTCGCGGCACTTGAGCTAGAAAGGCACTTGAGCTAGAACGCCATTCCTGTC	ACTCGCTGGTCACTTTCTAGCTCAAGTG
		**TENM2-3**	CACCCATTCGCTTGGCACTTGAGCTAGAAAGGCACTTGAGCTAGAAGTACTGAACGCTC	AGTCCGTCGTAACCTTCTAGCTCAAGTG
		**TENM2-4**	TTACTCCGCTGTCGGCACTTGAGCTAGAAAGGCACTTGAGCTAGAAGAAACGGTCACGT	GGAGAGCTGGCTGCTTCTAGCTCAAGTG
	TENM2	**TENM2-5**	TGGTAACAGCGTGGGCACTTGAGCTAGAAAGGCACTTGAGCTAGAAGCAACACTAGGCA	ACATGCTGTGCCGTTTCTAGCTCAAGTG
		**TENM2-6**	TCCCTCCATCTCCGGCACTTGAGCTAGAAAGGCACTTGAGCTAGAAGCGTCCACTGCTT	GGCTCATTAGGGTATTCTAGCTCAAGTG
		**TENM2-7**	TCCGCAAACATGCGGCACTTGAGCTAGAAAGGCACTTGAGCTAGAACCAAACCTTCCCA	AGGTAGGTGTAGCATTCTAGCTCAAGTG
		**TENM2-8**	CTCCTCGTCCAGCGGCACTTGAGCTAGAAAGGCACTTGAGCTAGAAACACTCTCGCCTT	CCGAGCCTGGTCTTTTCTAGCTCAAGTG
		**TENM2-9**	AGGCAGCCAAAGAGGCACTTGAGCTAGAAAGGCACTTGAGCTAGAACCAGACCACTGCT	CACGTTGACAGCACTTCTAGCTCAAGTG
		**TENM2-10**	GACCTCCACACGAGGCACTTGAGCTAGAAAGGCACTTGAGCTAGAAATGCAGGAACCGT	CACAGTTCCCTTCCTTCTAGCTCAAGTG
		**DCN-1**	AGGGTTGCTGAAGATGCTGCTGTAGTACTAATGCTGCTGTAGTACTCCCAGTATTGCAC	AGAGGGCTGGATTAAGTACTACAGCAGC
		**DCN-2**	AGGTCTTTTGGCAATGCTGCTGTAGTACTAATGCTGCTGTAGTACTTGTATCAGGGGGA	AAGTCCAGCAGAGAAGTACTACAGCAGC
		**DCN-3**	GAAGAGGAGAGCCATGCTGCTGTAGTACTAATGCTGCTGTAGTACTGCAGCAGTAGGAT	GGCCAAGCATACTCAGTACTACAGCAGC
		**DCN-4**	TGGCCCAAATCCAATGCTGCTGTAGTACTAATGCTGCTGTAGTACTGGAAGGGGCACAC	ATGGCACTGGCAGGAGTACTACAGCAGC
	DCN	**DCN-5**	AAAGAGGCCCATCATGCTGCTGTAGTACTAATGCTGCTGTAGTACTCCAGCATAAAGTC	TGACCCCTCATCTAAGTACTACAGCAGC
		**DCN-6**	TCCTTCTTTAATTATGCTGCTGTAGTACTAATGCTGCTGTAGTACTAGTTTTTGAAATC	ATGAAGATTCTTCTAGTACTACAGCAGC
		**DCN-7**	AACAAGGATTAATATGCTGCTGTAGTACTAATGCTGCTGTAGTACTTGATTTTGTTGTT	TGGGCTTATTTTGGAGTACTACAGCAGC
		**DCN-8**	TTTCTTCAGAGGAATGCTGCTGTAGTACTAATGCTGCTGTAGTACTAAAGTCTTTCCAG	ATTTTTGGACAGGAAGTACTACAGCAGC
		**DCN-9**	GTTTTCTGGAAGTATGCTGCTGTAGTACTAATGCTGCTGTAGTACTGAGACTTTGGCAT	CCGTATCTCTTGATAGTACTACAGCAGC

		**DCN-10**	TAAAGACTGCTTTATGCTGCTGTAGTACTAATGCTGCTGTAGTACTTGATTCAGTCCAT	CTAAGACAATCACAAGTACTACAGCAGC
	TENM4	**TENM4-1**	AGCCTGAGAGAGCGGAGACTGATTACACGAGGAGACTGATTACACGATTGGCAAGCATC	GCCACCTCCATGCGCGTGTAATCAGTCT
		**TENM4-2**	TGTAGCTGGAACCGGAGACTGATTACACGAGGAGACTGATTACACGGGGGATGACGTTG	GGCTTGGGGTAGCGCGTGTAATCAGTCT
		**TENM4-3**	TACACAGTGCTGGGGAGACTGATTACACGAGGAGACTGATTACACGAGGGGGAGGTGAG	CTAGGAAGGGGCCACGTGTAATCAGTCT
		**TENM4-4**	AGCAATGGCAGGTGGAGACTGATTACACGAGGAGACTGATTACACGAGAAAGCCAAGGA	CACAGTCAGGGCCGCGTGTAATCAGTCT
		**TENM4-5**	TTCCACTGCACAGGGAGACTGATTACACGAGGAGACTGATTACACGATGTACTGCCCAT	AGCACCTCCCTTTGCGTGTAATCAGTCT
		**TENM4-6**	AGGTCAACCTGGAGGAGACTGATTACACGAGGAGACTGATTACACGACCCGATTGCTGT	GCACCATGTCTTTCCGTGTAATCAGTCT
		**TENM4-7**	GTGAAGTGCAAGTGGAGACTGATTACACGAGGAGACTGATTACACGCACCCCGTCGATG	AAGTAGTGCGTGTGCGTGTAATCAGTCT
		**TENM4-8**	TCCATCAAGGAGCGGAGACTGATTACACGAGGAGACTGATTACACGTTAGCAGGCGTCT	TCTTGCCTCCTGCCCGTGTAATCAGTCT
		**TENM4-9**	GTTGCGTCCCCAAGGAGACTGATTACACGAGGAGACTGATTACACGGCCCAGACTTGGT	TAAGCAGGAGCTGGCGTGTAATCAGTCT
		**TENM4-10**	CAATCTGCTGCTGGGAGACTGATTACACGAGGAGACTGATTACACGCGTTGACCCCATT	TGGGGAATAGGTGTCGTGTAATCAGTCT
	INHBA	**INHBA-1**	TCTTTCCCCTCCCCCTCGATTAACATCGCACCTCGATTAACATCGCTCCAGCATCCTTT	TCTCCTGGGACTTGGCGATGTTAATCGA
		**INHBA-2**	AGCATGTTCCACCCCTCGATTAACATCGCACCTCGATTAACATCGCAGAGCCACACTTC	GGAGACCTTCAGGTGCGATGTTAATCGA
		**INHBA-3**	AGGCAATCCGCACCCTCGATTAACATCGCACCTCGATTAACATCGCTGGCACAGGTCAC	TTGCCCCAGTCTCAGCGATGTTAATCGA
		**INHBA-4**	GAAAGATGTGCCACCTCGATTAACATCGCACCTCGATTAACATCGCCTGCTGGAGACAG	GGAGTCTCTGGACTGCGATGTTAATCGA
		**INHBA-5**	CAAGTGCAACATGCCTCGATTAACATCGCACCTCGATTAACATCGCTAGGTCTGTCCCT	CGGCTGGGTGATAAGCGATGTTAATCGA
		**INHBA-6**	ACAGGCGGATGGTCCTCGATTAACATCGCACCTCGATTAACATCGCCGCTGCTGTTGAA	TGCCTTTTGGCTGGGCGATGTTAATCGA
		**INHBA-7**	TGGGGAACTCCTCCCTCGATTAACATCGCACCTCGATTAACATCGCCGGATCCTGGGGT	TGAACTGTGCCCCAGCGATGTTAATCGA
		**INHBA-8**	GAGAGCGTGGCAACCTCGATTAACATCGCACCTCGATTAACATCGCGGGCACATCCTTC	TCAGGCTGTGAGCAGCGATGTTAATCGA
		**INHBA-9**	GTTTTCTTGGGTGCCTCGATTAACATCGCACCTCGATTAACATCGCCTCAAAGTGCAAT	CCTTCCTTGGAAAAGCGATGTTAATCGA
		**INHBA-10**	CCTTTCACCTTTCCCTCGATTAACATCGCACCTCGATTAACATCGCTCAGAGTCTCACT	TGCCTTTTCTGAATGCGATGTTAATCGA
	G0S2	**G0S2-1**	CACACCGAAGAAGGGCACTTGAGCTAGAAAATGCTGCTGTAGTACTCCAGACCGATGAC	GCACACTGCCTCCTAGTACTACAGCAGC
		**G0S2-2**	AGCCTCCCTTCGGGGCACTTGAGCTAGAAAATGCTGCTGTAGTACTCTCCTCCTCCTCT	GGGGCAGGTTTCTAAGTACTACAGCAGC
		**G0S2-3**	ATGCATGGTTTCCGGCACTTGAGCTAGAAAATGCTGCTGTAGTACTAGGGGATCAGCTC	CAGTTCTTTGGCATAGTACTACAGCAGC
		**G0S2-4**	TCCTGTTGGGCTTGGCACTTGAGCTAGAAAATGCTGCTGTAGTACTAGCTTGACCATTT	TGCCCAGCATGTAGAGTACTACAGCAGC
		**G0S2-5**	TGAGGAAGTGTTTGGCACTTGAGCTAGAAAATGCTGCTGTAGTACTATCCTCCTGTTTC	TTTTCCAAGATCATAGTACTACAGCAGC
		**G0S2-6**	CGCTGCATCACCAGGCACTTGAGCTAGAAAATGCTGCTGTAGTACTGGTCACCAGGGCC	GAGGCGTGCTGCCAAGTACTACAGCAGC
	CSF3R	**CSF3R-1**	GTAGCGCTGGAAGGGCACTTGAGCTAGAAAGGAGACTGATTACACGACACCGAGATGTT	CTTGTAGAGGGGGGCGTGTAATCAGTCT
		**CSF3R-2**	TCGATGAGGGGCTGGCACTTGAGCTAGAAAGGAGACTGATTACACGCAGCAGGGGGAAG	ATCTGCAGGCCCTCCGTGTAATCAGTCT
		**CSF3R-3**	TGAGCAGGACGAAGGCACTTGAGCTAGAAAGGAGACTGATTACACGATCATCAGGGCTG	TCTTCTGGAAGCATCGTGTAATCAGTCT
		**CSF3R-4**	CACCCAGCACCACGGCACTTGAGCTAGAAAGGAGACTGATTACACGTCCCGTTCCACTC	AACCCGCTGCTTGCCGTGTAATCAGTCT
		**CSF3R-5**	CACCGTGACGGTAGGCACTTGAGCTAGAAAGGAGACTGATTACACGCTGCTCTTTCCAG	TGCCTGCTTCCCTCCGTGTAATCAGTCT

		**CSF3R-6**	TGCAGACAGTCGGGGCACTTGAGCTAGAAAGGAGACTGATTACACGGTGTCAGAGGTGT	TGAAGTCGCAGTGCCGTGTAATCAGTCT
		**CSF3R-7**	TCCAGGAGATCCGGGCACTTGAGCTAGAAAGGAGACTGATTACACGTCGTTGTCCAGCA	TCCCAGCCACTGTGCGTGTAATCAGTCT
		**CSF3R-8**	AGCCATCCCCTGCGGCACTTGAGCTAGAAAGGAGACTGATTACACGTGCTGGCCCTTGT	GACACGGGACGTGGCGTGTAATCAGTCT
		**CSF3R-9**	GAGGGCTTGGCAGGGCACTTGAGCTAGAAAGGAGACTGATTACACGGCAGCTGAGGTTG	CTGAGGTTGAGCACCGTGTAATCAGTCT
		**CSF3R-10**	GTTCACCTGCGGGGGCACTTGAGCTAGAAAGGAGACTGATTACACGACCCAGAGGCTTC	GTGCCTCCCAGTGGCGTGTAATCAGTCT
		**IL2RB-1**	TCCCCTCCATCACGGCACTTGAGCTAGAAACCTCGATTAACATCGCCCTGCCTTCCCAC	GATCAGTCGGAAGCGCGATGTTAATCGA
		**IL2RB-2**	TTGTGGGACAACAGGCACTTGAGCTAGAAACCTCGATTAACATCGCGCAGTGAAGGAAG	TGGATCCCCTTGATGCGATGTTAATCGA
		**IL2RB-3**	AGACCTGACAGGGGGCACTTGAGCTAGAAACCTCGATTAACATCGCTCGTACGTGAAGT	CCTGGGTGAAAGGGGCGATGTTAATCGA
		**IL2RB-4**	ACAAGCAAGACTGGGCACTTGAGCTAGAAACCTCGATTAACATCGCGCGAGTCATACCA	AAAGATTGCTGCCGGCGATGTTAATCGA
	IL2RB	**IL2RB-5**	AGGCACTTGAGTTGGCACTTGAGCTAGAAACCTCGATTAACATCGCCACACGCCTTTGT	TGCTCCAGTCACTGGCGATGTTAATCGA
		**IL2RB-6**	CAGAAGCTGGGCTGGCACTTGAGCTAGAAACCTCGATTAACATCGCGTCCCTTGGAAAG	TTCAGGAGTCAGGCGCGATGTTAATCGA
		**IL2RB-7**	GTTTGGGATCTGGGGCACTTGAGCTAGAAACCTCGATTAACATCGCGATAGGCACCGGT	GTCCCTCAGAGAATGCGATGTTAATCGA
		**IL2RB-8**	ACTCGCTGTCCTCGGCACTTGAGCTAGAAACCTCGATTAACATCGCAGAGCGTGGTAAG	GGTCTGGGGAGGGCGCGATGTTAATCGA
		**IL2RB-9**	TTGACGGGAAAGCGGCACTTGAGCTAGAAACCTCGATTAACATCGCGGGGTGGTTTCAC	GAACTGGTTTCGGAGCGATGTTAATCGA
		**IL2RB-10**	CAGGTTGTGAAGGGGCACTTGAGCTAGAAACCTCGATTAACATCGCCACAGGGCCTCAG	CTTTGTCCAGCTGCGCGATGTTAATCGA
		**BCL11B-1**	TGGTGATGCGTGGATGCTGCTGTAGTACTAGGAGACTGATTACACGAAAGGAGGAGGGA	CAGTCTCTGGTCCGCGTGTAATCAGTCT
		**BCL11B-2**	TGGCTCTGACACTATGCTGCTGTAGTACTAGGAGACTGATTACACGTCCCGATTTCCAC	GGGTGTGACTTGGTCGTGTAATCAGTCT
		**BCL11B-3**	TGGGTCCTGTCATATGCTGCTGTAGTACTAGGAGACTGATTACACGTGTTCACGCAAGA	CACCAAAGCCTGGCCGTGTAATCAGTCT
		**BCL11B-4**	ACCTGCCTGTCATATGCTGCTGTAGTACTAGGAGACTGATTACACGAGTCCATCGTCTG	AACTCGTGGTGGAGCGTGTAATCAGTCT
	BCL11B	**BCL11B-5**	TCTGGCAAATGTCATGCTGCTGTAGTACTAGGAGACTGATTACACGACACTGAAGGGCA	CCAGGGTGCTGTATCGTGTAATCAGTCT
		**BCL11B-6**	CTCGAGGACACAAATGCTGCTGTAGTACTAGGAGACTGATTACACGCTCGGACAGAGGT	CATCACCTGTGTCACGTGTAATCAGTCT
		**BCL11B-7**	TCCATTGACCGTCATGCTGCTGTAGTACTAGGAGACTGATTACACGGGCCAAAGTTTCT	GAAGGGCTCACCTCCGTGTAATCAGTCT
		**BCL11B-8**	TGGGAGTAAACGTATGCTGCTGTAGTACTAGGAGACTGATTACACGCCCTACCAGCCAC	CTCGATGCTGCGTTCGTGTAATCAGTCT
		**BCL11B-9**	AACCAAGCGCTATATGCTGCTGTAGTACTAGGAGACTGATTACACGATGCTGAAGCAAG	TGTGTGTTCTGGGGCGTGTAATCAGTCT
		**BCL11B-10**	TCGAAGCCTTCTGATGCTGCTGTAGTACTAGGAGACTGATTACACGTTCCTGCCAGCTC	AGGGGTGGAACTGACGTGTAATCAGTCT
		**NDST4-1**	ATAACTGGCACCAATGCTGCTGTAGTACTACCTCGATTAACATCGCTTCCCTCCTTCCA	CCAGGCACTTTGTAGCGATGTTAATCGA
		**NDST4-2**	GTGTCAGGCACCTATGCTGCTGTAGTACTACCTCGATTAACATCGCGCATACCATCCAG	GTTCGATGTGGACTGCGATGTTAATCGA
		**NDST4-3**	AGCTGGTGTACAAATGCTGCTGTAGTACTACCTCGATTAACATCGCATCTCCTGCTTGT	TTTCTGCAAGGGCGGCGATGTTAATCGA
	NDST4	**NDST4-4**	CACGTTGTGGTCTATGCTGCTGTAGTACTACCTCGATTAACATCGCGCTTGGACAGTTC	CAGCCTATGCAGCTGCGATGTTAATCGA
		**NDST4-5**	TCGCAAAGGTGTAATGCTGCTGTAGTACTACCTCGATTAACATCGCAAGCTGGCCAGGT	TCGAGCTCTTCACTGCGATGTTAATCGA
		**NDST4-6**	ACCCTTGGTGCTTATGCTGCTGTAGTACTACCTCGATTAACATCGCTGGGCCTTTCTCA	TCTTCGCCAGGCACGCGATGTTAATCGA
		**NDST4-7**	TGTACCGTGCAGGATGCTGCTGTAGTACTACCTCGATTAACATCGCATGAAGCCCCGTC	TGATGCCATTGTGCGCGATGTTAATCGA

		**NDST4-8**	GACGGTTGCATACATGCTGCTGTAGTACTACCTCGATTAACATCGCCCAAGTCCTGGAT	CCCATCATGAAGCGGCGATGTTAATCGA
		**NDST4-9**	GAACAGGAGGACAATGCTGCTGTAGTACTACCTCGATTAACATCGCATTGGCTTTCCAC	TCCCAGCTGGGAGAGCGATGTTAATCGA
		**NDST4-10**	AAACTTCTGCGGAATGCTGCTGTAGTACTACCTCGATTAACATCGCAACAAGTGTTCTG	GTAGCCAGAAGAAAGCGATGTTAATCGA
	TFEC	**TFEC-1**	ACAGCACTGAGTGGGAGACTGATTACACGACCTCGATTAACATCGCCGCAGCTAAATCT	TGCTTGATGACCTCGCGATGTTAATCGA
		**TFEC-2**	GTGCCTCTGGCTTGGAGACTGATTACACGACCTCGATTAACATCGCACTGCTTCACCTG	GTCAAGGGTCAGAAGCGATGTTAATCGA
		**TFEC-3**	CTCCTGCTGCTCTGGAGACTGATTACACGACCTCGATTAACATCGCGCTAAAGCTGCTT	TCTCCGTCGTCTGAGCGATGTTAATCGA
		**TFEC-4**	TGCTCCAAATGGTGGAGACTGATTACACGACCTCGATTAACATCGCACAAGAGTGGGTC	TGGGGATGTGGAACGCGATGTTAATCGA
		**TFEC-5**	AGGGAGAGGGTCTGGAGACTGATTACACGACCTCGATTAACATCGCGTGGGAGTGGGAC	GCAGGCTGGATTGAGCGATGTTAATCGA
		**TFEC-6**	AGTCCAGTCAGTGGGAGACTGATTACACGACCTCGATTAACATCGCGAGCTGAAACTGT	CGTAGCTTTCCTCAGCGATGTTAATCGA
		**TFEC-7**	CACTTCTTCTTGAGGAGACTGATTACACGACCTCGATTAACATCGCAATAACGTGCATG	CATATGGCACTGTCGCGATGTTAATCGA
		**TFEC-8**	TTGAACCCAGGATGGAGACTGATTACACGACCTCGATTAACATCGCCGGAAAAAAGCCA	AAGTGTGTTCCCACGCGATGTTAATCGA
		**TFEC-9**	AAGTGTGTTTGTAGGAGACTGATTACACGACCTCGATTAACATCGCAGACTGATTTTCC	TGGATATGGTATCAGCGATGTTAATCGA
		**TFEC-10**	GCATGTGTCCGTTGGAGACTGATTACACGACCTCGATTAACATCGCTCACTGACGGGAG	CTGGTGTGCCAATTGCGATGTTAATCGA
	ANGPTL4	**ANGPTL4-1**	CACATGCTCCTTCGTGCTTGAACTGGTCTAGTGCTTGAACTGGTCTCCTTGGTCCTGTC	CTCCCGCATCTGCGAGACCAGTTCAAGC
		**ANGPTL4-2**	ACGATCCCGAGTGGTGCTTGAACTGGTCTAGTGCTTGAACTGGTCTTGAGGTCATGGTC	GTTTGTGTCAGCTAAGACCAGTTCAAGC
		**ANGPTL4-3**	GATGCCGCTGCTTGTGCTTGAACTGGTCTAGTGCTTGAACTGGTCTGCTGCACCTGGAA	CTGAGACCCCGAGCAGACCAGTTCAAGC
		**ANGPTL4-4**	TGTCCTTCAGGTGGTGCTTGAACTGGTCTAGTGCTTGAACTGGTCTGACTGCGTTTTGT	TCTTCCACTTCGGTAGACCAGTTCAAGC
		**ANGPTL4-5**	GTCCAGCCACCTTGTGCTTGAACTGGTCTAGTGCTTGAACTGGTCTCCTCTGGATCACT	GAGCCATCTGTGCCAGACCAGTTCAAGC
		**ANGPTL4-6**	CTGCTGCTGCTTGGTGCTTGAACTGGTCTAGTGCTTGAACTGGTCTTGTCCAGCTTGTA	CTGCAGATTCTGCAAGACCAGTTCAAGC
		**ANGPTL4-7**	AGGAGGTTGTACCGTGCTTGAACTGGTCTAGTGCTTGAACTGGTCTCTCCAGCTCGATC	TTTCCCTCCCAGTGAGACCAGTTCAAGC
		**ANGPTL4-8**	CTGGGCATCCATCGTGCTTGAACTGGTCTAGTGCTTGAACTGGTCTCGATTCGGGAGTT	CTGCAAGAGCTCCAAGACCAGTTCAAGC
		**ANGPTL4-9**	TTCATGGTGGTGGGTGCTTGAACTGGTCTAGTGCTTGAACTGGTCTTGTGGGTTGGATT	TCTGCTTCCAGTGGAGACCAGTTCAAGC
		**ANGPTL4-10**	CTGAACCACCAGCGTGCTTGAACTGGTCTAGTGCTTGAACTGGTCTATGGCCACAGGTG	CCGTTGAGGTTGGGAGACCAGTTCAAGC
	SDC4	**SDC4-1**	TCCAGAACCAGGGCTGACTAACGACACCAACTGACTAACGACACCACATCTGGCAGGTC	GCCAATGTCTTCAATGGTGTCGTTAGTC
		**SDC4-2**	TCGGTTTCTCTCACTGACTAACGACACCAACTGACTAACGACACCATGCATCCATGGTC	TAGTCGAGCCATCCTGGTGTCGTTAGTC
		**SDC4-3**	TGTTGGACAGGTTCTGACTAACGACACCAACTGACTAACGACACCAGCCATGGAGATCT	TGTTGGCTGTGCTATGGTGTCGTTAGTC
		**SDC4-4**	AGGAGGGCGACGCCTGACTAACGACACCAACTGACTAACGACACCAGAGGGCGAGCAGG	GCGGAGGCCAGGACTGGTGTCGTTAGTC
		**SDC4-5**	TAACCCATCGGAACTGACTAACGACACCAACTGACTAACGACACCATCTCATCTGTATC	TCCAGAACCAGACCTGGTGTCGTTAGTC
		**SDC4-6**	ATAGCATCTTCAGCTGACTAACGACACCAACTGACTAACGACACCAGGTGGTCAGATAT	AGAGGAGTATCCAATGGTGTCGTTAGTC
		**SDC4-7**	TAGTTGTCAGATACTGACTAACGACACCAACTGACTAACGACACCAATCTCCAGGGATA	ATCTTTCTCTCCCATGGTGTCGTTAGTC
		**SDC4-8**	CATCGTGTTTTTCCTGACTAACGACACCAACTGACTAACGACACCATTTCATTGTCCAG	TTTGTCTGGAATGTTGGTGTCGTTAGTC
		**SDC4-9**	CCCCTCCTGCAATCTGACTAACGACACCAACTGACTAACGACACCAAGGAGGCCAACTG	GGAAGACAGCAAAGTGGTGTCGTTAGTC

		**SDC4-10**	CTTCATGCGATAGCTGACTAACGACACCAACTGACTAACGACACCACTTCGTCCTTTTT	AAGGTCGTAACTGGTGGTGTCGTTAGTC
	SDC2	**SDC2-1**	CTTTCTCATGCGAACTTGGATCCACTCGTAACTTGGATCCACTCGTTGCCTTCATCCTT	ACCAAGGTCGTAAGACGAGTGGATCCAA
		**SDC2-2**	TTTCACCGTGGTGACTTGGATCCACTCGTAACTTGGATCCACTCGTTCTTGGTCTGCGT	TGTTTGTGCAGGCTACGAGTGGATCCAA
		**SDC2-3**	TCCCCTGGCTCATACTTGGATCCACTCGTAACTTGGATCCACTCGTCACGTCAGTGTCA	TGCTTTTCGGTGATACGAGTGGATCCAA
		**SDC2-4**	CTAGCCACACGCCACTTGGATCCACTCGTAACTTGGATCCACTCGTCTGAGGGCTAGCA	CACAGGCCACGAACACGAGTGGATCCAA
		**SDC2-5**	GCCAGCAATAACAACTTGGATCCACTCGTAACTTGGATCCACTCGTAACCGATAACTCC	GATTGCGAAAAGATACGAGTGGATCCAA
		**SDC2-6**	GGATGGTTTACGTACTTGGATCCACTCGTAACTTGGATCCACTCGTGATAGGCAGCACT	AGTAGGTGCCTTTAACGAGTGGATCCAA
		**SDC2-7**	ATCGATTTCTTCGACTTGGATCCACTCGTAACTTGGATCCACTCGTGCCTTTCCTCTTT	GGTATCCACCTCACACGAGTGGATCCAA
		**SDC2-8**	CCTCATCTTCTTCACTTGGATCCACTCGTAACTTGGATCCACTCGTATCACTGTGCTAT	TTCTGGATGTTGTAACGAGTGGATCCAA
		**SDC2-9**	TGGTAACTTTGGAACTTGGATCCACTCGTAACTTGGATCCACTCGTCATCACTTGTTGT	CTCAGCTCTAGATCACGAGTGGATCCAA
		**SDC2-10**	TCAGATCTGCTCTACTTGGATCCACTCGTAACTTGGATCCACTCGTTCCTTATCAGAGG	TGTCCAGGTATAATACGAGTGGATCCAA
	TLR4	**TLR4-1**	CAGCACCAAGAAGGATCTCGAGAACGTCAAGATCTCGAGAACGTCATACACCCACTGAG	TTCTGCAGAGTGTATGACGTTCTCGAGA
		**TLR4-2**	ATCGGTAGGTCAGGATCTCGAGAACGTCAAGATCTCGAGAACGTCAATGCAAGTGTCCA	AGCTCCTGTAGGGATGACGTTCTCGAGA
		**TLR4-3**	ACAGGATCGAAGGGATCTCGAGAACGTCAAGATCTCGAGAACGTCATGGCTCGTAGACT	GTGAGAGCTTGGACTGACGTTCTCGAGA
		**TLR4-4**	TGTGGCCCAAATTGATCTCGAGAACGTCAAGATCTCGAGAACGTCAGAAGCAATGCTGT	TGGGAAGCTTCAAATGACGTTCTCGAGA
		**TLR4-5**	TCAGACCGTGAAAGATCTCGAGAACGTCAAGATCTCGAGAACGTCAACTTGCAGAGAGT	CTGCCATCTTGAGGTGACGTTCTCGAGA
		**TLR4-6**	CTGCCTCAGCAAGGATCTCGAGAACGTCAAGATCTCGAGAACGTCAACAGCCCCAGCTT	CCTGAGGTATCGGCTGACGTTCTCGAGA
		**TLR4-7**	AGAAGTGCGCATGGATCTCGAGAACGTCAAGATCTCGAGAACGTCACCAGGCCGTGAAG	GACCTGTAGCCCTCTGACGTTCTCGAGA
		**TLR4-8**	TCACAGCAGGCACGATCTCGAGAACGTCAAGATCTCGAGAACGTCAAAGAGGGACAGCT	CTCTCAGCTCCTTTTGACGTTCTCGAGA
		**TLR4-9**	CAACAGGACACTCGATCTCGAGAACGTCAAGATCTCGAGAACGTCACCAGGGCTGAGTC	AGCAGGCAGGATTATGACGTTCTCGAGA
		**TLR4-10**	TGCCACCATGCCTGATCTCGAGAACGTCAAGATCTCGAGAACGTCATGACCACTGAGCA	TGCAGCAAGCAATTTGACGTTCTCGAGA
	CD226	**CD226-1**	CAGTGCACGCTTTGTGCTTGAACTGGTCTACTGACTAACGACACCACAGAGCAATCCAC	GGAGCTTGCTTGAATGGTGTCGTTAGTC
		**CD226-2**	TCCATAGGAAAACGTGCTTGAACTGGTCTACTGACTAACGACACCAGCGATGCAGATAA	TGTCATAAGCAGTCTGGTGTCGTTAGTC
		**CD226-3**	GCGAAGTCAGAGTGTGCTTGAACTGGTCTACTGACTAACGACACCAACAGCGATATGTT	CTTCCAGTAGCCAGTGGTGTCGTTAGTC
		**CD226-4**	TTTGTTTCTTCCAGTGCTTGAACTGGTCTACTGACTAACGACACCATCATCACATAGGT	AAGCATGGTGAAAGTGGTGTCGTTAGTC
		**CD226-5**	TGAATTACACTGGGTGCTTGAACTGGTCTACTGACTAACGACACCAGTTGCCCTCCTAA	ATCTGCACCAAAGGTGGTGTCGTTAGTC
		**CD226-6**	CTTCCTTCTGCATGTGCTTGAACTGGTCTACTGACTAACGACACCATGAATCCACAAAC	GAAAGCTTTACAAATGGTGTCGTTAGTC
		**CD226-7**	ATATGCATTCCAGGTGCTTGAACTGGTCTACTGACTAACGACACCAGCTTTTTTTGGAT	TCTGGATTATCACTTGGTGTCGTTAGTC
		**CD226-8**	ATTATGTTTCATCGTGCTTGAACTGGTCTACTGACTAACGACACCACTTTATAAGTTAC	GACAGCTATATTTATGGTGTCGTTAGTC
	COL6A3	**COL6A3-1**	AACTCCAACAGCAGTGCTTGAACTGGTCTAGATCTCGAGAACGTCATATCCGCATTCCT	TTCAAGCTCTGCCATGACGTTCTCGAGA
		**COL6A3-2**	TCGTCCGTCCGTTGTGCTTGAACTGGTCTAGATCTCGAGAACGTCACATCATCCTGGGA	TGATGGCAGAGCCTTGACGTTCTCGAGA
		**COL6A3-3**	TCGAAGCCAAGTAGTGCTTGAACTGGTCTAGATCTCGAGAACGTCAGACGTCTGTCACA	TTCTGGCCGTTCCATGACGTTCTCGAGA

		**COL6A3-4**	AATTGAAGGCGGAGTGCTTGAACTGGTCTAGATCTCGAGAACGTCAAATGGCTTGCATG	AGCACTTCAGGAACTGACGTTCTCGAGA
		**COL6A3-5**	GTCCCCTCTTCCTGTGCTTGAACTGGTCTAGATCTCGAGAACGTCAGAAGTCCCTGCAG	TACCACTTCAGCAGTGACGTTCTCGAGA
		**COL6A3-6**	CTGGTGCTTACGGGTGCTTGAACTGGTCTAGATCTCGAGAACGTCAACTTTGCGTGGTG	GGCTTGGTGCTGGATGACGTTCTCGAGA
		**COL6A3-7**	TCCCAATGCTCCAGTGCTTGAACTGGTCTAGATCTCGAGAACGTCATGGAAATGCTCCT	ACTCTCGAACGAGATGACGTTCTCGAGA
		**COL6A3-8**	CTTATGCCCGGTGGTGCTTGAACTGGTCTAGATCTCGAGAACGTCAGCCTTGCTCTCCT	CTGGGCCCAGGAAGTGACGTTCTCGAGA
		**COL6A3-9**	AGCACTTGCAATGGTGCTTGAACTGGTCTAGATCTCGAGAACGTCACCTTCCTGCCCTG	GCGGCCCTCTTTCATGACGTTCTCGAGA
		**COL6A3-10**	CATCACCTCCTCGGTGCTTGAACTGGTCTAGATCTCGAGAACGTCACGAACTCCAGCTG	GGTGAAACCCCGCGTGACGTTCTCGAGA
	COL6A1	**COL6A1-1**	CTTCTTCTTCGCCCTGACTAACGACACCAAACTTGGATCCACTCGTAAAACACCAGCAC	CGAGTTGCCATCACACGAGTGGATCCAA
		**COL6A1-2**	AGCTGATGGCCGACTGACTAACGACACCAAACTTGGATCCACTCGTCCCTTCCCGATGT	AGTCGGTGTGGGTCACGAGTGGATCCAA
		**COL6A1-3**	AGTCCTCTCGATGCTGACTAACGACACCAAACTTGGATCCACTCGTGAACCCCCTGCAC	GATACCGGCCTGCAACGAGTGGATCCAA
		**COL6A1-4**	TGCCCTTCTCACCCTGACTAACGACACCAAACTTGGATCCACTCGTTCGATCCCACGCT	TGCCATCAATGCCCACGAGTGGATCCAA
		**COL6A1-5**	GACACGGACATTGCTGACTAACGACACCAAACTTGGATCCACTCGTTCCCTGTGACCAG	GTAGTCTGCGCTCAACGAGTGGATCCAA
		**COL6A1-6**	TTACGCTGAAACGCTGACTAACGACACCAAACTTGGATCCACTCGTGATGACCGTGTAG	TCGATGGCTTTGGGACGAGTGGATCCAA
		**COL6A1-7**	TACTGGACCACCGCTGACTAACGACACCAAACTTGGATCCACTCGTGTTTTTCCCGCTG	TCGACCTTCTGCTCACGAGTGGATCCAA
		**COL6A1-8**	GTAGCCCGGGGTACTGACTAACGACACCAAACTTGGATCCACTCGTCCTTTGCAGCCAG	CGAATCCAGGAGACACGAGTGGATCCAA
		**COL6A1-9**	CAGGCTTTCCATCCTGACTAACGACACCAAACTTGGATCCACTCGTATCCCCTGCCGTC	CAGGACTGCCAGGCACGAGTGGATCCAA
		**COL6A1-10**	TGTACCCCTCCAACTGACTAACGACACCAAACTTGGATCCACTCGTCCACAGGGCTCCT	CATCATCCAGGCCTACGAGTGGATCCAA
	CD44	**CD44-1**	ATGACGTGTGCCACTGACTAACGACACCAAGATCTCGAGAACGTCAAGGAGTCTCCACT	CACACCTGAGGTTCTGACGTTCTCGAGA
		**CD44-2**	TCGTGTGAGACTGCTGACTAACGACACCAAGATCTCGAGAACGTCACAGCTGCTTCAGT	AGCTCTGCAAAGGATGACGTTCTCGAGA
		**CD44-3**	ACACCGTAGGGTACTGACTAACGACACCAAGATCTCGAGAACGTCATCCCATCCTGGAA	TGGCCACACTTATTTGACGTTCTCGAGA
		**CD44-4**	ATCCAAAAGTTGCCTGACTAACGACACCAAGATCTCGAGAACGTCATTCAGCAAACATA	CTGTTAGACAAAGGTGACGTTCTCGAGA
		**CD44-5**	TATCTGCAACTTACTGACTAACGACACCAAGATCTCGAGAACGTCAAAAAACTCCTCTA	TTTTTTTCCACATCTGACGTTCTCGAGA
		**CD44-6**	GTGTTGACAAGGTCTGACTAACGACACCAAGATCTCGAGAACGTCATCCAGCTGTTCTA	GTTCGTGAGCTTTATGACGTTCTCGAGA
		**CD44-7**	ACTACAAAACCATCTGACTAACGACACCAAGATCTCGAGAACGTCAAACAATATACCCC	TTGATTCGTGGGAATGACGTTCTCGAGA
		**CD44-8**	TTTGCTGCACAAACTGACTAACGACACCAAGATCTCGAGAACGTCAAATTCCAGTATGA	GCTGAAAGTTTATTTGACGTTCTCGAGA
		**CD44-9**	GCCTGTTGTATTTCTGACTAACGACACCAAGATCTCGAGAACGTCAATGCATCATAACG	TGCATTGTAACAAATGACGTTCTCGAGA
		**CD44-10**	AGTGTCTATTCTTCTGACTAACGACACCAAGATCTCGAGAACGTCATACTGAGAAAAGA	TATTTCACCCTGAATGACGTTCTCGAGA
	COL6A2	**COL6A2-1**	AGCTCCTAGGTTCACTTGGATCCACTCGTAGATCTCGAGAACGTCACCCTGCCACAAAG	ATTGACGAGCACAATGACGTTCTCGAGA
		**COL6A2-2**	CTCTCTCACTGCCACTTGGATCCACTCGTAGATCTCGAGAACGTCAACCACGTCCTGCT	AGGTCAGTGGGAACTGACGTTCTCGAGA
		**COL6A2-3**	GATCACCACAGCAACTTGGATCCACTCGTAGATCTCGAGAACGTCAAGCGCCCATCTGT	ATCACGAGGGTCAATGACGTTCTCGAGA
		**COL6A2-4**	GACCACCGCAAACACTTGGATCCACTCGTAGATCTCGAGAACGTCAGGCCATCAGTGAT	ACTGCCAGTGACGATGACGTTCTCGAGA
		**COL6A2-5**	CTACTCCAACACGACTTGGATCCACTCGTAGATCTCGAGAACGTCATGACTGTACTGCA	CAAAGGTCCCCTCTTGACGTTCTCGAGA

		**COL6A2-6**	CTGAATAGCAGCCACTTGGATCCACTCGTAGATCTCGAGAACGTCACCCGGCCAAGGTA	GTCTGTGAAGGTGGTGACGTTCTCGAGA
		**COL6A2-7**	GTCCAATTCGACCACTTGGATCCACTCGTAGATCTCGAGAACGTCATTGCAGCCTGGTG	CACCACGGTCTCCGTGACGTTCTCGAGA
		**COL6A2-8**	CAGACGCTTCACTACTTGGATCCACTCGTAGATCTCGAGAACGTCACAGCAATCCACTC	TGTCCAGGTGCCTGTGACGTTCTCGAGA
		**COL6A2-9**	TCTTTCAGAGCCAACTTGGATCCACTCGTAGATCTCGAGAACGTCATCTGCTCCCCGAT	GGCCCTGTGGAAAATGACGTTCTCGAGA
		**COL6A2-10**	GATCTCCACGTCGACTTGGATCCACTCGTAGATCTCGAGAACGTCACCCTTCGTCCCTG	GACTGCCTTTGCTCTGACGTTCTCGAGA
	COL4A1	**COL4A1-1**	GTCCAACAGCACCAGACGTAATGACAGCGATCCAGTACGTCGACTACCTATACCCGGCT	GAGGCCCAGGGAAGTAGTCGACGTACTG
		**COL4A1-2**	ACCCCTTTTCTCCAGACGTAATGACAGCGATCCAGTACGTCGACTATACTCCCCACGTA	TTCCTGGTTCTCCTTAGTCGACGTACTG
		**COL4A1-3**	CTCTCCTTTGGCCAGACGTAATGACAGCGATCCAGTACGTCGACTACTTCGCTCCCCTG	AGTCCCAGGCCAGGTAGTCGACGTACTG
		**COL4A1-4**	AATGAAGGGAGCGAGACGTAATGACAGCGATCCAGTACGTCGACTAGGCCATGGCATTC	ATTGCAGGTGCCCGTAGTCGACGTACTG
		**COL4A1-5**	CTGGTGGGCCCATAGACGTAATGACAGCGATCCAGTACGTCGACTAACAGATGCTGCAC	GGAAGCCATGAGCTTAGTCGACGTACTG
		**COL4A1-6**	GACTACCAGGCGGAGACGTAATGACAGCGATCCAGTACGTCGACTACCATCAAACCCTG	TTTGCCCAGGTTTCTAGTCGACGTACTG
		**COL4A1-7**	AGGCGTGGACAACAGACGTAATGACAGCGATCCAGTACGTCGACTATTGGCATGGGTTC	AGGAGCCATACTCTTAGTCGACGTACTG
		**COL4A1-8**	TTAGGGCCCTCCAAGACGTAATGACAGCGATCCAGTACGTCGACTATCCTCTGTCTCCT	GGTTGACCTTGGGCTAGTCGACGTACTG
		**COL4A1-9**	CATCGCCTTTCGCAGACGTAATGACAGCGATCCAGTACGTCGACTACCTGGGAAACCCA	GACTTCCTGGGGAATAGTCGACGTACTG
		**COL4A1-10**	GACCAGGGAATCCAGACGTAATGACAGCGATCCAGTACGTCGACTATCAGGACCAGGTG	CCCCAGGCAAACCATAGTCGACGTACTG
	COL4A2	**COL4A2-1**	CTTTCAGGCCCACAGACGTAATGACAGCGAGCAATTAGCGATGGCATCACCAGACAGAC	CCGTTTCACCTTTCTGCCATCGCTAATT
		**COL4A2-2**	CAGCAGGACCAGCAGACGTAATGACAGCGAGCAATTAGCGATGGCAACAGCACCACCTC	TACTCTGACGGCTCTGCCATCGCTAATT
		**COL4A2-3**	TGTCCTCCACCTTAGACGTAATGACAGCGAGCAATTAGCGATGGCAAGAAACCAGCGAC	AGGCAGCTCCCAGCTGCCATCGCTAATT
		**COL4A2-4**	GAAATTCCCGGGGAGACGTAATGACAGCGAGCAATTAGCGATGGCAACCAAAATGGCCT	TGGGAGCCAGGAGATGCCATCGCTAATT
		**COL4A2-5**	CTCTTCCTCCACAAGACGTAATGACAGCGAGCAATTAGCGATGGCACCTCCACTGCAGT	GAAAACACTGGCAATGCCATCGCTAATT
		**COL4A2-6**	ACCAGGAAGCCCAAGACGTAATGACAGCGAGCAATTAGCGATGGCACTCCCCCTCTGAT	GCCTGGAAAACCAATGCCATCGCTAATT
		**COL4A2-7**	GTGGCCTTACACAAGACGTAATGACAGCGAGCAATTAGCGATGGCATGGGACCCCTTTG	GTCCTGGCTTGCCATGCCATCGCTAATT
		**COL4A2-8**	TTTGTTCATGCCGAGACGTAATGACAGCGAGCAATTAGCGATGGCAACCCACTCCAGAG	GTACAGGAGGCTGATGCCATCGCTAATT
		**COL4A2-9**	ACCCATCAGACCAAGACGTAATGACAGCGAGCAATTAGCGATGGCAGTGATCCTGGCAA	TCCGTGTGTTCCTCTGCCATCGCTAATT
		**COL4A2-10**	TTCCCCCTTGATCAGACGTAATGACAGCGAGCAATTAGCGATGGCAATCATGACCCGGT	GTCCCGTTACAGCGTGCCATCGCTAATT
	SNX10	**SNX10-1**	ATTCTCGAAAGCGTCCAGTACGTCGACTAAGCAATTAGCGATGGCACGAAGCCAAACAA	TCTGAAGCCTCTGGTGCCATCGCTAATT
		**SNX10-2**	CACGTGATGCGGATCCAGTACGTCGACTAAGCAATTAGCGATGGCAGCCGACGATGGTC	TTCTTGCAGACCCATGCCATCGCTAATT
		**SNX10-3**	CCCGGAGGATGAATCCAGTACGTCGACTAAGCAATTAGCGATGGCACACTAGGTCCAAG	AGAAATGCTGTCAATGCCATCGCTAATT
		**SNX10-4**	TAATGCATTCTGTTCCAGTACGTCGACTAAGCAATTAGCGATGGCATGTCTGAAAGCAA	GAGGTGAAGCCTAGTGCCATCGCTAATT
		**SNX10-5**	CTTAGCTGAGTCTTCCAGTACGTCGACTAAGCAATTAGCGATGGCACATATCTTCTGGG	CATACACAAGCTTGTGCCATCGCTAATT
		**SNX10-6**	GCGACAGAATATTTCCAGTACGTCGACTAAGCAATTAGCGATGGCAATGAATTGCATCA	AAAGAGGCAAACTATGCCATCGCTAATT
		**SNX10-7**	CTCCTCATCTTCTTCCAGTACGTCGACTAAGCAATTAGCGATGGCATCCCTTTTTTTCC	GTCTGCATCGTTTATGCCATCGCTAATT

		**SNX10-8**	CACATACTGTTTGTCCAGTACGTCGACTAAGCAATTAGCGATGGCATTTCCTTGTGAAG	CTAACACAGGATGATGCCATCGCTAATT
		**SNX10-9**	GGTCTCGCACCAATCCAGTACGTCGACTAAGCAATTAGCGATGGCATTCTGCGTCCTGG	GCCATGAGTCTTCATGCCATCGCTAATT
	IFIT5	**IFIT5-1**	ACTCCCATCTGGTTATGTGACCGGGTACAATATGTGACCGGGTACATGCTCTGTAGCTG	AACTGCTTCAGCTATGTACCCGGTCACA
**Viral genes**	**PB2**	**PB2-1**	CAACACATTTGCCTATCTGACGCGAACTGAAGACGTAATGACAGCGCTCCTTGCCCTAT	TACCAACACCACGACGCTGTCATTACGT
		**PB2-2**	CTTTCCCGAGAACTATCTGACGCGAACTGAAGACGTAATGACAGCGAATGCACCTGCGT	CCGGATCTTCTGTACGCTGTCATTACGT
		**PB2-3**	AATGCAACACCTCTATCTGACGCGAACTGAAGACGTAATGACAGCGGTCCCTTGGGTCA	TCTGTTCCCAACATCGCTGTCATTACGT
		**PB2-4**	AAACCCATTGCTGTATCTGACGCGAACTGAAGACGTAATGACAGCGTGAGCTGATCCTC	CCGAAACTGAAGGTCGCTGTCATTACGT
		**PB2-5**	ATATCCACCCGGCTATCTGACGCGAACTGAAGACGTAATGACAGCGGTGACCAGGGTTT	GCACTGAGGTCCGGCGCTGTCATTACGT
		**PB2-6**	AAGTGTCTGCCCTTATCTGACGCGAACTGAAGACGTAATGACAGCGTTGTCTTGCTCCA	TGATCCAGCATCAACGCTGTCATTACGT
		**PB2-7**	TGCGGGACTGTGATATCTGACGCGAACTGAAGACGTAATGACAGCGAGTATCTCGCGAG	CGGTGGTCTTTGTACGCTGTCATTACGT
		**PB2-8**	TTGATGCATGGGGTATCTGACGCGAACTGAAGACGTAATGACAGCGAGTGCCTCAGTAG	TGCATCCTTTTGGTCGCTGTCATTACGT
		**PB2-9**	TGGCAACAGCTTTTATCTGACGCGAACTGAAGACGTAATGACAGCGGGGCTGCTGCAAA	ACTCTGCTCTGGTCCGCTGTCATTACGT
		**PB2-10**	TCTGGCTGTCAGTTATCTGACGCGAACTGAAGACGTAATGACAGCGCTTTTGGTCGCTG	TGGCCATCCGAATACGCTGTCATTACGT
		**PB2-11**	GATTTGTGTGCTATATCTGACGCGAACTGAAGACGTAATGACAGCGTCCTTATTCCACC	AAGGATATCCACCTCGCTGTCATTACGT
		**PB2-12**	TCAATTGAATCAGTATCTGACGCGAACTGAAGACGTAATGACAGCGCTTCCACTTACTA	TTGATTGTTCATCGCGCTGTCATTACGT
		**PB2-13**	TGAAAGTGAAACCTATCTGACGCGAACTGAAGACGTAATGACAGCGCCACTTGTTCTTT	TCTTCACAGATGAGCGCTGTCATTACGT
		**PB2-14**	TAGTGTTTGAAGGTATCTGACGCGAACTGAAGACGTAATGACAGCGGCACTCTTATTCT	TTCATACCCTTCAACGCTGTCATTACGT
		**PB2-15**	AAGCACTGATTCATATCTGACGCGAACTGAAGACGTAATGACAGCGGGTAAGTGTTGAC	CCTGATGATCCATACGCTGTCATTACGT
	**PB1**	**PB1-1**	TGGCCTCCACCATTATCTGACGCGAACTGATCCAGTACGTCGACTAGCCCTAGACACCA	GTGCGTCAATTCGCTAGTCGACGTACTG
		**PB1-2**	TGAAATTGGCCACTATCTGACGCGAACTGATCCAGTACGTCGACTAGGCAGTTCCATAC	ACACTCCAAAGCTCTAGTCGACGTACTG
		**PB1-3**	GAGGGTCTCCAGTTATCTGACGCGAACTGATCCAGTACGTCGACTACCATGGCTGTATG	ATCCTGTTCCCGTATAGTCGACGTACTG
		**PB1-4**	GTGTGGTTGCAACTATCTGACGCGAACTGATCCAGTACGTCGACTAGGGATCCATGAGT	AGCGGTTCCTTTTTAGTCGACGTACTG
		**PB1-5**	TGCGTCCGTAGCTTATCTGACGCGAACTGATCCAGTACGTCGACTATTCTGCCGGTATT	ATGTTTGCAAGCAATAGTCGACGTACTG
		**PB1-6**	CTACAGCGTTGTTTATCTGACGCGAACTGATCCAGTACGTCGACTATGGGCTGGCATTA	TCTTAGCTGGACCTTAGTCGACGTACTG
		**PB1-7**	ATTGCCCGCCTTTTATCTGACGCGAACTGATCCAGTACGTCGACTATCCGGGTGTAGCA	CCCCTGATTTGCAATAGTCGACGTACTG
		**PB1-8**	GAGGTTCCGGATATATCTGACGCGAACTGATCCAGTACGTCGACTACCTCCGGAATGTG	CCACTTCAAGCAGTTAGTCGACGTACTG
		**PB1-9**	AGACACGAGTTTTTATCTGACGCGAACTGATCCAGTACGTCGACTATTCCATCGTTTCA	GTTTGCTGAACAAATAGTCGACGTACTG
		**PB1-10**	GTGGAACAGATCTTATCTGACGCGAACTGATCCAGTACGTCGACTAGAGCTCTTCAATG	TATTTTTGCCGTCATAGTCGACGTACTG
		**PB1-11**	TTGTGTGATCATTTATCTGACGCGAACTGATCCAGTACGTCGACTATTCCTATTGTCCT	TCTTTGCTTTTTCATAGTCGACGTACTG
		**PB1-12**	GGTTCAGGATTGATATCTGACGCGAACTGATCCAGTACGTCGACTACTCTTCTGTCCAA	TGGTTTTGGTGTAATAGTCGACGTACTG
		**PB1-13**	TAGTTTGCAAGTCTATCTGACGCGAACTGATCCAGTACGTCGACTATGTTGATCCCAAT	TTTCTTCTTGCTCTTAGTCGACGTACTG
		**PB1-14**	CCAGGACTCAATGTATCTGACGCGAACTGATCCAGTACGTCGACTAGCCCATCATCATT	AGCATGTTGAACAATAGTCGACGTACTG

		**PB1-15**	GCTCTTATTAGGTTATCTGACGCGAACTGATCCAGTACGTCGACTAGTTCAGTGTTAAG	TCTTTTGTCATTGATAGTCGACGTACTG
		**PA-1**	AGCCAGGAGGTAATATCTGACGCGAACTGAGCAATTAGCGATGGCAGCACCTGTTTCCA	TTGGAGTTCTGCTTTGCCATCGCTAATT
		**PA-2**	AGTGCCTGAACAATATCTGACGCGAACTGAGCAATTAGCGATGGCACAGGTTGTCCCTA	AAGGTTCCAGGTTGTGCCATCGCTAATT
		**PA-3**	CTGGATCCAGCTTTATCTGACGCGAACTGAGCAATTAGCGATGGCATGTTGAACTCGCT	CAATTCACAGGCCATGCCATCGCTAATT
		**PA-4**	TCCACATAGGCTCTATCTGACGCGAACTGAGCAATTAGCGATGGCACGGTTTGAATCCA	TCAATGCAGCCGTATGCCATCGCTAATT
		**PA-5**	TTTGGTCGGCAAGTATCTGACGCGAACTGAGCAATTAGCGATGGCATTCGGTGGGAGAC	CAAGGCTGGAGAACTGCCATCGCTAATT
		**PA-6**	ATTGCAGTCCGCATATCTGACGCGAACTGAGCAATTAGCGATGGCATGACACTTGGCCT	AGGAACATGGGCCATGCCATCGCTAATT
		**PA-7**	CAGCCGAAAAATGTATCTGACGCGAACTGAGCAATTAGCGATGGCAGGTGGGCTCTTTC	TGTGGTTTGACGAGTGCCATCGCTAATT
	**PA**	**PA-8**	CAACAGGGCTGTGTATCTGACGCGAACTGAGCAATTAGCGATGGCACACAGGATGCATT	GTCATCCATGGCTCTGCCATCGCTAATT
		**PA-9**	ATCTAAGAGGGCGTATCTGACGCGAACTGAGCAATTAGCGATGGCAGGCCCATCAGGTA	GCTGAGAGCAGGGTTGCCATCGCTAATT
		**PA-10**	TTCCCCAATTGGCTATCTGACGCGAACTGAGCAATTAGCGATGGCACTCCTTTGGGTGA	TGAGCCTTCTTCCTTGCCATCGCTAATT
		**PA-11**	GTTTCGATTTTCGTATCTGACGCGAACTGAGCAATTAGCGATGGCAGGCAAATTTGTTC	TGTGTGCATATTGGTGCCATCGCTAATT
		**PA-12**	GATCTTGGAAGTTTATCTGACGCGAACTGAGCAATTAGCGATGGCACCCATTTCATCTT	TCTCATTTCCATGGTGCCATCGCTAATT
		**PA-13**	CGAAAGGAATCCCTATCTGACGCGAACTGAGCAATTAGCGATGGCATCTTTCGGACTGA	ATTGTCTCTTCGCGTGCCATCGCTAATT
		**PA-14**	GGTATCATTCCTCTATCTGACGCGAACTGAGCAATTAGCGATGGCAAGTTTACCACATC	TTCCATACTCACATTGCCATCGCTAATT
		**PA-15**	ACTTTCTCTGGTGTATCTGACGCGAACTGAGCAATTAGCGATGGCAGTCCTCAAAGTCT	CTGACATCTTTGCTTGCCATCGCTAATT
		**HA-1**	TGGAGCACATCCATATCTGACGCGAACTGATATCTGACGCGAACTGTGTAACGACCCAT	TGCAAATTCTGCAGCAGTTCGCGTCAGA
		**HA-2**	ATGGTGGTACCCATATCTGACGCGAACTGATATCTGACGCGAACTGCCTGCTCATTGCT	AGCGTACCCACTCGCAGTTCGCGTCAGA
		**HA-3**	TTGGGTTTCCGAGTATCTGACGCGAACTGATATCTGACGCGAACTGAATTCGTCGCACA	CCGGCACTCTGATCCAGTTCGCGTCAGA
		**HA-4**	AAGCTGCGCTCACTATCTGACGCGAACTGATATCTGACGCGAACTGCCCTGGTATGGAC	AAAAGGAGGGCGCACAGTTCGCGTCAGA
		**HA-5**	ACTCCATCTATTGTATCTGACGCGAACTGATATCTGACGCGAACTGGACCTTATTGGTA	TCAATGATTGAGTACAGTTCGCGTCAGA
		**HA-6**	AAATCTAGAGTCCTATCTGACGCGAACTGATATCTGACGCGAACTGATTTGAATCATGG	TAAAGGTTCTTGAGCAGTTCGCGTCAGA
		**HA-7**	TCTGATCACTCTTTATCTGACGCGAACTGATATCTGACGCGAACTGTAACCAATGCAAA	AATTGTTTGCATGCCAGTTCGCGTCAGA
	**HA**	**HA-8**	GGTCATTAGCTGGTATCTGACGCGAACTGATATCTGACGCGAACTGCCTGGGTAACAGA	AGTCATTGAGGCTGCAGTTCGCGTCAGA
		**HA-9**	GACATCTAGGAATTATCTGACGCGAACTGATATCTGACGCGAACTGCATTATAGGTCCA	AACTAGAAGTTCACCAGTTCGCGTCAGA
		**HA-10**	TCATCCGGTTTTATATCTGACGCGAACTGATATCTGACGCGAACTGGAAATGGATTGCA	TTTCCGTTACTCTGCAGTTCGCGTCAGA
		**HA-11**	TCTTCTGAATACTTATCTGACGCGAACTGATATCTGACGCGAACTGTTTTAATCTTGCT	CTTATTTCCTCTCACAGTTCGCGTCAGA
		**HA-12**	AGTGGCCCATATTTATCTGACGCGAACTGATATCTGACGCGAACTGCATTTGGTGTTGC	CTACTGGGGTTTGTCAGTTCGCGTCAGA
		**HA-13**	CCTCTGGTTTAAATATCTGACGCGAACTGATATCTGACGCGAACTGTTTTTGGTACCAA	GGATCTAGTAGCTTCAGTTCGCGTCAGA
		**HA-14**	TATCGTGTCAACTTATCTGACGCGAACTGATATCTGACGCGAACTGCGTTCTTTTCCAT	ATGTGTAACAGTGTCAGTTCGCGTCAGA
		**HA-15**	ACCACATTTCTGATATCTGACGCGAACTGATATCTGACGCGAACTGTTTGATAAGCCAC	TATGCATCGTTCTACAGTTCGCGTCAGA
	**NP**	**NP-1**	GATGCTCGCTGCTTAGTCGCAACTCTGAAATAGTCGCAACTCTGAAAATCTGCCCTGCA	GTGGGTTGAACACATTCAGAGTTGCGAC

		**NP-2**	AAATTCCGGTCGTTAGTCGCAACTCTGAAATAGTCGCAACTCTGAATTCGCCTCTCCAG	GTTCTCCGTCCATATTCAGAGTTGCGAC
		**NP-3**	CAAGCAGGCAAGCTAGTCGCAACTCTGAAATAGTCGCAACTCTGAAAAGTCCGTACACA	CCACTGGCCACTGGTTCAGAGTTGCGAC
		**NP-4**	CTTGGCGCCAAATTAGTCGCAACTCTGAAATAGTCGCAACTCTGAATCTCCGTTGTTTG	CAGCAGTTGCGTCGTTCAGAGTTGCGAC
		**NP-5**	GATCTTCTCGGGATAGTCGCAACTCTGAAATAGTCGCAACTCTGAAACCAGCAGCTCCA	CCCTTCACTGCTGGTTCAGAGTTGCGAC
		**NP-6**	ACGAGAGCTCTTGTAGTCGCAACTCTGAAATAGTCGCAACTCTGAACATTCCGGTACGA	CACATCCTGGGGTGTTCAGAGTTGCGAC
		**NP-7**	GAAGACTCCCCGCTAGTCGCAACTCTGAAATAGTCGCAACTCTGAACGTCCGAGAGCTC	GCTCGTTGCCTTTATTCAGAGTTGCGAC
		**NP-8**	GAACGAGAGCTCTTAGTCGCAACTCTGAAATAGTCGCAACTCTGAATCCATTCCGGTAC	AGCACATCCTGGGCTTCAGAGTTGCGAC
		**NP-9**	ACCGTGCCAGAAATAGTCGCAACTCTGAAATAGTCGCAACTCTGAAAGGATGAGTGCAG	CCACTGATCCTCTGTTCAGAGTTGCGAC
		**NP-10**	TTTCCCCTTGAGGTAGTCGCAACTCTGAAATAGTCGCAACTCTGAACAGCTGTTTGGAA	CATTGCTCGTTGTCTTCAGAGTTGCGAC
		**NP-11**	GTCGCTGAGTTTGTAGTCGCAACTCTGAAATAGTCGCAACTCTGAAGTCTCCCTTCGTA	GCTGTTCTGGATCTTTCAGAGTTGCGAC
		**NP-12**	GCTATTTTGAAGCTAGTCGCAACTCTGAAATAGTCGCAACTCTGAAGACTAAAGACCTG	ATTTGGCCTAATGTTTCAGAGTTGCGAC
		**NP-13**	GTACCATTCTCTCTAGTCGCAACTCTGAAATAGTCGCAACTCTGAATCAAATGCAGAAA	TGTTCCTTCTTTCTTTCAGAGTTGCGAC
		**NP-14**	AATTGGACCTCCATAGTCGCAACTCTGAAATAGTCGCAACTCTGAACTCTCCTCCGATA	AATCCATTTCCCGATTCAGAGTTGCGAC
		**NP-15**	CTCGCACTTGATCTAGTCGCAACTCTGAAATAGTCGCAACTCTGAAGGATTCCTGCTCT	TCTCAGCATTCCCTTTCAGAGTTGCGAC
	**NA**	**NA-1**	TGTTGTGCCCTGAAGACGTAATGACAGCGAATGAGATCGTCATCGTGTTGCCCTCTGAG	GGTTCCATTCGCAAACGATGACGATCTC
		**NA-2**	TATTGGCGCATCAAGACGTAATGACAGCGAATGAGATCGTCATCGTGGTTCCCTCCTTT	TACCCCGGGGTCTCACGATGACGATCTC
		**NA-3**	TCCCAATCGTTCCAGACGTAATGACAGCGAATGAGATCGTCATCGTGCCAGGACCATGA	TTCTGCACCATCAAACGATGACGATCTC
		**NA-4**	TGGAAGTCCACAGAGACGTAATGACAGCGAATGAGATCGTCATCGTGCAACCATGCTAC	TGGATCCACAGAGTACGATGACGATCTC
		**NA-5**	GACAGGTAAGGCTAGACGTAATGACAGCGAATGAGATCGTCATCGTCTGTGGATCACAG	AACATTCTGCAGCGACGATGACGATCTC
		**NA-6**	ATGCATTCGACCCAGACGTAATGACAGCGAATGAGATCGTCATCGTGCTTGACCATCCT	TCATGGCATGACGAACGATGACGATCTC
		**NA-7**	TGTCGATTTCTGCAGACGTAATGACAGCGAATGAGATCGTCATCGTGGCCCTGATTGAG	GCTGGTACGAGATAACGATGACGATCTC
		**NA-8**	TGACTACTGGGCAAGACGTAATGACAGCGAATGAGATCGTCATCGTGGACCATCTGTCA	CCTTGTTGTCTGCAACGATGACGATCTC
		**NA-9**	AGGAATGCAAACCAGACGTAATGACAGCGAATGAGATCGTCATCGTGTTCTCCCCGTCT	CTTCCAAGCCATGTACGATGACGATCTC
		**NA-10**	TTTAGGCCCAAATAGACGTAATGACAGCGAATGAGATCGTCATCGTGTGTAGTCCGATA	TCACTCACTTTGTTACGATGACGATCTC
		**NA-11**	ATTTGCTTGTGTGAGACGTAATGACAGCGAATGAGATCGTCATCGTTTCGAACACAAGT	TGTCGGTTAAGACAACGATGACGATCTC
		**NA-12**	TCTGTTACTGGTCAGACGTAATGACAGCGAATGAGATCGTCATCGTCCATGATGGGATT	AGAATGTTCCCTGGACGATGACGATCTC
		**NA-13**	CTTTCGACAGAATAGACGTAATGACAGCGAATGAGATCGTCATCGTCTTATCGCATTGT	CATCCTCACCTATAACGATGACGATCTC
		**NA-14**	TATAGTGTTAGTGAGACGTAATGACAGCGAATGAGATCGTCATCGTCGTTTTTATTCAT	AAATGTTTTTTCTAACGATGACGATCTC
		**NA-15**	TCTGCATACACATAGACGTAATGACAGCGAATGAGATCGTCATCGTCCTTCCAATTGTC	TGGTCTATTTGCCGACGATGACGATCTC
	**M**	**M-1**	CTGCTCCATAGCCTCCAGTACGTCGACTAATCCAGTACGTCGACTATCGATCCAGCCAT	CGCTGCTTGCTCAGTAGTCGACGTACTG
		**M-2**	TGCACTCCCATCCTCCAGTACGTCGACTAATCCAGTACGTCGACTAGAATCGCTGCAGT	CGAGAGGATCACTATAGTCGACGTACTG
		**M-3**	AAGGCCCTCTTTTTCCAGTACGTCGACTAATCCAGTACGTCGACTAACTCCTTCCGTAG	TCATGGACTCAGGATAGTCGACGTACTG

		**M-4**	ATGCCACTTCTGCTCCAGTACGTCGACTAATCCAGTACGTCGACTACACACTAGGCCCA	GCTCACAGGTGGCTTAGTCGACGTACTG
		**M-5**	TCCCTGCAAAGACTCCAGTACGTCGACTAATCCAGTACGTCGACTAAGATCGGTGTTCT	CCATGAGAGCCTCTTAGTCGACGTACTG
		**M-6**	TTCGACCTCGGTTTCCAGTACGTCGACTAATCCAGTACGTCGACTAAGAGAACGTACGT	TGACGGGACGATACTAGTCGACGTACTG
		**M-7**	CTGTTCCTGCCGGTCCAGTACGTCGACTAATCCAGTACGTCGACTACCACAGCACTCTG	ACCATCGTCAACAATAGTCGACGTACTG
		**M-8**	GACTGGCAACCTCTCCAGTACGTCGACTAATCCAGTACGTCGACTAATCTGCCTAGCCT	TCATCGCCTGCACGTAGTCGACGTACTG
		**M-9**	ACTGAGTGCAACTTCCAGTACGTCGACTAATCCAGTACGTCGACTACACCGGTTGAGTA	ACAACTGGCAAGTCTAGTCGACGTACTG
		**M-10**	TCTCTCTCTTCAGTCCAGTACGTCGACTAATCCAGTACGTCGACTACCATGGAATGTTA	CAACTTCTTTAGCGTAGTCGACGTACTG
		**M-11**	TTGGGTCTCCATTTCCAGTACGTCGACTAATCCAGTACGTCGACTACTGTCCATGTTGT	ACAGTTTGACTGCGTAGTCGACGTACTG
		**M-12**	AAGATCCCAATGATCCAGTACGTCGACTAATCCAGTACGTCGACTACAATATCAAGTGC	CGATCAAGAATCCTTAGTCGACGTACTG
		**M-13**	GTCTTTAGCCATTTCCAGTACGTCGACTAATCCAGTACGTCGACTACAGGATTGGTCTT	TTAGTCAGAGGTGTTAGTCGACGTACTG
		**M-14**	TGTCTGTGAGACCTCCAGTACGTCGACTAATCCAGTACGTCGACTAGGTGGTTGCTATC	ATTAGTGGGTTGGATAGTCGACGTACTG
		**M-15**	AGGAGATCATCTCTCCAGTACGTCGACTAATCCAGTACGTCGACTACTGCAAATTTTCA	CGTTTTTGGTAGGGTAGTCGACGTACTG
	**NS**	**NS-1**	GTCATTTTGAGCGGCAATTAGCGATGGCAAGCAATTAGCGATGGCACACGGAAGCAATG	TAGCGCGAAGCAGCTGCCATCGCTAATT
		**NS-2**	GTGGCTGTTTCGAGCAATTAGCGATGGCAAGCAATTAGCGATGGCACTTTCCCGAACGA	CGCTCCACTATCTCTGCCATCGCTAATT
		**NS-3**	AGGGACTTGGGATGCAATTAGCGATGGCAAGCAATTAGCGATGGCAGCCTCTTCCTCTC	AGACCAAGAGTGCATGCCATCGCTAATT
		**NS-4**	TTGGAGGGAGTGGGCAATTAGCGATGGCAAGCAATTAGCGATGGCATTCCGTTTCTGCT	TTGTTCTCGCCATATGCCATCGCTAATT
		**NS-5**	TGAGCATGACCCAGCAATTAGCGATGGCAAGCAATTAGCGATGGCATTCTGCTTGGGCA	GGGAACCTGCTACATGCCATCGCTAATT
		**NS-6**	TGCCAAAGAAAGCGCAATTAGCGATGGCAAGCAATTAGCGATGGCATCGTTTGCGGACA	TCTTGGTCTGCAATTGCCATCGCTAATT
		**NS-7**	TAAATAAGCTGAAGCAATTAGCGATGGCAAGCAATTAGCGATGGCAGTGTTTTTATCAT	ATAGTAGAACAAGCTGCCATCGCTAATT
		**NS-8**	AGTATGTCCTGGAGCAATTAGCGATGGCAAGCAATTAGCGATGGCATGACATCCTCATC	CCCAATTGCATTTATGCCATCGCTAATT
		**NS-9**	ATTCCATTCAAGTGCAATTAGCGATGGCAAGCAATTAGCGATGGCAGAACTGTGTTATC	AGTTTCAGAGATTGTGCCATCGCTAATT
		**NS-10**	CTATTATTGCCTGGCAATTAGCGATGGCAAGCAATTAGCGATGGCAATGATGTTTTTGT	AATTTGCTTTCAAATGCCATCGCTAATT
		**NS-11**	TTCTGTGAAAGCTGCAATTAGCGATGGCAAGCAATTAGCGATGGCACAATTGCTCCTTC	TGAGATTTCTCCCTTGCCATCGCTAATT
		**NS-12**	CACTTCTTCAATCGCAATTAGCGATGGCAAGCAATTAGCGATGGCATCAACTTATGTCG	GTTCTCTGTGATTATGCCATCGCTAATT
		**NS-13**	AGGCTTGCAAAAAGCAATTAGCGATGGCAAGCAATTAGCGATGGCAAGCAATAGTTGCA	CTTGCTCCACTTCTTGCCATCGCTAATT
**Mouse**	**Viral genes with mutations**	**PB2-627E**	CTGGTGGGGCTGCATGAGATCGTCATCGTAATGAGATCGTCATCGTATTCTACTCTGCT	GAGAAGAAAATTGGACGATGACGATCTC
		**PB2-E627K**	TTGGTGGGGCTGCTAGTCGCAACTCTGAAAATGAGATCGTCATCGTATTCTACTCTGCT	GAGAAGAAAATTGGACGATGACGATCTC
		**PB2-192E**	CTTCCTTTTTCTCAGACGTAATGACAGCGAGCAATTAGCGATGGCACAATCCTGGAGCT	AGGGAGCAATCTTTTGCCATCGCTAATT
		**PB2-E192K**	TTTCCTTTTTCTCTCCAGTACGTCGACTAAGCAATTAGCGATGGCACAATCCTGGAGCT	AGGGAGCAATCTTTTGCCATCGCTAATT
		**PB2-220V**	CTGGTAGAAATCTCAGATAGGACTAGTCAACAGATAGGACTAGTCAGTTCCGCCTGCTA	TGTATACACTACTATGACTAGTCCTATC
		**PB2-V220I**	TTGGTAGAAATCTTAGTCGCAACTCTGAAATGATCTTCCGATCTAAGTTCCGCCTGCTA	TGTATACACTACTATTAGATCGGAAGAT
		**PA-97T**	GTGTTGCAGATACTGATCTTCCGATCTAAATGATCTTCCGATCTAAGTCGACTCCTGTG	AGGAATTTAGGCTATTAGATCGGAAGAT

		**PA-T97I**	ATGTTGCAGATACTAGTCGCAACTCTGAAACAGATAGGACTAGTCAGTCGACTCCTGTG	AGGAATTTAGGCTATGACTAGTCCTATC
**Chicken**	**Viral genes with mutations**	**PB1-14A**	GCTGGAACTTTCAATGAGATCGTCATCGTAATGAGATCGTCATCGTTATAGCATTTTGC	GGGAATGTAGTACAACGATGACGATCTC
		**PB1-A14V**	ACTGGAACTTTCATAGTCGCAACTCTGAAAATGAGATCGTCATCGTTATAGCATTTTGC	GGGAATGTAGTACAACGATGACGATCTC
		**PB1-533N**	TTGTTCTTTATCAATGAGATCGTCATCGTATGATCTTCCGATCTAAATTGTTTATCATA	GCTGGTCCAAGGTGTTAGATCGGAAGAT
		**PB1-N533S**	CTGTTCTTTATCATAGTCGCAACTCTGAAATGATCTTCCGATCTAAATTGTTTATCATA	GCTGGTCCAAGGTGTTAGATCGGAAGAT
		**PA-528T**	GTAAGAGAGAATTTGATCTTCCGATCTAAATGATCTTCCGATCTAACAGCCTCGGGTCA	CACTTGTGTGGCTGTTAGATCGGAAGAT
		**PA-T528I**	ATAAGAGAGAATTTAGTCGCAACTCTGAAACAGATAGGACTAGTCACAGCCTCGGGTCA	CACTTGTGTGGCTGTGACTAGTCCTATC
		**NA-330I**	TTGGCGCATCACACAGATAGGACTAGTCAACAGATAGGACTAGTCATTCCCTCCTTTTA	CCCCGGGGTCTGGCTGACTAGTCCTATC
		**NA-I330L**	ATGGCGCATCACAATGAGATCGTCATCGTACAGATAGGACTAGTCATTCCCTCCTTTTA	CCCCGGGGTCTGGCTGACTAGTCCTATC
