# Supplementary material for: Heterogeneous viral-host response at single-cell resolution and viral adaptive mutational atlas in birds and mammals upon H5 influenza virus infection
Source: Natl Sci Rev. 2026 Jun 25;13(14):nwag380. doi: 10.1093/nsr/nwag380 (PMC13458601; doi:10.1093/nsr/nwag380)
Supplement: Supplementary file 1 [file nwag380_supplemental_files.zip › Supplementary Data 4.pdf]

Supplementary Data 4. The primers of host genes used for qPCR of HD11, 3D4/21, and MH-S cells. For represents forward primers, and rev represents reward primers.

Cell line	Gene name	Oligonucleotides
HD11	ANXA1-for	tgtttctgctttggataaagct
HD11	ANXA1-rev	tgacgttgagcatttgttct
HD11	BST2-for	tccatctctgagatgcacaag
HD11	BST2-rev	tcttgcagttgcttgacca
HD11	DDX60-for	tacaagtgacagggatgaagag
HD11	DDX60-rev	caccataatccgggtcttca
HD11	DHX58-for	tgctgagttacctccagaag
HD11	DHX58-rev	gcacagatctgcaggatgt
HD11	EEF1D-for	tggtgaccaaaatgaacttct
HD11	EEF1D-rev	cttgaagatggccttctgga
HD11	EPSTI1-for	tcctgtcagactgctaattgt
HD11	EPSTI1-rev	atcttctgttttcatcctctcga
HD11	IFI30-for	tgttggtgtcacatgaggag
HD11	IFI30-rev	ctgagagccagtgcagaga
HD11	IFI35-for	tggacatactgctgccatct
HD11	IFI35-rev	cttgtccaataggatgtcctcag
HD11	IFIH1-for	tggttgggattgagactgt
HD11	IFIH1-rev	agaaccagtccttcttctgca
HD11	NMI-for	tgagctgacagagctaaatgac
HD11	NMI-rev	tcttcctcataggcagcctta
HD11	PARP9-for	tggtctataaggatgatctcacaag
HD11	PARP9-rev	gctaaagcaagagctcctga
HD11	PARP14-for	tcactcaatggaacaccgat
HD11	PARP14-rev	tggcccttcctttccgtata
HD11	PML-for	tggagaacaacctggaggag
HD11	PML-rev	ttcagcagcttgggtgaga
HD11	RNF19A-for	tgatcgcatttggatgtgc
HD11	RNF19A-rev	ggtttggatgccaaatctgctta
HD11	RSAD2-for	tgcaaagaggagctgaaact
HD11	RSAD2-rev	ttgaaccaccgttccctga
HD11	STAT1-for	ttgatgcaaaagttaaggctg
HD11	STAT1-rev	cttacattcctcttgtgatgca
HD11	UBE2L6-for	ccttcctgcaccaggca
HD11	UBE2L6-rev	aggagtgaggtaggagcttta
HD11	USP18-for	tgtacaacattggacagacct
HD11	USP18-rev	tccttcgcagtatctttgtga
HD11	ZNFX1-for	tagaagaaaccaaggtggacc

HD11	ZNFX1-rev	accttcttccccttccatca
HD11	β actin-for	tgggtatggagtcctgtggt
HD11	β actin-rev	ctgtcagcaatgccagggta
MH-S	Anxa1-for	cctcatcttcgcagagtgtttcag
MH-S	Anxa1-rev	cttctcaatgtcacccttcagttcc
MH-S	Bst2-for	agaatgaagtcacgaagctgaacc
MH-S	Bst2-rev	gacactttgagcaccagtaggc
MH-S	Ddx60-for	agaaaggggcattggctacc
MH-S	Ddx60-rev	gctgtcaccaccctgatgaa
MH-S	Dhx58-for	aattctcgctgattgtggtggac
MH-S	Dhx58-rev	cggctcaagatggtgttgtagac
MH-S	Eef1d-for	agtgacgaggaggaagaagataagg
MH-S	Eef1d-rev	ttctctgcgtactggcgtagc
MH-S	Epsti1-for	aagaactggcagacctggagag
MH-S	Epsti1-rev	cgttgtggcaccaagtagactg
MH-S	Ifi30-for	ctgggtgctggtcaatgagaaac
MH-S	Ifi30-rev	tcggcaatggaggagcagatg
MH-S	Ifi35-for	tgtccagccagccagataacc
MH-S	Ifi35-rev	ttcctcttcactcagcctaagtcc
MH-S	Ifi44-for	tctgctgctgggtcctattgg
MH-S	Ifi44-rev	tgtgtgatgctgccccttgaac
MH-S	Ifih1-for	gccttgtcacgaacgagatagc
MH-S	Ifih1-rev	tcggtaactcctgagccactg
MH-S	Ifit1-for	tgagatggactgtgaggaaggc
MH-S	Ifit1-rev	tcttggcgataggctacgactg
MH-S	Ifit2-for	gagtgaaggtgtgtttccagaagg
MH-S	Ifit2-rev	catccagacggtagttcgcaatg
MH-S	Ifit3-for	gatccacagtgaacaacagtcctc
MH-S	Ifit3-rev	tcgtctcagttctgccatcctc
MH-S	Isg15-for	tcctggtgtccgtgactaactc
MH-S	Isg15-rev	taagaccgtcctggagcactg
MH-S	Nmi-for	tggacgatatgagaggcgaacag
MH-S	Nmi-rev	tccgactgcaattcagcttcaag
MH-S	Oas2-for	tcctctacgctccaatgagaatcc
MH-S	Oas2-rev	gtgtggtgaagcagtctgagaac
MH-S	Parp9-for	gcaggtggagcagatagacaataag
MH-S	Parp9-rev	atccctttggcgtcctctcttc
MH-S	Parp14-for	acgctactgcctacgggaaag
MH-S	Parp14-rev	attggtgtctggtctggaatatgtg
MH-S	Pml-for	ccaataccaaccaccgcaacc
MH-S	Pml-rev	aatatcgcaatggagatggctgtg
MH-S	Psme2-for	ggtggcaattcaggagaaggtg
MH-S	Psme2-rev	cttggaaatggttgtctggaaagc
MH-S	Rnf19a-for	gtgactgtaggcattggtgttcc

MH-S	Rnf19a-rev	cccacttcggcagagagaaattg
MH-S	Rsad2-for	ctctgtgagcatagtgagcaatgg
MH-S	Rsad2-rev	gtcgcaggagatagcaagaatgtc
MH-S	Stat1-for	tggaggagttggaacagaaattcac
MH-S	Stat1-rev	gctgctggaagaggaggaagg
MH-S	Ube2l6-for	aagccttacaccaagccttatcaag
MH-S	Ube2l6-rev	cagcaagttccagacgcacag
MH-S	Usp18-for	cgatgctgctcaactctaccttac
MH-S	Usp18-rev	tcctgcgtccagatggtgaac
MH-S	Znfx1-for	tctatccgcttctatgaccacttgg
MH-S	Znfx1-rev	tcctcacgctctgctgttctc
MH-S	β actin-for	ggctgtattcccctccatcg
MH-S	β actin-rev	ccagttggtaacaatgccatgt
3D4/21	ANXA1-for	gcacagcgtcagcagatcaag
3D4/21	ANXA1-rev	tttcagcaaagccaaagcaacttc
3D4/21	BST2-for	agactgtggagaacctacgagattc
3D4/21	BST2-rev	ccttagtcgctccgcctcttc
3D4/21	DDX60-for	acgctacctggtggatctgattac
3D4/21	DDX60-rev	cgatgttggtgttgtgttgaagatg
3D4/21	DHX58-for	tacgagcagcaagtggtggag
3D4/21	DHX58-rev	gttgtagcgacgcaggtgaag
3D4/21	EEF1D-for	gaggacgacgaggacgatgac
3D4/21	EEF1D-rev	cagcaggatggaggacttggc
3D4/21	EPSTI1-for	ttaaacgacagcaacaggaggaag
3D4/21	EPSTI1-rev	ctggttgactttcgccttggag
3D4/21	IFI30-for	aaacgcccaggaacgaaatgtc
3D4/21	IFI30-rev	gcctccaccttgttcatcttgc
3D4/21	IFI35-for	ggtgctggtgctcaacattcc
3D4/21	IFI35-rev	cacaactgtcacggcttccac
3D4/21	IFI44-for	cgtggacaccttggatctgattac
3D4/21	IFI44-rev	cttgagtttcacaggcacacatttg
3D4/21	IFIH1-for	gcttctggttaccgatgtcttgg
3D4/21	IFIH1-rev	gctcccttactcctgattcatttcc
3D4/21	IFIT1-for	agatggactgtgaggaaggatgg
3D4/21	IFIT1-rev	gtctaggcgatagatggtgattgc
3D4/21	IFIT2-for	cgcagaagacggcagagaatg
3D4/21	IFIT2-rev	aggcgagataggagcagacaag
3D4/21	IFIT3-for	gccattgagttgagccctgac
3D4/21	IFIT3-rev	cactgcggaggacatctgtttg
3D4/21	ISG15-for	gcagcacagtcctgttgatgg
3D4/21	ISG15-rev	tgcgtcagccagacctcatag
3D4/21	OAS2-for	ctctgaagaactccgatgctgac
3D4/21	OAS2-rev	ctgcttgtggatttccttgatgatg
3D4/21	PARP9-for	ctcggtggcggttaatggatag

3D4/21	PARP9-rev	agtgctggaattgctactgtcttg
3D4/21	PARP14-for	aggaactgaagatagtcggacaagg
3D4/21	PARP14-rev	atgaagcacgcattggcaagg
3D4/21	PSME2-for	gaaaaggtgctggagagggtaaatg
3D4/21	PSME2-rev	gtacttggaaatggtggtctggaag
3D4/21	RNF19A-for	aaggcaacagtatggaggttcaag
3D4/21	RNF19A-rev	catcgtctacactgctgcttcc
3D4/21	RSAD2-for	ccactagcgtcaattaccacttcac
3D4/21	RSAD2-rev	ttcagcagccacaagcctctc
3D4/21	STAT1-for	accttcagcctcttccagcag
3D4/21	STAT1-rev	acttcaccaacagtctcaacttcac
3D4/21	UBE2I6-for	cccggaggagtacccattcaag
3D4/21	UBE2I6-rev	ttccagttctcgttgctgatgatg
3D4/21	USP18-for	ctctacctcaccgtctggaacc
3D4/21	USP18-rev	catgctccgtggtacattcaagg
3D4/21	ZNFX1-for	gcggctagaagagattgaagtcg
3D4/21	ZNFX1-rev	ttaagtcgttcctggctgattgc
3D4/21	NMI-for	ccaccacaaactcccagattcac
3D4/21	NMI-rev	actgtctgtcactctcaggattctc
3D4/21	PML-for	gtgtatgcctactccatcaaagacc
3D4/21	PML-rev	cctcctccacttctgactccatc
3D4/21	β actin-for	gctcgttgtagaaggtgtggtg
3D4/21	β actin-rev	cctgaccctcaagtacccca
