# Supplementary material for: Heterogeneous viral-host response at single-cell resolution and viral adaptive mutational atlas in birds and mammals upon H5 influenza virus infection
Source: Natl Sci Rev. 2026 Jun 25;13(14):nwag380. doi: 10.1093/nsr/nwag380 (PMC13458601; doi:10.1093/nsr/nwag380)
Supplement: Supplementary file 1 [file nwag380_supplemental_files.zip › Supplementary Data 5.pdf]

Supplementary Data 5. The primers of 21957-1/H5N6 mutants using site-directed mutagenesis PCR. For represents forward primers, and rev represents reward primers.

Primer name	Oligonucleotides
PB2-G74R-for	cagaaagaaatgaacaaaggcagacactttggagcaagac
PB2-G74R-rev	gtcttgctccaaagtgtctgcctttgttcatttctttctg
PB2-E192K-for	caataacaaaggagaaaaaggaaaagctccaggattgtaagattgctc
PB2-E192K-rev	gagcaatcttacaatcctggagcttttccttttctcctttgttattg
PB2-V220I-for	ctggtccgcaaaaccagatttctaccaatagcaggcggaacaagtagtg
PB2-V220I-rev	cactacttgttccgcctgctattggtagaaatctggttttgcggaccag
PB2-E627K-for	gccatttgcagcagccccaccaaagcagagtagaatgcaattttcttc
PB2-E627K-rev	gaagaaaattgcattctactctgctttggtggggctgctgcaaatggc
PB1-A14V-for	gttccagtgcaaaatgctataagtactacattcccttatactggagac
PB1-A14V-rev	gtctccagtataagggaatgtagtacttatagcattttgcactggaac
PB1-T123S-for	cagcaaacaagagtggataaactgagccaaggtcgccagacctatg
PB1-T123S-rev	cataggtctggcgaccttggctcagtttatccactcttgtttgctg
PB1-N533S-for	gtgttacagtgataaagaacagtatgataaacaatgaccttggaccag
PB1-N533S-rev	ctggtccaaggtcattgtttatcatactgttctttatcactgtaacac
PB1-H562Q-for	gactacagatacacttatcggtgccaaaggggtgatacgc
PB1-H562Q-rev	gcgtatcacccctttggcaccgataagtgtatctgtagtc
PA-K34R-for	gaacagatttgccgcaatatgcacacacctagaagtctgtttcatg
PA-K34R-rev	catgaaacagacttctaggtgtgtgcatattgcggcaaatctgttc
PA-T97I-for	ctggacagtggtaaacagtatctgcaacatcacaggagtcgacaagcc
PA-T97I-rev	ggcttgtcgactcctgtgatgttgcagatactgtttaccactgtccag
PA-I145V-for	gcagagaggacacacgttcacatattctcattcactggtg
PA-I145V-rev	caccagtgaatgagaatatgtgaacgtgtgtcctctctgc
PA-M485I-for	gtgcagccatggatgactttcaactgattccaataataagcaaatgc
PA-M485I-rev	gcatttgcttattattggaatcagttgaaagtcatccatggctgcac
PA-T528I-for	gtgagtatggaattctctcttattgacccgaggctggagccacacaag
PA-T528I-rev	cttgtgtggctccagcctcgggtcaataagagagaattccatactcac
HA-S233P-for	ggtaccaaaaatagctactagaccccaagtaaacgggcaacgtggaag
HA-S233P-rev	cttccacgttgcccgtttacttggggtctagtagctatttttggtacc
HA-D465G-for	gtcaagaacctttacggcaaagtcagactacagcttaggg
HA-D465G-rev	ccctaagctgtagtctgactttgccgtaaaggttcttgac
NA-N49T-for	ccaaccatgaatgagaccaacccaacaacaacaaacatcactaacac
NA-N49T-rev	gtgttagtgatgtttgttgttgttgggttggtctcattcatggttgg
NA-I330L-for	caatgggaactgtgatgcgccattaaaaggagggaacccagaccccgg
NA-I330L-rev	ccggggtctgggttccctccttttaatggcgcatcacagttcccattg
M2-E70K-for	cttctacggaaggagtacctaagtccatgagggaagagta
M2-E70K-rev	tactcttccctcatggacttaggtactccttccgtagaag
NS-P216S-for	cttggagaagcagtaatgagaatgggggacctccactcccttcaaagc
NS-P216S-rev	gctttgaagggagtggaggtcccccattctcattactgcttctccaag
