# Supplementary material for: Heterogeneous viral-host response at single-cell resolution and viral adaptive mutational atlas in birds and mammals upon H5 influenza virus infection
Source: Natl Sci Rev. 2026 Jun 25;13(14):nwag380. doi: 10.1093/nsr/nwag380 (PMC13458601; doi:10.1093/nsr/nwag380)
Supplement: Supplementary file 1 [file nwag380_supplemental_files.zip › Supplementary Data 6.pdf]

Supplementary Data 6. Mutation rates of the 19 non-synonymous viral mutations in the lungs of different species after infection.

Gene	Mutations	Nucleotide position	Mutation rate
PB2	E192K	547	0.184475323
	E627K	1879	0.296891585
	V220I	685	0.134020619
	G74R	220	0.581442974
PB1	A14V	41	0.212874442
	N533S	1598	0.14547711
	T123S	368	0.017072703
	H562Q	1686	0.243083837
PA	T97I	290	0.210180863
	T528I	1583	0.127939142
	M485I	1455	0.069627193
	K34R	101	0.354383814
	I145V	433	0.336650315
HA	S233P	697	0.112206638
	D465G	1394	0.347460369
NA	I330L	988	0.239182187
	N49T	146	0.096396132
M2	E69K	896	0.177312213
NS1	P216S	646	0.017629775
