# Supplementary material for: Heterogeneous viral-host response at single-cell resolution and viral adaptive mutational atlas in birds and mammals upon H5 influenza virus infection
Source: Natl Sci Rev. 2026 Jun 25;13(14):nwag380. doi: 10.1093/nsr/nwag380 (PMC13458601; doi:10.1093/nsr/nwag380)
Supplement: Supplementary file 1 [file nwag380_supplemental_files.zip › Supplementary Data 7.pdf]

Supplementary Data 7. The raw data of the viral titers of infected MDCK cells used in this study.

	12 hpi (TCID50/mL)	36 hpi (TCID50/mL)
WT	4.67	8.61
	4.33	8.57
	4.67	8.59
PB2-G74R	4.67	8.50
	4.61	8.43
	4.80	8.20
PB2-E192K	5.33	8.57
	5.41	8.59
	5.33	8.50
PB2-V220I	3.59	7.81
	3.57	7.67
	2.90	7.67
PB2-E627K	6.41	9.20
	5.75	8.67
	6.29	9.00
PB1-H562Q	2.64	7.57
	3.57	7.75
	2.90	7.67
PA-T97I	5.43	8.57
	5.50	8.50
	5.59	8.20
PA-I145V	1.67	7.12
	2.12	7.59
	2.12	7.59
HA-D465G	3.75	8.33
	3.50	8.20
	3.43	8.29
M2-E70K	2.57	7.80
	2.80	7.80
	2.67	7.81
