# Supplementary material for: Heterogeneous viral-host response at single-cell resolution and viral adaptive mutational atlas in birds and mammals upon H5 influenza virus infection
Source: Natl Sci Rev. 2026 Jun 25;13(14):nwag380. doi: 10.1093/nsr/nwag380 (PMC13458601; doi:10.1093/nsr/nwag380)
Supplement: Supplementary file 1 [file nwag380_supplemental_files.zip › Supplementary Data 8.pdf]

Supplementary Data 8. The raw data of the viral titers of infected DF1 cells used in this study.

	12 hpi (TCID50/mL)	36 hpi (TCID50/mL)
WT	7.50	8.67
	7.00	8.80
	7.39	8.49
PB1-A14V	5.50	8.39
	5.43	8.80
	5.50	8.75
PB1-T123S	6.23	8.57
	5.90	9.23
	6.50	9.23
PB1-N533S	6.20	8.80
	6.43	8.61
	6.00	8.57
PA-K34R	5.50	8.33
	5.50	8.67
	5.50	8.20
PA-M485I	5.50	8.49
	5.64	8.20
	6.20	8.41
PA-T528I	6.29	8.57
	6.43	8.50
	5.80	8.50
HA-S233P	6.29	8.67
	6.20	8.50
	6.41	8.80
NA-N49T	5.67	8.67
	5.67	9.00
	5.67	8.75
NA-I330L	5.50	8.57
	5.41	8.67
	5.39	8.80
NS1-P216S	6.50	9.12
	6.59	8.50
	6.50	8.59
