# Supplementary material for: Heterogeneous viral-host response at single-cell resolution and viral adaptive mutational atlas in birds and mammals upon H5 influenza virus infection
Source: Natl Sci Rev. 2026 Jun 25;13(14):nwag380. doi: 10.1093/nsr/nwag380 (PMC13458601; doi:10.1093/nsr/nwag380)
Supplement: Supplementary file 1 [file nwag380_supplemental_files.zip › Supplementary Data 9.pdf]

Supplementary Data 9. The raw data of the firefly luciferase activities and Renilla luciferase activities in DF1 cells after transfected with plasmids. FI represents firefly luciferase activities, and TK represents Renilla luciferase activities.

Mutations	WT			PB1-T123S		
FI	169066	188550	175856	207460	189848	210778
TK	3220	4238	4450	4916	3547	3299
FI/TK	52.50497	44.49033	39.5182	42.20098	53.52354	63.89148
FI	168571	189278	175047	246662	196208	216877
TK	4871	4995	5411	7775	4055	5063
FI/TK	34.60706	37.89349	32.35021	31.72502	48.38668	42.83567
FI	167276	185215	170008	251854	197833	208183
TK	5728	6453	6313	6319	5246	4989
FI/TK	29.20321	28.70215	26.92983	39.85662	37.71121	41.7284
Mutations	PB1-N533S			PA-K34R		
FI	149341	156599	153240	279529	260034	304413
TK	2942	4565	3223	8897	7451	4529
FI/TK	50.76173	34.30427	47.54576	31.41834	34.89921	67.21418
FI	154454	158190	155865	264430	257389	295338
TK	5287	5034	4865	6867	7404	8274
FI/TK	29.21392	31.42431	32.03803	38.50735	34.76351	35.69471
FI	155708	157255	156845	265163	252092	288761
TK	5741	5444	4500	7548	8840	9636
FI/TK	27.1221	28.88593	34.85444	35.13023	28.51719	29.96689
Mutations	PA-M485I			PA-T528I		
FI	135444	169589	171363	167360	167510	185181
TK	5165	5426	4937	5426	5481	5529
FI/TK	26.22343	31.25488	34.70995	30.84408	30.56194	33.49267
FI	136074	163839	171990	160902	157120	182620
TK	5404	5376	5728	5464	4308	5125
FI/TK	25.18024	30.476	30.02619	29.44766	36.47168	35.63317
FI	134797	162570	168259	159870	150929	178259
TK	5382	6265	5530	4464	4926	5222
FI/TK	25.04589	25.94892	30.42658	35.81317	30.63926	34.13615
