# Supplementary material for: Heterogeneous viral-host response at single-cell resolution and viral adaptive mutational atlas in birds and mammals upon H5 influenza virus infection
Source: Natl Sci Rev. 2026 Jun 25;13(14):nwag380. doi: 10.1093/nsr/nwag380 (PMC13458601; doi:10.1093/nsr/nwag380)
Supplement: Supplementary file 1 [file nwag380_supplemental_files.zip › Supplementary Figures.docx]

**Supplementary Figure 1.** Phylogenetic analysis of the HA gene sequences of the H5 subtype influenza viruses. Maximum likelihood (ML) phylogenies for the codon alignment of HA gene segments were estimated using the TIM nucleotide substitution model in the IQ-TREE. Node support was determined by nonparametric bootstrapping with 1,000 replicates. The phylogenetic tree was graphically illustrated in the FigTree (version 1.4.3) program (<http://tree.bio.ed.ac.uk/software/figtree/>). In HA phylogenetic tree, 21957-1/H5N6 isolated in the study was marked with red color, and H5N1 influenza viruses isolated from dairy cows were marked with blue color.

**Supplementary Figure 2*.*** Histopathological analysis of lungs in uninfected chickens (A) and infected chickens (B). 1# represents the first chicken, and 2# represents the second chicken, and 3# represents the third chicken.

**Supplementary Figure 3*.*** Histopathological analysis of lungs in uninfected ducks (A) and infected ducks (B). 1# represents the first duck, and 2# represents the second duck, and 3# represents the third duck.

**Supplementary Figure 4*.*** Histopathological analysis of lungs in uninfected pigeons (A) and infected pigeons (B). 1# represents the first pigeon, and 2# represents the second pigeon, and 3# represents the third pigeon.

**Supplementary Figure 5*.*** Histopathological analysis of lungs in uninfected mice (A) and infected mice (B). 1# represents the first mice, and 2# represents the second mice, and 3# represents the third mice.

**Supplementary Figure 6*.*** Histopathological analysis of lungs in uninfected pigs (A) and infected pigs (B). 1# represents the first pig, and 2# represents the second pig, and 3# represents the third pig.

**Supplementary Figure 7.** Quality check of scRNA-seq in different groups. (A) The number of cells in different groups. (B) The average gene number of each cell in different groups. (C) Percentage of mitochondrial genes (Percent.mt), ribosomal genes (Percent.hb), and red blood cell genes (Percent.Htt) in different groups. (D) The cell density of the number of UMIs and gene numbers per cell in different groups. (E) Cell density of the number of UMIs in different groups. (F) Cell density of the number of genes in different groups. (F) Cell density of percentage of mitochondrial genes in different groups. (G) The correlation between number of genes and number of UMIs per cell in different groups. (H) Heatmap of the expression level of top marker genes in different cell type. Violin plot of the expression level of marker genes in each cell types of chickens (I), ducks (J), pigeons (K), mice (L), and pigs (M).

**Supplementary Figure 8*.*** Violin plot showing the fold change of DEGs in T cells (A) and NK cells (B) across five species upon infection.

**Supplementary Figure 9*.*** Violin plot showing the fold change of DEGs in macrophages (A) and neutrophils (B) across five species upon infection.

**Supplementary Figure 10*.*** Violin plot showing the fold change of DEGs in B cells (A) and dendritic cells (B) across five species upon infection.

**Supplementary Figure 11*.*** Violin plot showing the fold change of DEGs in fibroblasts (A) and endothelial cells (B) across five species upon infection.

**Supplementary Figure 12*.*** GO enrichment analysis of species-specific up-regulated DEGs in all subtype cells of chickens upon infection.

**Supplementary Figure 13*.*** GO enrichment analysis of species-specific up-regulated DEGs in all subtype cells of ducks upon infection.

**Supplementary Figure 14*.*** GO enrichment analysis of species-specific up-regulated DEGs in all subtype cells of pigeons upon infection.

**Supplementary Figure 15*.*** GO enrichment analysis of species-specific up-regulated DEGs in all subtype cells of mice upon infection.

**Supplementary Figure 16*.*** GO enrichment analysis of species-specific up-regulated DEGs in all subtype cells of pigs upon infection.

**Supplementary Figure 17*.*** Gene-level heatmaps showing log_2_ fold changes in gene module scores of all cell types across species upon infection.

**Supplementary Figure 18*.*** Gene-level heatmaps showing log_2_ fold changes in gene module scores of fibroblasts across species upon infection. (A) UMAP plot for three subtypes of fibroblasts in lungs of different species. (B) Dotplot revealing the viral genes expression level in fibroblasts of infected lungs in chickens, ducks, and mice. (C) Gene-level heatmaps showing log_2_ fold changes in gene module scores of fibroblasts across species upon infection.

**Supplementary Figure 19.** The proportions of the subtypes of T cells and GO enrichment analysis of the CD4^+^ and CD8^+^ T cells in different species. (A) The proportion of different subtypes of T cells in UI and IN species. (B) Dotplot revealing the expression level of marker genes of different subtype cells.

**Supplementary Figure 20.** Gene-level heatmaps showing log_2_ fold changes in NETosis-related genes of neutrophils across all species.

**Supplementary Figure 21.** The changes of the mRNA level in immune-related genes of HD11, 3D4/21, and MH-S cells after infection. (A-D) The transcriptional level changes of immune-related genes in HD11 cells after infection at 6 hpi (A), in MH-S cells after infection at 6 hpi (B), and in 3D4/21 cells after infection at 6 hpi (C) and at 24 hpi (D). Relative expression of mRNA levels of different host genes was indicated. The mRNA expression levels of different host genes are expressed as fold changes in relation to the infected groups. Data are represented as means ± SEM. The independent-sample (n=9) t-test was used for analysis. Statistical significance was indicated as ∗ (p < 0.05), ∗∗ (p < 0.01), ∗∗∗ (p < 0.001), ∗∗∗∗ (p < 0.0001).

**Supplementary Figure 22.** Dot plot showing the expression level of marker genes of single-cell spatial transcriptomic data of five species. The size of the dot represents the proportion of the cells in which genes are detected. The color of the dot indicates the gene expression level.

**Supplementary Figure 23.** The spatial single-cell atlas showing the location of RNA of the marker genes of each cell type in uninfected (UI) and infected (IN) chickens. 1# represents the first animal, and 2# represents the second animal, and 3# represents the third animal.

**Supplementary Figure 24.** The spatial single-cell atlas showing the location of RNA of the marker genes of each cell type in uninfected (UI) and infected (IN) ducks. 1# represents the first animal, and 2# represents the second animal, and 3# represents the third animal.

**Supplementary Figure 25.** The spatial single-cell atlas showing the location of RNA of the marker genes of each cell type in uninfected (UI) and infected (IN) pigeons. 1# represents the first animal, and 2# represents the second animal, and 3# represents the third animal.

**Supplementary Figure 26.** The spatial single-cell atlas showing the location of RNA of the marker genes of each cell type in uninfected (UI) and infected (IN) mice. 1# represents the first animal, and 2# represents the second animal, and 3# represents the third animal.

**Supplementary Figure 27.** The spatial single-cell atlas showing the location of RNA of the marker genes of each cell type in uninfected (UI) and infected (IN) pigs. 1# represents the first animal, and 2# represents the second animal, and 3# represents the third animal.

**Supplementary Figure 28.** Spatial localization of different cell types in uninfected (UI) and infected (IN) lungs of chickens, ducks, pigeons, mice, and pigs. 2# represents the second animal, and 3# represents the third animal.

**Supplementary Figure 29.** Spatial localization of different cells with different infection levels (Bystander, Low, and High) in chickens (A), ducks (B), pigeons (C), mice (D), and pigs (E). 2# represents the second animal, and 3# represents the third animal. 2# represents the second animal, and 3# represents the third animal.

**Supplementary Figure 30.** The spatial distance and Moran’s I of each celltype different species upon infection. (A) Heatmaps showing log_2_ fold changes of the spatial distance within different cell types of infected and uninfected species using Sopa. (B) Moran’s I of each cell type across infected and uninfected species. UI represents uninfected species, and IN represents infected species.

**Supplementary Figure 31.** Immune microenvironment of the lungs in various species after infection. Spatial single-cell atlas showing the location of RNA of viral genes and immune-related genes in chickens (A) and mice (B). (C) Relative expression levels of immune-related genes in bystander cells (Bystander), low infected cells (Low), and high infected cells (High) using spatial single-cell transcriptomic data.

**Supplementary Figure 32.** Percentage of specific amino acid diversity in this study of H5N6 influenza viruses in birds and humans in GISAID Epiflu’s Database.

**Supplementary Figure 33.** The transcriptional level changes of the immune-related genes in the MH-S cells and NIH/3T3 cells after virus infection. (A) The mRNA level changes of immune-related genes in MH-S cells infected with wild-type (WT) virus and different mutants after 24 hpi. Relative expression levels of different host genes mRNA were indicated. The mRNA expression levels of different host genes are expressed as fold changes of different mutants in relation to WT. (B) The expression level changes of immune-related genes in NIH/3T3 cells infected with wild-type and different mutants after 24 hpi. Data are represented as means ± SEM. The independent-sample (n=3) t-test was used for analysis. Statistical significance was indicated as ∗ (p < 0.05), ∗∗ (p < 0.01), ∗∗∗ (p < 0.001), ∗∗∗∗ (p < 0.0001).
